# A Proteomic Approach to Investigate the Drought Response in the Orphan Crop *Eragrostis tef*

**DOI:** 10.3390/proteomes5040032

**Published:** 2017-11-15

**Authors:** Rizqah Kamies, Jill M. Farrant, Zerihun Tadele, Gina Cannarozzi, Mohammed Suhail Rafudeen

**Affiliations:** 1 Department of Molecular and Cell Biology, University of Cape Town, Private Bag X3, Rondebosch 7701, South Africa; rizqahkamies@gmail.com (R.K.); Jill.farrant@uct.ac.za (J.M.F.); 2 Institute of Plant Sciences, University of Bern, Altenbergrain 21, 3013 Bern, Switzerland; zerihun.tadele@ips.unibe.ch (Z.T.); gina@cannarozzi.com (G.C.)

**Keywords:** tef, drought-stress, functional enrichment analysis, GO-term, iTRAQ, physiological characterisation, quantitative proteomics, stress-responsive proteins

## Abstract

The orphan crop, *Eragrostis tef*, was subjected to controlled drought conditions to observe the physiological parameters and proteins changing in response to dehydration stress. Physiological measurements involving electrolyte leakage, chlorophyll fluorescence and ultra-structural analysis showed tef plants tolerated water loss to 50% relative water content (RWC) before adverse effects in leaf tissues were observed. Proteomic analysis using isobaric tag for relative and absolute quantification (iTRAQ) mass spectrometry and appropriate database searching enabled the detection of 5727 proteins, of which 211 proteins, including a number of spliced variants, were found to be differentially regulated with the imposed stress conditions. Validation of the iTRAQ dataset was done with selected stress-related proteins, fructose-bisphosphate aldolase (FBA) and the protective antioxidant proteins, monodehydroascorbate reductase (MDHAR) and peroxidase (POX). Western blot analyses confirmed protein presence and showed increased protein abundance levels during water deficit while enzymatic activity for FBA, MDHAR and POX increased at selected RWC points. Gene ontology (GO)-term enrichment and analysis revealed terms involved in biotic and abiotic stress response, signaling, transport, cellular homeostasis and pentose metabolic processes, to be enriched in tef upregulated proteins, while terms linked to reactive oxygen species (ROS)-producing processes under water-deficit, such as photosynthesis and associated light harvesting reactions, manganese transport and homeostasis, the synthesis of sugars and cell wall catabolism and modification, to be enriched in tef downregulated proteins.

## 1. Introduction 

Water-deficit stress, as a consequence of drought, has been proposed to be the most important abiotic factor in limiting crop plant growth, development and productivity [1,2]. Furthermore, climate change models predict increasing periods of extended drought over much of Africa, where the bulk of agriculture is rainfed, rendering conventional cropping practises ineffective [3,4]. *Eragrostis tef* (Zucc.) Trotter, commonly known as tef, is an indigenous African grass that has been cultivated in the Horn of Africa for some 2000 years [5]. The grain is a source of income to many resource-poor subsistence farmers and a staple food source for many low-income consumers [6]. Tef is a versatile crop able to grow under a wide range of soil types, climatic conditions and at differing altitudes ranging from 1000 to 2500 m above sea level [7,8,9]. The major abiotic stress factors affecting its growth and production include drought, soil salinity and acidity [10]. While some research has shown that different varieties of tef exhibit relative tolerance to increased salinity [11] and soil acidity [12], the majority of studies have reported on tef tolerance to drought stress [13,14,15,16,17]. Although tef is well suited to growth and development in semi-arid areas often prone to drought conditions [18,19,20], prolonged drought is still a major factor limiting tef productivity [13,14]. 

While generally considered to be an under-researched or ‘orphan crop’ in terms of genetic manipulation and improvement [6], a diverse array of genetic studies on tef have provided information on phylogeny [21], phenotypic and genetic diversity [22,23,24,25,26,27,28,29] as well as other molecular characteristics [30,31]. Most of these studies, however, have been tailored to the generation of molecular tools for marker assisted breeding projects for tef growth and improvement under a variety of growth limiting conditions. The recent sequencing of the genome and transcriptome of tef variety Tsedey (DZ-Cr-37) has provided a valuable resource for further “omic” studies aimed towards ultimate enhancement of tef productivity for food security purposes [18,32]. Insights gained from understanding which genes, proteins and metabolites, and their respective roles in stress response, could facilitate enhancement of tef tolerance to abiotic stress factors. In particular, high throughput proteomic techniques in combination with this genome resource are a powerful tool for the identification and characterisation of proteins associated with drought stress. The use of quantitative proteomic methods have become a powerful and widely-used technique in the field of crop stress tolerance research, as it has the ability to identify and quantify changing stress-related proteins and compare proteomic profiles of stress-sensitive to stress-tolerant crops [33]. This approach is strengthened by having the most comprehensive database, as it facilitates further downstream bioinformatics analyses [33,34,35,36]. This information could, in turn, be utilized to select for tef varieties with improved tolerance to water-deficit stress. To date, there has been no published data on the use of high throughput proteomics or comparative proteomics studies on vegetative tissues of such varieties in response to abiotic or biotic stress. Previous protein studies conducted on tef were mostly targeted to the amino acid composition of tef seeds [37] and the characterisation of albumin, globulin and prolamin contents in relation to nutritional quality during tef grain consumption [38,39].

In this study, pre-flowering *E. tef* plants propagated from a “brown seeded” variety were subjected to controlled dehydration stress conditions. Selected physiological parameters (electrolyte leakage and photosynthetic activity) in combination with ultra-structural observations of leaf tissues during dehydration were chosen to determine critical water contents at which stress associated damages are initiated for further proteomic investigation using an iTRAQ approach. Changes in abundance of proteins during dehydration were noted and these were validated for selected proteins by use of Western blotting. Enzyme assays associated with such proteins were conducted to characterise their activity profiles during water deficit stress. Bioinformatics analysis was employed for further characterisation of stress responsive proteins. 

This study, to our knowledge, is the first proteomic analysis of leaf tissues of a tef variety in response to water-deficit stress brought about as a consequence of drought conditions.

## 2. Materials and Methods

### 2.1. Plant Material and Growth Conditions 

Tef plantlets were germinated from seed (brown seed, local market variety purchased in Ethiopia) into 6 trays (length = 30 cm, width = 27 cm and depth = 11 cm) each containing 4 kg soil mix (2.5 parts potting soil, 2 parts peat vermiculite mix (Sunshine mix 1, SunGro Horticulture, Agawam, MA, USA) and 1 part quartz sand). The soil was hydrated to field capacity before sowing seeds onto the top layer of soil. Seeds were sprayed with water using a spray canister until well moistened, followed by an additional spray with 0.114% (*w*/*v*) phostrogen (Bayer, Leverkusen, Germany) to further aid seed germination. Trays were covered with plastic wrap to prevent moisture loss and left to germinate under plant growth room conditions (16 h light and 8 h dark, temperature of 25 °C, relative humidity of 45–50% and light intensities ranging from 135–150 μmol·m^−2^·s^−1^) for one week before the plastic wrap was removed. Following one week of germination, tef plantlets were watered twice weekly to allow adequate plant growth and development for at least 6 weeks before imposing dehydration stress. Plants were fertilised twice with 0.114% (*w*/*v*) phostrogen during the plant growth period before initiating stress treatment. 

### 2.2. Sampling and Dehydration Stress Treatment 

Prior to initiating dehydration stress, six week-old tef plants were moved to a plant growth chamber (Percival Intellus control system) and incubated under controlled conditions of 25 °C, 14 h day with light intensities of approximately 153–163 μmol·m^−2^·s^−1^; 17 °C, 10 h night. During a 10-day acclimation period, tef plants were watered every 2 days with 500 mL water. Subsequent to acclimation, dehydration stress was imposed by withholding water for a period of 20 days from 3 trays of tef plants designated D1 to D3 (Dehydrated experimental biological repeats), while the remaining 3 trays designated H1 to H3 (Hydrated control biological repeats) were maintained hydrated by addition of 500 mL water every second day. Previous experiments had established that each tray contained sufficient material to act as a biological repeat of pooled plants for all further testing. Leaf tissues were sampled at regular intervals for determination of water content and for physiological and proteome investigations outlined below. Following the 20-day dehydration treatment, trays D1-D3 were rehydrated with 500 mL water to observe tef plant recovery. At each sampling time point during dehydration, tef leaf material (randomly selected) was flash frozen in liquid nitrogen and stored at −80 °C until further use in total protein extractions and biological validation procedures.

#### Absolute Water Content (AWC) and Relative Water Content (RWC) Determination 

Leaf AWC and RWC measurements were determined for six randomly sampled leaves from D1–D3 and H1–H3 according to the standard protocol: AWC (gH_2_O·gdw^−1^) = (fresh weight − dry weight)/dry weight; RWC (%) = (AWC_sample_/AWC_full turgor_) × 100, where AWC_full turgor_ was determined by floating leaves in water in the dark for 24 h for maximal water uptake.

### 2.3. Electrolyte Leakage 

The rate of electrolyte leakage from leaves of tef plants during dehydration stress was measured using a CM 100-2 Multiple Cell Conductivity Meter (Reid & Associates, Durban, South Africa). Leaves (*n* = 3) were sampled from D1–D3 replicate trays, cut into 1.5 cm long segments and placed in separate wells. In addition, 1.5 mL ultrapure water was added to each well and conductivity was measured immediately and subsequently every min over a 20 min period. Leaf samples were then oven dried (70 °C for 48 h) to obtain leaf dry mass. The rate of electrolyte leakage was calculated by plotting the change in electrolyte leakage values over time and used in the following equation: rate of leakage/ dry weight of leaf segments, where the rate of leakage was expressed as μS∙min^−1^∙gdw^−1^. 

### 2.4. Chlorophyll Fluorescence 

Chlorophyll fluorescence measurements were performed according to Maxwell and Johnson [40] using a portable PAM-2100 Chlorophyll fluorometer (Walz, Effeltrich, Germany). Approximately three tef leaves were aligned in order to cover the area of the dark adaption clips (4 mm in diameter). Leaves were dark-adapted for 15 min before maximum quantum yield of photosystem PS II (*F_v_*/*F_m_*) values were calculated using the standard formula: *F_v_*/*F_m_* = (*F_m_* − *F*_0_)/*F_m_*, where *F_m_* is the maximum fluorescence yield of PS II after a saturating light pulse and *F*_0_ is the baseline fluorescence of dark adapted leaves. Chlorophyll fluorescence measurements were performed in triplicate on plants from each of the replicate trays D1-D3. 

### 2.5. Transmission Electron Microscopy (TEM) 

The ultrastructure of leaf mesophyll cells situated immediately above the basal meristem was examined at various stages of dehydration. This zone was selected as this tissue is most likely to retain viability relative to older and thus more naturally senescent leaf tips. A band of tissue one cm from the leaf base was removed from randomly selected leaves from three plants and these were further dissected into smaller segments (1–2 mm). These were fixed and embedded according to the method of Sherwin and Farrant [41]. Embedded samples were sectioned at 95 nm using a Diatome diamond knife (Diatome, Nidau, Switzerland) on a Reichert Ultracut S Ultra-microtome (Leica, Wien, Austria) and mounted onto copper grids. Sections were stained with uranyl acetate and lead citrate as described by Reynolds [42] for 10 min each before being viewed with a FEI Tecnai T20 microscope (Thermo Fisher Scientific, Waltham, MA, USA).

### 2.6. Plant Protein Material and iTRAQ Experimental Design 

Leaf tissues harvested at full turgor (80% RWC) and dehydrated to 50% RWC were utilized for protein extraction and used in an 8-plex iTRAQ analysis as described in Figure S1. Three biological replicates of each treatment (hydrated control and dehydrated to 50% RWC) as well as two labels used for internal controls were subjected to the protein preparation procedures according to Figure S1. 

### 2.7. Protein Extraction and Quantification

Total leaf proteins were extracted according to the method of Isaacson, et al. [43] with a few modifications. Leaf tissue, to which 1% (*w*/*w*) insoluble polyvinylpolypyrrolidone (PVPP) was added, was ground in liquid nitrogen and aliquoted into 2 mL centrifuge tubes. Ice-cold extraction buffer (0.7 M sucrose, 0.1 M KCl, 0.5 M Tris-HCl, pH 7.5, and 50 mM EDTA) to the volume of 1 mL together with 1 mL Tris (0.5 M, pH 8.0)-saturated phenol was added to samples and mixed by vortexing for 15 min at 4 °C, followed by centrifugation at 12,000× *g* for 10 min at 4 °C to allow phase separation. A protease inhibitor tablet (1 Roche Complete Mini tablet per 50 mL volume of extraction buffer) and the reducing reagent dithiothreitol (DTT) as well as serine protease inhibitor phenylmethylsulfonyl fluoride (PMSF) was added to the extraction buffer (just before use) at a final concentration of 2% (*w*/*v*) and 1 mM, respectively. Subsequent to phase separation, the upper phenolic phase containing all phenol soluble protein was collected and re-extracted with an equal volume of extraction buffer using the same mixing and centrifugation conditions described above. Proteins were precipitated by addition of 5 volumes (relative to that of the collected phenolic phase) of cold 0.1 M ammonium acetate in methanol followed by incubation at −20 °C for 16 h. Protein pellets were recovered by centrifugation at 12,000× *g* for 15 min at 4 °C and washed once with 1 mL 100% methanol at 12,000× *g* for 5 min at 4 °C and once with 80% (*v*/*v*) acetone, before being air-dried under a fume hood for 5 min. Protein sample re-suspension occurred in 2% (*w*/*v*) sodium dodecyl sulfate and was mixed by vortexing for 15 min at room temperature. Additionally, samples were placed on a heating block at 90 °C for 3–5 min to facilitate dissolving of the pellet. Proteins were quantified using the Pierce BCA protein assay kit (Thermo Fisher Scientific, Waltham, MA, USA) according to the manufacturer’s instructions and concentrations determined via a standard curve with bovine serum albumin as a standard. 

### 2.8. Protein Preparation and Tryptic Digests

The filter assisted sample preparation procedure (FASP) was used as described in Wisniewski, et al. [44]. A starting protein concentration of 300 μg per sample was utilised, to which 0.1 volumes of 50 mM Tris (2-carboxylethyl)-phosphine hydrochloride (TCEP) was added, followed by incubation on a heating block at 60 °C for 1 h to reduce cysteine disulphide bonds. The reduced protein samples were transferred to a 30 kDa molecular weight cut off centrifugal Amicon filter (Merck, Darmstadt, Germany) and volumes were reduced to 30 μL by centrifugation at 10,000× *g*. To block cysteine residues, samples were incubated in 100 μL of 8 M urea in 0.5 M triethylammonium bicarbonate (TEAB), pH 8.5 containing 15 mM methyl methanethiosulfonate (MMTS) at room temperature for 15 min. Subsequently, four washes with 8 M urea in 0.5 M TEAB were performed to reduce the concentration of SDS, followed by two washes with 0.5 M TEAB to reduce the concentration of urea to an acceptable level (approximately 1 M). For digestion of protein to peptides, proteomics-grade modified trypsin (Trypsin Gold, mass spectrometry grade, Promega, Madison, WI, USA) in 40 μL of 0.5 M TEAB was added to samples at a trypsin: protein ratio of 1:100 (*v*/*v*). Tryptic digests were allowed to proceed for 16 h at 37 °C in a temperature incubator under sealed air-tight conditions to prevent evaporation. Subsequent to incubation, protein digests were collected through centrifugation at 10,000× *g* and concentrated down to 20 μL using a Savant SC110 Speed-Vac (Thermo Fisher Scientific, Thermo Fisher Scientific, Waltham, MA, USA).

### 2.9. iTRAQ Labelling 

For labelling of digested peptide, an 8-plex iTRAQ system was used (AbSciEx, Foster City, CA, USA), where the iTRAQ tags (113, 114, 115, 116, 117, 118, 119 and 121) were used according to Figure S1. The labels were reconstituted with proteomics grade isopropanol and added to each sample, mixed by vortexing and left to incubate at room temperature for 2 h. Subsequent to labelling, the contents for each labelled peptide sample were pooled together and reduced to approximately 30 μL by vacuum concentration before for de-salting and purification on Pierce C-18 Spin Columns (Thermo Fisher Scientific, Waltham, MA, USA) according to the manufacturer’s guide. The purified peptide samples were then dried by vacuum concentration and subjected to OFFGEL fractionation. 

### 2.10. Peptide Purification and OFFGEL Fractionation 

For separation of labelled peptide samples according to their isoelectric points (p*I*), the 3100 OFFGEL fractionator (Agilent Technologies Santa Clara, CA, USA) with a 12-well setup was used. Dry peptide samples were dissolved in 1.25X peptide OFFGEL rehydration solution 6% (*v*/*v*) glycerol, 1.25% (*v*/*v*) carrier ampholytes, at pH 3-10 (Sigma, St. Louis, MO, USA) and loaded onto 13 cm immobilized pH gradient (IPG) strips (GE Healthcare, Little Chalfont, UK), with a linear pH 3–10 range, previously rehydrated with 1.25X peptide OFFGEL rehydration solution according to the Agilent 3100 Quick Start Guide. Peptide electro-focusing was then performed using the pre-loaded OGPE12 program for peptide fractionation until a voltage of 20 kV∙h^−1^ was reached. After electro-focusing, fractions were collected and purified using C-18 columns as described above to remove all traces of glycerol and contaminating substances and re-quantified in preparation for analysis by ESI-Q-tof-MS/MS mass spectrometry. 

### 2.11. Mass Spectrometry Settings 

MS/MS analysis was carried out on the purified peptide fractions using an Agilent 6530 quadrupole-time of flight (Q-TOF) mass spectrometer fitted with a Polaris HR 3 μm C18 high pressure liquid chromatography (HPLC)-Chip Cube source (Agilent Technologies Santa Clara, CA, USA). The chip was equipped with a 75 μm × 150 mm analytical column and a 360 nL Zorbax enrichment column connected online to the HPLC (1200 Series nanoflow) via an orthogonal spray (HPLC-Chip/MS interface, Agilent Technologies Santa Clara, CA, USA). Peptide samples (2 μg) in 1% (*v*/*v*) acetonitrile (ACN) and 0.1% (*v*/*v*) formic acid (FA) were loaded onto the column and separation was achieved through the mobile phases A (1% (*v*/*v*) ACN, 0.1% (*v*/*v*) FA) and B (90% (*v*/*v*) ACN, 0.1% FA) during a 1 h increasing gradient. The flow rate was constant at 1.6 μL∙min^−1^. The mass spectrometer was run in positive ion mode, with MS scans running over a range of *m*/*z* 200 to 1700 at a rate of seven spectra∙sec^−1^. MS/MS scans were run over a range of *m*/*z* 90 to 1700 at a scan rate of 2.50 spectra∙sec^−1^ and a narrow (~1.3 amu) isolation width. Precursor ions were selected for auto MS/MS at an absolute threshold of 1000 and a relative threshold of 0.001, with a maximum of ten precursors per cycle, and active exclusion set at 1 spectrum and released after 1.5 min. Precursor charge-state selection and preference was set to 2+, 3+, and >3+, and precursors were sorted by abundance only. 

### 2.12. Mass Spectra Data Preparation 

The raw mass spectra data files (d format) were firstly converted to mzML format followed by conversion to mgf file formats, using the open source software, MSConvert available from the ProteoWizard version 1.6.0 package [45]. Files were imported into PEAKS Studio version 6.0 (Bioinformatics Solutions Inc., Waterloo, ON, Canada) and the ‘data refine’ tool with default parameters (parent ion *m*/*z* tolerance at 0.1, retention time tolerance window of 30 s, precursor charge correction, no merged scans and no filtering) was used to produce improved fragmentation, better signal-to-noise ratio and enhance reporter ion intensities. 

### 2.13. Database Selection and Searching 

The Tef Extended (TE) transcriptome database converted to protein sequences in FASTA format, available from the Tef Improvement Project (accessed in August 2013; [18]), was used to match protein sequences to the iTRAQ generated mass spectra. In addition, tef proteins were searched against the *Liliopsida* (all monocotyledonous plants) database available from UniProtKB SwissProt/TREMBL. Database searching was employed with the following parameters (parent mass error tolerance of 20.0 ppm, fragment mass error tolerance of 0.1 Da, pre-cursor mass search type set as monoisotopic, selection of trypsin as enzyme used, maximum missed cleavages per peptide set at 2, fixed modifications set at iTRAQ 8-plex (K, N-term) and beta-methylthiolation, variable modifications set at iTRAQ 8-plex (Y) and Oxidation (M) with max variable post translational modification (PTM) per peptide set at 3. A concatenated decoy database was automatically generated by PEAKS Studio 6.0 when searches were implemented and further used to determine false discovery rates Quantification results were filtered to false discovery rate (FDR < 0.01; peptide and protein -10logP score > 20) and only considering proteins with 2 unique peptides.

The mass spectrometry proteomics data have been deposited to the ProteomeXchange Consortium [46] via the PRIDE [47] partner repository with the dataset identifier PXD007907.

### 2.14. iTRAQ Data Processing—Protein Quantitation and Statistical Analysis 

iTRAQ data was manually edited and refined before being statistically analysed as shown in Figure S2. 

#### 2.14.1. Data Refinement 

Data refinement was conducted as follows: firstly, the peptide output list generated from PEAKS database searching was used, where the list of peptides corresponds to the proteins identified from database searching with appropriate FDRs and threshold scores (−10logP scores). Secondly, using a BioPerl script [48], peptides with only 1 or 2 expression values present (of the 3 for each treatment) in the labelled channels (115 to 121) were removed. Peptides with a zero value were kept if the zero values were found consecutively in the hydrated (115–117) or dehydrated (118–121) labelled channels, respectively. Values of zero in the labelled channels 113 and 114, used as internal controls to observe technical variance between samples, were also retained. In order to avoid problems with numerical computations in the downstream analysis, the remaining zeros were changed to 1 (excluding labelled channels 113 and 114). These changes resulted in a refined peptide list with non-normalised quantitative expression values (Figure S2A). Thirdly, quantile normalisation was employed using the R-Bioconductor program [49], for normalisation of peptide quantitative expression data (Figure S2B). 

#### 2.14.2. protViz: For Visualising and Analysis of Proteomic Mass Spectrometry Data

To observe whether peptide intensities were in an acceptable format for statistical analysis, the normalised peptide list was then transformed using an inverse hyperbolic sine function (arcsinh) (Figure S2B,C) and statistically analysed using the R-Bioconductor program with the protViz package [50]. Subsequent to normalisation and transformation, peptide quantitative expression data was laid out in a two-group comparison manner using the ‘iTRAQ two-group analysis’ function in protViz. In this comparison, the expression values from the labelled channels were placed in two groups, whereby group 1 consisted of all the hydrated labelled channels (115–117) and group 2 consisted of all the dehydrated labelled channels (118–121). The averaged quantitative expression values were then calculated from each group and used in the ‘two-group’ comparison. Statistical testing was performed through an independent samples *t*-test (unpaired) that assigns a *p*-value to each individual peptide identity and tests for a significant difference (*p*-value ≤ 0.05) through the two-group comparison test between hydrated (control) and dehydrated (experimental) quantitative expression values (Figure S2D). While the analysis was performed on each individual peptide identity, the output was given in such a manner that statistical significance for change in quantitative expression is observed in protein form. Thus, the peptides corresponding to the designated proteins were then stacked together through a ‘weighted sum’ approach to provide the overall change in quantitative expression between individual proteins (Figure S2E).

### 2.15. Western Blot Analyses 

For confirmation of protein presence, tissues at RWCs similar to those used in iTRAQ analysis (Figure S1) were chosen, viz. 92% RWC (as a hydrated-control) and 55%, 52% and 50% RWC (as dehydrated-experimental repeats, designated D1, D2 and D3, respectively). These were subjected to PAGE separation and subsequent Western blot analyses using commercial polyclonal antibody against fructose-bisphosphate aldolase (As08294, Agrisera, Vännäs, Sweden) for immunodetection and quantification. Total protein extracts (15 μg) and Fermentas molecular weight marker (Thermo Fisher Scientific, Waltham, MA, USA) were loaded onto 12% SDS polyacrylamide gels and subjected to 1D-PAGE separation at a constant voltage of 100 V for 2 h before transfer of proteins to pure nitrocellulose membranes (Pall Life Sciences, Port Washington, NY, USA) at 100 V for 1 h at 4 °C. Probing with primary antibodies occurred at a dilution ratio of 1:5000 for FBA for 16 h at 4 °C before incubation with goat anti-rabbit peroxidase conjugated secondary antibody (Agrisera, AS09 602) at a dilution of 1:5000 for 1 h at 4 °C. Detection and visualisation of protein expression was initiated using the WesternBright ECL HRP chemiluminescent detection kit (Advansta, Menlo Park, CA, USA) according to the manufacturer’s instructions. Images were visualised by chemiluminescence using the ChemiDoc™ XRS imager installed with ImageLab software version 4.1 (Biorad, Hercules, CA, USA) where the relative quantification of detected protein band intensities were made relative to the hydrated-control. A one-way ANOVA statistical test was performed with the relative quantification values in Graph Pad Prism 6.0, (La Jolla, CA, USA) where significance was based on *p*-value (*p*-value < 0.05). 

### 2.16. Enzyme Assays 

For testing of enzyme activities, leaf tissues in the RWC ranges: 90–95%, 75–80%, 60–65%, 50–55%, 35–40% and 25–30% RWC, were selected and assayed using spectrophotometric methods (described below). Prior to enzyme assay procedures, the total protein content of all extracted samples were determined using the Bradford assay at 595 nm following the manufacturer’s instructions (Quick Start Bradford Protein Assay, Biorad, Hercules, CA, USA). GraphPad Prism 6.0 software was used for graph generation and statistical analysis by one-way ANOVA, where significance was based on the *p*-value (*p*-value < 0.05) of changing enzyme activities at differing RWC ranges. 

#### 2.16.1. Monodehydroascorbate Reductase (MDHAR, EC: 1.6.5.4) 

Enzyme extraction was performed according to Valyova et al. [51] before de-salting on a PD-10 de-salting column (GE Healthcare, Little Chalfont, UK) following the manufacturer’s instructions. MDHAR enzyme activity was determined as originally described by Miyake and Asada [52] and further employed by Kingston-Smith and Foyer [53], by following the decrease in absorbance at 340 nm due to the oxidation of NADH. Total enzyme activity was calculated using the extinction coefficient of NADH (6.22 mM^−1^·cm^−1^) and measuring the rate of change over time, while specific activity was given as enzyme units∙min^−1^∙mg protein^−1^. 

#### 2.16.2. Fructose-Bisphosphate Aldolase (FBA, EC: 4.1.2.13) 

Extraction and analysis of FBA enzyme activity was performed according to [54] with modifications. Approximately 0.25 g leaf tissue was ground in liquid nitrogen to a fine powder and 0.2% (*w*/*w*) insoluble PVPP was added. In addition, 2.5 mL extraction buffer (0.05M KH_2_PO_4_ buffer, pH 7.0, 4 mM MgCl_2_, 1 mM EDTA, 10% (*v*/*v*) glycerol, 5 mM dithiothreitol (DTT)) was added to ground material and mixed by vortexing before centrifugation at 12,000× *g* at 4 °C. The resulting supernatant was passed through a PD-10 de-salting column and quantified for total protein concentration. FBA enzyme activity was measured in a combined reaction with glycerol-3-phosphate dehydrogenase (G-3-P) (EC: 1.1.1.8, Sigma, St. Louis, MO, USA) and triose-phosphate-isomerase (T-P-I) (EC: 5.3.1.1, St. Louis, MO, USA) in the forward reaction at 22 °C by observing the decrease in absorbance at 340 nm due to the oxidation of NADH. A reaction mixture of 1 mL containing (50 mM Hepes-KOH buffer, pH 7.3, 0.1 mM NADH, 1 mM EDTA, 0.75 units G-3-P and 10 units T-P-I) was reconstituted and sample extract (100 μL) was added. To start the reaction, 4 mM fructose-bisphosphate (Sigma-Aldrich, Inc.) substrate was added and total enzyme activity was calculated by measuring the rate of change over time using the extinction coefficient of NADH (6.22 mM^−1^·cm^−1^). One unit of activity was defined as the amount of enzyme required for the oxidation of 2 μMol NADH at 22 °C and specific activity was given by enzyme units∙min^−1^∙mg protein^−1^.

#### 2.16.3. Peroxidase (POX, EC: 1.11.1.7) 

Extraction and assay of total POX activity was performed according to [55], by the determining the rate of guaiacol oxidation [56]. Total enzyme activity was calculated by measuring the rate of change over time using the extinction coefficient of tetraguaiacol (26.6 mM^−1^·cm^−1^), where a unit of peroxidase activity was expressed as the amount of enzyme required to catalyse the conversion of 1 mMol H_2_O_2_, with guaiacol as hydrogen donor, per min under specified conditions, while specific activity was given as enzyme units∙min^−1^∙mg protein^−1^.

### 2.17. Blast2GO for Protein Identification, Annotation and Functional Enrichment Analysis 

All proteins matched to the TE database (from here on referred to as the TE dataset) and the *Liliopsida* database (from here on referred to as the MU dataset) with PEAKS Studio 6.0 were annotated using Blast2GO version 2.8 [57]. Analysis included both the tef foreground (differentially regulated) and the tef background proteins (all proteins identified). 

#### 2.17.1. Protein Identification, Annotation and GO-Term Retrieval

To provide protein descriptions, the dataset (in FASTA format) was searched against the UNIPROTKB/SwissProt database using the BLASTP algorithm with the following parameters: report a maximum of twenty blast hits, with a blast expect value of 1e^−3^ and minimum high scoring segment pairs (HSPs) length equal to 33. FASTA sequences for the datasets were retrieved from either database using an in-house shell script written for extracting FASTA files. Subsequent to BLAST steps, the steps to mapping and annotation were initiated for GO-term retrieval using the Blast2GO default parameters (*E*-value filter if 1e^−6^, an hsp-hit coverage cut-off of 0, annotation cut-off of 55, and GO weight of 5) with the September 2014 database.

#### 2.17.2. Functional Enrichment Analysis 

Subsequent to tef protein annotation and classification, functional enrichment of GO-terms was initiated using the Fisher’s exact test for statistical significance [58] in Blast2GO. For input, both tef foreground and background annotation files were merged as one file (.annot), which was then used as a reference set. A list of protein identifiers containing individually named contigs from the foreground was used as a test set. For enrichment of up and downregulated proteins, the test set (foreground) was separated into two lists, one containing upregulated protein identifiers and the other containing downregulated protein identifiers. Fisher’s exact test was employed for up and downregulated proteins to show both over and under-represented GO-terms. A two-sided Fisher’s exact test, using a term filter of 0.05 and term filter mode set as false discovery rate (FDR) with the removal duplicate IDs, was utilized. The graph generated to better display functional enrichment of GO-terms for tef foreground-upregulated proteins and tef foreground-downregulated proteins vs. tef background proteins was performed using GraphPad Prism 6.0 software. 

## 3. Results

### 3.1. Physiological Characterisation 

**Figure 1:**
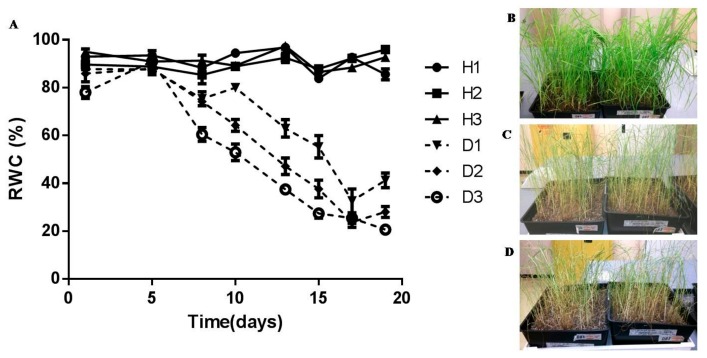
Tef plants subjected to 20 day dehydration treatment: (**A**) leaf relative water content (RWC) of tef plants maintained at full hydration (solid lines, H1–H3) and subjected to dehydration (dashed lines, D1–D3), values are means of five replicates and error bars represent standard error between replicates; (**B**) fully hydrated tef plants (~85% RWC, ~3 g H_2_O∙gdw^−1^); (**C**) plants after 13 days of withholding water (~50% RWC,~1.5 g H_2_O∙gdw^−1^); (**D**) after 17 days of no water (~25% RWC,~1 g H_2_O∙gdw^−1^).

In order to determine critical water contents at which dehydration stress is invoked, tef plants were dehydrated over a period of 20 days and effects on leaf tissue membrane integrity and photosynthetic activity were determined. Leaves maintained a water content of between 80% and 90% RWC for six days before a gradual loss of water was observed, reaching 50% RWC by 13 days and circa 25% by 17 days after withholding water (Figure 1A). Plants were able to lose up to 65% of their water before loss of viability was observed based on previous dehydration treatments with tef plants (data not shown) and physiological characterisation procedures described below. 

**Figure 2:**
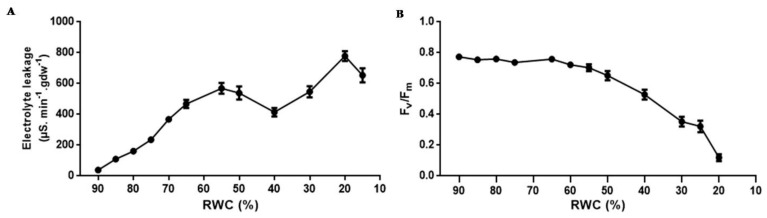
Tef leaf membrane permeability and photosynthetic potential during dehydration. (**A**) changes in electrolyte leakage and (**B**) quantum efficiency of PS II (*Fv*/*Fm*) during photosynthesis. Electrolyte leakage values (*n* ≥ 6) were pooled from dehydrated plants at designated RWCs and *Fv*/*Fm* measurements were conducted in triplicate at each RWC point (*n* ≥ 3). Error bars denote standard error between replicates.

As water deficit in desiccation sensitive plants is known to affect membrane integrity, the effects of progressive dehydration on electrical conductivity (an indirect measure of plasmalemma integrity) and photosynthetic efficiency of photosystem II (*F_v_*/*F_m_*) was investigated (Figure 2). Upon dehydration, there was a progressive increase in electrolyte leakage with values reaching 570 μS∙min^−1^∙gdw^−1^ at 50% RWC with further increases occurring below this RWC reaching a maximum of 780 μS∙min^−1^∙gdw^−1^ at low tissue RWCs (Figure 2A). *F_v_*/*F_m_* was maintained at values of approximately 0.75% until 55% RWC, below which quantum efficiency of electron transport through PS II declined significantly (Figure 2B), suggesting shut down or progressive damage to the photosynthetic apparatus.

**Figure 3:**
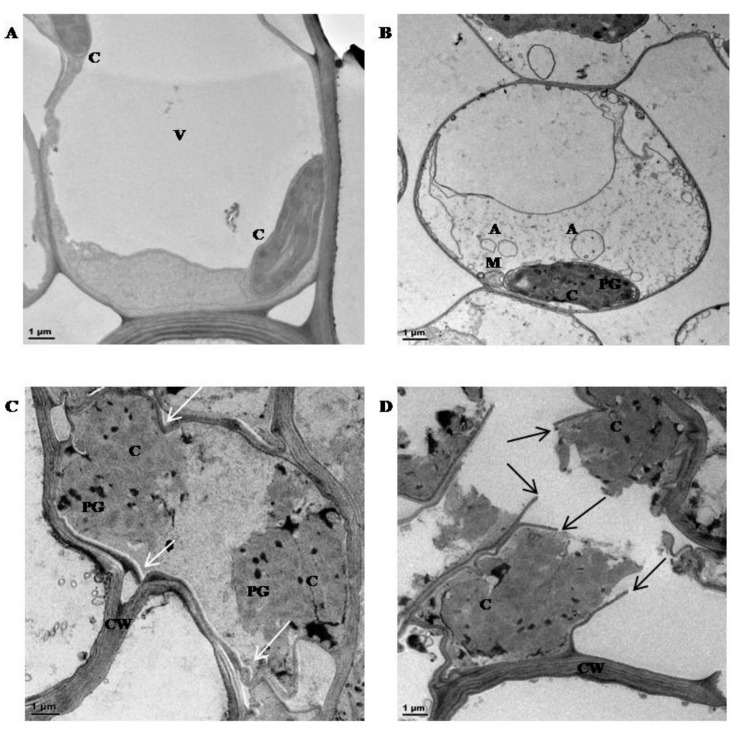
Transmission Electron Micrographs (TEM) of mesophyll cells from tef leaf tissues: (**A**) in the hydrated state at 87% RWC; (**B**) 50% RWC; (**C**) 30% RWC and (**D**) 20% RWC. C = chloroplast, V = vacuole, A = autophagosomes; M = mitochondria, CW = cell wall, and PG = plastoglobuli. White arrows = cell wall folding, black arrows = cell wall breakage. Scale bar = 1 μm.

TEM investigations were conducted in order to assess ultra-structural changes in mesophyll cells during dehydration (Figure 3). Cells from hydrated tissues were typical of a metabolically active state with a large central vacuole and peripherally located organelles (Figure 3A). Dehydration to 50% RWC (Figure 3B) resulted in reduction in primary vacuolar area, some plasma membrane withdrawal, evidence of potential autophagosome formation and chloroplasts containing evidence of plastoglobuli formation. The formation of plastoglobuli are often indicative of desiccation stress [59]. Further dehydration to 30% RWC and below (Figure 3C,D) resulted in increased evidence of stress-induced injury, with compaction of organelles and evidence of cell wall folding (white arrows) and ultimately breakage (black arrows). These data suggest that under the experimental conditions utilized, six-week-old pre-flowering tef plants were able to survive dehydration to 50% RWC, below which there is increasing evidence of subcellular damage.

### 3.2. Tef Proteomic Analysis

iTRAQ was used to observe differential regulation of the tef proteome in response to dehydration to 50% RWC. Tef protein mass spectra were searched against the TE and *Liliopsida* databases using PEAKS studio 6.0 with more than two peptide matches. The generated peptide output was subjected to rigorous manual editing and filtering of data to reduce the occurrence of false positives (Figure S2) before proceeding to statistical analysis with protViz (see input File S1). A sanity check of theoretical quantiles plotted against the sample quantiles (Q-Q plots) showed intensities to be normally distributed after quantile normalisation (Figure S3) and a correlation test (cluster analysis) showed the respective reporter ion channels (113–121) to cluster according to experimental design (Figure S4). 

#### Identification of Differentially Regulated Proteins

Through the use of protViz [50], a total of 211 out of 5727 identified proteins from the TE database (the TE dataset) were found to be differentially regulated, where 97 proteins were upregulated and 114 proteins were downregulated in response to dehydration stress (Table 1 and Table 2, respectively). These differentially regulated proteins in the TE dataset were categorised as significantly different in protein expression according to statistical testing using a *p*-value (*p*-value ≤ 0.05), while differentially regulated proteins showing largely significant changes in protein expression based on both fold change (values >2 and <0.5) and statistical testing using a *p*-value (*p*-value ≤ 0.05) are depicted in Table S1. 

As the generated peptide output was used for quantification, the problem of protein inference (shared peptides) was addressed by using appropriate FDR thresholds. However, to further ensure that all proteins identified from the Tef database were valid entities, two additional differentially regulated datasets, the tef extended unique (TEU) and monocot unique (MU) datasets were generated based on peptides that were unique to those identified proteins. 

For proteins matched against the TE database using unique peptides alone (TEU dataset), a total of 111 out of 2656 identified proteins were statistically significant with 44 upregulated proteins and 67 downregulated proteins (Tables S2 and S3, respectively). A significant proportion of proteins identified from the TE database were unidentifiable and did not have protein descriptions. Approximately 67% and 63% of proteins were annotated and identified with Blast2GO tools [57,60] for TE up and downregulated proteins, respectively (Table 1 and Table 2) and 72 and 63% of proteins had descriptions for TEU up and downregulated proteins, respectively (Tables S2 and S3). In addition, a number of proteins with the same protein description and quantification values are repeated within the TE dataset (Table 1 and Table 2, e.g., CL1Contig3395, CL1Contig3396, and CL1Contig3397 code for endoglucanase 7). These proteins may have arisen through alternative splicing and could be spliced variants of the same protein. In addition, a suitably large number of proteins were found to be commonly identified in both the TE (Table 1 and Table 2) and TEU datasets (Tables S2 and S3). To name a few: 40S ribosomal protein S28, fructose-bisphosphate aldolase, monodehydroascorbate reductase, gras family protein 2 and leucoanthocyanidin dioxygenase found in upregulated proteins (Table 1 and Table S2) and 2-methyl-6-phytyl-hydroquinone methyltransferase, chlorophyll a-b binding protein, alliin lyase 1, protein dek and polyamine oxidase found in downregulated proteins (Table 2 and Table S3). Furthermore, a number of interesting proteins were shown to have large increases in fold change, mainly gras family protein 2, monodehydroascorbate isoform 2, peroxidase 3-rare cold-inducible protein, poly polymerase, leucoanthocyanidin dioxygenase and cyclin-p4 (Table S1). These proteins and their roles in biological processes of interest are discussed later with the bioinformatic analyses. 

For tef proteins matched against the *Liliopsida* database, using unique peptides alone, a total of 174 out of 4328 identified proteins were statistically significant with 85 upregulated and 89 downregulated proteins (Supplementary Tables S4 and S5, respectively). The text files of all identified proteins, box-plots and quality control figures for these protein datasets can be found under Supplementary Materials (Files S2–S4 referring to the TE, TEU and MU datasets, respectively). 

### 3.3. Tef Biological Validation

#### Western Blots and Enzyme Assays

**Figure 4:**
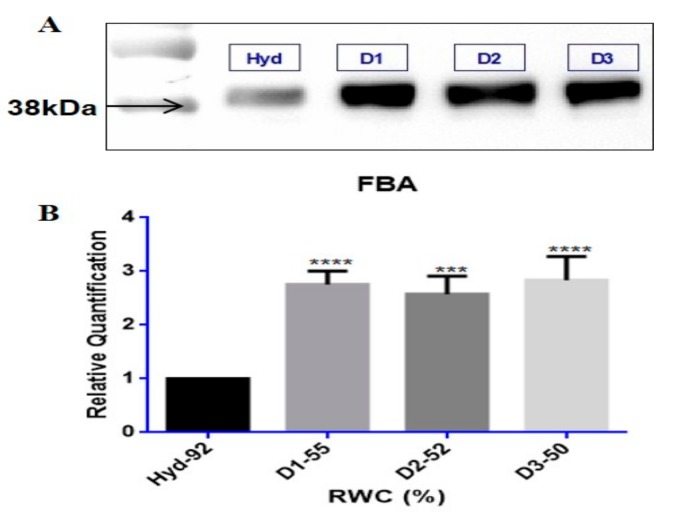
Western blot validation of the upregulated protein FBA; (**A**) protein band intensities are at the correct molecular weights (kDa) for fructose bisphosphate (FBA) tested at hydrated (Hyd-92% RWC, control) and dehydrated conditions (D1–55, D2–52 and D3—50% RWC); (**B**) relative quantification of band intensities (*n* ≥ 5) were performed and analysed for statistical significance (*p*-values ≤ 0.05) using one-way ANOVA, shown by asterisks (*** *p*-value ≤ 0.001; **** *p*-value ≤ 0.0001) placed on RWC points significant to control. Error bars denote standard error between tested replicates.

In order to validate whether the differentially regulated proteins identified from iTRAQ analyses were upregulated during dehydration, Western blotting was conducted on fructose bisphosphate aldolase (FBA) retrieved from the TE upregulated protein dataset, shown in Figure 4. Immunodetection showed an increase in protein abundance levels in response to dehydration stress for FBA in all samples tested (Figure 4A). Protein band intensities were enhanced by at least a 2-fold significant increase in relative quantification values (*p*-value < 0.05) for FBA at 38 kDa (Figure 4B).

**Figure 5:**
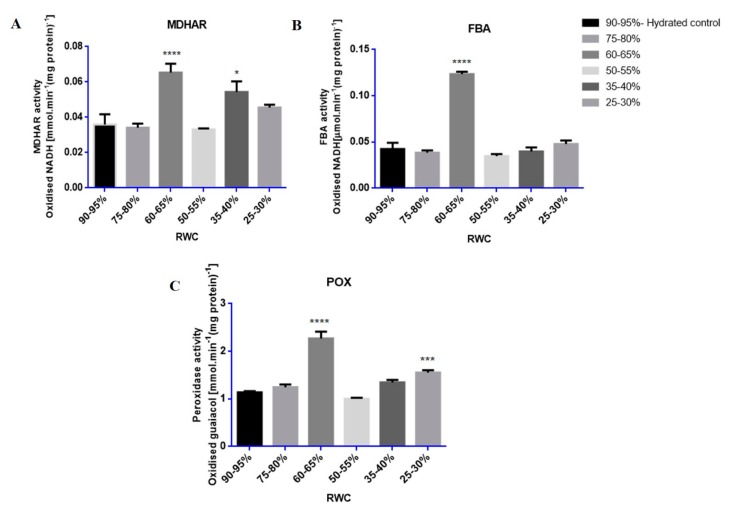
Relative enzyme activities of selected upregulated proteins: (**A**) monodehydroascorbate reductase (MDHAR); (**B**) fructose bisphosphate aldolase (FBA) and (**C**) peroxidase (POX). Enzymes were assayed from each RWC range (90–95% to 25–30% RWC) where enzyme activities (*n* ≥ 10), displayed as specific activity (enzyme units. mg·protein^−1^) were measured in tef leaves throughout dehydration stress. Statistical significance (*p*-values < 0.05) was done by one-way ANOVA with hydrated as control, shown by asterisks (* *p*-value ≤ 0.05; *** *p*-value ≤ 0.001; **** *p*-value ≤ 0.0001). Error bars denote standard error between tested replicates.

Enzyme assays showed that the activities of MDHAR, POX and FBA were strongly induced at specific RWCs during dehydration stress with nearly all increasing at 60–65% RWC (Figure 5A–C).

### 3.4. Functional Enrichment Analysis (Fisher’s Exact Test) 

**Figure 6:**
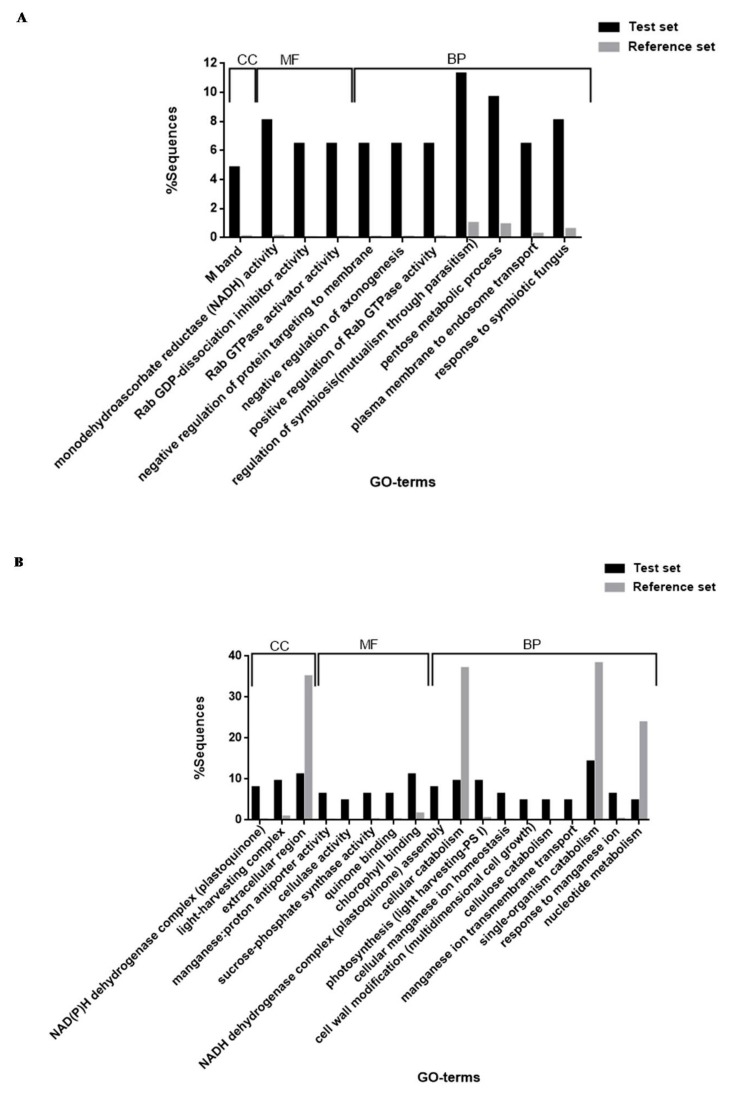
Histograms showing GO-term processes allocated to tef foreground (test set) vs. tef background (reference set) in response to dehydration stress: (**A**) tef upregulated proteins; (**B**) tef downregulated proteins. GO-term results are summarised according to the categories cellular component (CC), molecular function (MF) and biological process (BP), where terms have been reduced to the most specific GO (FDR, 0.05).

In order to observe functional GO-term enrichment of differentially regulated tef proteins in response to dehydration stress, a Fisher’s exact test for statistical significance in Blast2GO was used. A total of 50 GO-term processes were functionally enriched, of which 22 GO-terms were found for upregulated proteins (Table S6A) and 28 GO-terms were found for downregulated proteins (Table S6B). All of these belonged to the classification categories (ontologies) of cellular component (CC), molecular function (MF) and biological process (BP). To summarise the findings, GO-terms were filtered and reduced to the most specific annotations (most specific GO, FDR < 0.05) and represented as histograms for both up and downregulated proteins, shown in Figure 6. The enriched GO-terms shown were reduced to 29 most specific terms in total of which 11 GO-terms were found for upregulated proteins (Figure 6A) and 18 GO-terms for downregulated proteins (Figure 6B) in the classification categories CC, MF and BP. 

## 4. Discussion

### 4.1. Physiological Characterisation

Tef has previously been classified as moderately drought tolerant in comparison to species within the *Eragrostis* genus [61]. Ginbot and Farrant [15] have confirmed that this species has some measure of tolerance to water-deficit under drought stress, with brown seeded varieties (as used in this study) being tolerant of slightly higher amounts of water loss than white seeded varieties. In the current study, similar trends were observed in pre-flowering, brown seeded tef plants, where dehydration to below 40% RWC (Figure 1A) resulted in increased electrolyte leakage rates (Figure 2A) and considerable evidence of subcellular damage (Figure 3). Increased membrane permeability with continuous dehydration stress has been linked to the enhanced synthesis of reactive oxygen species (ROS), a consequence of metabolic processes in chloroplasts, mitochondria and peroxisomes in particular, which can cause the breakdown of proteins, membrane lipids and photosynthetic pigments that function in maintaining cell membrane stability [62,63]. Photosynthesis is particularly susceptible to excess ROS formation under water deficit conditions and this has frequently been cited as a primary cause of damage and resultant plant death in most species [64,65]. The sensitivity of PS II activity to abiotic and biotic factors has resulted in the use of chlorophyll fluorescence, and particularly the measure of quantum efficiency of PS II (*F_v_*/*F_m_*) as an indicator of how plants respond to environmental change. Data from Figure 2B show maintenance of *F_v_*/*F_m_* at values indicative of healthy, non-stressed leaves until 50–55% RWC, with a sharp drop (values declining below 0.4, indicative of possible damage related to photosynthetic shutdown) below 30% RWC. Ultrastructural analysis showed considerable decline in vacuolar area as water was lost from tissues, with some evidence of plasmalemma withdrawal and autophagosome formation upon dehydration to 50% RWC (Figure 3B). Autophagy has been associated with cellular survival by removal of damaged organelles and cellular toxins and recycling of the breakdown products for the maintenance of cellular homeostasis. Furthermore, it has been proposed as being essential for drought stress tolerance [66,67]. We propose that tef is able to survive loss of up to 50% RWC, in part, due to such a strategy. However, drying to lower RWC, suggested increased evidence of subcellular damage, including breakage of cell walls, plasmalemma and loss of integrity of organelles (Figure 3C,D) all further signs of stress-induced injury. 

In summary, physiological studies performed here indicate that six week-old plants from a brown seeded tef variety are able to tolerate drying to ca 50% RWC (loss of 1.5 g H_2_O) before irreversible damage is initiated. We were thus interested in understanding the nature of protection afforded during initial drying to 50%, by investigating the tef proteome changing in response to dehydration stress.

### 4.2. Tef Proteomics

The starting point of the iTRAQ analysis was the examination and refinement of the list of peptides generated from database searching as opposed to the list of generated proteins. This is not an uncommon approach and has been used by many researchers in the field of mass spectrometry-based proteomics [50,68,69,70]. A potential concern with working with a list of peptides instead of proteins is the challenge of protein inference [71], where the generated list contains both unique and non-unique (shared) peptides matched against the chosen database for protein identification. This concern is adequately addressed by using appropriate FDR thresholds and employing stringent estimation of error rates, so that only valid peptide identities meeting the FDR threshold requirements are detected and used for subsequent protein analysis [69,72]. Furthermore, the analysis of both unique and non-unique peptide mass spectra scans that meet FDR thresholds would be more representative of the proteins changing in a particular study. Interestingly, a total of 57 out of the 211 proteins (27%) found to be differentially regulated within the TE dataset (Table 1 and Table 2) were spliced variants arising from the alternative splicing of 25 potential splice events (genes). During this regulatory mechanism, primary transcripts or precursor-mRNAs with introns undergo alternative splicing to produce multiple transcripts from a single gene within the genome by using differential splice sites [73]. In this regard, the functional complexity of the transcriptome and diversity of the proteome are increased between plant cells and tissues [73,74], particularly during plant development and in response to environmental stimuli, such as biotic and abiotic stress conditions [75,76]. In the TEU differentially regulated datasets (Tables S2 and S3) and MU differentially regulated datasets (Tables S4 and S5), however, no occurrences of spliced variants were present, presumably because only uniquely-matched peptides were used for protein identification, resulting in only one definitive protein entity per entry. Because iTRAQ experiments on the whole do not usually produce large amounts of peptide reads per protein [69], the use and manipulation of only uniquely scanned peptides for protein identification has been shown to drastically limit the number of confidently proteins identified [71,72]. This is especially evident by the marginal difference observed in the amount of proteins identified between the TE and TEU differentially regulated datasets, 211 and 111 proteins, respectively. Because tef is considered to be a non-model crop species whose genome has only been recently sequenced [18], the amount of annotated information therein cannot compare to that of model plant organisms. It is important to note that the tef genome and transcriptome have only been moderately-annotated, and this consequently, would lead to not all tef proteins being identified during database searching (as shown in Table 1 and Table 2; Tables S2 and S3). Nevertheless, a significant amount of proteins within the TE and TEU datasets do contain protein annotations and therefore can be used to make protein inferences through bioinformatics analyses, while those unidentified proteins may lead to discovery of some unique new targets within the tef genome.

It could be suggested that a ‘cross-species identification’ approach would be better for non-model plant systems such as tef, where a generic (non-specific plant species) but well-annotated database is used for protein identification [77,78,79]. This would potentially increase the amount of annotated and identified proteins, as in the case with the proteins identified by use of the *Liliopsida* database (the MU dataset) available from UniprotKB, where 4328 tef proteins were identified during database searching, and 174 proteins were found to be differentially regulated (File S3, Tables S4 and S5). Although this approach is widely used for non-model plant systems [77,78] such as tef and many others [80,81], using the same approach is not ideal as the number and confidence of identified proteins is reduced [79]. The MU dataset was generated using only uniquely scanned peptides during database searching and contained more proteins with usable descriptions and annotations for bioinformatics inference (Tables S4 and S5). The use of the TE database, however, provided identification of 5727 tef proteins in total (File S1), of which 211 were differentially regulated. The difference in the total amount of proteins detected can be explained by the fact that either some species-specific proteins will not be present during cross-species identification or those homologous proteins that are present will show small evolutionary differences in their sequences [79]. Thus, the use of a very specific but moderately-annotated database (the TE database), would detect more proteins present, potentially highlight more proteo-bioinformatics changes that are unique to the organism under study, and also improve annotation and curation within the existing tef database.

### 4.3. Tef Protein Validation

Biological validation of the upregulated protein FBA by Western blotting, showed increased protein accumulation and band intensity with dehydration stress to 50% RWC (Figure 4). Although FBA displayed negligible increases in fold change in the iTRAQ data (Table 1 and Table S2, fold change = 1.02), statistical testing based on *p*-value showed it to be highly significant (FBA, *p*-value = 0.005 in Table 1 and Table S2). Since an overall increase in protein accumulation is observed with dehydration stress, this result supports the iTRAQ findings and show that protein change is due to a biological consequence and not experimental variation. 

Proteins tested for biological validation by enzymatic methods, MDHAR, POX and FBA, all showed increased enzyme activities at 60–65% RWC in response to dehydration stress (Figure 5). FBA catalyses the reversible conversion of glyceraldehyde-3-phosphate and dihydroxyacetone phosphate to fructose-1, 6-bisphosphate during glycolysis/gluconeogenesis or in the reaction where erythrose-4-phosphate and dihydroxyacetone phosphate is converted to sedoheptulose-1,7-bisphosphate in the Calvin cycle [82,83]. Furthermore, FBA has been classified as one of the six non-regulated enzymes in the Calvin cycle that have been suggested to have a potential role in controlling photosynthetic carbon flux through the Calvin cycle [83]. A significant increase in FBA activity was observed in tef at 60–65% RWC (Figure 5B). It has been proposed that increased activity of FBA may function in the regeneration of ribulose 1,5 bisphosphate and increased CO_2_ fixation, contributing to enhanced photosynthesis, increased growth rates and biomass yields [83]. Furthermore, an increase in FBA activity has been observed in stress response for various other crop plants such as rice, in response to drought stress and increased salinity [84,85]; wheat seedlings, in response to anaerobic conditions [86,87]; wheat roots, in response to increased aluminium concentrations[87,88] and in Indian mustard, in response to increased cadmium concentrations [89]. 

The stress responsive antioxidant enzymes known to offer protection against free radical accumulation, MDHAR and POX [90,91,92,93], displayed a large increase in enzymatic activity at 60–65% RWC (Figure 5A,C). A second increase in enzyme activity was observed by both MDHAR and POX at 35–40% and 25–30% RWC, respectively, towards the latter stages of dehydration (Figure 5A,C). These results support protein presence and accumulation according to iTRAQ findings for MDHAR (Table 1 and Table S2, fold change value = 1.03, *p*-value = 0.027 and 0.04) and POX (Table 1, fold change value = 1.08, *p*-value = 0.002; Table S4, fold change = 1.02, *p*-value = 0.011) and also confirms that the large increases in quantitative expression observed in these protein isoforms in Table 1 (fold change = 6.54, *p*-value = 0.006) for MDHAR and POX (fold change = 6.82, *p*-value = 0.008) are due to a biological change in response to dehydration stress and not experimental error. An increase in POX activity has been related to many oxidative and abiotic stresses [94,95], particularly in response to dehydration stress conditions in the crop plants wheat [93,96], oilseed rape [97], sunflower [98], horse gram beans [99] as well as in response to salt stress in fox-tail millet and rice [55,95]. The increased production of free radicals as a consequence of stress conditions has been proposed to be the main reason for membrane lipid peroxidation, whereby the extent of peroxidation-induced damage is regulated by the antioxidative peroxidase enzyme system [92,95]. This could be due, in part, to the ability of POX acting on increased levels of H_2_O_2_ in cells as dehydration stress proceeds, even towards the final stages of dehydration stress (25–30% RWC) (Figure 5C). The free radical, H_2_O_2_, has been postulated to have a dual role in plant cells, by either acting as a signalling molecule at low concentrations during non-stress conditions or as an activator of programmed cell death (PCD) at high concentrations during stressed conditions [90,100]. Dehydration to 50% RWC resulted in evidence of autophagy (Figure 3B) and increased electrolyte leakage measurements (Figure 2A) at RWCs below this, suggesting increased membrane damage. This is perhaps due to the extenuating effects of H_2_O_2_ build-up.

### 4.4. Tef Bioinformatic Analysis

Functional enrichment analysis of the GO-terms of tef proteins regulated in response to dehydration stress yielded a wealth of protein ontological information (Table S6; Figure 6A). Monodehydroascorbate reductase (NADH) activity was the most significantly changed GO-term in the category MF (Figure 6A; 8.1% protein sequences). MDHAR was also significantly increased in quantitative expression during iTRAQ analysis in response to dehydration stress in the TE (Table 1, *p*-value = 0.006) and TEU datasets (Table S2, *p*-value = 0.027) and showed a considerable increase in enzymatic activity at low RWCs (35–40%) (Figure 5A). MDHAR is one of the key enzymes involved in ascorbate reduction [101] and functions in reducing the oxidised form of ascorbate (monodehydroascorbate) before being returned to the ascorbate pool [91,101]. MDHAR has been proposed to be an indicator of oxidative stress within plant tissues, playing an important role against accumulation of ROS due to increasing stress conditions [90,91]. This suggests that the generation of ascorbate as well as the regulation and maintenance of the ascorbate–glutathione cycle are important in the response to initial dehydration stress. 

The Rab family of cellular processes active in the regulation of vesicular membrane traffic [102] and regulatory membrane protein transport processes were equally over-represented in response to dehydration stress (Figure 6A). The flow of membrane constituents between endomembrane structures and the plasmalemma is critical for the maintenance of cellular homeostasis in response to signal transduction [103]. This is also important in autophagy, which has been linked to the restoration and maintenance of cellular homeostasis through the recycling and removal of damaged cellular constituents through protein degradation [66,67], where drought has been reported to induce PCD [67]. Furthermore, GO-terms allocated to biological processes responsible for regulating membrane trafficking and the flow of proteins and other macromolecules to numerous endpoints inside and outside the cell through a signalling cascade[104,105] were over-represented in response to dehydration stress (Figure 6A). These Rab family of small GTP-binding proteins function as molecular alterations that cycle between ‘active’ and ‘inactive’ states within the cell through the binding and hydrolysis of GTP [105], thereby controlling the endocytic network in plants [106]. Interestingly, the stress-inducible small GTP-binding protein Rab7 gene (*PgRab7*) isolated from *Pennisetum glaucum*, a relatively drought-stress tolerant food grain crop grown in India, has been reported to increase tolerance to abiotic stresses such as drought and increased salinity in transgenic tobacco[106]. Similarly, the Rab7 gene (*TaRab7*) isolated from wheat leaves infected with the wheat stripe rust pathogen (*Puccinia striiformis* f. sp. *tritici*), was proposed to play an important role in early stages of wheat-stripe rust fungus interaction and stress tolerance [107]. In tef, the regulation of autophagy with dehydration stress may enhance drought stress tolerance until plant viability is compromised and PCD pathways are triggered.

During dehydration stress, tef responses to biotic challenges such as fungal or bacterial infections are also important, as the GO-terms response to symbiont and symbiotic fungus and regulation of symbiosis encompassing mutualism through parasitism were highly over-represented (Figure 6A; 8.1% and 11.3% protein sequences, respectively). Although tef has been proposed to be relatively resistant to damage by insects or competition from weeds [108], at least 22 species of fungi and three pathogenic nematodes have been previously associated with tef [38,108]. The GO-term pentose metabolic process was also significantly over-represented in response to dehydration stress (Figure 6A; 9.7% protein sequences). The pentose phosphate pathway has been reported to have a dual role in oxidative stress response in plants [109]. Firstly, by providing an available source of soluble-sugars that can either be involved in ROS-producing metabolic pathways [109,110] or, alternatively, by being involved in the active production of NADPH, a major co-factor required in the antioxidant ascorbate-glutahione cycle [90,109]. In addition, these soluble sugars have been proposed to act as nutrient and metabolite signalling molecules that activate specific signalling pathways leading to imperative gene modification and proteomic changes in response to a number of stresses [109].

A substantial amount of GO-terms were enriched in tef downregulated proteins (Table S6B; Figure 6B). The functional enrichment of GO-terms found to be over-represented in downregulated proteins, were commonly linked to quinone cycling in the plastoquinone pool during oxidative phosphorylation (Figure 6B). The complexes NADH dehydrogenase and NAD(P)H dehydrogenase, both function in reducing plastoquinones during the flow of electrons when ATP is generated [111,112]. While NADH dehydrogenase functions in cellular respiration in the mitochondria [112], NAD(P)H dehydrogenase is localised in the thylakoid membranes of chloroplasts, participating in cyclic electron transport reactions around photosystem I and chlororespiration (interactions linking respiratory electron transport chain and photosynthetic electron transport chain in thylakoid membranes of chloroplasts) [113,114]. NAD(P)H, in particular, has been proposed to lessen oxidative stress in plants [114]. Increased supplying of ATP for photosynthesis has been reported during environmental stress conditions, particularly during drought stress [115]. However, since photosynthetic metabolism under water-deficit stress is reported to be responsible for the production of large amounts of free radicals [90], these processes, in effect, are decreased in tef in an attempt to perhaps minimise ROS production. In further support that reduced ROS production is important in the tef dehydration stress response, GO-terms involved in photosynthetic processes such as light harvesting and chlorophyll binding as well as GO-terms linked to ROS-producing processes through the generation of additional ATP, such as the transfusion of solutes in the form of cations and protons across membranes, were over-represented in downregulated proteins (Figure 6B).

The categories, transport and response of metal ions in the form of manganese, were well over-represented in downregulated proteins (Figure 6B). The positively charged micronutrient, manganese, is required during the splitting of water in photosystem II, when photosynthesis occurs [116,117] and has been reported to play important roles as a co-factor and activator of enzymes in various sub-cellular compartments [103,116]. To avoid toxicity within cell tissues, cytosolic manganese concentrations need to be kept low [116] and are usually transported out of the cytosol by metal transporters where they are either localised to the plant cell membrane or to the vacuolar membrane where metals are sequestered into large moderately inert compartments [117]. If manganese concentrations are not carefully monitored in plant cells, toxicity is usually indicated by chlorosis, brown specks, necrosis and crinkled leaves, which arise due to the inhibition of chlorophyll synthesis [117]. The disruption of manganese ion transport and homeostasis and consequent decreased protein abundance in tef, comes as no surprise in response to dehydration stress as photosynthetic potential has been shown to decrease at water contents below 55% RWC (Figure 2B). The decrease in photosynthesis and inhibition of chlorophyll synthesis, would ultimately lead to increased manganese concentrations and toxicity within tef plant cells due to metal transport and cellular manganese homeostasis disruption. 

Potential modification of the cell wall, particularly in the form of the terms cellulase activity, cellulose catabolism, beta-glucan catabolism and cell wall modification involved in multidimensional cell growth, were over-represented in tef downregulated proteins (Figure 6B). The effect of cell wall re-structuring and modification during stress conditions is a common phenomenon in plant cells [118] as a consequence of turgor loss during dehydration stress [119,120]. Many plants curtail the growth of their stems and leaves when subjected to low water potential [121] and continue to elongate the root tissues for deeper soil penetration and water mining as a result of adapting to drought conditions [120,121]. Previous observations in tef with regards to increased primary root lengths and decreased shoot growth in response to drought conditions have been reported [13] and have been proposed to be an adaptive morphological response of tef in water-limiting environments [13]. Lastly, the GO-term, sucrose-phosphate synthase activity, was over-represented in downregulated proteins (Figure 6B). Sucrose phosphate synthase (EC 2.4.1.14) plays an important role in the synthesis of sucrose using substrates derived from glycolysis such as fructose-6-phosphate and UDP-glucose. In correlation to being functionally enriched in downregulated tef proteins (Figure 6B), the enzyme has been previously shown to decrease in activity in the leaves of other C4 species as well, such as maize [122] and sugarcane [123], in response to dehydration stress. The decline in sucrose accumulation has been proposed to be due to the decline in readily available photosynthetic triose phosphate, which ultimately leads to a decline in the enzyme activity of sucrose phosphate synthase [124].

## 5. Conclusions

In summary, an in-depth proteomic analyses in tef leaf tissues was conducted, during hydrated, non-stressed conditions at approximately 80% RWC and at the previously established critical water content stages in a range of 50% RWC, where tef was shown to be physiologically affected by the imposed stress conditions. iTRAQ mass spectrometry and appropriate database searching enabled the detection of 5727 proteins, of which 211 proteins were found to be differentially regulated in response to dehydration stress. A considerable number of identified proteins (57 in total) were generated through alternative splicing of the tef genome. Proteins arising from alternative splicing are potential isoforms and altered proteins, known to potentially assist in tolerance to various abiotic stresses [125], particularly in response to drought [126]. In tef, alternative splicing of the genome can be proposed as a regulatory mechanism that enhances adaptation to stress, by providing multiple transcripts and proteins that aid in tolerance to drought. These would include the stress-responsive proteins generated through alternative splicing, MDHAR, POX, and FBA. Validation of these protein targets by means of Western blotting and enzymatic assay confirmed protein presence according to iTRAQ findings and showed increased protein concentrations and relative enzymatic activities in response to dehydration stress. GO-term evaluation and enrichment analysis revealed terms involved in biotic and abiotic stress response, signalling, transport, cellular homeostasis and pentose metabolic processes, enriched in tef upregulated proteins, while terms linked to ROS-producing processes under water-deficit conditions, such as photosynthesis and associated light harvesting reactions as well as cell wall catabolism, manganese transport and homeostasis, the synthesis of sugars and cell wall modification, were enriched in tef downregulated proteins. Furthermore, an overall subtle shift in the proteome of tef occurs with dehydration stress, where proteins functioning in stress response, antioxidant protection mechanisms, autophagy and those active in maintaining crucial plant cell maintenance processes are accumulated. Interestingly, abiotic stresses such as drought conditions occur in tandem with an increase in biotic stress factors, where tef showed increased susceptibility to symbiotic relationships involving parasitism and fungal responses. These results show that abiotic stress factors do not occur in isolation [127] and that biotic stress factors should be taken into account when observing plant response to adverse changes in the environment. Lastly, enrichment of terms associated with the decrease of predominantly ROS-producing processes through those generated from photosynthetic reactions and metal transport were observed in an attempt to minimise ROS proliferation associated with internal water loss. 

This study, to our knowledge, is the first reported comparative proteomic analyses of the tef proteome in response to dehydration stress as a consequence of drought conditions and could serve as a basis for future studies and for further characterisation of tef ‘omic’ resources. 

## Figures and Tables

**Table 1:** TE upregulated proteins in response to dehydration stress *.

Protein ID	Protein Description	Hydrated (Group 1)	Dehydrated (Group 2)	Fold Change	*p*-Value
CL1Contig10009	---NA---	7.22	8.09	1.12	0.001
CL5492Contig2	fructose-bisphosphate aldolase cytoplasmic isozyme	10.70	10.86	1.02	0.001
CL856Contig3	40s ribosomal protein s28	9.17	9.33	1.02	0.001
CL856Contig4	40s ribosomal protein s28	9.17	9.33	1.02	0.001
CL68Contig25	peroxidase 3-rare cold-inducible protein	7.14	7.73	1.08	0.002
CL4104Contig2	gras family protein 2	0.69	4.68	6.75	0.003
CL3156Contig1	---NA---	0.69	6.05	8.73	0.004
CL6974Contig4	---NA---	0.69	6.05	8.73	0.004
CL715Contig2	protease includes: reverse transcriptase inlcudes: endonuclease	9.03	9.43	1.04	0.004
Locus_2136_1_5	ENO2_ERATE, Enolase 2	11.01	11.18	1.02	0.004
CL8690Contig2	---NA---	0.69	5.34	7.70	0.005
CL1942Contig1	---NA---	0.69	4.53	6.54	0.006
CL2699Contig6	monodehydroascorbate cytoplasmic isoform 2	0.69	4.53	6.54	0.006
isotig02308	probable monodehydroascorbate cytoplasmic isoform 2	0.69	4.53	6.54	0.006
CL2976Contig3	---NA---	0.69	4.94	7.12	0.007
CL68Contig6	peroxidase 3-rare cold-inducible protein	0.66	4.49	6.82	0.008
CL799Contig2	hua2-like protein 2	9.35	9.58	1.02	0.008
CL8983Contig3	---NA---	0.66	4.49	6.82	0.008
CL2991Contig2	hydroxyphenylpyruvate reductase	10.22	10.29	1.01	0.009
CL3629Contig1	poly polymerase i	0.69	5.13	7.41	0.013
CL3629Contig2	poly polymerase i	0.69	5.13	7.41	0.013
CL7746Contig3	---NA---	0.69	5.04	7.28	0.013
CL873Contig3	cyclin-p4-1	0.69	5.04	7.28	0.013
CL2289Contig1	---NA---	6.60	7.55	1.14	0.014
CL2761Contig4	red chlorophyll catabolite reductase	8.78	9.12	1.04	0.014
CL5492Contig1	fructose-bisphosphate aldolase cytoplasmic isozyme	10.66	10.84	1.02	0.014
CL1Contig421	calcium-dependent protein kinase 5	7.83	8.40	1.07	0.015
CL1Contig7756	serine carboxypeptidase-like 51	9.51	9.77	1.03	0.015
CL3374Contig5	---NA---	0.69	5.41	7.80	0.016
CL57Contig23	---NA---	0.69	5.41	7.80	0.016
CL4404Contig1	---NA---	4.75	6.49	1.37	0.017
CL8759Contig1	proteasome subunit beta type-3	4.75	6.49	1.37	0.017
CL8759Contig2	proteasome subunit beta type-3	4.75	6.49	1.37	0.017
CL1Contig7029	protease includes: reverse transcriptase includes:endonuclease	6.94	7.76	1.12	0.018
CL1Contig4553	chlorophyll a-b binding protein chloroplastic	7.16	7.82	1.09	0.019
CL2228Contig1	s phase cyclin a-associated protein in the endoplasmic reticulum	9.04	9.19	1.02	0.019
CL124Contig7	---NA---	8.82	9.24	1.05	0.02
CL415Contig1	glutathione hydrolase 3	6.58	7.51	1.14	0.02
CL124Contig2	---NA---	8.30	8.72	1.05	0.021
CL3894Contig4	leucoanthocyanidin dioxygenase	0.69	5.49	7.92	0.021
CL1498Contig7	guanosine nucleotide diphosphate dissociation inhibitor 2	5.56	6.77	1.22	0.022
CL413Contig11	u-box domain-containing protein 4	7.18	7.96	1.11	0.022
CL8890Contig3	ENO3_ERATE, Enolase 3	10.96	11.08	1.01	0.022
CL1888Contig1	glycerophosphodiester phosphodiesterase gdpdl3	11.40	11.61	1.02	0.023
CL1888Contig2	glycerophosphodiester phosphodiesterase gdpdl3	11.40	11.61	1.02	0.023
CL1498Contig4	guanosine nucleotide diphosphate dissociation inhibitor 1	6.53	7.06	1.08	0.024
CL1498Contig5	guanosine nucleotide diphosphate dissociation inhibitor 2	6.53	7.06	1.08	0.024
CL5028Contig3	plant intracellular ras-group-related lrr protein 6	8.98	9.21	1.03	0.024
isotig02787	guanosine nucleotide diphosphate dissociation inhibitor 2	6.53	7.06	1.08	0.024
CL3527Contig4	nucleolar complex protein 2 homolog	6.24	7.62	1.22	0.027
CL1Contig6763	---NA---	5.57	6.63	1.19	0.028
CL1224Contig6	gtp-binding protein sar1a	7.63	8.20	1.07	0.029
CL90Contig16	---NA---	7.59	8.02	1.06	0.029
CL18849Contig1	---NA---	5.79	6.54	1.13	0.031
CL4737Contig2	acetohydroxy-acid reductoisomerase	9.65	9.89	1.03	0.032
CL577Contig14	ubiquinol oxidase chloroplastic chromoplastic	9.39	9.56	1.02	0.032
CL24657Contig1	fructokinase-1	7.48	7.92	1.06	0.036
CL546Contig2	f-box protein skip24	5.26	6.16	1.17	0.036
CL7996Contig1	fructokinase-1	7.48	7.92	1.06	0.036
CL1073Contig1	peptidyl-prolyl cis-trans isomerase d	7.15	7.79	1.09	0.037
CL3347Contig4	delta-aminolevulinic acid chloroplastic	8.68	9.00	1.04	0.037
CL5Contig21	probable wrky transcription factor 19	7.72	8.19	1.06	0.037
CL7405Contig3	chlorophyll a-b binding protein cp24 chloroplastic s	10.71	10.85	1.01	0.038
CL136Contig17	---NA---	7.58	8.24	1.09	0.04
CL1Contig5054	f-box only protein 8	7.30	7.68	1.05	0.04
CL326Contig6	---NA---	4.92	6.56	1.33	0.04
CL3687Contig5	---NA---	4.92	6.56	1.33	0.04
CL4000Contig1	monodehydroascorbate reductase	9.99	10.26	1.03	0.04
CL4000Contig2	monodehydroascorbate reductase	9.99	10.26	1.03	0.04
CL4000Contig3	monodehydroascorbate reductase	9.99	10.26	1.03	0.04
CL4207Contig1	---NA---	4.92	6.56	1.33	0.04
CL445Contig4	elongator complex protein 6	4.92	6.56	1.33	0.04
CL445Contig6	elongator complex protein 7	4.92	6.56	1.33	0.04
CL4771Contig3	---NA---	4.92	6.56	1.33	0.04
CL4956Contig4	premnaspirodiene oxygenase	4.92	6.56	1.33	0.04
CL522Contig8	---NA---	4.92	6.56	1.33	0.04
CL6050Contig1	---NA---	4.92	6.56	1.33	0.04
CL7668Contig1	nadh dehydrogenase complex assembly factor 6	6.77	7.66	1.13	0.04
CL7668Contig2	nadh dehydrogenase complex assembly factor 6	6.77	7.66	1.13	0.04
CL837Contig7	cell division cycle protein 48 homolog	10.78	10.96	1.02	0.04
CL102Contig20	---NA---	6.34	7.08	1.12	0.041
CL5577Contig3	---NA---	7.34	7.84	1.07	0.041
CL1Contig7889	---NA---	8.42	9.03	1.07	0.042
CL2761Contig3	red chlorophyll catabolite reductase	8.59	8.97	1.04	0.042
CL836Contig11	probable polyamine transporter	7.78	8.38	1.08	0.042
CL4591Contig2	phosphatidylinositol n-acetylglucosaminyltransferase subunit a	8.95	9.18	1.03	0.043
CL61Contig20	probable ufm1-specific protease	8.15	8.64	1.06	0.043
CL61Contig7	probable ufm1-specific protease	8.15	8.64	1.06	0.043
CL4852Contig2	---NA---	9.18	9.68	1.05	0.046
CL2637Contig1	peroxisome biogenesis protein 6 (PEX6)	9.85	10.06	1.02	0.047
CL680Contig10	elongation factor tu gtp-binding domain-containing protein 2	7.43	8.05	1.08	0.048
CL680Contig5	elongation factor tu gtp-binding domain-containing protein 2	7.43	8.05	1.08	0.048
CL7065Contig1	---NA---	5.58	6.49	1.16	0.048
CL7065Contig2	---NA---	5.58	6.49	1.16	0.048
CL140Contig10	npk1-activating kinesin-1	8.98	9.27	1.03	0.049
CL4289Contig6	---NA---	8.29	8.58	1.03	0.049
isotig08284	protein disulfide isomerase-like 1-1	10.76	10.98	1.02	0.049

* Protein ID: protein identifier; protein description: identified protein; hydrated: averaged quantitative expression value of group 1 (hydrated labels 115–117); dehydrated: averaged quantitative expression value of group 2 (dehydrated labels 118–121); fold change: change in quantitative expression of proteins with dehydration stress (group 2/group 1), where values >1 display an increase in protein expression; *p*-value: associated *p*-value during two-group analysis for statistical significance (*p*-value ≤ 0.05).

**Table 2:** TE downregulated proteins in response to dehydration stress *.

Protein ID	Protein Description	Hydrated (Group 1)	Dehydrated (Group 2)	Fold Change	*p*-Value
CL5604Contig1	2-methyl-6-phytyl-hydroquinone methyltransferase chloroplastic	9.36	8.94	0.96	0.001
CL977Contig4	---NA---	7.70	7.08	0.92	0.001
CL2349Contig3	protein dek	9.77	9.35	0.96	0.002
CL36Contig35	nad-dependent malic enzyme 59 kda mitochondrial	10.52	10.36	0.99	0.002
CL5457Contig2	---NA---	9.09	8.91	0.98	0.002
CL11972Contig1	metal tolerance protein 5	6.23	1.19	0.19	0.004
CL700Contig3	nad-dependent malic enzyme 62 kda mitochondrial	9.37	9.12	0.97	0.005
CL236Contig5	probable sucrose-phosphate synthase 2	9.46	9.21	0.97	0.006
CL236Contig6	probable sucrose-phosphate synthase 2	9.44	9.18	0.97	0.006
CL1456Contig11	s-adenosylmethionine decarboxylase proenzyme	5.33	0.69	0.13	0.007
CL1456Contig8	s-adenosylmethionine decarboxylase proenzyme	5.33	0.69	0.13	0.007
CL2948Contig2	haloalkane dehalogenase	9.85	9.63	0.98	0.007
isotig10649	s-adenosylmethionine decarboxylase proenzyme	5.33	0.69	0.13	0.007
CL13Contig40	rhodanese-like domain-containing protein 10	8.66	7.80	0.90	0.009
CL1595Contig2	---NA---	4.99	0.69	0.14	0.009
CL1042Contig2	alpha-glucan water chloroplastic	9.41	9.13	0.97	0.01
CL19Contig25	---NA---	8.92	8.29	0.93	0.01
CL7534Contig1	cellulose synthase-like protein a9	7.36	6.55	0.89	0.01
CL7716Contig2	---NA---	7.78	7.25	0.93	0.013
CL7716Contig3	Putative uncharacterized protein CysX	7.78	7.25	0.93	0.013
CL7582Contig1	ribosomal rna processing protein 36 homolog	8.47	7.75	0.92	0.014
CL14686Contig1	alliin lyase 1	5.51	0.69	0.13	0.015
CL2382Contig6	chlorophyll a-b binding chloroplastic	9.39	8.99	0.96	0.015
Locus_49_75_82	---NA---	11.12	10.92	0.98	0.015
CL1759Contig3	---NA---	7.61	7.19	0.94	0.016
CL1Contig8969	---NA---	10.54	10.40	0.99	0.016
CL14672Contig1	---NA---	8.33	7.87	0.94	0.017
CL16131Contig1	---NA---	8.33	7.87	0.94	0.017
CL456Contig16	---NA---	10.12	9.95	0.98	0.017
CL7059Contig2	cytochrome b561 and domon domain-containing protein	8.00	7.36	0.92	0.017
CL9348Contig2	ras-related protein raba5c; ras-related protein ara-4; ras-related protein rab11f	9.07	8.73	0.96	0.017
CL1630Contig1	nad h azoreductase	9.33	9.12	0.98	0.018
CL3227Contig1	---NA---	5.69	0.69	0.12	0.018
CL3227Contig2	---NA---	5.69	0.69	0.12	0.018
CL58Contig14	---NA---	5.69	0.69	0.12	0.018
CL58Contig2	---NA---	5.69	0.69	0.12	0.018
CL467Contig12	---NA---	9.78	9.57	0.98	0.02
isotig23406	photosystem ii protein d1	10.59	10.33	0.98	0.02
CL1Contig3562	---NA---	8.20	7.53	0.92	0.022
CL3294Contig3	nad h-quinone oxidoreductase subunit chloroplastic	9.37	9.17	0.98	0.022
CL3294Contig4	nad h-quinone oxidoreductase subunit chloroplastic	9.37	9.17	0.98	0.022
CL3294Contig5	nad h-quinone oxidoreductase subunit chloroplastic	9.37	9.17	0.98	0.022
CL3294Contig6	nad h-quinone oxidoreductase subunit chloroplastic	9.37	9.17	0.98	0.022
CL6495Contig2	polyamine oxidase	8.91	8.42	0.95	0.022
comp294_c0_seq1	nad h-quinone oxidoreductase subunit chloroplastic	9.37	9.17	0.98	0.022
CL5963Contig1	60s ribosomal protein l5-1	10.95	10.86	0.99	0.023
CL8805Contig2	---NA---	9.60	9.14	0.95	0.023
CL5672Contig2	---NA---	8.49	8.03	0.95	0.024
CL6932Contig1	---NA---	9.87	9.60	0.97	0.024
Locus_954_4_4	---NA---	9.87	9.60	0.97	0.024
CL4237Contig3	SYN3_ERATE, Asparagine tRNA ligase 3	9.96	9.63	0.97	0.024
CL4237Contig2	SYN8_ERATE, Asparagine tRNA ligase 8	9.96	9.63	0.97	0.024
CL1805Contig10	protein dj-1 homolog b	8.06	7.57	0.94	0.025
CL1805Contig2	protein dj-1 homolog b	8.06	7.57	0.94	0.025
CL1Contig3266	---NA---	7.90	7.43	0.94	0.025
CL327Contig3	cbs domain-containing protein cbsppr1	7.85	7.37	0.94	0.025
Locus_2288_7_9	---NA---	9.38	9.05	0.97	0.025
CL2320Contig2	---NA---	7.27	6.85	0.94	0.026
CL977Contig1	---NA---	7.27	6.85	0.94	0.026
CL10226Contig1	---NA---	8.21	7.20	0.88	0.027
CL2336Contig7	---NA---	9.33	9.15	0.98	0.027
CL1Contig5286	metal tolerance protein 5	5.00	0.69	0.14	0.029
CL1Contig5699	metal tolerance protein 6	5.00	0.69	0.14	0.029
CL1Contig8303	metal tolerance protein 5	5.00	0.69	0.14	0.029
CL73Contig10	clathrin heavy chain 1	11.05	10.94	0.99	0.03
CL3204Contig2	---NA---	6.50	5.77	0.89	0.031
CL10162Contig3	---NA---	7.16	6.23	0.87	0.032
CL349Contig4	homeobox-leucine zipper protein roc6	7.24	6.70	0.93	0.033
CL349Contig7	homeobox-leucine zipper protein roc6	7.24	6.70	0.93	0.033
CL5826Contig1	long chain acyl- synthetase 4	10.35	10.18	0.98	0.033
CL5826Contig2	long chain acyl- synthetase 4	10.35	10.18	0.98	0.033
CL5942Contig6	---NA---	9.19	8.87	0.97	0.033
CL6511Contig2	v-type proton atpase subunit g1	9.82	9.58	0.98	0.033
CL7Contig43	---NA---	7.48	7.28	0.97	0.033
comp13984_c0_se	---NA---	8.33	7.85	0.94	0.035
CL1Contig3395	endoglucanase 7	8.19	7.50	0.92	0.036
CL1Contig3396	endoglucanase 7	8.19	7.50	0.92	0.036
CL1Contig3397	endoglucanase 7	8.19	7.50	0.92	0.036
CL3496Contig11	chlorophyll a-b binding protein 1b- chloroplastic -light-harvesting complex i	10.21	9.97	0.98	0.036
CL154Contig2	ankyrin repeat domain-containing protein chloroplastic	9.95	9.70	0.97	0.038
CL1Contig4279	---NA---	9.23	8.89	0.96	0.038
CL4622Contig2	rubredoxin	11.01	10.88	0.99	0.038
CL1Contig4299	histone-lysine n-methyltransferase setd3	8.28	7.84	0.95	0.039
CL1Contig4635	protease do-like 14	6.90	5.87	0.85	0.039
CL8953Contig2	---NA---	9.05	8.80	0.97	0.039
CL5563Contig3	---NA---	9.08	8.84	0.97	0.04
CL1Contig242	bax inhibitor 1	9.79	9.67	0.99	0.041
CL2736Contig1	ubiquinol-cytochrome-c reductase subunit ii	9.80	9.66	0.99	0.041
CL3528Contig3	golgin candidate 4	9.24	8.95	0.97	0.041
CL5380Contig1	glutathione s-transferase t3	8.68	8.50	0.98	0.041
CL94Contig6	---NA---	9.49	9.33	0.98	0.041
CL5774Contig2	r60s acidic ribosomal protein p0	9.92	9.78	0.99	0.042
CL3496Contig14	chlorophyll a-b binding protein 1b- chloroplastic -light-harvesting complex i	10.11	9.95	0.98	0.043
CL3496Contig15	chlorophyll a-b binding protein 1b- chloroplastic -light-harvesting complex i	10.11	9.95	0.98	0.043
CL811Contig3	myb-like transcription factor 1	7.47	6.96	0.93	0.043
CL3168Contig2	---NA---	8.47	8.06	0.95	0.044
Locus_393_4_9	---NA---	10.96	10.81	0.99	0.044
CL1Contig492	---NA---	9.24	8.99	0.97	0.045
CL1Contig6871	chlorophyll a-b binding protein chloroplastic	11.19	10.99	0.98	0.045
CL1Contig7112	hexose carrier protein hex6	8.75	8.57	0.98	0.045
CL185Contig19	probable disease resistance protein rf45	9.19	8.93	0.97	0.046
CL885Contig1	formin-like protein 3	9.72	9.54	0.98	0.046
Locus_61_5_6	---NA---	10.11	9.92	0.98	0.046
CL3496Contig13	chlorophyll a-b binding protein 1b- chloroplastic -light-harvesting complex i	10.10	9.88	0.98	0.048
CL3496Contig17	chlorophyll a-b binding protein 1b- chloroplastic -light-harvesting complex i	10.10	9.88	0.98	0.048
CL321Contig12	---NA---	8.27	7.66	0.93	0.049
CL785Contig5	---NA---	7.16	6.68	0.93	0.049
CL94Contig5	choline chloroplastic	9.49	9.35	0.99	0.049
CL131Contig9	---NA---	7.77	7.25	0.93	0.05
CL19309Contig1	---NA---	8.53	8.27	0.97	0.05
CL236Contig2	---NA---	8.87	8.56	0.97	0.05
CL236Contig9	---NA---	8.87	8.56	0.97	0.05
CL305Contig27	---NA---	8.53	8.27	0.97	0.05
CL7612Contig2	---NA---	8.53	8.27	0.97	0.05

* Protein ID: protein identifier; protein description: identified protein; hydrated: averaged quantitative expression value of group 1 (hydrated labels 115–117); dehydrated: averaged quantitative expression value of group 2 (dehydrated labels 118–121); fold change: change in quantitative expression with dehydration stress (group 2/group 1), where values <1 display a decrease in protein expression; *p*-value: associated *p*-value during two-group analysis for statistical significance (*p*-value ≤ 0.05).

## Supplementary Materials

The following figures, tables and supplementary data files are available online at www.mdpi.com/2227-7382/5/4/32/s1. Figure S1: iTRAQ analysis workflow of tef protein samples in preparation for MS analysis. Figure S2: Steps to tef iTRAQ data pre-processing, refinement and statistical analysis. Figure S3: Sanity check (Q-Q plots) of all labels (113–121) used in iTRAQ analysis. Figure S4: Heat map (cluster analysis) of the labelled channels in iTRAQ analysis after data refinement steps. Table S1 Tef differentially regulated proteins of highly significant fold change and *p*-values *. Table S2 TEU upregulated proteins in response to dehydration stress. Table S3 TEU downregulated proteins in response to dehydration stress. Table S4 MU upregulated proteins in response to dehydration stress. Table S5 MU downregulated proteins in response to dehydration stress. Table S6 Functional enrichment analysis of GO-terms. File S1 Refined and processed list of tef proteins-peptides for protViz File S2.1 TE dataset (all 5727 proteins with expression values, fold changes and *p*-values). File S2.2 TE—two-group analysis (box-plots). File S2.3 TE—Sanity Check (quality check 1). File S2.4 TE—Heat map (quality check 2). File S3.1 TEU dataset (all 2656 proteins with expression values, fold changes and *p*-values). File S3.2 TEU—two-group analysis (box-plots). File S3.3 TEU—Sanity Check (quality check 1). File S3.4 TEU—Heat map (quality check 2). File S4.1 MU dataset (all 4328 proteins with expression values, fold changes and *p*-values). File S4.2 MU—two-group analysis (box-plots). File S4.3 MU—Sanity Check (quality check 1). File S4.4 MU—Heat map (quality check 2).

## Abbreviations

AWC	Absolute water content
FASP	Filter assisted sample preparation procedure
FBA	Fructose bisphosphate aldolase
FDR	False discovery rate
GO	Gene ontology
GTP iTRAQ	Guanosine triphosphate Isobaric tag for relative and absolute quantitation
MDHAR	Monodehydroascorbate reductase
MS	Mass spectrometry
MS/MS	Tandem mass spectrometry
MU	Monocot unique
NAD POX	Nicotinamide adenine dinucleotide Peroxidase
ROS	Reactive oxygen species
RWC	Relative water content
TE	Tef Extended
TEM	Transmission electron microscopy
TEU	Tef Extended unique
