# Supplementary material for: A Proteomic Approach to Investigate the Drought Response in the Orphan Crop Eragrostis tef
Source: Proteomes. 2017 Nov 15;5(4):32. doi: 10.3390/proteomes5040032 (PMC5748567; doi:10.3390/proteomes5040032)
Supplement: Supplementary file 1 [file proteomes-05-00032-s001.zip › proteomes-208511 supplementary final/Summary page of TEF serach with all 12 fractions.pdf]

1. Notes

2. Result Statistics

Figure 1: The false discovery rate (FDR) curve. X-axis is the number of peptide-spectrum matches (PSM) being kept and the y-axis is the FDR.

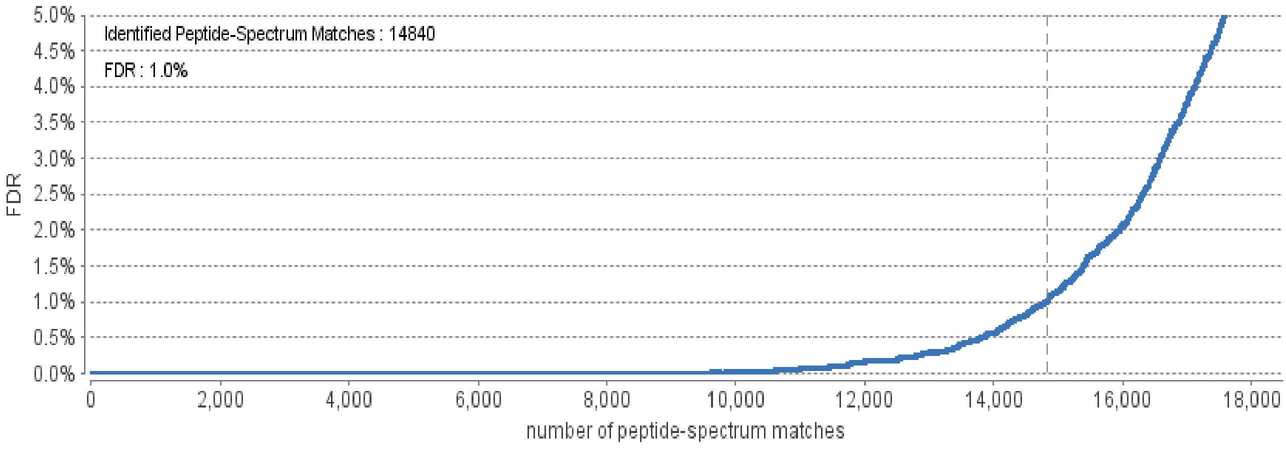

Figure 2: The distribution of PEAKS peptide score (-10lgP): **(a)** histogram of score; **(b)** The plot of precursor mass error vs. score.

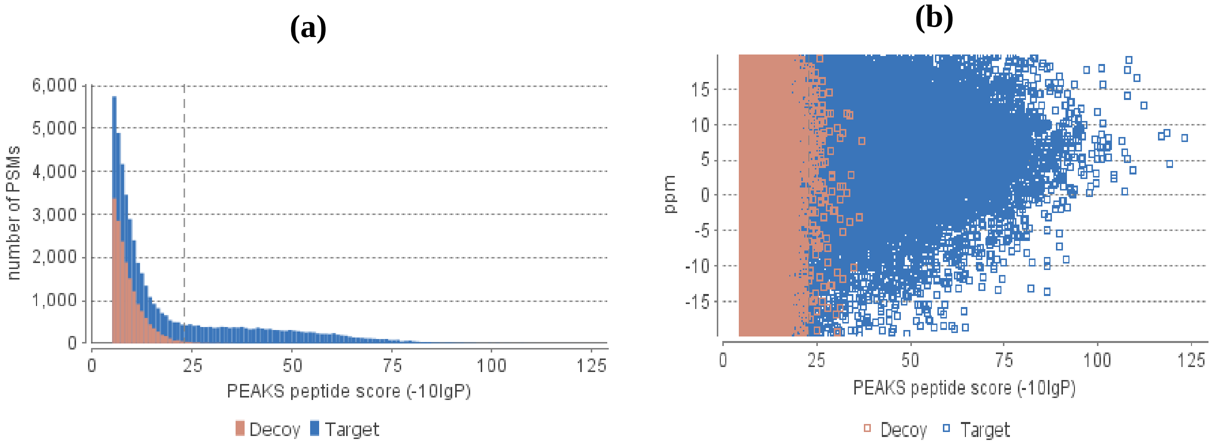

Table 1. Statistics of data.

# of MS Scans	76564
# of MS/MS Scans	102696

Table 2. Result filtration parameters.

Peptide -10lgP	≥23	De Novo TLC	≥3
Protein -10lgP	≥20		
Proteins Unique Peptides	≥2	De Novo ALC	≥50%

Table 3. Statistics of filtered result.

Peptide-Spectrum Matches	14840
Peptide Sequences	6244
Protein Groups	570
Proteins	774
Proteins (#Unique Peptides)	503 (>2); 271 (=2); 0 (=1);
FDR (Peptide-Spectrum Matches)	1.0%
FDR (Peptide Sequences)	2.3%
FDR (Protein)	0.0%
De Novo Only	51022

Table 4. PTM Profile.

**Name**	**ΔMass**	**#PSM**	**Position**
iTRAQ-8plex (K)	304.20	14840	K,N-term
Beta-methylthiolation	45.99	1338	C
Oxidation	15.99	653	M
iTRAQ-8plex (Y)	304.20	649	Y

3. Experiment Control

Figure 3: The precursor mass error (observed m/z - theoretical m/z) distribution of the PSMs in filtered result. **(a)** Each bar indicates the number of PSMs (y-axis) with a certain precursor error (x-axis); **(b)** Each data point represents a PSM. x-axis is the theoretical m/z and y-axis is the precursor mass error

(a) (b)

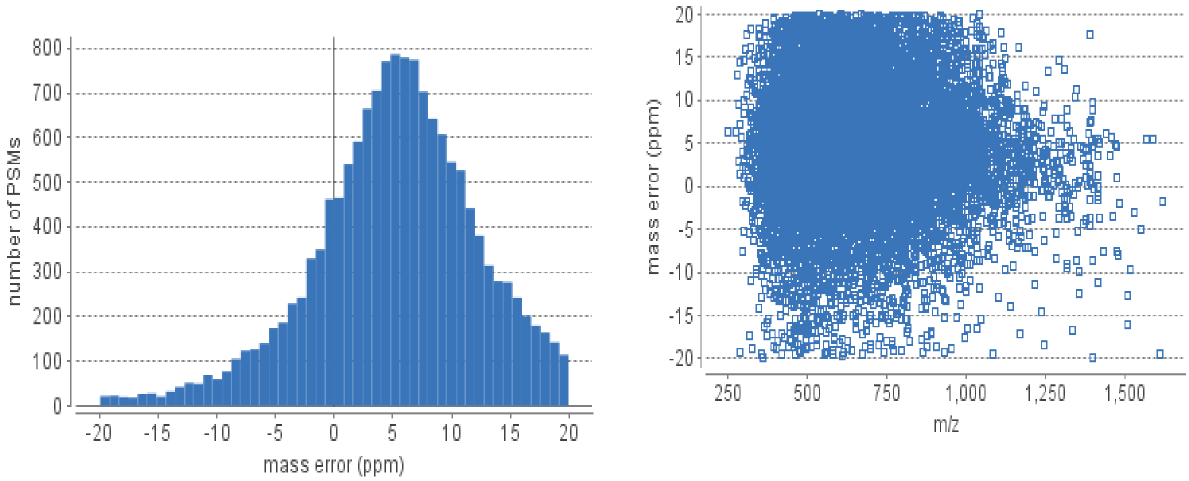

Table 5. Number of peptides by number of missed cleavages

Missed Cleavages	0	1	2	3	4+
Tef extended db search 6 fract1-12	5795	431	18	0	0

4. Other Information

Table 6. Search parameters.

Search Engine Name: PEAKS 6.0
Parent Mass Error Tolerance: 20.0 ppm
Fragment Mass Error Tolerance: 0.1 Da
Precursor Mass Search Type: monoisotopic
Enzyme: Trypsin
Max Missed Cleavages: 2
Non-specific Cleavage: one
Fixed Modifications:
Beta-methylthiolation: 45.99

Table 7. Instrument parameters.

Fractions: Riz_April8_2013_TEF_iTRAQF1.mzML,Riz_April8_2013_[illegible]
_iTRAQF10.mzML,Riz_April8_2013_TEF_iTRAQF11.mzML,Riz_Ap[illegible]
013_TEF_iTRAQF12.mzML,Riz_April8_2013_TEF_iTRAQF2.mzML[illegible]
ril8_2013_TEF_iTRAQF3.mzML,Riz_April8_2013_TEF_iTRAQF4.m[illegible]
iz_April8_2013_TEF_iTRAQF5.mzML,Riz_April8_2013_TEF_iTRA[illegible]
zML,Riz_April8_2013_TEF_iTRAQF7.mzML,Riz_April8_2013_TEF_[illegible]
QF8.mzML,Riz_April8_2013_TEF_iTRAQF9.mzML
Ion Source: ESI(nano-spray)
Fragmentation Mode: CID, CAD, IRMPD (y and b ions)
MS Scan Mode: Quadrupole
MS/MS Scan Mode: Time of Fligh

iTRAQ 8plex (K, N-term): 304.20
Variable Modifications:
iTRAQ 8plex (Y): 304.20
Oxidation (M): 15.99
Max variable PTM per peptide: 3
Database: Tef extended sp out validated
Taxon: All
Searched Entry: 68593
Decoy DB Size: 1
Merge Options: no merge
Precursor Options: corrected
Charge Options: no correction
Filter Options: no filter
Process: true
