# Supplementary figures and images for: A Proteomic Approach to Investigate the Drought Response in the Orphan Crop Eragrostis tef
Source: Proteomes. 2017 Nov 15;5(4):32. doi: 10.3390/proteomes5040032 (PMC5748567; doi:10.3390/proteomes5040032)

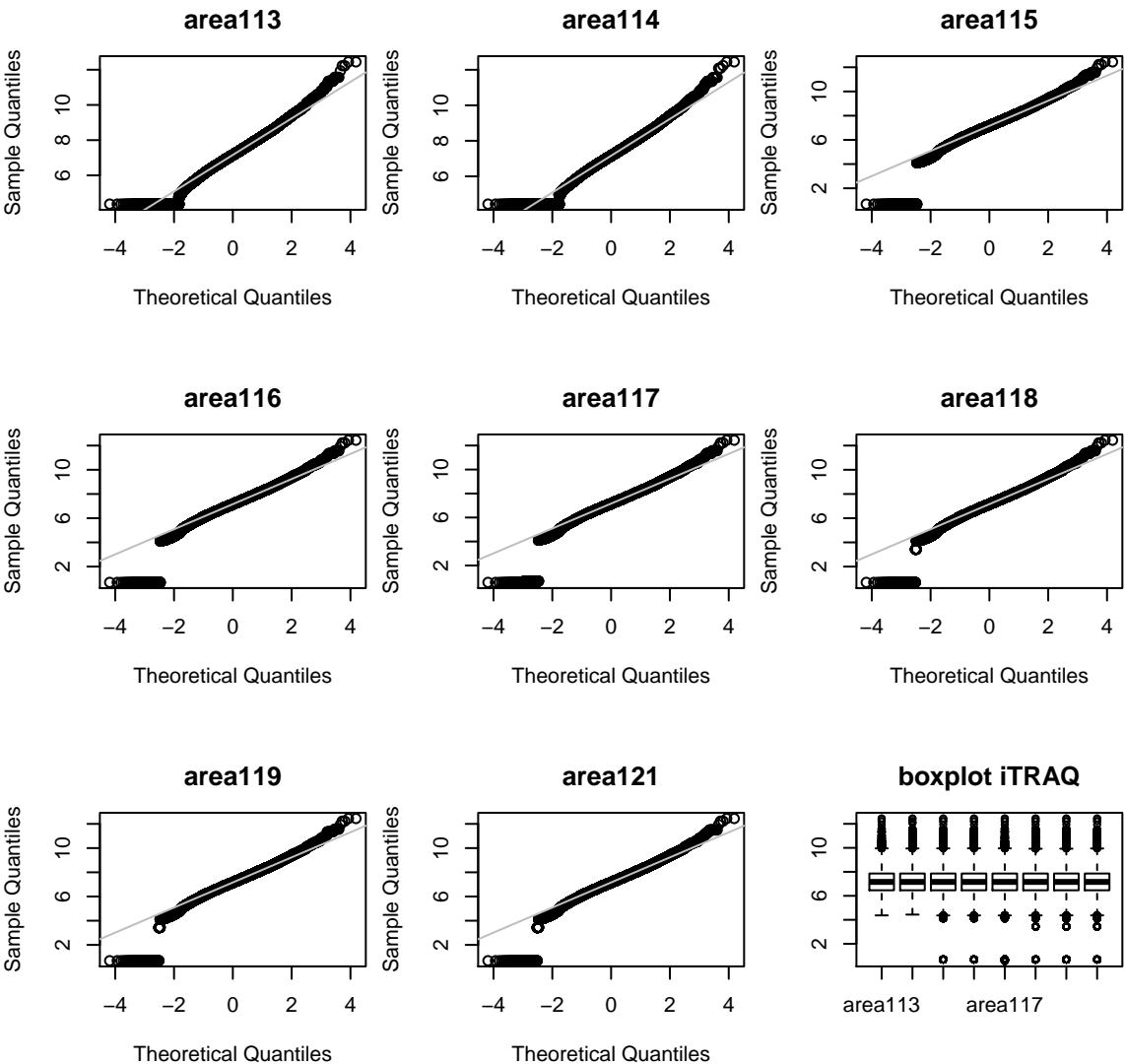

Supplement: Supplementary file 1 [file proteomes-05-00032-s001.zip › proteomes-208511 supplementary final/S2.3 TE-Sanity Check (quality check1).pdf]

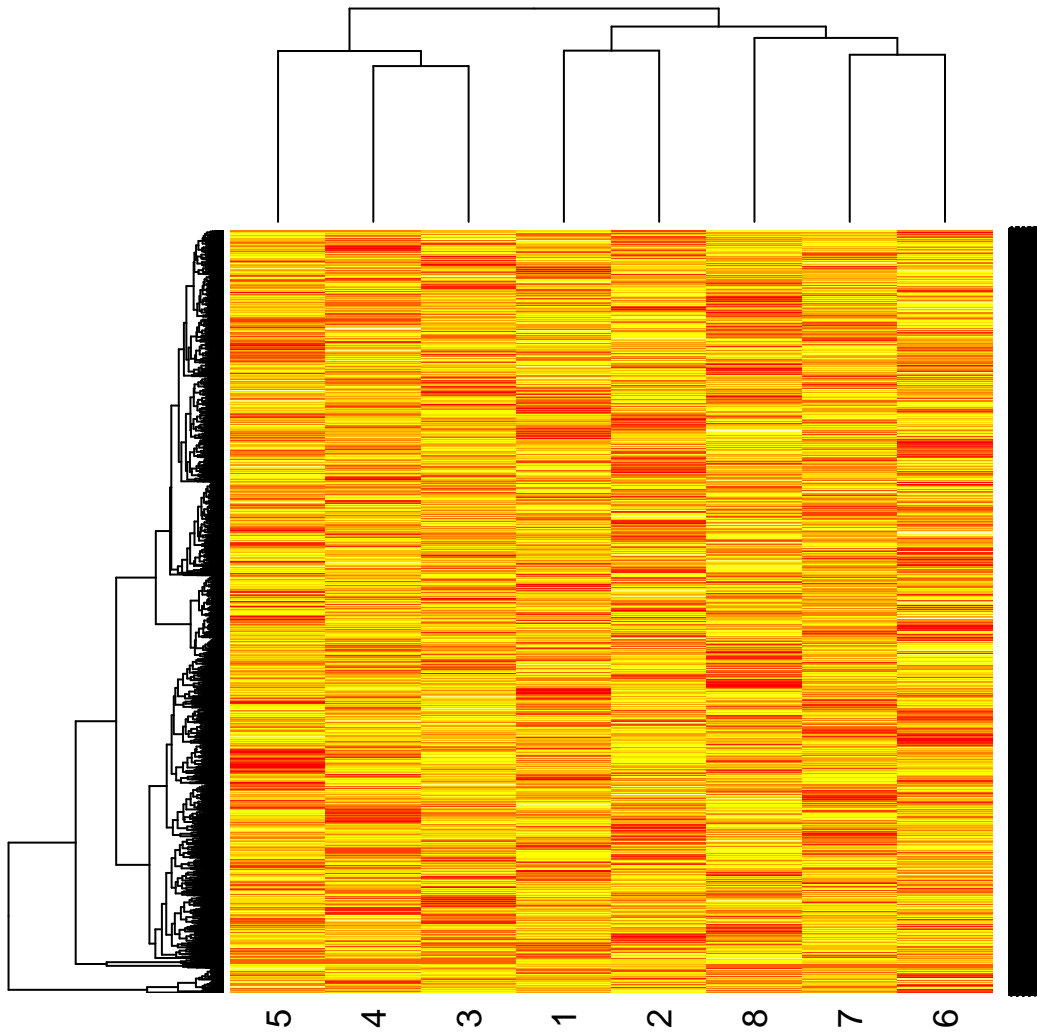

Supplement: Supplementary file 1 [file proteomes-05-00032-s001.zip › proteomes-208511 supplementary final/S2.4 TE-Heatmap- (quality check2).pdf]

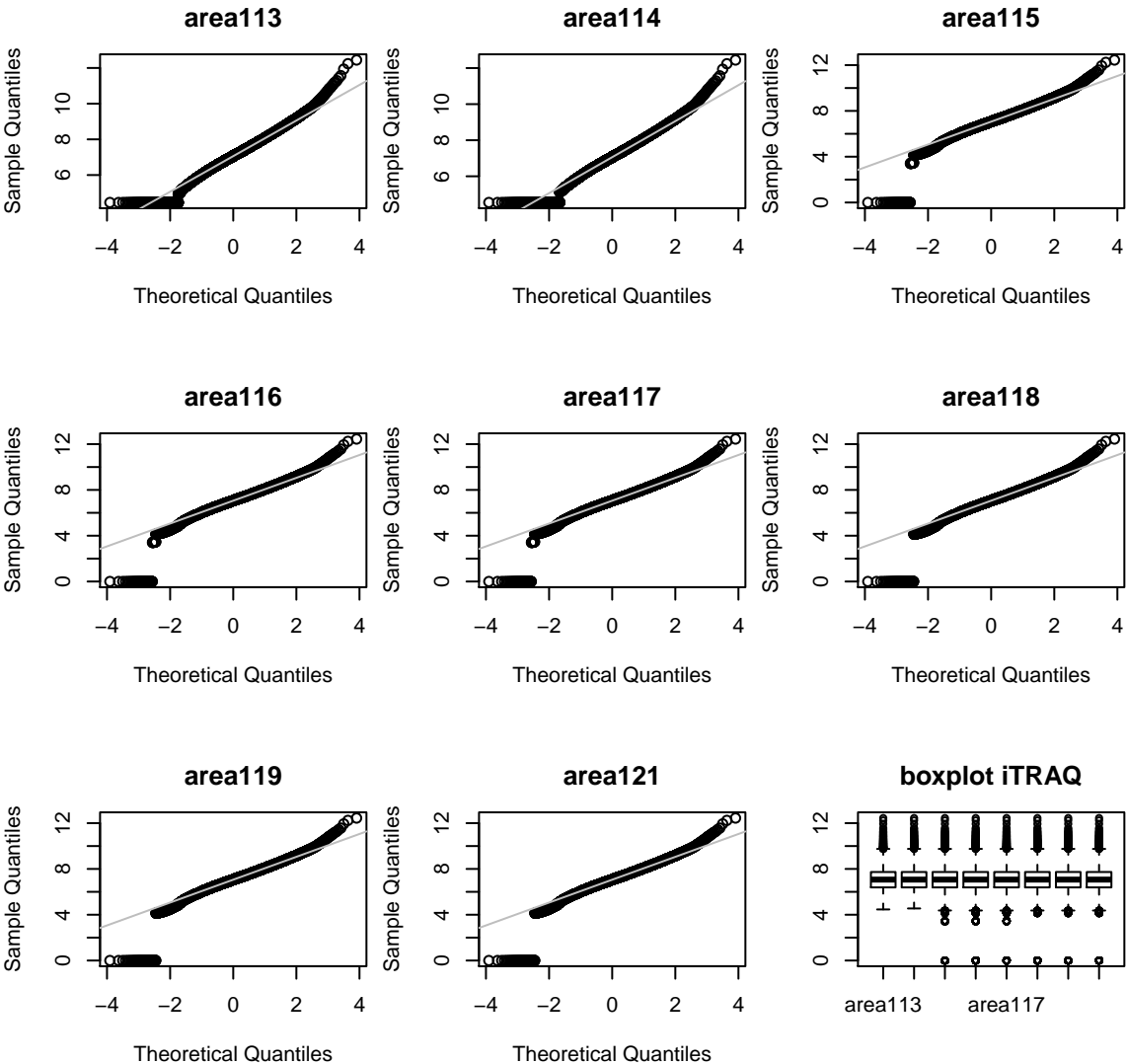

Supplement: Supplementary file 1 [file proteomes-05-00032-s001.zip › proteomes-208511 supplementary final/S3.3 TEU-Sanity Check (quality check1).pdf]

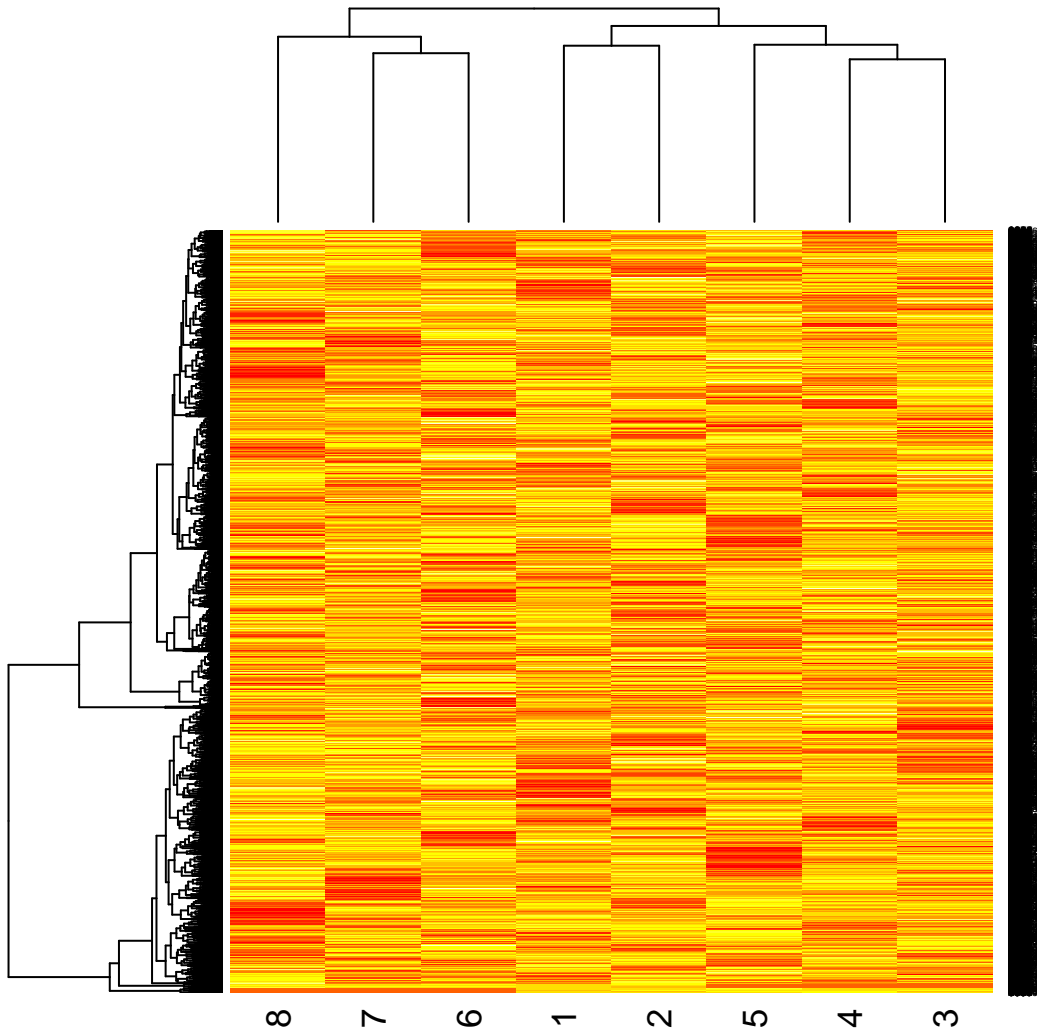

Supplement: Supplementary file 1 [file proteomes-05-00032-s001.zip › proteomes-208511 supplementary final/S3.4 TEU-Heatmap(quality check2).pdf]

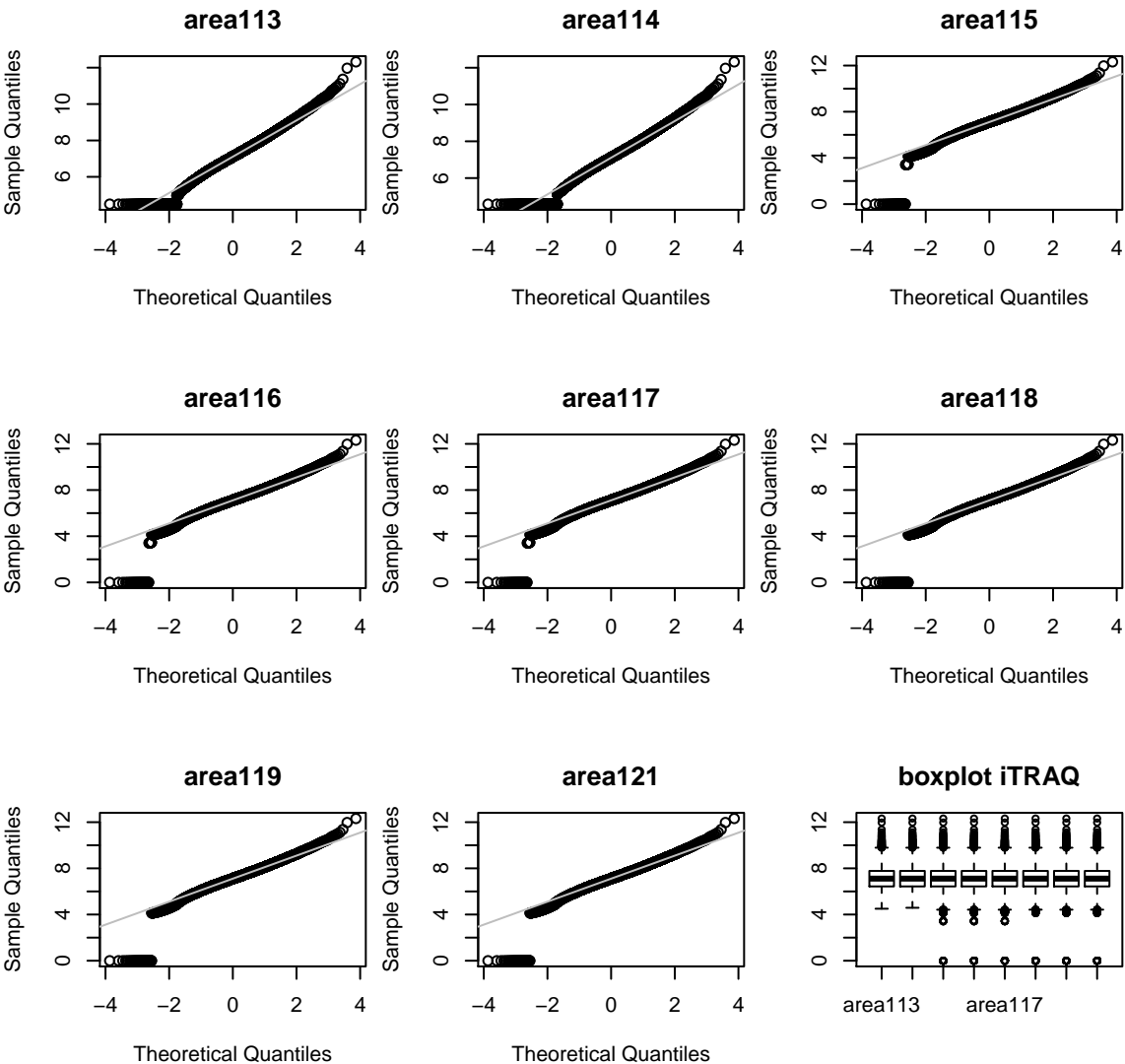

Supplement: Supplementary file 1 [file proteomes-05-00032-s001.zip › proteomes-208511 supplementary final/S4.3 MU-Sanity Check (quality check1).pdf]

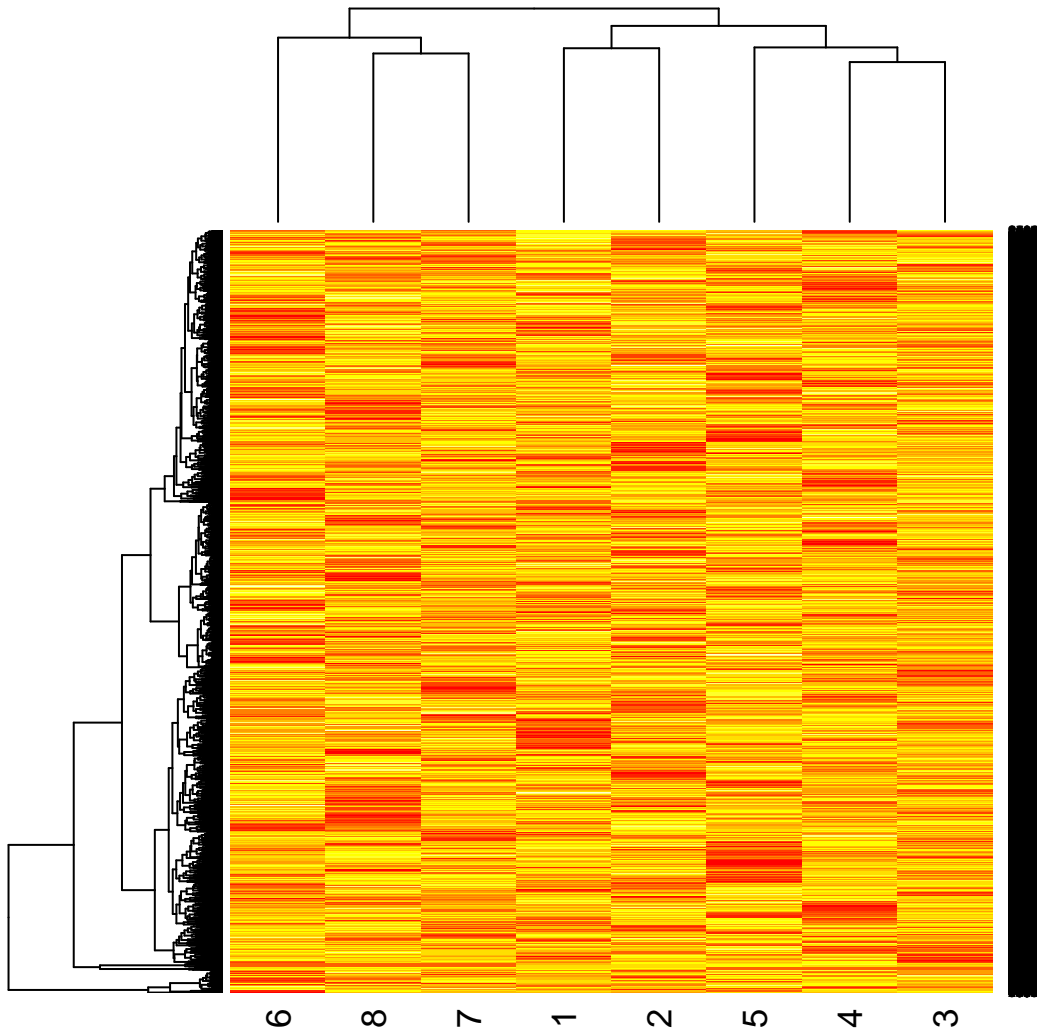

Supplement: Supplementary file 1 [file proteomes-05-00032-s001.zip › proteomes-208511 supplementary final/S4.4 MU-Heatmap (quality check2).pdf]
