# Supplementary material for: A Proteomic Approach to Investigate the Drought Response in the Orphan Crop Eragrostis tef
Source: Proteomes. 2017 Nov 15;5(4):32. doi: 10.3390/proteomes5040032 (PMC5748567; doi:10.3390/proteomes5040032)
Supplement: Supplementary file 1 [file S2.2 TE two group analysis (box-plots).pdf › proteomes-208511 supplementary final/S2.2 TE two group analysis (box-plots).pdf]

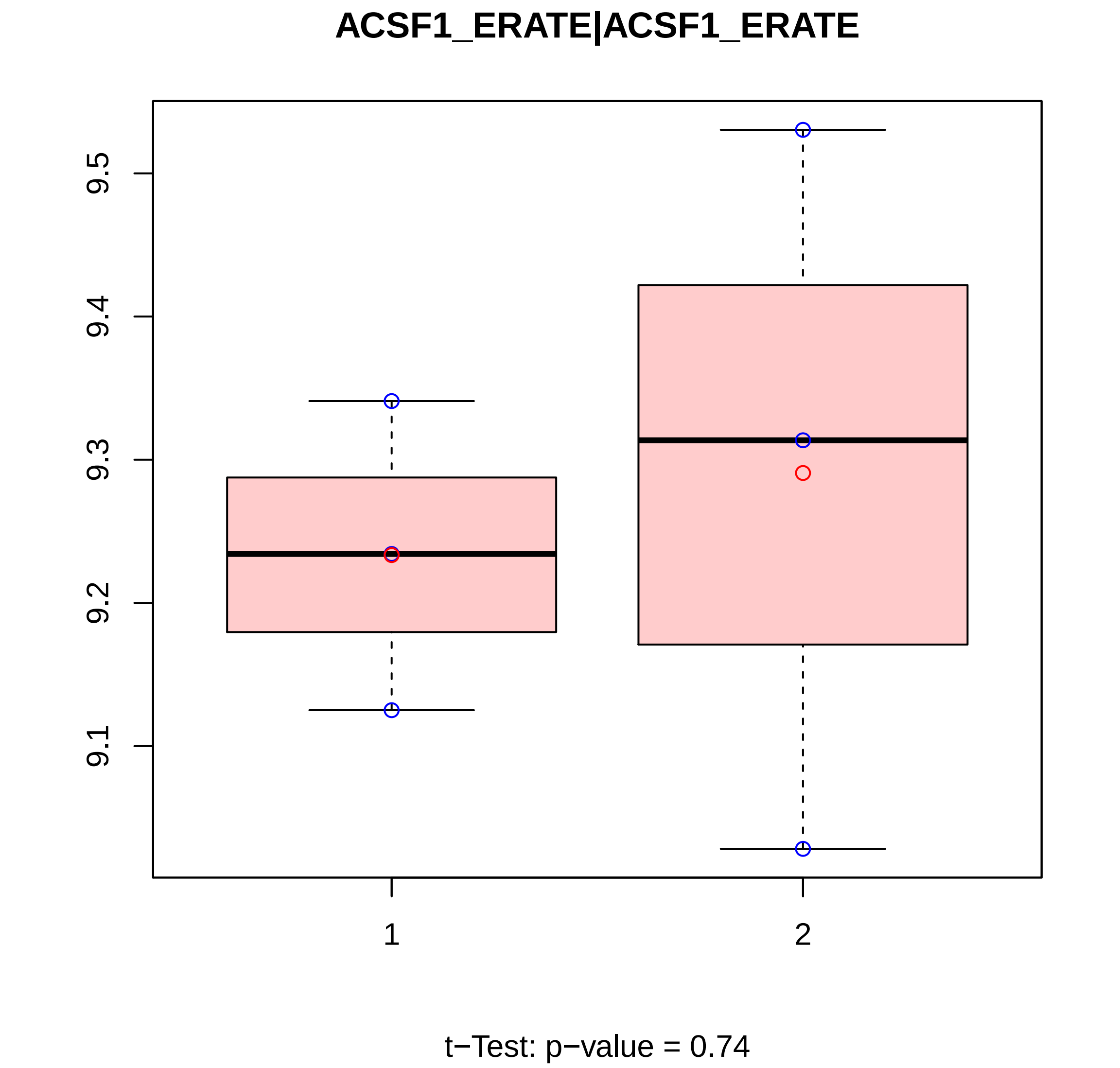
ACSF1_ERATE|ACSF1_ERATE
9.1
9.2
9.3
9.4
9.5
1
2
t−Test: p−value = 0.74

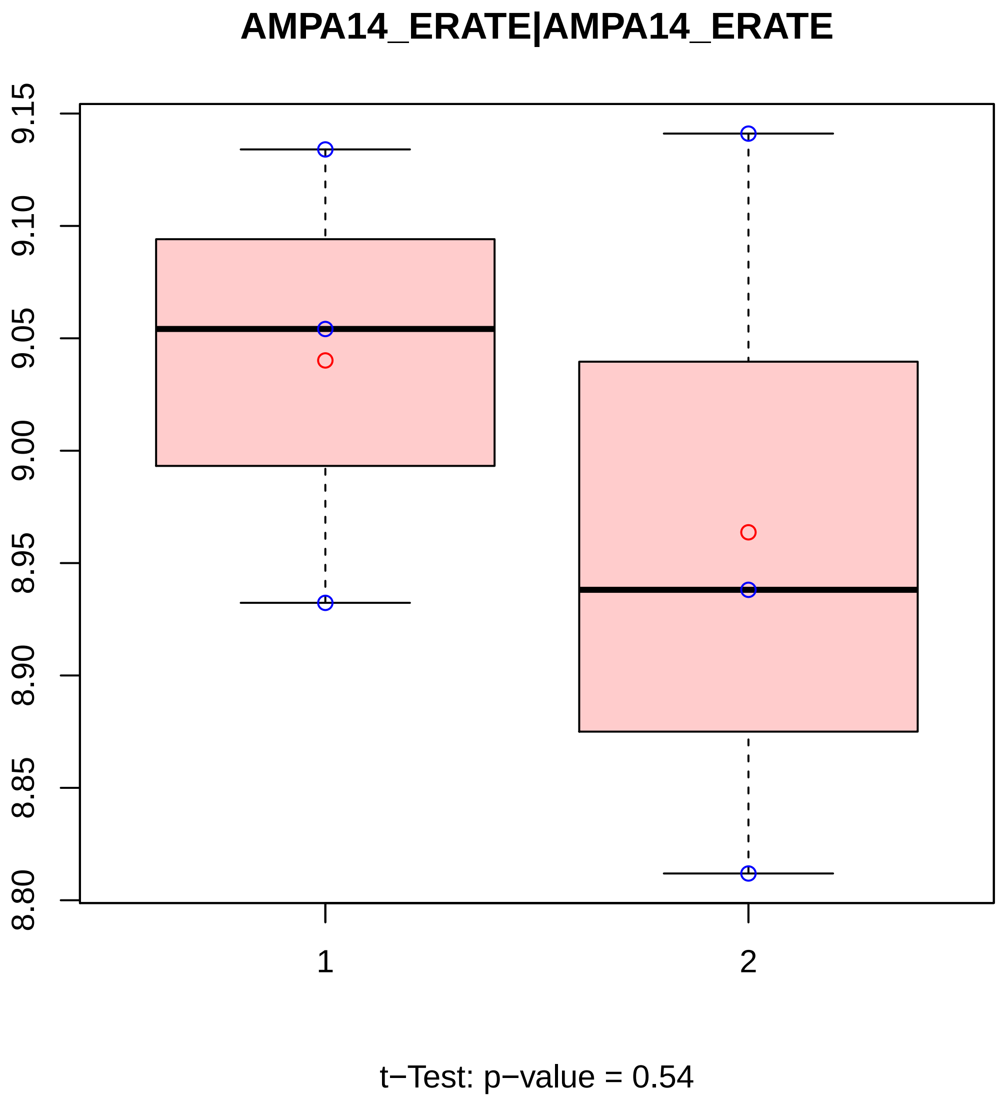
AMPA14_ERATE|AMPA14_ERATE
9.15
9.10
9.05
9.00
8.95
8.90
8.85
8.80
1
2
t−Test: p−value = 0.54

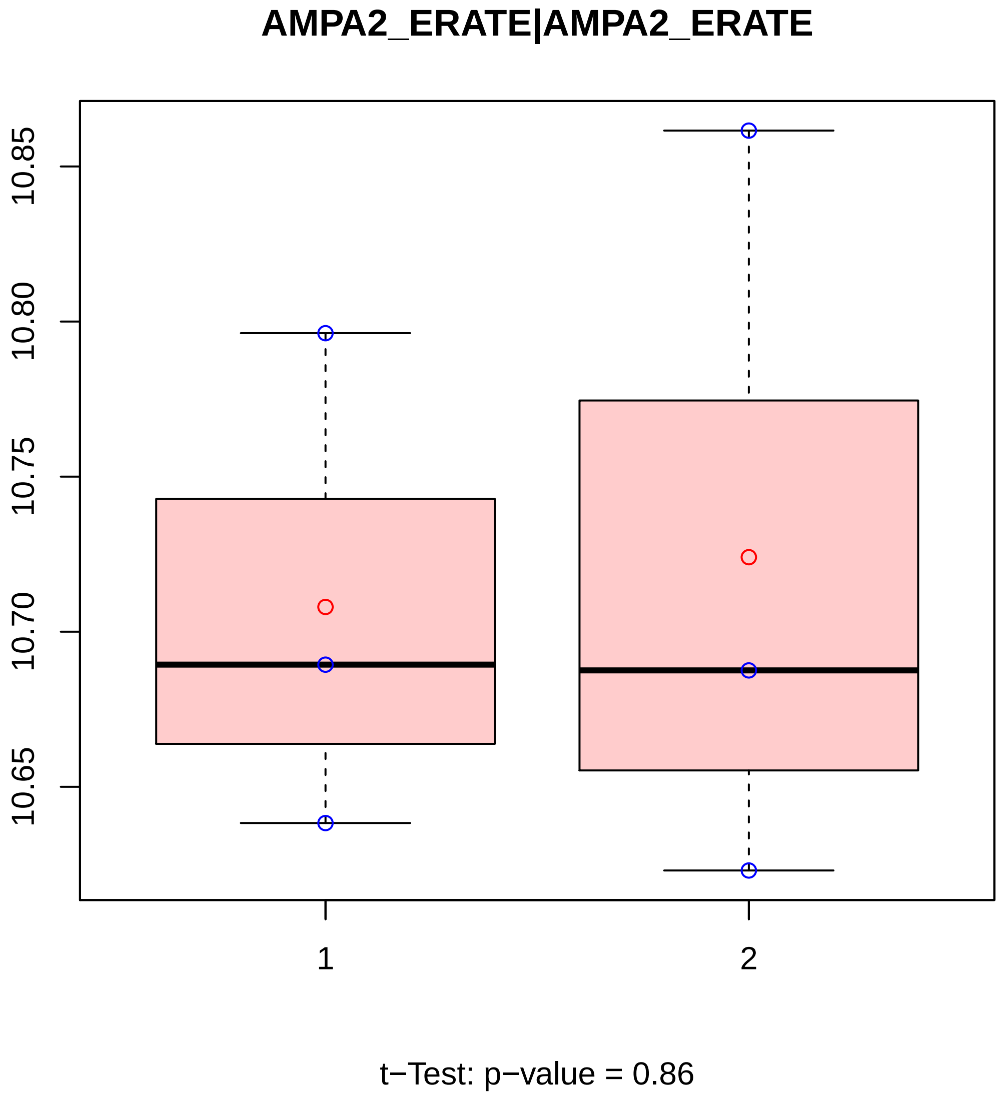

AMPA2_ERATE|AMPA2_ERATE
10.65
10.70
10.75
10.80
10.85
1
2
t−Test: p−value = 0.86

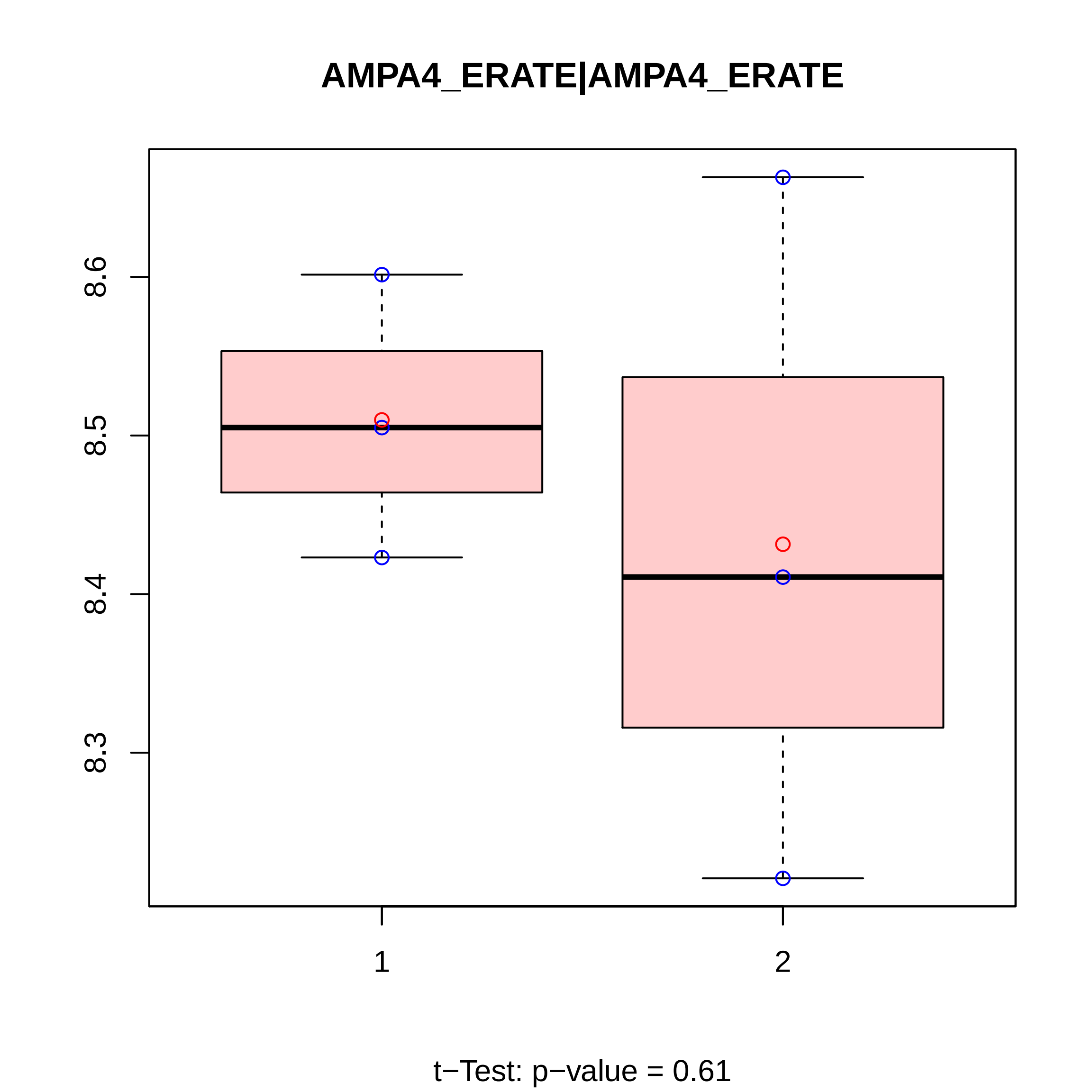
AMPA4_ERATE|AMPA4_ERATE
8.3
8.4
8.5
8.6
1
2
t−Test: p−value = 0.61

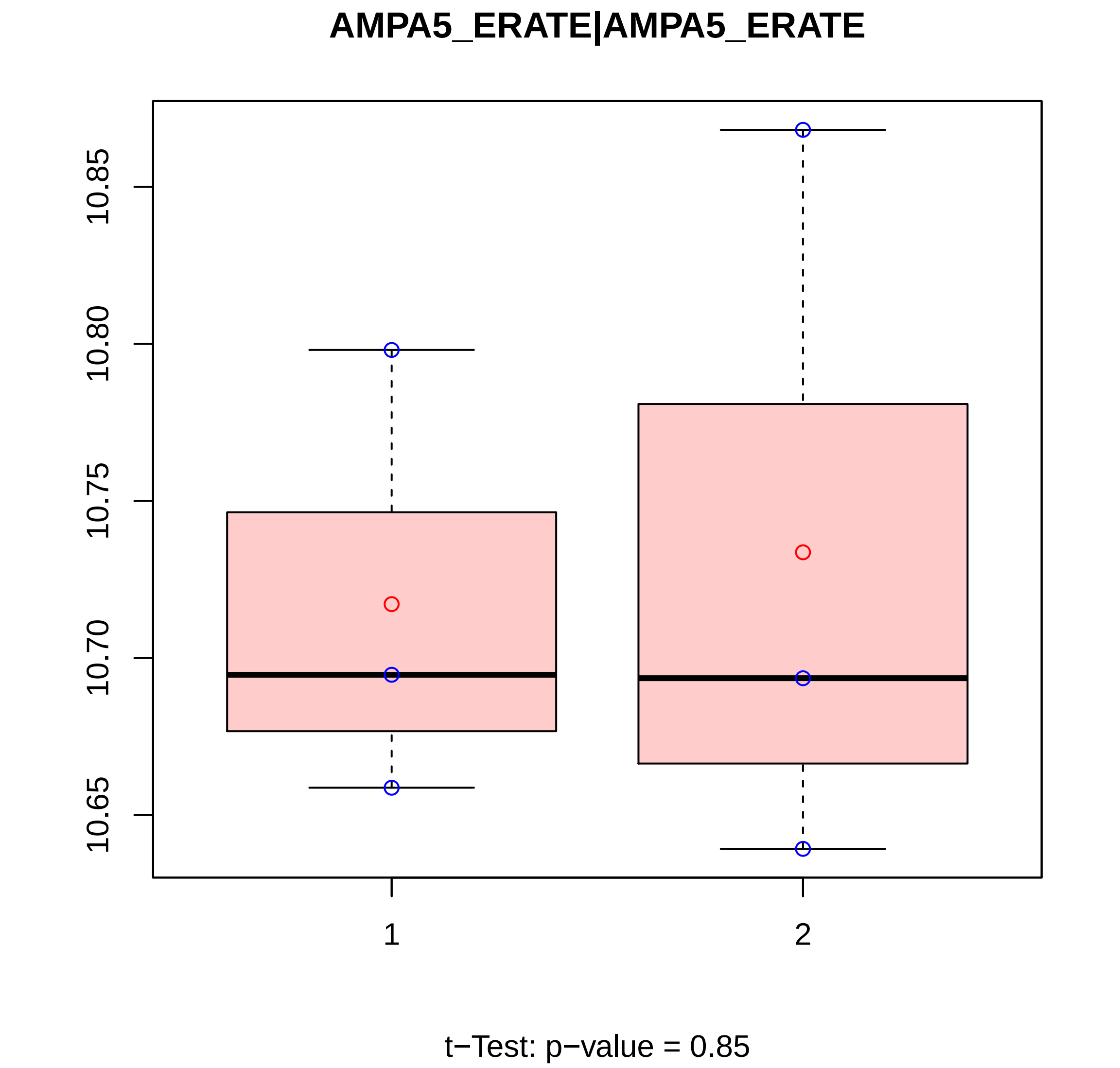
AMPA5_ERATE|AMPA5_ERATE
10.65
10.70
10.75
10.80
10.85
1
2
t−Test: p−value = 0.85

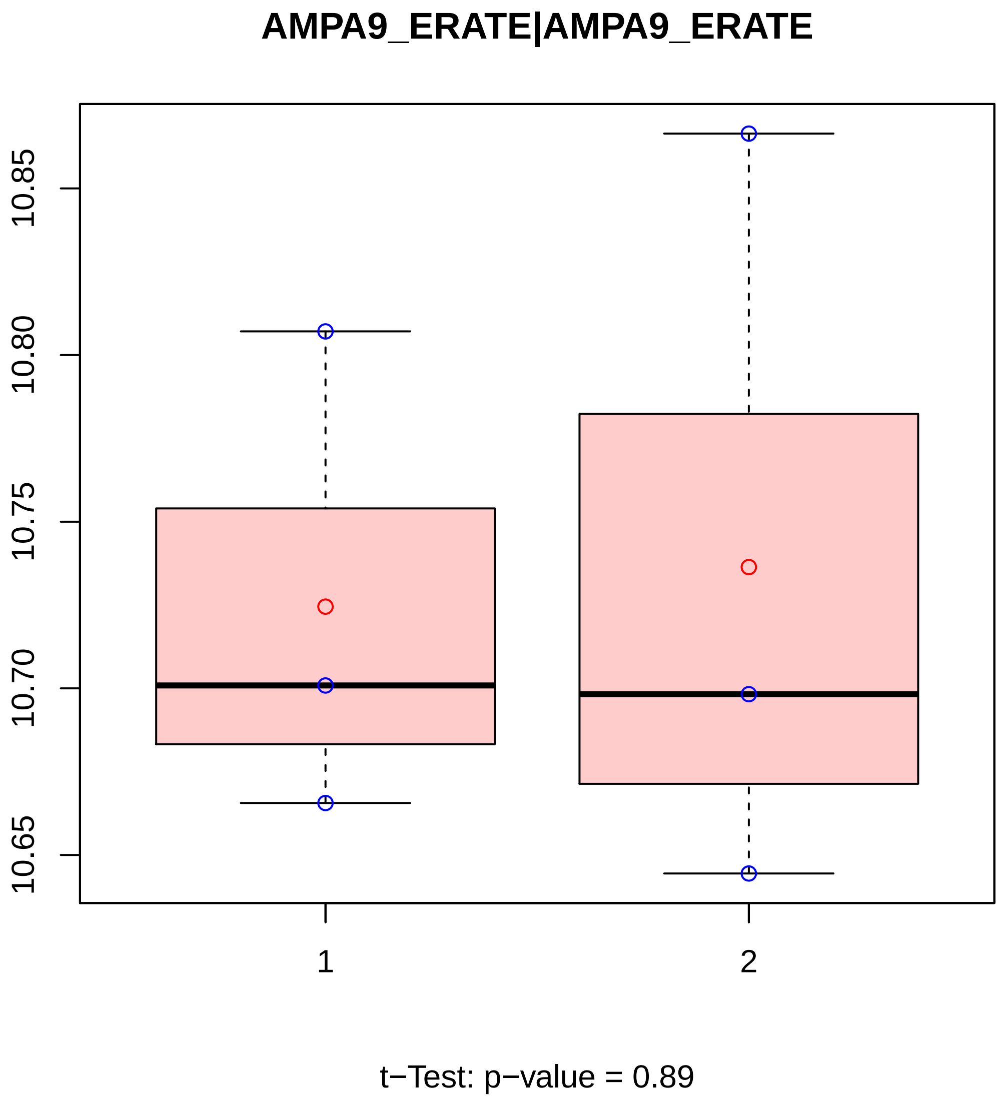
AMPA9_ERATE|AMPA9_ERATE
10.65
10.70
10.75
10.80
10.85
1
2
t−Test: p−value = 0.89

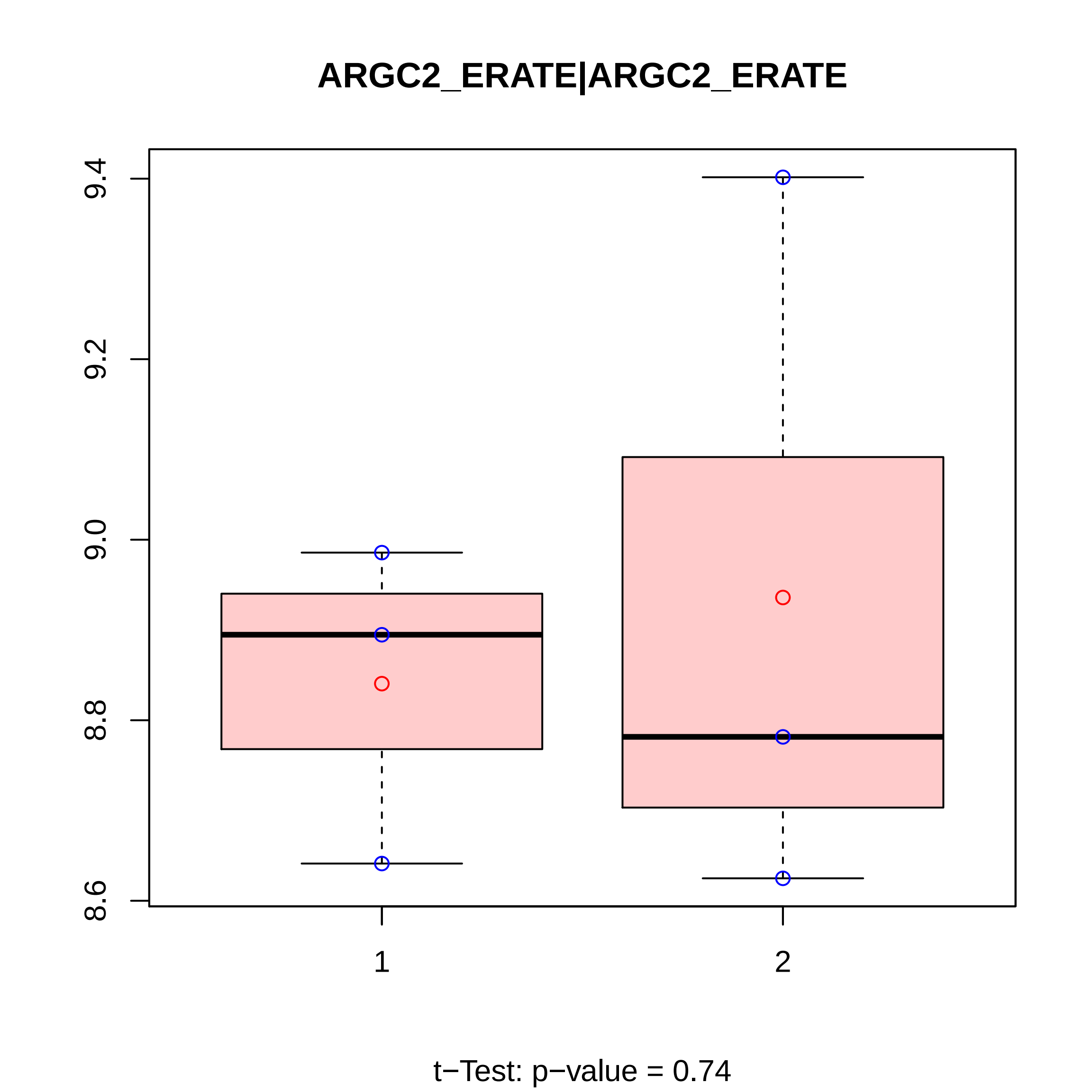

ARGC2_ERATE|ARGC2_ERATE
9.4
9.2
9.0
8.8
8.6
1
2
t−Test: p−value = 0.74

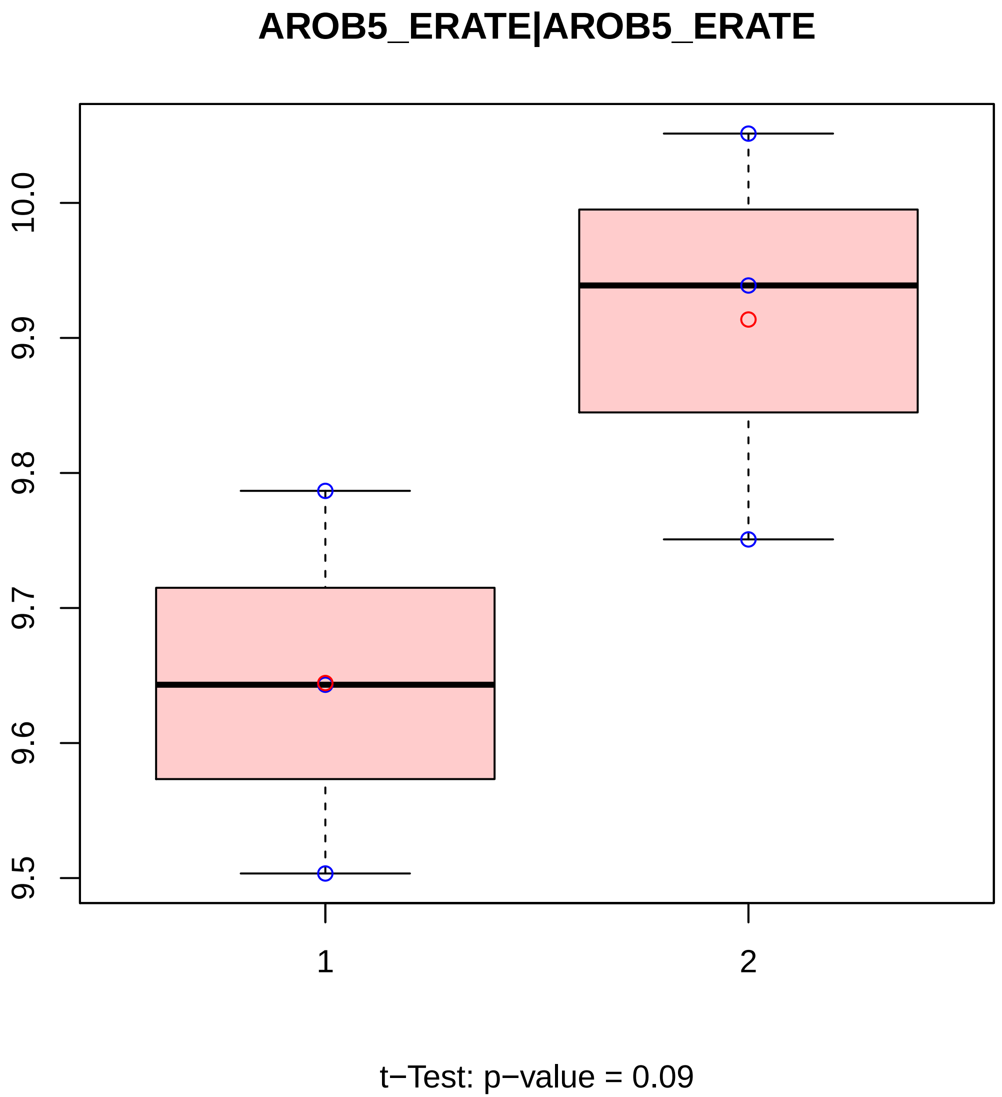
AROB5_ERATE|AROB5_ERATE
10.0
9.9
9.8
9.7
9.6
9.5
1
2
t−Test: p−value = 0.09

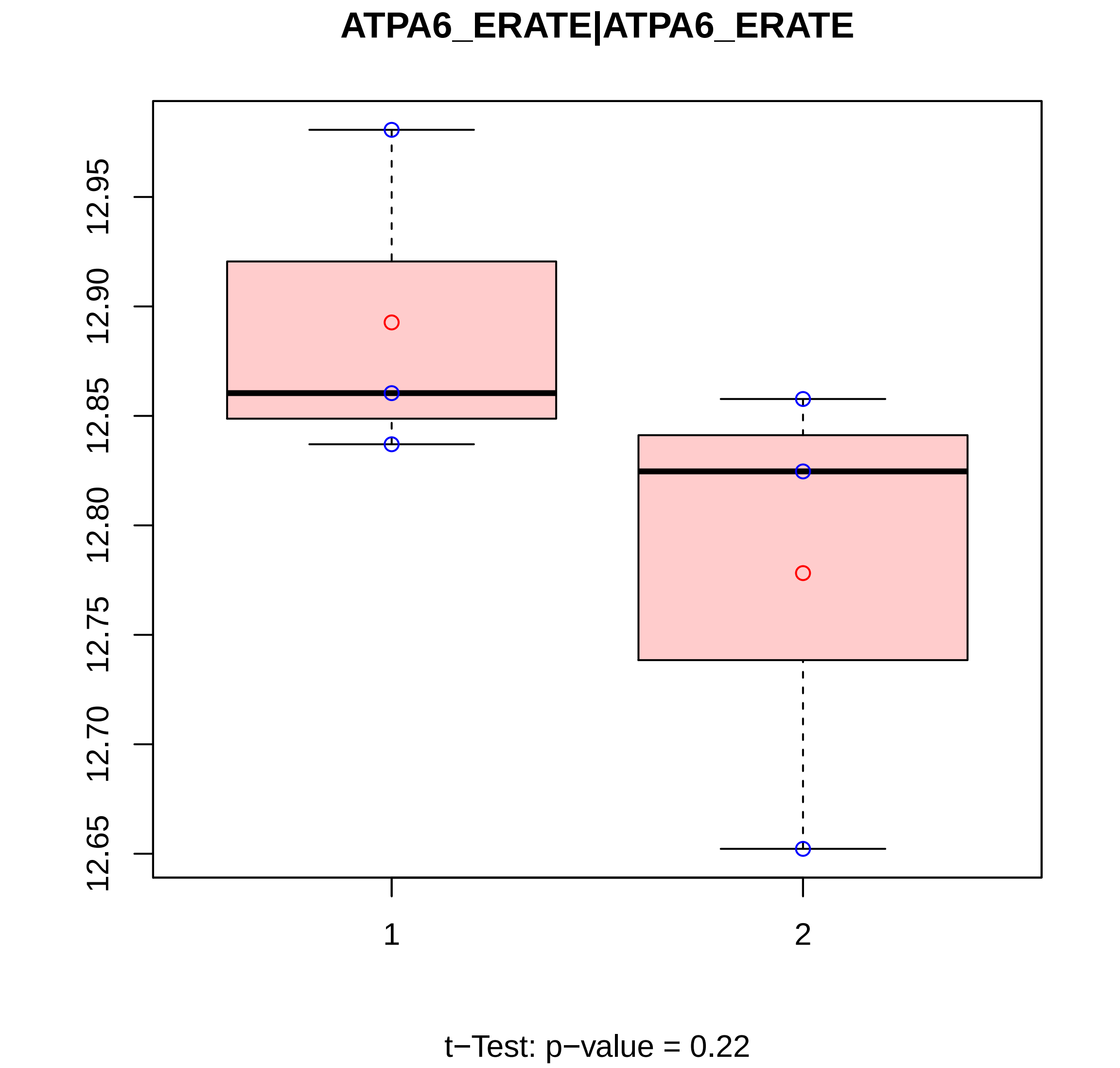
ATPA6_ERATE|ATPA6_ERATE
12.65
12.70
12.75
12.80
12.85
12.90
12.95
1
2
t−Test: p−value = 0.22

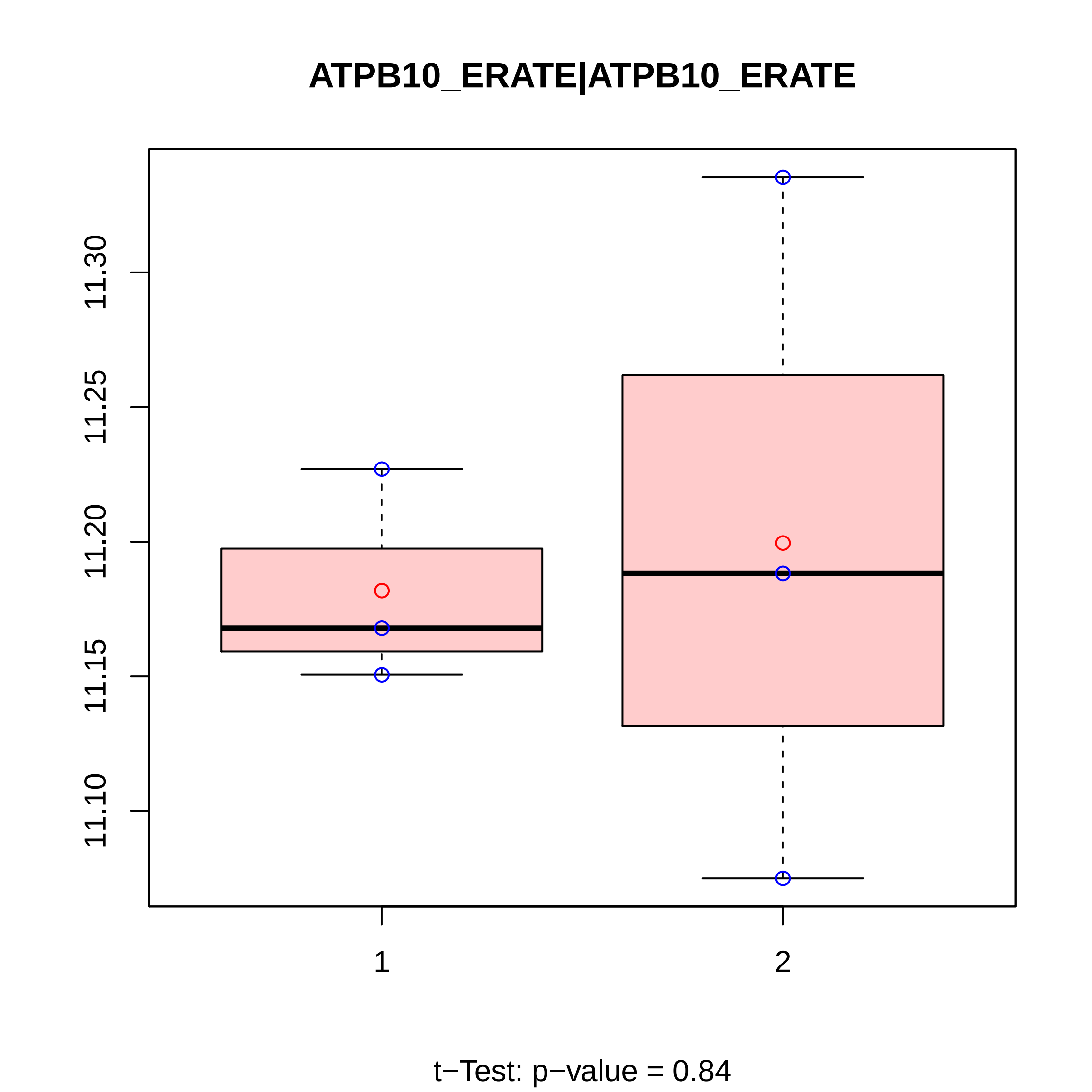
ATPB10_ERATE|ATPB10_ERATE
11.10
11.15
11.20
11.25
11.30
1
2
t−Test: p−value = 0.84

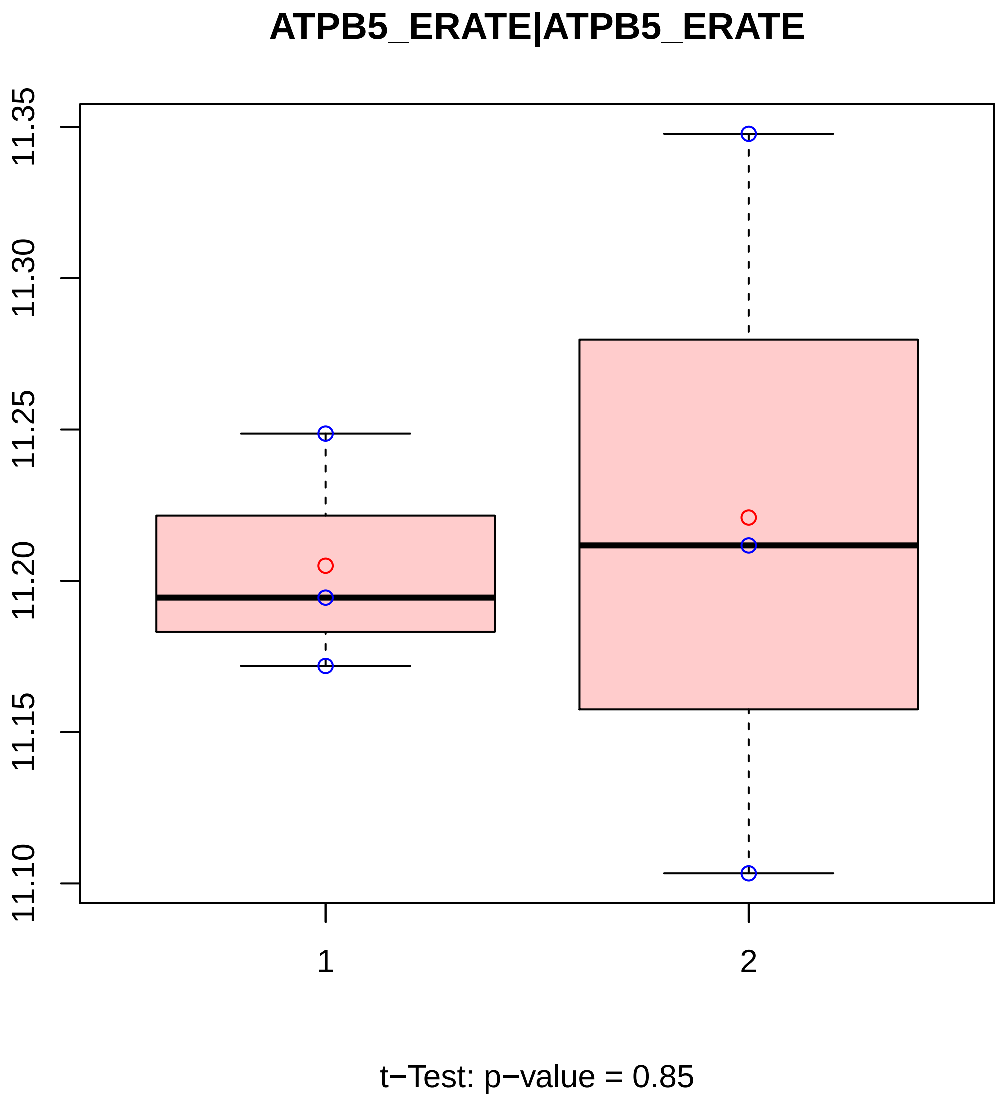
ATPB5_ERATE|ATPB5_ERATE
11.35
11.30
11.25
11.20
11.15
11.10
1
2
t−Test: p−value = 0.85

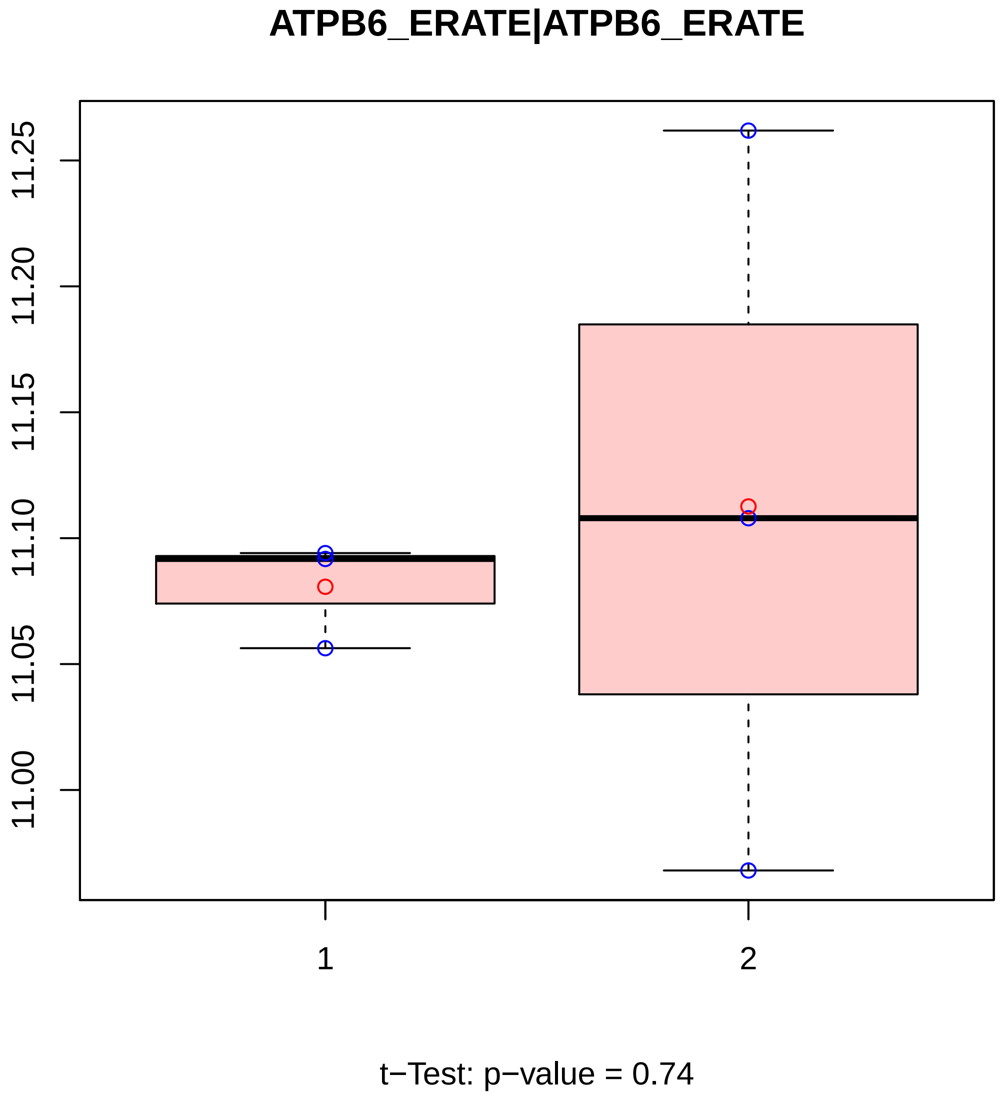
ATPB6_ERATE|ATPB6_ERATE
11.25
11.20
11.15
11.10
11.05
11.00
1
2
t−Test: p−value = 0.74

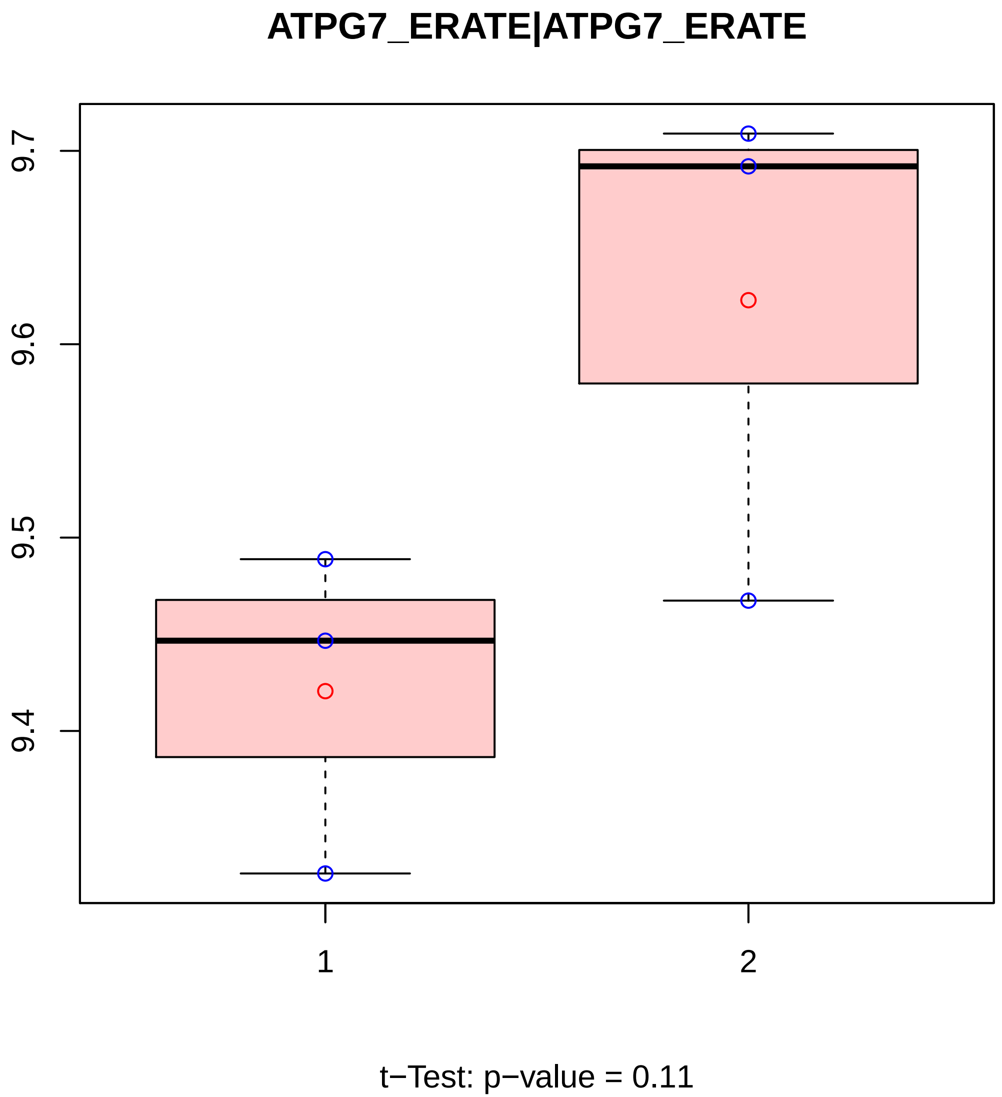
ATPG7_ERATE|ATPG7_ERATE
9.7
9.6
9.5
9.4
1
2
t−Test: p−value = 0.11

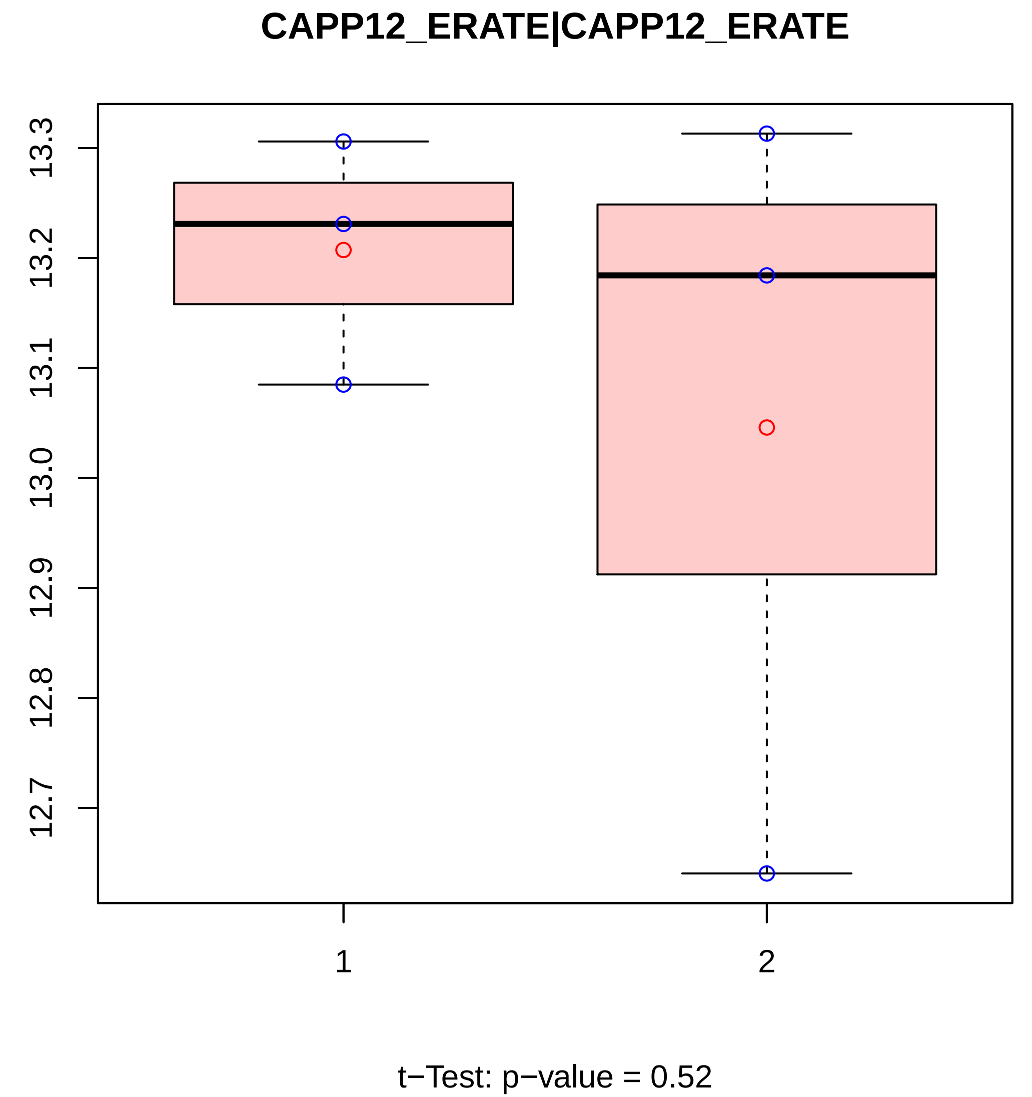
CAPP12_ERATE|CAPP12_ERATE
13.3
13.2
13.1
13.0
12.9
12.8
12.7
1
2
t−Test: p−value = 0.52

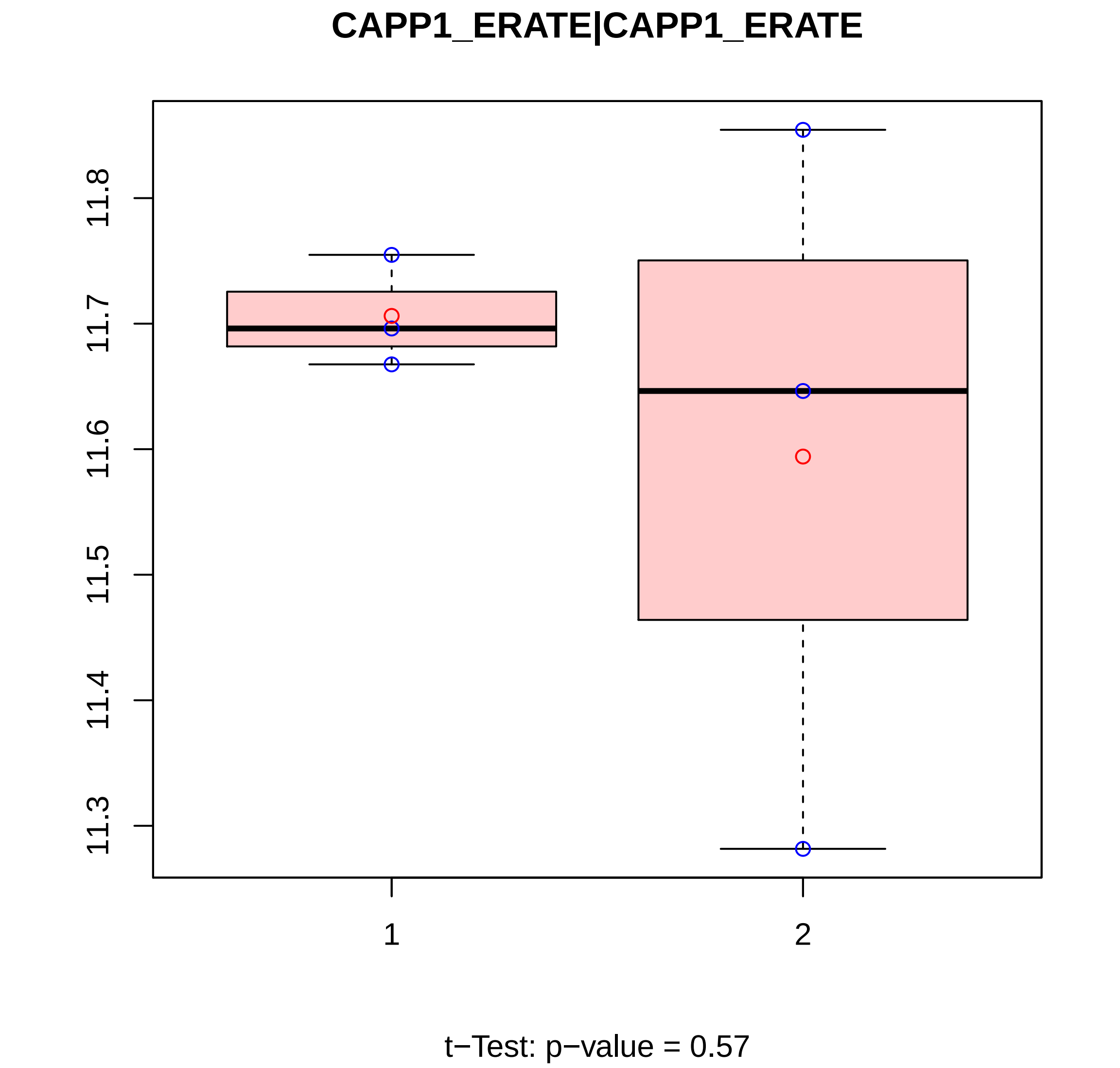
CAPP1_ERATE|CAPP1_ERATE
11.3
11.4
11.5
11.6
11.7
11.8
1
2
t−Test: p−value = 0.57

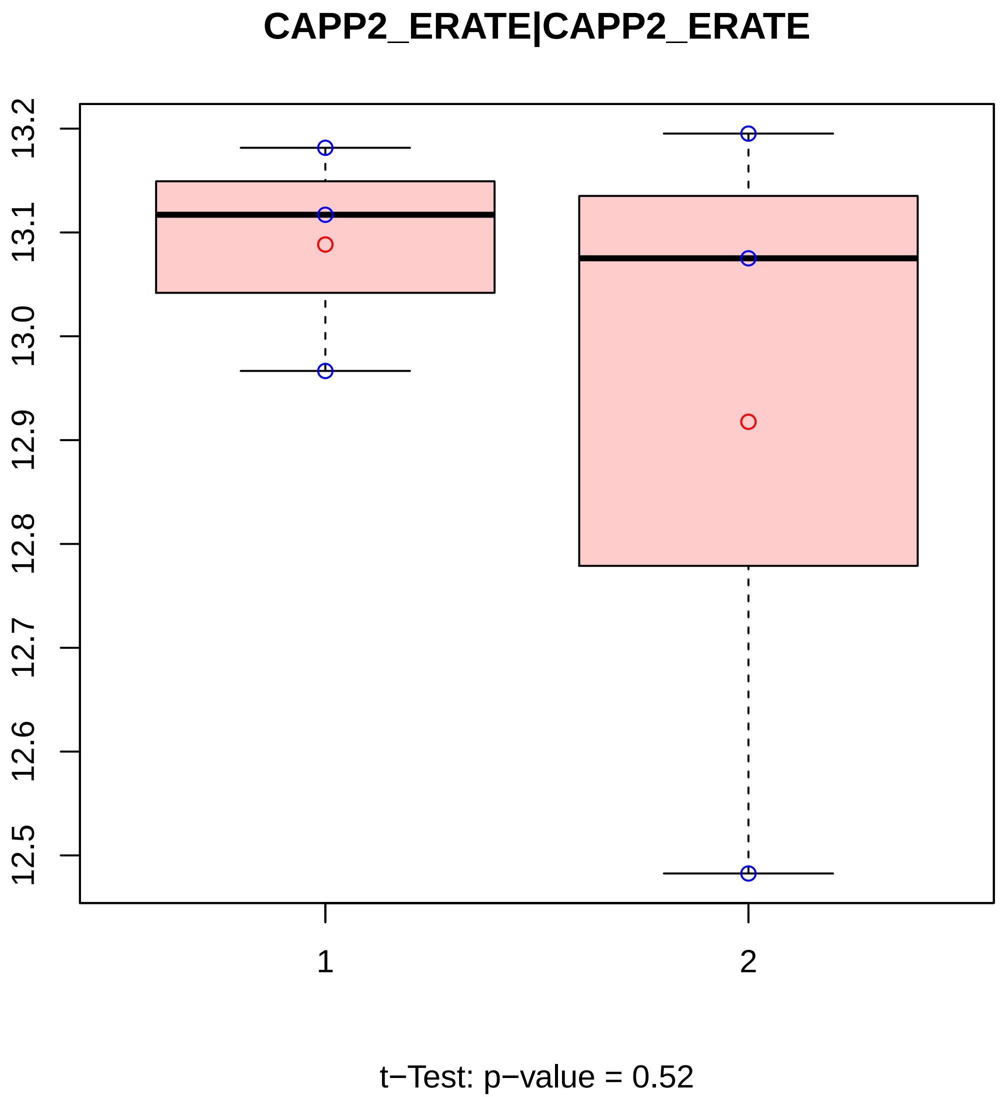
CAPP2_ERATE|CAPP2_ERATE
13.2
13.1
13.0
12.9
12.8
12.7
12.6
12.5
1
2
t−Test: p−value = 0.52

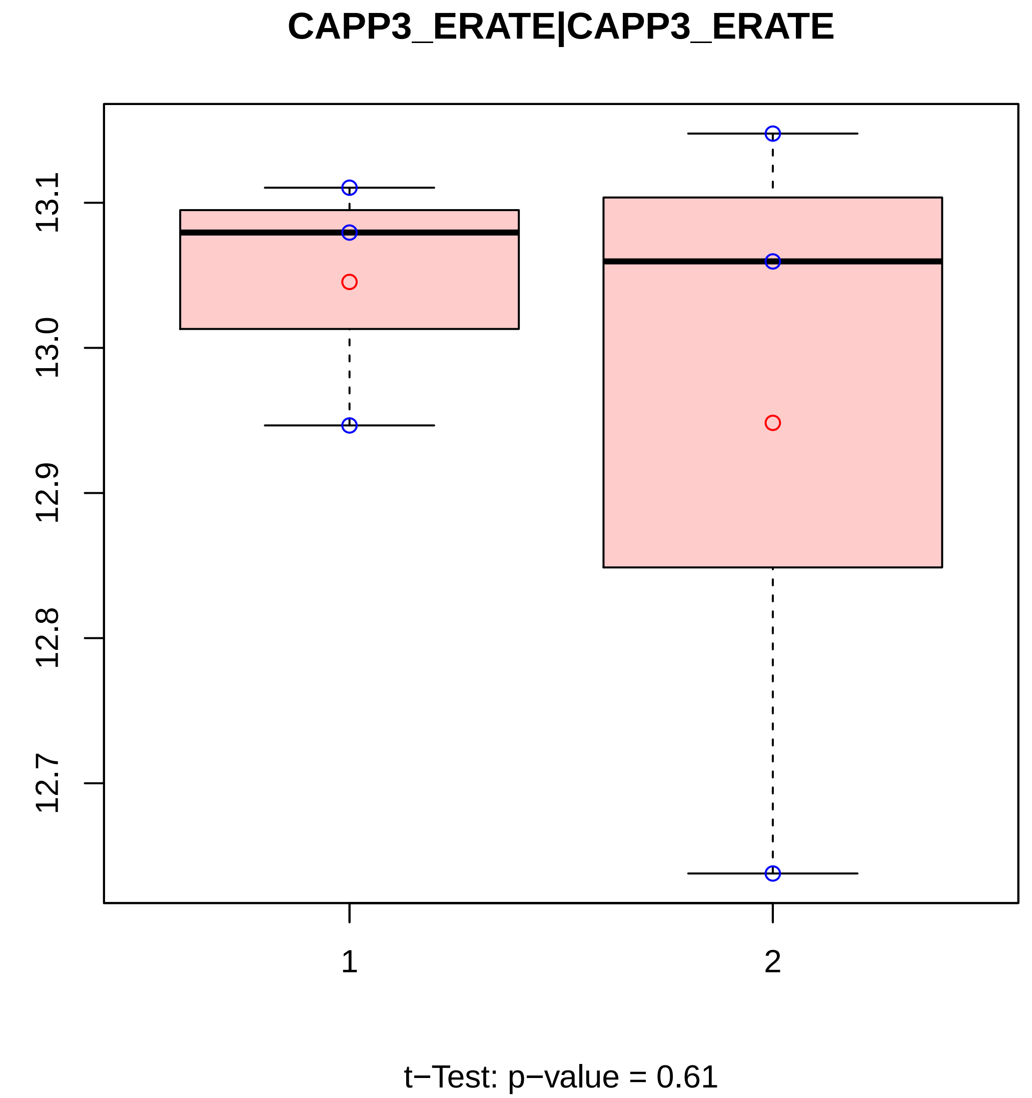
CAPP3_ERATE|CAPP3_ERATE
13.1
13.0
12.9
12.8
12.7
1
2
t−Test: p−value = 0.61

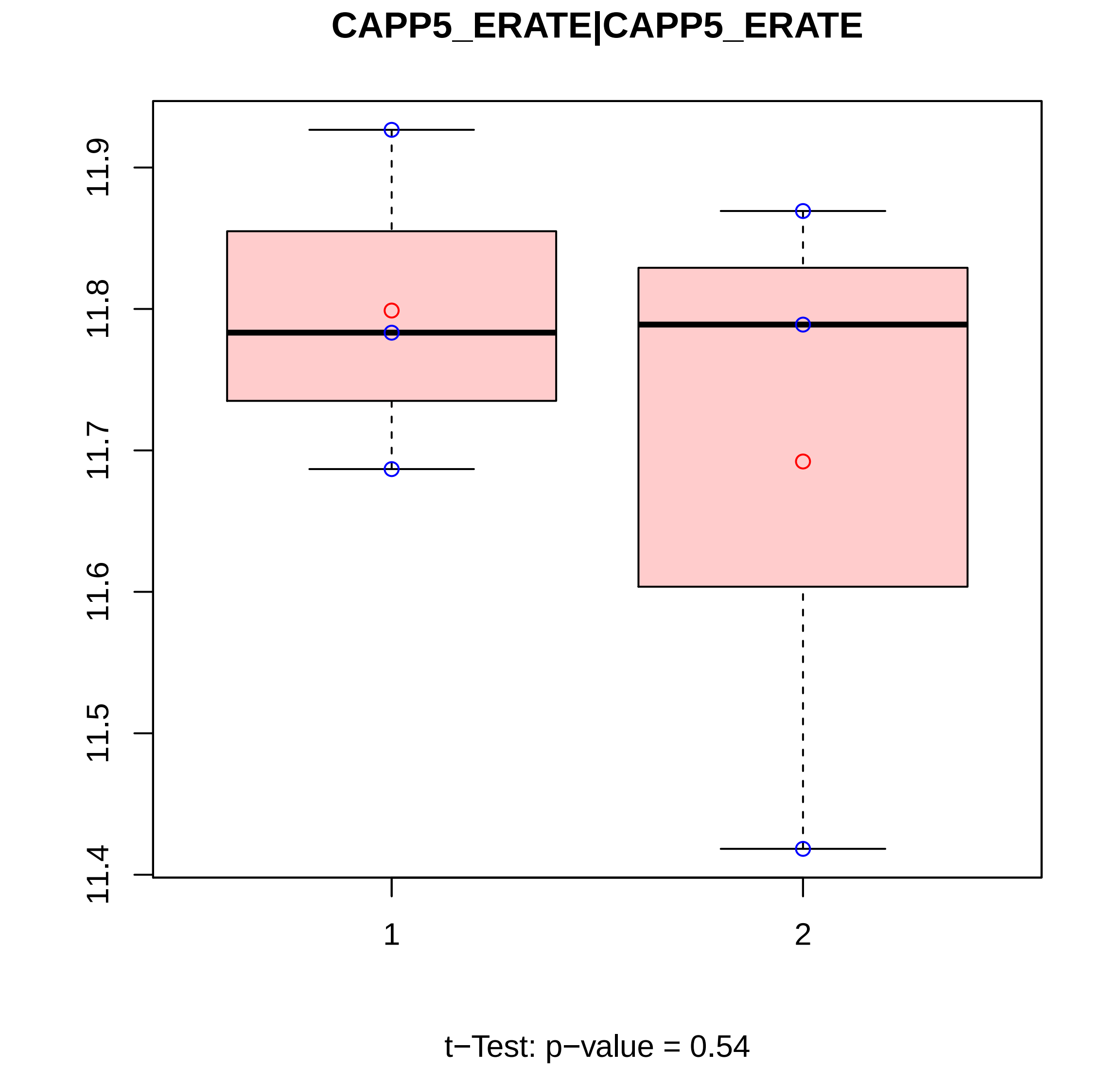

CAPP5_ERATE|CAPP5_ERATE
11.4
11.5
11.6
11.7
11.8
11.9
1
2
t−Test: p−value = 0.54

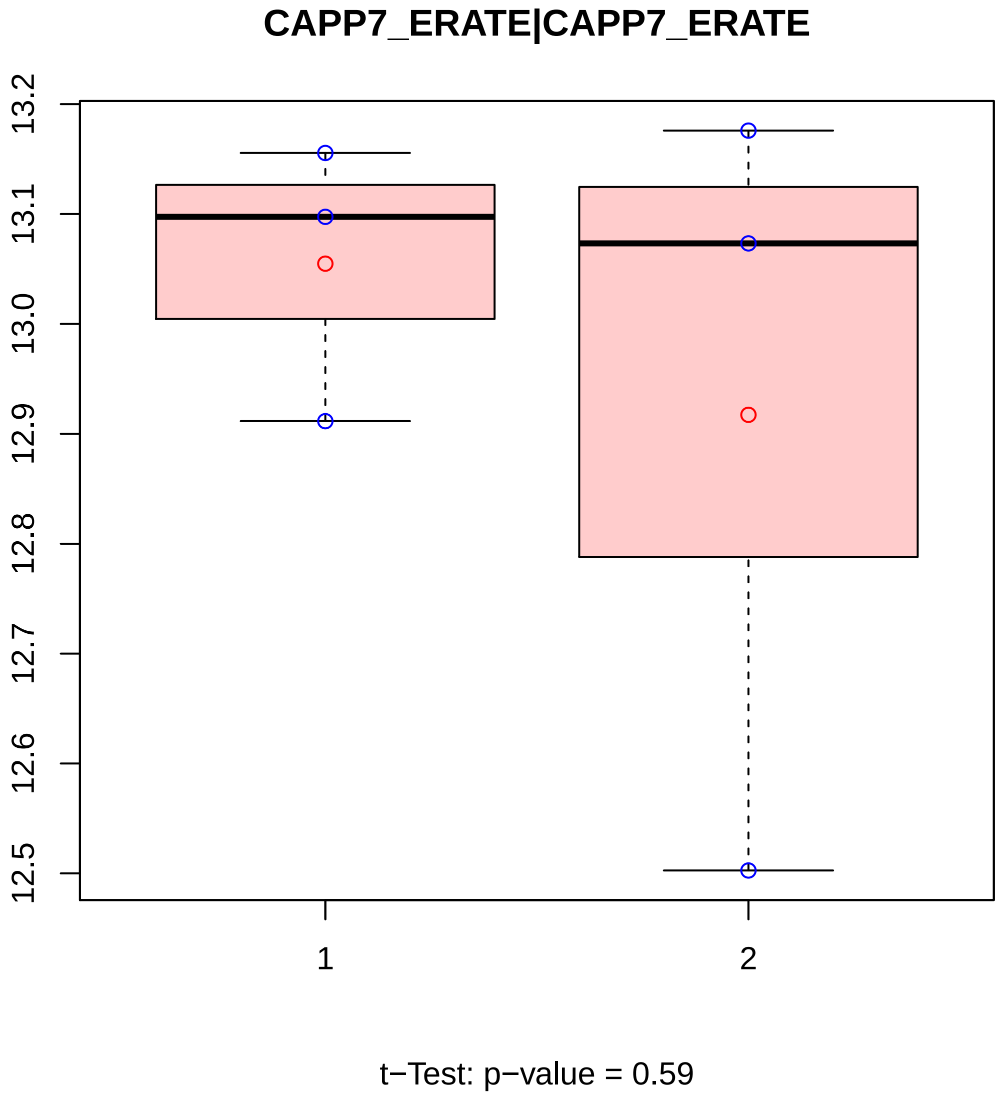
CAPP7_ERATE|CAPP7_ERATE
13.2
13.1
13.0
12.9
12.8
12.7
12.6
12.5
1
2
t−Test: p−value = 0.59

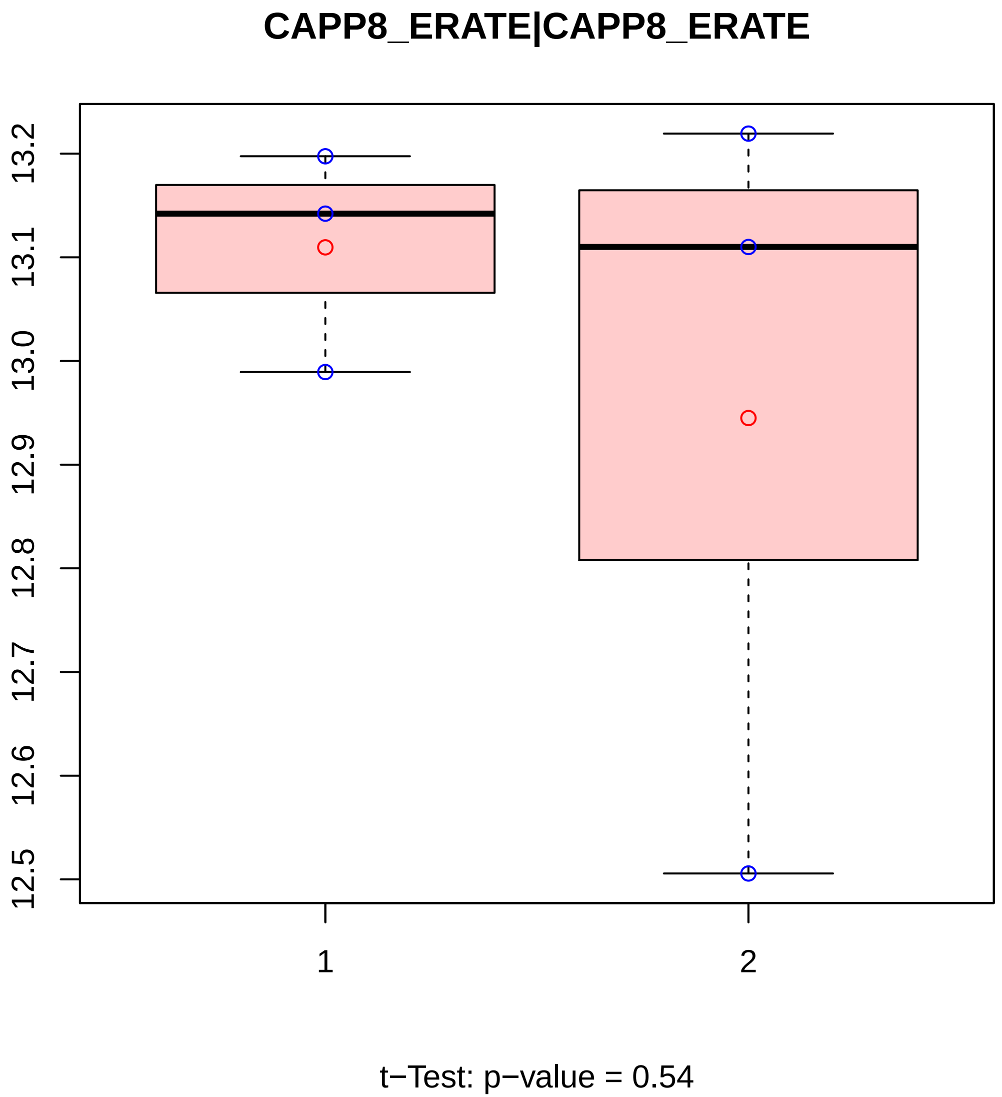

CAPP8_ERATE|CAPP8_ERATE
13.2
13.1
13.0
12.9
12.8
12.7
12.6
12.5
1
2
t−Test: p−value = 0.54

# CH601_ERATE|CH601_ERATE

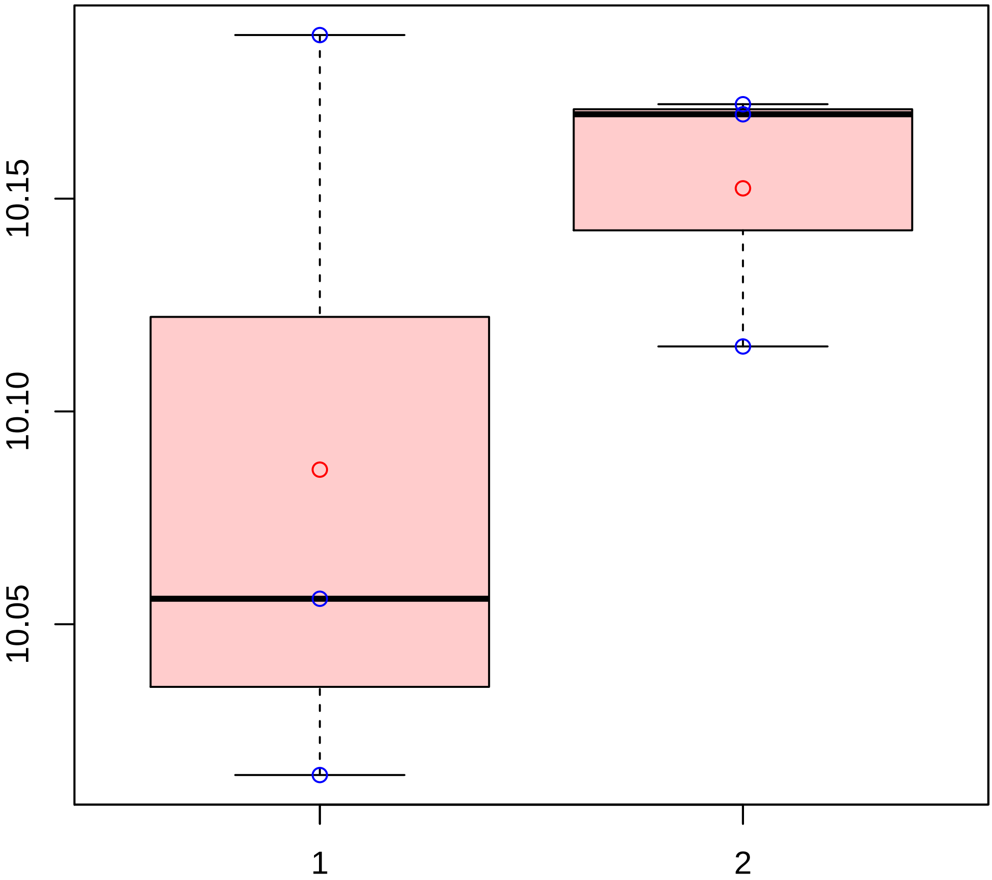


t−Test: p−value = 0.34

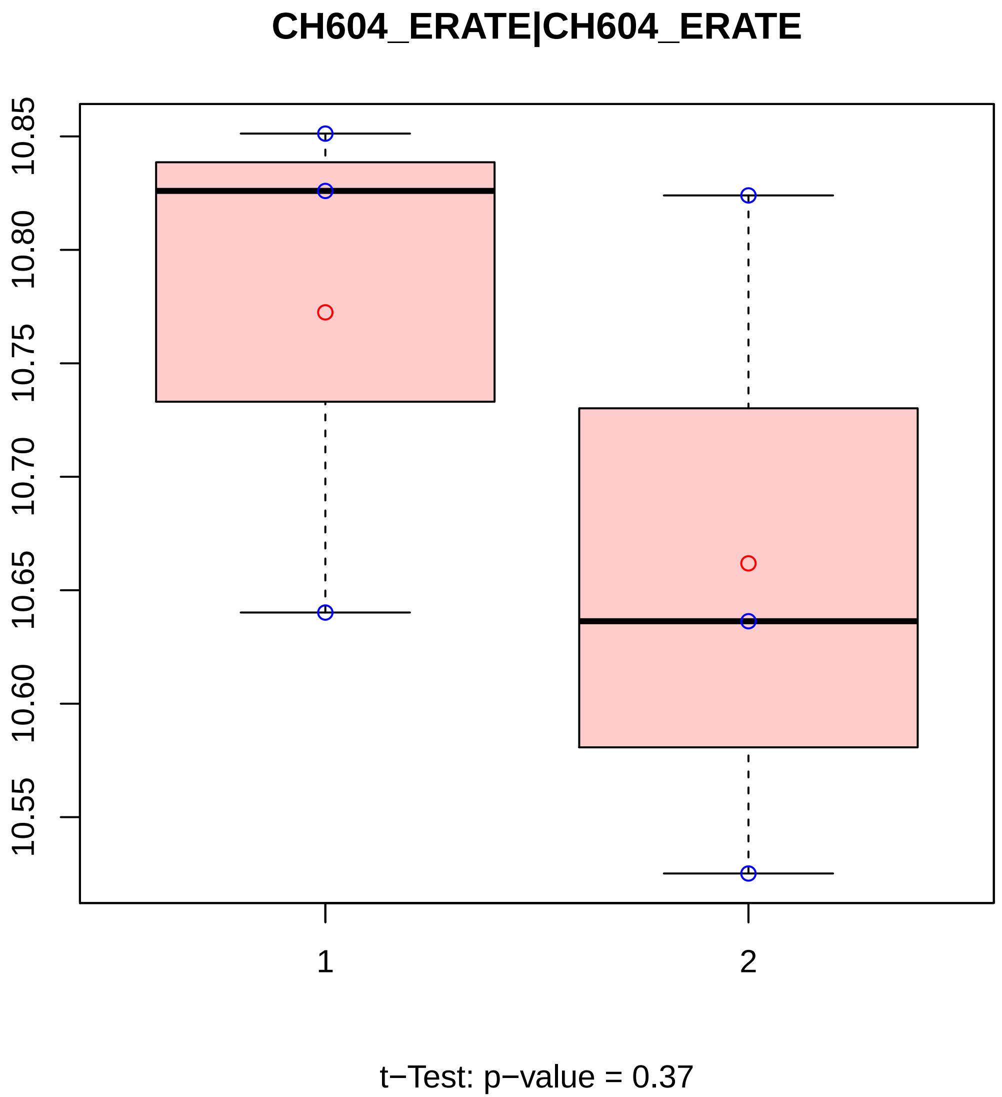

CH604_ERATE|CH604_ERATE
10.55
10.60
10.65
10.70
10.75
10.80
10.85
1
2
t−Test: p−value = 0.37

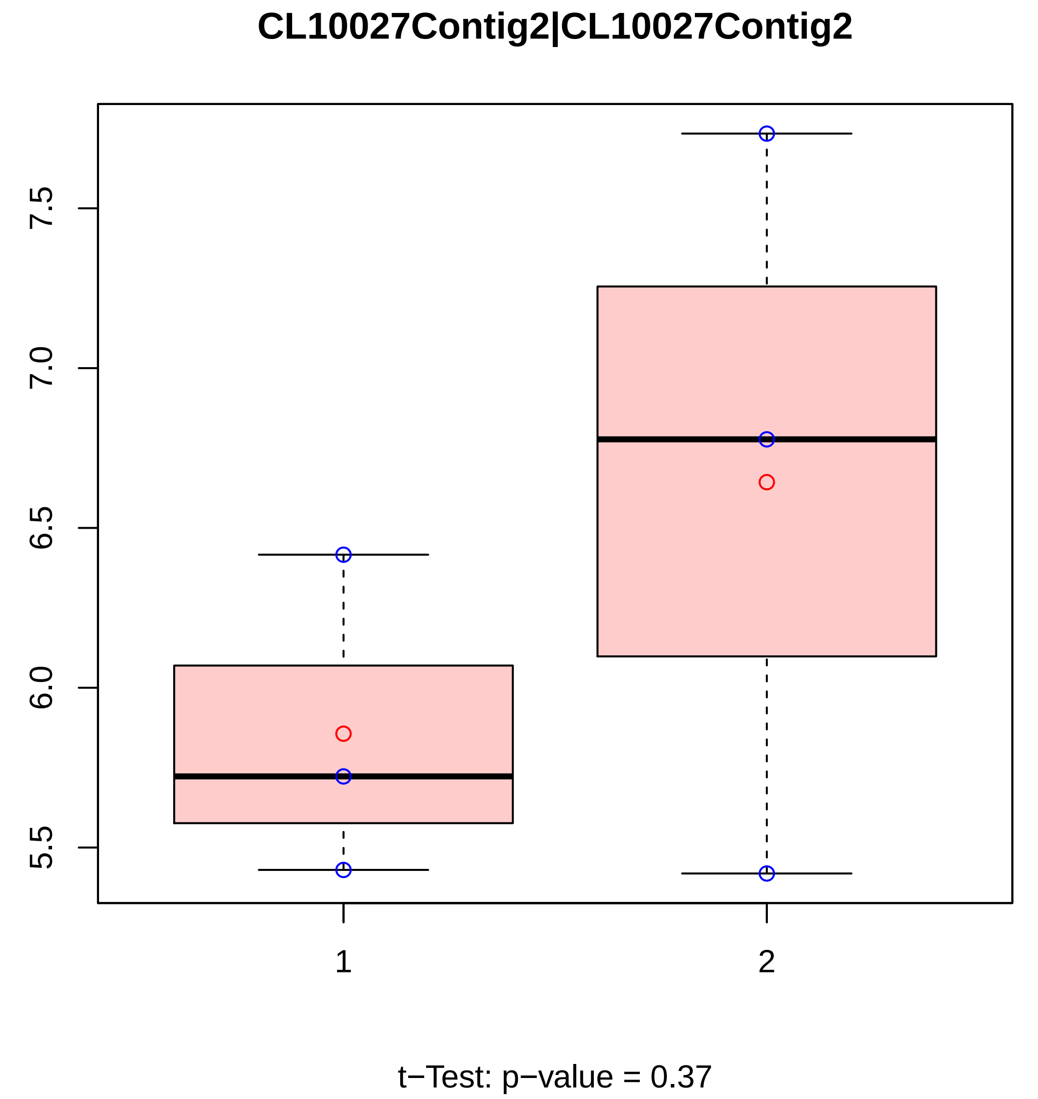

CL10027Contig2|CL10027Contig2
7.5
7.0
6.5
6.0
5.5
1
2
t−Test: p−value = 0.37

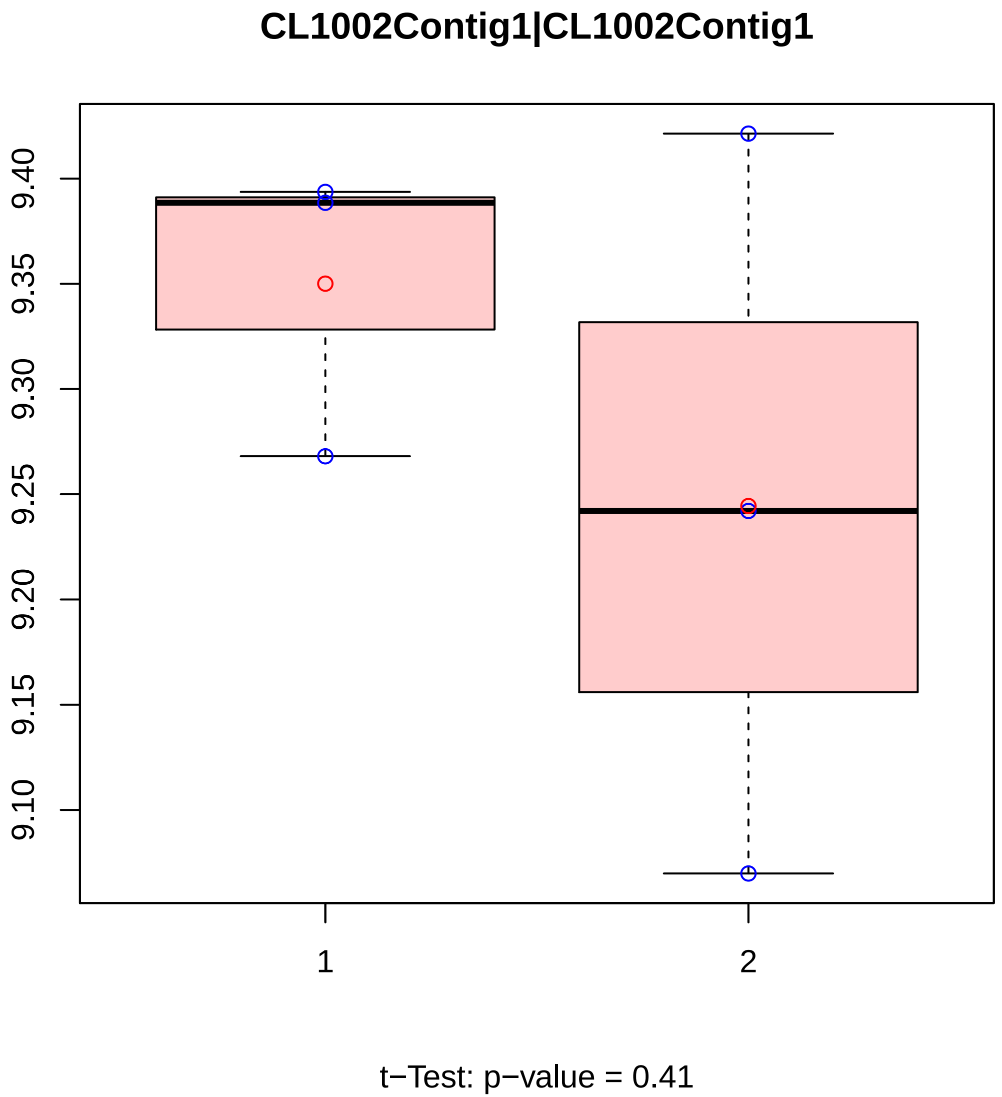
CL1002Contig1|CL1002Contig1
9.10
9.15
9.20
9.25
9.30
9.35
9.40
1
2
t−Test: p−value = 0.41

# CL1002Contig4|CL1002Contig4

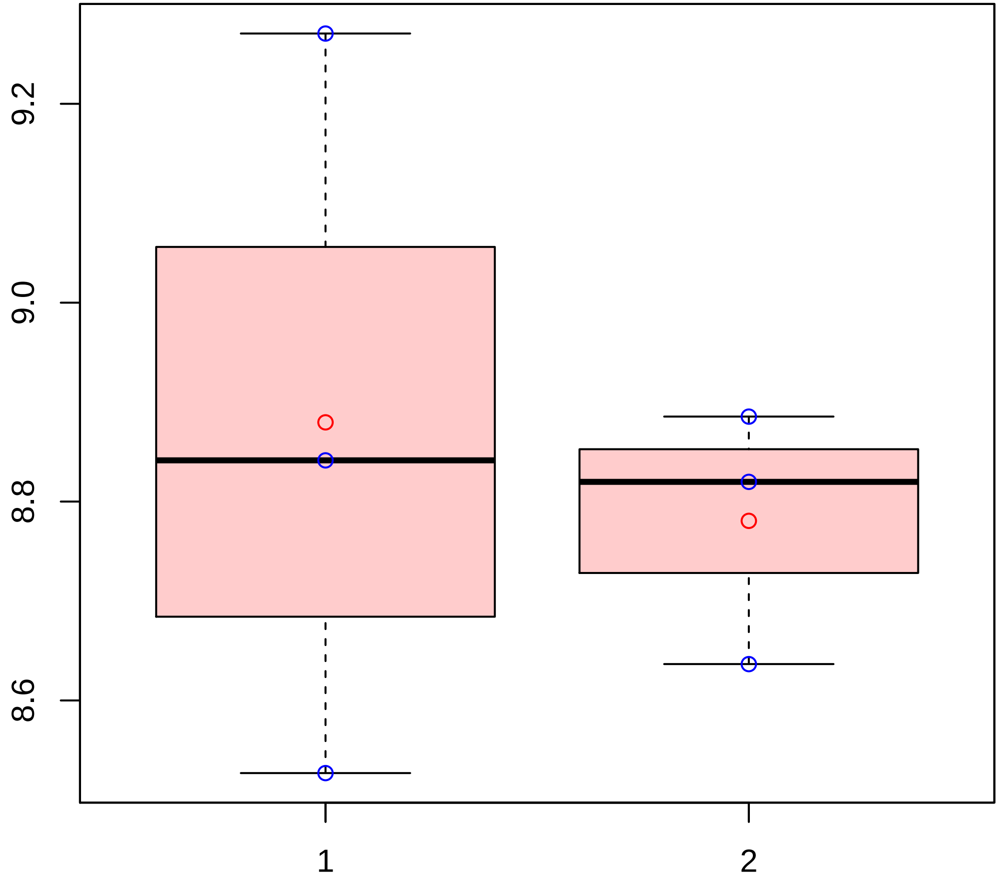


t−Test: p−value = 0.7

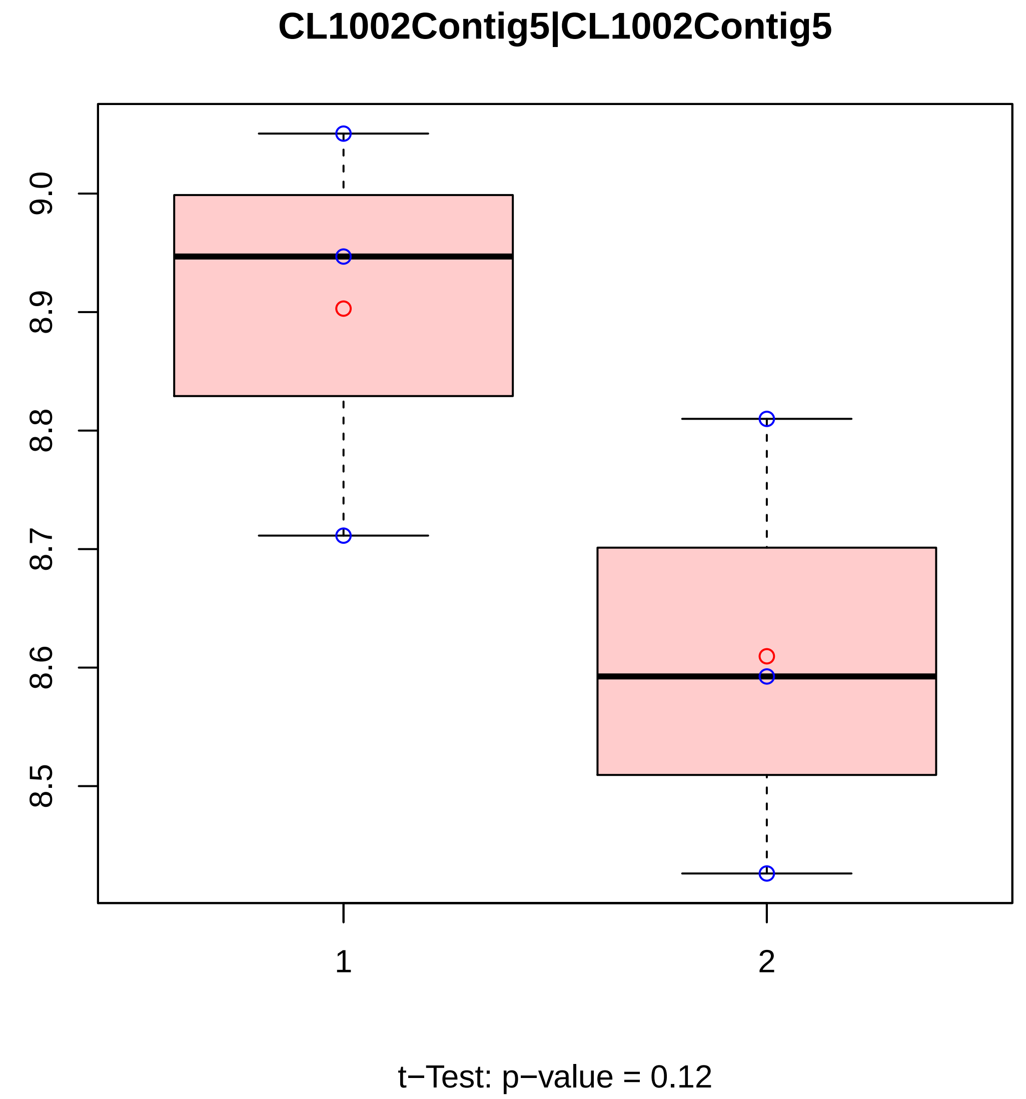
CL1002Contig5|CL1002Contig5
9.0
8.9
8.8
8.7
8.6
8.5
1
2
t−Test: p−value = 0.12

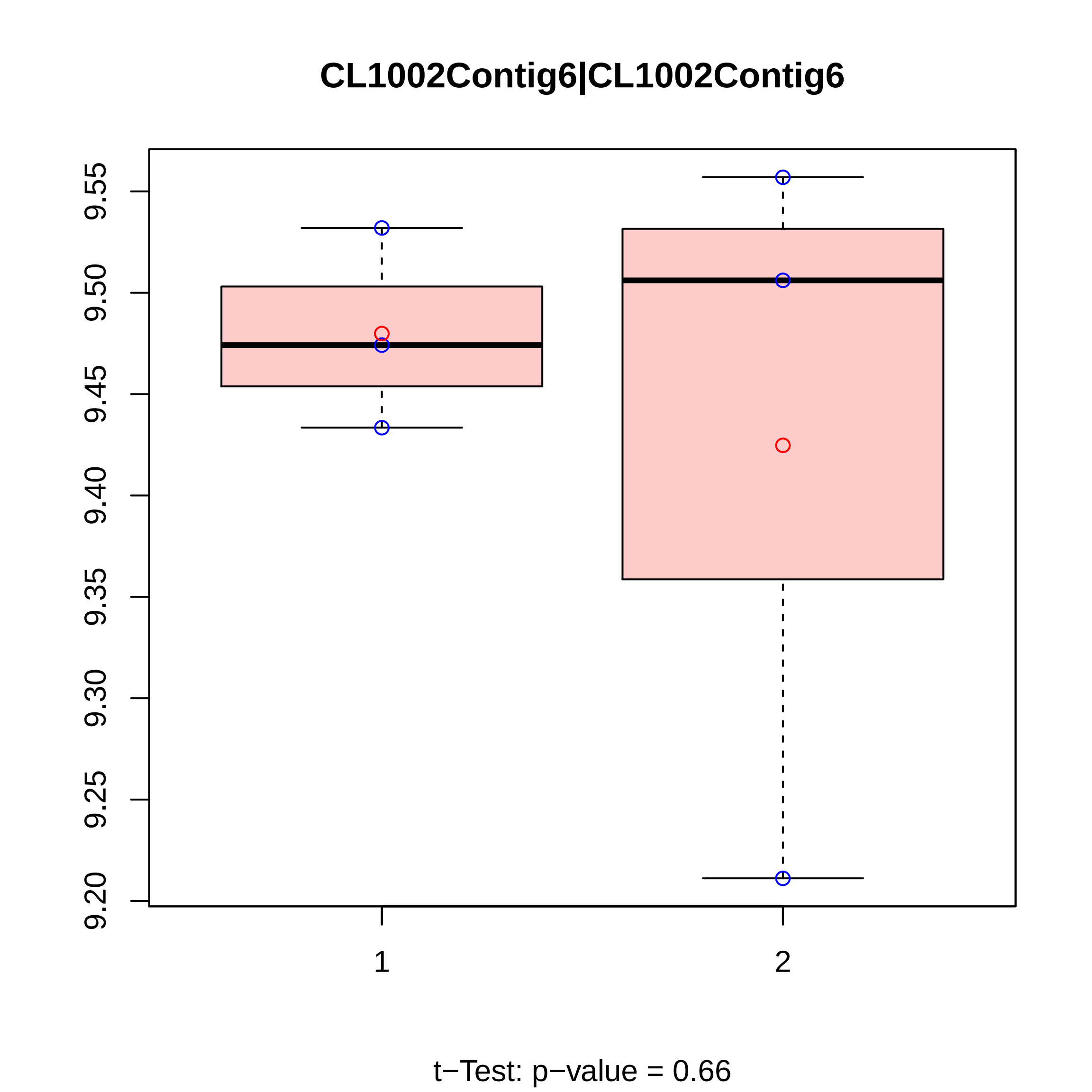
CL1002Contig6|CL1002Contig6
9.20
9.25
9.30
9.35
9.40
9.45
9.50
9.55
1
2
t−Test: p−value = 0.66

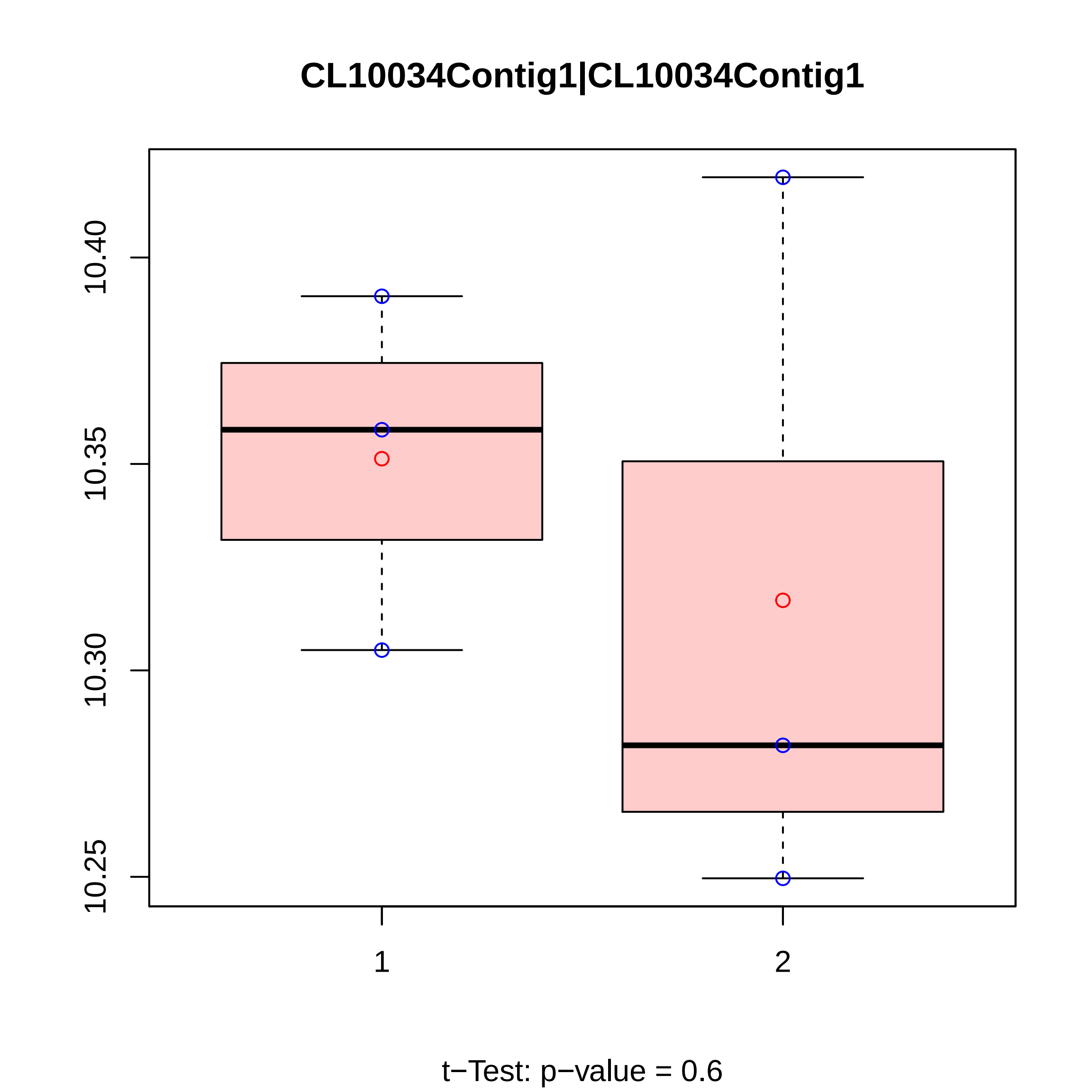
CL10034Contig1|CL10034Contig1
10.40
10.35
10.30
10.25
1
2
t−Test: p−value = 0.6

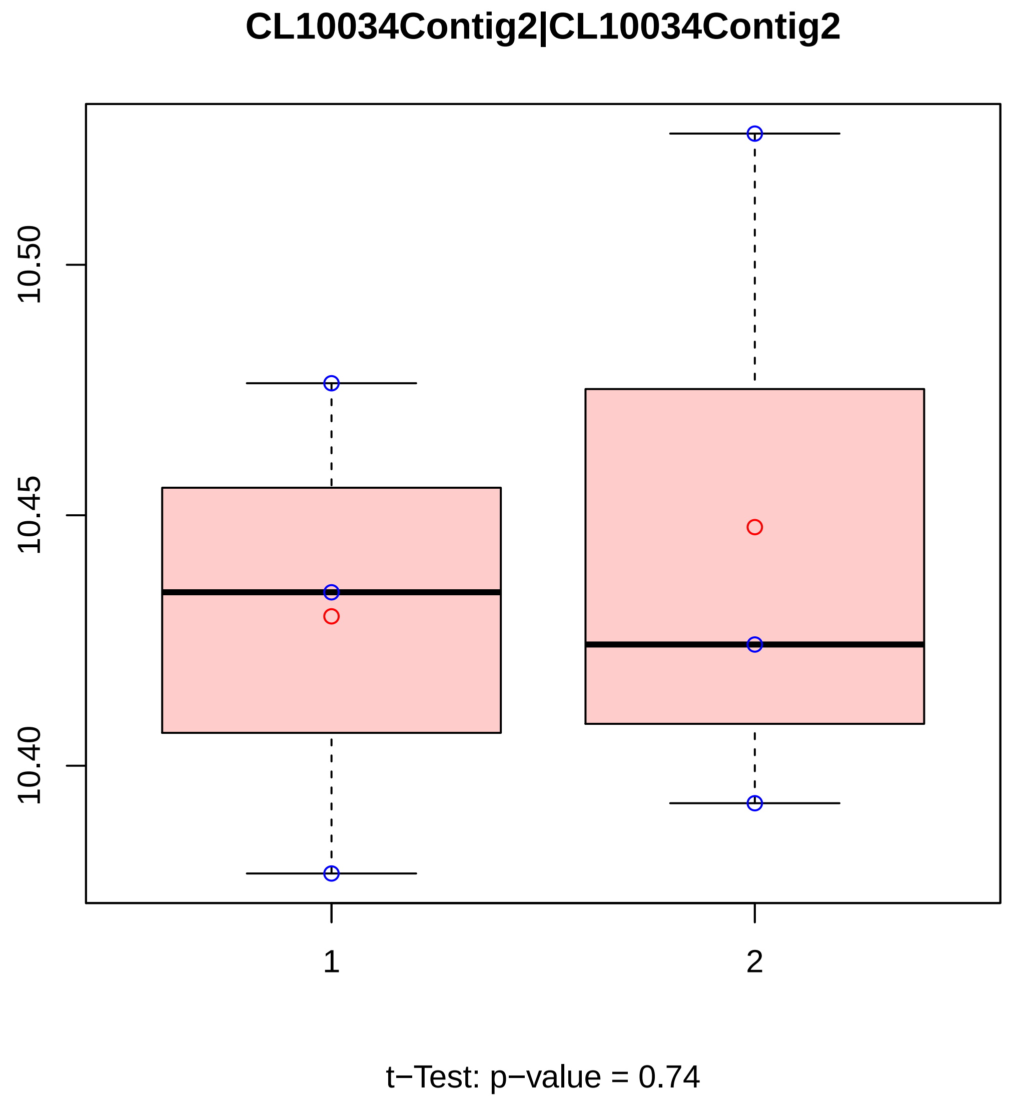
CL10034Contig2|CL10034Contig2
10.40
10.45
10.50
1
2
t−Test: p−value = 0.74

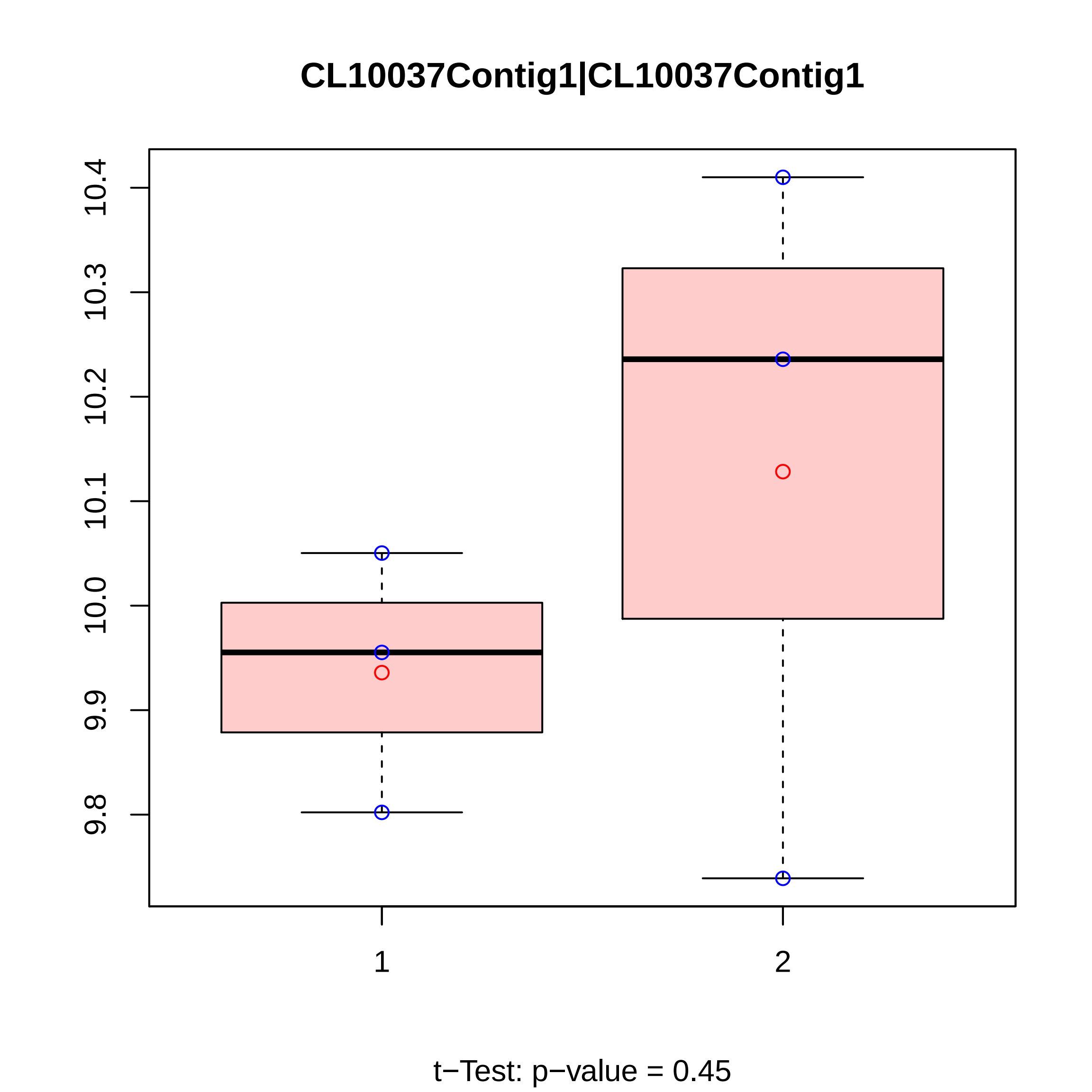

CL10037Contig1|CL10037Contig1
10.4
10.3
10.2
10.1
10.0
9.9
9.8
1
2
t−Test: p−value = 0.45

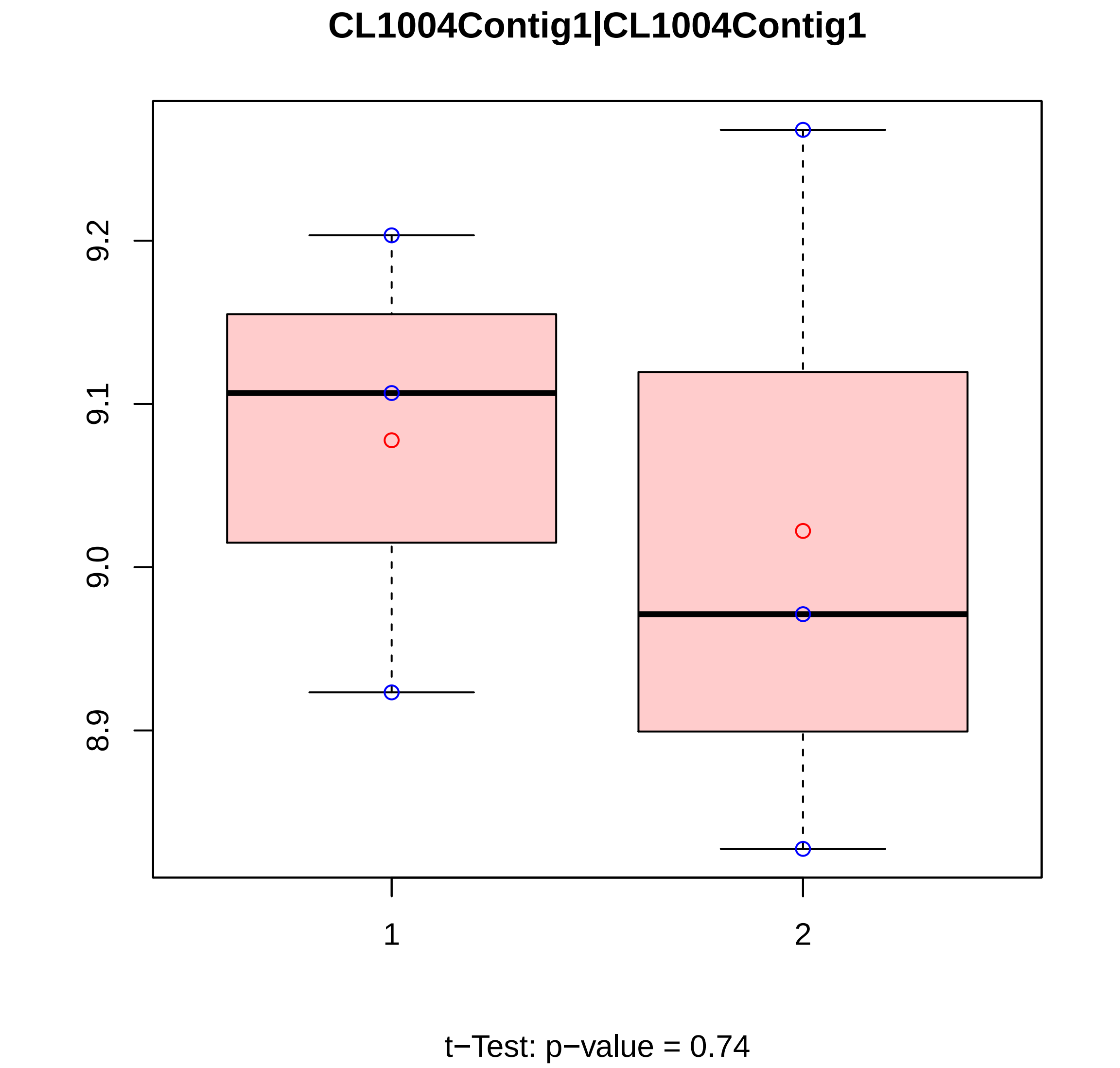
CL1004Contig1|CL1004Contig1
9.2
9.1
9.0
8.9
1
2
t−Test: p−value = 0.74

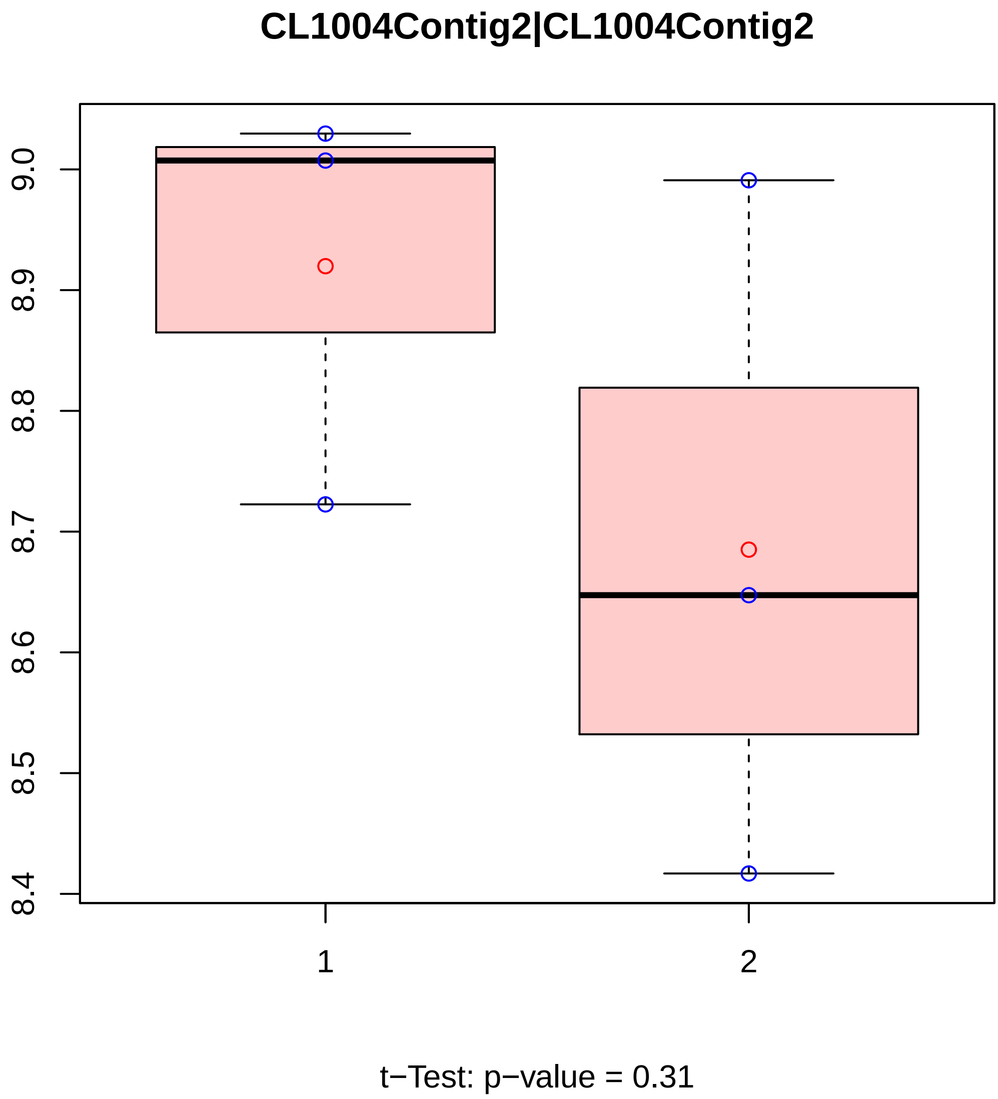

CL1004Contig2|CL1004Contig2
9.0
8.9
8.8
8.7
8.6
8.5
8.4
1
2
t−Test: p−value = 0.31

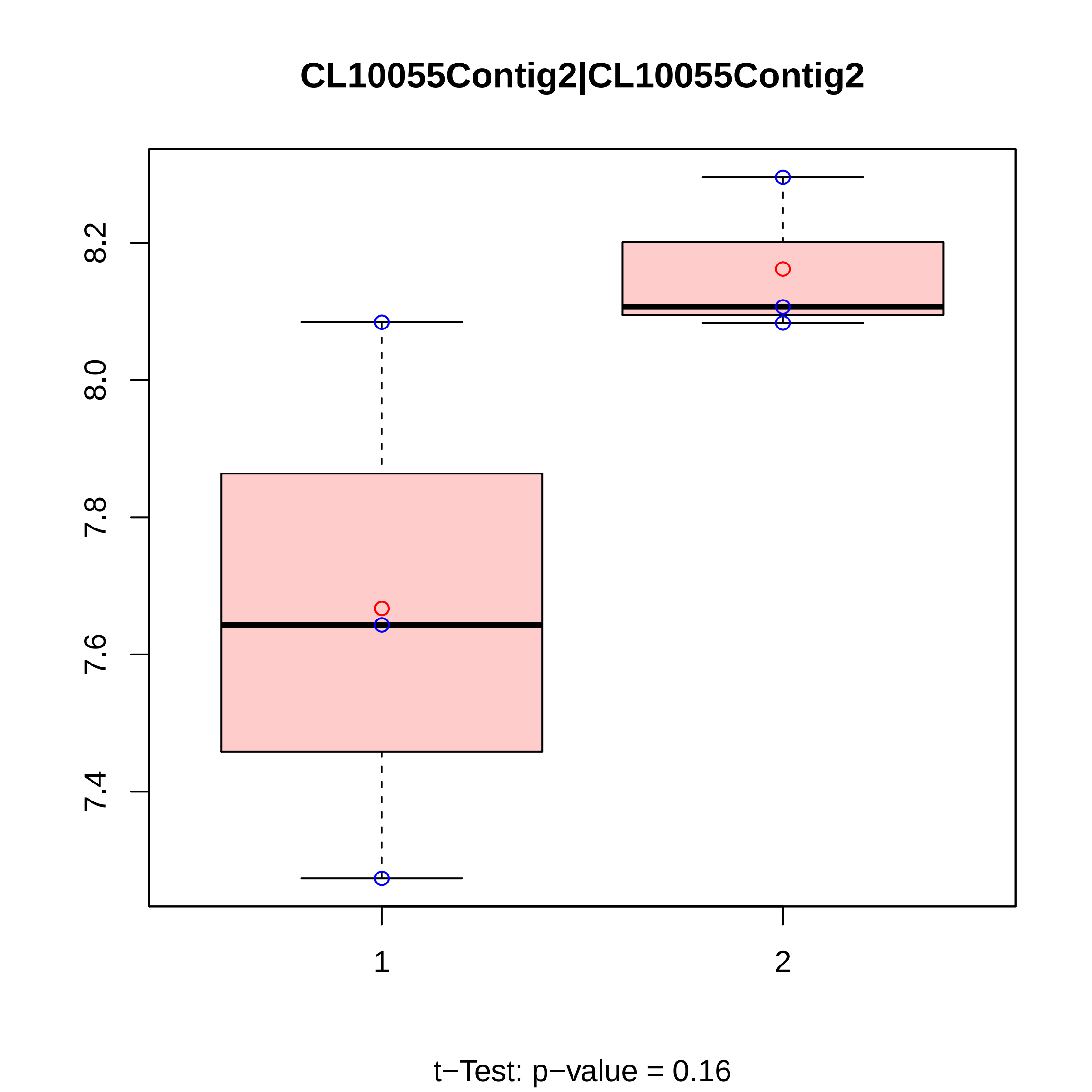
CL10055Contig2|CL10055Contig2
8.2
8.0
7.8
7.6
7.4
1
2
t−Test: p−value = 0.16

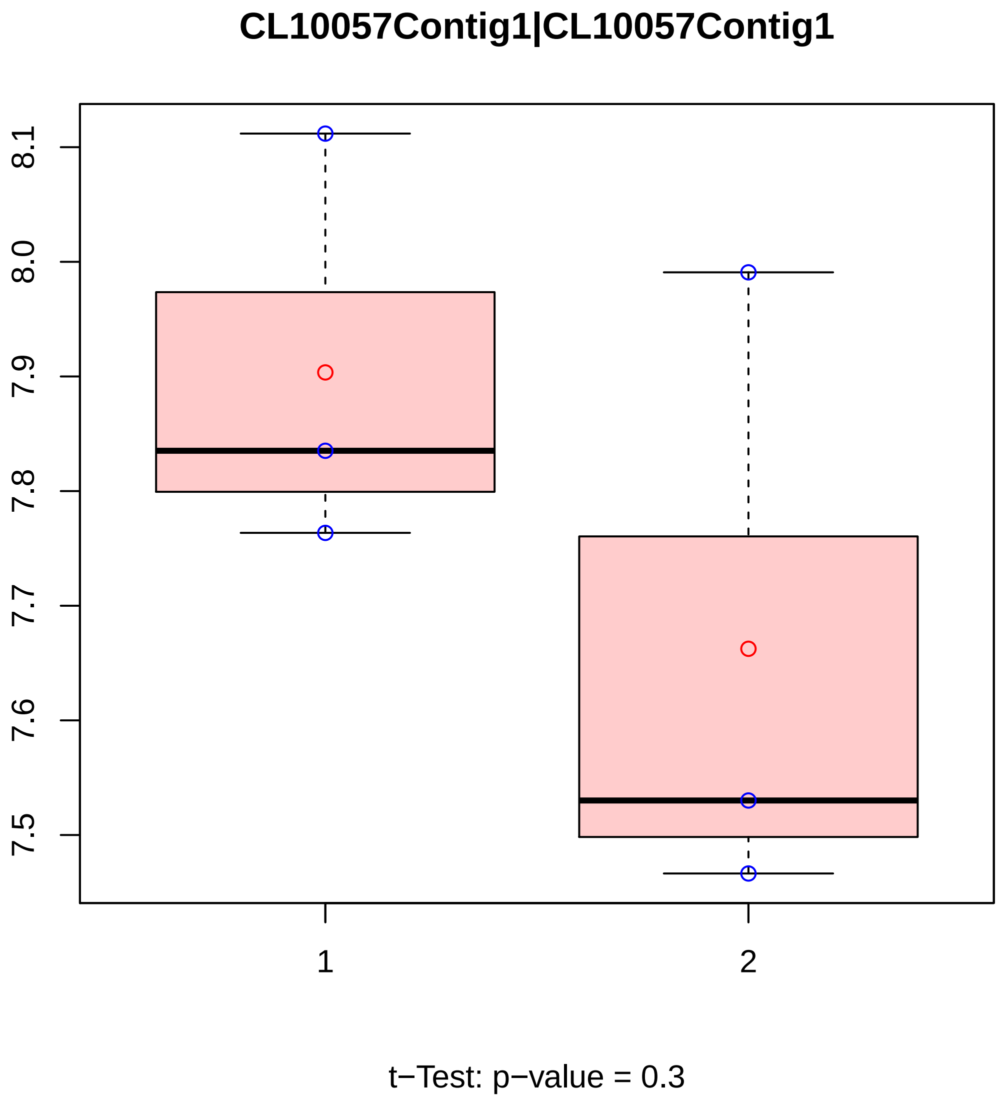

CL10057Contig1|CL10057Contig1
8.1
8.0
7.9
7.8
7.7
7.6
7.5
1
2
t−Test: p−value = 0.3

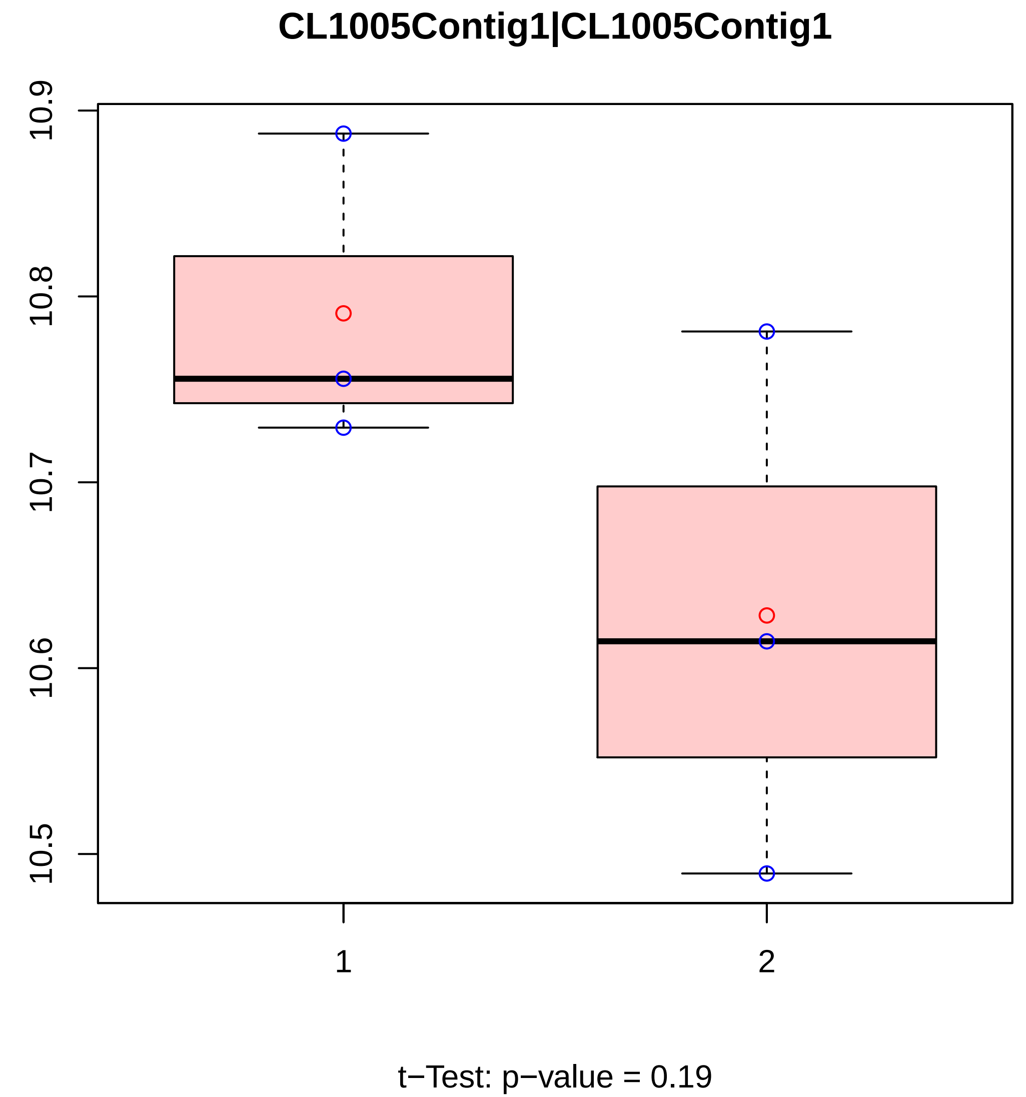
CL1005Contig1|CL1005Contig1
10.9
10.8
10.7
10.6
10.5
1
2
t−Test: p−value = 0.19

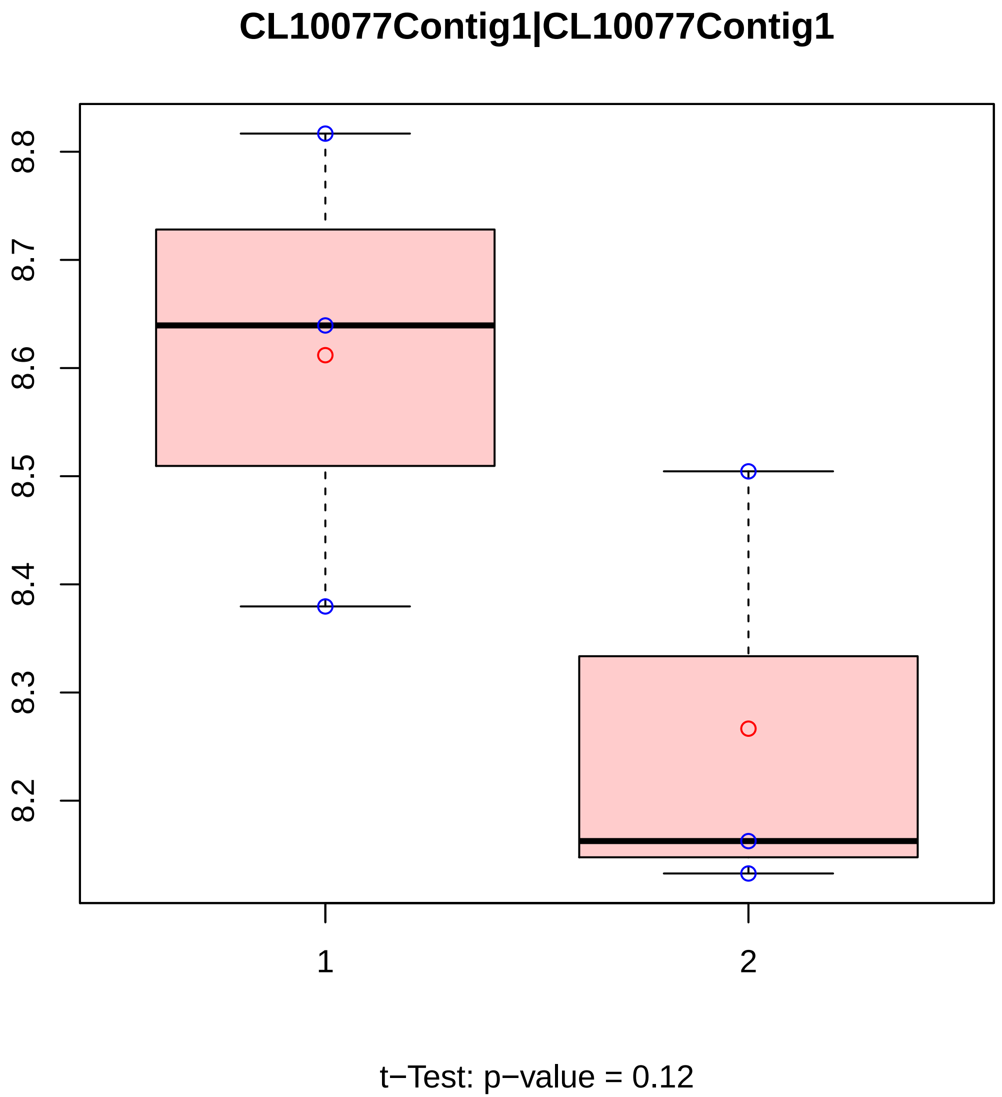

CL10077Contig1|CL10077Contig1
8.2
8.3
8.4
8.5
8.6
8.7
8.8
1
2
t−Test: p−value = 0.12

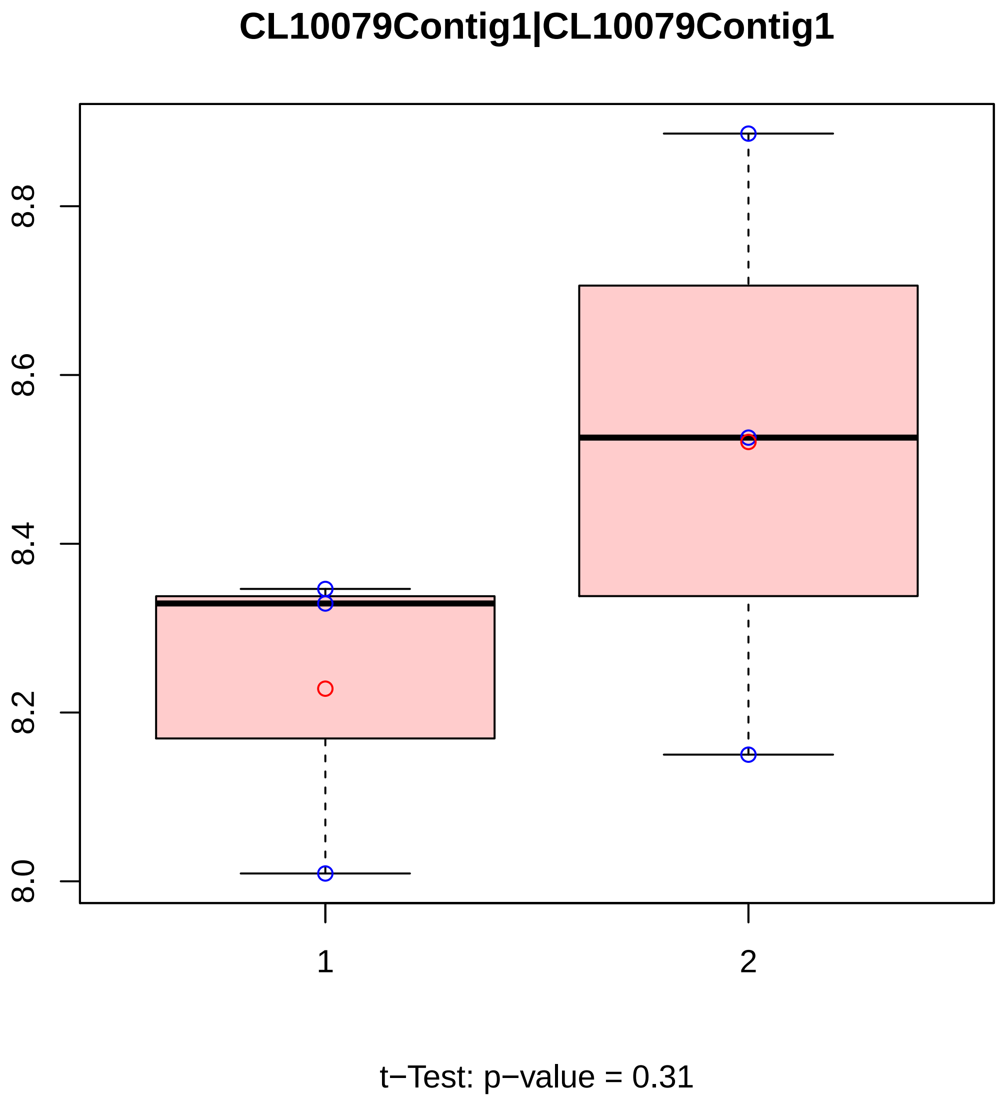
CL10079Contig1|CL10079Contig1
8.0
8.2
8.4
8.6
8.8
1
2
t−Test: p−value = 0.31

## CL100Contig17|CL100Contig17

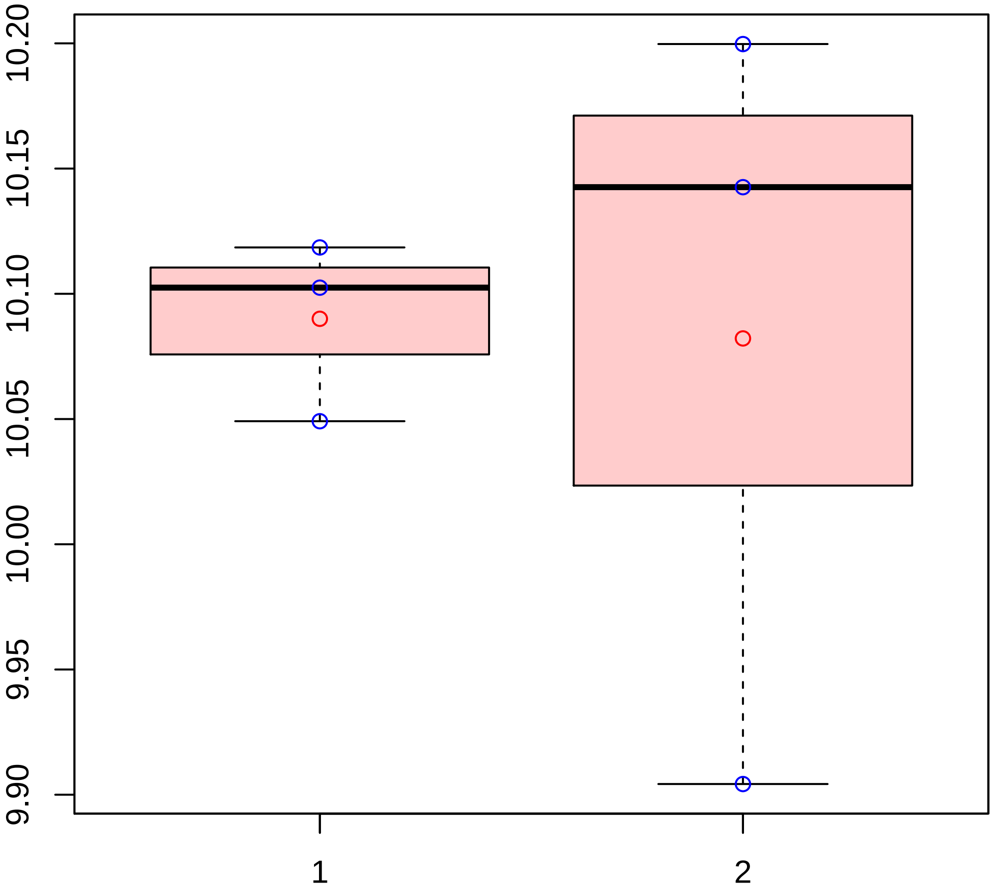


t−Test: p−value = 0.94

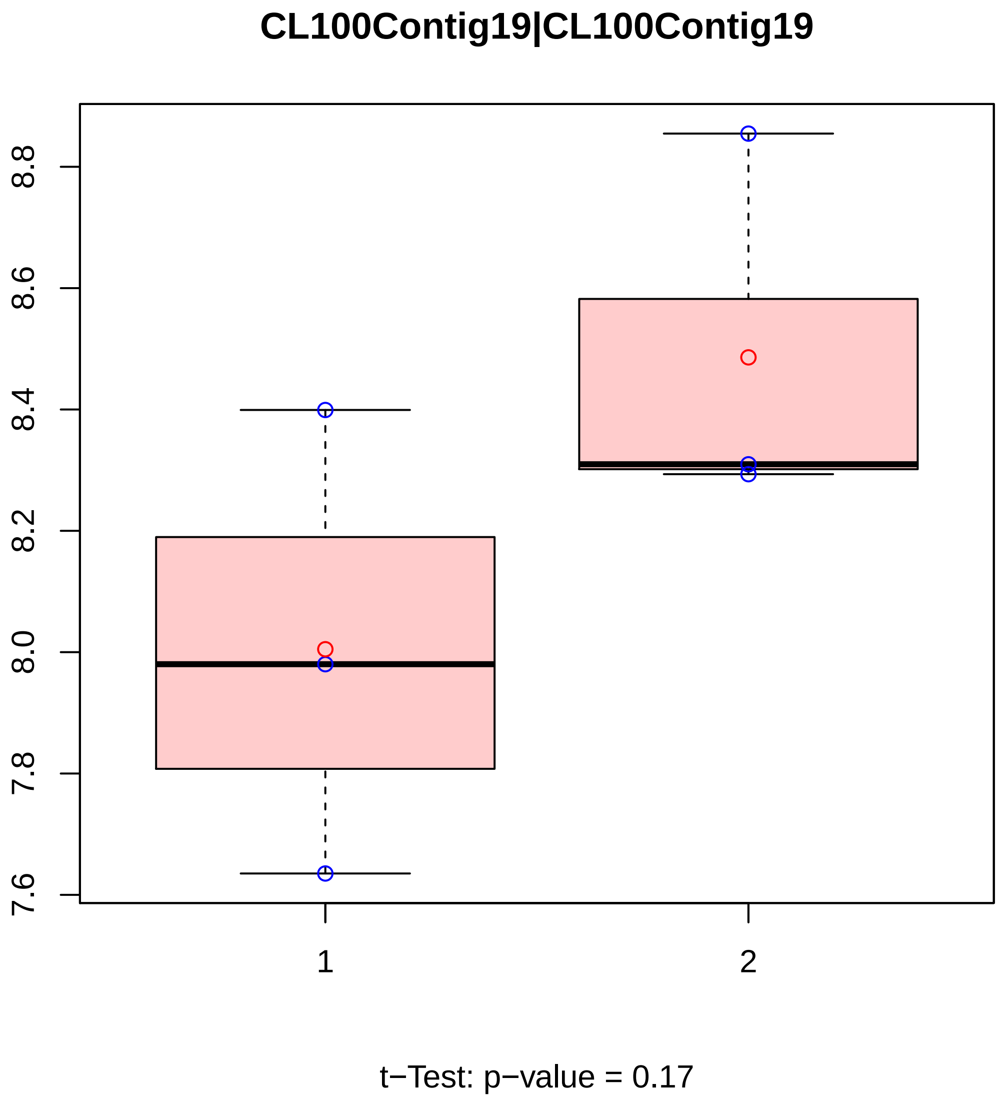
CL100Contig19|CL100Contig19
8.8
8.6
8.4
8.2
8.0
7.8
7.6
1
2
t−Test: p−value = 0.17

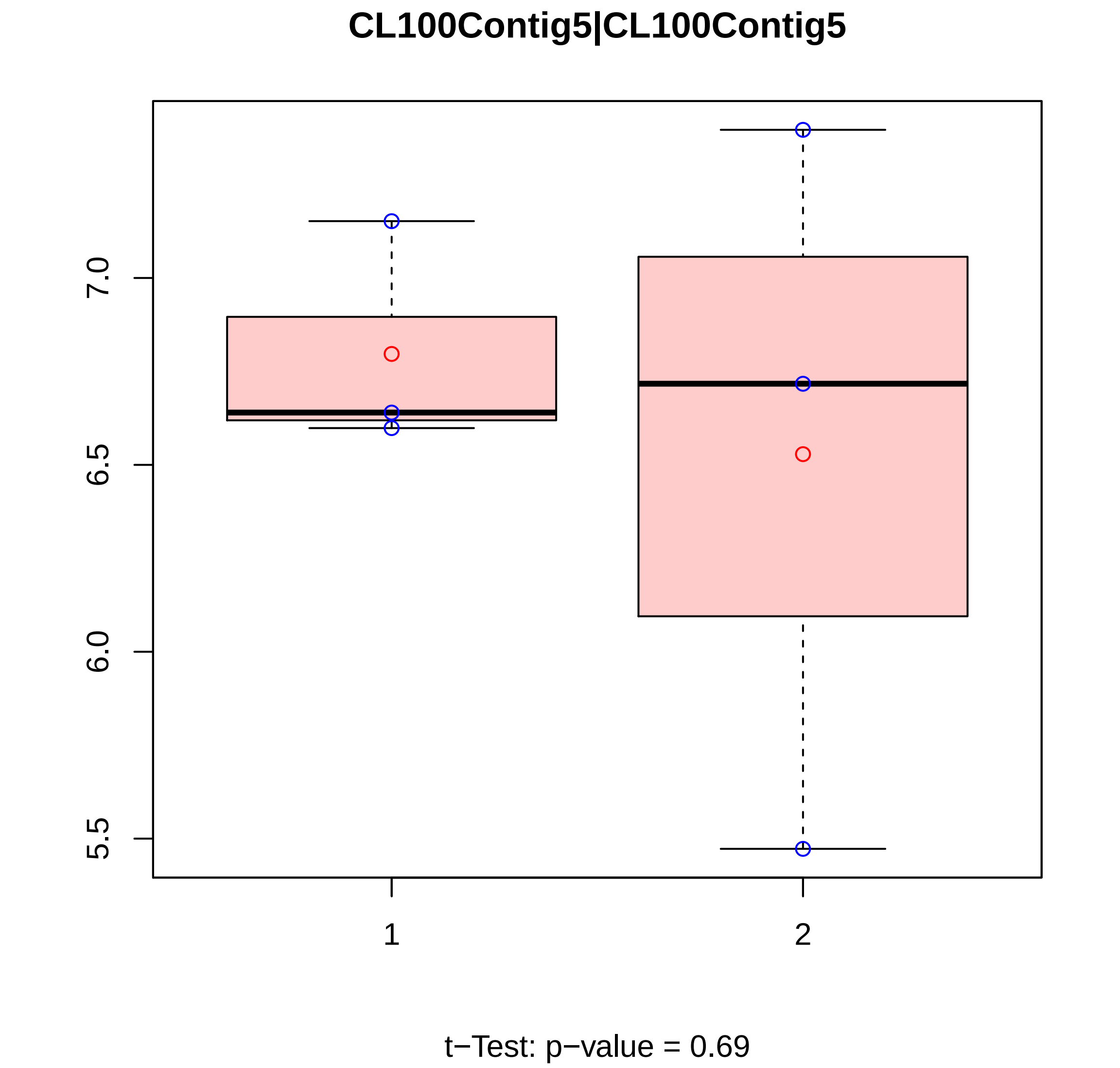
CL100Contig5|CL100Contig5
7.0
6.5
6.0
5.5
1
2
t−Test: p−value = 0.69

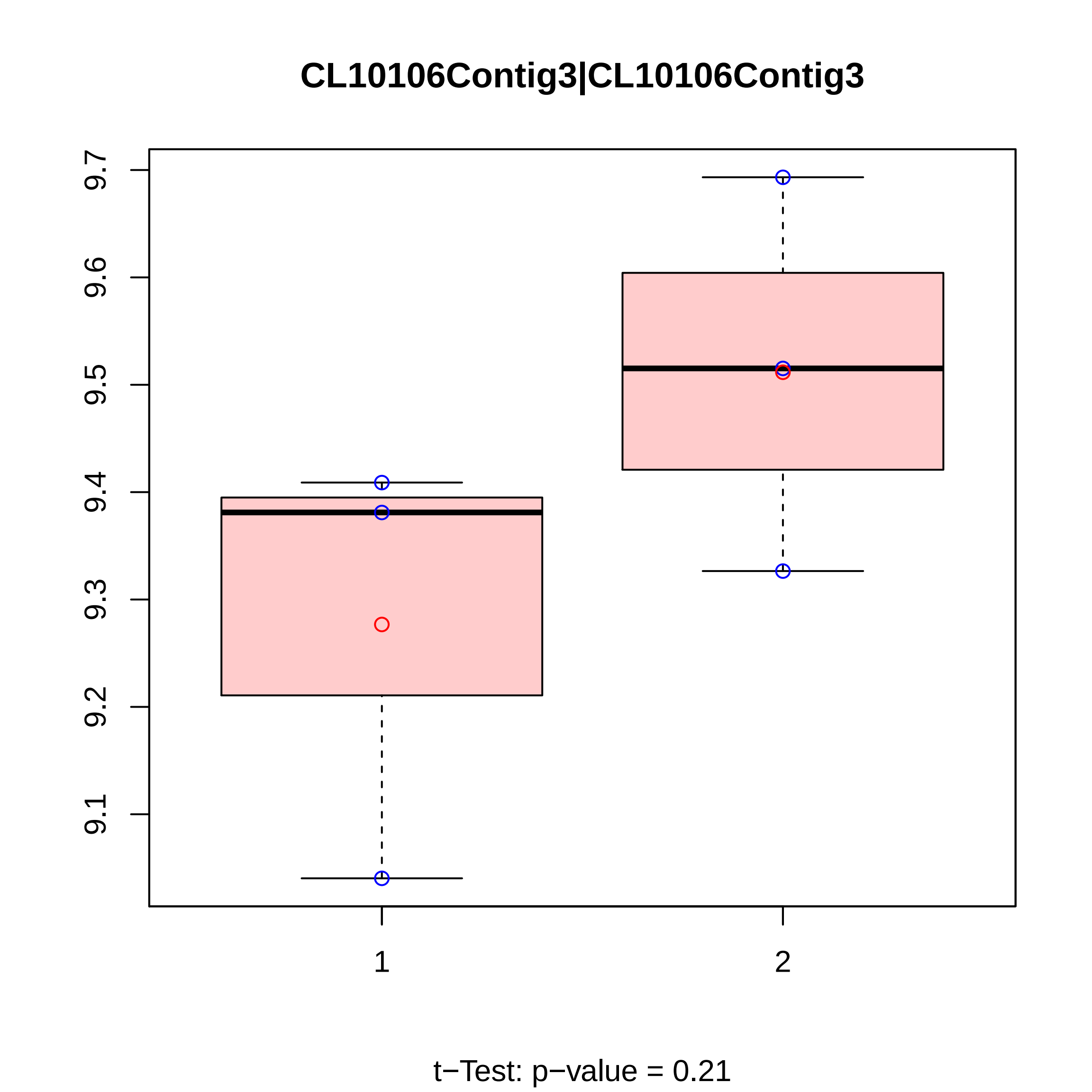
CL10106Contig3|CL10106Contig3
9.1
9.2
9.3
9.4
9.5
9.6
9.7
1
2
t−Test: p−value = 0.21

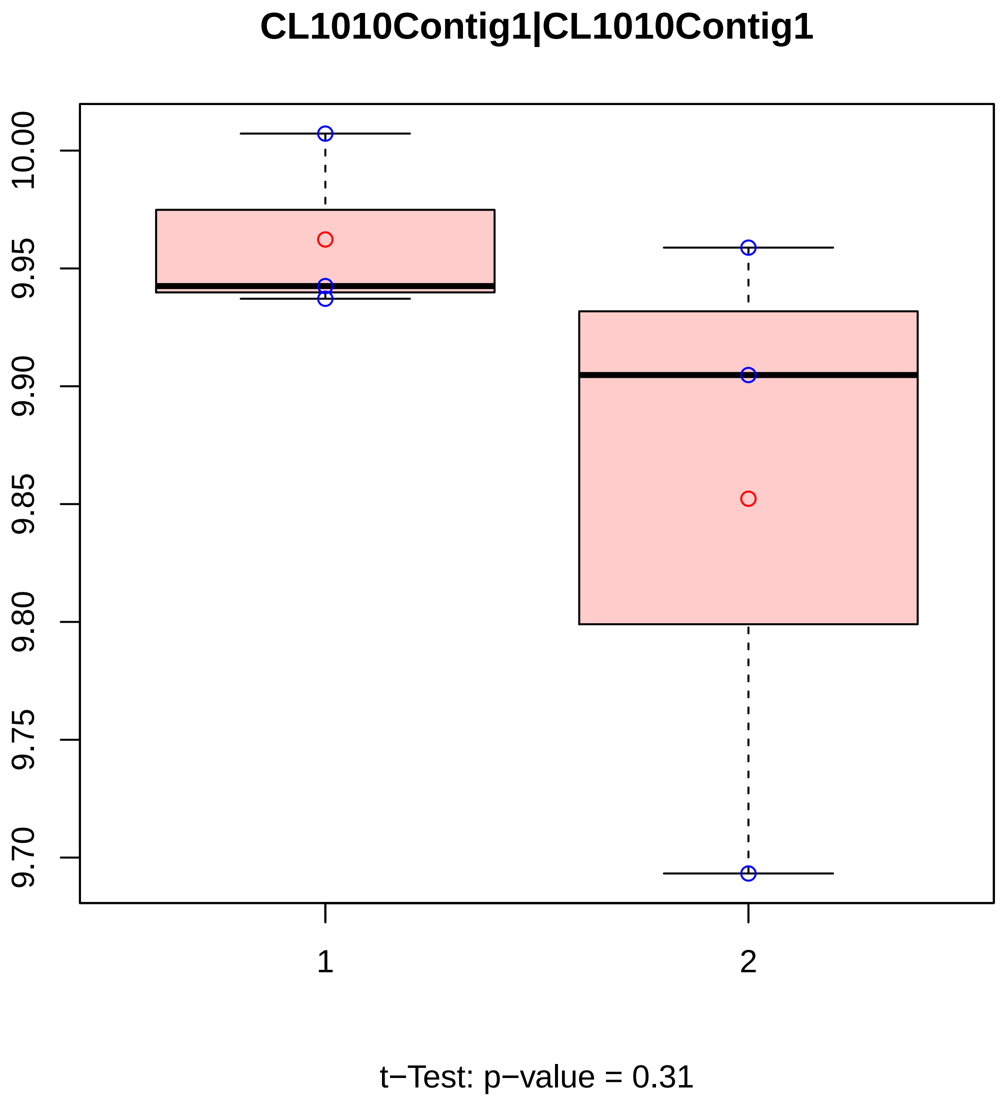
CL1010Contig1|CL1010Contig1
10.00
9.95
9.90
9.85
9.80
9.75
9.70
1
2
t−Test: p−value = 0.31

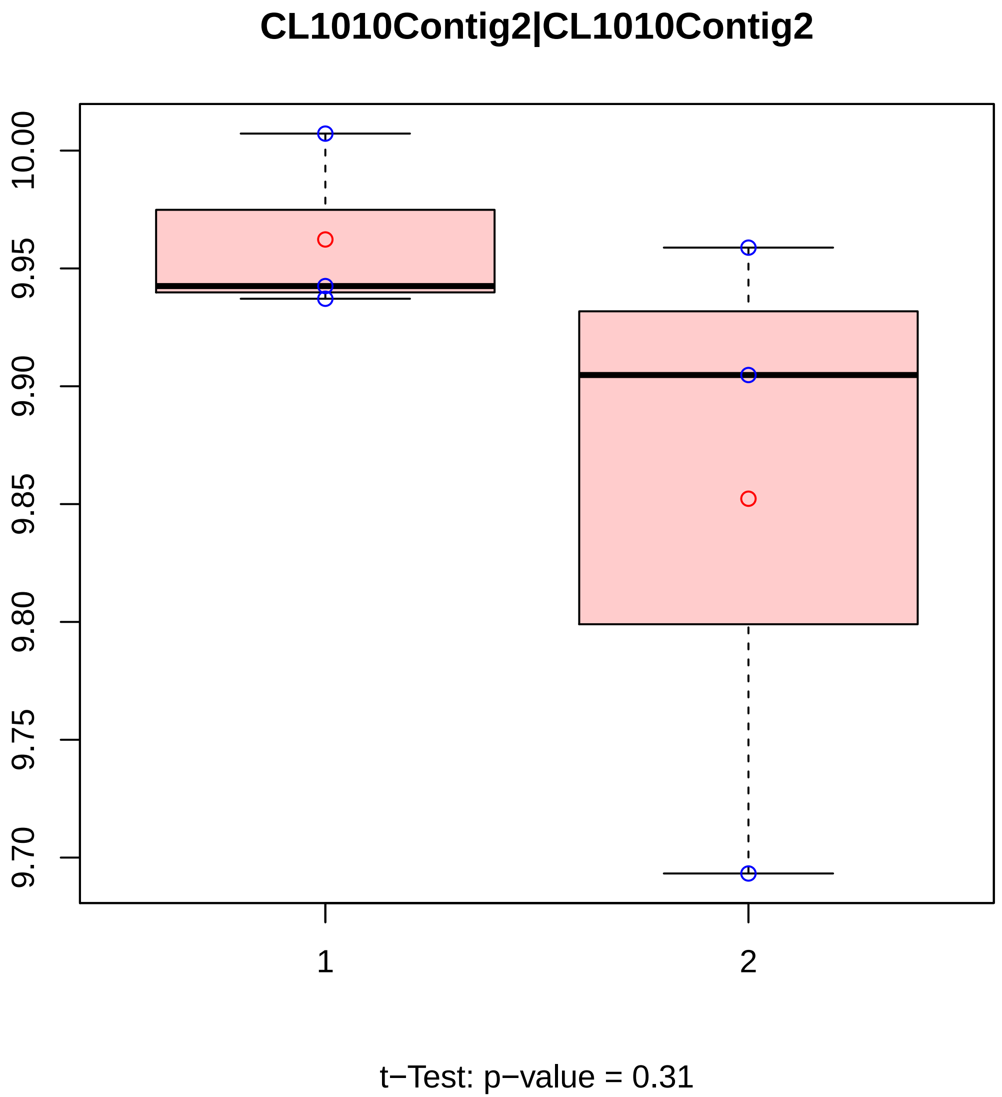
CL1010Contig2|CL1010Contig2
10.00
9.95
9.90
9.85
9.80
9.75
9.70
1
2
t−Test: p−value = 0.31

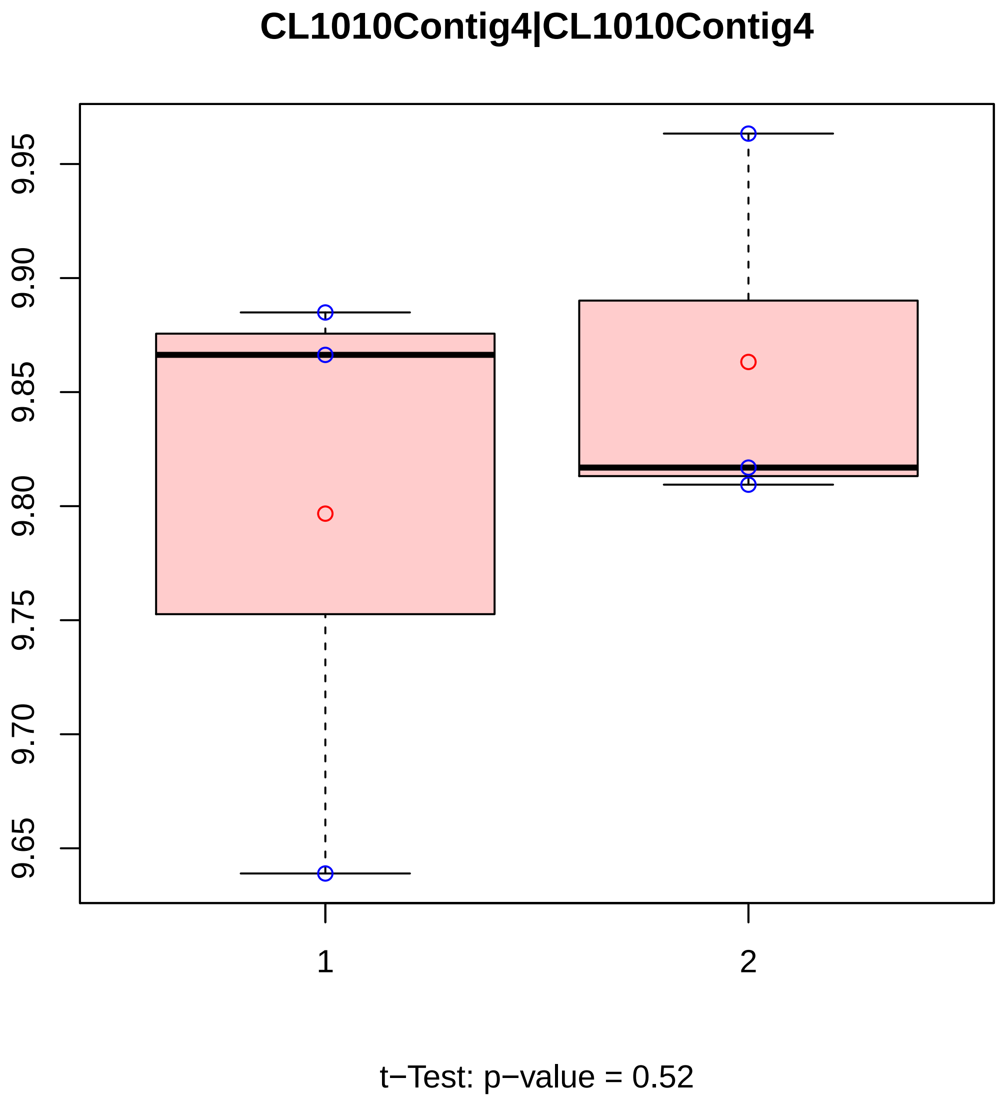
CL1010Contig4|CL1010Contig4
9.65
9.70
9.75
9.80
9.85
9.90
9.95
1
2
t−Test: p−value = 0.52

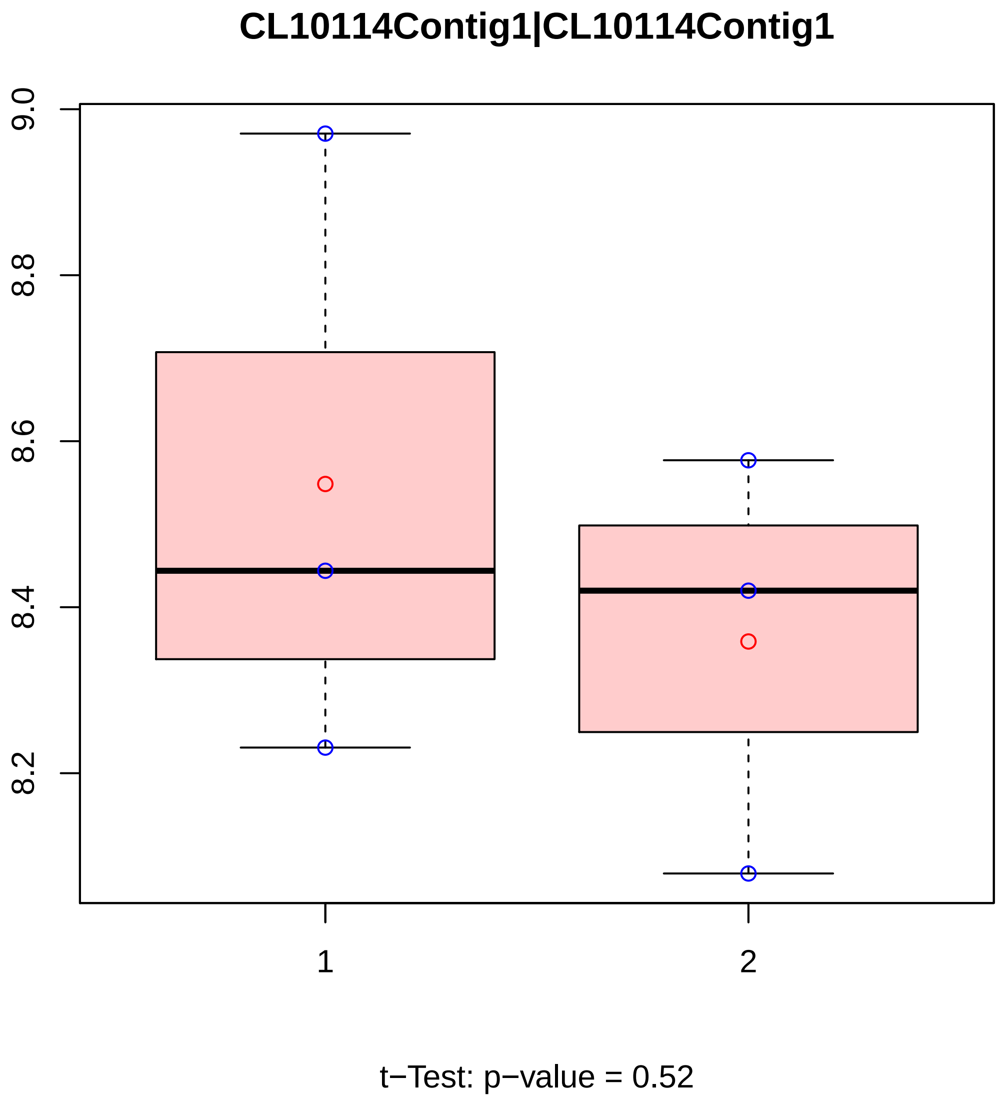
CL10114Contig1|CL10114Contig1
9.0
8.8
8.6
8.4
8.2
1
2
t−Test: p−value = 0.52

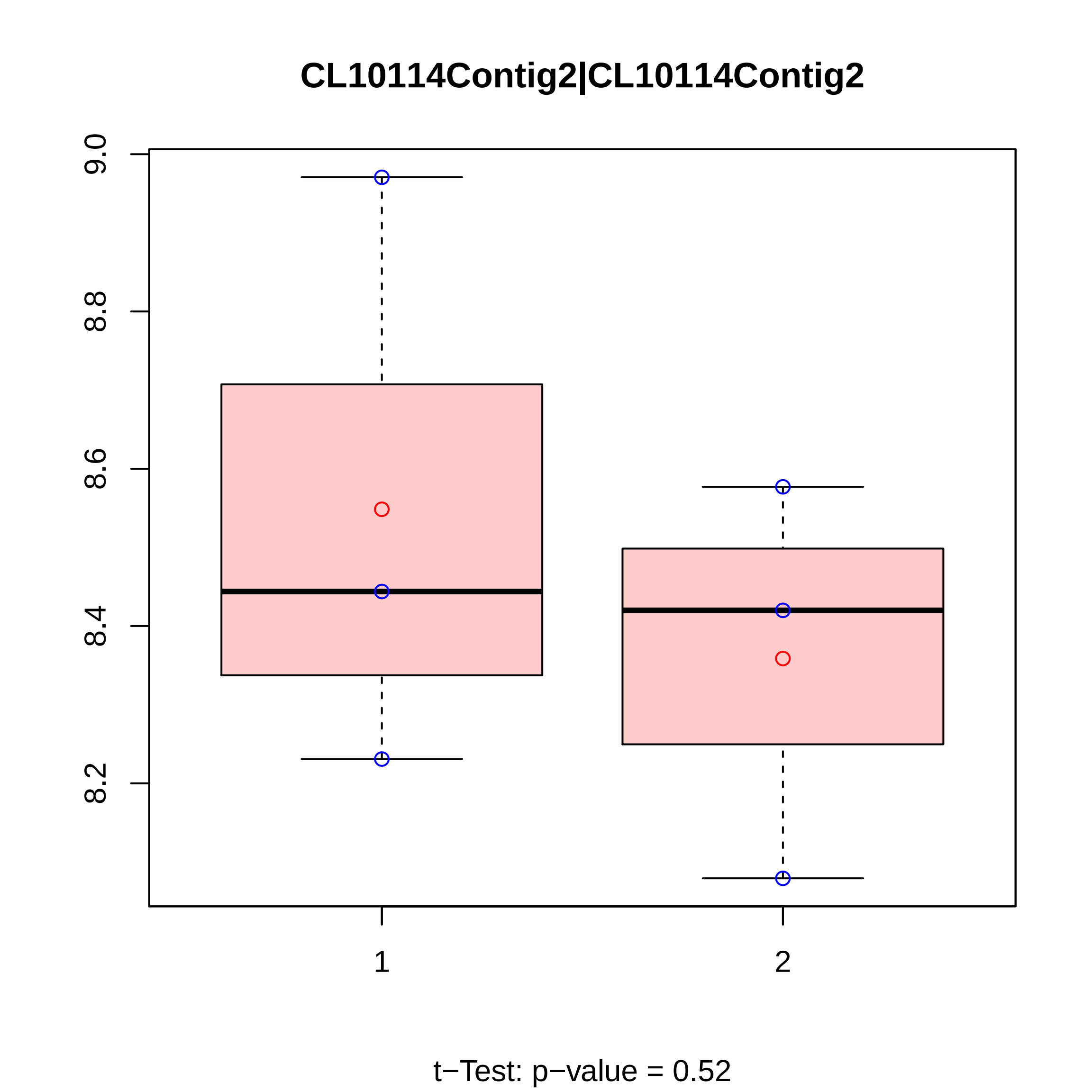
CL10114Contig2|CL10114Contig2
9.0
8.8
8.6
8.4
8.2
1
2
t−Test: p−value = 0.52

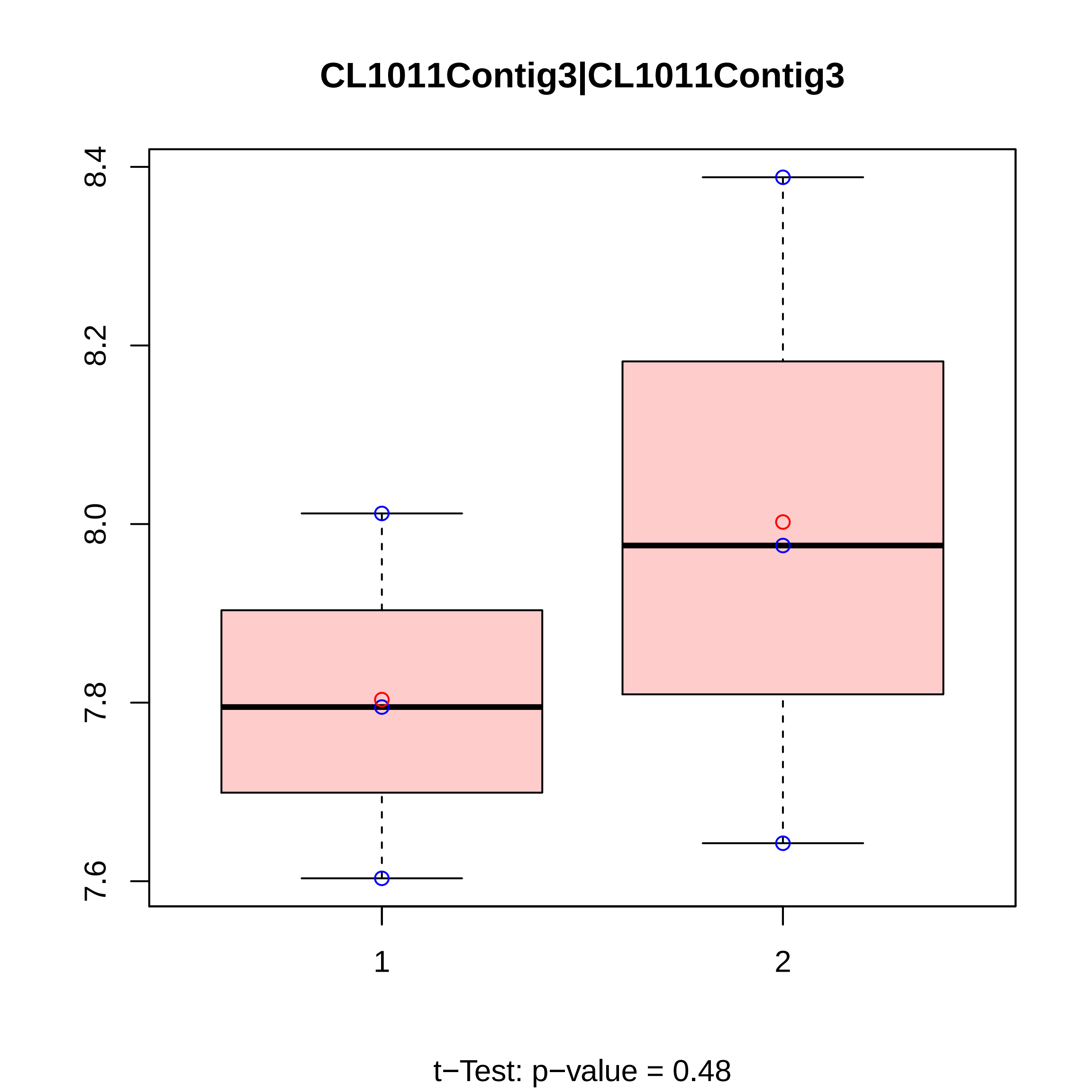
CL1011Contig3|CL1011Contig3
8.4
8.2
8.0
7.8
7.6
1
2
t−Test: p−value = 0.48

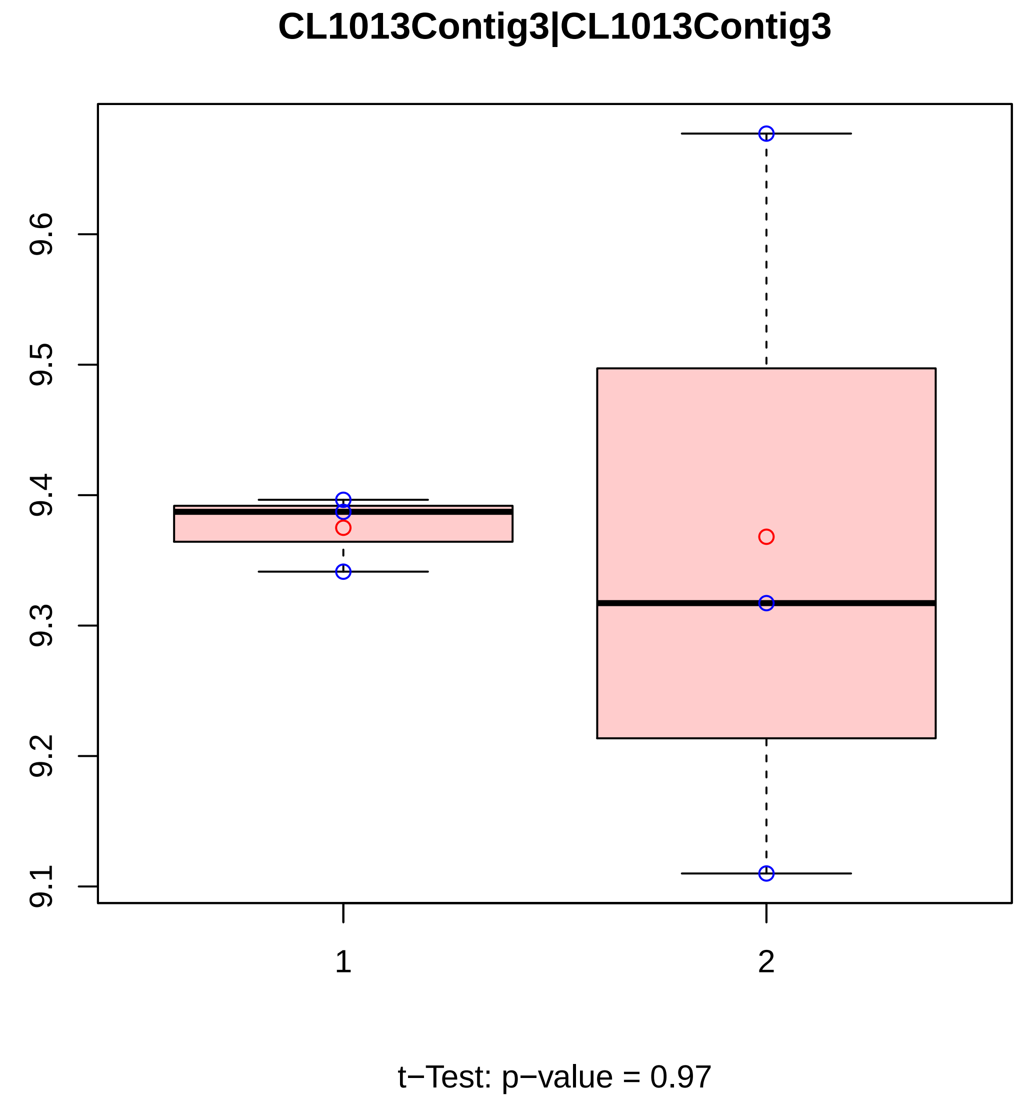
CL1013Contig3|CL1013Contig3
9.1
9.2
9.3
9.4
9.5
9.6
1
2
t−Test: p−value = 0.97

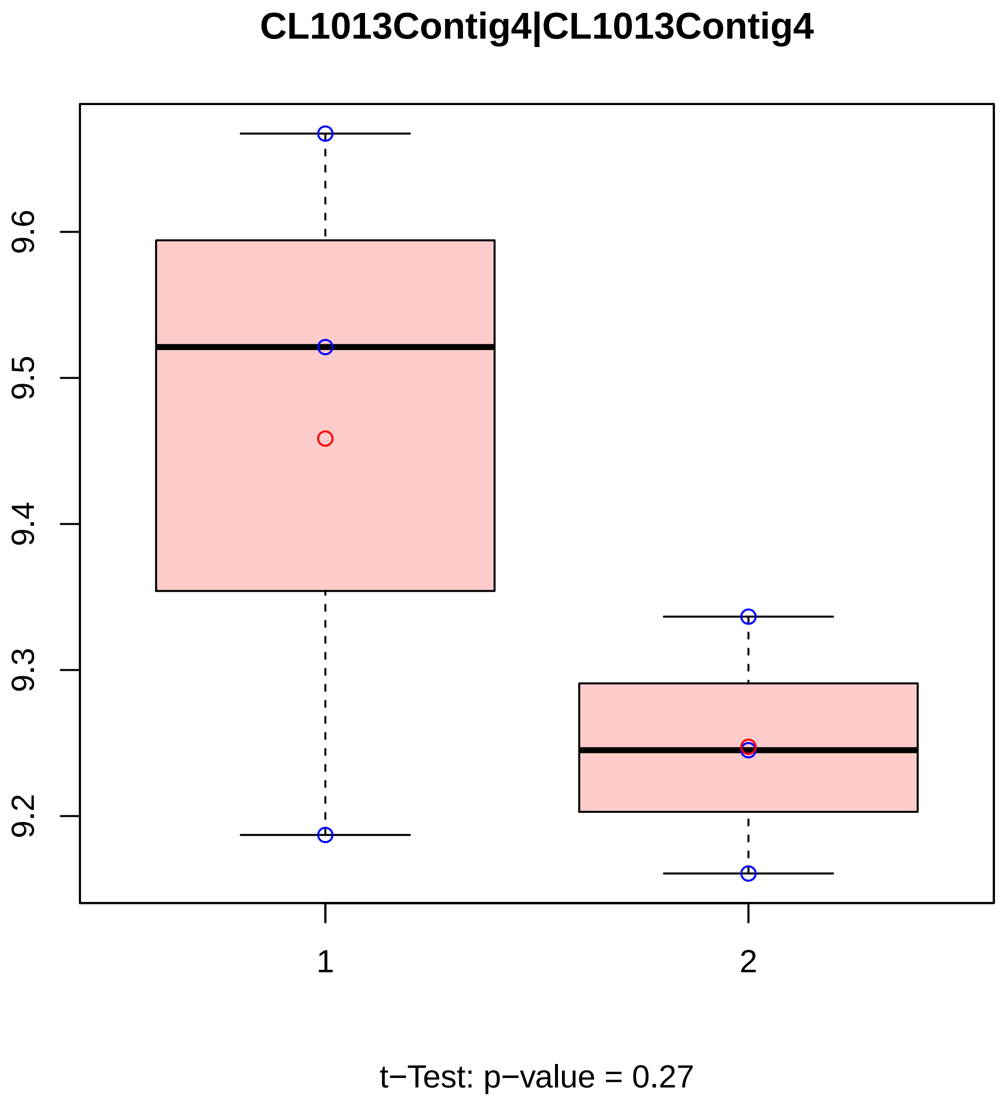
CL1013Contig4|CL1013Contig4
9.2
9.3
9.4
9.5
9.6
1
2
t−Test: p−value = 0.27

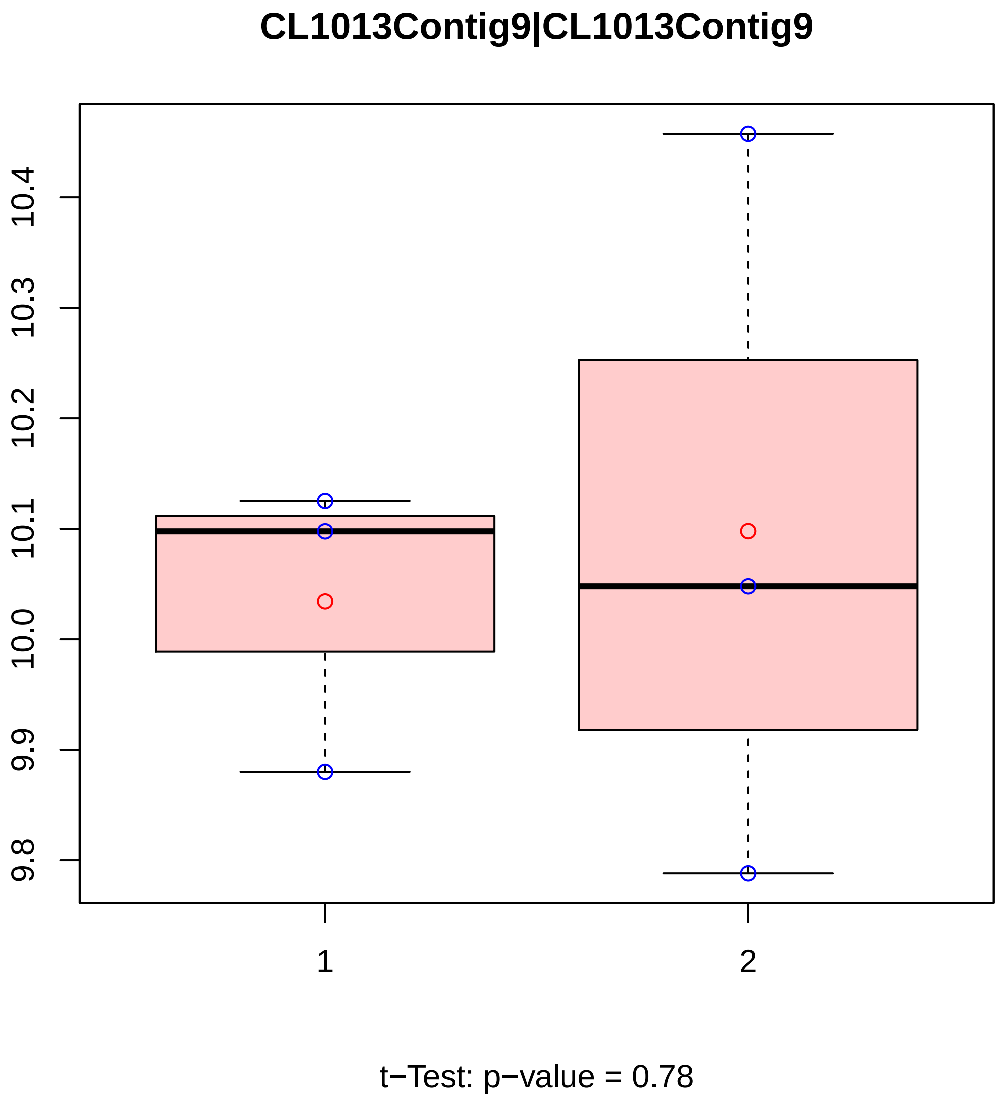
CL1013Contig9|CL1013Contig9
10.4
10.3
10.2
10.1
10.0
9.9
9.8
1
2
t−Test: p−value = 0.78

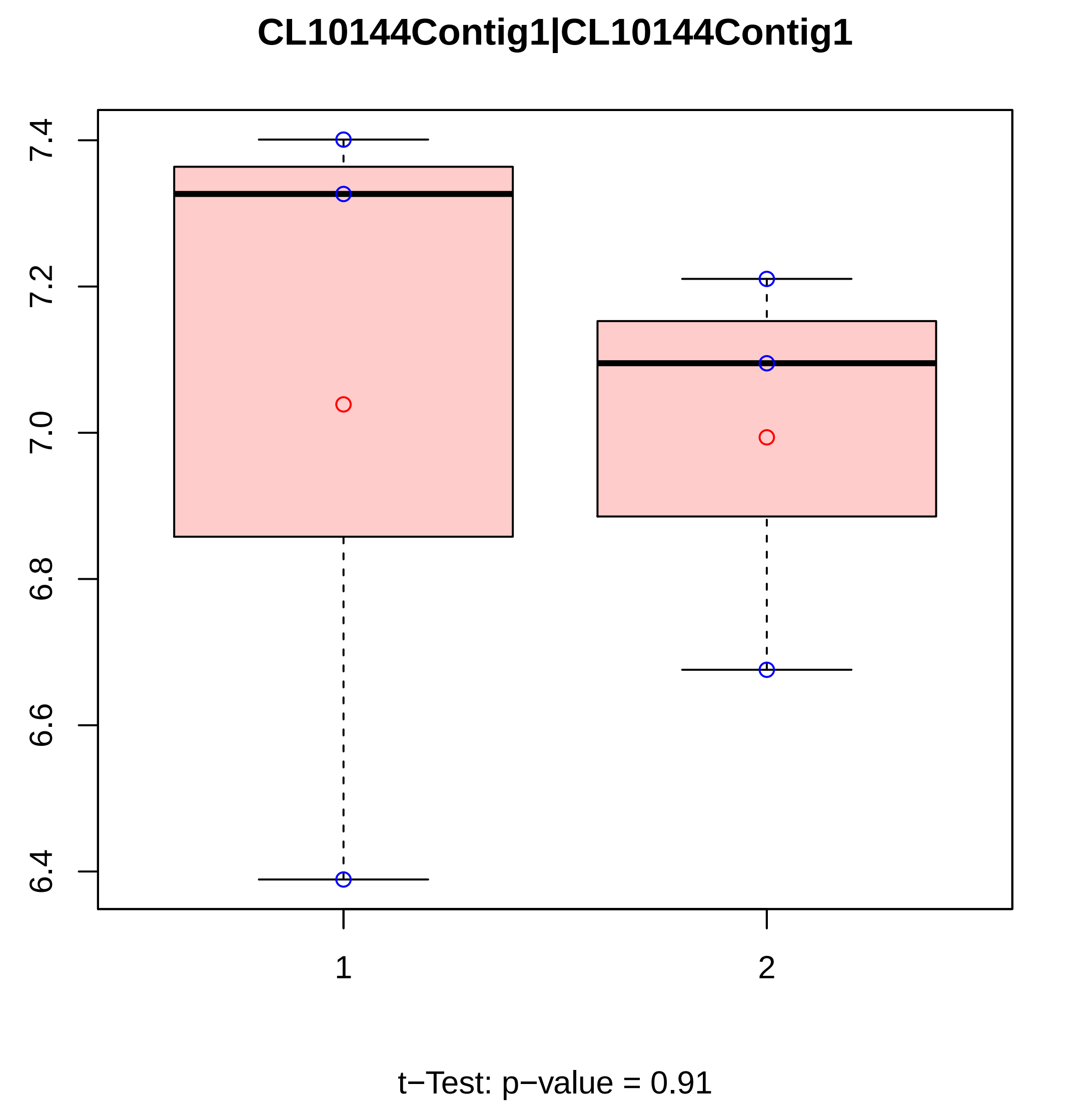
CL10144Contig1|CL10144Contig1
7.4
7.2
7.0
6.8
6.6
6.4
1
2
t−Test: p−value = 0.91

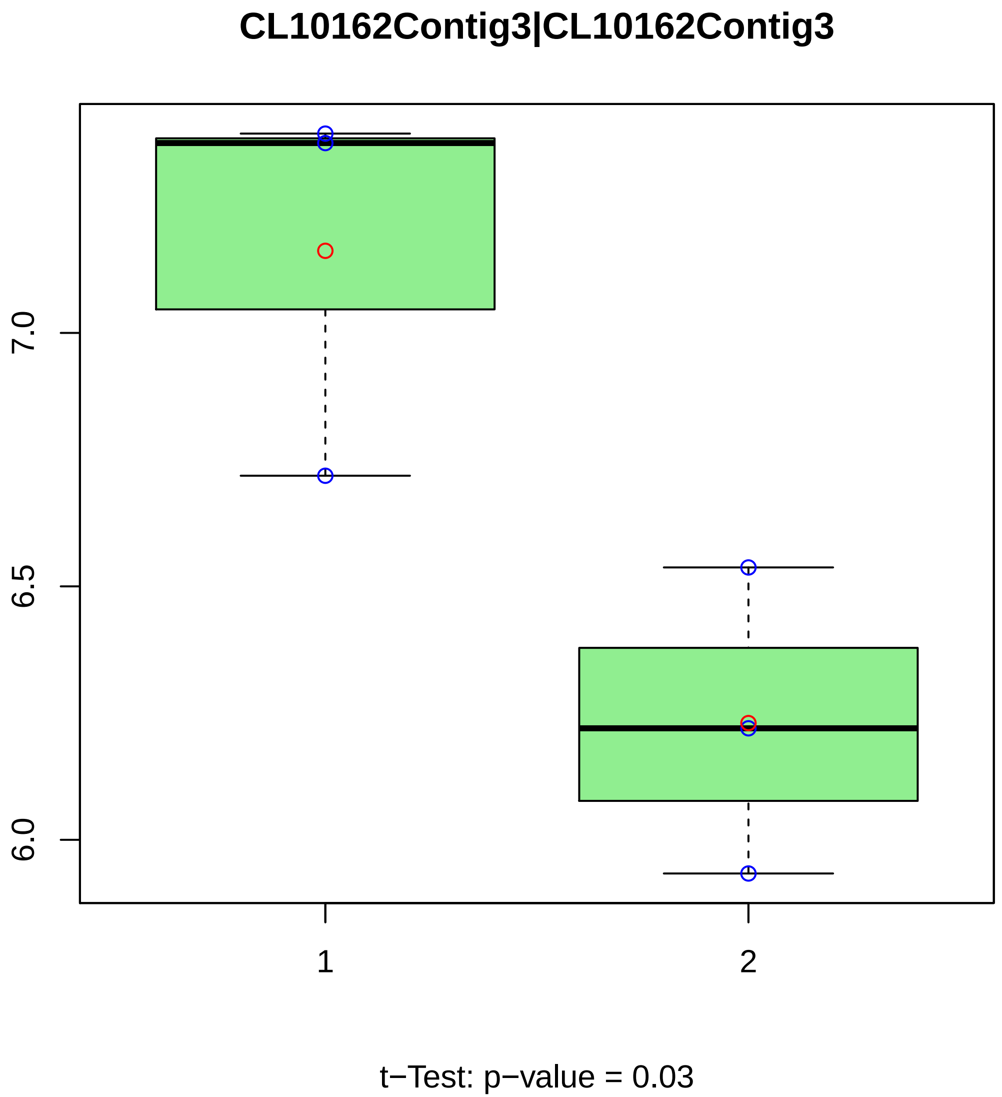
CL10162Contig3|CL10162Contig3
7.0
6.5
6.0
1
2
t−Test: p−value = 0.03

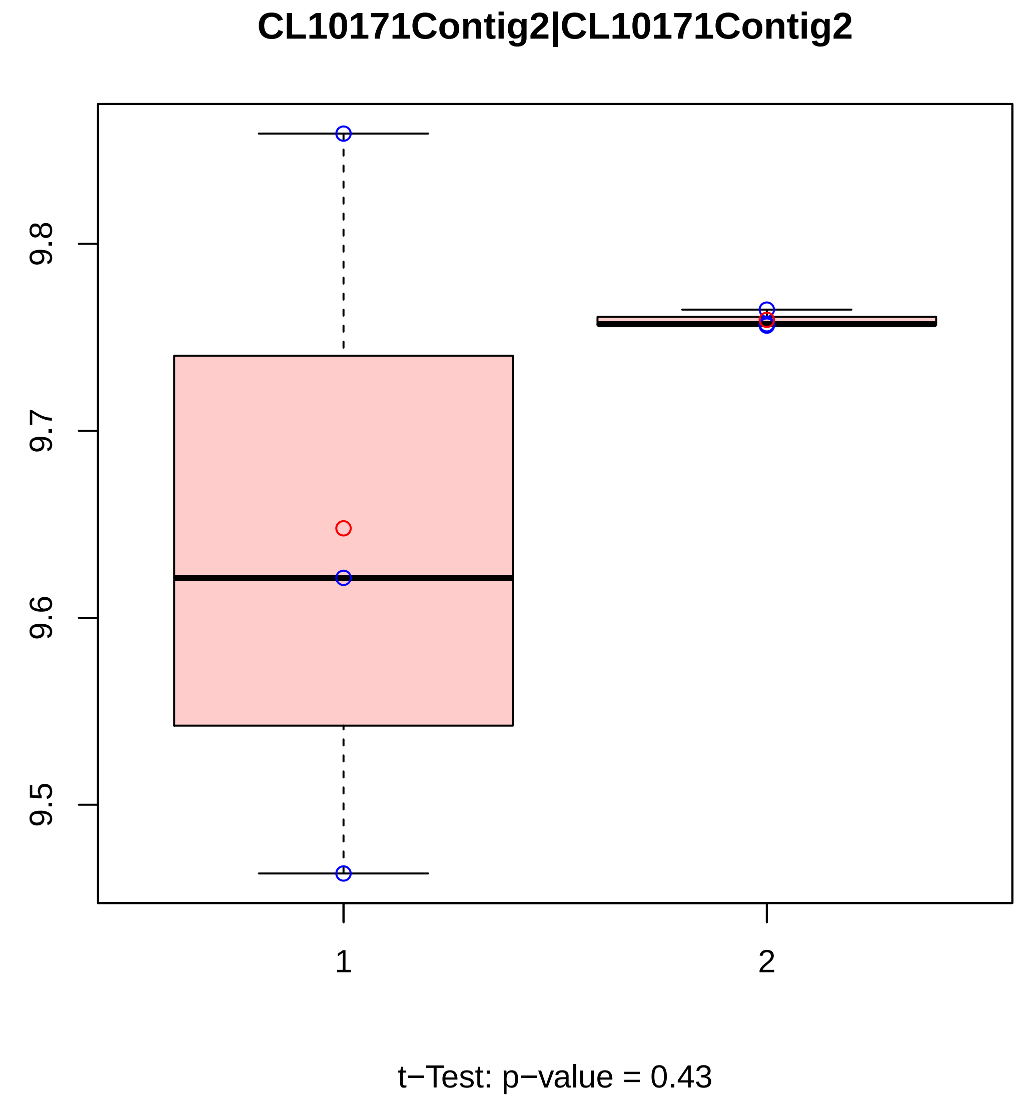
CL10171Contig2|CL10171Contig2
9.8
9.7
9.6
9.5
1
2
t−Test: p−value = 0.43

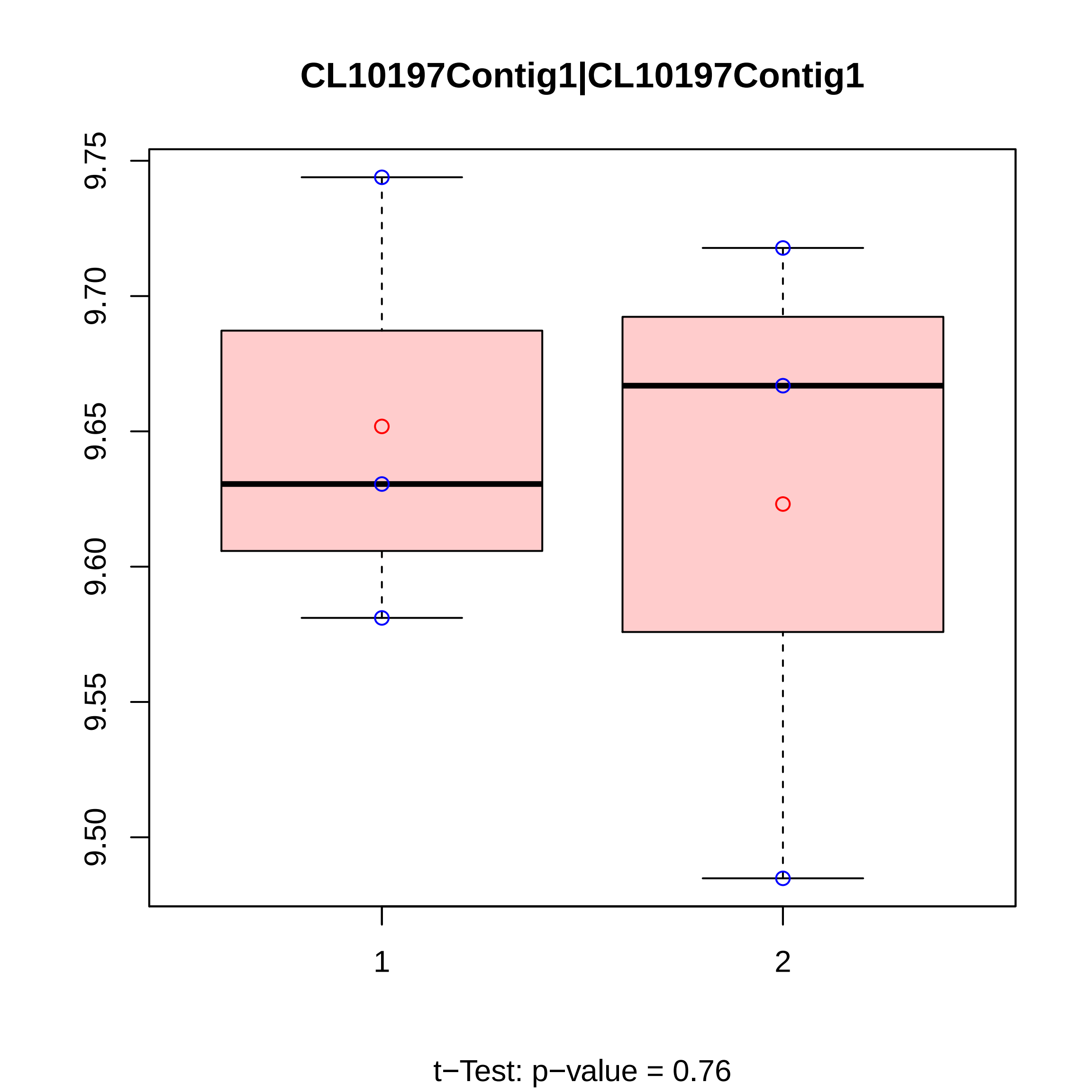

CL10197Contig1|CL10197Contig1
9.50
9.55
9.60
9.65
9.70
9.75
1
2
t−Test: p−value = 0.76

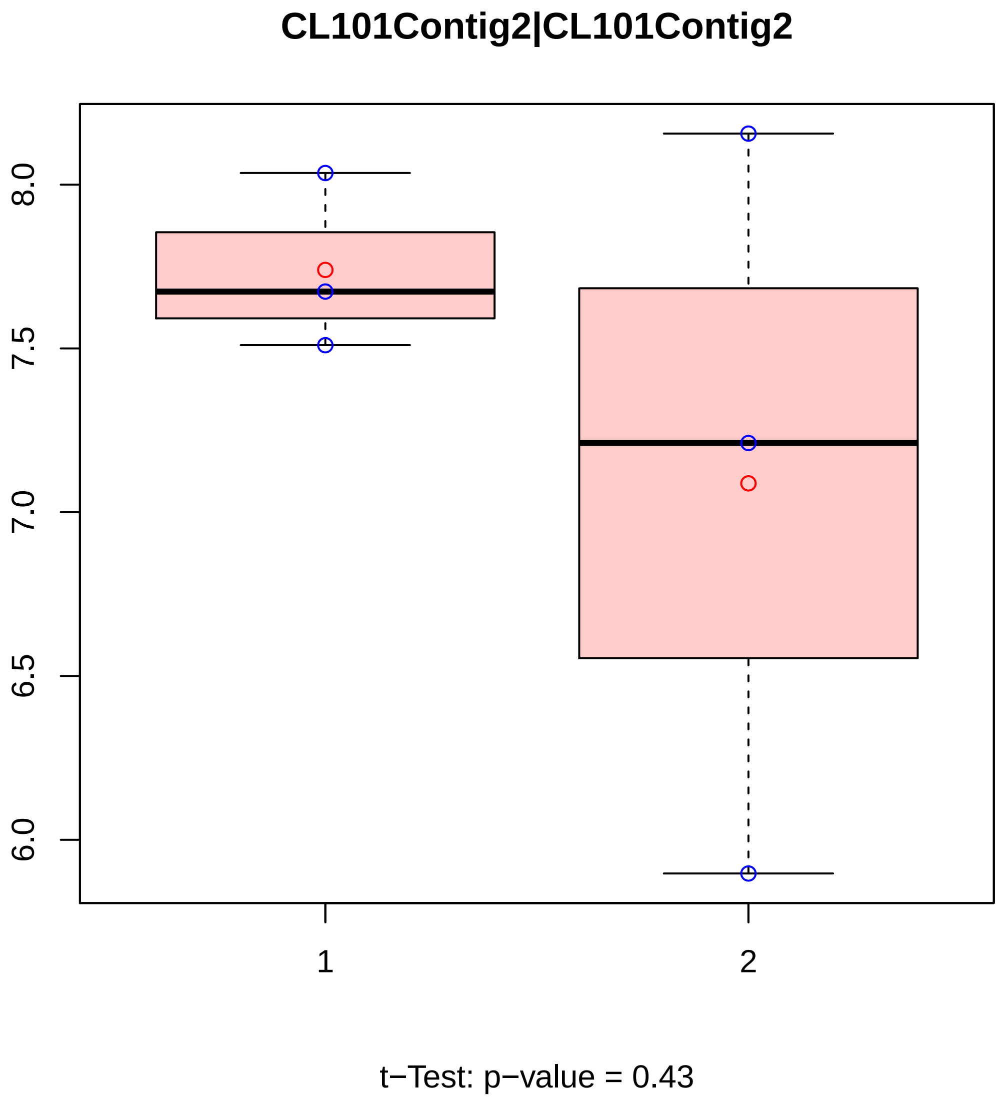
CL101Contig2|CL101Contig2
8.0
7.5
7.0
6.5
6.0
1
2
t−Test: p−value = 0.43

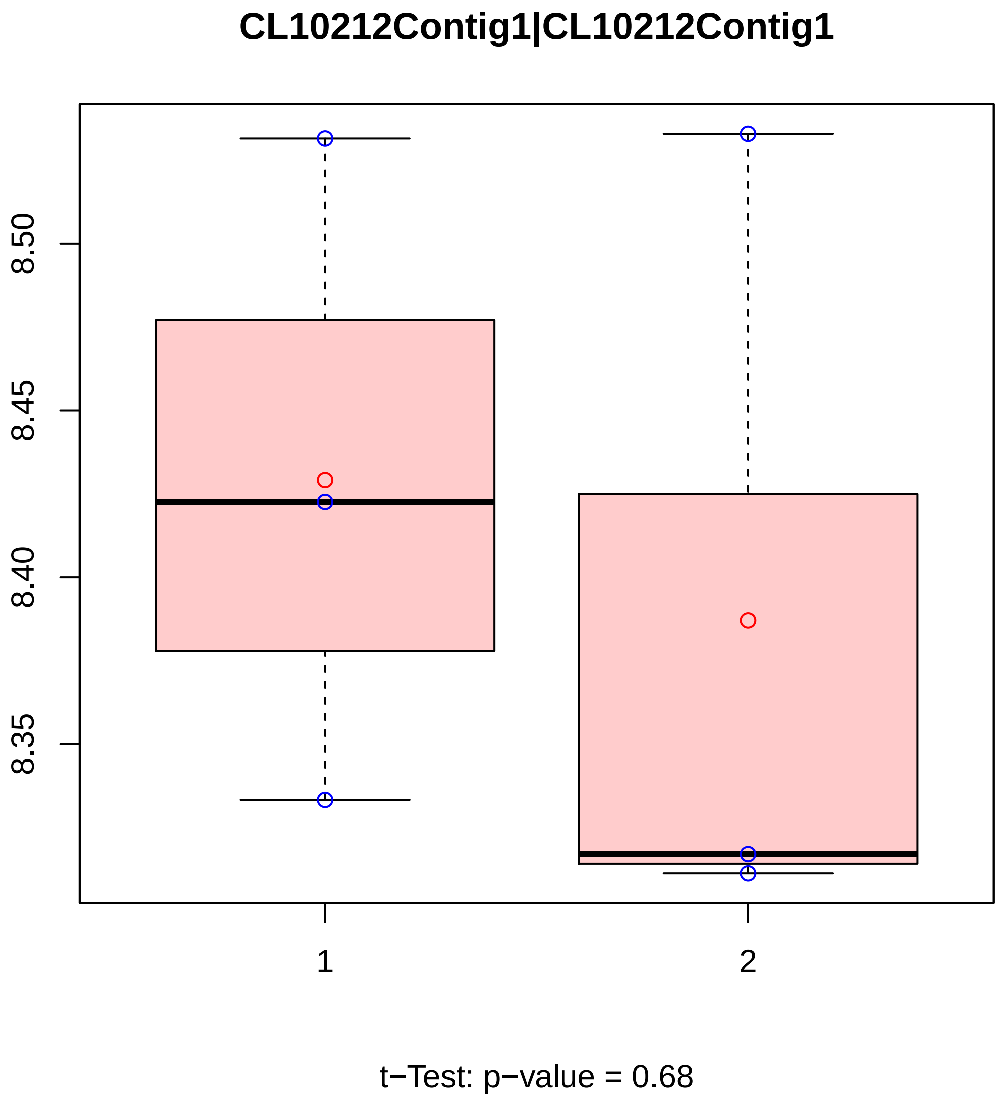

CL10212Contig1|CL10212Contig1
8.35
8.40
8.45
8.50
1
2
t−Test: p−value = 0.68

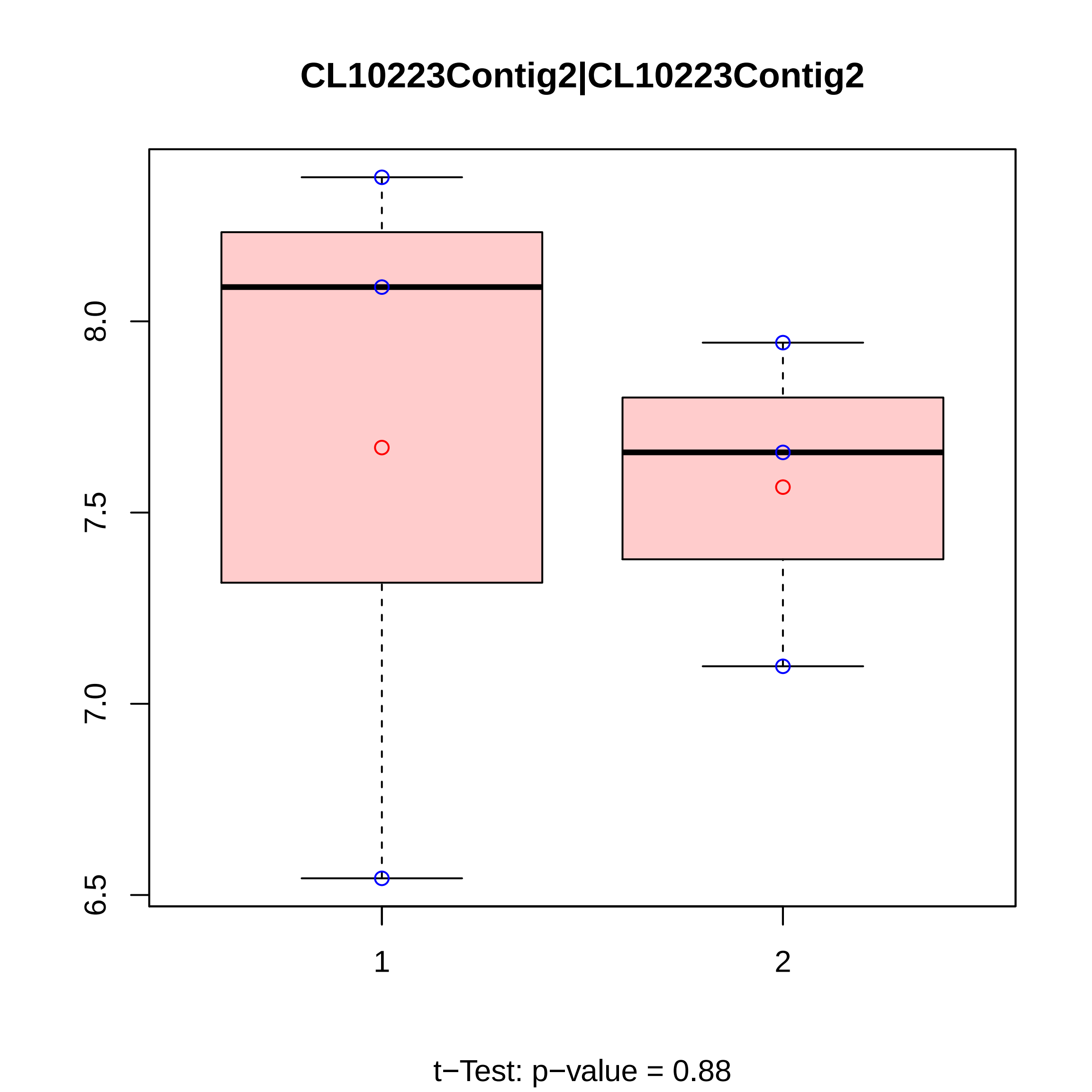
CL10223Contig2|CL10223Contig2
8.0
7.5
7.0
6.5
1
2
t−Test: p−value = 0.88

# CL10223Contig3|CL10223Contig3

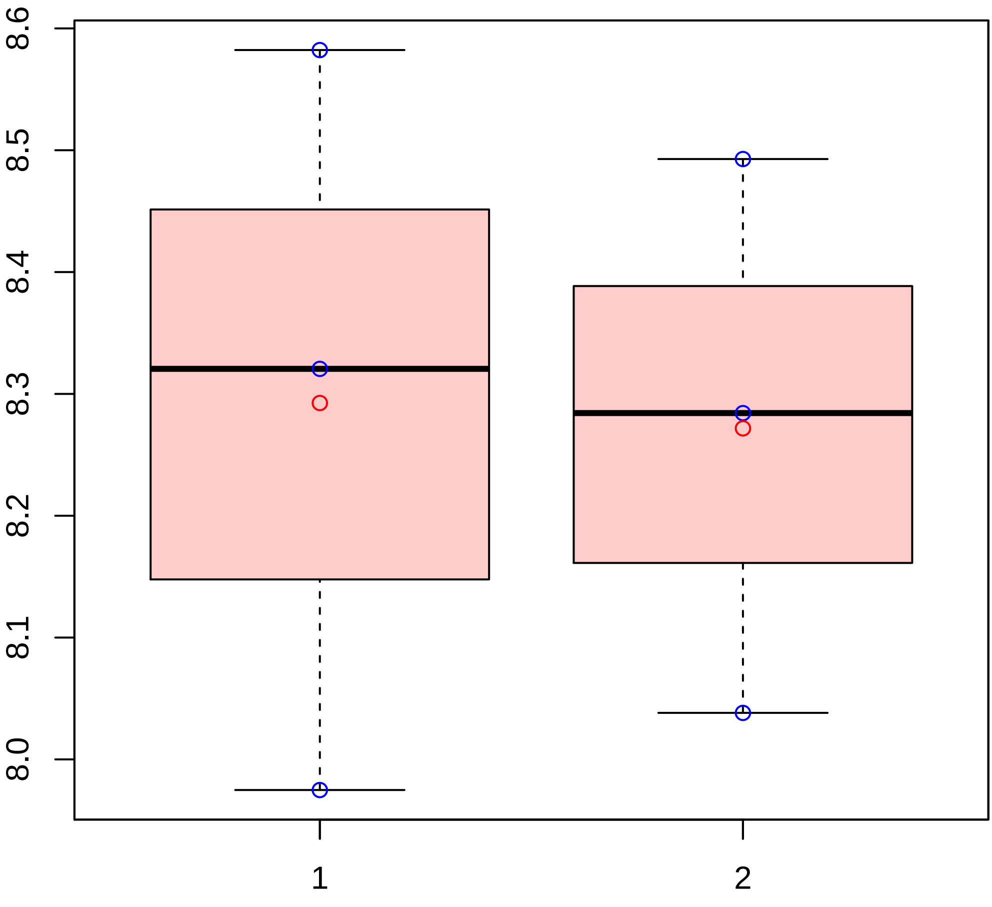


t−Test: p−value = 0.93

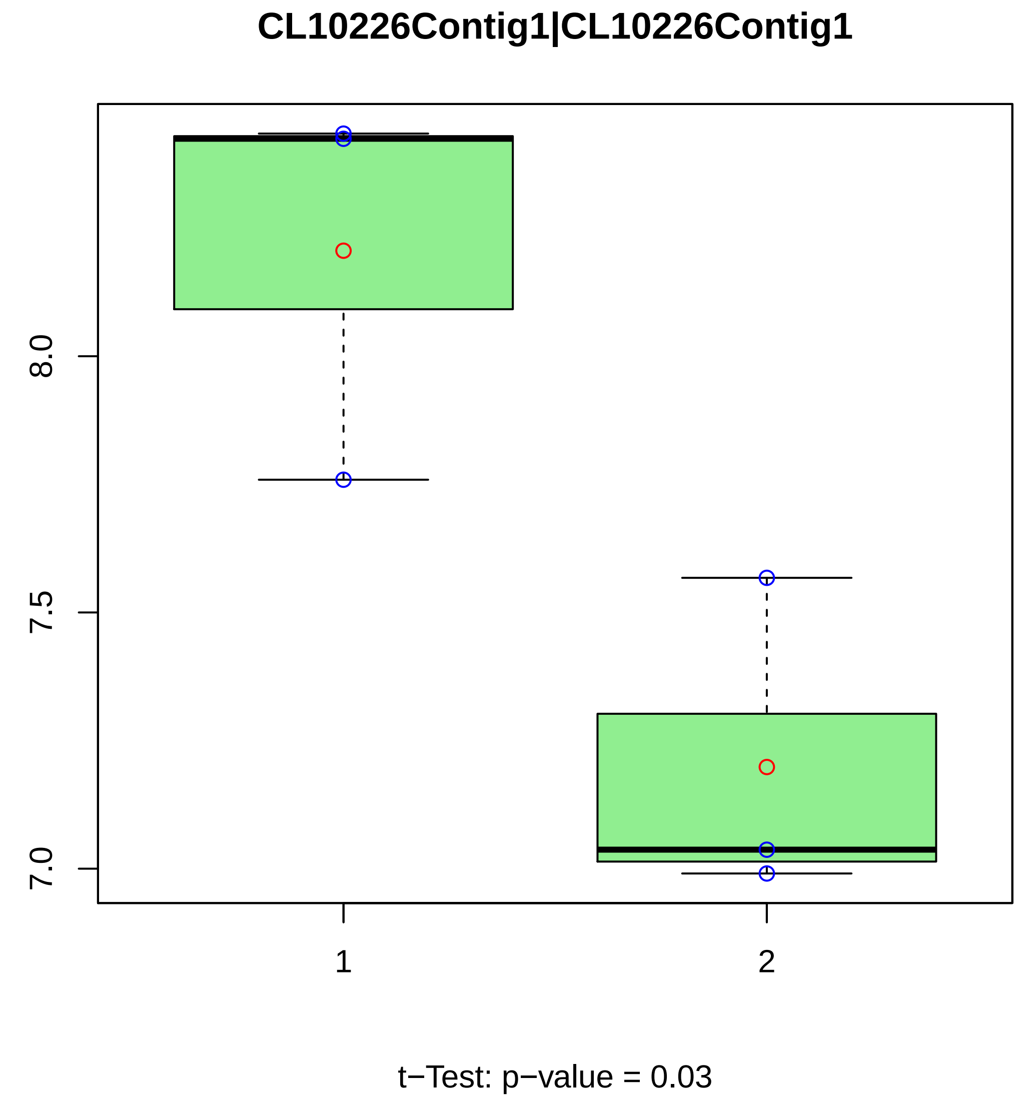
CL10226Contig1|CL10226Contig1
8.0
7.5
7.0
1
2
t−Test: p−value = 0.03

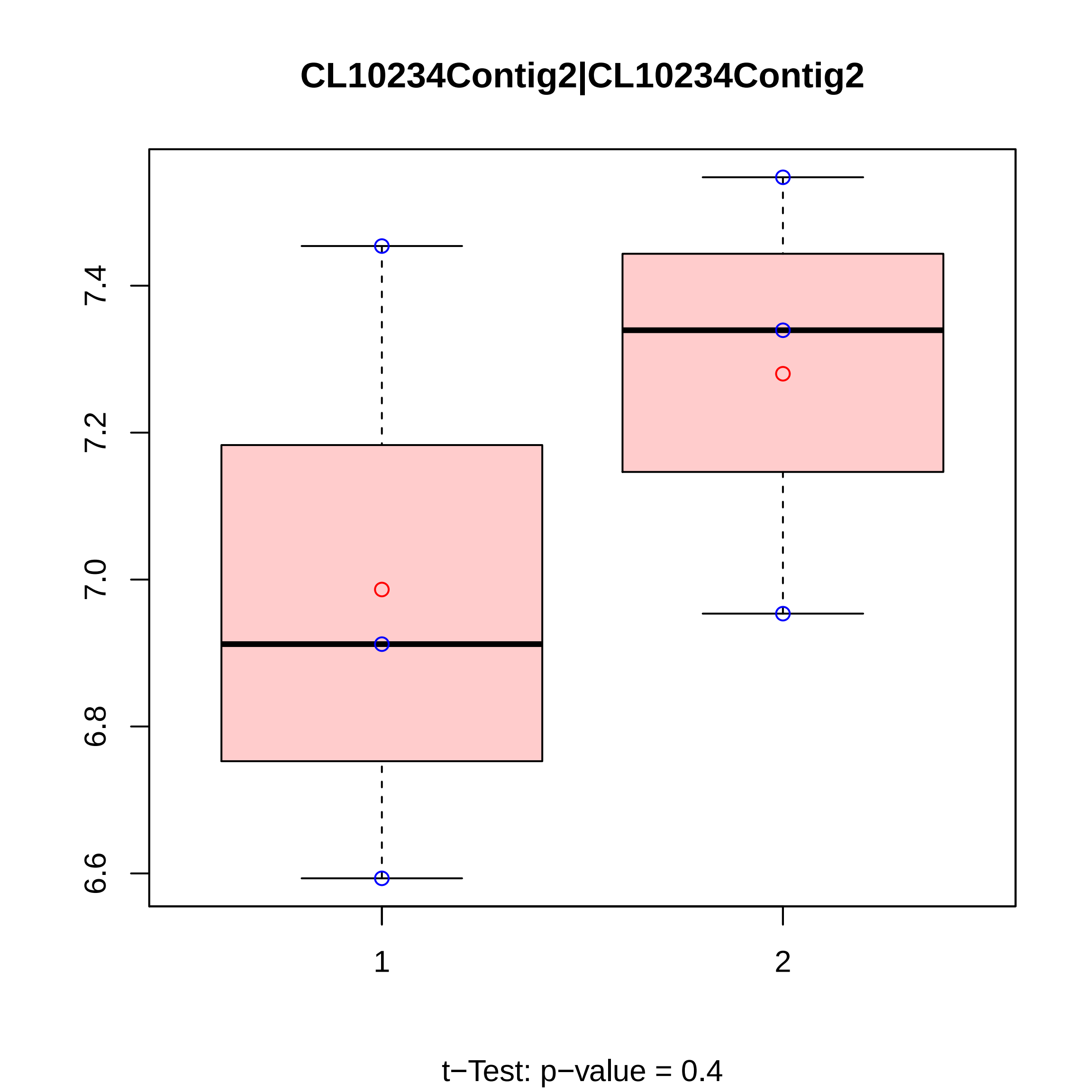
CL10234Contig2|CL10234Contig2
7.4
7.2
7.0
6.8
6.6
1
2
t−Test: p−value = 0.4

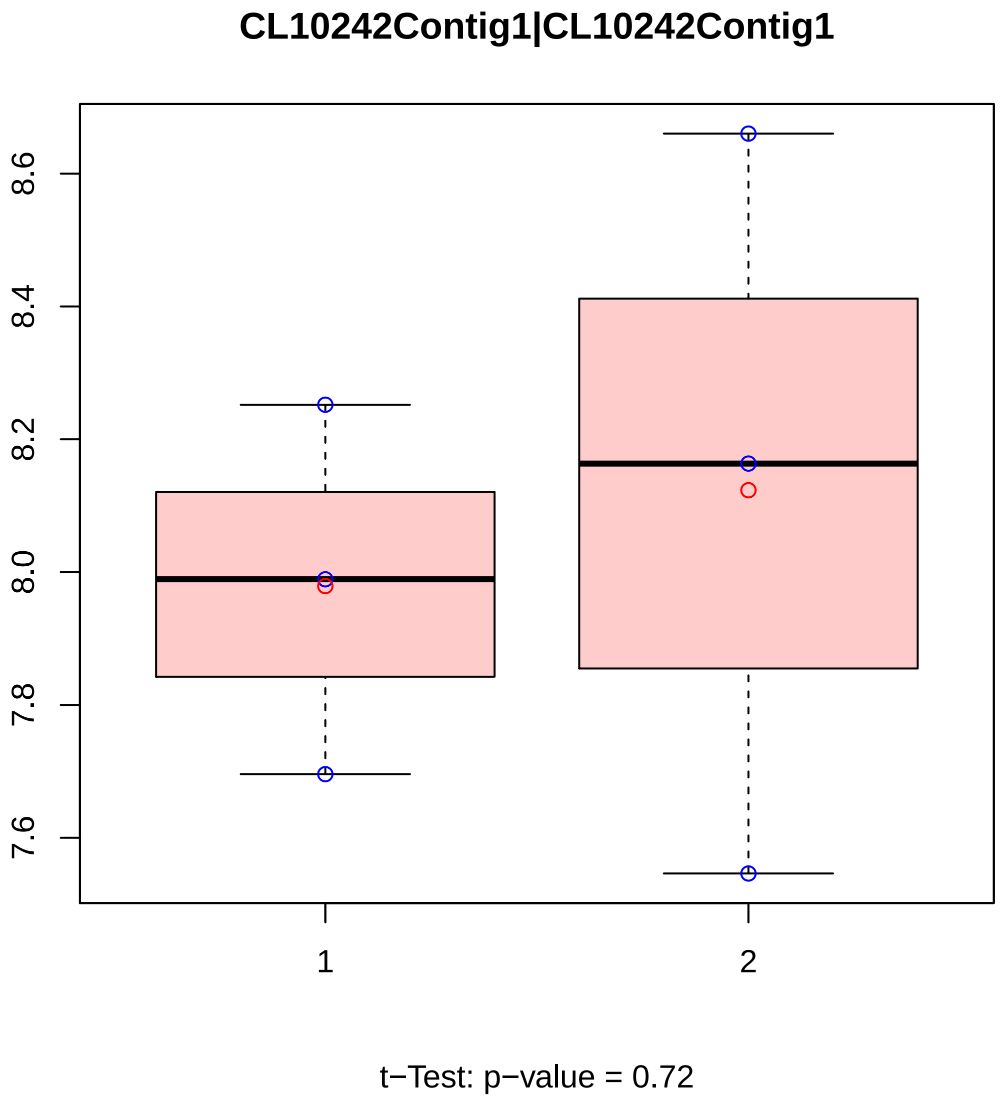
CL10242Contig1|CL10242Contig1
8.6
8.4
8.2
8.0
7.8
7.6
1
2
t−Test: p−value = 0.72

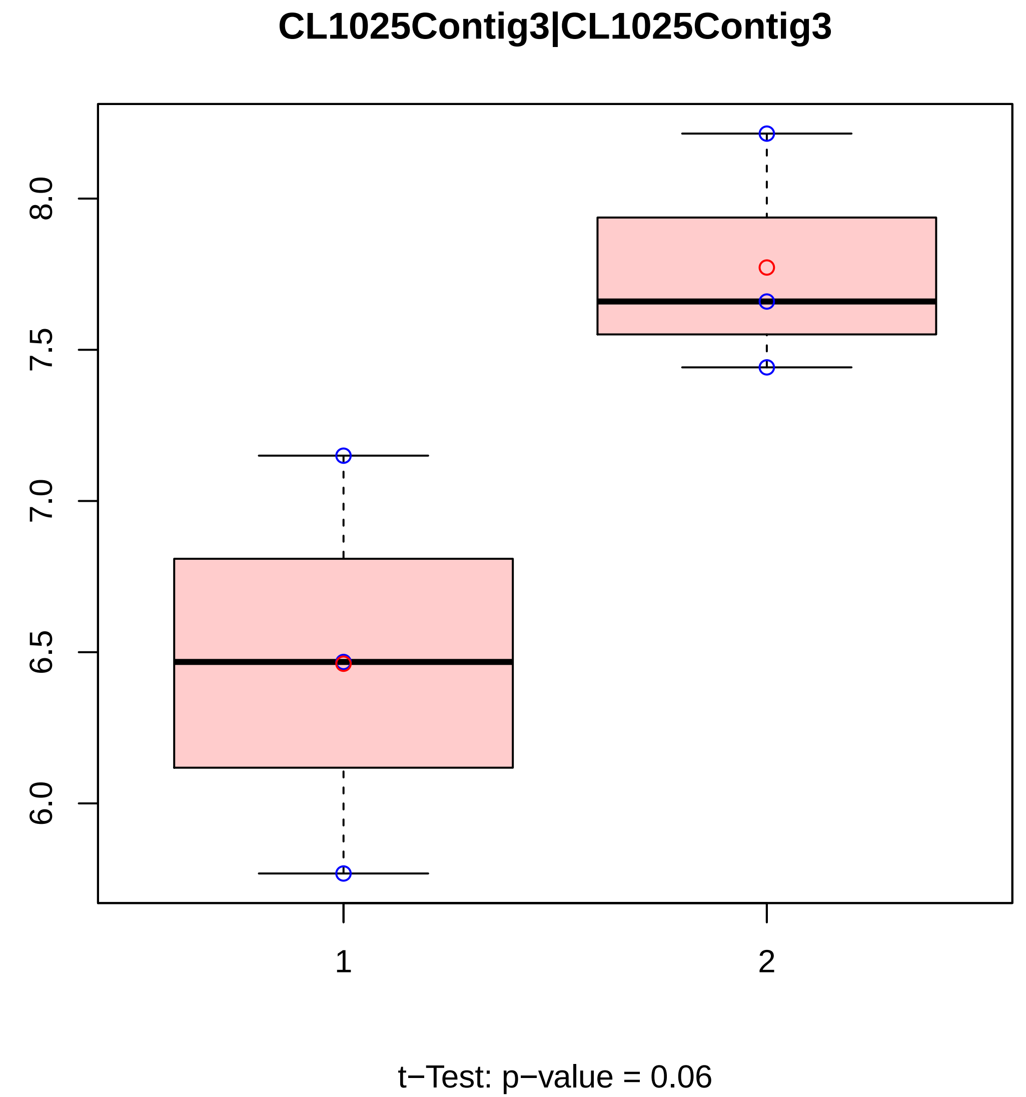
CL1025Contig3|CL1025Contig3
6.0
6.5
7.0
7.5
8.0
1
2
t−Test: p−value = 0.06

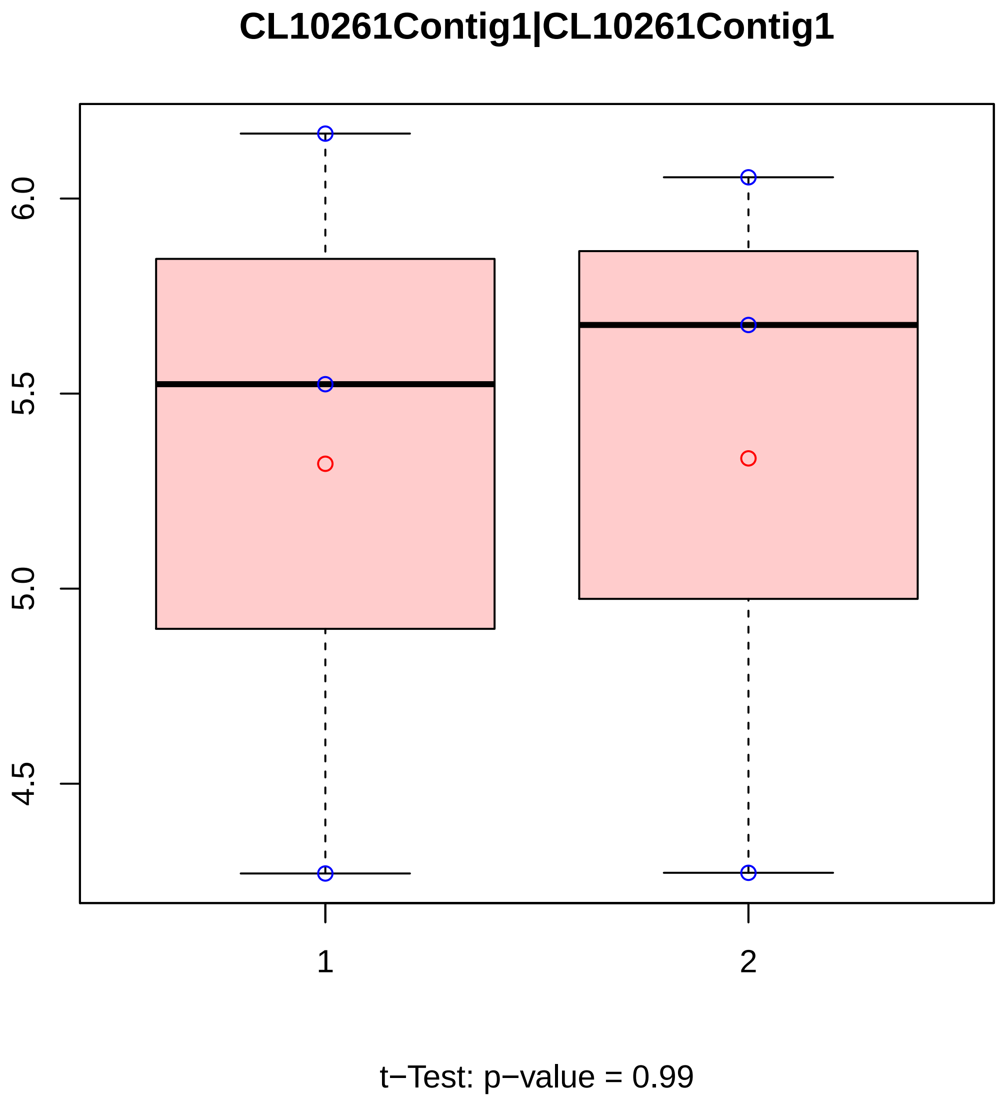
CL10261Contig1|CL10261Contig1
6.0
5.5
5.0
4.5
1
2
t−Test: p−value = 0.99

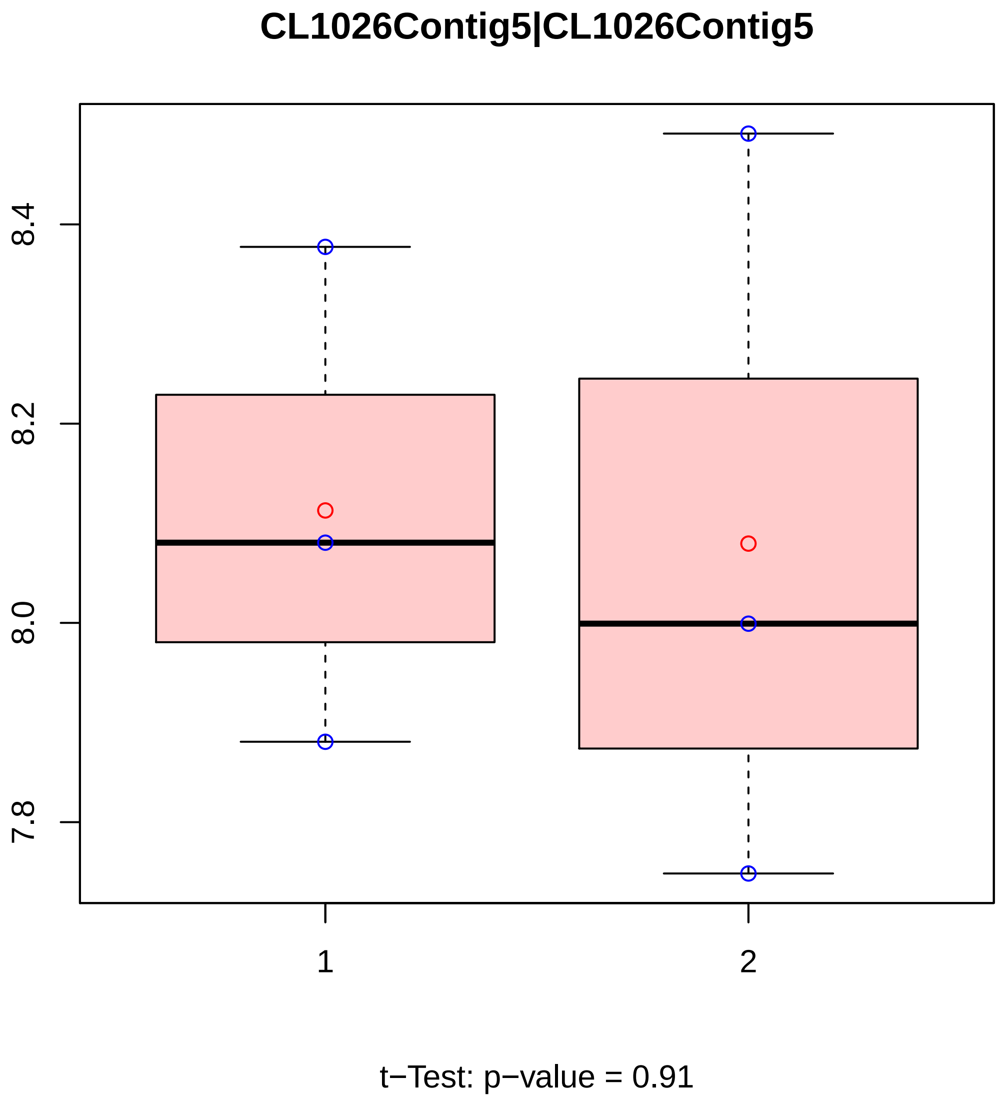
CL1026Contig5|CL1026Contig5
8.4
8.2
8.0
7.8
1
2
t−Test: p−value = 0.91

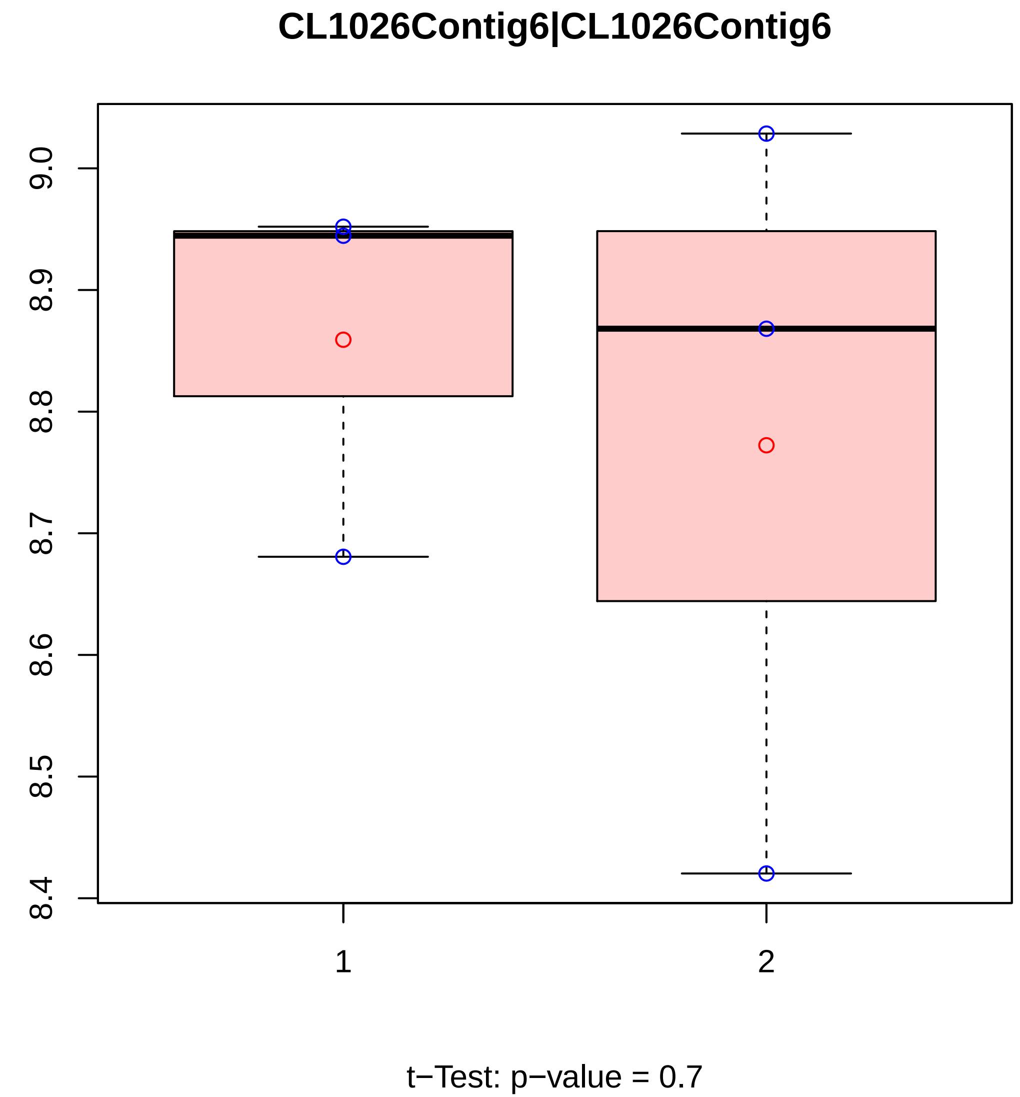

CL1026Contig6|CL1026Contig6
9.0
8.9
8.8
8.7
8.6
8.5
8.4
1
2
t−Test: p−value = 0.7

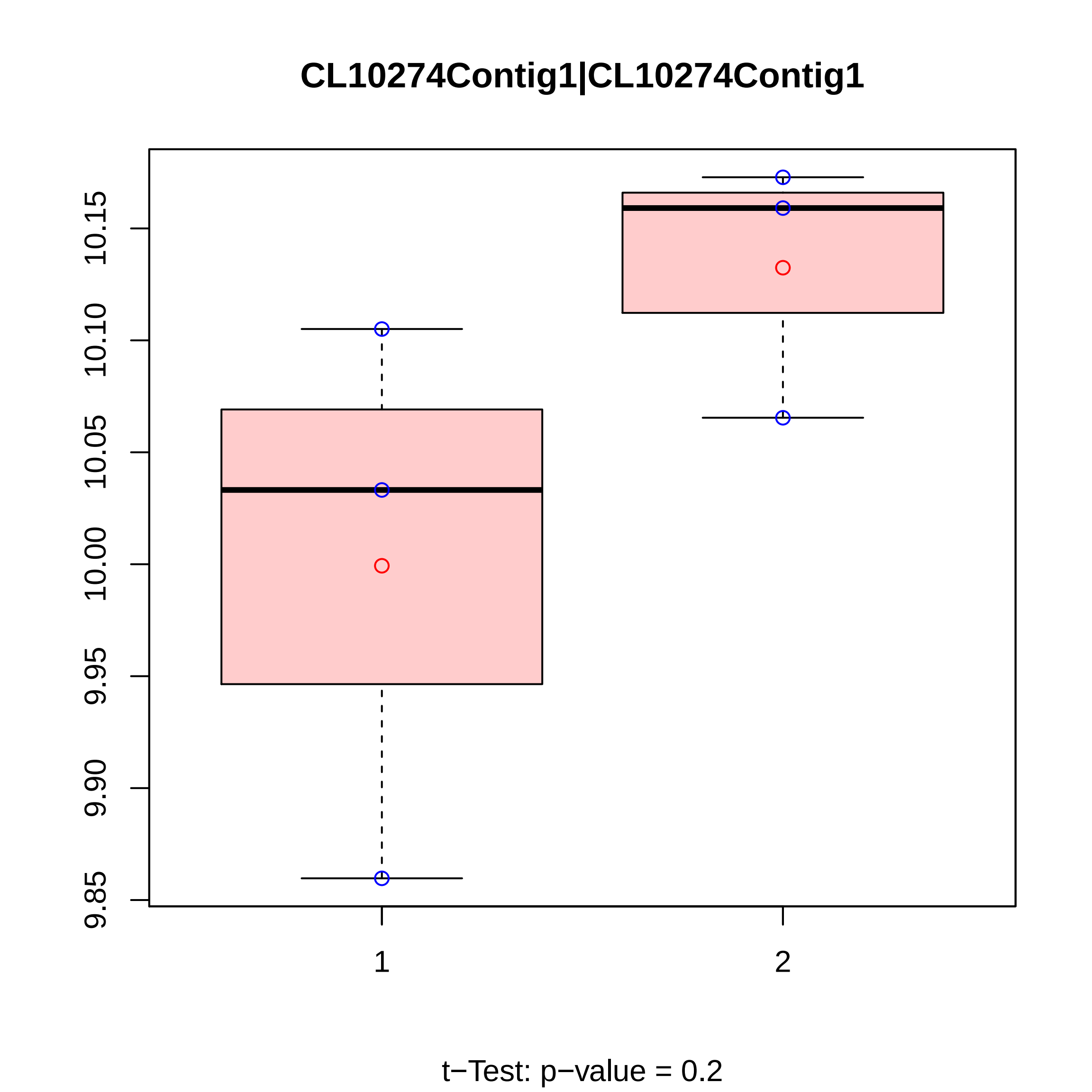
CL10274Contig1|CL10274Contig1
9.85
9.90
9.95
10.00
10.05
10.10
10.15
1
2
t−Test: p−value = 0.2

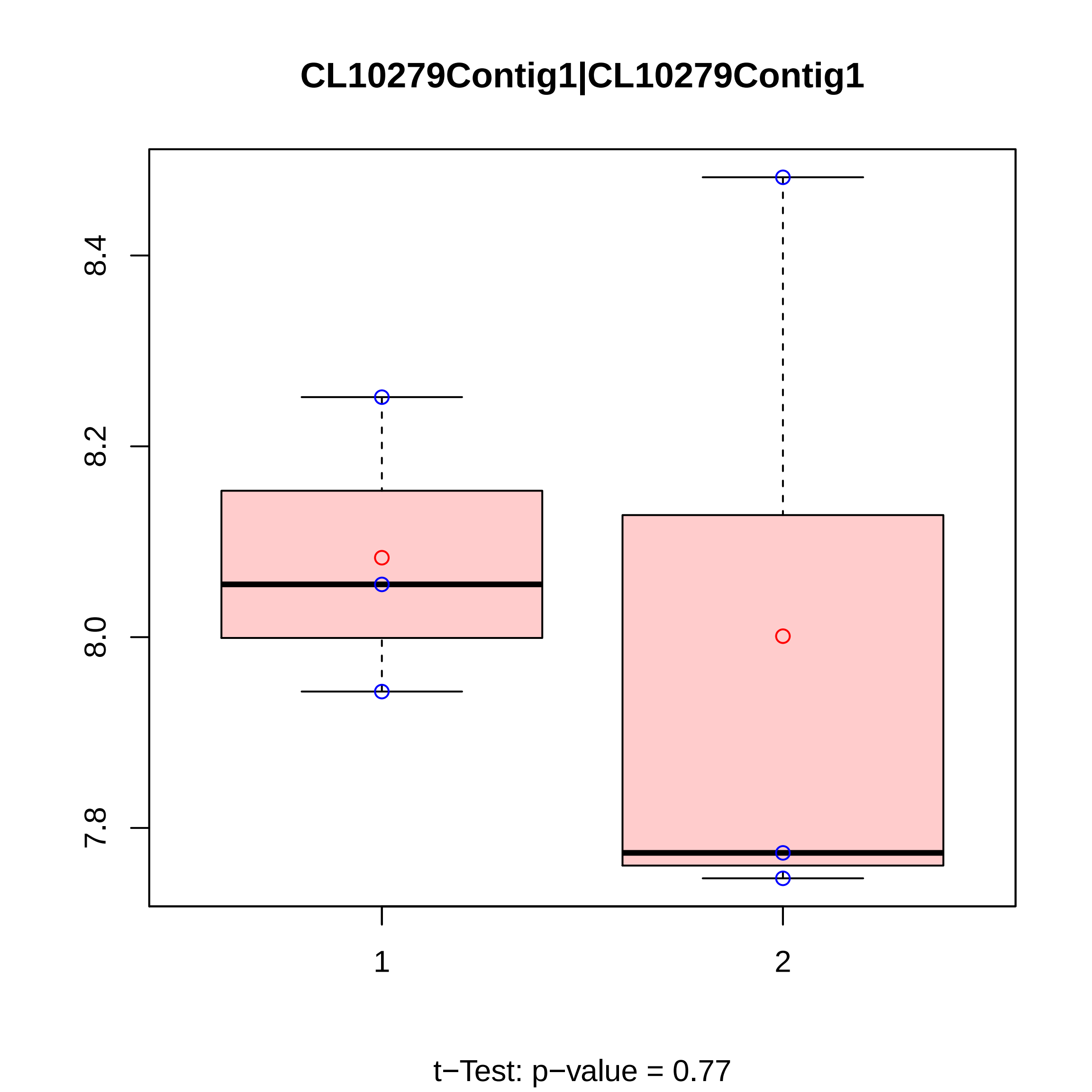

CL10279Contig1|CL10279Contig1
8.4
8.2
8.0
7.8
1
2
t−Test: p−value = 0.77

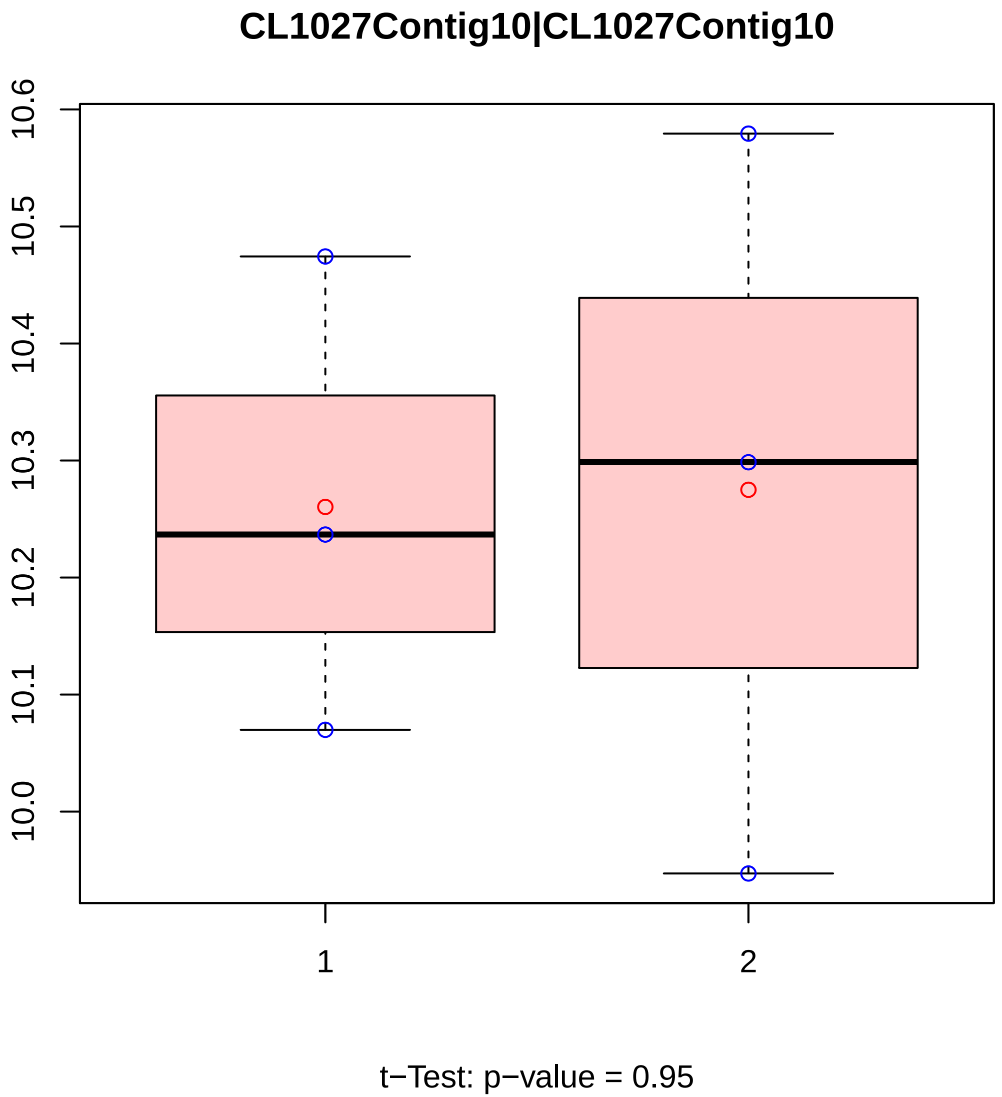
CL1027Contig10|CL1027Contig10
10.0
10.1
10.2
10.3
10.4
10.5
10.6
1
2
t−Test: p−value = 0.95

## CL1027Contig3|CL1027Contig3

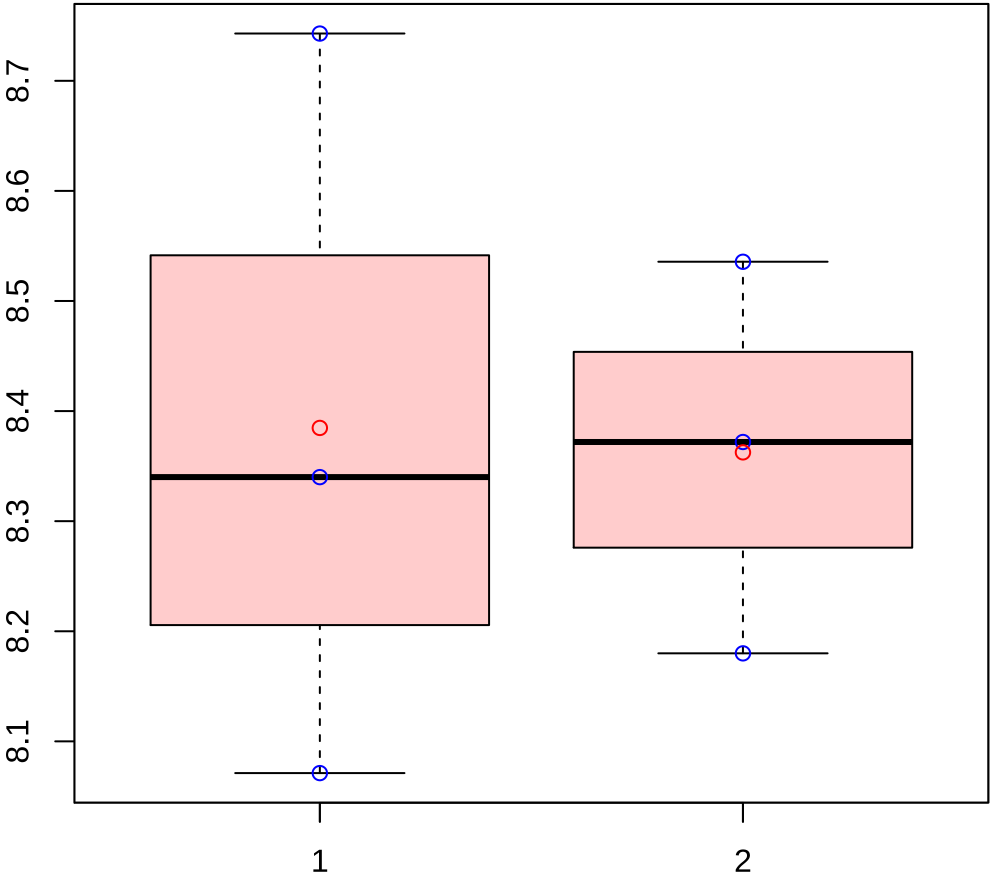


t−Test: p−value = 0.93

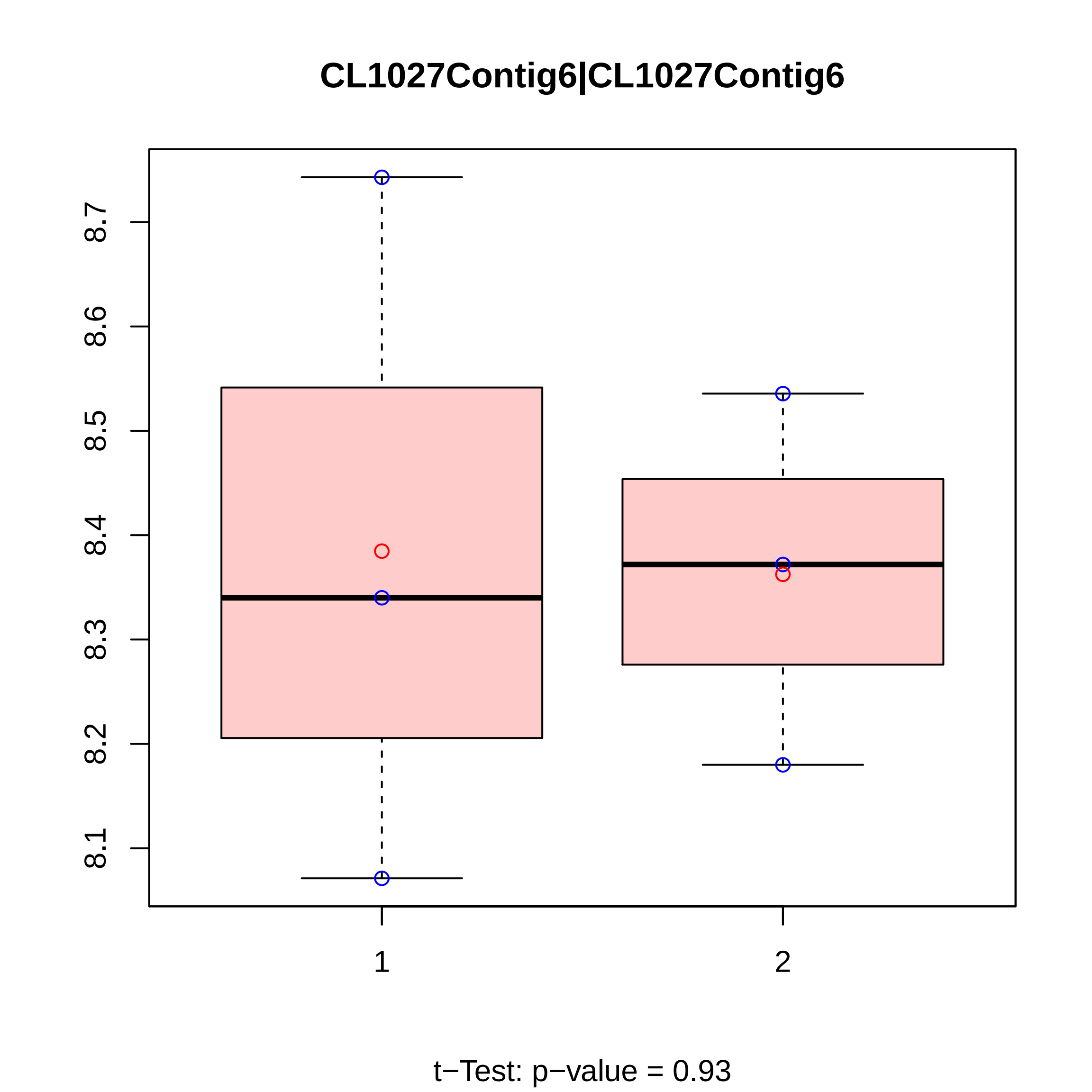

CL1027Contig6|CL1027Contig6
8.1
8.2
8.3
8.4
8.5
8.6
8.7
1
2
t−Test: p−value = 0.93

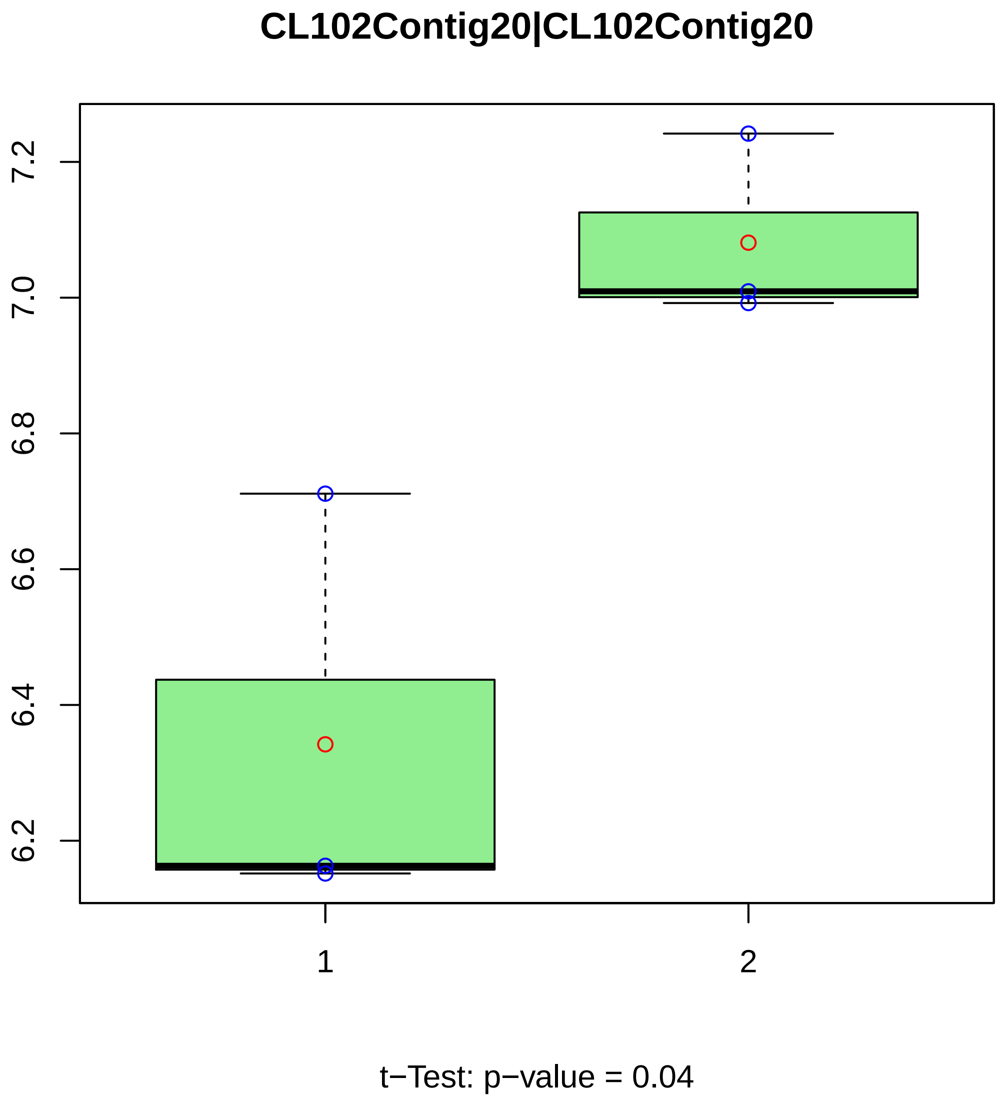

CL102Contig20|CL102Contig20
7.2
7.0
6.8
6.6
6.4
6.2
1
2
t−Test: p−value = 0.04

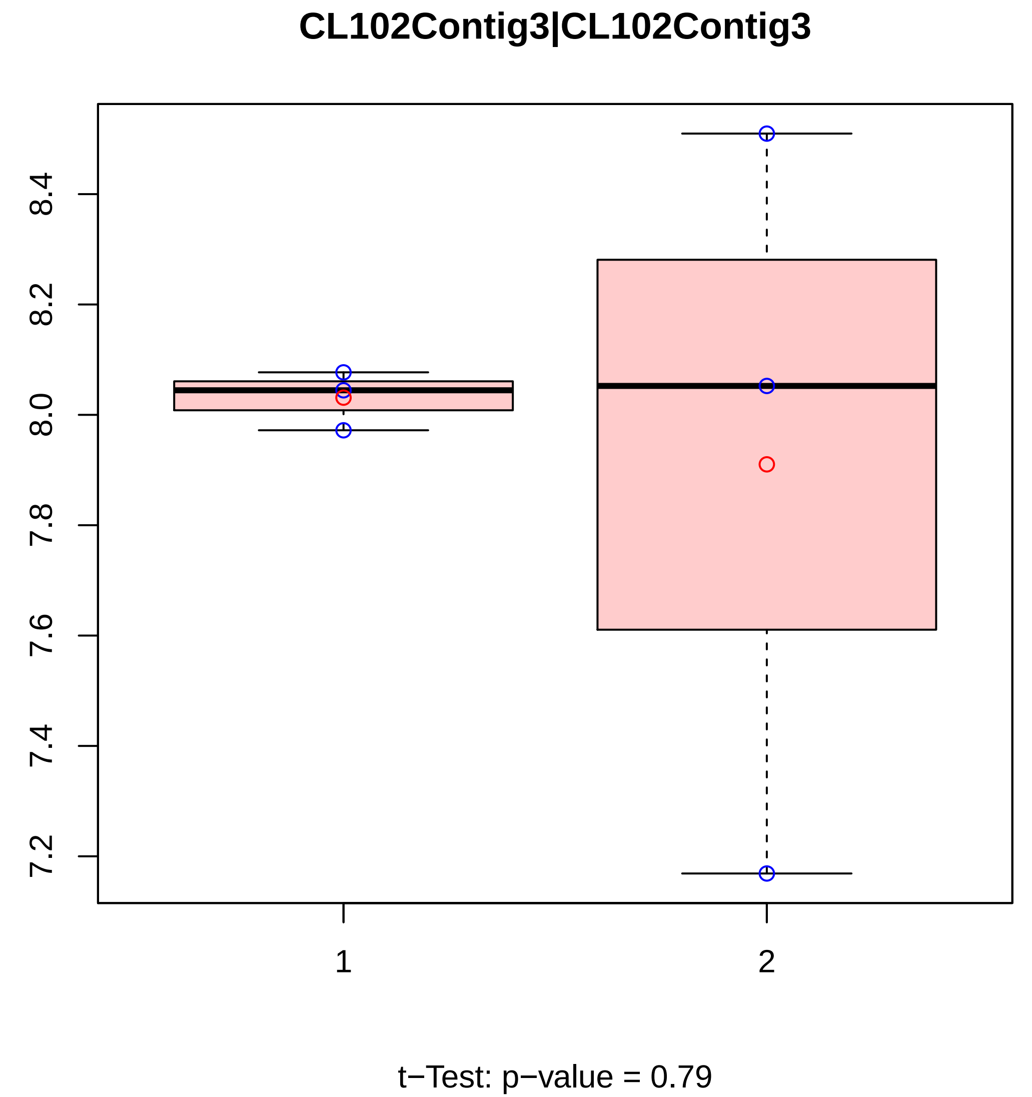
CL102Contig3|CL102Contig3
8.4
8.2
8.0
7.8
7.6
7.4
7.2
1
2
t−Test: p−value = 0.79

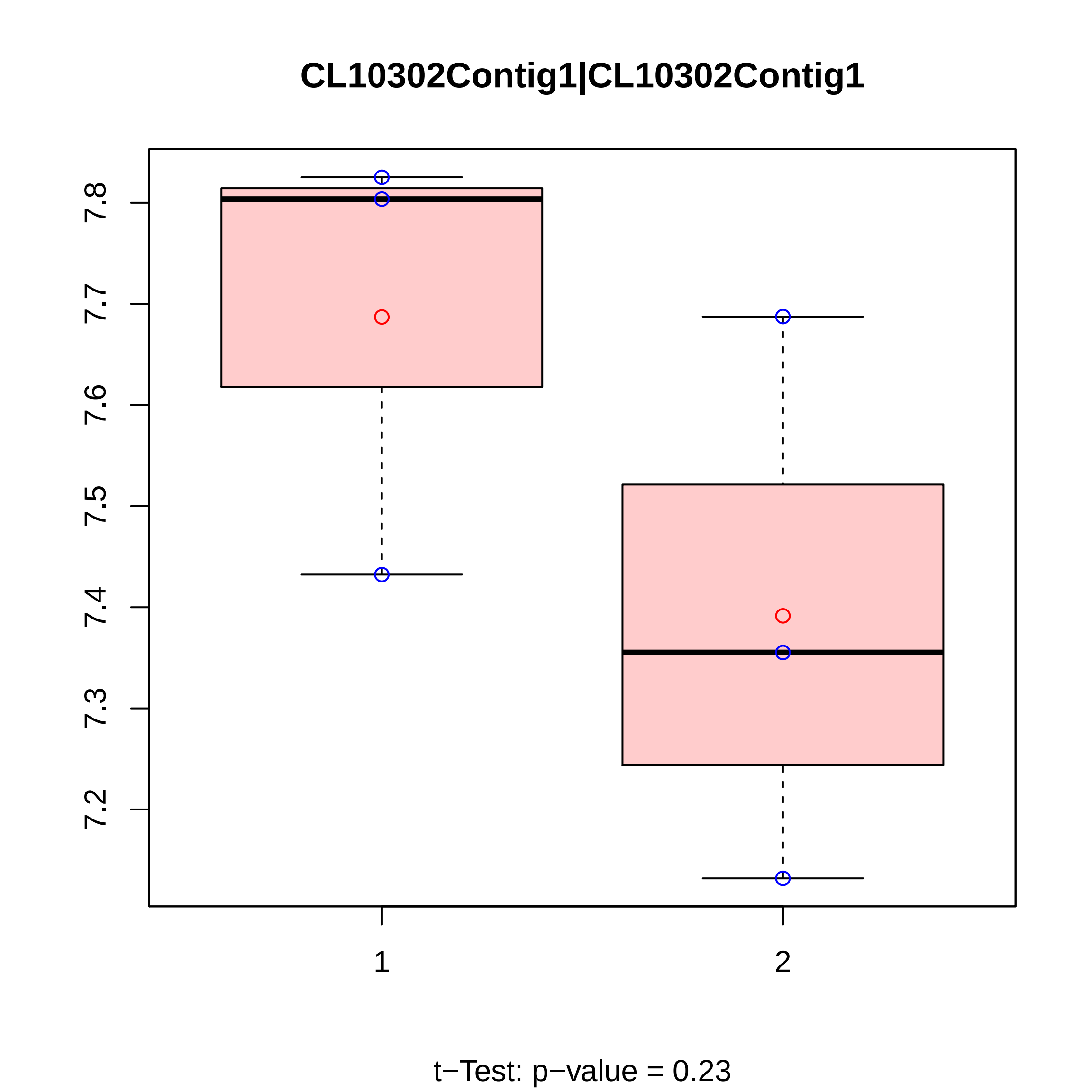
CL10302Contig1|CL10302Contig1
7.2
7.3
7.4
7.5
7.6
7.7
7.8
1
2
t−Test: p−value = 0.23

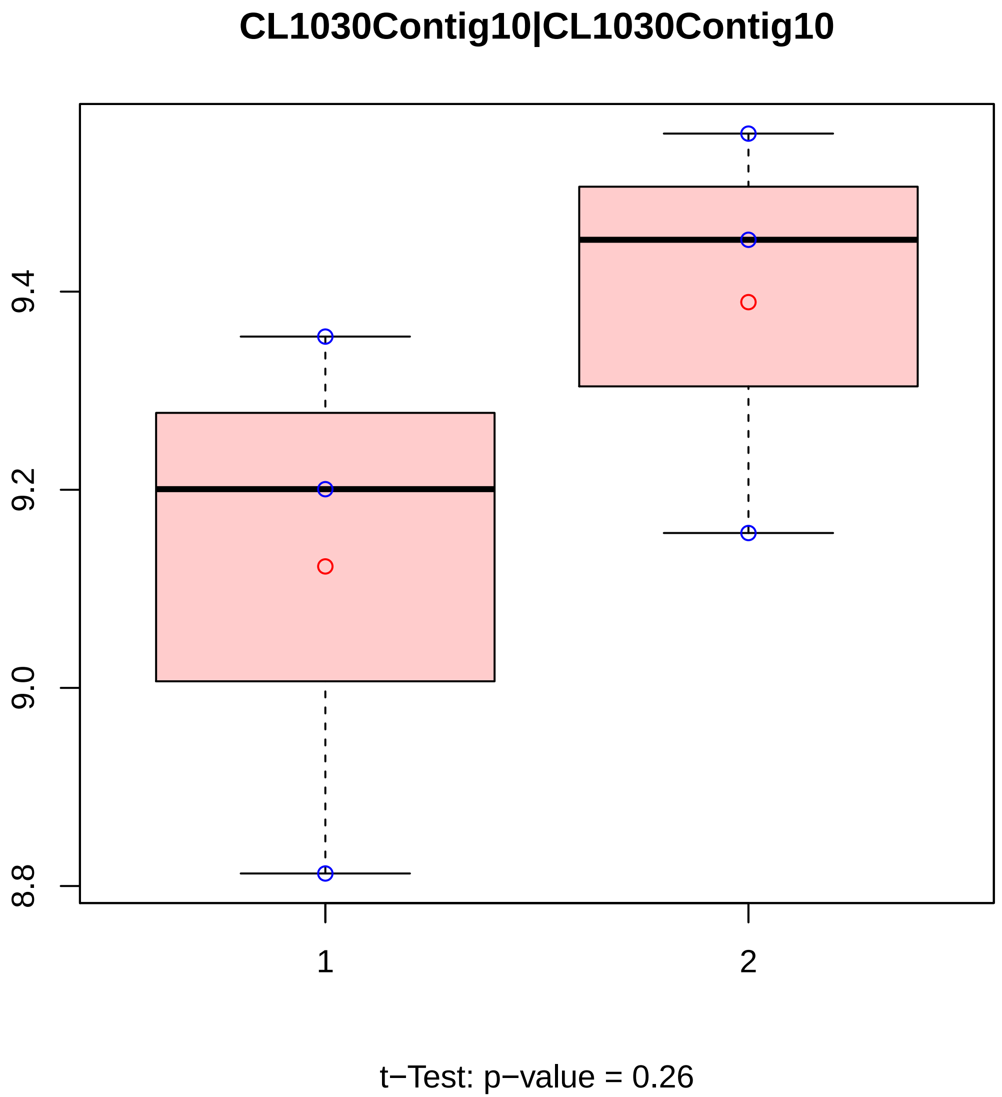
CL1030Contig10|CL1030Contig10
9.4
9.2
9.0
8.8
1
2
t−Test: p−value = 0.26

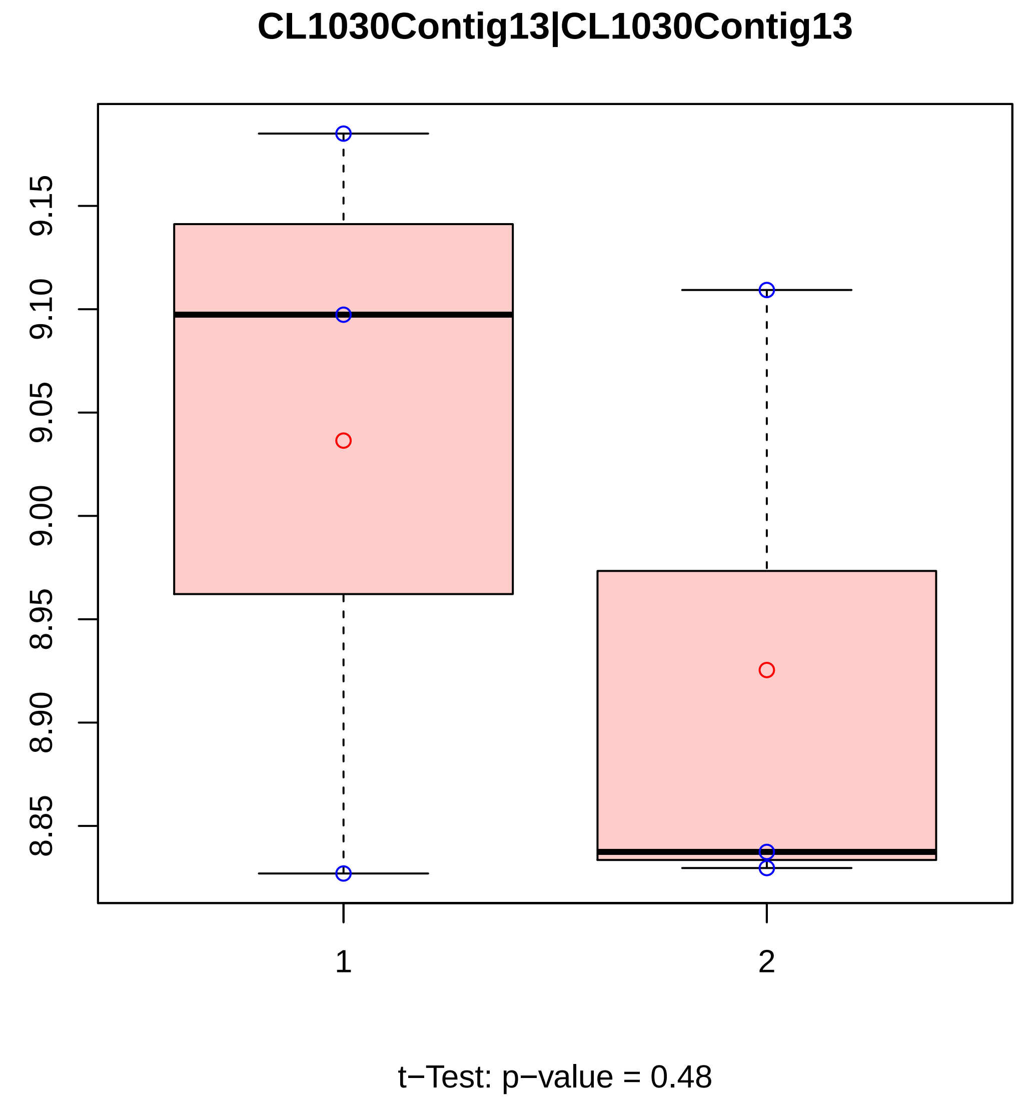

CL1030Contig13|CL1030Contig13
9.15
9.10
9.05
9.00
8.95
8.90
8.85
1
2
t−Test: p−value = 0.48

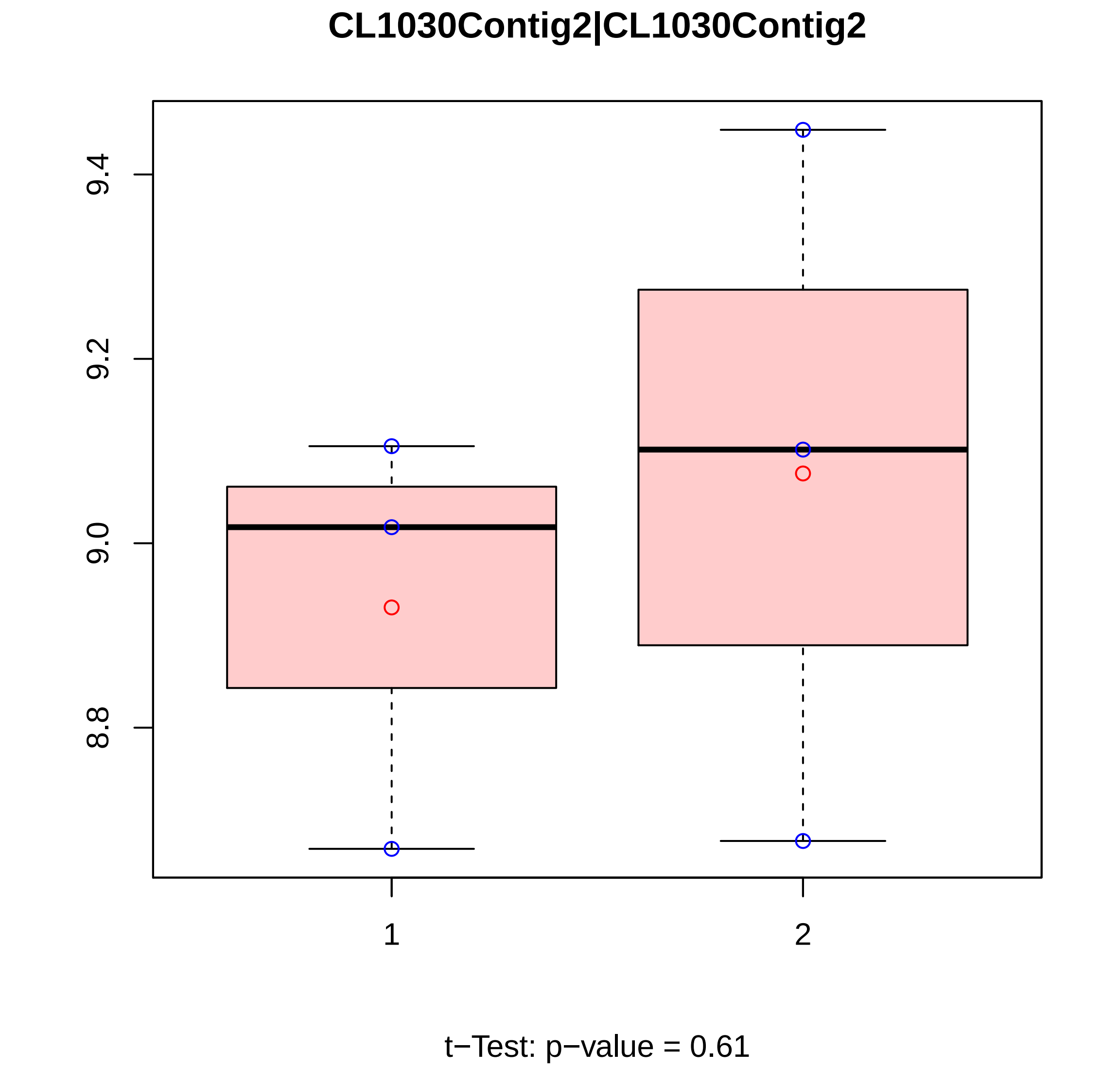
CL1030Contig2|CL1030Contig2
9.4
9.2
9.0
8.8
1
2
t−Test: p−value = 0.61

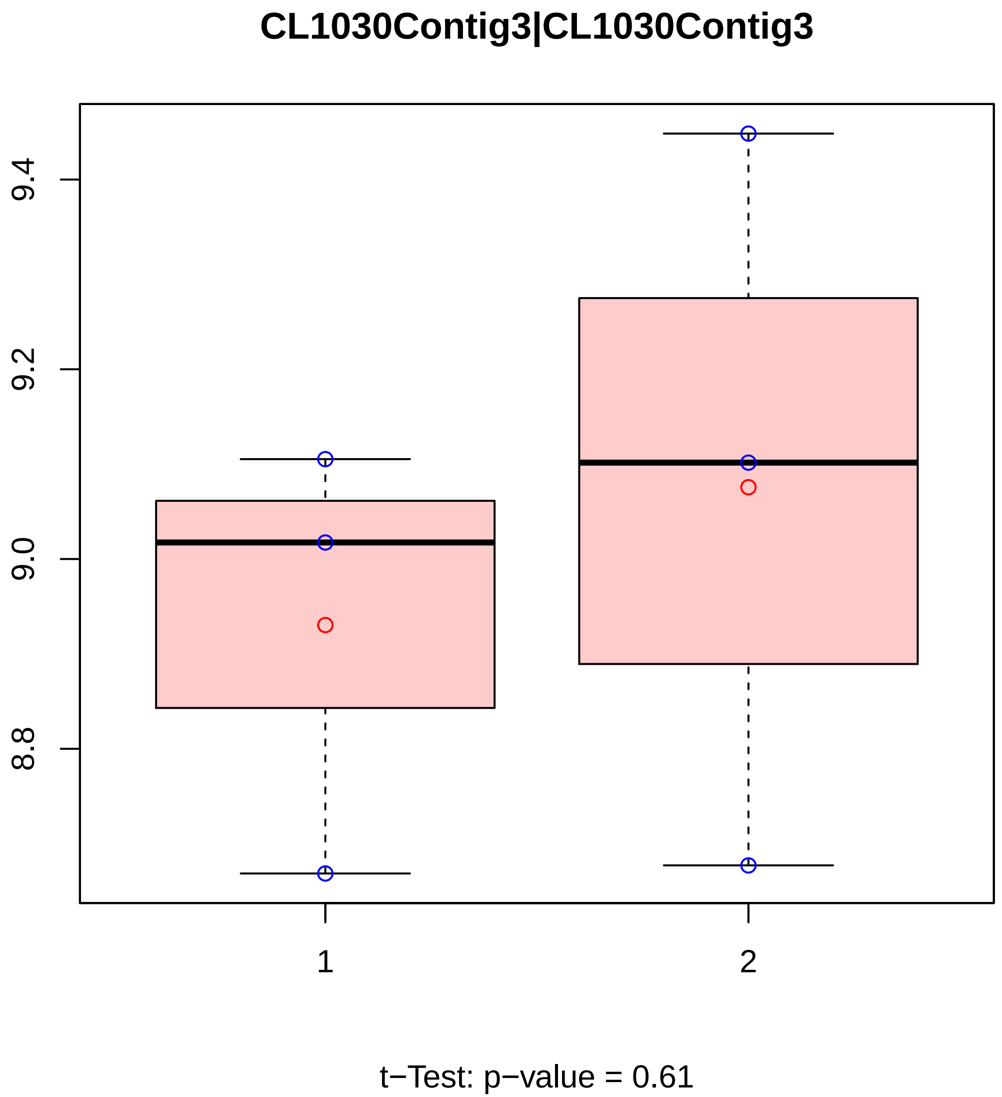
CL1030Contig3|CL1030Contig3
9.4
9.2
9.0
8.8
1
2
t−Test: p−value = 0.61

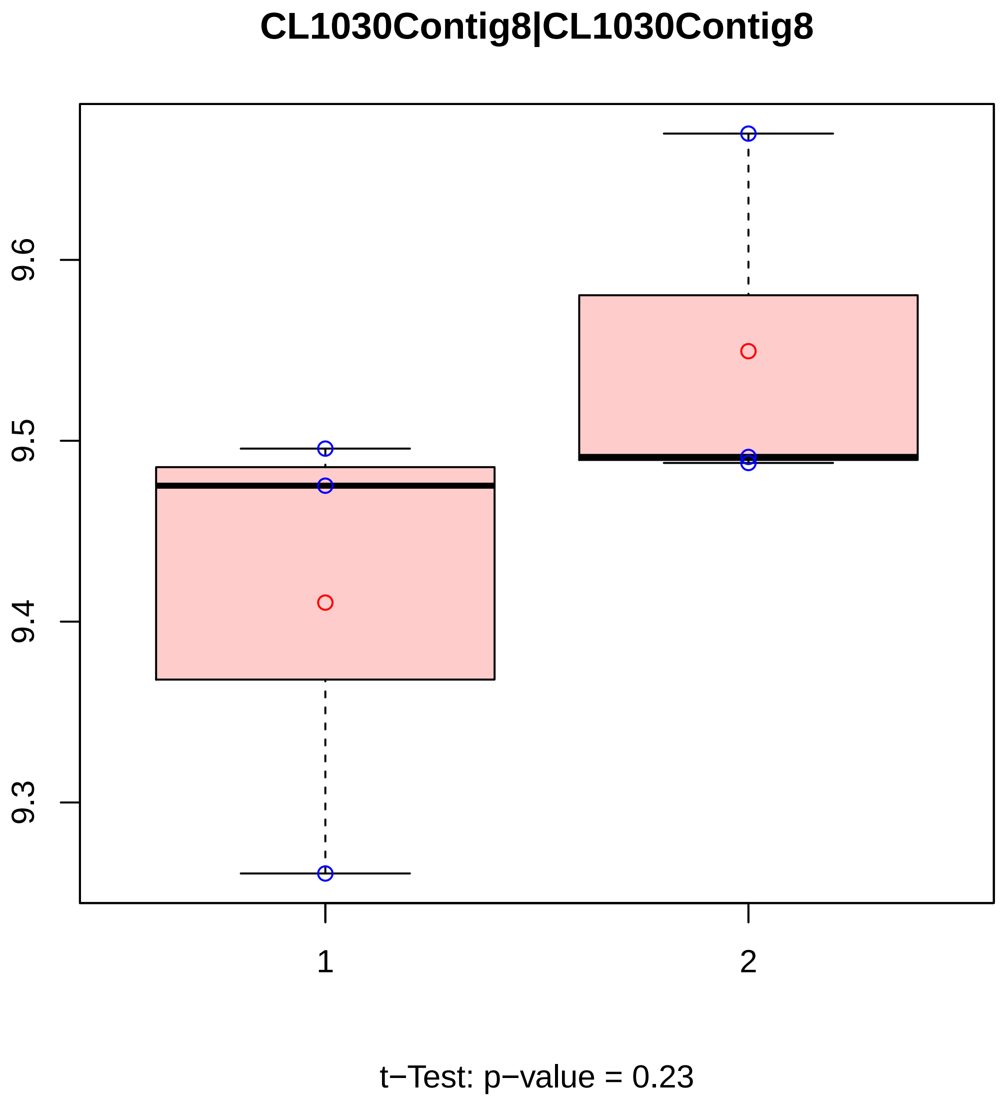
CL1030Contig8|CL1030Contig8
9.3
9.4
9.5
9.6
1
2
t−Test: p−value = 0.23

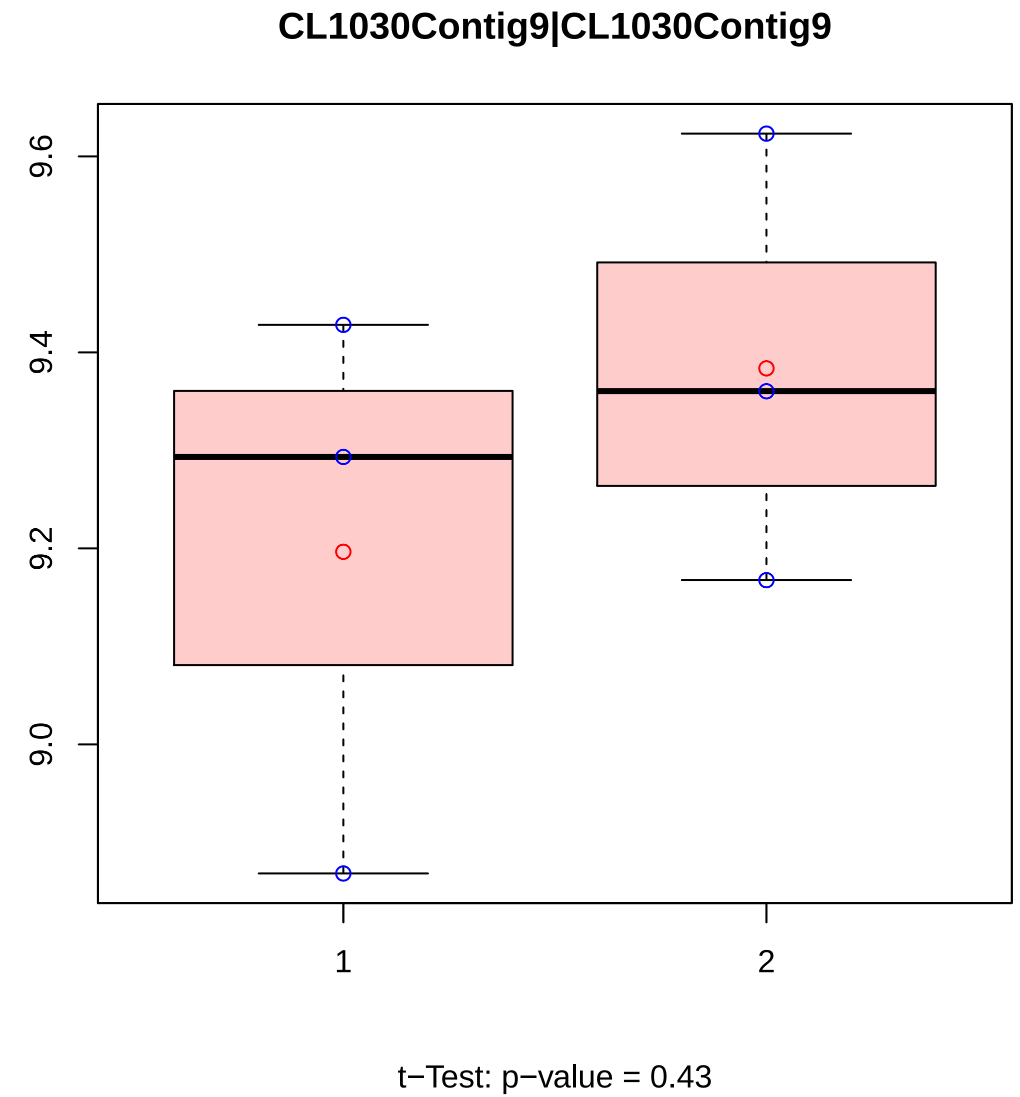
CL1030Contig9|CL1030Contig9
9.6
9.4
9.2
9.0
1
2
t−Test: p−value = 0.43

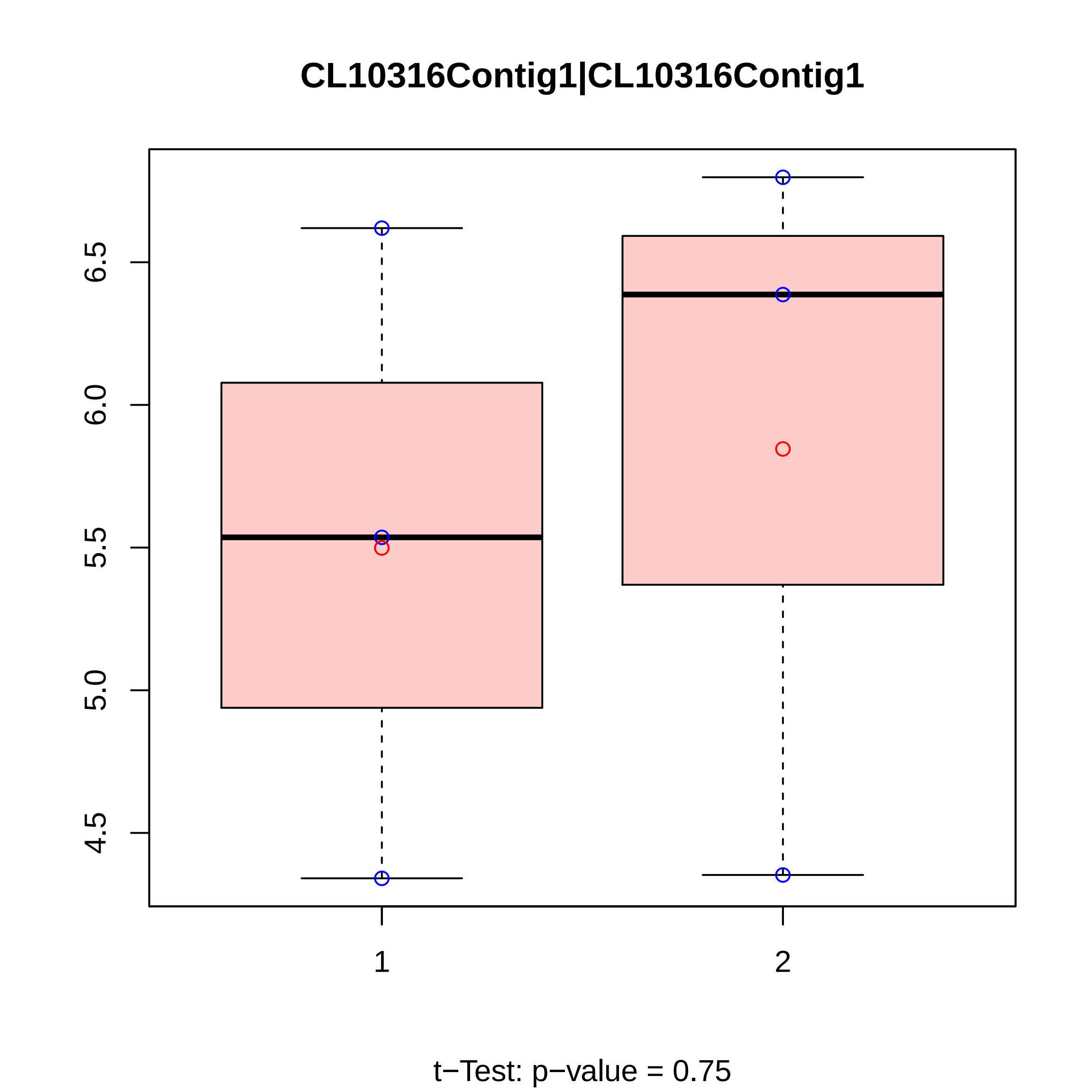
CL10316Contig1|CL10316Contig1
6.5
6.0
5.5
5.0
4.5
1
2
t−Test: p−value = 0.75

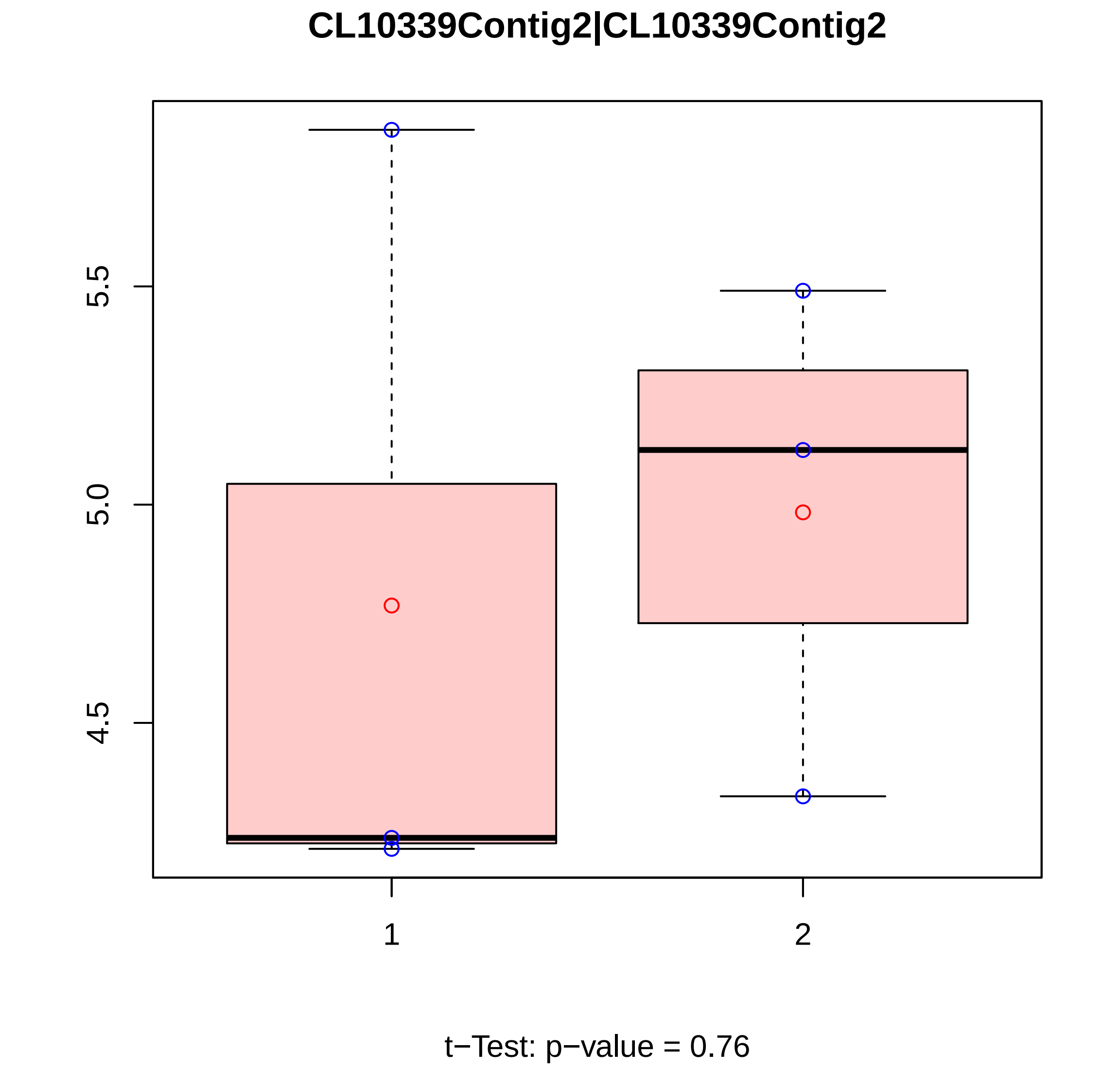

CL10339Contig2|CL10339Contig2
5.5
5.0
4.5
1
2
t−Test: p−value = 0.76

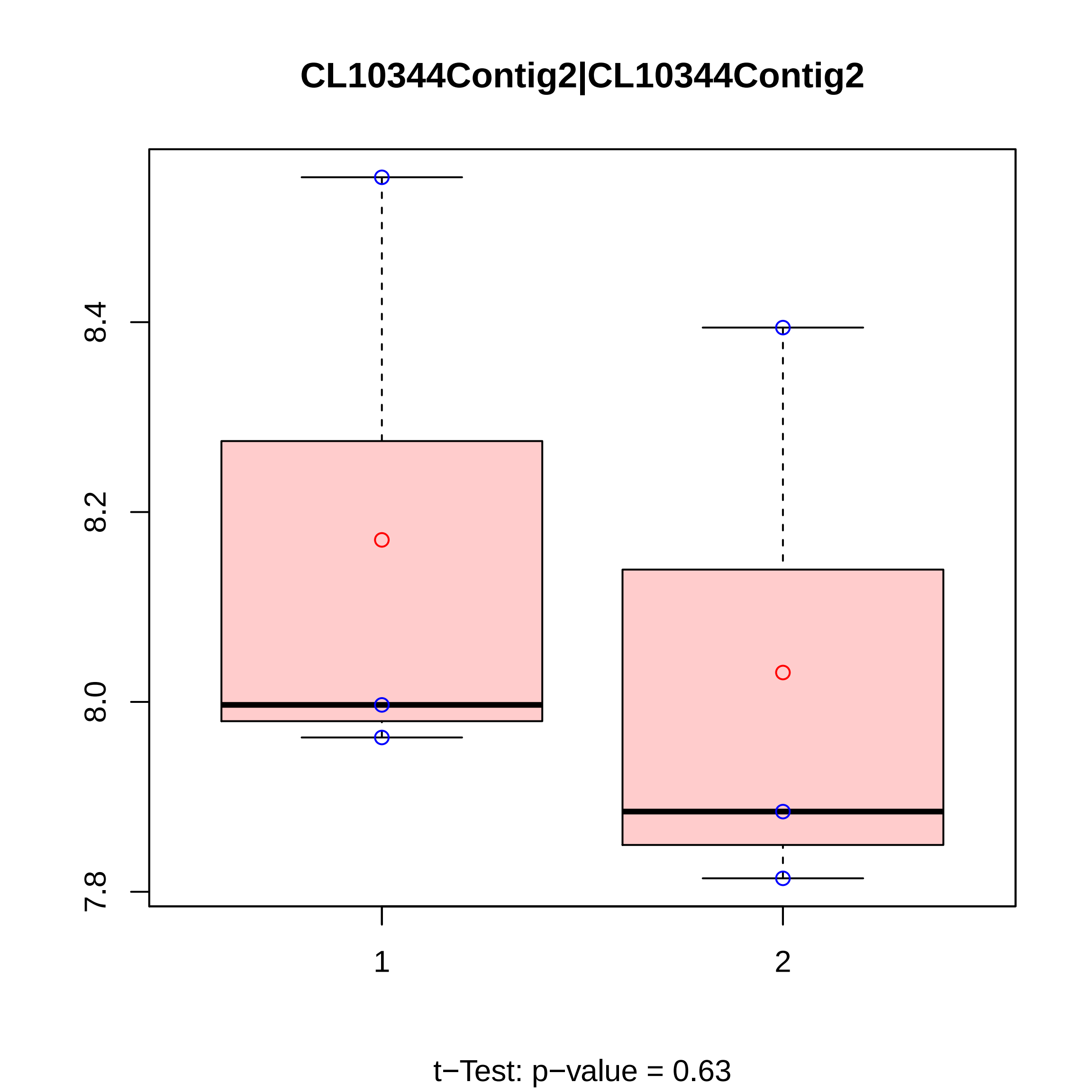
CL10344Contig2|CL10344Contig2
8.4
8.2
8.0
7.8
1
2
t−Test: p−value = 0.63

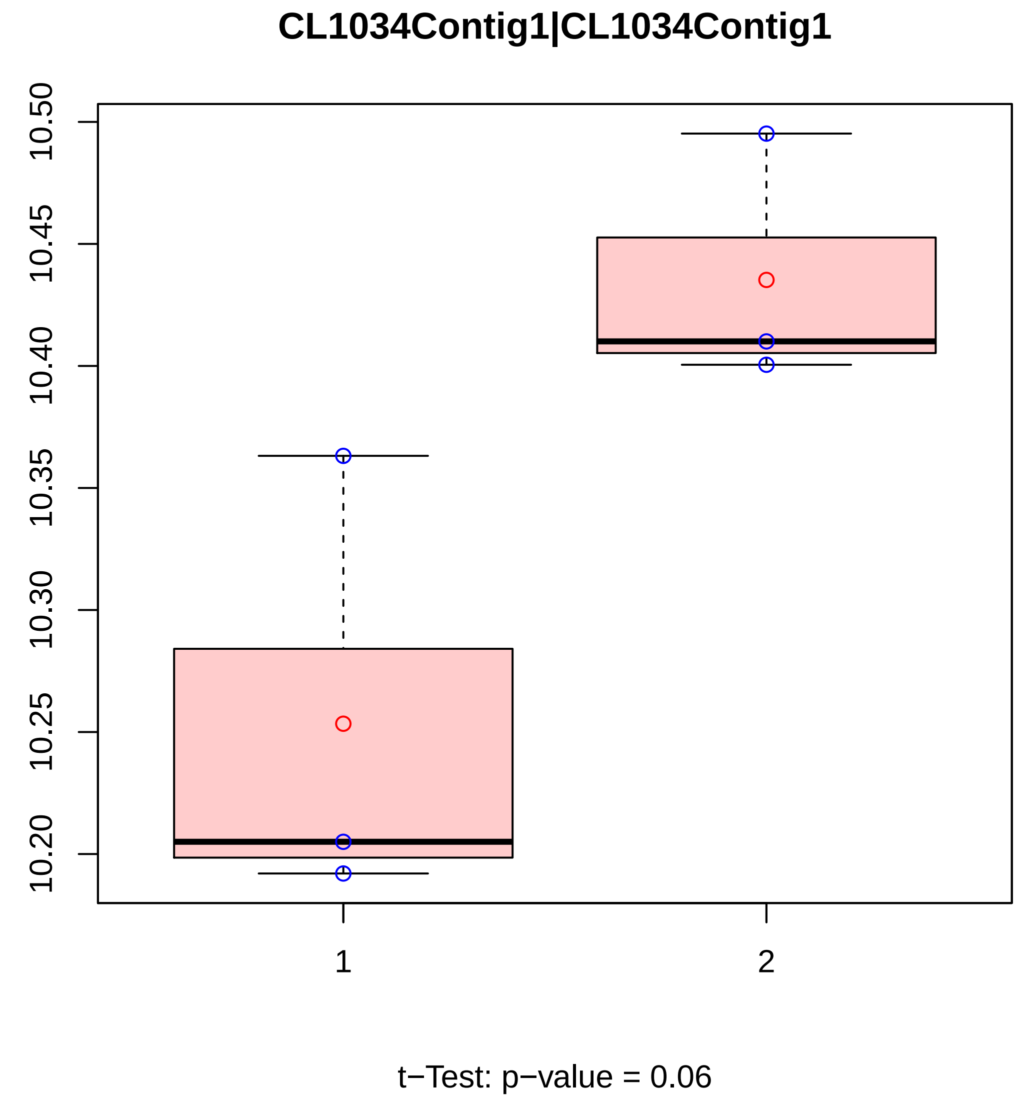
CL1034Contig1|CL1034Contig1
10.20
10.25
10.30
10.35
10.40
10.45
10.50
1
2
t−Test: p−value = 0.06

**CL1034Contig2|CL1034Contig2**

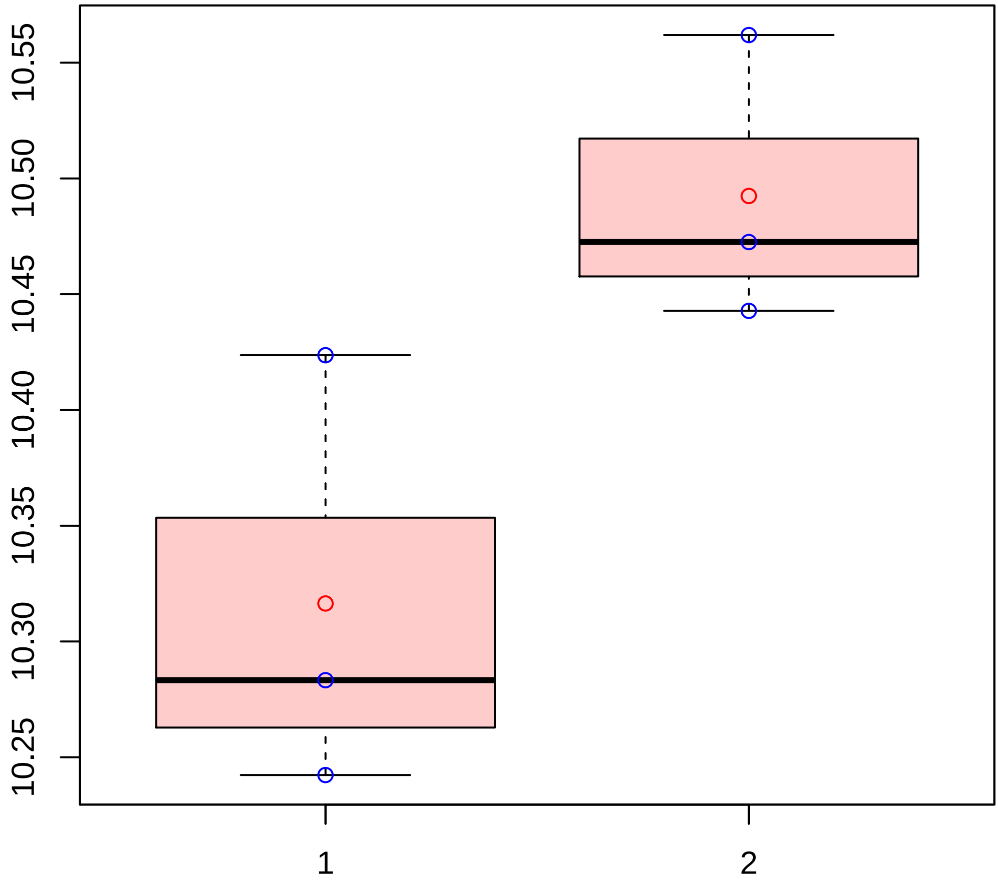


t−Test: p−value = 0.06

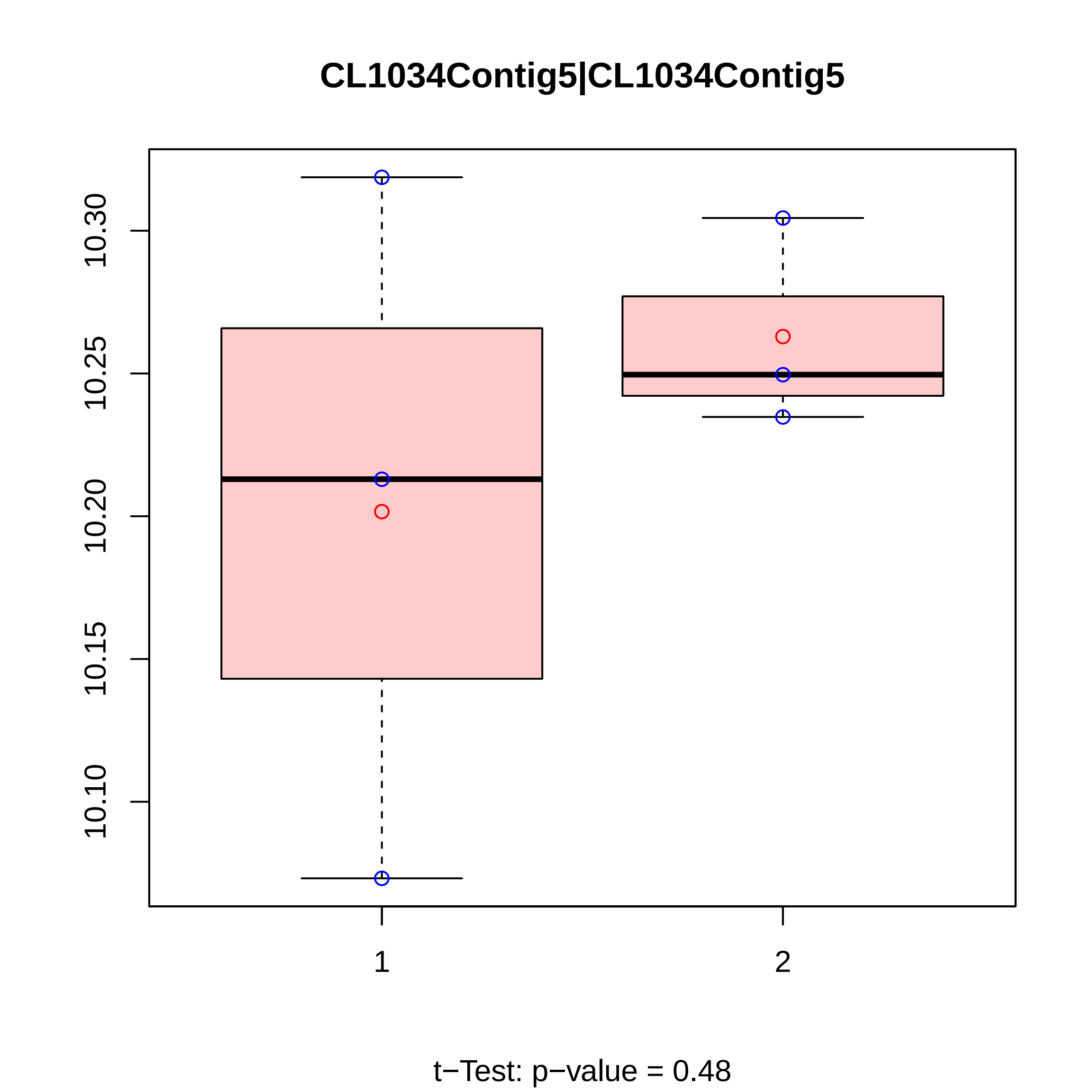
CL1034Contig5|CL1034Contig5
10.10
10.15
10.20
10.25
10.30
1
2
t−Test: p−value = 0.48

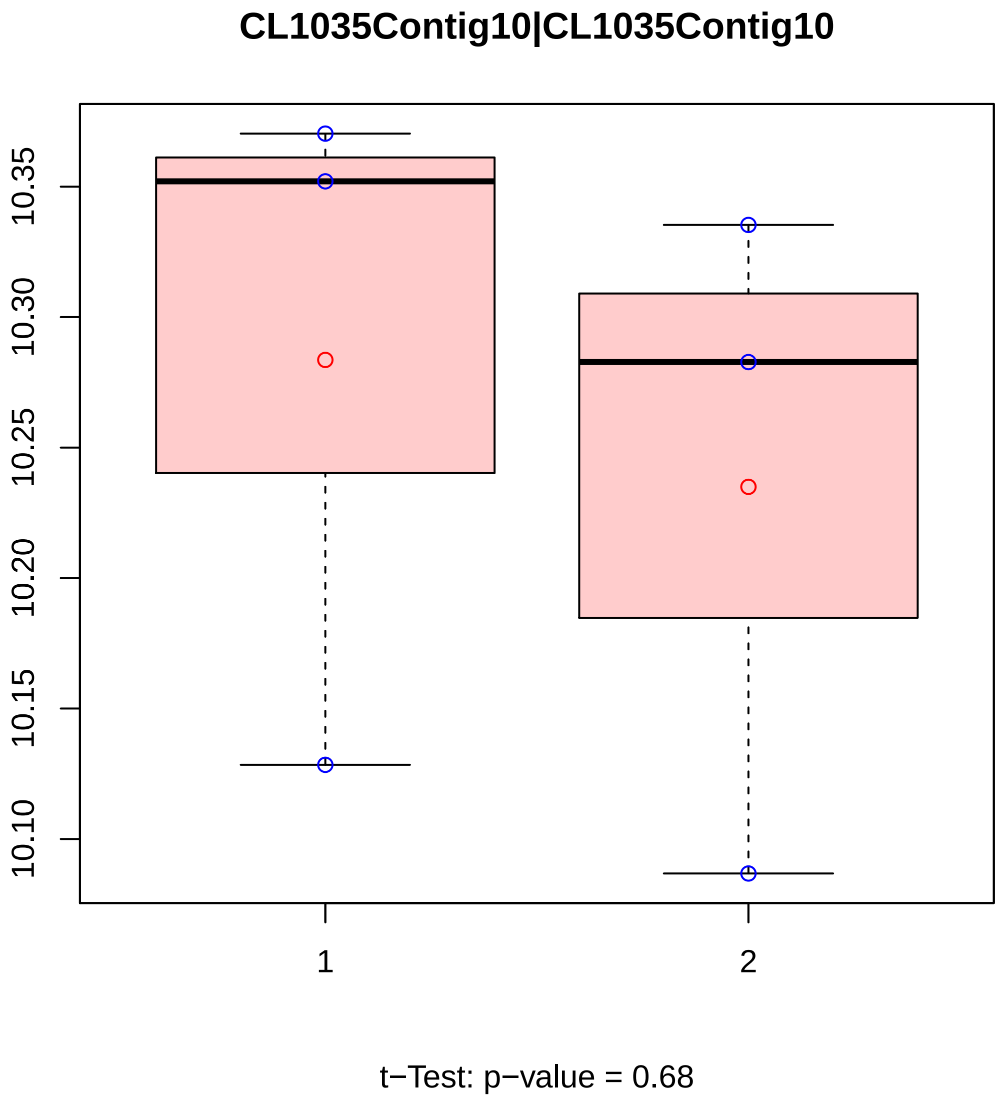
CL1035Contig10|CL1035Contig10
10.35
10.30
10.25
10.20
10.15
10.10
1
2
t−Test: p−value = 0.68

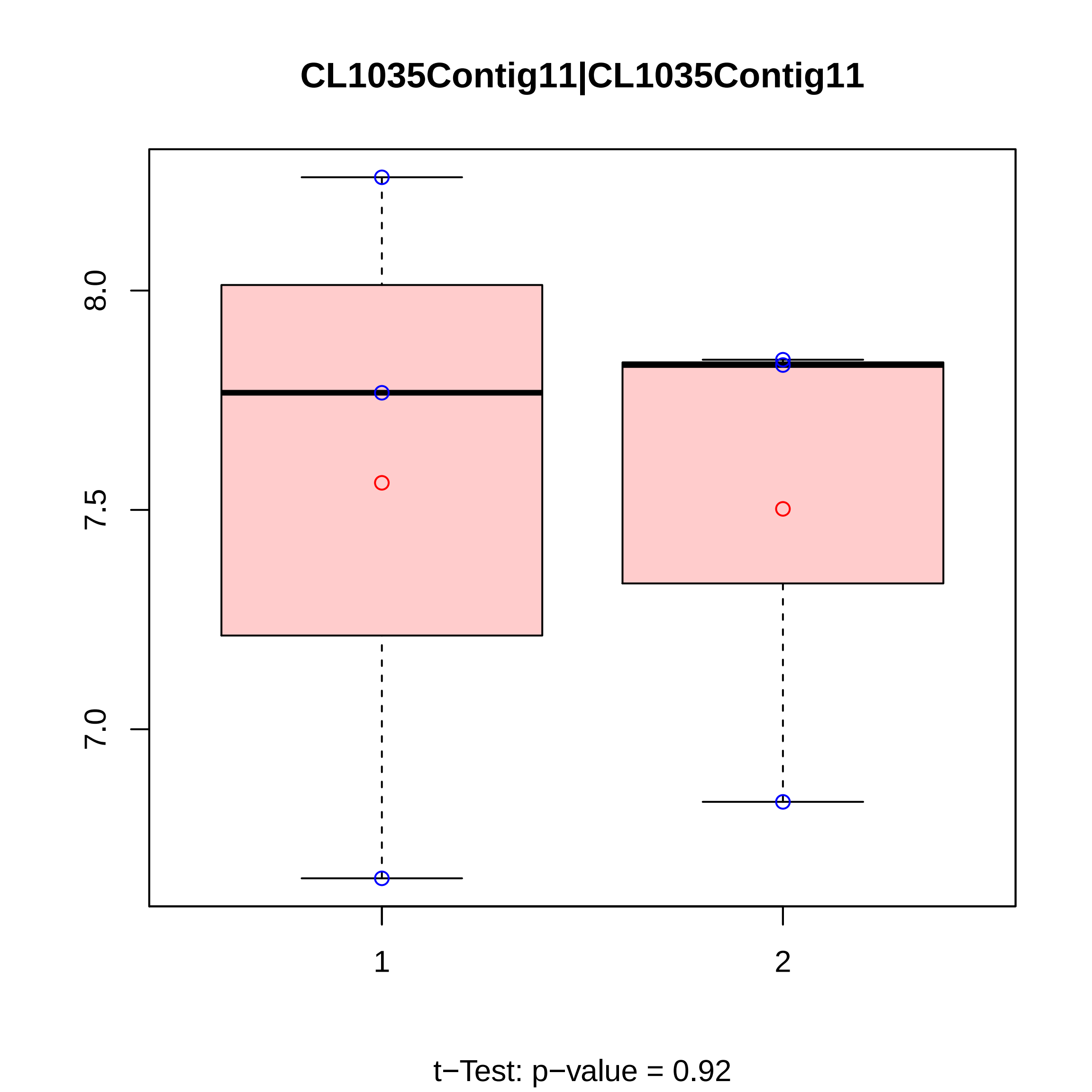

CL1035Contig11|CL1035Contig11
8.0
7.5
7.0
1
2
t−Test: p−value = 0.92

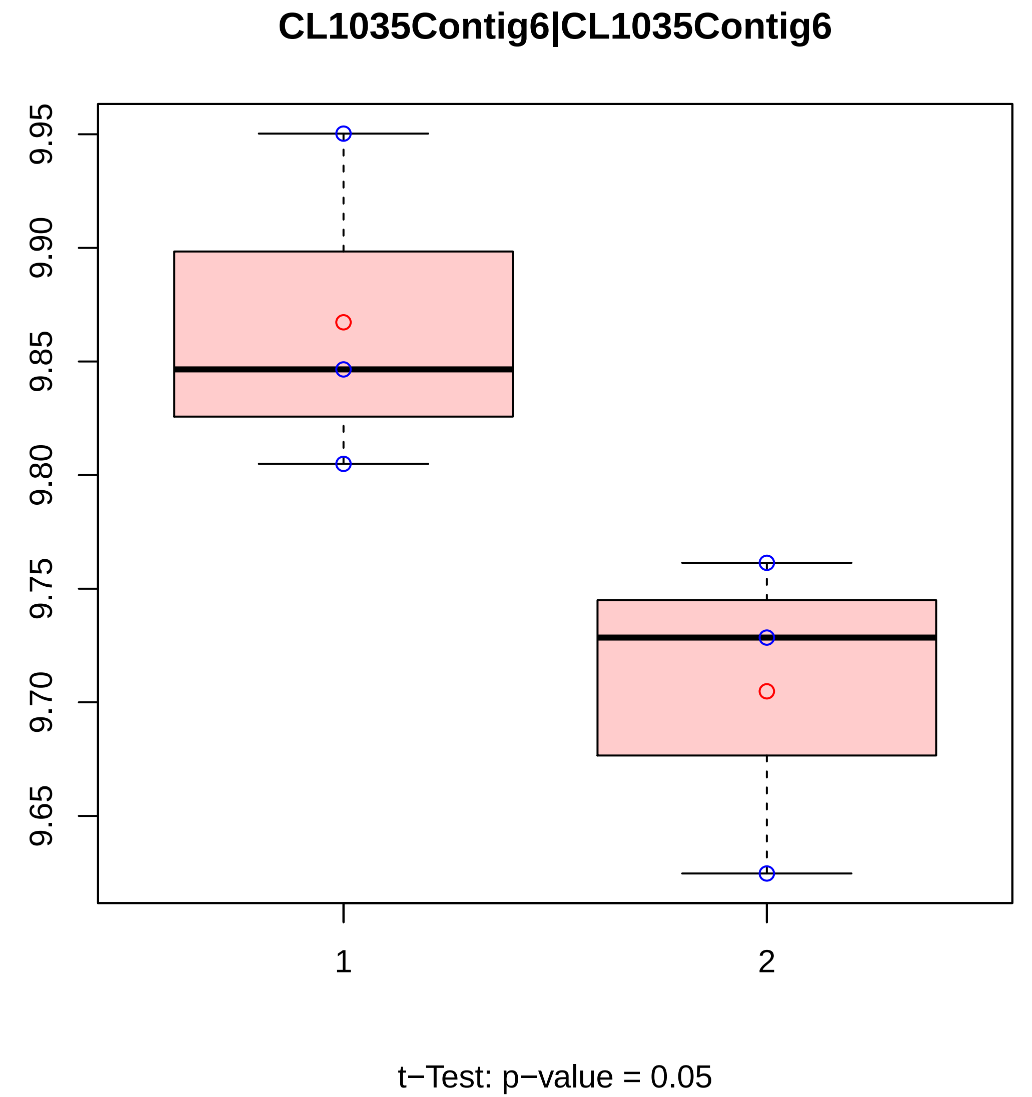

CL1035Contig6|CL1035Contig6
9.65
9.70
9.75
9.80
9.85
9.90
9.95
1
2
t−Test: p−value = 0.05

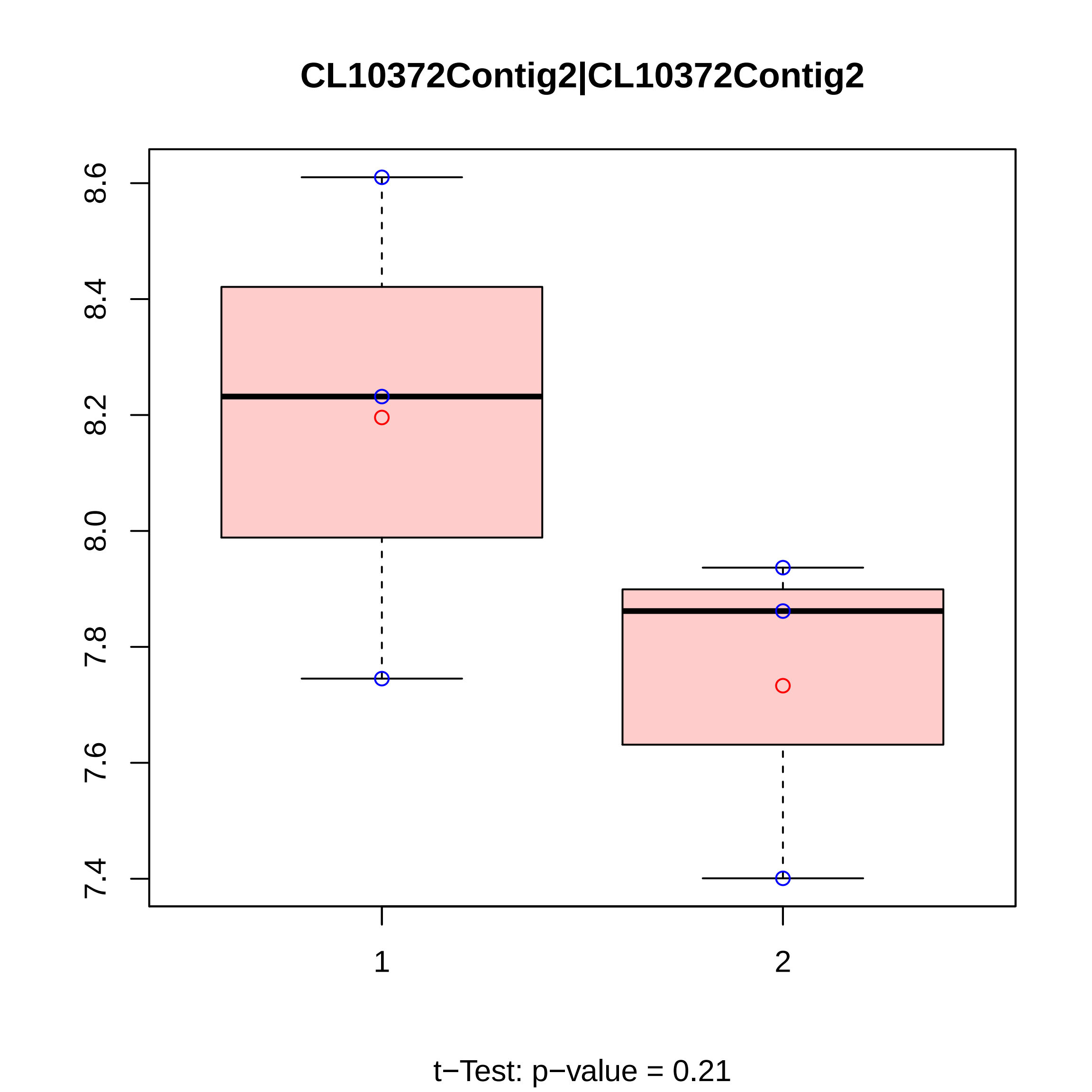

CL10372Contig2|CL10372Contig2
8.6
8.4
8.2
8.0
7.8
7.6
7.4
1
2
t−Test: p−value = 0.21

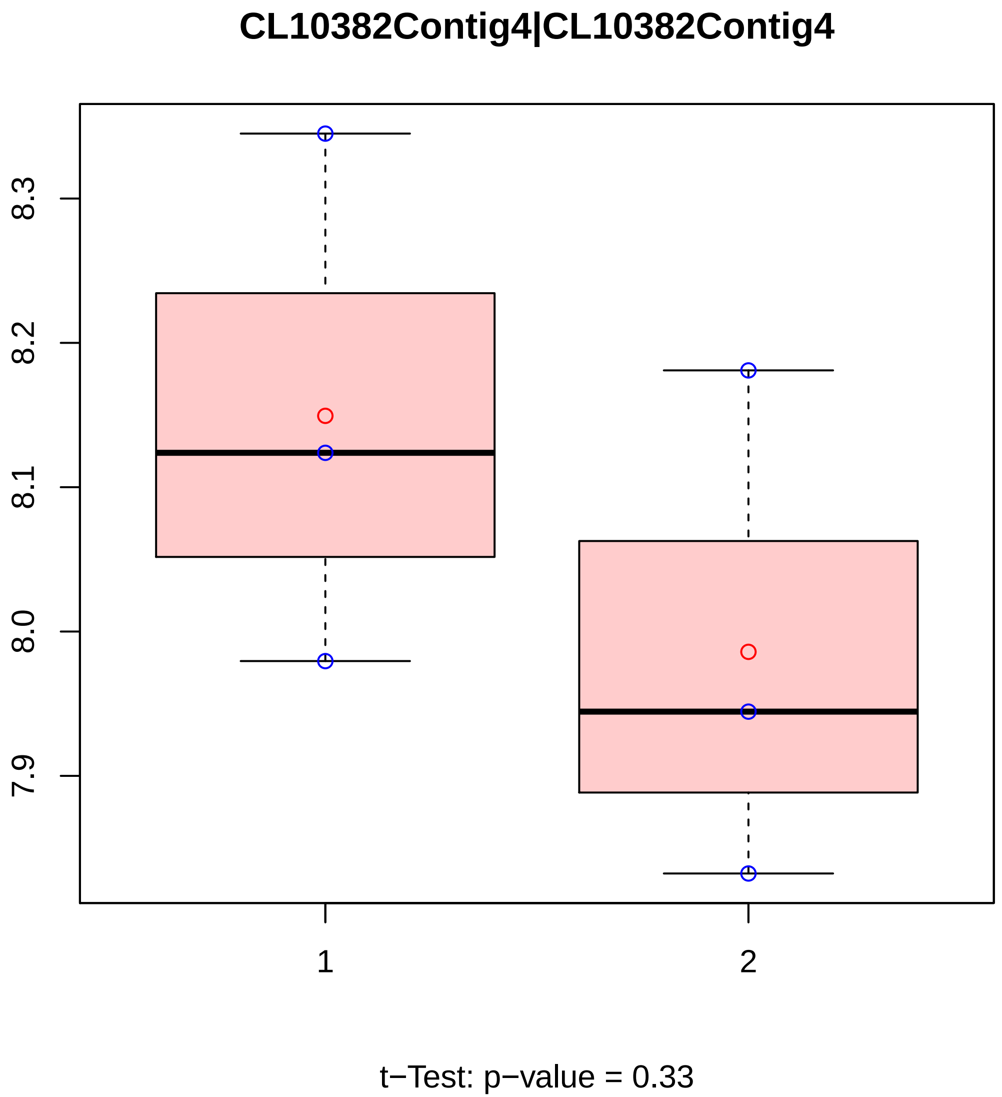
CL10382Contig4|CL10382Contig4
8.3
8.2
8.1
8.0
7.9
1
2
t−Test: p−value = 0.33

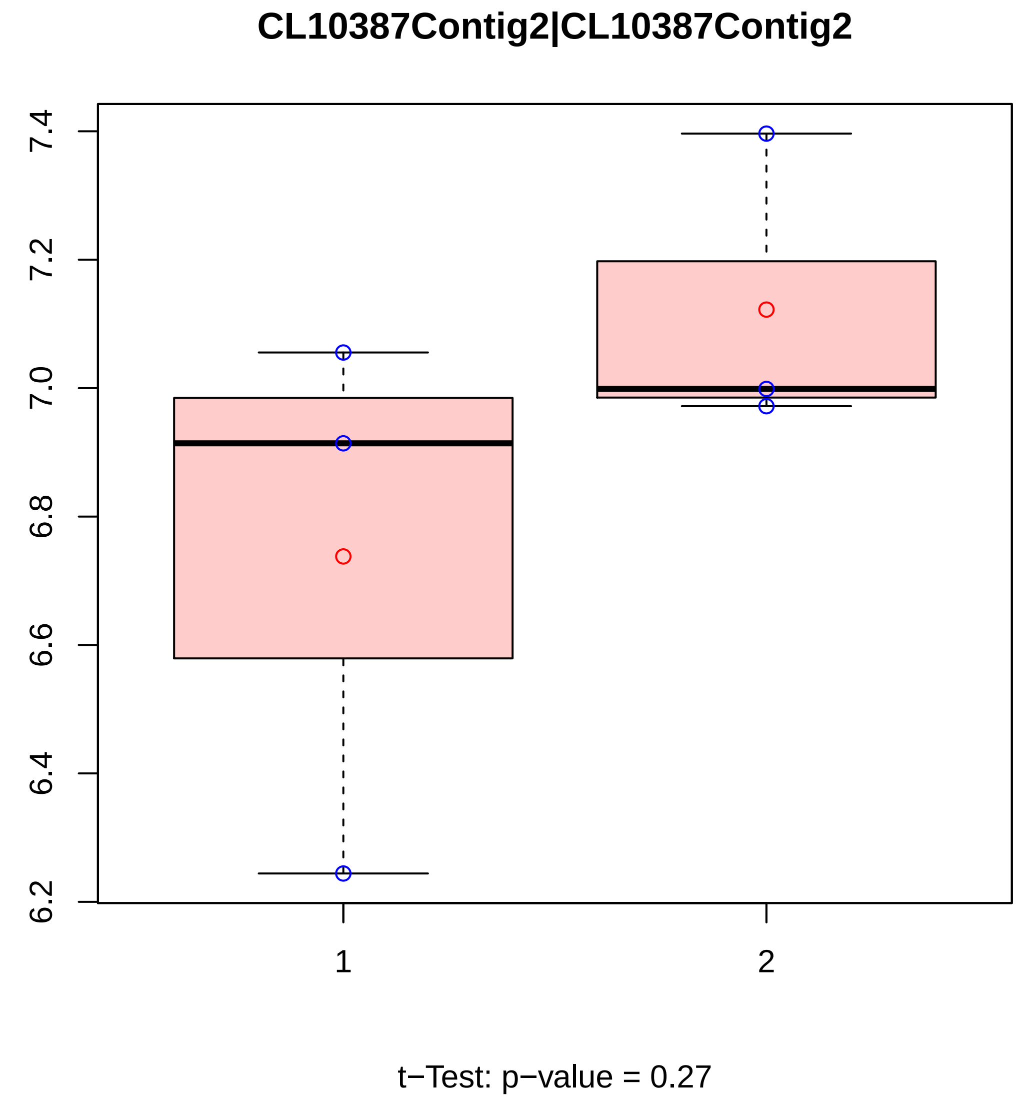

CL10387Contig2|CL10387Contig2
7.4
7.2
7.0
6.8
6.6
6.4
6.2
1
2
t−Test: p−value = 0.27

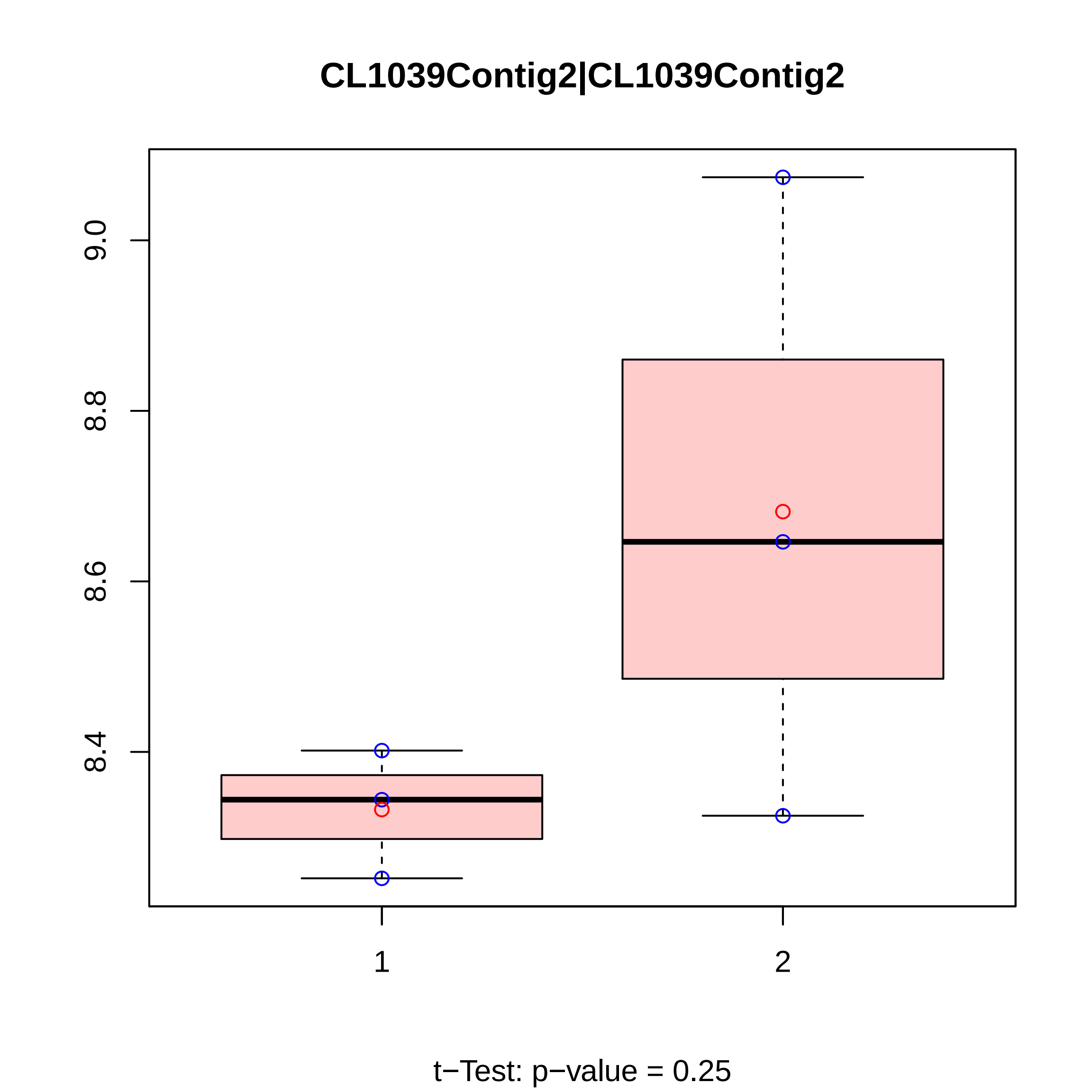
CL1039Contig2|CL1039Contig2
9.0
8.8
8.6
8.4
1
2
t−Test: p−value = 0.25

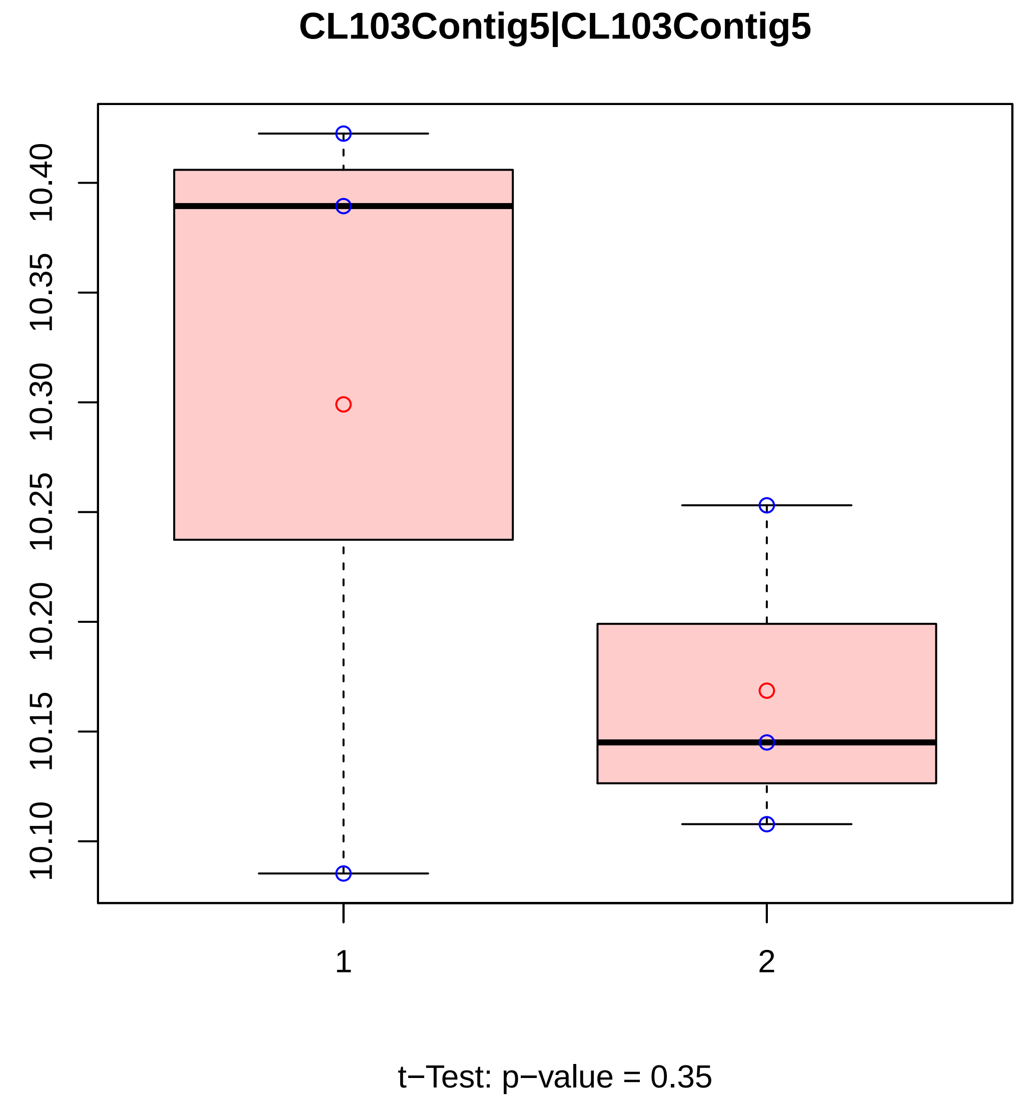
CL103Contig5|CL103Contig5
10.10
10.15
10.20
10.25
10.30
10.35
10.40
1
2
t−Test: p−value = 0.35

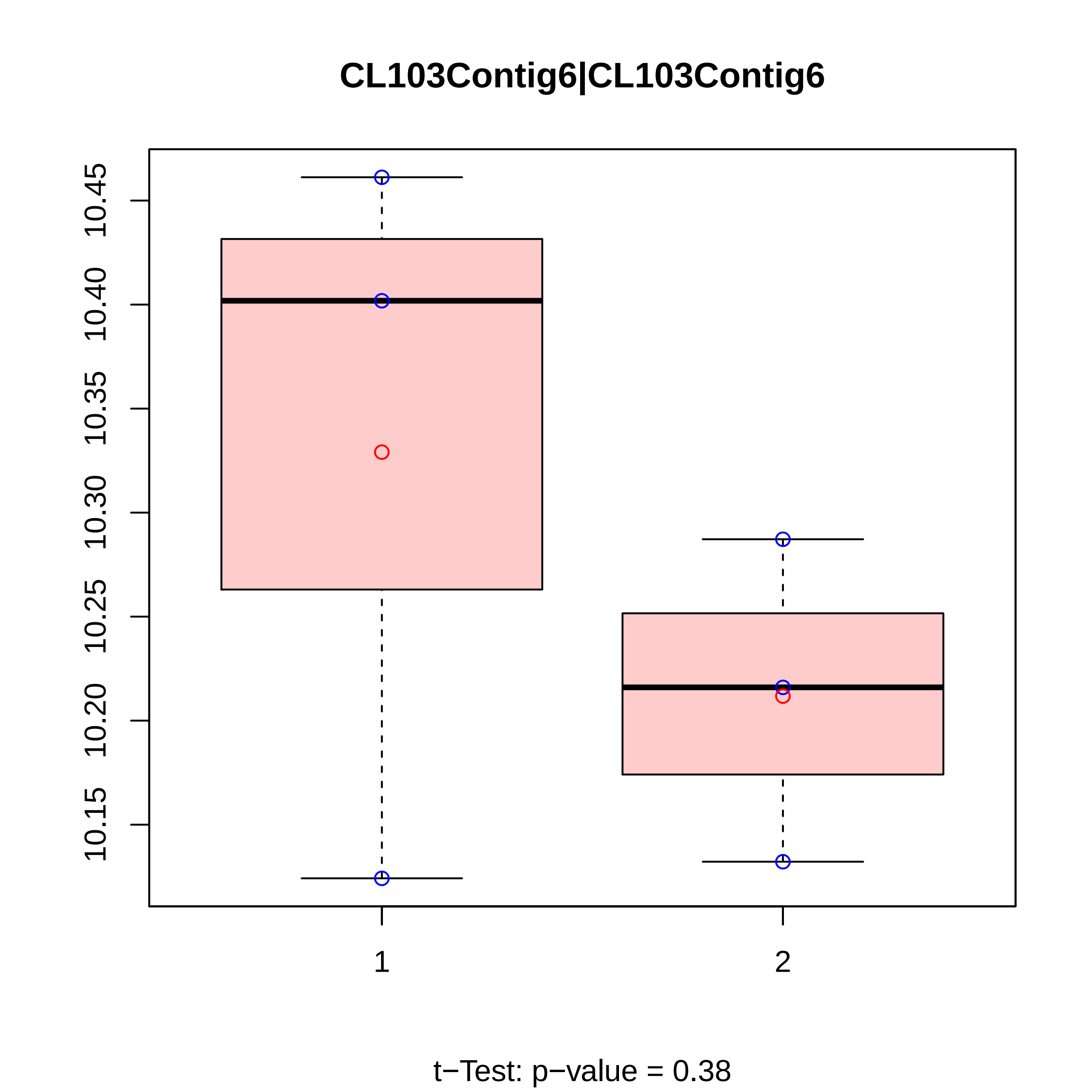
CL103Contig6|CL103Contig6
10.15
10.20
10.25
10.30
10.35
10.40
10.45
1
2
t−Test: p−value = 0.38

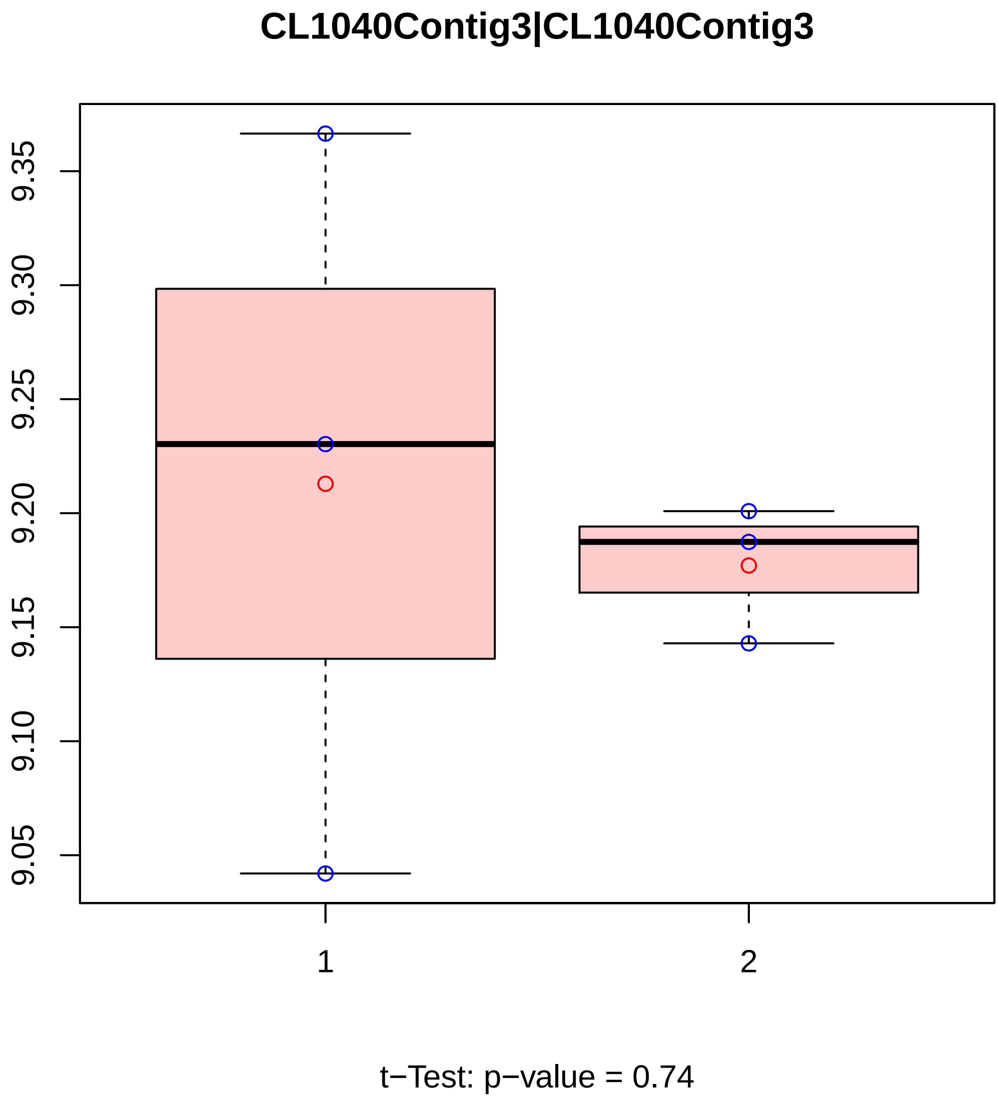
CL1040Contig3|CL1040Contig3
9.05
9.10
9.15
9.20
9.25
9.30
9.35
1
2
t−Test: p−value = 0.74

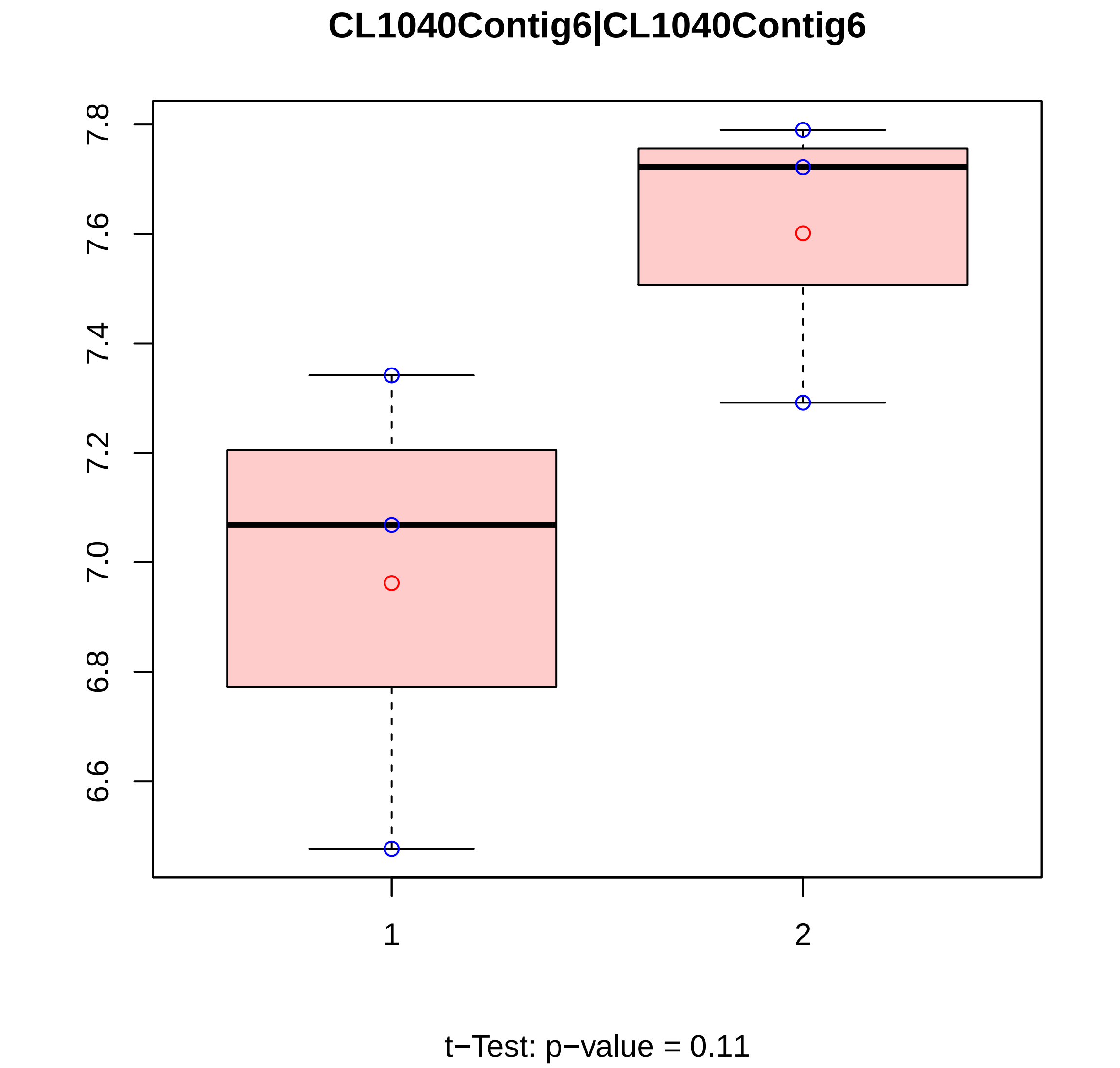
CL1040Contig6|CL1040Contig6
7.8
7.6
7.4
7.2
7.0
6.8
6.6
1
2
t−Test: p−value = 0.11

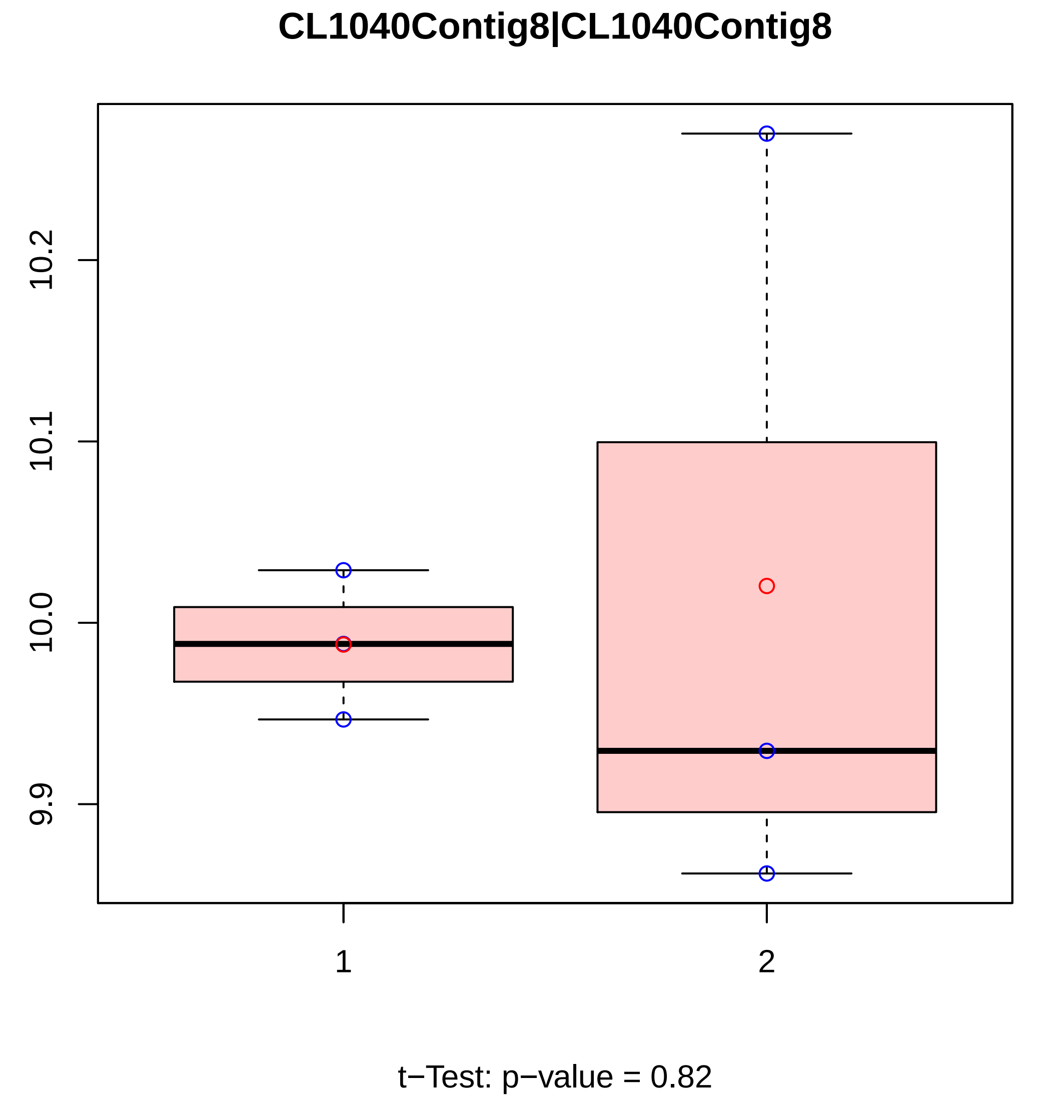
CL1040Contig8|CL1040Contig8
10.2
10.1
10.0
9.9
1
2
t−Test: p−value = 0.82

# CL10414Contig2|CL10414Contig2

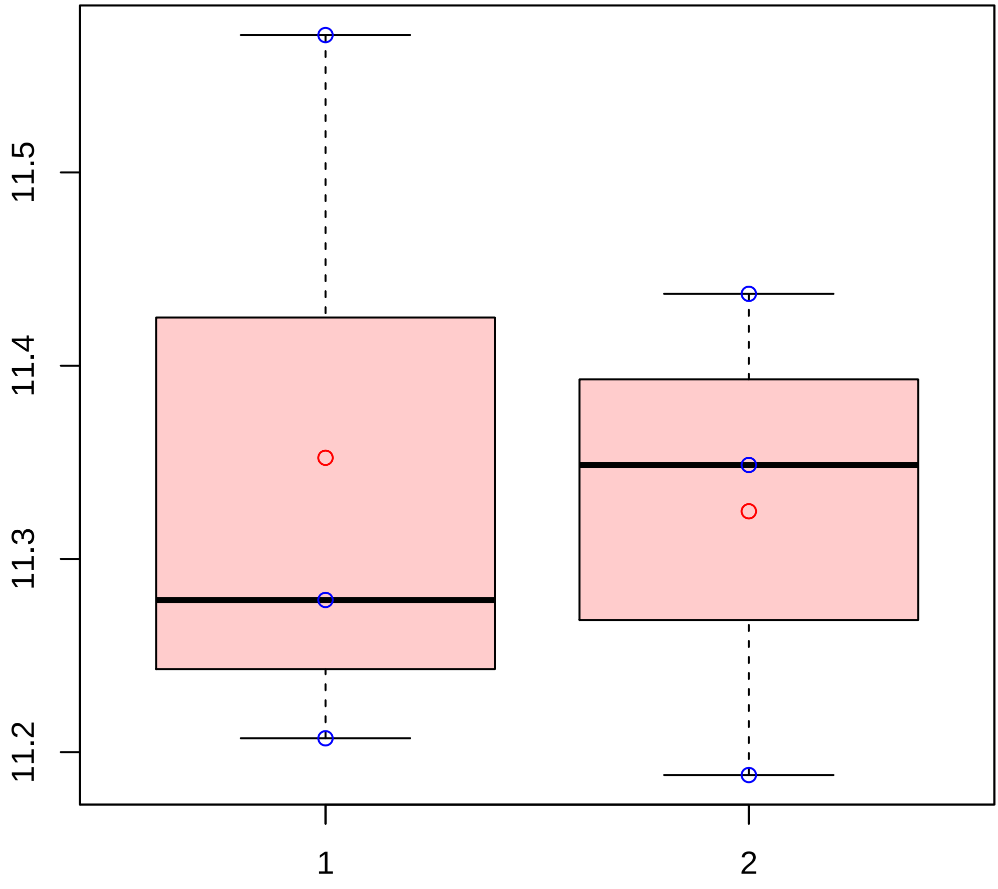


t−Test: p−value = 0.85

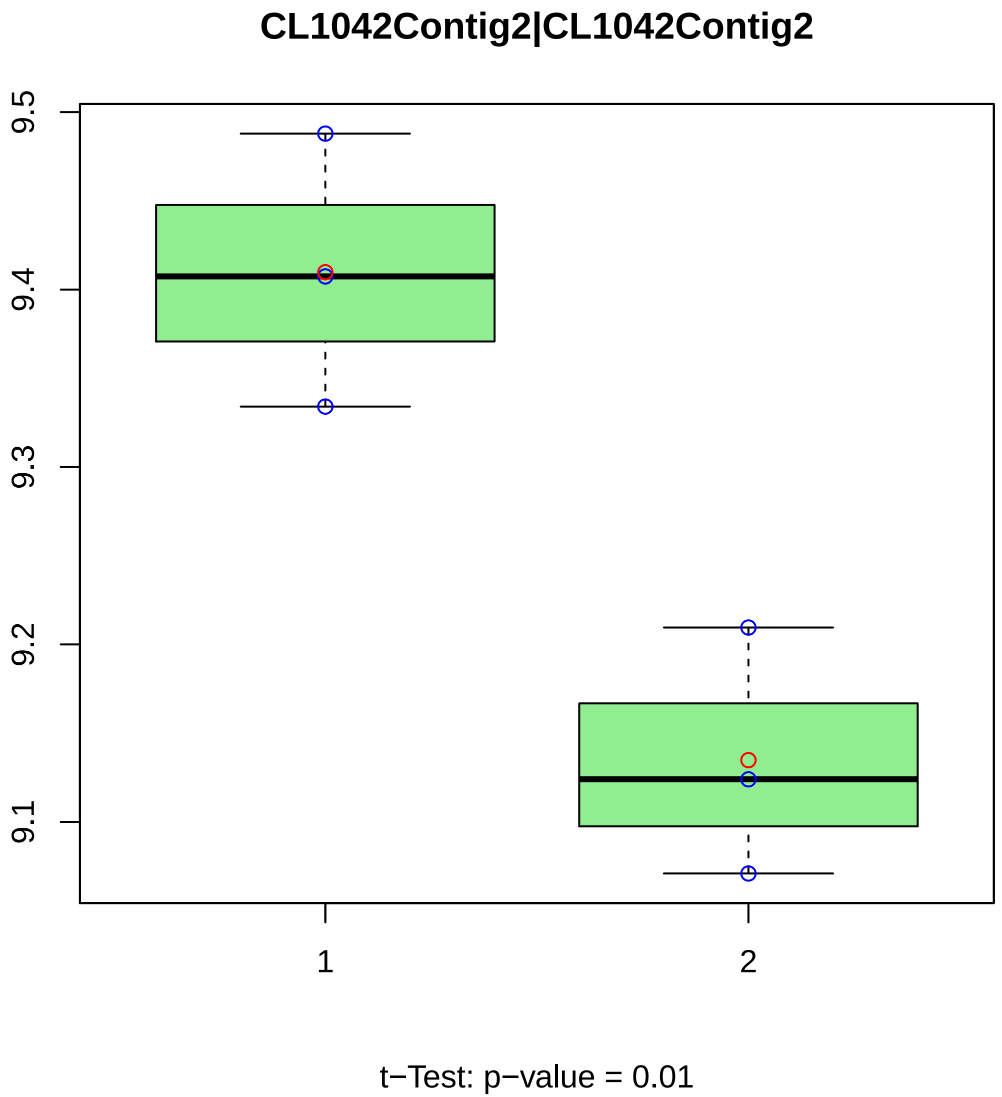
CL1042Contig2|CL1042Contig2
9.5
9.4
9.3
9.2
9.1
1
2
t−Test: p−value = 0.01

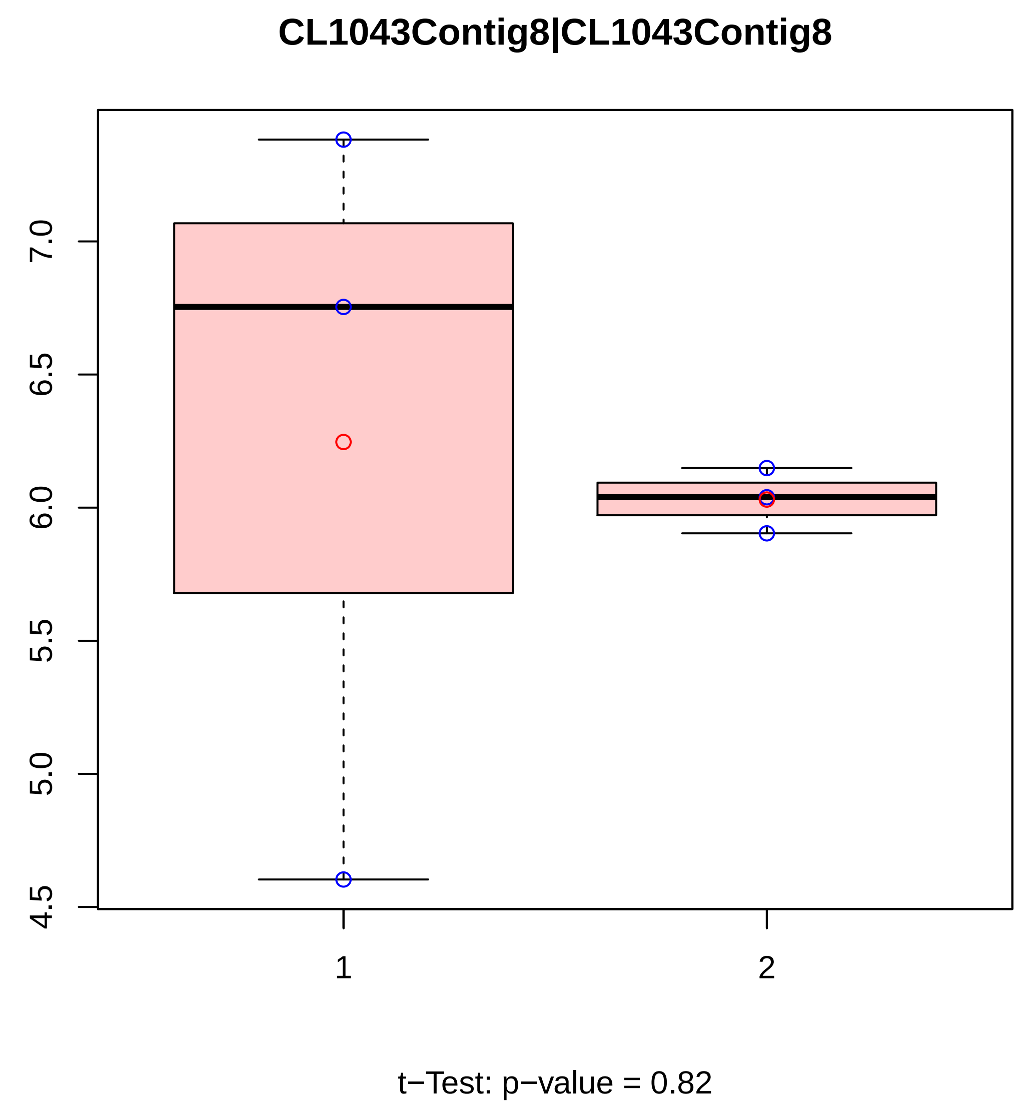
CL1043Contig8|CL1043Contig8
7.0
6.5
6.0
5.5
5.0
4.5
1
2
t−Test: p−value = 0.82

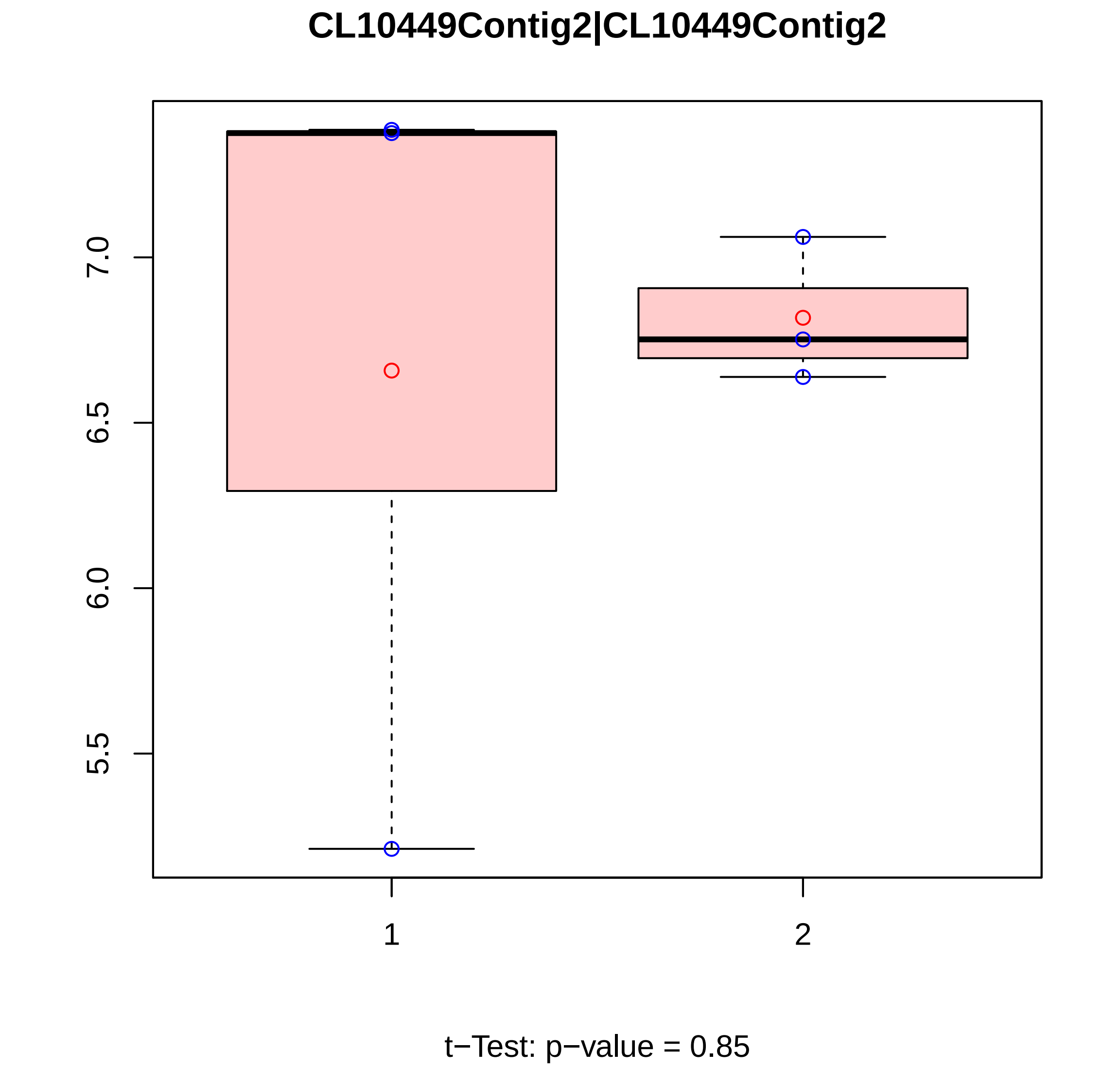
CL10449Contig2|CL10449Contig2
7.0
6.5
6.0
5.5
1
2
t−Test: p−value = 0.85

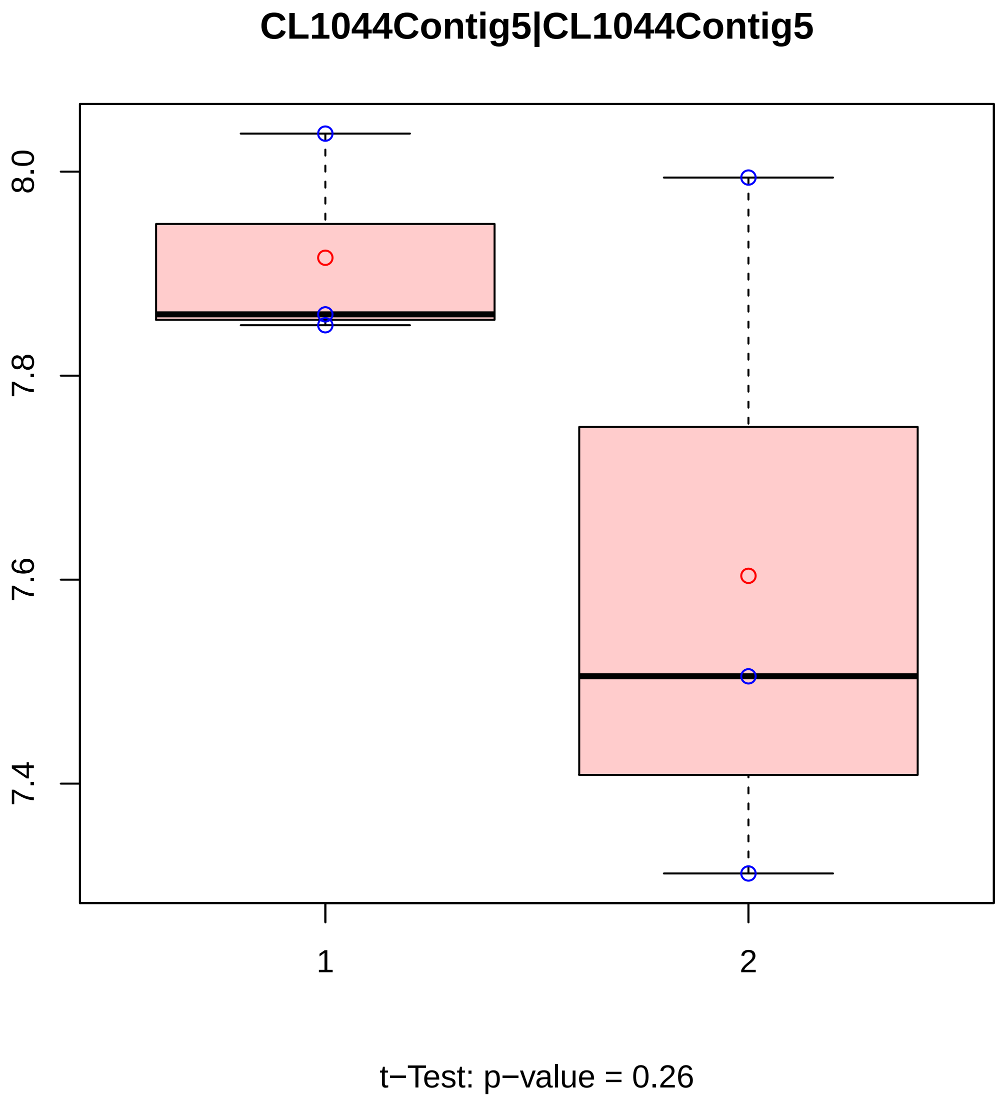
CL1044Contig5|CL1044Contig5
8.0
7.8
7.6
7.4
1
2
t−Test: p−value = 0.26

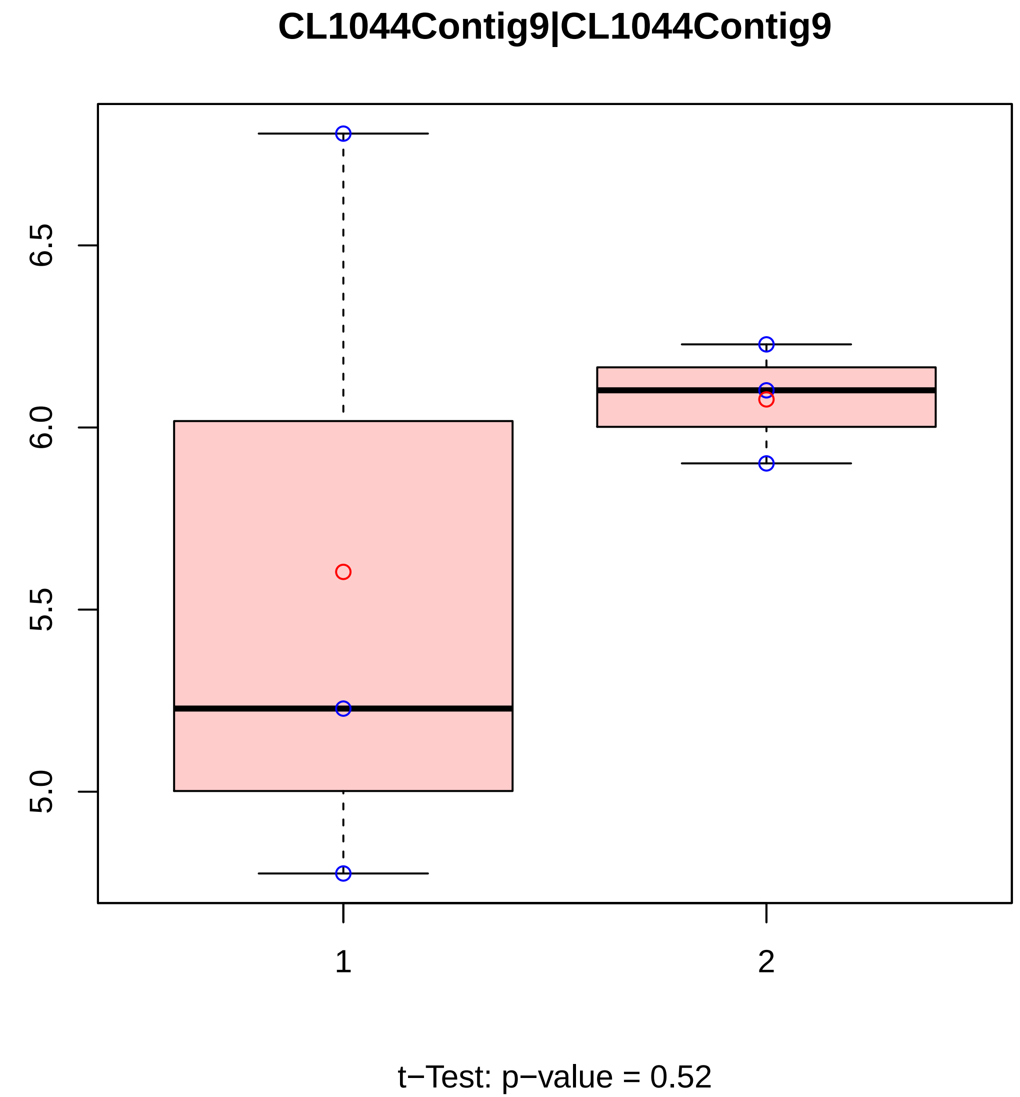
CL1044Contig9|CL1044Contig9
6.5
6.0
5.5
5.0
1
2
t−Test: p−value = 0.52

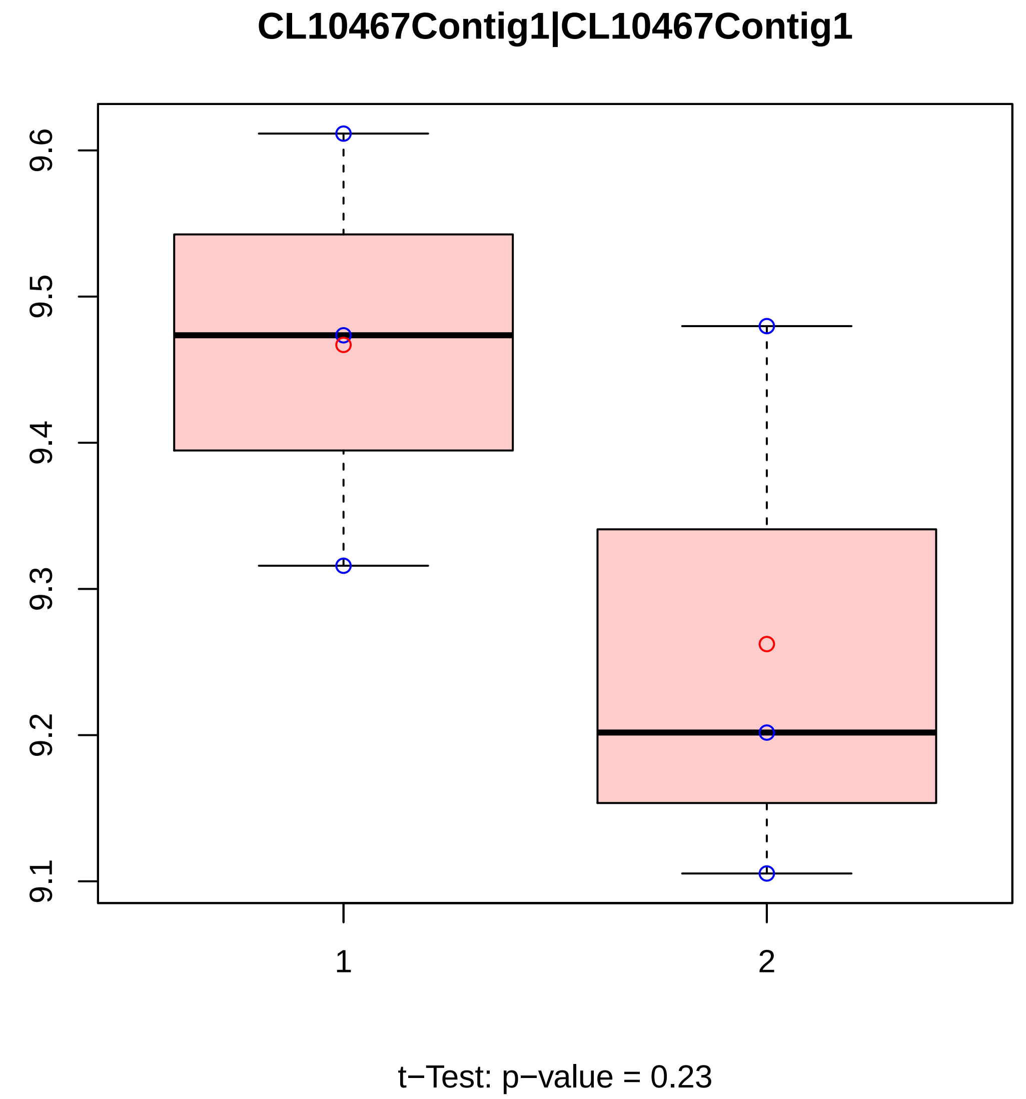

CL10467Contig1|CL10467Contig1
9.1
9.2
9.3
9.4
9.5
9.6
1
2
t−Test: p−value = 0.23

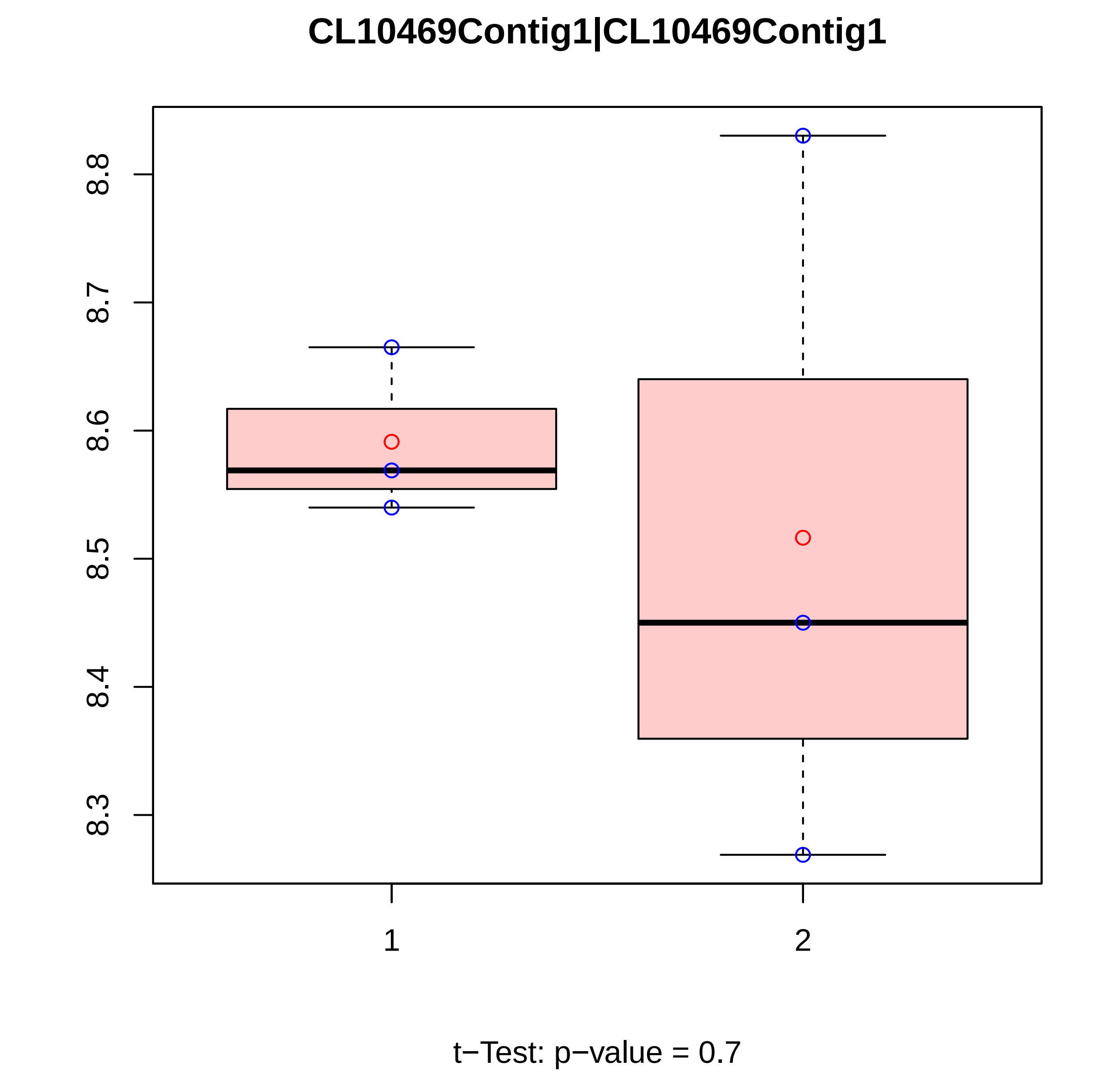

CL10469Contig1|CL10469Contig1
8.3
8.4
8.5
8.6
8.7
8.8
1
2
t−Test: p−value = 0.7

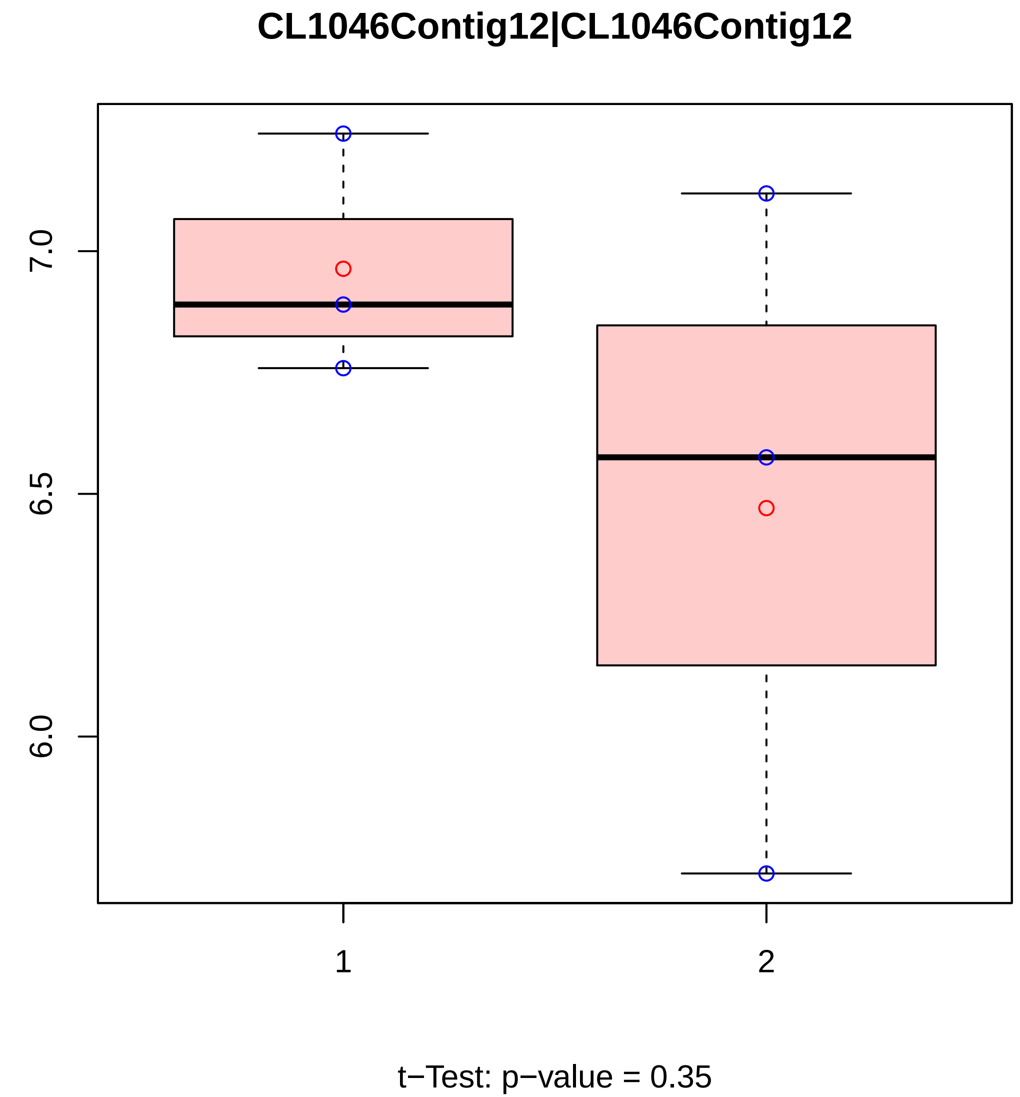
CL1046Contig12|CL1046Contig12
7.0
6.5
6.0
1
2
t−Test: p−value = 0.35

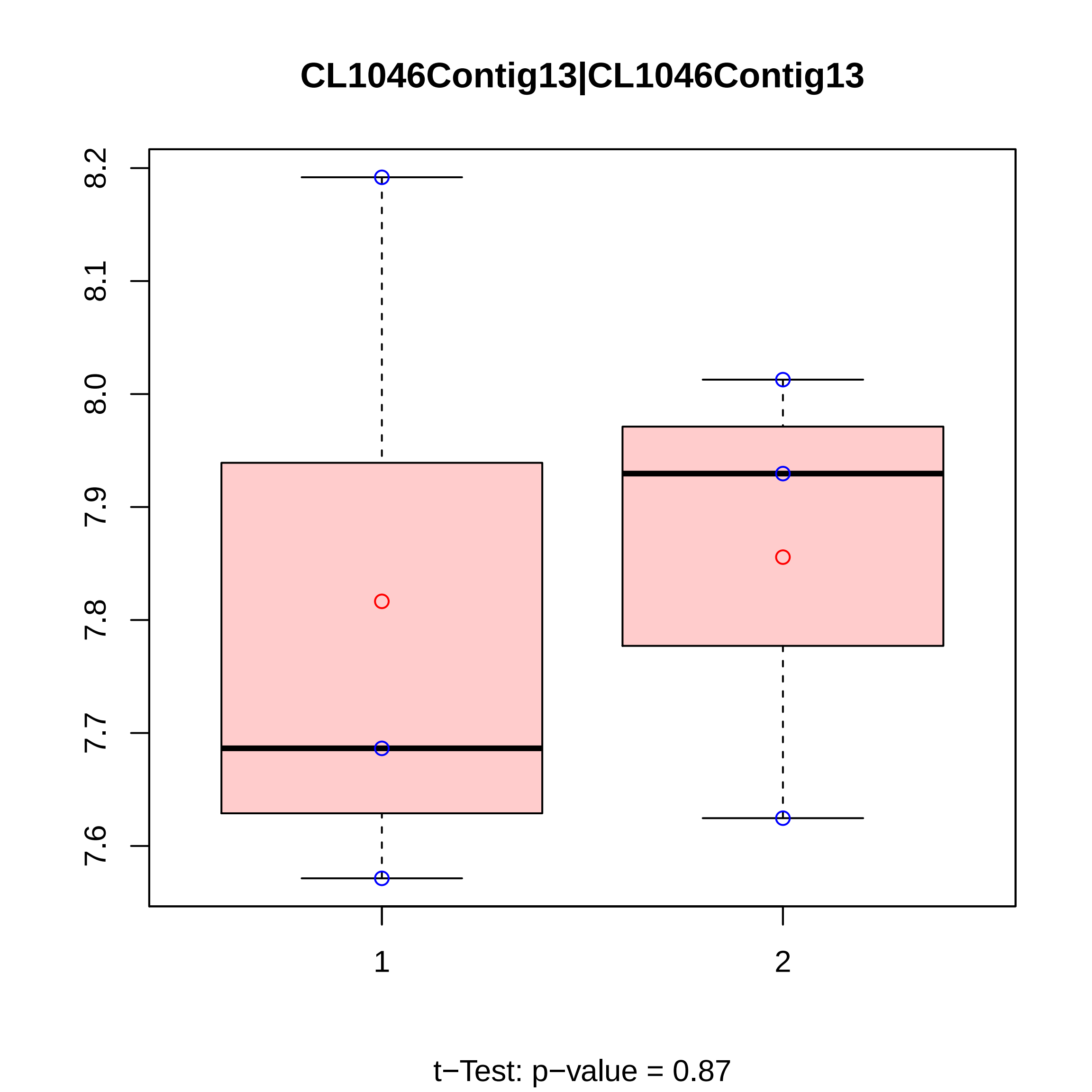
CL1046Contig13|CL1046Contig13
8.2
8.1
8.0
7.9
7.8
7.7
7.6
1
2
t−Test: p−value = 0.87

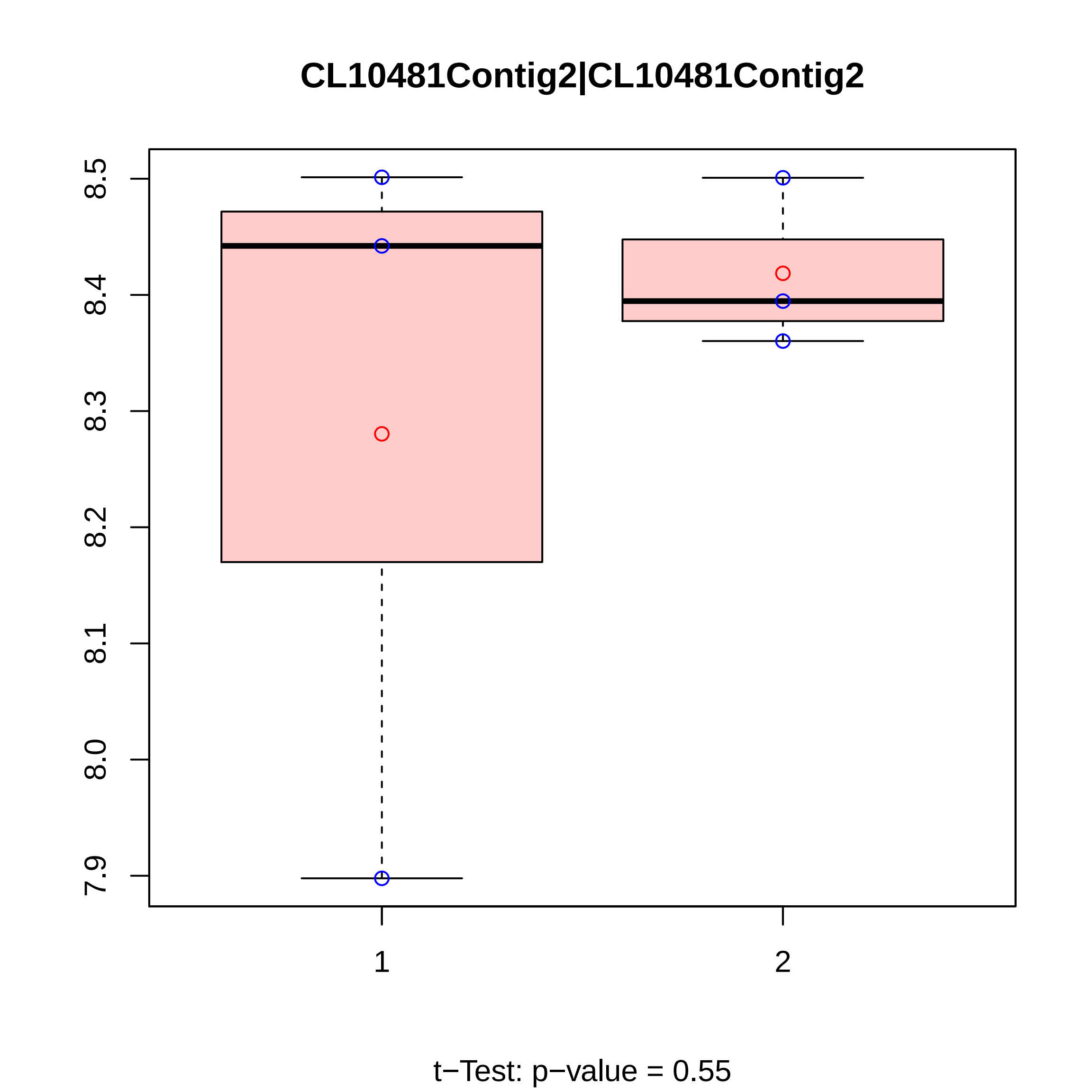

CL10481Contig2|CL10481Contig2
8.5
8.4
8.3
8.2
8.1
8.0
7.9
1
2
t−Test: p−value = 0.55

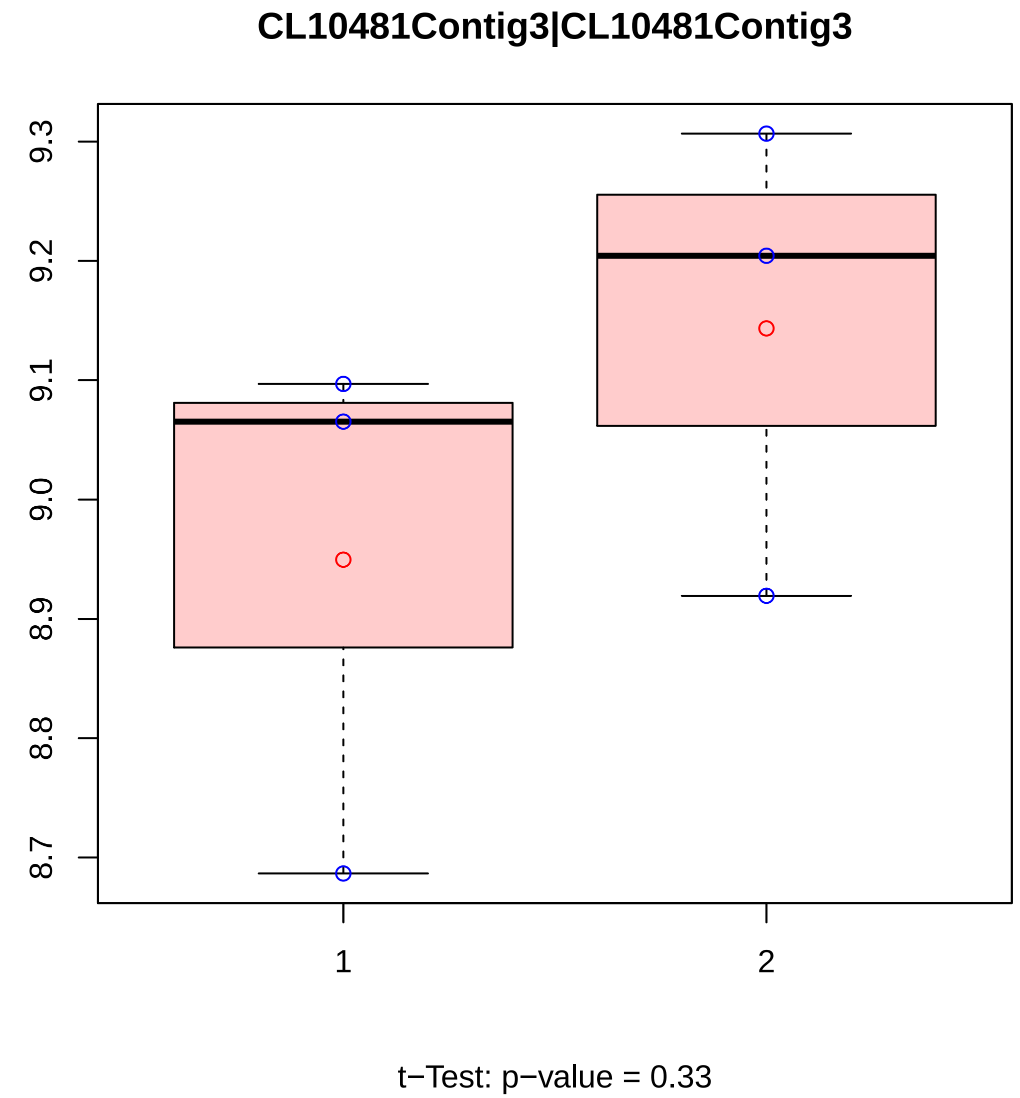
CL10481Contig3|CL10481Contig3
9.3
9.2
9.1
9.0
8.9
8.8
8.7
1
2
t−Test: p−value = 0.33

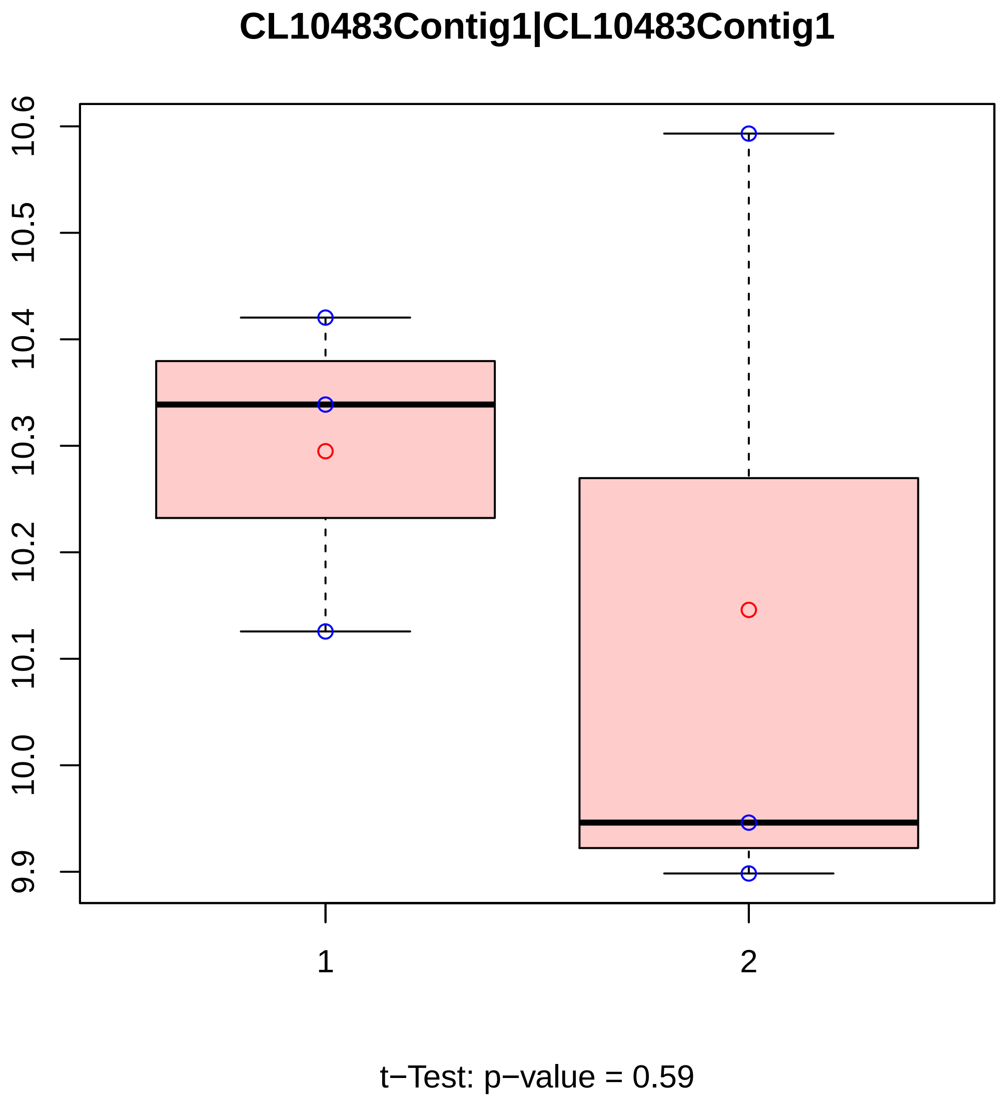
CL10483Contig1|CL10483Contig1
10.6
10.5
10.4
10.3
10.2
10.1
10.0
9.9
1
2
t−Test: p−value = 0.59

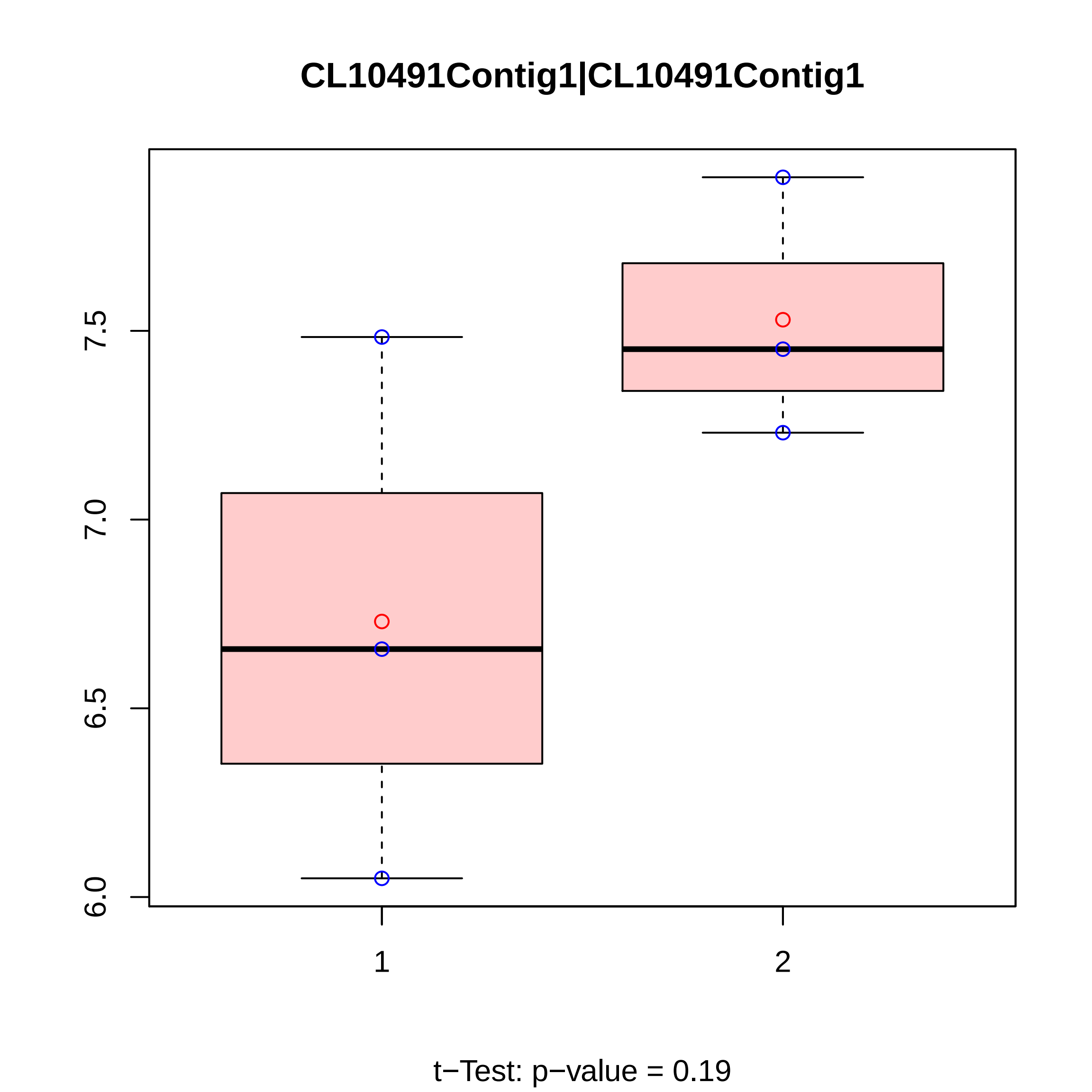
CL10491Contig1|CL10491Contig1
7.5
7.0
6.5
6.0
1
2
t−Test: p−value = 0.19

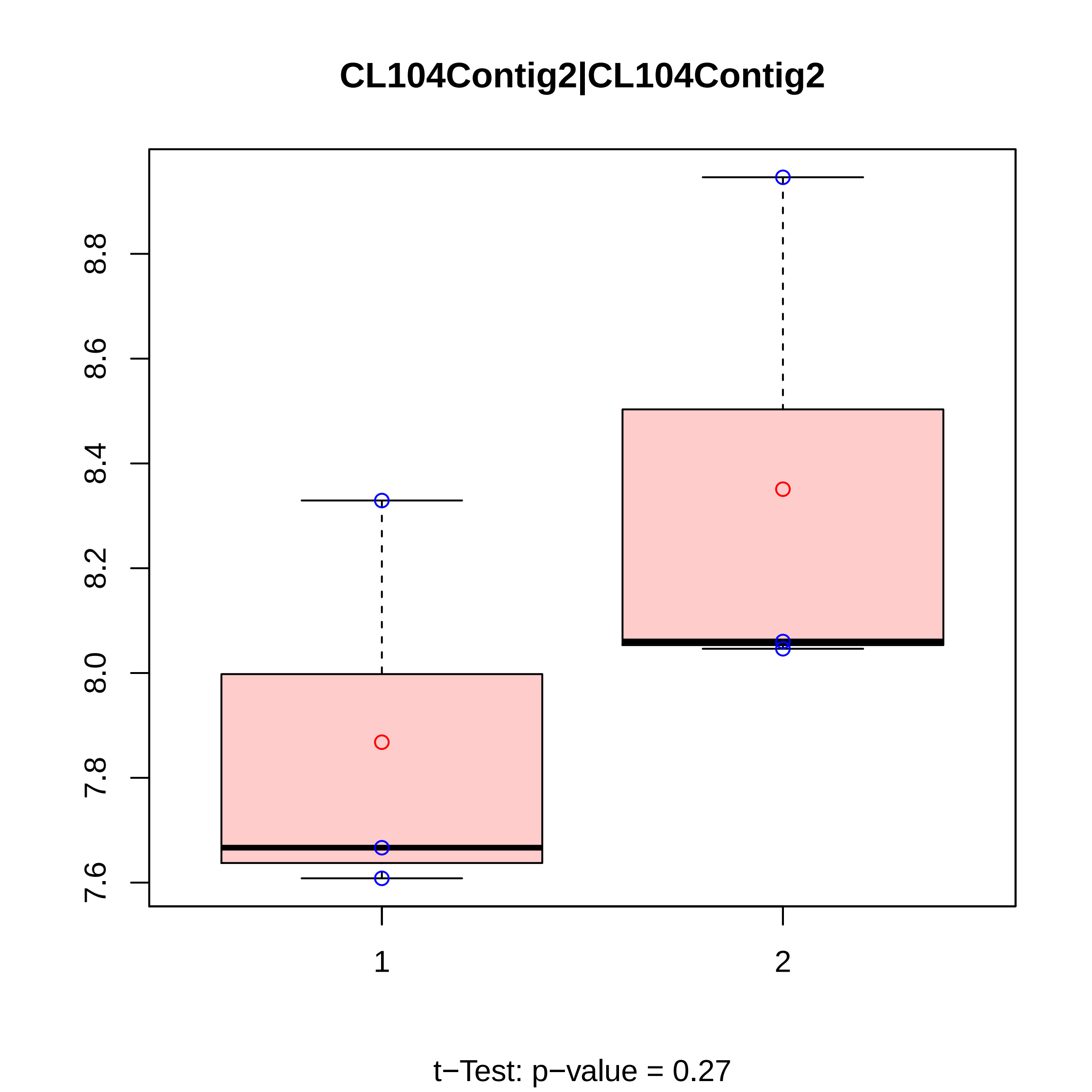
CL104Contig2|CL104Contig2
7.6
7.8
8.0
8.2
8.4
8.6
8.8
1
2
t−Test: p−value = 0.27

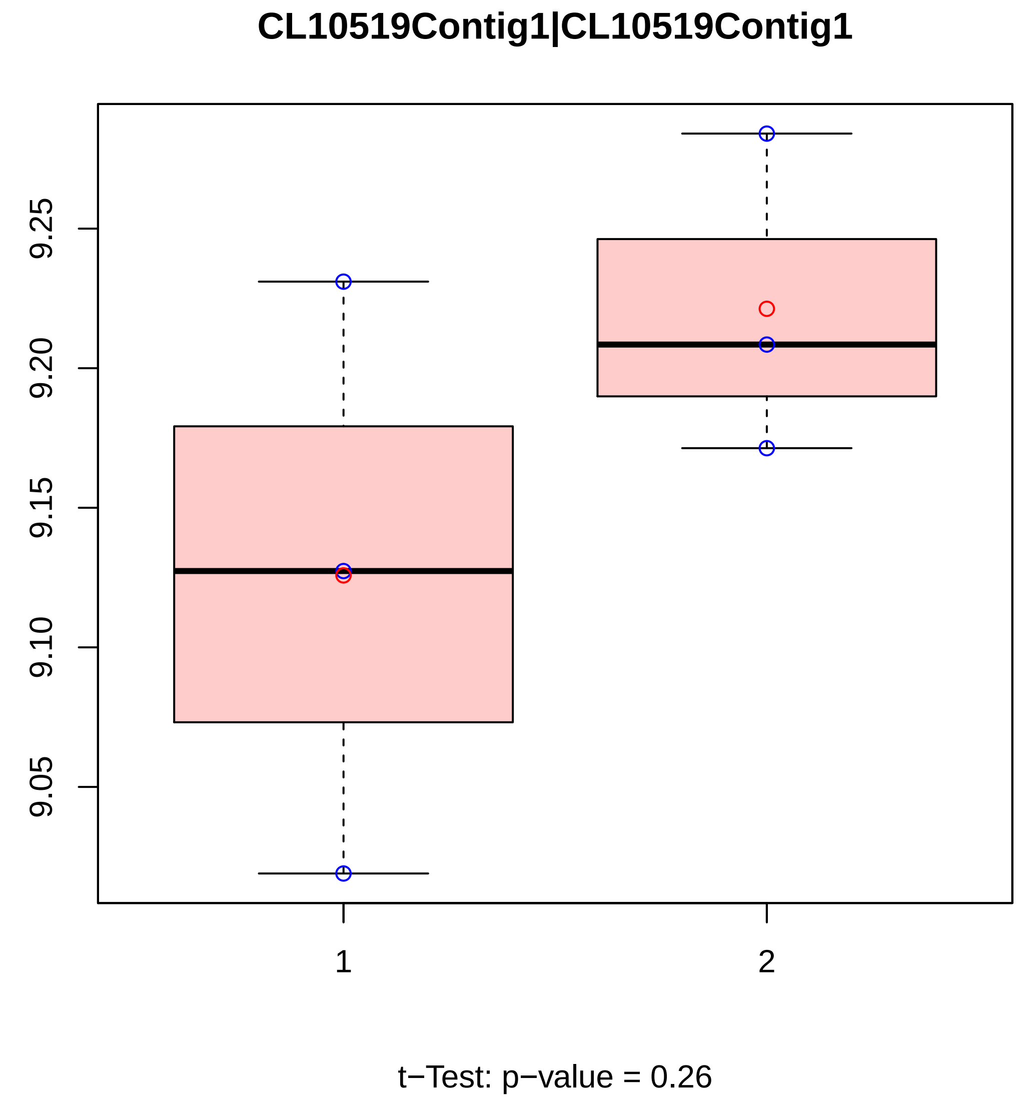
CL10519Contig1|CL10519Contig1
9.05
9.10
9.15
9.20
9.25
1
2
t−Test: p−value = 0.26

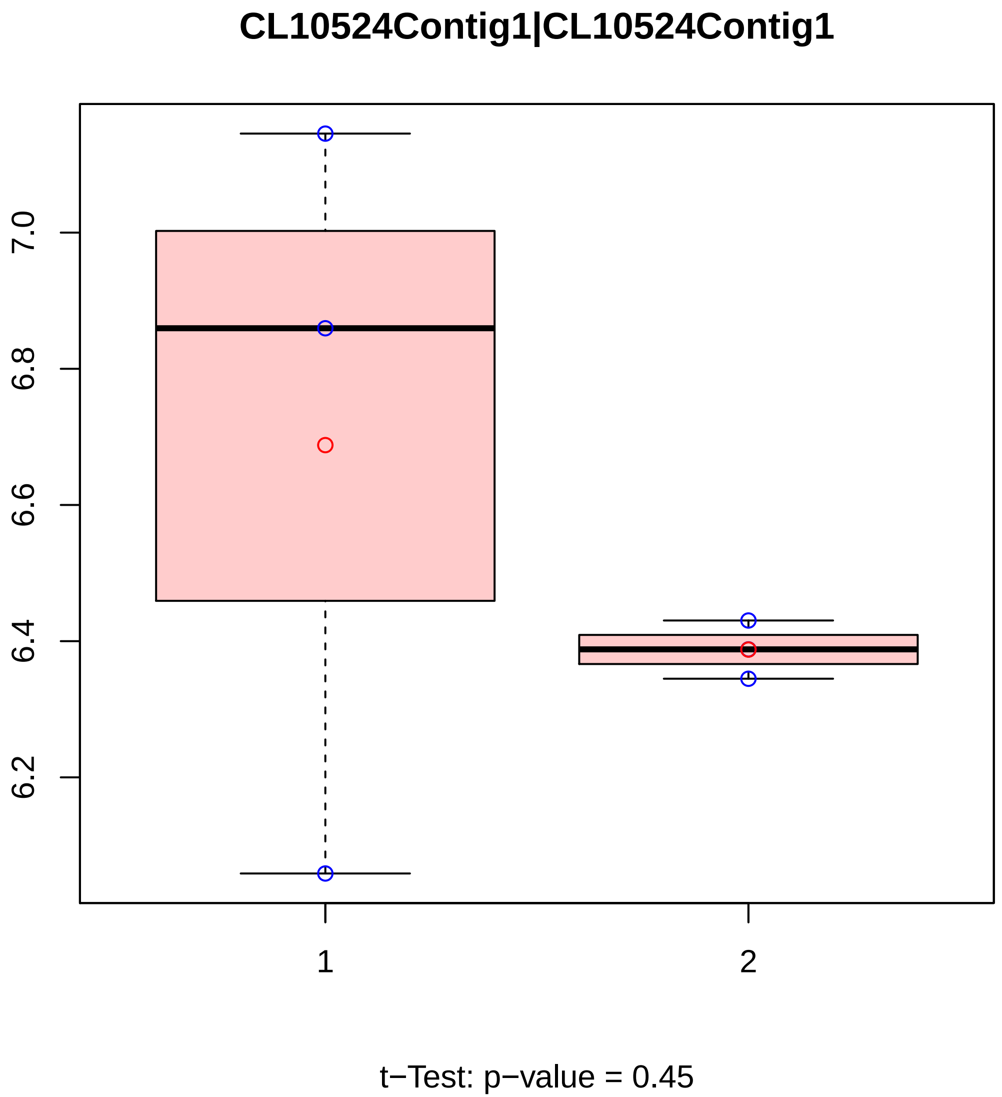

CL10524Contig1|CL10524Contig1
7.0
6.8
6.6
6.4
6.2
1
2
t−Test: p−value = 0.45

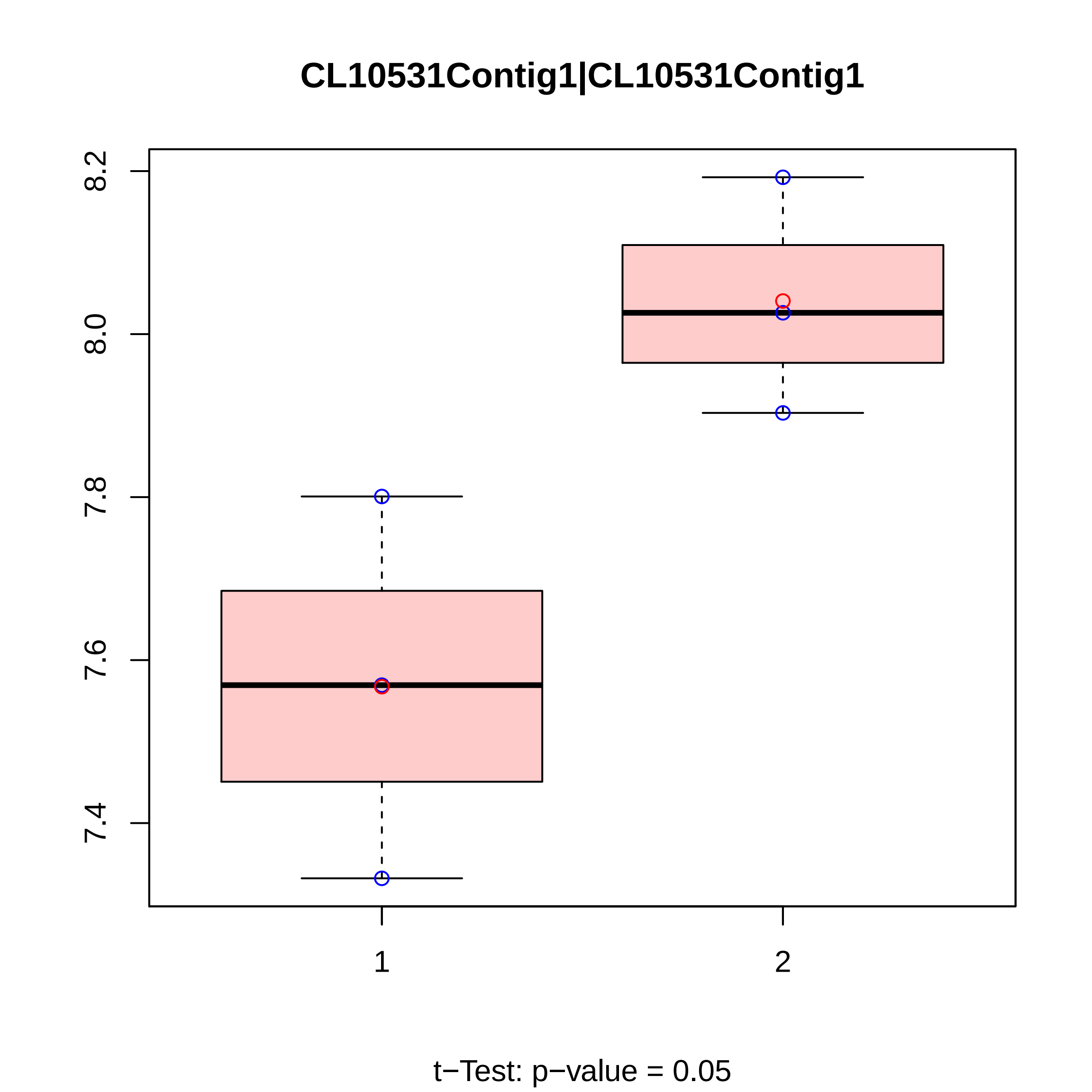
CL10531Contig1|CL10531Contig1
8.2
8.0
7.8
7.6
7.4
1
2
t−Test: p−value = 0.05

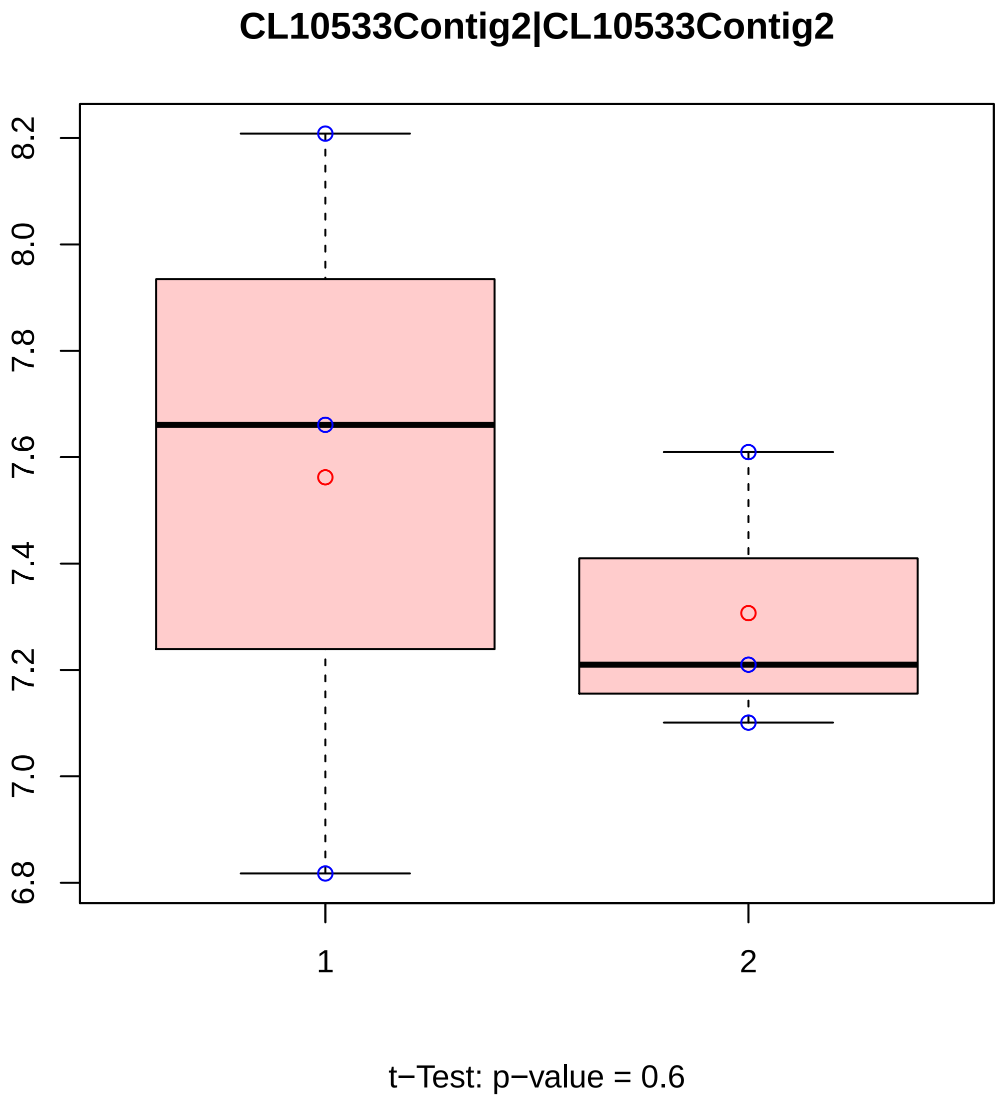
CL10533Contig2|CL10533Contig2
8.2
8.0
7.8
7.6
7.4
7.2
7.0
6.8
1
2
t−Test: p−value = 0.6

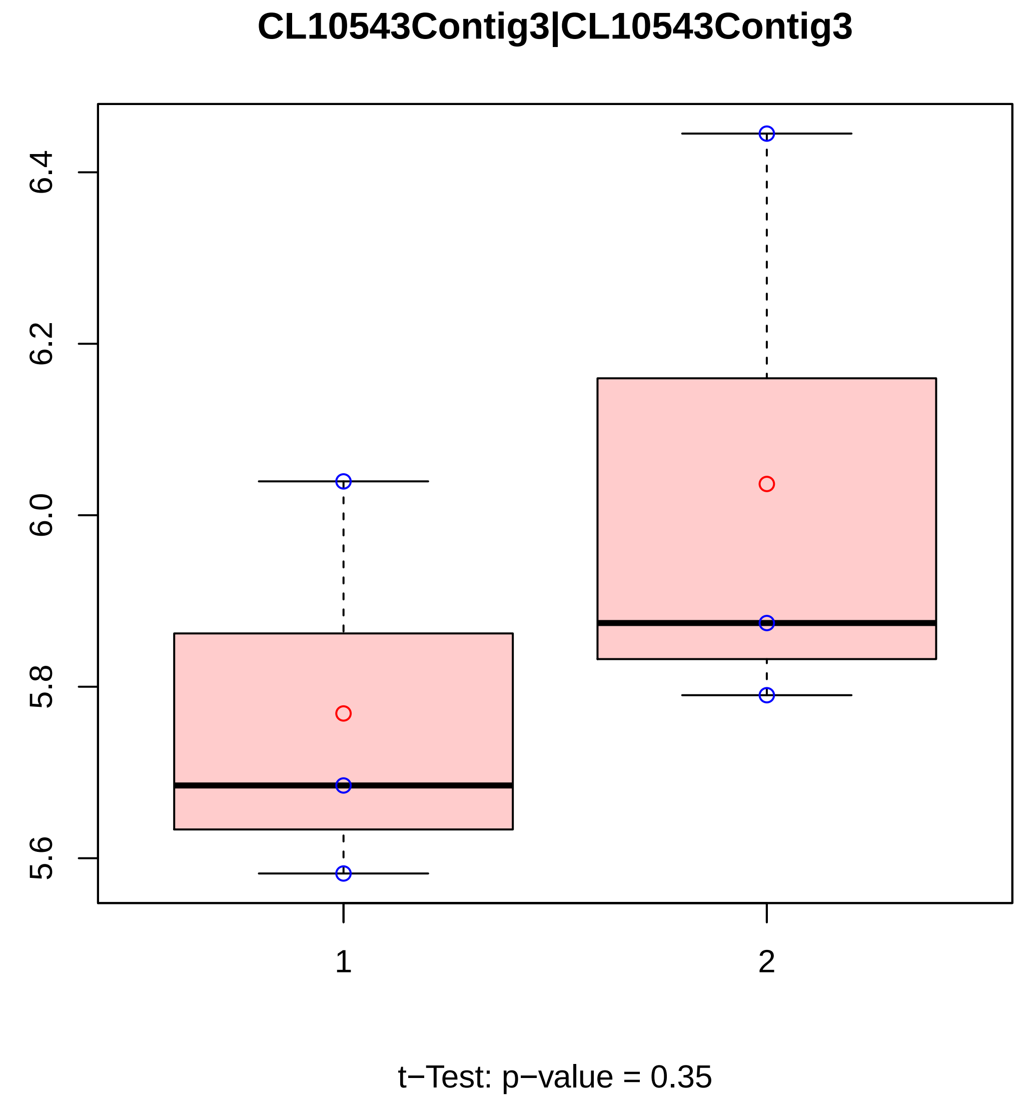
CL10543Contig3|CL10543Contig3
6.4
6.2
6.0
5.8
5.6
1
2
t−Test: p−value = 0.35

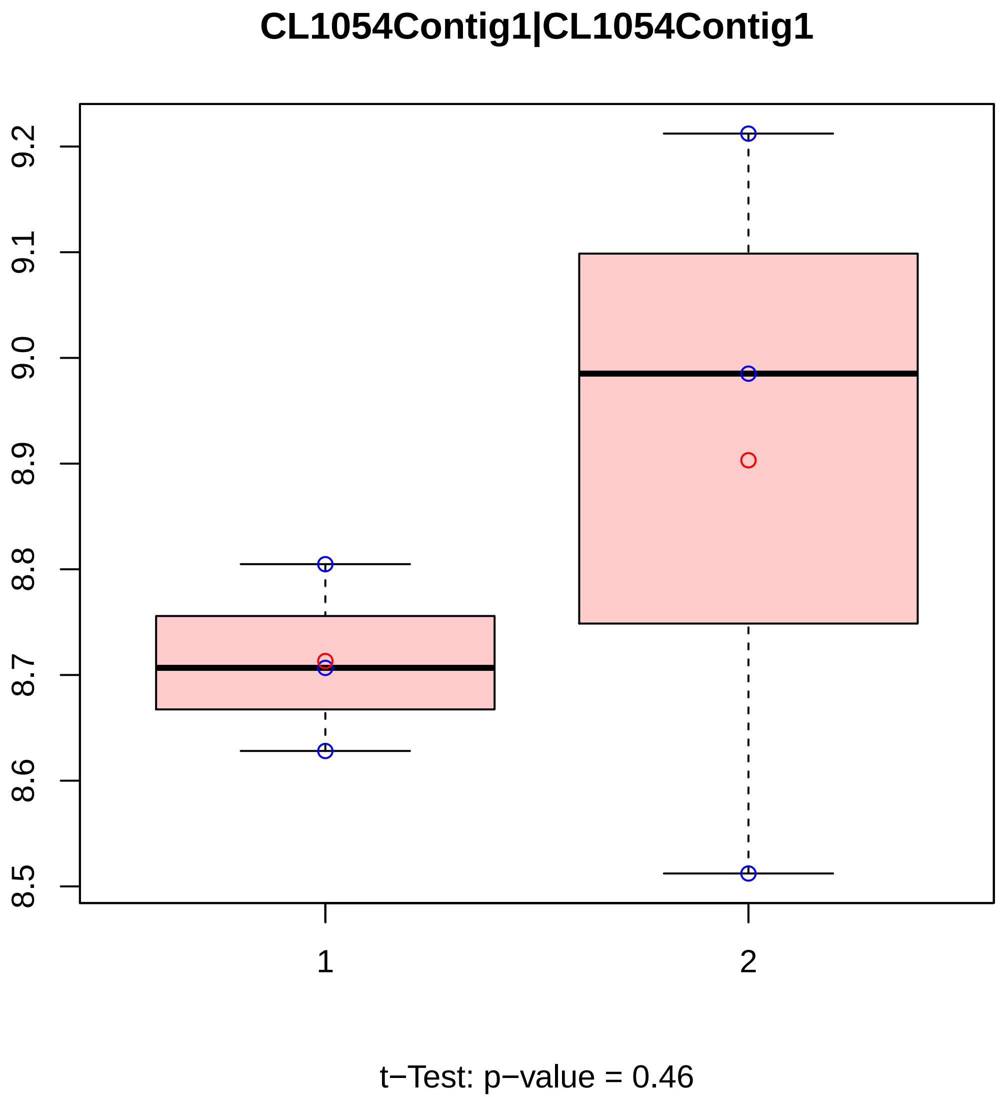

CL1054Contig1|CL1054Contig1
9.2
9.1
9.0
8.9
8.8
8.7
8.6
8.5
1
2
t−Test: p−value = 0.46

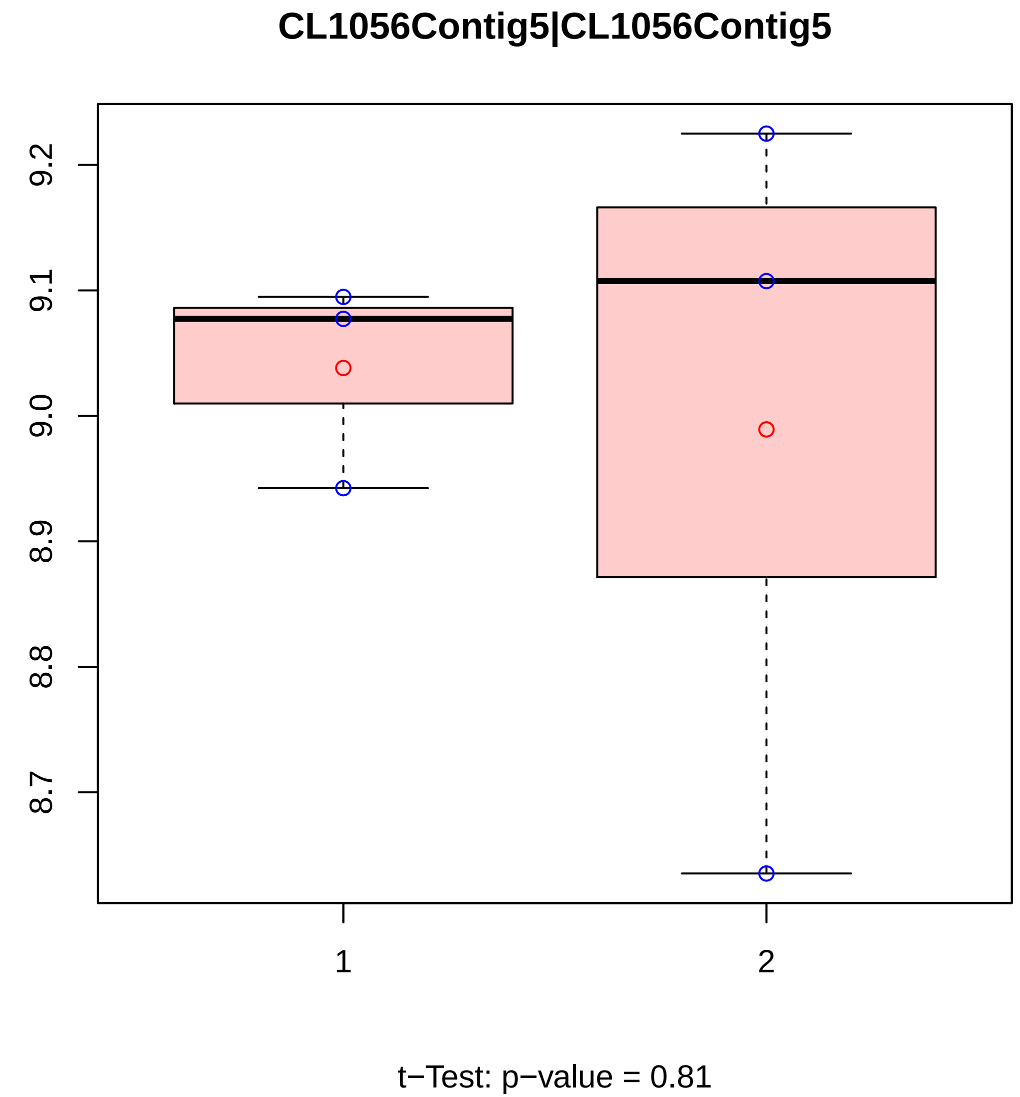
CL1056Contig5|CL1056Contig5
9.2
9.1
9.0
8.9
8.8
8.7
1
2
t−Test: p−value = 0.81

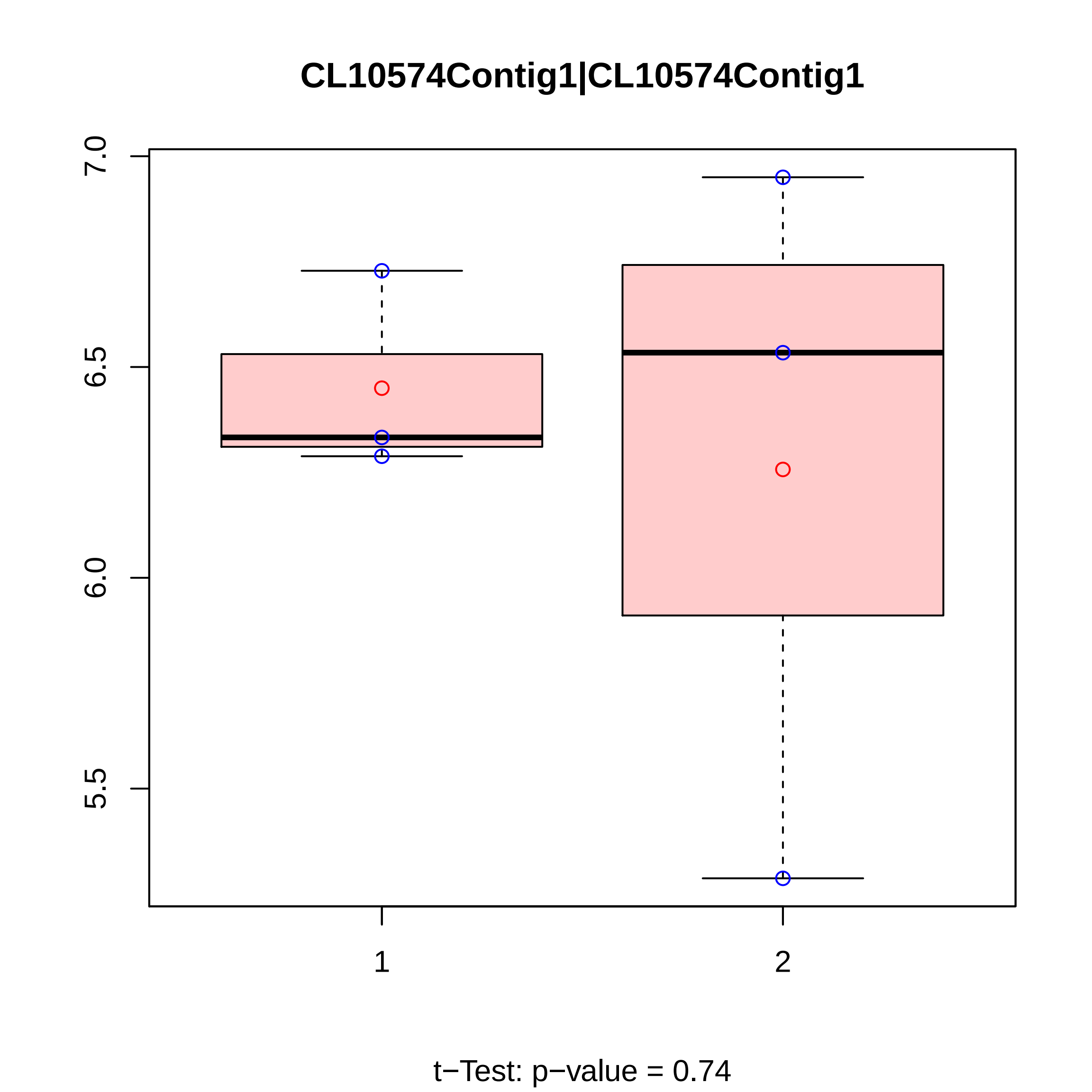
CL10574Contig1|CL10574Contig1
7.0
6.5
6.0
5.5
1
2
t−Test: p−value = 0.74

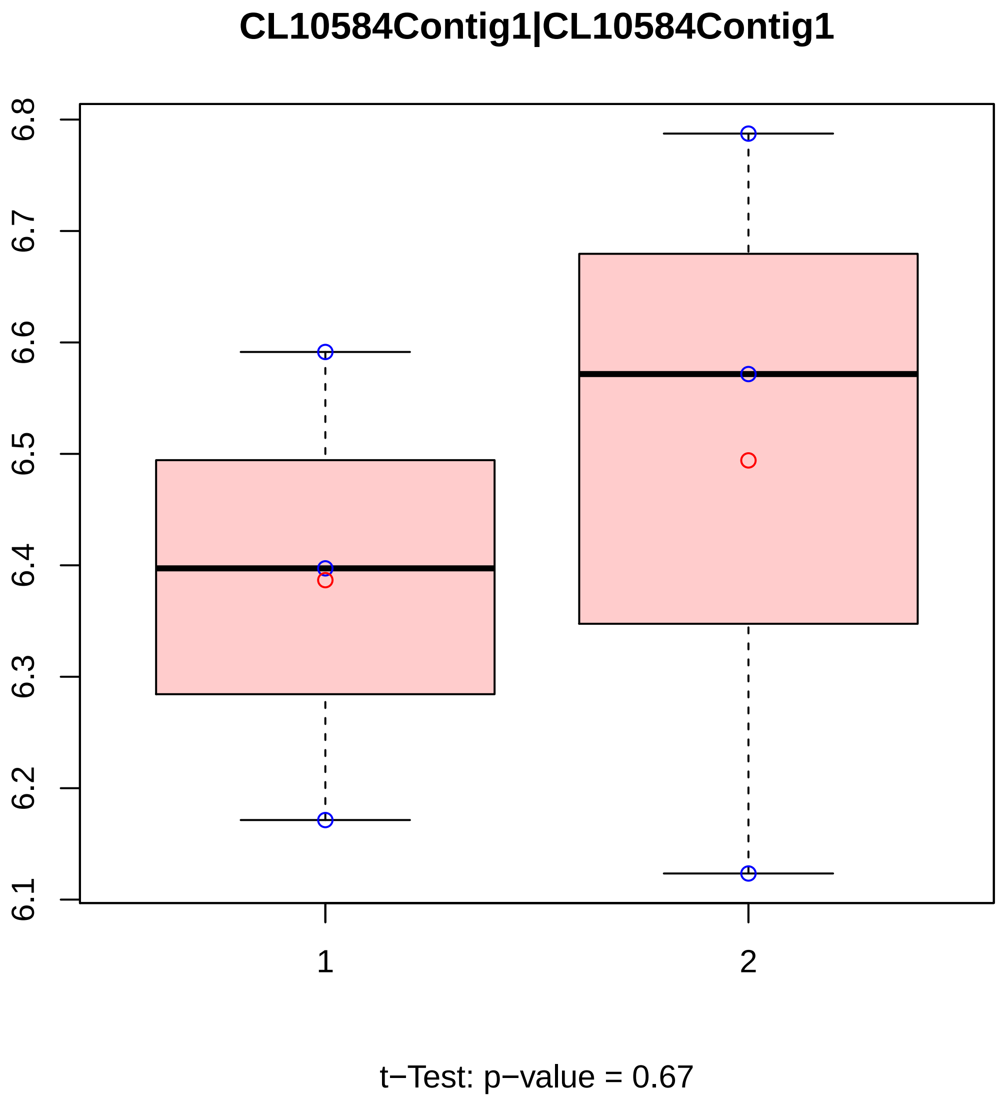
CL10584Contig1|CL10584Contig1
6.1
6.2
6.3
6.4
6.5
6.6
6.7
6.8
1
2
t−Test: p−value = 0.67

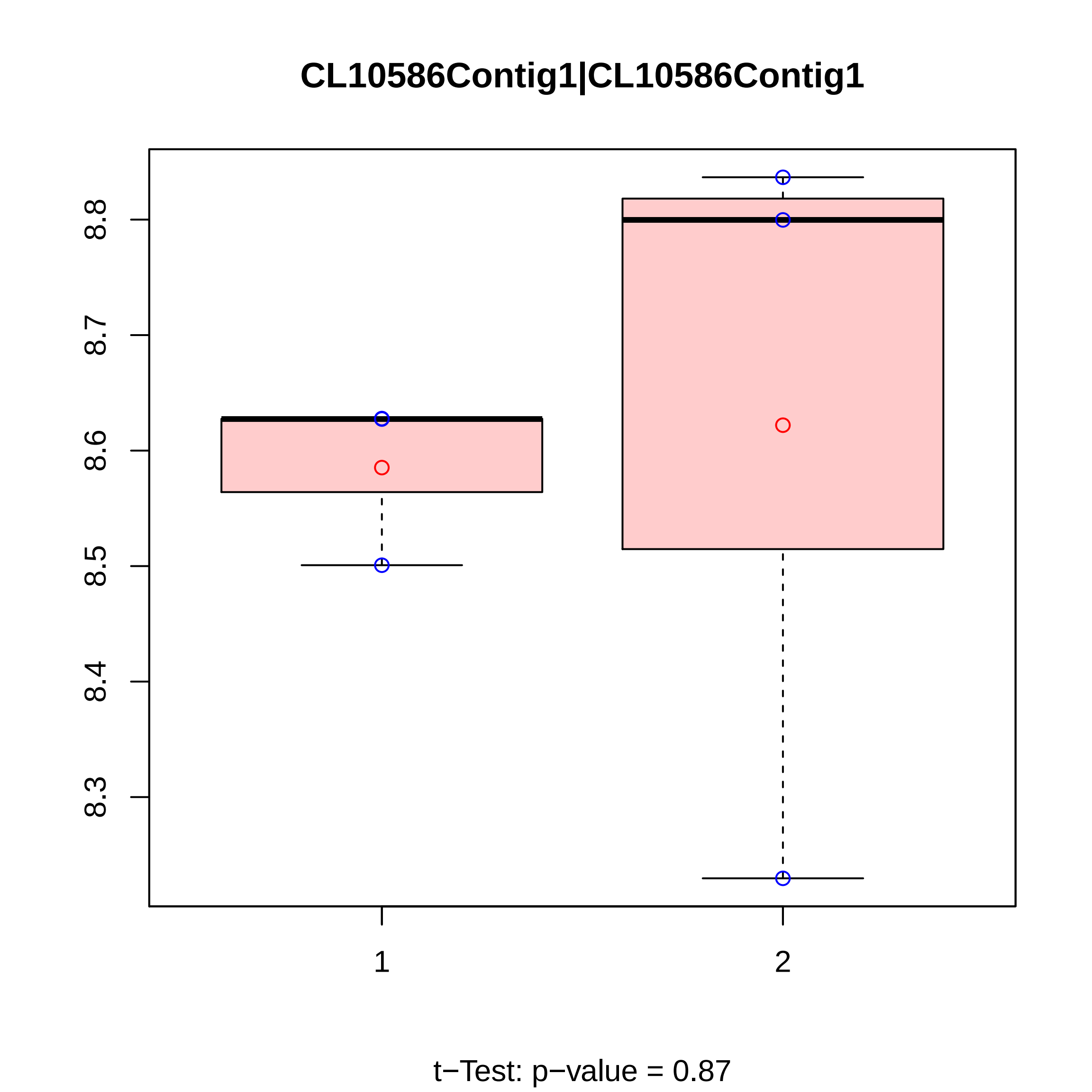
CL10586Contig1|CL10586Contig1
8.3
8.4
8.5
8.6
8.7
8.8
1
2
t−Test: p−value = 0.87

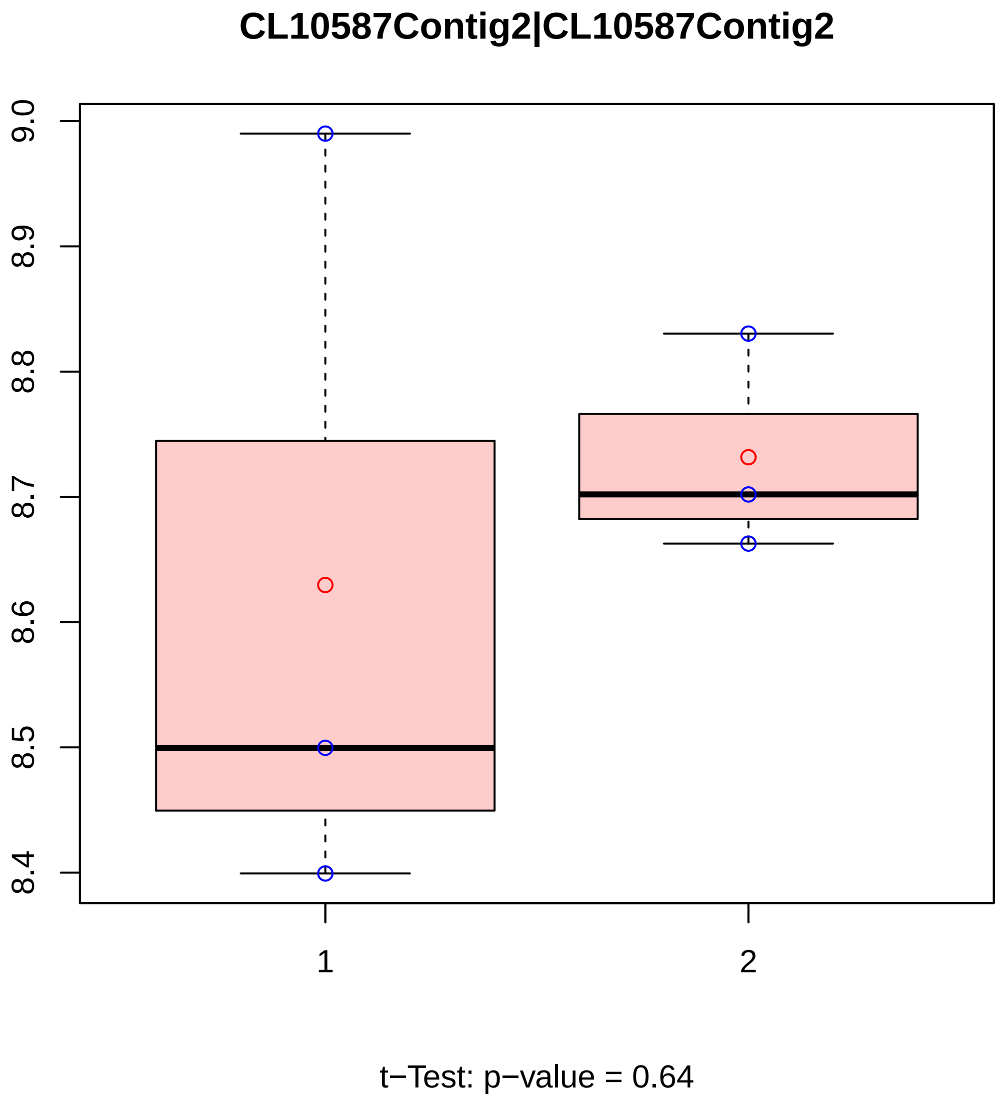

CL10587Contig2|CL10587Contig2
9.0
8.9
8.8
8.7
8.6
8.5
8.4
1
2
t−Test: p−value = 0.64

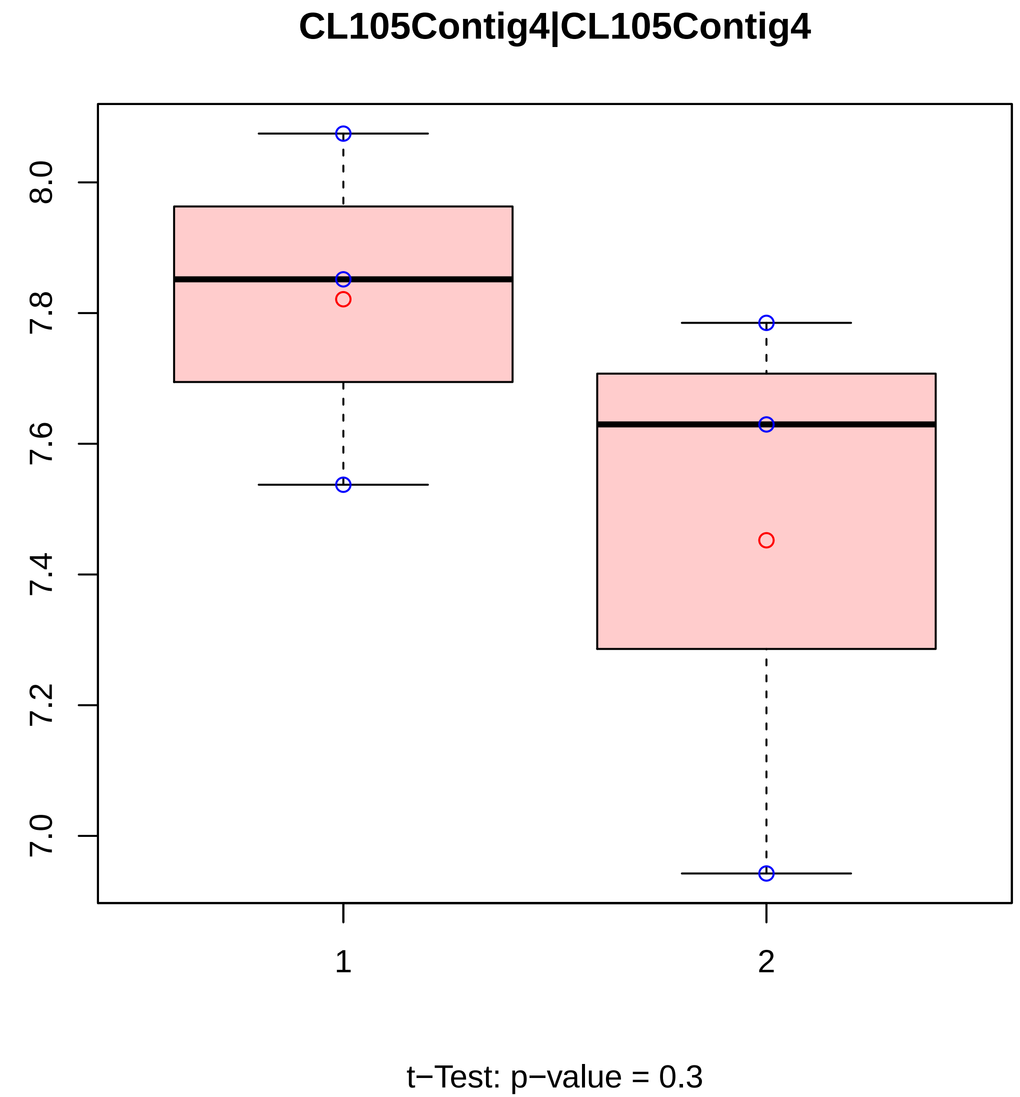

CL105Contig4|CL105Contig4
8.0
7.8
7.6
7.4
7.2
7.0
1
2
t−Test: p−value = 0.3

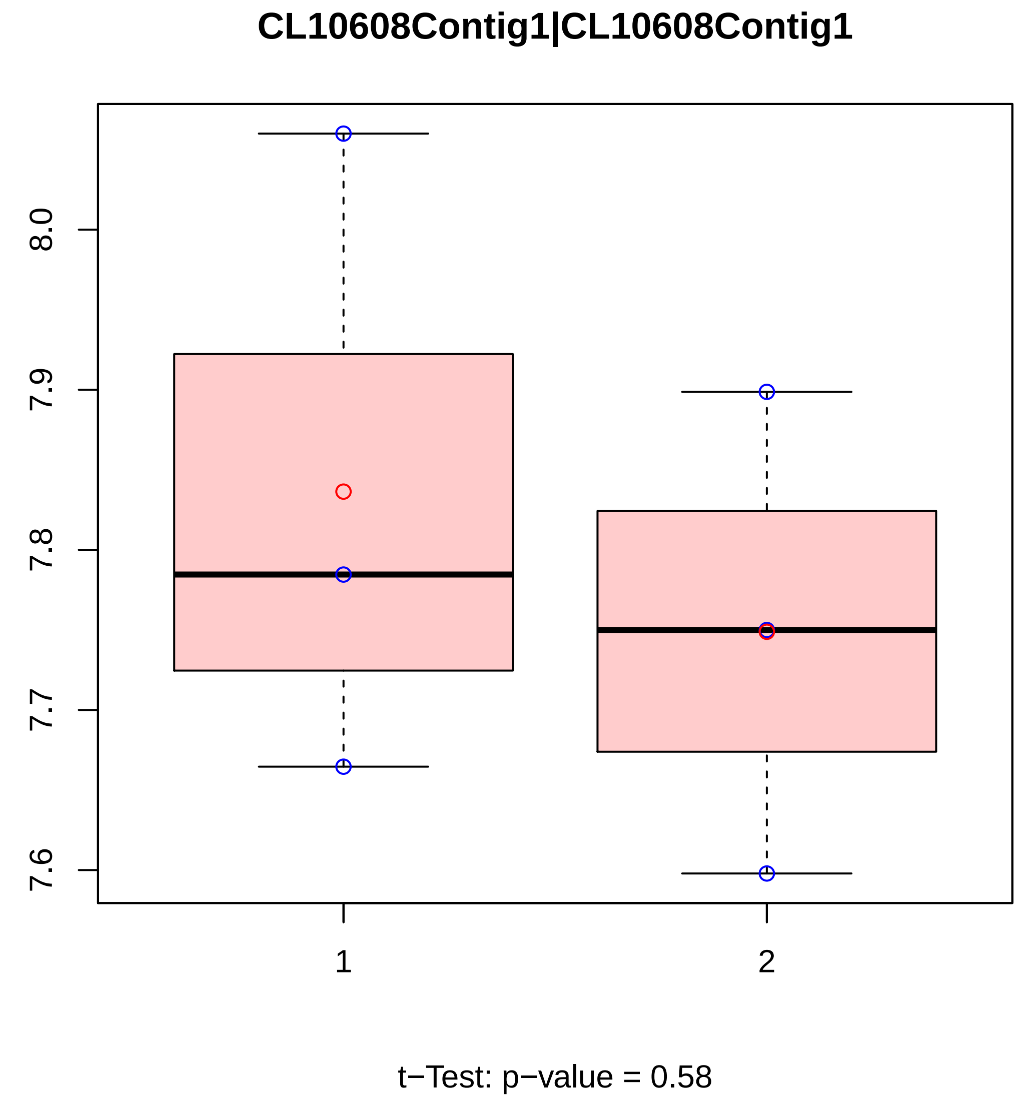
CL10608Contig1|CL10608Contig1
8.0
7.9
7.8
7.7
7.6
1
2
t−Test: p−value = 0.58

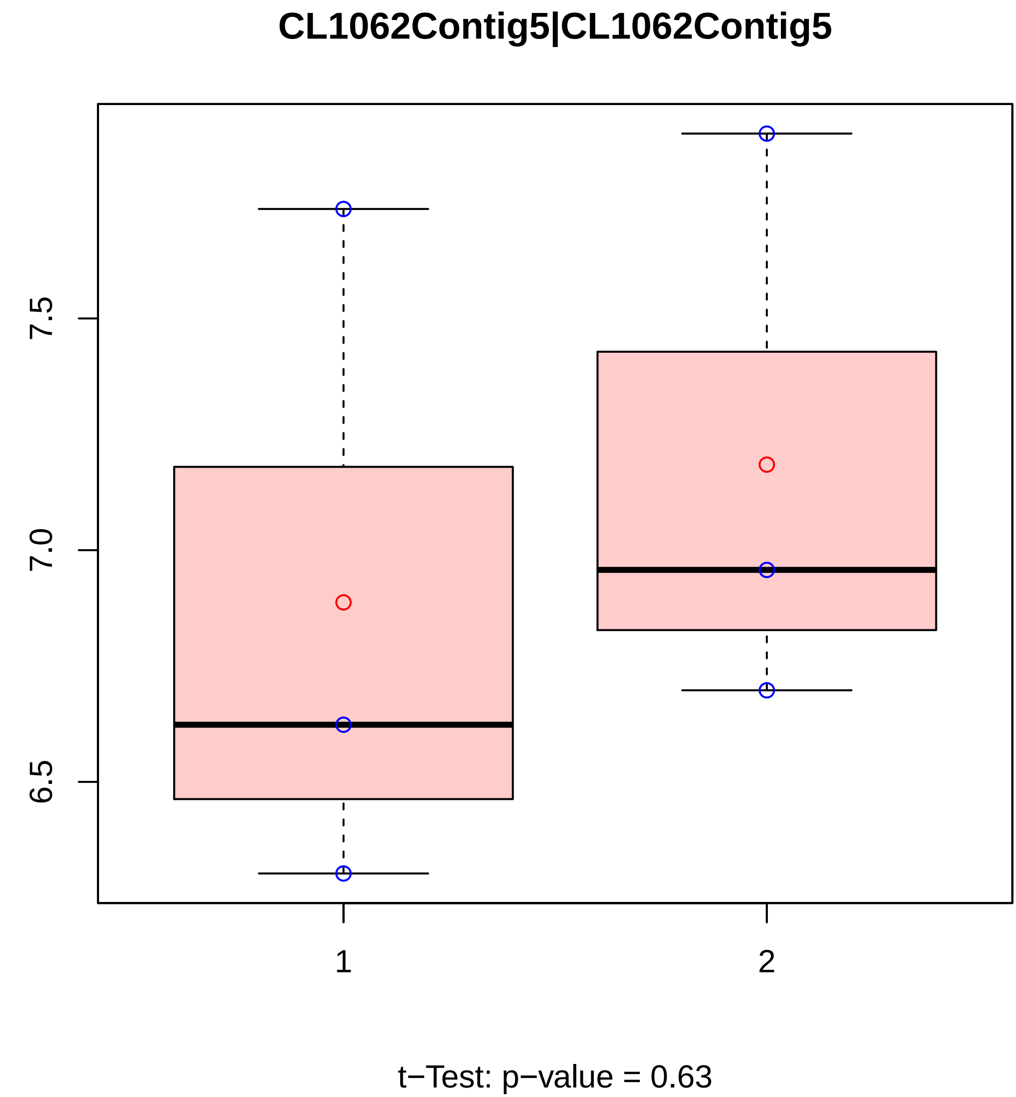
CL1062Contig5|CL1062Contig5
7.5
7.0
6.5
1
2
t−Test: p−value = 0.63

# CL10636Contig1|CL10636Contig1

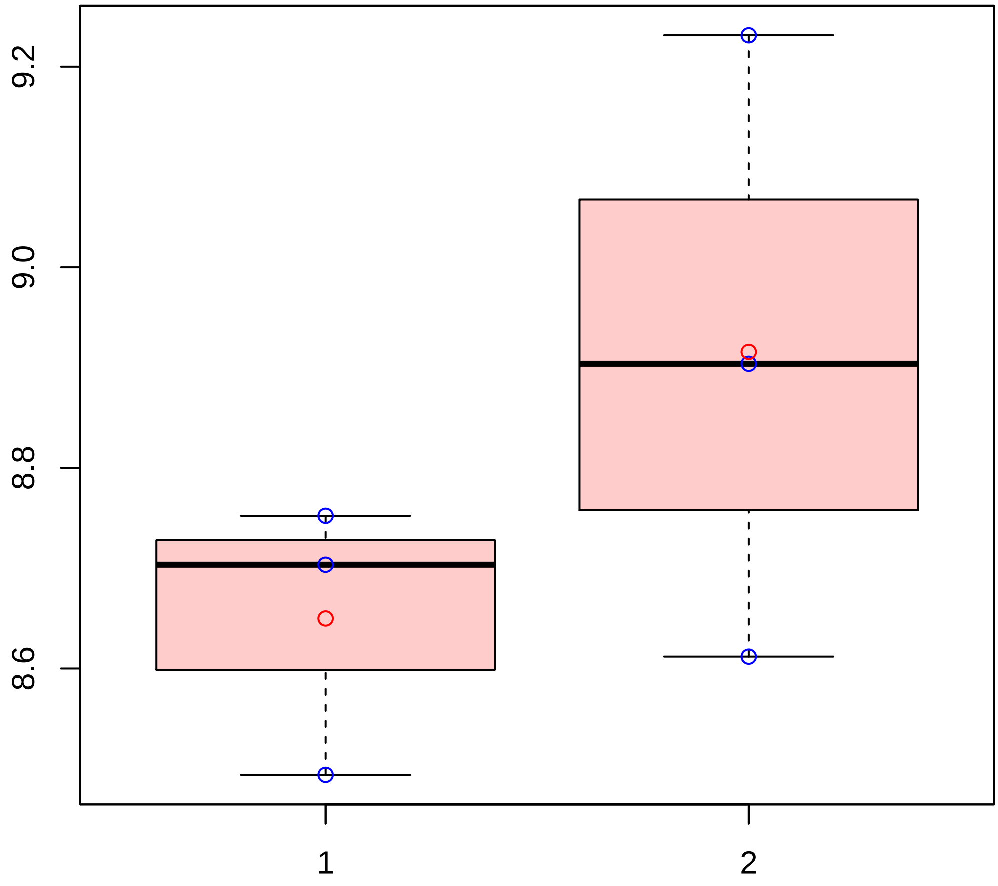


t−Test: p−value = 0.27

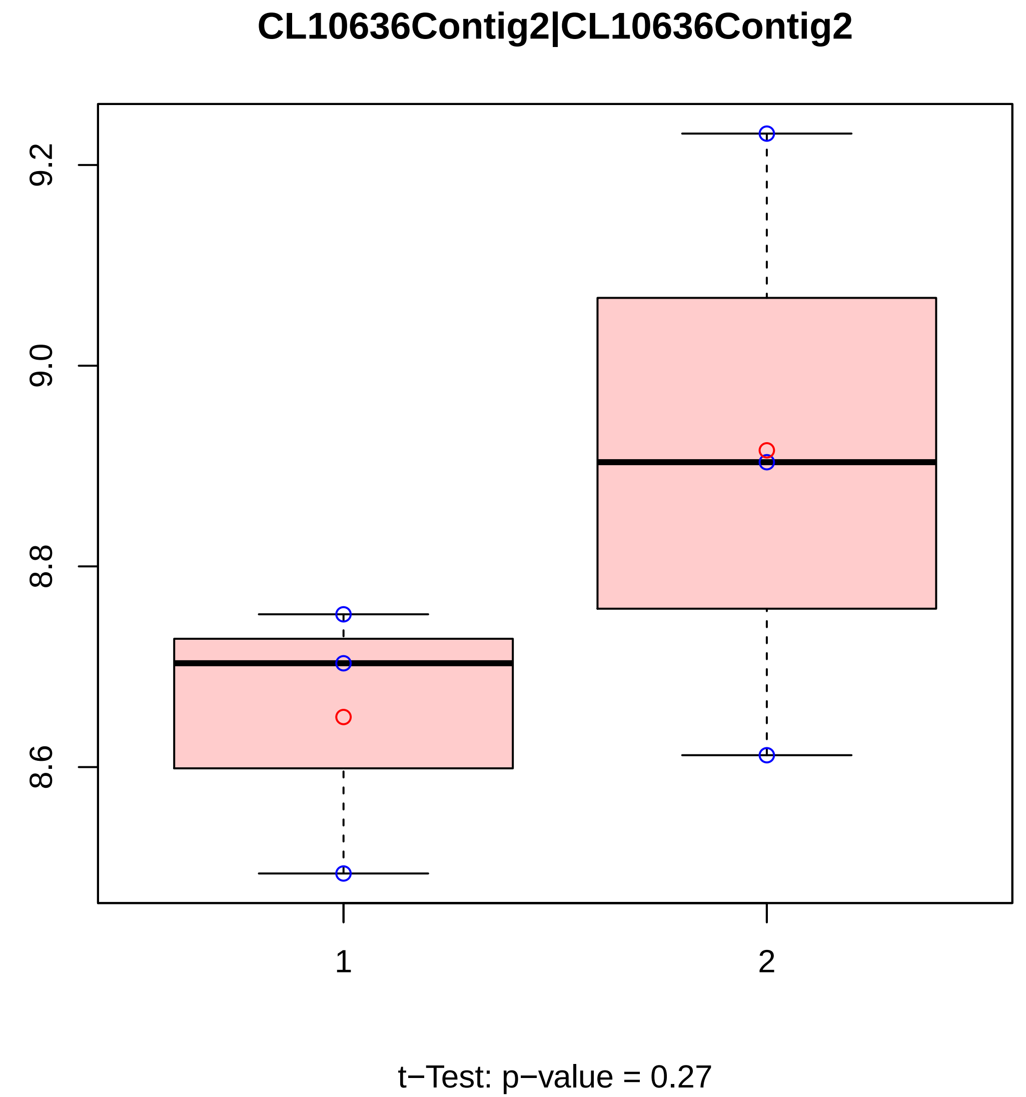
CL10636Contig2|CL10636Contig2
9.2
9.0
8.8
8.6
1
2
t−Test: p−value = 0.27

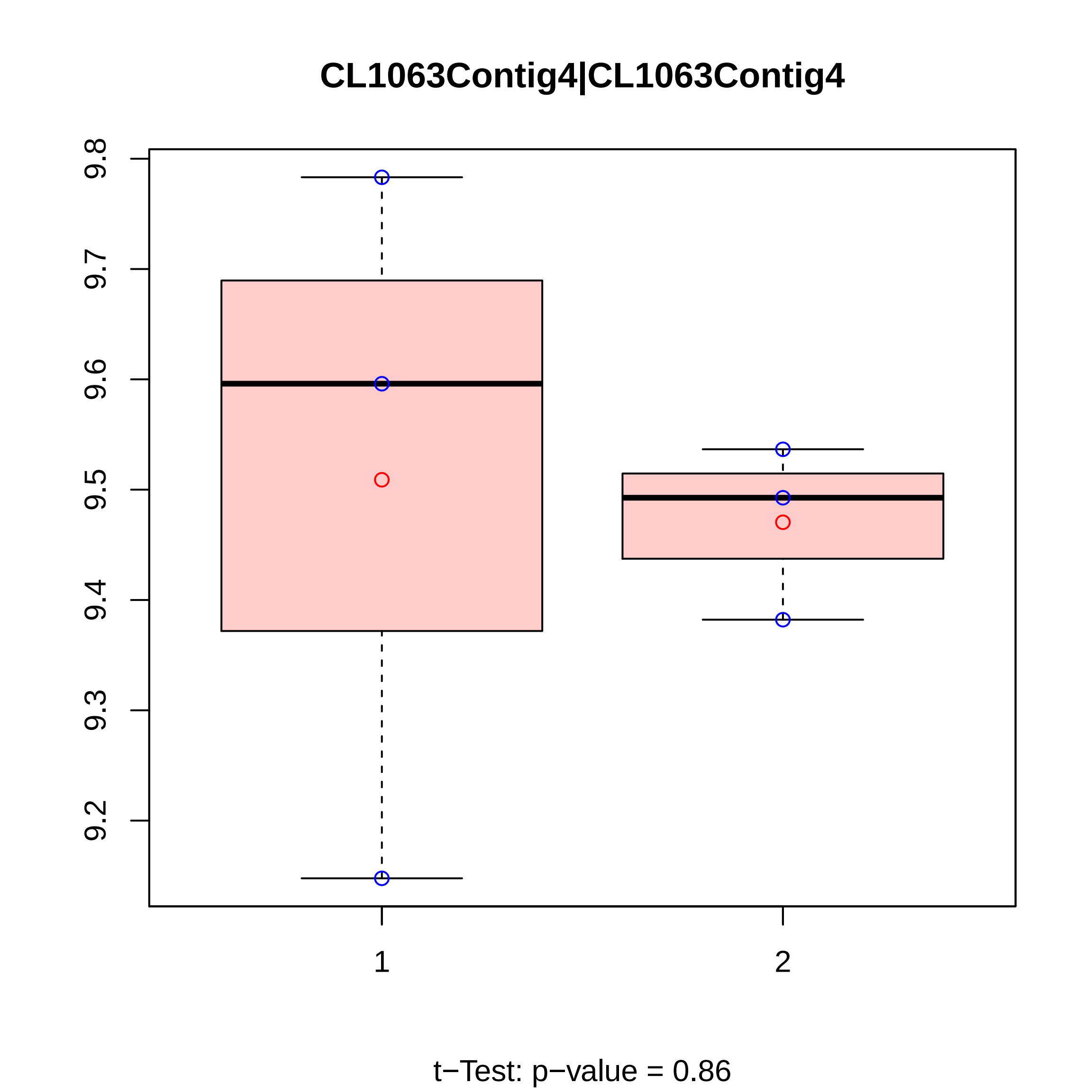
CL1063Contig4|CL1063Contig4
9.2
9.3
9.4
9.5
9.6
9.7
9.8
1
2
t−Test: p−value = 0.86

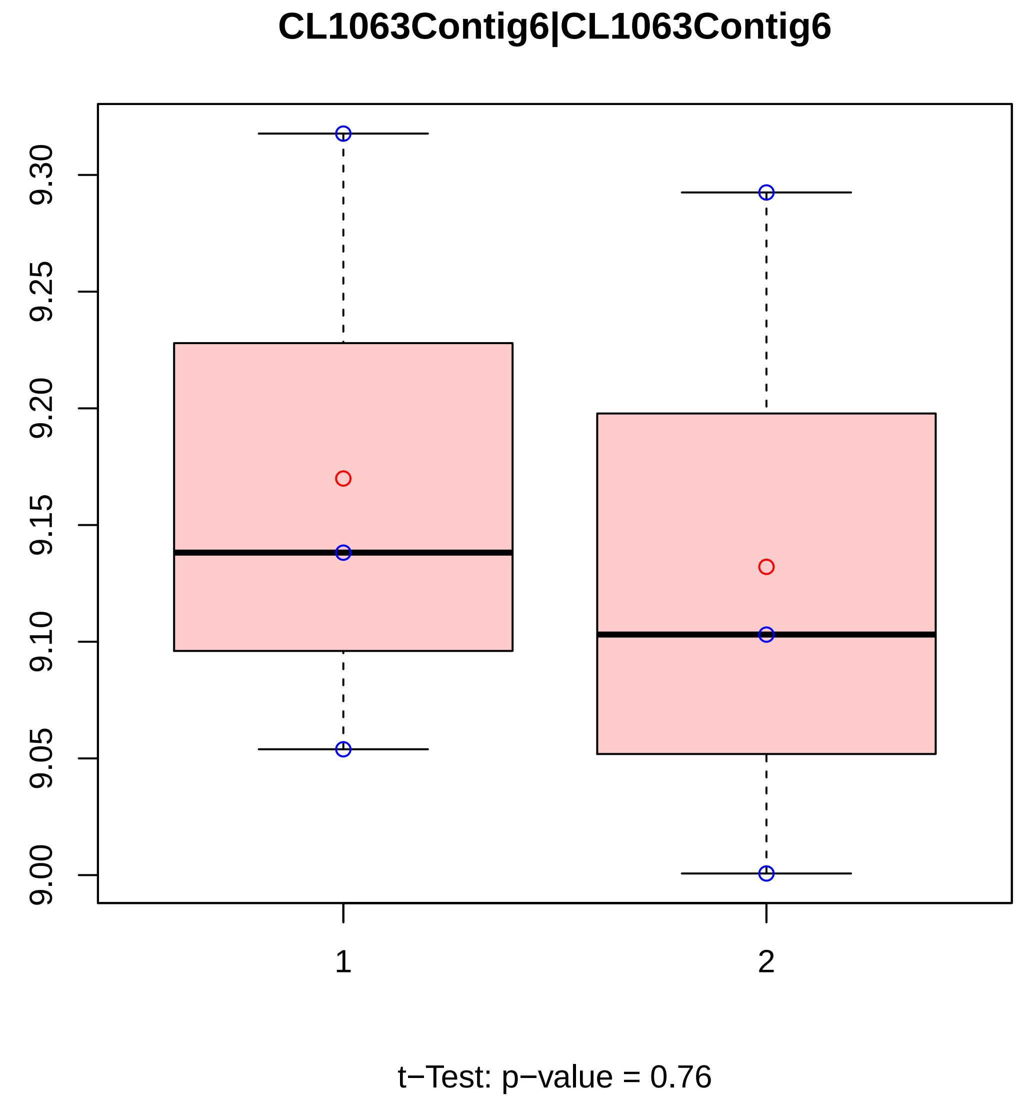
CL1063Contig6|CL1063Contig6
9.00
9.05
9.10
9.15
9.20
9.25
9.30
1
2
t−Test: p−value = 0.76

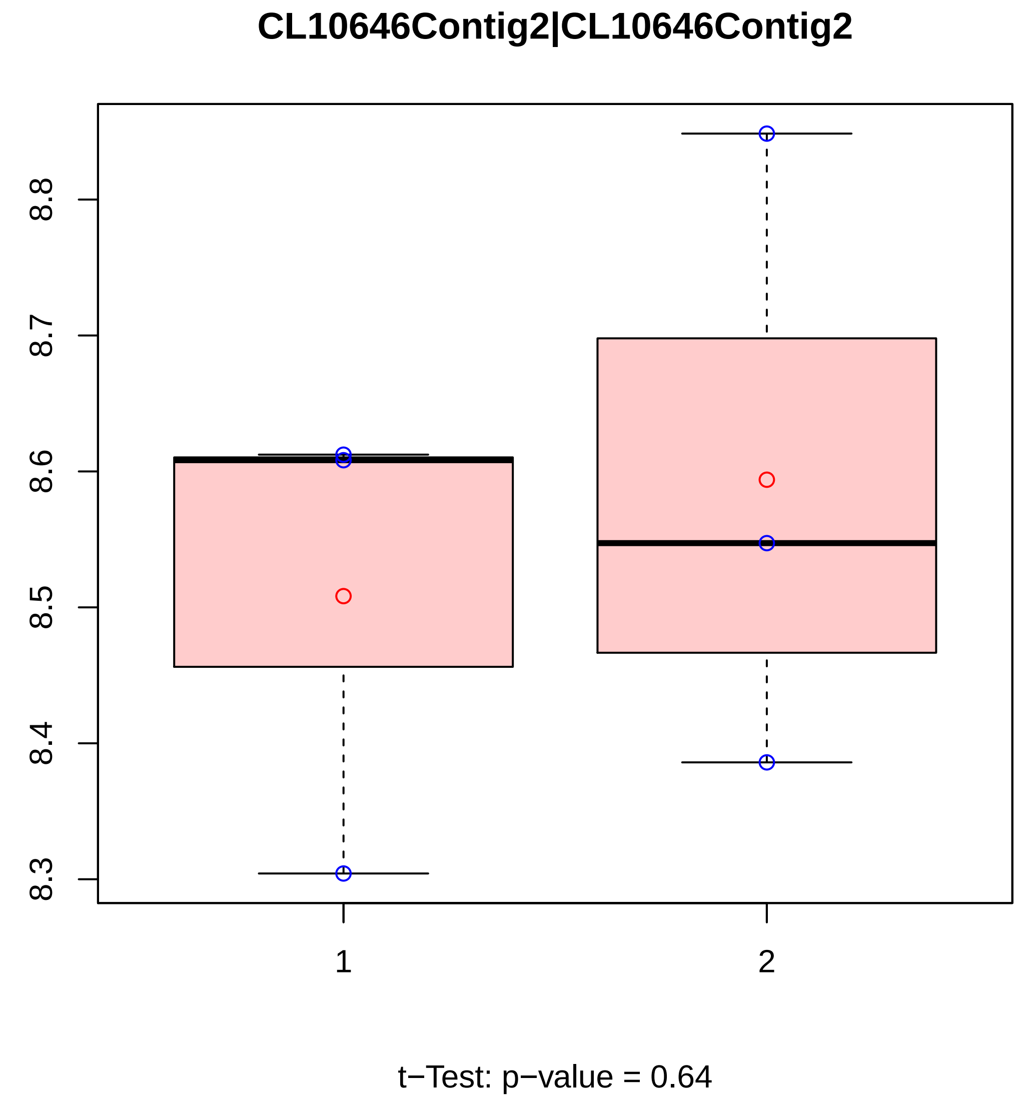
CL10646Contig2|CL10646Contig2
8.3
8.4
8.5
8.6
8.7
8.8
1
2
t−Test: p−value = 0.64

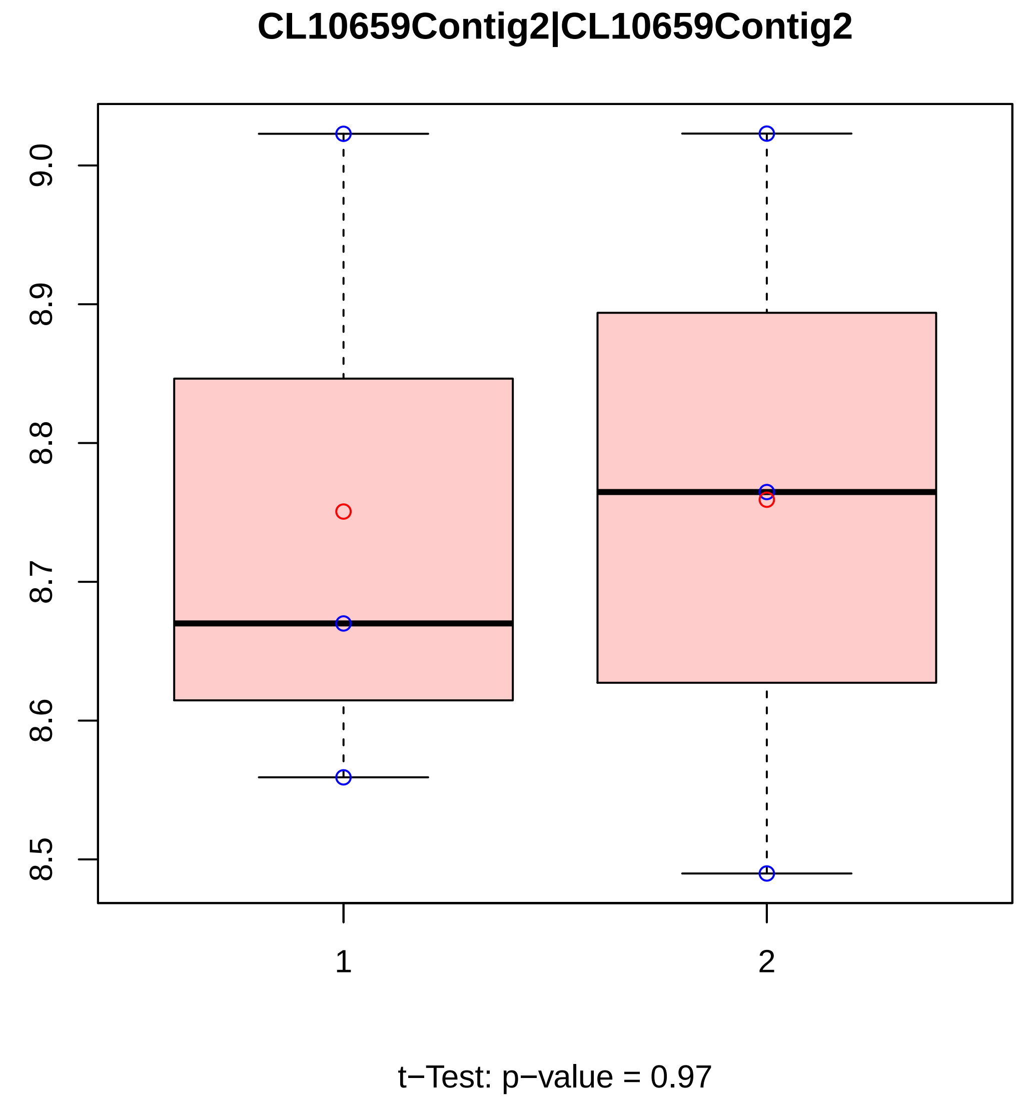
CL10659Contig2|CL10659Contig2
9.0
8.9
8.8
8.7
8.6
8.5
1
2
t−Test: p−value = 0.97

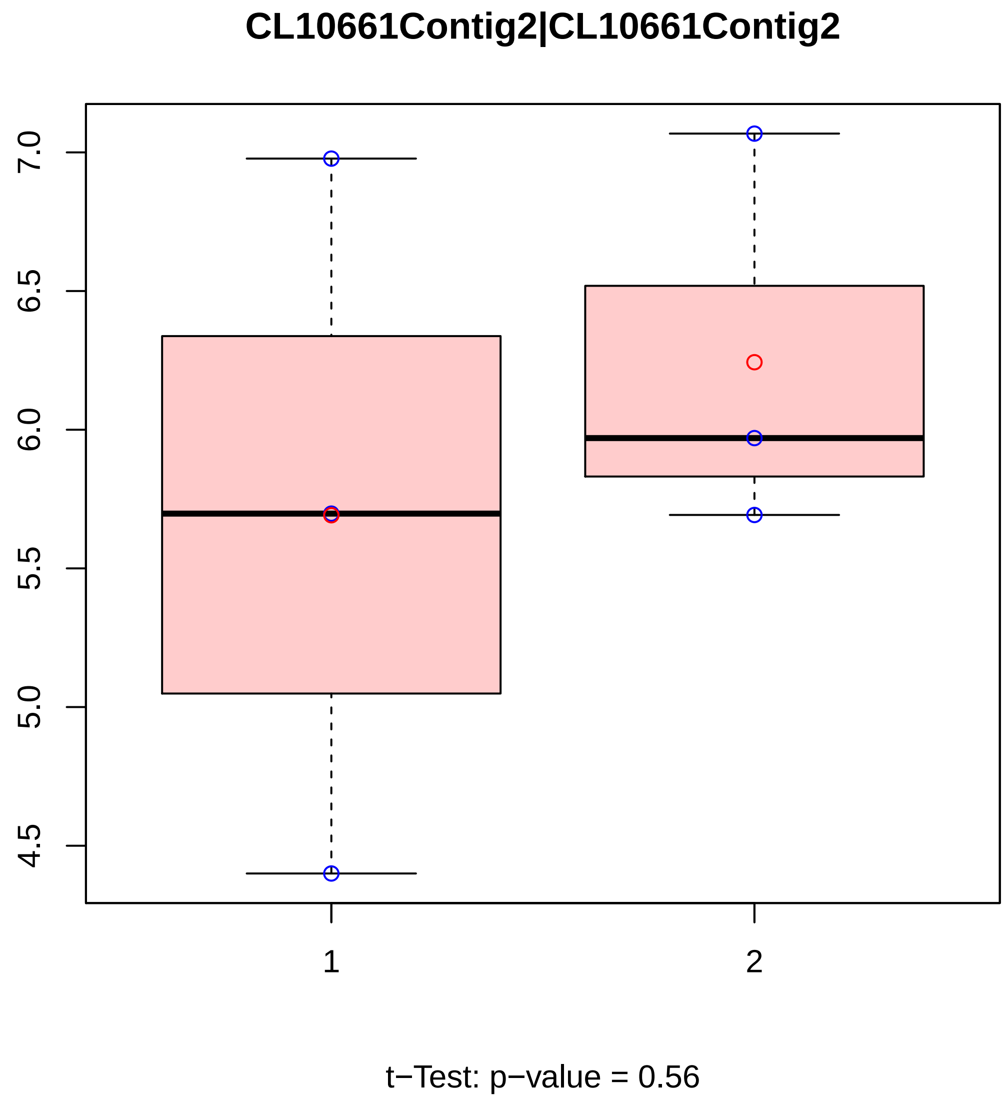
CL10661Contig2|CL10661Contig2
7.0
6.5
6.0
5.5
5.0
4.5
1
2
t−Test: p−value = 0.56

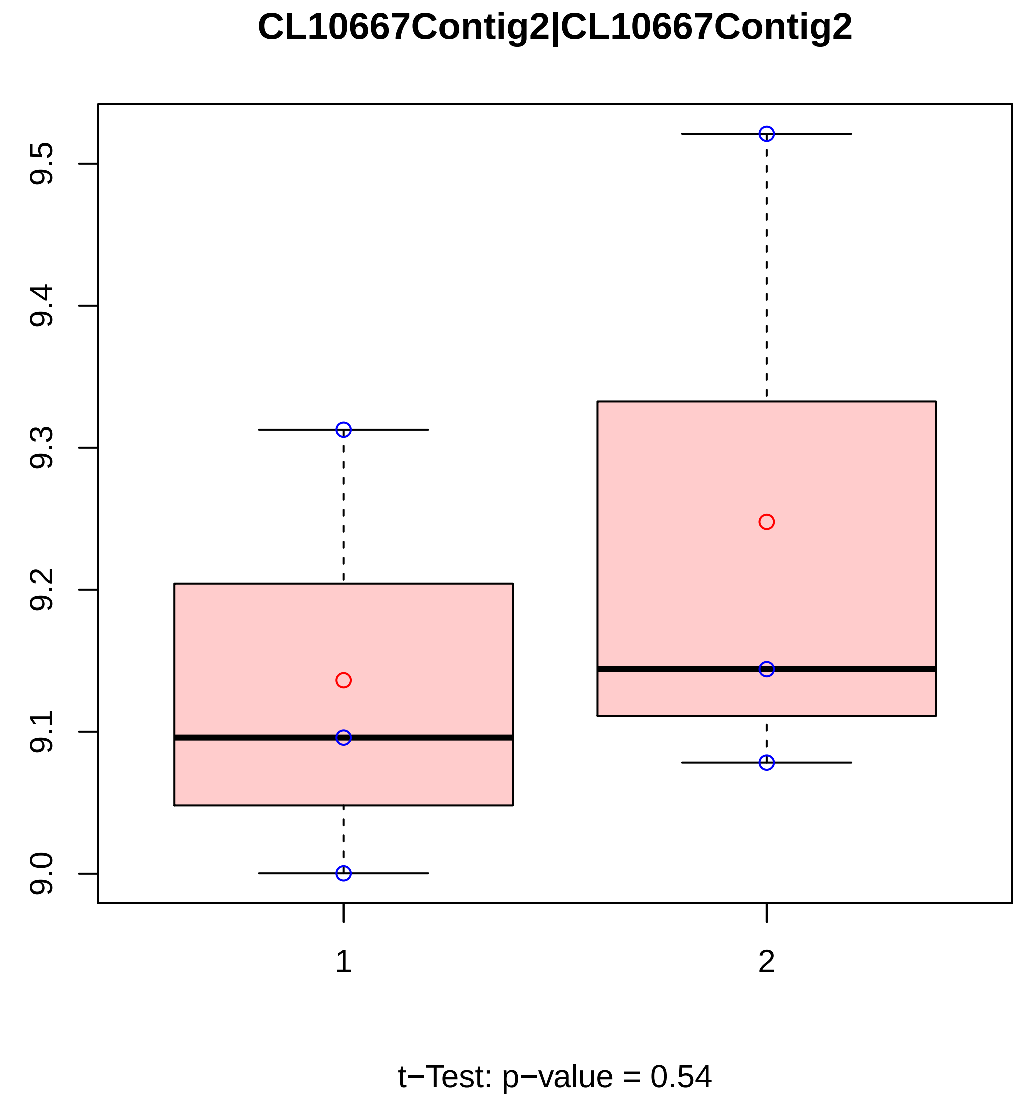
CL10667Contig2|CL10667Contig2
9.0
9.1
9.2
9.3
9.4
9.5
1
2
t−Test: p−value = 0.54

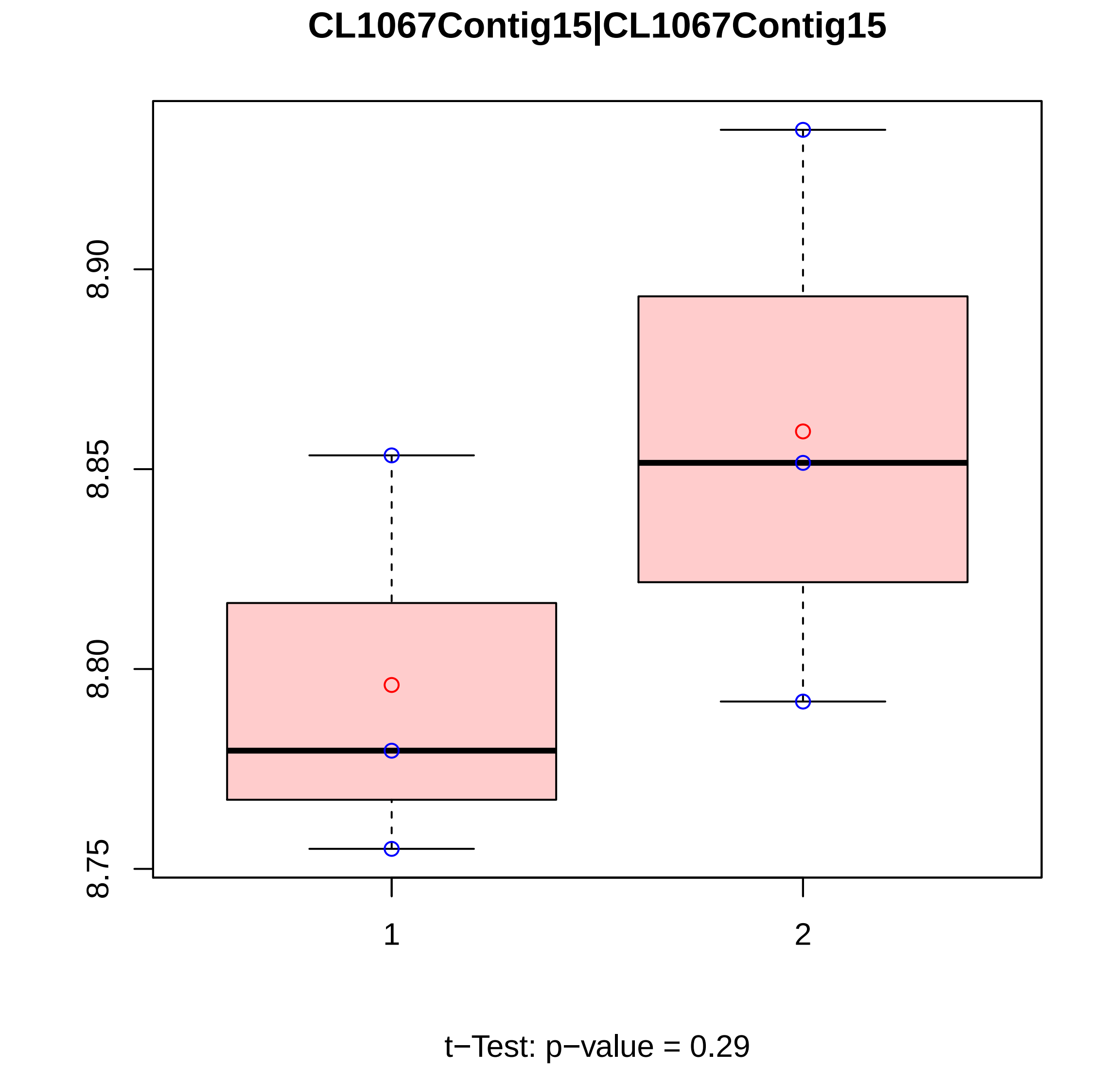
CL1067Contig15|CL1067Contig15
8.75
8.80
8.85
8.90
1
2
t−Test: p−value = 0.29

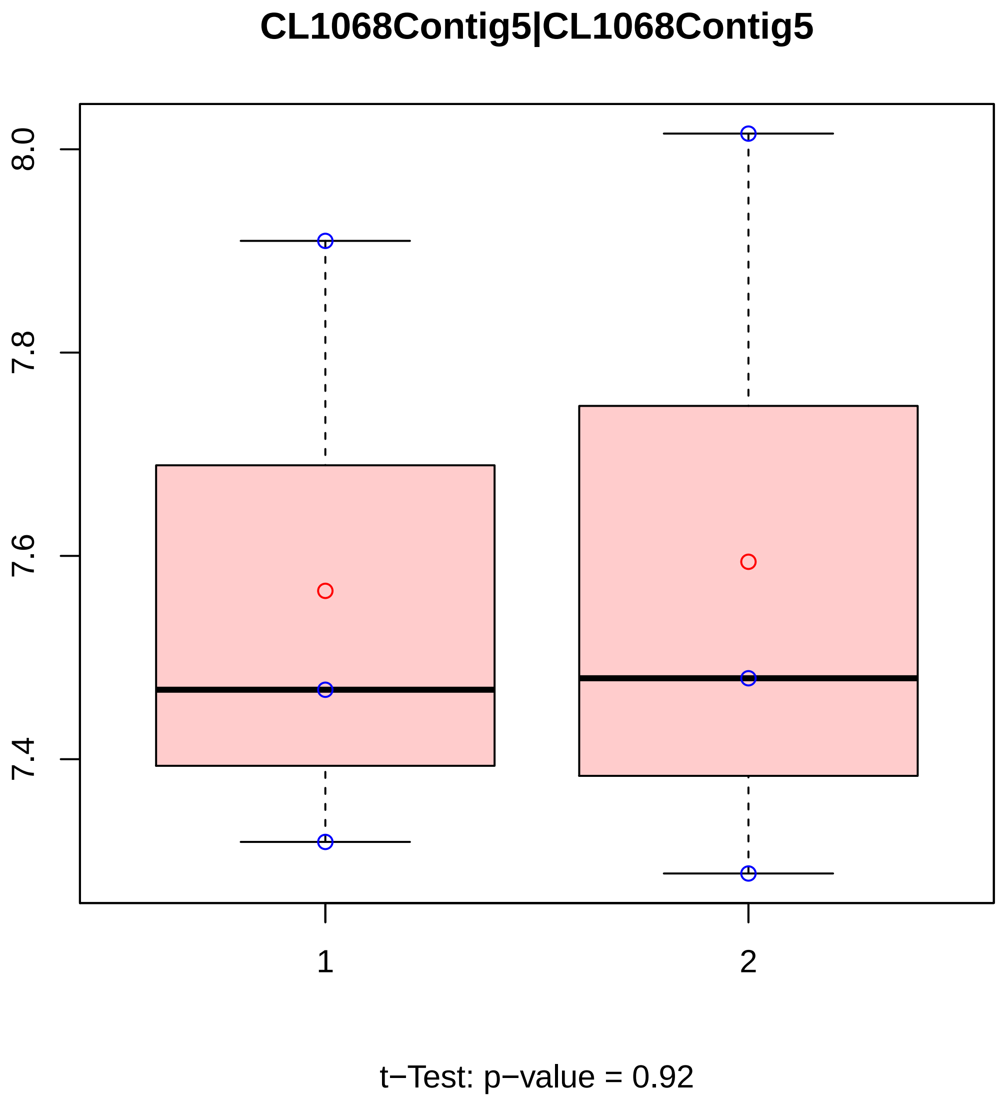
CL1068Contig5|CL1068Contig5
8.0
7.8
7.6
7.4
1
2
t−Test: p−value = 0.92

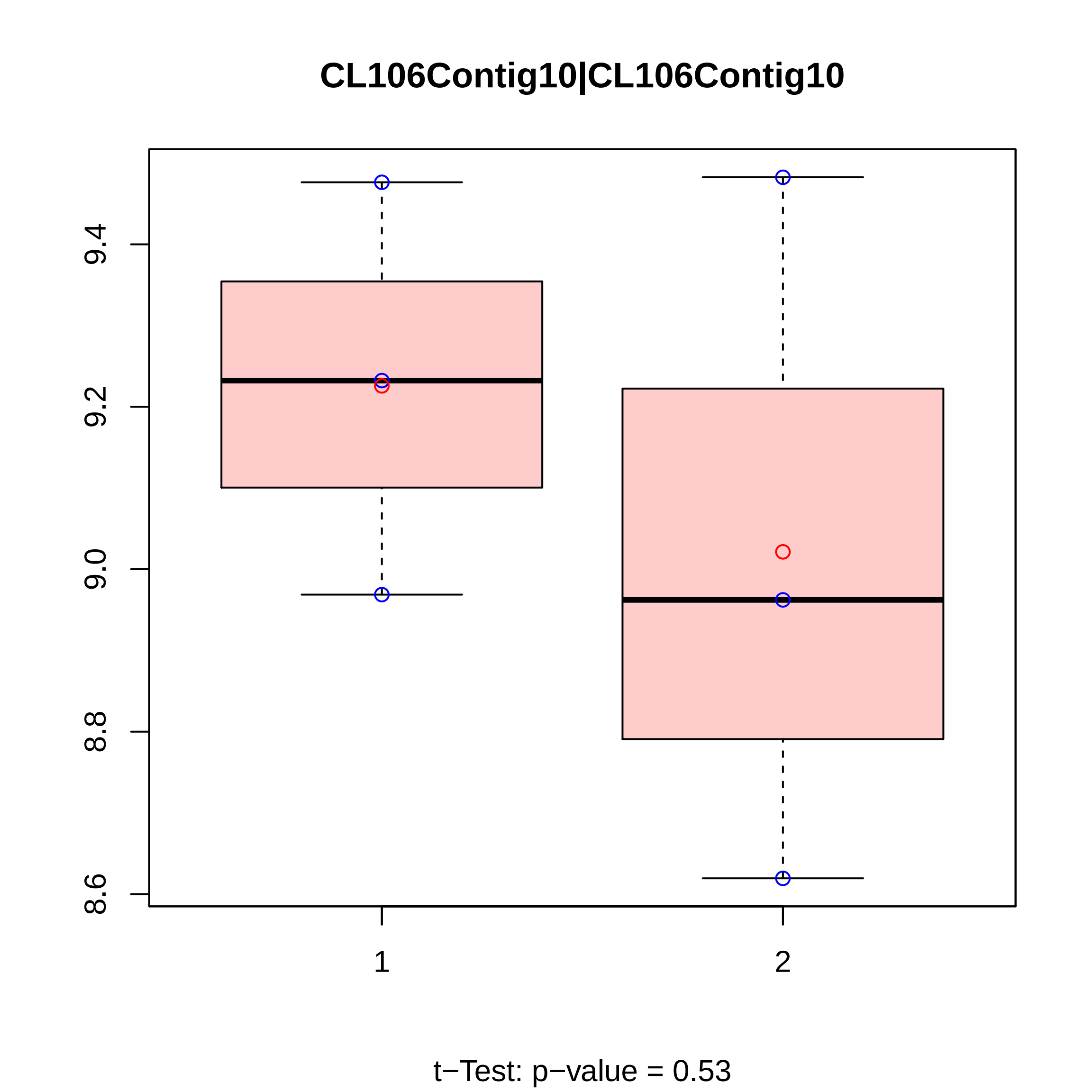

CL106Contig10|CL106Contig10
9.4
9.2
9.0
8.8
8.6
1
2
t−Test: p−value = 0.53

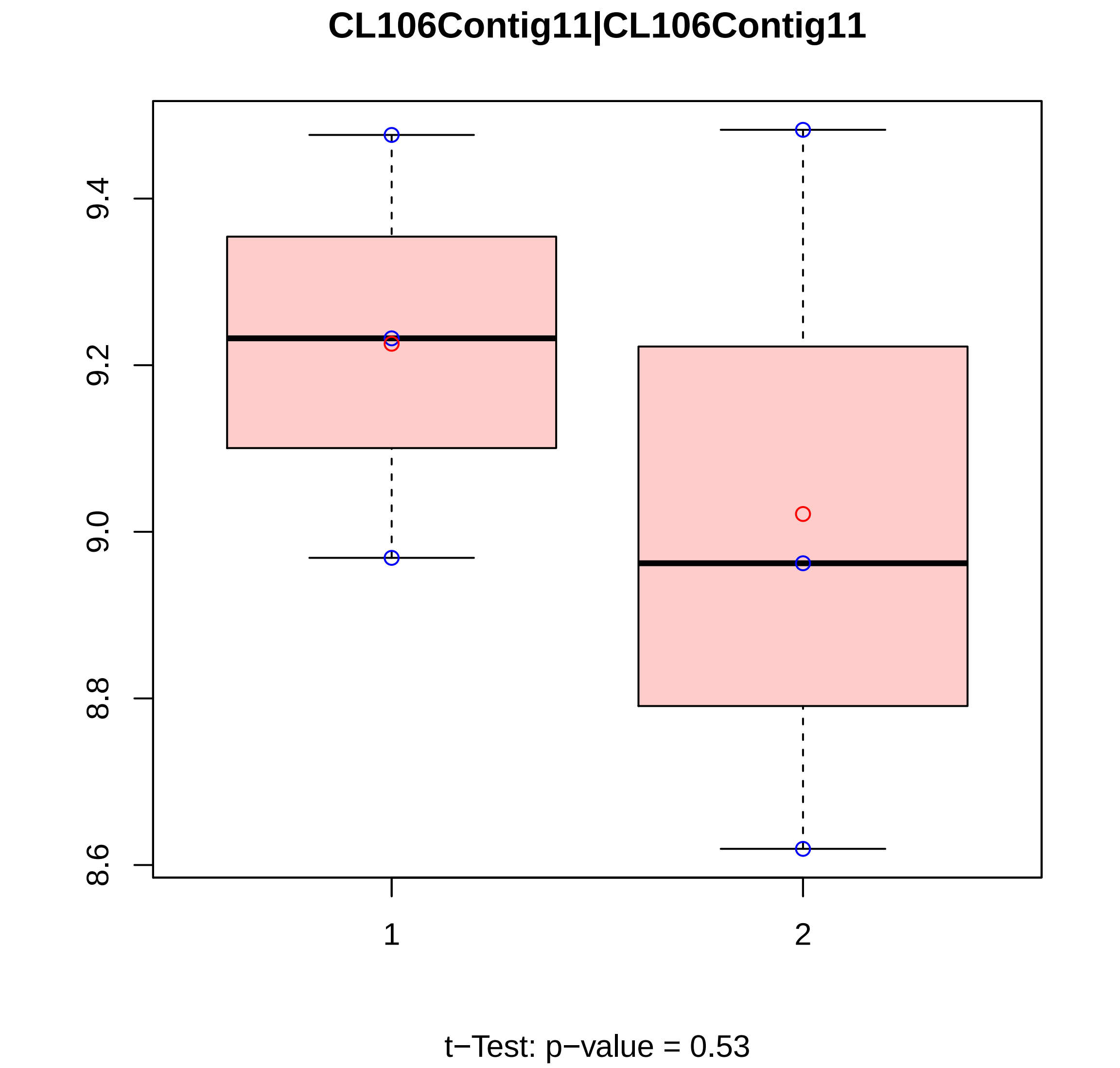
CL106Contig11|CL106Contig11
9.4
9.2
9.0
8.8
8.6
1
2
t−Test: p−value = 0.53

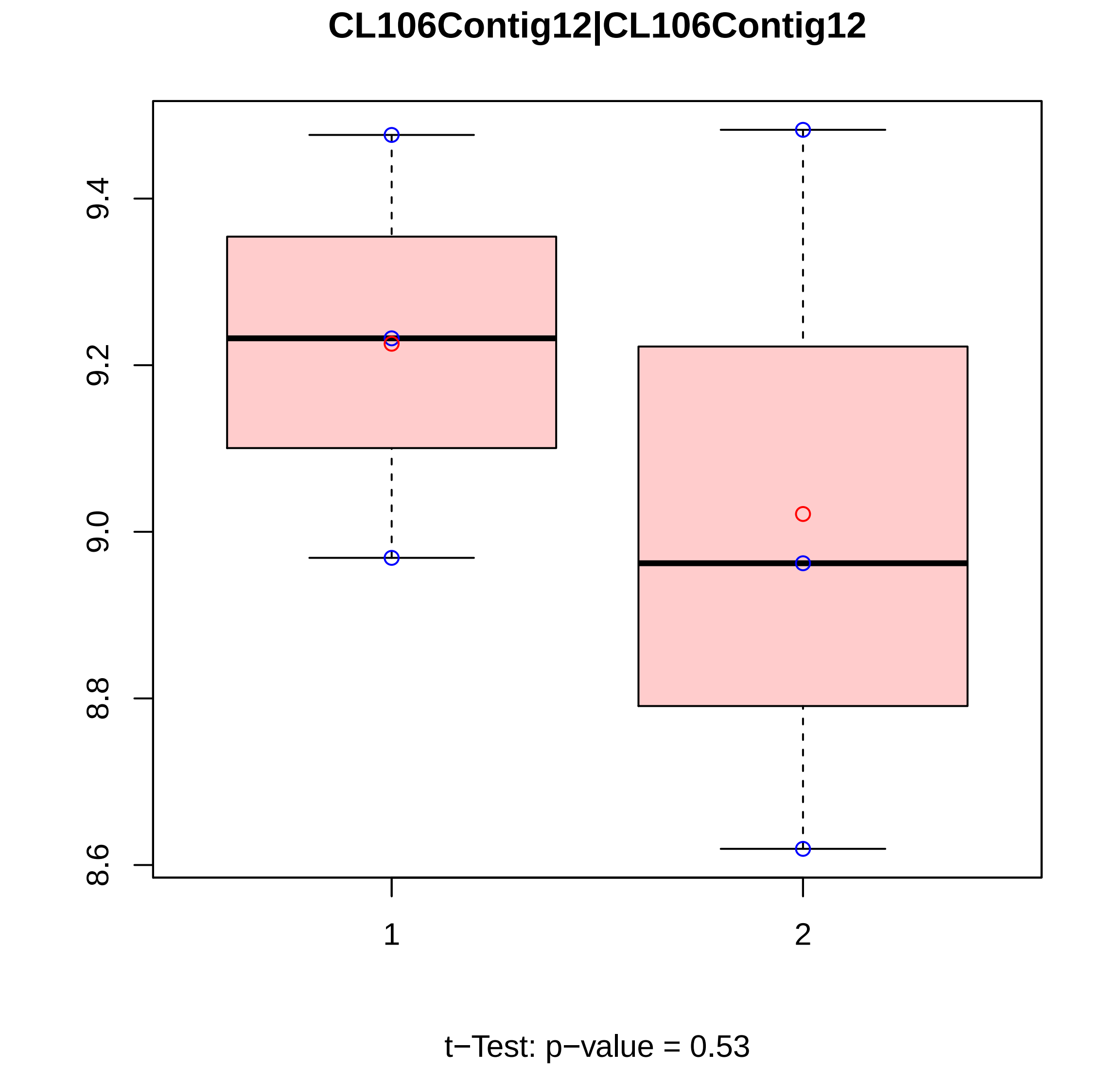
CL106Contig12|CL106Contig12
9.4
9.2
9.0
8.8
8.6
1
2
t−Test: p−value = 0.53

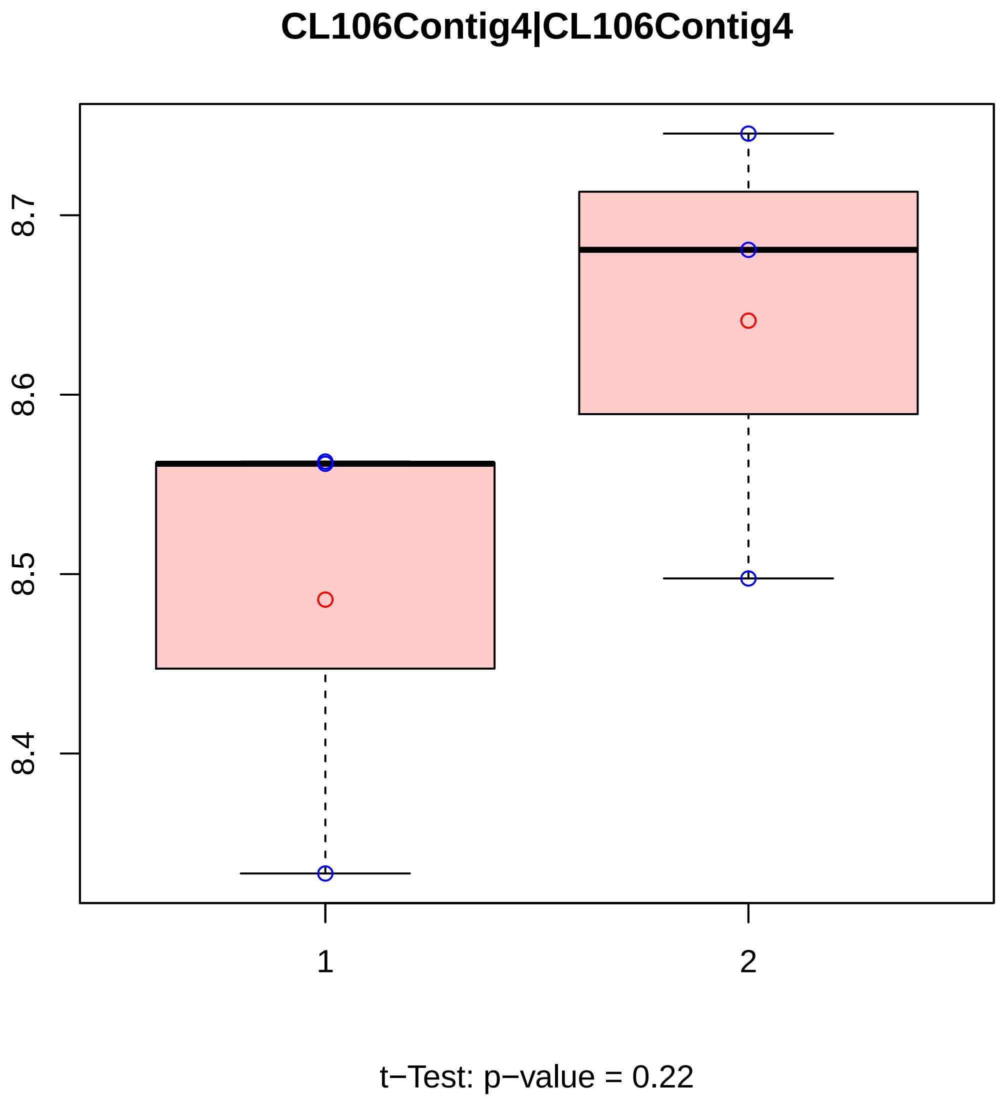
CL106Contig4|CL106Contig4
8.4
8.5
8.6
8.7
1
2
t−Test: p−value = 0.22

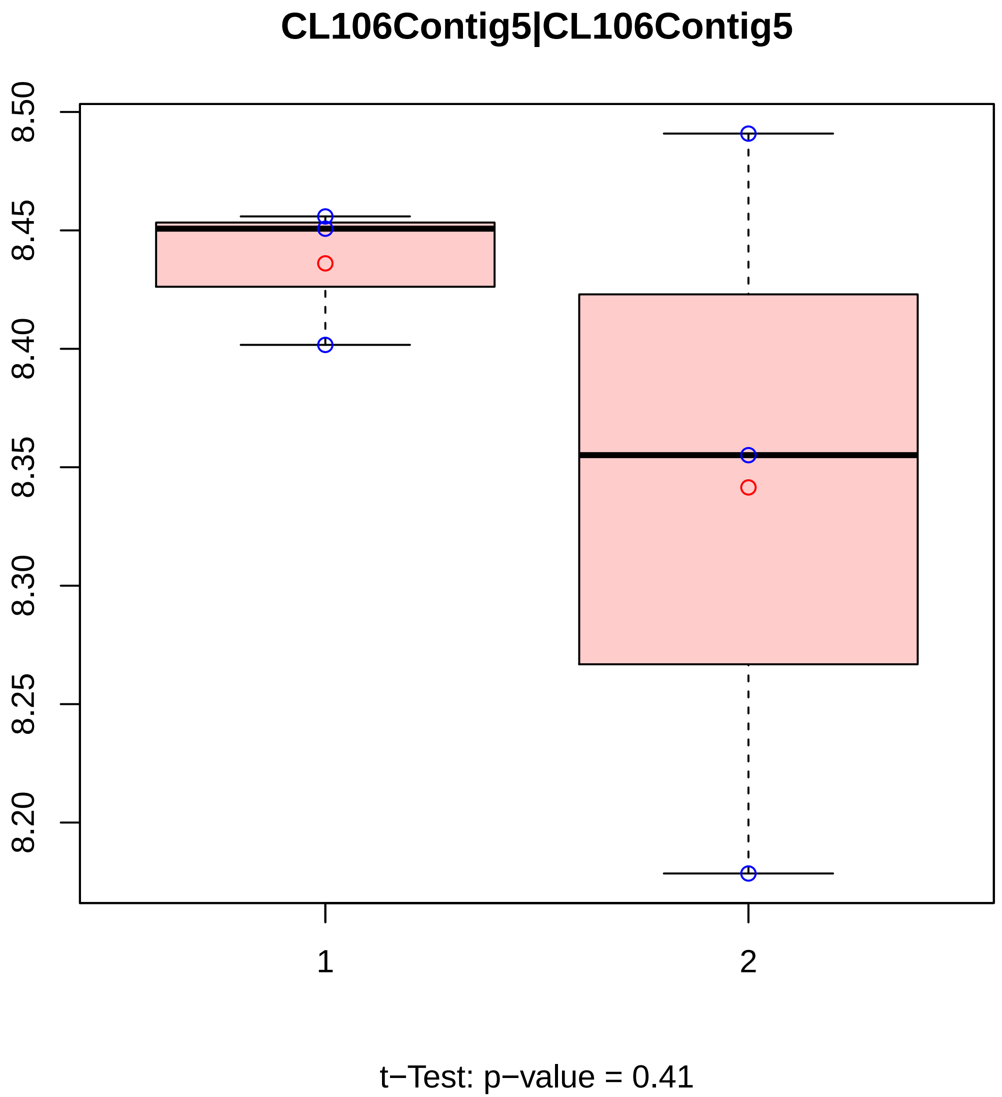
CL106Contig5|CL106Contig5
8.50
8.45
8.40
8.35
8.30
8.25
8.20
1
2
t−Test: p−value = 0.41

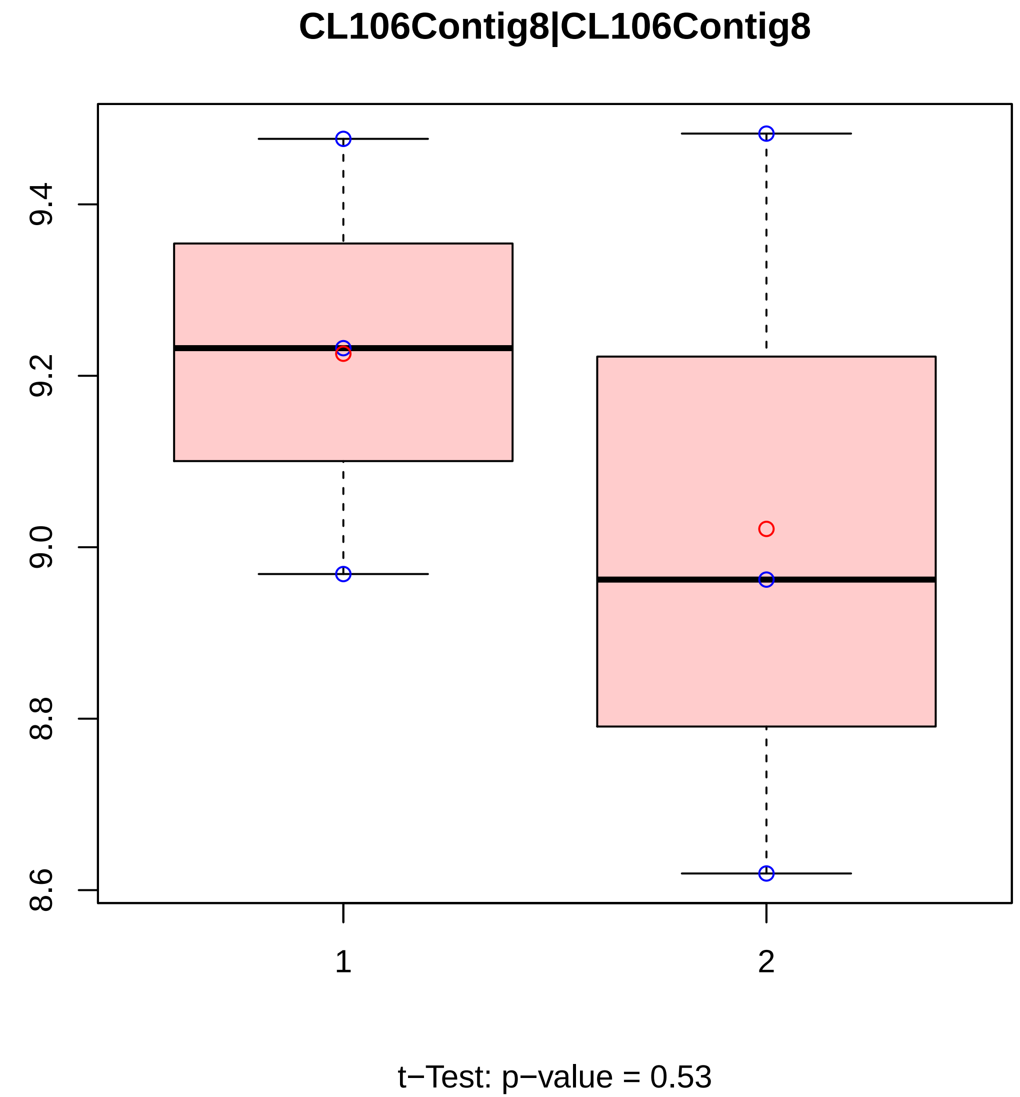
CL106Contig8|CL106Contig8
9.4
9.2
9.0
8.8
8.6
1
2
t−Test: p−value = 0.53

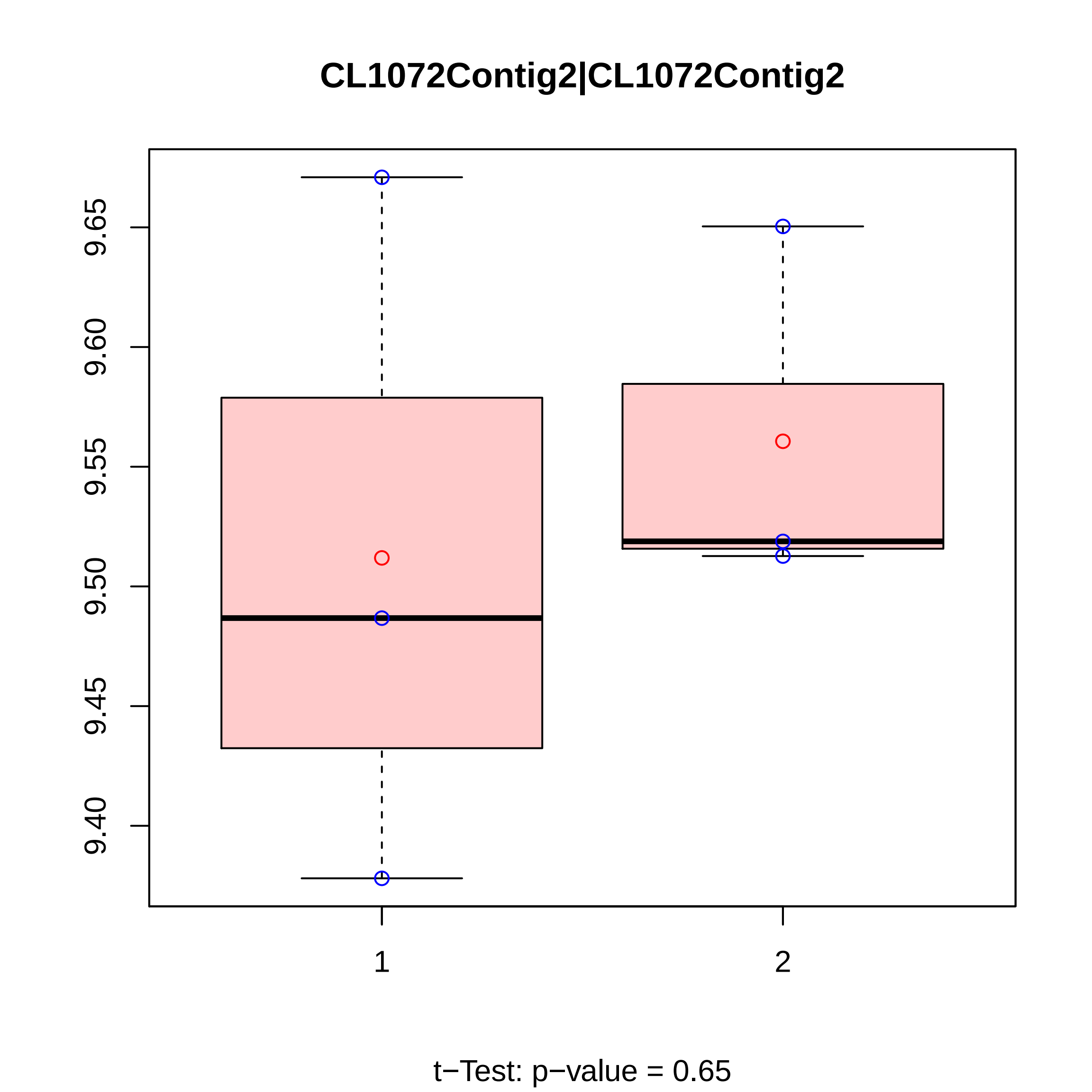
CL1072Contig2|CL1072Contig2
9.40
9.45
9.50
9.55
9.60
9.65
1
2
t−Test: p−value = 0.65

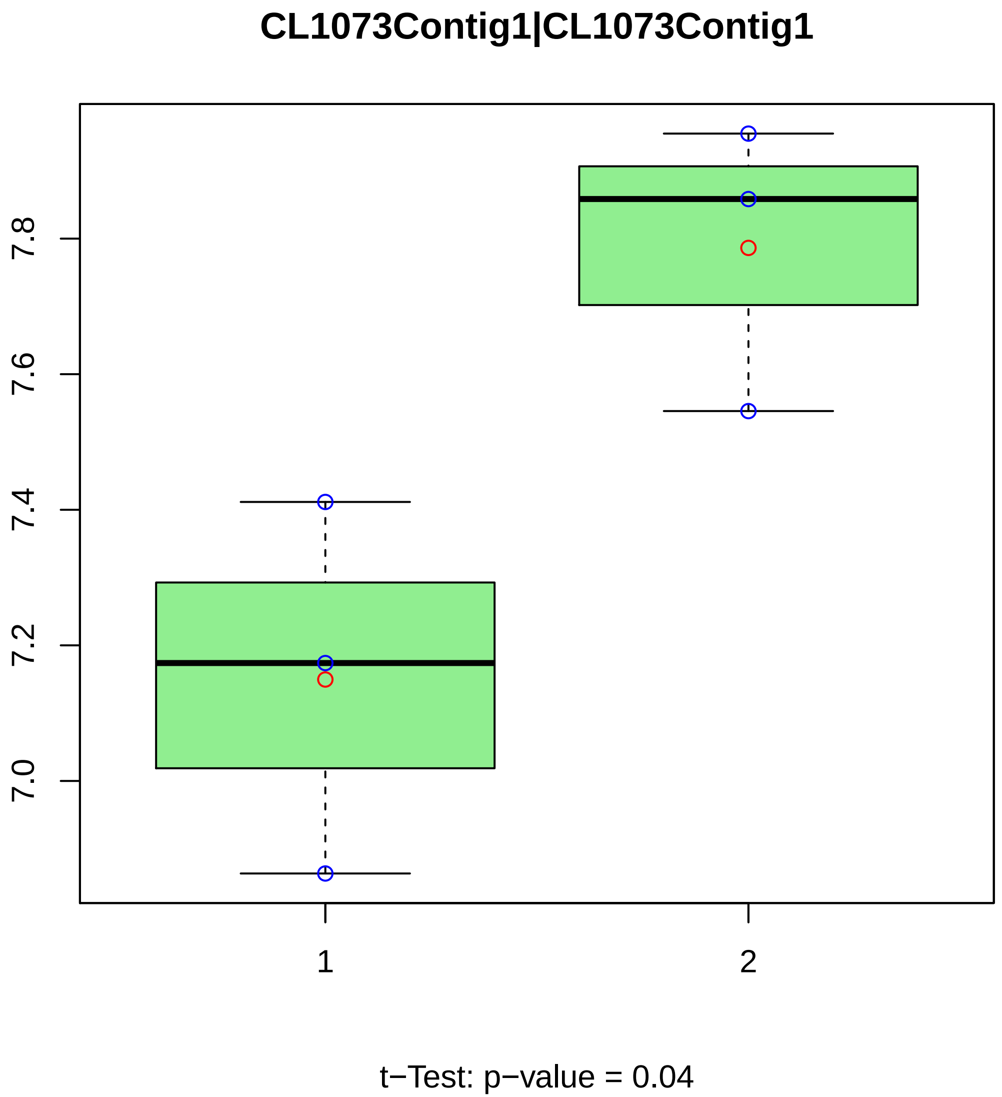
CL1073Contig1|CL1073Contig1
7.0
7.2
7.4
7.6
7.8
1
2
t−Test: p−value = 0.04

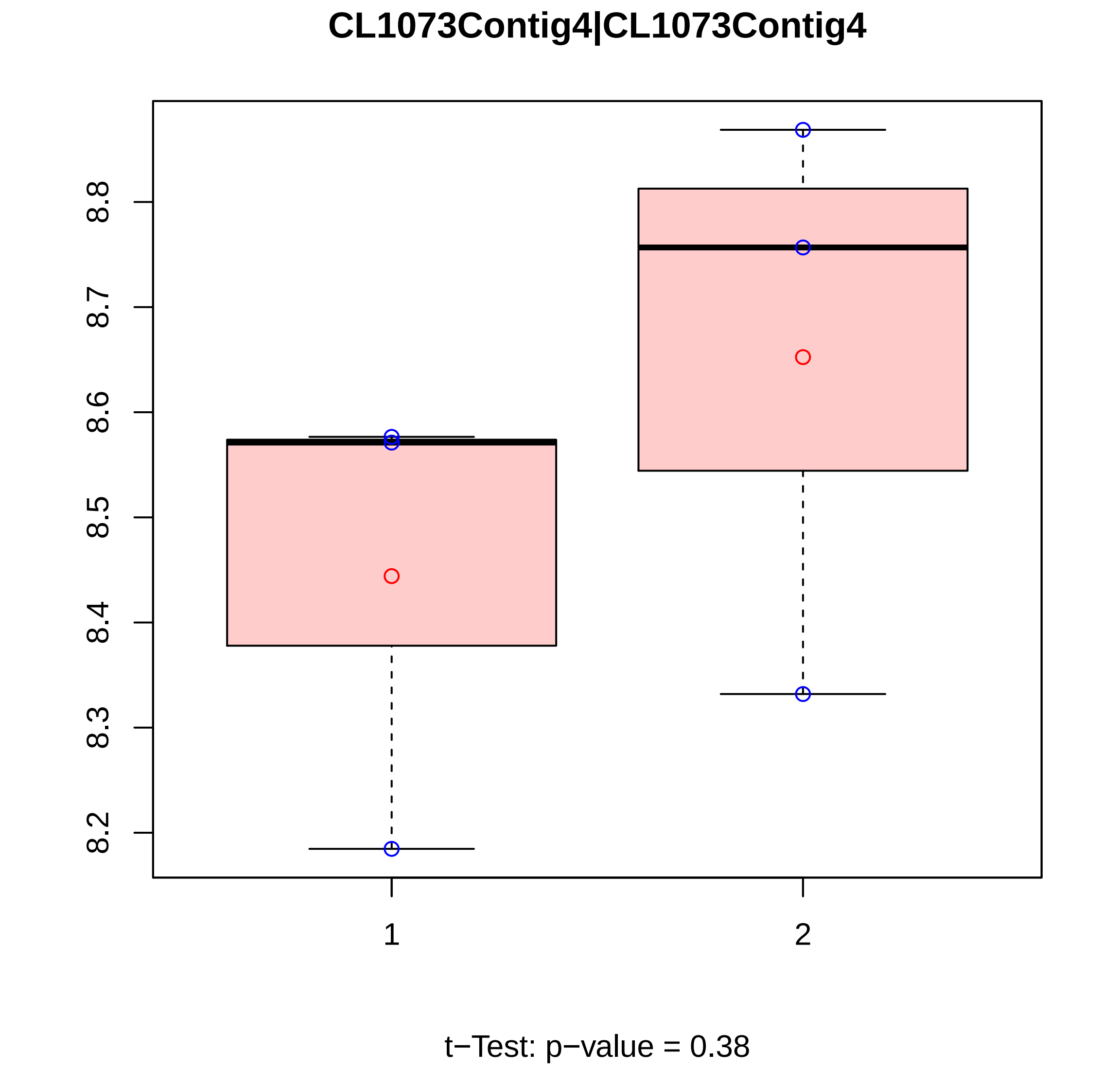
CL1073Contig4|CL1073Contig4
8.2
8.3
8.4
8.5
8.6
8.7
8.8
1
2
t−Test: p−value = 0.38

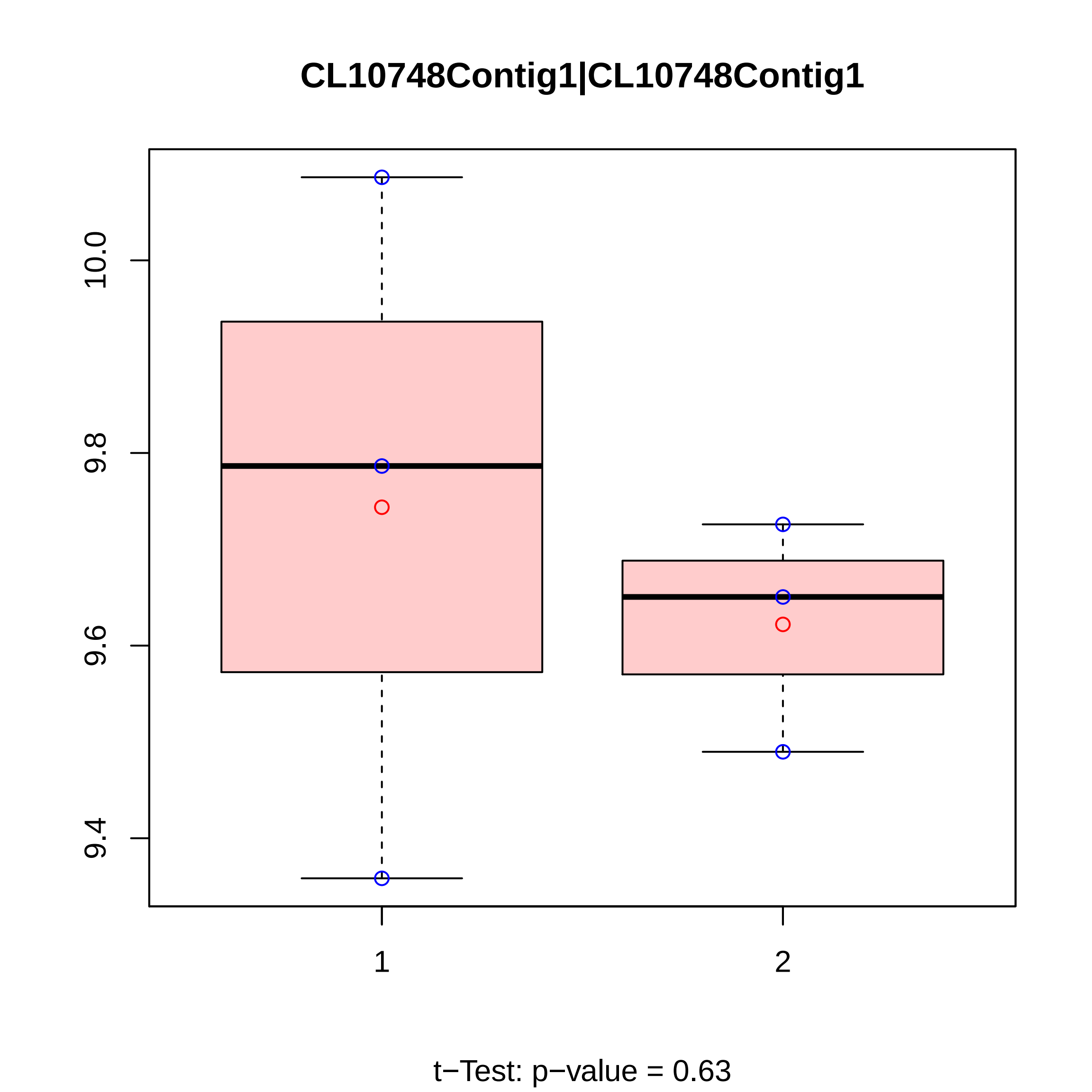
CL10748Contig1|CL10748Contig1
10.0
9.8
9.6
9.4
1
2
t−Test: p−value = 0.63

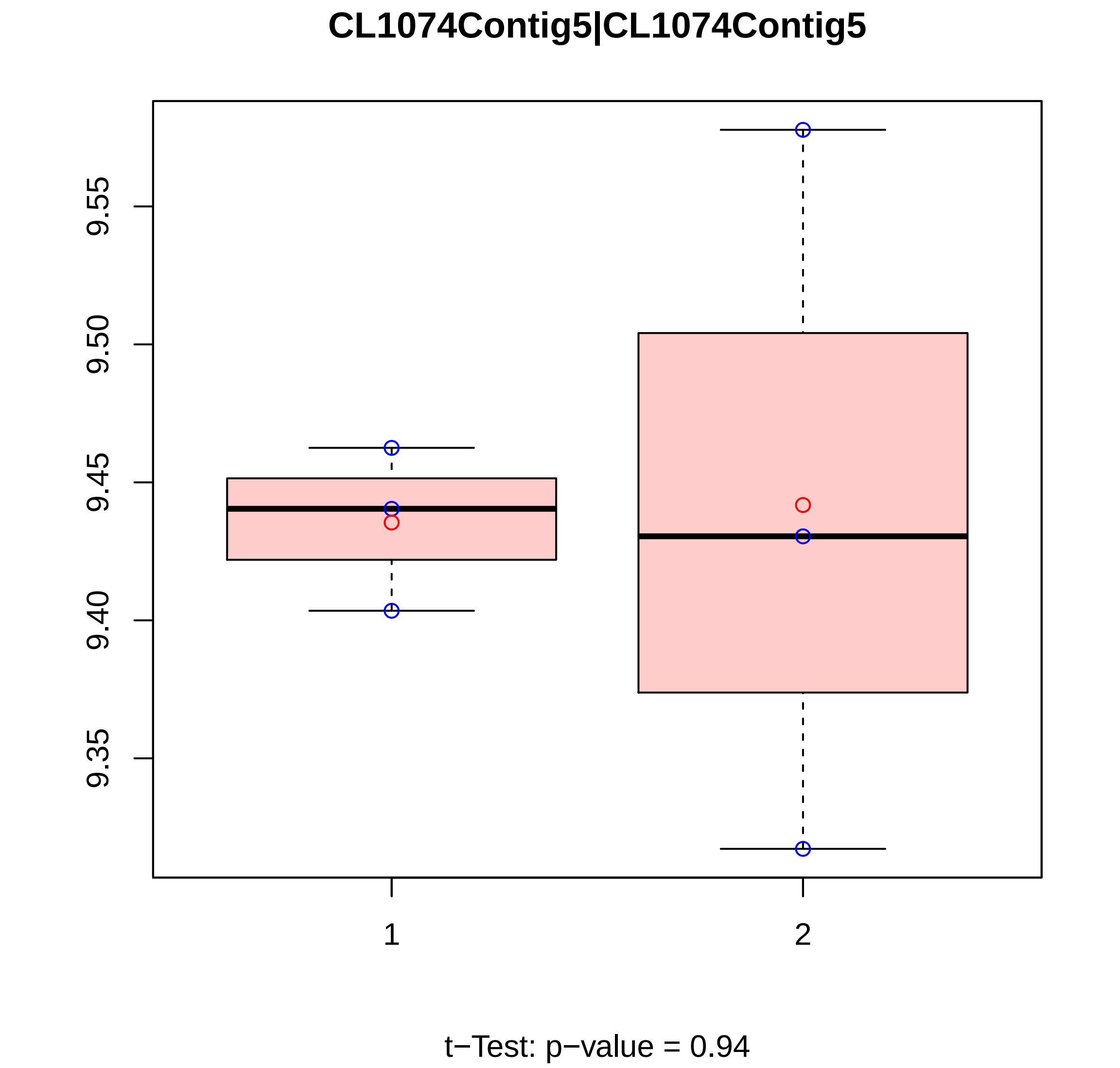

CL1074Contig5|CL1074Contig5
9.35
9.40
9.45
9.50
9.55
1
2
t−Test: p−value = 0.94

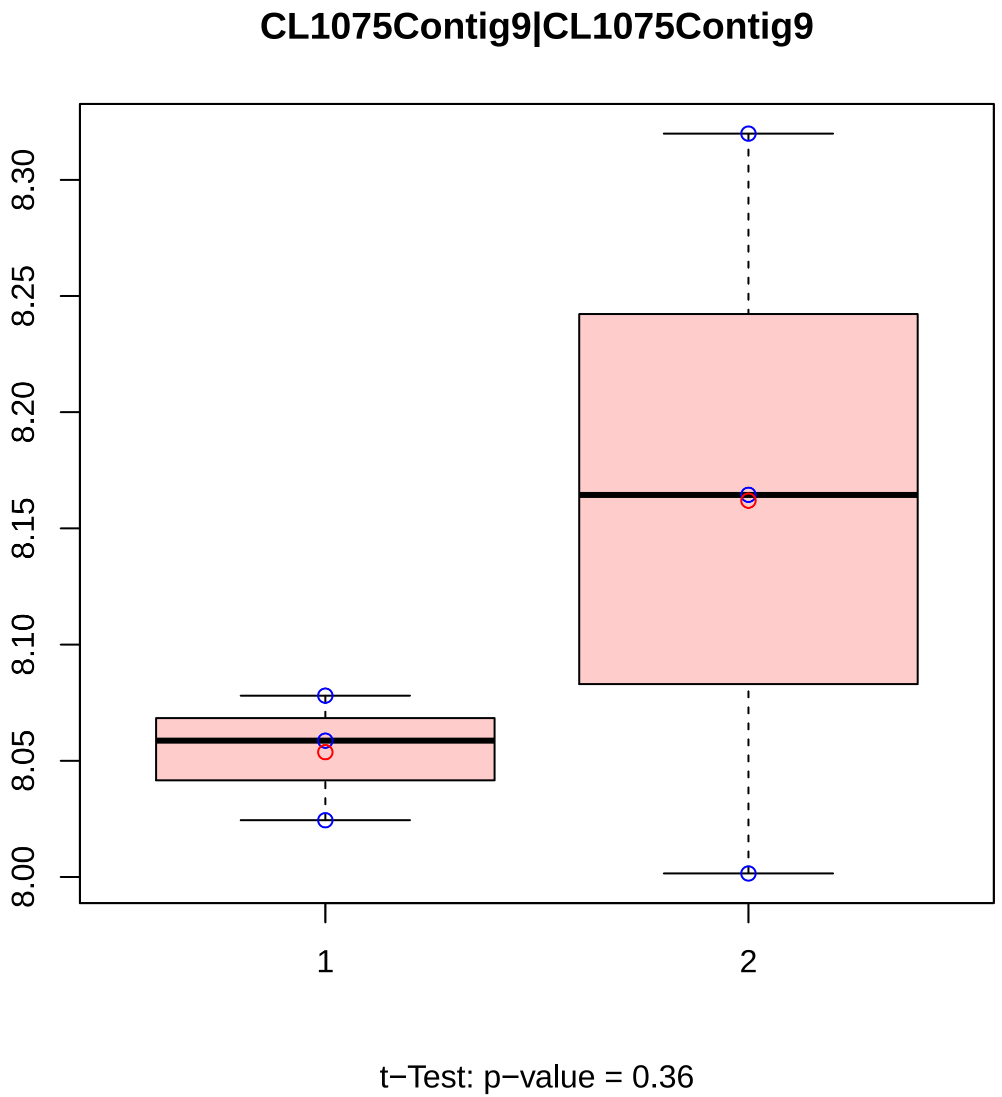
CL1075Contig9|CL1075Contig9
8.00
8.05
8.10
8.15
8.20
8.25
8.30
1
2
t−Test: p−value = 0.36

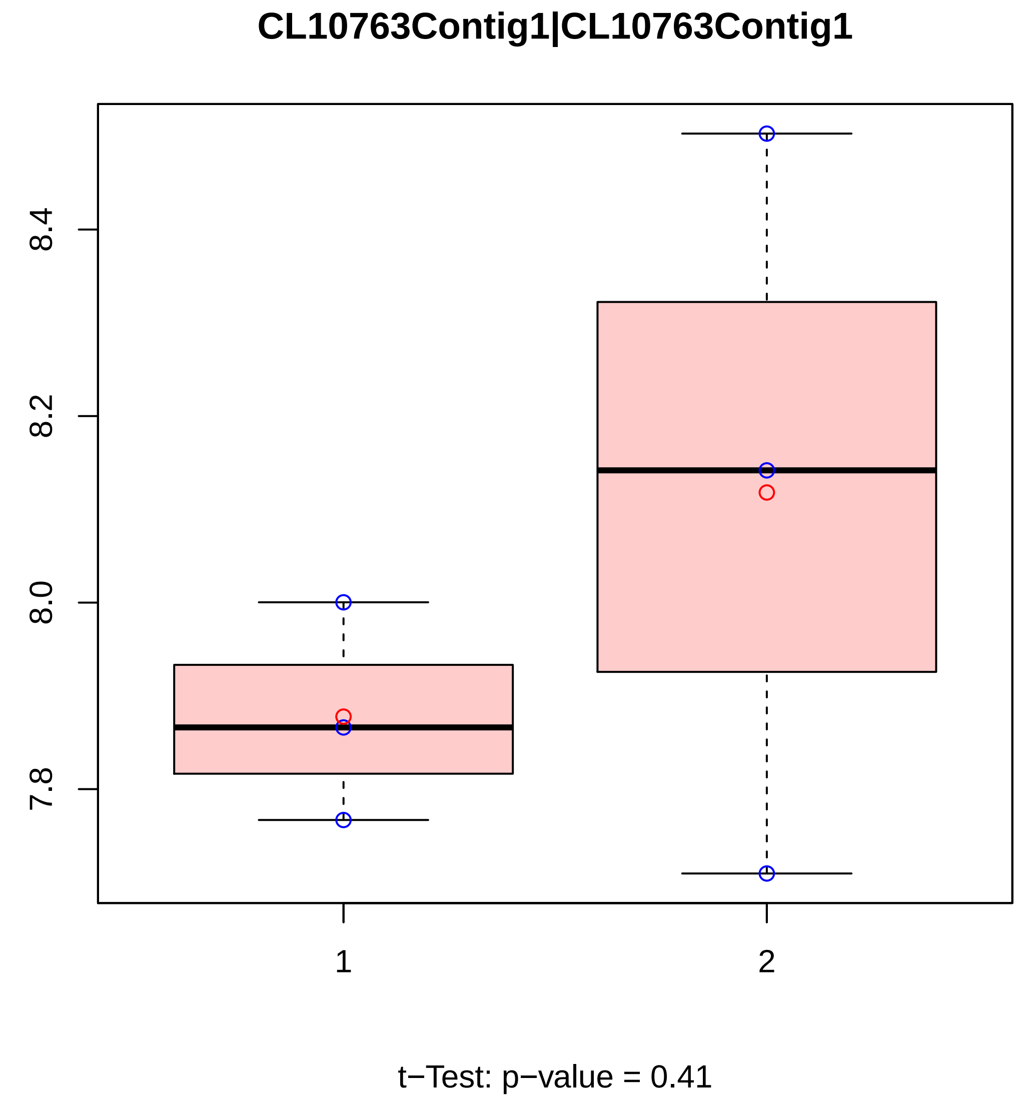
CL10763Contig1|CL10763Contig1
8.4
8.2
8.0
7.8
1
2
t−Test: p−value = 0.41

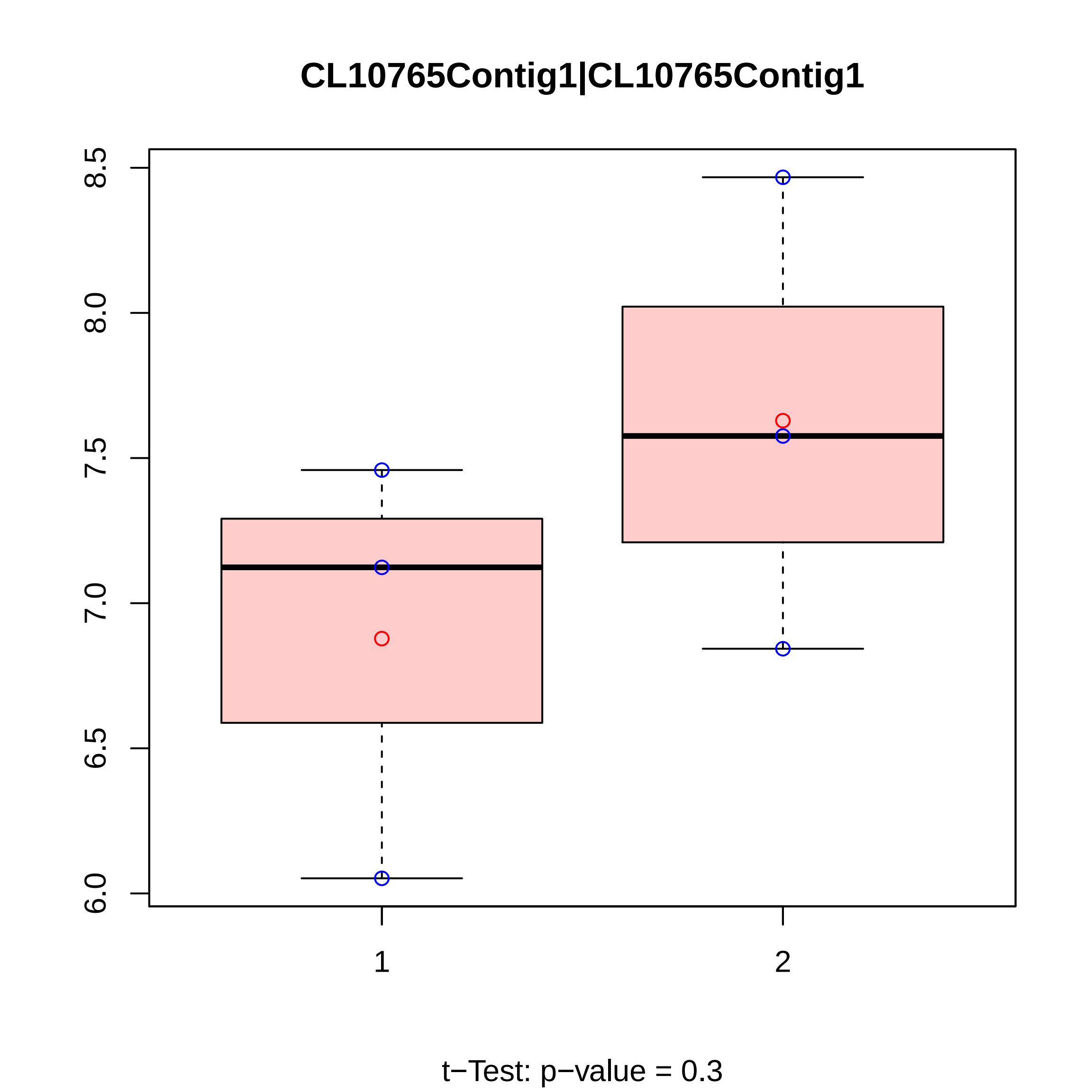
CL10765Contig1|CL10765Contig1
8.5
8.0
7.5
7.0
6.5
6.0
1
2
t−Test: p−value = 0.3

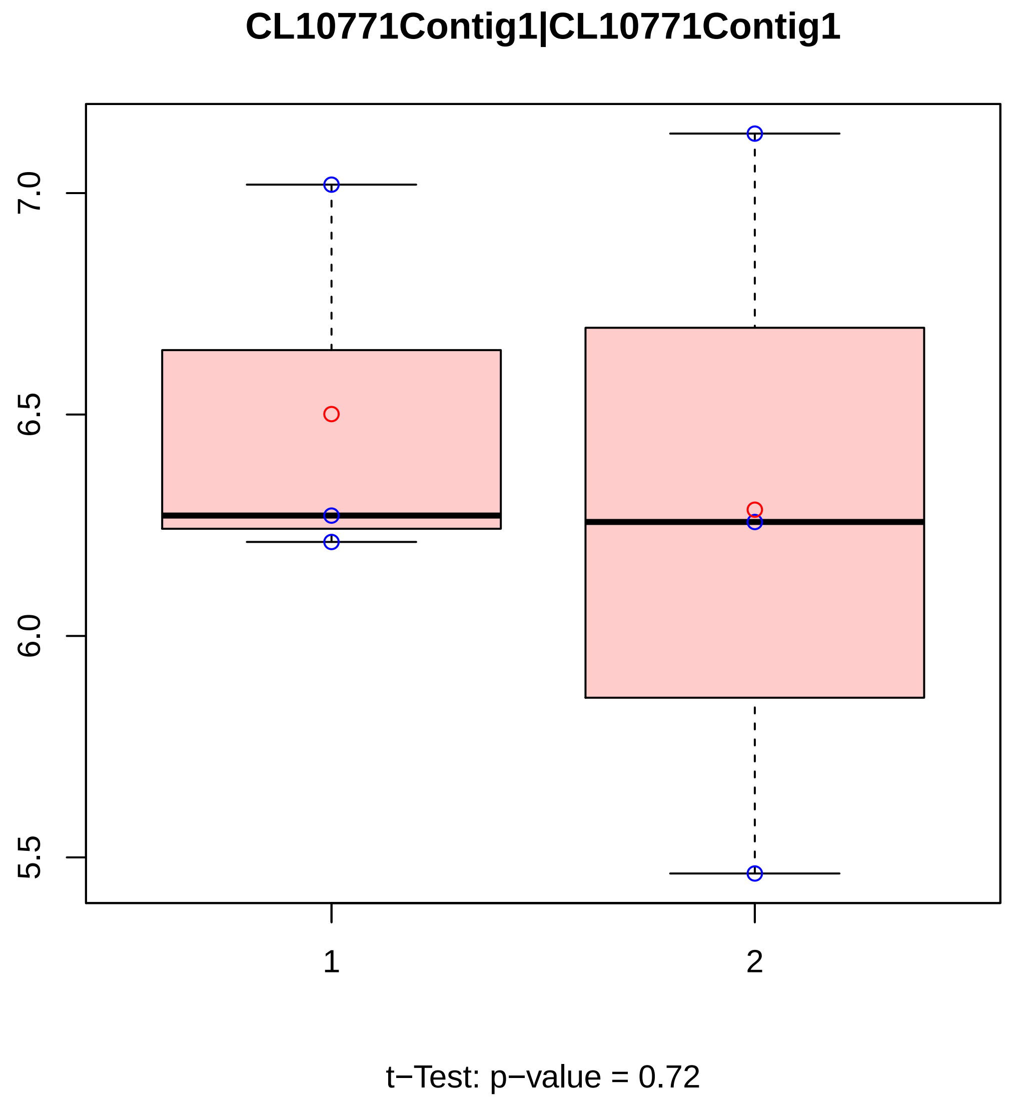
CL10771Contig1|CL10771Contig1
7.0
6.5
6.0
5.5
1
2
t−Test: p−value = 0.72

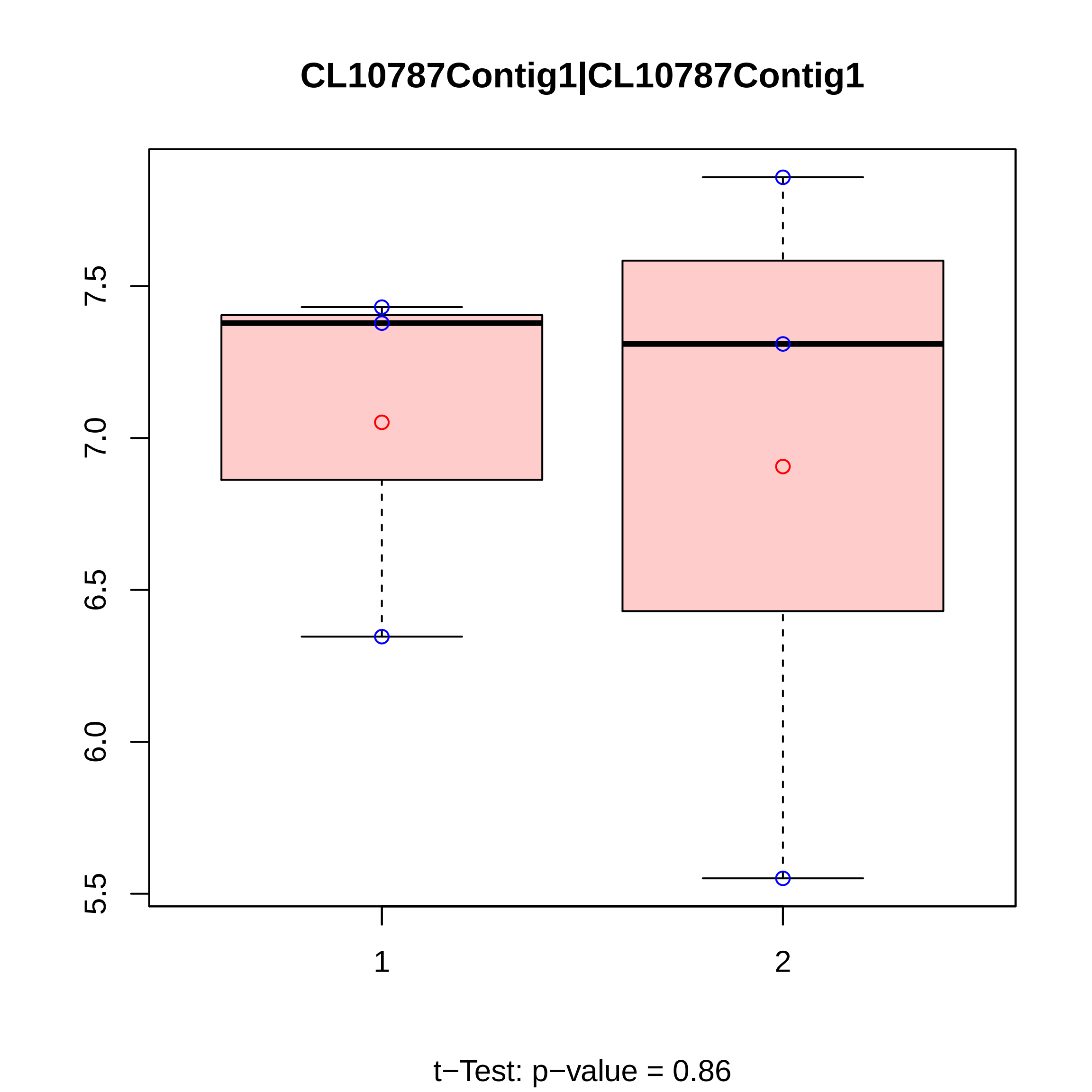
CL10787Contig1|CL10787Contig1
7.5
7.0
6.5
6.0
5.5
1
2
t−Test: p−value = 0.86

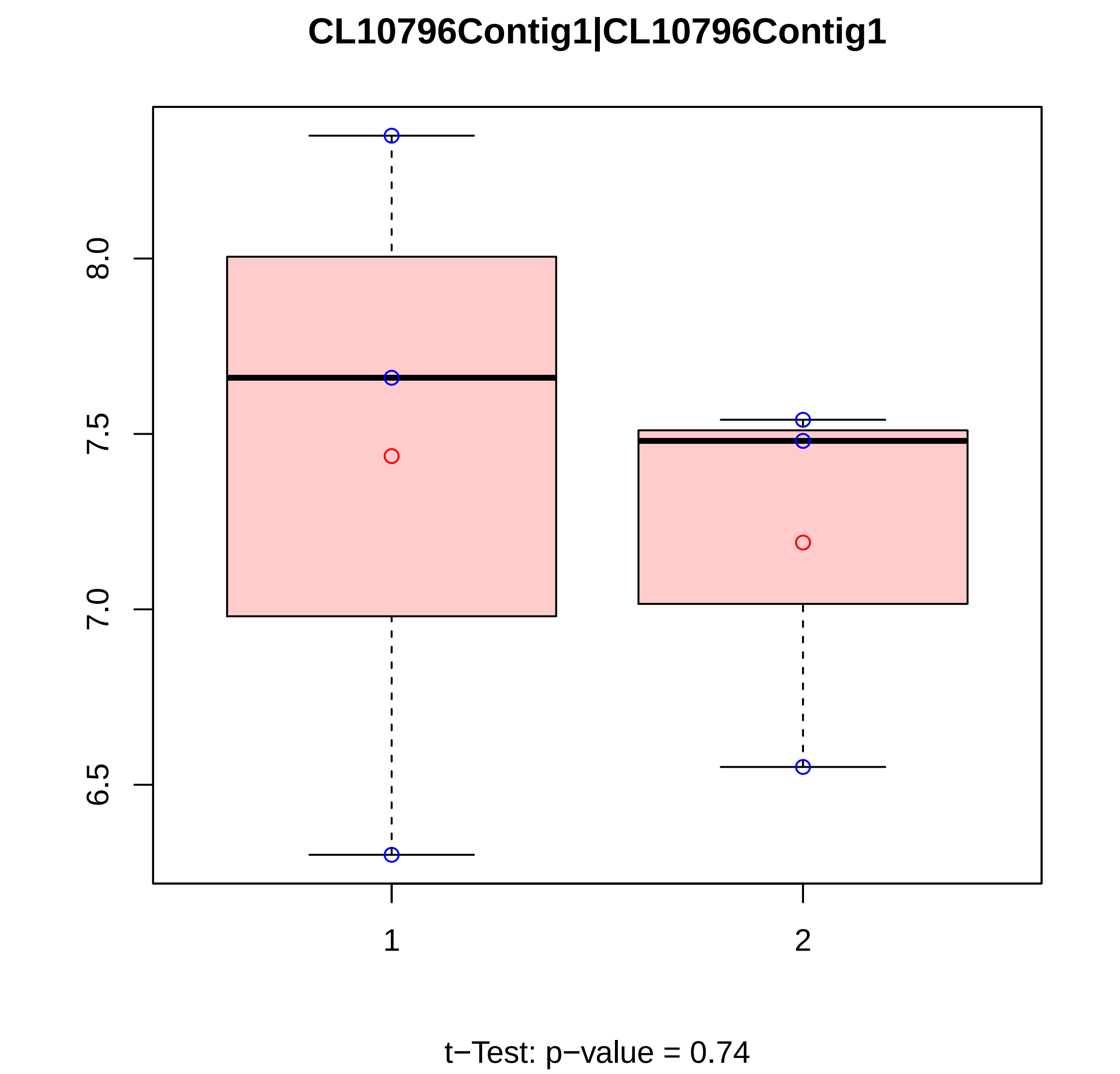
CL10796Contig1|CL10796Contig1
8.0
7.5
7.0
6.5
1
2
t−Test: p−value = 0.74

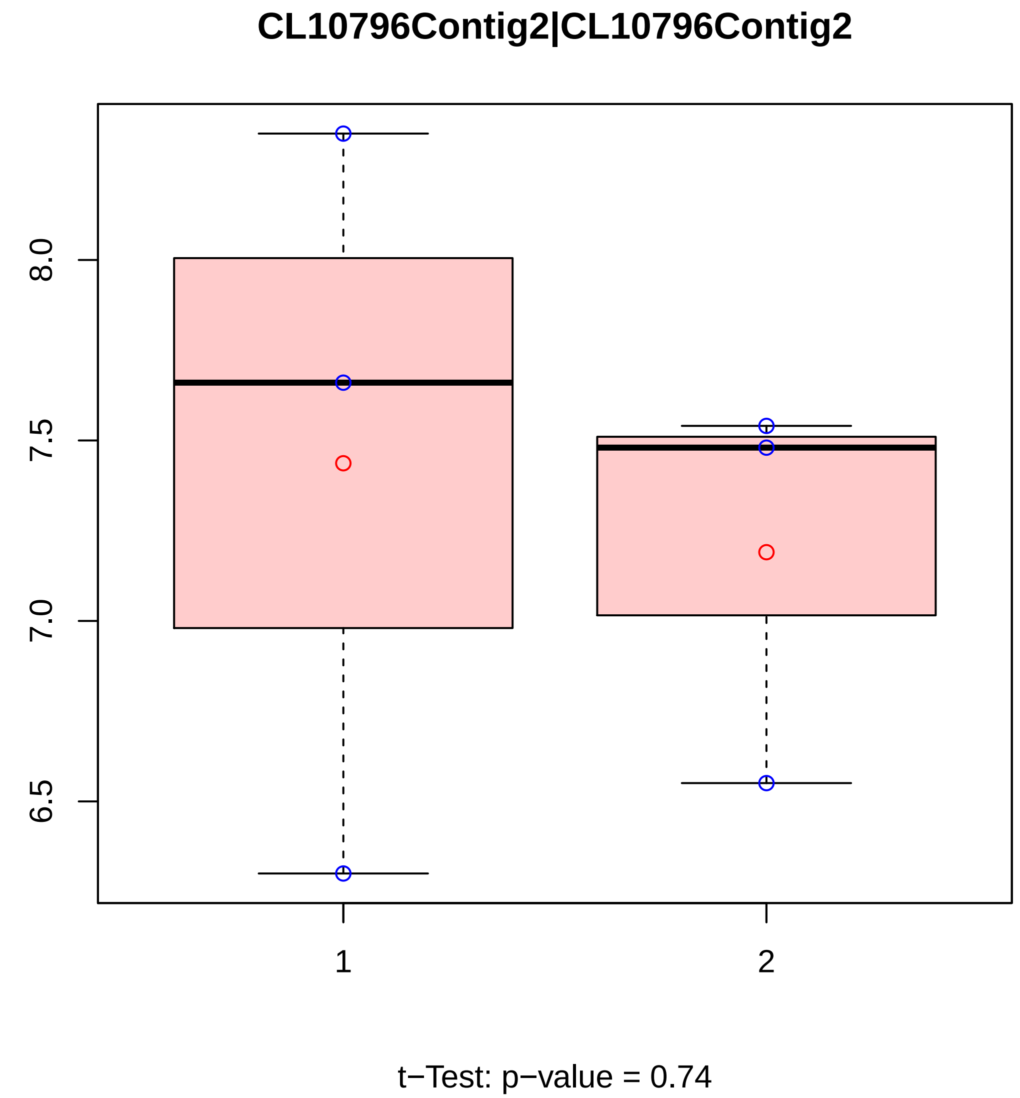
CL10796Contig2|CL10796Contig2
8.0
7.5
7.0
6.5
1
2
t−Test: p−value = 0.74

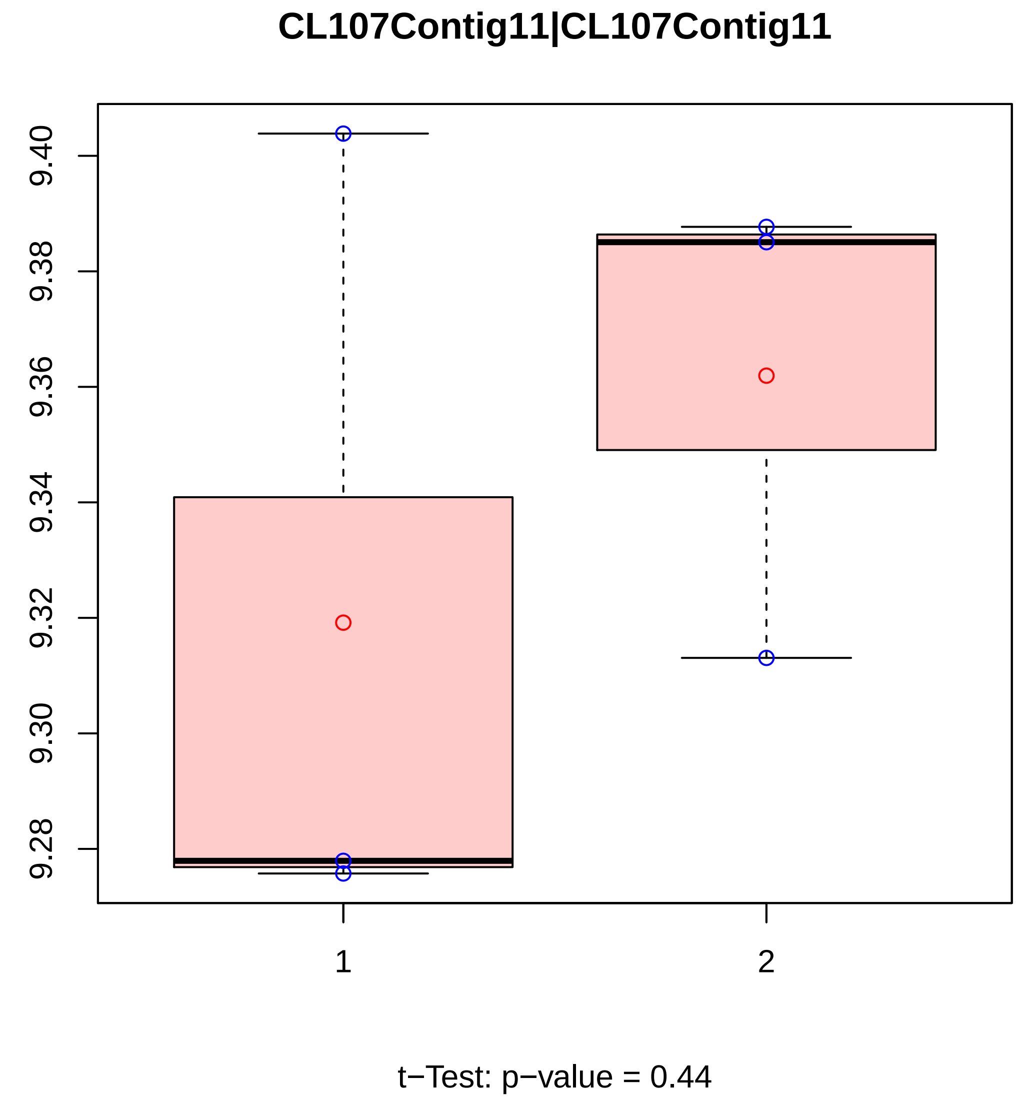
CL107Contig11|CL107Contig11
9.28
9.30
9.32
9.34
9.36
9.38
9.40
1
2
t−Test: p−value = 0.44

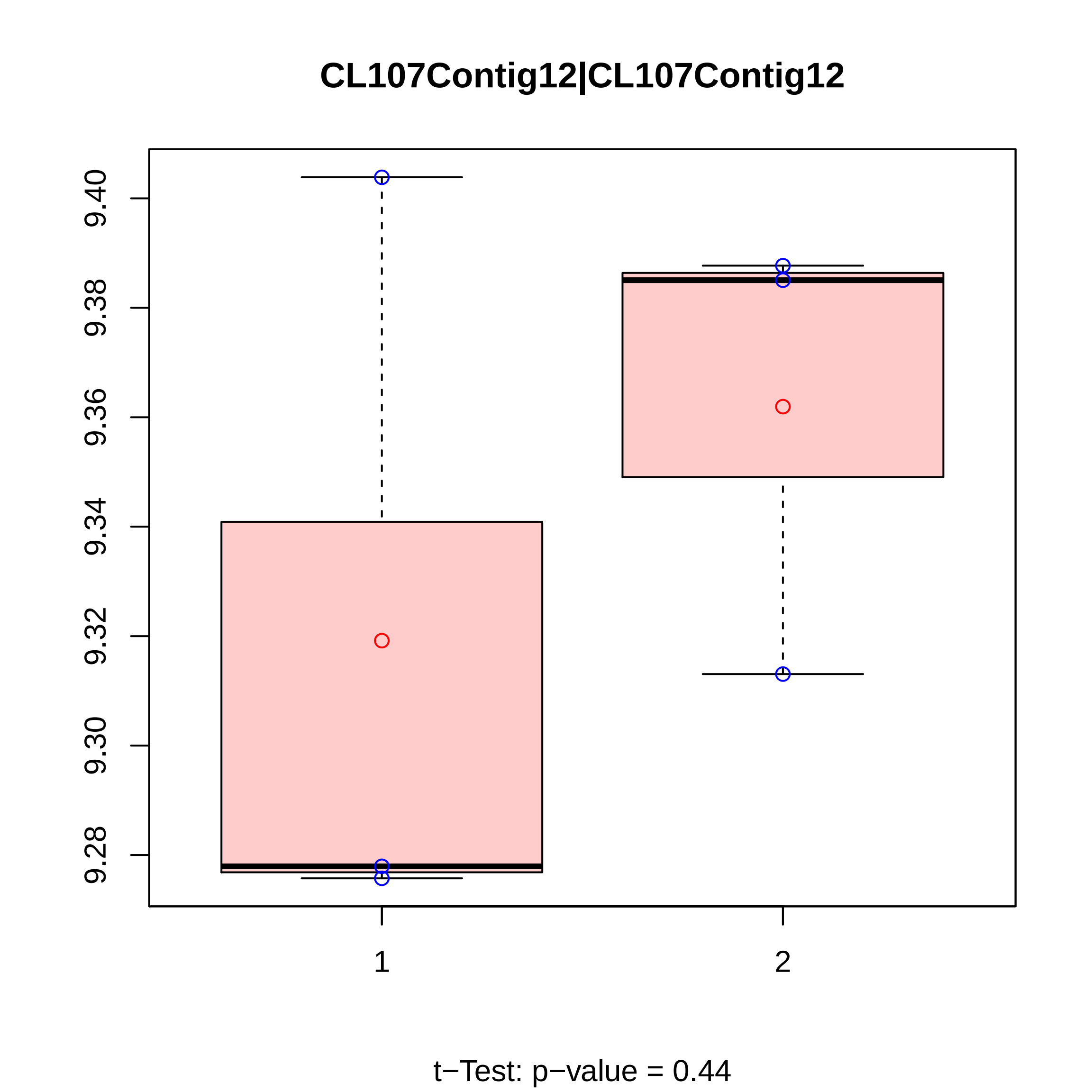
CL107Contig12|CL107Contig12
9.28
9.30
9.32
9.34
9.36
9.38
9.40
1
2
t−Test: p−value = 0.44

## CL107Contig15|CL107Contig15

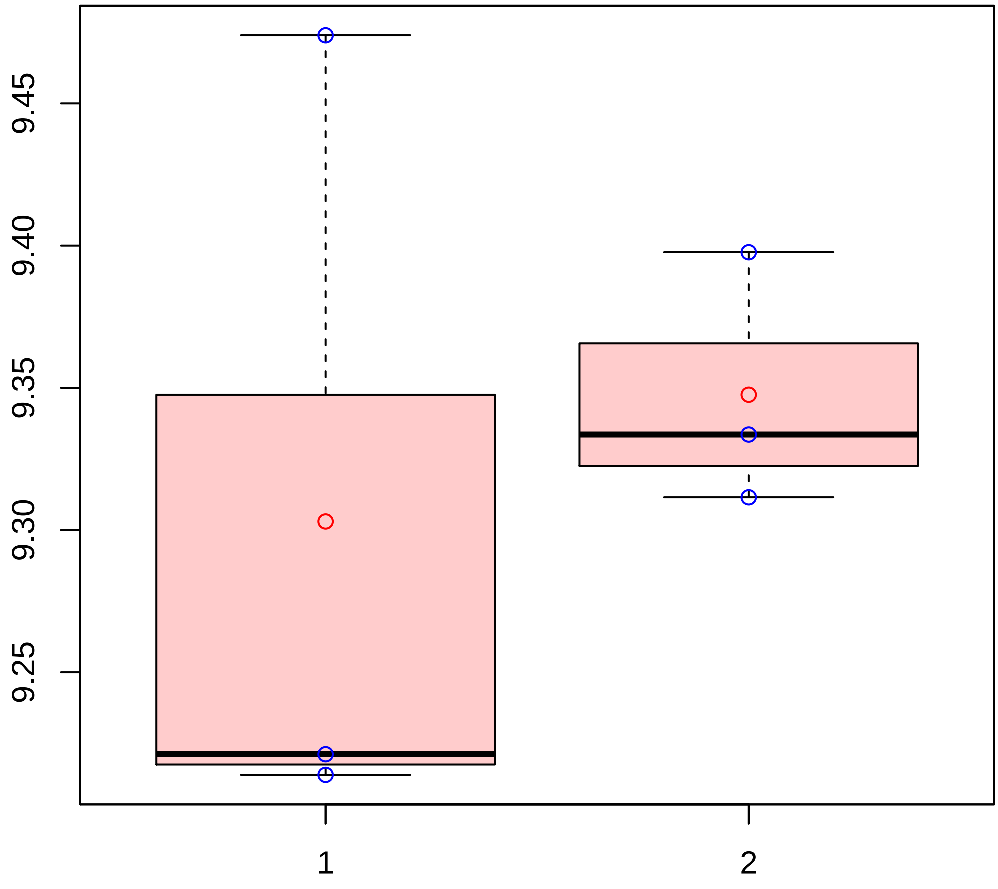


t−Test: p−value = 0.66

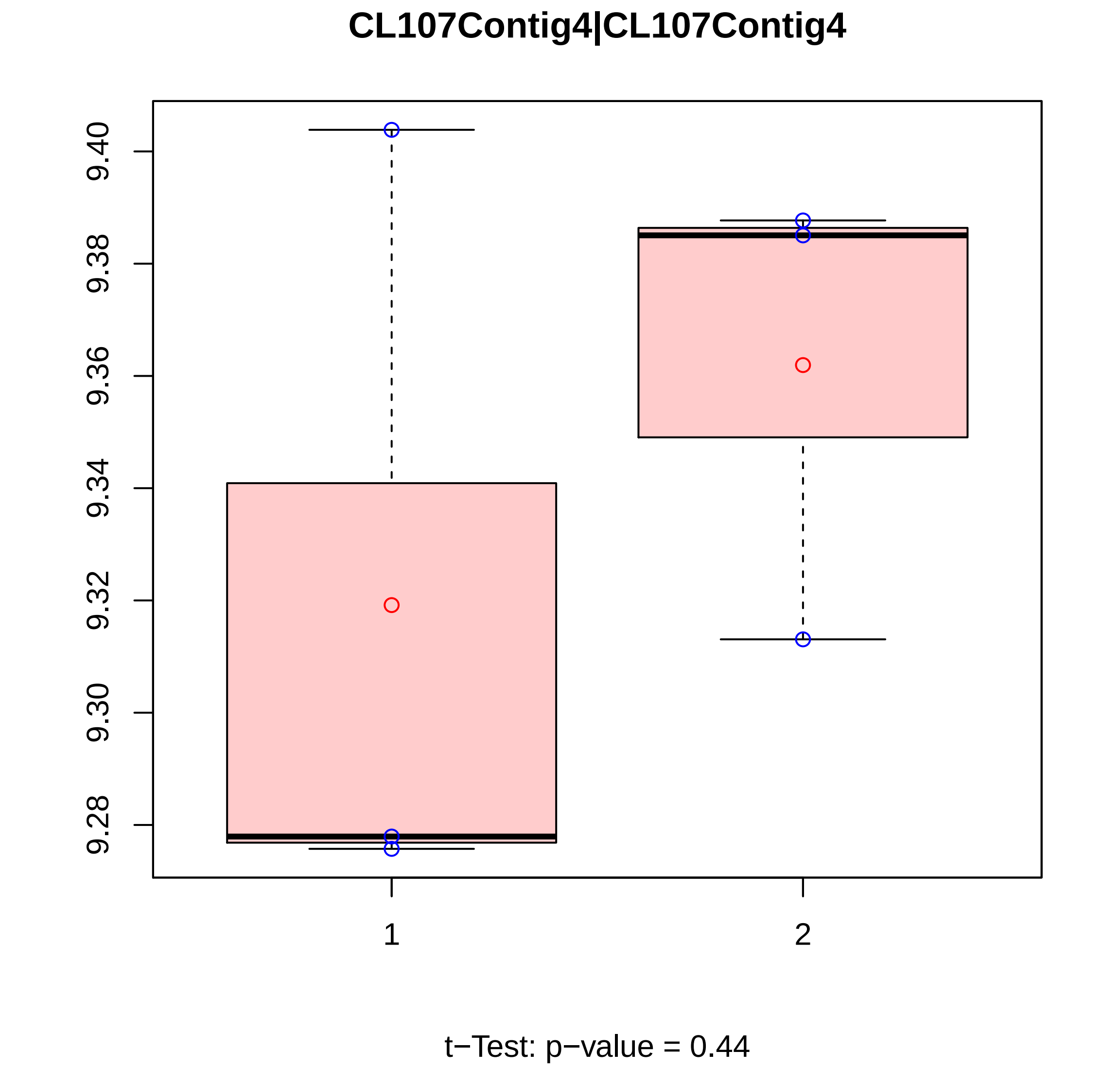
CL107Contig4|CL107Contig4
9.28
9.30
9.32
9.34
9.36
9.38
9.40
1
2
t−Test: p−value = 0.44

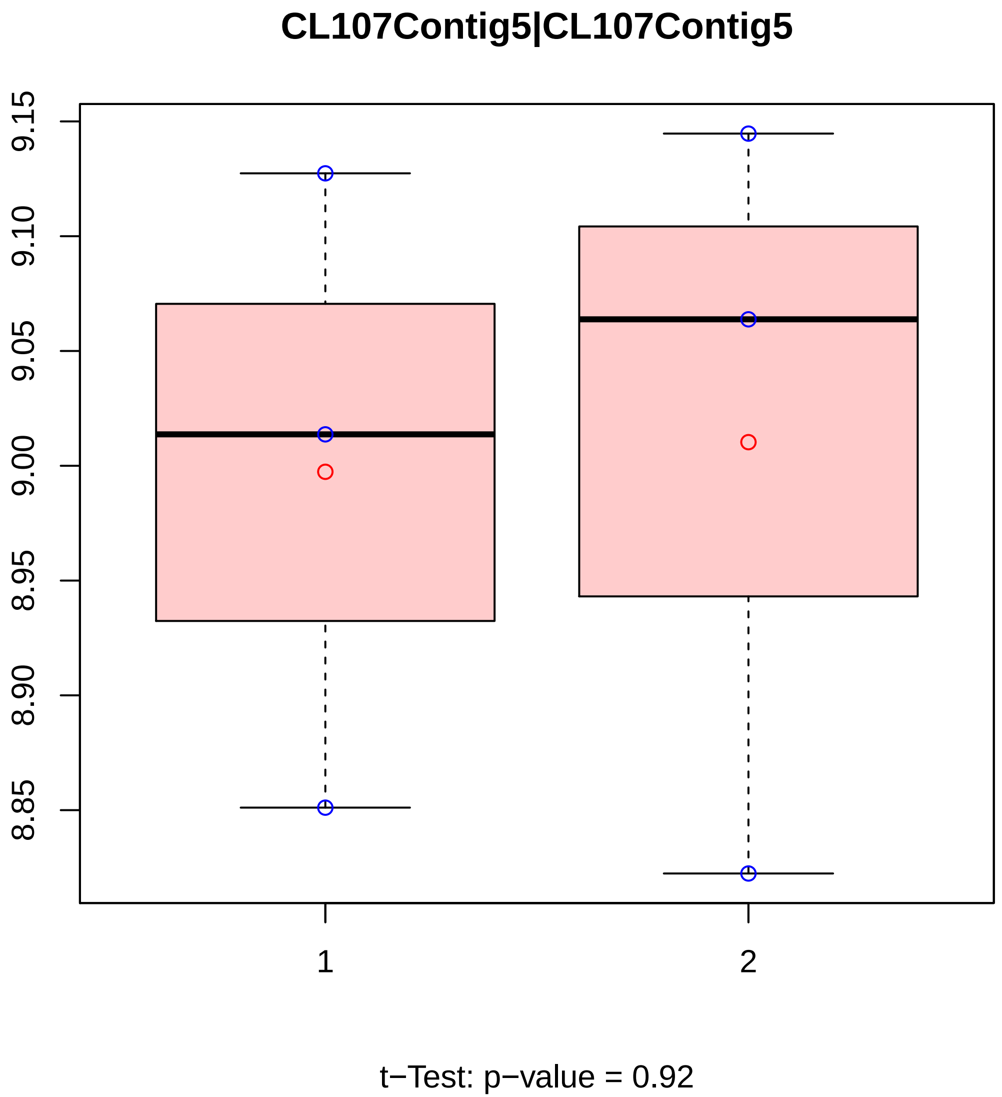
CL107Contig5|CL107Contig5
9.15
9.10
9.05
9.00
8.95
8.90
8.85
1
2
t−Test: p−value = 0.92

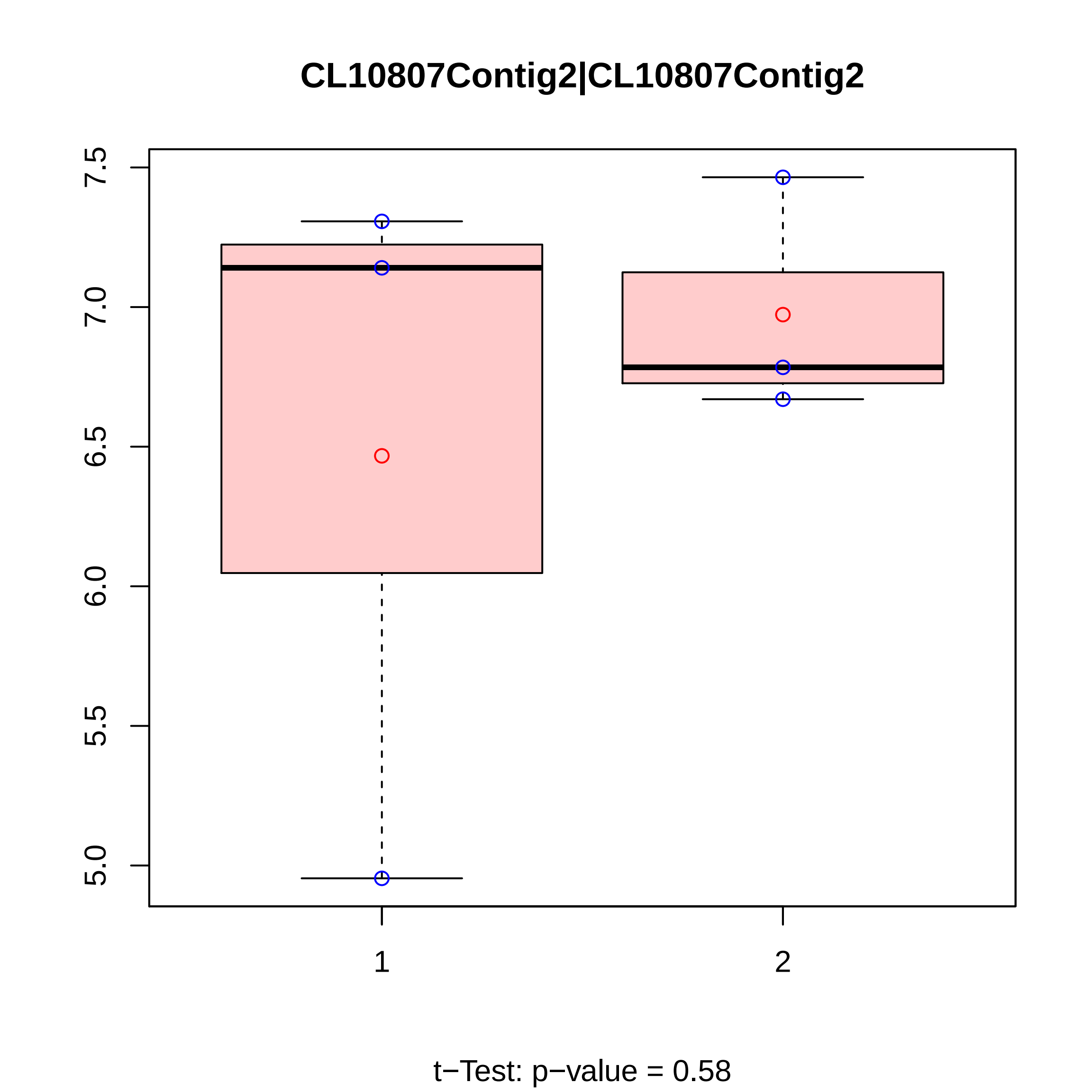
CL10807Contig2|CL10807Contig2
7.5
7.0
6.5
6.0
5.5
5.0
1
2
t−Test: p−value = 0.58

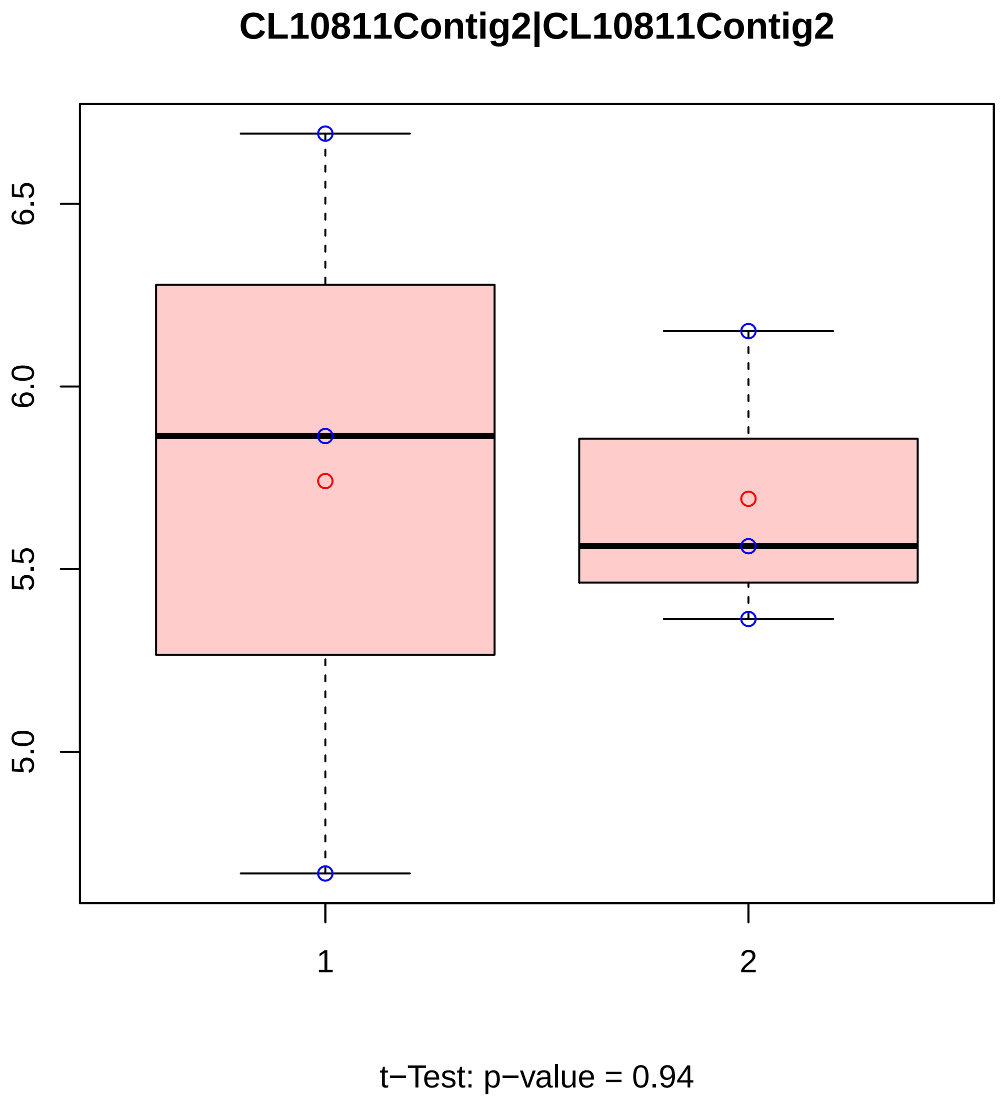
CL10811Contig2|CL10811Contig2
6.5
6.0
5.5
5.0
1
2
t−Test: p−value = 0.94

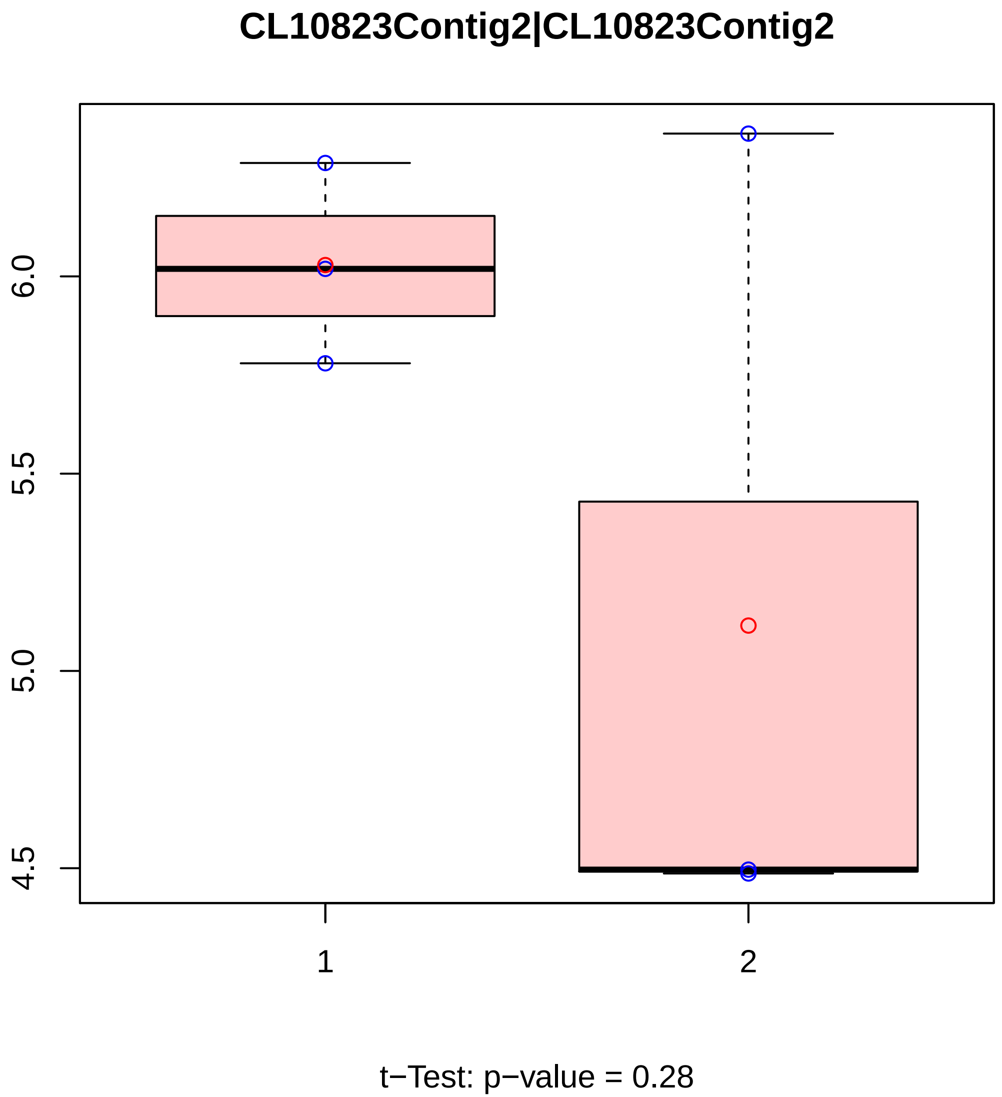
CL10823Contig2|CL10823Contig2
6.0
5.5
5.0
4.5
1
2
t−Test: p−value = 0.28

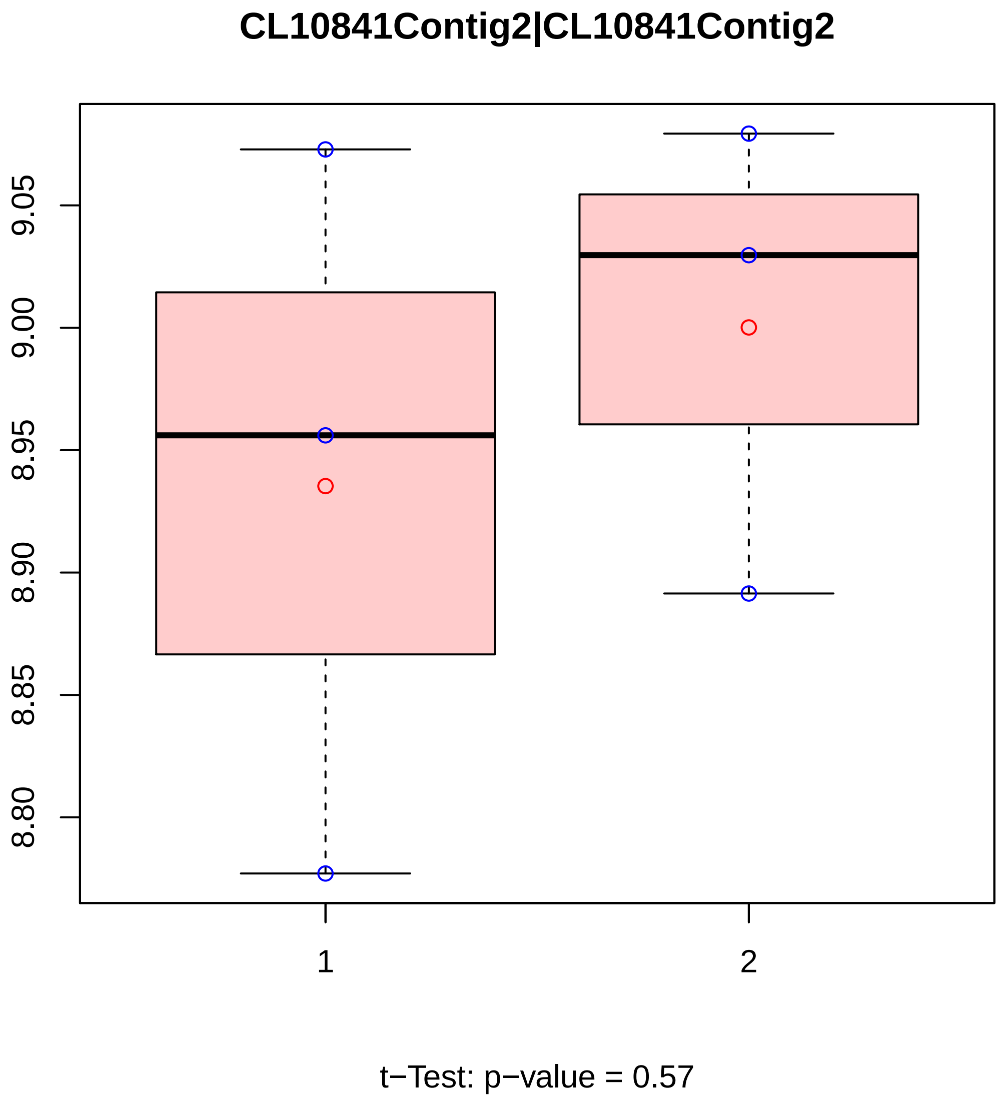
CL10841Contig2|CL10841Contig2
9.05
9.00
8.95
8.90
8.85
8.80
1
2
t−Test: p−value = 0.57

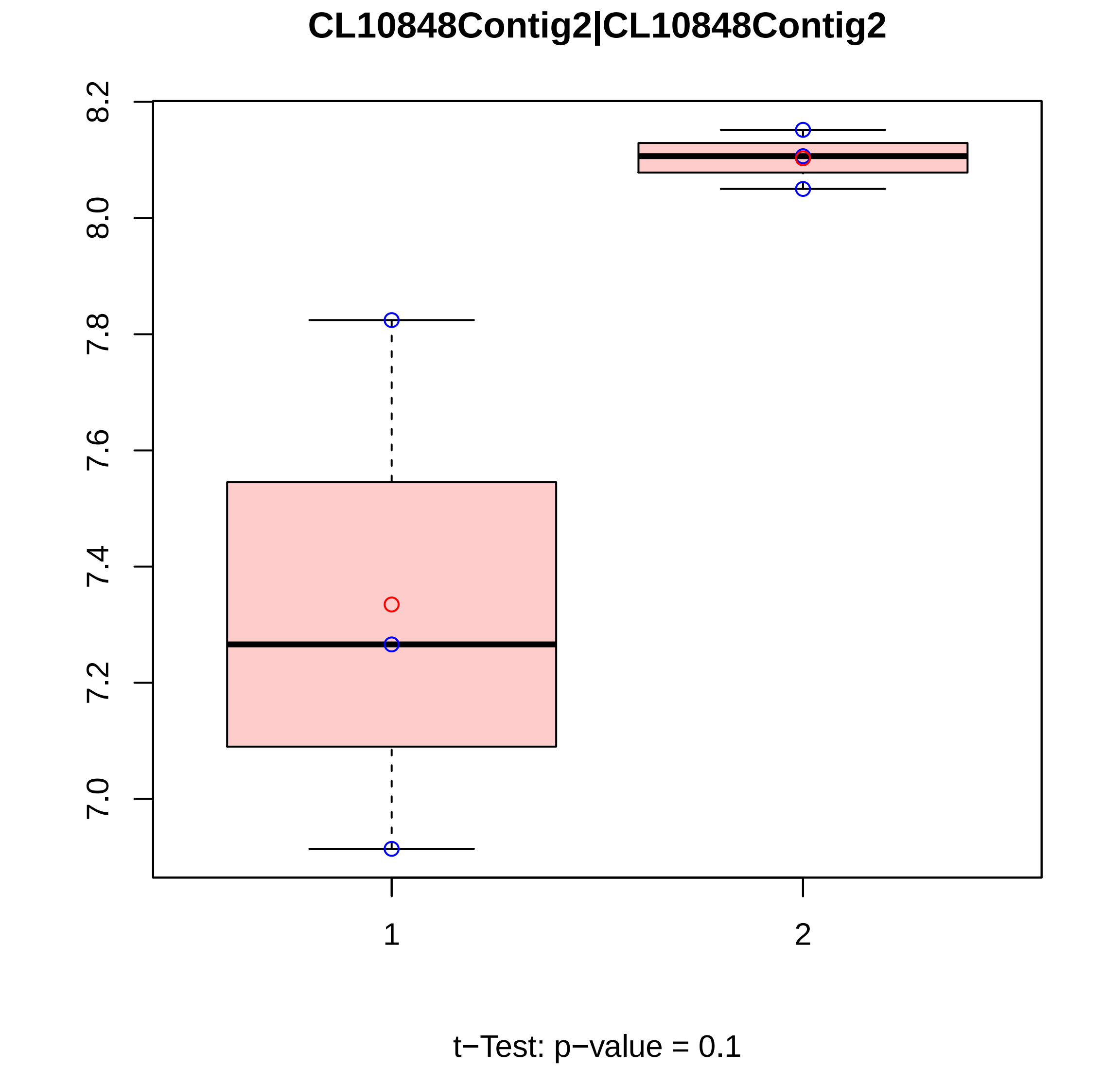

CL10848Contig2|CL10848Contig2
8.2
8.0
7.8
7.6
7.4
7.2
7.0
1
2
t−Test: p−value = 0.1

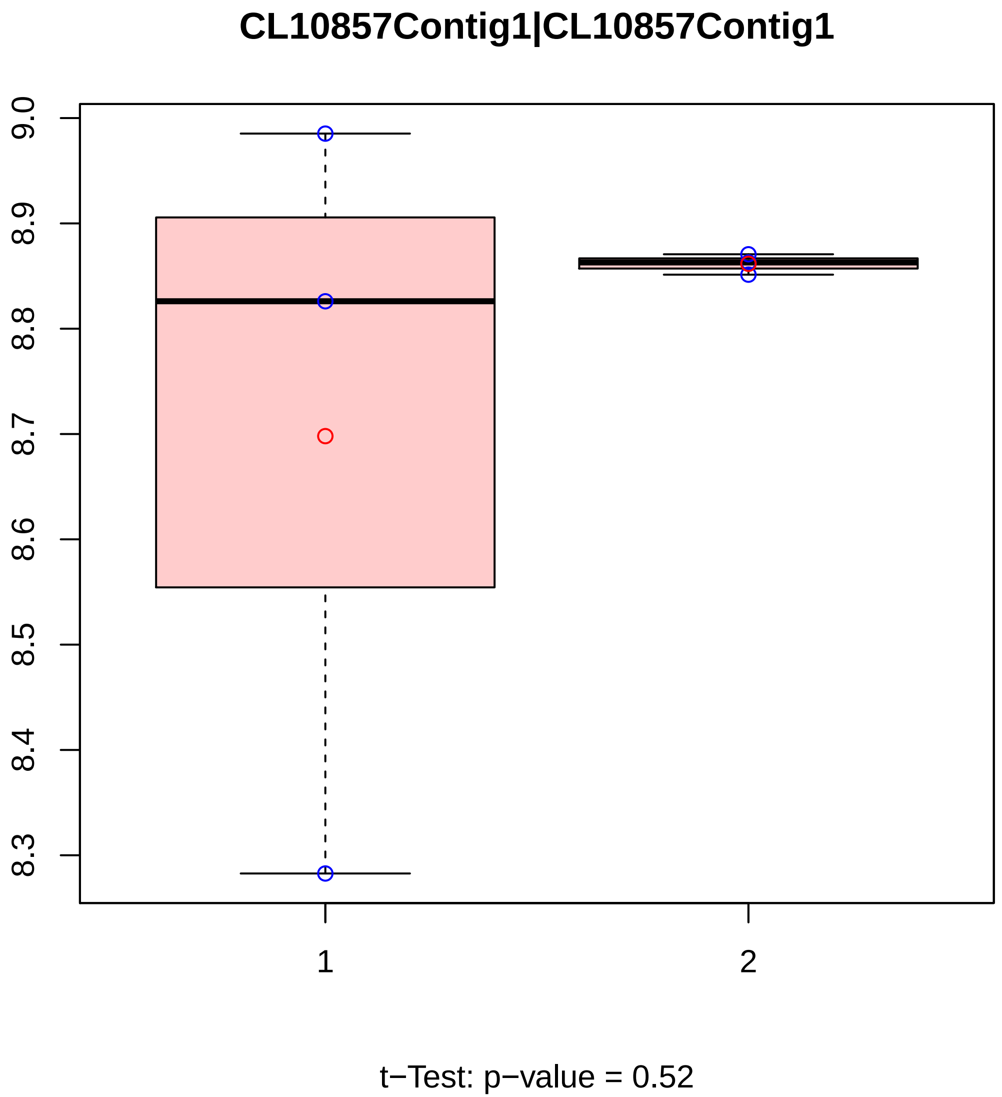
CL10857Contig1|CL10857Contig1
9.0
8.9
8.8
8.7
8.6
8.5
8.4
8.3
1
2
t−Test: p−value = 0.52

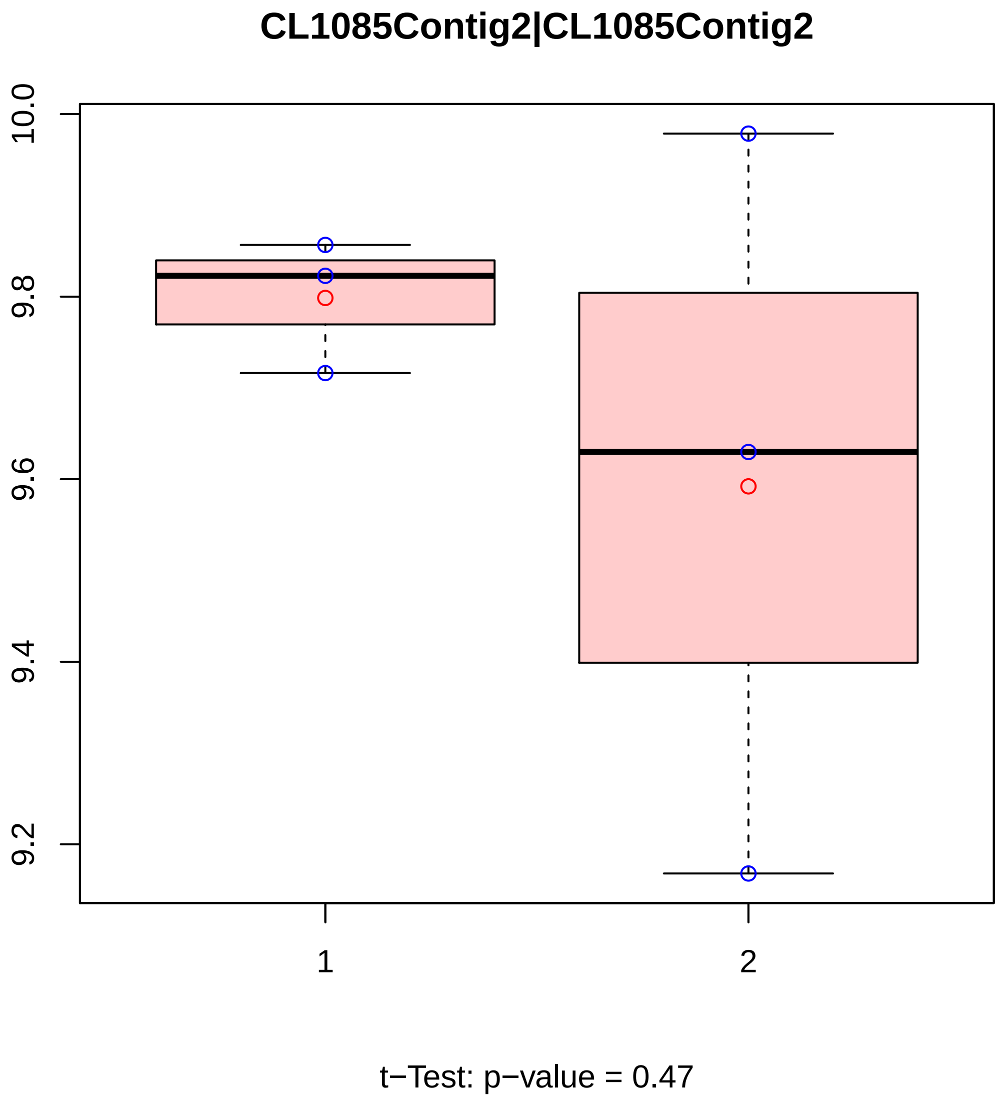

CL1085Contig2|CL1085Contig2
10.0
9.8
9.6
9.4
9.2
1
2
t−Test: p−value = 0.47

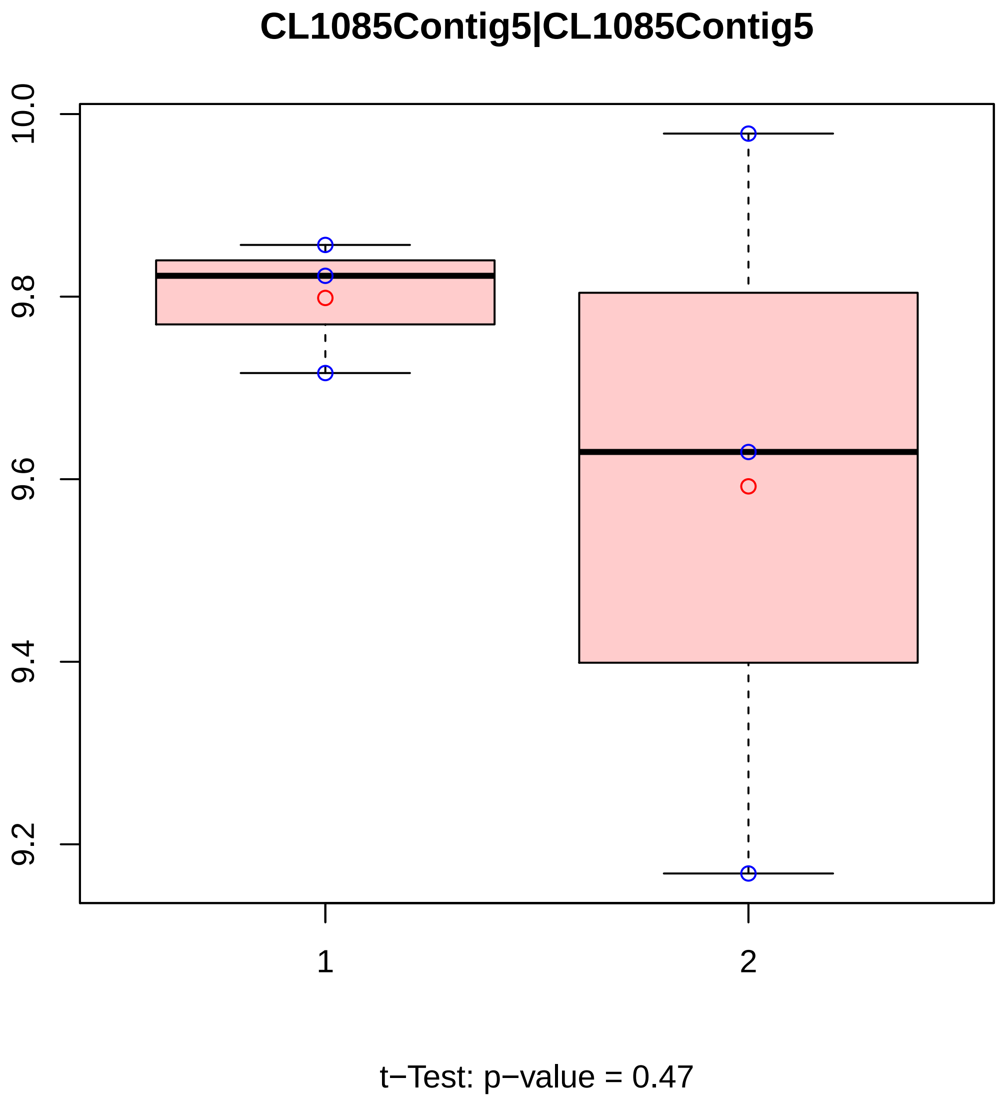
CL1085Contig5|CL1085Contig5
10.0
9.8
9.6
9.4
9.2
1
2
t−Test: p−value = 0.47

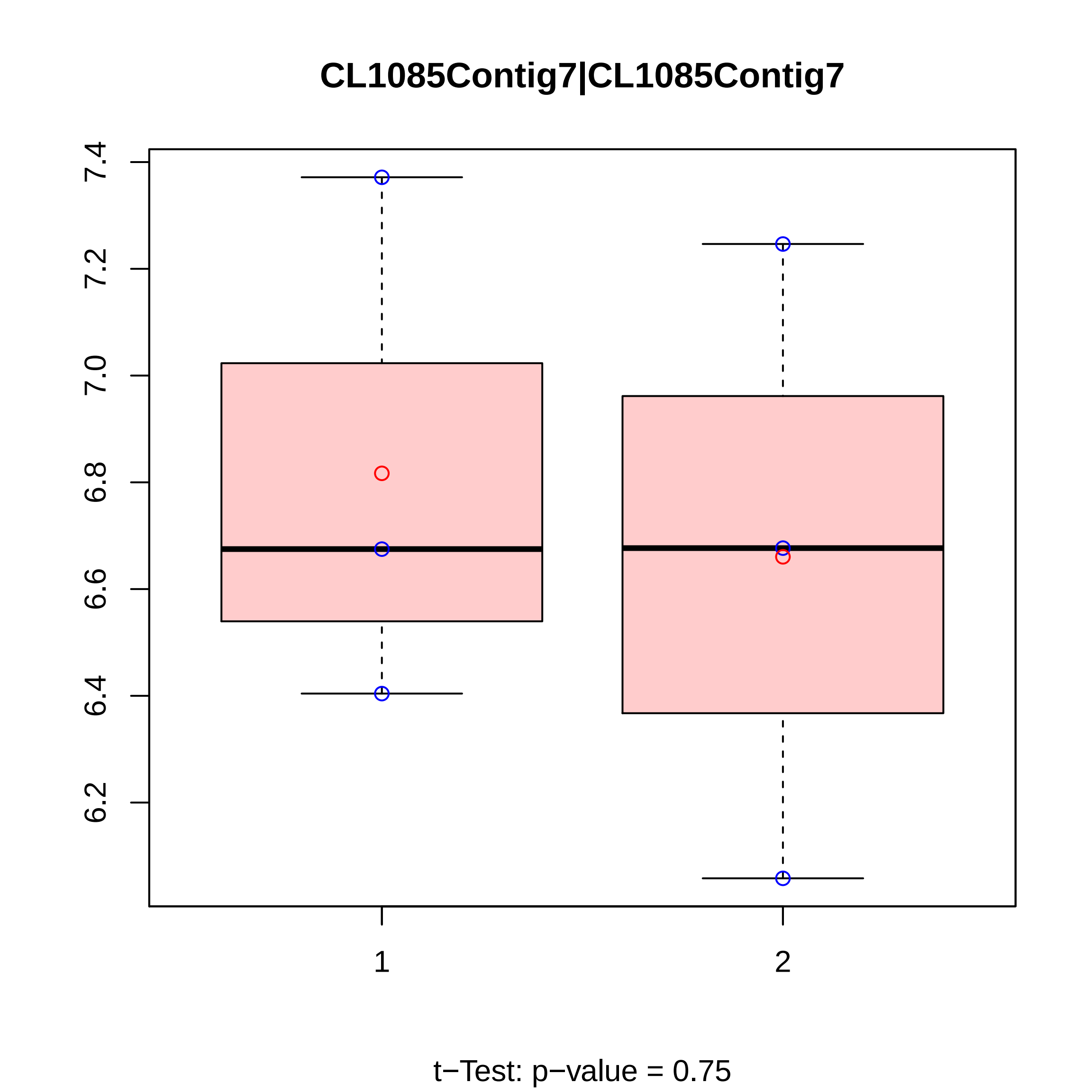

CL1085Contig7|CL1085Contig7
7.4
7.2
7.0
6.8
6.6
6.4
6.2
1
2
t−Test: p−value = 0.75

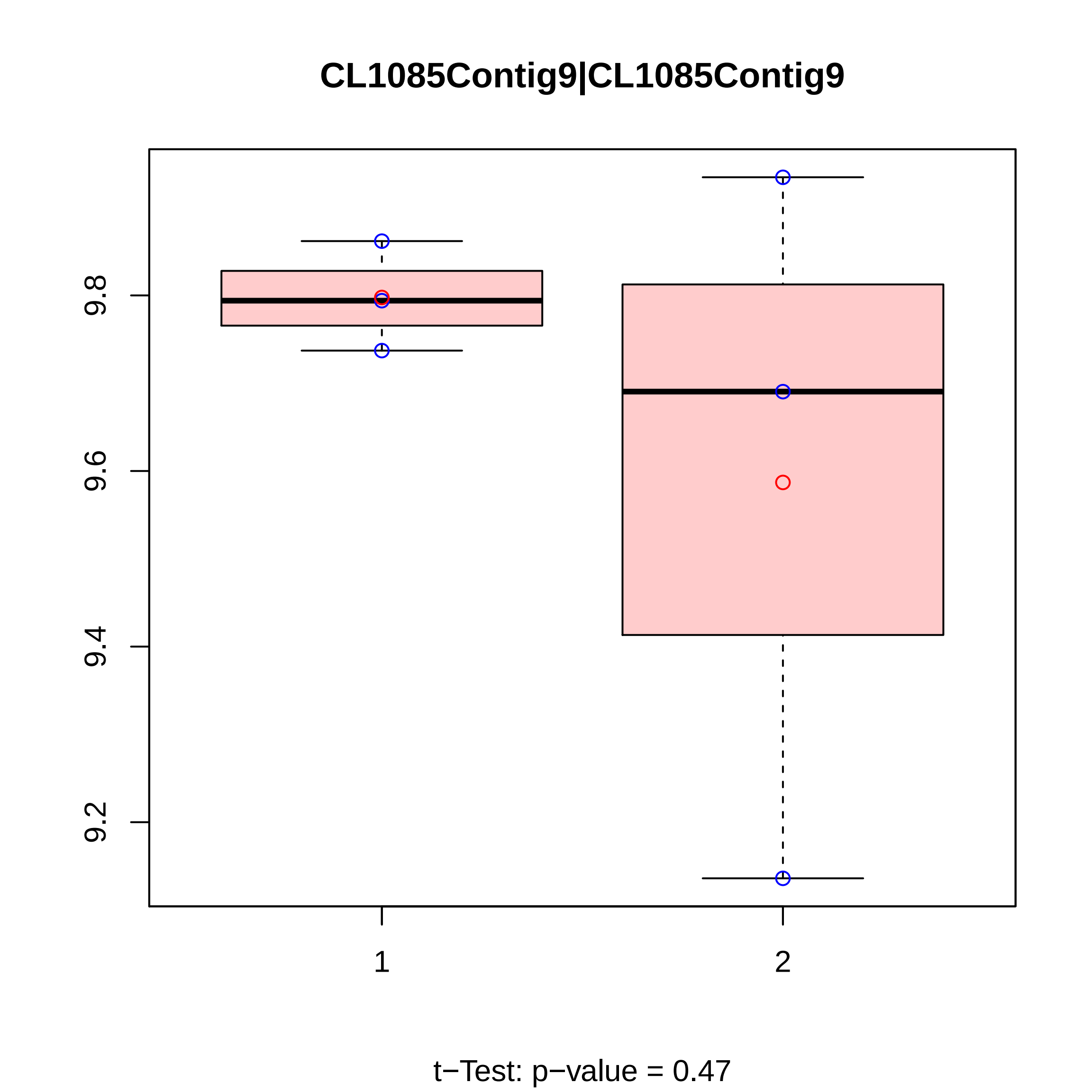
CL1085Contig9|CL1085Contig9
9.2
9.4
9.6
9.8
1
2
t−Test: p−value = 0.47

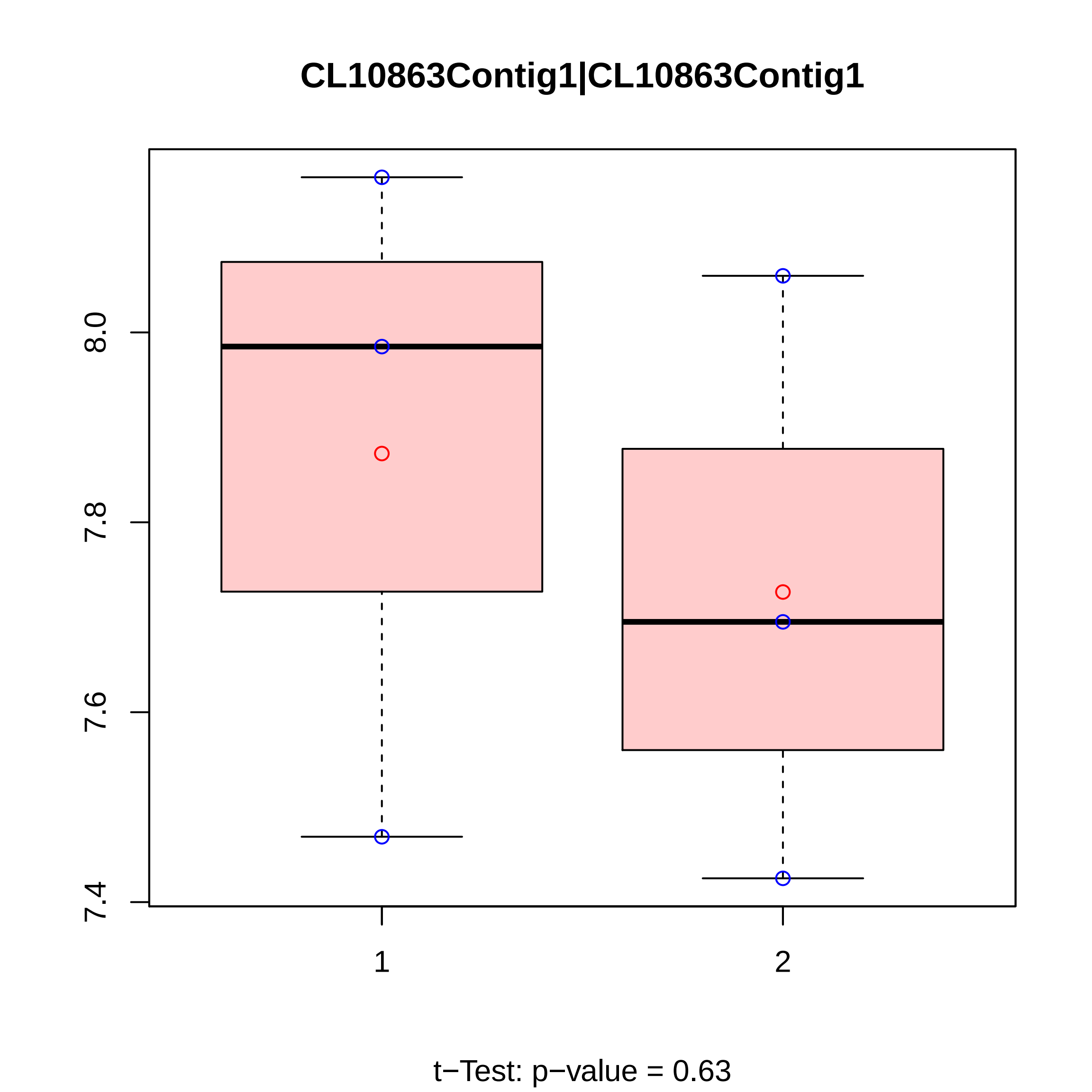
CL10863Contig1|CL10863Contig1
8.0
7.8
7.6
7.4
1
2
t−Test: p−value = 0.63

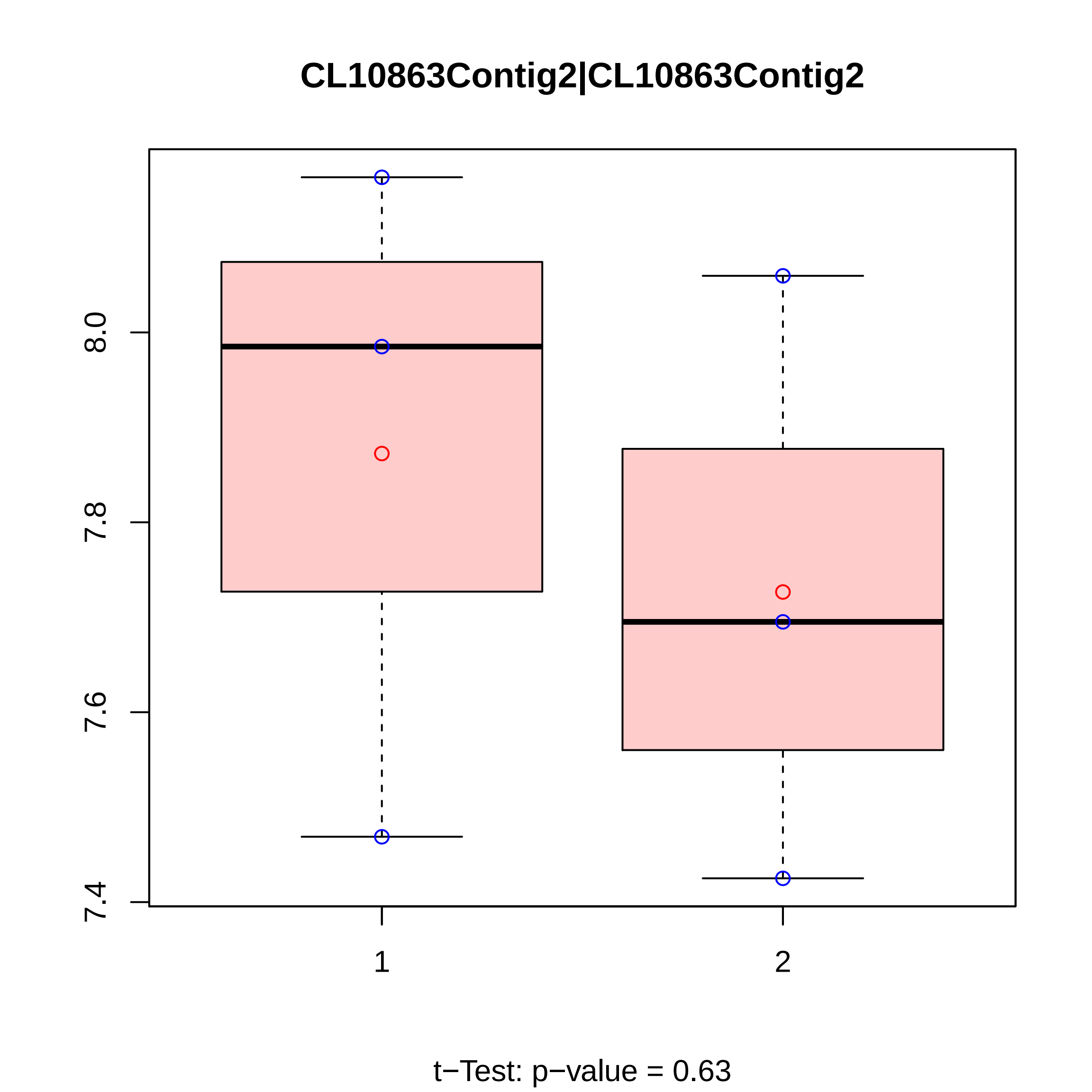
CL10863Contig2|CL10863Contig2
8.0
7.8
7.6
7.4
1
2
t−Test: p−value = 0.63

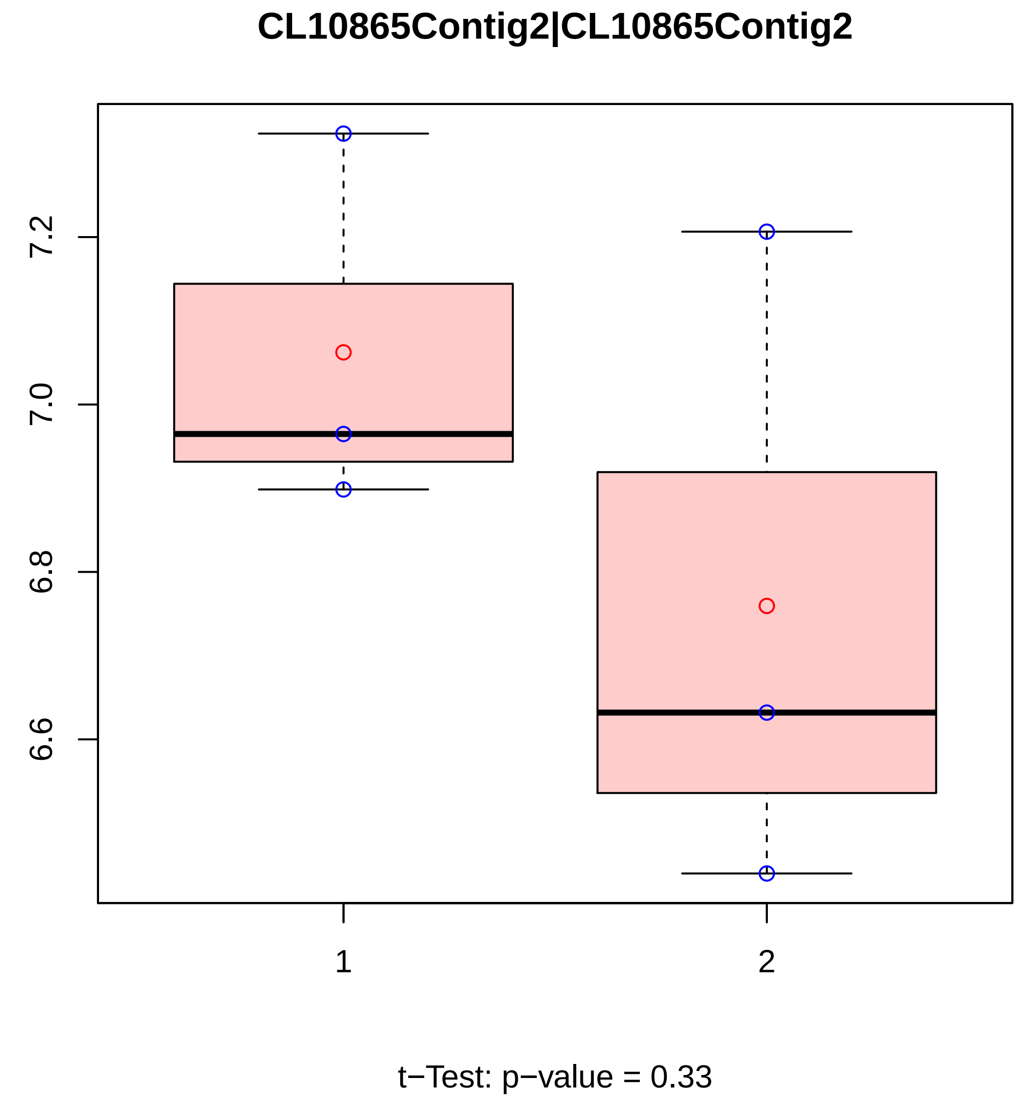
CL10865Contig2|CL10865Contig2
7.2
7.0
6.8
6.6
1
2
t−Test: p−value = 0.33

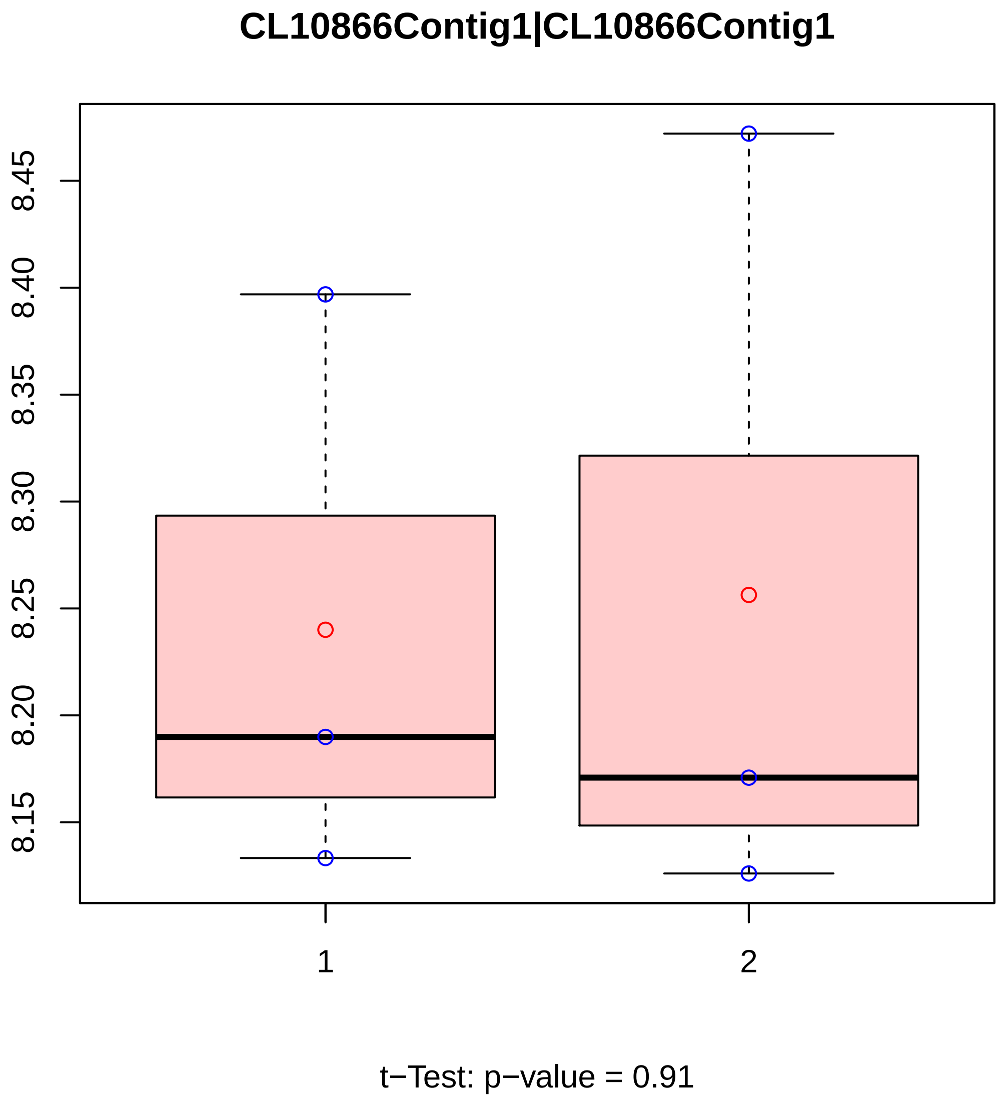
CL10866Contig1|CL10866Contig1
8.15
8.20
8.25
8.30
8.35
8.40
8.45
1
2
t−Test: p−value = 0.91

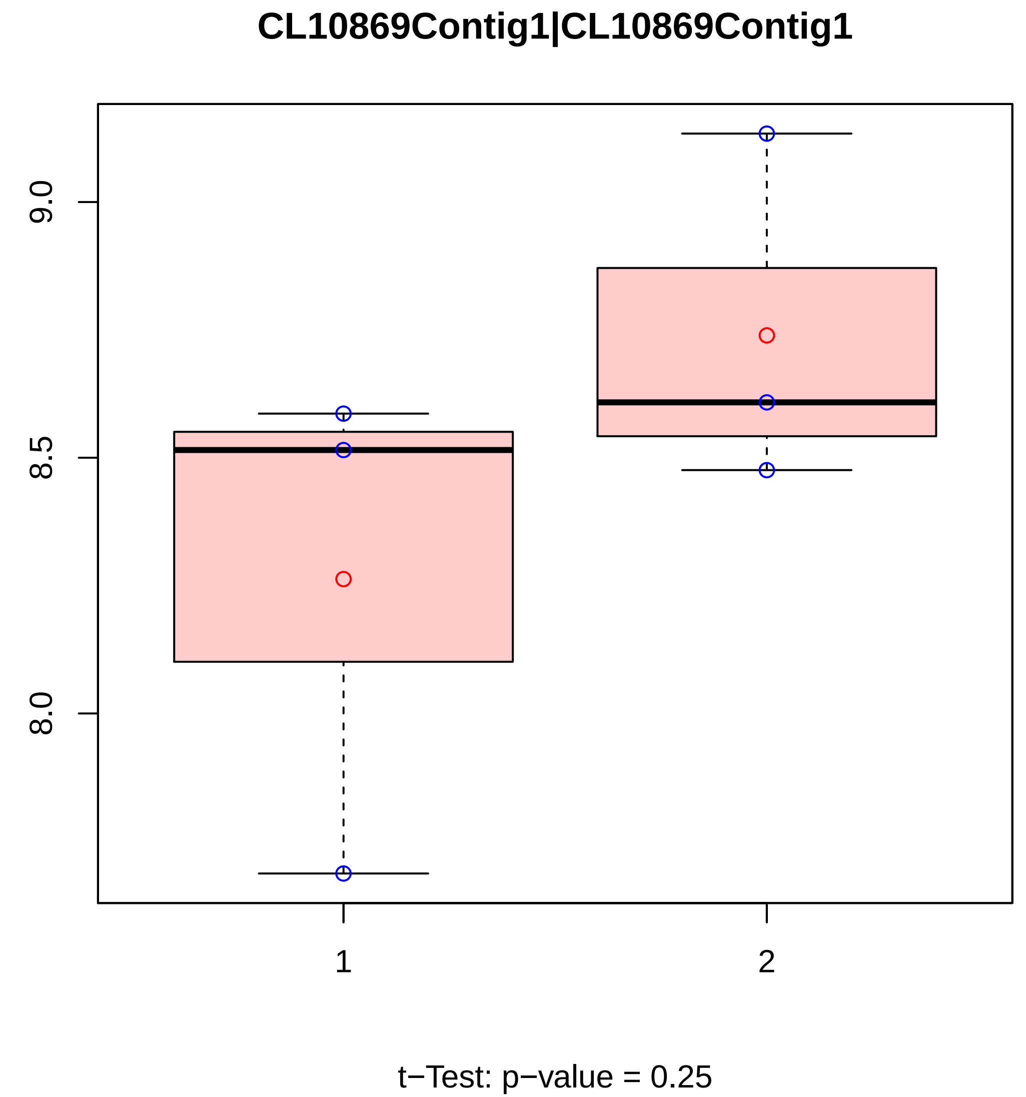
CL10869Contig1|CL10869Contig1
9.0
8.5
8.0
1
2
t−Test: p−value = 0.25

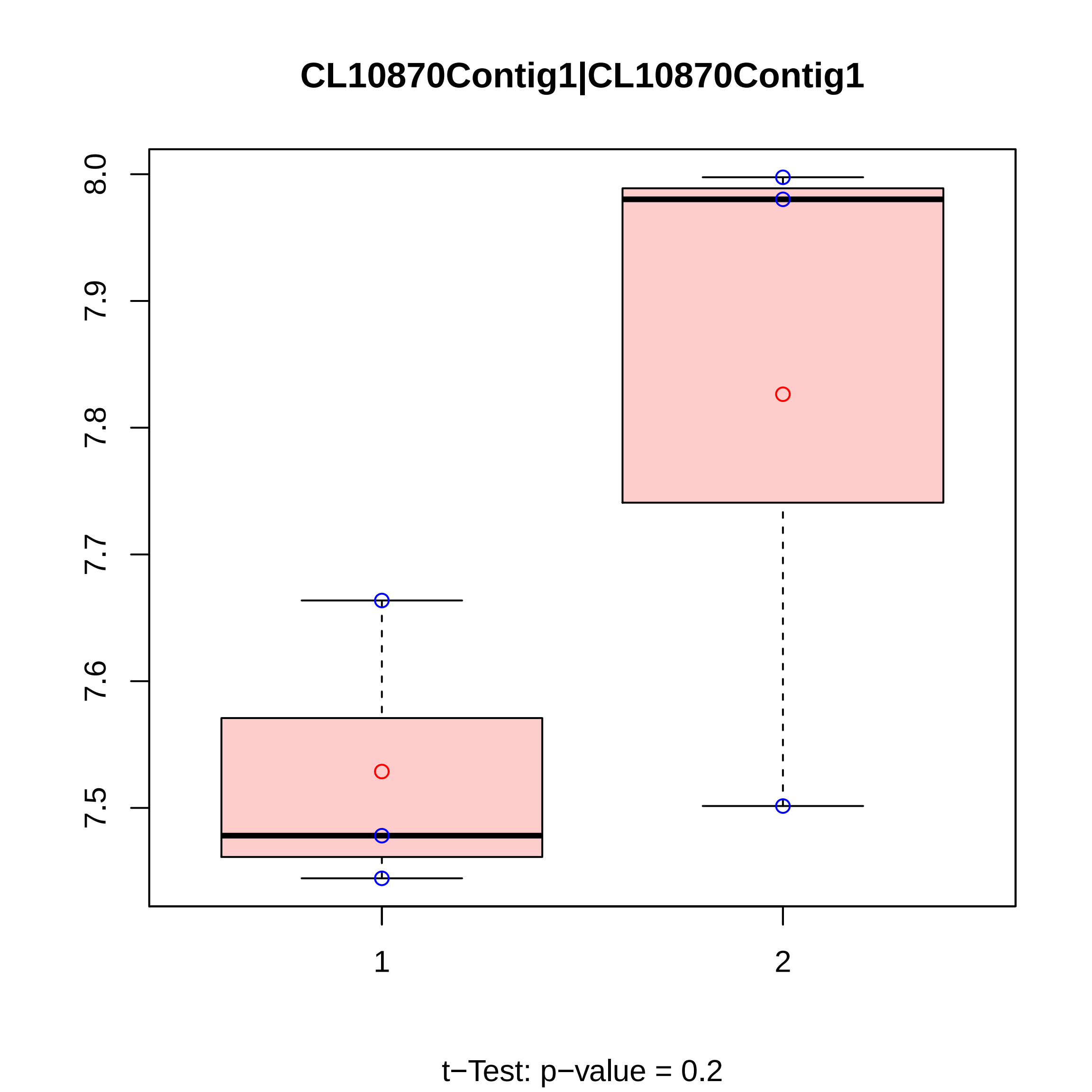
CL10870Contig1|CL10870Contig1
8.0
7.9
7.8
7.7
7.6
7.5
1
2
t−Test: p−value = 0.2

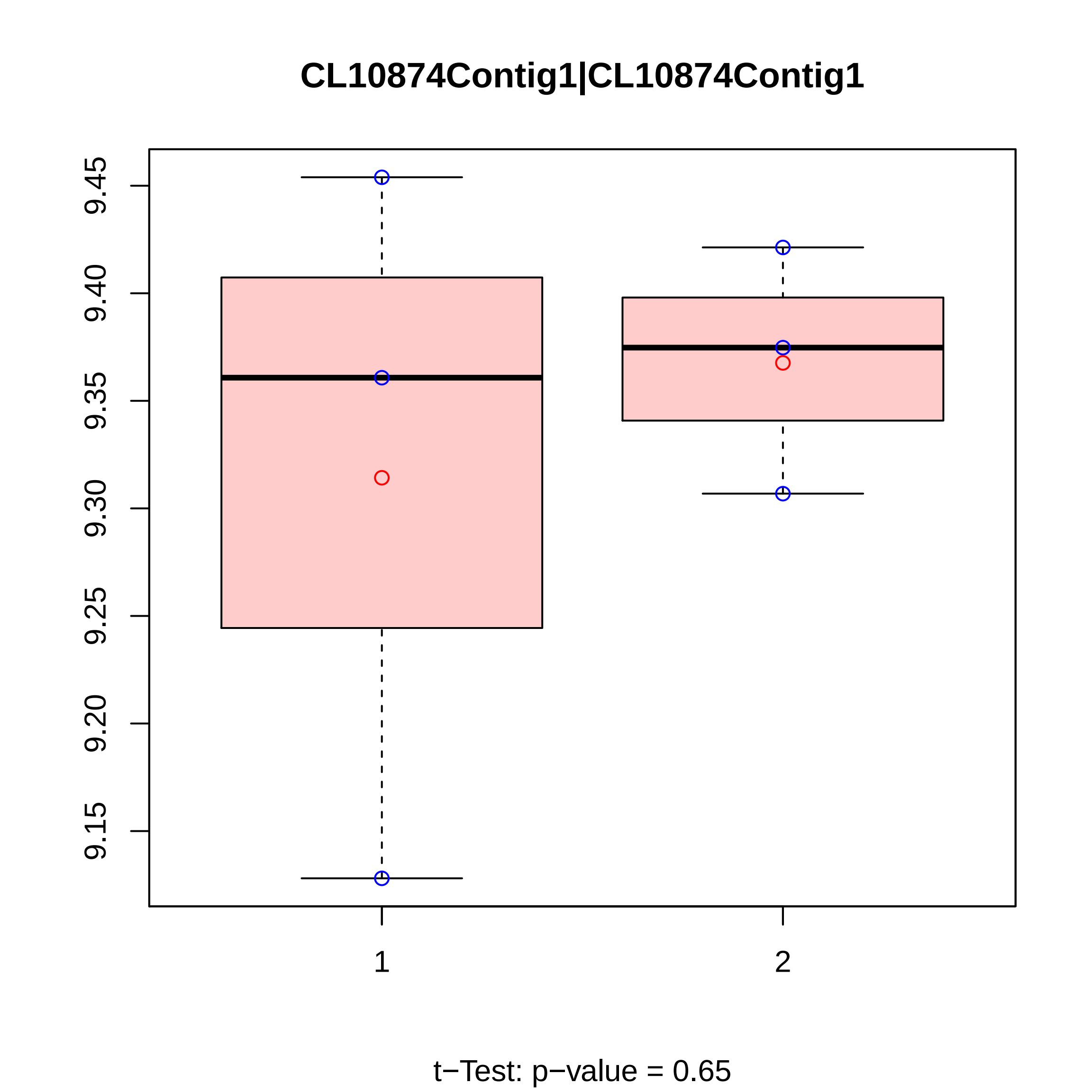
CL10874Contig1|CL10874Contig1
9.15
9.20
9.25
9.30
9.35
9.40
9.45
1
2
t−Test: p−value = 0.65

## CL1087Contig13|CL1087Contig13

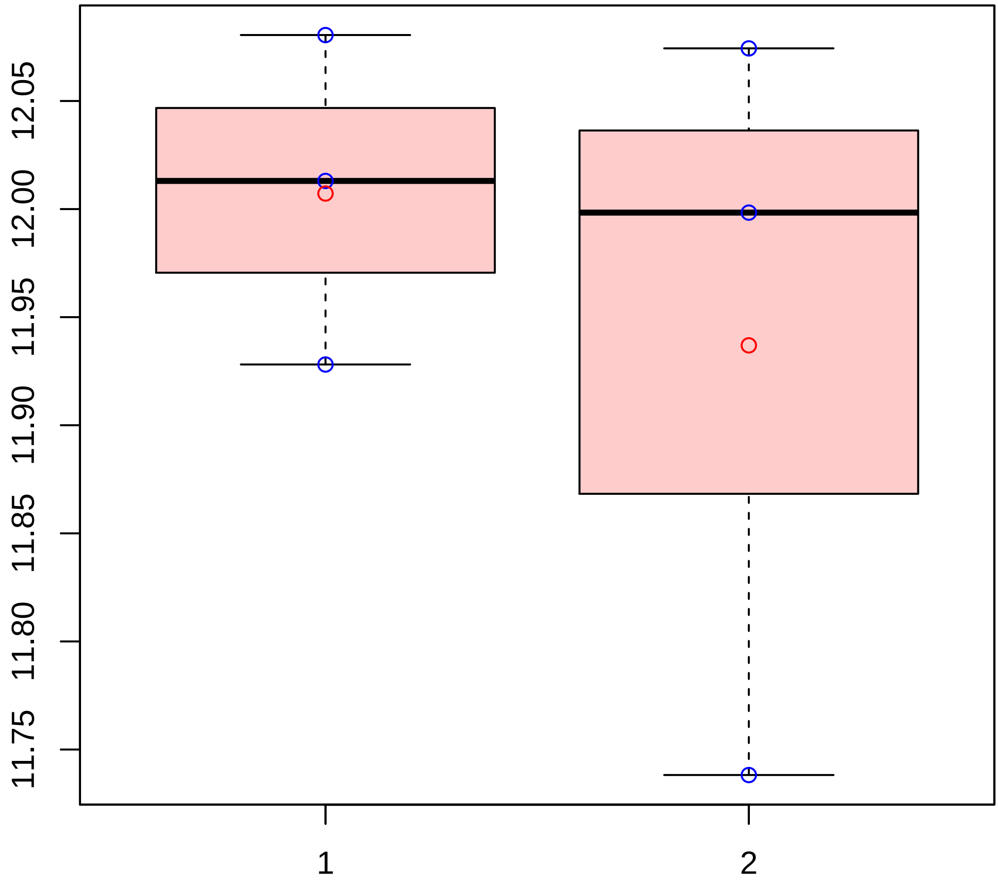


t−Test: p−value = 0.58

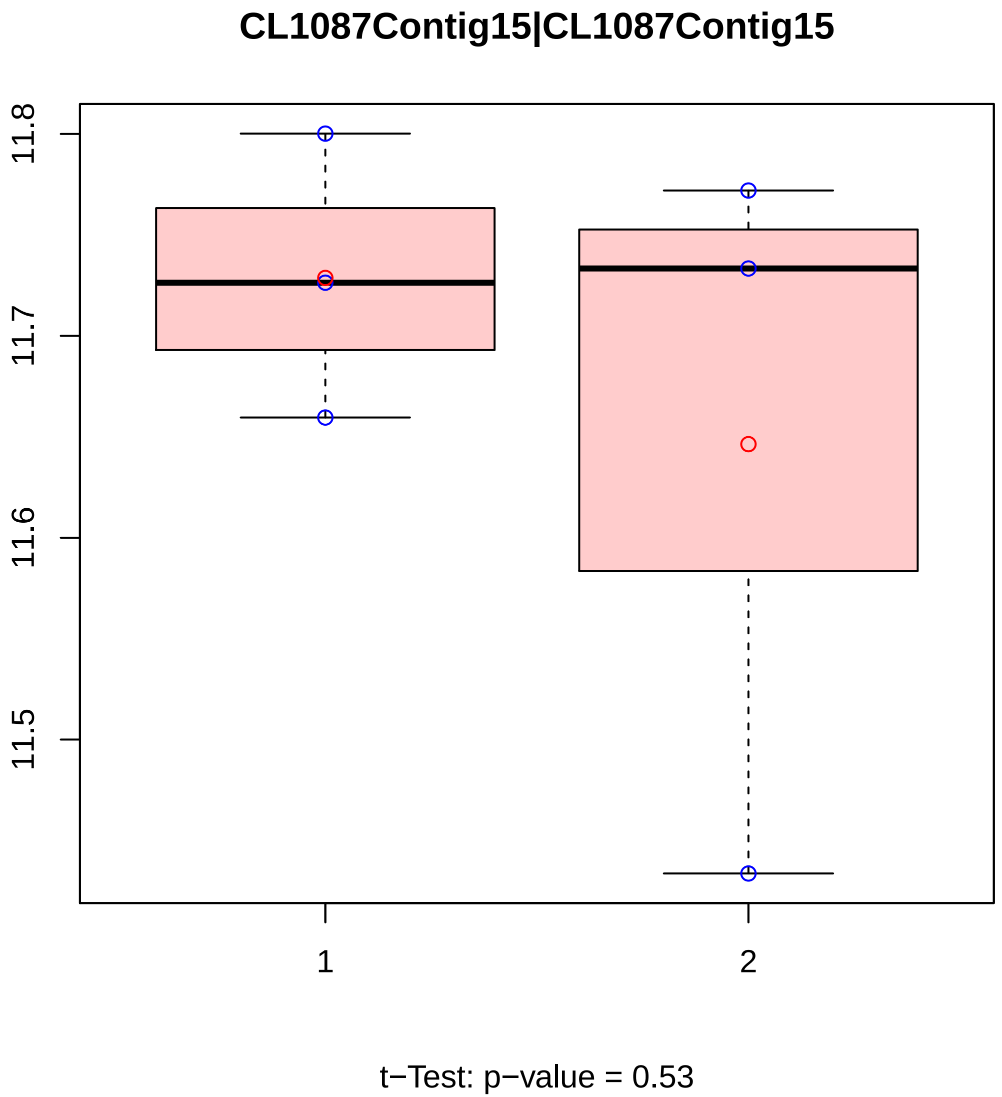
CL1087Contig15|CL1087Contig15
11.8
11.7
11.6
11.5
1
2
t−Test: p−value = 0.53

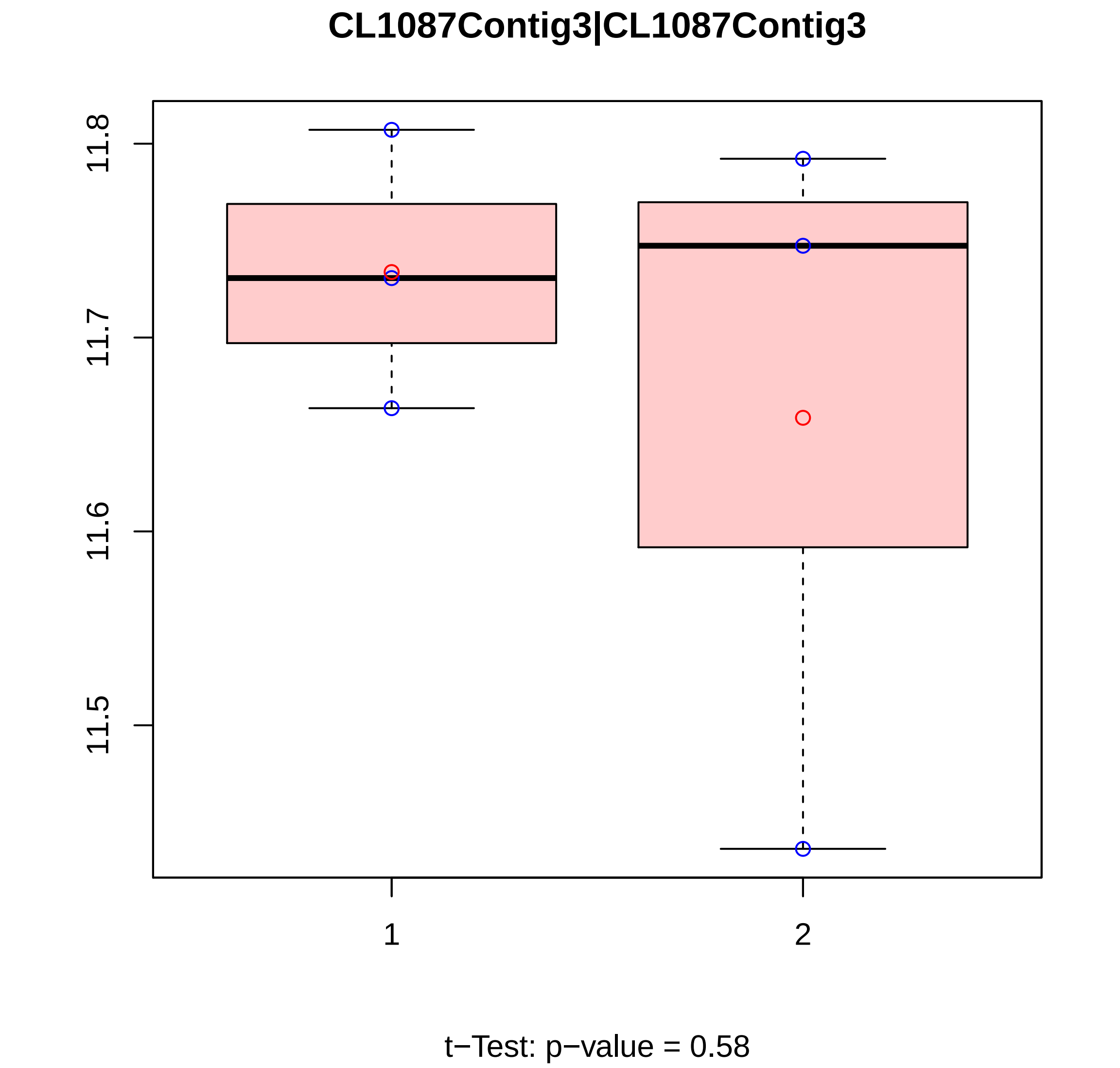
CL1087Contig3|CL1087Contig3
11.8
11.7
11.6
11.5
1
2
t−Test: p−value = 0.58

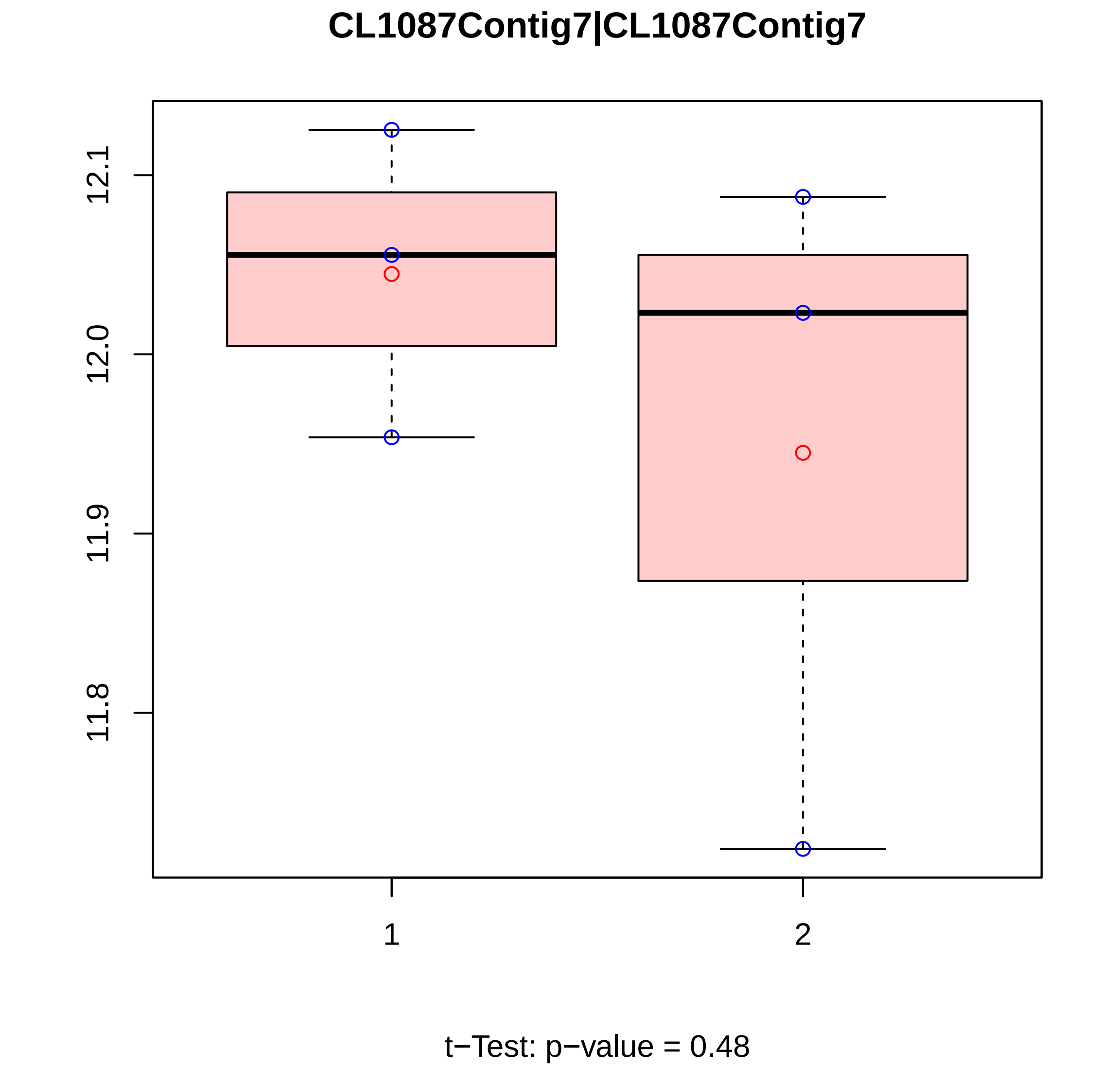
CL1087Contig7|CL1087Contig7
12.1
12.0
11.9
11.8
1
2
t−Test: p−value = 0.48

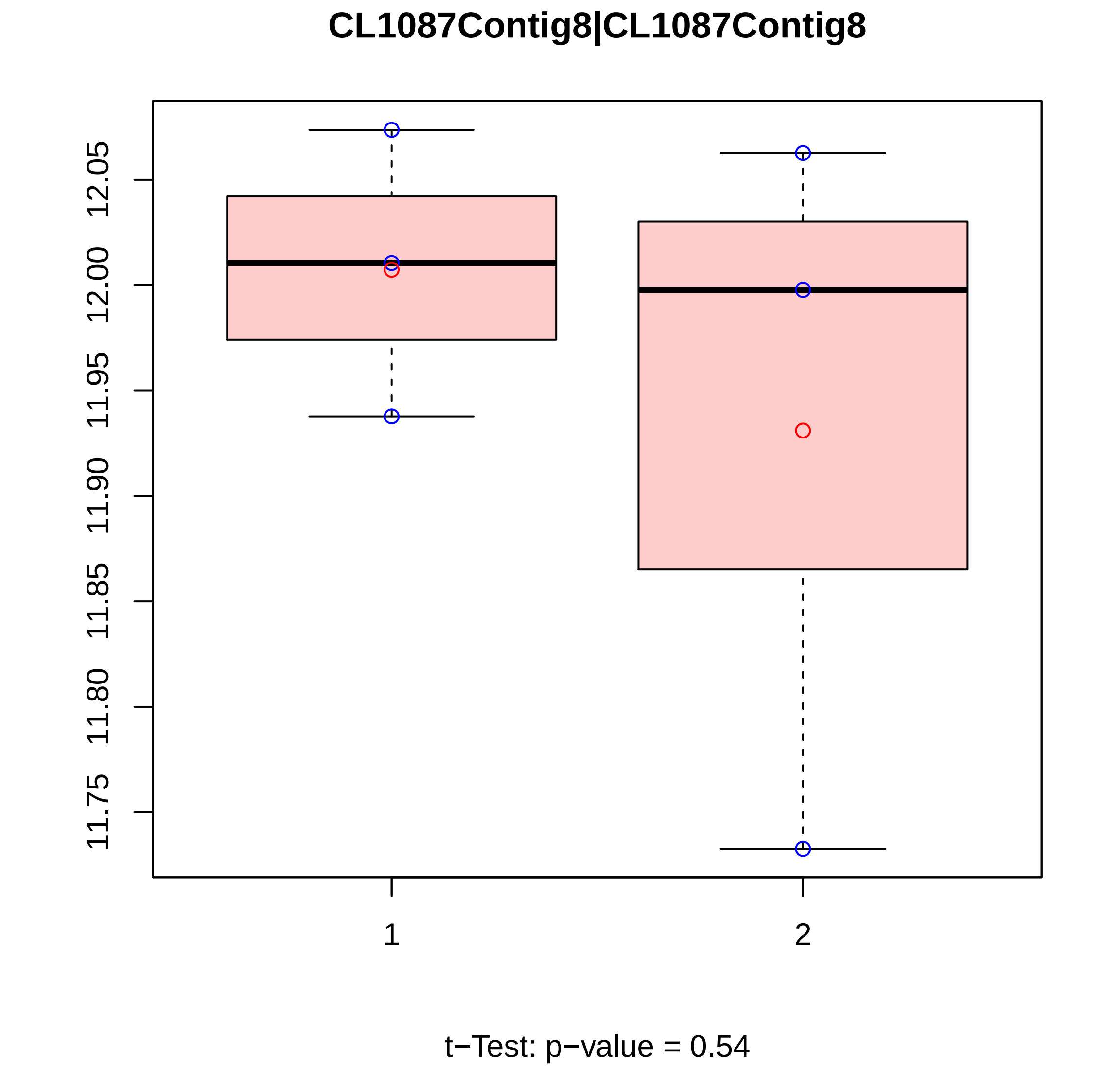
CL1087Contig8|CL1087Contig8
12.05
12.00
11.95
11.90
11.85
11.80
11.75
1
2
t−Test: p−value = 0.54

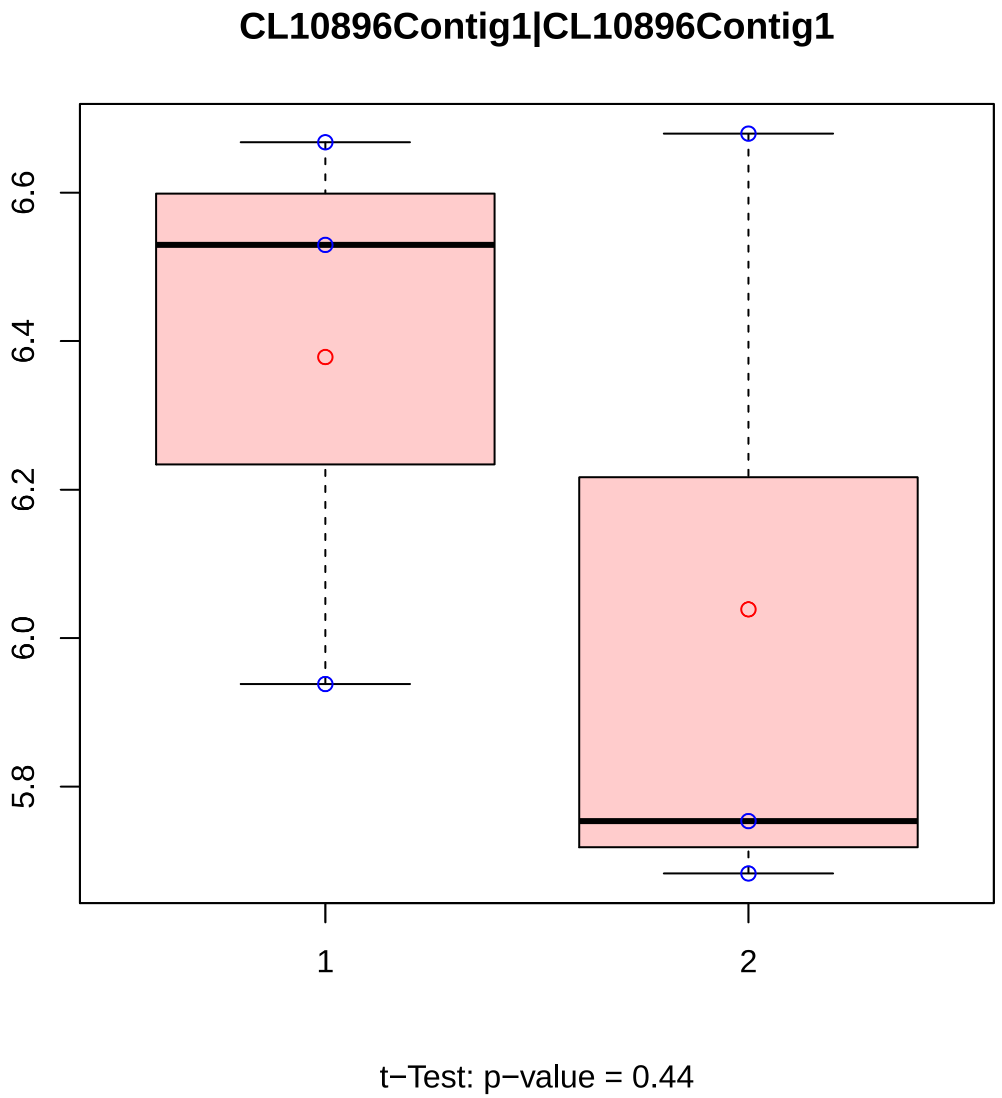
CL10896Contig1|CL10896Contig1
6.6
6.4
6.2
6.0
5.8
1
2
t−Test: p−value = 0.44

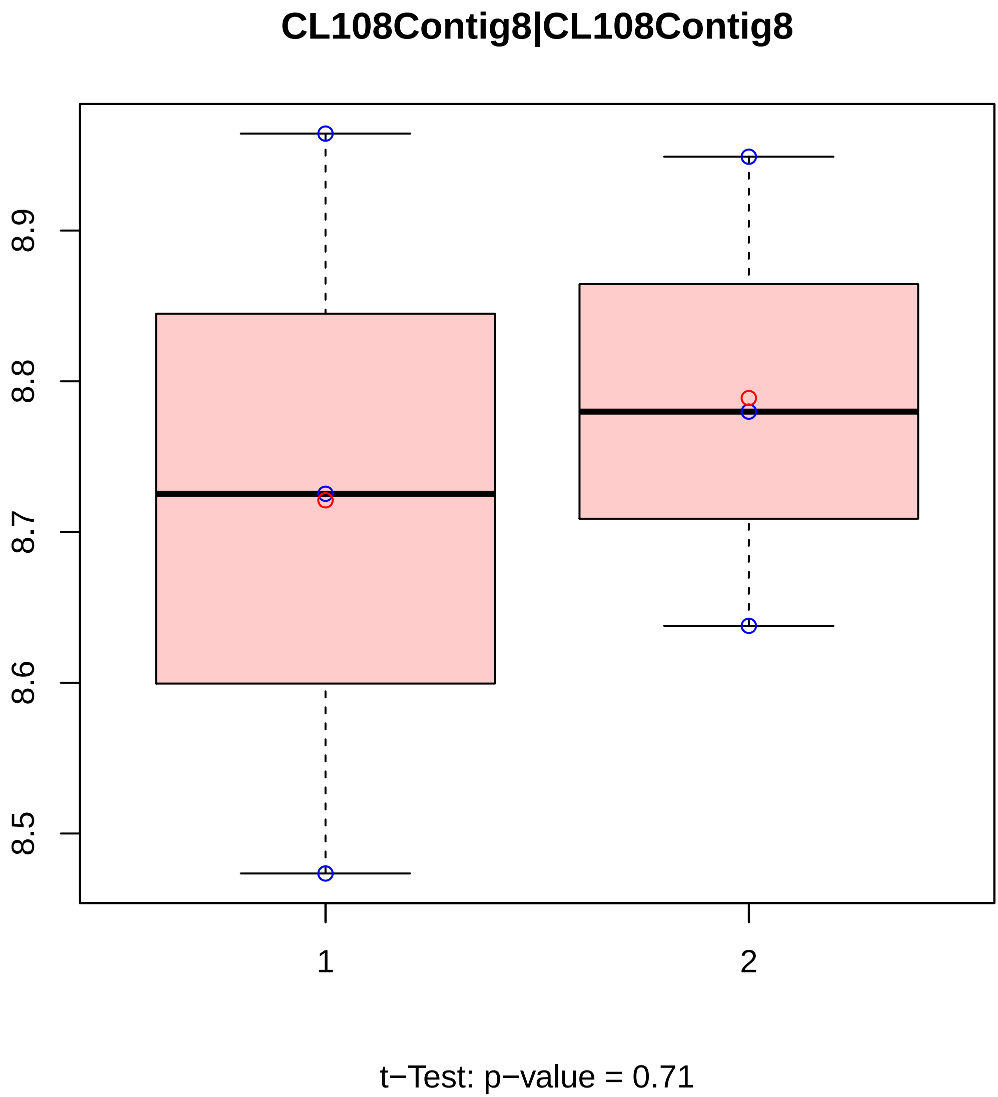
CL108Contig8|CL108Contig8
8.5
8.6
8.7
8.8
8.9
1
2
t−Test: p−value = 0.71

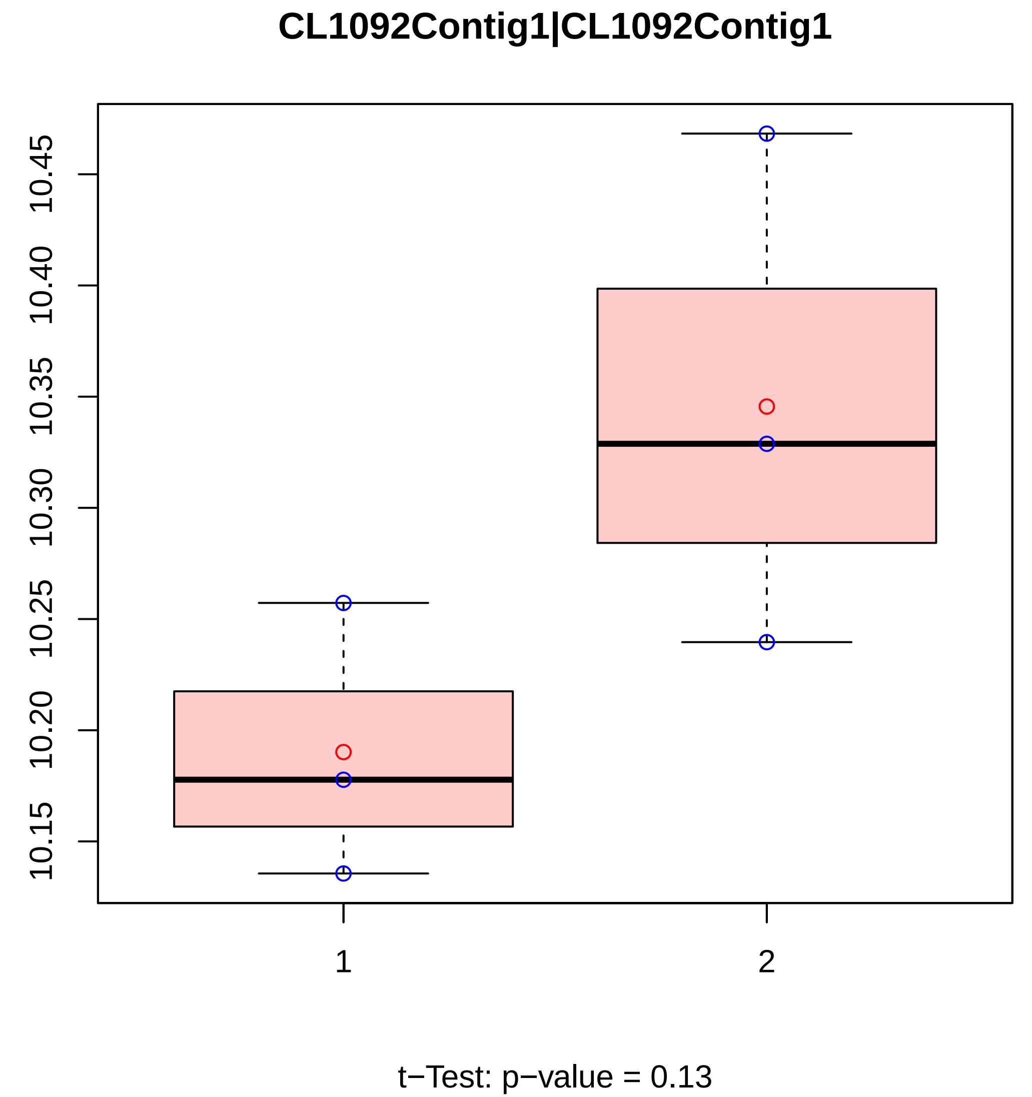
CL1092Contig1|CL1092Contig1
10.15
10.20
10.25
10.30
10.35
10.40
10.45
1
2
t−Test: p−value = 0.13

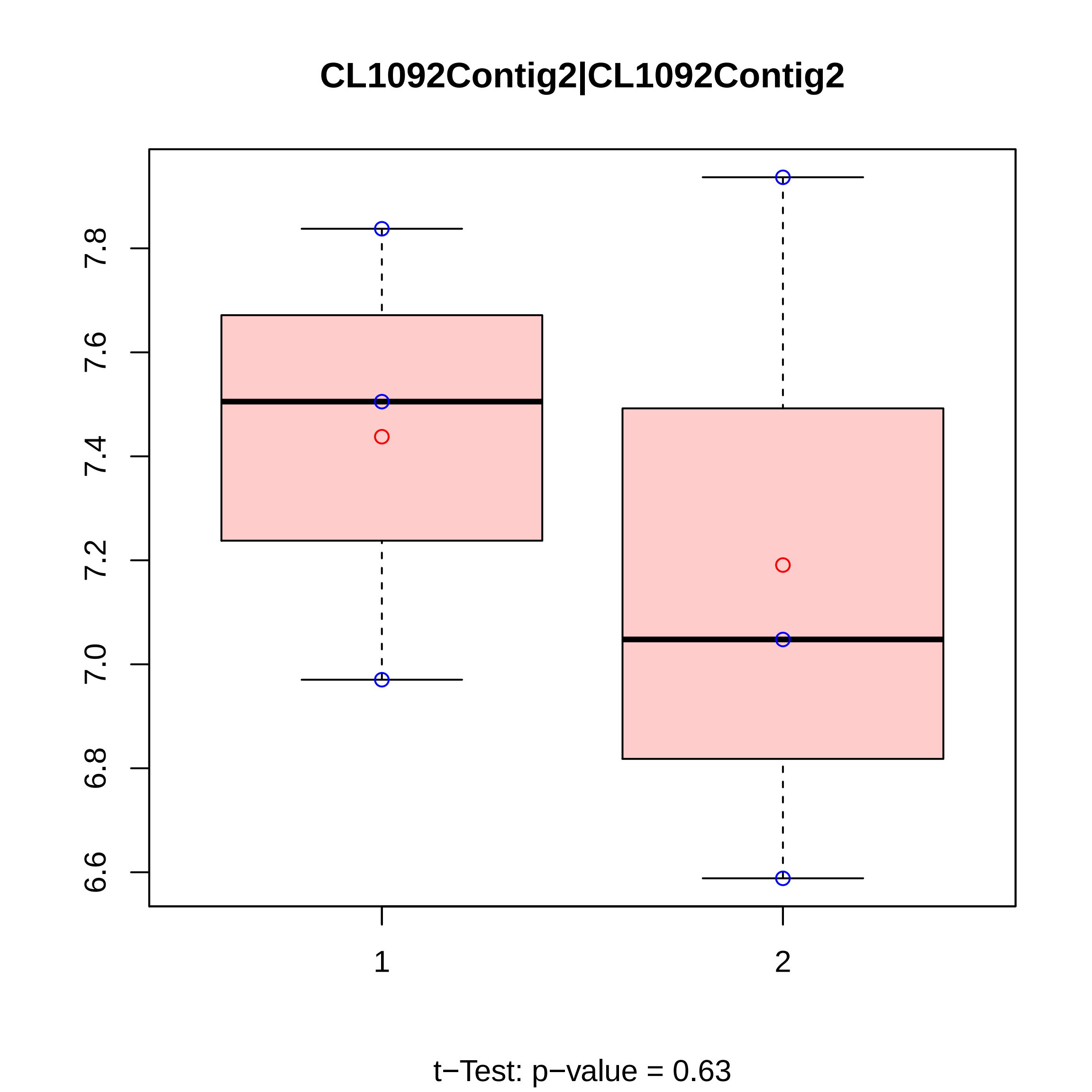
CL1092Contig2|CL1092Contig2
6.6
6.8
7.0
7.2
7.4
7.6
7.8
1
2
t−Test: p−value = 0.63

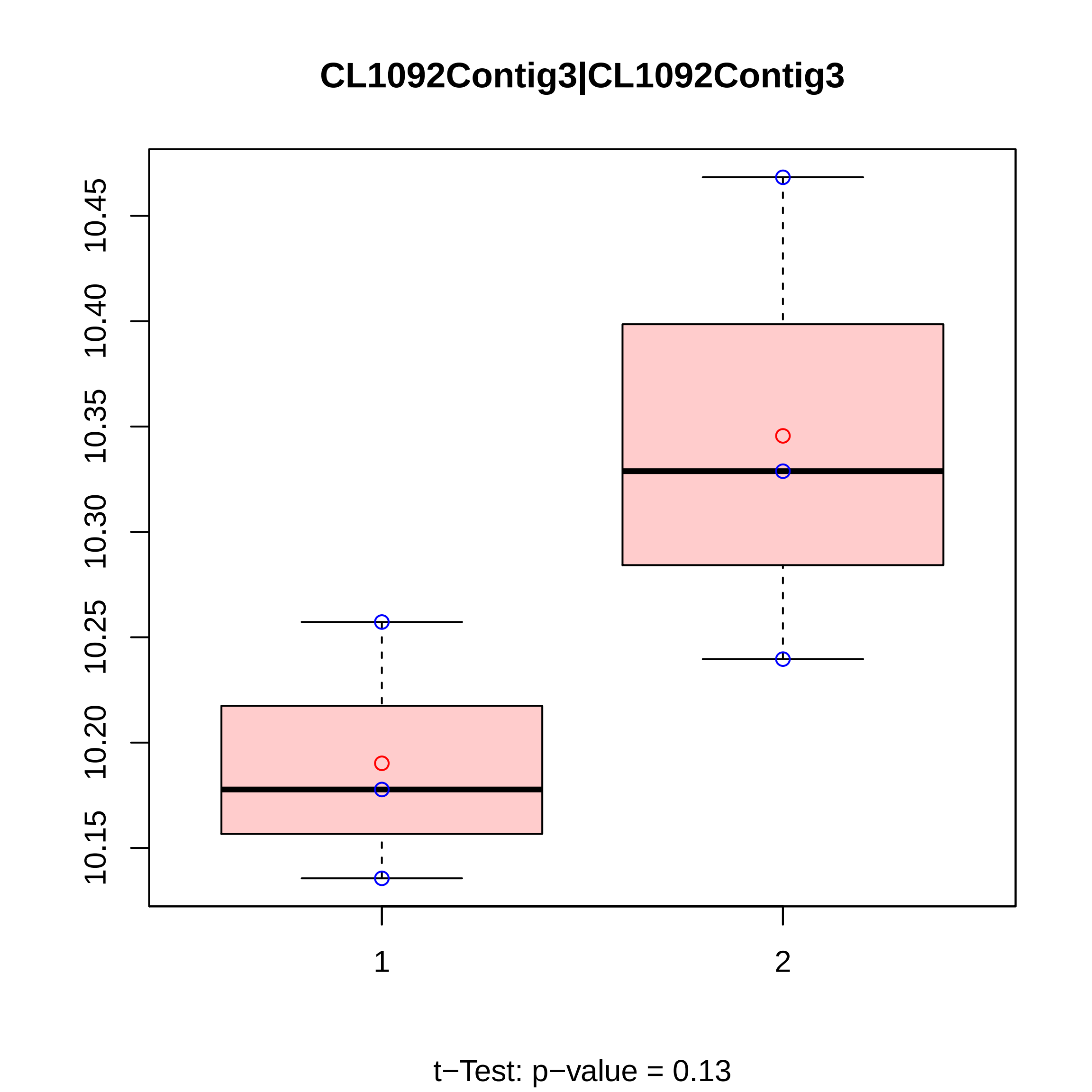

CL1092Contig3|CL1092Contig3
10.15
10.20
10.25
10.30
10.35
10.40
10.45
1
2
t−Test: p−value = 0.13

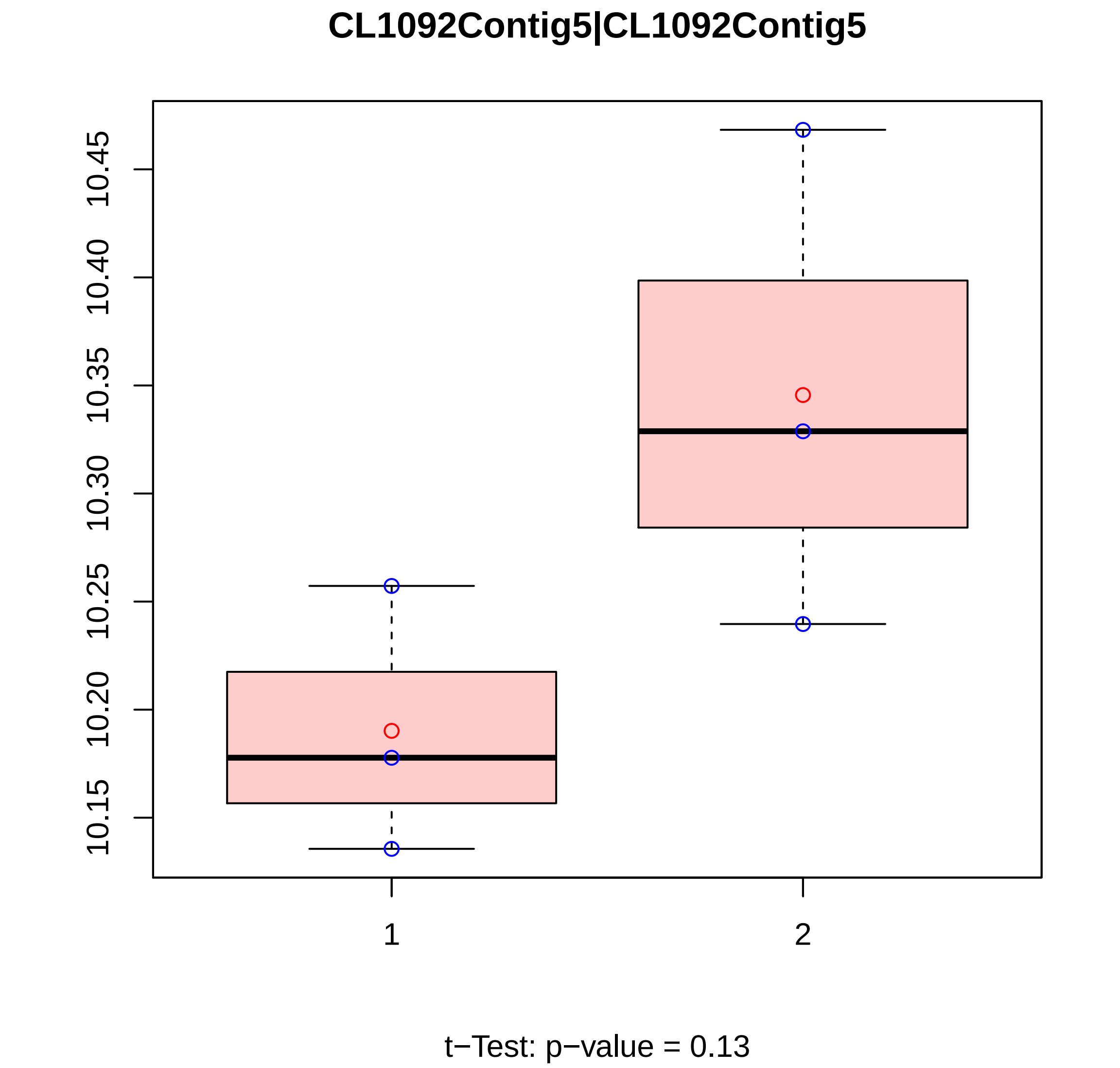

CL1092Contig5|CL1092Contig5
10.15
10.20
10.25
10.30
10.35
10.40
10.45
1
2
t−Test: p−value = 0.13

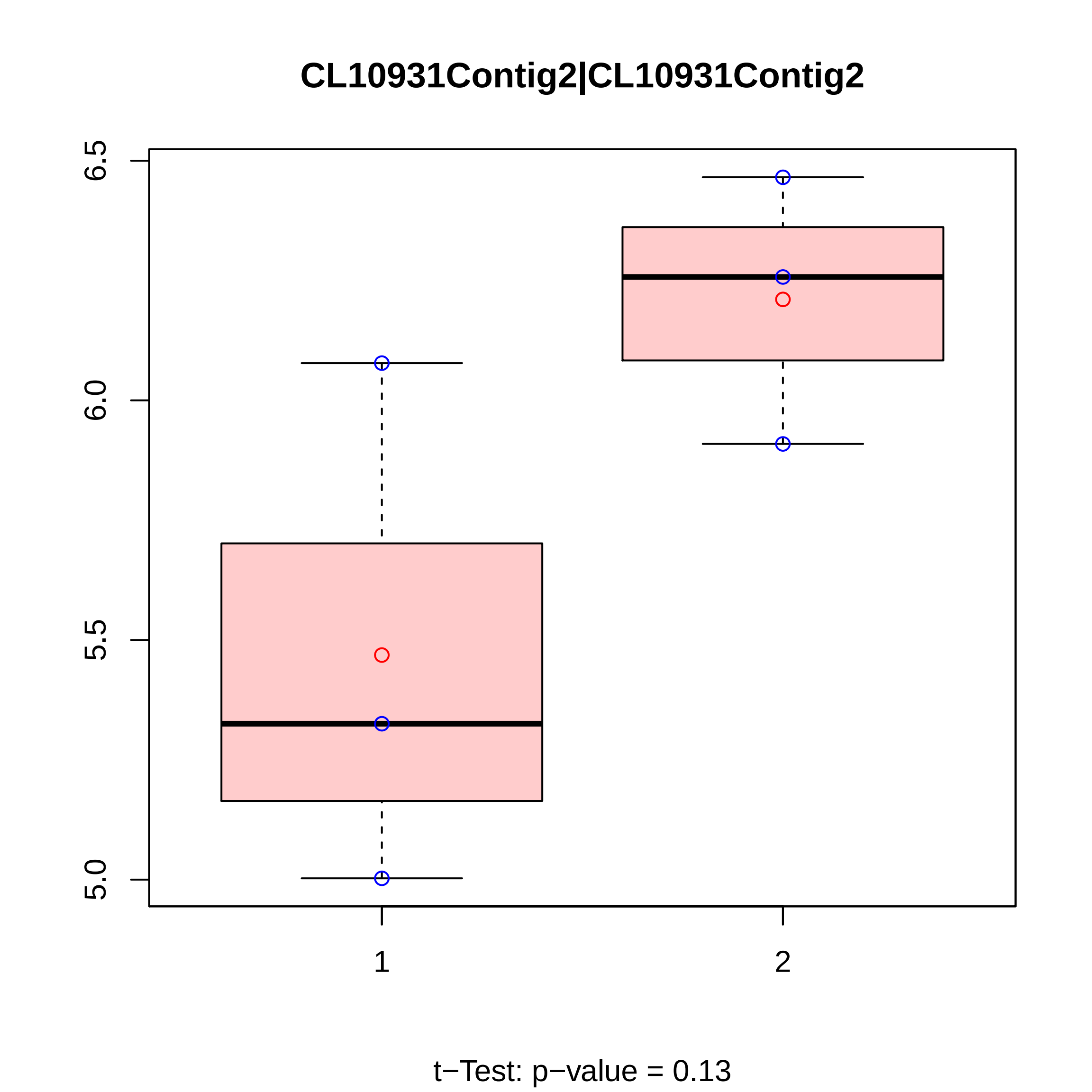
CL10931Contig2|CL10931Contig2
6.5
6.0
5.5
5.0
1
2
t−Test: p−value = 0.13

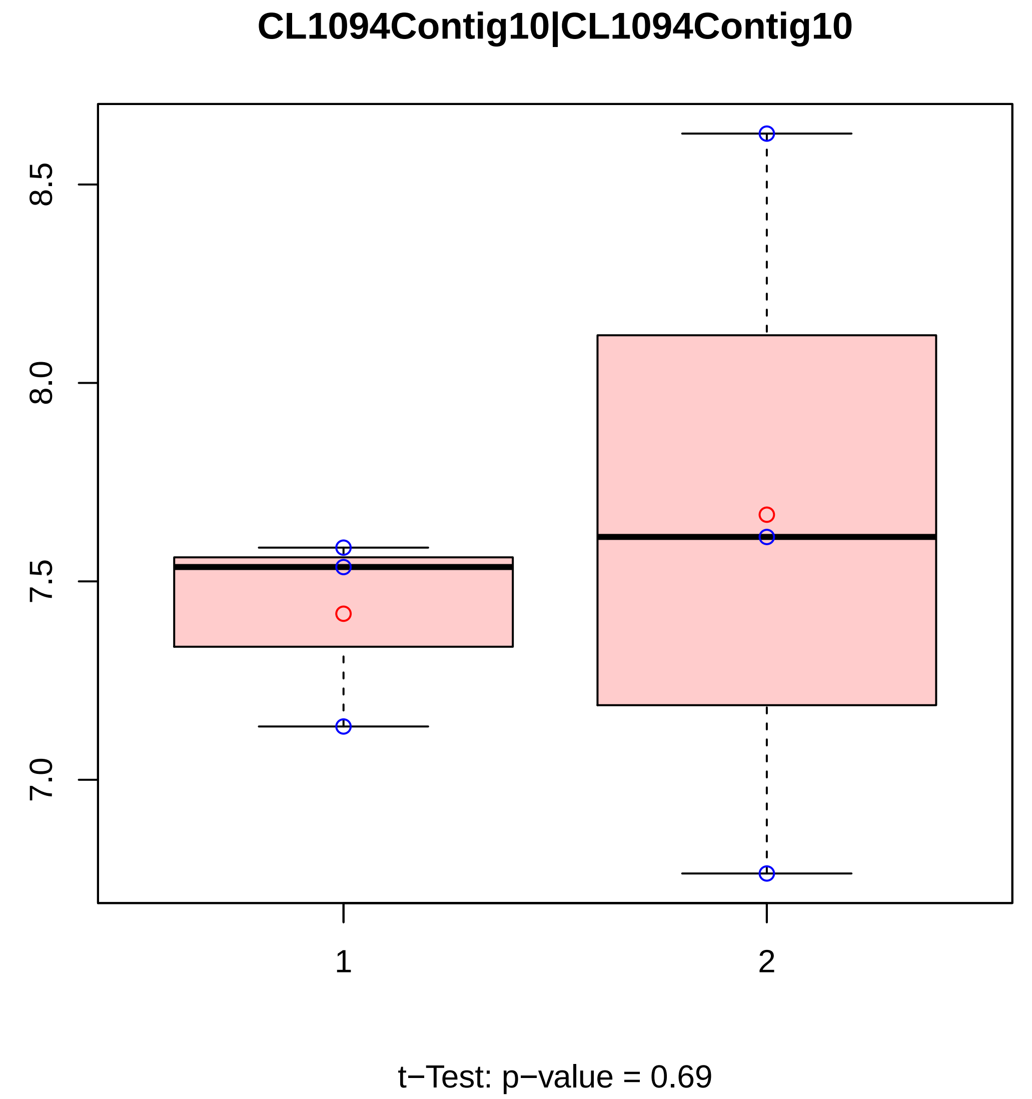
CL1094Contig10|CL1094Contig10
8.5
8.0
7.5
7.0
1
2
t−Test: p−value = 0.69

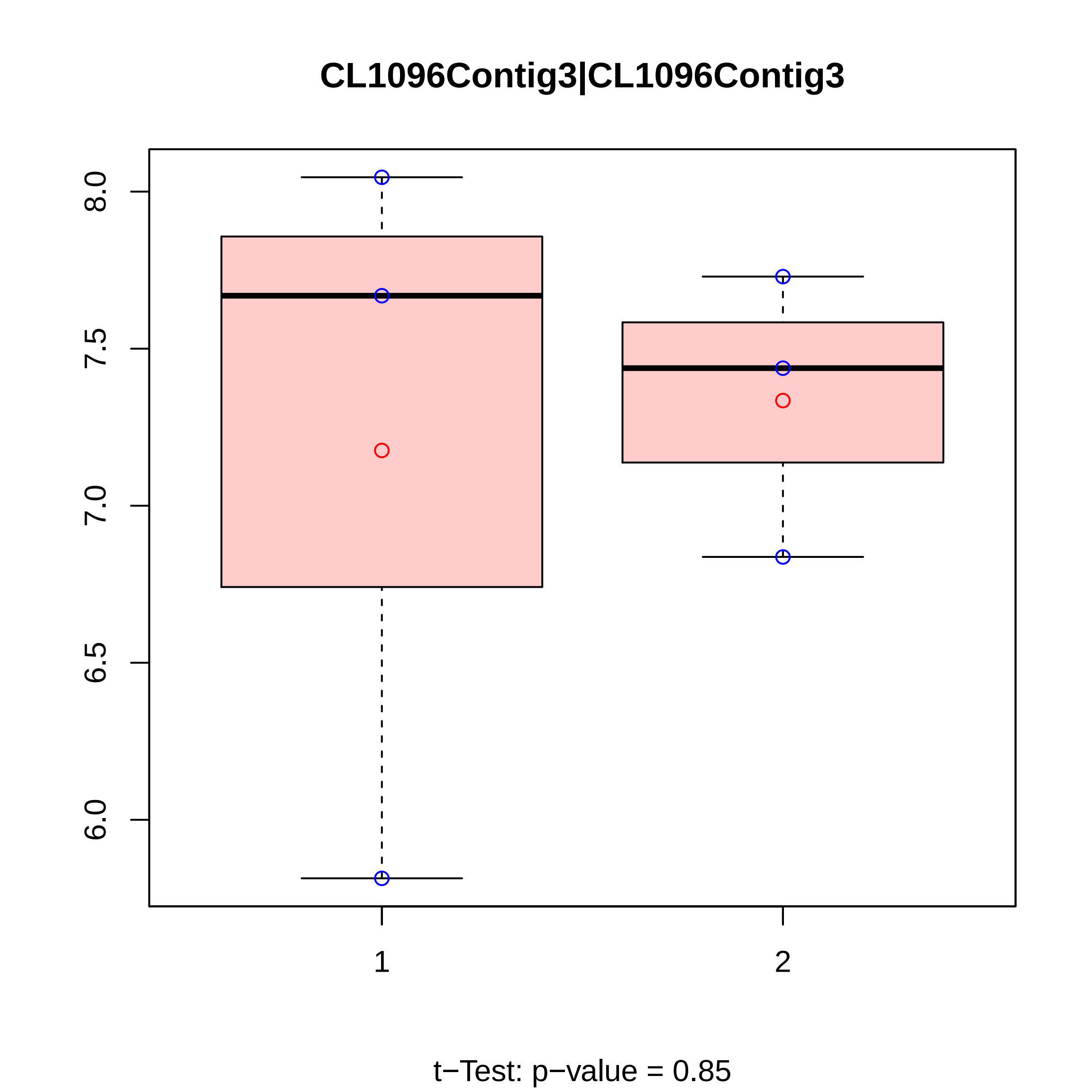
CL1096Contig3|CL1096Contig3
8.0
7.5
7.0
6.5
6.0
1
2
t−Test: p−value = 0.85

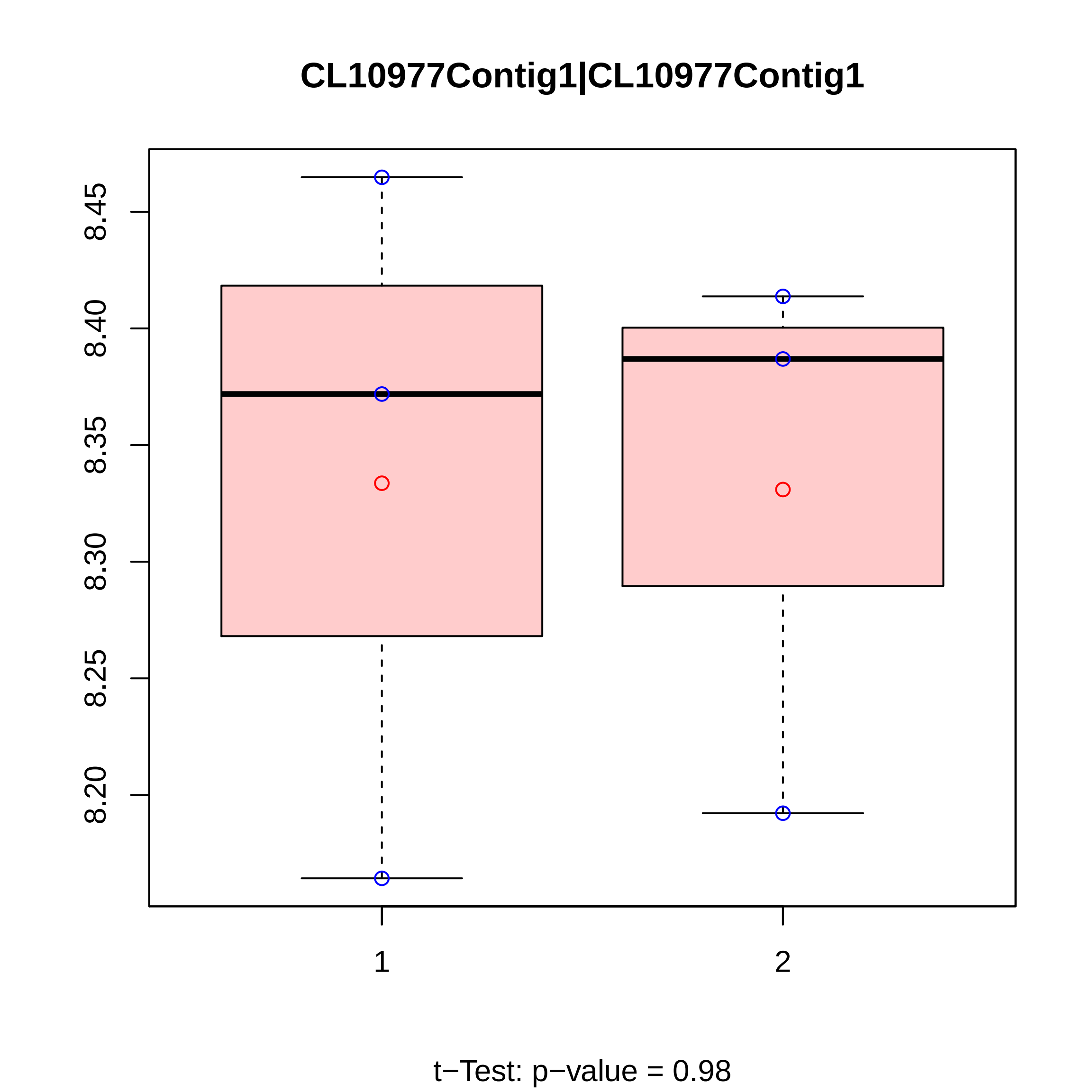

CL10977Contig1|CL10977Contig1
8.20
8.25
8.30
8.35
8.40
8.45
1
2
t−Test: p−value = 0.98

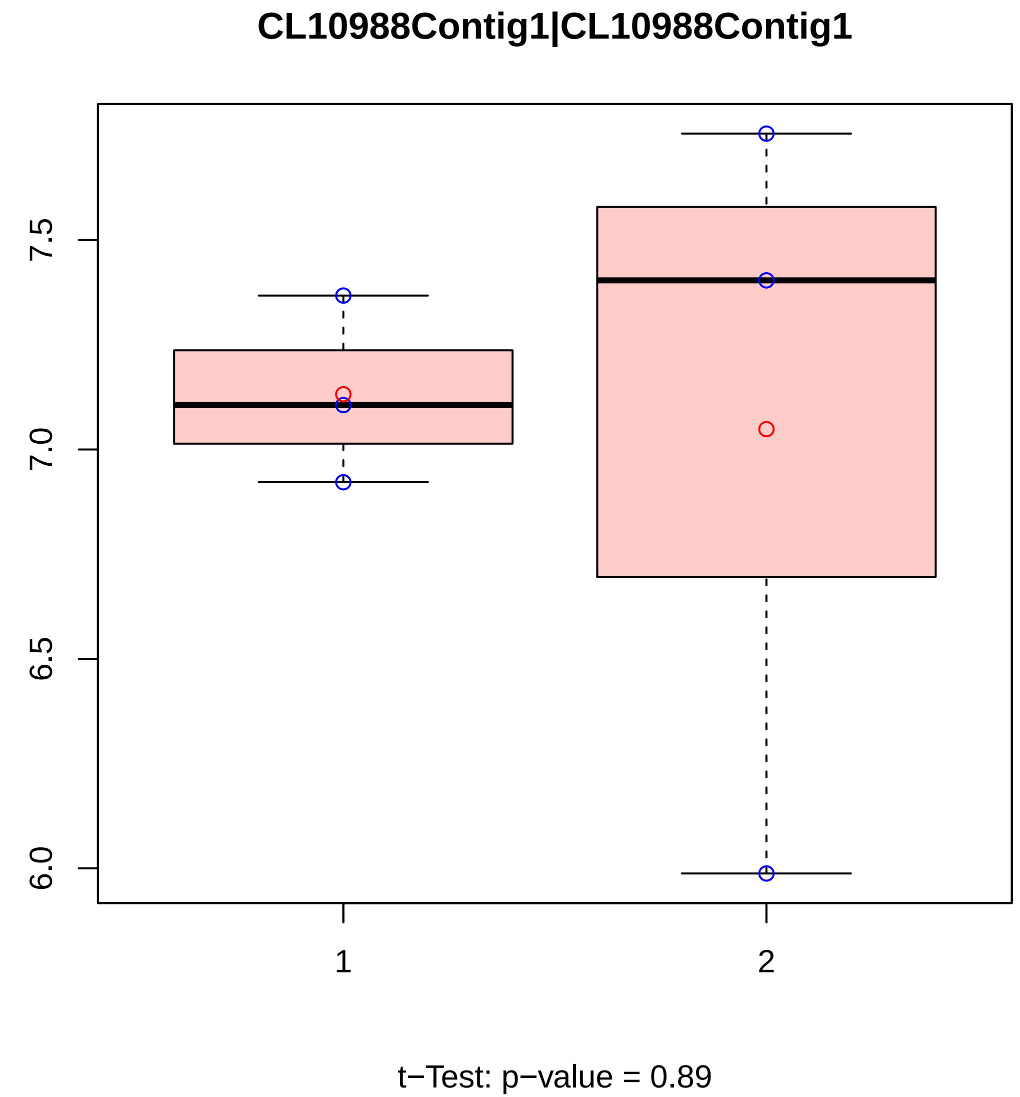
CL10988Contig1|CL10988Contig1
7.5
7.0
6.5
6.0
1
2
t−Test: p−value = 0.89

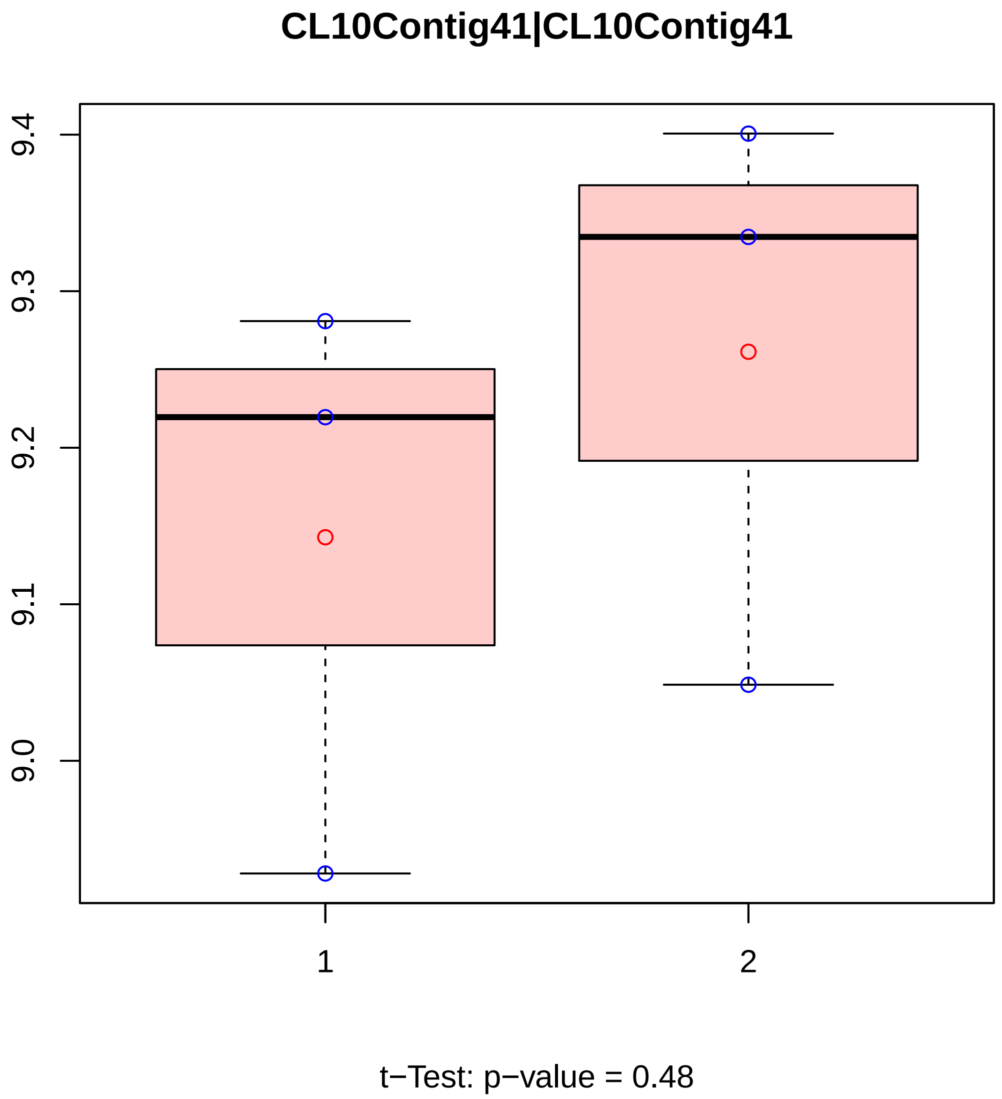
CL10Contig41|CL10Contig41
9.4
9.3
9.2
9.1
9.0
1
2
t−Test: p−value = 0.48

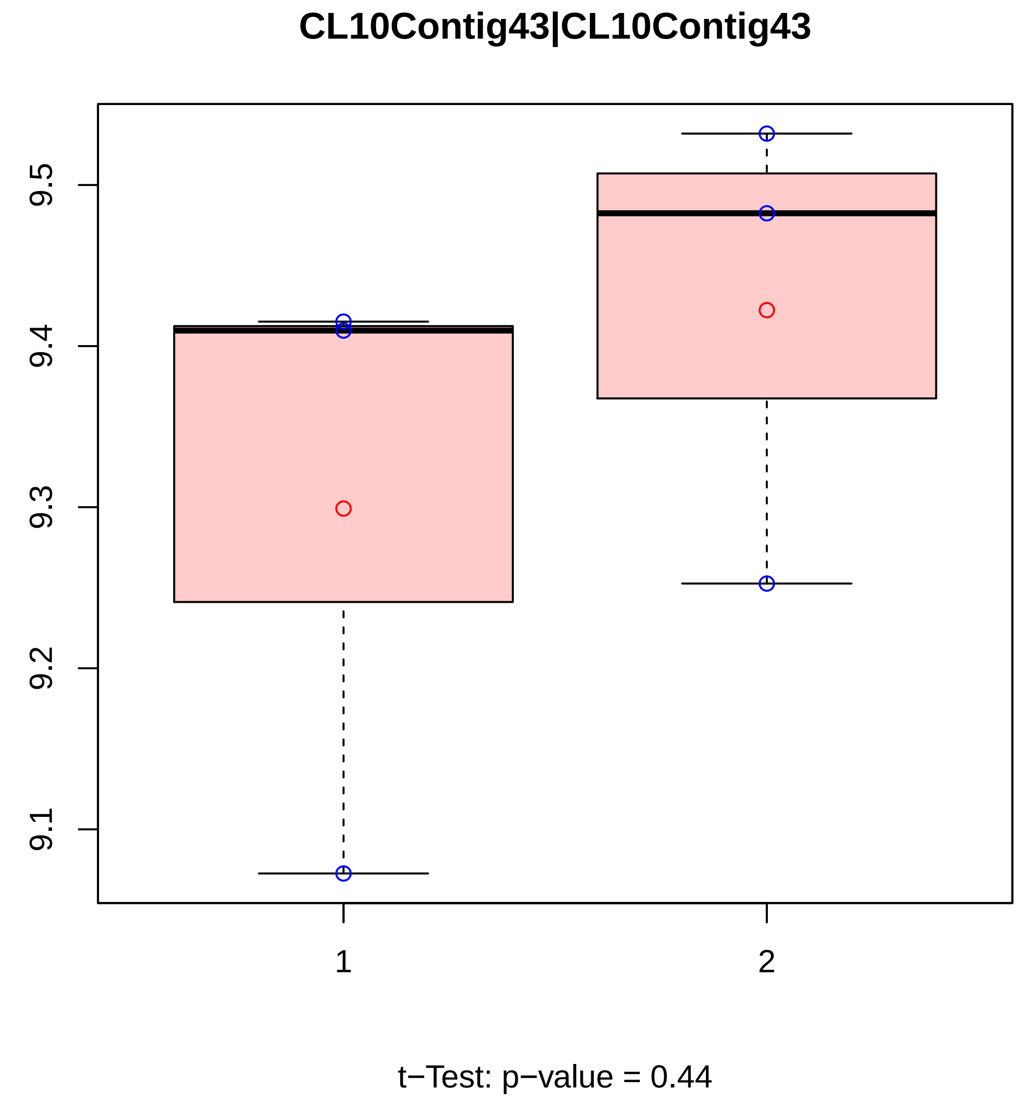
CL10Contig43|CL10Contig43
9.1
9.2
9.3
9.4
9.5
1
2
t−Test: p−value = 0.44

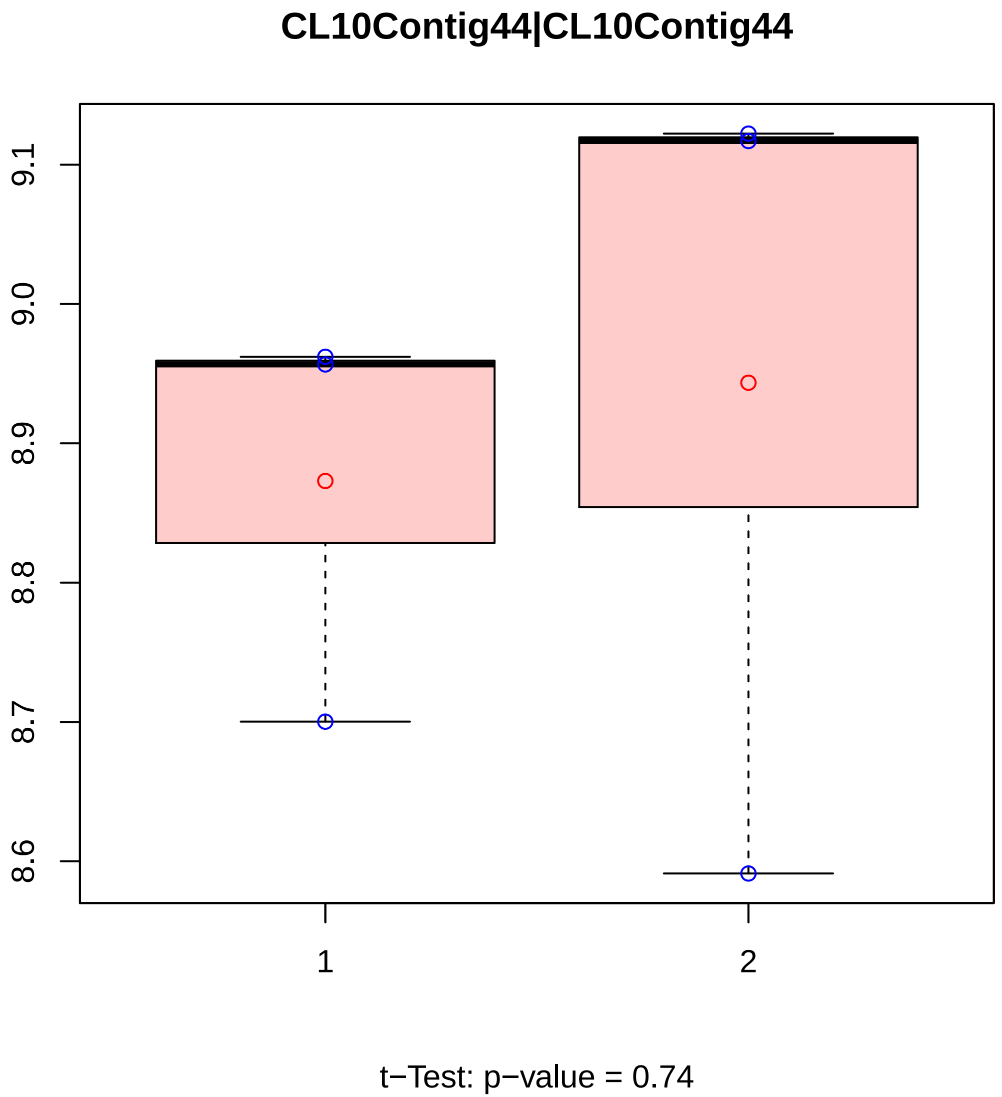

CL10Contig44|CL10Contig44
9.1
9.0
8.9
8.8
8.7
8.6
1
2
t−Test: p−value = 0.74

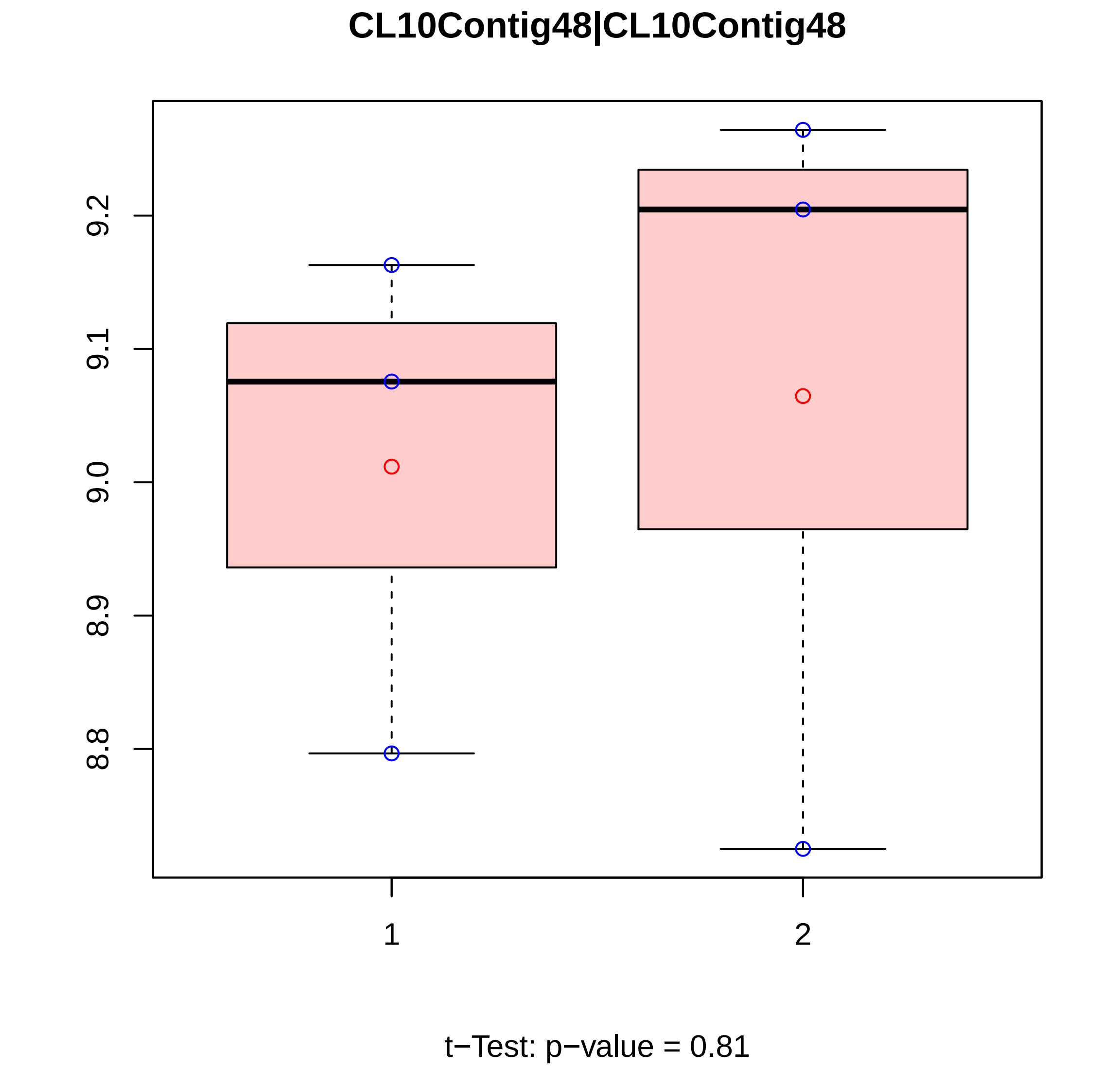
CL10Contig48|CL10Contig48
9.2
9.1
9.0
8.9
8.8
1
2
t−Test: p−value = 0.81

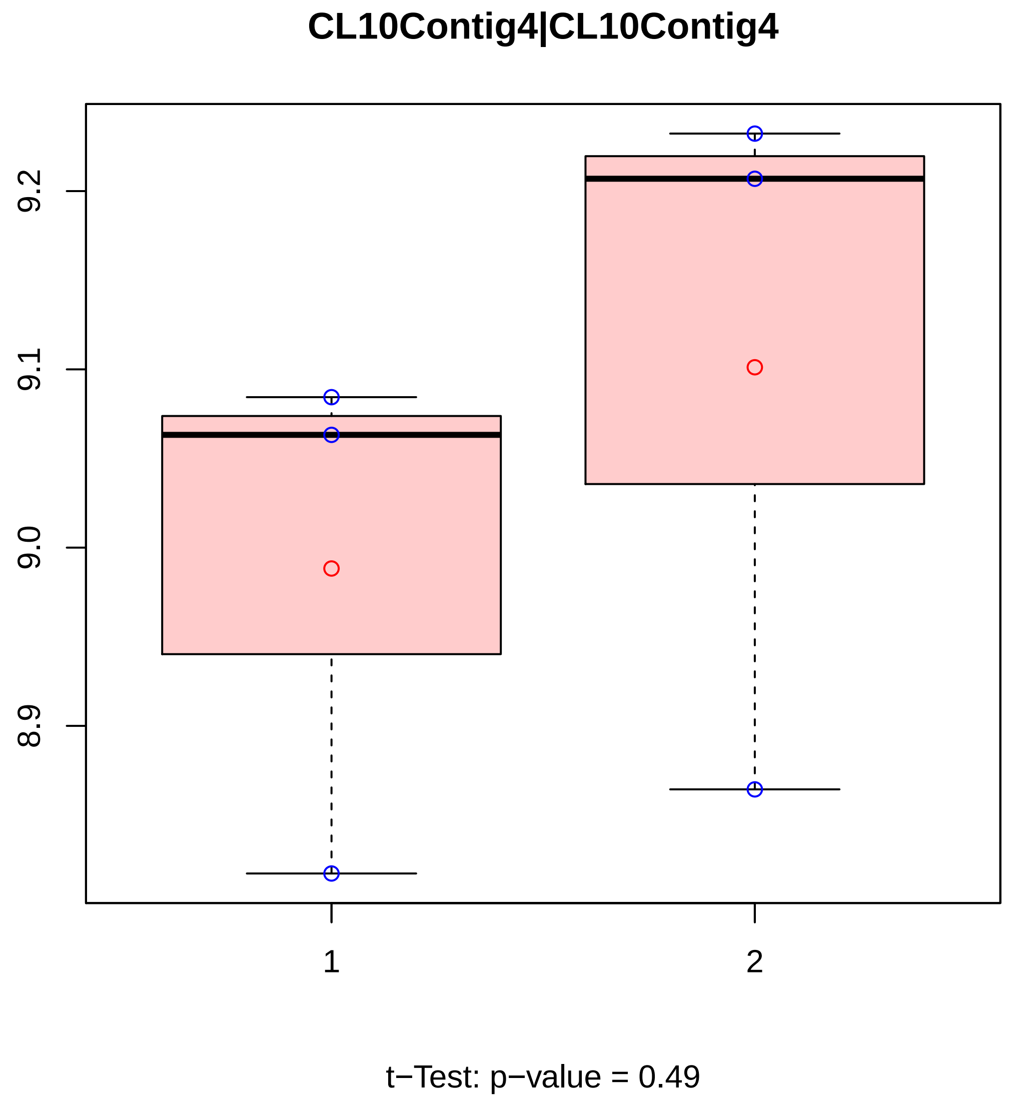
CL10Contig4|CL10Contig4
9.2
9.1
9.0
8.9
1
2
t−Test: p−value = 0.49

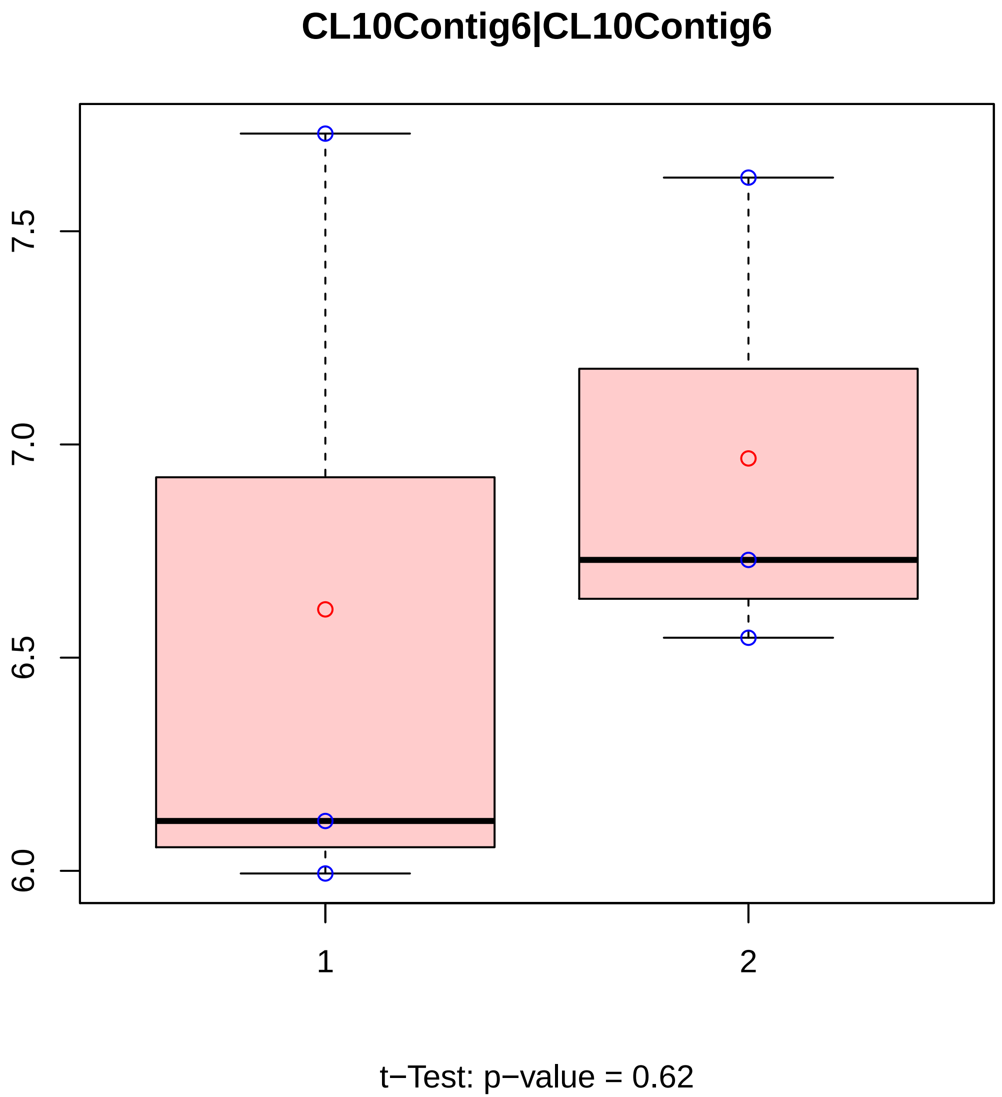
CL10Contig6|CL10Contig6
7.5
7.0
6.5
6.0
1
2
t−Test: p−value = 0.62

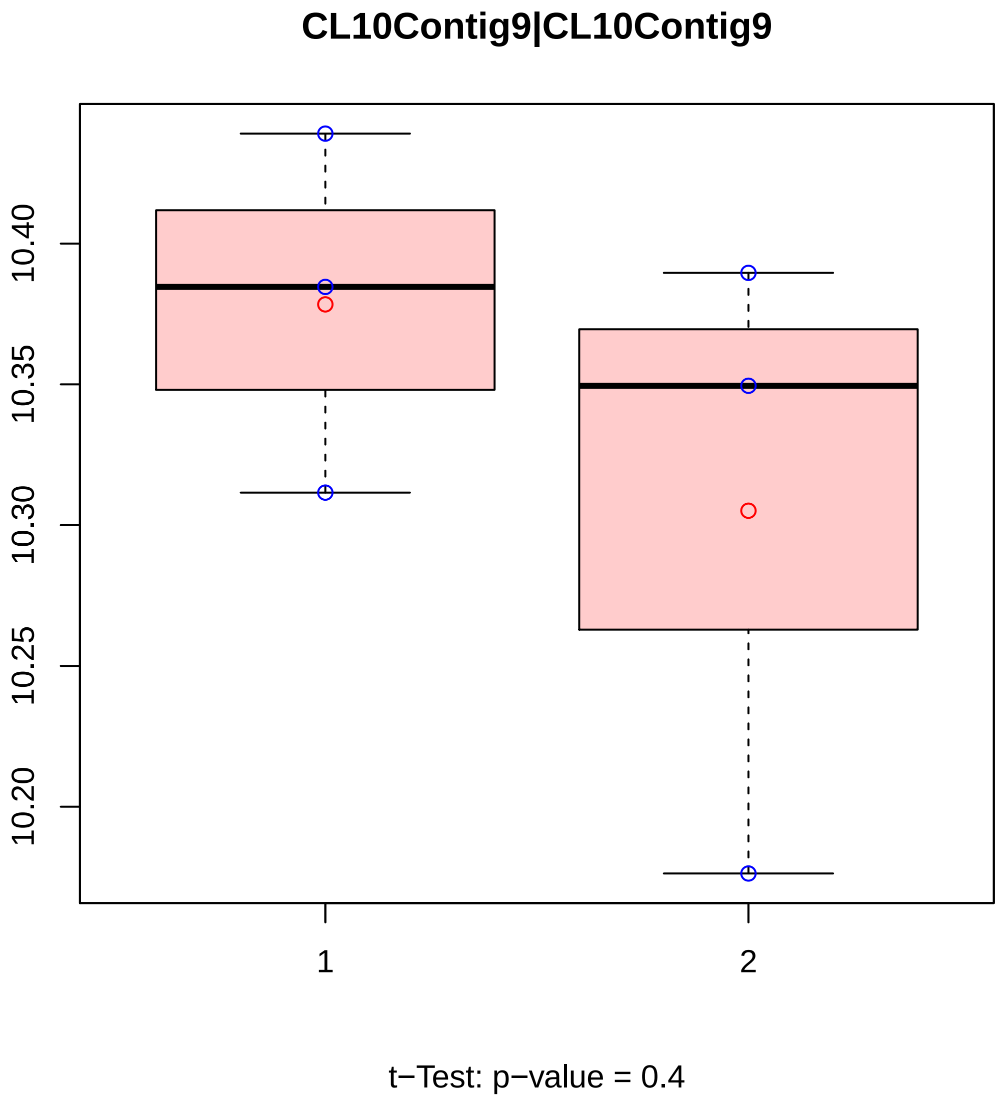
CL10Contig9|CL10Contig9
10.40
10.35
10.30
10.25
10.20
1
2
t−Test: p−value = 0.4

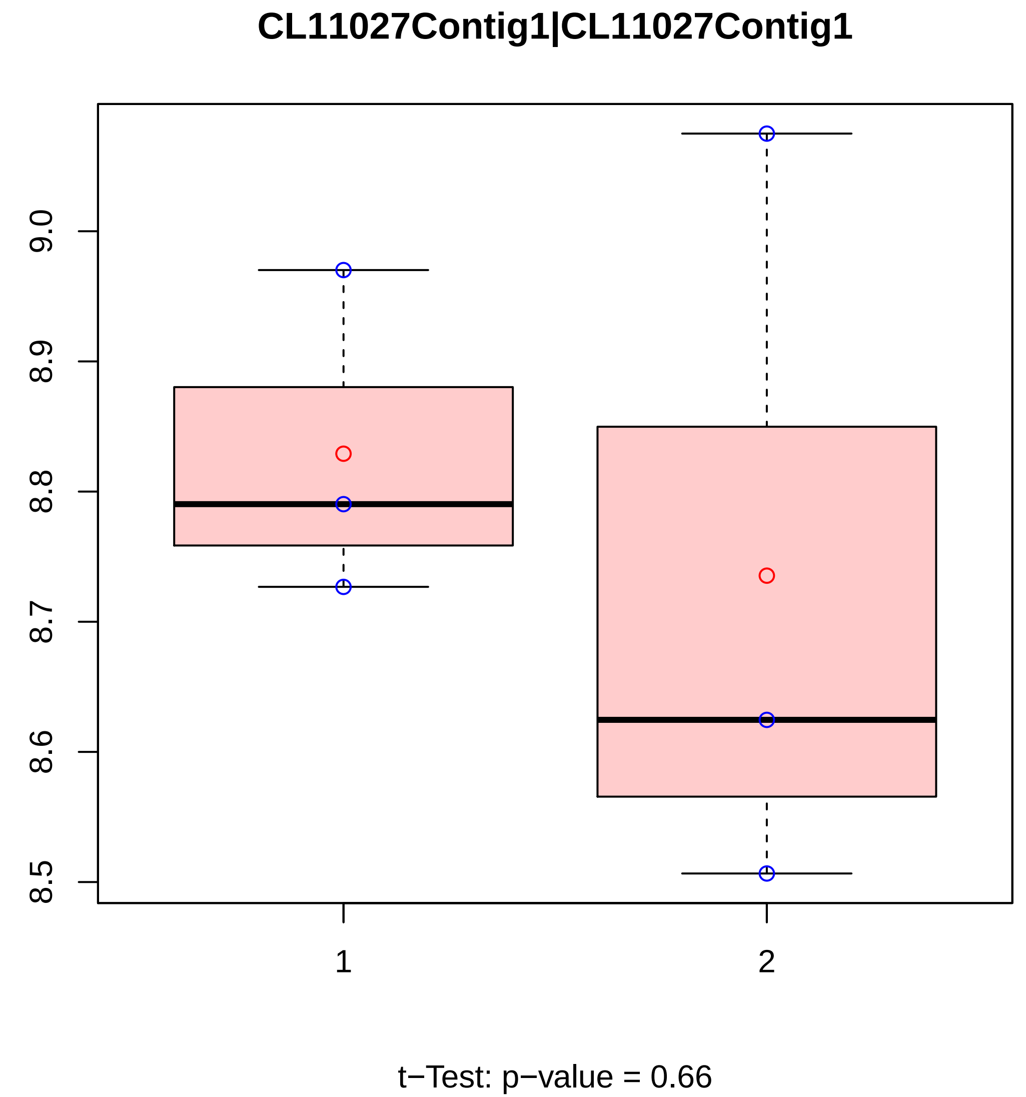
CL11027Contig1|CL11027Contig1
9.0
8.9
8.8
8.7
8.6
8.5
1
2
t−Test: p−value = 0.66

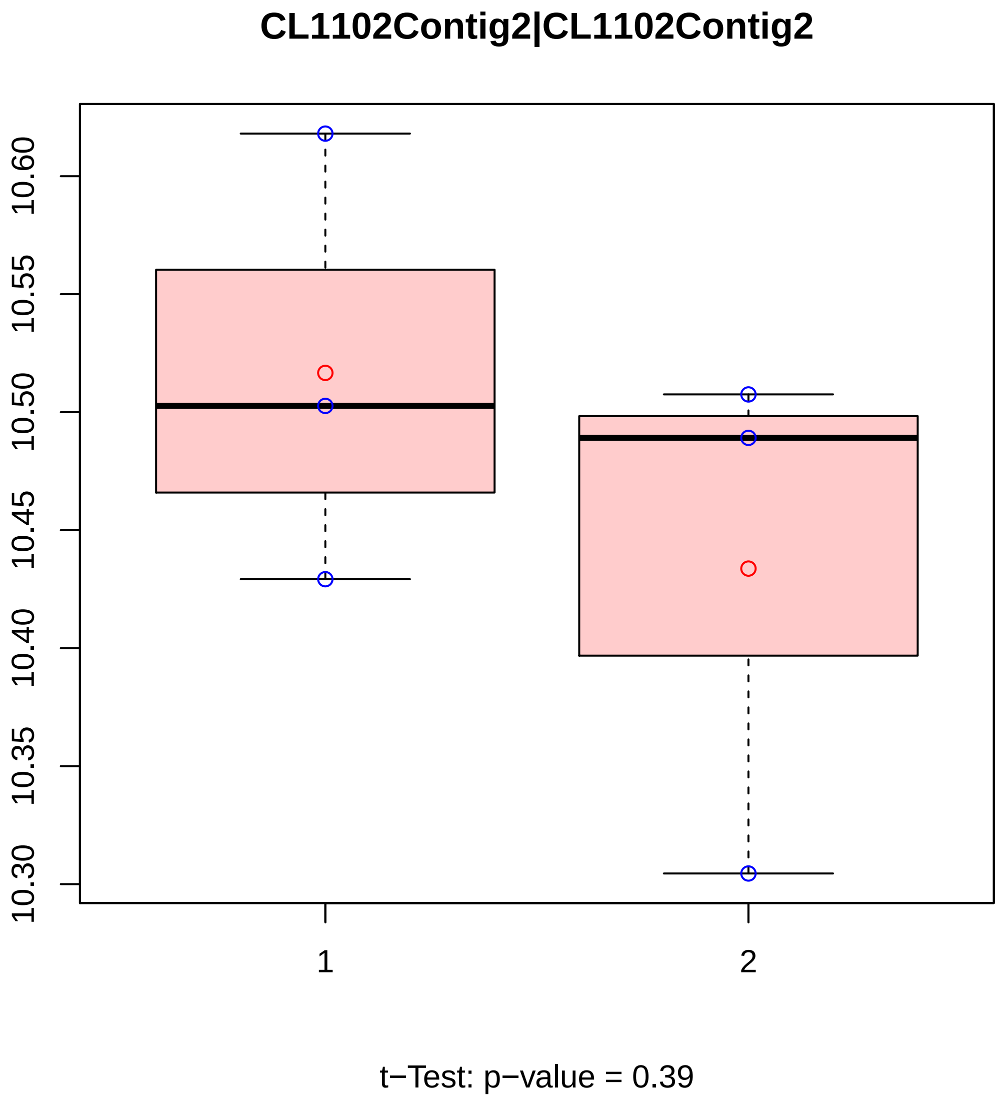

CL1102Contig2|CL1102Contig2
10.30
10.35
10.40
10.45
10.50
10.55
10.60
1
2
t−Test: p−value = 0.39

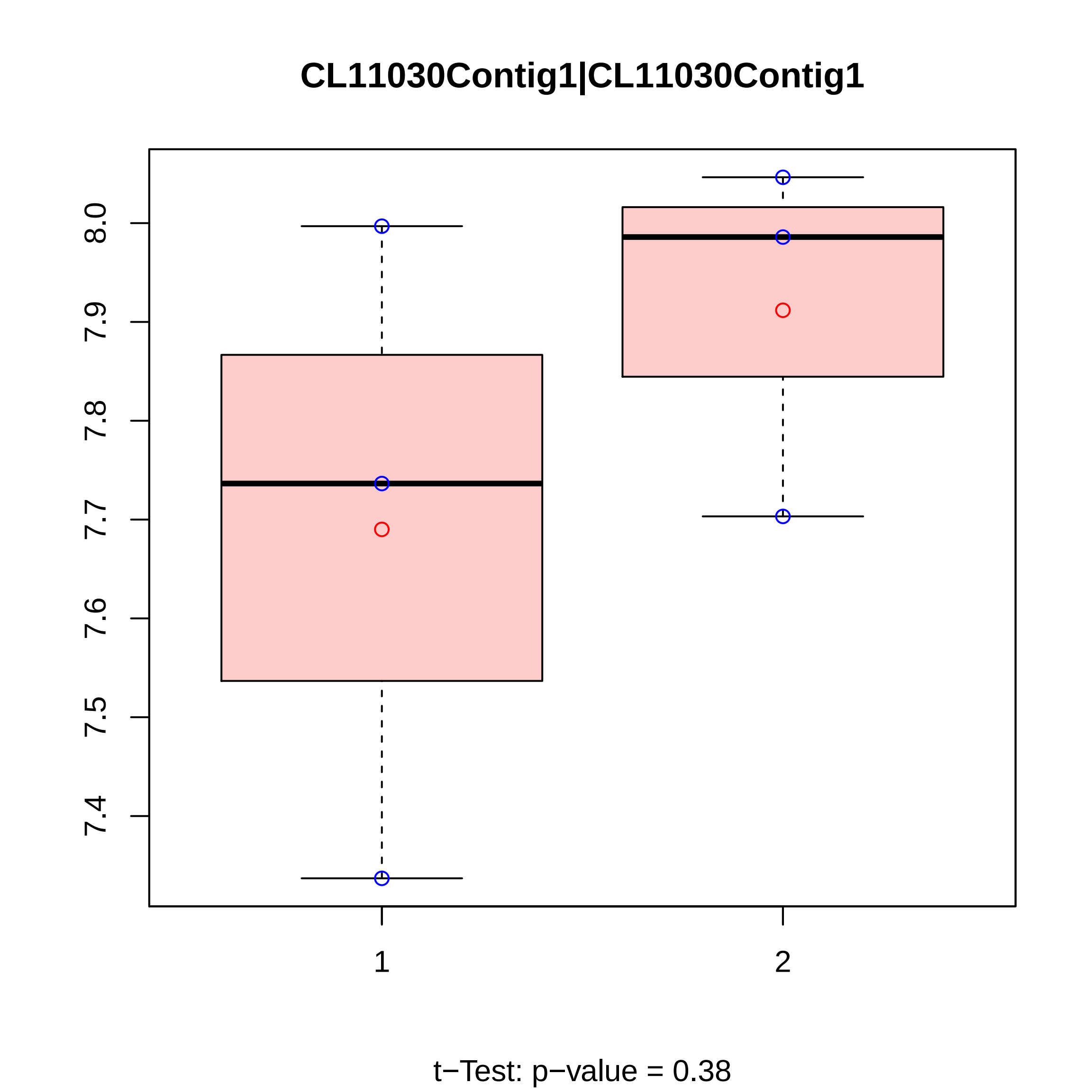

CL11030Contig1|CL11030Contig1
8.0
7.9
7.8
7.7
7.6
7.5
7.4
1
2
t−Test: p−value = 0.38

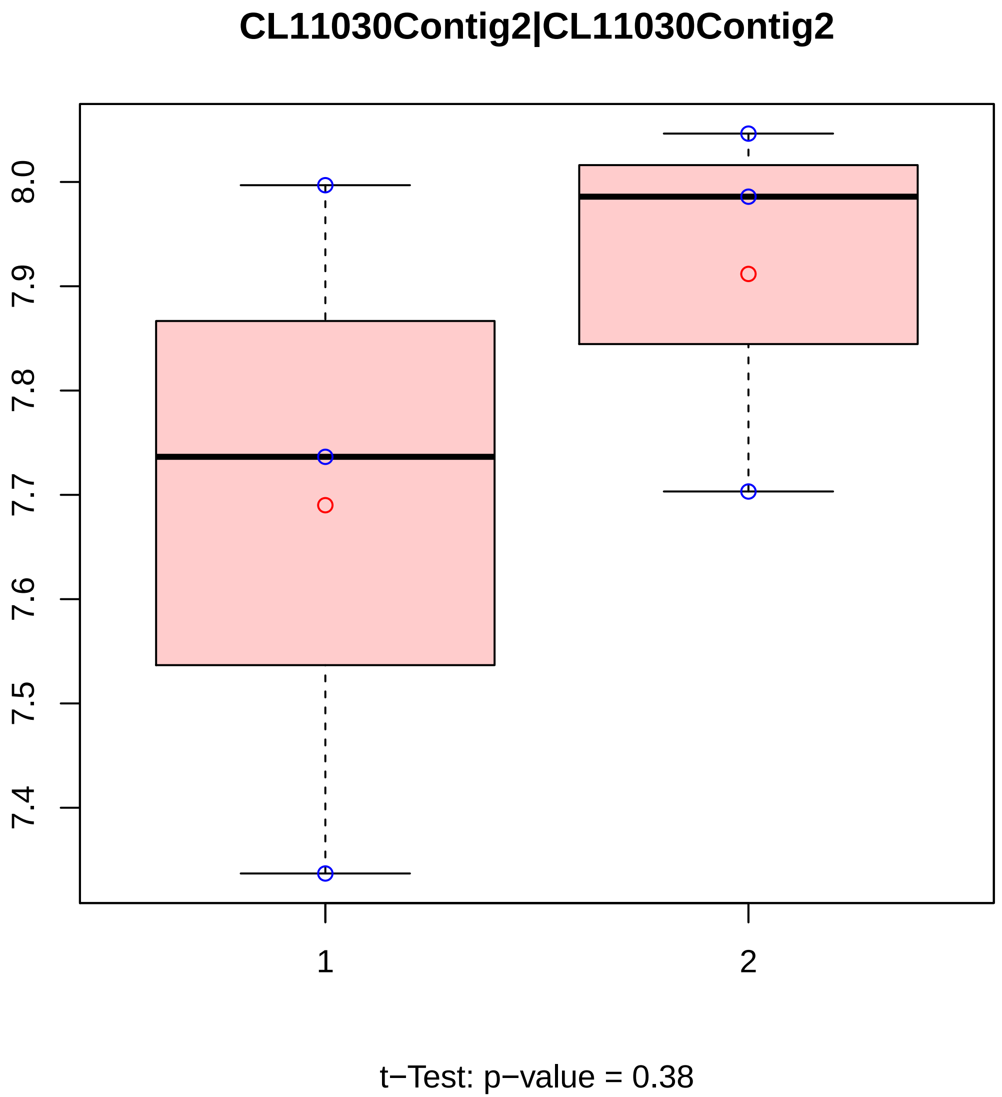
CL11030Contig2|CL11030Contig2
8.0
7.9
7.8
7.7
7.6
7.5
7.4
1
2
t−Test: p−value = 0.38

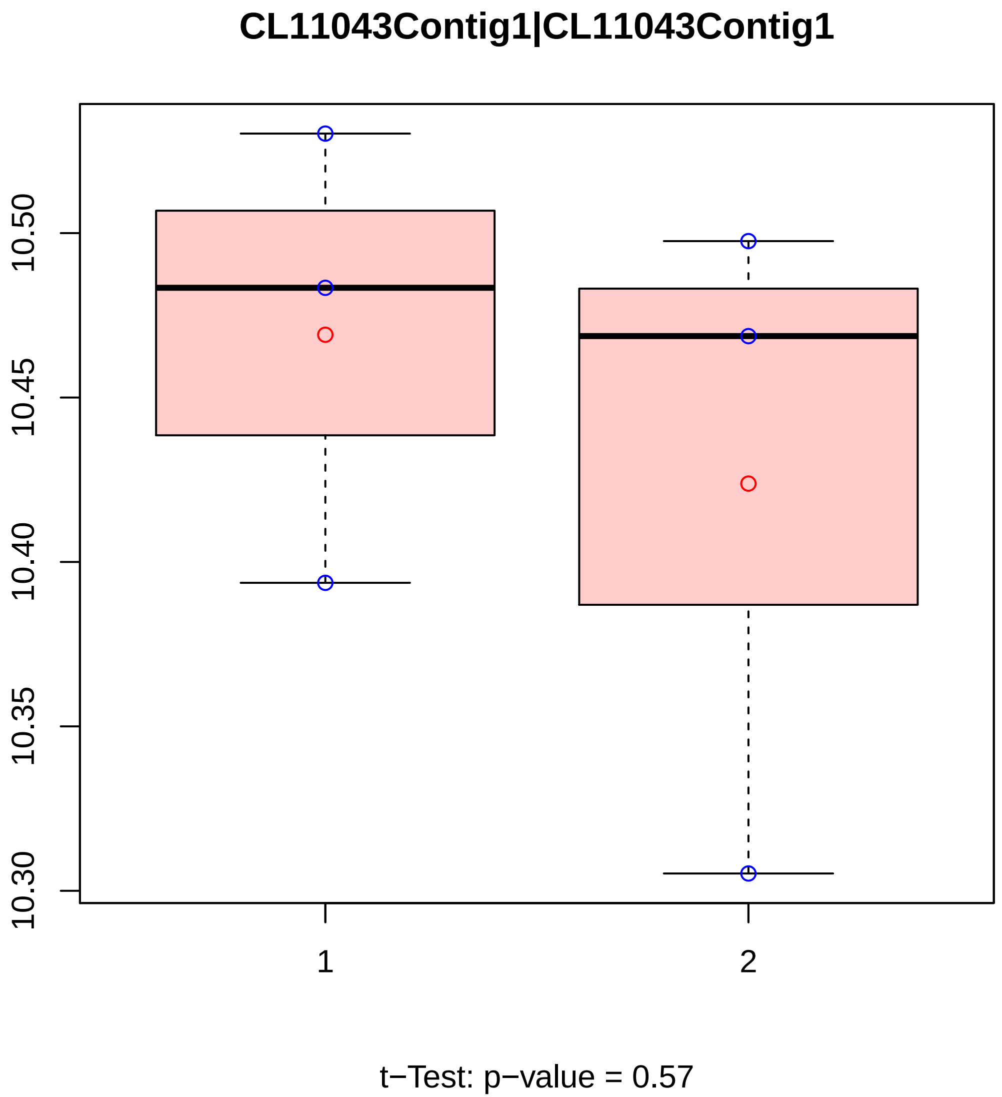
CL11043Contig1|CL11043Contig1
10.30
10.35
10.40
10.45
10.50
1
2
t−Test: p−value = 0.57

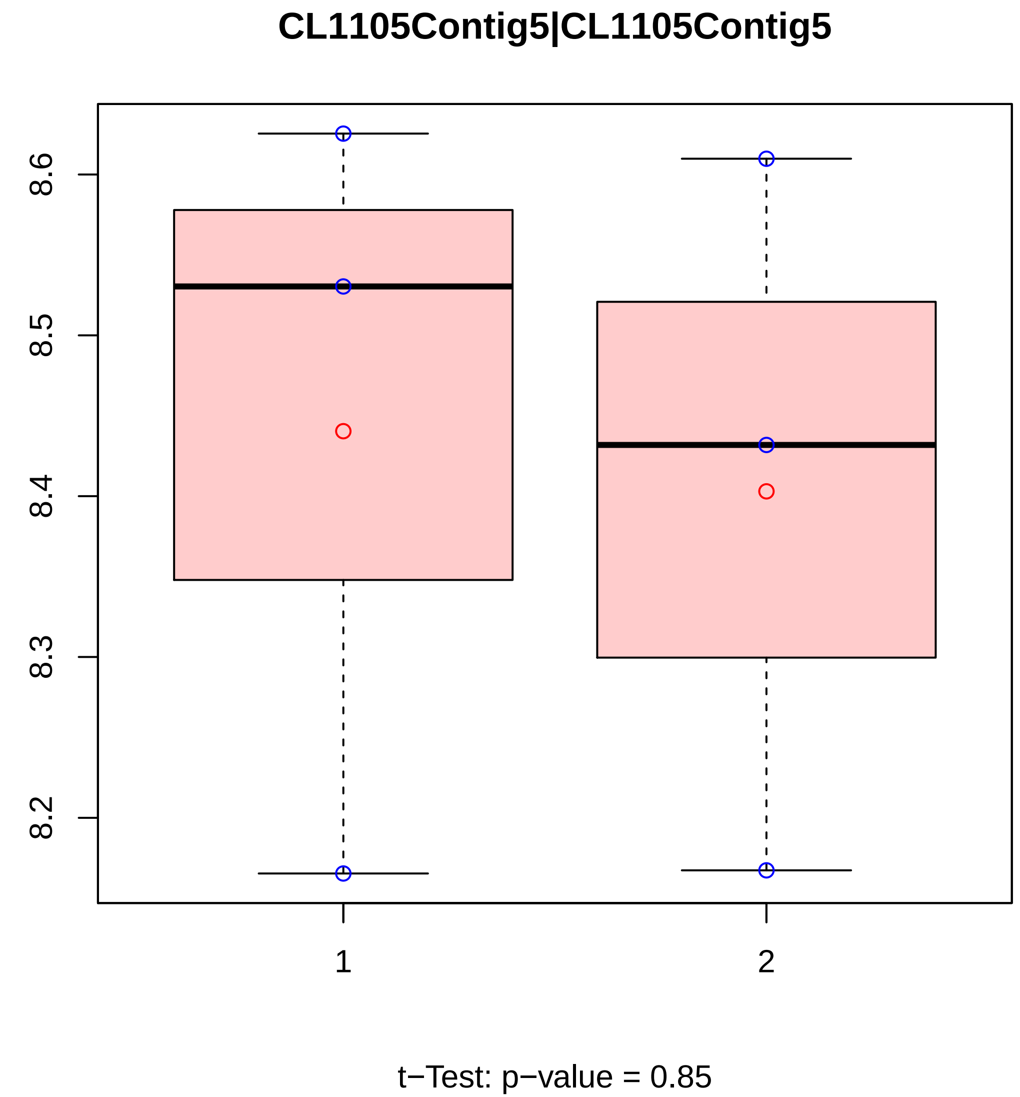
CL1105Contig5|CL1105Contig5
8.2
8.3
8.4
8.5
8.6
1
2
t−Test: p−value = 0.85

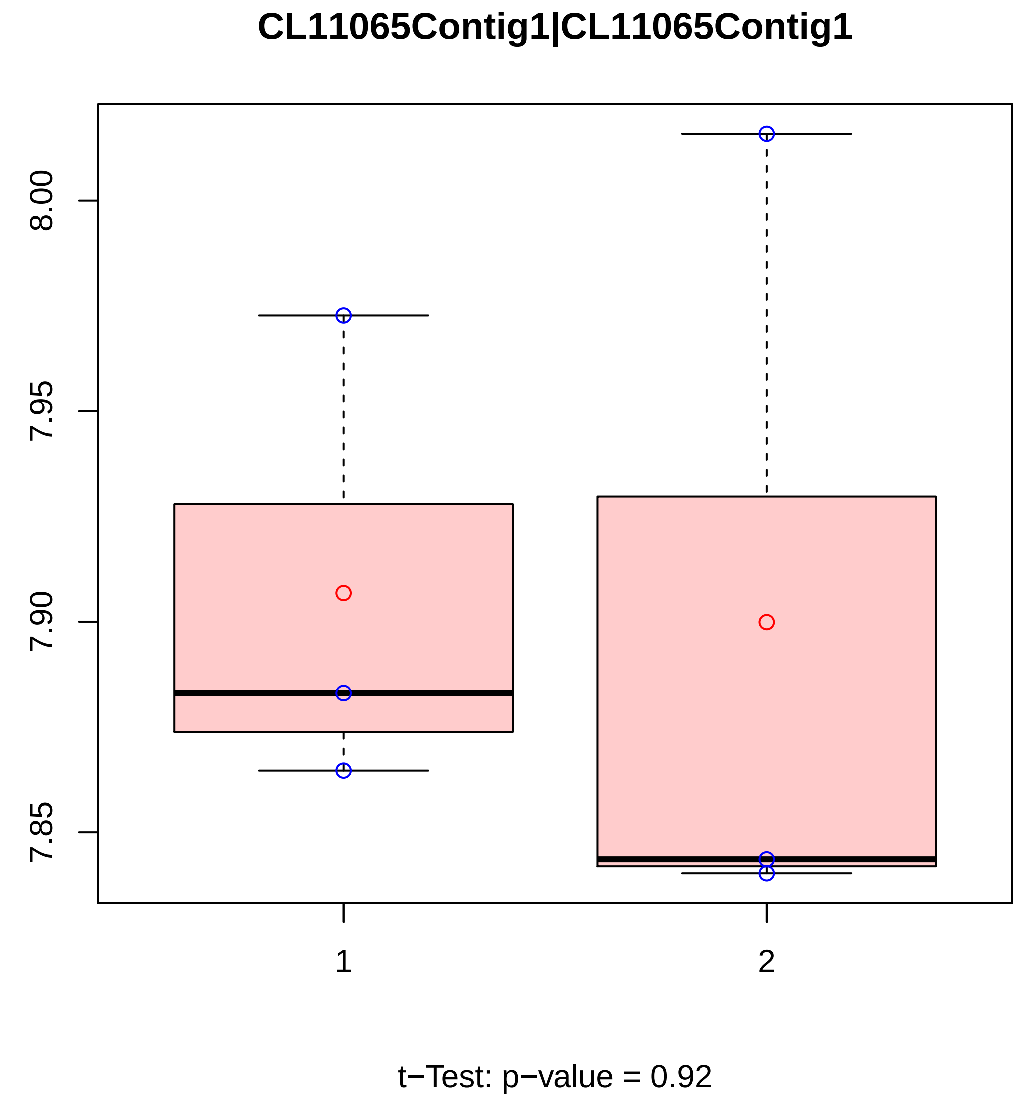
CL11065Contig1|CL11065Contig1
8.00
7.95
7.90
7.85
1
2
t−Test: p−value = 0.92

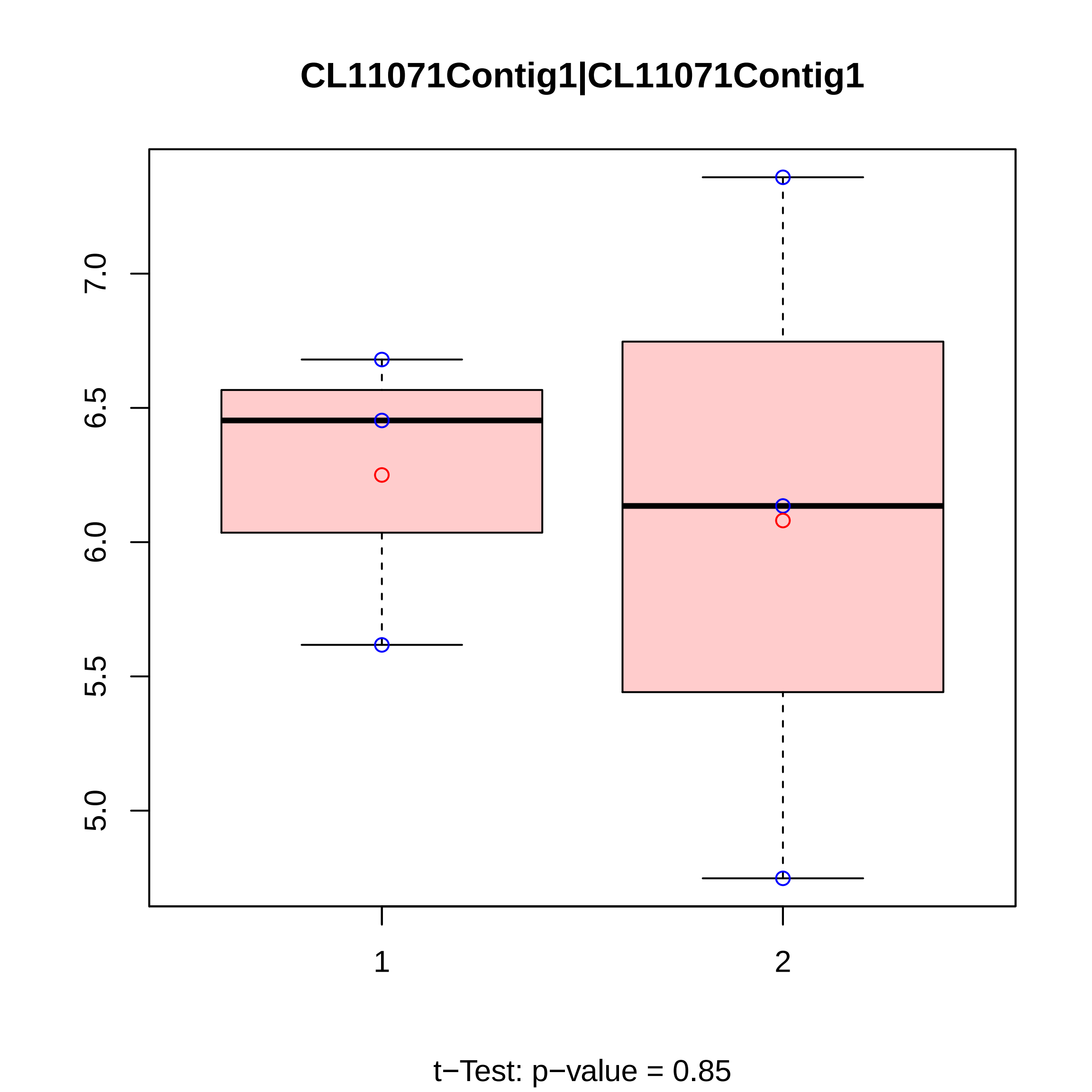
CL11071Contig1|CL11071Contig1
7.0
6.5
6.0
5.5
5.0
1
2
t−Test: p−value = 0.85

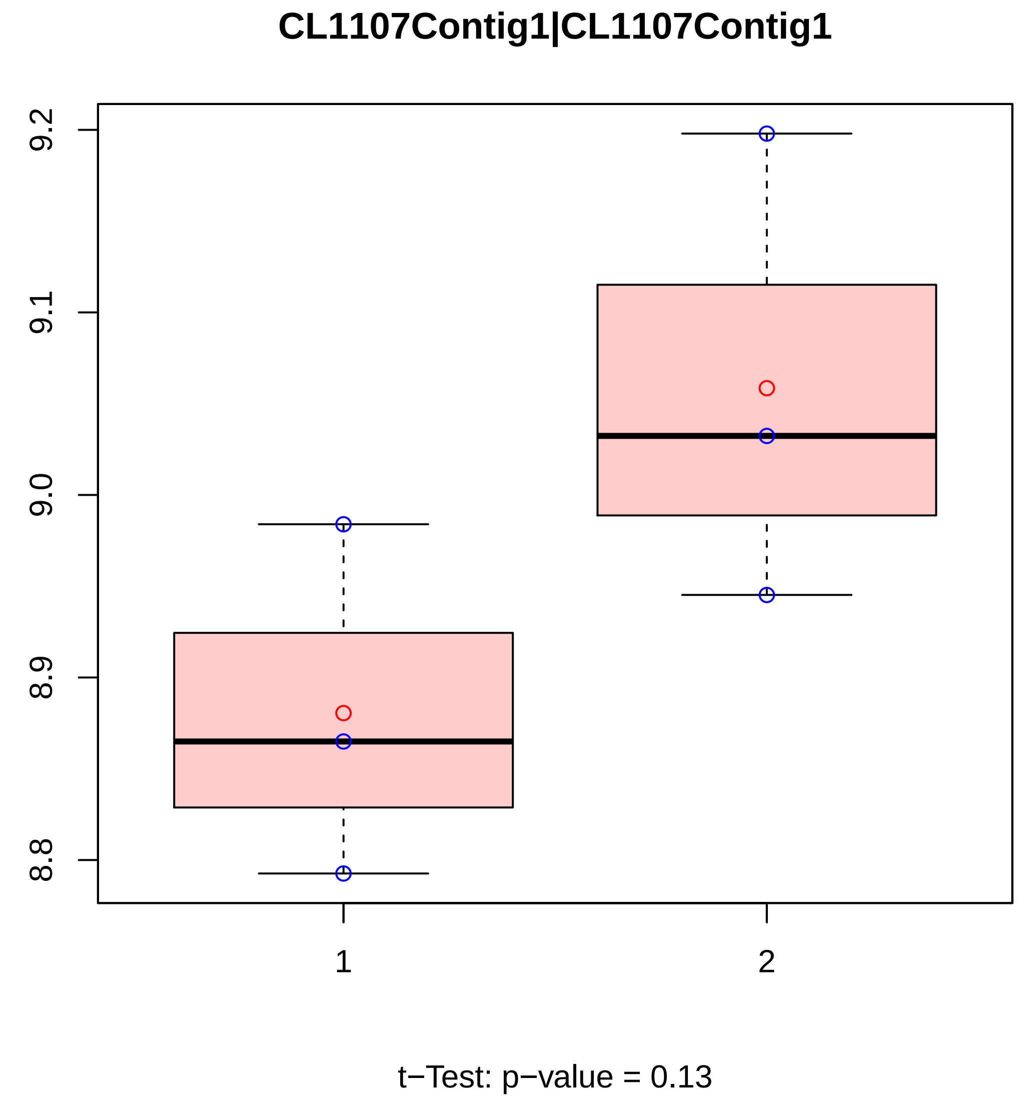

CL1107Contig1|CL1107Contig1
9.2
9.1
9.0
8.9
8.8
1
2
t−Test: p−value = 0.13

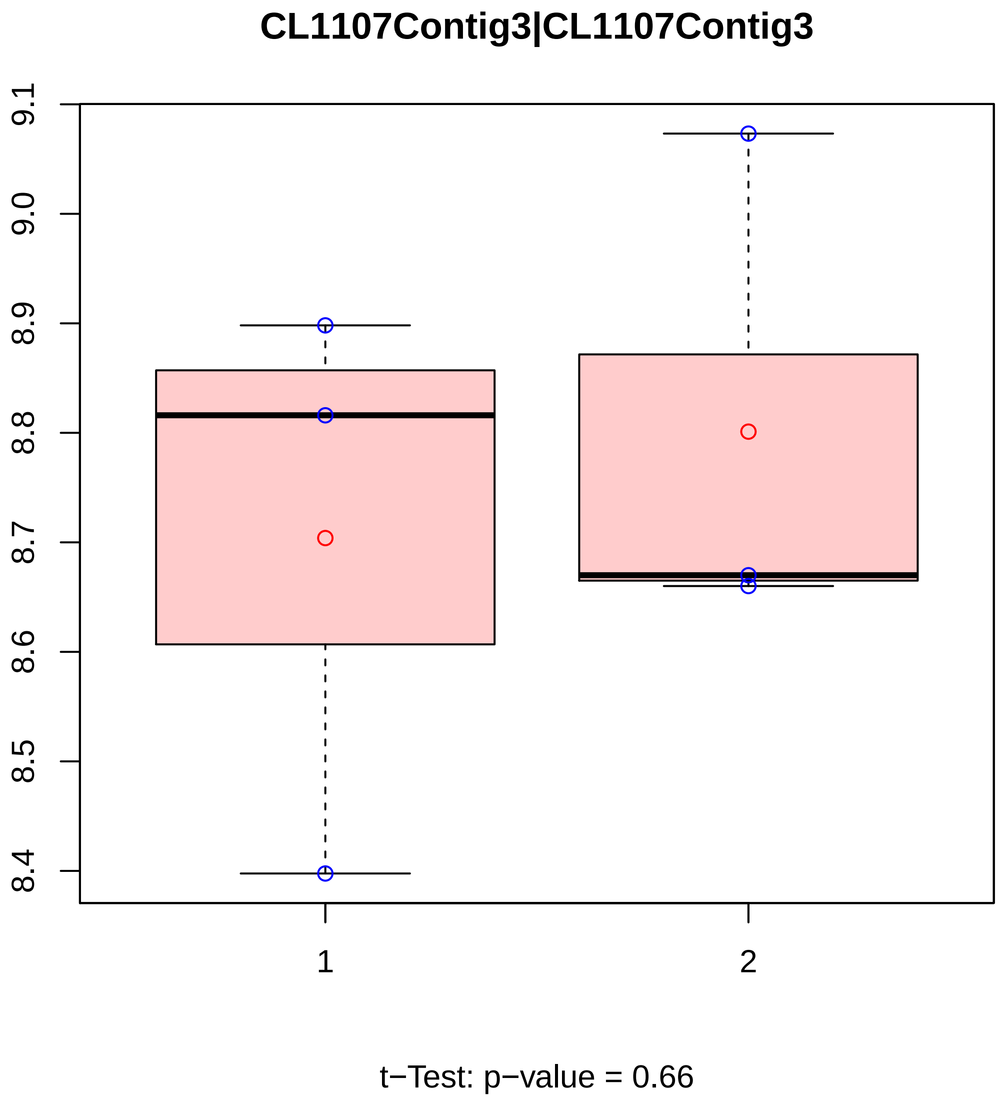

CL1107Contig3|CL1107Contig3
9.1
9.0
8.9
8.8
8.7
8.6
8.5
8.4
1
2
t−Test: p−value = 0.66

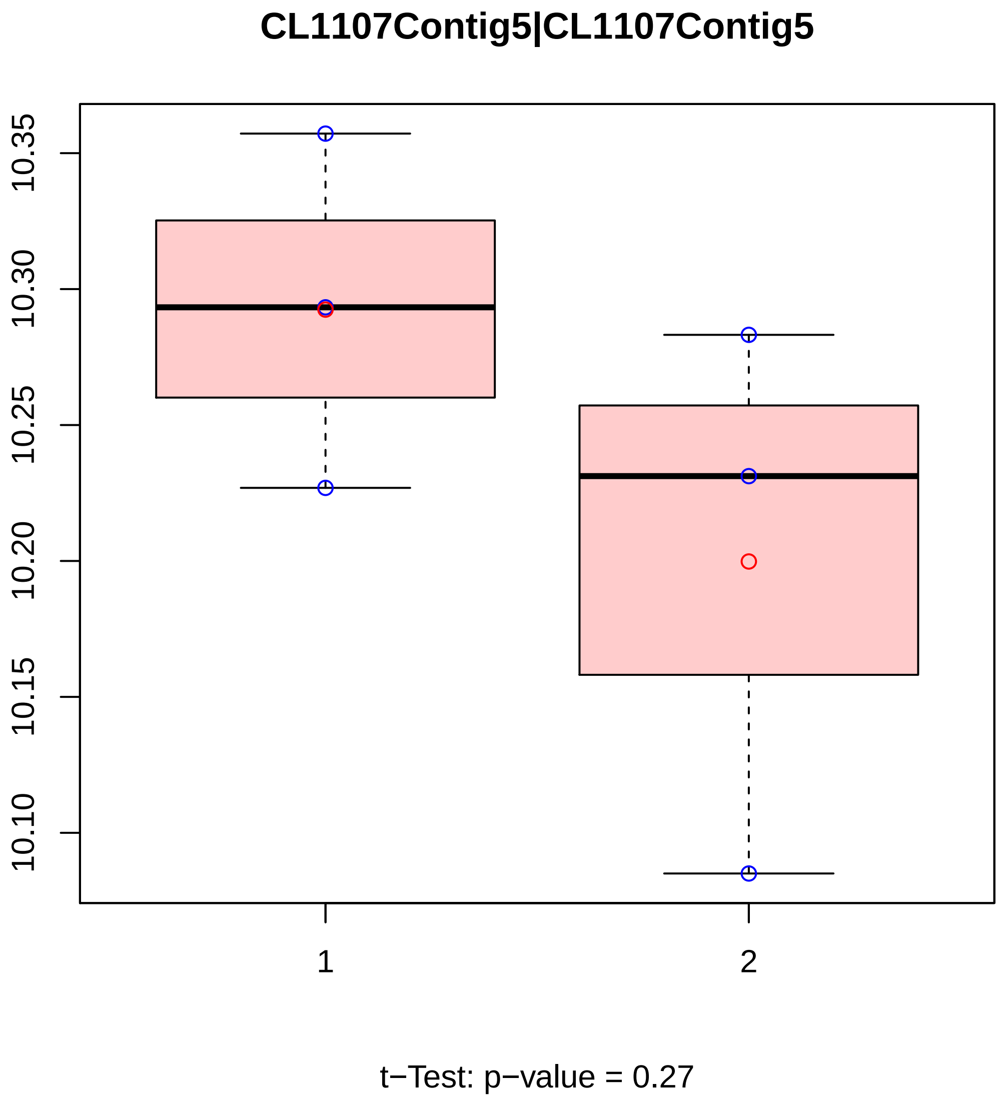
CL1107Contig5|CL1107Contig5
10.35
10.30
10.25
10.20
10.15
10.10
1
2
t−Test: p−value = 0.27

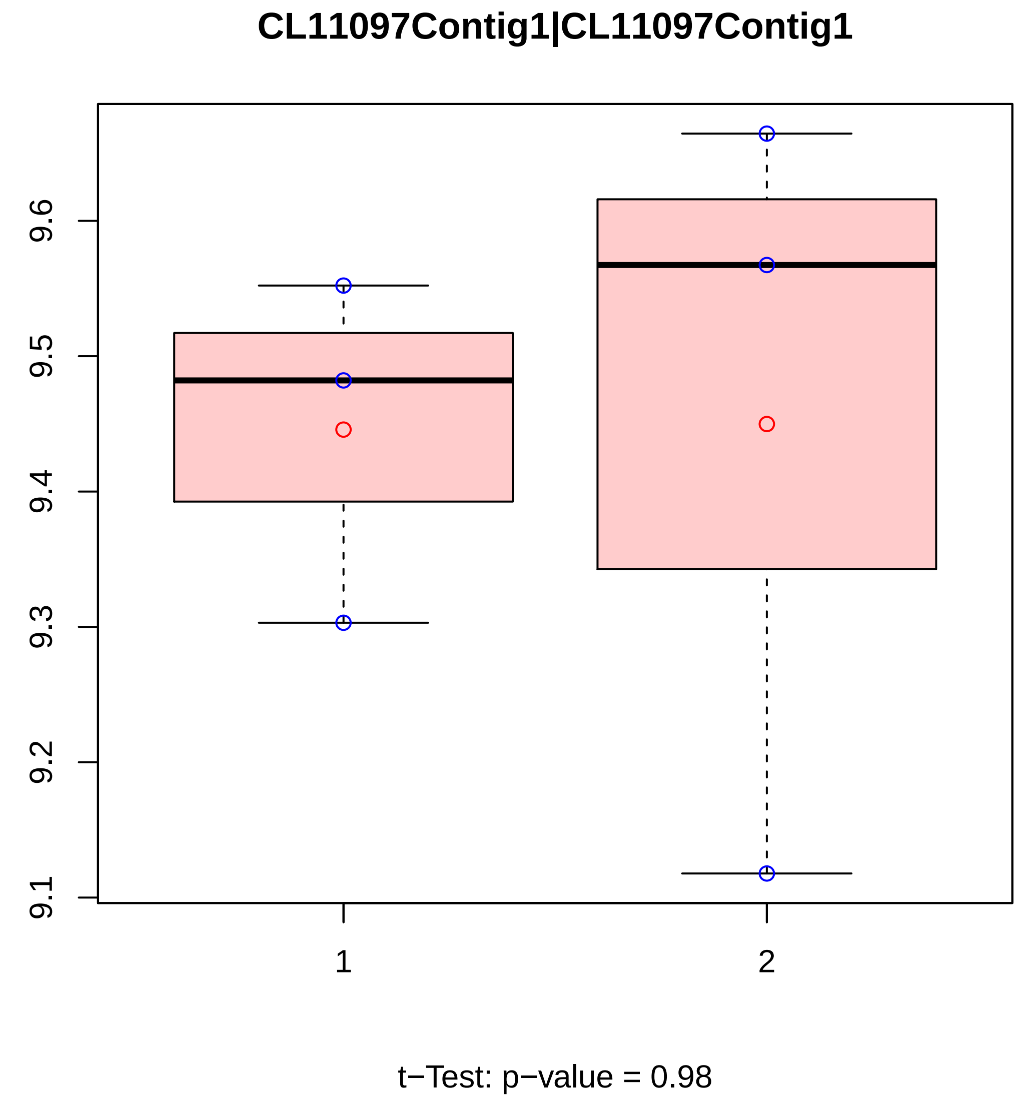
CL11097Contig1|CL11097Contig1
9.1
9.2
9.3
9.4
9.5
9.6
1
2
t−Test: p−value = 0.98

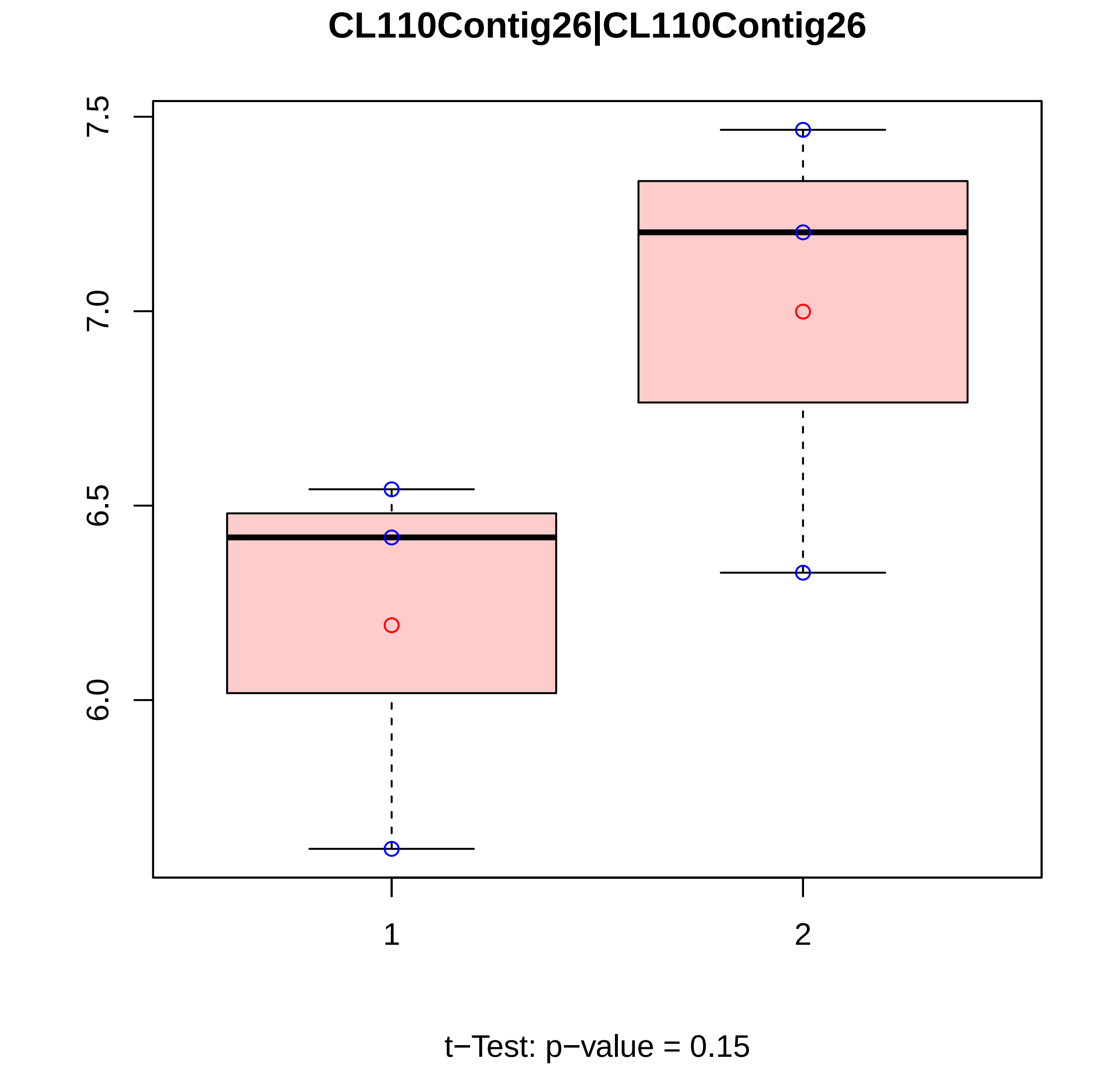
CL110Contig26|CL110Contig26
7.5
7.0
6.5
6.0
1
2
t−Test: p−value = 0.15

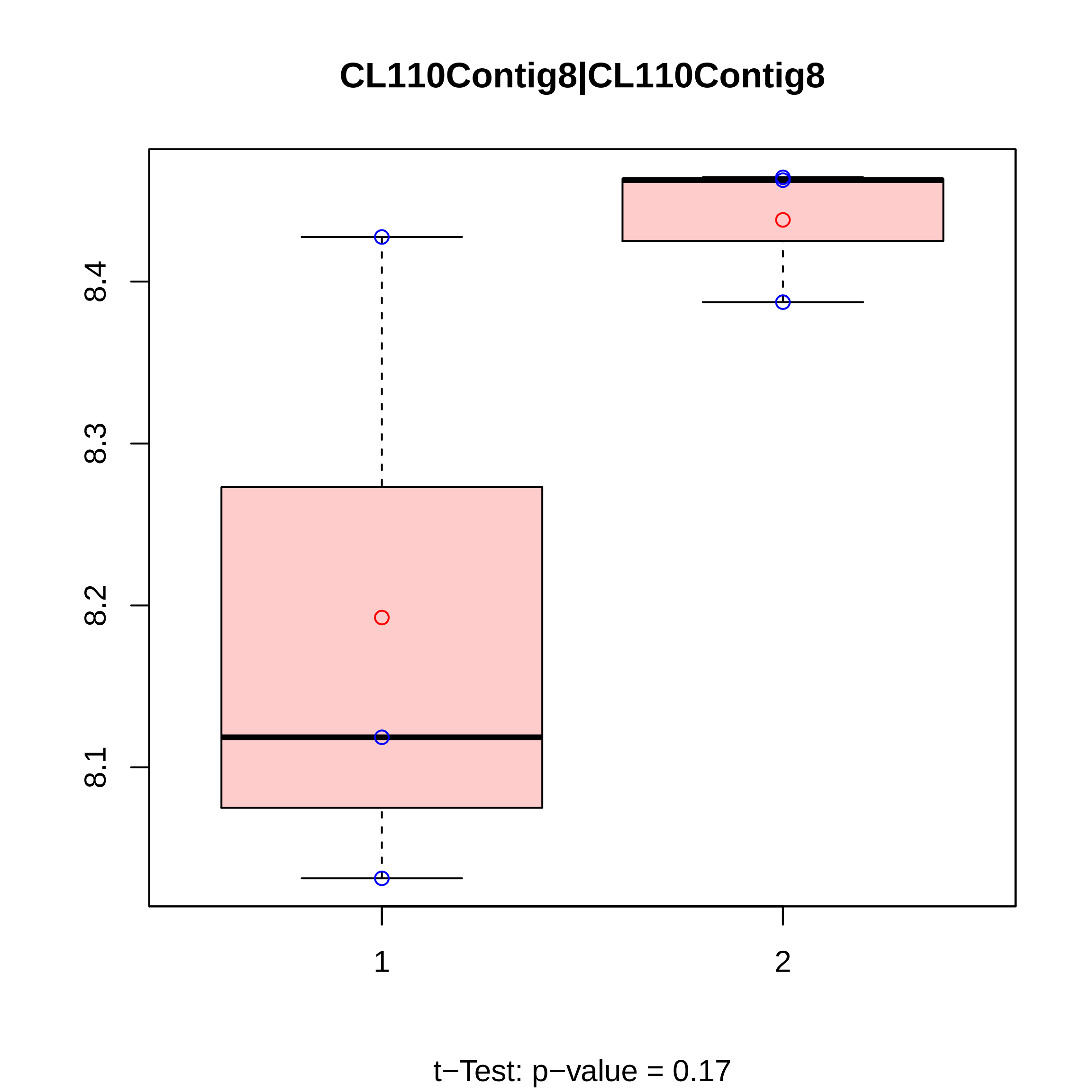
CL110Contig8|CL110Contig8
8.1
8.2
8.3
8.4
1
2
t−Test: p−value = 0.17

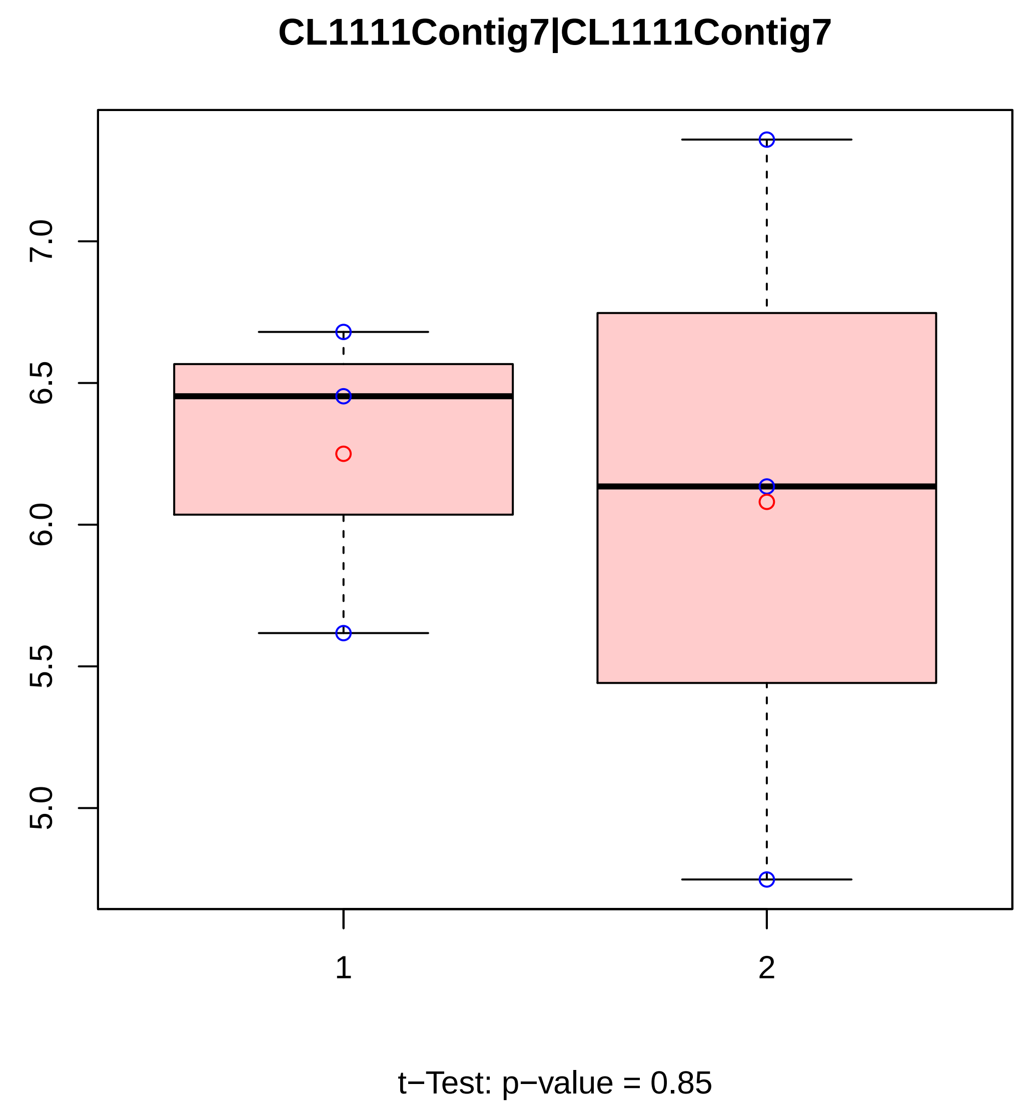
CL1111Contig7|CL1111Contig7
7.0
6.5
6.0
5.5
5.0
1
2
t−Test: p−value = 0.85

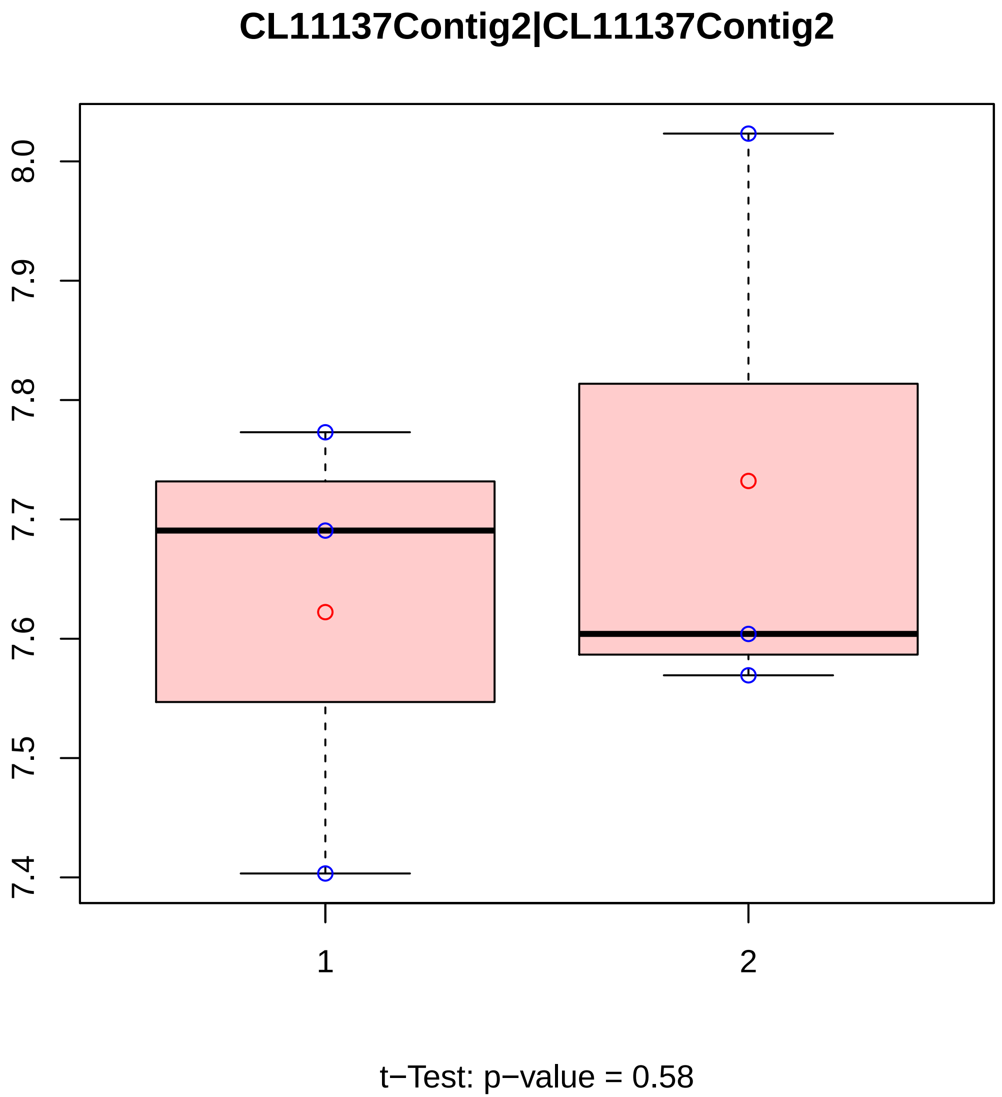
CL11137Contig2|CL11137Contig2
8.0
7.9
7.8
7.7
7.6
7.5
7.4
1
2
t−Test: p−value = 0.58

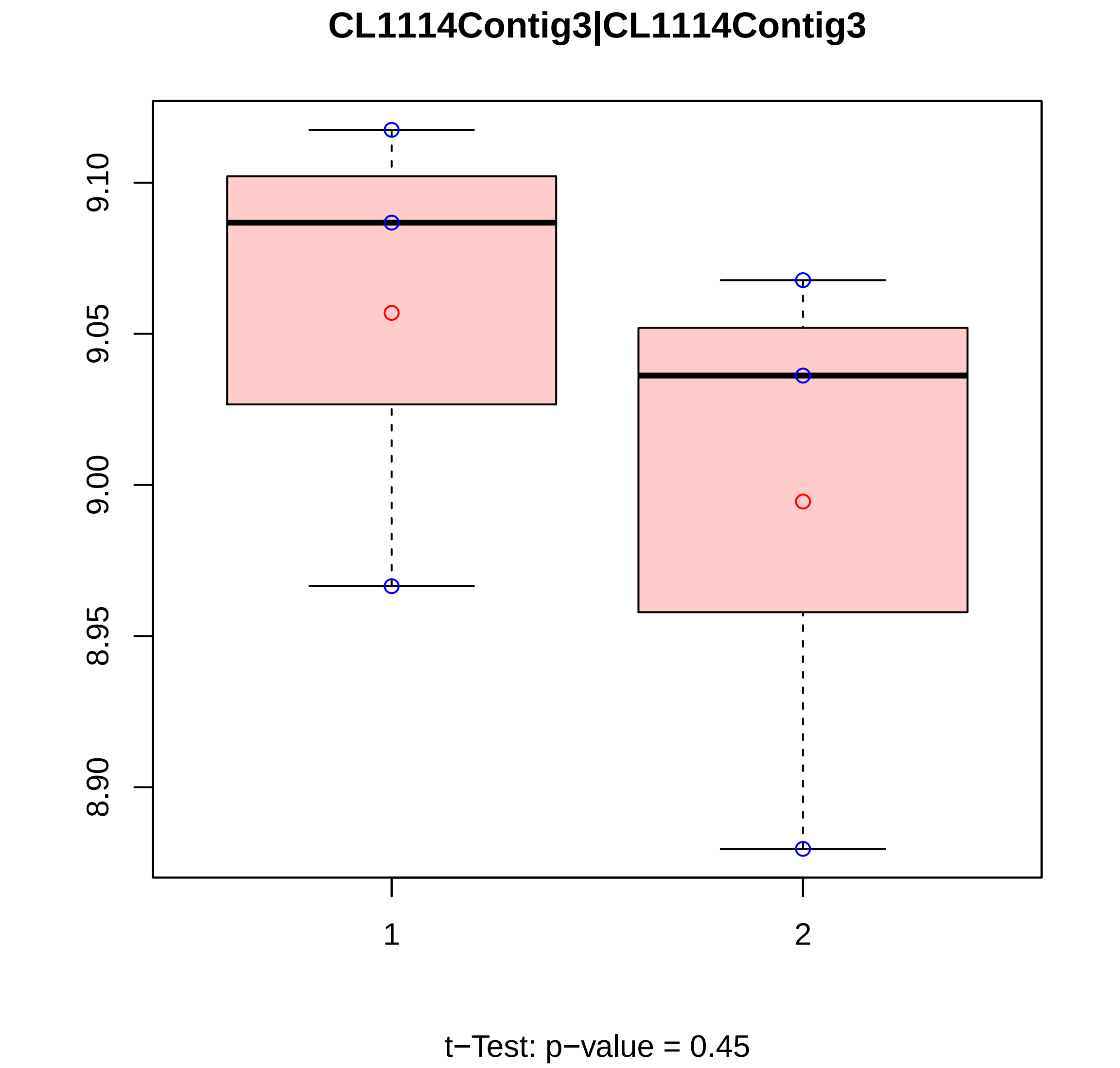

CL1114Contig3|CL1114Contig3
9.10
9.05
9.00
8.95
8.90
1
2
t−Test: p−value = 0.45

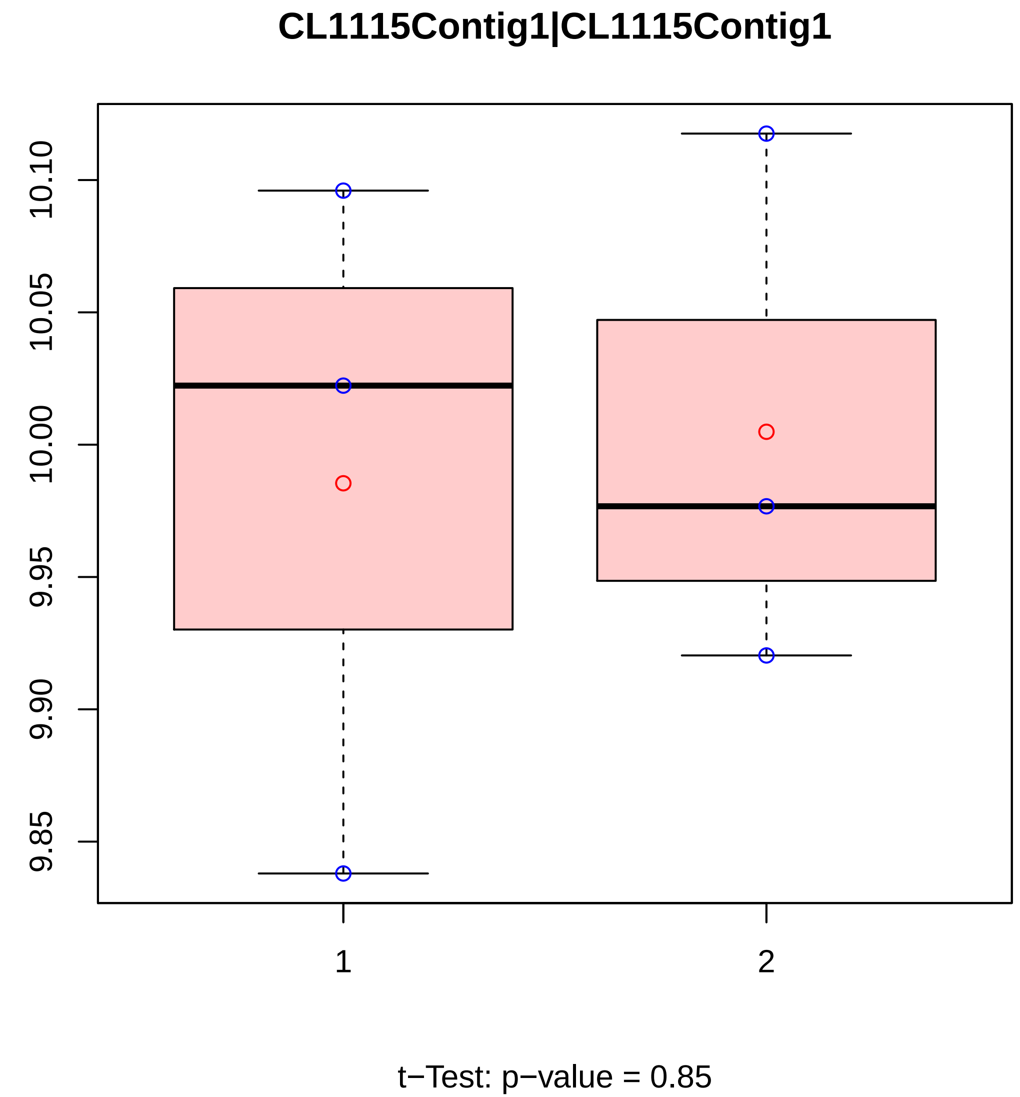
CL1115Contig1|CL1115Contig1
10.10
10.05
10.00
9.95
9.90
9.85
1
2
t−Test: p−value = 0.85

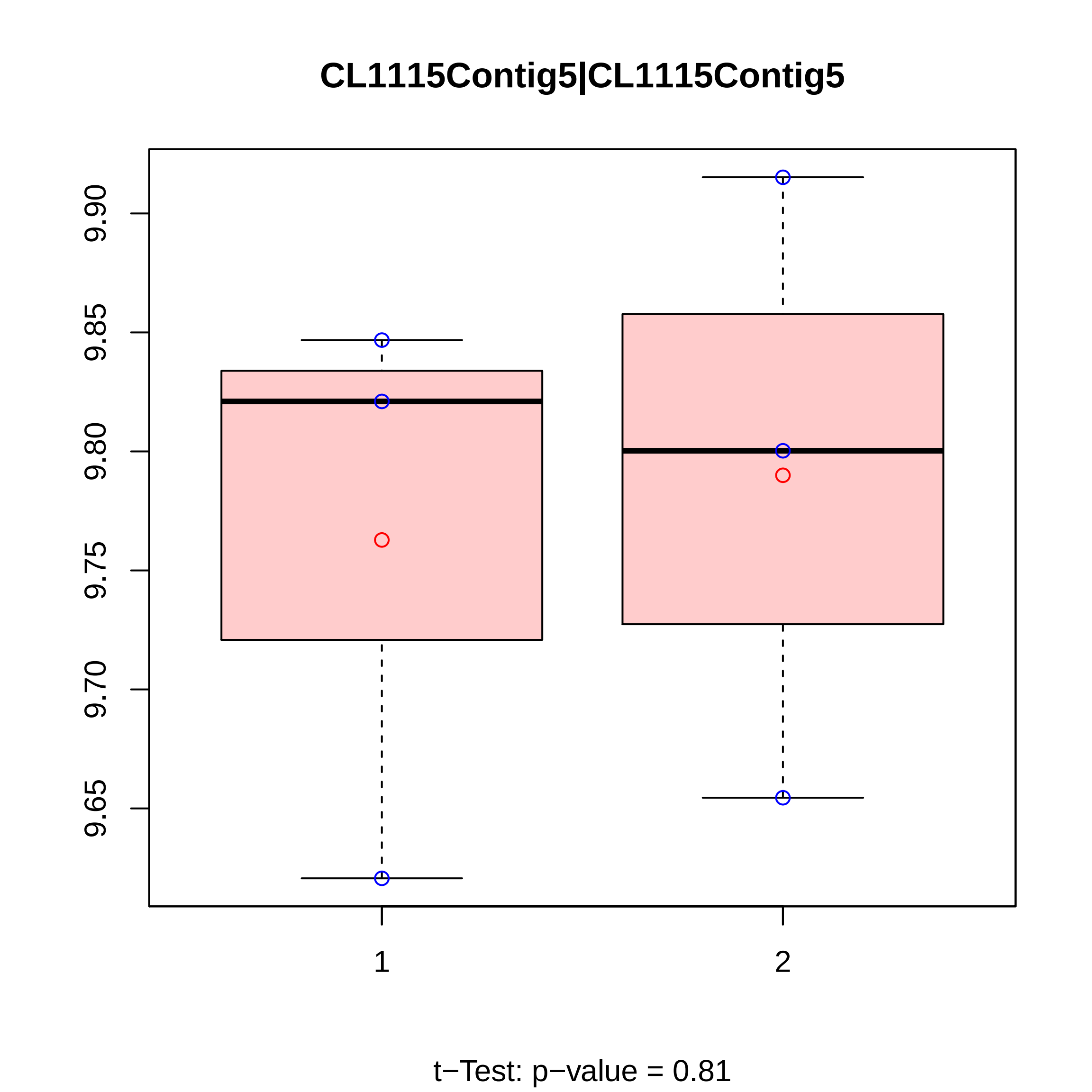
CL1115Contig5|CL1115Contig5
9.65
9.70
9.75
9.80
9.85
9.90
1
2
t−Test: p−value = 0.81

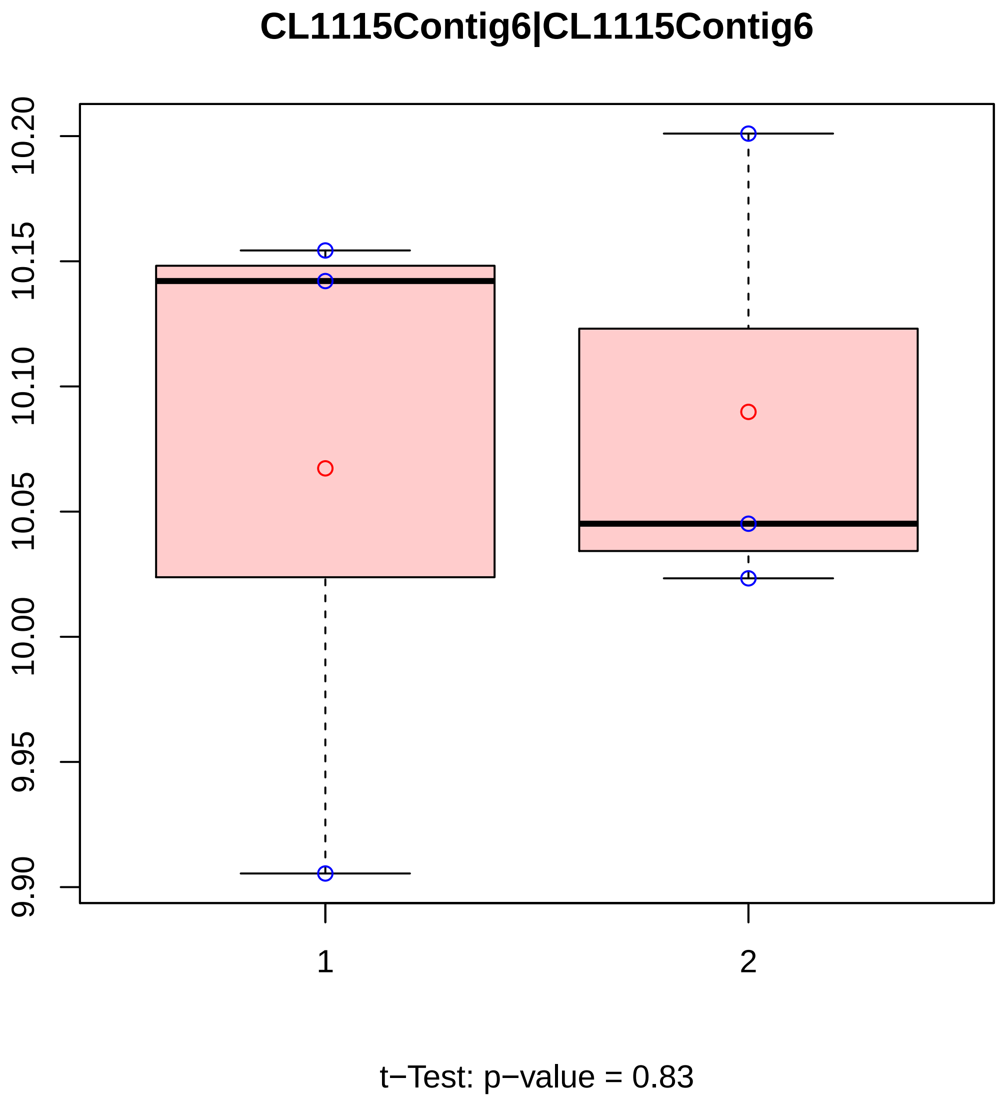
CL1115Contig6|CL1115Contig6
10.20
10.15
10.10
10.05
10.00
9.95
9.90
1
2
t−Test: p−value = 0.83

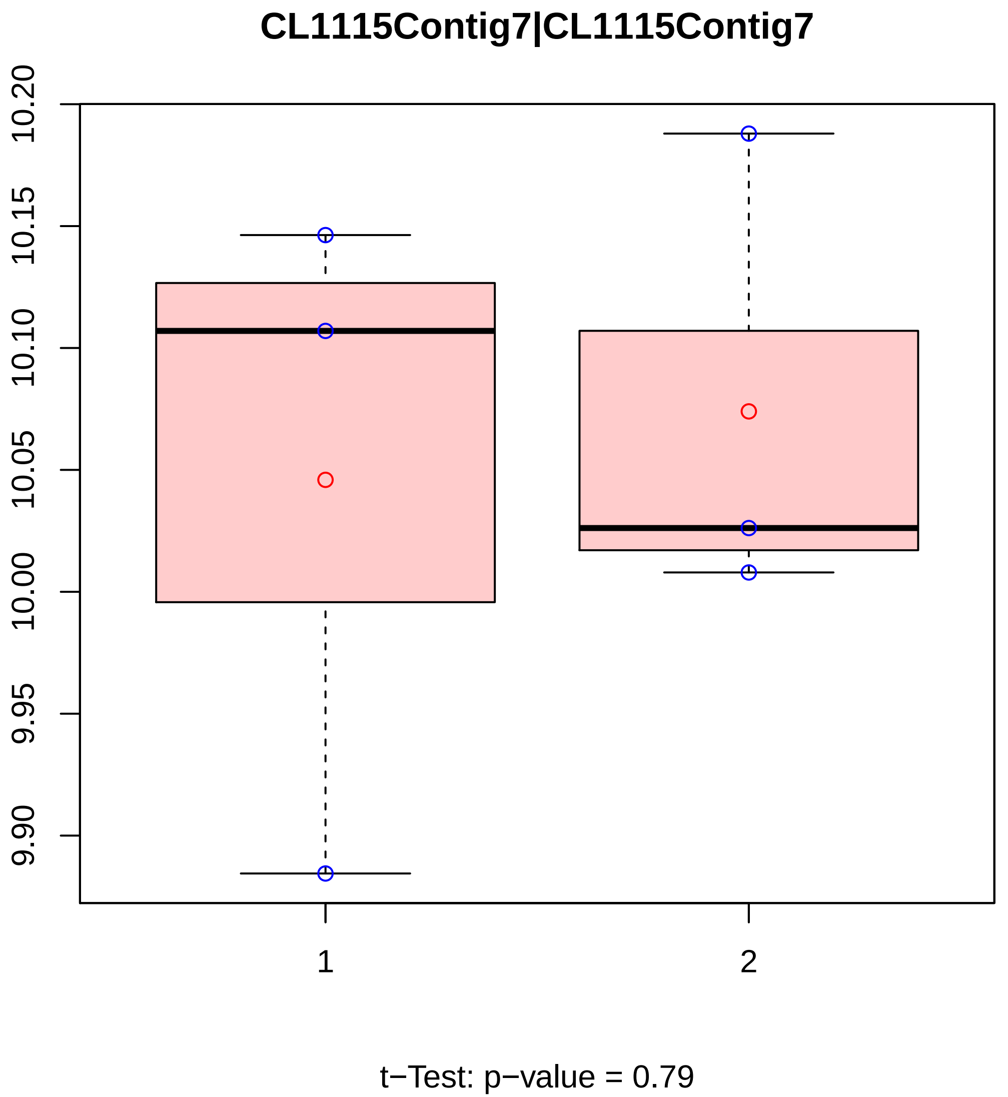
CL1115Contig7|CL1115Contig7
10.20
10.15
10.10
10.05
10.00
9.95
9.90
1
2
t−Test: p−value = 0.79

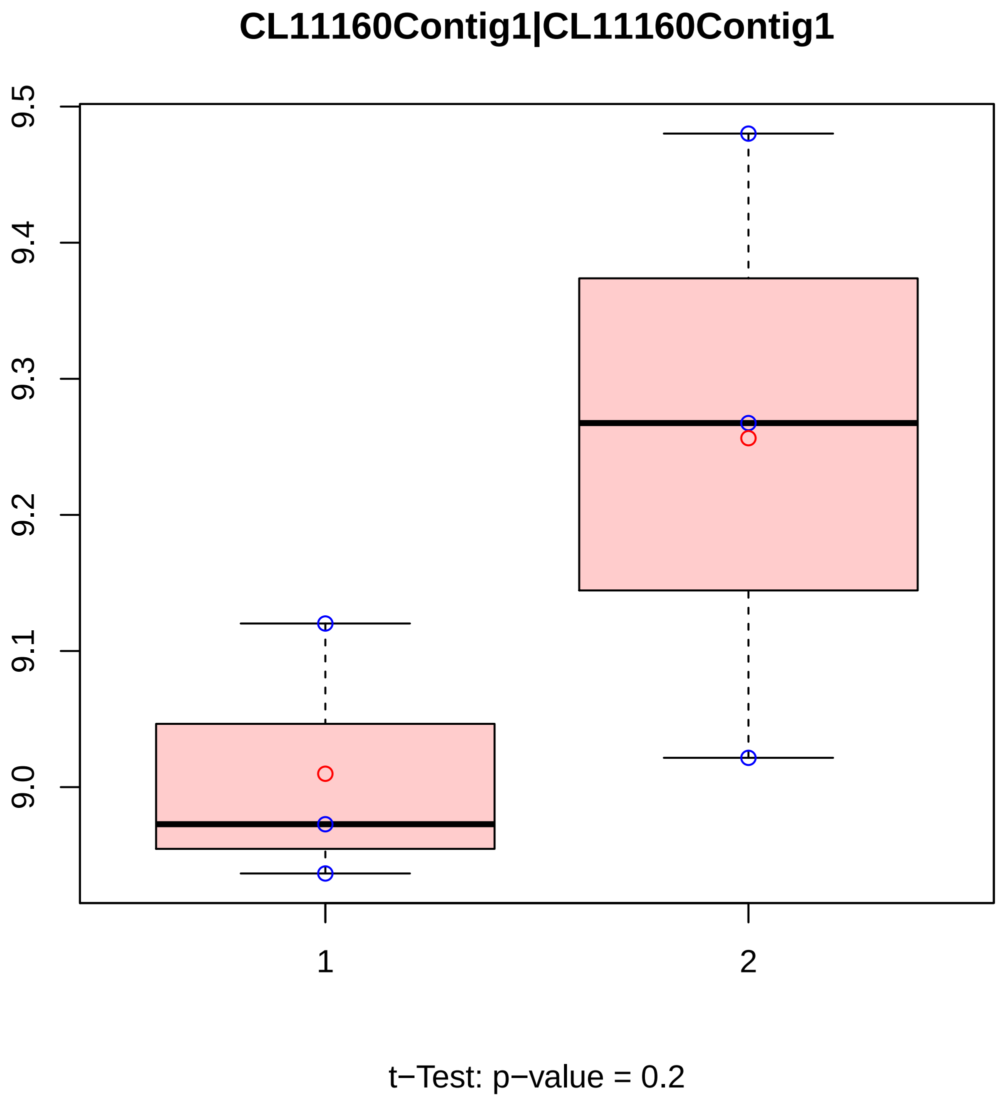
CL11160Contig1|CL11160Contig1
9.0
9.1
9.2
9.3
9.4
9.5
1
2
t−Test: p−value = 0.2

# CL11170Contig2|CL11170Contig2

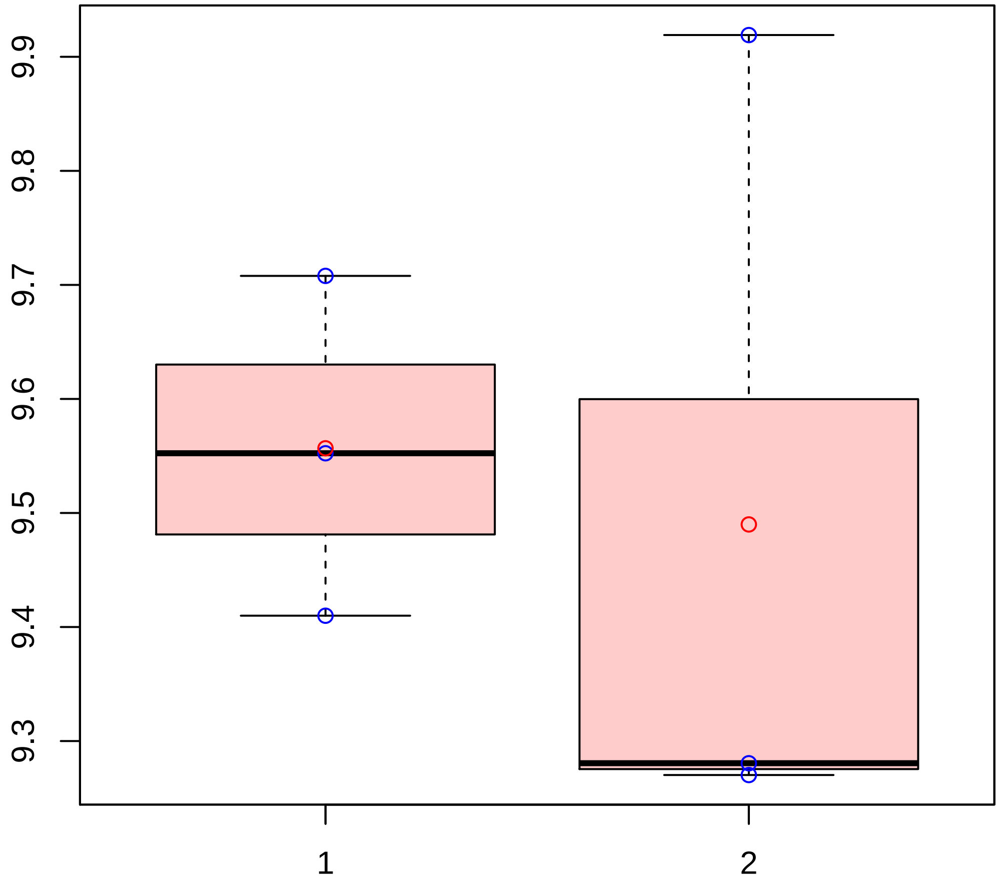


t−Test: p−value = 0.79

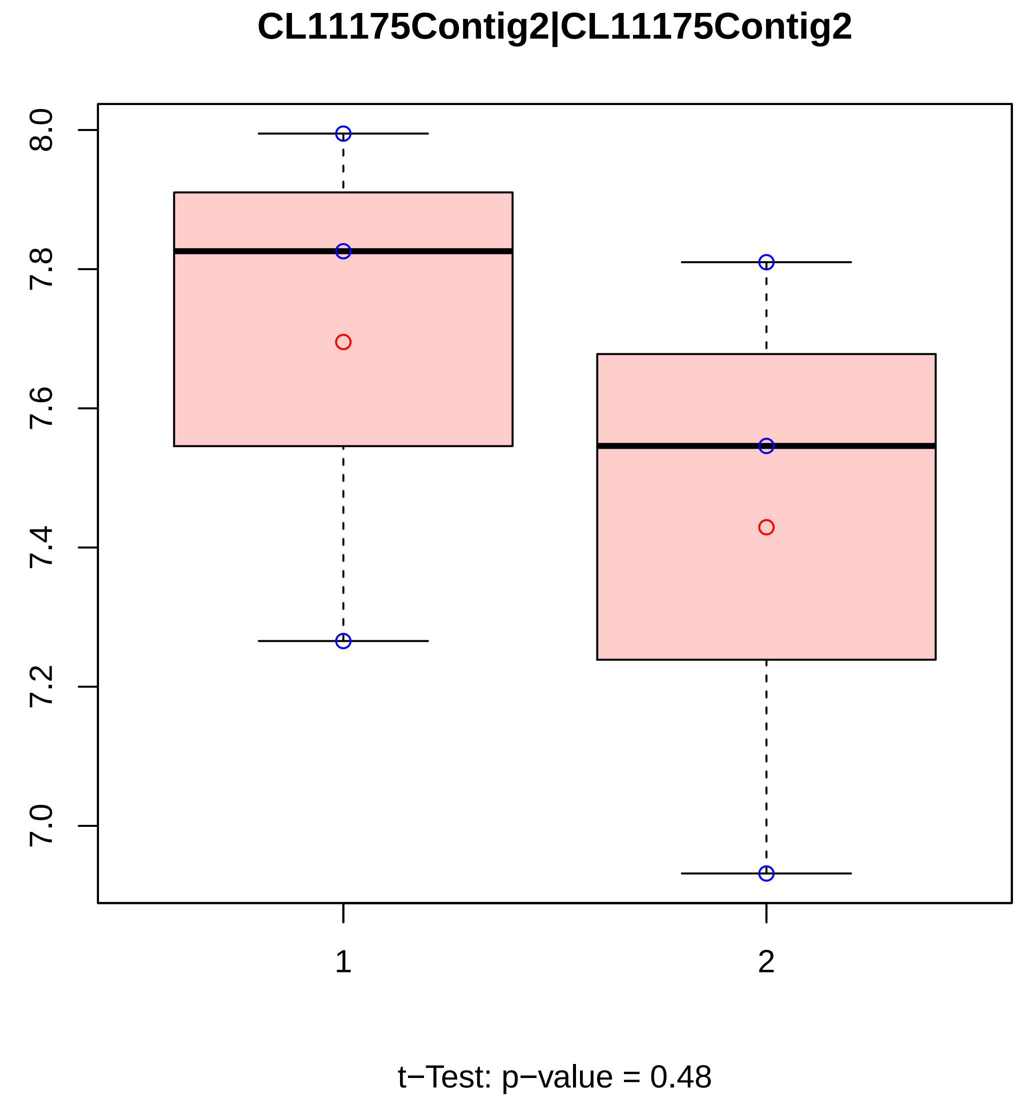

CL11175Contig2|CL11175Contig2
8.0
7.8
7.6
7.4
7.2
7.0
1
2
t−Test: p−value = 0.48

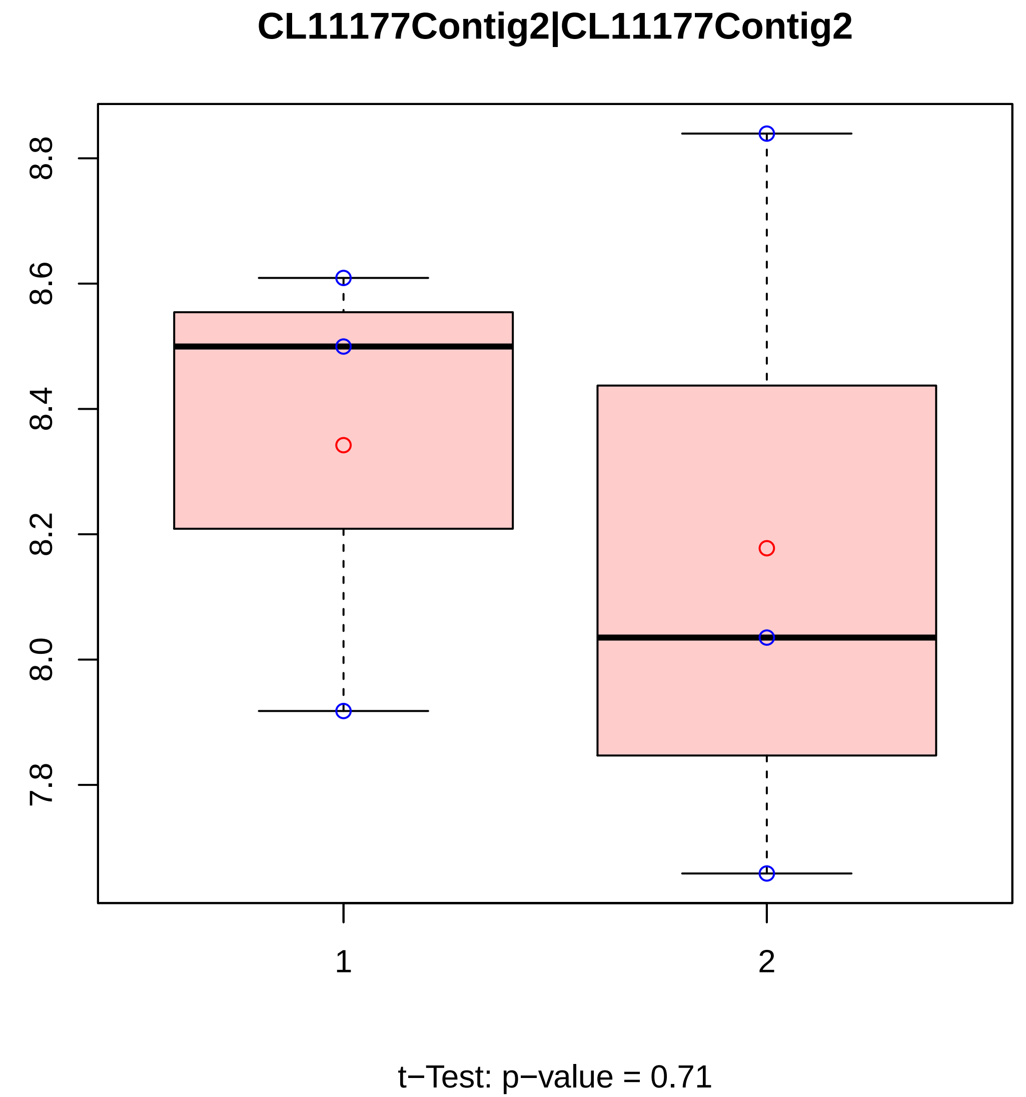

CL11177Contig2|CL11177Contig2
8.8
8.6
8.4
8.2
8.0
7.8
1
2
t−Test: p−value = 0.71

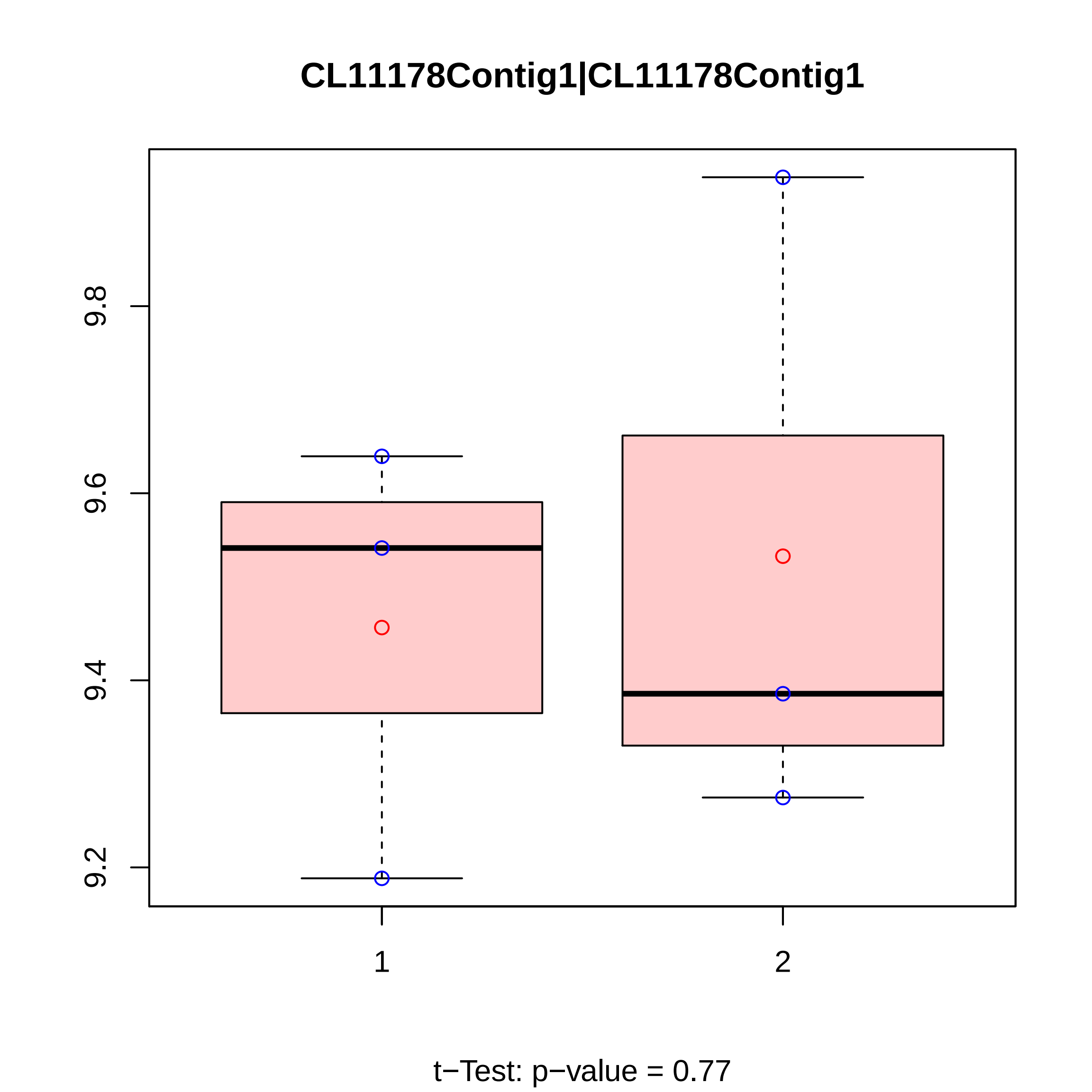

CL11178Contig1|CL11178Contig1
9.2
9.4
9.6
9.8
1
2
t−Test: p−value = 0.77

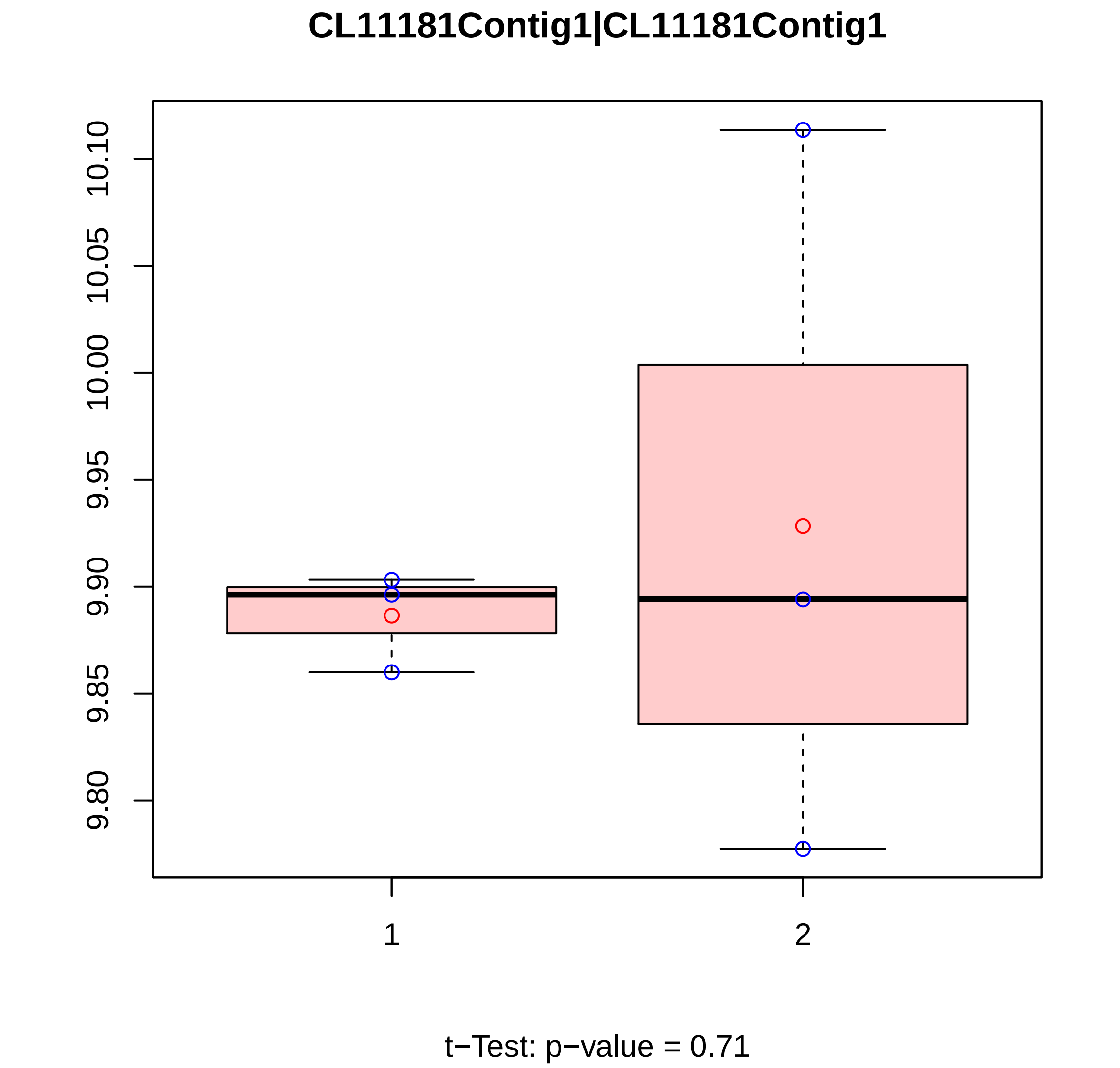
CL11181Contig1|CL11181Contig1
10.10
10.05
10.00
9.95
9.90
9.85
9.80
1
2
t−Test: p−value = 0.71

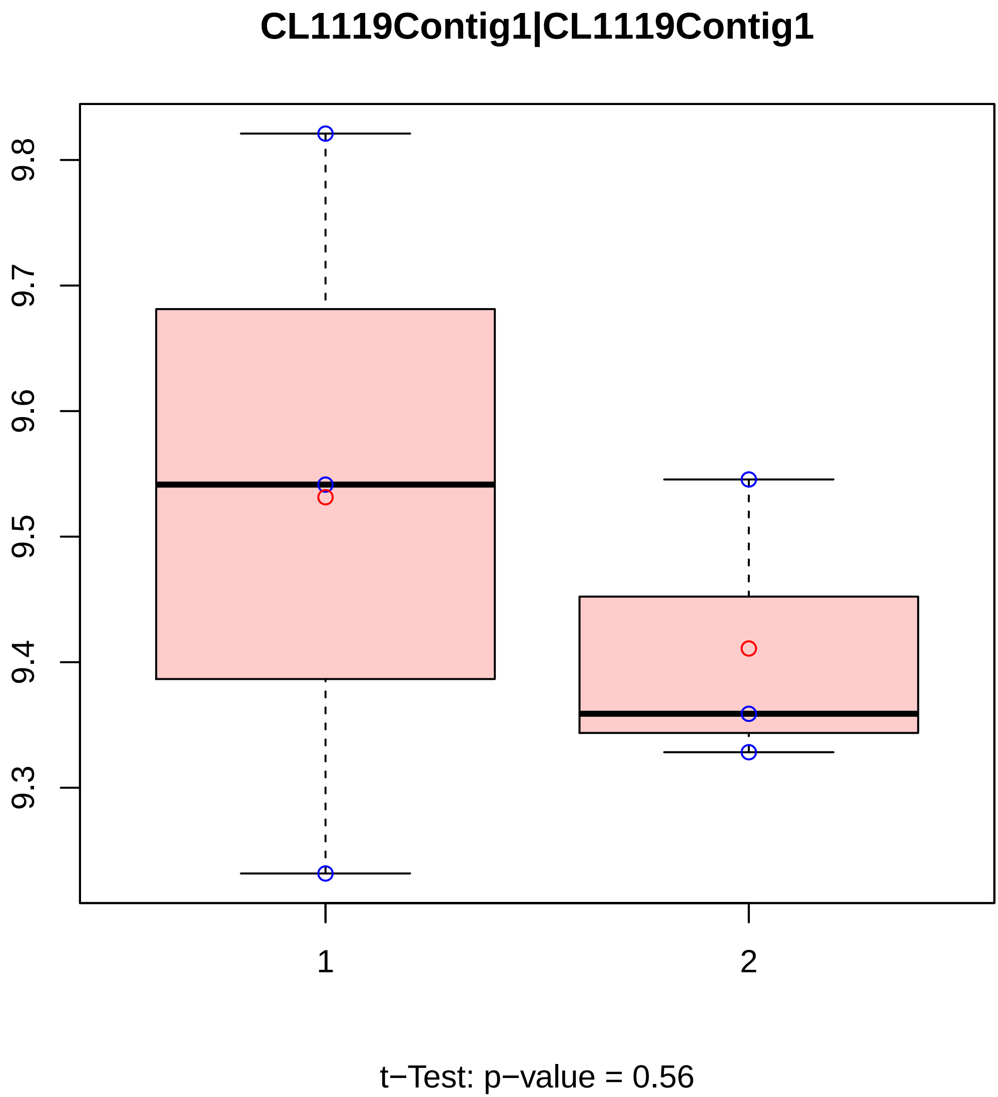
CL1119Contig1|CL1119Contig1
9.3
9.4
9.5
9.6
9.7
9.8
1
2
t−Test: p−value = 0.56

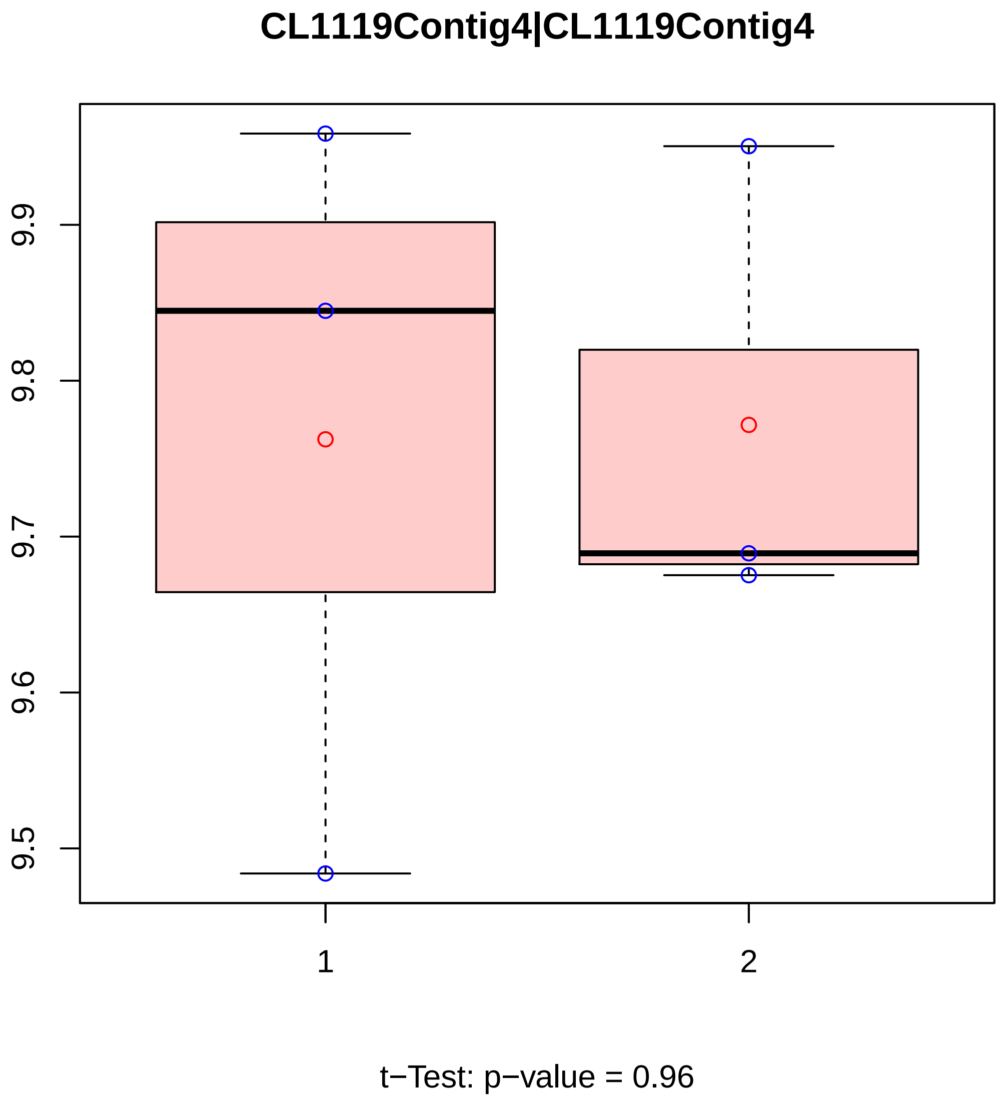

CL1119Contig4|CL1119Contig4
9.5
9.6
9.7
9.8
9.9
1
2
t−Test: p−value = 0.96

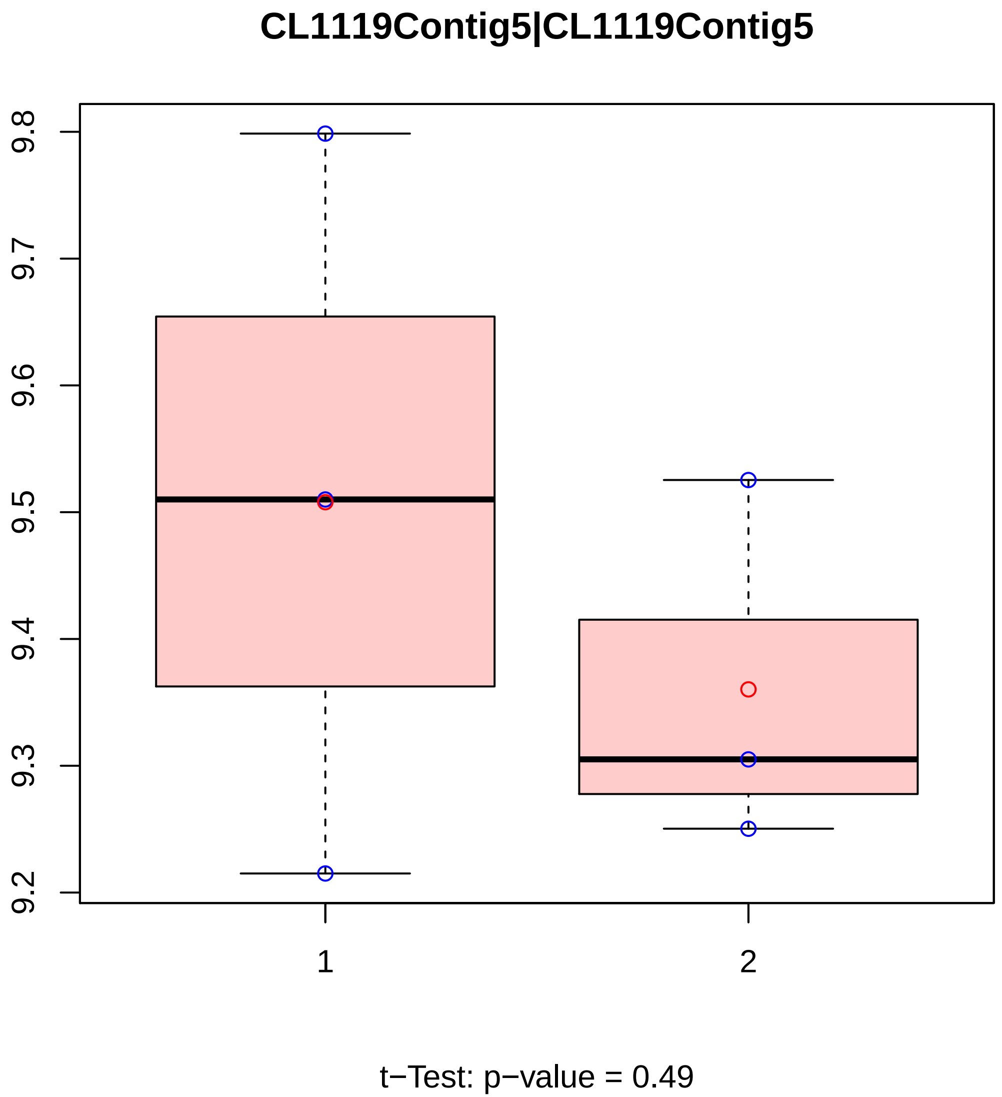

CL1119Contig5|CL1119Contig5
9.2
9.3
9.4
9.5
9.6
9.7
9.8
1
2
t−Test: p−value = 0.49

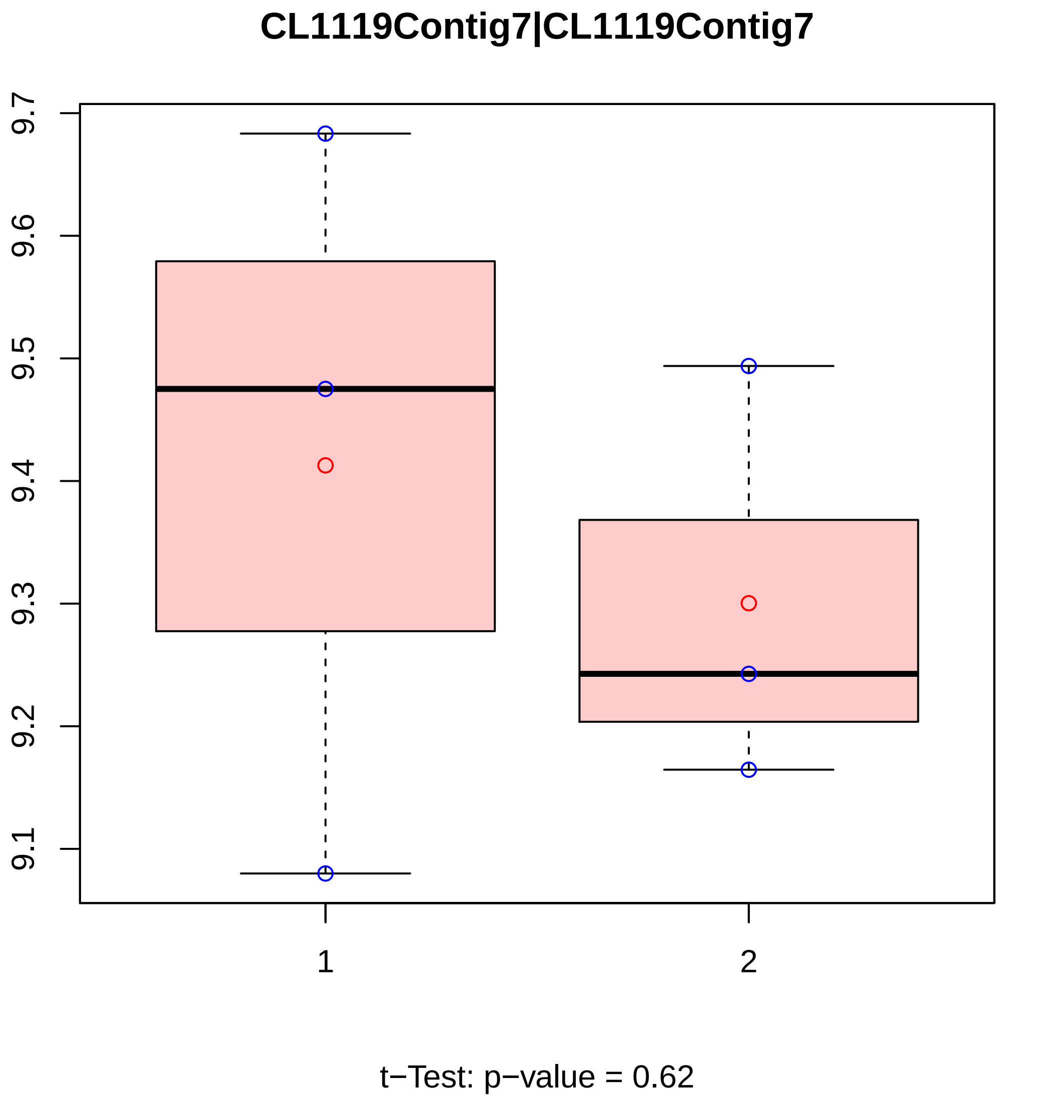
CL1119Contig7|CL1119Contig7
9.1
9.2
9.3
9.4
9.5
9.6
9.7
1
2
t−Test: p−value = 0.62

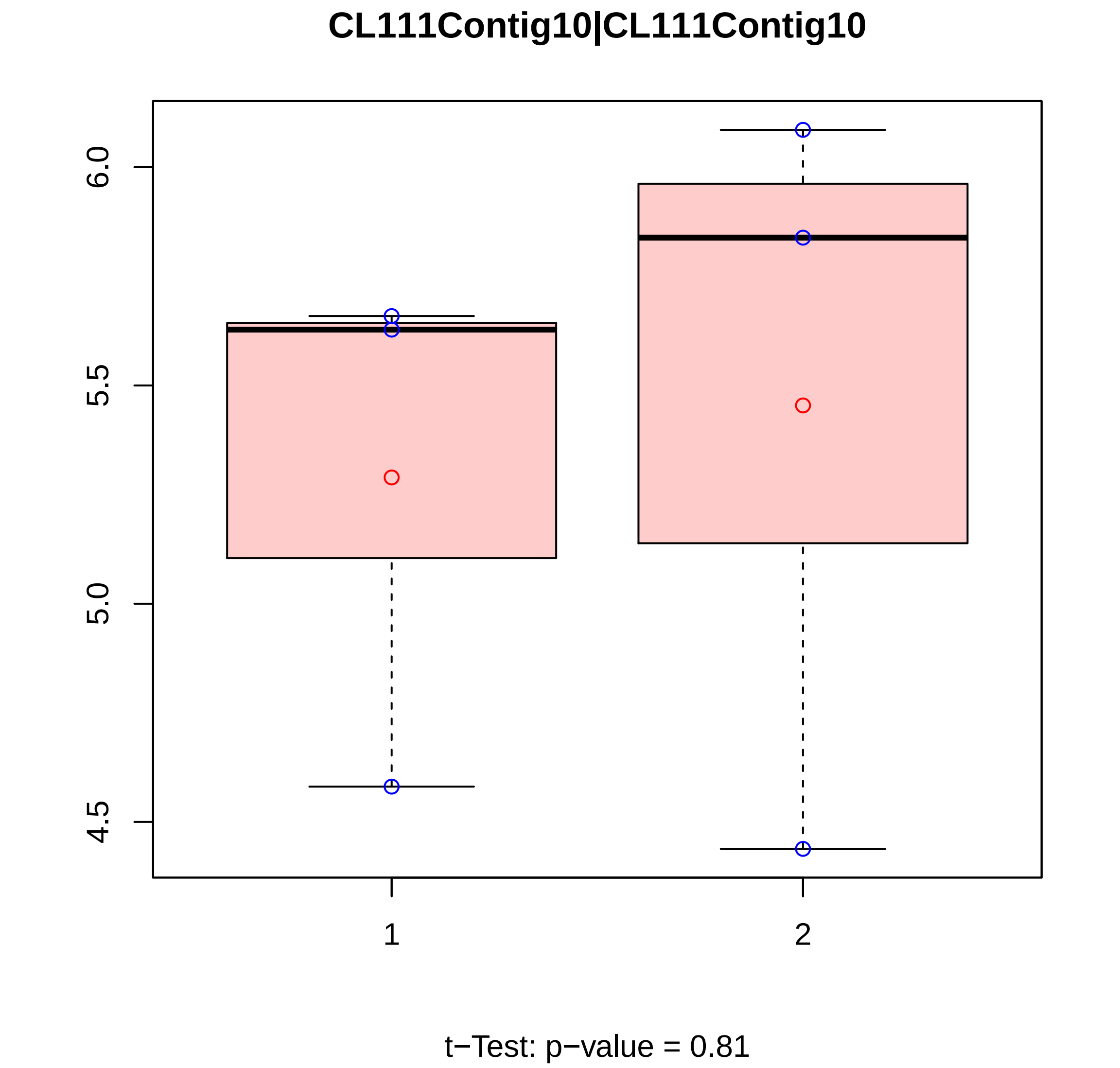
CL111Contig10|CL111Contig10
6.0
5.5
5.0
4.5
1
2
t−Test: p−value = 0.81

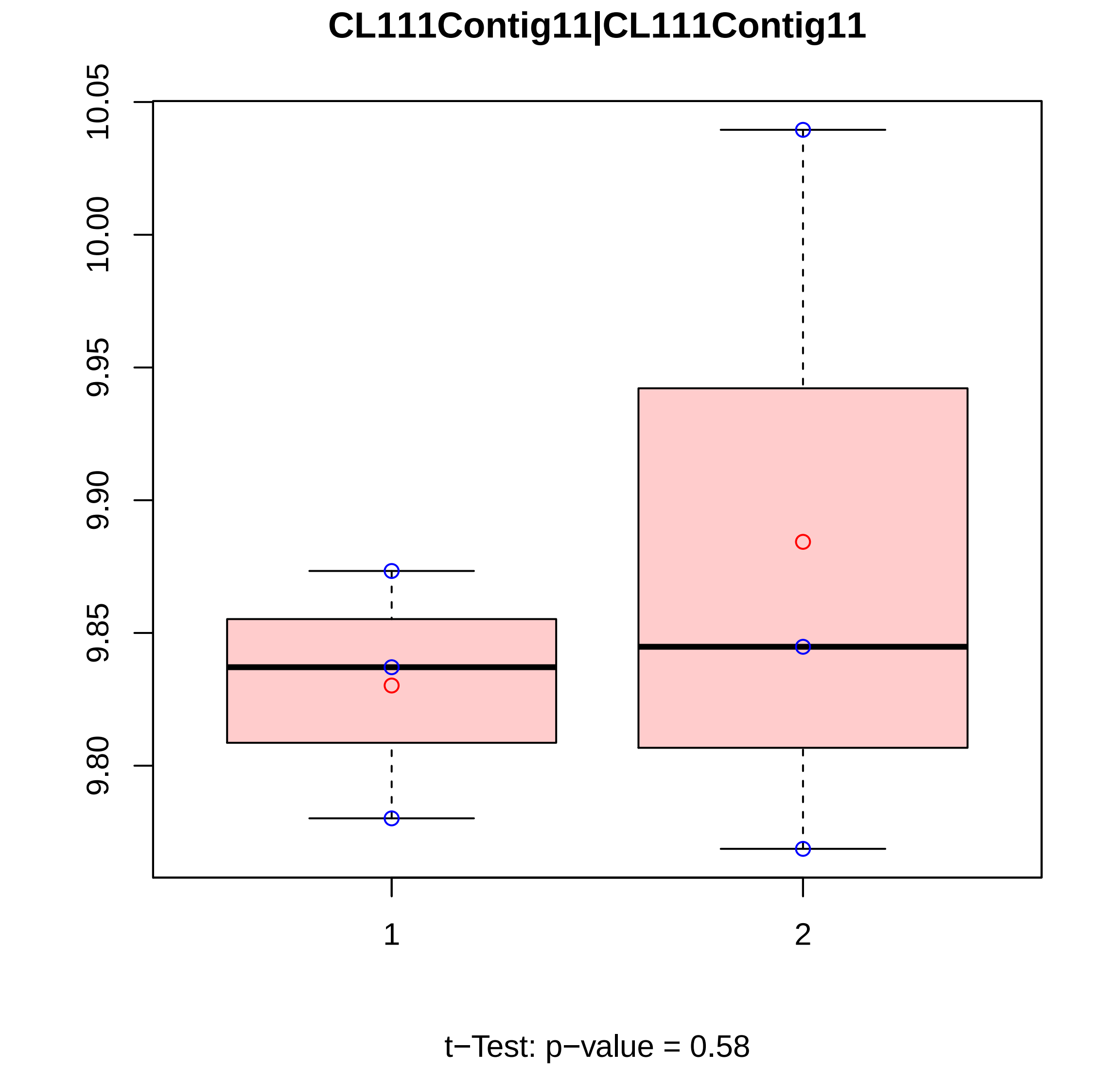
CL111Contig11|CL111Contig11
10.05
10.00
9.95
9.90
9.85
9.80
1
2
t−Test: p−value = 0.58

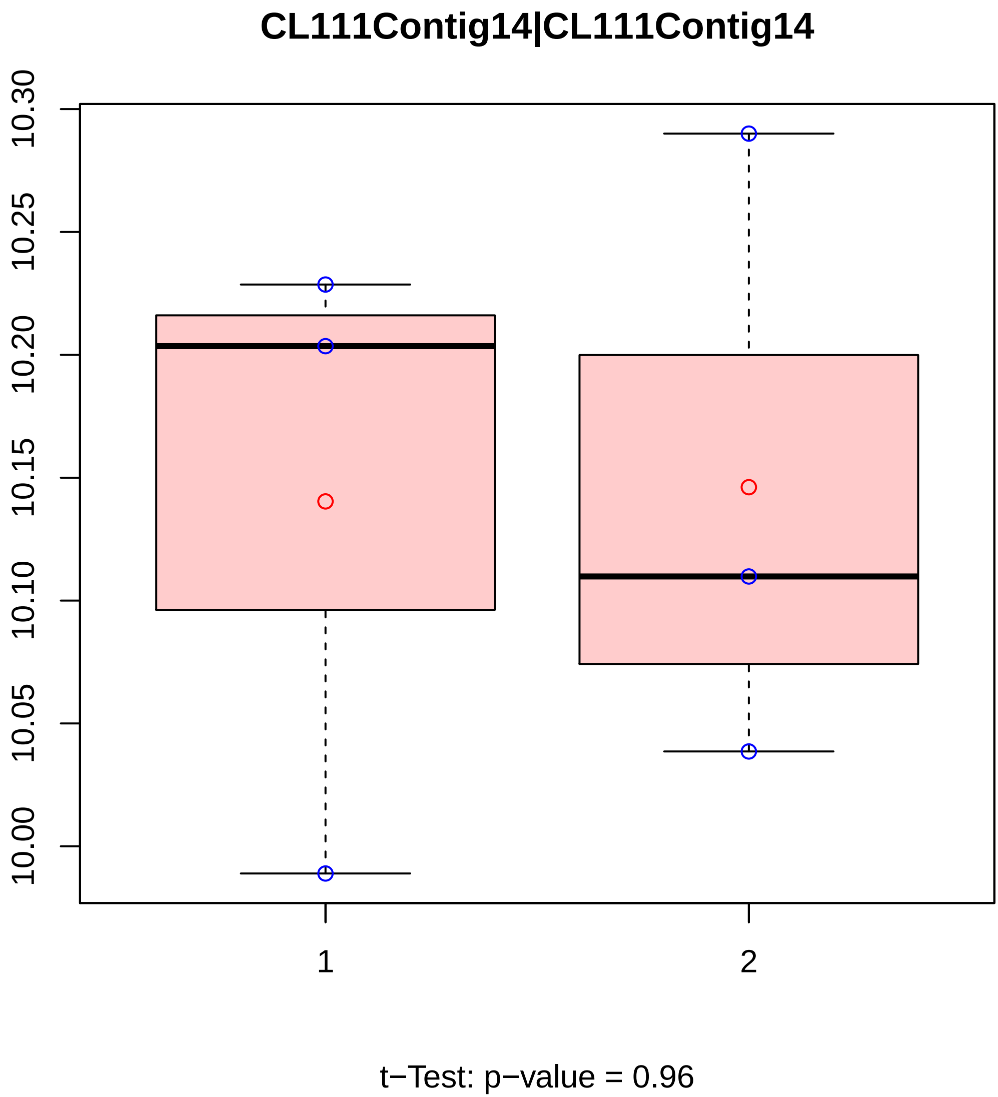
CL111Contig14|CL111Contig14
10.00
10.05
10.10
10.15
10.20
10.25
10.30
1
2
t−Test: p−value = 0.96

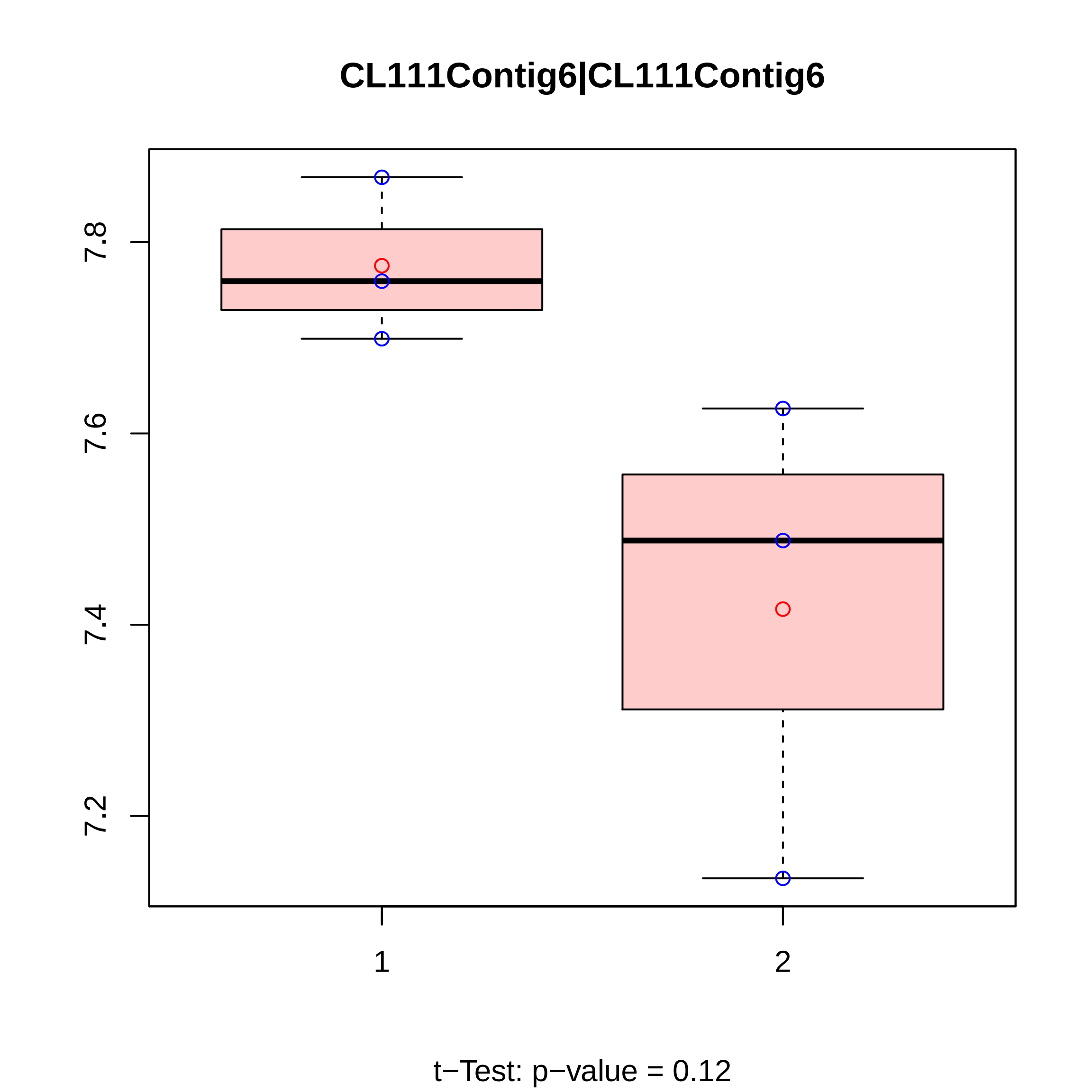
CL111Contig6|CL111Contig6
7.2
7.4
7.6
7.8
1
2
t−Test: p−value = 0.12

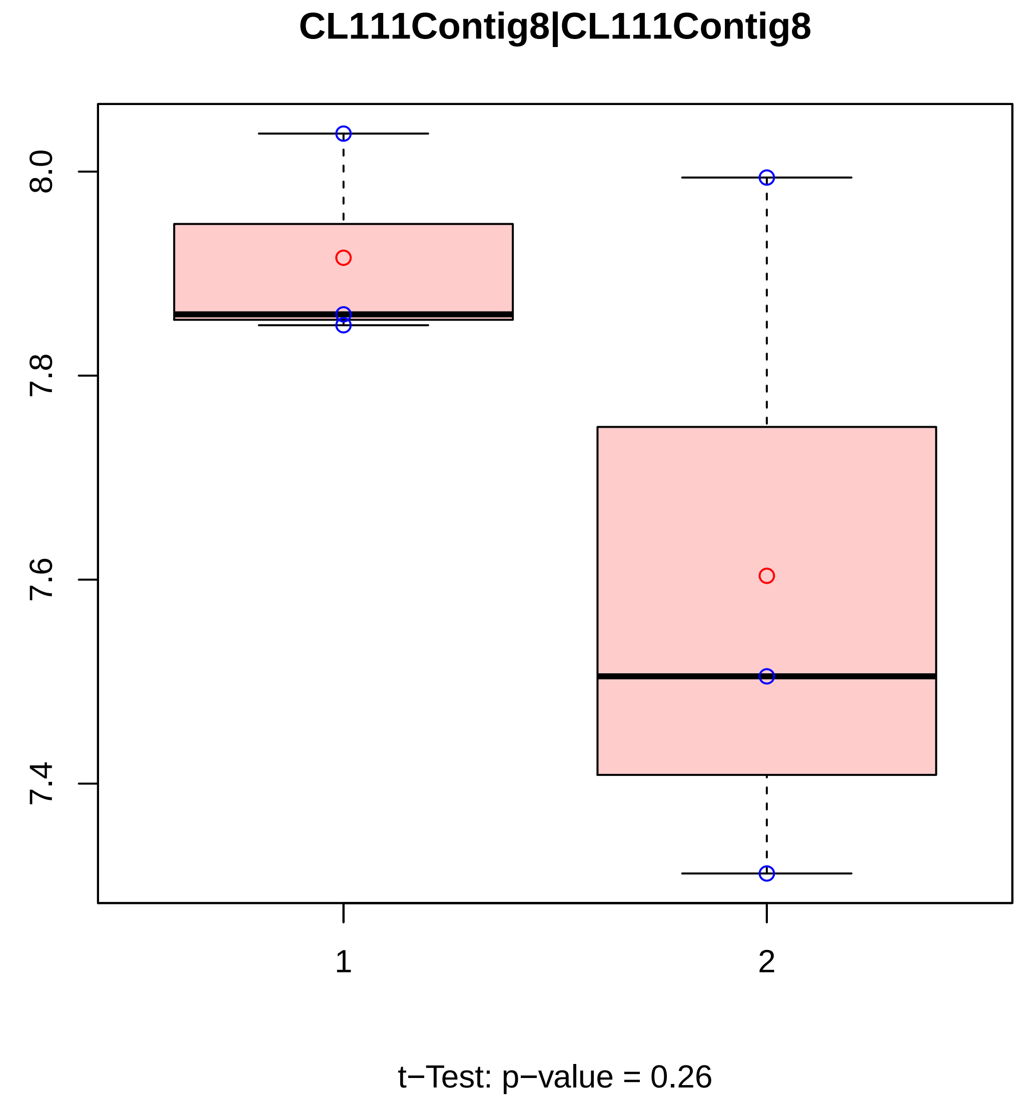
CL111Contig8|CL111Contig8
8.0
7.8
7.6
7.4
1
2
t−Test: p−value = 0.26

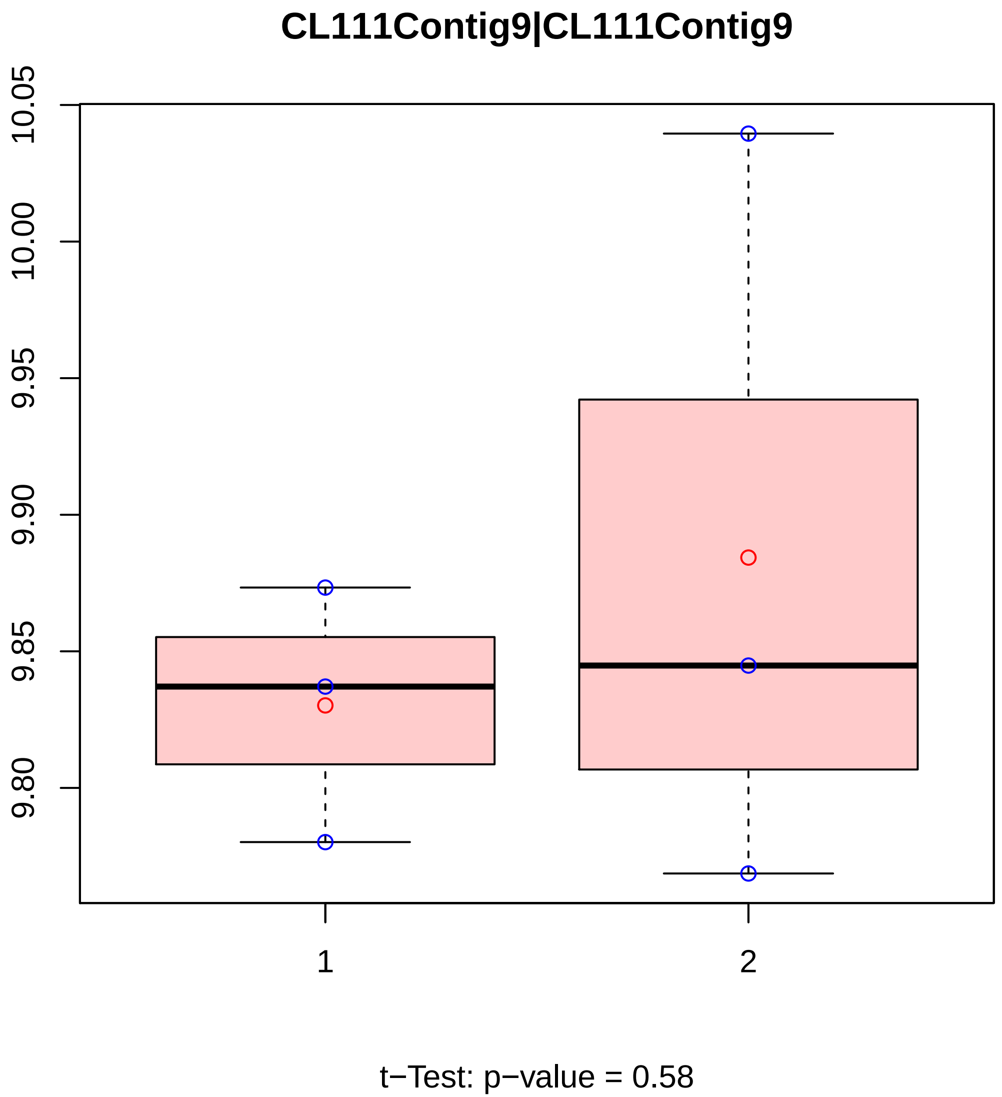
CL111Contig9|CL111Contig9
10.05
10.00
9.95
9.90
9.85
9.80
1
2
t−Test: p−value = 0.58

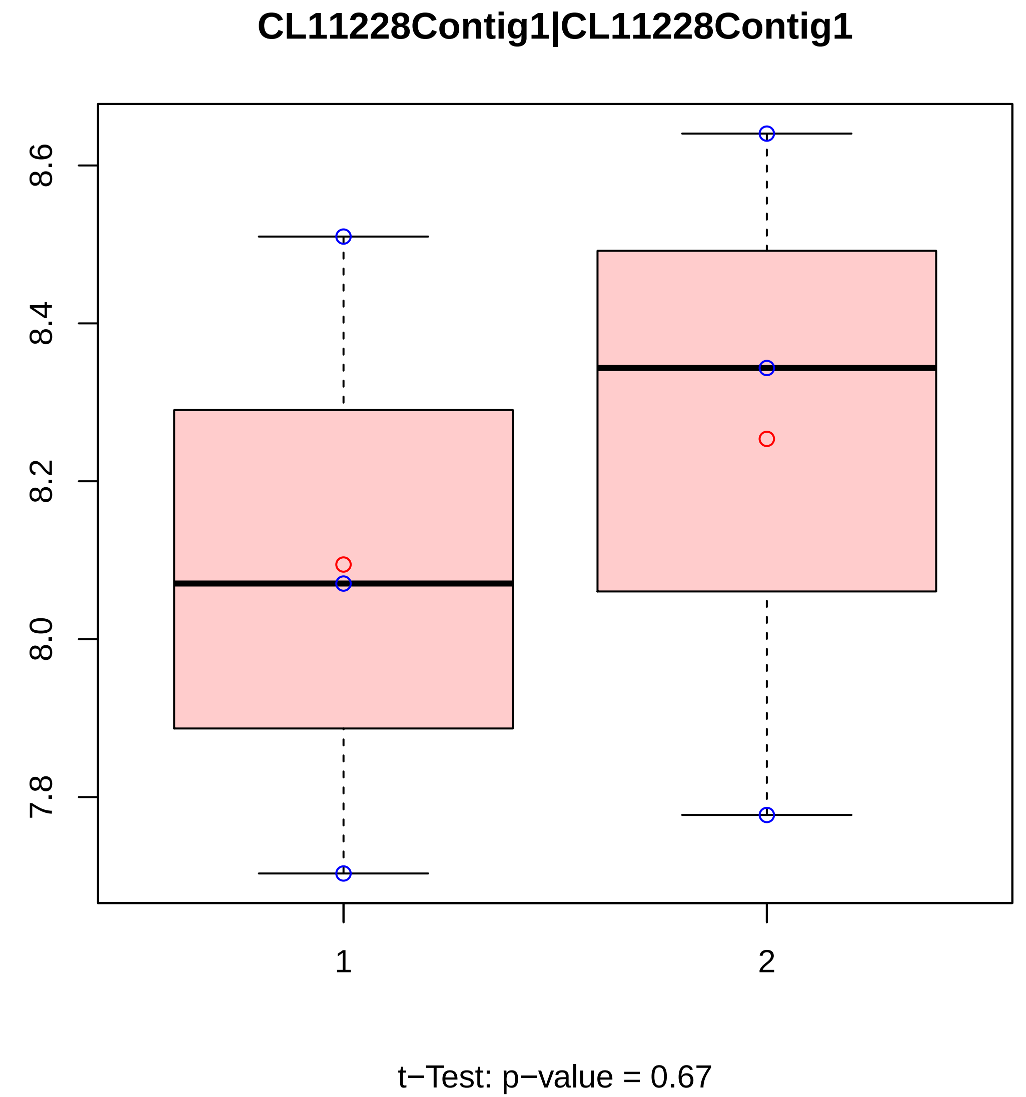
CL11228Contig1|CL11228Contig1
8.6
8.4
8.2
8.0
7.8
1
2
t−Test: p−value = 0.67

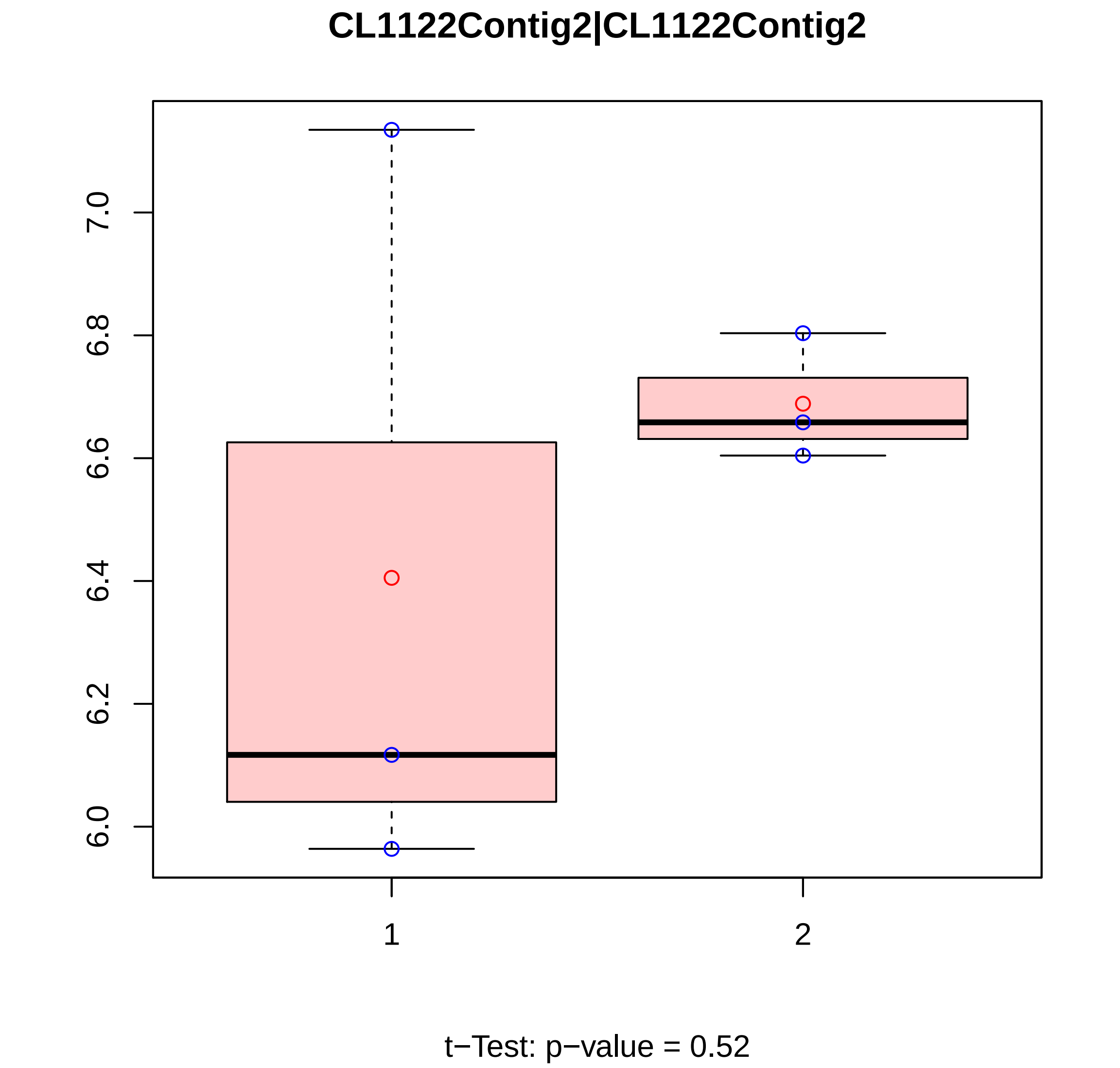
CL1122Contig2|CL1122Contig2
7.0
6.8
6.6
6.4
6.2
6.0
1
2
t−Test: p−value = 0.52

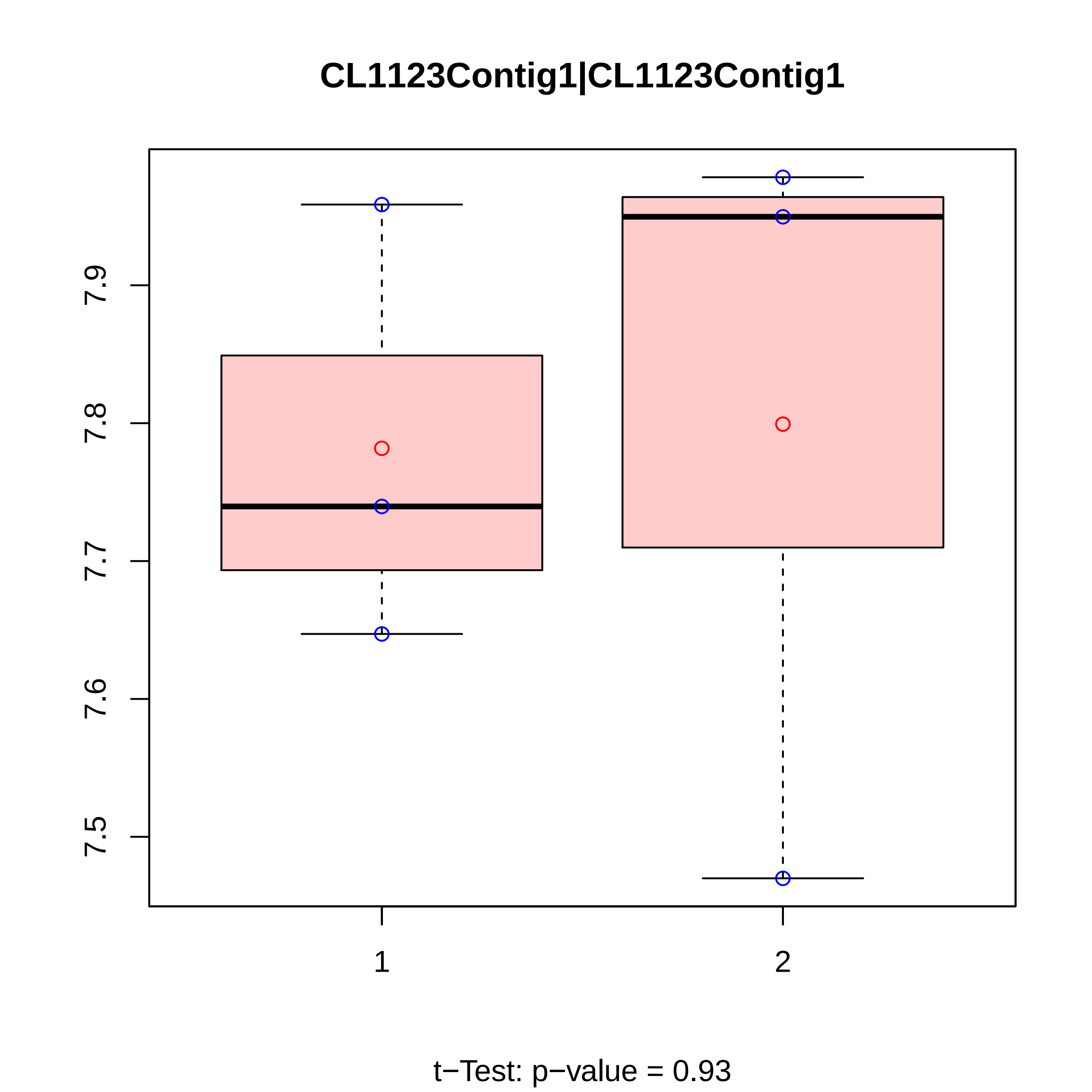
CL1123Contig1|CL1123Contig1
7.5
7.6
7.7
7.8
7.9
1
2
t−Test: p−value = 0.93

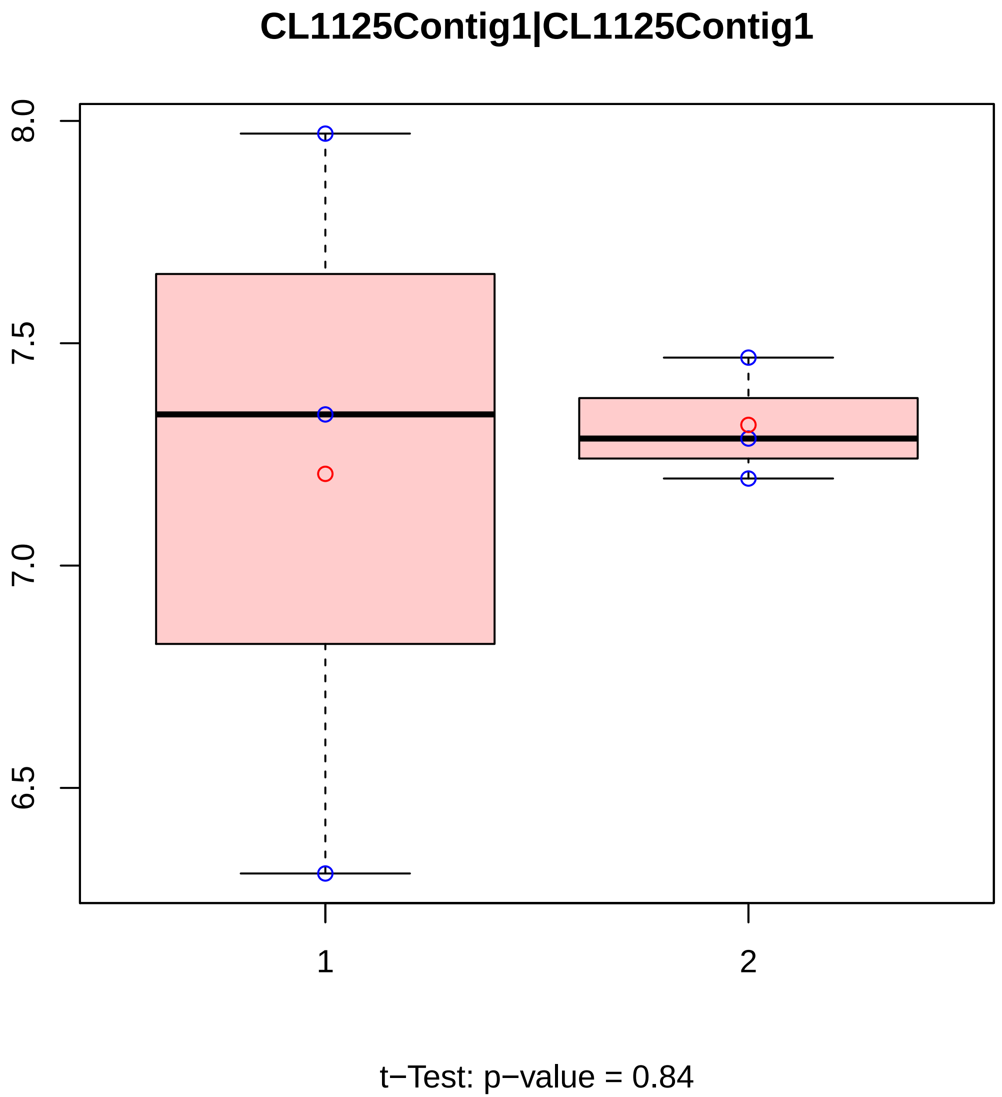

CL1125Contig1|CL1125Contig1
8.0
7.5
7.0
6.5
1
2
t−Test: p−value = 0.84

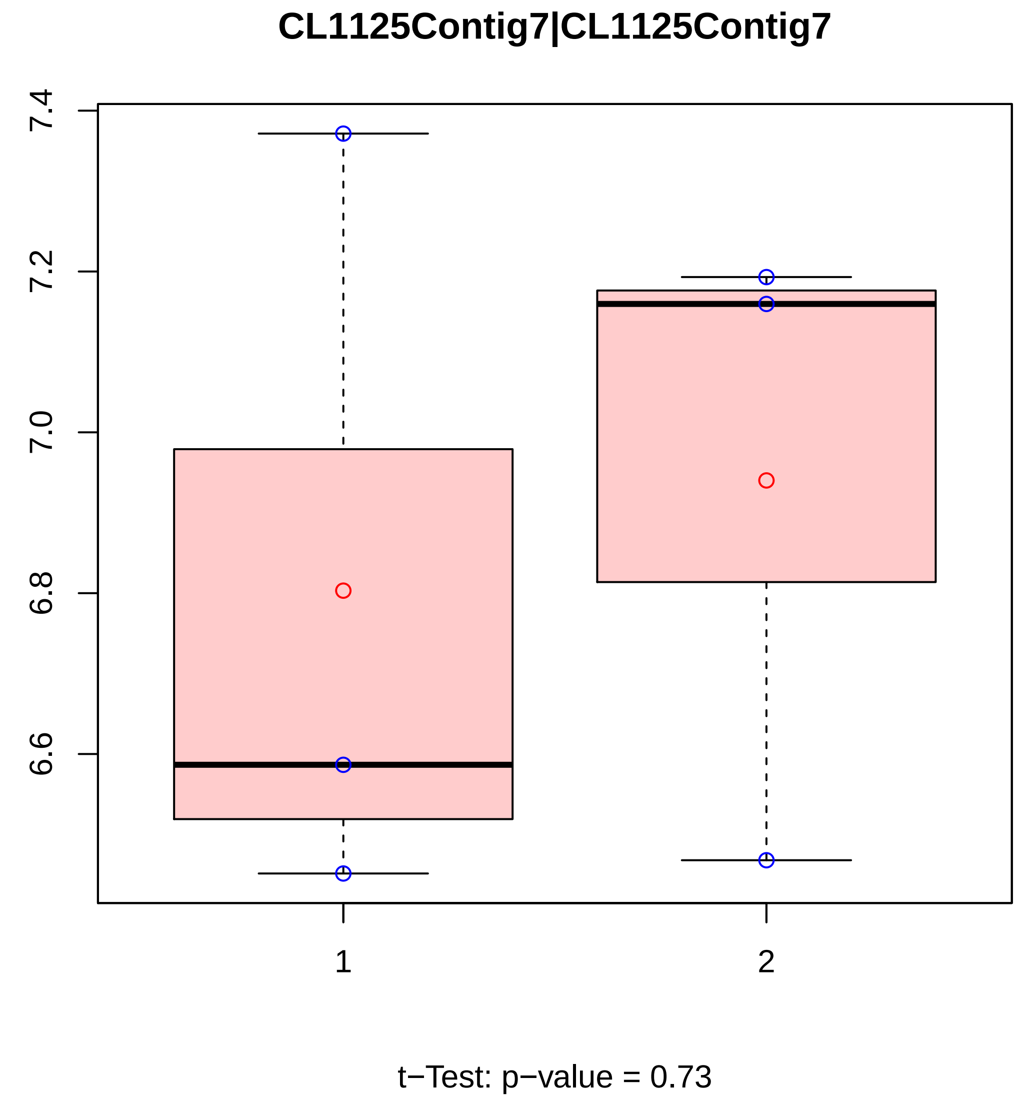
CL1125Contig7|CL1125Contig7
7.4
7.2
7.0
6.8
6.6
1
2
t−Test: p−value = 0.73

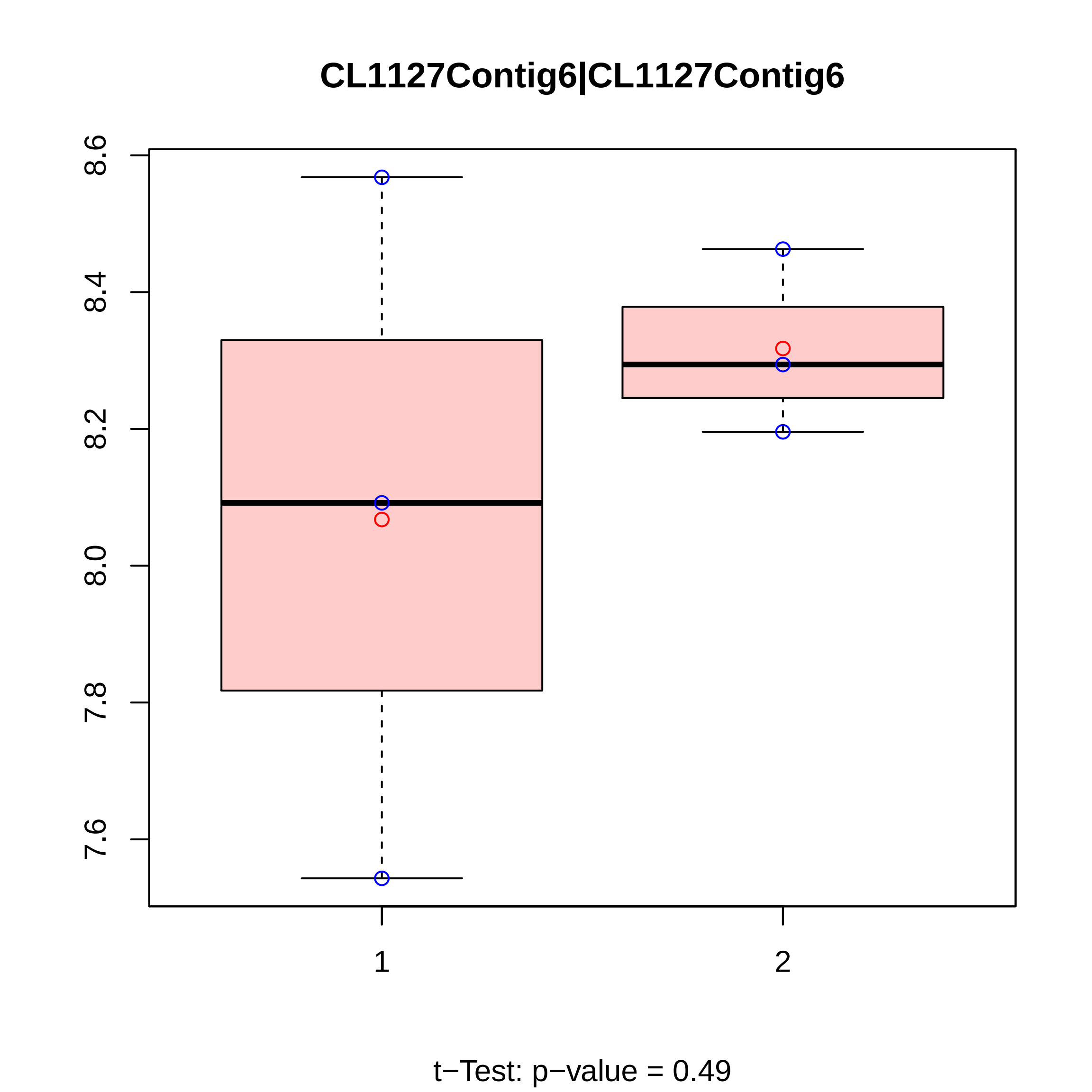
CL1127Contig6|CL1127Contig6
8.6
8.4
8.2
8.0
7.8
7.6
1
2
t−Test: p−value = 0.49

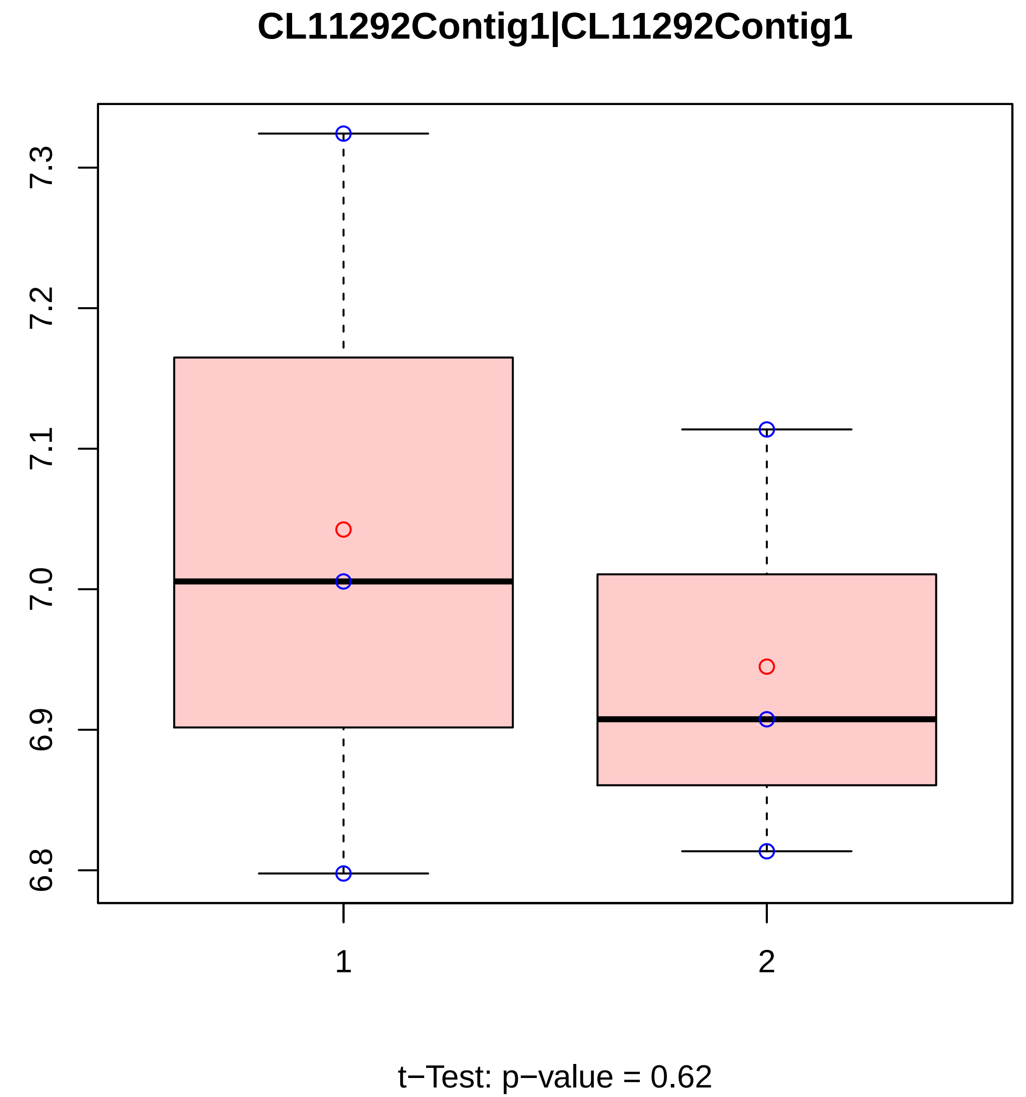
CL11292Contig1|CL11292Contig1
7.3
7.2
7.1
7.0
6.9
6.8
1
2
t−Test: p−value = 0.62

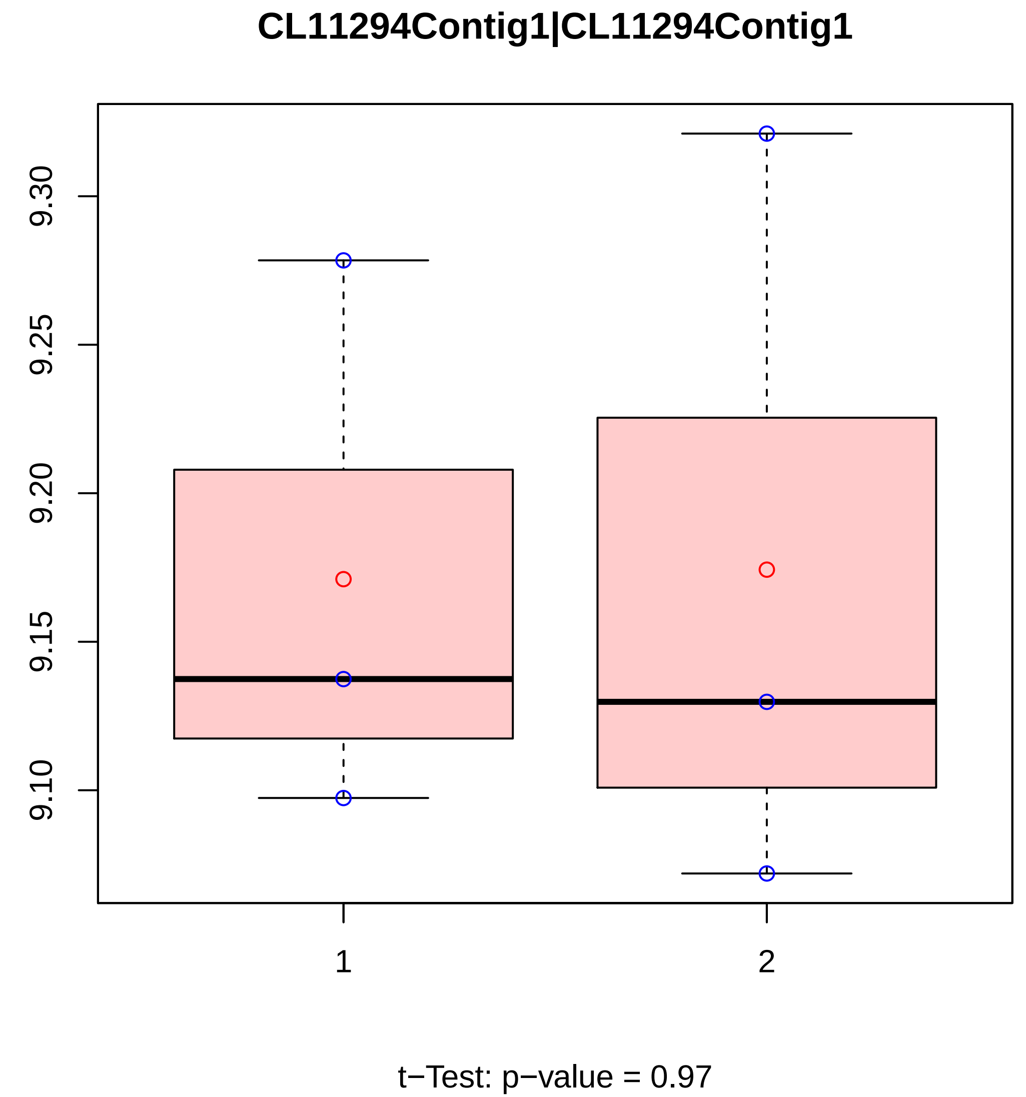
CL11294Contig1|CL11294Contig1
9.10
9.15
9.20
9.25
9.30
1
2
t−Test: p−value = 0.97

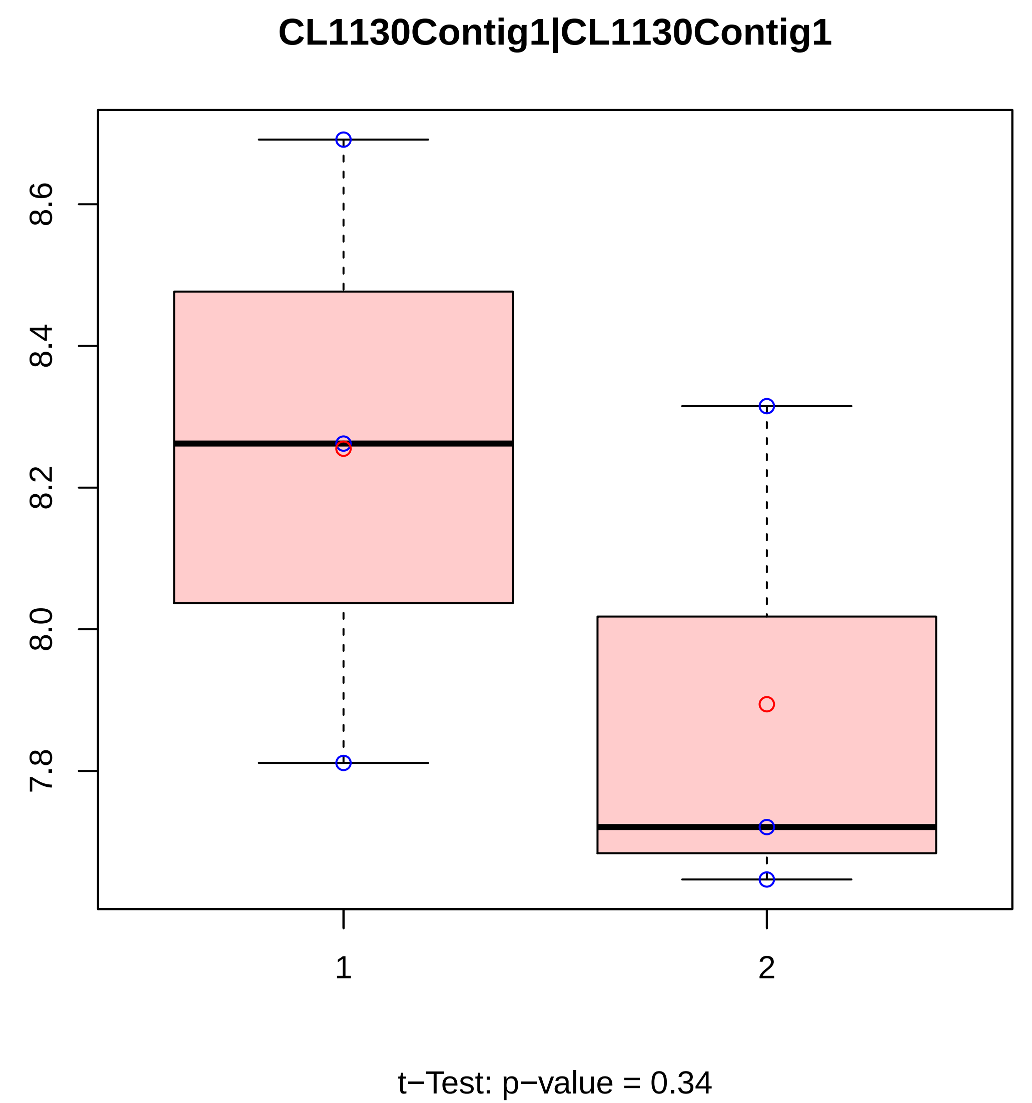
CL1130Contig1|CL1130Contig1
8.6
8.4
8.2
8.0
7.8
1
2
t−Test: p−value = 0.34

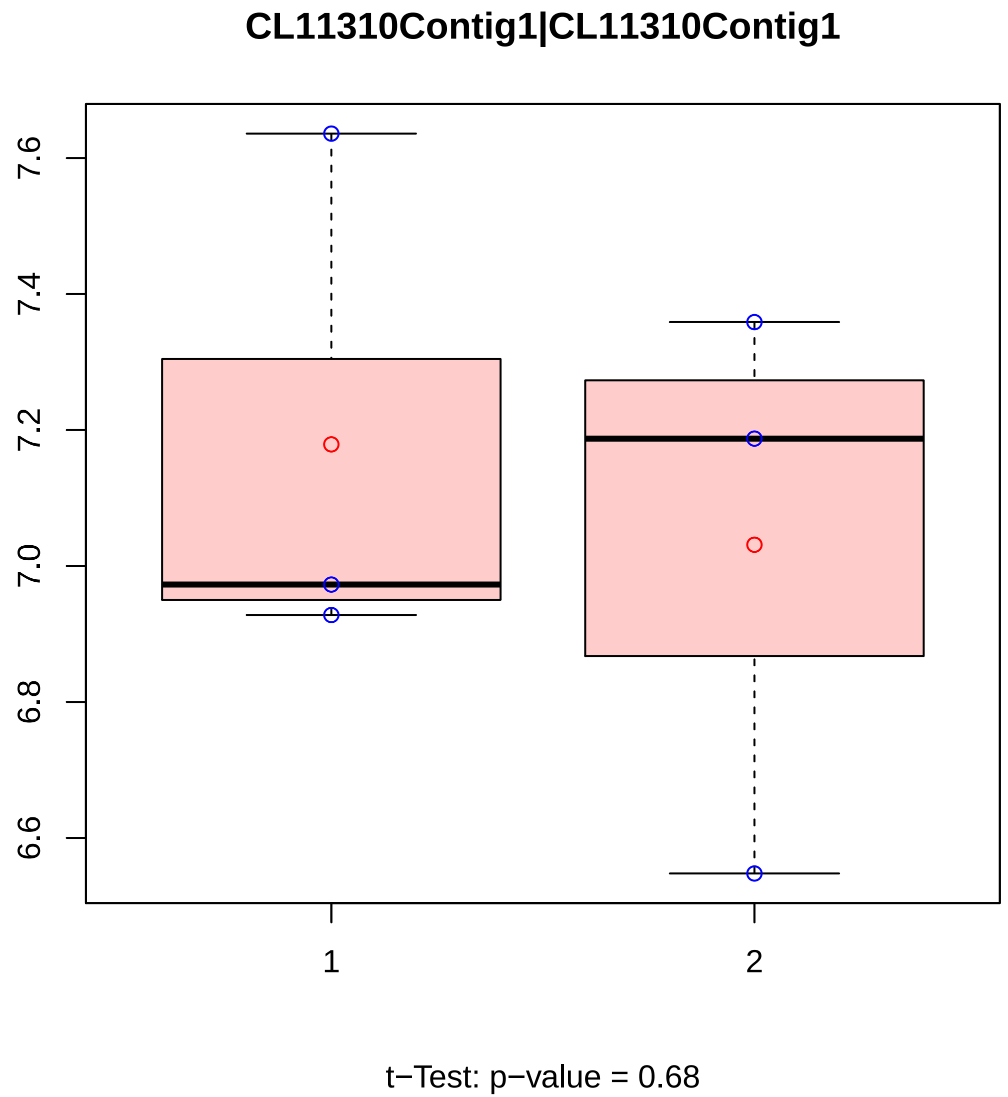
CL11310Contig1|CL11310Contig1
7.6
7.4
7.2
7.0
6.8
6.6
1
2
t−Test: p−value = 0.68

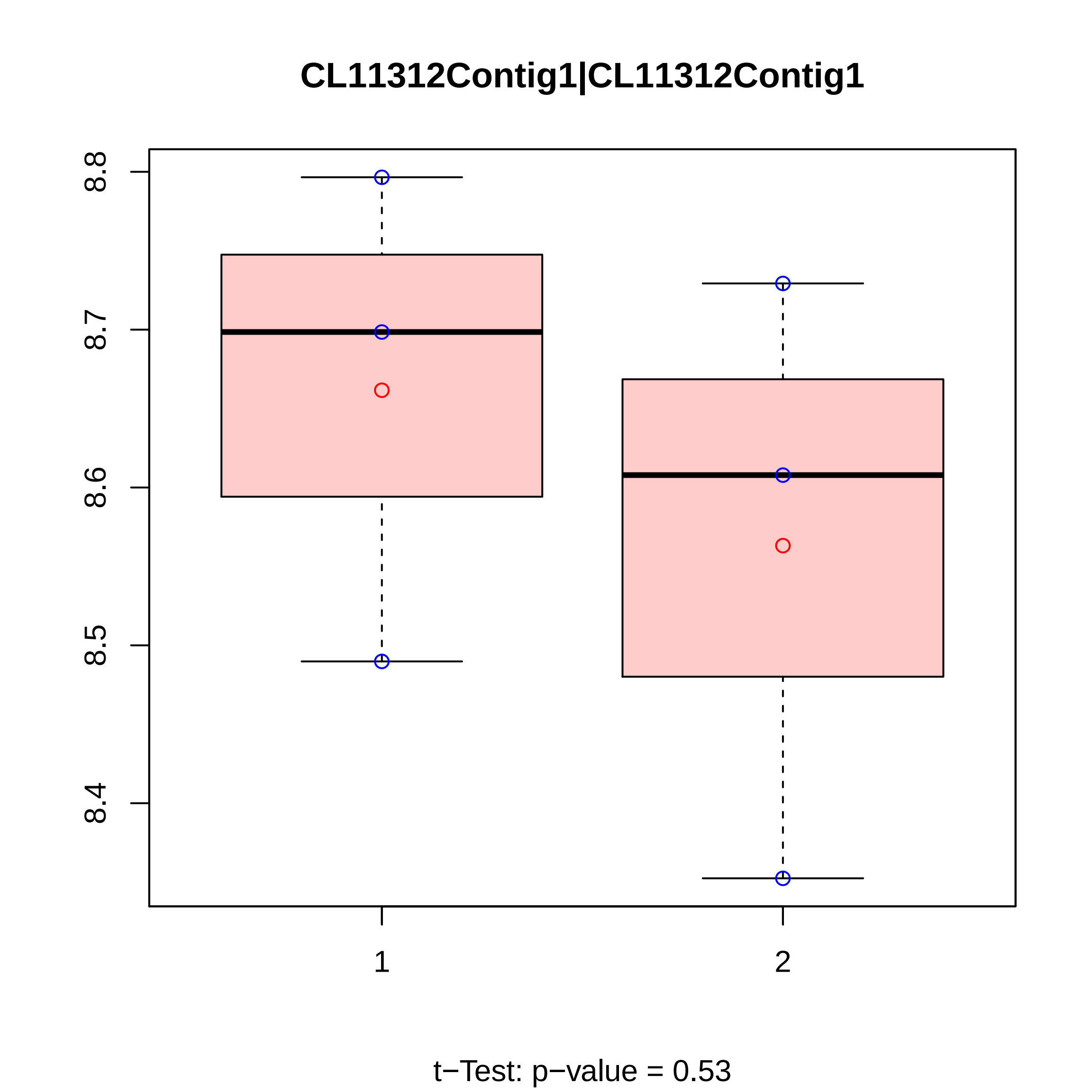
CL11312Contig1|CL11312Contig1
8.4
8.5
8.6
8.7
8.8
1
2
t−Test: p−value = 0.53

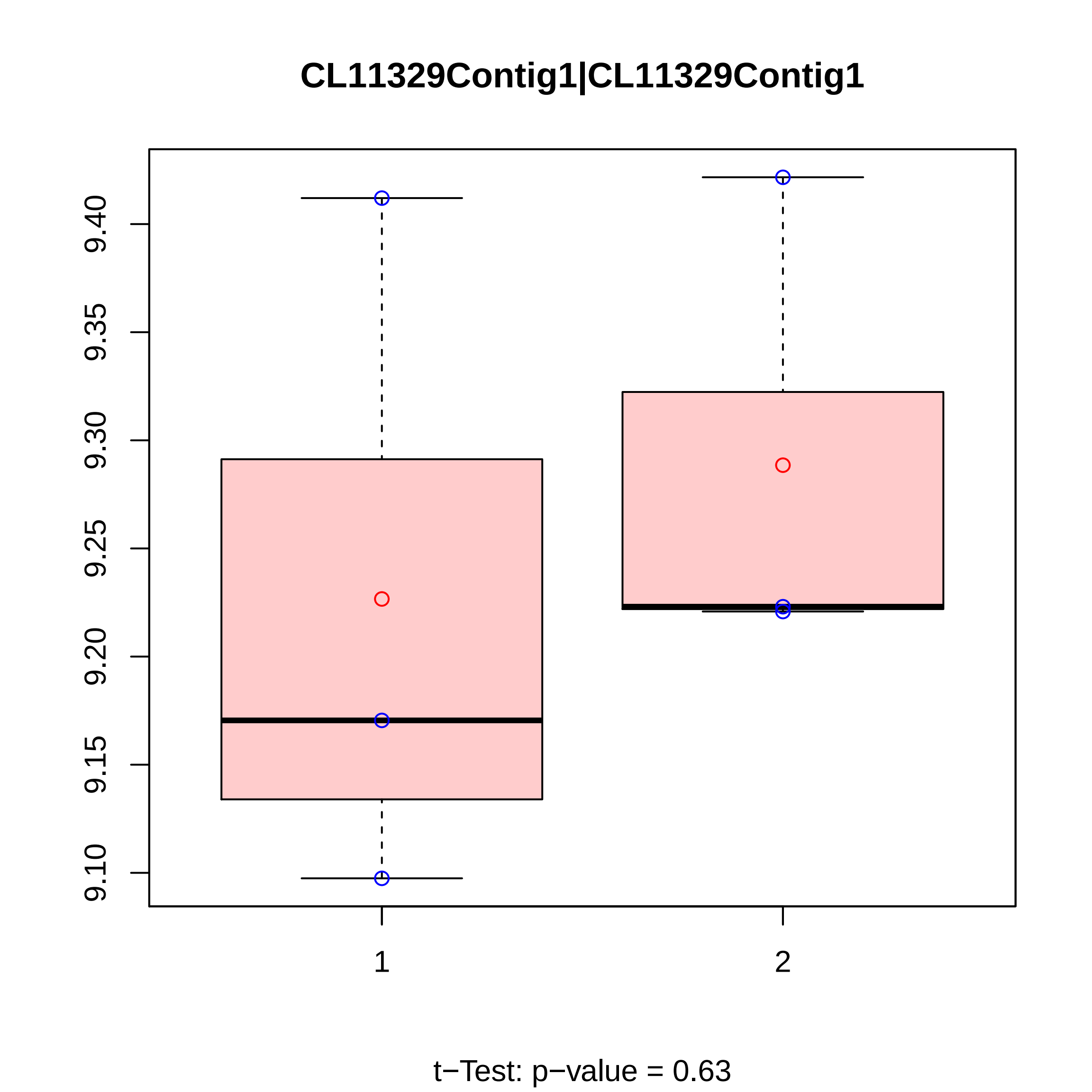
CL11329Contig1|CL11329Contig1
9.10
9.15
9.20
9.25
9.30
9.35
9.40
1
2
t−Test: p−value = 0.63

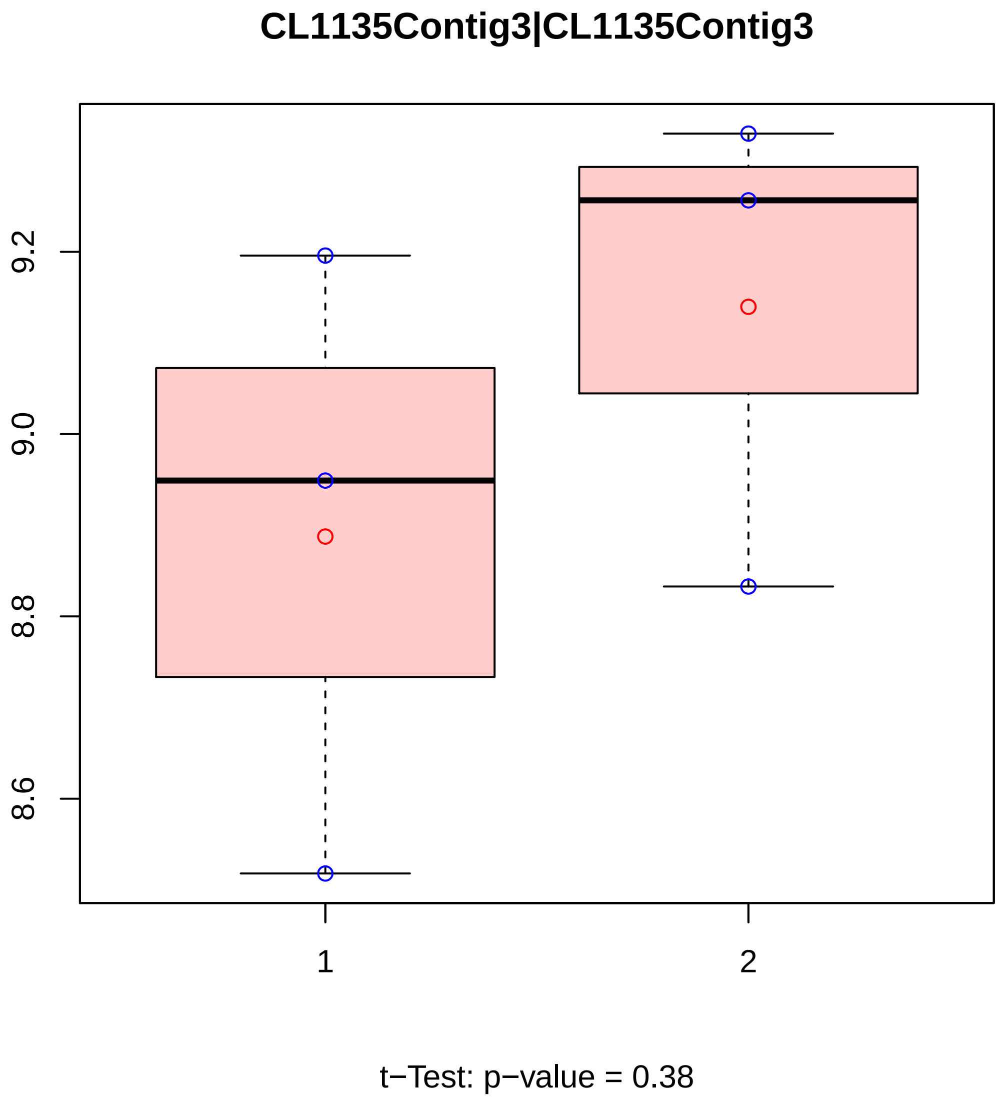

CL1135Contig3|CL1135Contig3
9.2
9.0
8.8
8.6
1
2
t−Test: p−value = 0.38

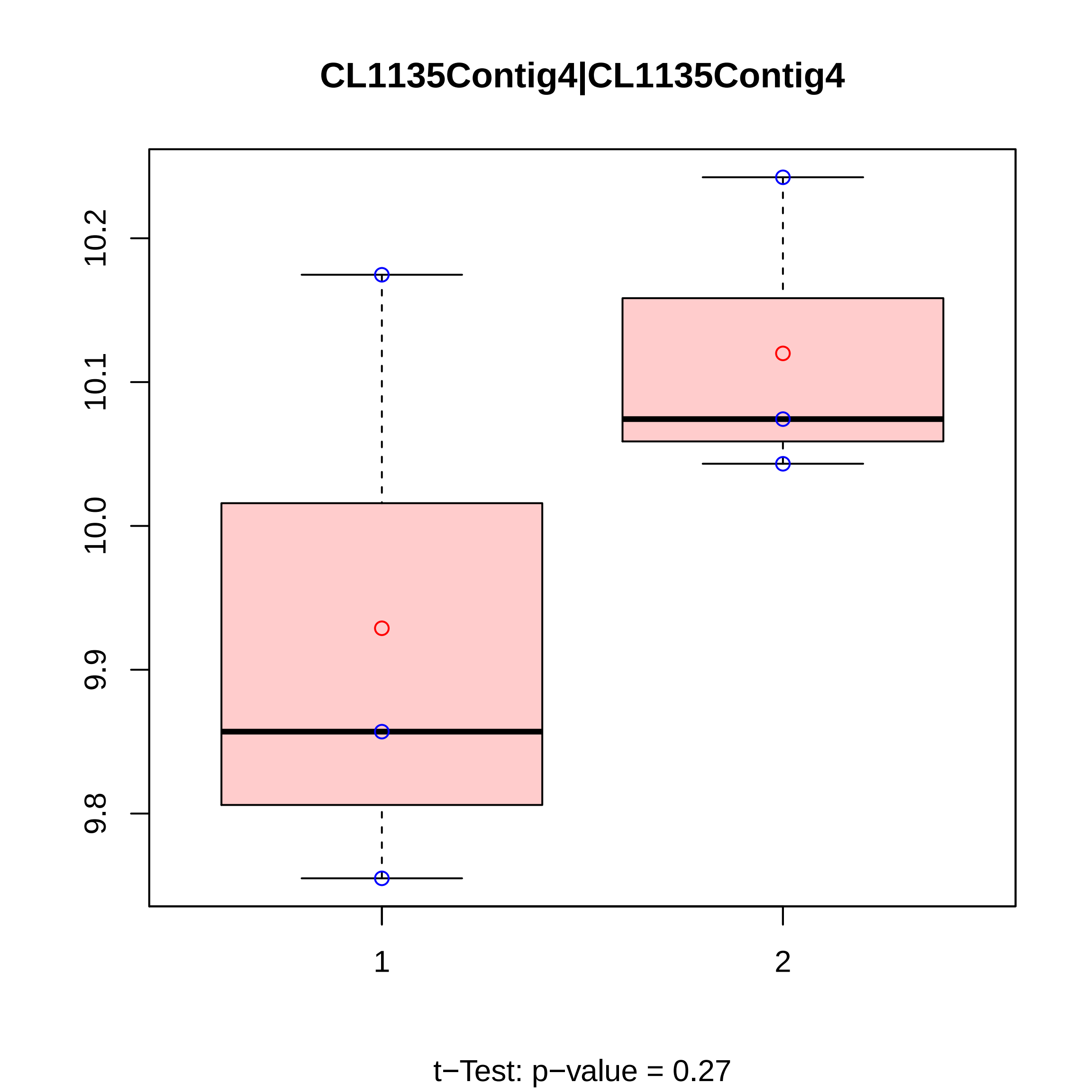
CL1135Contig4|CL1135Contig4
10.2
10.1
10.0
9.9
9.8
1
2
t−Test: p−value = 0.27

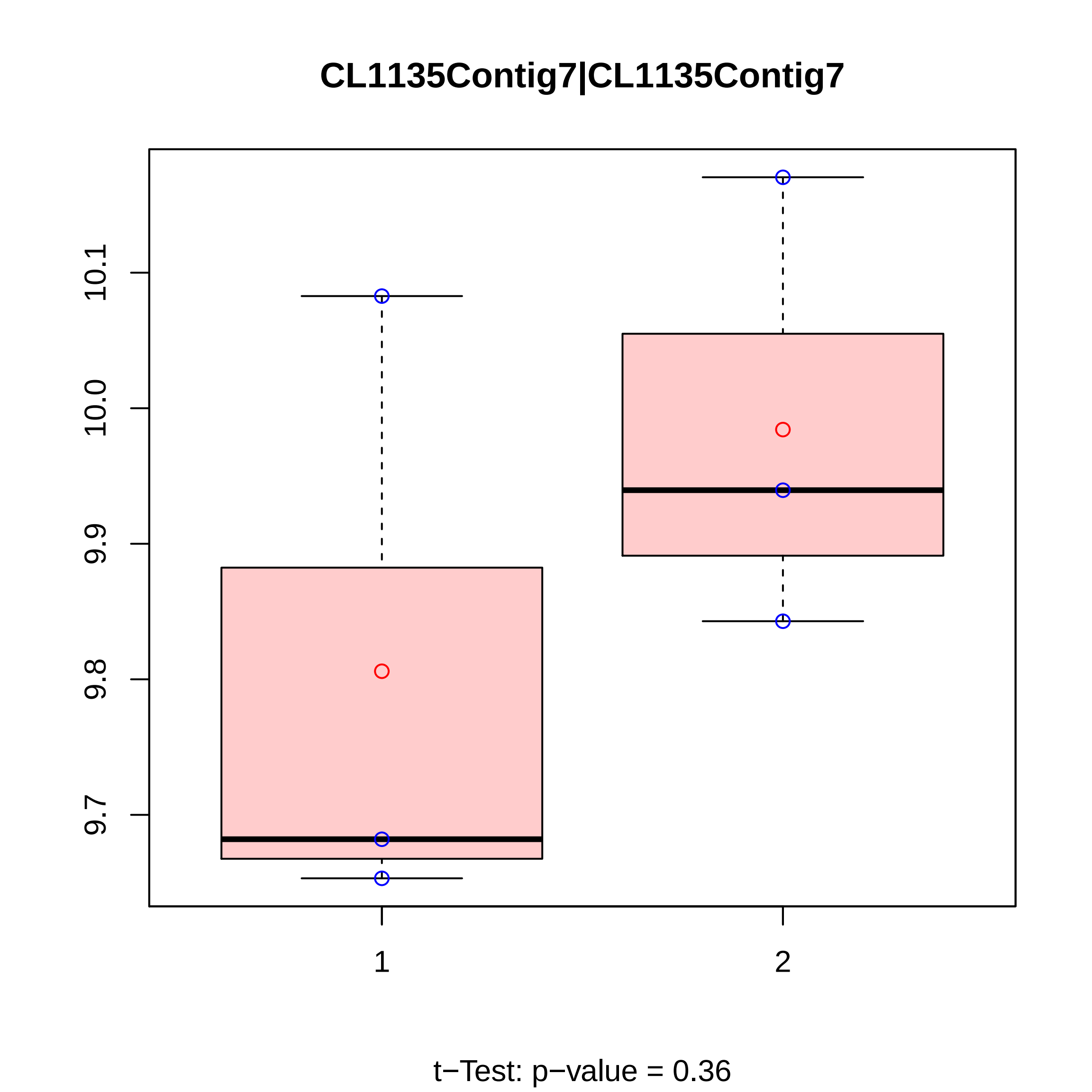
CL1135Contig7|CL1135Contig7
10.1
10.0
9.9
9.8
9.7
1
2
t−Test: p−value = 0.36

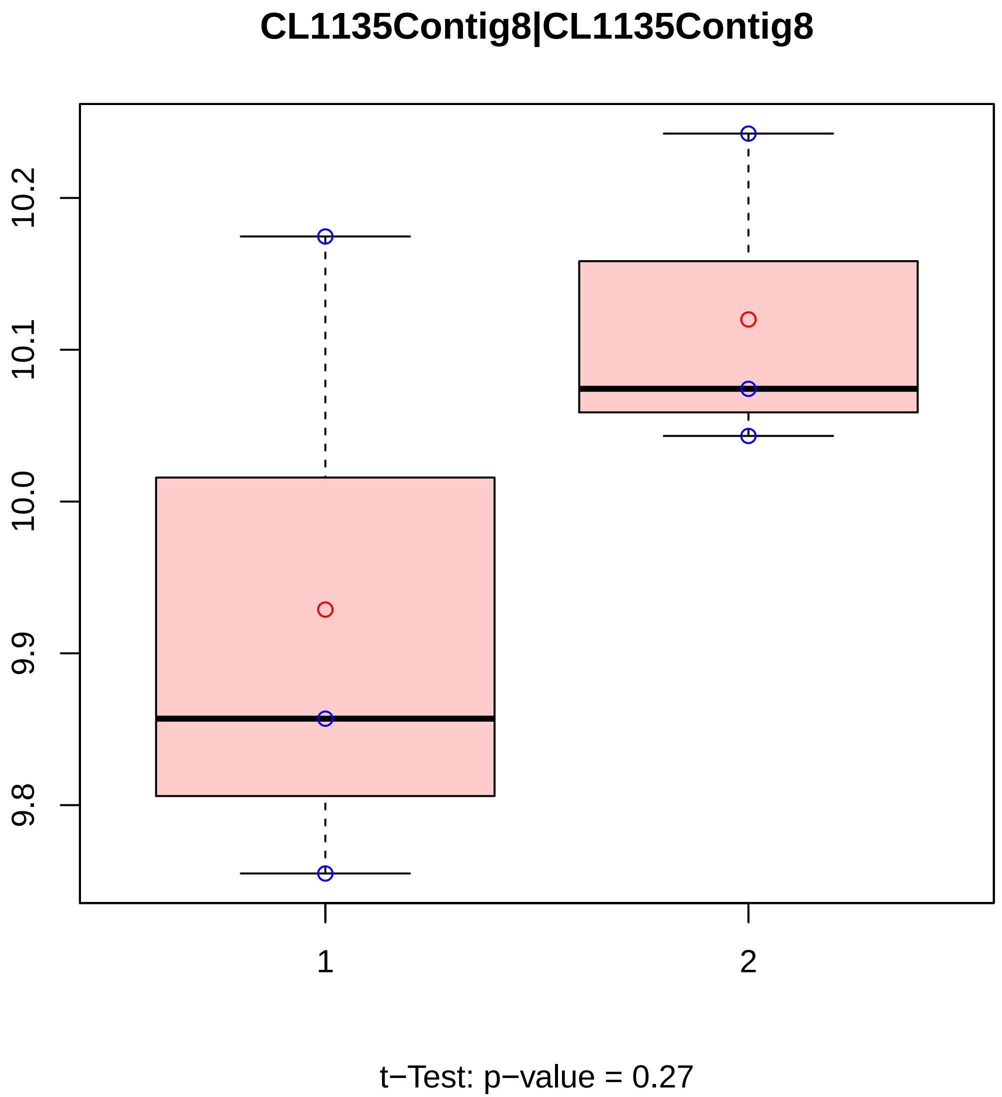
CL1135Contig8|CL1135Contig8
10.2
10.1
10.0
9.9
9.8
1
2
t−Test: p−value = 0.27

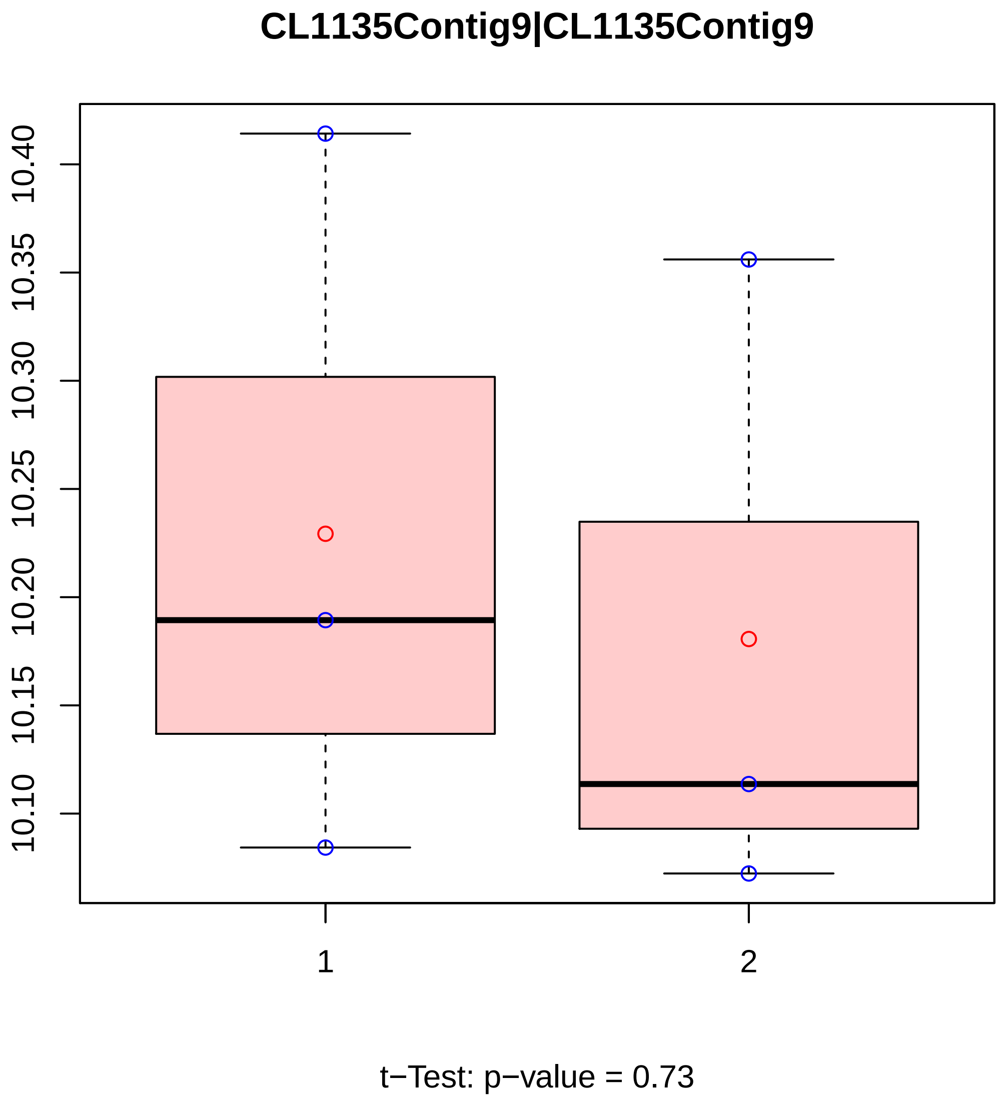
CL1135Contig9|CL1135Contig9
10.40
10.35
10.30
10.25
10.20
10.15
10.10
1
2
t−Test: p−value = 0.73

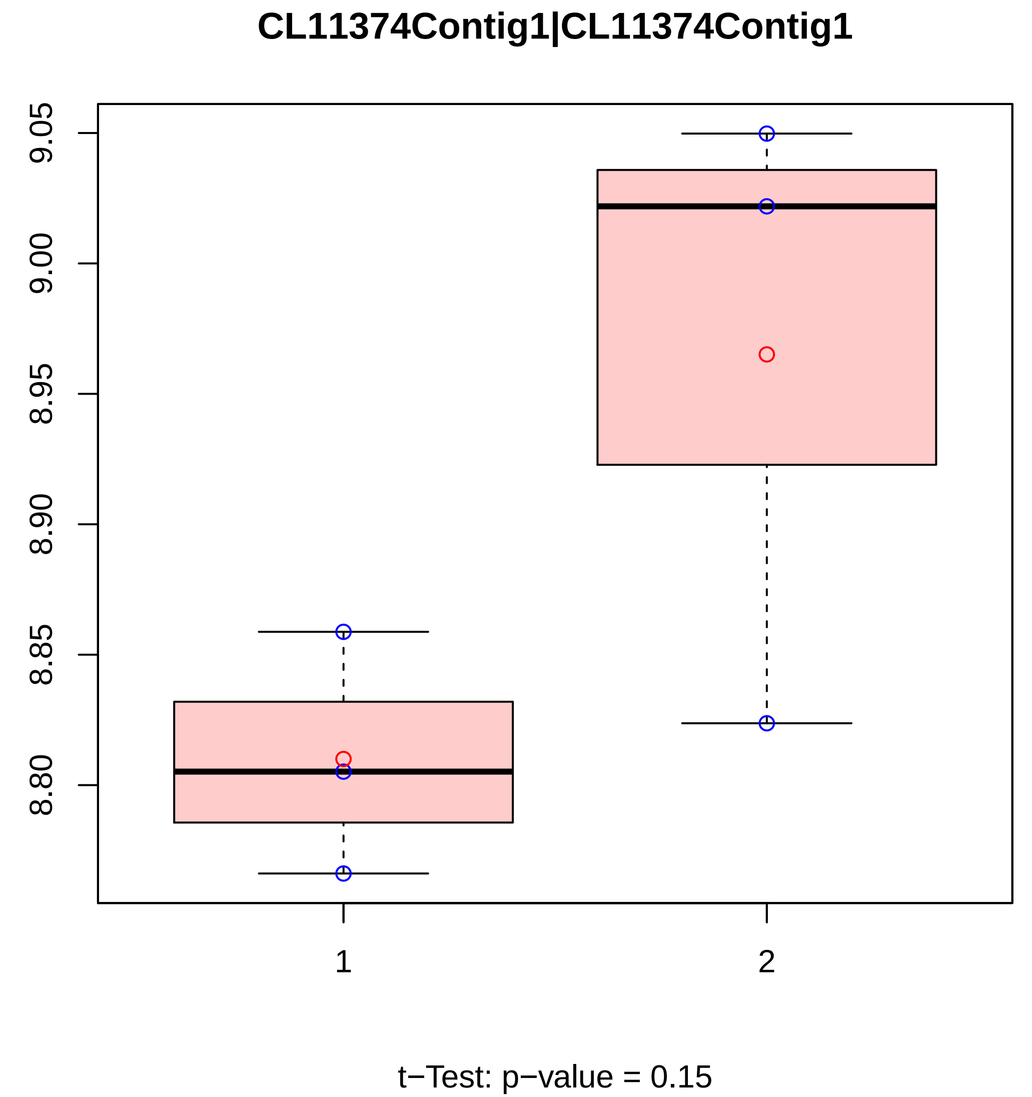
CL11374Contig1|CL11374Contig1
9.05
9.00
8.95
8.90
8.85
8.80
1
2
t−Test: p−value = 0.15

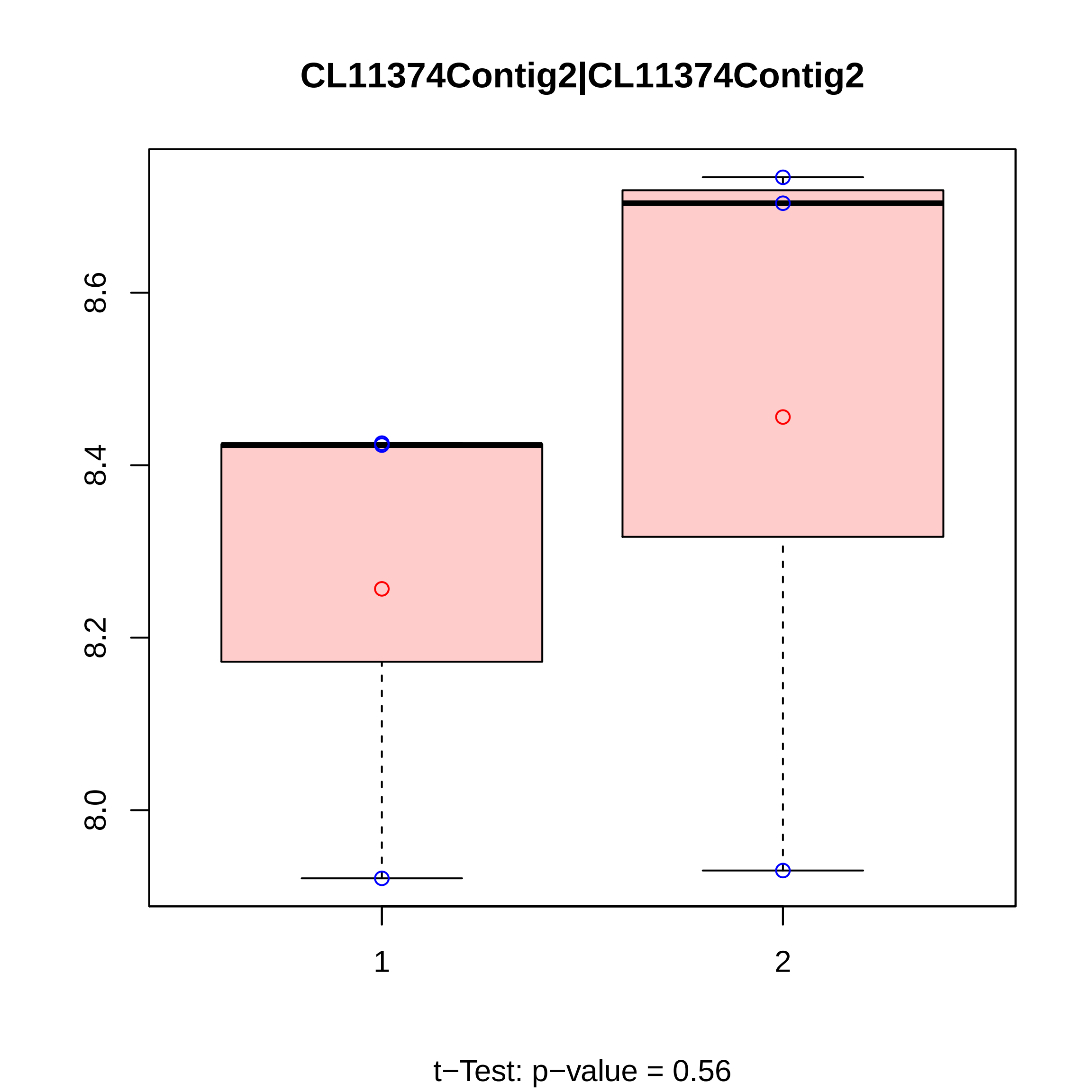
CL11374Contig2|CL11374Contig2
8.0
8.2
8.4
8.6
1
2
t−Test: p−value = 0.56

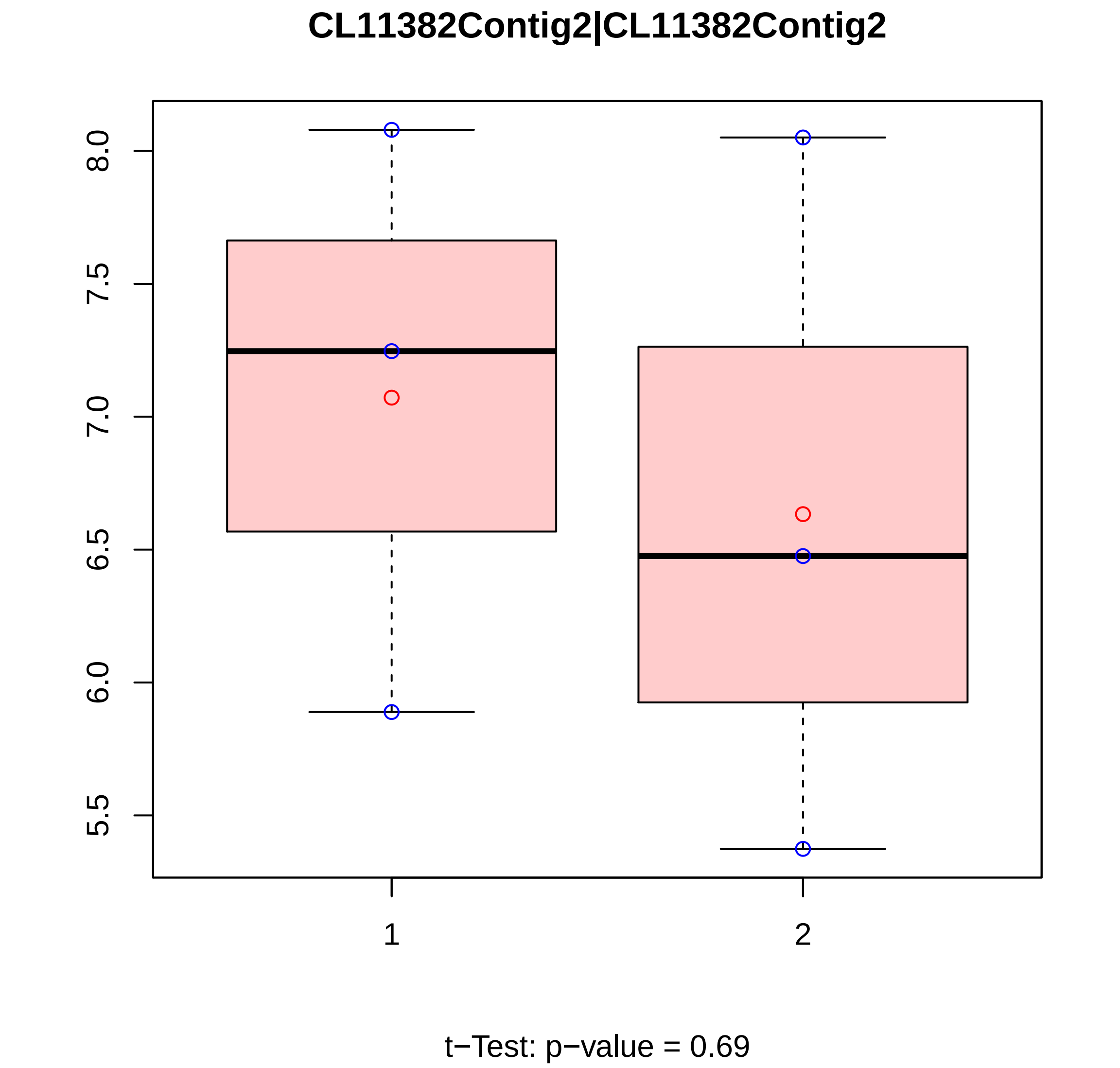
CL11382Contig2|CL11382Contig2
8.0
7.5
7.0
6.5
6.0
5.5
1
2
t−Test: p−value = 0.69

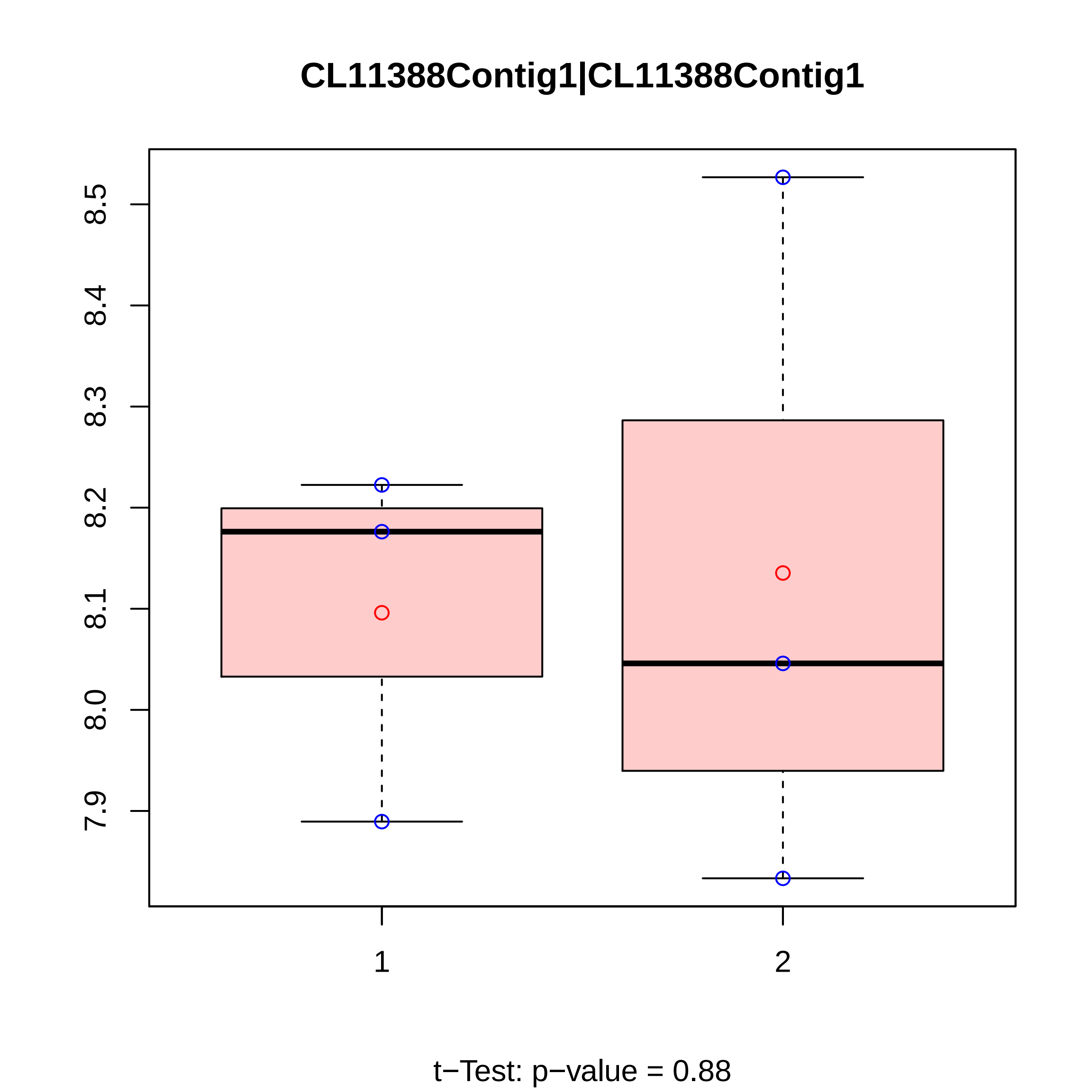
CL11388Contig1|CL11388Contig1
8.5
8.4
8.3
8.2
8.1
8.0
7.9
1
2
t−Test: p−value = 0.88

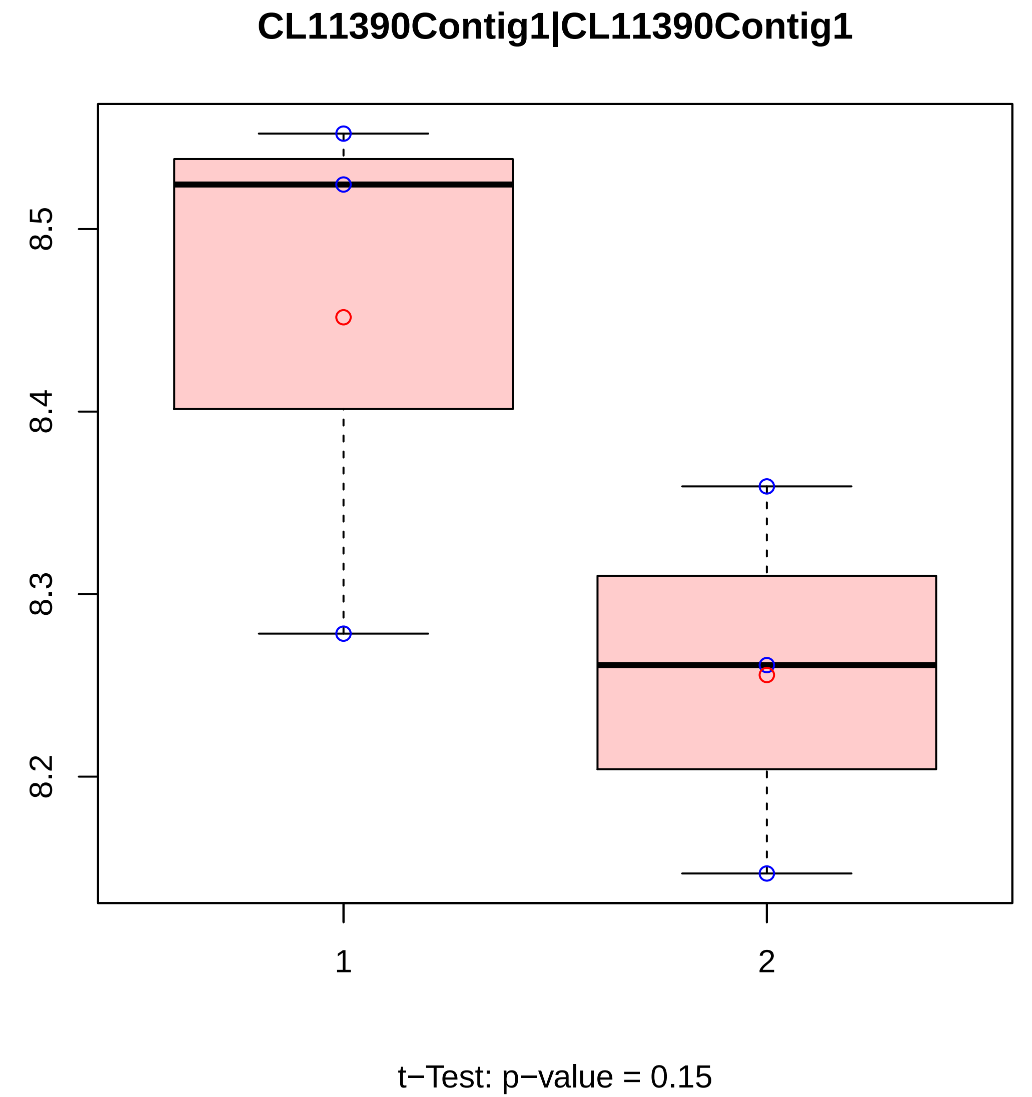
CL11390Contig1|CL11390Contig1
8.5
8.4
8.3
8.2
1
2
t−Test: p−value = 0.15

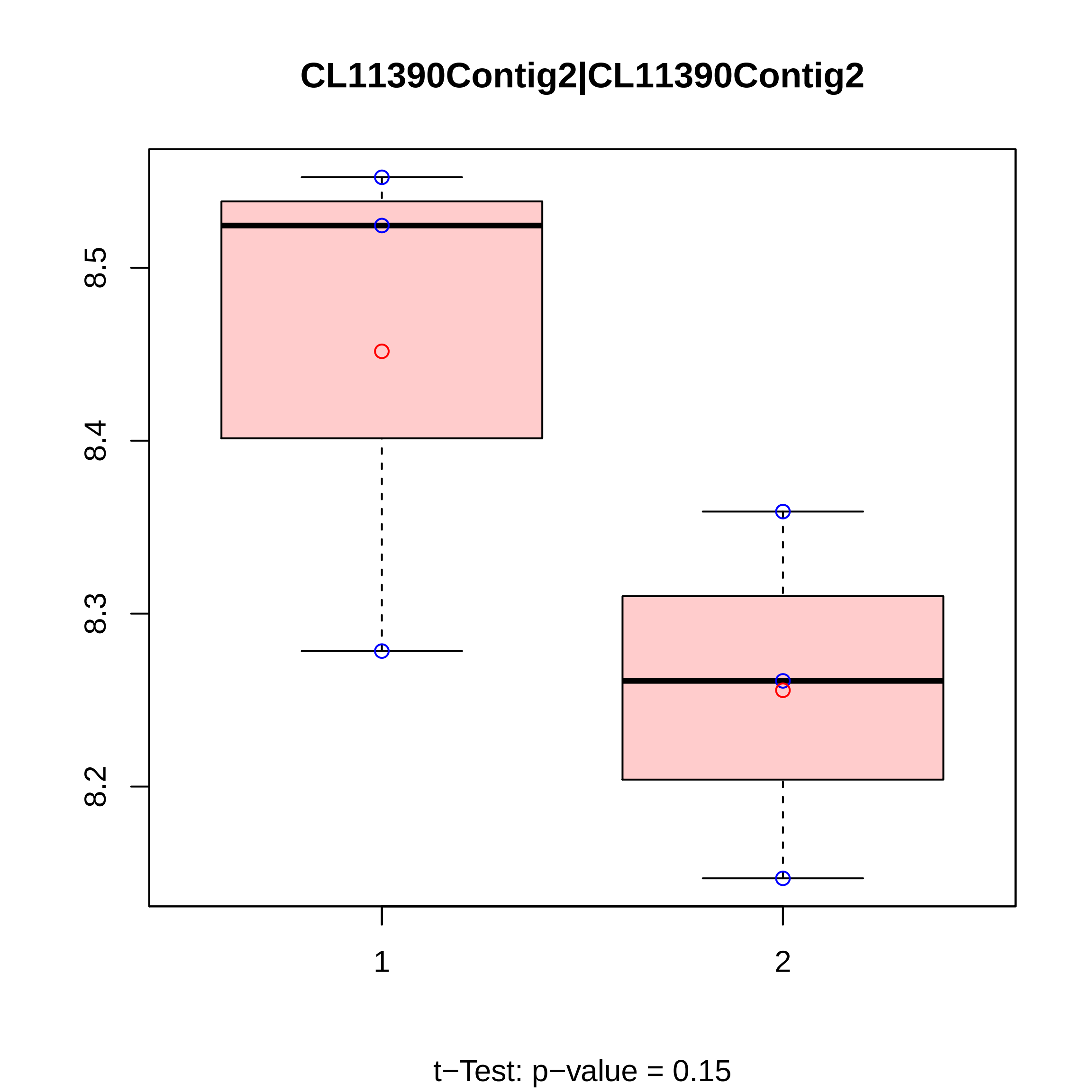
CL11390Contig2|CL11390Contig2
8.2
8.3
8.4
8.5
1
2
t−Test: p−value = 0.15

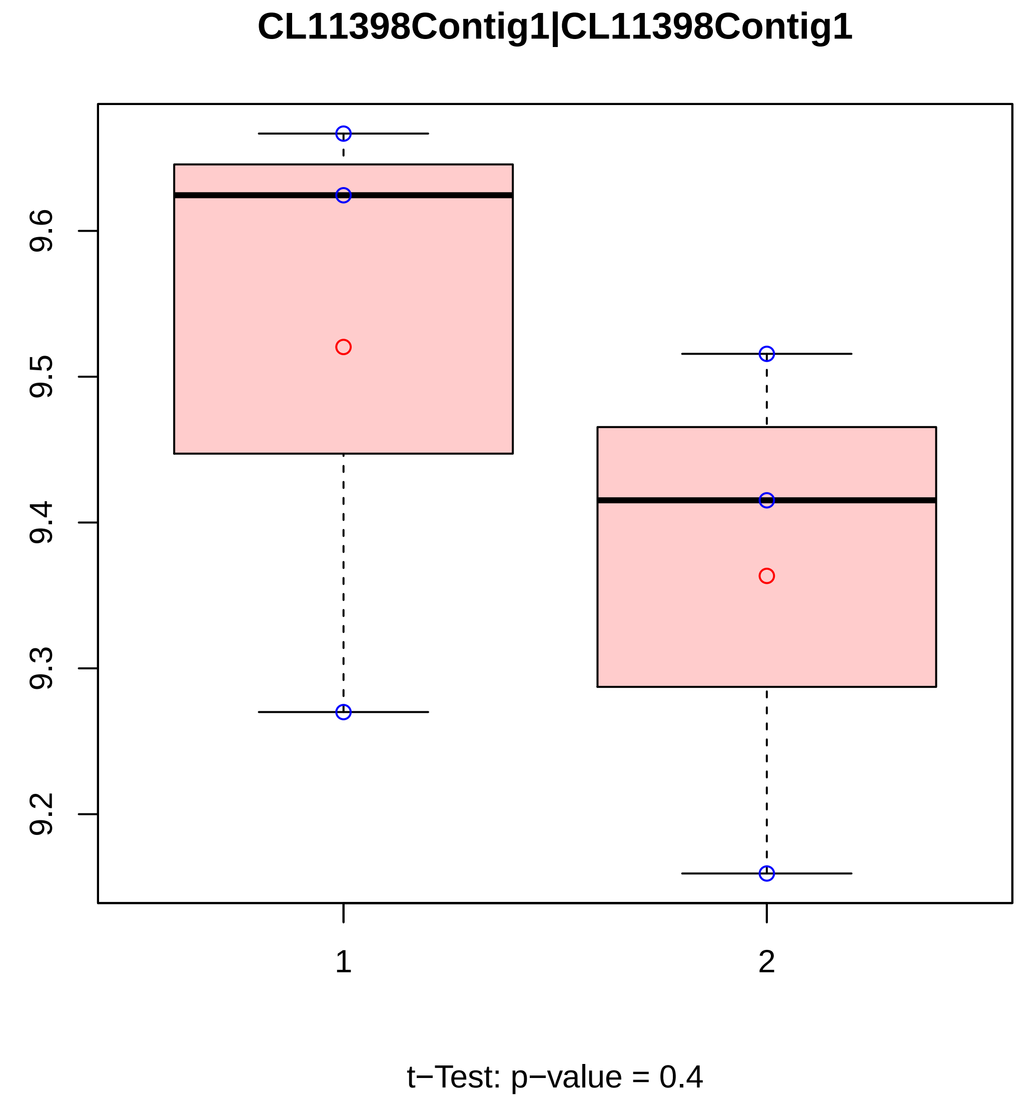

CL11398Contig1|CL11398Contig1
9.2
9.3
9.4
9.5
9.6
1
2
t−Test: p−value = 0.4

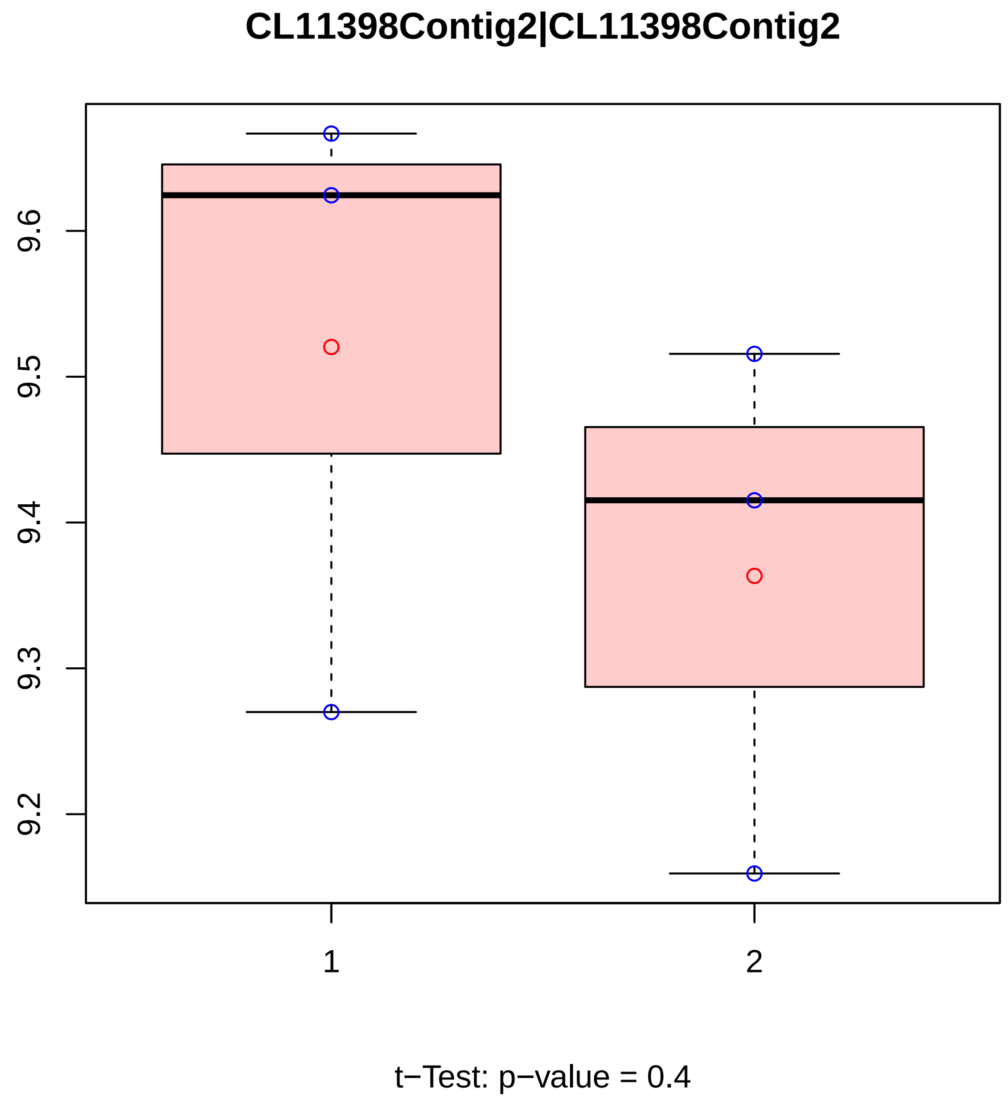

CL11398Contig2|CL11398Contig2
9.2
9.3
9.4
9.5
9.6
1
2
t−Test: p−value = 0.4

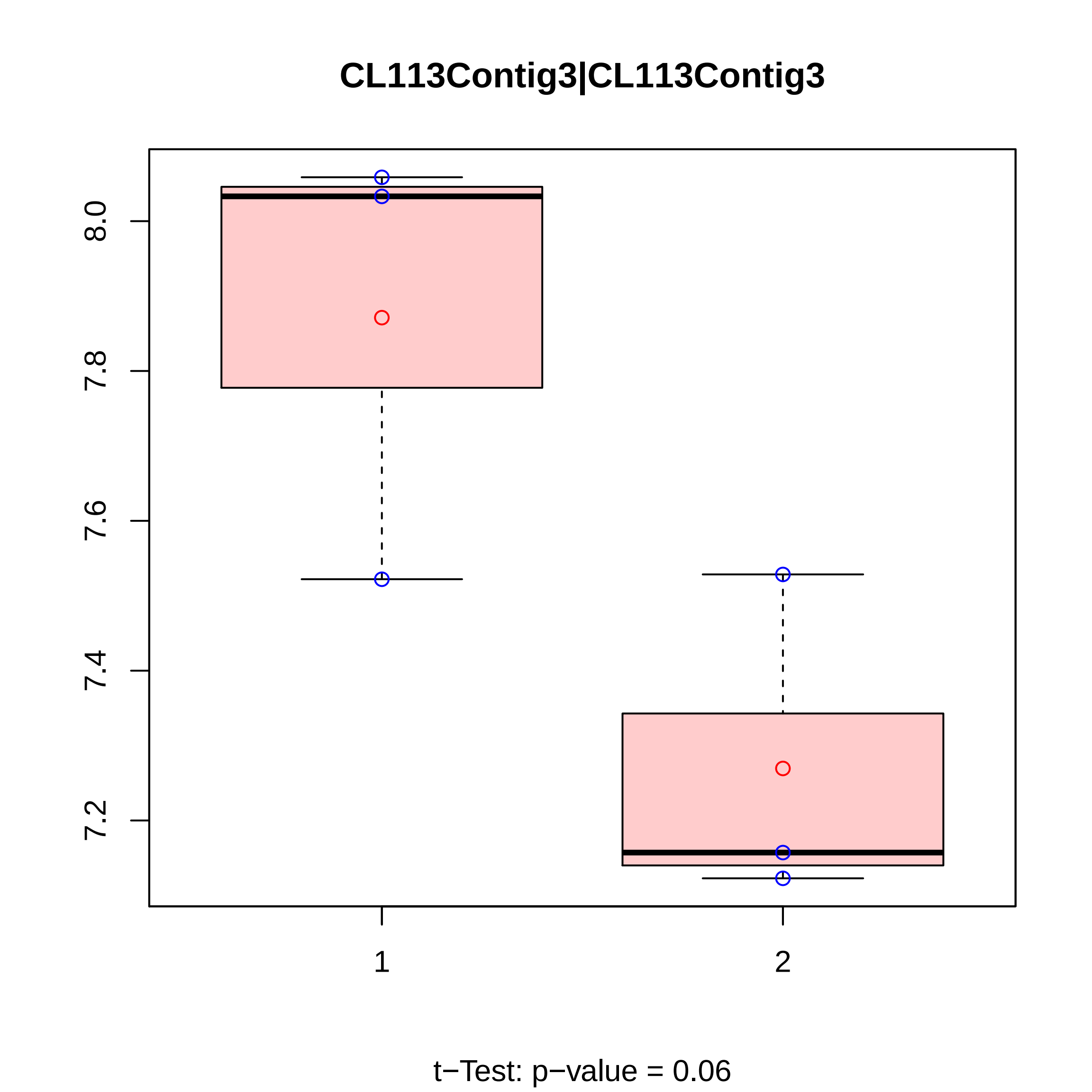
CL113Contig3|CL113Contig3
8.0
7.8
7.6
7.4
7.2
1
2
t−Test: p−value = 0.06

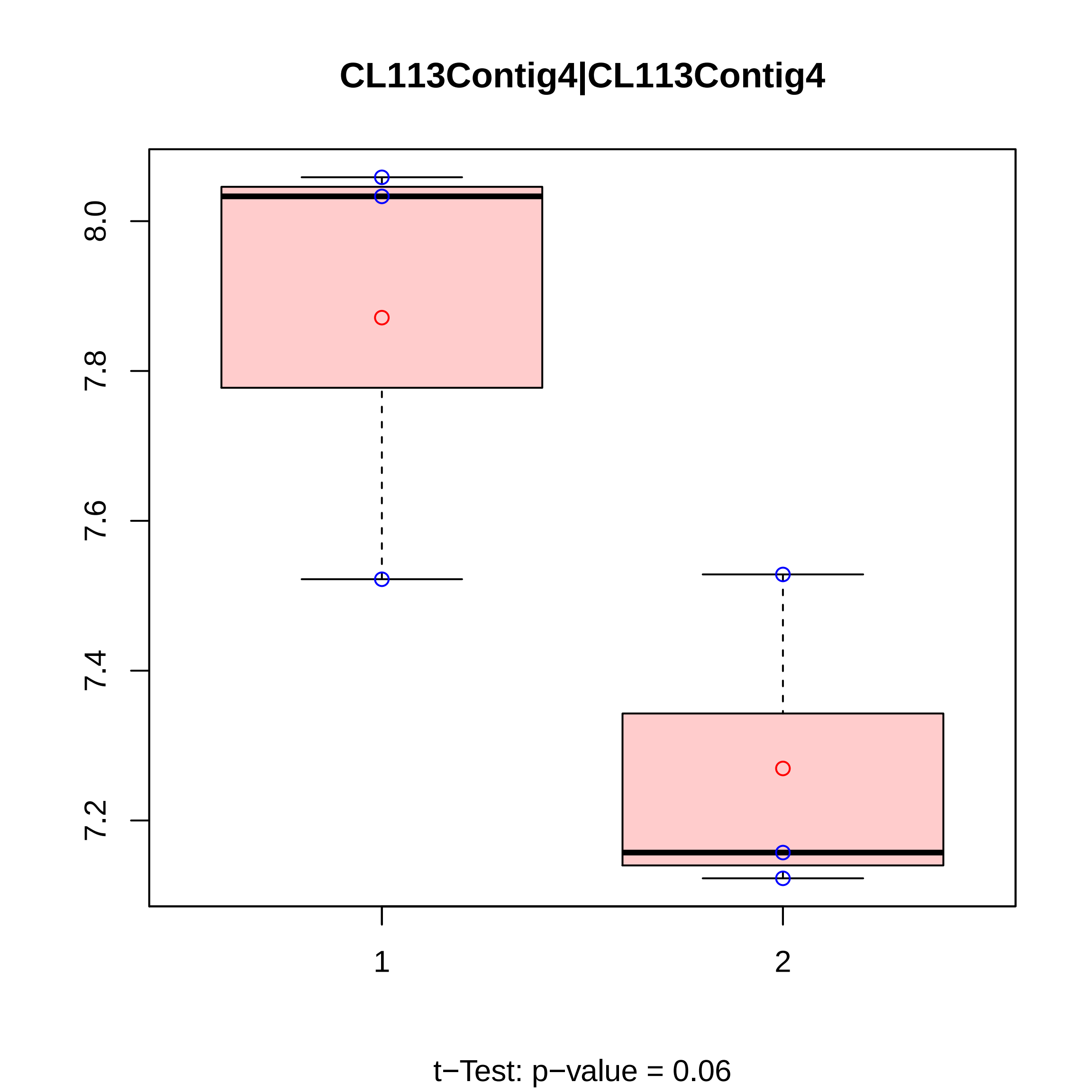
CL113Contig4|CL113Contig4
8.0
7.8
7.6
7.4
7.2
1
2
t−Test: p−value = 0.06

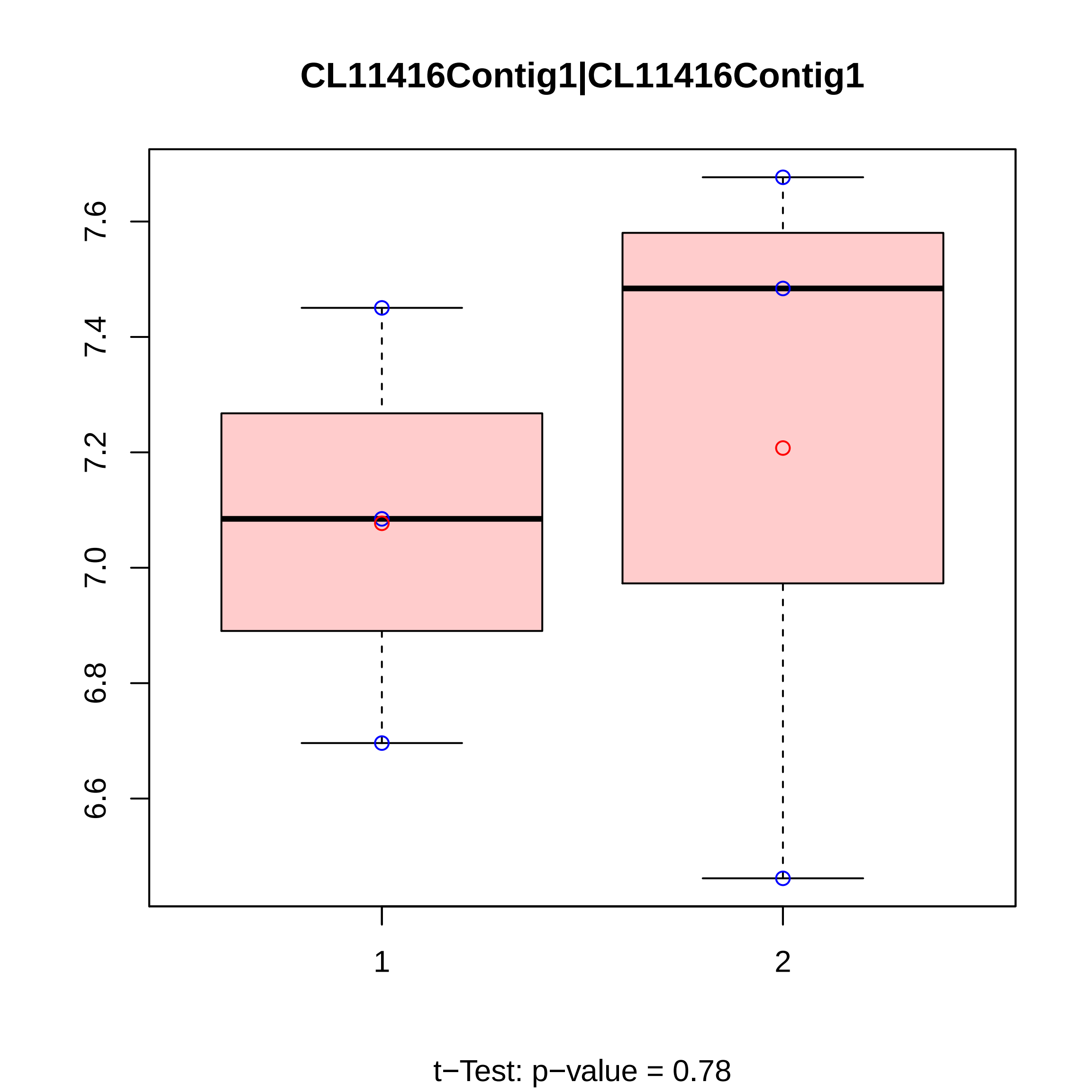
CL11416Contig1|CL11416Contig1
7.6
7.4
7.2
7.0
6.8
6.6
1
2
t−Test: p−value = 0.78

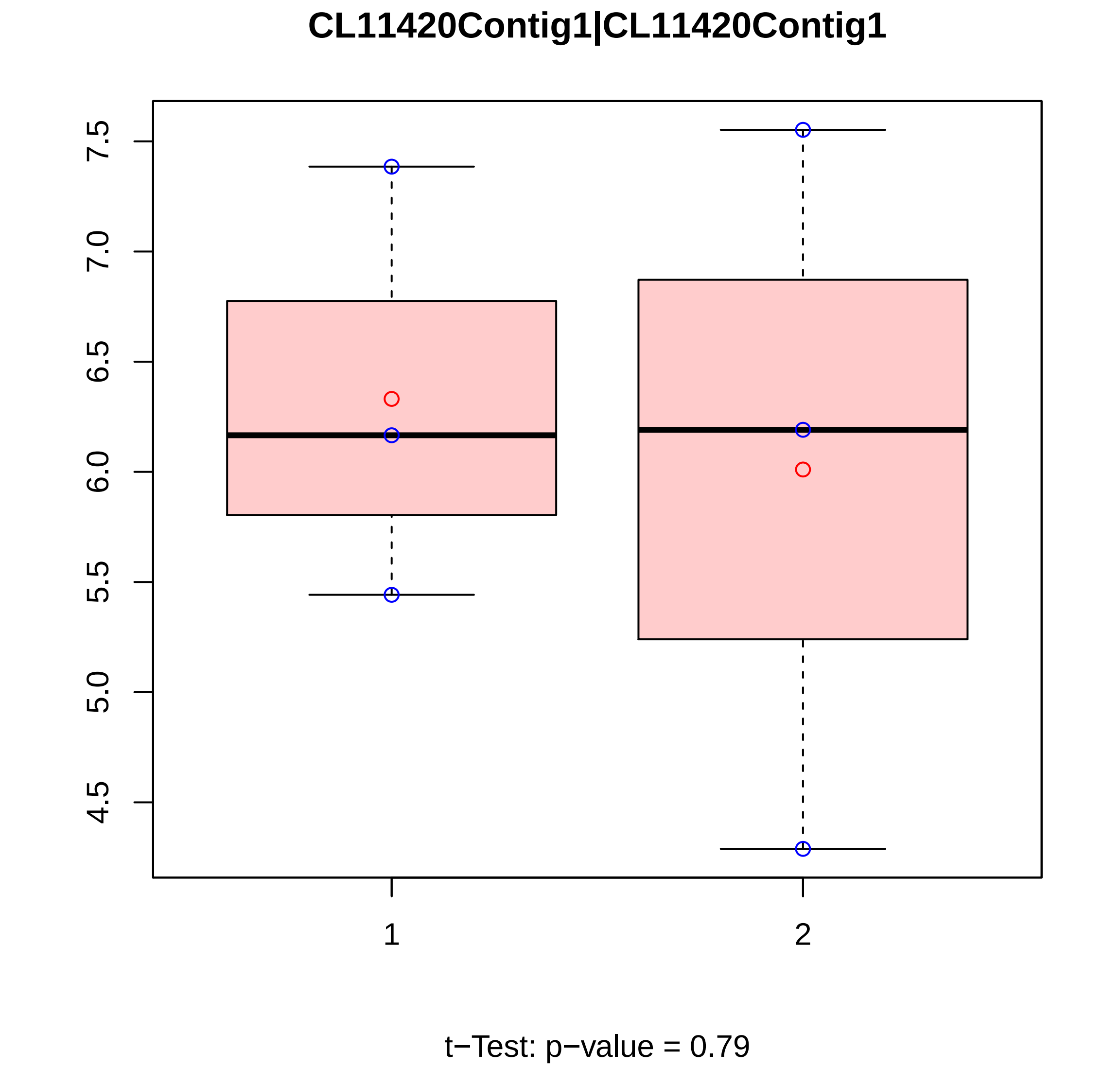
CL11420Contig1|CL11420Contig1
7.5
7.0
6.5
6.0
5.5
5.0
4.5
1
2
t−Test: p−value = 0.79

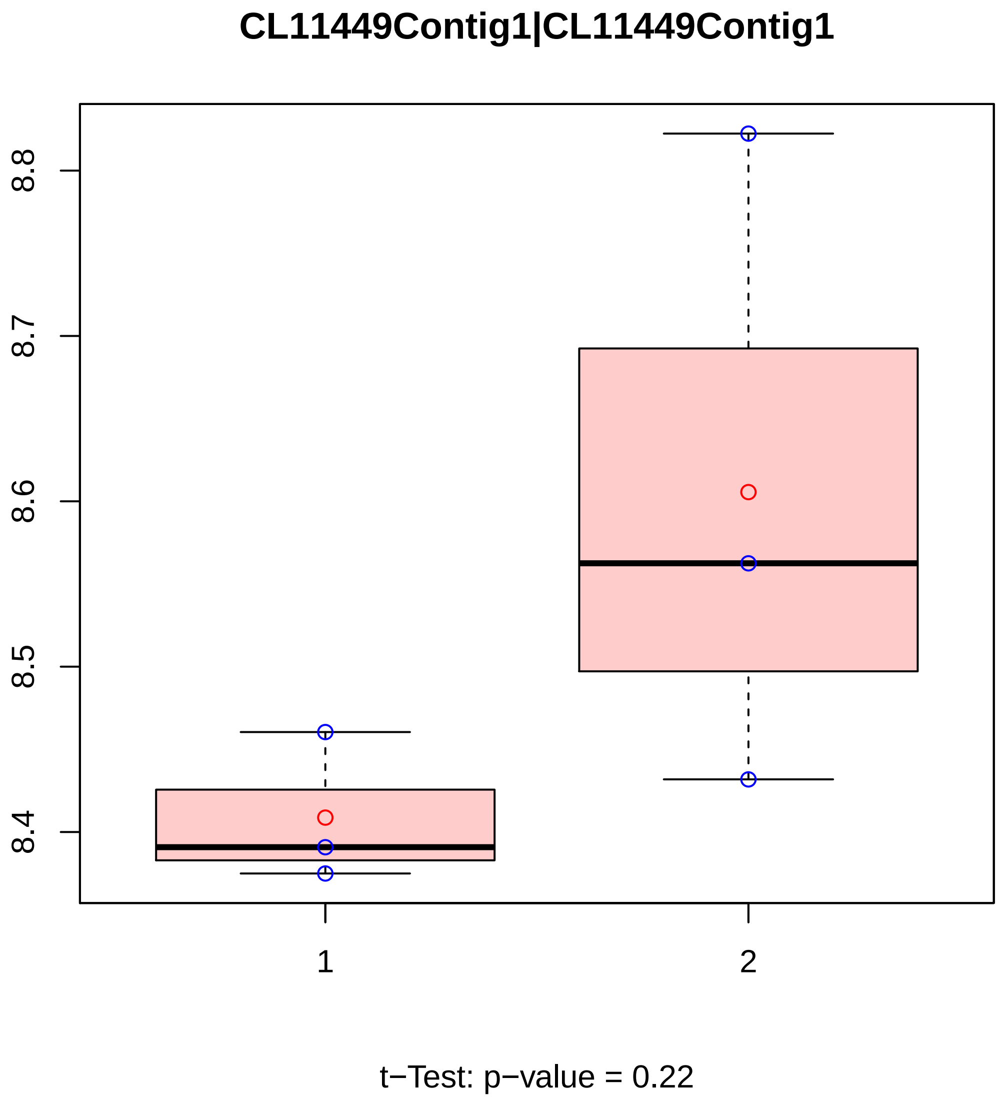
CL11449Contig1|CL11449Contig1
8.4
8.5
8.6
8.7
8.8
1
2
t−Test: p−value = 0.22

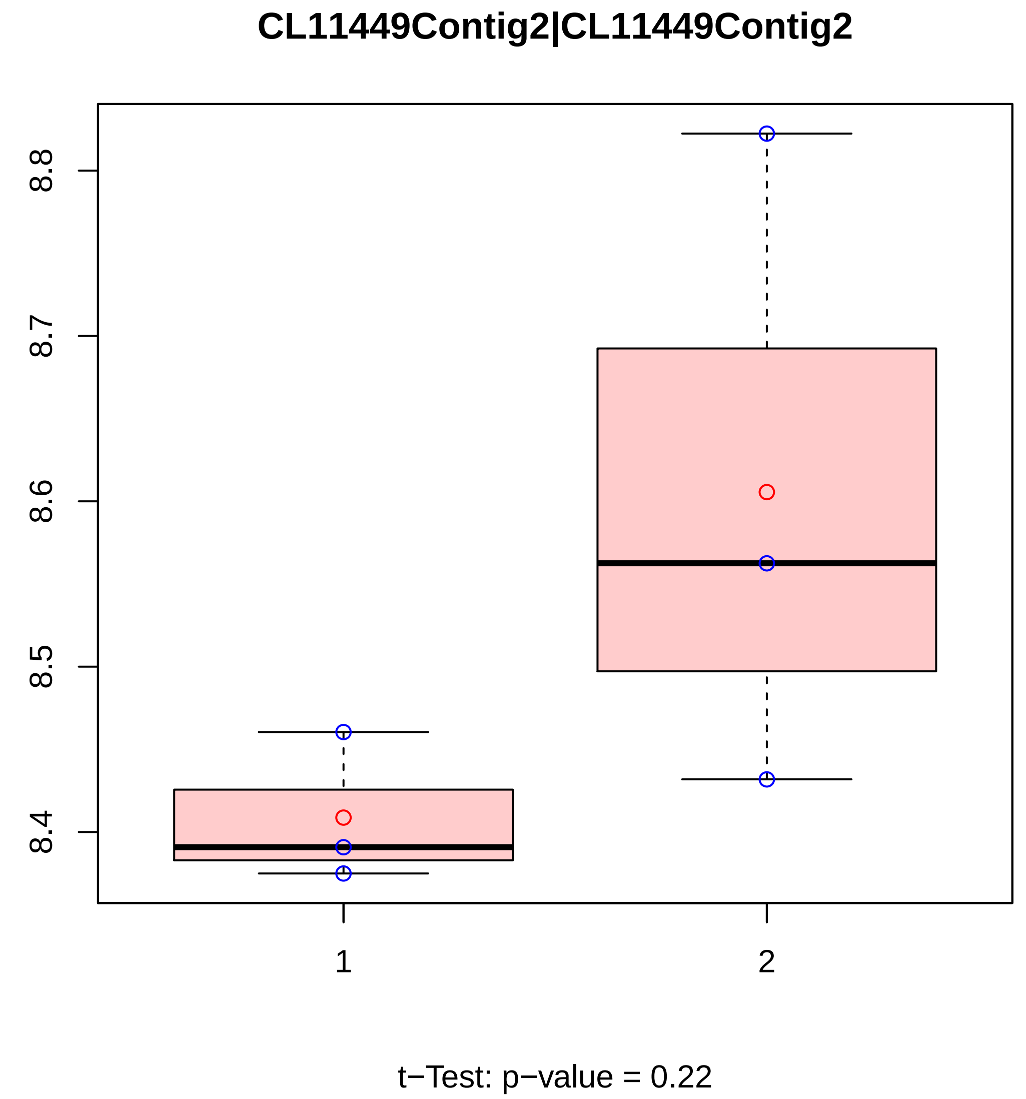
CL11449Contig2|CL11449Contig2
8.4
8.5
8.6
8.7
8.8
1
2
t−Test: p−value = 0.22

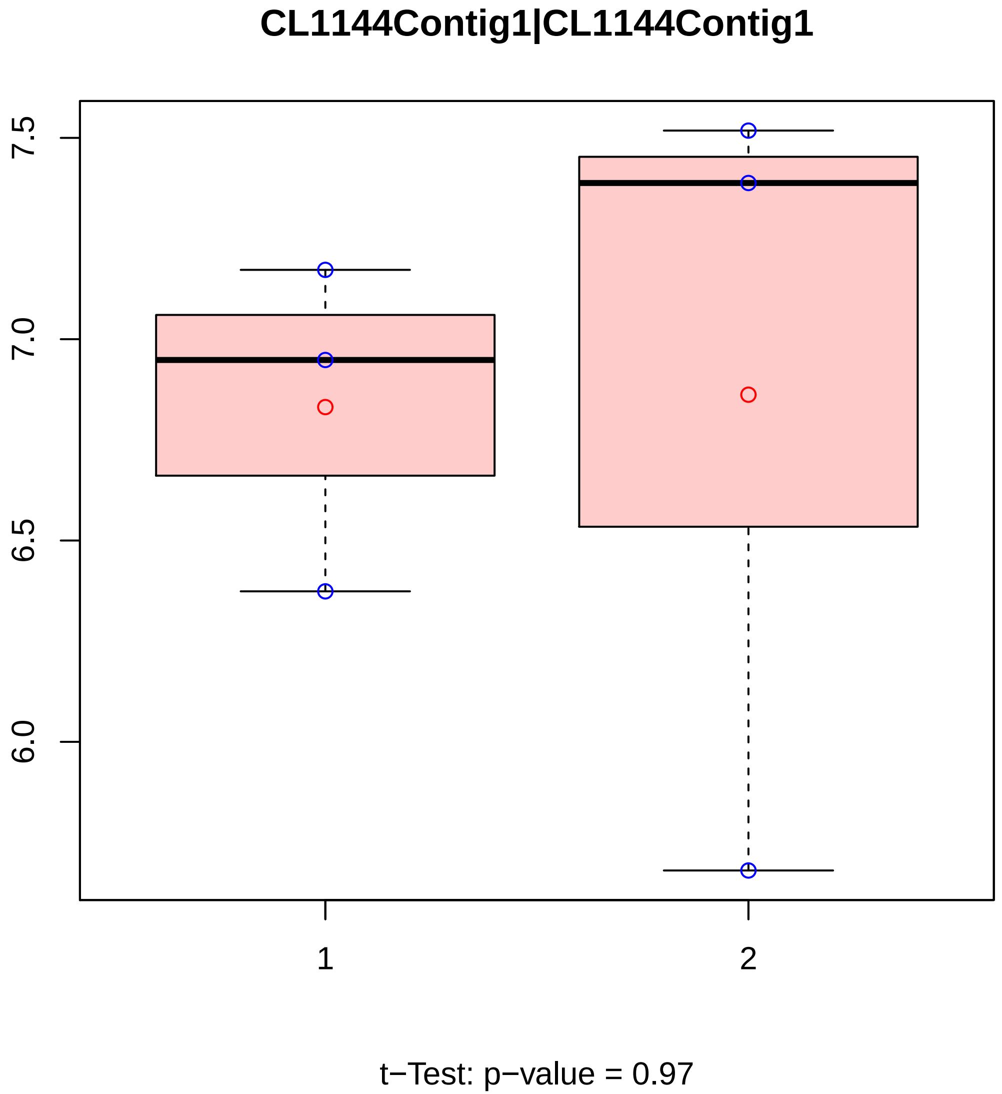
CL1144Contig1|CL1144Contig1
7.5
7.0
6.5
6.0
1
2
t−Test: p−value = 0.97

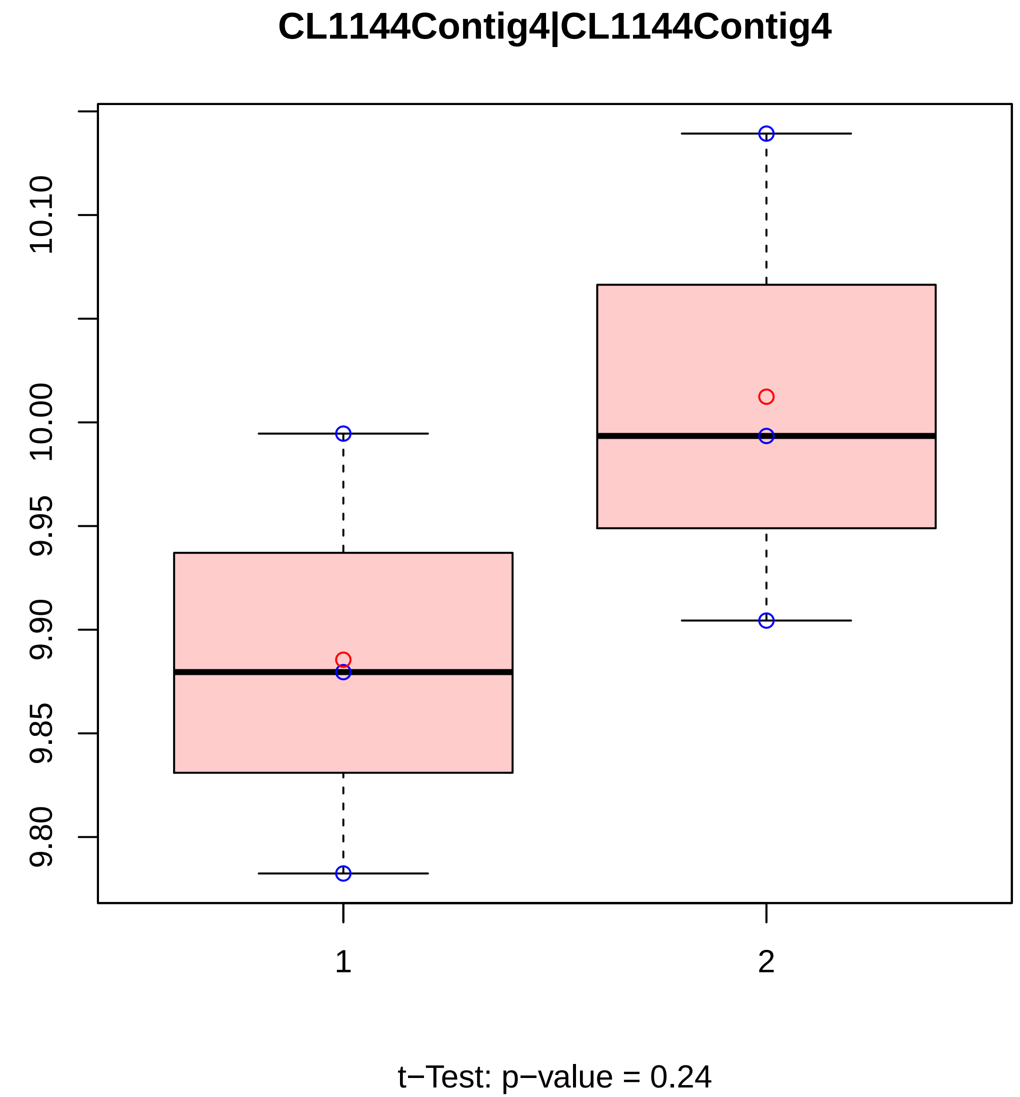
CL1144Contig4|CL1144Contig4
10.10
10.00
9.95
9.90
9.85
9.80
1
2
t−Test: p−value = 0.24

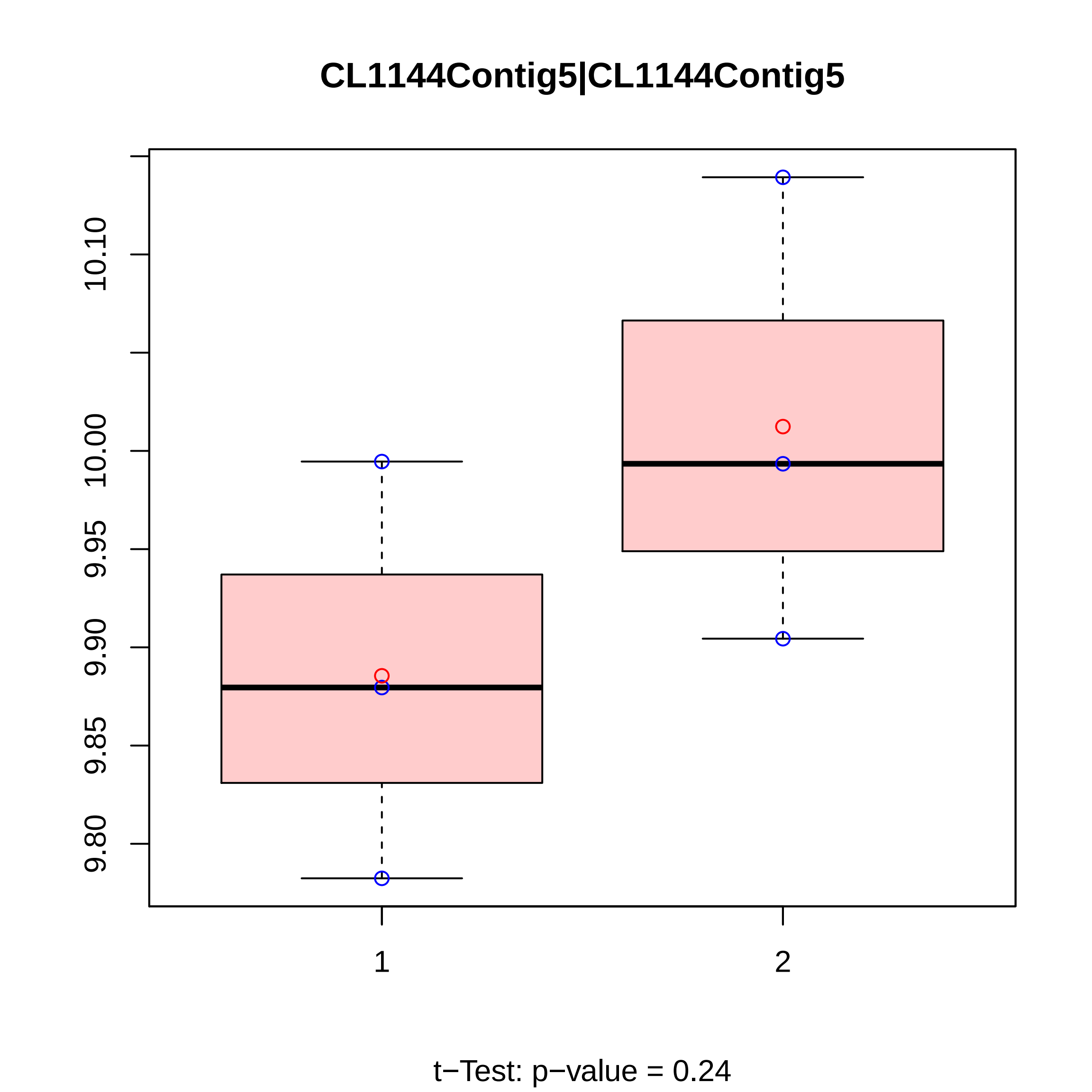

CL1144Contig5|CL1144Contig5
10.10
10.00
9.95
9.90
9.85
9.80
1
2
t−Test: p−value = 0.24

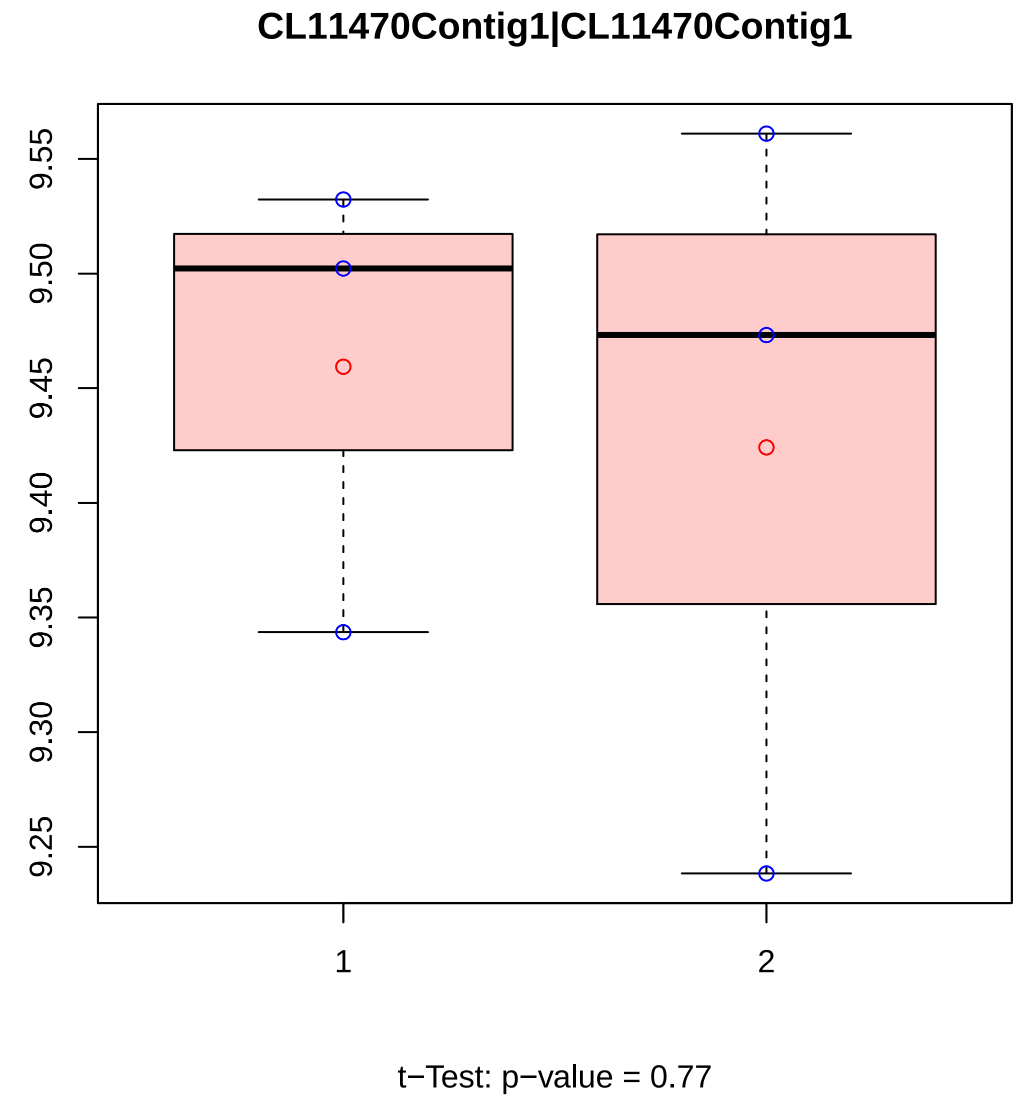

CL11470Contig1|CL11470Contig1
9.25
9.30
9.35
9.40
9.45
9.50
9.55
1
2
t−Test: p−value = 0.77

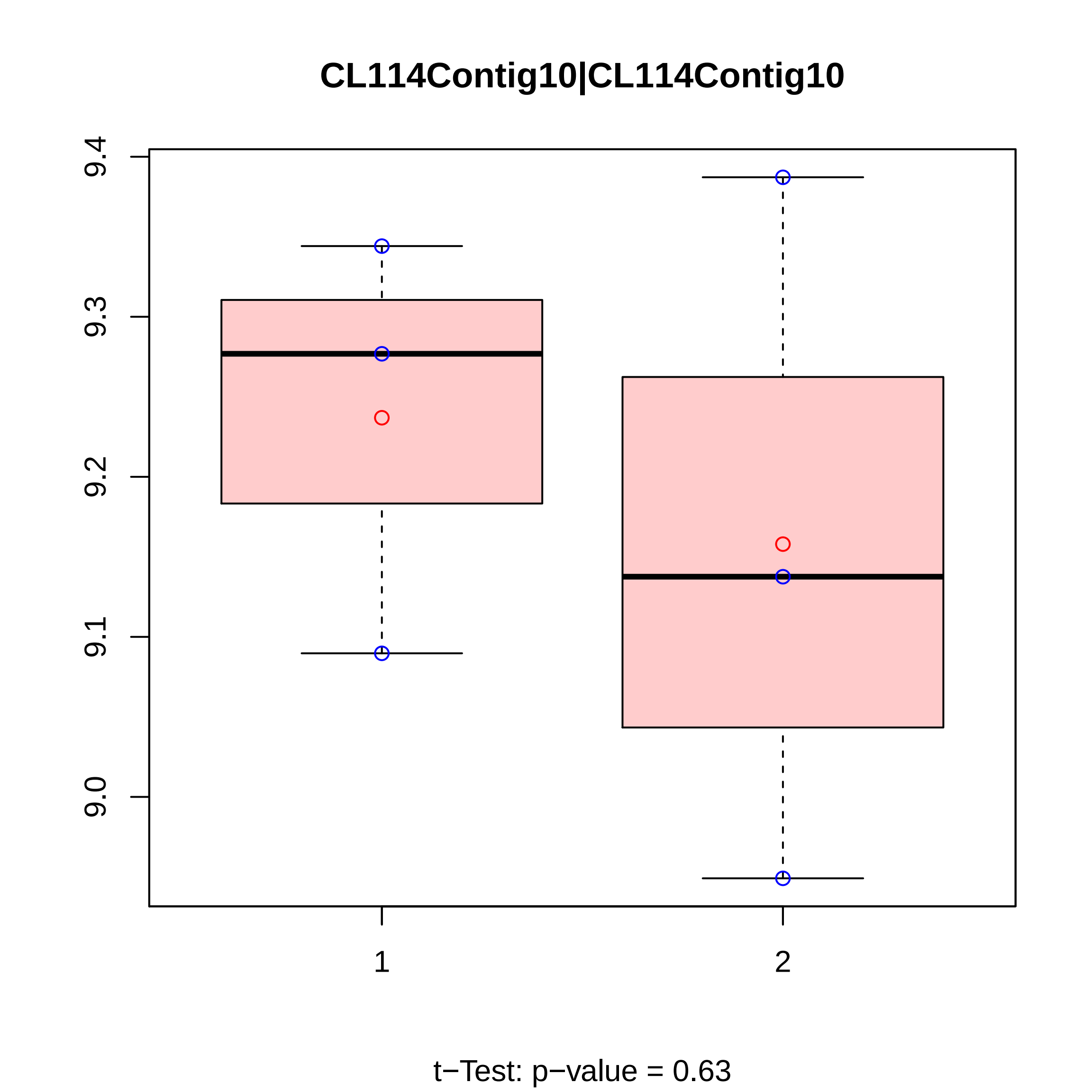
CL114Contig10|CL114Contig10
9.0
9.1
9.2
9.3
9.4
1
2
t−Test: p−value = 0.63

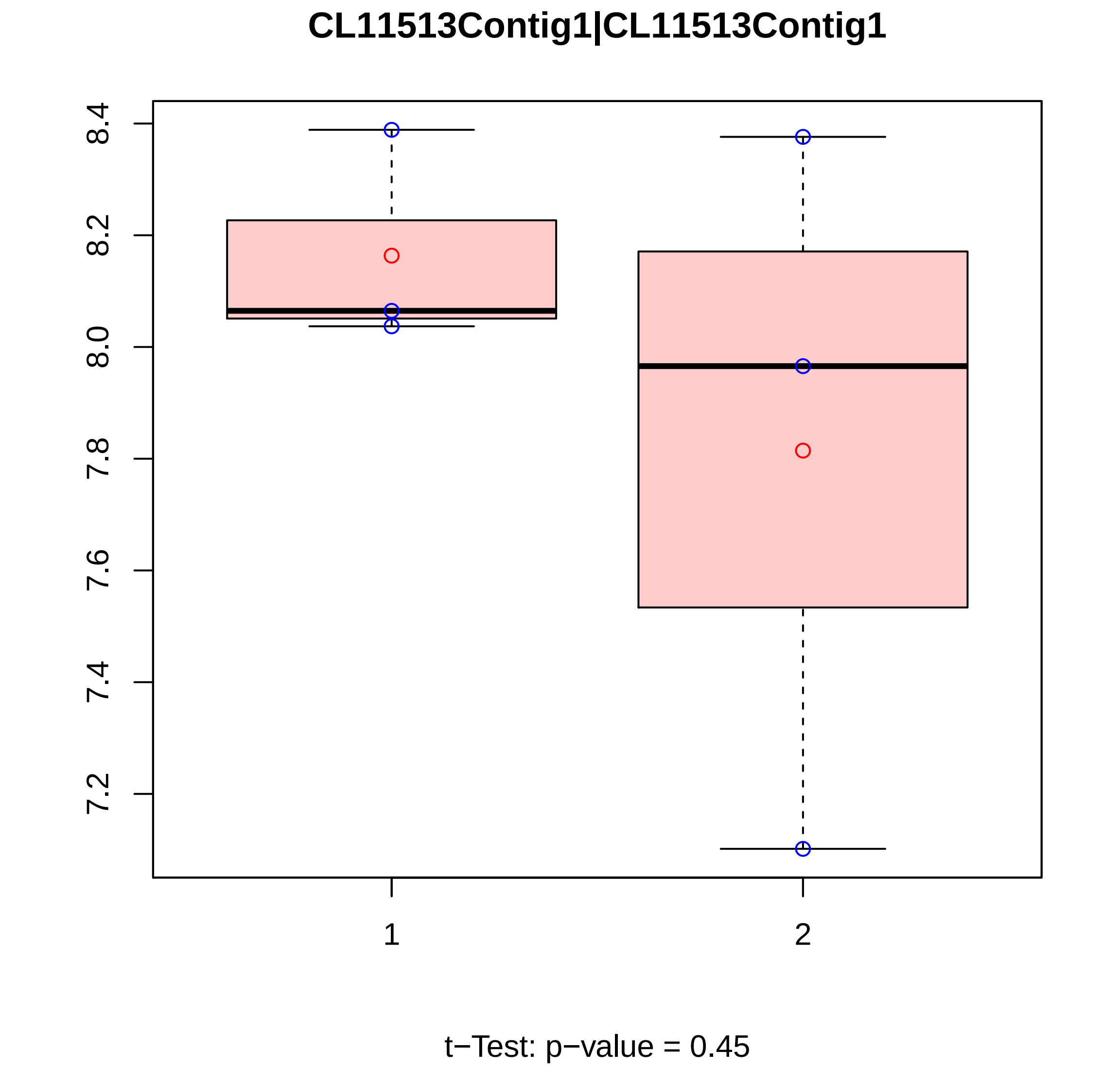
CL11513Contig1|CL11513Contig1
8.4
8.2
8.0
7.8
7.6
7.4
7.2
1
2
t−Test: p−value = 0.45

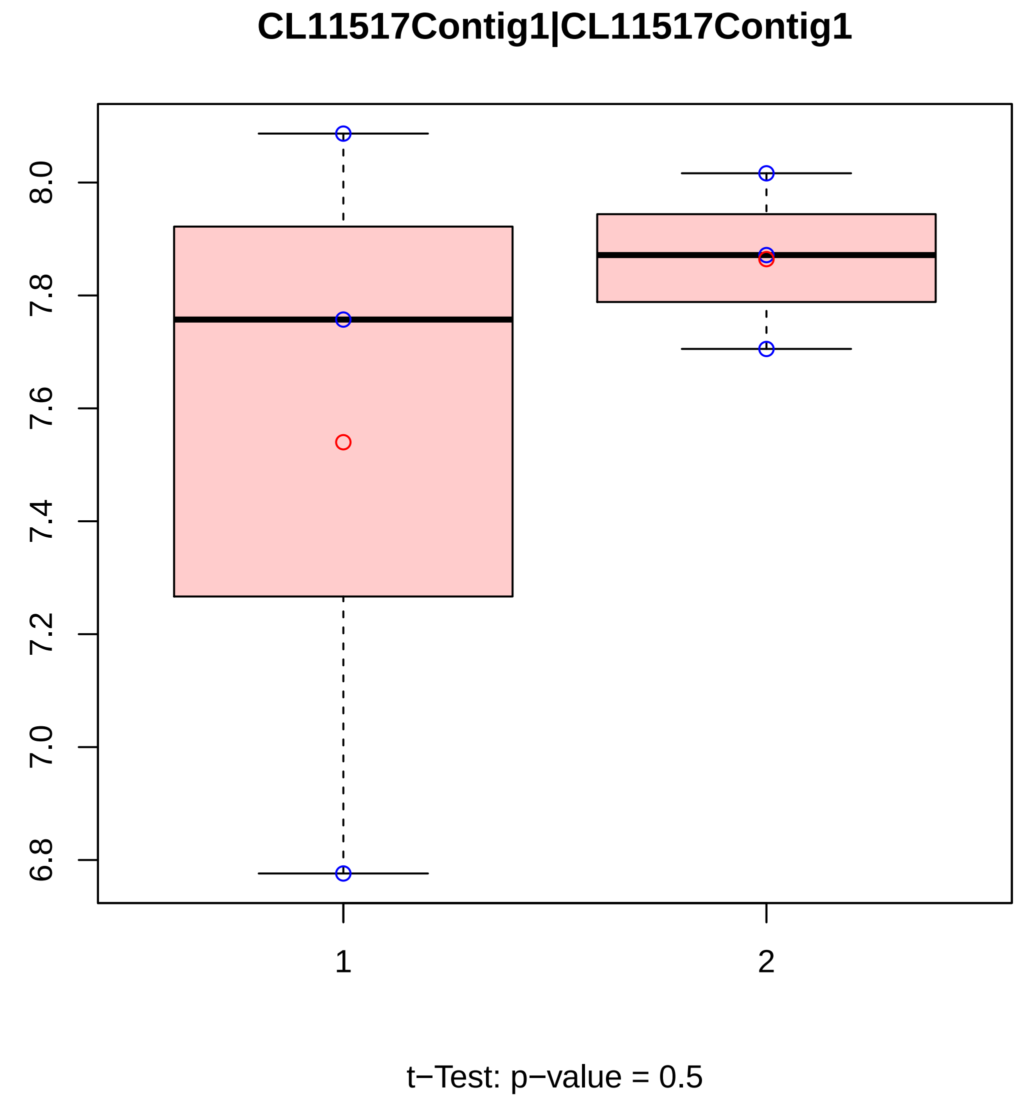

CL11517Contig1|CL11517Contig1
8.0
7.8
7.6
7.4
7.2
7.0
6.8
1
2
t−Test: p−value = 0.5

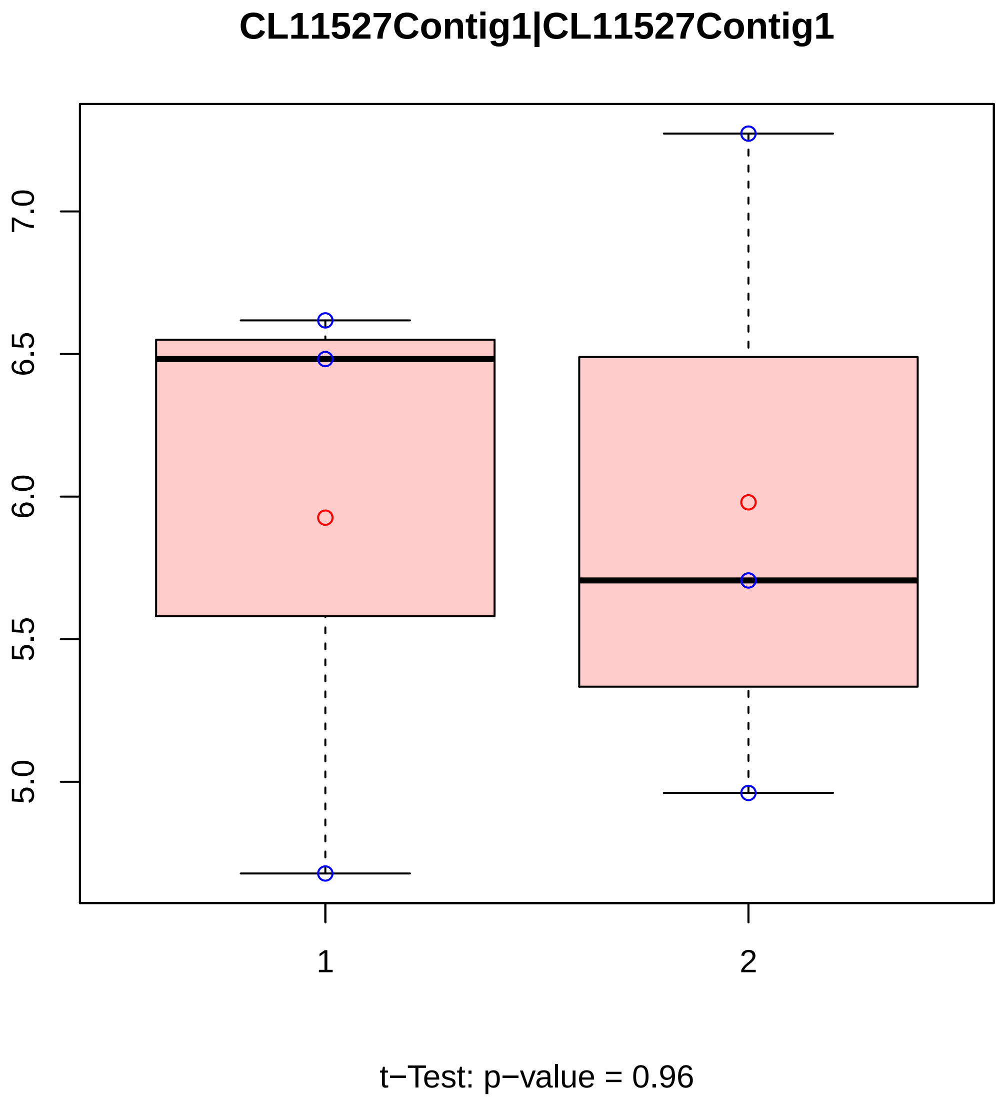
CL11527Contig1|CL11527Contig1
7.0
6.5
6.0
5.5
5.0
1
2
t−Test: p−value = 0.96

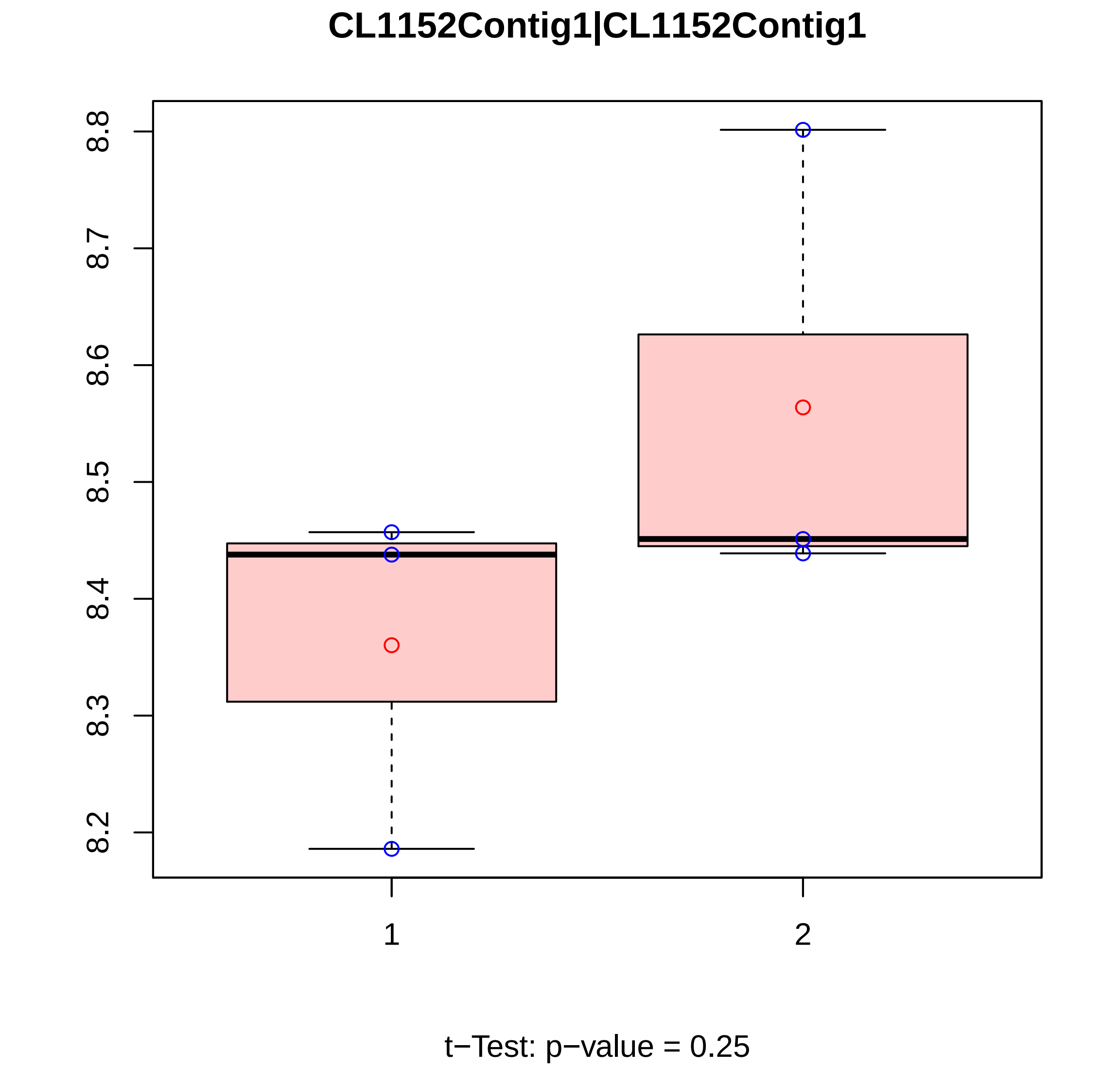
CL1152Contig1|CL1152Contig1
8.2
8.3
8.4
8.5
8.6
8.7
8.8
1
2
t−Test: p−value = 0.25

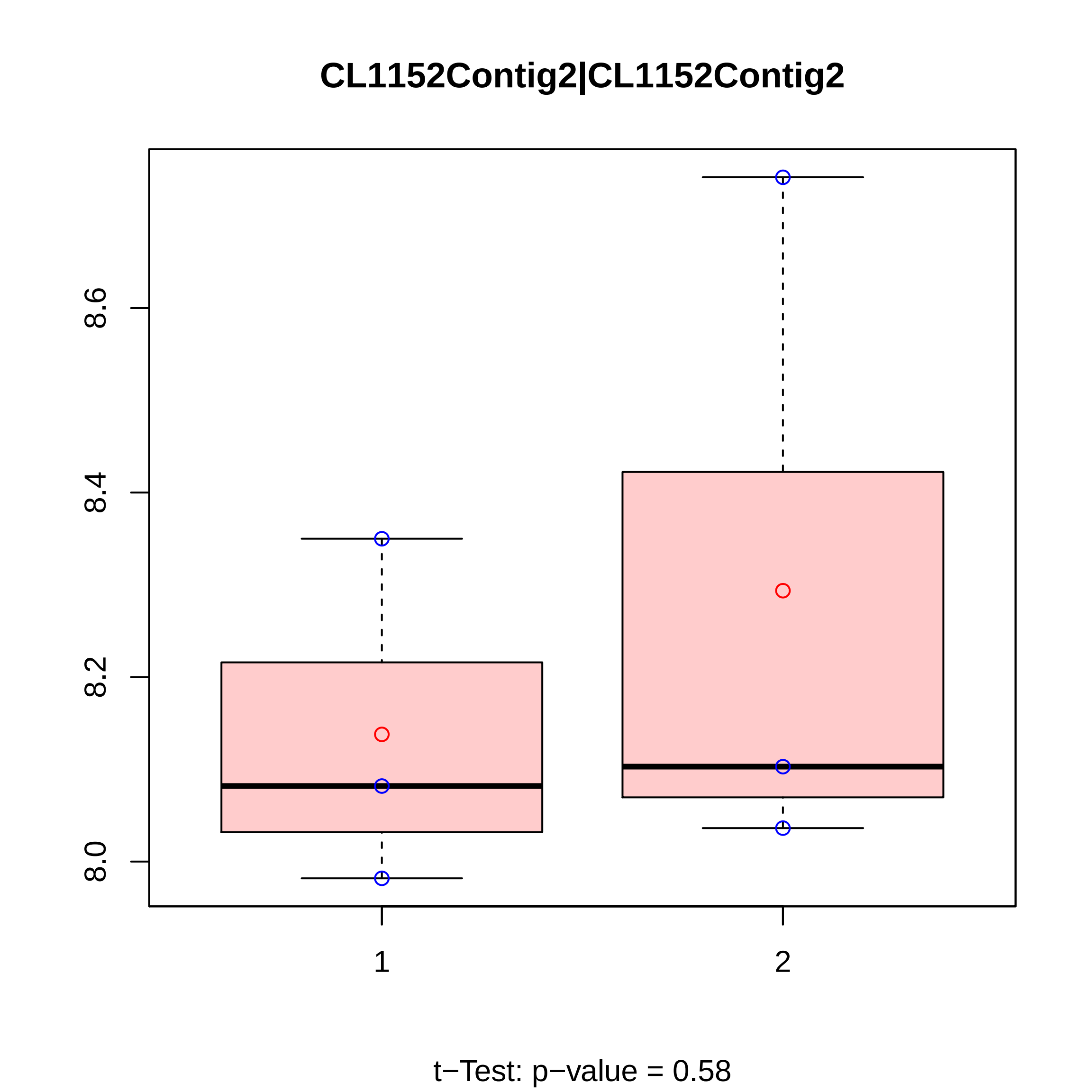

CL1152Contig2|CL1152Contig2
8.0
8.2
8.4
8.6
1
2
t−Test: p−value = 0.58

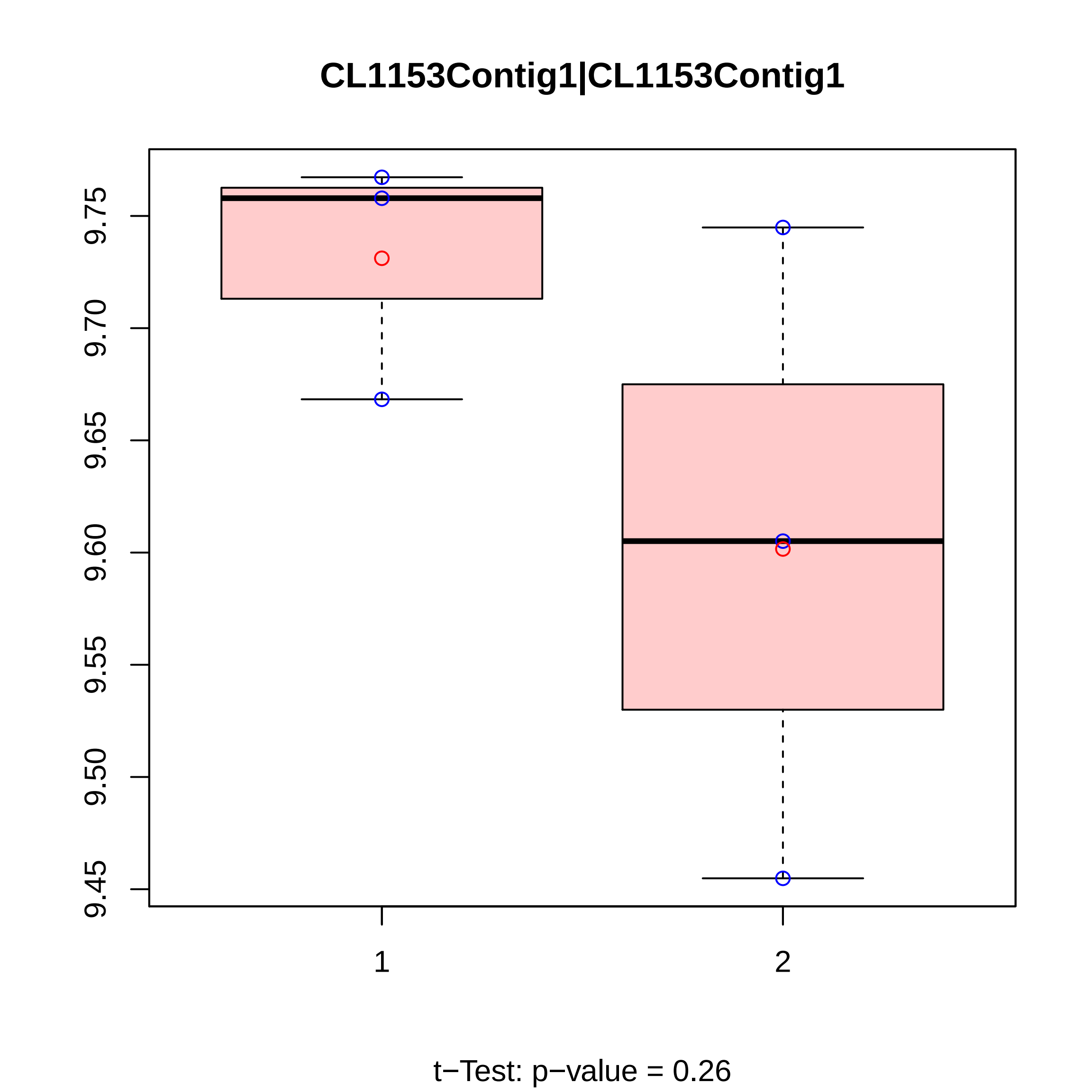
CL1153Contig1|CL1153Contig1
9.45
9.50
9.55
9.60
9.65
9.70
9.75
1
2
t−Test: p−value = 0.26

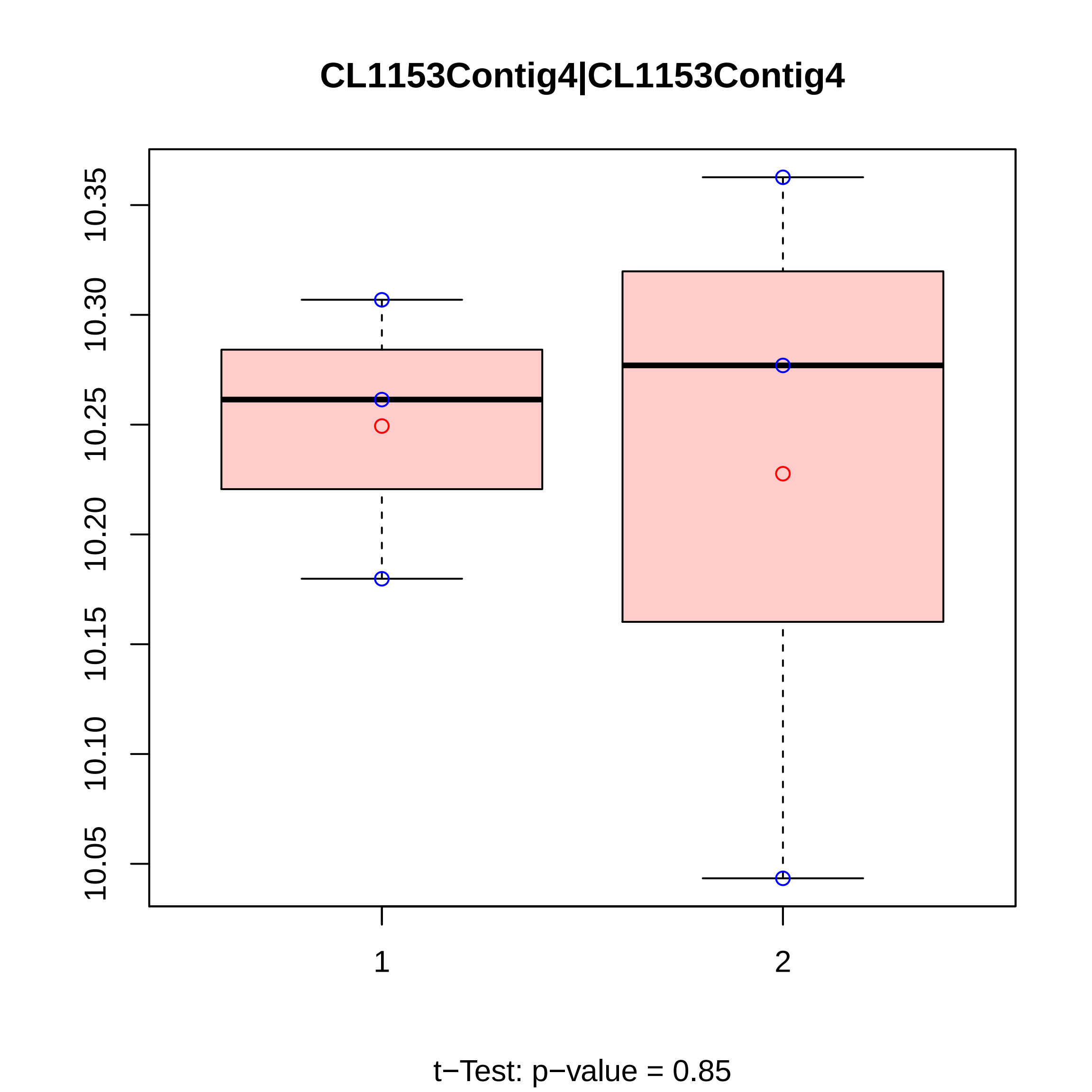
CL1153Contig4|CL1153Contig4
10.05
10.10
10.15
10.20
10.25
10.30
10.35
1
2
t−Test: p−value = 0.85

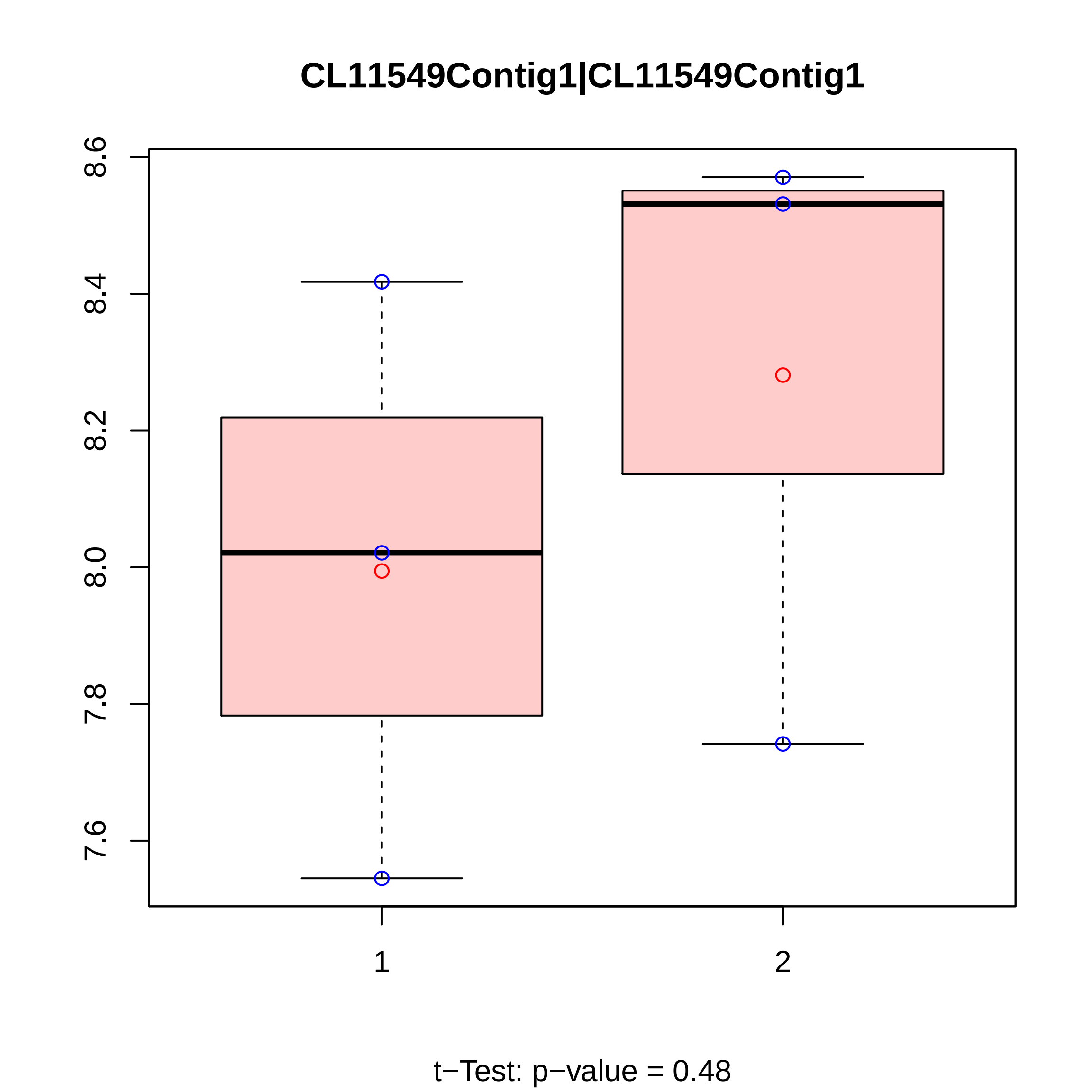

CL11549Contig1|CL11549Contig1
8.6
8.4
8.2
8.0
7.8
7.6
1
2
t−Test: p−value = 0.48

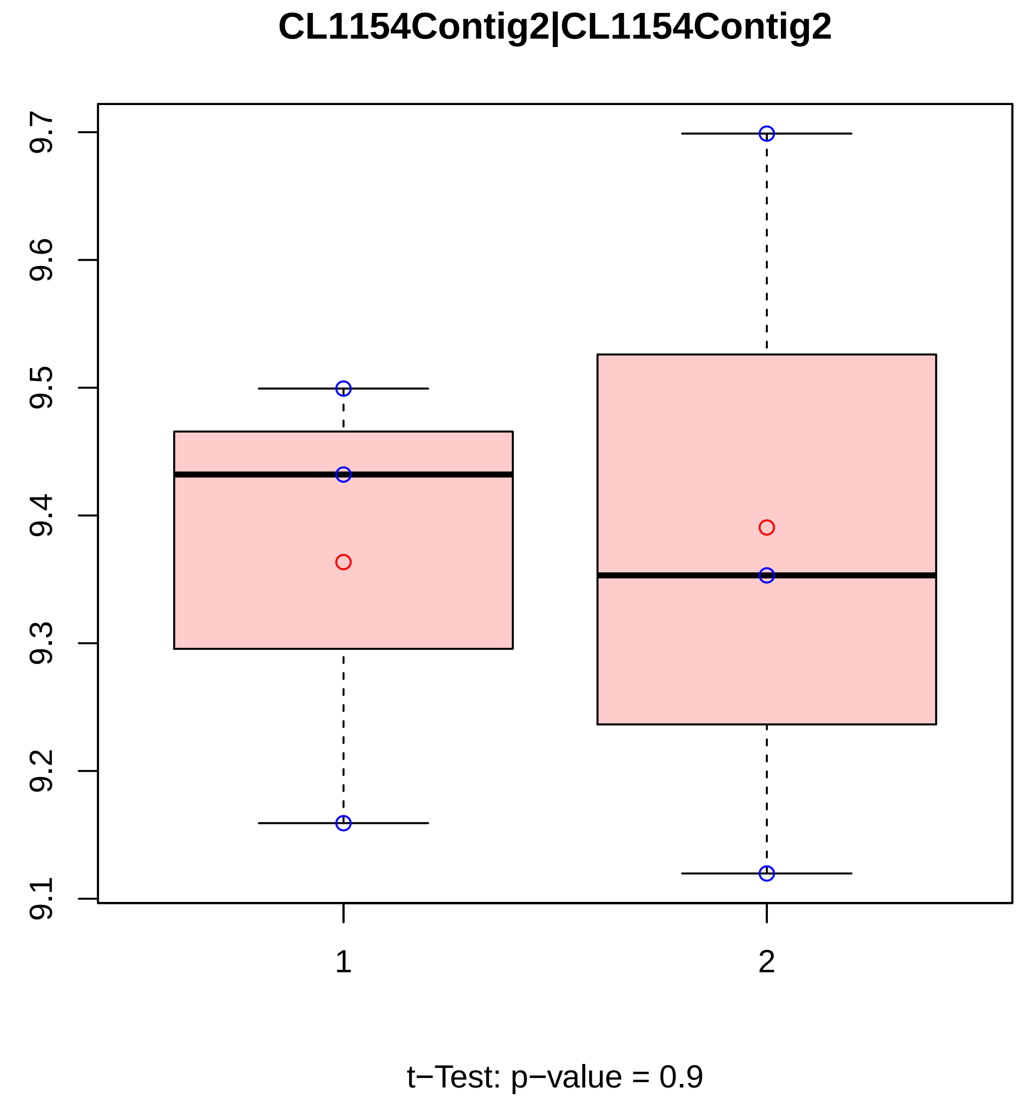
CL1154Contig2|CL1154Contig2
9.1
9.2
9.3
9.4
9.5
9.6
9.7
1
2
t−Test: p−value = 0.9

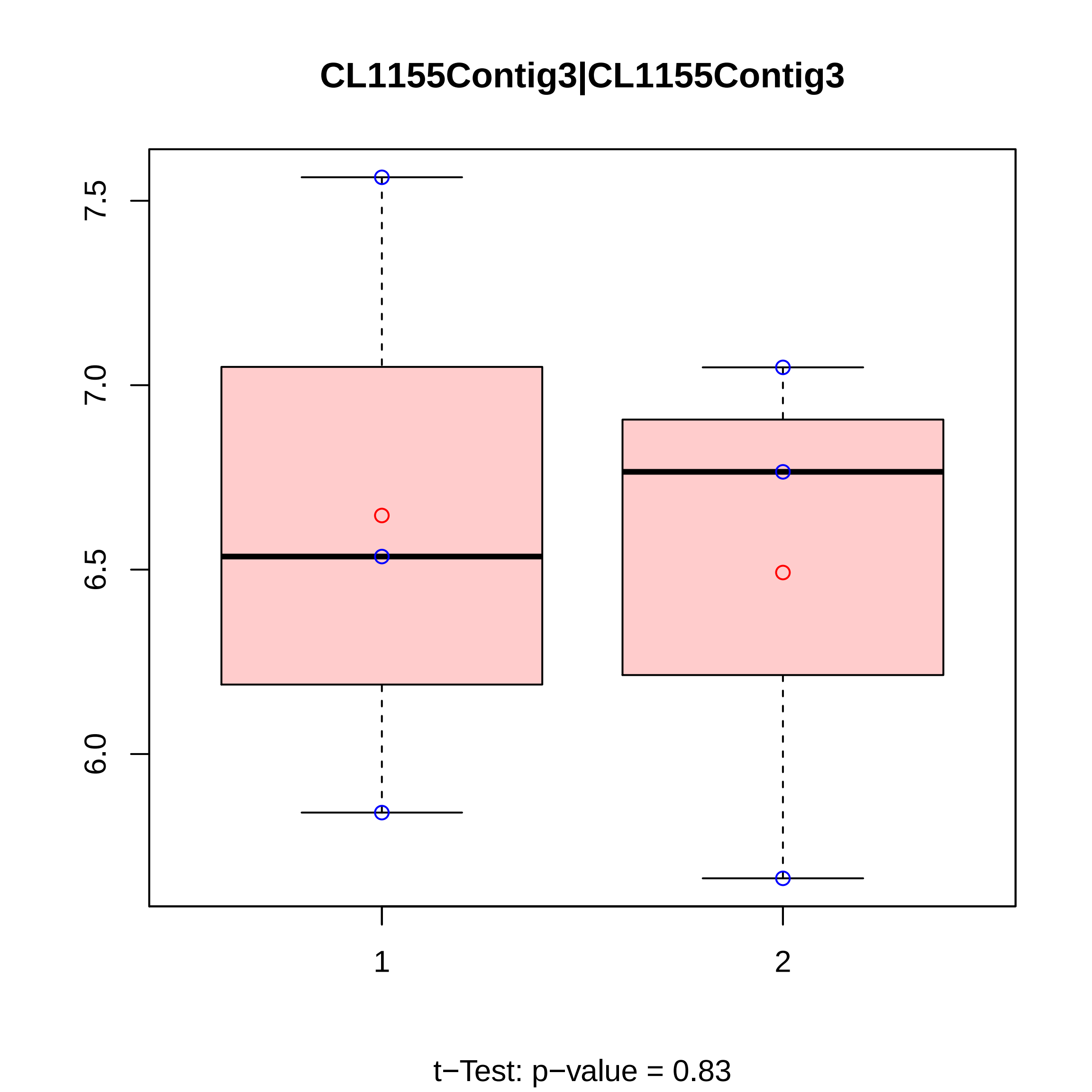
CL1155Contig3|CL1155Contig3
7.5
7.0
6.5
6.0
1
2
t−Test: p−value = 0.83

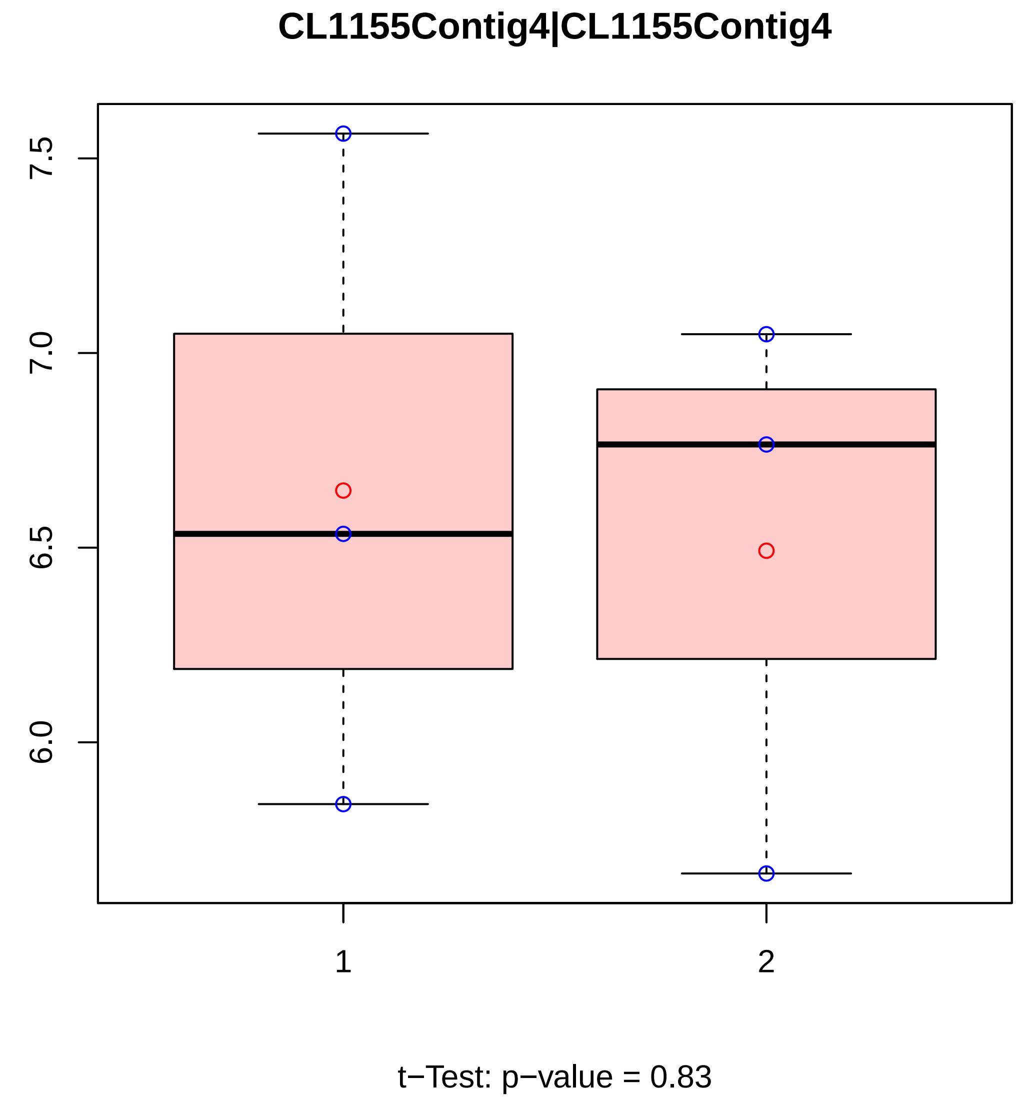
CL1155Contig4|CL1155Contig4
7.5
7.0
6.5
6.0
1
2
t−Test: p−value = 0.83

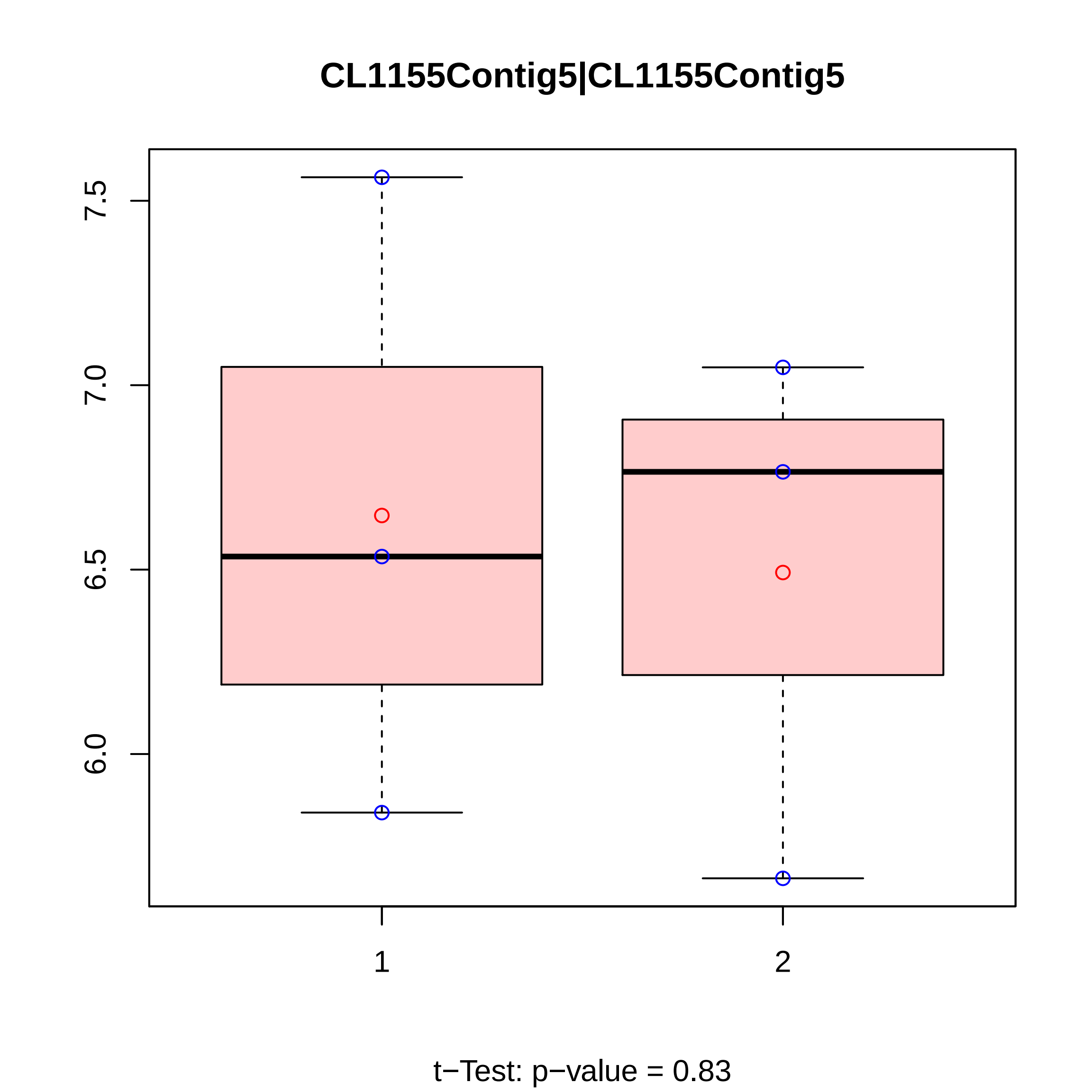
CL1155Contig5|CL1155Contig5
7.5
7.0
6.5
6.0
1
2
t−Test: p−value = 0.83

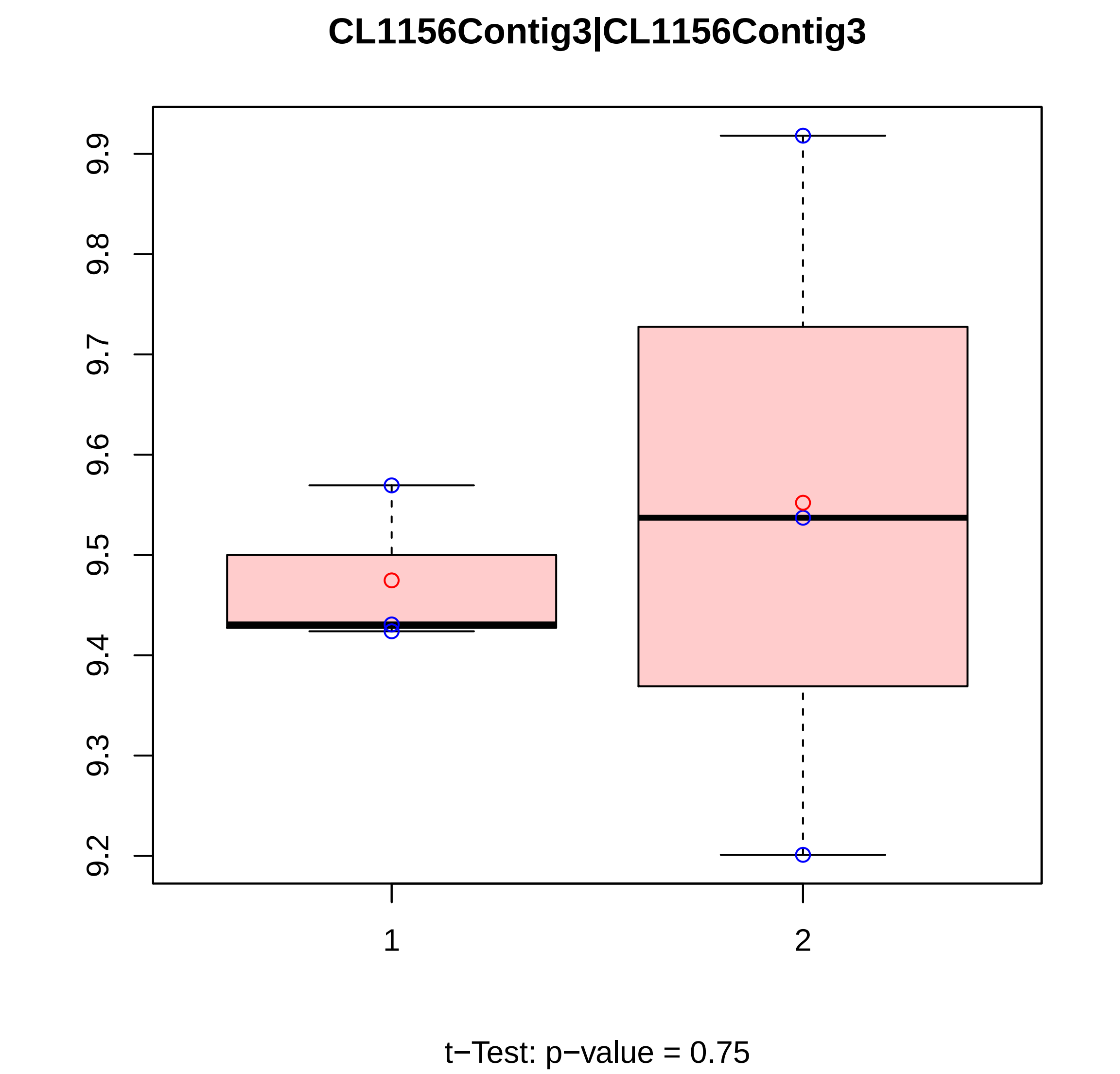
CL1156Contig3|CL1156Contig3
9.2
9.3
9.4
9.5
9.6
9.7
9.8
9.9
1
2
t−Test: p−value = 0.75

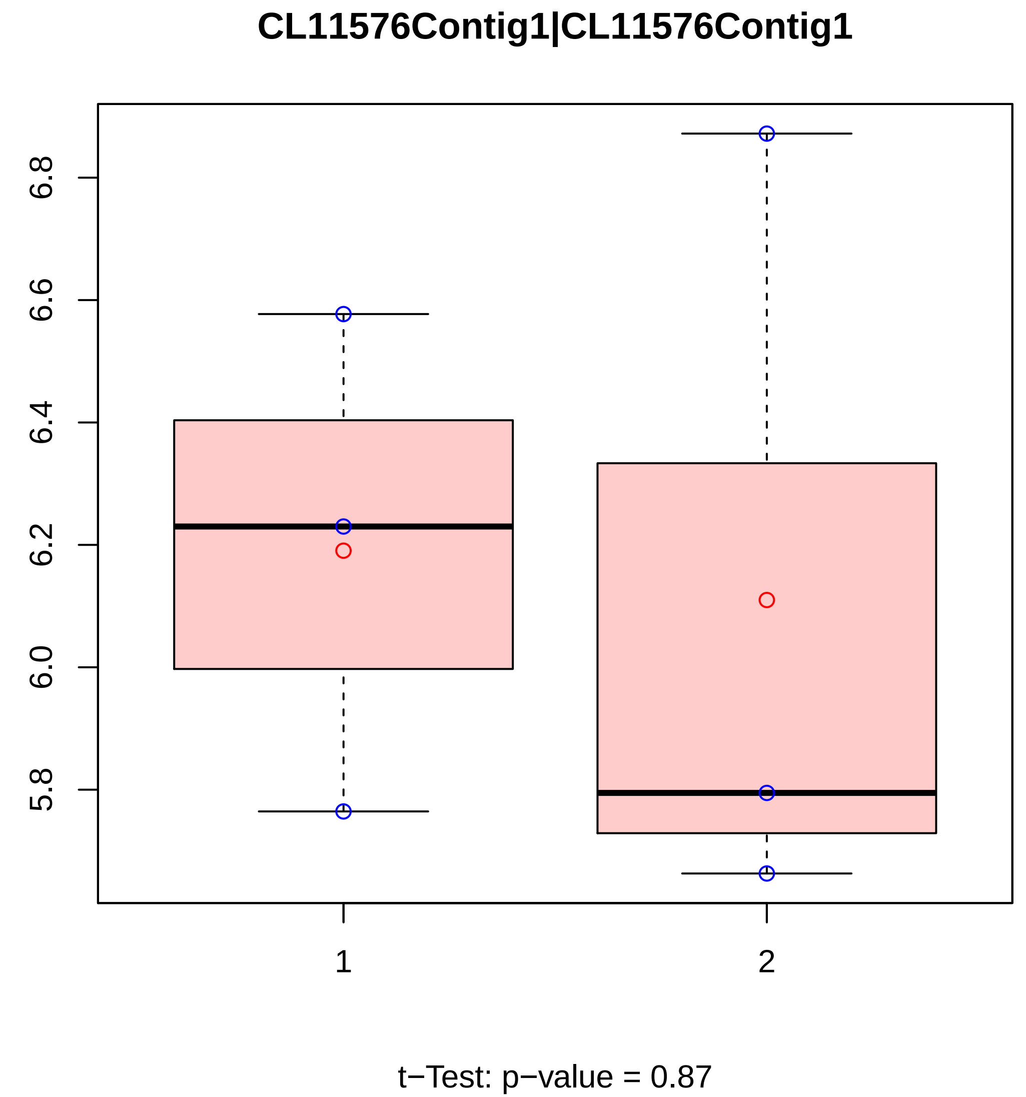
CL11576Contig1|CL11576Contig1
6.8
6.6
6.4
6.2
6.0
5.8
1
2
t−Test: p−value = 0.87

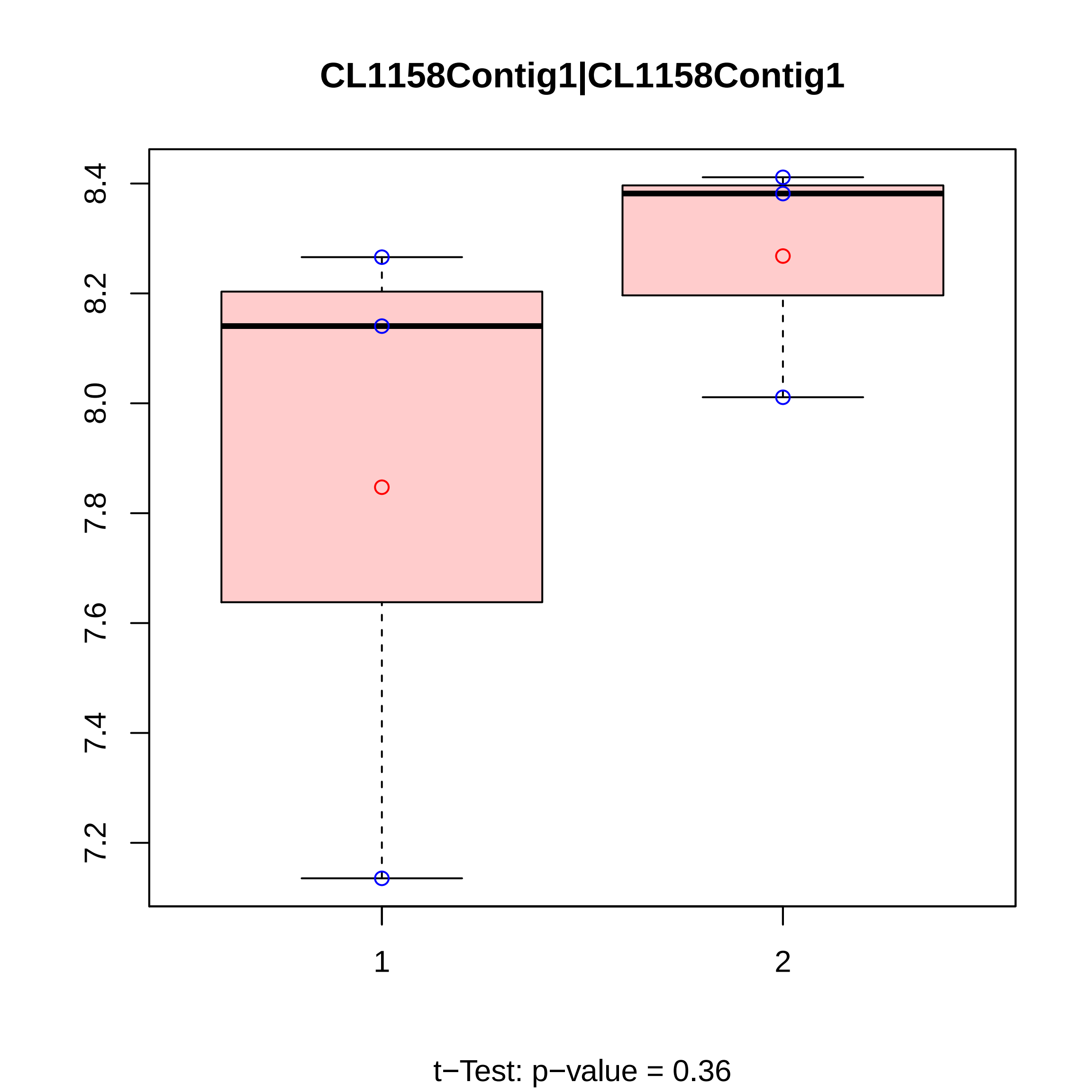
CL1158Contig1|CL1158Contig1
8.4
8.2
8.0
7.8
7.6
7.4
7.2
1
2
t−Test: p−value = 0.36

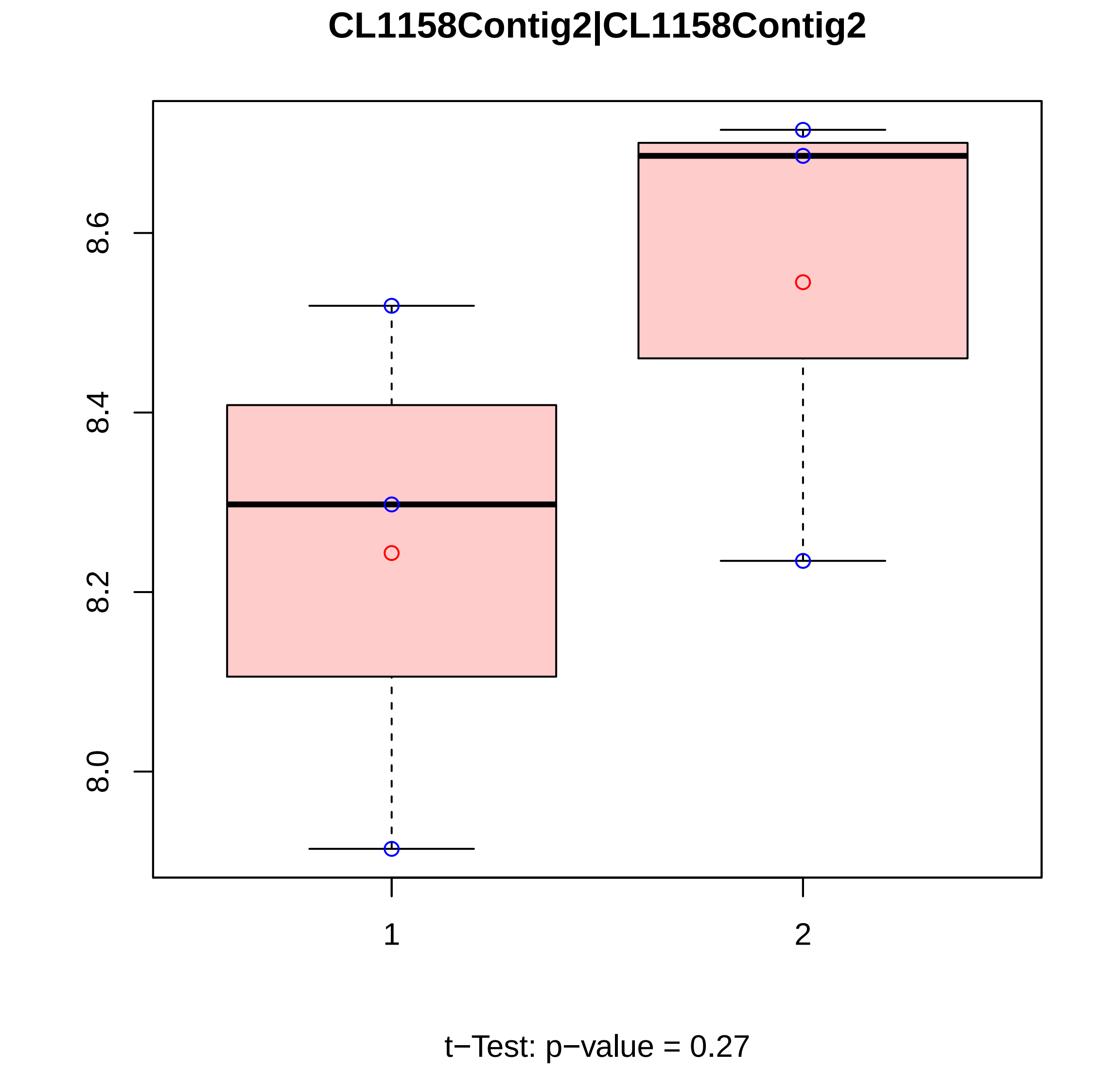
CL1158Contig2|CL1158Contig2
8.0
8.2
8.4
8.6
1
2
t−Test: p−value = 0.27

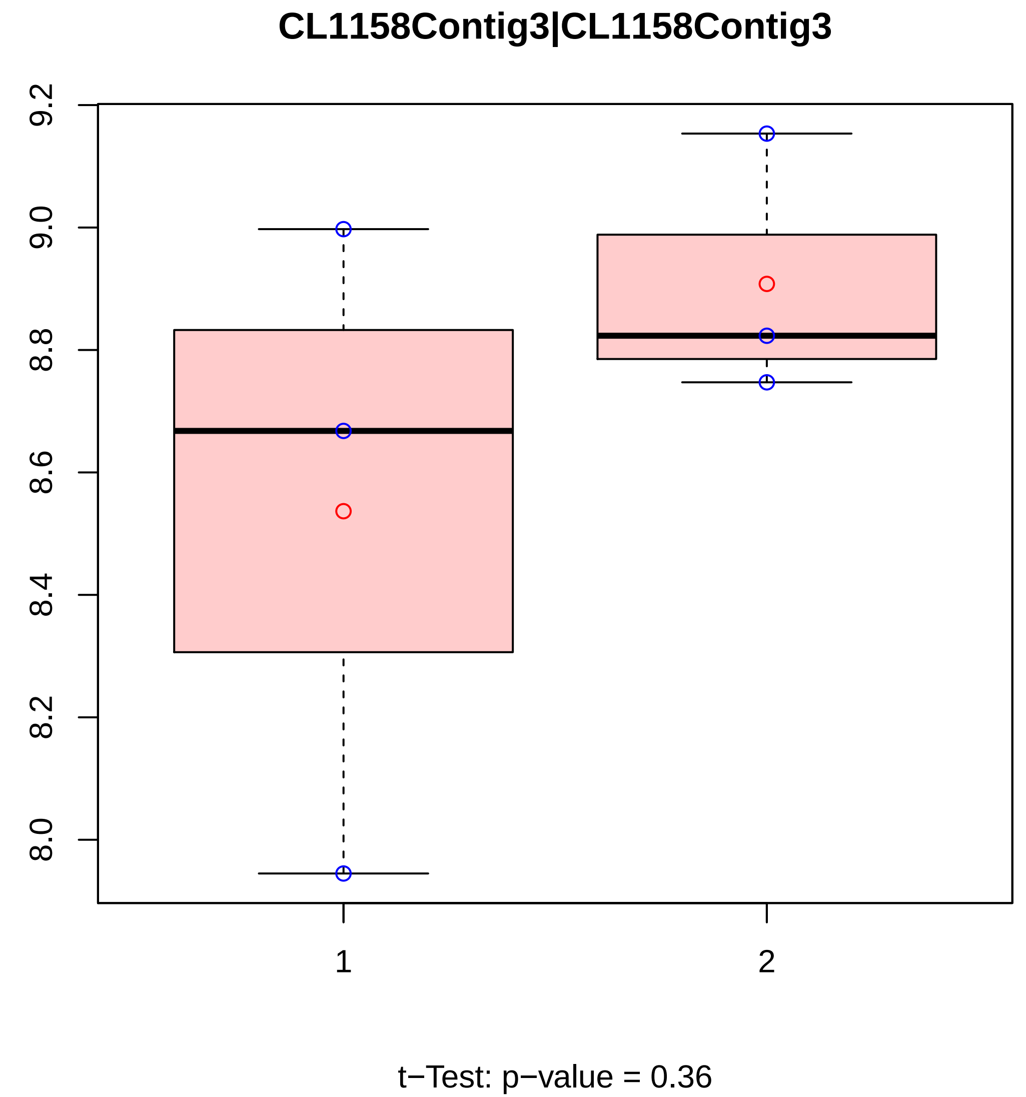

CL1158Contig3|CL1158Contig3
9.2
9.0
8.8
8.6
8.4
8.2
8.0
1
2
t−Test: p−value = 0.36

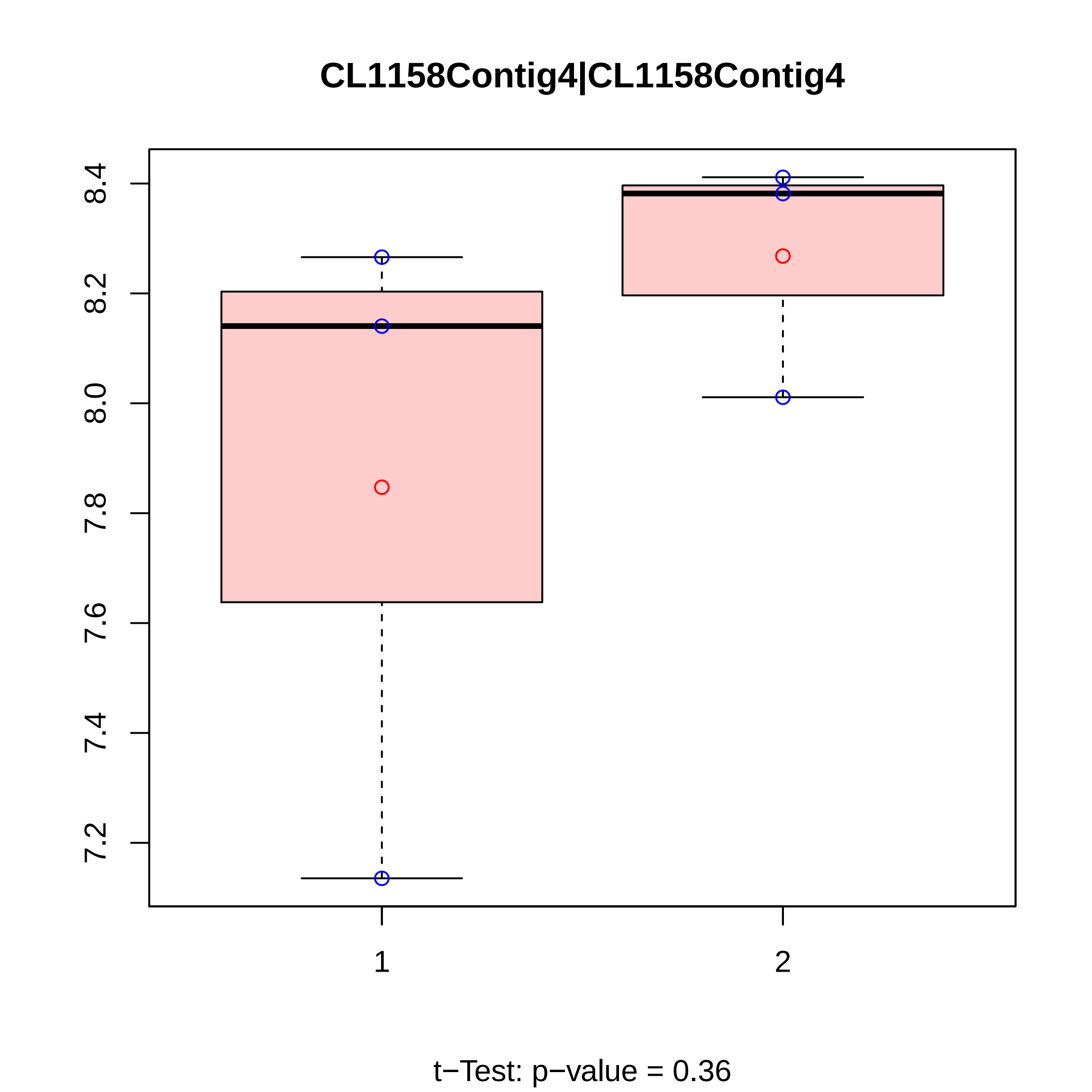

CL1158Contig4|CL1158Contig4
8.4
8.2
8.0
7.8
7.6
7.4
7.2
1
2
t−Test: p−value = 0.36

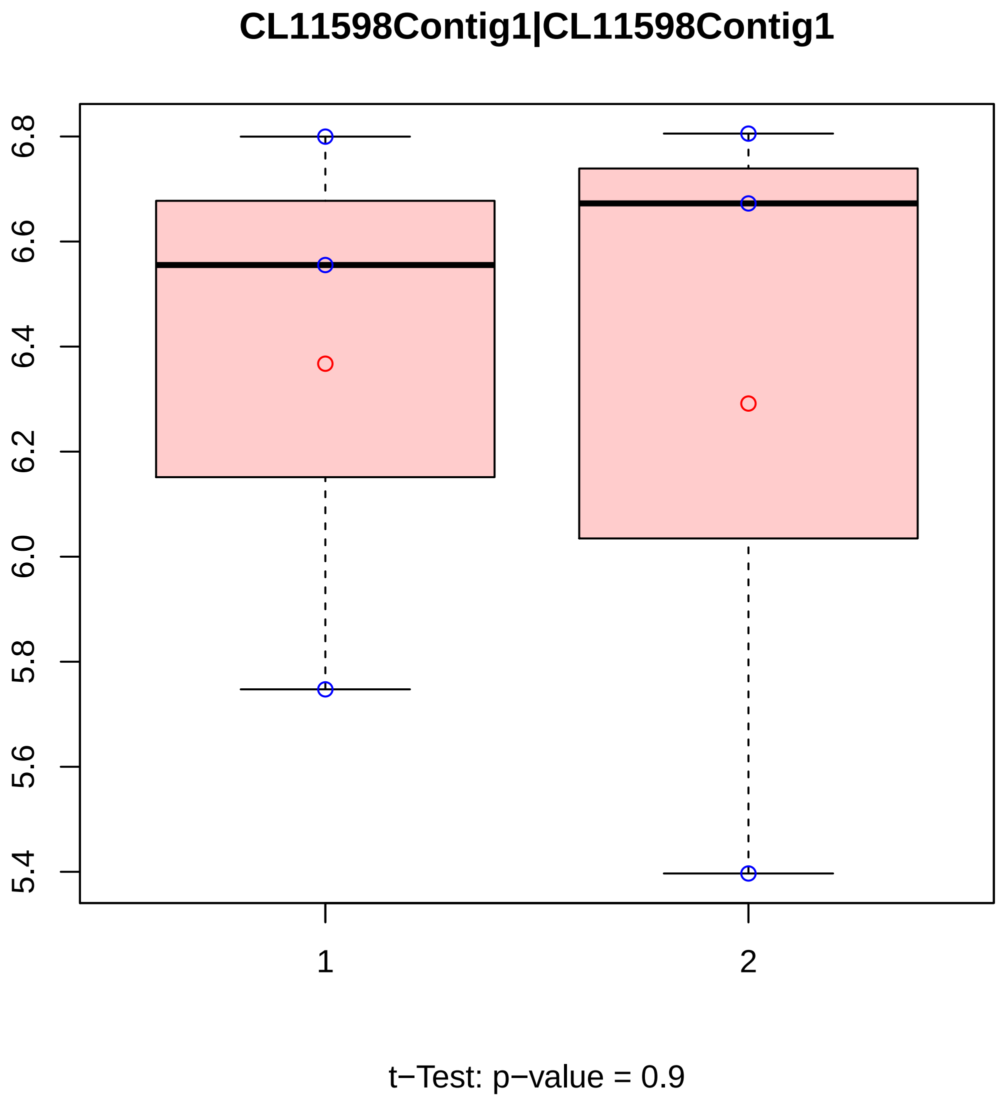
CL11598Contig1|CL11598Contig1
6.8
6.6
6.4
6.2
6.0
5.8
5.6
5.4
1
2
t−Test: p−value = 0.9

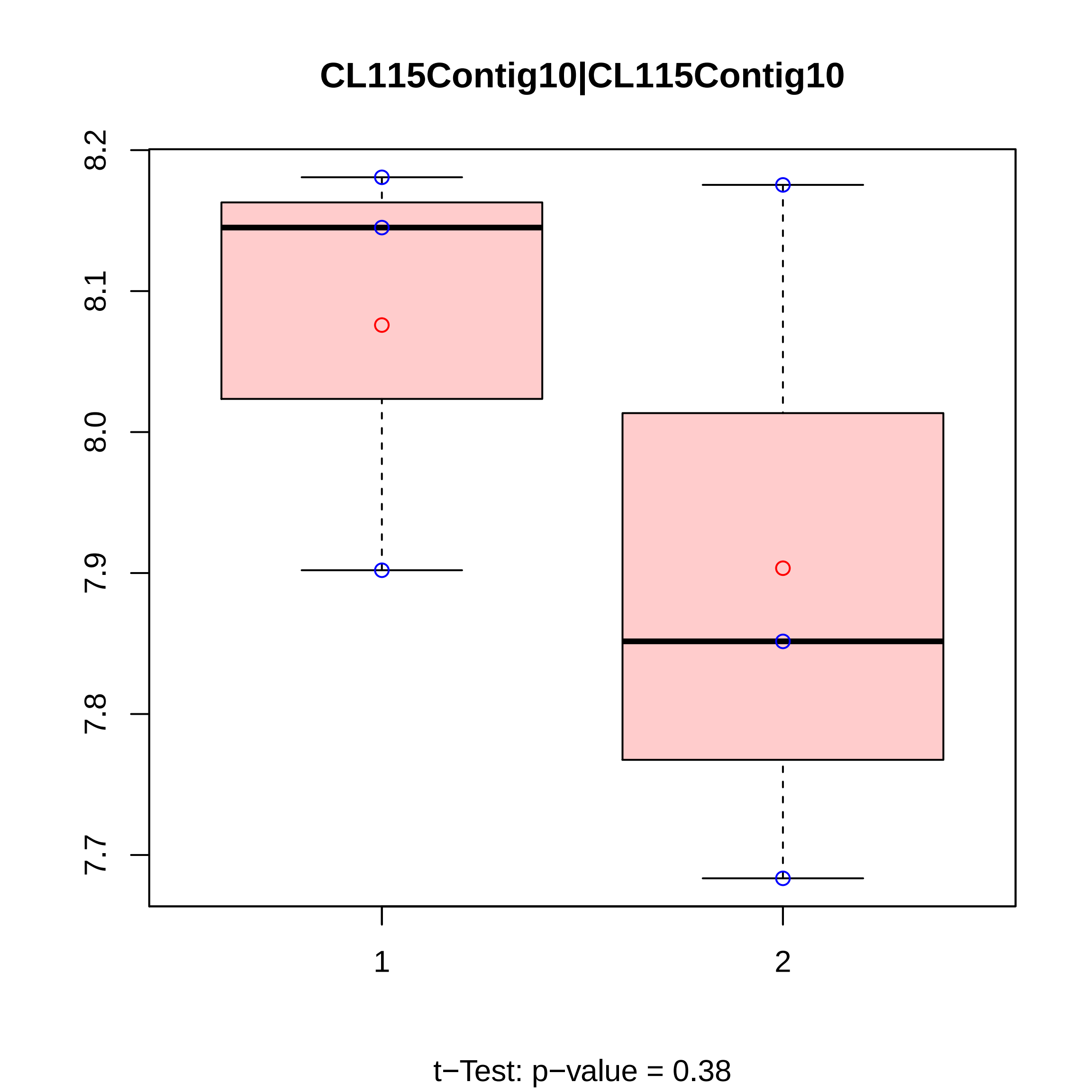
CL115Contig10|CL115Contig10
8.2
8.1
8.0
7.9
7.8
7.7
1
2
t−Test: p−value = 0.38

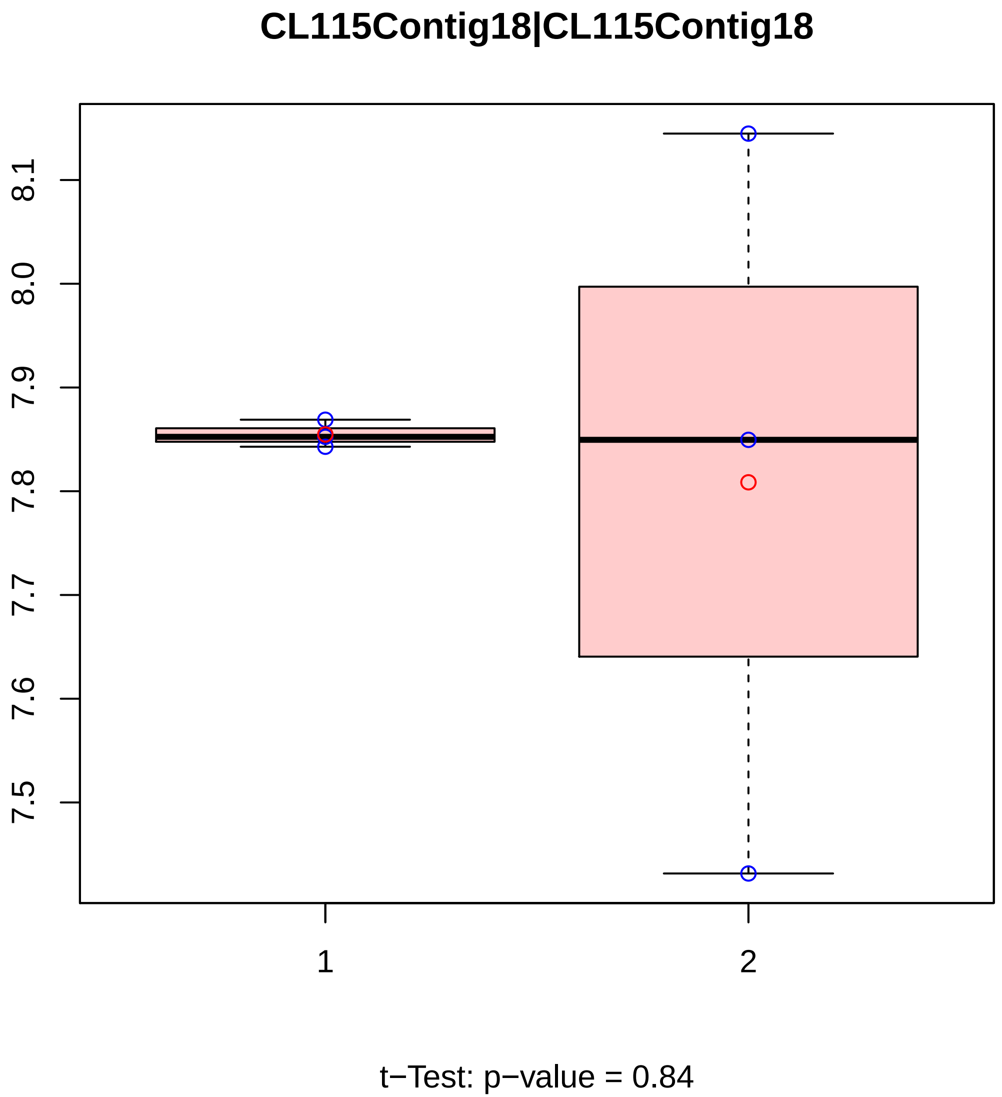
CL115Contig18|CL115Contig18
8.1
8.0
7.9
7.8
7.7
7.6
7.5
1
2
t−Test: p−value = 0.84

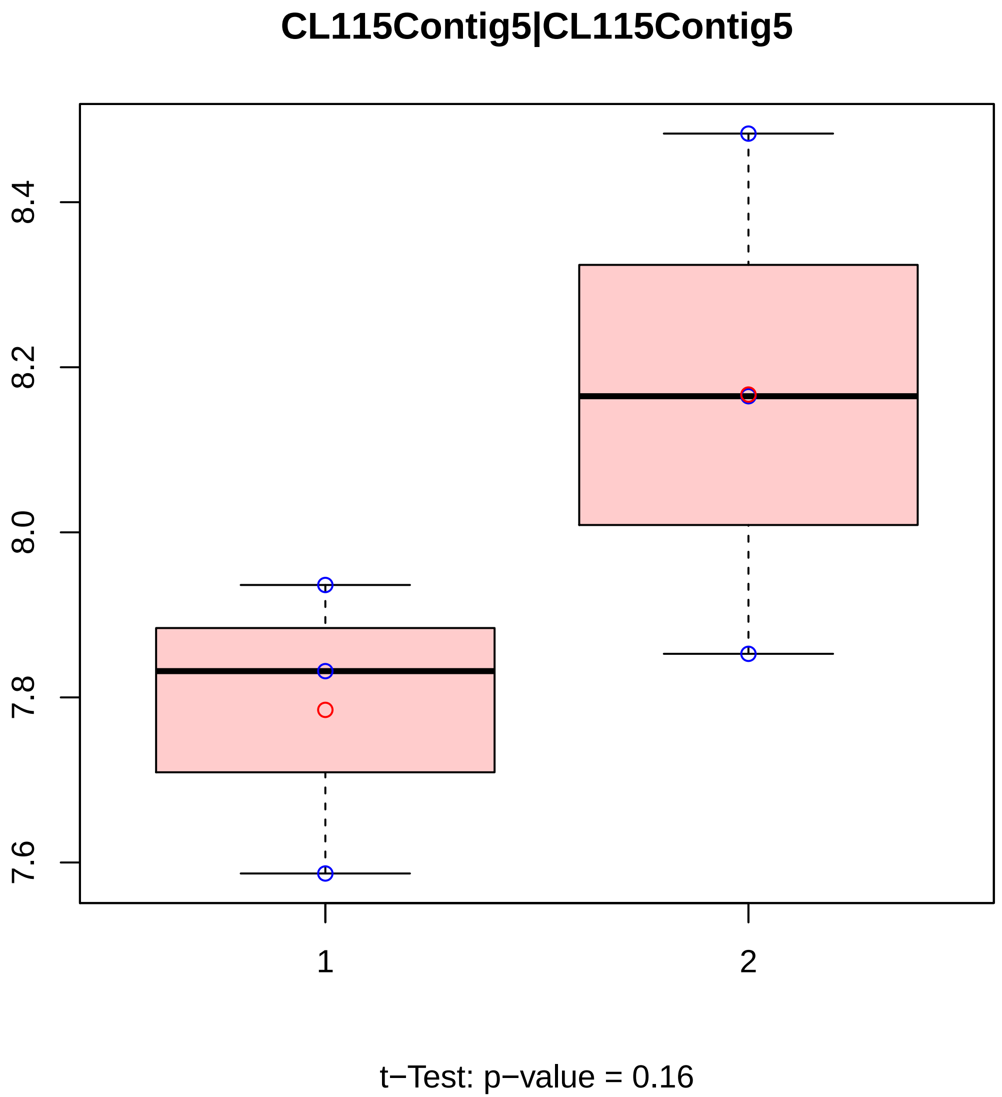
CL115Contig5|CL115Contig5
7.6
7.8
8.0
8.2
8.4
1
2
t−Test: p−value = 0.16

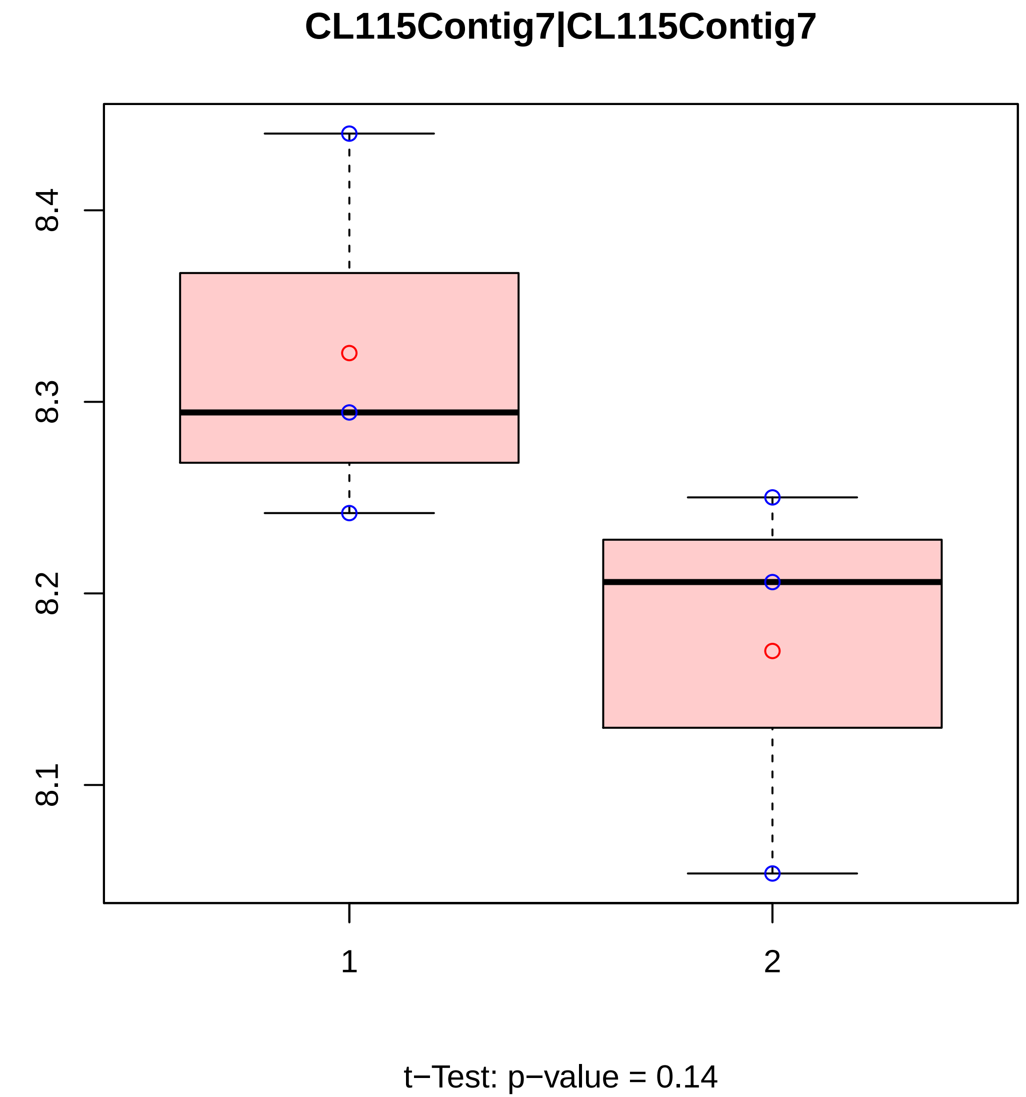
CL115Contig7|CL115Contig7
8.1
8.2
8.3
8.4
1
2
t−Test: p−value = 0.14

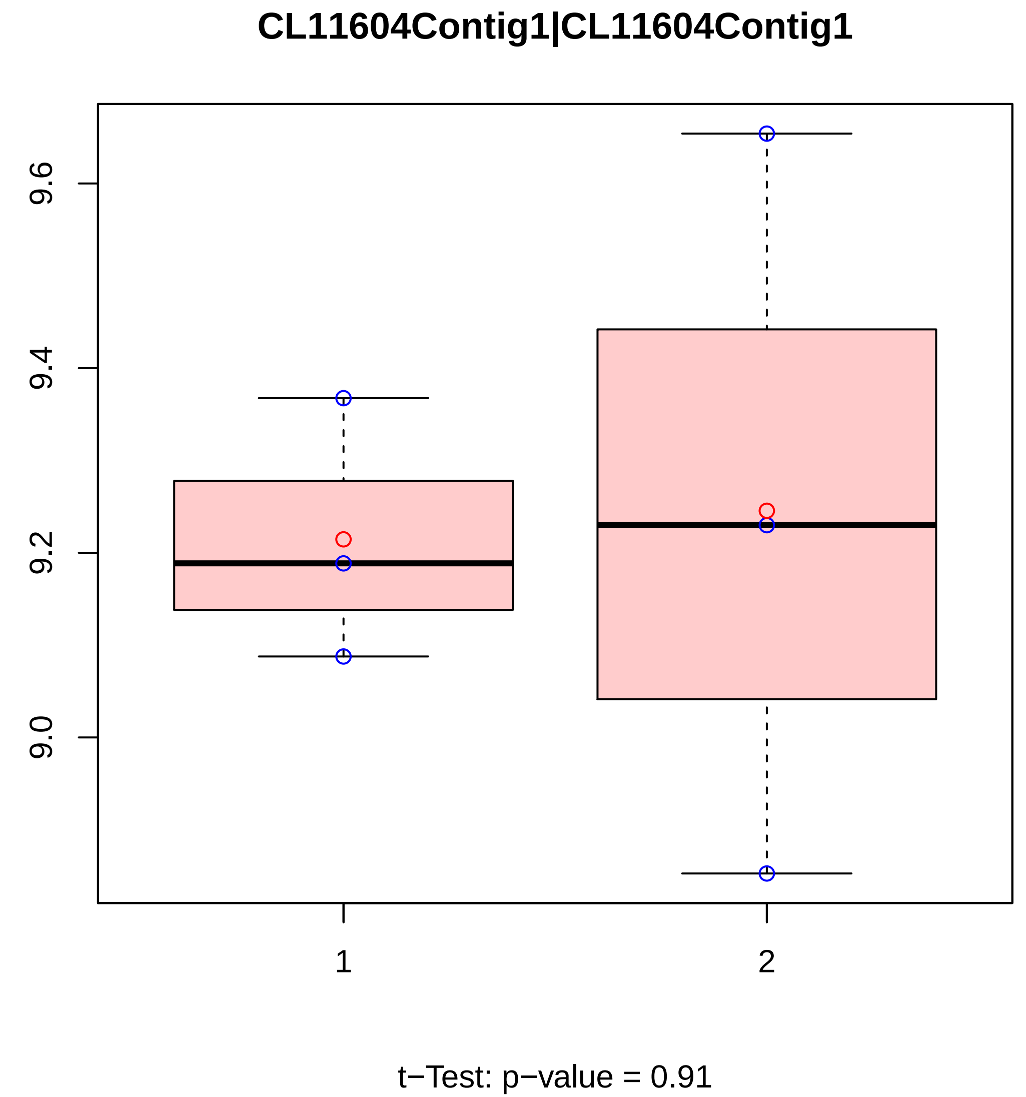

CL11604Contig1|CL11604Contig1
9.0
9.2
9.4
9.6
1
2
t−Test: p−value = 0.91

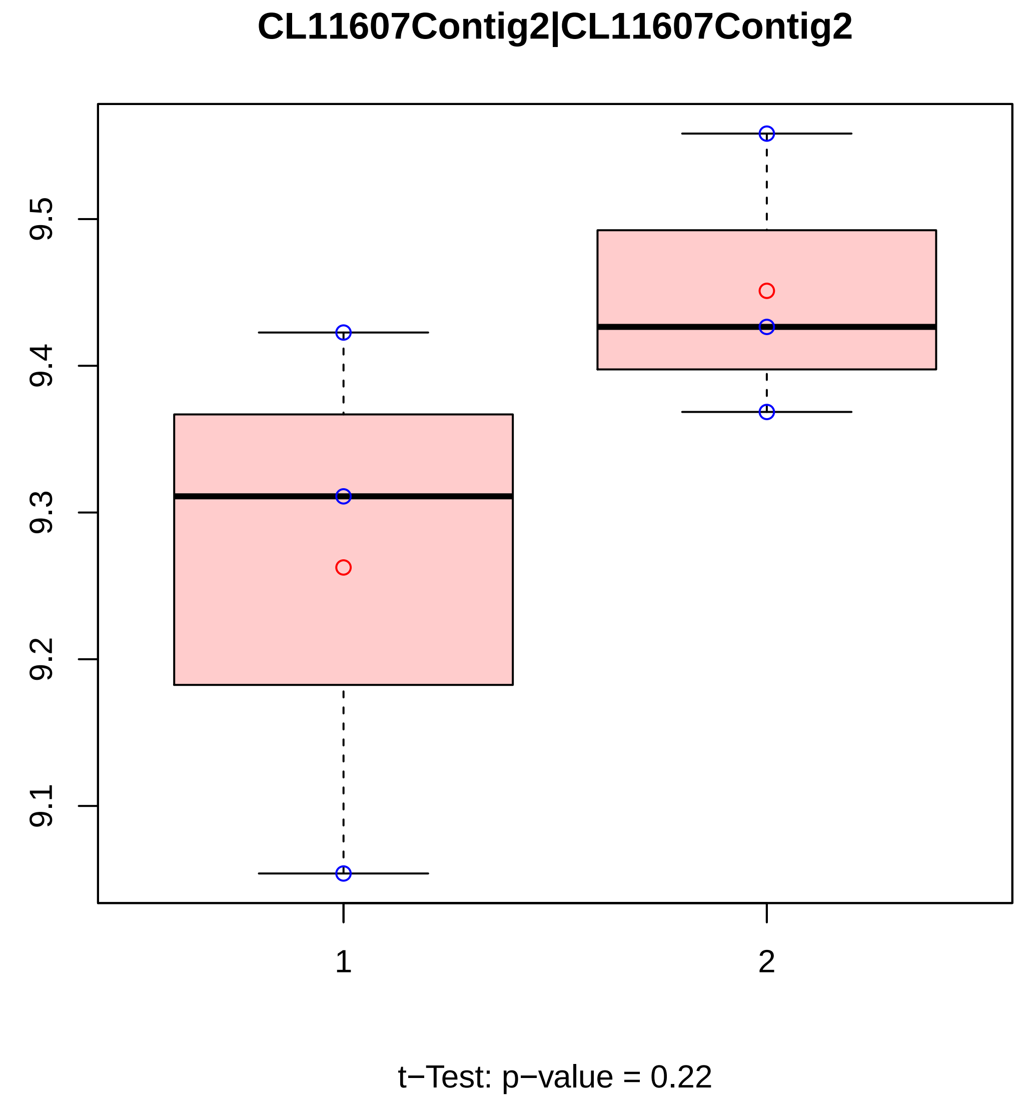
CL11607Contig2|CL11607Contig2
9.1
9.2
9.3
9.4
9.5
1
2
t−Test: p−value = 0.22

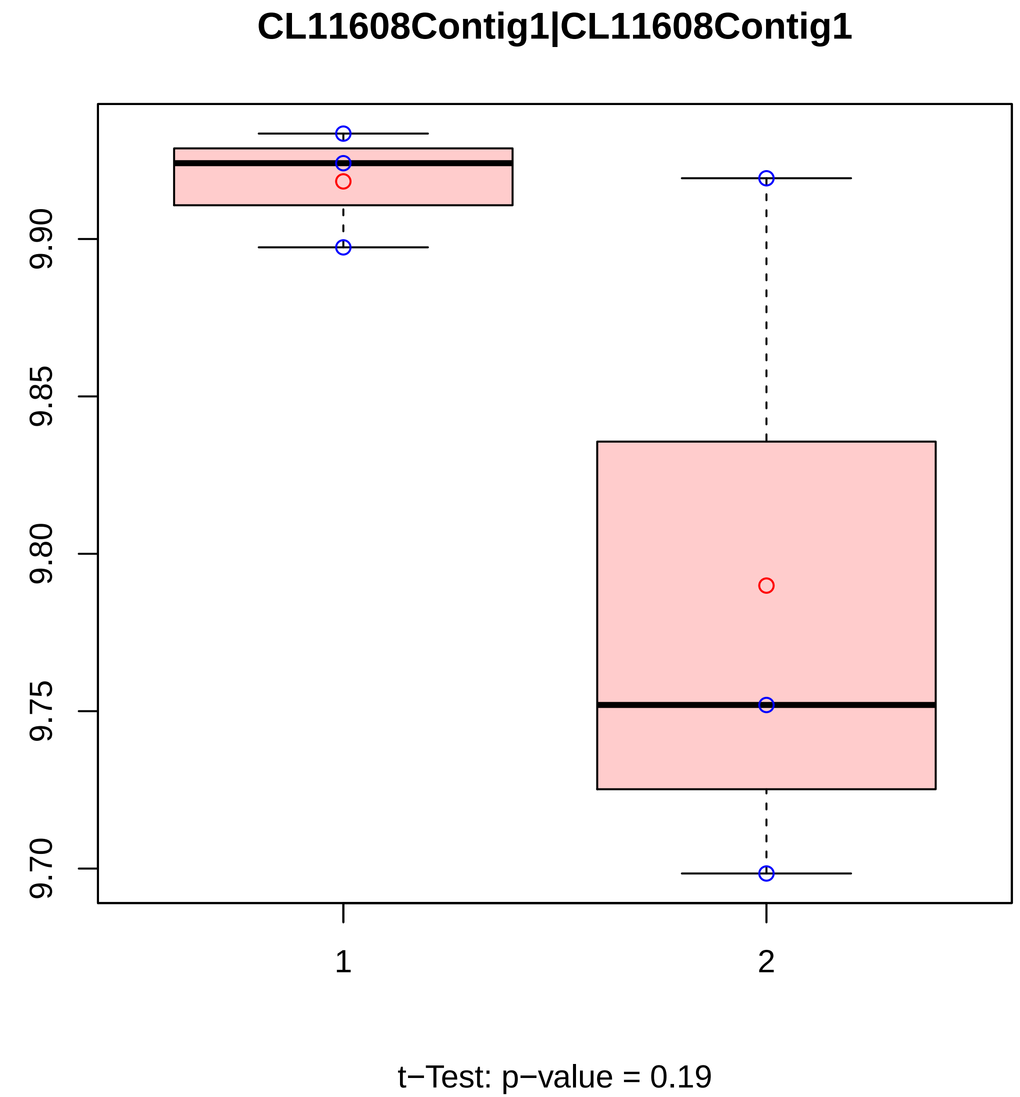
CL11608Contig1|CL11608Contig1
9.70
9.75
9.80
9.85
9.90
1
2
t−Test: p−value = 0.19

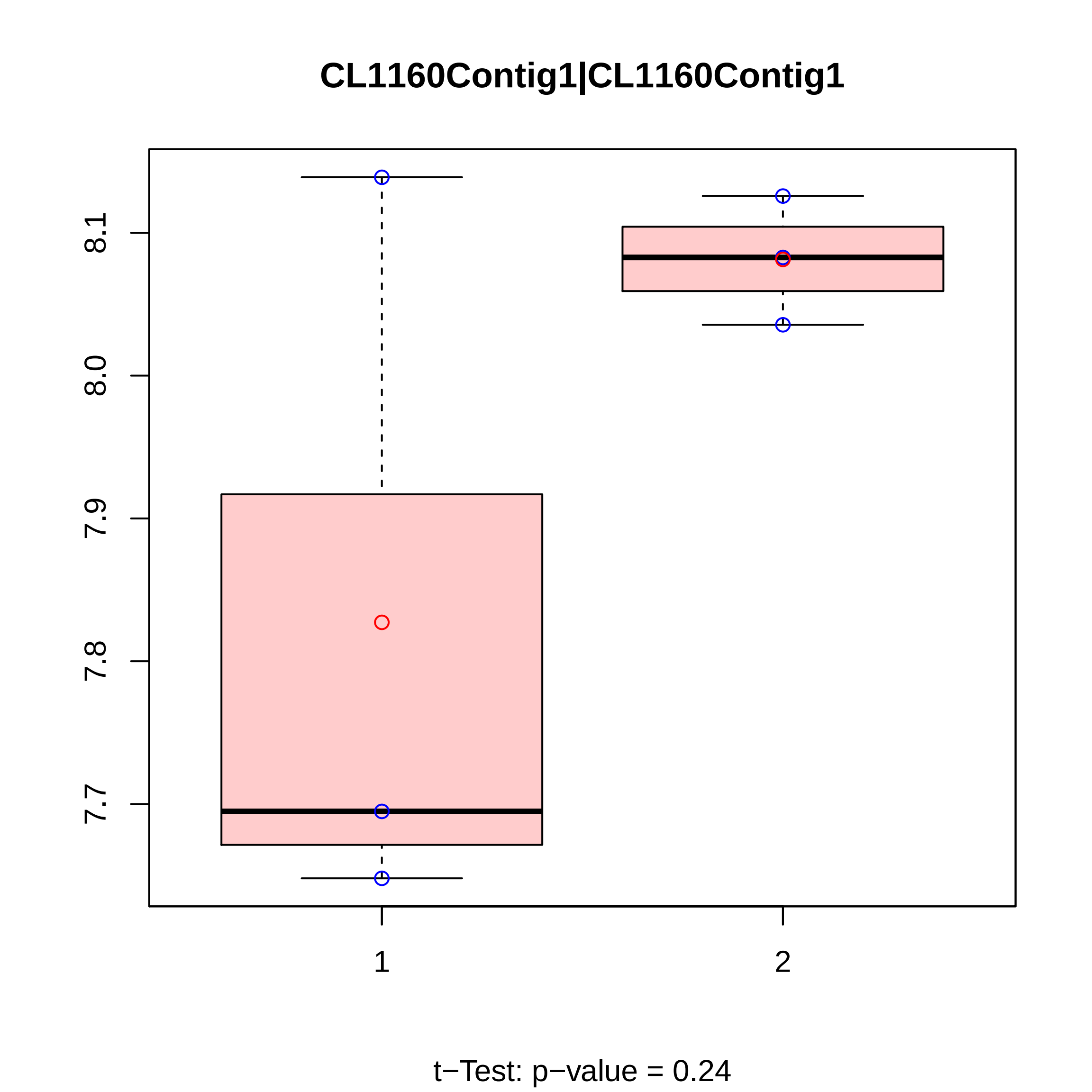
CL1160Contig1|CL1160Contig1
8.1
8.0
7.9
7.8
7.7
1
2
t−Test: p−value = 0.24

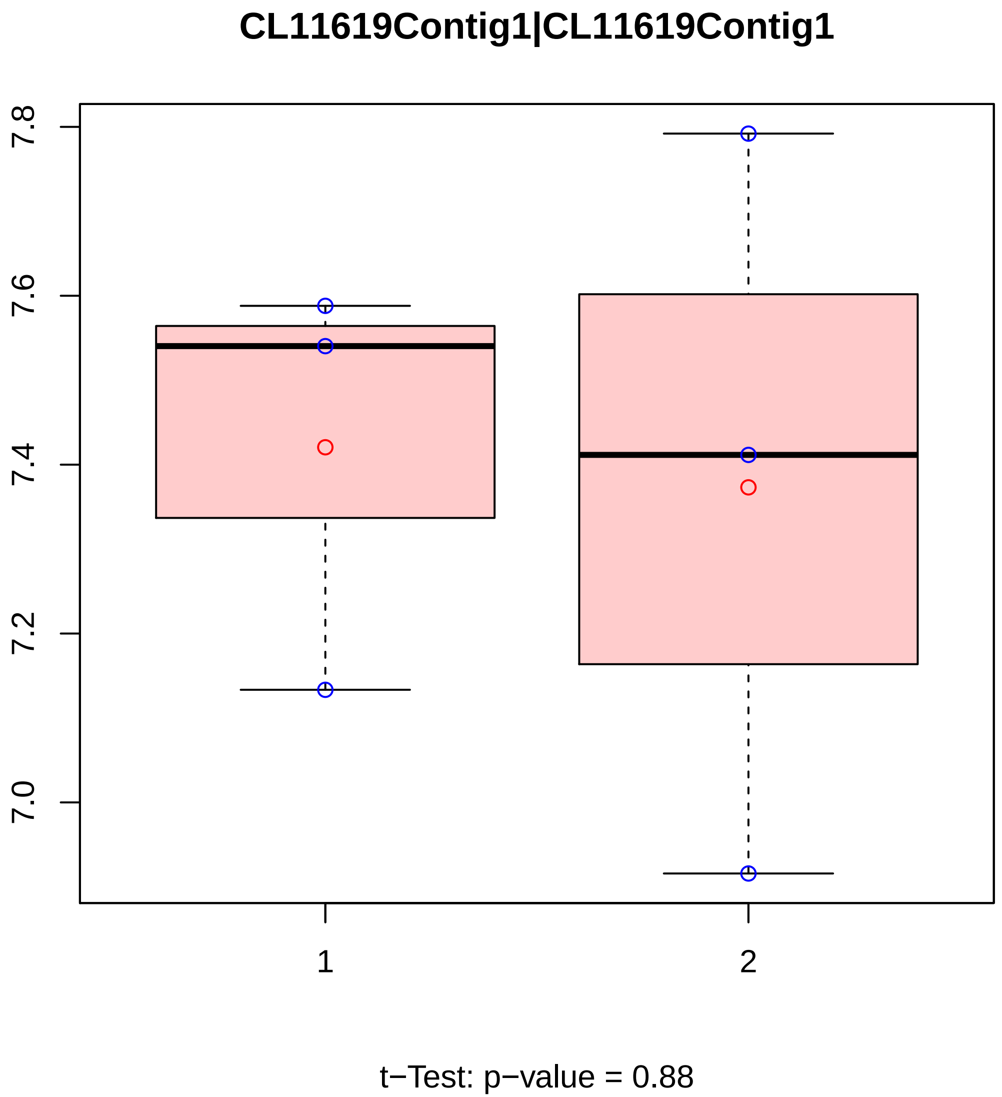
CL11619Contig1|CL11619Contig1
7.8
7.6
7.4
7.2
7.0
1
2
t−Test: p−value = 0.88

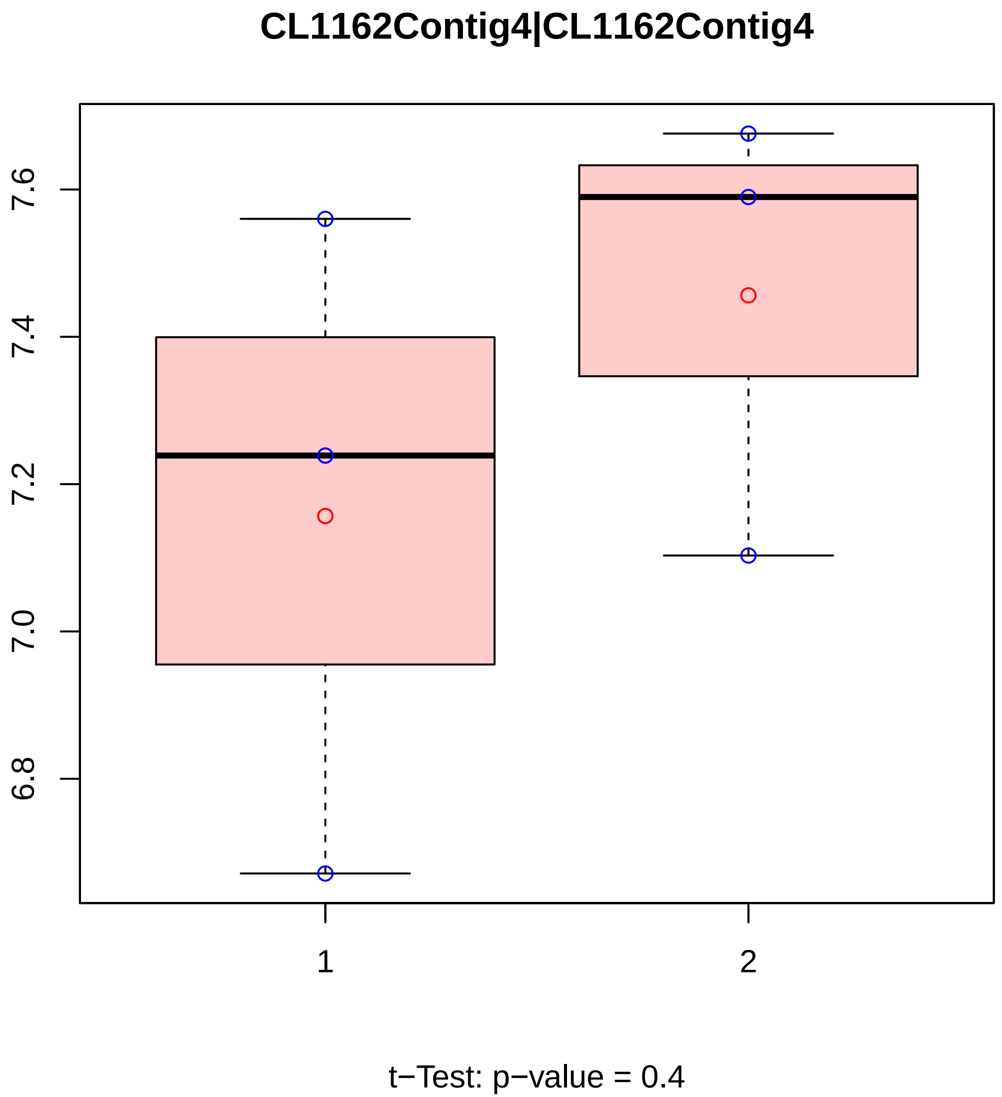
CL1162Contig4|CL1162Contig4
6.8
7.0
7.2
7.4
7.6
1
2
t−Test: p−value = 0.4

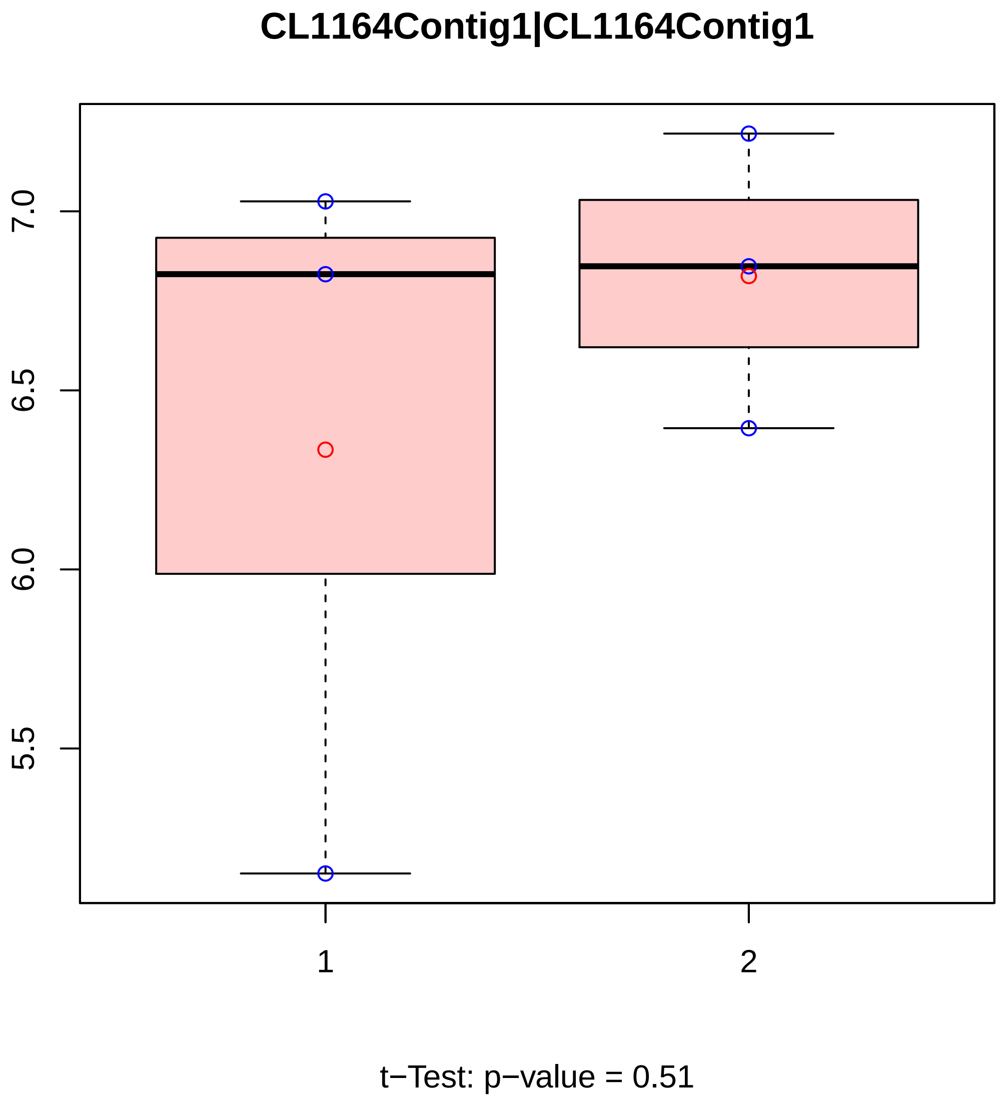
CL1164Contig1|CL1164Contig1
7.0
6.5
6.0
5.5
1
2
t−Test: p−value = 0.51

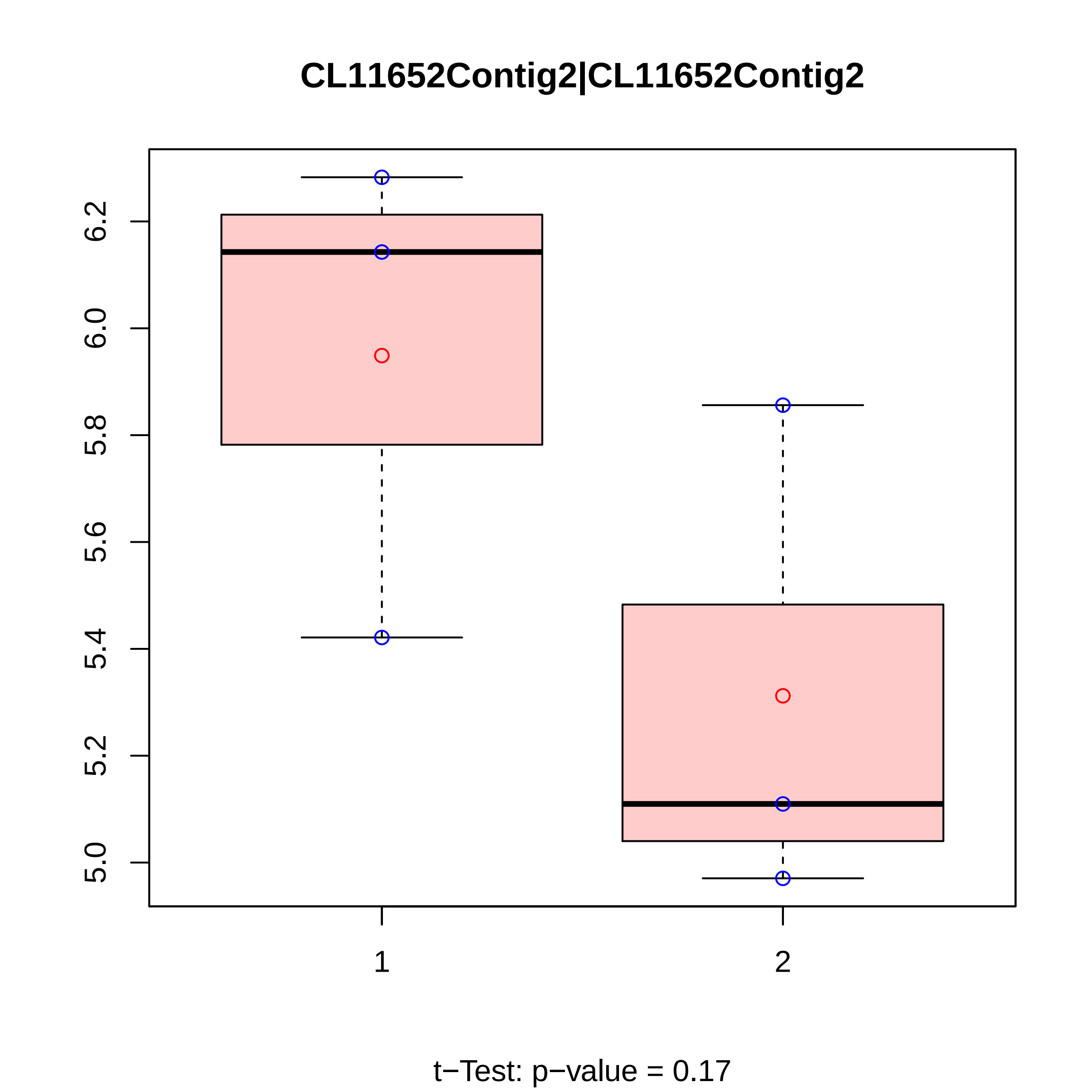
CL11652Contig2|CL11652Contig2
6.2
6.0
5.8
5.6
5.4
5.2
5.0
1
2
t−Test: p−value = 0.17

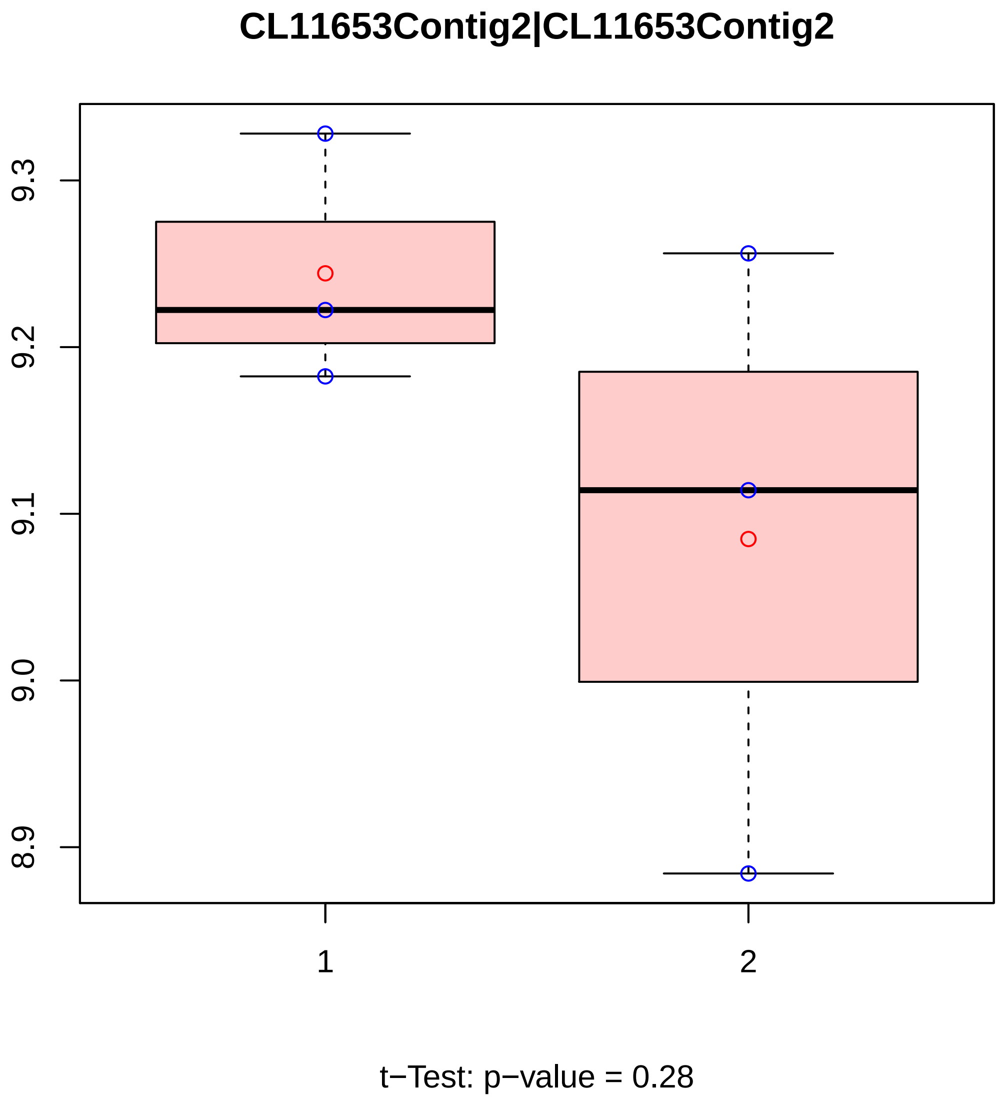
CL11653Contig2|CL11653Contig2
9.3
9.2
9.1
9.0
8.9
1
2
t−Test: p−value = 0.28

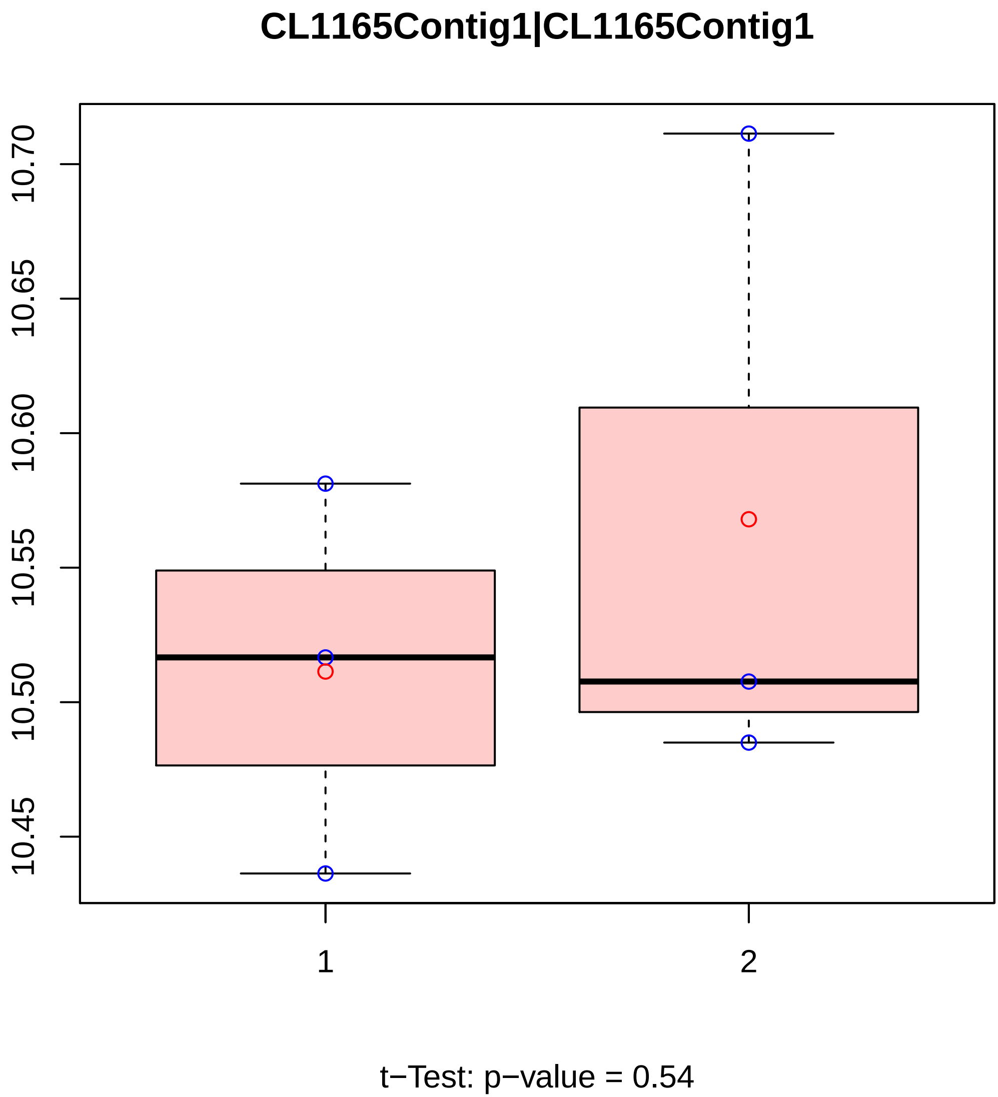
CL1165Contig1|CL1165Contig1
10.45
10.50
10.55
10.60
10.65
10.70
1
2
t−Test: p−value = 0.54

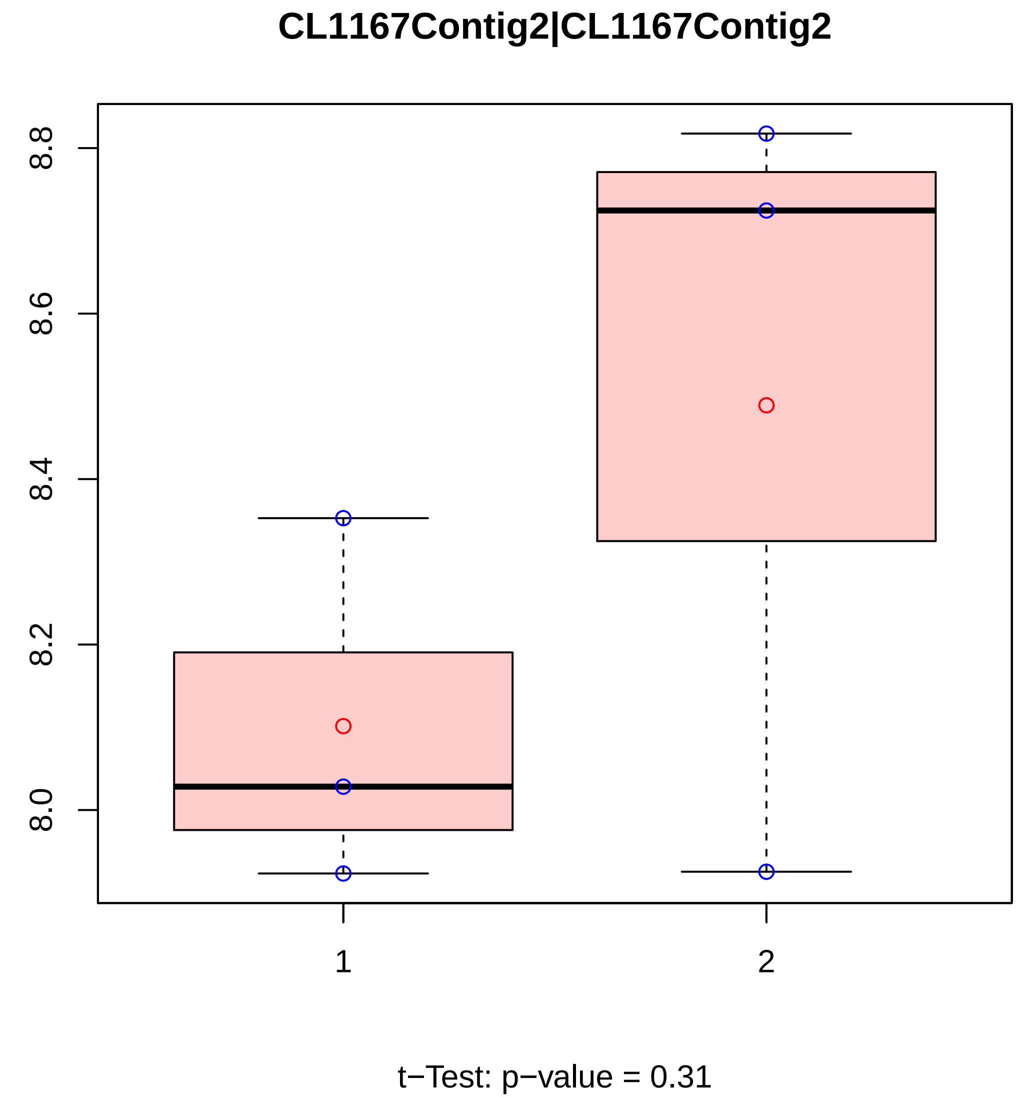
CL1167Contig2|CL1167Contig2
8.0
8.2
8.4
8.6
8.8
1
2
t−Test: p−value = 0.31

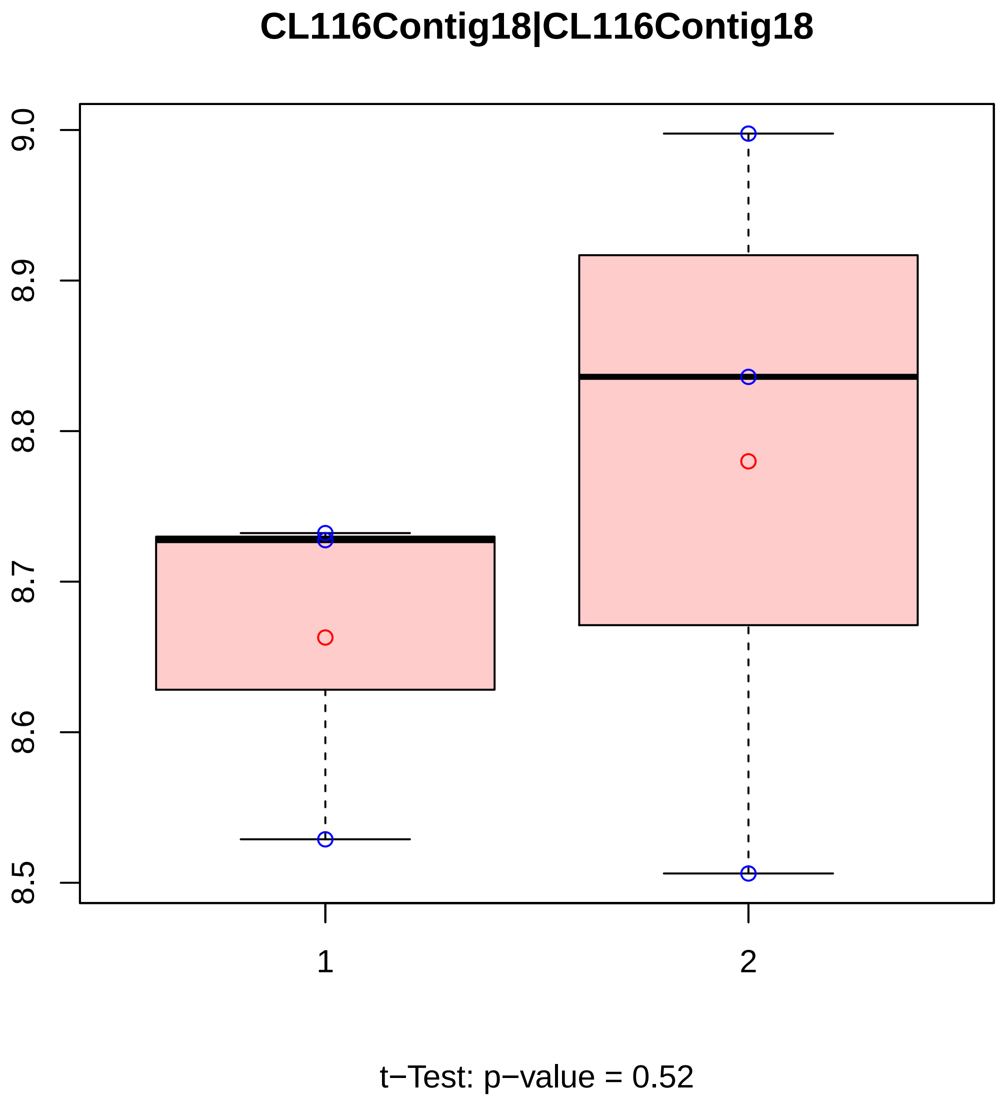

CL116Contig18|CL116Contig18
9.0
8.9
8.8
8.7
8.6
8.5
1
2
t−Test: p−value = 0.52

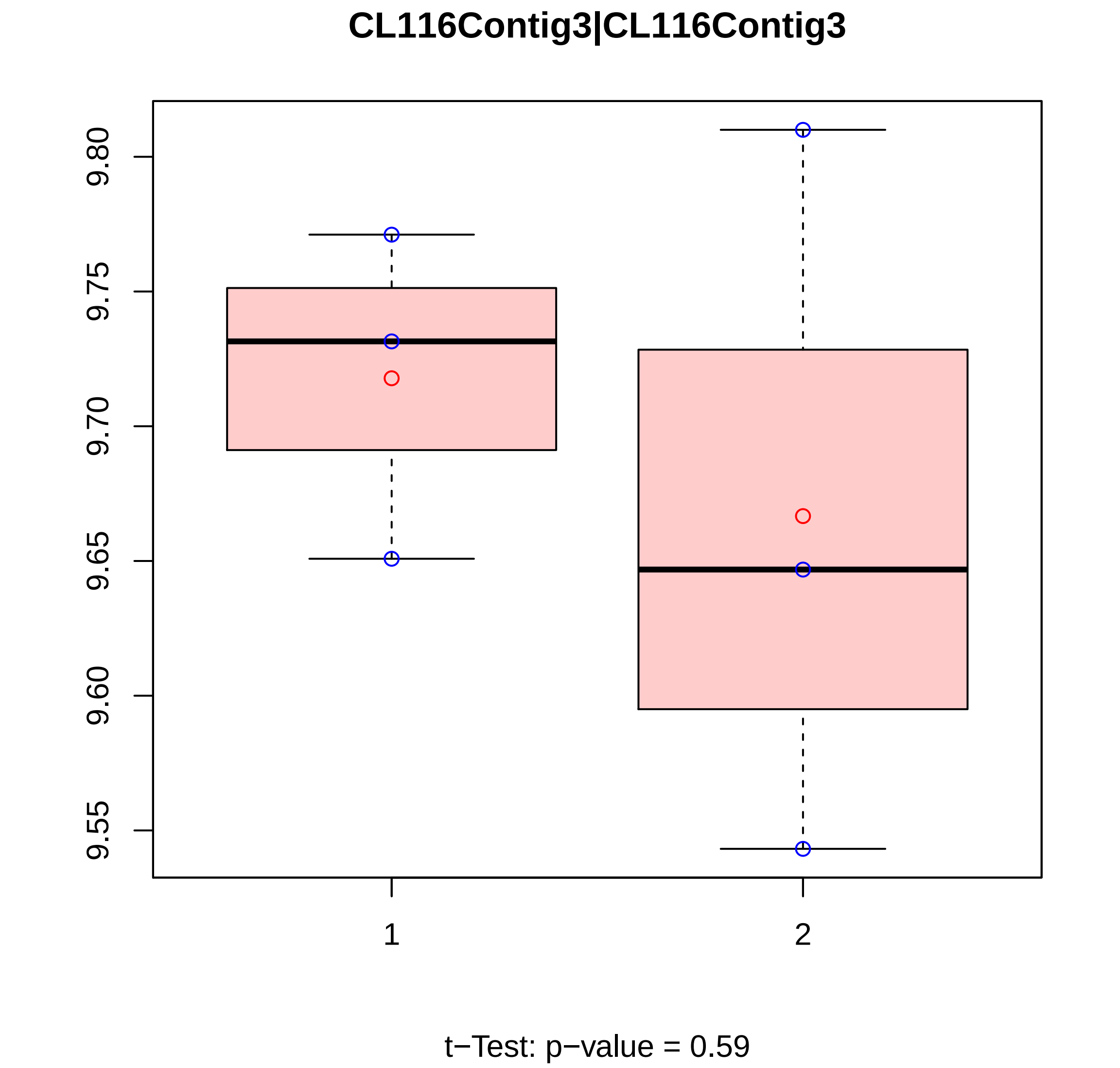
CL116Contig3|CL116Contig3
9.55
9.60
9.65
9.70
9.75
9.80
1
2
t−Test: p−value = 0.59

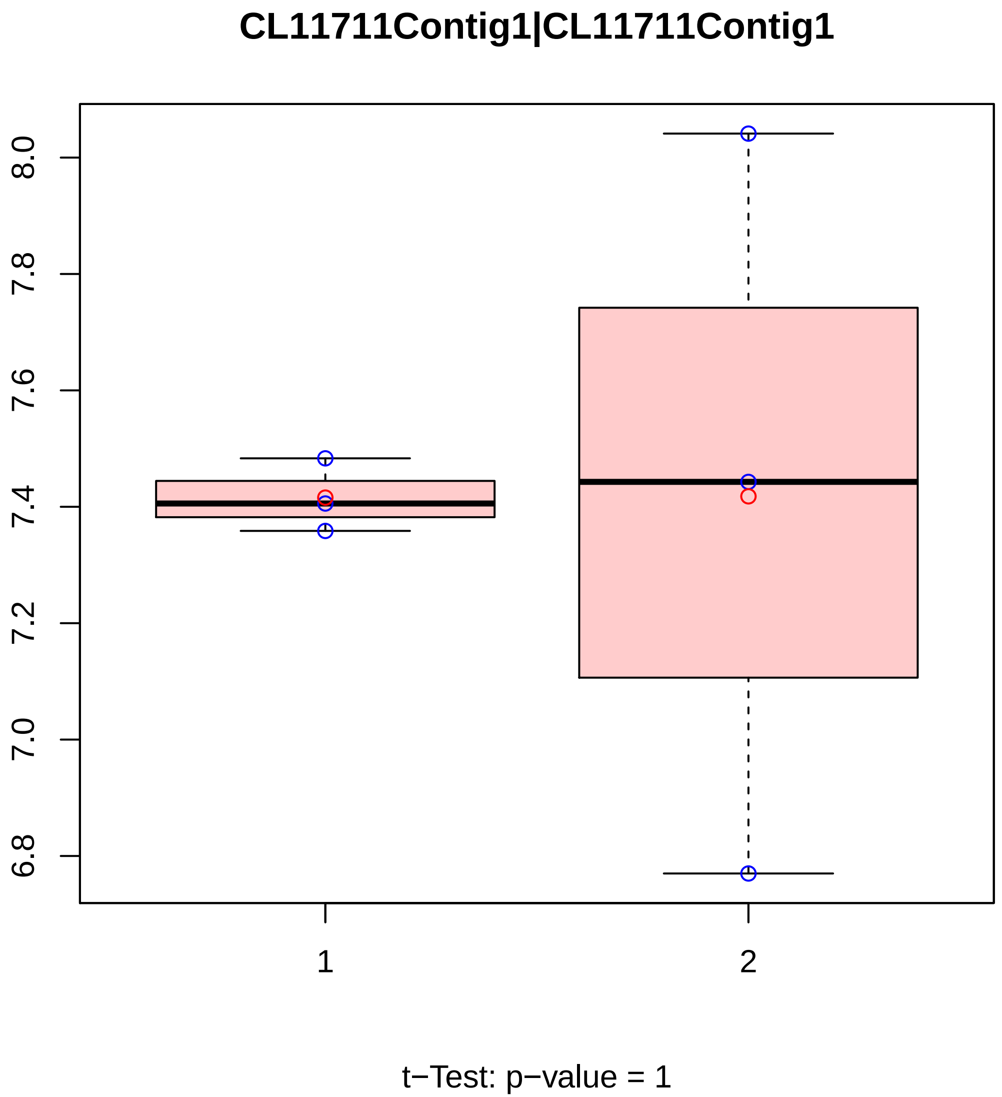
CL11711Contig1|CL11711Contig1
8.0
7.8
7.6
7.4
7.2
7.0
6.8
1
2
t−Test: p−value = 1

## CL1172Contig2|CL1172Contig2

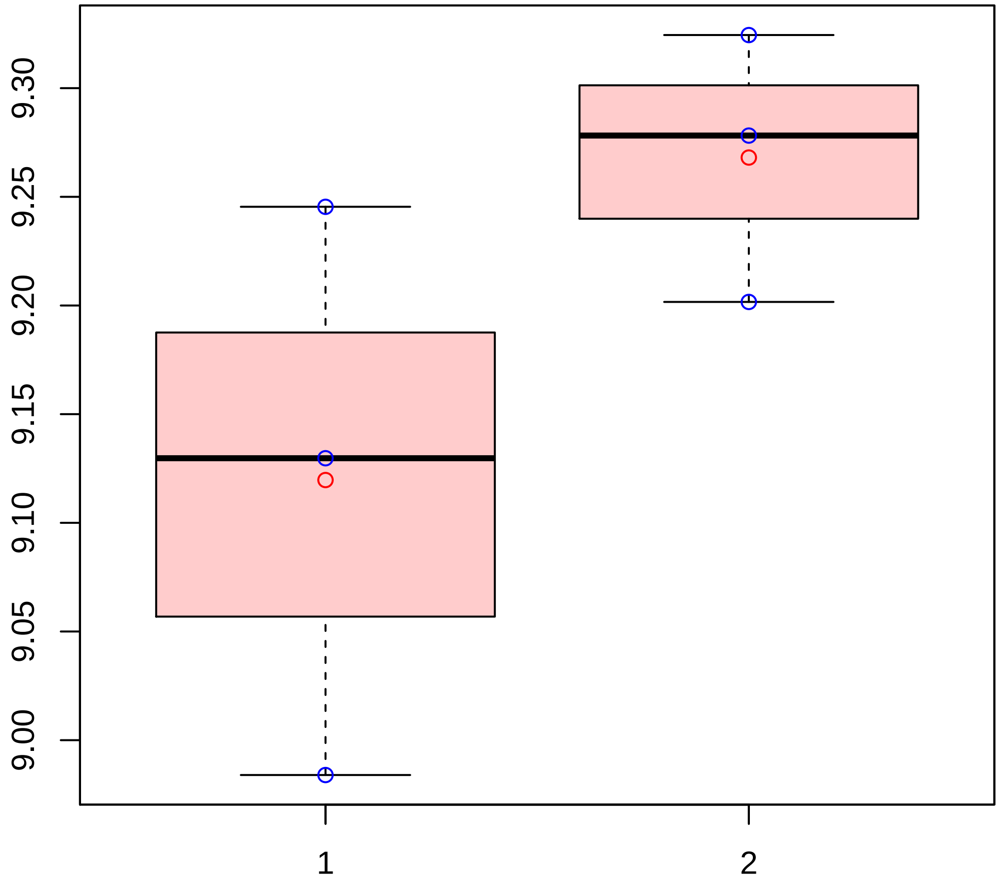


t−Test: p−value = 0.18

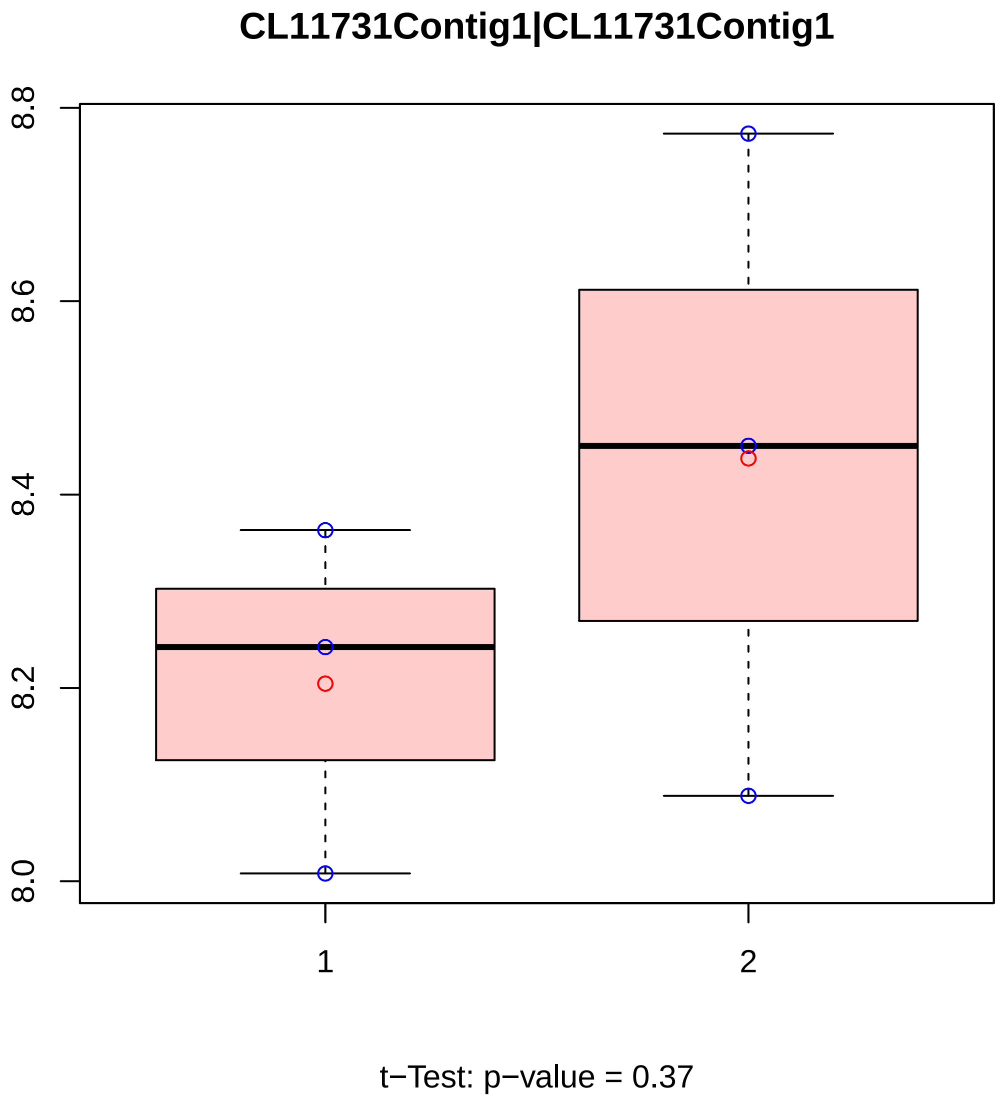

CL11731Contig1|CL11731Contig1
8.0
8.2
8.4
8.6
8.8
1
2
t−Test: p−value = 0.37

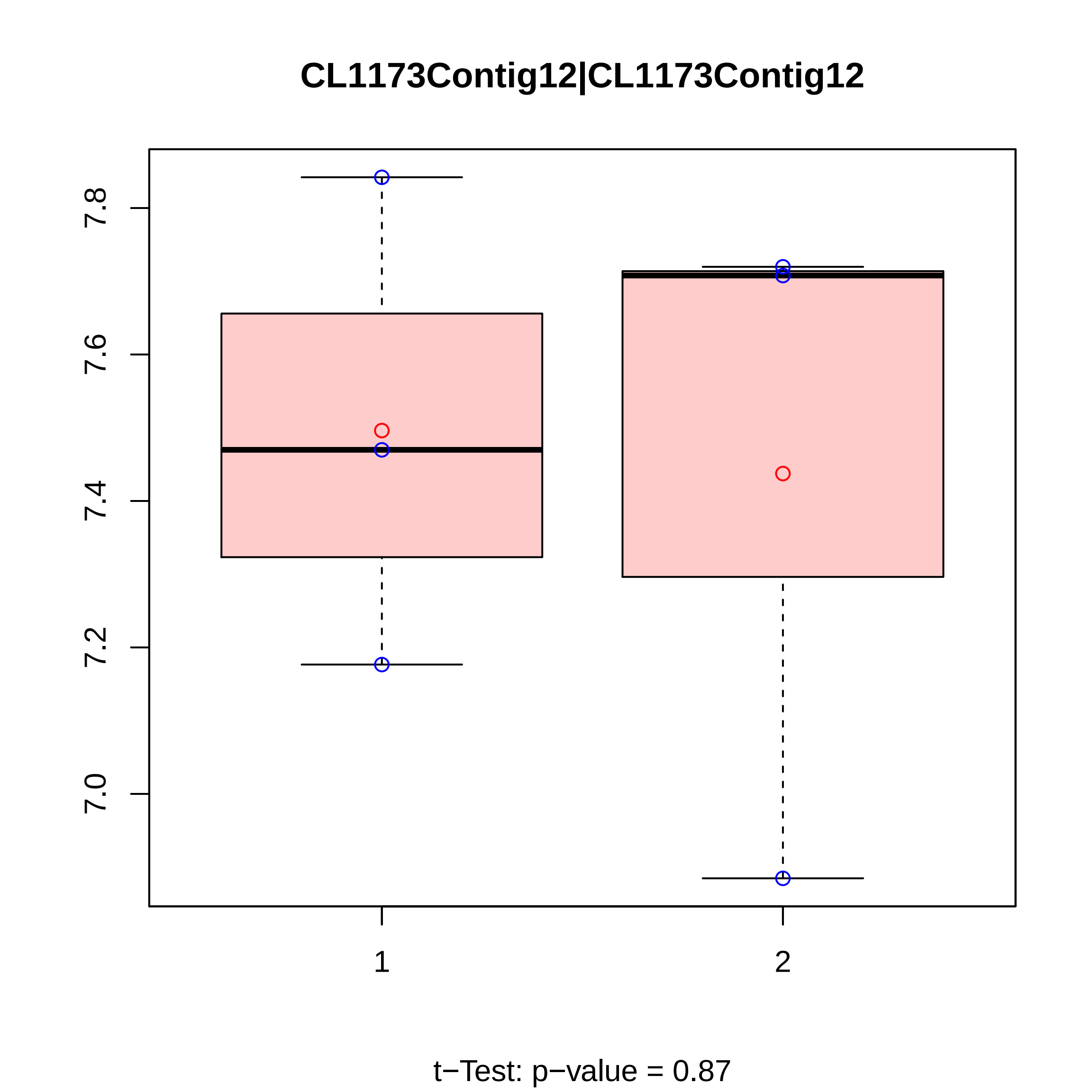
CL1173Contig12|CL1173Contig12
7.8
7.6
7.4
7.2
7.0
1
2
t−Test: p−value = 0.87

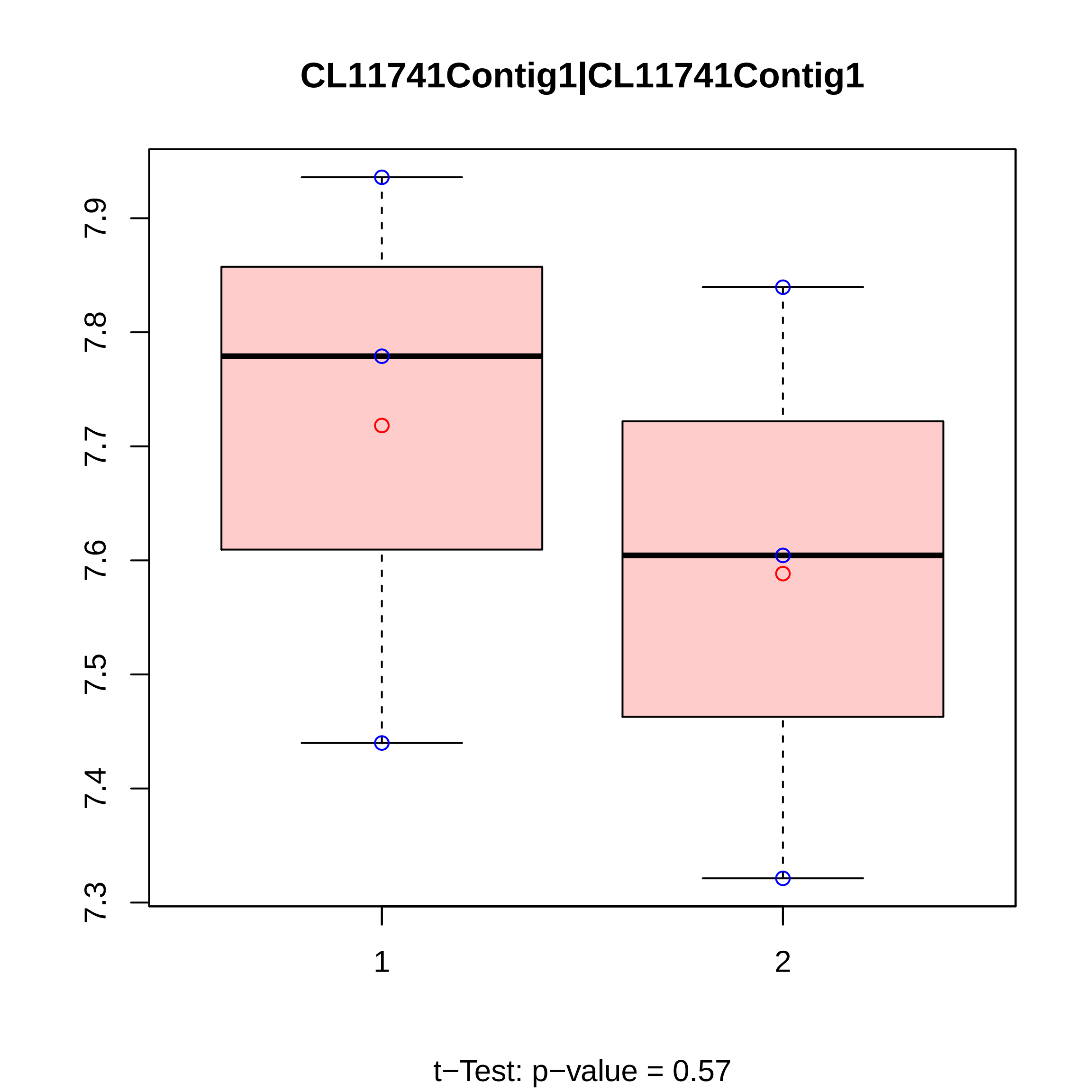

CL11741Contig1|CL11741Contig1
7.3
7.4
7.5
7.6
7.7
7.8
7.9
1
2
t−Test: p−value = 0.57

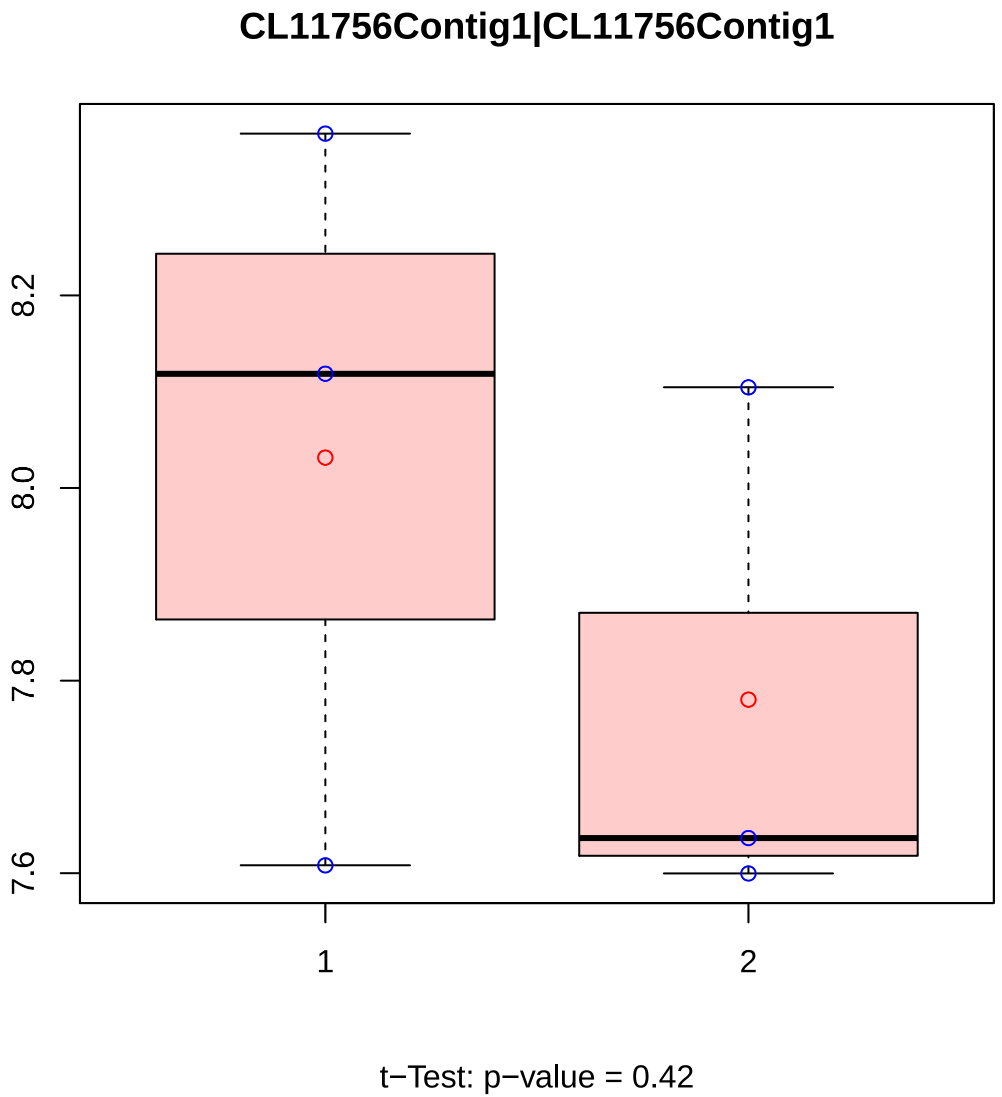

CL11756Contig1|CL11756Contig1
8.2
8.0
7.8
7.6
1
2
t−Test: p−value = 0.42

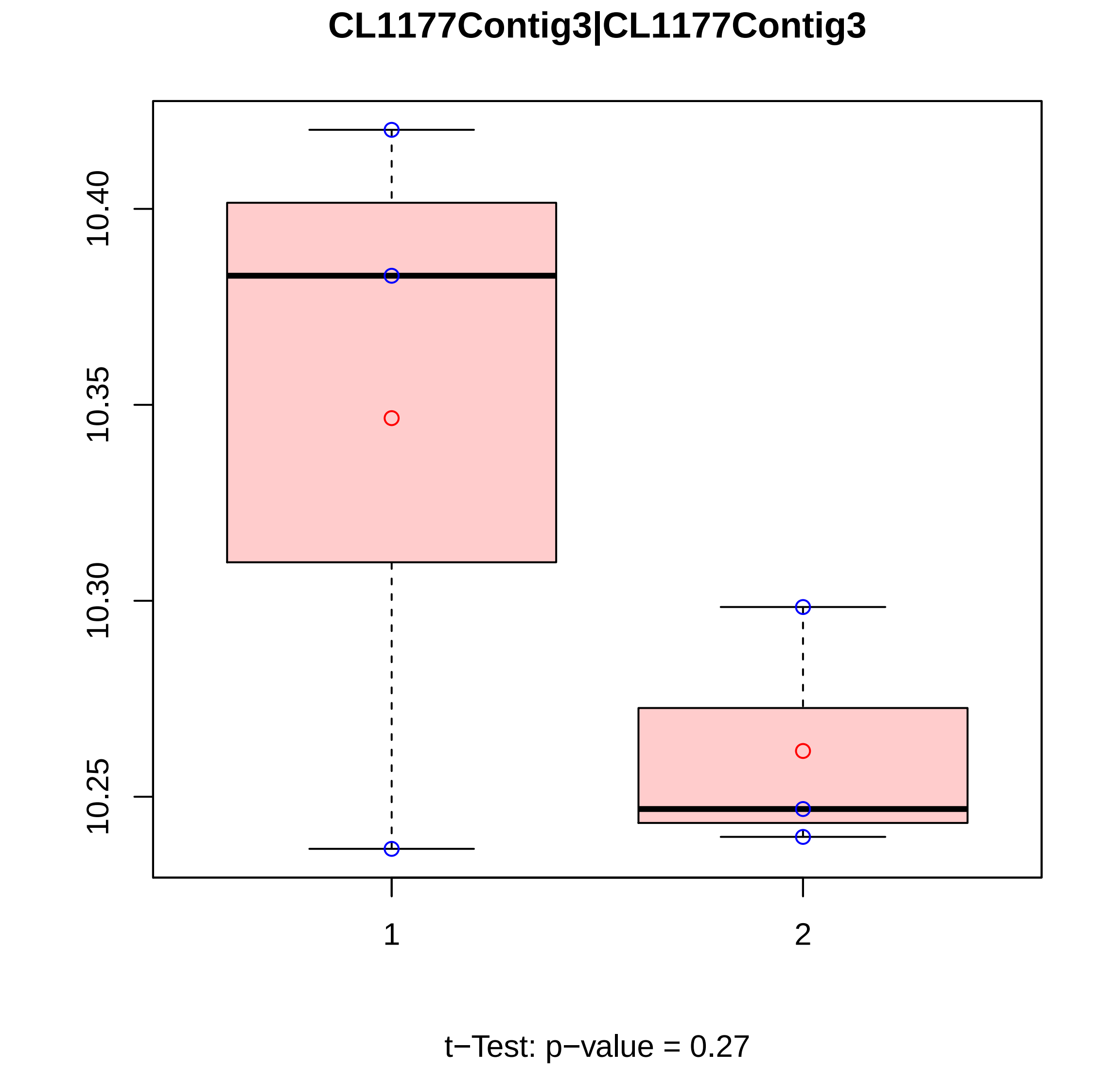
CL1177Contig3|CL1177Contig3
10.40
10.35
10.30
10.25
1
2
t−Test: p−value = 0.27

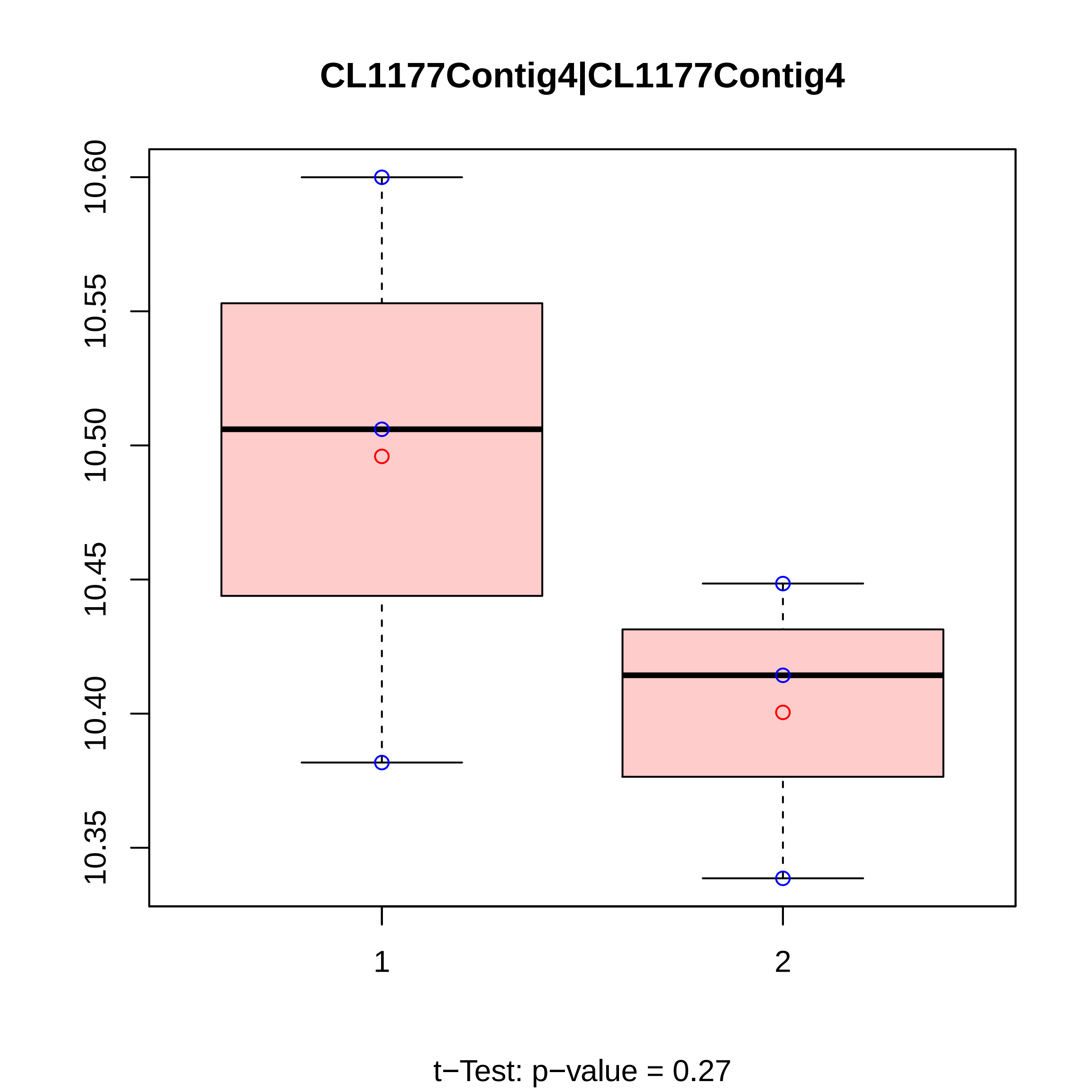
CL1177Contig4|CL1177Contig4
10.60
10.55
10.50
10.45
10.40
10.35
1
2
t−Test: p−value = 0.27

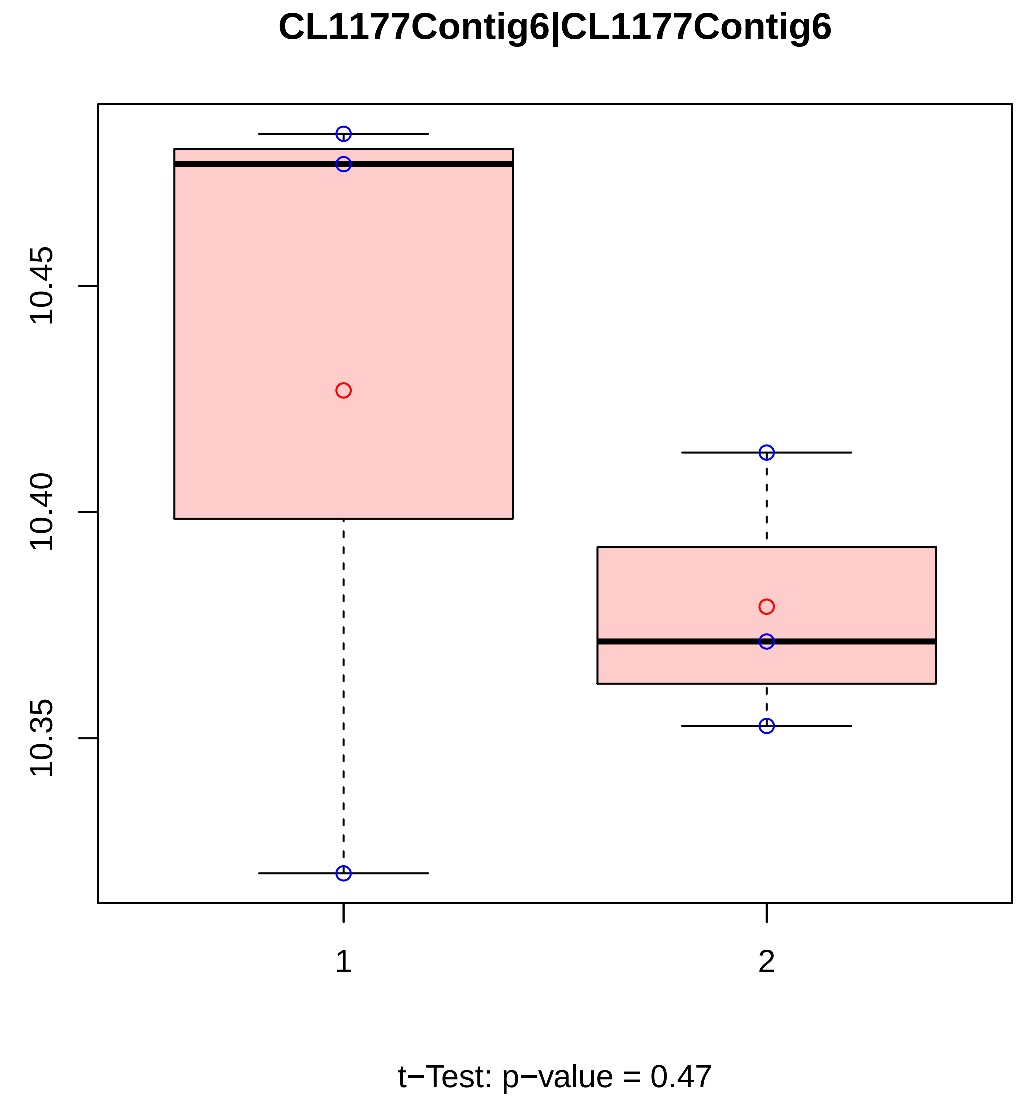
CL1177Contig6|CL1177Contig6
10.45
10.40
10.35
1
2
t−Test: p−value = 0.47

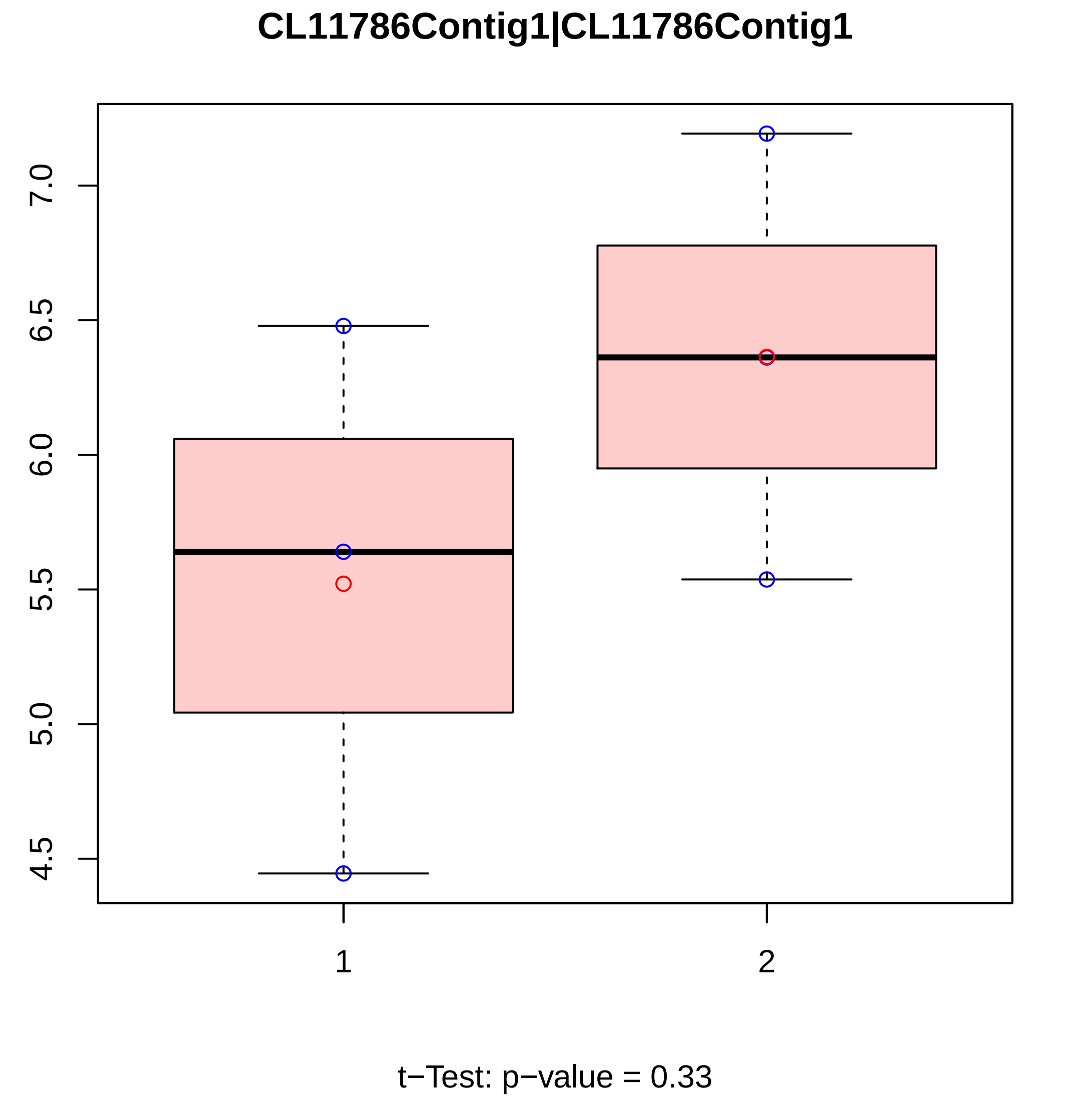
CL11786Contig1|CL11786Contig1
7.0
6.5
6.0
5.5
5.0
4.5
1
2
t−Test: p−value = 0.33

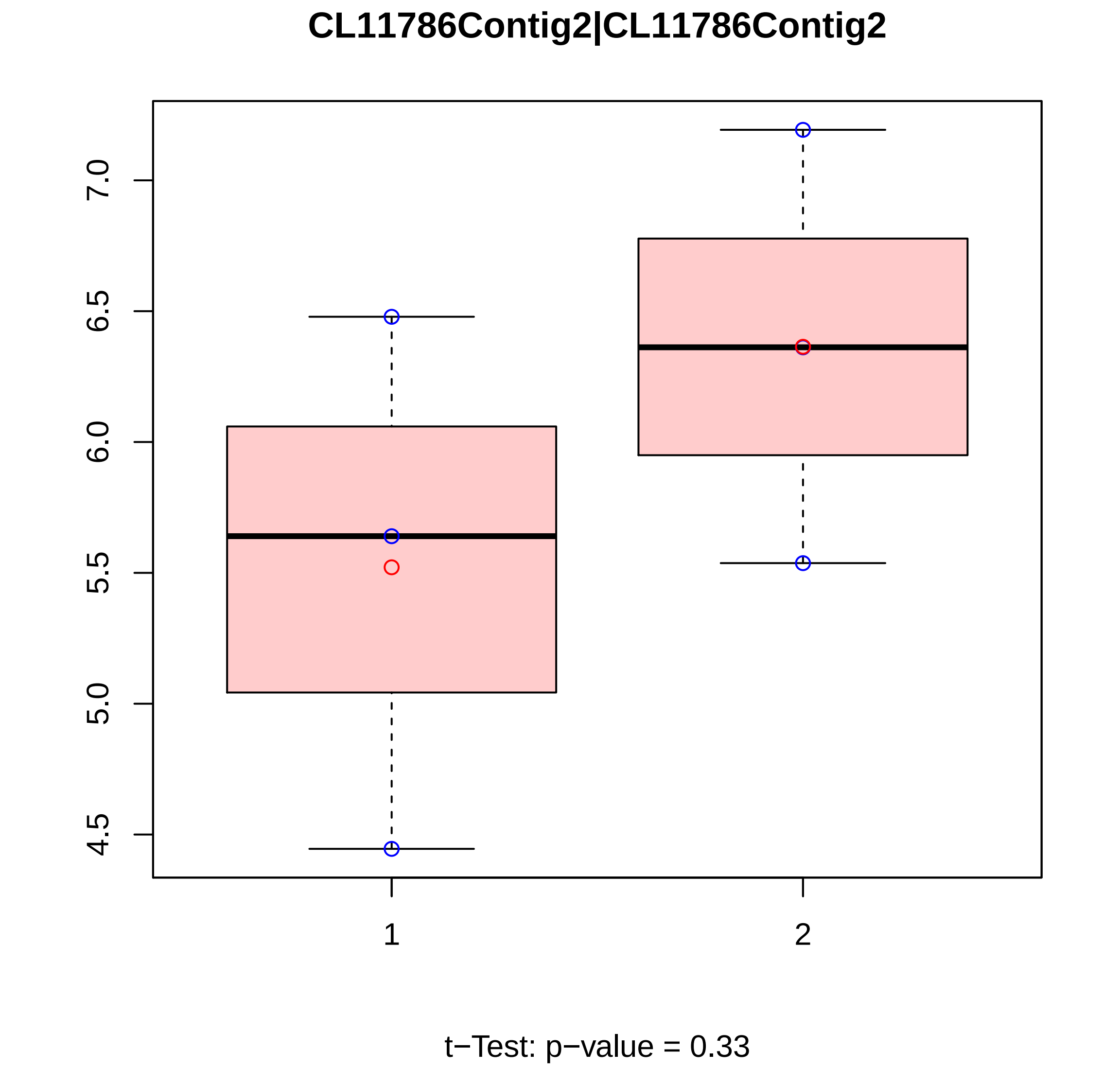
CL11786Contig2|CL11786Contig2
4.5
5.0
5.5
6.0
6.5
7.0
1
2
t−Test: p−value = 0.33

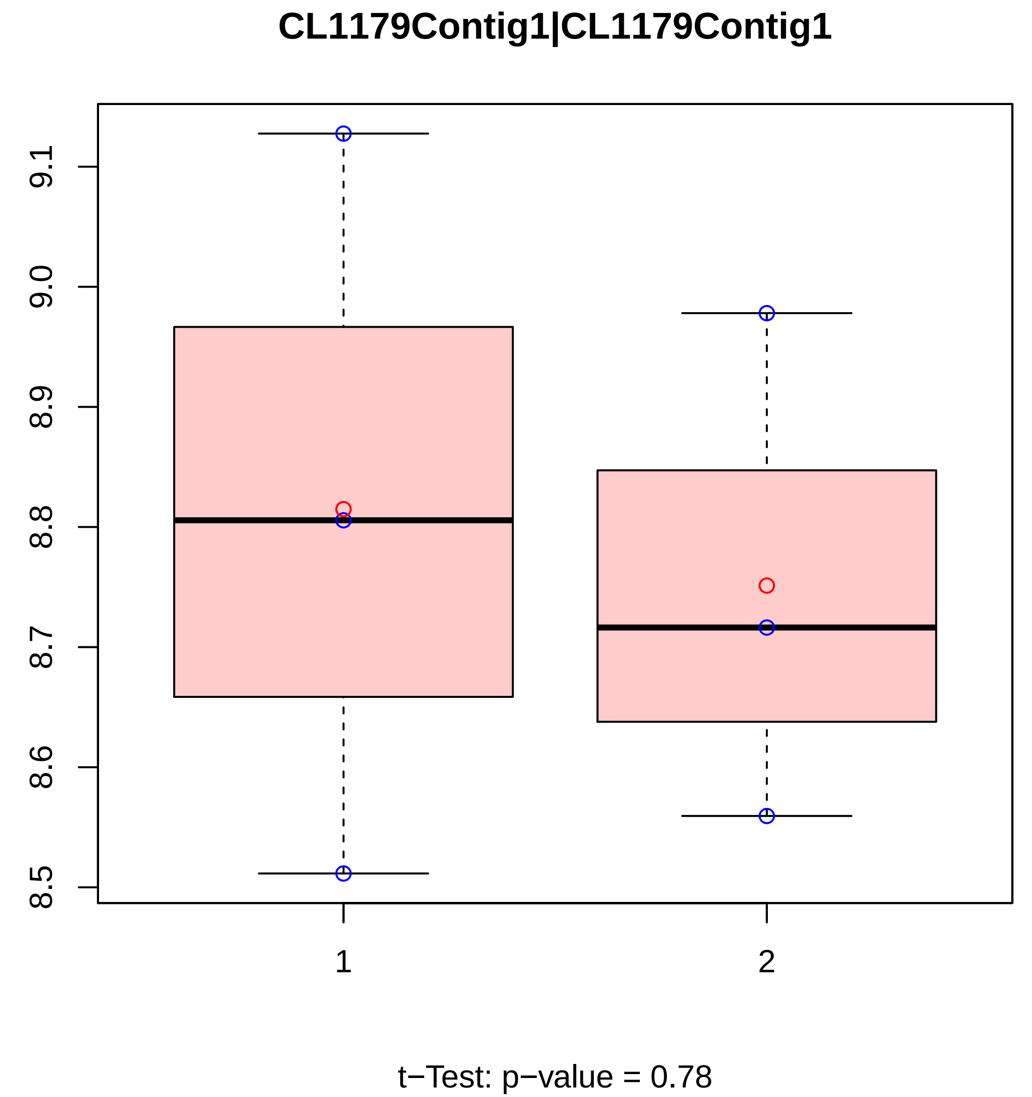
CL1179Contig1|CL1179Contig1
9.1
9.0
8.9
8.8
8.7
8.6
8.5
1
2
t−Test: p−value = 0.78

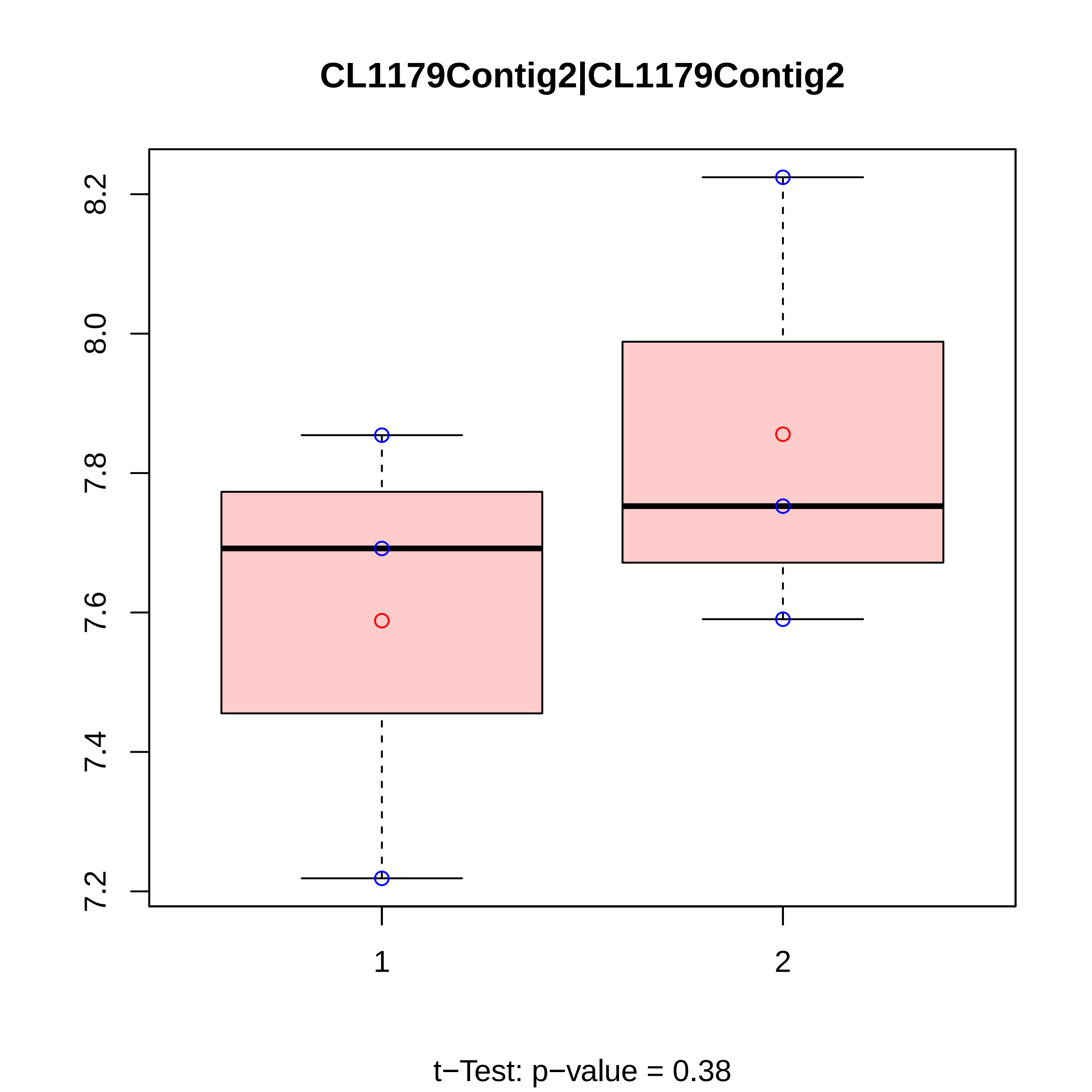

CL1179Contig2|CL1179Contig2
8.2
8.0
7.8
7.6
7.4
7.2
1
2
t−Test: p−value = 0.38

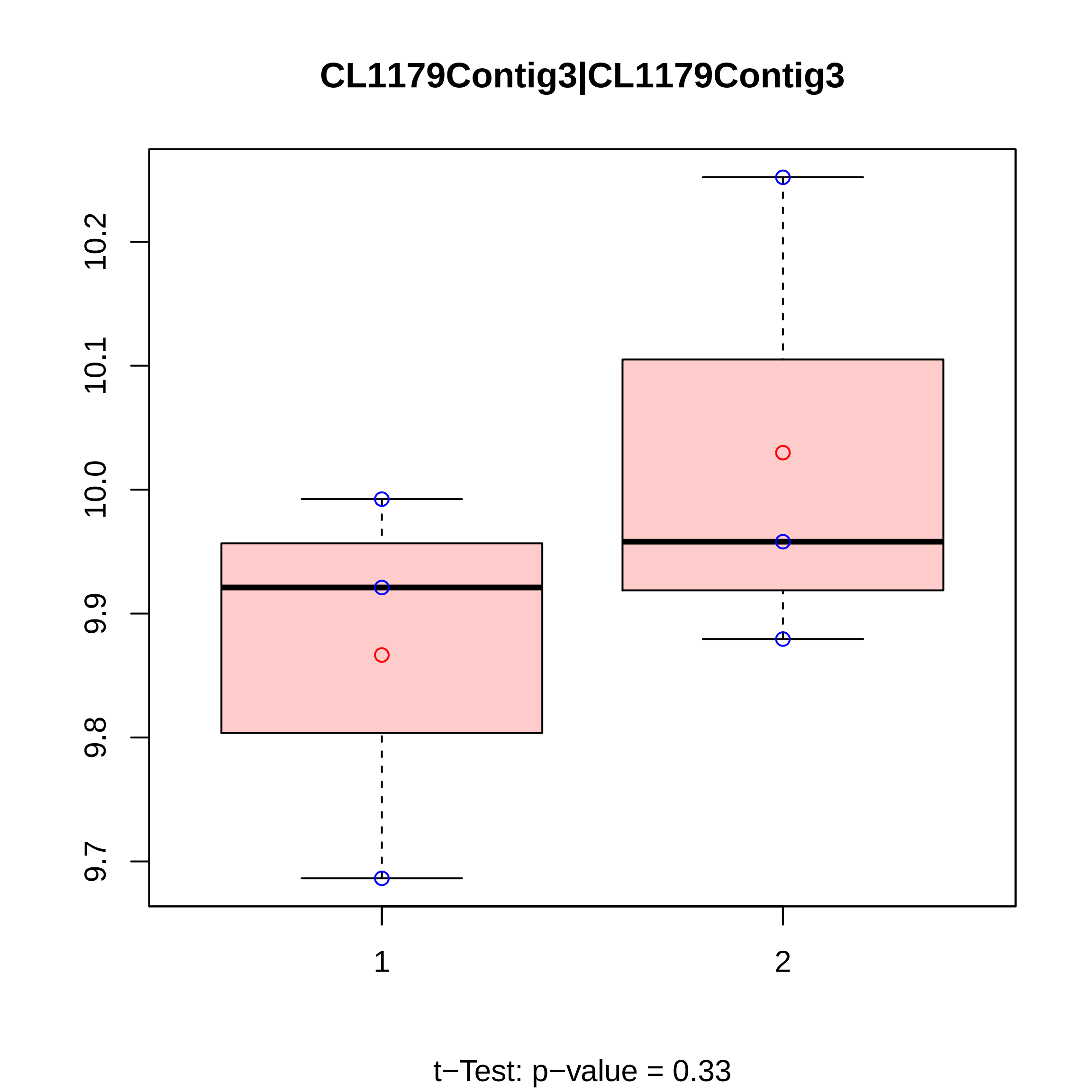
CL1179Contig3|CL1179Contig3
10.2
10.1
10.0
9.9
9.8
9.7
1
2
t−Test: p−value = 0.33

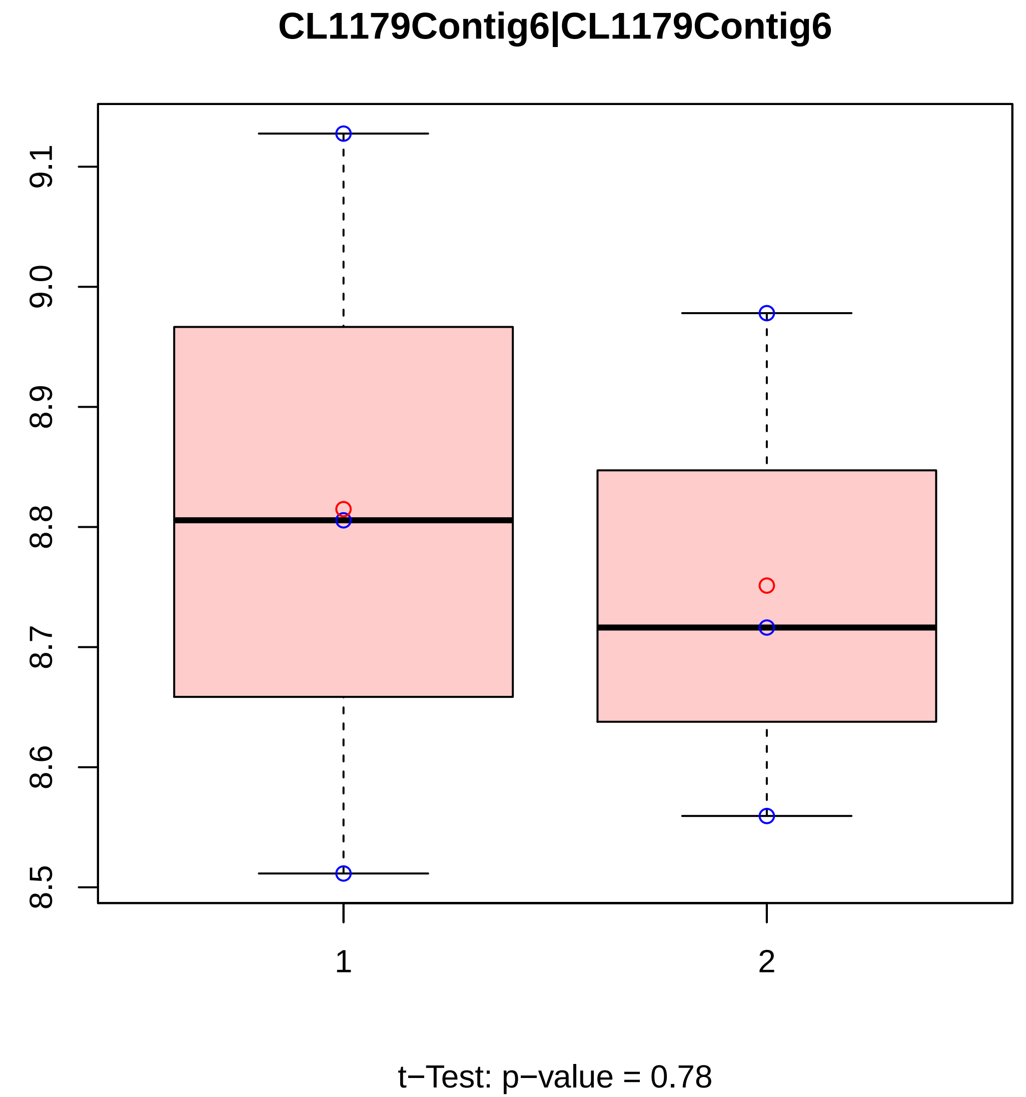
CL1179Contig6|CL1179Contig6
9.1
9.0
8.9
8.8
8.7
8.6
8.5
1
2
t−Test: p−value = 0.78

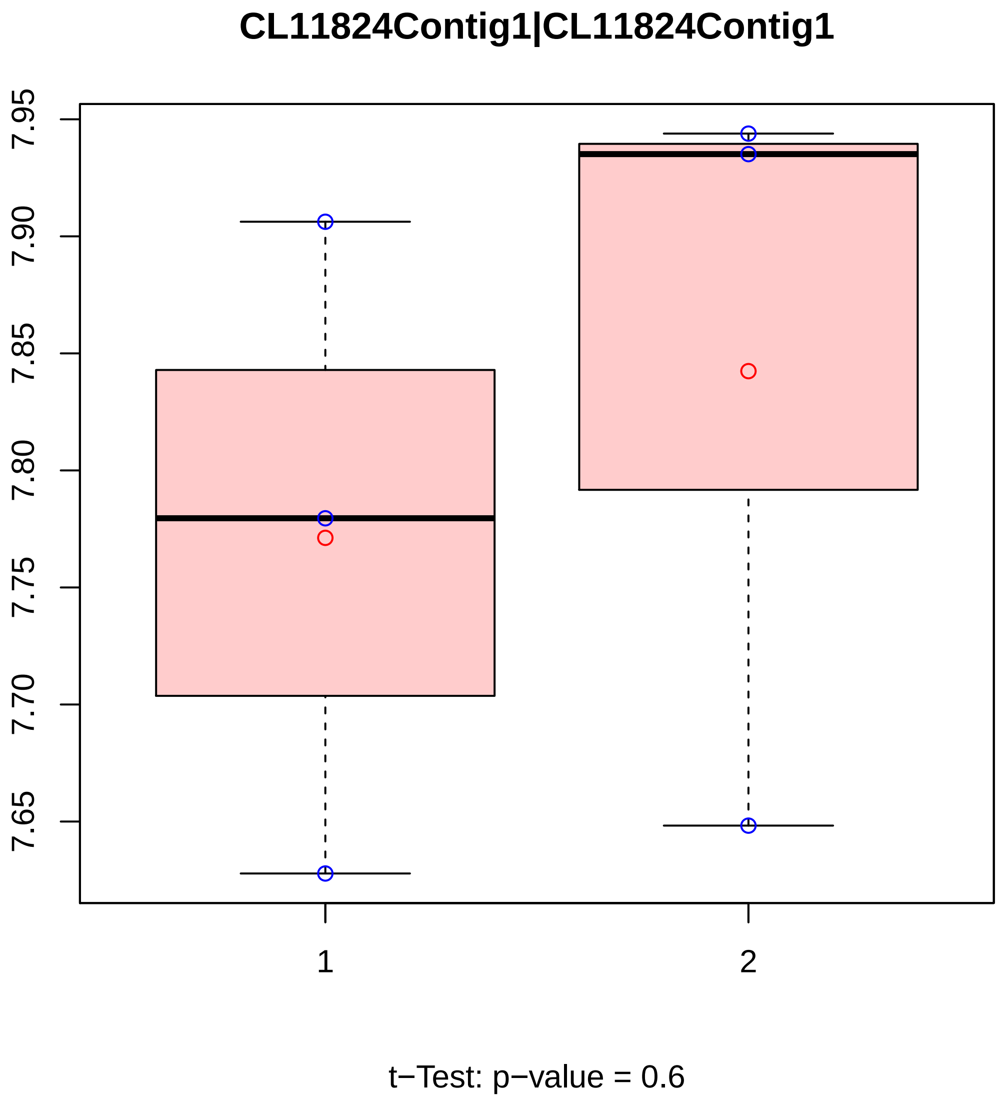
CL11824Contig1|CL11824Contig1
7.65
7.70
7.75
7.80
7.85
7.90
7.95
1
2
t−Test: p−value = 0.6

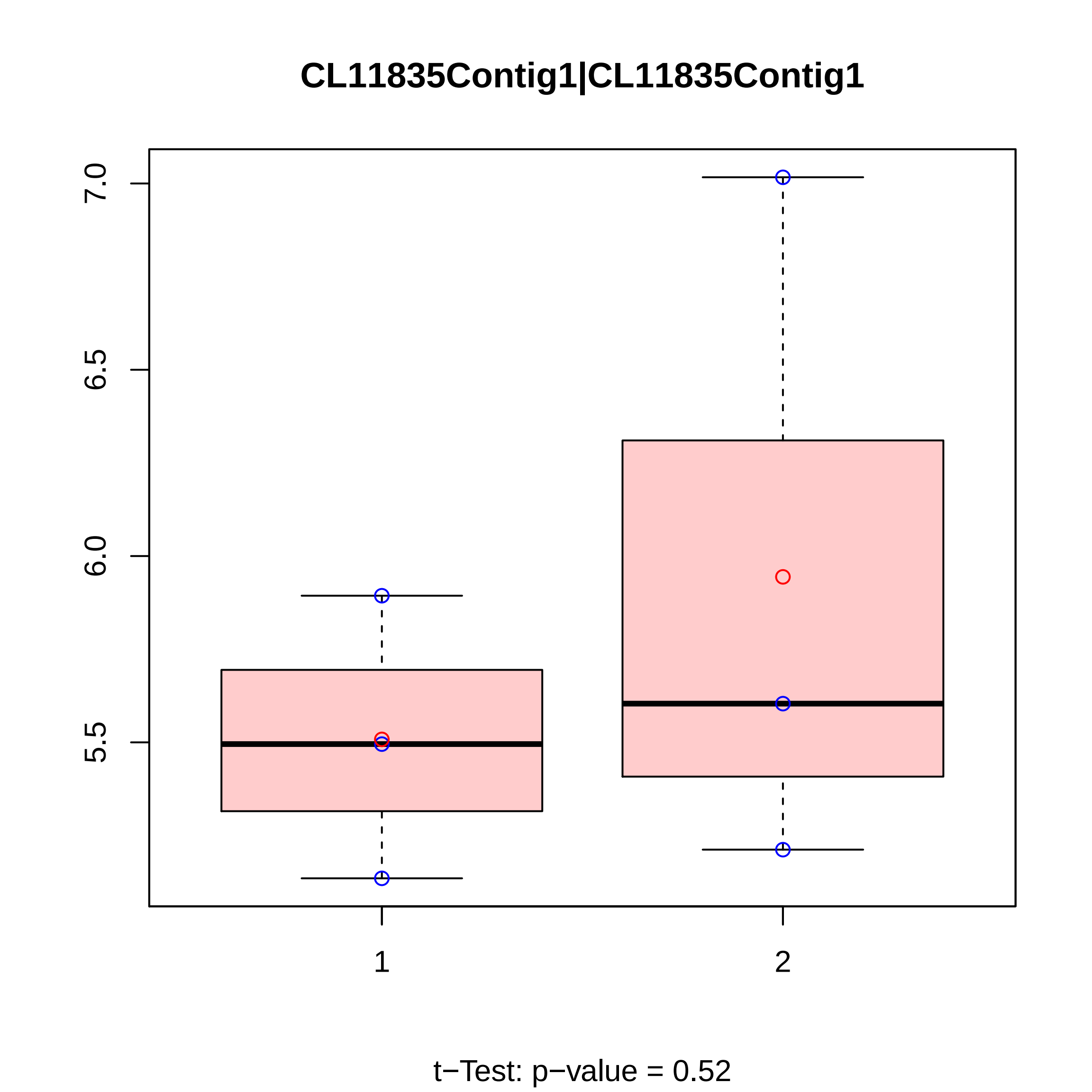
CL11835Contig1|CL11835Contig1
7.0
6.5
6.0
5.5
1
2
t−Test: p−value = 0.52

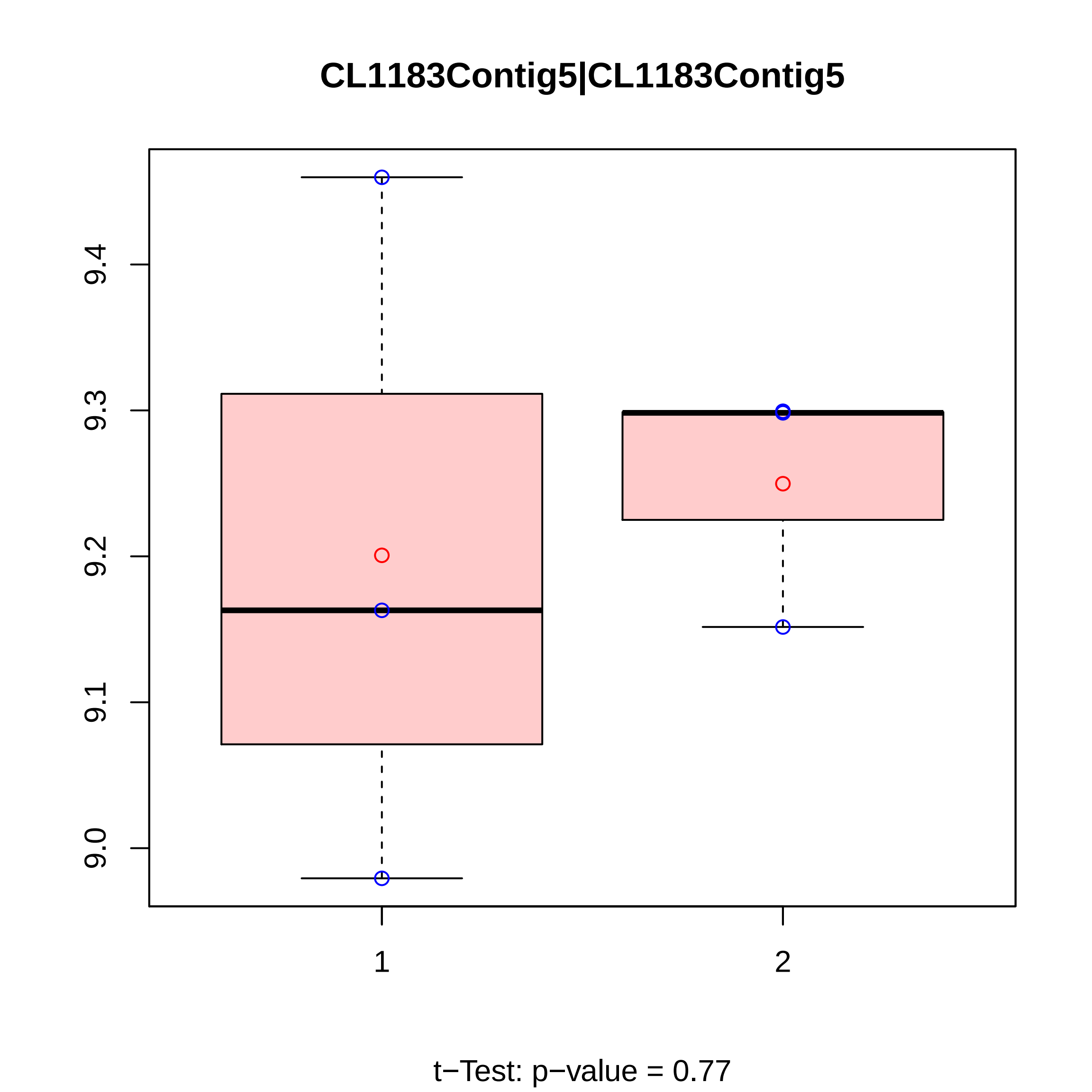
CL1183Contig5|CL1183Contig5
9.0
9.1
9.2
9.3
9.4
1
2
t−Test: p−value = 0.77

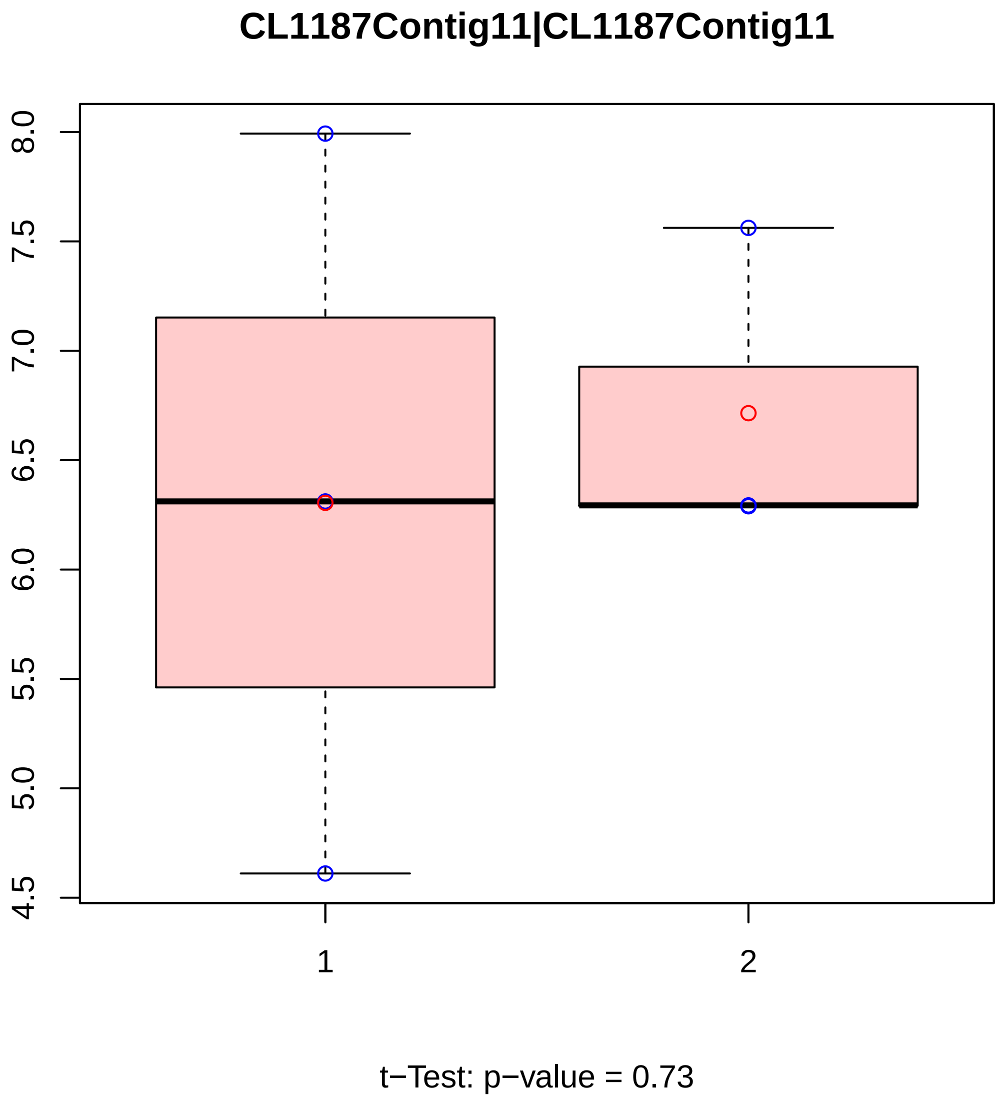
CL1187Contig11|CL1187Contig11
8.0
7.5
7.0
6.5
6.0
5.5
5.0
4.5
1
2
t−Test: p−value = 0.73

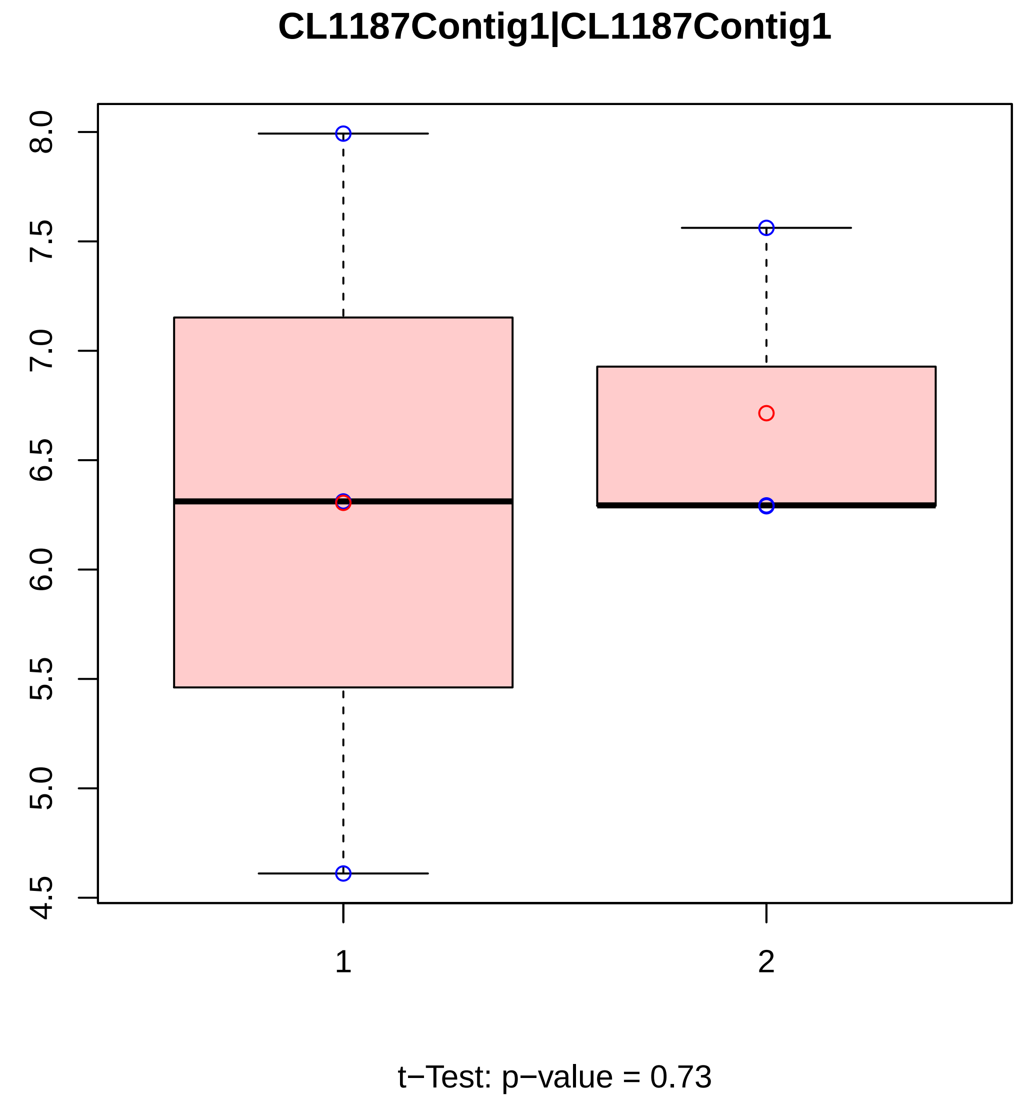
CL1187Contig1|CL1187Contig1
8.0
7.5
7.0
6.5
6.0
5.5
5.0
4.5
1
2
t−Test: p−value = 0.73

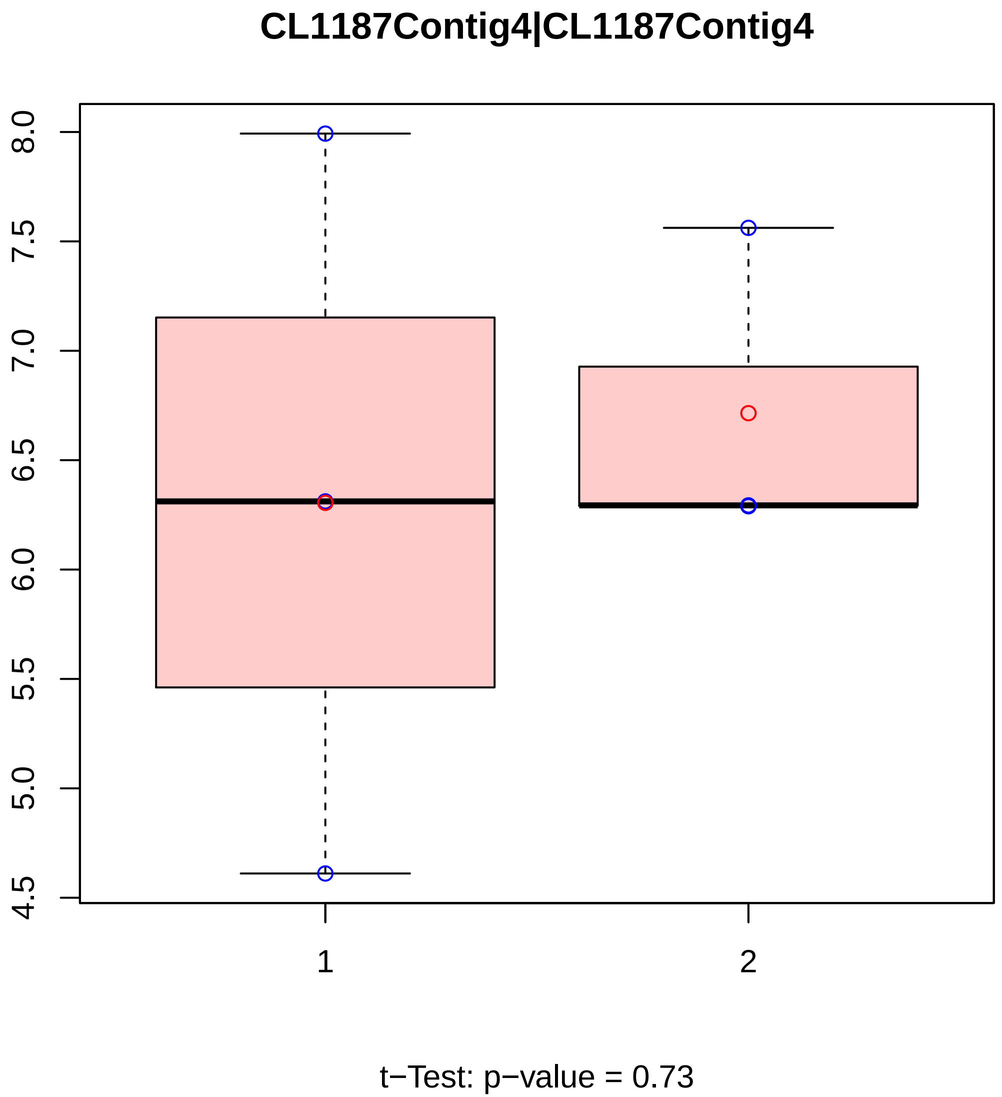
CL1187Contig4|CL1187Contig4
8.0
7.5
7.0
6.5
6.0
5.5
5.0
4.5
1
2
t−Test: p−value = 0.73

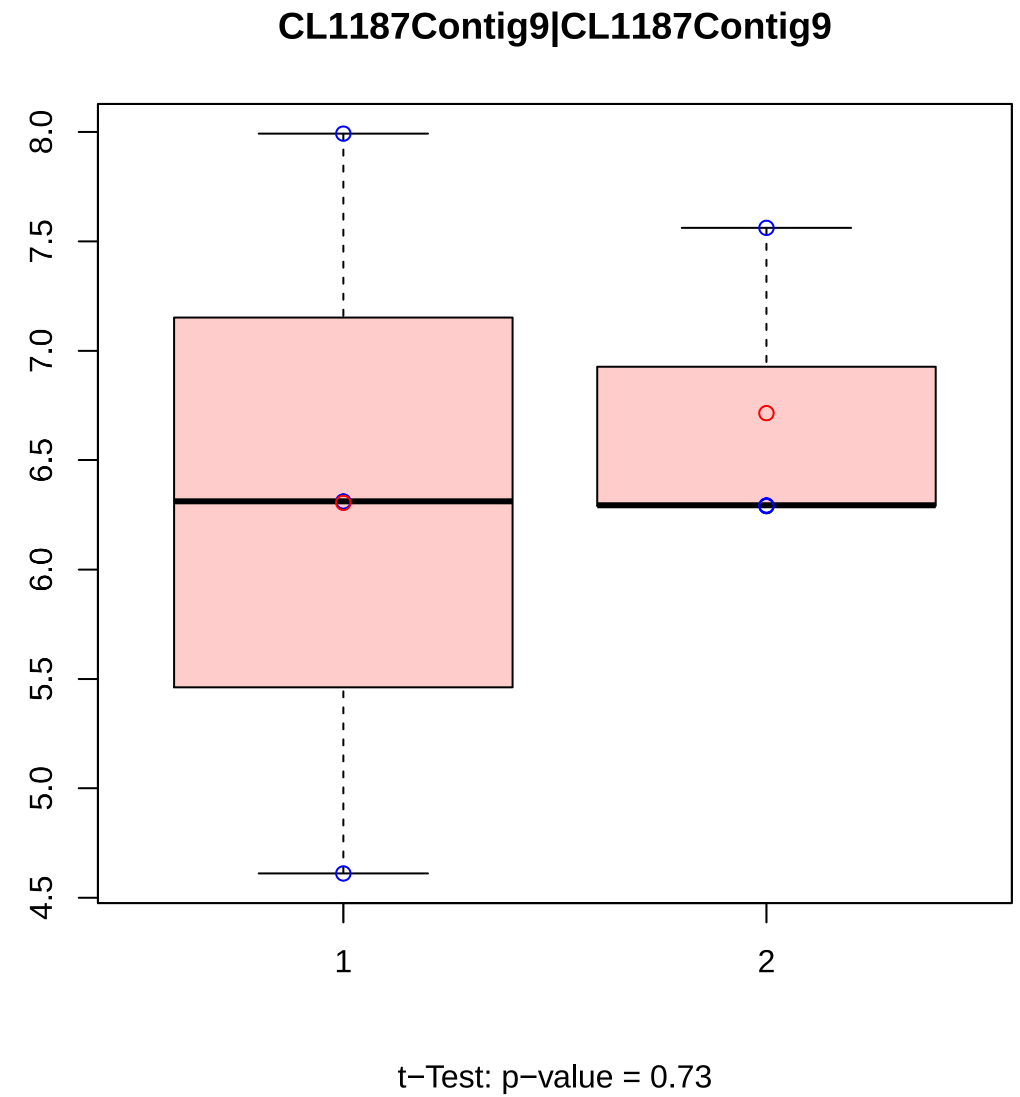
CL1187Contig9|CL1187Contig9
8.0
7.5
7.0
6.5
6.0
5.5
5.0
4.5
1
2
t−Test: p−value = 0.73

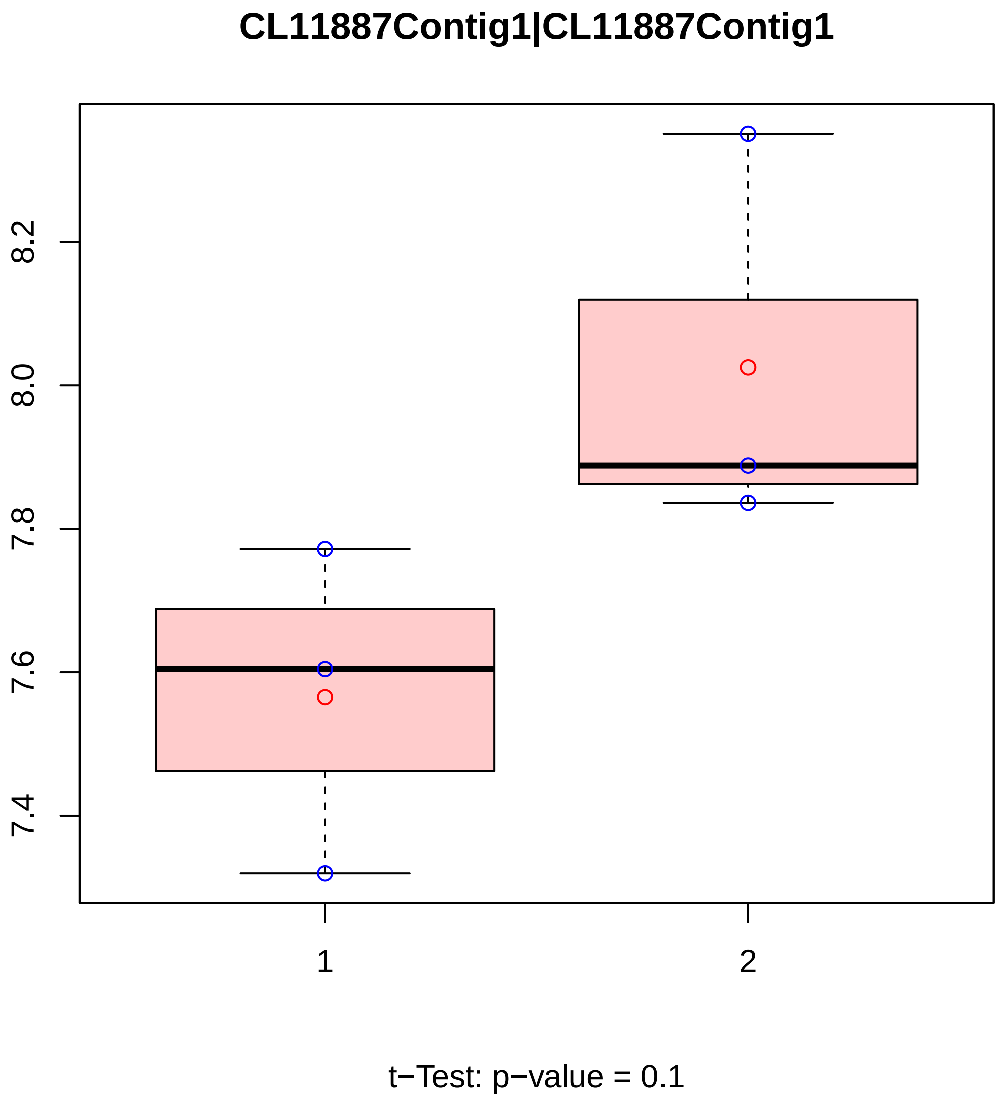

CL11887Contig1|CL11887Contig1
8.2
8.0
7.8
7.6
7.4
1
2
t−Test: p−value = 0.1

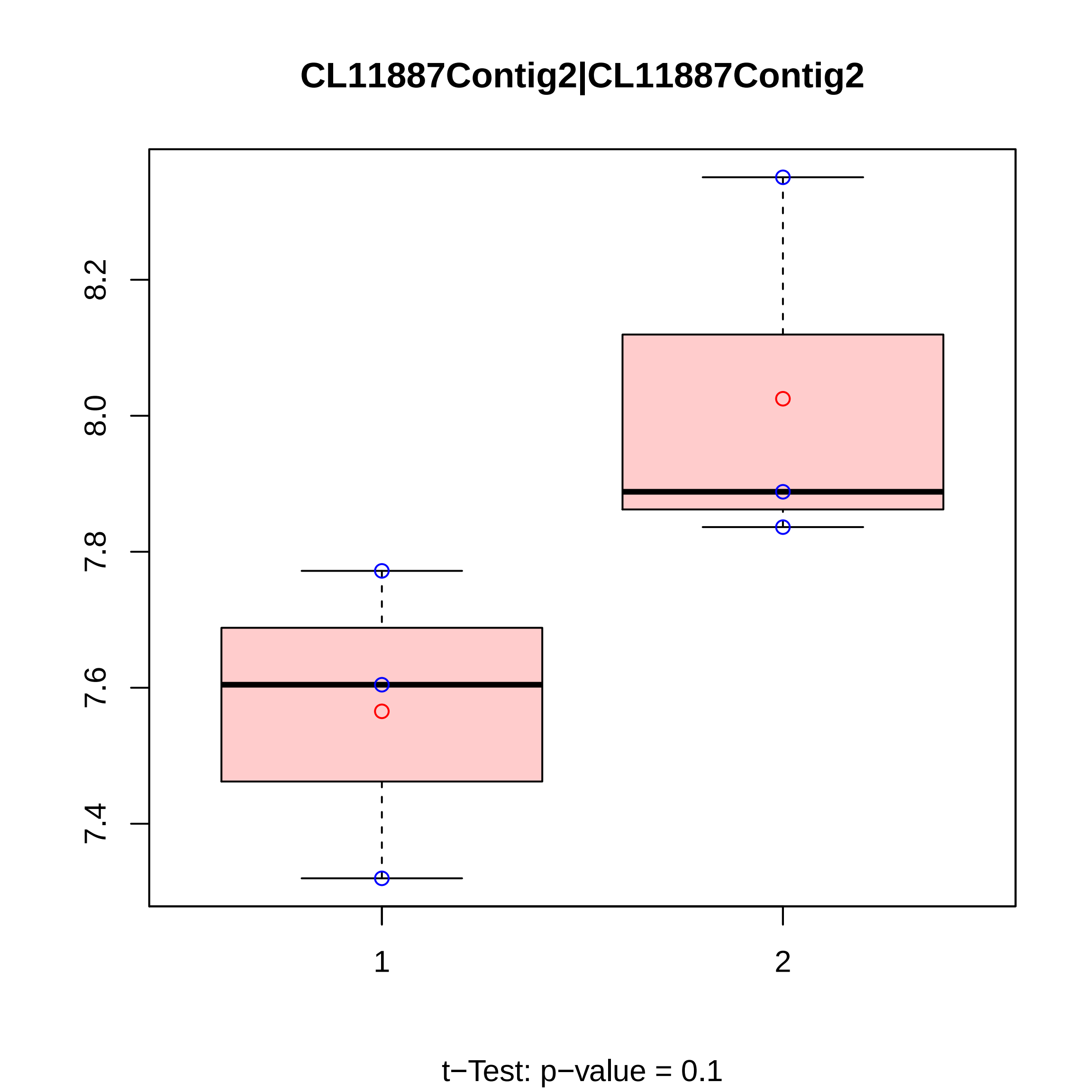
CL11887Contig2|CL11887Contig2
7.4
7.6
7.8
8.0
8.2
1
2
t−Test: p−value = 0.1

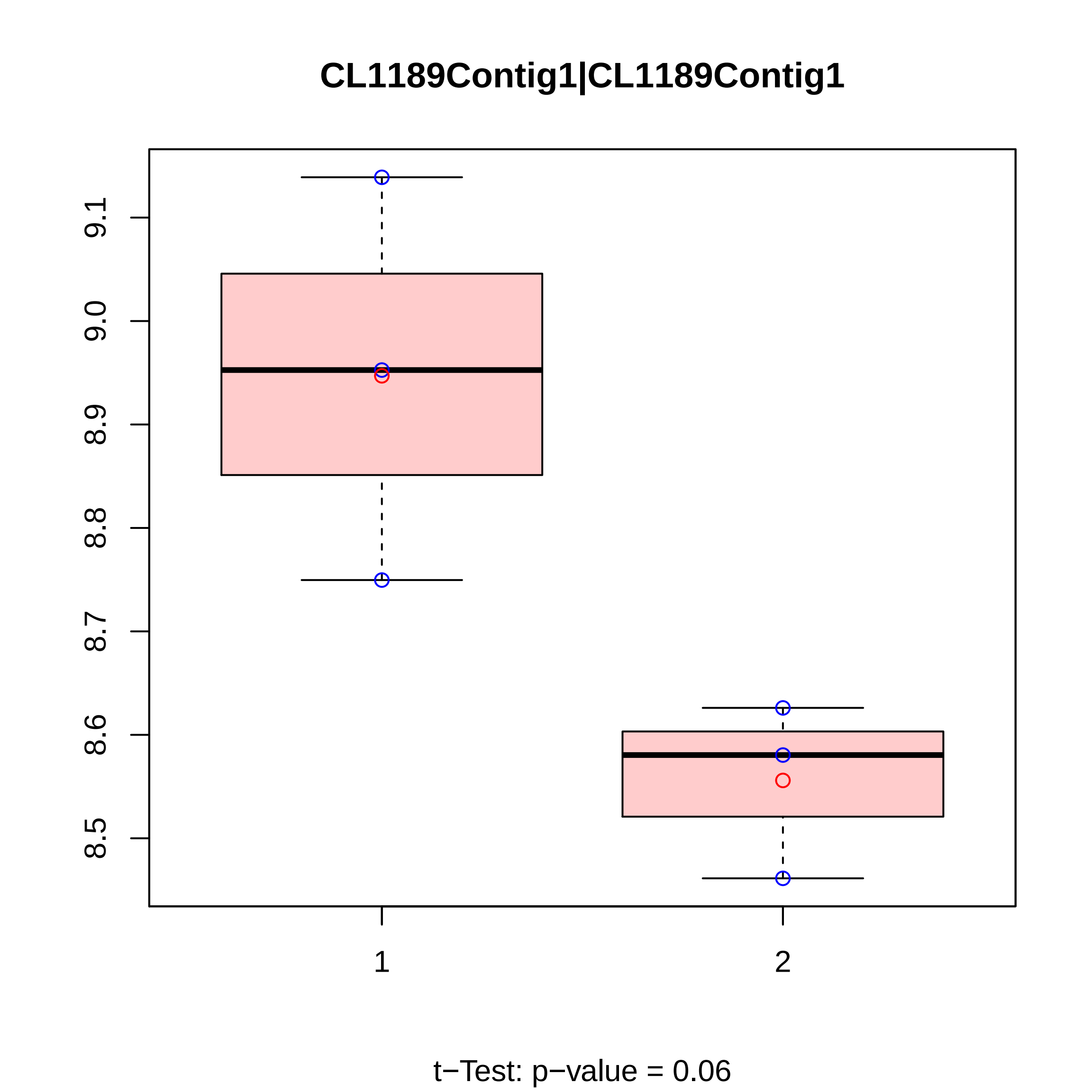
CL1189Contig1|CL1189Contig1
9.1
9.0
8.9
8.8
8.7
8.6
8.5
1
2
t−Test: p−value = 0.06

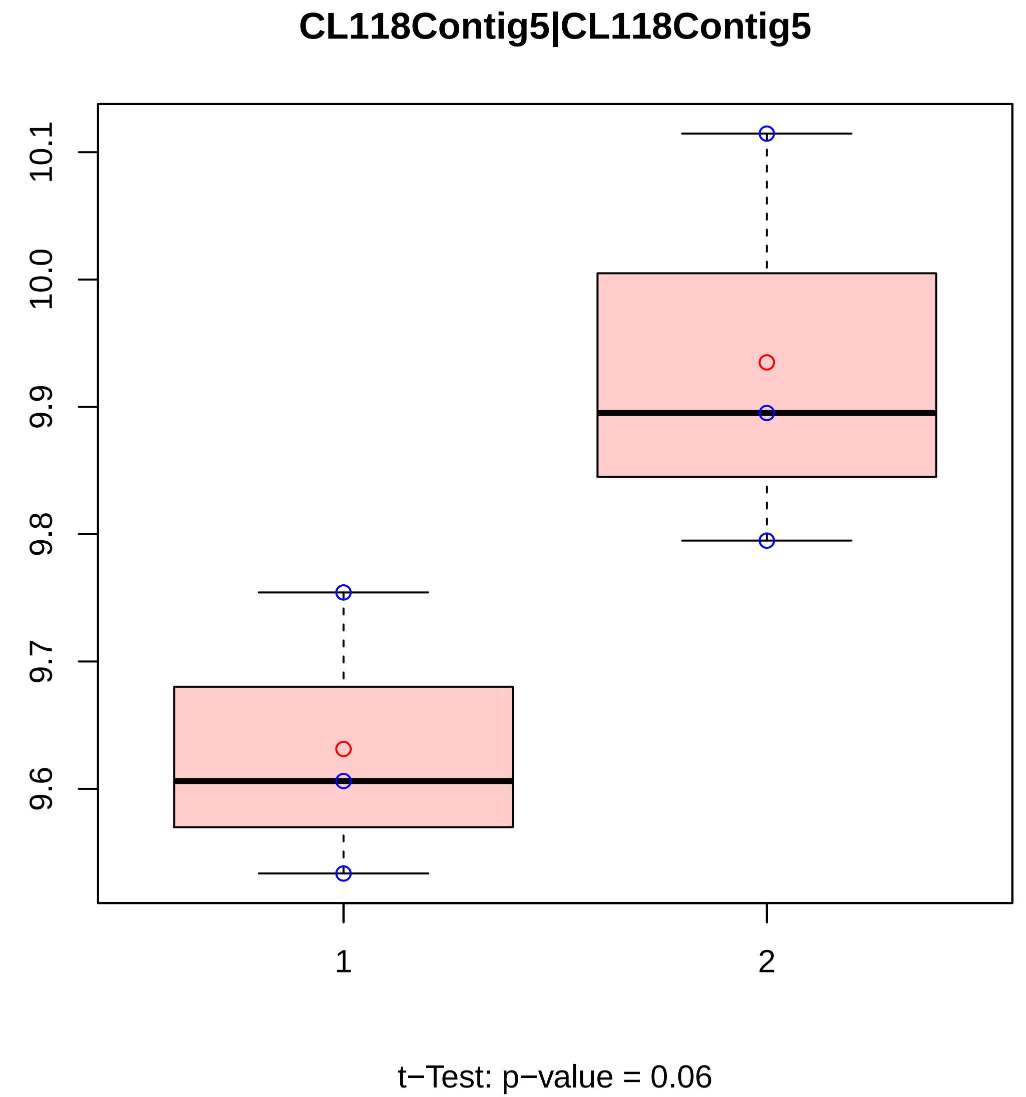

CL118Contig5|CL118Contig5
10.1
10.0
9.9
9.8
9.7
9.6
1
2
t−Test: p−value = 0.06

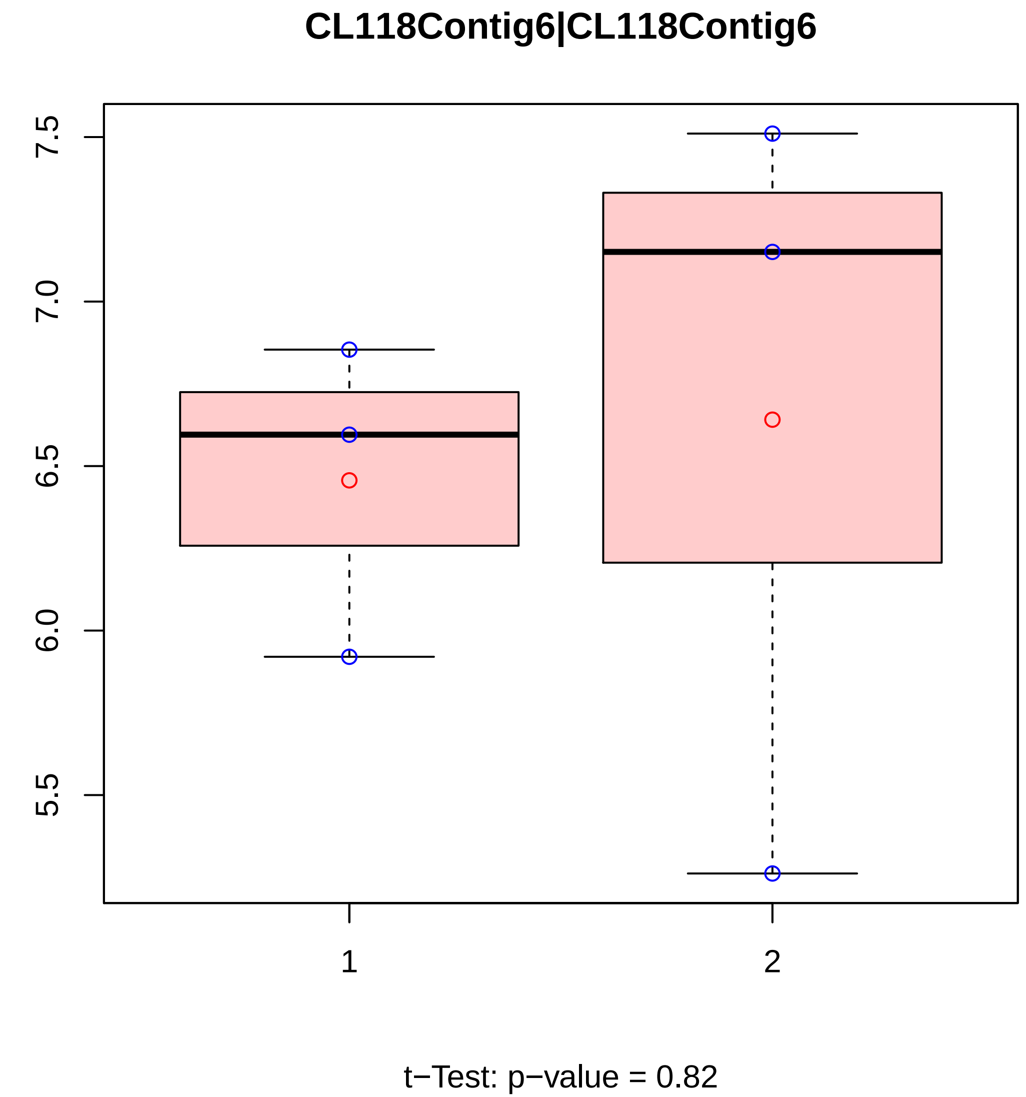
CL118Contig6|CL118Contig6
7.5
7.0
6.5
6.0
5.5
1
2
t−Test: p−value = 0.82

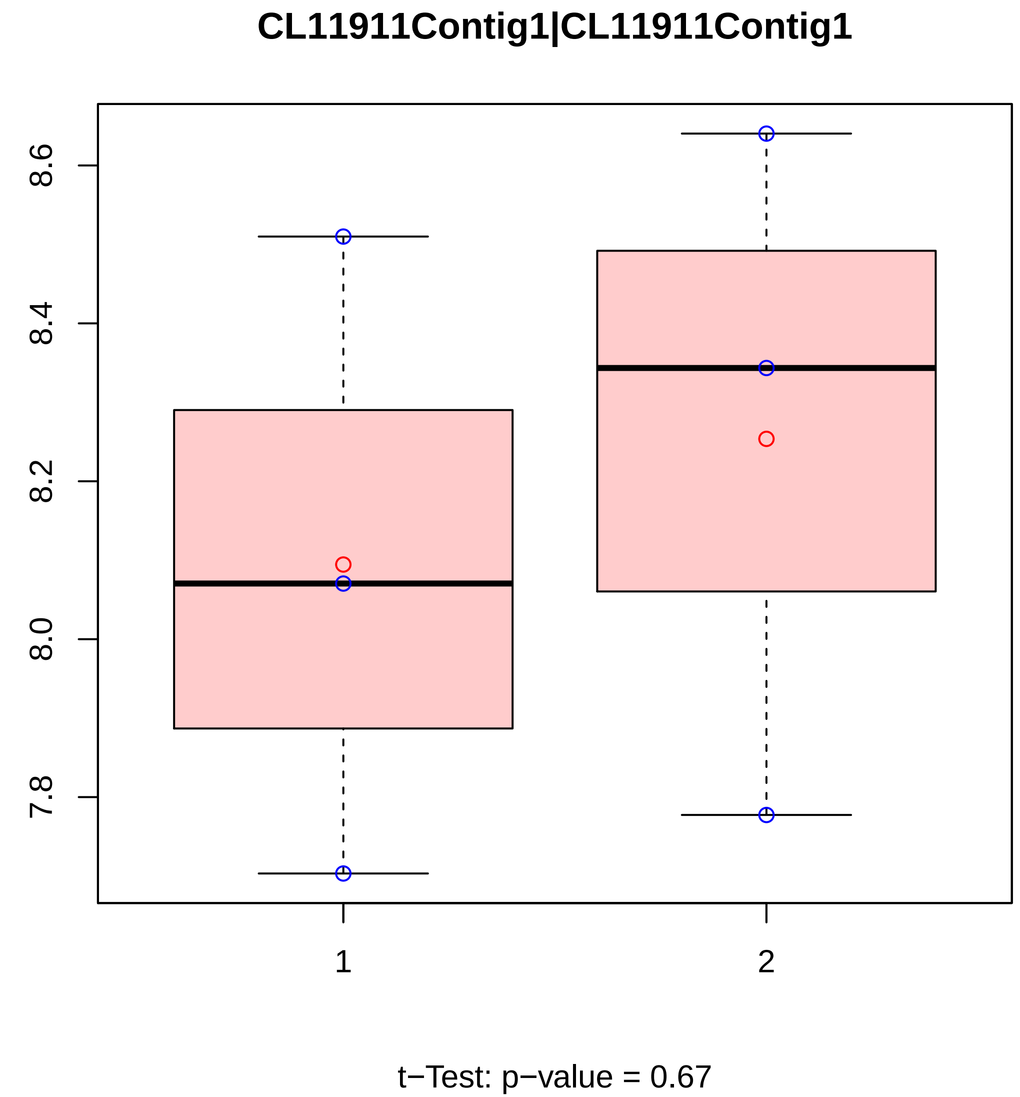
CL11911Contig1|CL11911Contig1
8.6
8.4
8.2
8.0
7.8
1
2
t−Test: p−value = 0.67

**CL11931Contig1|CL11931Contig1**

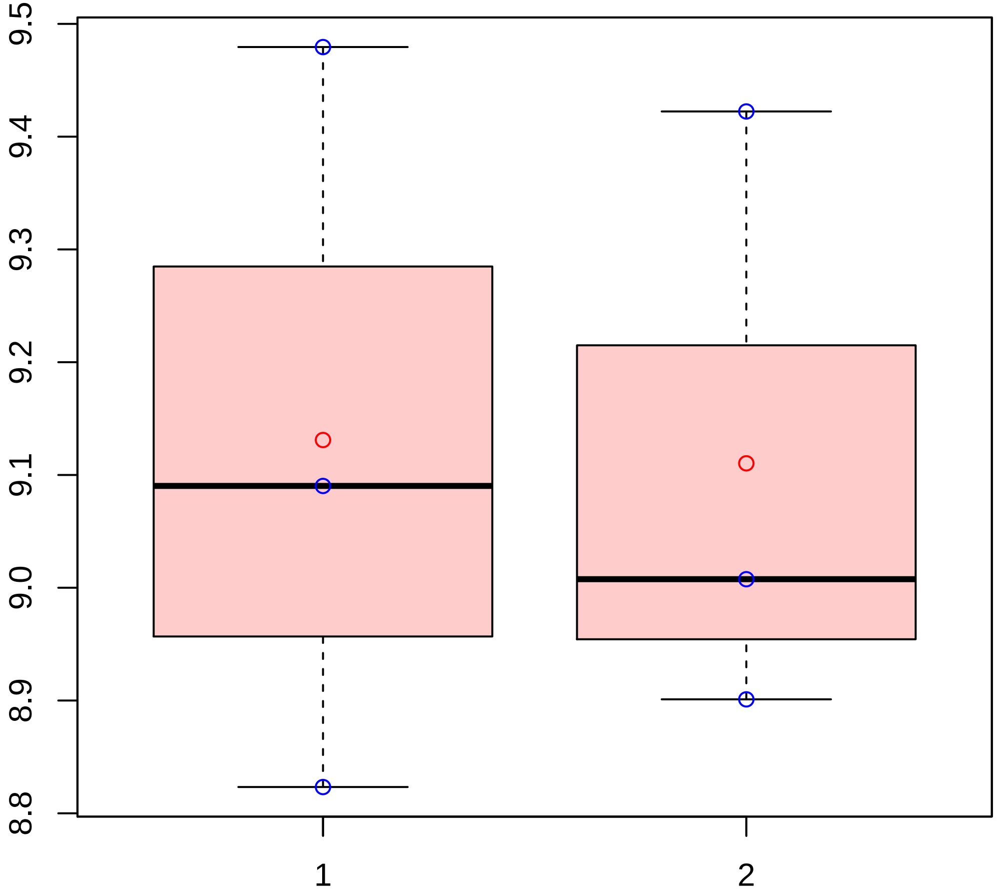


t−Test: p−value = 0.94

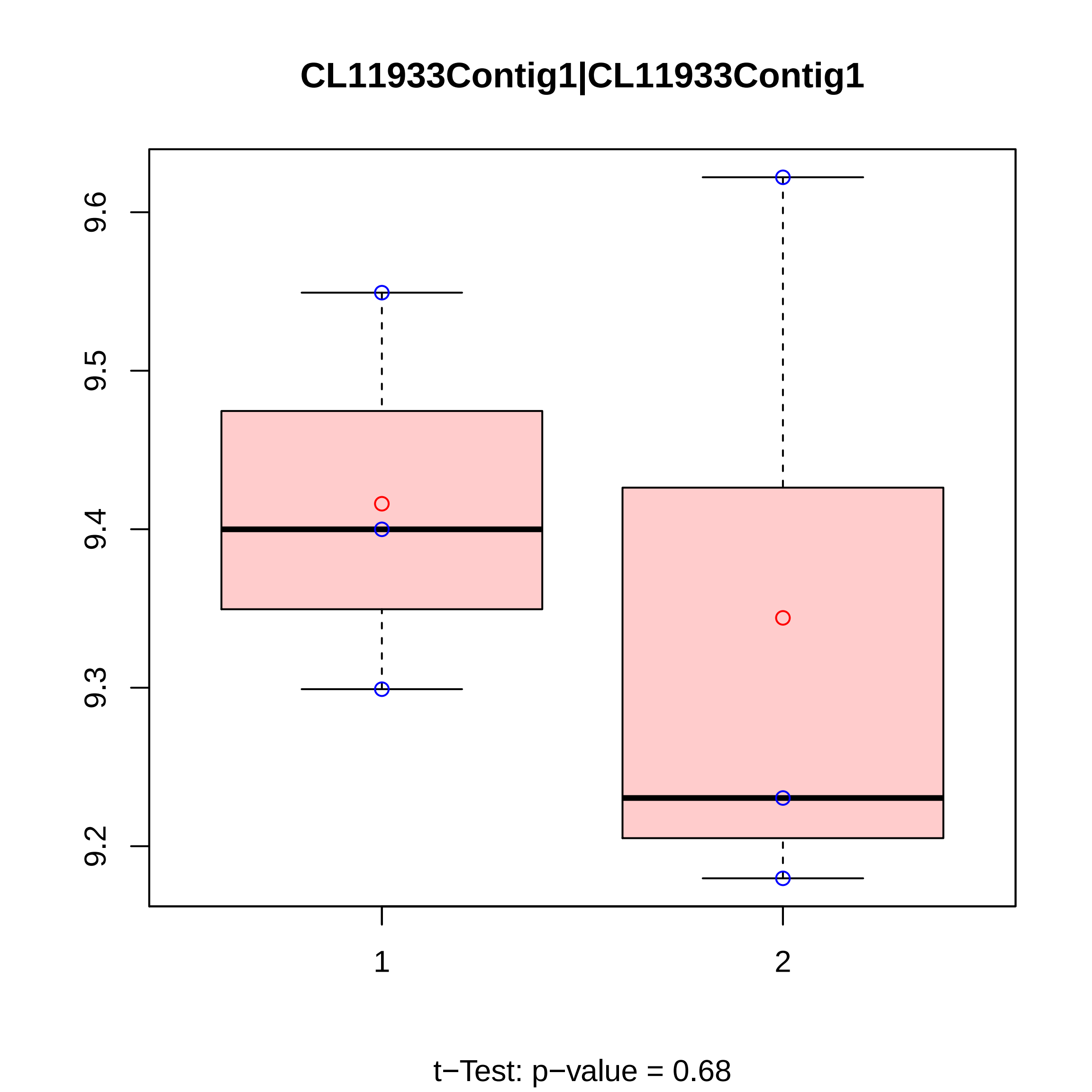
CL11933Contig1|CL11933Contig1
9.2
9.3
9.4
9.5
9.6
1
2
t−Test: p−value = 0.68

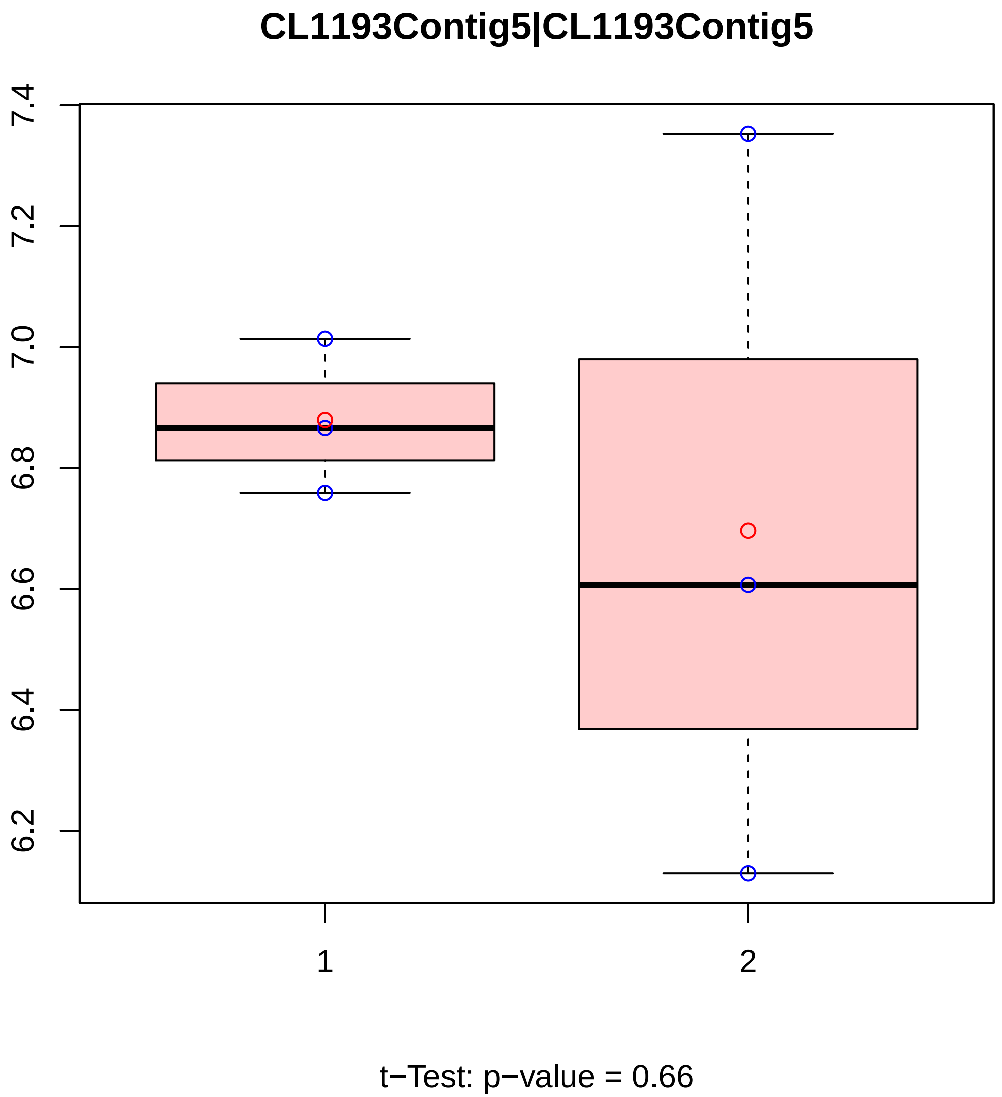
CL1193Contig5|CL1193Contig5
7.4
7.2
7.0
6.8
6.6
6.4
6.2
1
2
t−Test: p−value = 0.66

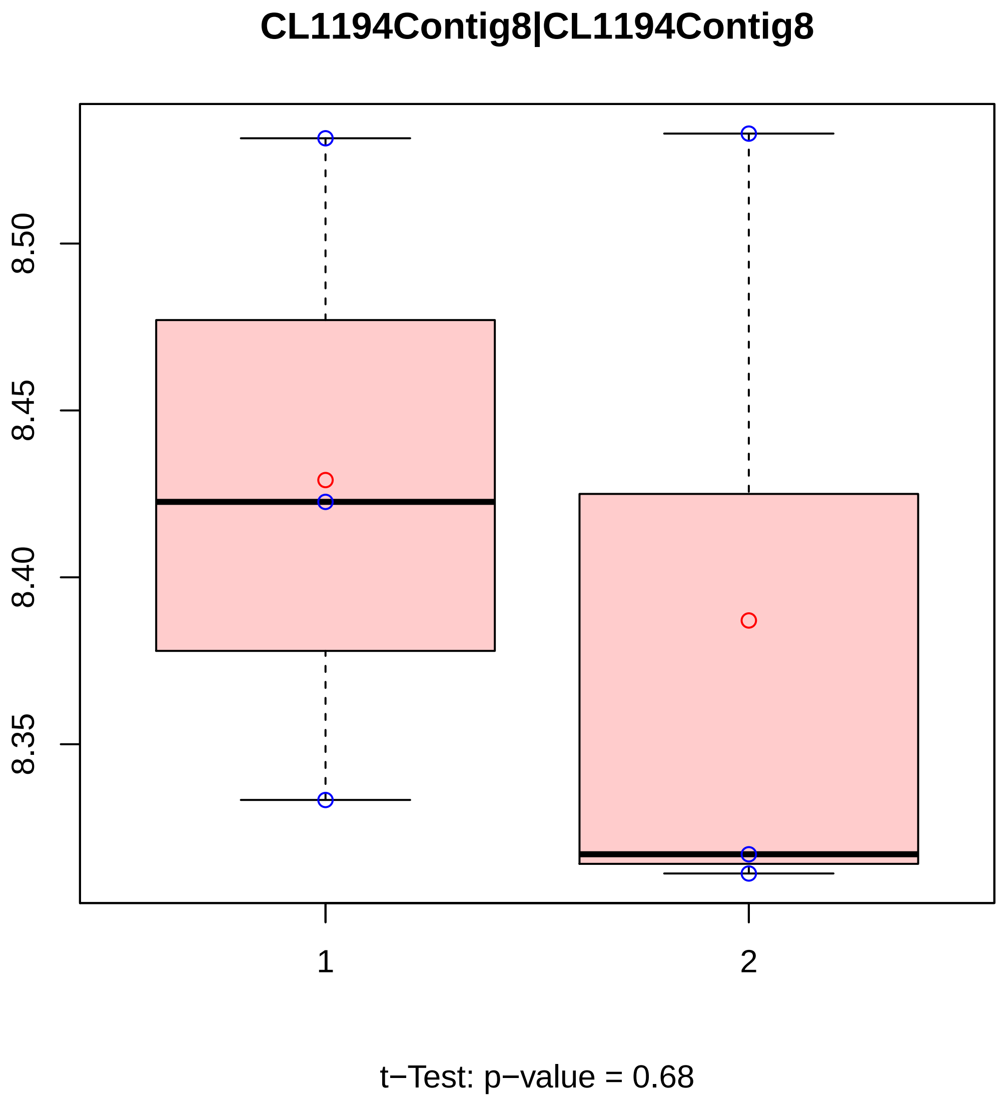

CL1194Contig8|CL1194Contig8
8.35
8.40
8.45
8.50
1
2
t−Test: p−value = 0.68

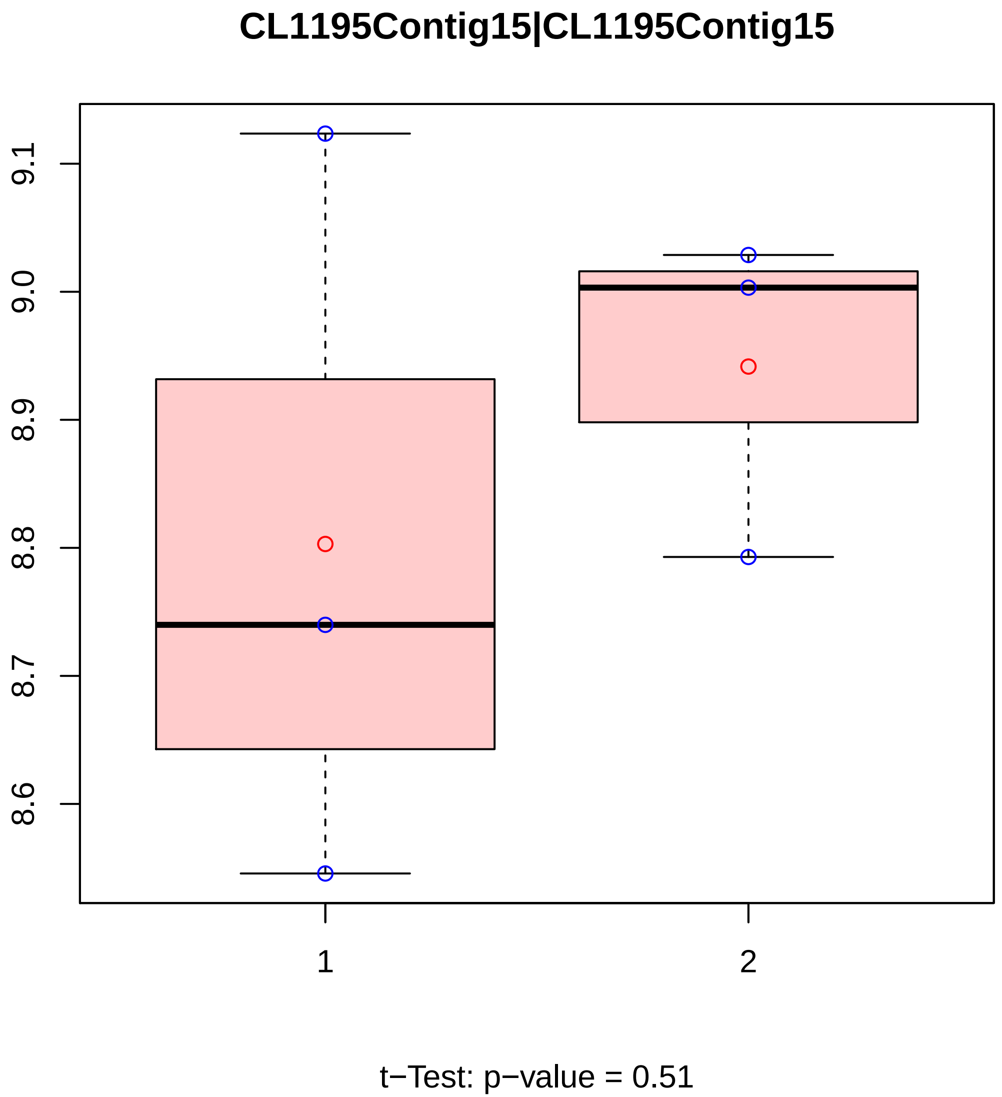
CL1195Contig15|CL1195Contig15
9.1
9.0
8.9
8.8
8.7
8.6
1
2
t−Test: p−value = 0.51

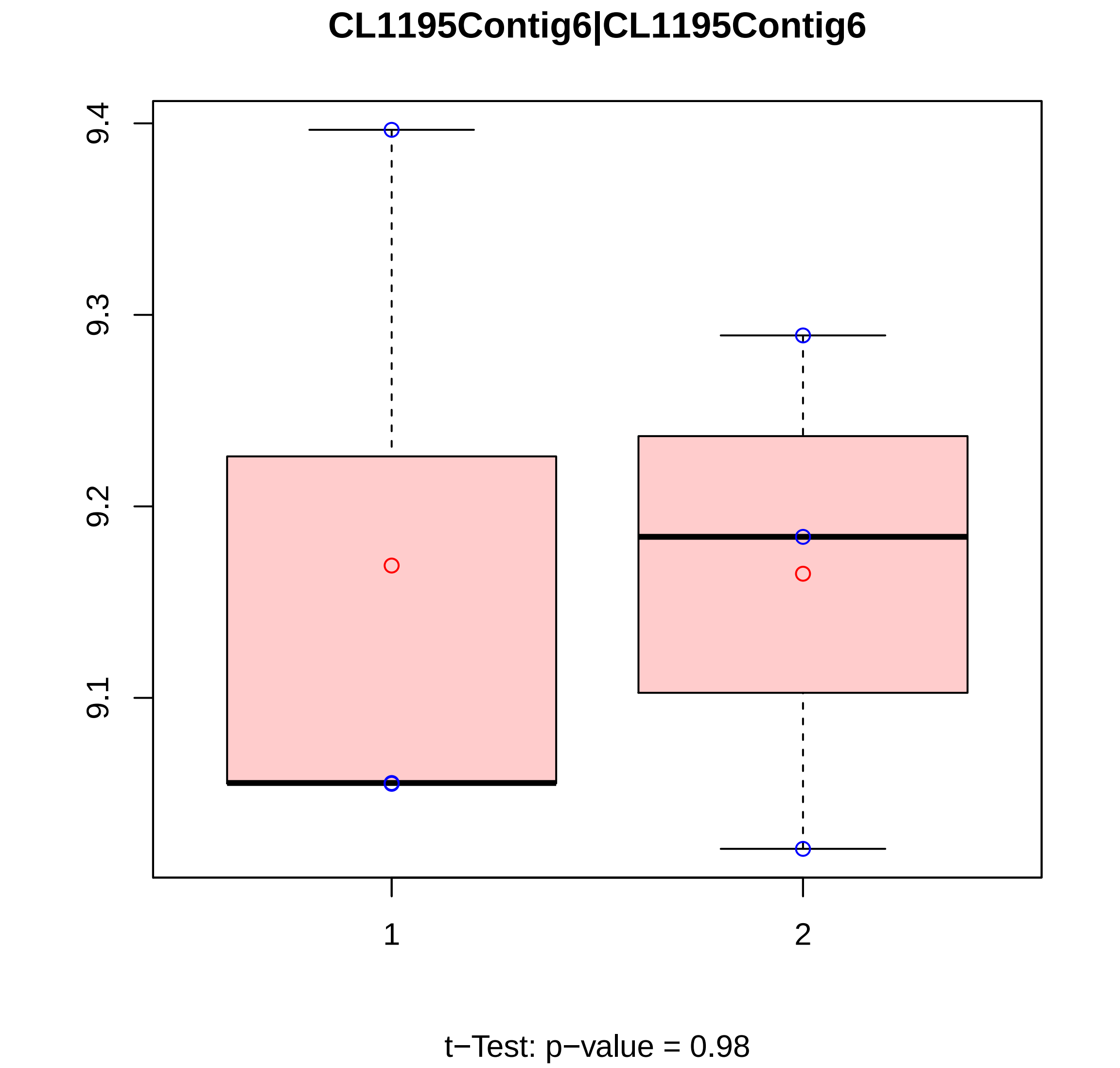

CL1195Contig6|CL1195Contig6
9.4
9.3
9.2
9.1
1
2
t−Test: p−value = 0.98

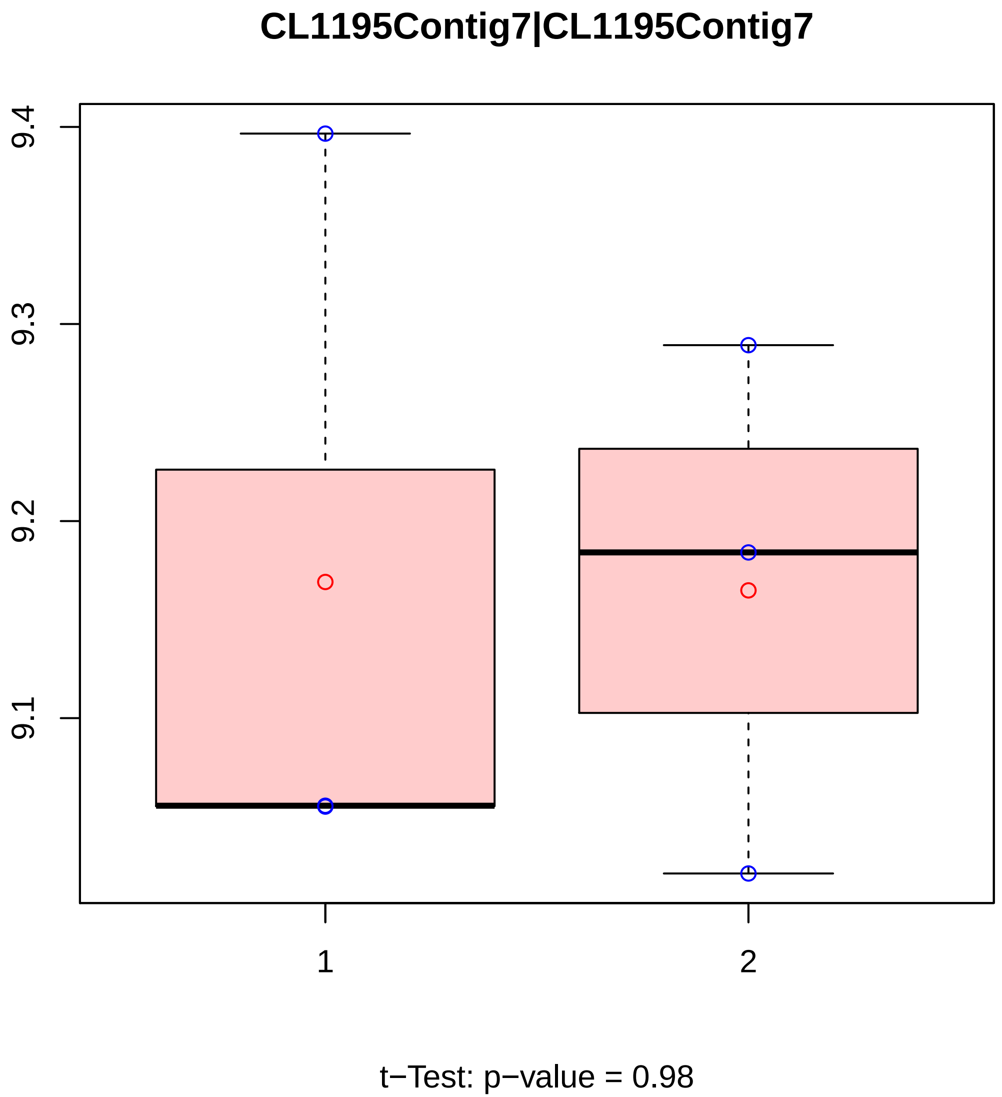
CL1195Contig7|CL1195Contig7
9.4
9.3
9.2
9.1
1
2
t−Test: p−value = 0.98

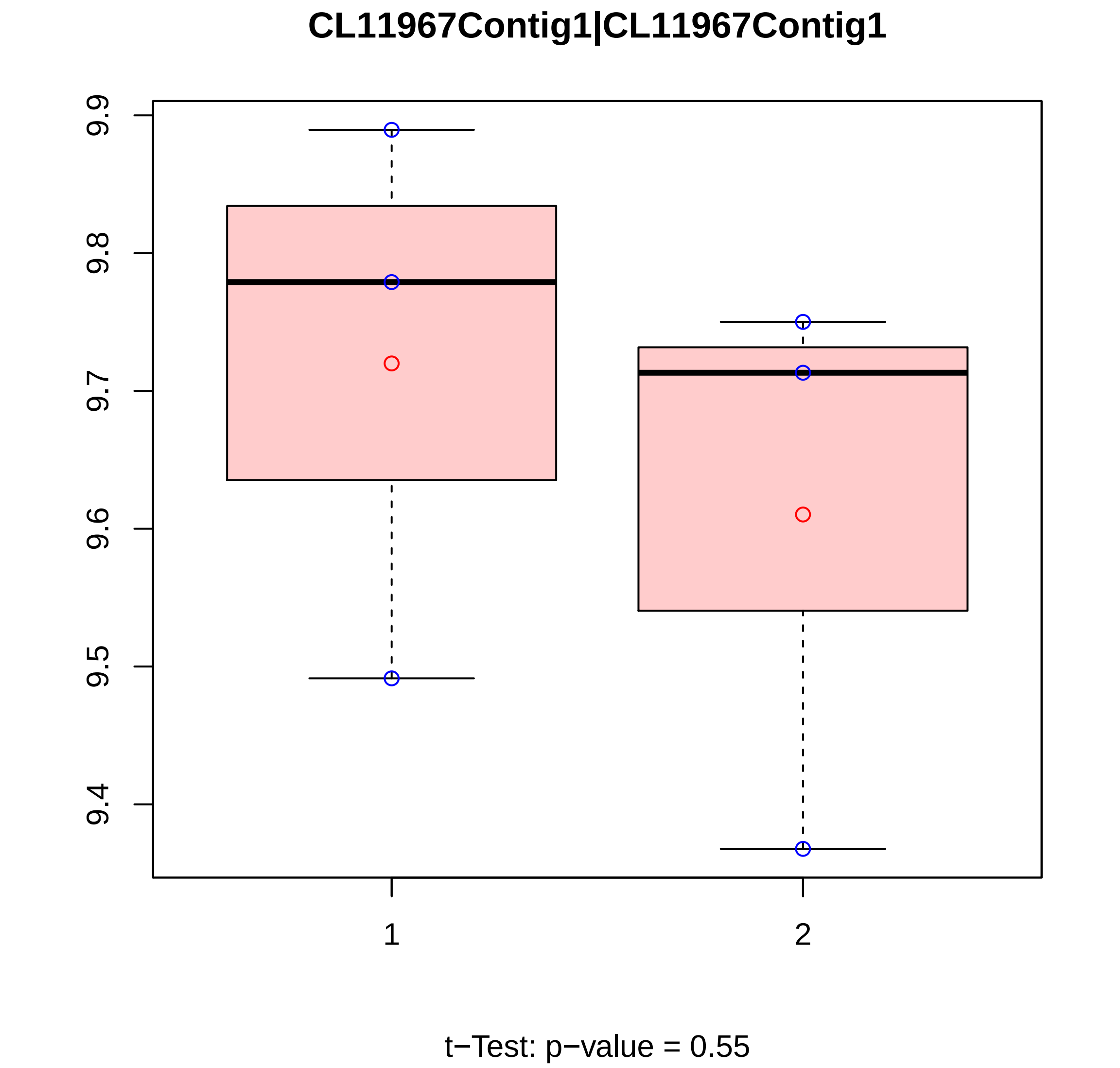
CL11967Contig1|CL11967Contig1
9.4
9.5
9.6
9.7
9.8
9.9
1
2
t−Test: p−value = 0.55

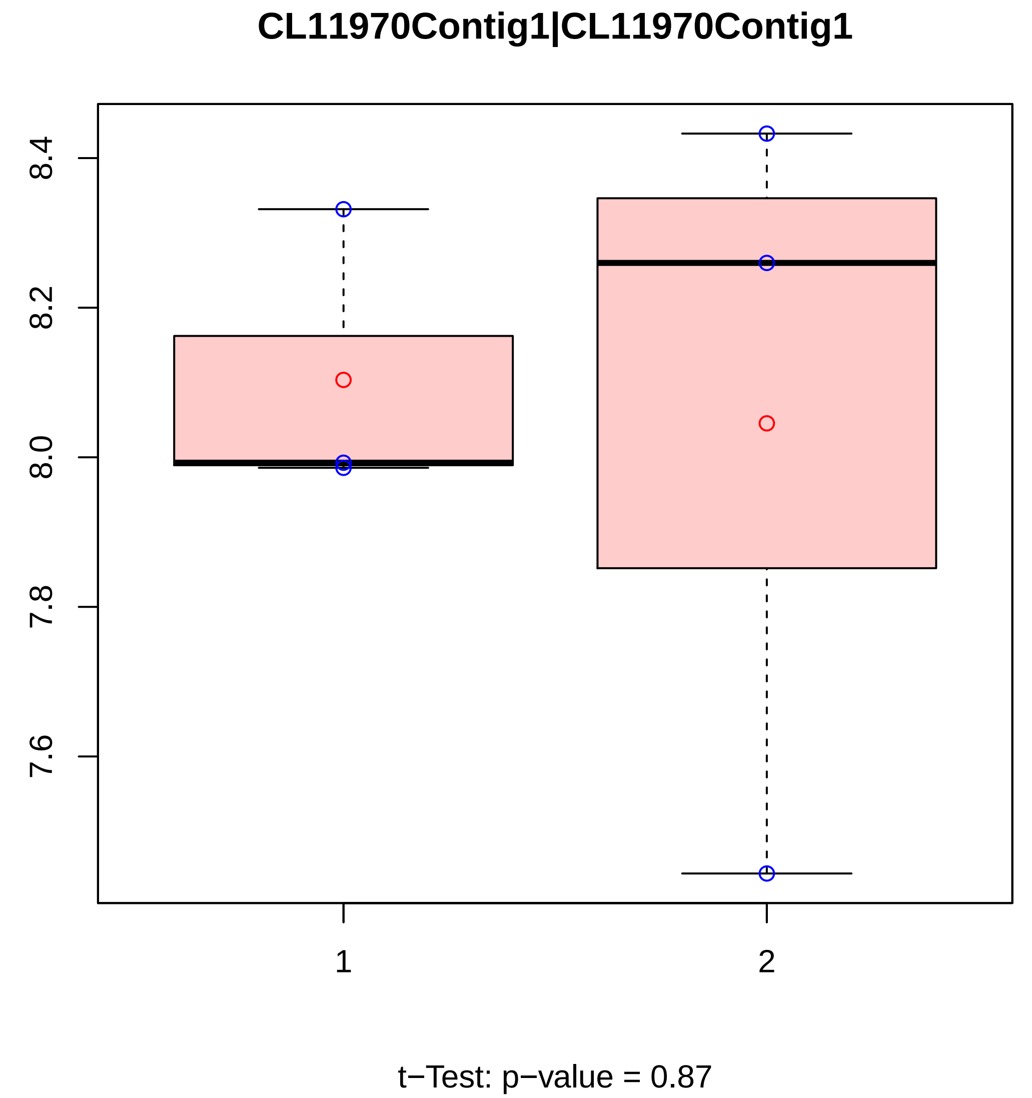
CL11970Contig1|CL11970Contig1
8.4
8.2
8.0
7.8
7.6
1
2
t−Test: p−value = 0.87

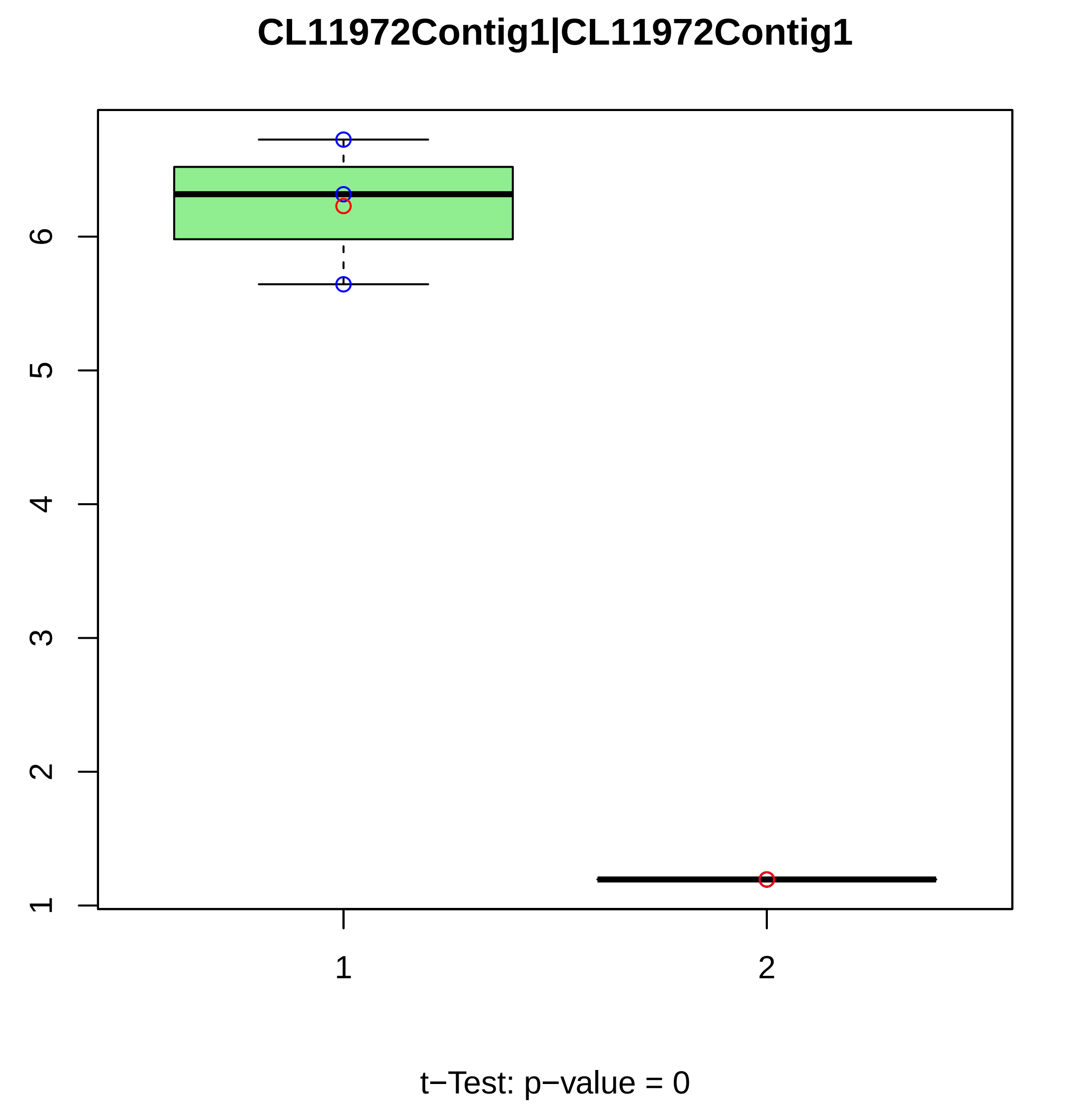

CL11972Contig1|CL11972Contig1
1
2
3
4
5
6
1
2
t−Test: p−value = 0

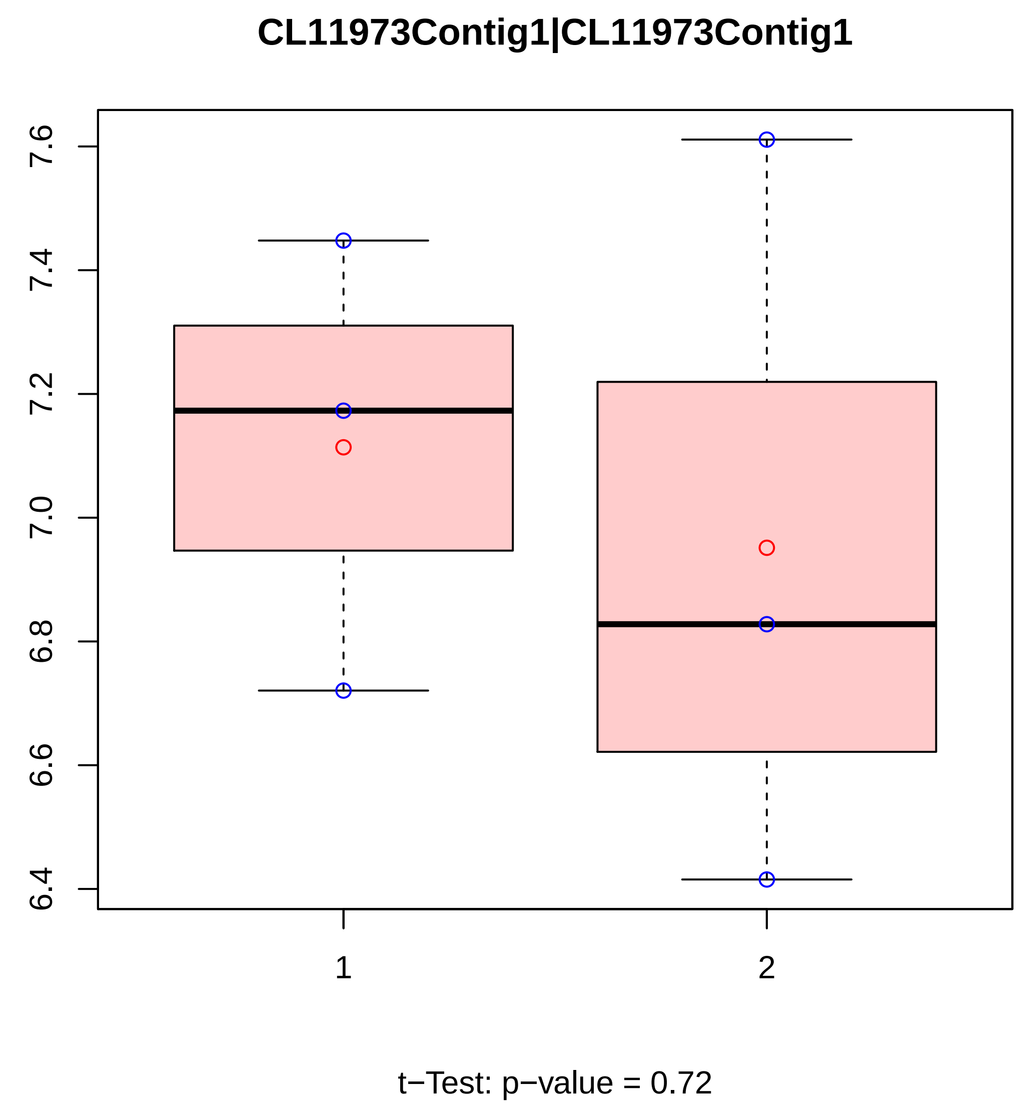
CL11973Contig1|CL11973Contig1
7.6
7.4
7.2
7.0
6.8
6.6
6.4
1
2
t−Test: p−value = 0.72

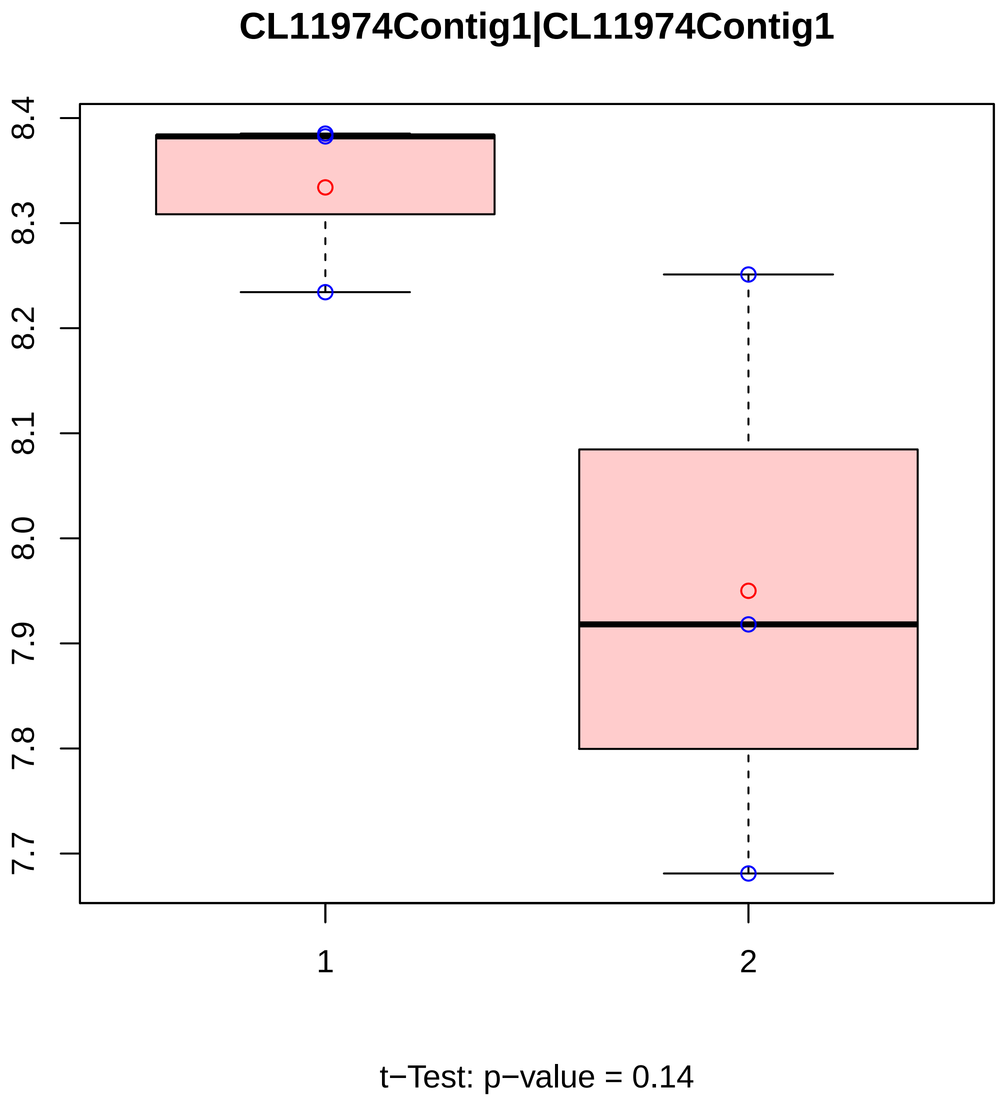
CL11974Contig1|CL11974Contig1
8.4
8.3
8.2
8.1
8.0
7.9
7.8
7.7
1
2
t−Test: p−value = 0.14

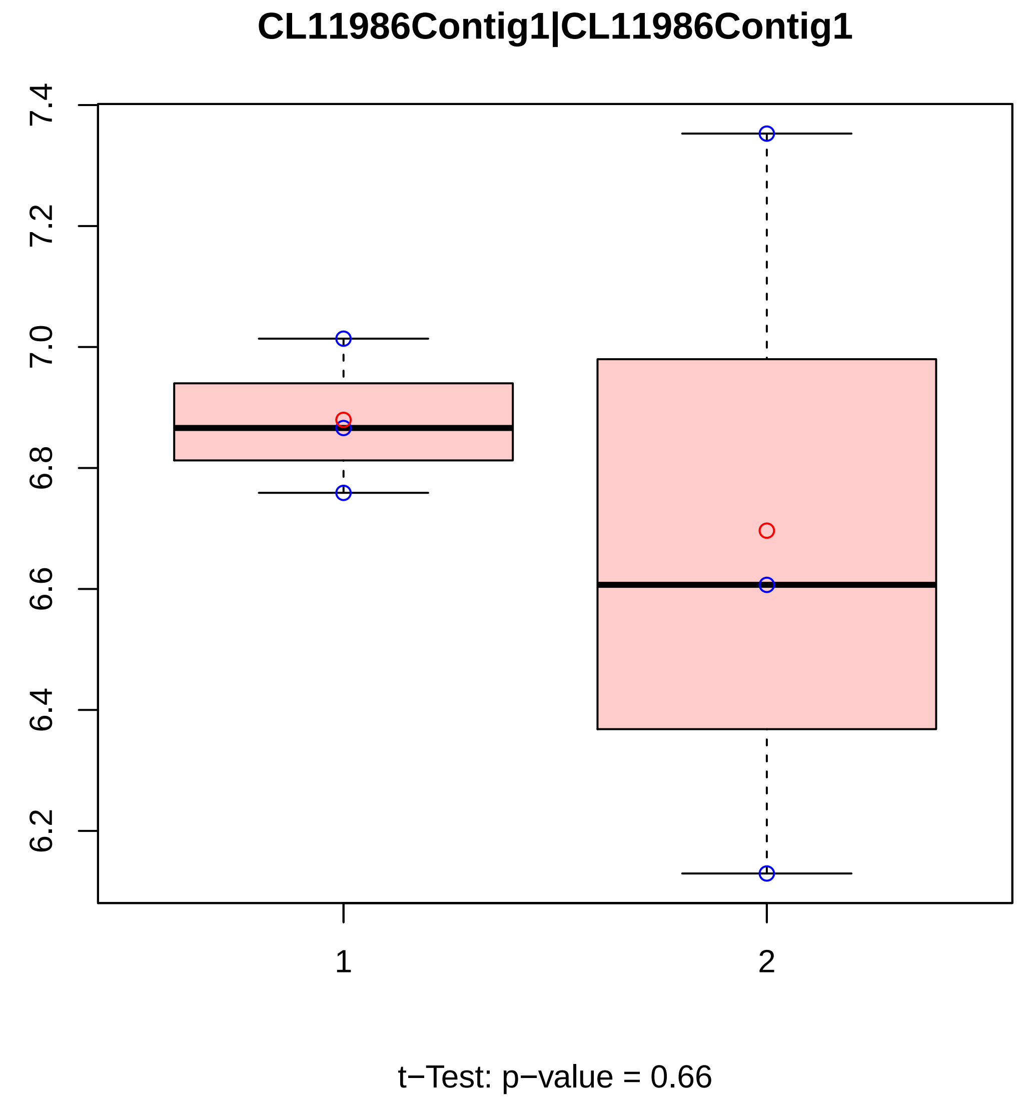

CL11986Contig1|CL11986Contig1
7.4
7.2
7.0
6.8
6.6
6.4
6.2
1
2
t−Test: p−value = 0.66

# CL119Contig20|CL119Contig20

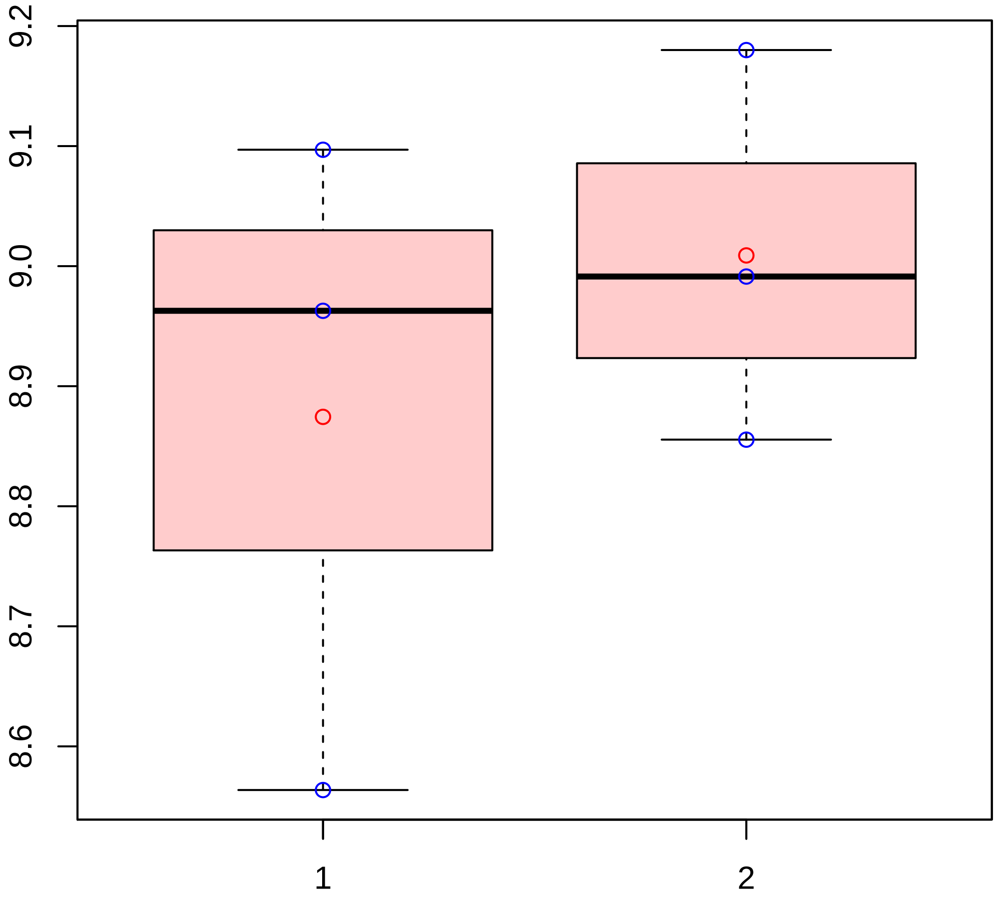


t−Test: p−value = 0.52

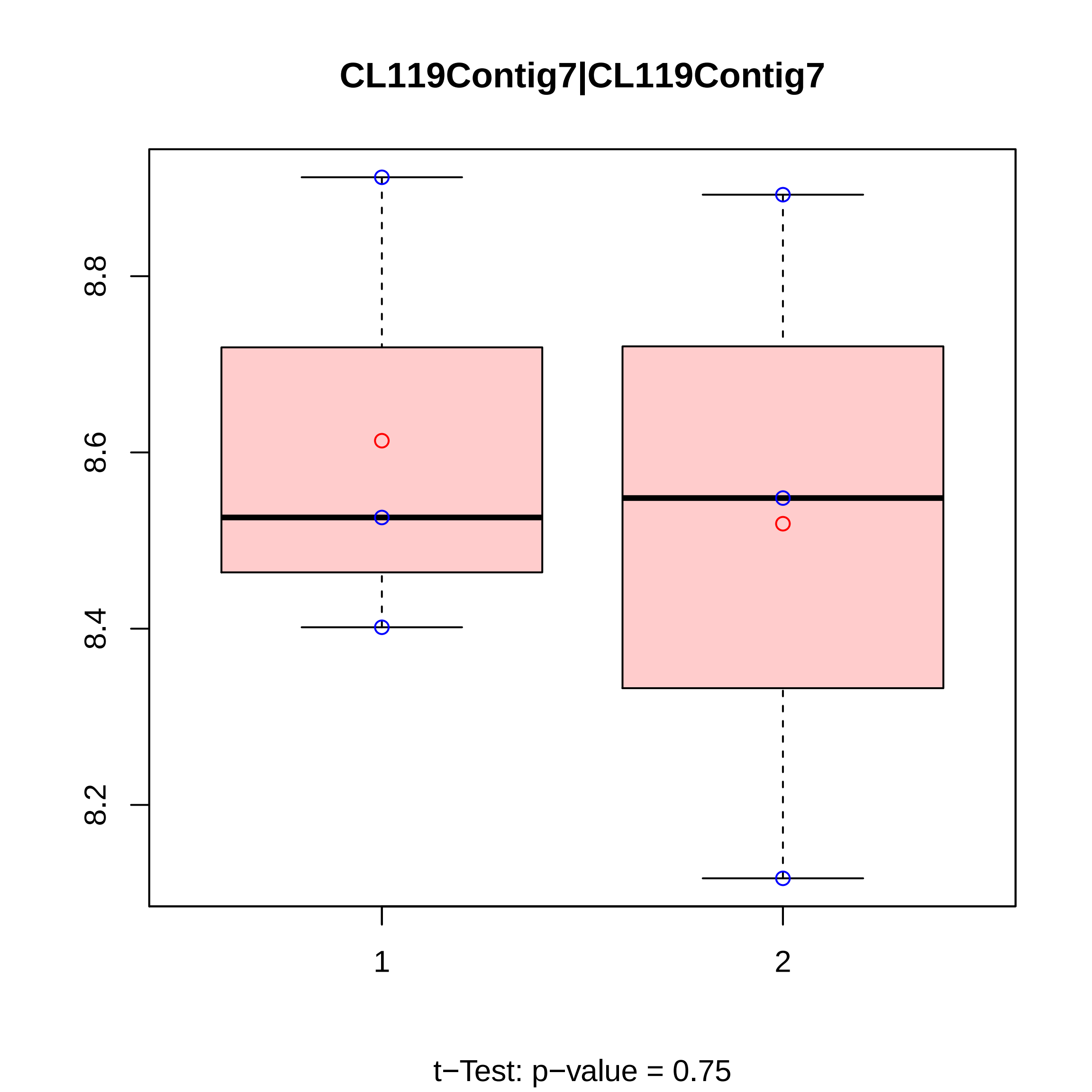
CL119Contig7|CL119Contig7
8.2
8.4
8.6
8.8
1
2
t−Test: p−value = 0.75

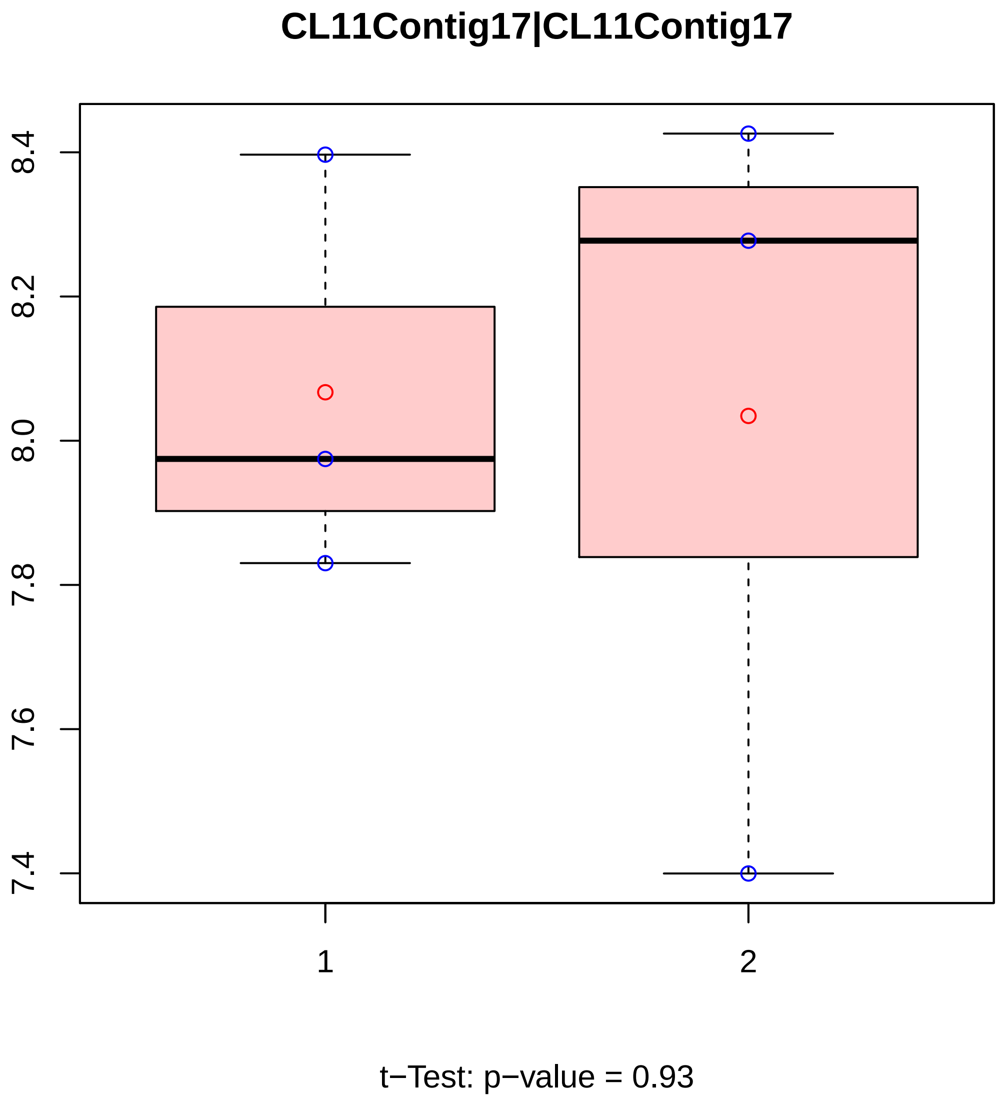

CL11Contig17|CL11Contig17
8.4
8.2
8.0
7.8
7.6
7.4
1
2
t−Test: p−value = 0.93

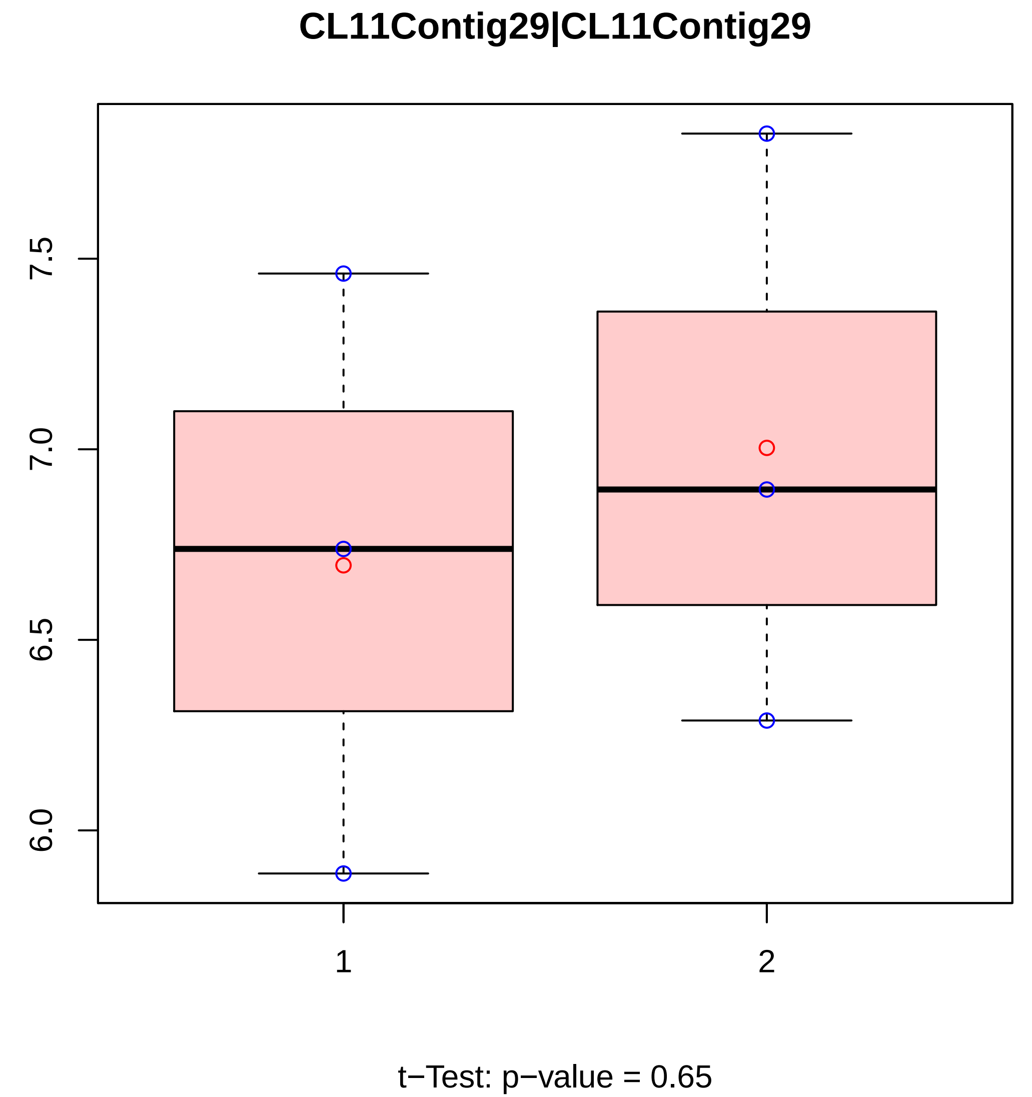
CL11Contig29|CL11Contig29
7.5
7.0
6.5
6.0
1
2
t−Test: p−value = 0.65

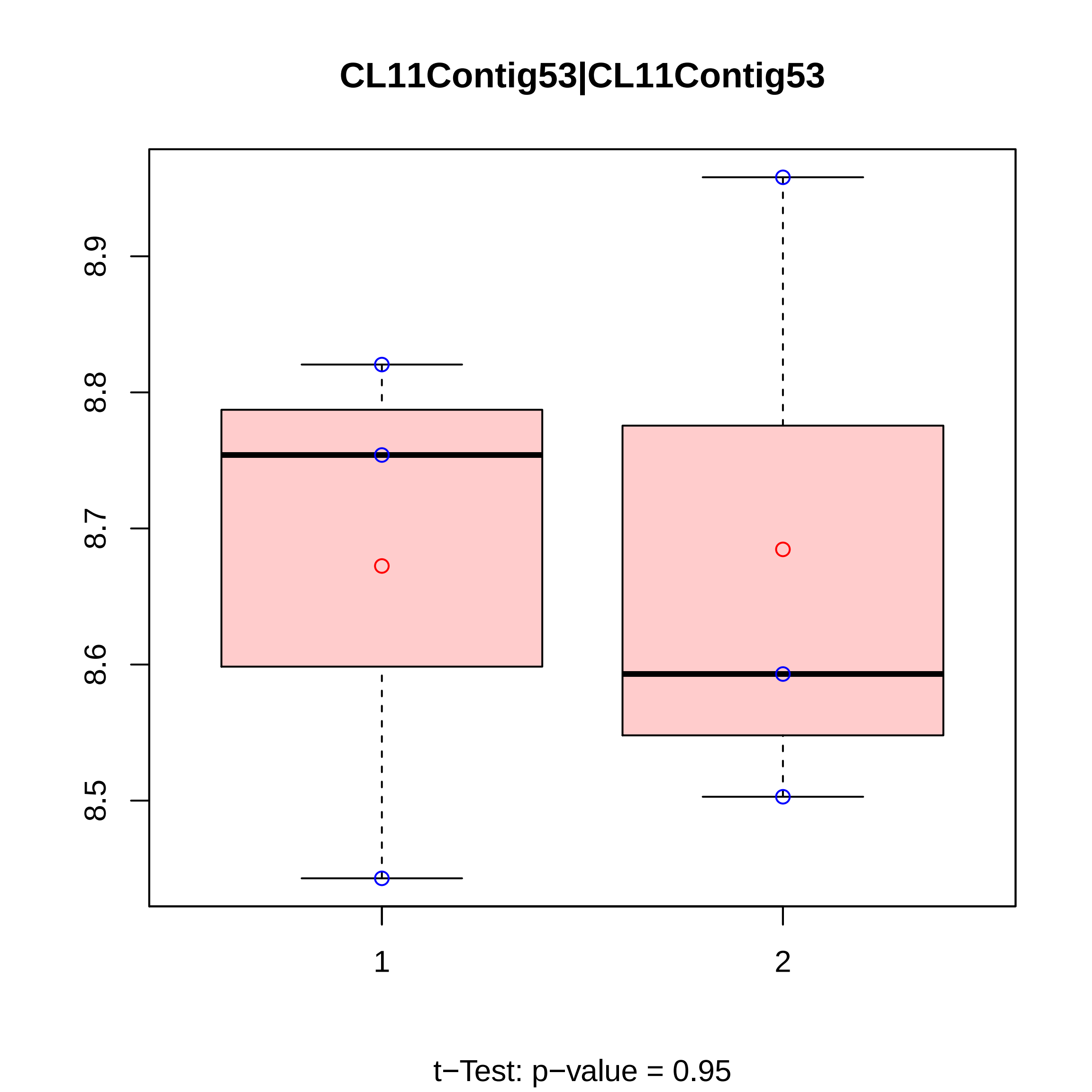
CL11Contig53|CL11Contig53
8.5
8.6
8.7
8.8
8.9
1
2
t−Test: p−value = 0.95

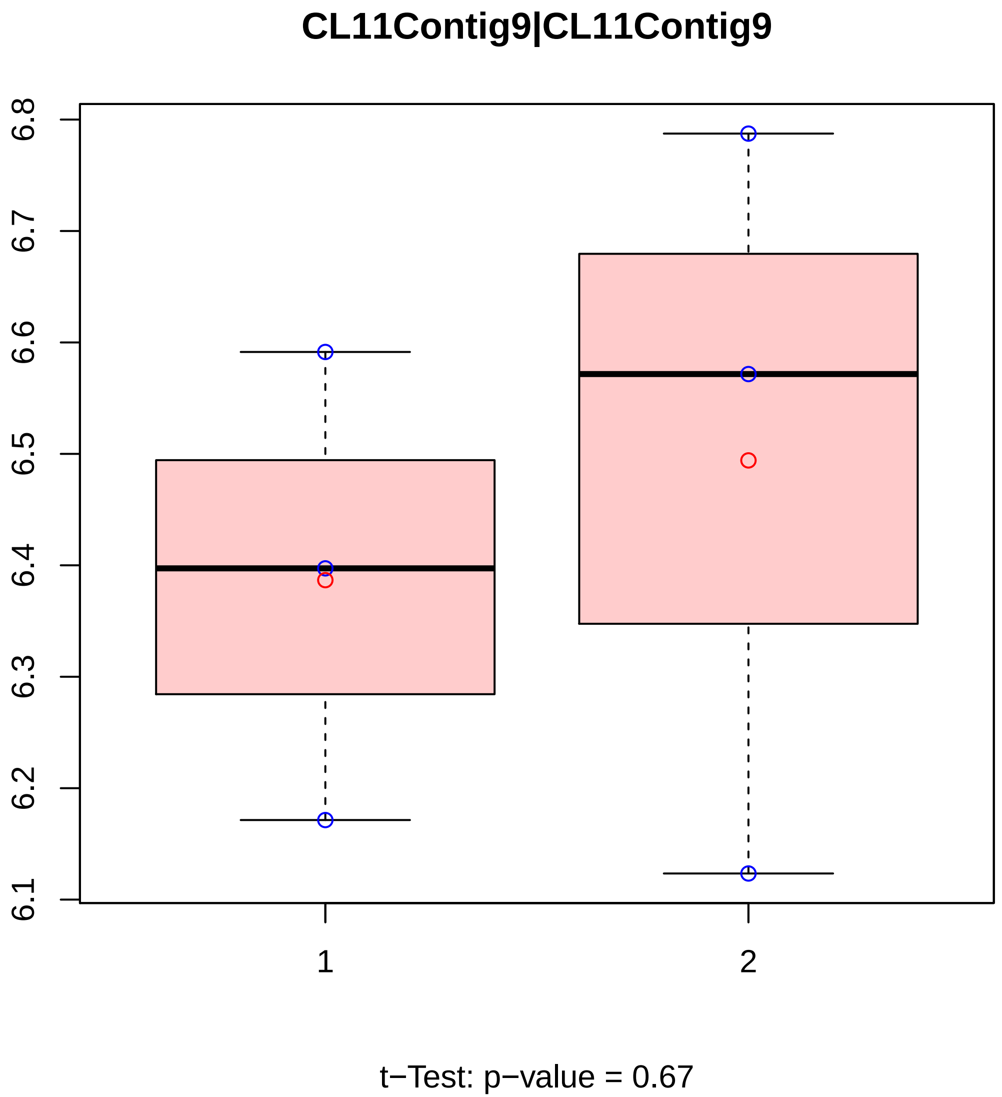
CL11Contig9|CL11Contig9
6.1
6.2
6.3
6.4
6.5
6.6
6.7
6.8
1
2
t−Test: p−value = 0.67

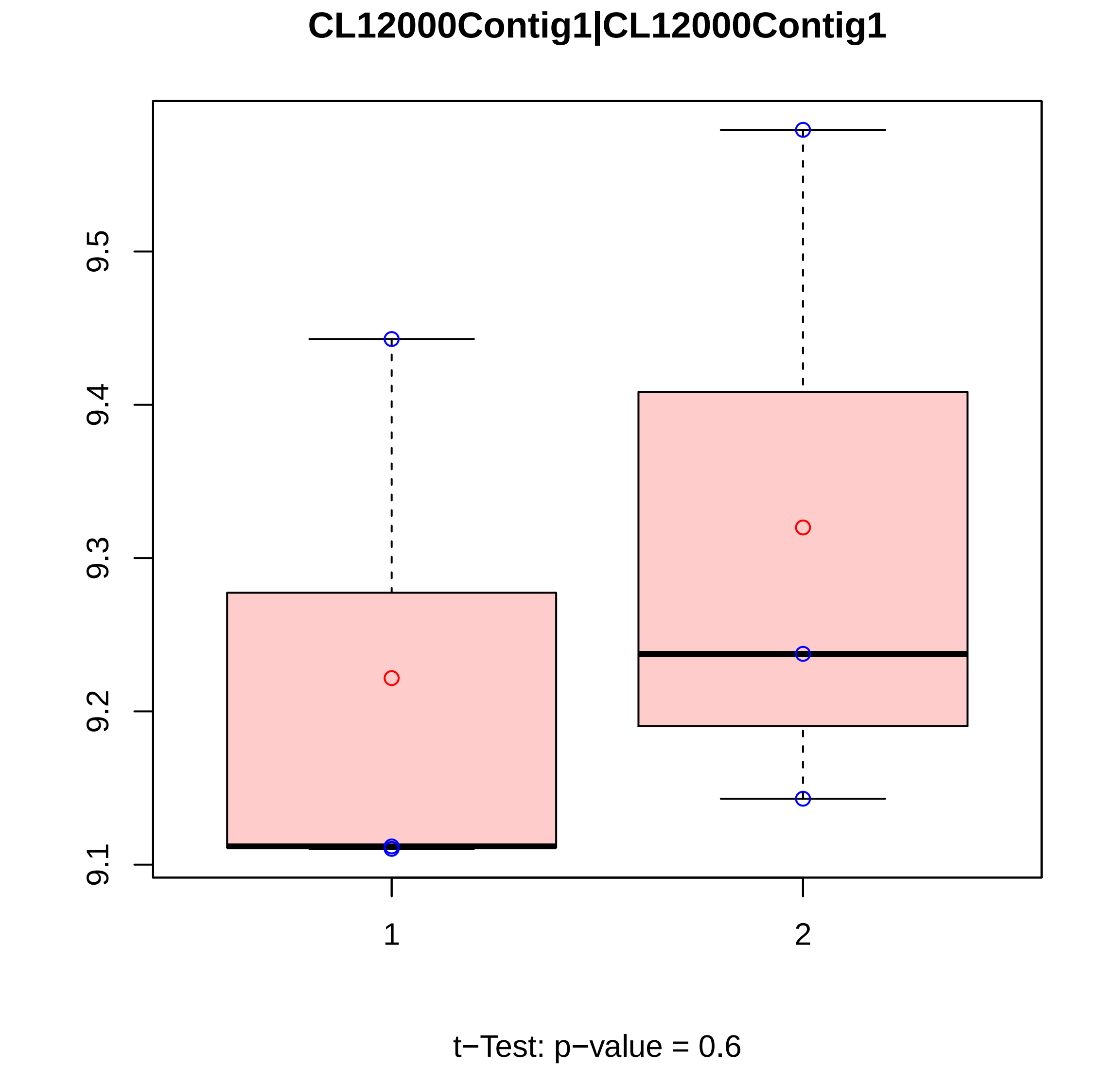
CL12000Contig1|CL12000Contig1
9.1
9.2
9.3
9.4
9.5
1
2
t−Test: p−value = 0.6

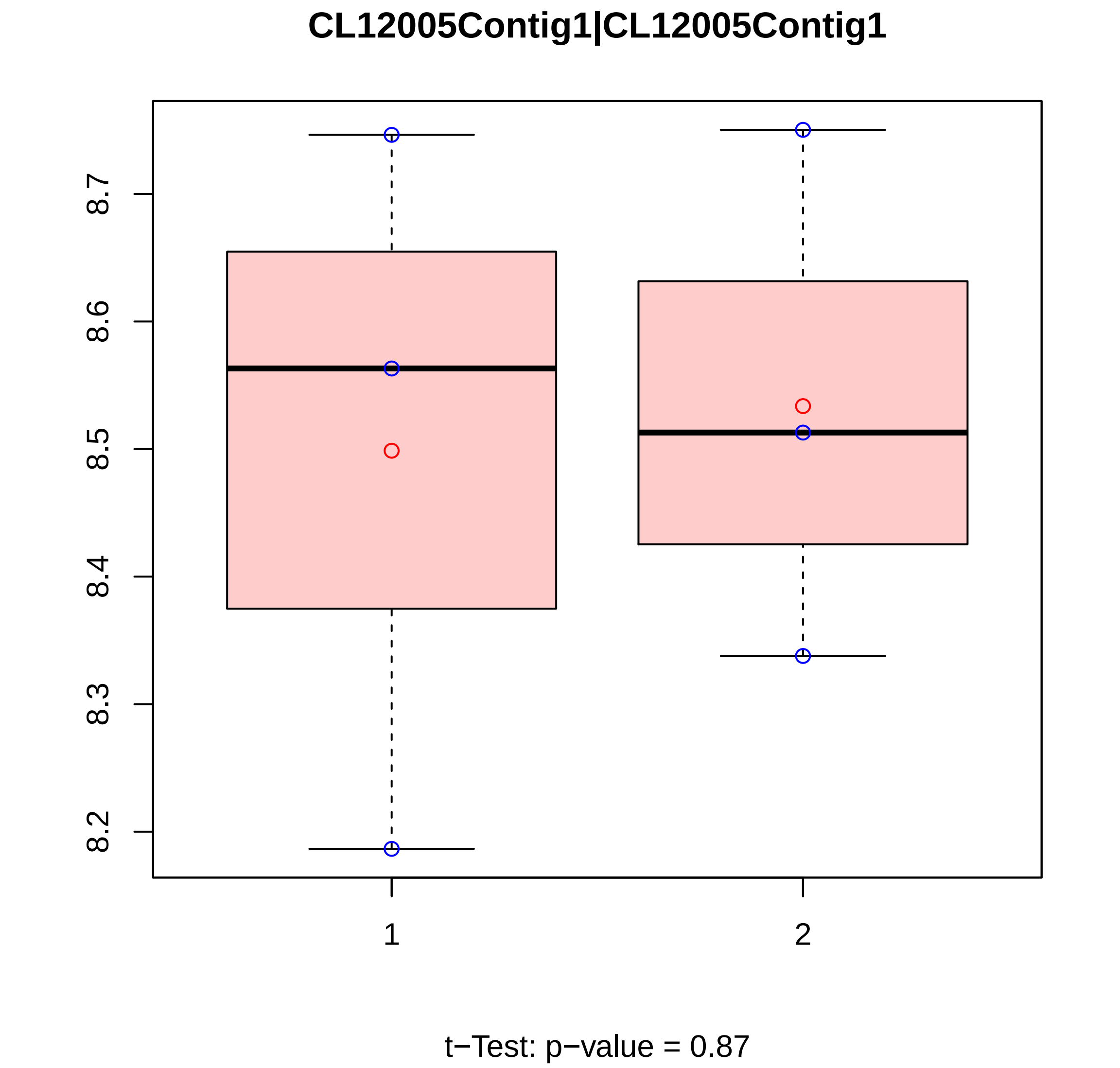
CL12005Contig1|CL12005Contig1
8.2
8.3
8.4
8.5
8.6
8.7
1
2
t−Test: p−value = 0.87

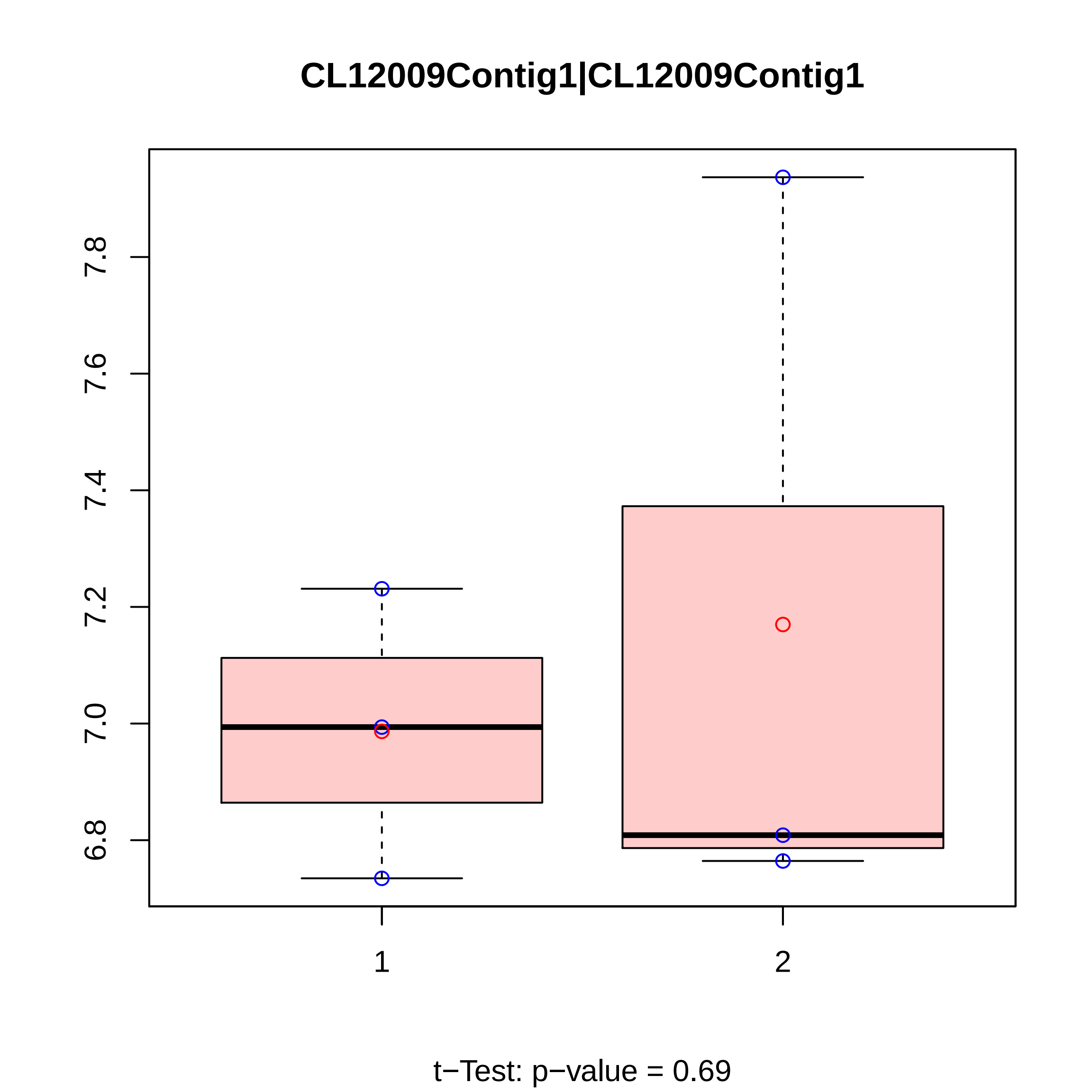

CL12009Contig1|CL12009Contig1
7.8
7.6
7.4
7.2
7.0
6.8
1
2
t−Test: p−value = 0.69

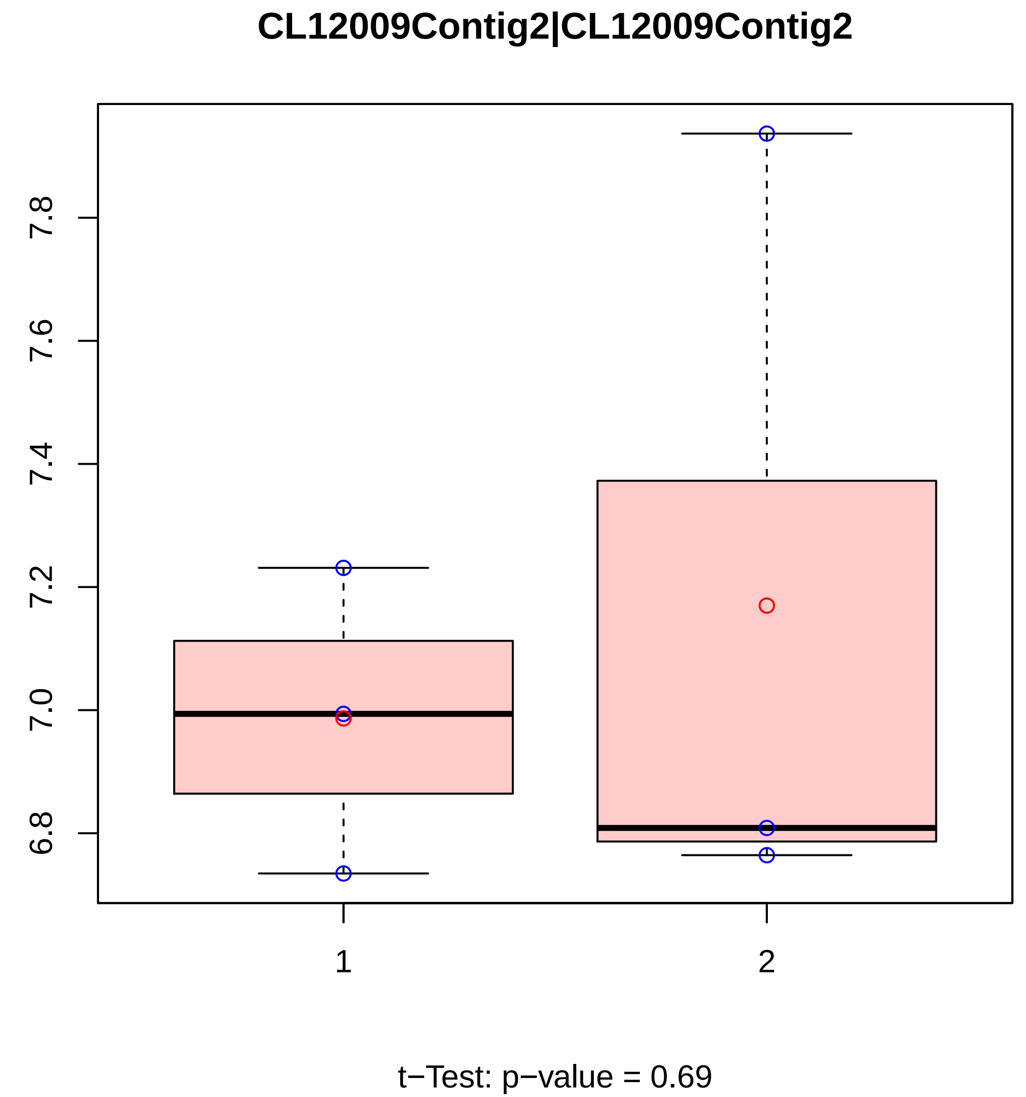
CL12009Contig2|CL12009Contig2
7.8
7.6
7.4
7.2
7.0
6.8
1
2
t−Test: p−value = 0.69

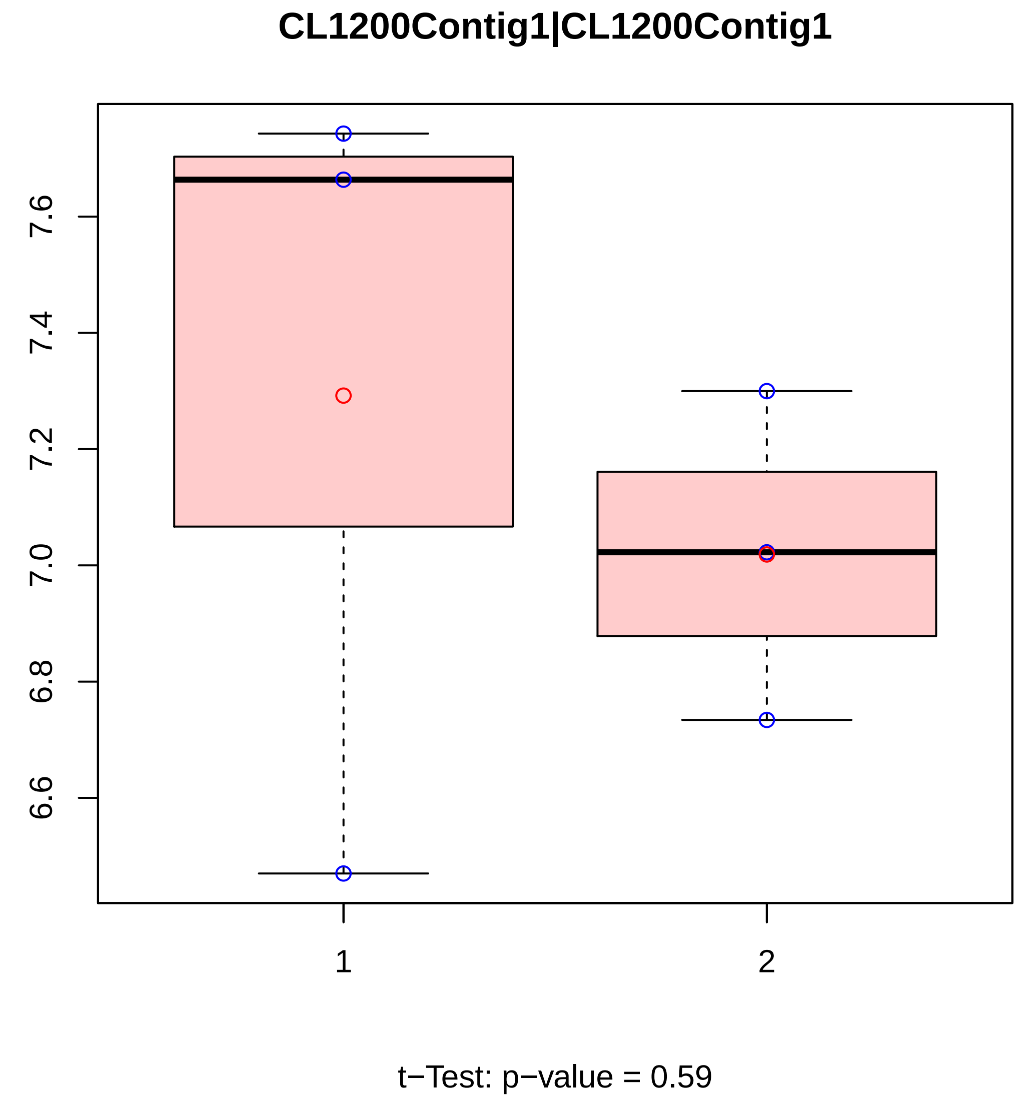
CL1200Contig1|CL1200Contig1
7.6
7.4
7.2
7.0
6.8
6.6
1
2
t−Test: p−value = 0.59

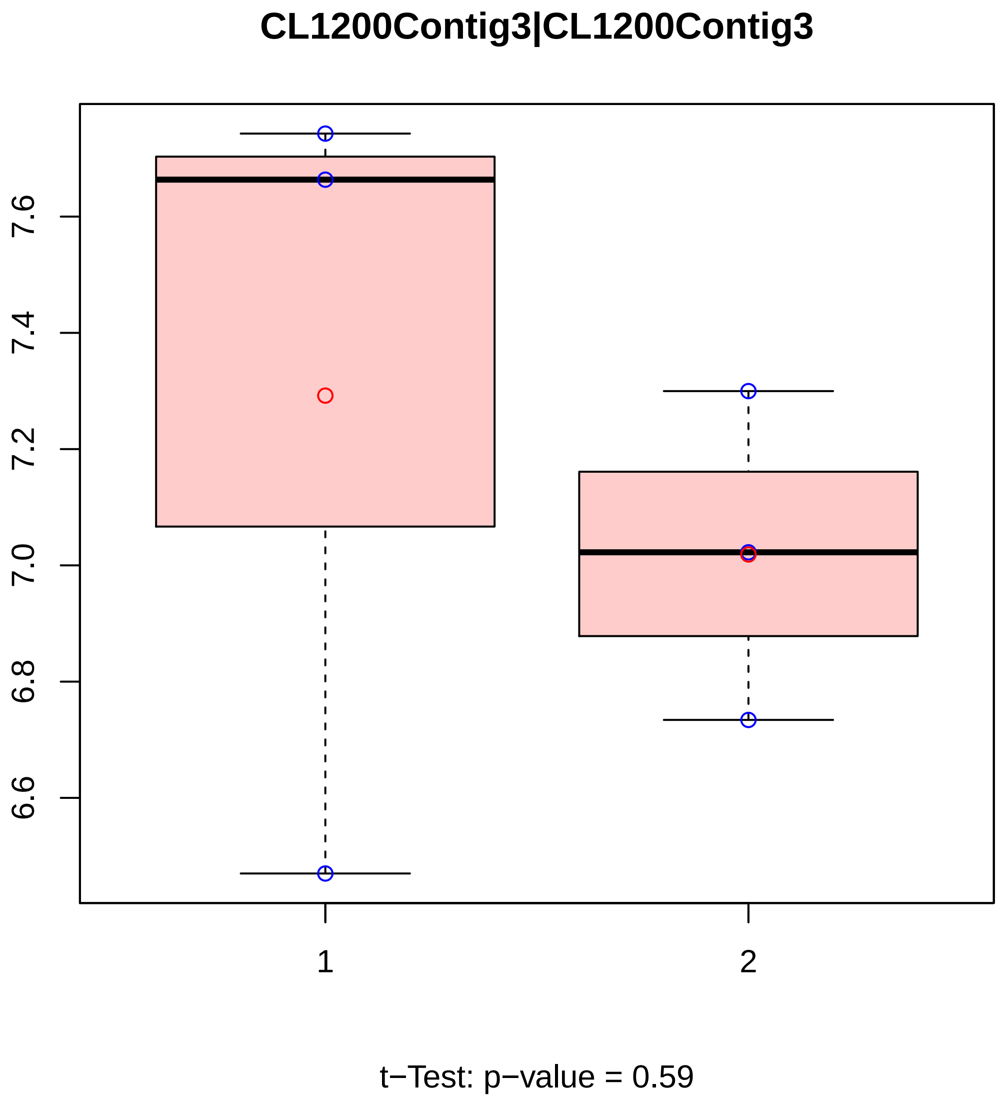
CL1200Contig3|CL1200Contig3
7.6
7.4
7.2
7.0
6.8
6.6
1
2
t−Test: p−value = 0.59

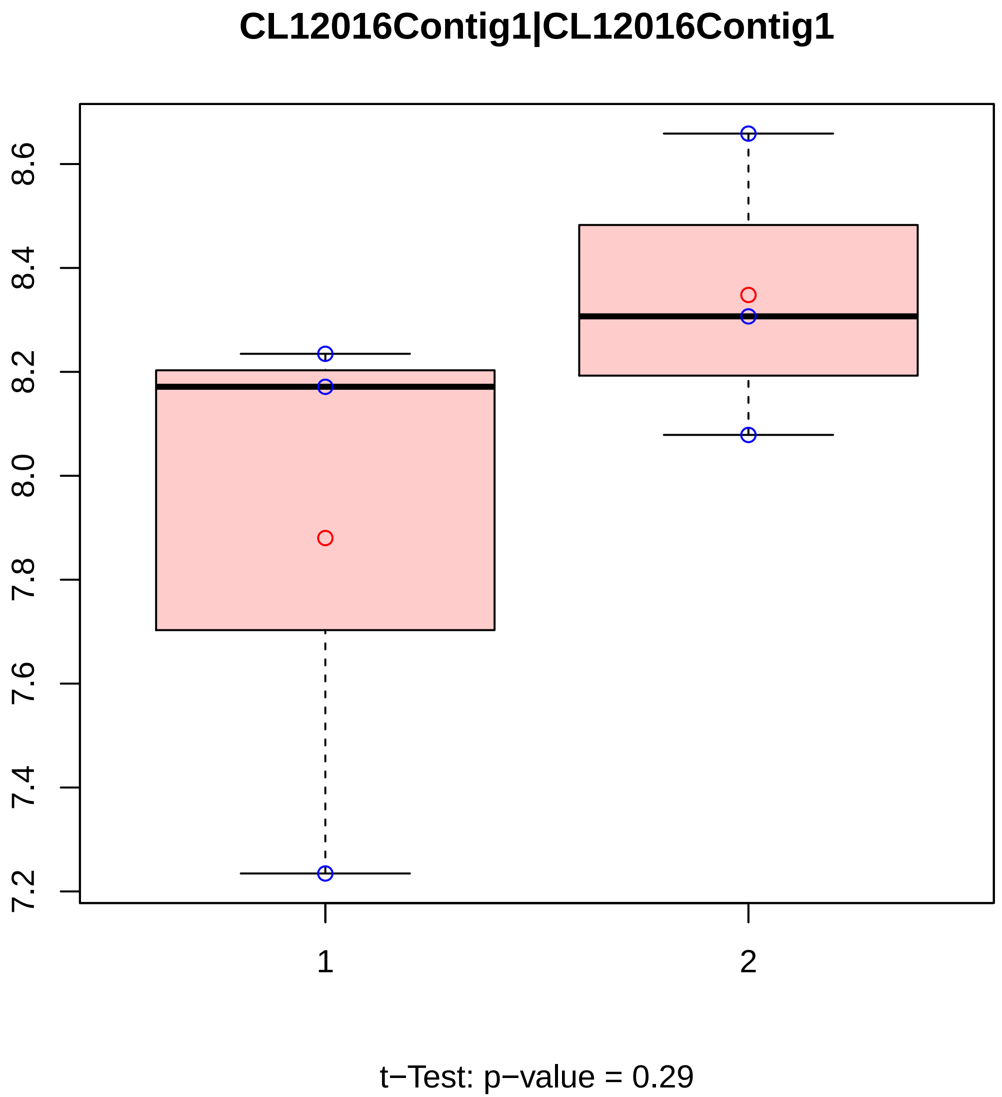
CL12016Contig1|CL12016Contig1
8.6
8.4
8.2
8.0
7.8
7.6
7.4
7.2
1
2
t−Test: p−value = 0.29

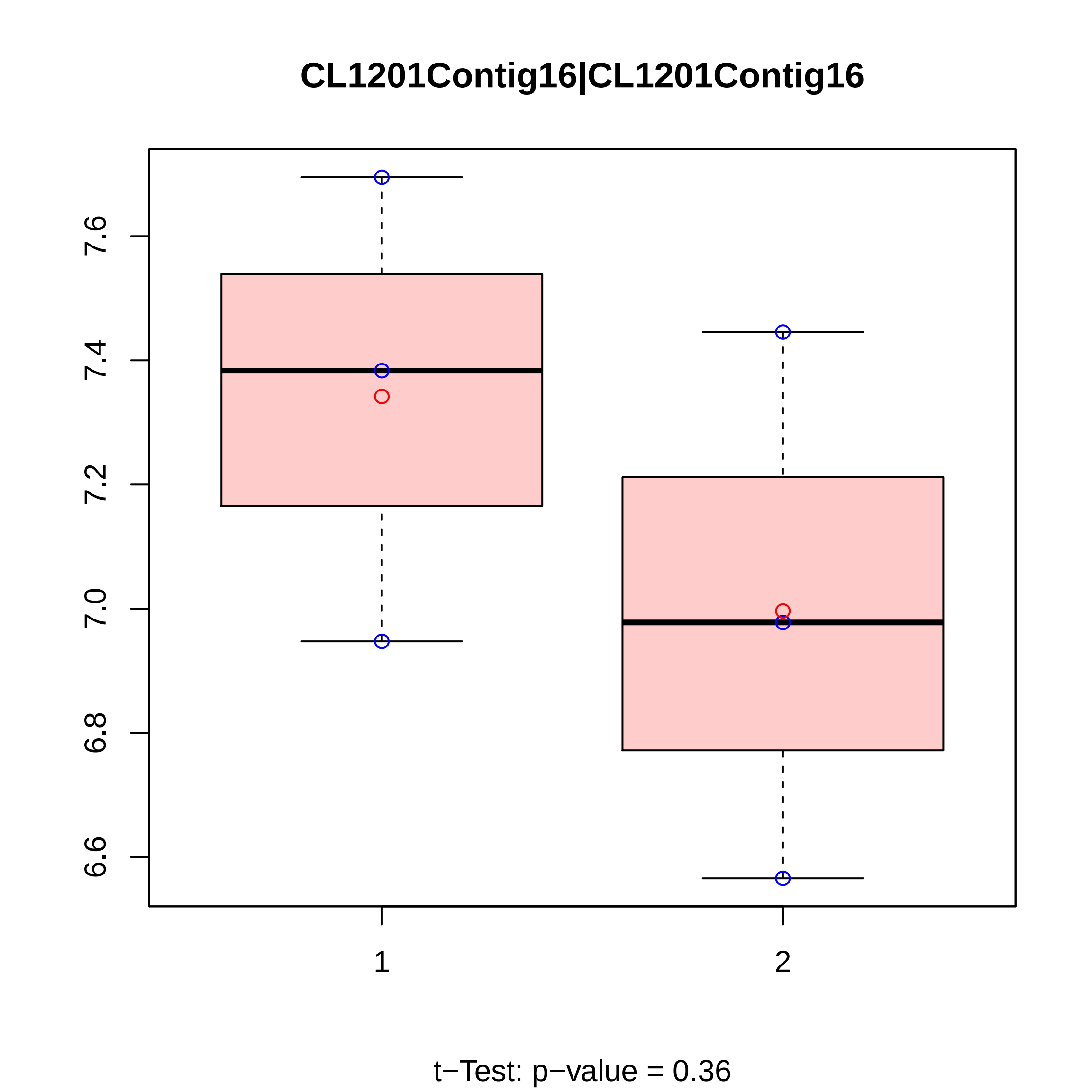
CL1201Contig16|CL1201Contig16
7.6
7.4
7.2
7.0
6.8
6.6
1
2
t−Test: p−value = 0.36

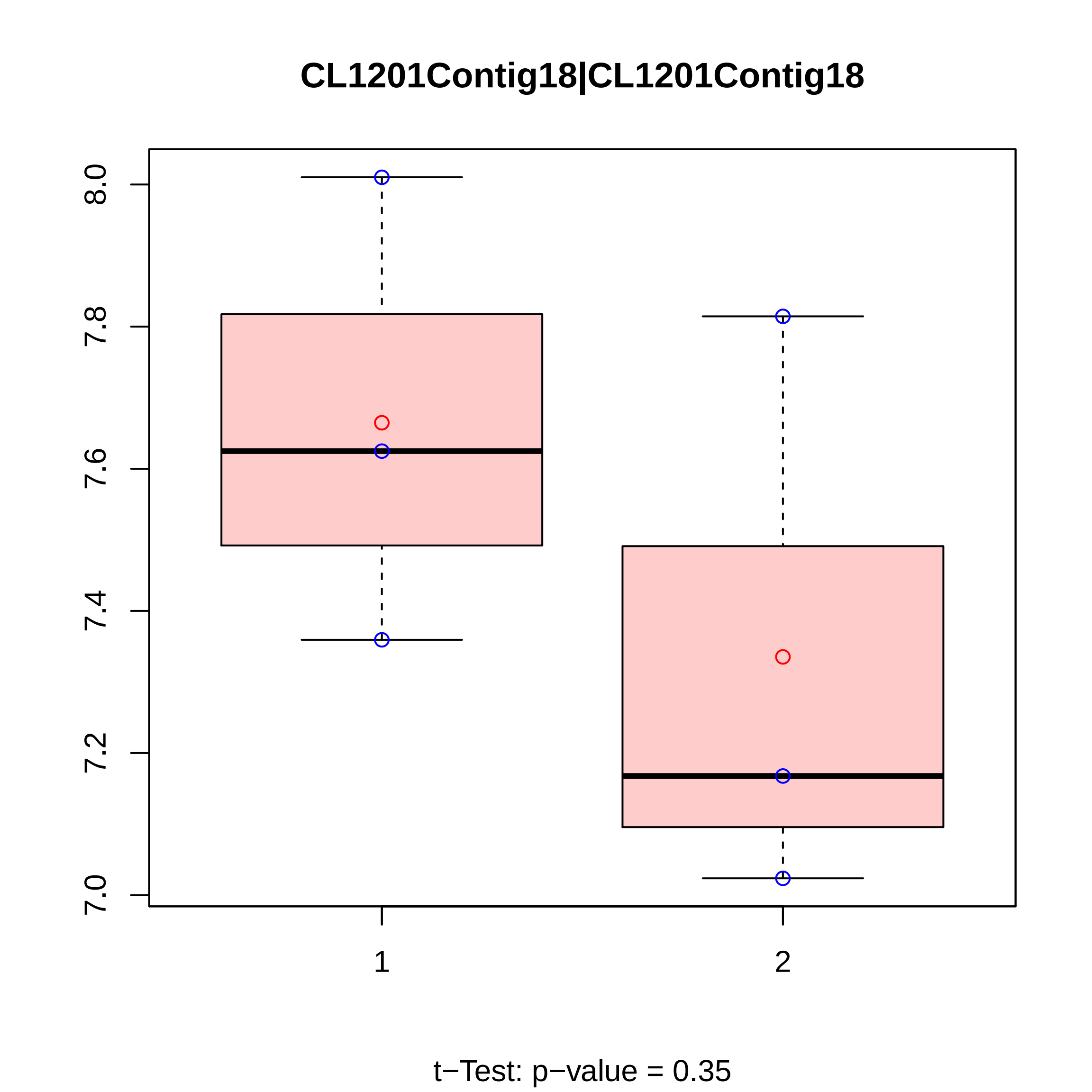

CL1201Contig18|CL1201Contig18
8.0
7.8
7.6
7.4
7.2
7.0
1
2
t−Test: p−value = 0.35

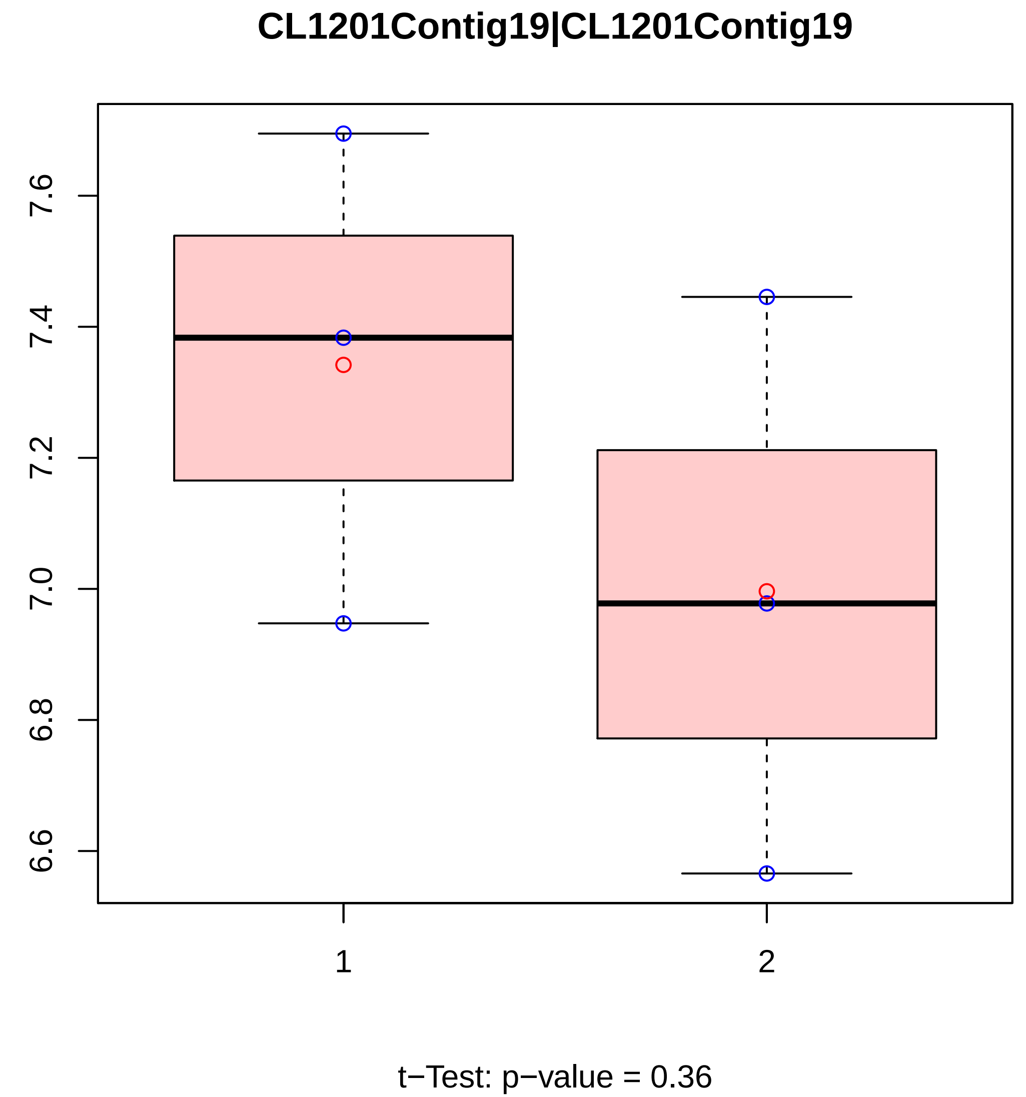
CL1201Contig19|CL1201Contig19
7.6
7.4
7.2
7.0
6.8
6.6
1
2
t−Test: p−value = 0.36

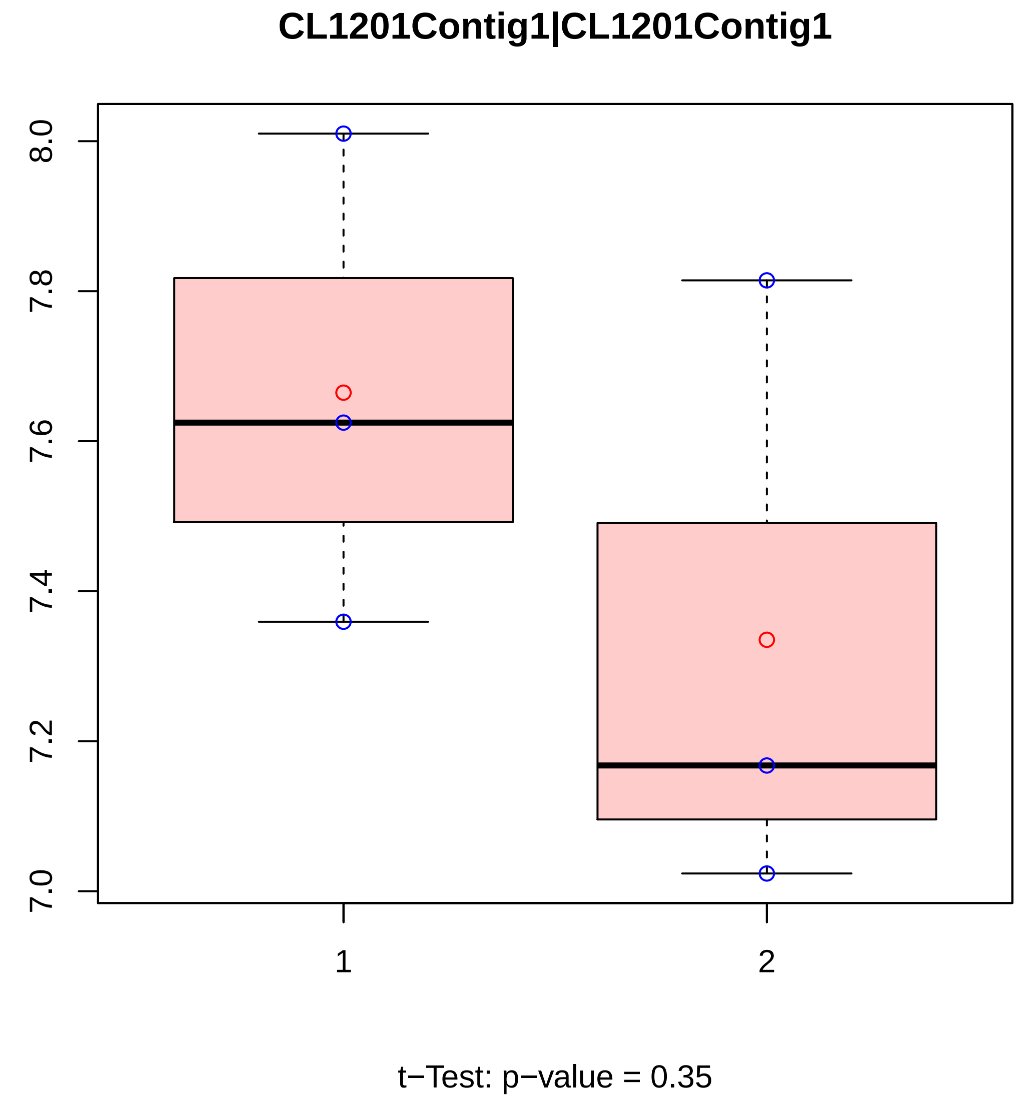

CL1201Contig1|CL1201Contig1
8.0
7.8
7.6
7.4
7.2
7.0
1
2
t−Test: p−value = 0.35

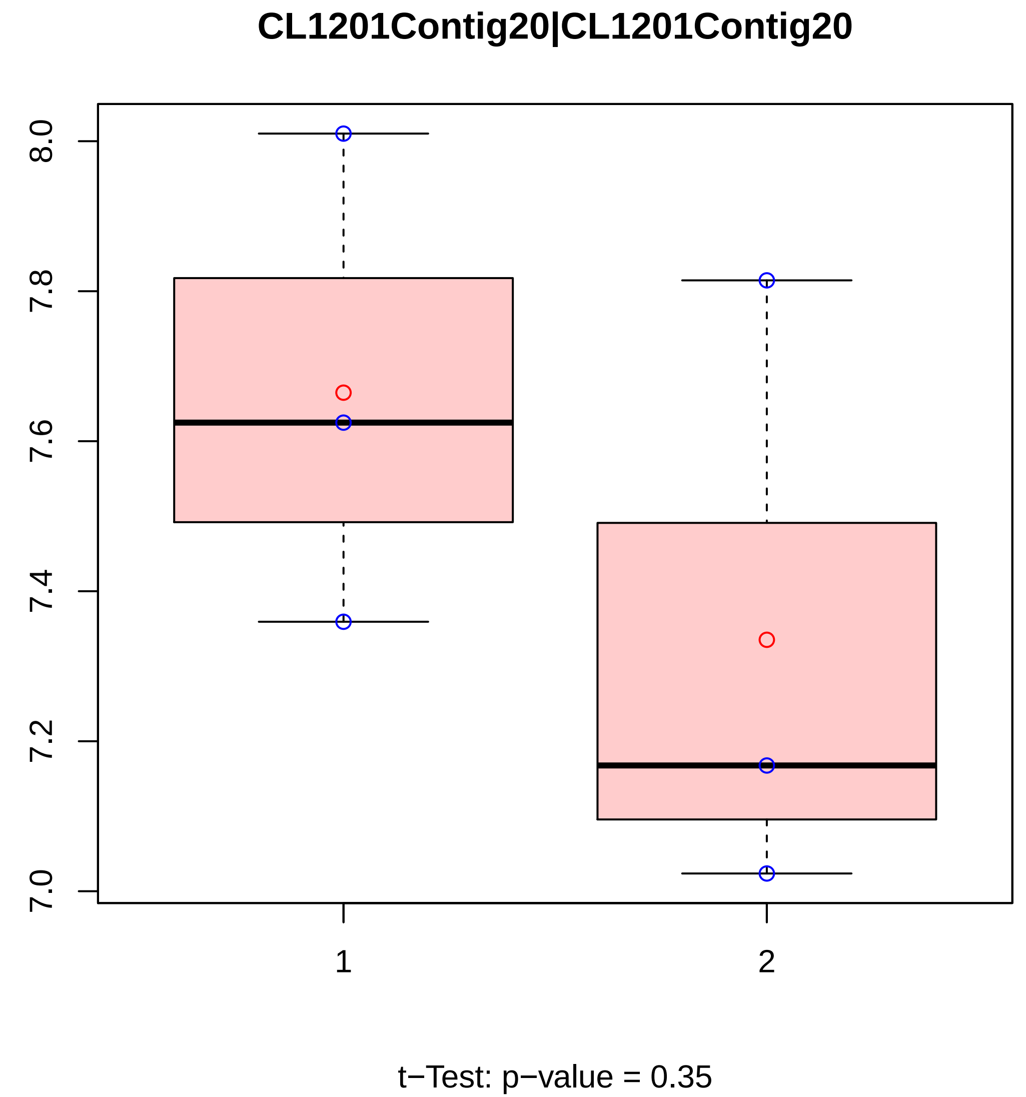
CL1201Contig20|CL1201Contig20
8.0
7.8
7.6
7.4
7.2
7.0
1
2
t−Test: p−value = 0.35

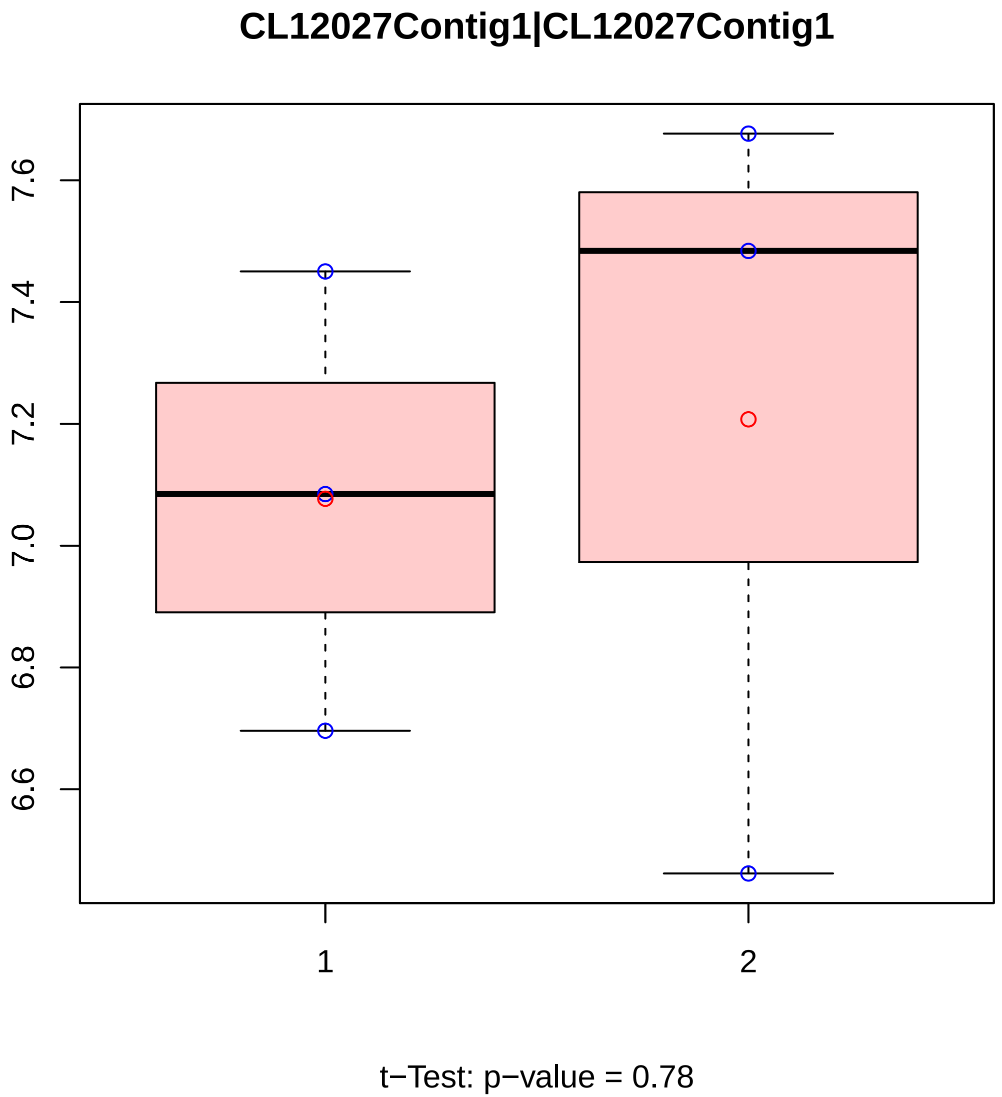

CL12027Contig1|CL12027Contig1
7.6
7.4
7.2
7.0
6.8
6.6
1
2
t−Test: p−value = 0.78

## CL1202Contig3|CL1202Contig3

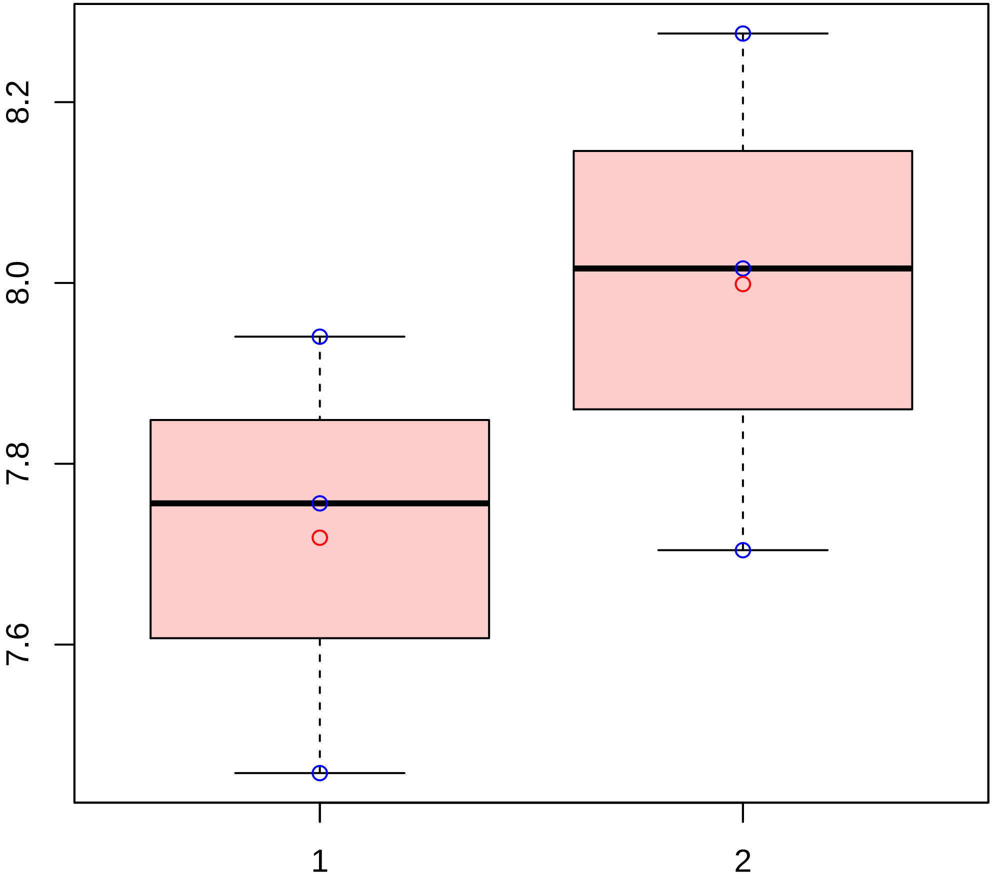


t−Test: p−value = 0.27

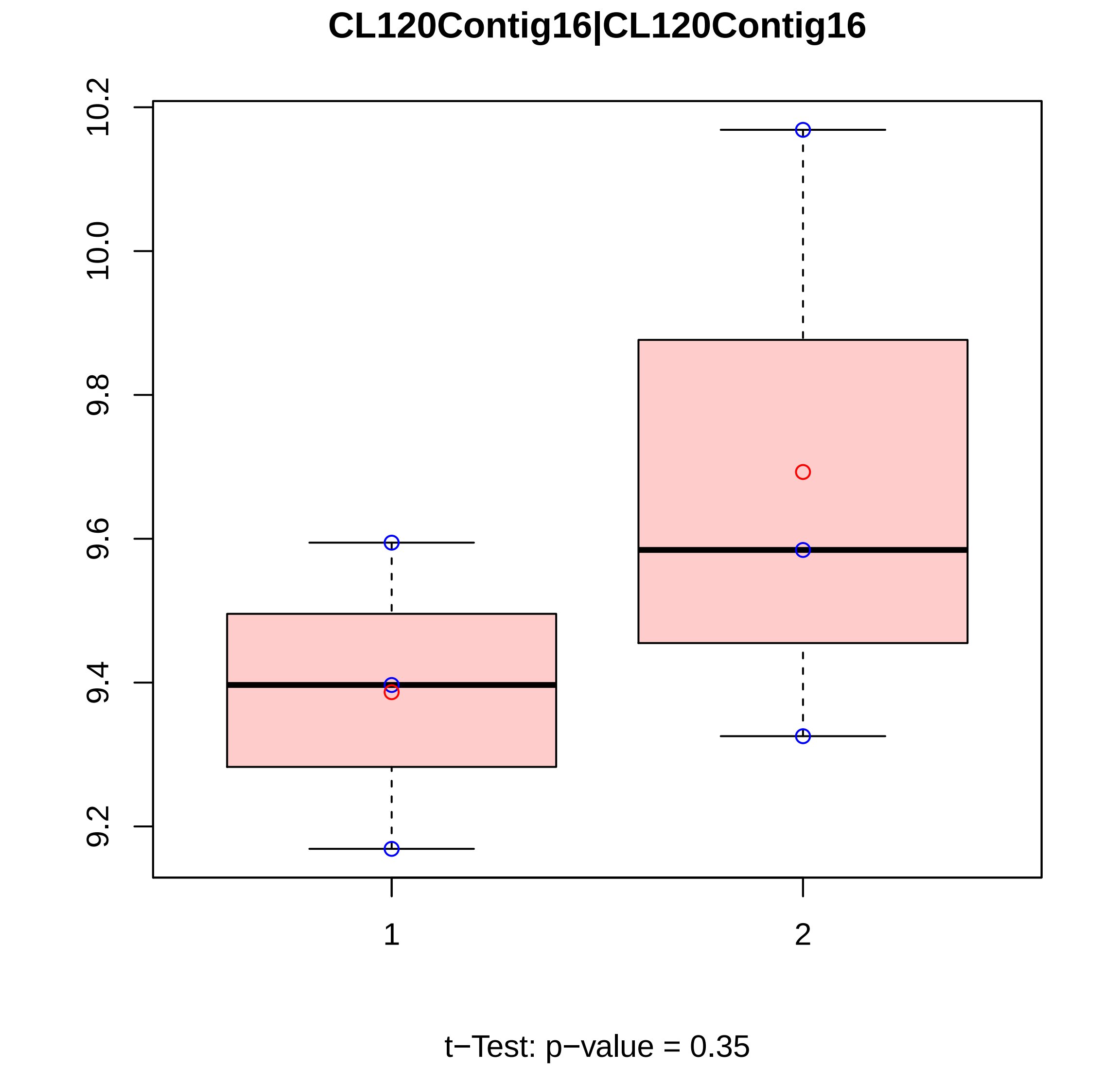
CL120Contig16|CL120Contig16
10.2
10.0
9.8
9.6
9.4
9.2
1
2
t−Test: p−value = 0.35

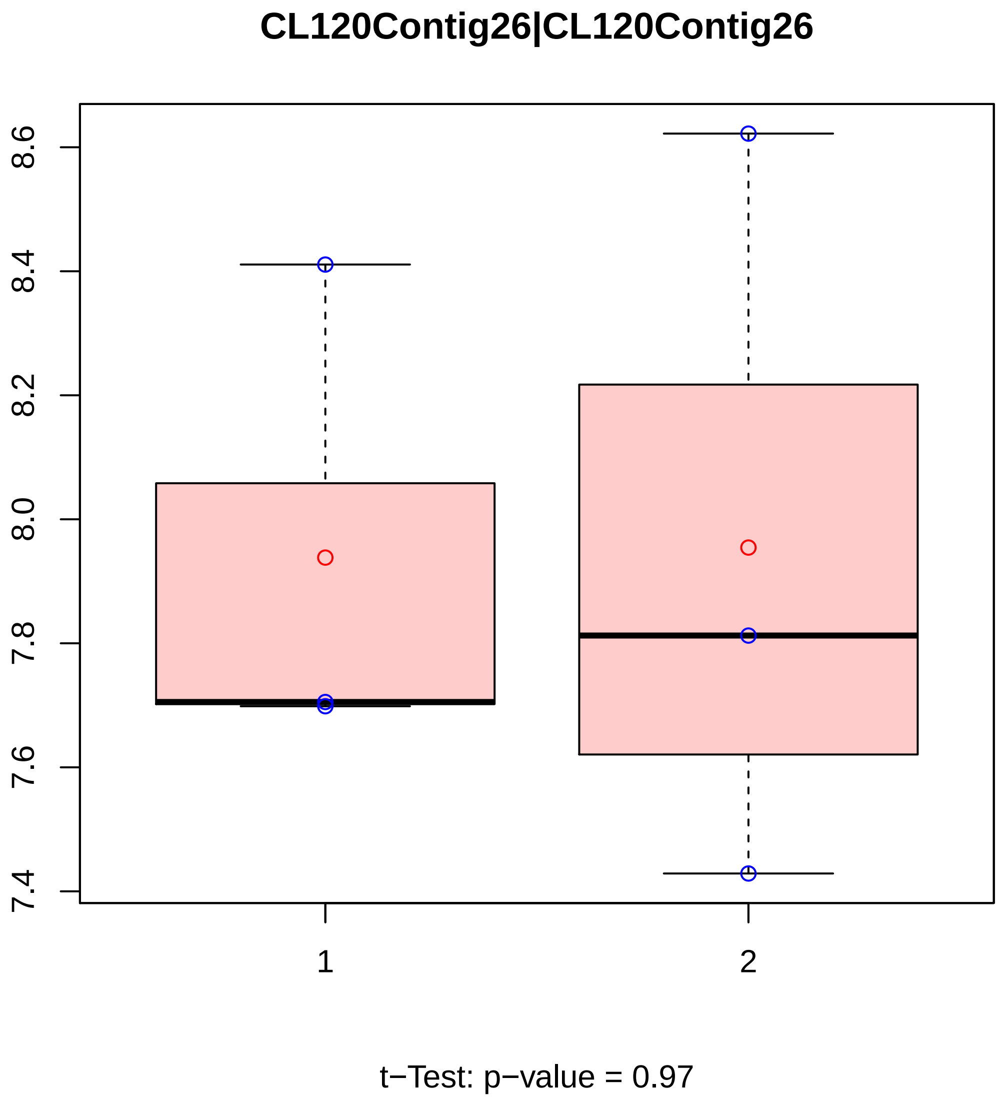
CL120Contig26|CL120Contig26
8.6
8.4
8.2
8.0
7.8
7.6
7.4
1
2
t−Test: p−value = 0.97

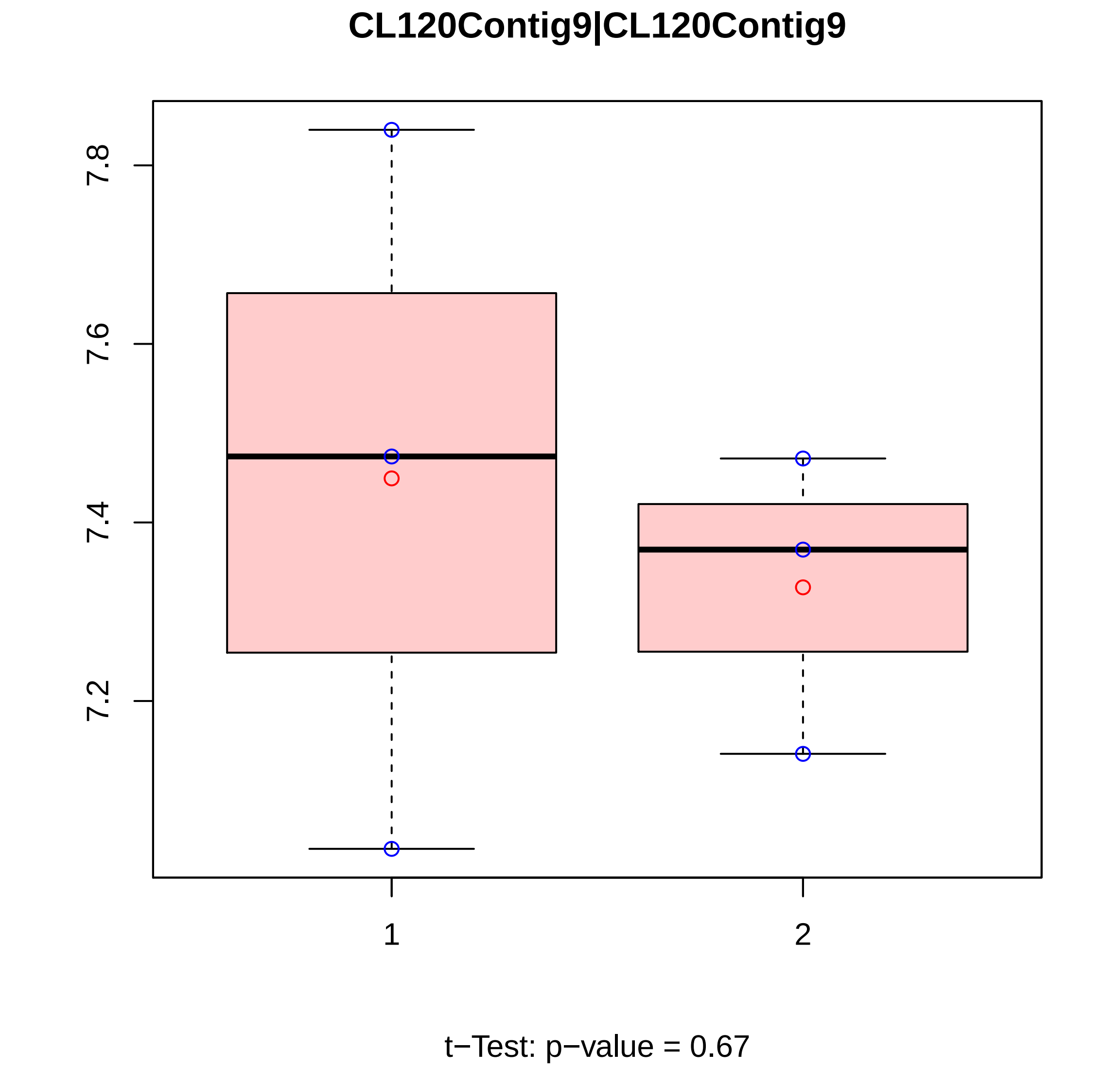
CL120Contig9|CL120Contig9
7.2
7.4
7.6
7.8
1
2
t−Test: p−value = 0.67

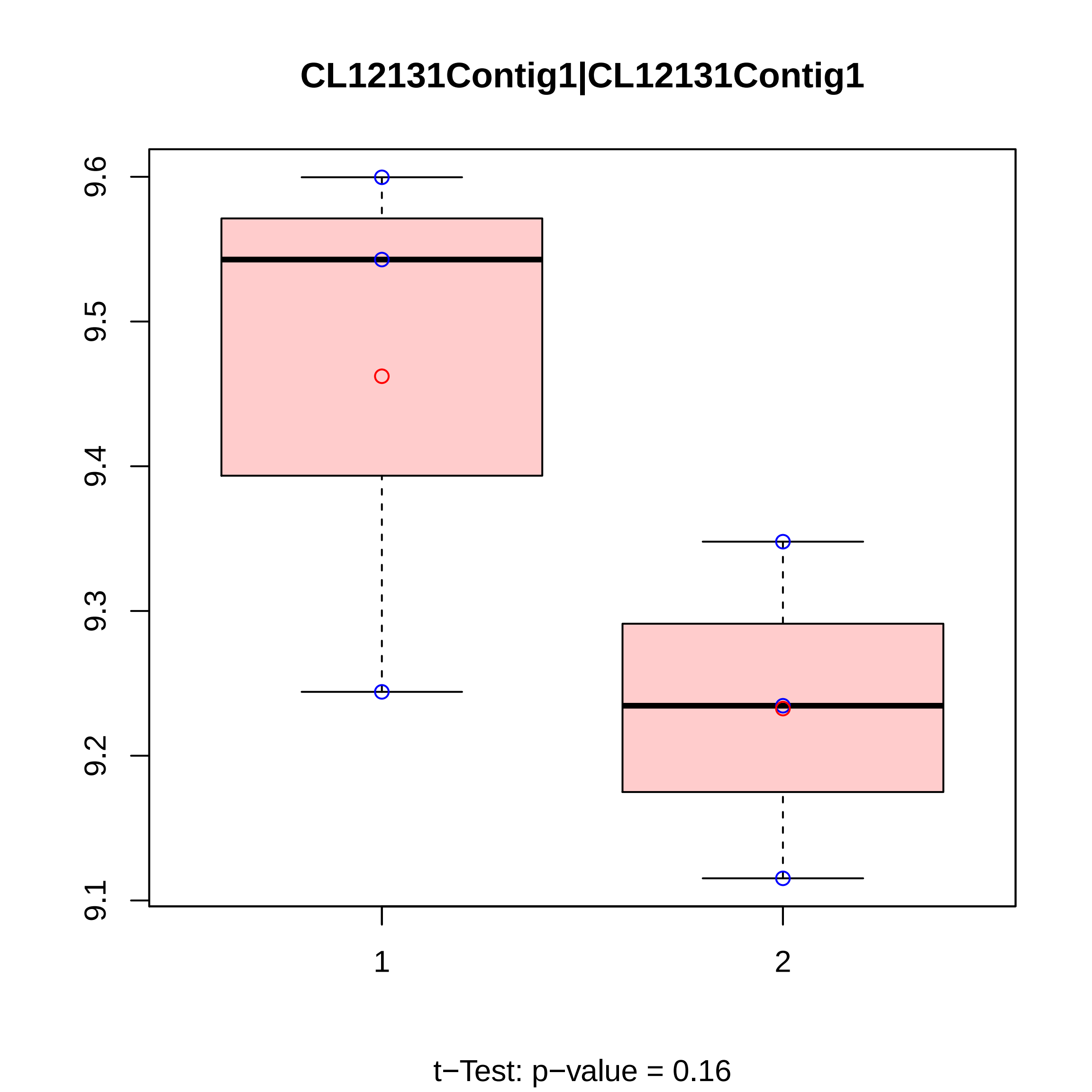

CL12131Contig1|CL12131Contig1
9.1
9.2
9.3
9.4
9.5
9.6
1
2
t−Test: p−value = 0.16

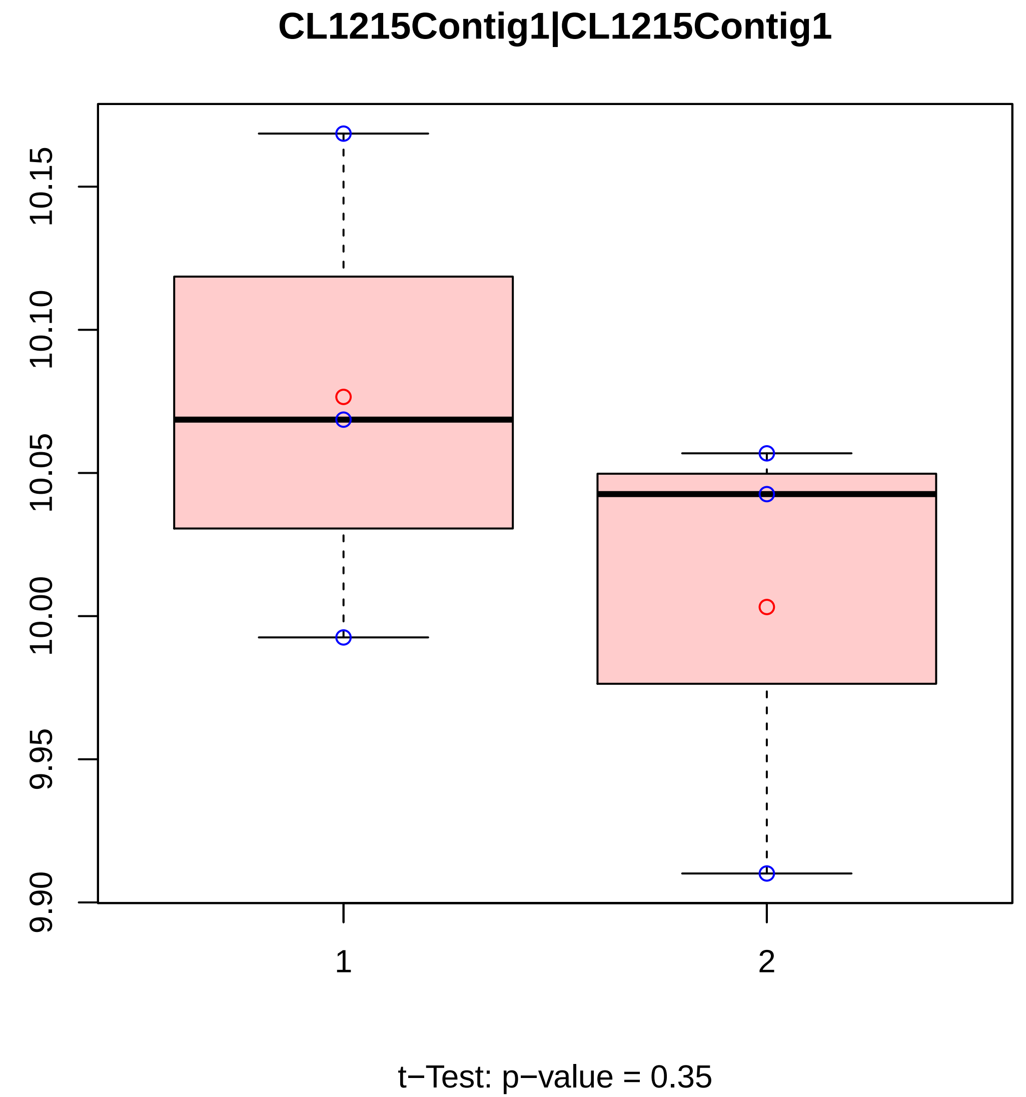
CL1215Contig1|CL1215Contig1
10.15
10.10
10.05
10.00
9.95
9.90
1
2
t−Test: p−value = 0.35

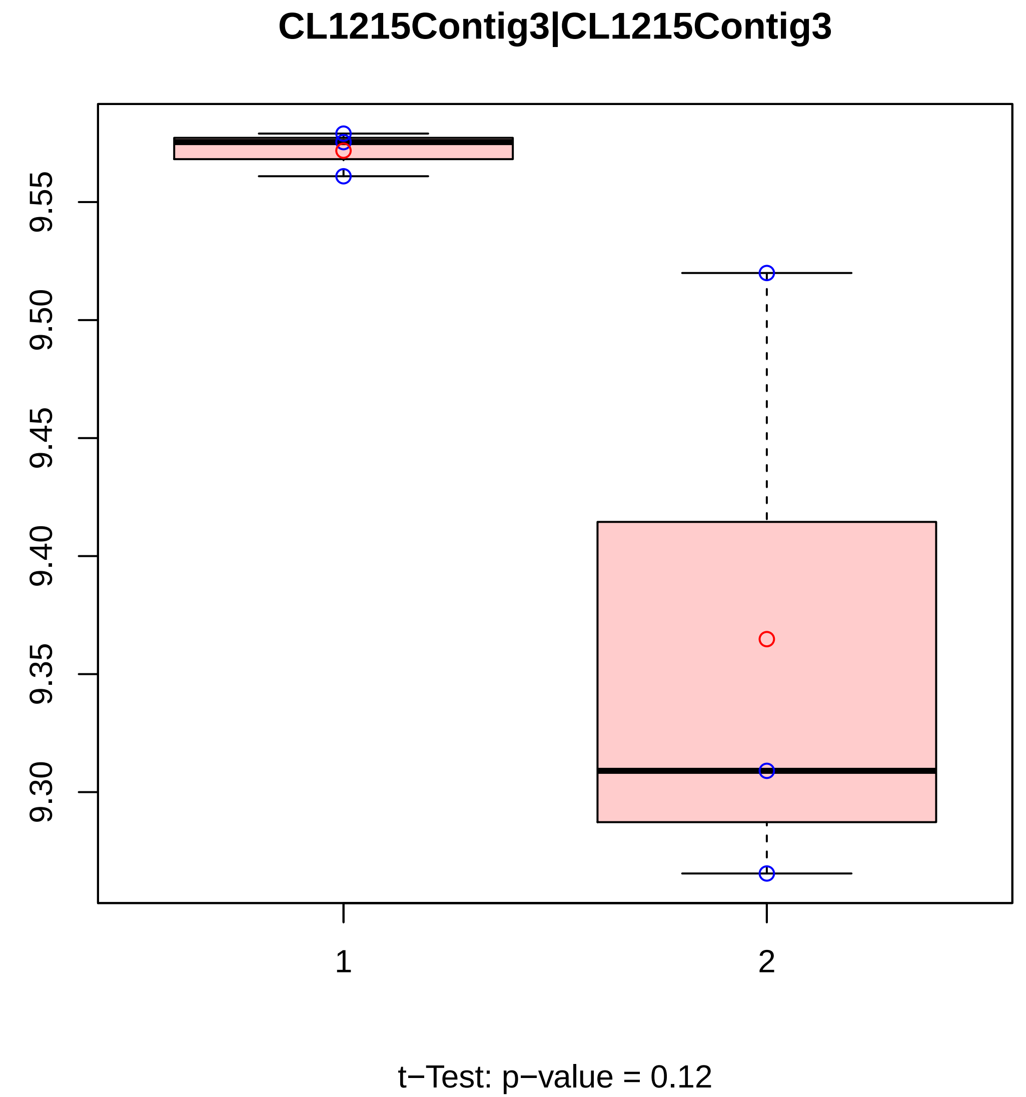
CL1215Contig3|CL1215Contig3
9.30
9.35
9.40
9.45
9.50
9.55
1
2
t−Test: p−value = 0.12

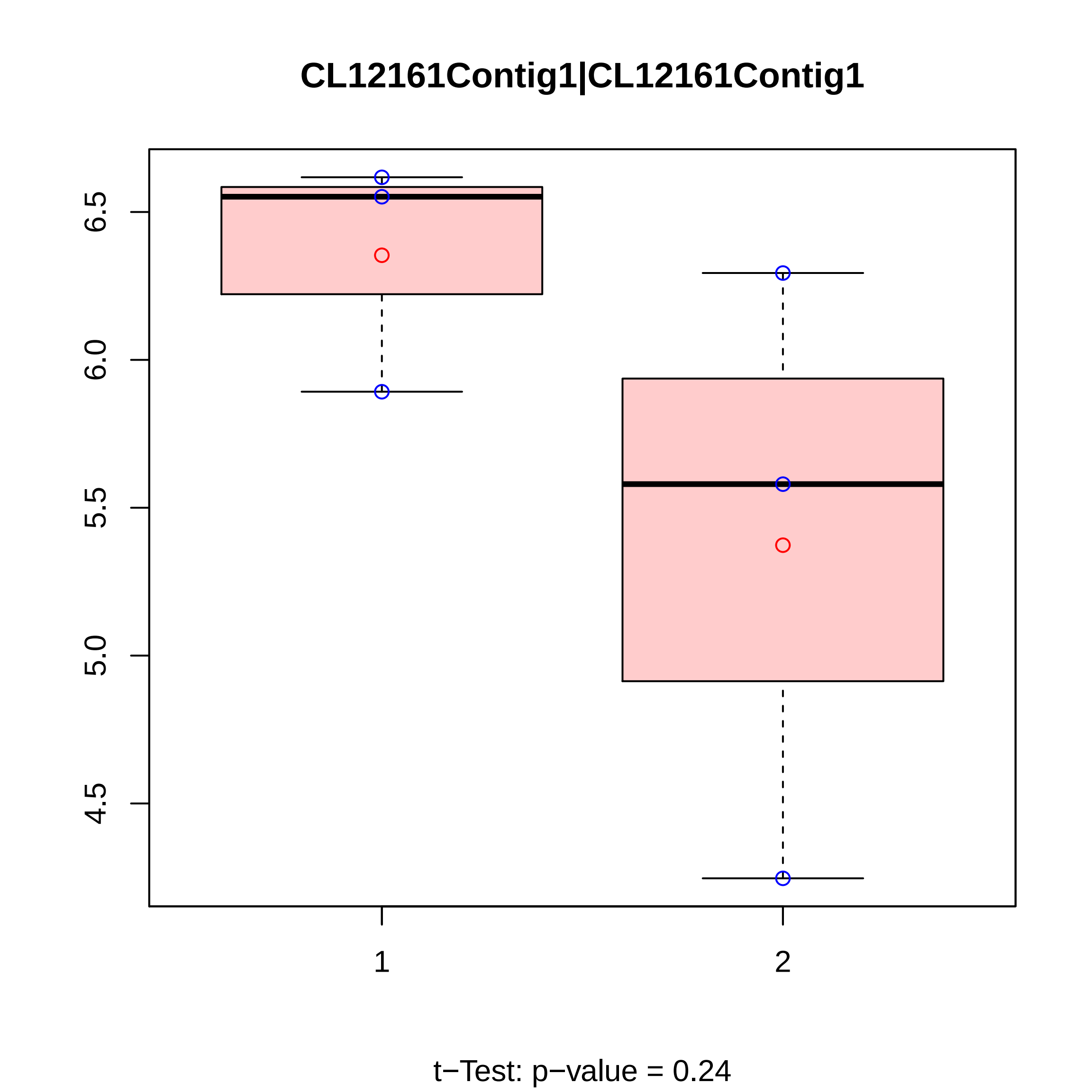
CL12161Contig1|CL12161Contig1
6.5
6.0
5.5
5.0
4.5
1
2
t−Test: p−value = 0.24

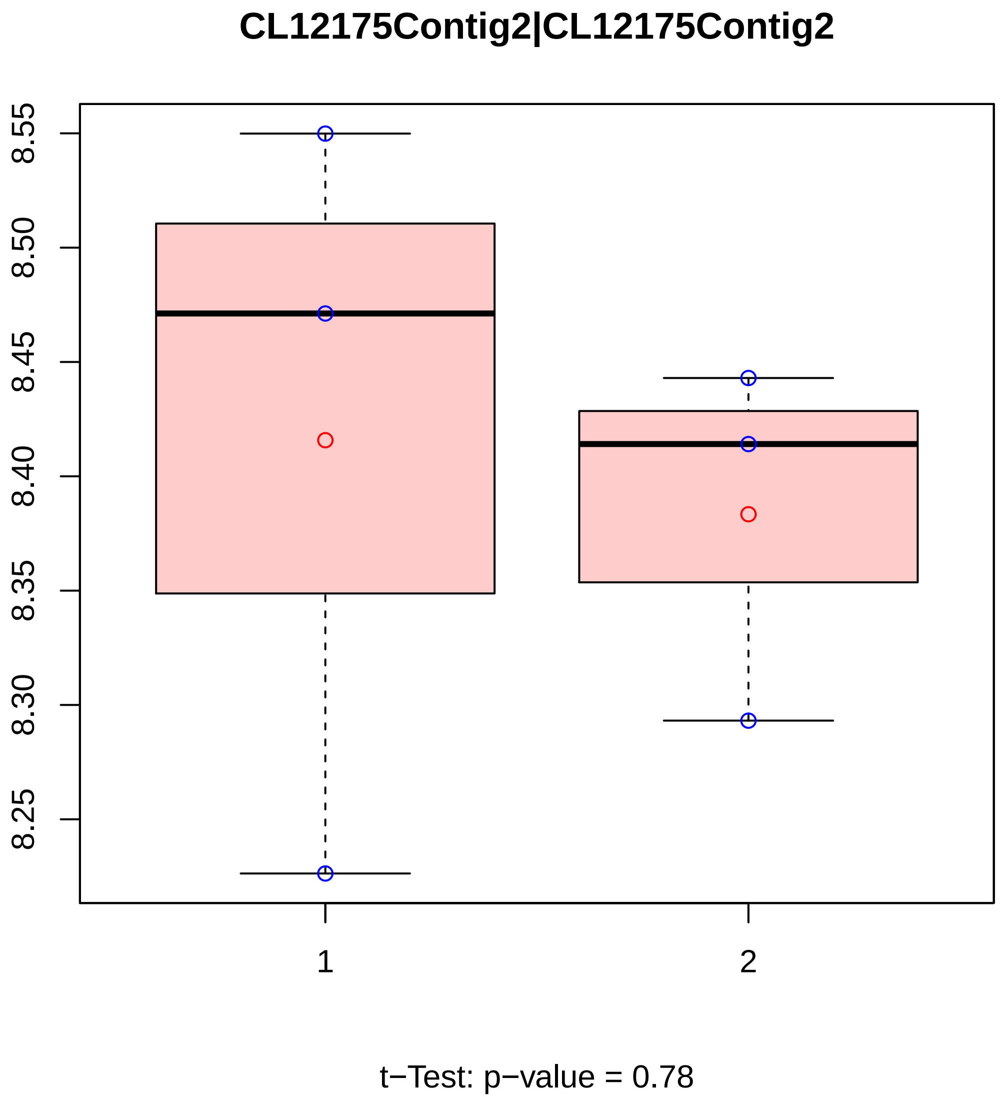
CL12175Contig2|CL12175Contig2
8.25
8.30
8.35
8.40
8.45
8.50
8.55
1
2
t−Test: p−value = 0.78

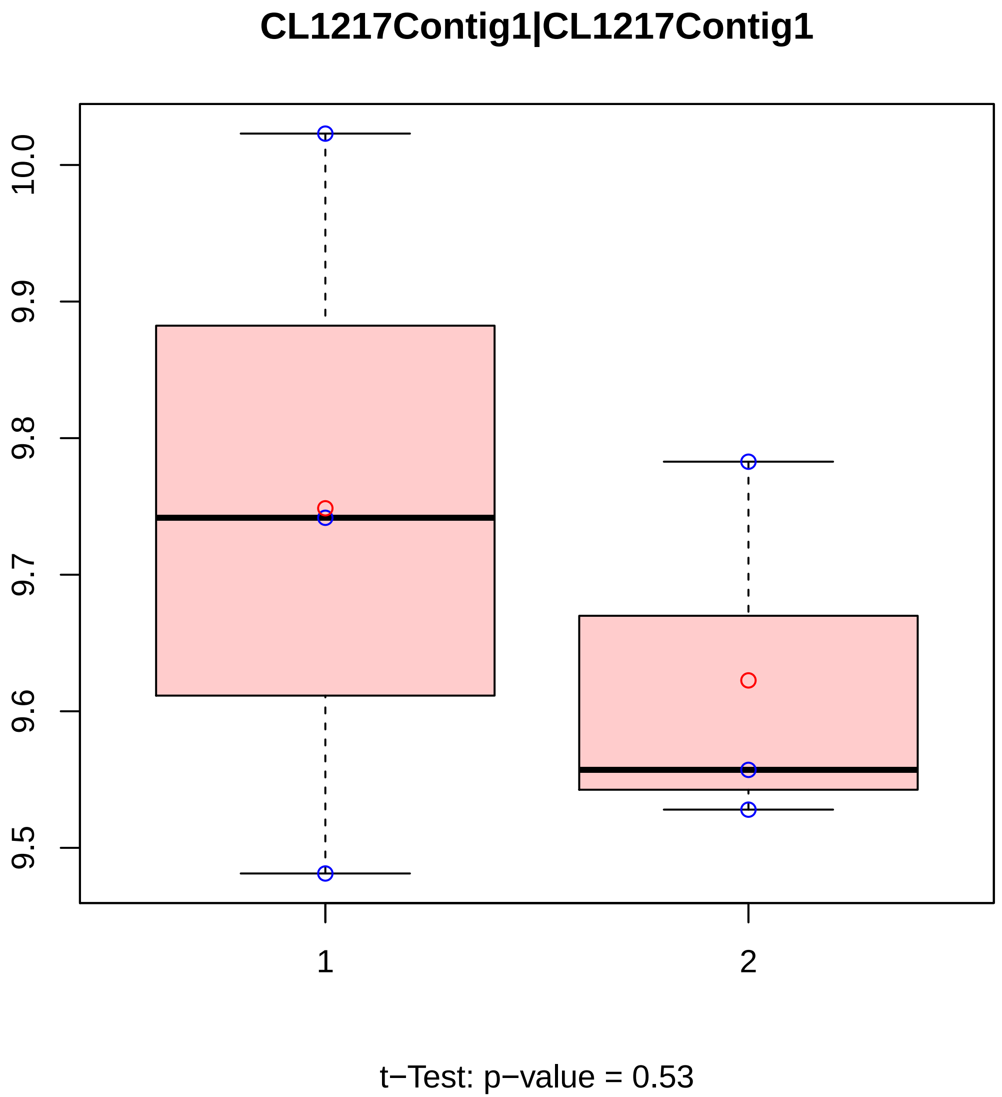
CL1217Contig1|CL1217Contig1
10.0
9.9
9.8
9.7
9.6
9.5
1
2
t−Test: p−value = 0.53

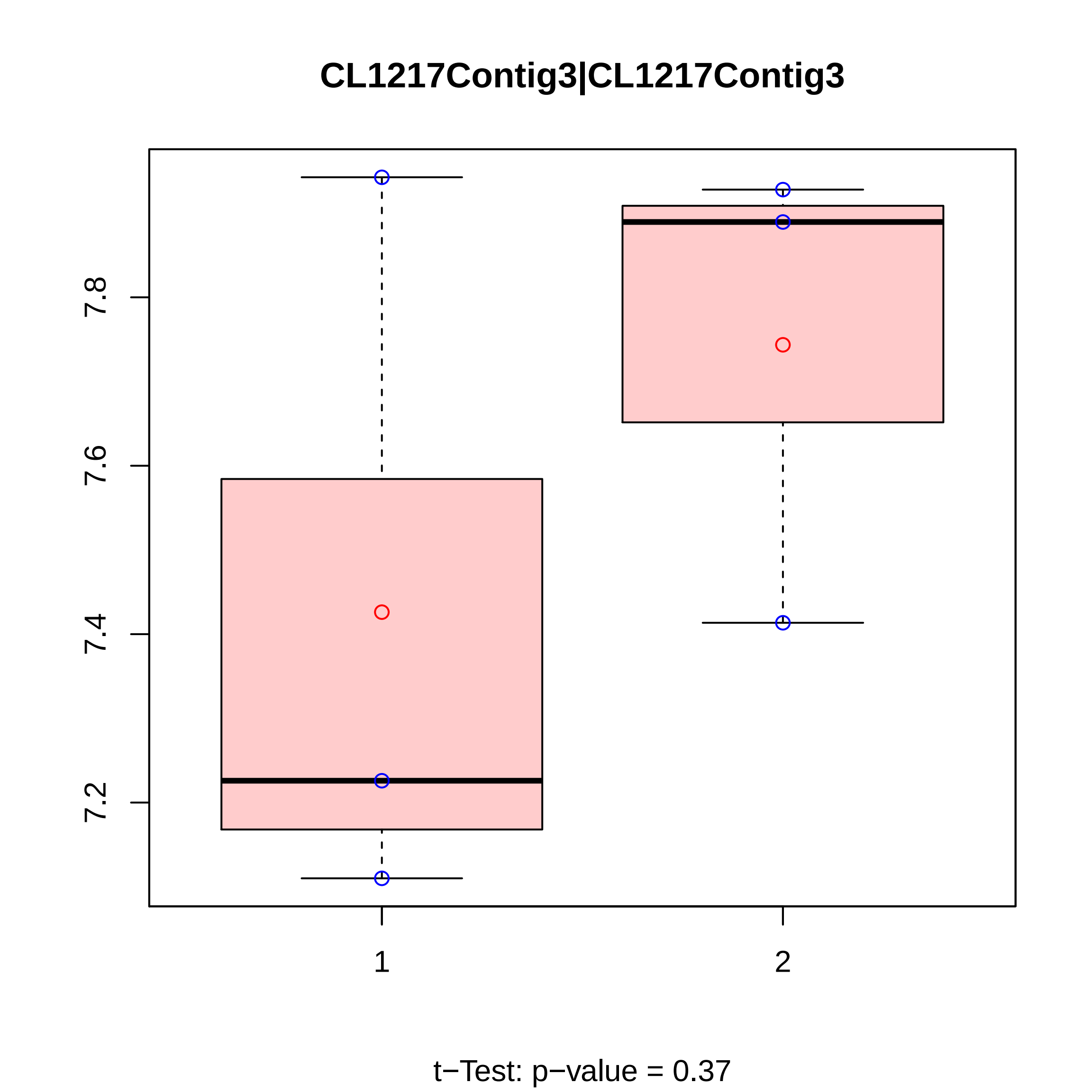

CL1217Contig3|CL1217Contig3
7.2
7.4
7.6
7.8
1
2
t−Test: p−value = 0.37

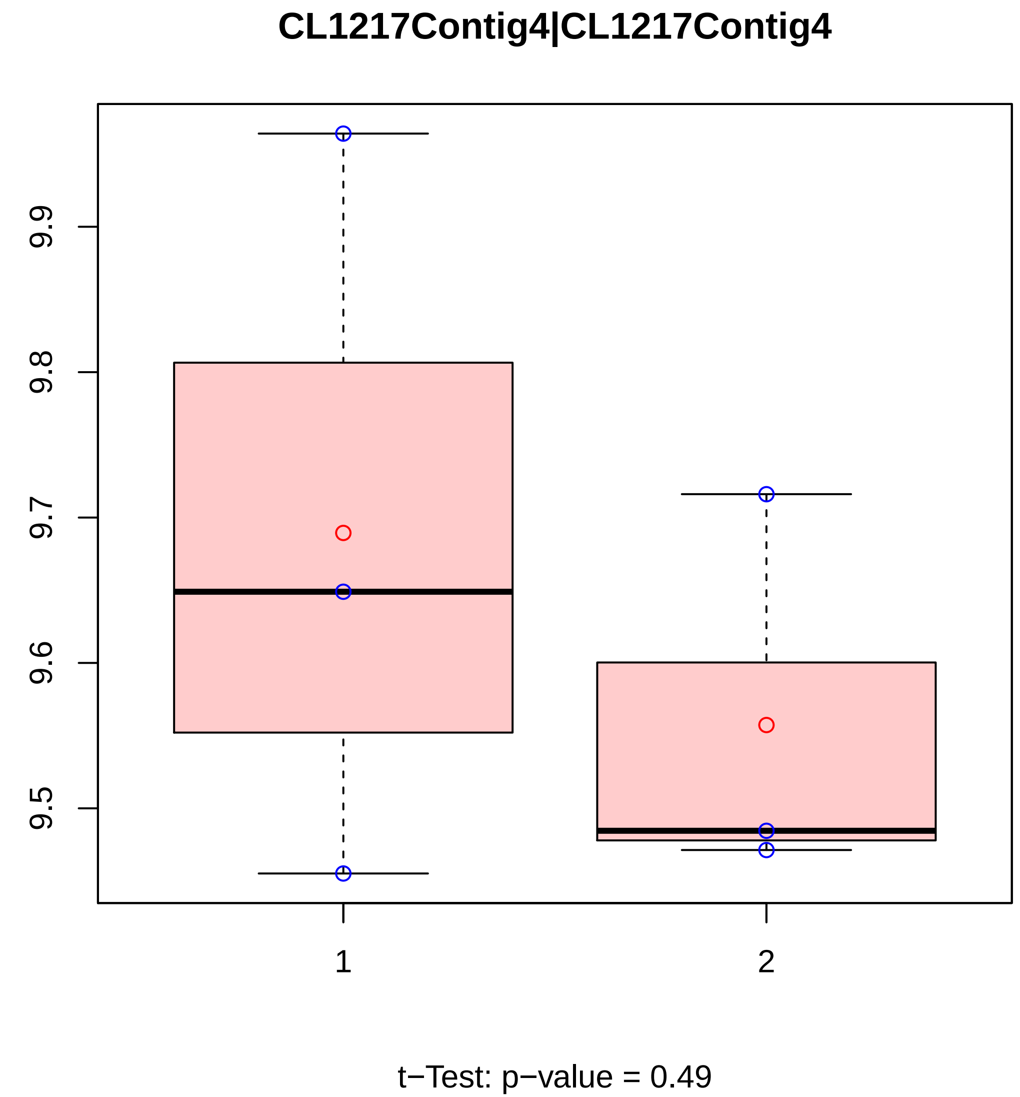
CL1217Contig4|CL1217Contig4
9.5
9.6
9.7
9.8
9.9
1
2
t−Test: p−value = 0.49

## CL12182Contig1|CL12182Contig1

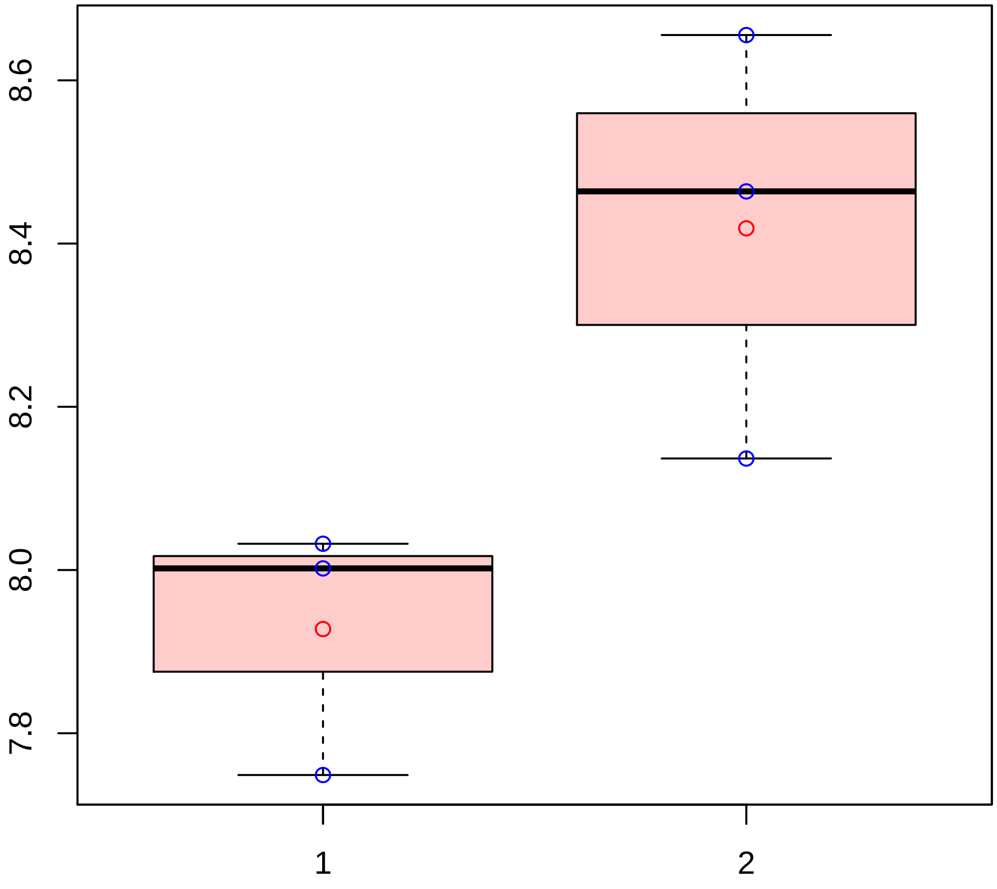


t−Test: p−value = 0.06

# CL1218Contig1|CL1218Contig1

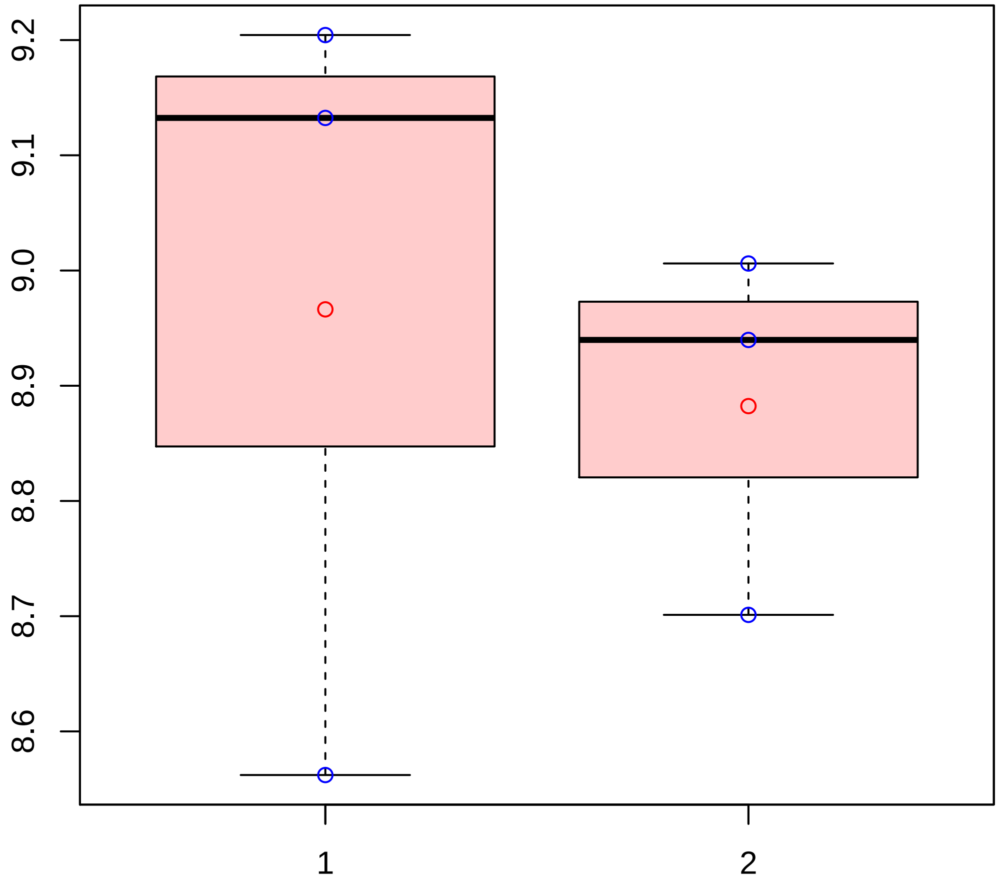


t−Test: p−value = 0.73

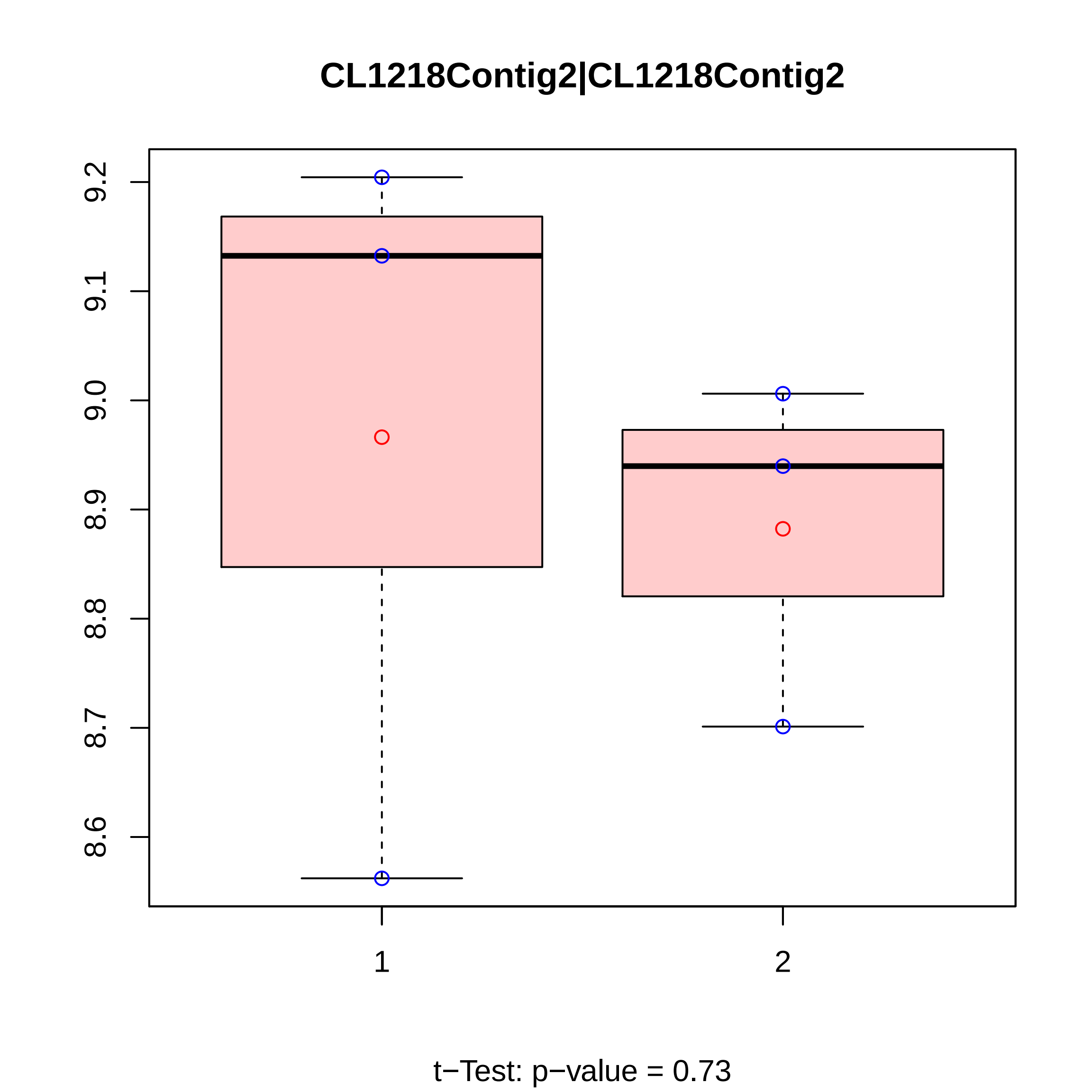

CL1218Contig2|CL1218Contig2
9.2
9.1
9.0
8.9
8.8
8.7
8.6
1
2
t−Test: p−value = 0.73

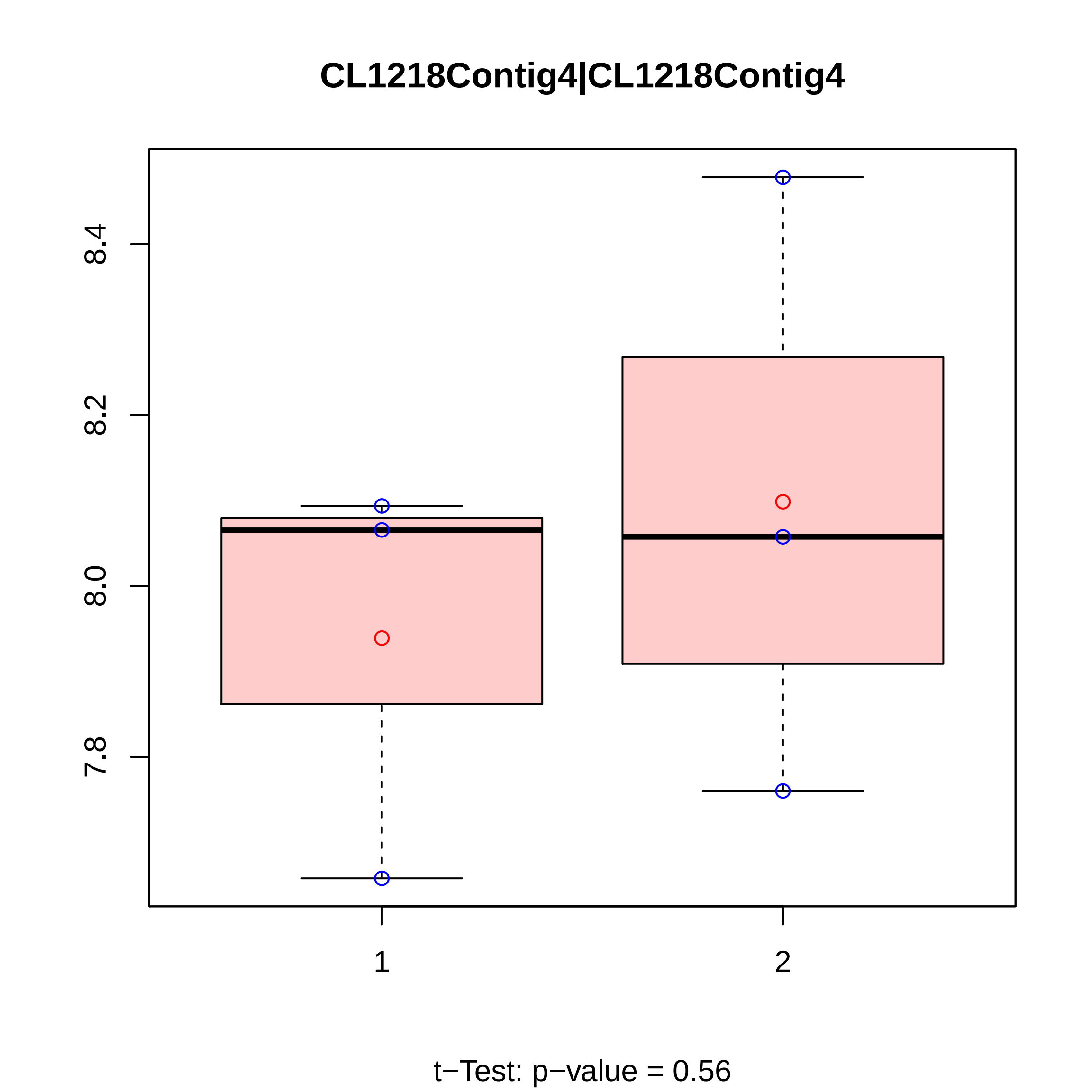
CL1218Contig4|CL1218Contig4
8.4
8.2
8.0
7.8
1
2
t−Test: p−value = 0.56

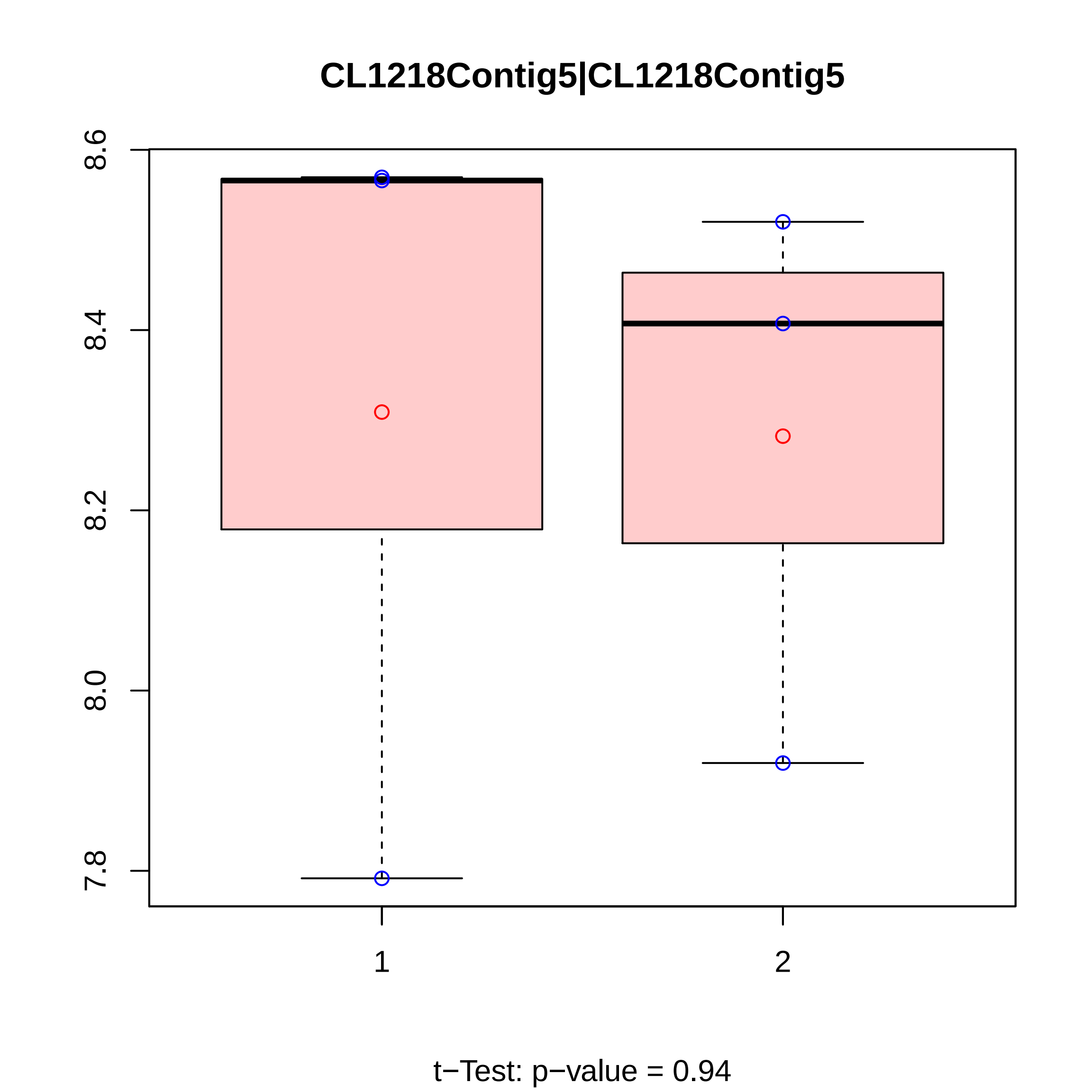
CL1218Contig5|CL1218Contig5
8.6
8.4
8.2
8.0
7.8
1
2
t−Test: p−value = 0.94

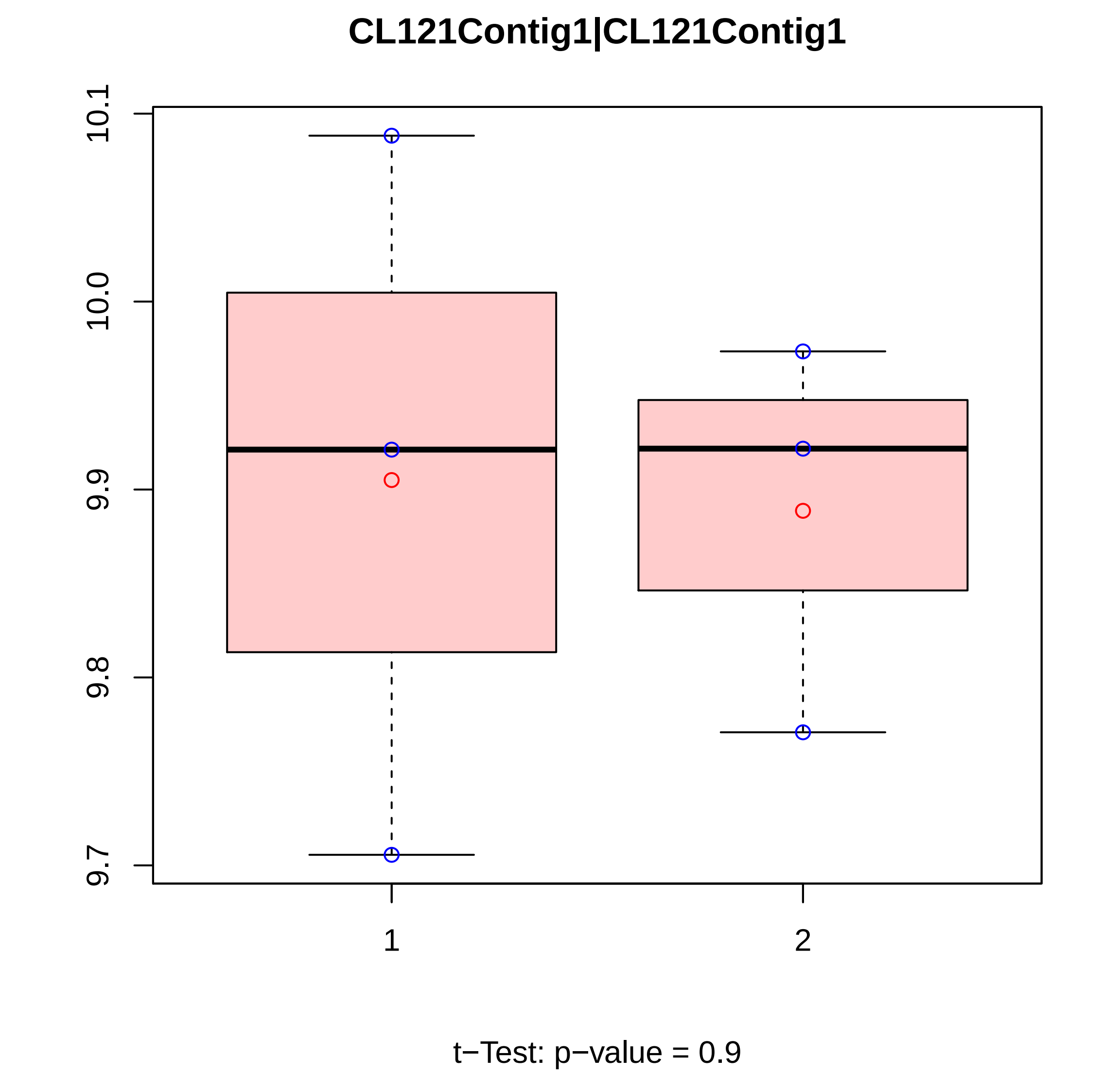
CL121Contig1|CL121Contig1
10.1
10.0
9.9
9.8
9.7
1
2
t−Test: p−value = 0.9

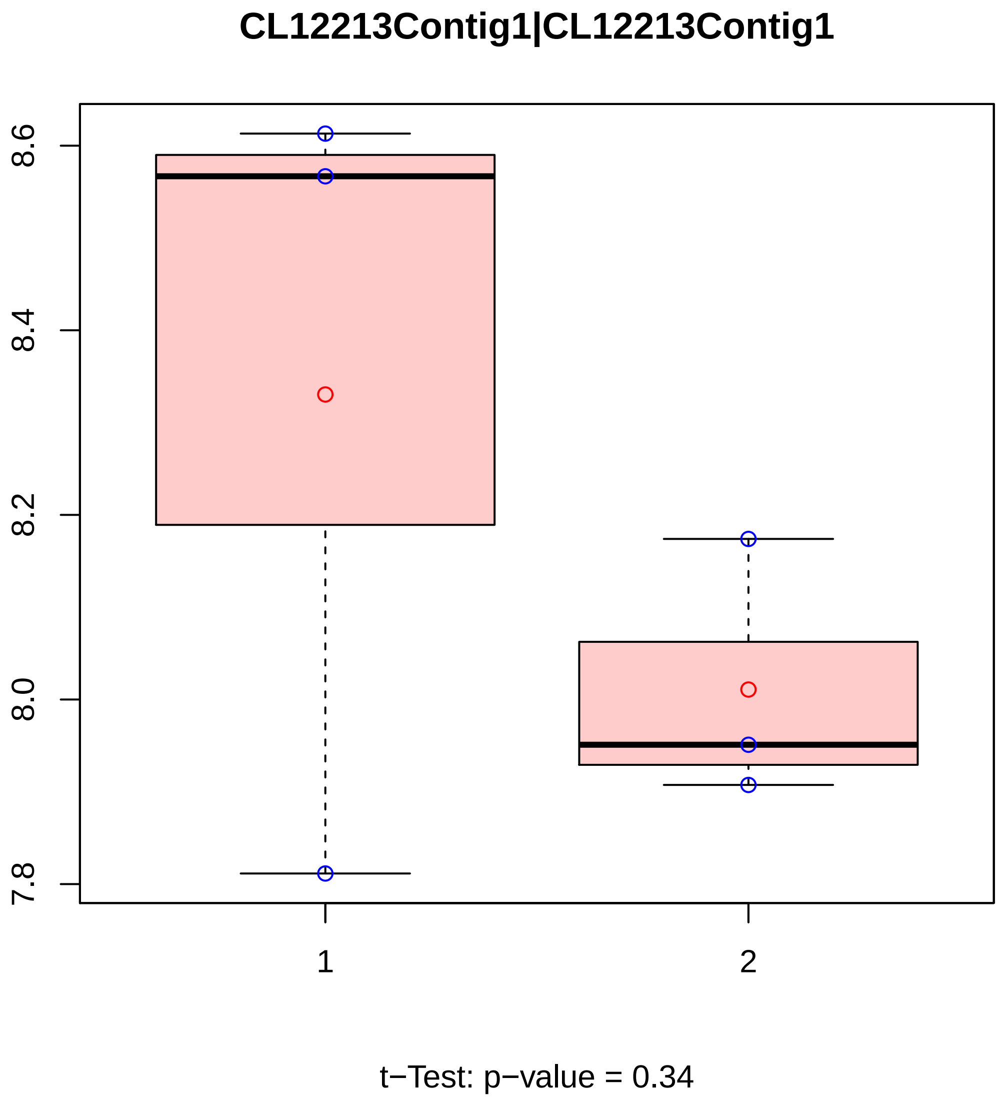
CL12213Contig1|CL12213Contig1
8.6
8.4
8.2
8.0
7.8
1
2
t−Test: p−value = 0.34

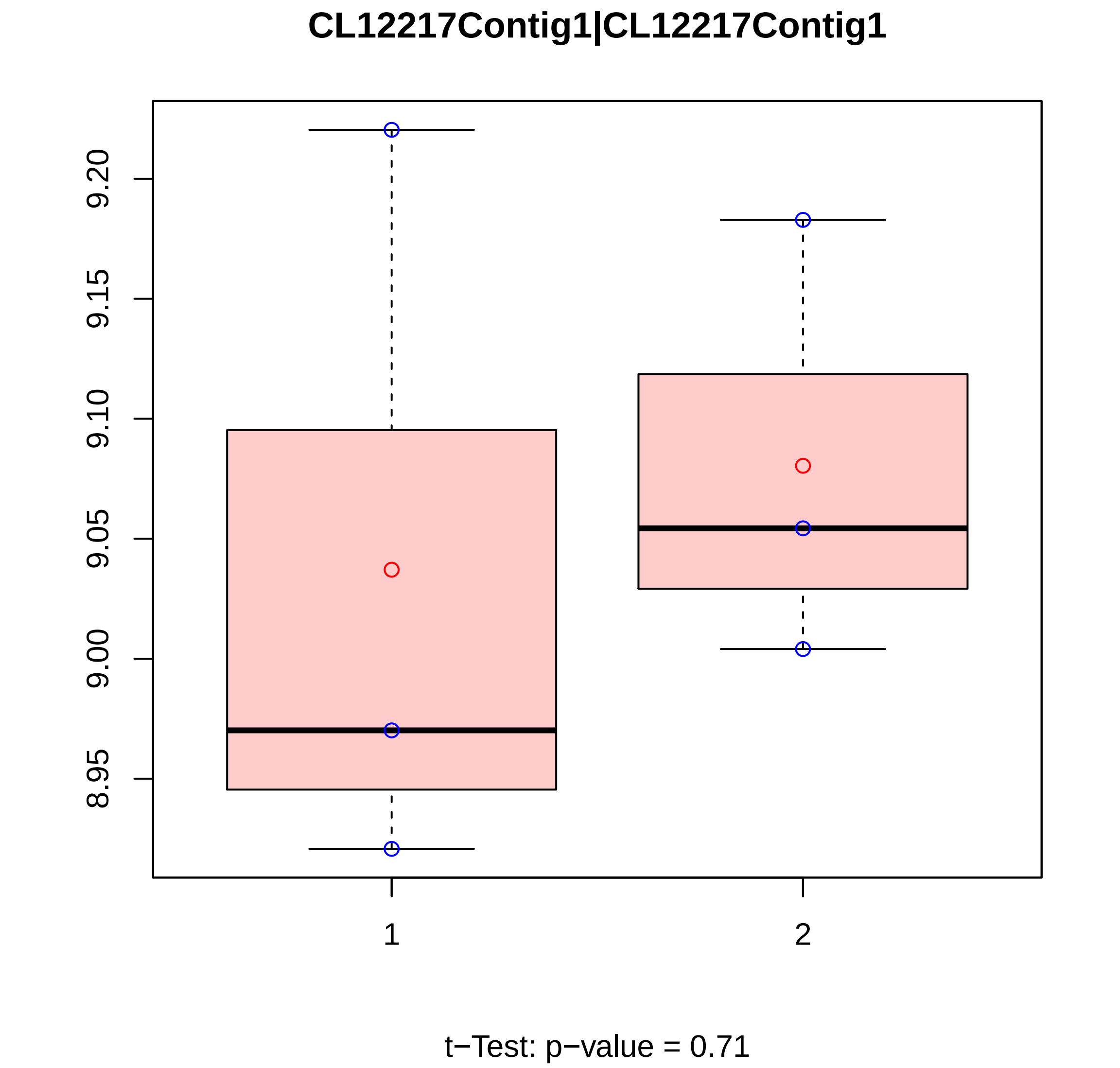
CL12217Contig1|CL12217Contig1
9.20
9.15
9.10
9.05
9.00
8.95
1
2
t−Test: p−value = 0.71

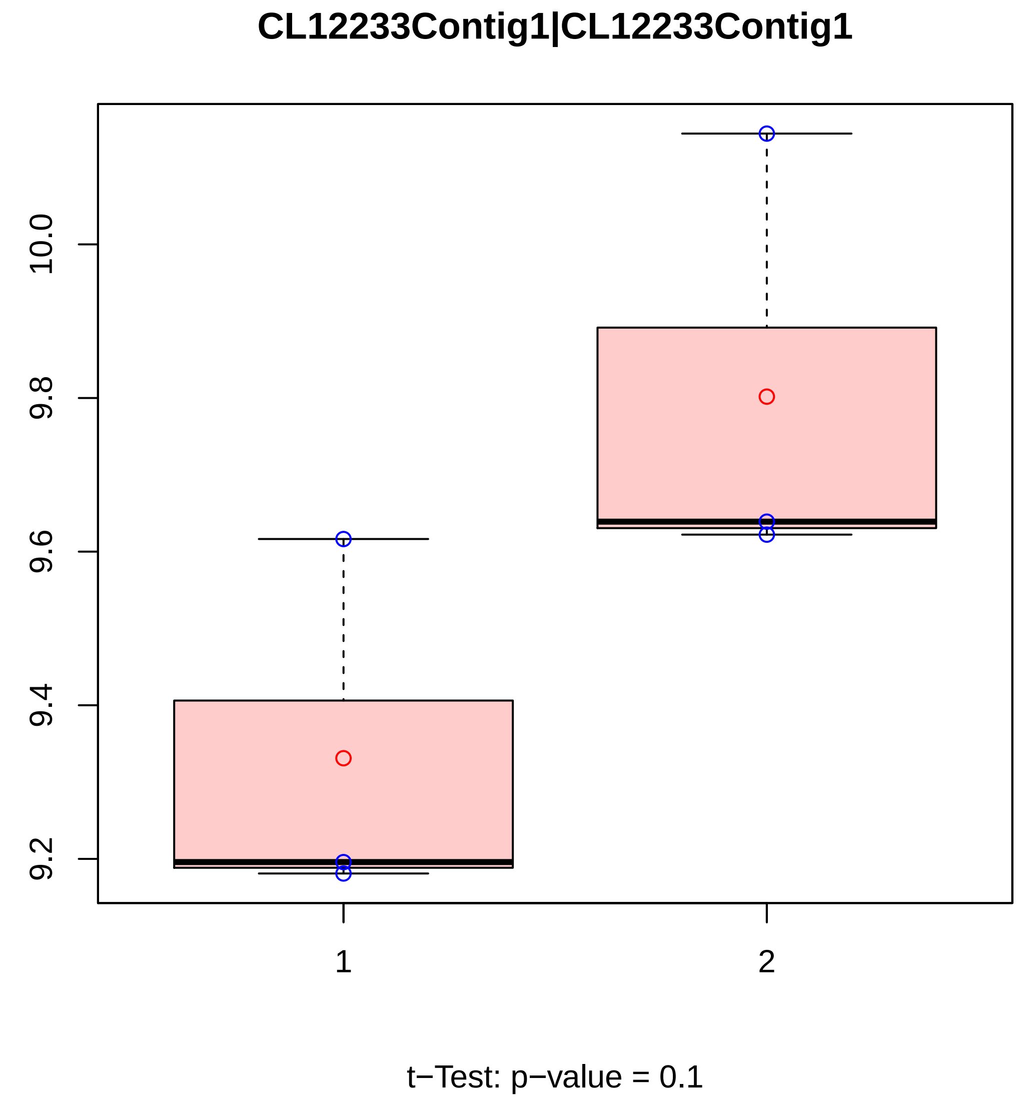

CL12233Contig1|CL12233Contig1
10.0
9.8
9.6
9.4
9.2
1
2
t−Test: p−value = 0.1

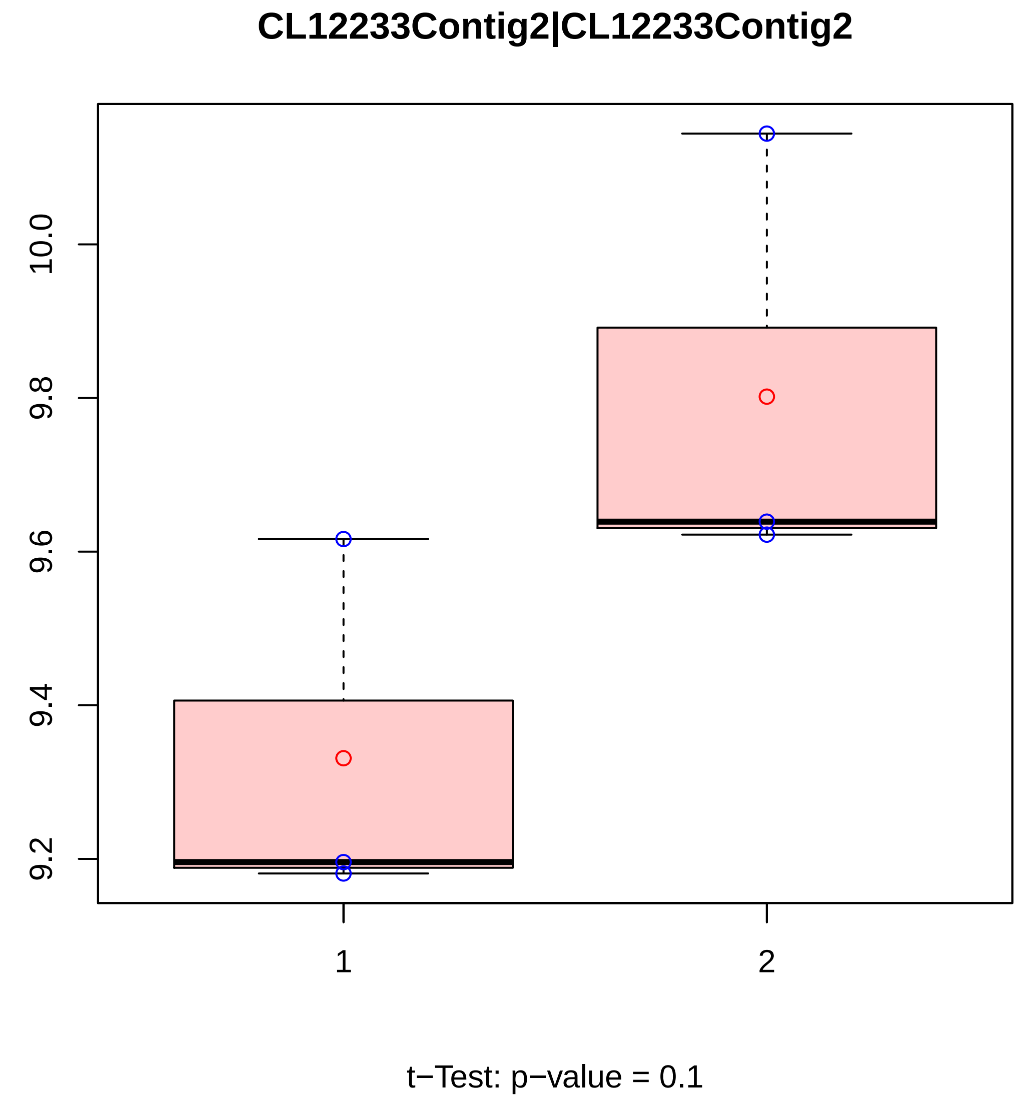

CL12233Contig2|CL12233Contig2
10.0
9.8
9.6
9.4
9.2
1
2
t−Test: p−value = 0.1

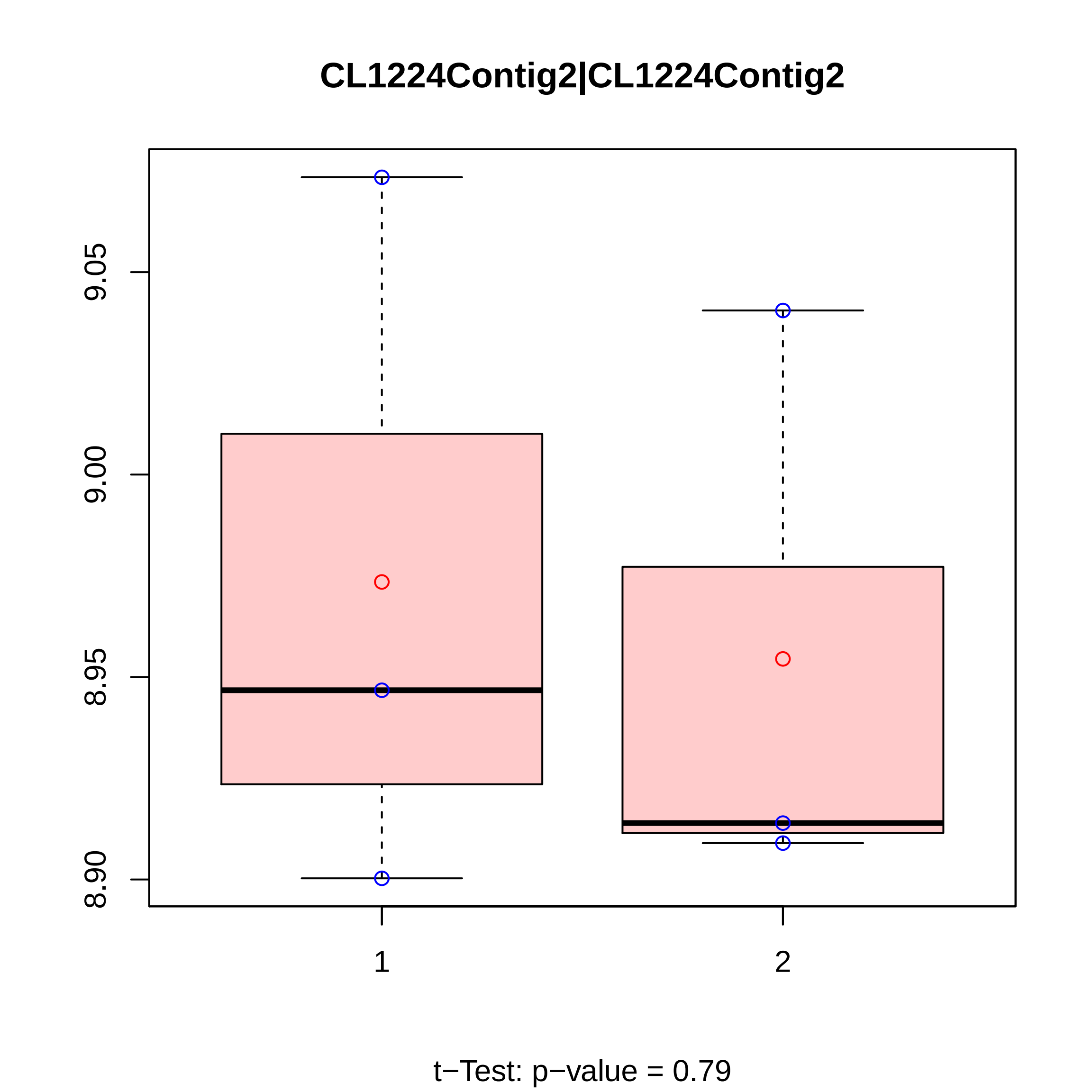
CL1224Contig2|CL1224Contig2
9.05
9.00
8.95
8.90
1
2
t−Test: p−value = 0.79

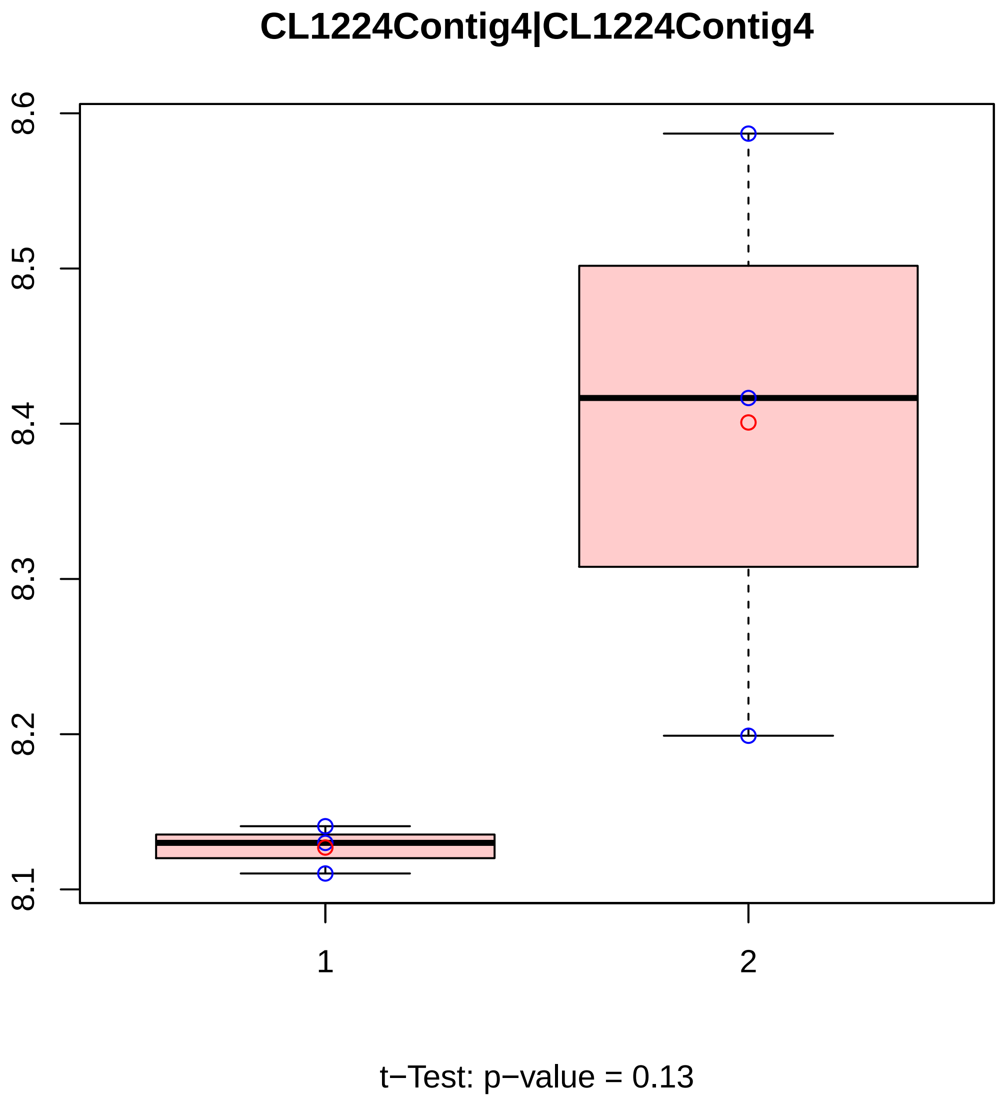
CL1224Contig4|CL1224Contig4
8.1
8.2
8.3
8.4
8.5
8.6
1
2
t−Test: p−value = 0.13

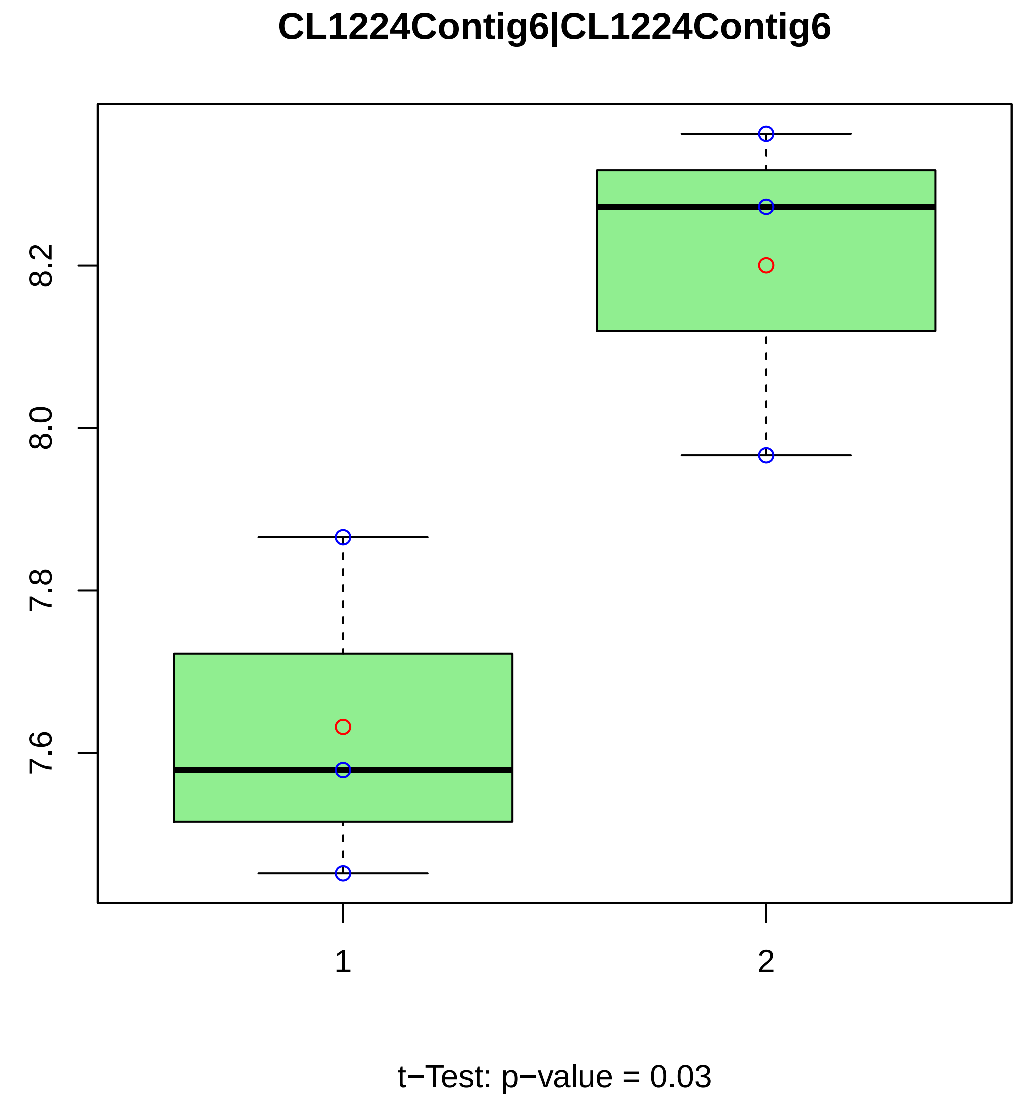
CL1224Contig6|CL1224Contig6
8.2
8.0
7.8
7.6
1
2
t−Test: p−value = 0.03

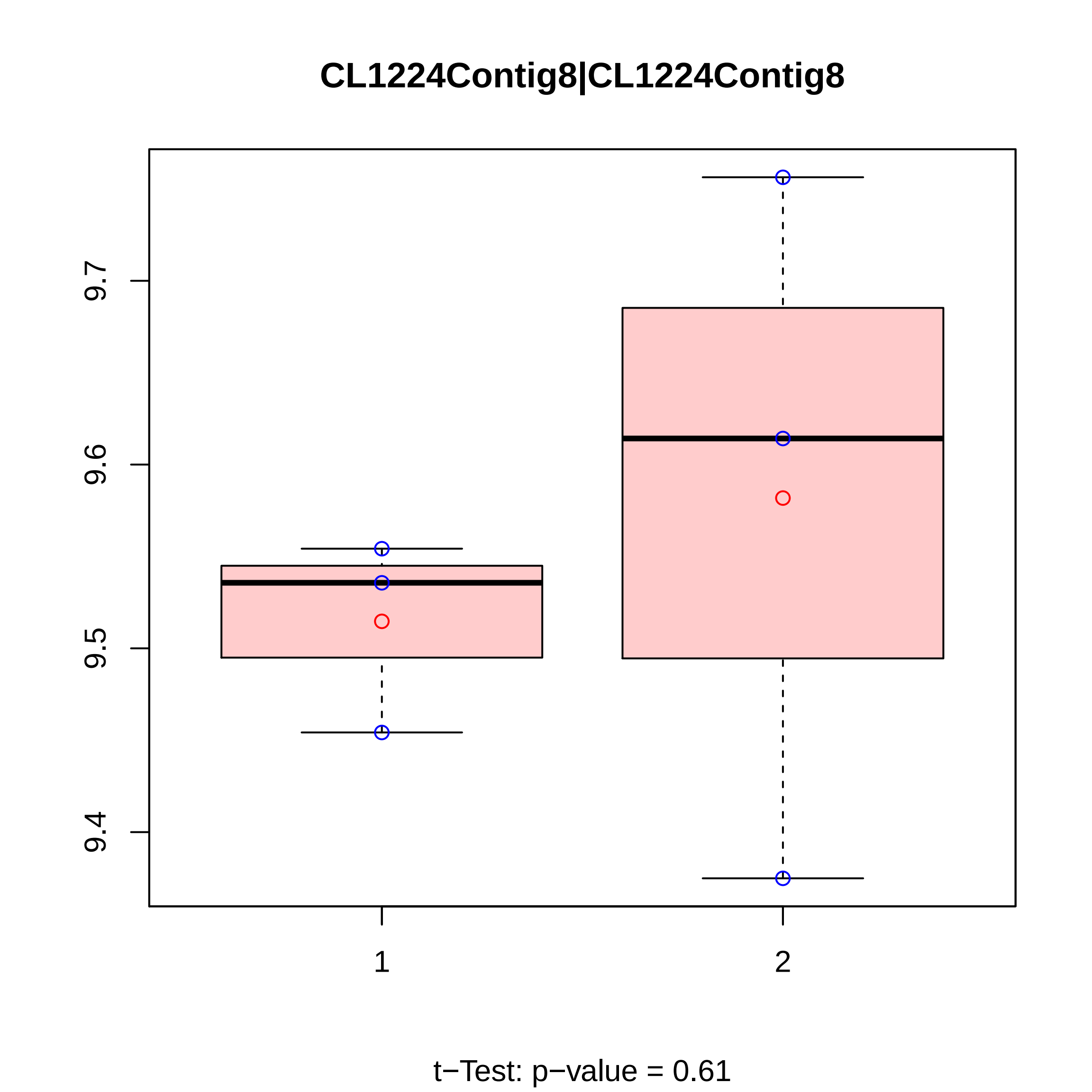
CL1224Contig8|CL1224Contig8
9.4
9.5
9.6
9.7
1
2
t−Test: p−value = 0.61

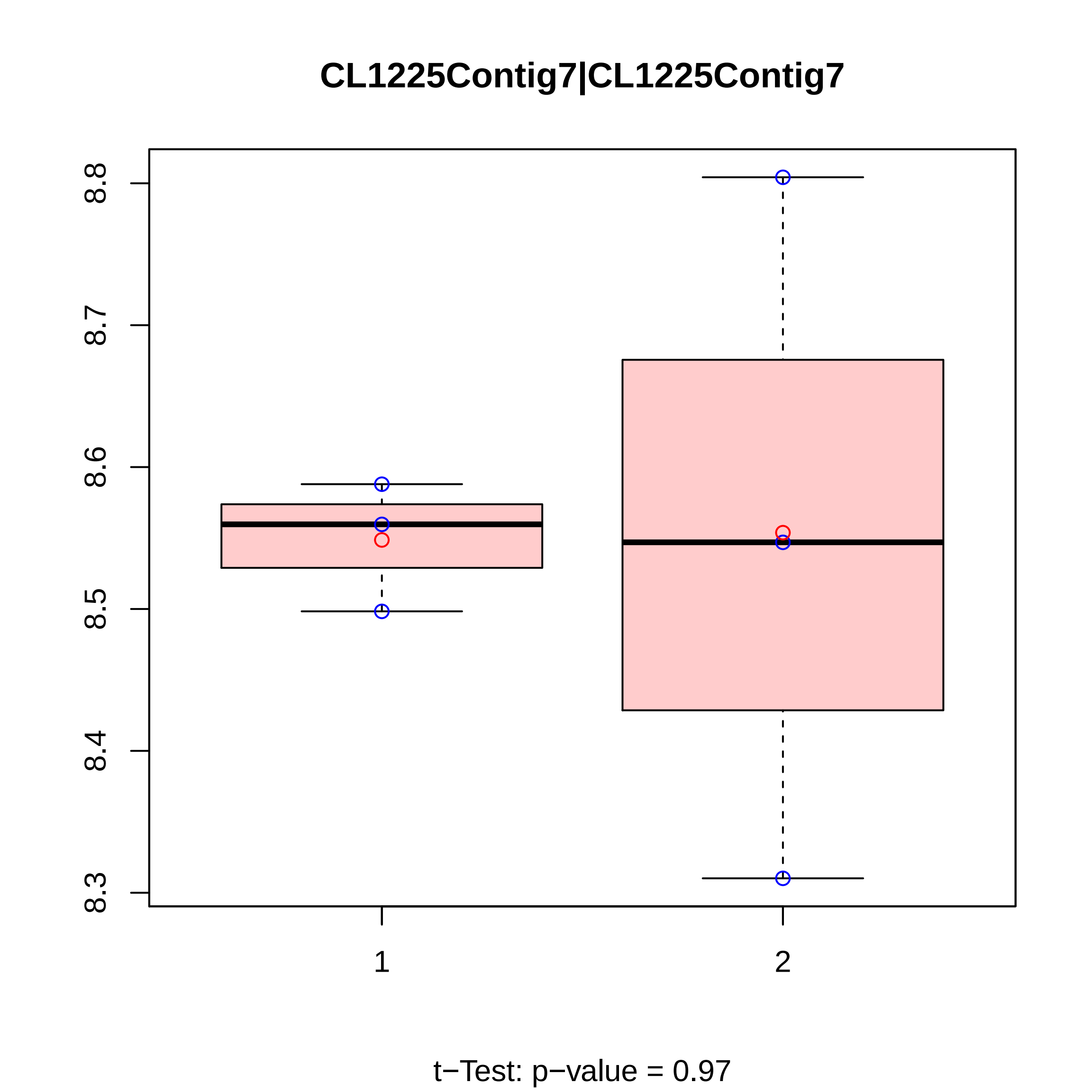

CL1225Contig7|CL1225Contig7
8.8
8.7
8.6
8.5
8.4
8.3
1
2
t−Test: p−value = 0.97

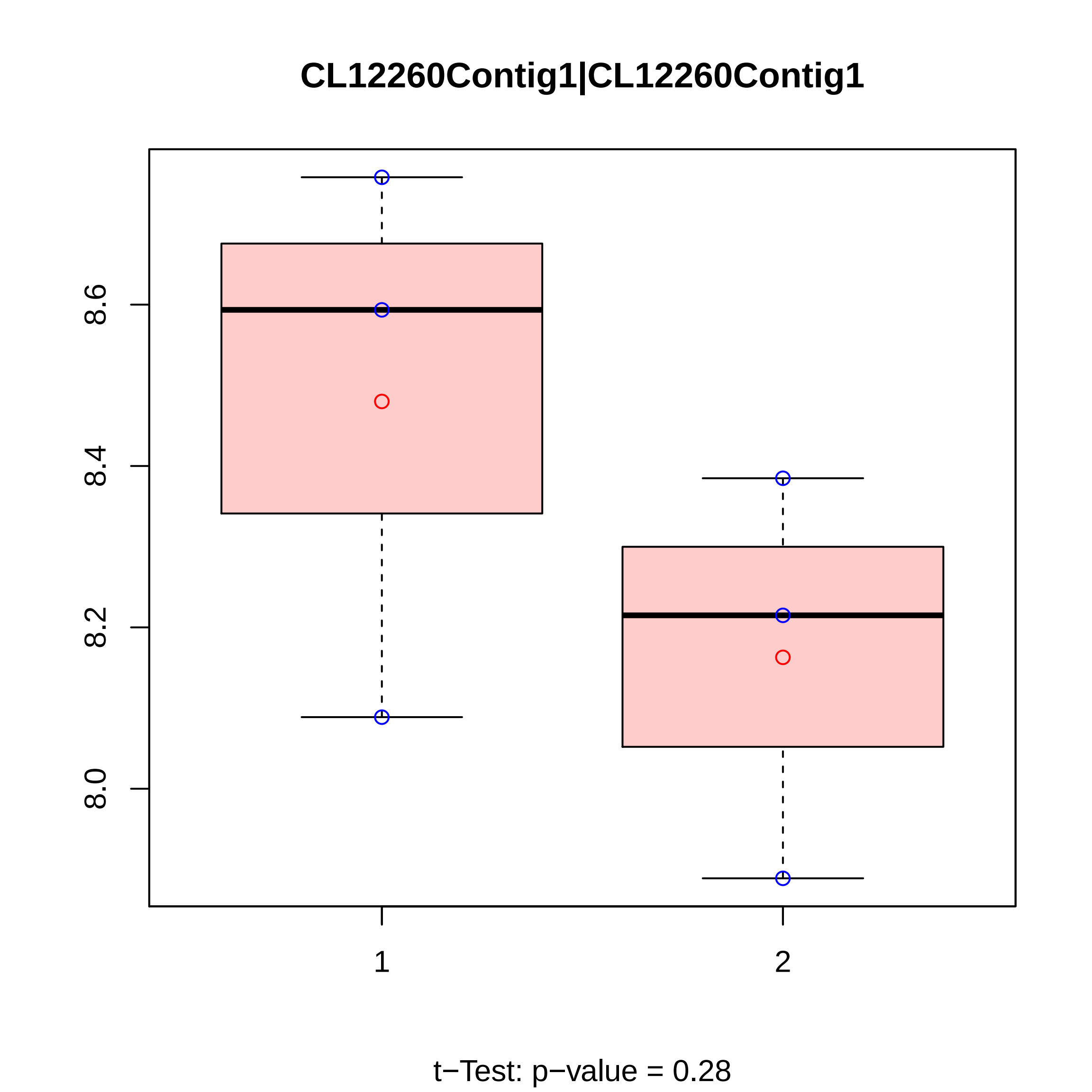
CL12260Contig1|CL12260Contig1
8.0
8.2
8.4
8.6
1
2
t−Test: p−value = 0.28

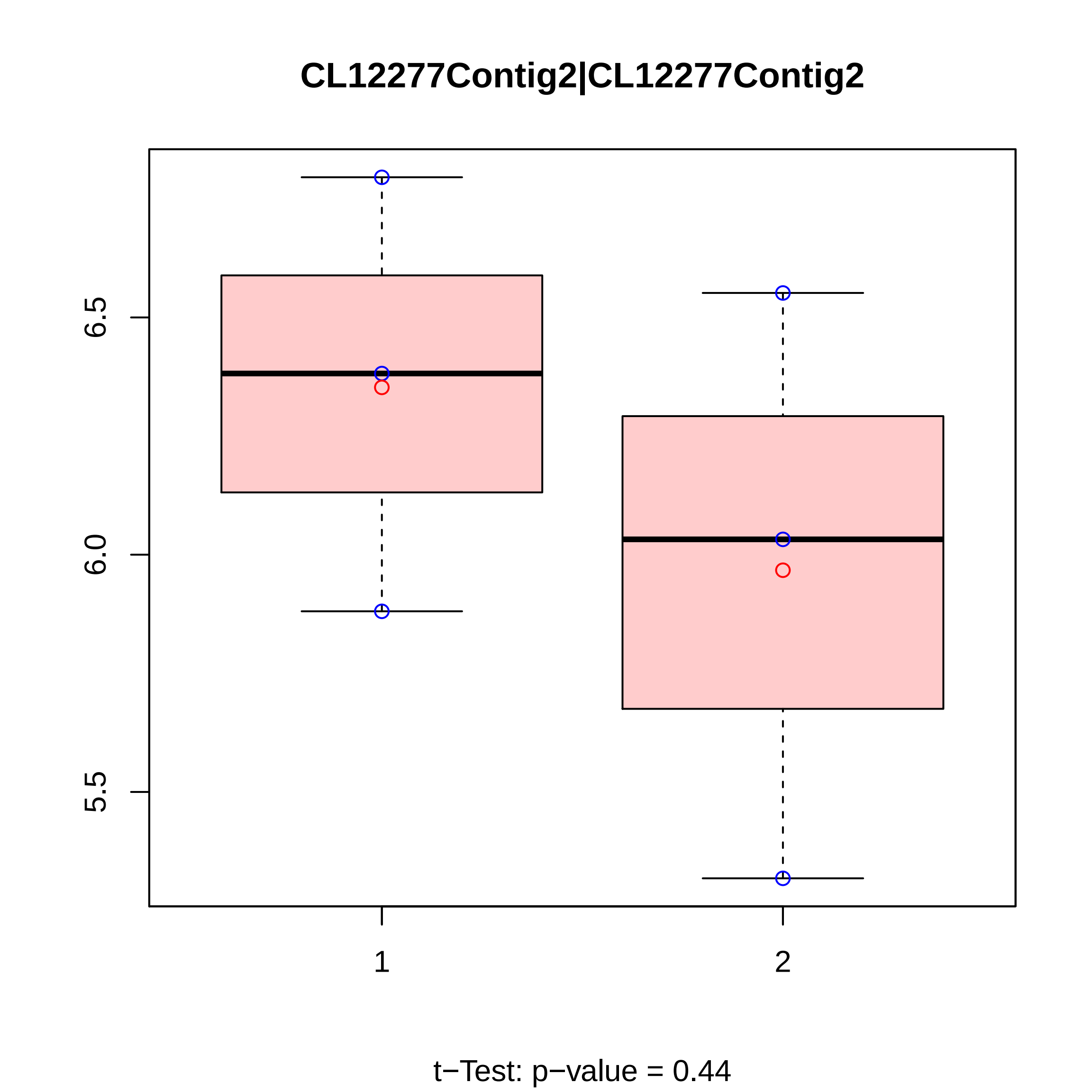
CL12277Contig2|CL12277Contig2
6.5
6.0
5.5
1
2
t−Test: p−value = 0.44

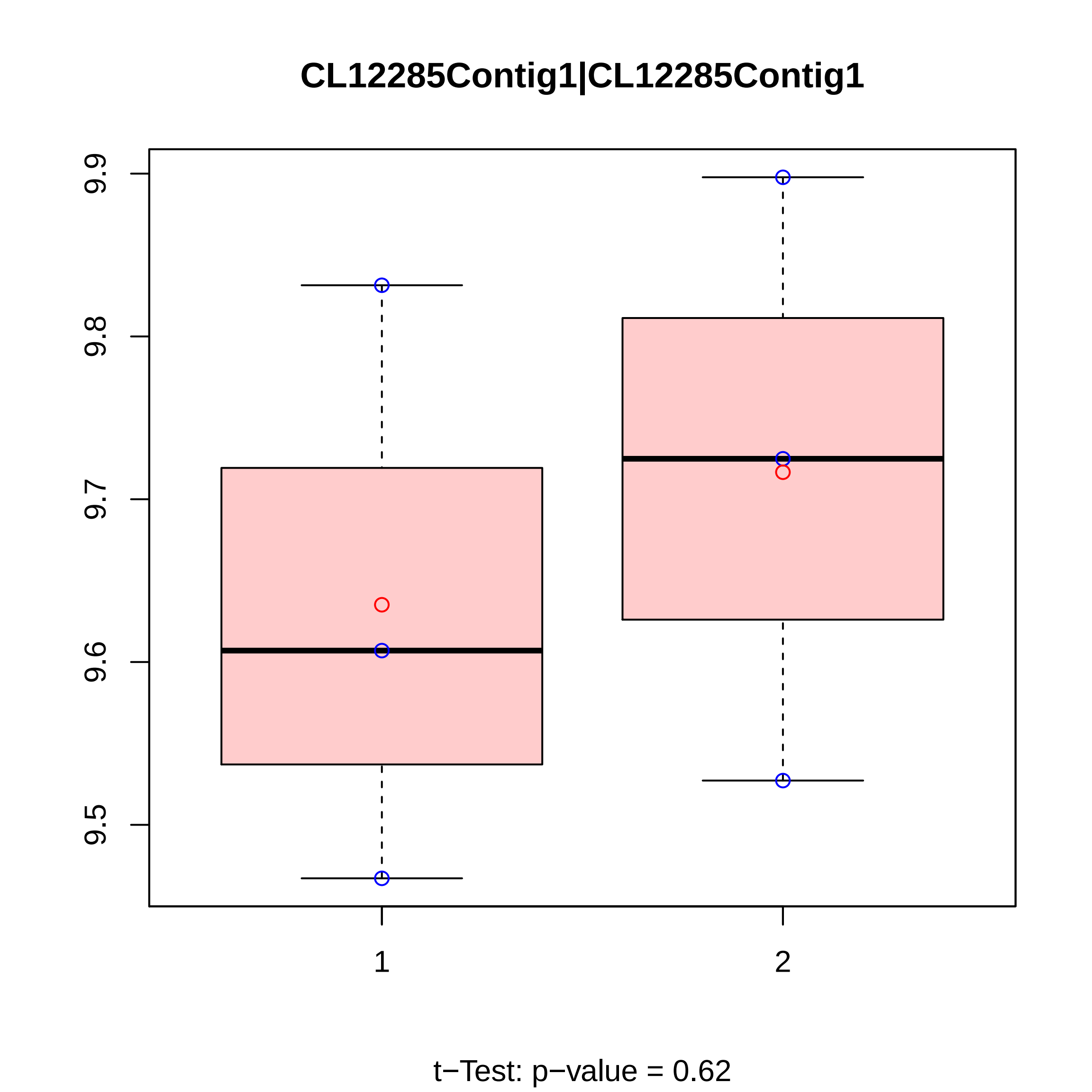
CL12285Contig1|CL12285Contig1
9.5
9.6
9.7
9.8
9.9
1
2
t−Test: p−value = 0.62

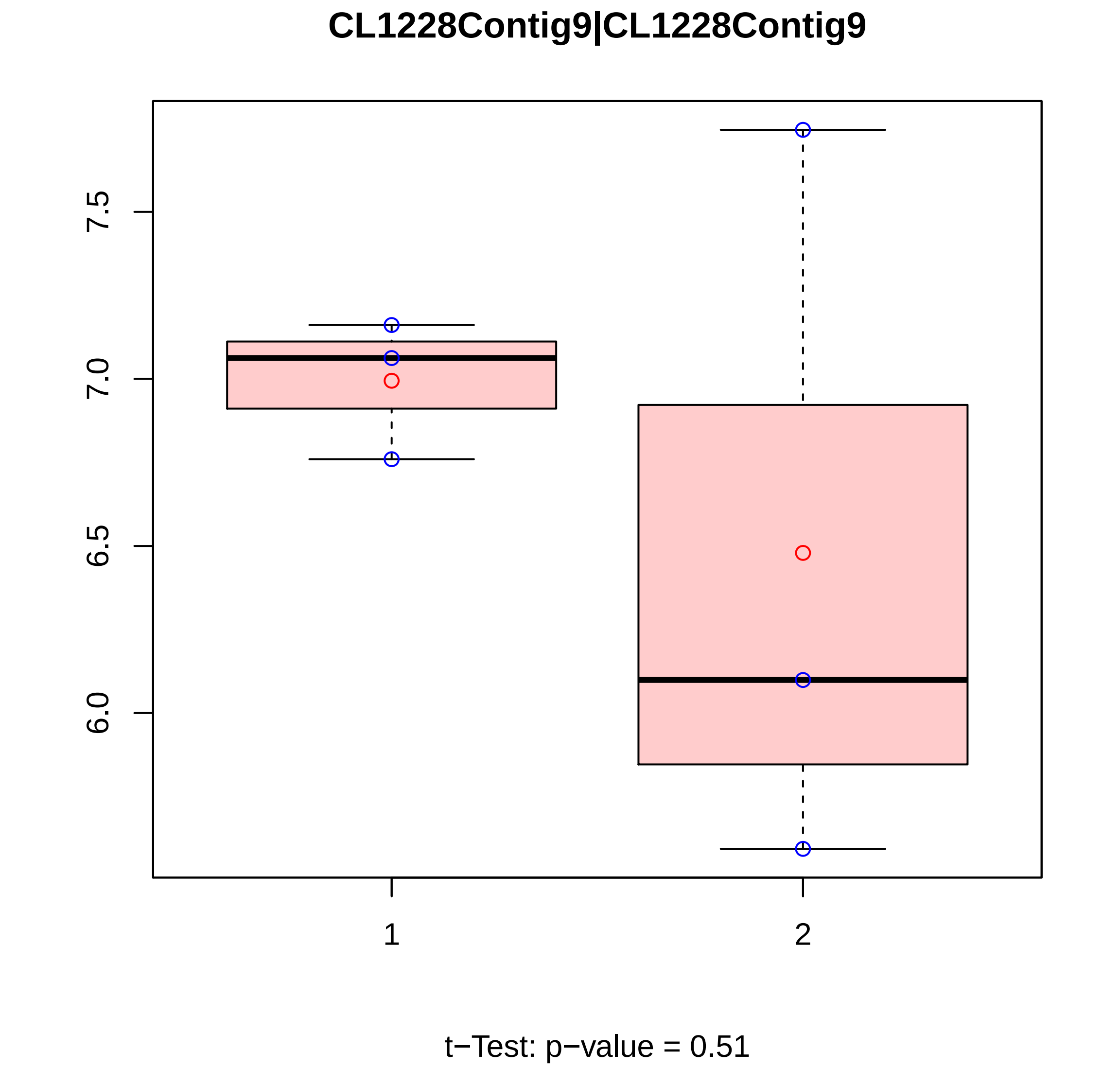

CL1228Contig9|CL1228Contig9
7.5
7.0
6.5
6.0
1
2
t−Test: p−value = 0.51

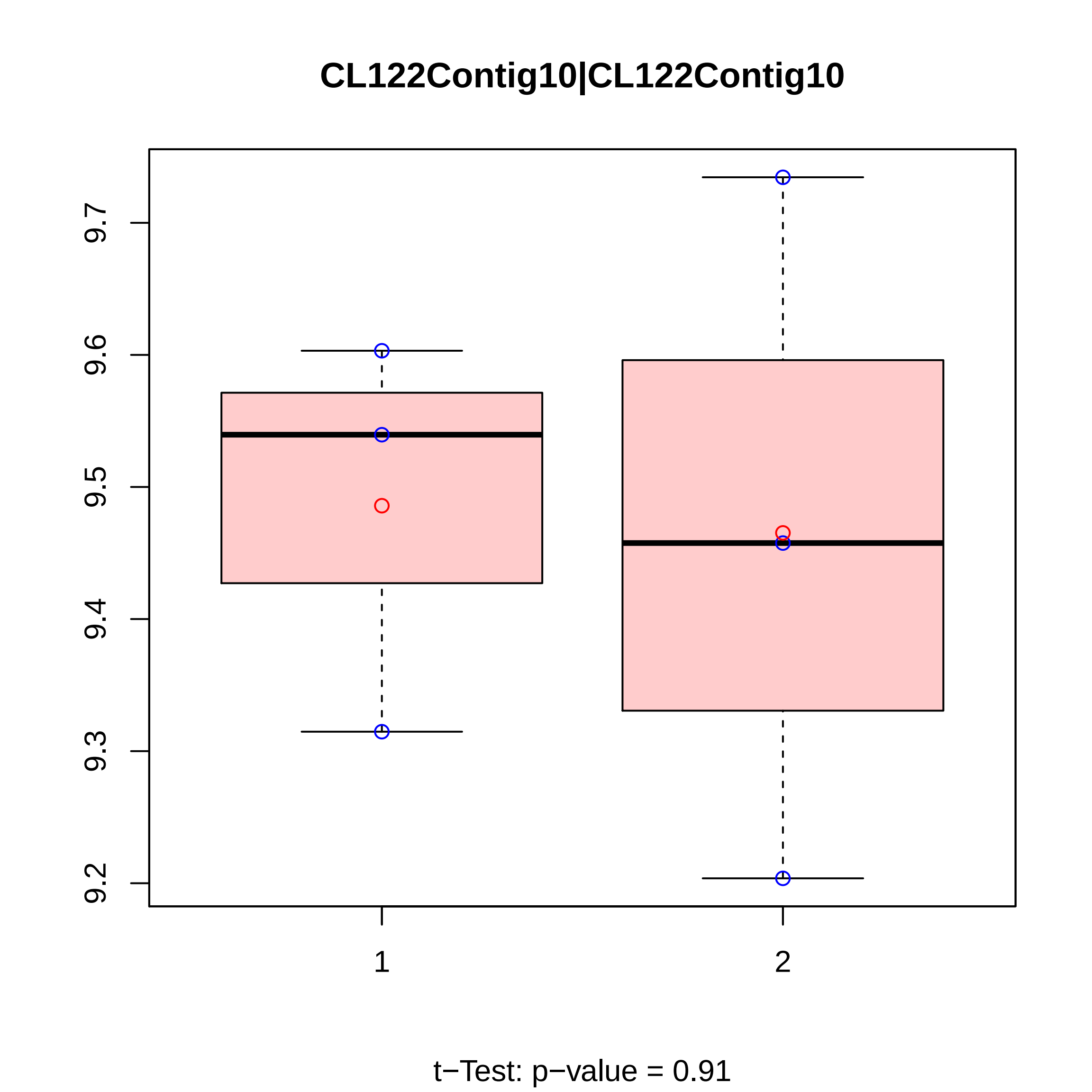
CL122Contig10|CL122Contig10
9.2
9.3
9.4
9.5
9.6
9.7
1
2
t−Test: p−value = 0.91

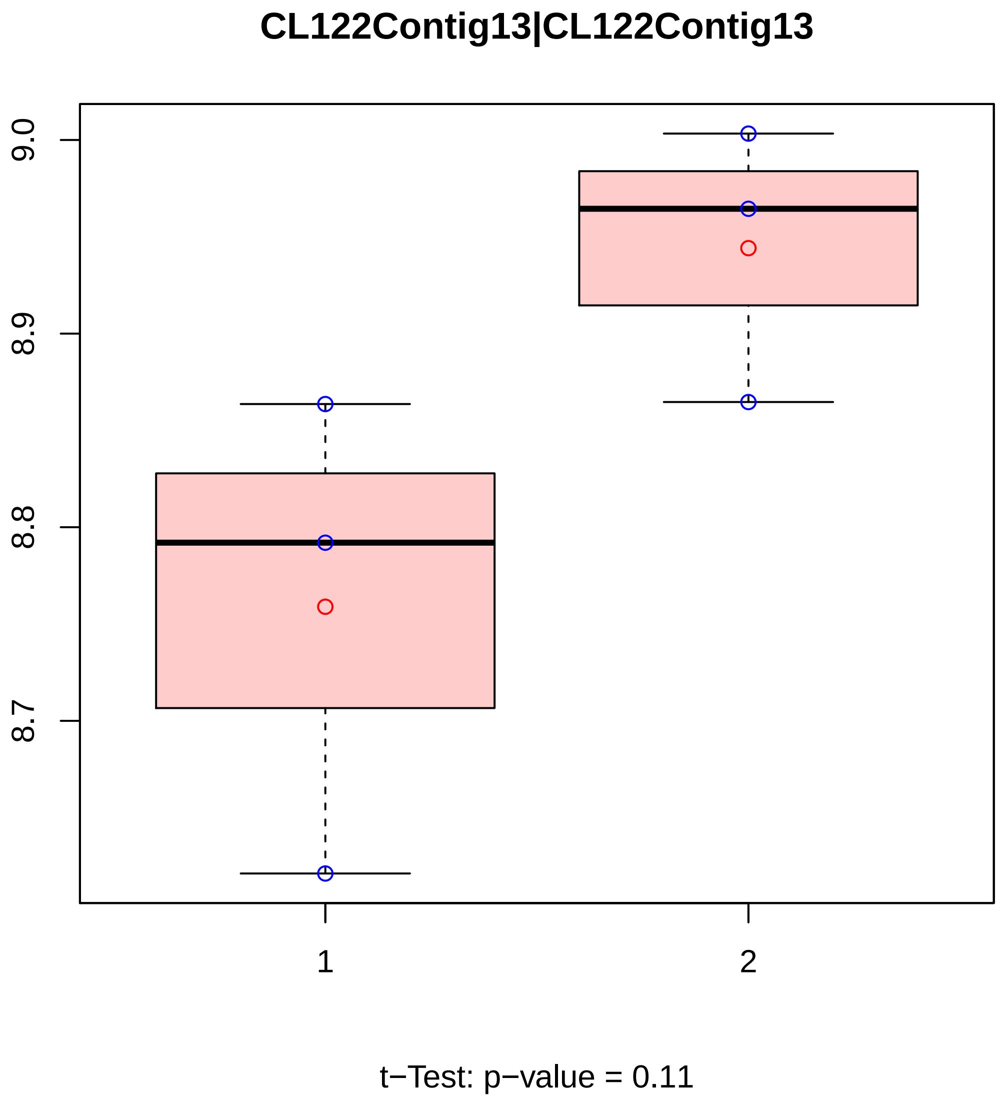
CL122Contig13|CL122Contig13
9.0
8.9
8.8
8.7
1
2
t−Test: p−value = 0.11

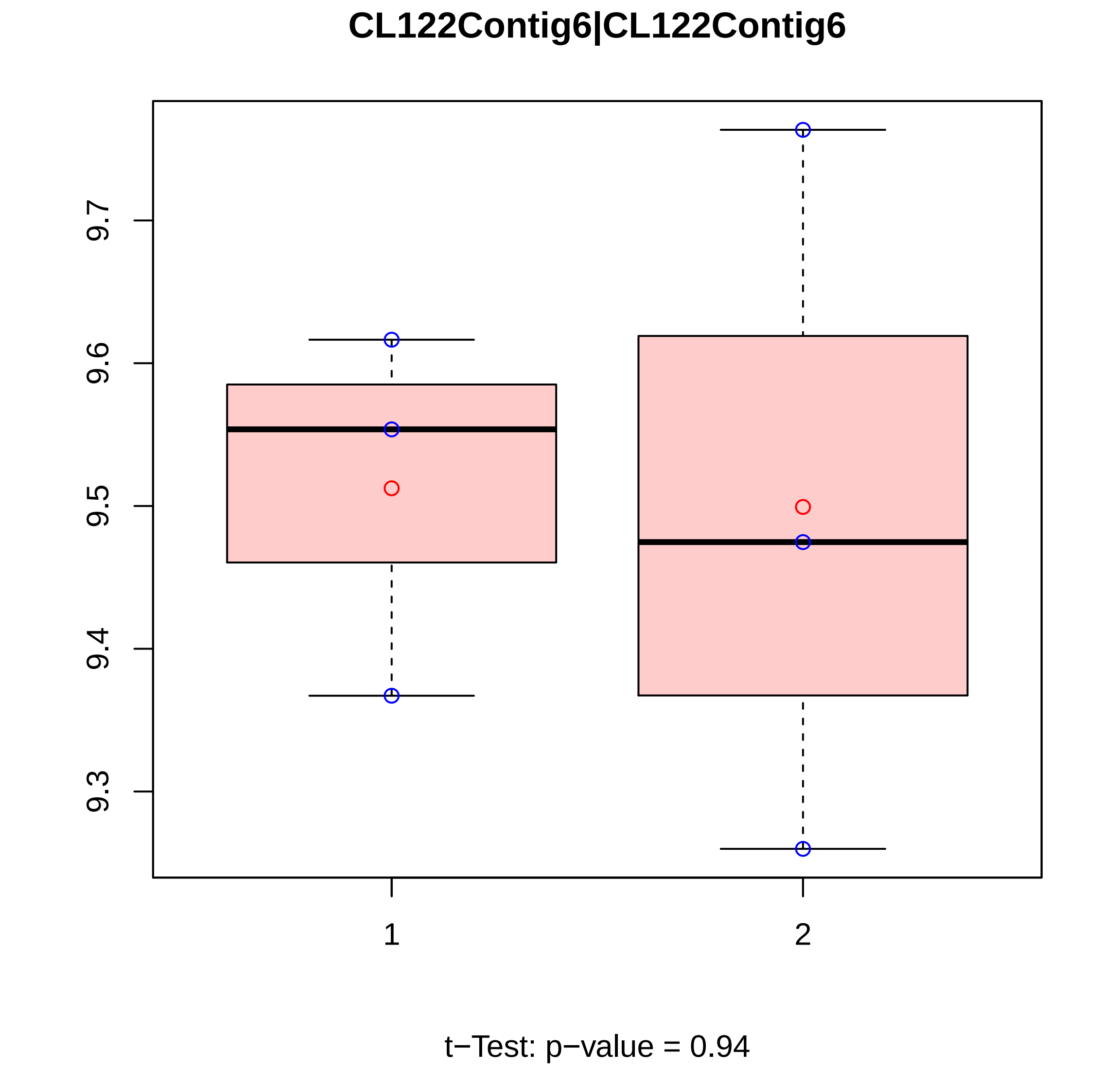
CL122Contig6|CL122Contig6
9.3
9.4
9.5
9.6
9.7
1
2
t−Test: p−value = 0.94

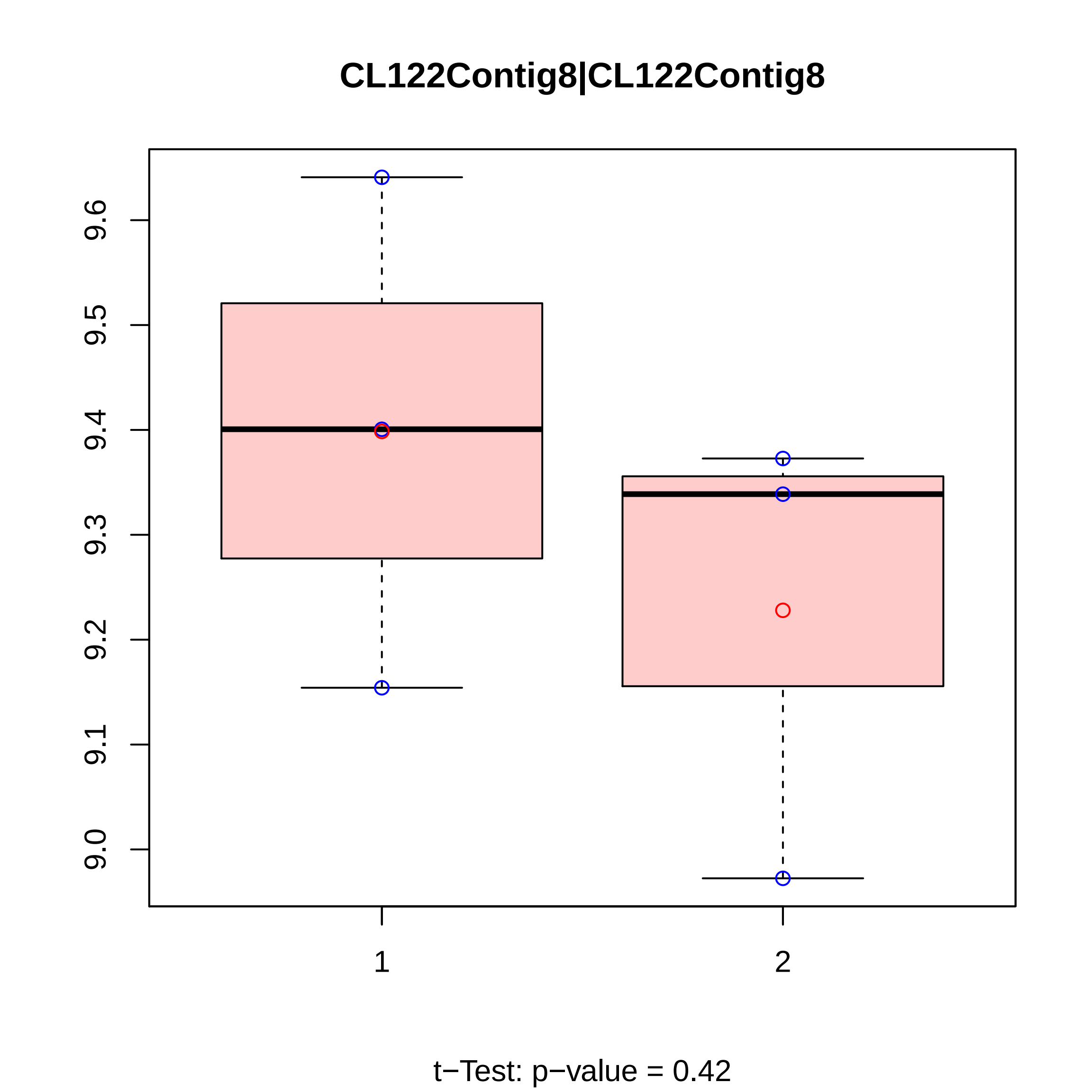
CL122Contig8|CL122Contig8
9.0
9.1
9.2
9.3
9.4
9.5
9.6
1
2
t−Test: p−value = 0.42

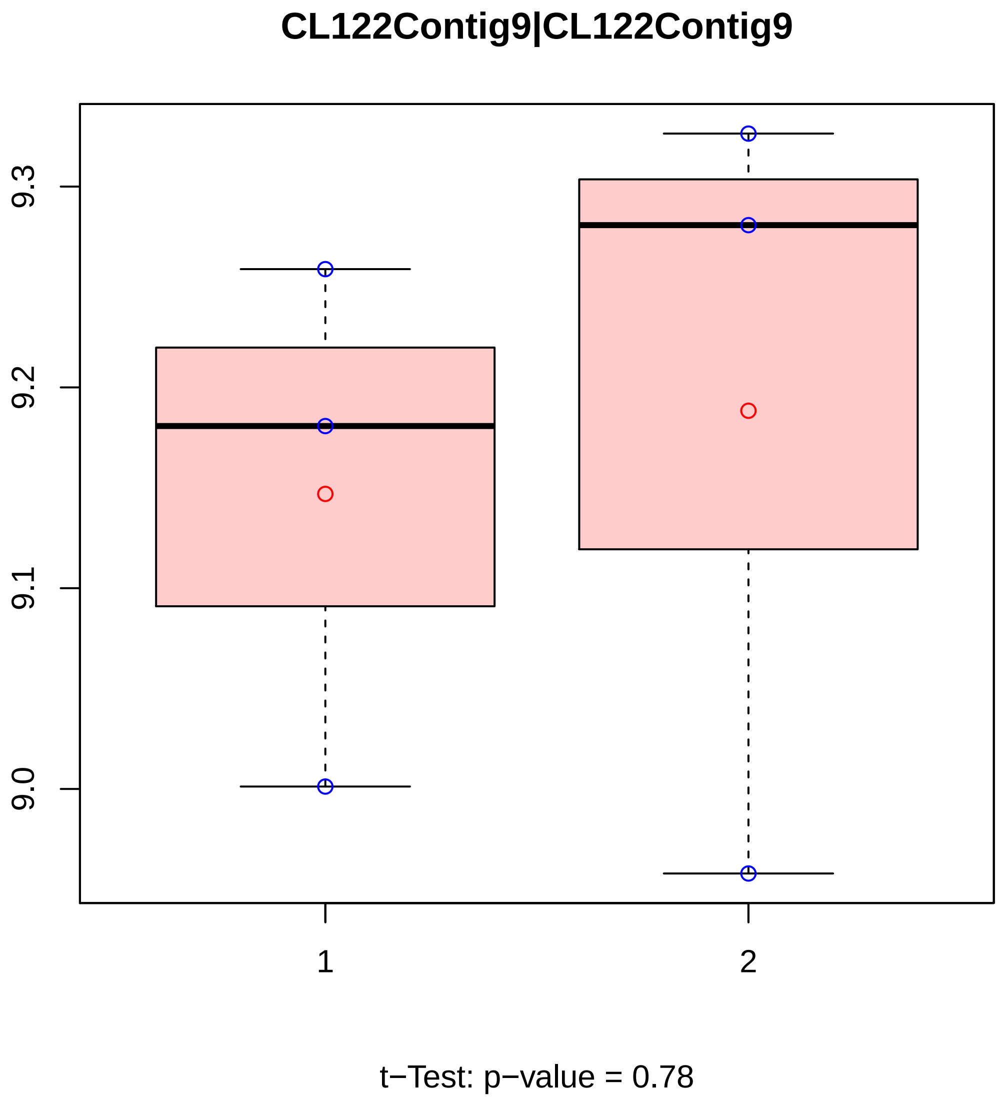
CL122Contig9|CL122Contig9
9.0
9.1
9.2
9.3
1
2
t−Test: p−value = 0.78

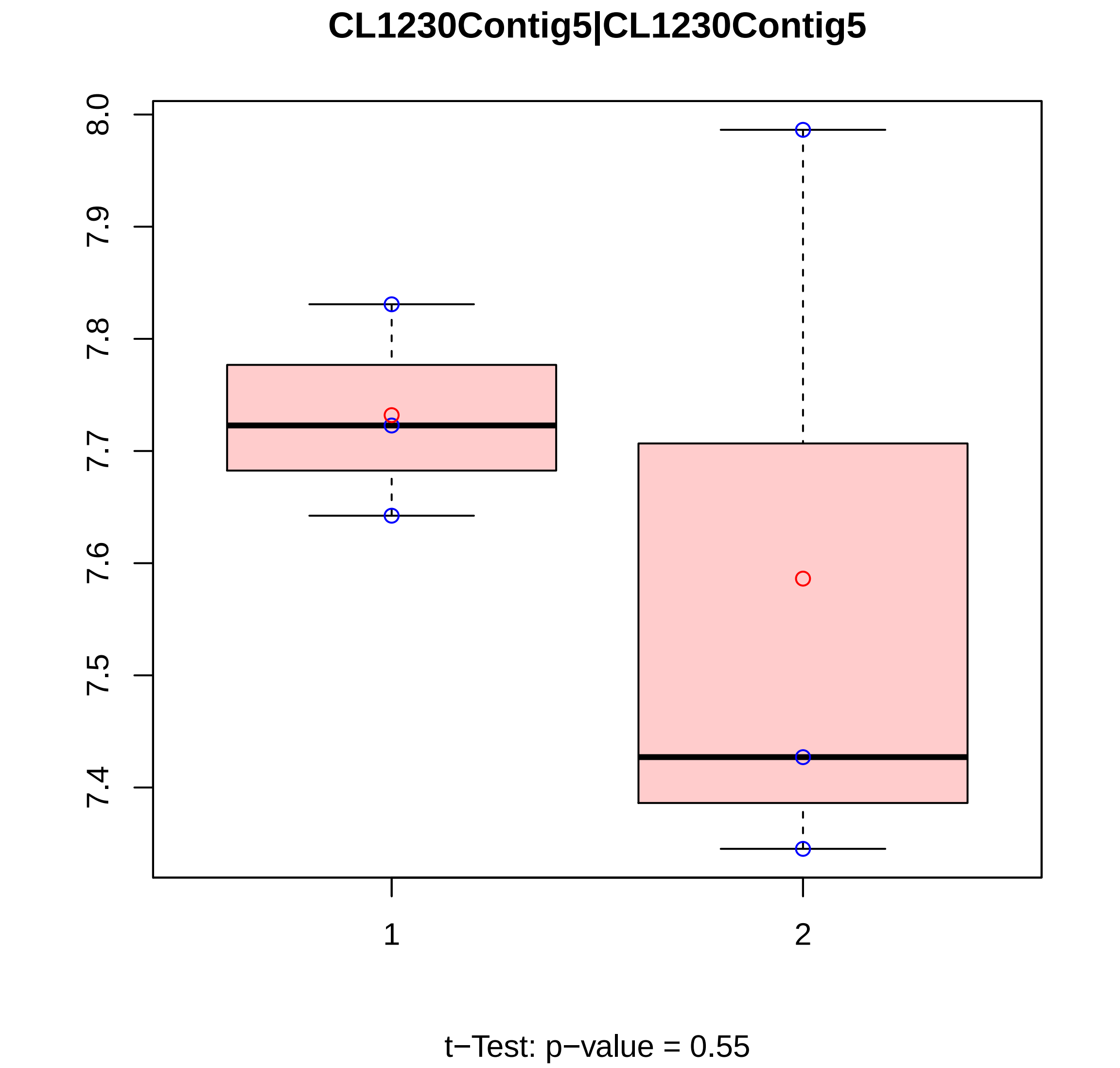
CL1230Contig5|CL1230Contig5
8.0
7.9
7.8
7.7
7.6
7.5
7.4
1
2
t−Test: p−value = 0.55

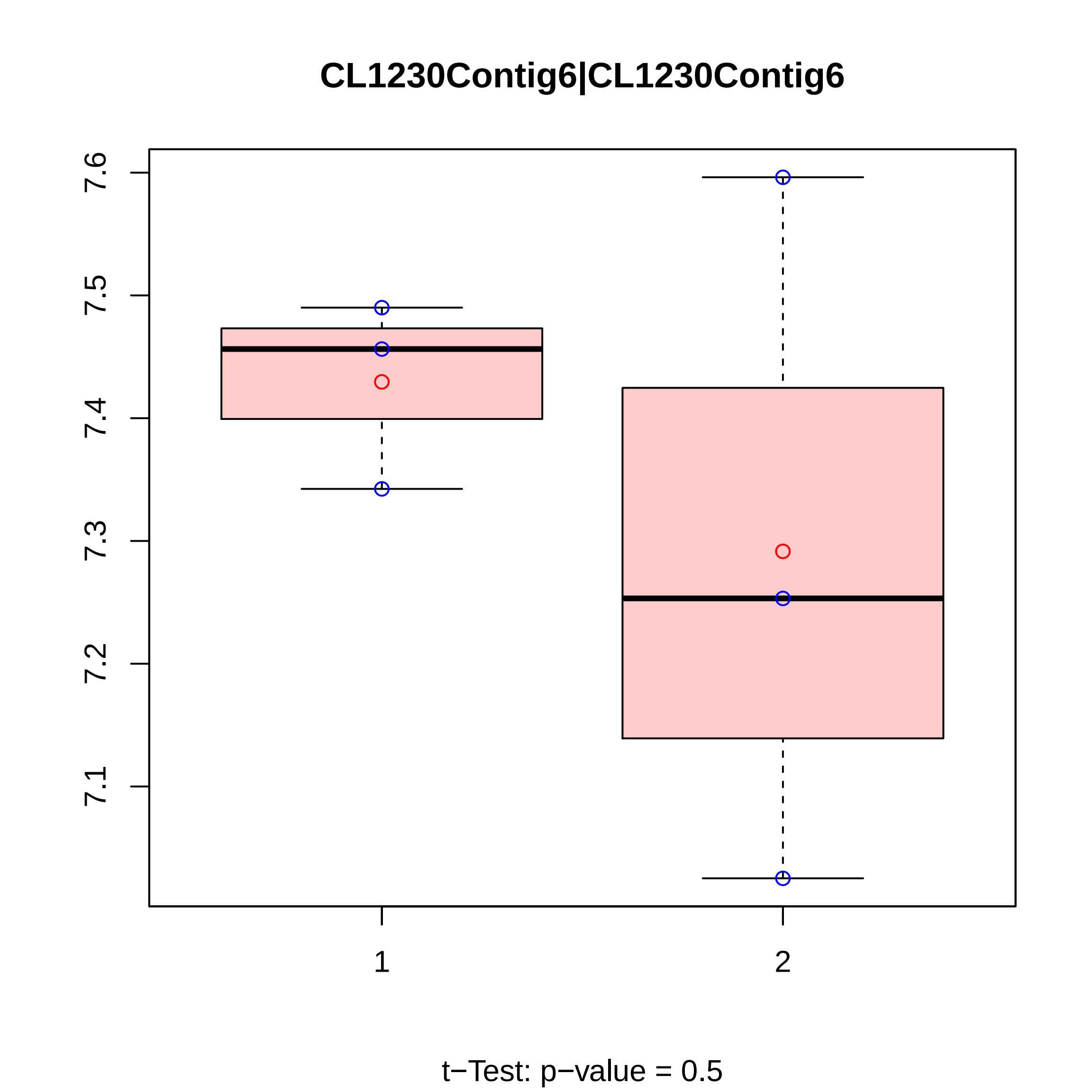
CL1230Contig6|CL1230Contig6
7.6
7.5
7.4
7.3
7.2
7.1
1
2
t−Test: p−value = 0.5

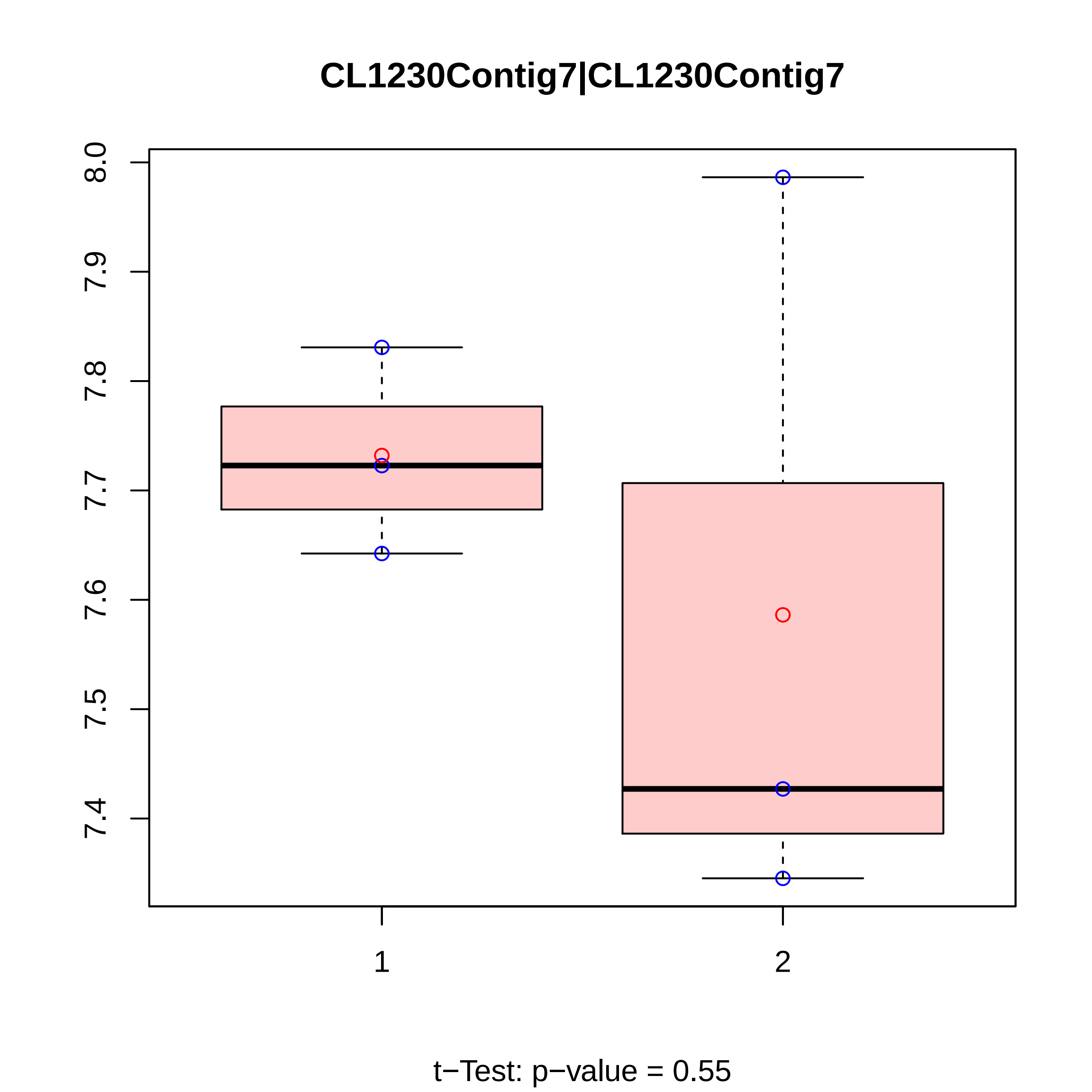
CL1230Contig7|CL1230Contig7
8.0
7.9
7.8
7.7
7.6
7.5
7.4
1
2
t−Test: p−value = 0.55

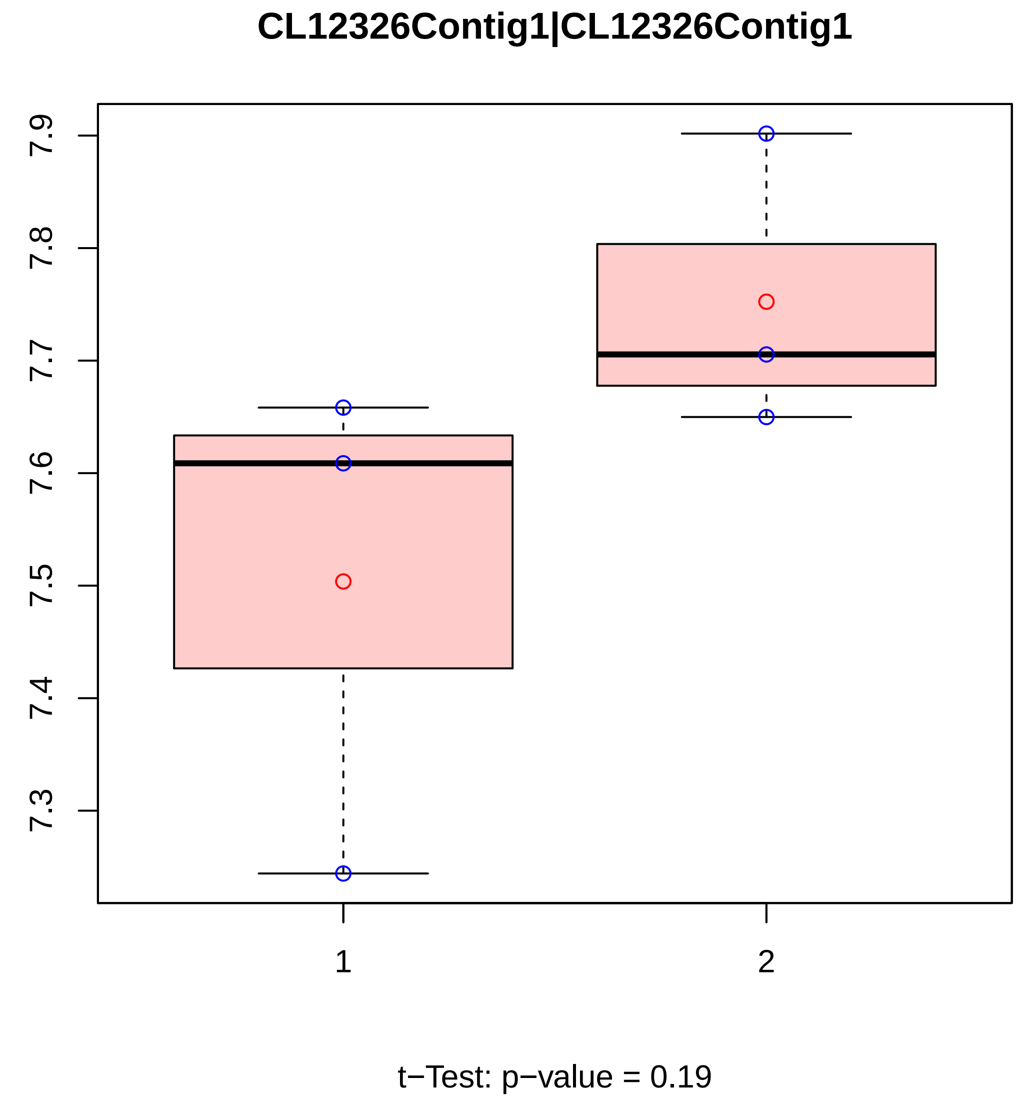
CL12326Contig1|CL12326Contig1
7.3
7.4
7.5
7.6
7.7
7.8
7.9
1
2
t−Test: p−value = 0.19

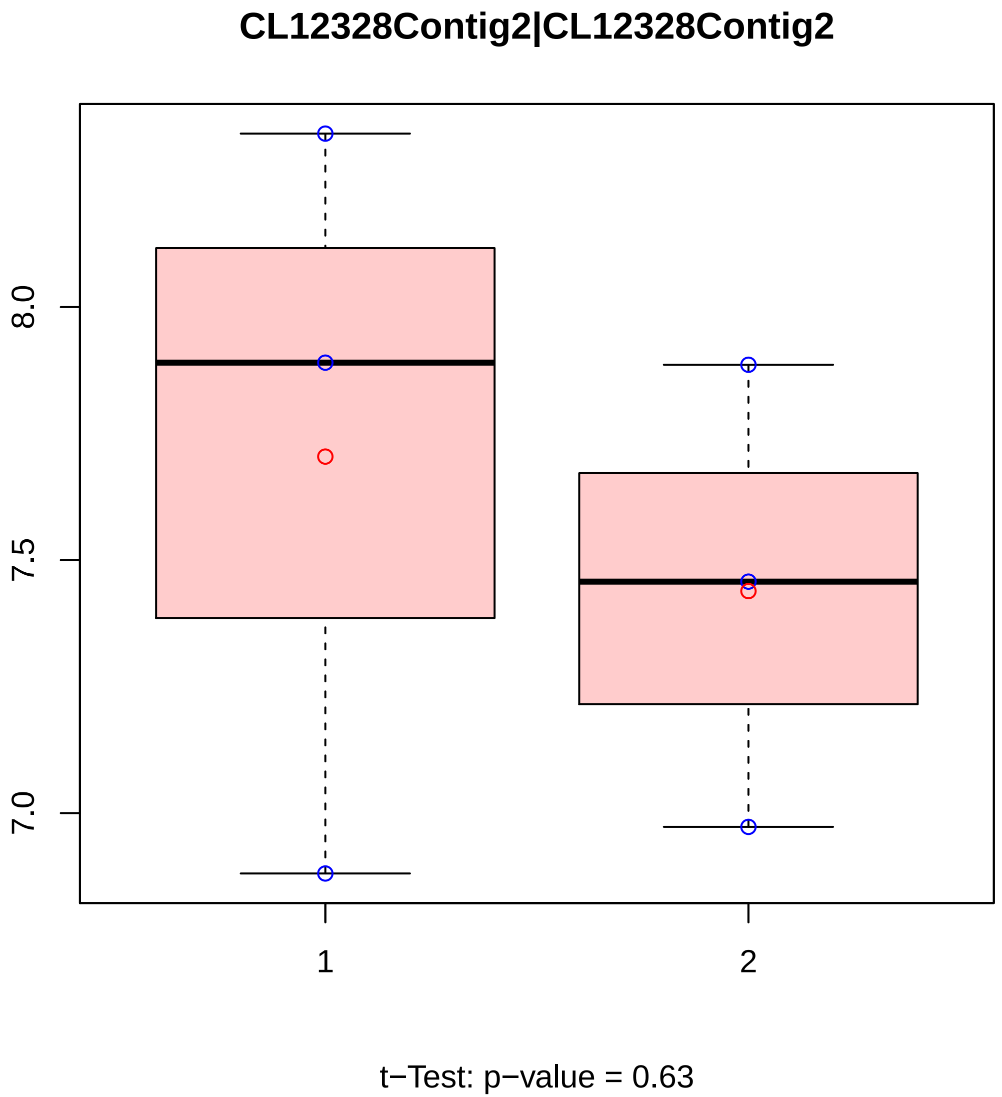
CL12328Contig2|CL12328Contig2
8.0
7.5
7.0
1
2
t−Test: p−value = 0.63

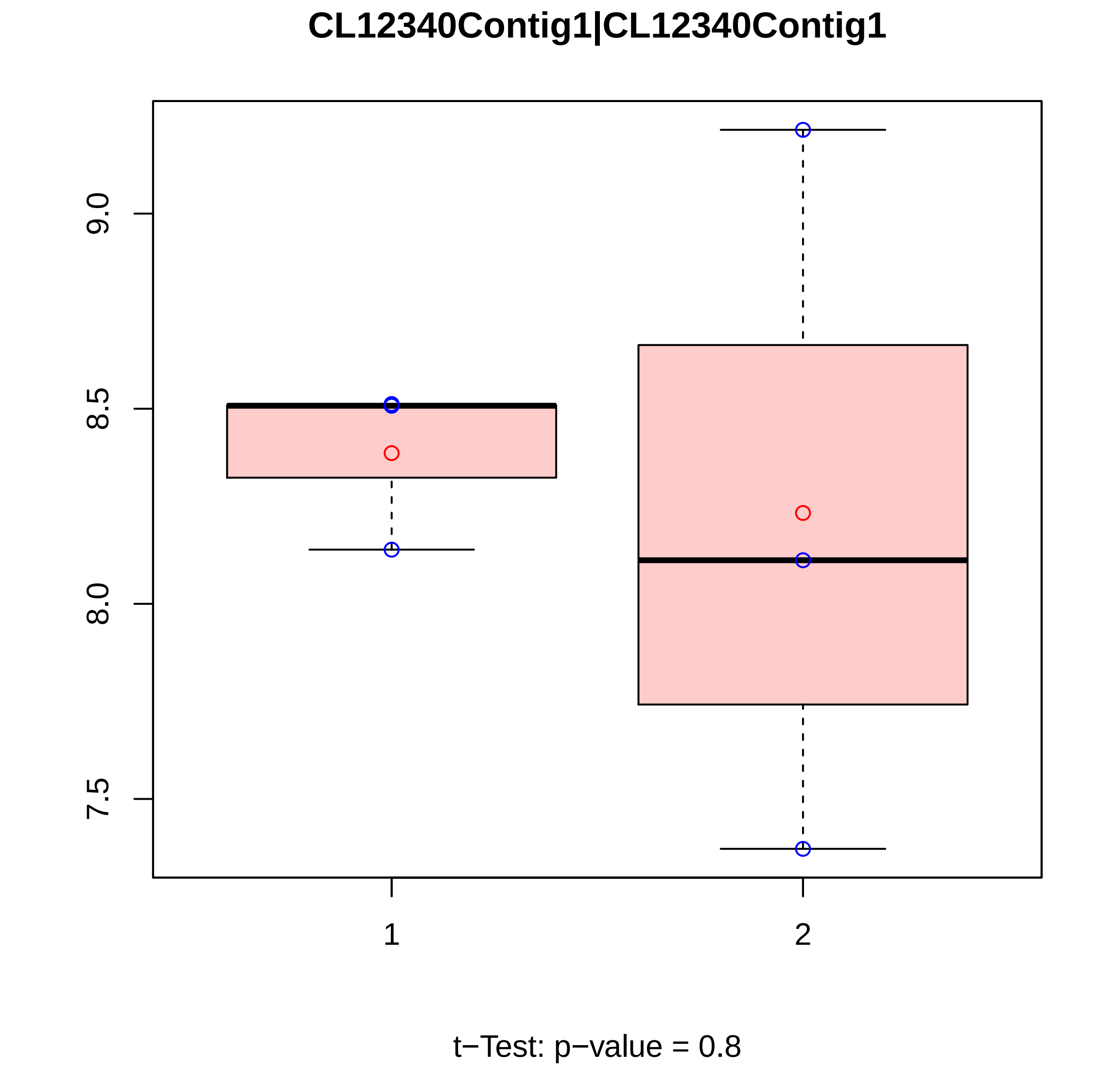

CL12340Contig1|CL12340Contig1
9.0
8.5
8.0
7.5
1
2
t−Test: p−value = 0.8

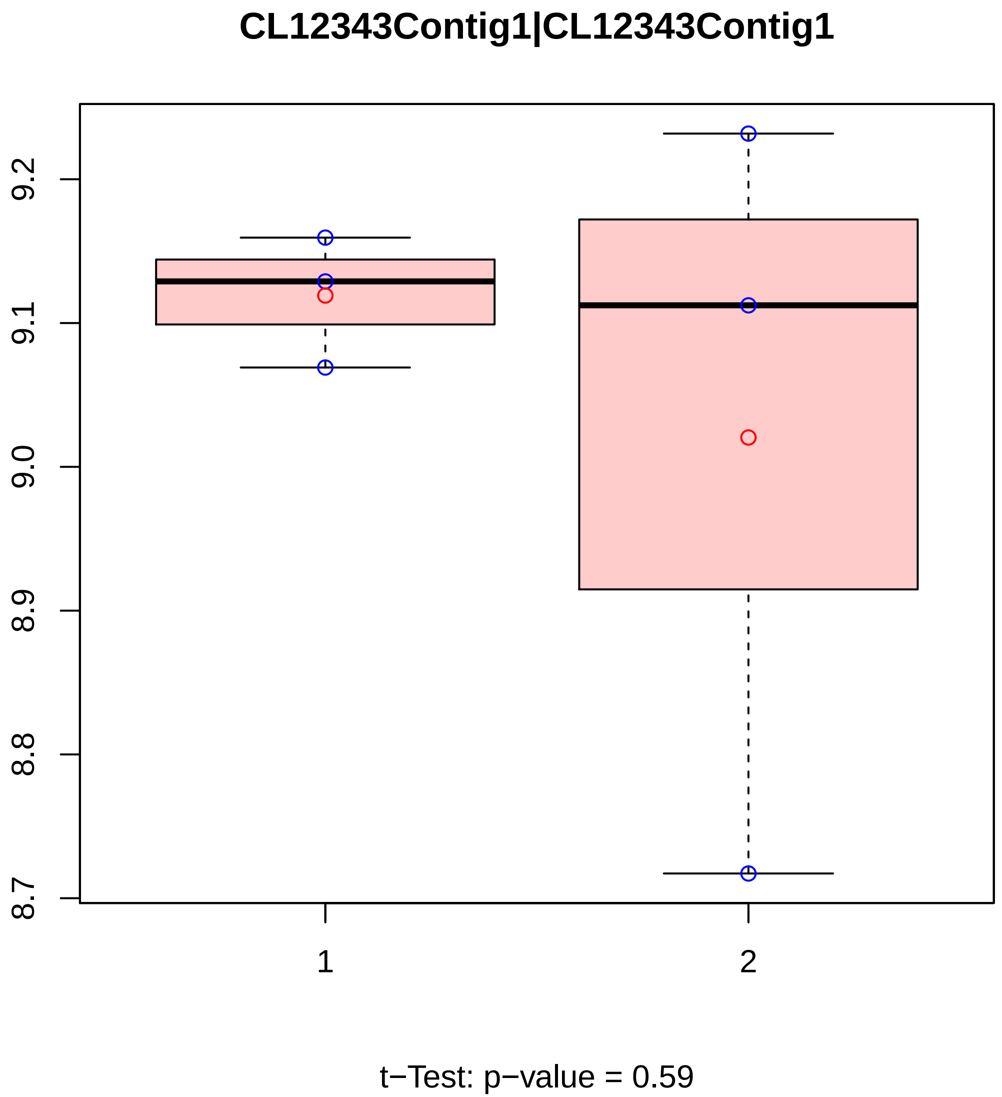

CL12343Contig1|CL12343Contig1
9.2
9.1
9.0
8.9
8.8
8.7
1
2
t−Test: p−value = 0.59

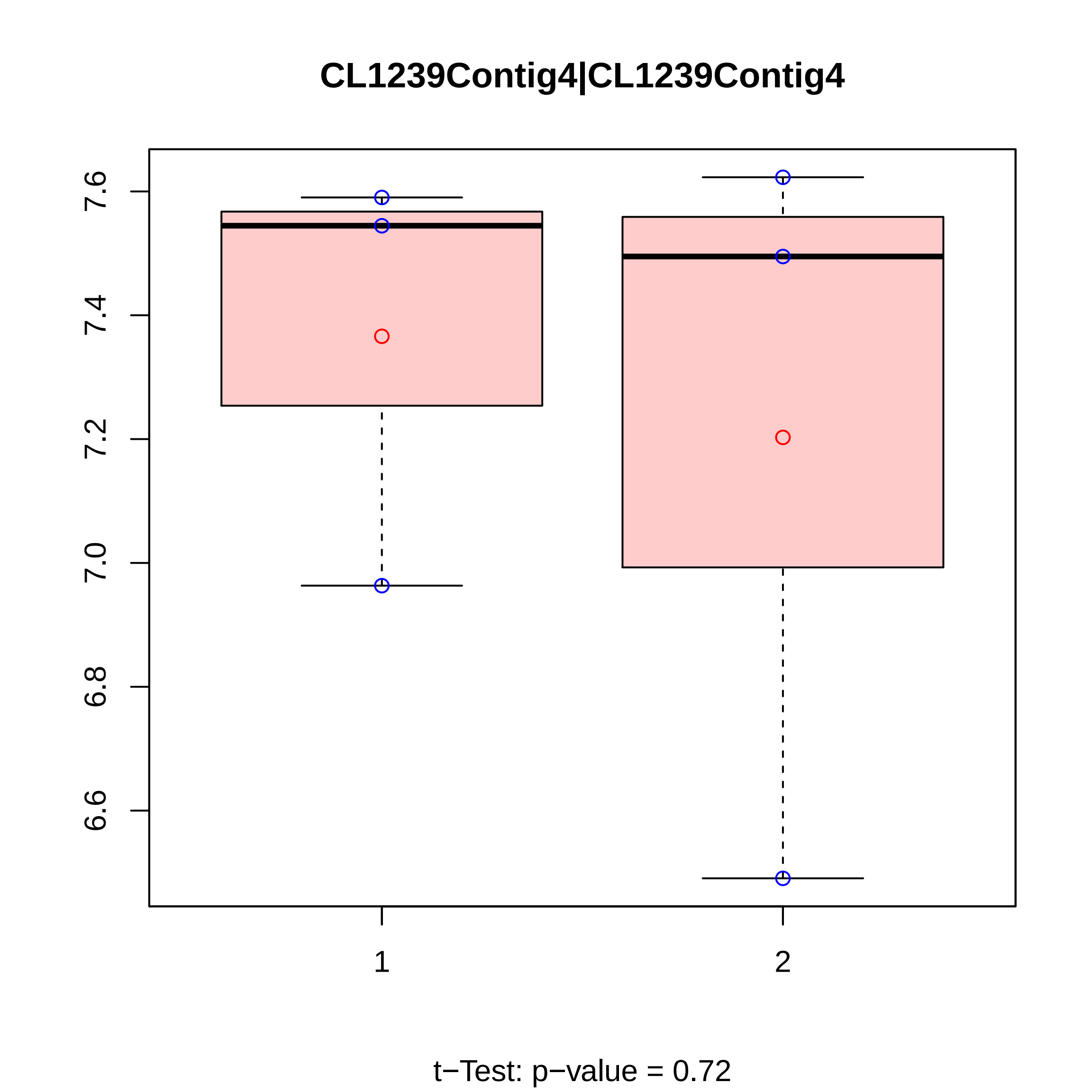
CL1239Contig4|CL1239Contig4
7.6
7.4
7.2
7.0
6.8
6.6
1
2
t−Test: p−value = 0.72

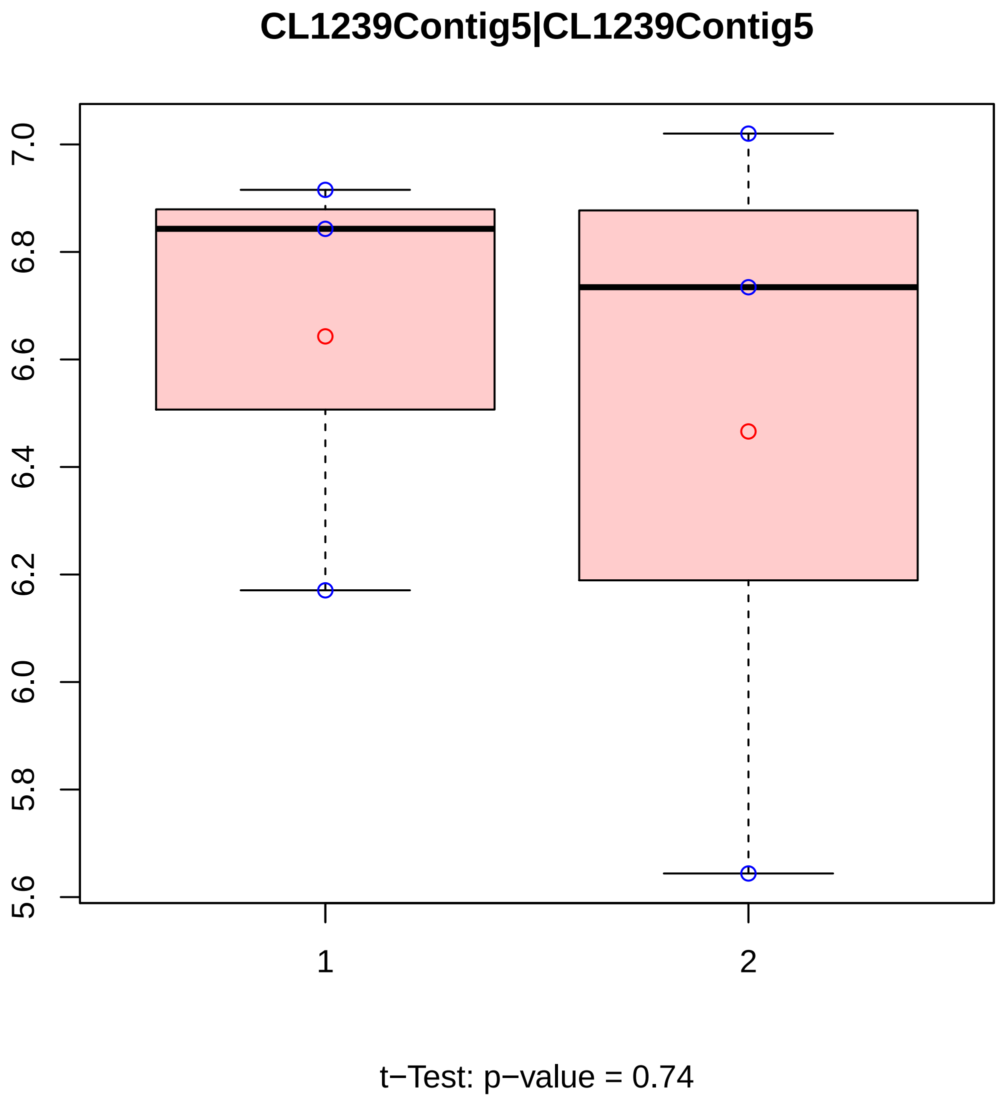
CL1239Contig5|CL1239Contig5
7.0
6.8
6.6
6.4
6.2
6.0
5.8
5.6
1
2
t−Test: p−value = 0.74

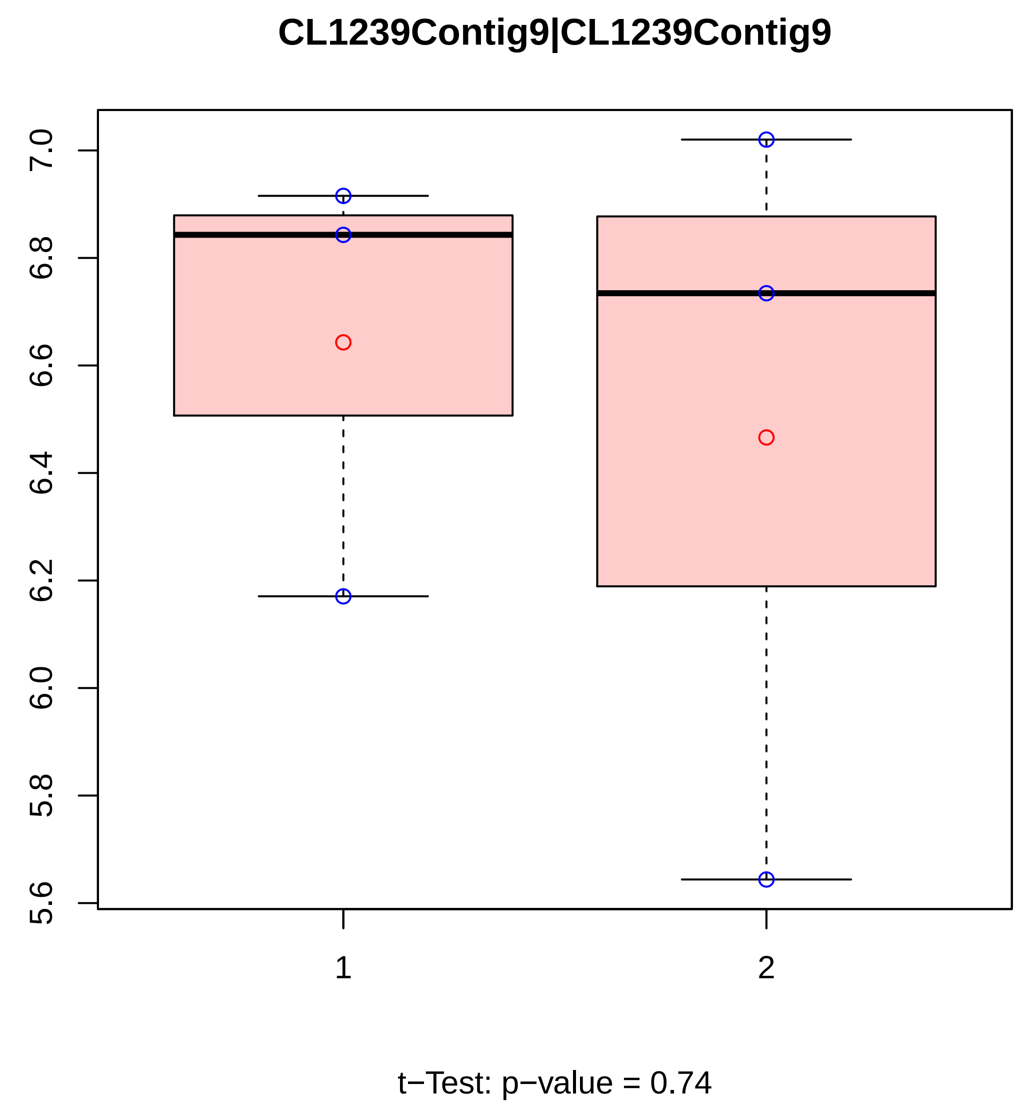
CL1239Contig9|CL1239Contig9
7.0
6.8
6.6
6.4
6.2
6.0
5.8
5.6
1
2
t−Test: p−value = 0.74

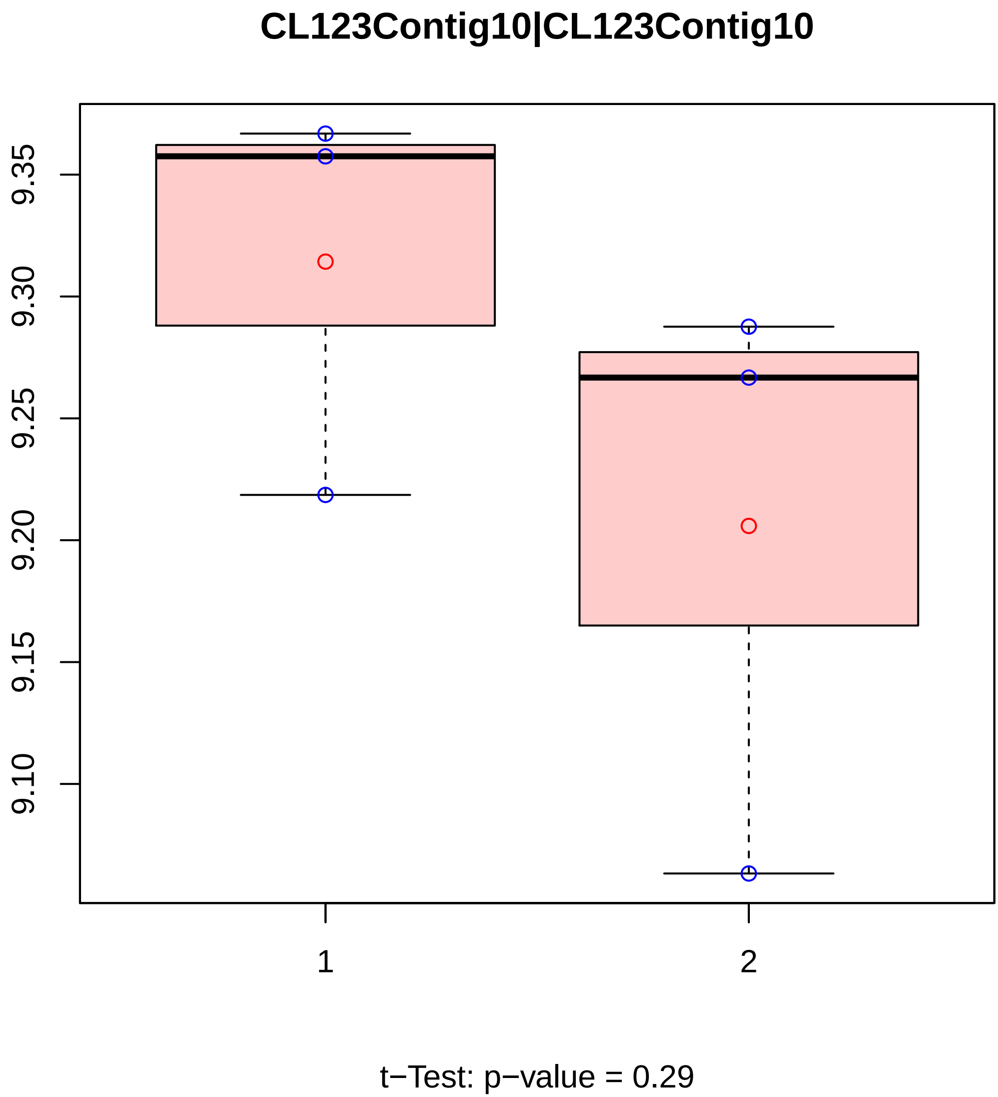
CL123Contig10|CL123Contig10
9.10
9.15
9.20
9.25
9.30
9.35
1
2
t−Test: p−value = 0.29

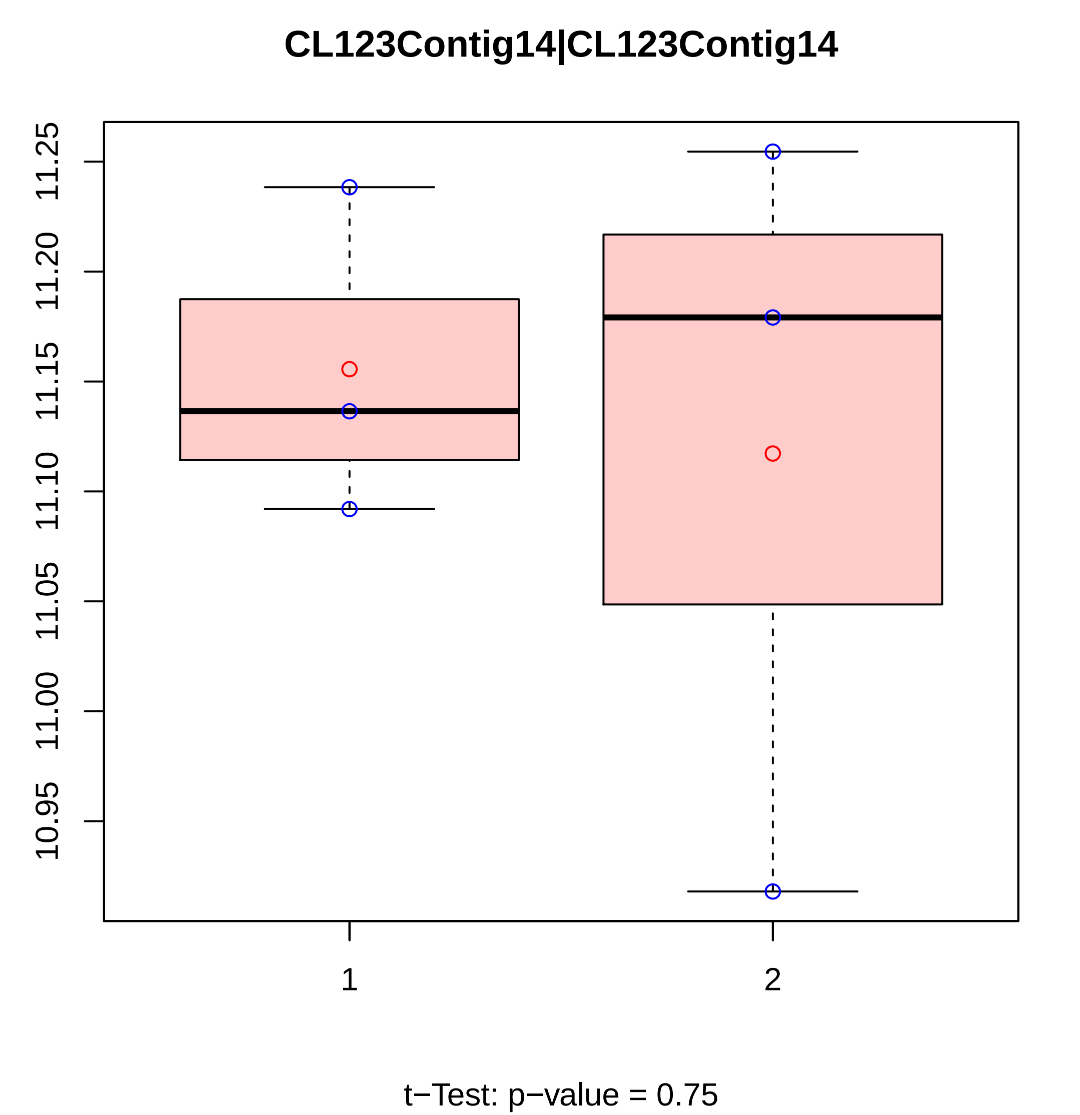
CL123Contig14|CL123Contig14
11.25
11.20
11.15
11.10
11.05
11.00
10.95
1
2
t−Test: p−value = 0.75

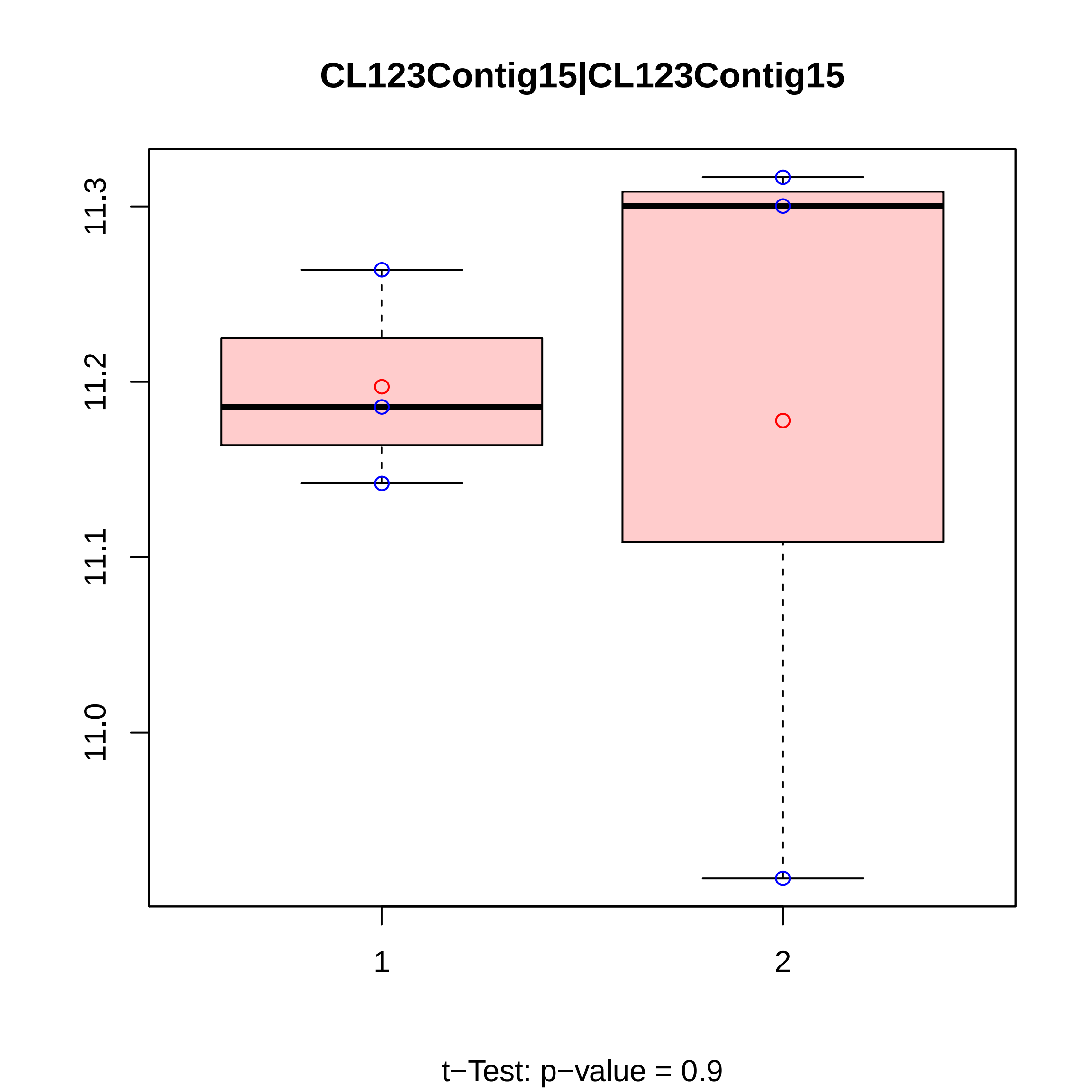

CL123Contig15|CL123Contig15
11.0
11.1
11.2
11.3
1
2
t−Test: p−value = 0.9

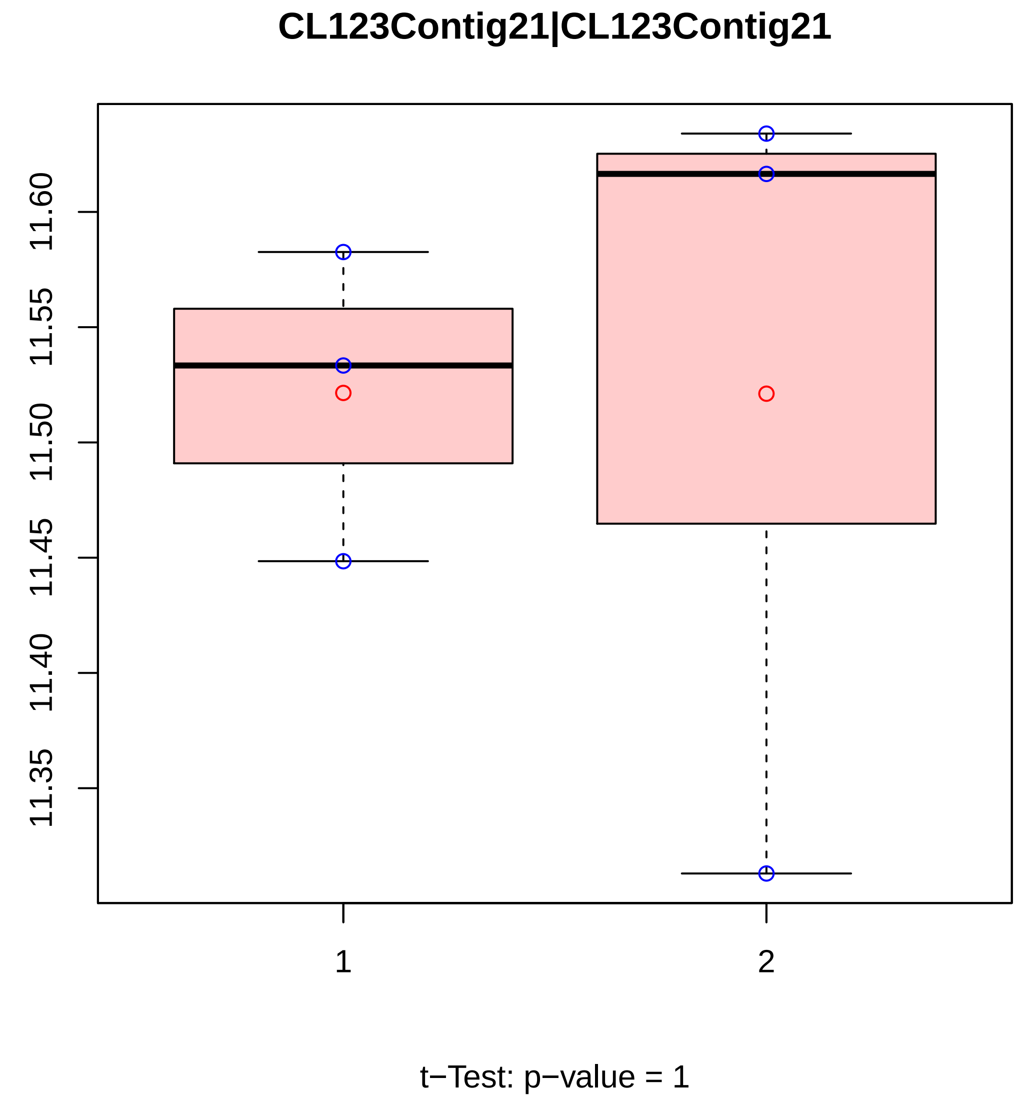
CL123Contig21|CL123Contig21
11.35
11.40
11.45
11.50
11.55
11.60
1
2
t−Test: p−value = 1

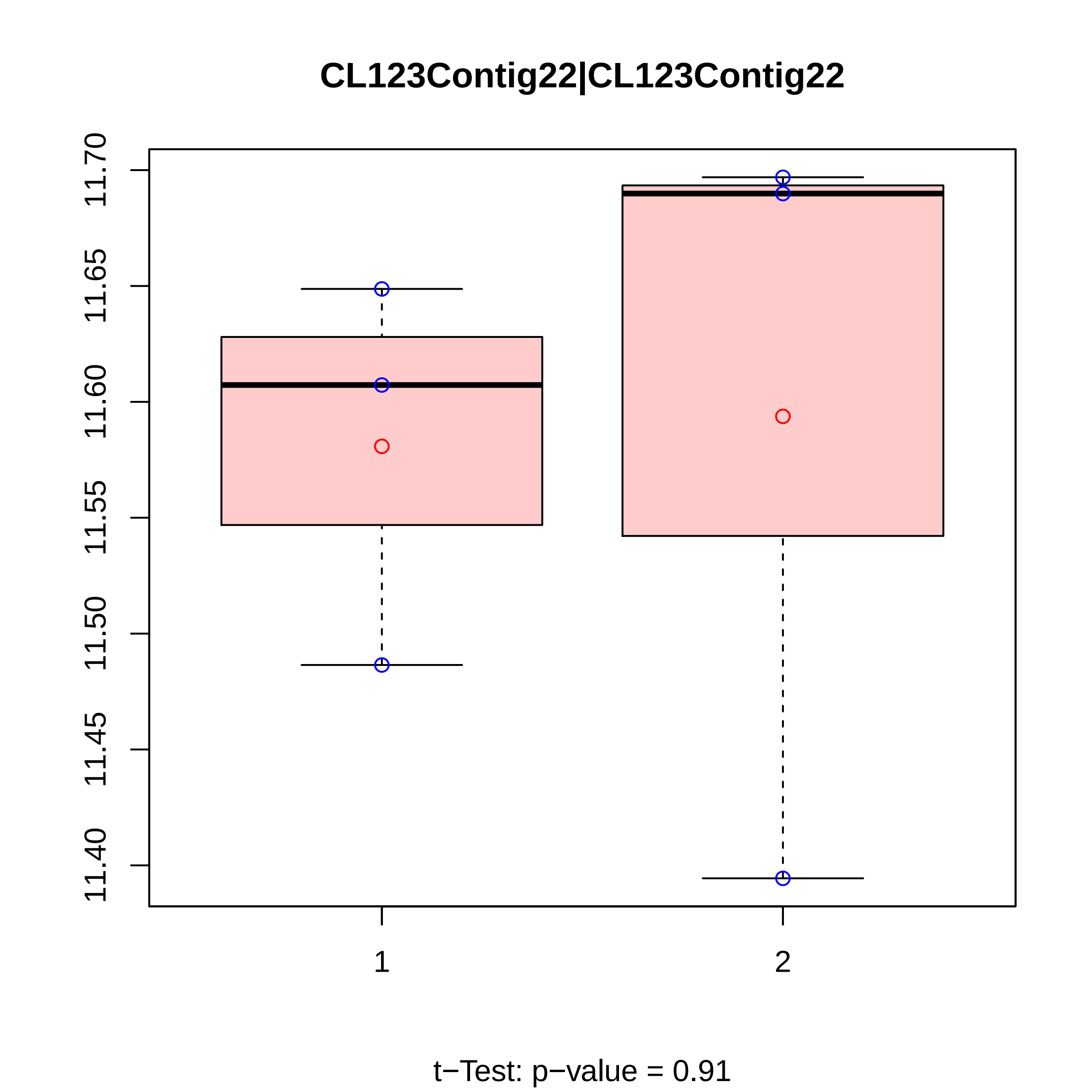
CL123Contig22|CL123Contig22
11.70
11.65
11.60
11.55
11.50
11.45
11.40
1
2
t−Test: p−value = 0.91

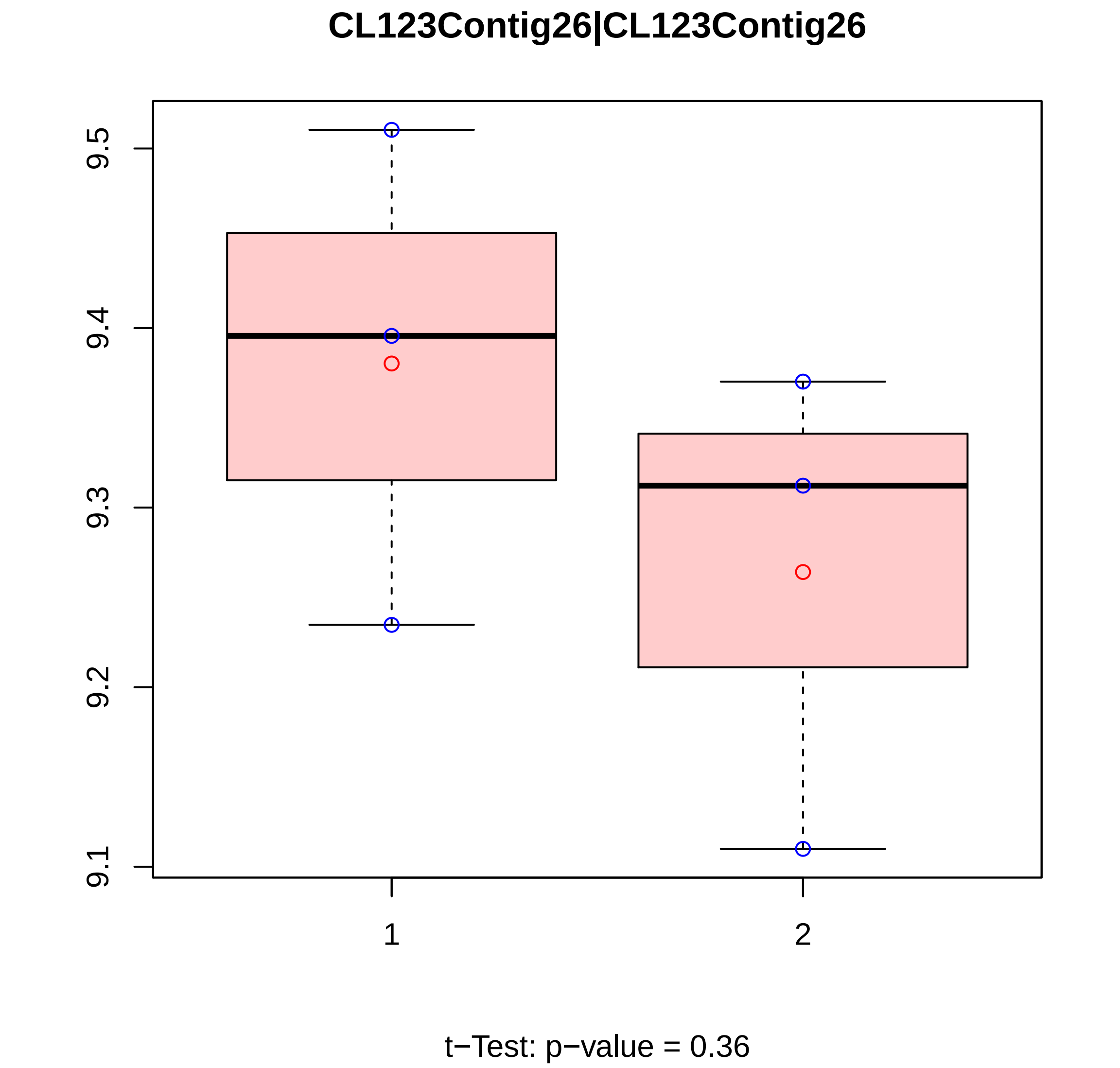
CL123Contig26|CL123Contig26
9.1
9.2
9.3
9.4
9.5
1
2
t−Test: p−value = 0.36

# CL123Contig27|CL123Contig27

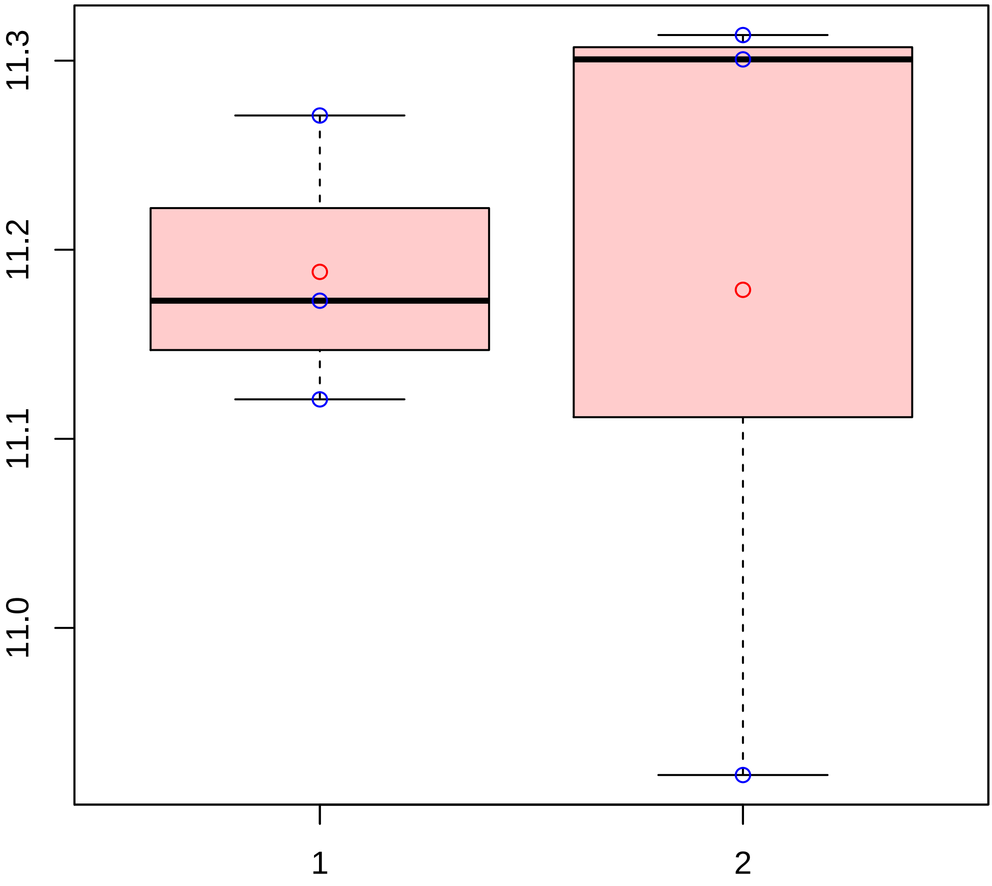


t−Test: p−value = 0.95

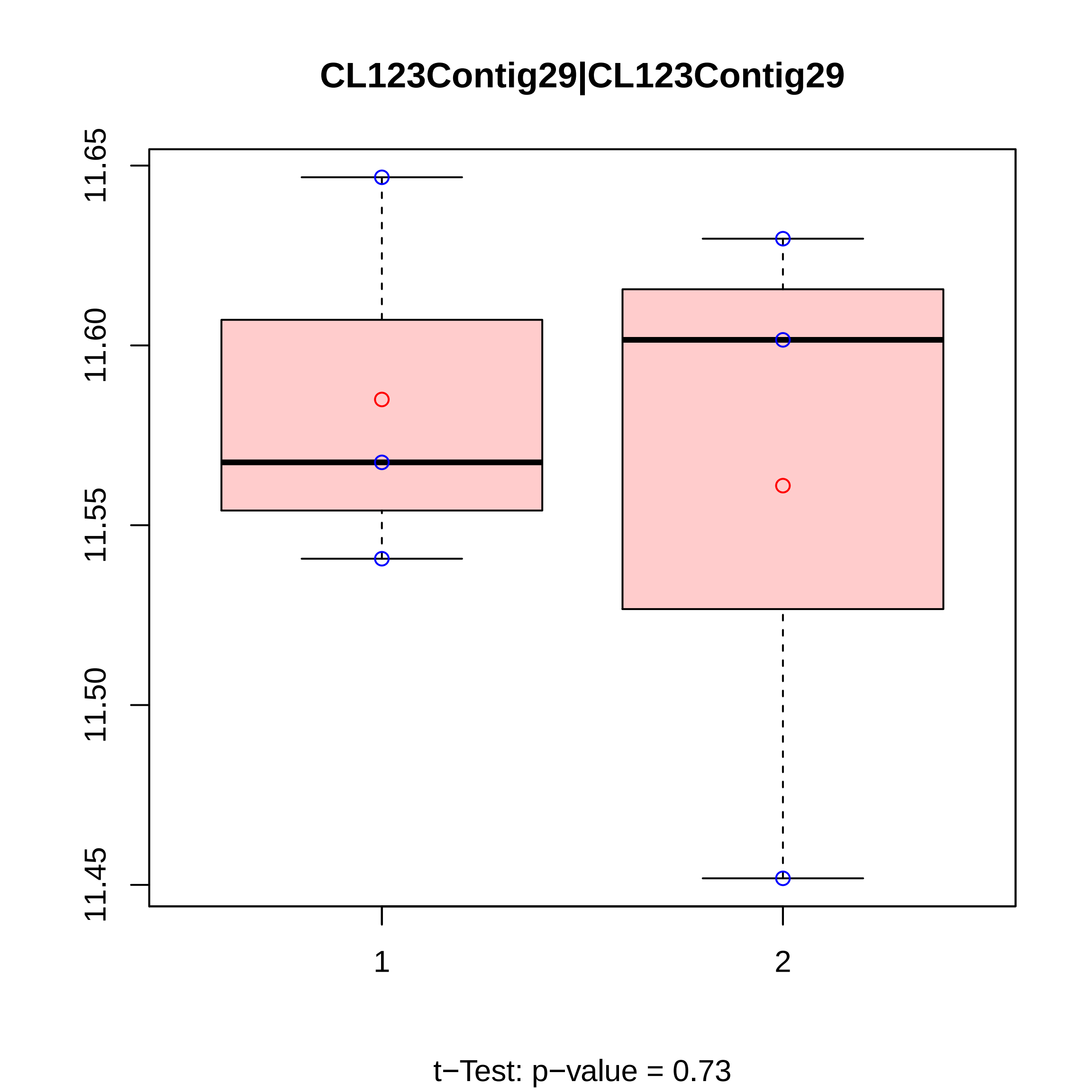
CL123Contig29|CL123Contig29
11.65
11.60
11.55
11.50
11.45
1
2
t−Test: p−value = 0.73

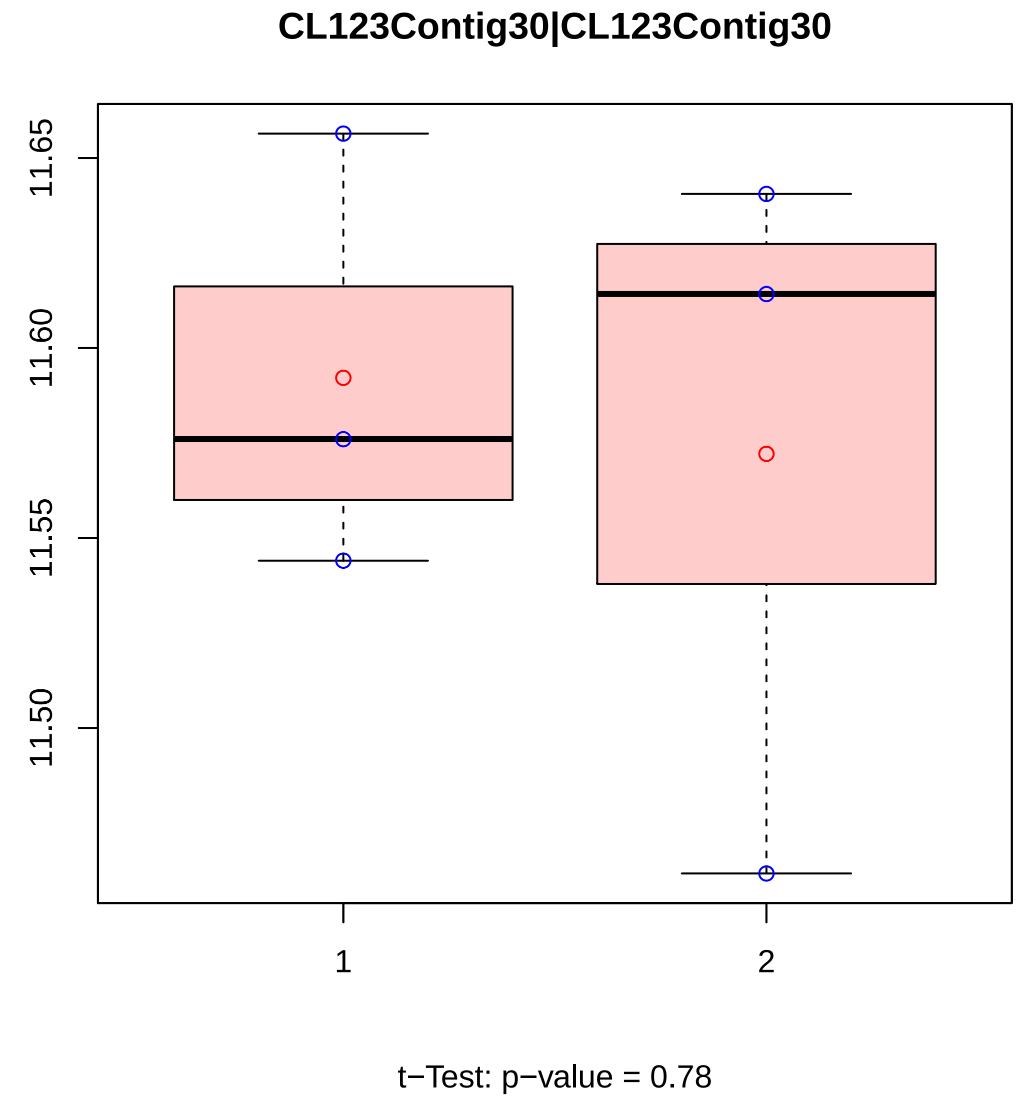
CL123Contig30|CL123Contig30
11.65
11.60
11.55
11.50
1
2
t−Test: p−value = 0.78

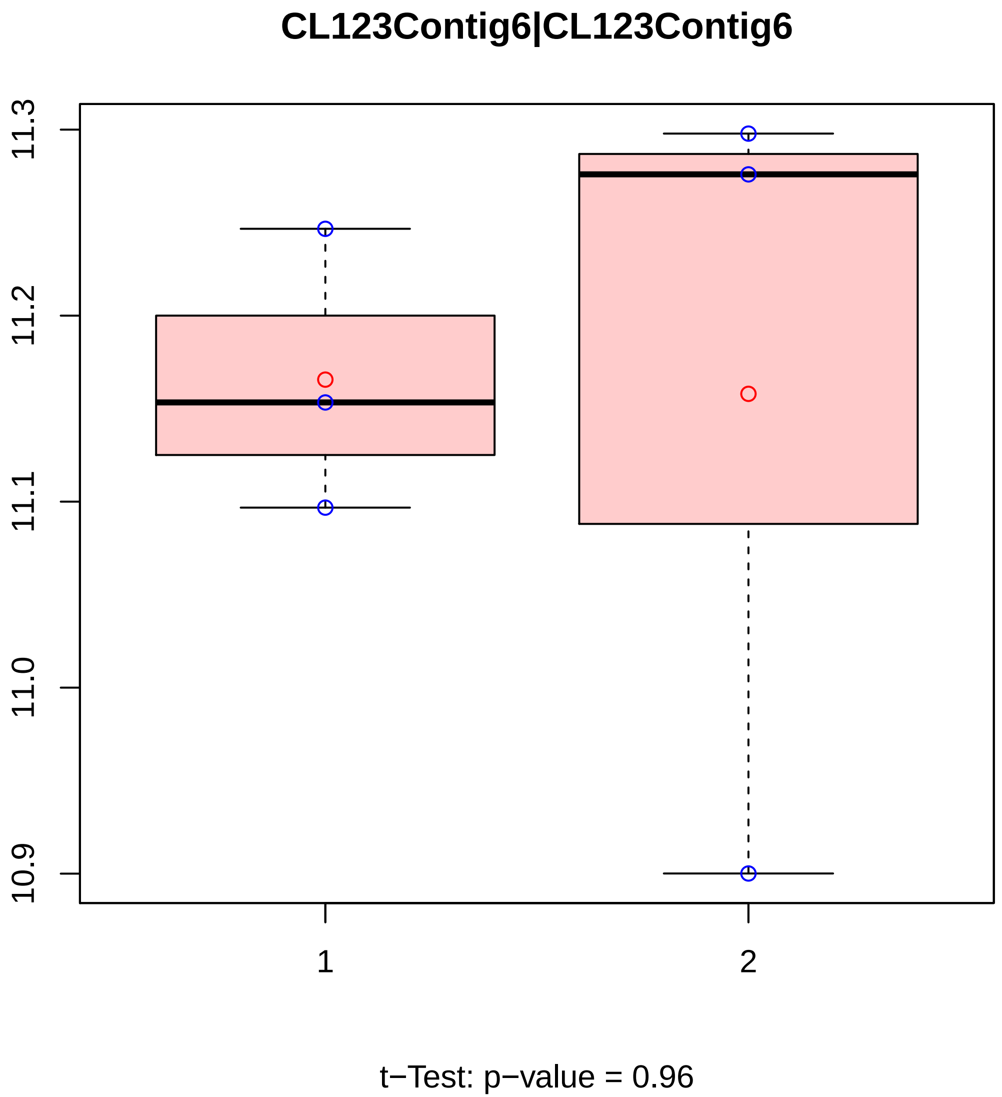
CL123Contig6|CL123Contig6
11.3
11.2
11.1
11.0
10.9
1
2
t−Test: p−value = 0.96

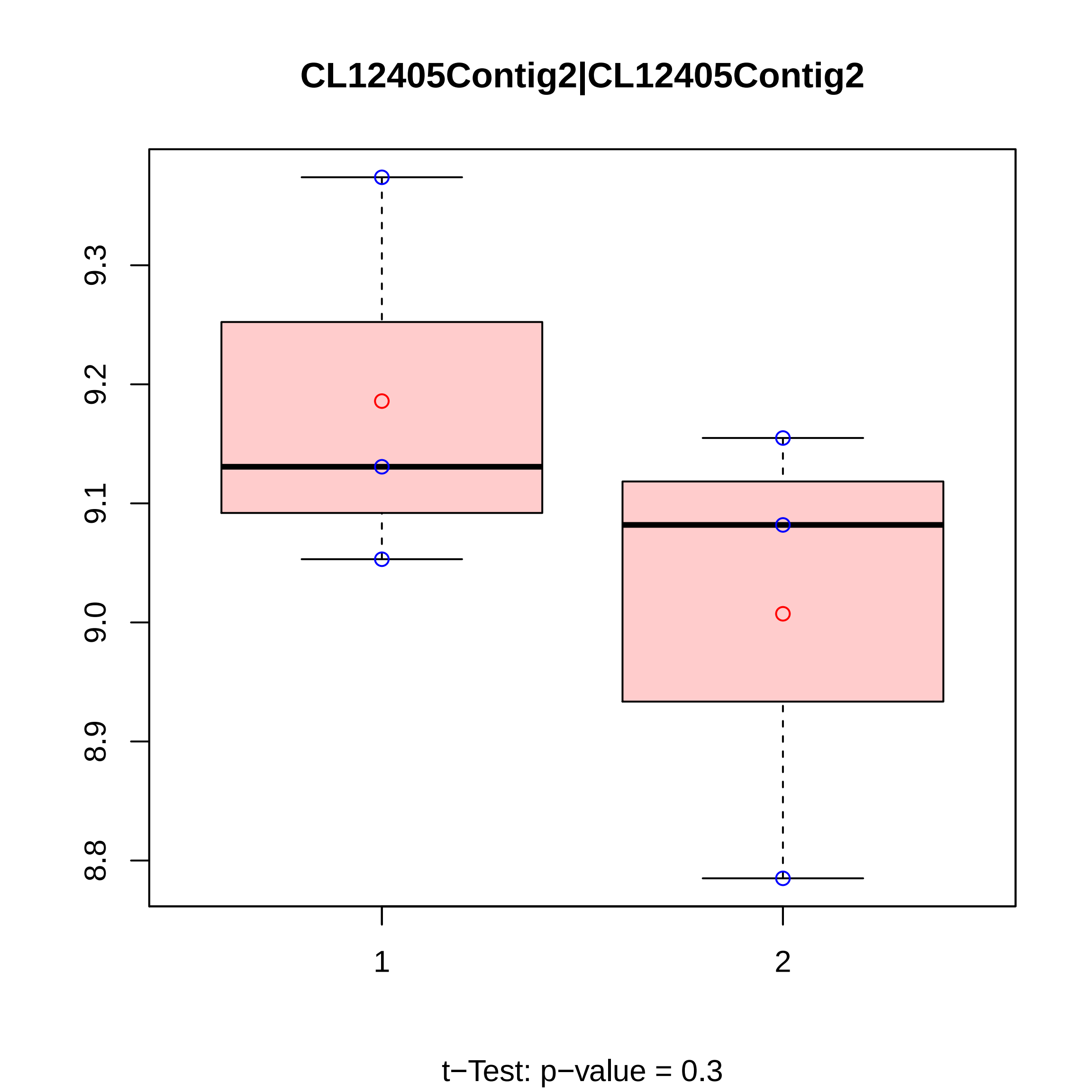

CL12405Contig2|CL12405Contig2
9.3
9.2
9.1
9.0
8.9
8.8
1
2
t−Test: p−value = 0.3

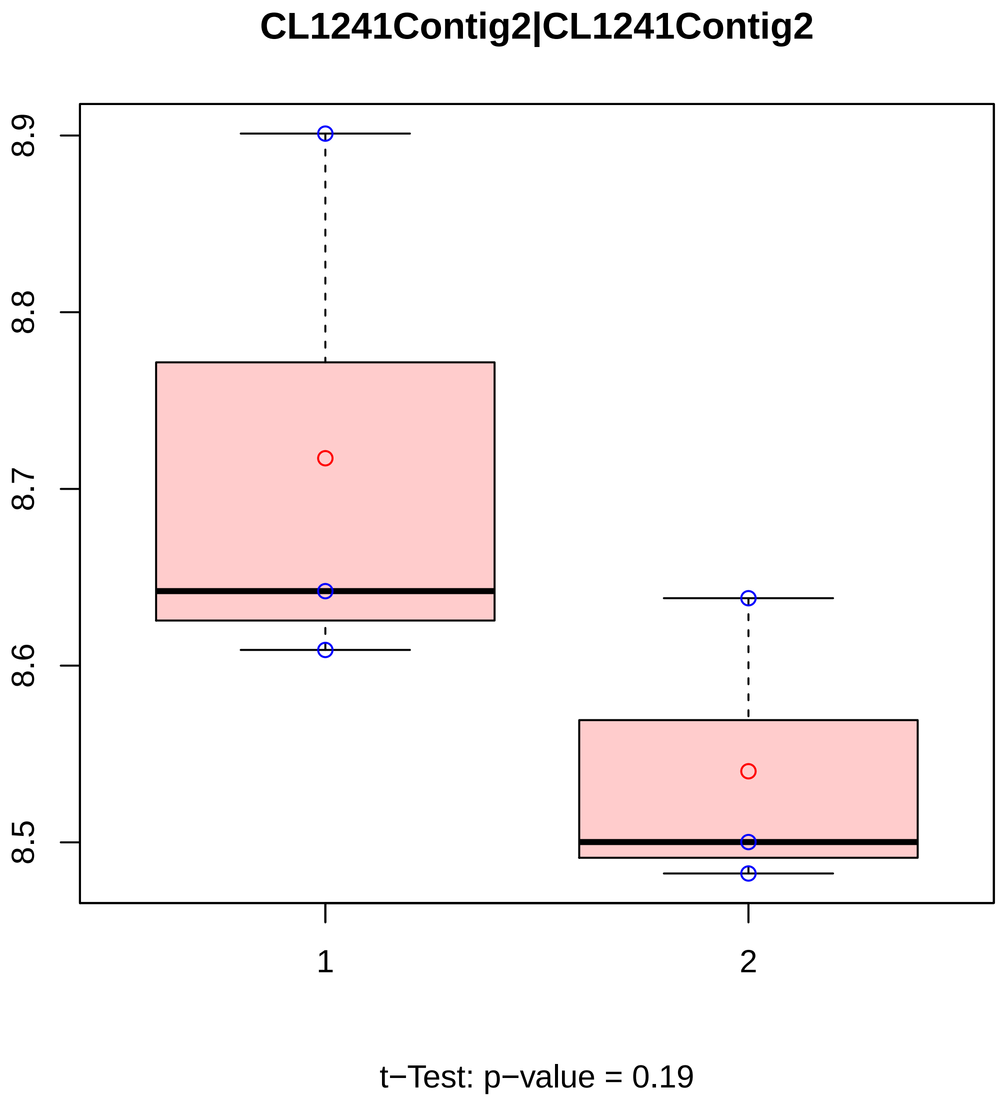

CL1241Contig2|CL1241Contig2
8.5
8.6
8.7
8.8
8.9
1
2
t−Test: p−value = 0.19

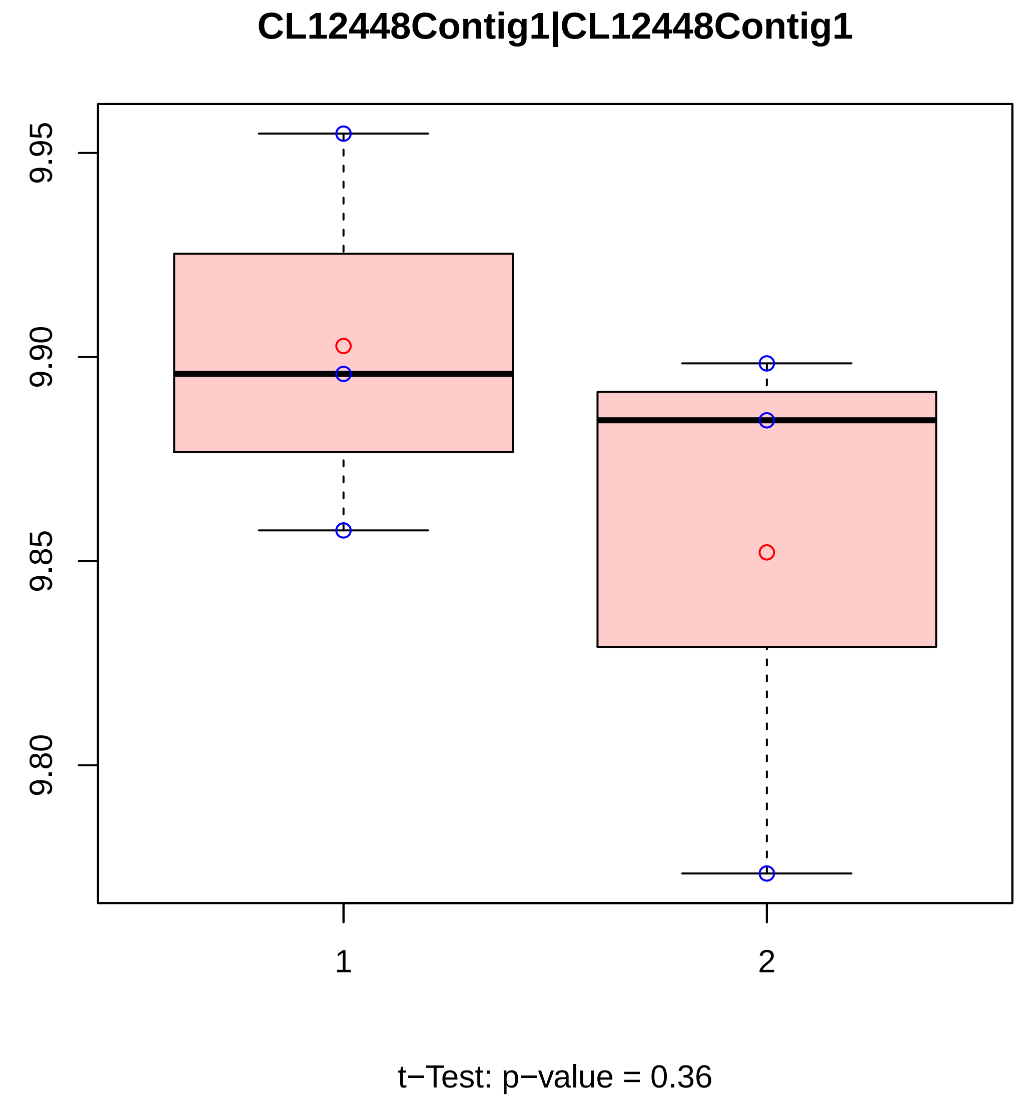
CL12448Contig1|CL12448Contig1
9.80
9.85
9.90
9.95
1
2
t−Test: p−value = 0.36

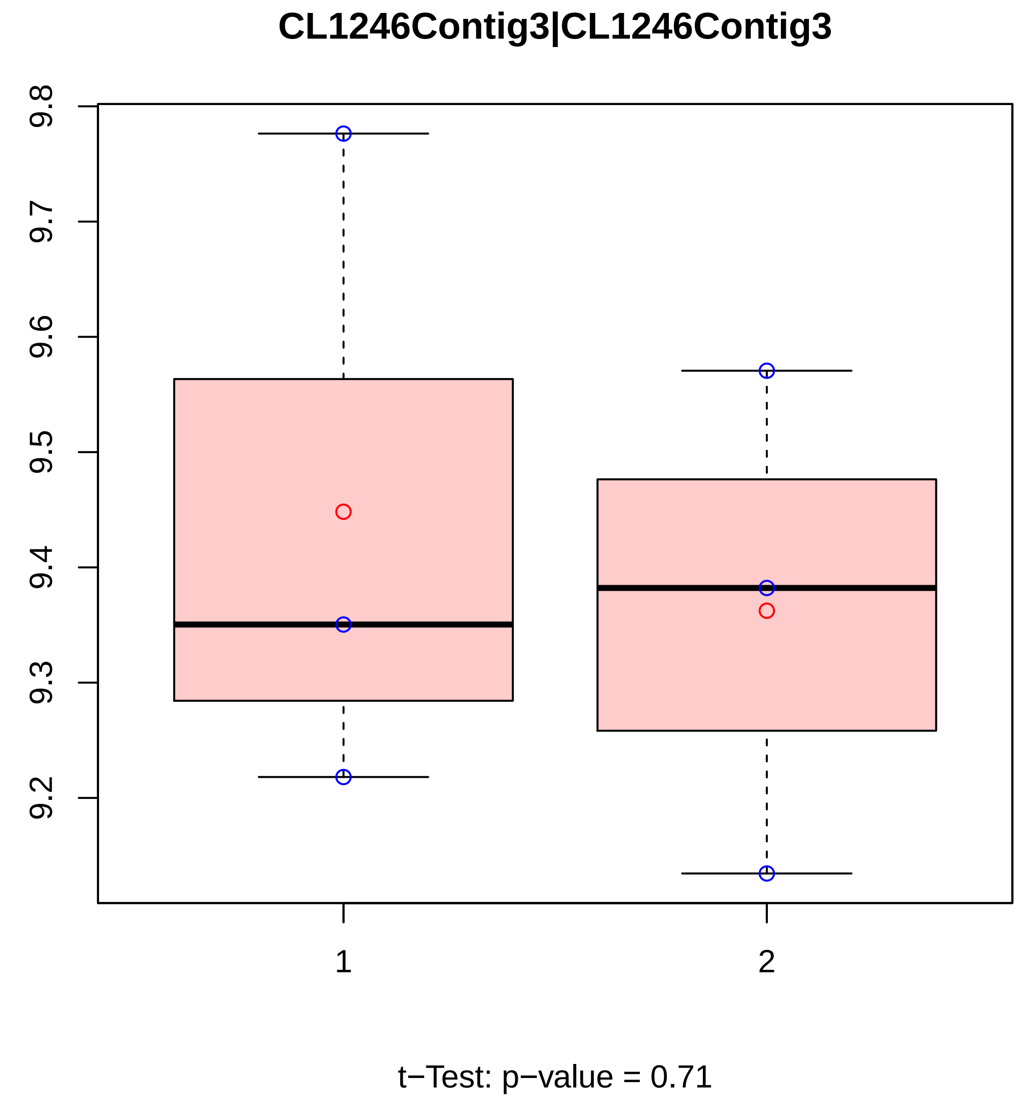
CL1246Contig3|CL1246Contig3
9.8
9.7
9.6
9.5
9.4
9.3
9.2
1
2
t−Test: p−value = 0.71

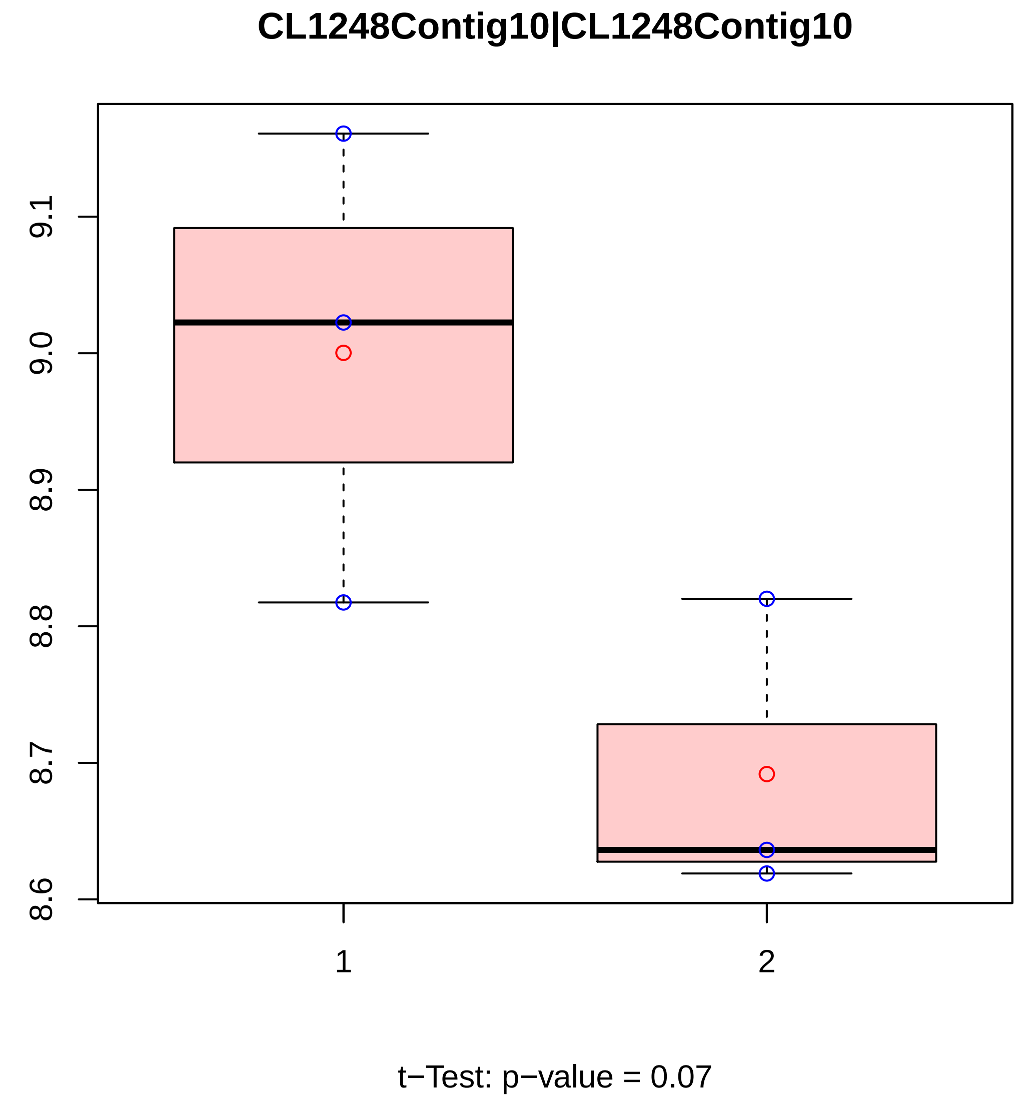

CL1248Contig10|CL1248Contig10
8.6
8.7
8.8
8.9
9.0
9.1
1
2
t−Test: p−value = 0.07

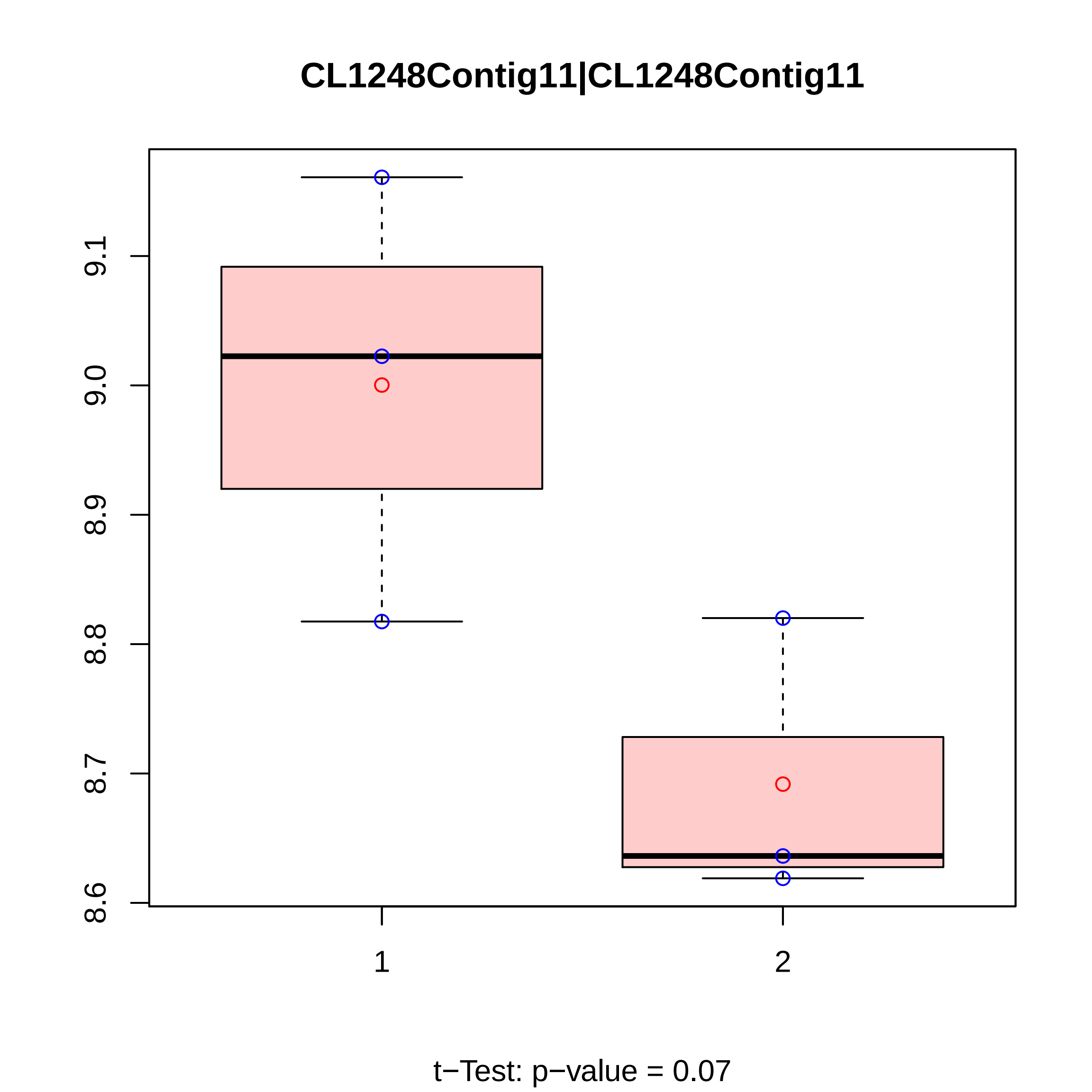
CL1248Contig11|CL1248Contig11
9.1
9.0
8.9
8.8
8.7
8.6
1
2
t−Test: p−value = 0.07

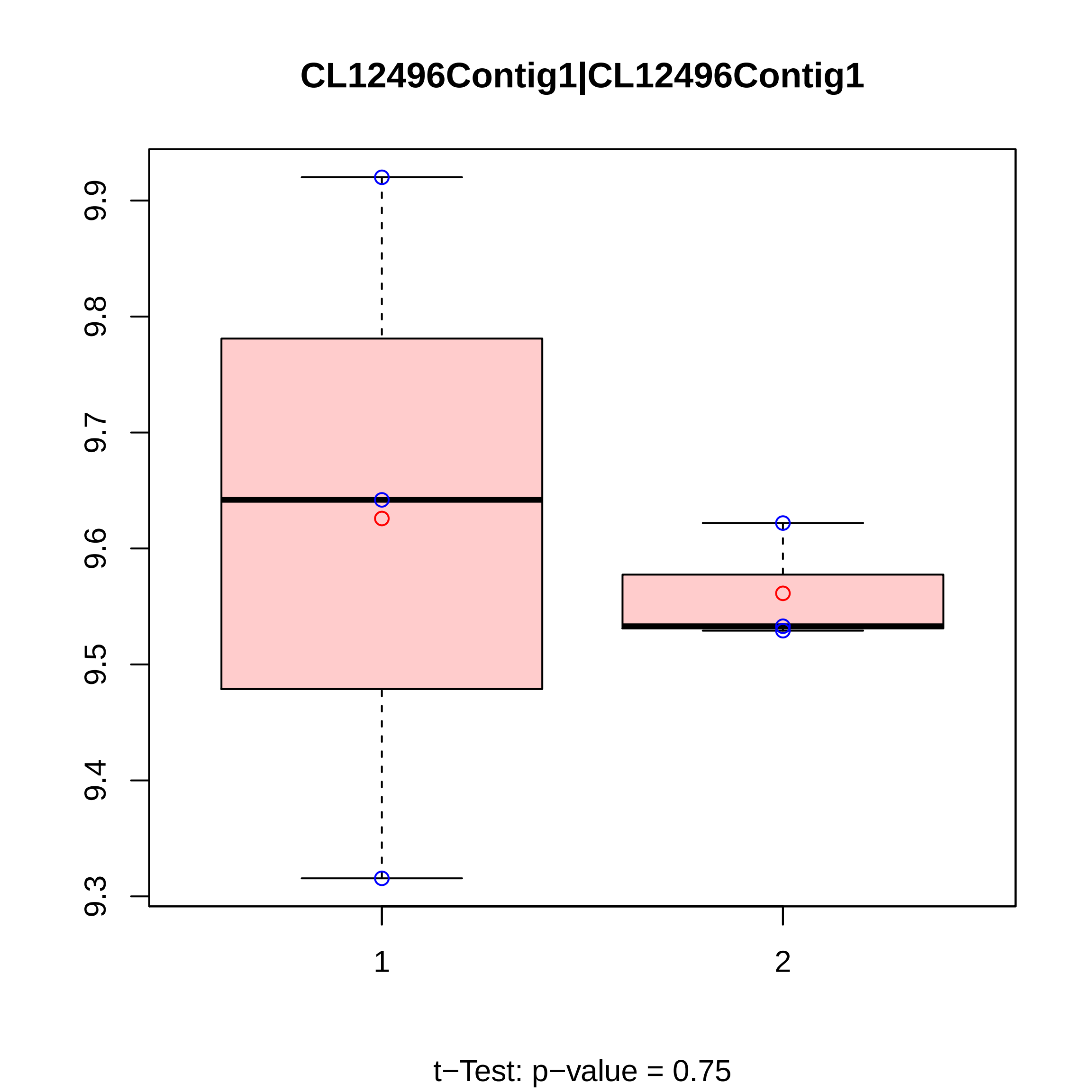
CL12496Contig1|CL12496Contig1
9.3
9.4
9.5
9.6
9.7
9.8
9.9
1
2
t−Test: p−value = 0.75

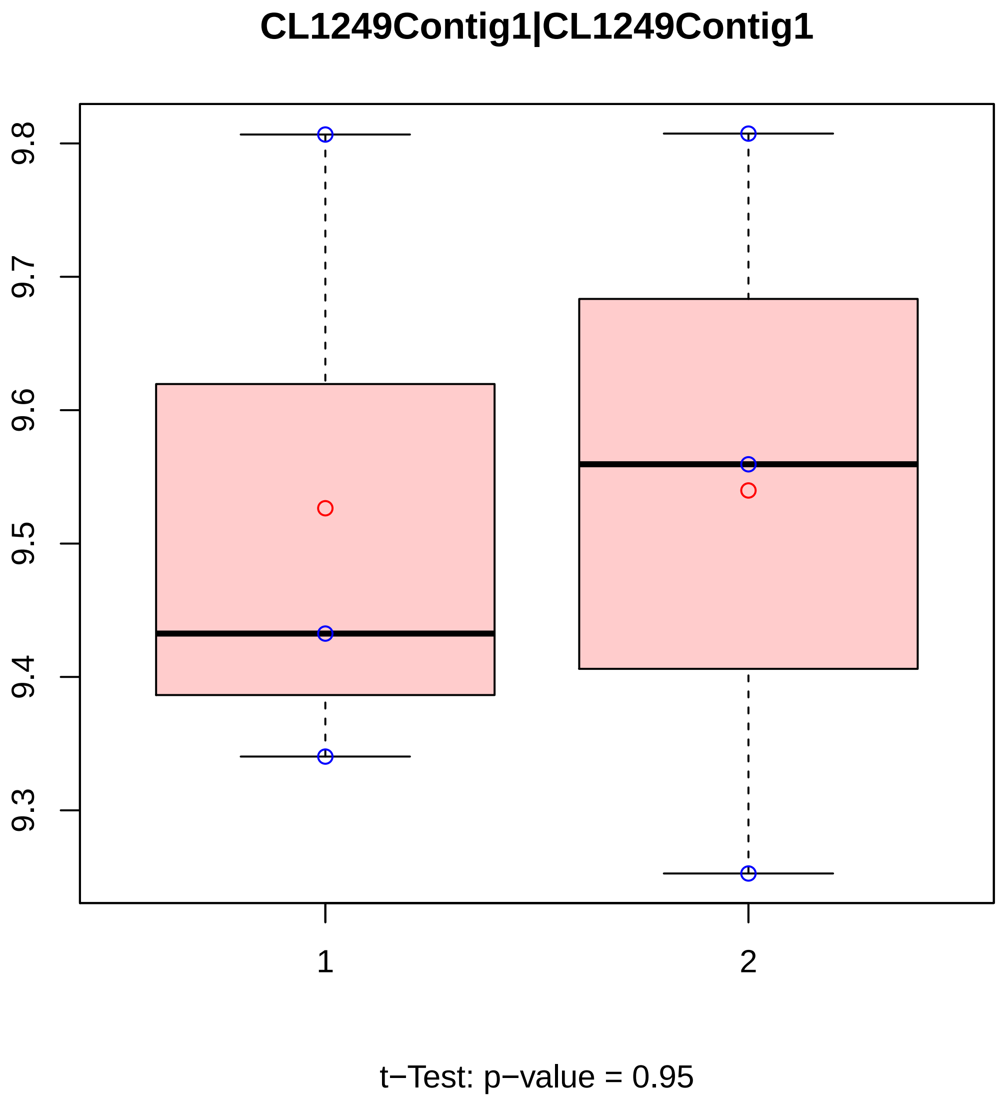
CL1249Contig1|CL1249Contig1
9.3
9.4
9.5
9.6
9.7
9.8
1
2
t−Test: p−value = 0.95

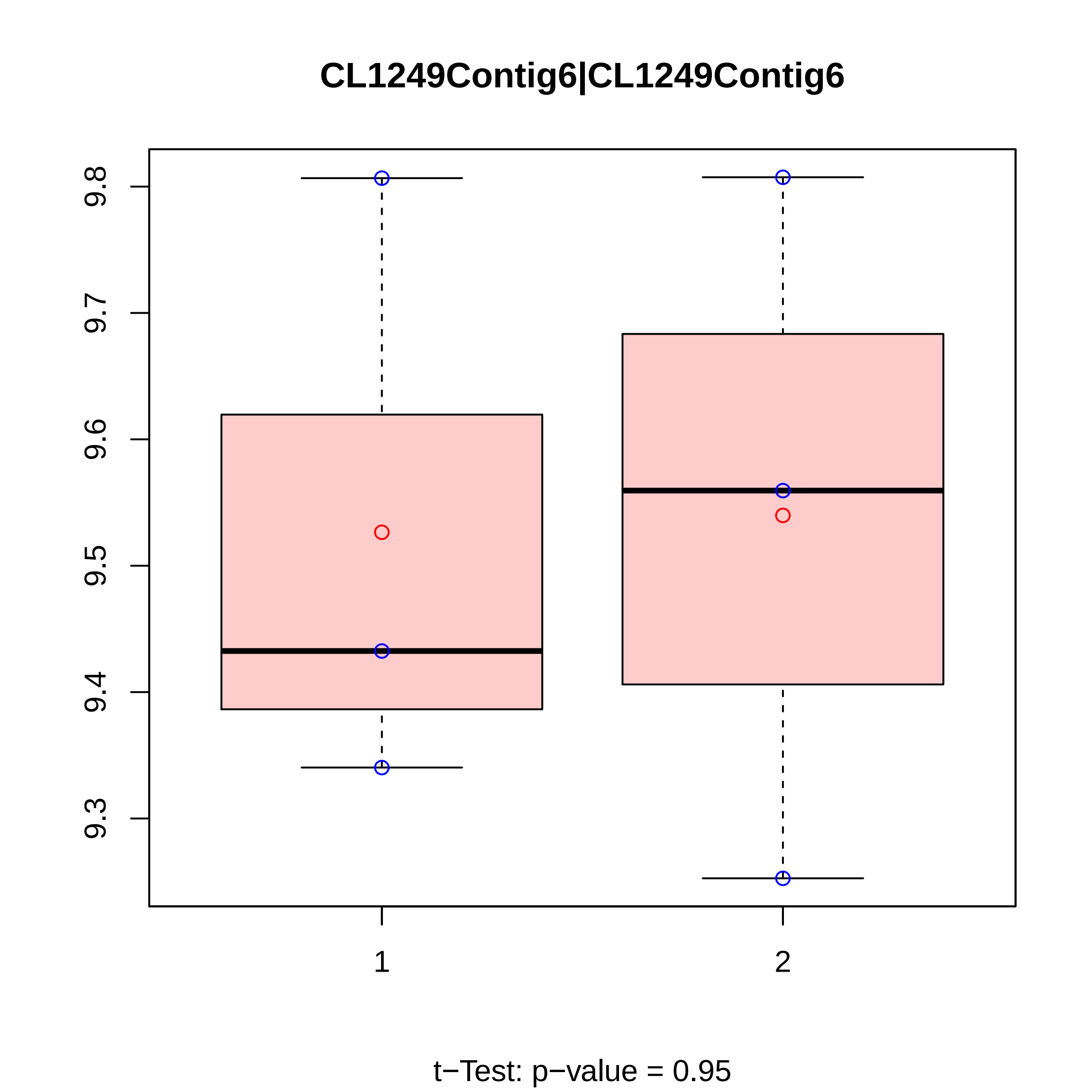
CL1249Contig6|CL1249Contig6
9.3
9.4
9.5
9.6
9.7
9.8
1
2
t−Test: p−value = 0.95

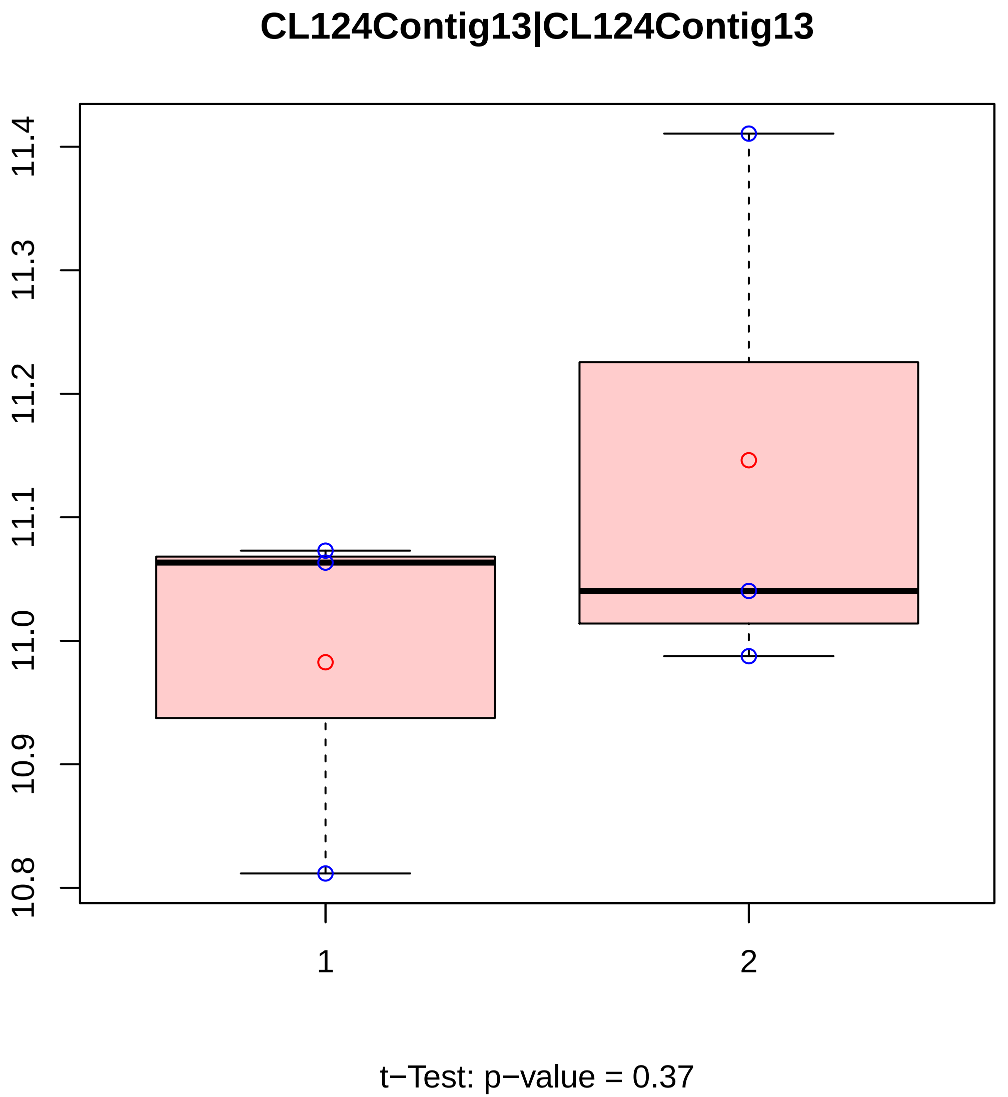
CL124Contig13|CL124Contig13
11.4
11.3
11.2
11.1
11.0
10.9
10.8
1
2
t−Test: p−value = 0.37

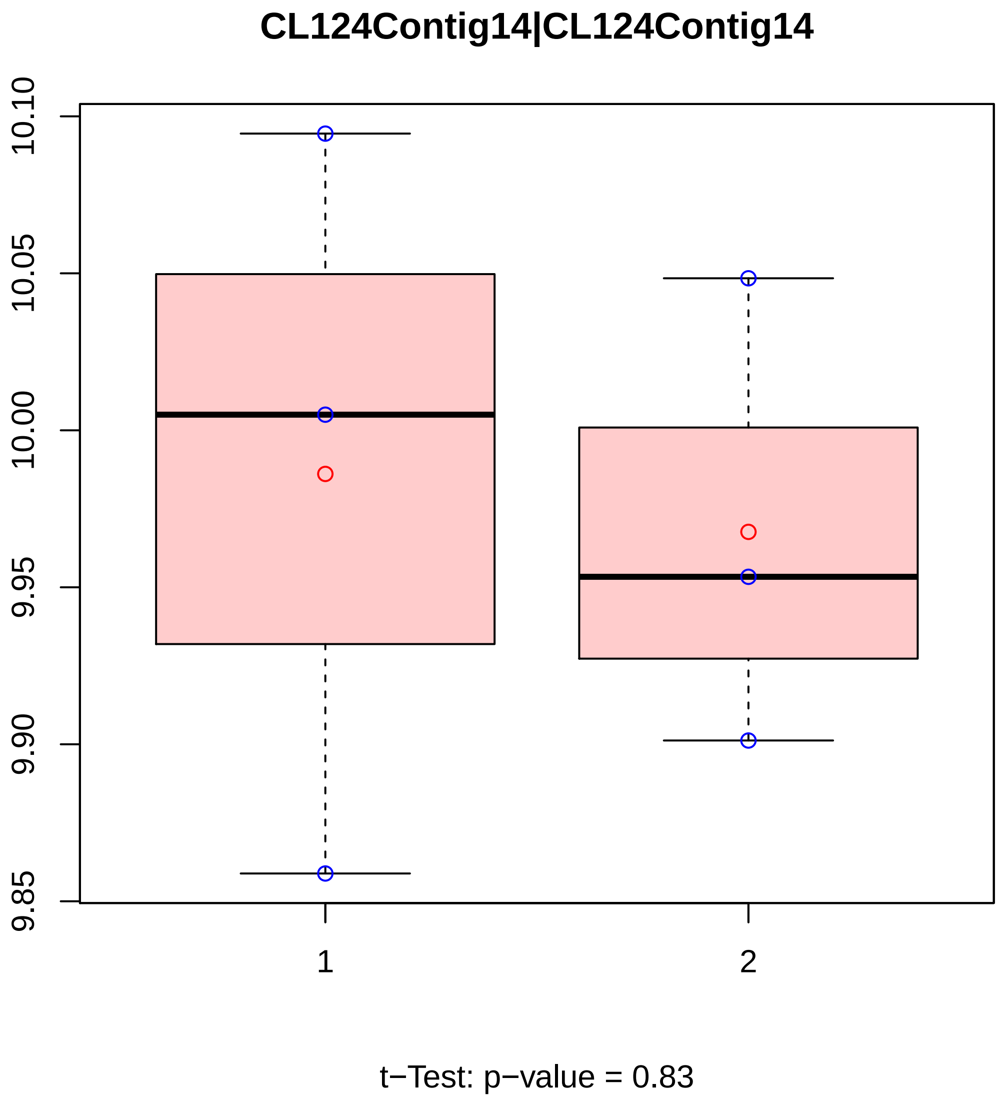
CL124Contig14|CL124Contig14
10.10
10.05
10.00
9.95
9.90
9.85
1
2
t−Test: p−value = 0.83

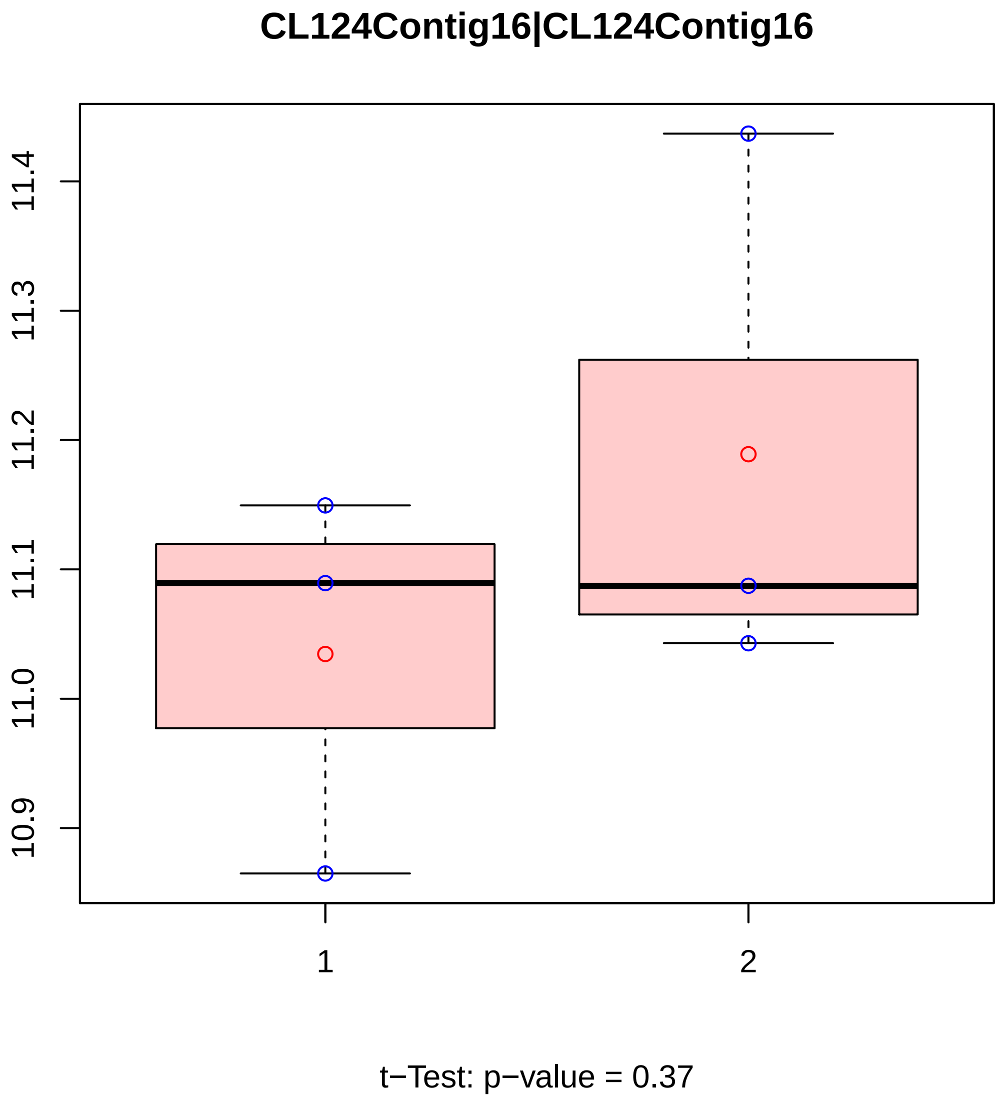

CL124Contig16|CL124Contig16
11.4
11.3
11.2
11.1
11.0
10.9
1
2
t−Test: p−value = 0.37

# CL124Contig17|CL124Contig17

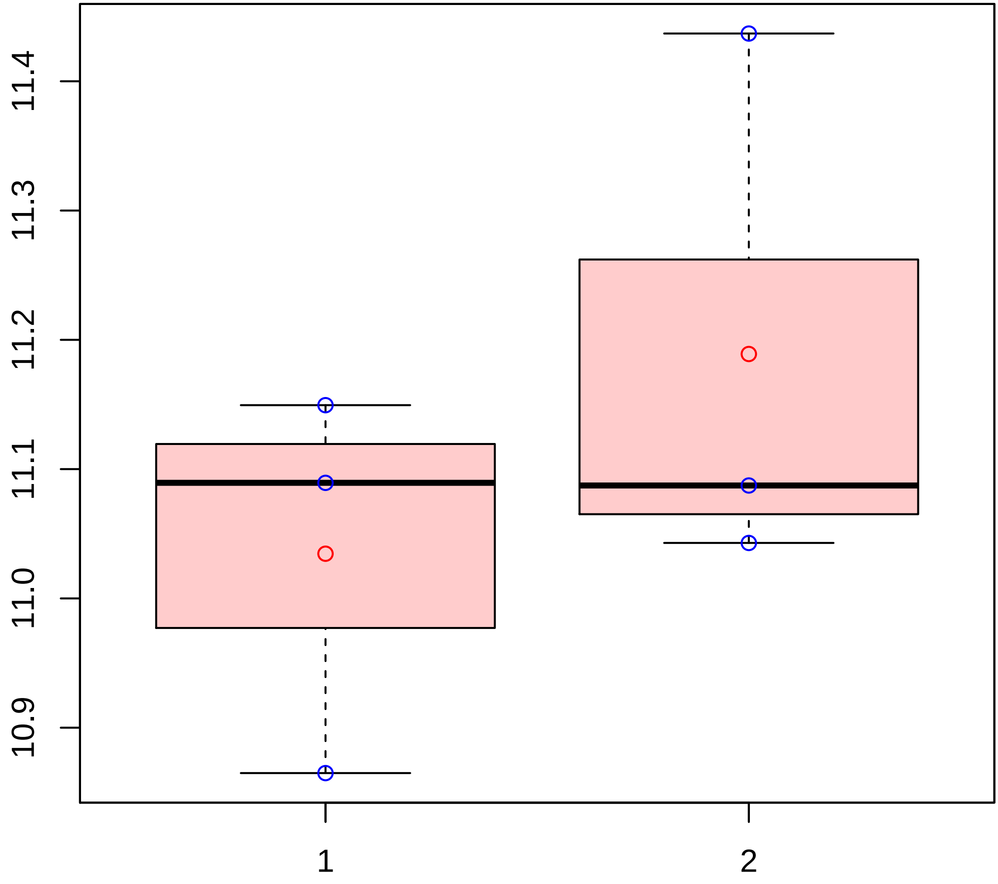


t−Test: p−value = 0.37

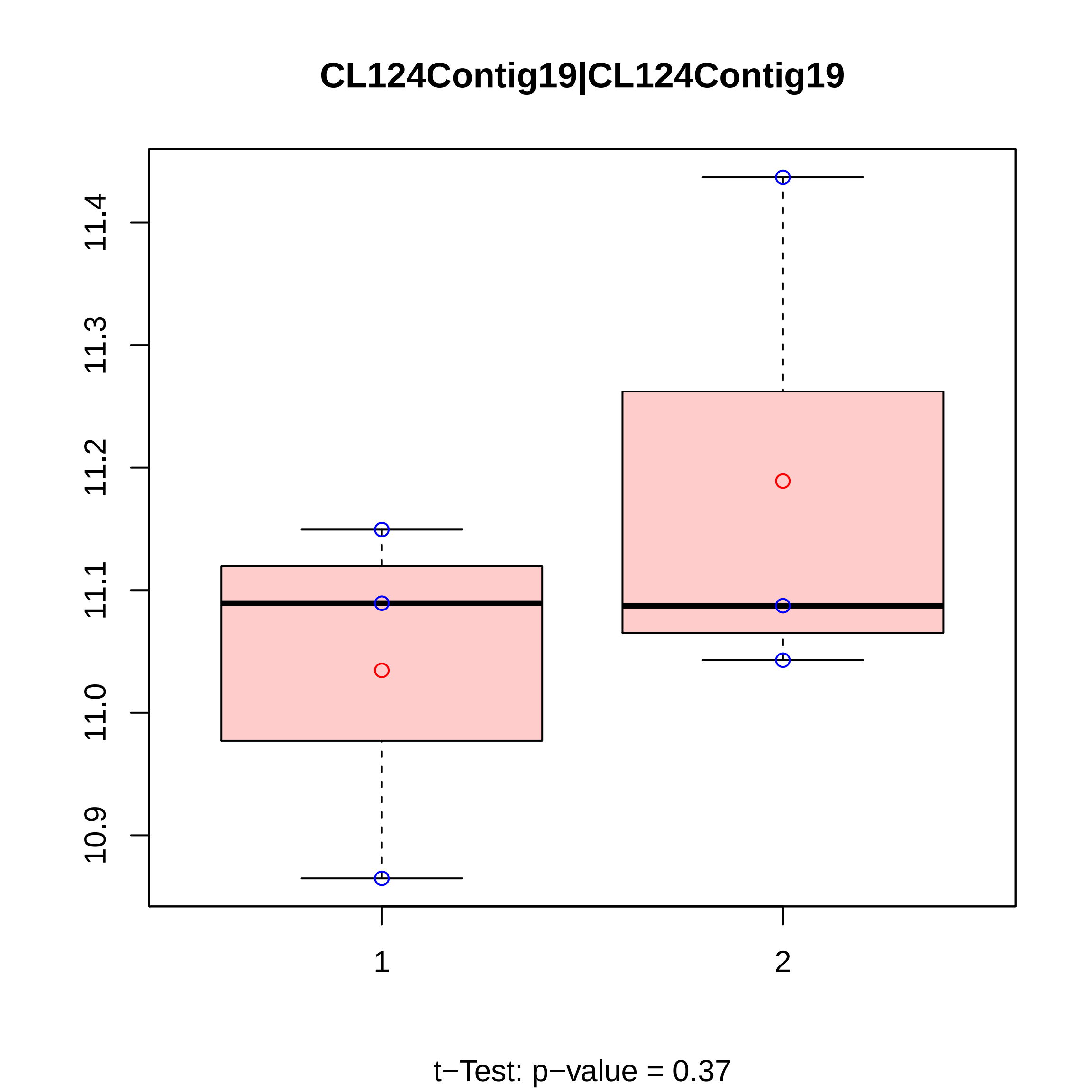

CL124Contig19|CL124Contig19
11.4
11.3
11.2
11.1
11.0
10.9
1
2
t−Test: p−value = 0.37

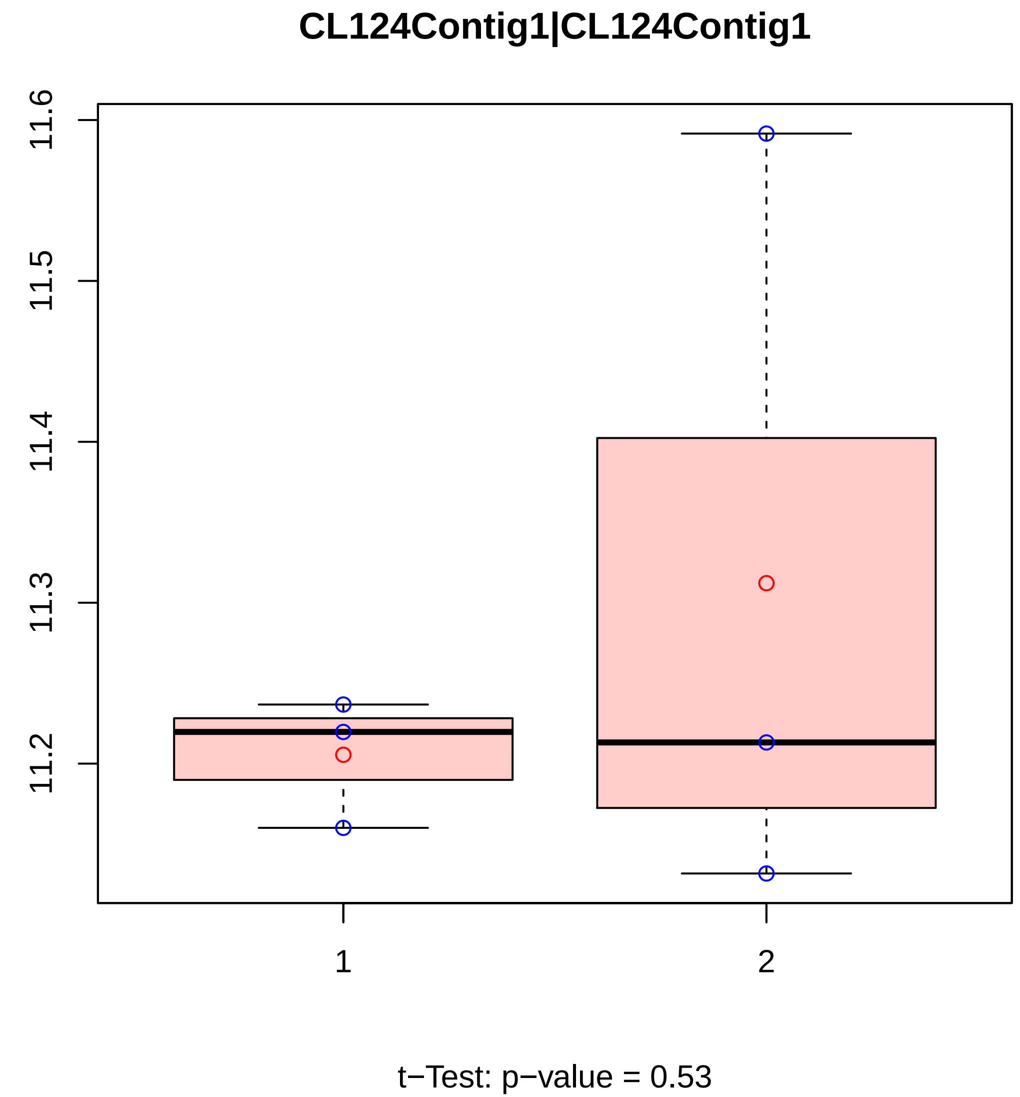
CL124Contig1|CL124Contig1
11.2
11.3
11.4
11.5
11.6
1
2
t−Test: p−value = 0.53

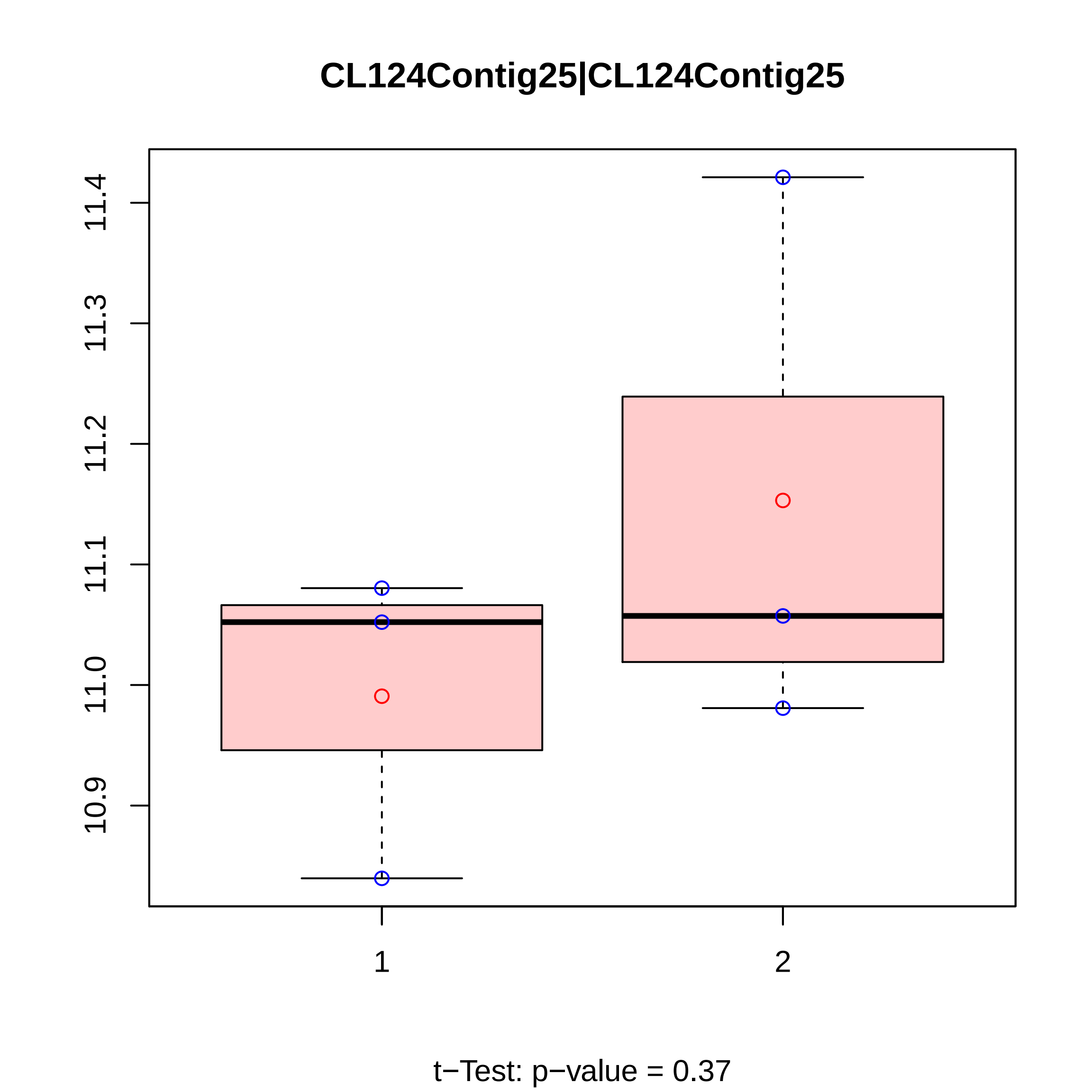
CL124Contig25|CL124Contig25
11.4
11.3
11.2
11.1
11.0
10.9
1
2
t−Test: p−value = 0.37

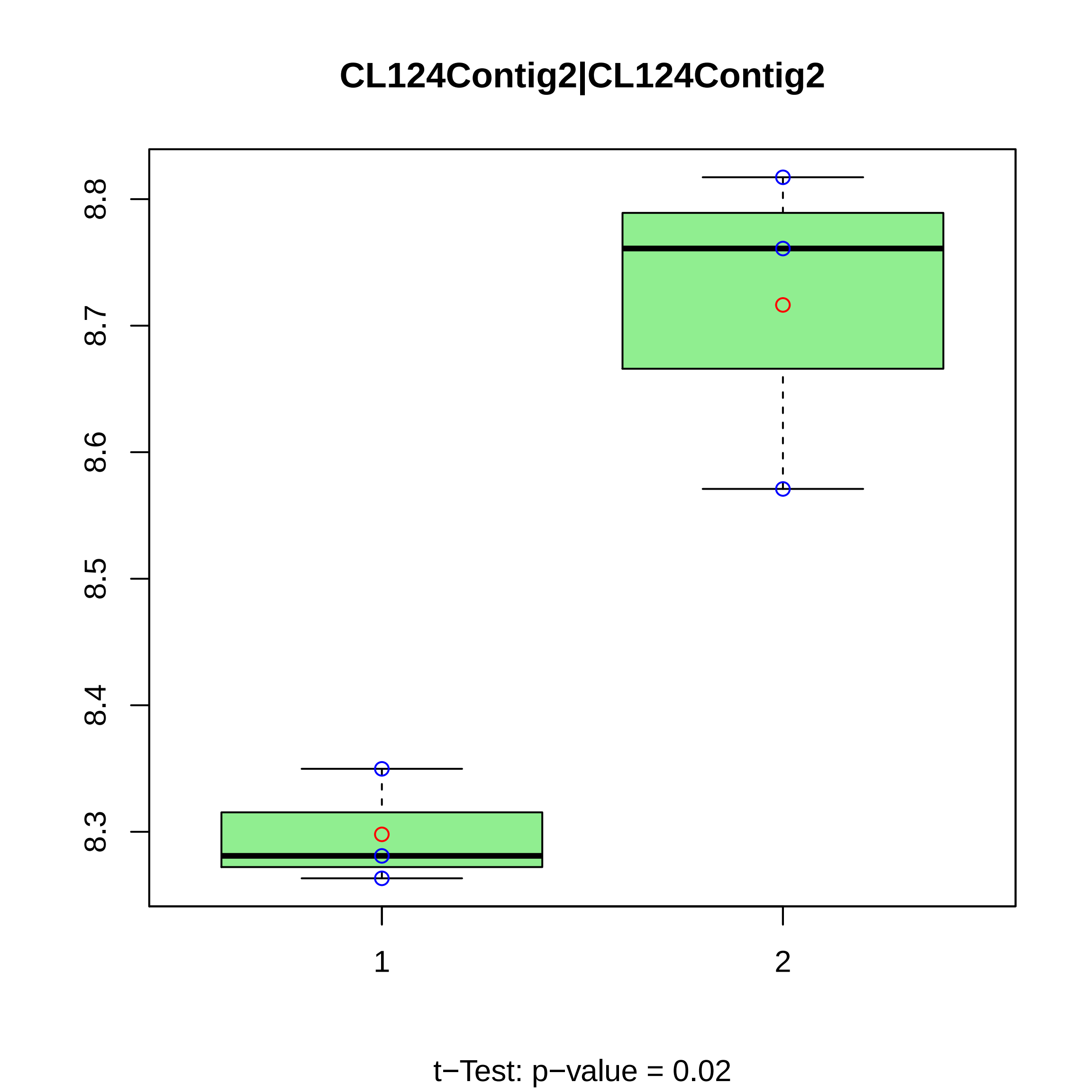
CL124Contig2|CL124Contig2
8.3
8.4
8.5
8.6
8.7
8.8
1
2
t−Test: p−value = 0.02

## CL124Contig6|CL124Contig6

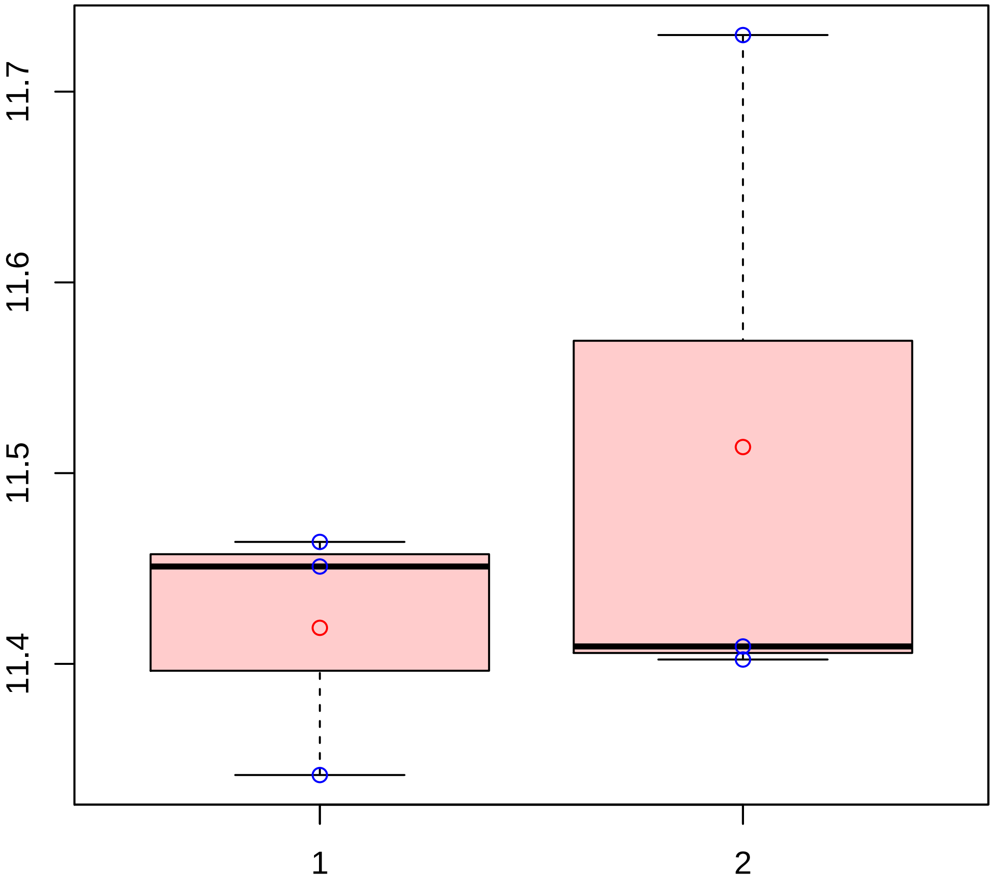


t−Test: p−value = 0.48

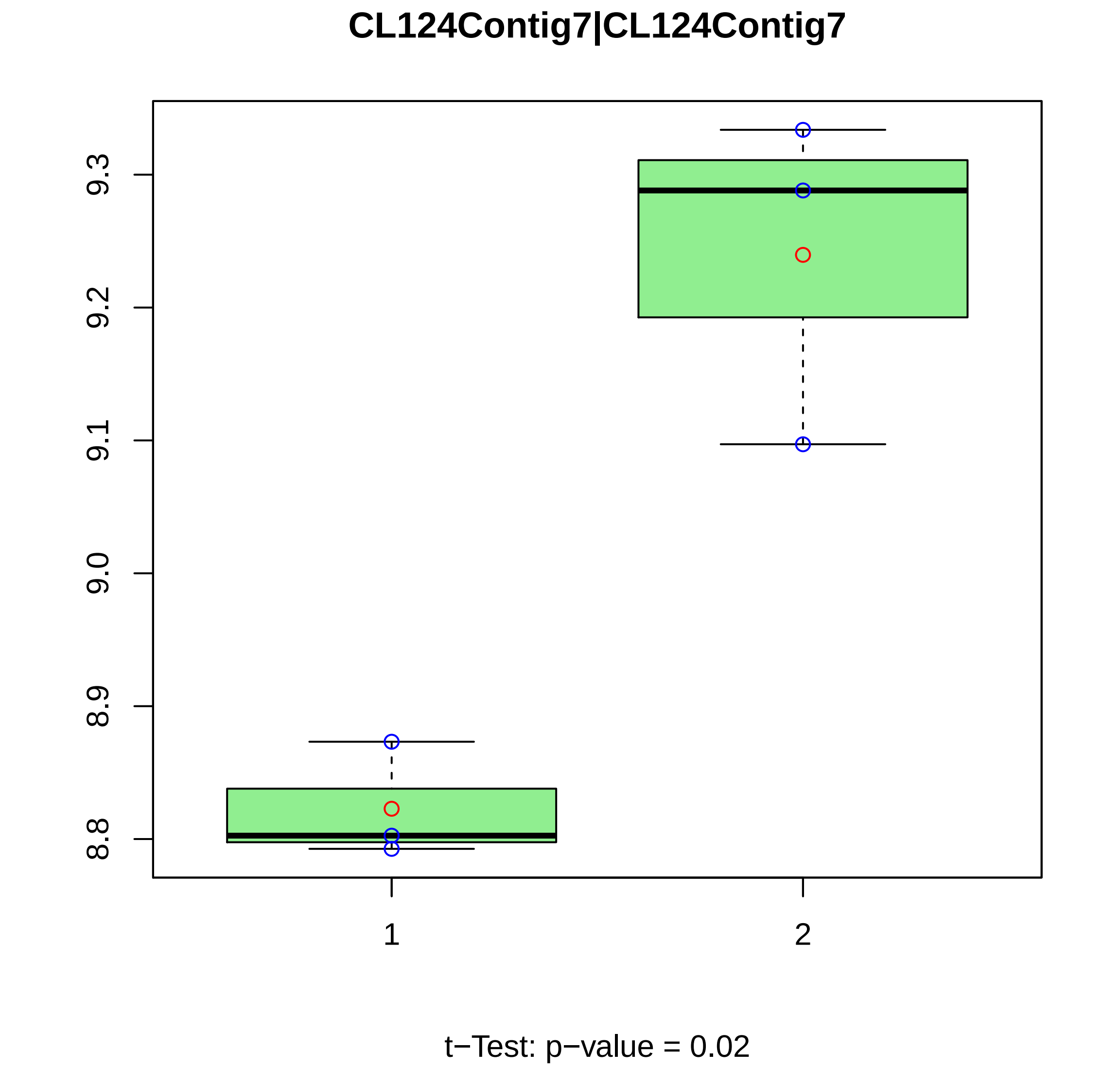
CL124Contig7|CL124Contig7
9.3
9.2
9.1
9.0
8.9
8.8
1
2
t−Test: p−value = 0.02

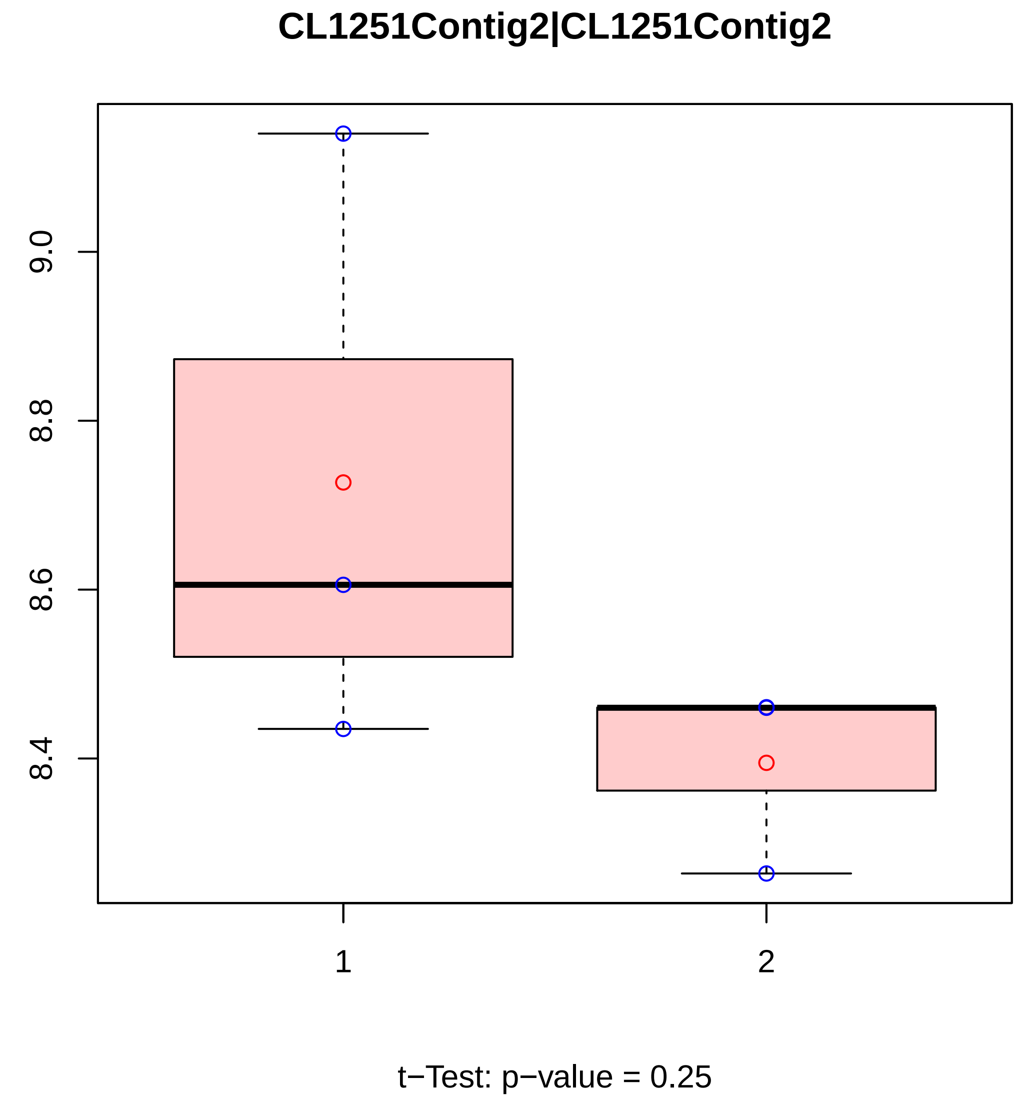

CL1251Contig2|CL1251Contig2
9.0
8.8
8.6
8.4
1
2
t−Test: p−value = 0.25

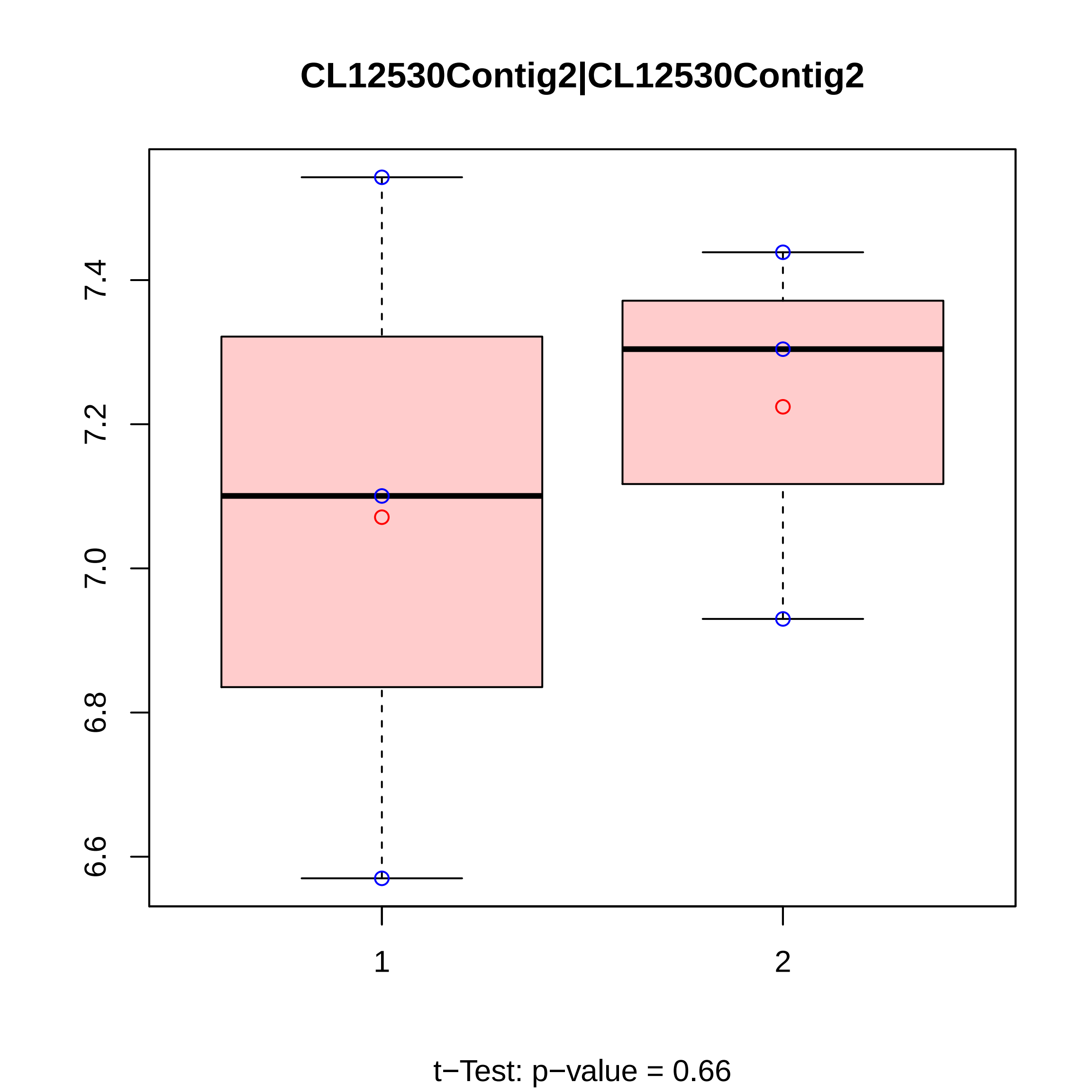
CL12530Contig2|CL12530Contig2
7.4
7.2
7.0
6.8
6.6
1
2
t−Test: p−value = 0.66

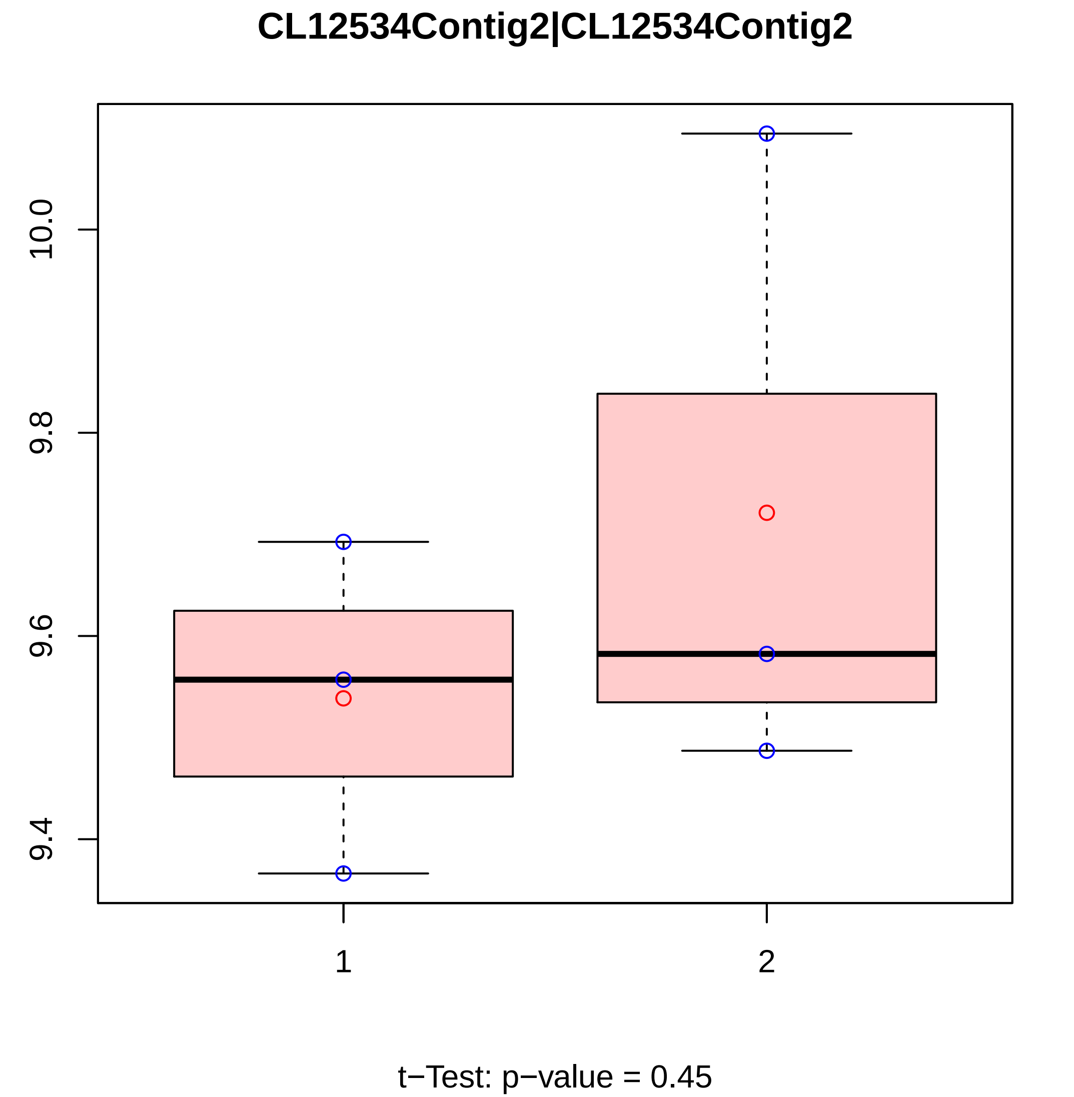
CL12534Contig2|CL12534Contig2
10.0
9.8
9.6
9.4
1
2
t−Test: p−value = 0.45

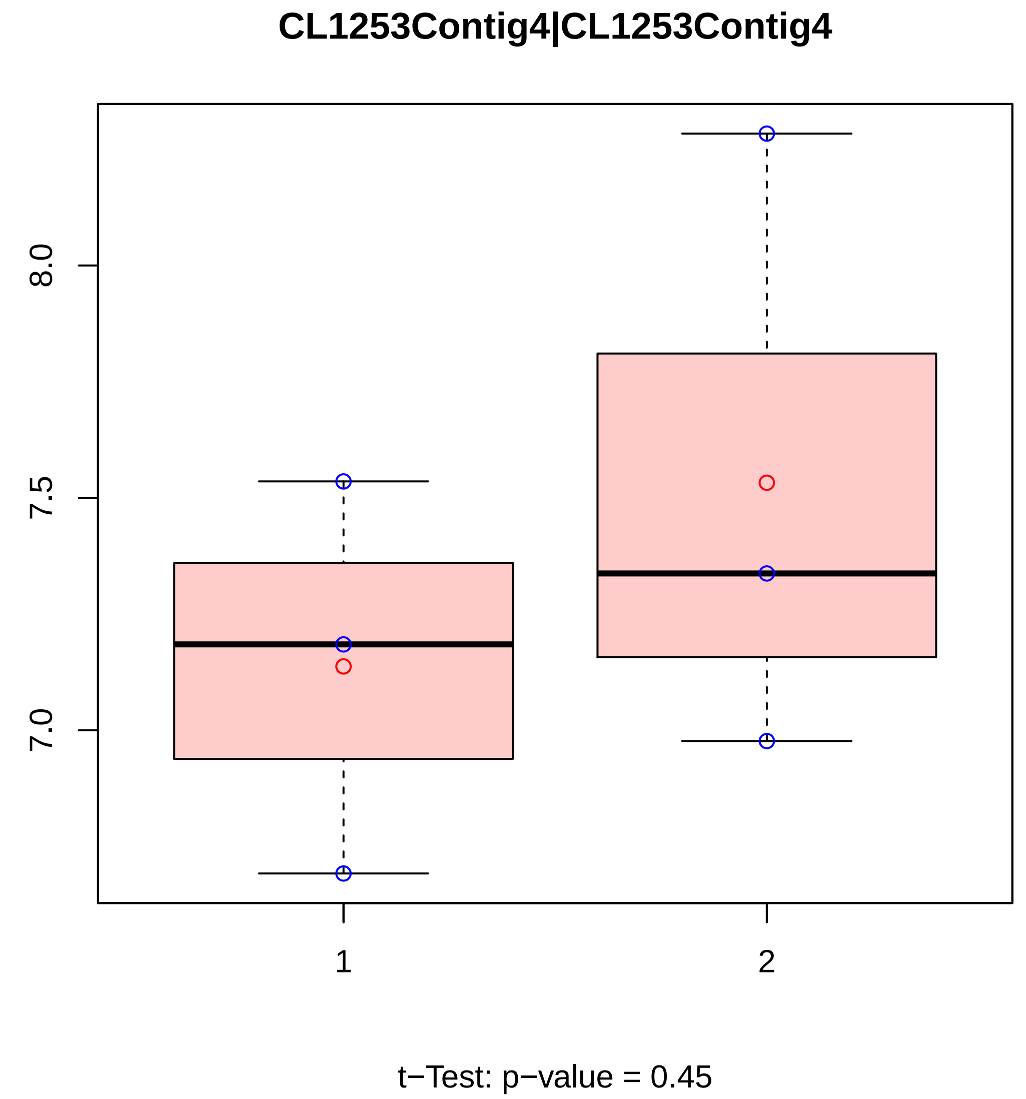
CL1253Contig4|CL1253Contig4
8.0
7.5
7.0
1
2
t−Test: p−value = 0.45

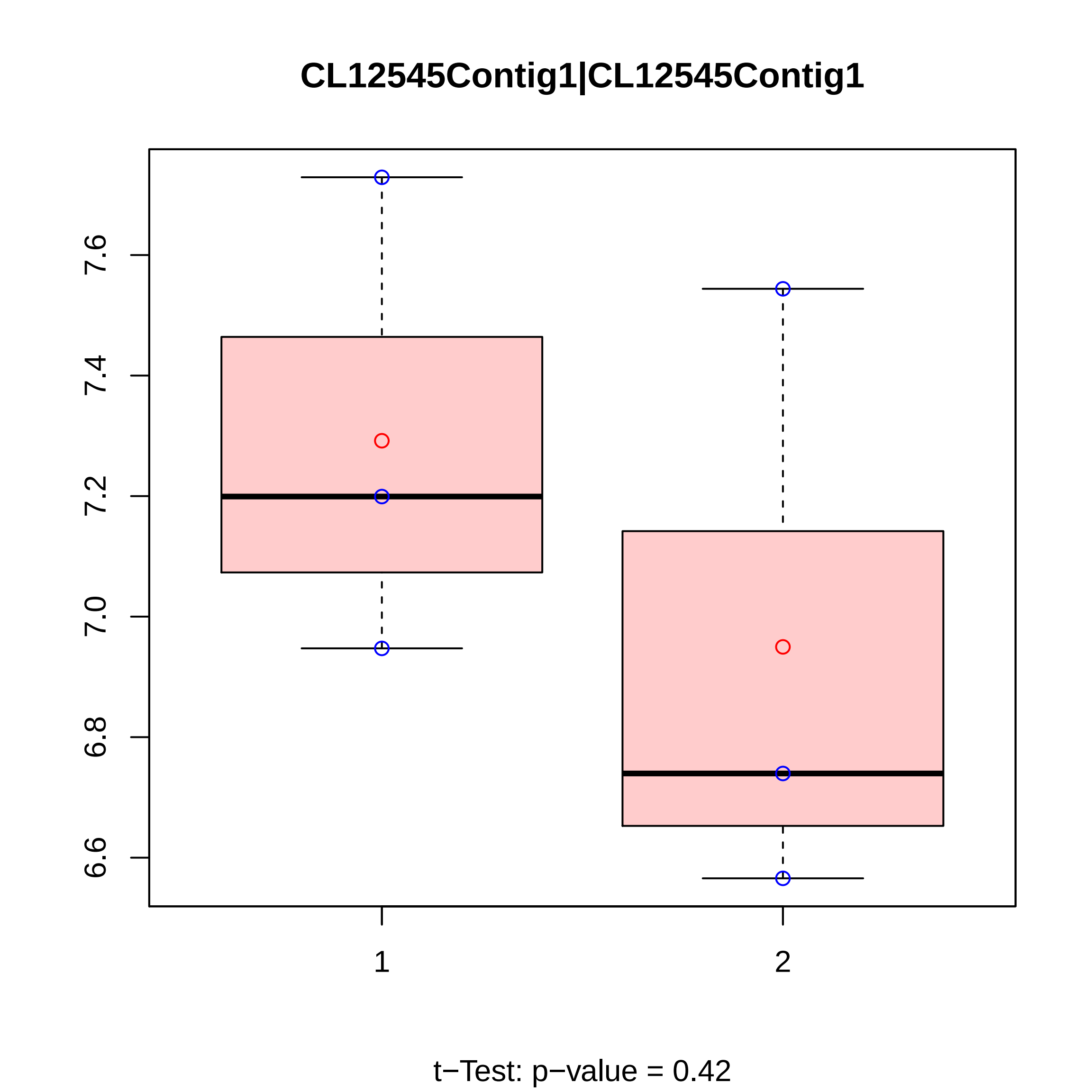

CL12545Contig1|CL12545Contig1
7.6
7.4
7.2
7.0
6.8
6.6
1
2
t−Test: p−value = 0.42

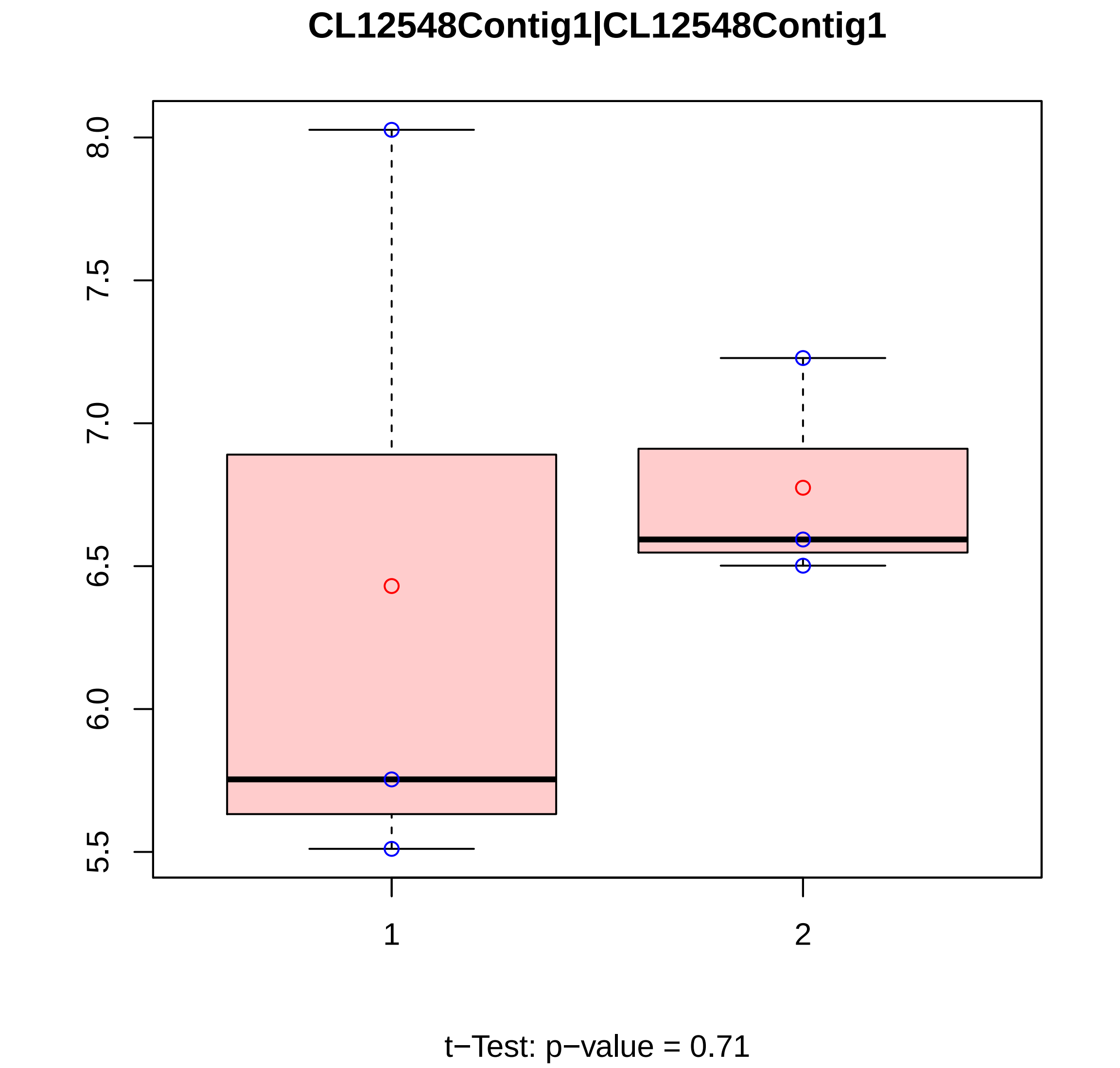
CL12548Contig1|CL12548Contig1
8.0
7.5
7.0
6.5
6.0
5.5
1
2
t−Test: p−value = 0.71

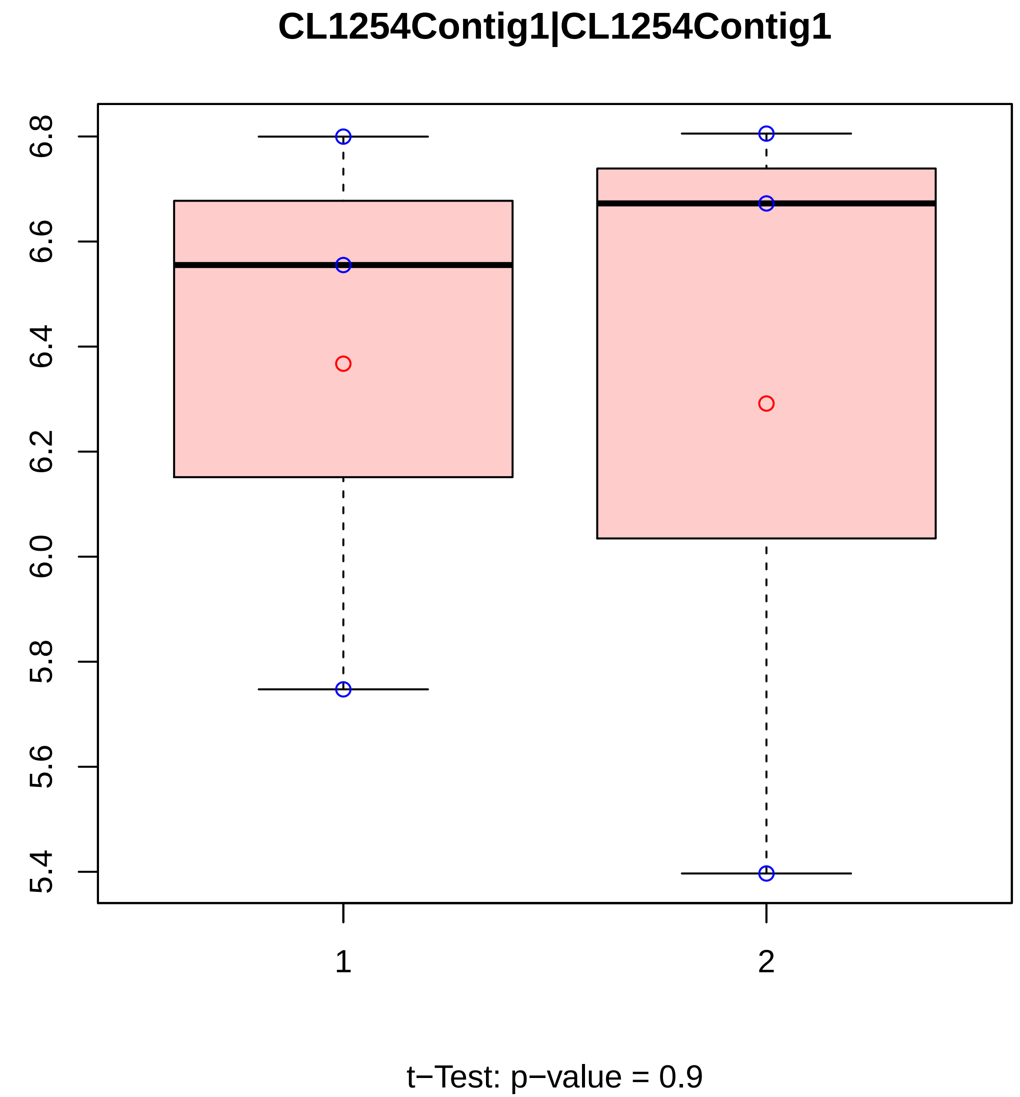
CL1254Contig1|CL1254Contig1
6.8
6.6
6.4
6.2
6.0
5.8
5.6
5.4
1
2
t−Test: p−value = 0.9

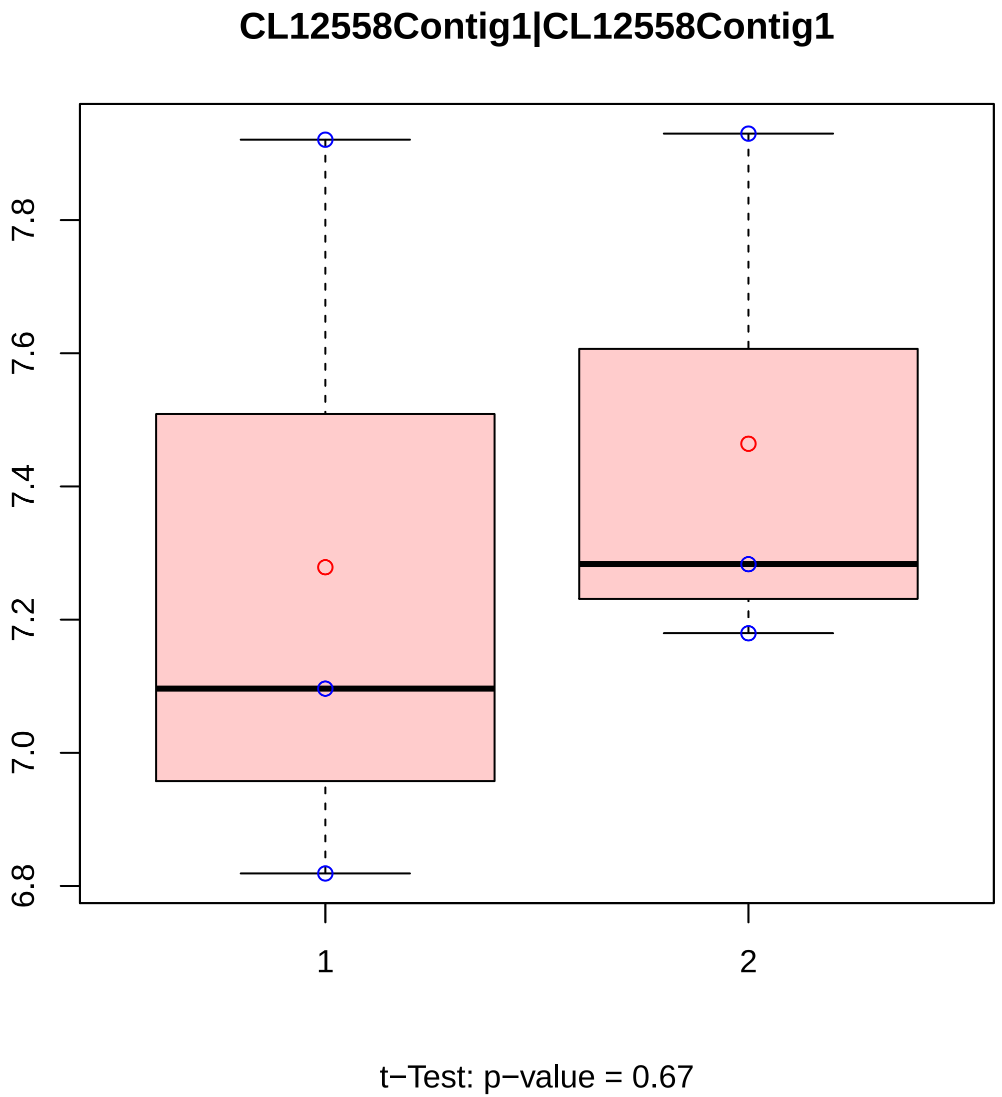
CL12558Contig1|CL12558Contig1
7.8
7.6
7.4
7.2
7.0
6.8
1
2
t−Test: p−value = 0.67

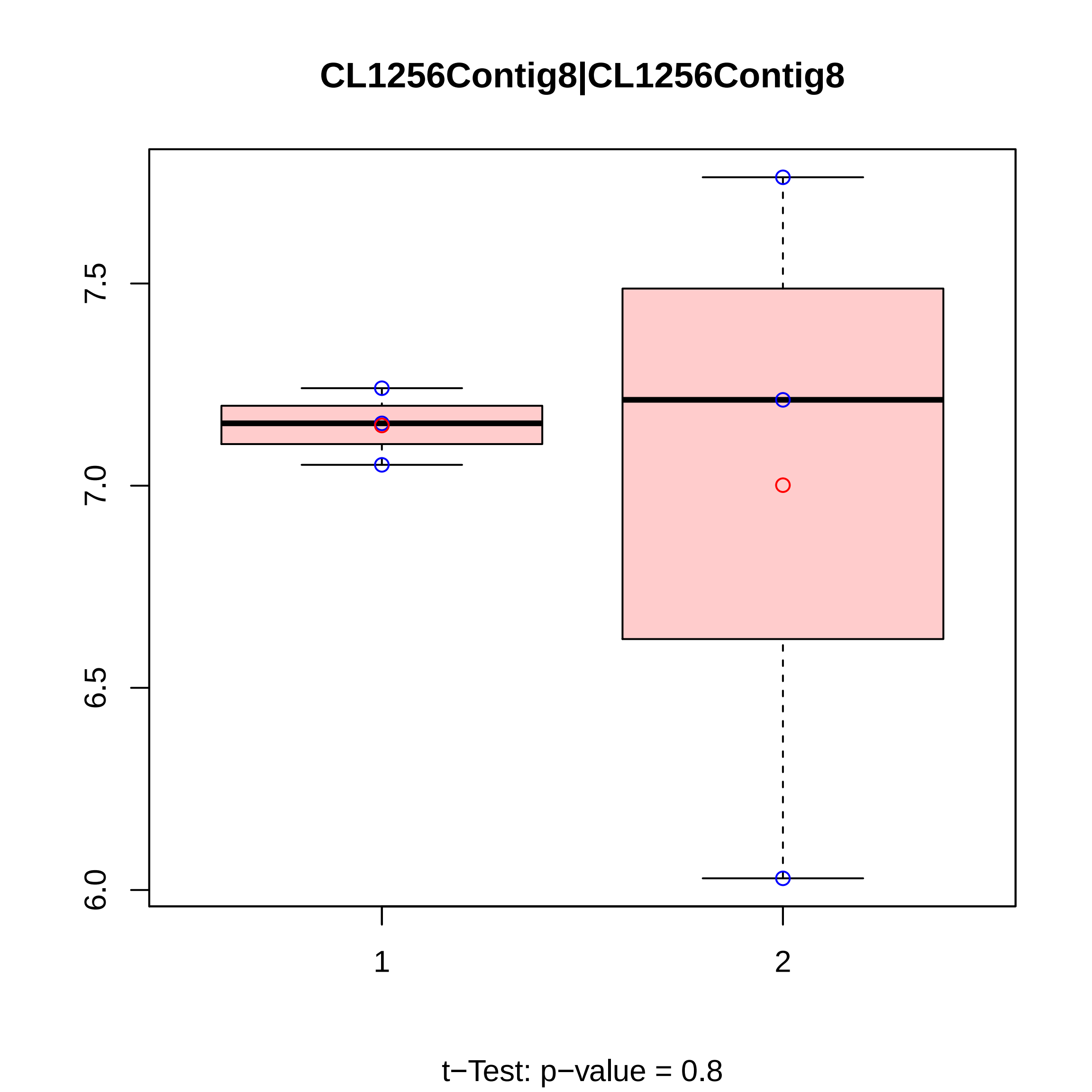

CL1256Contig8|CL1256Contig8
7.5
7.0
6.5
6.0
1
2
t−Test: p−value = 0.8

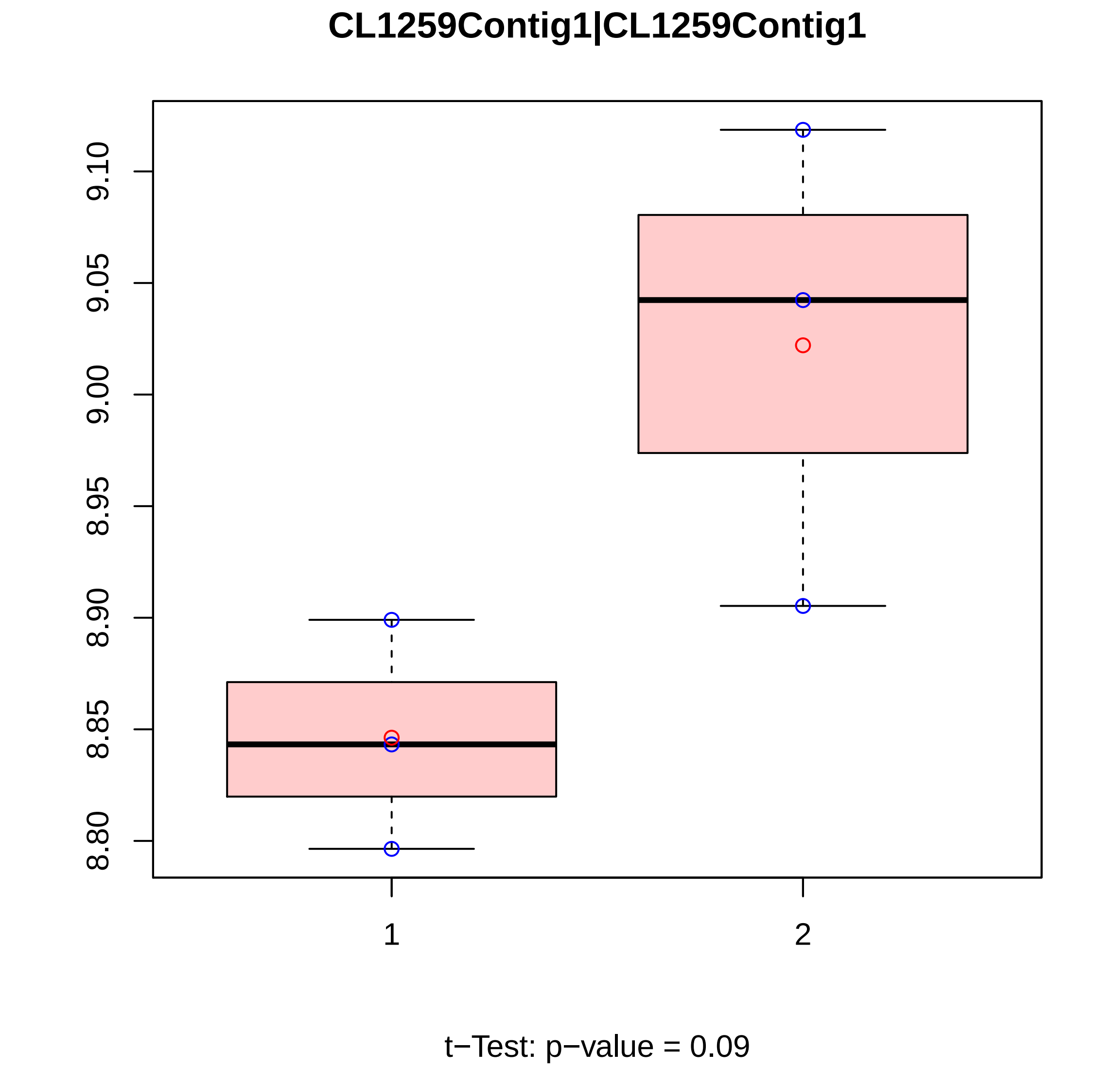
CL1259Contig1|CL1259Contig1
9.10
9.05
9.00
8.95
8.90
8.85
8.80
1
2
t−Test: p−value = 0.09

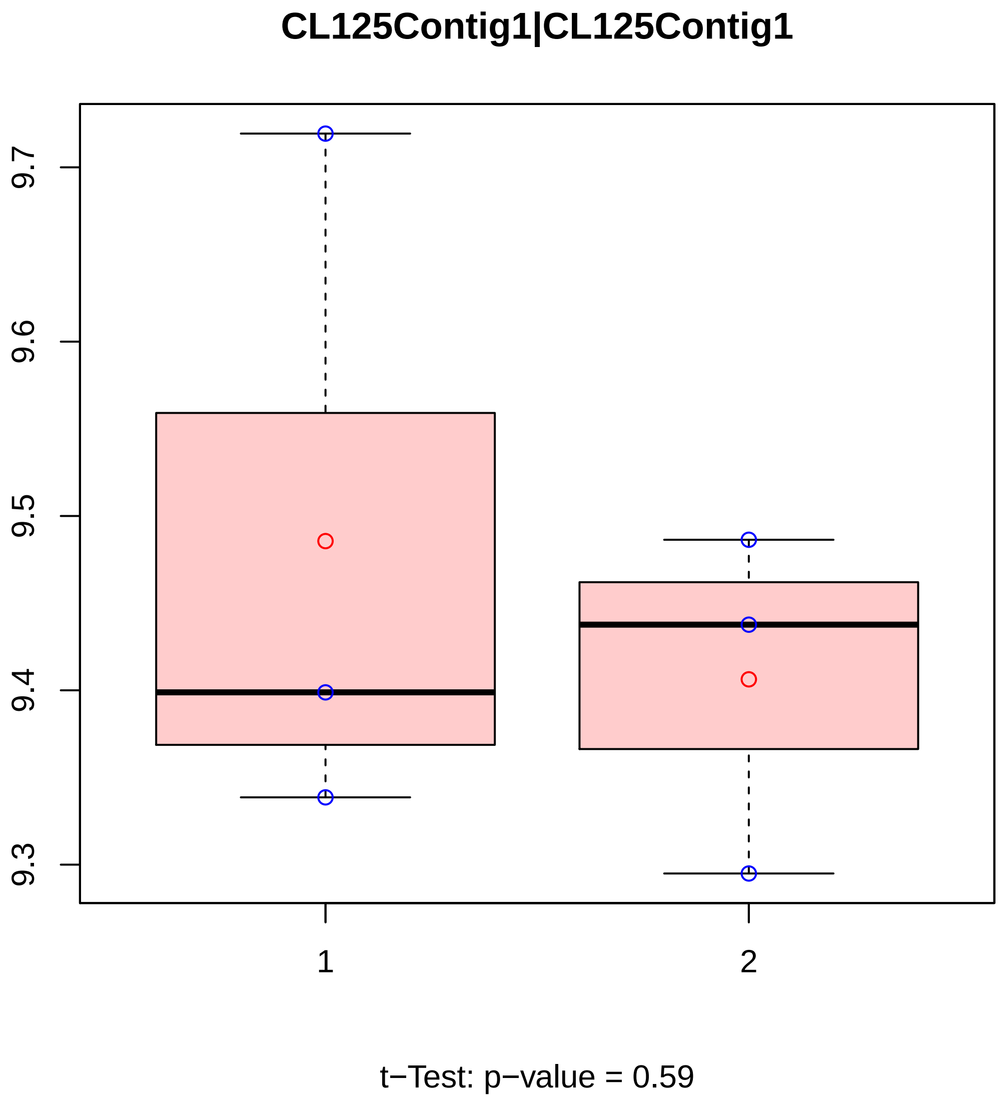

CL125Contig1|CL125Contig1
9.3
9.4
9.5
9.6
9.7
1
2
t−Test: p−value = 0.59

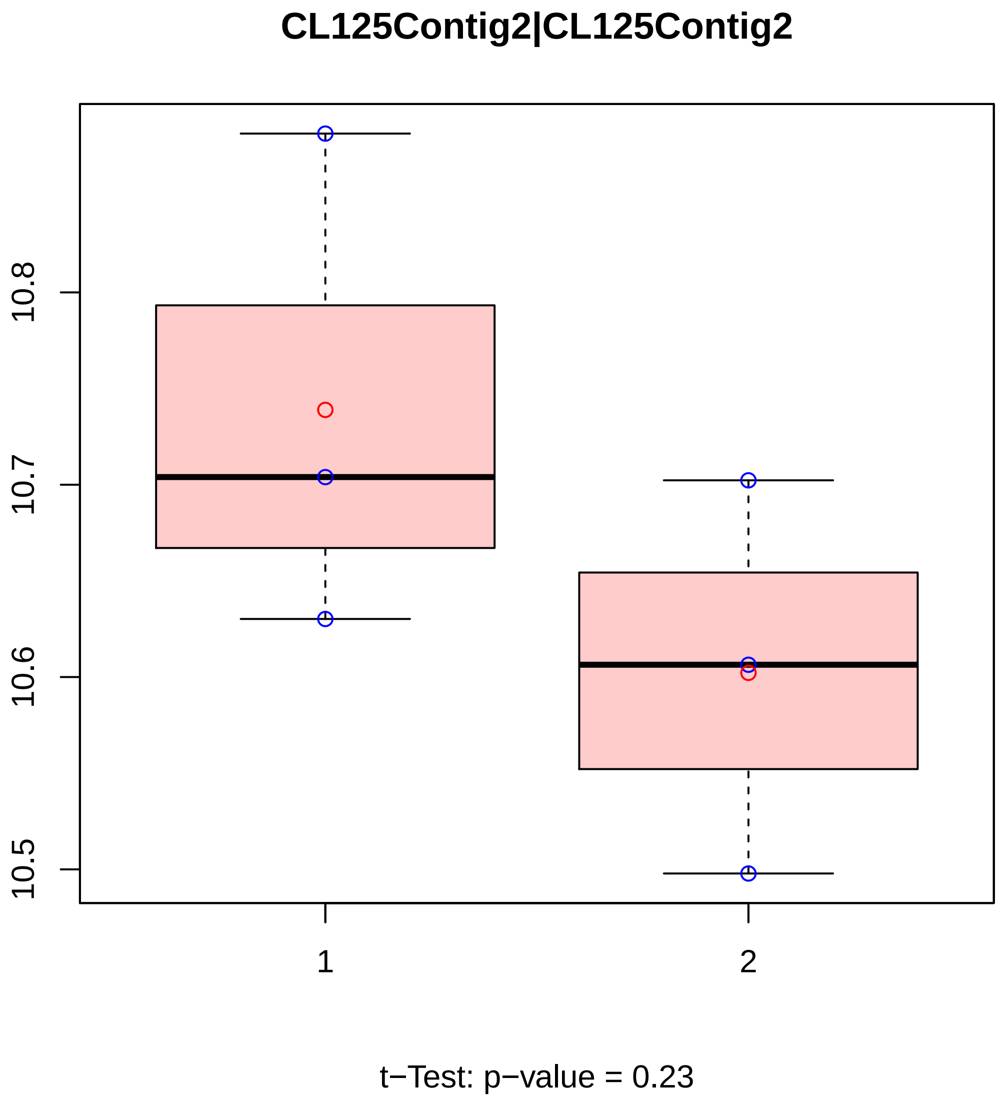
CL125Contig2|CL125Contig2
10.5
10.6
10.7
10.8
1
2
t−Test: p−value = 0.23

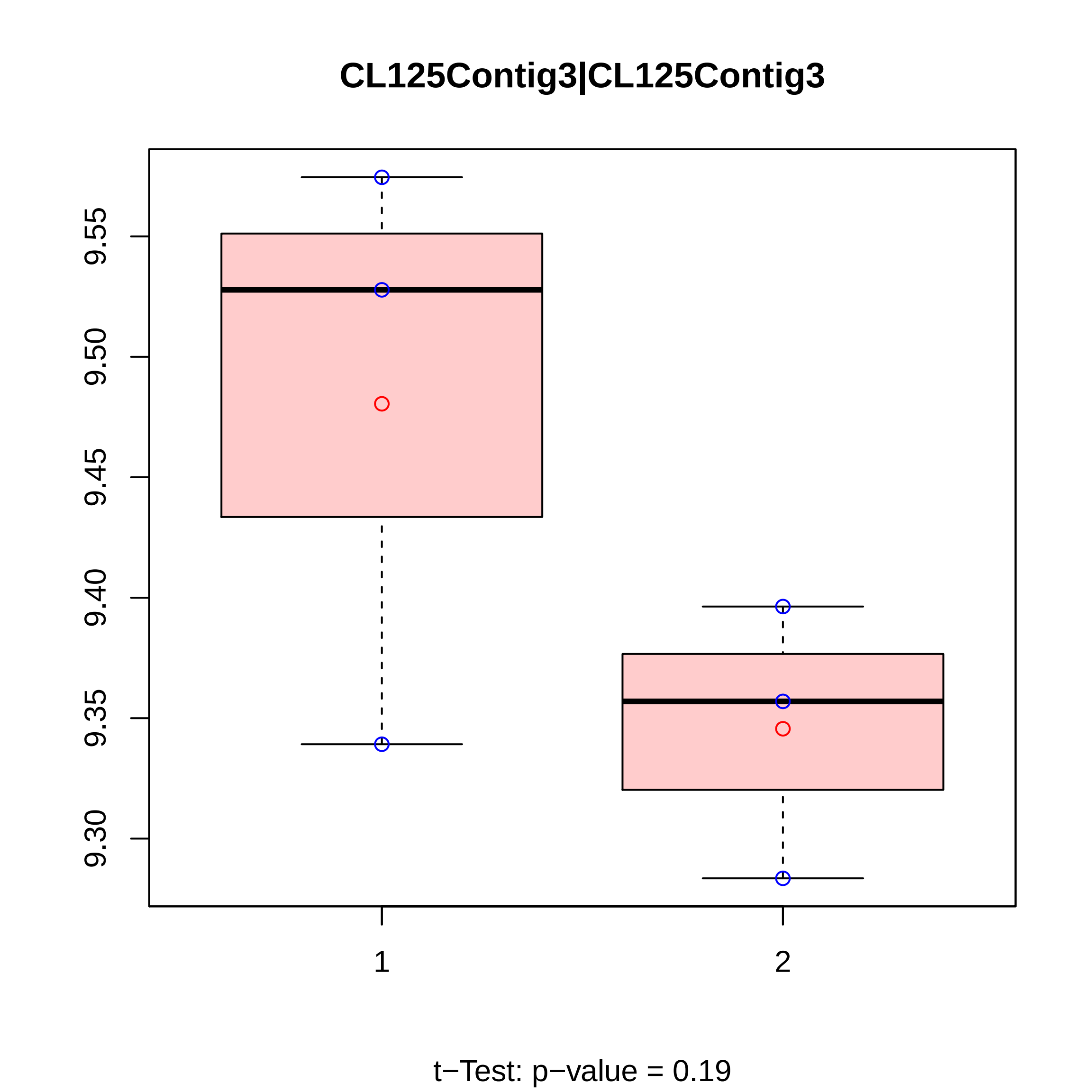

CL125Contig3|CL125Contig3
9.30
9.35
9.40
9.45
9.50
9.55
1
2
t−Test: p−value = 0.19

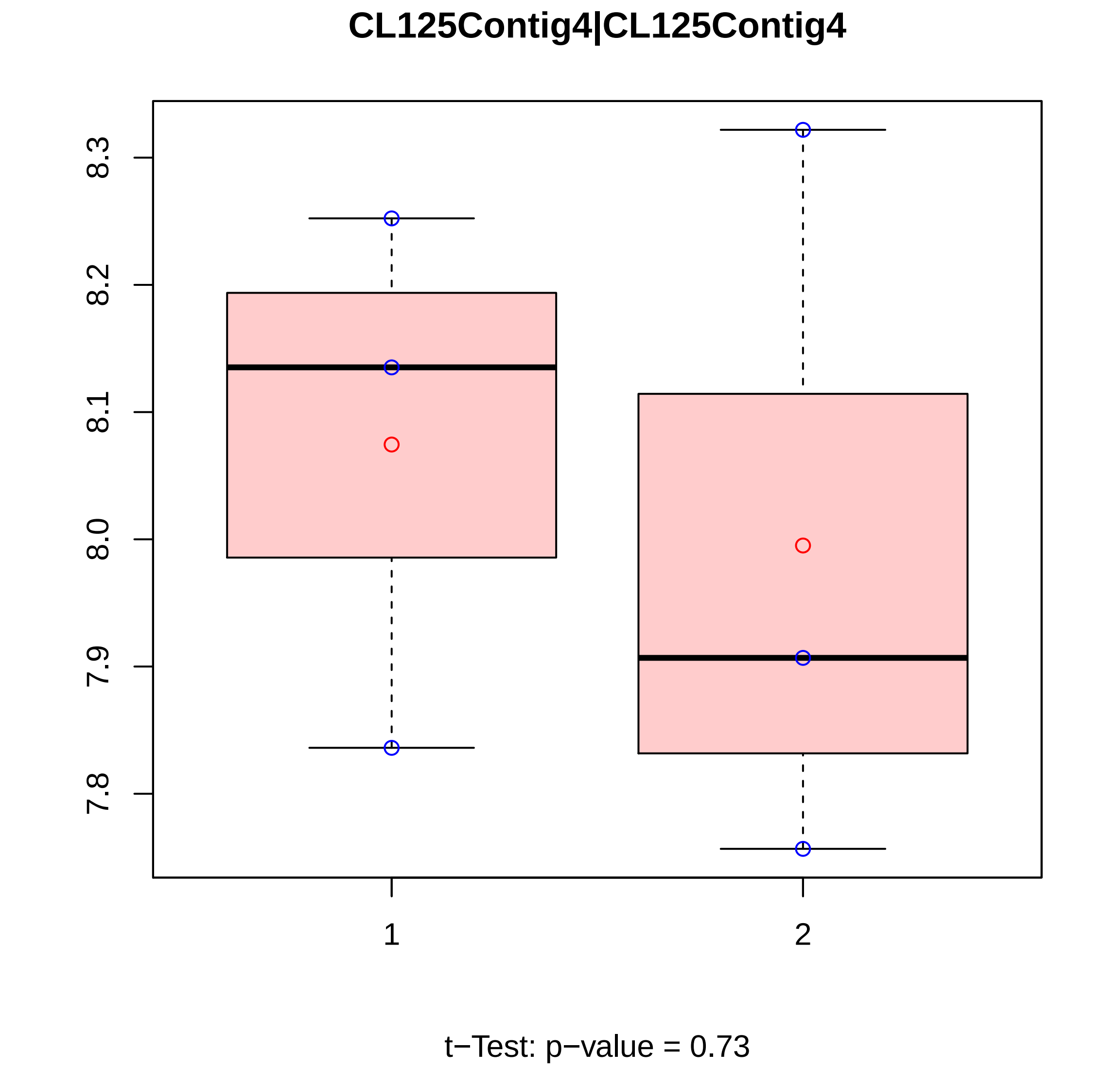
CL125Contig4|CL125Contig4
8.3
8.2
8.1
8.0
7.9
7.8
1
2
t−Test: p−value = 0.73

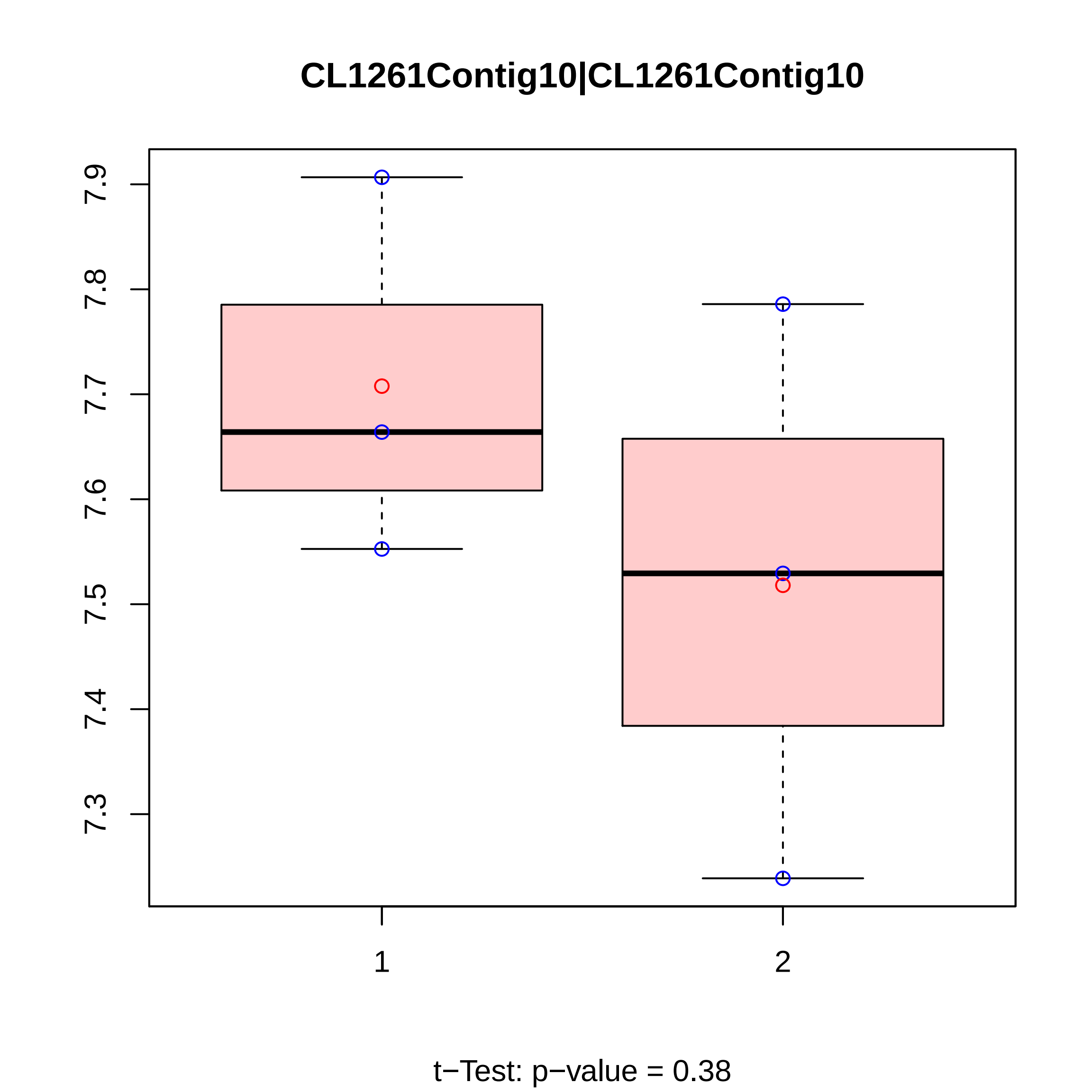
CL1261Contig10|CL1261Contig10
7.9
7.8
7.7
7.6
7.5
7.4
7.3
1
2
t−Test: p−value = 0.38

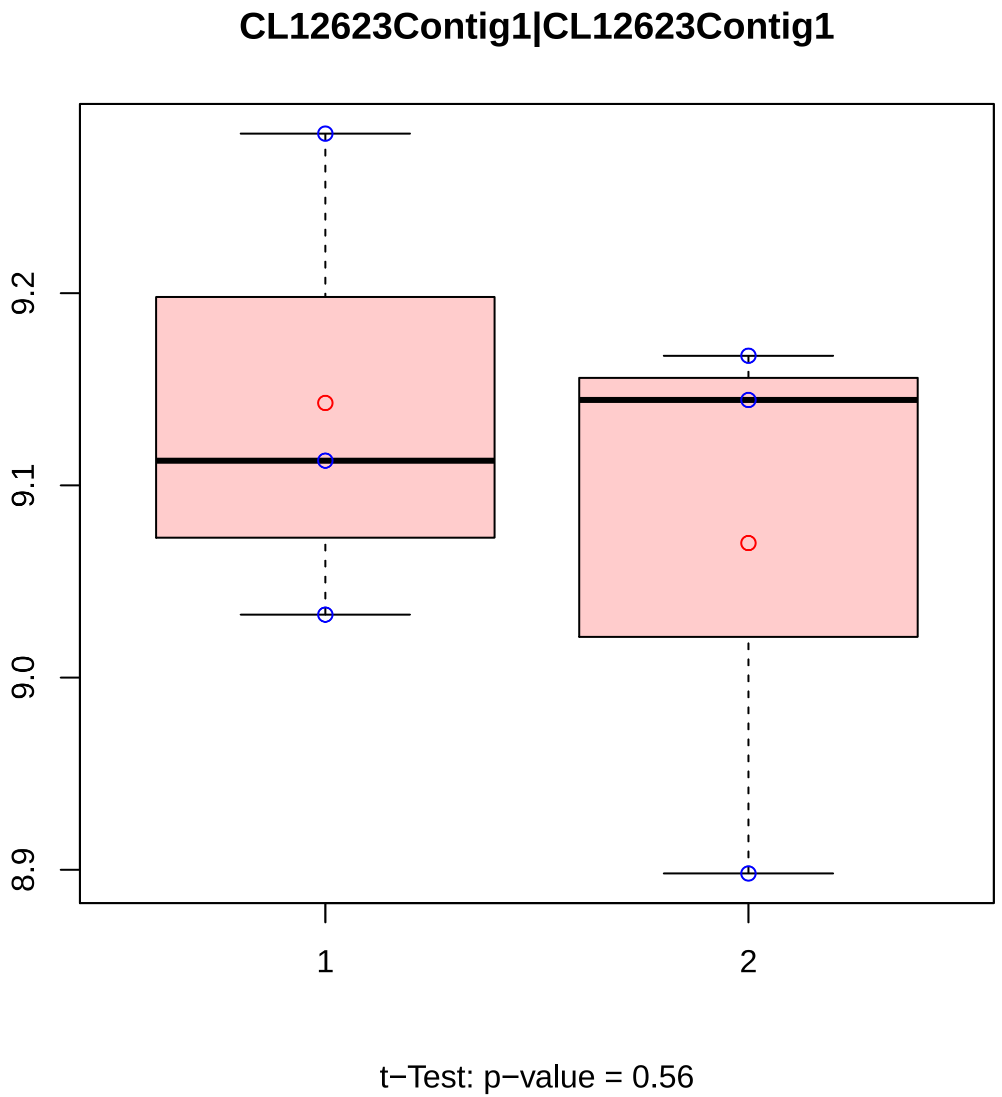
CL12623Contig1|CL12623Contig1
9.2
9.1
9.0
8.9
1
2
t−Test: p−value = 0.56

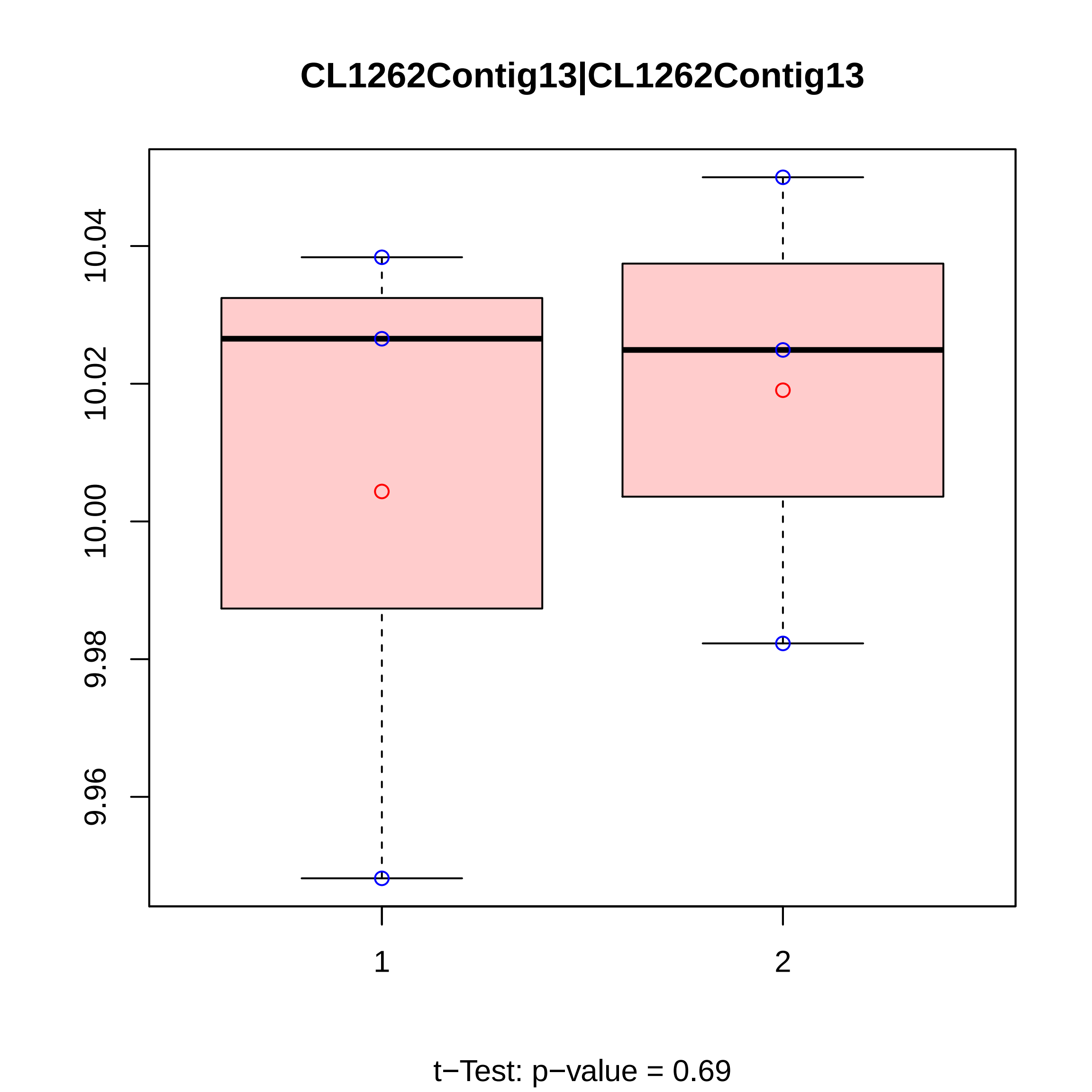
CL1262Contig13|CL1262Contig13
10.04
10.02
10.00
9.98
9.96
1
2
t−Test: p−value = 0.69

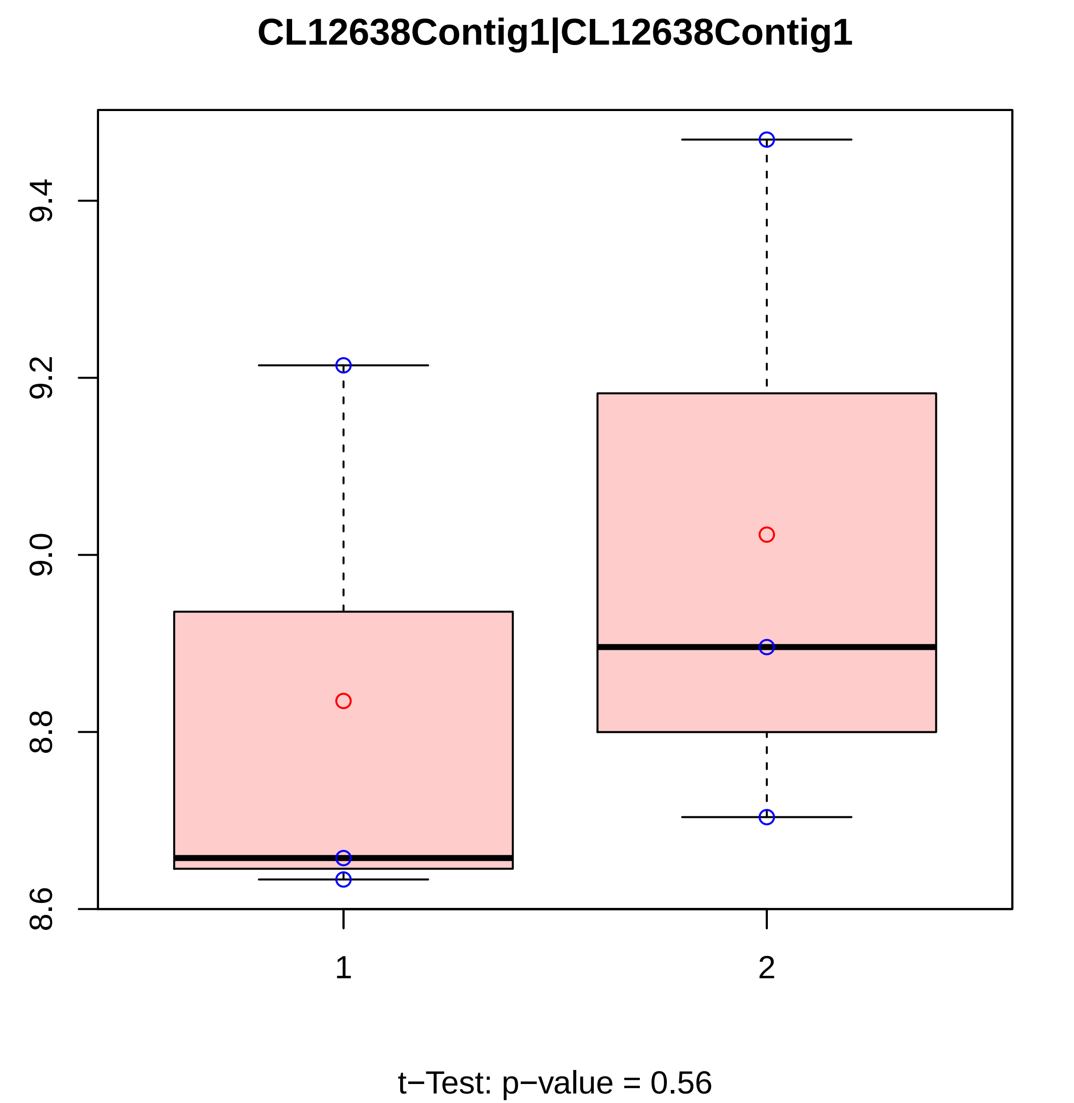
CL12638Contig1|CL12638Contig1
9.4
9.2
9.0
8.8
8.6
1
2
t−Test: p−value = 0.56

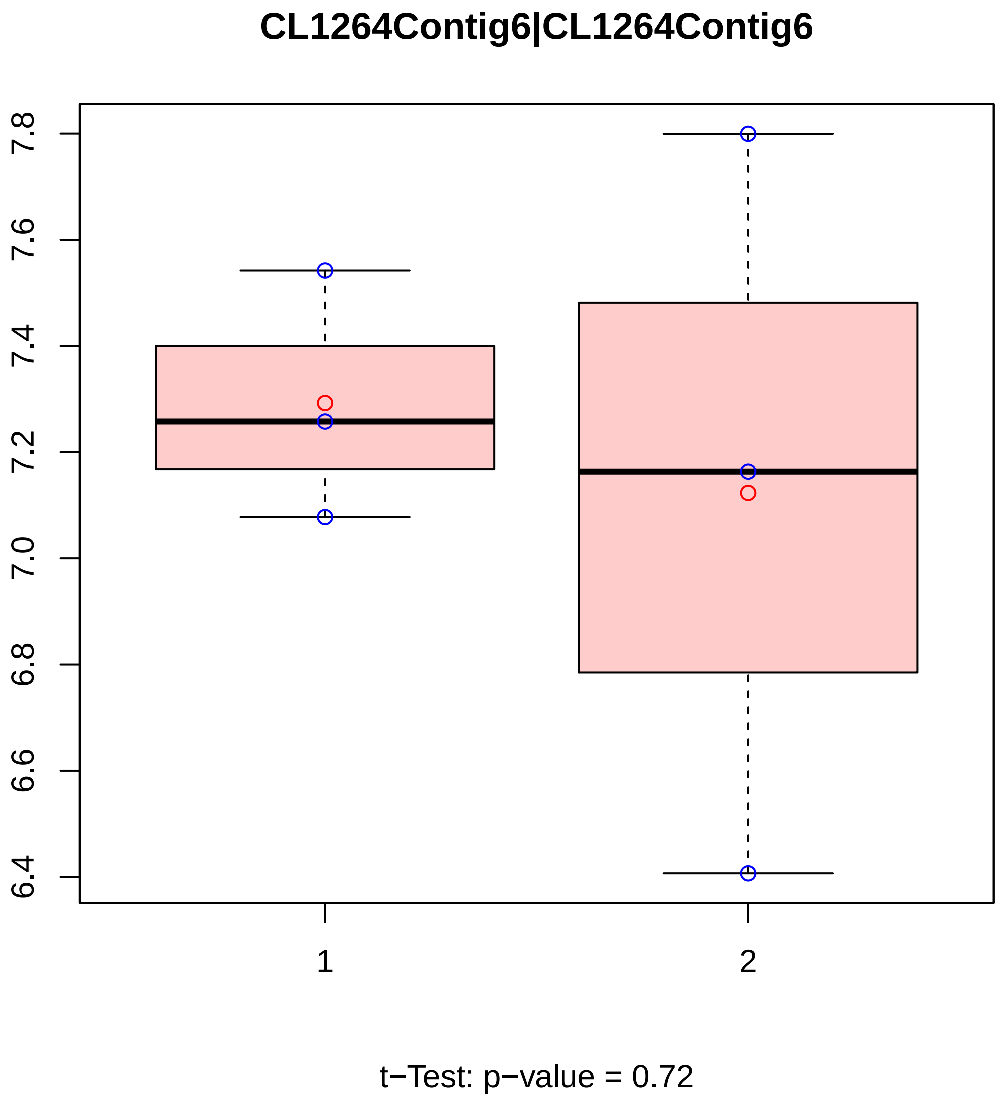
CL1264Contig6|CL1264Contig6
7.8
7.6
7.4
7.2
7.0
6.8
6.6
6.4
1
2
t−Test: p−value = 0.72

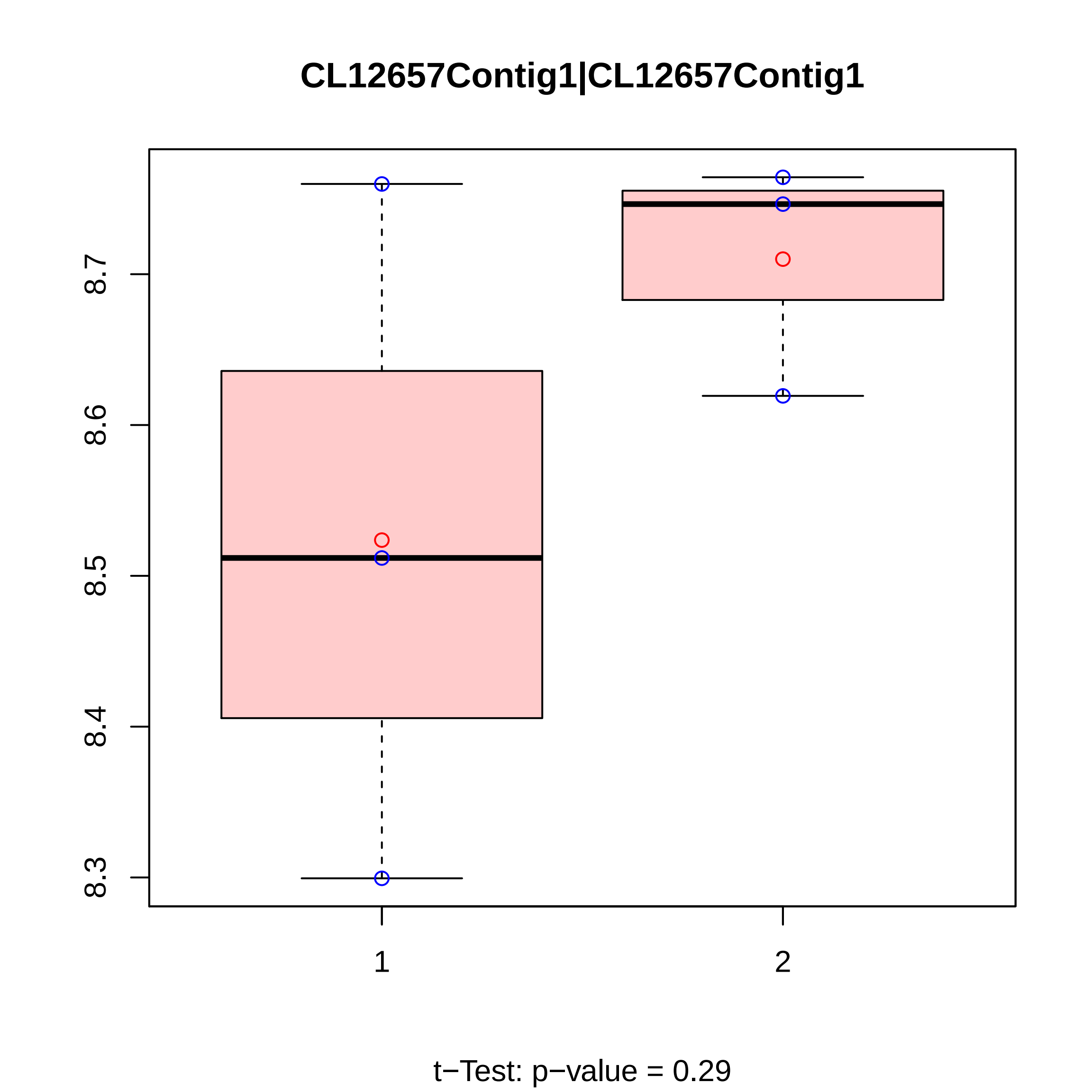
CL12657Contig1|CL12657Contig1
8.3
8.4
8.5
8.6
8.7
1
2
t−Test: p−value = 0.29

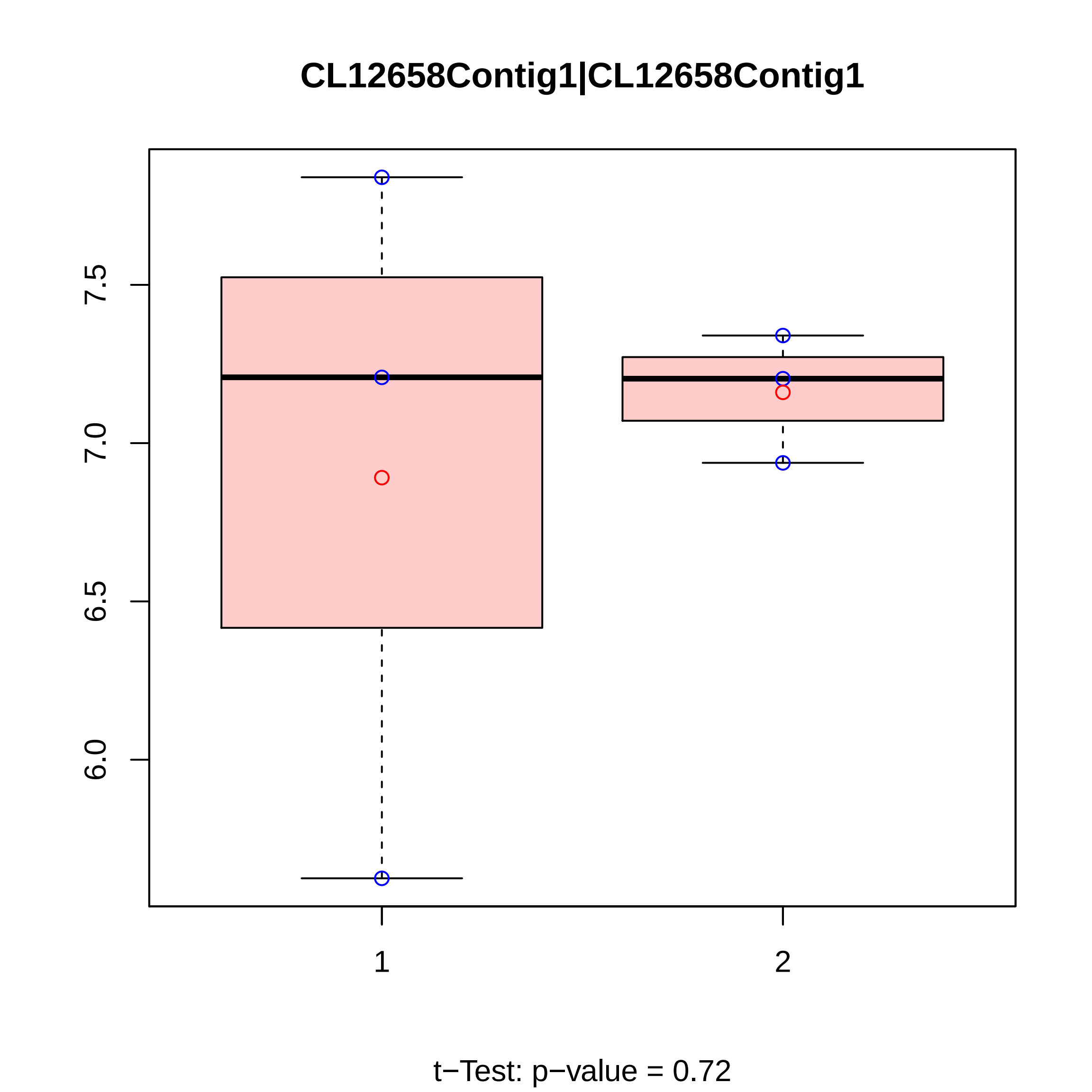
CL12658Contig1|CL12658Contig1
7.5
7.0
6.5
6.0
1
2
t−Test: p−value = 0.72

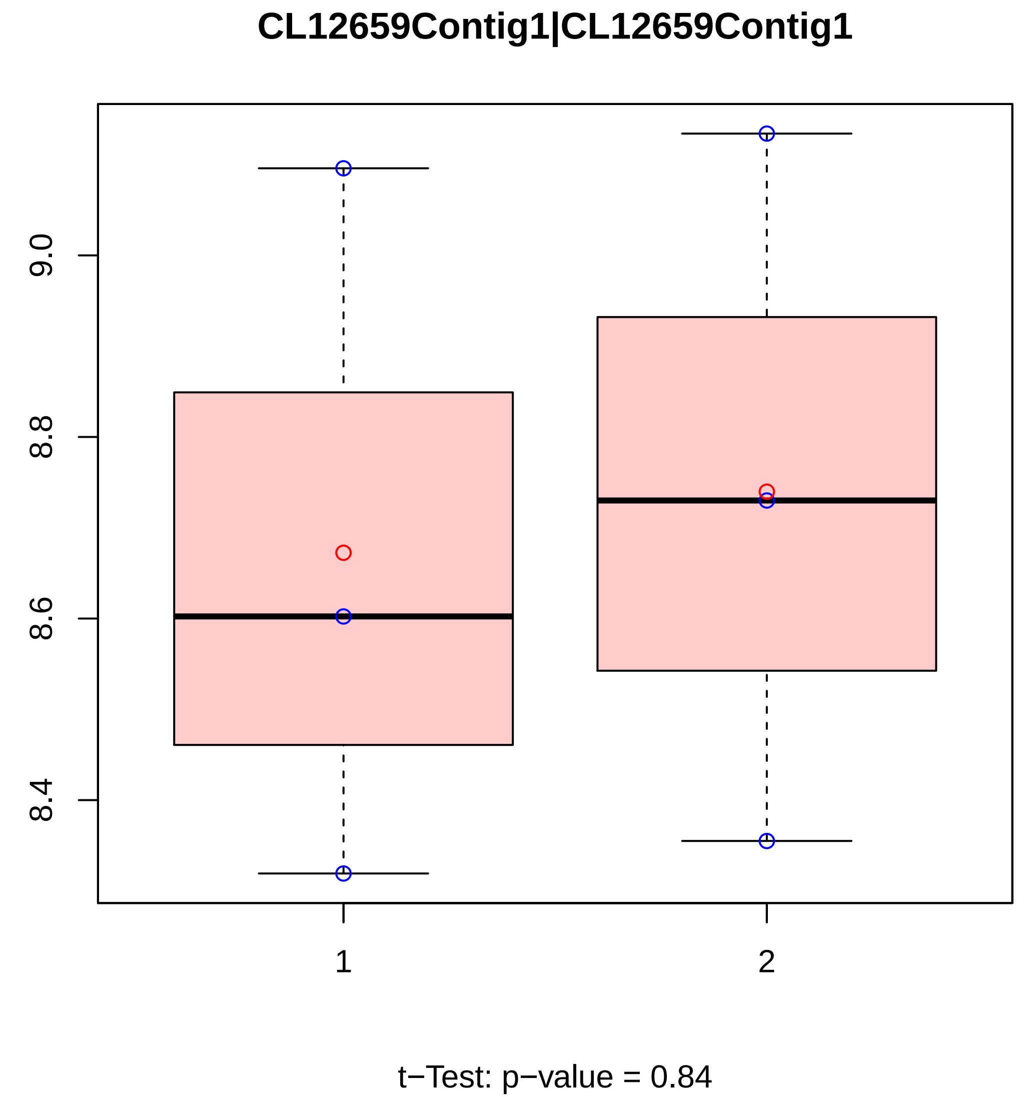
CL12659Contig1|CL12659Contig1
9.0
8.8
8.6
8.4
1
2
t−Test: p−value = 0.84

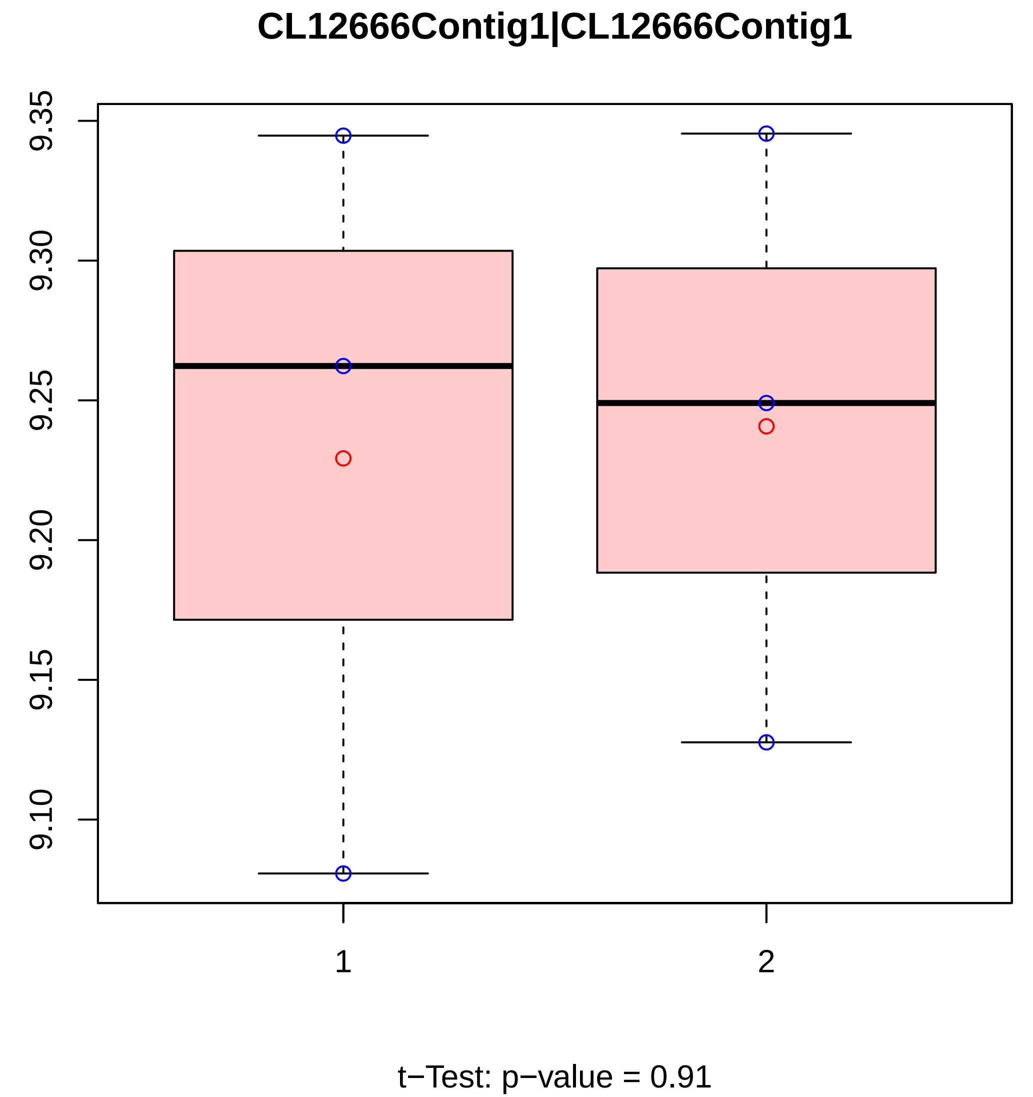
CL12666Contig1|CL12666Contig1
9.35
9.30
9.25
9.20
9.15
9.10
1
2
t−Test: p−value = 0.91

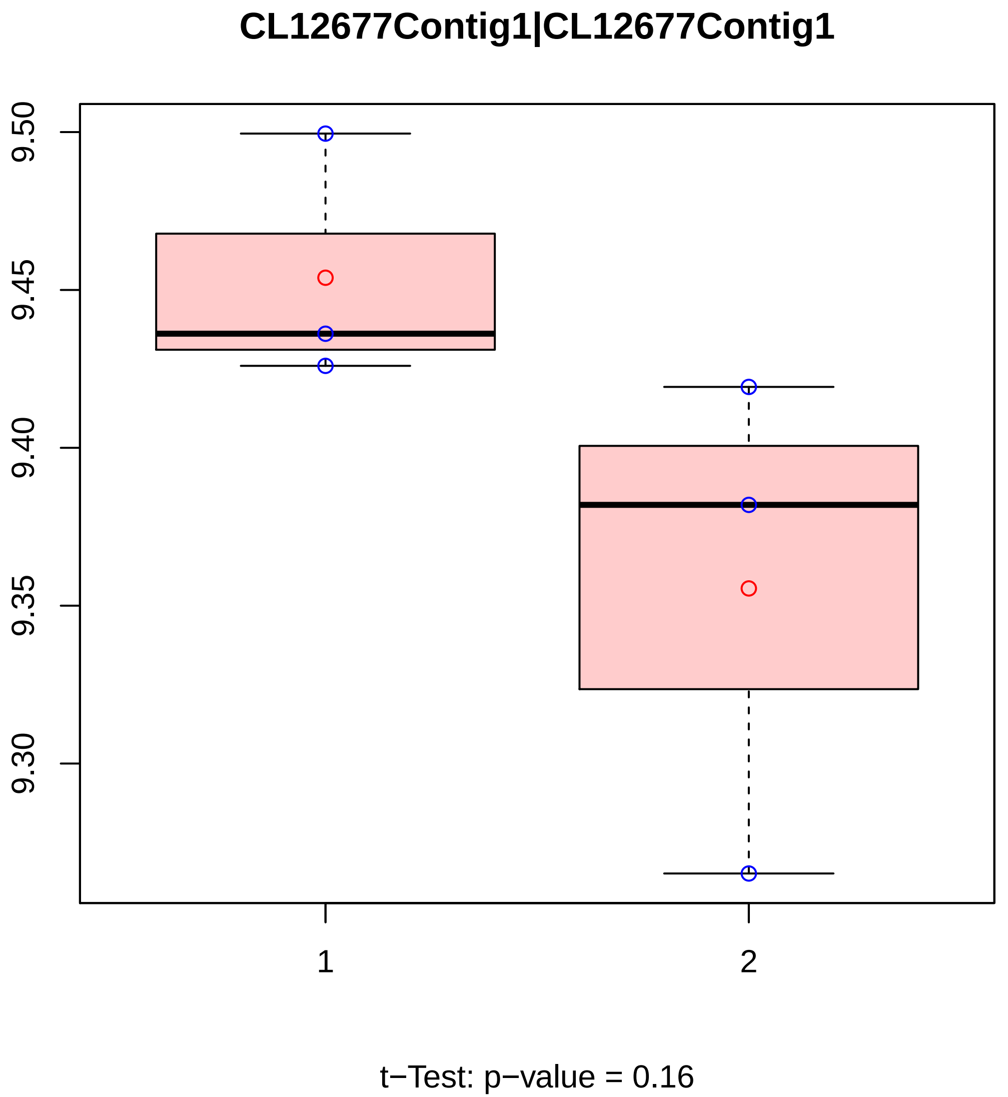
CL12677Contig1|CL12677Contig1
9.50
9.45
9.40
9.35
9.30
1
2
t−Test: p−value = 0.16

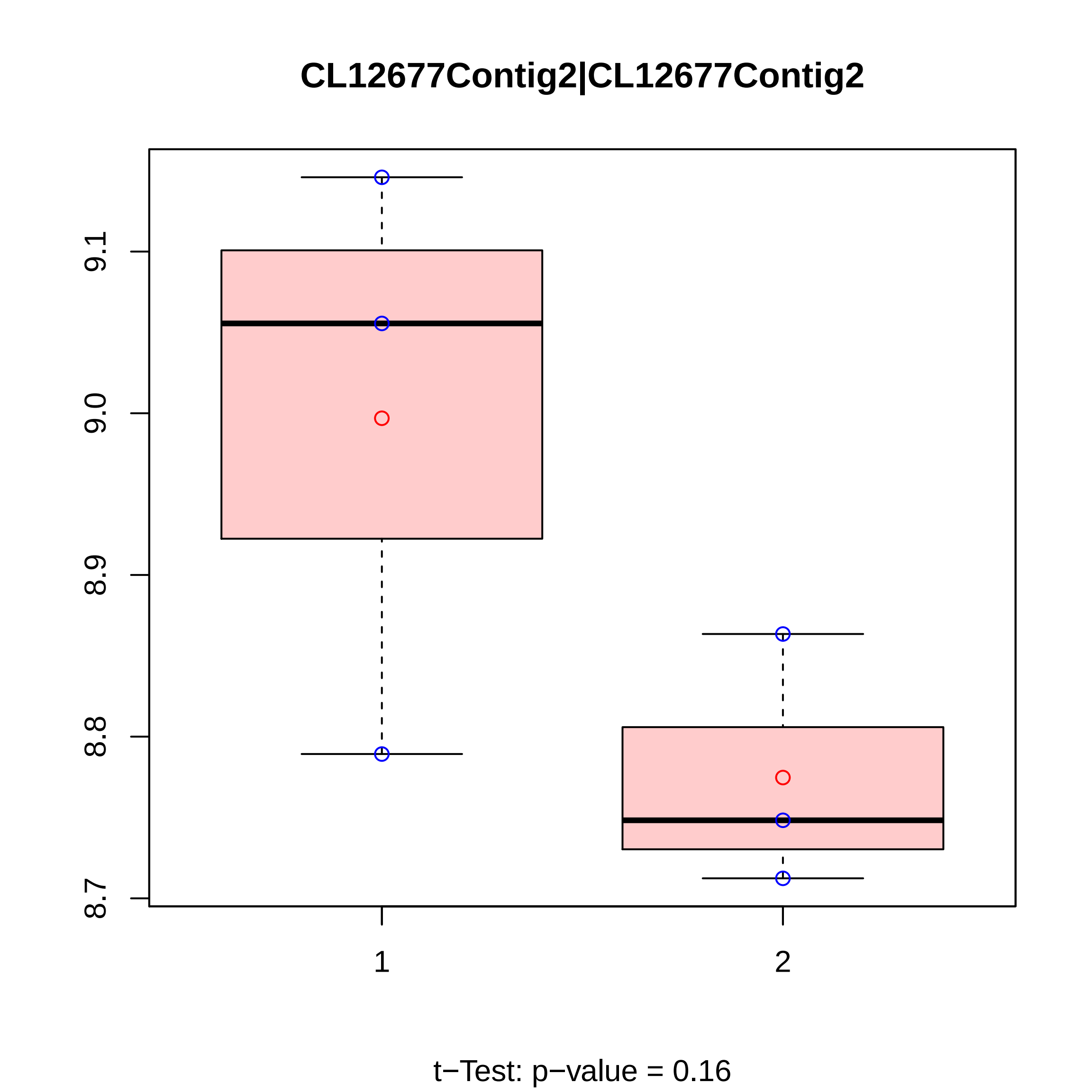

CL12677Contig2|CL12677Contig2
9.1
9.0
8.9
8.8
8.7
1
2
t−Test: p−value = 0.16

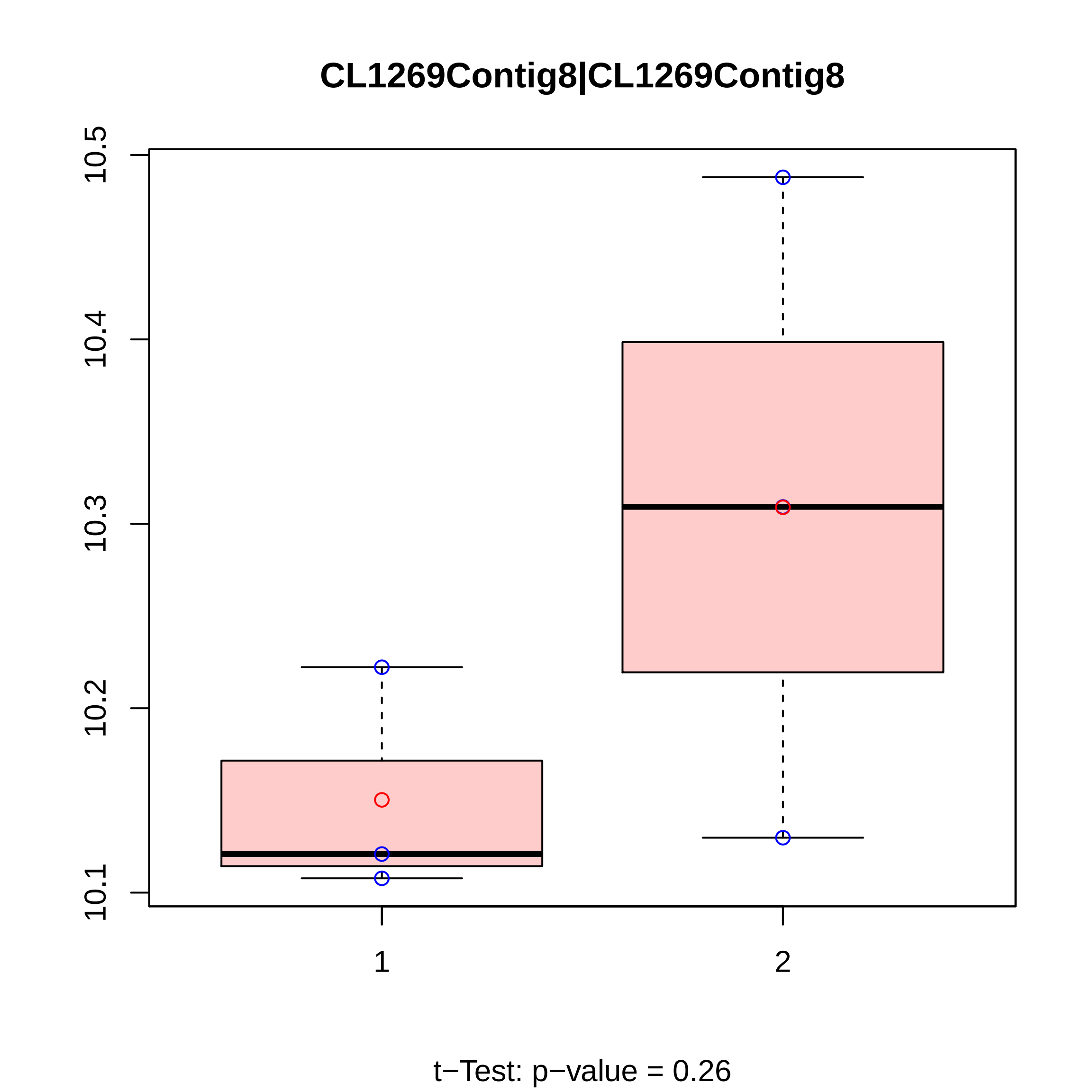
CL1269Contig8|CL1269Contig8
10.1
10.2
10.3
10.4
10.5
1
2
t−Test: p−value = 0.26

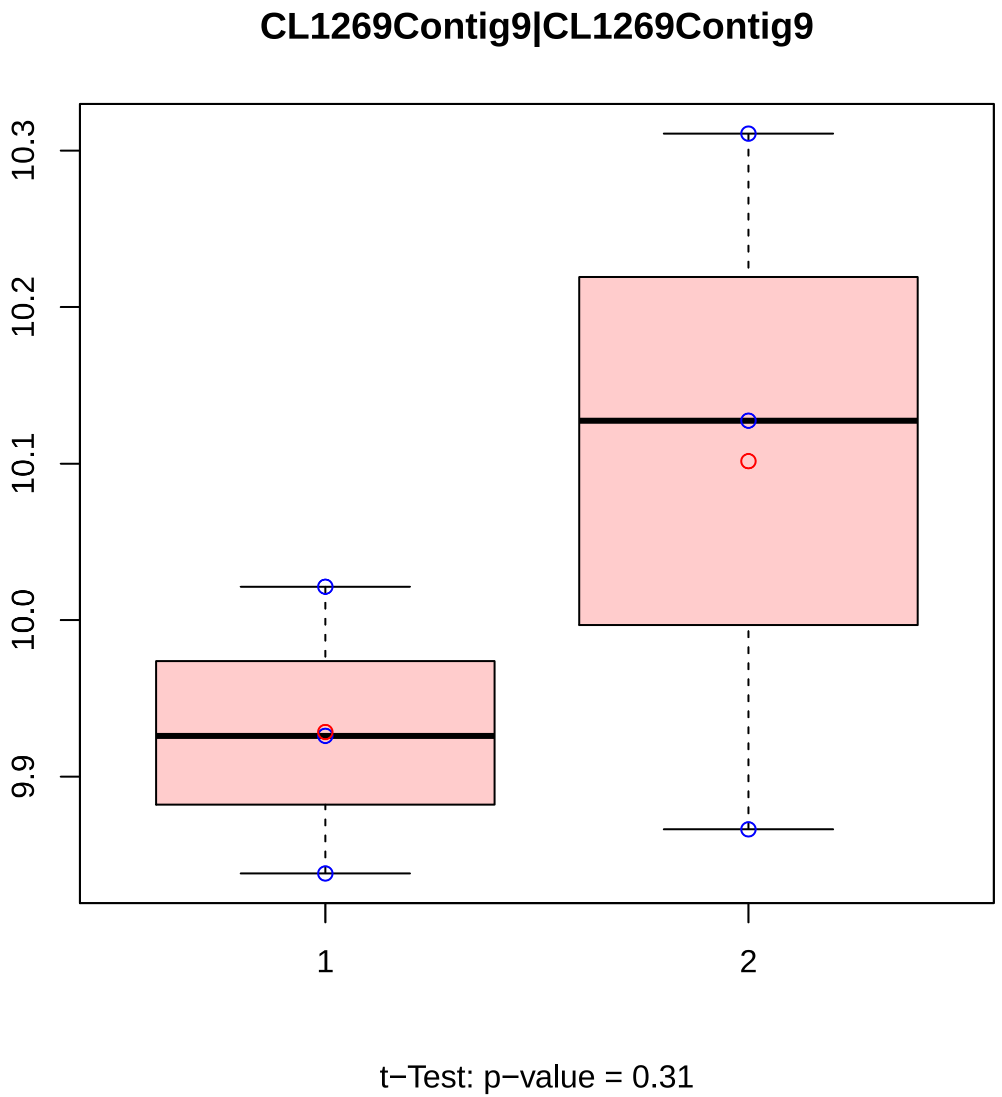
CL1269Contig9|CL1269Contig9
10.3
10.2
10.1
10.0
9.9
1
2
t−Test: p−value = 0.31

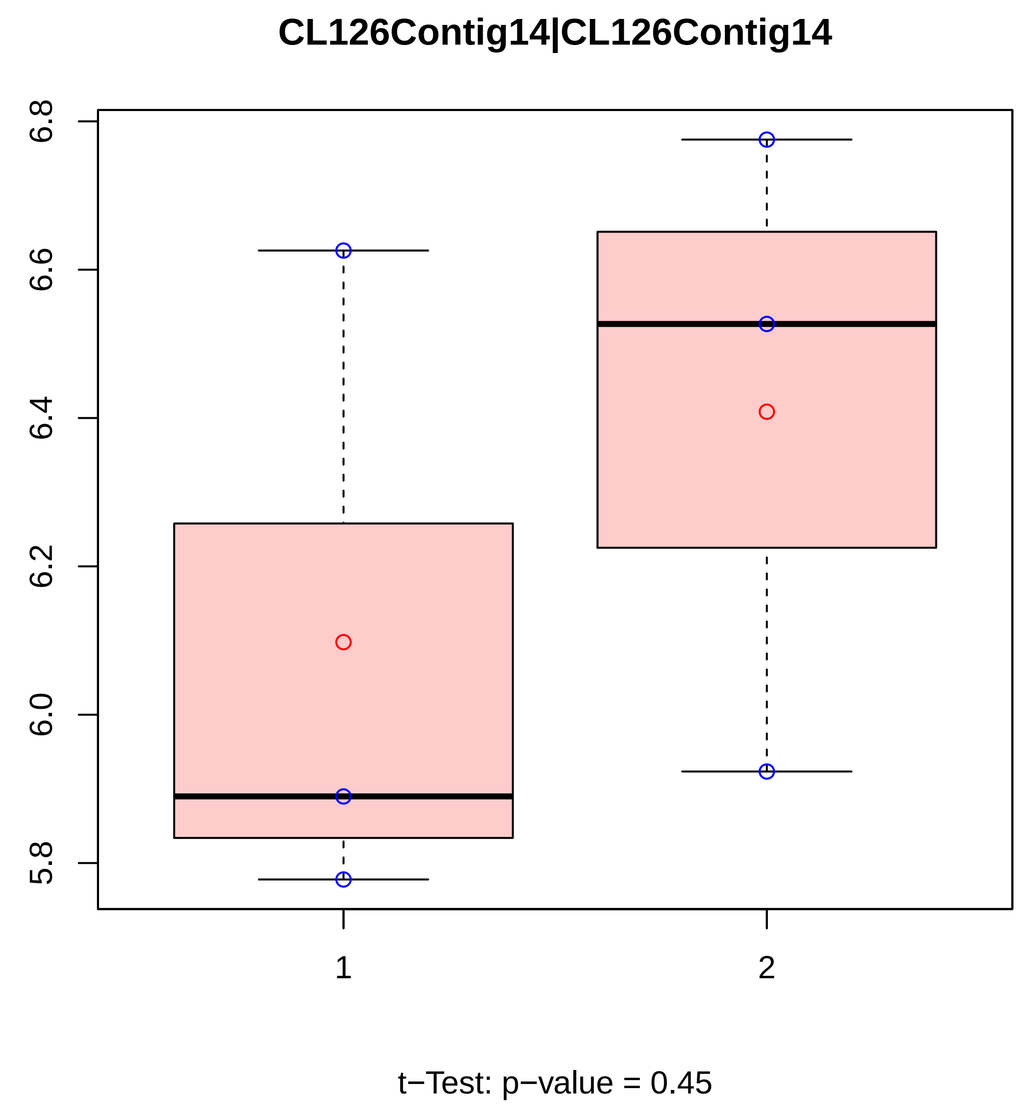

CL126Contig14|CL126Contig14
6.8
6.6
6.4
6.2
6.0
5.8
1
2
t−Test: p−value = 0.45

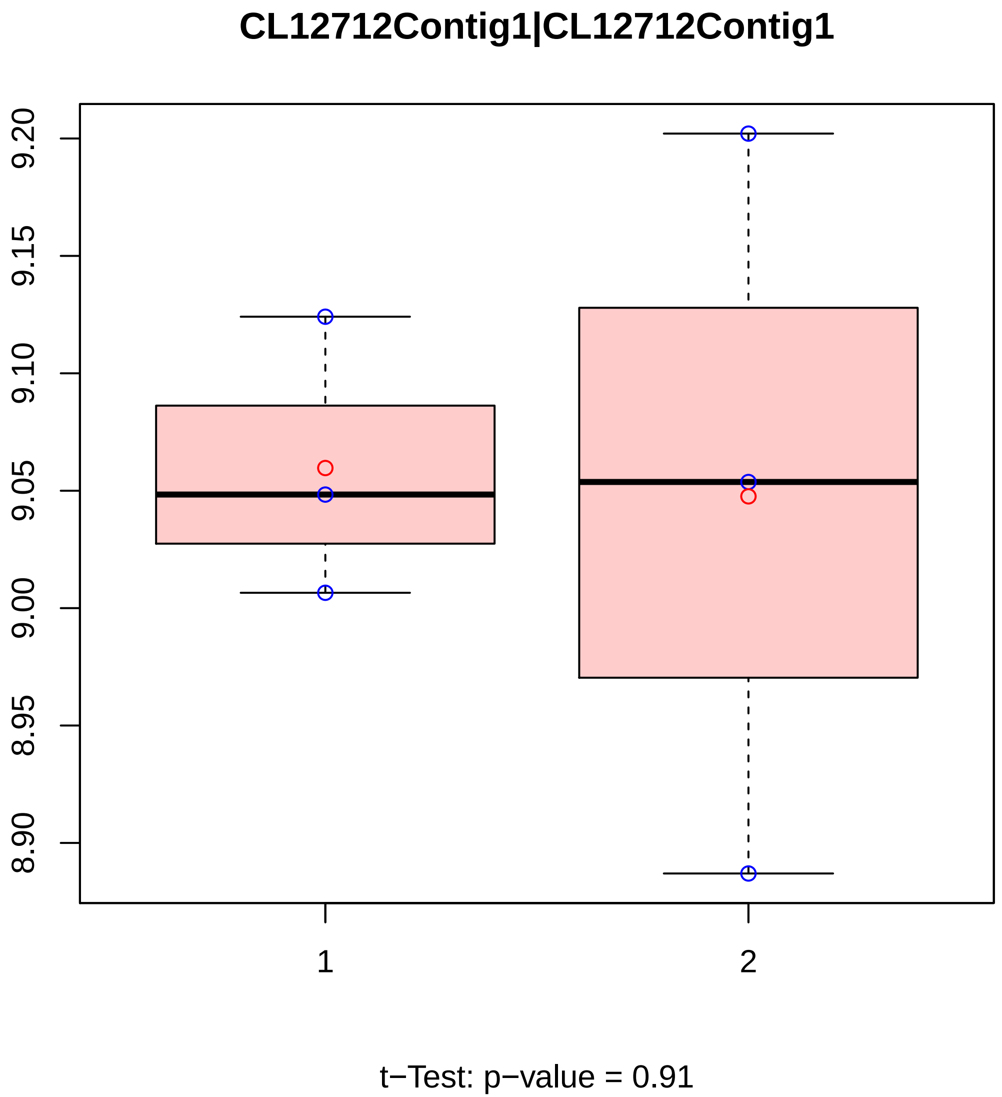
CL12712Contig1|CL12712Contig1
9.20
9.15
9.10
9.05
9.00
8.95
8.90
1
2
t−Test: p−value = 0.91

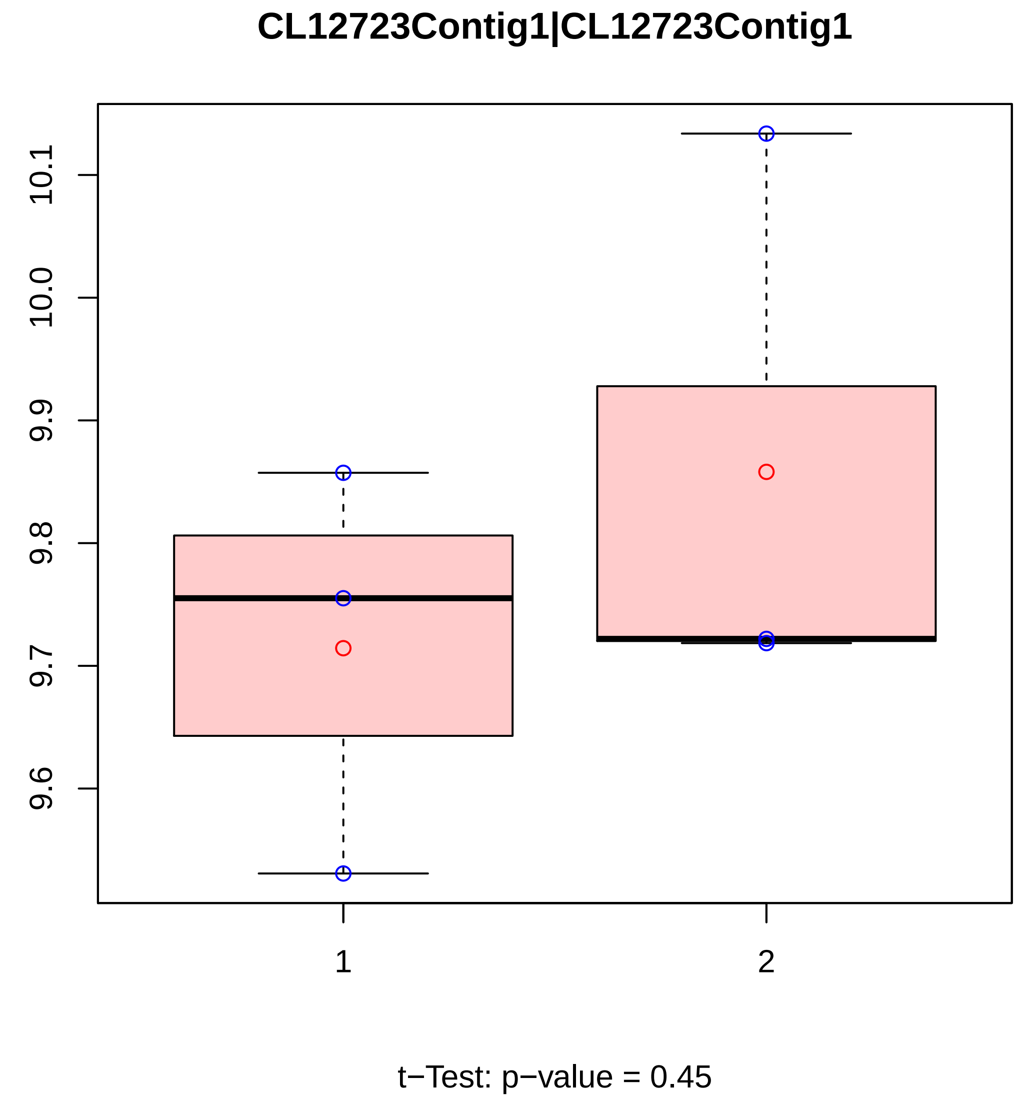
CL12723Contig1|CL12723Contig1
10.1
10.0
9.9
9.8
9.7
9.6
1
2
t−Test: p−value = 0.45

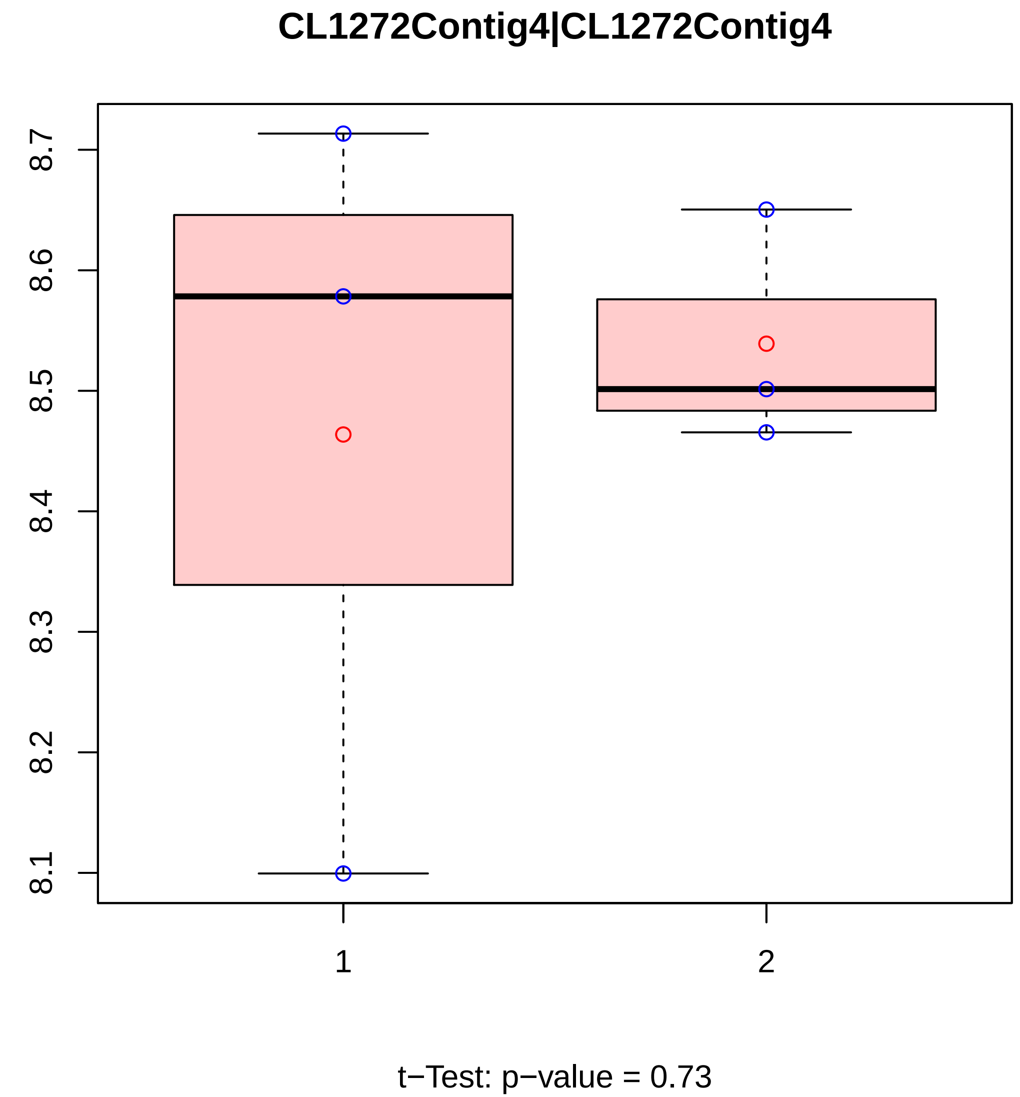
CL1272Contig4|CL1272Contig4
8.1
8.2
8.3
8.4
8.5
8.6
8.7
1
2
t−Test: p−value = 0.73

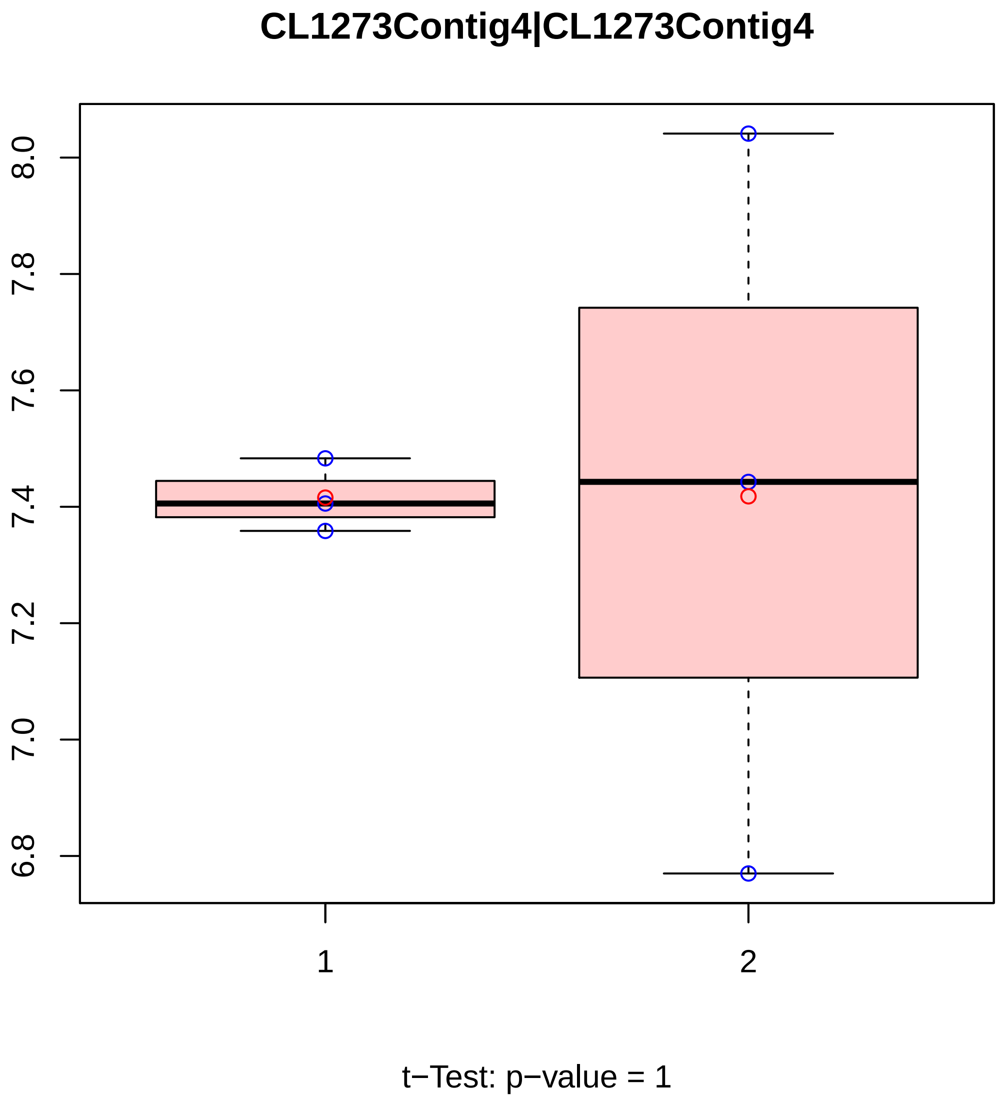

CL1273Contig4|CL1273Contig4
8.0
7.8
7.6
7.4
7.2
7.0
6.8
1
2
t−Test: p−value = 1

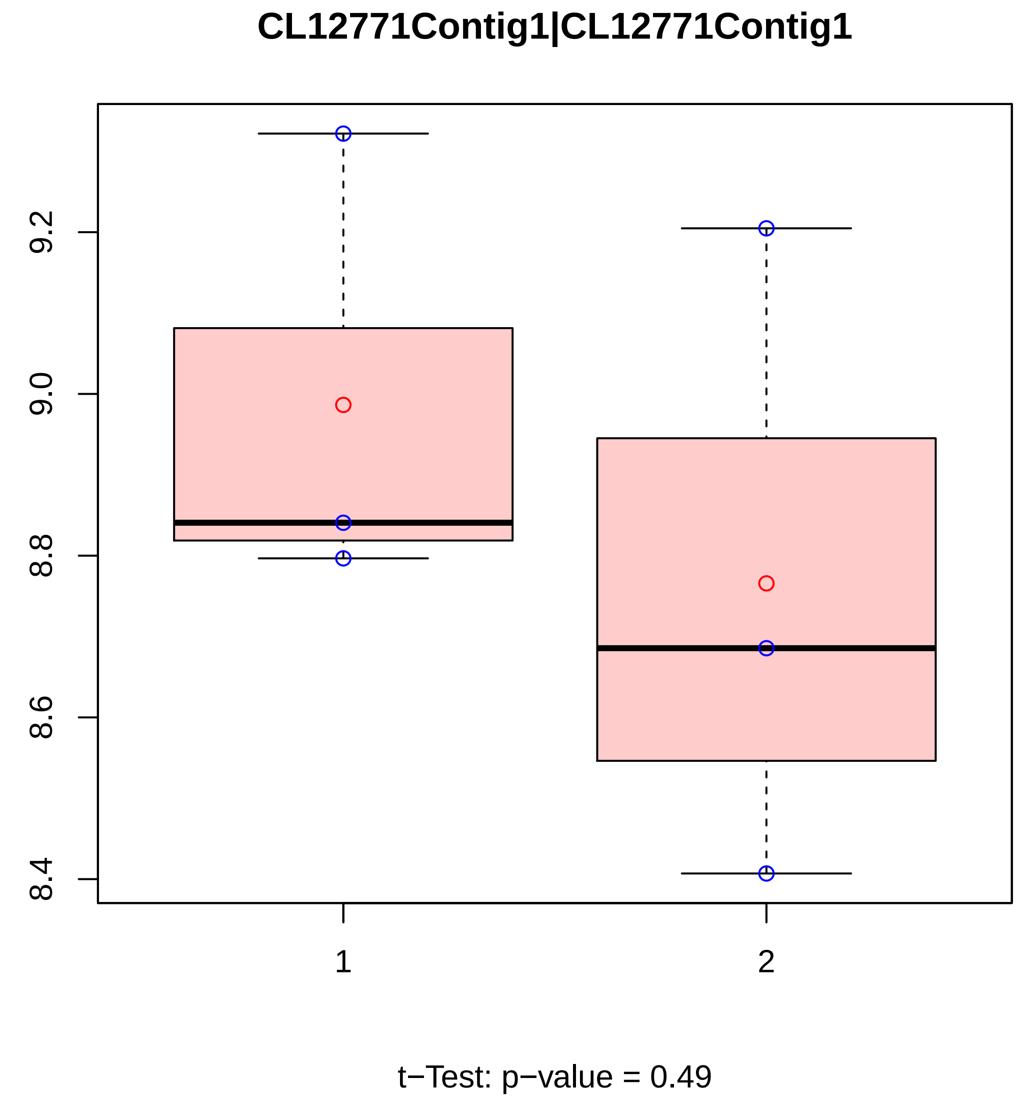

CL12771Contig1|CL12771Contig1
9.2
9.0
8.8
8.6
8.4
1
2
t−Test: p−value = 0.49

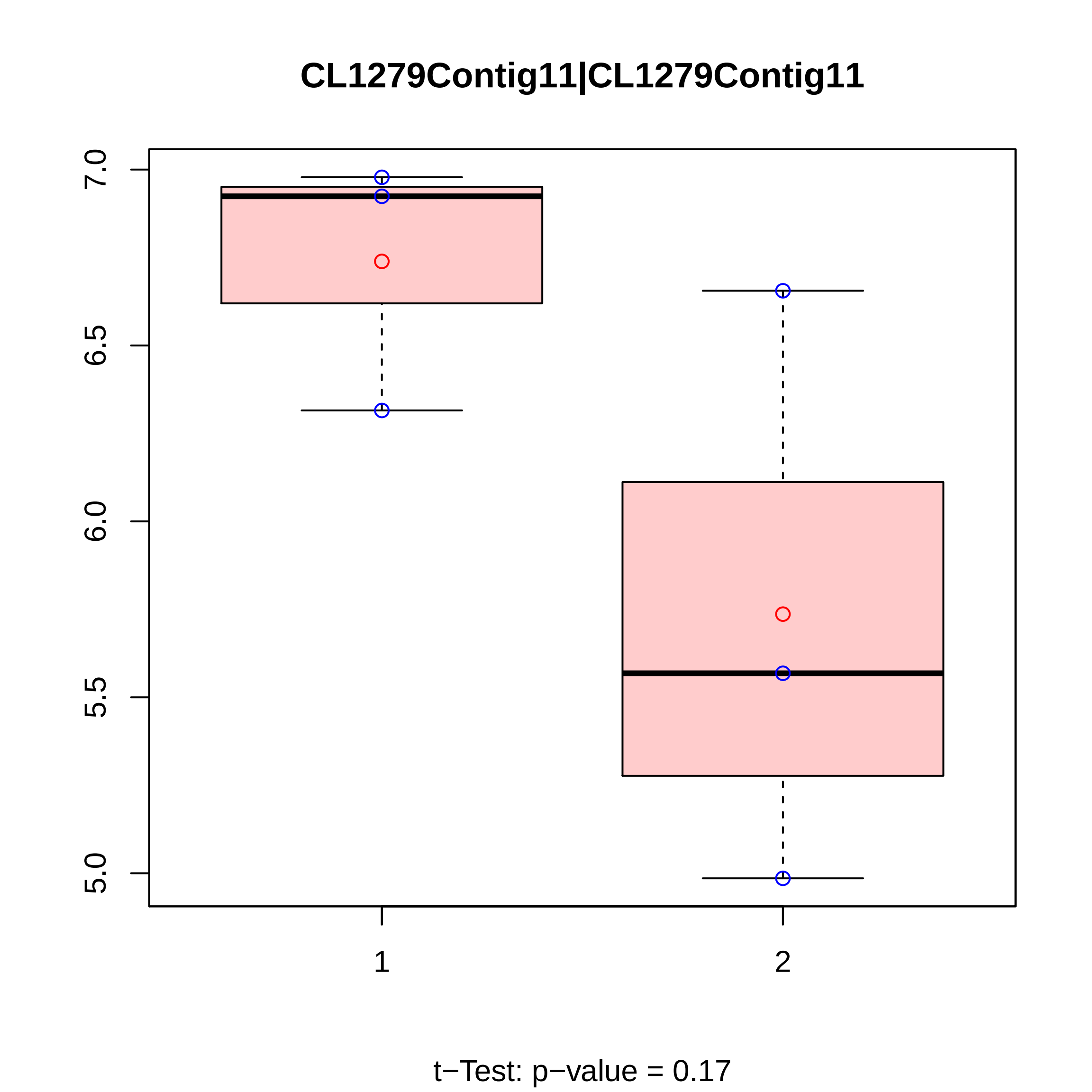
CL1279Contig11|CL1279Contig11
7.0
6.5
6.0
5.5
5.0
1
2
t−Test: p−value = 0.17

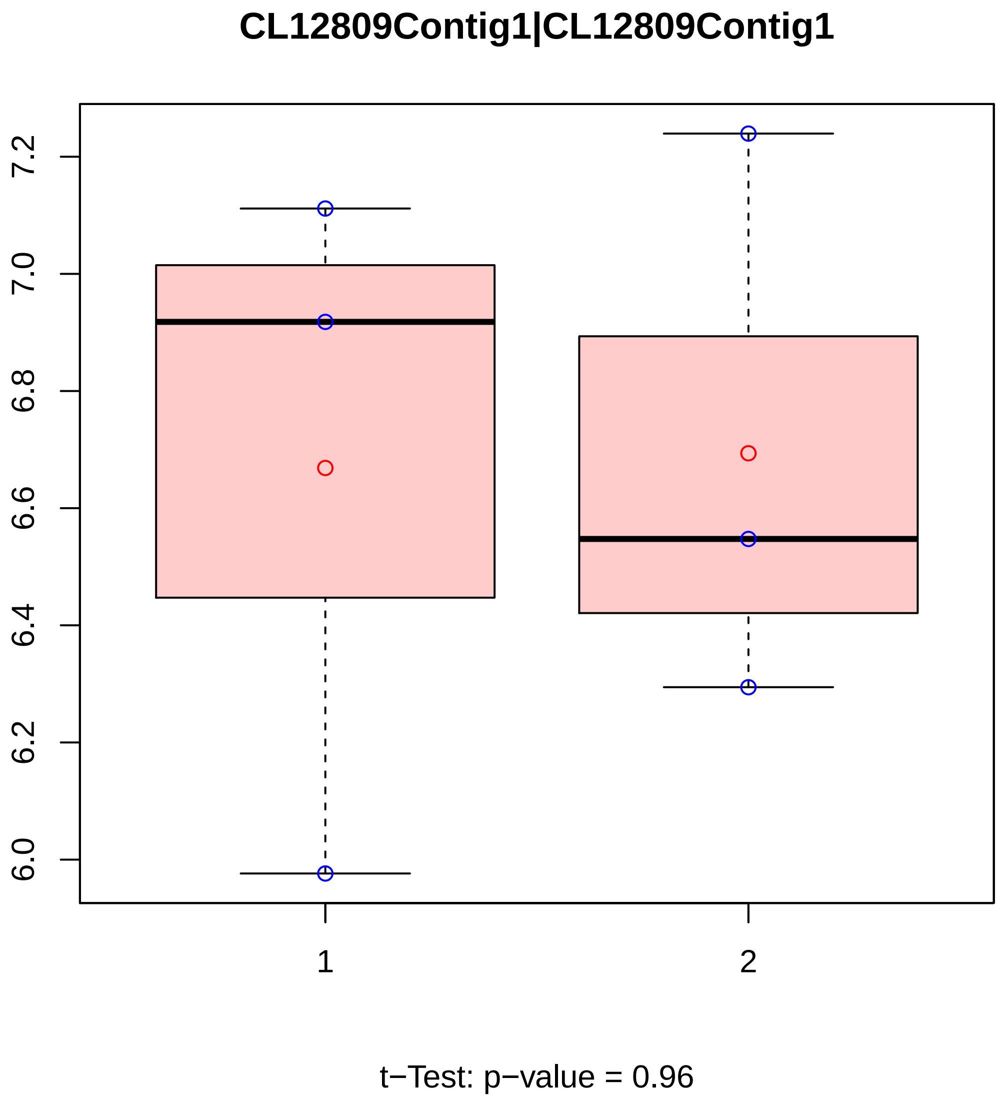
CL12809Contig1|CL12809Contig1
7.2
7.0
6.8
6.6
6.4
6.2
6.0
1
2
t−Test: p−value = 0.96

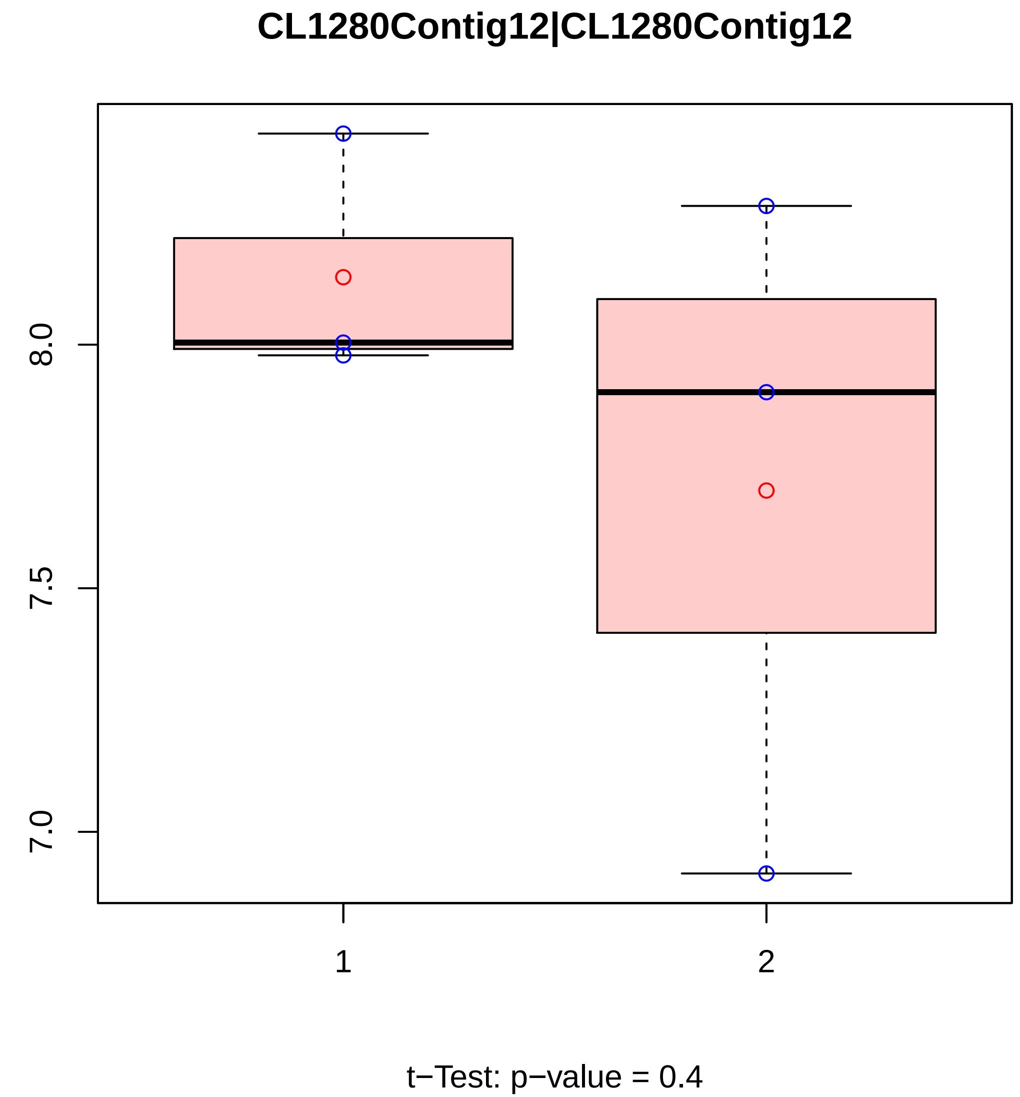
CL1280Contig12|CL1280Contig12
8.0
7.5
7.0
1
2
t−Test: p−value = 0.4

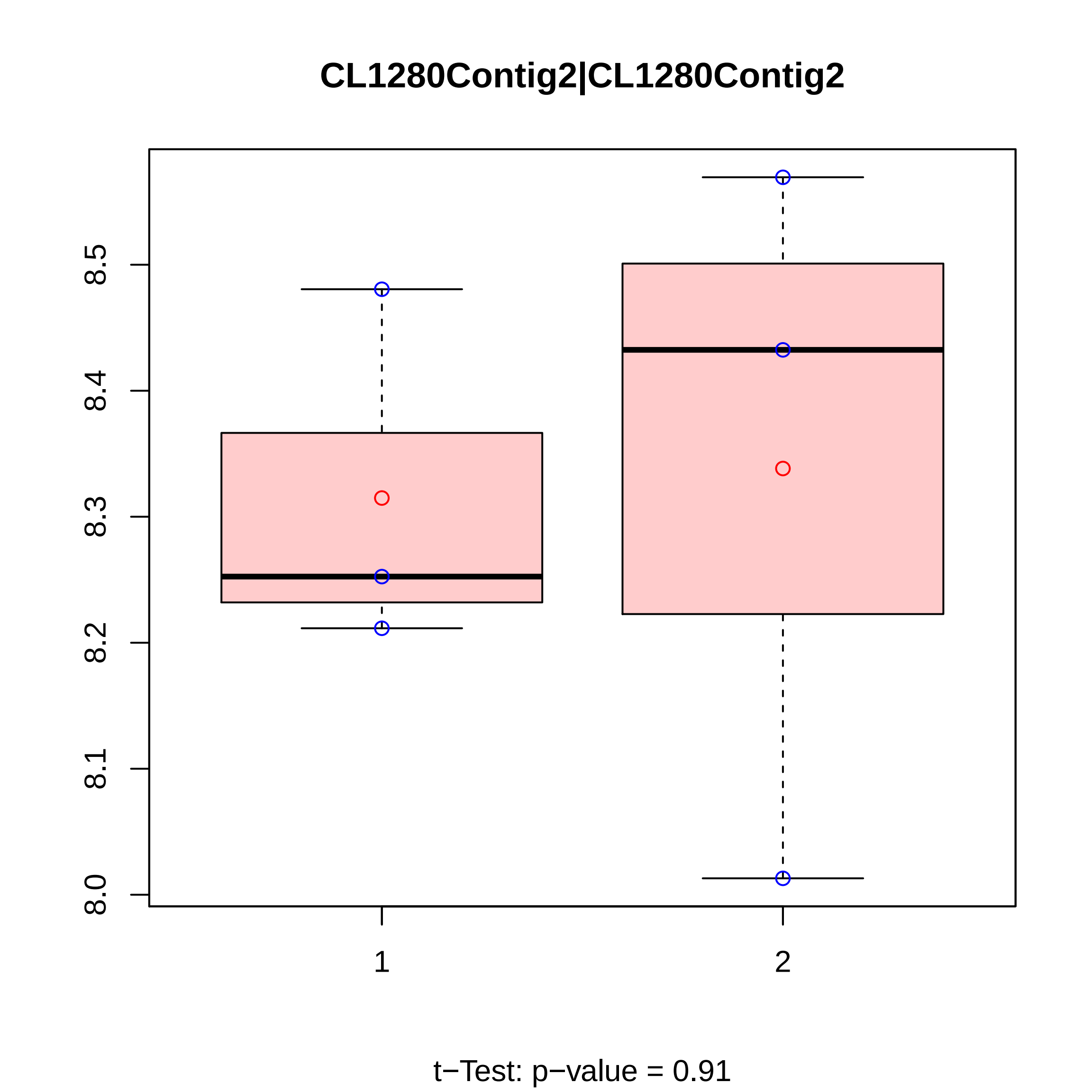

CL1280Contig2|CL1280Contig2
8.0
8.1
8.2
8.3
8.4
8.5
1
2
t−Test: p−value = 0.91

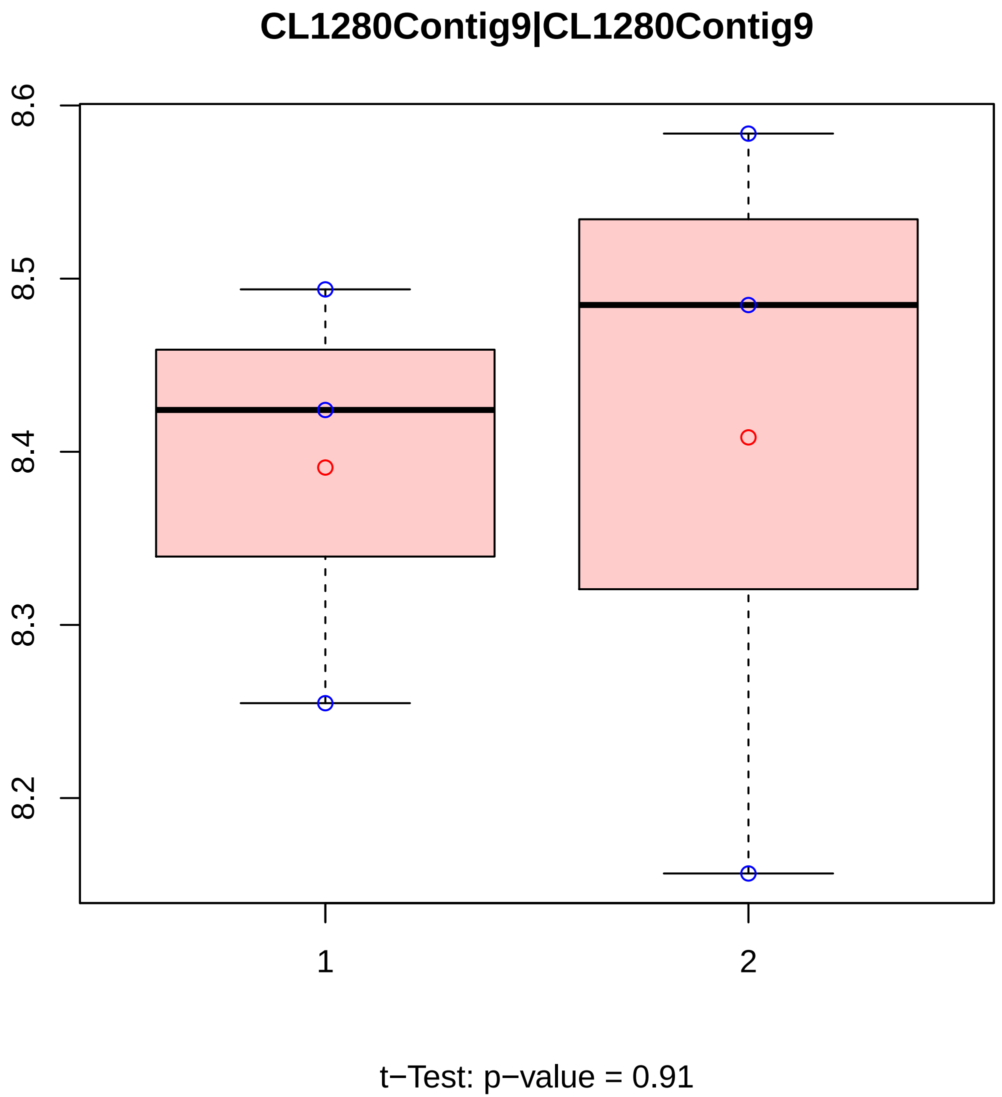

CL1280Contig9|CL1280Contig9
8.2
8.3
8.4
8.5
8.6
1
2
t−Test: p−value = 0.91

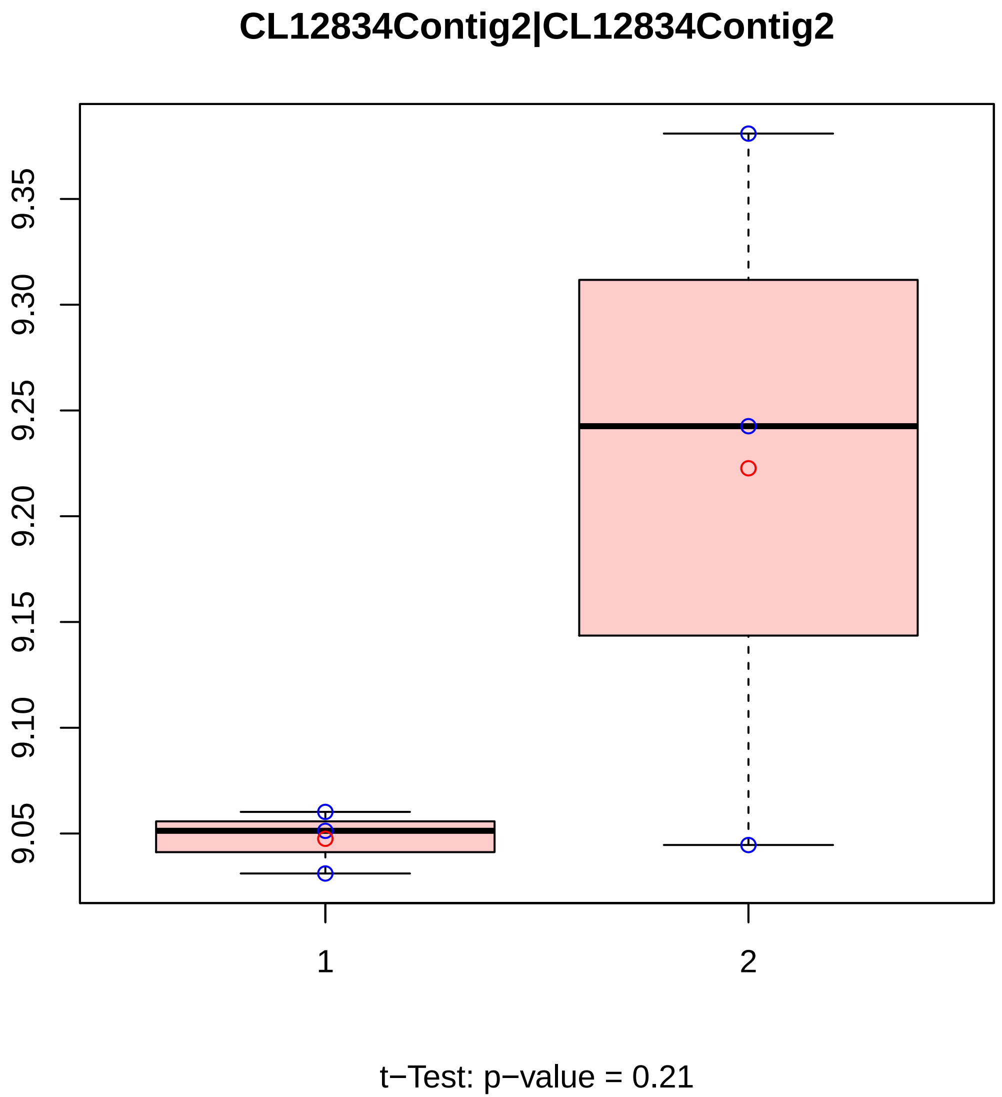
CL12834Contig2|CL12834Contig2
9.05
9.10
9.15
9.20
9.25
9.30
9.35
1
2
t−Test: p−value = 0.21

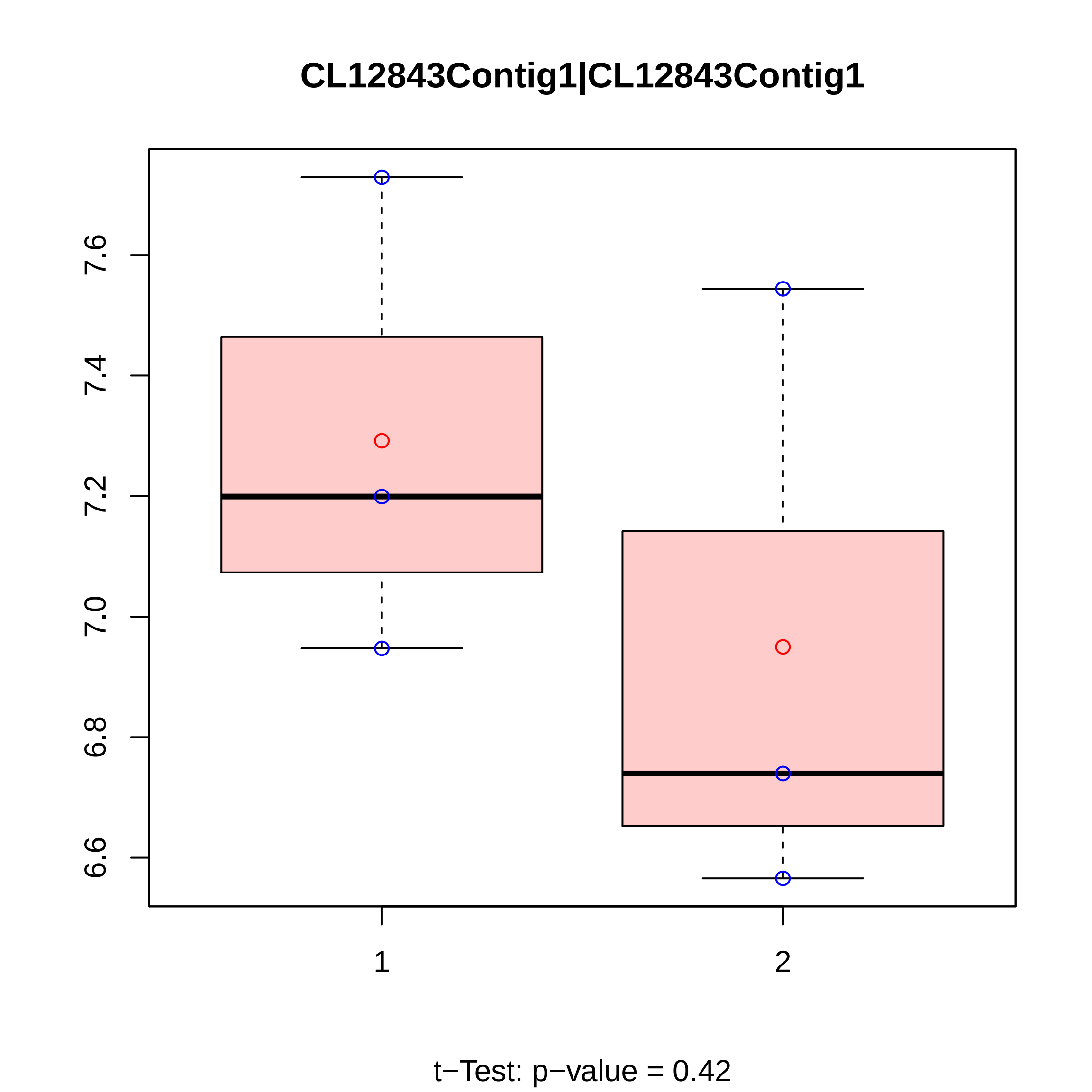
CL12843Contig1|CL12843Contig1
7.6
7.4
7.2
7.0
6.8
6.6
1
2
t−Test: p−value = 0.42

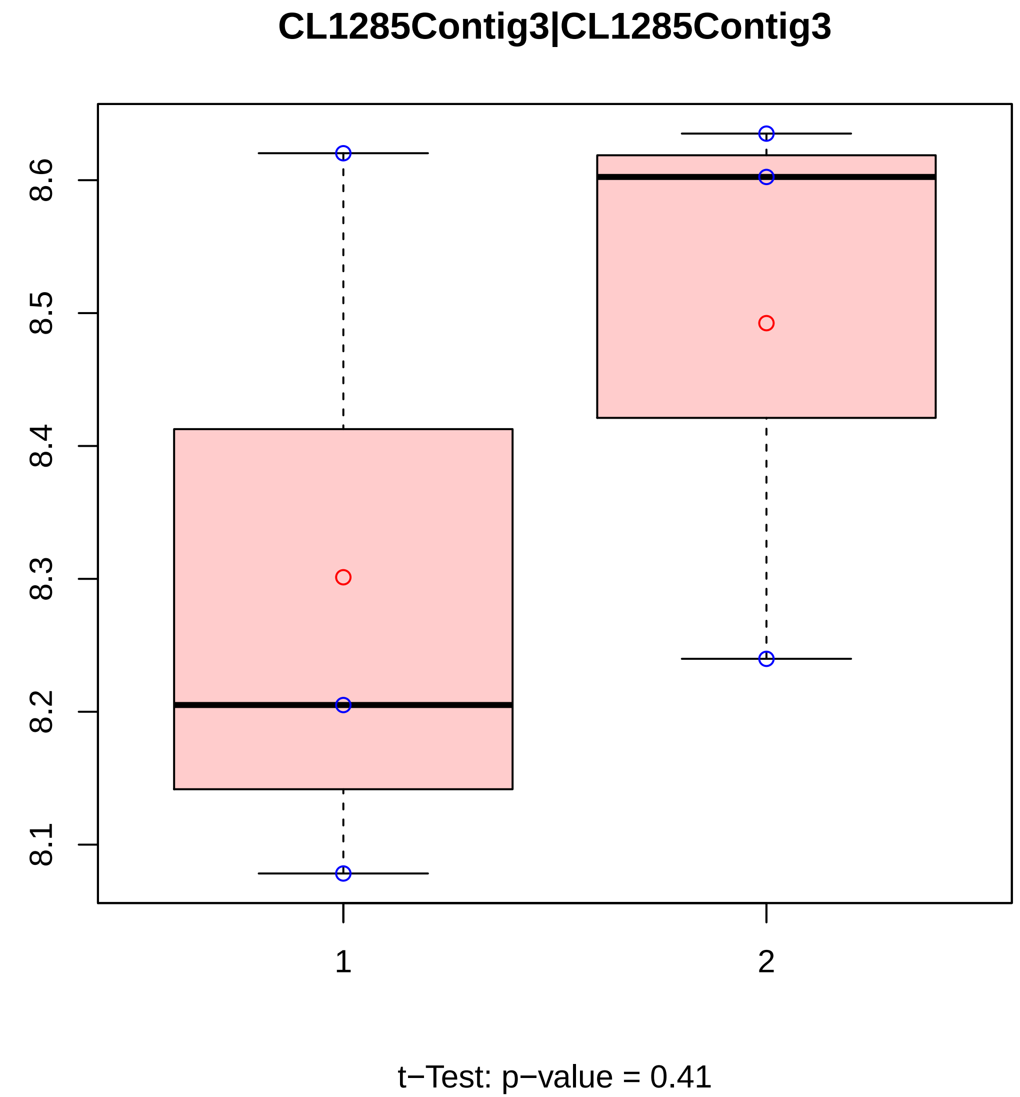

CL1285Contig3|CL1285Contig3
8.1
8.2
8.3
8.4
8.5
8.6
1
2
t−Test: p−value = 0.41

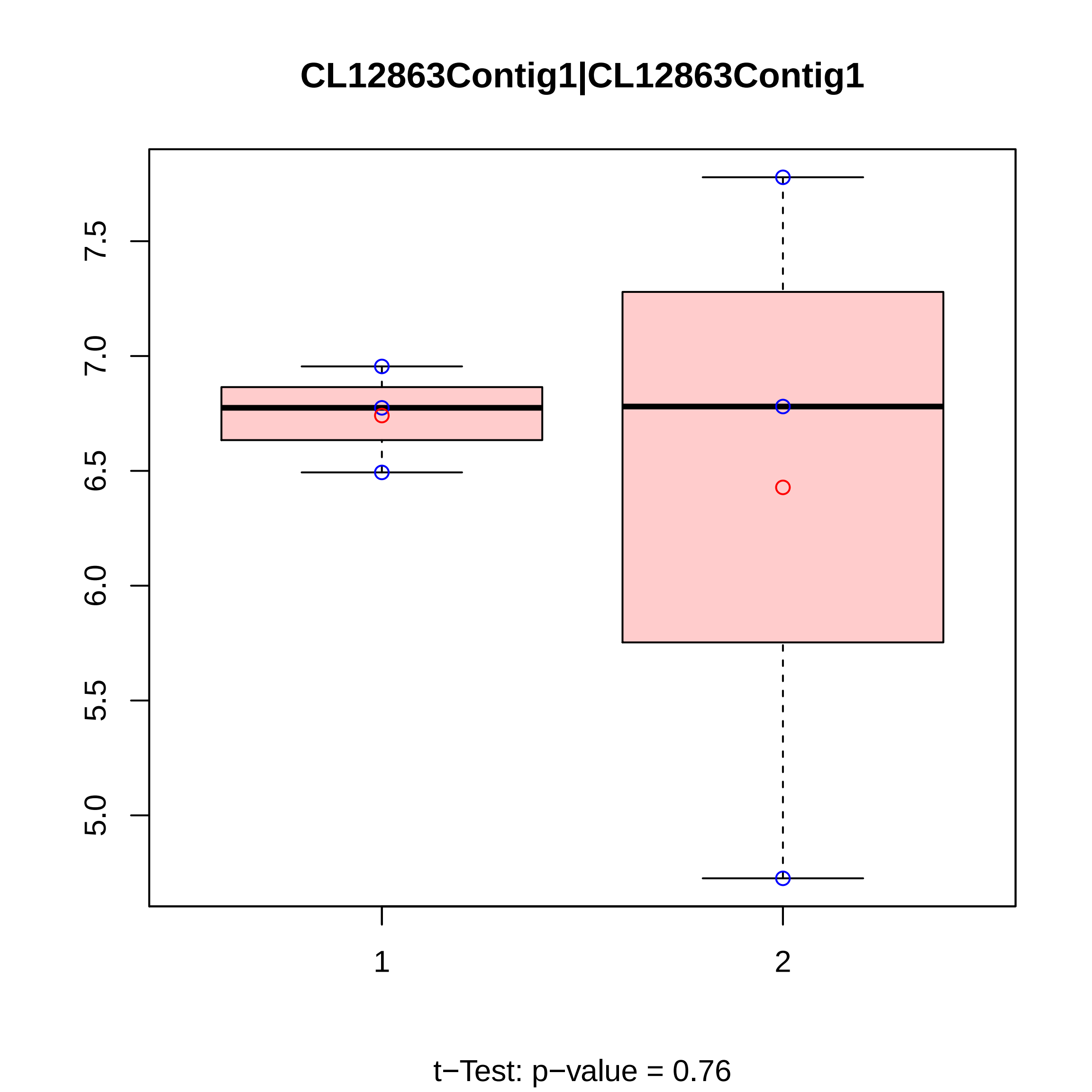
CL12863Contig1|CL12863Contig1
7.5
7.0
6.5
6.0
5.5
5.0
1
2
t−Test: p−value = 0.76

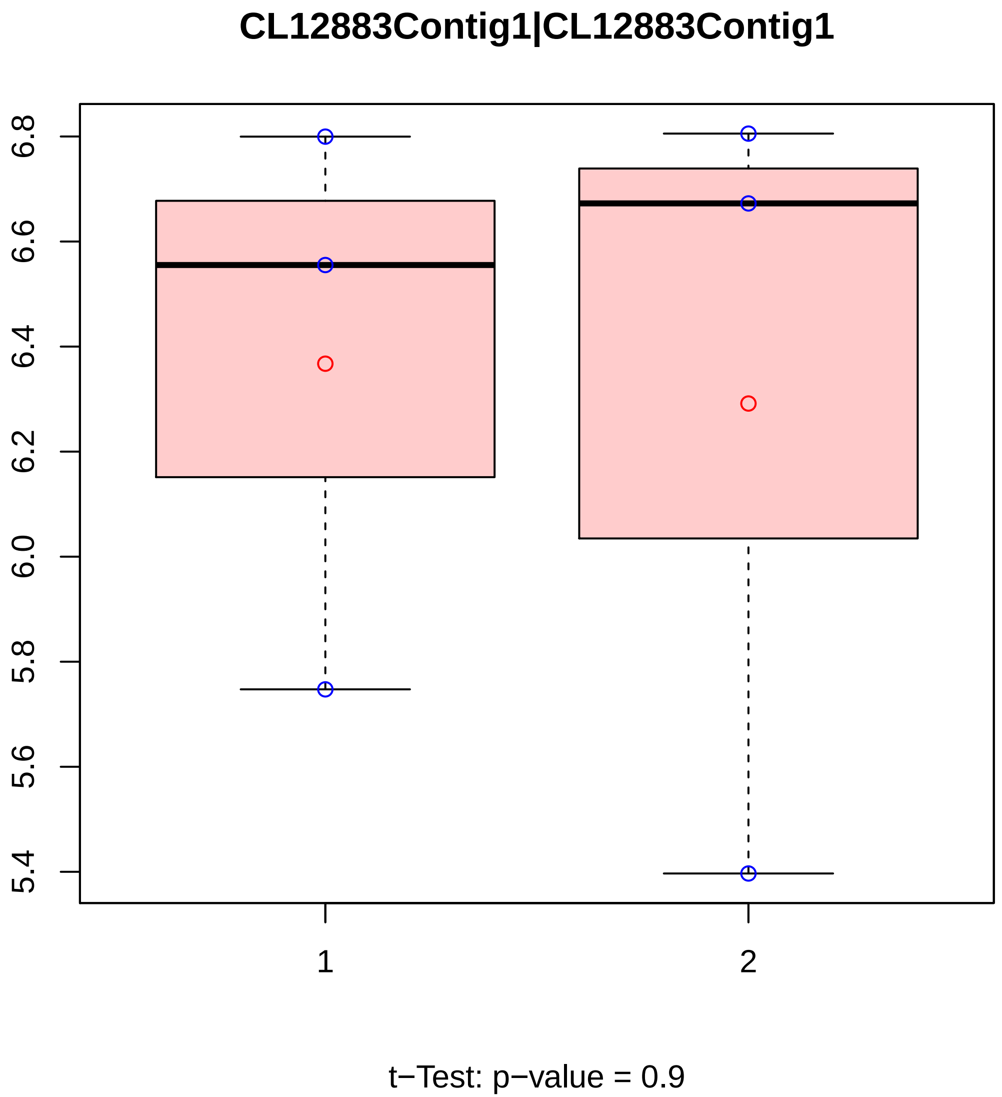

CL12883Contig1|CL12883Contig1
6.8
6.6
6.4
6.2
6.0
5.8
5.6
5.4
1
2
t−Test: p−value = 0.9

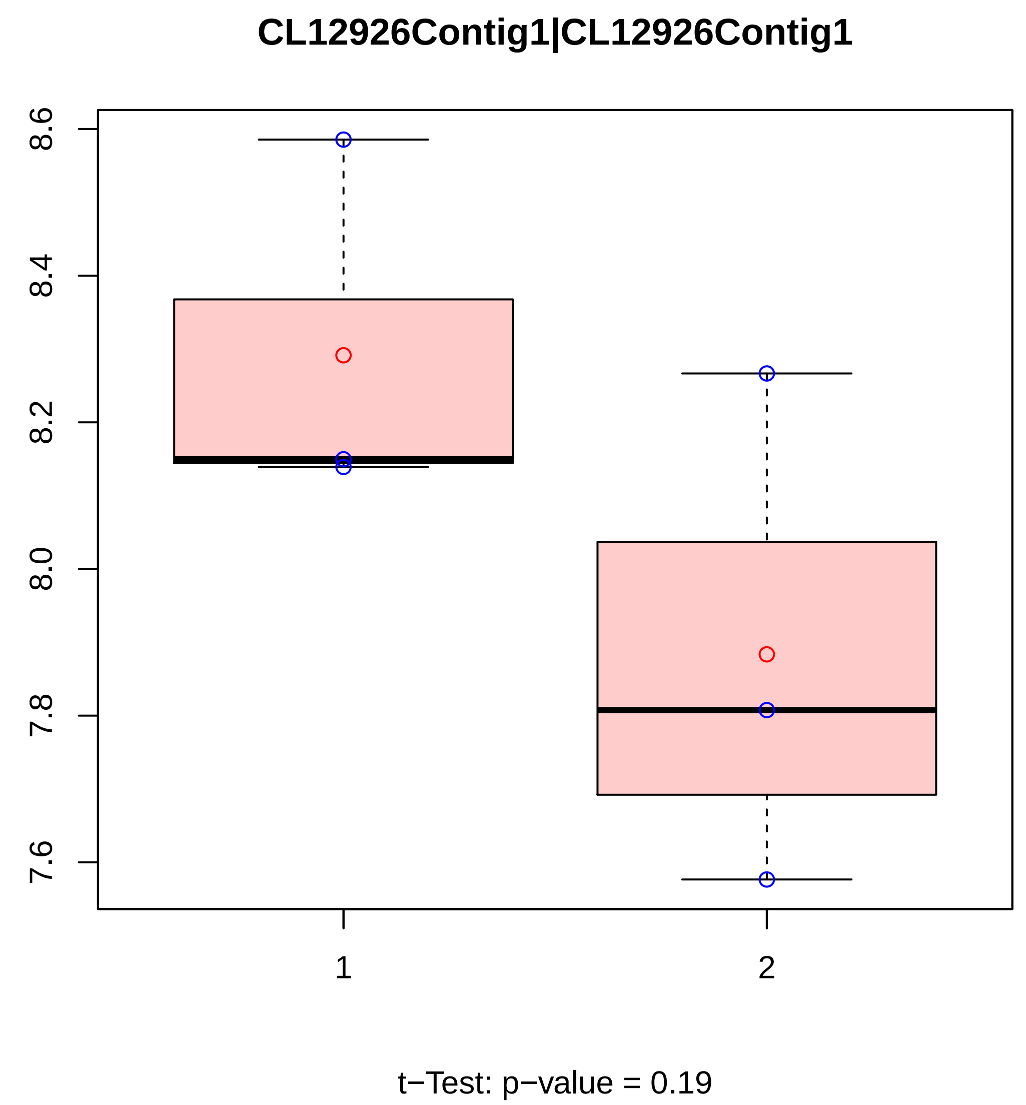
CL12926Contig1|CL12926Contig1
8.6
8.4
8.2
8.0
7.8
7.6
1
2
t−Test: p−value = 0.19

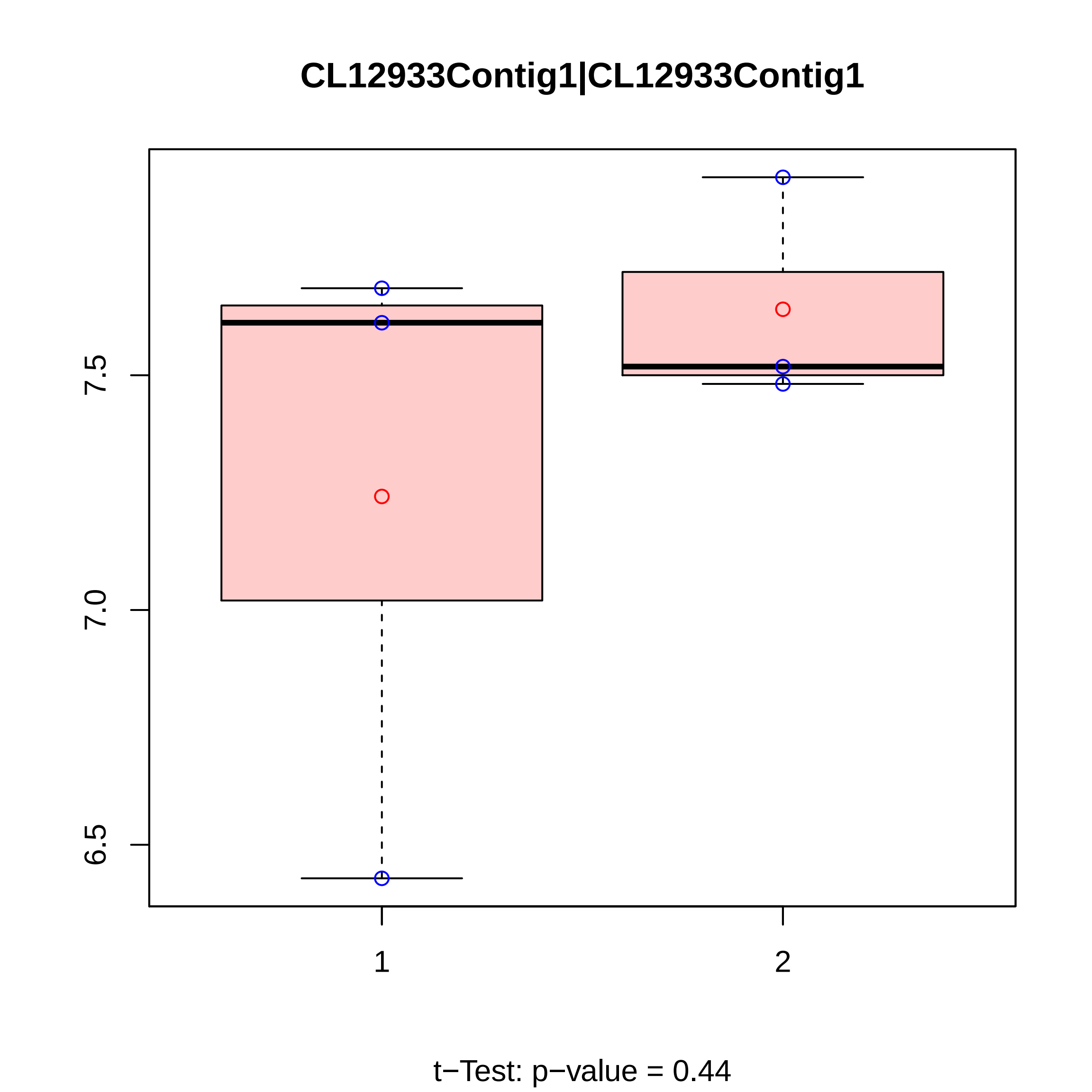
CL12933Contig1|CL12933Contig1
7.5
7.0
6.5
1
2
t−Test: p−value = 0.44

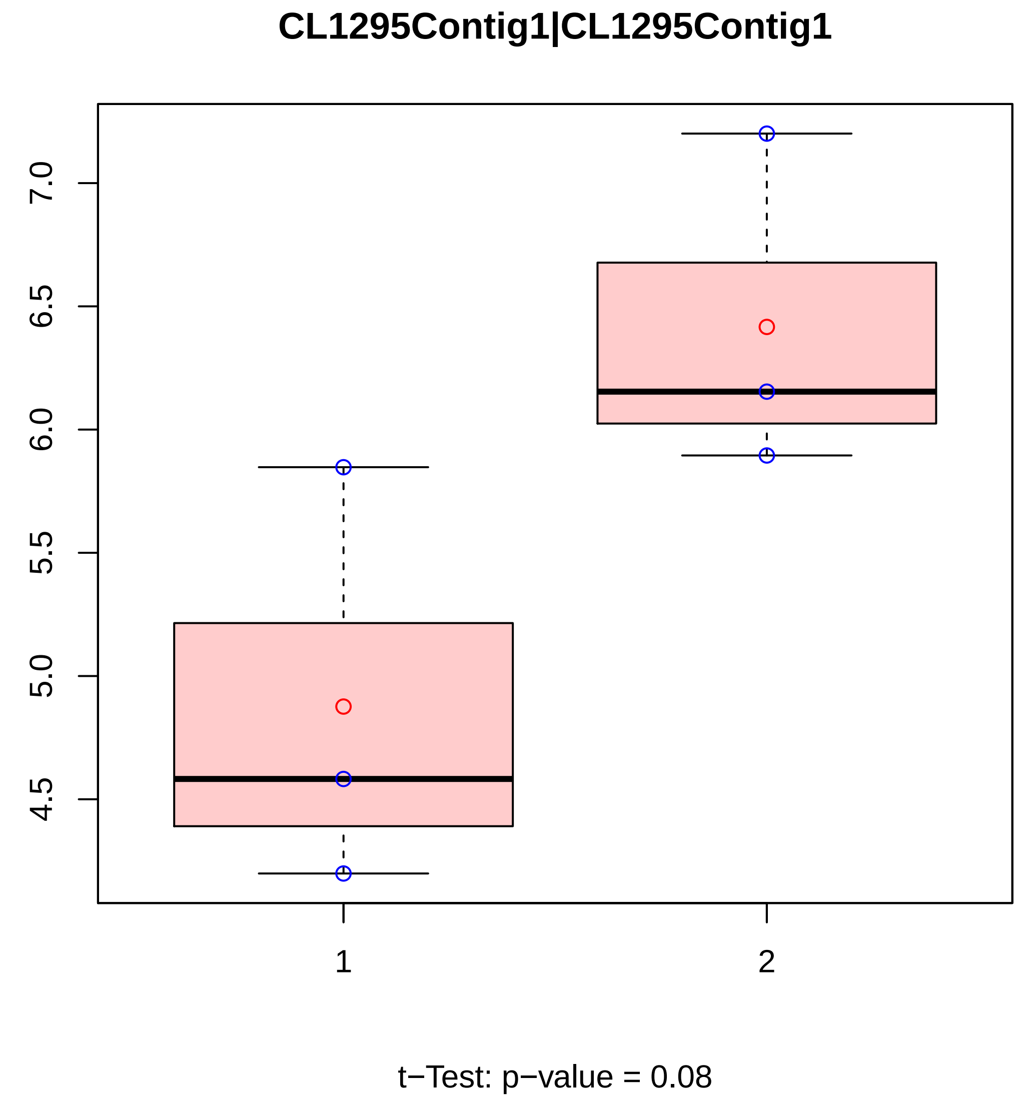

CL1295Contig1|CL1295Contig1
7.0
6.5
6.0
5.5
5.0
4.5
1
2
t−Test: p−value = 0.08

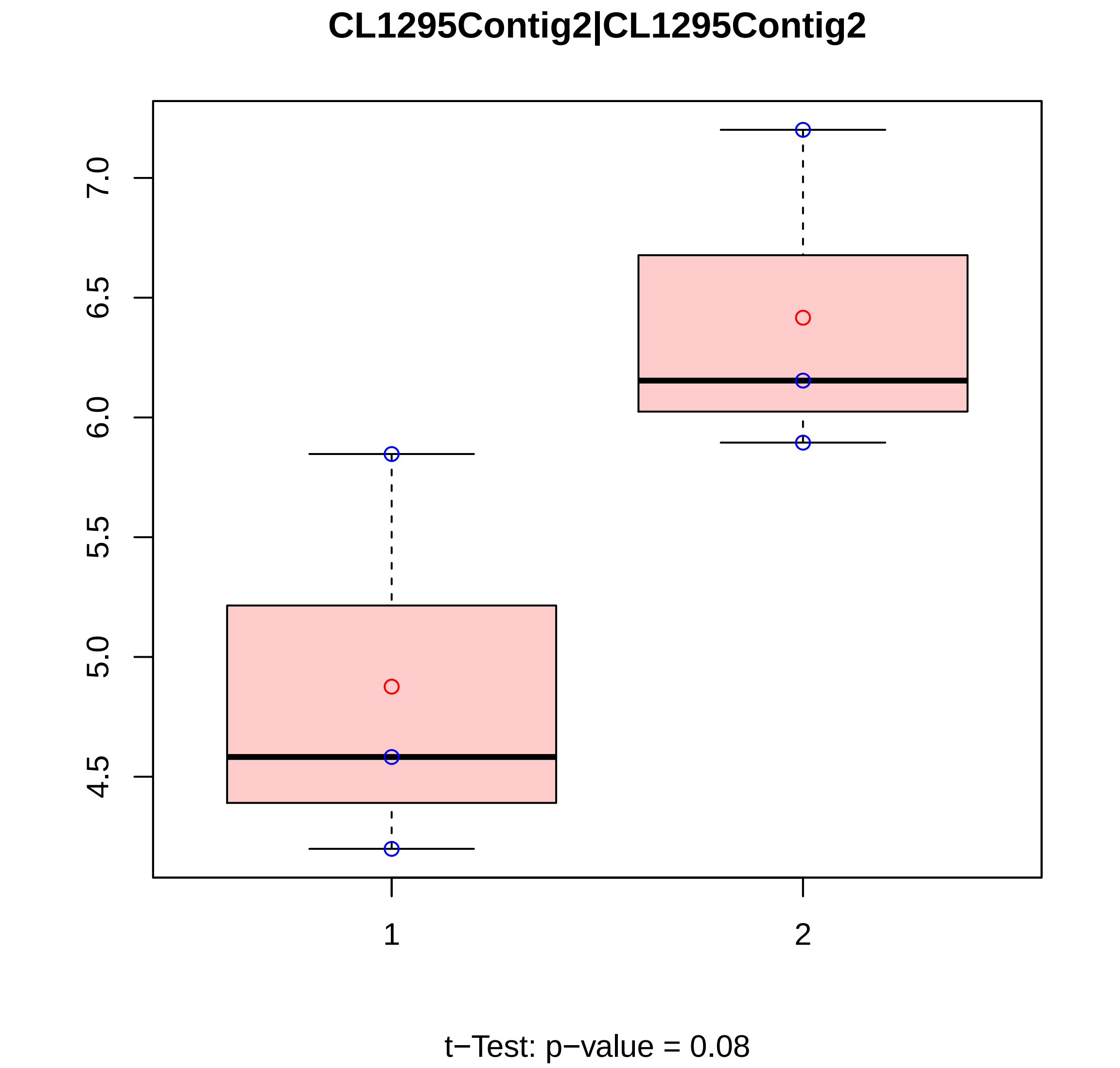
CL1295Contig2|CL1295Contig2
7.0
6.5
6.0
5.5
5.0
4.5
1
2
t−Test: p−value = 0.08

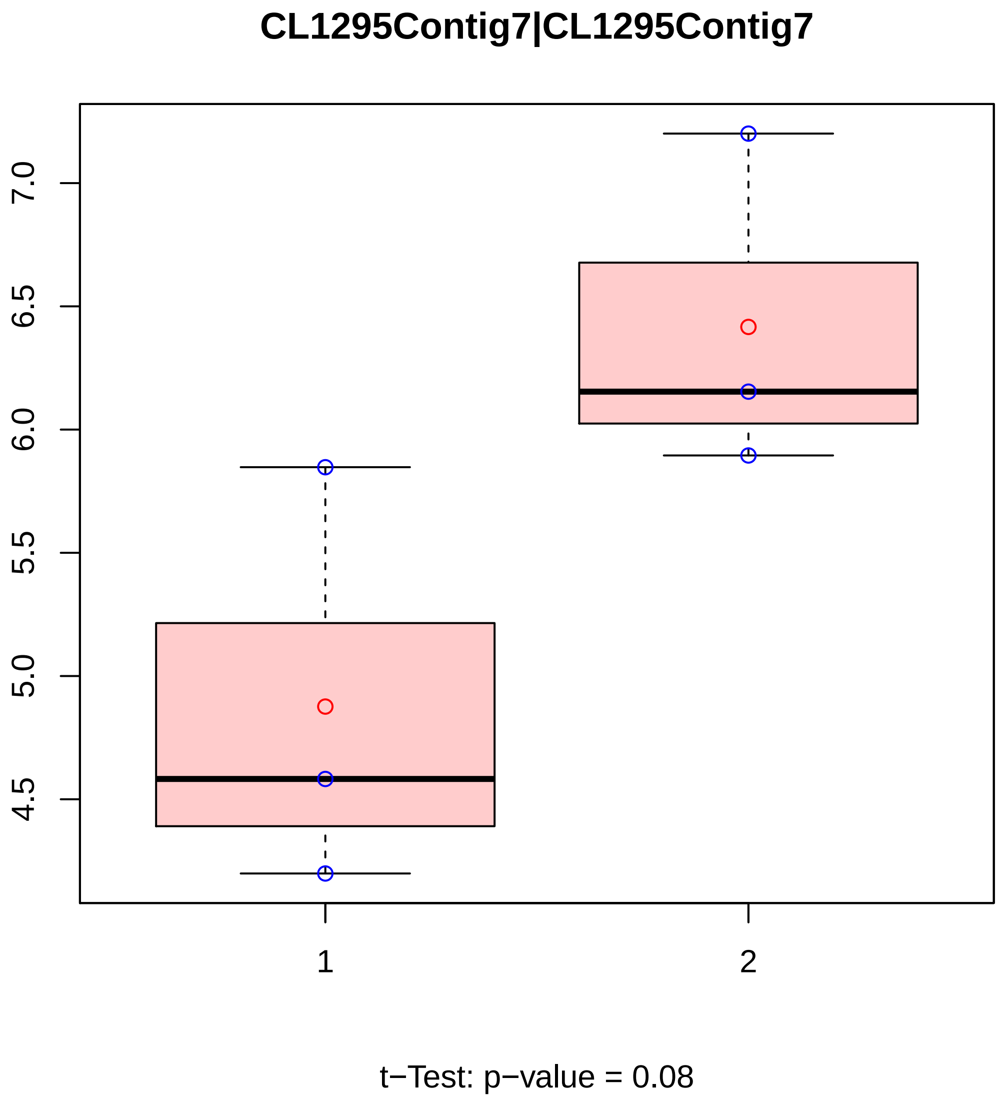
CL1295Contig7|CL1295Contig7
7.0
6.5
6.0
5.5
5.0
4.5
1
2
t−Test: p−value = 0.08

CL12967Contig1|CL12967Contig1

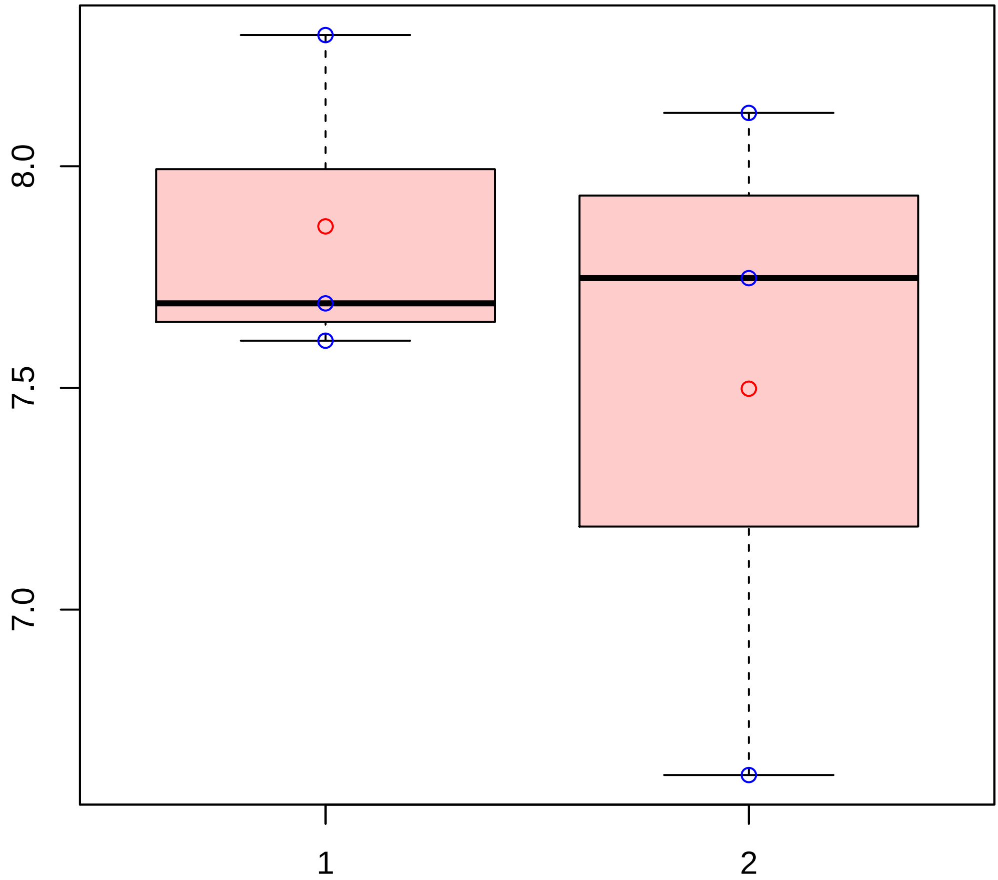


t−Test: p−value = 0.52

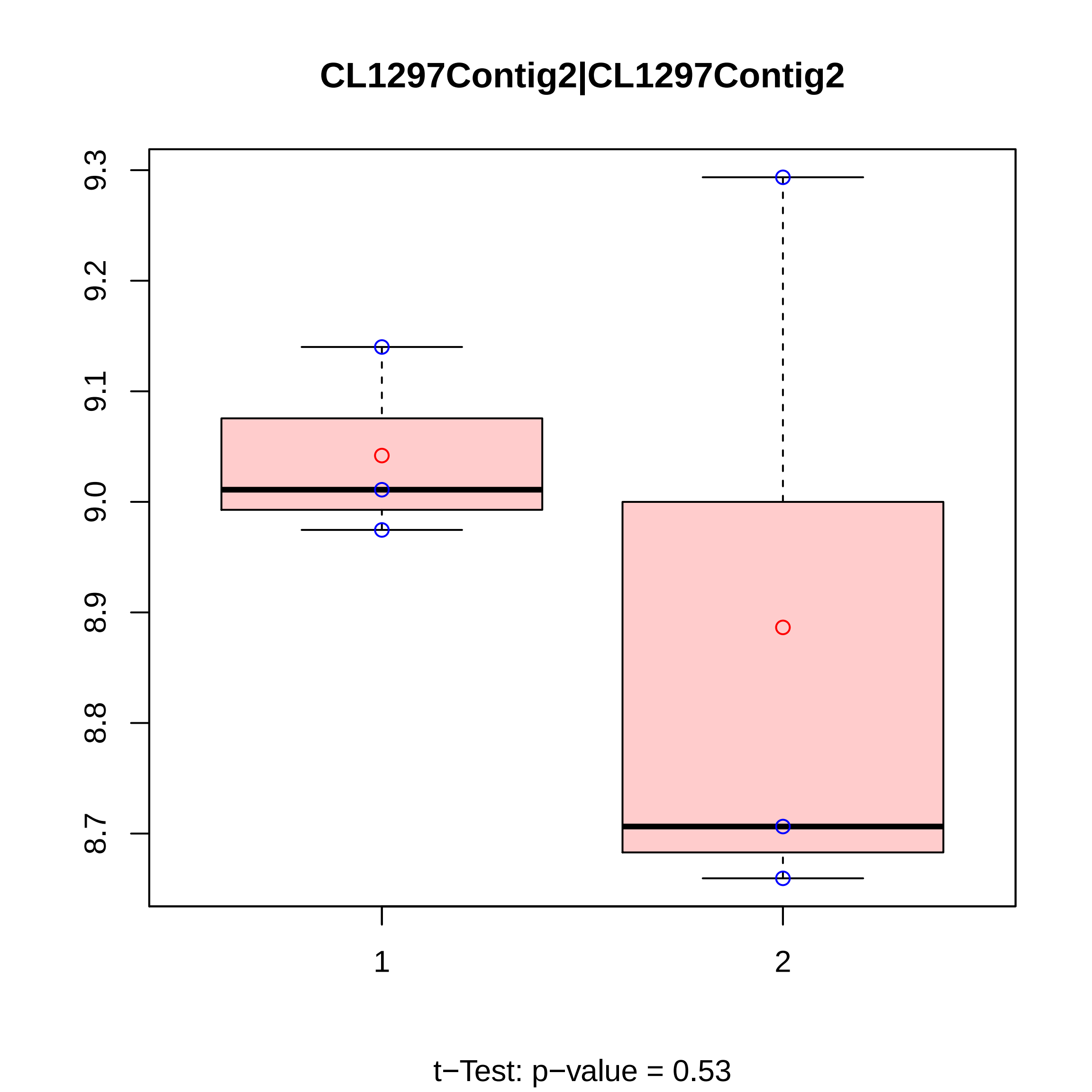
CL1297Contig2|CL1297Contig2
9.3
9.2
9.1
9.0
8.9
8.8
8.7
1
2
t−Test: p−value = 0.53

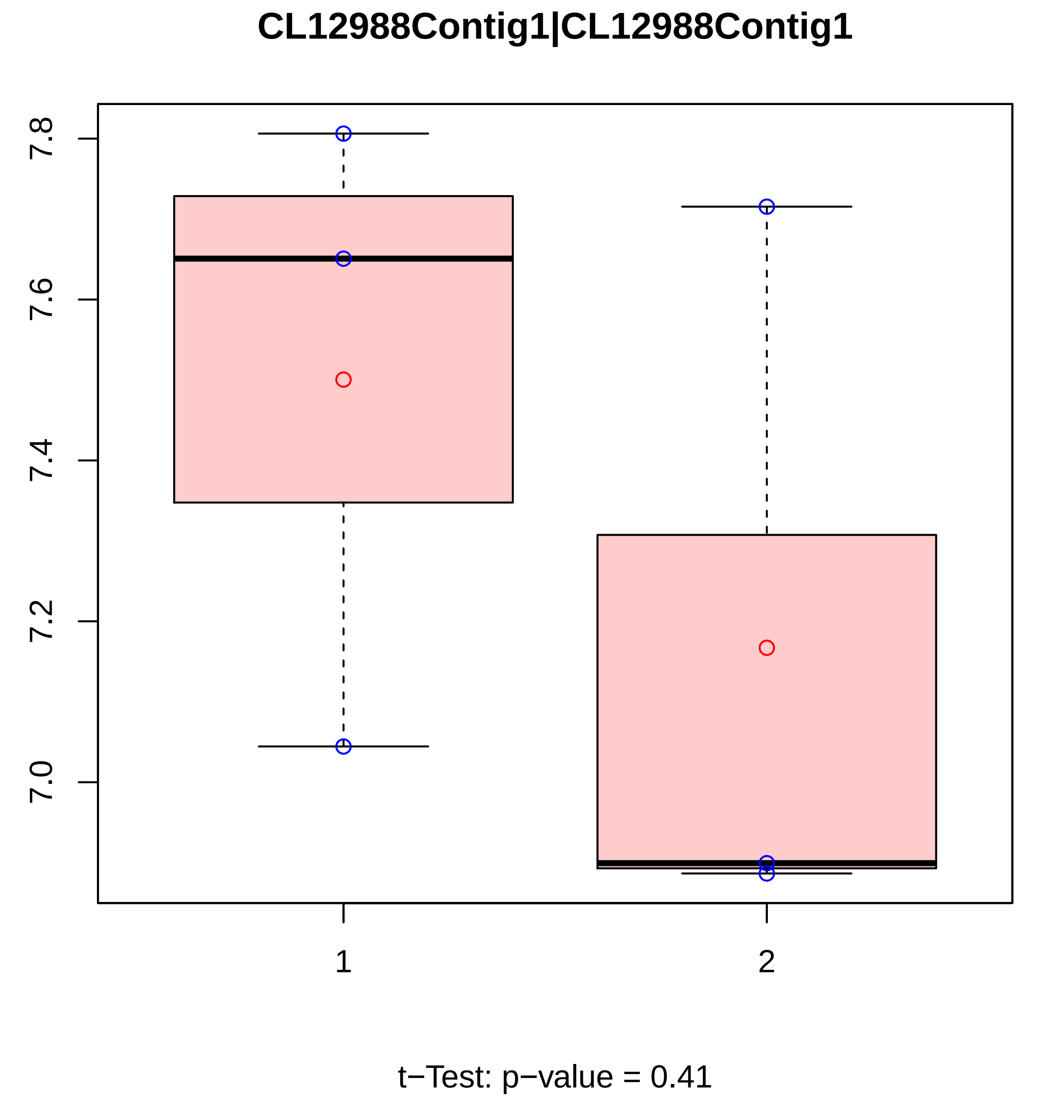
CL12988Contig1|CL12988Contig1
7.8
7.6
7.4
7.2
7.0
1
2
t−Test: p−value = 0.41

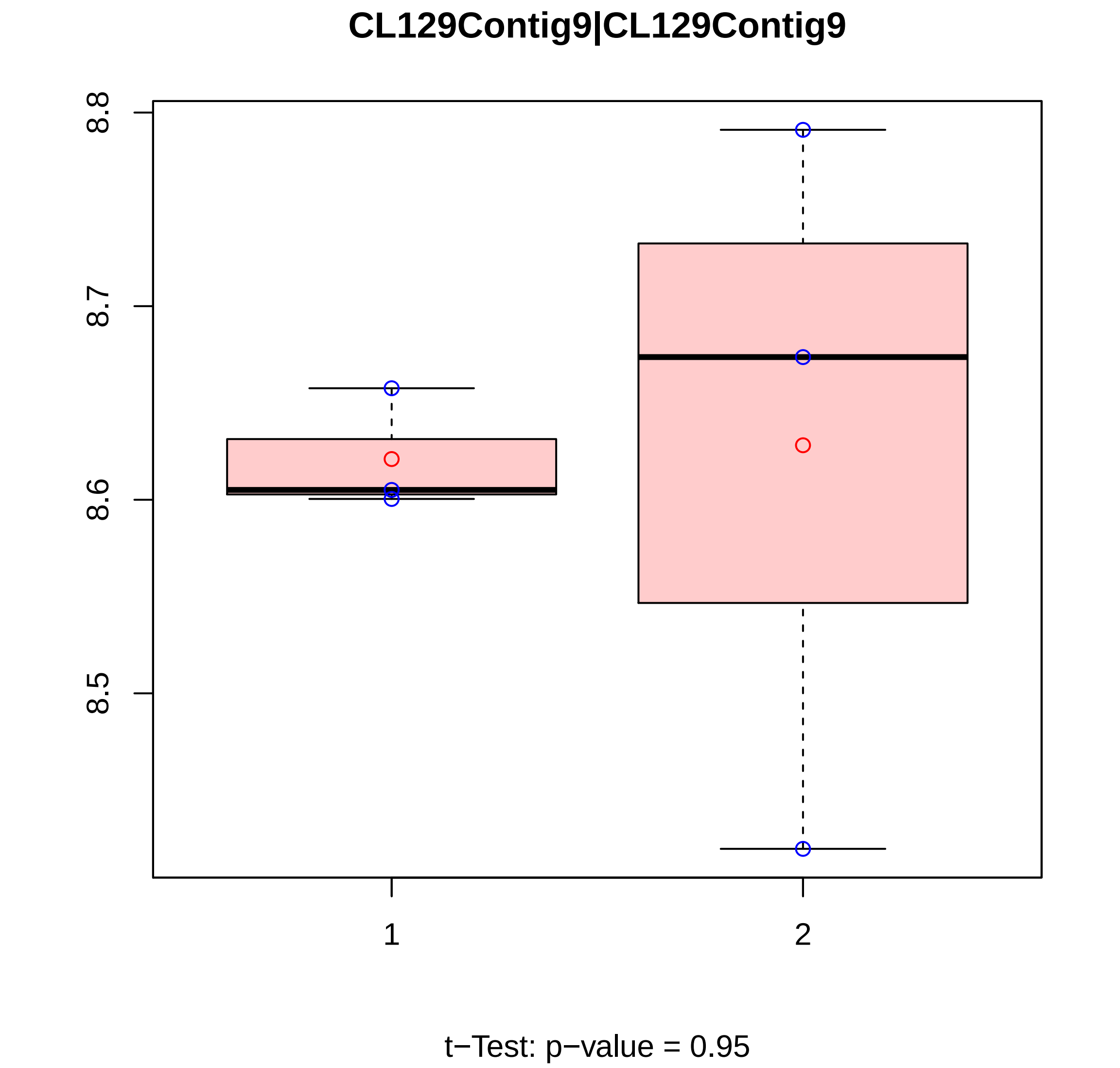
CL129Contig9|CL129Contig9
8.5
8.6
8.7
8.8
1
2
t−Test: p−value = 0.95

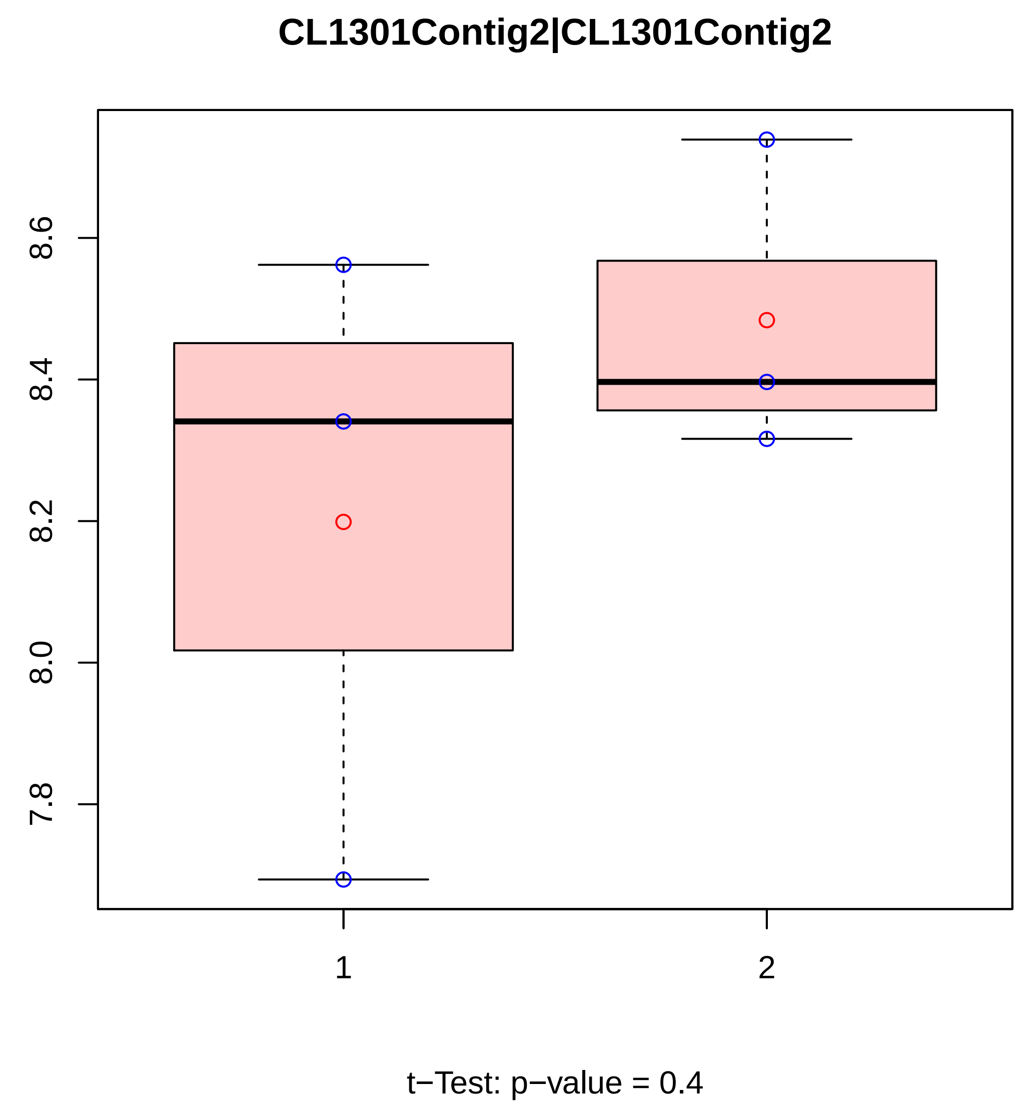
CL1301Contig2|CL1301Contig2
8.6
8.4
8.2
8.0
7.8
1
2
t−Test: p−value = 0.4

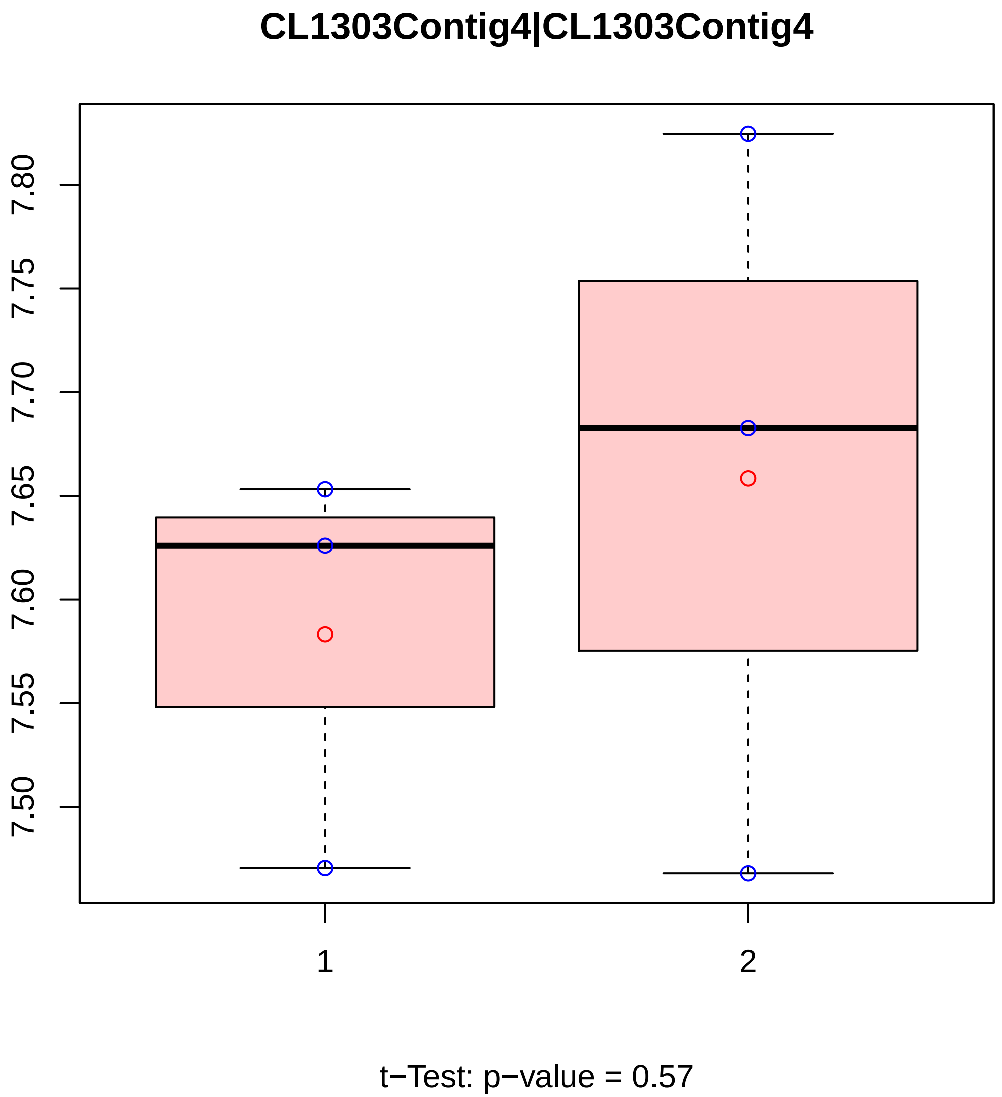
CL1303Contig4|CL1303Contig4
7.50
7.55
7.60
7.65
7.70
7.75
7.80
1
2
t−Test: p−value = 0.57

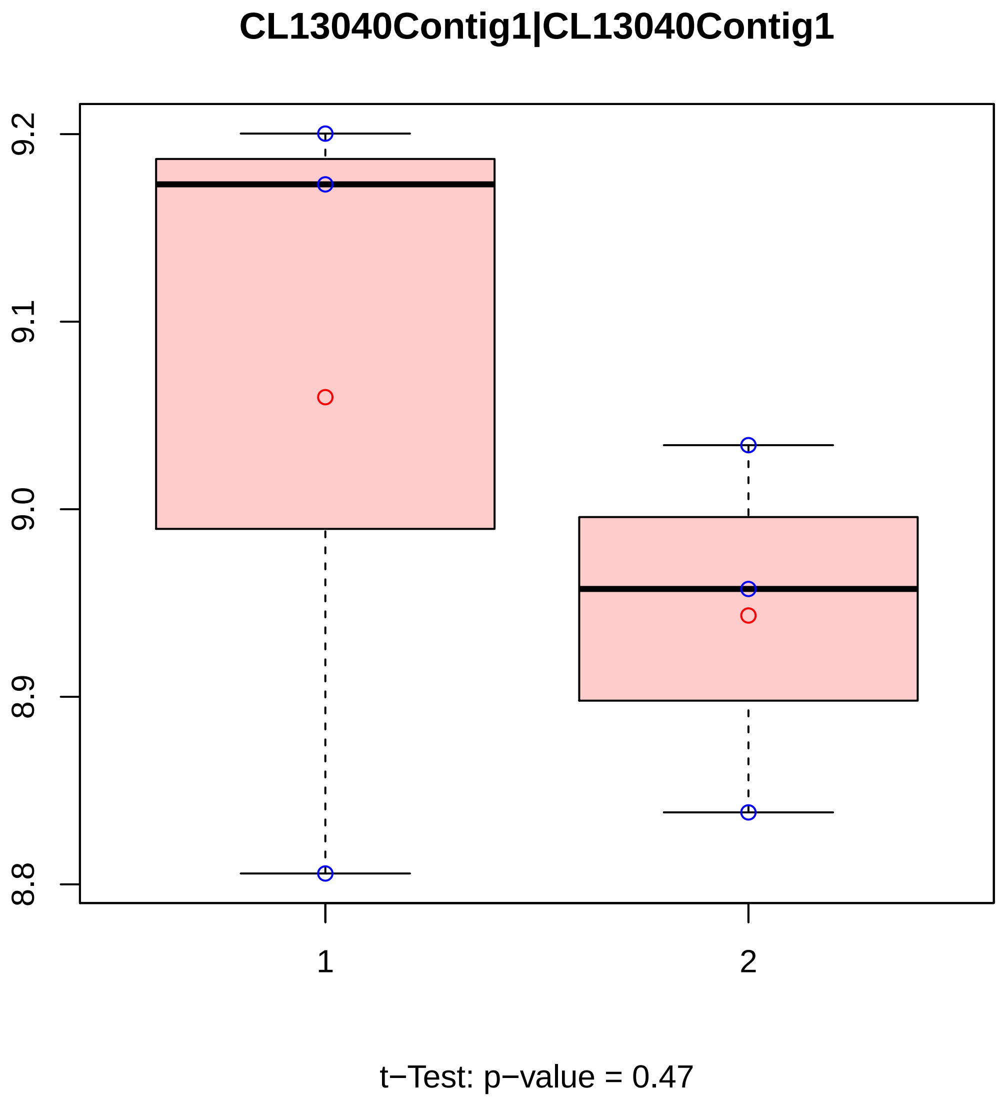
CL13040Contig1|CL13040Contig1
9.2
9.1
9.0
8.9
8.8
1
2
t−Test: p−value = 0.47

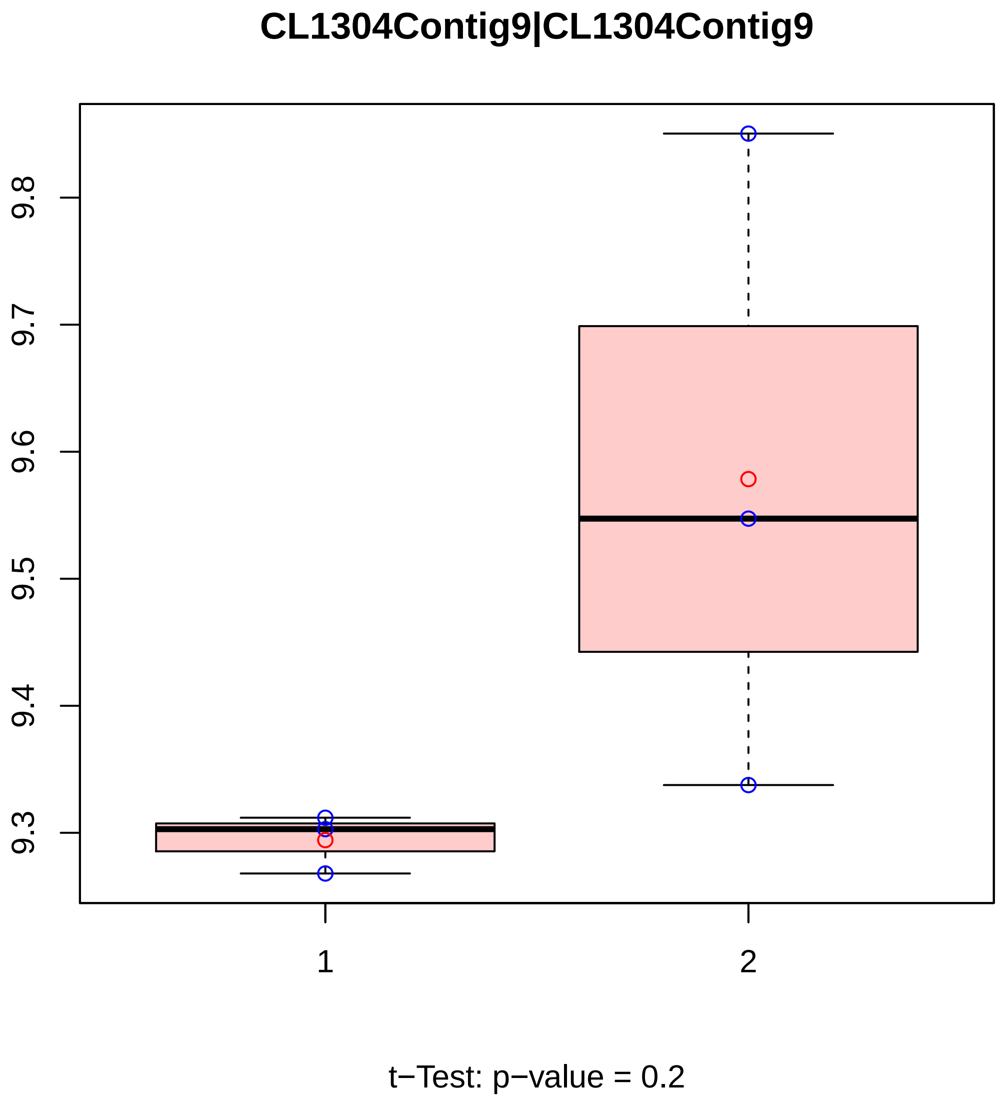
CL1304Contig9|CL1304Contig9
9.3
9.4
9.5
9.6
9.7
9.8
1
2
t−Test: p−value = 0.2

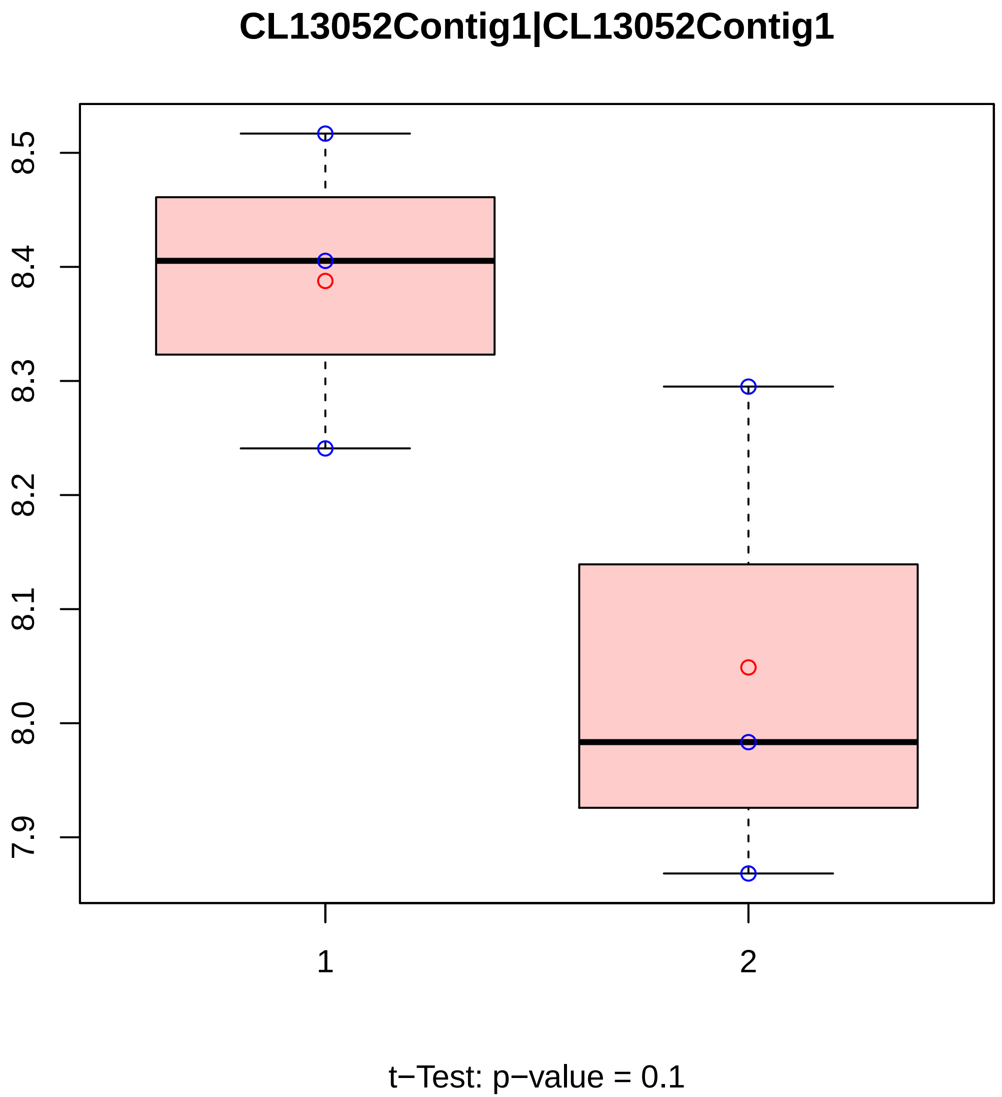
CL13052Contig1|CL13052Contig1
8.5
8.4
8.3
8.2
8.1
8.0
7.9
1
2
t−Test: p−value = 0.1

## CL1305Contig4|CL1305Contig4

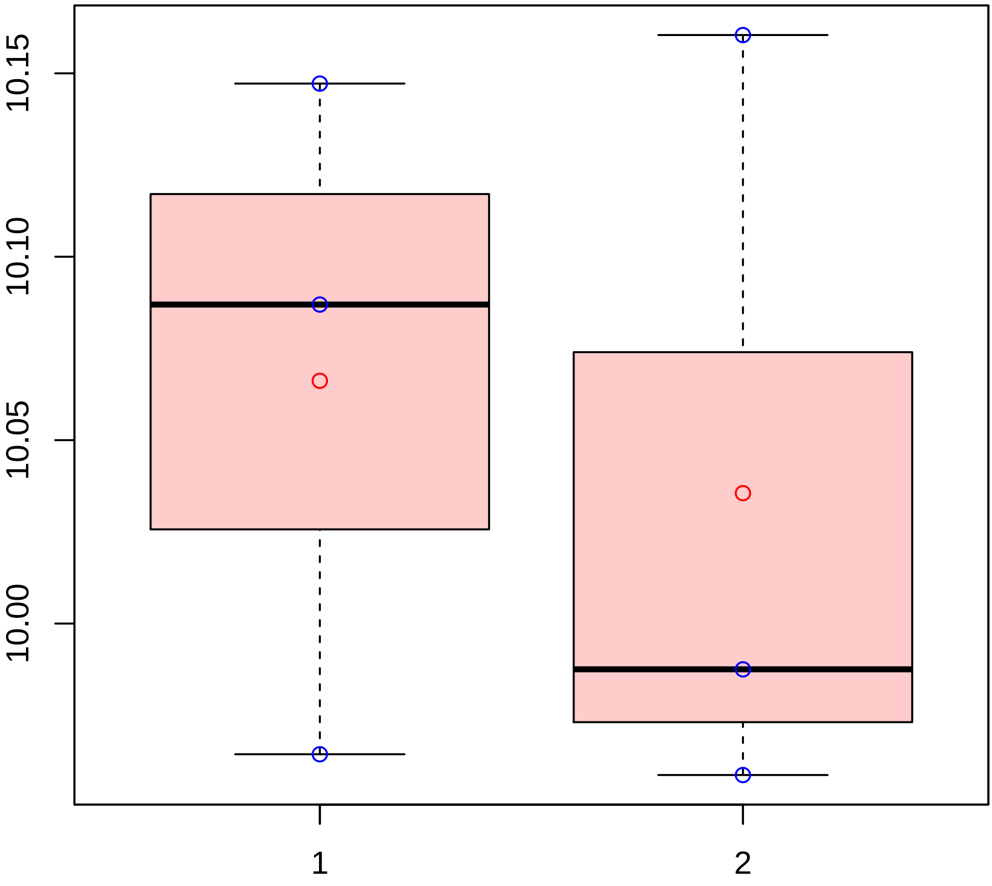


t−Test: p−value = 0.73

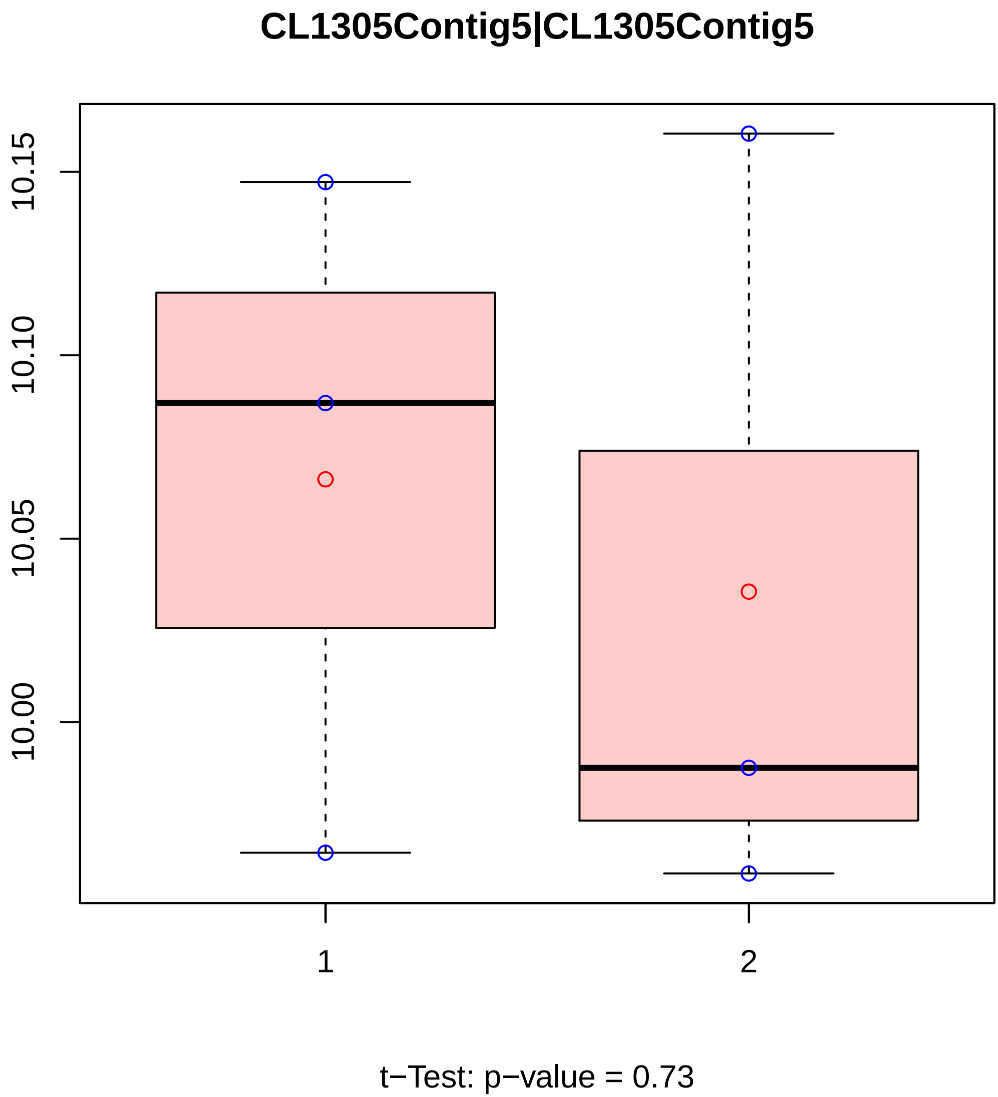

CL1305Contig5|CL1305Contig5
10.00
10.05
10.10
10.15
1
2
t−Test: p−value = 0.73

# CL1307Contig3|CL1307Contig3

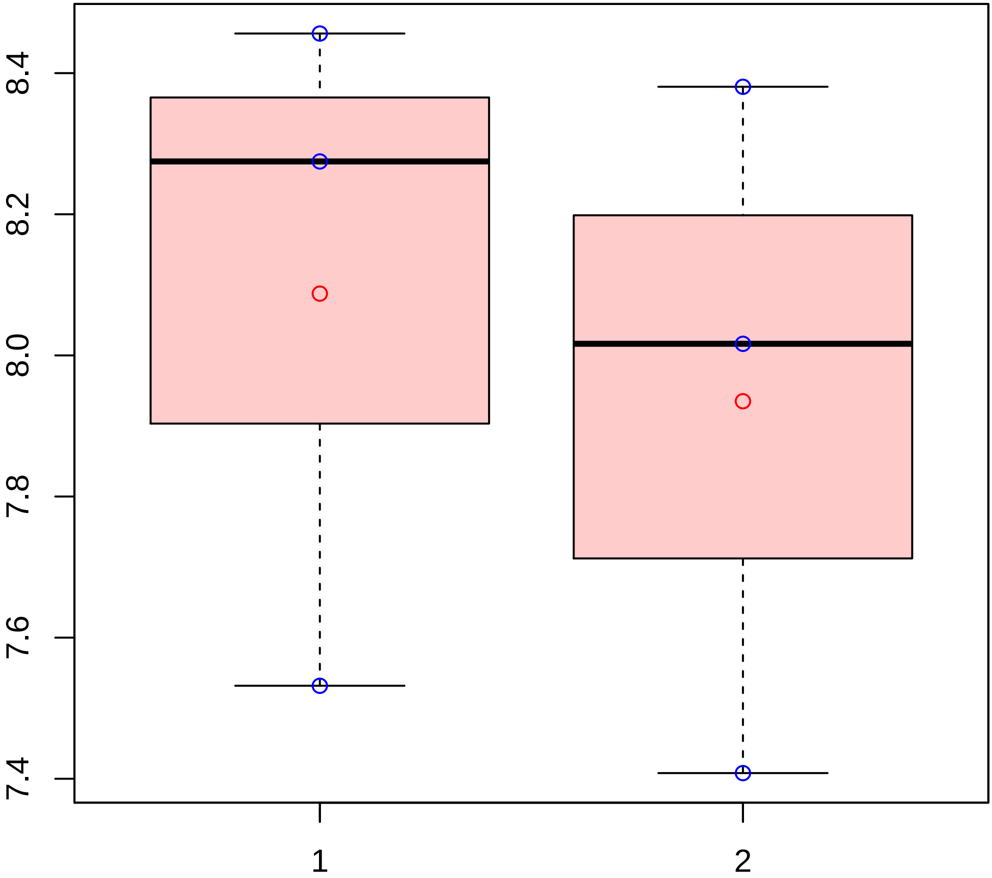


t−Test: p−value = 0.72

# CL1309Contig10|CL1309Contig10

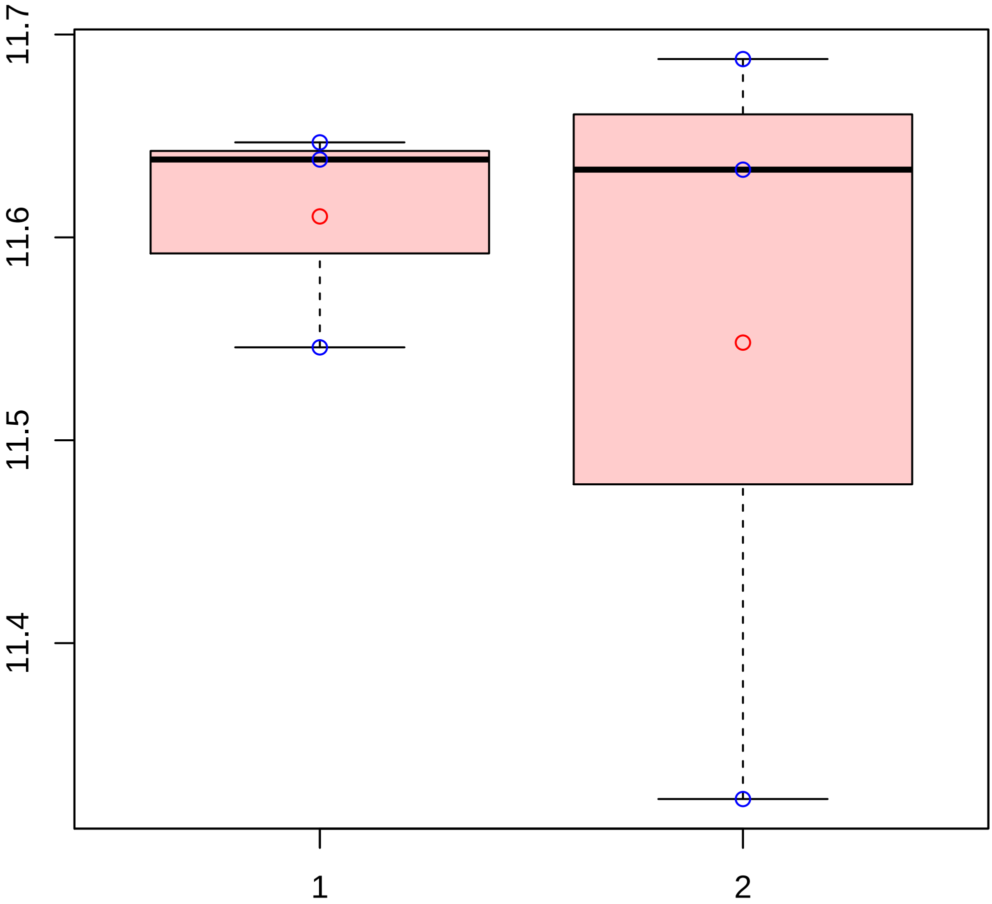


t−Test: p−value = 0.64

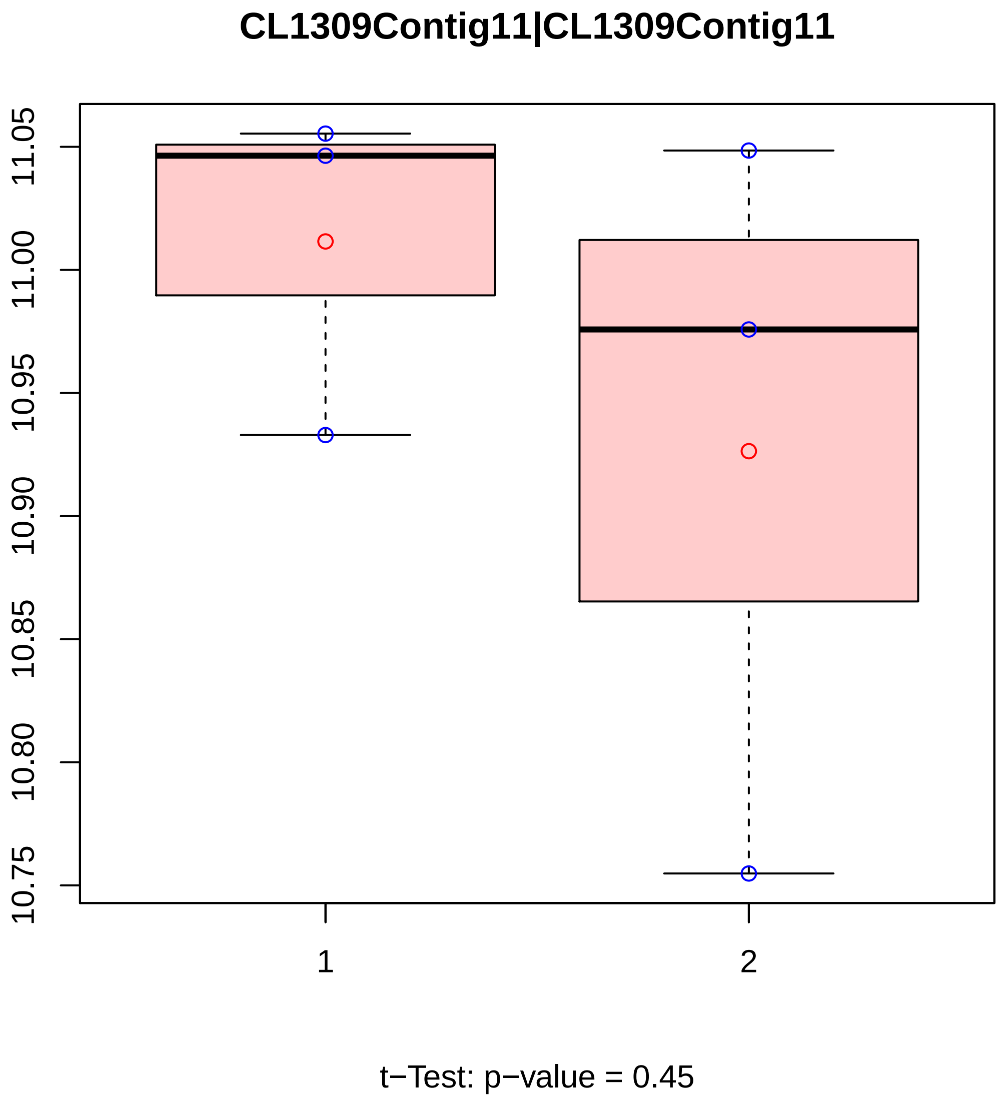
CL1309Contig11|CL1309Contig11
11.05
11.00
10.95
10.90
10.85
10.80
10.75
1
2
t−Test: p−value = 0.45

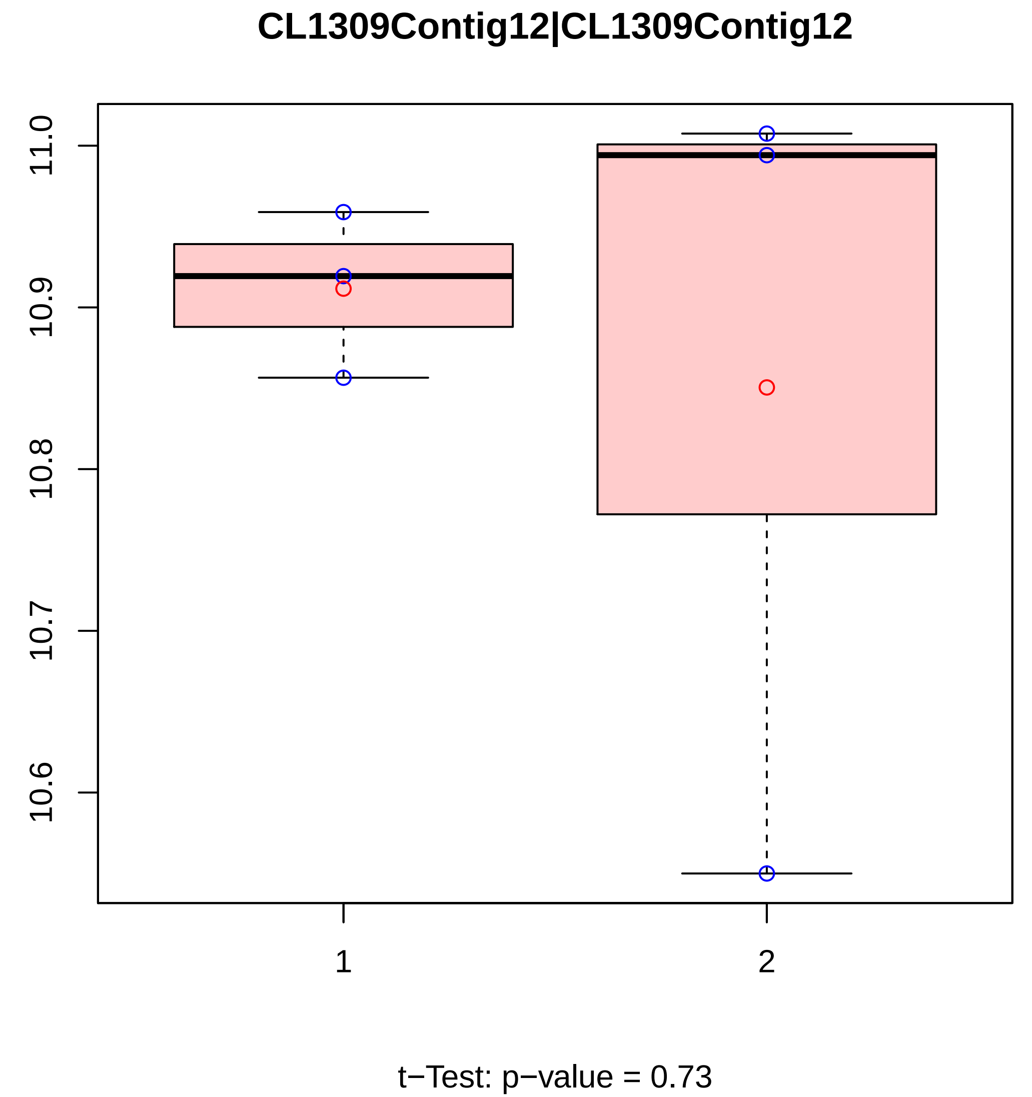
CL1309Contig12|CL1309Contig12
11.0
10.9
10.8
10.7
10.6
1
2
t−Test: p−value = 0.73

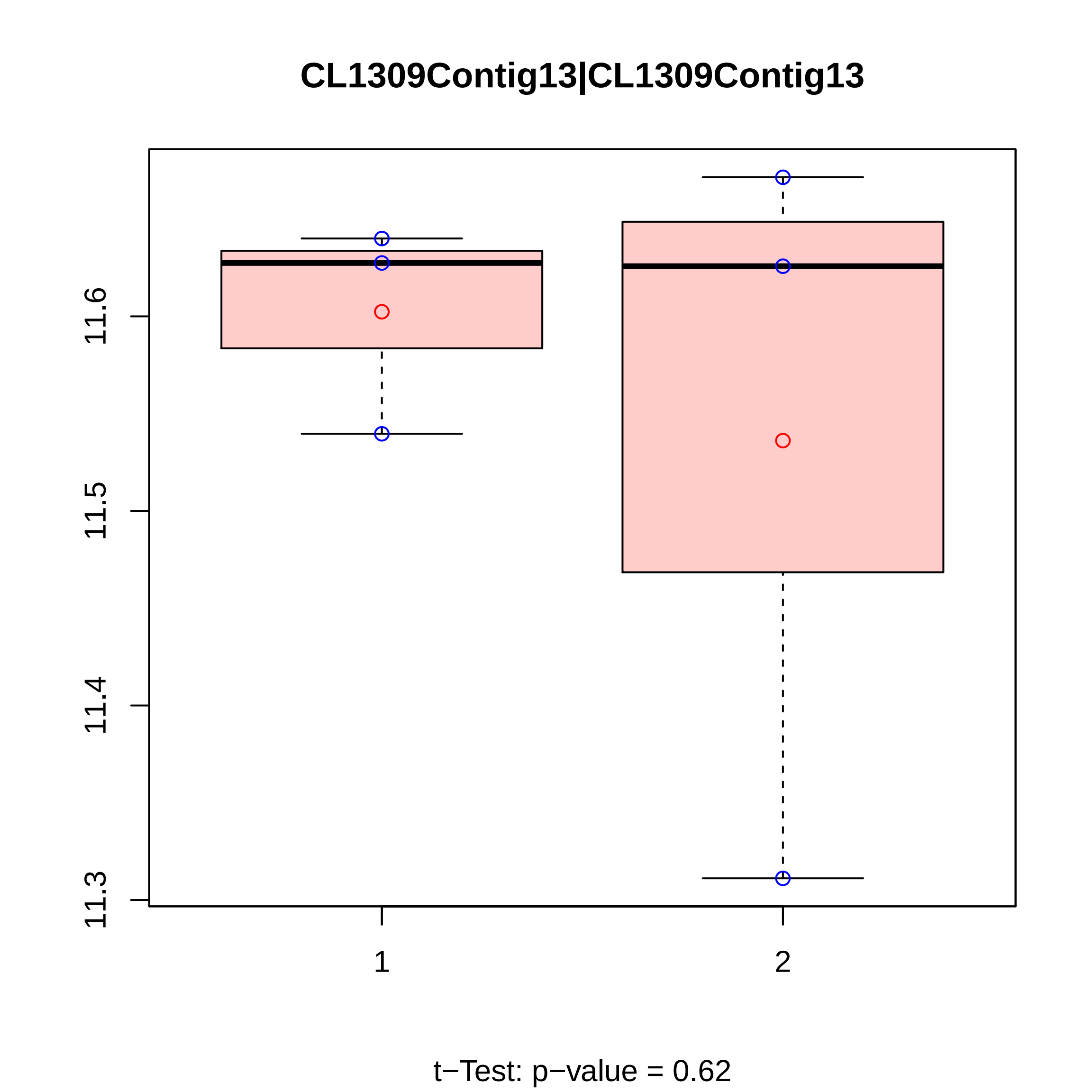
CL1309Contig13|CL1309Contig13
11.3
11.4
11.5
11.6
1
2
t−Test: p−value = 0.62

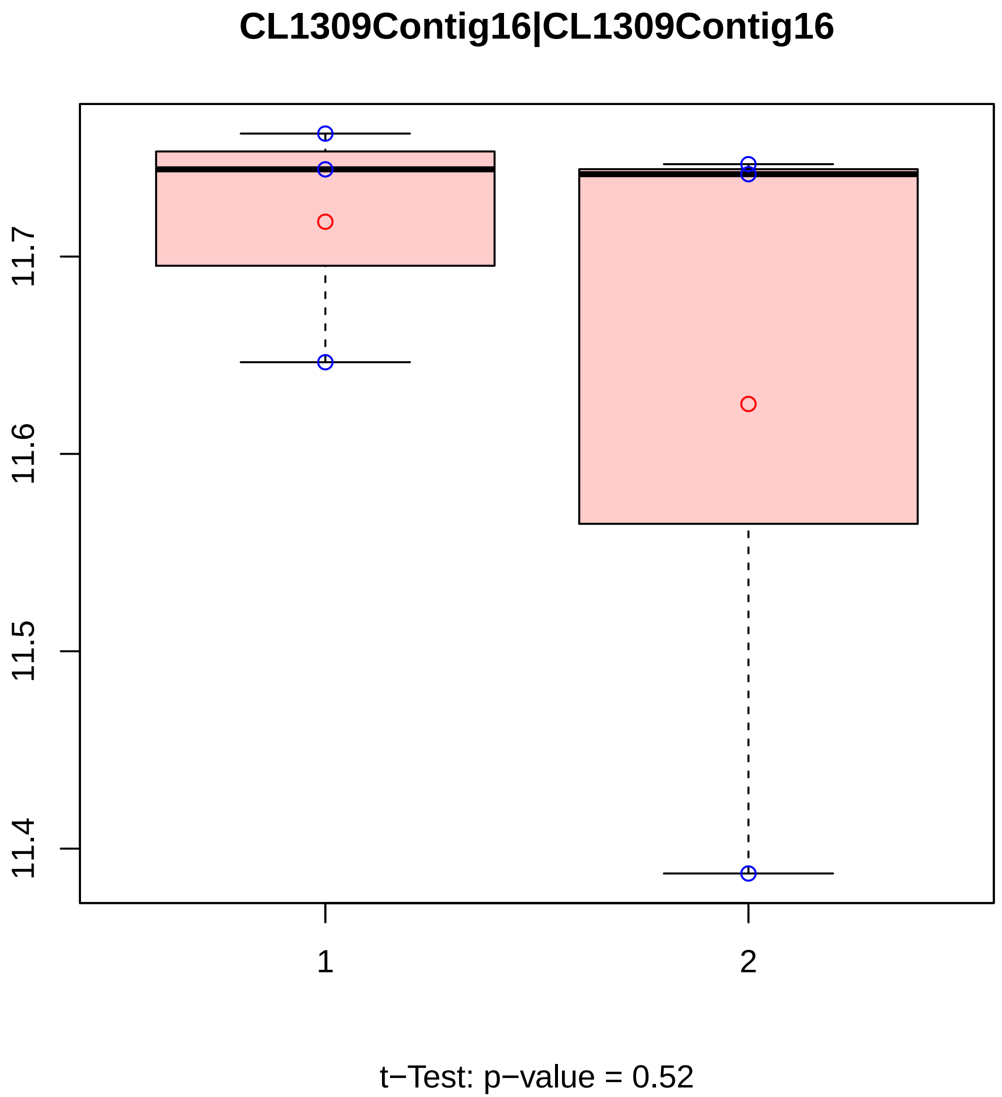

CL1309Contig16|CL1309Contig16
11.4
11.5
11.6
11.7
1
2
t−Test: p−value = 0.52

# CL1309Contig17|CL1309Contig17

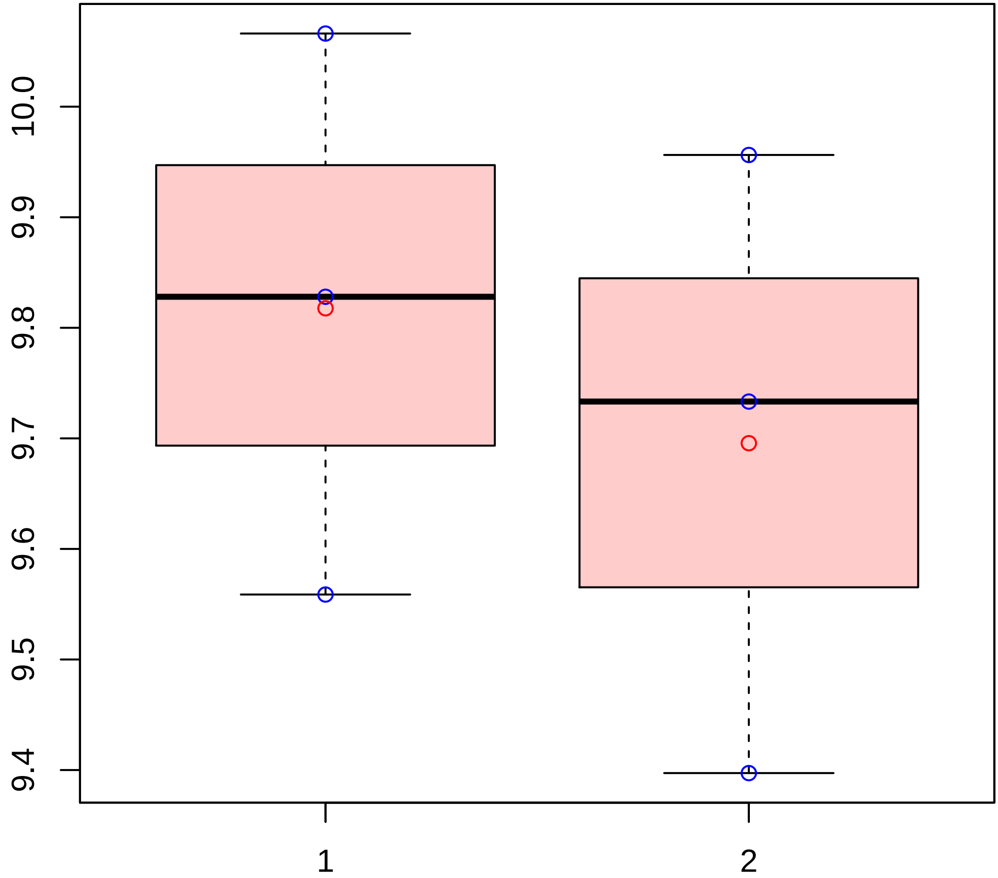


t−Test: p−value = 0.61

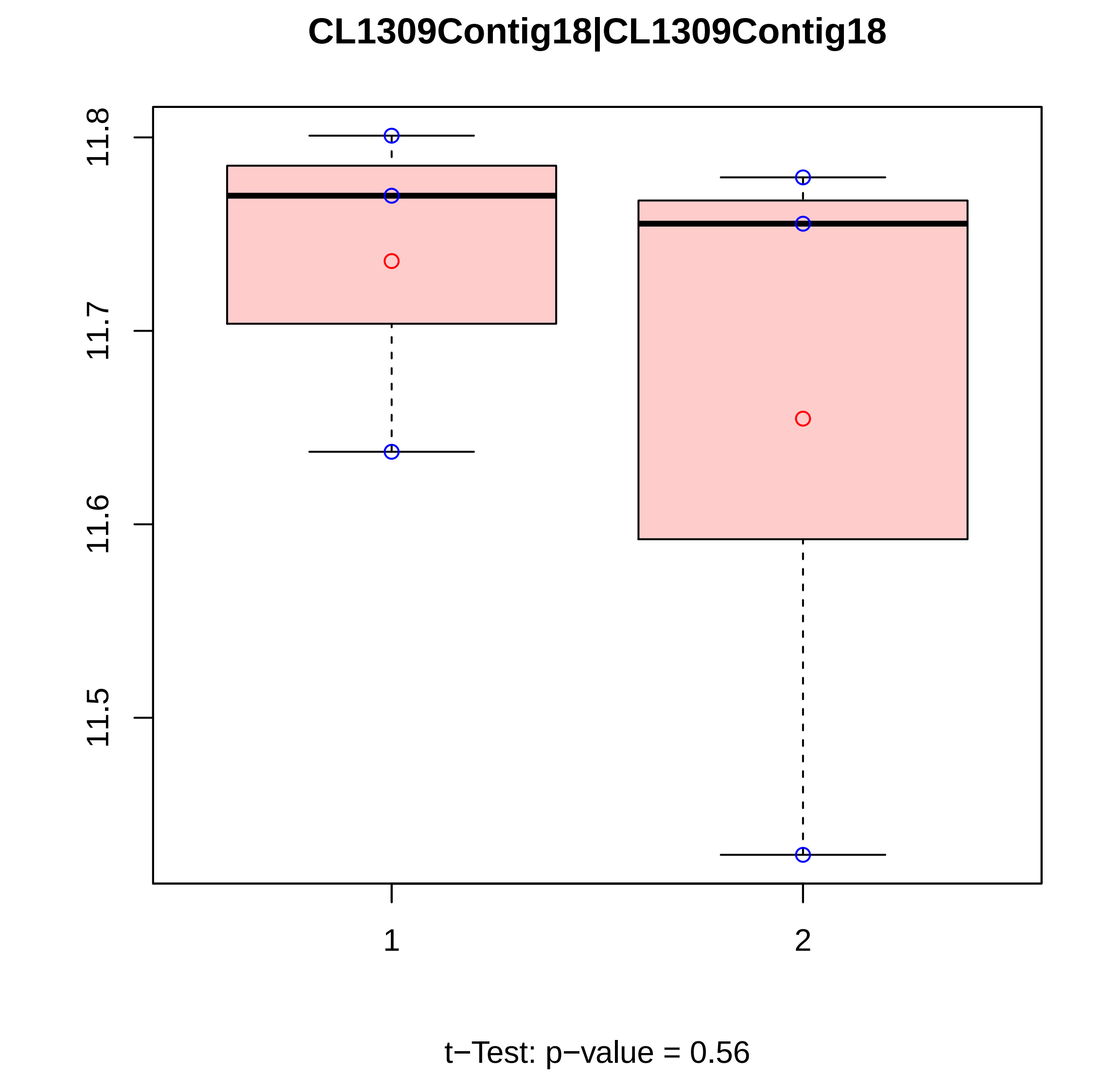
CL1309Contig18|CL1309Contig18
11.8
11.7
11.6
11.5
1
2
t−Test: p−value = 0.56

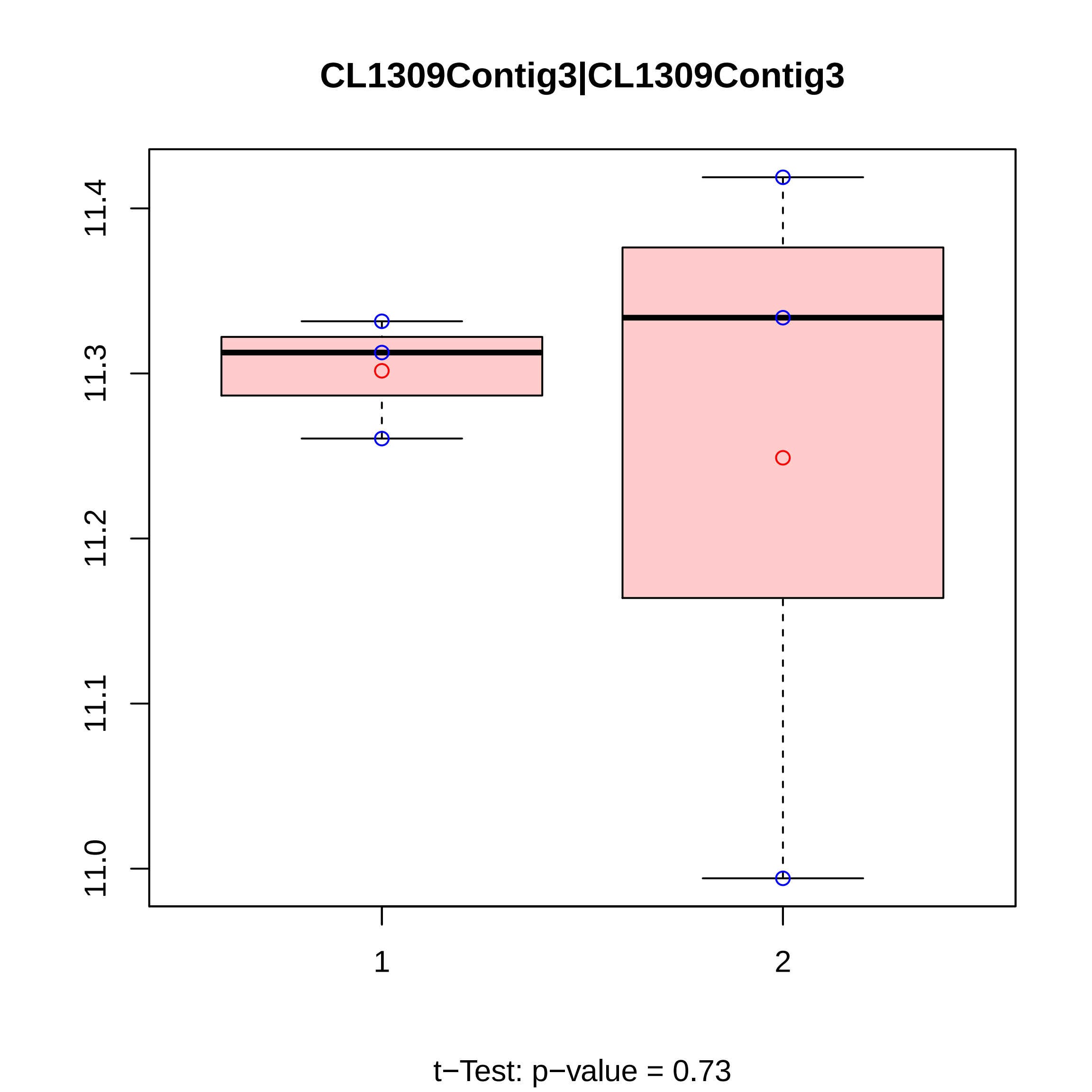
CL1309Contig3|CL1309Contig3
11.0
11.1
11.2
11.3
11.4
1
2
t−Test: p−value = 0.73

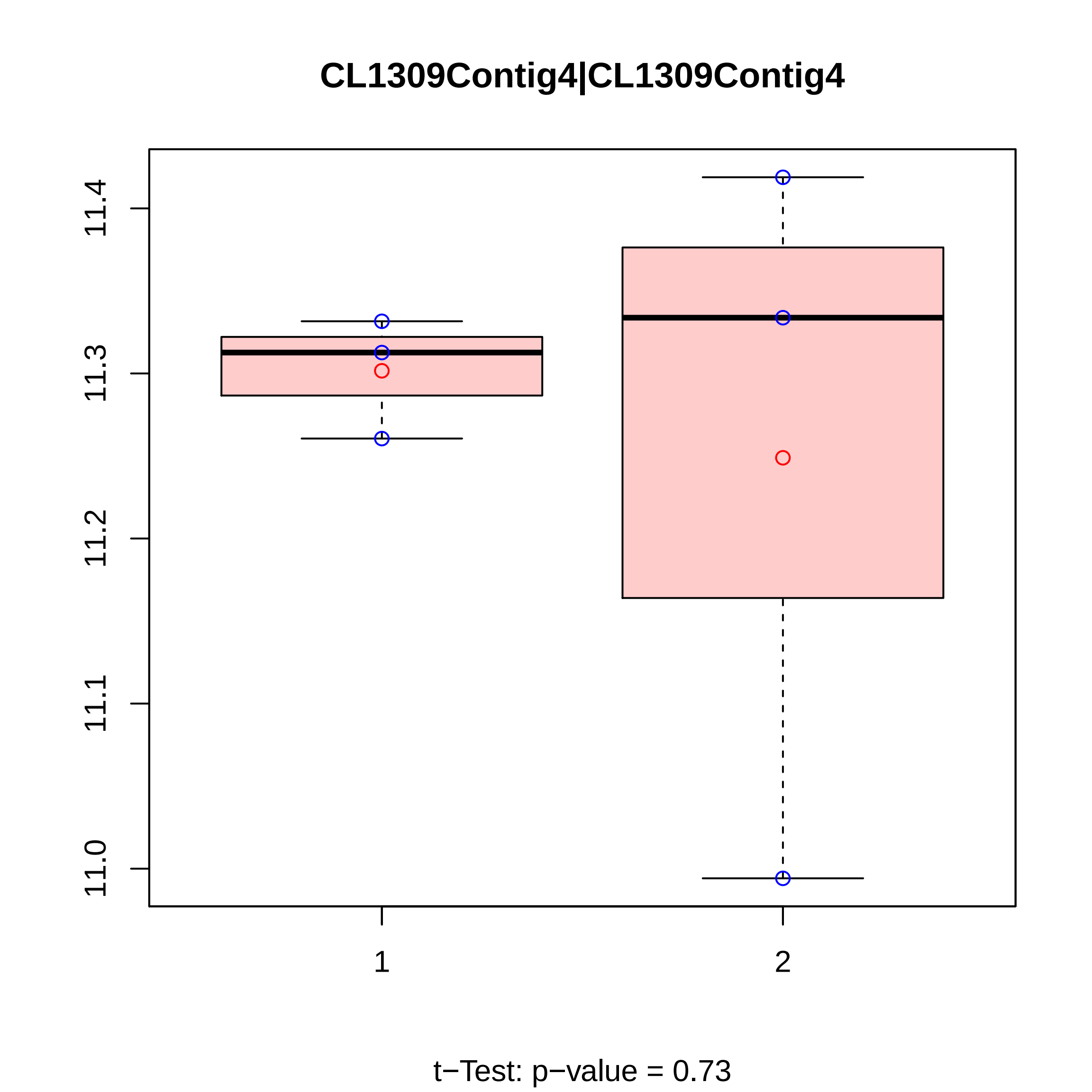
CL1309Contig4|CL1309Contig4
11.0
11.1
11.2
11.3
11.4
1
2
t−Test: p−value = 0.73

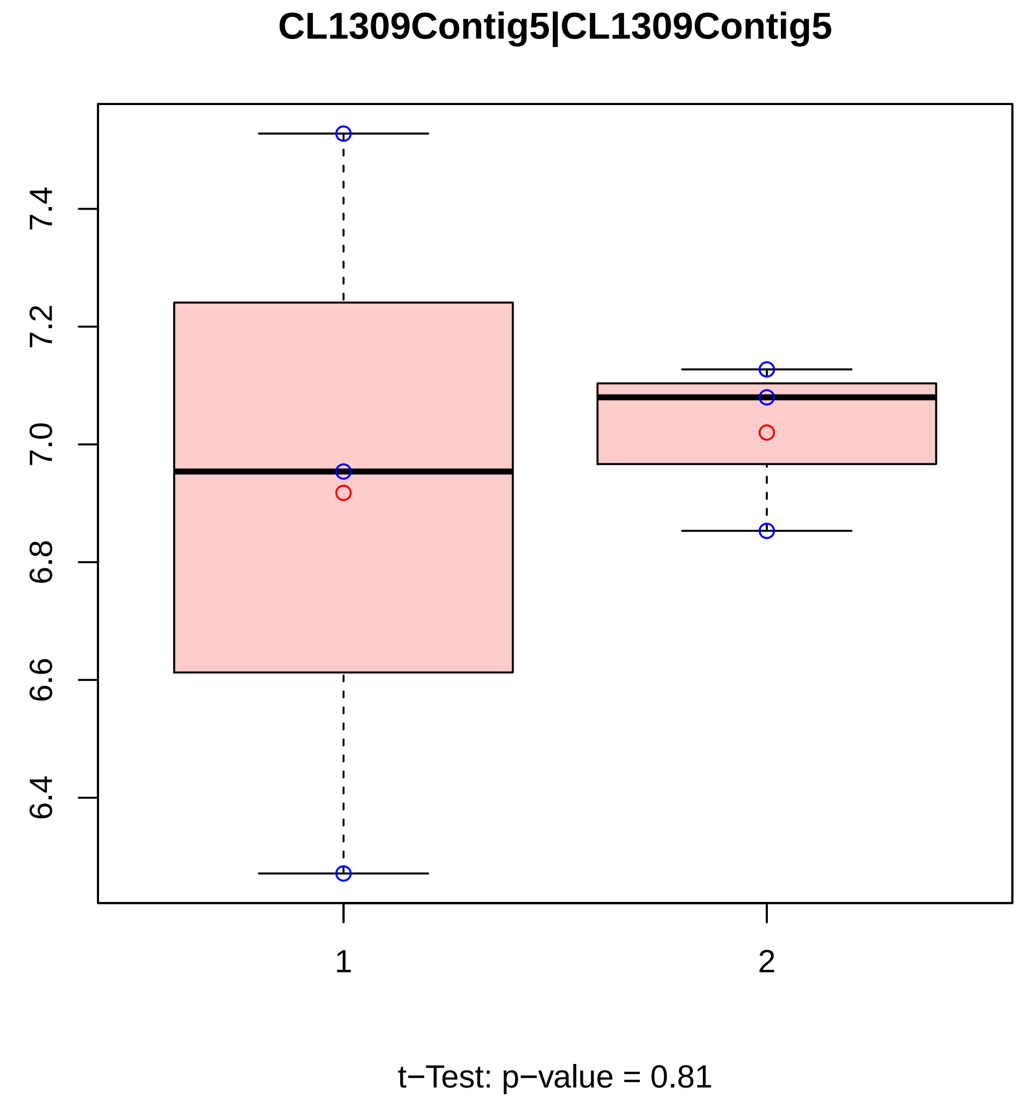
CL1309Contig5|CL1309Contig5
7.4
7.2
7.0
6.8
6.6
6.4
1
2
t−Test: p−value = 0.81

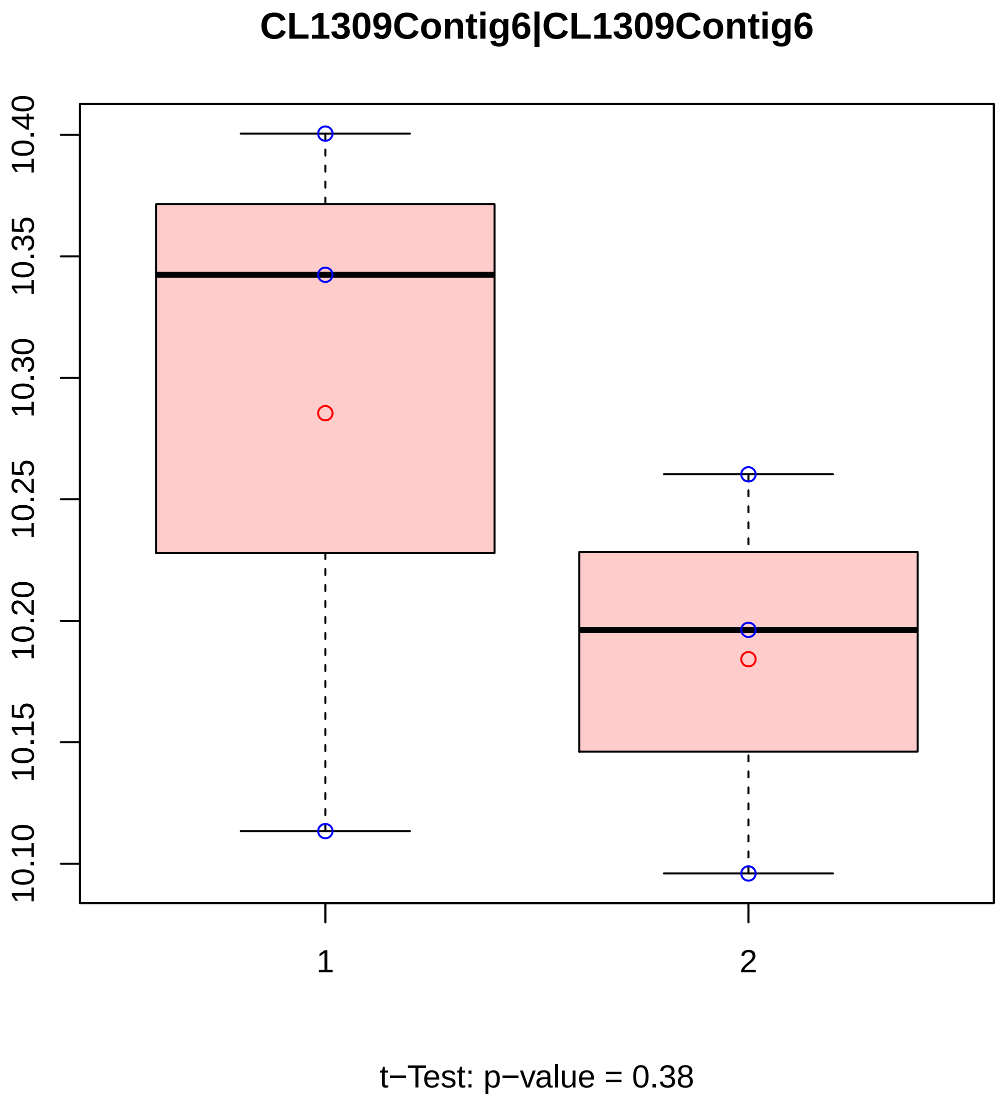
CL1309Contig6|CL1309Contig6
10.40
10.35
10.30
10.25
10.20
10.15
10.10
1
2
t−Test: p−value = 0.38

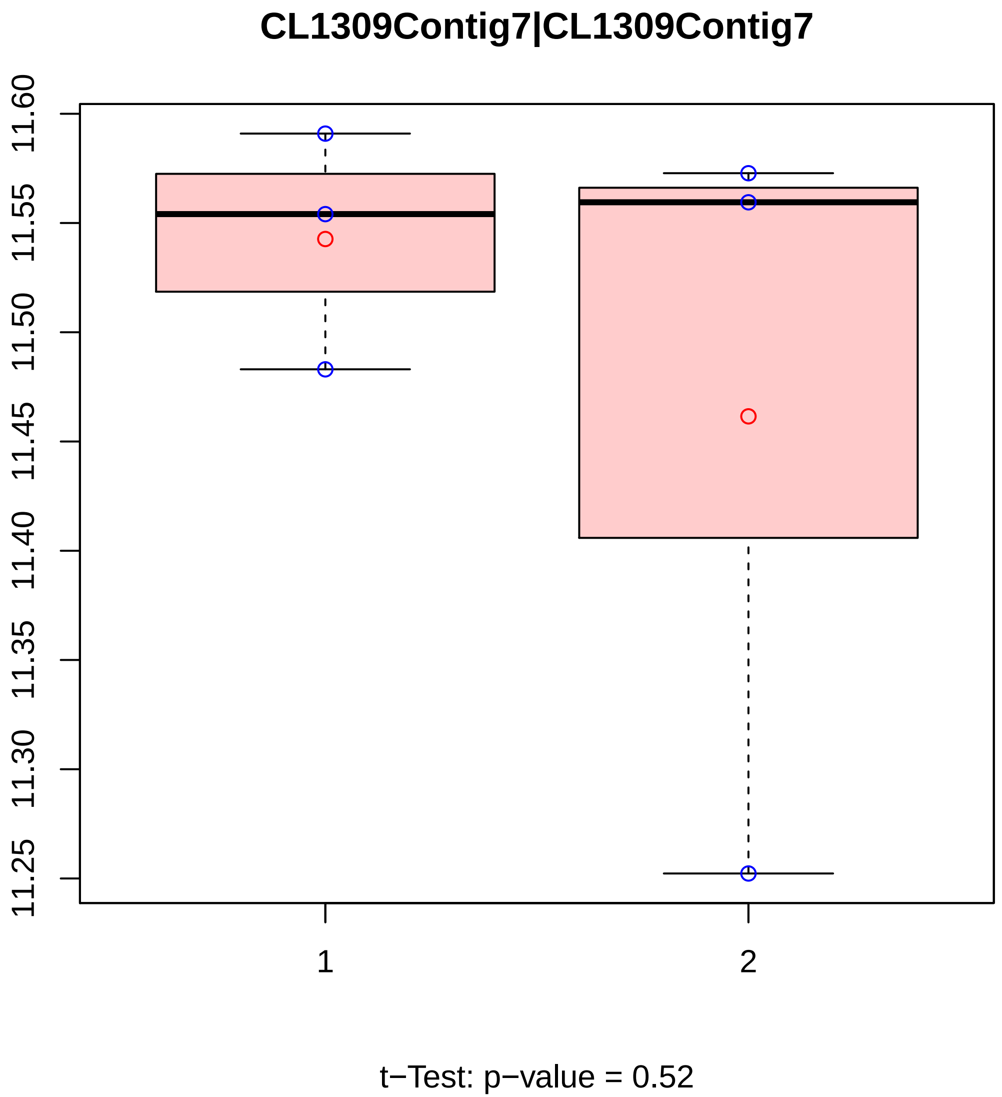
CL1309Contig7|CL1309Contig7
11.60
11.55
11.50
11.45
11.40
11.35
11.30
11.25
1
2
t−Test: p−value = 0.52

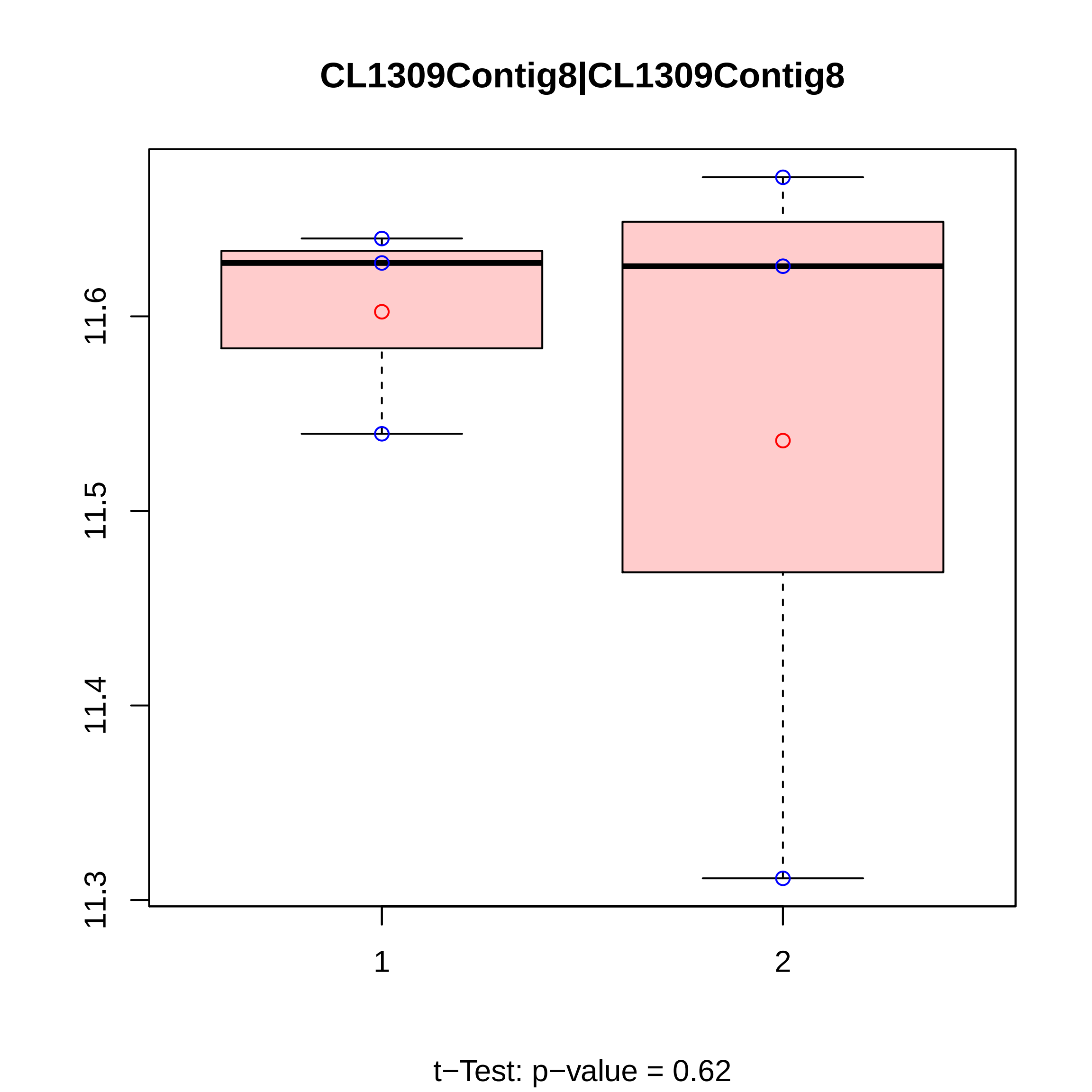
CL1309Contig8|CL1309Contig8
11.3
11.4
11.5
11.6
1
2
t−Test: p−value = 0.62

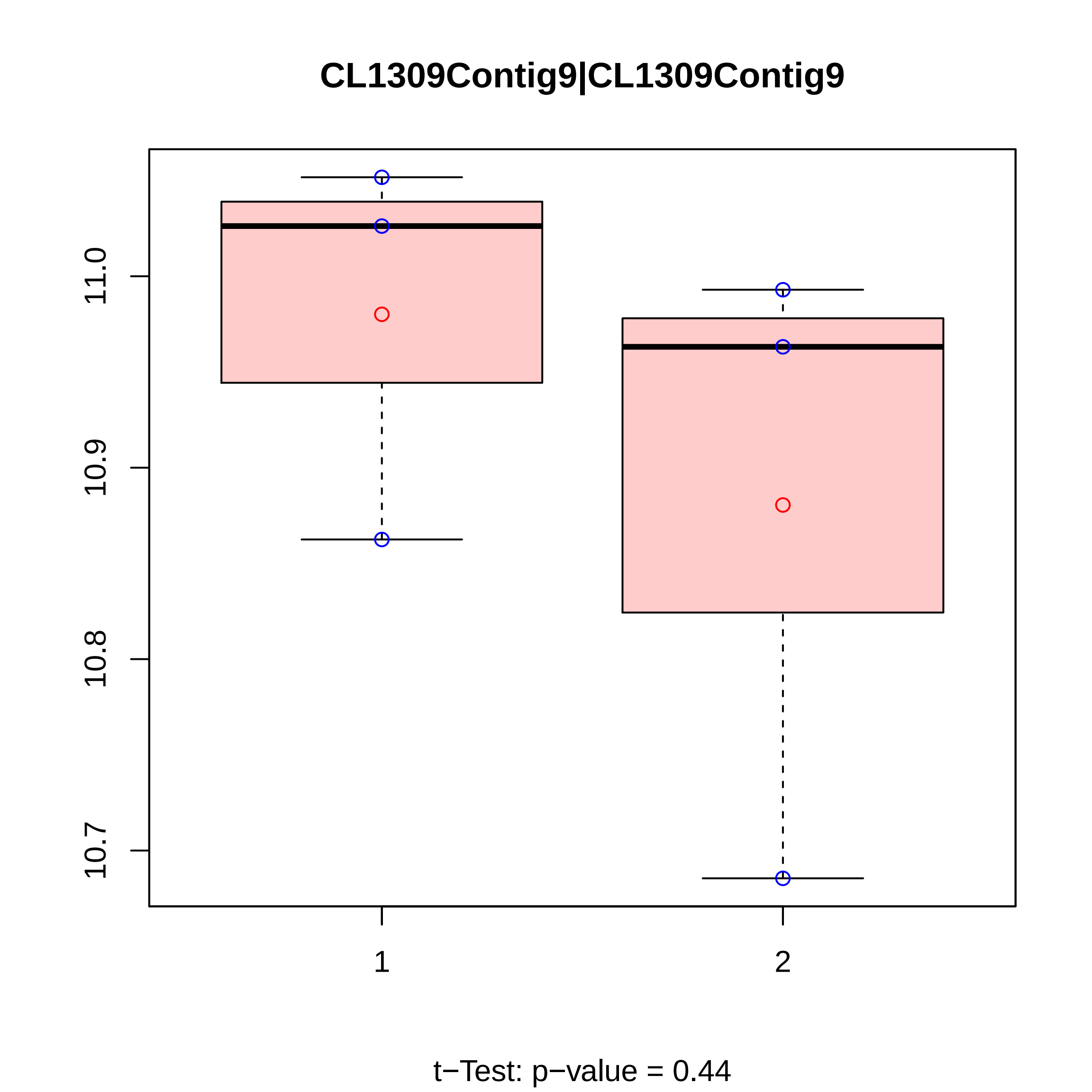
CL1309Contig9|CL1309Contig9
11.0
10.9
10.8
10.7
1
2
t−Test: p−value = 0.44

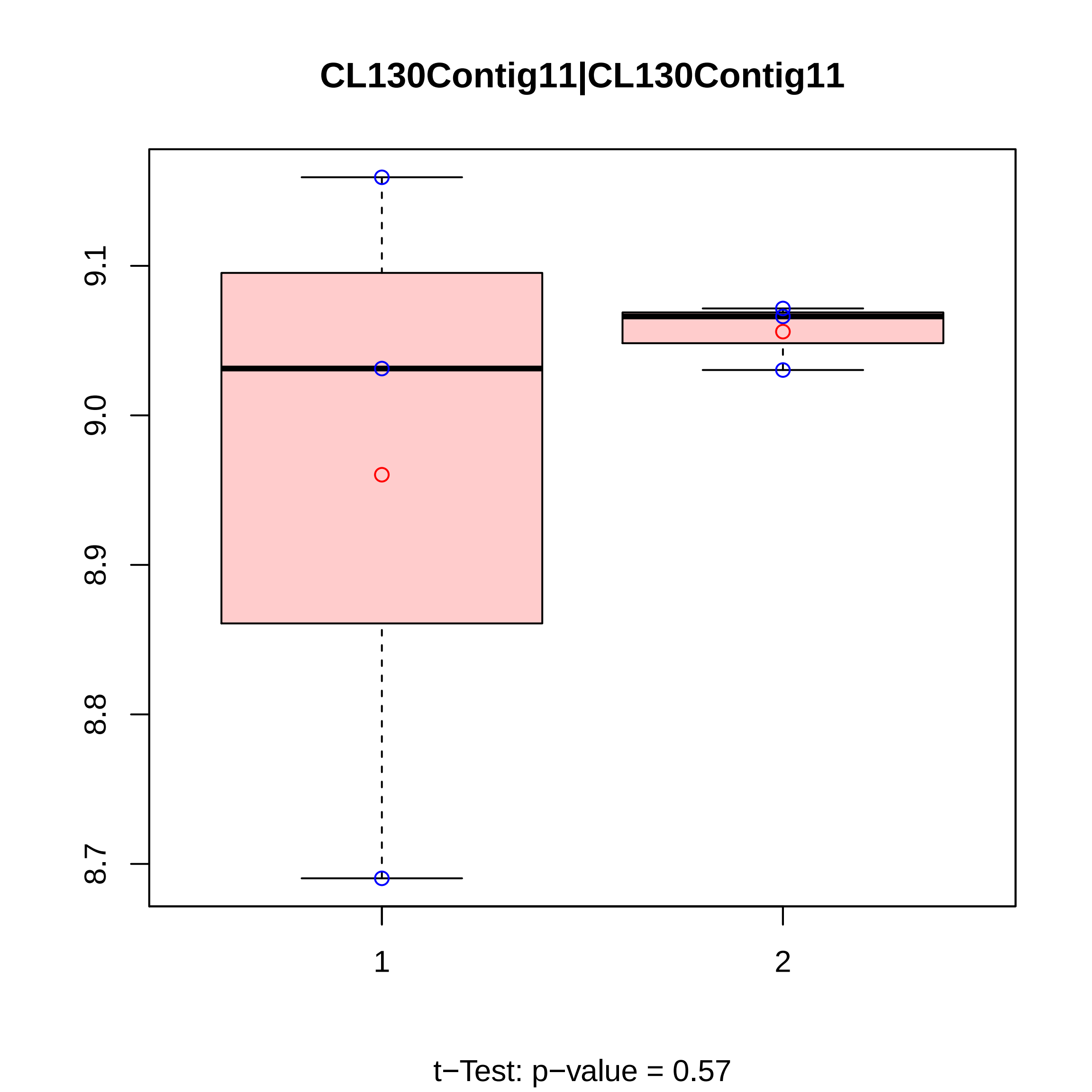
CL130Contig11|CL130Contig11
9.1
9.0
8.9
8.8
8.7
1
2
t−Test: p−value = 0.57

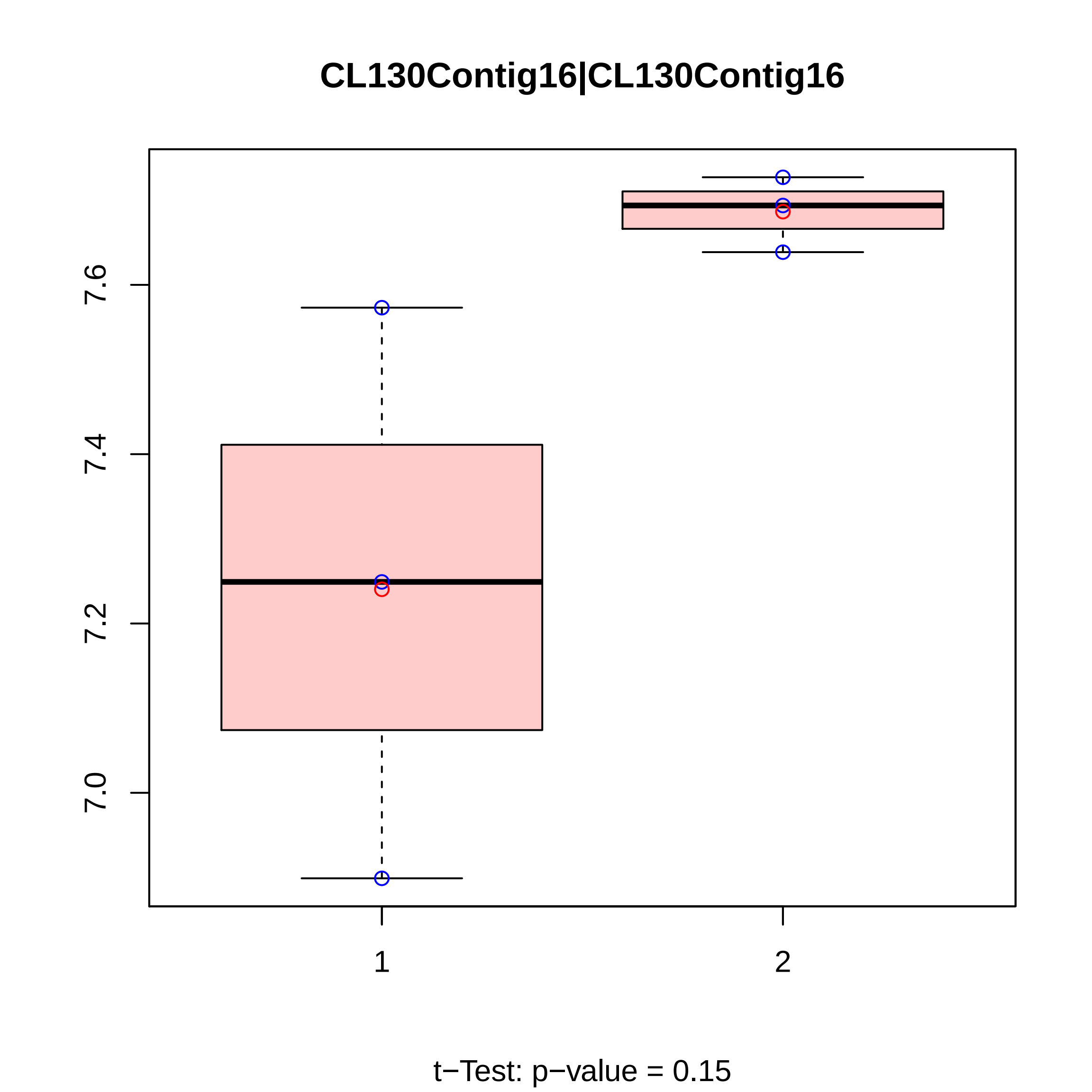

CL130Contig16|CL130Contig16
7.0
7.2
7.4
7.6
1
2
t−Test: p−value = 0.15

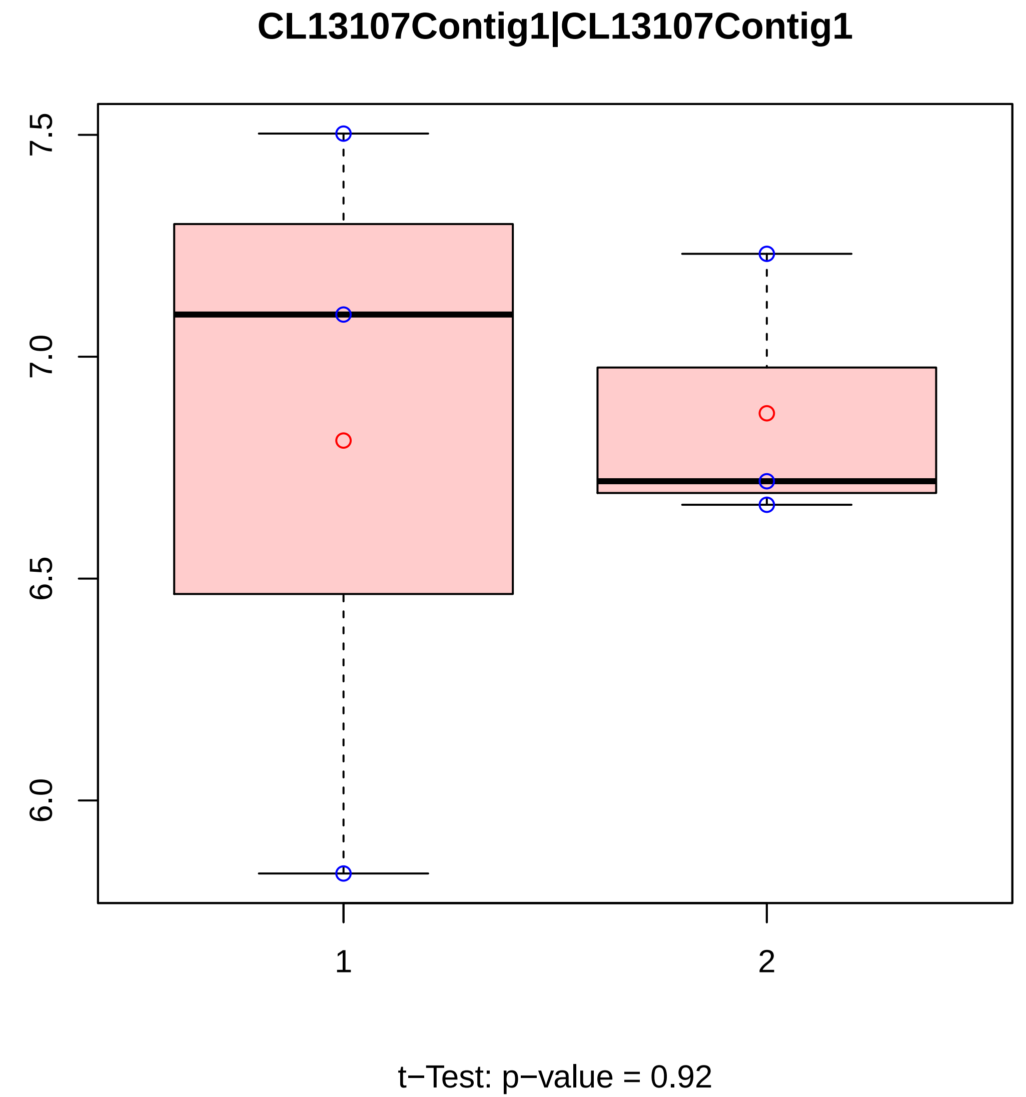
CL13107Contig1|CL13107Contig1
7.5
7.0
6.5
6.0
1
2
t−Test: p−value = 0.92

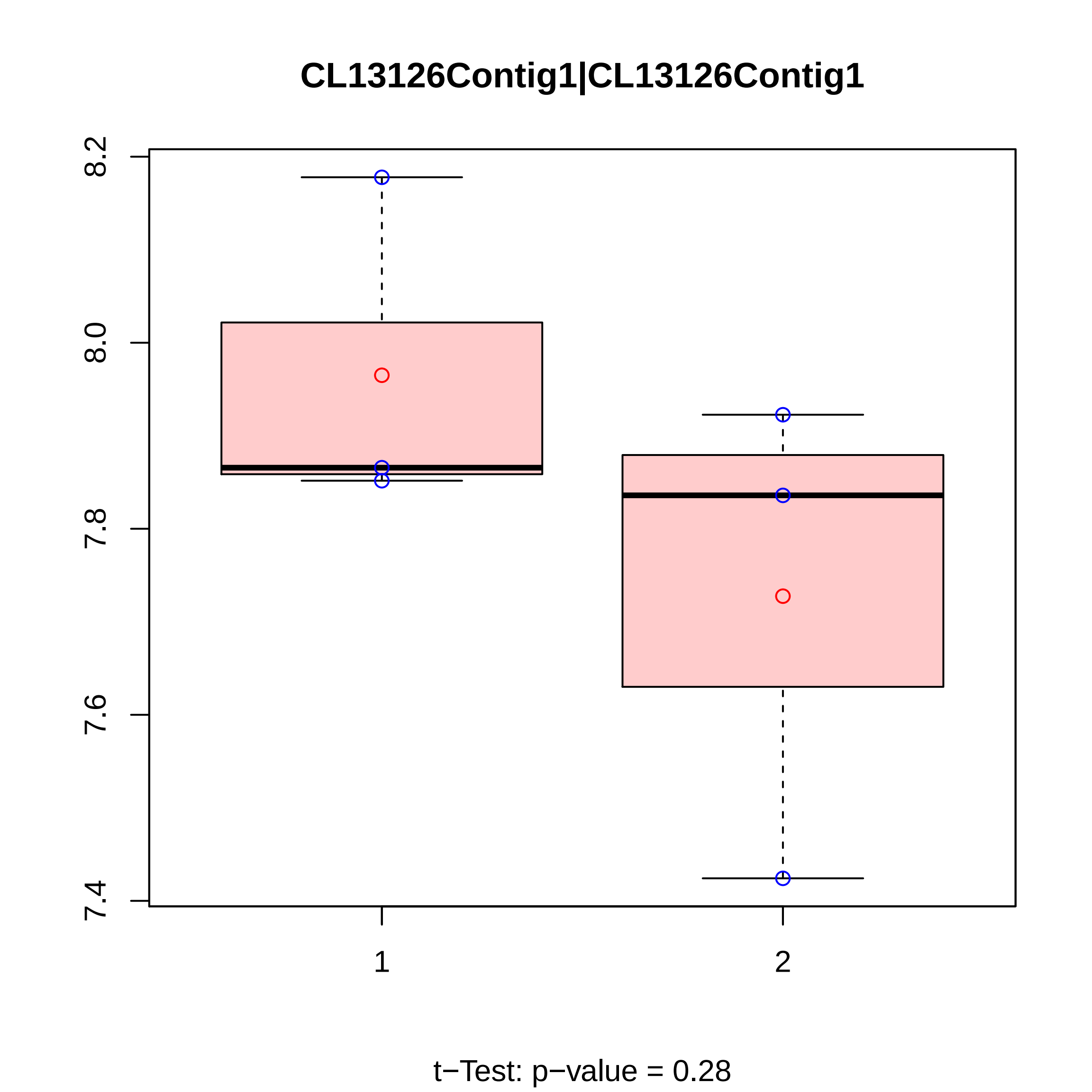
CL13126Contig1|CL13126Contig1
8.2
8.0
7.8
7.6
7.4
1
2
t−Test: p−value = 0.28

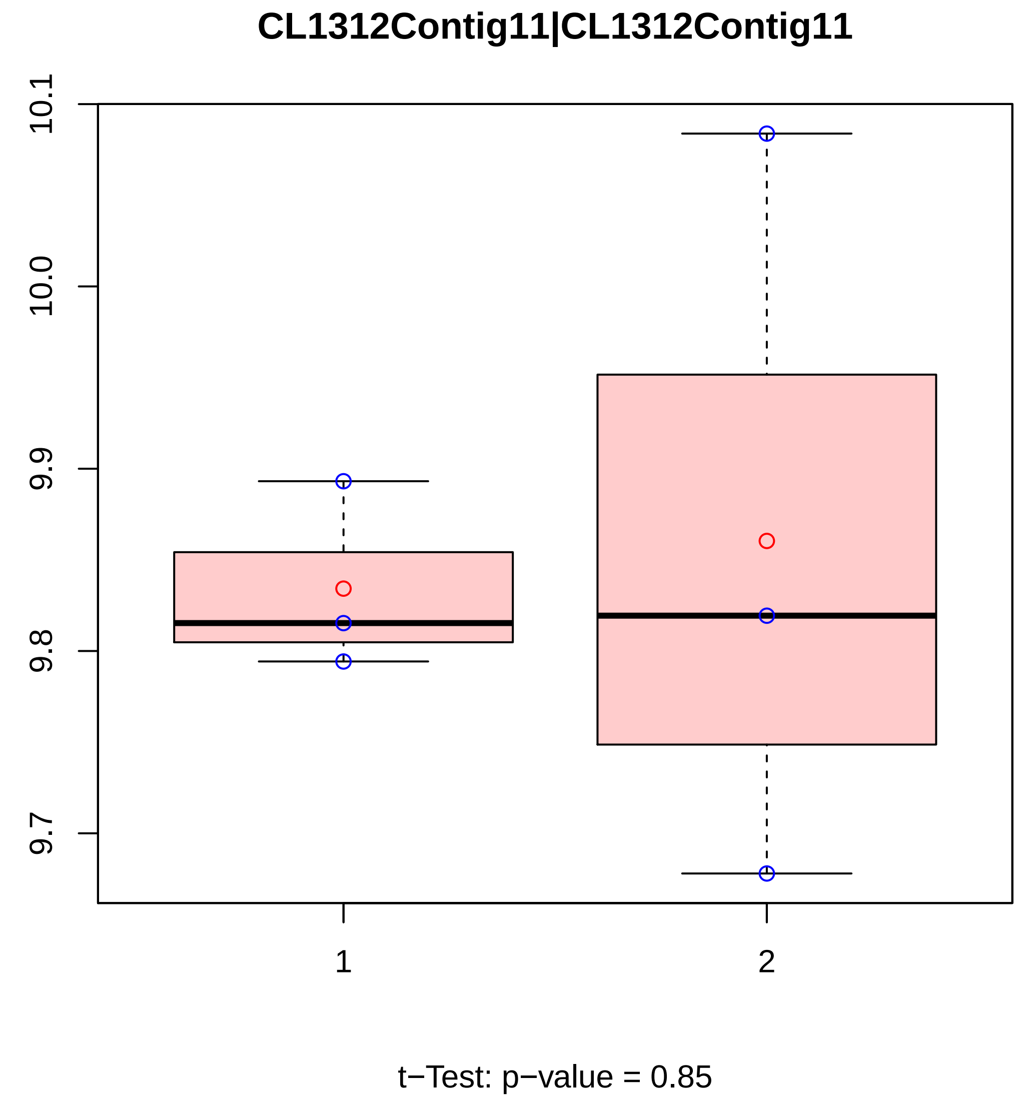
CL1312Contig11|CL1312Contig11
10.1
10.0
9.9
9.8
9.7
1
2
t−Test: p−value = 0.85

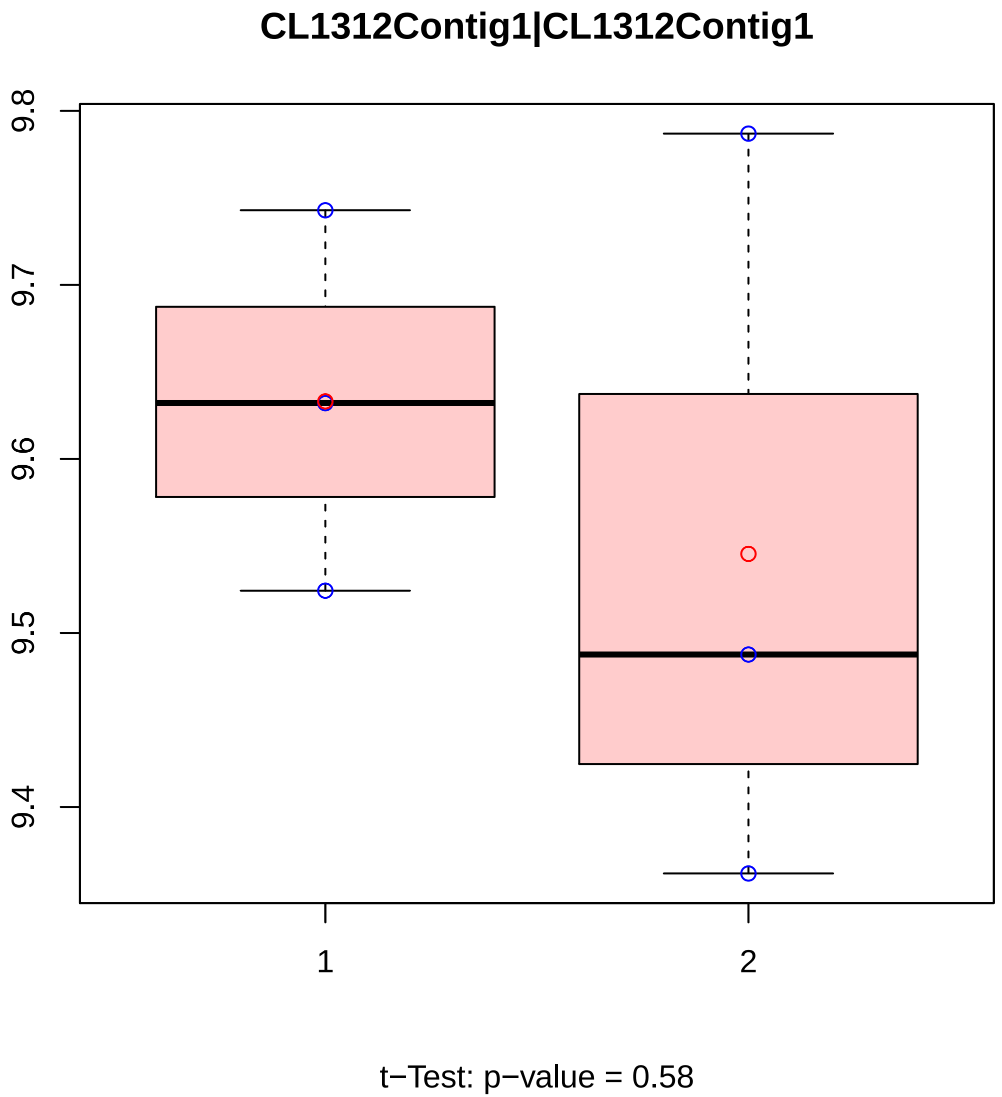
CL1312Contig1|CL1312Contig1
9.8
9.7
9.6
9.5
9.4
1
2
t−Test: p−value = 0.58

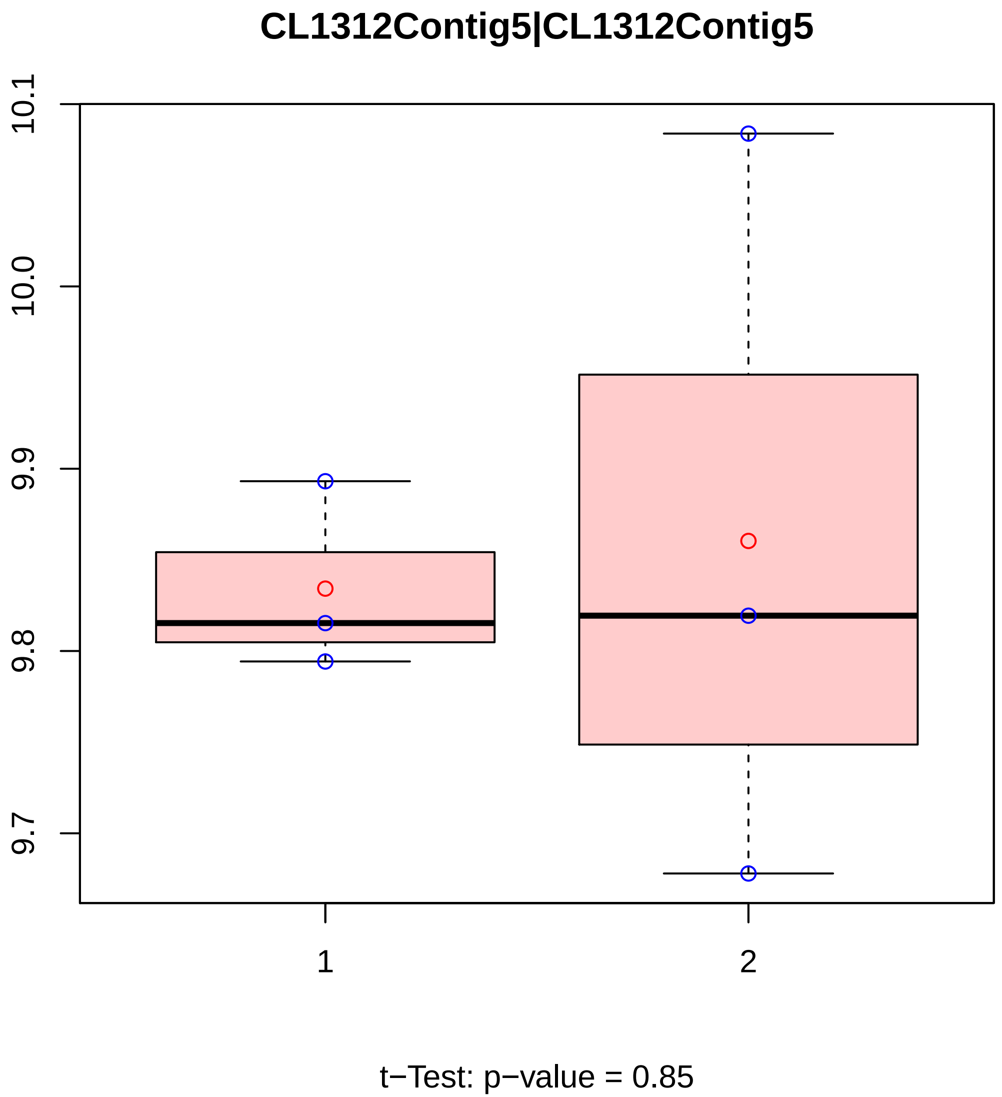
CL1312Contig5|CL1312Contig5
10.1
10.0
9.9
9.8
9.7
1
2
t−Test: p−value = 0.85

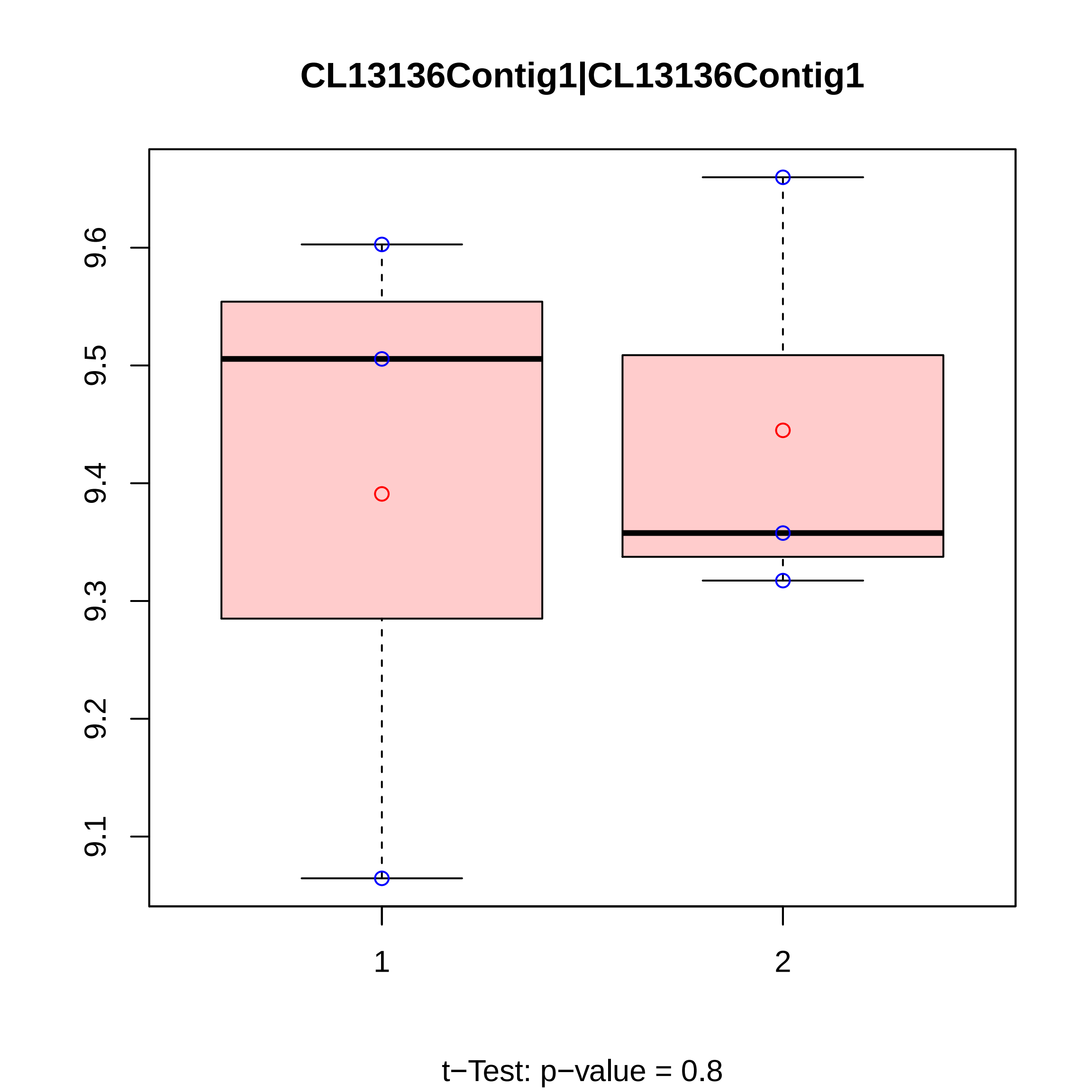
CL13136Contig1|CL13136Contig1
9.1
9.2
9.3
9.4
9.5
9.6
1
2
t−Test: p−value = 0.8

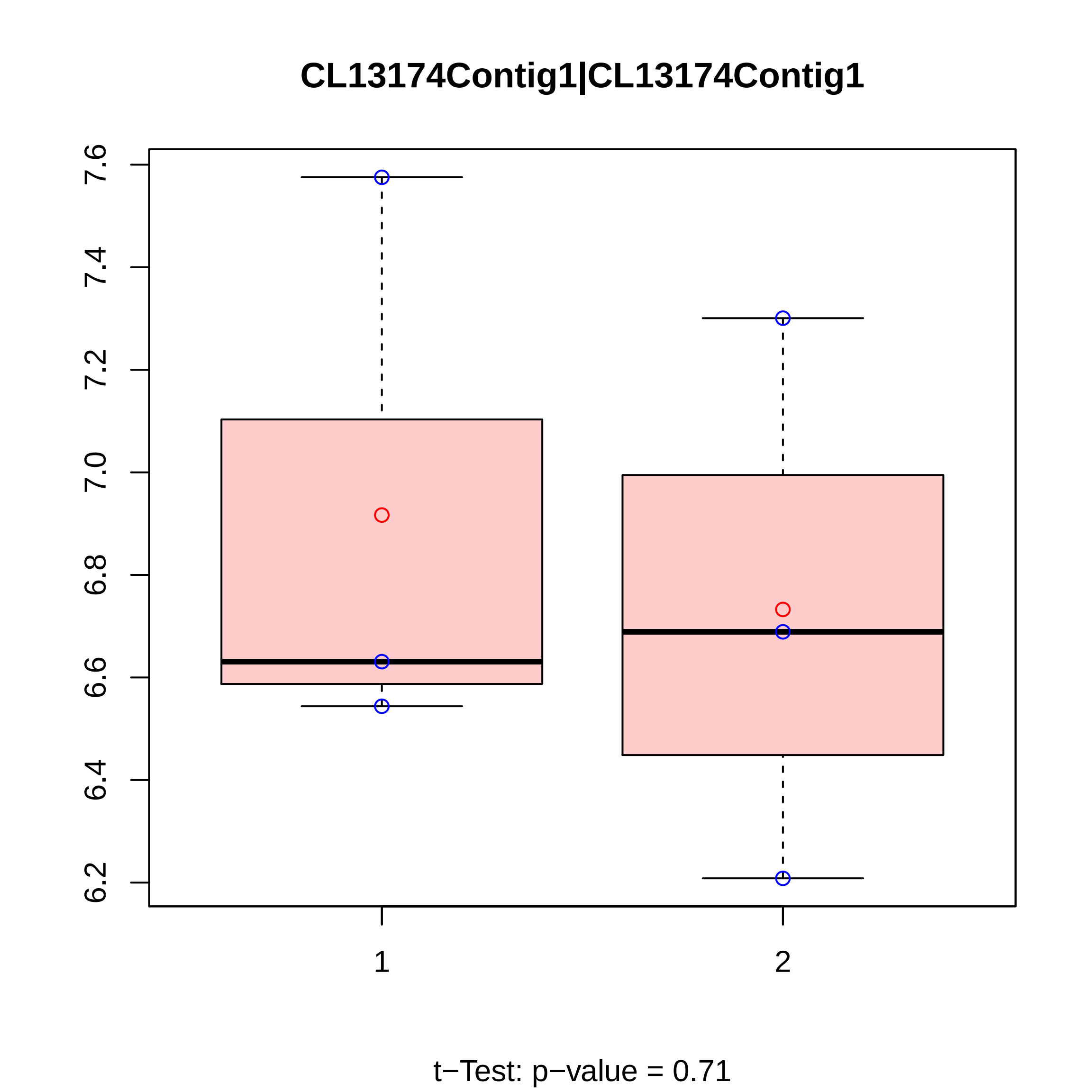
CL13174Contig1|CL13174Contig1
7.6
7.4
7.2
7.0
6.8
6.6
6.4
6.2
1
2
t−Test: p−value = 0.71

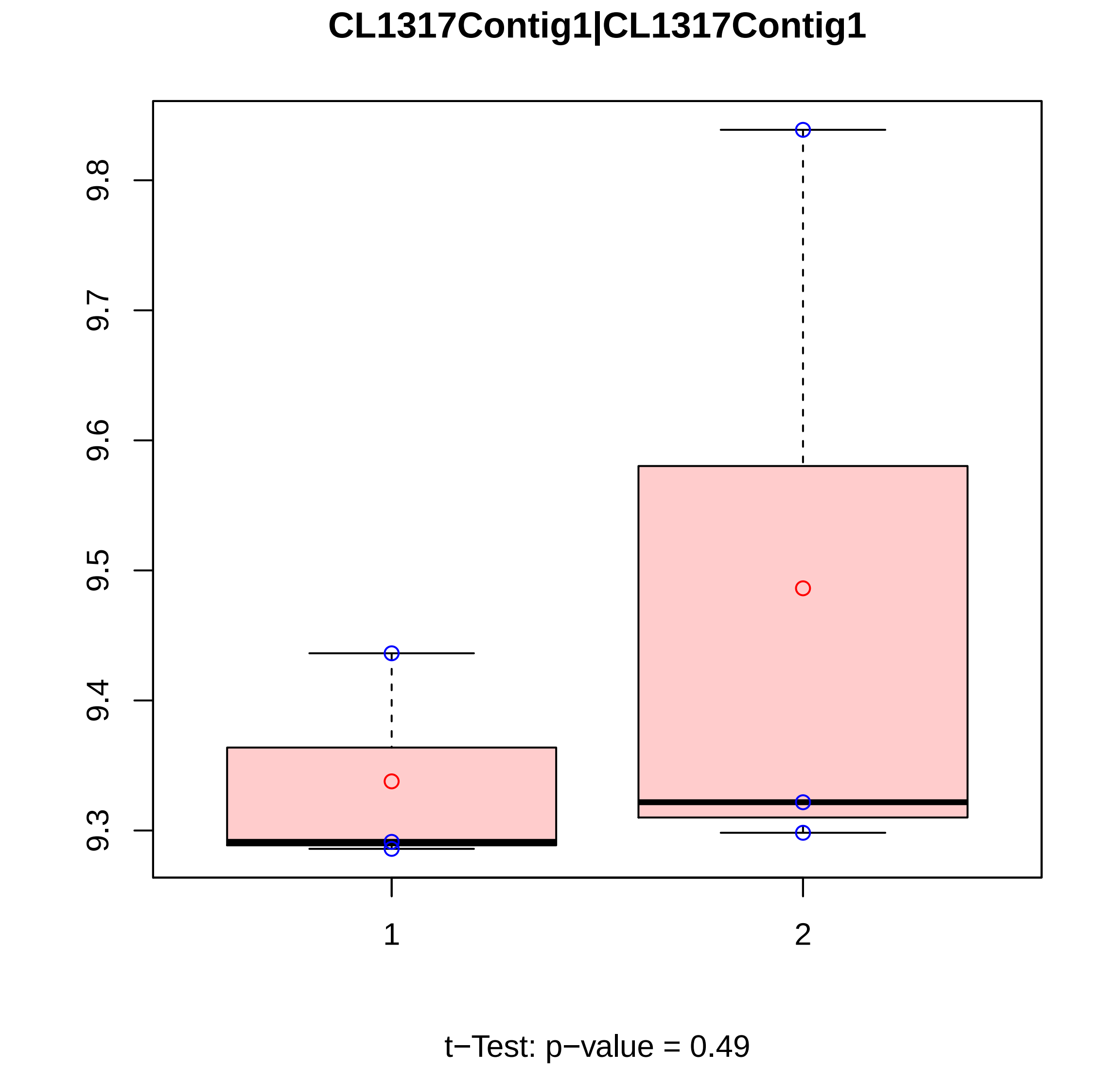
CL1317Contig1|CL1317Contig1
9.3
9.4
9.5
9.6
9.7
9.8
1
2
t−Test: p−value = 0.49

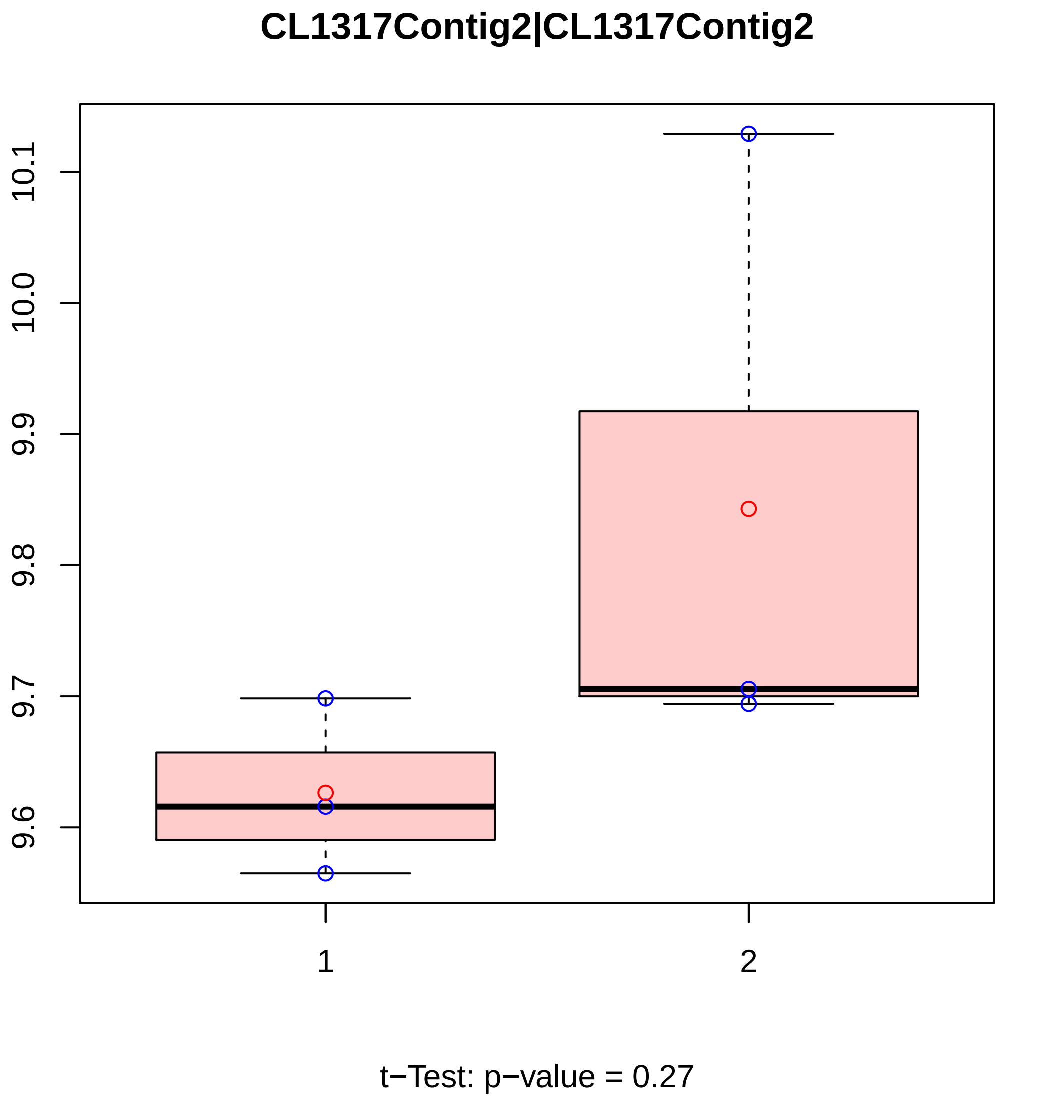

CL1317Contig2|CL1317Contig2
10.1
10.0
9.9
9.8
9.7
9.6
1
2
t−Test: p−value = 0.27

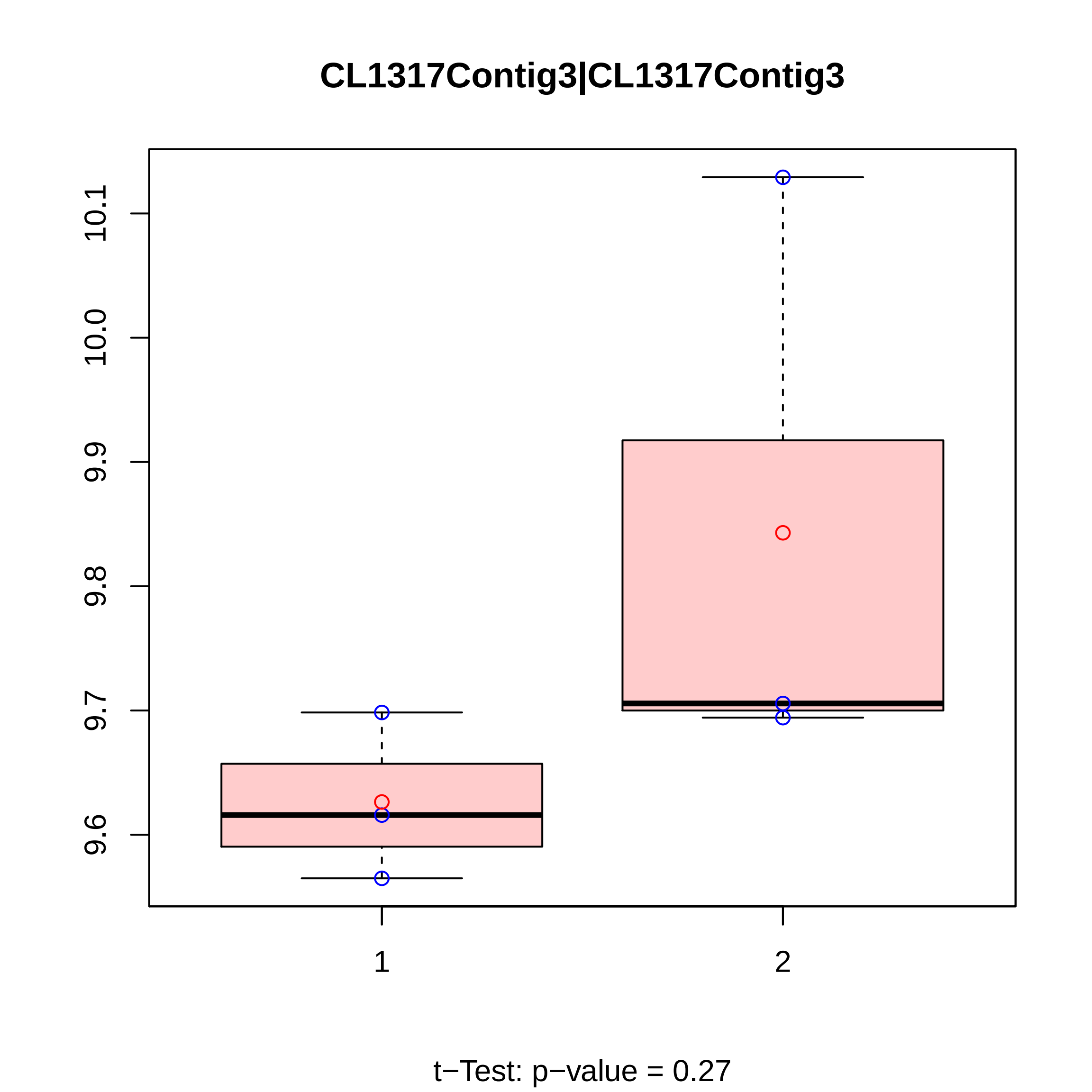
CL1317Contig3|CL1317Contig3
10.1
10.0
9.9
9.8
9.7
9.6
1
2
t−Test: p−value = 0.27

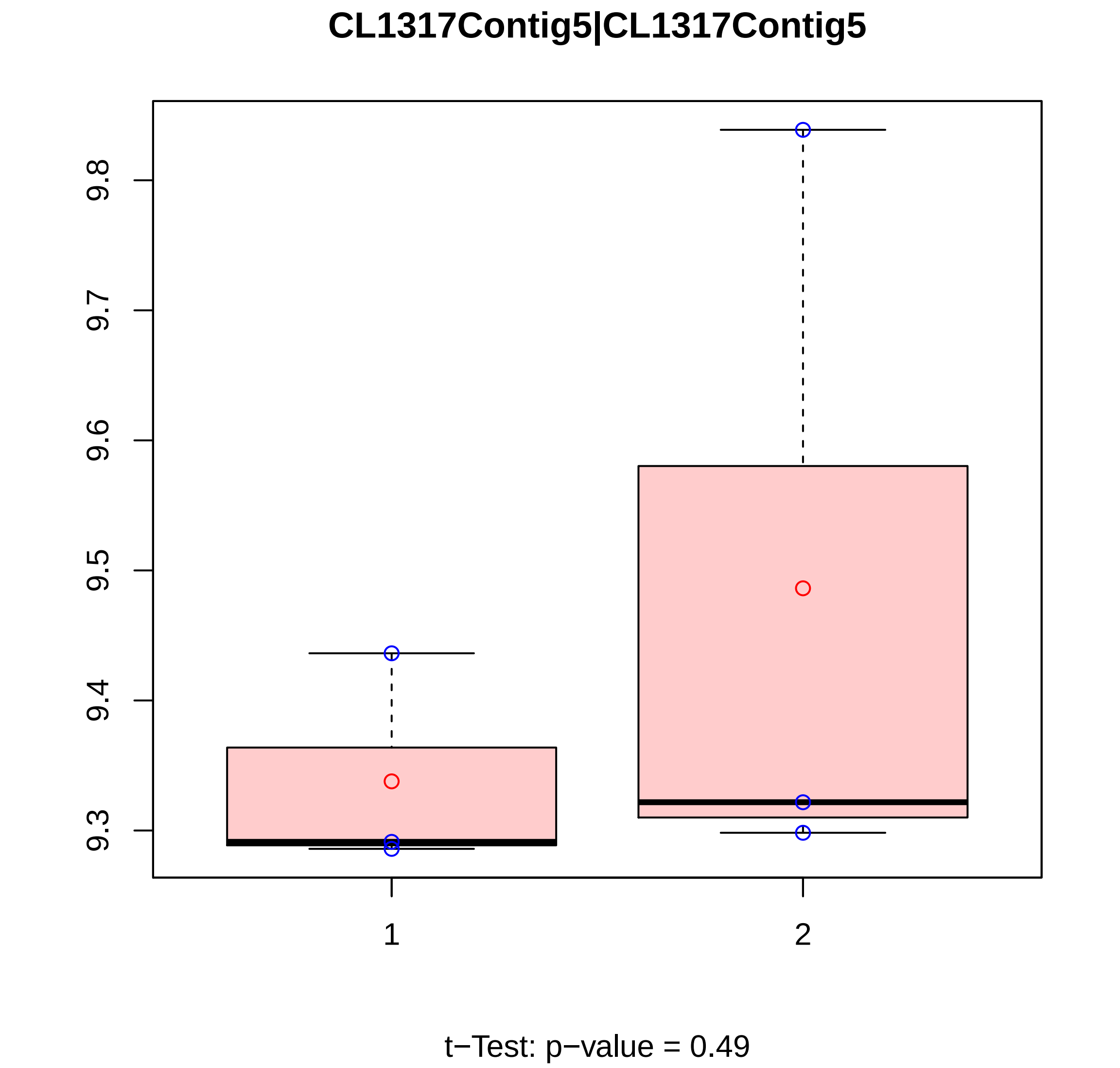
CL1317Contig5|CL1317Contig5
9.3
9.4
9.5
9.6
9.7
9.8
1
2
t−Test: p−value = 0.49

# CL1317Contig6|CL1317Contig6

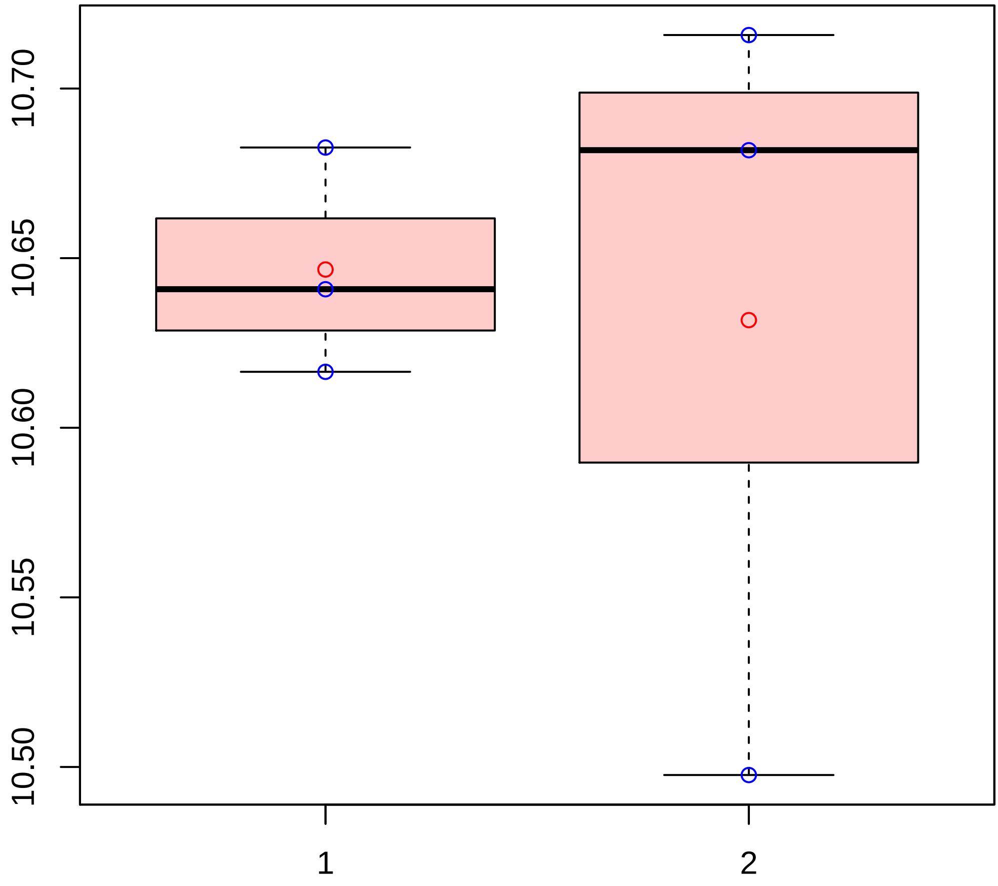


t−Test: p−value = 0.85

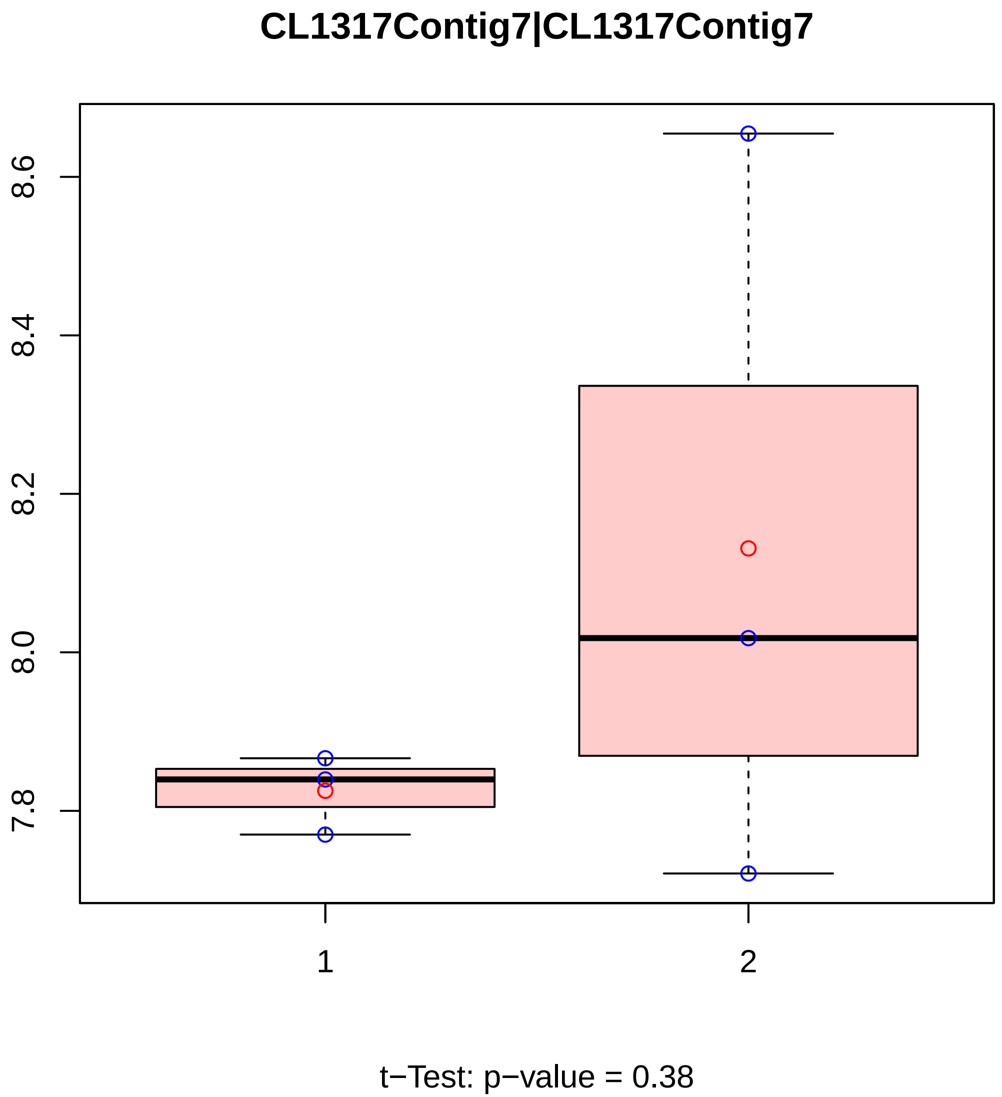
CL1317Contig7|CL1317Contig7
8.6
8.4
8.2
8.0
7.8
1
2
t−Test: p−value = 0.38

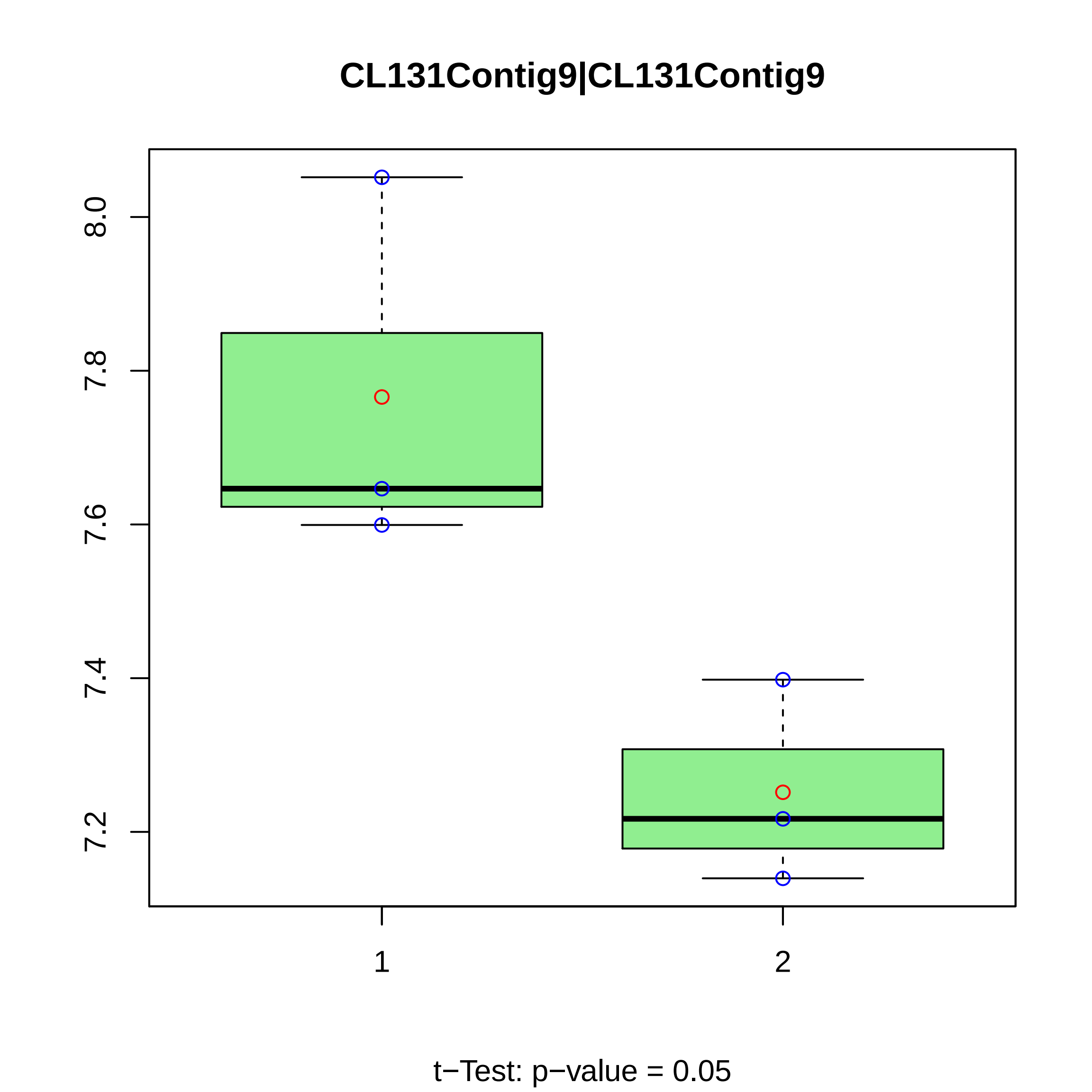

CL131Contig9|CL131Contig9
8.0
7.8
7.6
7.4
7.2
1
2
t−Test: p−value = 0.05

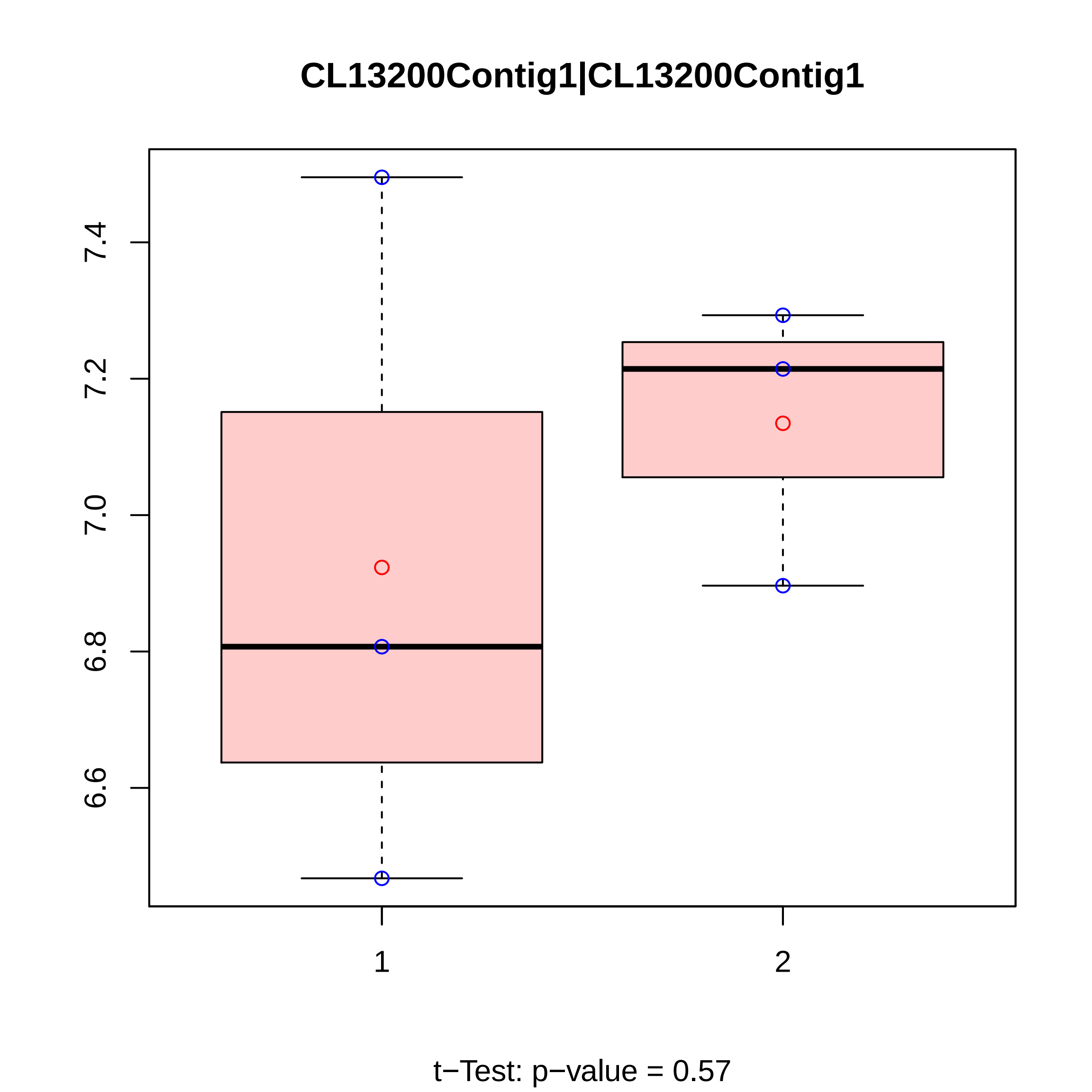
CL13200Contig1|CL13200Contig1
7.4
7.2
7.0
6.8
6.6
1
2
t−Test: p−value = 0.57

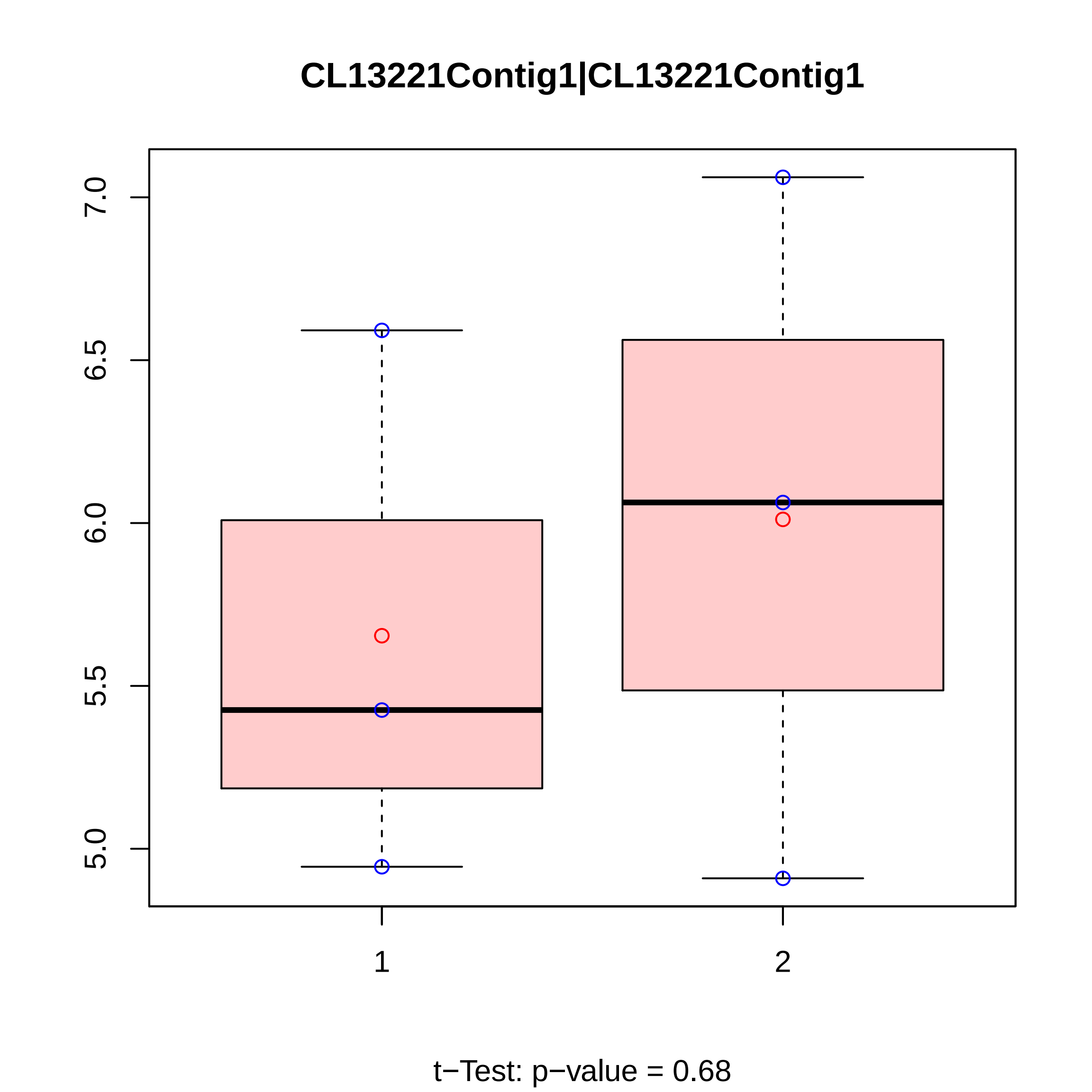

CL13221Contig1|CL13221Contig1
7.0
6.5
6.0
5.5
5.0
1
2
t−Test: p−value = 0.68

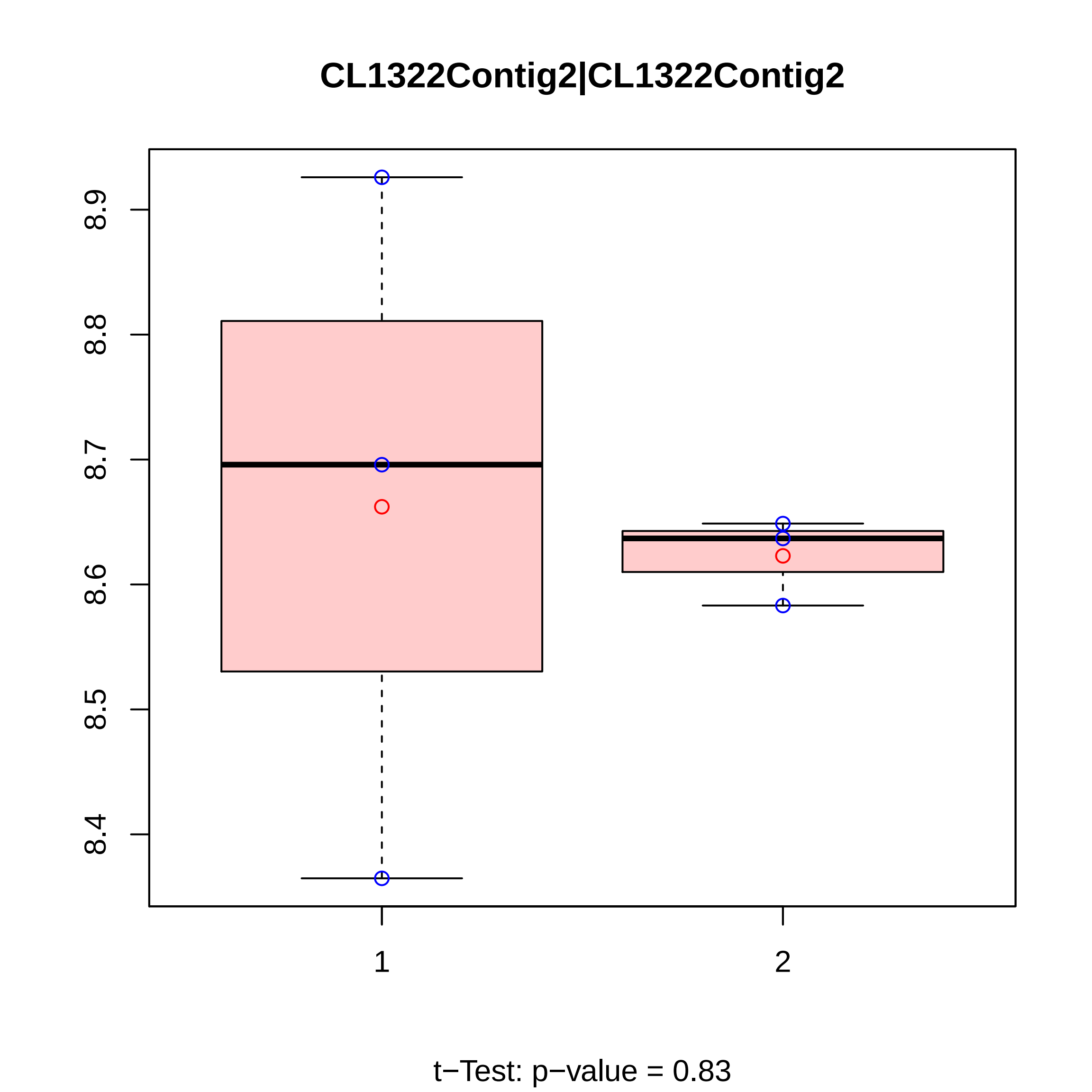
CL1322Contig2|CL1322Contig2
8.4
8.5
8.6
8.7
8.8
8.9
1
2
t−Test: p−value = 0.83

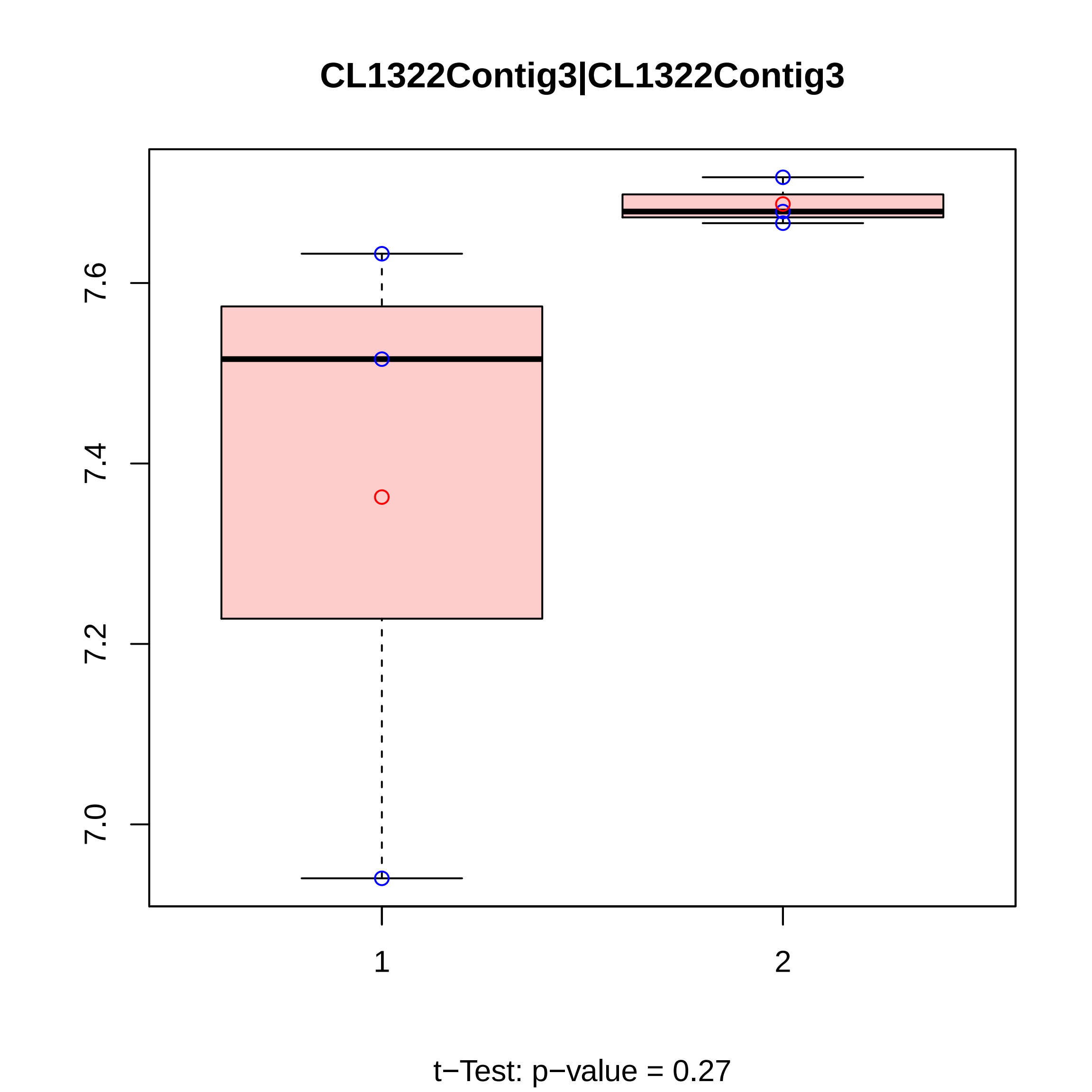
CL1322Contig3|CL1322Contig3
7.6
7.4
7.2
7.0
1
2
t−Test: p−value = 0.27

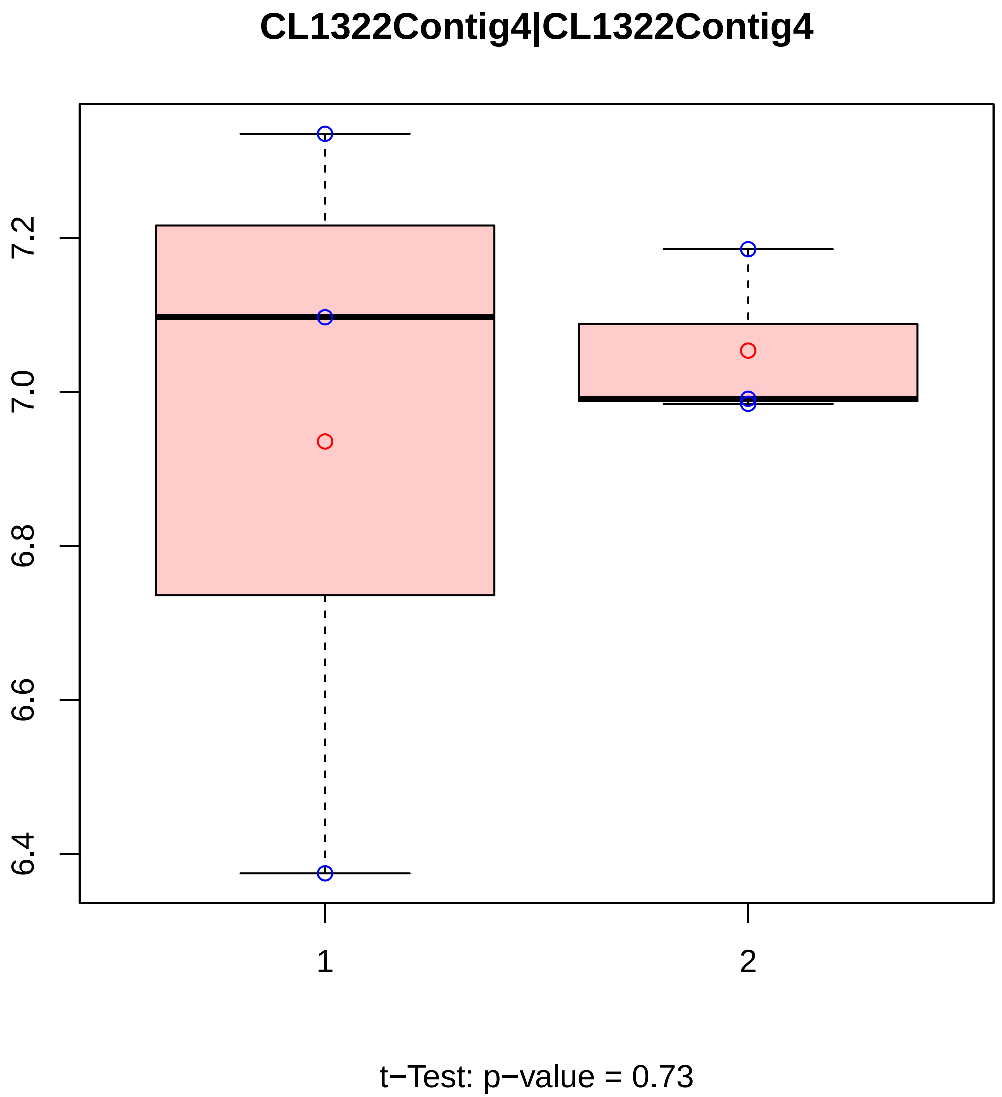
CL1322Contig4|CL1322Contig4
7.2
7.0
6.8
6.6
6.4
1
2
t−Test: p−value = 0.73

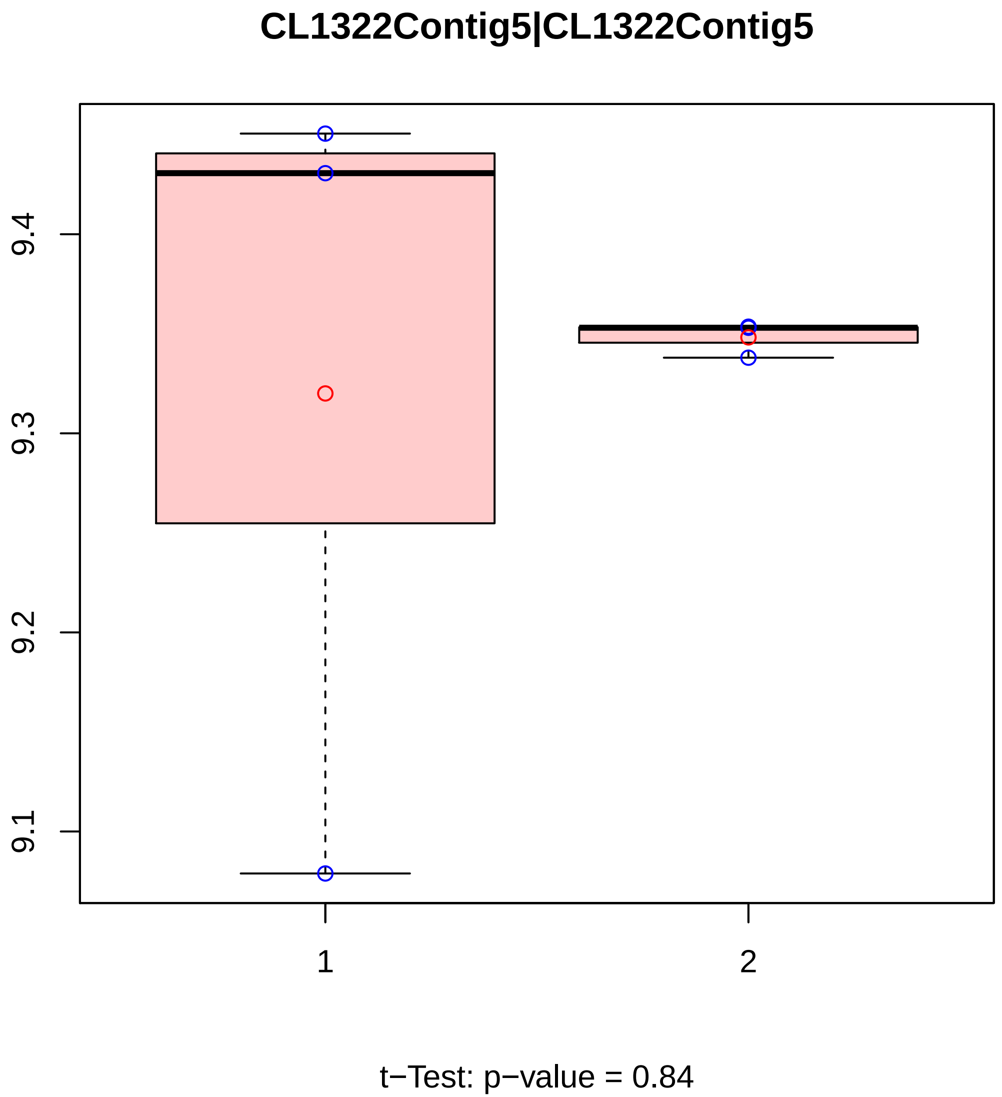
CL1322Contig5|CL1322Contig5
9.1
9.2
9.3
9.4
1
2
t−Test: p−value = 0.84

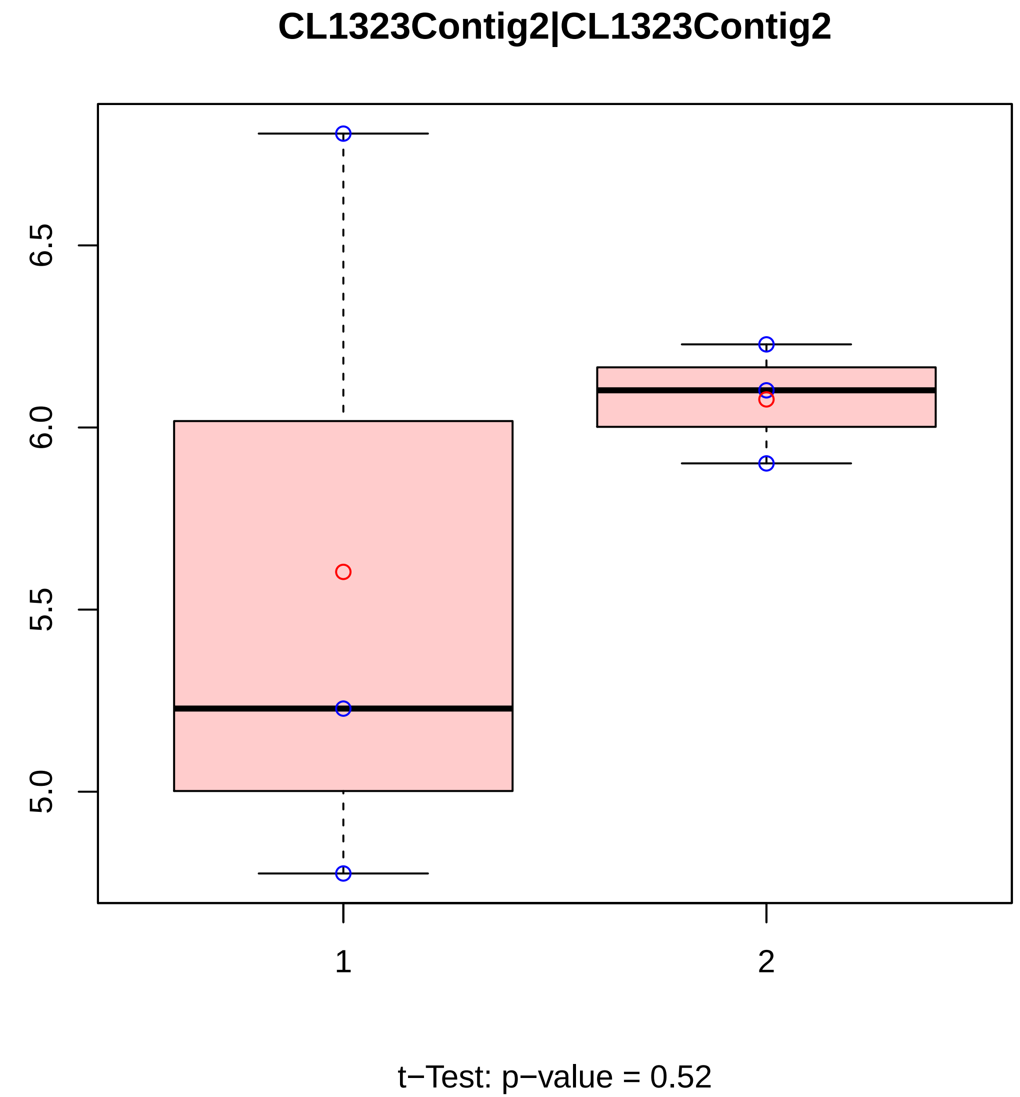
CL1323Contig2|CL1323Contig2
6.5
6.0
5.5
5.0
1
2
t−Test: p−value = 0.52

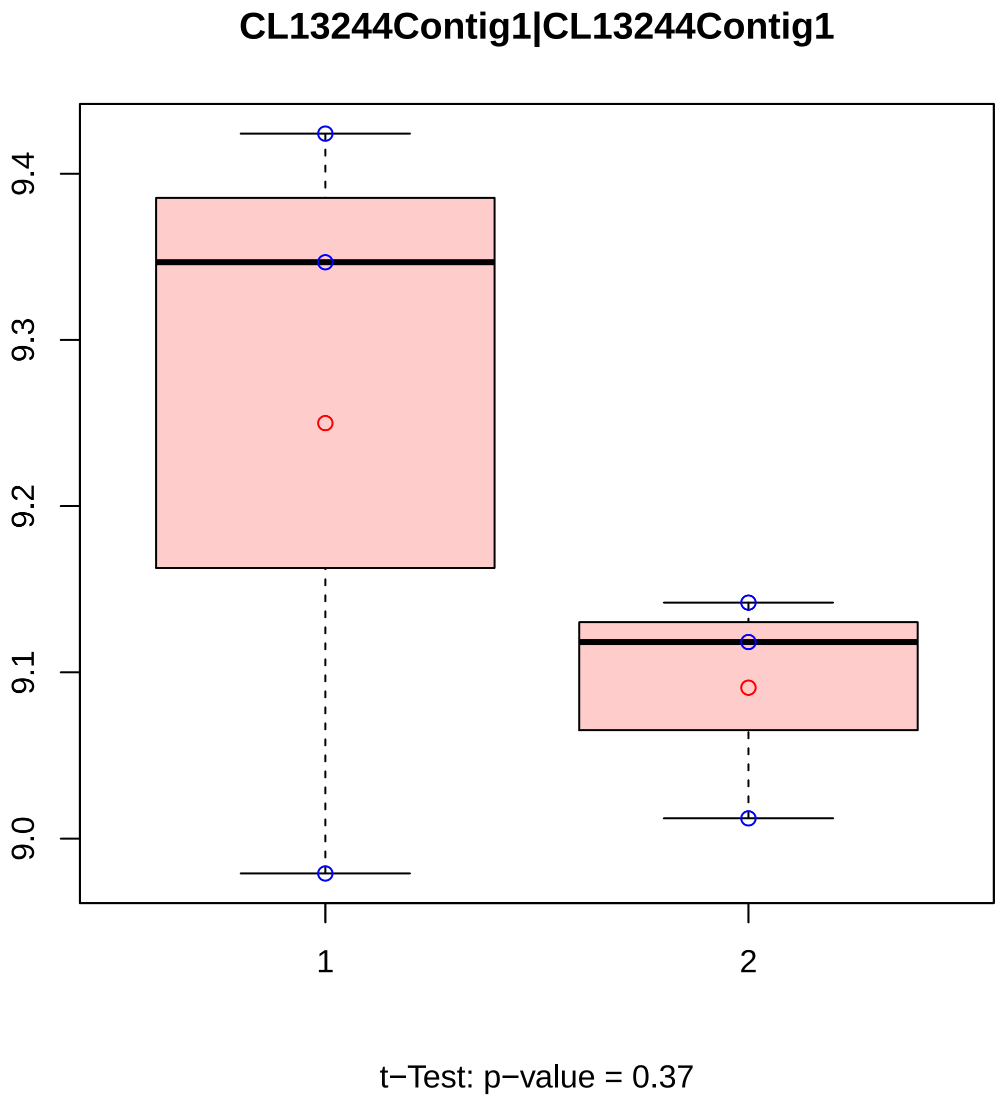
CL13244Contig1|CL13244Contig1
9.0
9.1
9.2
9.3
9.4
1
2
t−Test: p−value = 0.37

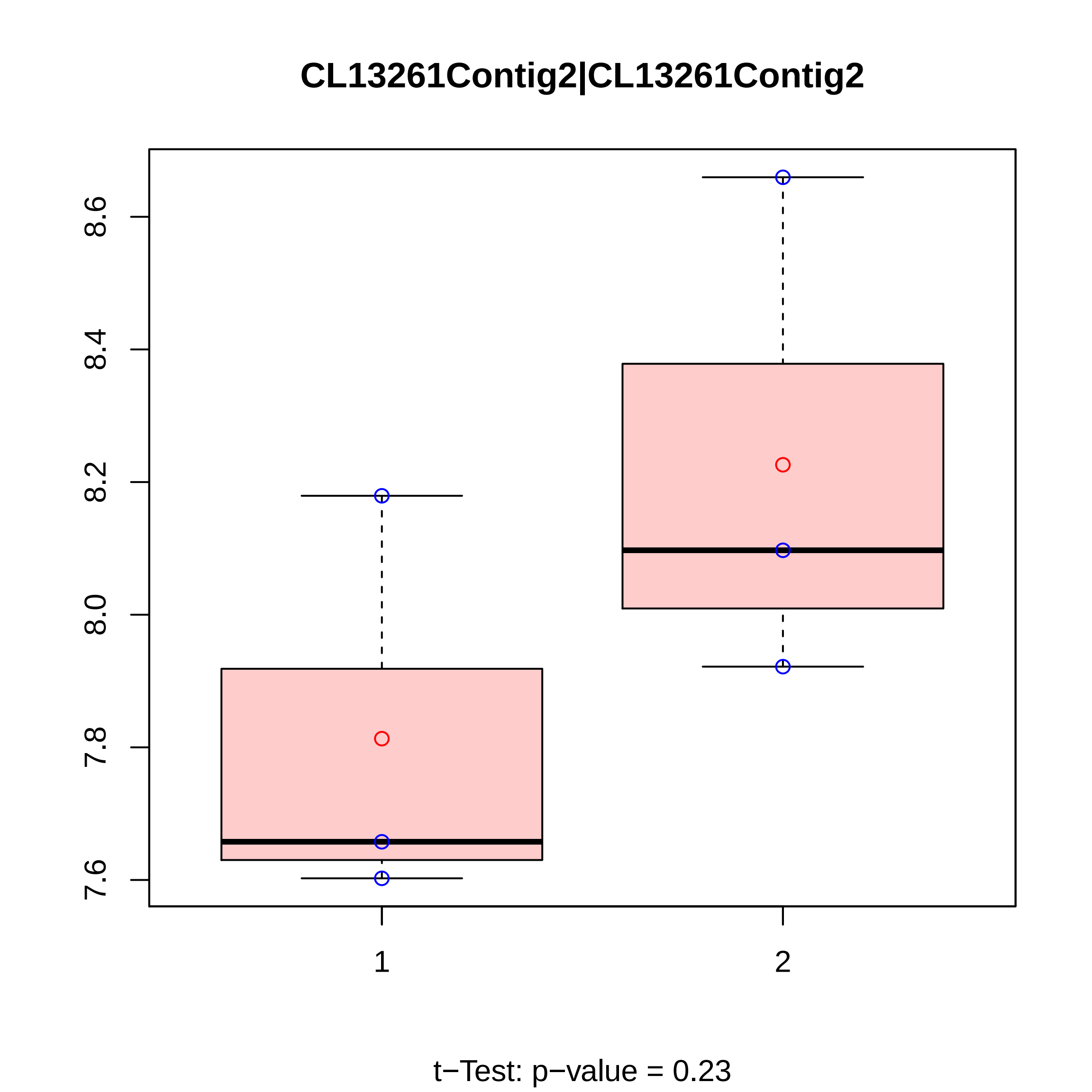

CL13261Contig2|CL13261Contig2
8.6
8.4
8.2
8.0
7.8
7.6
1
2
t−Test: p−value = 0.23

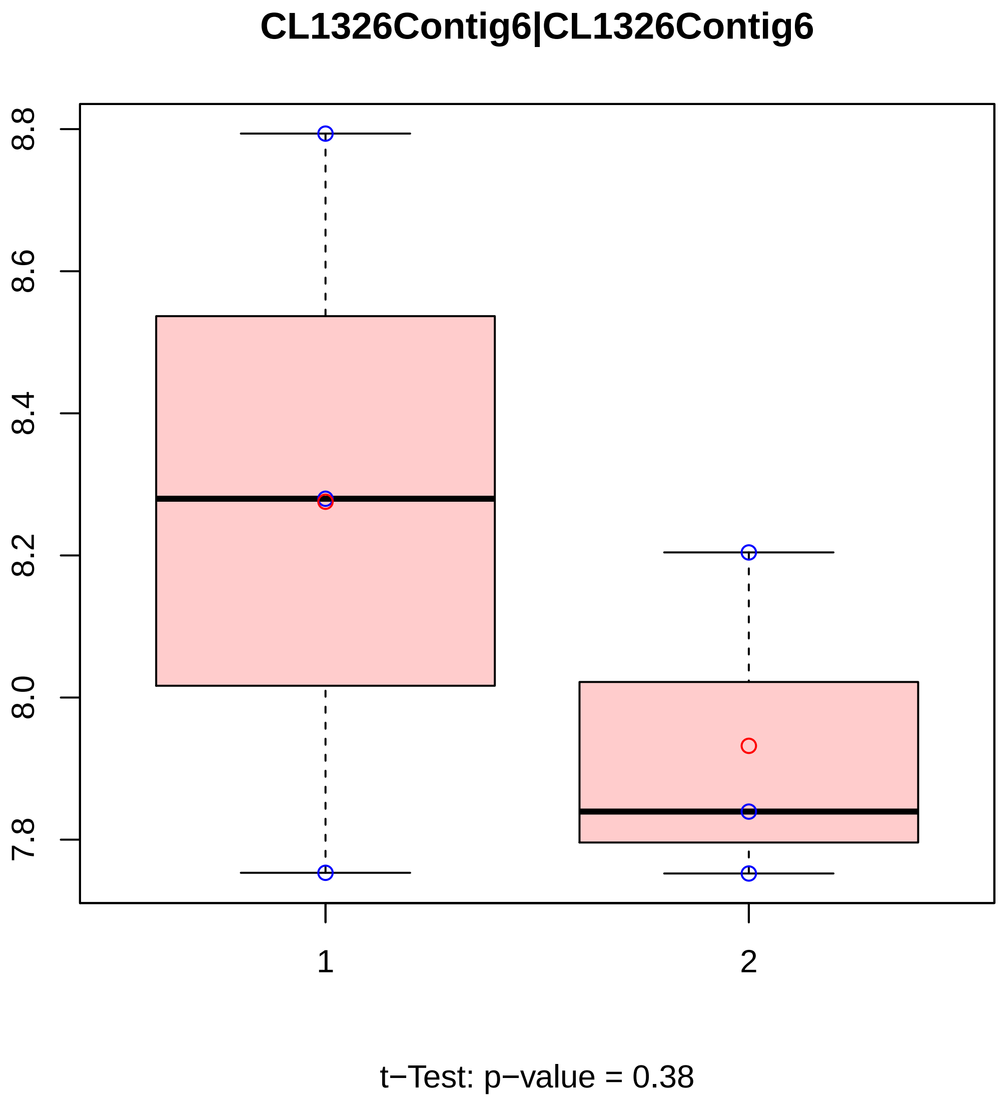
CL1326Contig6|CL1326Contig6
8.8
8.6
8.4
8.2
8.0
7.8
1
2
t−Test: p−value = 0.38

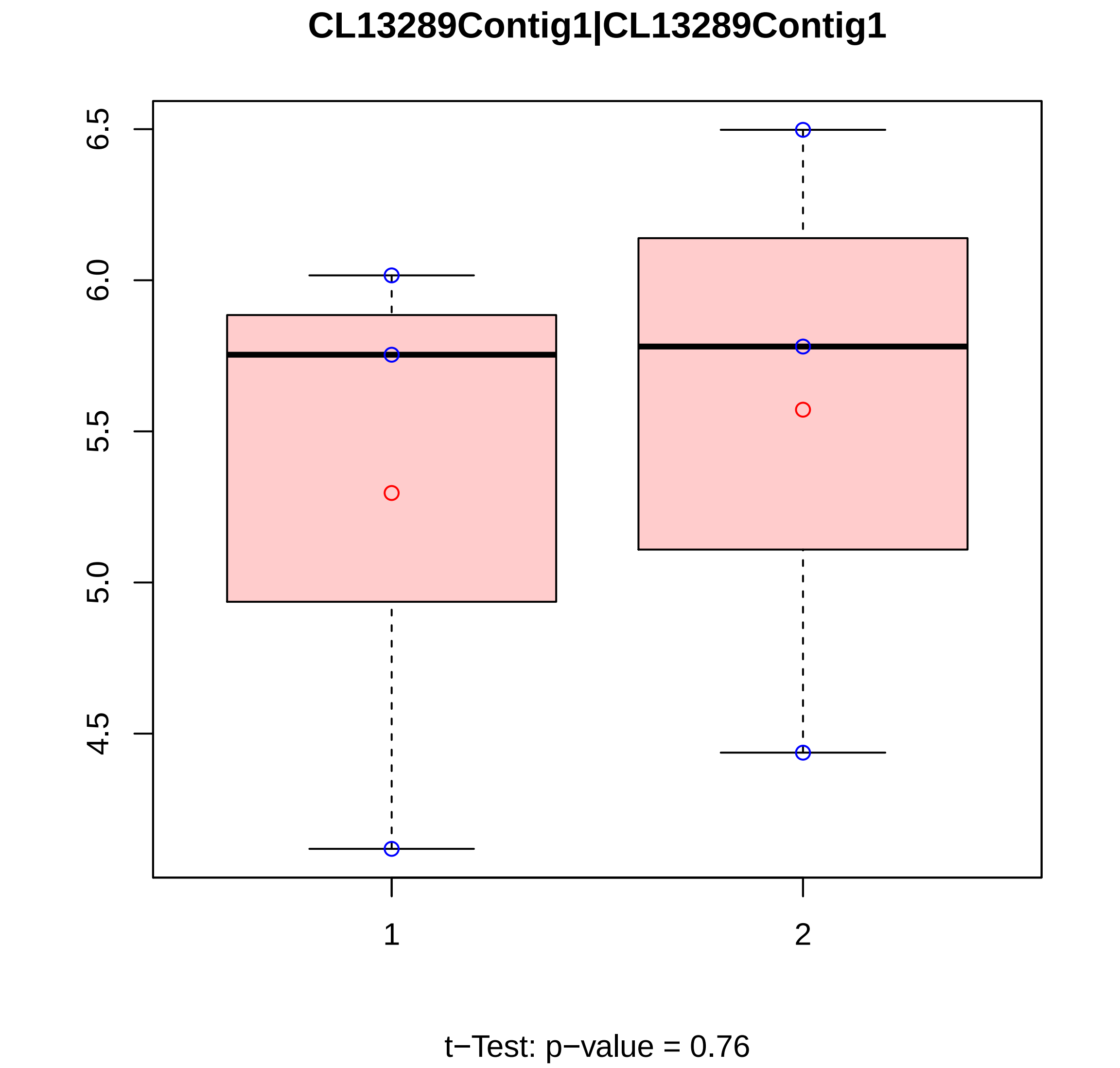
CL13289Contig1|CL13289Contig1
6.5
6.0
5.5
5.0
4.5
1
2
t−Test: p−value = 0.76

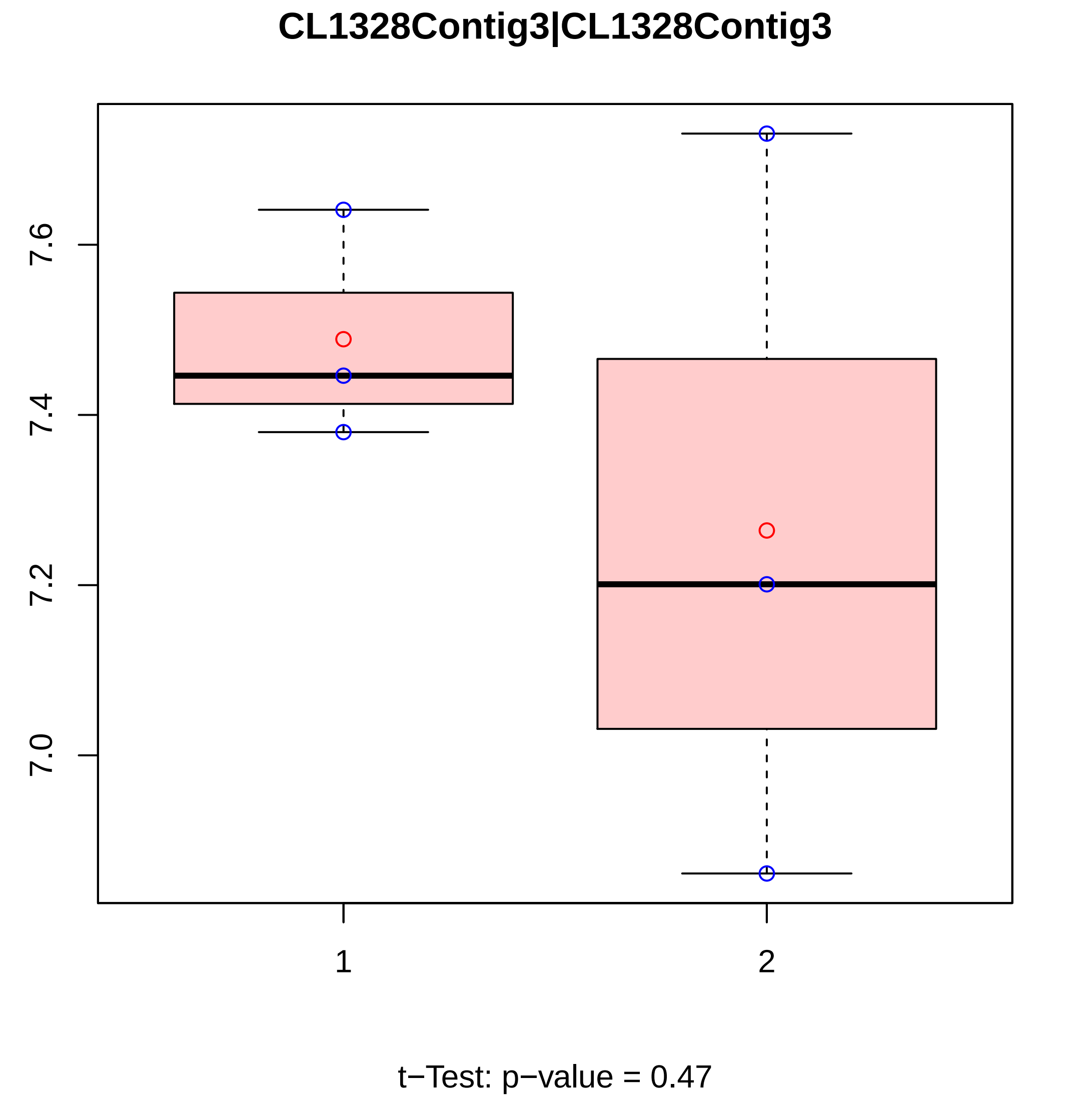
CL1328Contig3|CL1328Contig3
7.0
7.2
7.4
7.6
1
2
t−Test: p−value = 0.47

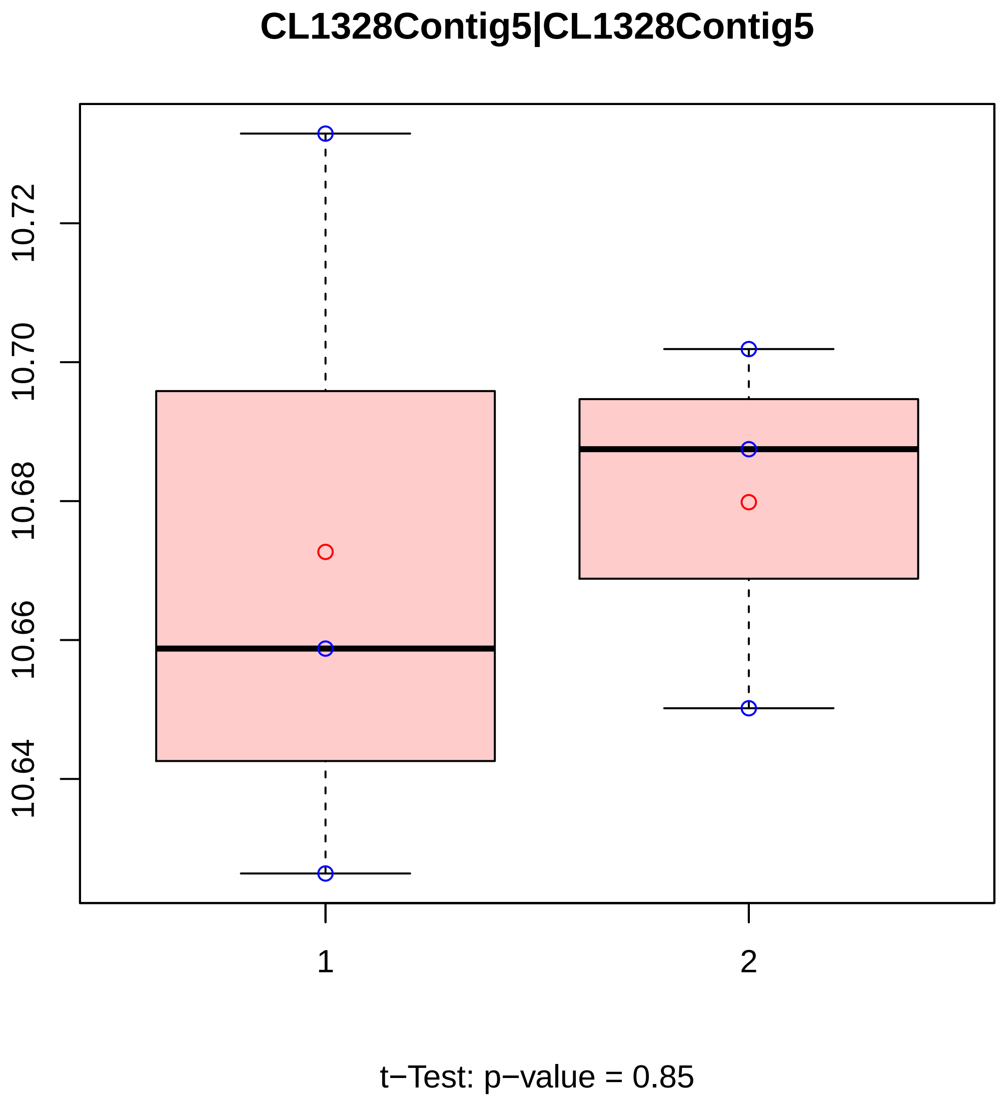
CL1328Contig5|CL1328Contig5
10.64
10.66
10.68
10.70
10.72
1
2
t−Test: p−value = 0.85

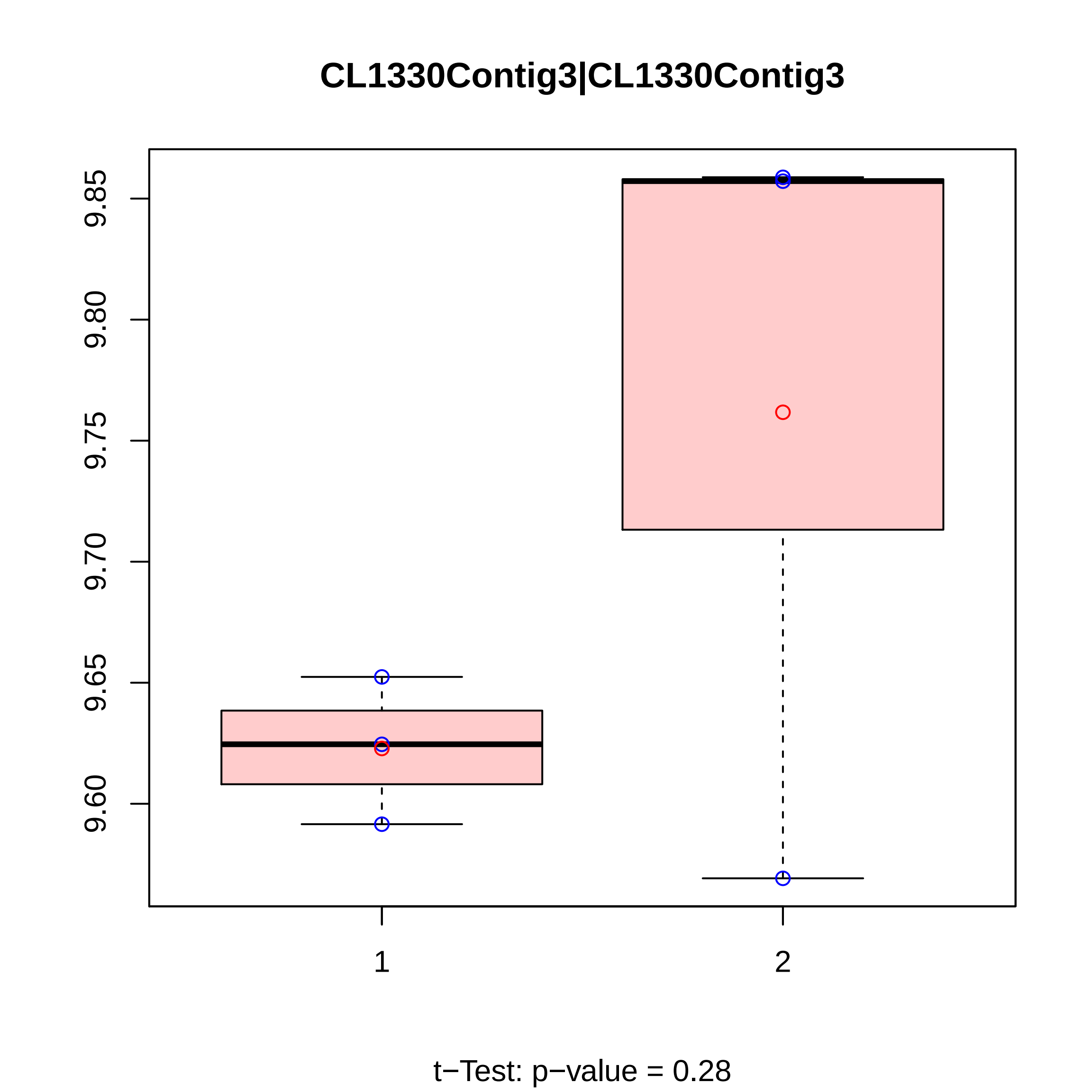
CL1330Contig3|CL1330Contig3
9.60
9.65
9.70
9.75
9.80
9.85
1
2
t−Test: p−value = 0.28

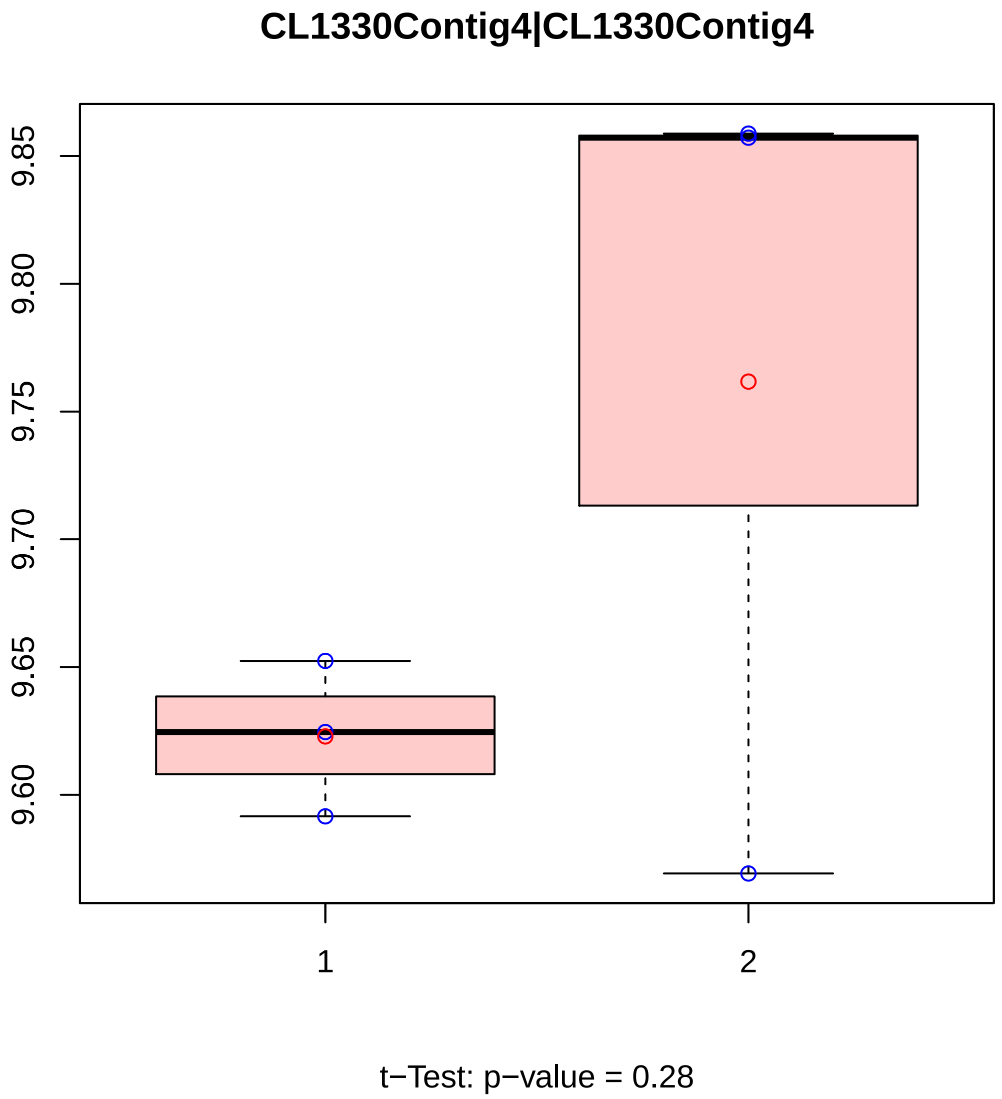
CL1330Contig4|CL1330Contig4
9.60
9.65
9.70
9.75
9.80
9.85
1
2
t−Test: p−value = 0.28

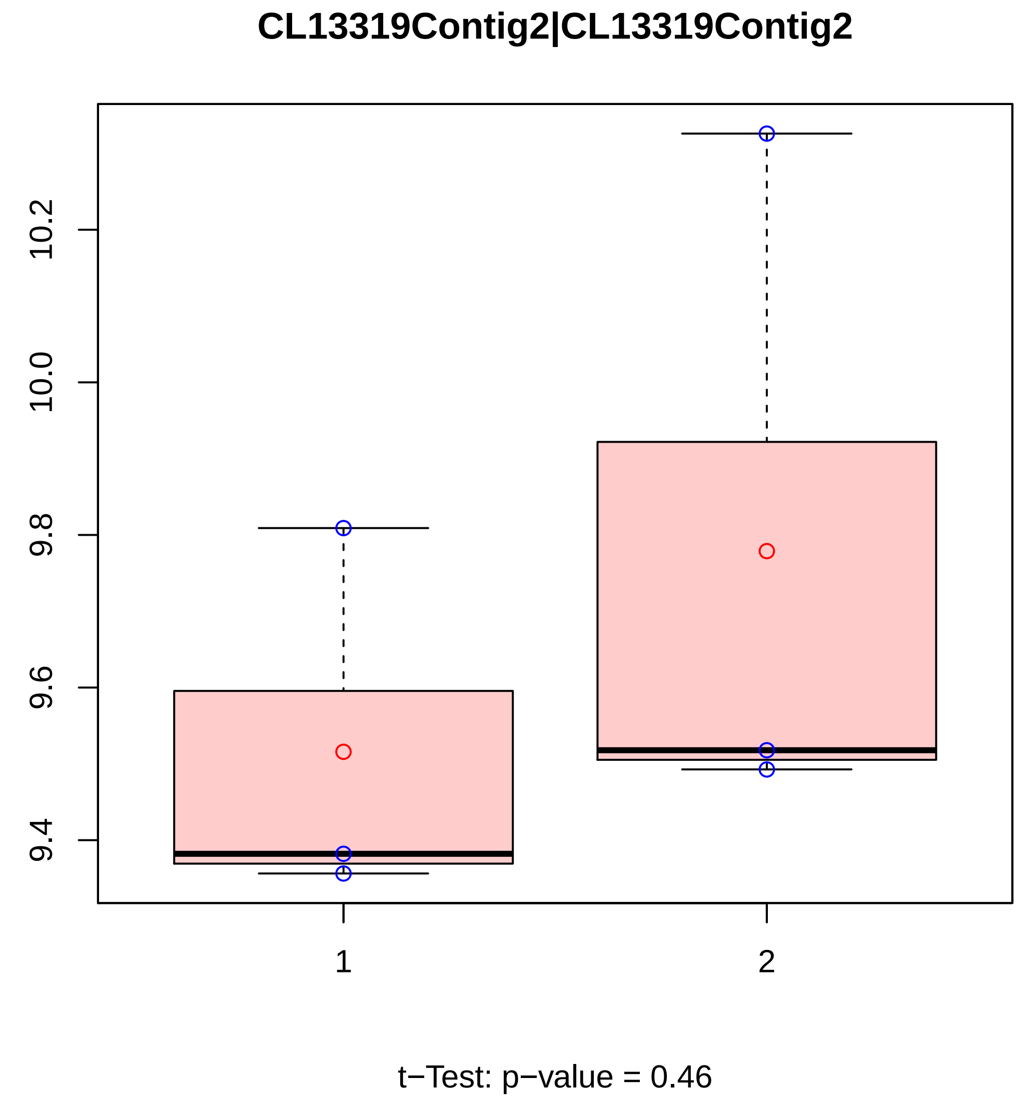

CL13319Contig2|CL13319Contig2
10.2
10.0
9.8
9.6
9.4
1
2
t−Test: p−value = 0.46

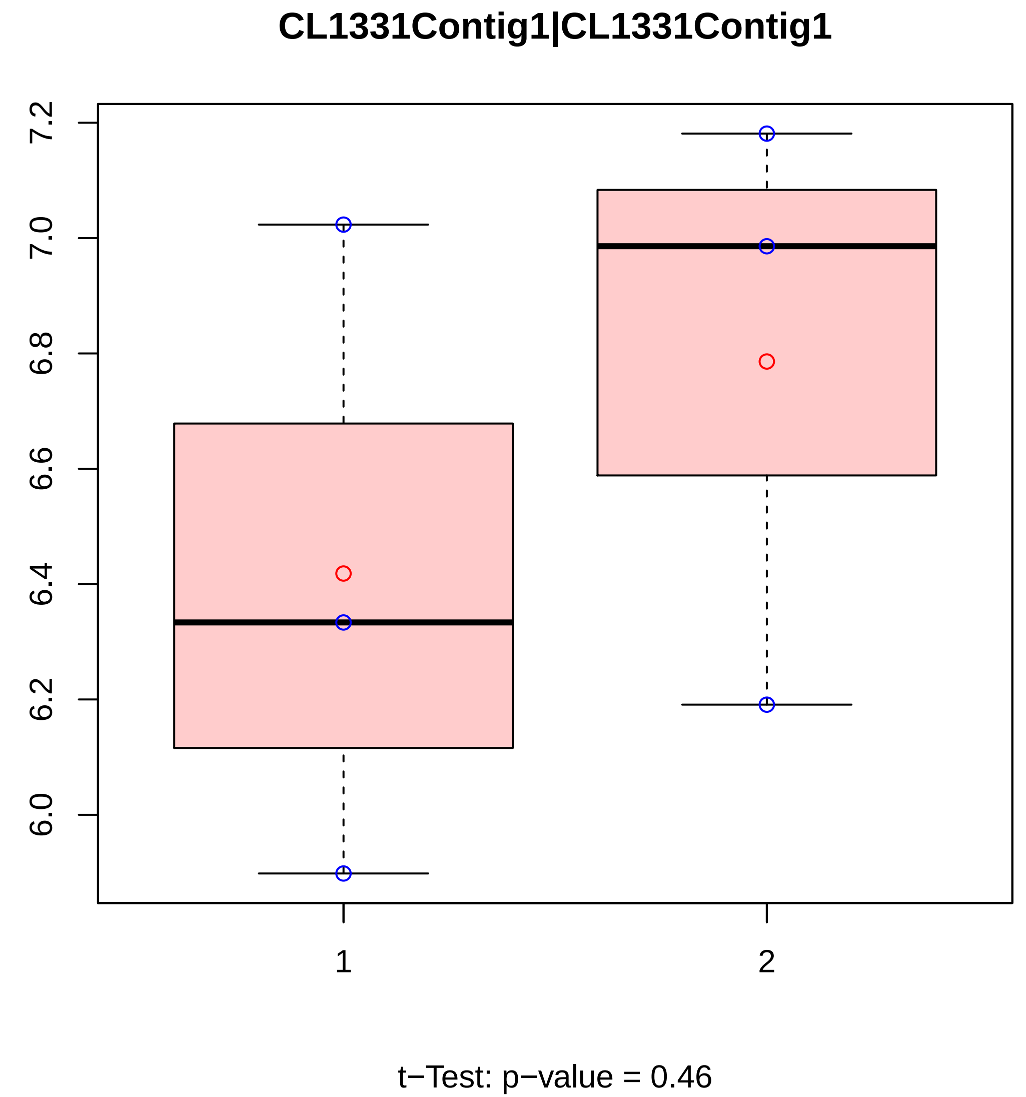
CL1331Contig1|CL1331Contig1
7.2
7.0
6.8
6.6
6.4
6.2
6.0
1
2
t−Test: p−value = 0.46

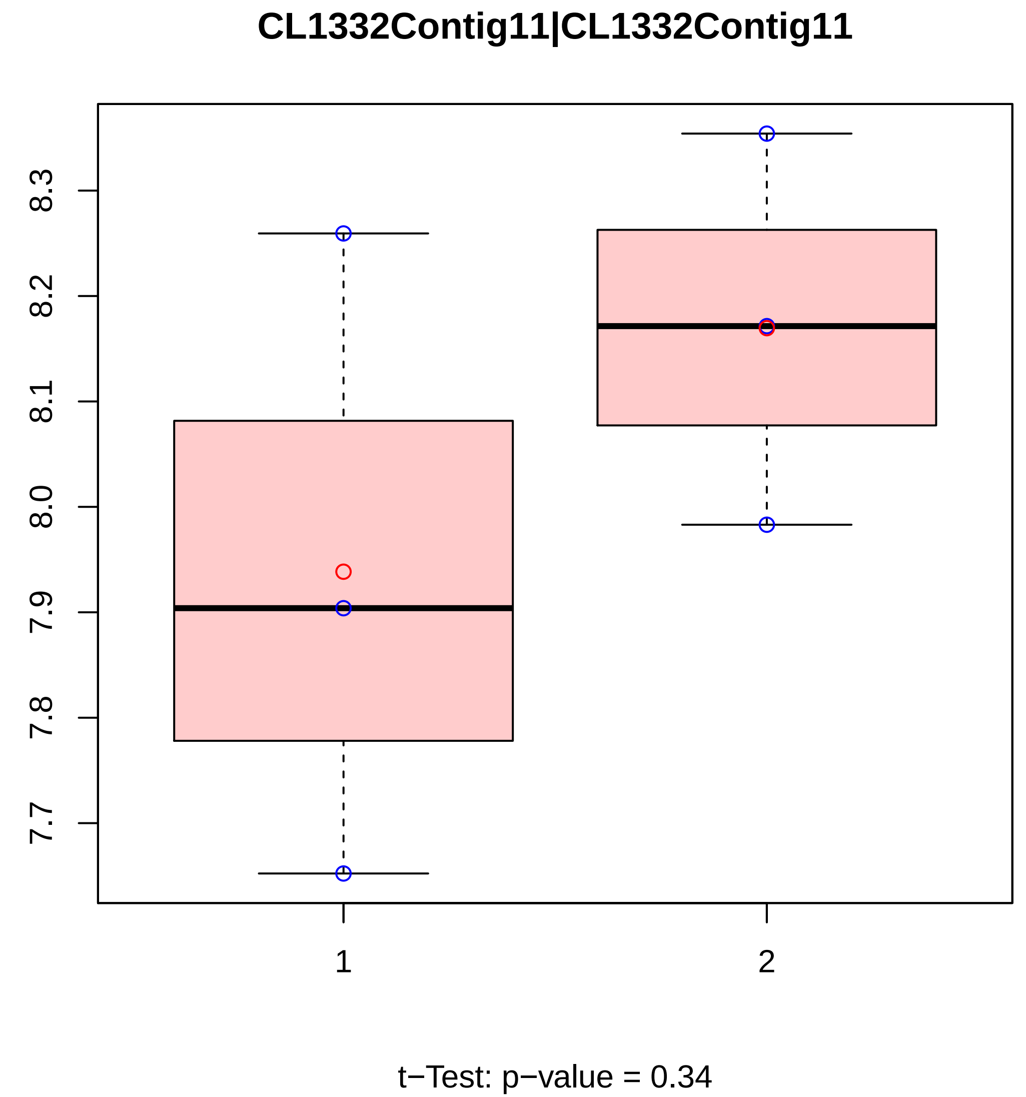
CL1332Contig11|CL1332Contig11
8.3
8.2
8.1
8.0
7.9
7.8
7.7
1
2
t−Test: p−value = 0.34

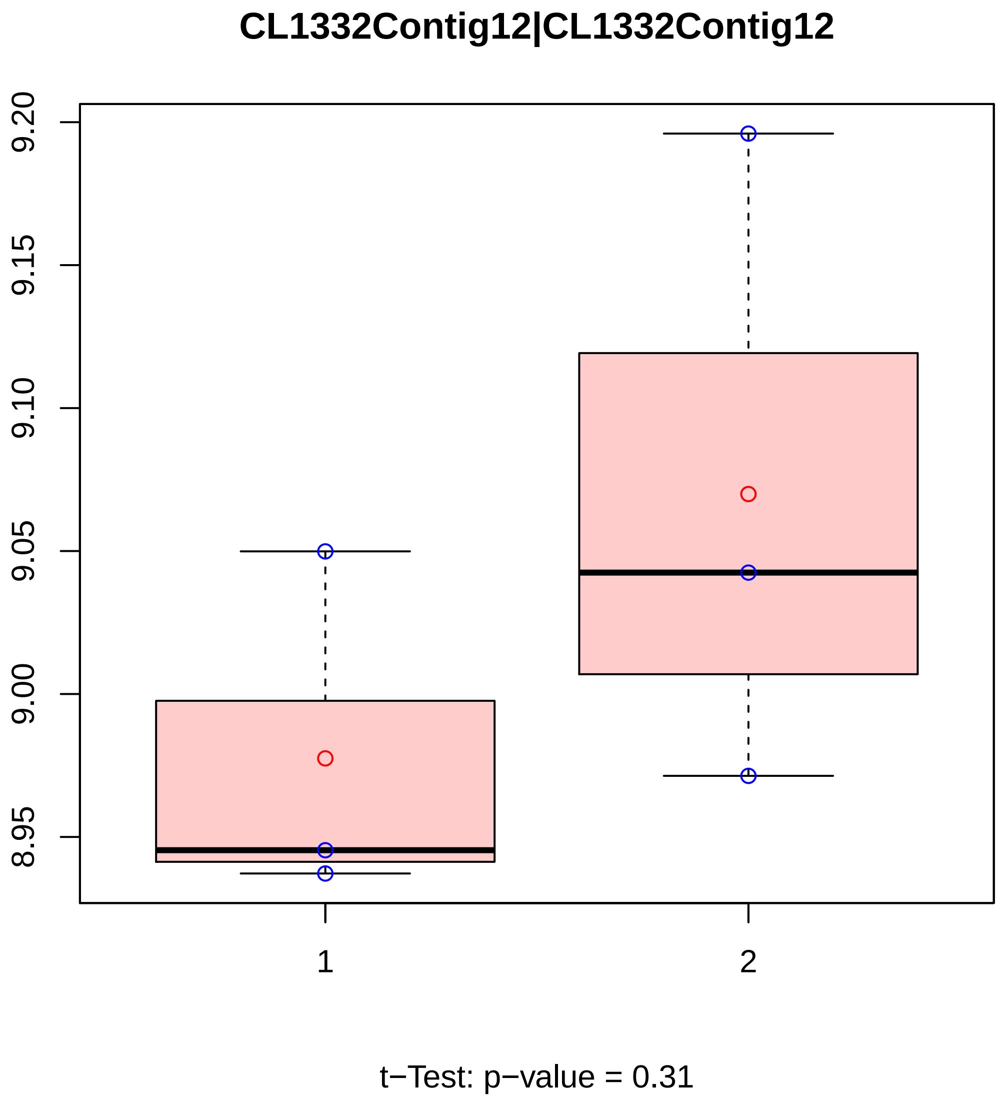
CL1332Contig12|CL1332Contig12
9.20
9.15
9.10
9.05
9.00
8.95
1
2
t−Test: p−value = 0.31

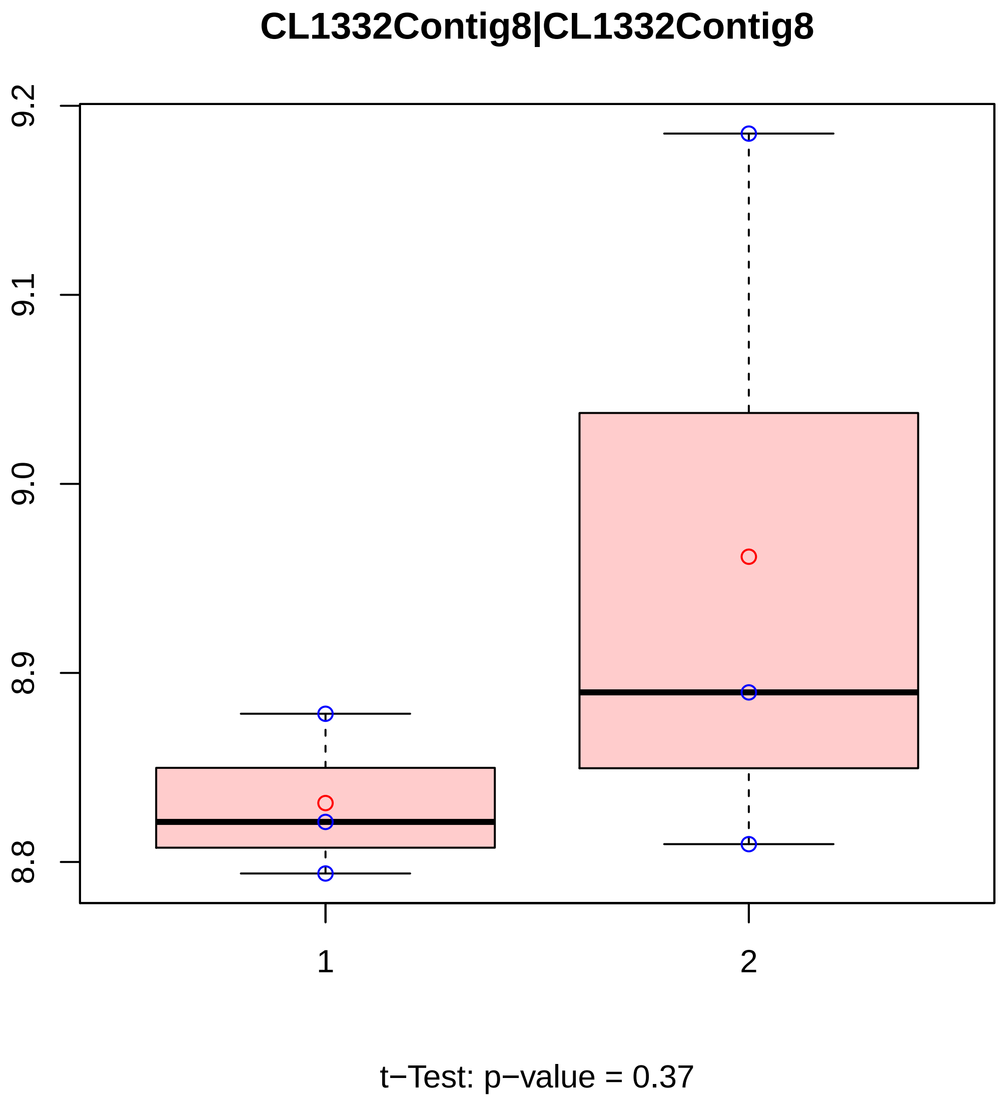
CL1332Contig8|CL1332Contig8
9.2
9.1
9.0
8.9
8.8
1
2
t−Test: p−value = 0.37

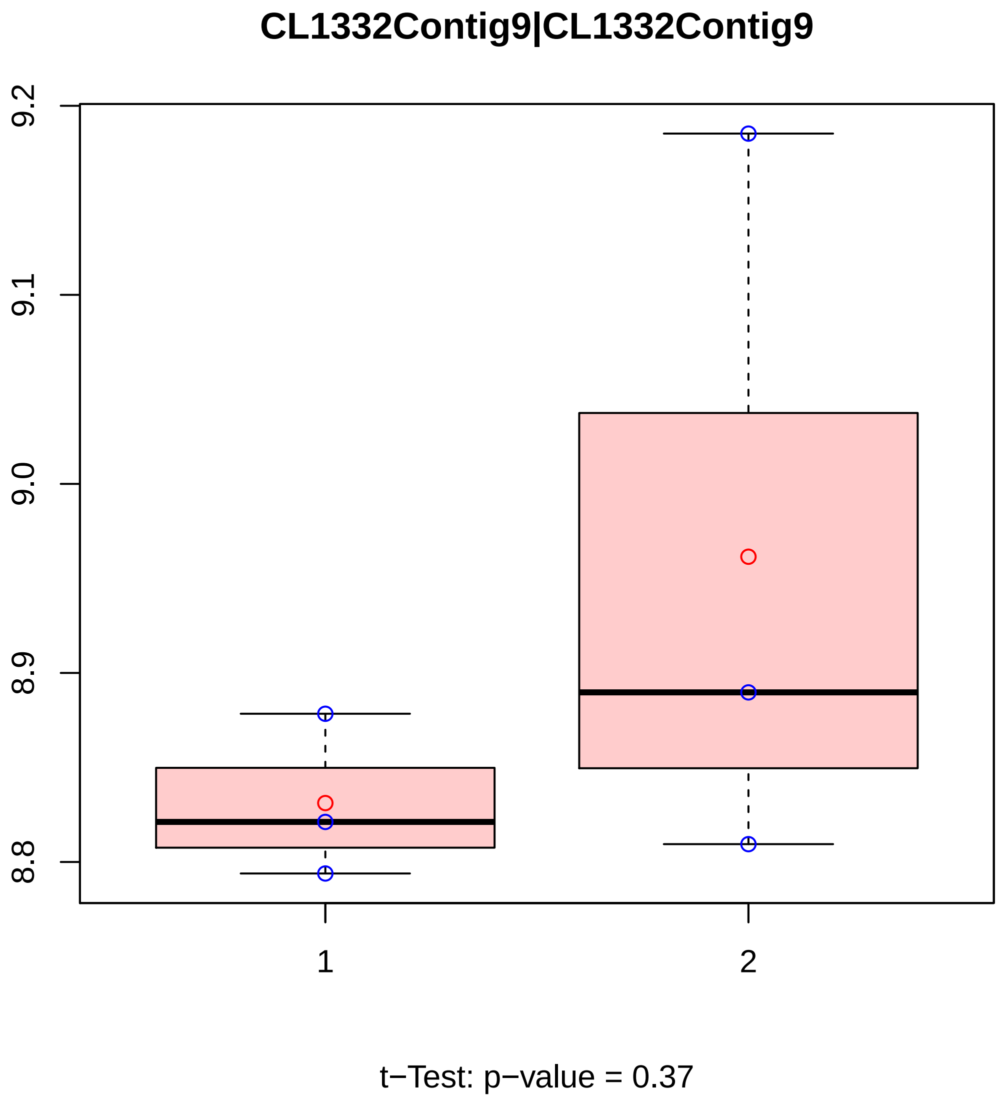
CL1332Contig9|CL1332Contig9
9.2
9.1
9.0
8.9
8.8
1
2
t−Test: p−value = 0.37

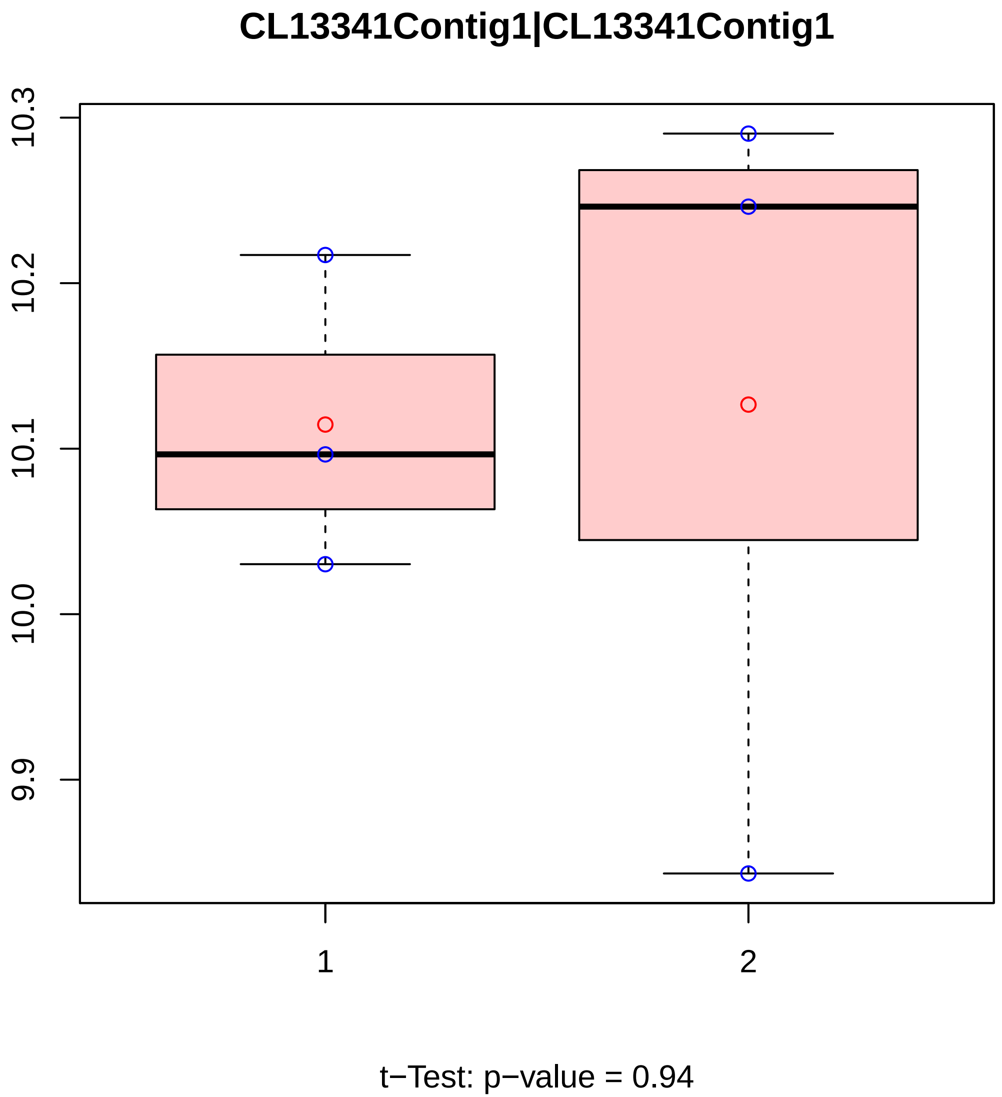
CL13341Contig1|CL13341Contig1
10.3
10.2
10.1
10.0
9.9
1
2
t−Test: p−value = 0.94

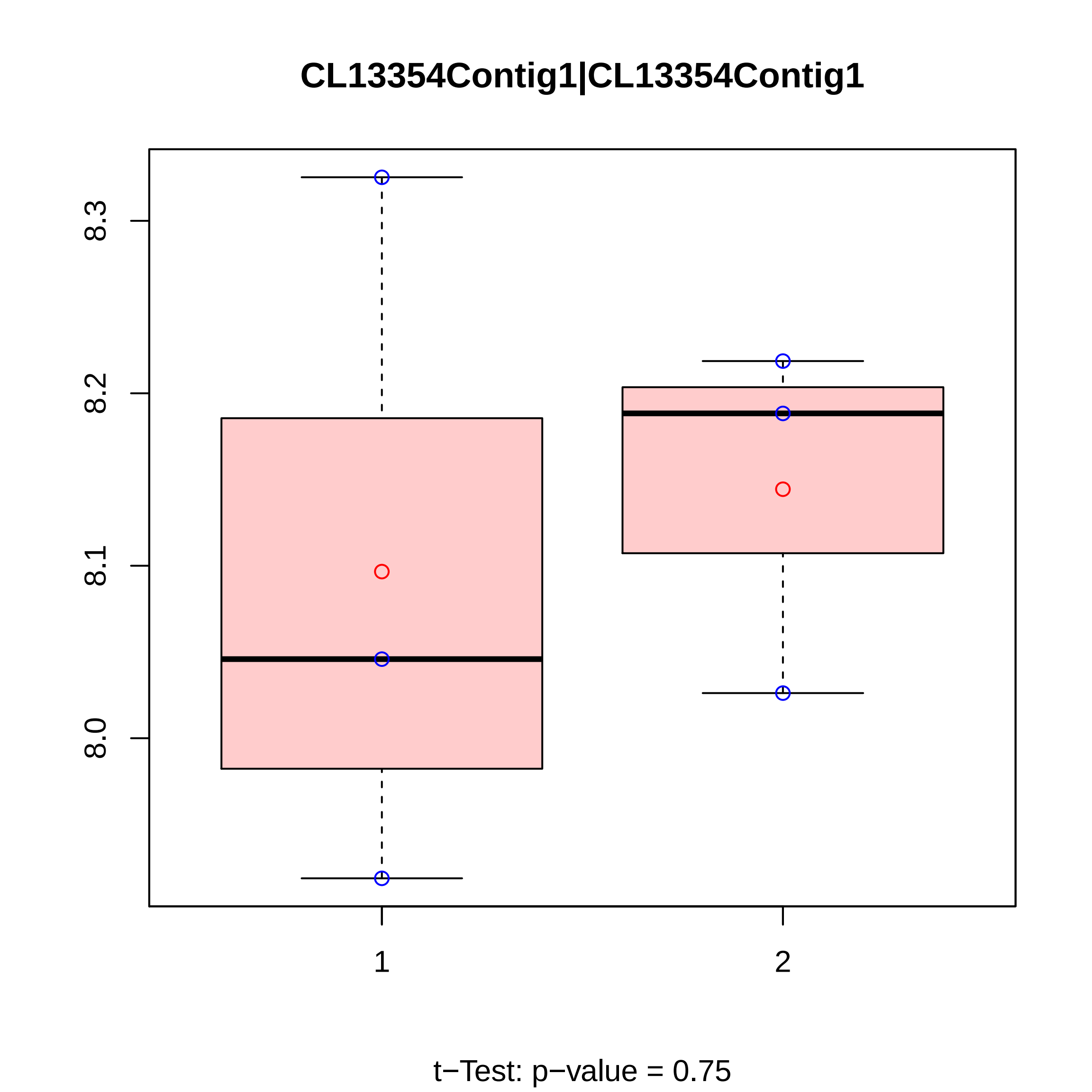
CL13354Contig1|CL13354Contig1
8.3
8.2
8.1
8.0
1
2
t−Test: p−value = 0.75

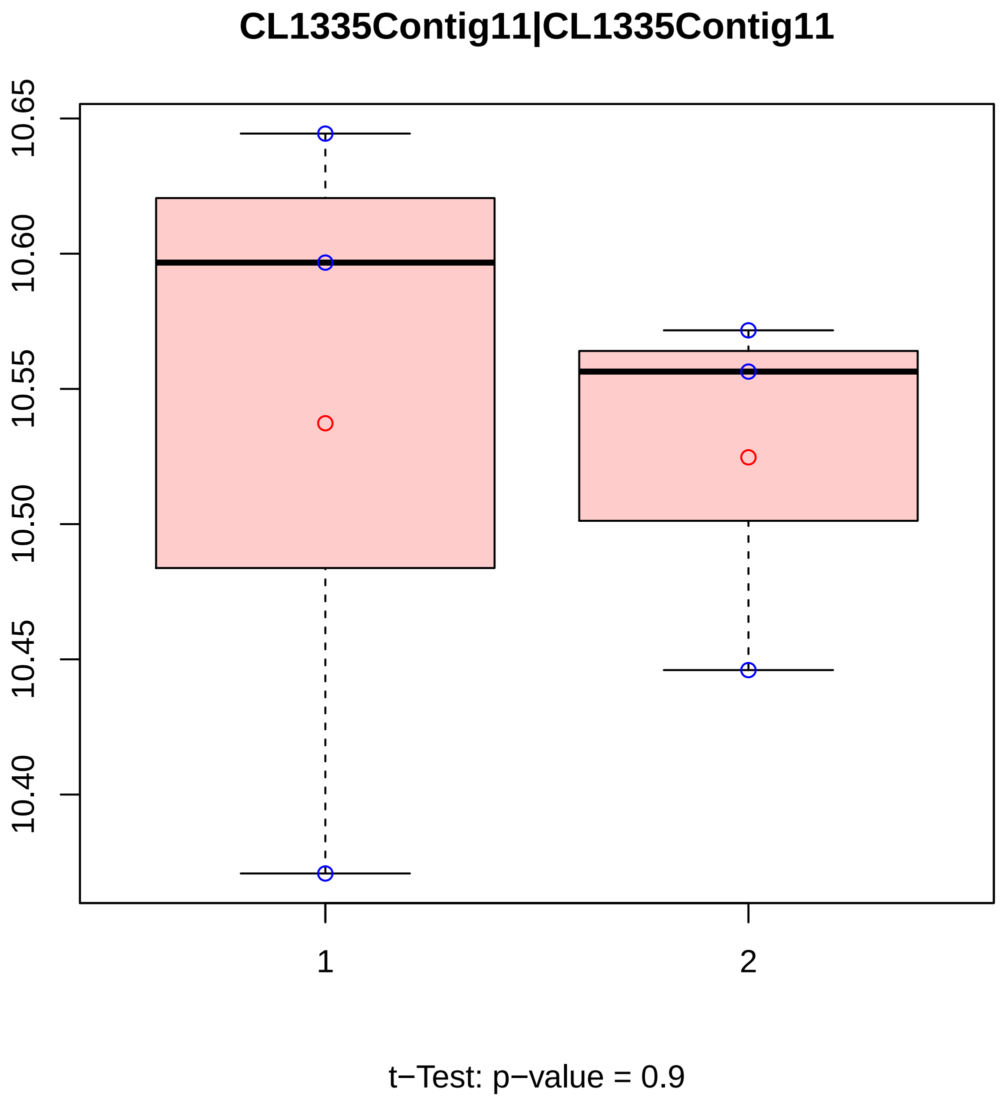
CL1335Contig11|CL1335Contig11
10.65
10.60
10.55
10.50
10.45
10.40
1
2
t−Test: p−value = 0.9

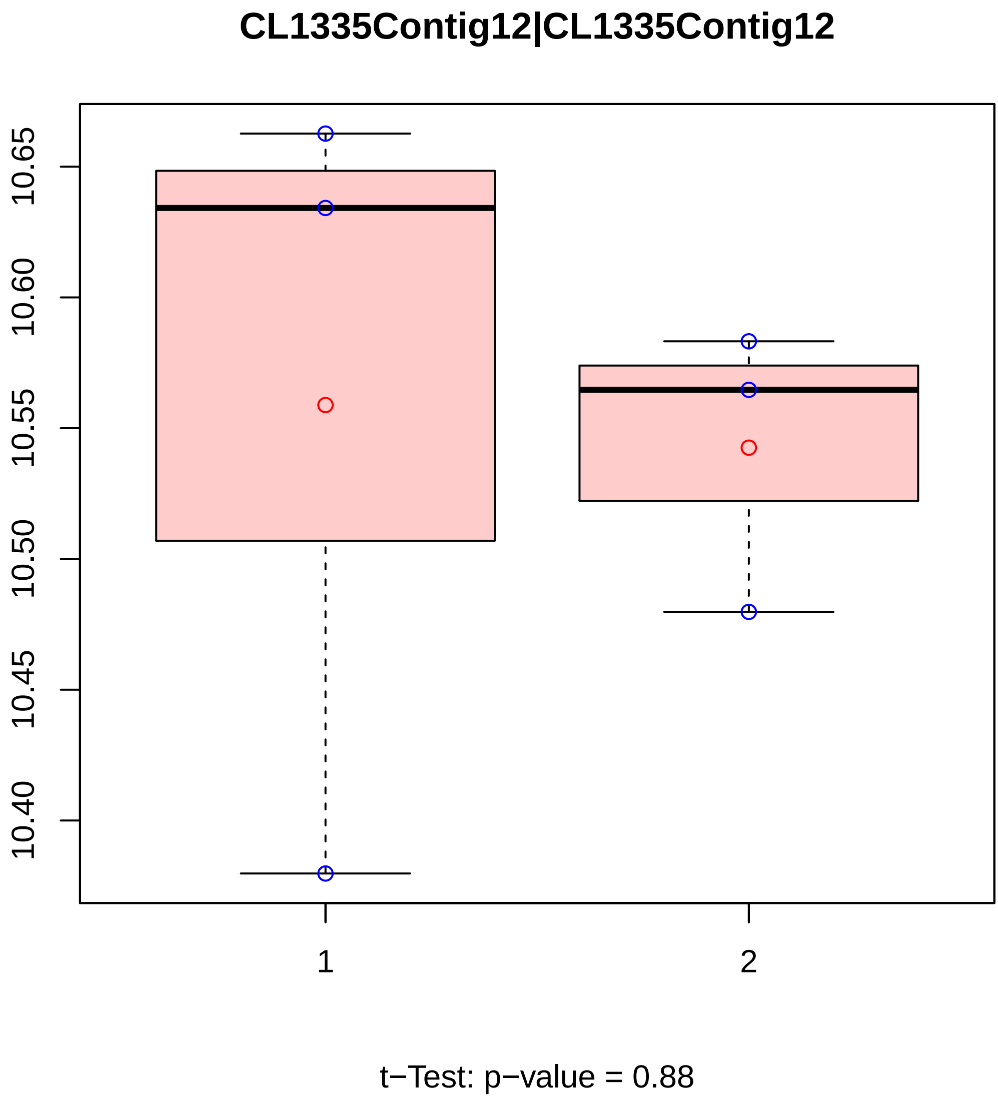

CL1335Contig12|CL1335Contig12
10.65
10.60
10.55
10.50
10.45
10.40
1
2
t−Test: p−value = 0.88

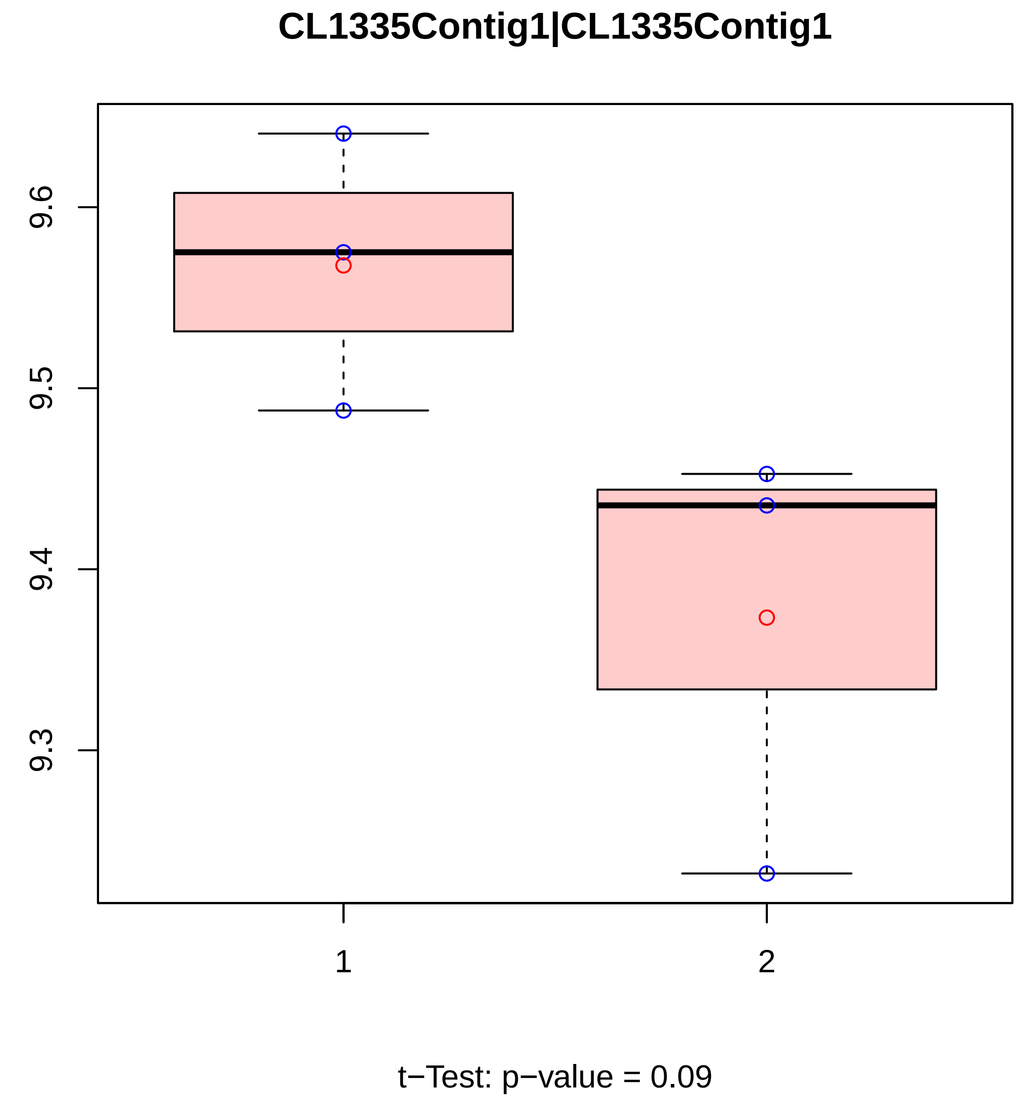

CL1335Contig1|CL1335Contig1
9.3
9.4
9.5
9.6
1
2
t−Test: p−value = 0.09

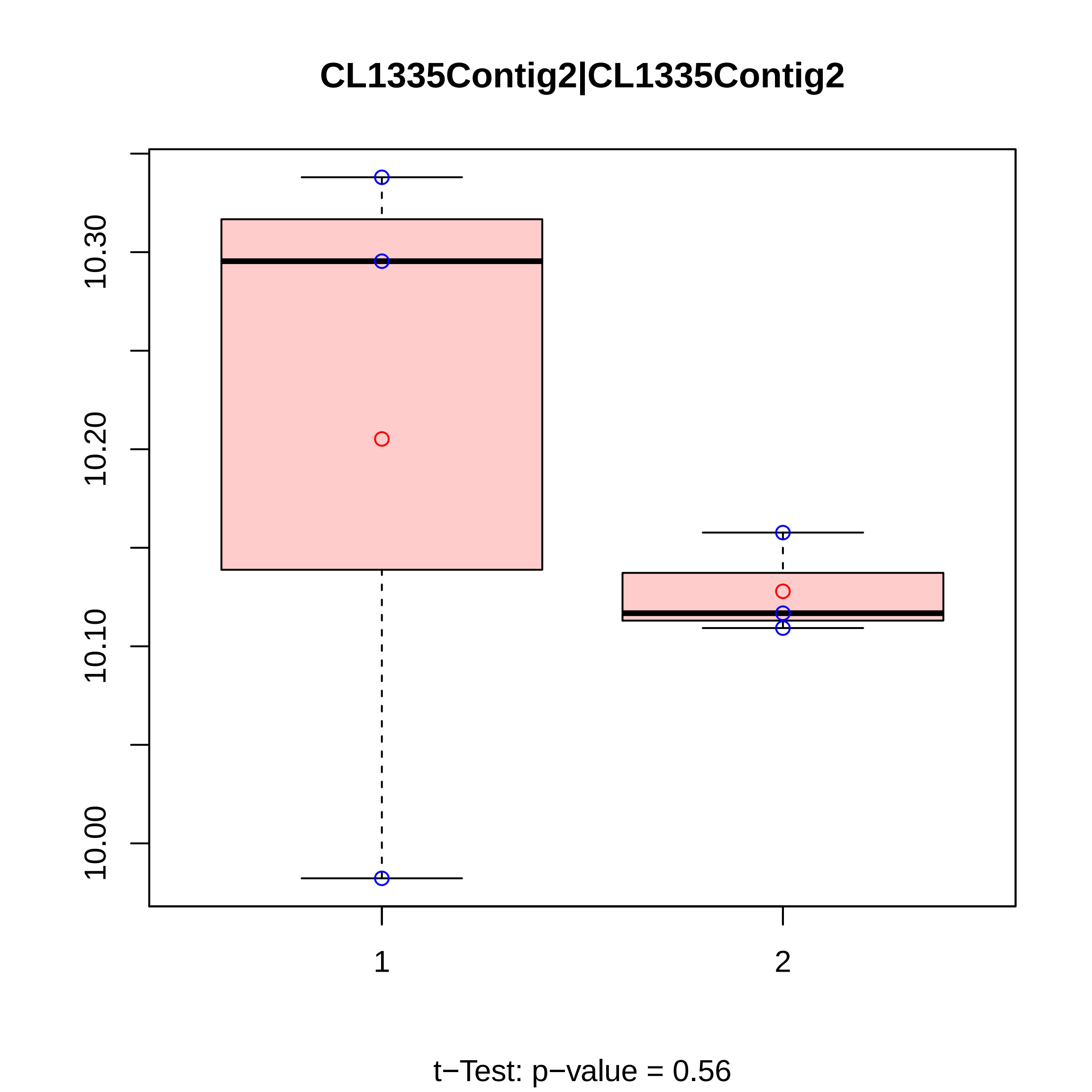
CL1335Contig2|CL1335Contig2
10.00
10.10
10.20
10.30
1
2
t−Test: p−value = 0.56

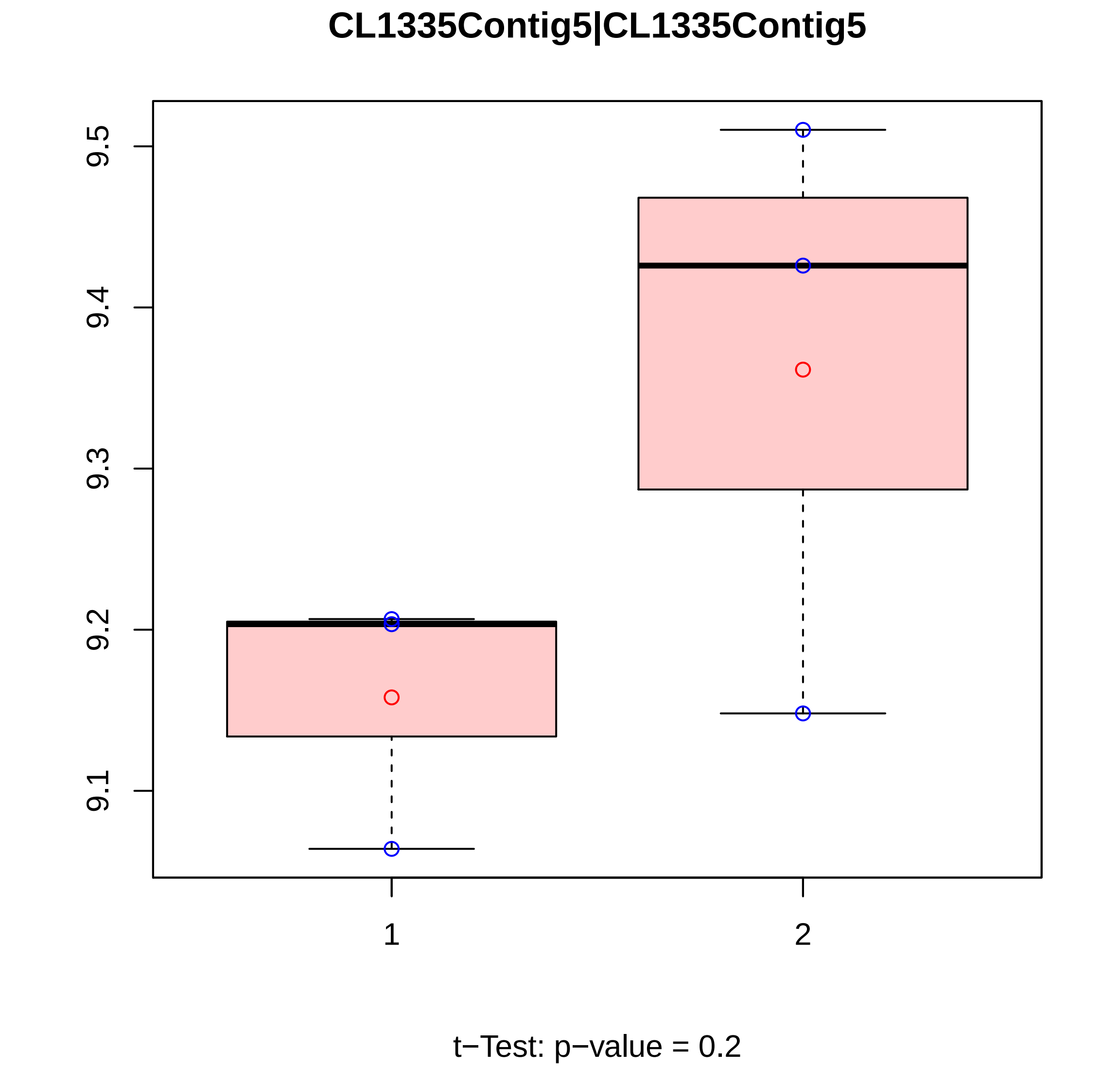
CL1335Contig5|CL1335Contig5
9.1
9.2
9.3
9.4
9.5
1
2
t−Test: p−value = 0.2

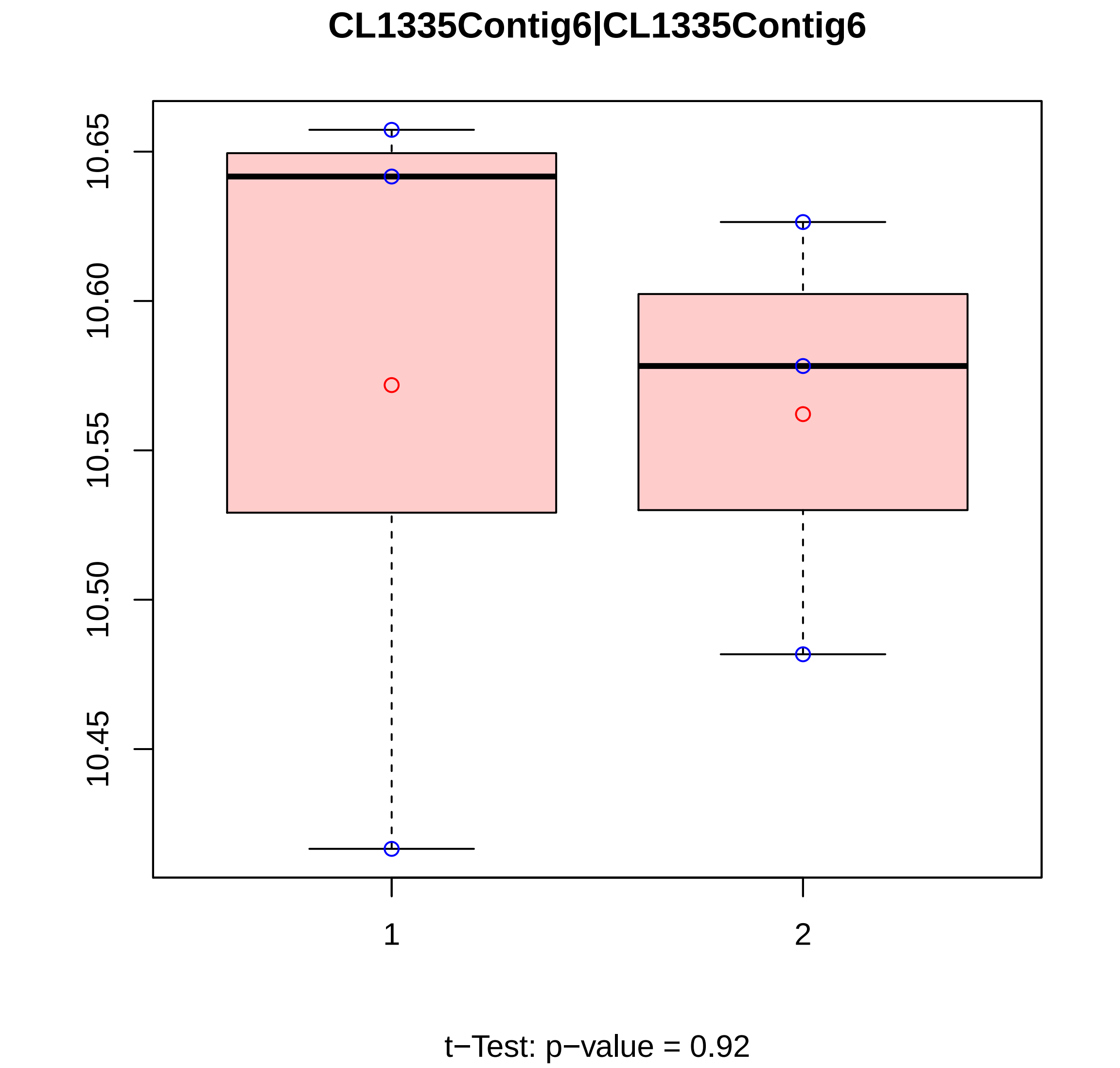
CL1335Contig6|CL1335Contig6
10.45
10.50
10.55
10.60
10.65
1
2
t−Test: p−value = 0.92

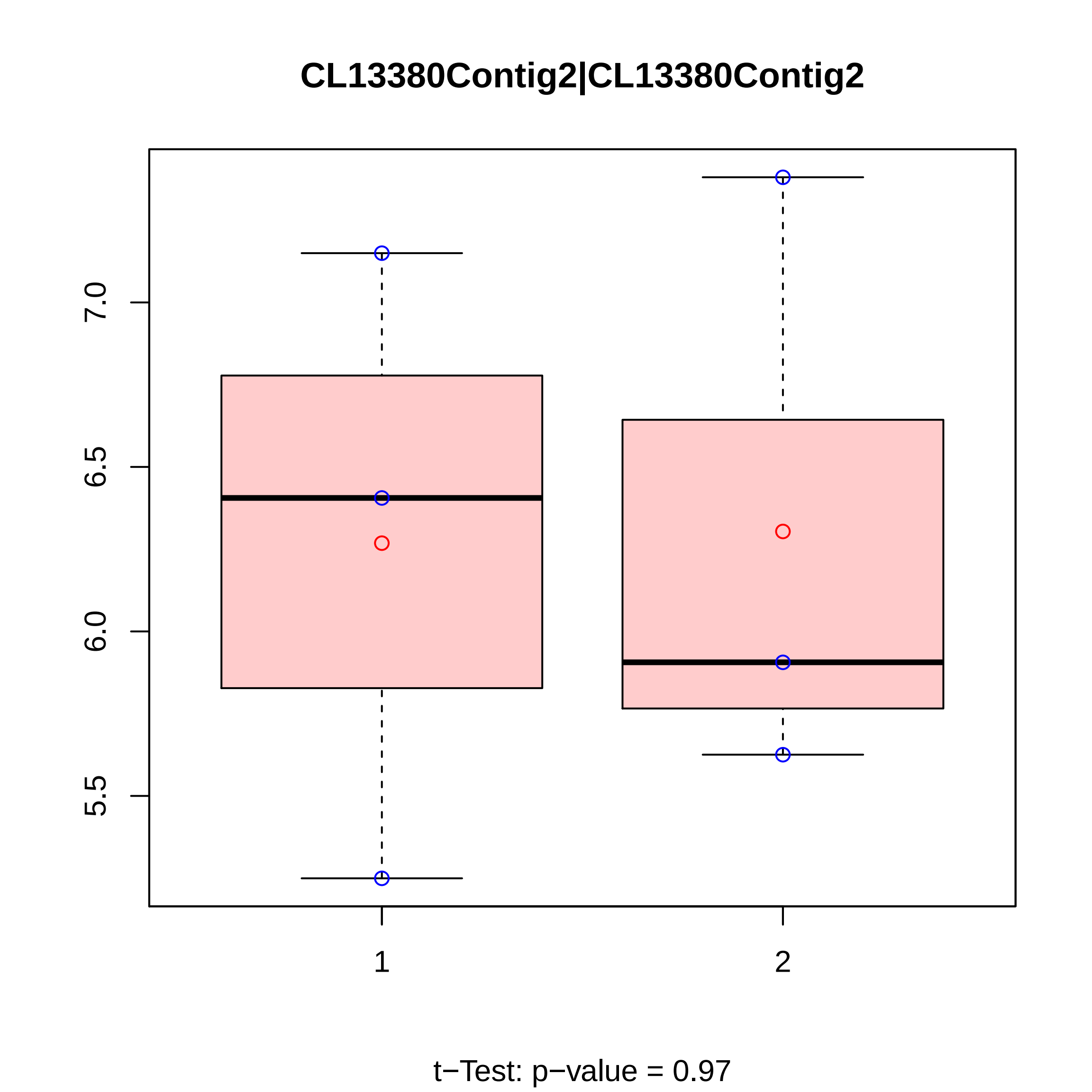
CL13380Contig2|CL13380Contig2
7.0
6.5
6.0
5.5
1
2
t−Test: p−value = 0.97

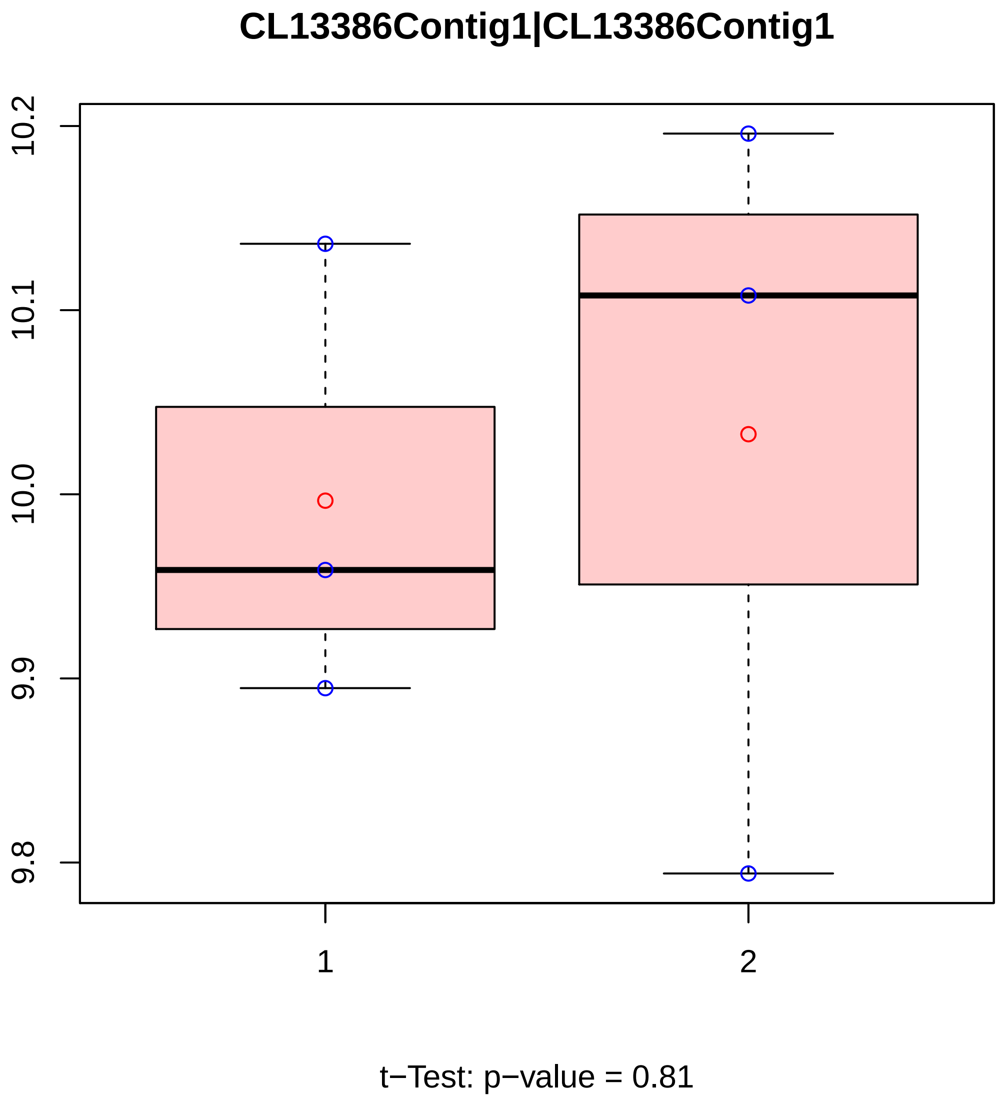
CL13386Contig1|CL13386Contig1
10.2
10.1
10.0
9.9
9.8
1
2
t−Test: p−value = 0.81

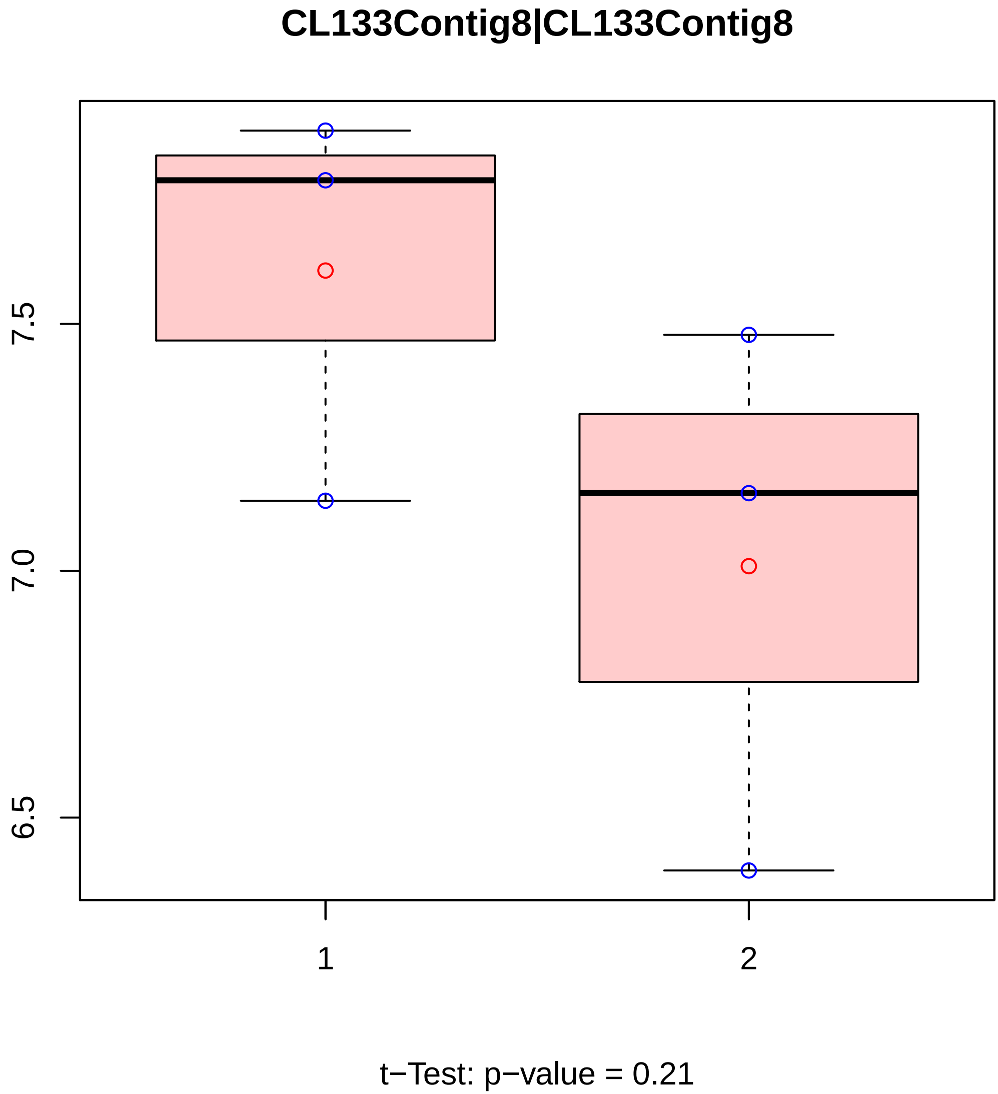
CL133Contig8|CL133Contig8
7.5
7.0
6.5
1
2
t−Test: p−value = 0.21

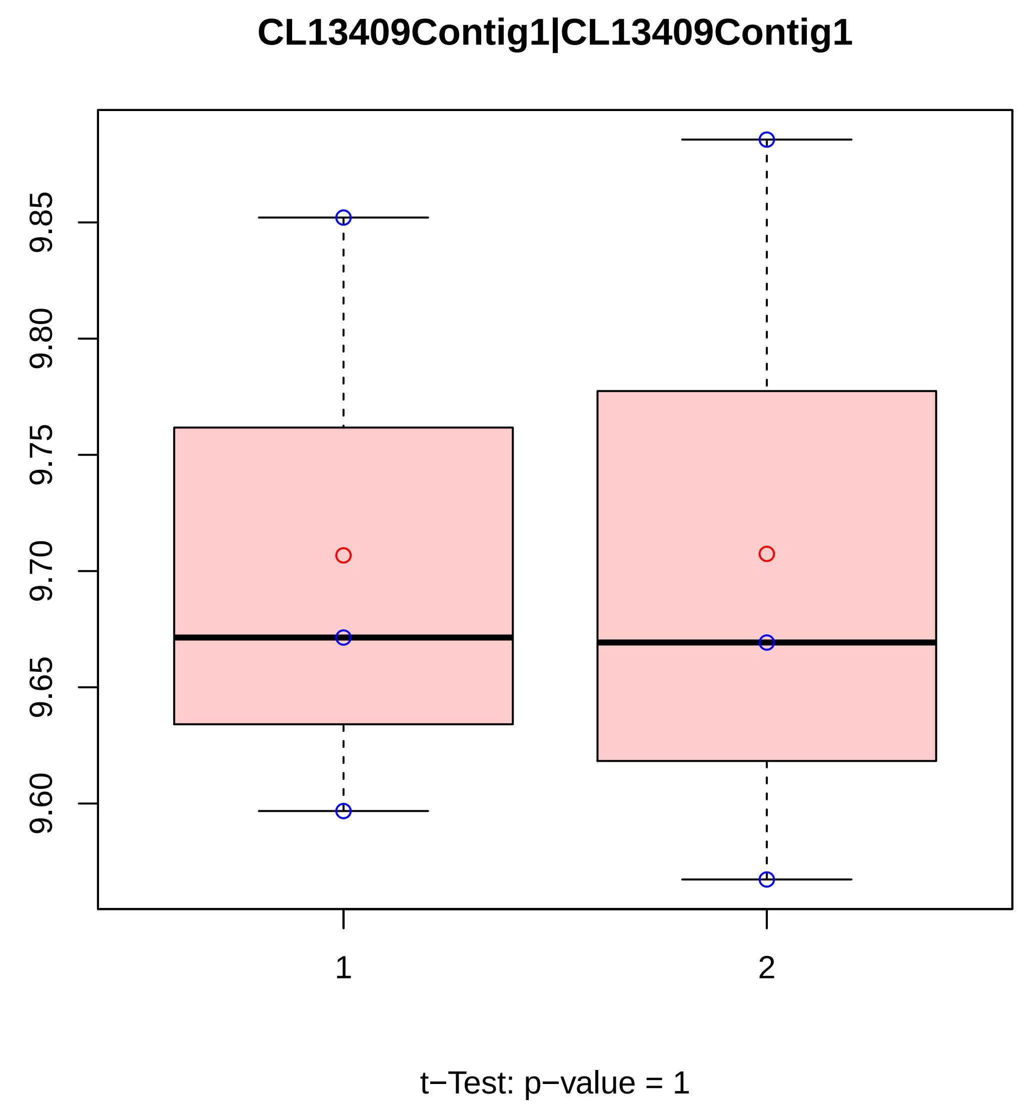
CL13409Contig1|CL13409Contig1
9.60
9.65
9.70
9.75
9.80
9.85
1
2
t−Test: p−value = 1

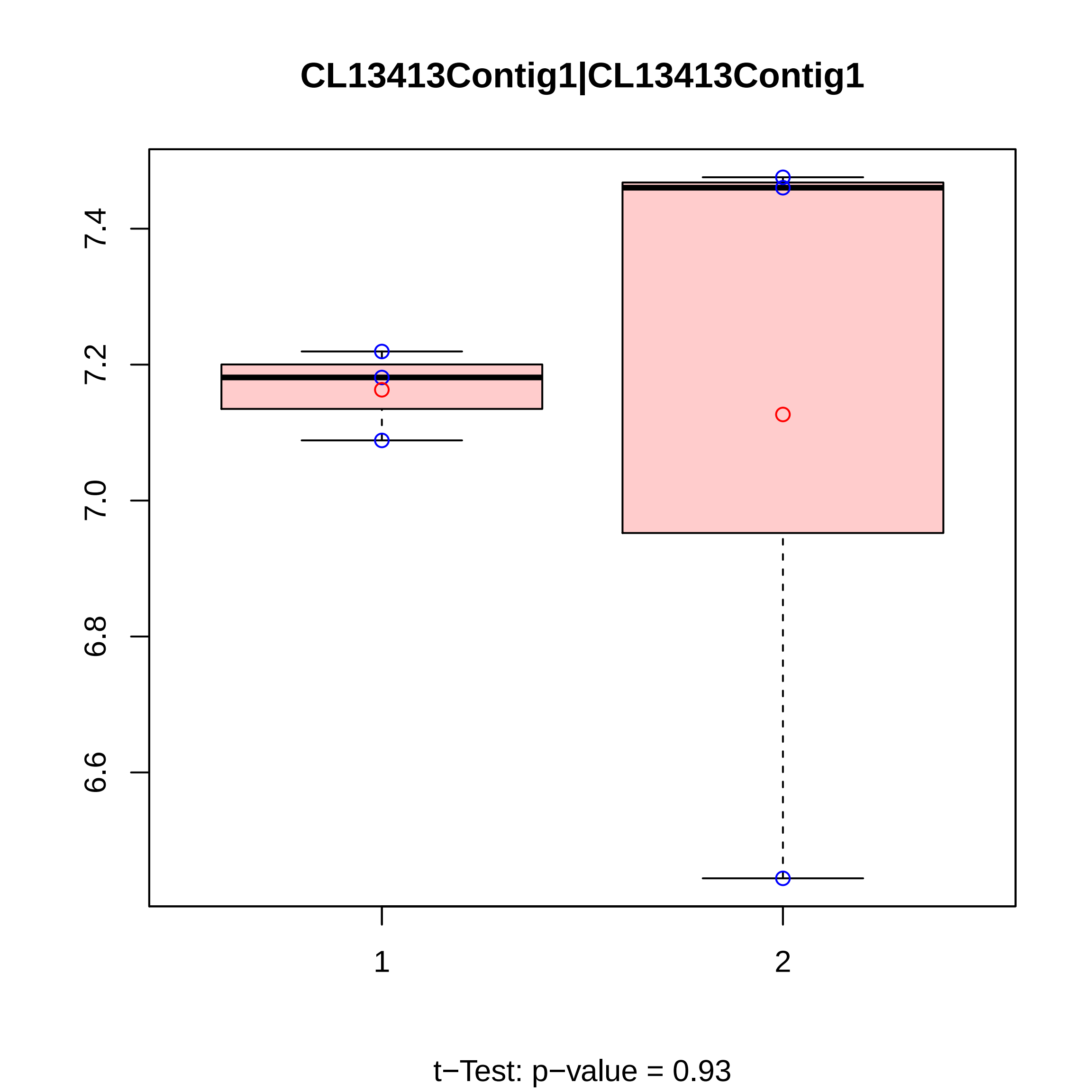
CL13413Contig1|CL13413Contig1
7.4
7.2
7.0
6.8
6.6
1
2
t−Test: p−value = 0.93

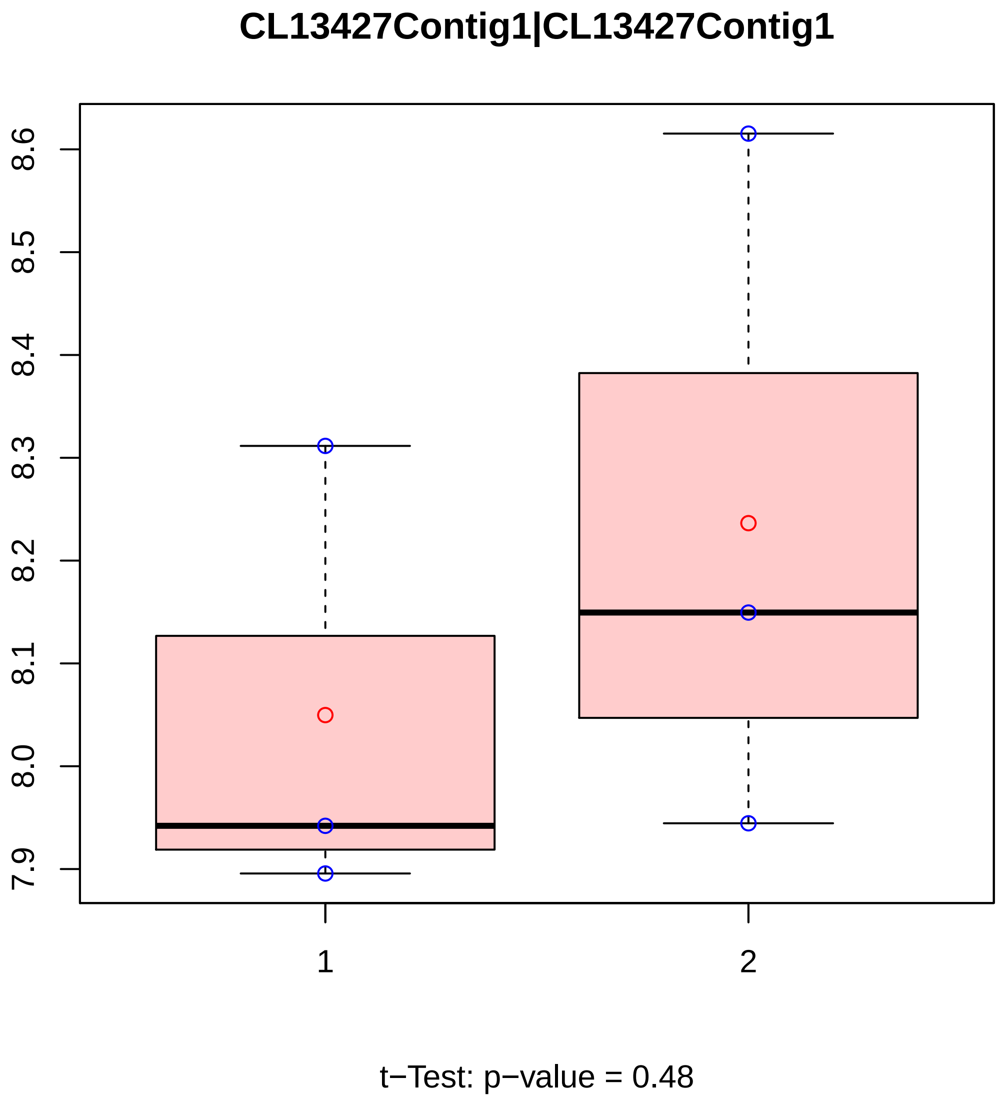
CL13427Contig1|CL13427Contig1
8.6
8.5
8.4
8.3
8.2
8.1
8.0
7.9
1
2
t−Test: p−value = 0.48

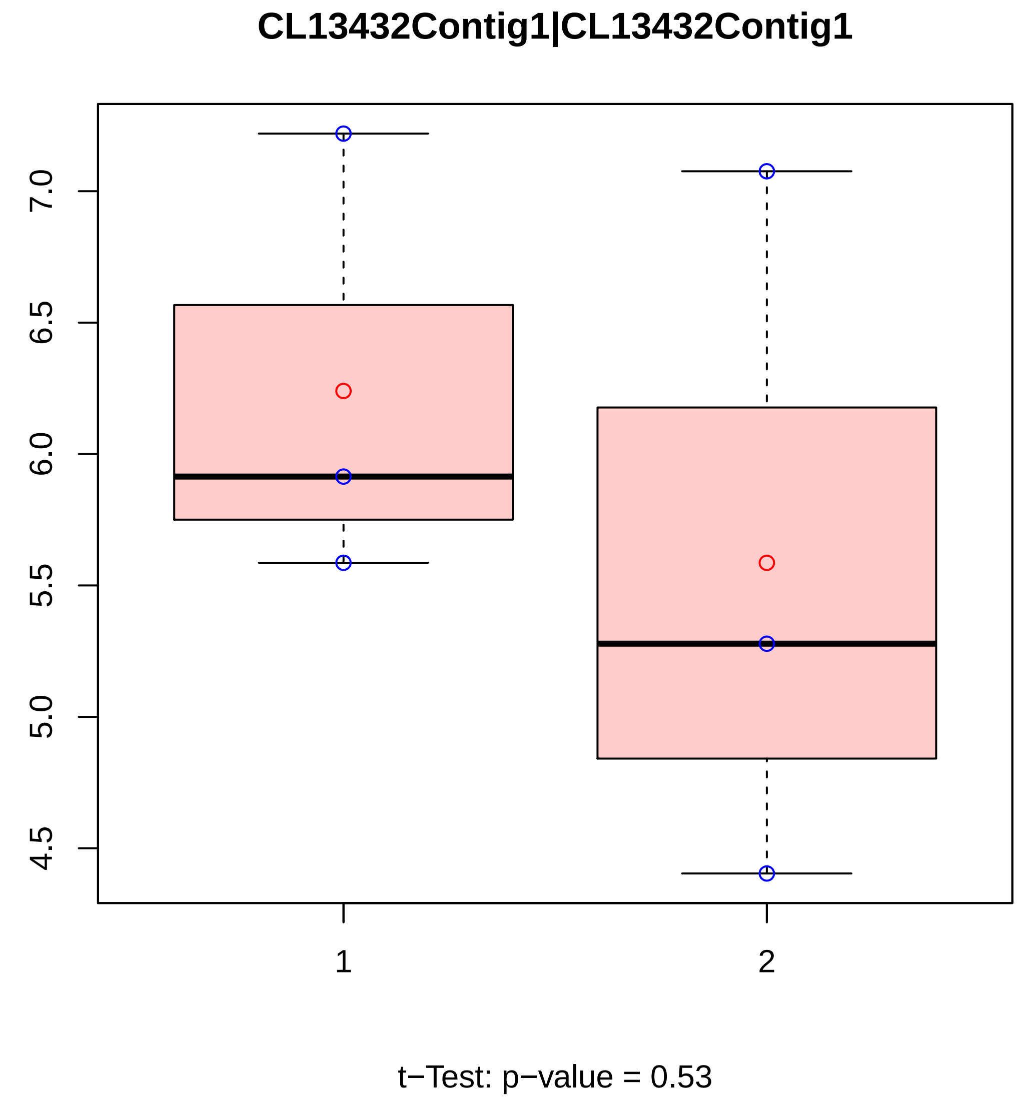
CL13432Contig1|CL13432Contig1
7.0
6.5
6.0
5.5
5.0
4.5
1
2
t−Test: p−value = 0.53

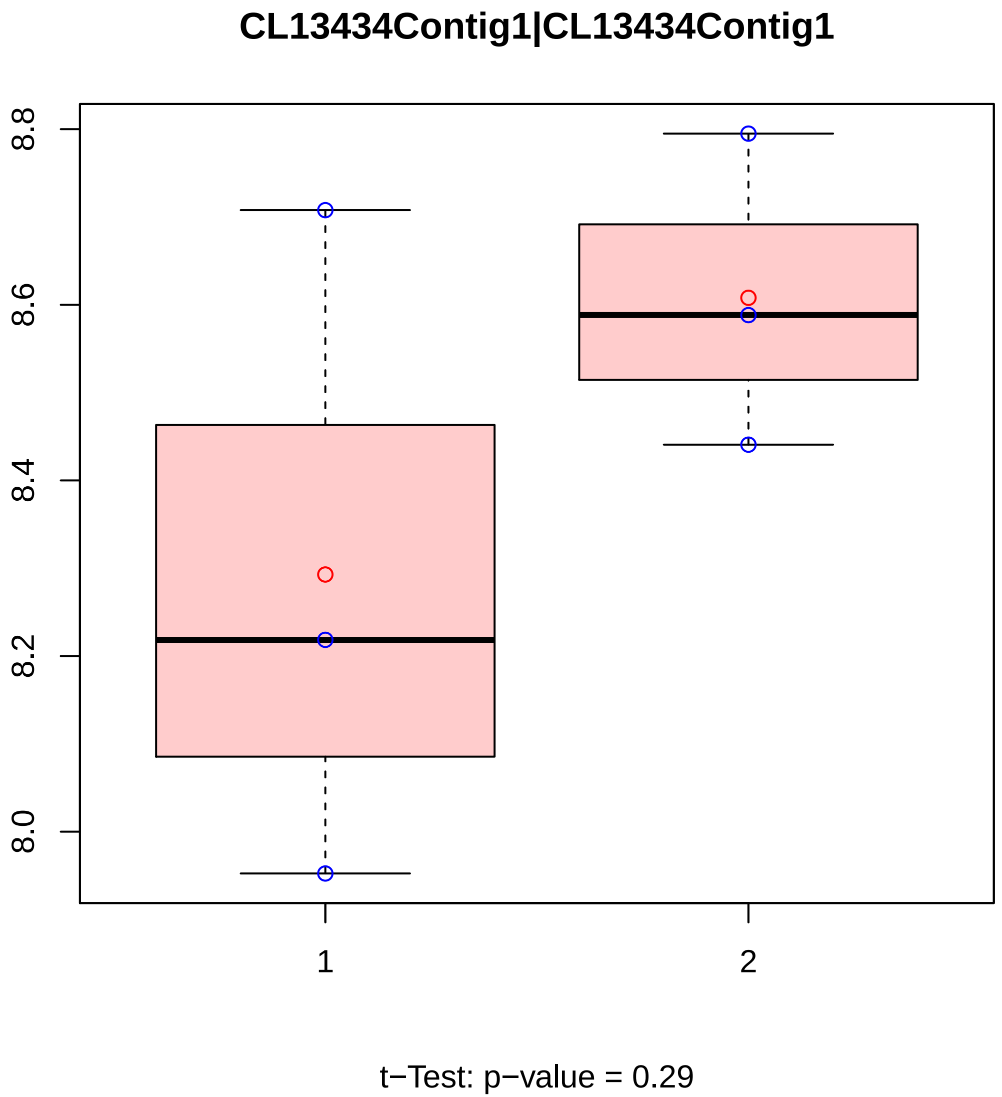
CL13434Contig1|CL13434Contig1
8.0
8.2
8.4
8.6
8.8
1
2
t−Test: p−value = 0.29

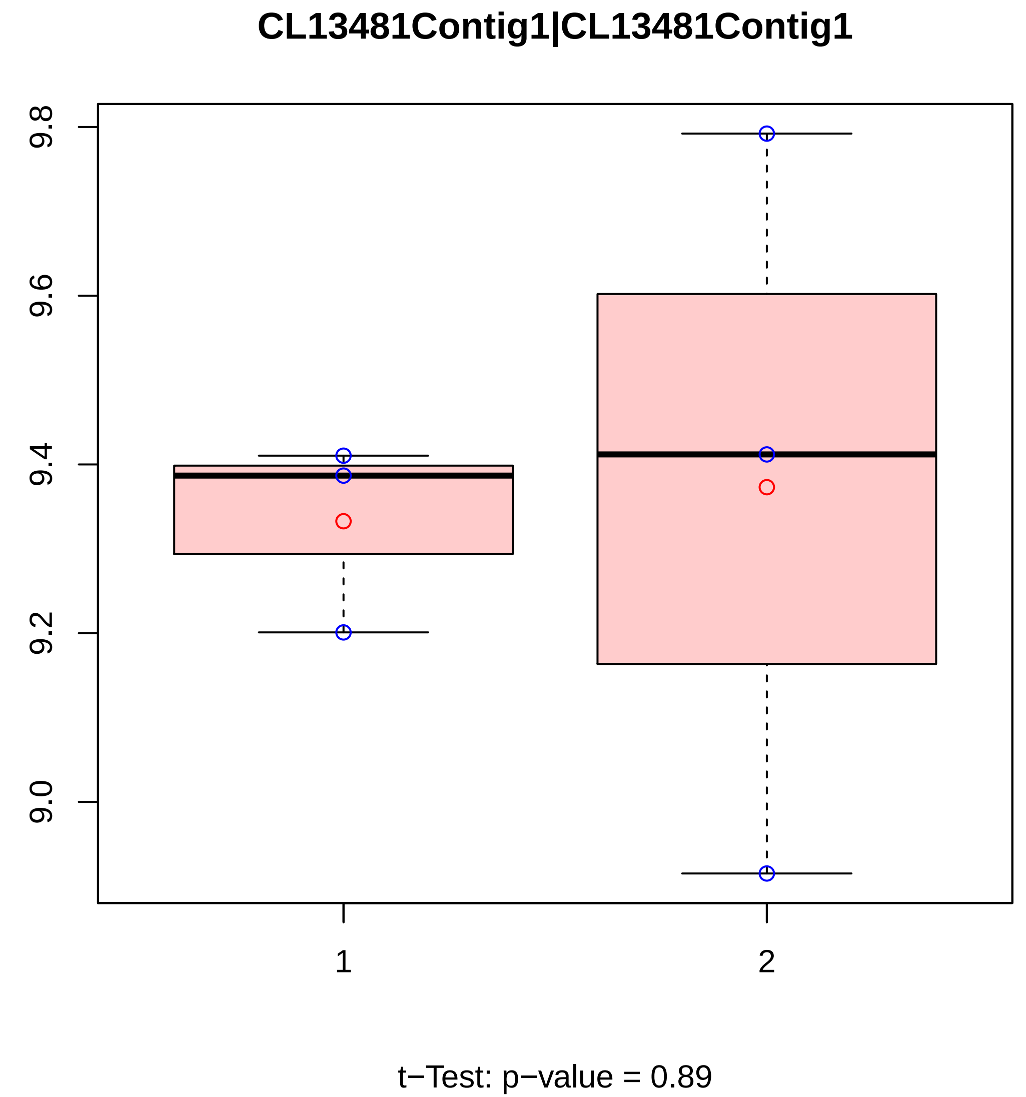

CL13481Contig1|CL13481Contig1
9.0
9.2
9.4
9.6
9.8
1
2
t−Test: p−value = 0.89

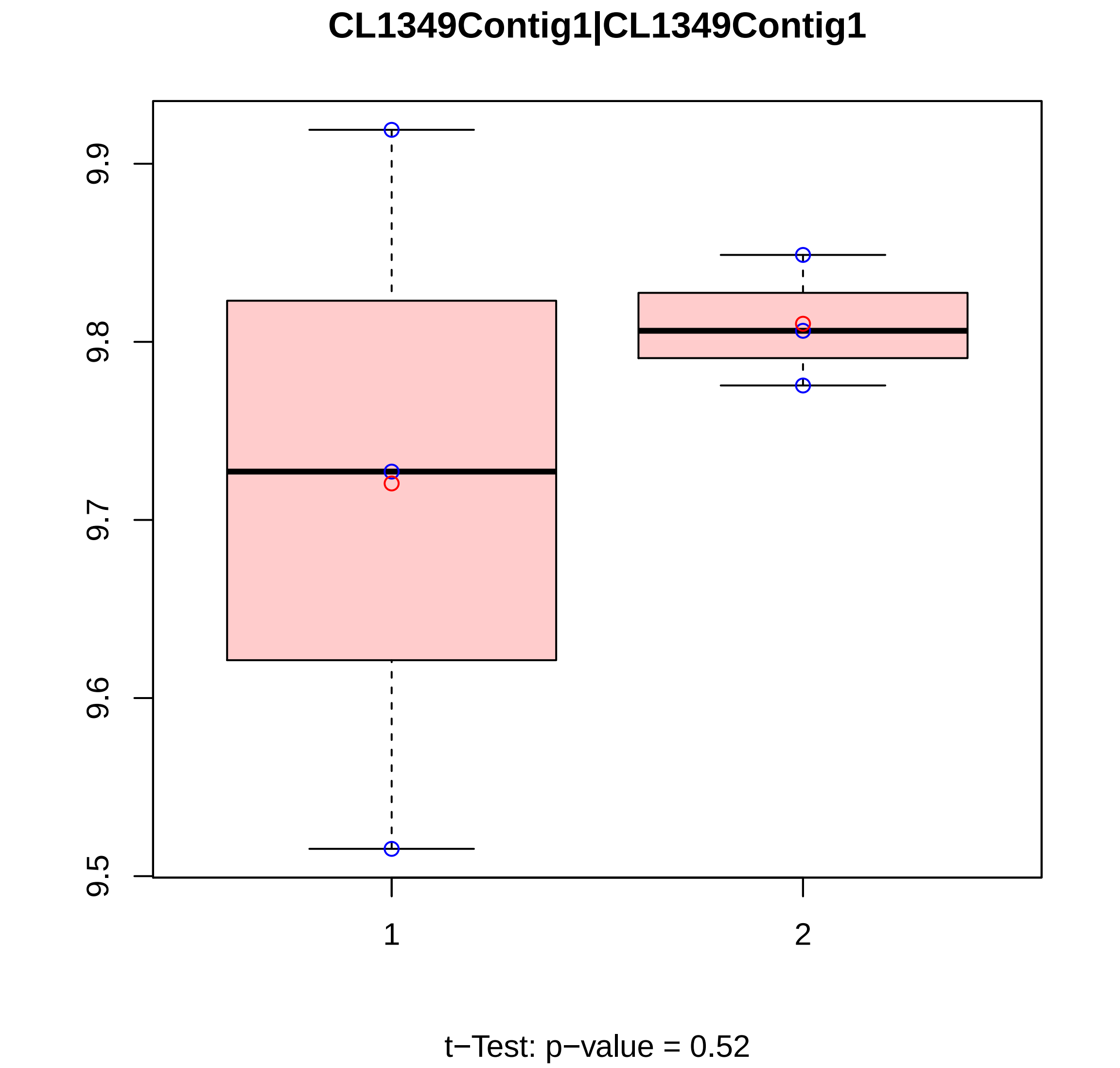
CL1349Contig1|CL1349Contig1
9.5
9.6
9.7
9.8
9.9
1
2
t−Test: p−value = 0.52

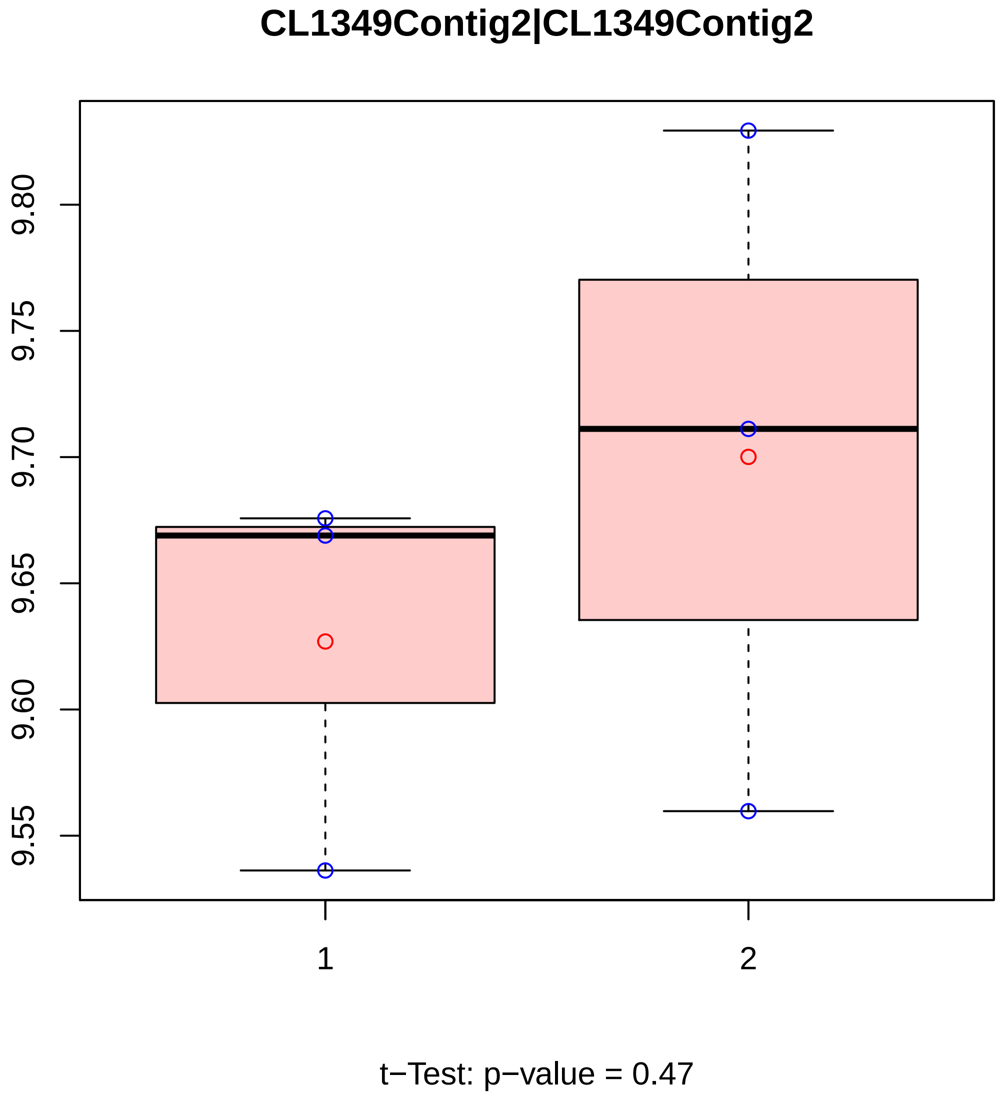

CL1349Contig2|CL1349Contig2
9.55
9.60
9.65
9.70
9.75
9.80
1
2
t−Test: p−value = 0.47

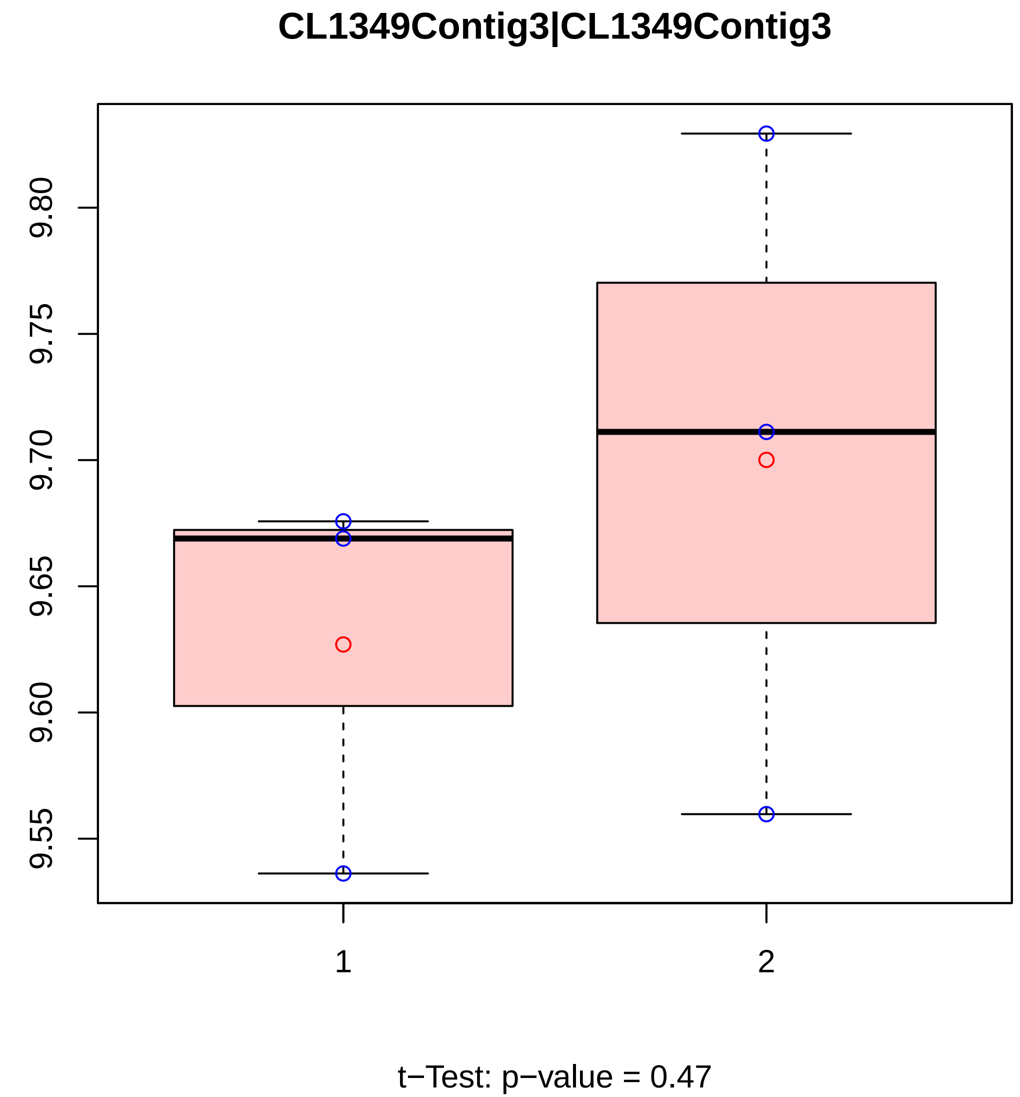
CL1349Contig3|CL1349Contig3
9.80
9.75
9.70
9.65
9.60
9.55
1
2
t−Test: p−value = 0.47

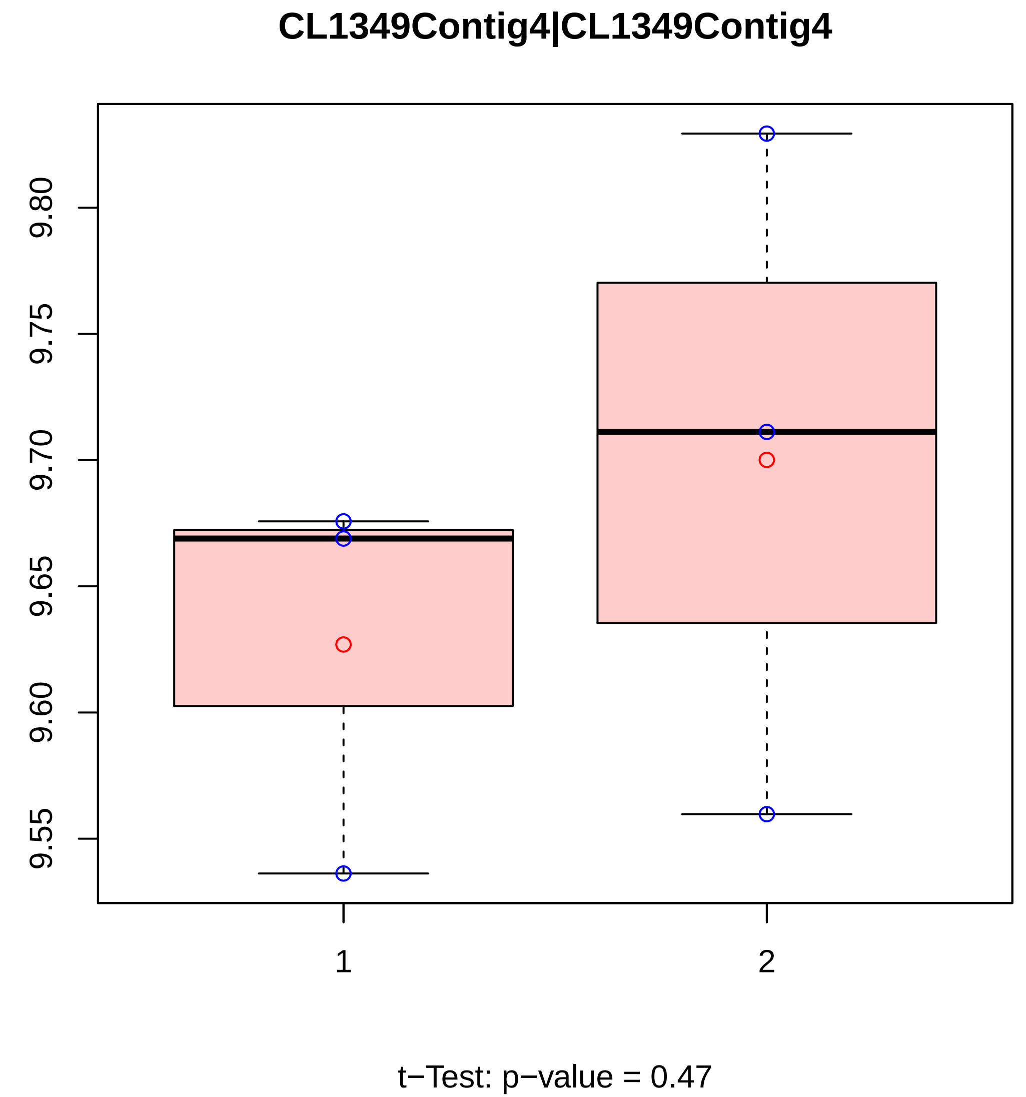
CL1349Contig4|CL1349Contig4
9.55
9.60
9.65
9.70
9.75
9.80
1
2
t−Test: p−value = 0.47

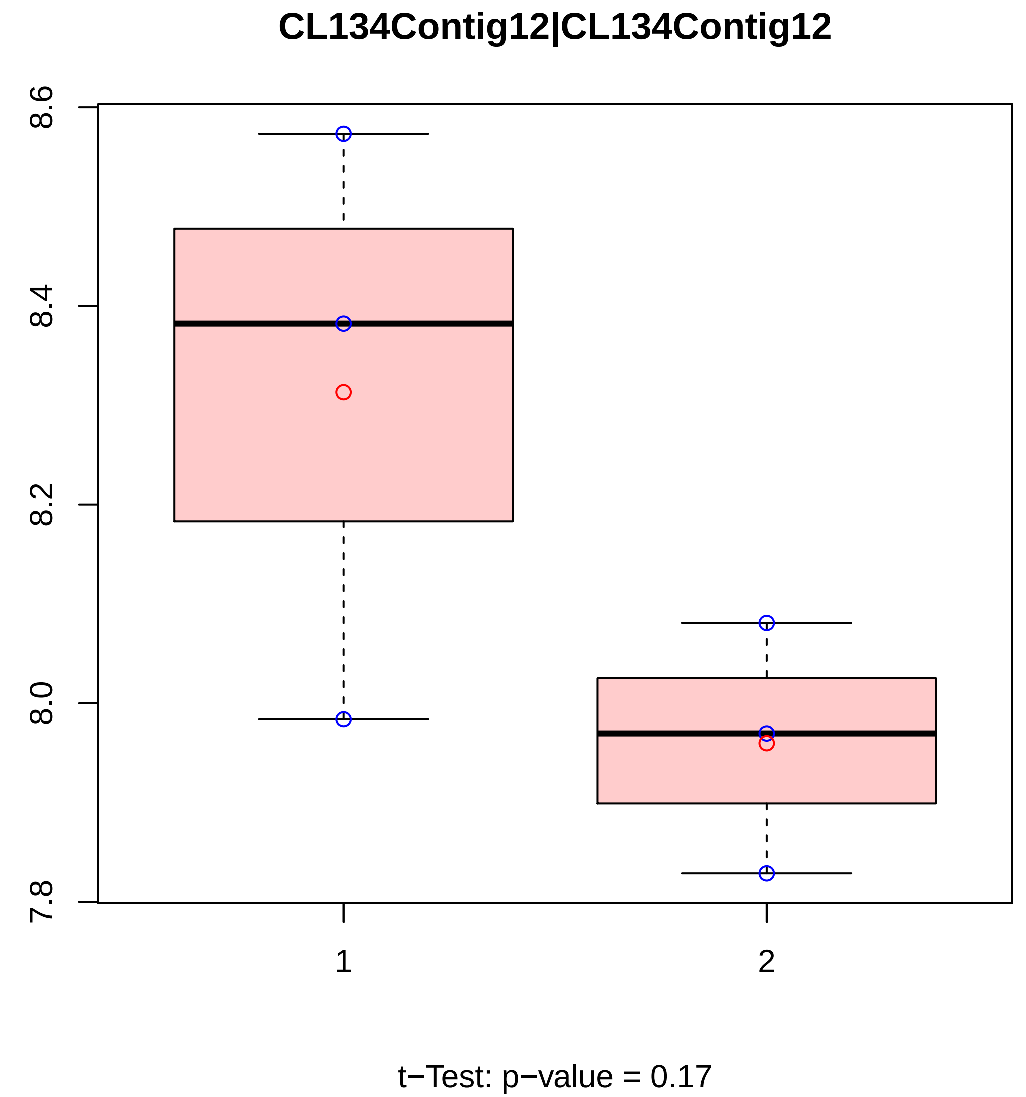
CL134Contig12|CL134Contig12
8.6
8.4
8.2
8.0
7.8
1
2
t−Test: p−value = 0.17

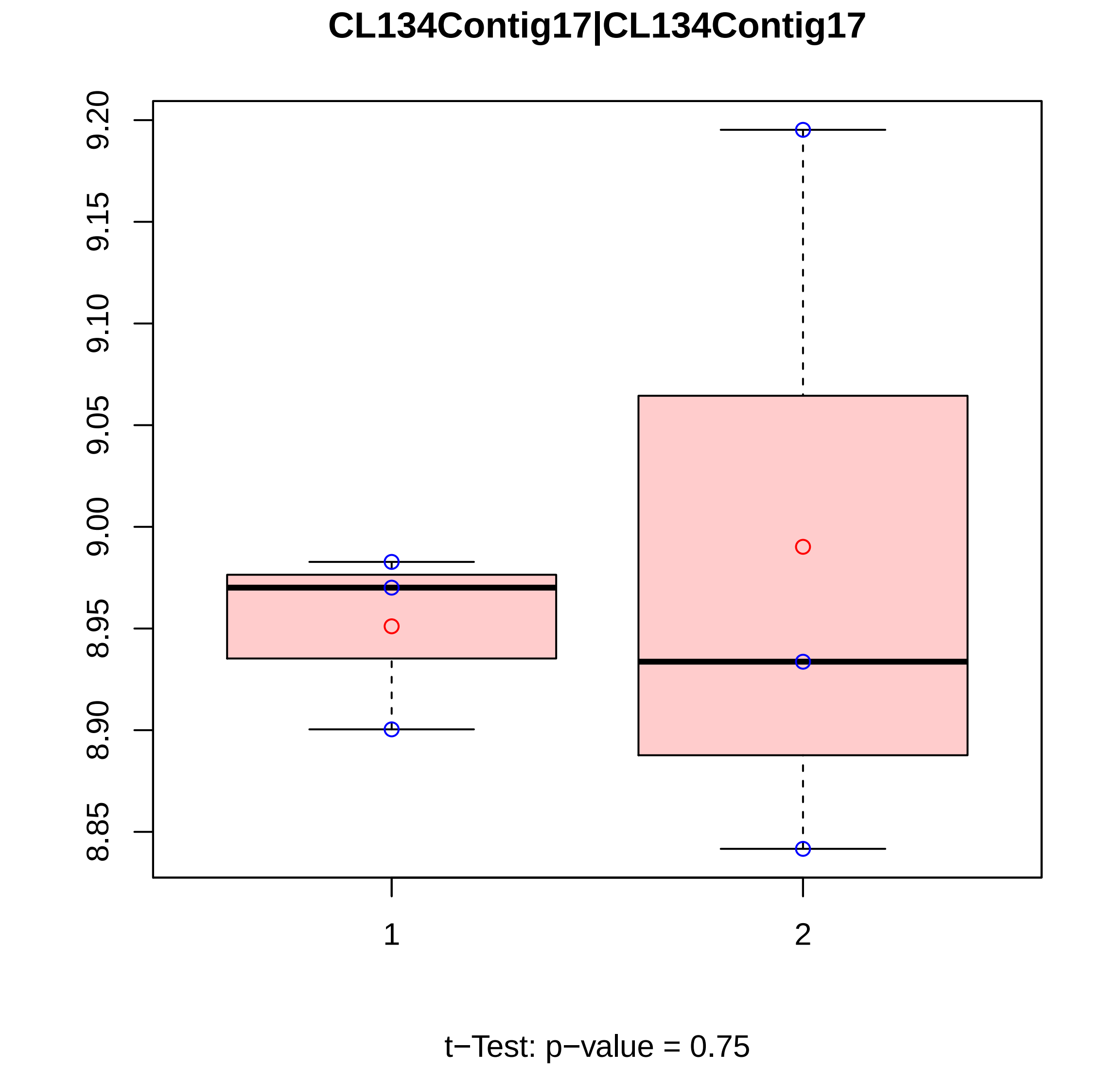
CL134Contig17|CL134Contig17
9.20
9.15
9.10
9.05
9.00
8.95
8.90
8.85
1
2
t−Test: p−value = 0.75

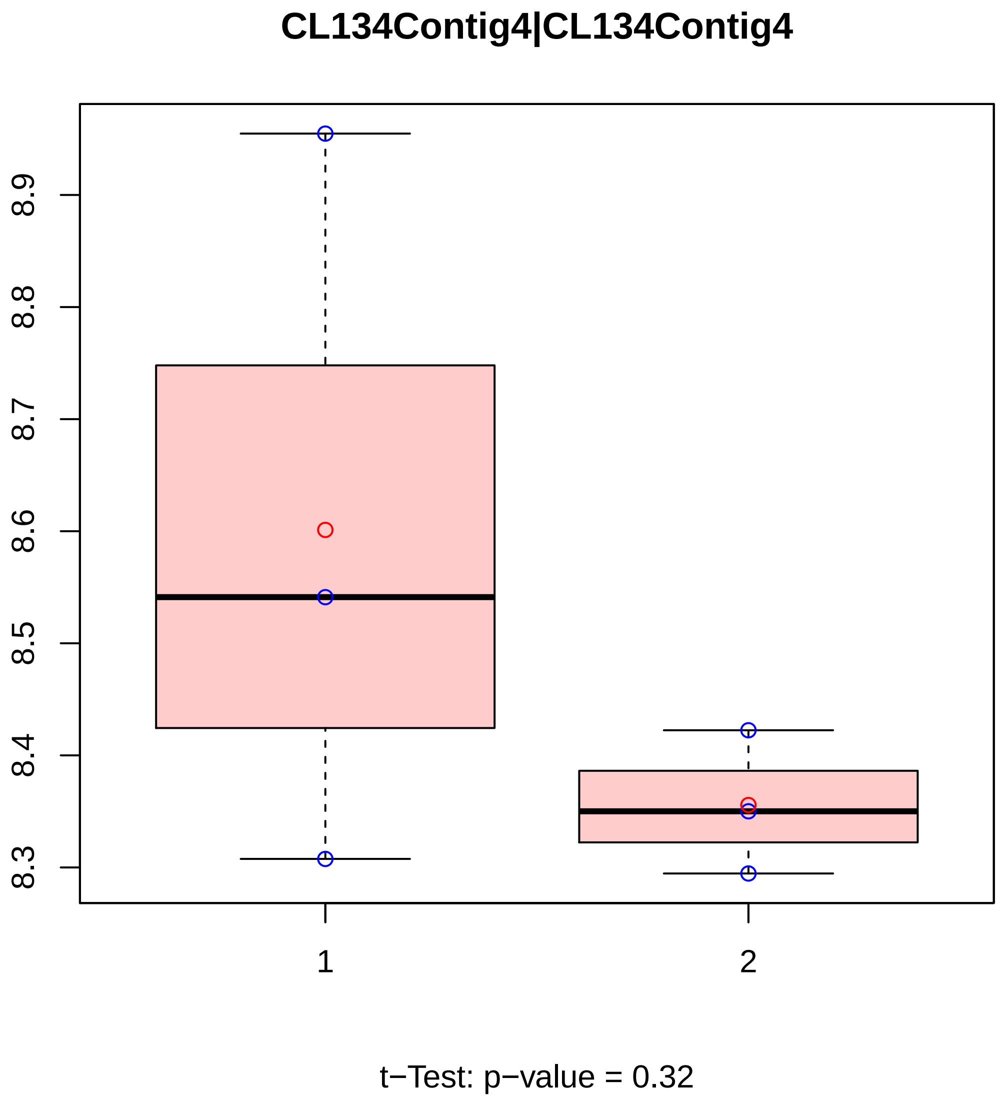
CL134Contig4|CL134Contig4
8.3
8.4
8.5
8.6
8.7
8.8
8.9
1
2
t−Test: p−value = 0.32

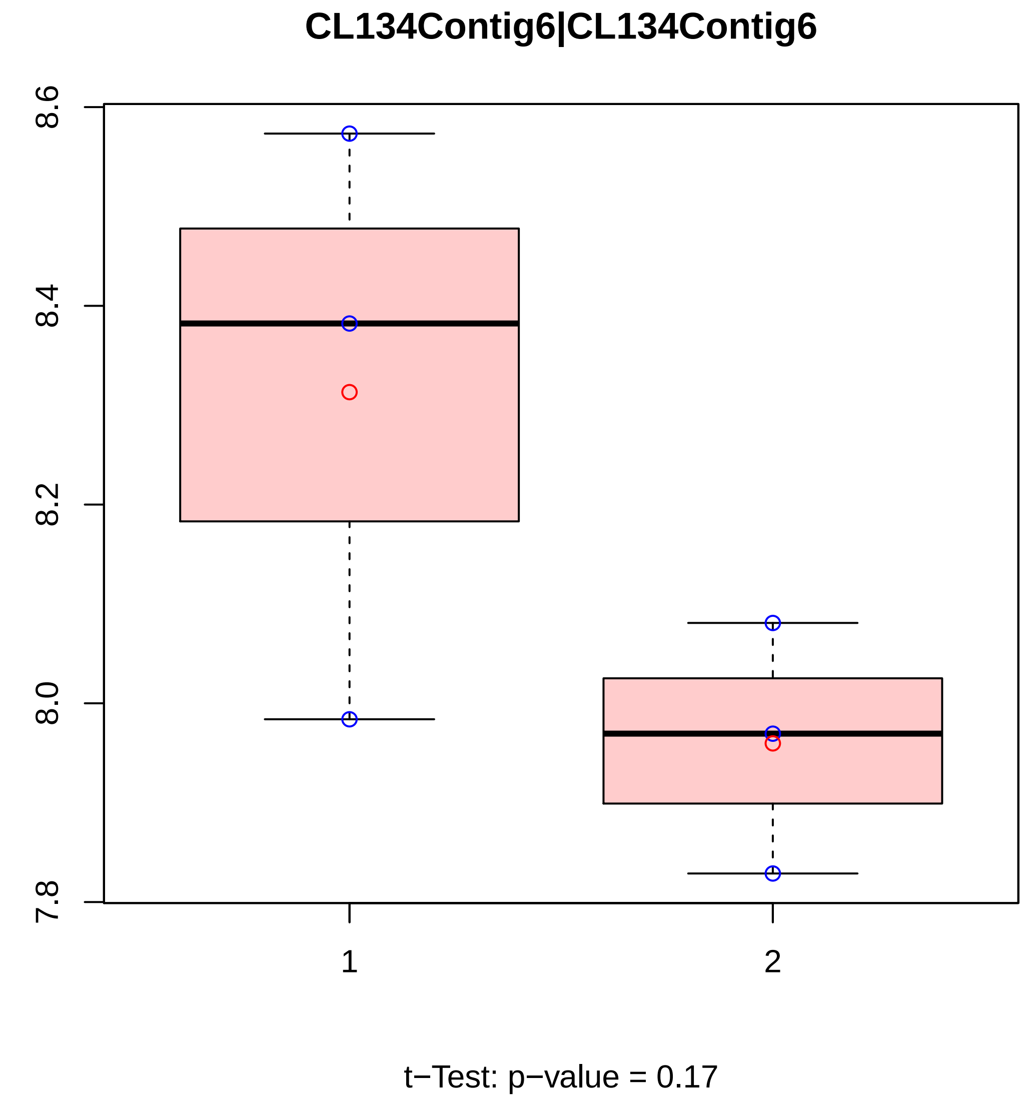
CL134Contig6|CL134Contig6
8.6
8.4
8.2
8.0
7.8
1
2
t−Test: p−value = 0.17

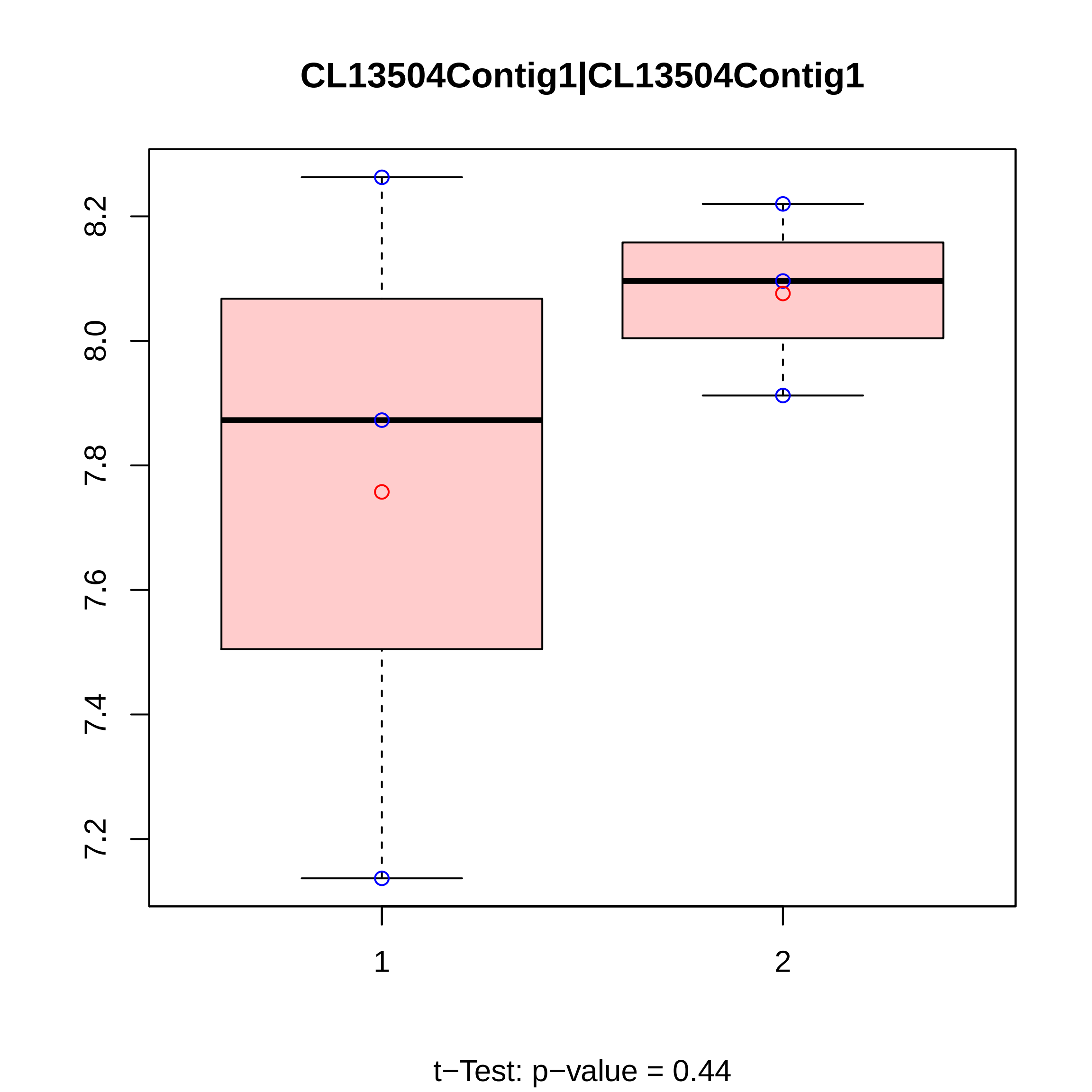
CL13504Contig1|CL13504Contig1
8.2
8.0
7.8
7.6
7.4
7.2
1
2
t−Test: p−value = 0.44

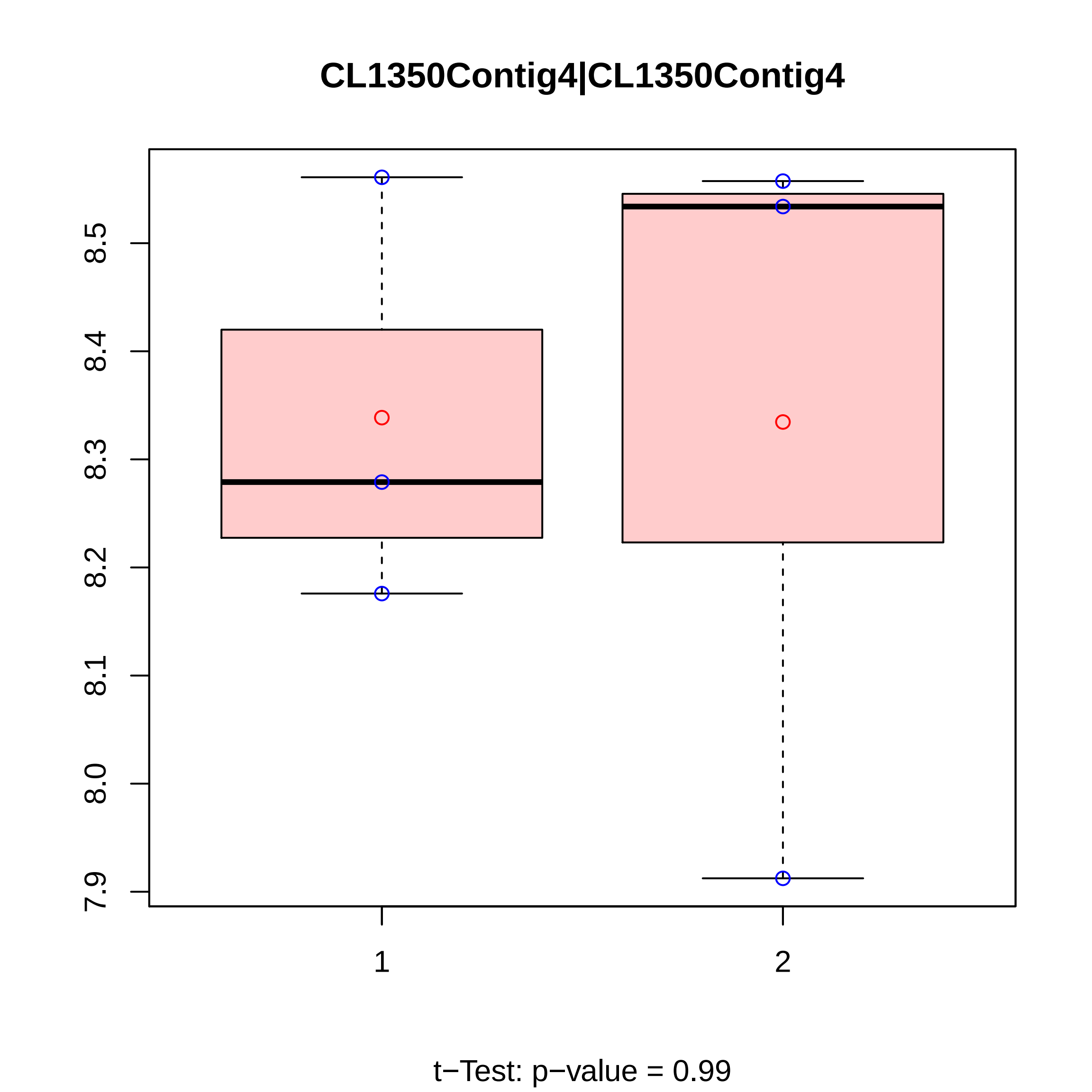

CL1350Contig4|CL1350Contig4
8.5
8.4
8.3
8.2
8.1
8.0
7.9
1
2
t−Test: p−value = 0.99

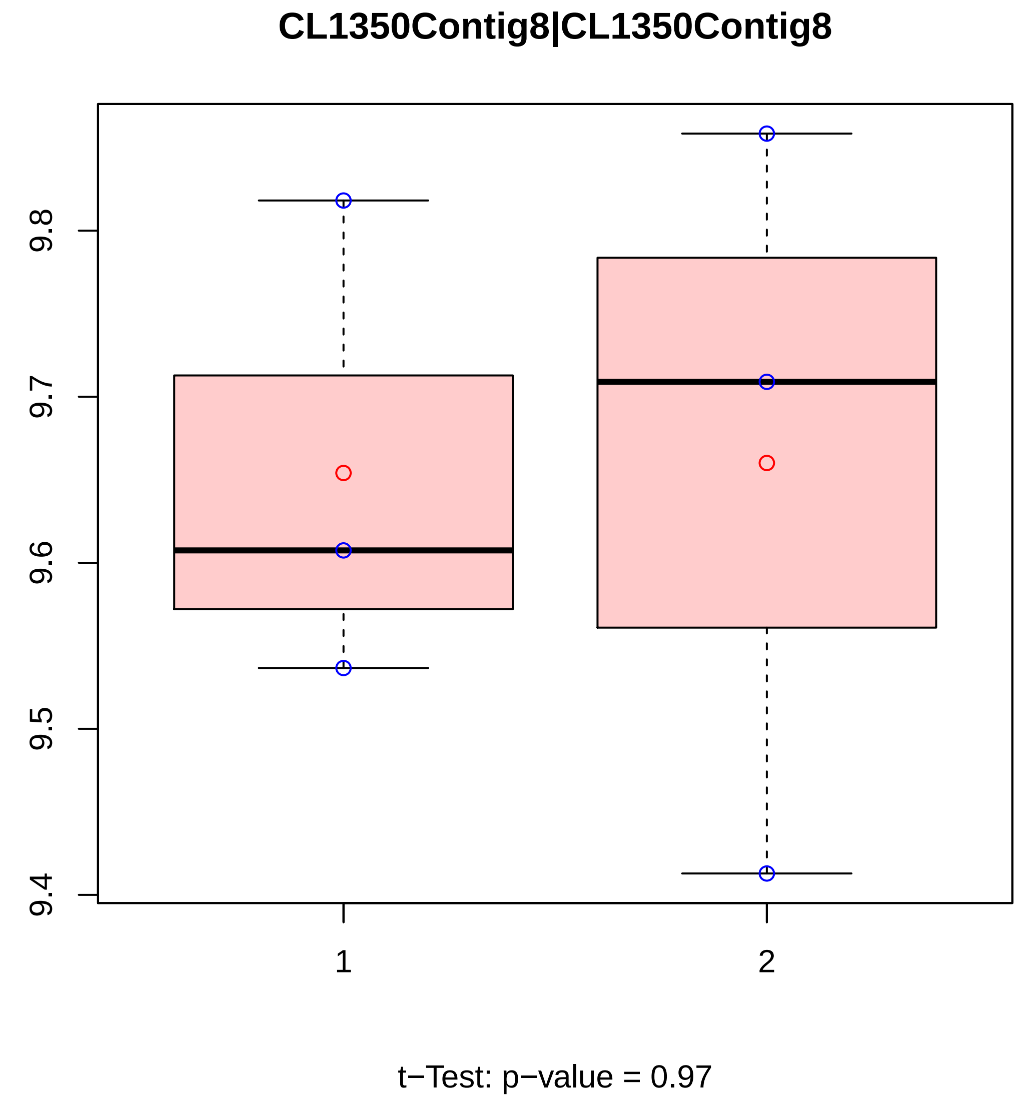
CL1350Contig8|CL1350Contig8
9.4
9.5
9.6
9.7
9.8
1
2
t−Test: p−value = 0.97

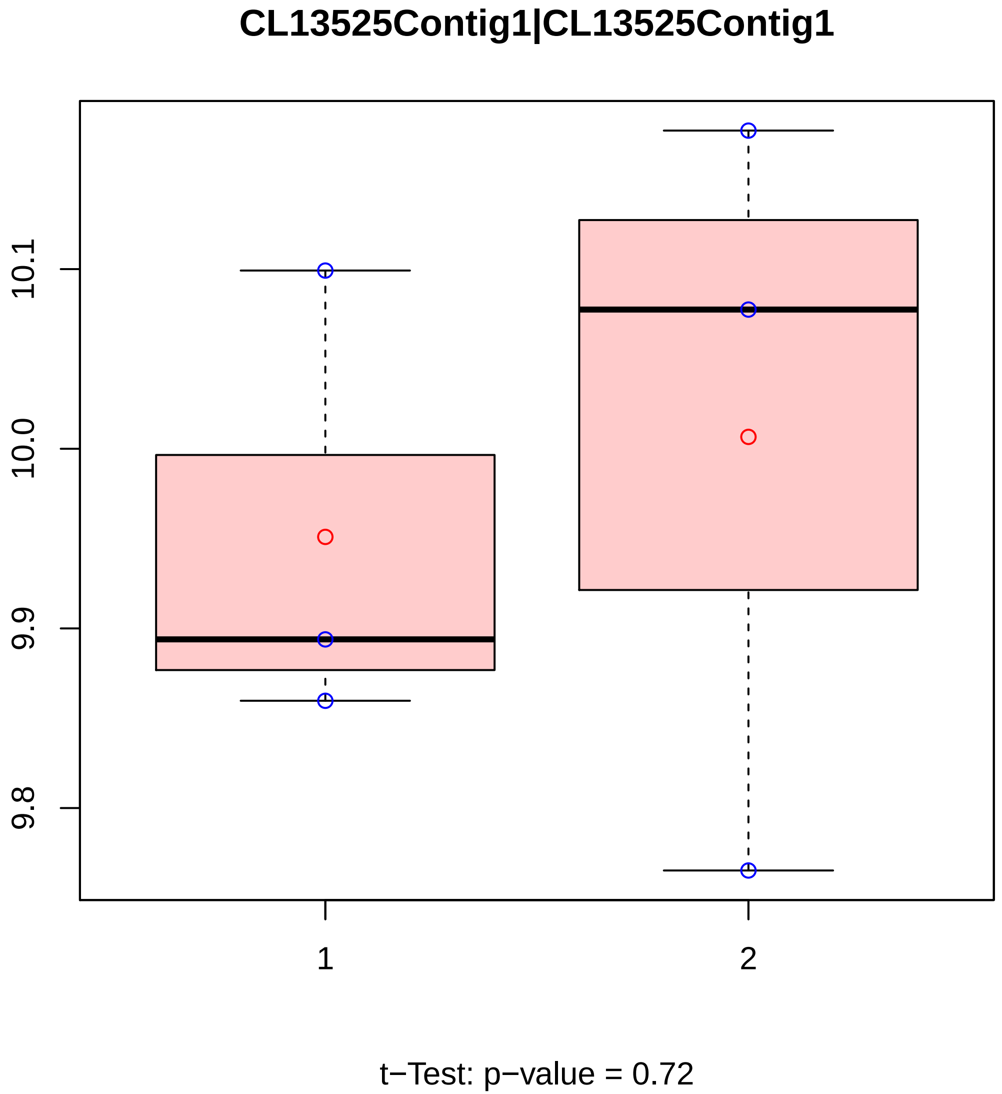
CL13525Contig1|CL13525Contig1
10.1
10.0
9.9
9.8
1
2
t−Test: p−value = 0.72

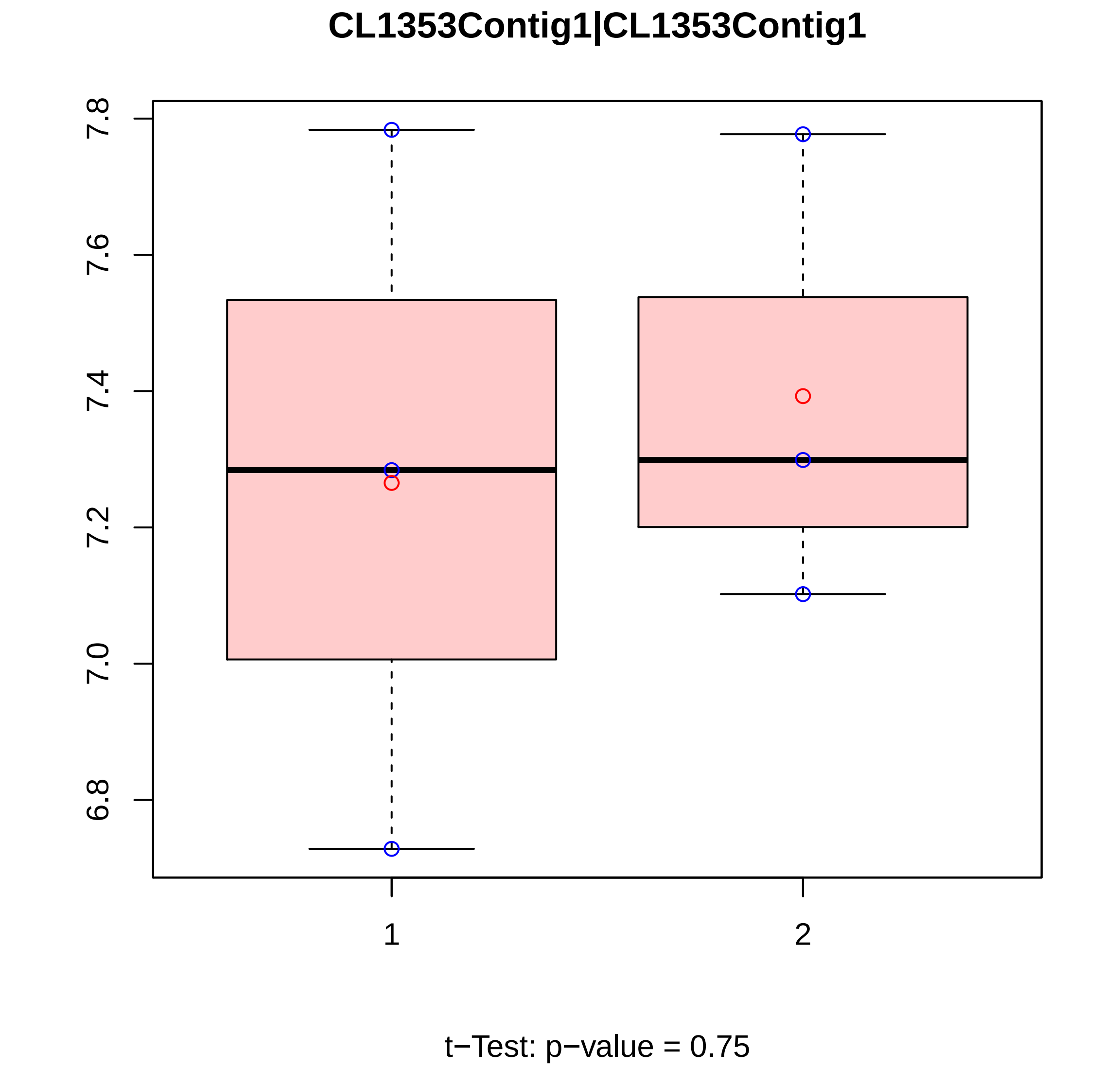
CL1353Contig1|CL1353Contig1
7.8
7.6
7.4
7.2
7.0
6.8
1
2
t−Test: p−value = 0.75

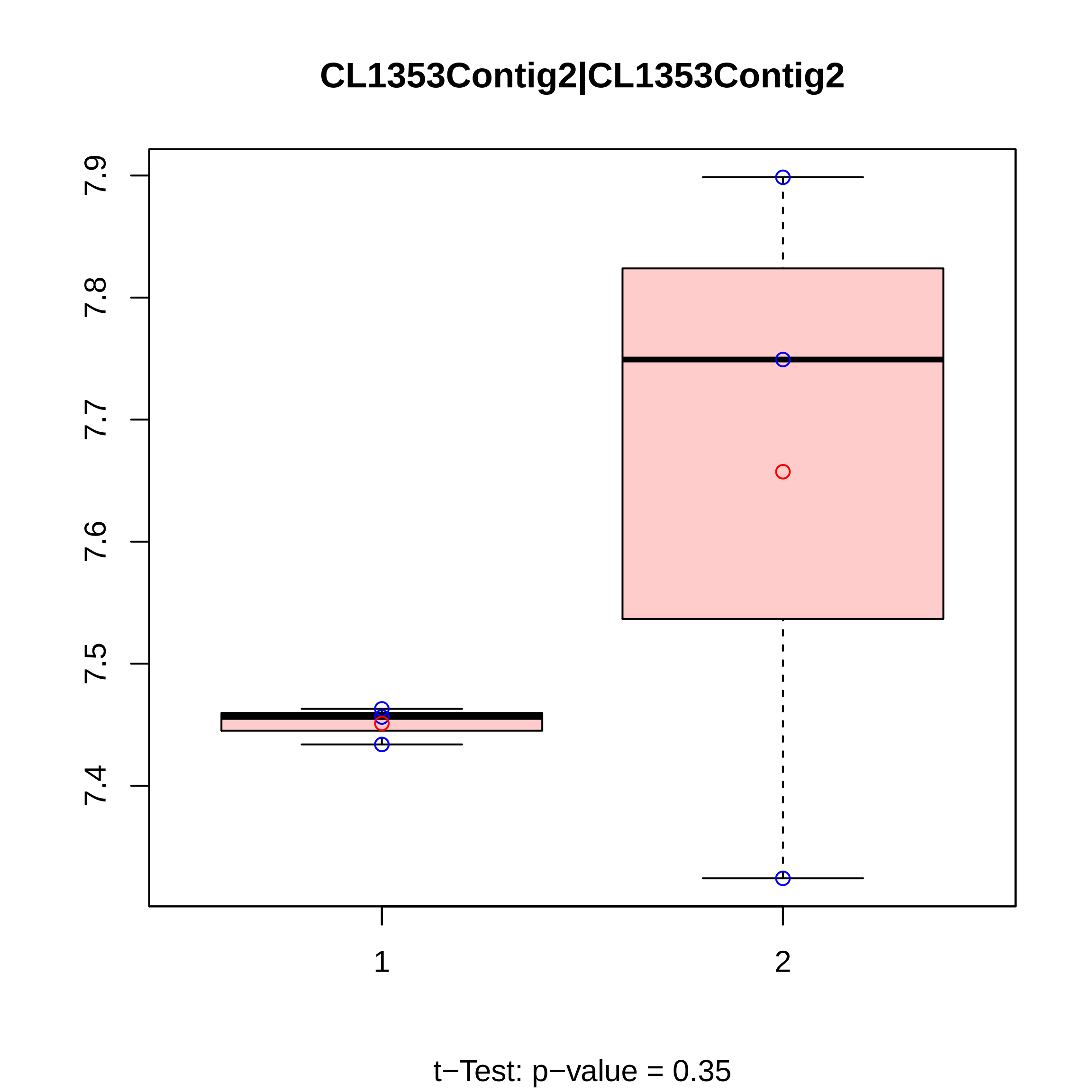
CL1353Contig2|CL1353Contig2
7.4
7.5
7.6
7.7
7.8
7.9
1
2
t−Test: p−value = 0.35

# CL1353Contig3|CL1353Contig3

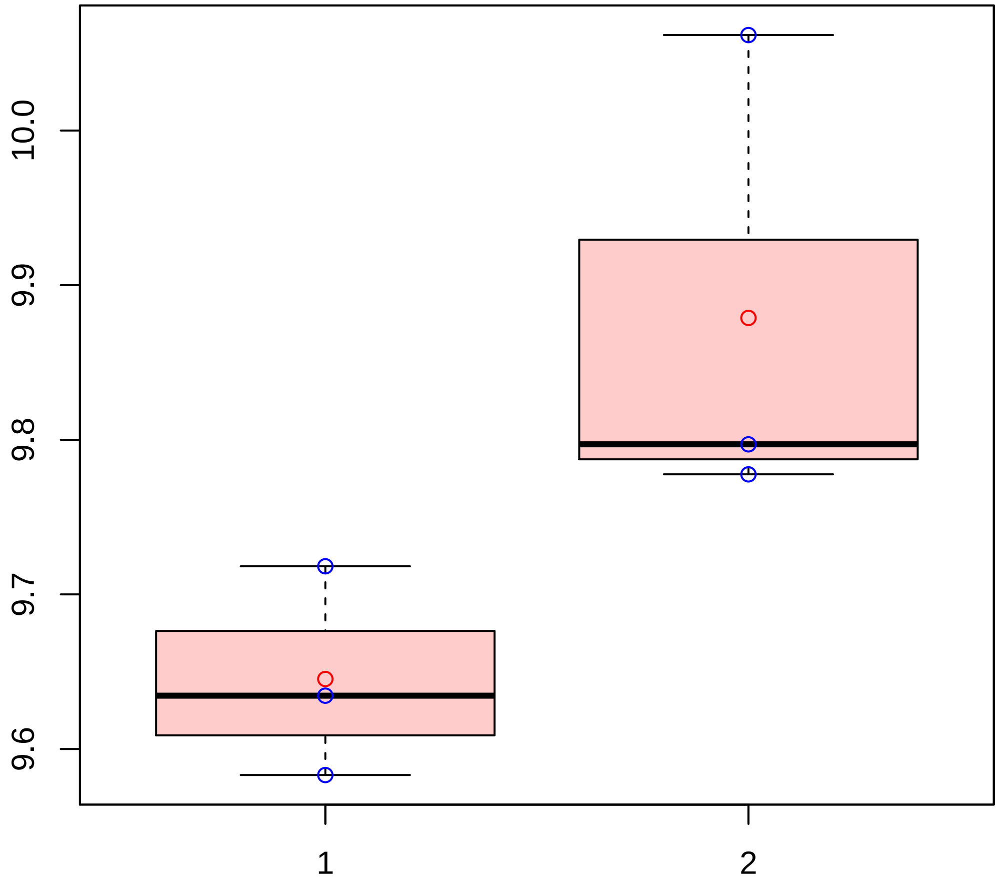


t−Test: p−value = 0.11

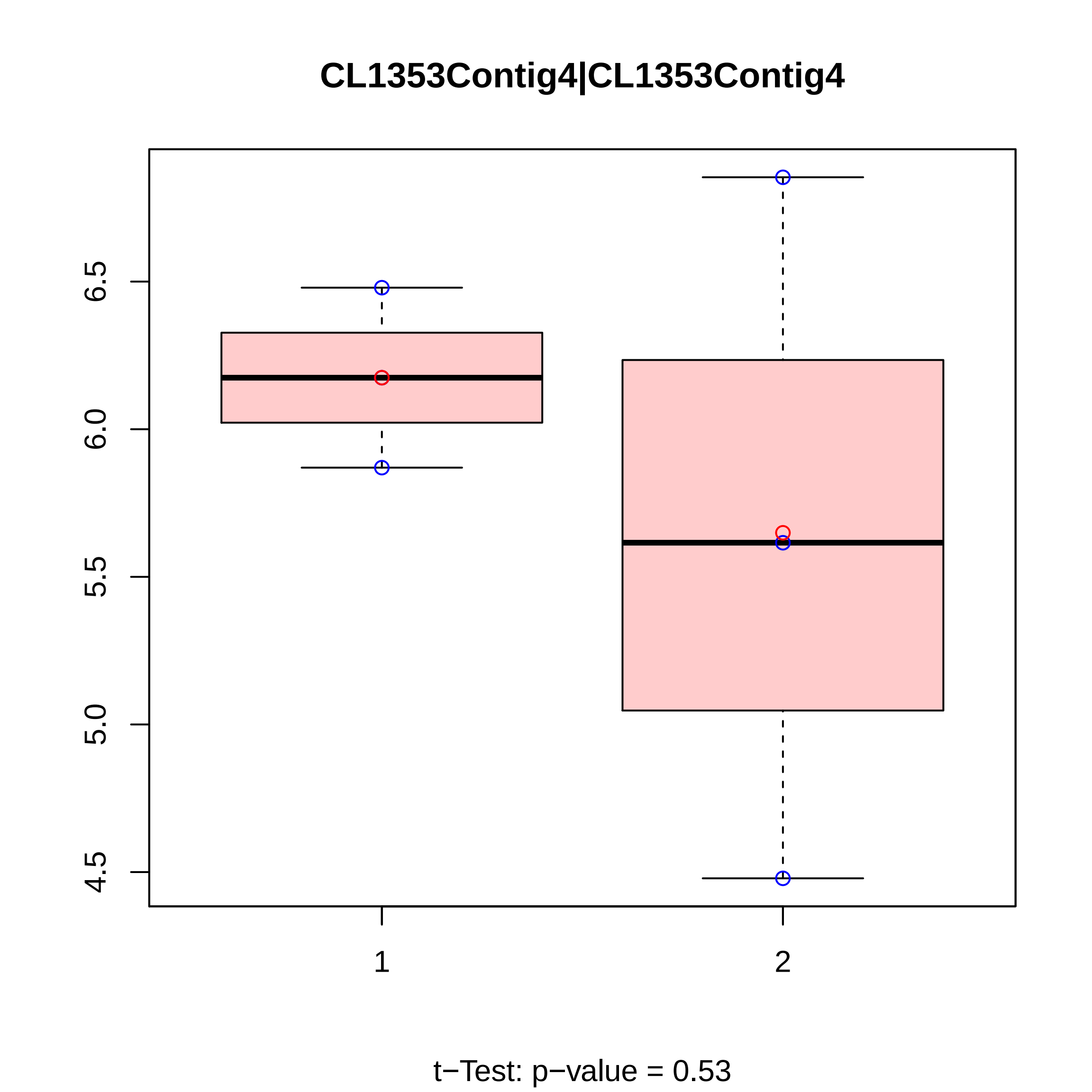

CL1353Contig4|CL1353Contig4
6.5
6.0
5.5
5.0
4.5
1
2
t−Test: p−value = 0.53

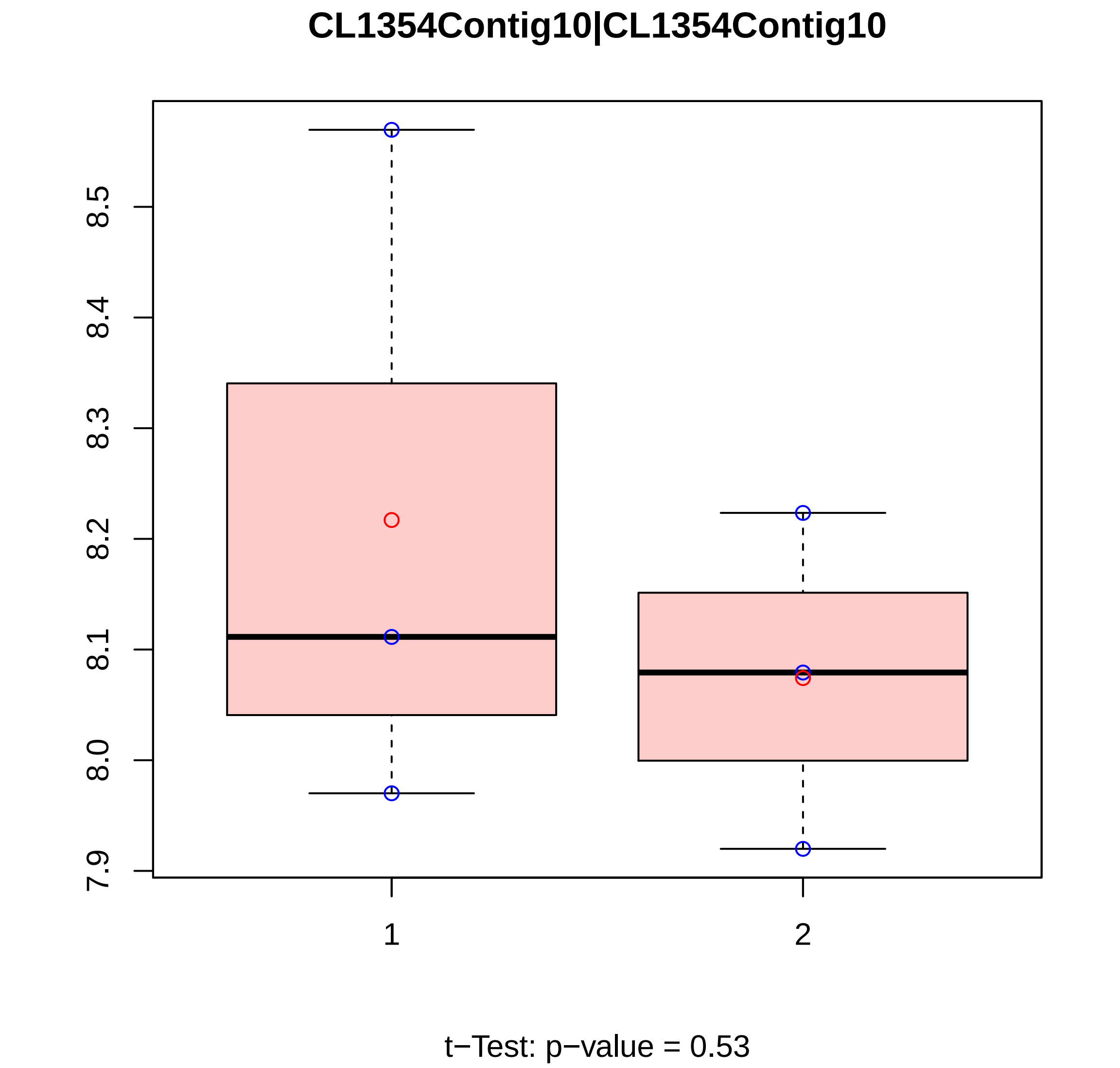
CL1354Contig10|CL1354Contig10
8.5
8.4
8.3
8.2
8.1
8.0
7.9
1
2
t−Test: p−value = 0.53

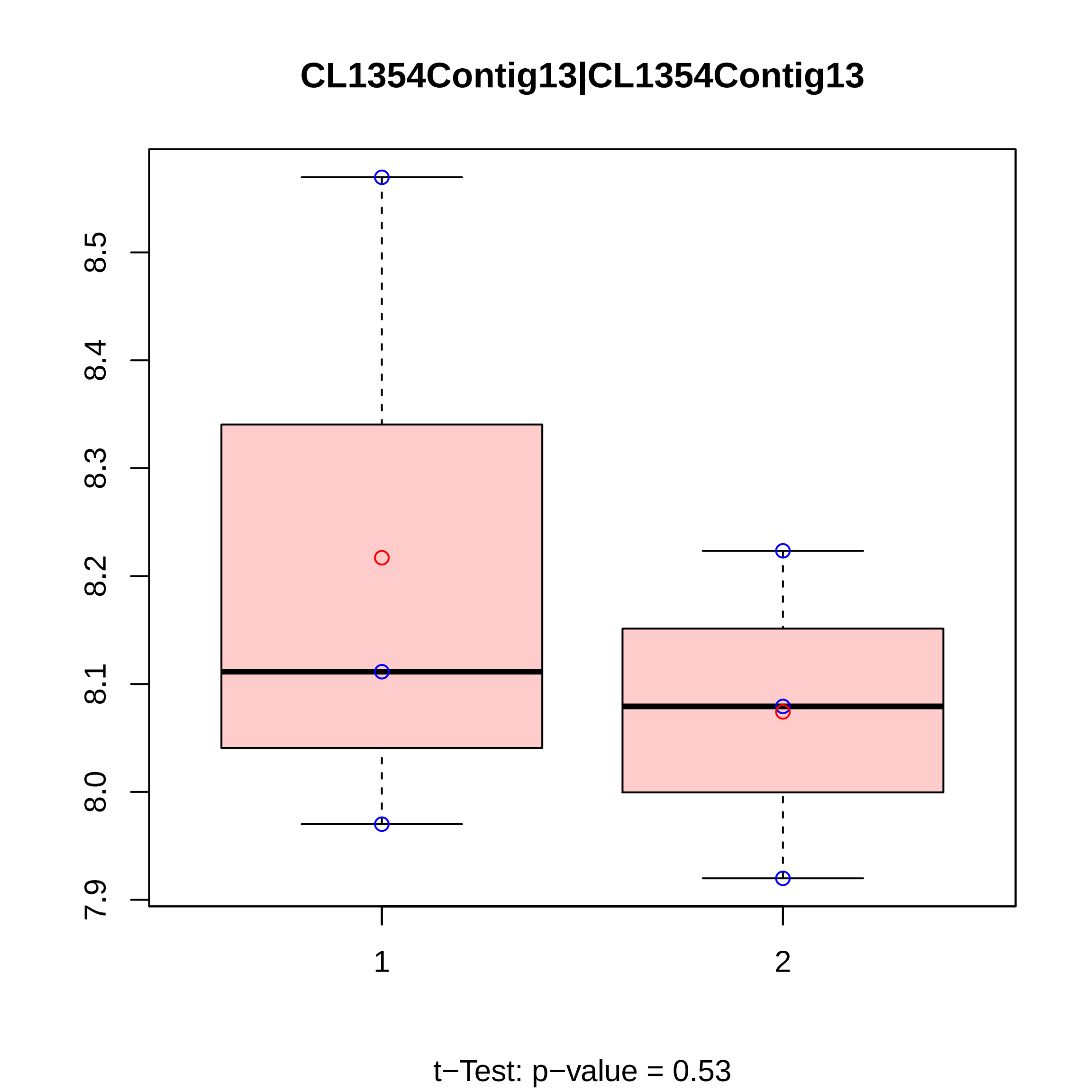
CL1354Contig13|CL1354Contig13
8.5
8.4
8.3
8.2
8.1
8.0
7.9
1
2
t−Test: p−value = 0.53

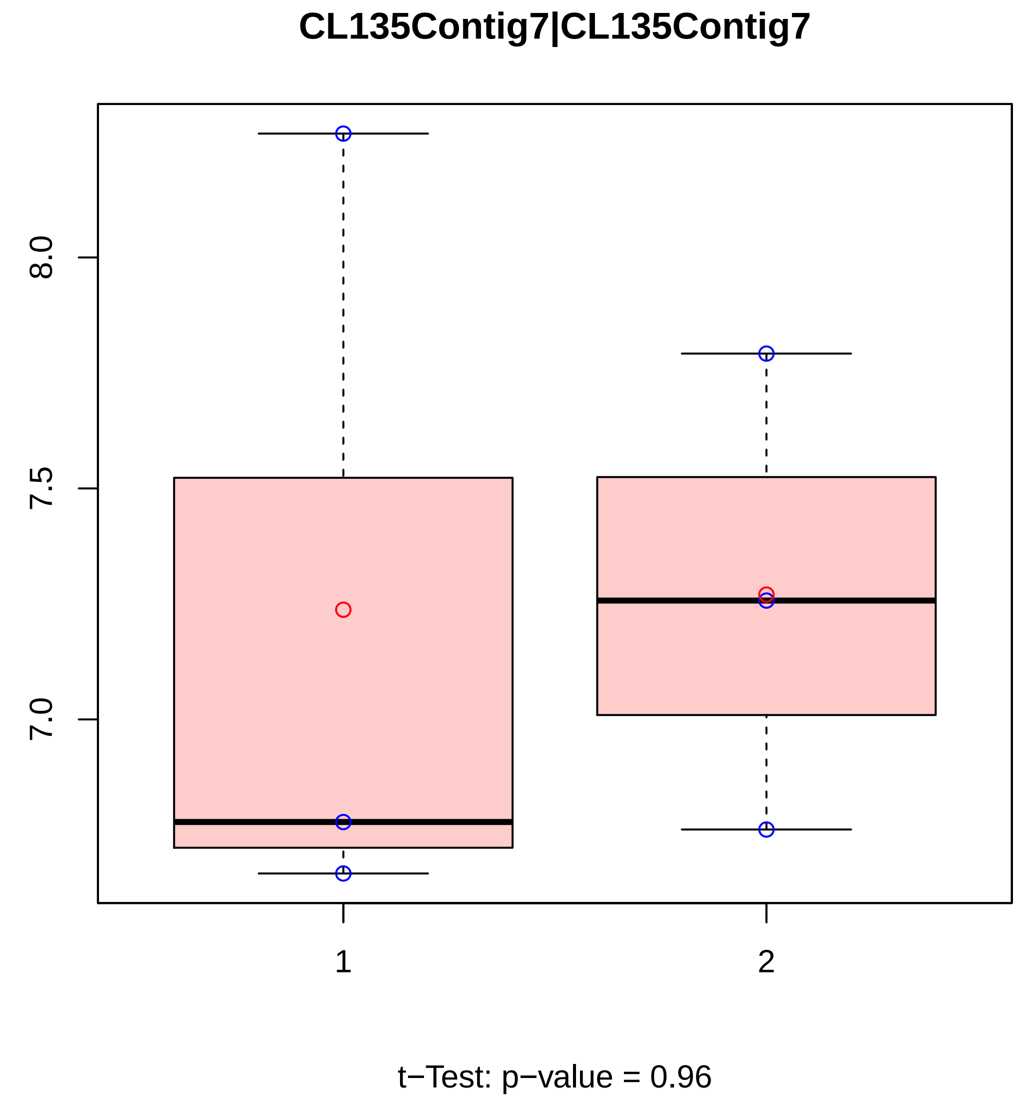

CL135Contig7|CL135Contig7
8.0
7.5
7.0
1
2
t−Test: p−value = 0.96

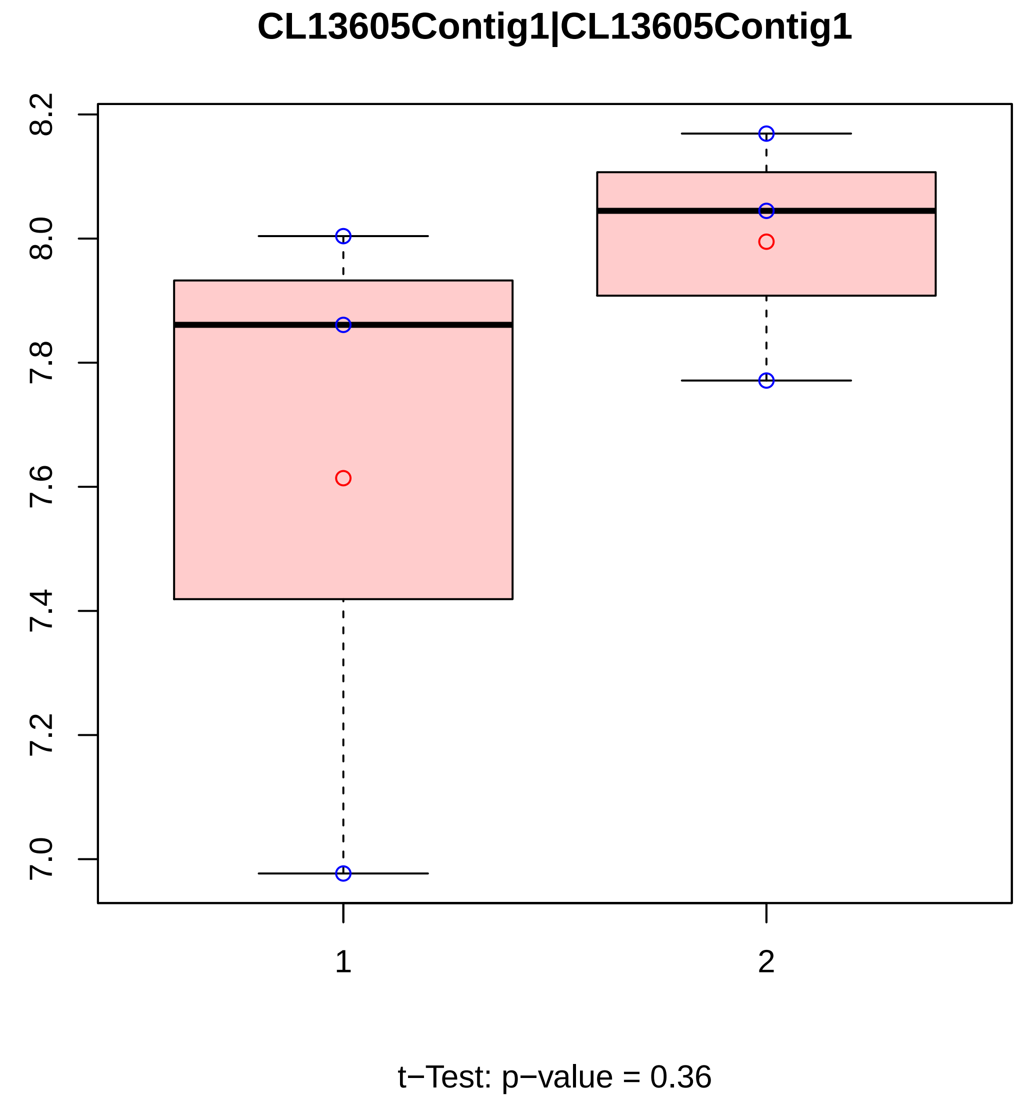
CL13605Contig1|CL13605Contig1
8.2
8.0
7.8
7.6
7.4
7.2
7.0
1
2
t−Test: p−value = 0.36

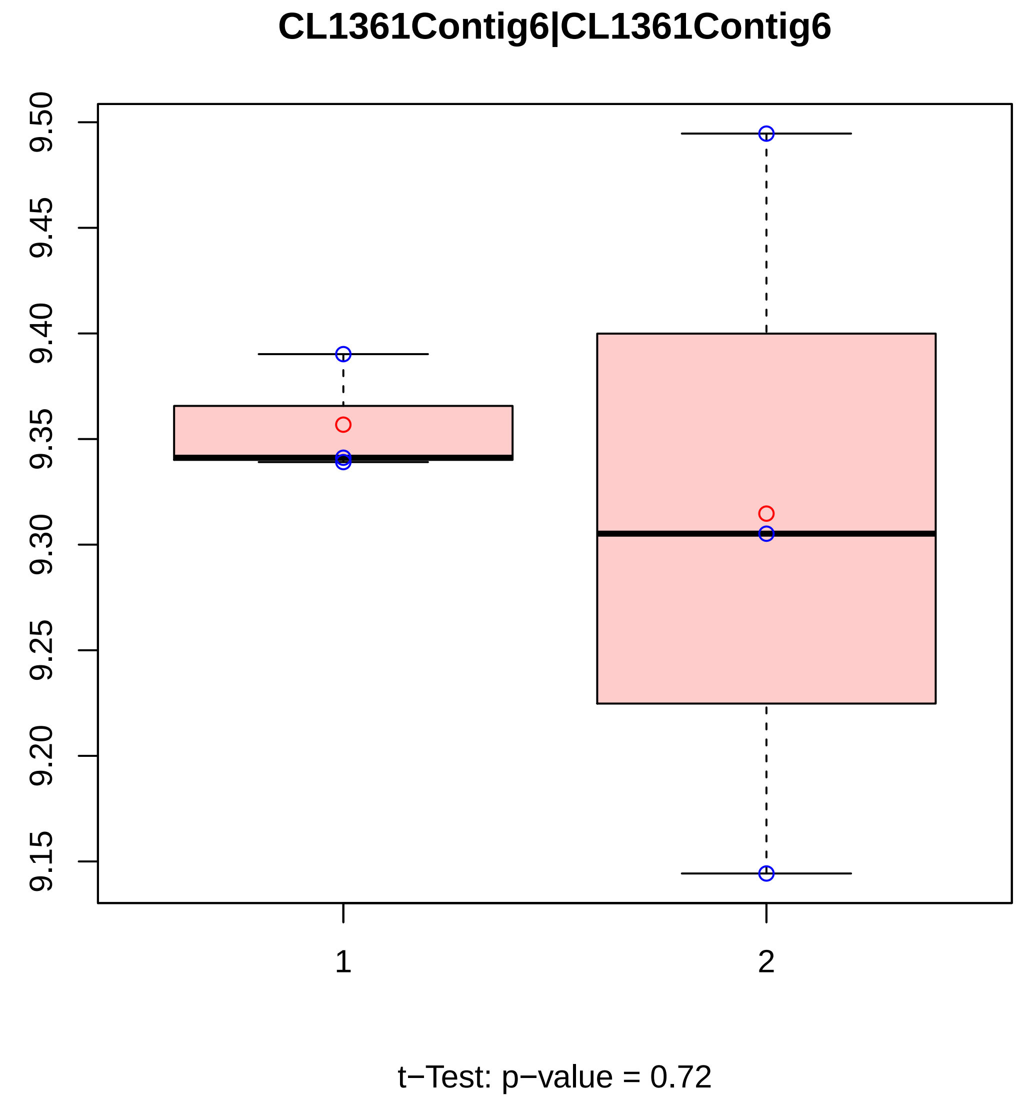
CL1361Contig6|CL1361Contig6
9.50
9.45
9.40
9.35
9.30
9.25
9.20
9.15
1
2
t−Test: p−value = 0.72

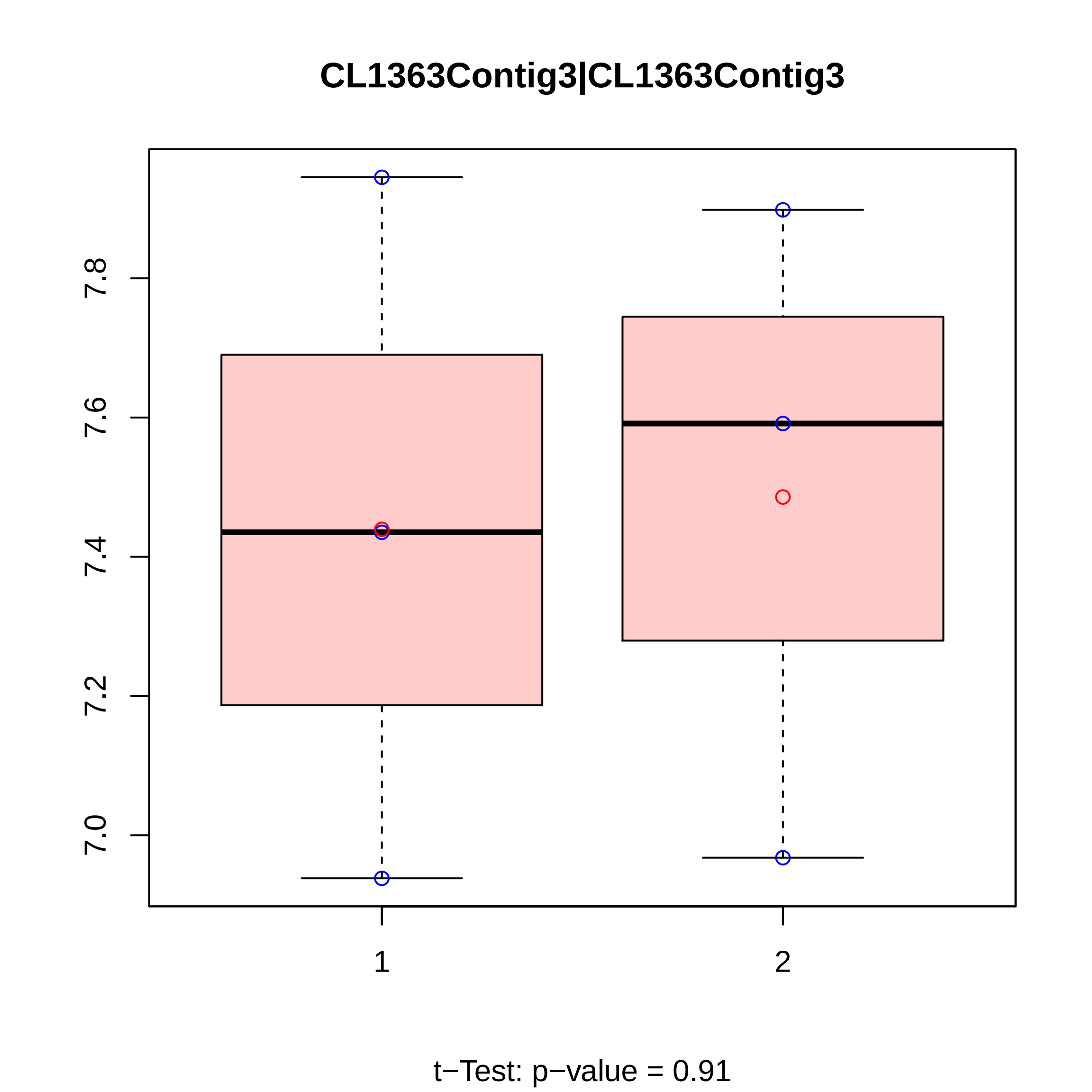
CL1363Contig3|CL1363Contig3
7.0
7.2
7.4
7.6
7.8
1
2
t−Test: p−value = 0.91

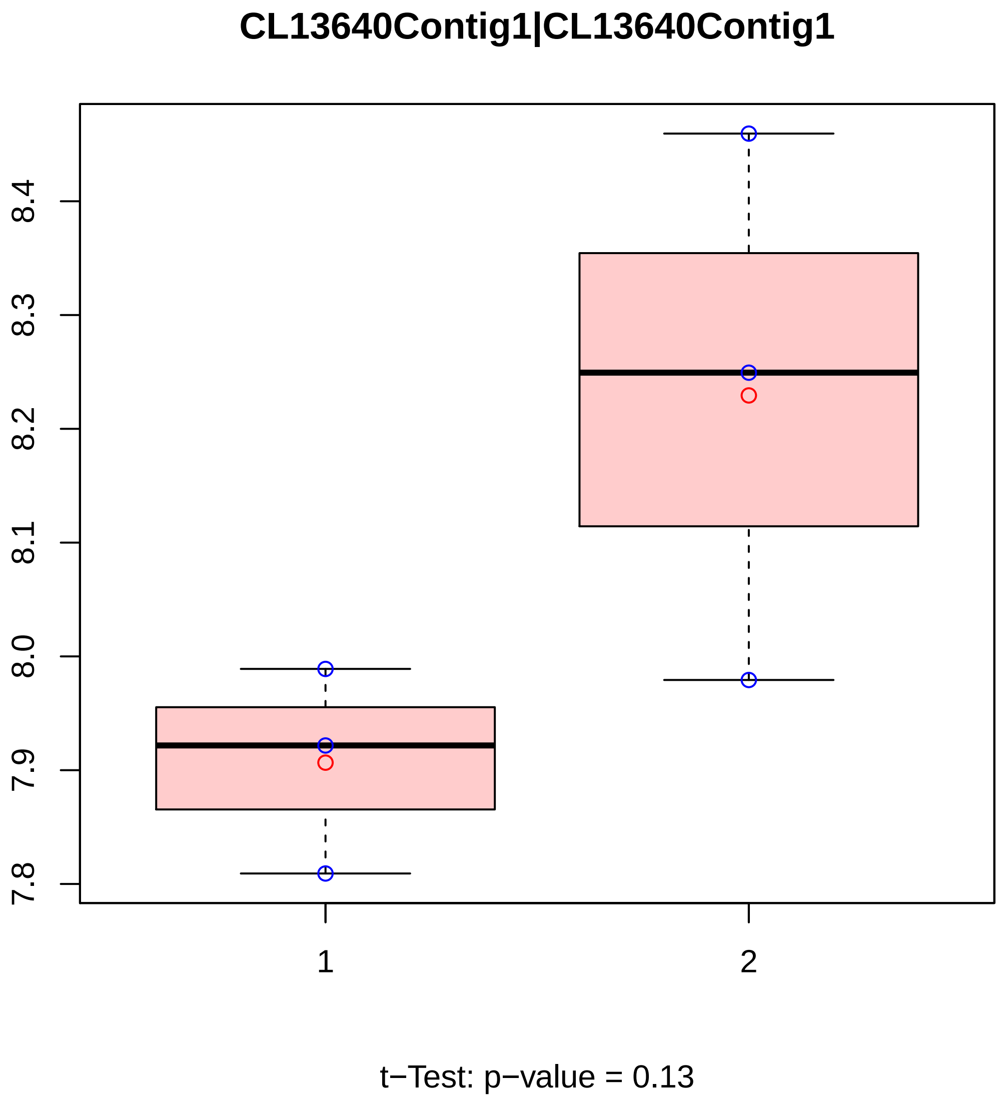
CL13640Contig1|CL13640Contig1
8.4
8.3
8.2
8.1
8.0
7.9
7.8
1
2
t−Test: p−value = 0.13

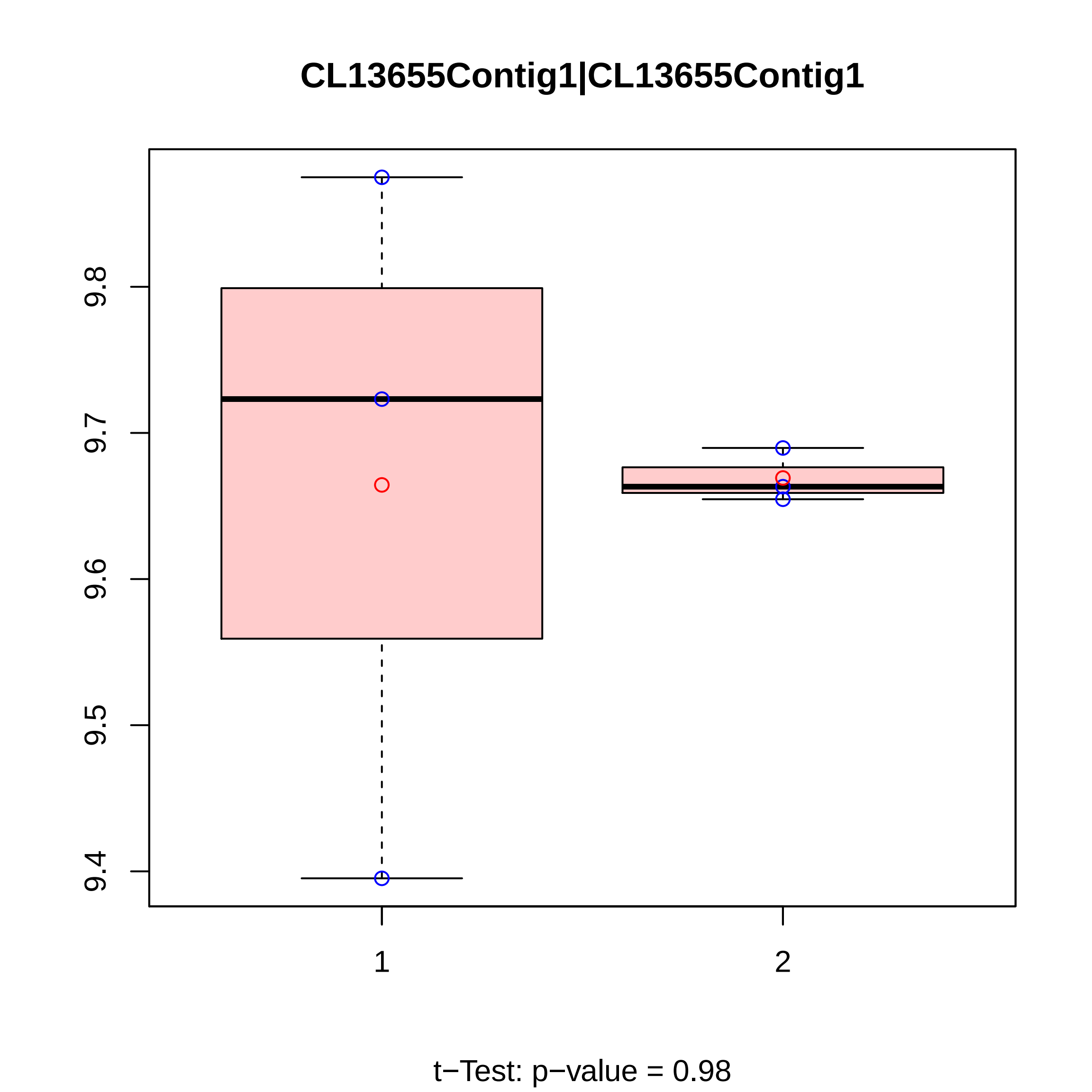
CL13655Contig1|CL13655Contig1
9.4
9.5
9.6
9.7
9.8
1
2
t−Test: p−value = 0.98

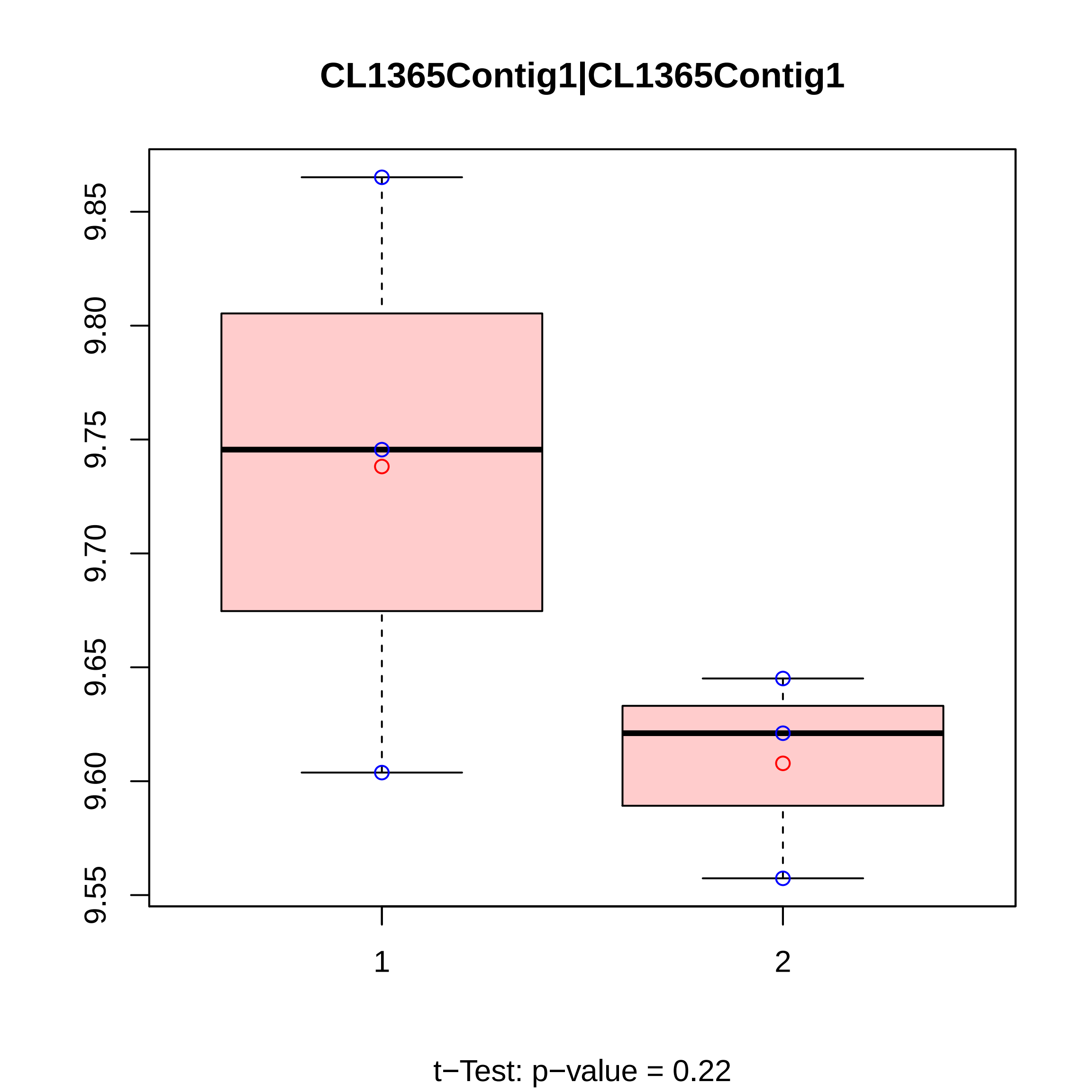
CL1365Contig1|CL1365Contig1
9.55
9.60
9.65
9.70
9.75
9.80
9.85
1
2
t−Test: p−value = 0.22

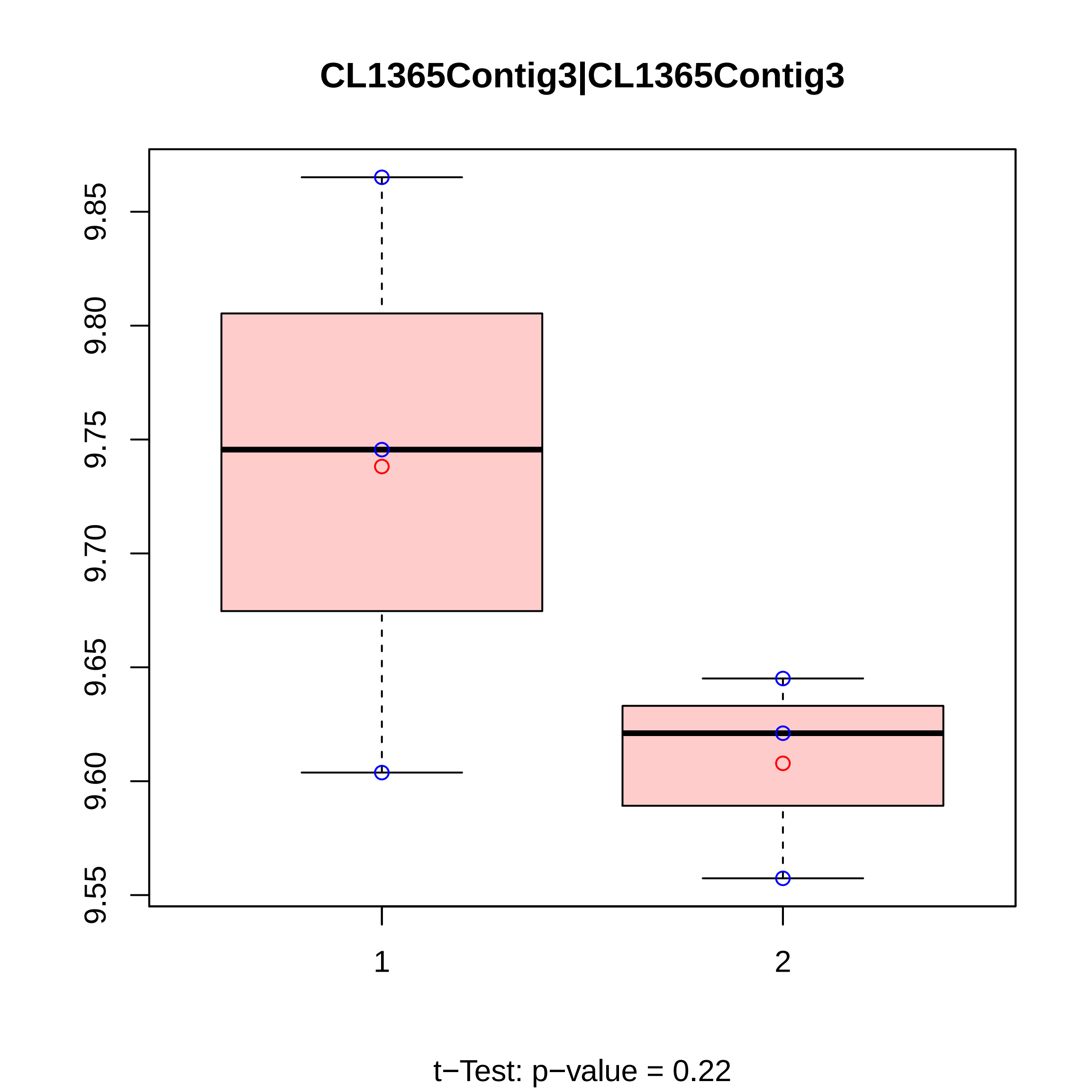
CL1365Contig3|CL1365Contig3
9.55
9.60
9.65
9.70
9.75
9.80
9.85
1
2
t−Test: p−value = 0.22

# CL1365Contig4|CL1365Contig4

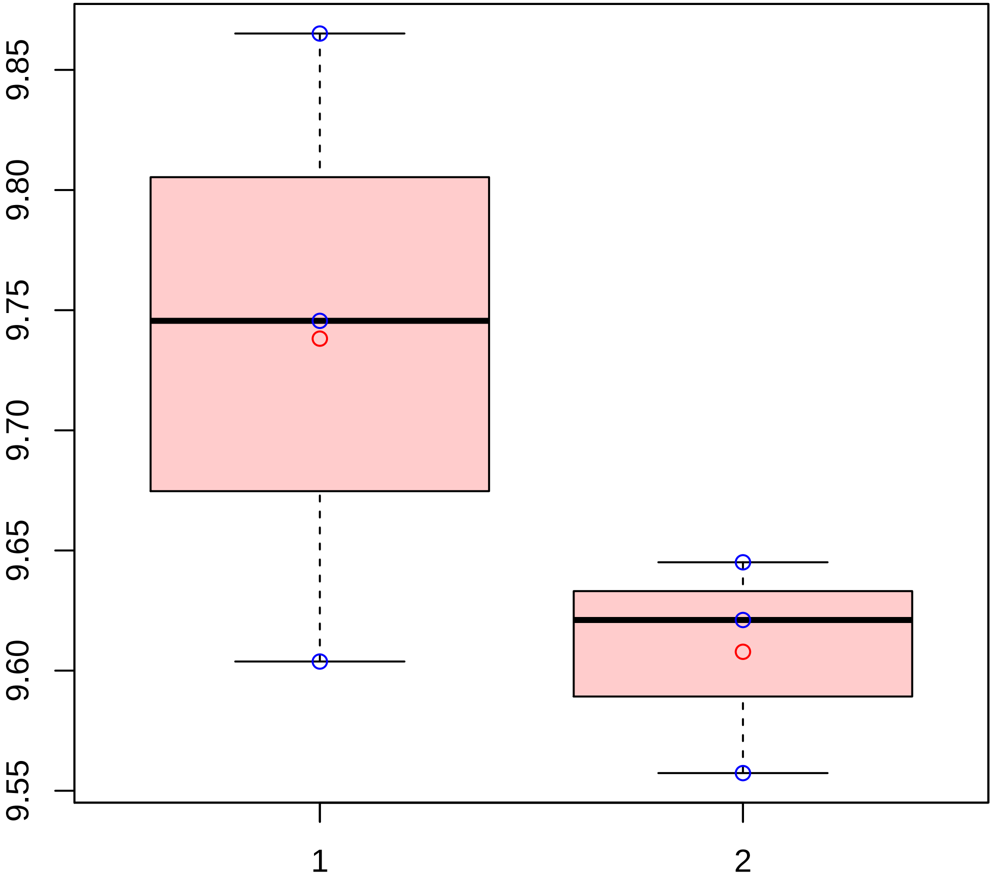


t−Test: p−value = 0.22

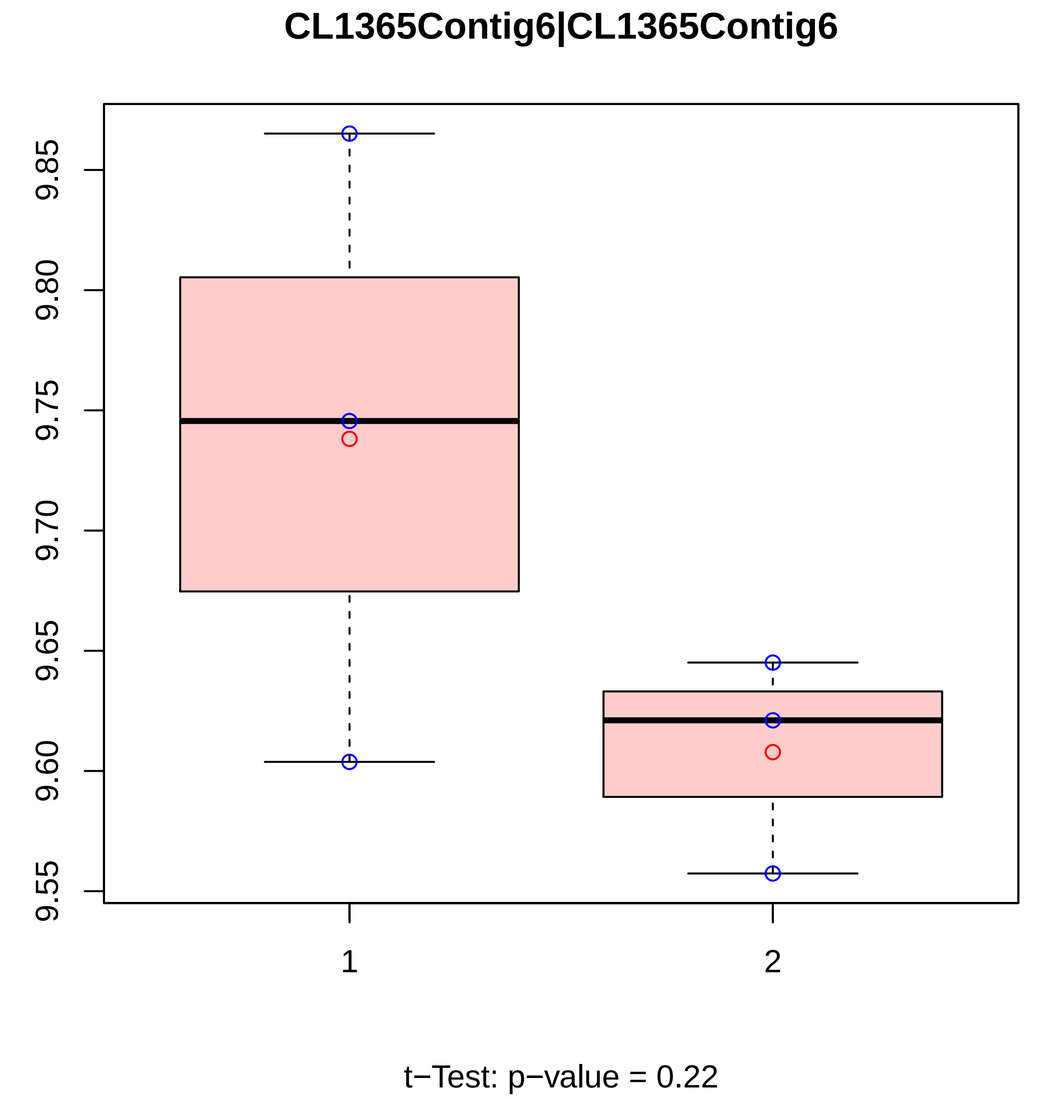
CL1365Contig6|CL1365Contig6
9.55
9.60
9.65
9.70
9.75
9.80
9.85
1
2
t−Test: p−value = 0.22

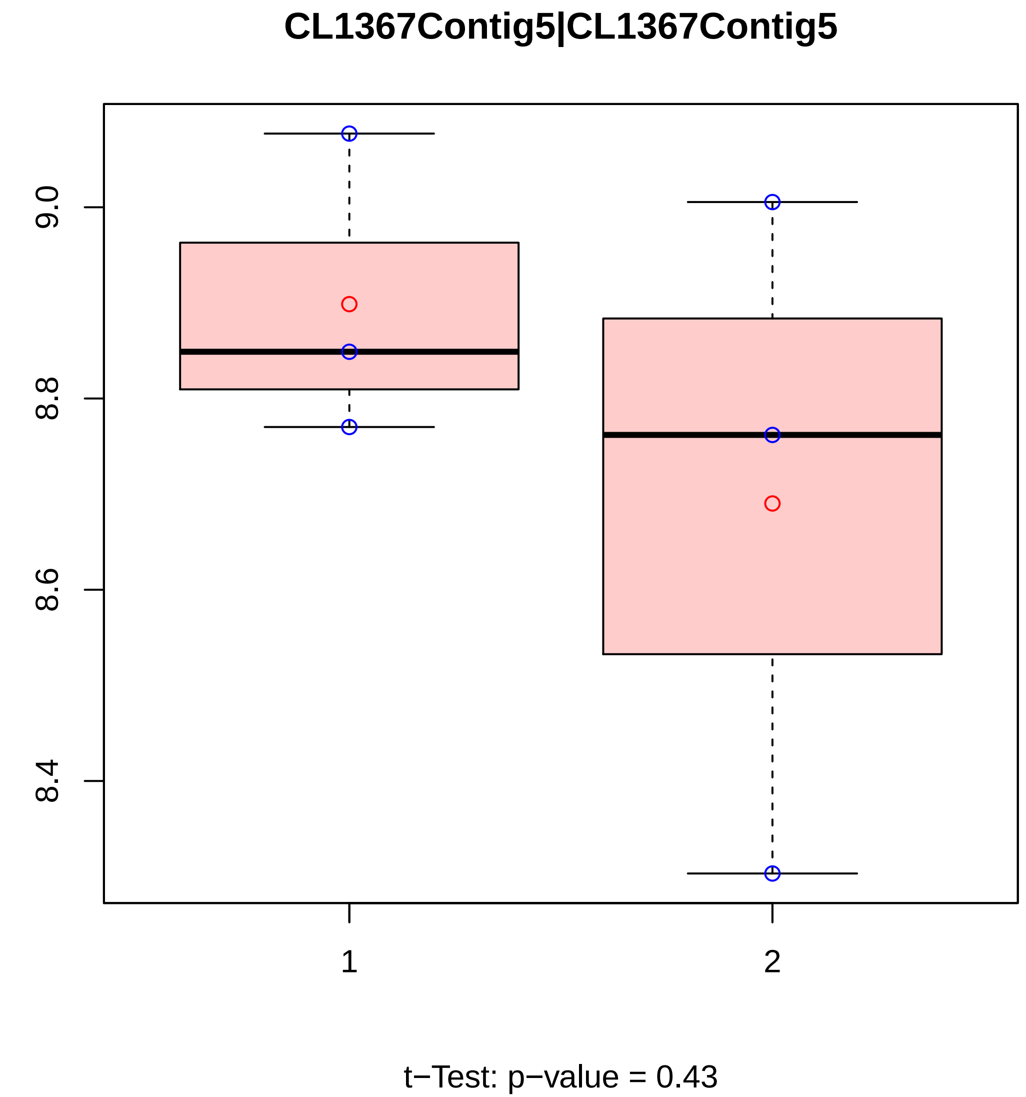
CL1367Contig5|CL1367Contig5
9.0
8.8
8.6
8.4
1
2
t−Test: p−value = 0.43

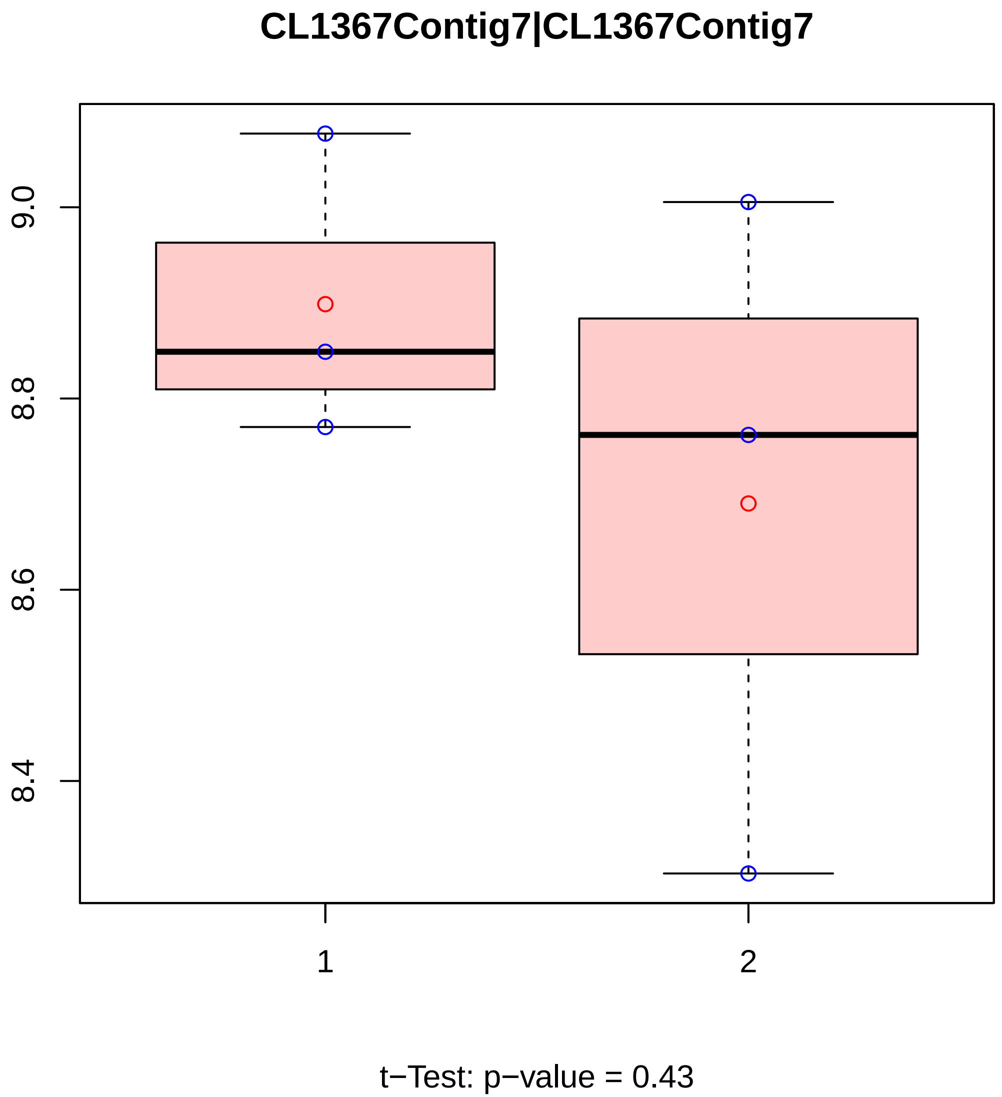
CL1367Contig7|CL1367Contig7
9.0
8.8
8.6
8.4
1
2
t−Test: p−value = 0.43

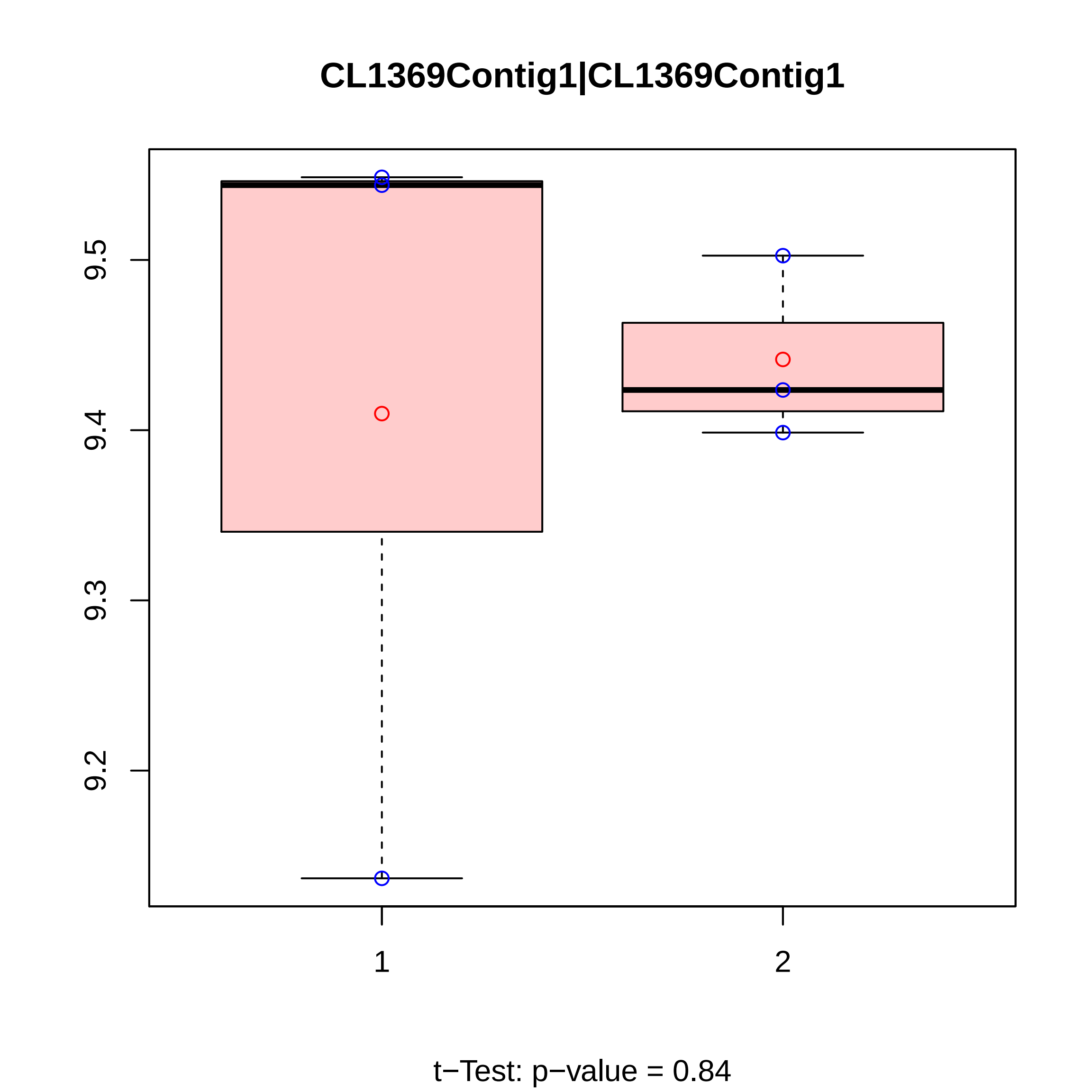
CL1369Contig1|CL1369Contig1
9.2
9.3
9.4
9.5
1
2
t−Test: p−value = 0.84

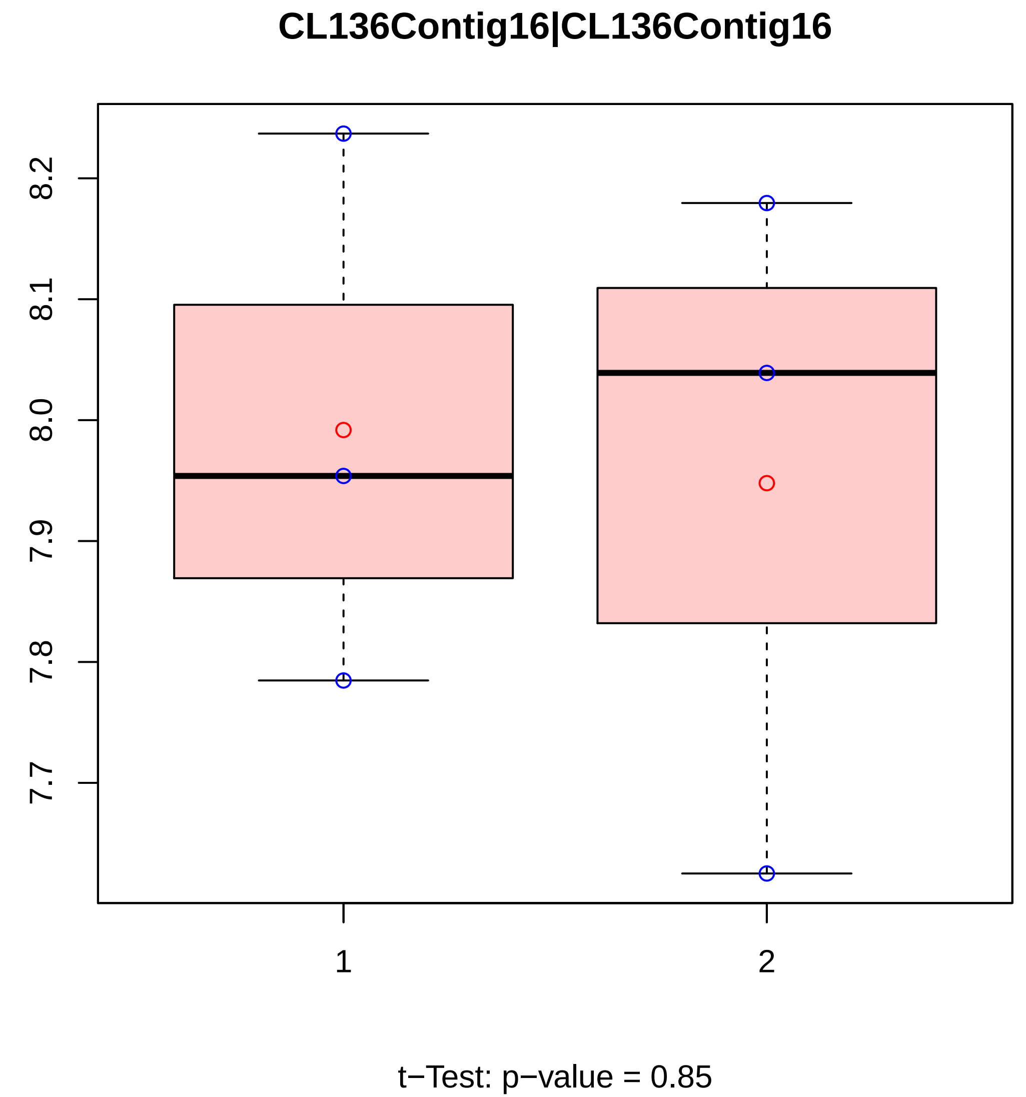
CL136Contig16|CL136Contig16
8.2
8.1
8.0
7.9
7.8
7.7
1
2
t−Test: p−value = 0.85

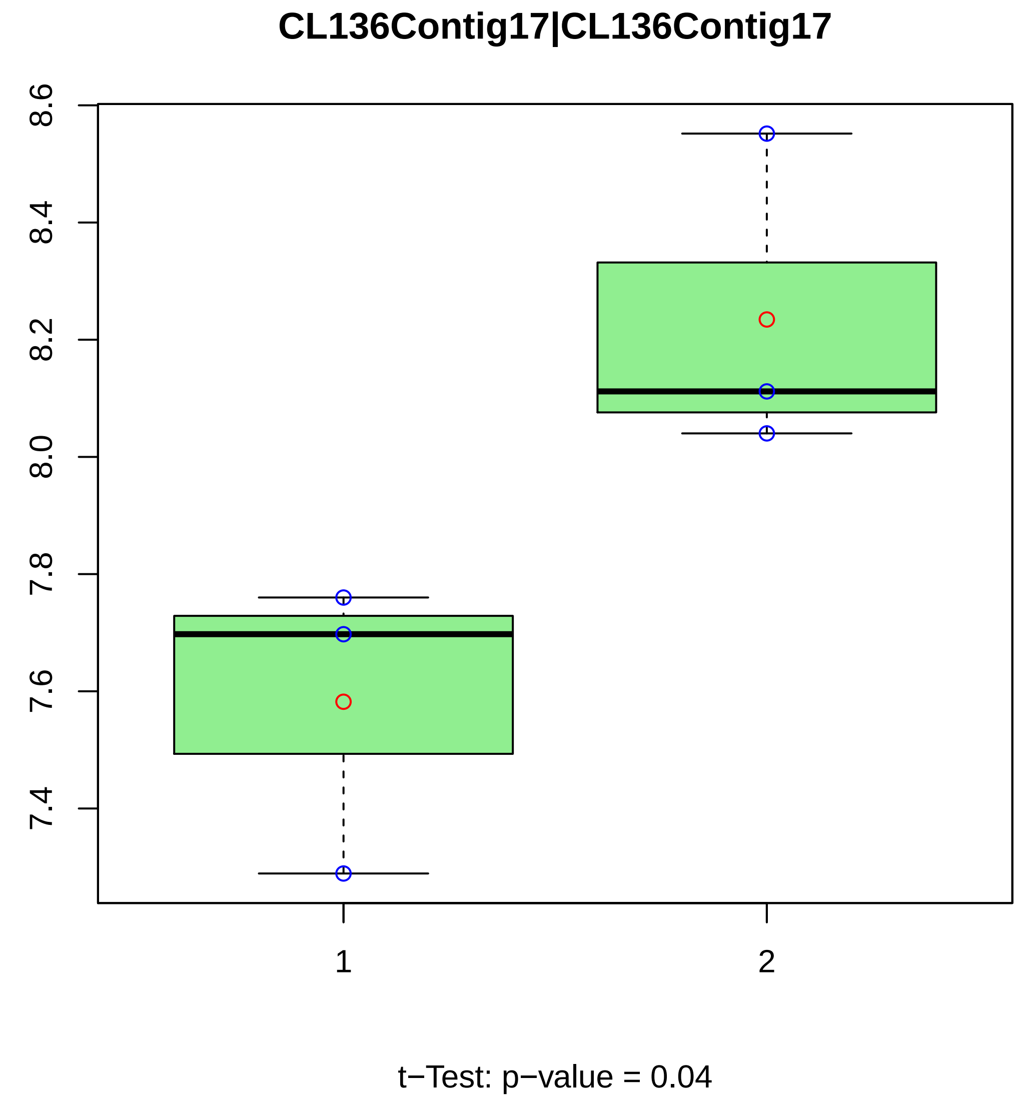
CL136Contig17|CL136Contig17
8.6
8.4
8.2
8.0
7.8
7.6
7.4
1
2
t−Test: p−value = 0.04

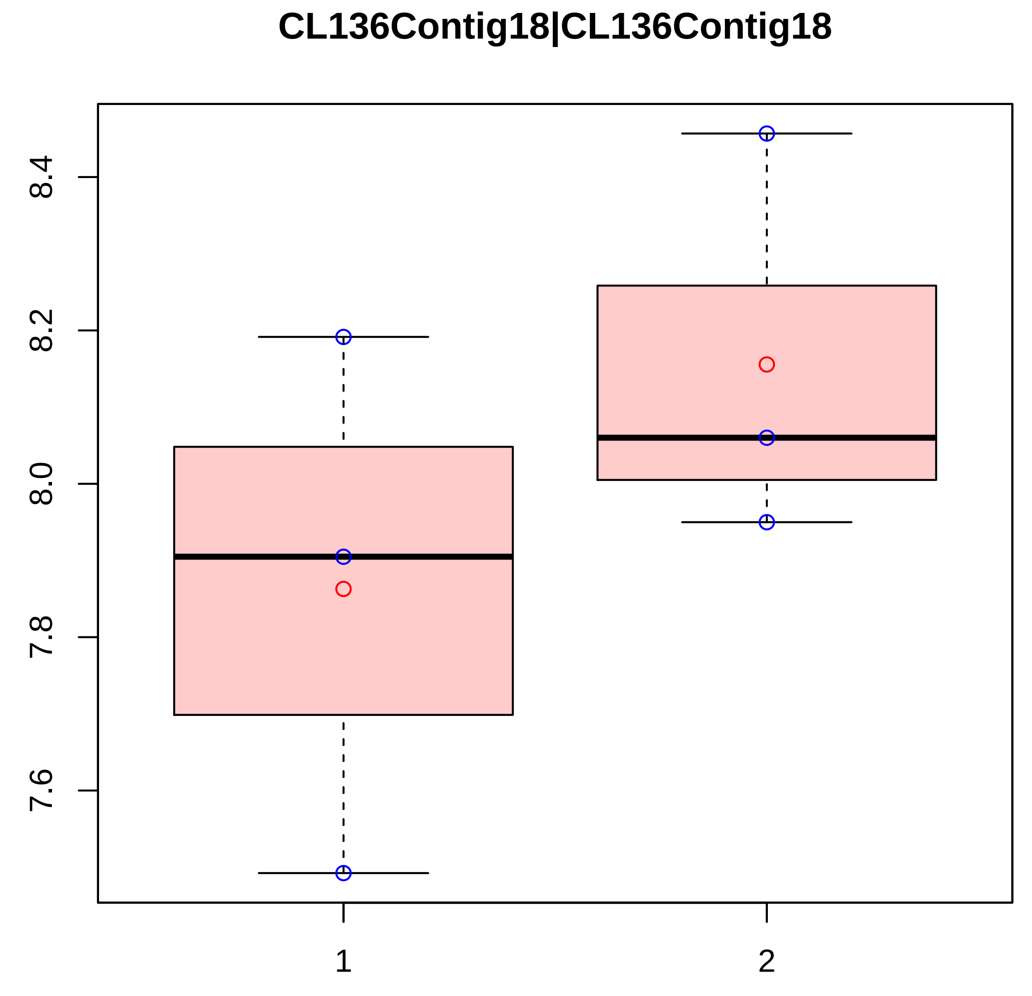


t−Test: p−value = 0.32

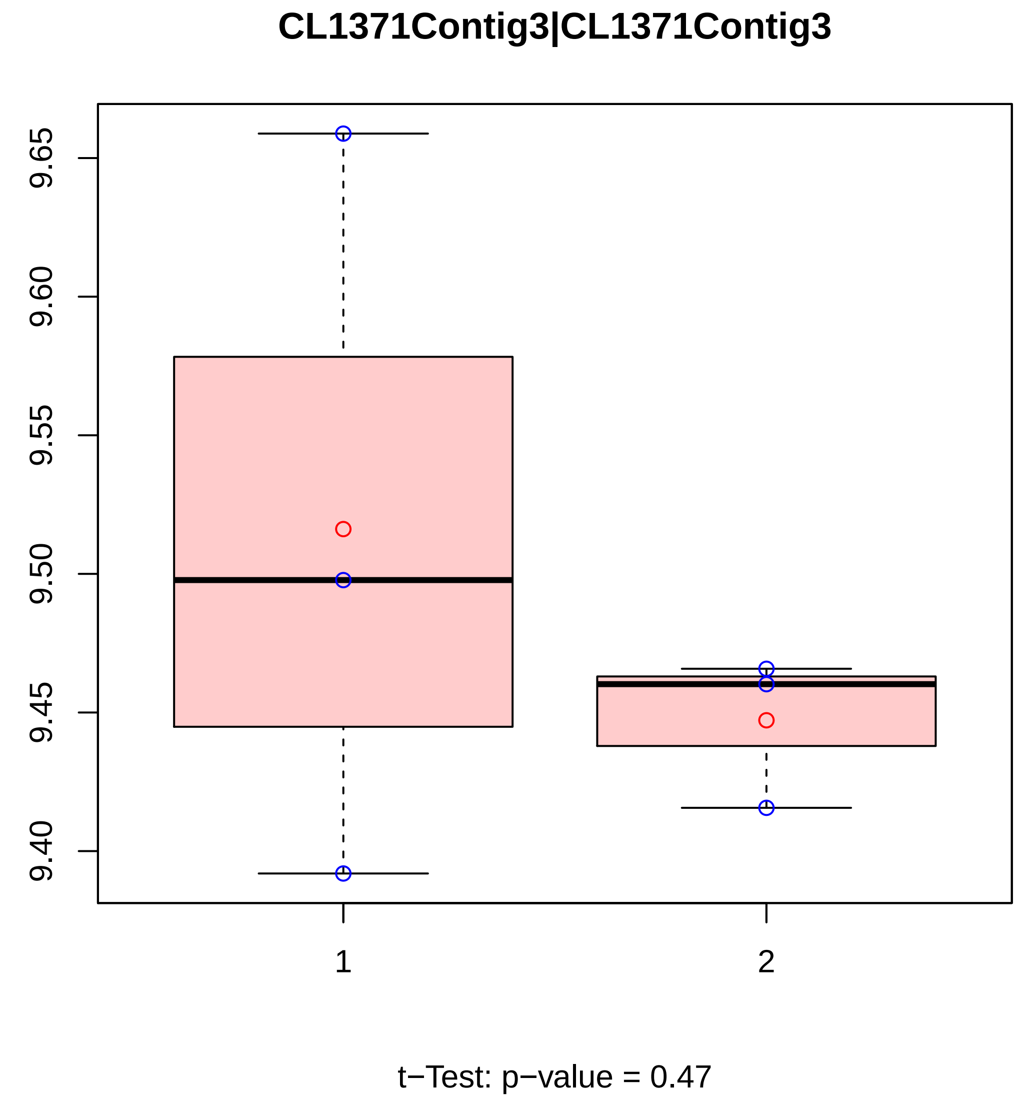
CL1371Contig3|CL1371Contig3
9.40
9.45
9.50
9.55
9.60
9.65
1
2
t−Test: p−value = 0.47

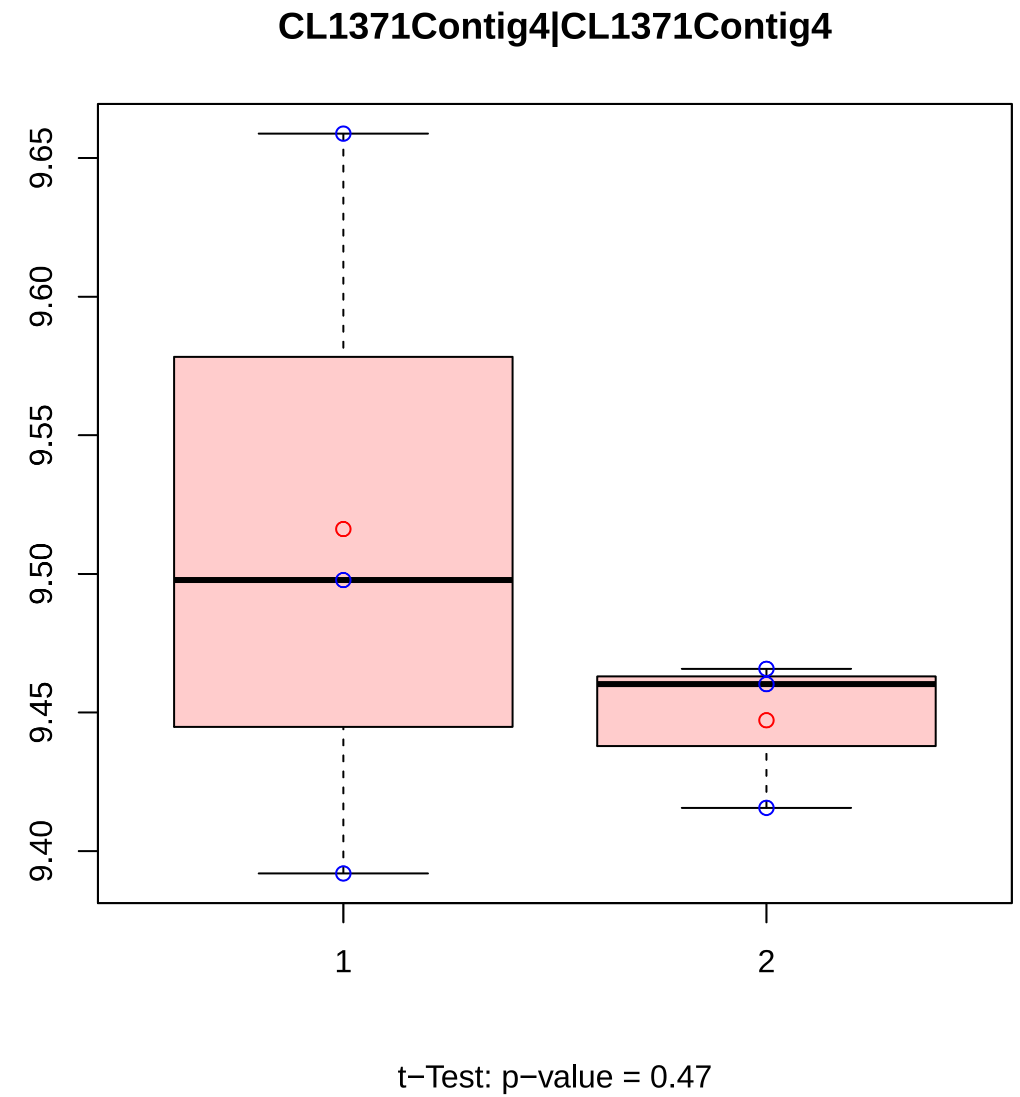
CL1371Contig4|CL1371Contig4
9.40
9.45
9.50
9.55
9.60
9.65
1
2
t−Test: p−value = 0.47

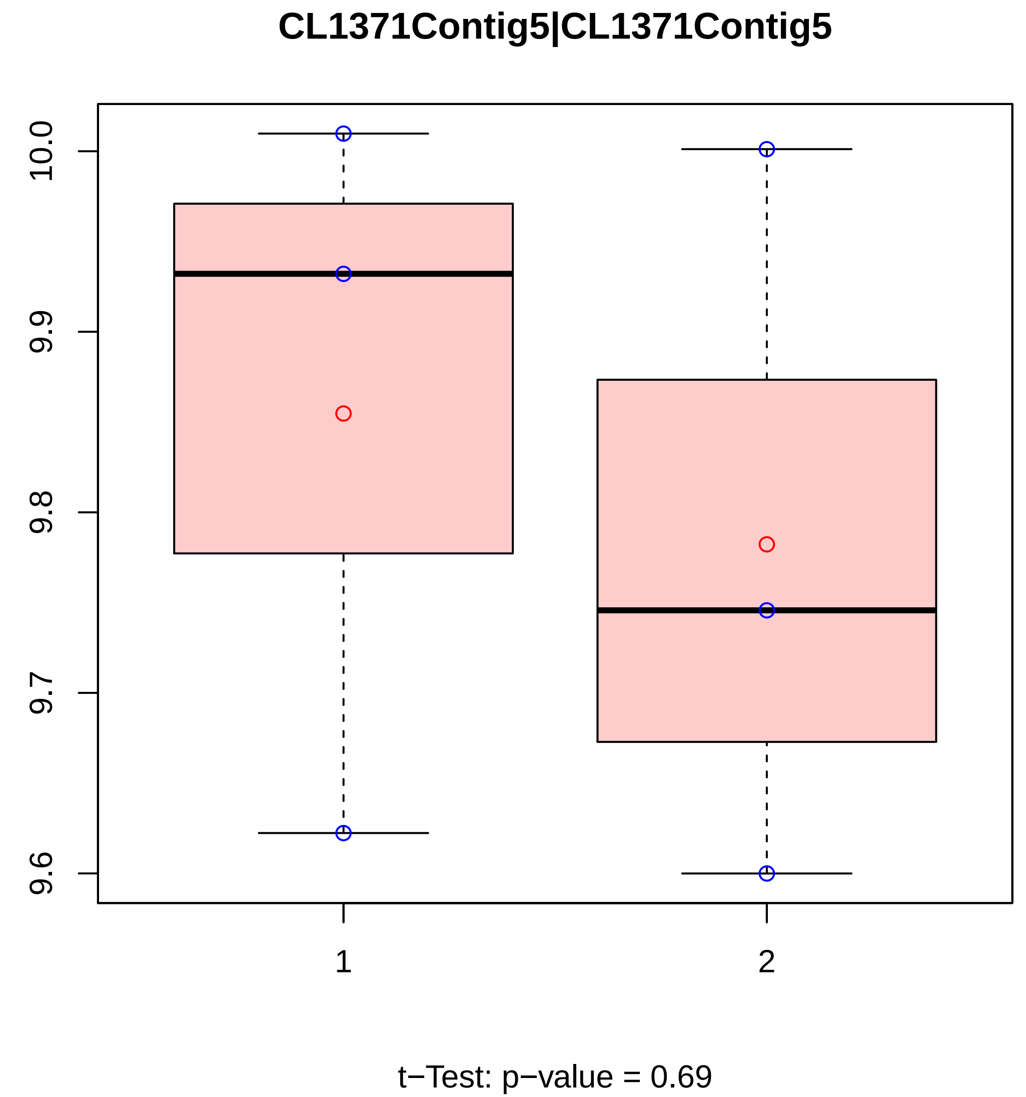
CL1371Contig5|CL1371Contig5
10.0
9.9
9.8
9.7
9.6
1
2
t−Test: p−value = 0.69

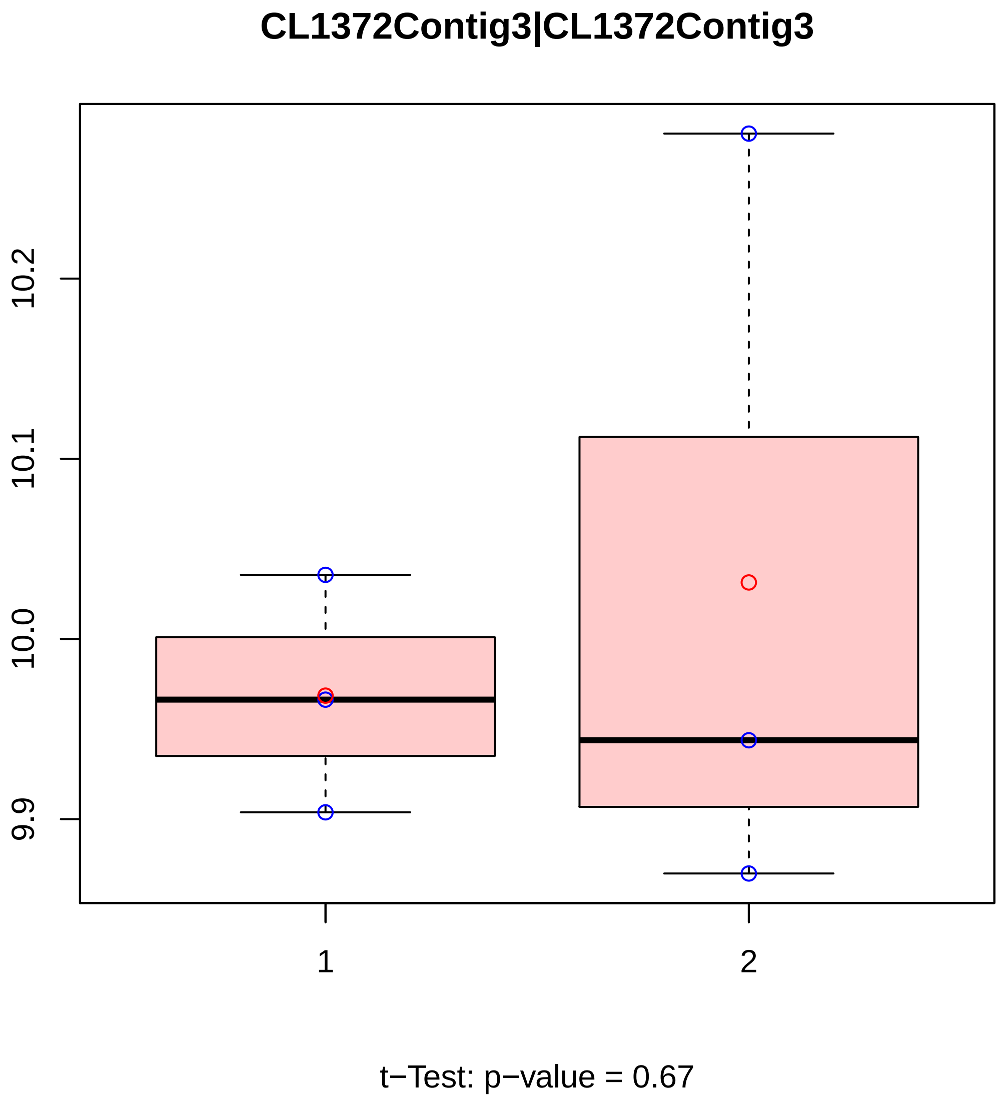
CL1372Contig3|CL1372Contig3
10.2
10.1
10.0
9.9
1
2
t−Test: p−value = 0.67

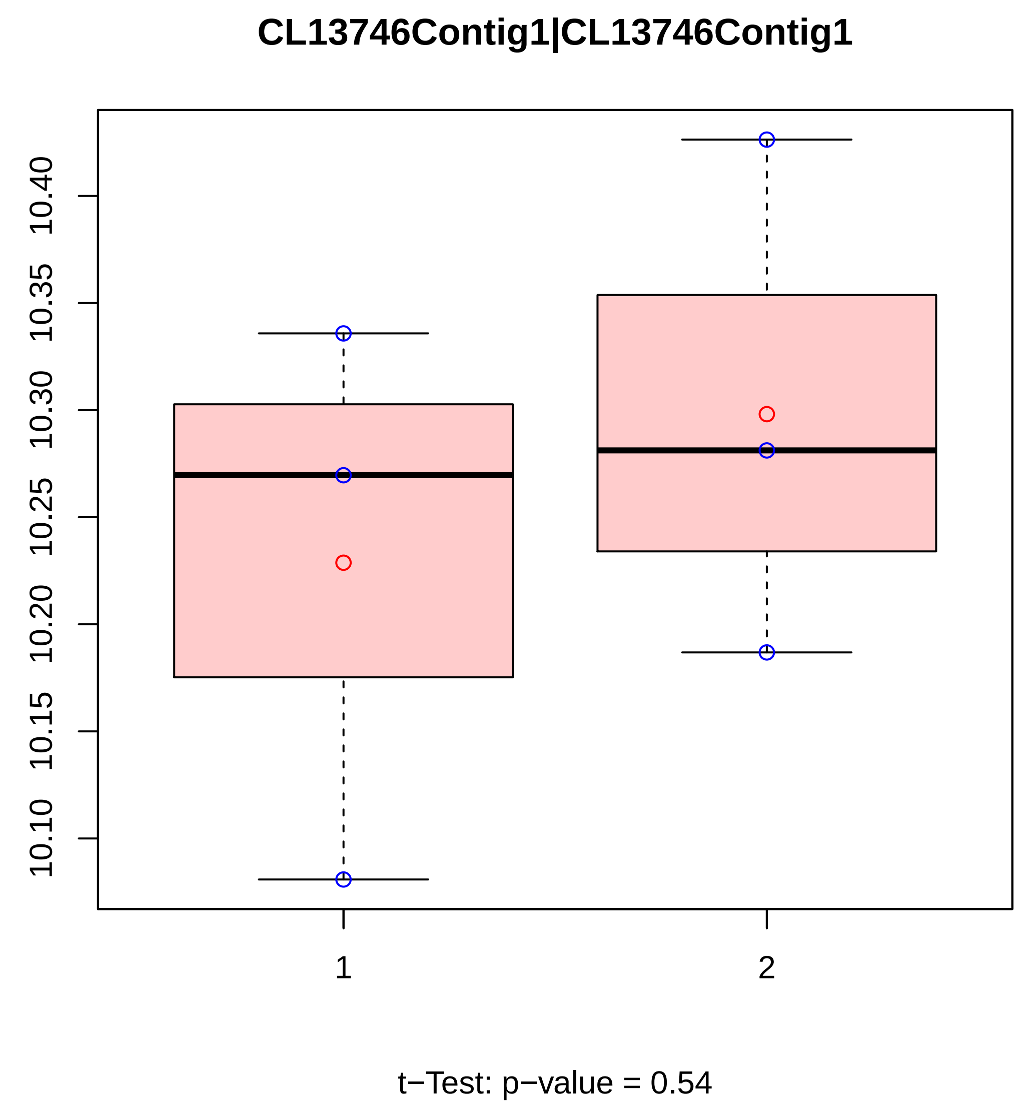
CL13746Contig1|CL13746Contig1
10.10
10.15
10.20
10.25
10.30
10.35
10.40
1
2
t−Test: p−value = 0.54

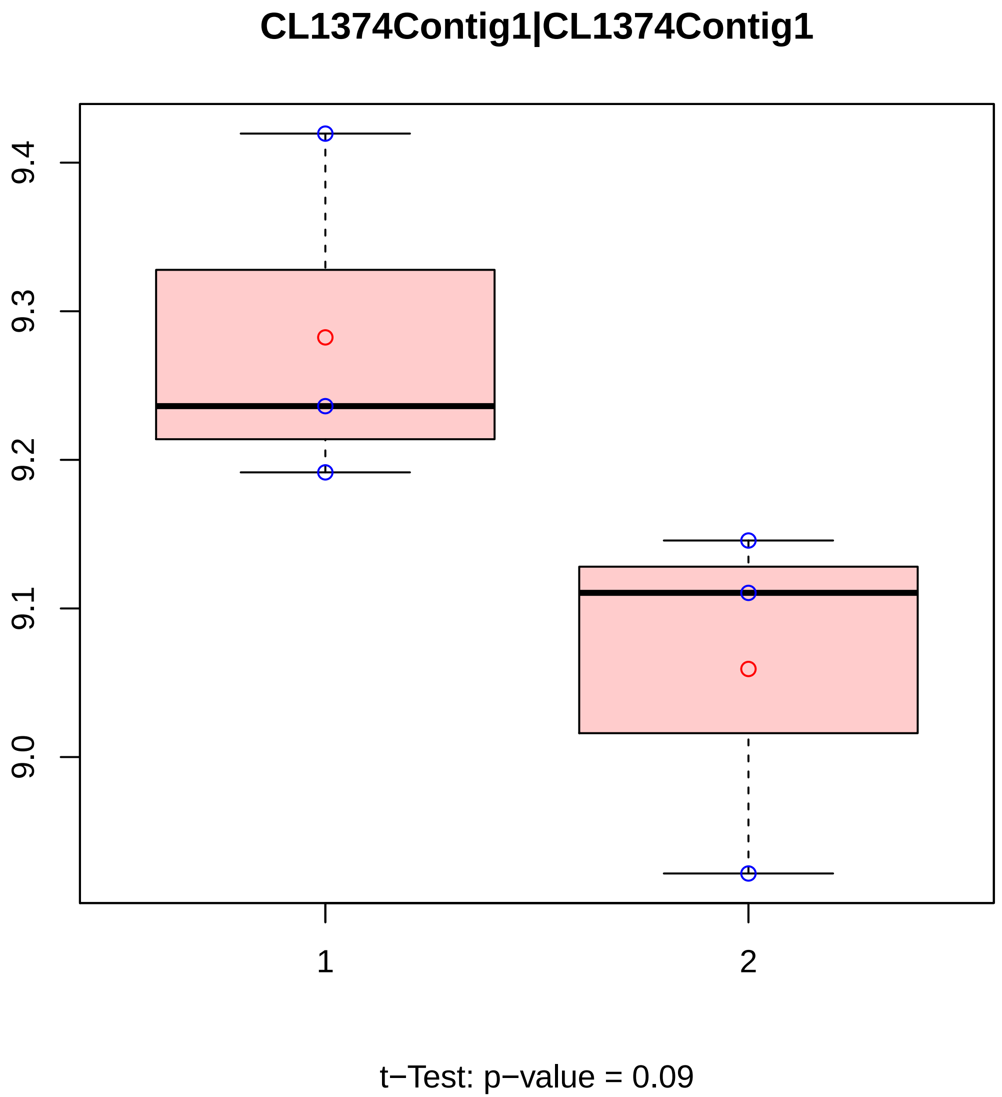

CL1374Contig1|CL1374Contig1
9.0
9.1
9.2
9.3
9.4
1
2
t−Test: p−value = 0.09

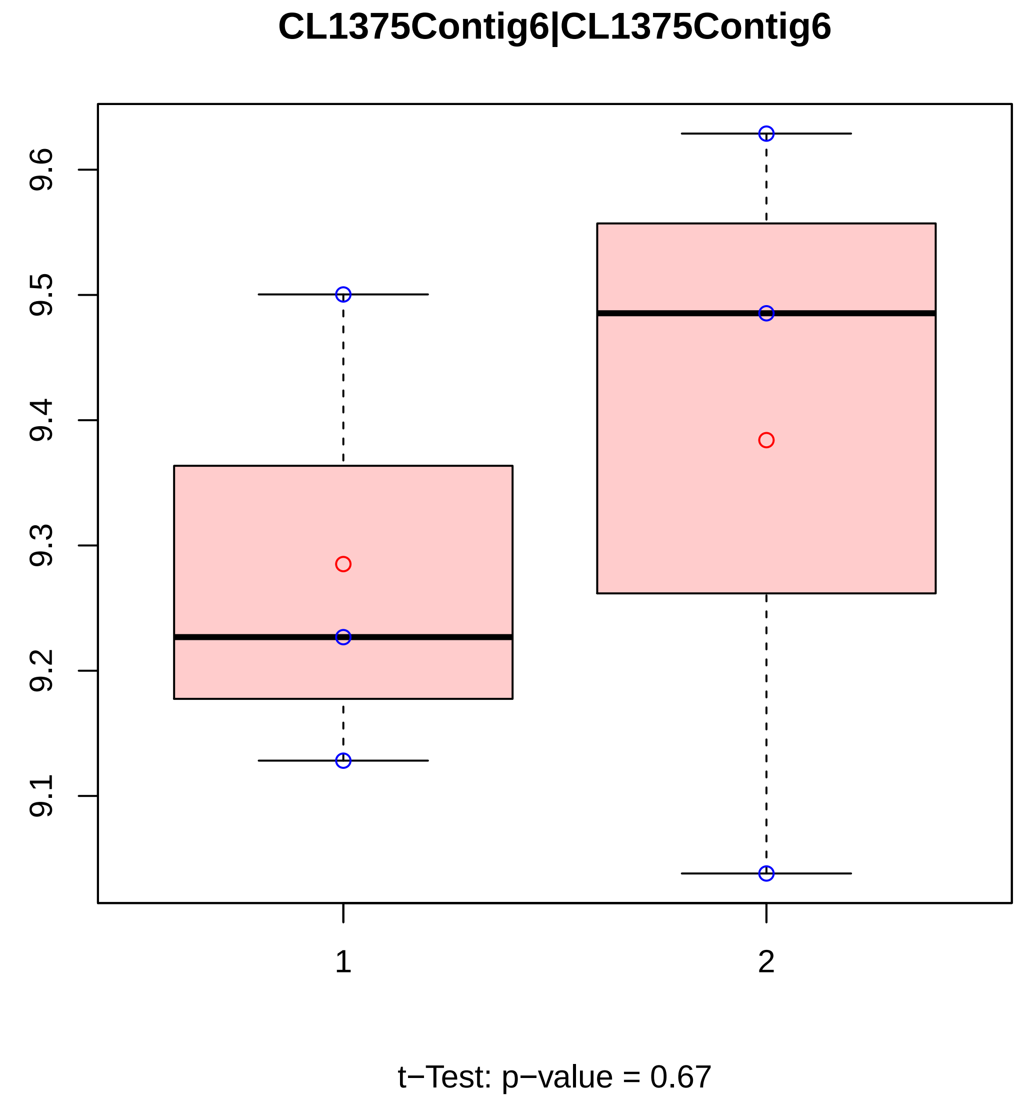
CL1375Contig6|CL1375Contig6
9.1
9.2
9.3
9.4
9.5
9.6
1
2
t−Test: p−value = 0.67

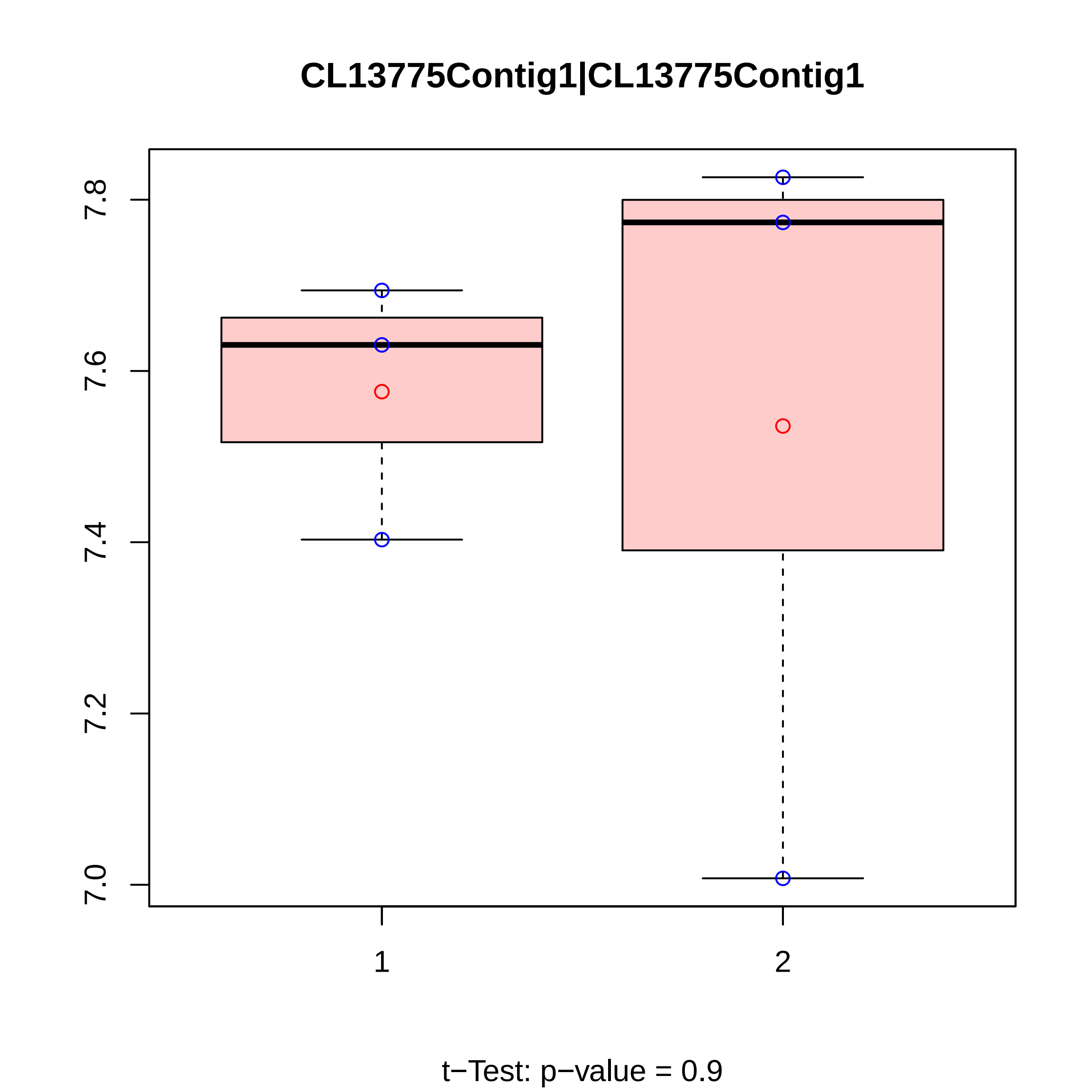
CL13775Contig1|CL13775Contig1
7.0
7.2
7.4
7.6
7.8
1
2
t−Test: p−value = 0.9

# CL1377Contig4|CL1377Contig4

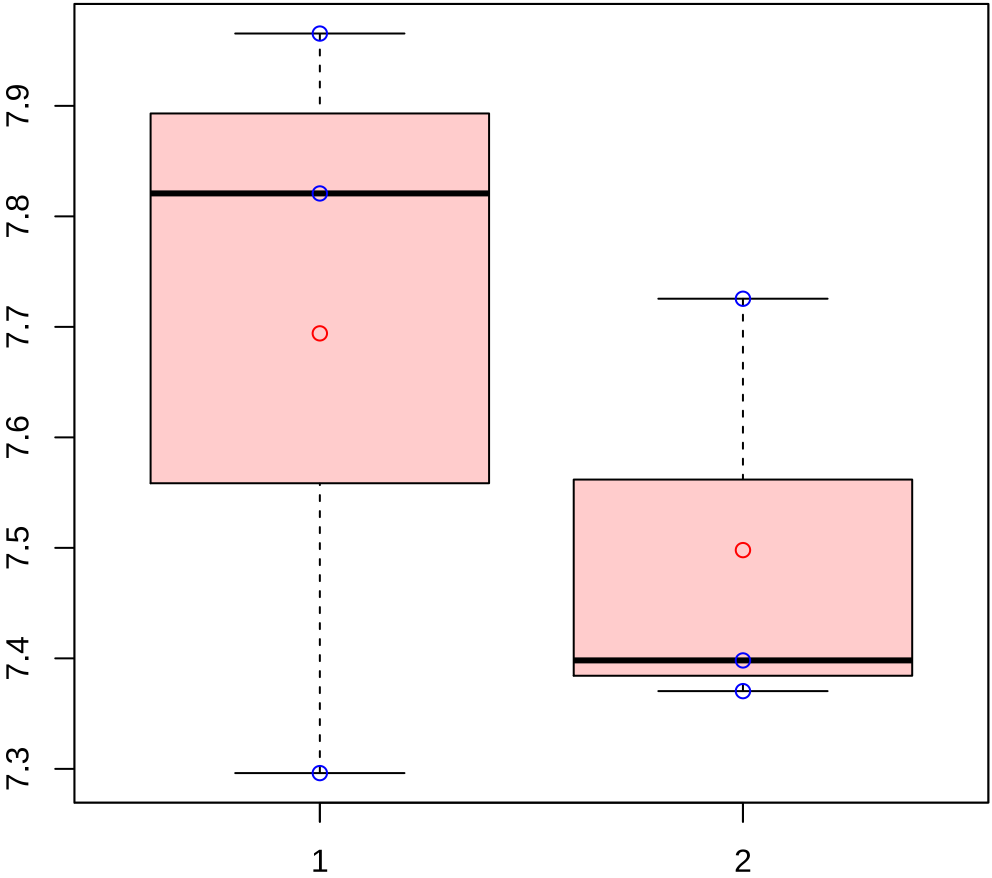


t−Test: p−value = 0.46

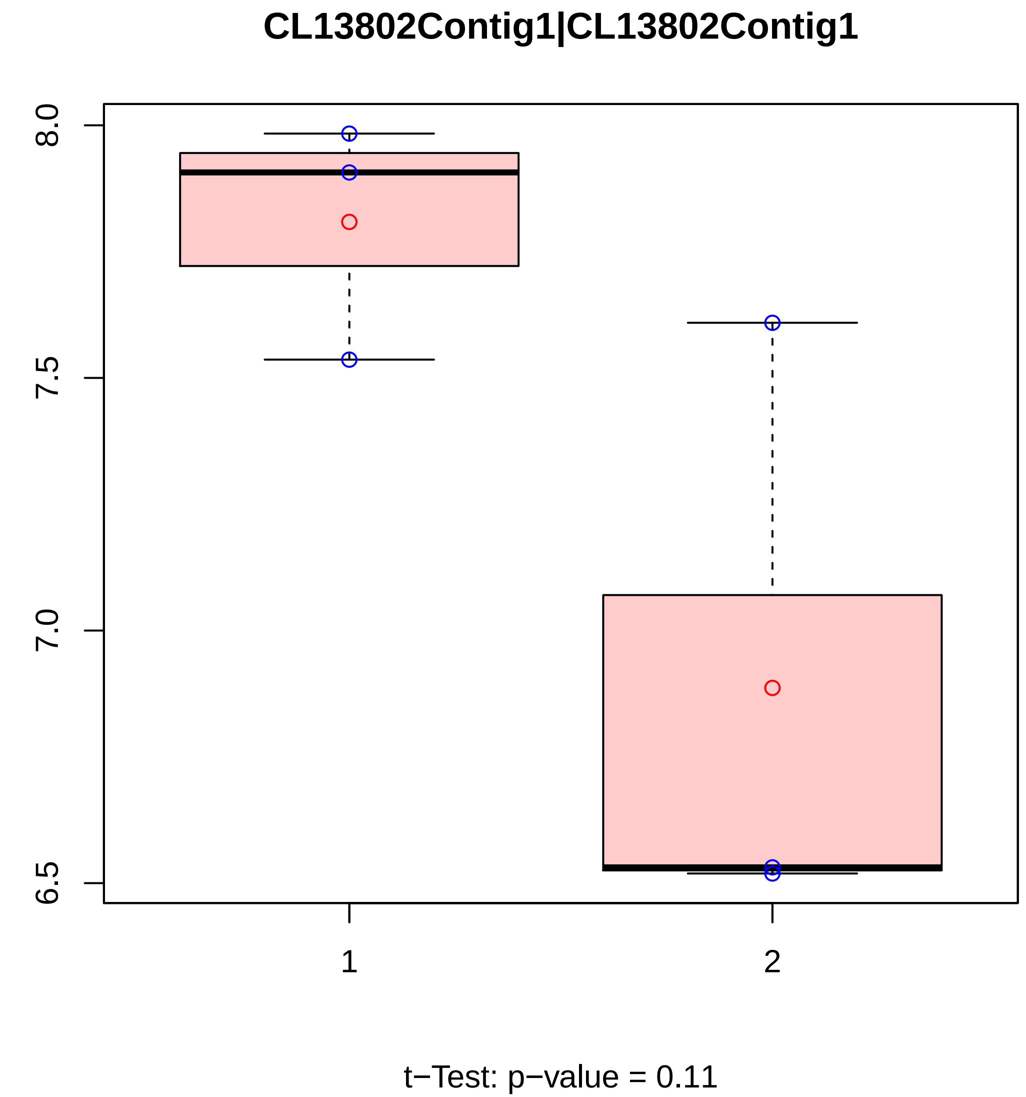

CL13802Contig1|CL13802Contig1
8.0
7.5
7.0
6.5
1
2
t−Test: p−value = 0.11

## CL1380Contig1|CL1380Contig1

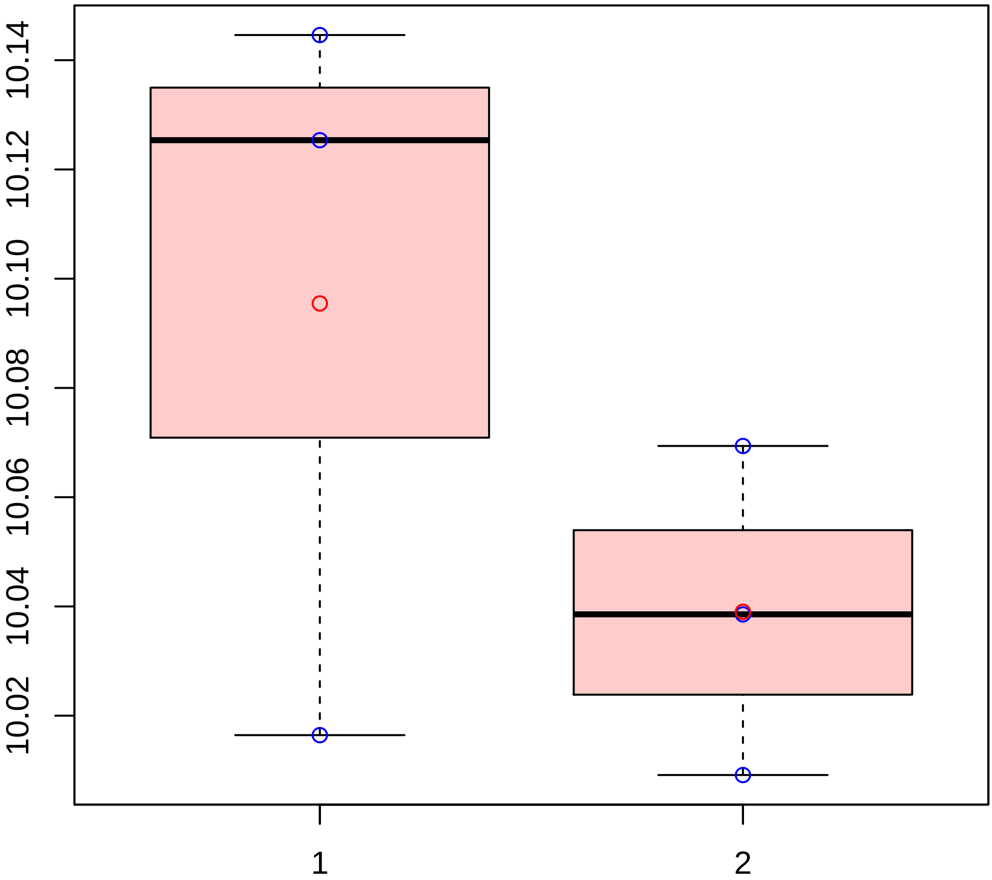


t−Test: p−value = 0.29

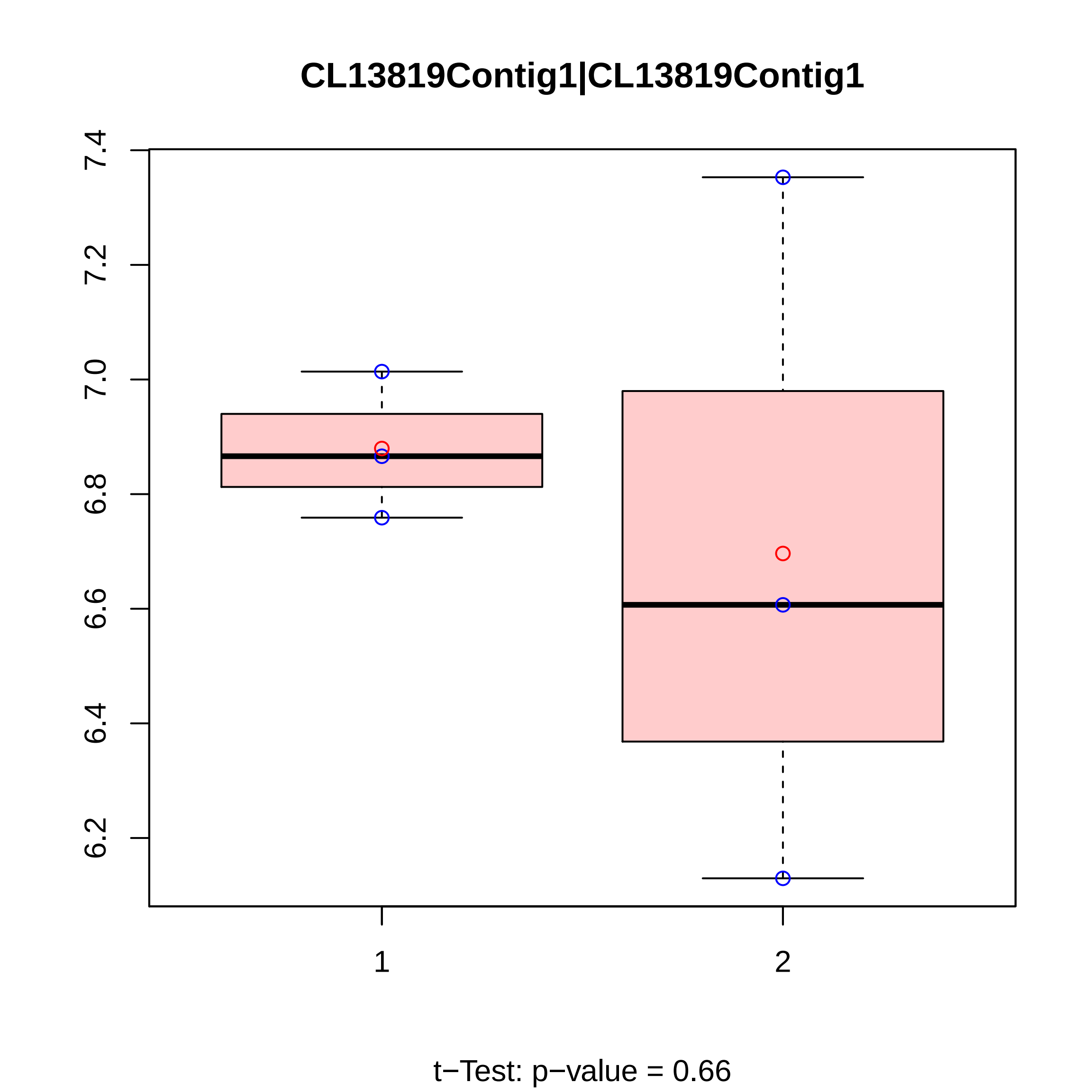
CL13819Contig1|CL13819Contig1
7.4
7.2
7.0
6.8
6.6
6.4
6.2
1
2
t−Test: p−value = 0.66

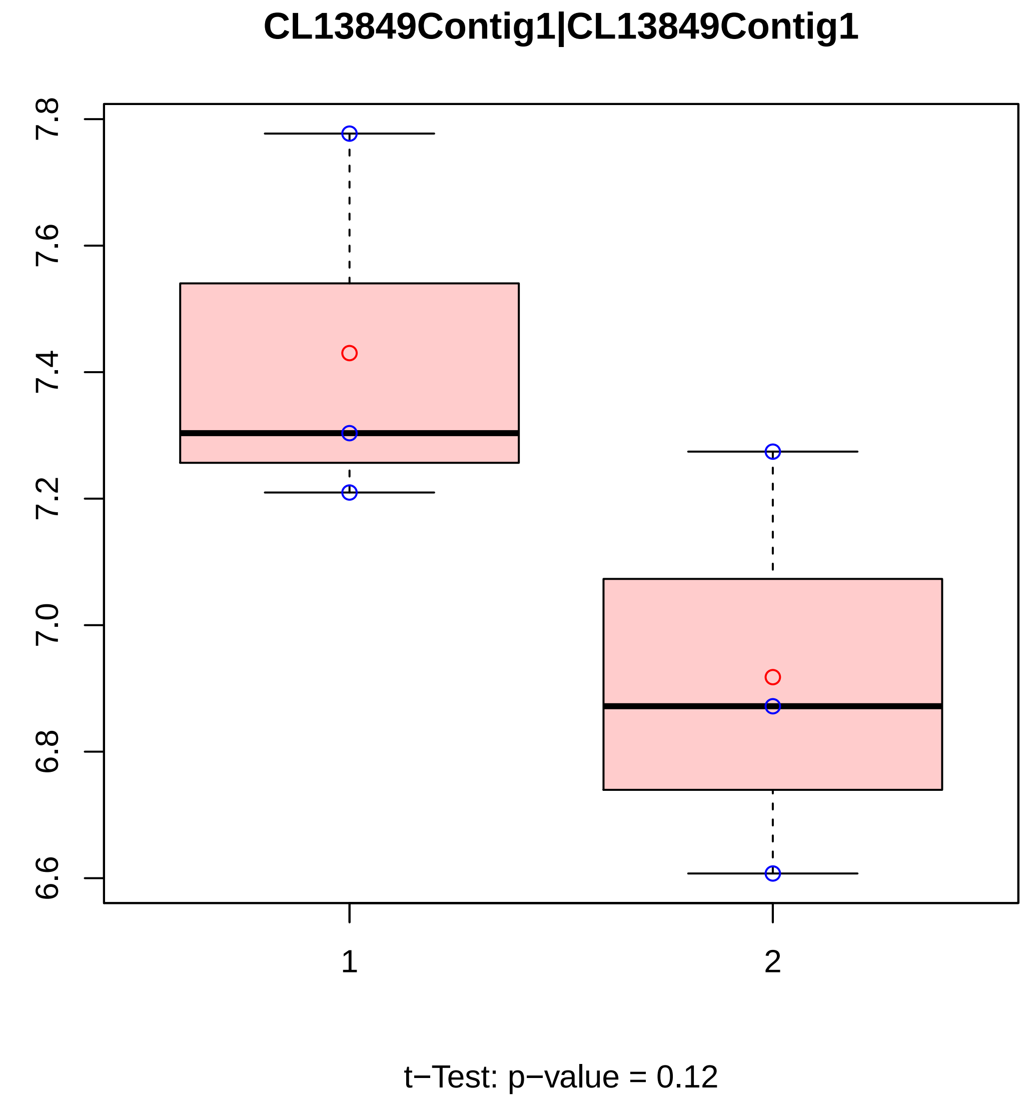
CL13849Contig1|CL13849Contig1
7.8
7.6
7.4
7.2
7.0
6.8
6.6
1
2
t−Test: p−value = 0.12

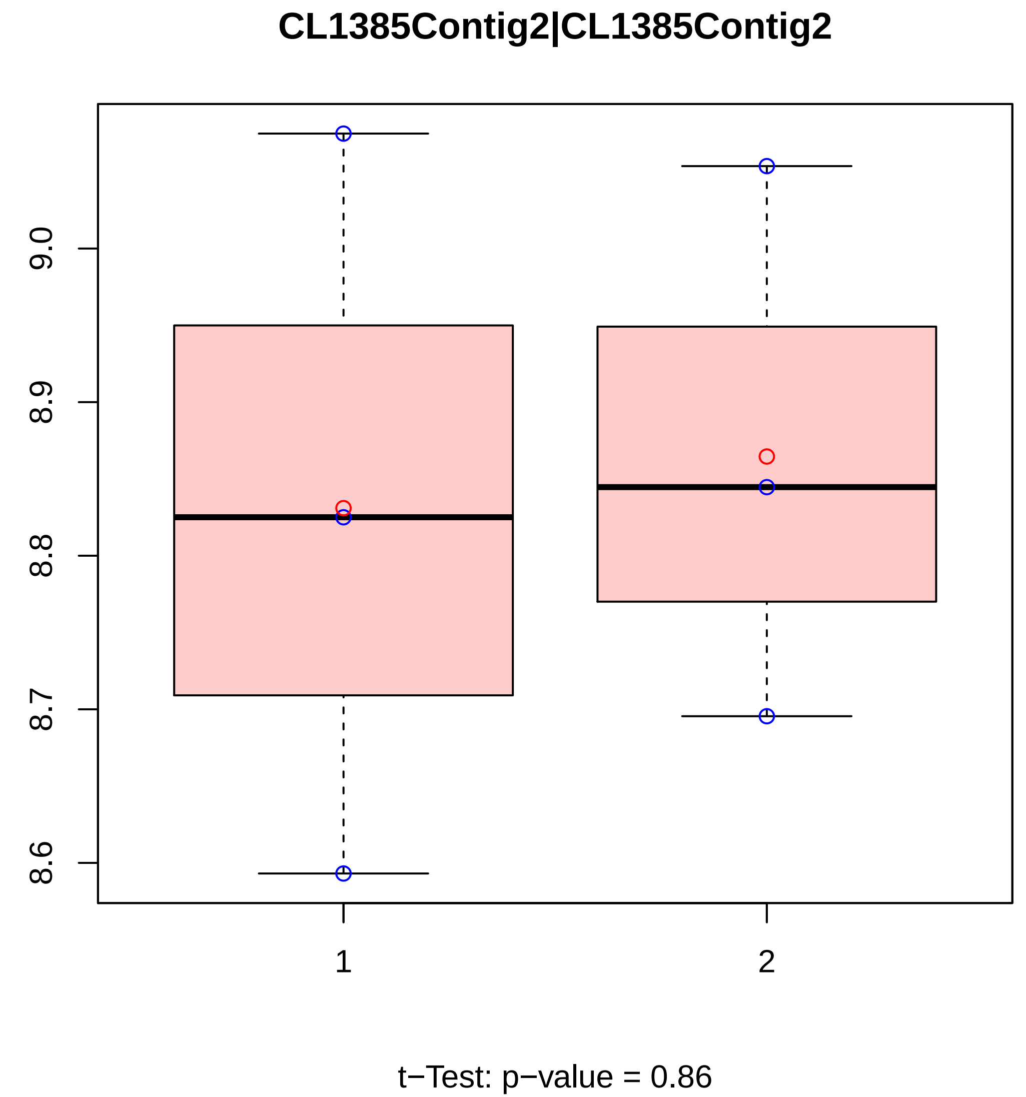
CL1385Contig2|CL1385Contig2
9.0
8.9
8.8
8.7
8.6
1
2
t−Test: p−value = 0.86

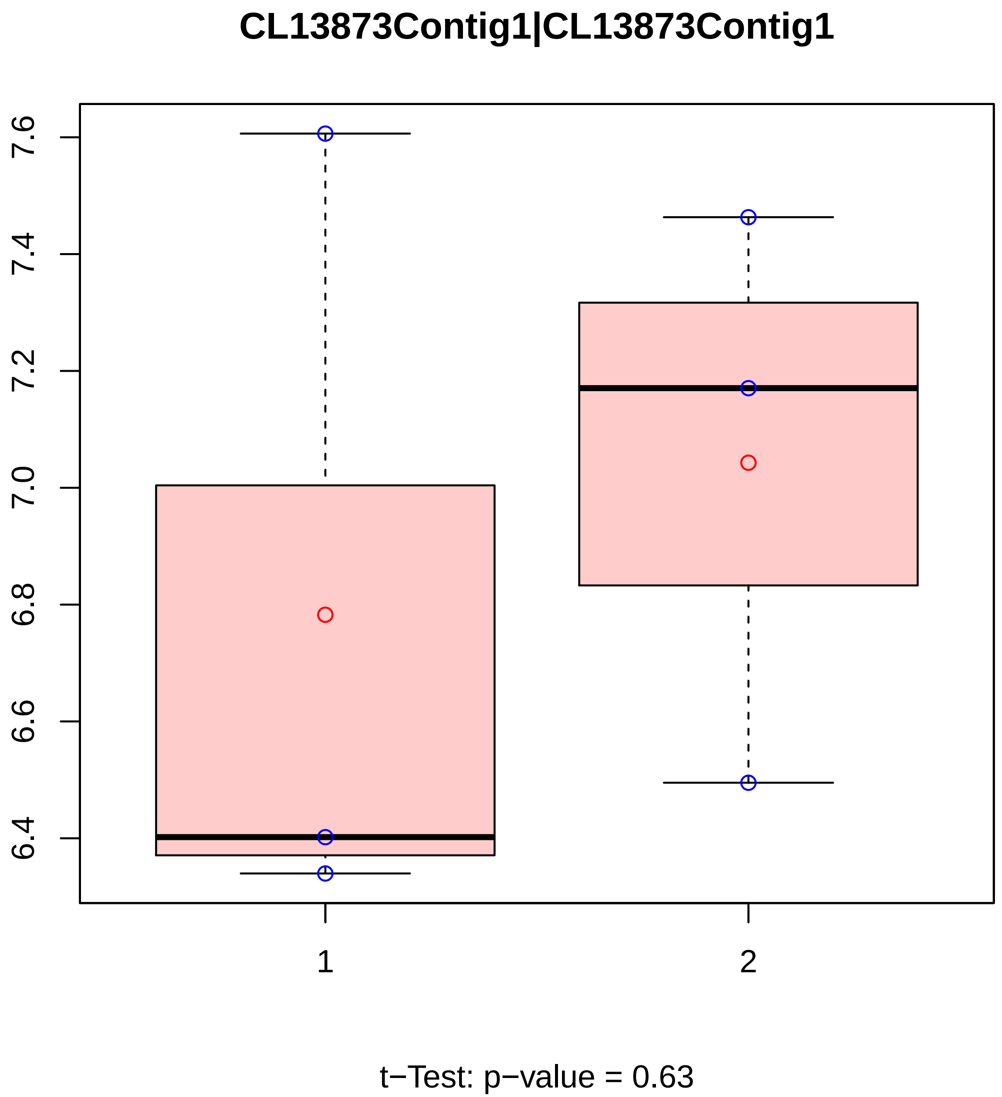
CL13873Contig1|CL13873Contig1
7.6
7.4
7.2
7.0
6.8
6.6
6.4
1
2
t−Test: p−value = 0.63

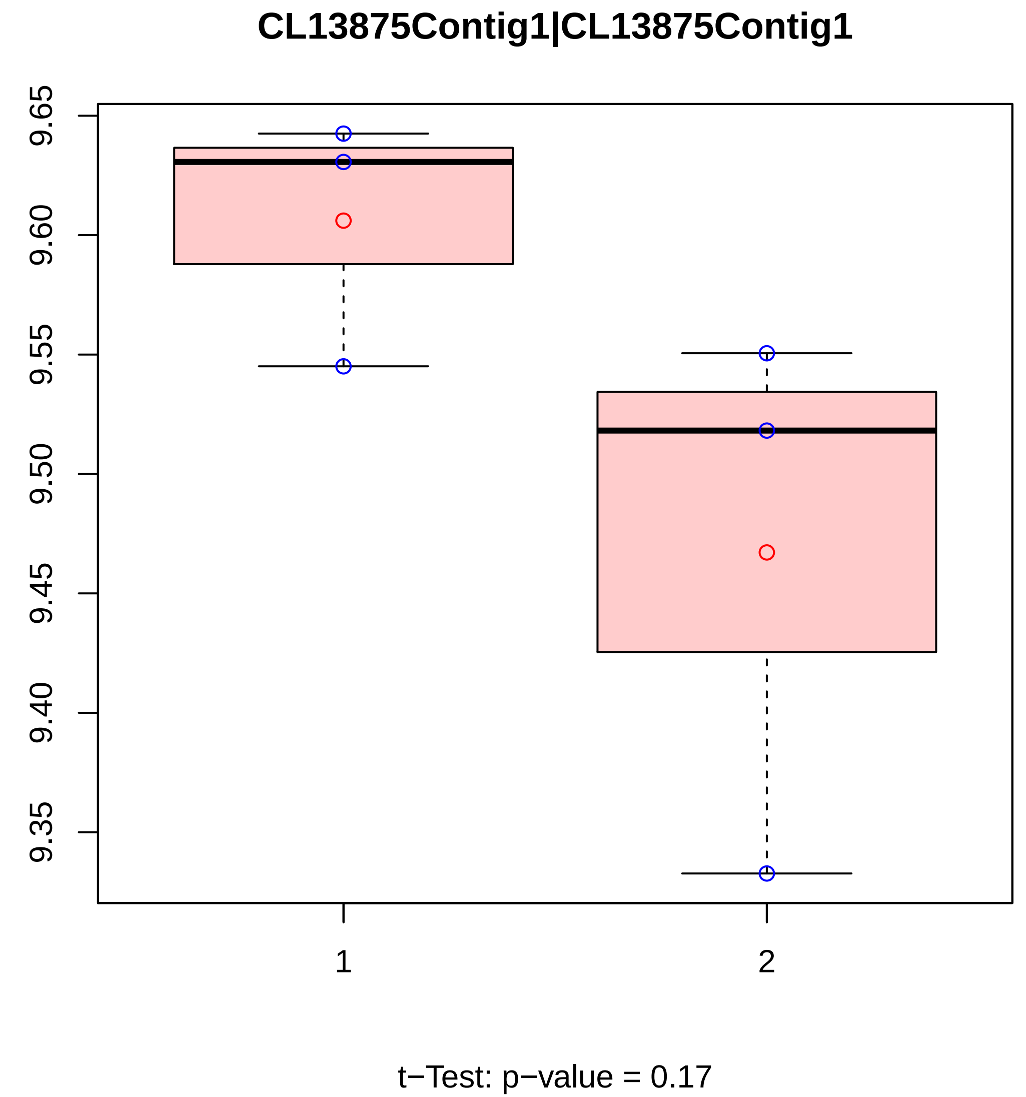
CL13875Contig1|CL13875Contig1
9.35
9.40
9.45
9.50
9.55
9.60
9.65
1
2
t−Test: p−value = 0.17

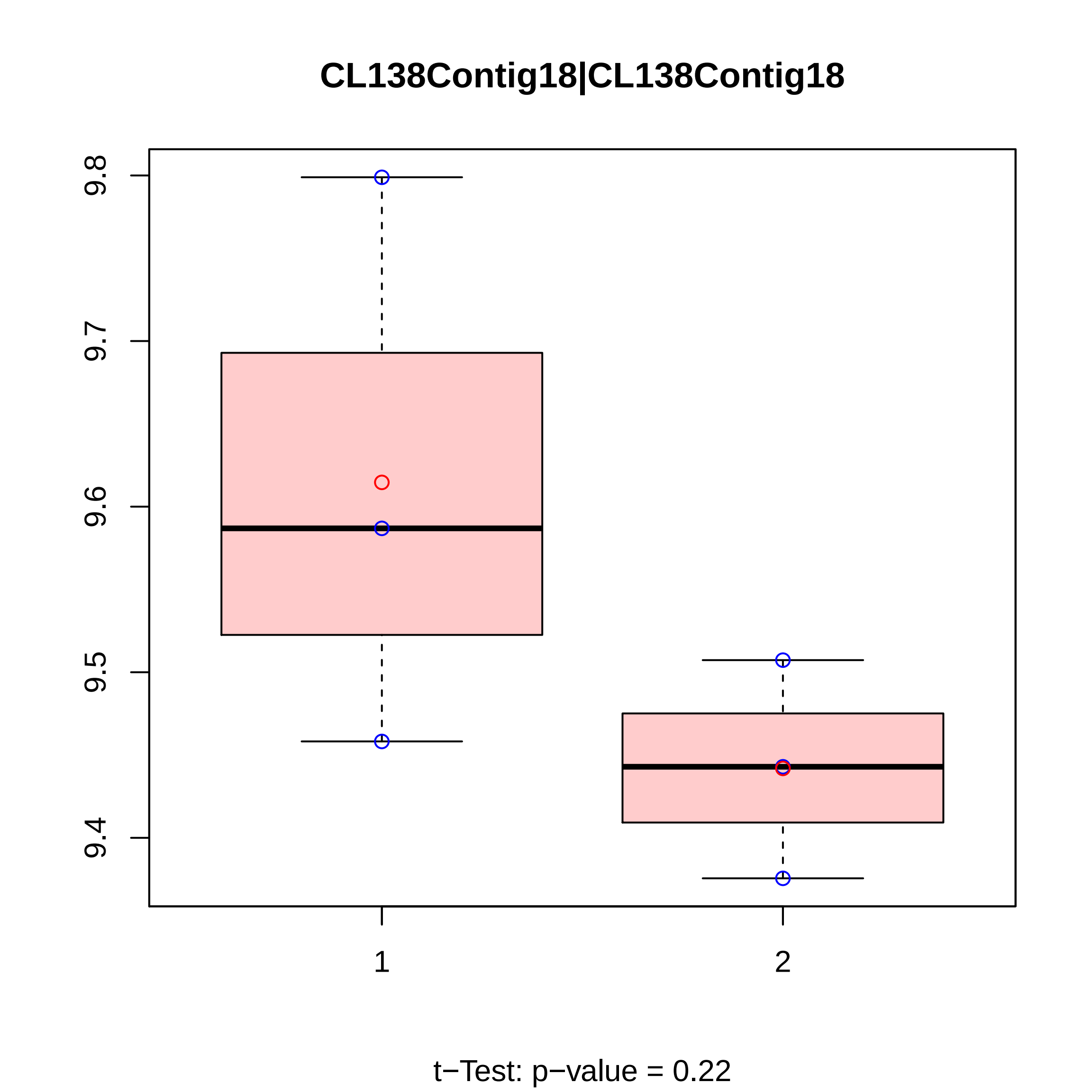

CL138Contig18|CL138Contig18
9.4
9.5
9.6
9.7
9.8
1
2
t−Test: p−value = 0.22

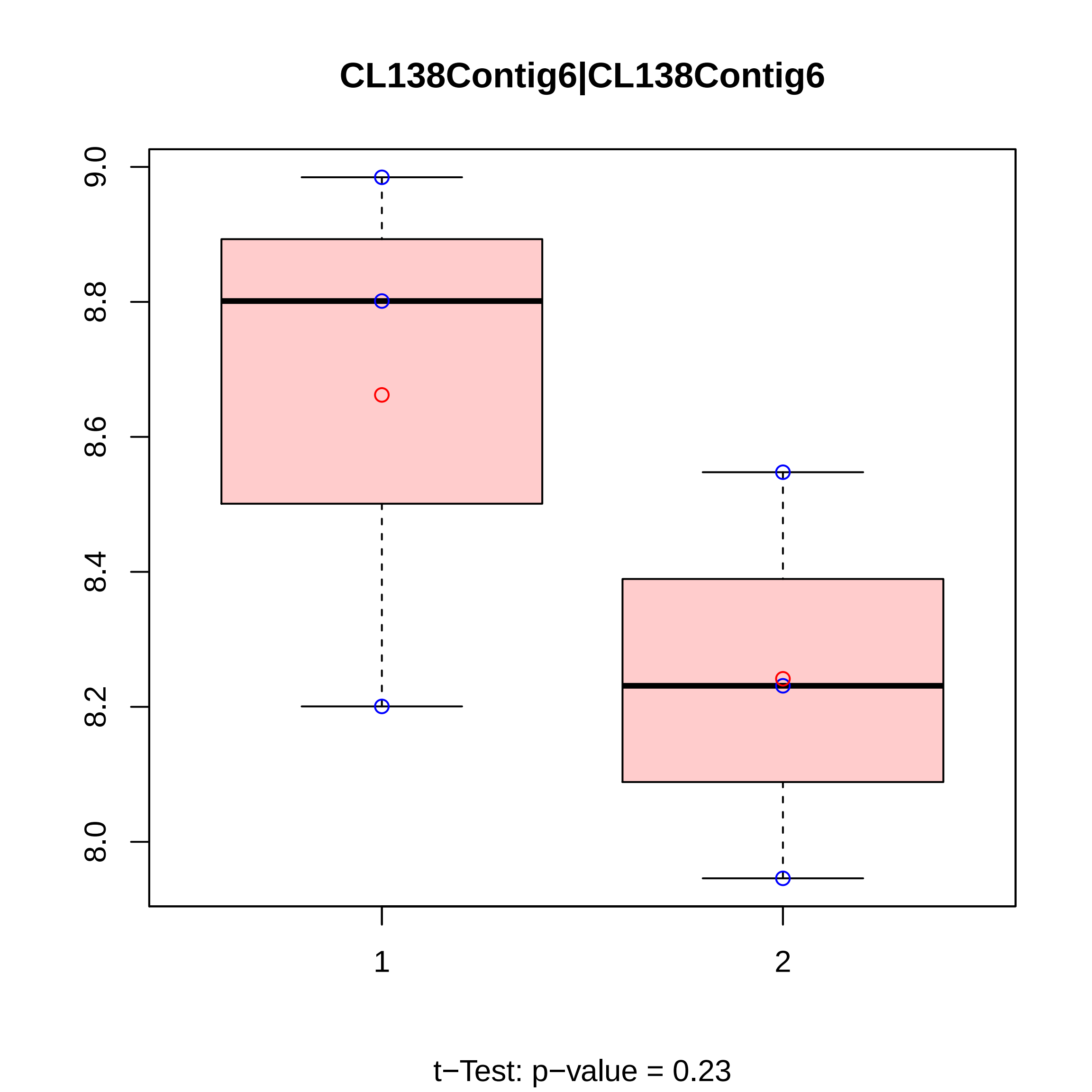
CL138Contig6|CL138Contig6
9.0
8.8
8.6
8.4
8.2
8.0
1
2
t−Test: p−value = 0.23

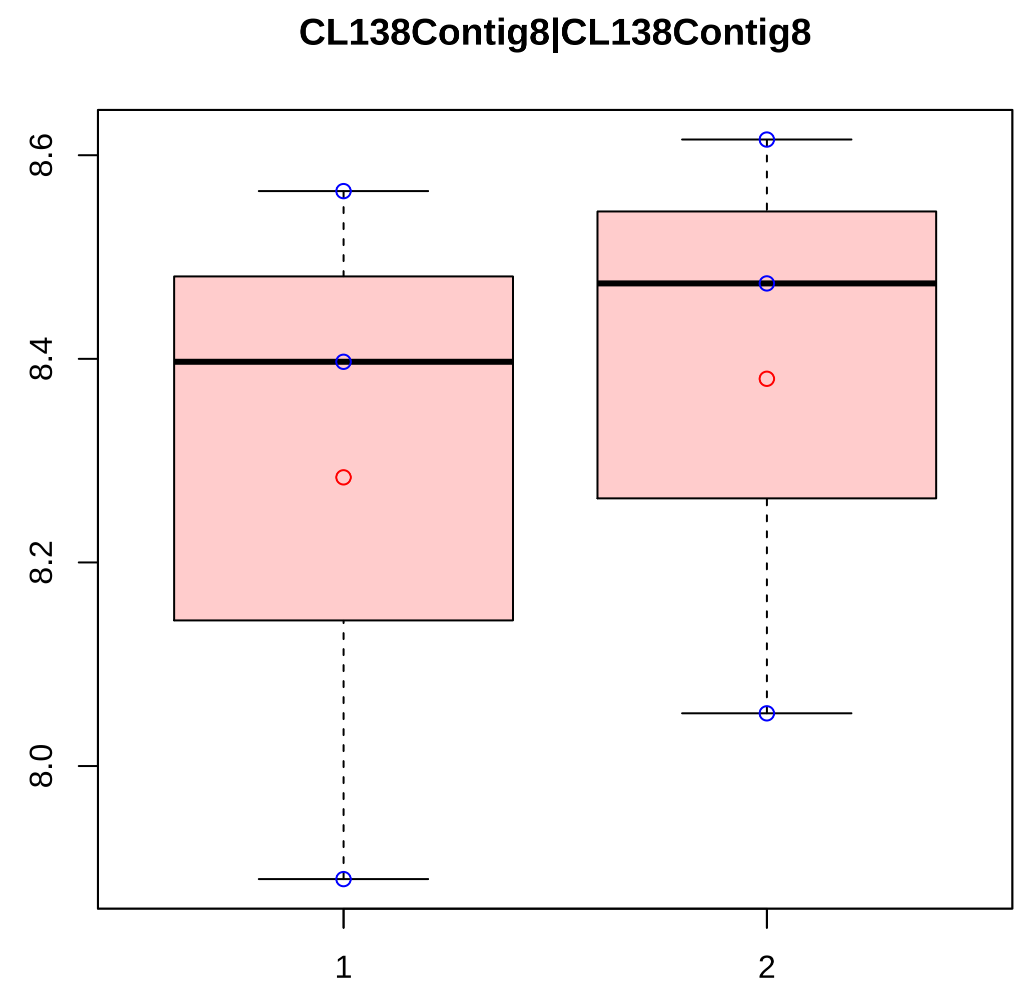


t−Test: p−value = 0.73

# CL138Contig9|CL138Contig9

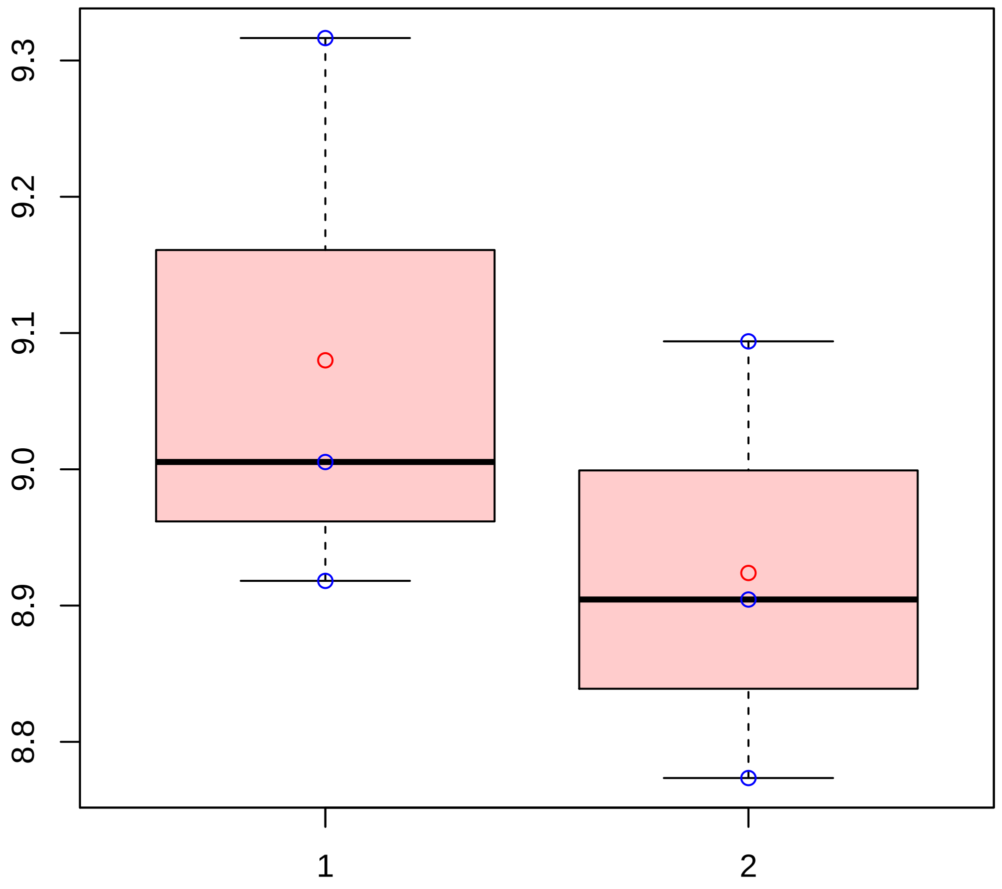


t−Test: p−value = 0.37

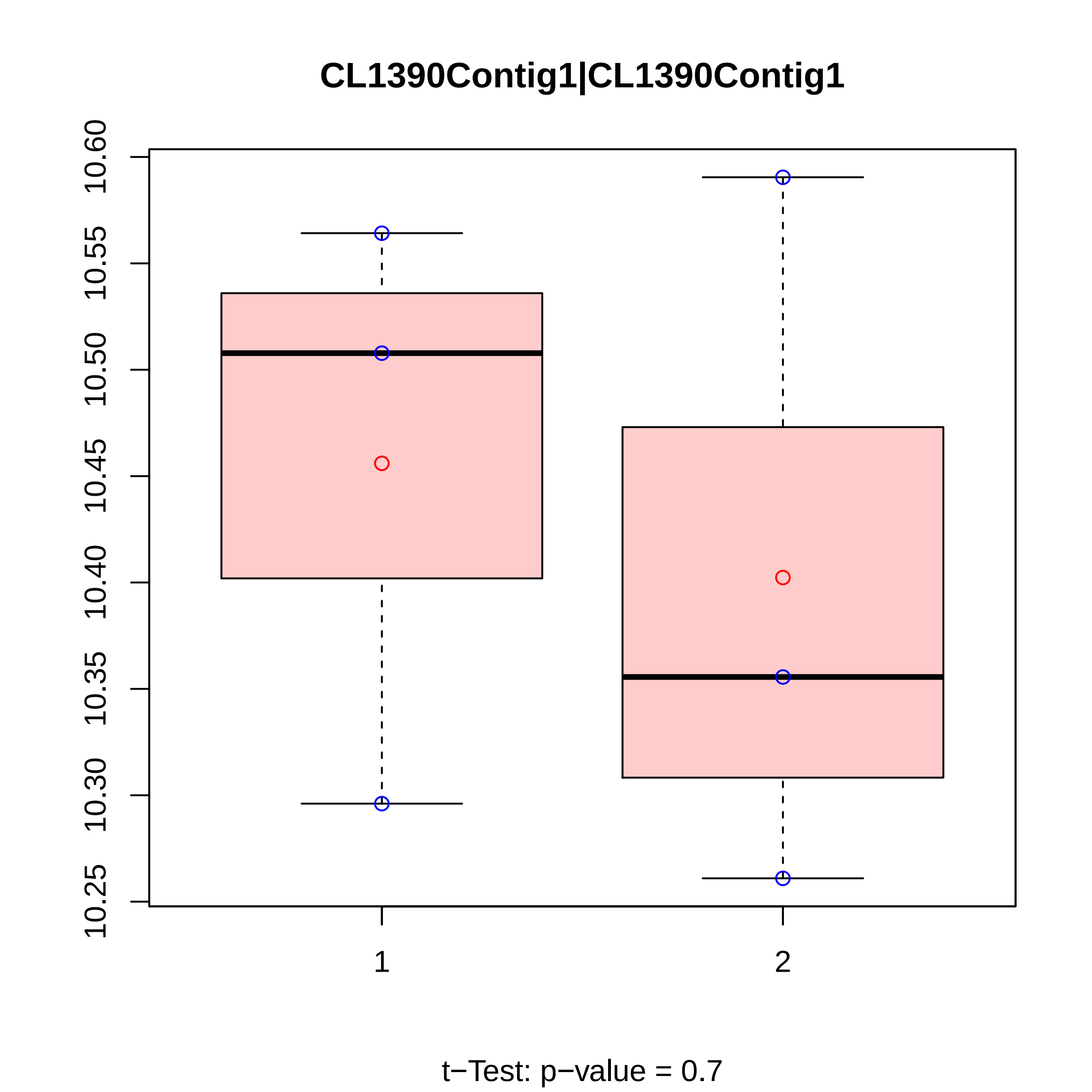
CL1390Contig1|CL1390Contig1
10.60
10.55
10.50
10.45
10.40
10.35
10.30
10.25
1
2
t−Test: p−value = 0.7

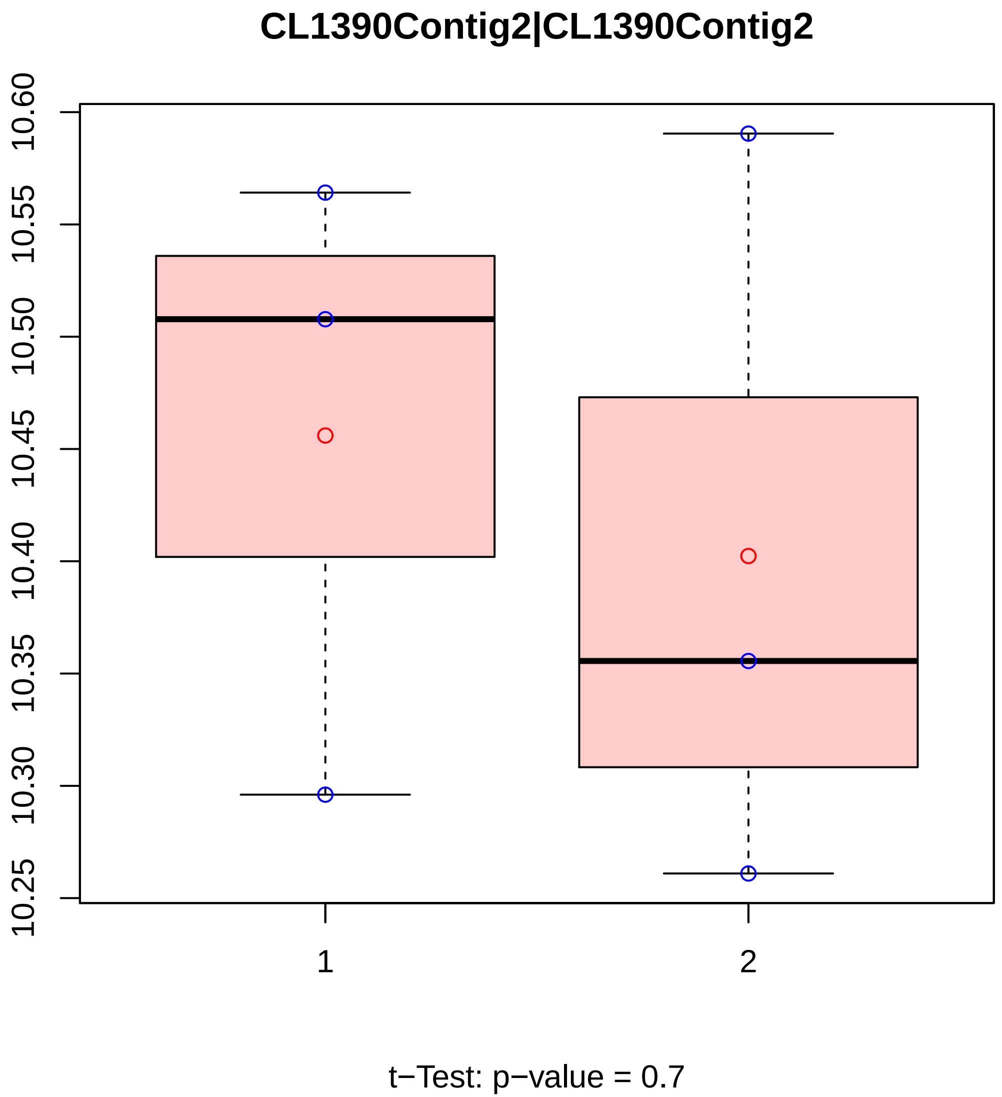
CL1390Contig2|CL1390Contig2
10.60
10.55
10.50
10.45
10.40
10.35
10.30
10.25
1
2
t−Test: p−value = 0.7

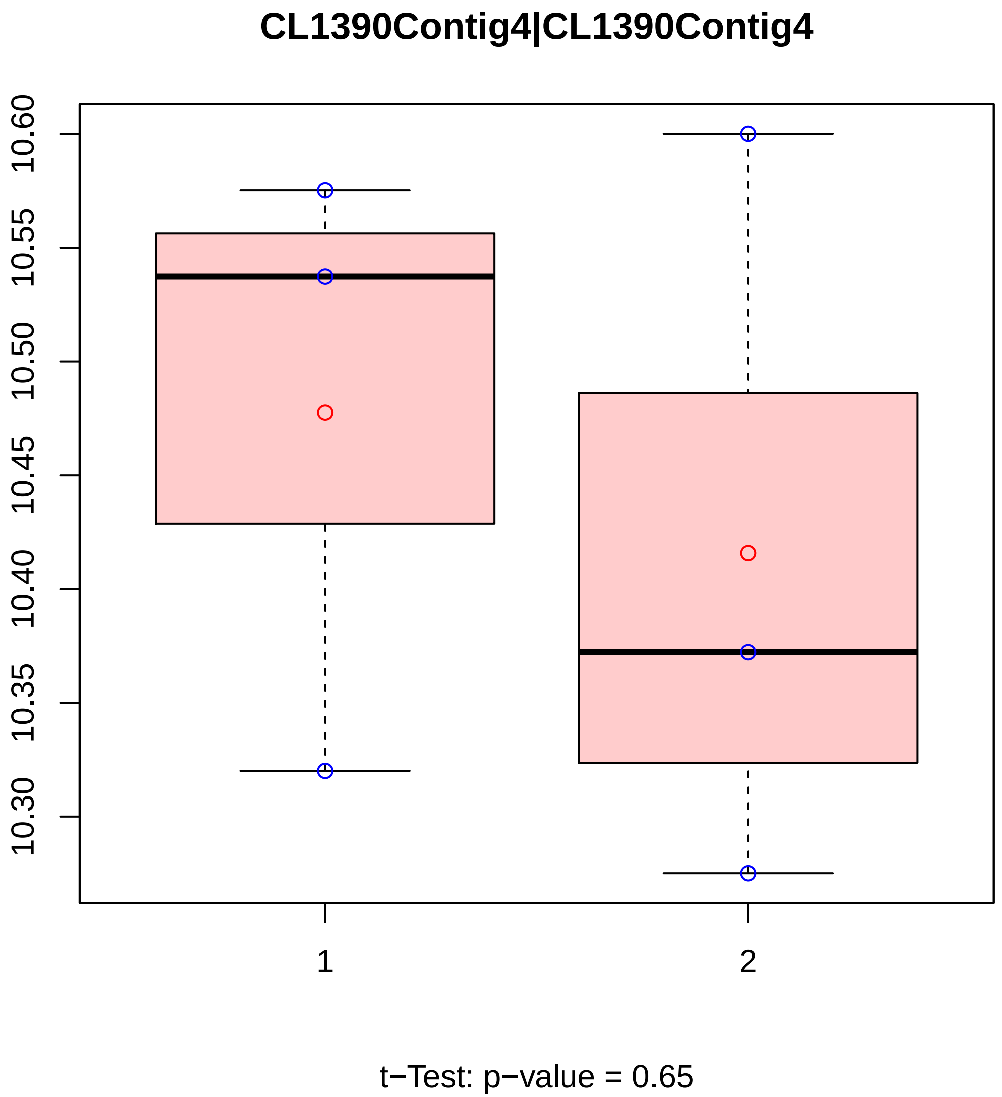
CL1390Contig4|CL1390Contig4
10.30
10.35
10.40
10.45
10.50
10.55
10.60
1
2
t−Test: p−value = 0.65

# CL1390Contig5|CL1390Contig5

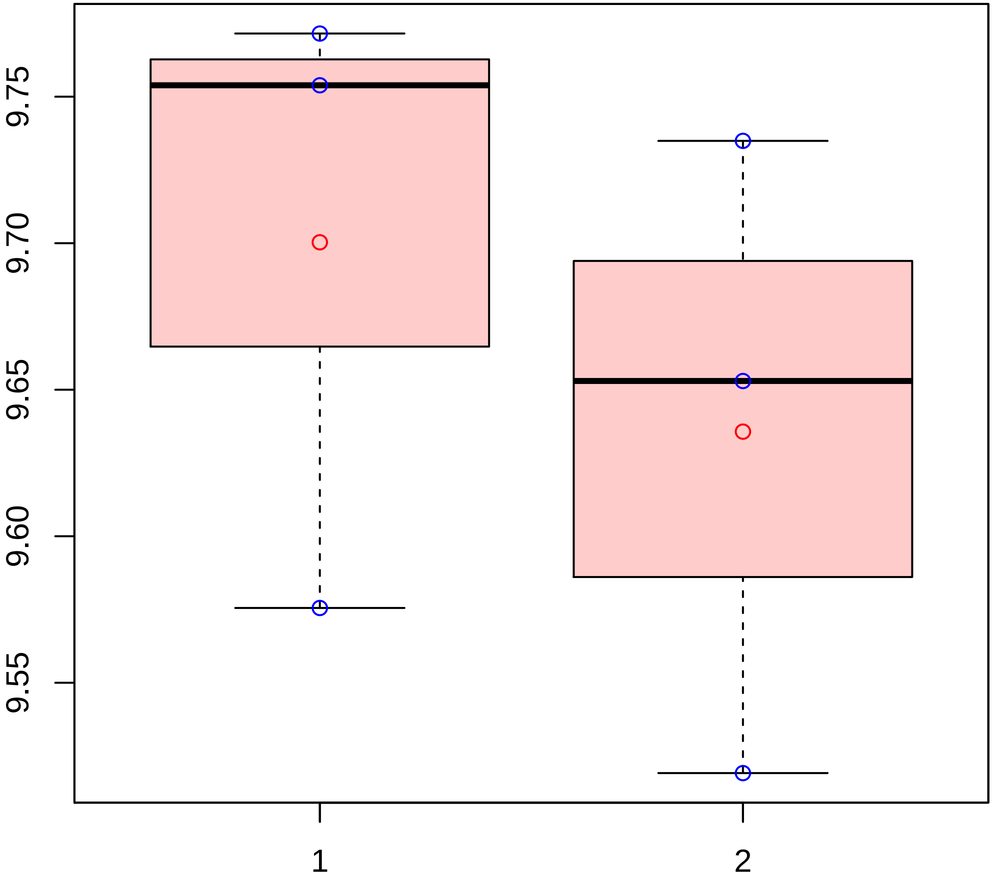


t−Test: p−value = 0.51

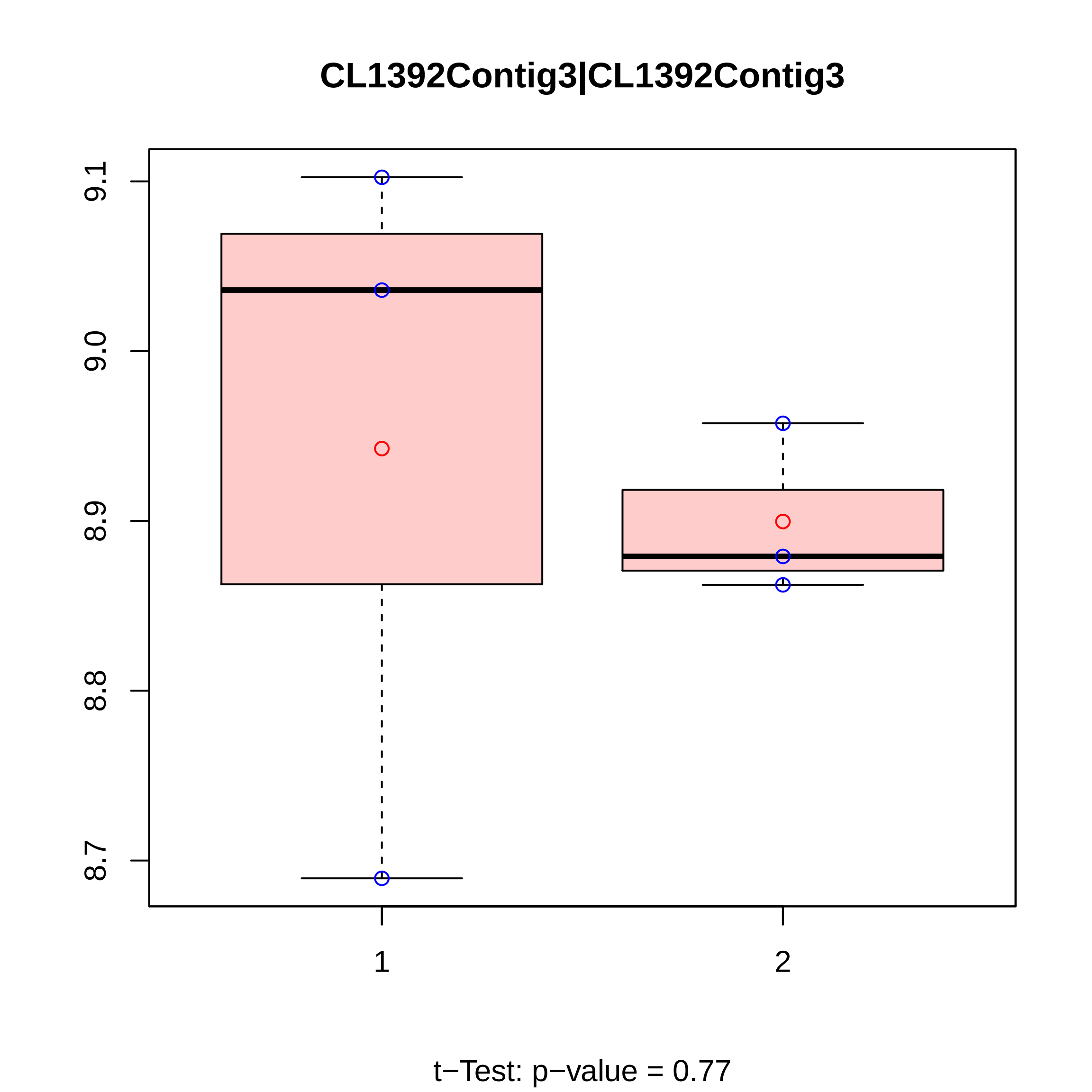
CL1392Contig3|CL1392Contig3
9.1
9.0
8.9
8.8
8.7
1
2
t−Test: p−value = 0.77

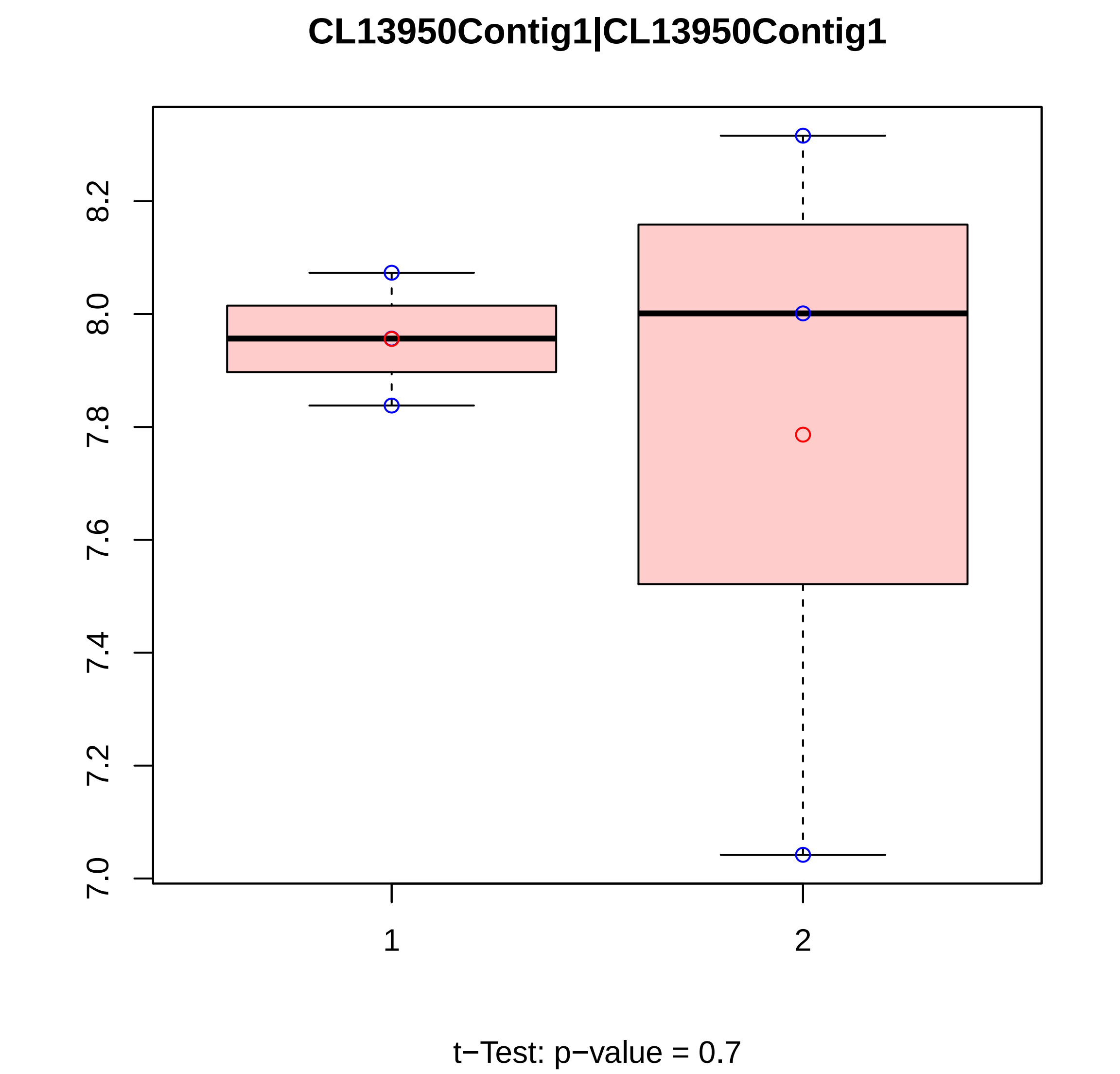

CL13950Contig1|CL13950Contig1
7.0
7.2
7.4
7.6
7.8
8.0
8.2
1
2
t−Test: p−value = 0.7

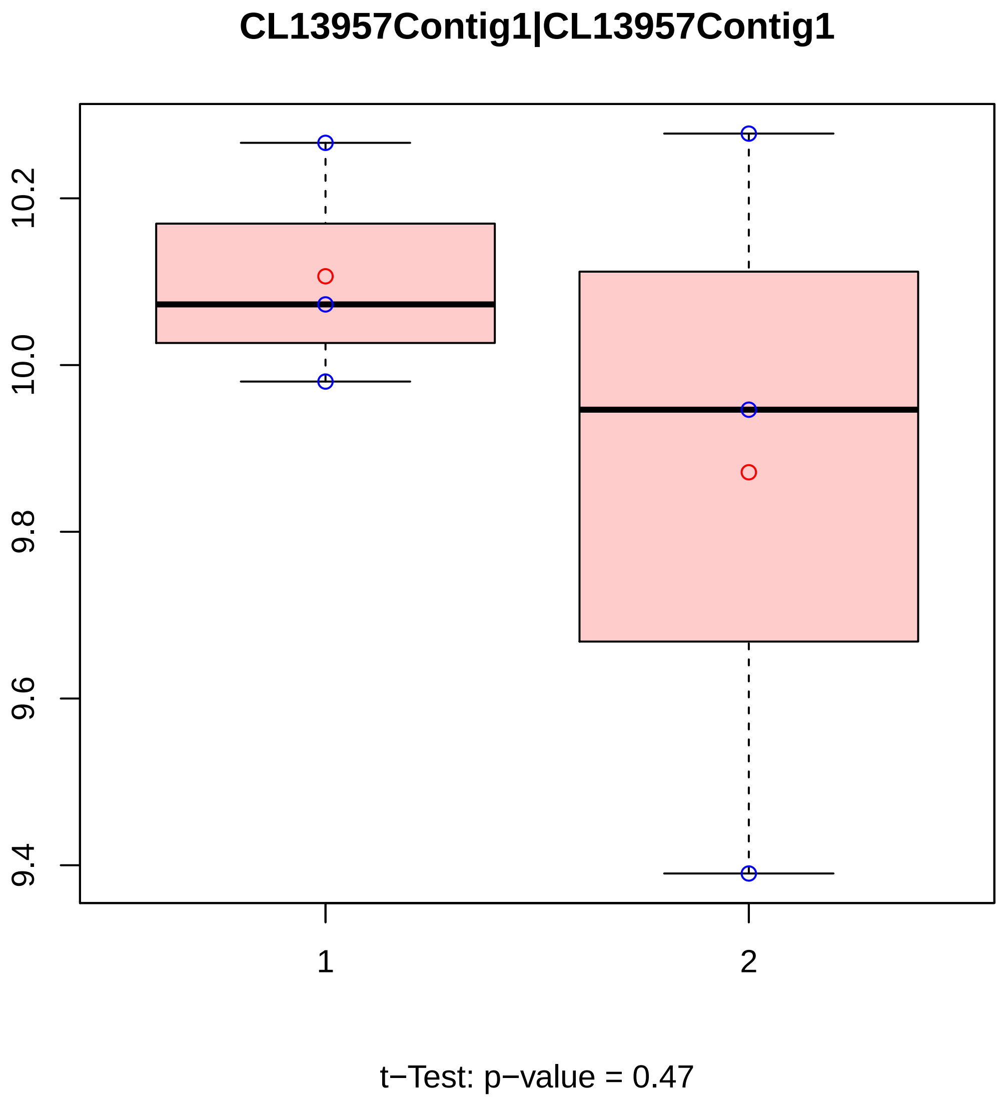
CL13957Contig1|CL13957Contig1
10.2
10.0
9.8
9.6
9.4
1
2
t−Test: p−value = 0.47

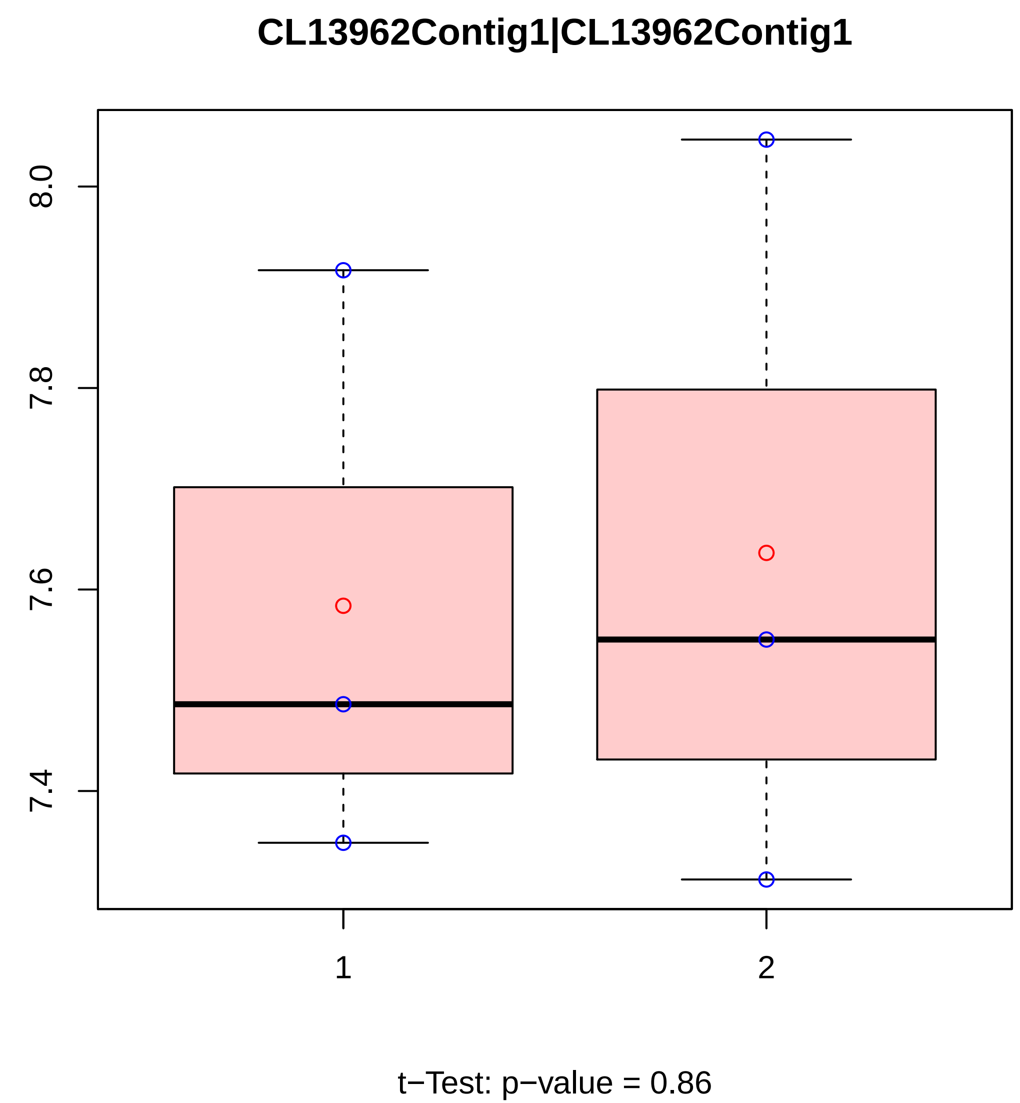
CL13962Contig1|CL13962Contig1
8.0
7.8
7.6
7.4
1
2
t−Test: p−value = 0.86

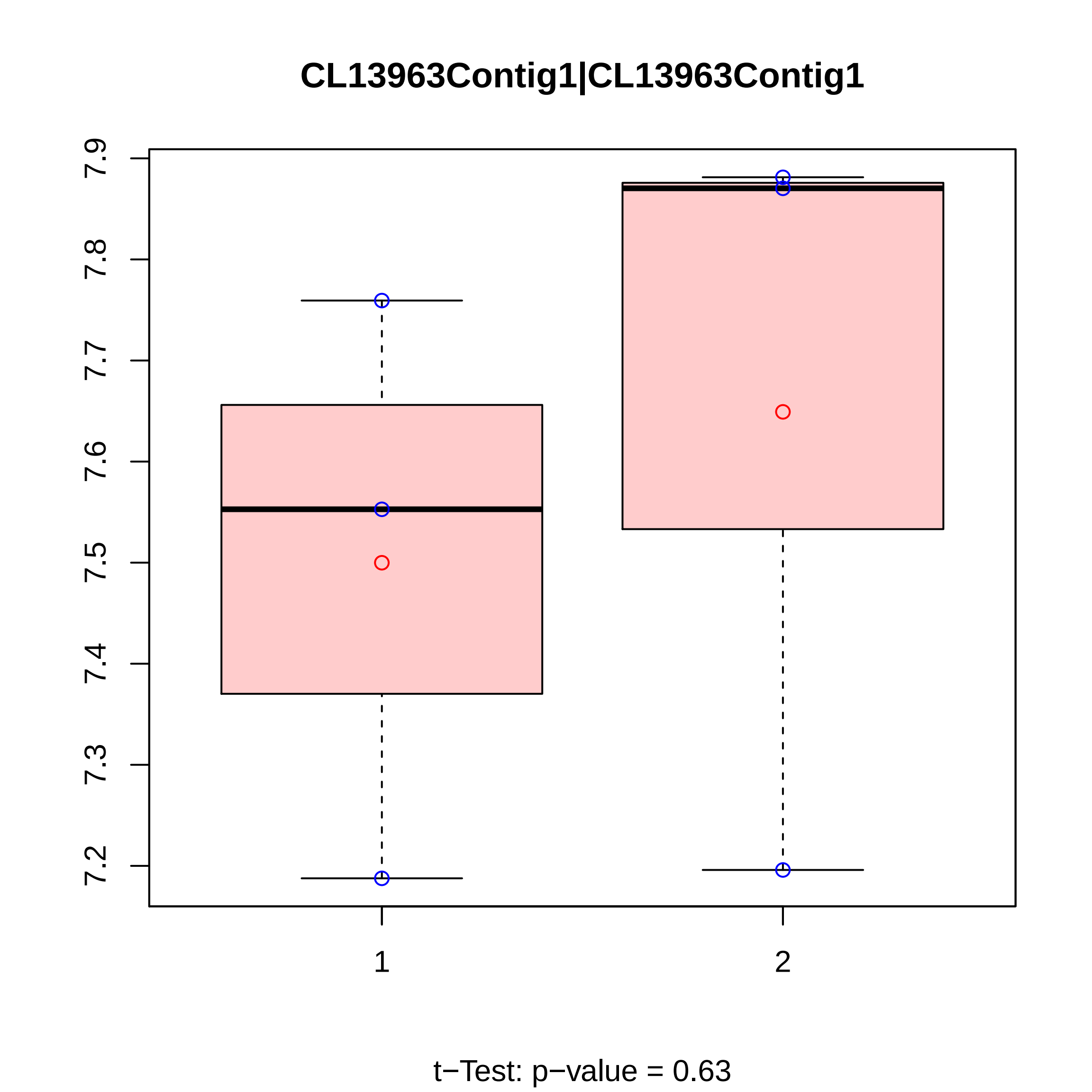
CL13963Contig1|CL13963Contig1
7.2
7.3
7.4
7.5
7.6
7.7
7.8
7.9
1
2
t−Test: p−value = 0.63

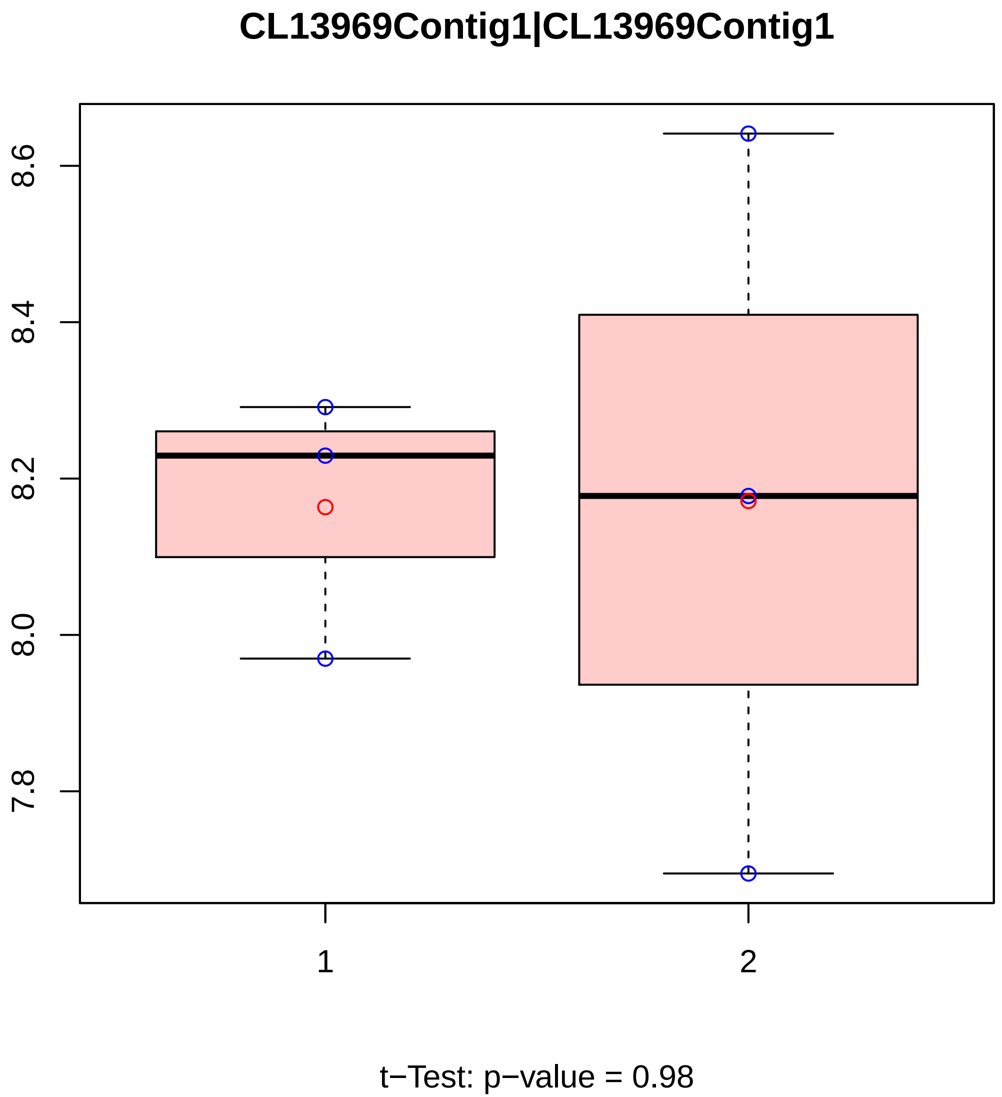

CL13969Contig1|CL13969Contig1
8.6
8.4
8.2
8.0
7.8
1
2
t−Test: p−value = 0.98

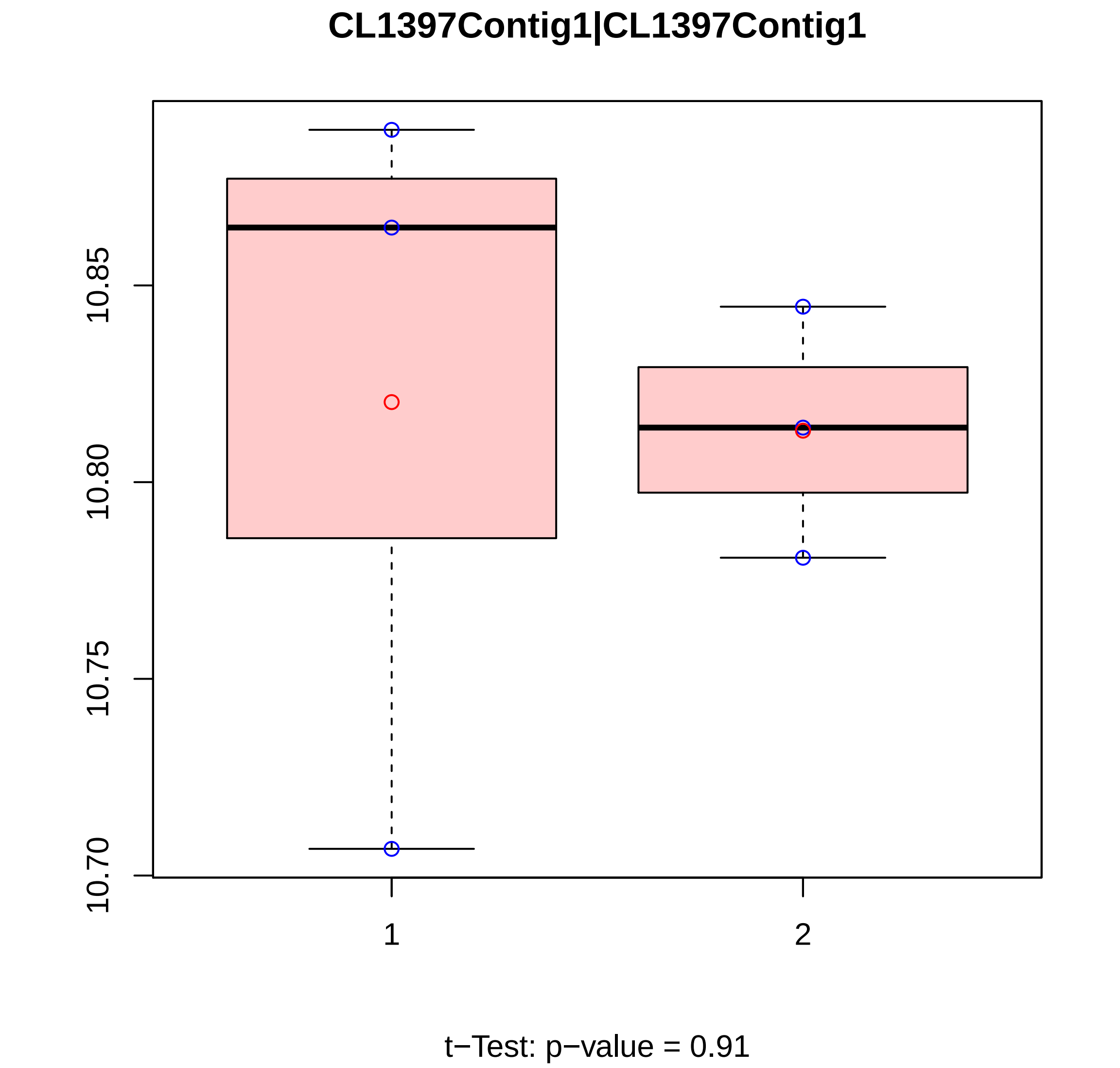

CL1397Contig1|CL1397Contig1
10.70
10.75
10.80
10.85
1
2
t−Test: p−value = 0.91

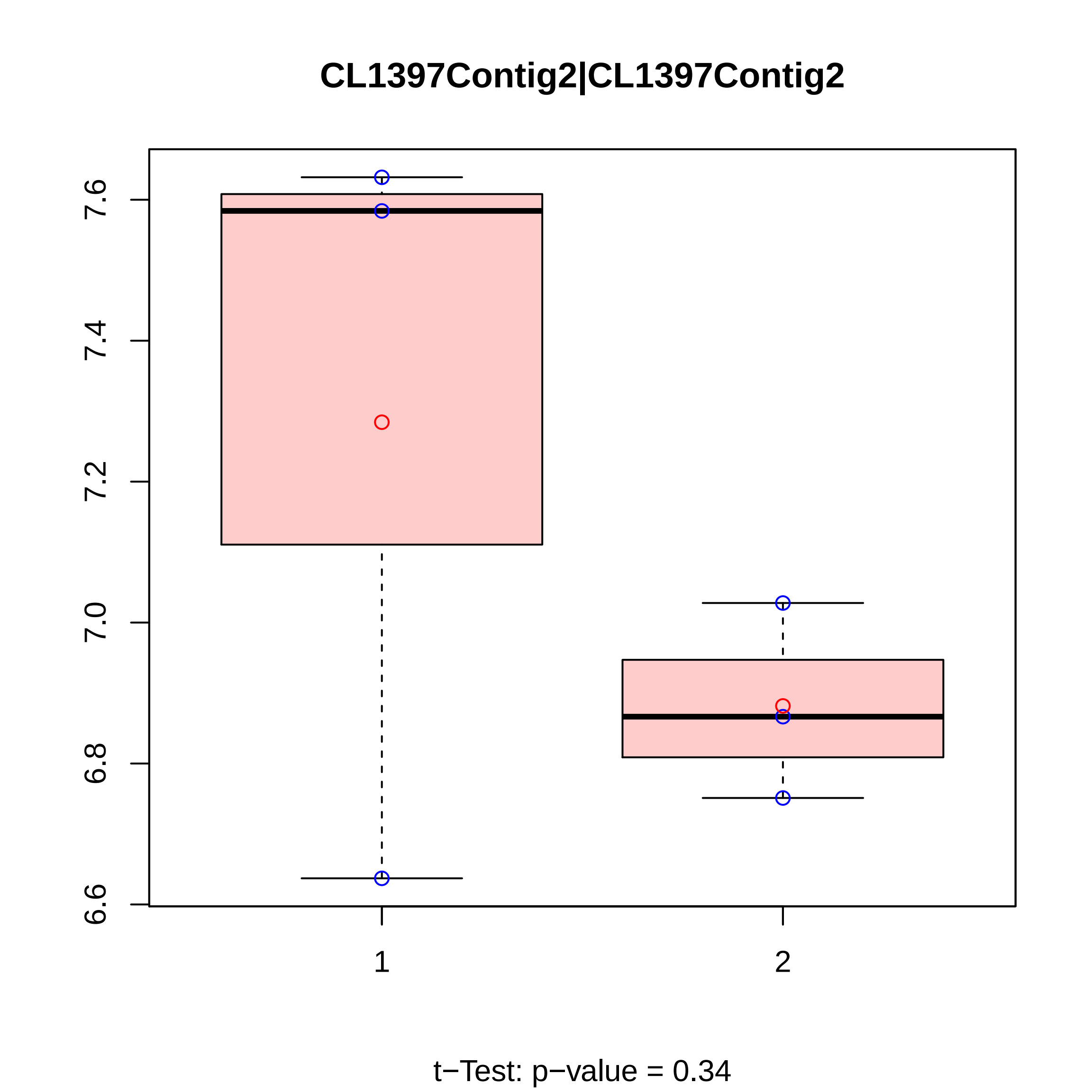
CL1397Contig2|CL1397Contig2
7.6
7.4
7.2
7.0
6.8
6.6
1
2
t−Test: p−value = 0.34

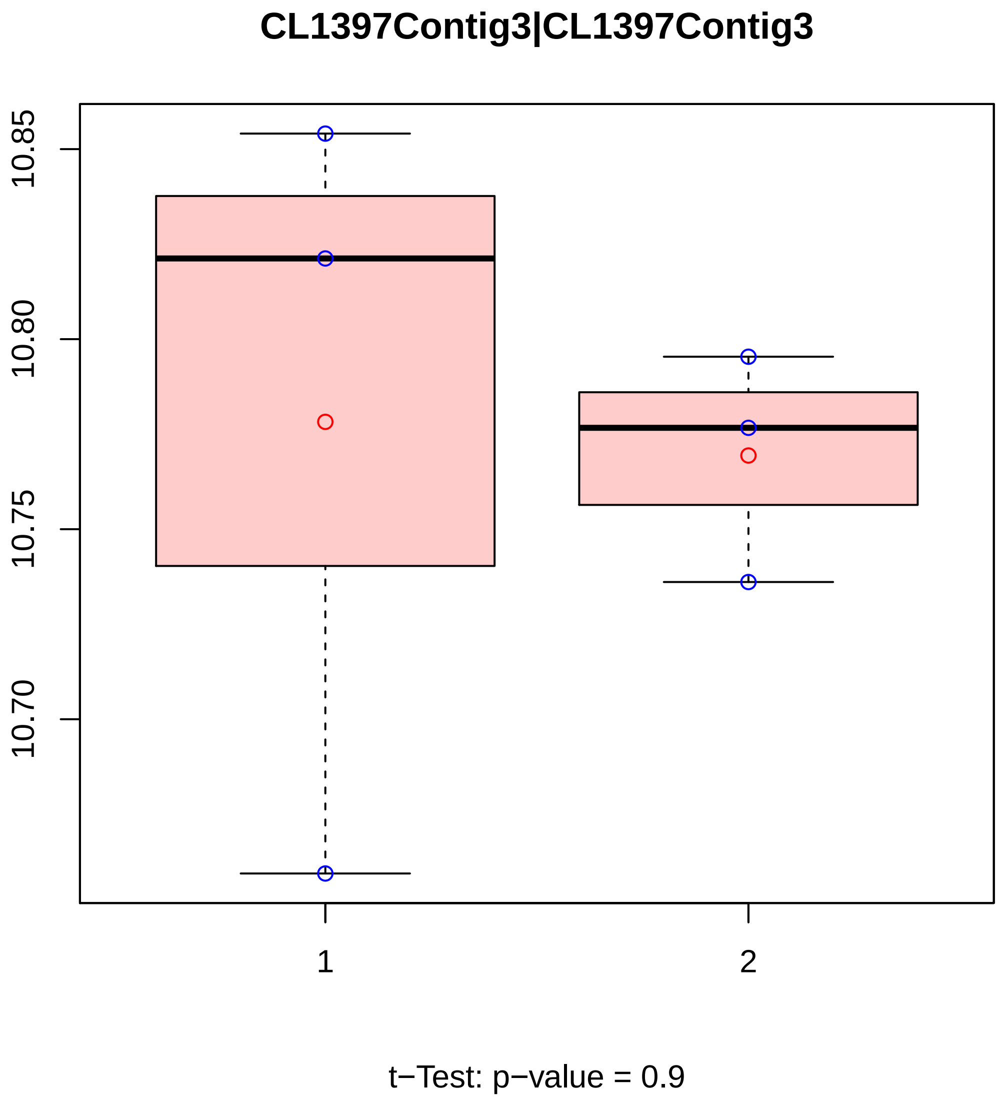
CL1397Contig3|CL1397Contig3
10.85
10.80
10.75
10.70
1
2
t−Test: p−value = 0.9

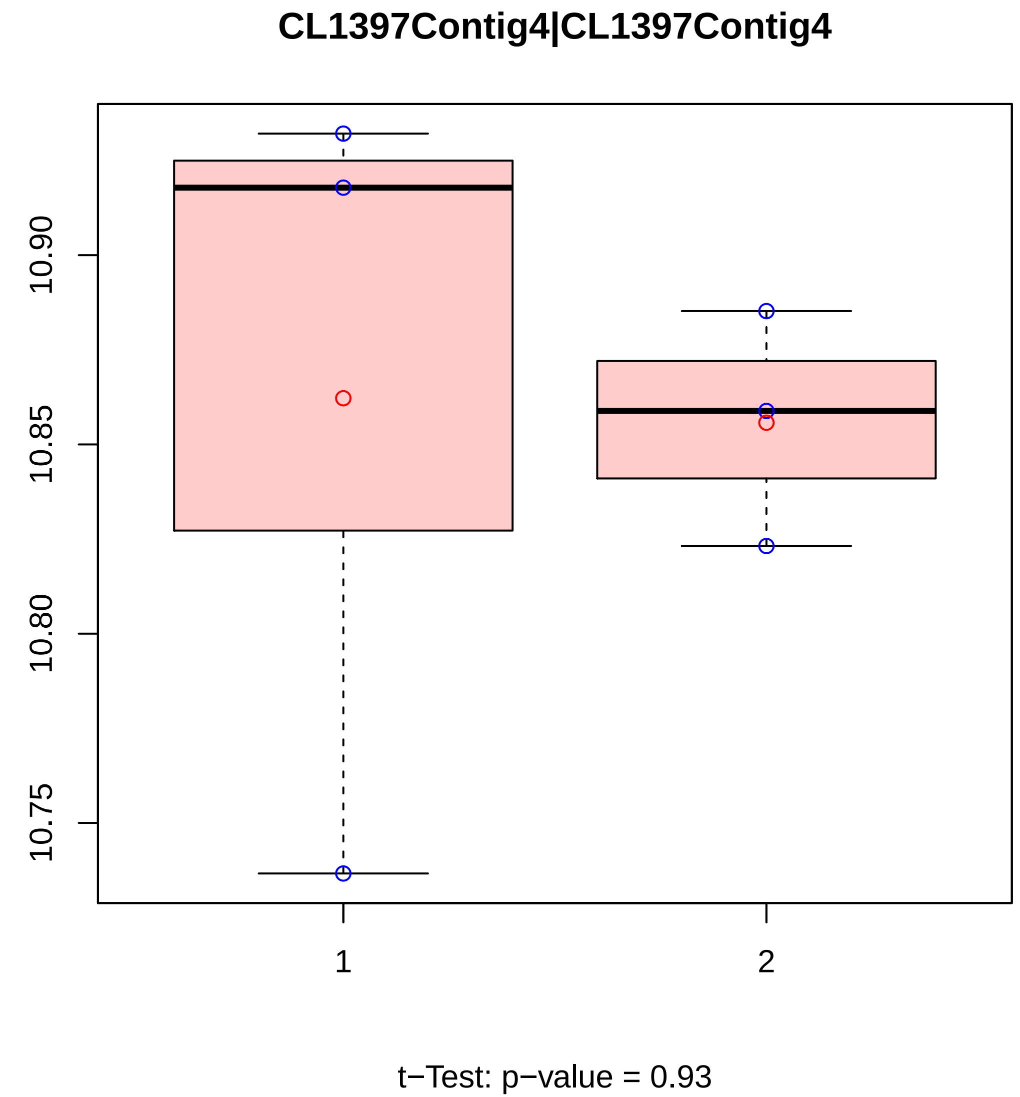

CL1397Contig4|CL1397Contig4
10.75
10.80
10.85
10.90
1
2
t−Test: p−value = 0.93

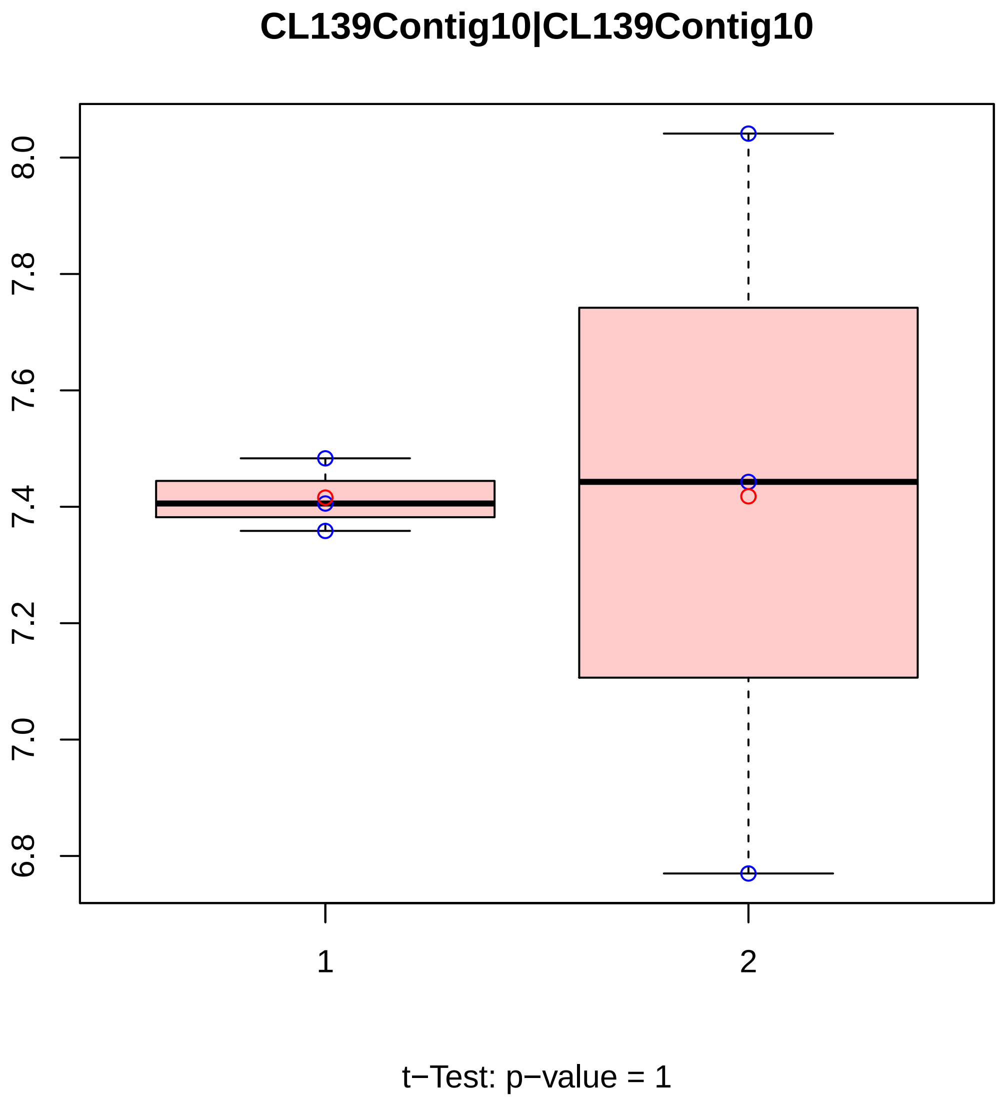
CL139Contig10|CL139Contig10
8.0
7.8
7.6
7.4
7.2
7.0
6.8
1
2
t−Test: p−value = 1

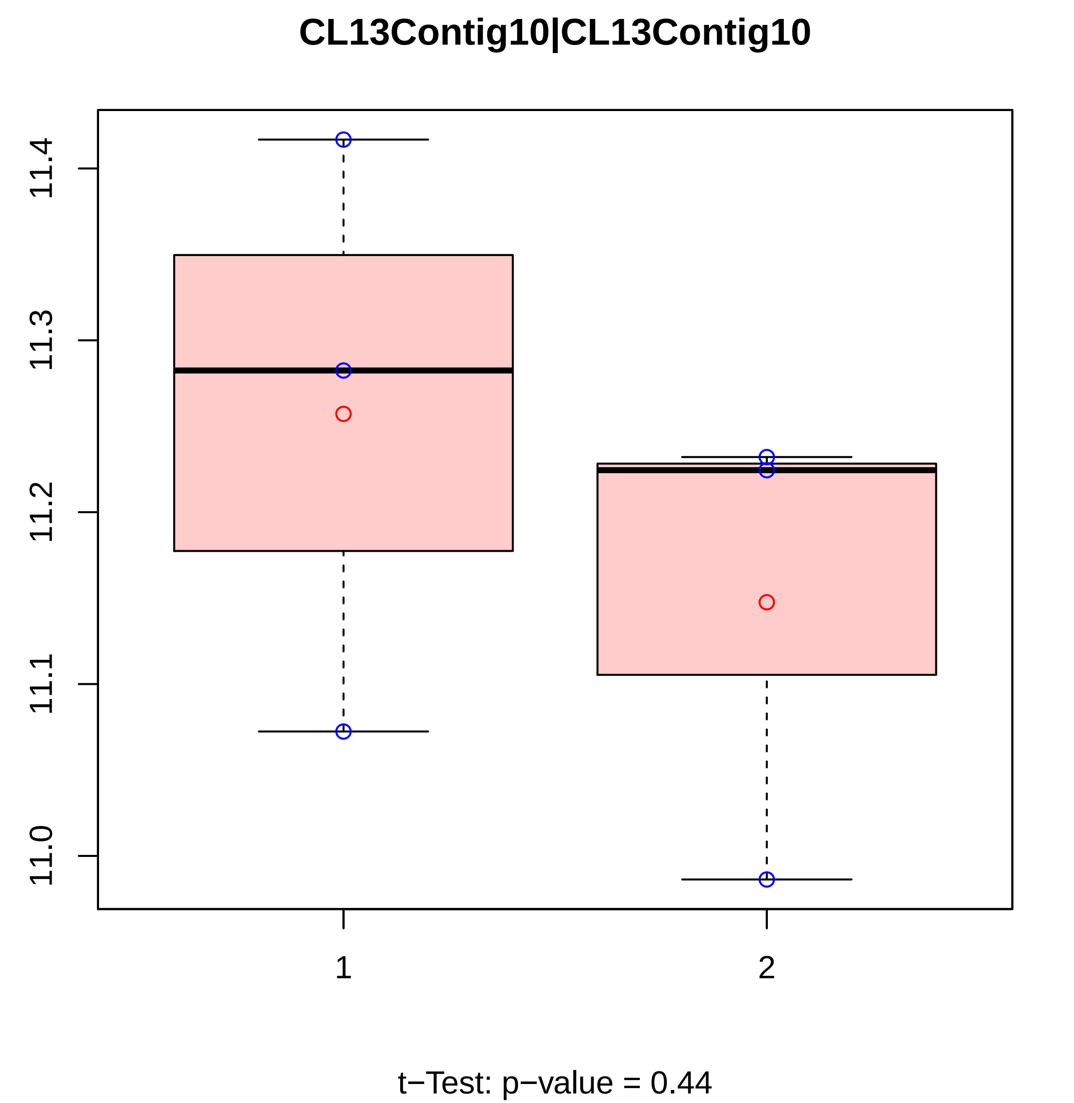
CL13Contig10|CL13Contig10
11.0
11.1
11.2
11.3
11.4
1
2
t−Test: p−value = 0.44

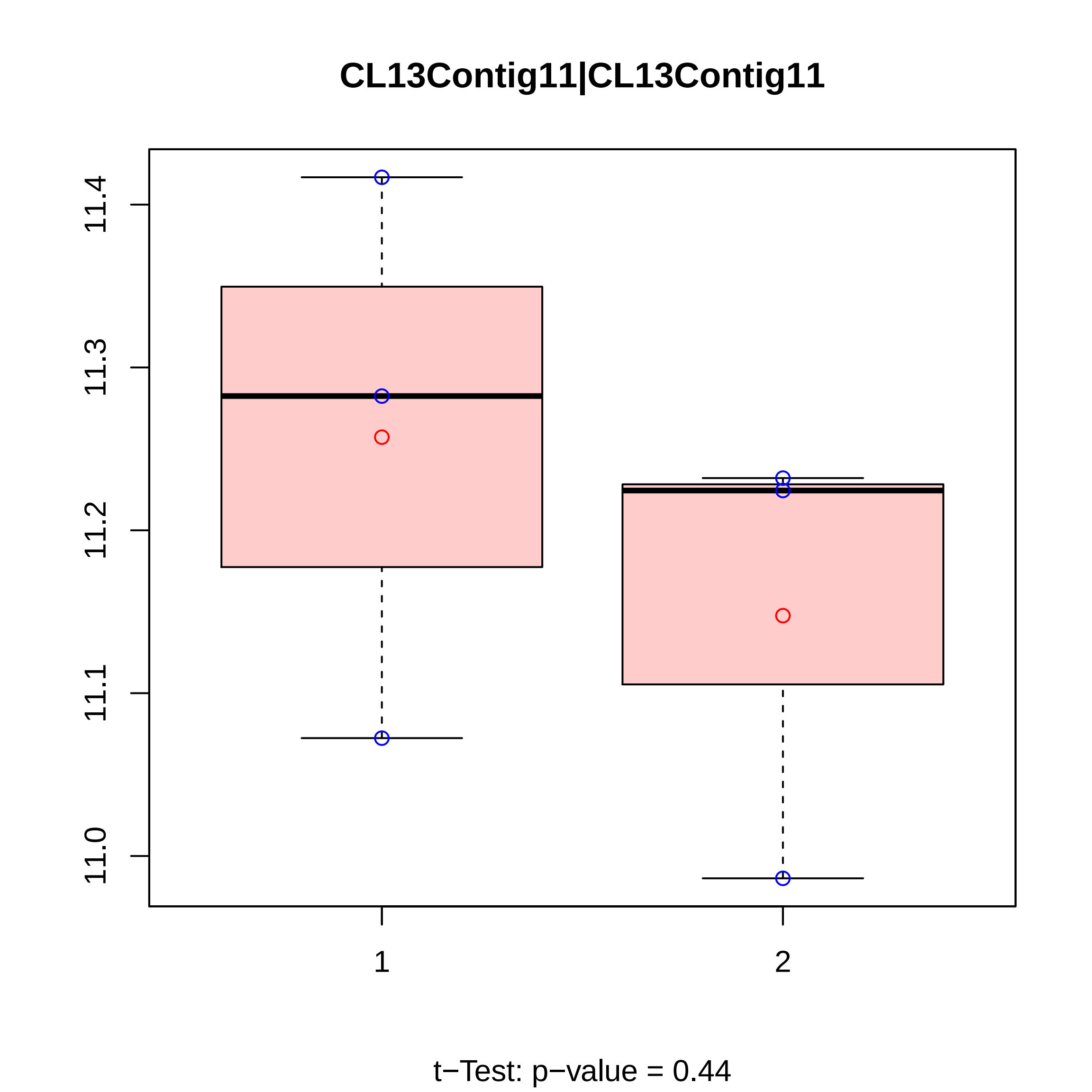
CL13Contig11|CL13Contig11
11.0
11.1
11.2
11.3
11.4
1
2
t−Test: p−value = 0.44

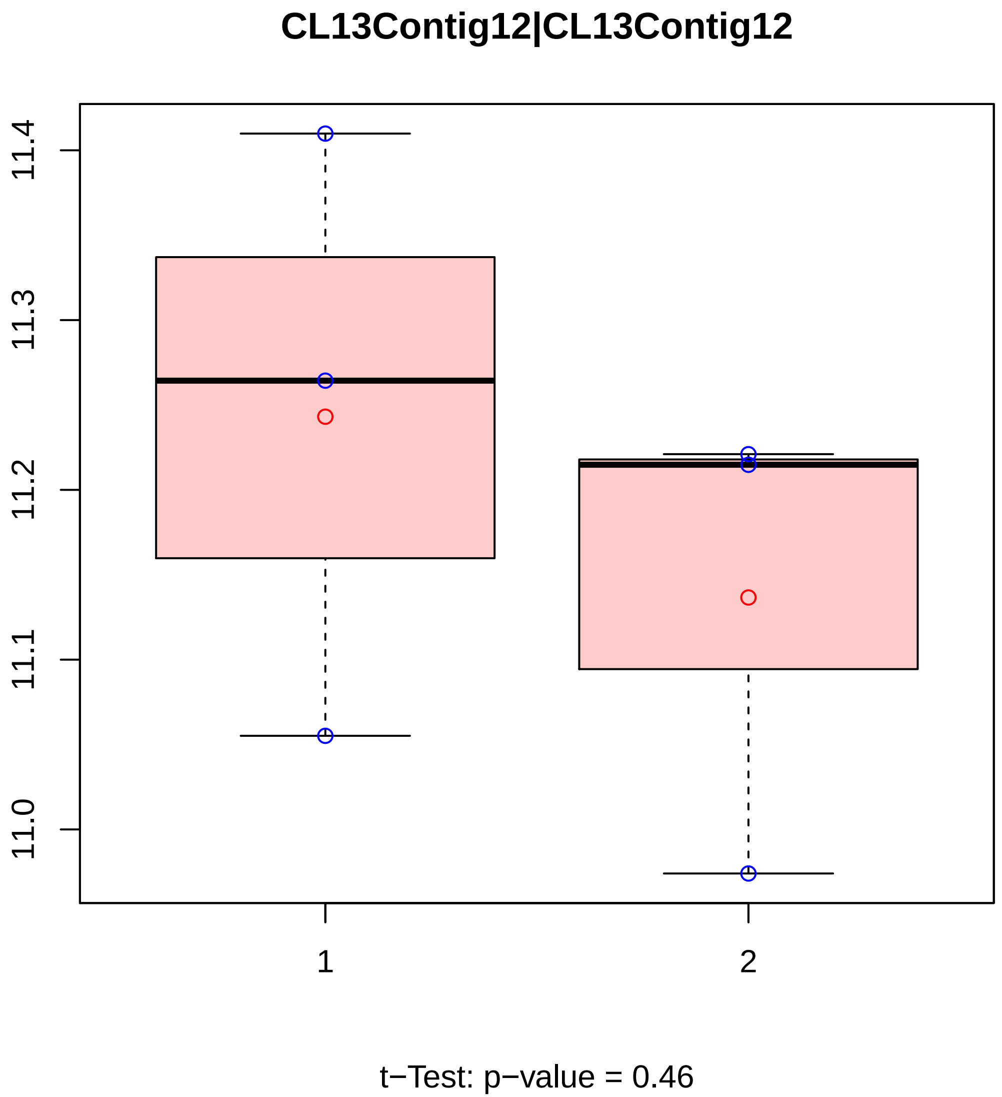
CL13Contig12|CL13Contig12
11.4
11.3
11.2
11.1
11.0
1
2
t−Test: p−value = 0.46

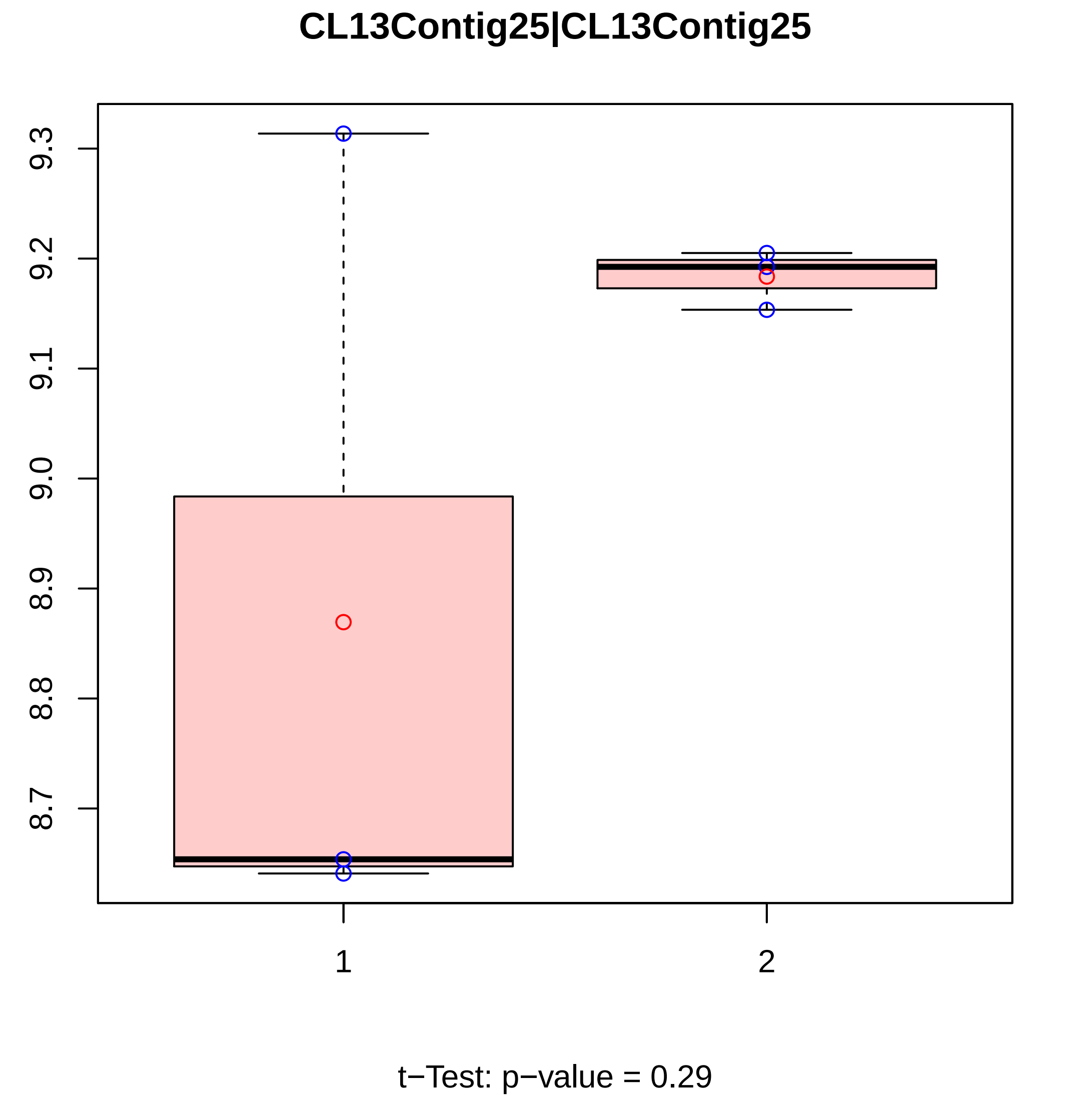
CL13Contig25|CL13Contig25
9.3
9.2
9.1
9.0
8.9
8.8
8.7
1
2
t−Test: p−value = 0.29

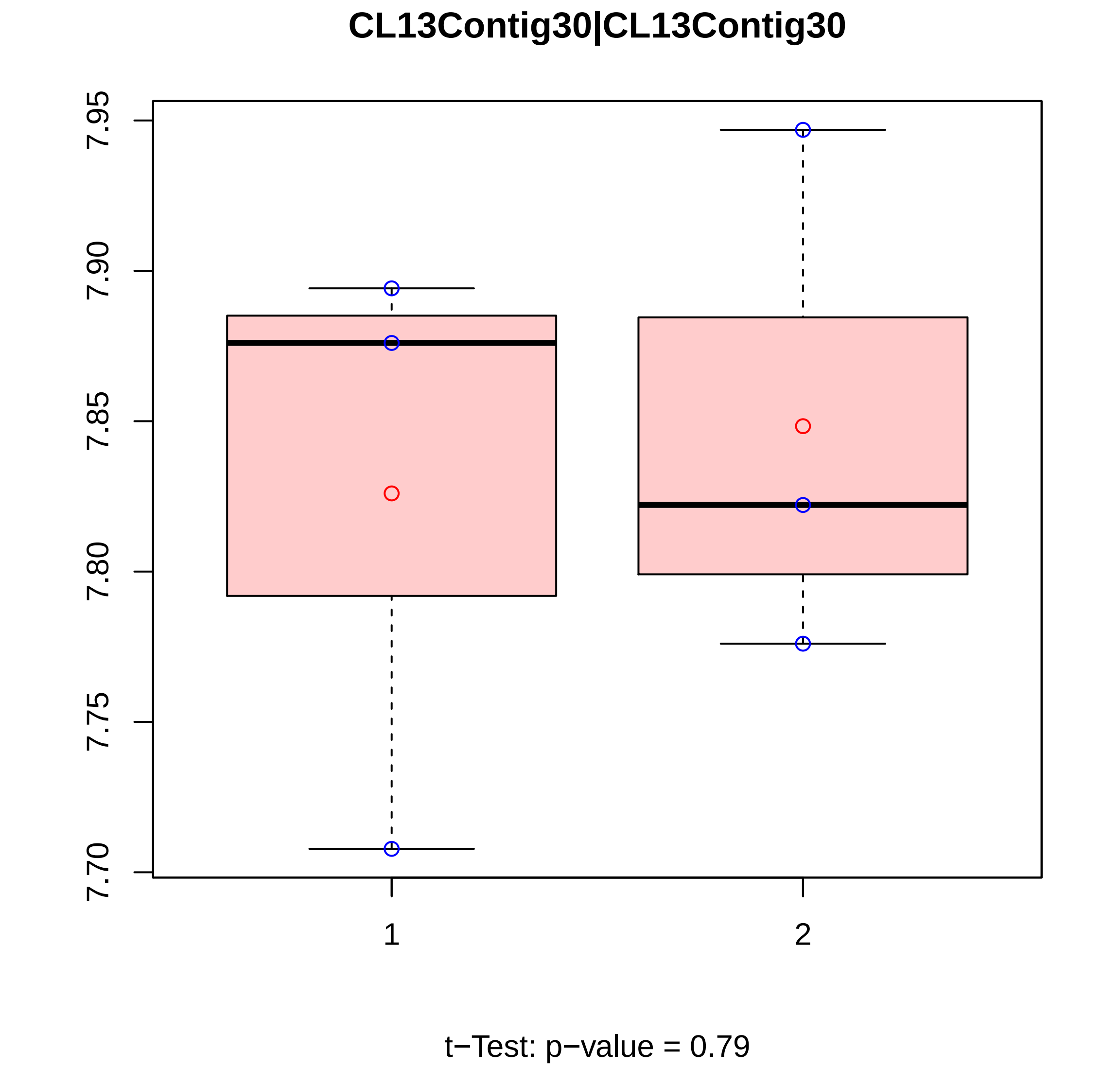
CL13Contig30|CL13Contig30
7.70
7.75
7.80
7.85
7.90
7.95
1
2
t−Test: p−value = 0.79

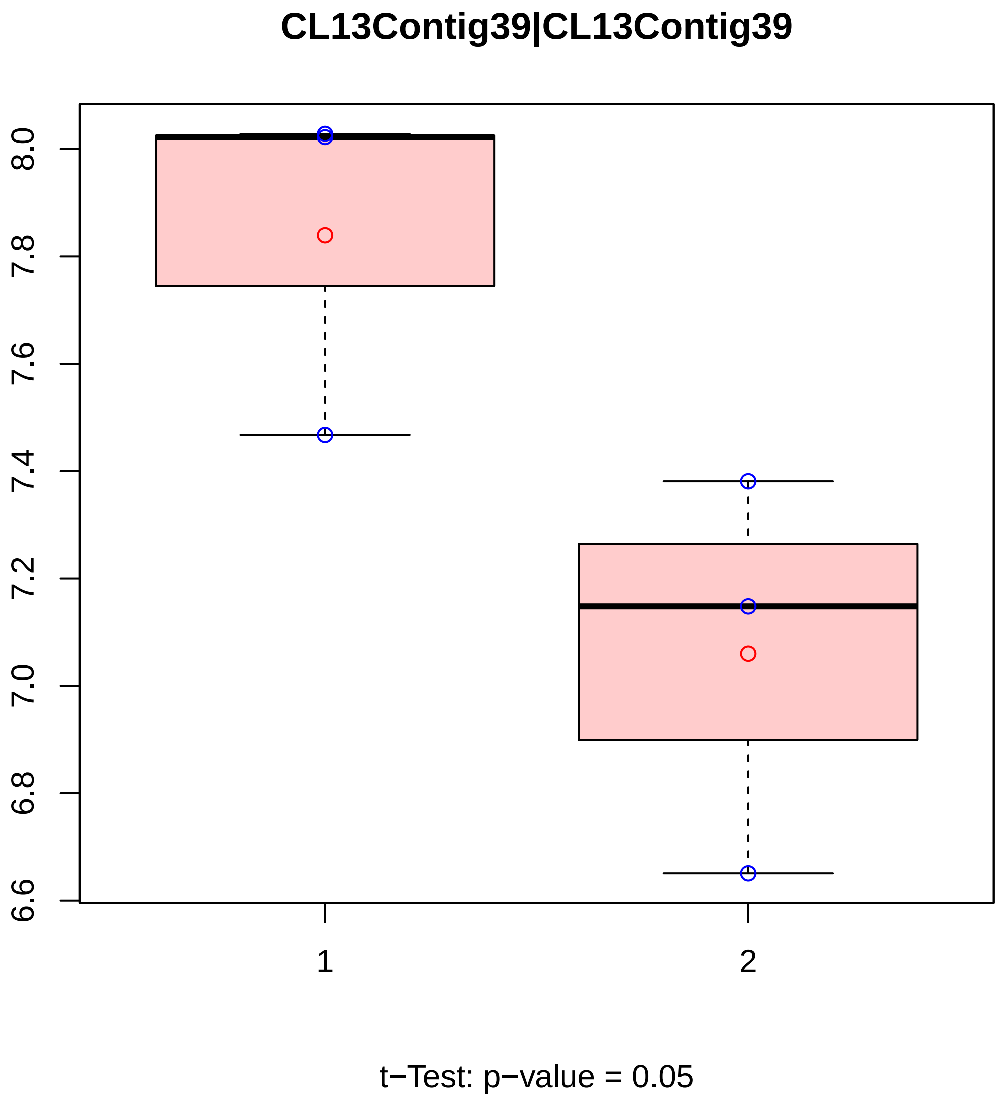
CL13Contig39|CL13Contig39
8.0
7.8
7.6
7.4
7.2
7.0
6.8
6.6
1
2
t−Test: p−value = 0.05

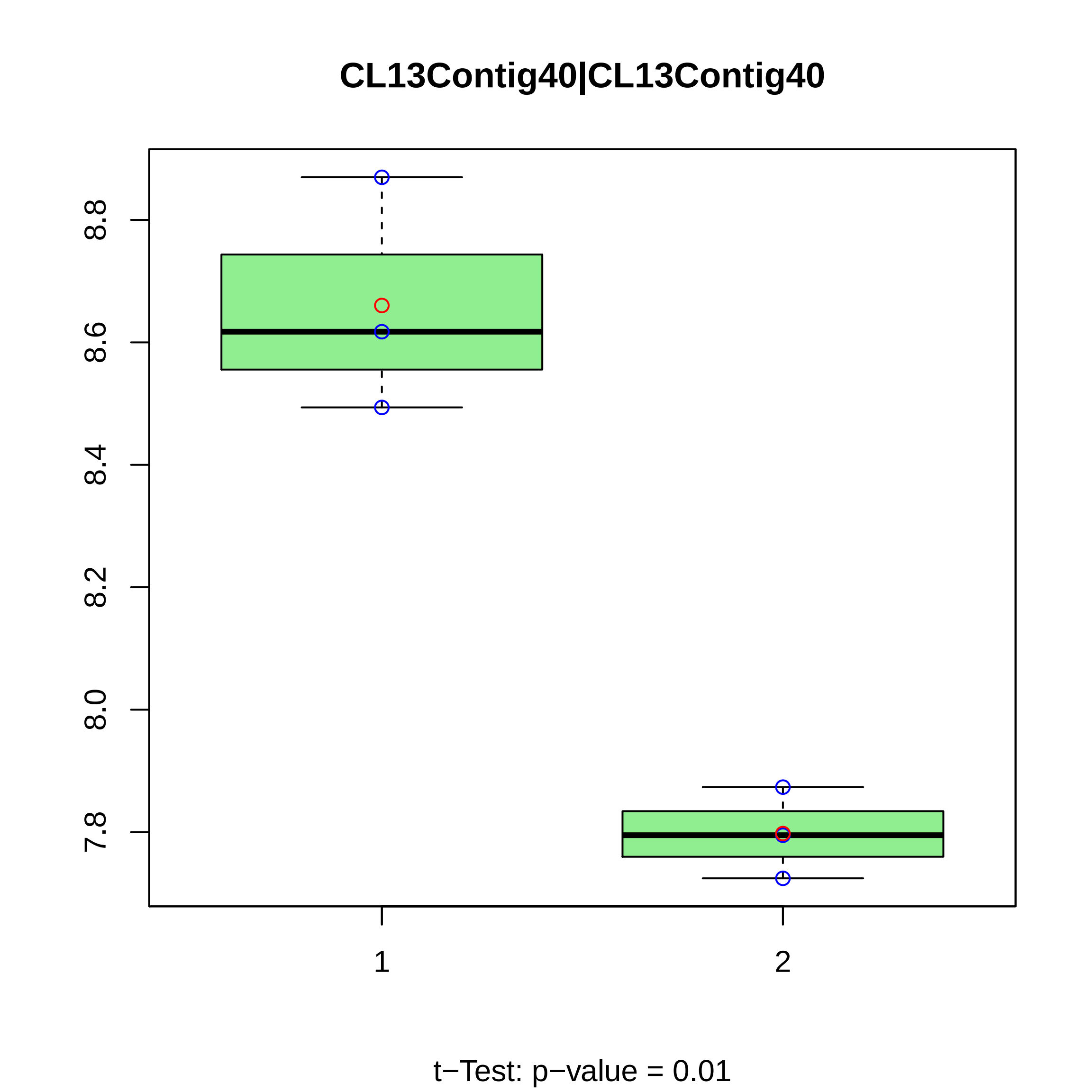
CL13Contig40|CL13Contig40
8.8
8.6
8.4
8.2
8.0
7.8
1
2
t−Test: p−value = 0.01

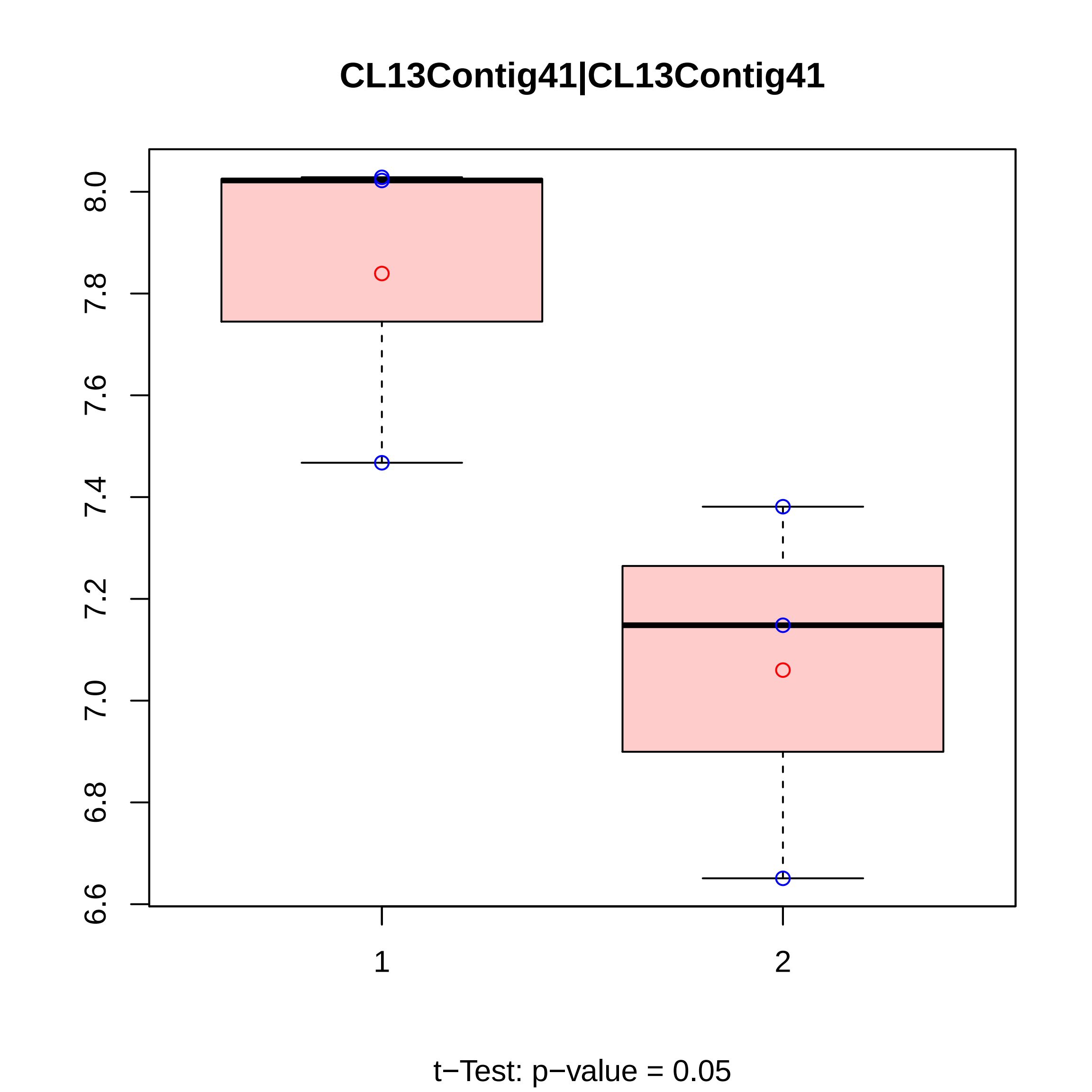
CL13Contig41|CL13Contig41
8.0
7.8
7.6
7.4
7.2
7.0
6.8
6.6
1
2
t−Test: p−value = 0.05

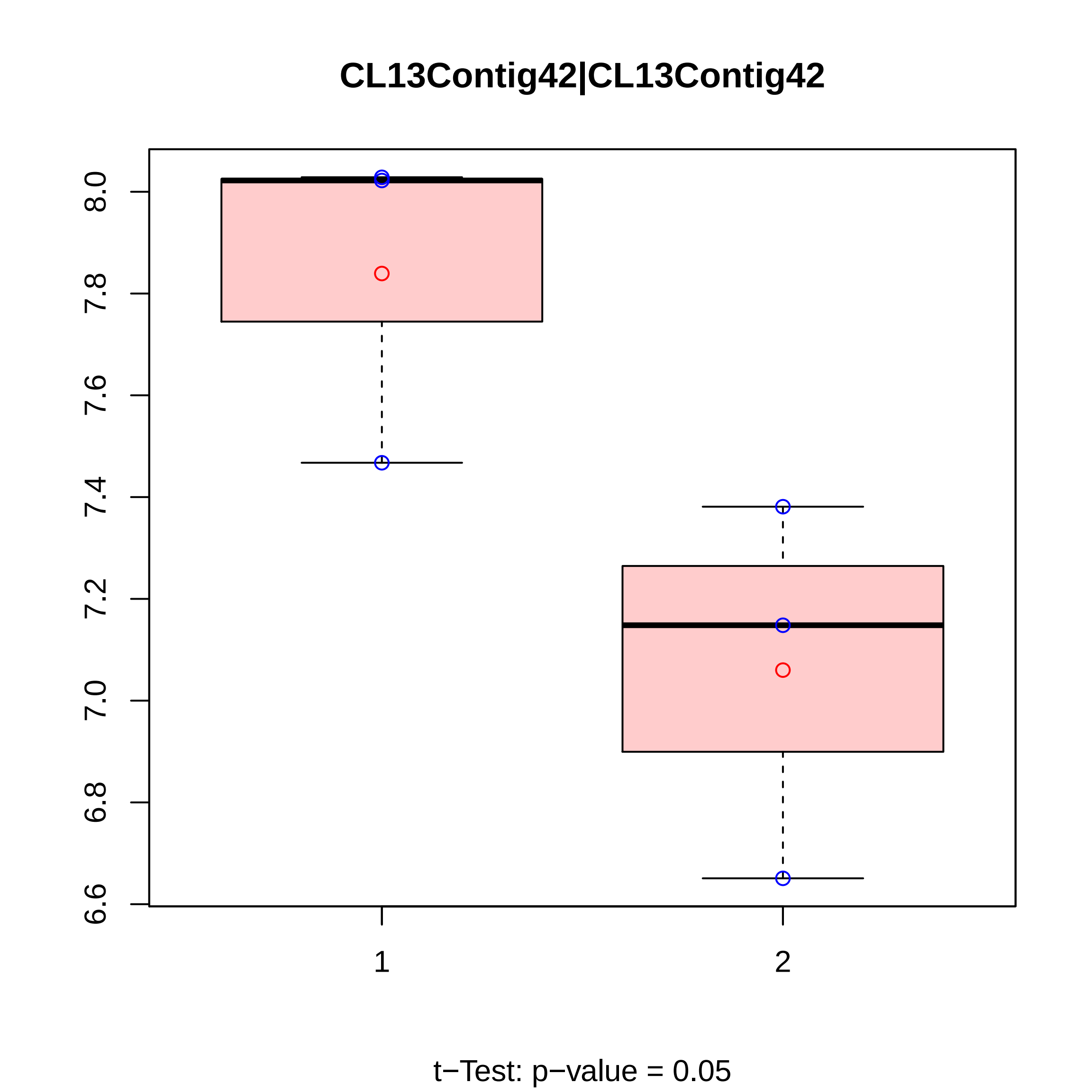

CL13Contig42|CL13Contig42
8.0
7.8
7.6
7.4
7.2
7.0
6.8
6.6
1
2
t−Test: p−value = 0.05

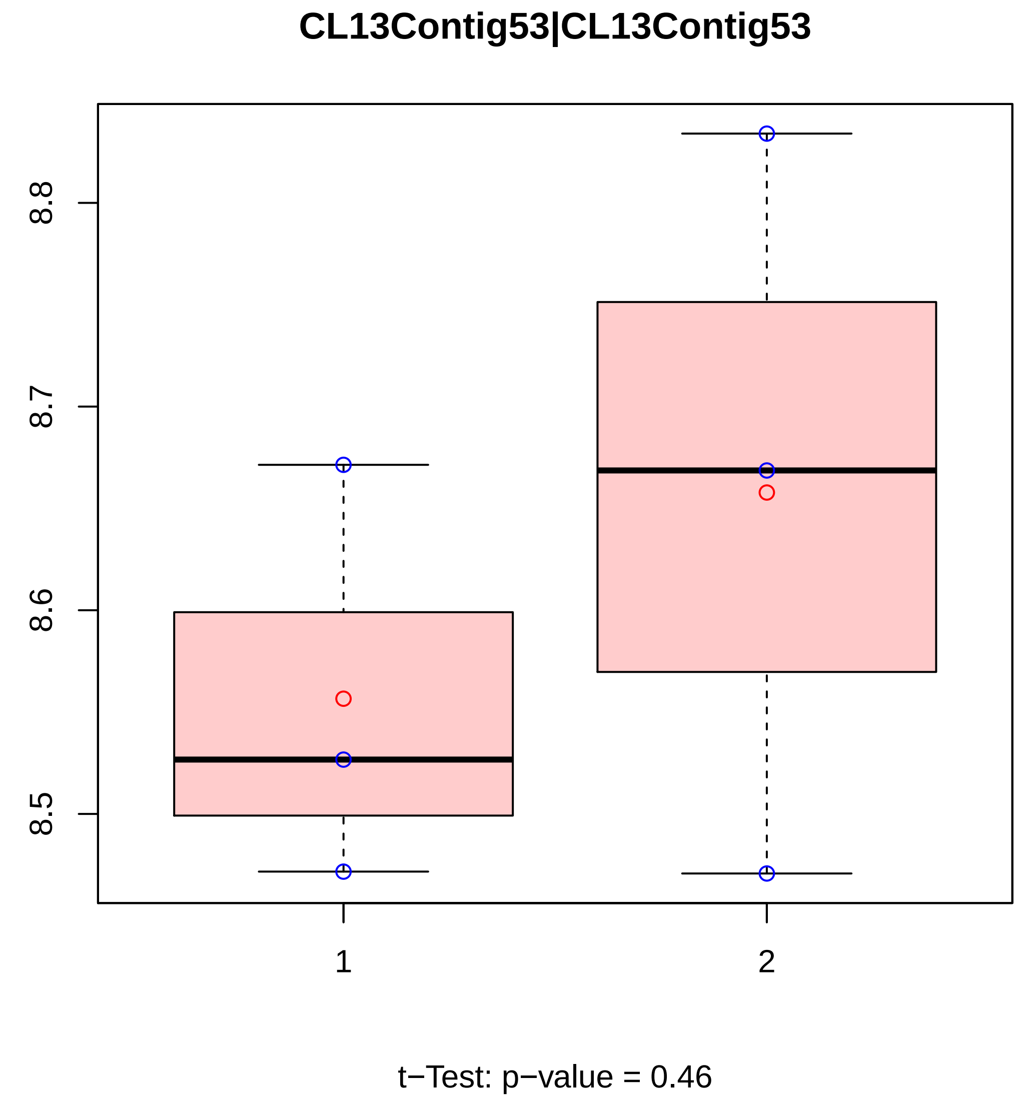
CL13Contig53|CL13Contig53
8.5
8.6
8.7
8.8
1
2
t−Test: p−value = 0.46

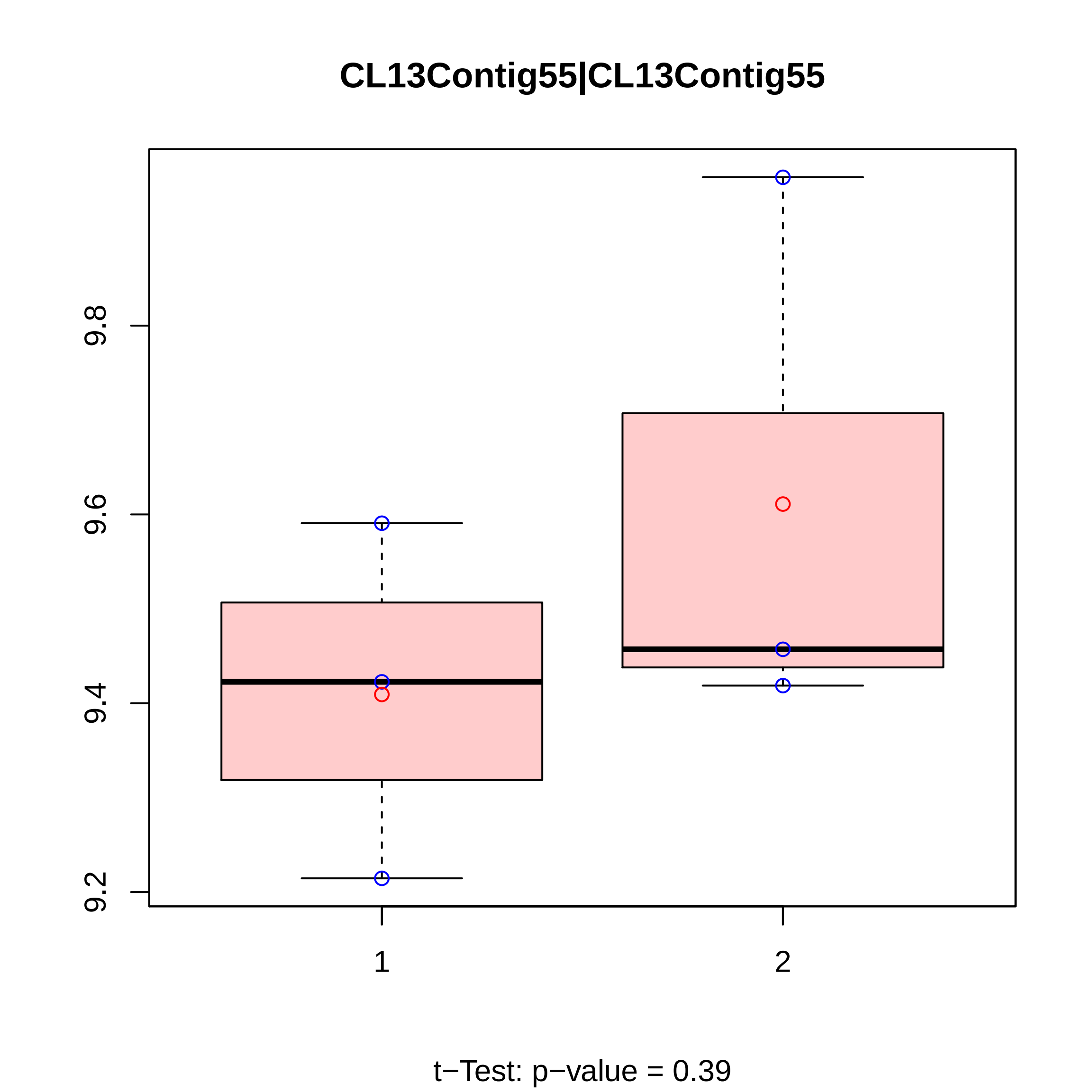
CL13Contig55|CL13Contig55
9.2
9.4
9.6
9.8
1
2
t−Test: p−value = 0.39

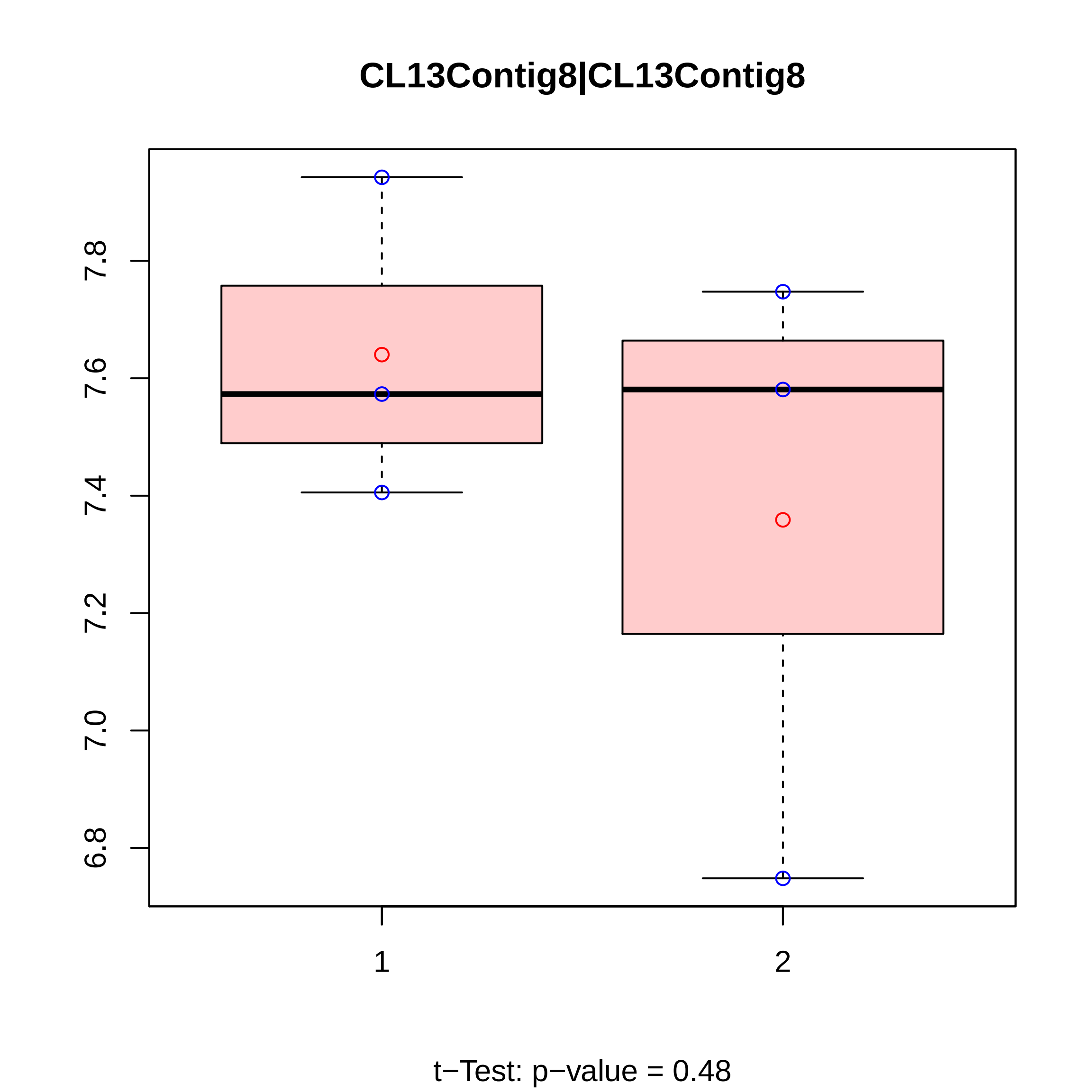
CL13Contig8|CL13Contig8
7.8
7.6
7.4
7.2
7.0
6.8
1
2
t−Test: p−value = 0.48

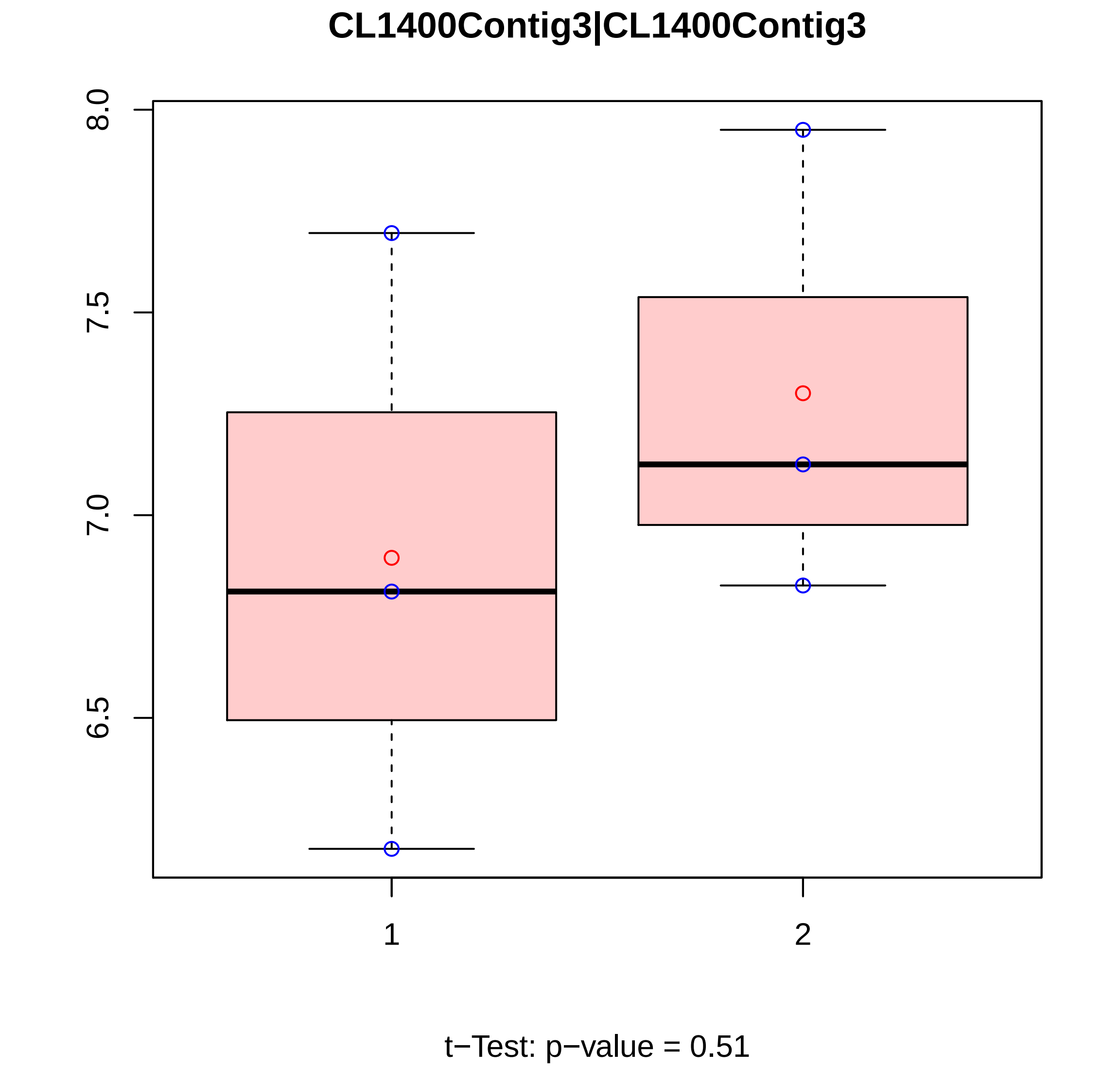
CL1400Contig3|CL1400Contig3
8.0
7.5
7.0
6.5
1
2
t−Test: p−value = 0.51

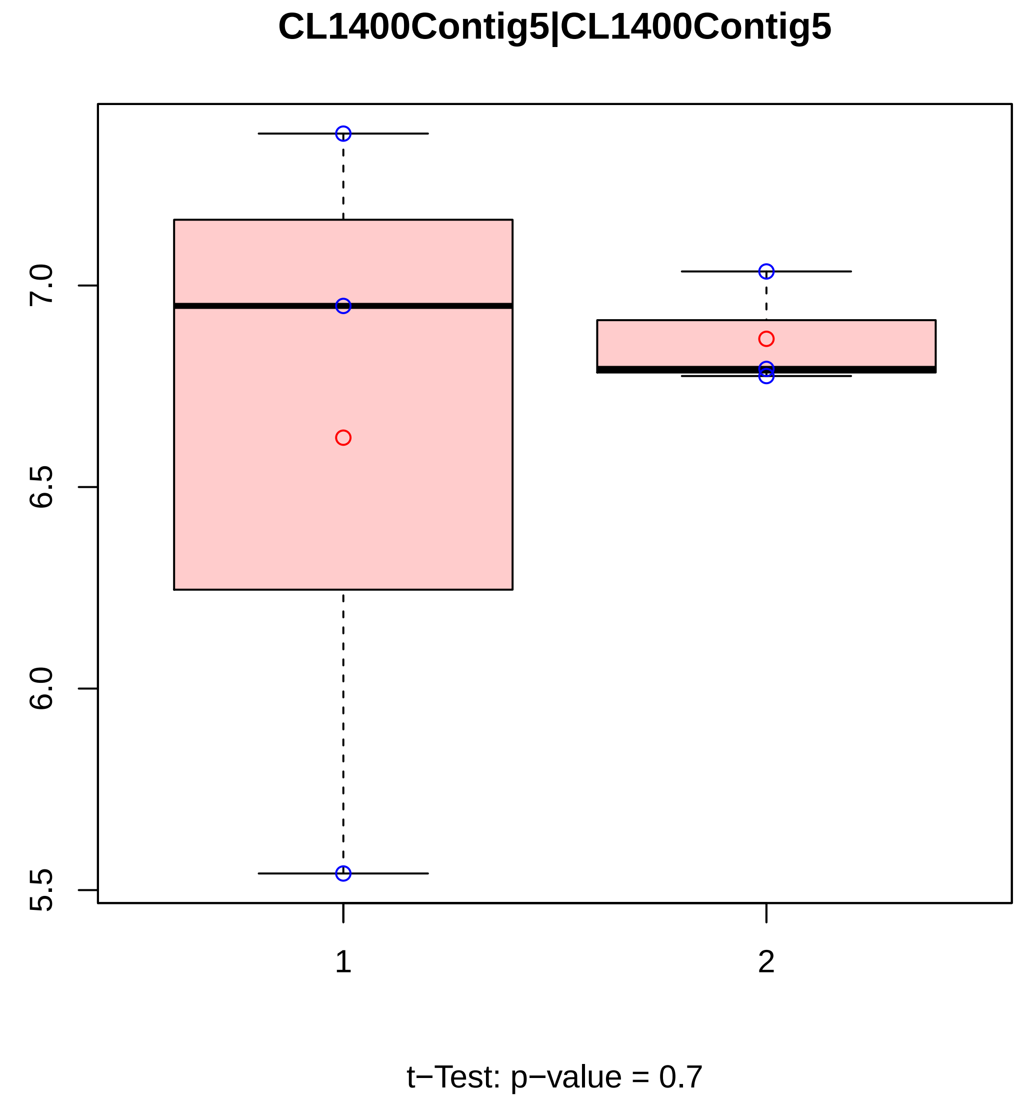
CL1400Contig5|CL1400Contig5
7.0
6.5
6.0
5.5
1
2
t−Test: p−value = 0.7

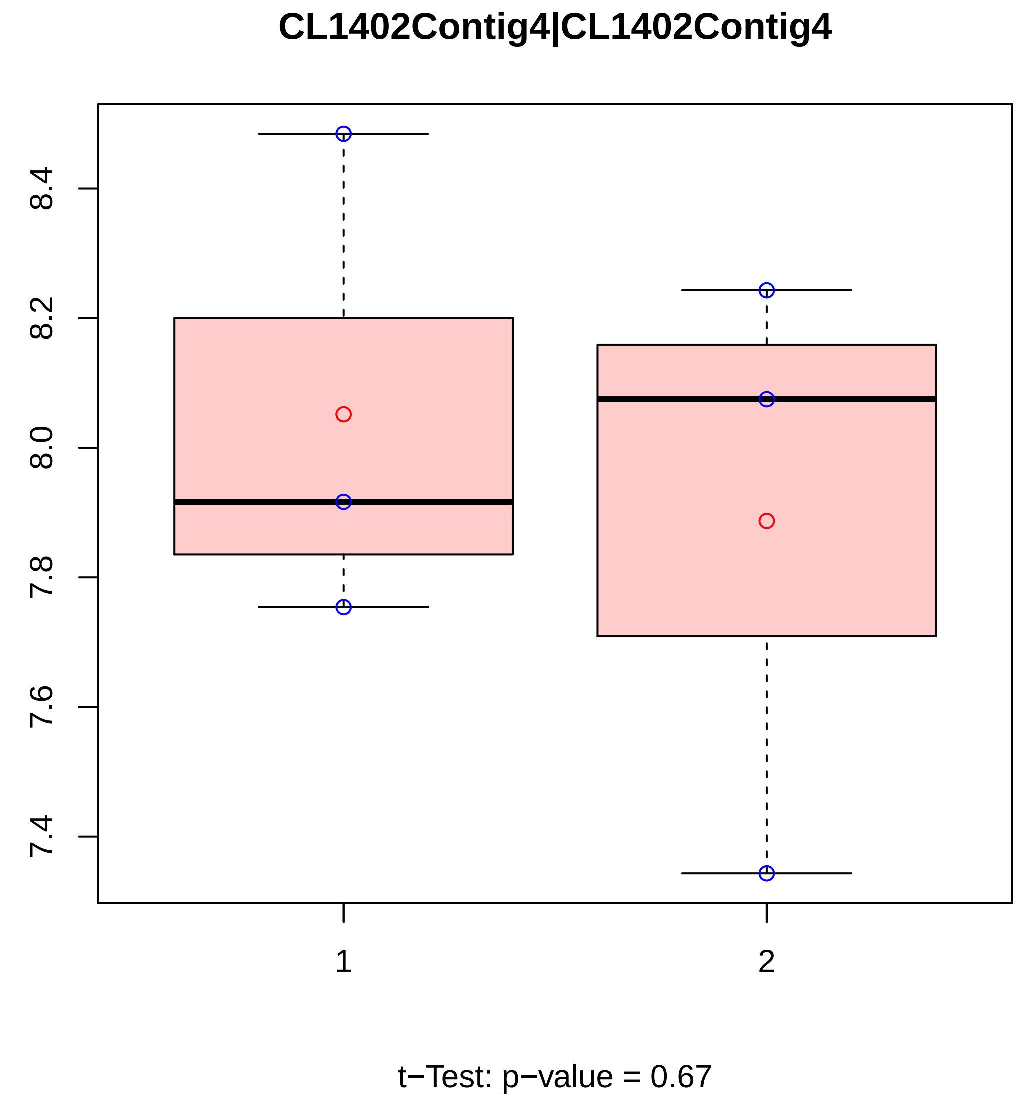
CL1402Contig4|CL1402Contig4
8.4
8.2
8.0
7.8
7.6
7.4
1
2
t−Test: p−value = 0.67

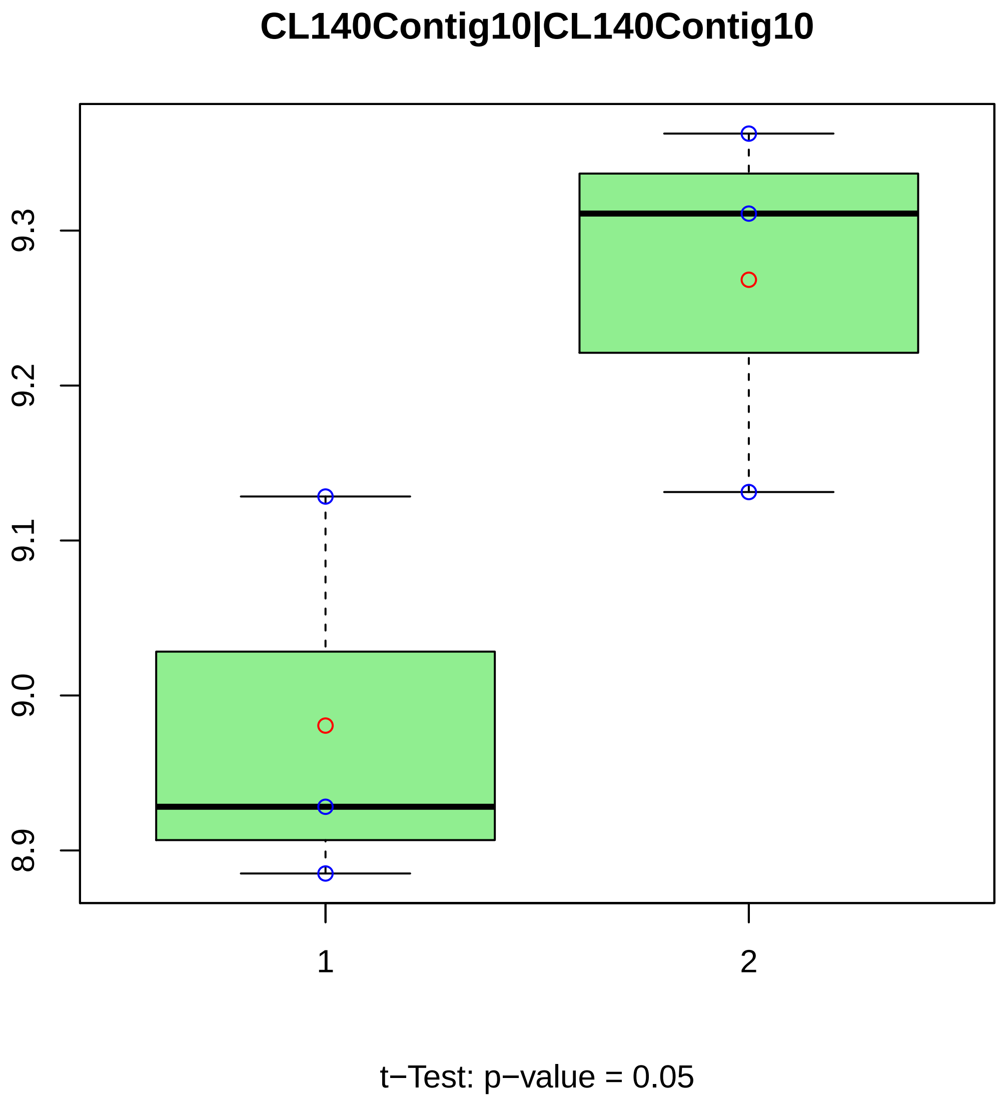
CL140Contig10|CL140Contig10
9.3
9.2
9.1
9.0
8.9
1
2
t−Test: p−value = 0.05

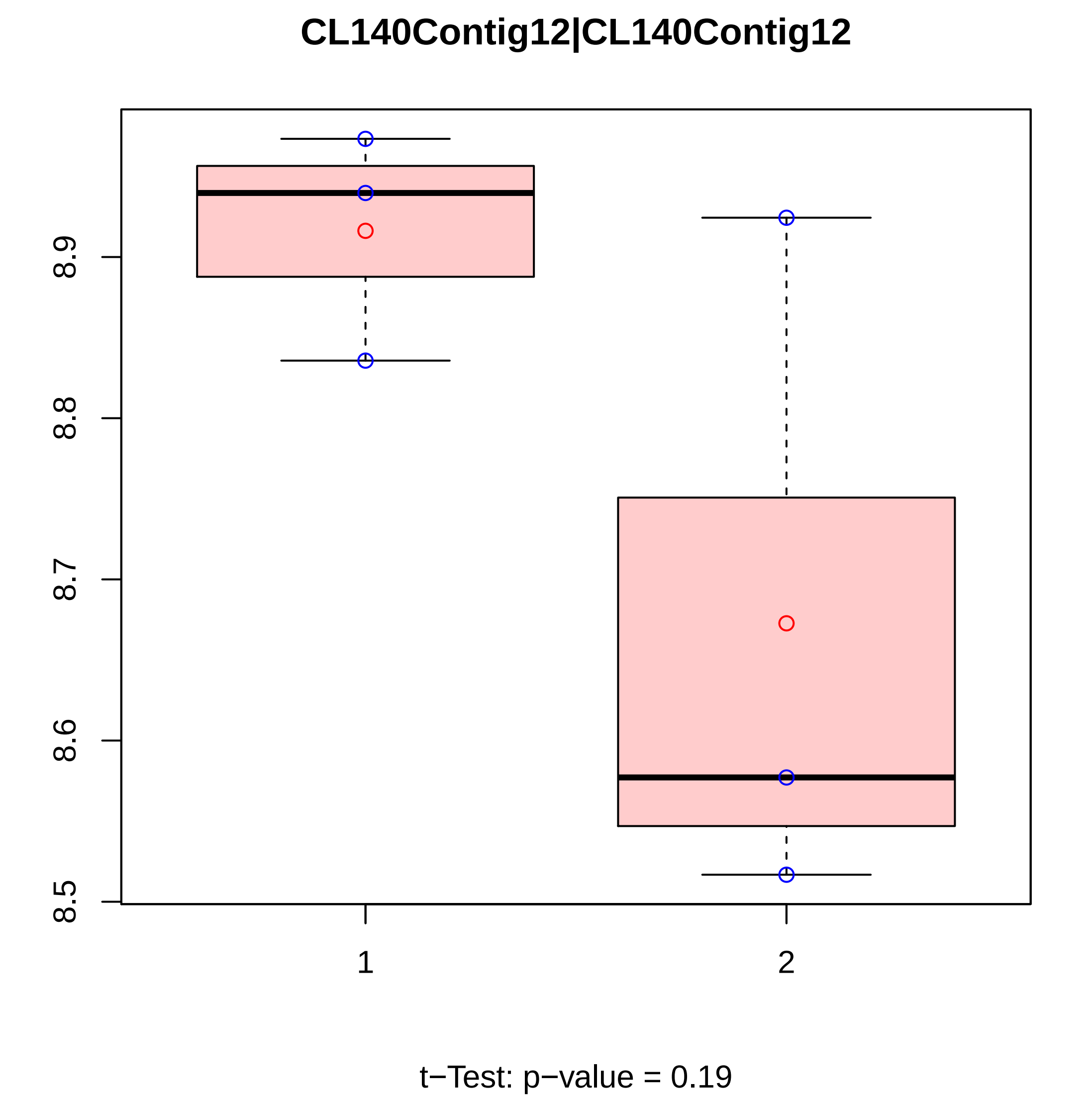
CL140Contig12|CL140Contig12
8.5
8.6
8.7
8.8
8.9
1
2
t−Test: p−value = 0.19

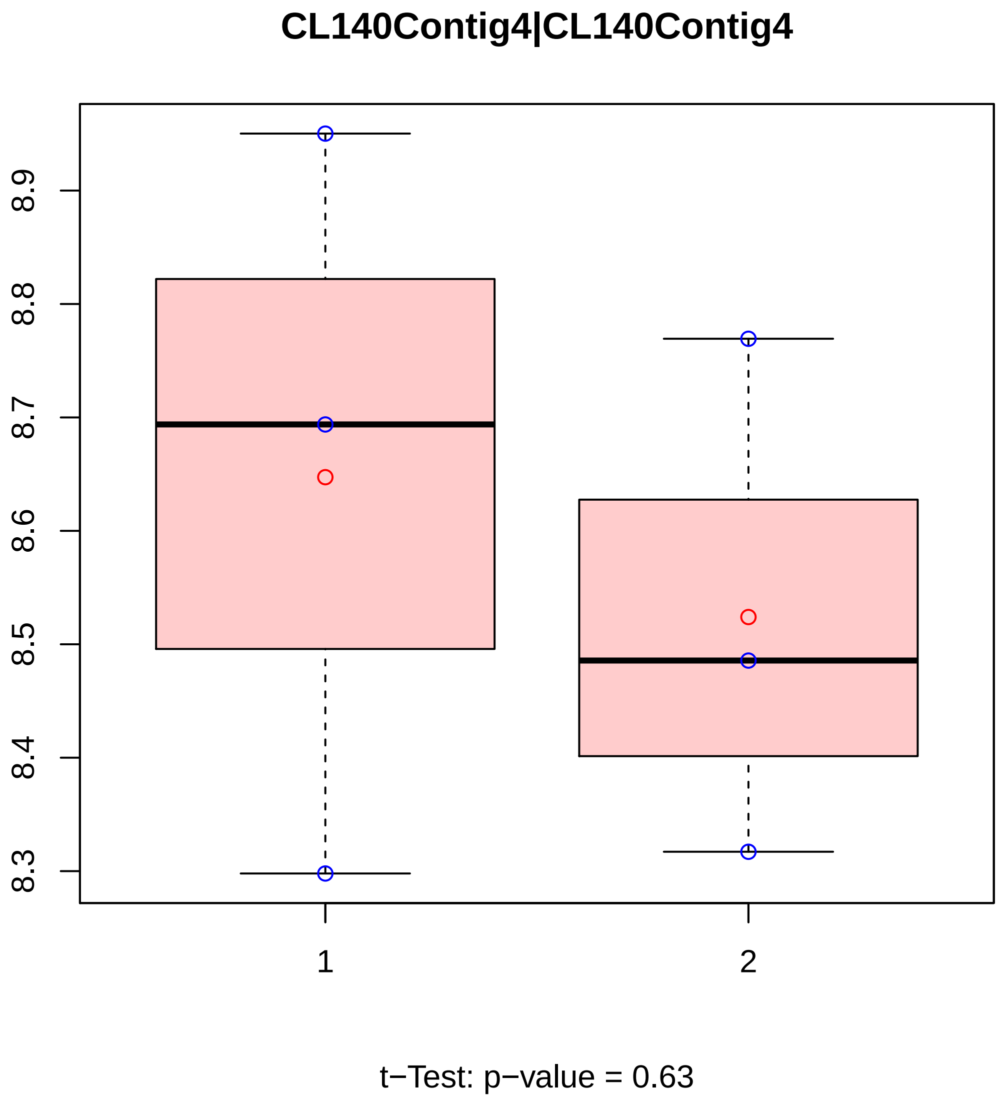
CL140Contig4|CL140Contig4
8.3
8.4
8.5
8.6
8.7
8.8
8.9
1
2
t−Test: p−value = 0.63

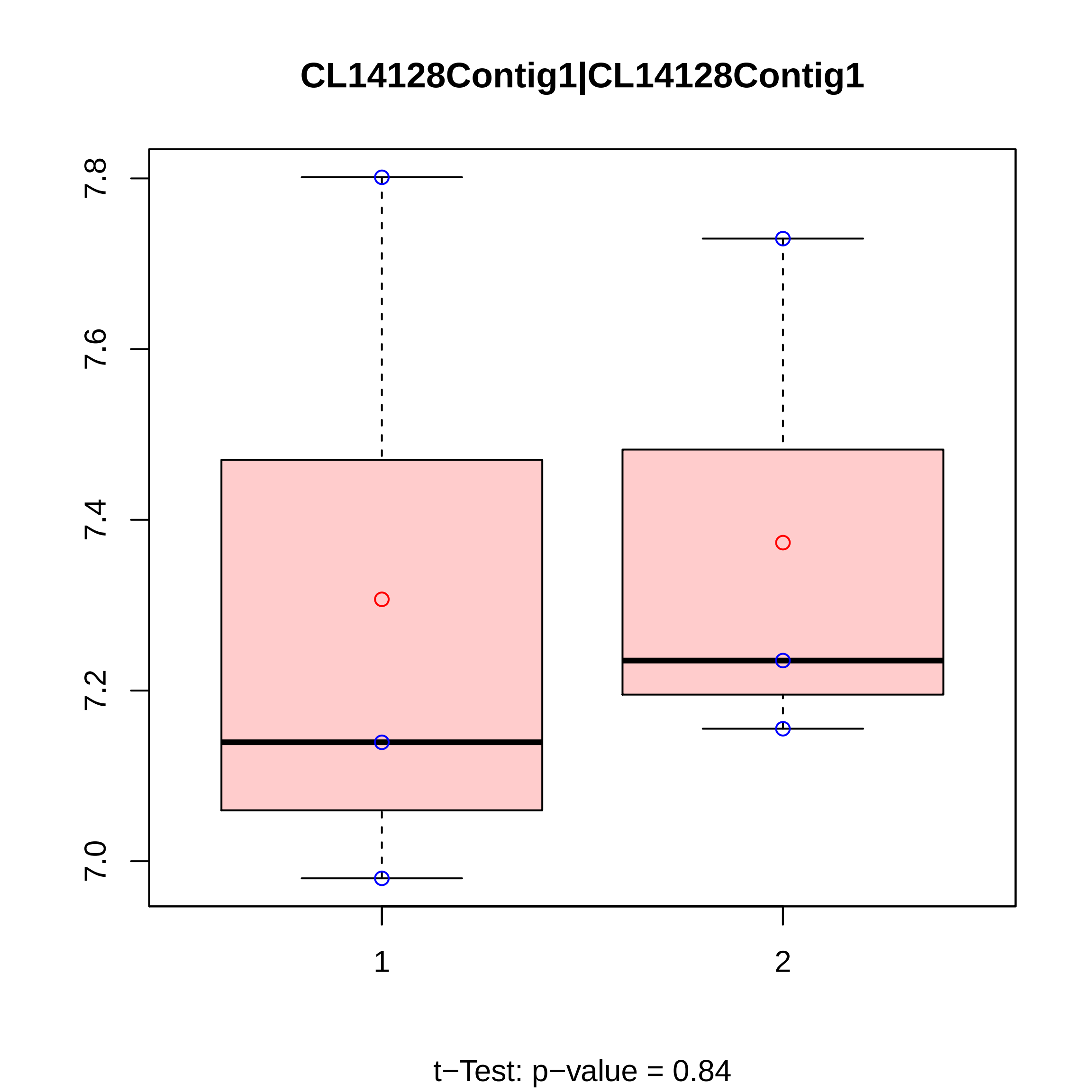
CL14128Contig1|CL14128Contig1
7.8
7.6
7.4
7.2
7.0
1
2
t−Test: p−value = 0.84

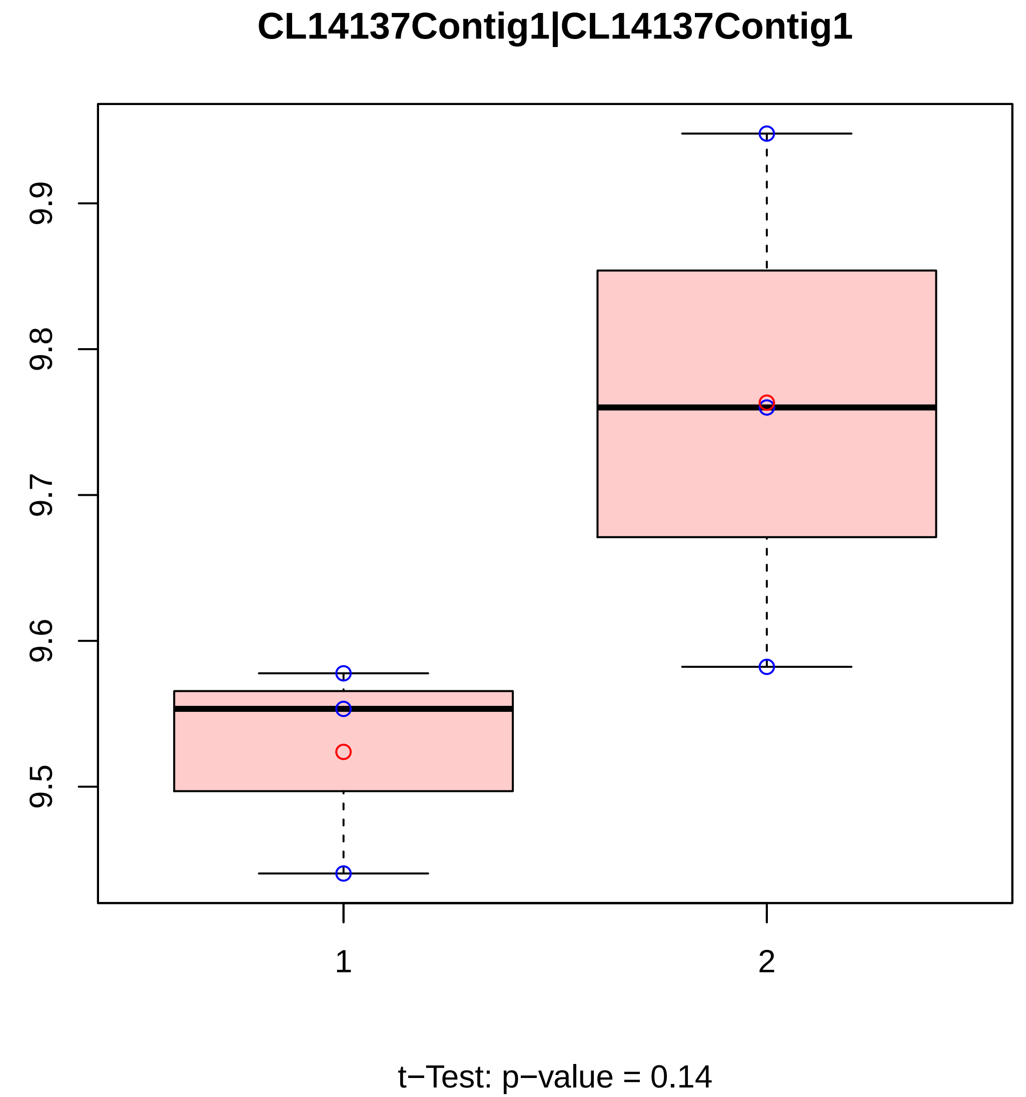
CL14137Contig1|CL14137Contig1
9.5
9.6
9.7
9.8
9.9
1
2
t−Test: p−value = 0.14

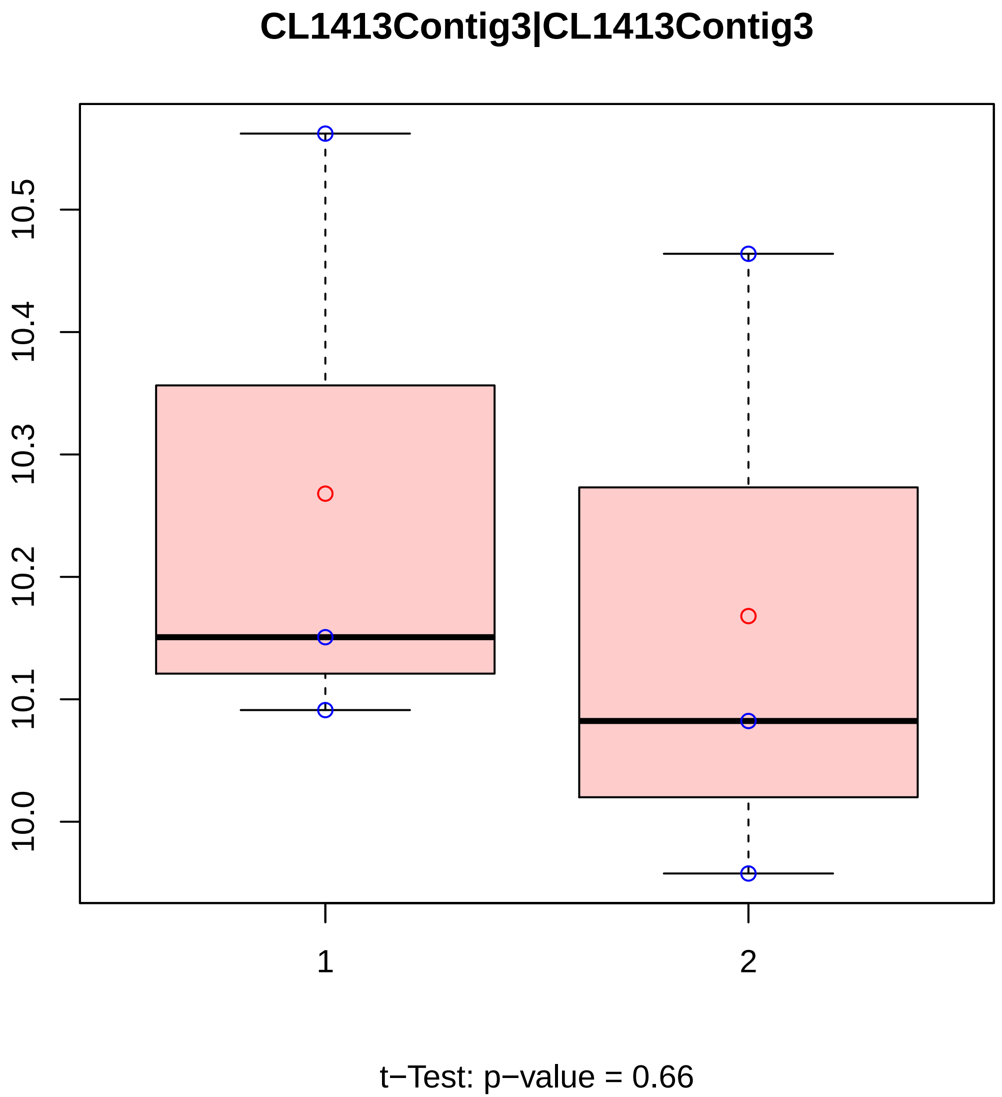

CL1413Contig3|CL1413Contig3
10.0
10.1
10.2
10.3
10.4
10.5
1
2
t−Test: p−value = 0.66

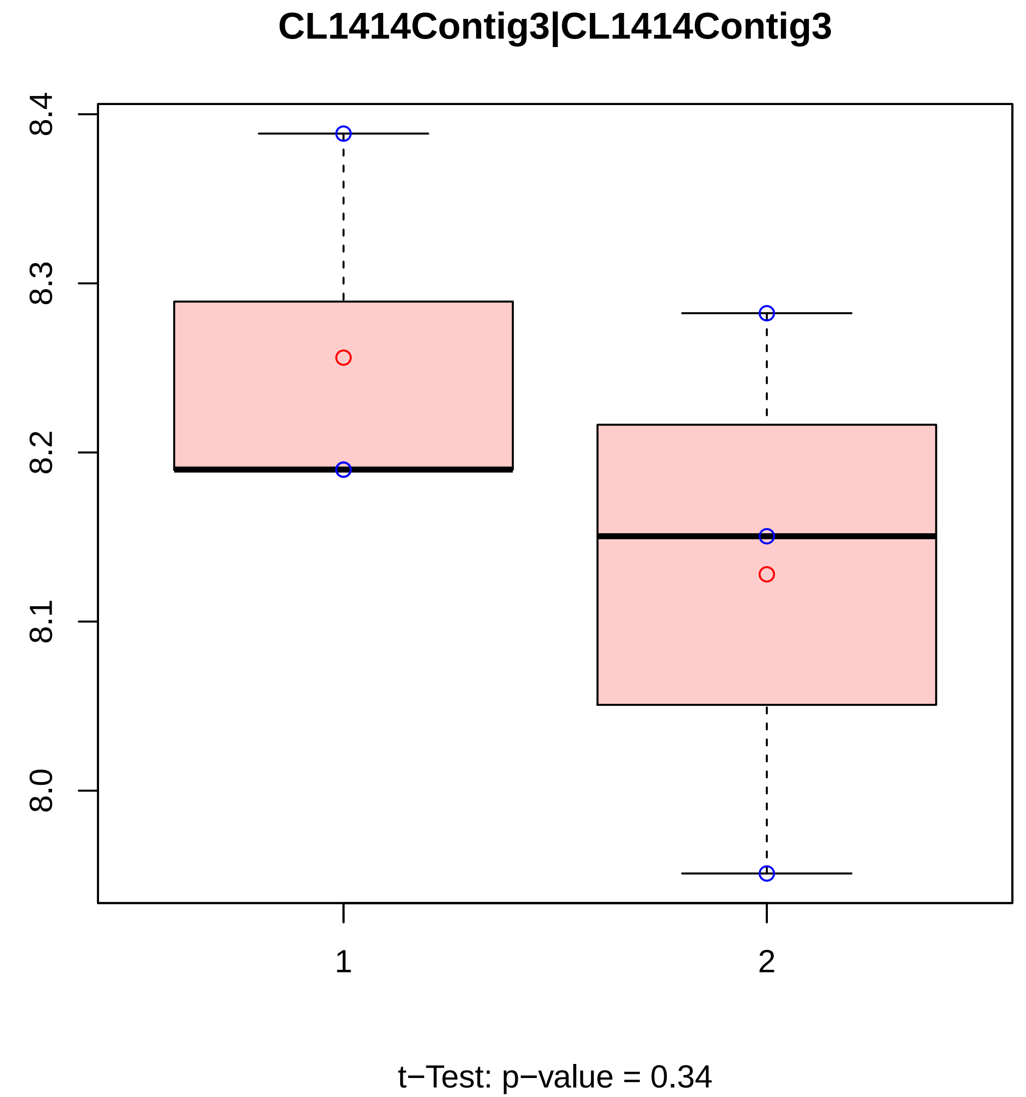
CL1414Contig3|CL1414Contig3
8.4
8.3
8.2
8.1
8.0
1
2
t−Test: p−value = 0.34

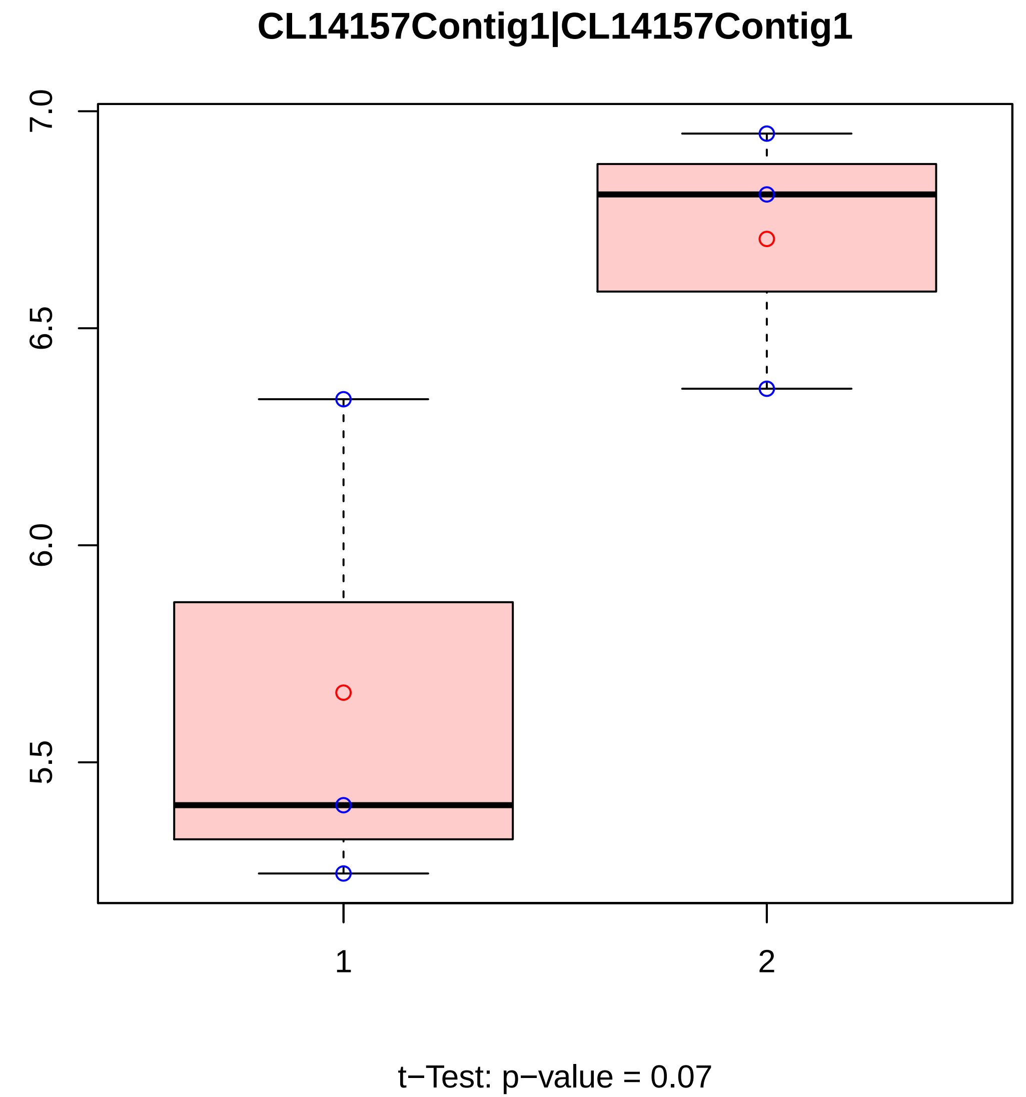
CL14157Contig1|CL14157Contig1
7.0
6.5
6.0
5.5
1
2
t−Test: p−value = 0.07

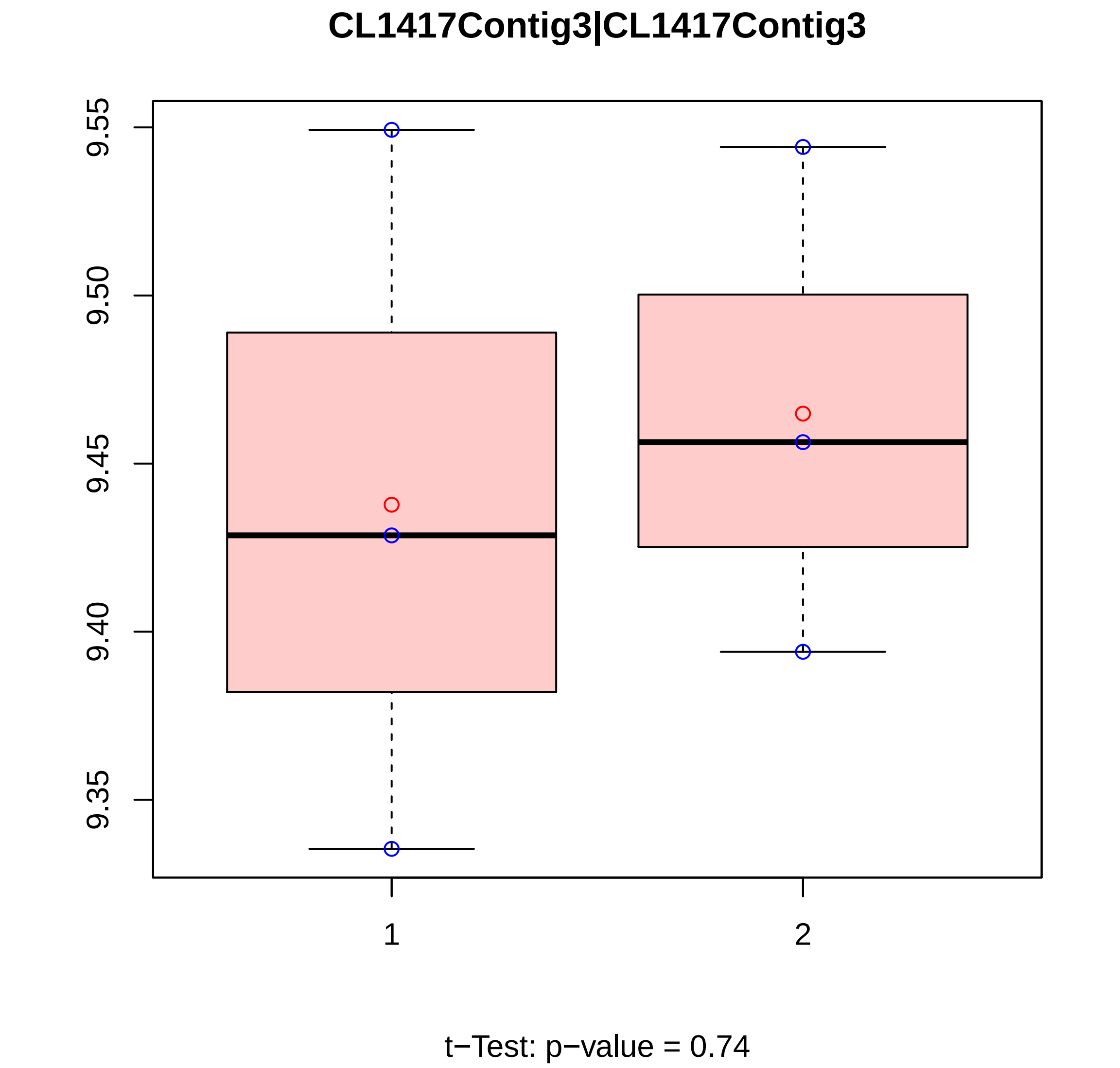
CL1417Contig3|CL1417Contig3
9.35
9.40
9.45
9.50
9.55
1
2
t−Test: p−value = 0.74

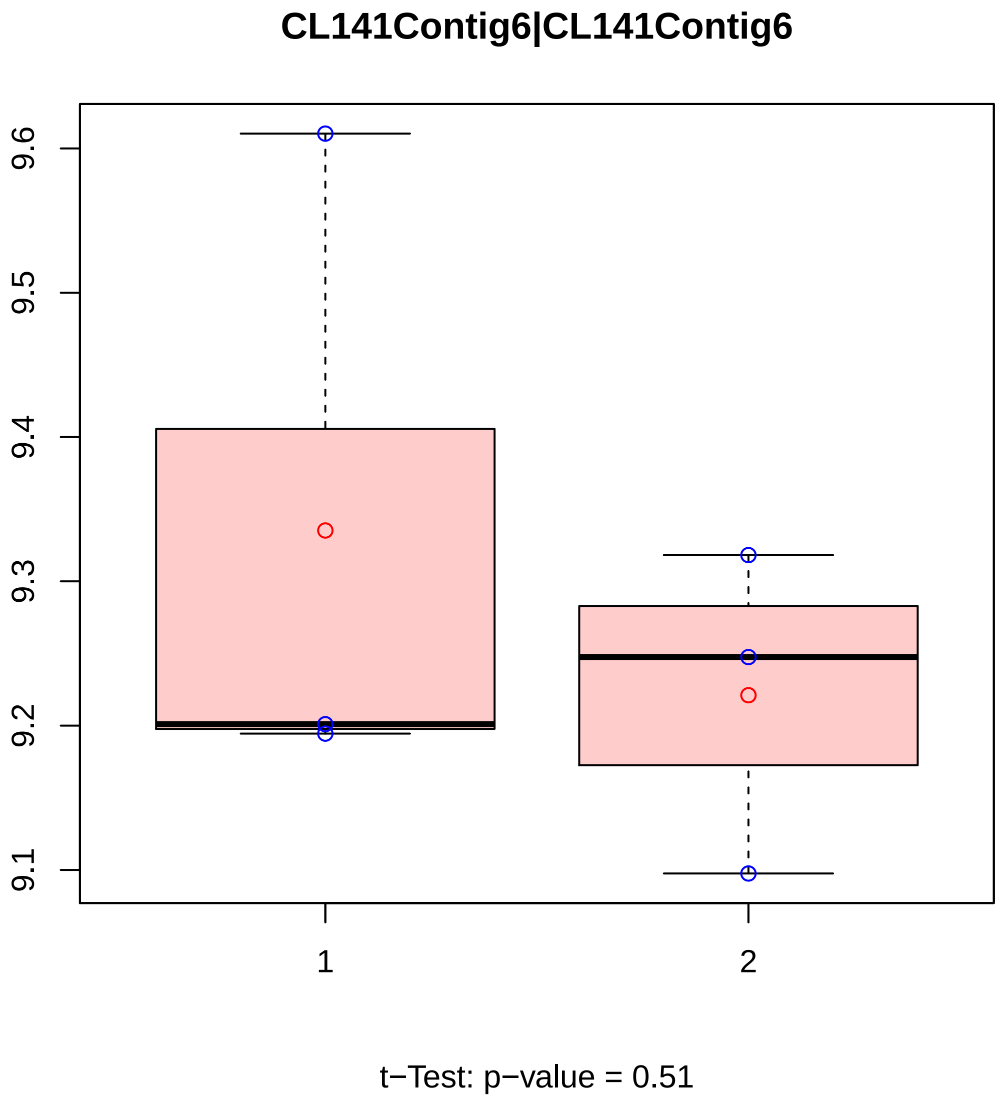
CL141Contig6|CL141Contig6
9.1
9.2
9.3
9.4
9.5
9.6
1
2
t−Test: p−value = 0.51

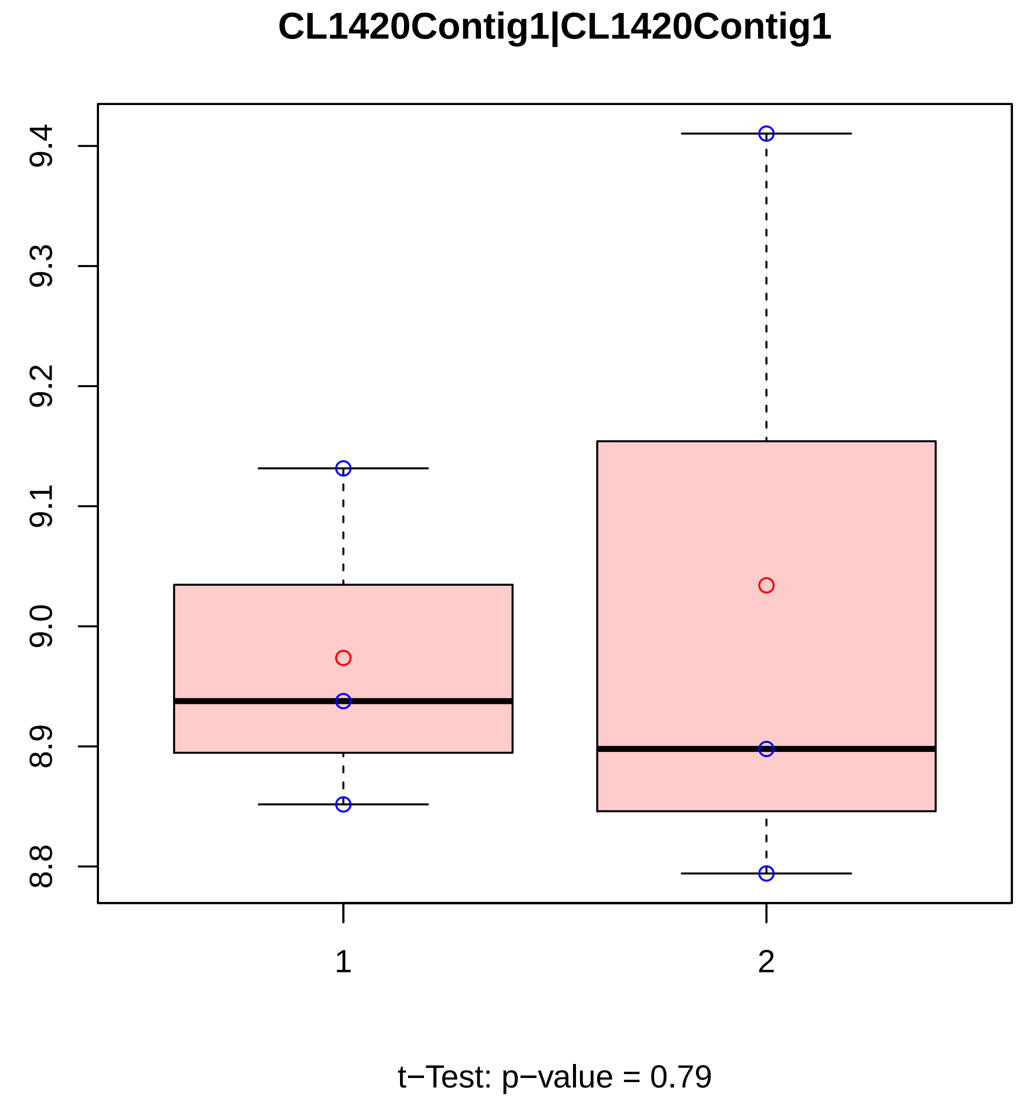
CL1420Contig1|CL1420Contig1
9.4
9.3
9.2
9.1
9.0
8.9
8.8
1
2
t−Test: p−value = 0.79

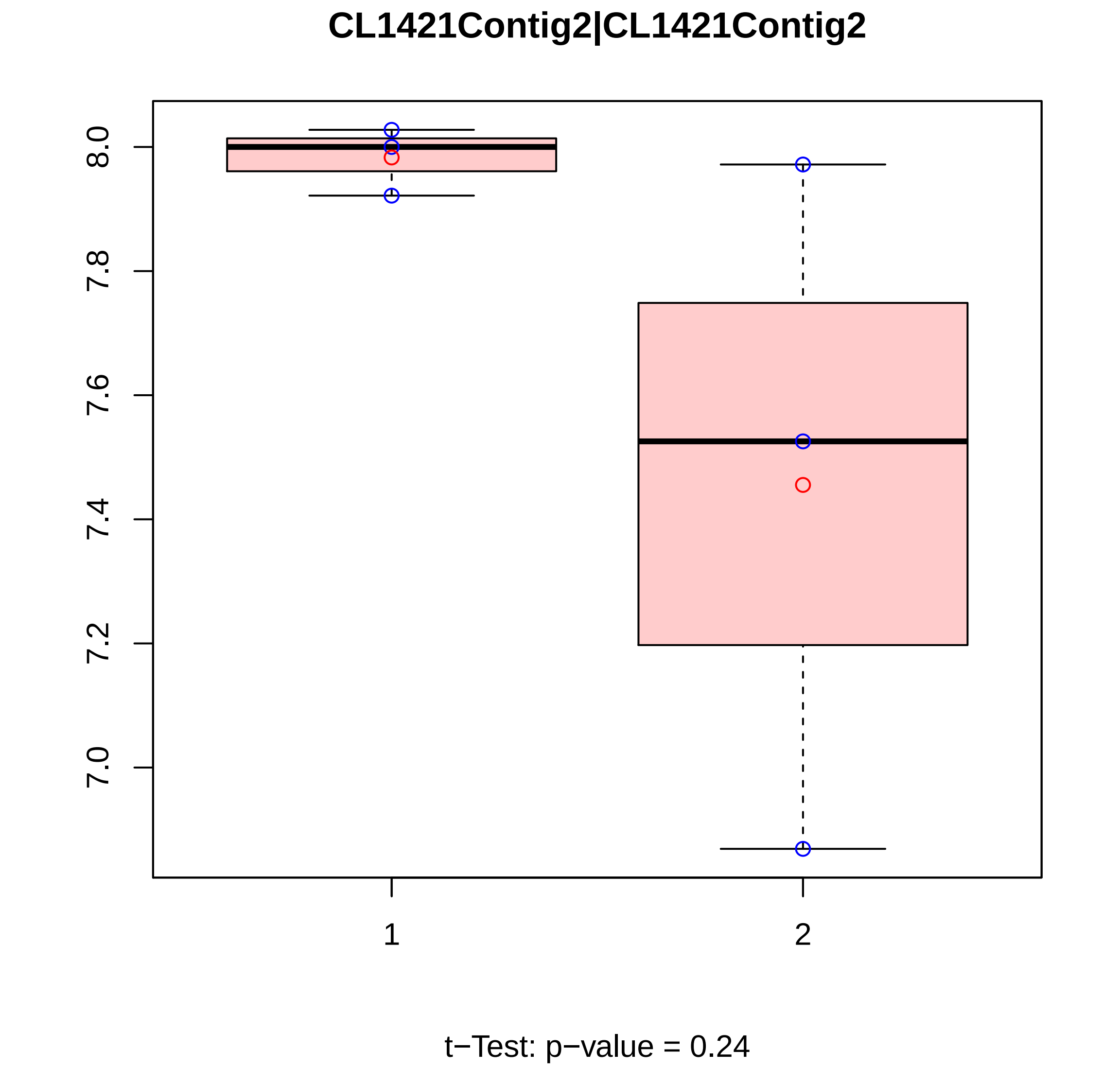
CL1421Contig2|CL1421Contig2
8.0
7.8
7.6
7.4
7.2
7.0
1
2
t−Test: p−value = 0.24

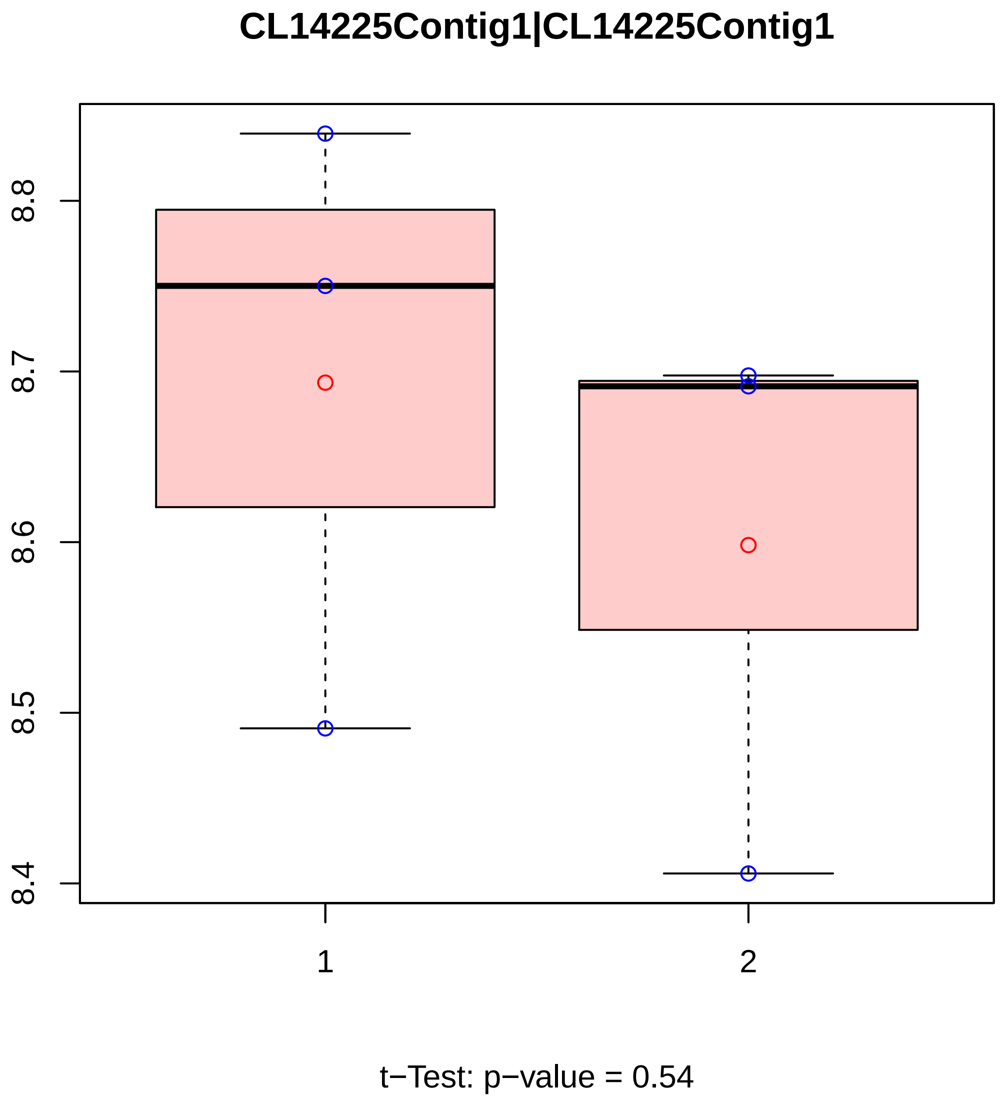
CL14225Contig1|CL14225Contig1
8.4
8.5
8.6
8.7
8.8
1
2
t−Test: p−value = 0.54

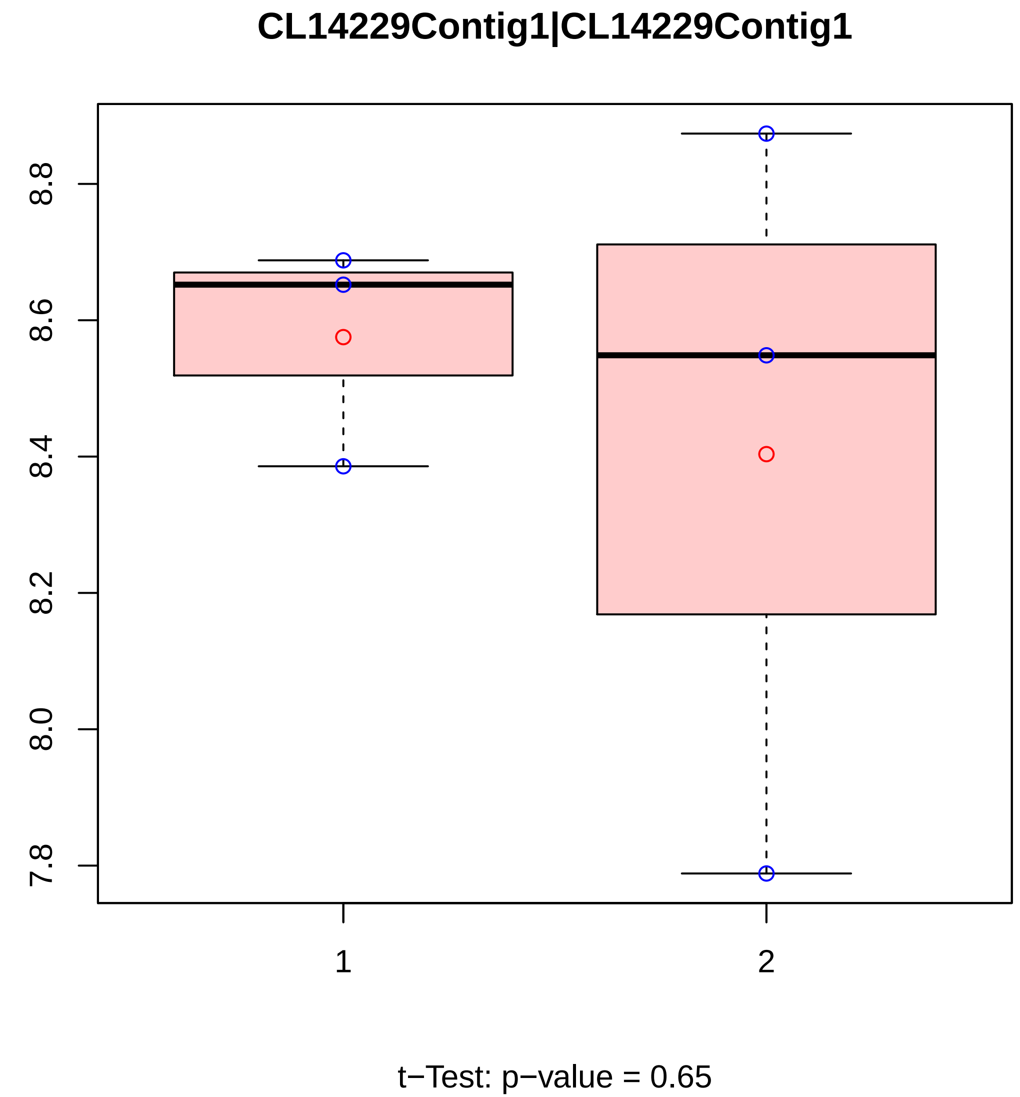

CL14229Contig1|CL14229Contig1
8.8
8.6
8.4
8.2
8.0
7.8
1
2
t−Test: p−value = 0.65

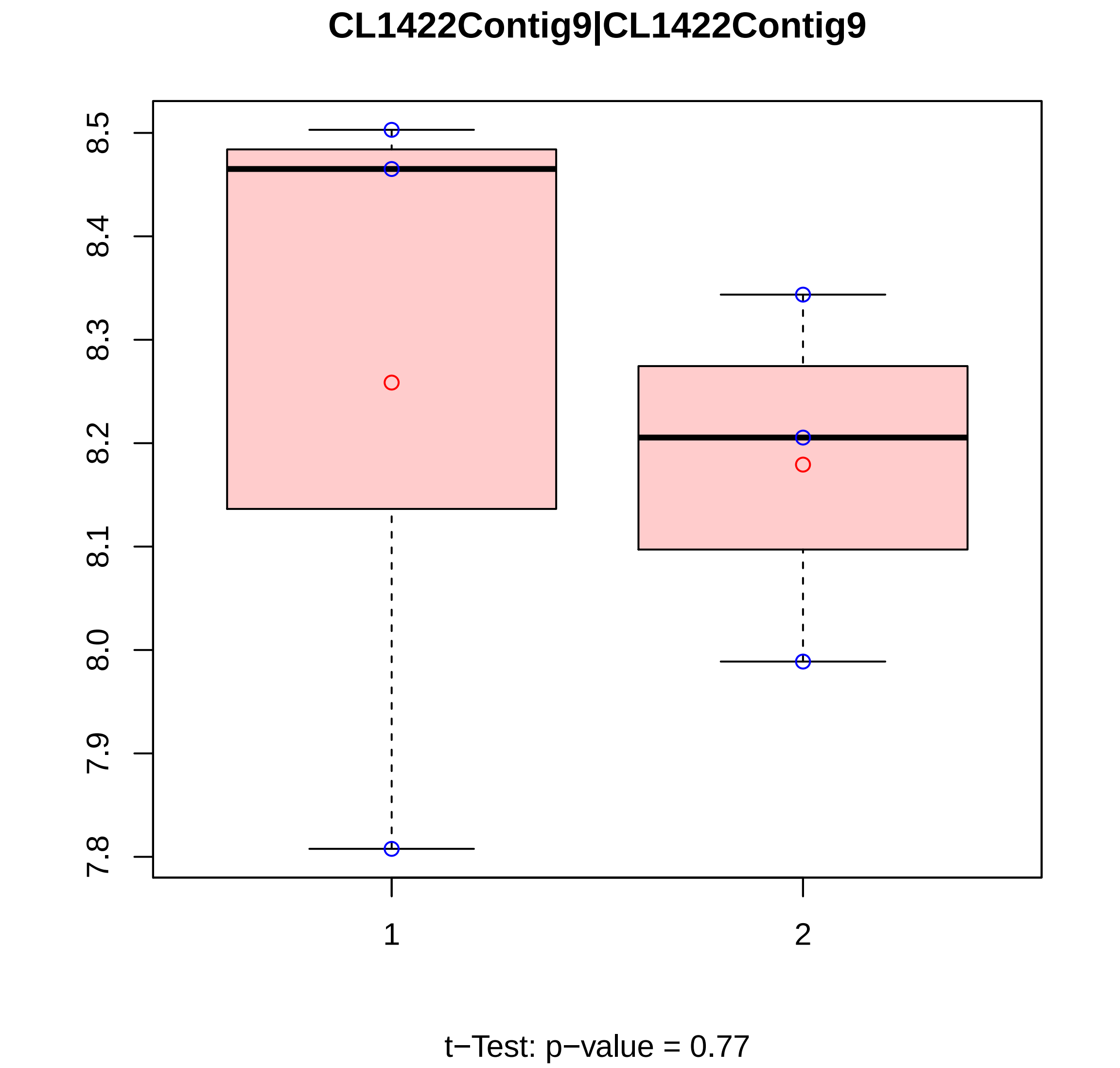
CL1422Contig9|CL1422Contig9
8.5
8.4
8.3
8.2
8.1
8.0
7.9
7.8
1
2
t−Test: p−value = 0.77

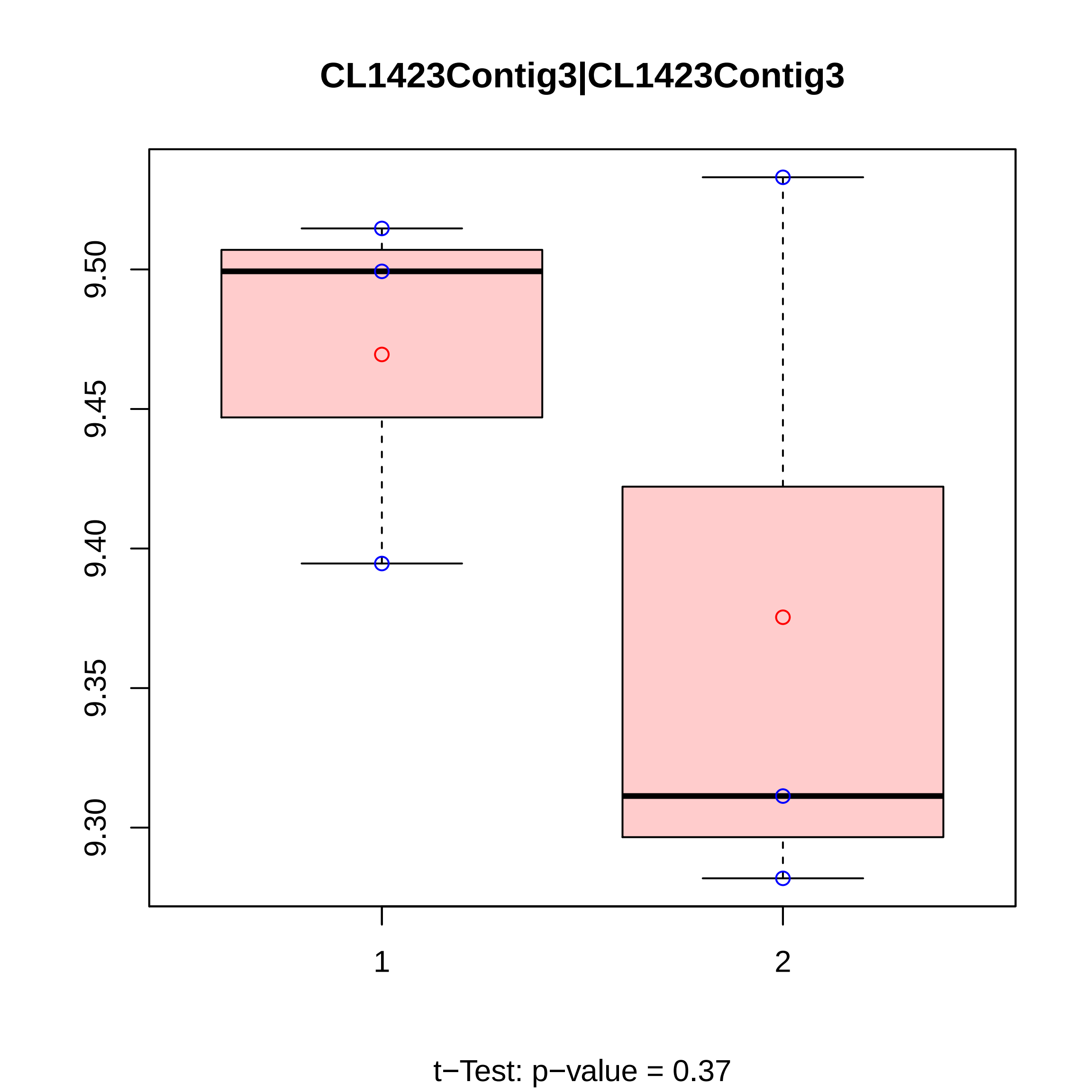
CL1423Contig3|CL1423Contig3
9.30
9.35
9.40
9.45
9.50
1
2
t−Test: p−value = 0.37

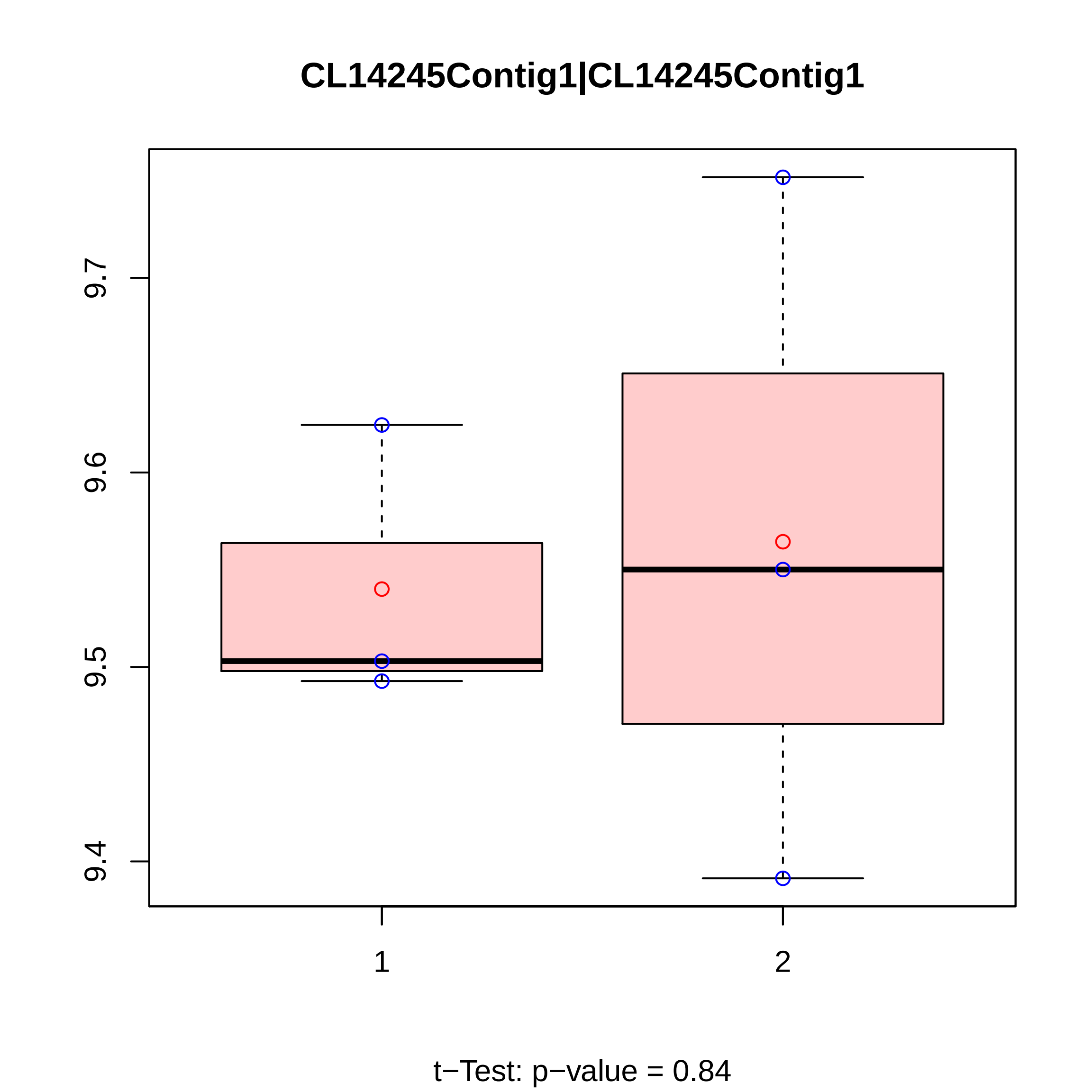
CL14245Contig1|CL14245Contig1
9.4
9.5
9.6
9.7
1
2
t−Test: p−value = 0.84

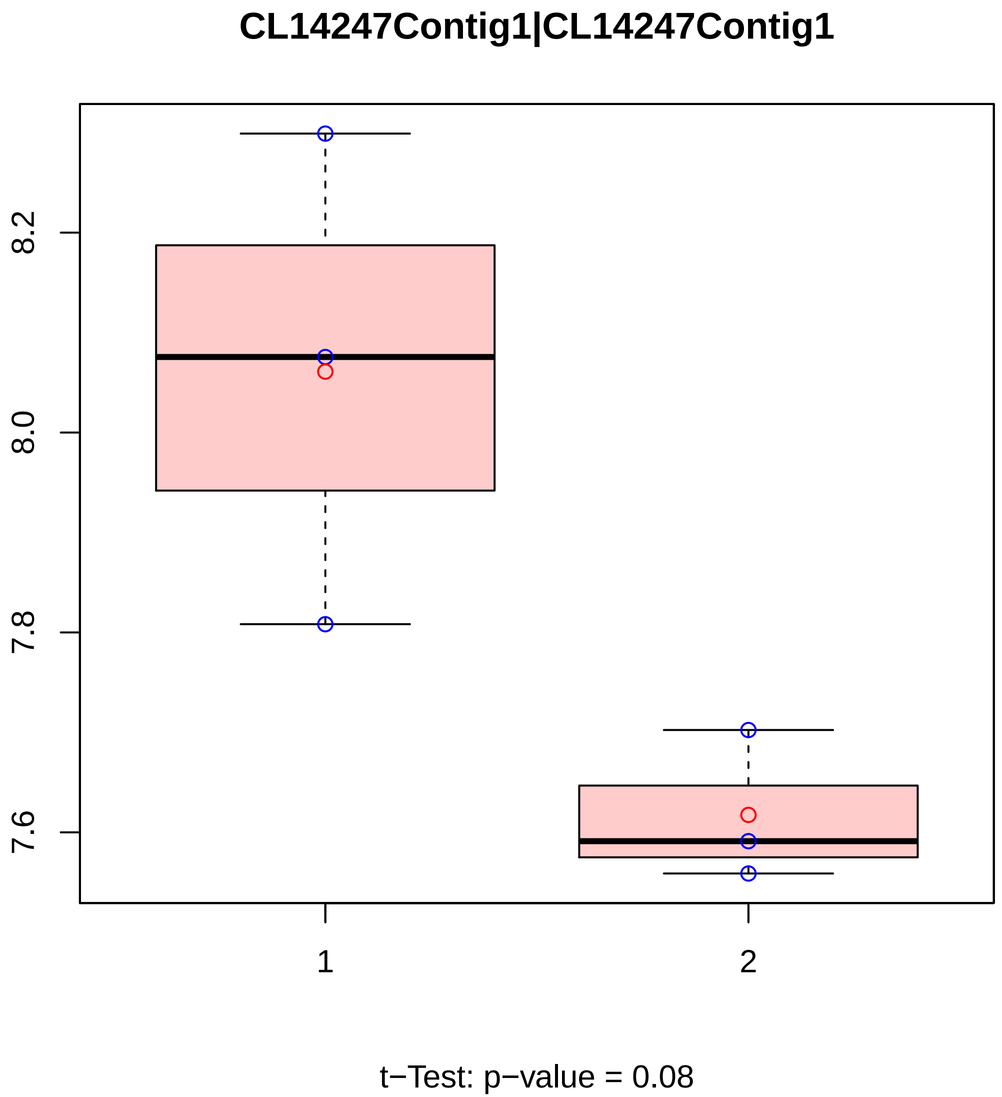
CL14247Contig1|CL14247Contig1
8.2
8.0
7.8
7.6
1
2
t−Test: p−value = 0.08

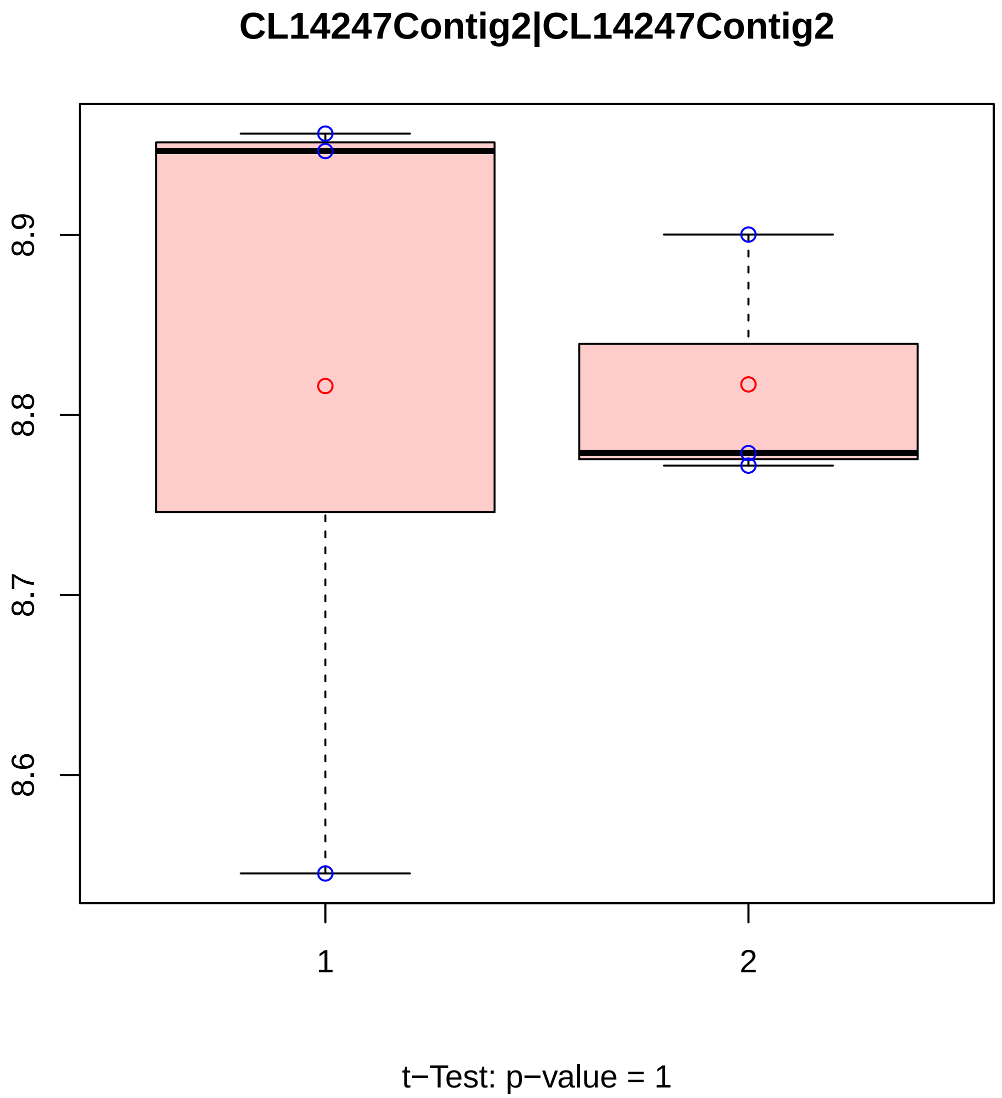
CL14247Contig2|CL14247Contig2
8.6
8.7
8.8
8.9
1
2
t−Test: p−value = 1

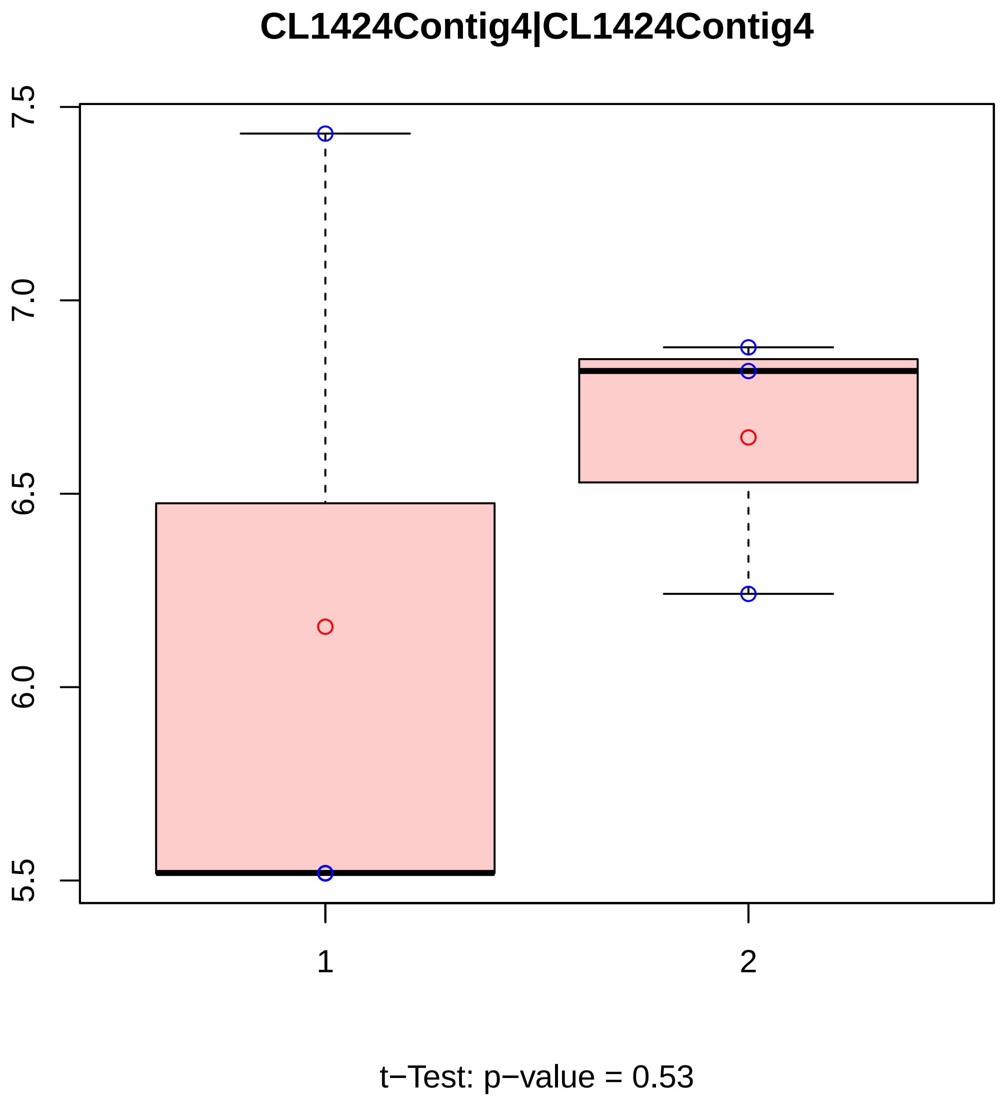
CL1424Contig4|CL1424Contig4
7.5
7.0
6.5
6.0
5.5
1
2
t−Test: p−value = 0.53

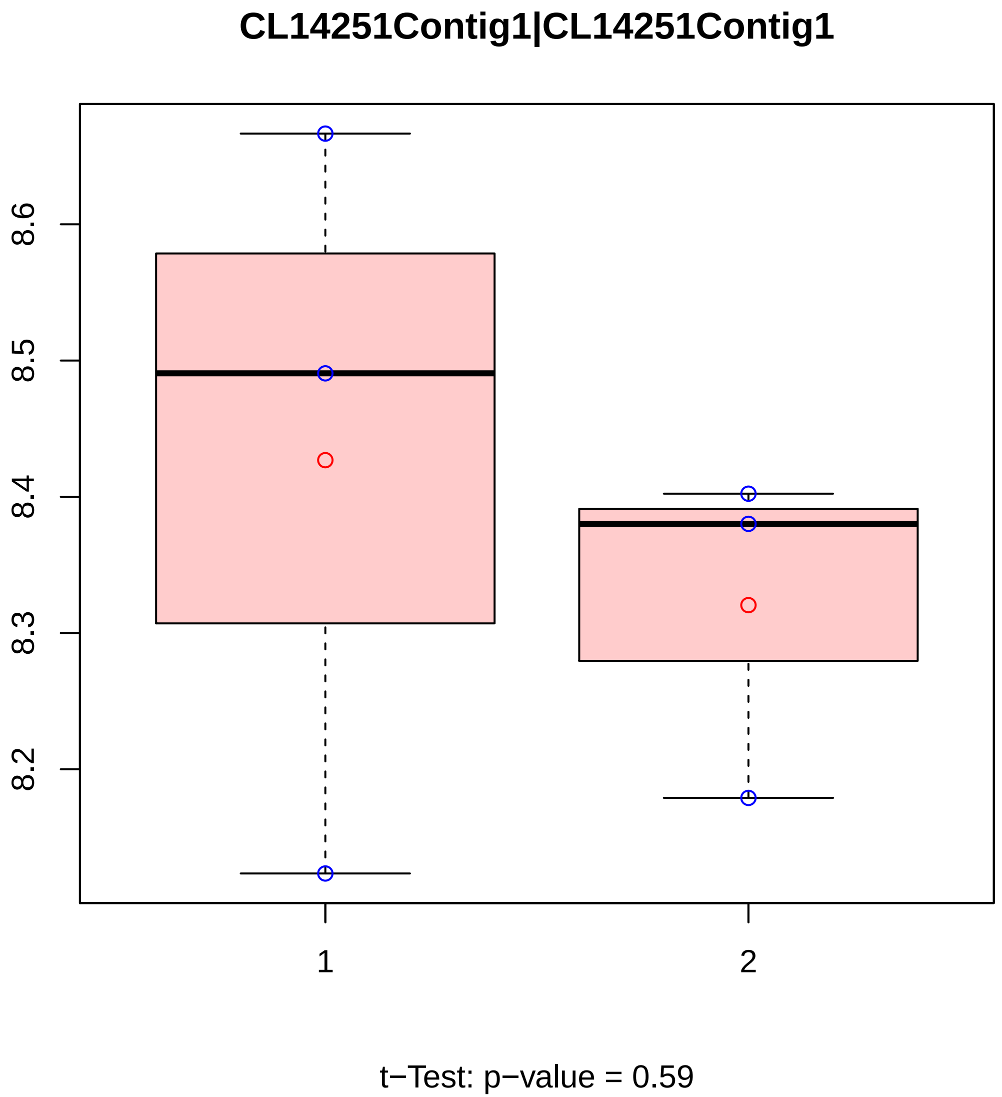
CL14251Contig1|CL14251Contig1
8.2
8.3
8.4
8.5
8.6
1
2
t−Test: p−value = 0.59

# CL14264Contig1|CL14264Contig1

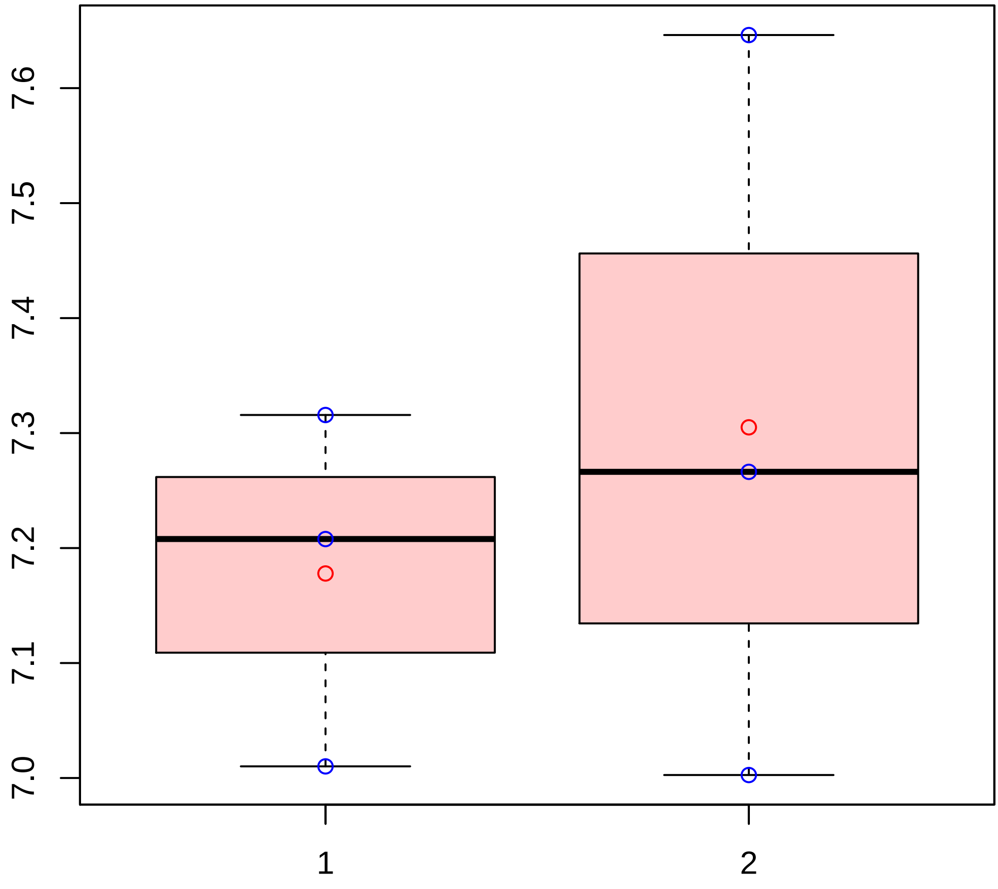


t−Test: p−value = 0.58

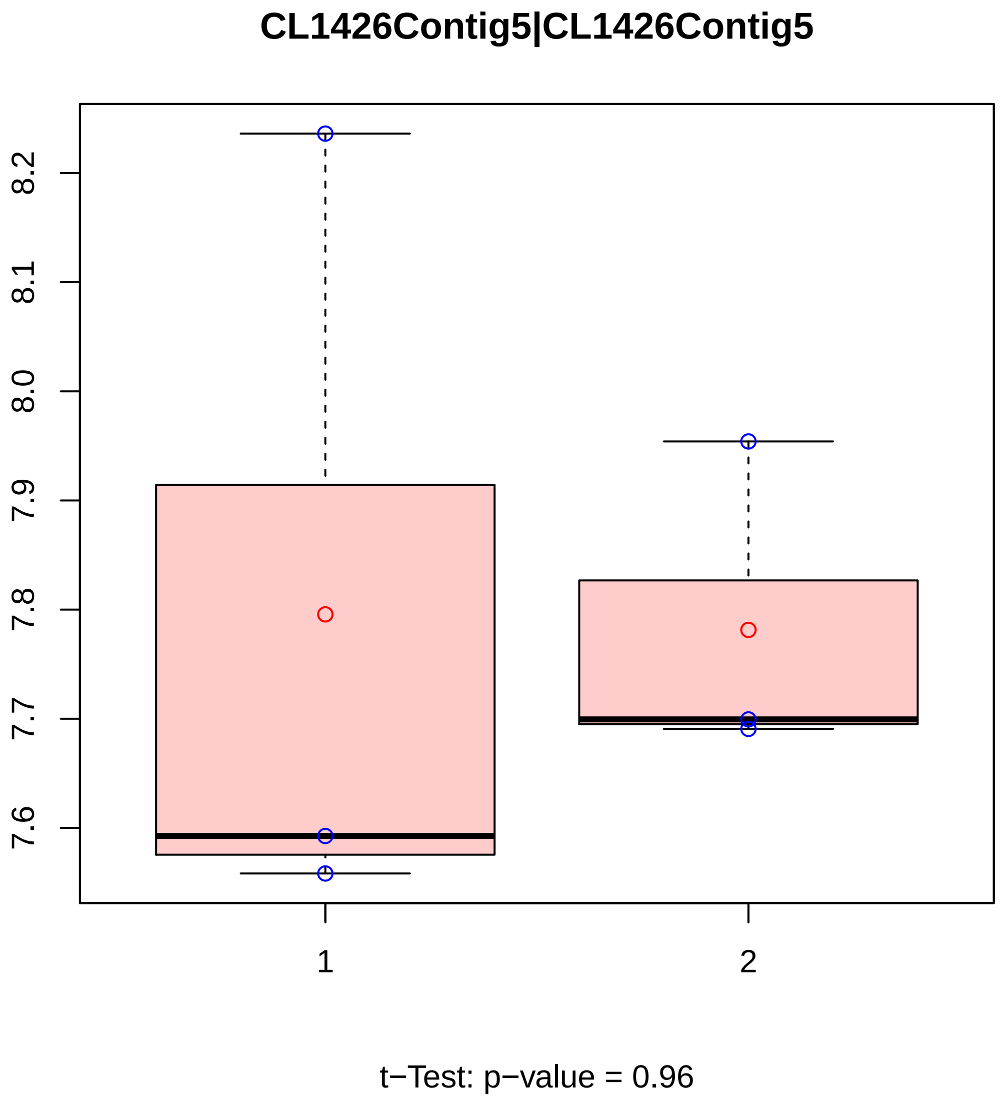
CL1426Contig5|CL1426Contig5
8.2
8.1
8.0
7.9
7.8
7.7
7.6
1
2
t−Test: p−value = 0.96

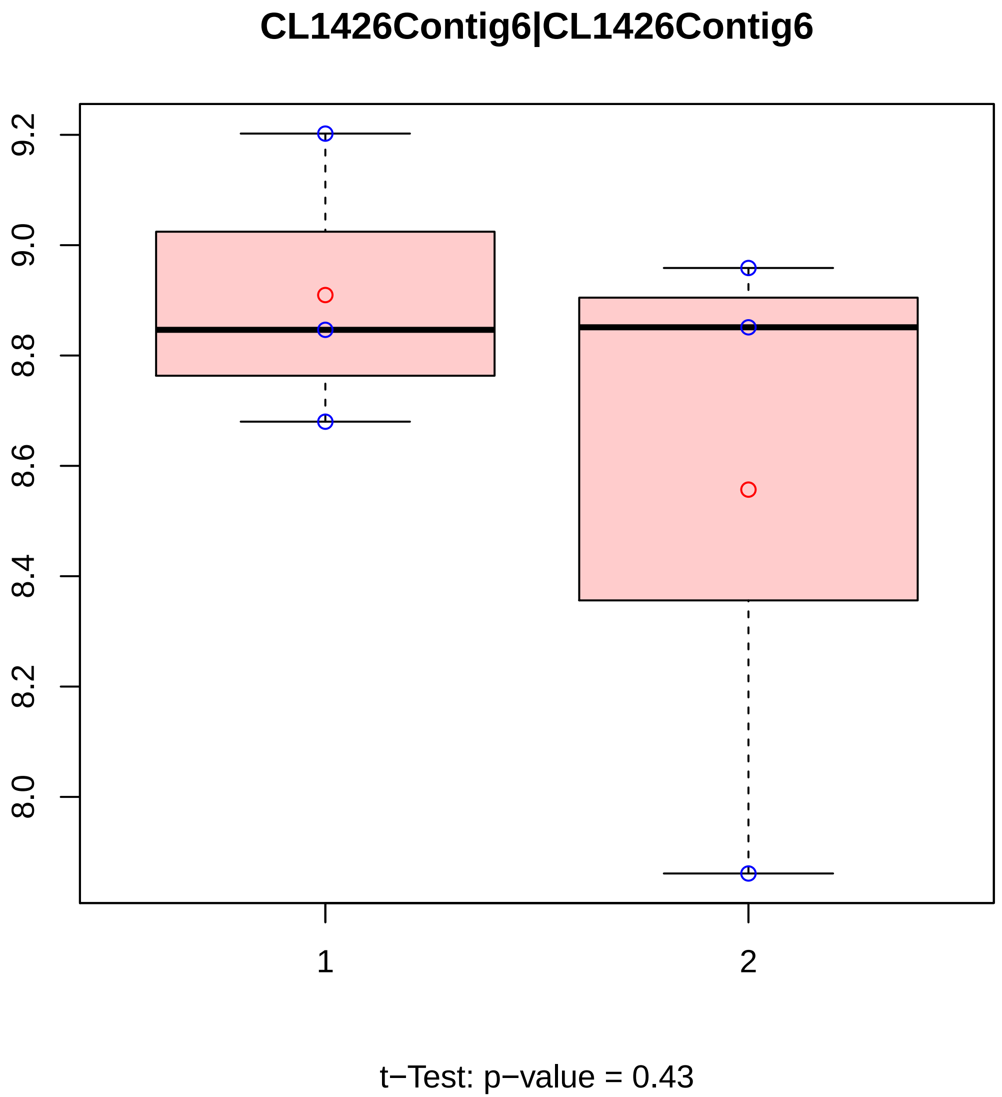
CL1426Contig6|CL1426Contig6
9.2
9.0
8.8
8.6
8.4
8.2
8.0
1
2
t−Test: p−value = 0.43

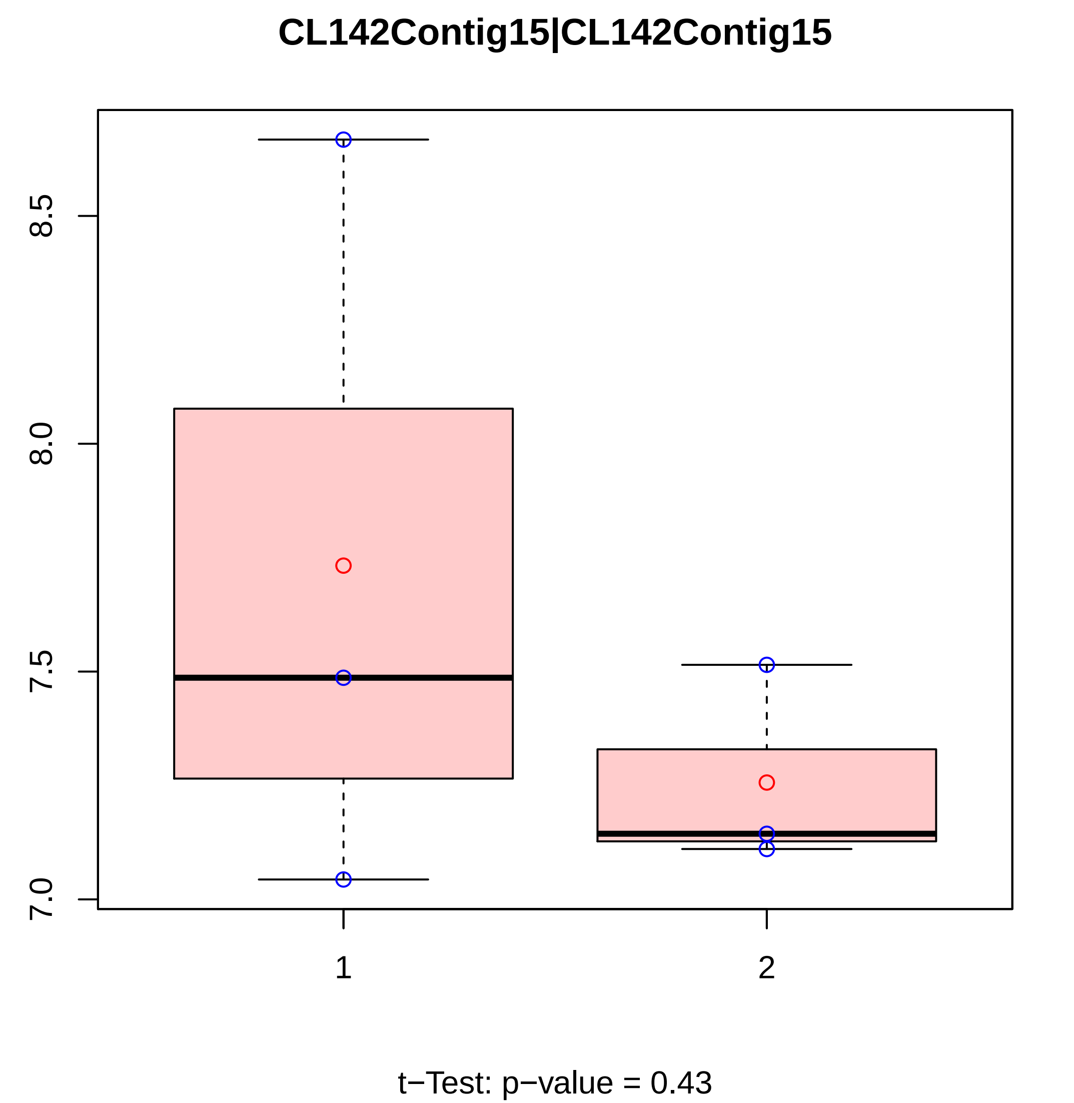
CL142Contig15|CL142Contig15
8.5
8.0
7.5
7.0
1
2
t−Test: p−value = 0.43

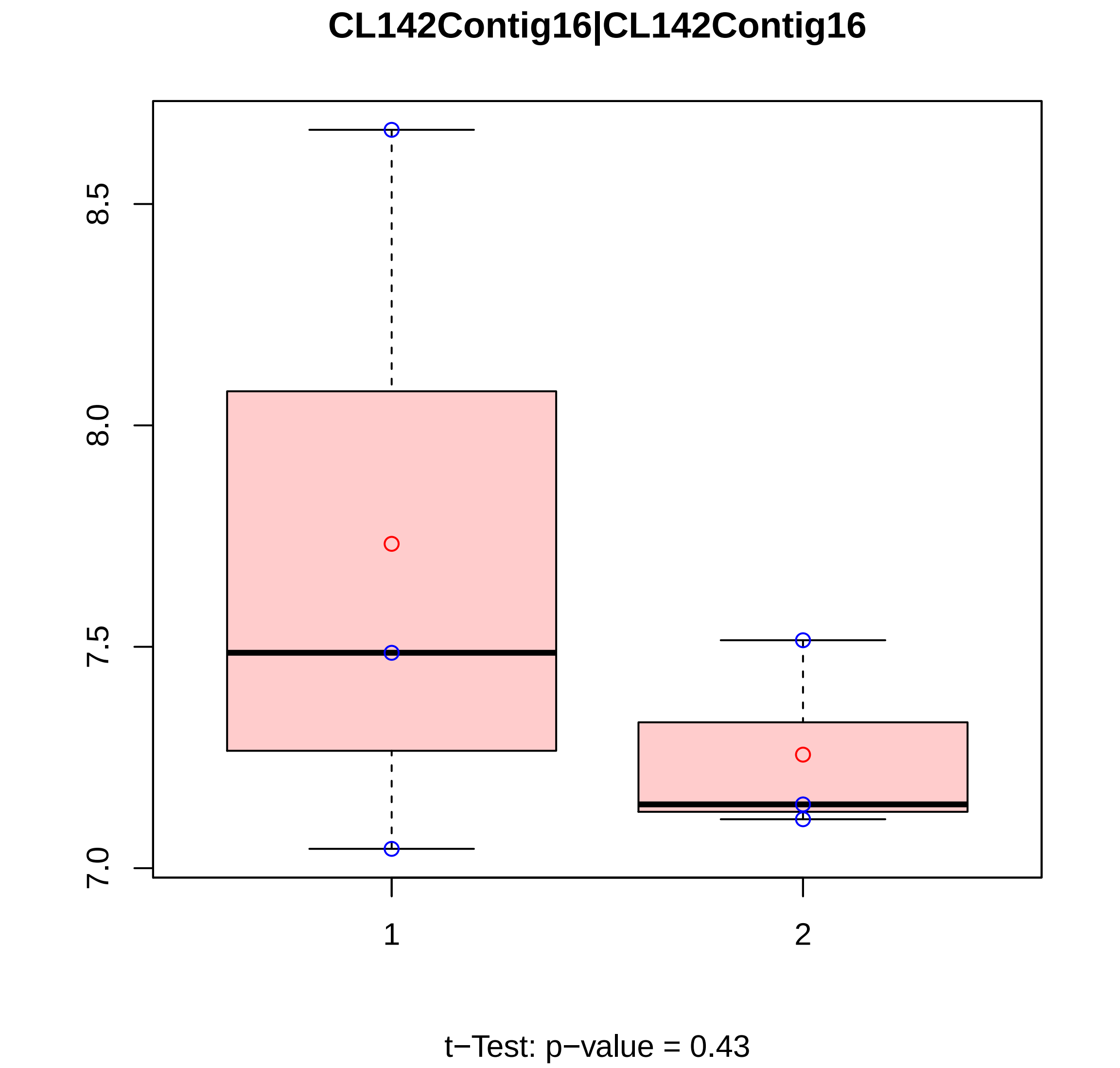

CL142Contig16|CL142Contig16
8.5
8.0
7.5
7.0
1
2
t−Test: p−value = 0.43

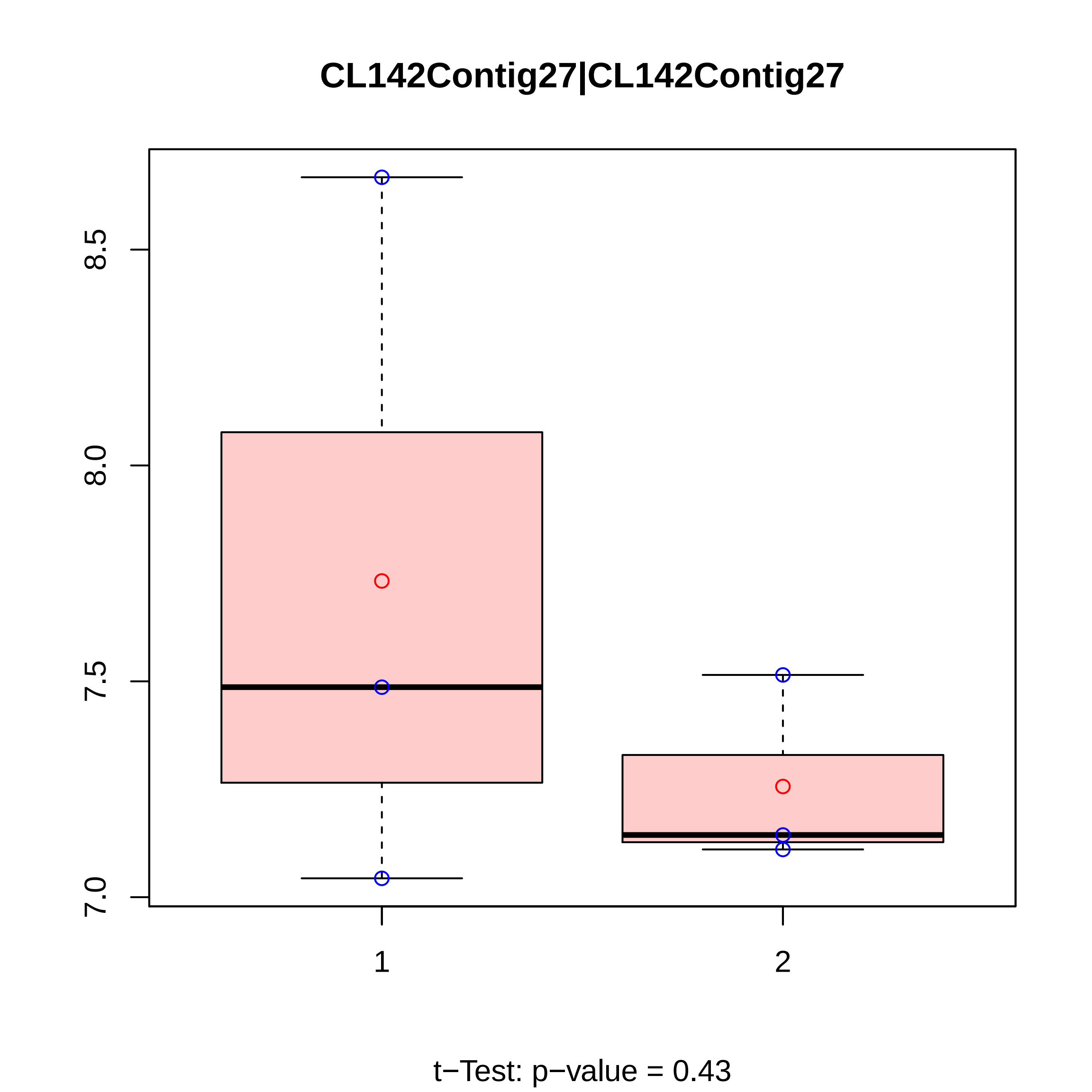
CL142Contig27|CL142Contig27
8.5
8.0
7.5
7.0
1
2
t−Test: p−value = 0.43

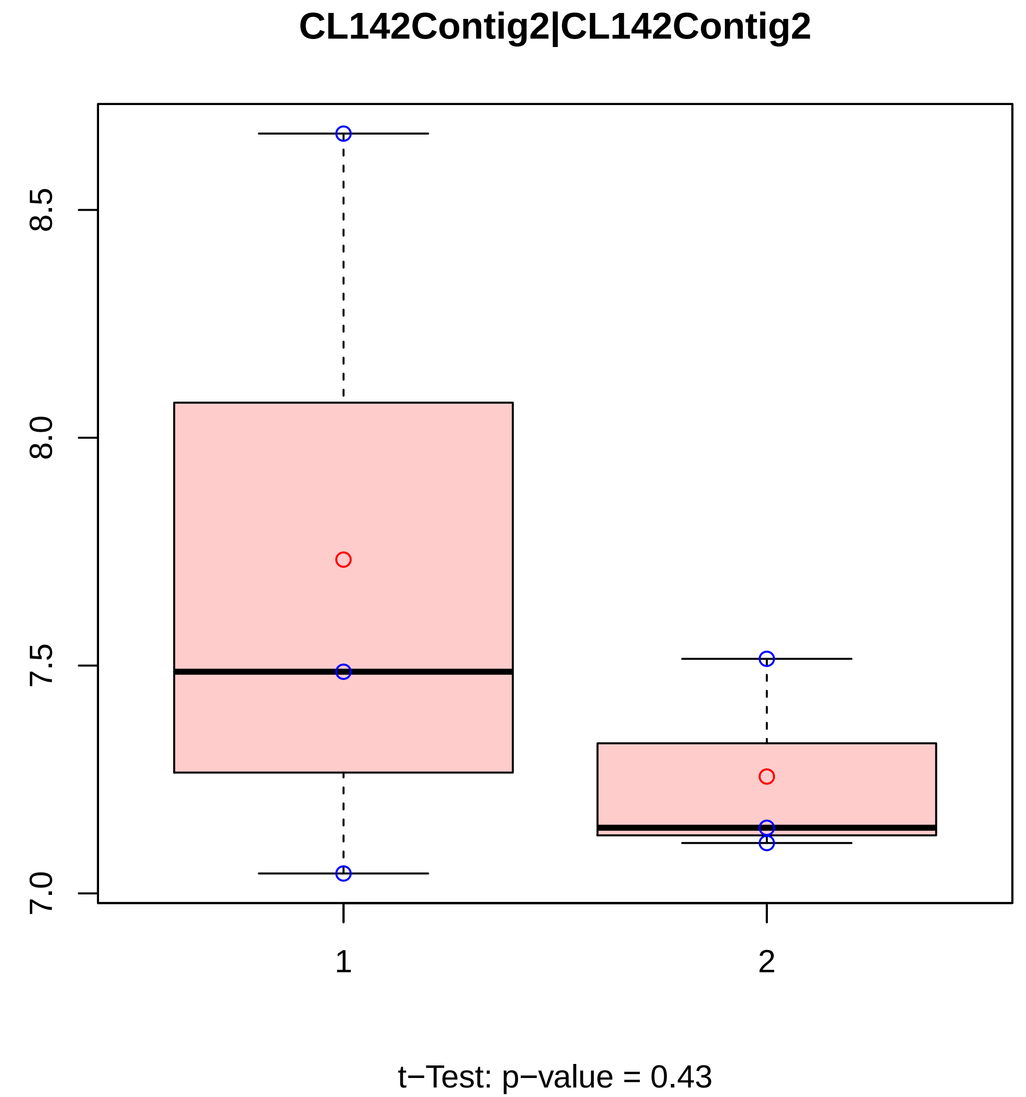
CL142Contig2|CL142Contig2
8.5
8.0
7.5
7.0
1
2
t−Test: p−value = 0.43

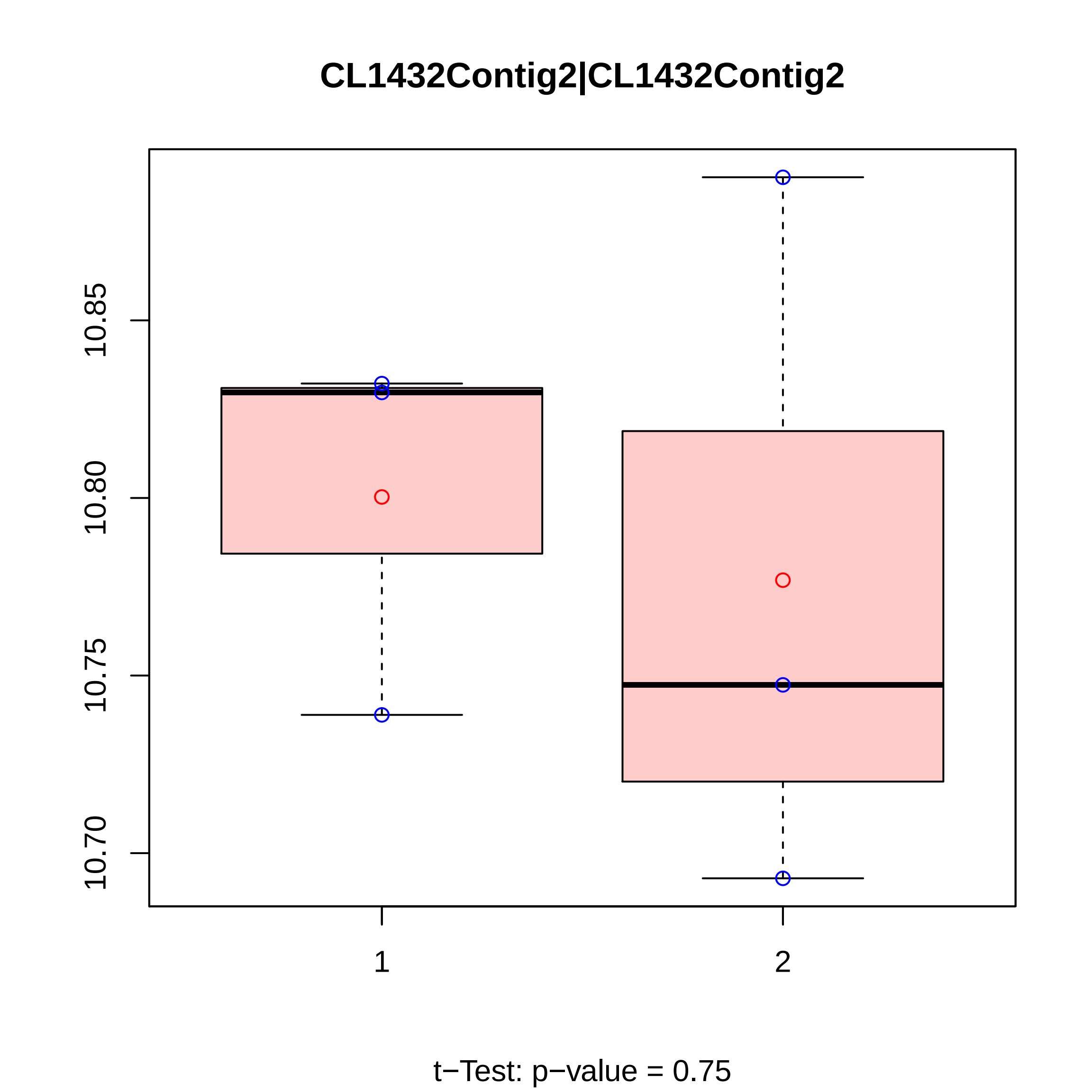

CL1432Contig2|CL1432Contig2
10.70
10.75
10.80
10.85
1
2
t−Test: p−value = 0.75

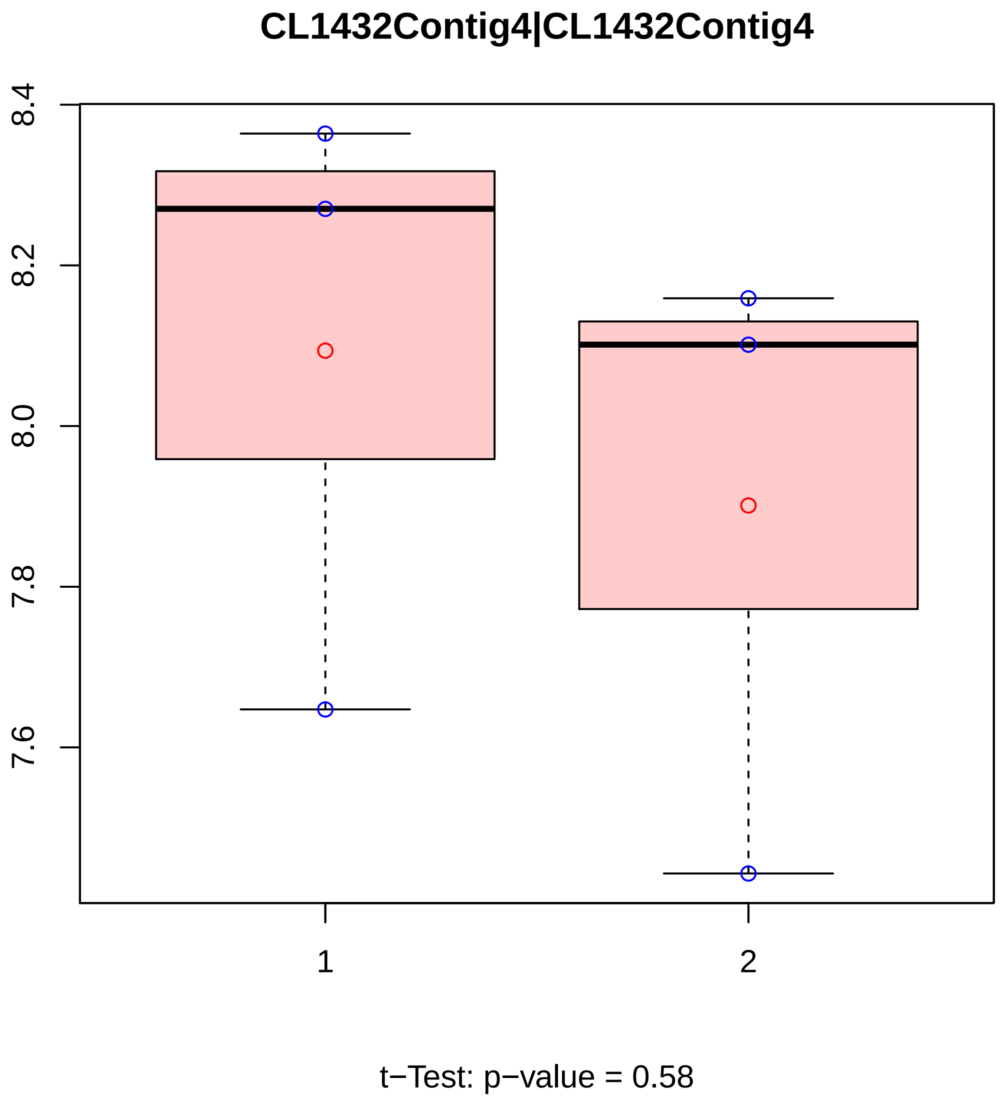
CL1432Contig4|CL1432Contig4
8.4
8.2
8.0
7.8
7.6
1
2
t−Test: p−value = 0.58

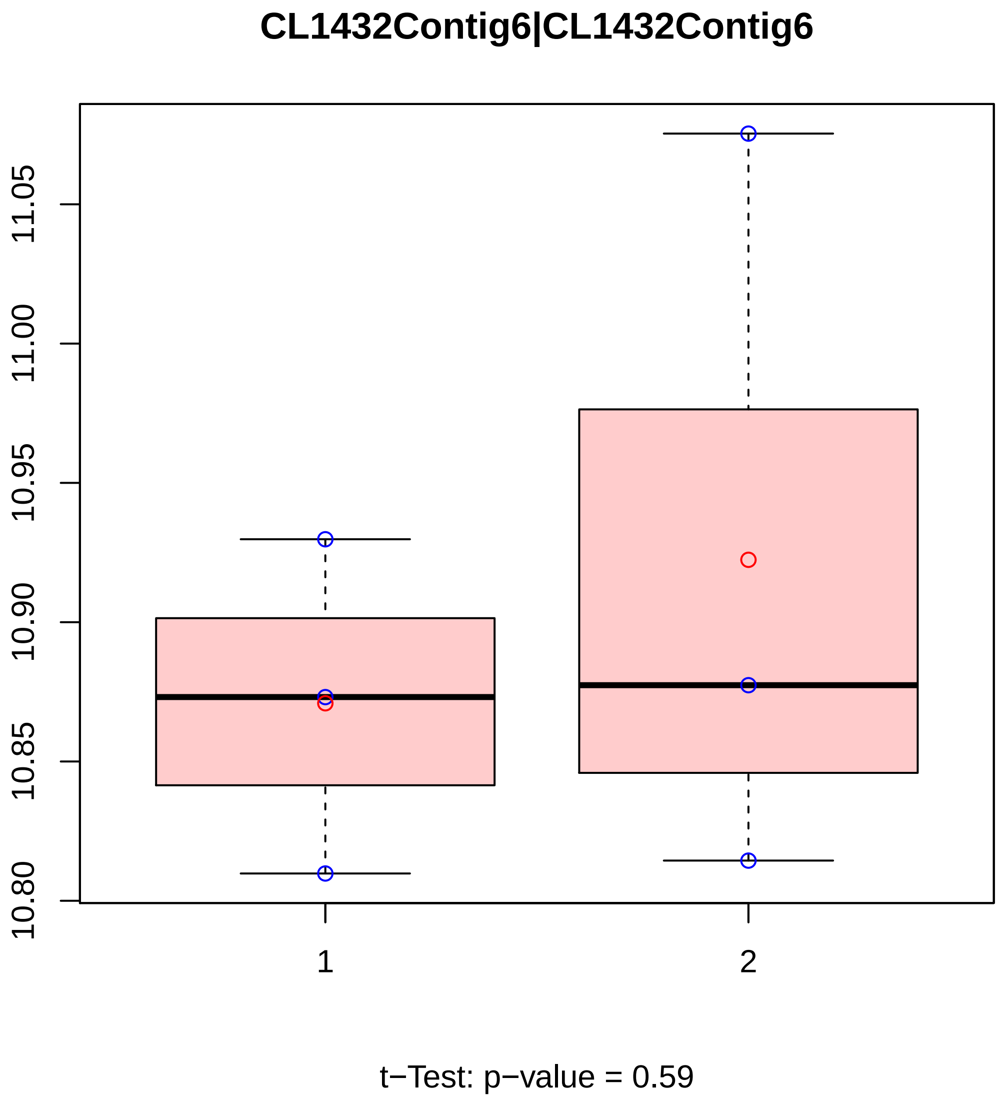
CL1432Contig6|CL1432Contig6
11.05
11.00
10.95
10.90
10.85
10.80
1
2
t−Test: p−value = 0.59

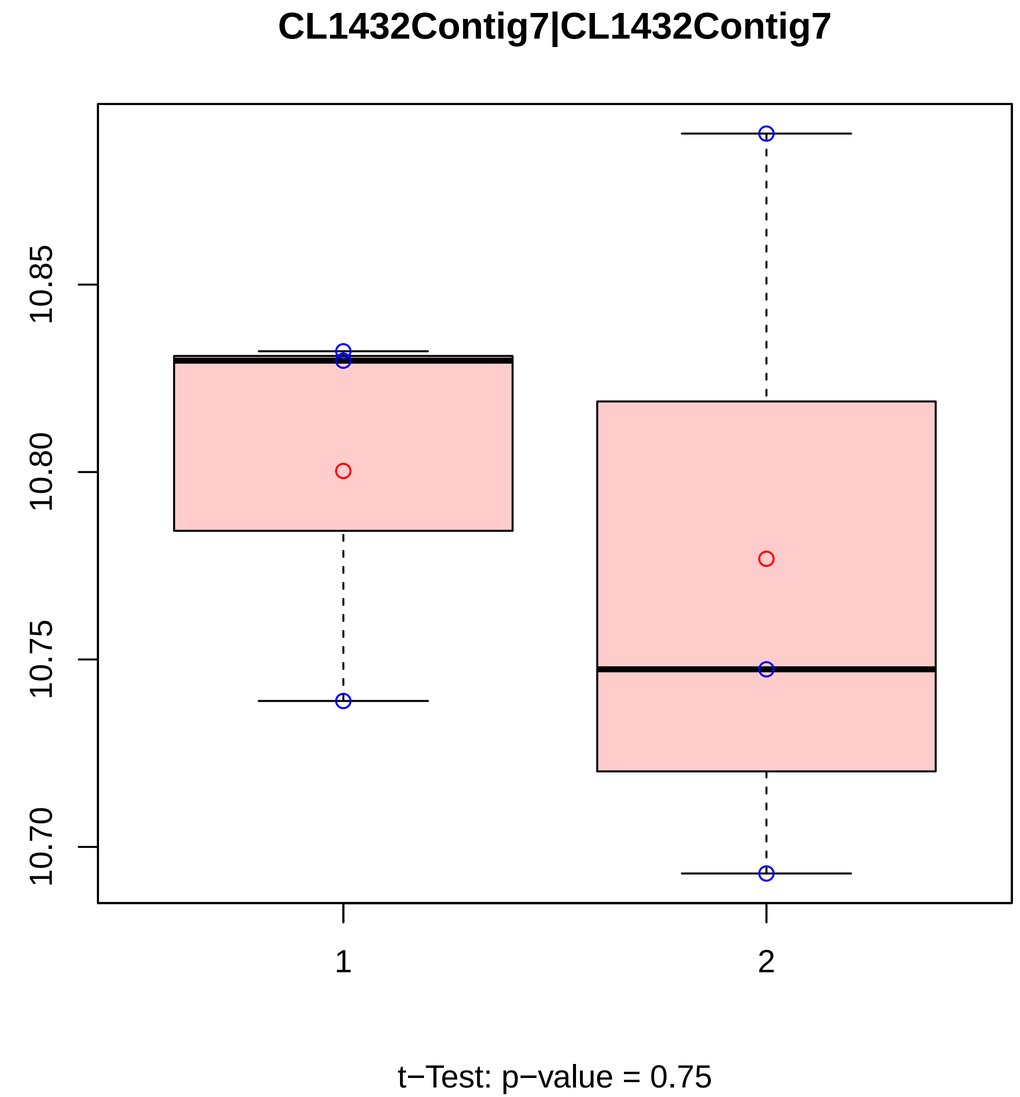
CL1432Contig7|CL1432Contig7
10.85
10.80
10.75
10.70
1
2
t−Test: p−value = 0.75

# CL1433Contig9|CL1433Contig9

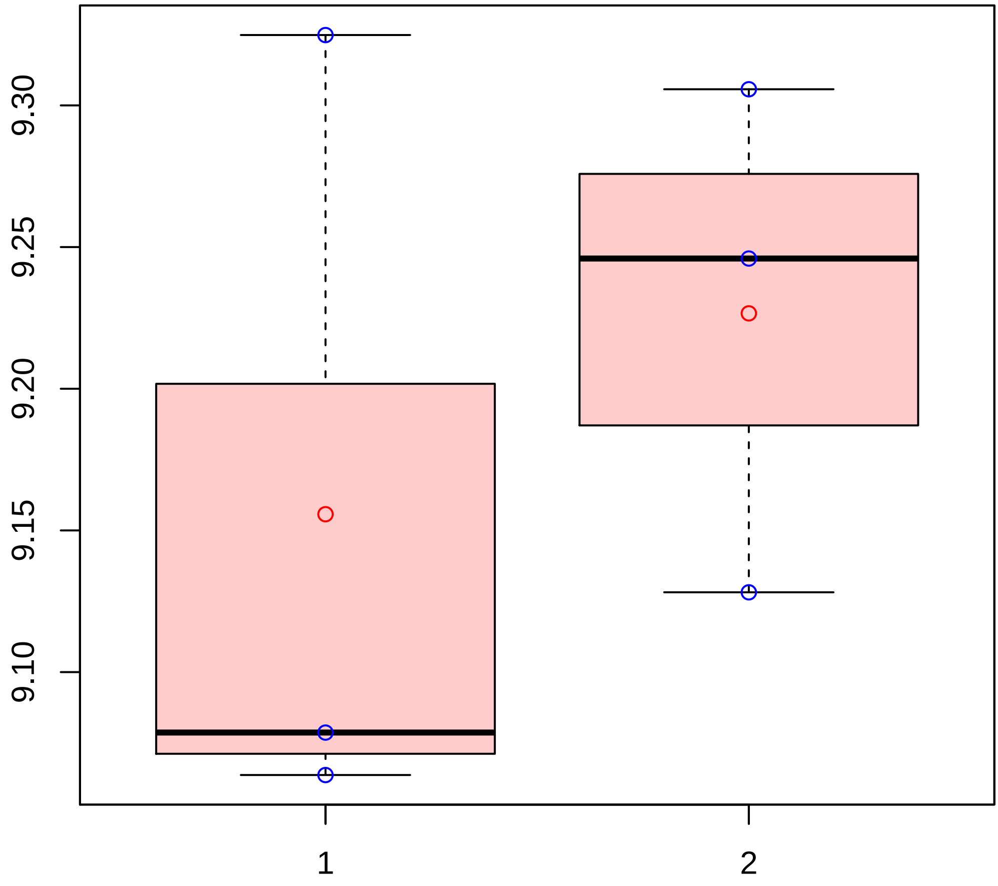


t−Test: p−value = 0.52

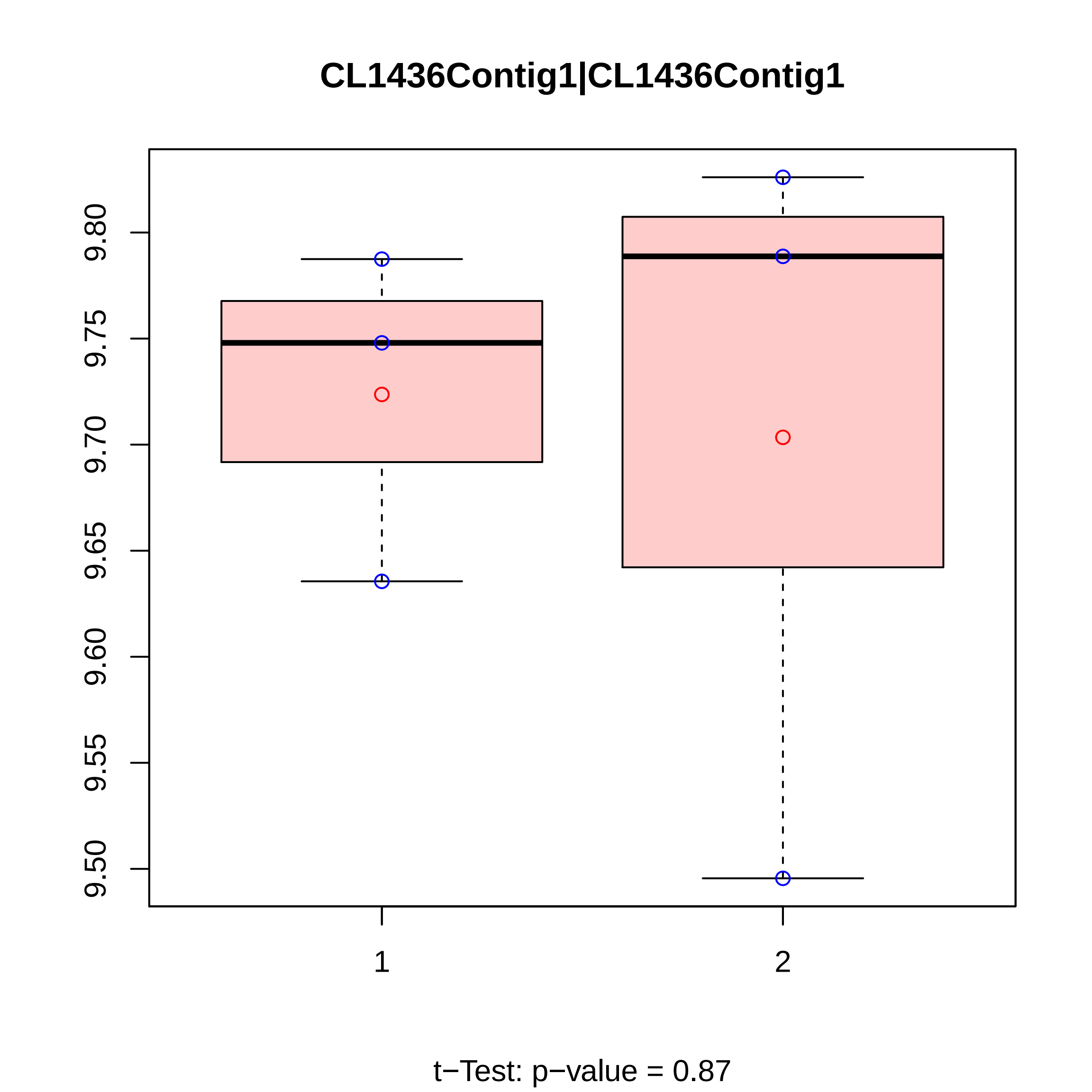
CL1436Contig1|CL1436Contig1
9.80
9.75
9.70
9.65
9.60
9.55
9.50
1
2
t−Test: p−value = 0.87

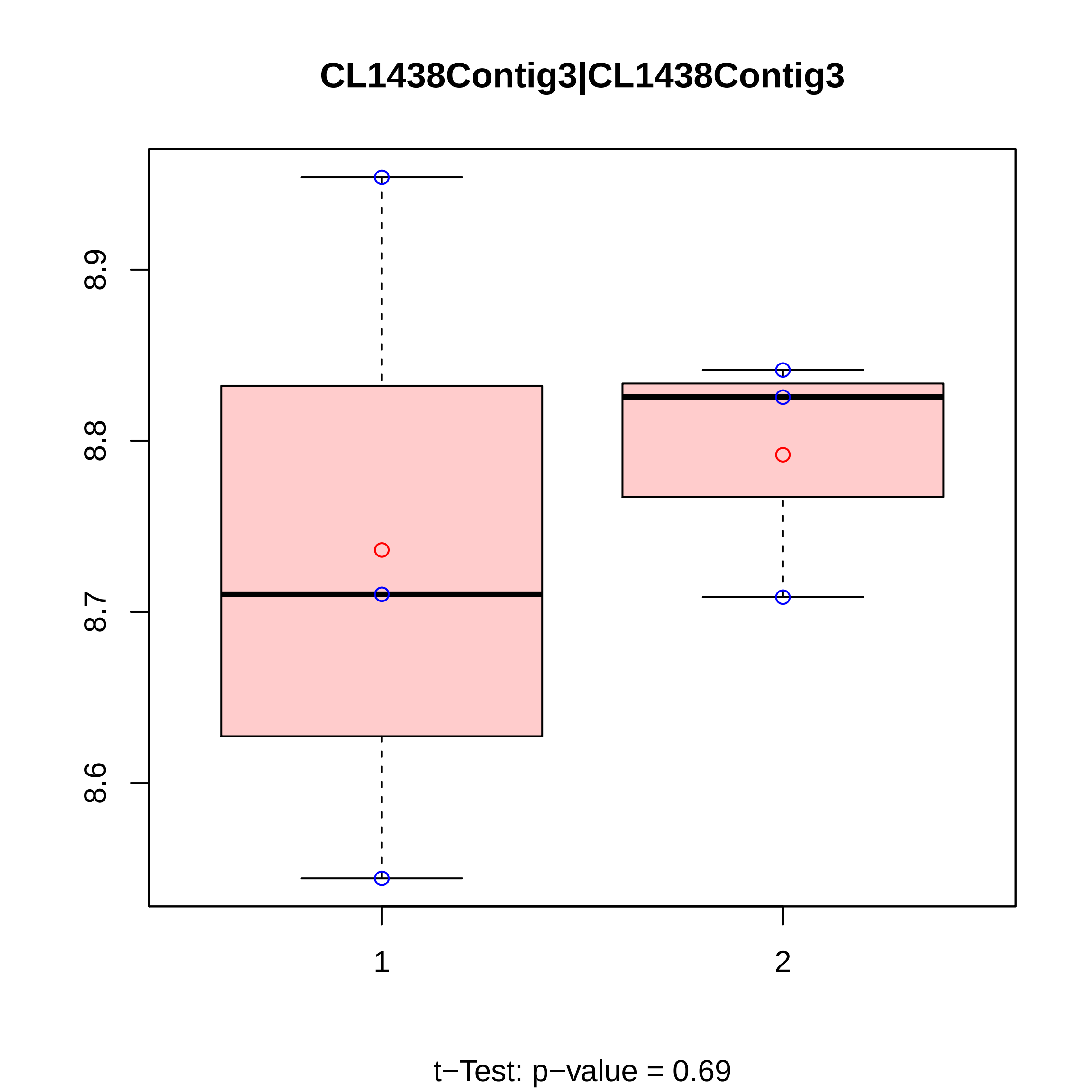

CL1438Contig3|CL1438Contig3
8.6
8.7
8.8
8.9
1
2
t−Test: p−value = 0.69

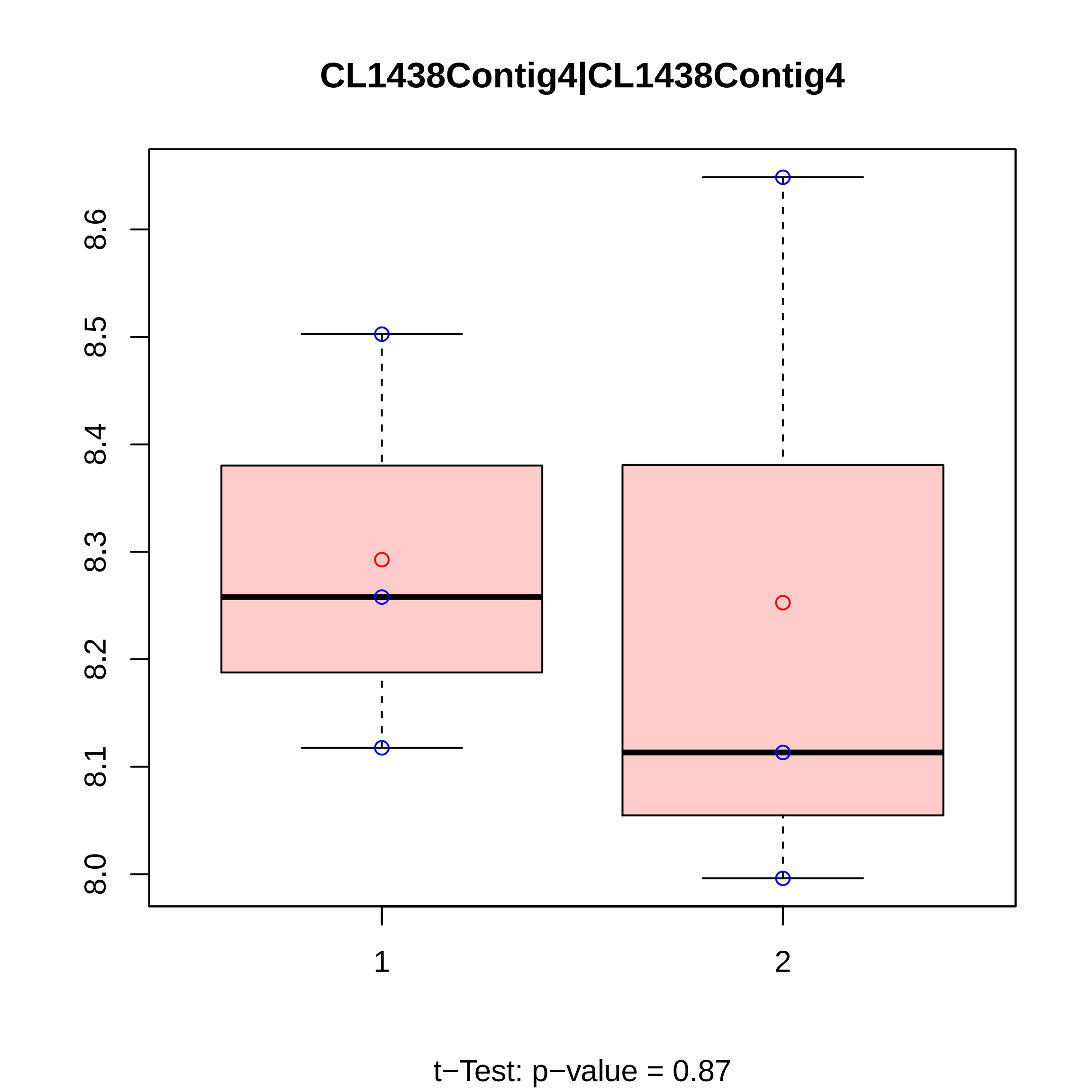
CL1438Contig4|CL1438Contig4
8.0
8.1
8.2
8.3
8.4
8.5
8.6
1
2
t−Test: p−value = 0.87

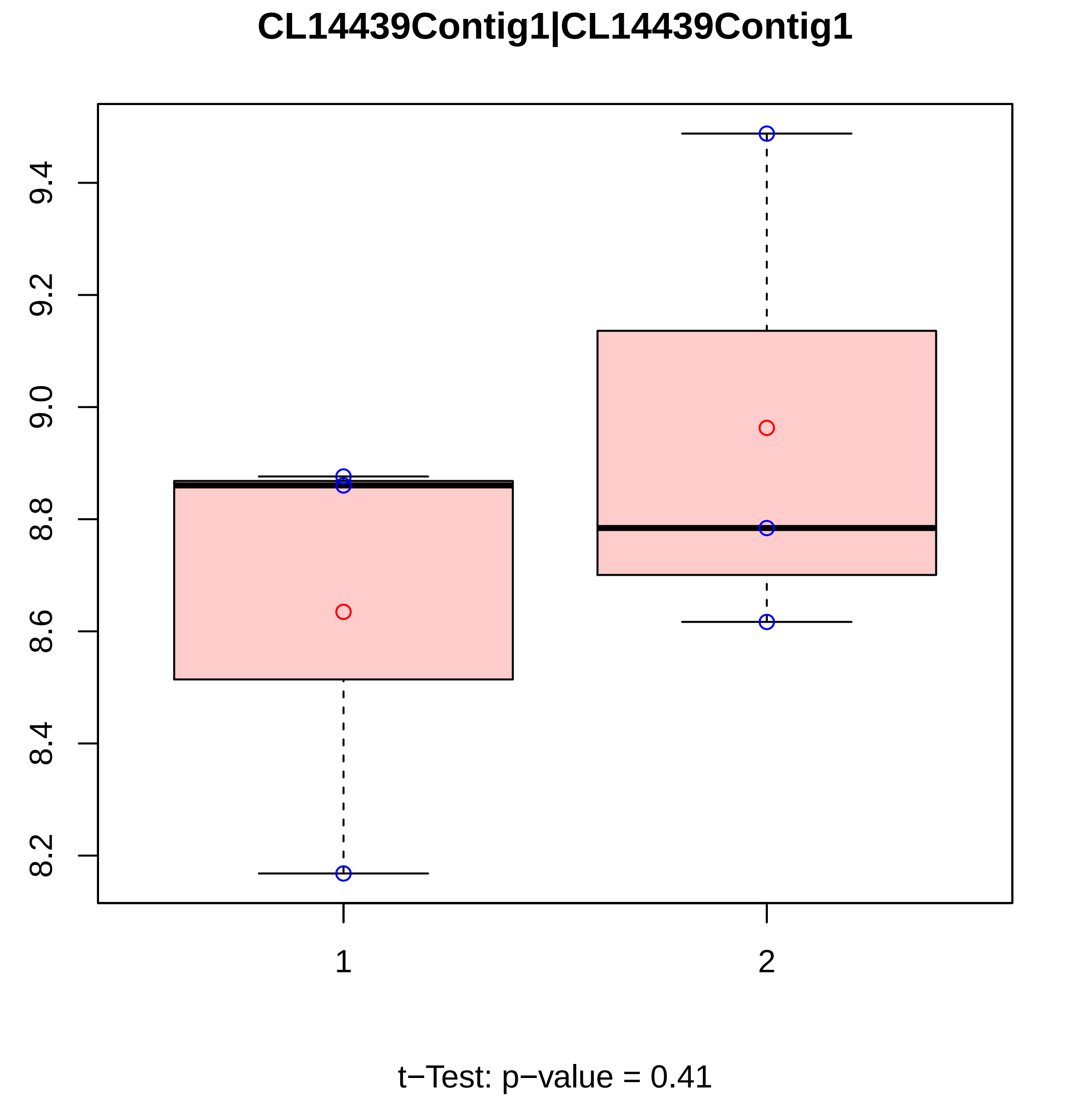
CL14439Contig1|CL14439Contig1
9.4
9.2
9.0
8.8
8.6
8.4
8.2
1
2
t−Test: p−value = 0.41

# CL14447Contig1|CL14447Contig1

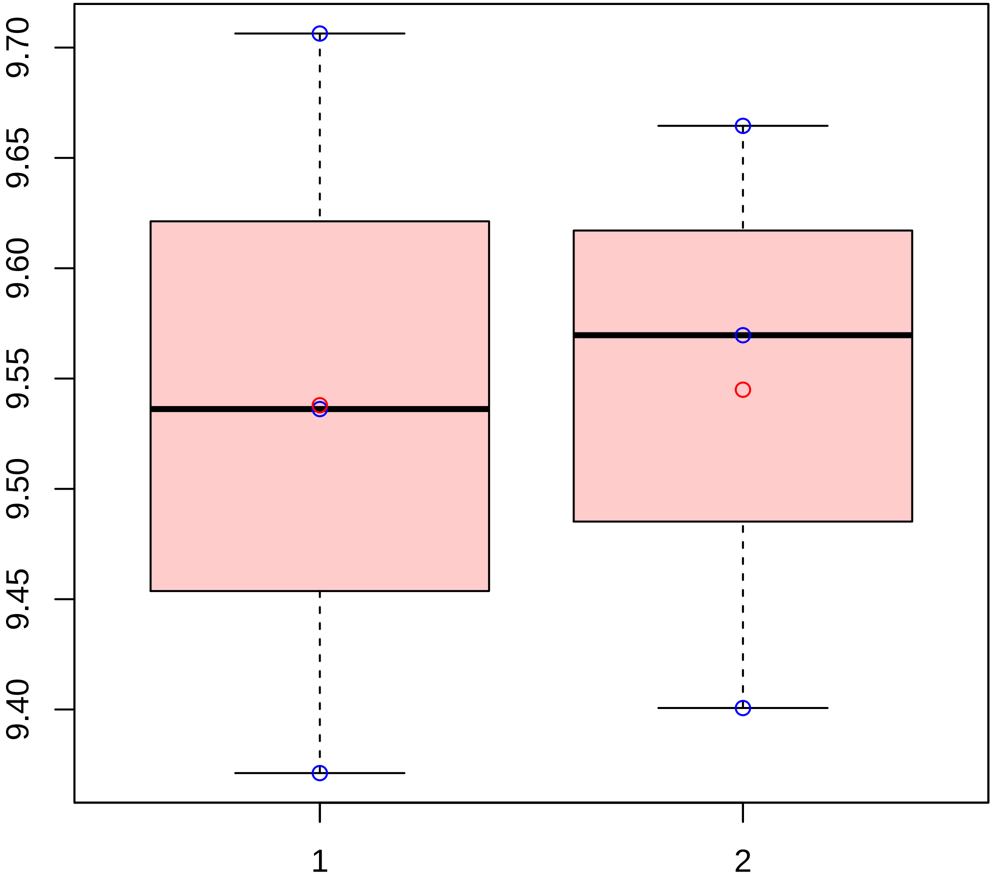


t−Test: p−value = 0.96

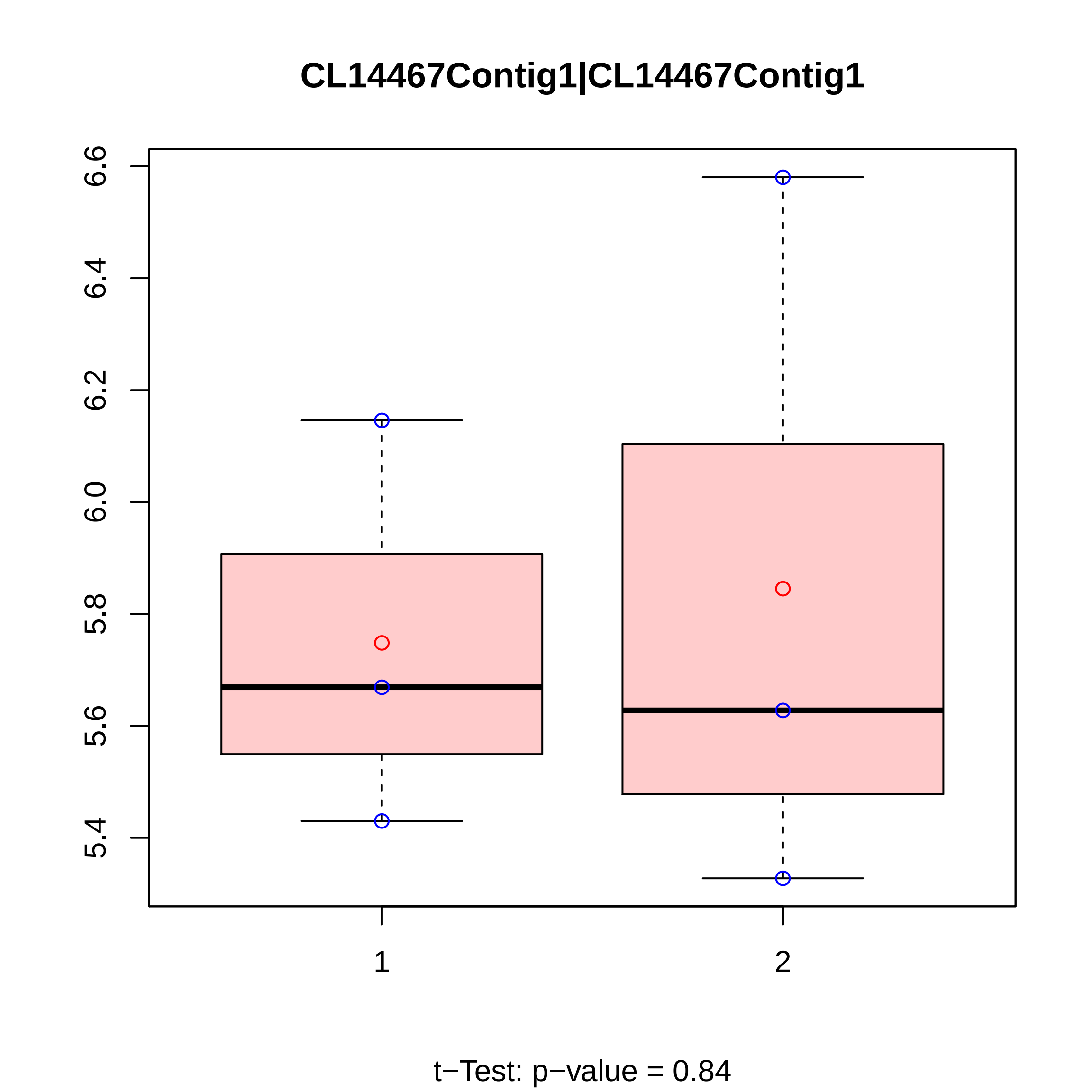

CL14467Contig1|CL14467Contig1
6.6
6.4
6.2
6.0
5.8
5.6
5.4
1
2
t−Test: p−value = 0.84

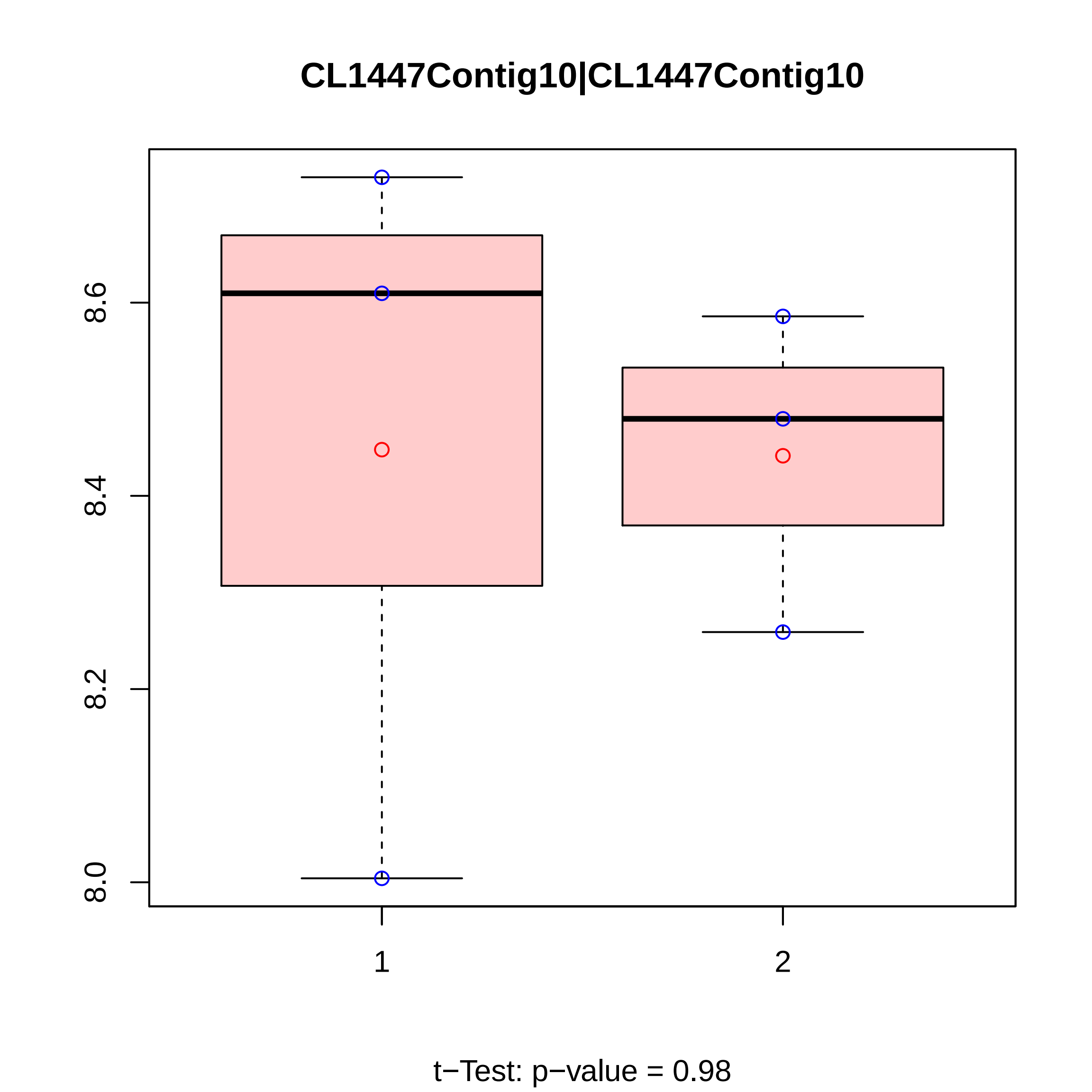
CL1447Contig10|CL1447Contig10
8.0
8.2
8.4
8.6
1
2
t−Test: p−value = 0.98

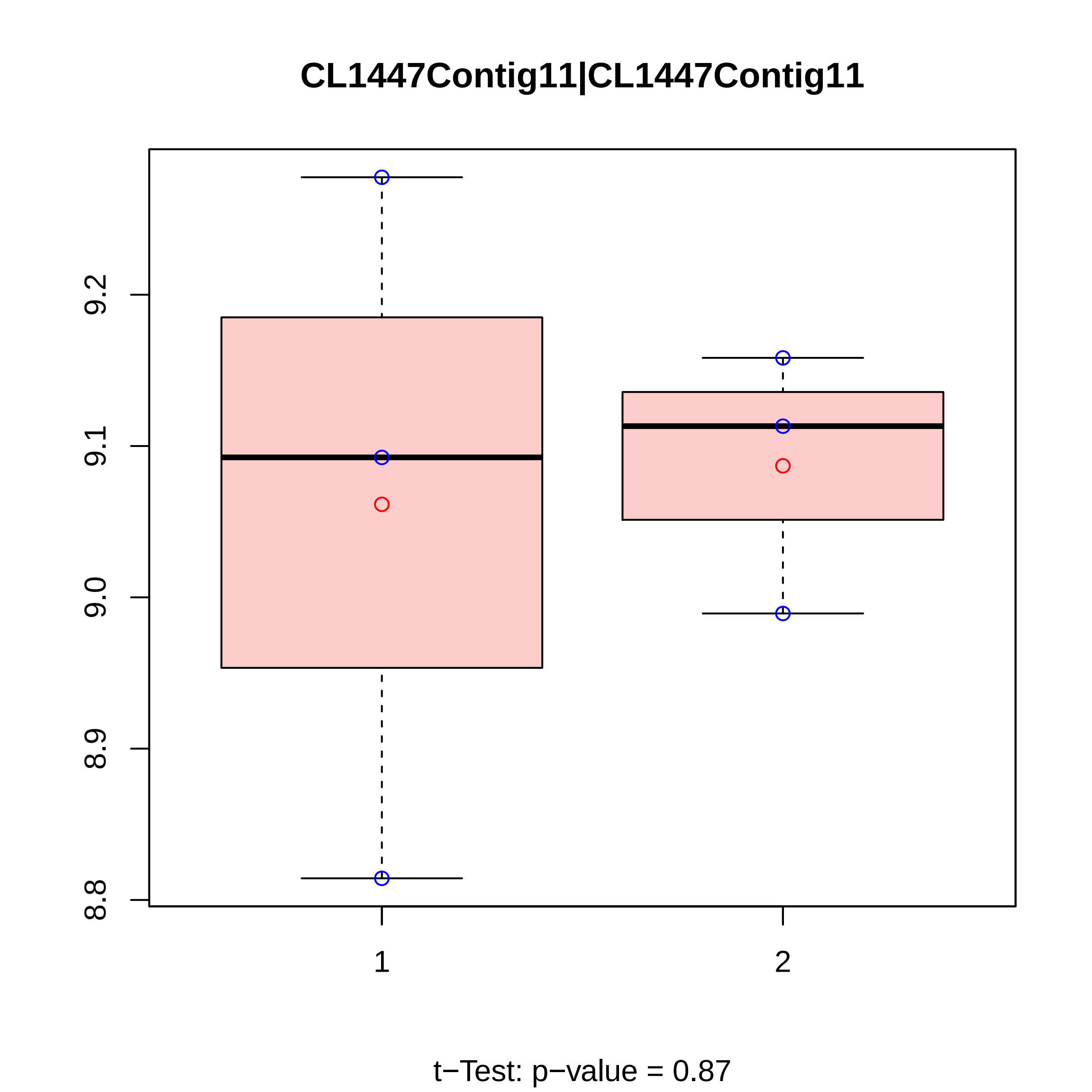
CL1447Contig11|CL1447Contig11
9.2
9.1
9.0
8.9
8.8
1
2
t−Test: p−value = 0.87

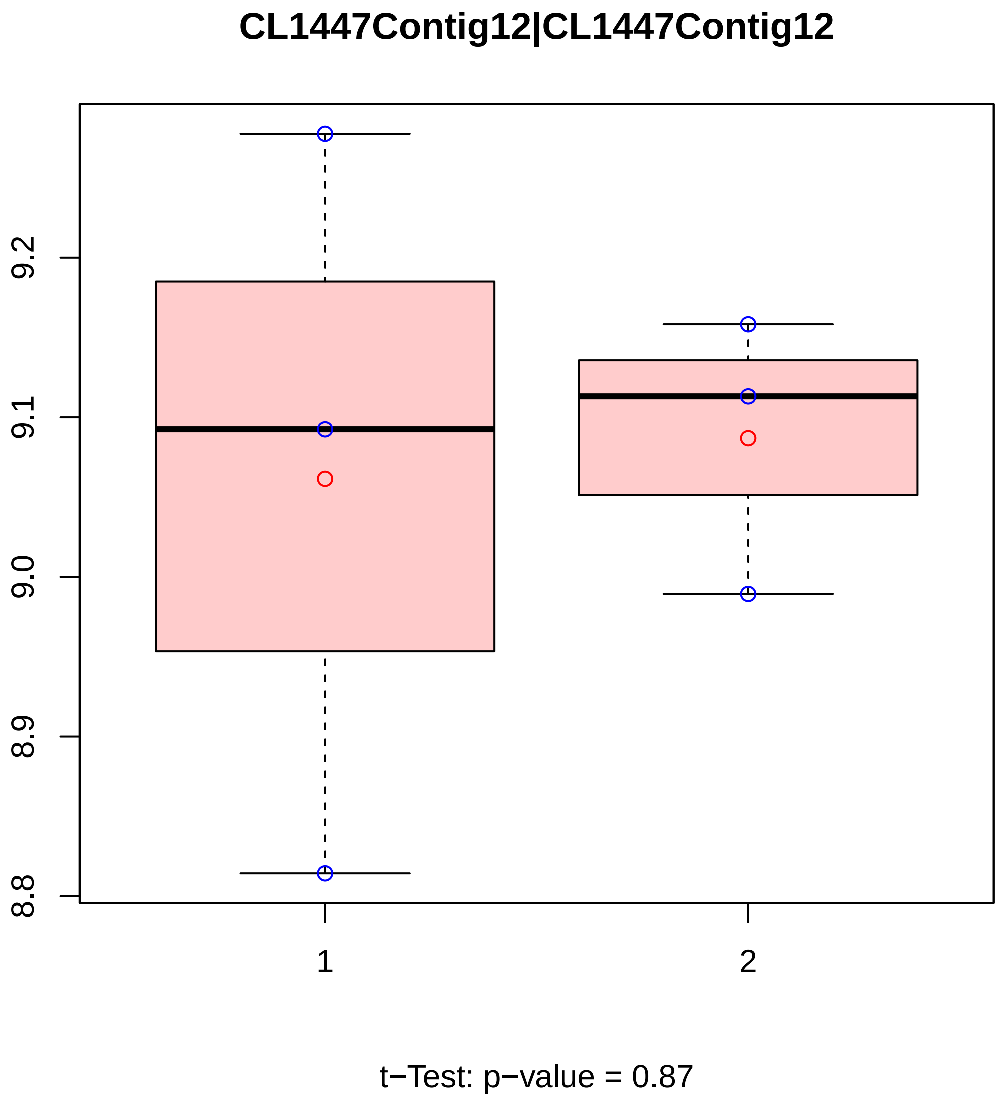
CL1447Contig12|CL1447Contig12
9.2
9.1
9.0
8.9
8.8
1
2
t−Test: p−value = 0.87

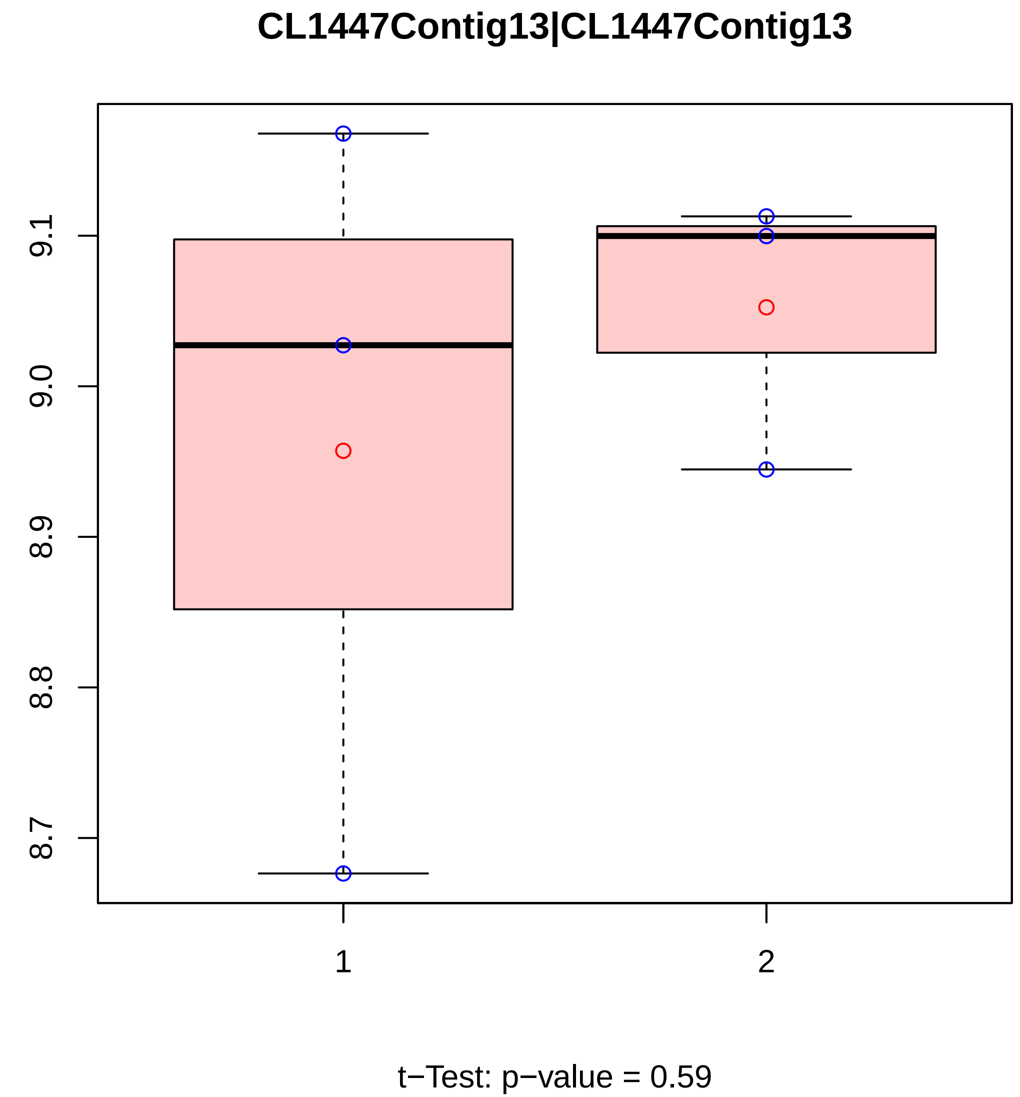
CL1447Contig13|CL1447Contig13
9.1
9.0
8.9
8.8
8.7
1
2
t−Test: p−value = 0.59

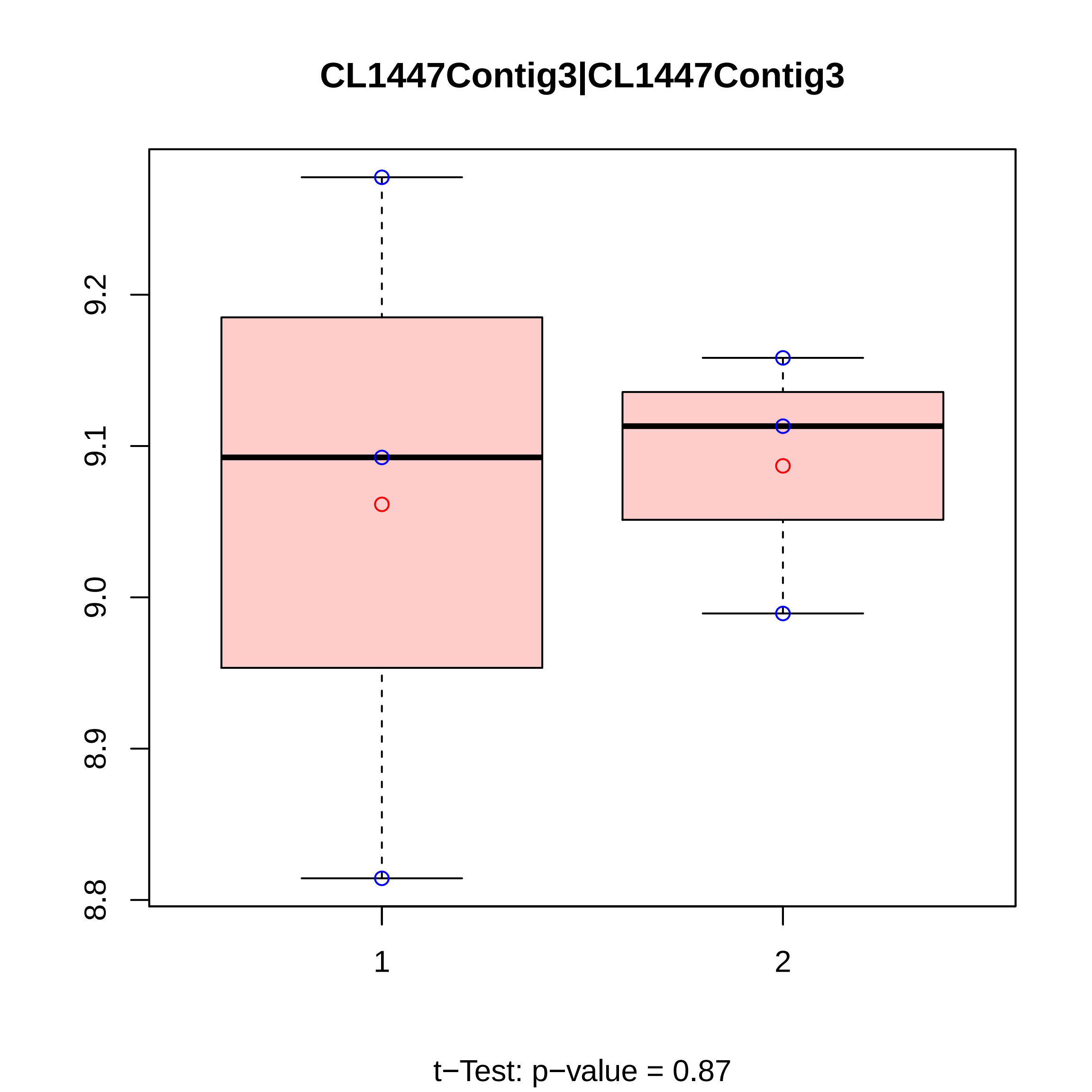

CL1447Contig3|CL1447Contig3
9.2
9.1
9.0
8.9
8.8
1
2
t−Test: p−value = 0.87

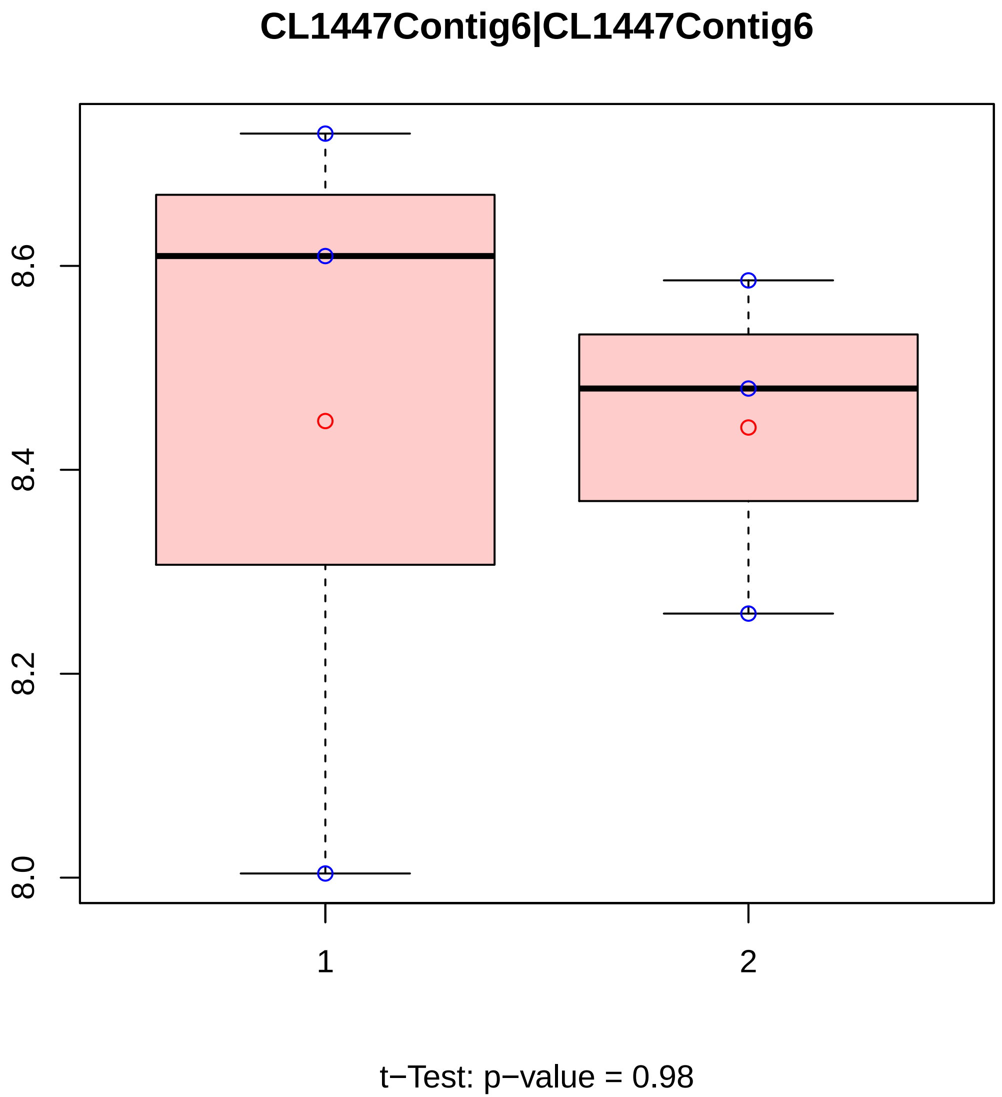

CL1447Contig6|CL1447Contig6
8.0
8.2
8.4
8.6
1
2
t−Test: p−value = 0.98

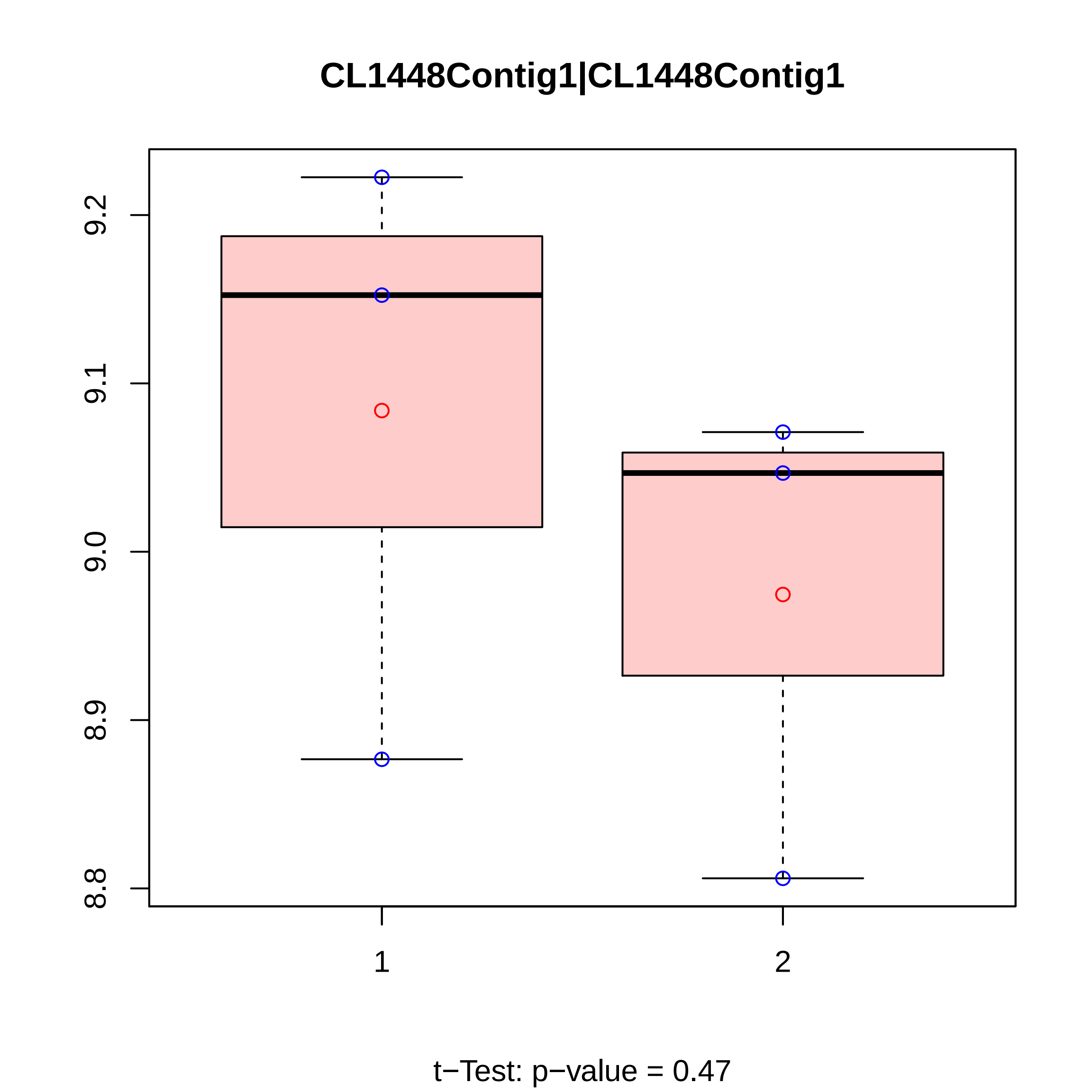
CL1448Contig1|CL1448Contig1
9.2
9.1
9.0
8.9
8.8
1
2
t−Test: p−value = 0.47

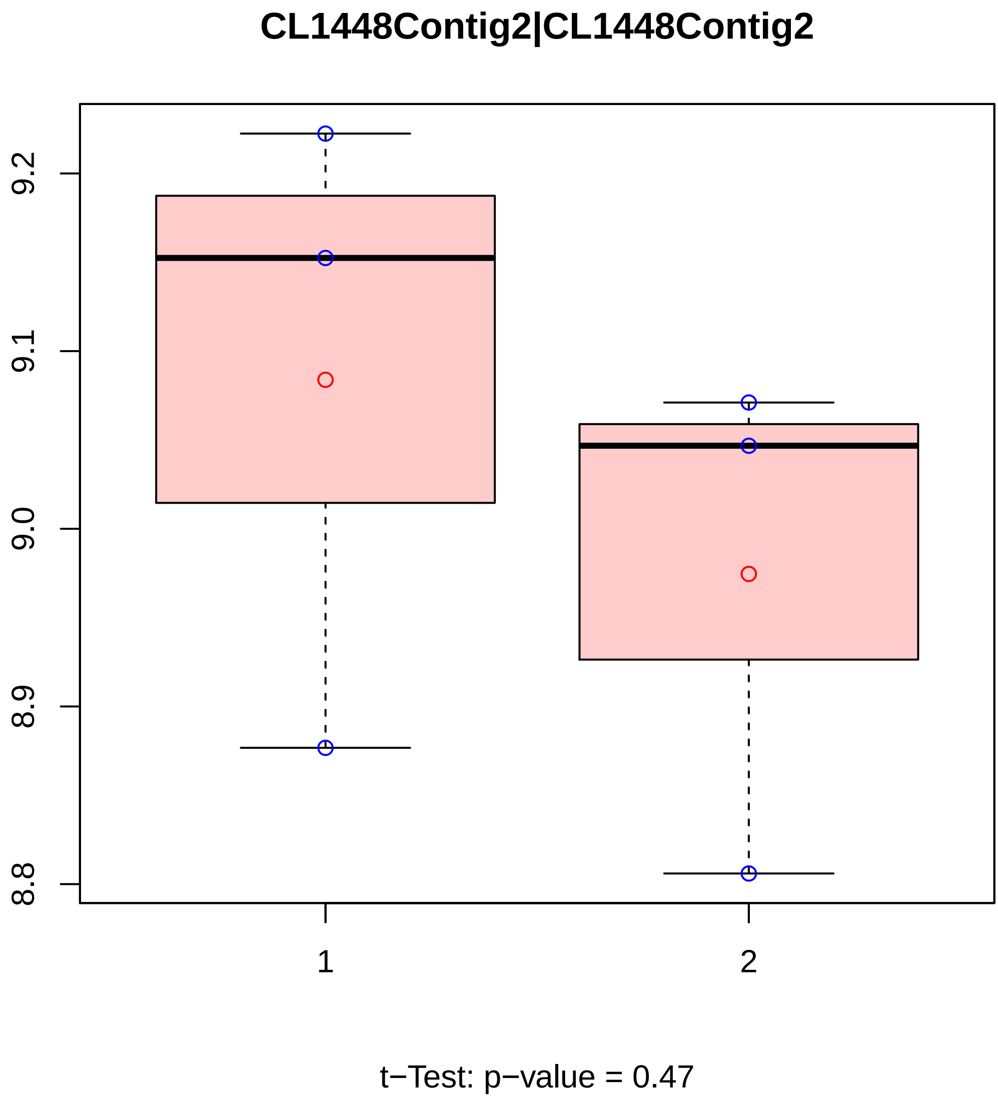

CL1448Contig2|CL1448Contig2
9.2
9.1
9.0
8.9
8.8
1
2
t−Test: p−value = 0.47

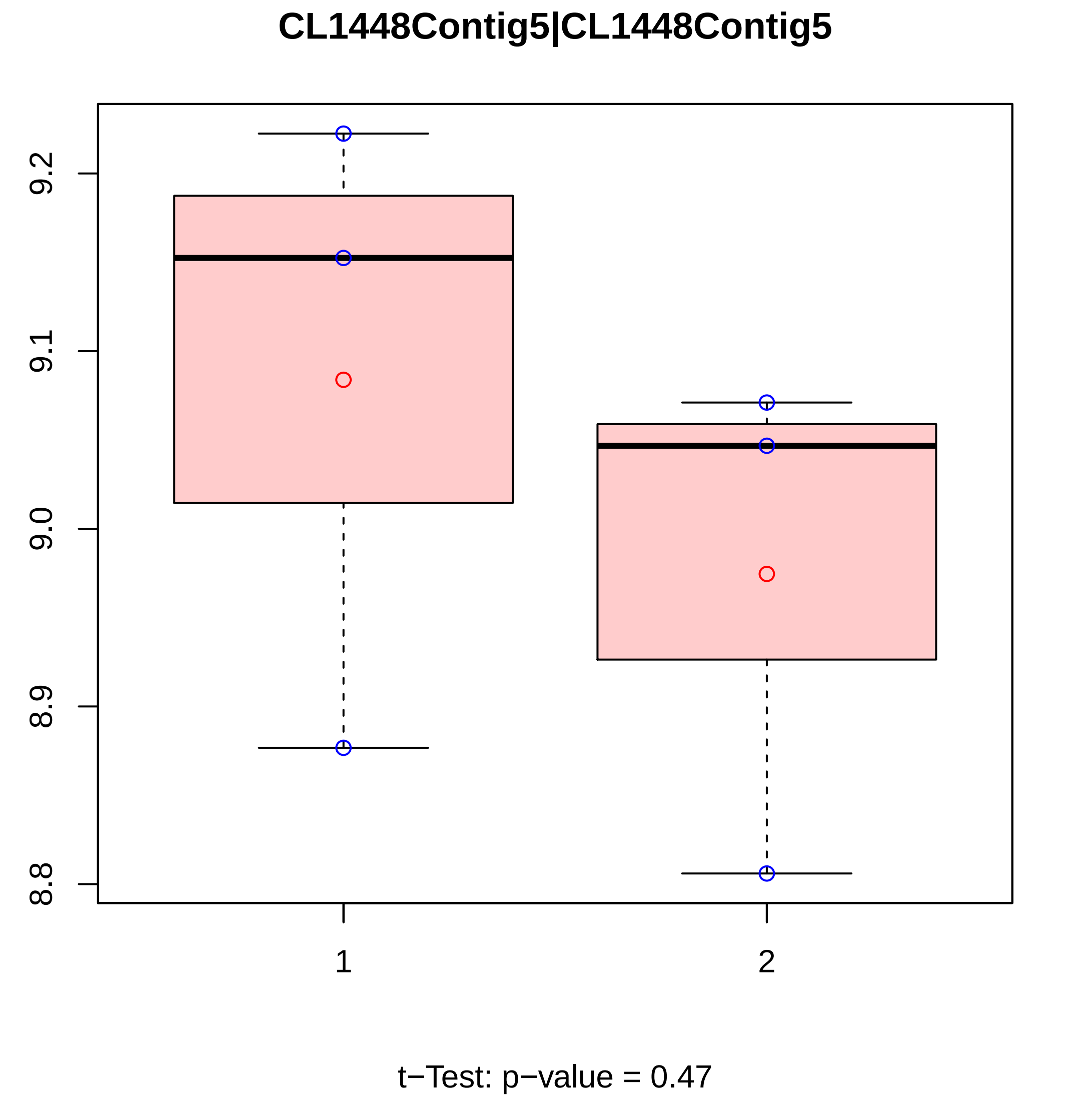
CL1448Contig5|CL1448Contig5
9.2
9.1
9.0
8.9
8.8
1
2
t−Test: p−value = 0.47

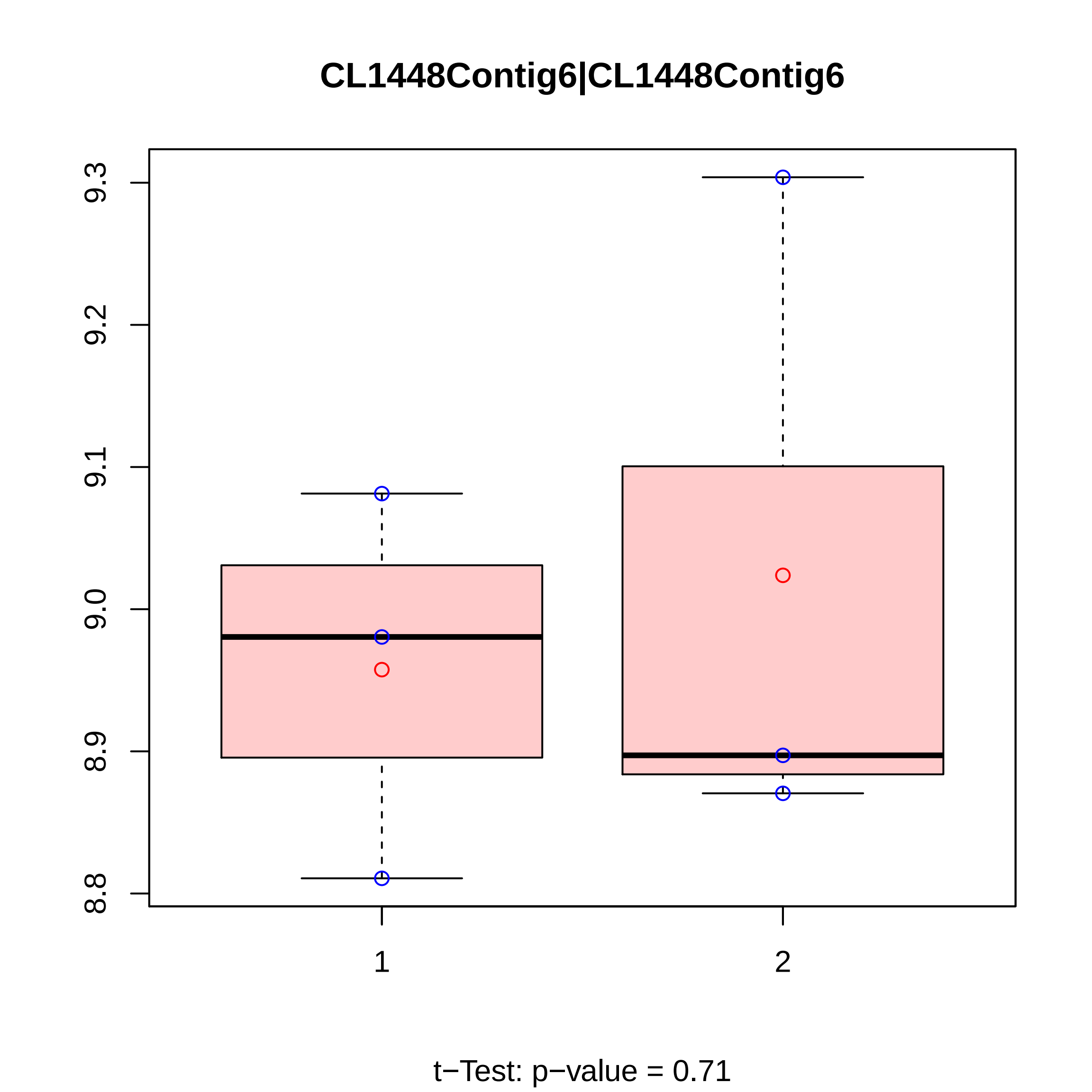
CL1448Contig6|CL1448Contig6
9.3
9.2
9.1
9.0
8.9
8.8
1
2
t−Test: p−value = 0.71

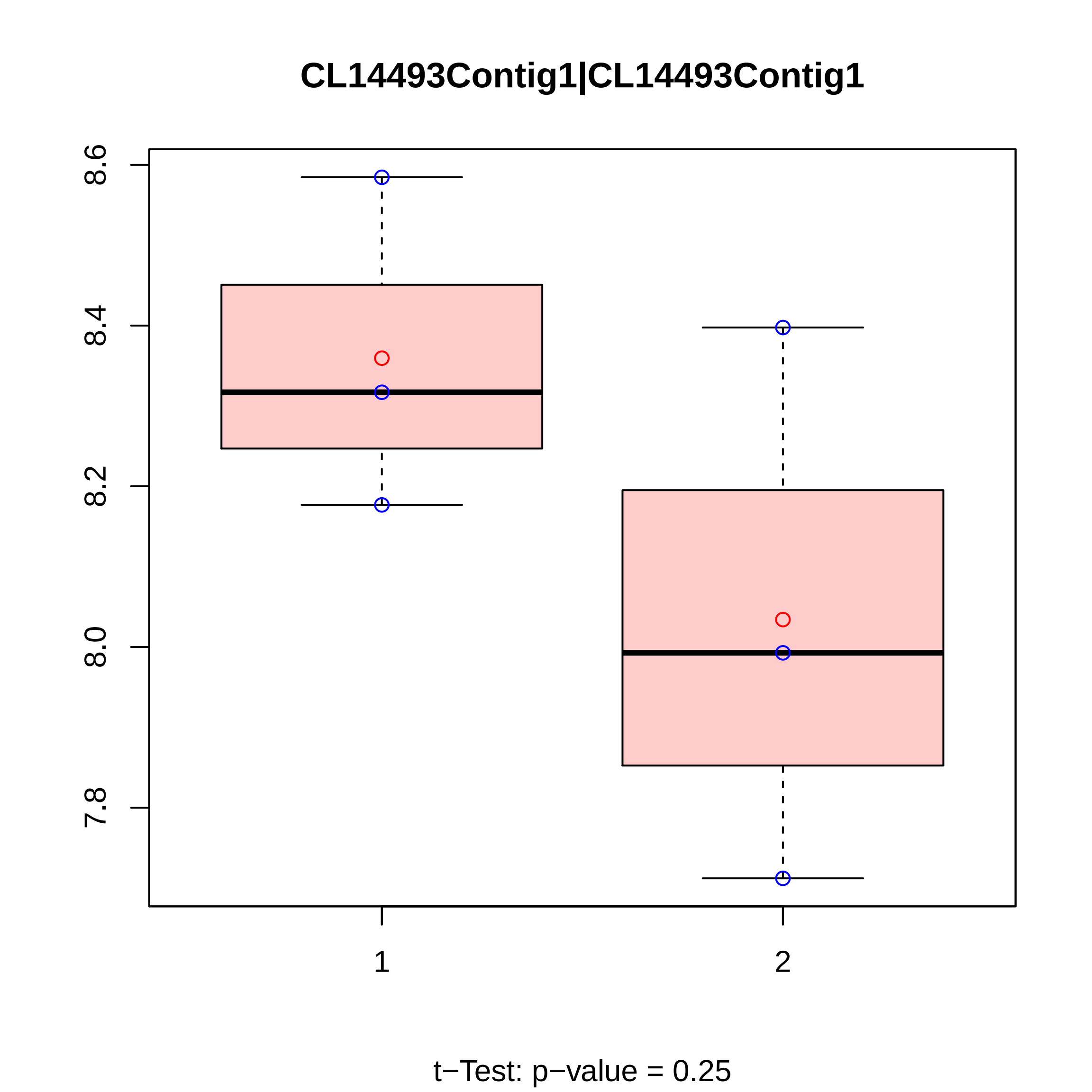
CL14493Contig1|CL14493Contig1
8.6
8.4
8.2
8.0
7.8
1
2
t−Test: p−value = 0.25

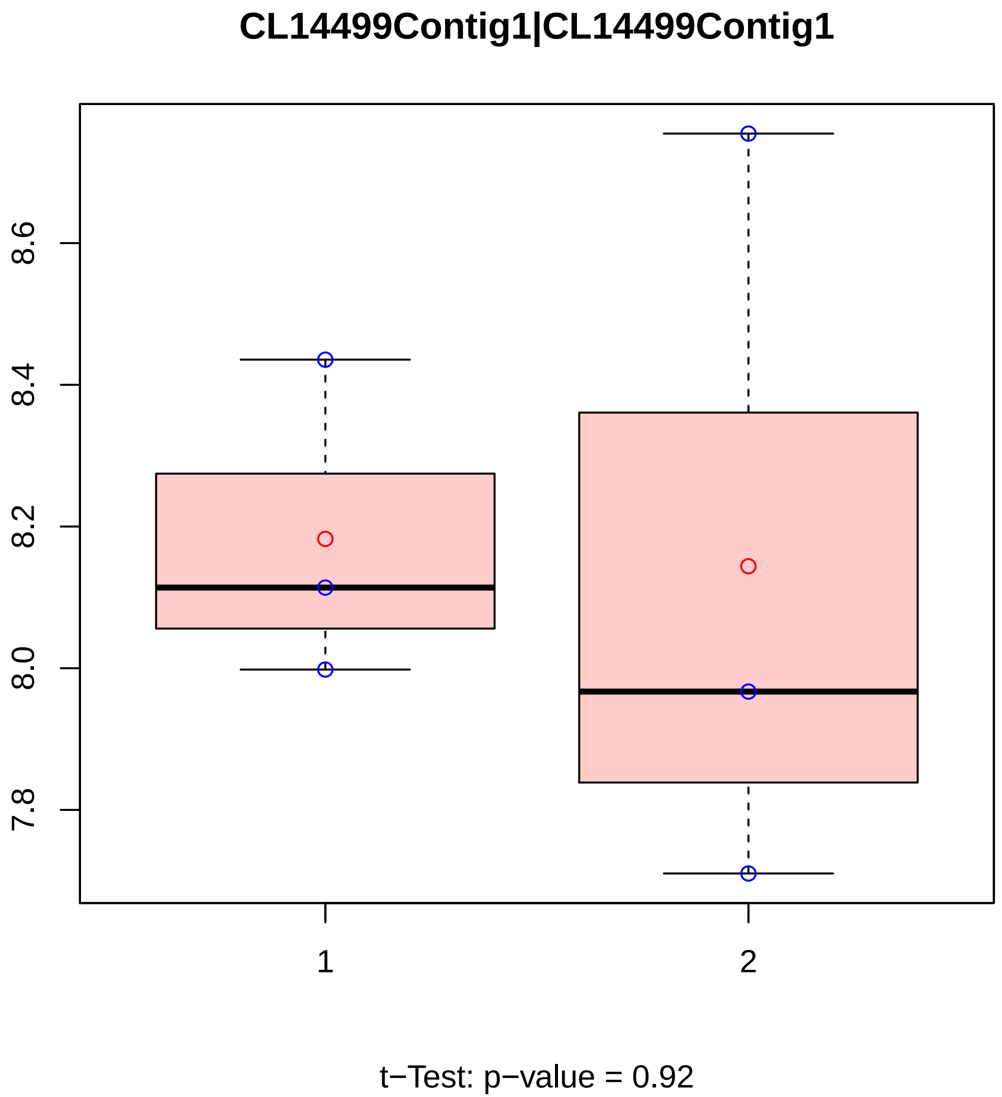
CL14499Contig1|CL14499Contig1
8.6
8.4
8.2
8.0
7.8
1
2
t−Test: p−value = 0.92

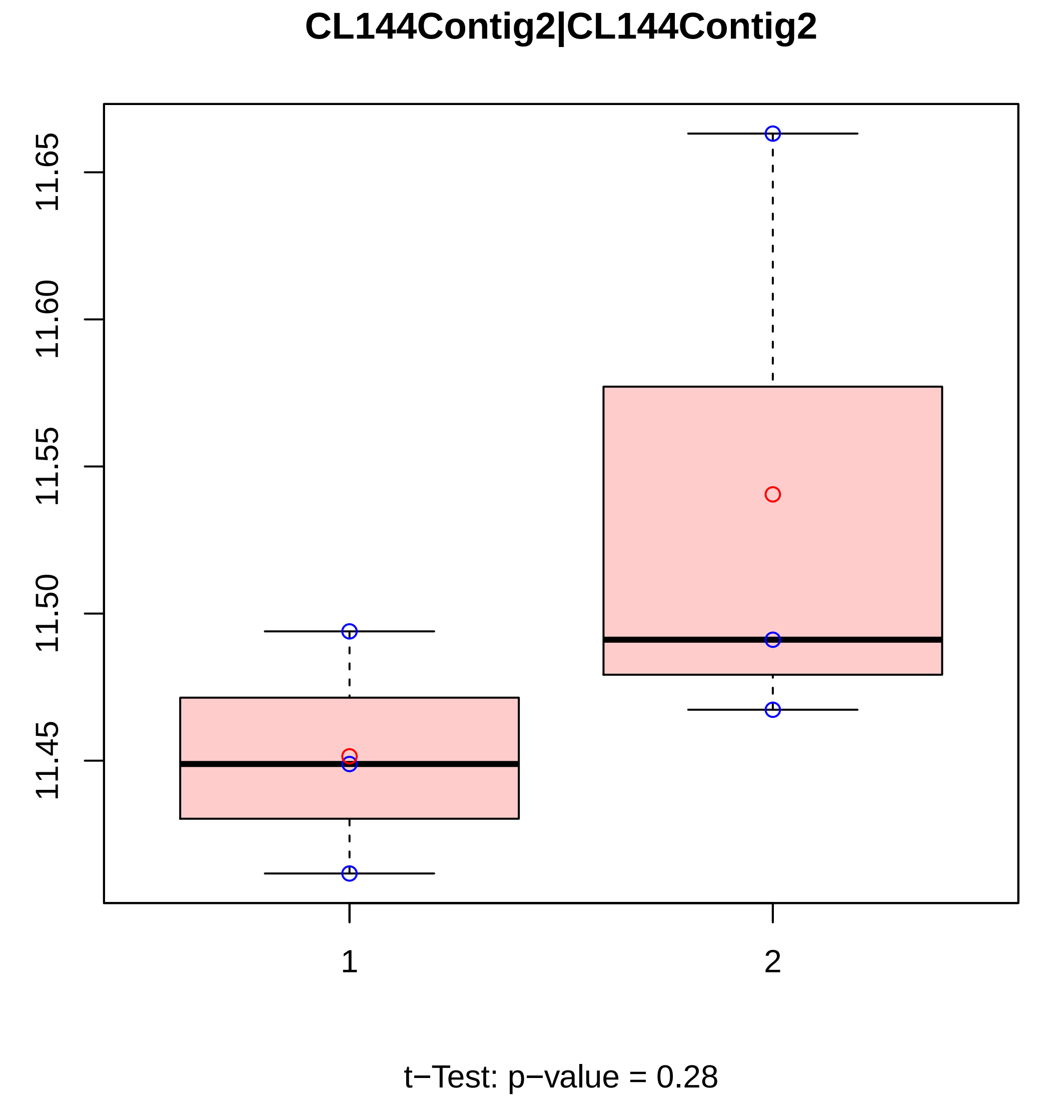
CL144Contig2|CL144Contig2
11.45
11.50
11.55
11.60
11.65
1
2
t−Test: p−value = 0.28

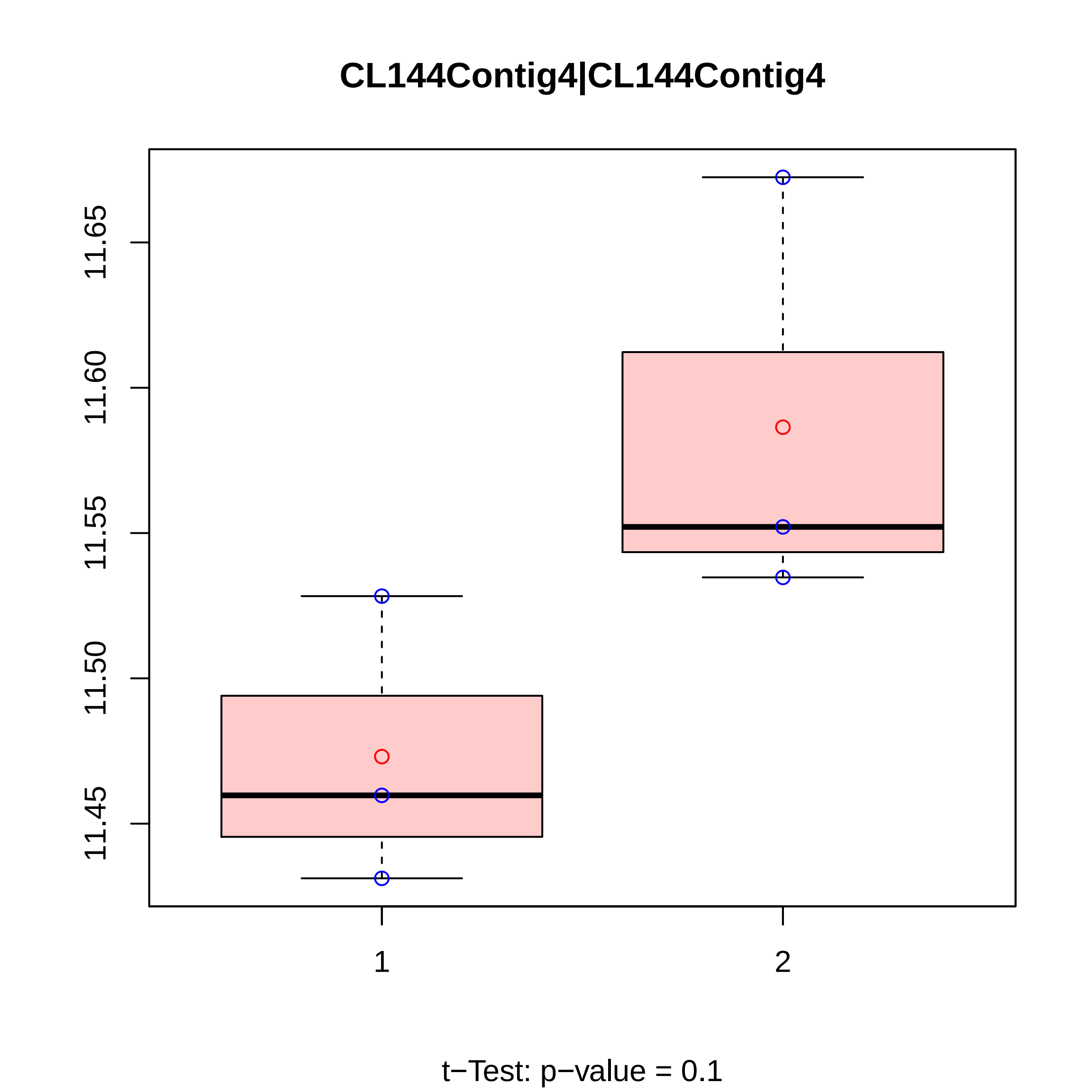
CL144Contig4|CL144Contig4
11.45
11.50
11.55
11.60
11.65
1
2
t−Test: p−value = 0.1

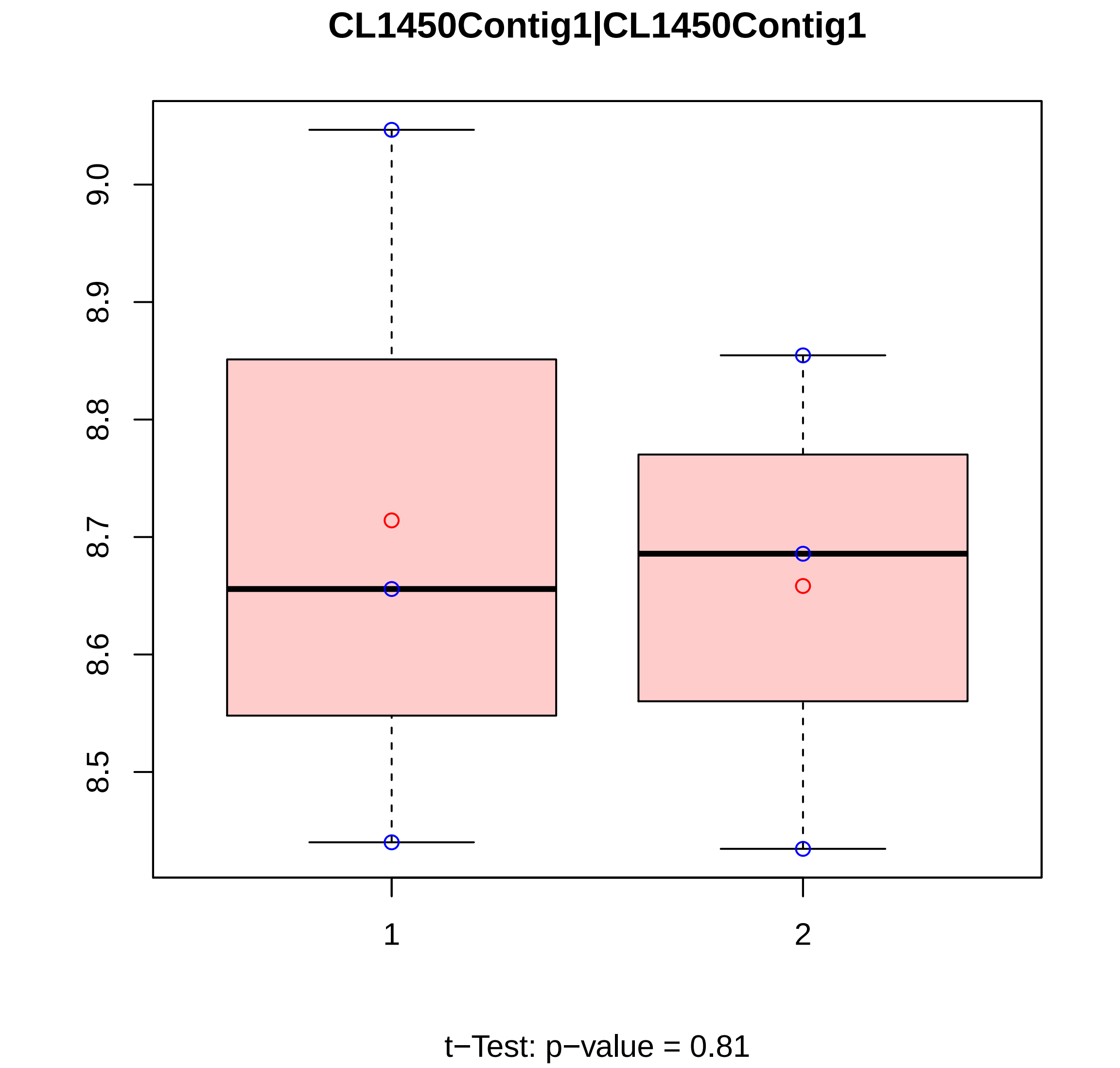
CL1450Contig1|CL1450Contig1
9.0
8.9
8.8
8.7
8.6
8.5
1
2
t−Test: p−value = 0.81

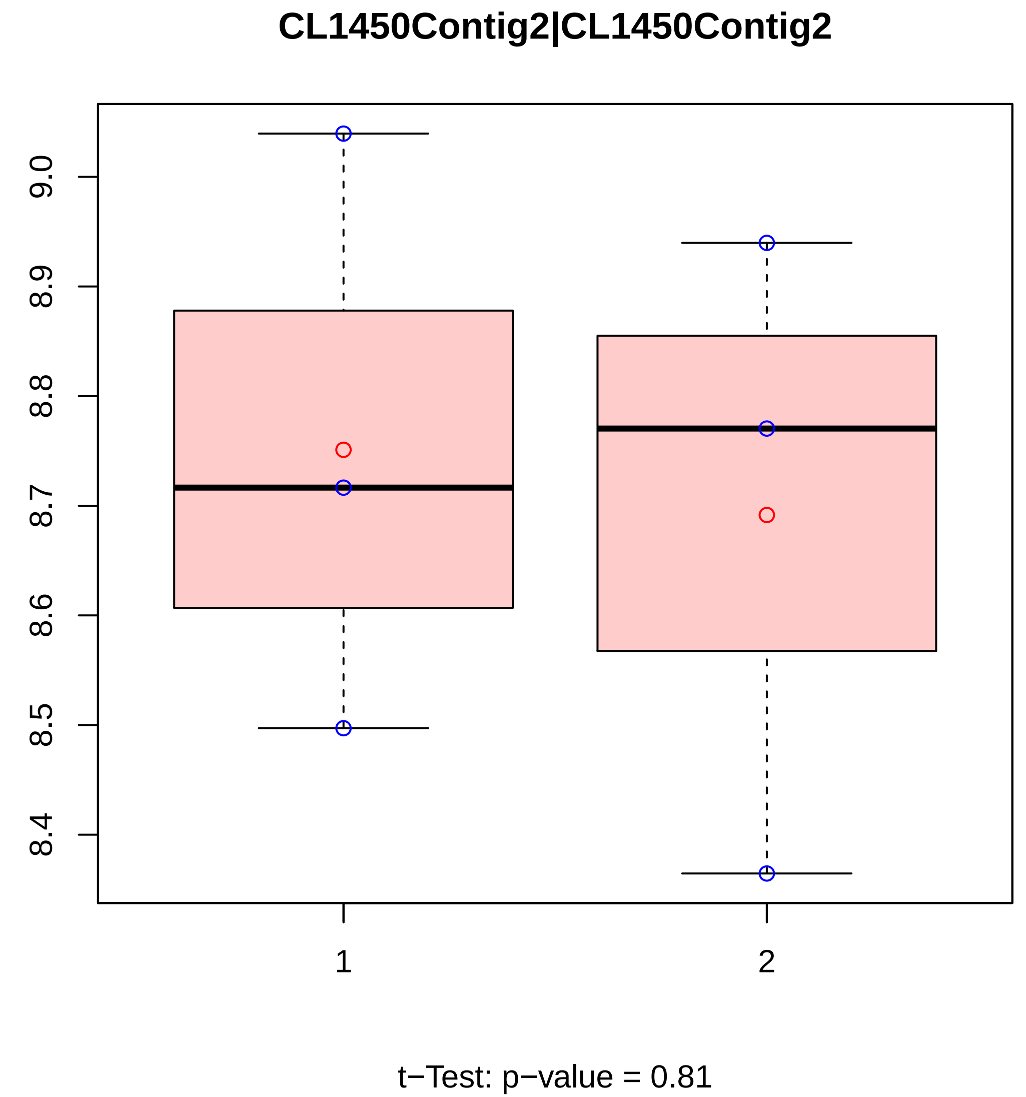
CL1450Contig2|CL1450Contig2
9.0
8.9
8.8
8.7
8.6
8.5
8.4
1
2
t−Test: p−value = 0.81

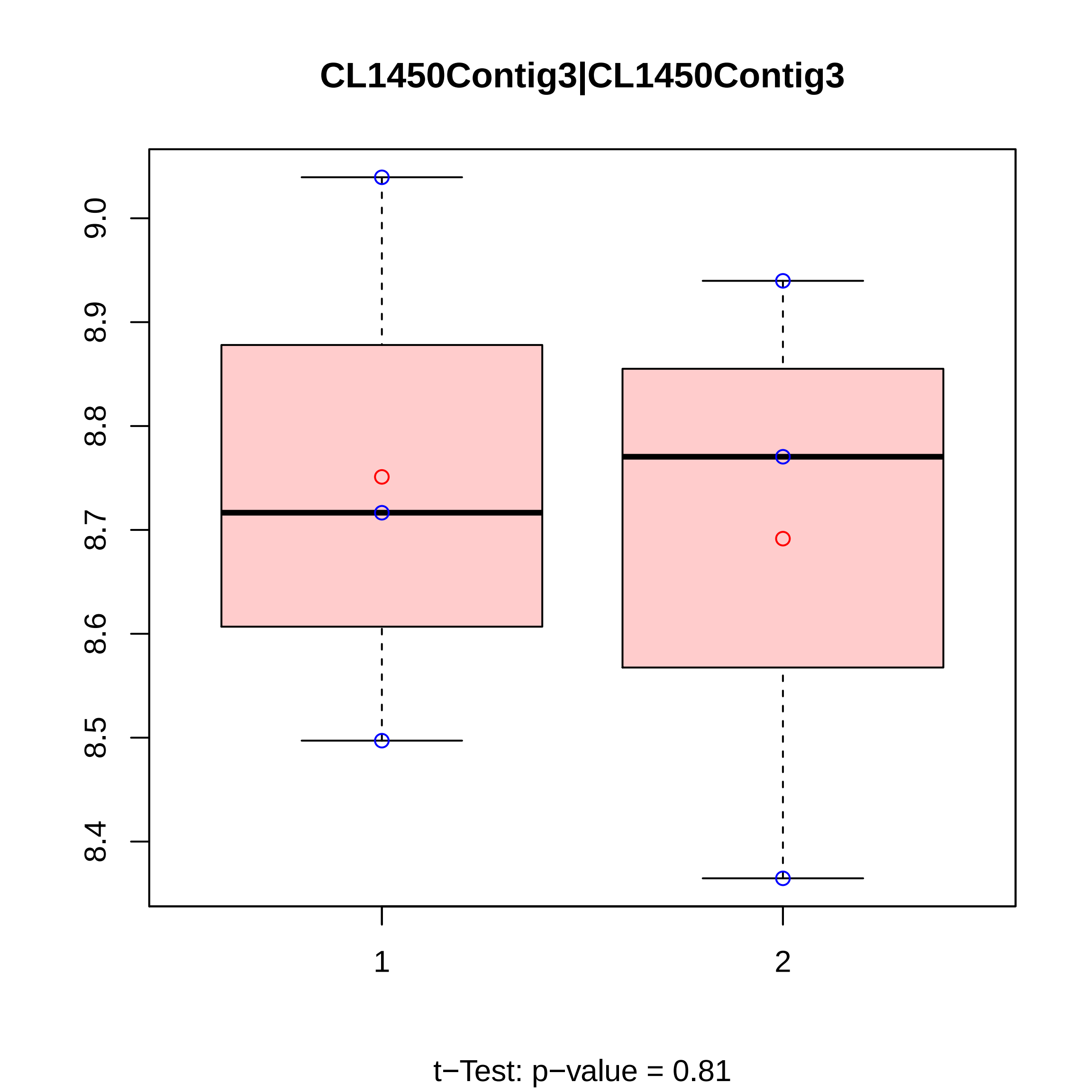
CL1450Contig3|CL1450Contig3
9.0
8.9
8.8
8.7
8.6
8.5
8.4
1
2
t−Test: p−value = 0.81

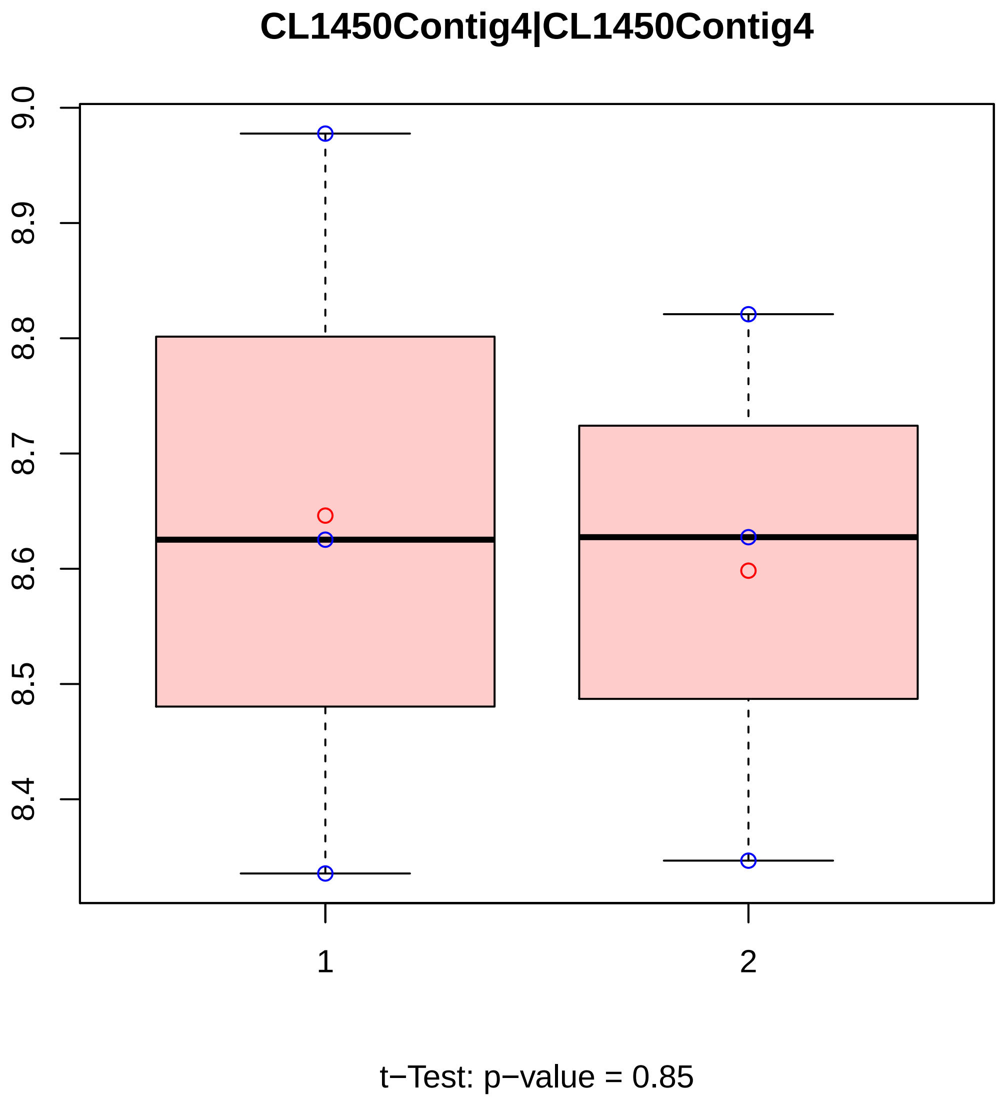
CL1450Contig4|CL1450Contig4
9.0
8.9
8.8
8.7
8.6
8.5
8.4
1
2
t−Test: p−value = 0.85

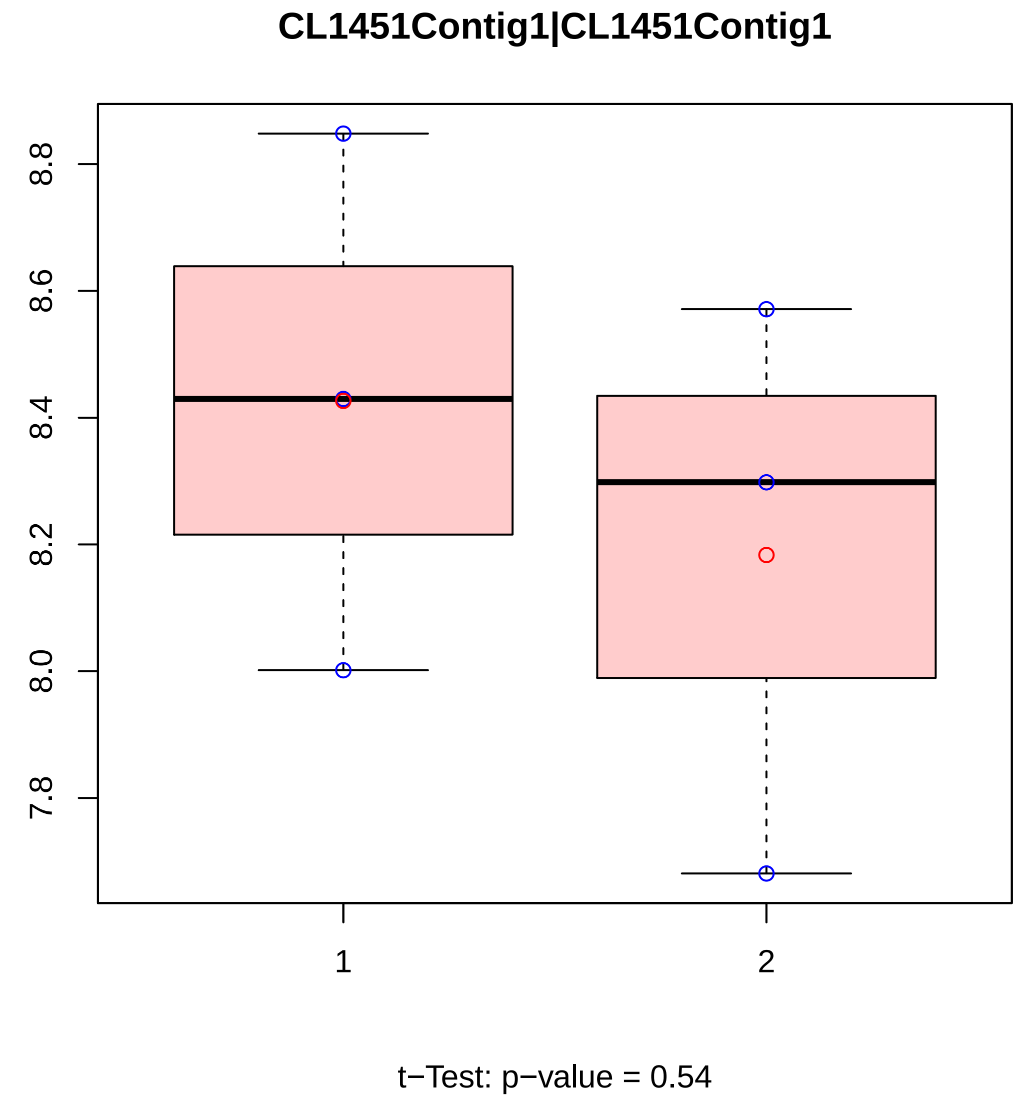
CL1451Contig1|CL1451Contig1
8.8
8.6
8.4
8.2
8.0
7.8
1
2
t−Test: p−value = 0.54

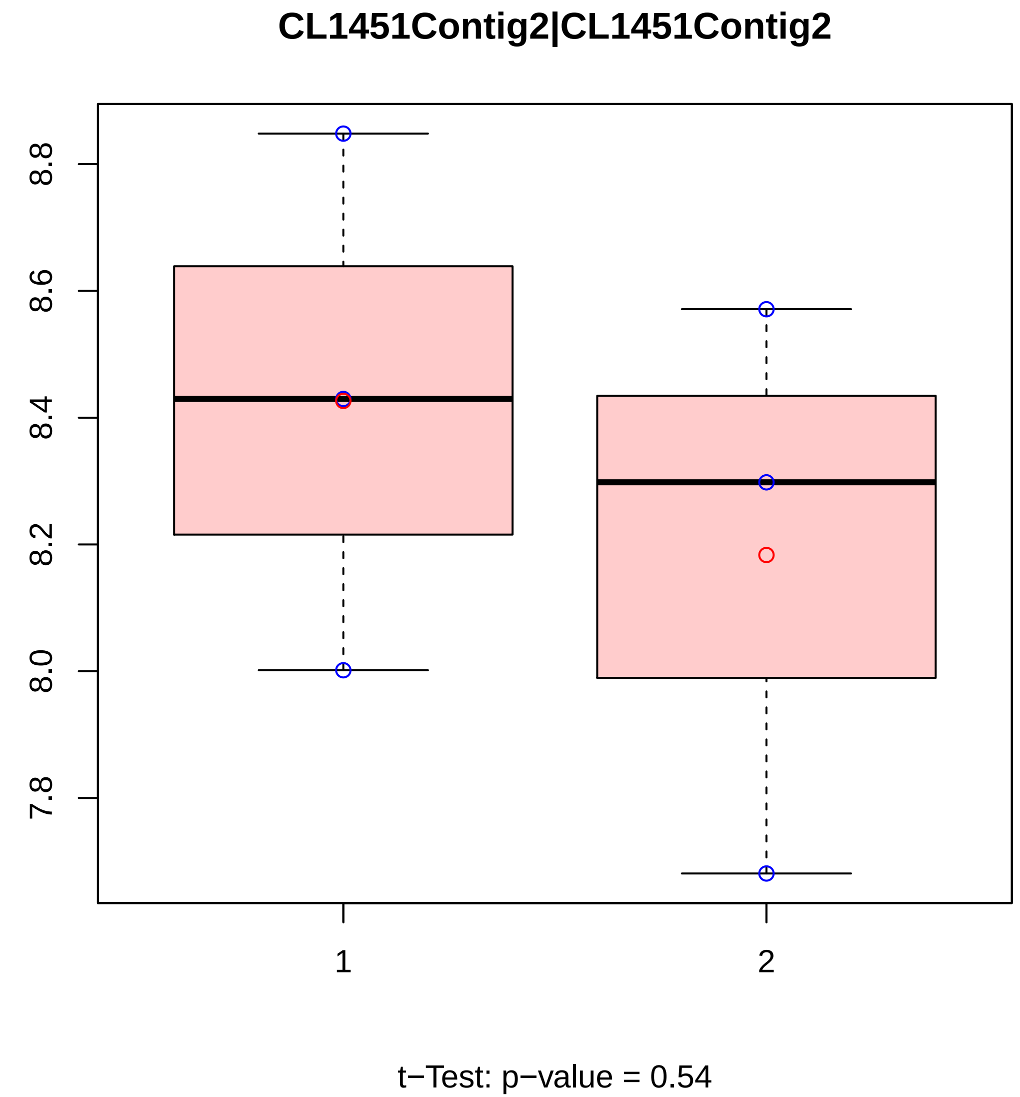
CL1451Contig2|CL1451Contig2
8.8
8.6
8.4
8.2
8.0
7.8
1
2
t−Test: p−value = 0.54

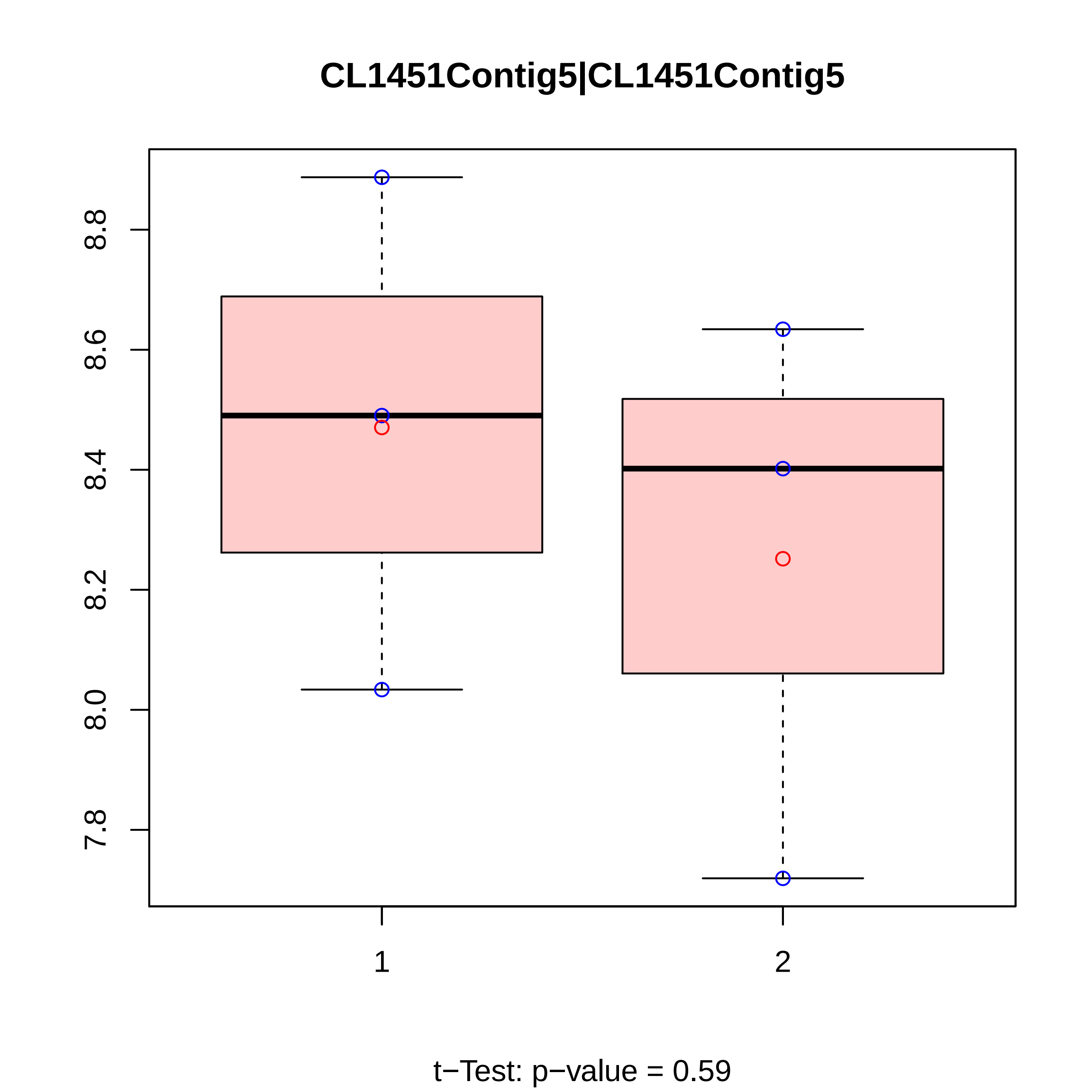
CL1451Contig5|CL1451Contig5
8.8
8.6
8.4
8.2
8.0
7.8
1
2
t−Test: p−value = 0.59

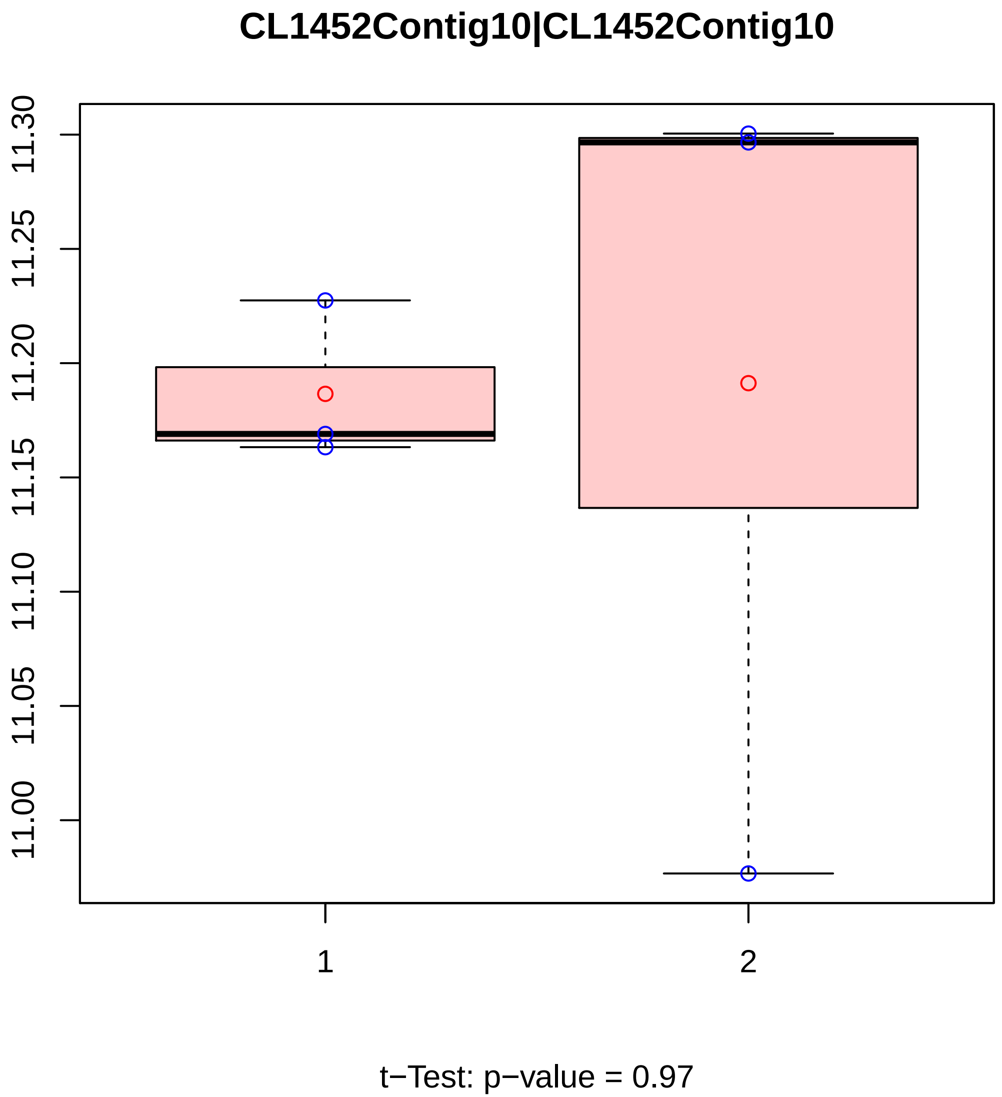

CL1452Contig10|CL1452Contig10
11.30
11.25
11.20
11.15
11.10
11.05
11.00
1
2
t−Test: p−value = 0.97

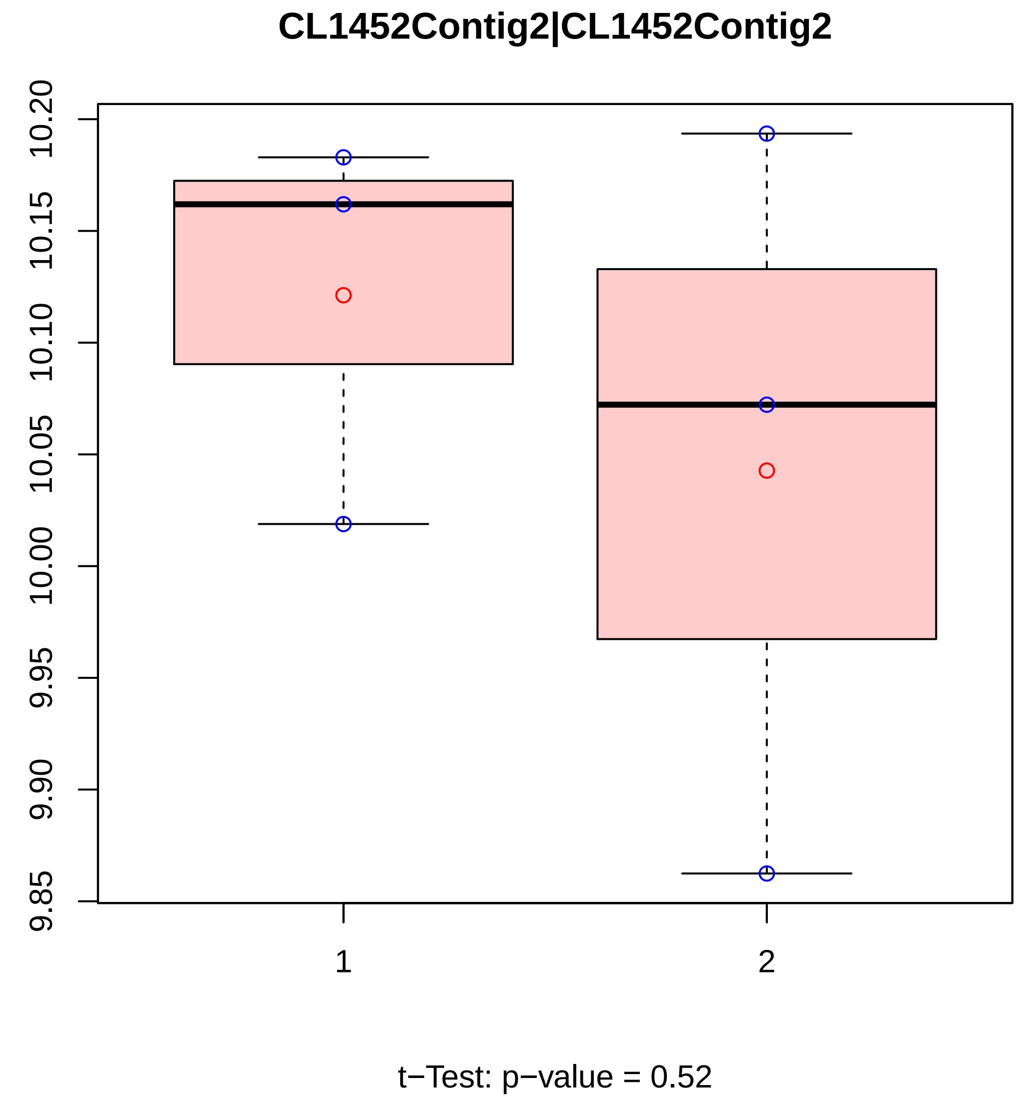
CL1452Contig2|CL1452Contig2
10.20
10.15
10.10
10.05
10.00
9.95
9.90
9.85
1
2
t−Test: p−value = 0.52

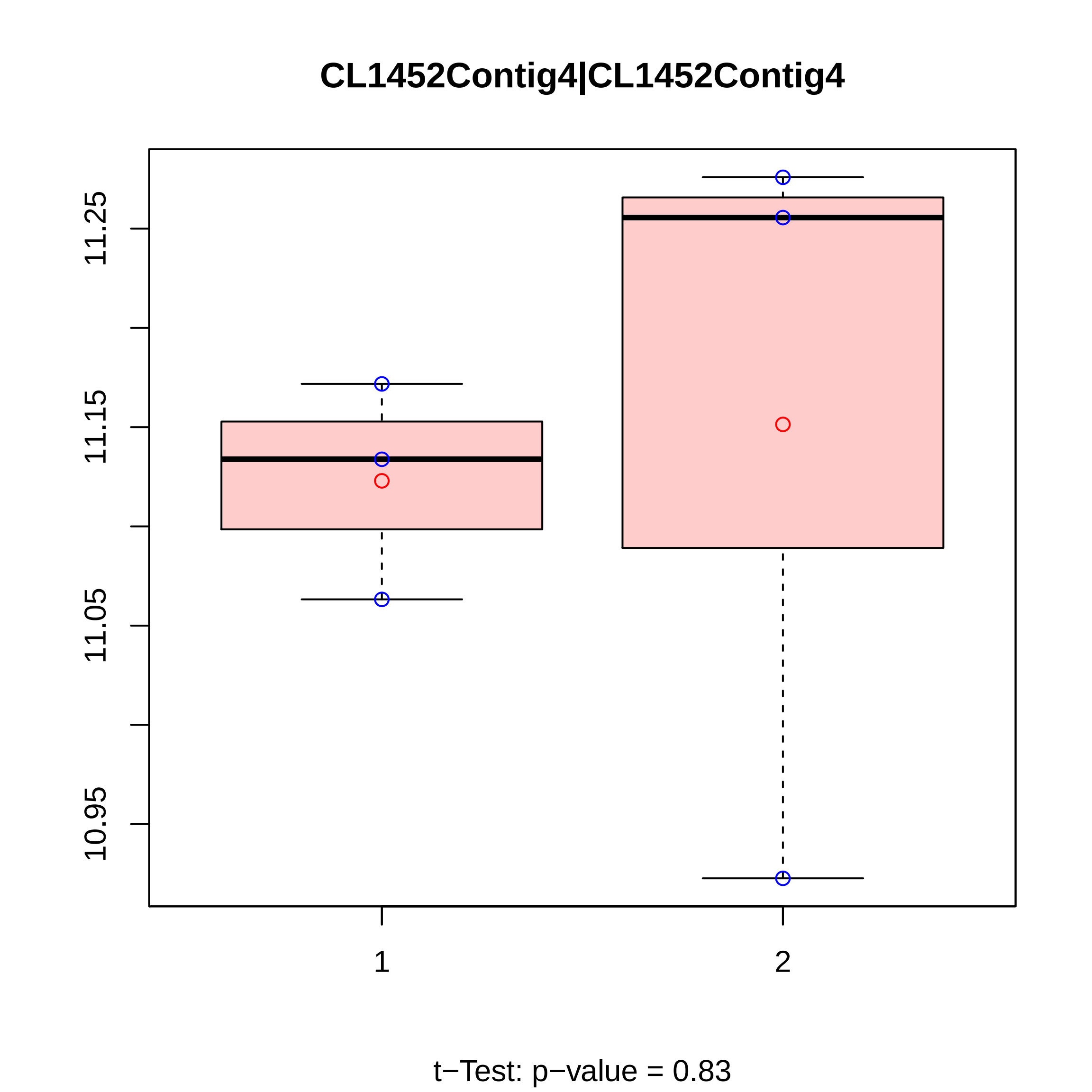
CL1452Contig4|CL1452Contig4
11.25
11.15
11.05
10.95
1
2
t−Test: p−value = 0.83

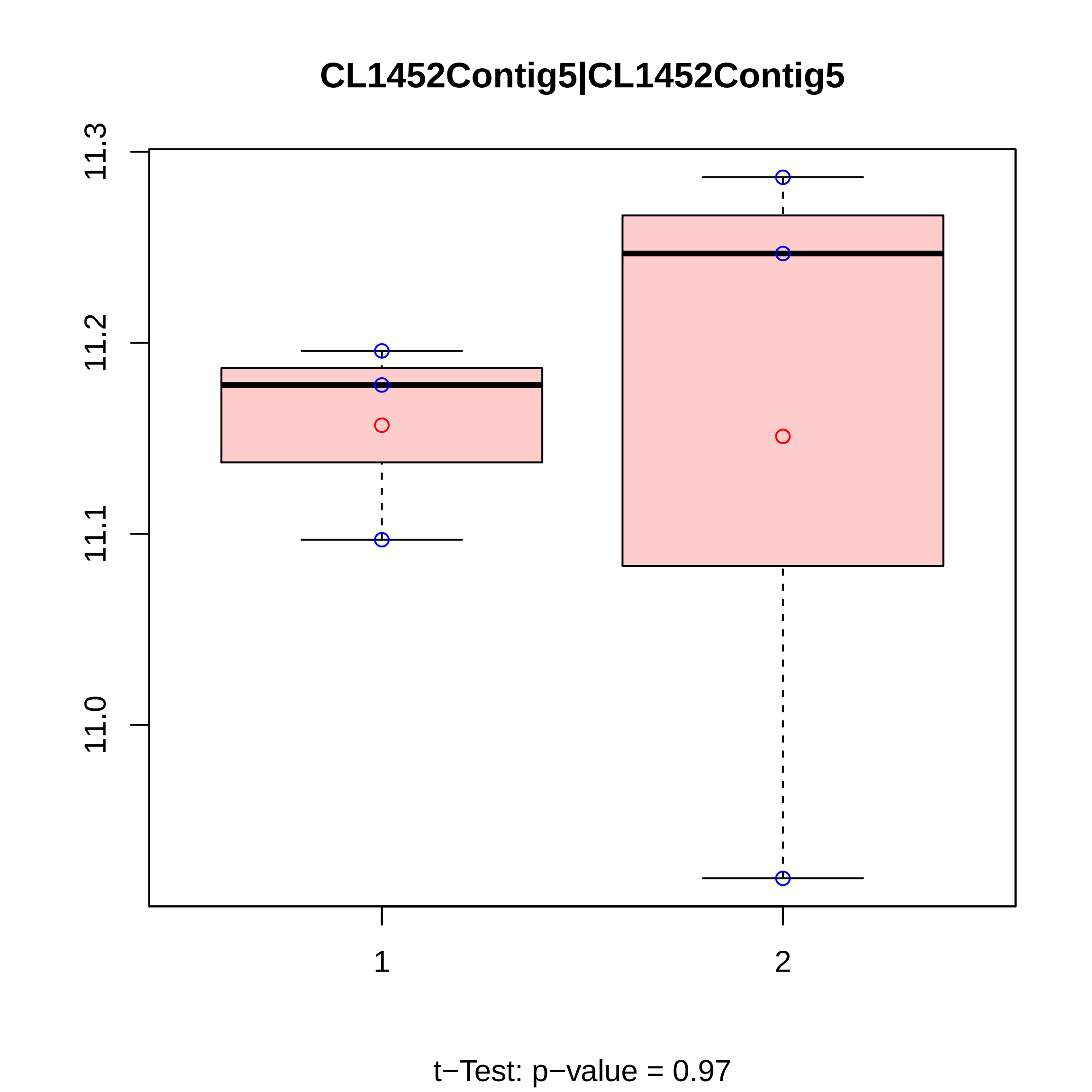
CL1452Contig5|CL1452Contig5
11.3
11.2
11.1
11.0
1
2
t−Test: p−value = 0.97

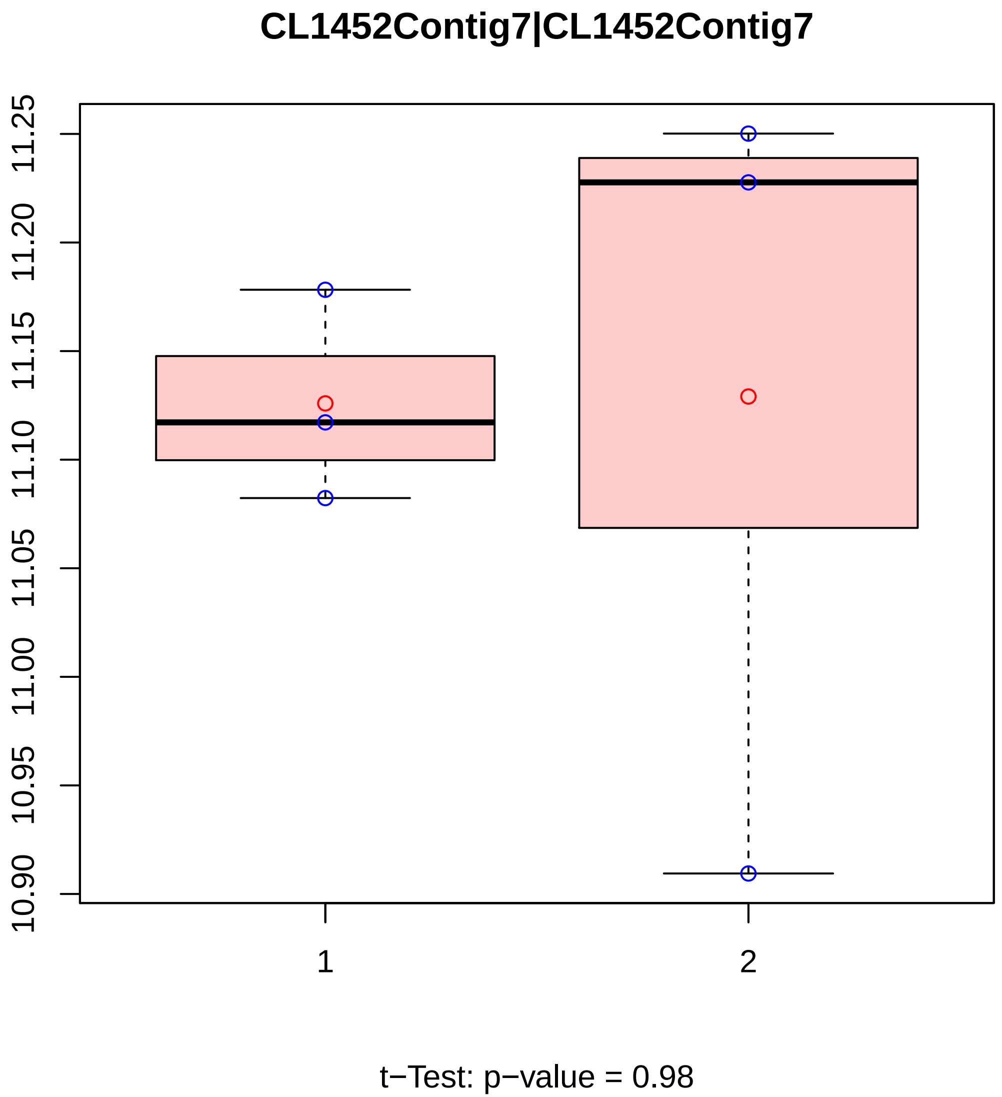
CL1452Contig7|CL1452Contig7
11.25
11.20
11.15
11.10
11.05
11.00
10.95
10.90
1
2
t−Test: p−value = 0.98

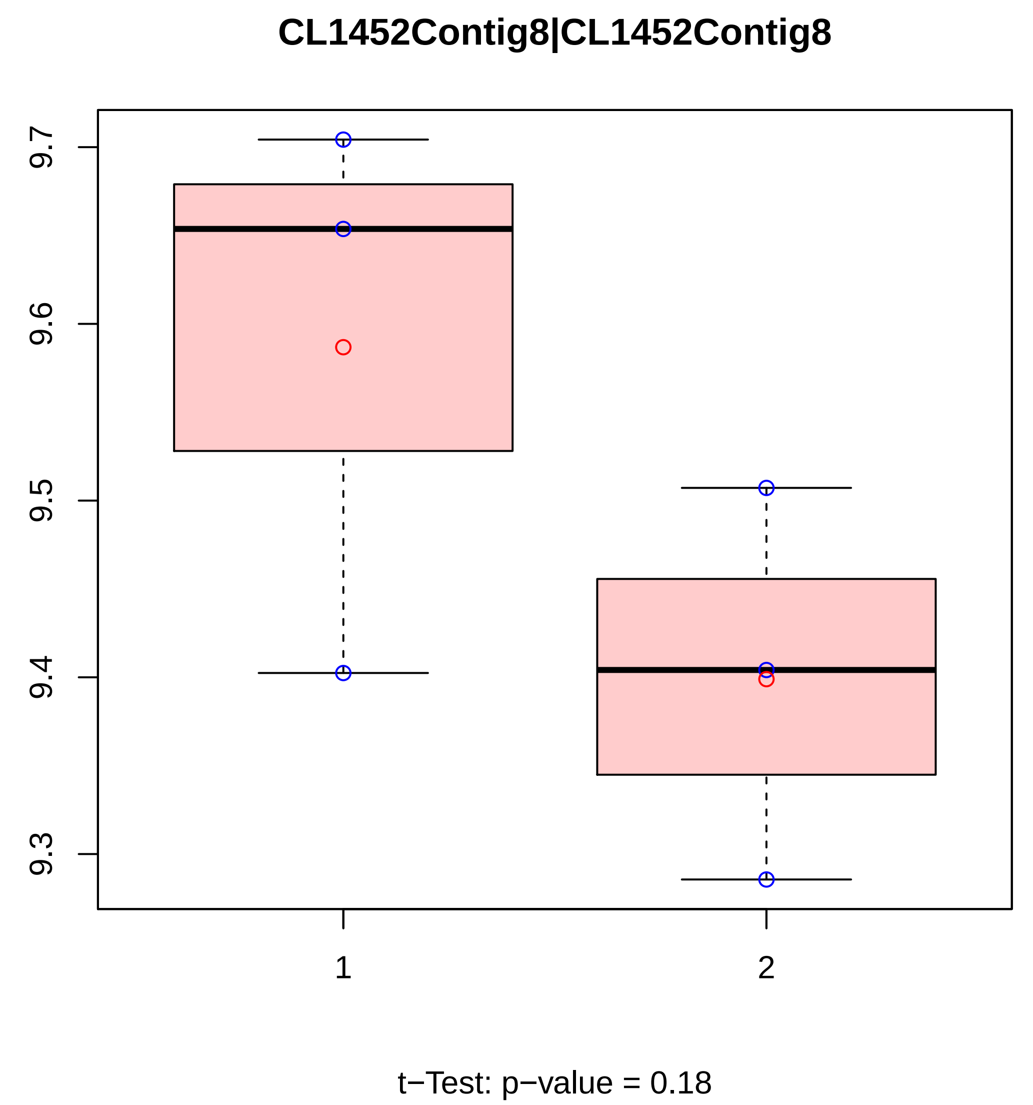
CL1452Contig8|CL1452Contig8
9.7
9.6
9.5
9.4
9.3
1
2
t−Test: p−value = 0.18

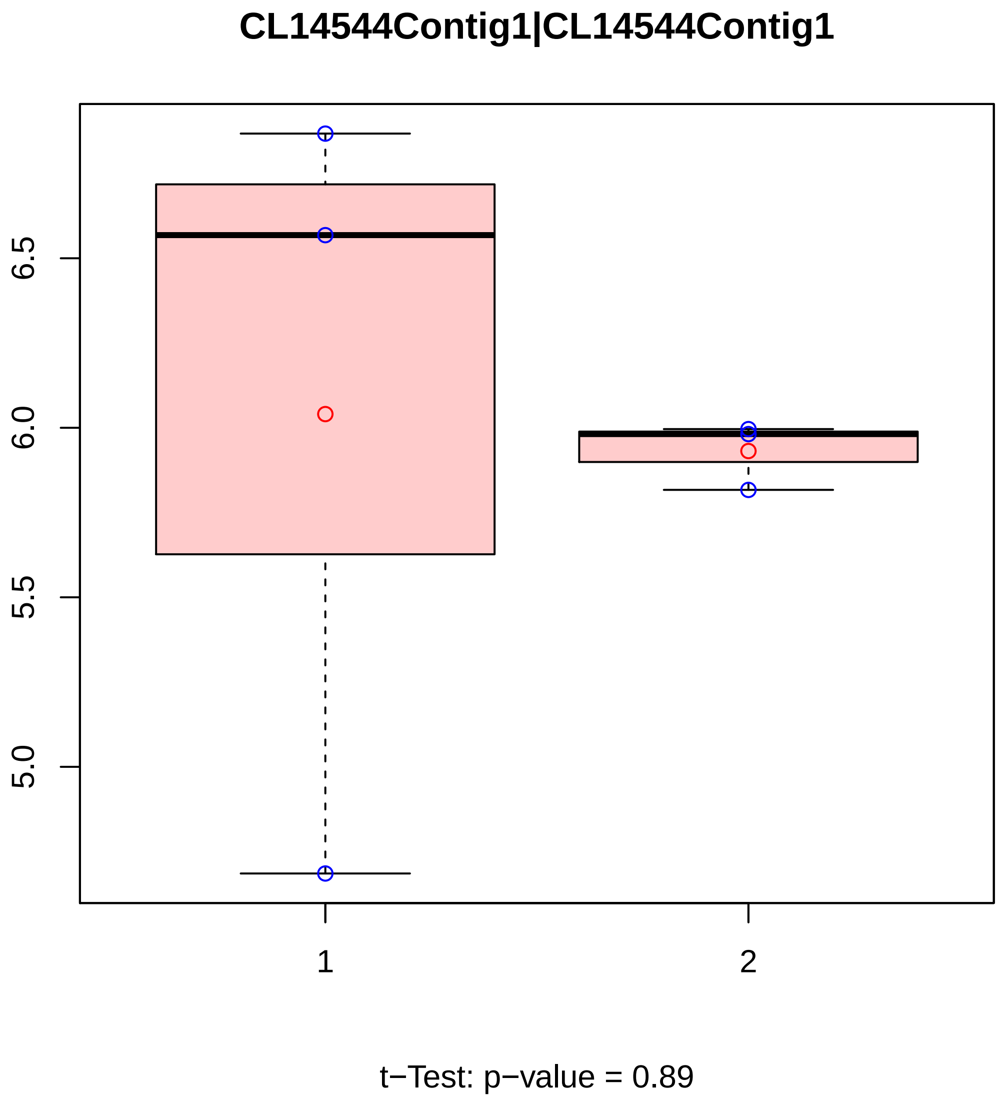
CL14544Contig1|CL14544Contig1
5.0
5.5
6.0
6.5
1
2
t−Test: p−value = 0.89

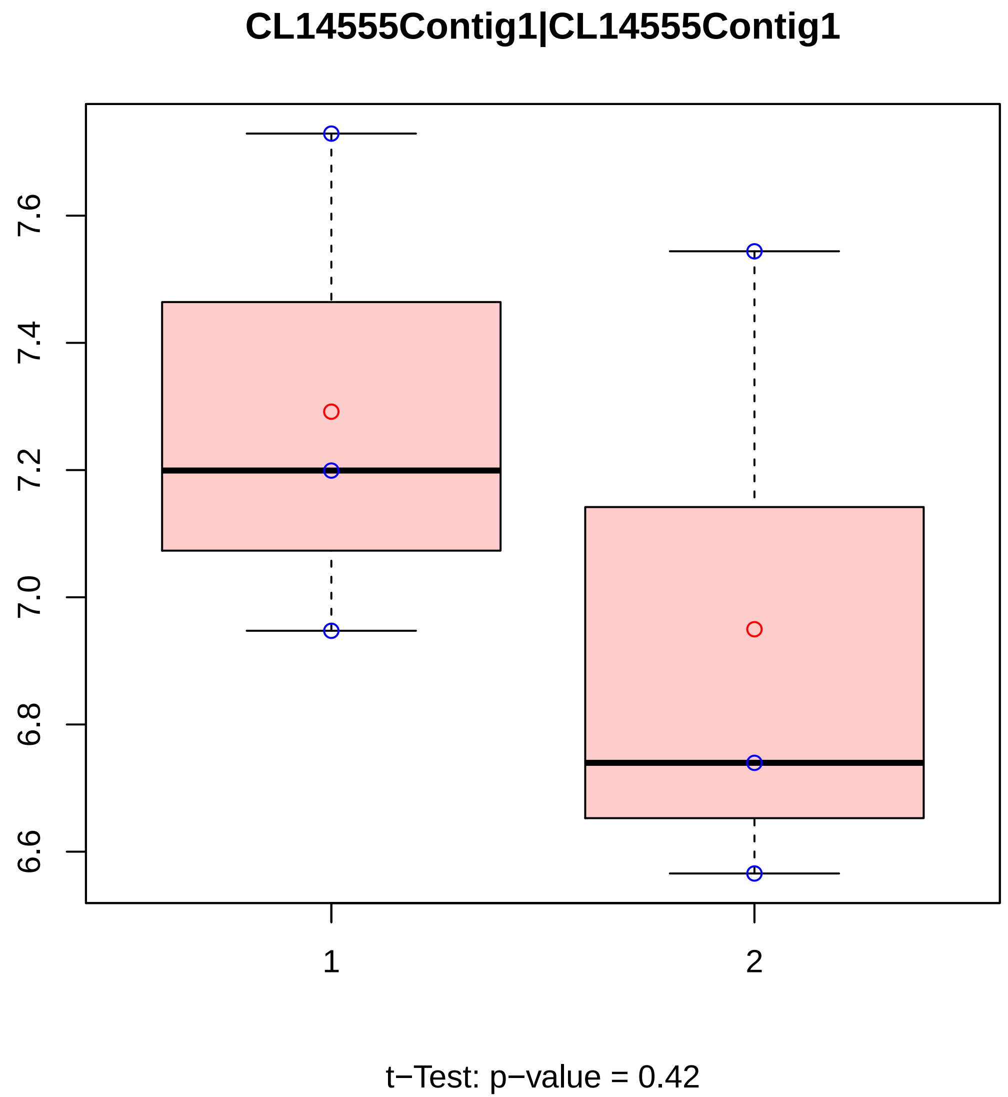

CL14555Contig1|CL14555Contig1
7.6
7.4
7.2
7.0
6.8
6.6
1
2
t−Test: p−value = 0.42

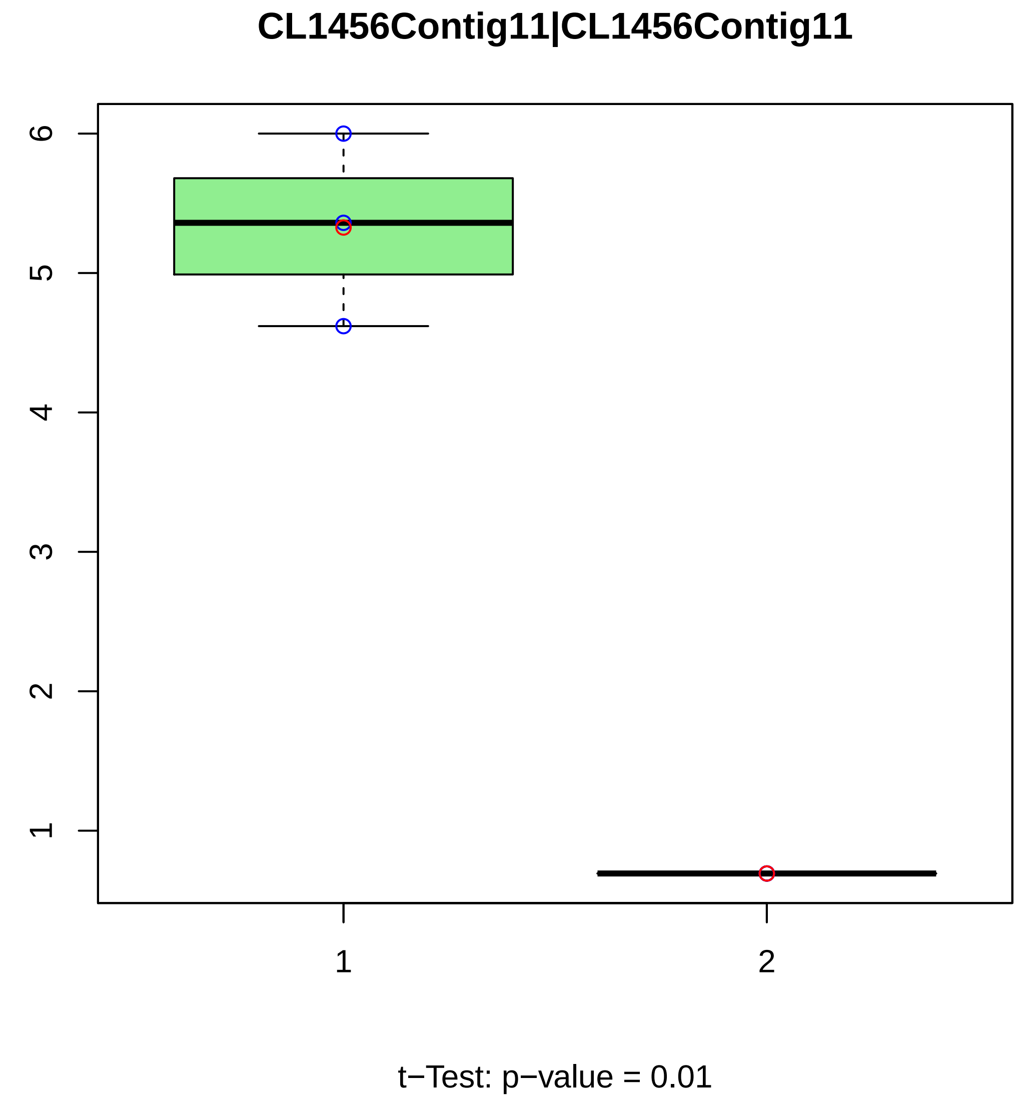
CL1456Contig11|CL1456Contig11
1
2
3
4
5
6
1
2
t−Test: p−value = 0.01

# CL1456Contig12|CL1456Contig12

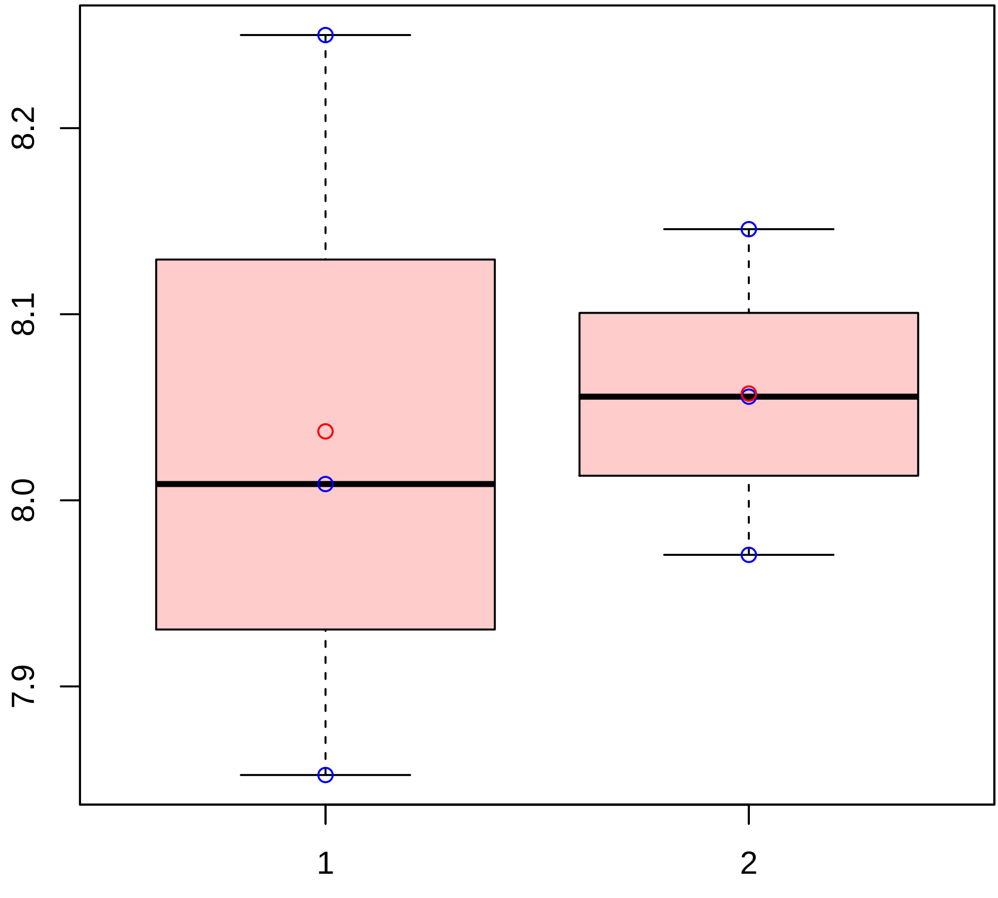


t−Test: p−value = 0.88

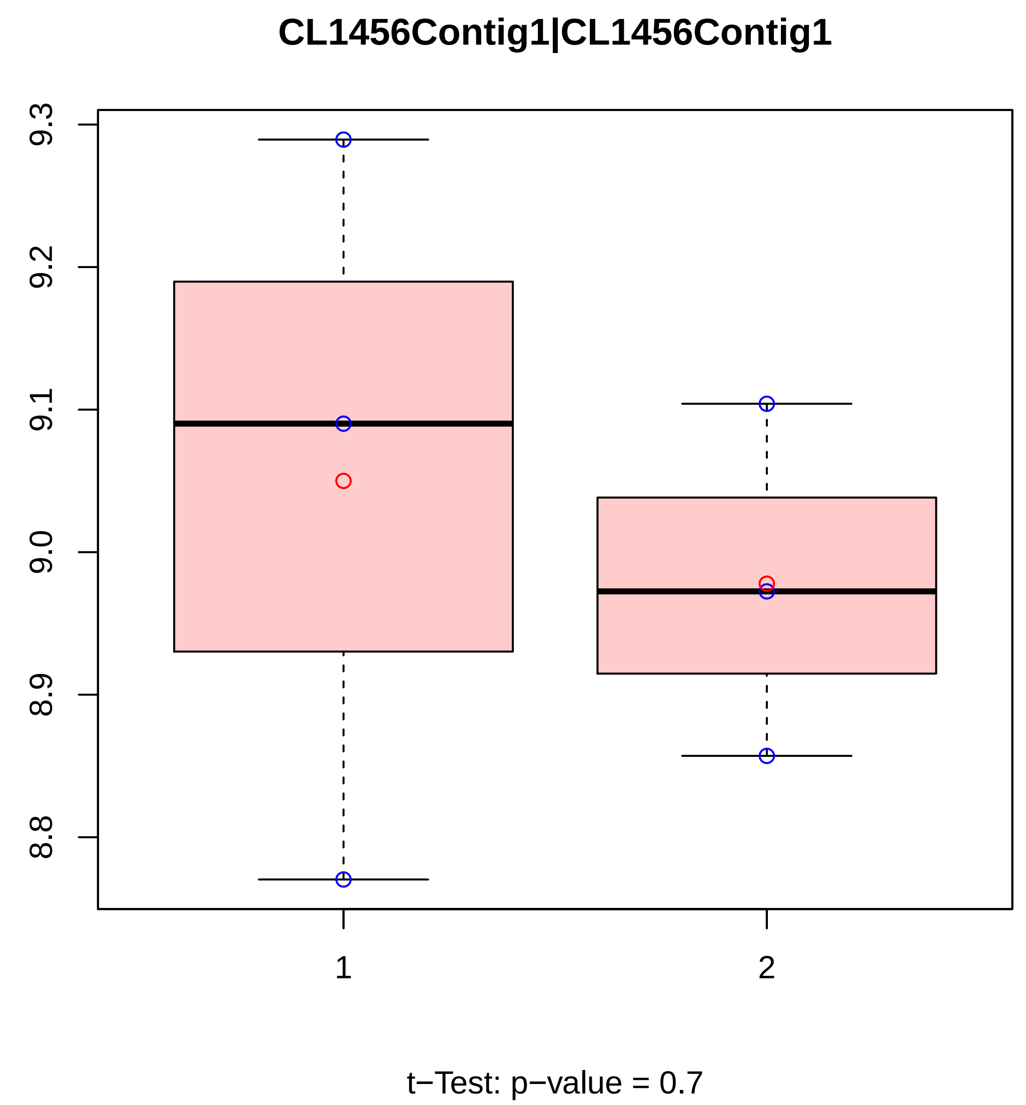
CL1456Contig1|CL1456Contig1
9.3
9.2
9.1
9.0
8.9
8.8
1
2
t−Test: p−value = 0.7

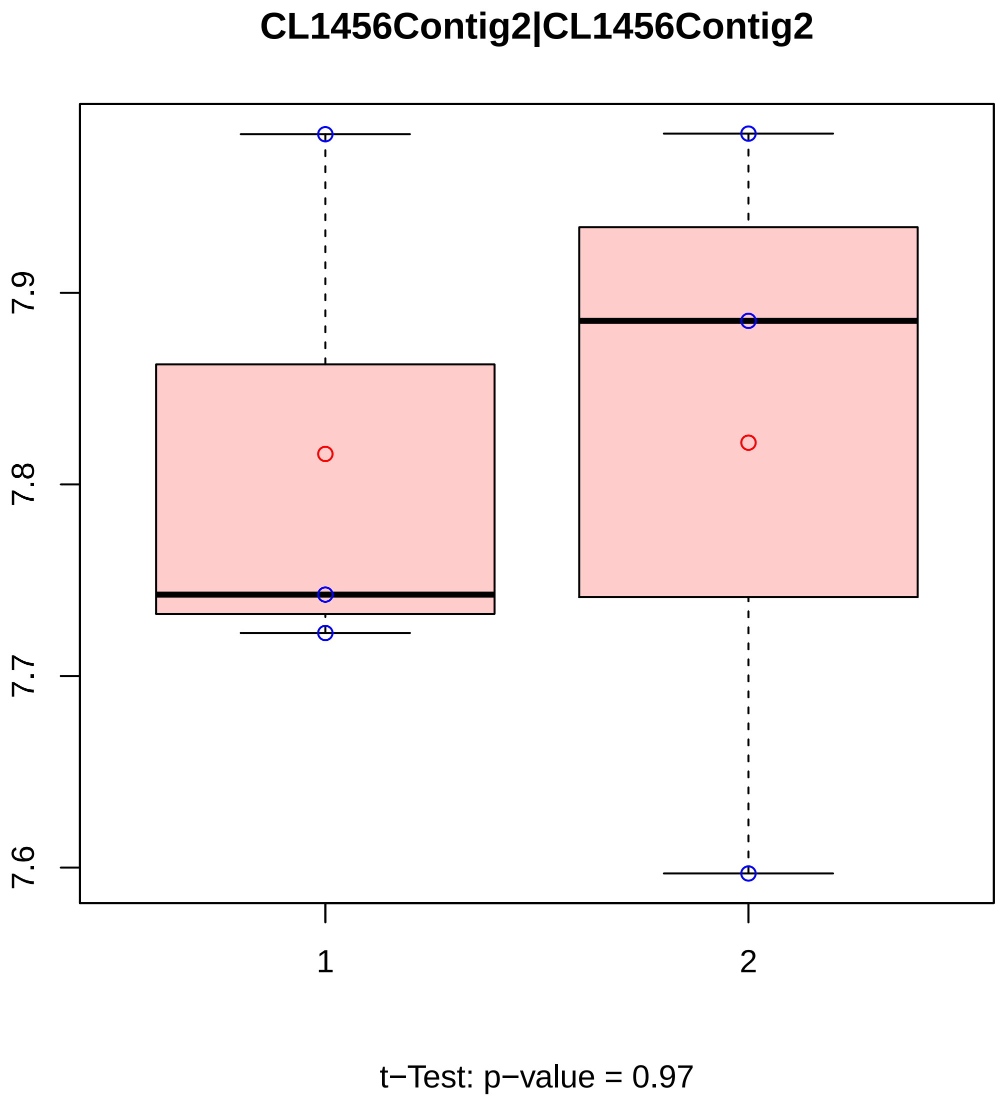

CL1456Contig2|CL1456Contig2
7.6
7.7
7.8
7.9
1
2
t−Test: p−value = 0.97

# CL1456Contig3|CL1456Contig3

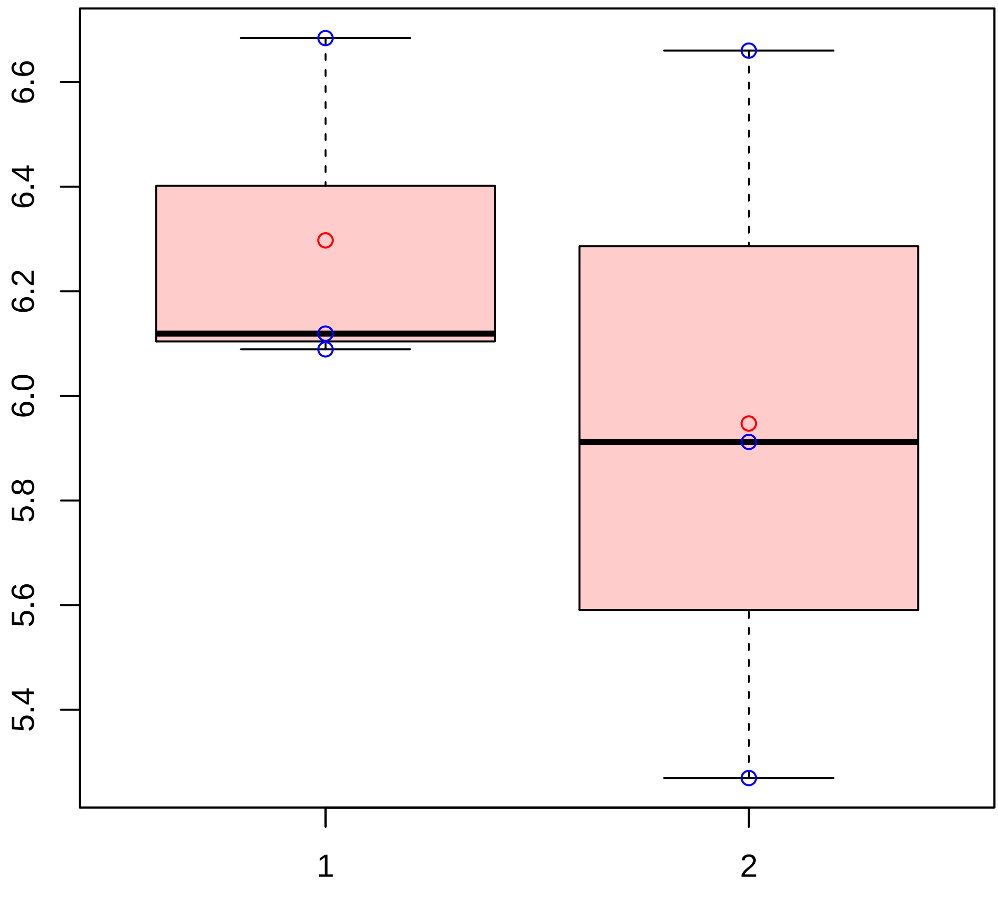


t−Test: p−value = 0.49

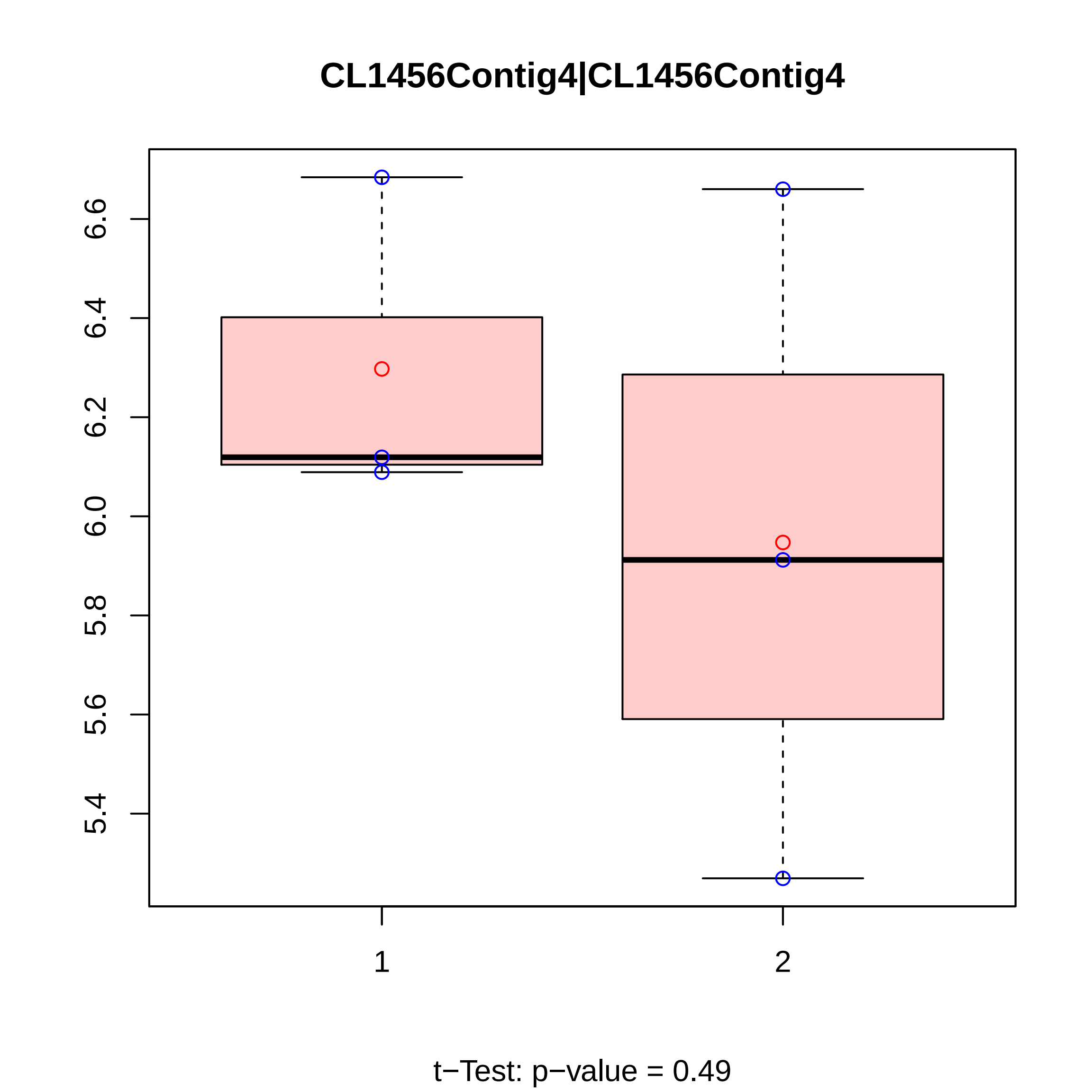
CL1456Contig4|CL1456Contig4
6.6
6.4
6.2
6.0
5.8
5.6
5.4
1
2
t−Test: p−value = 0.49

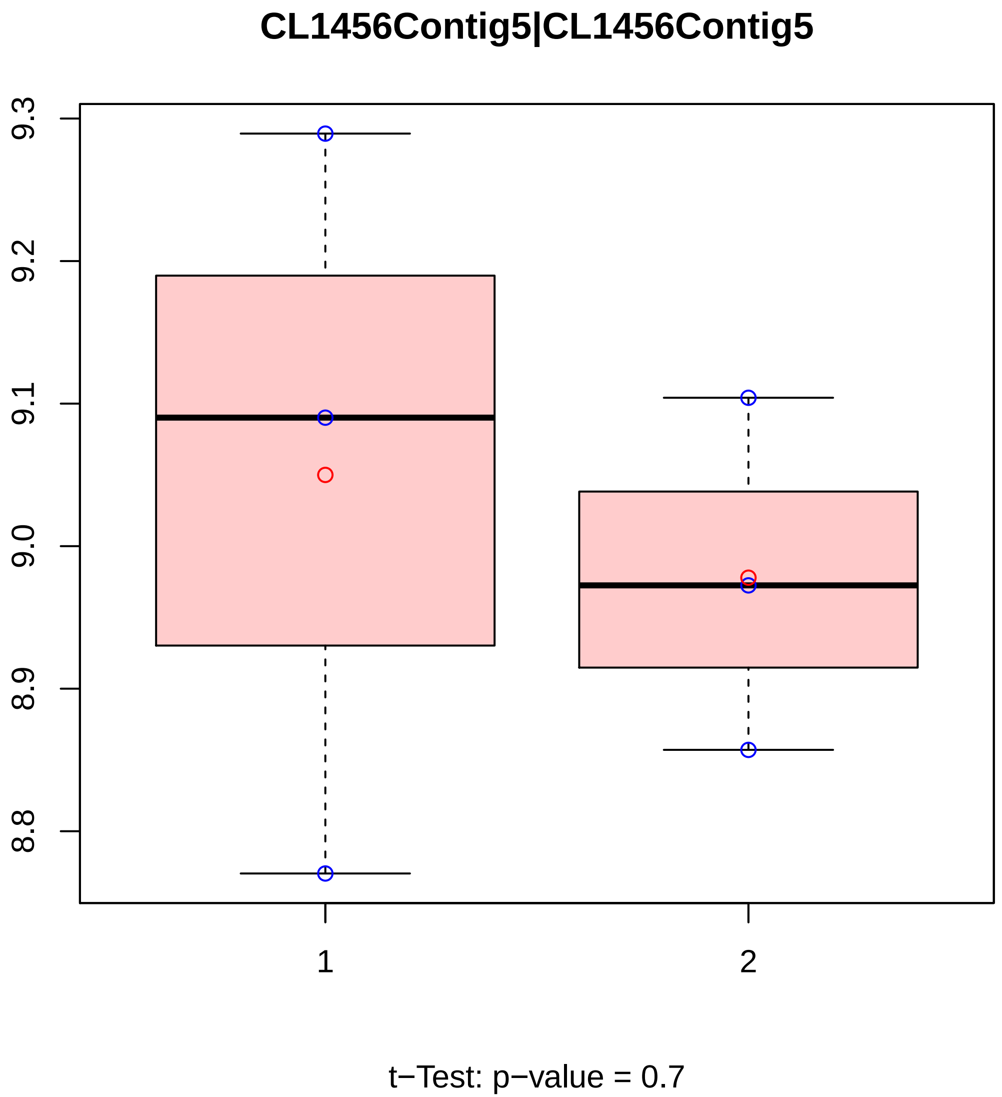
CL1456Contig5|CL1456Contig5
9.3
9.2
9.1
9.0
8.9
8.8
1
2
t−Test: p−value = 0.7

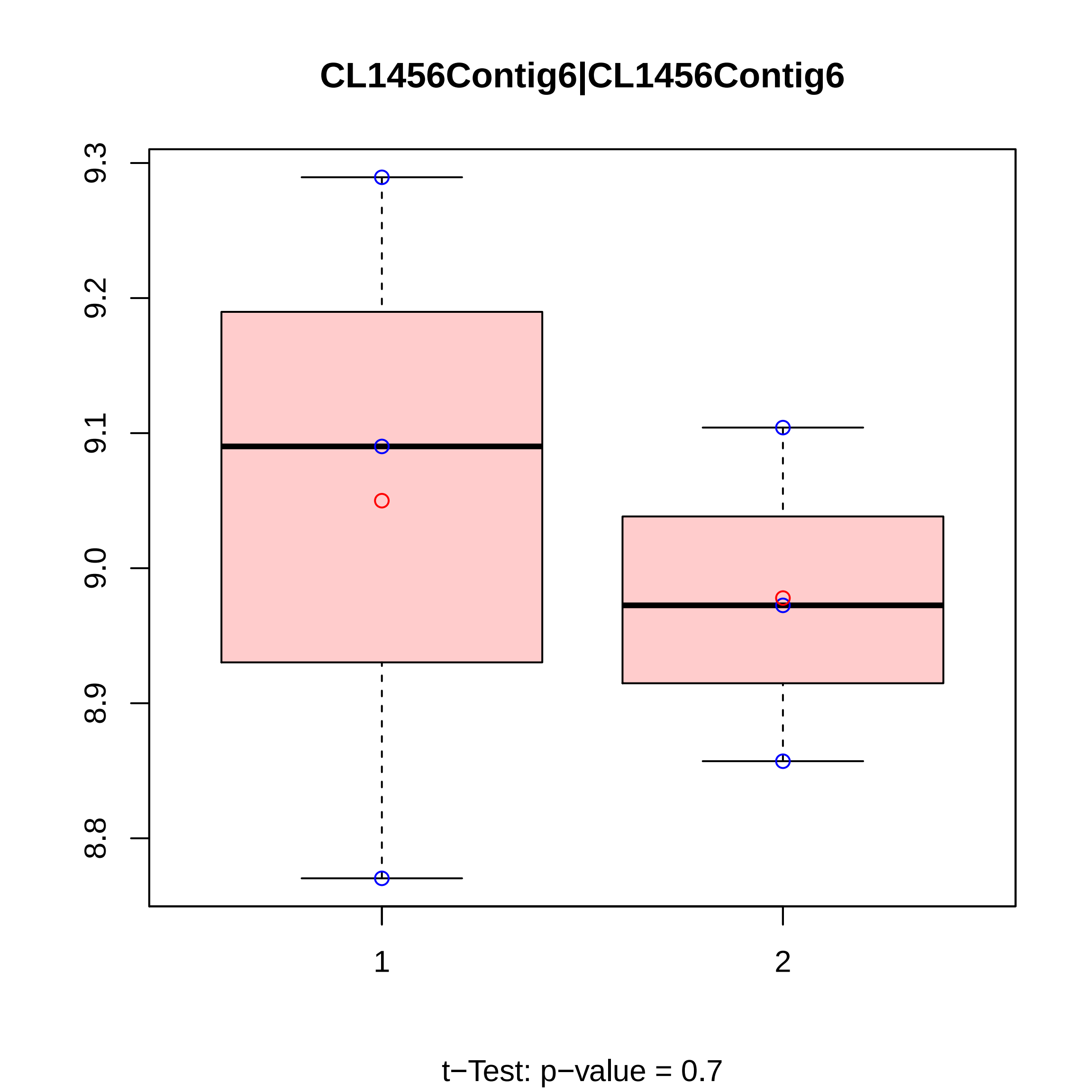

CL1456Contig6|CL1456Contig6
9.3
9.2
9.1
9.0
8.9
8.8
1
2
t−Test: p−value = 0.7

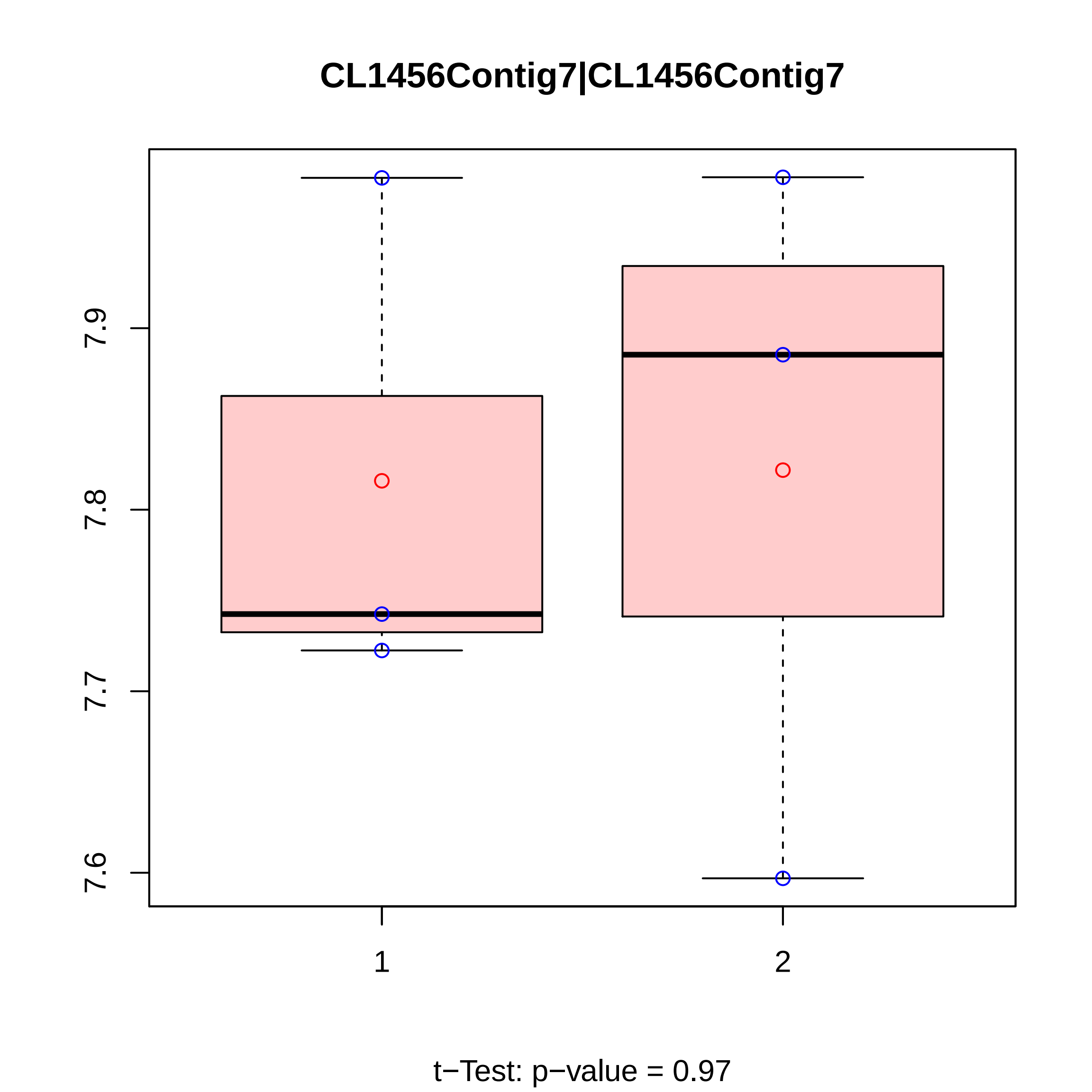
CL1456Contig7|CL1456Contig7
7.6
7.7
7.8
7.9
1
2
t−Test: p−value = 0.97

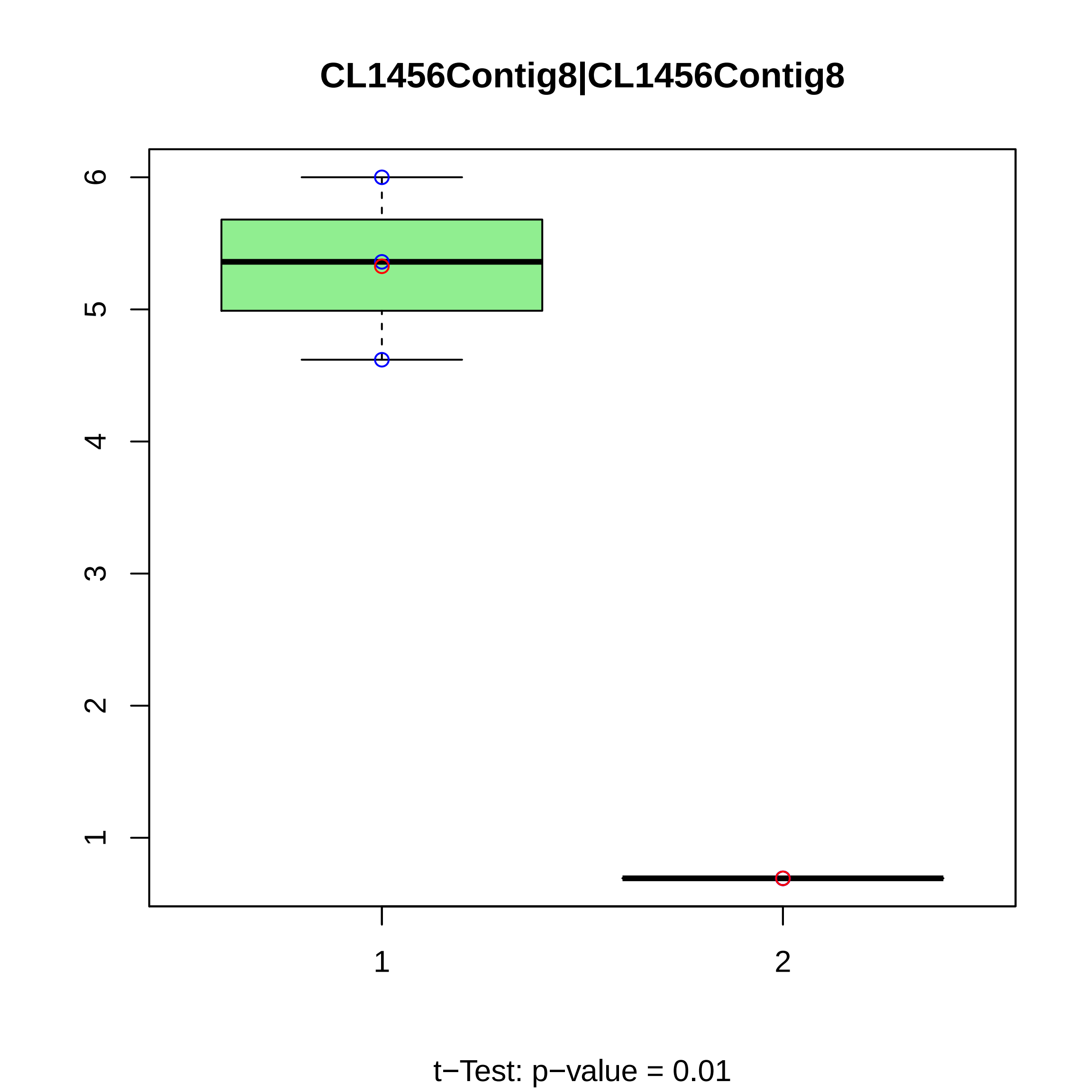
CL1456Contig8|CL1456Contig8
1
2
3
4
5
6
1
2
t−Test: p−value = 0.01

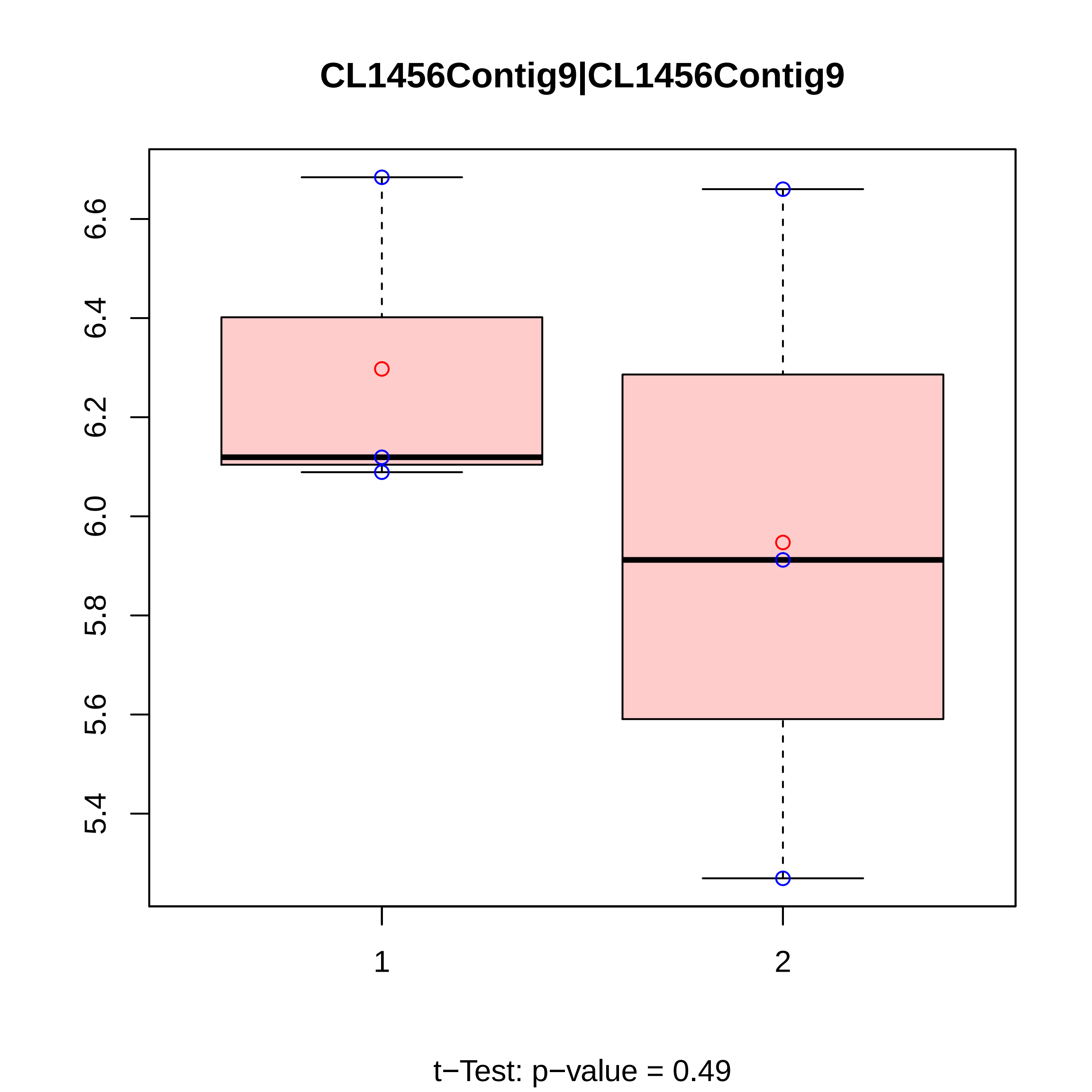

CL1456Contig9|CL1456Contig9
6.6
6.4
6.2
6.0
5.8
5.6
5.4
1
2
t−Test: p−value = 0.49

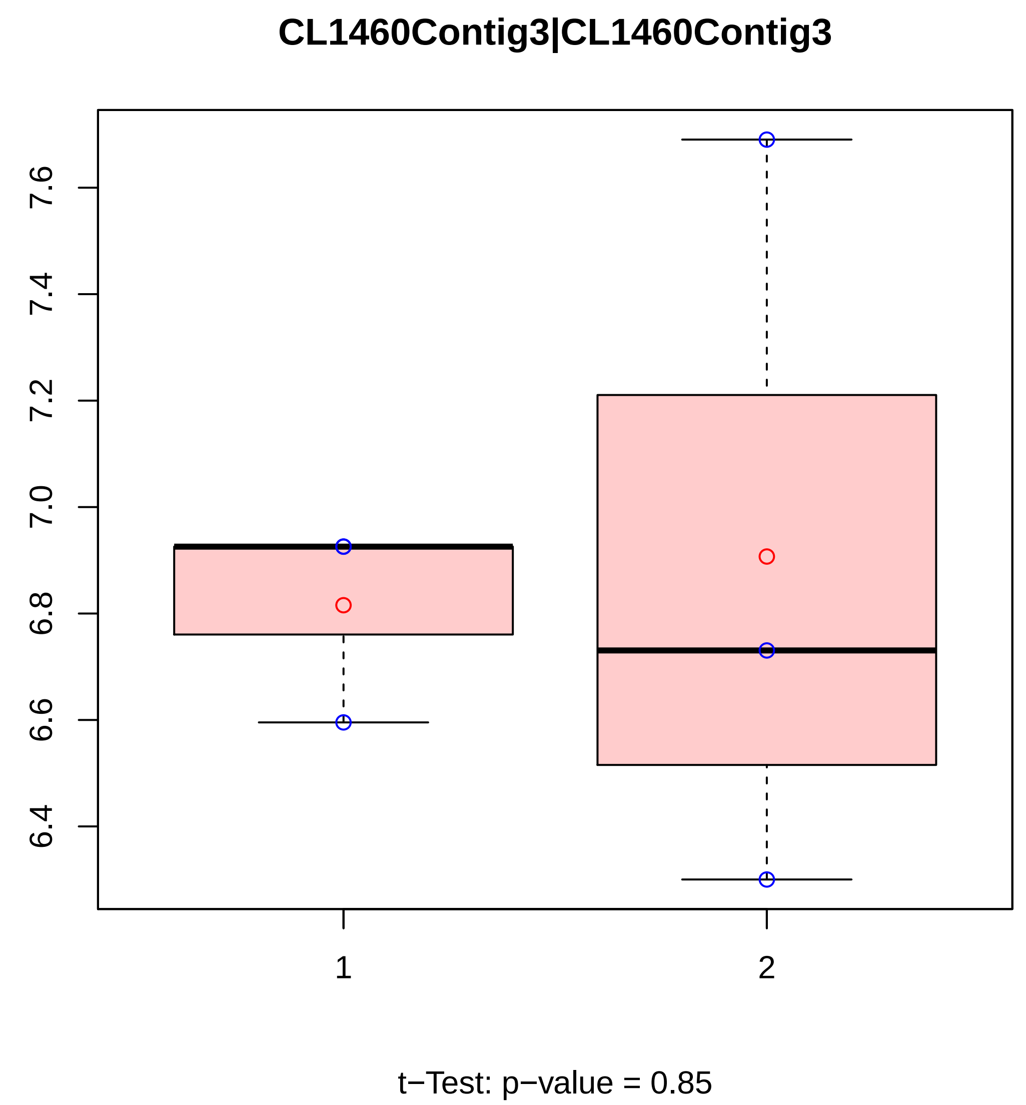
CL1460Contig3|CL1460Contig3
7.6
7.4
7.2
7.0
6.8
6.6
6.4
1
2
t−Test: p−value = 0.85

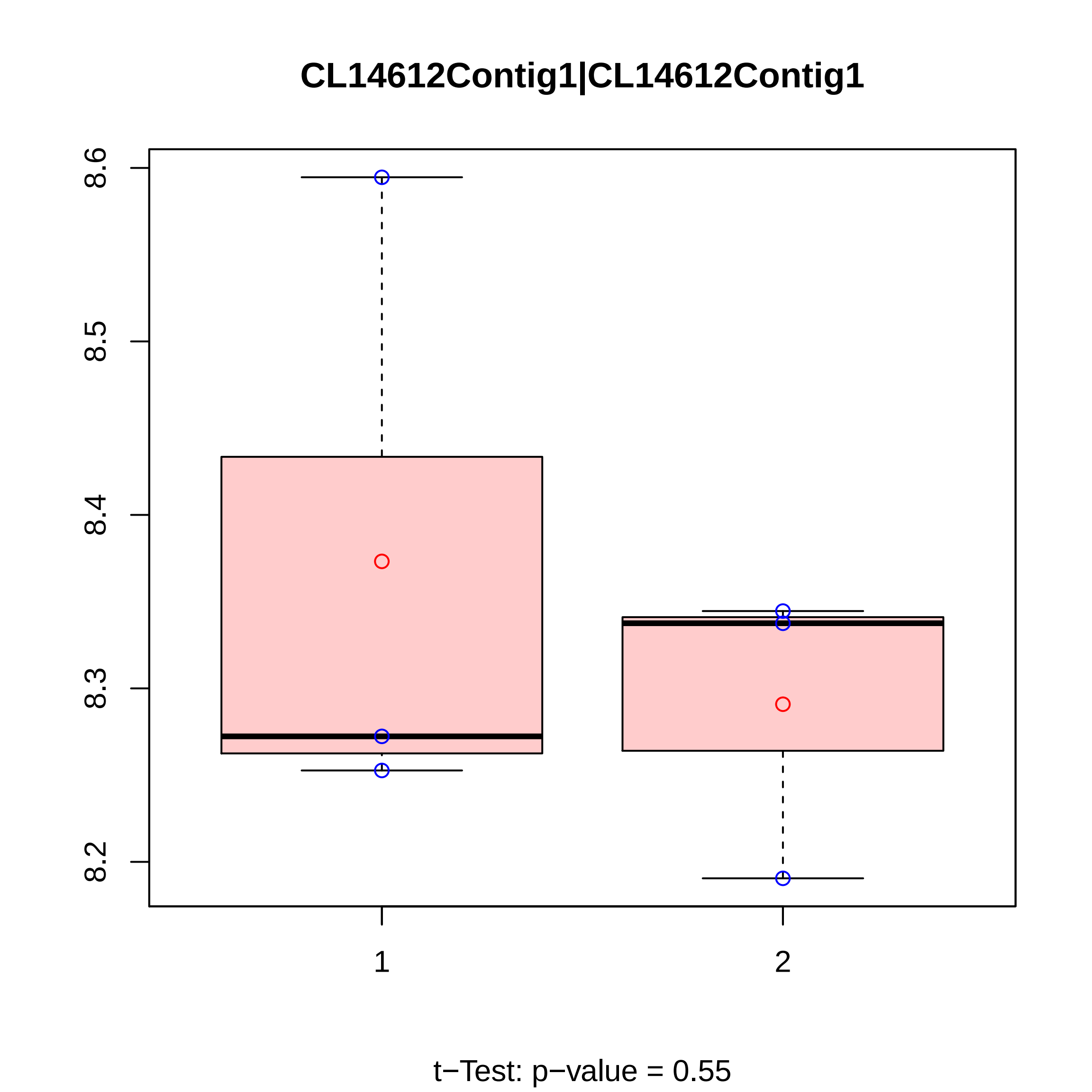
CL14612Contig1|CL14612Contig1
8.6
8.5
8.4
8.3
8.2
1
2
t−Test: p−value = 0.55

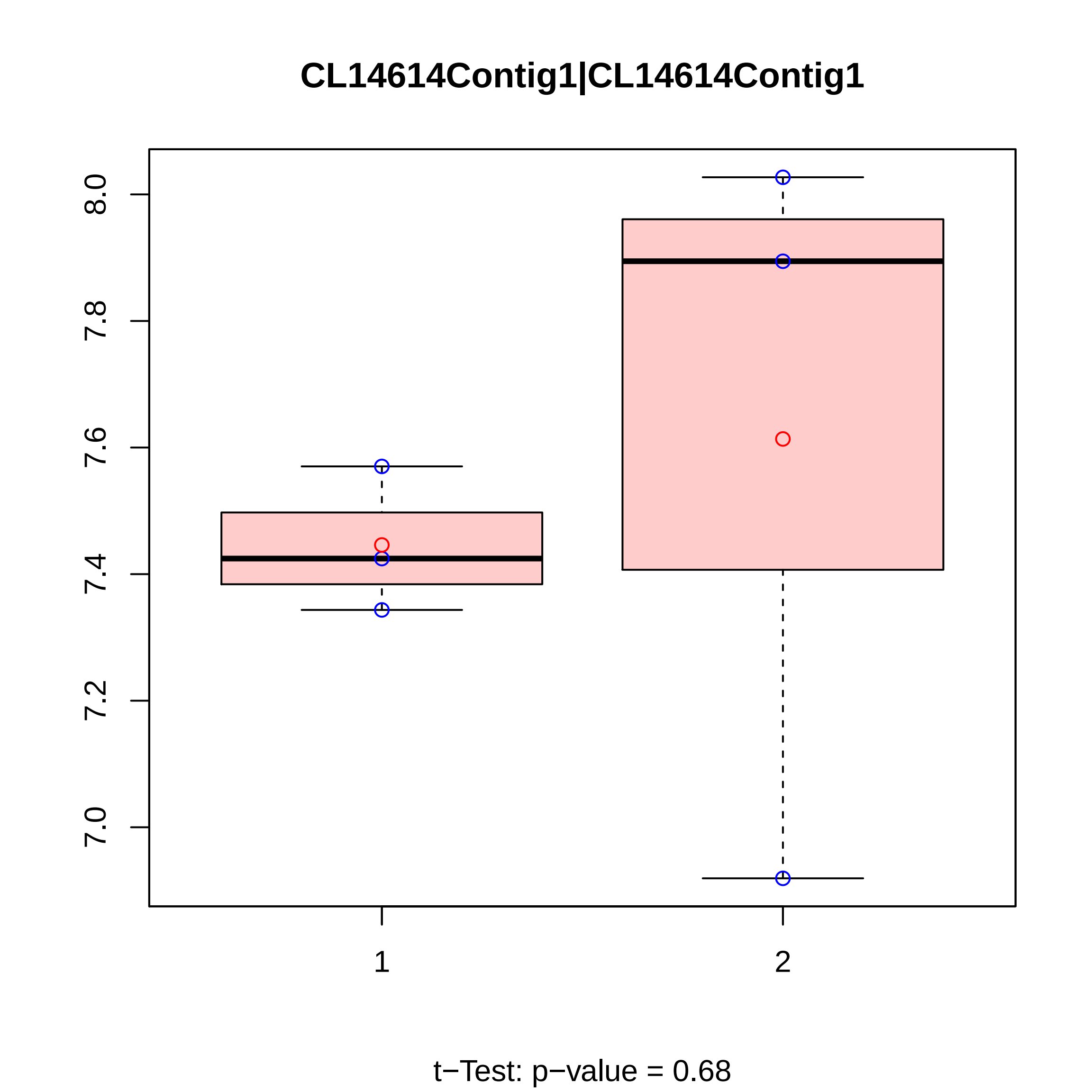

CL14614Contig1|CL14614Contig1
8.0
7.8
7.6
7.4
7.2
7.0
1
2
t−Test: p−value = 0.68

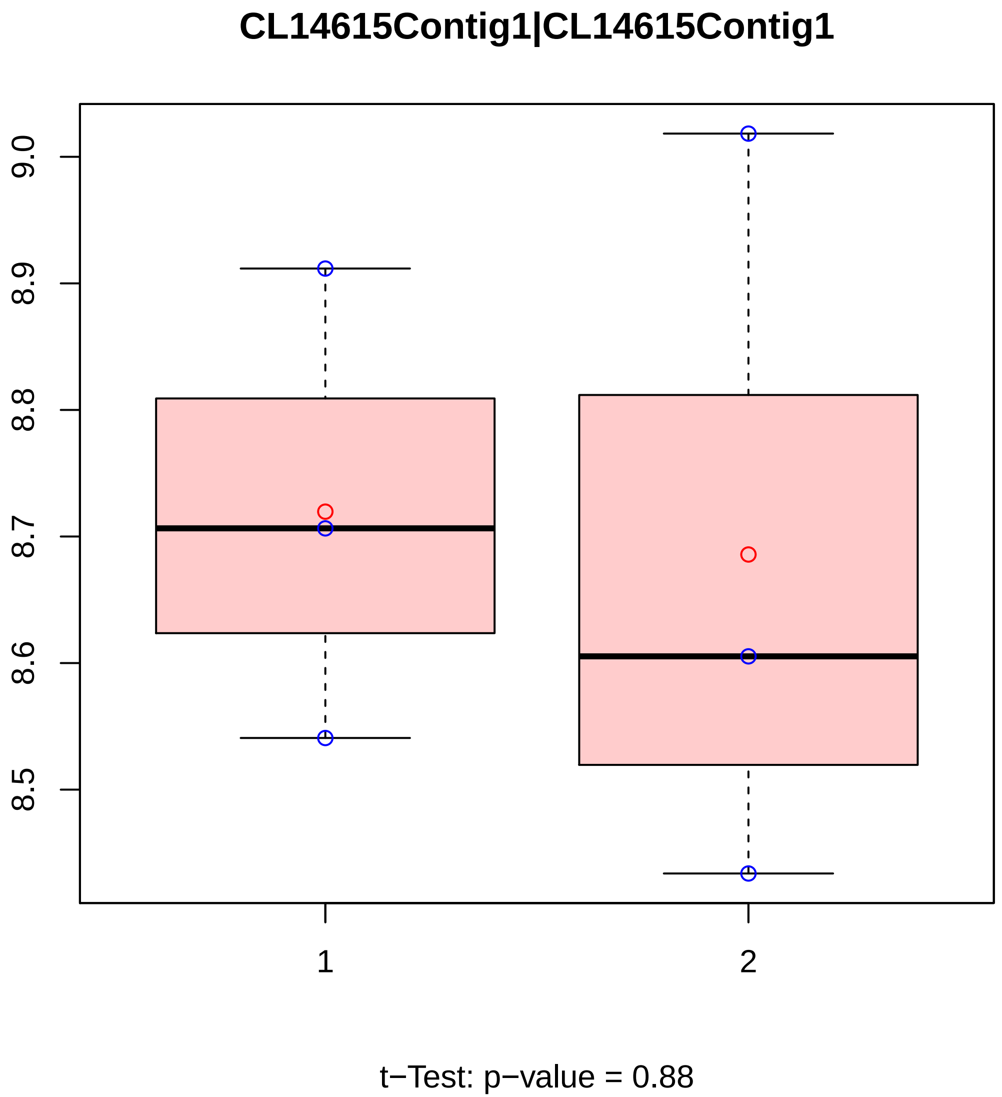
CL14615Contig1|CL14615Contig1
9.0
8.9
8.8
8.7
8.6
8.5
1
2
t−Test: p−value = 0.88

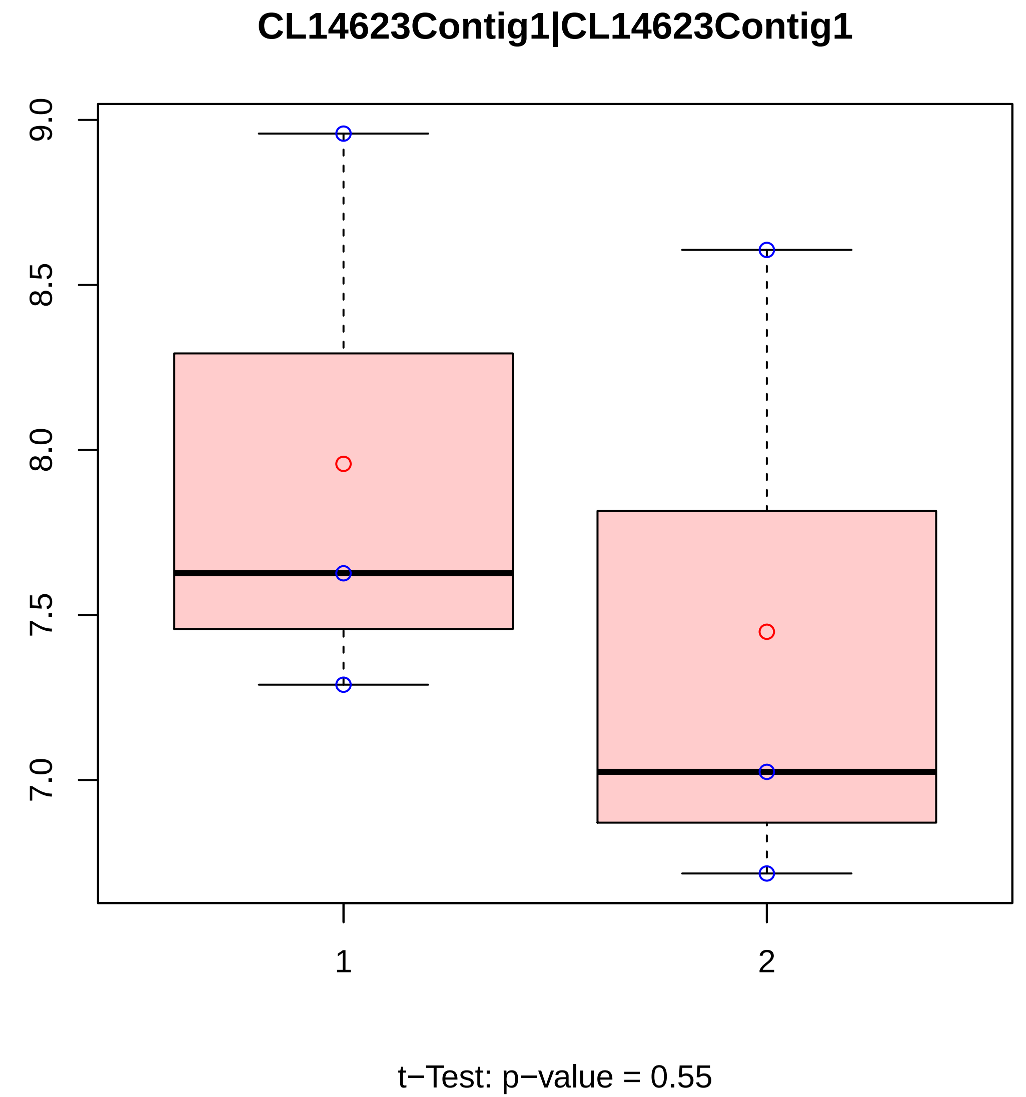
CL14623Contig1|CL14623Contig1
9.0
8.5
8.0
7.5
7.0
1
2
t−Test: p−value = 0.55

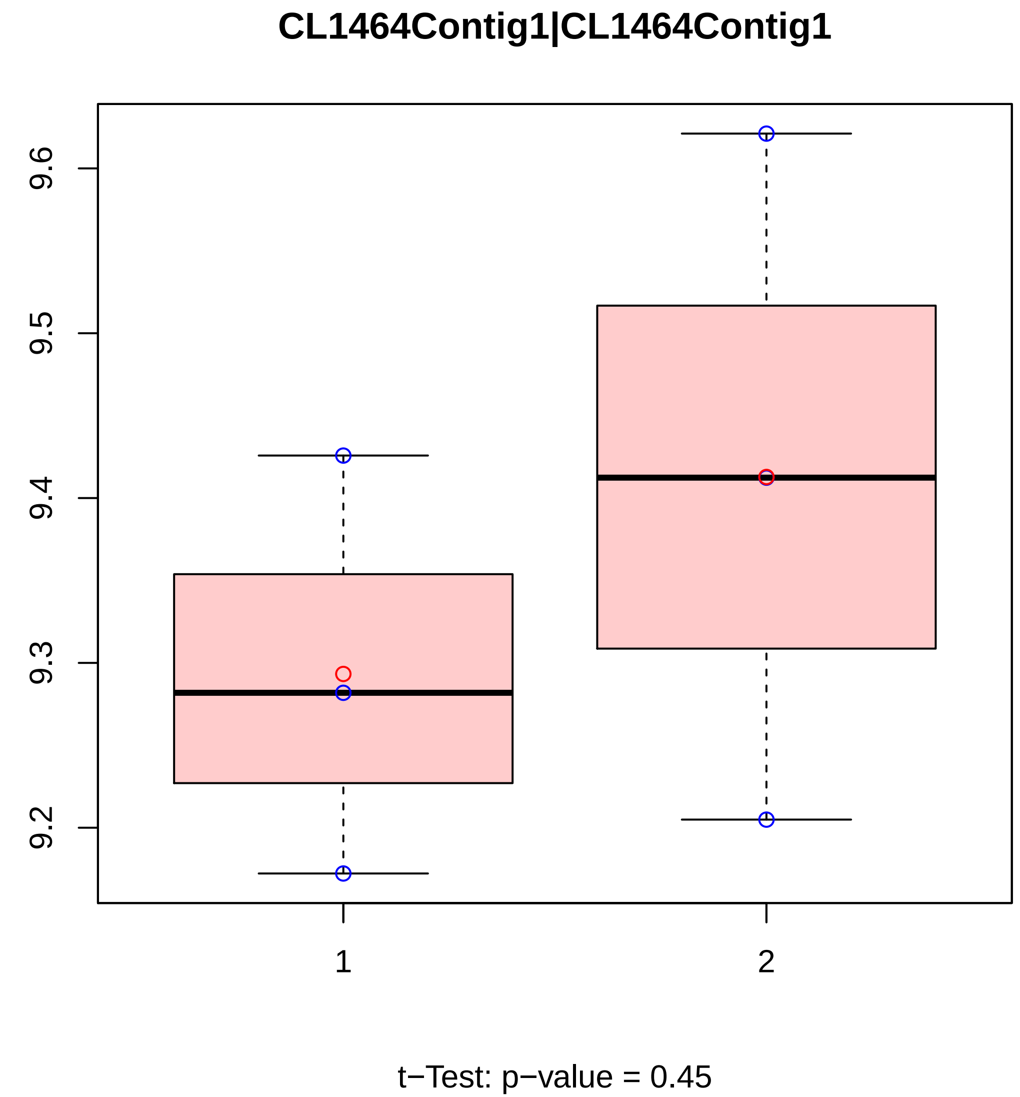
CL1464Contig1|CL1464Contig1
9.2
9.3
9.4
9.5
9.6
1
2
t−Test: p−value = 0.45

# CL1465Contig4|CL1465Contig4

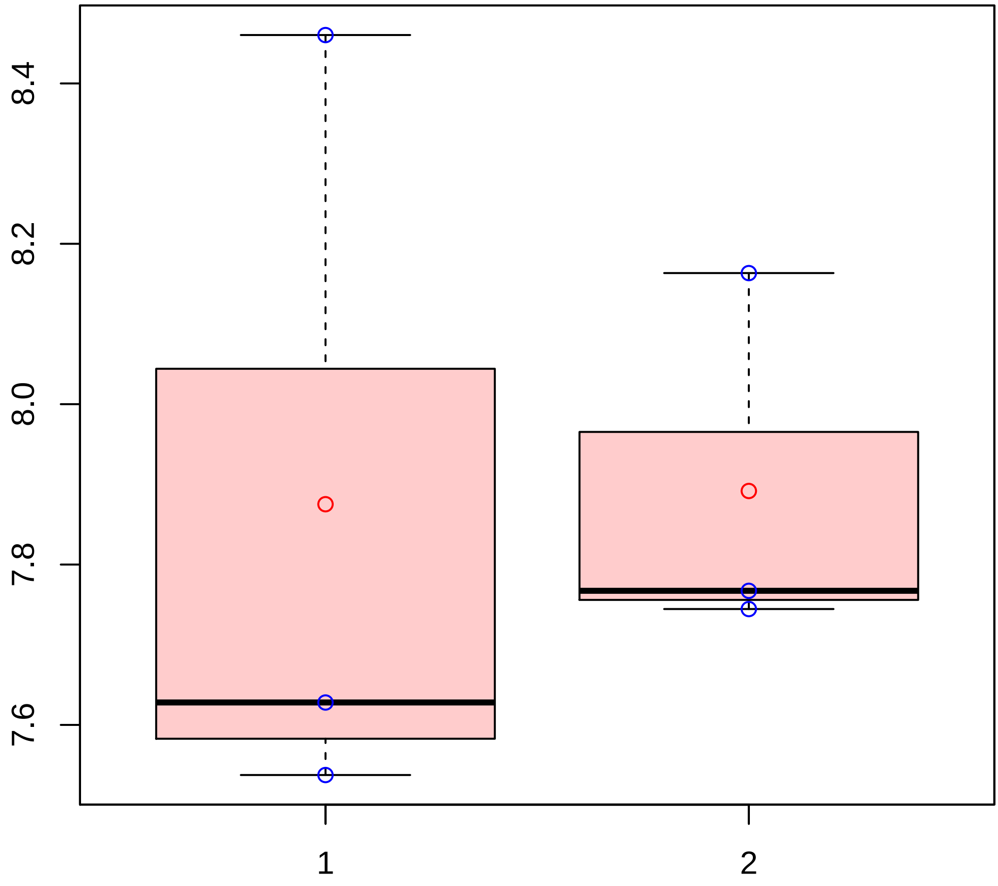


t−Test: p−value = 0.96

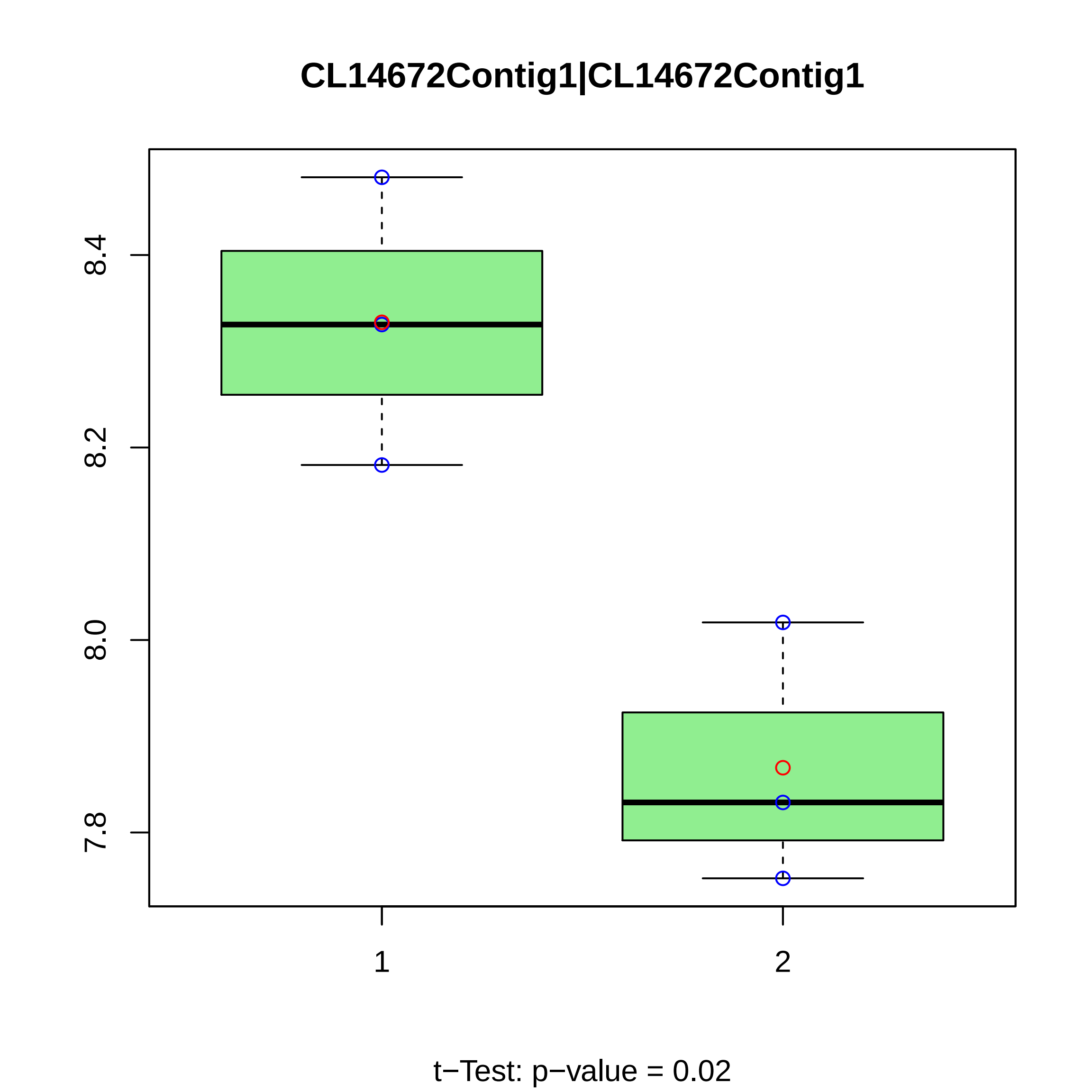

CL14672Contig1|CL14672Contig1
8.4
8.2
8.0
7.8
1
2
t−Test: p−value = 0.02

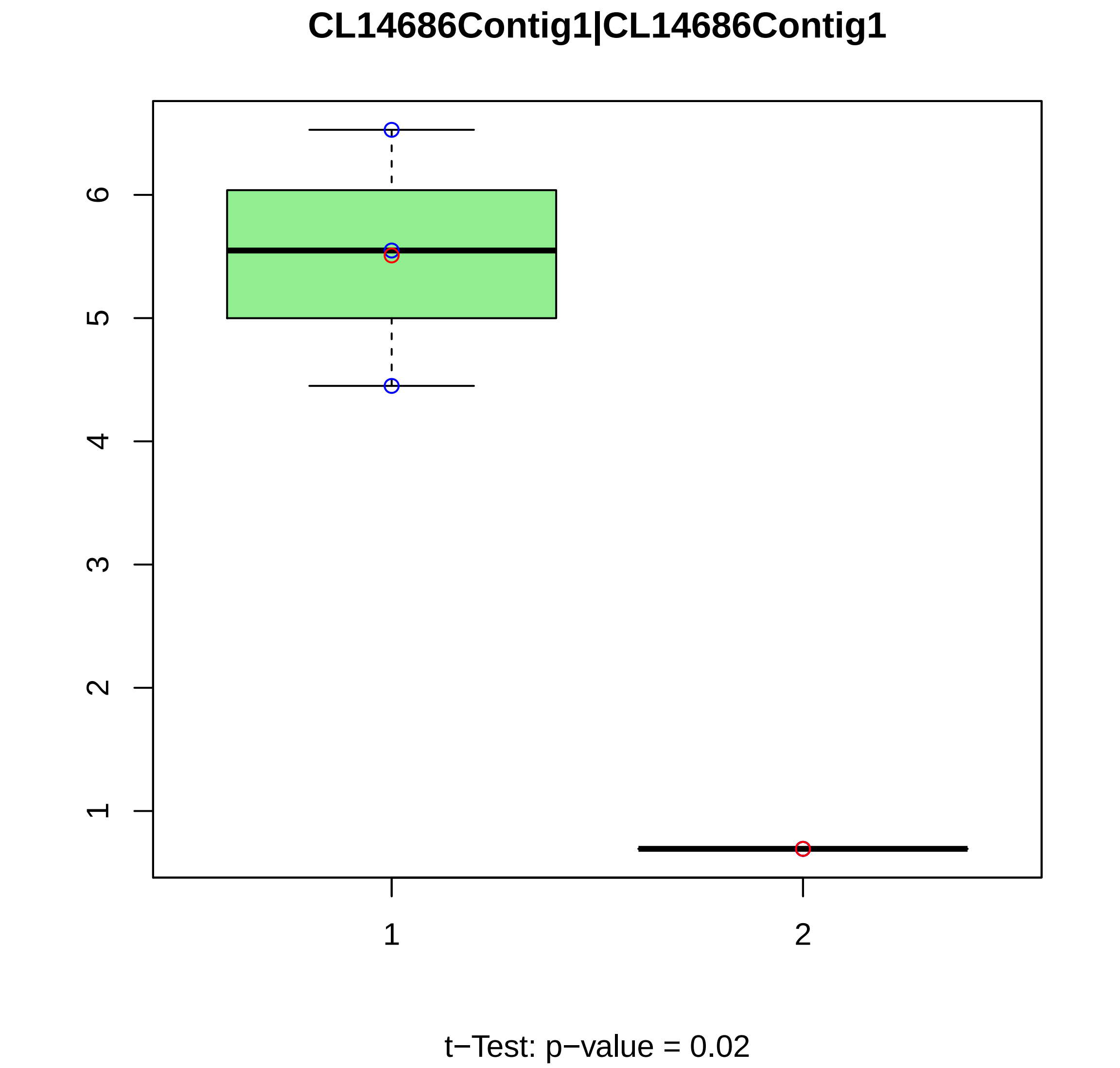
CL14686Contig1|CL14686Contig1
1
2
3
4
5
6
1
2
t−Test: p−value = 0.02

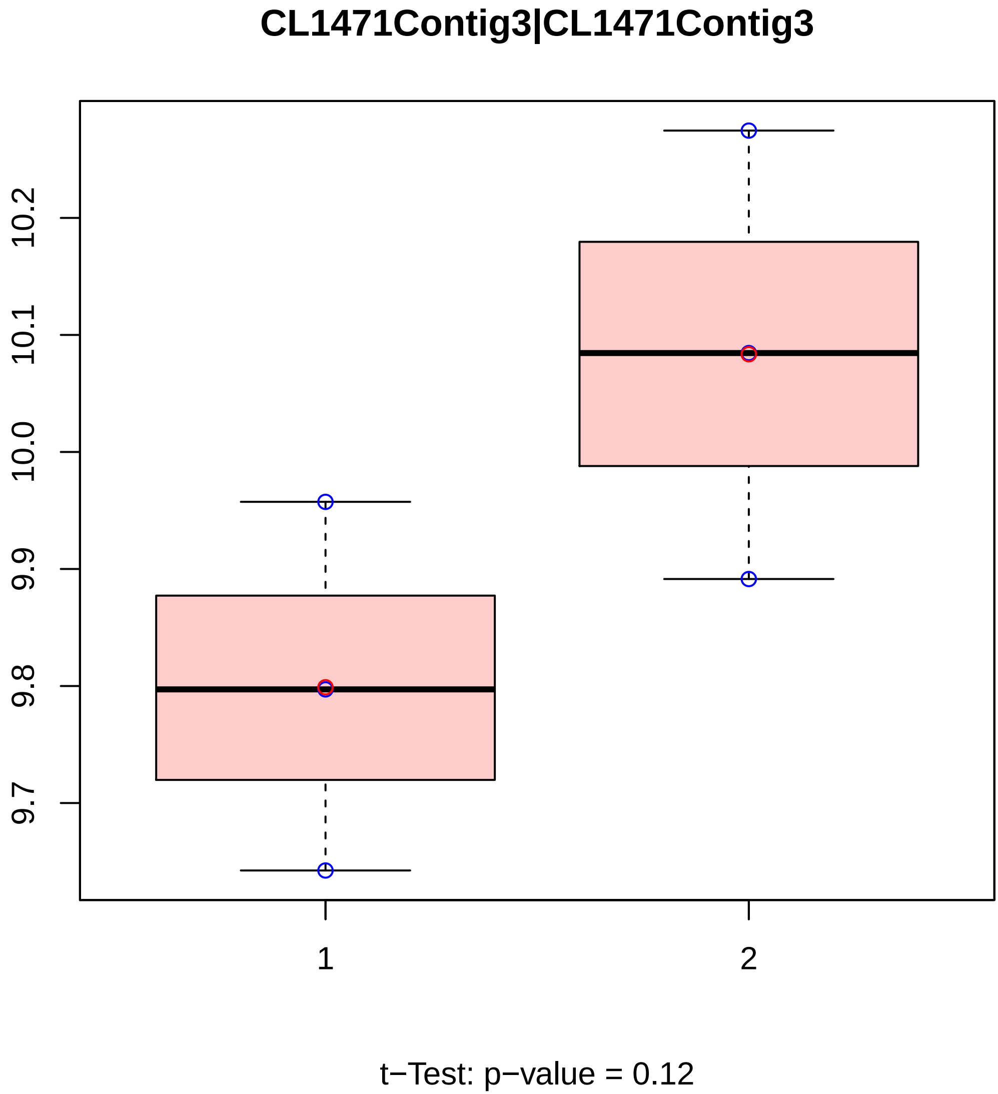
CL1471Contig3|CL1471Contig3
10.2
10.1
10.0
9.9
9.8
9.7
1
2
t−Test: p−value = 0.12

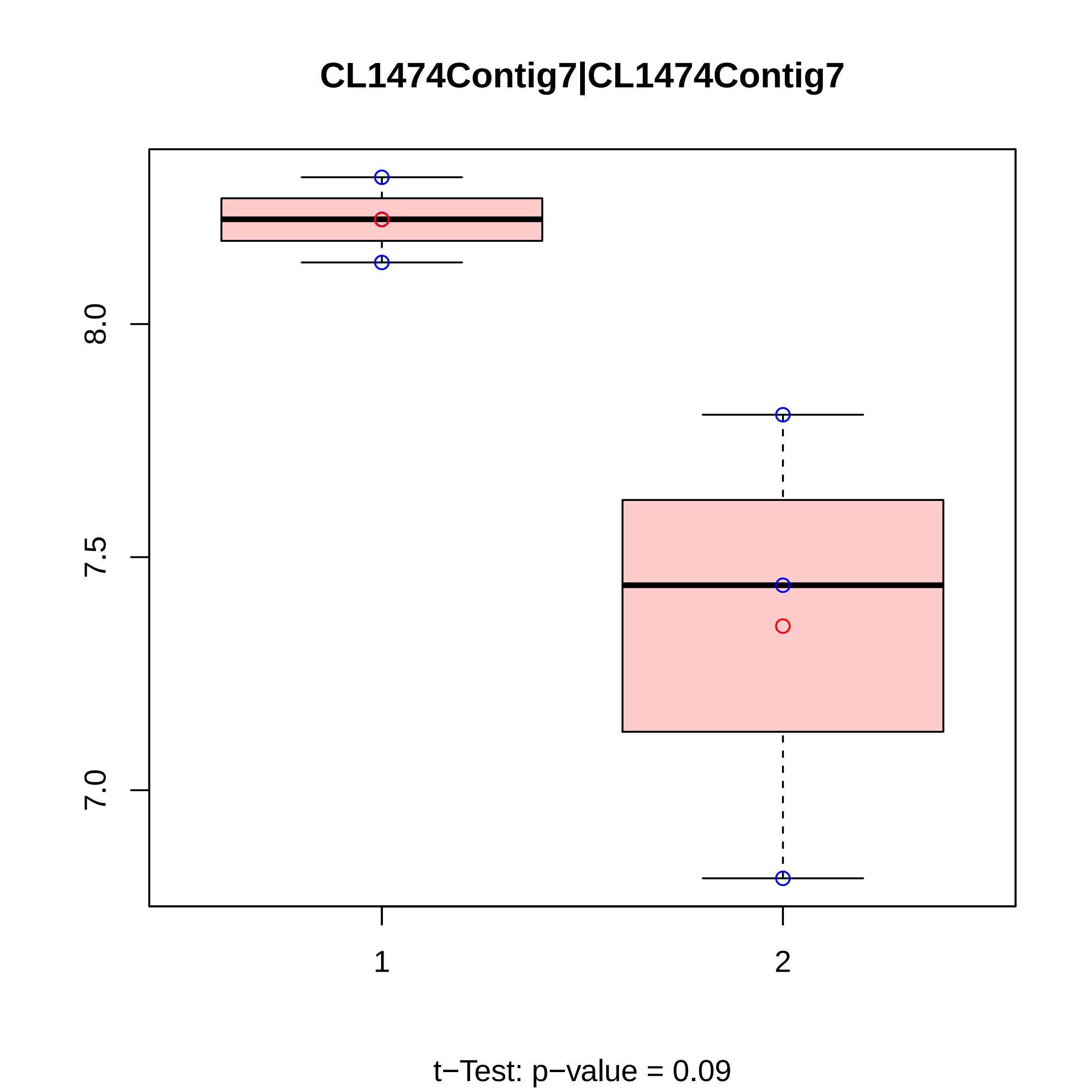

CL1474Contig7|CL1474Contig7
8.0
7.5
7.0
1
2
t−Test: p−value = 0.09

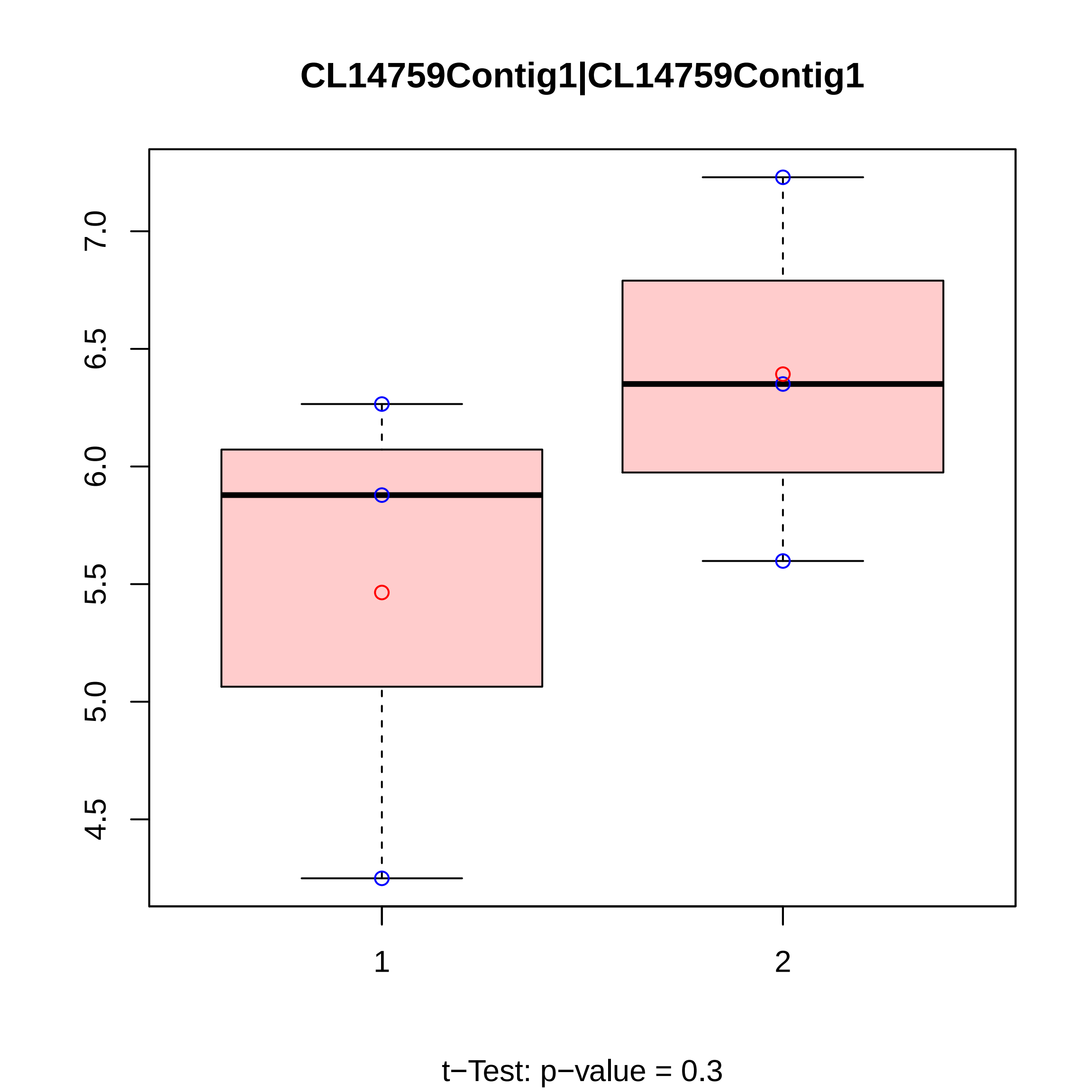

CL14759Contig1|CL14759Contig1
7.0
6.5
6.0
5.5
5.0
4.5
1
2
t−Test: p−value = 0.3

## CL1475Contig1|CL1475Contig1

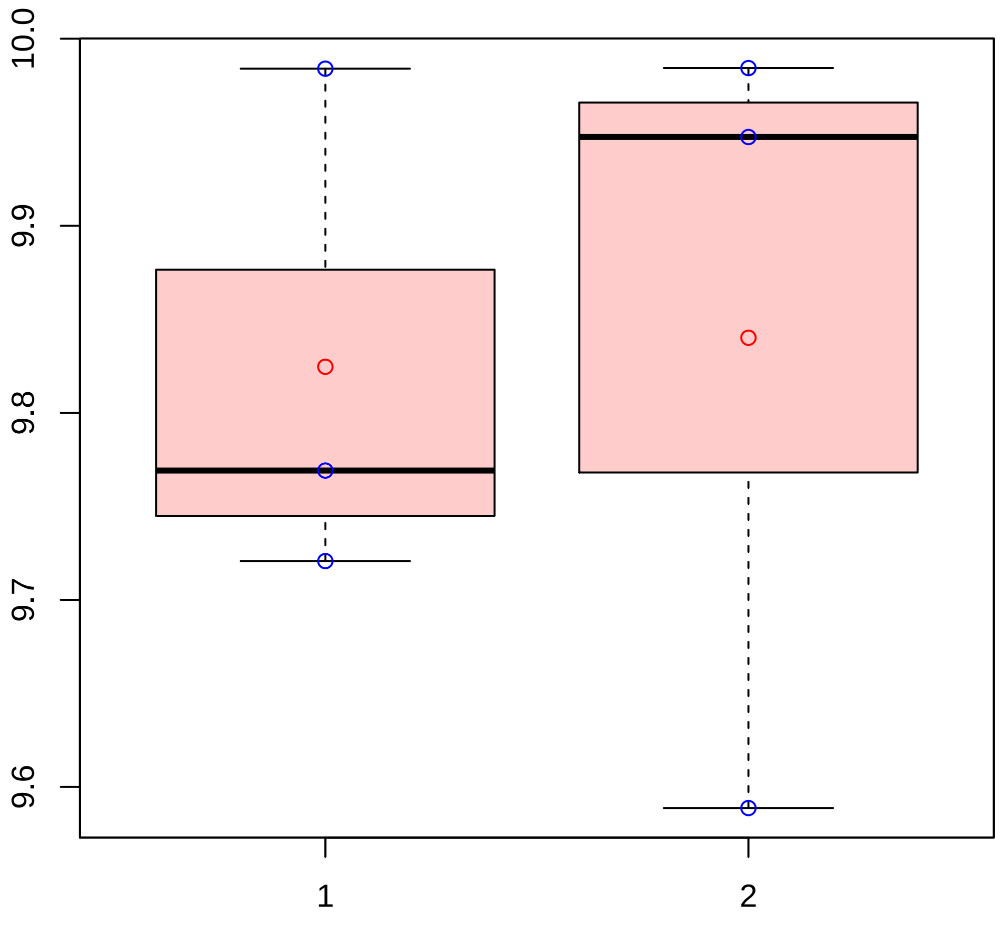


t−Test: p−value = 0.92

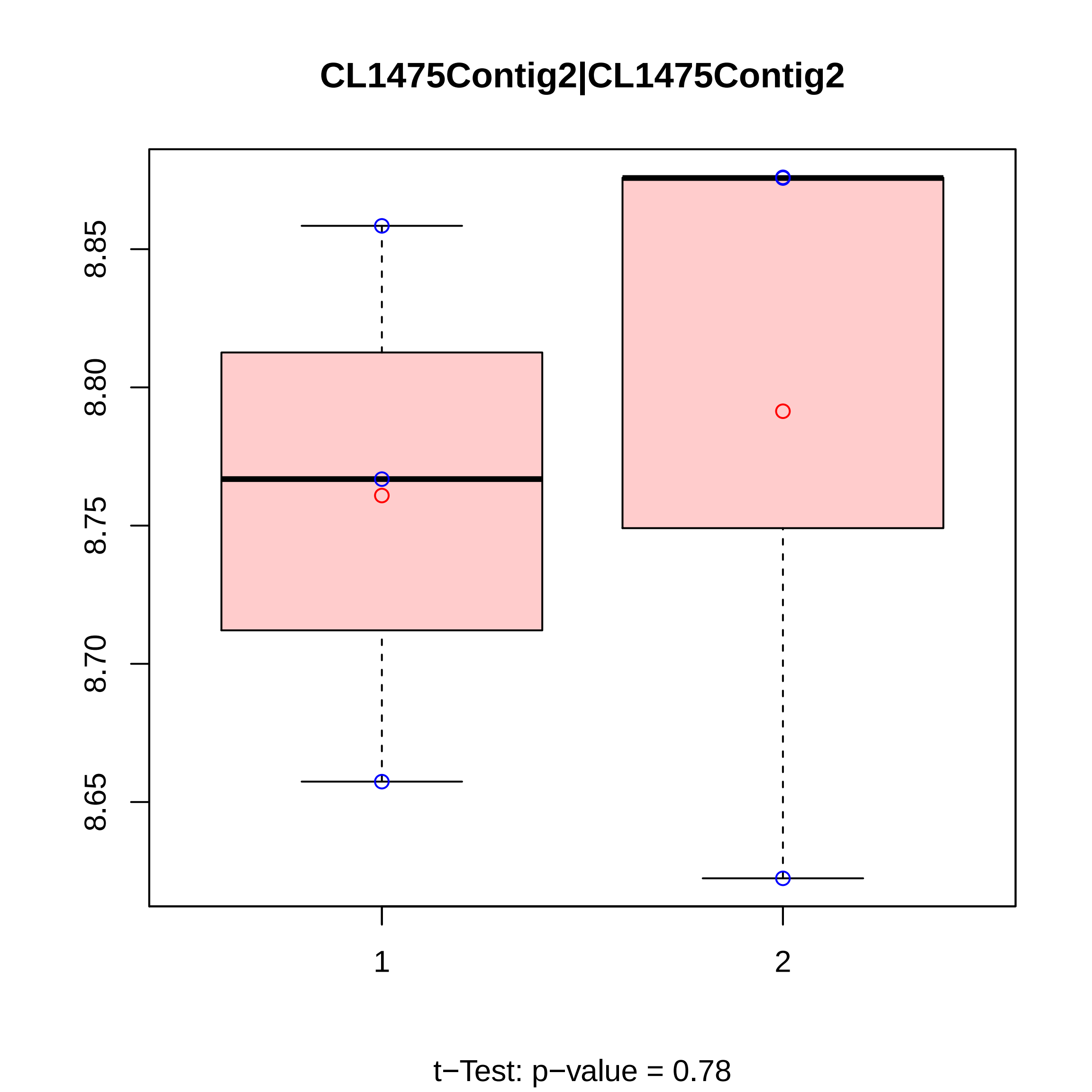
CL1475Contig2|CL1475Contig2
8.65
8.70
8.75
8.80
8.85
1
2
t−Test: p−value = 0.78

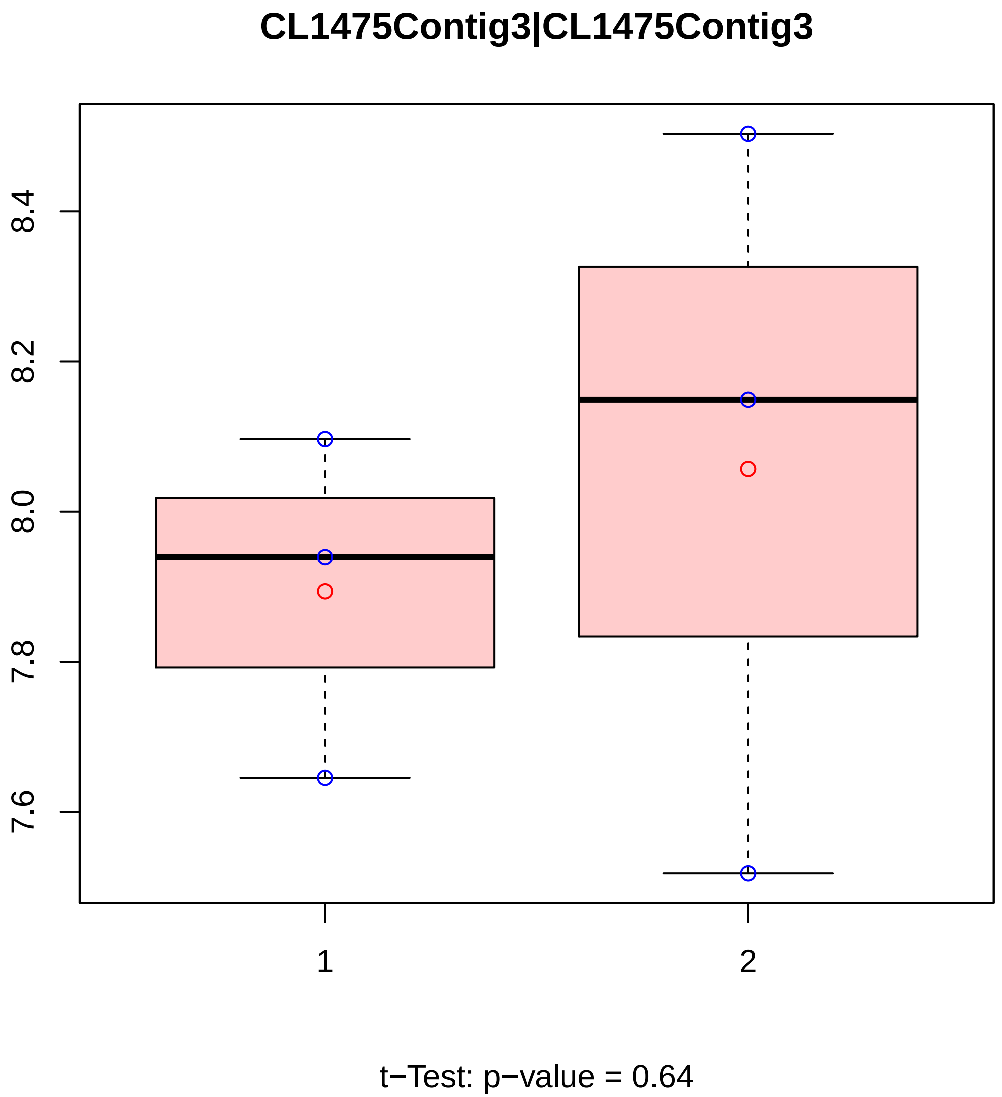

CL1475Contig3|CL1475Contig3
8.4
8.2
8.0
7.8
7.6
1
2
t−Test: p−value = 0.64

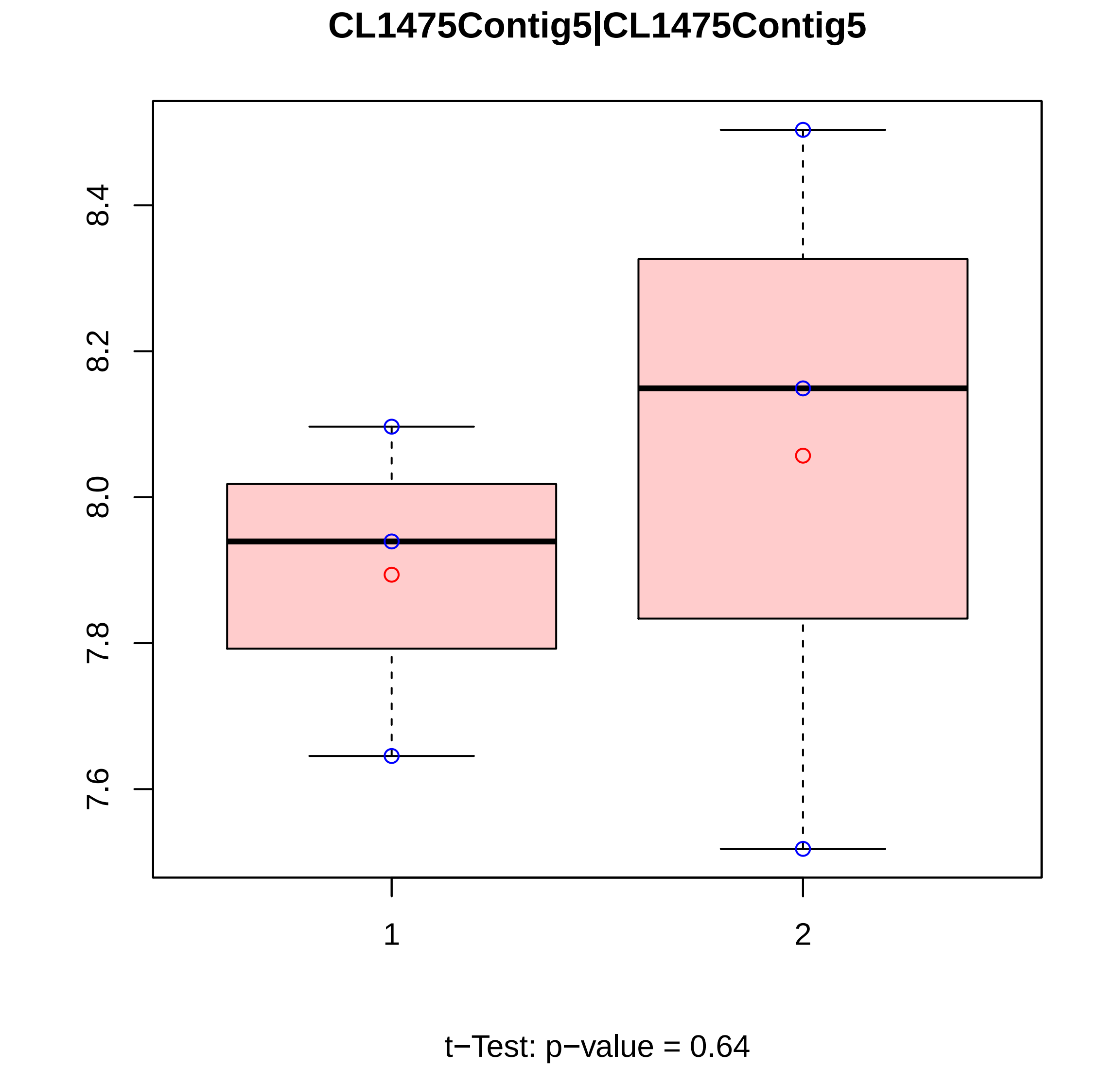
CL1475Contig5|CL1475Contig5
8.4
8.2
8.0
7.8
7.6
1
2
t−Test: p−value = 0.64

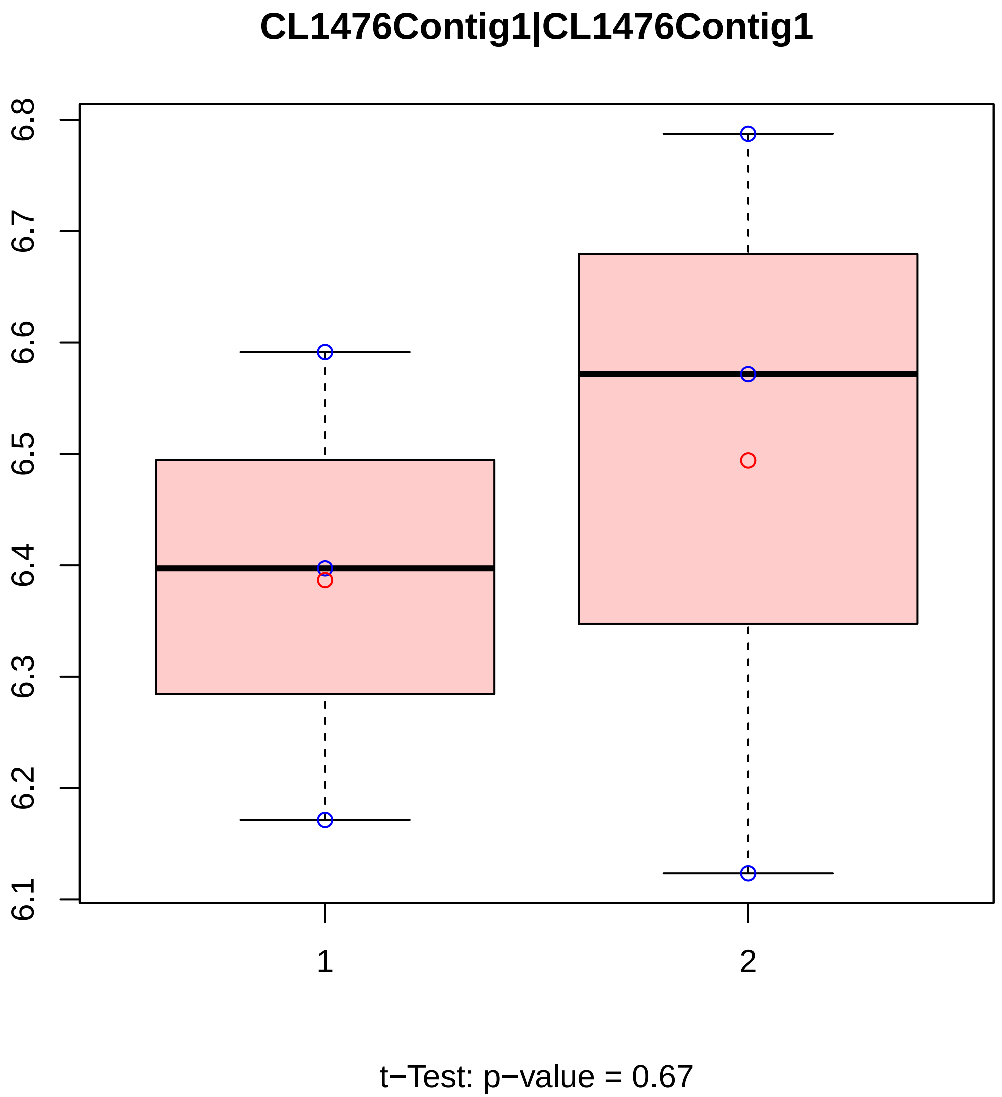

CL1476Contig1|CL1476Contig1
6.1
6.2
6.3
6.4
6.5
6.6
6.7
6.8
1
2
t−Test: p−value = 0.67

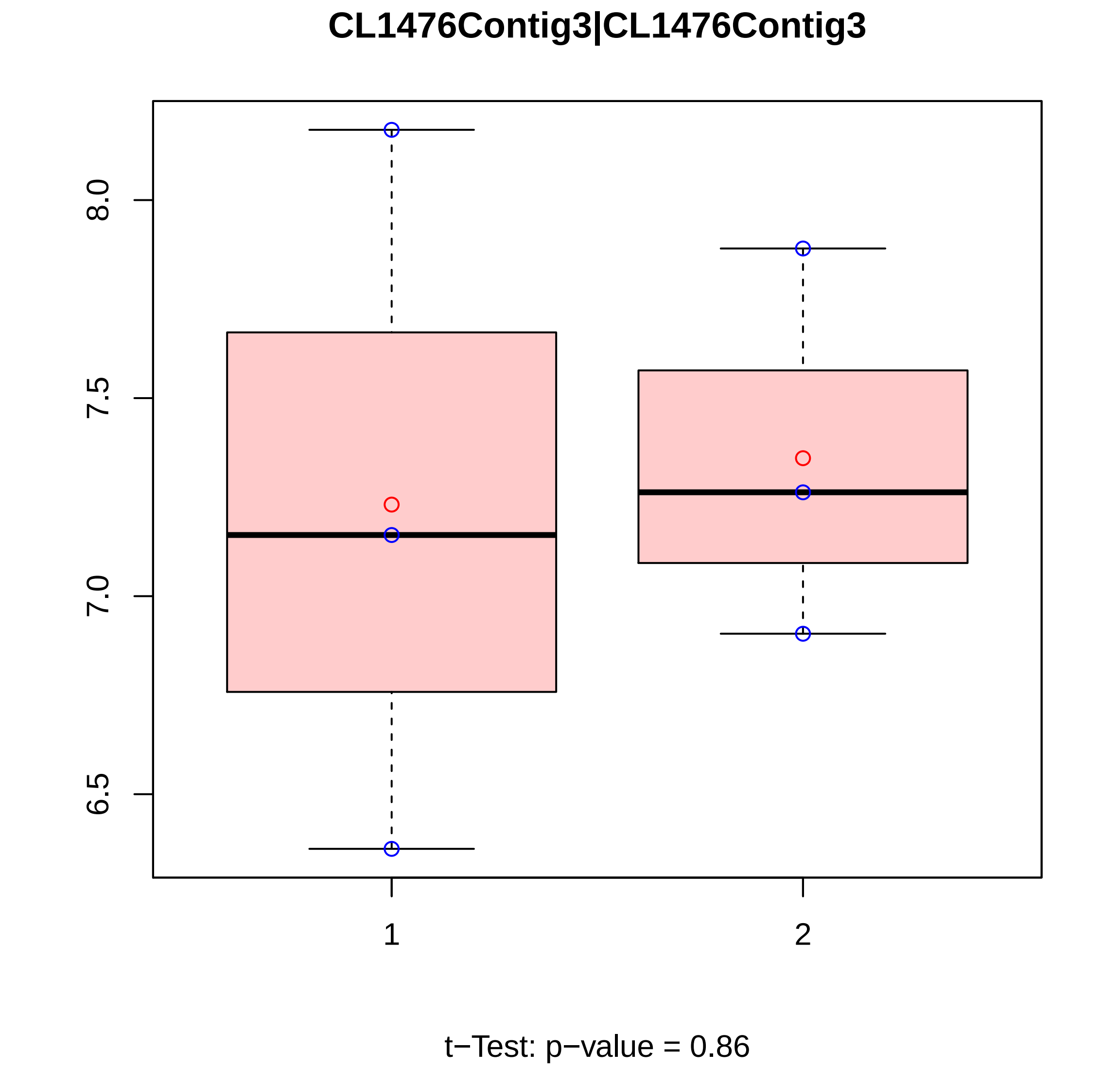
CL1476Contig3|CL1476Contig3
8.0
7.5
7.0
6.5
1
2
t−Test: p−value = 0.86

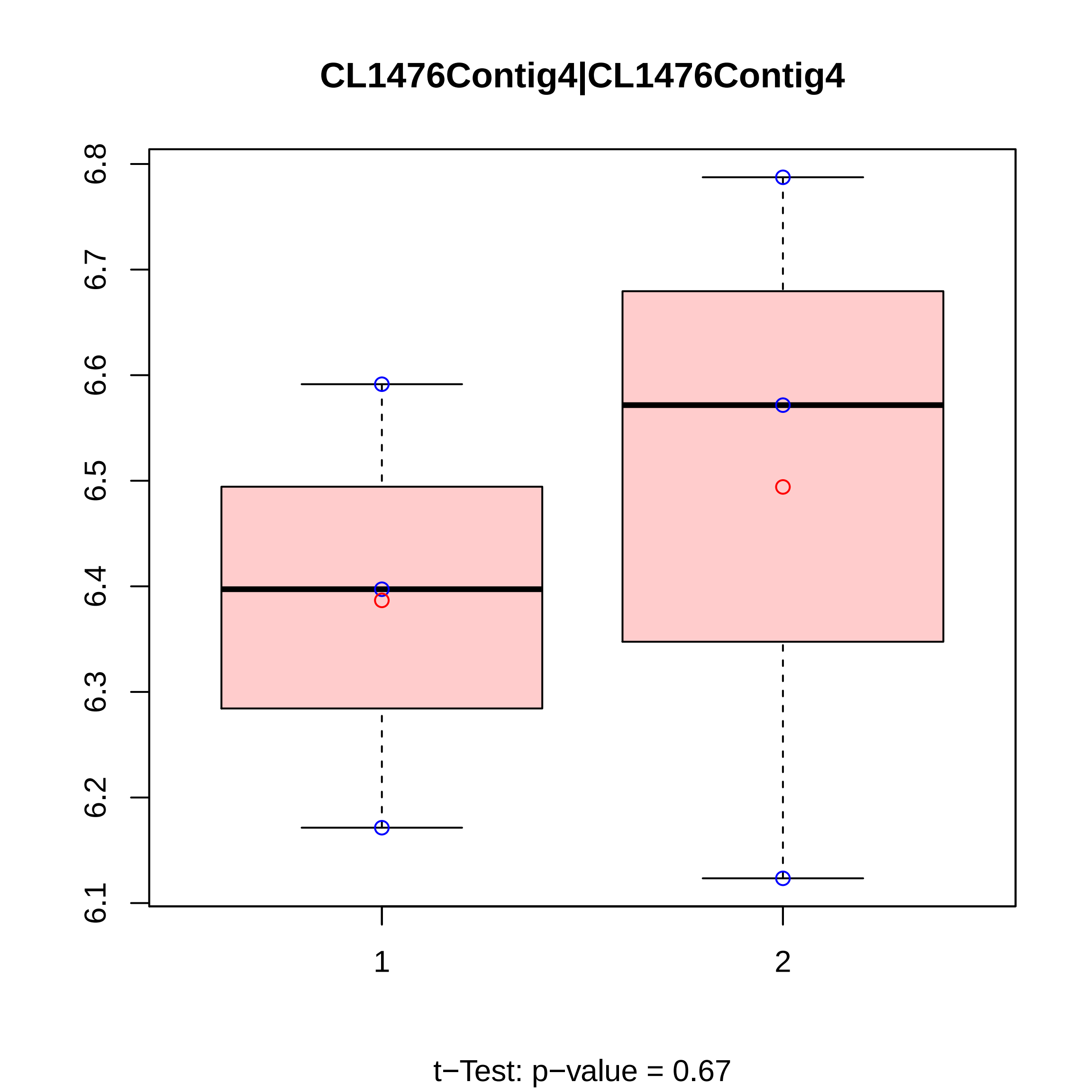

CL1476Contig4|CL1476Contig4
6.1
6.2
6.3
6.4
6.5
6.6
6.7
6.8
1
2
t−Test: p−value = 0.67

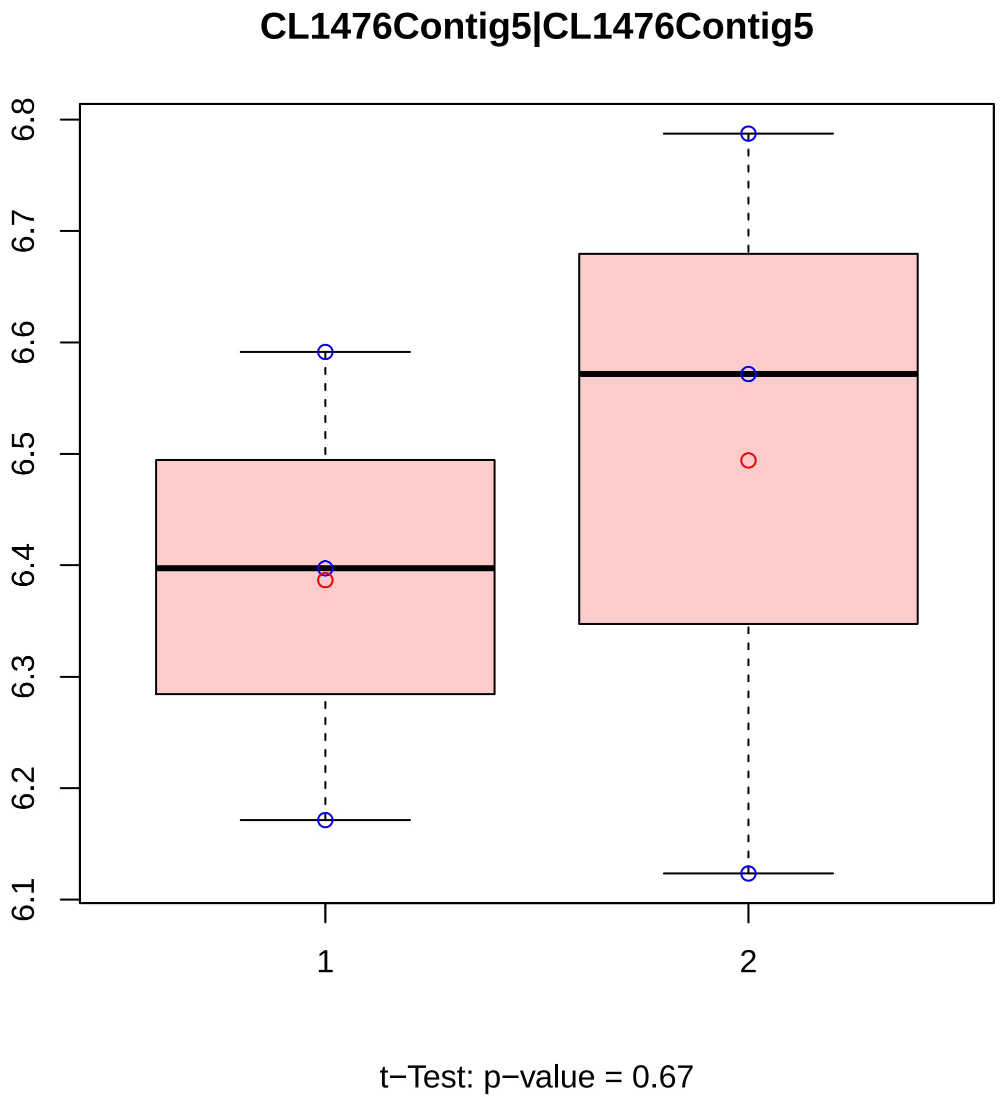
CL1476Contig5|CL1476Contig5
6.1
6.2
6.3
6.4
6.5
6.6
6.7
6.8
1
2
t−Test: p−value = 0.67

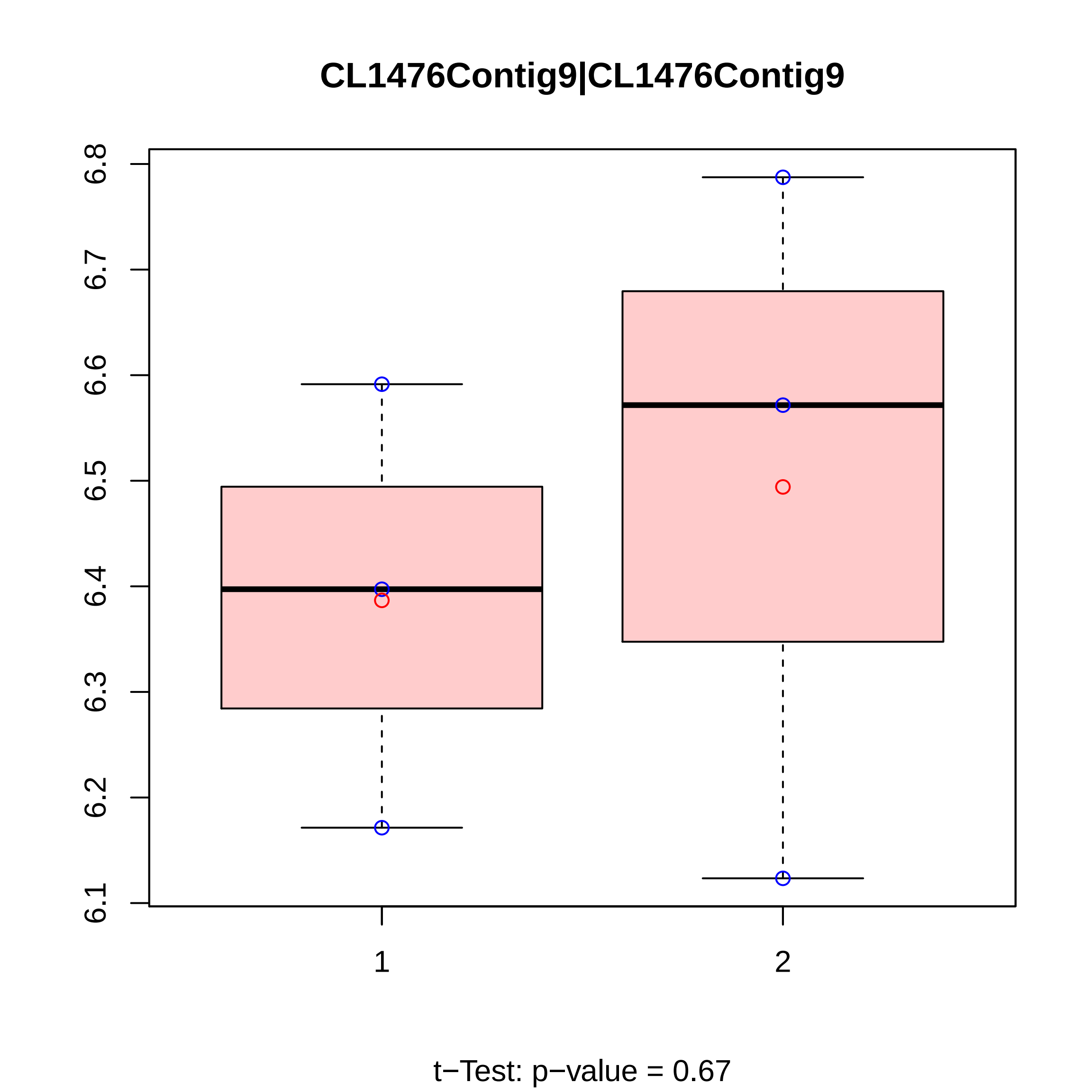
CL1476Contig9|CL1476Contig9
6.1
6.2
6.3
6.4
6.5
6.6
6.7
6.8
1
2
t−Test: p−value = 0.67

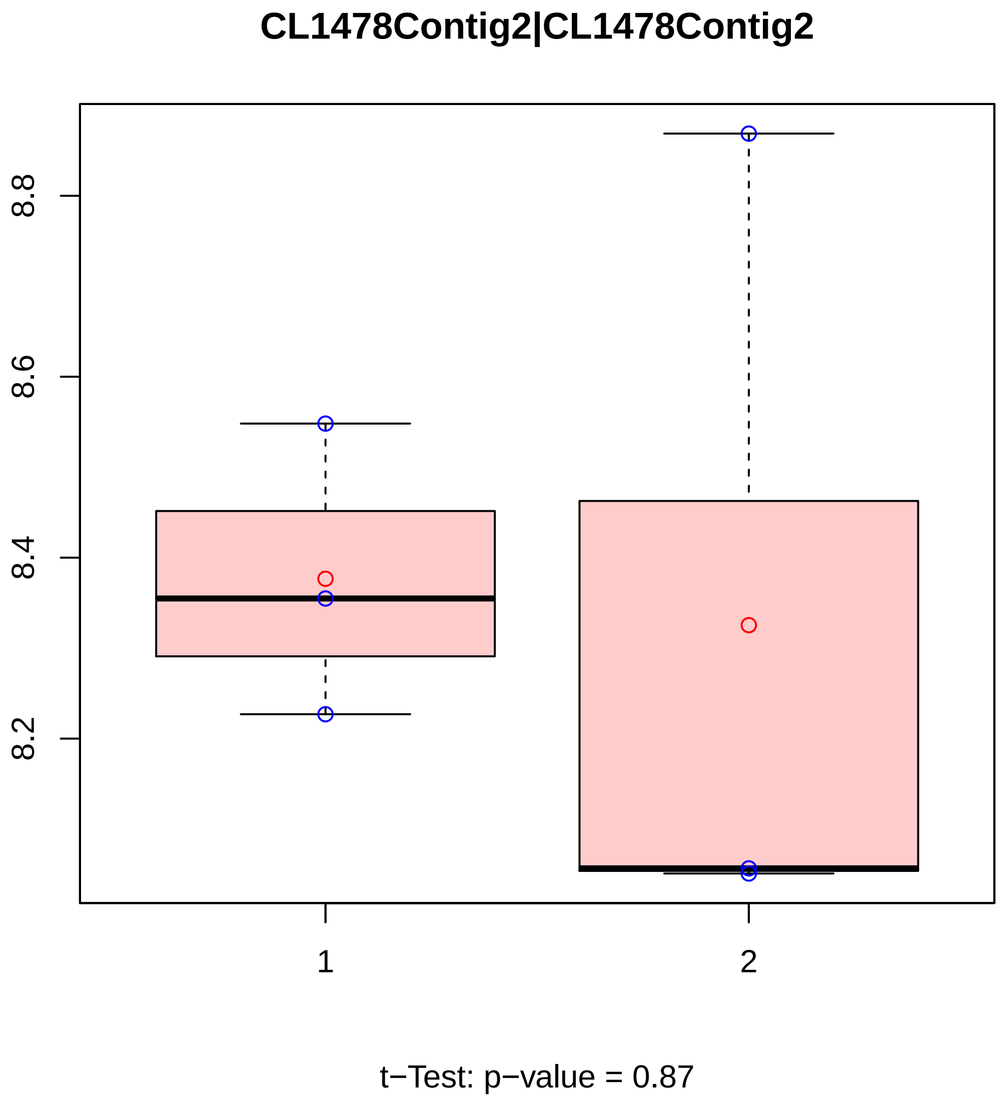
CL1478Contig2|CL1478Contig2
8.2
8.4
8.6
8.8
1
2
t−Test: p−value = 0.87

## CL147Contig10|CL147Contig10

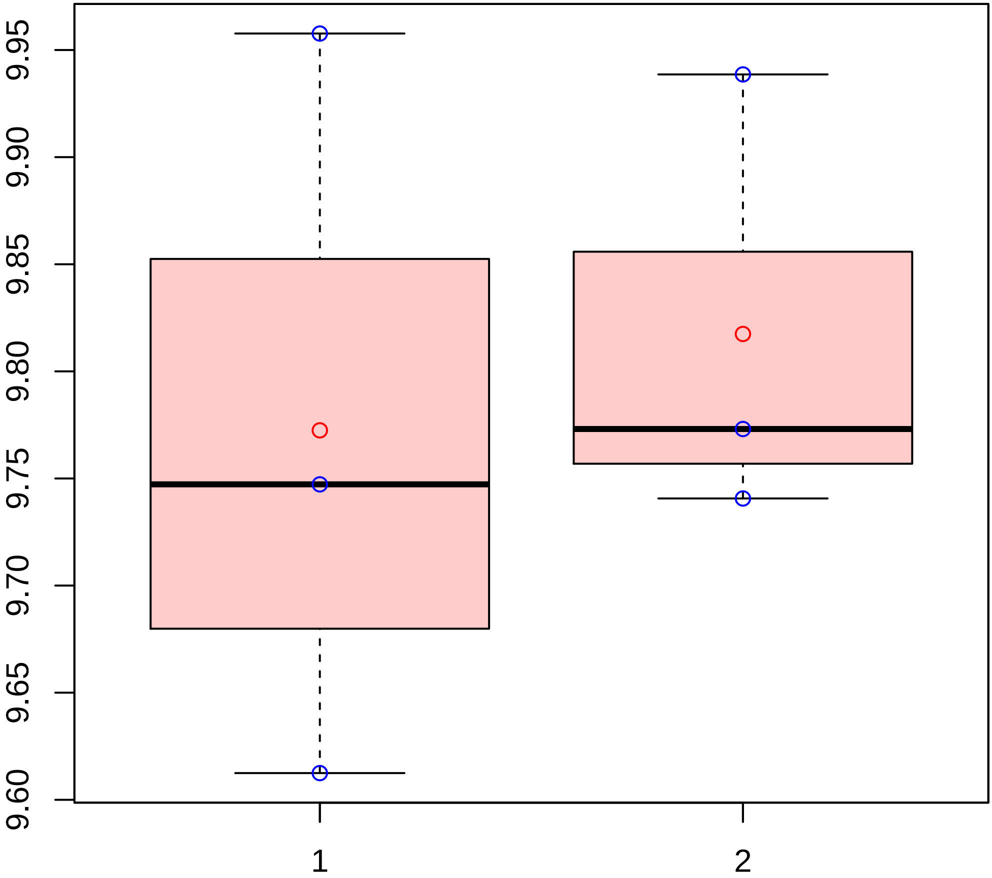


t−Test: p−value = 0.73

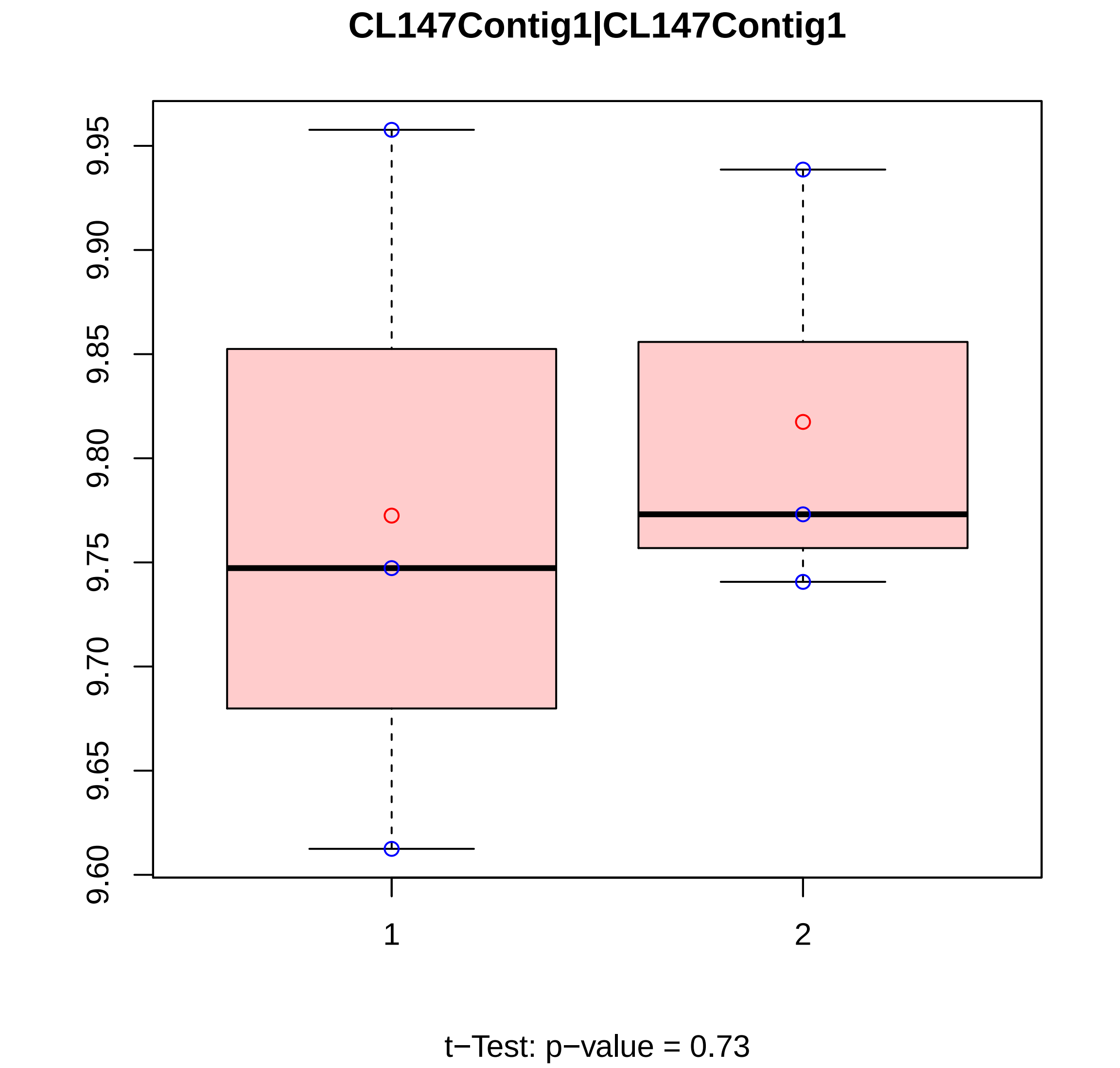

CL147Contig1|CL147Contig1
9.60
9.65
9.70
9.75
9.80
9.85
9.90
9.95
1
2
t−Test: p−value = 0.73

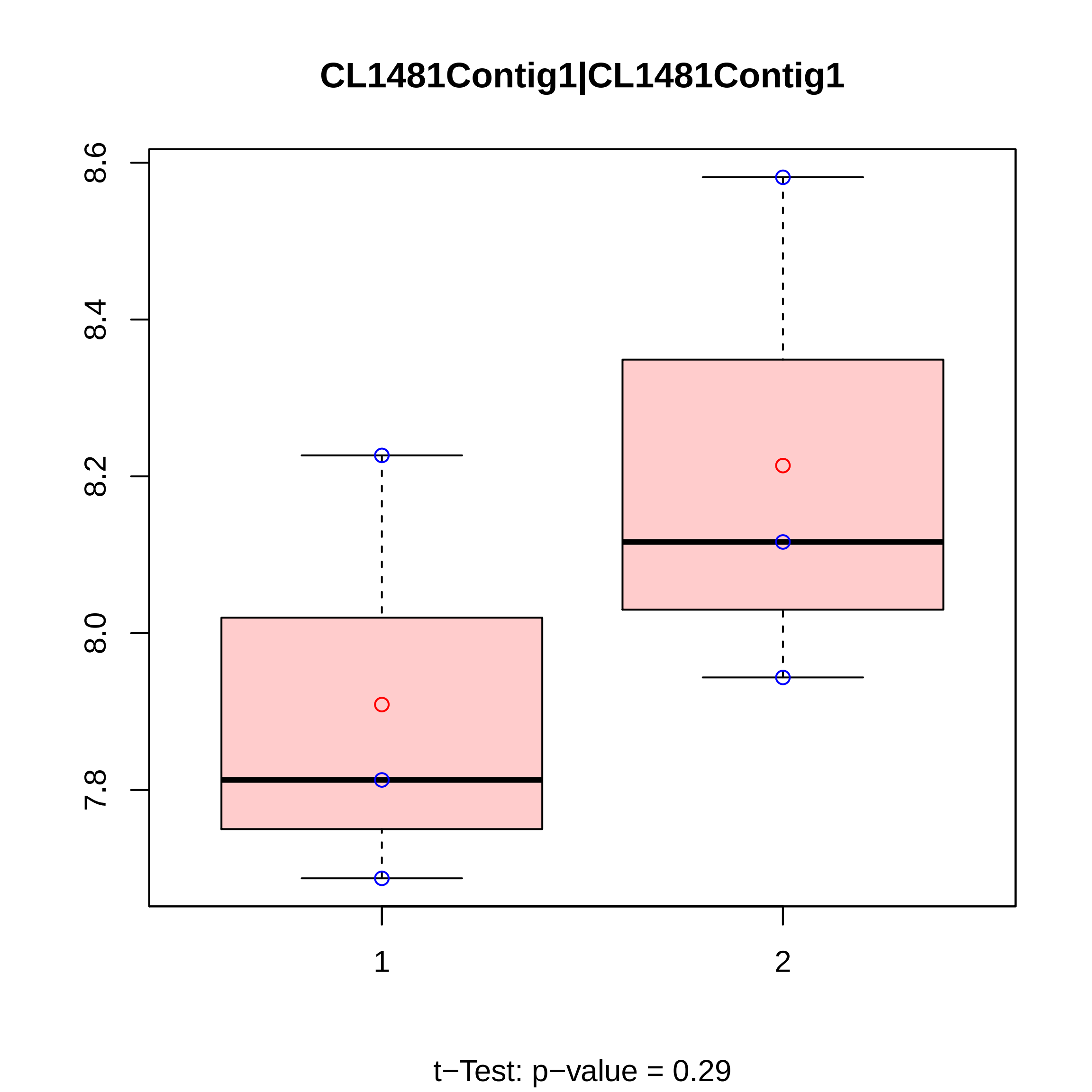
CL1481Contig1|CL1481Contig1
8.6
8.4
8.2
8.0
7.8
1
2
t−Test: p−value = 0.29

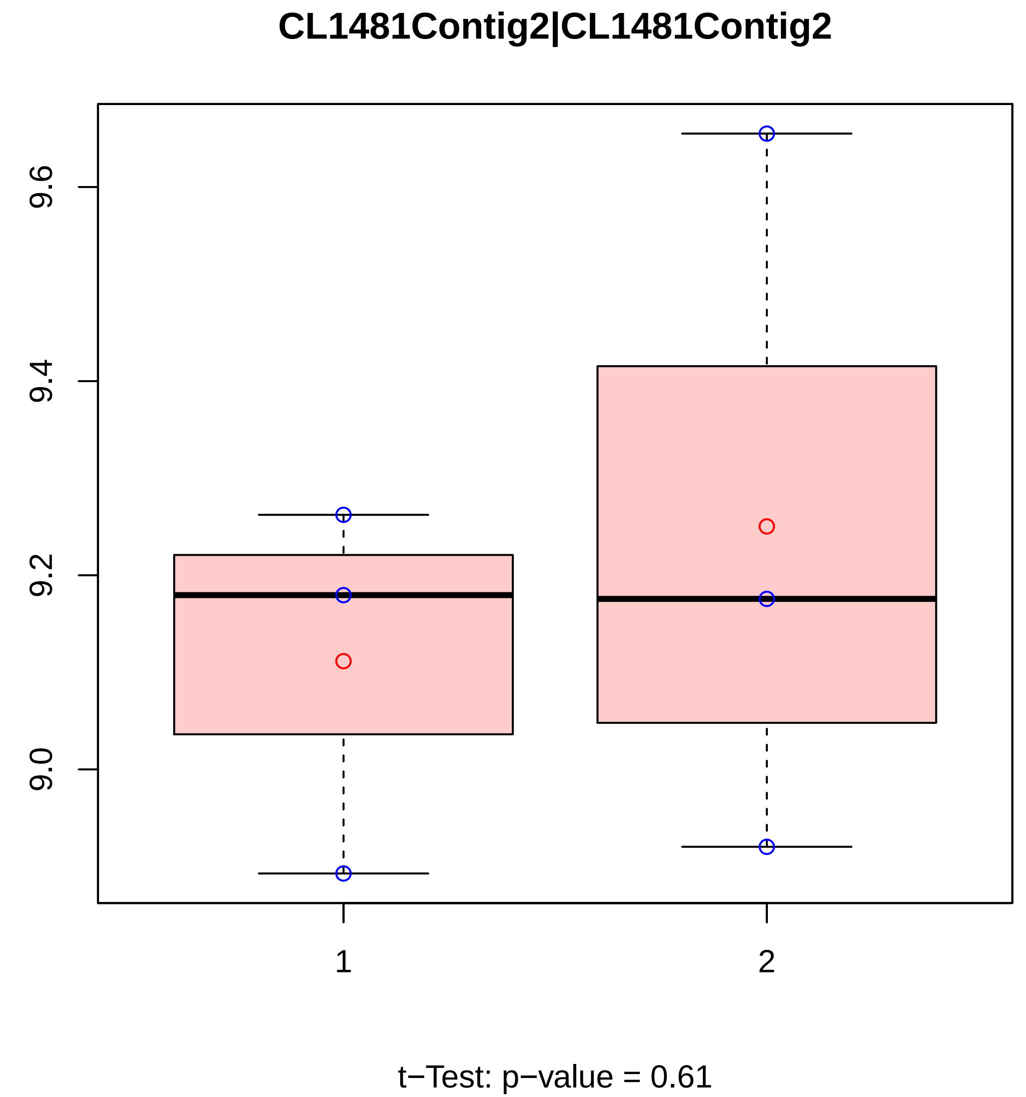
CL1481Contig2|CL1481Contig2
9.0
9.2
9.4
9.6
1
2
t−Test: p−value = 0.61

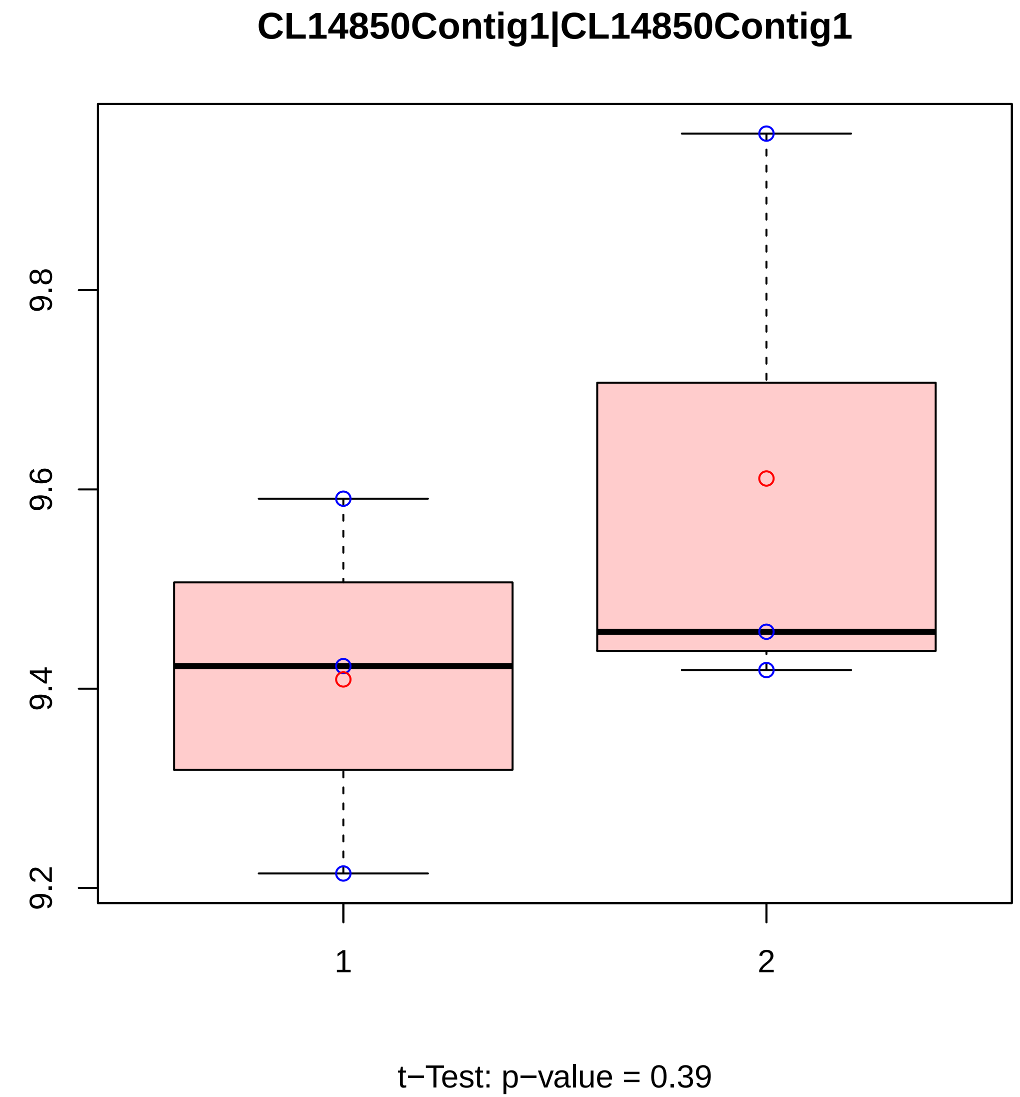
CL14850Contig1|CL14850Contig1
9.2
9.4
9.6
9.8
1
2
t−Test: p−value = 0.39

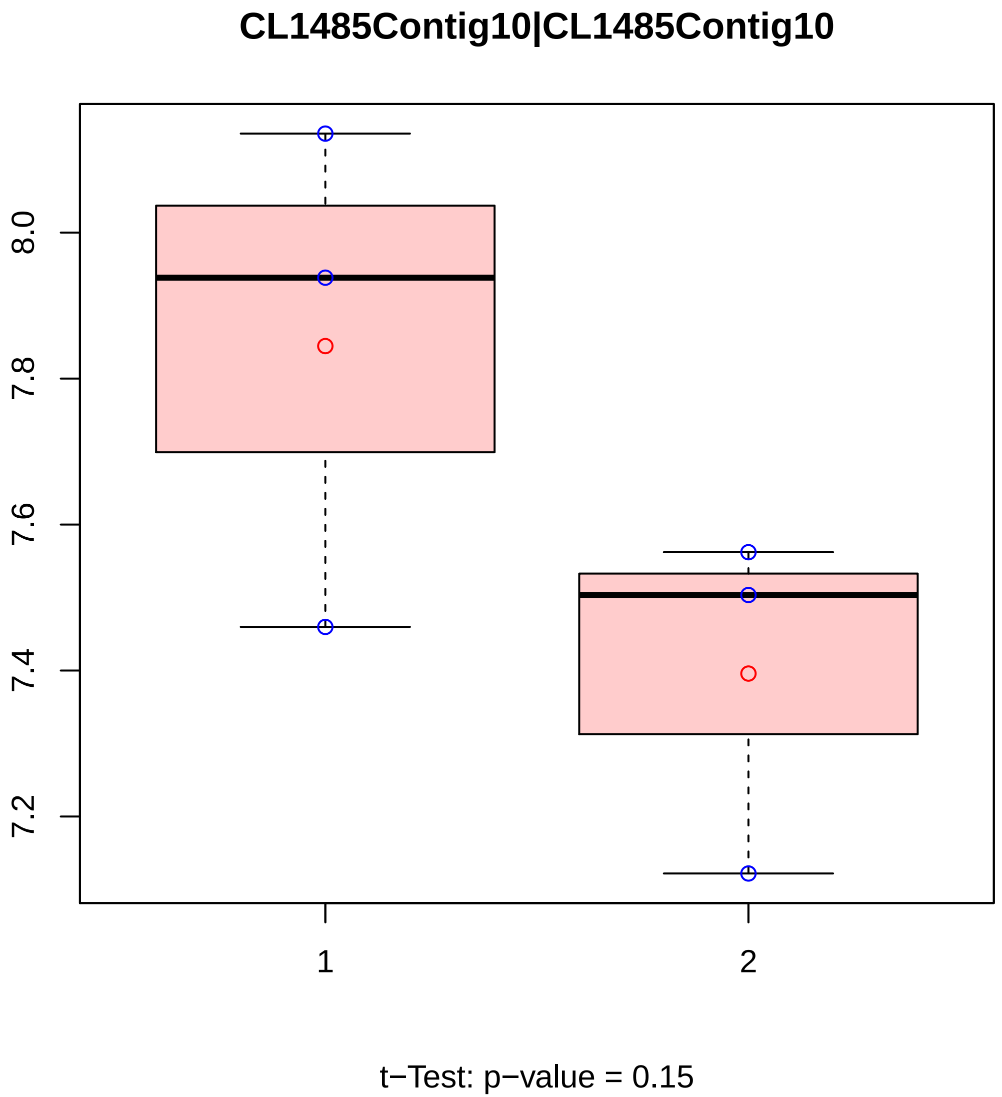

CL1485Contig10|CL1485Contig10
8.0
7.8
7.6
7.4
7.2
1
2
t−Test: p−value = 0.15

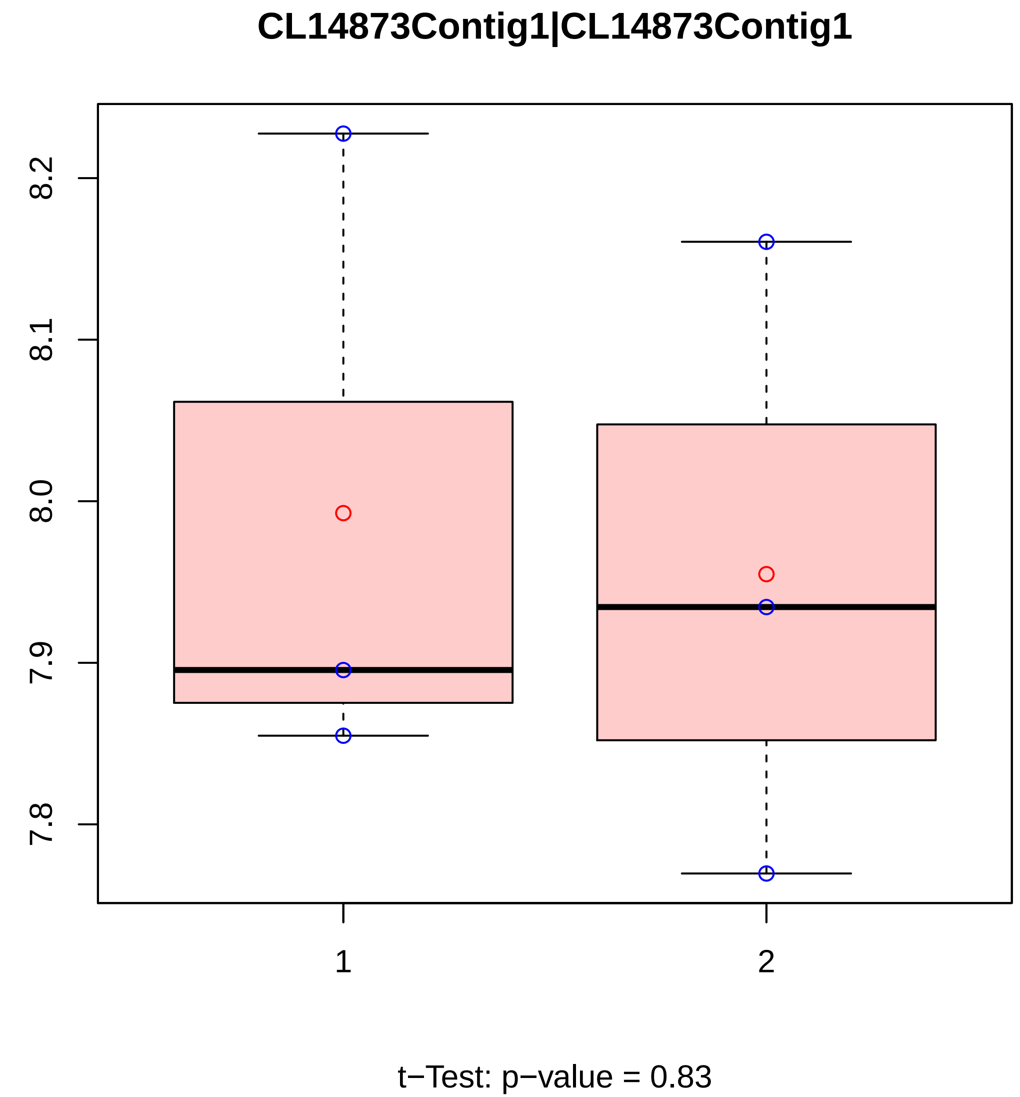
CL14873Contig1|CL14873Contig1
8.2
8.1
8.0
7.9
7.8
1
2
t−Test: p−value = 0.83

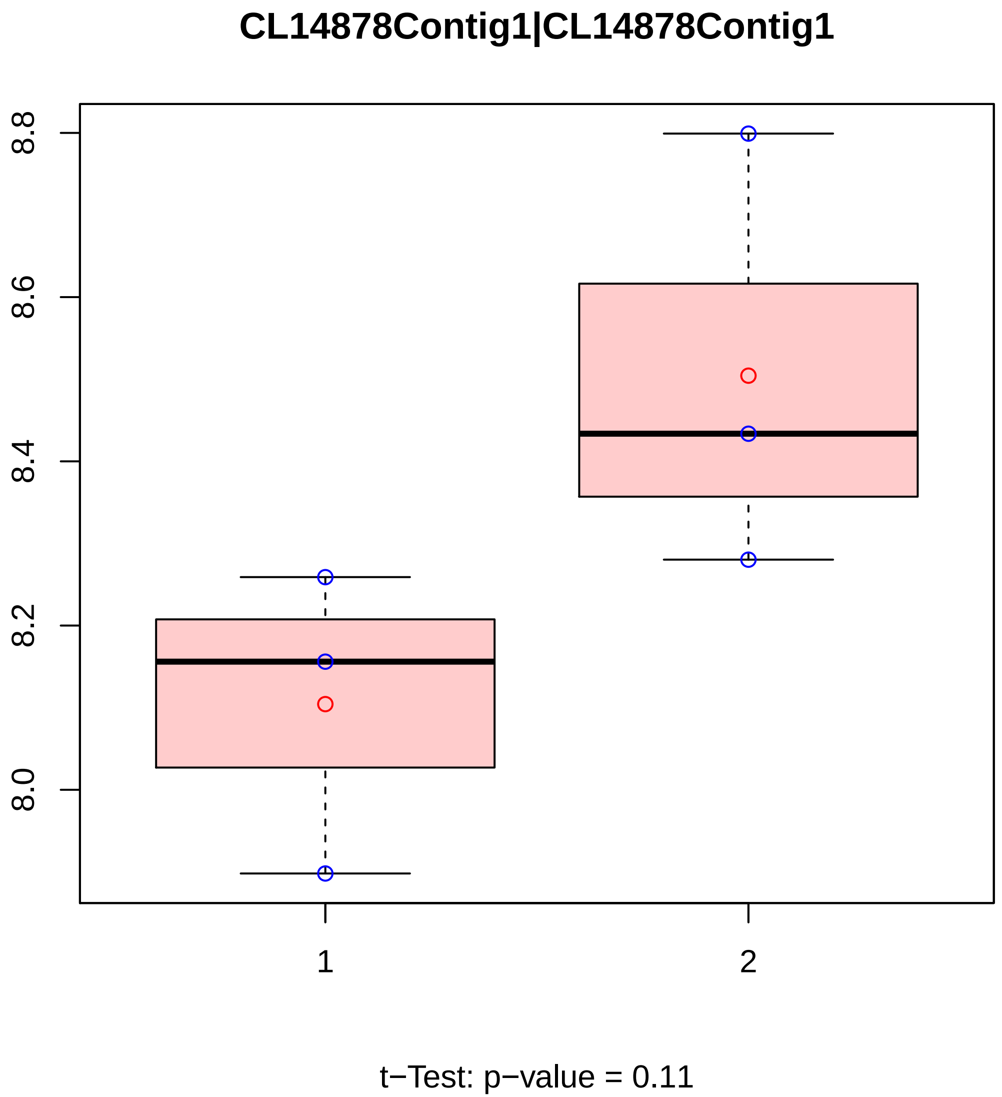

CL14878Contig1|CL14878Contig1
8.0
8.2
8.4
8.6
8.8
1
2
t−Test: p−value = 0.11

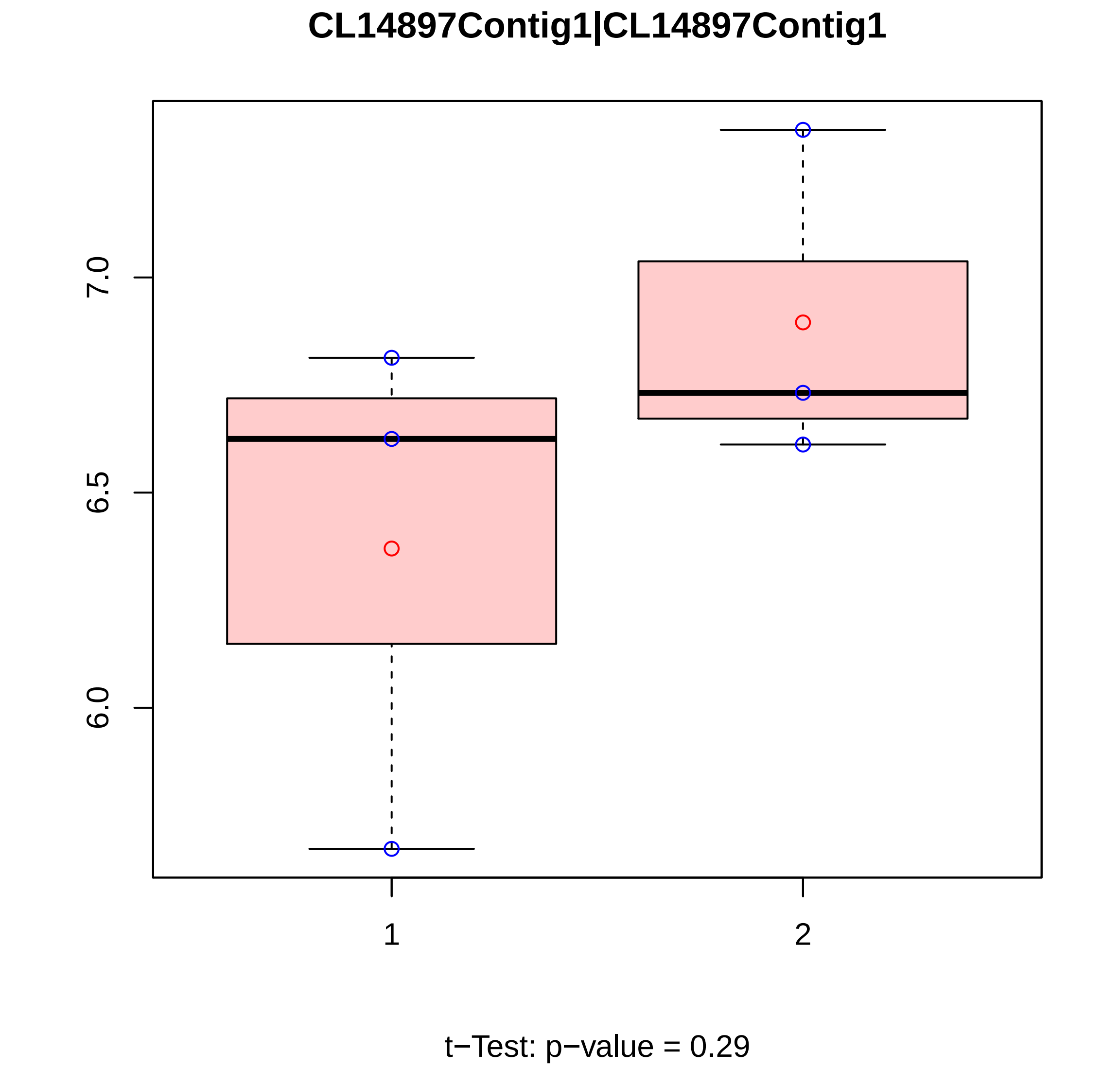
CL14897Contig1|CL14897Contig1
6.0
6.5
7.0
1
2
t−Test: p−value = 0.29

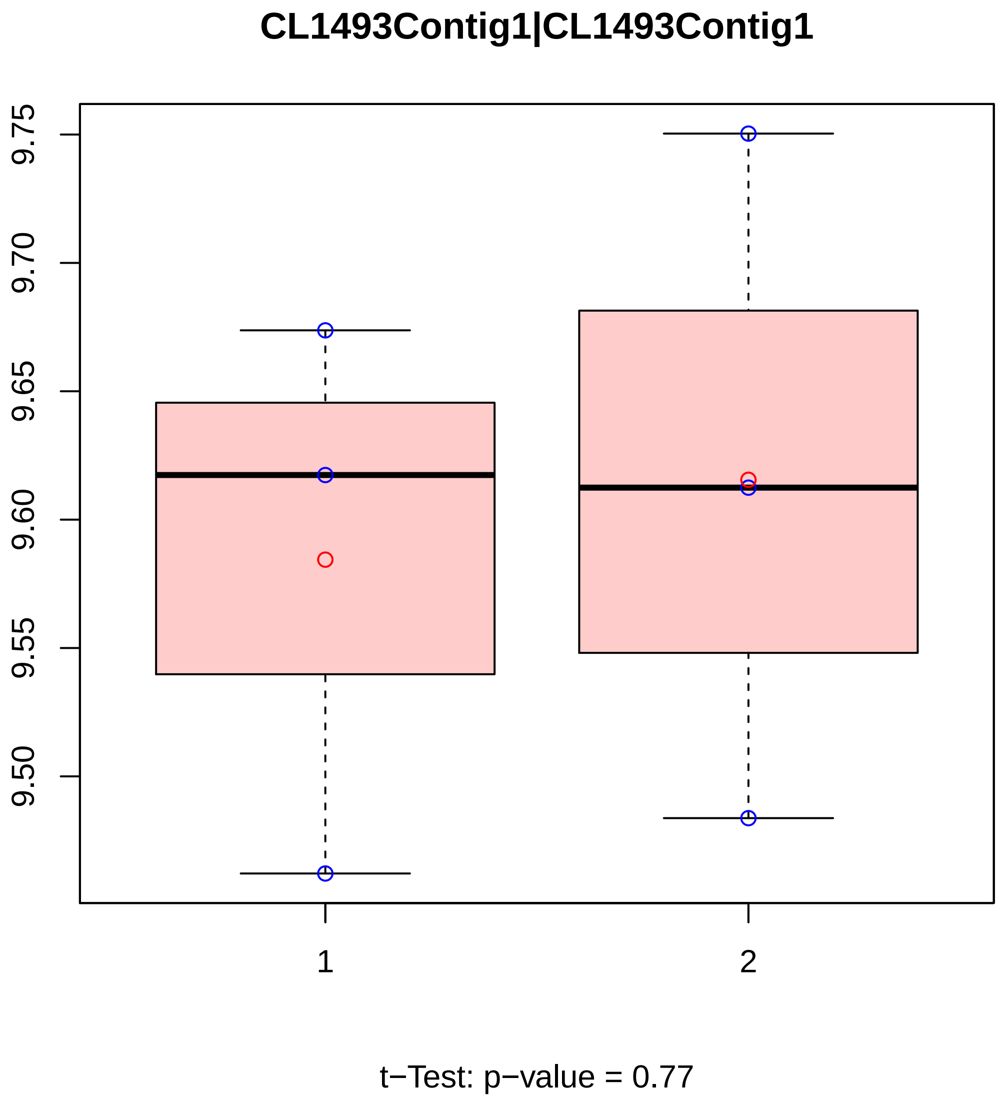
CL1493Contig1|CL1493Contig1
9.75
9.70
9.65
9.60
9.55
9.50
1
2
t−Test: p−value = 0.77

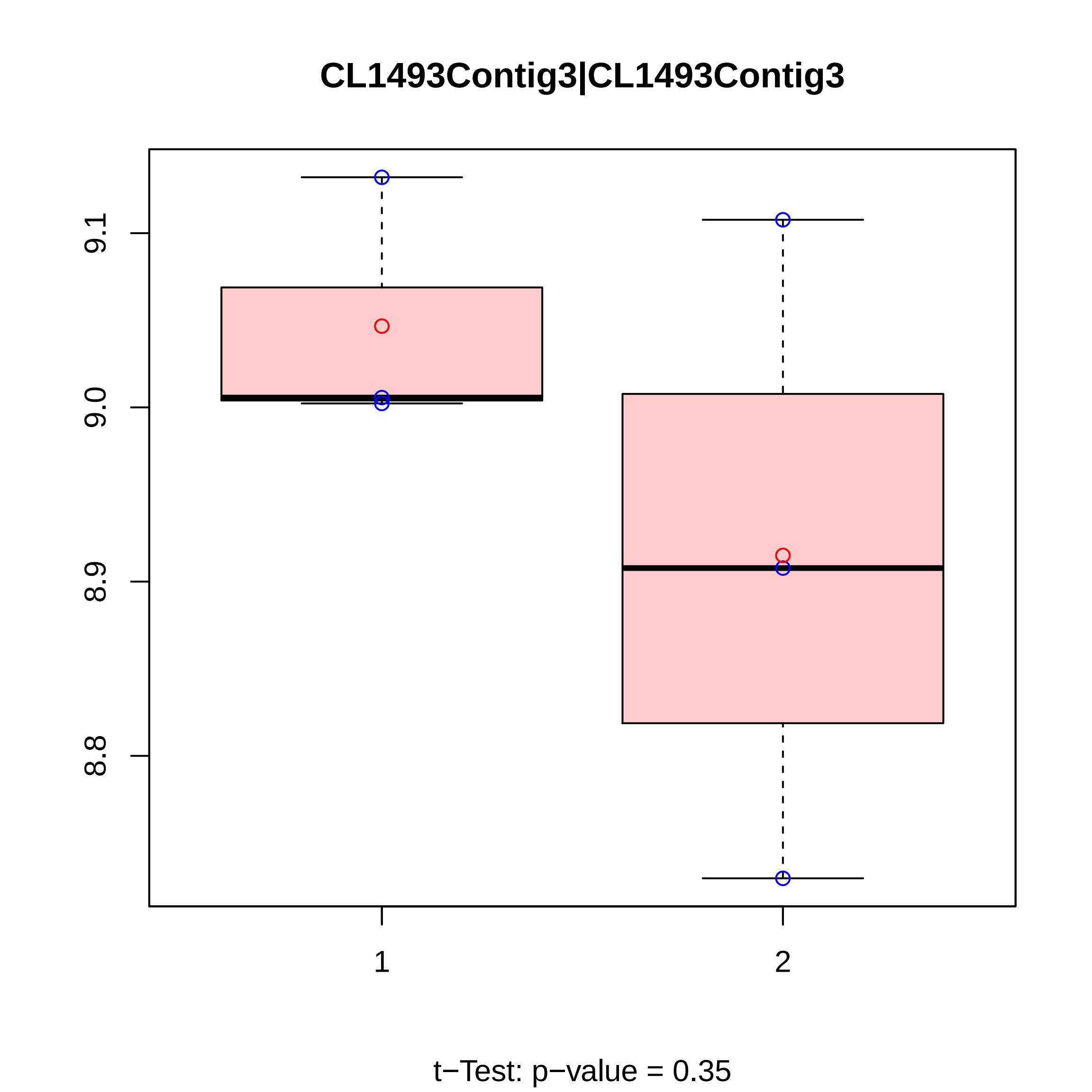

CL1493Contig3|CL1493Contig3
9.1
9.0
8.9
8.8
1
2
t−Test: p−value = 0.35

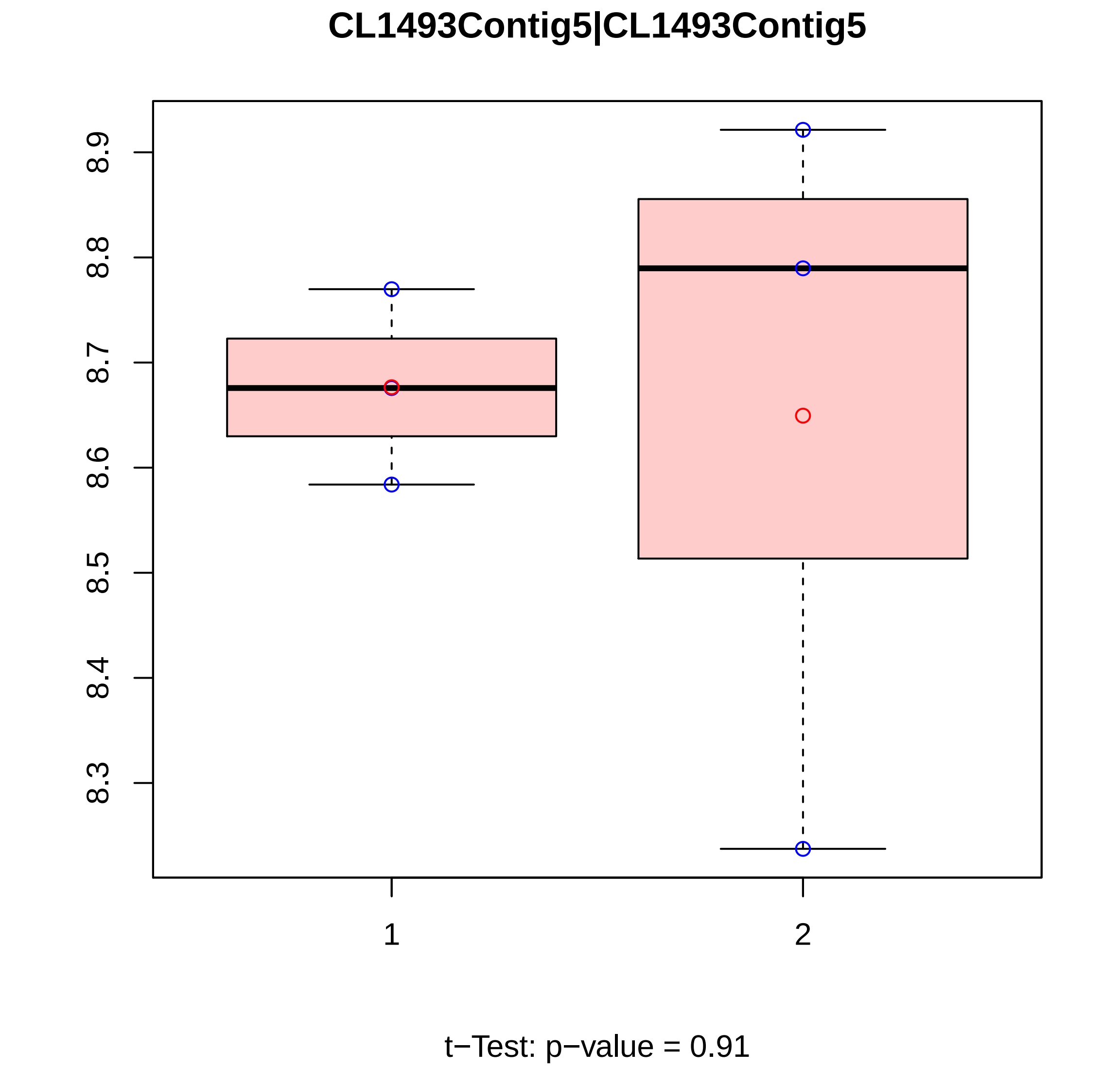
CL1493Contig5|CL1493Contig5
8.3
8.4
8.5
8.6
8.7
8.8
8.9
1
2
t−Test: p−value = 0.91

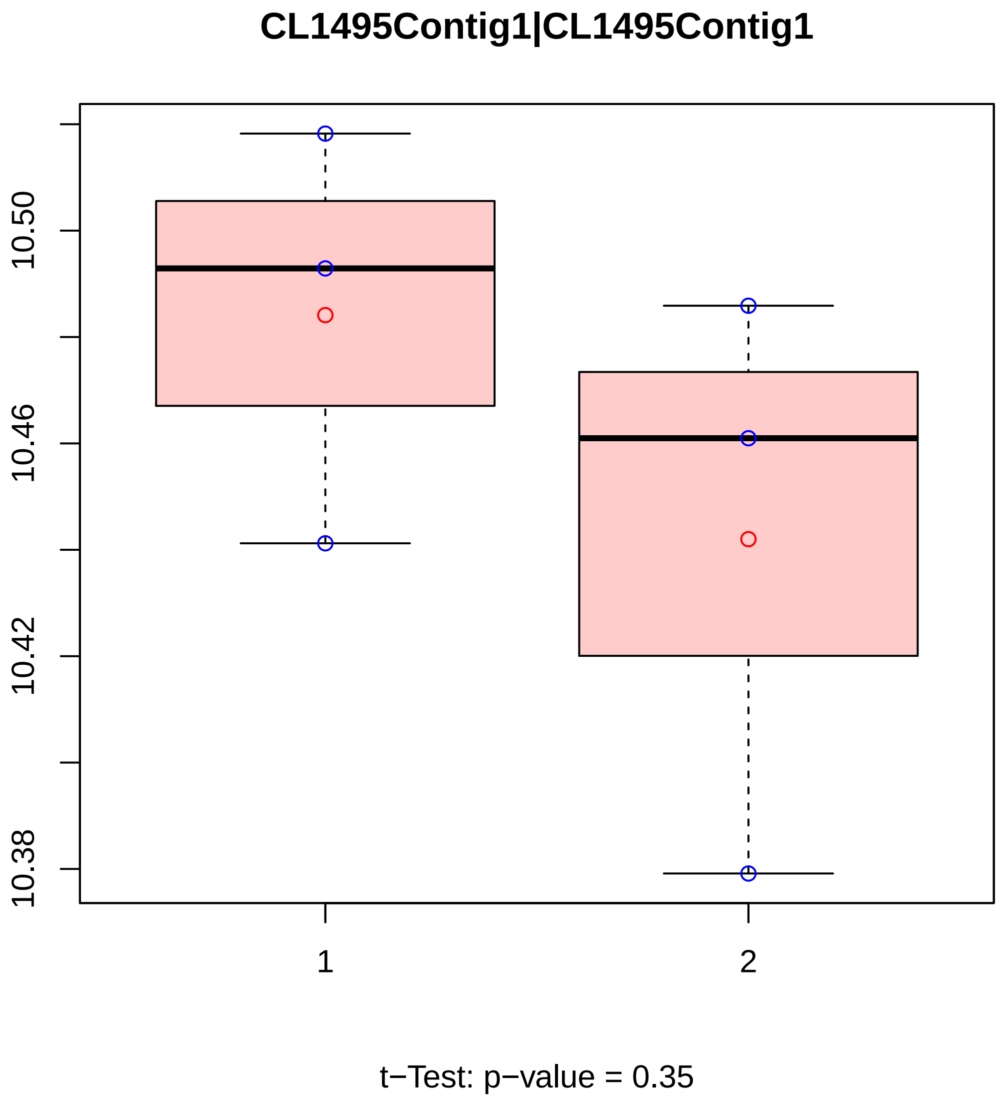
CL1495Contig1|CL1495Contig1
10.50
10.46
10.42
10.38
1
2
t−Test: p−value = 0.35

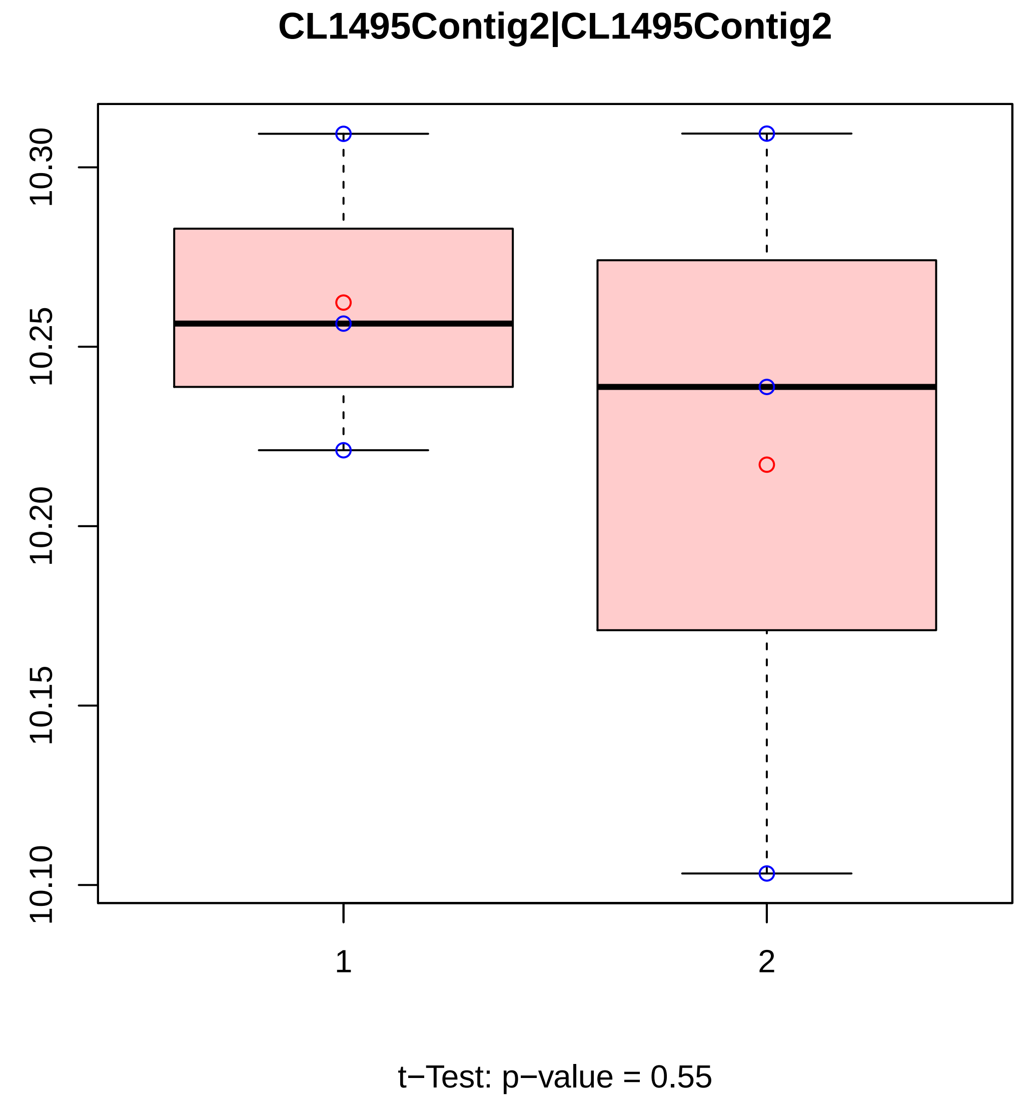

CL1495Contig2|CL1495Contig2
10.30
10.25
10.20
10.15
10.10
1
2
t−Test: p−value = 0.55

**CL1495Contig3|CL1495Contig3**

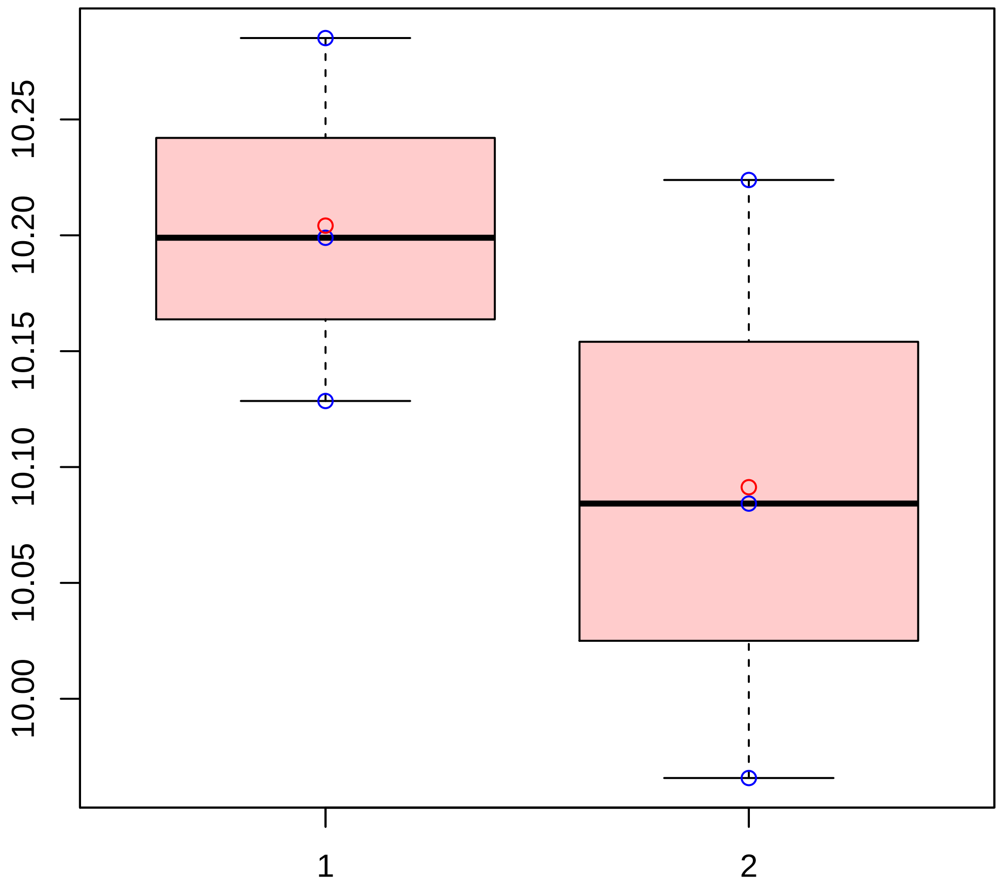


t−Test: p−value = 0.28

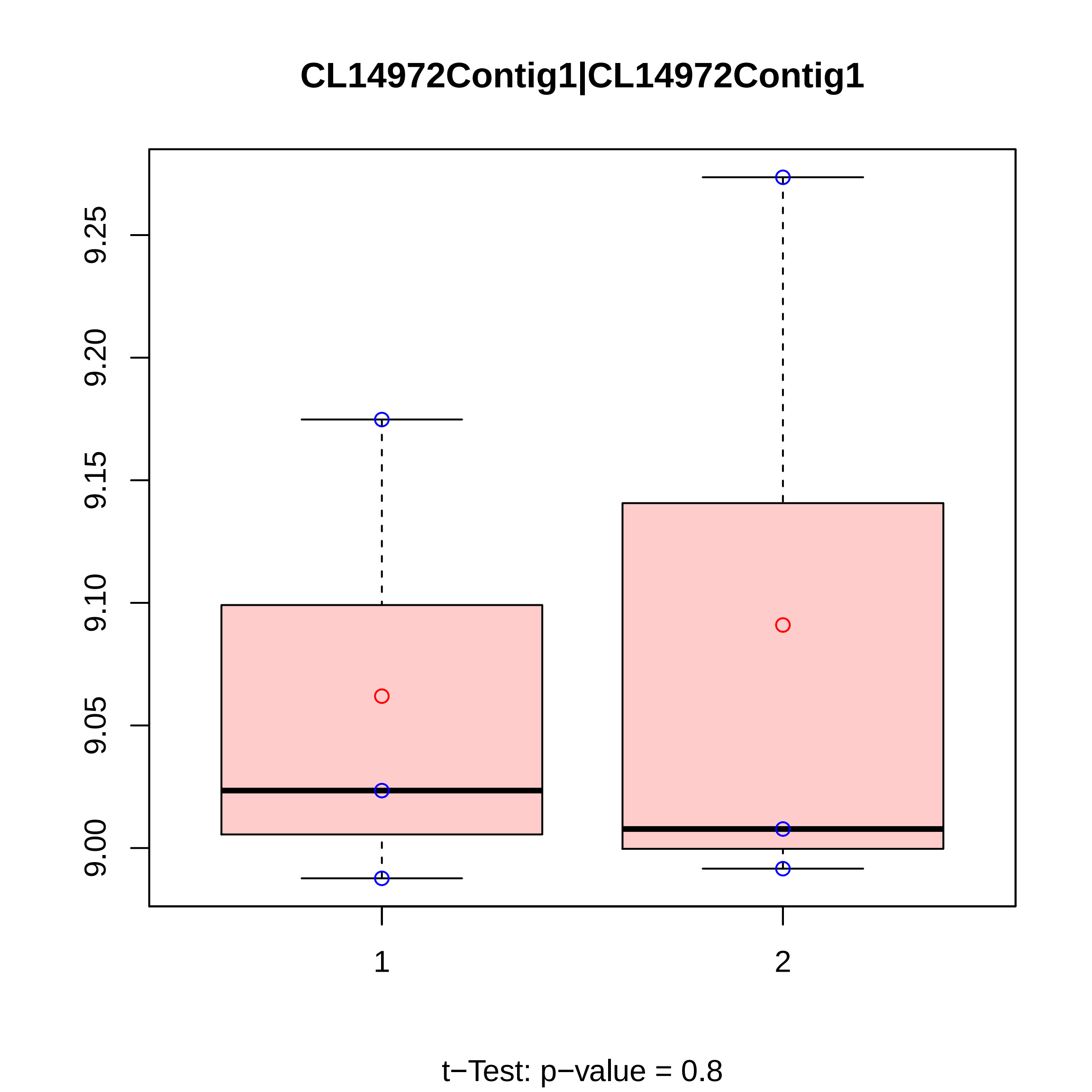
CL14972Contig1|CL14972Contig1
9.00
9.05
9.10
9.15
9.20
9.25
1
2
t−Test: p−value = 0.8

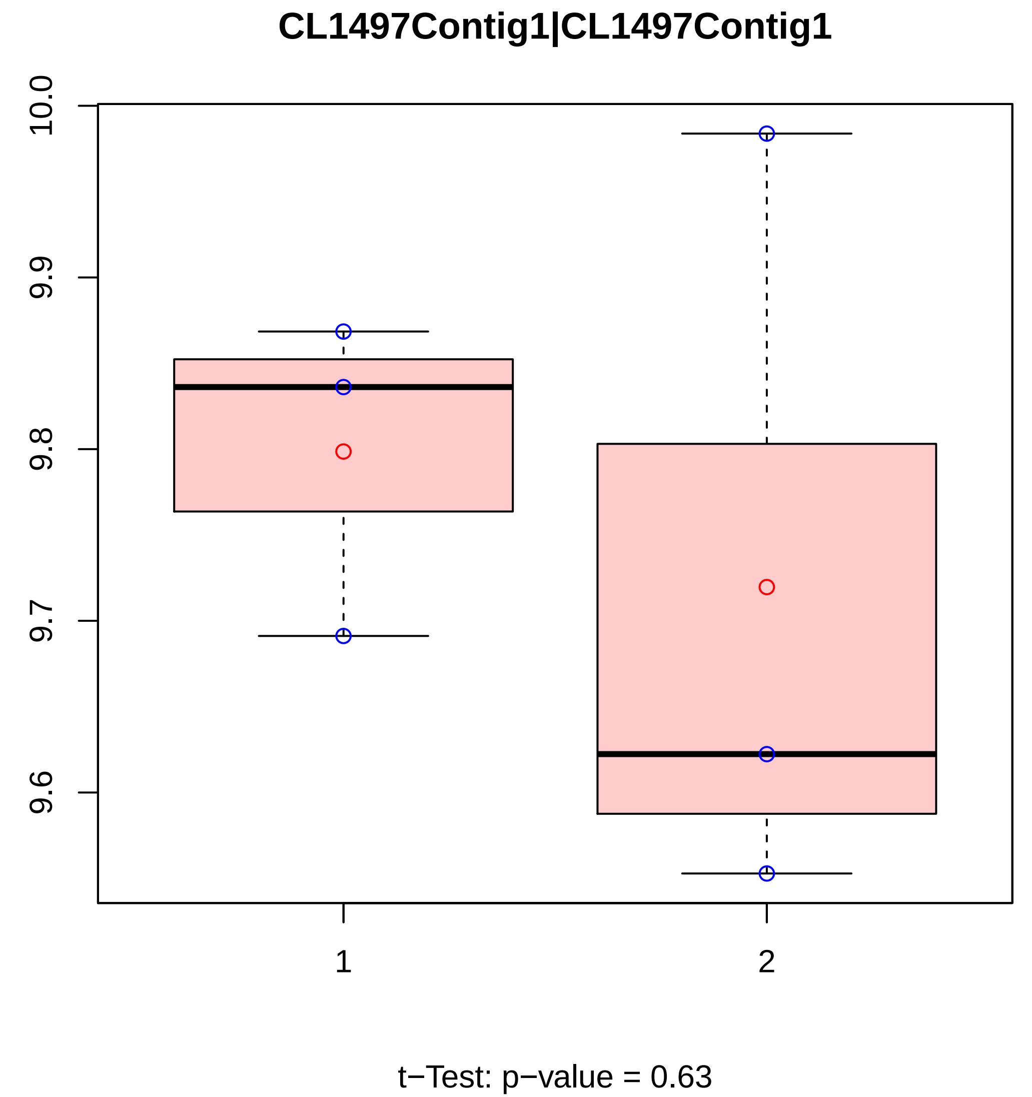
CL1497Contig1|CL1497Contig1
10.0
9.9
9.8
9.7
9.6
1
2
t−Test: p−value = 0.63

# CL1497Contig2|CL1497Contig2

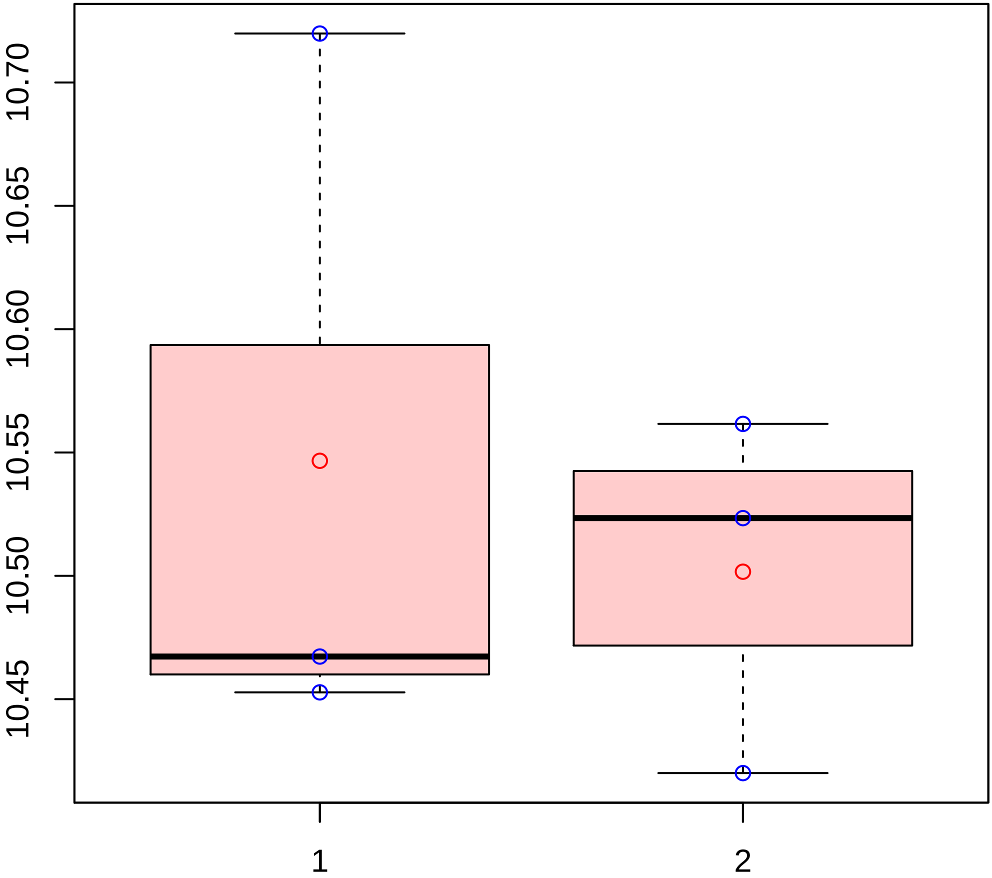


t−Test: p−value = 0.67

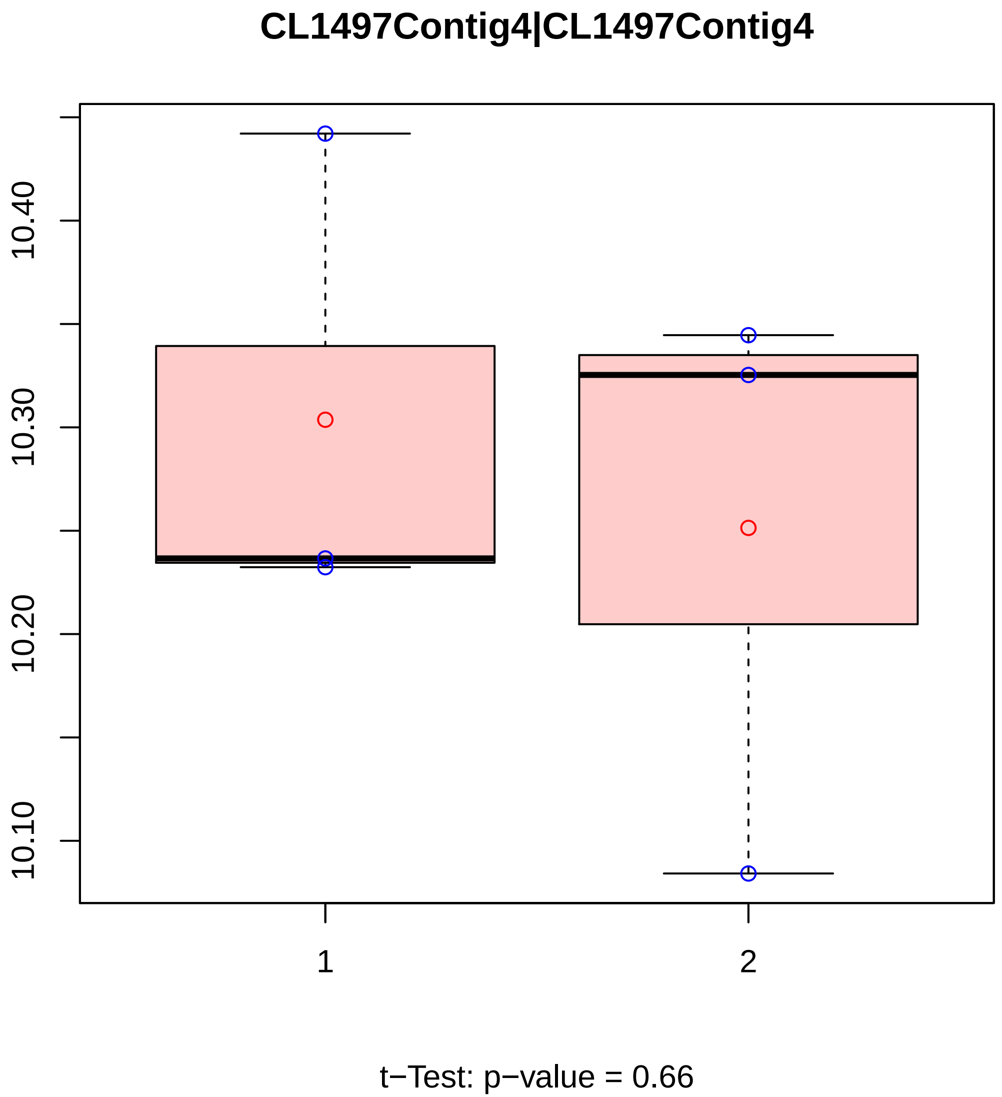
CL1497Contig4|CL1497Contig4
10.40
10.30
10.20
10.10
1
2
t−Test: p−value = 0.66

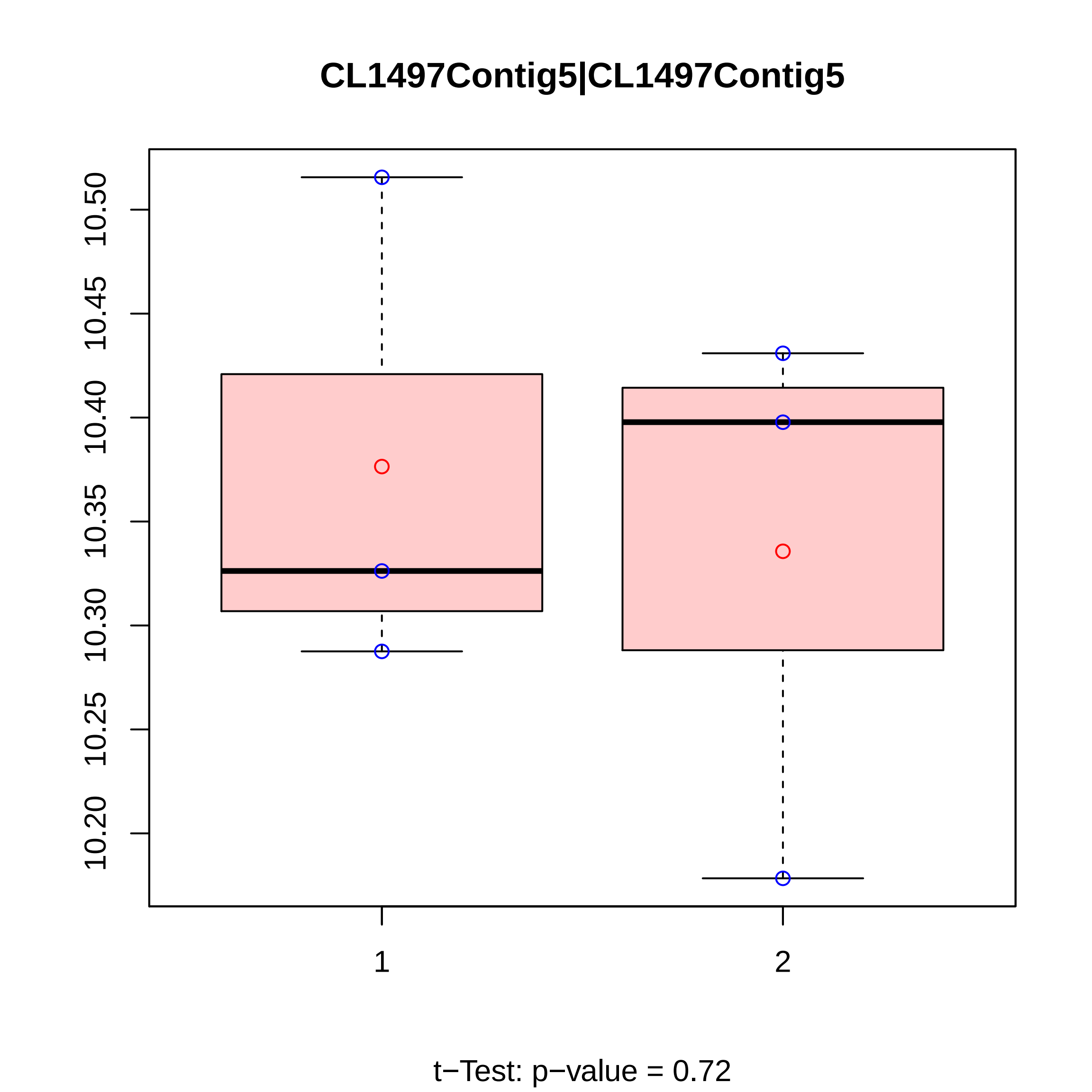
CL1497Contig5|CL1497Contig5
10.20
10.25
10.30
10.35
10.40
10.45
10.50
1
2
t−Test: p−value = 0.72

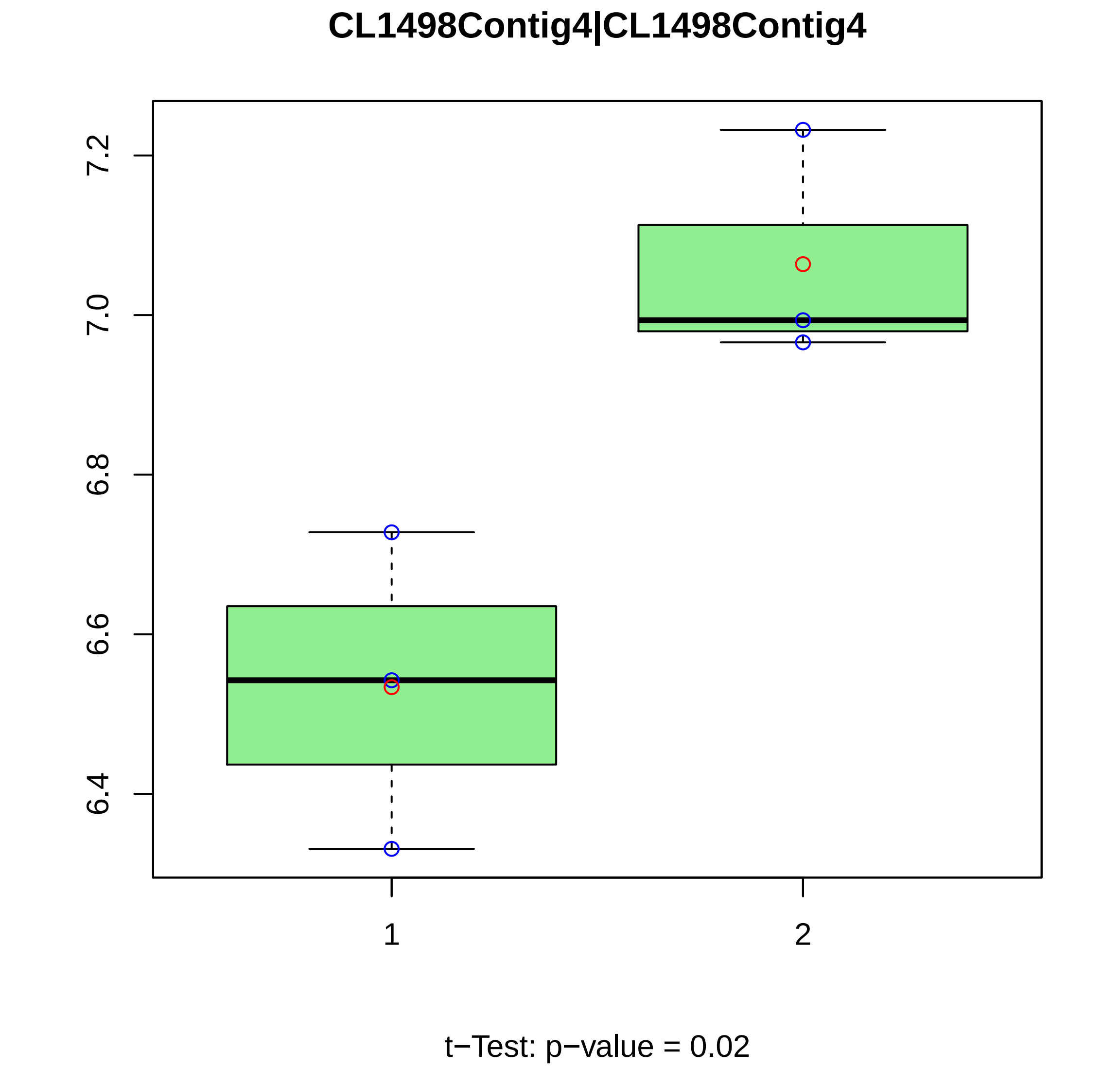
CL1498Contig4|CL1498Contig4
7.2
7.0
6.8
6.6
6.4
1
2
t−Test: p−value = 0.02

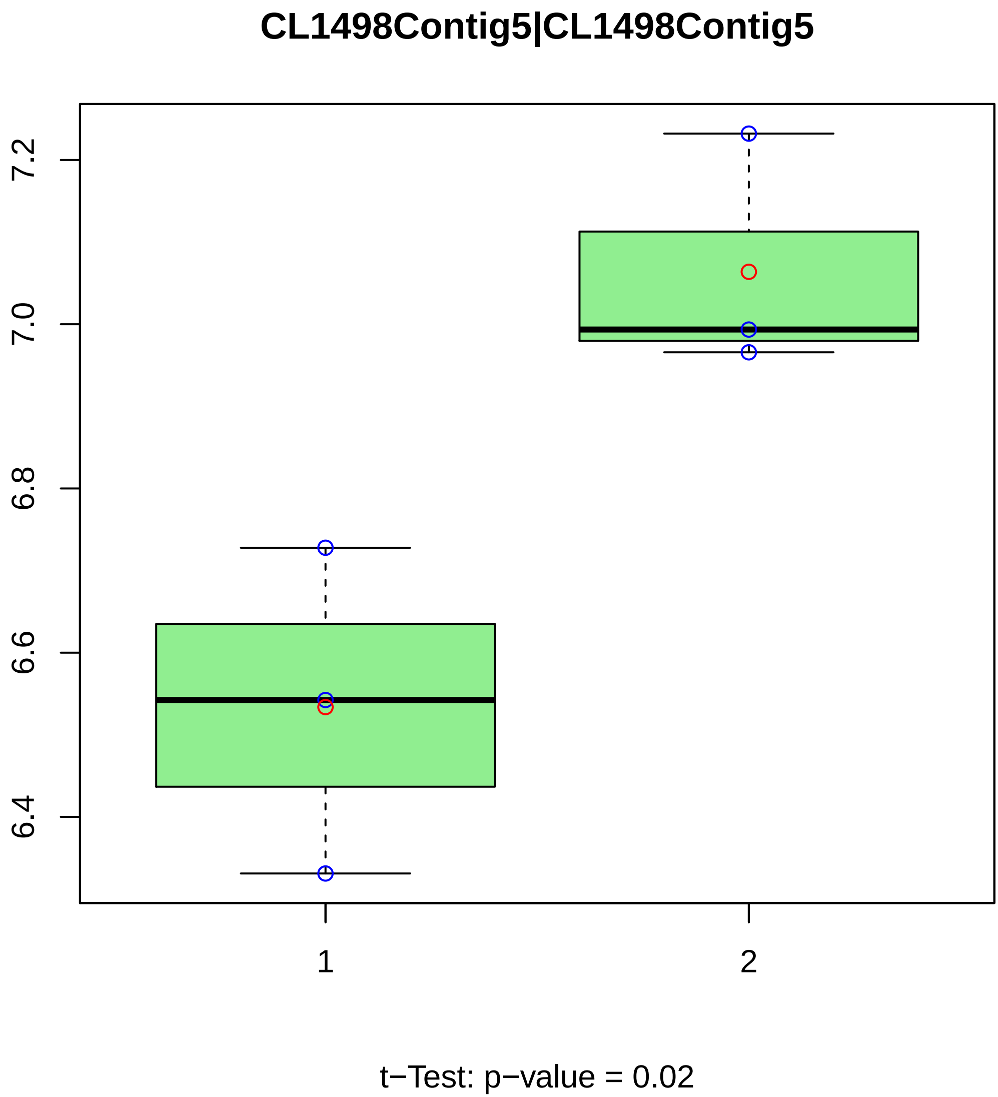
CL1498Contig5|CL1498Contig5
7.2
7.0
6.8
6.6
6.4
1
2
t−Test: p−value = 0.02

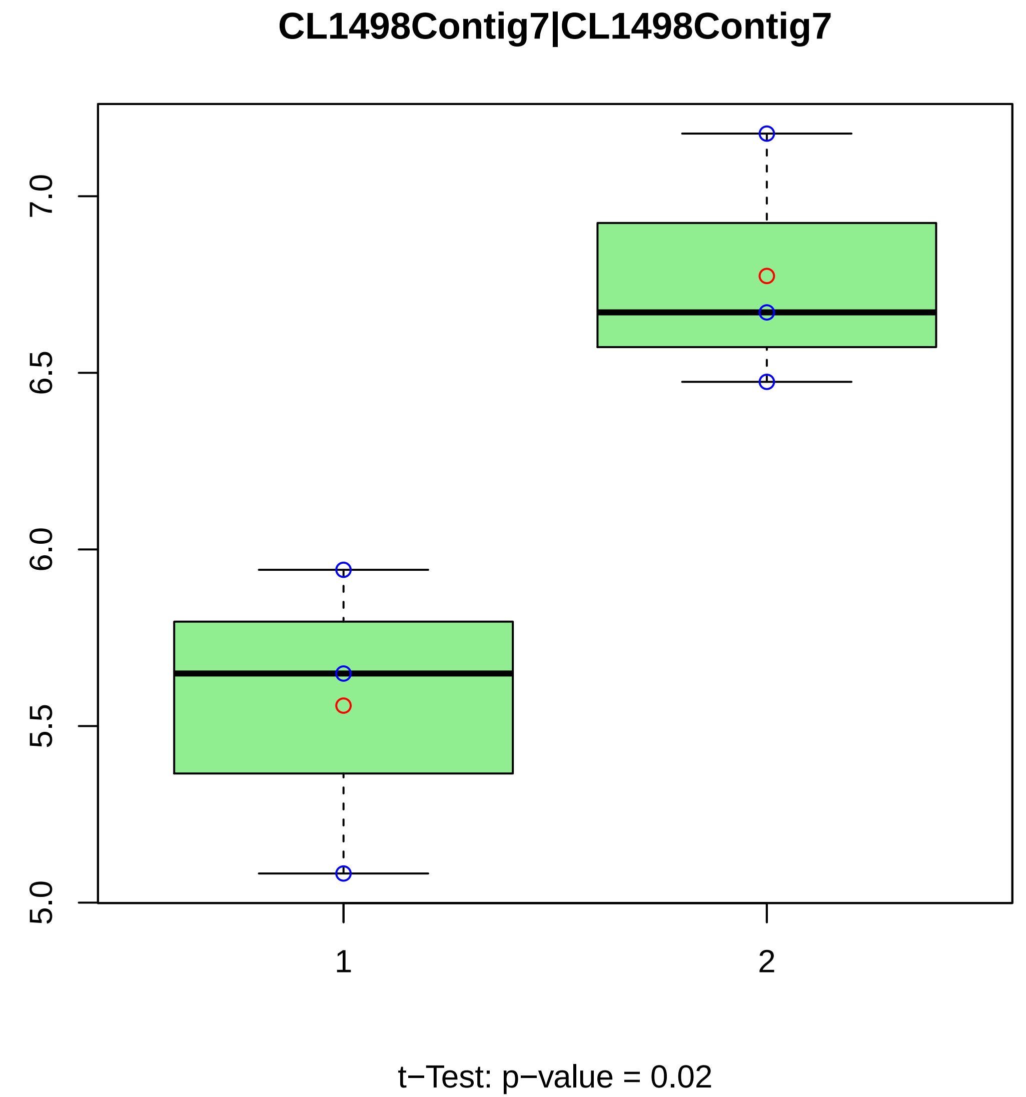
CL1498Contig7|CL1498Contig7
7.0
6.5
6.0
5.5
5.0
1
2
t−Test: p−value = 0.02

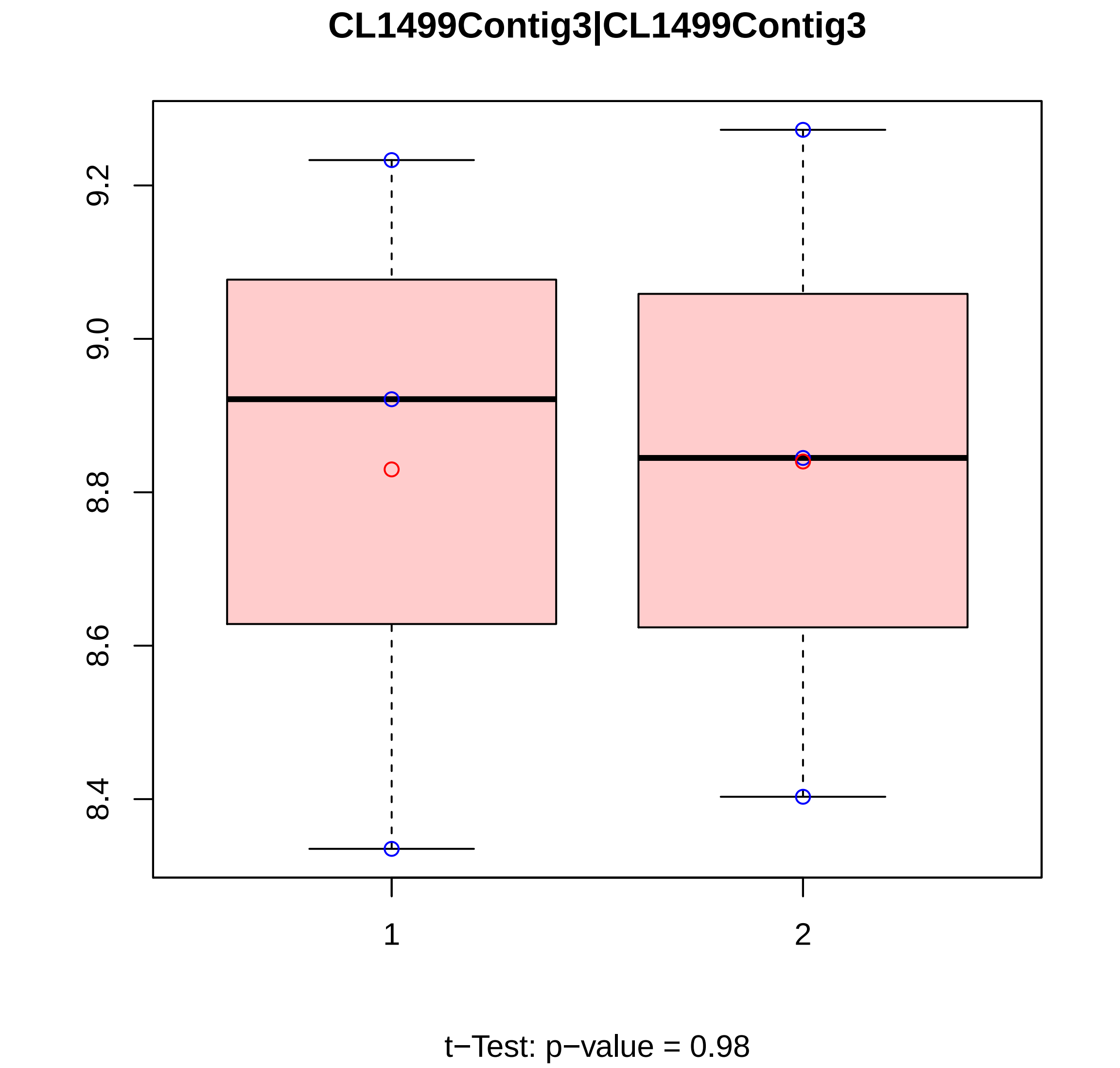
CL1499Contig3|CL1499Contig3
9.2
9.0
8.8
8.6
8.4
1
2
t−Test: p−value = 0.98

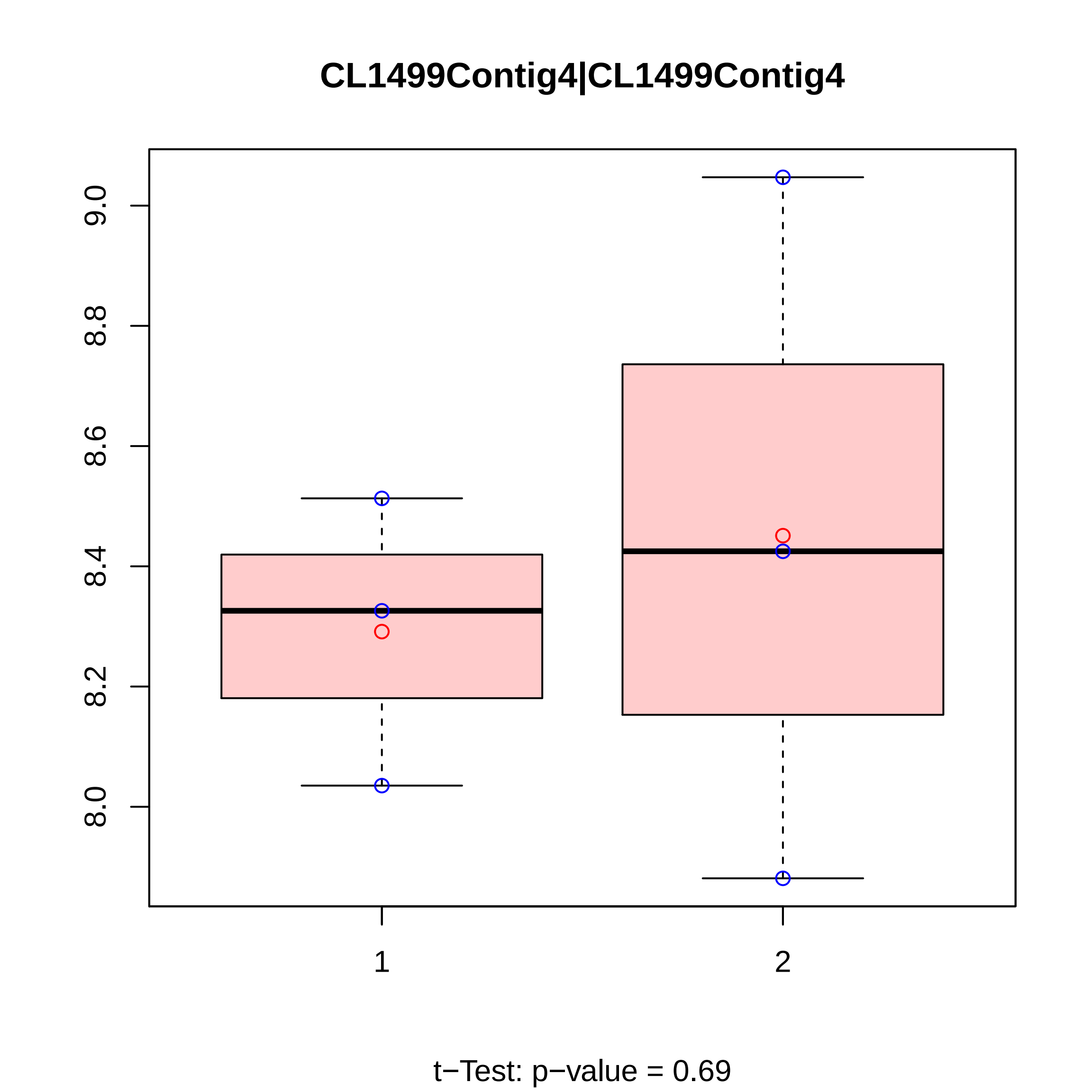

CL1499Contig4|CL1499Contig4
9.0
8.8
8.6
8.4
8.2
8.0
1
2
t−Test: p−value = 0.69

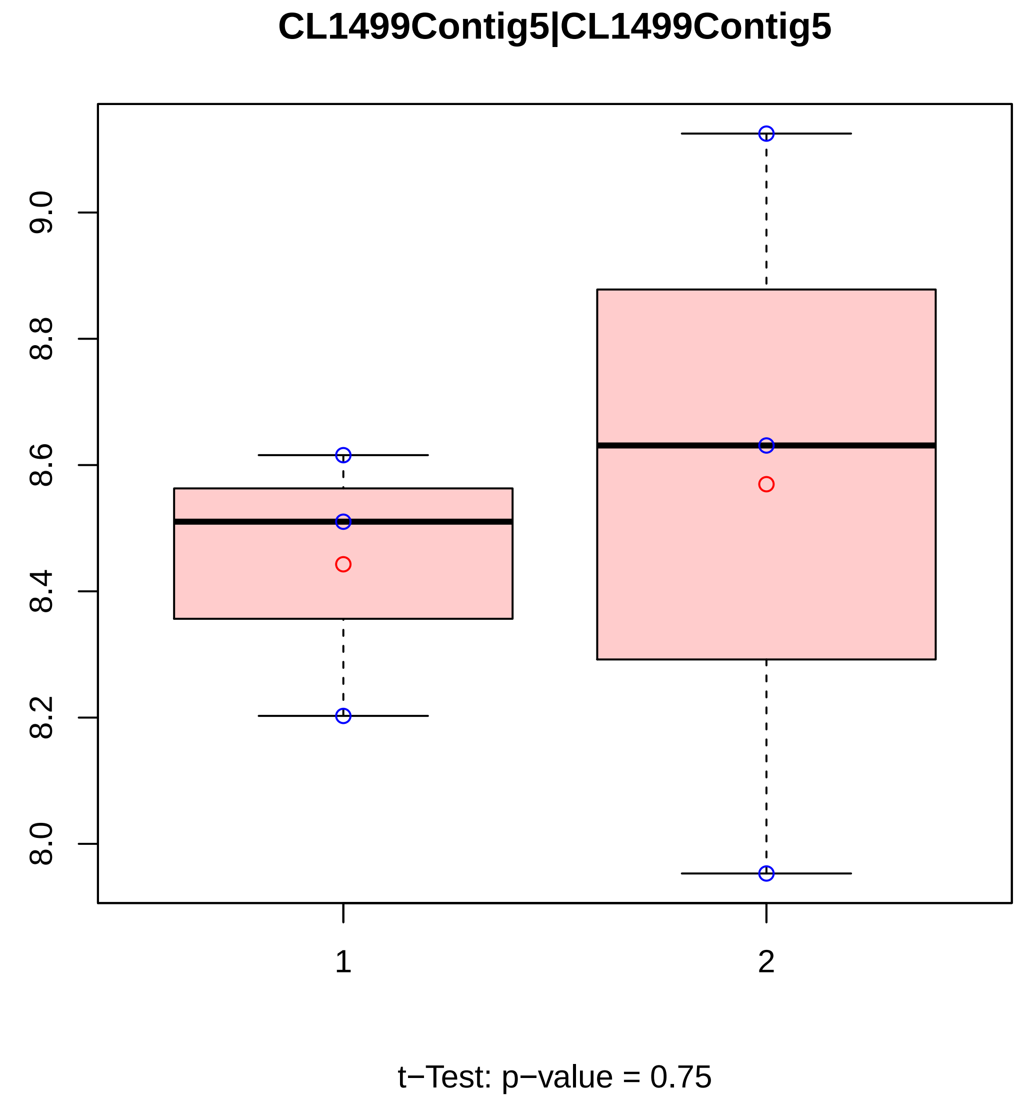
CL1499Contig5|CL1499Contig5
9.0
8.8
8.6
8.4
8.2
8.0
1
2
t−Test: p−value = 0.75

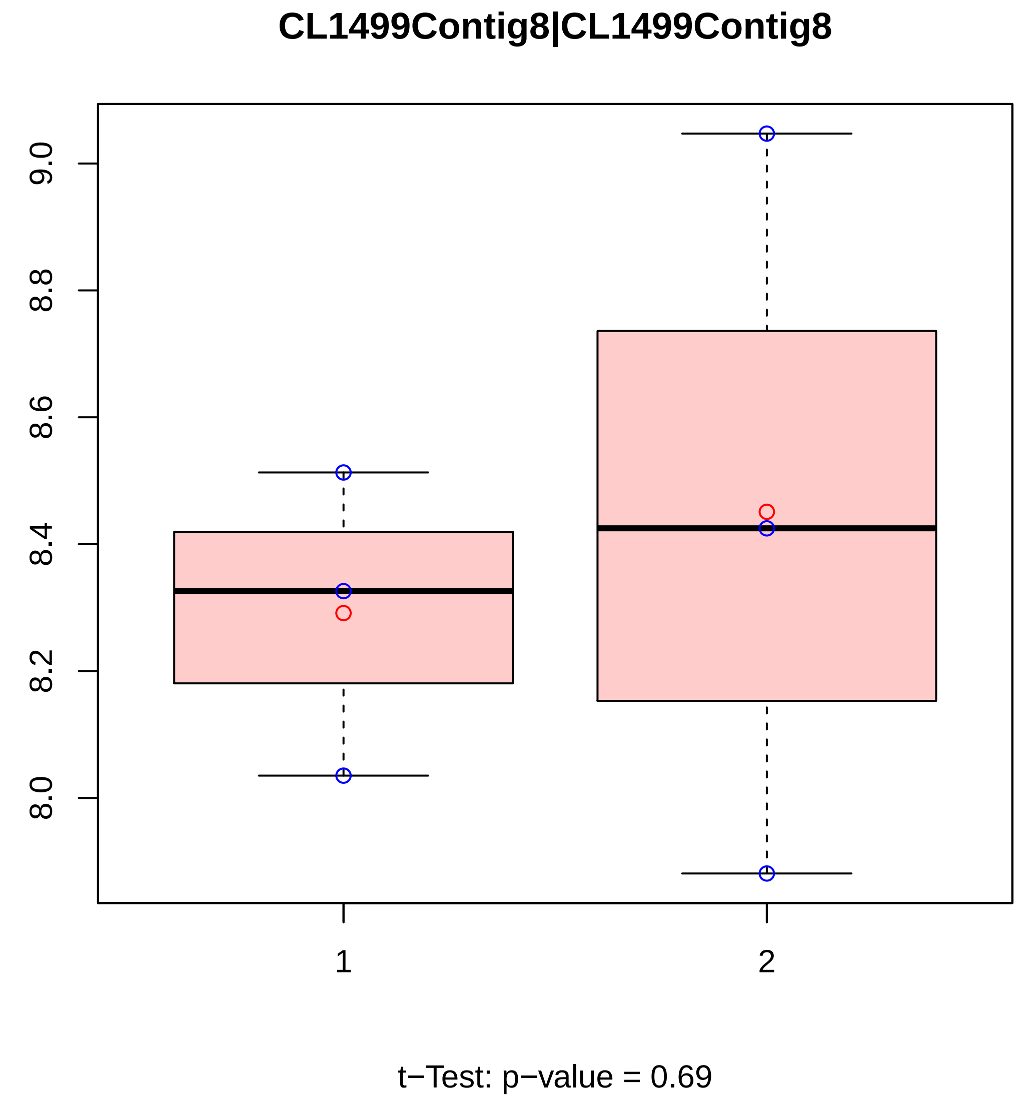
CL1499Contig8|CL1499Contig8
9.0
8.8
8.6
8.4
8.2
8.0
1
2
t−Test: p−value = 0.69

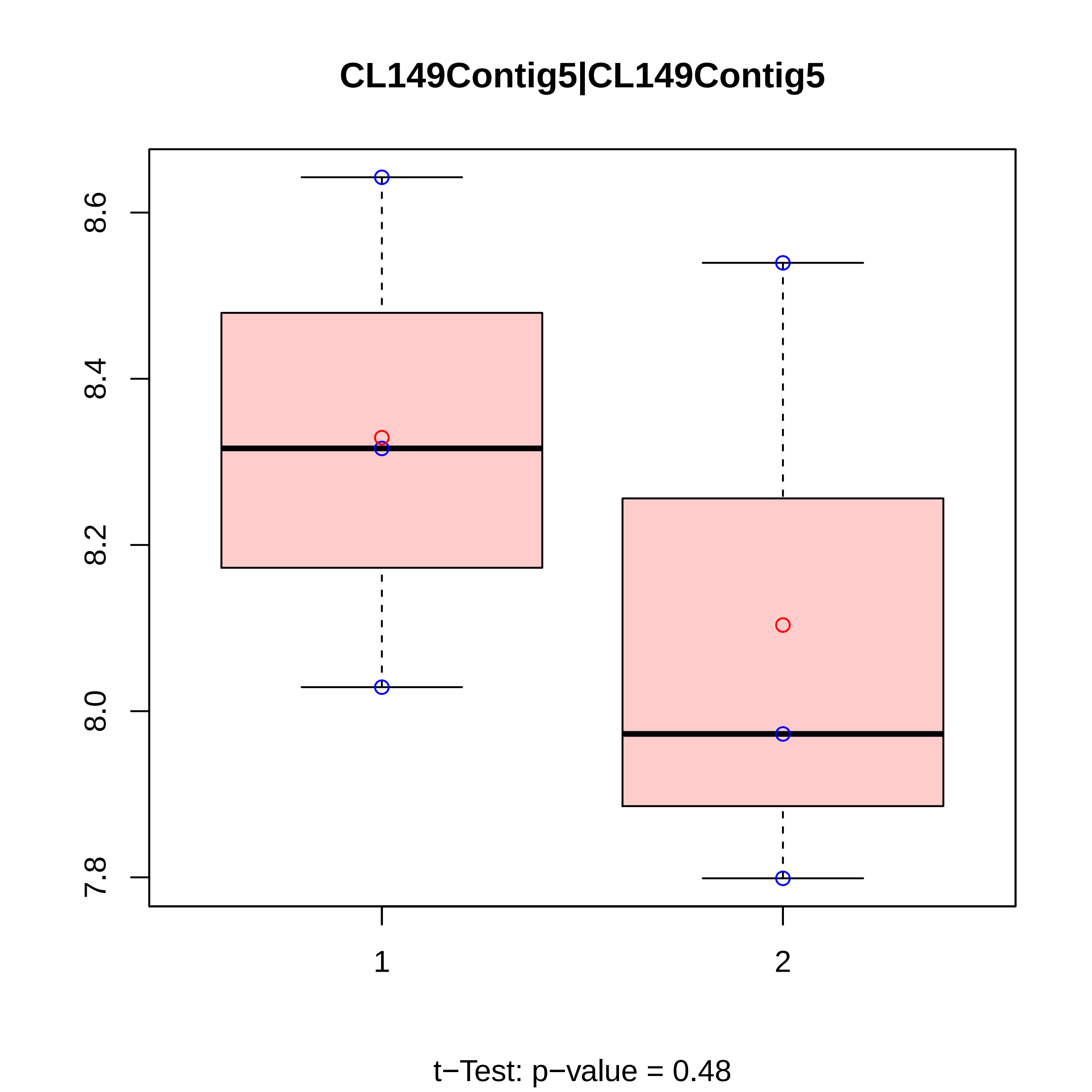
CL149Contig5|CL149Contig5
8.6
8.4
8.2
8.0
7.8
1
2
t−Test: p−value = 0.48

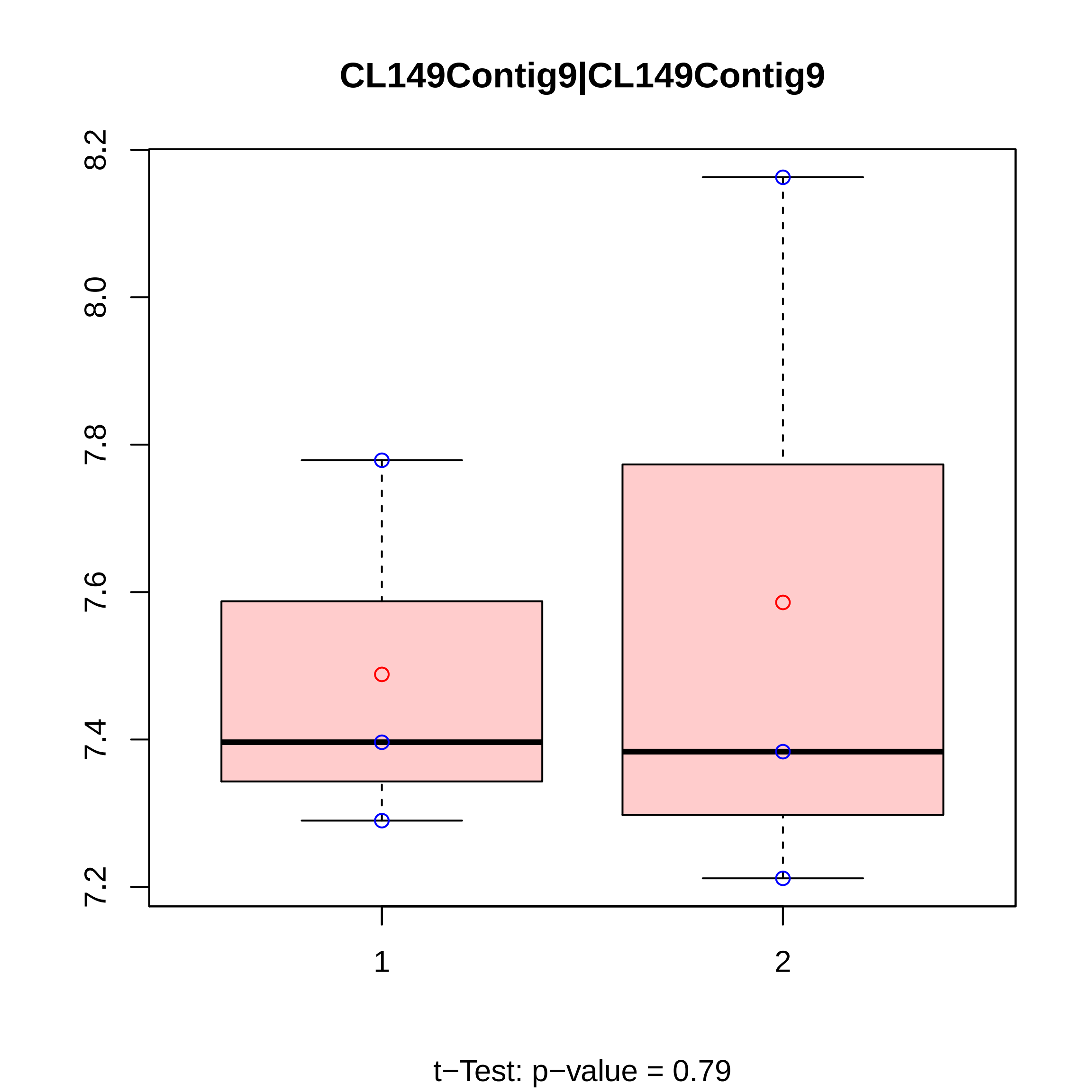

CL149Contig9|CL149Contig9
8.2
8.0
7.8
7.6
7.4
7.2
1
2
t−Test: p−value = 0.79

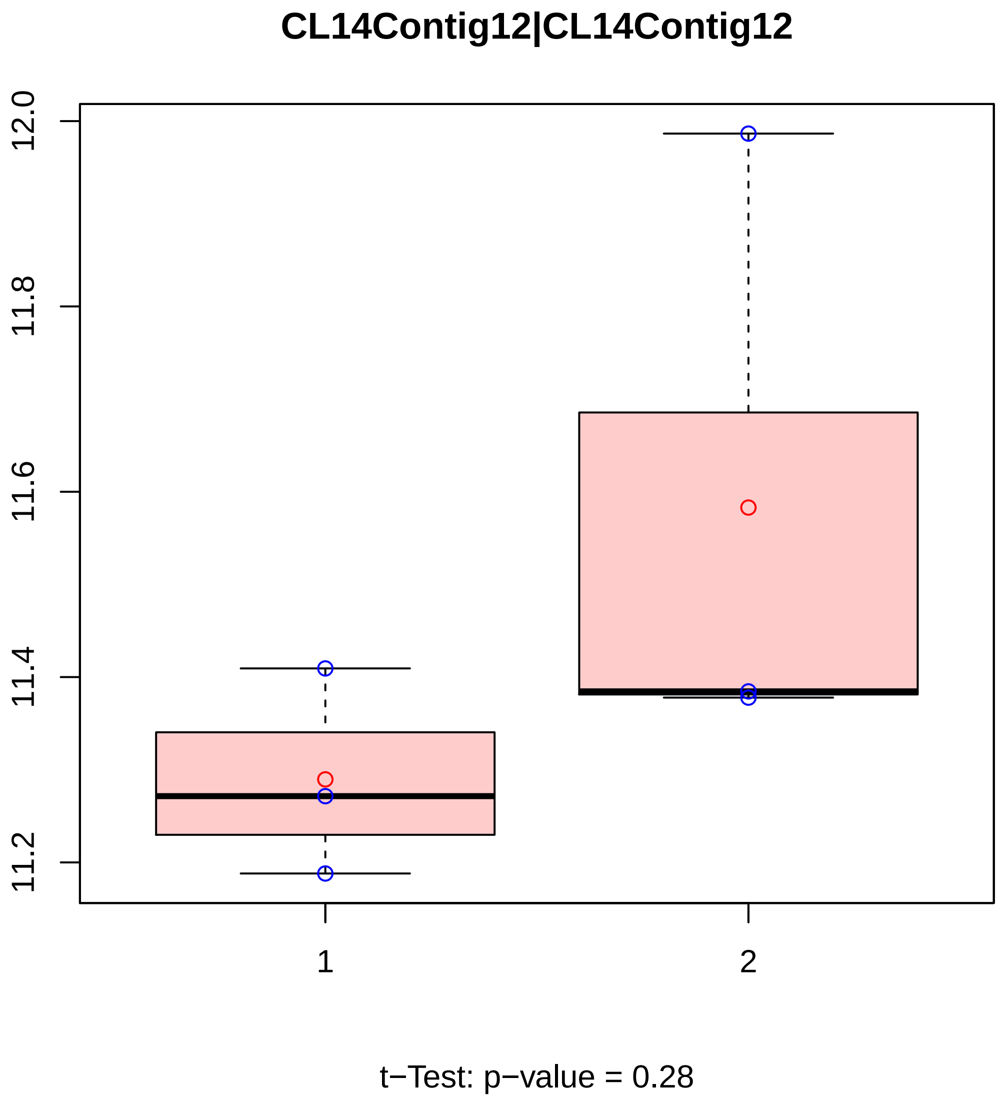
CL14Contig12|CL14Contig12
12.0
11.8
11.6
11.4
11.2
1
2
t−Test: p−value = 0.28

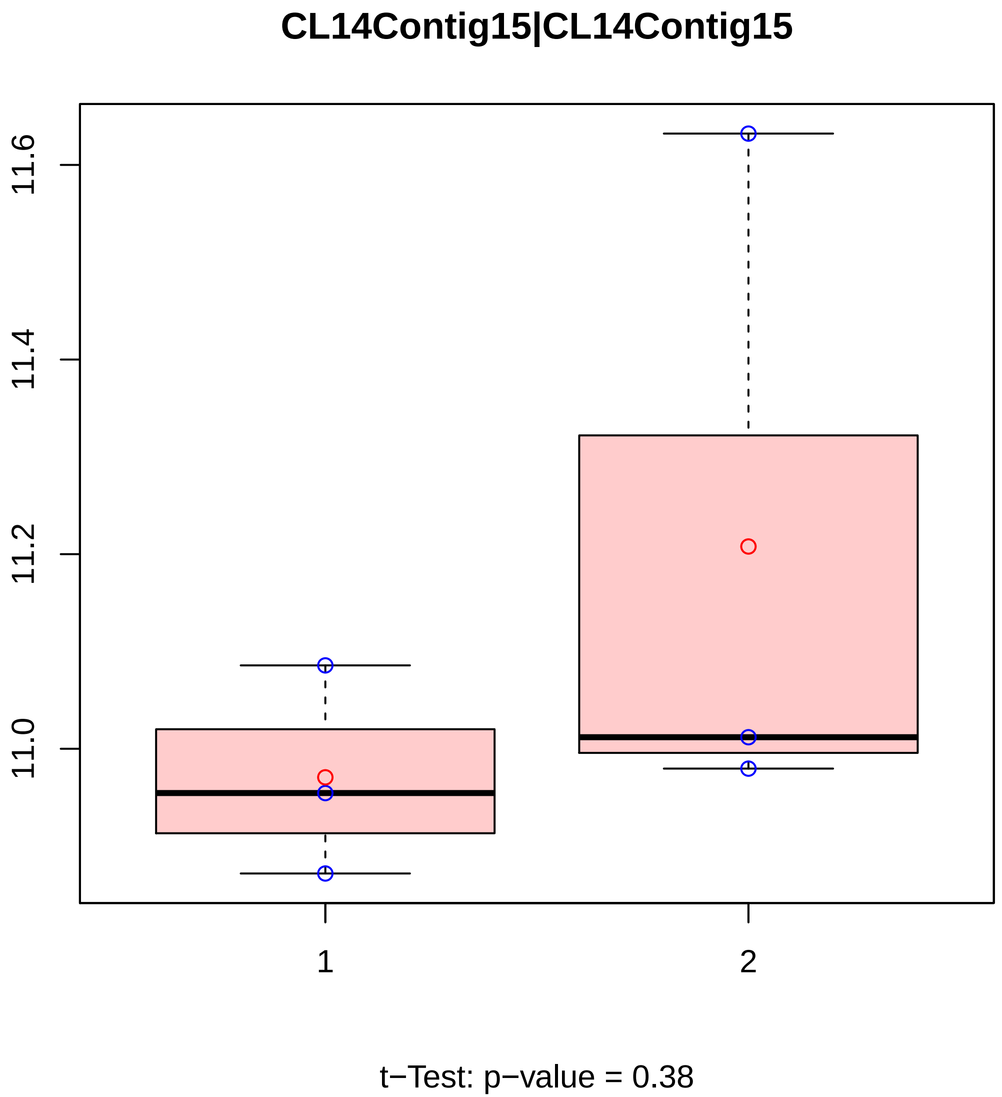
CL14Contig15|CL14Contig15
11.6
11.4
11.2
11.0
1
2
t−Test: p−value = 0.38

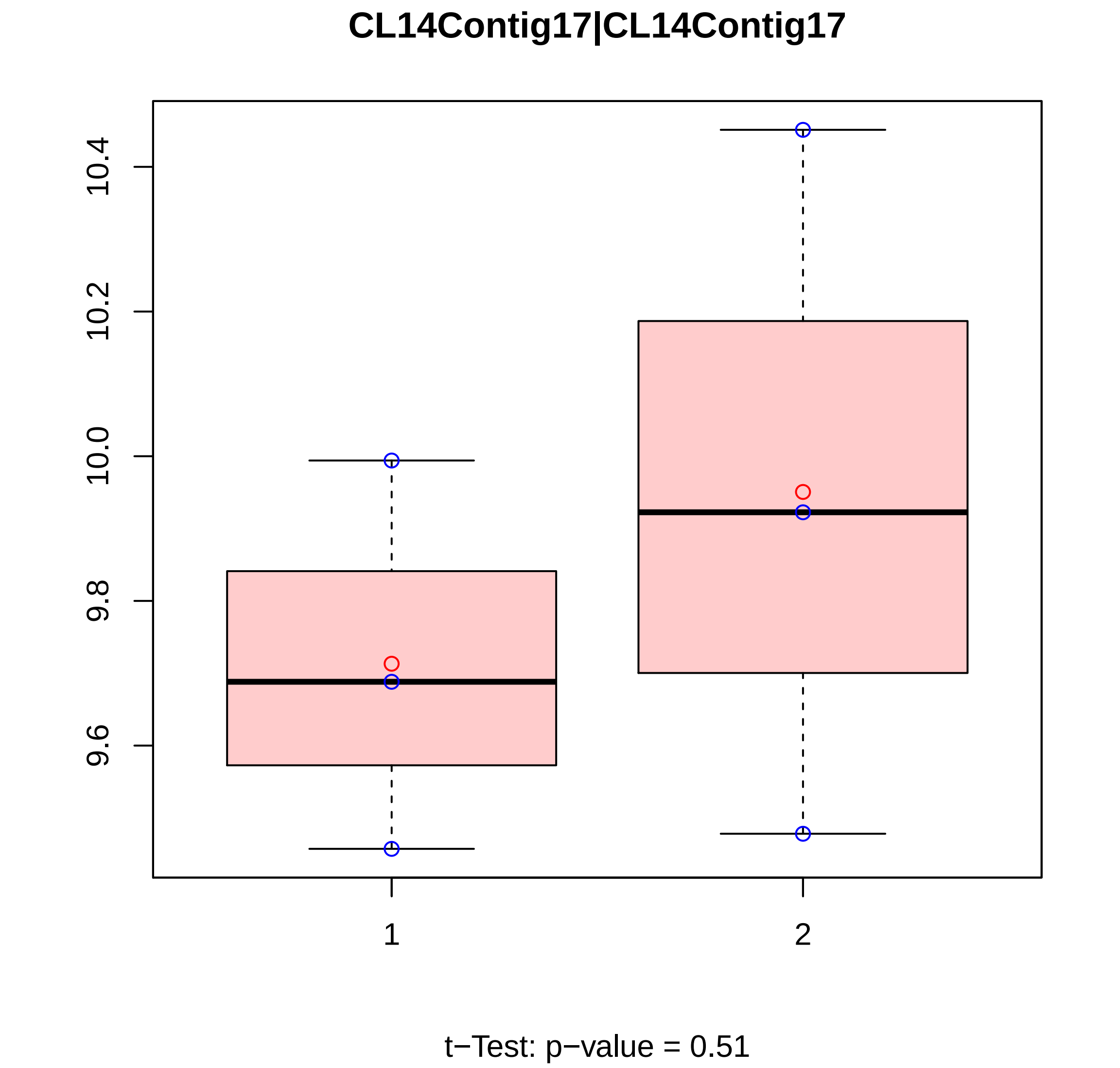
CL14Contig17|CL14Contig17
10.4
10.2
10.0
9.8
9.6
1
2
t−Test: p−value = 0.51

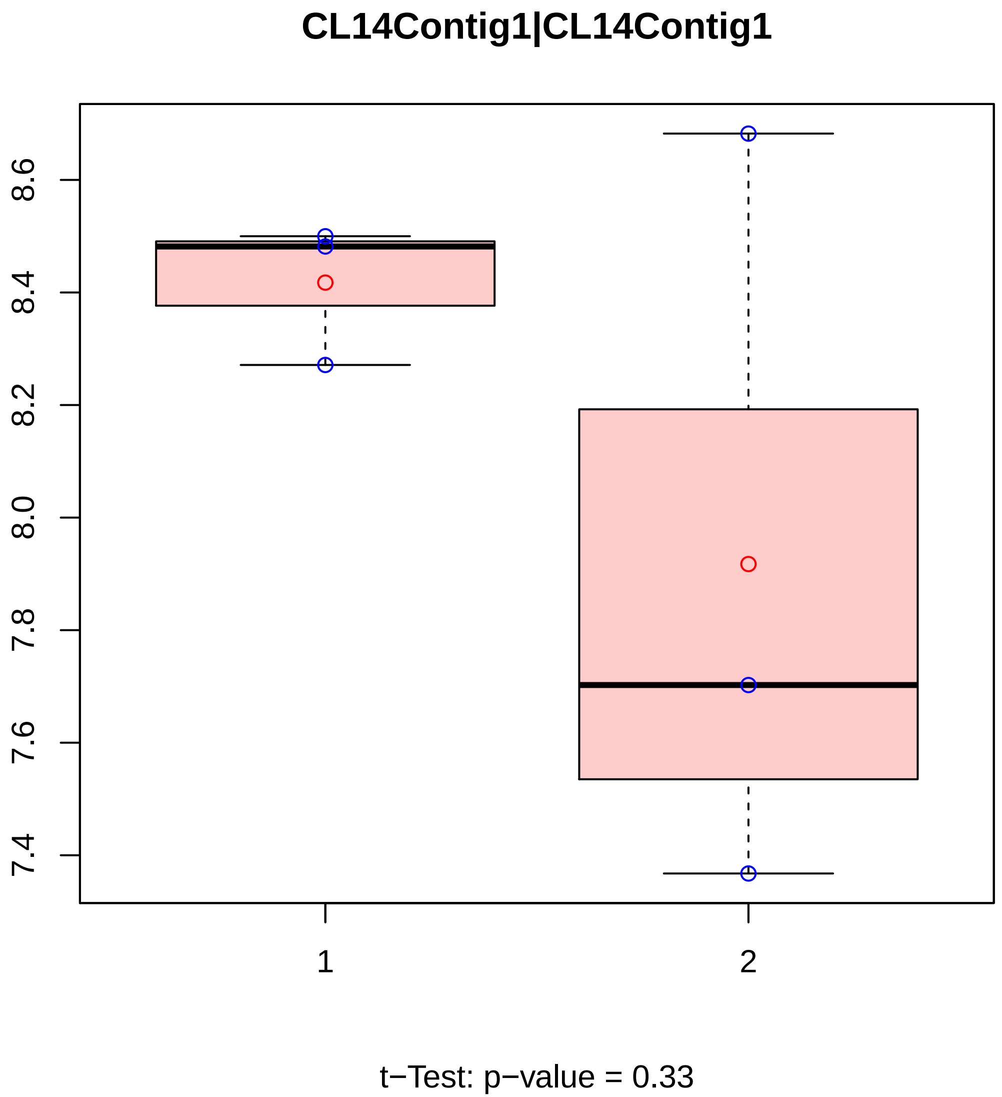

CL14Contig1|CL14Contig1
8.6
8.4
8.2
8.0
7.8
7.6
7.4
1
2
t−Test: p−value = 0.33

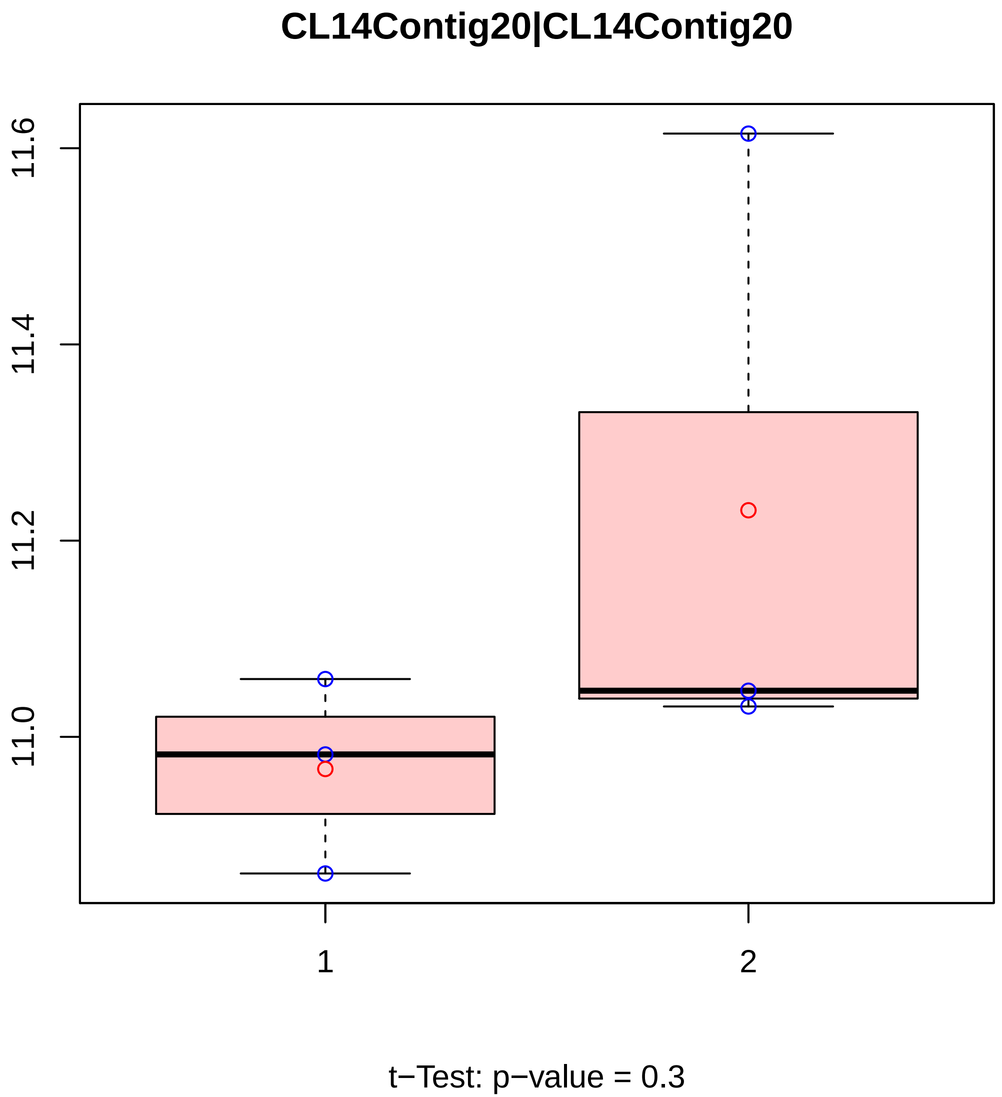

CL14Contig20|CL14Contig20
11.6
11.4
11.2
11.0
1
2
t−Test: p−value = 0.3

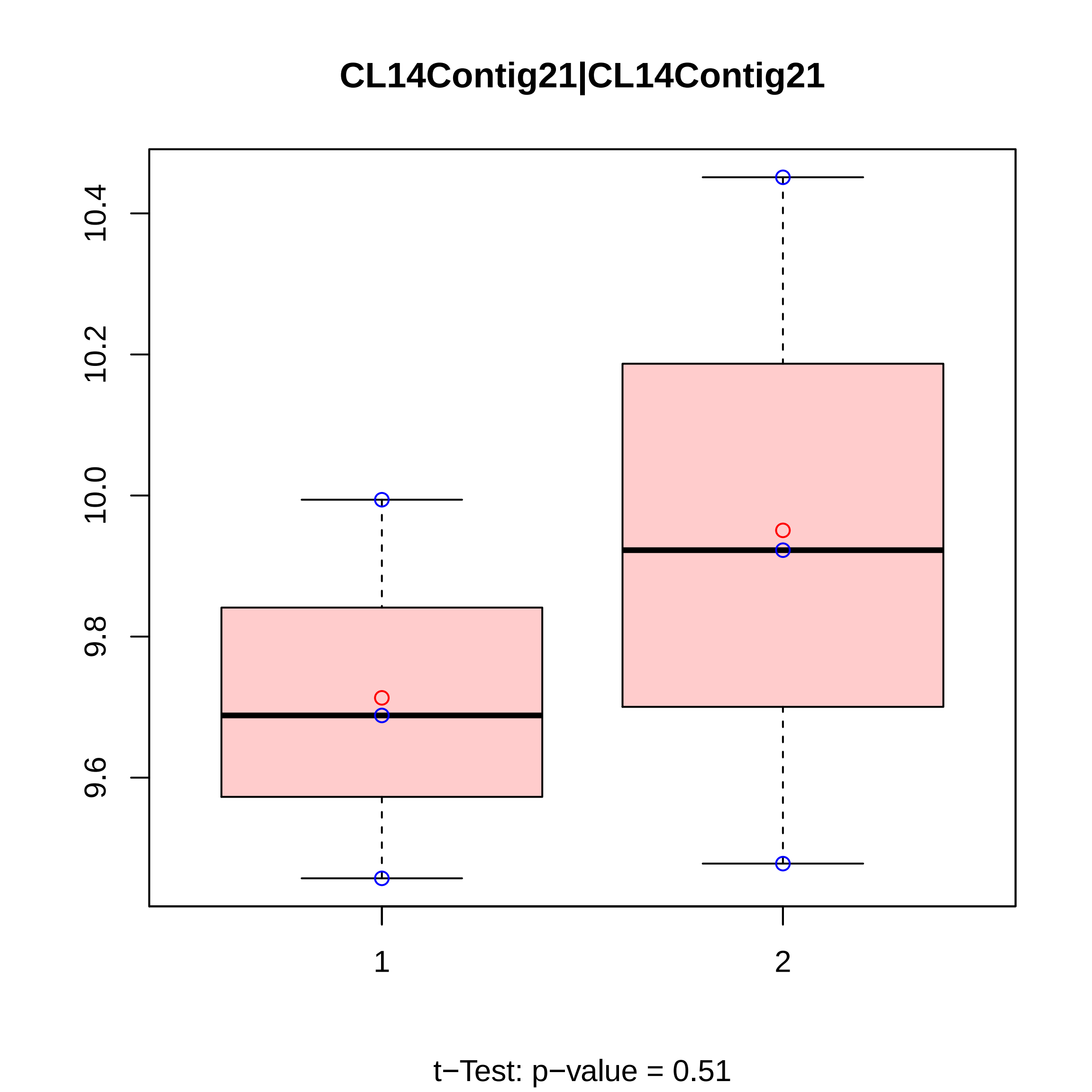
CL14Contig21|CL14Contig21
10.4
10.2
10.0
9.8
9.6
1
2
t−Test: p−value = 0.51

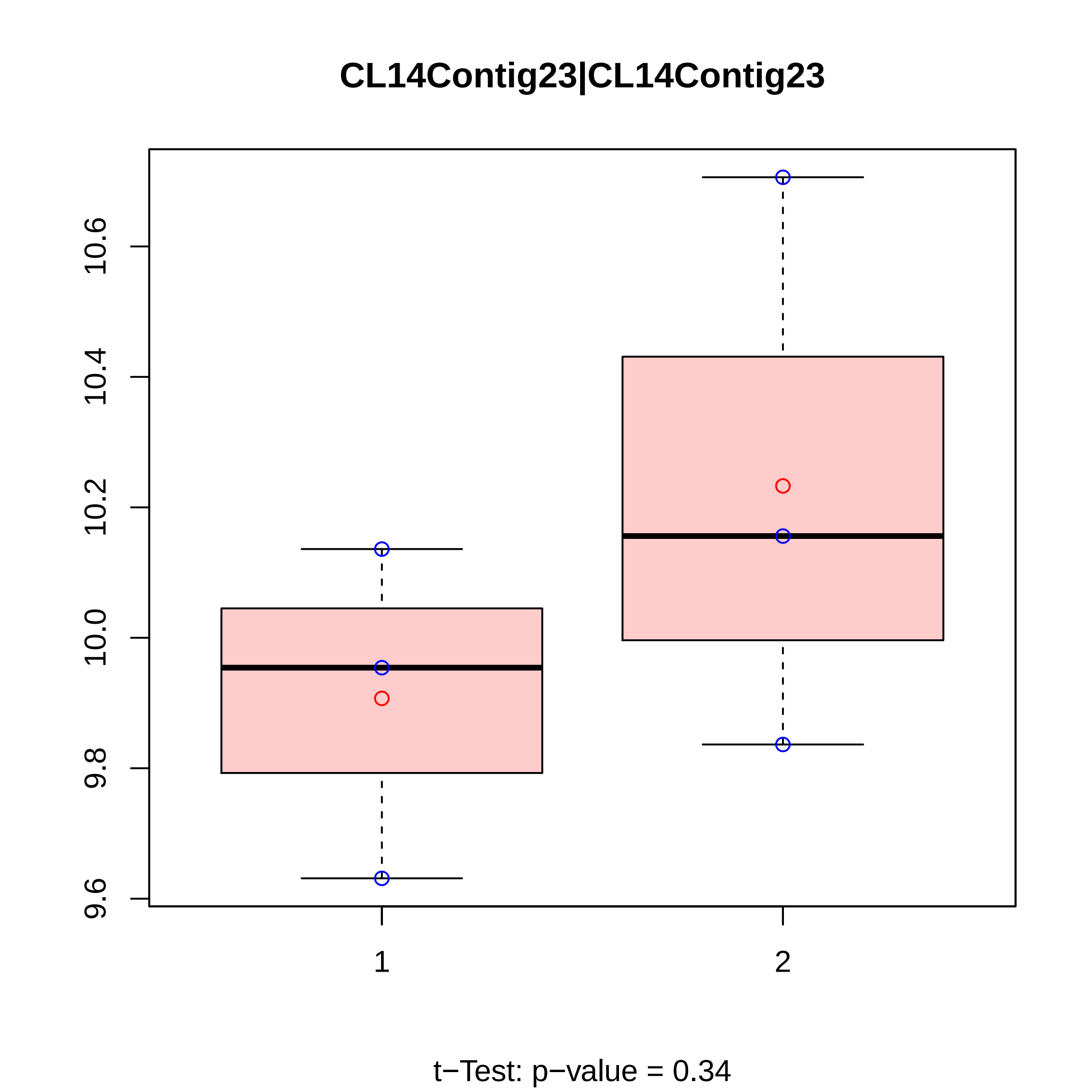
CL14Contig23|CL14Contig23
10.6
10.4
10.2
10.0
9.8
9.6
1
2
t−Test: p−value = 0.34

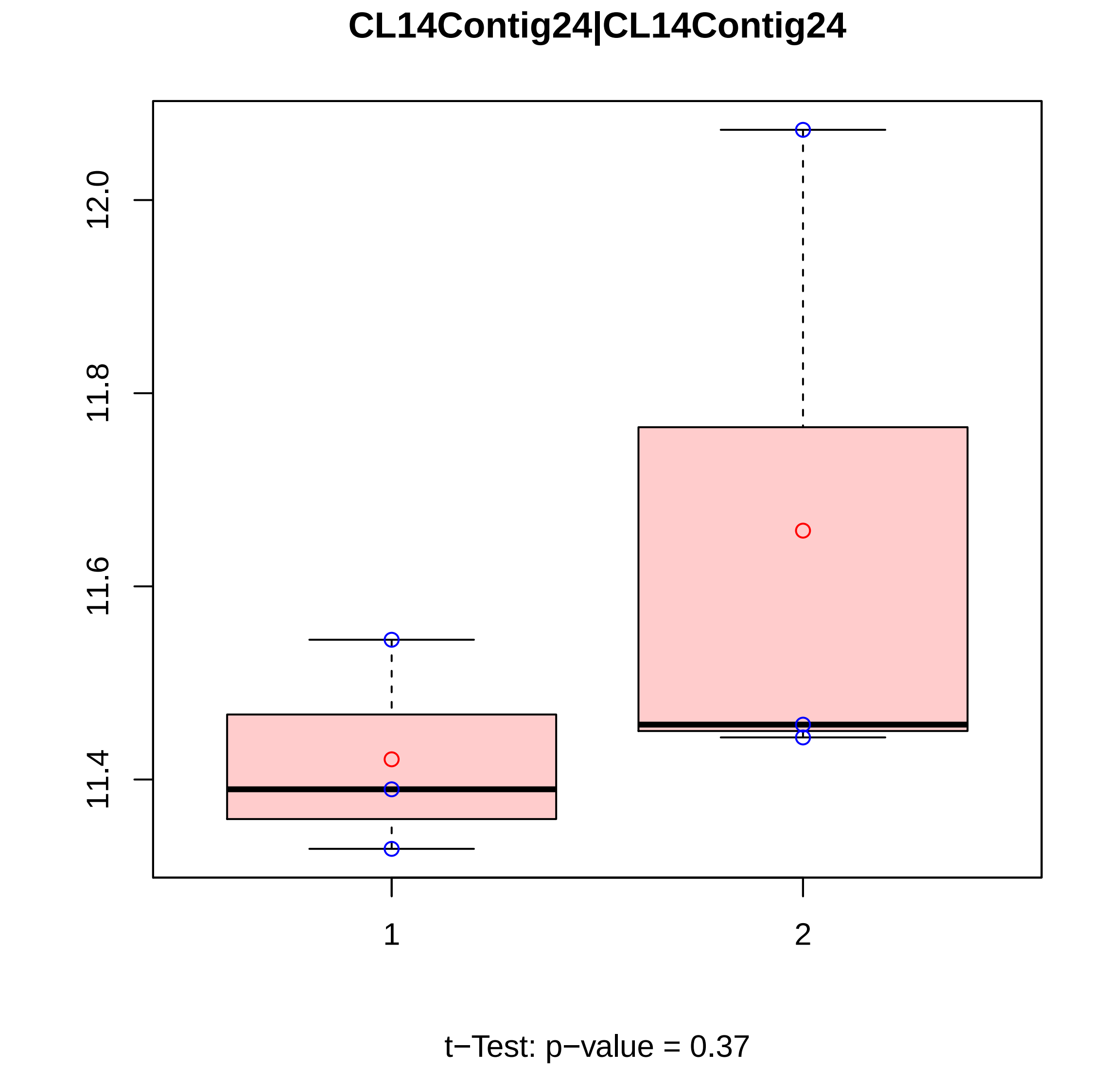
CL14Contig24|CL14Contig24
12.0
11.8
11.6
11.4
1
2
t−Test: p−value = 0.37

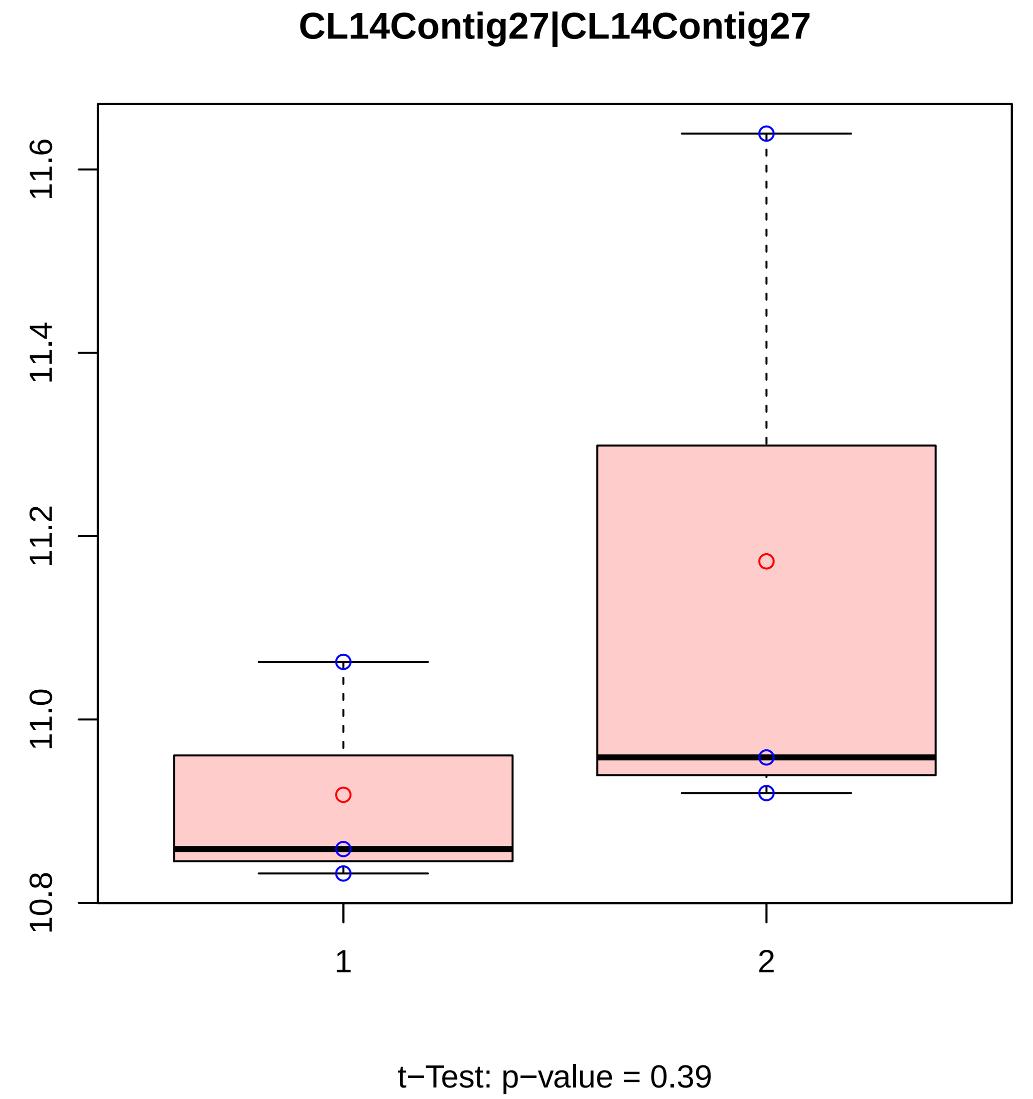

CL14Contig27|CL14Contig27
11.6
11.4
11.2
11.0
10.8
1
2
t−Test: p−value = 0.39

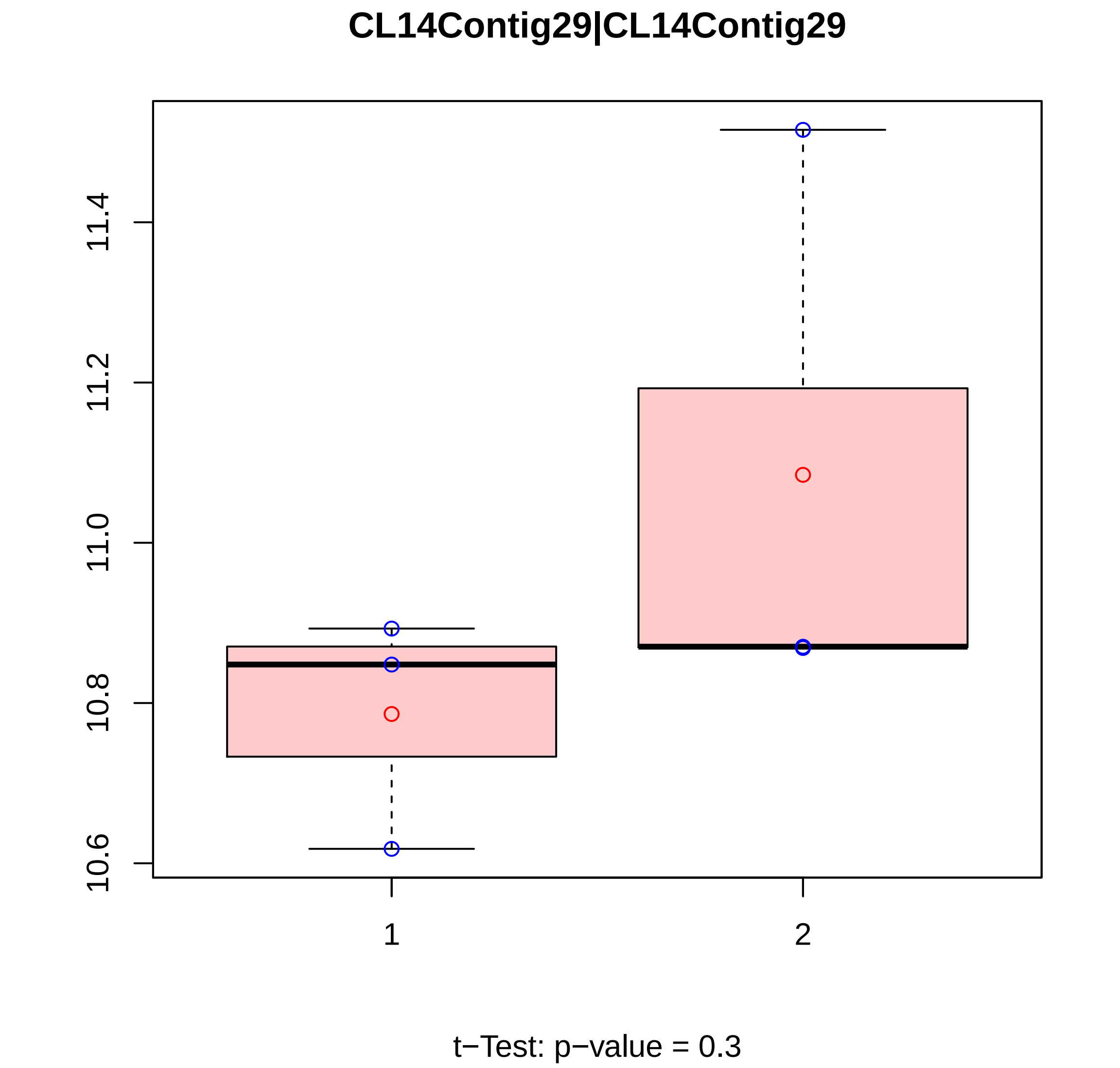

CL14Contig29|CL14Contig29
10.6
10.8
11.0
11.2
11.4
1
2
t−Test: p−value = 0.3

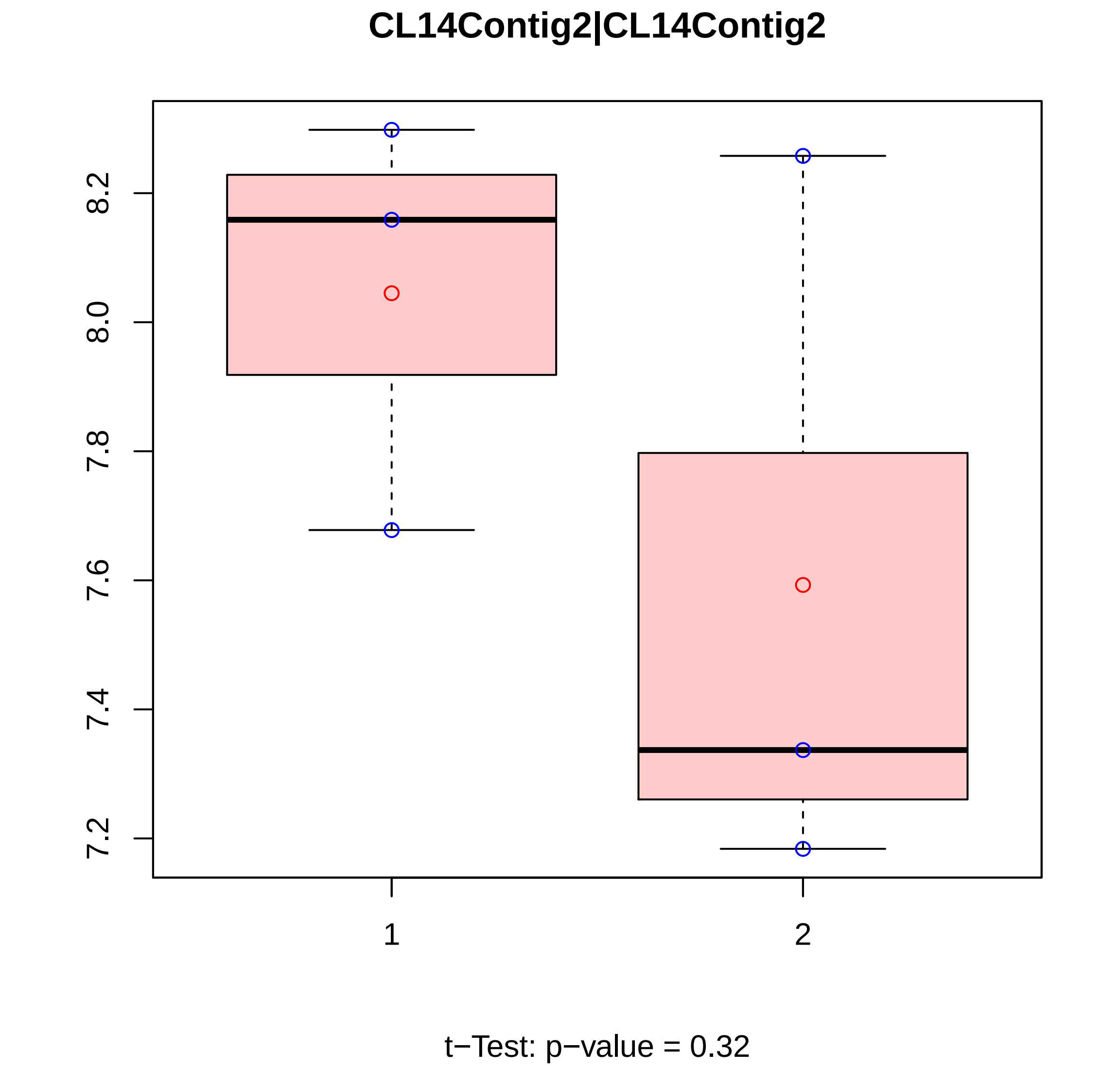
CL14Contig2|CL14Contig2
8.2
8.0
7.8
7.6
7.4
7.2
1
2
t−Test: p−value = 0.32

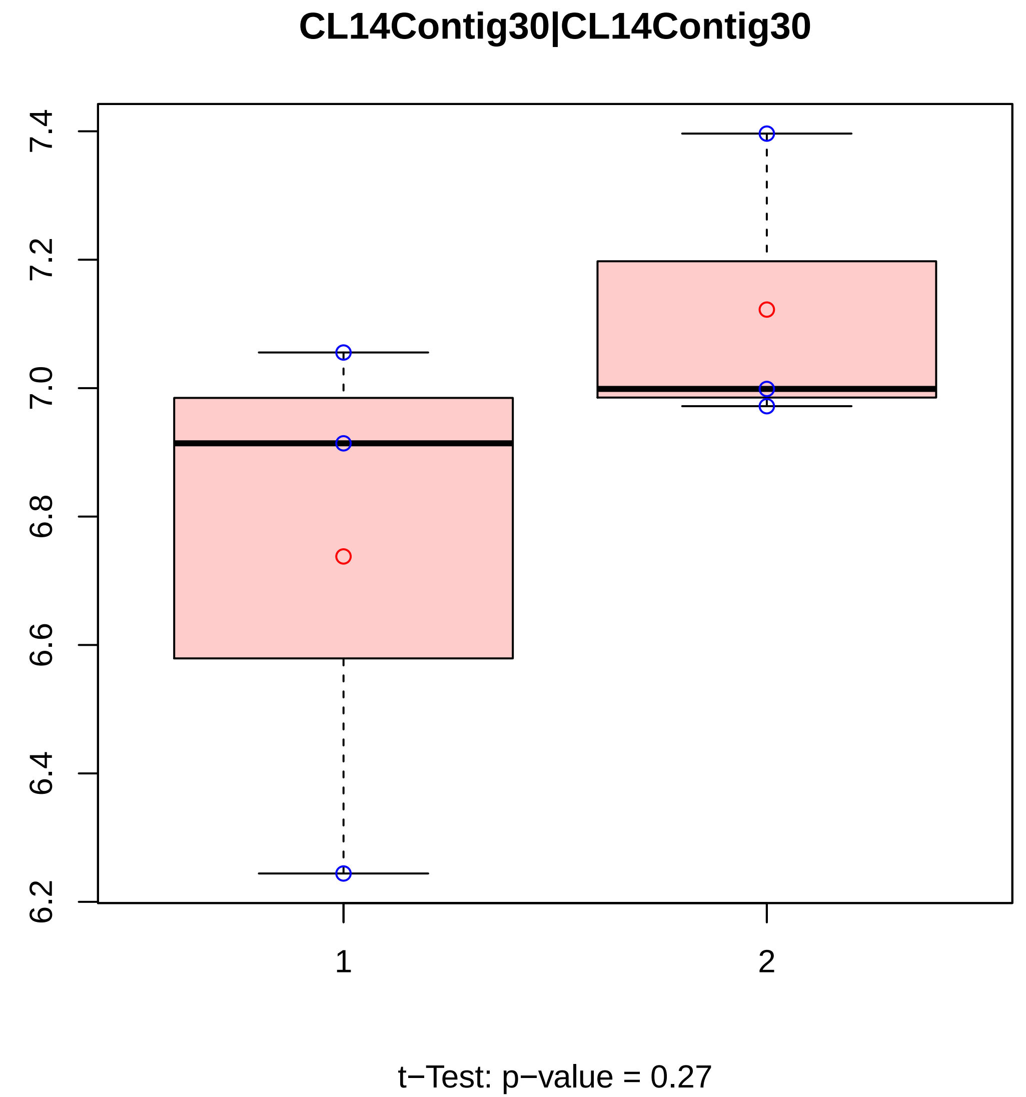
CL14Contig30|CL14Contig30
7.4
7.2
7.0
6.8
6.6
6.4
6.2
1
2
t−Test: p−value = 0.27

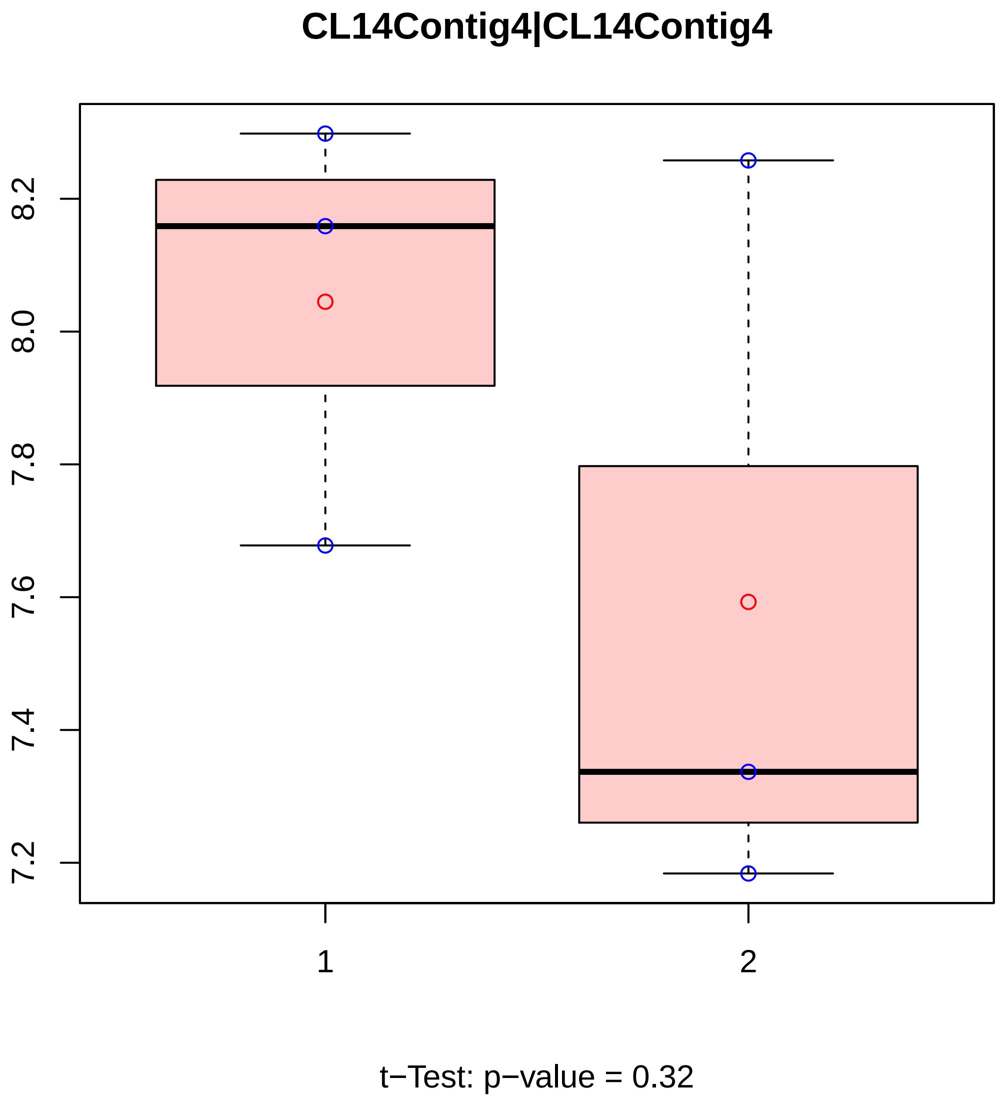

CL14Contig4|CL14Contig4
8.2
8.0
7.8
7.6
7.4
7.2
1
2
t−Test: p−value = 0.32

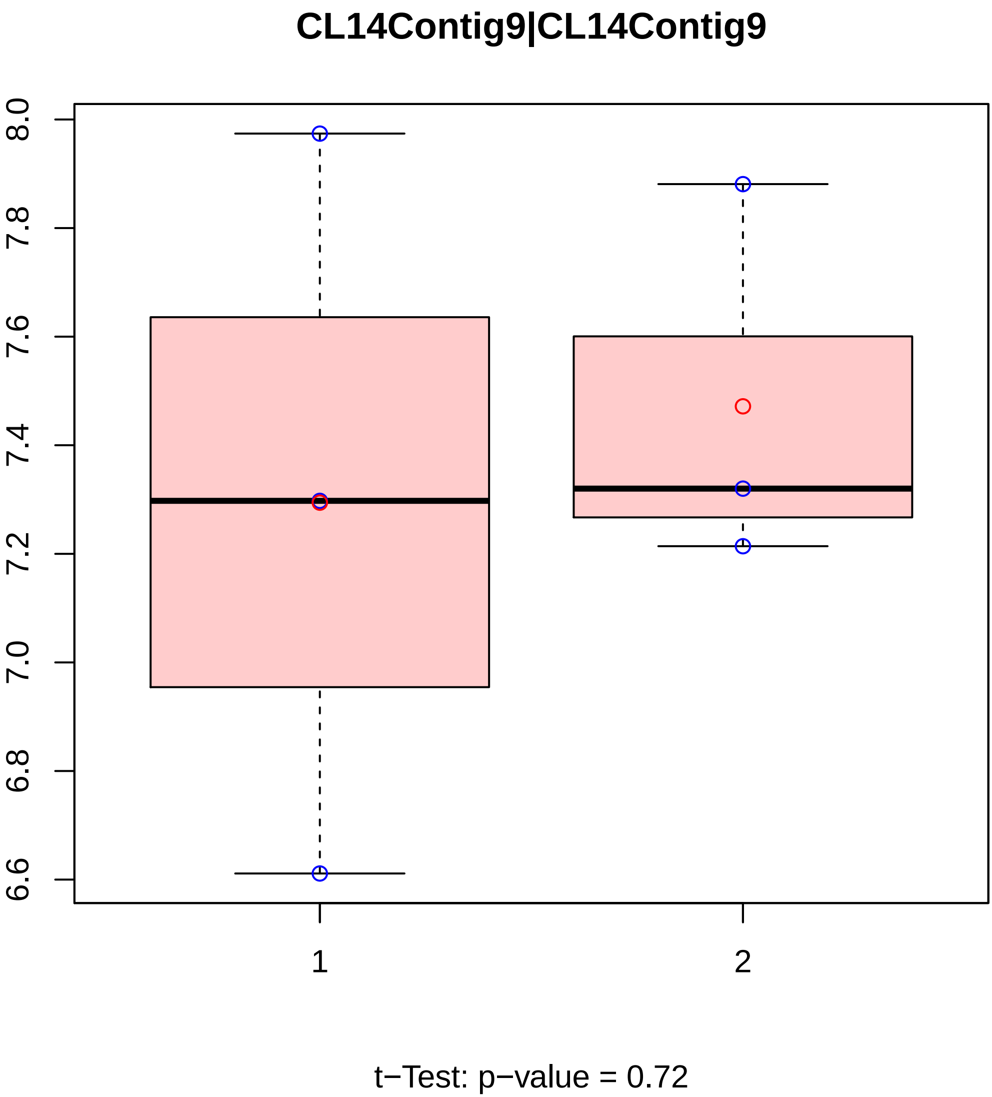
CL14Contig9|CL14Contig9
8.0
7.8
7.6
7.4
7.2
7.0
6.8
6.6
1
2
t−Test: p−value = 0.72

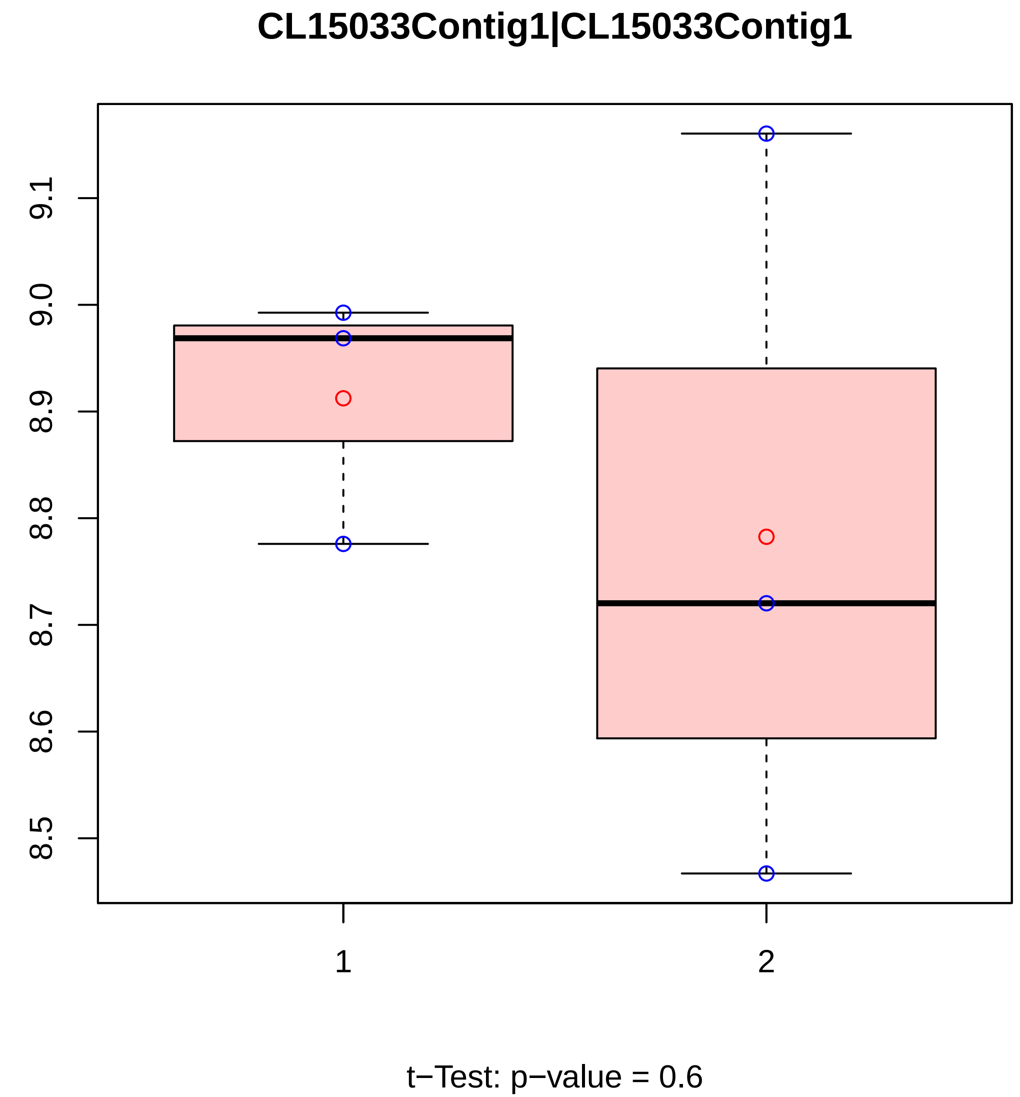
CL15033Contig1|CL15033Contig1
9.1
9.0
8.9
8.8
8.7
8.6
8.5
1
2
t−Test: p−value = 0.6

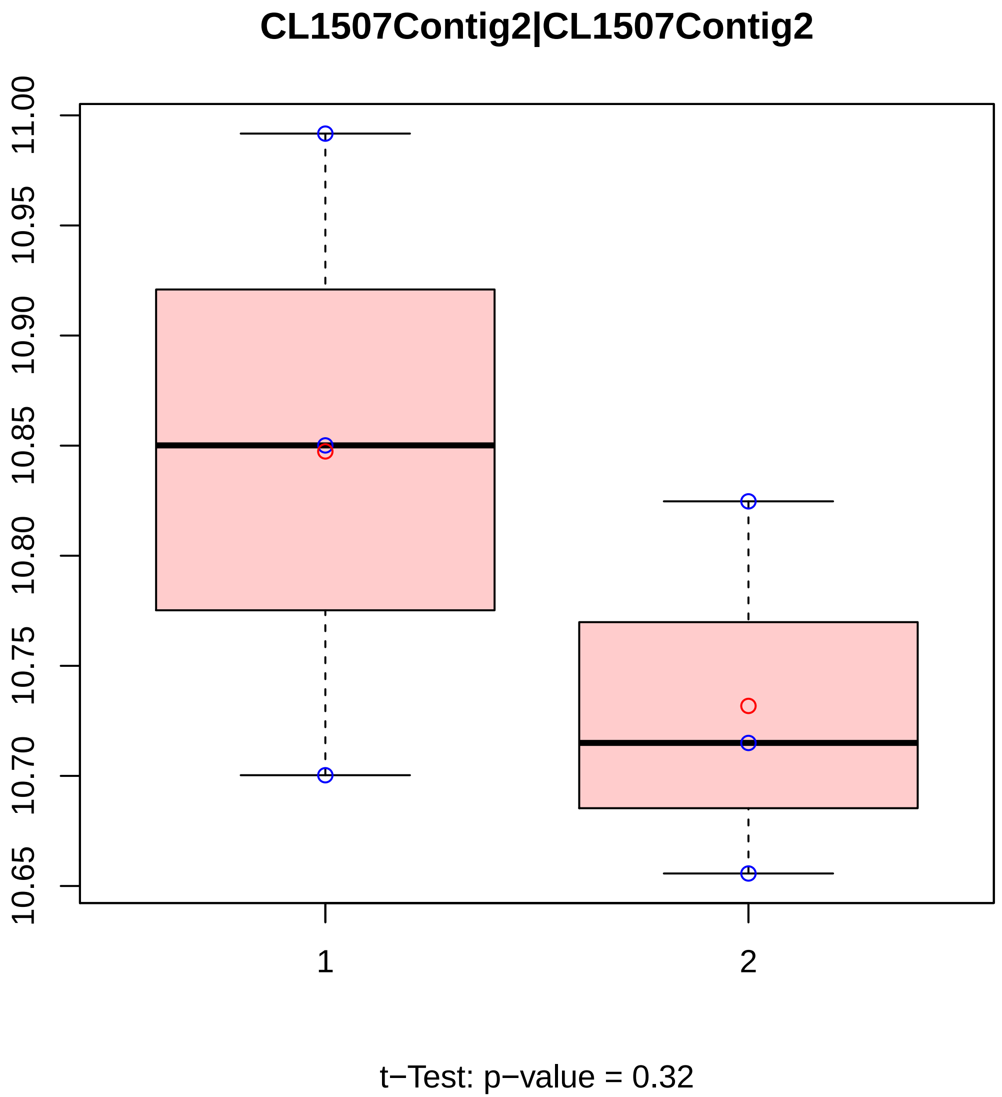
CL1507Contig2|CL1507Contig2
11.00
10.95
10.90
10.85
10.80
10.75
10.70
10.65
1
2
t−Test: p−value = 0.32

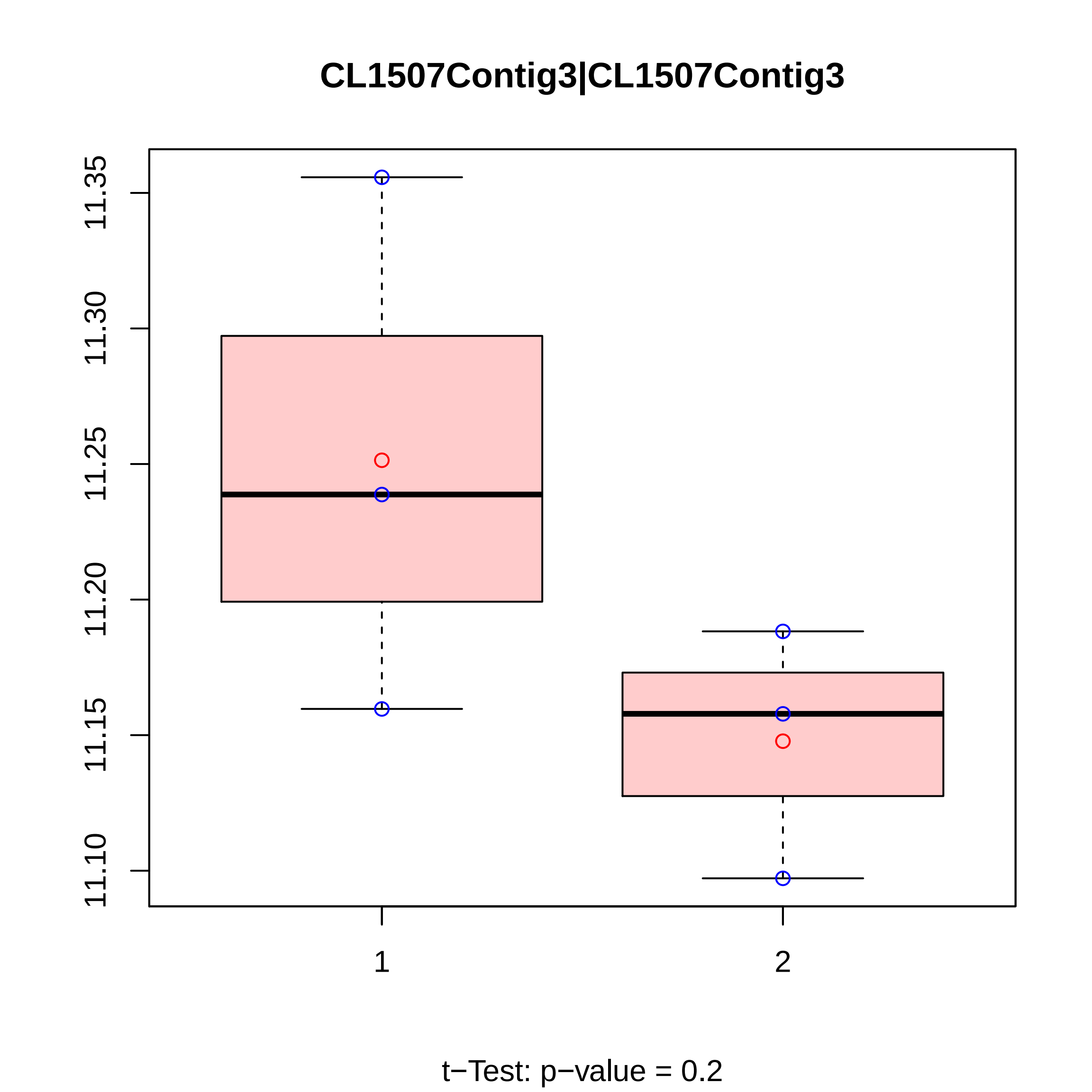
CL1507Contig3|CL1507Contig3
11.10
11.15
11.20
11.25
11.30
11.35
1
2
t−Test: p−value = 0.2

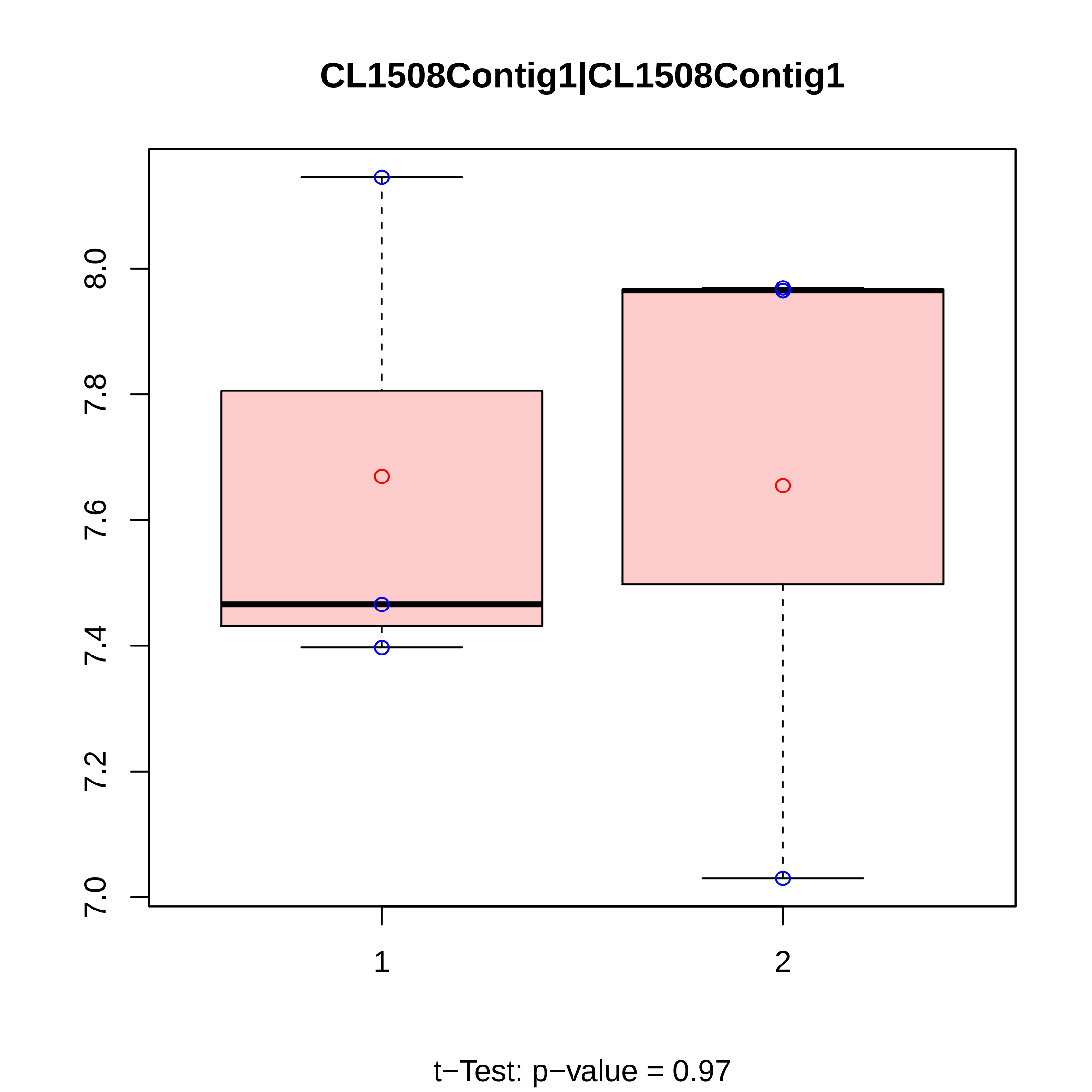
CL1508Contig1|CL1508Contig1
8.0
7.8
7.6
7.4
7.2
7.0
1
2
t−Test: p−value = 0.97

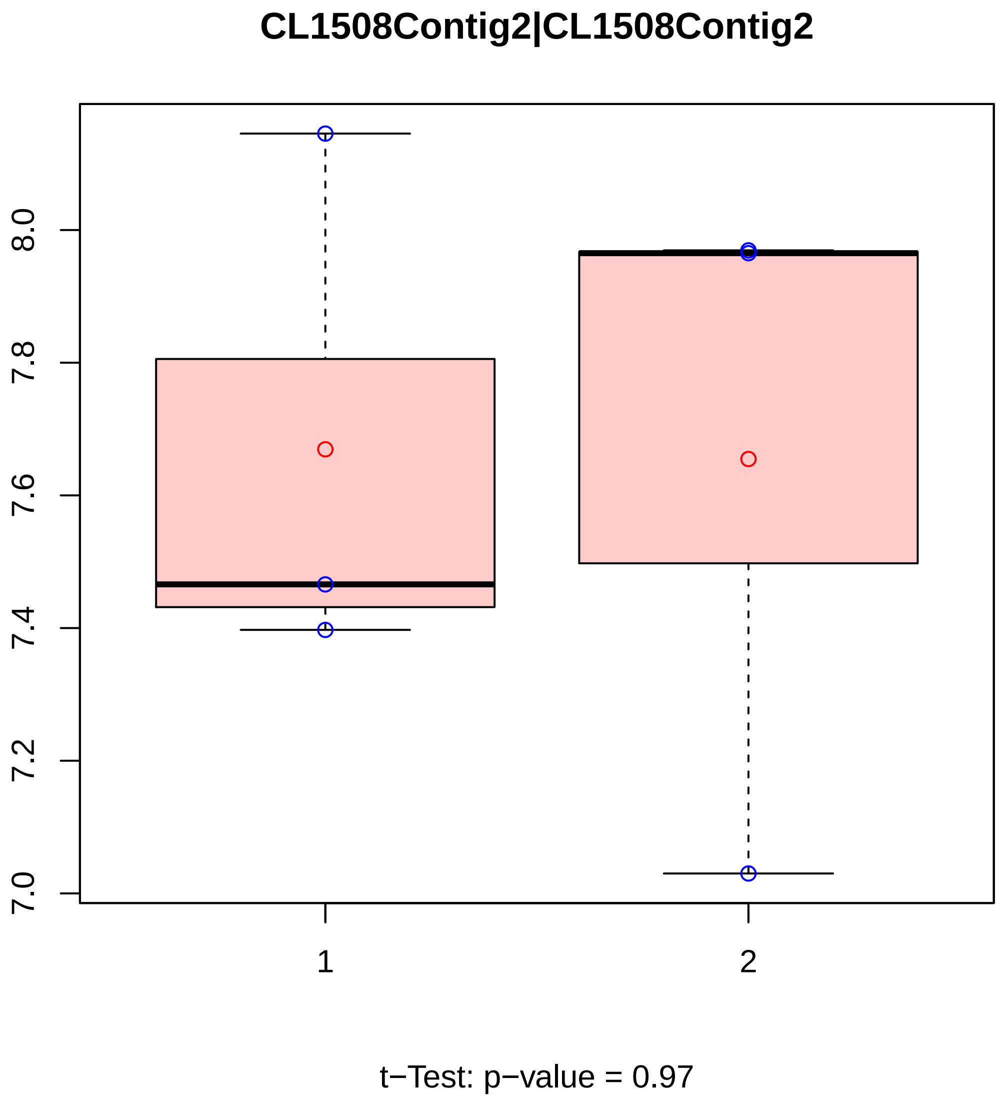
CL1508Contig2|CL1508Contig2
8.0
7.8
7.6
7.4
7.2
7.0
1
2
t−Test: p−value = 0.97

## CL1508Contig4|CL1508Contig4

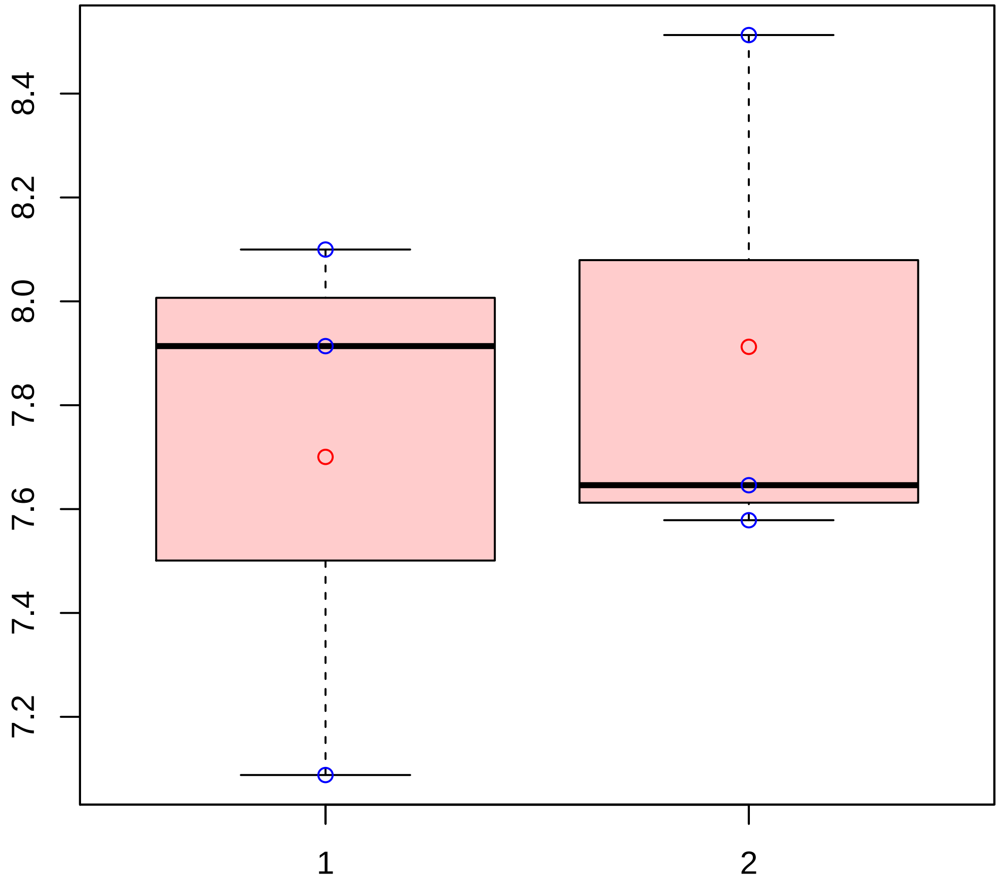


t−Test: p−value = 0.65

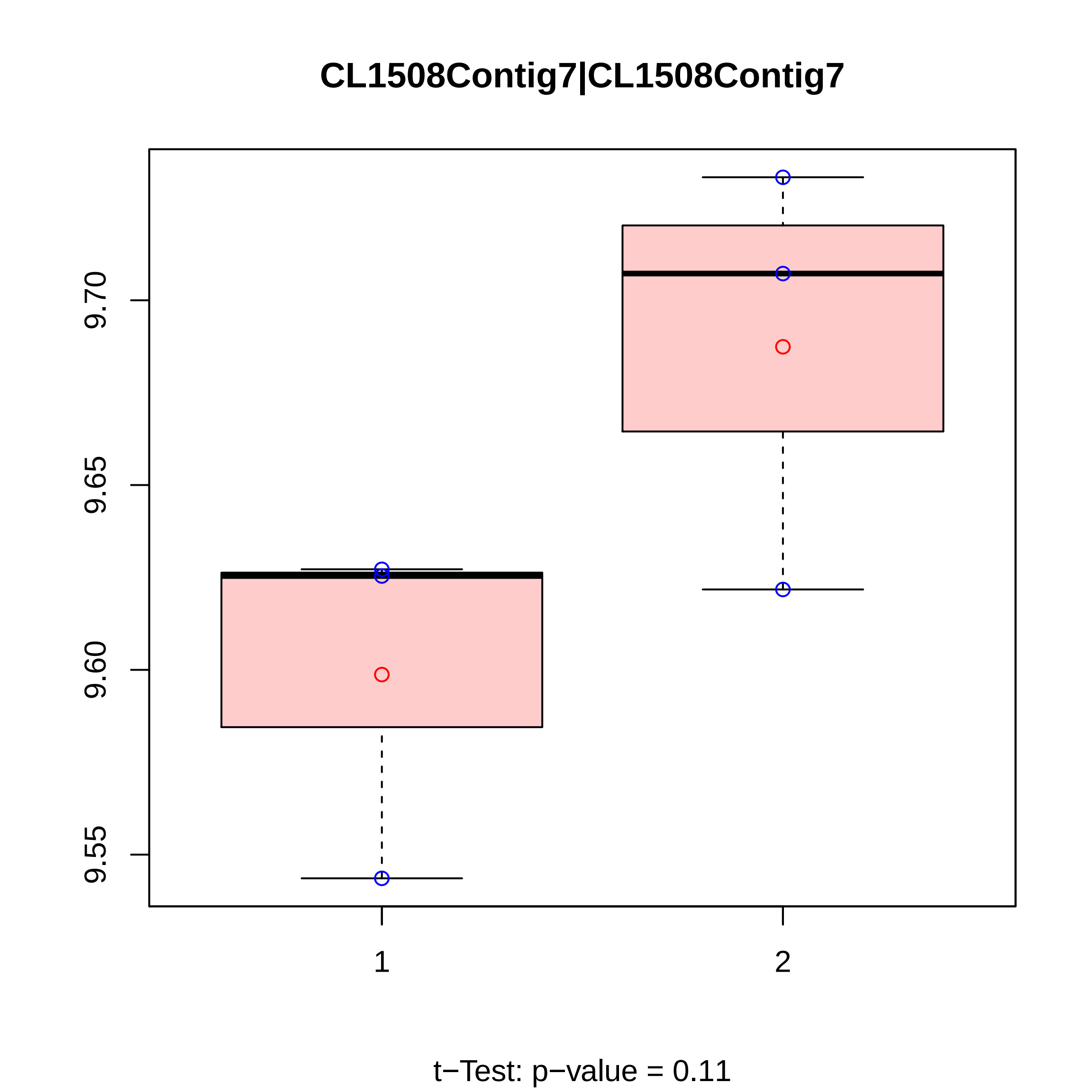
CL1508Contig7|CL1508Contig7
9.55
9.60
9.65
9.70
1
2
t−Test: p−value = 0.11

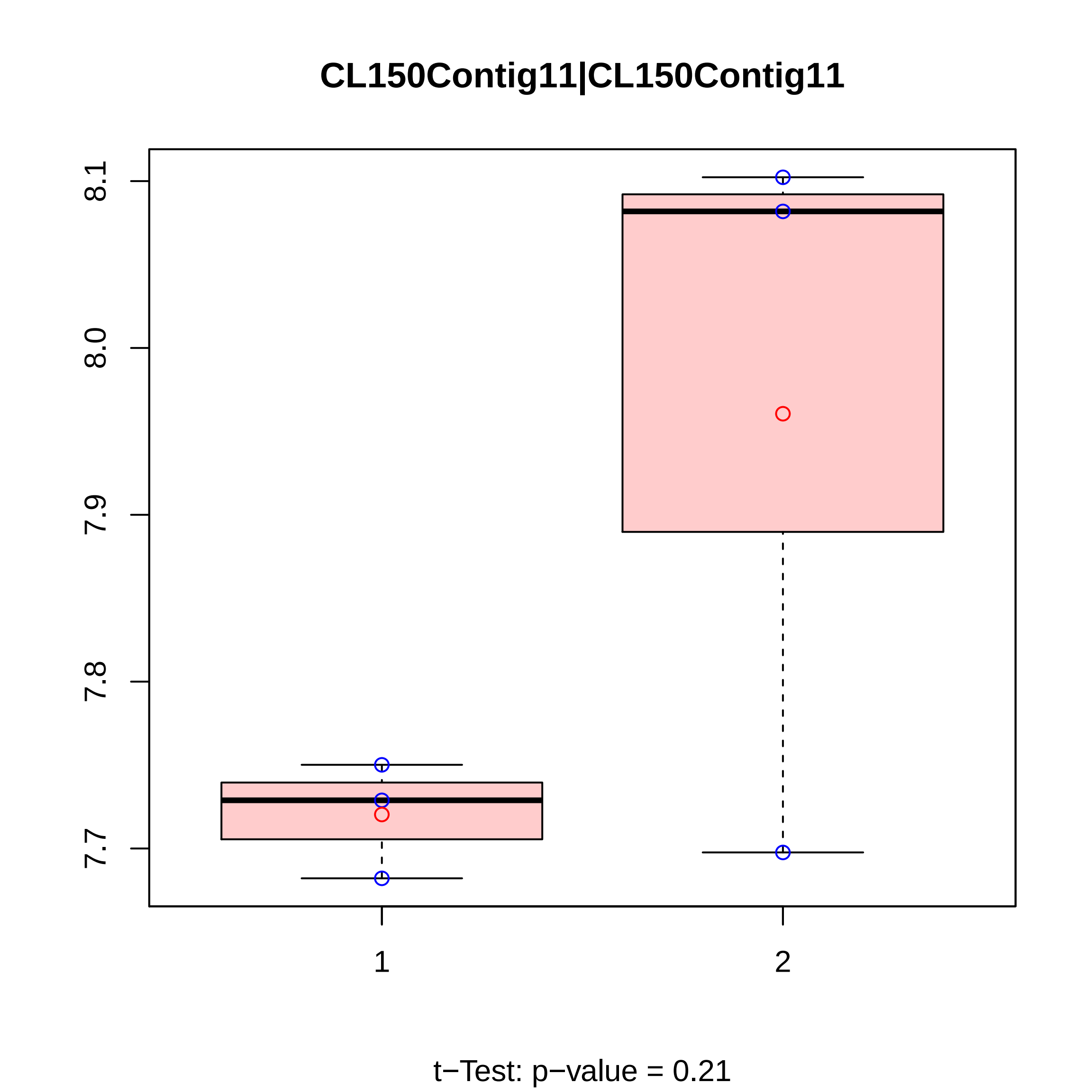
CL150Contig11|CL150Contig11
8.1
8.0
7.9
7.8
7.7
1
2
t−Test: p−value = 0.21

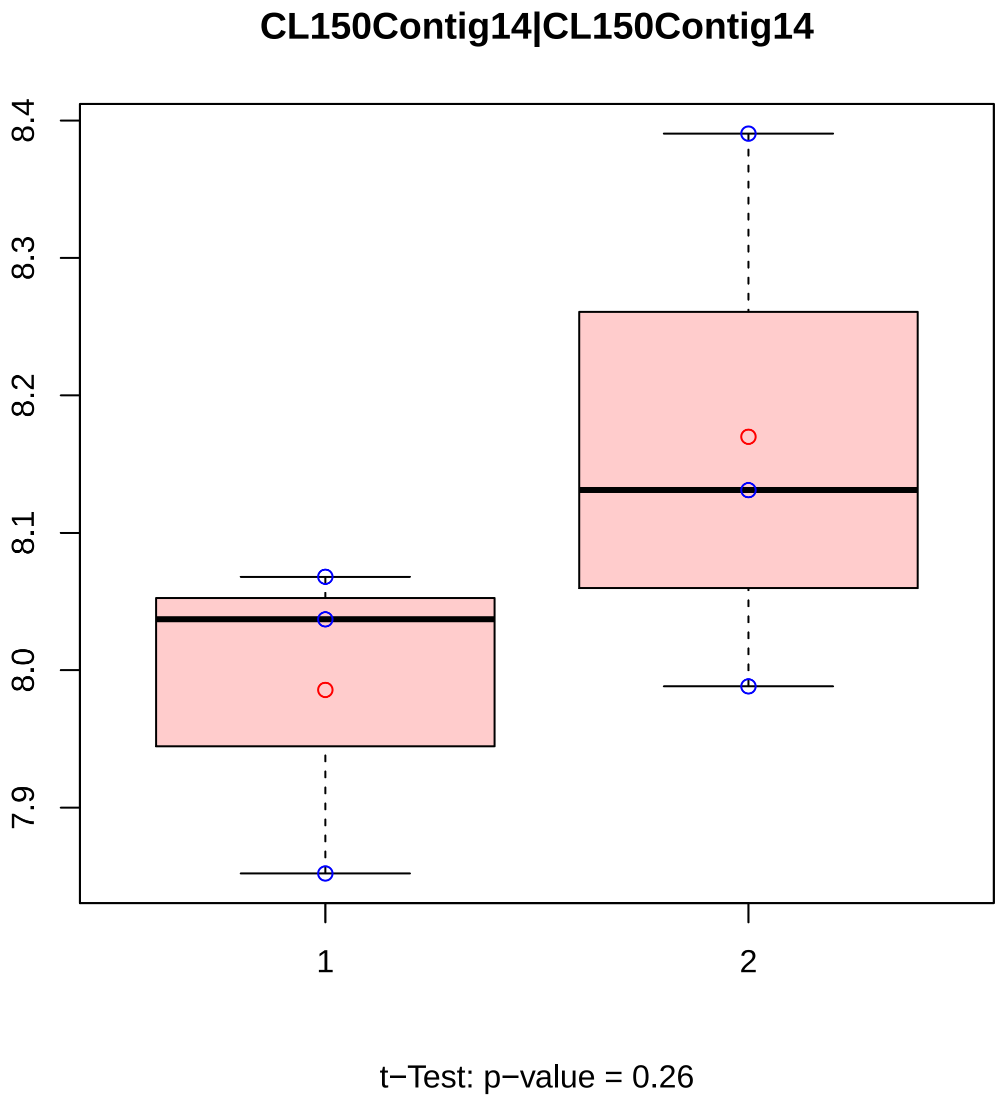
CL150Contig14|CL150Contig14
8.4
8.3
8.2
8.1
8.0
7.9
1
2
t−Test: p−value = 0.26

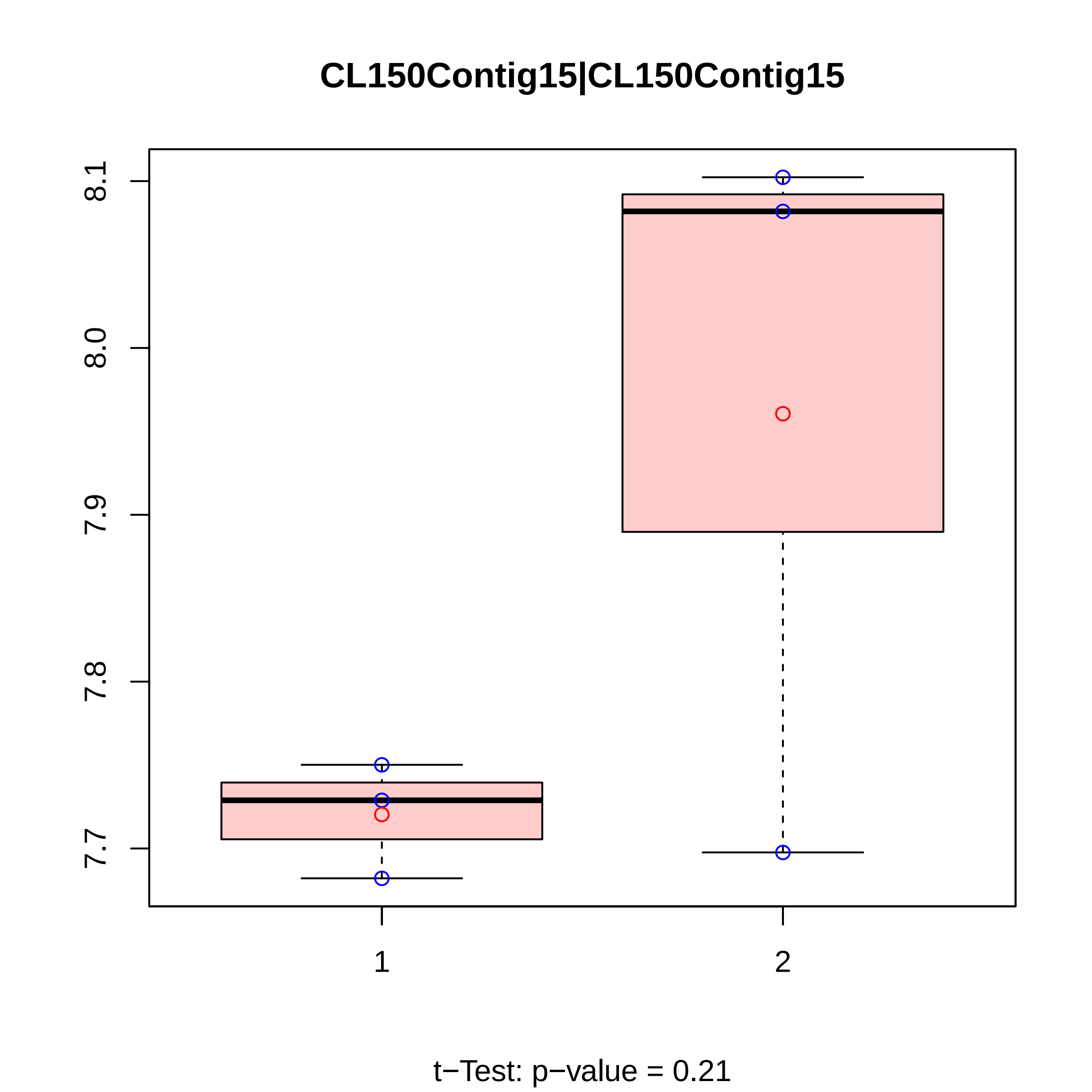

CL150Contig15|CL150Contig15
8.1
8.0
7.9
7.8
7.7
1
2
t−Test: p−value = 0.21

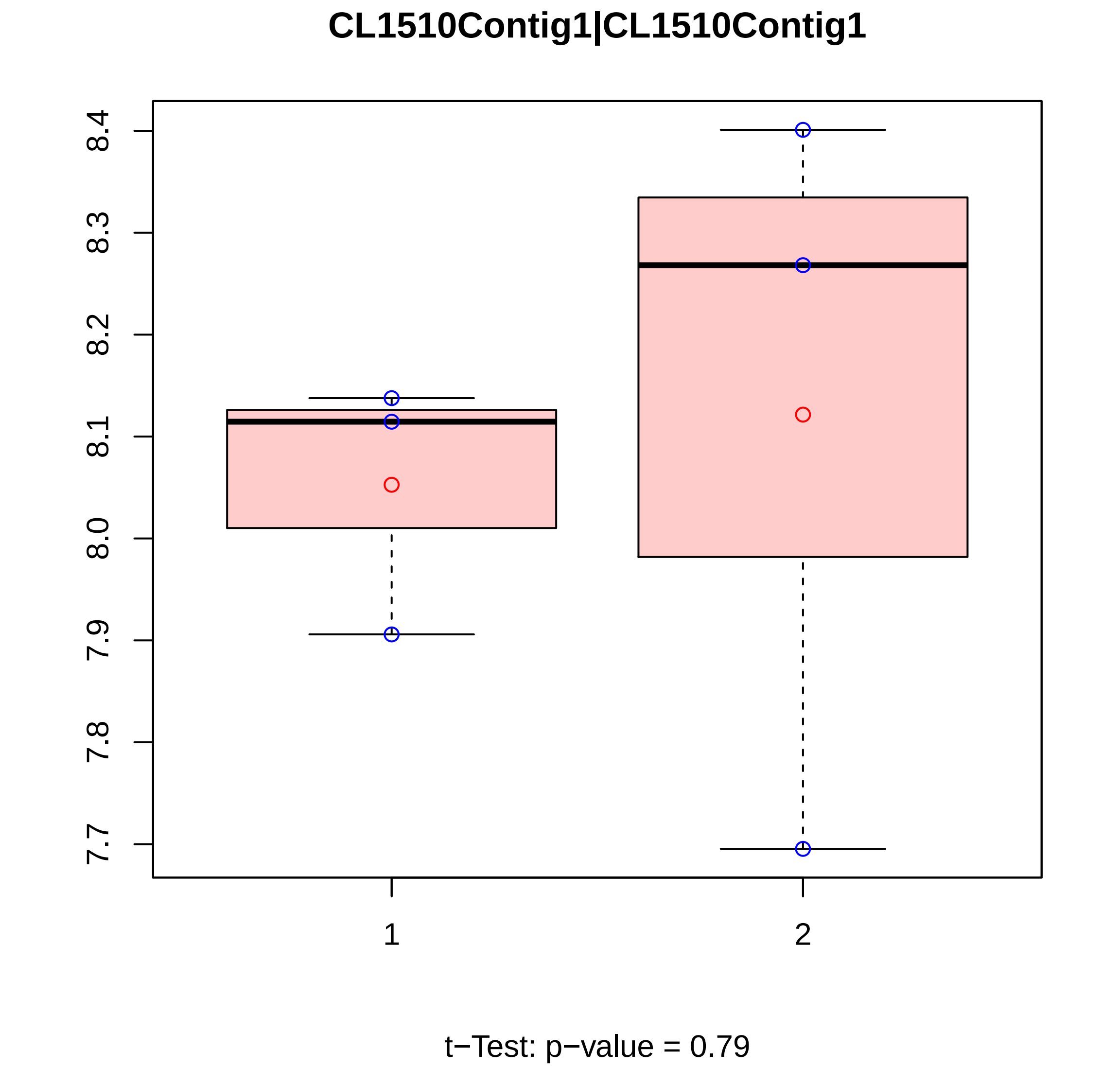
CL1510Contig1|CL1510Contig1
8.4
8.3
8.2
8.1
8.0
7.9
7.8
7.7
1
2
t−Test: p−value = 0.79

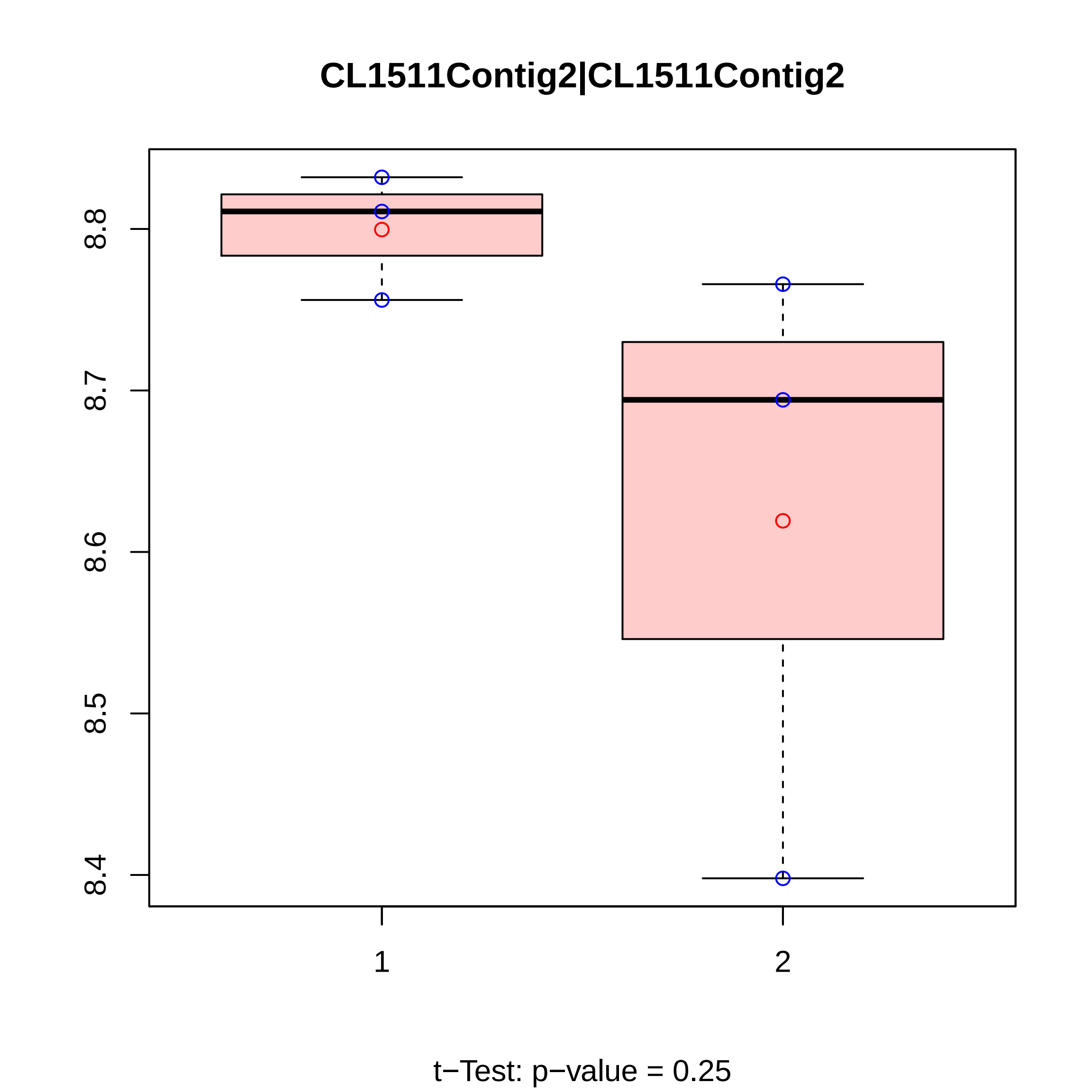
CL1511Contig2|CL1511Contig2
8.4
8.5
8.6
8.7
8.8
1
2
t−Test: p−value = 0.25

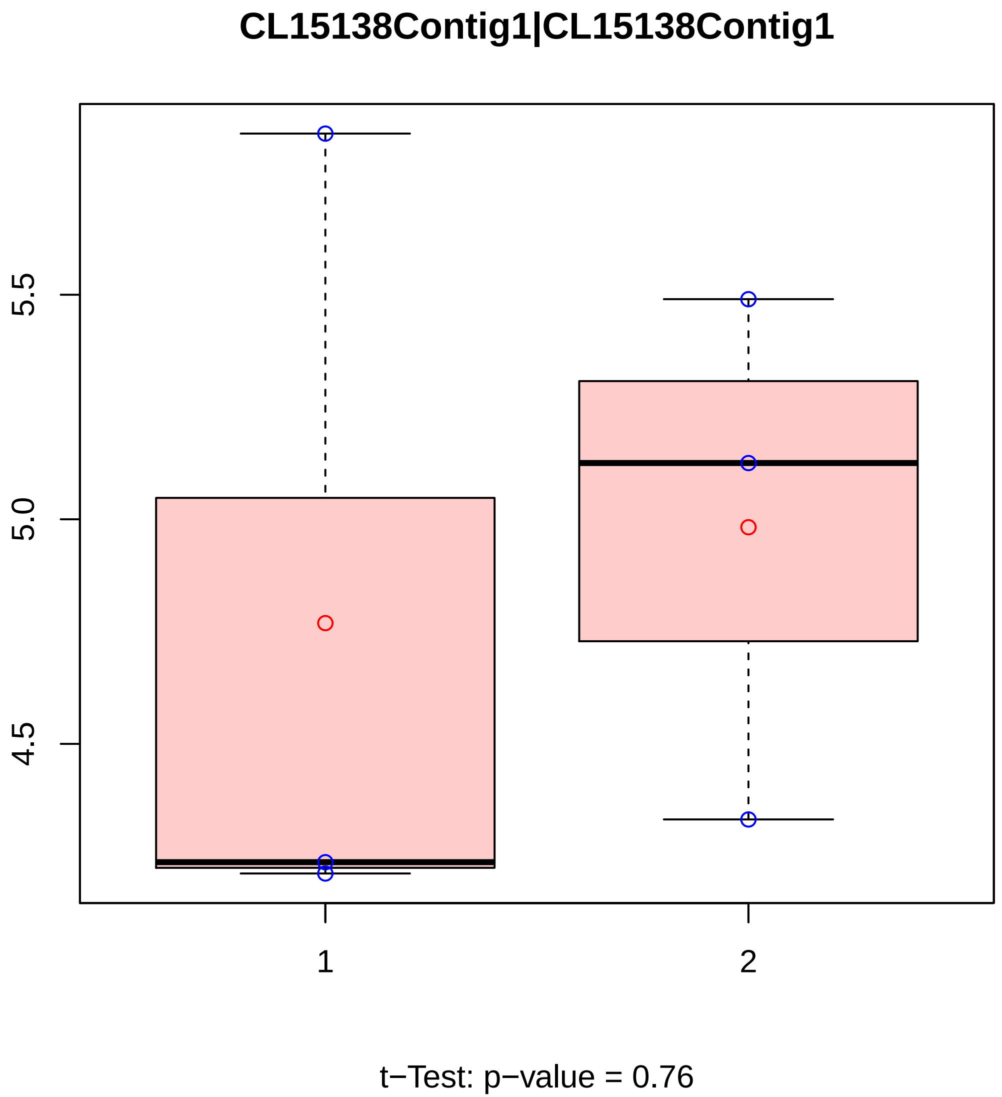
CL15138Contig1|CL15138Contig1
5.5
5.0
4.5
1
2
t−Test: p−value = 0.76

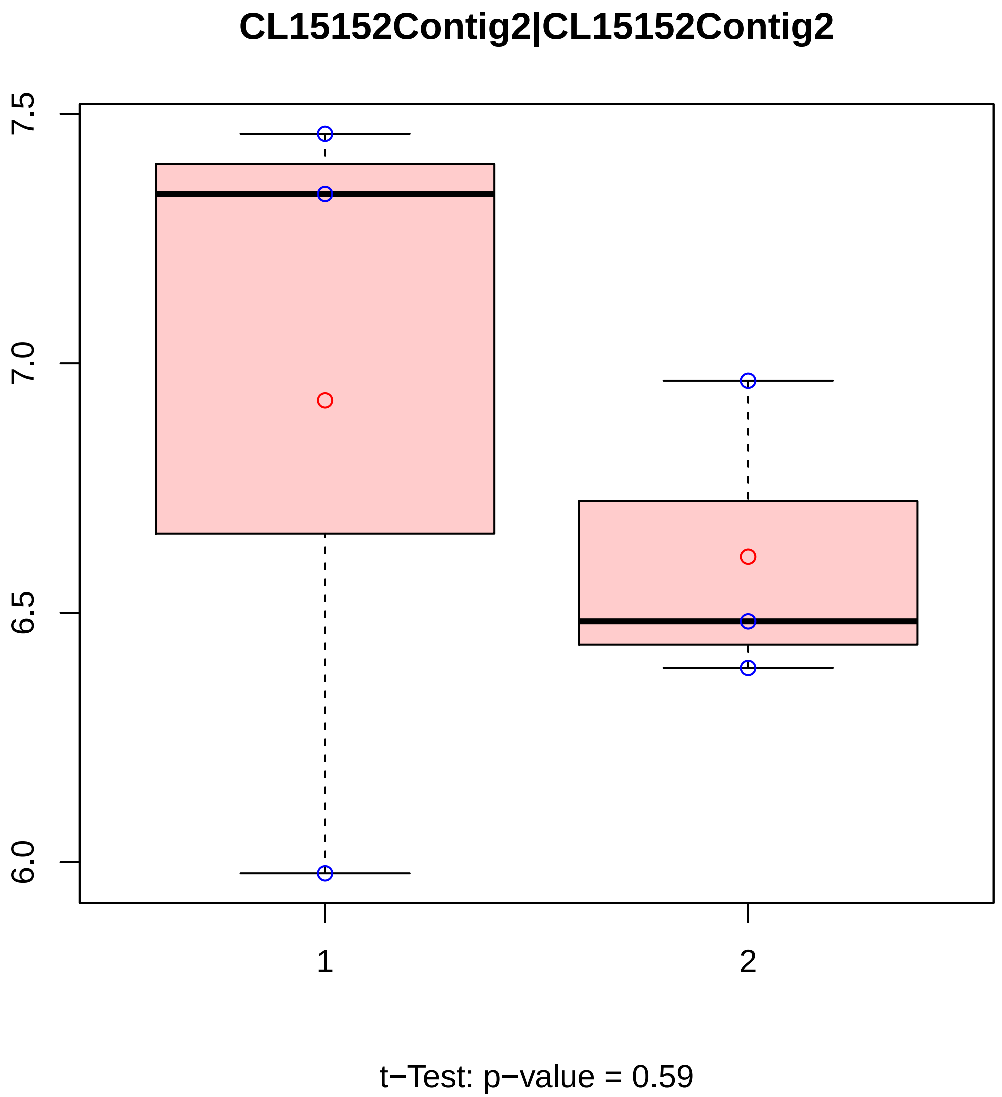

CL15152Contig2|CL15152Contig2
7.5
7.0
6.5
6.0
1
2
t−Test: p−value = 0.59

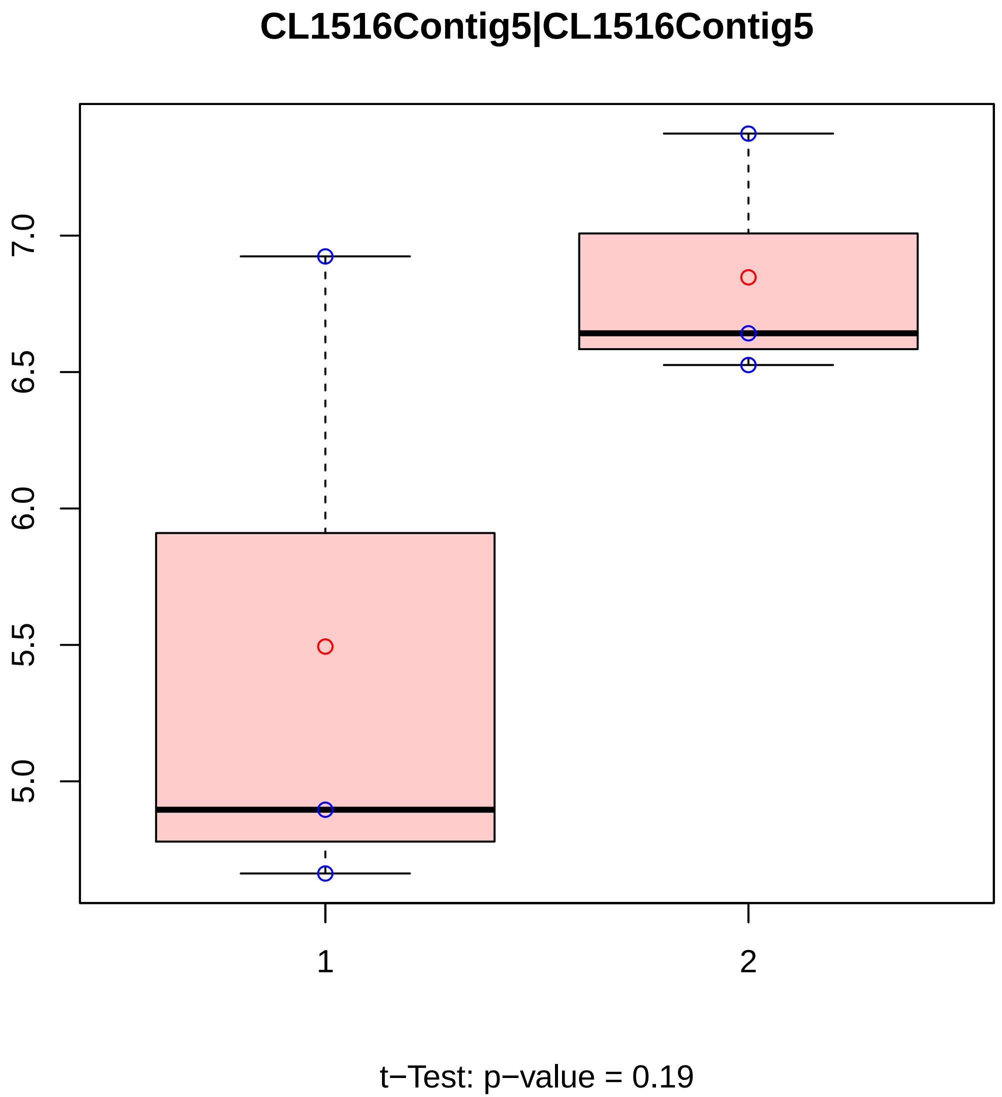
CL1516Contig5|CL1516Contig5
7.0
6.5
6.0
5.5
5.0
1
2
t−Test: p−value = 0.19

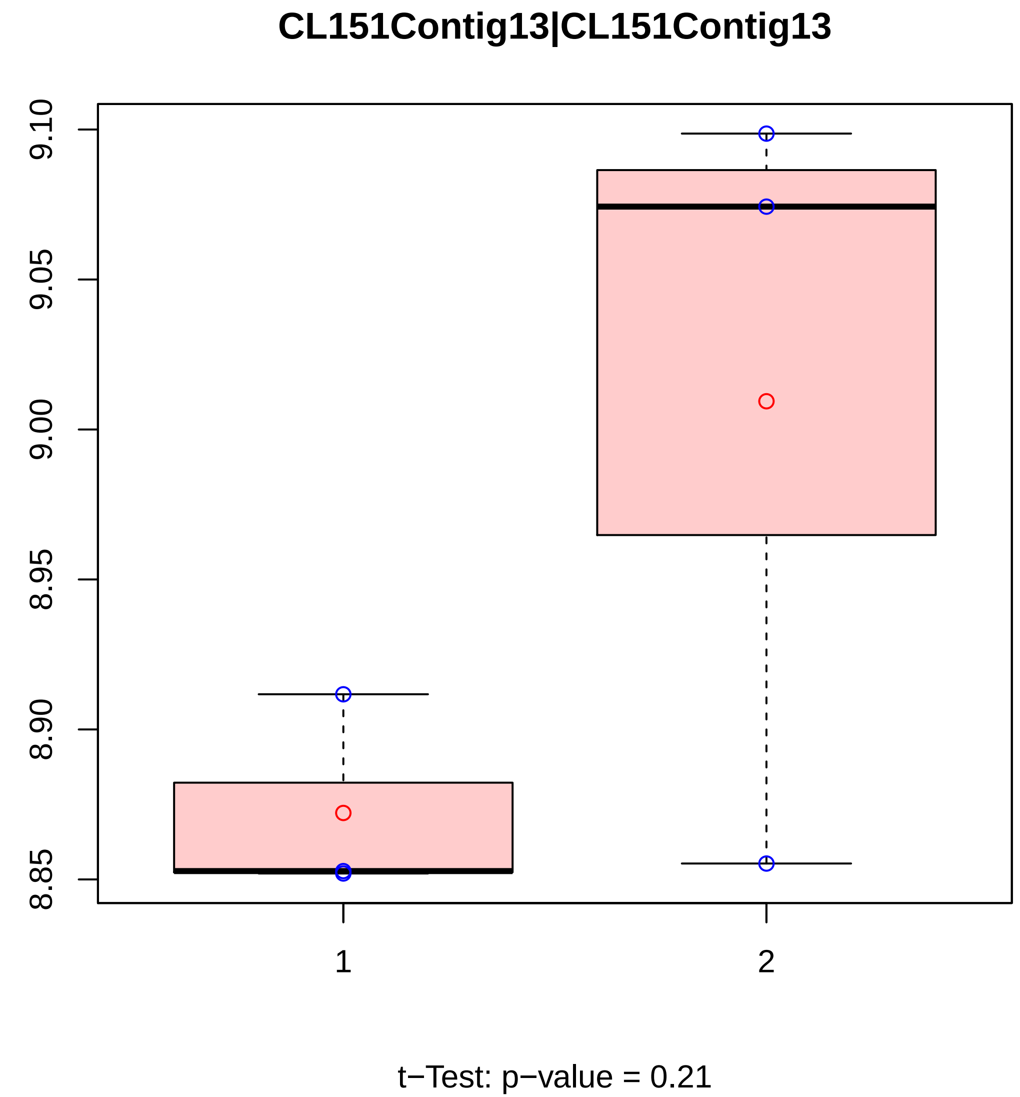
CL151Contig13|CL151Contig13
9.10
9.05
9.00
8.95
8.90
8.85
1
2
t−Test: p−value = 0.21

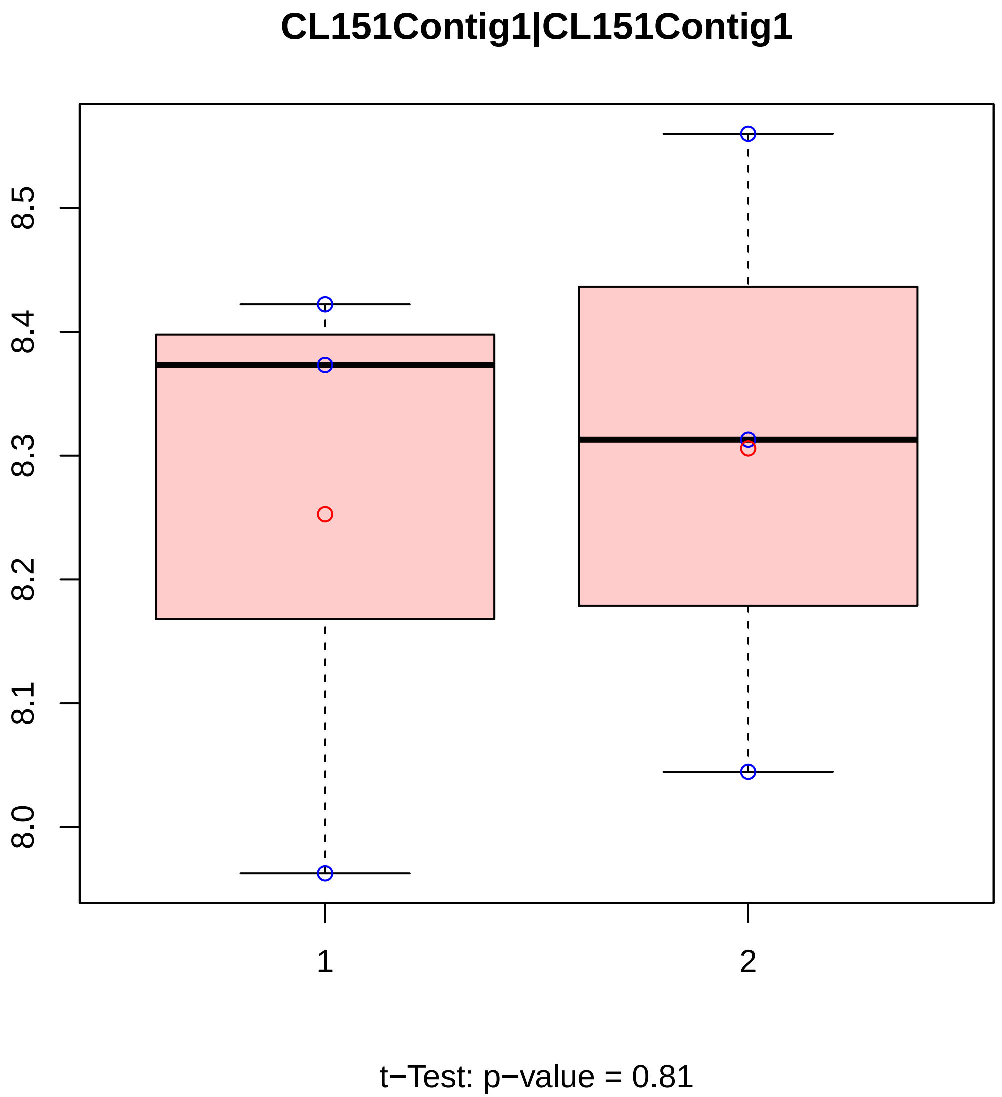
CL151Contig1|CL151Contig1
8.0
8.1
8.2
8.3
8.4
8.5
1
2
t−Test: p−value = 0.81

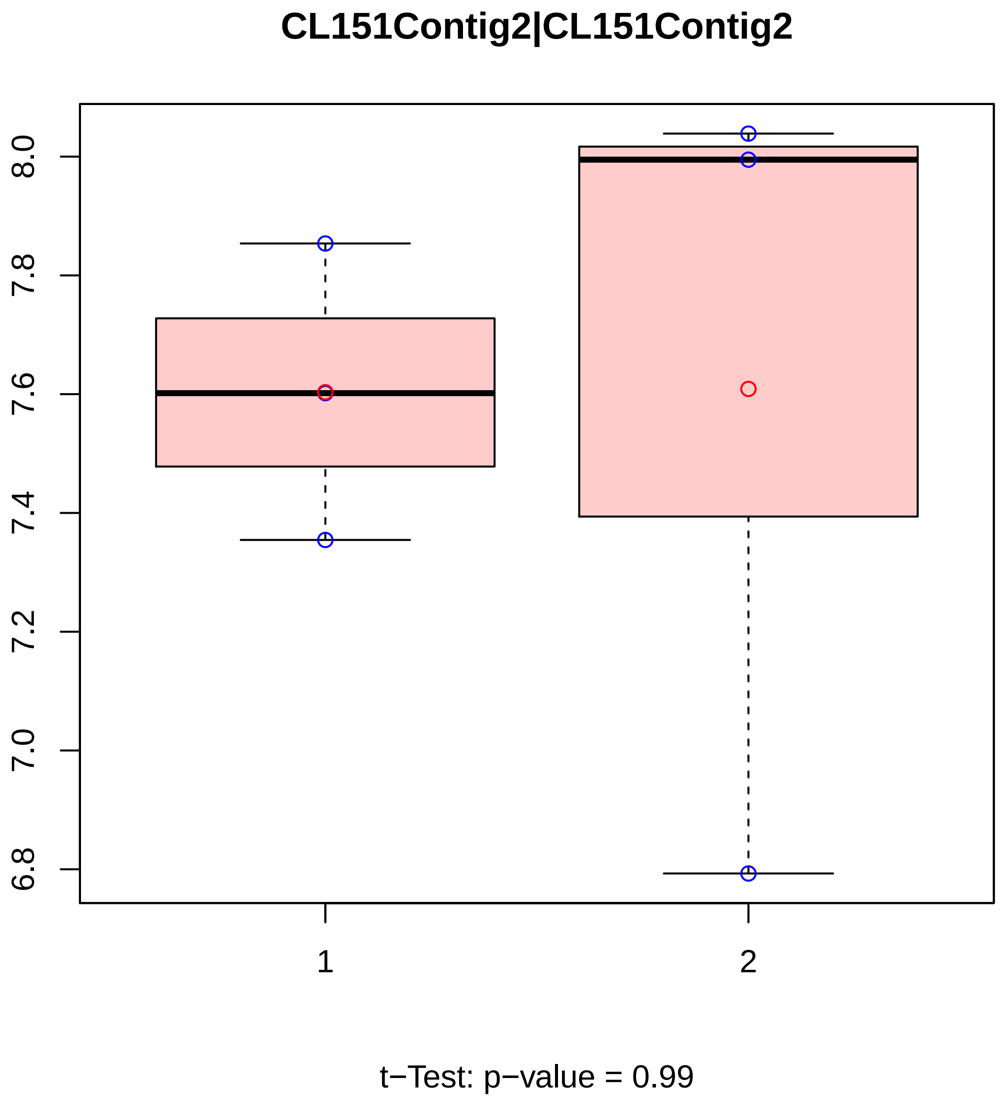
CL151Contig2|CL151Contig2
8.0
7.8
7.6
7.4
7.2
7.0
6.8
1
2
t−Test: p−value = 0.99

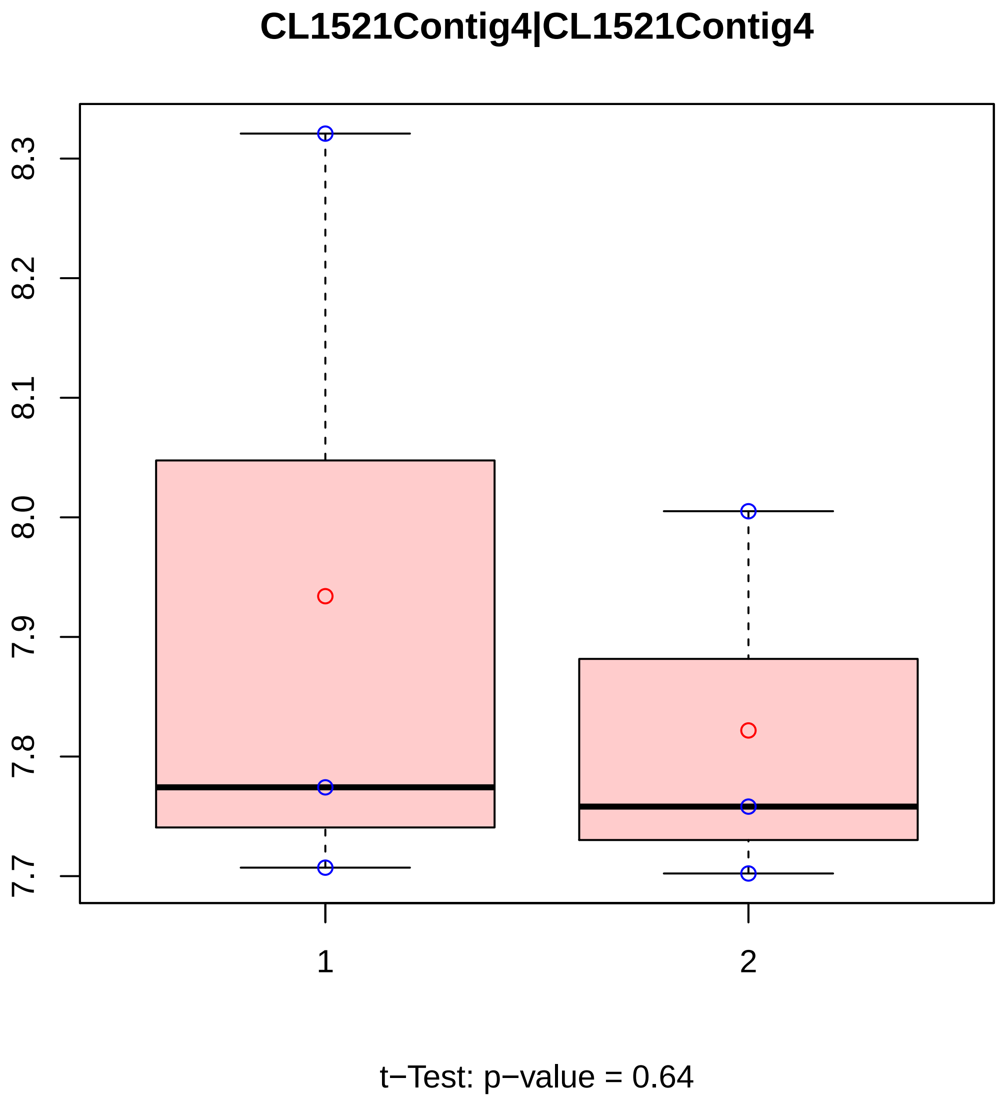
CL1521Contig4|CL1521Contig4
8.3
8.2
8.1
8.0
7.9
7.8
7.7
1
2
t−Test: p−value = 0.64

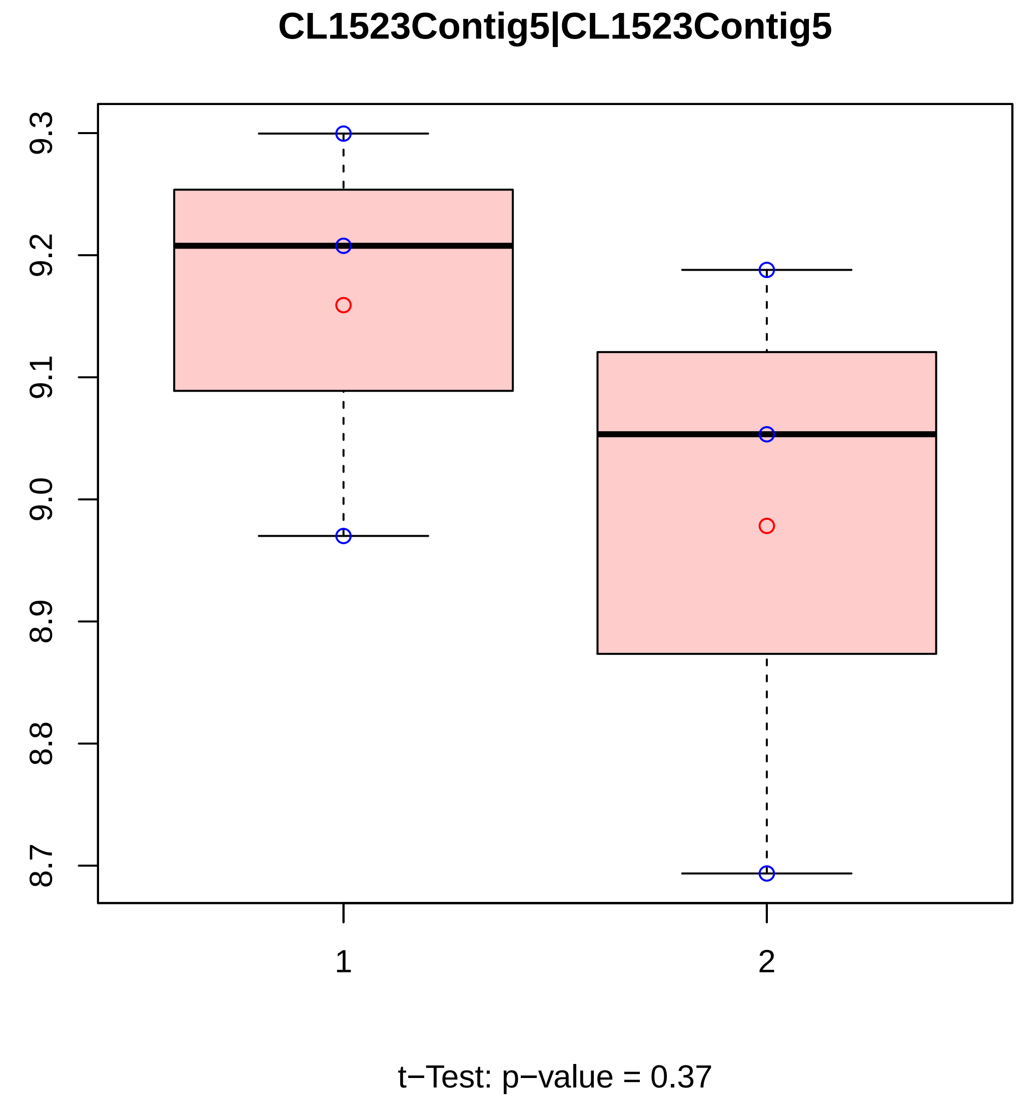
CL1523Contig5|CL1523Contig5
9.3
9.2
9.1
9.0
8.9
8.8
8.7
1
2
t−Test: p−value = 0.37

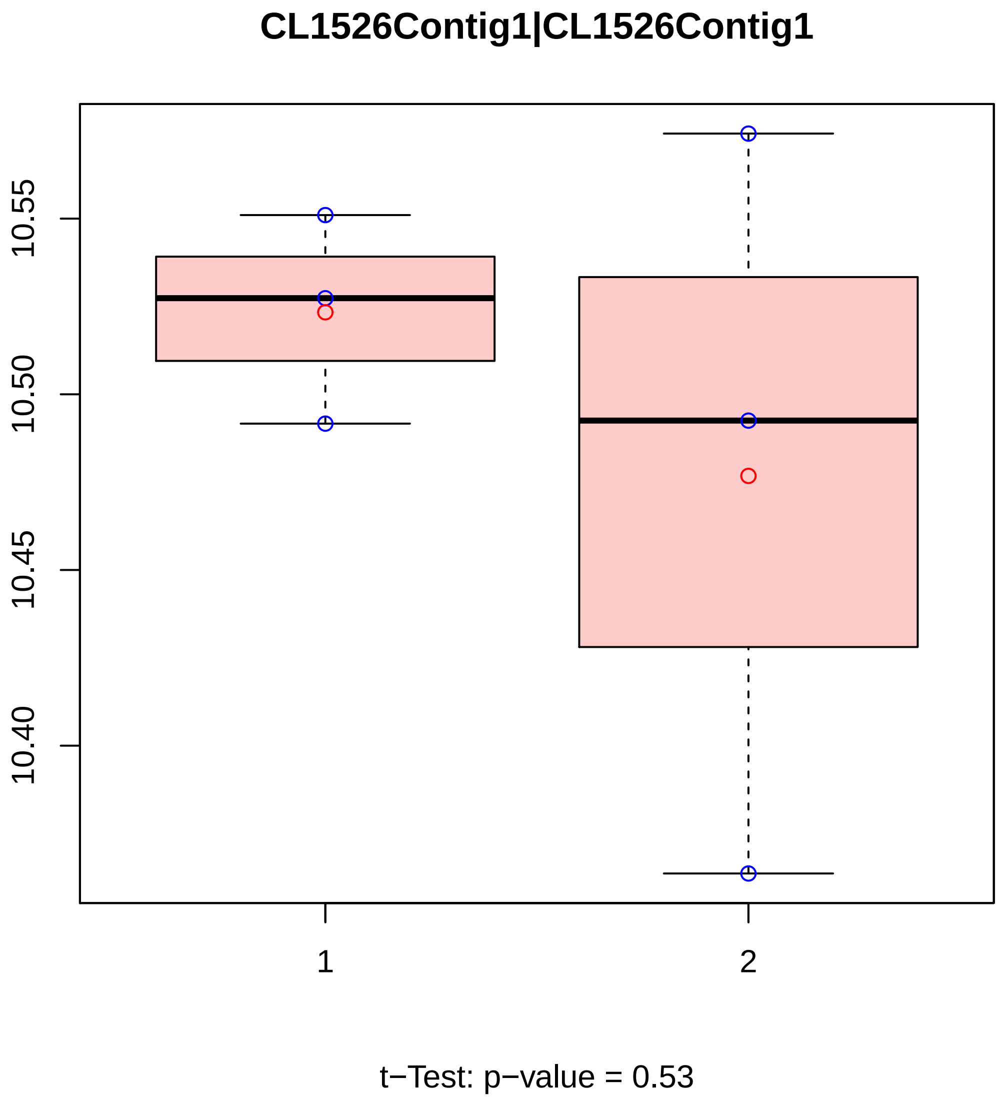
CL1526Contig1|CL1526Contig1
10.40
10.45
10.50
10.55
1
2
t−Test: p−value = 0.53

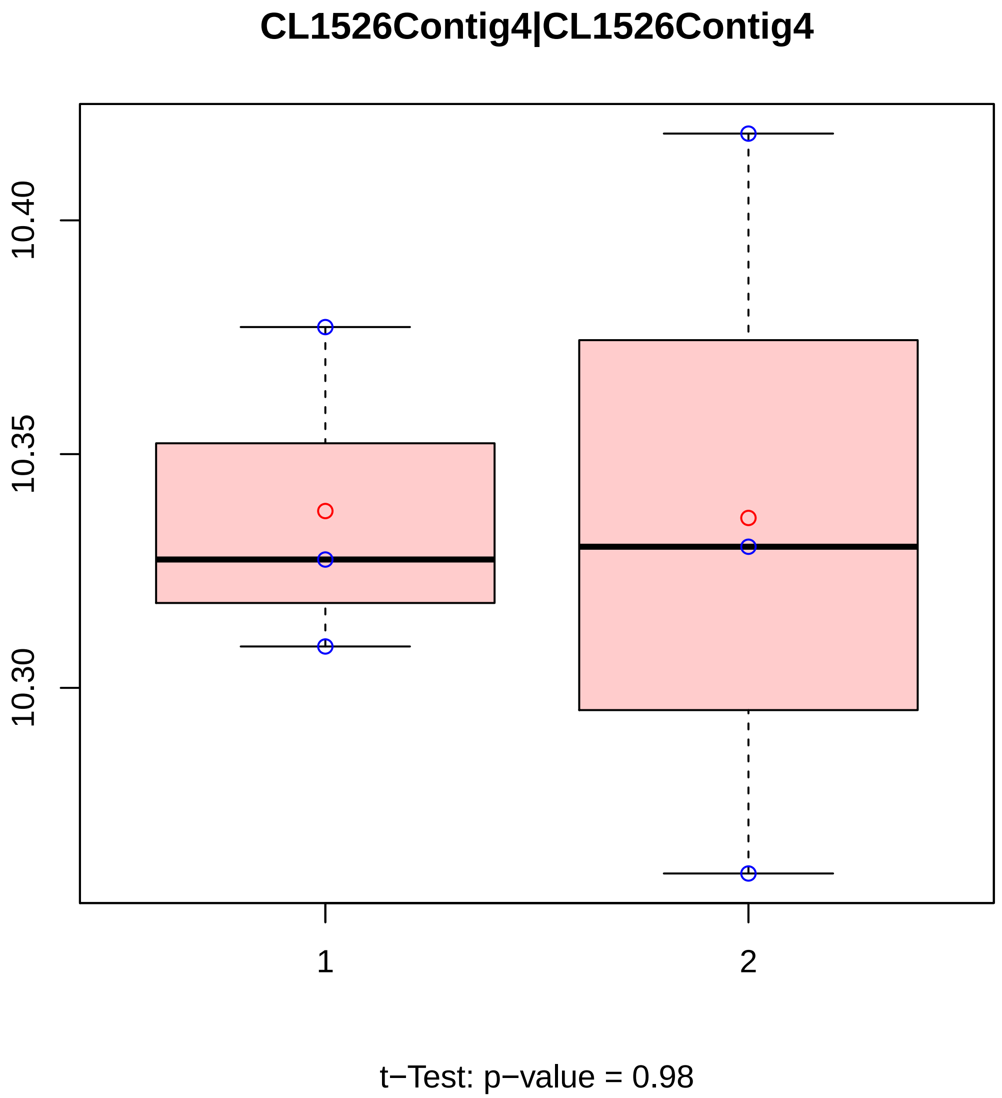
CL1526Contig4|CL1526Contig4
10.40
10.35
10.30
1
2
t−Test: p−value = 0.98

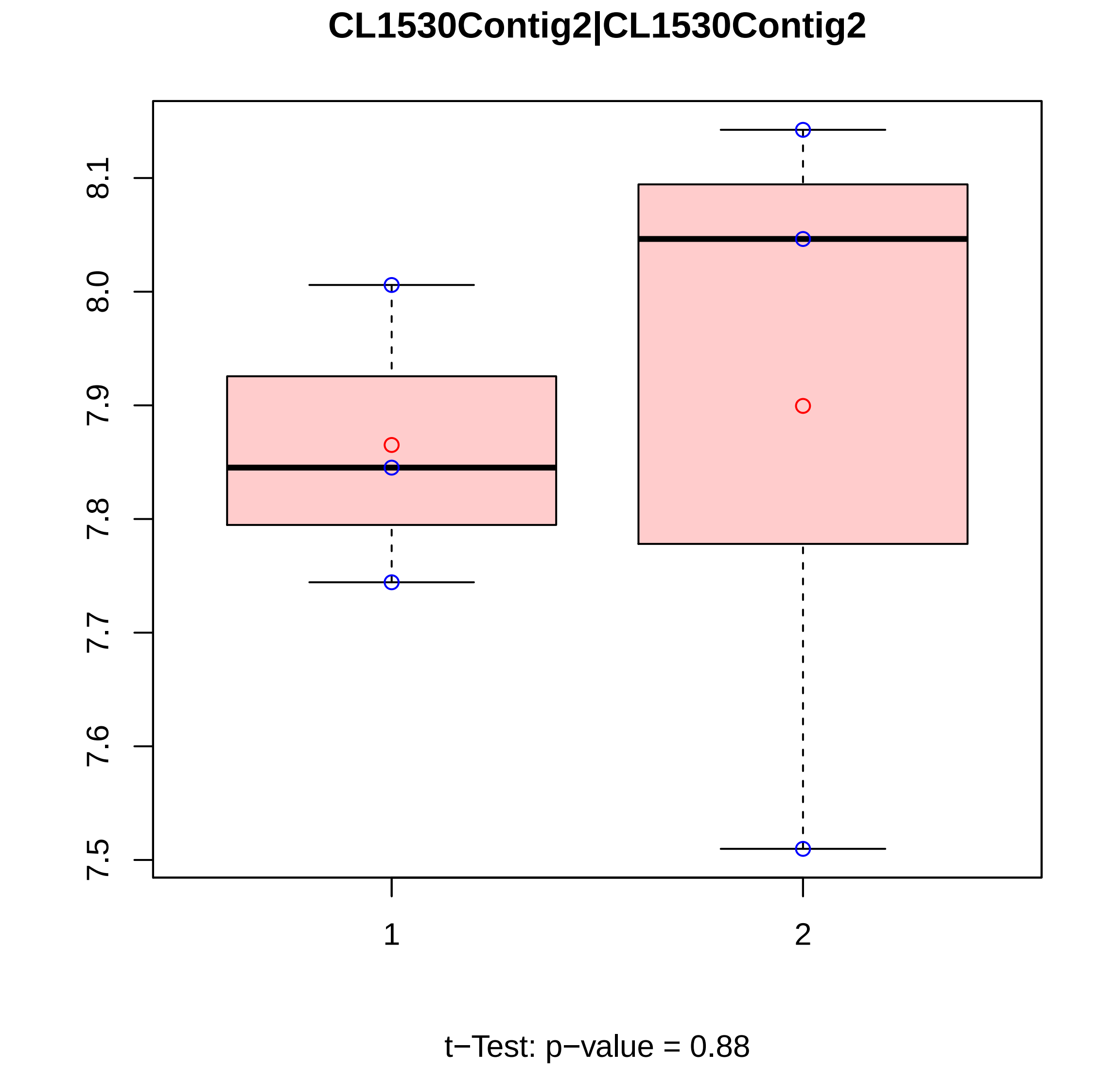
CL1530Contig2|CL1530Contig2
8.1
8.0
7.9
7.8
7.7
7.6
7.5
1
2
t−Test: p−value = 0.88

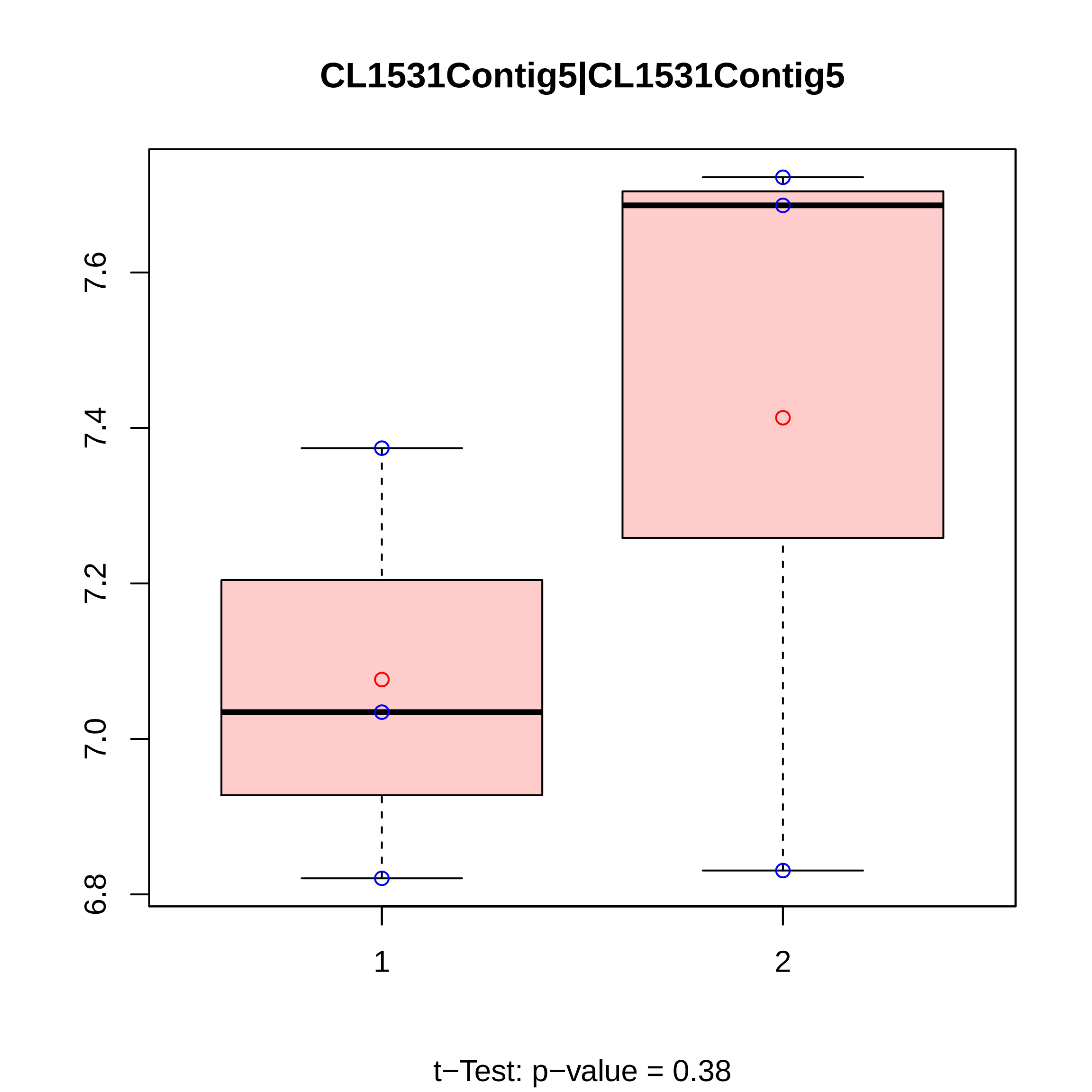
CL1531Contig5|CL1531Contig5
7.6
7.4
7.2
7.0
6.8
1
2
t−Test: p−value = 0.38

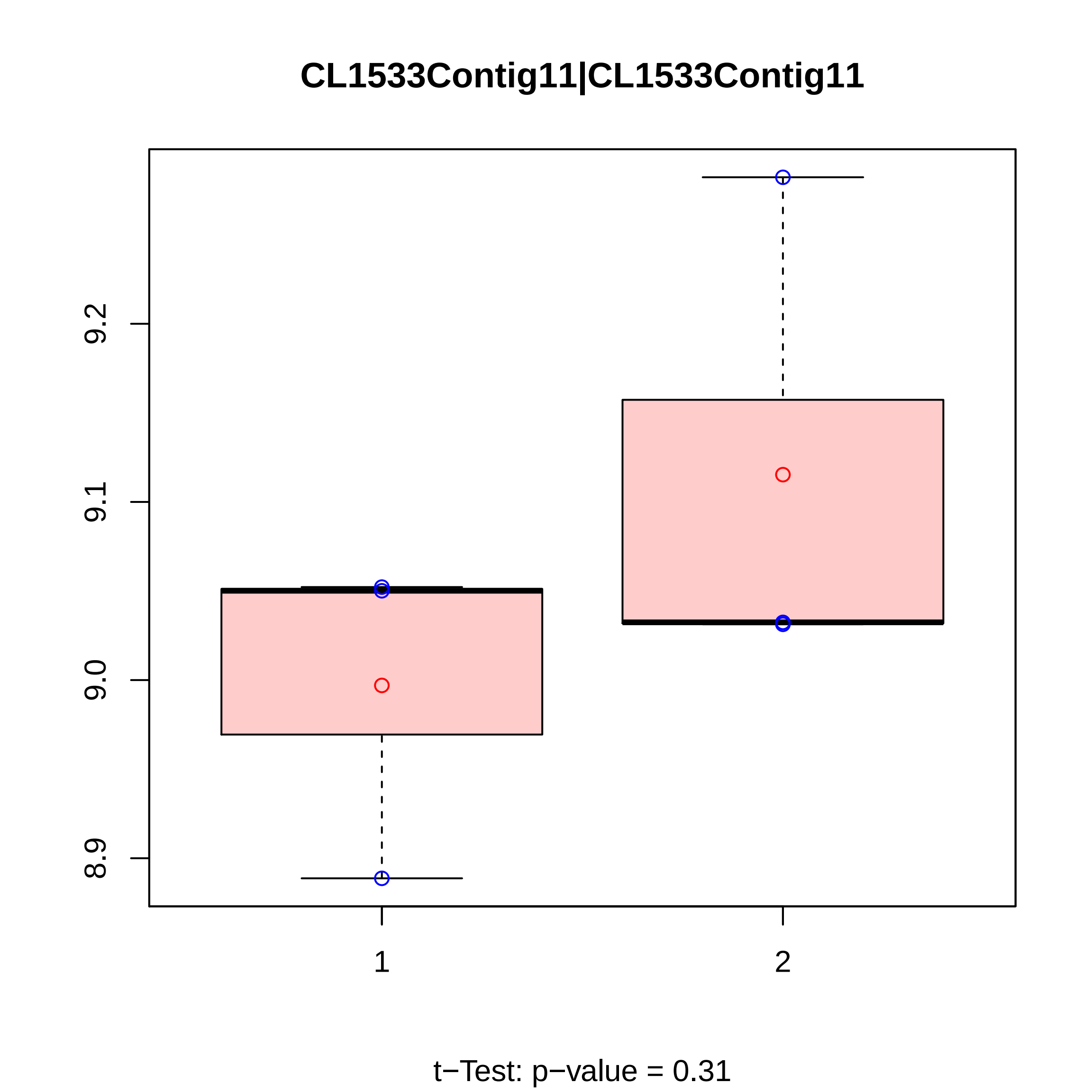
CL1533Contig11|CL1533Contig11
9.2
9.1
9.0
8.9
1
2
t−Test: p−value = 0.31

# CL1533Contig12|CL1533Contig12

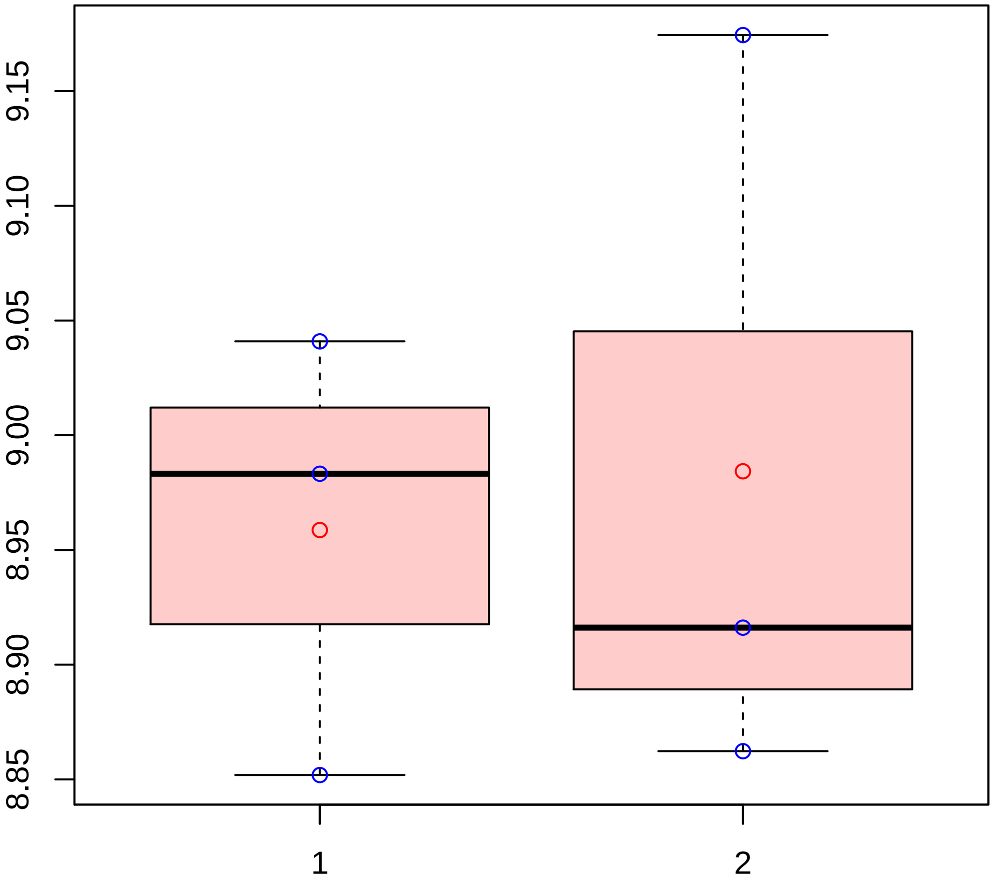


t−Test: p−value = 0.83

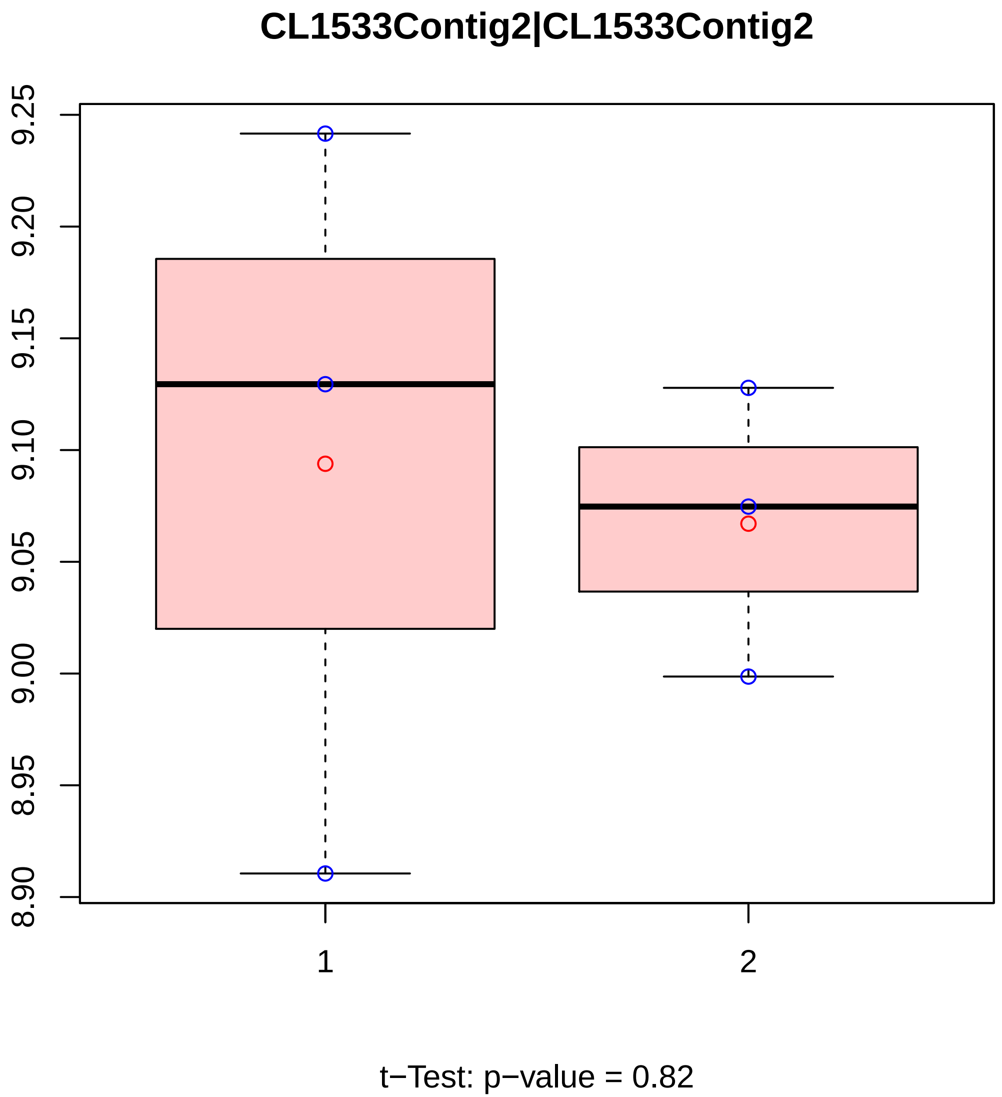
CL1533Contig2|CL1533Contig2
9.25
9.20
9.15
9.10
9.05
9.00
8.95
8.90
1
2
t−Test: p−value = 0.82

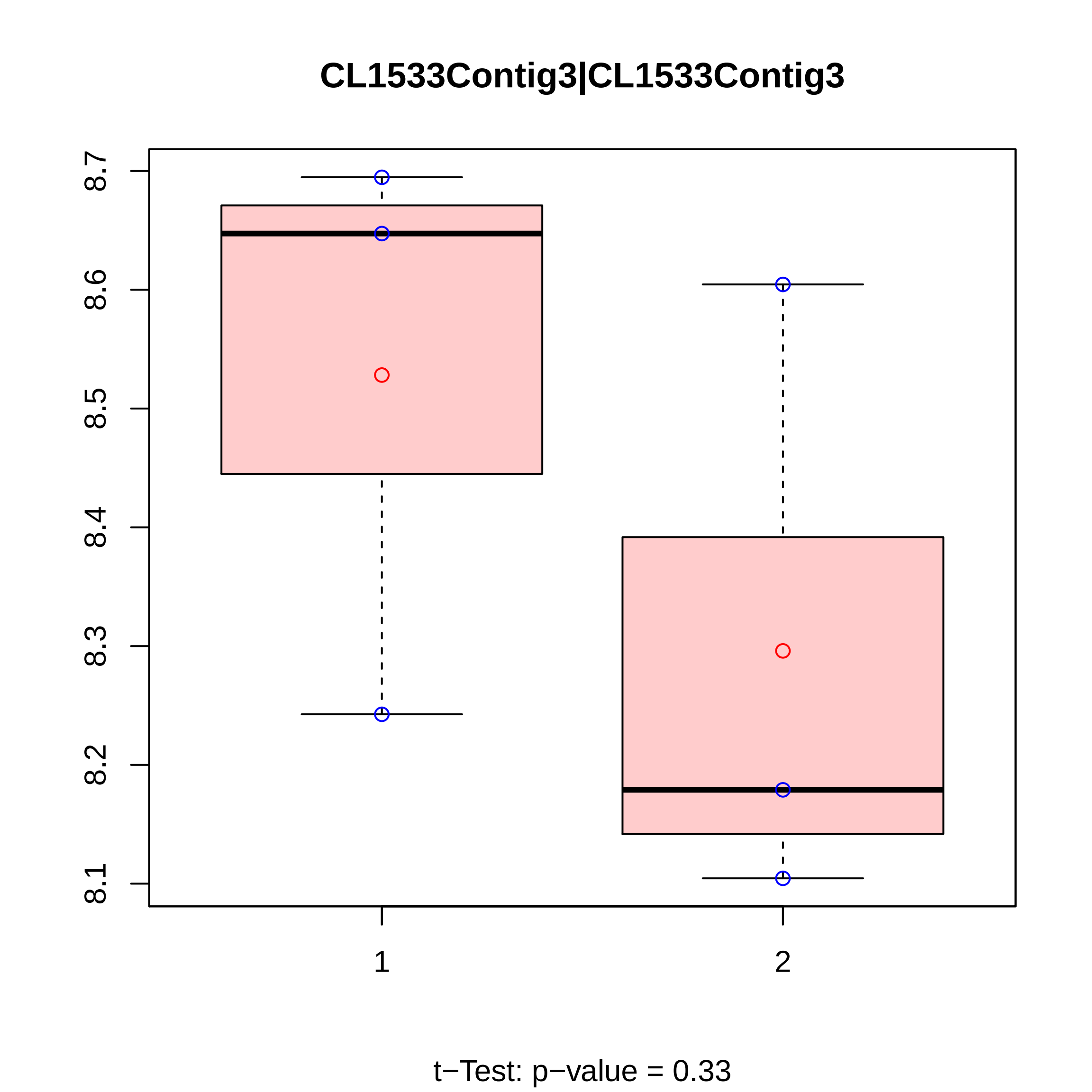
CL1533Contig3|CL1533Contig3
8.1
8.2
8.3
8.4
8.5
8.6
8.7
1
2
t−Test: p−value = 0.33

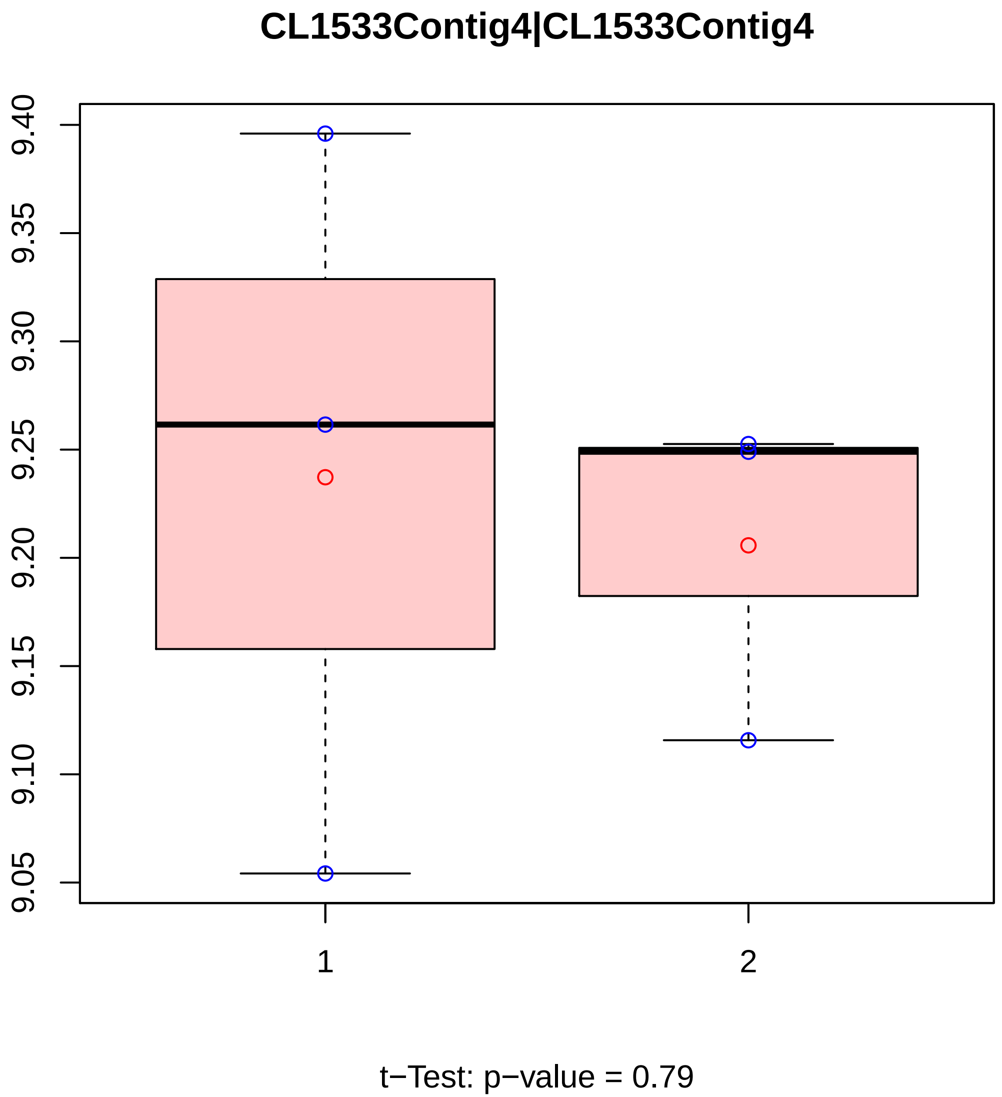
CL1533Contig4|CL1533Contig4
9.05
9.10
9.15
9.20
9.25
9.30
9.35
9.40
1
2
t−Test: p−value = 0.79

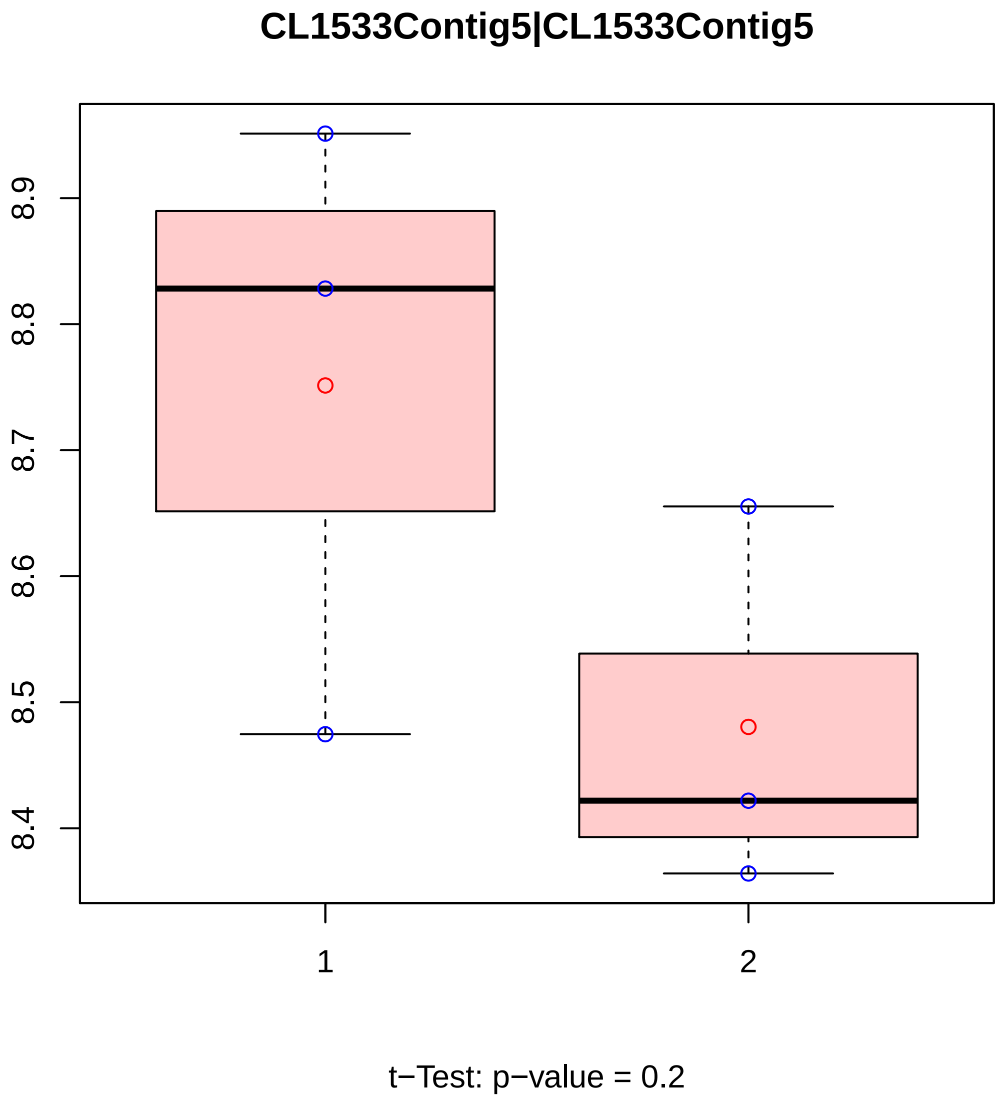

CL1533Contig5|CL1533Contig5
8.4
8.5
8.6
8.7
8.8
8.9
1
2
t−Test: p−value = 0.2

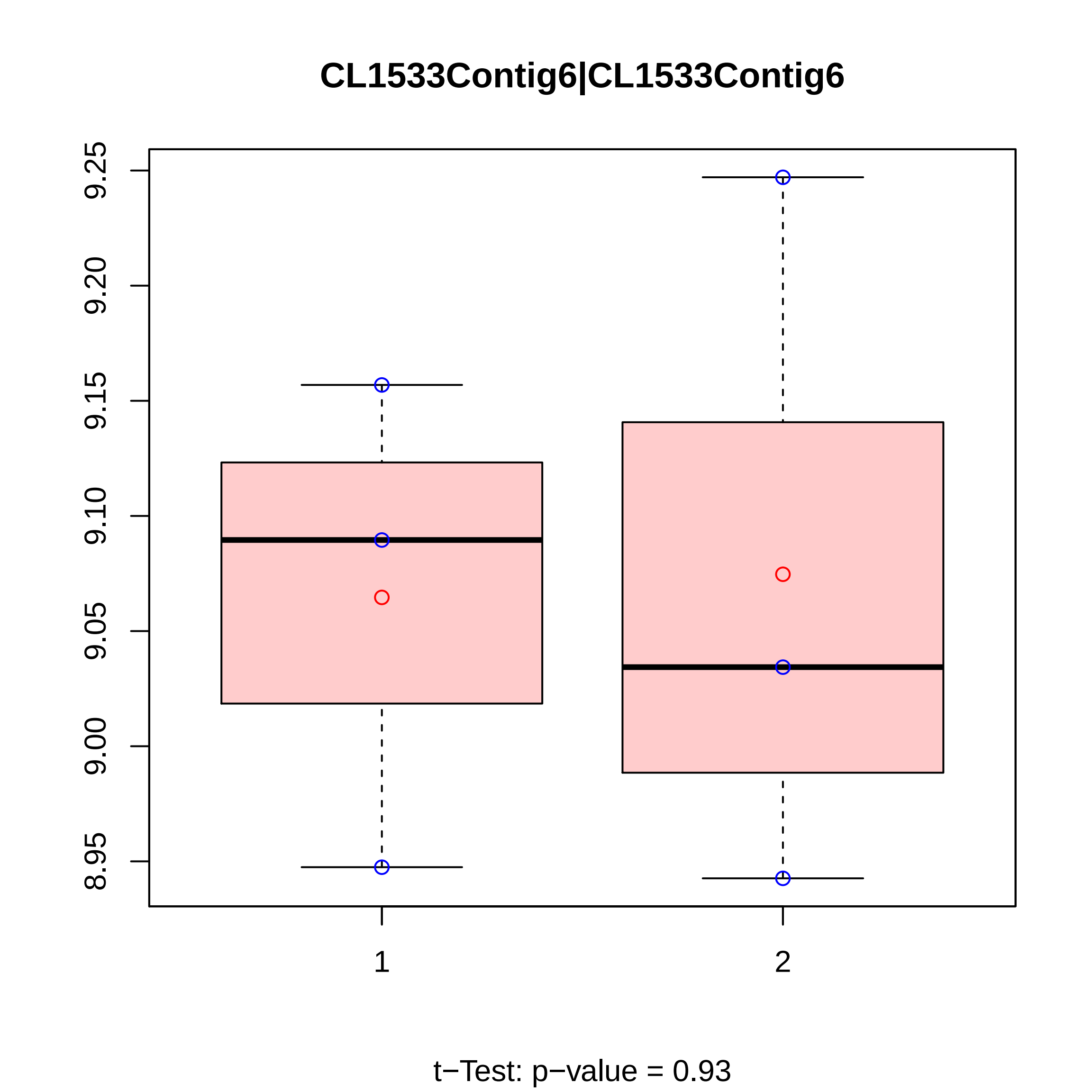
CL1533Contig6|CL1533Contig6
9.25
9.20
9.15
9.10
9.05
9.00
8.95
1
2
t−Test: p−value = 0.93

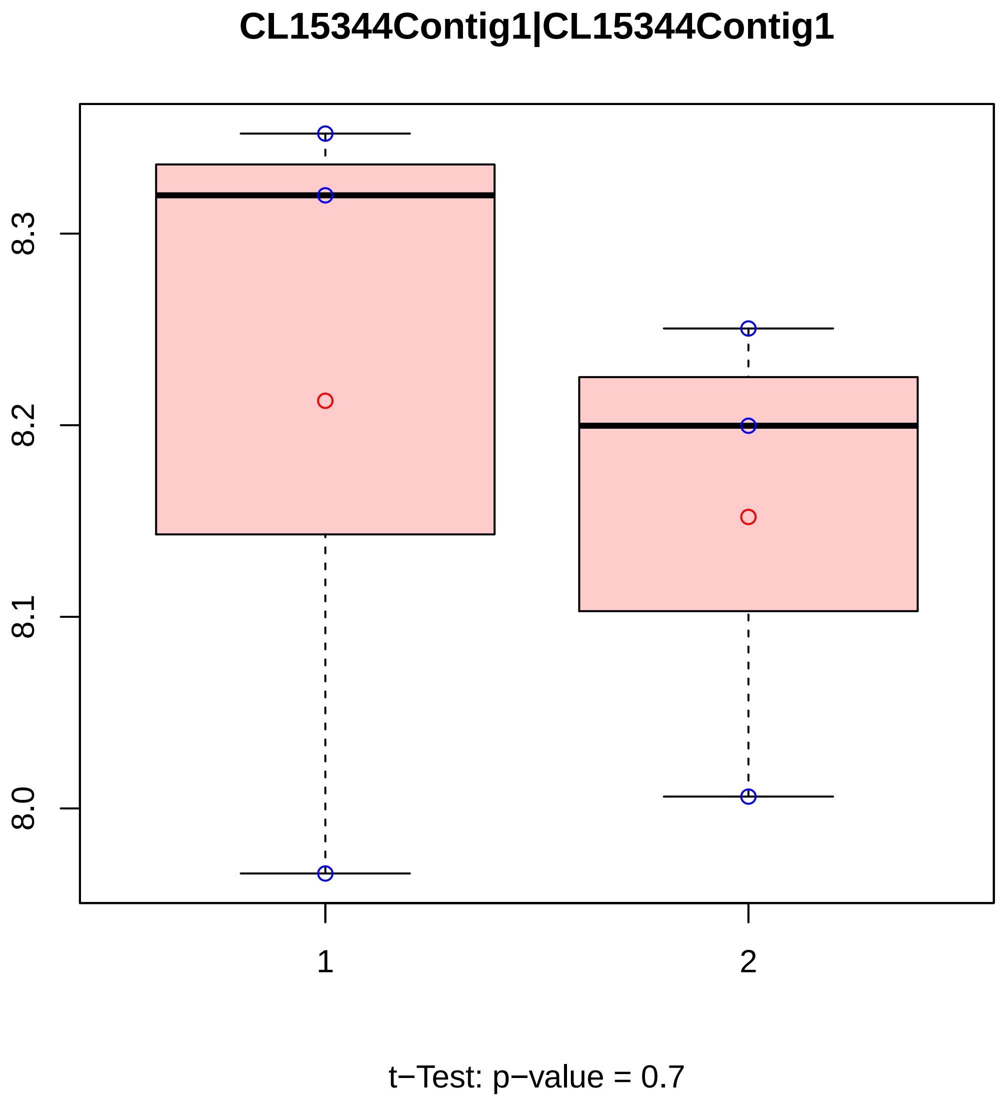
CL15344Contig1|CL15344Contig1
8.0
8.1
8.2
8.3
1
2
t−Test: p−value = 0.7

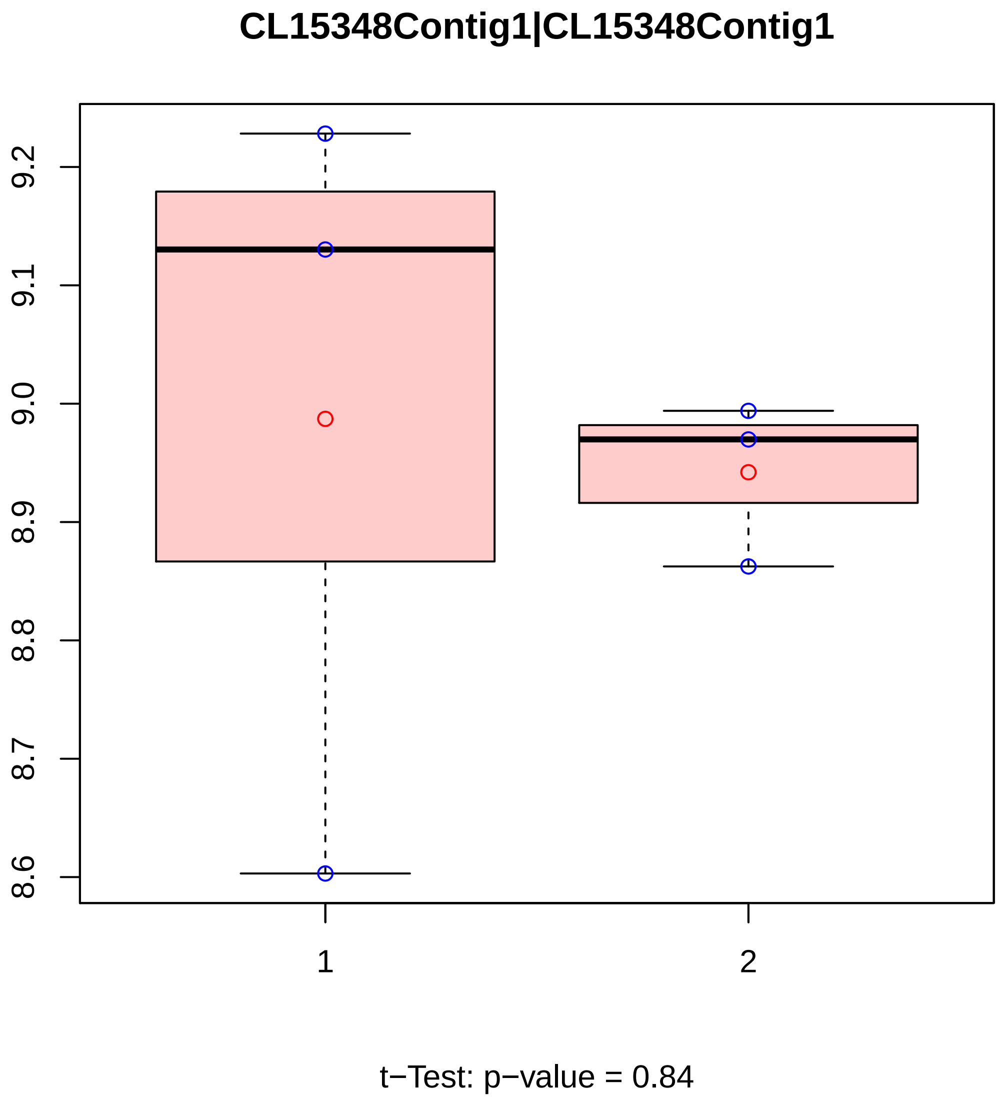
CL15348Contig1|CL15348Contig1
9.2
9.1
9.0
8.9
8.8
8.7
8.6
1
2
t−Test: p−value = 0.84

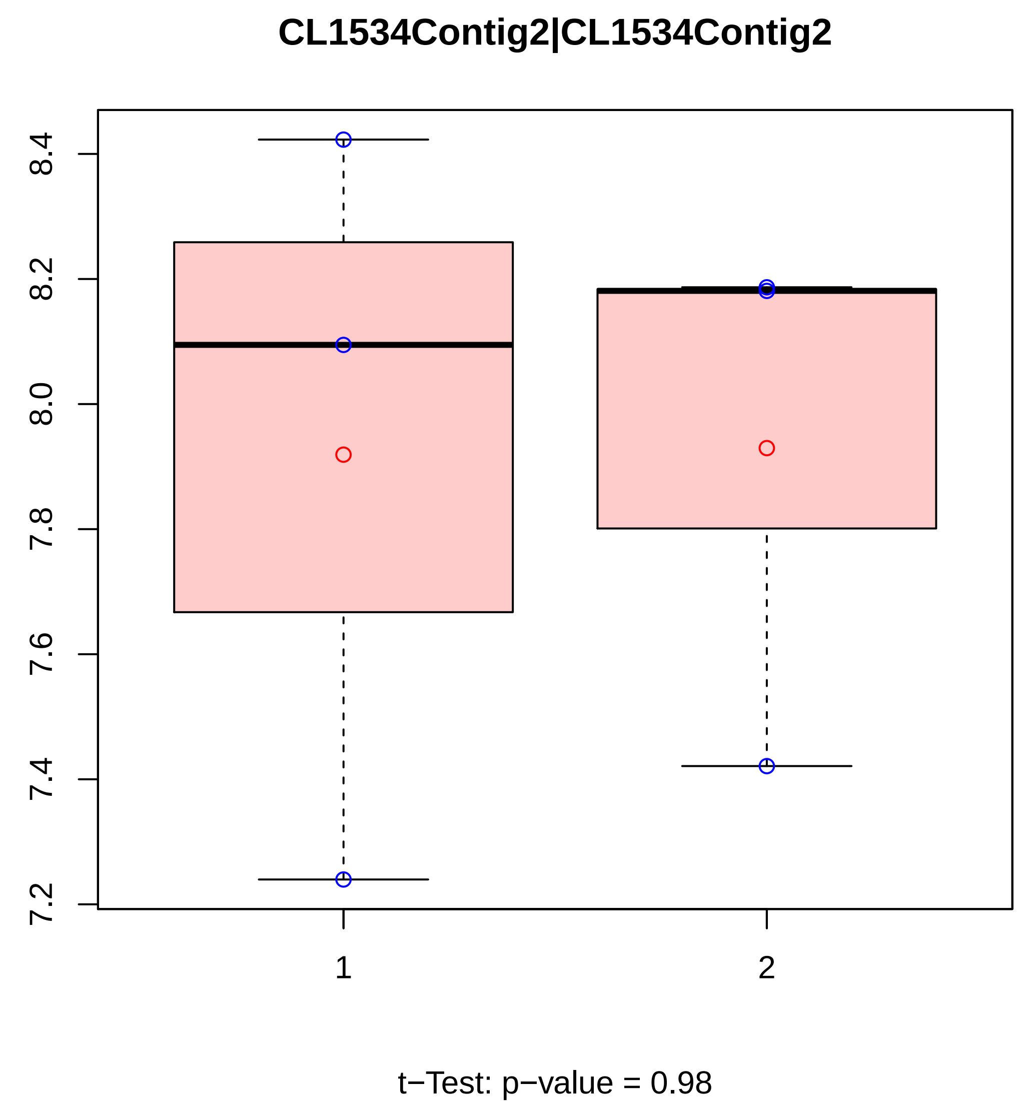
CL1534Contig2|CL1534Contig2
8.4
8.2
8.0
7.8
7.6
7.4
7.2
1
2
t−Test: p−value = 0.98

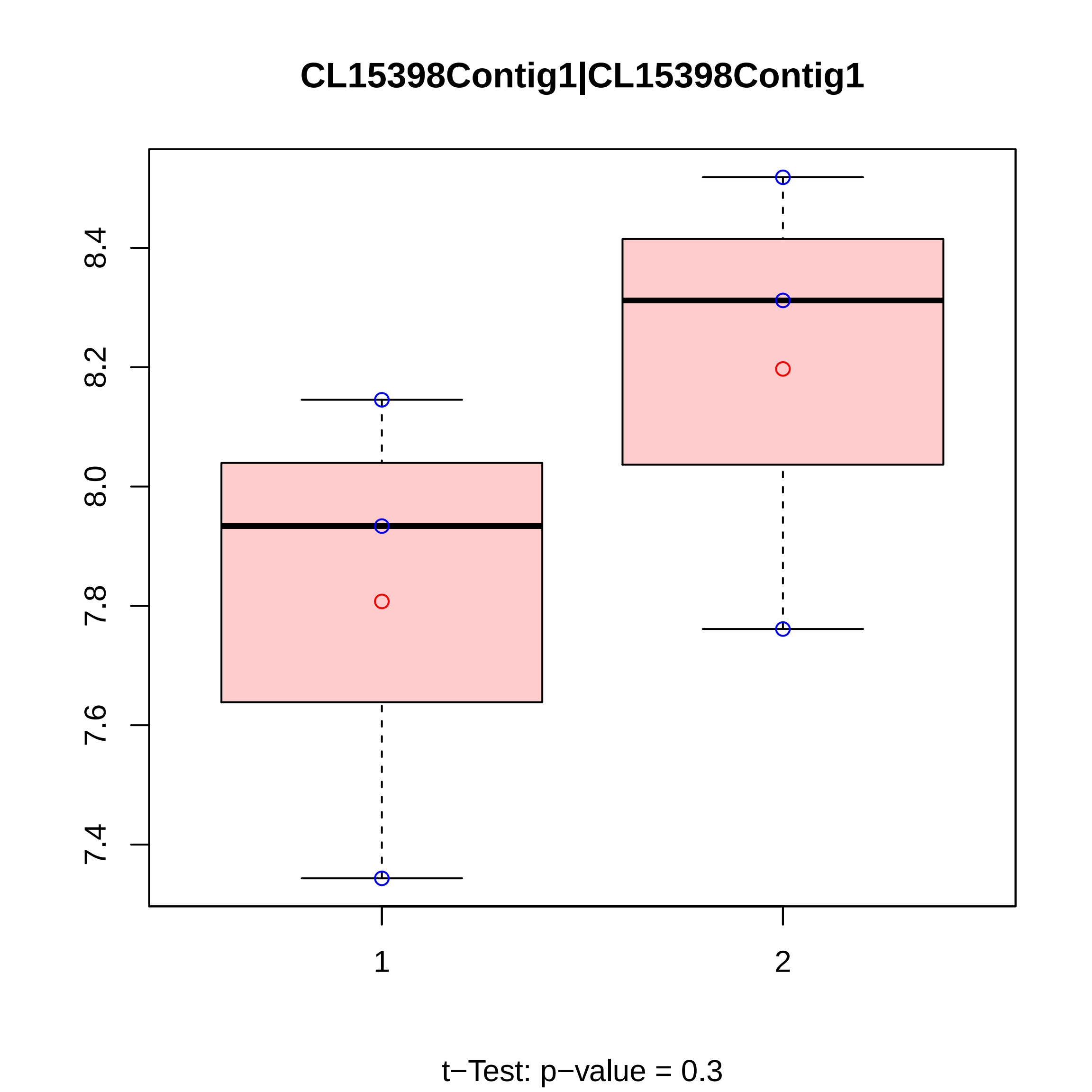
CL15398Contig1|CL15398Contig1
8.4
8.2
8.0
7.8
7.6
7.4
1
2
t−Test: p−value = 0.3

# CL153Contig9|CL153Contig9

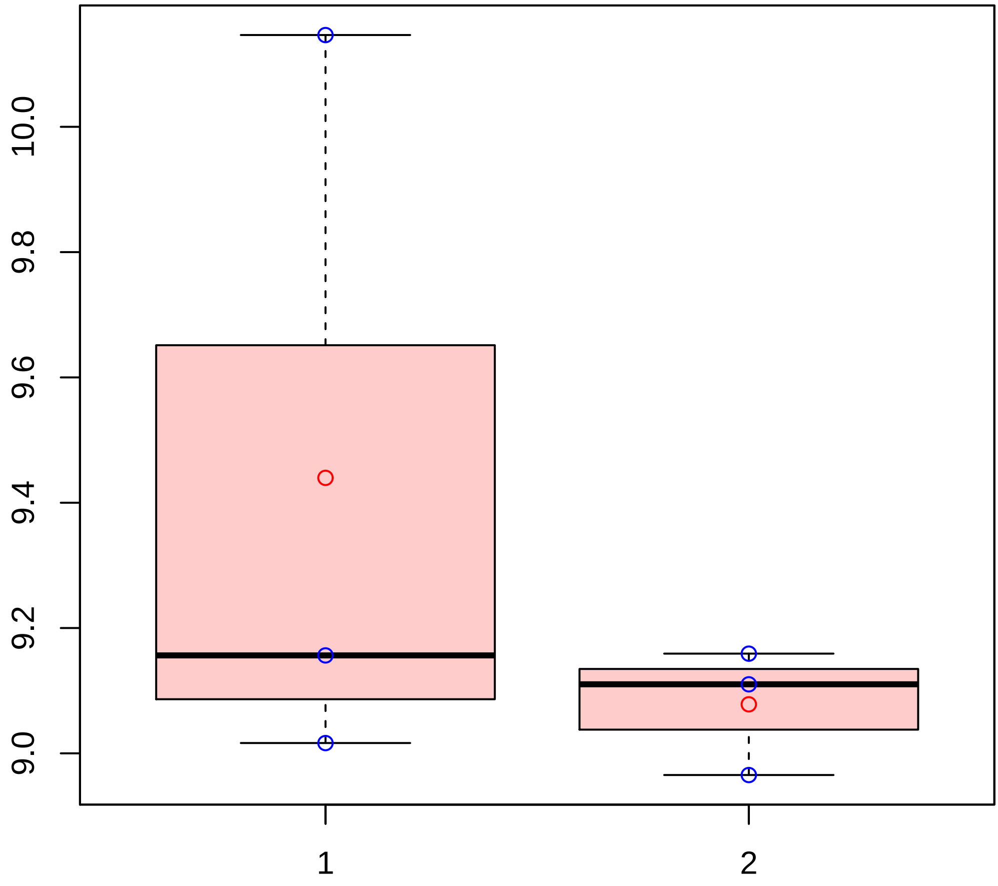


t−Test: p−value = 0.42

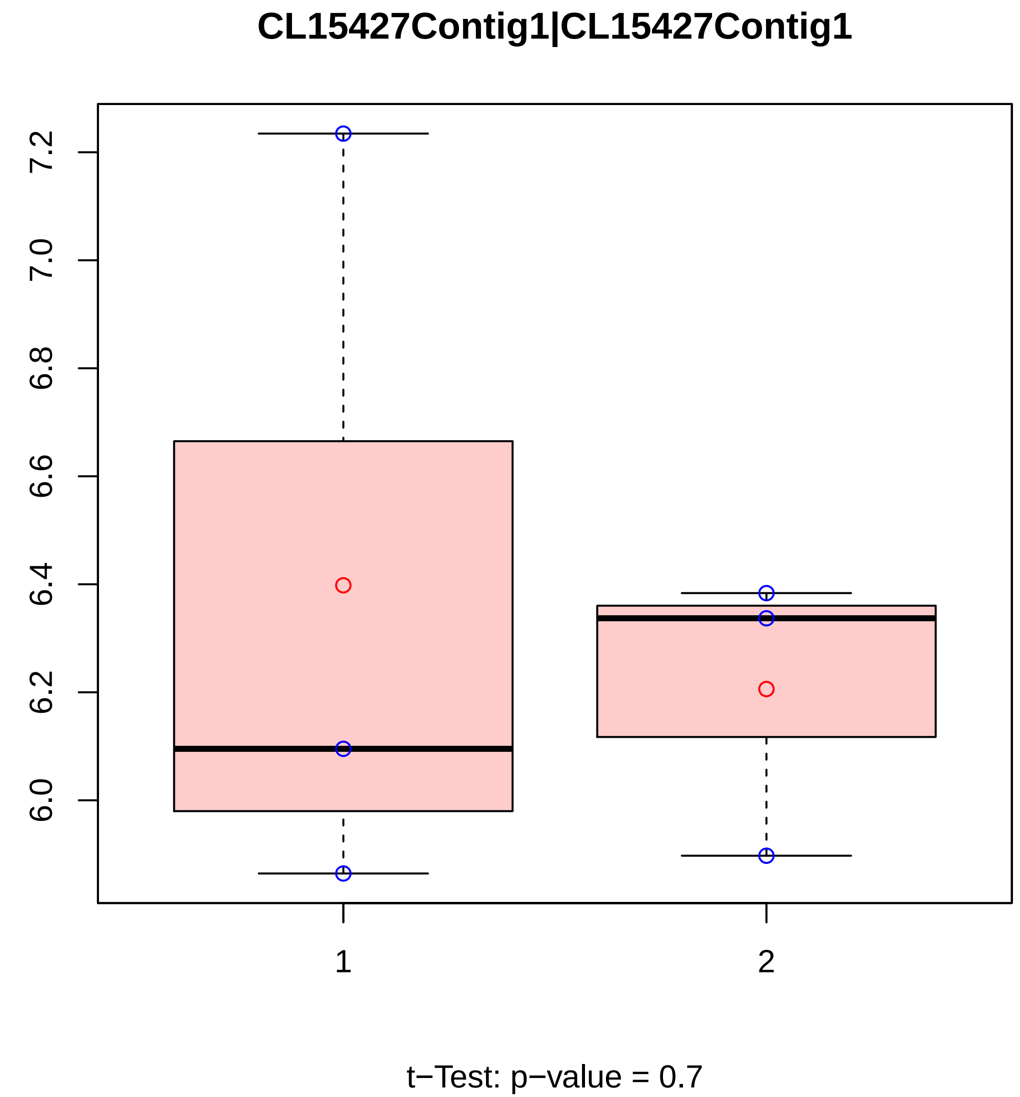

CL15427Contig1|CL15427Contig1
7.2
7.0
6.8
6.6
6.4
6.2
6.0
1
2
t−Test: p−value = 0.7

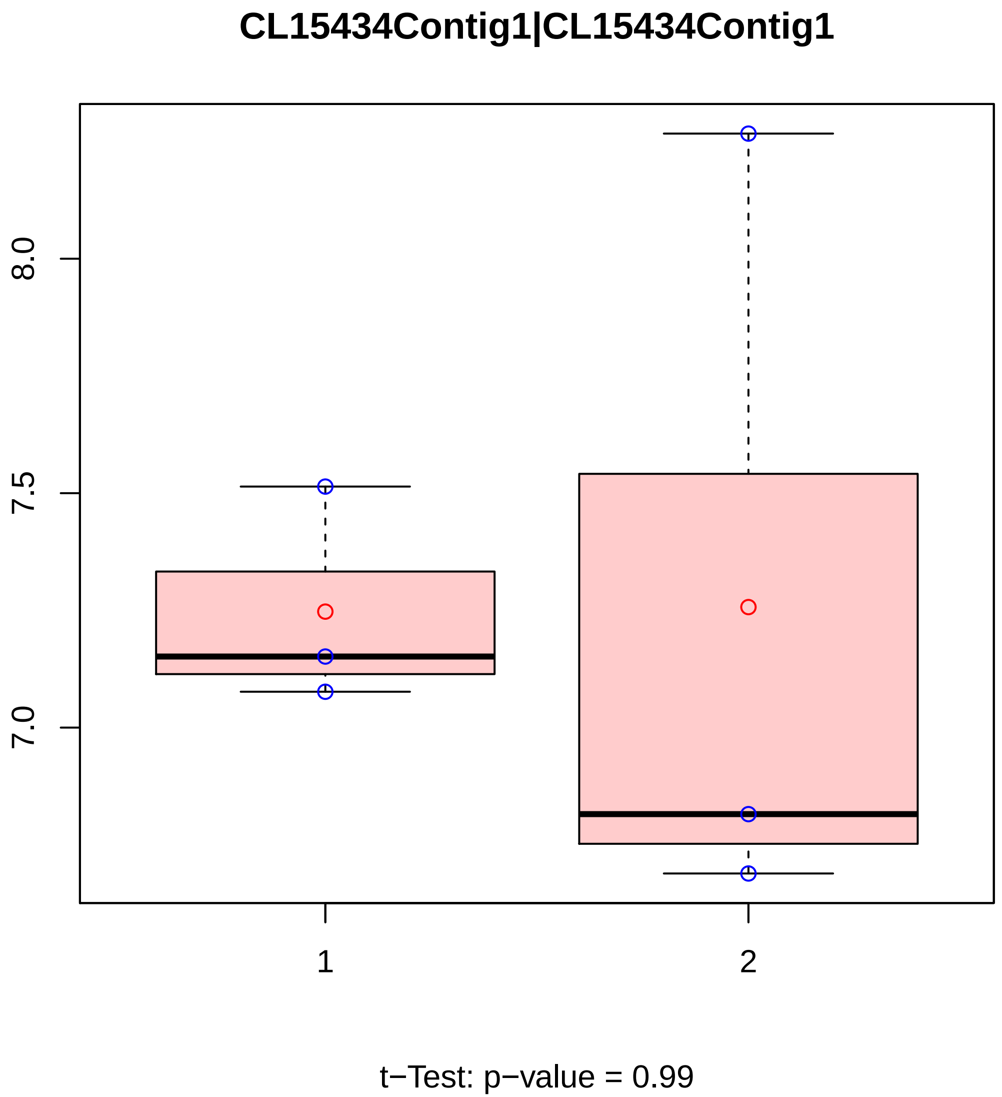
CL15434Contig1|CL15434Contig1
8.0
7.5
7.0
1
2
t−Test: p−value = 0.99

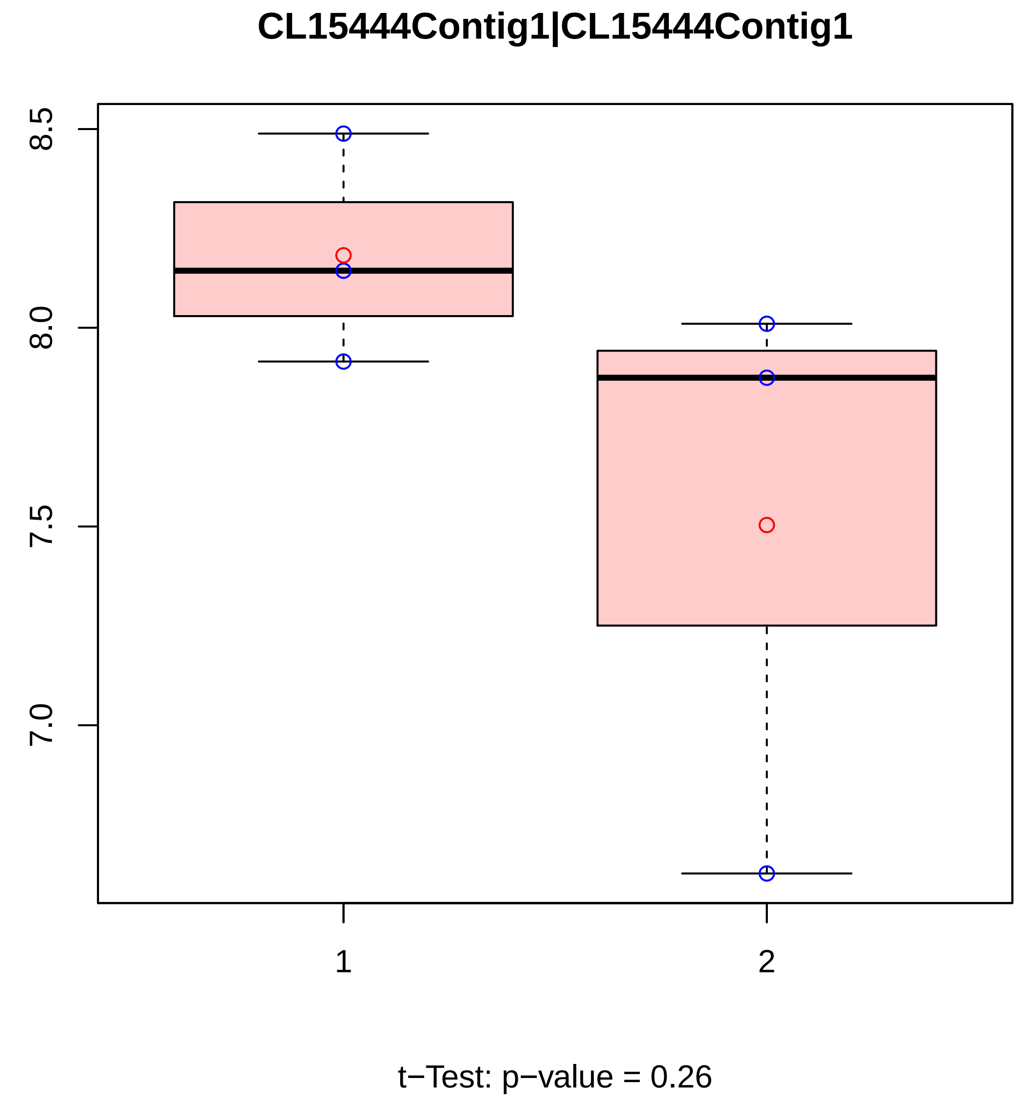
CL15444Contig1|CL15444Contig1
8.5
8.0
7.5
7.0
1
2
t−Test: p−value = 0.26

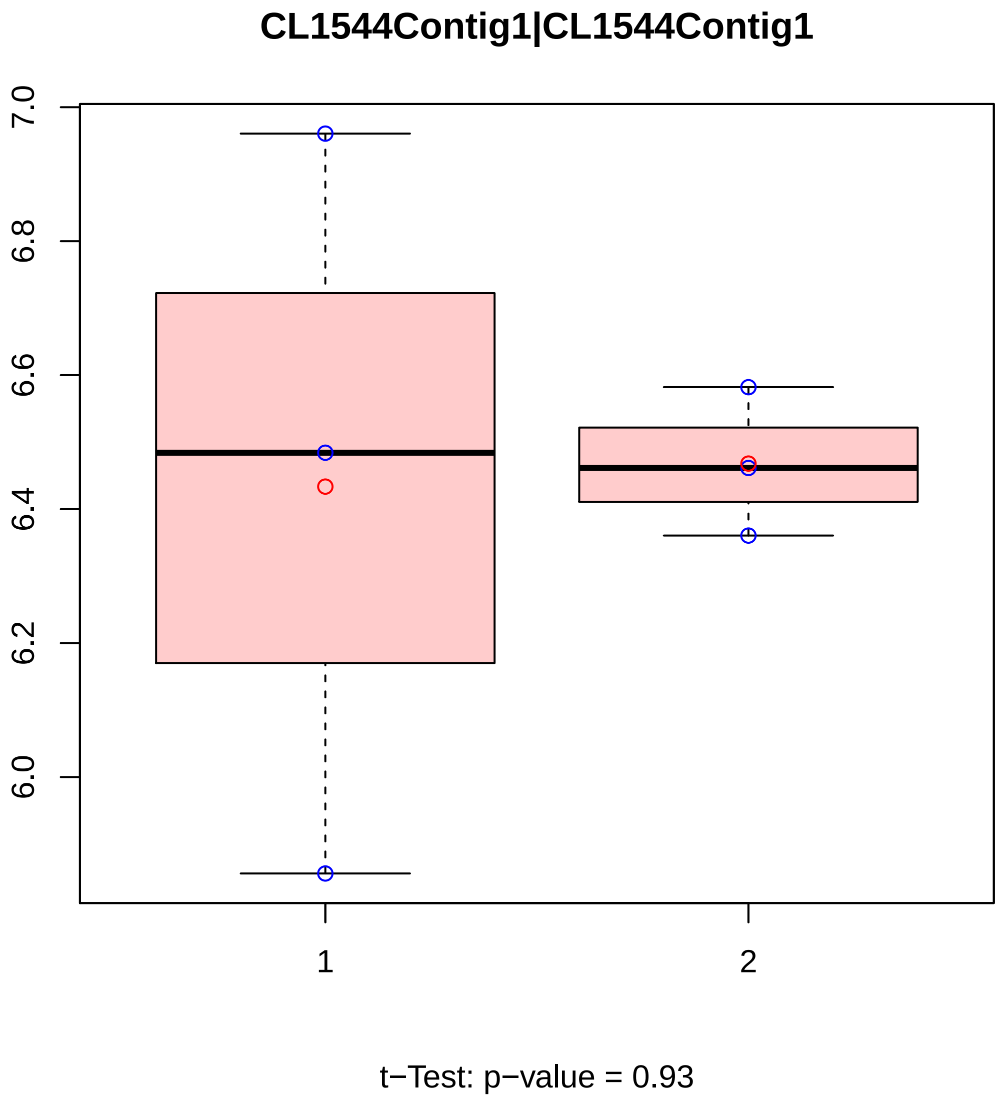
CL1544Contig1|CL1544Contig1
7.0
6.8
6.6
6.4
6.2
6.0
1
2
t−Test: p−value = 0.93

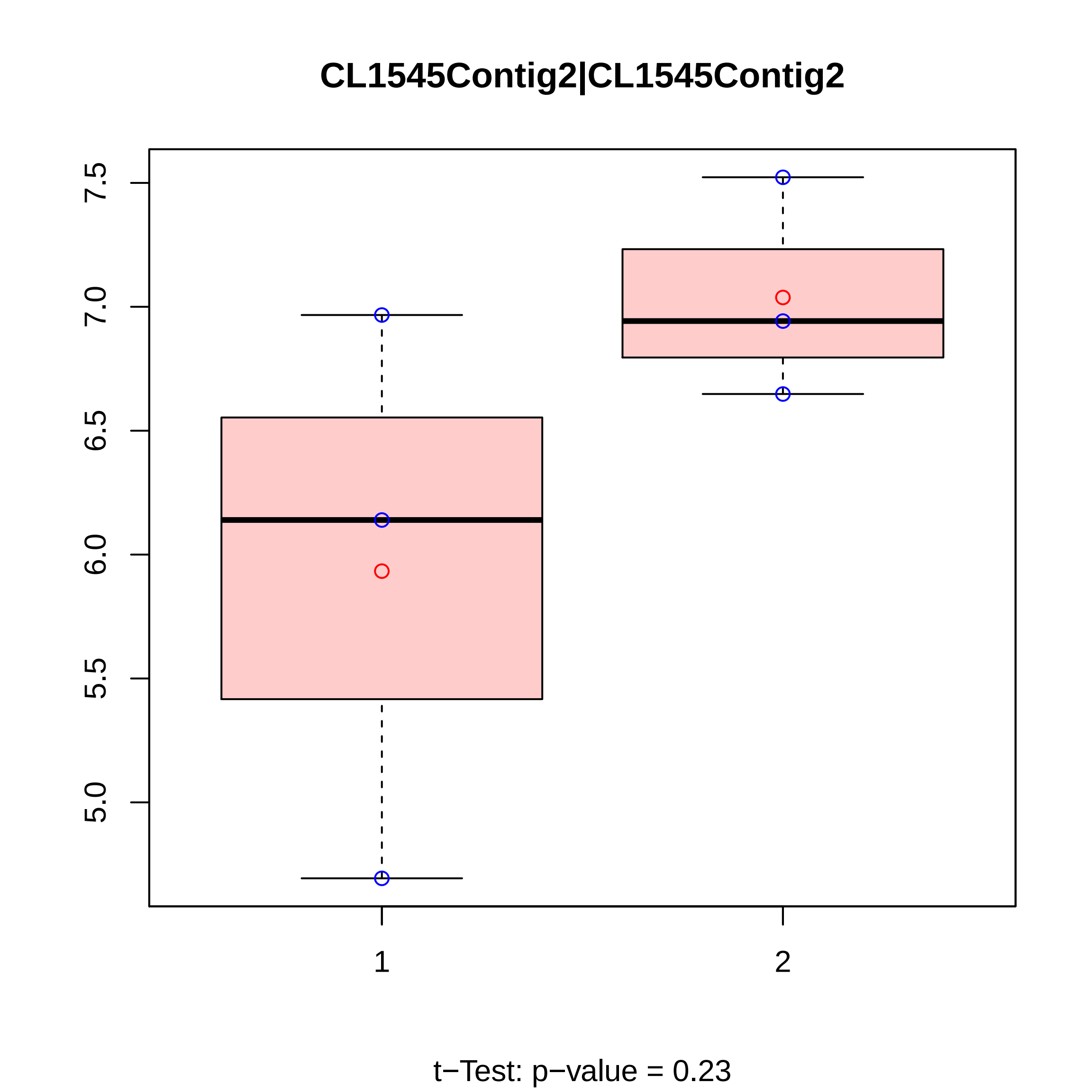
CL1545Contig2|CL1545Contig2
7.5
7.0
6.5
6.0
5.5
5.0
1
2
t−Test: p−value = 0.23

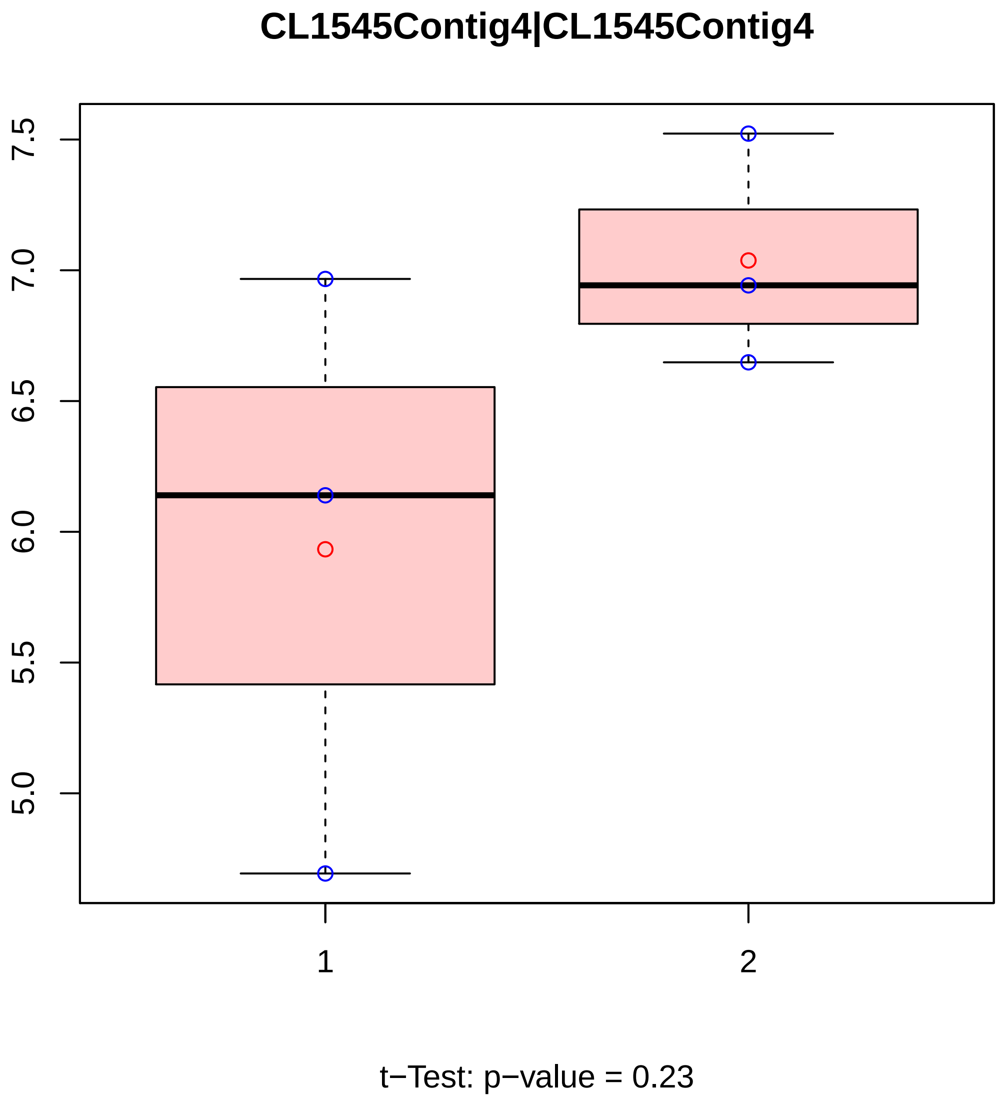

CL1545Contig4|CL1545Contig4
7.5
7.0
6.5
6.0
5.5
5.0
1
2
t−Test: p−value = 0.23

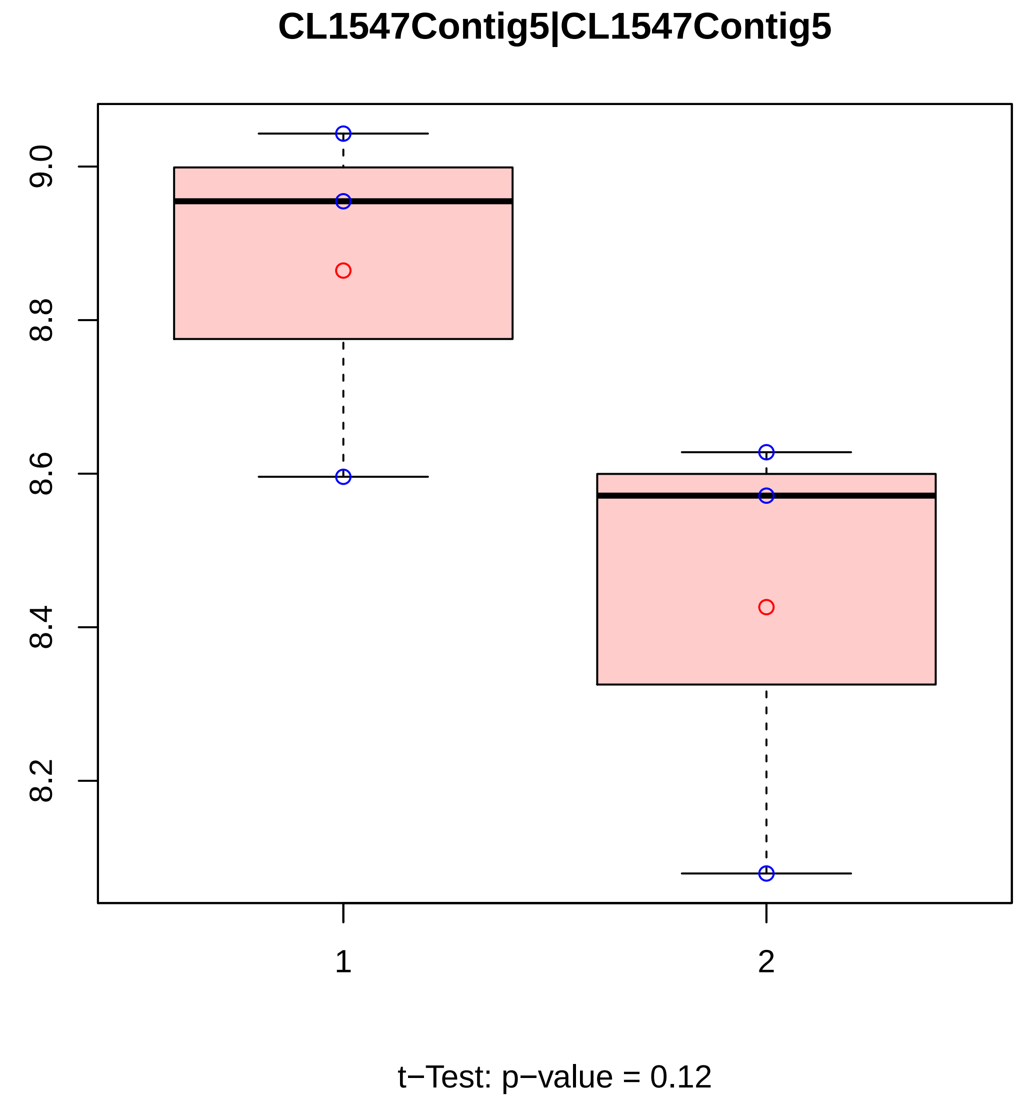
CL1547Contig5|CL1547Contig5
9.0
8.8
8.6
8.4
8.2
1
2
t−Test: p−value = 0.12

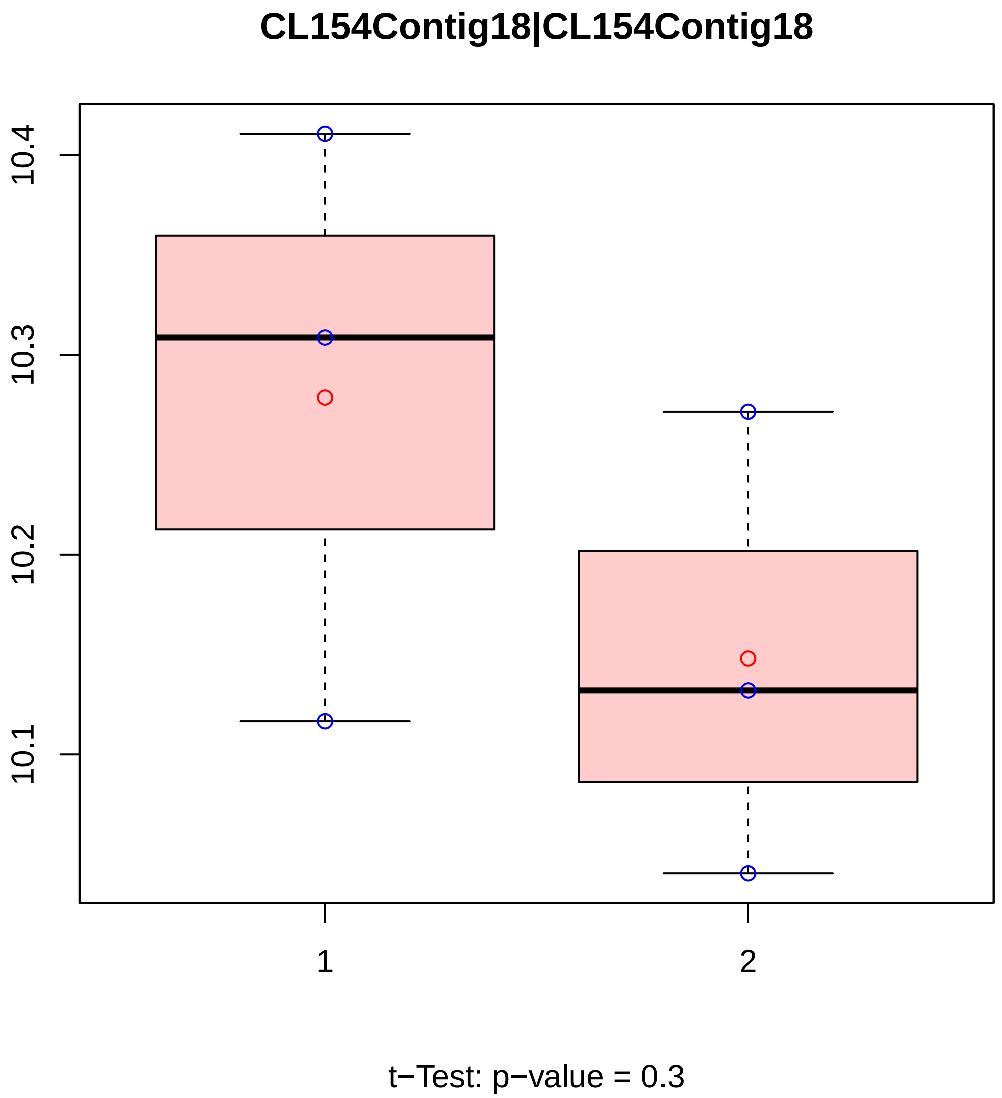
CL154Contig18|CL154Contig18
10.1
10.2
10.3
10.4
1
2
t−Test: p−value = 0.3

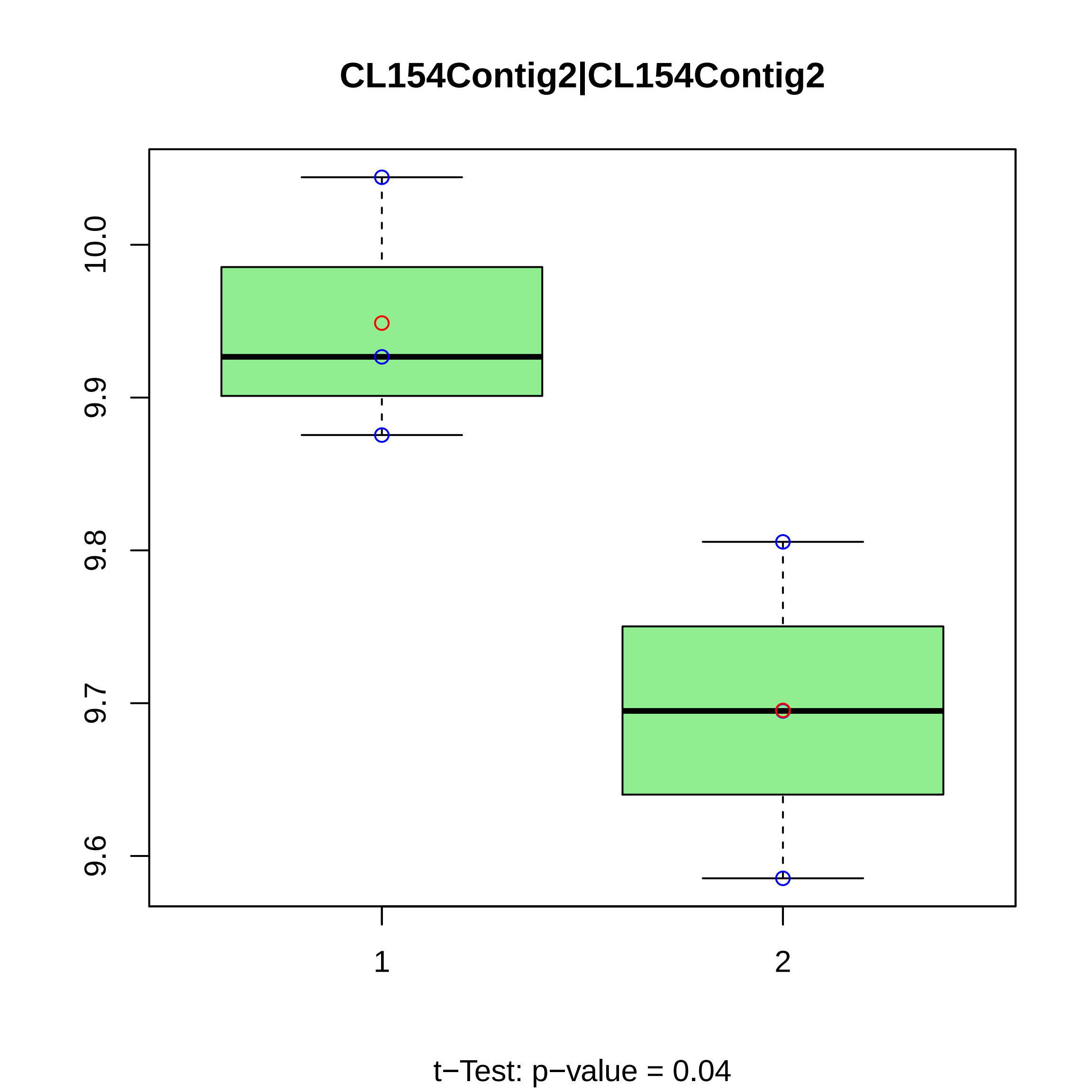
CL154Contig2|CL154Contig2
10.0
9.9
9.8
9.7
9.6
1
2
t−Test: p−value = 0.04

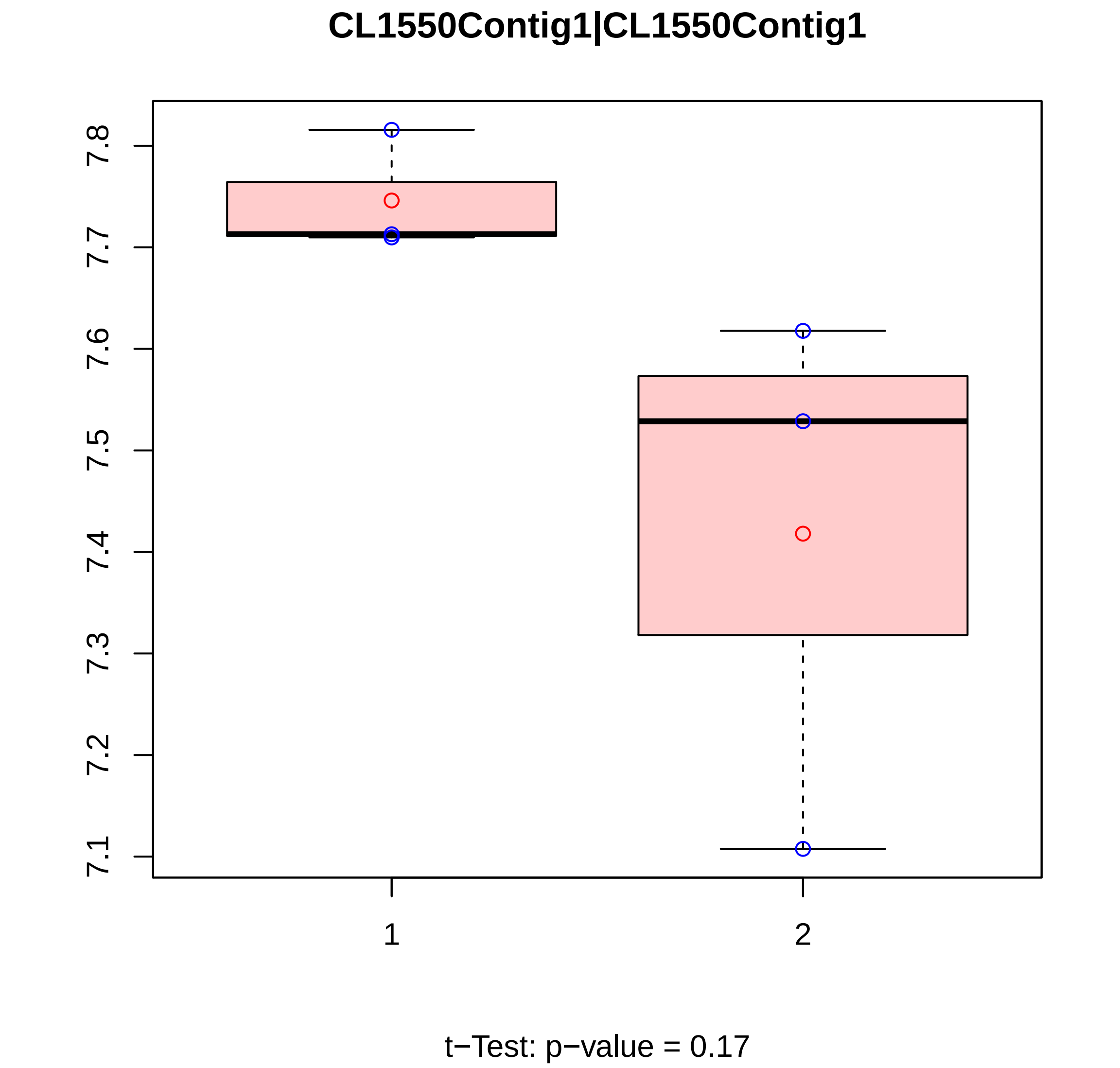
CL1550Contig1|CL1550Contig1
7.8
7.7
7.6
7.5
7.4
7.3
7.2
7.1
1
2
t−Test: p−value = 0.17

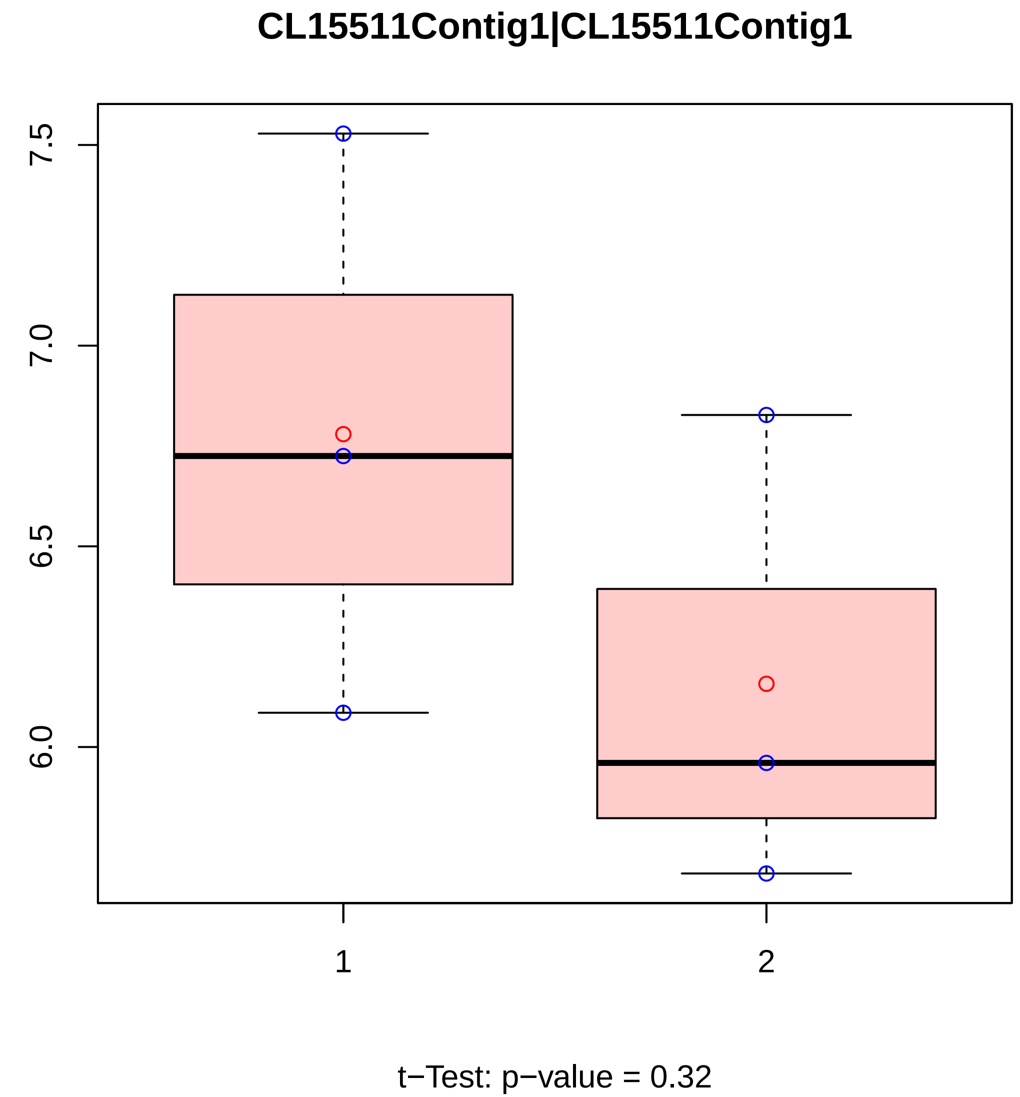
CL15511Contig1|CL15511Contig1
7.5
7.0
6.5
6.0
1
2
t−Test: p−value = 0.32

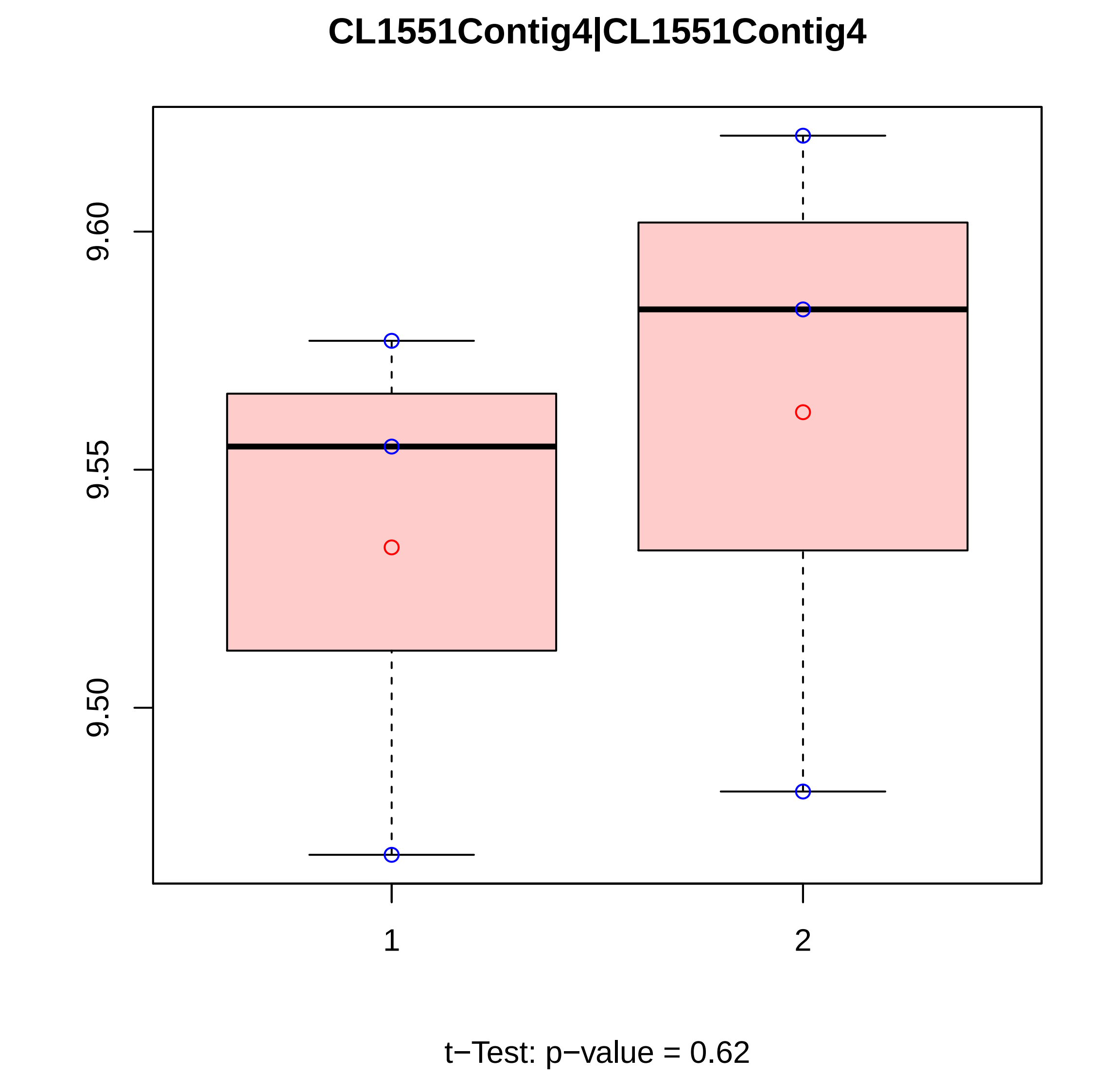
CL1551Contig4|CL1551Contig4
9.50
9.55
9.60
1
2
t−Test: p−value = 0.62

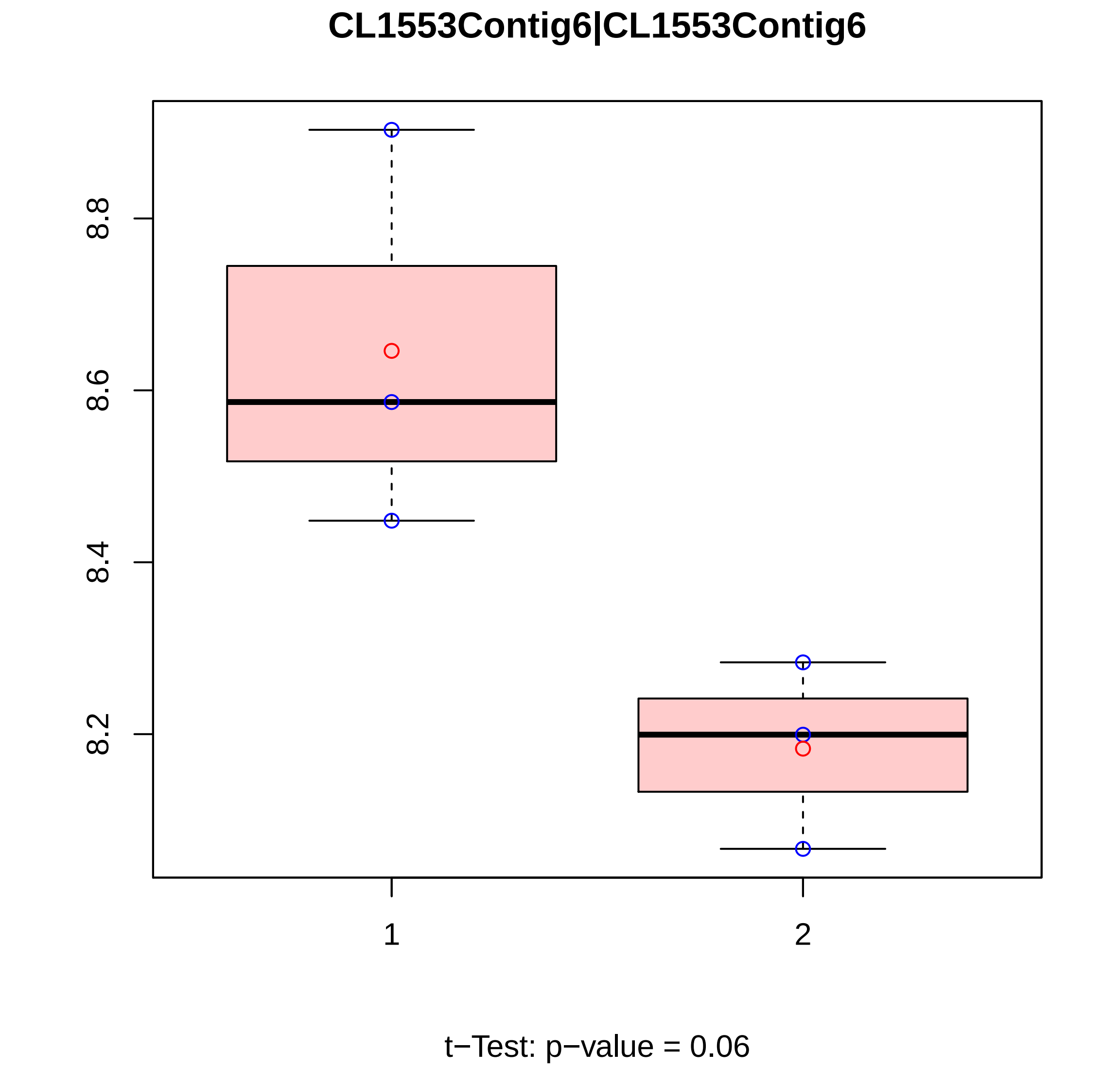
CL1553Contig6|CL1553Contig6
8.8
8.6
8.4
8.2
1
2
t−Test: p−value = 0.06

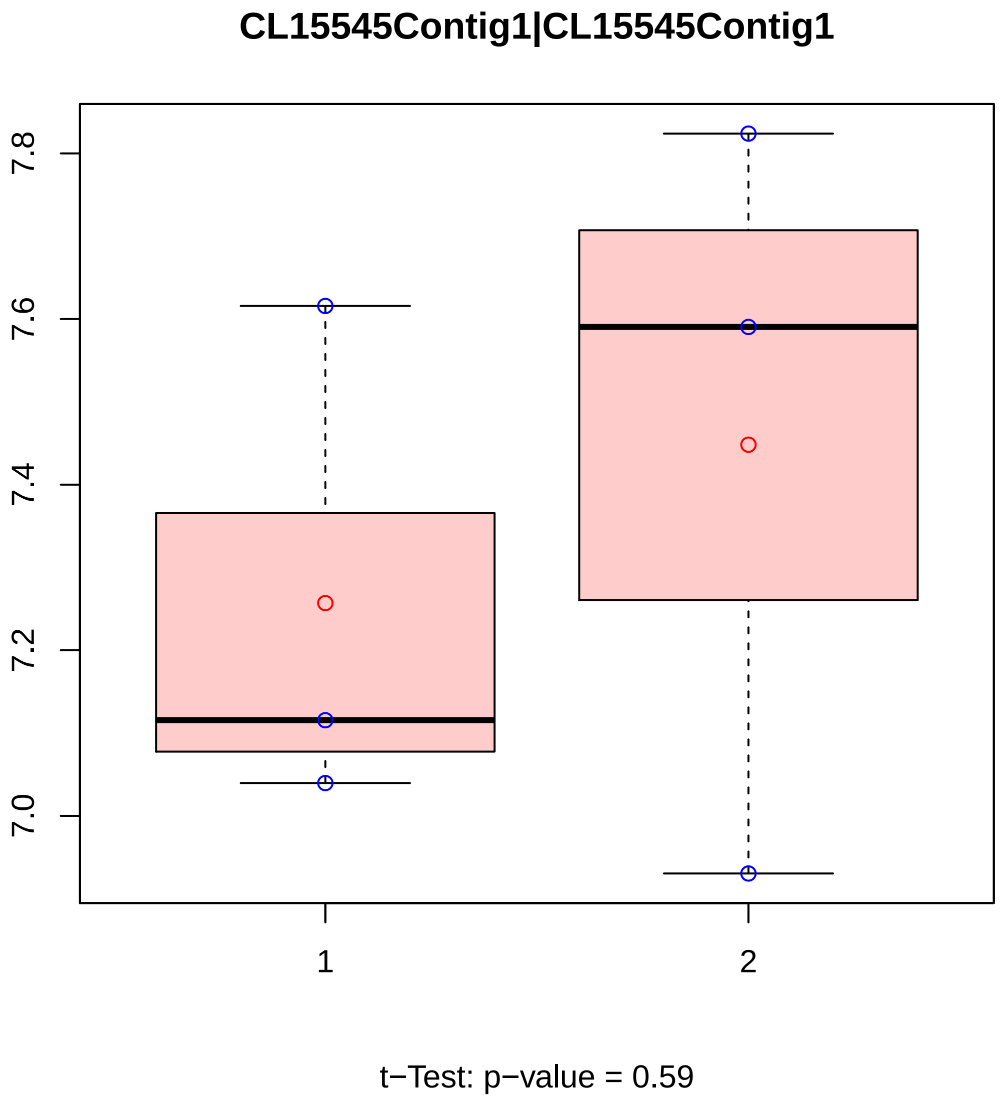

CL15545Contig1|CL15545Contig1
7.8
7.6
7.4
7.2
7.0
1
2
t−Test: p−value = 0.59

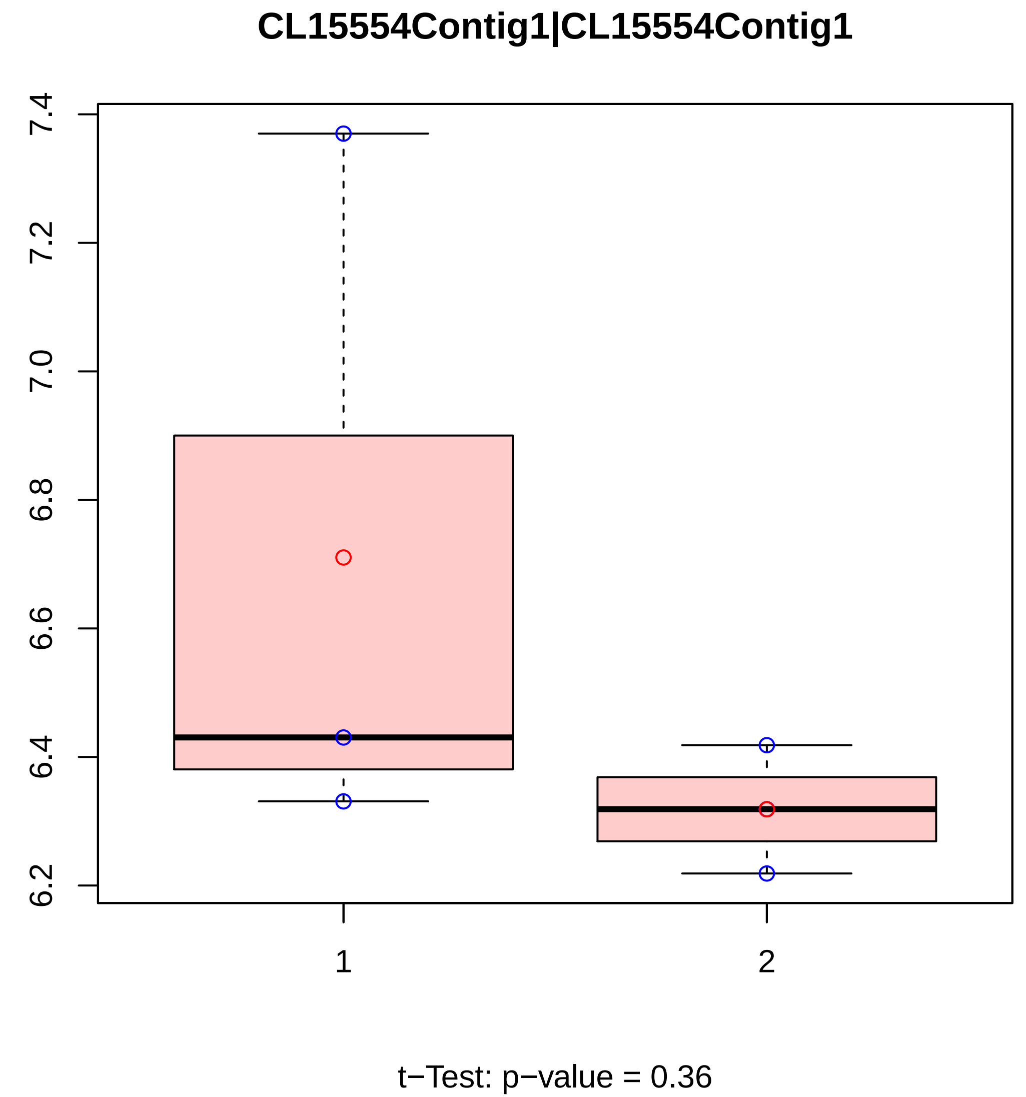
CL15554Contig1|CL15554Contig1
7.4
7.2
7.0
6.8
6.6
6.4
6.2
1
2
t−Test: p−value = 0.36

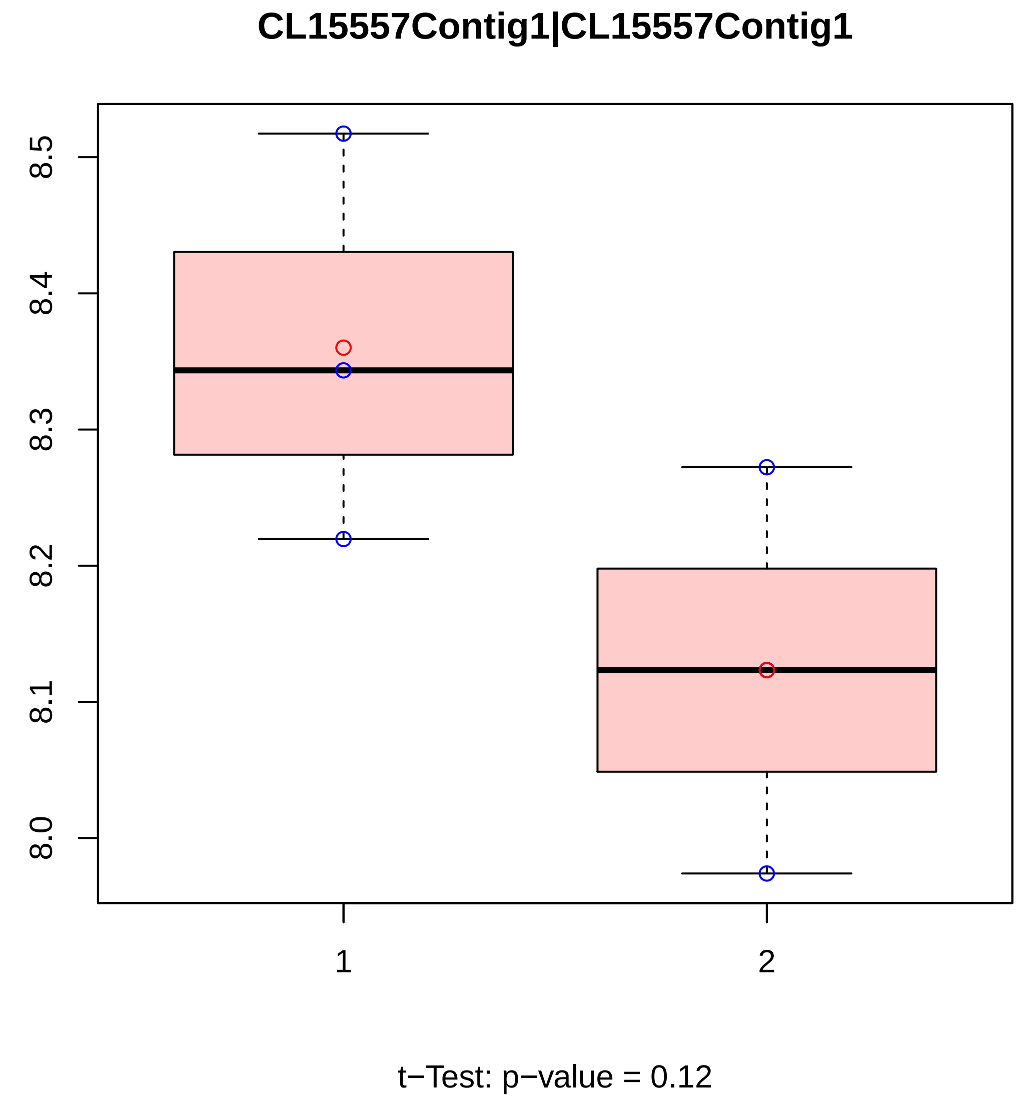
CL15557Contig1|CL15557Contig1
8.5
8.4
8.3
8.2
8.1
8.0
1
2
t−Test: p−value = 0.12

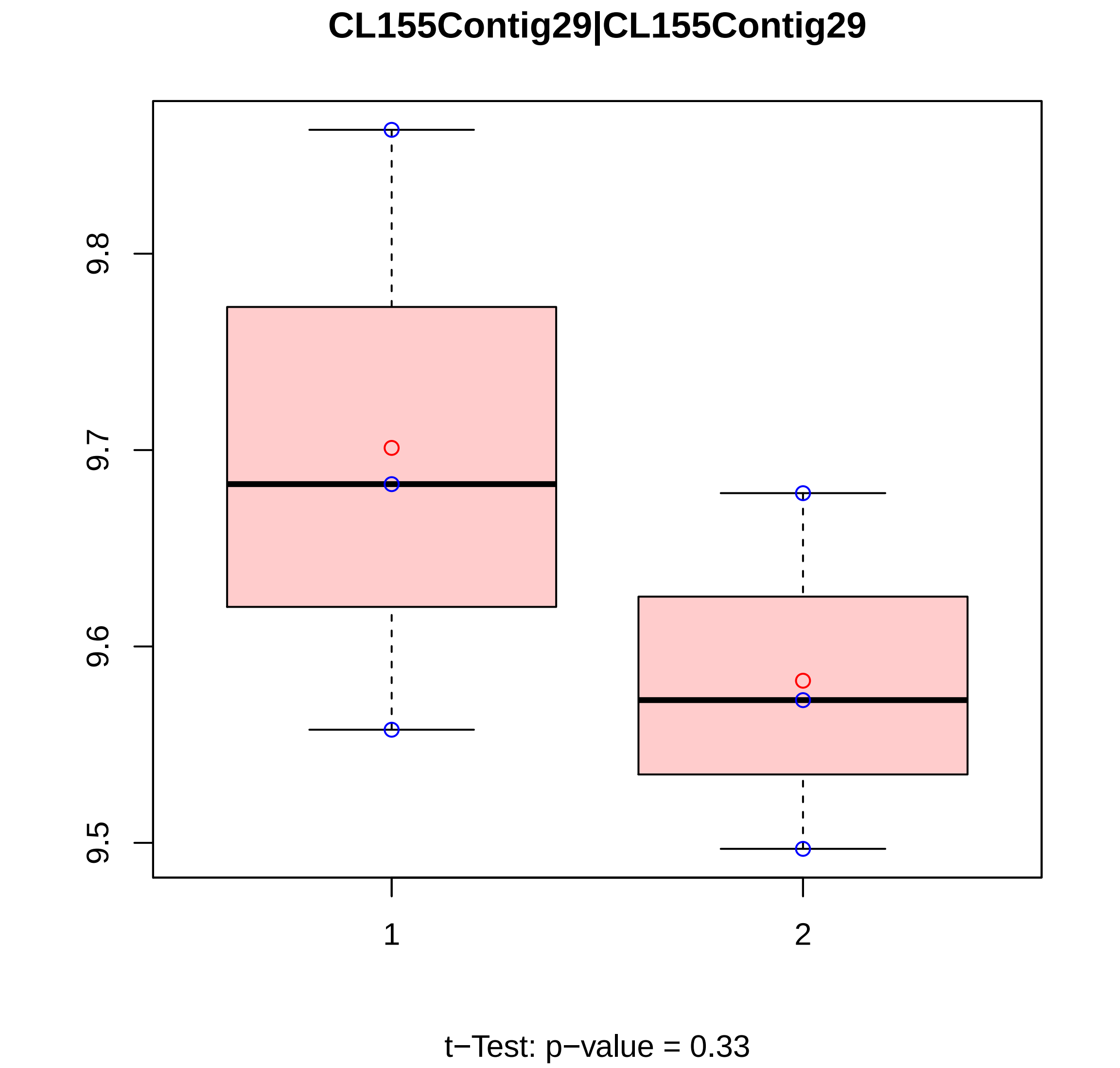
CL155Contig29|CL155Contig29
9.5
9.6
9.7
9.8
1
2
t−Test: p−value = 0.33

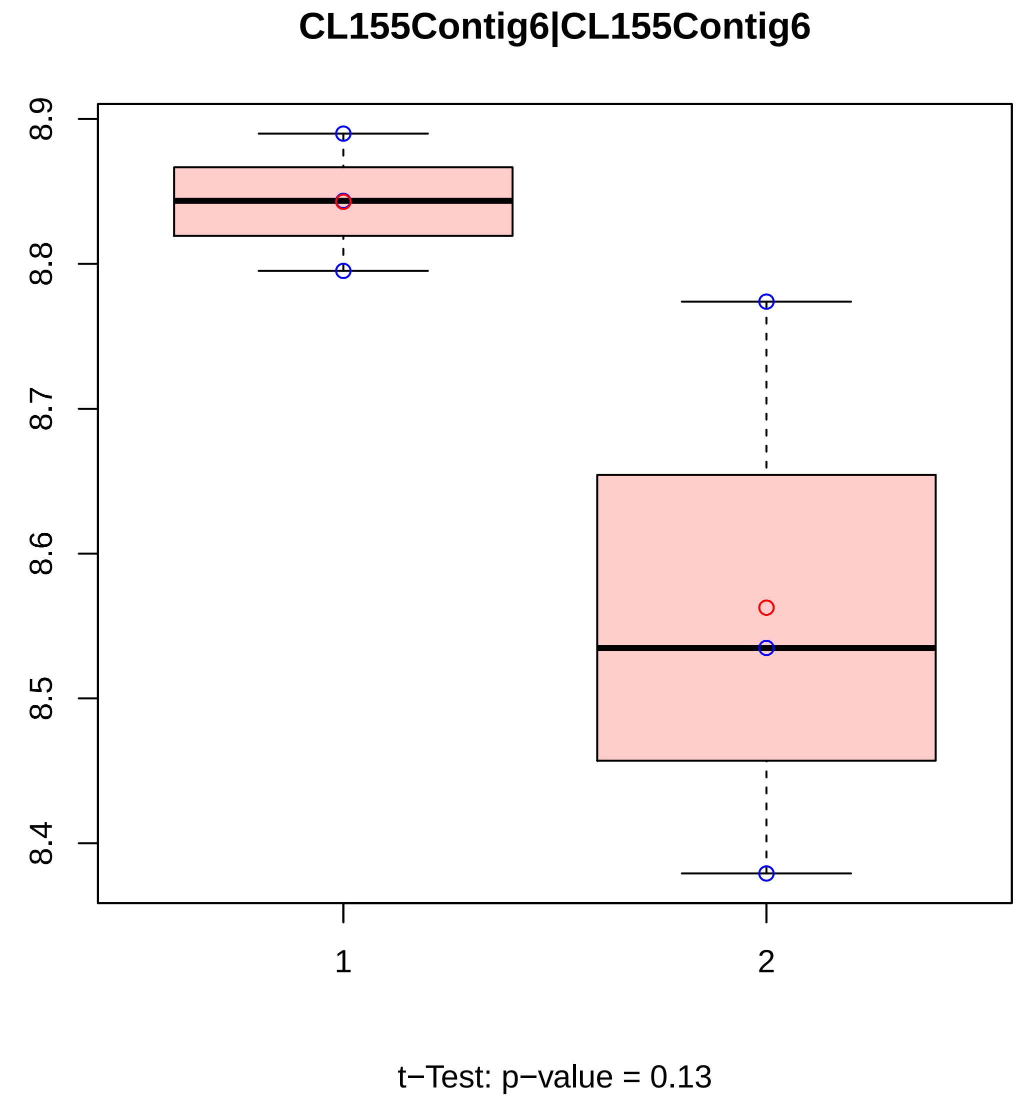
CL155Contig6|CL155Contig6
8.9
8.8
8.7
8.6
8.5
8.4
1
2
t−Test: p−value = 0.13

# CL155Contig7|CL155Contig7

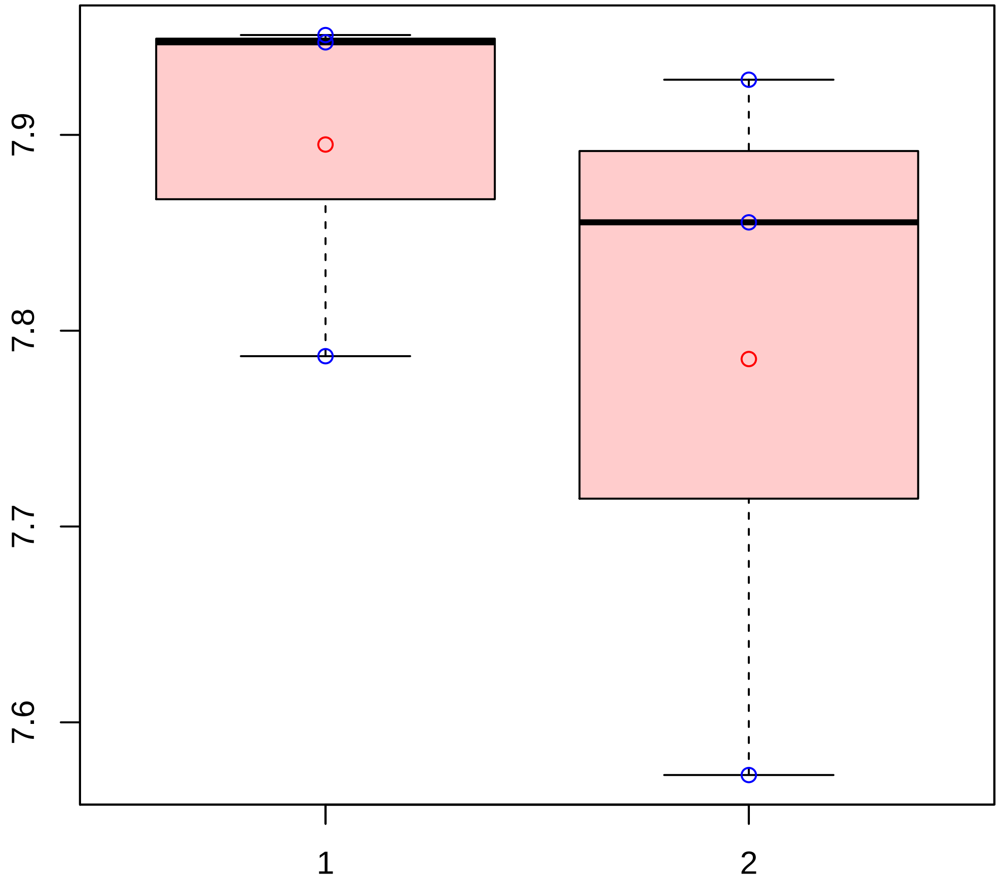


t−Test: p−value = 0.43

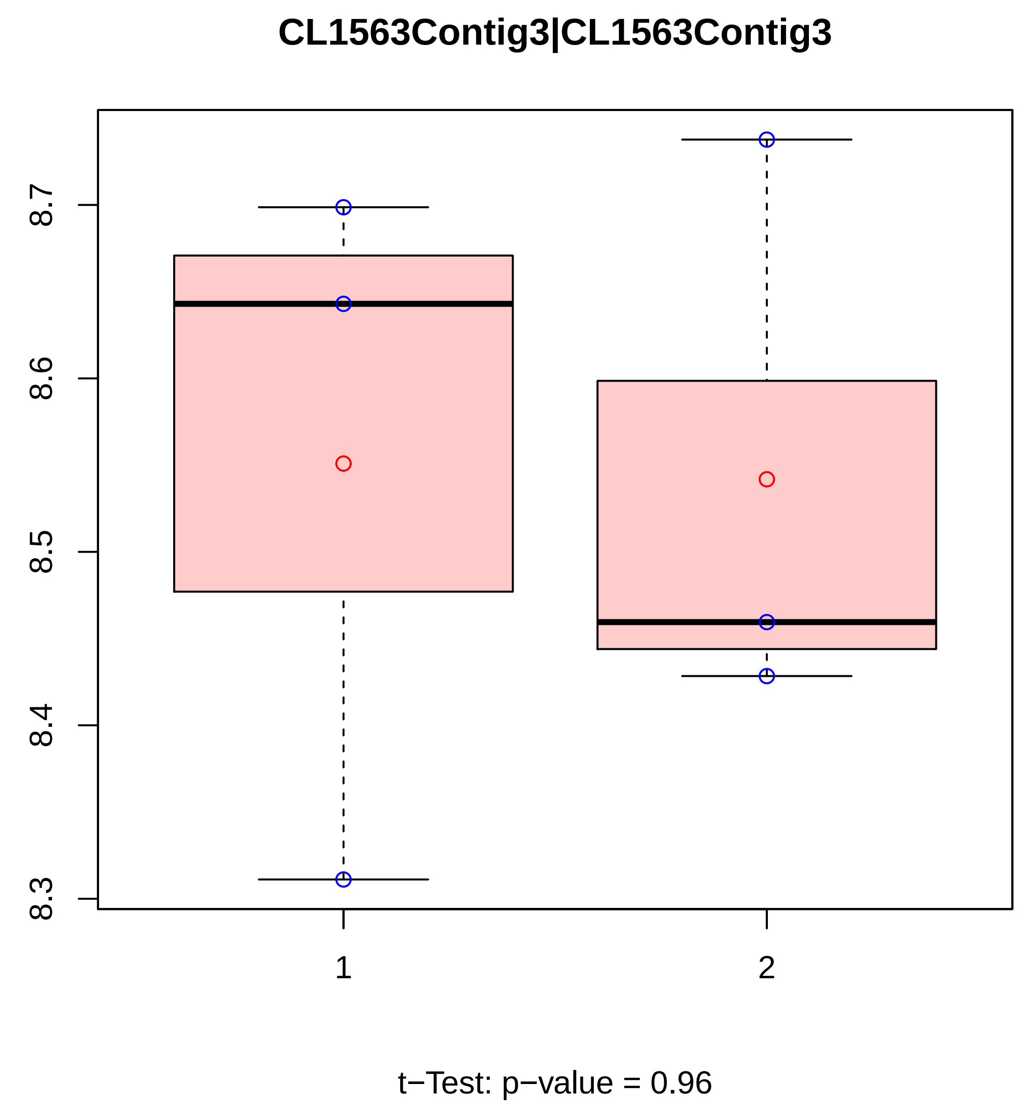
CL1563Contig3|CL1563Contig3
8.3
8.4
8.5
8.6
8.7
1
2
t−Test: p−value = 0.96

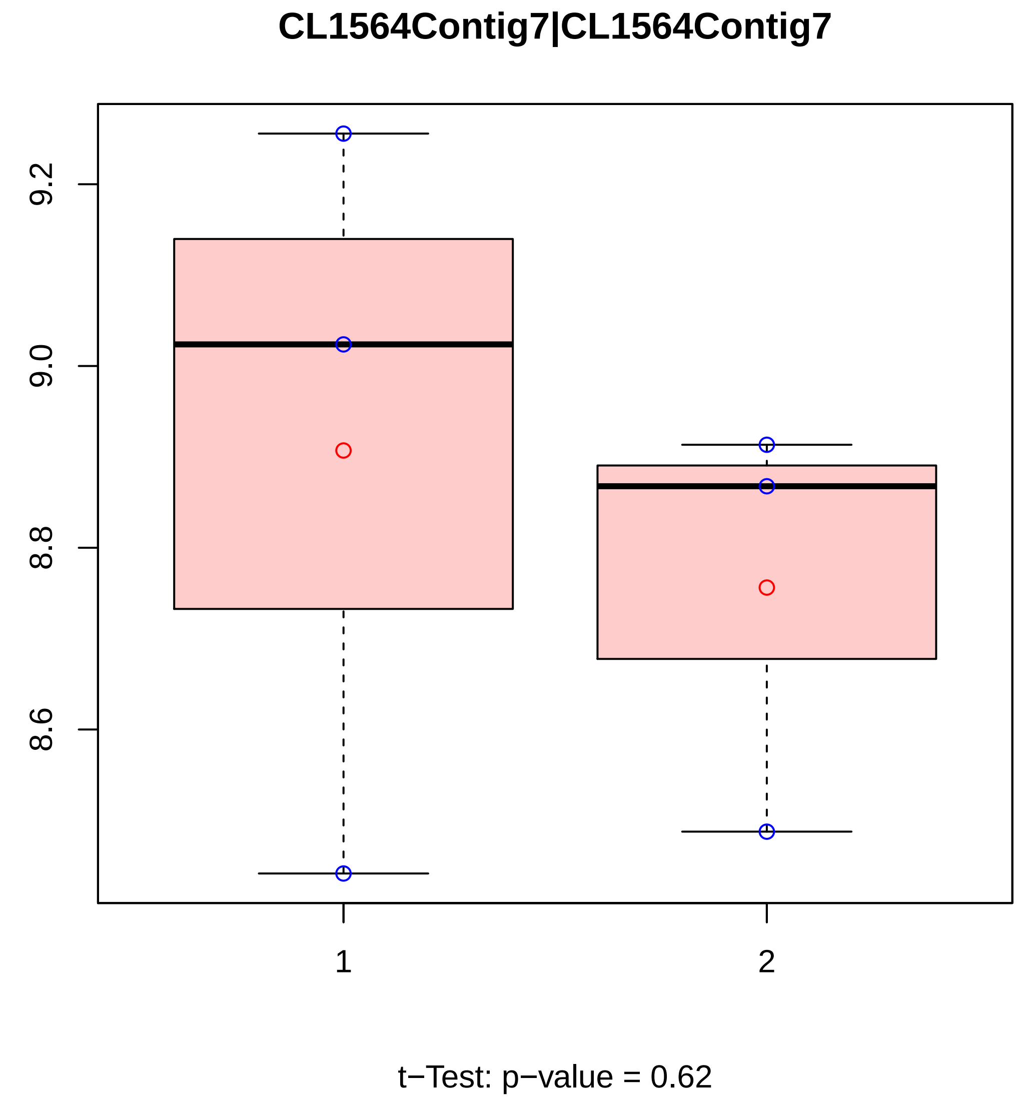
CL1564Contig7|CL1564Contig7
9.2
9.0
8.8
8.6
1
2
t−Test: p−value = 0.62

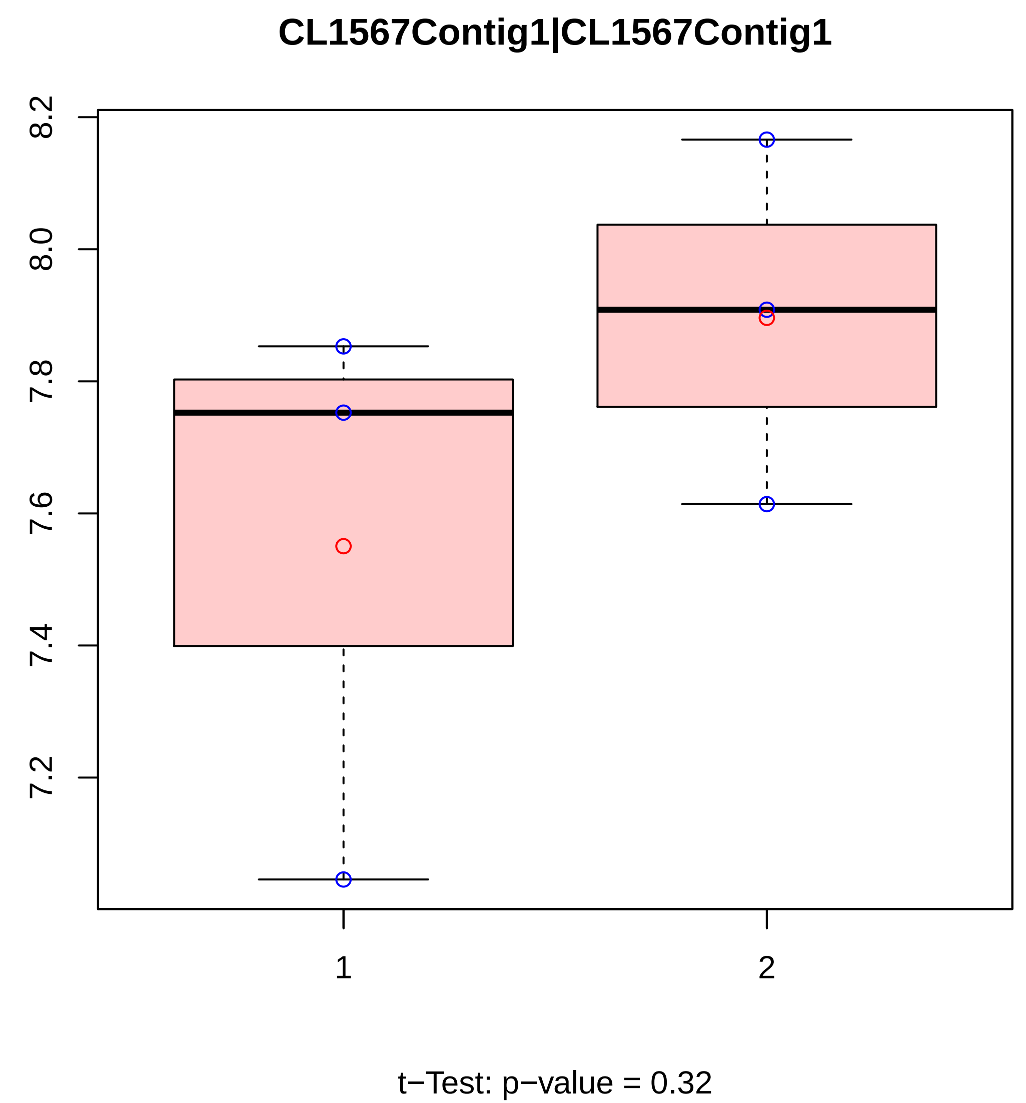

CL1567Contig1|CL1567Contig1
8.2
8.0
7.8
7.6
7.4
7.2
1
2
t−Test: p−value = 0.32

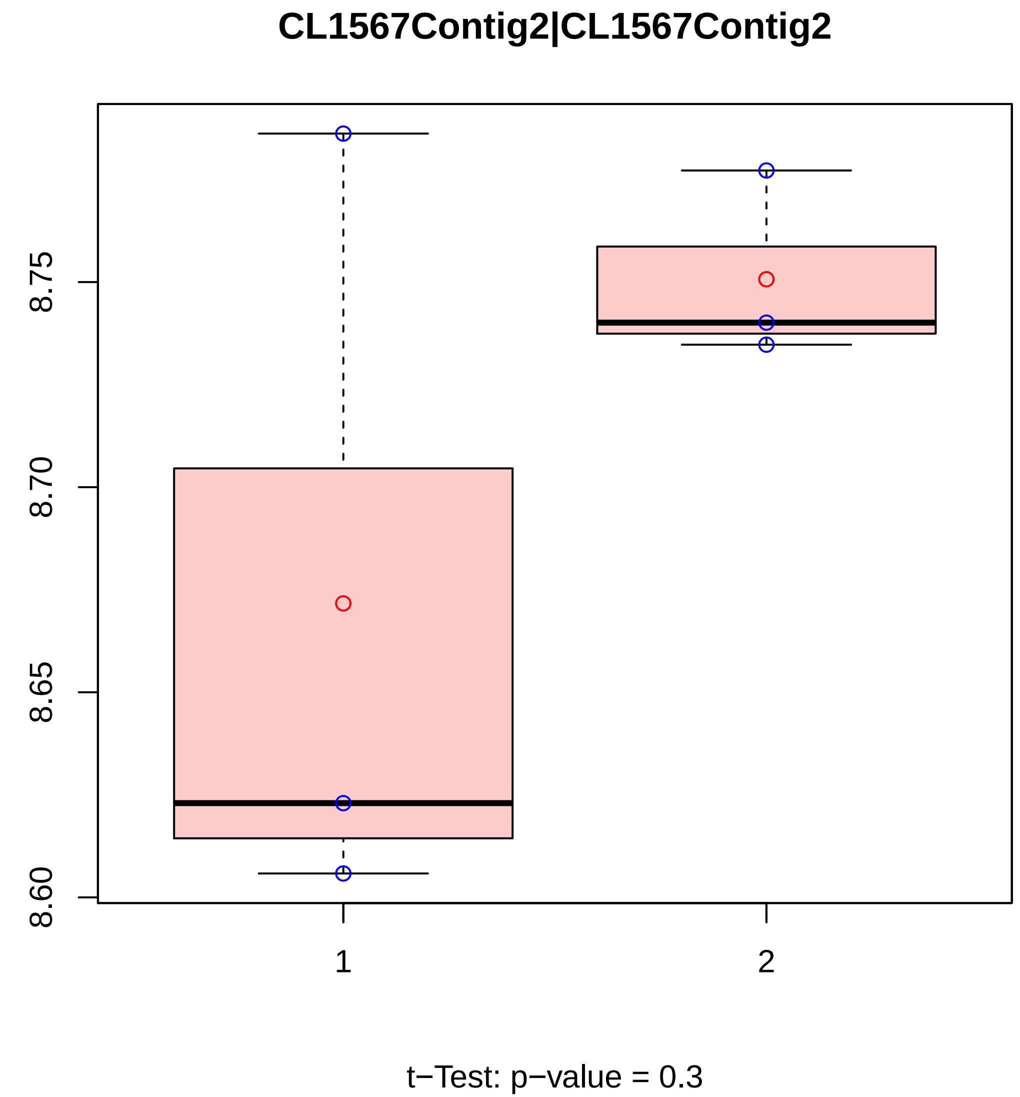

CL1567Contig2|CL1567Contig2
8.60
8.65
8.70
8.75
1
2
t−Test: p−value = 0.3

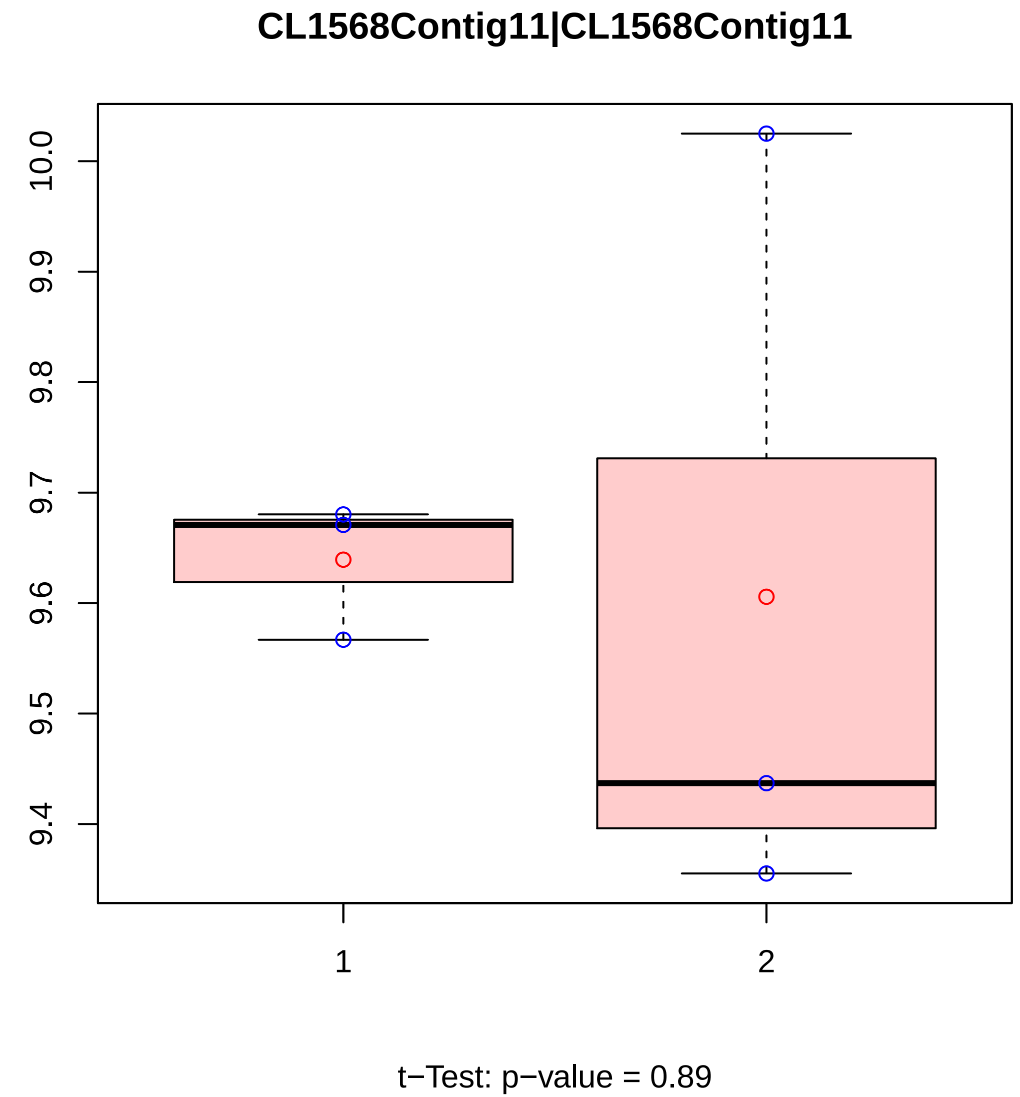
CL1568Contig11|CL1568Contig11
10.0
9.9
9.8
9.7
9.6
9.5
9.4
1
2
t−Test: p−value = 0.89

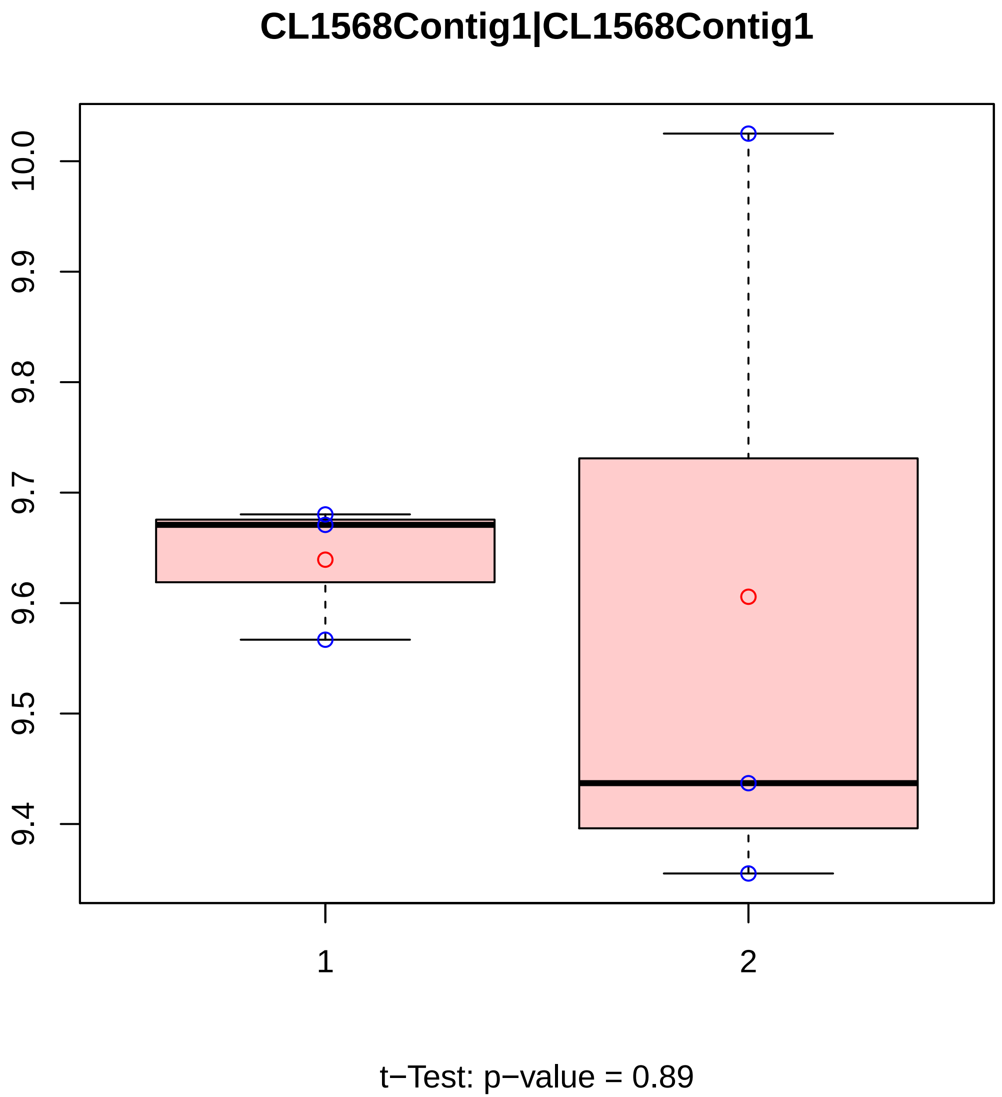
CL1568Contig1|CL1568Contig1
10.0
9.9
9.8
9.7
9.6
9.5
9.4
1
2
t−Test: p−value = 0.89

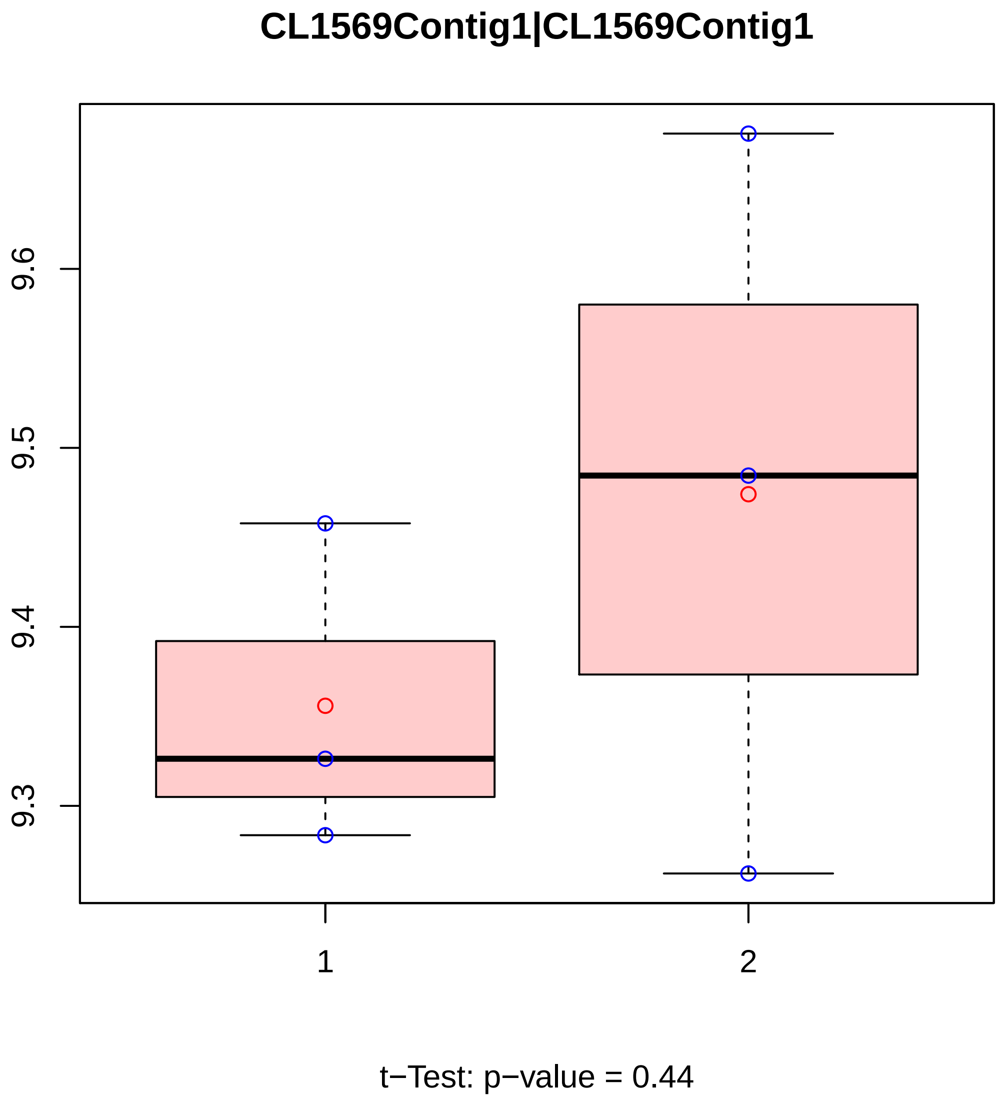
CL1569Contig1|CL1569Contig1
9.3
9.4
9.5
9.6
1
2
t−Test: p−value = 0.44

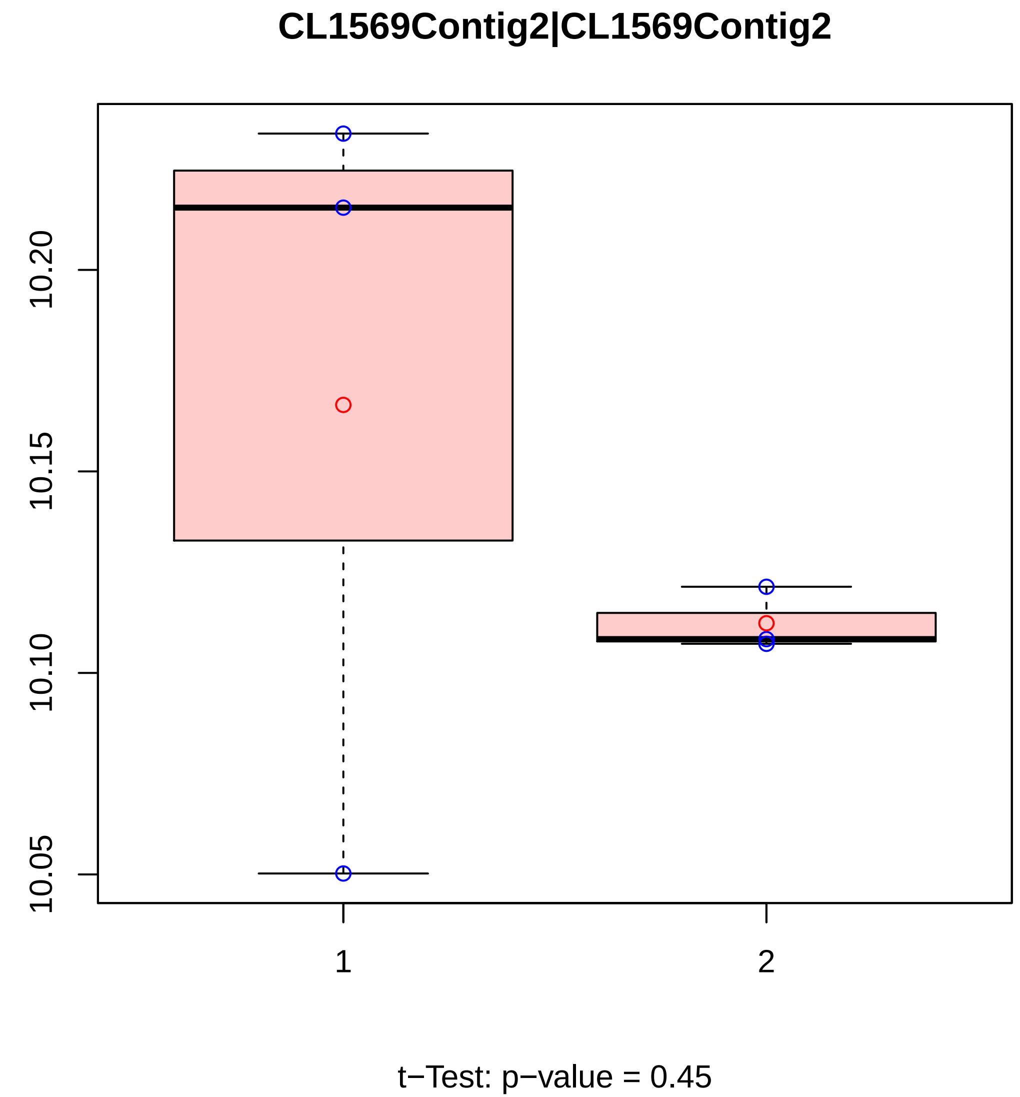
CL1569Contig2|CL1569Contig2
10.05
10.10
10.15
10.20
1
2
t−Test: p−value = 0.45

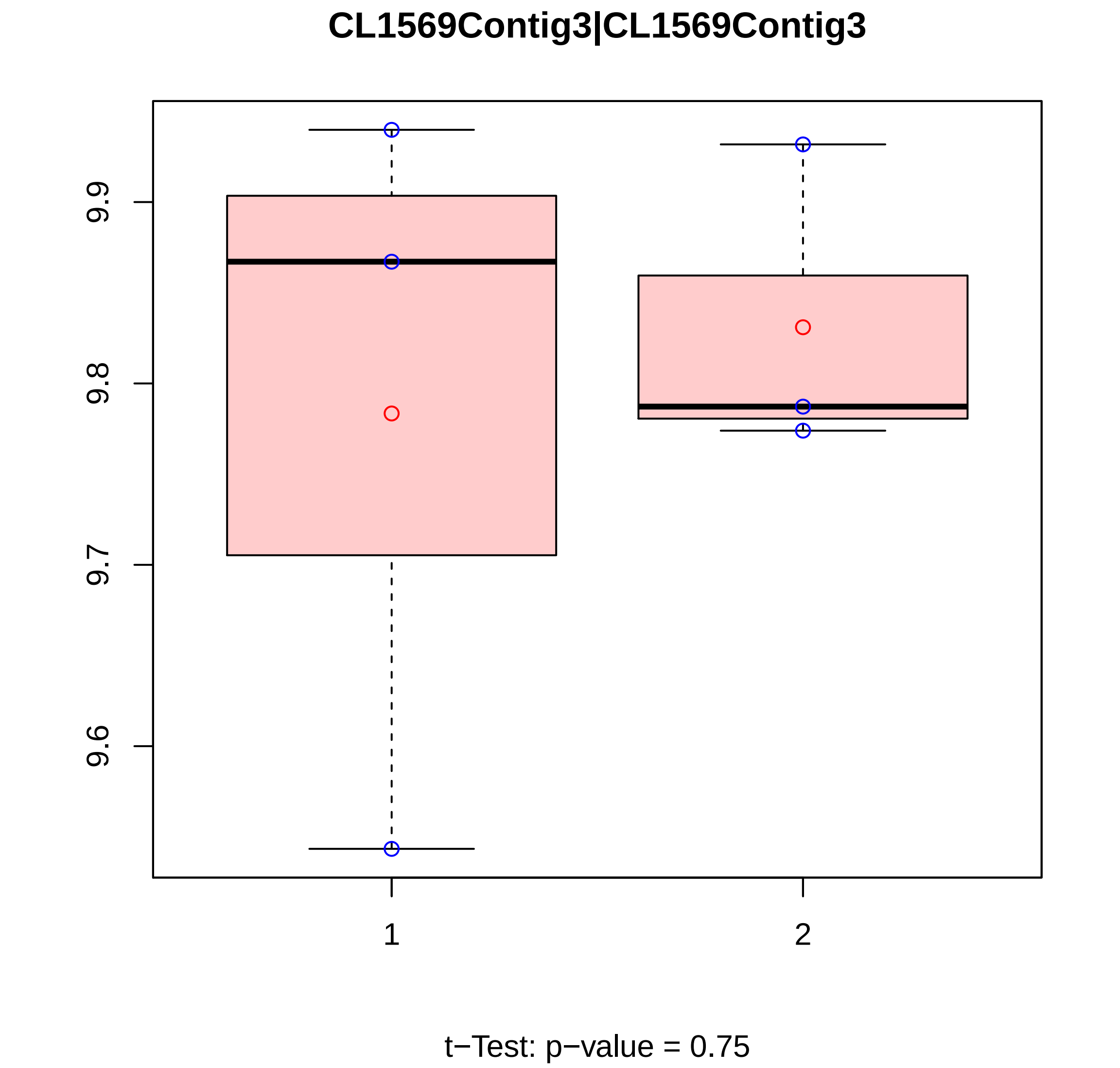
CL1569Contig3|CL1569Contig3
9.9
9.8
9.7
9.6
1
2
t−Test: p−value = 0.75

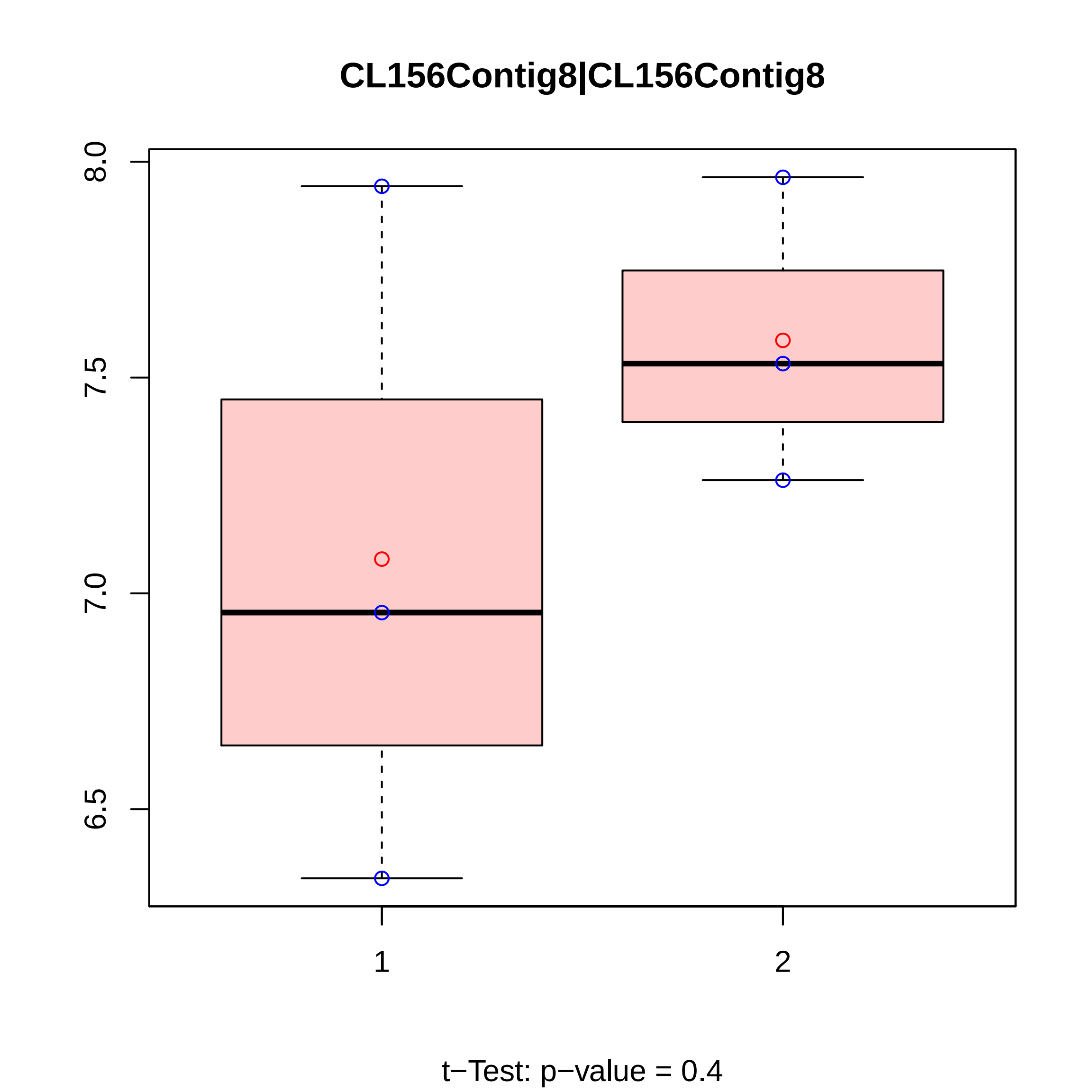
CL156Contig8|CL156Contig8
8.0
7.5
7.0
6.5
1
2
t−Test: p−value = 0.4

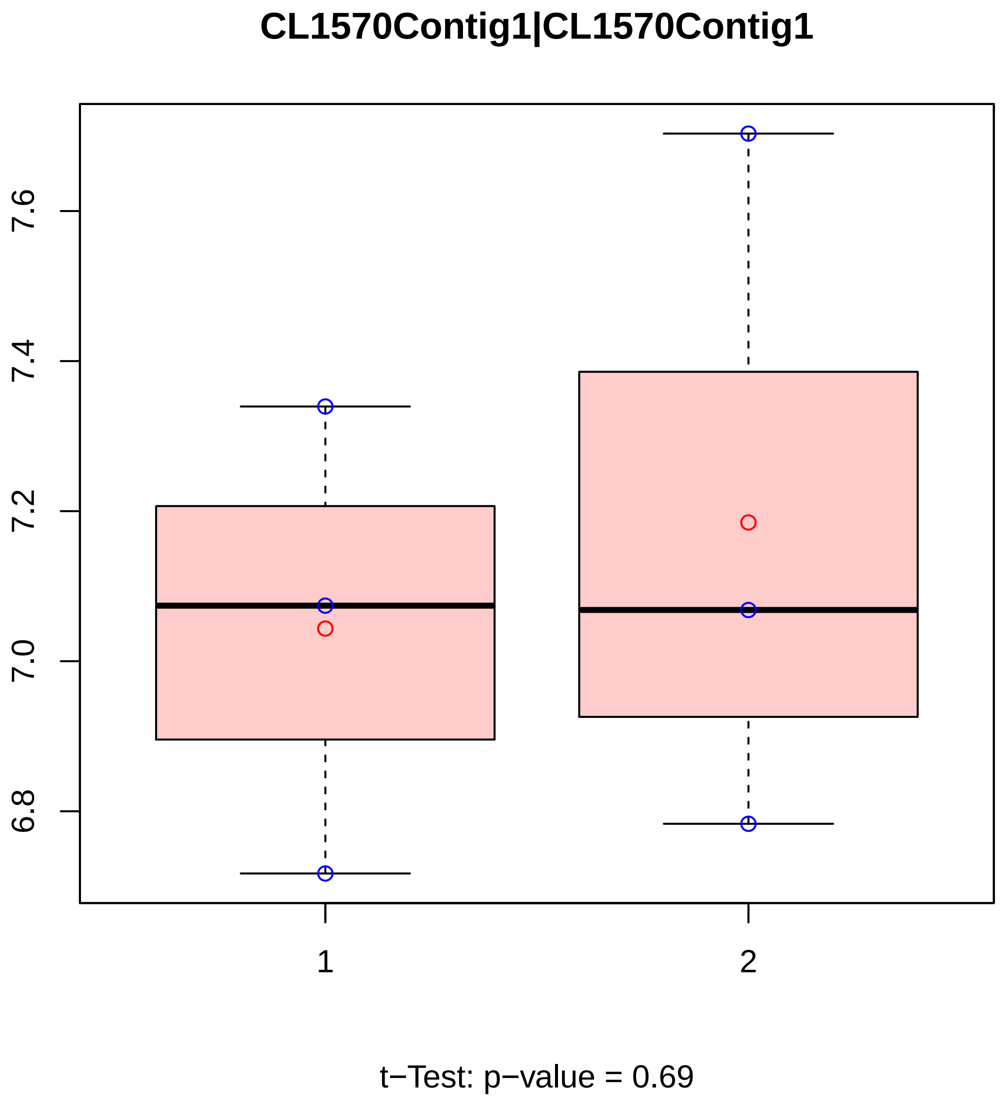
CL1570Contig1|CL1570Contig1
7.6
7.4
7.2
7.0
6.8
1
2
t−Test: p−value = 0.69

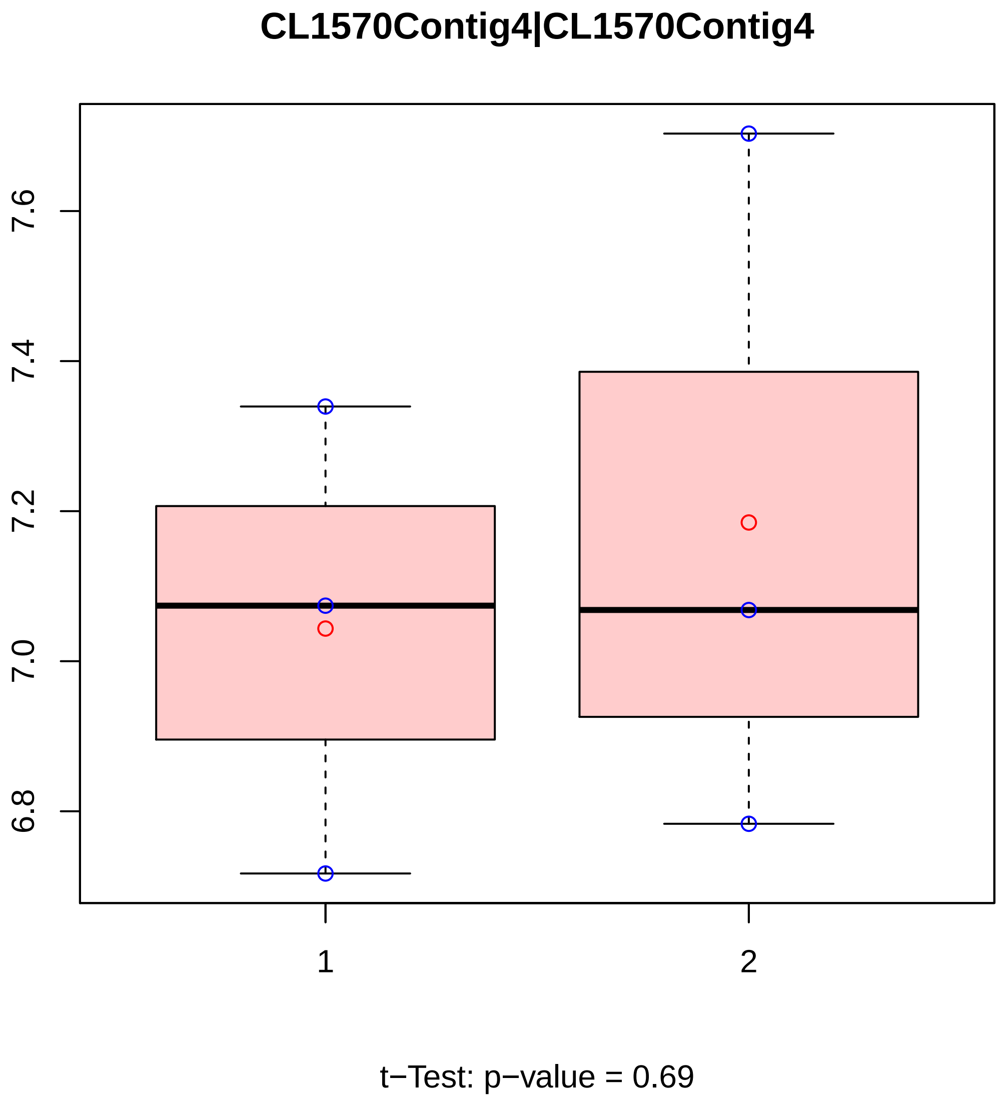
CL1570Contig4|CL1570Contig4
7.6
7.4
7.2
7.0
6.8
1
2
t−Test: p−value = 0.69

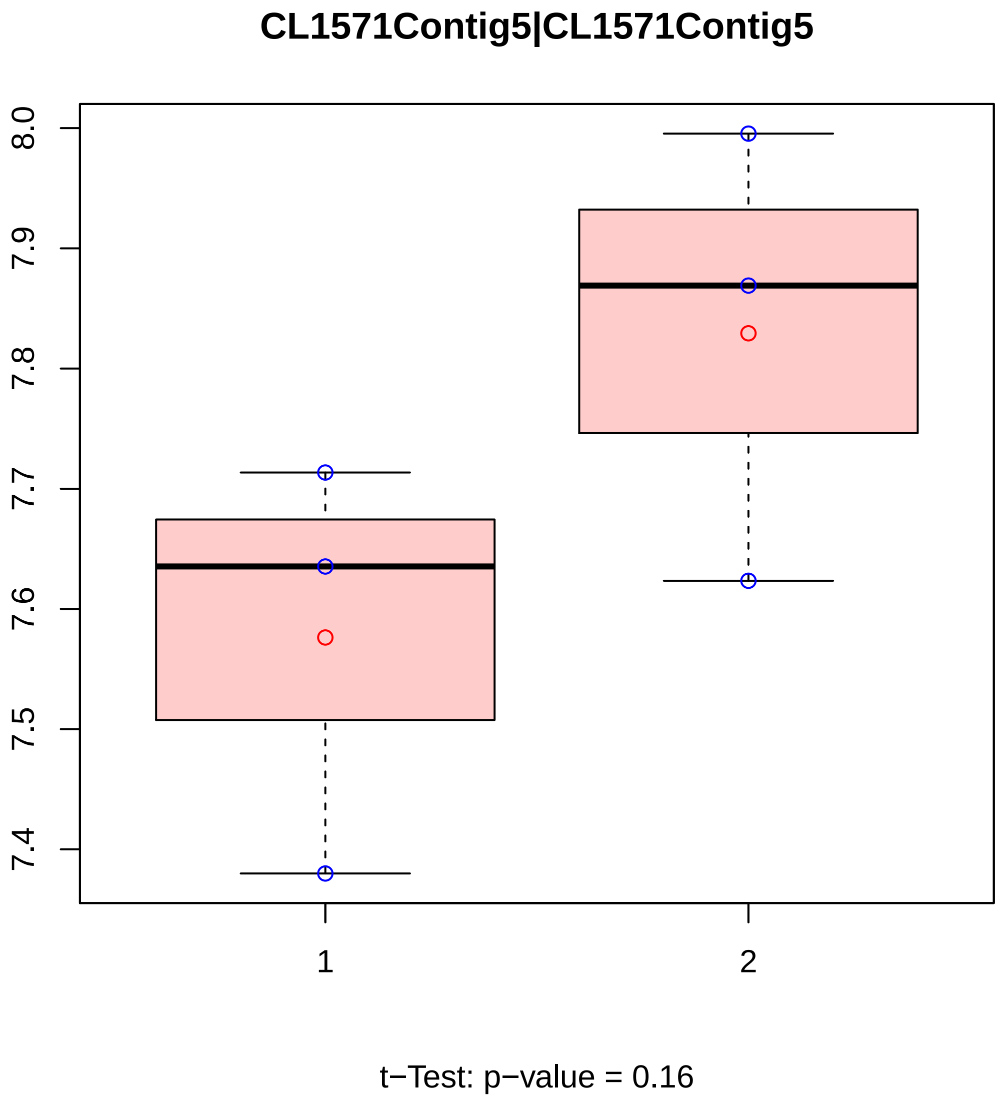

CL1571Contig5|CL1571Contig5
8.0
7.9
7.8
7.7
7.6
7.5
7.4
1
2
t−Test: p−value = 0.16

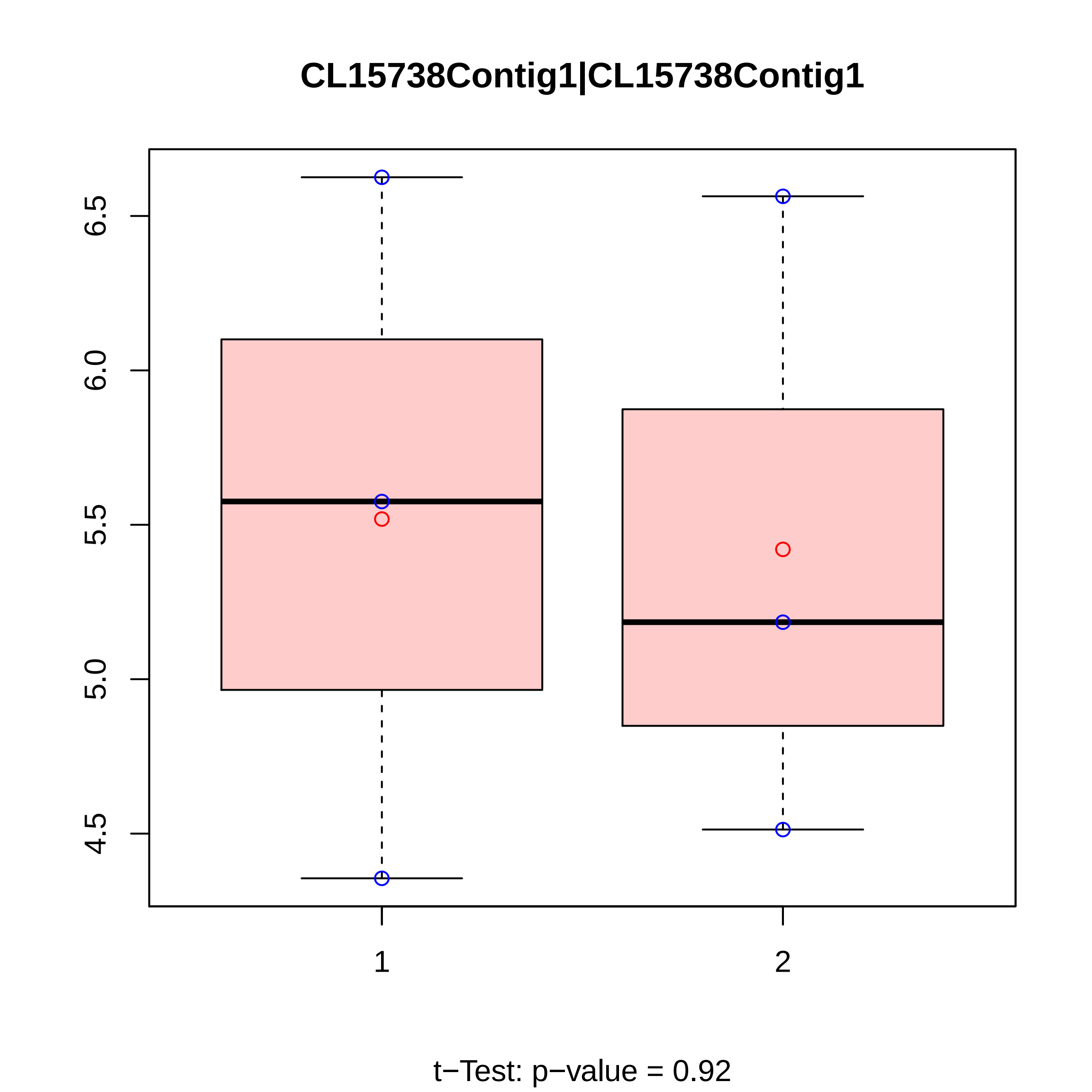

CL15738Contig1|CL15738Contig1
6.5
6.0
5.5
5.0
4.5
1
2
t−Test: p−value = 0.92

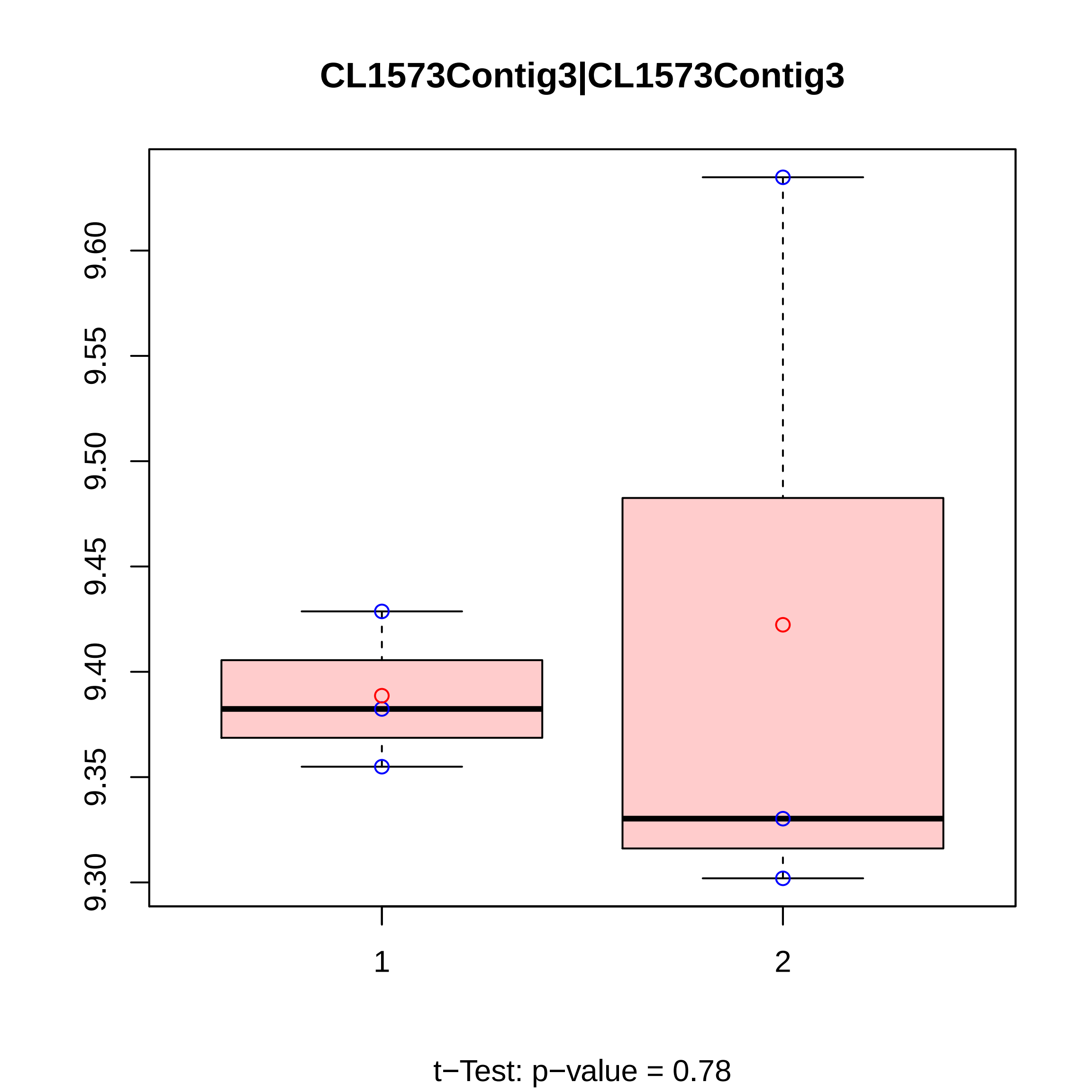
CL1573Contig3|CL1573Contig3
9.30
9.35
9.40
9.45
9.50
9.55
9.60
1
2
t−Test: p−value = 0.78

# CL15782Contig1|CL15782Contig1

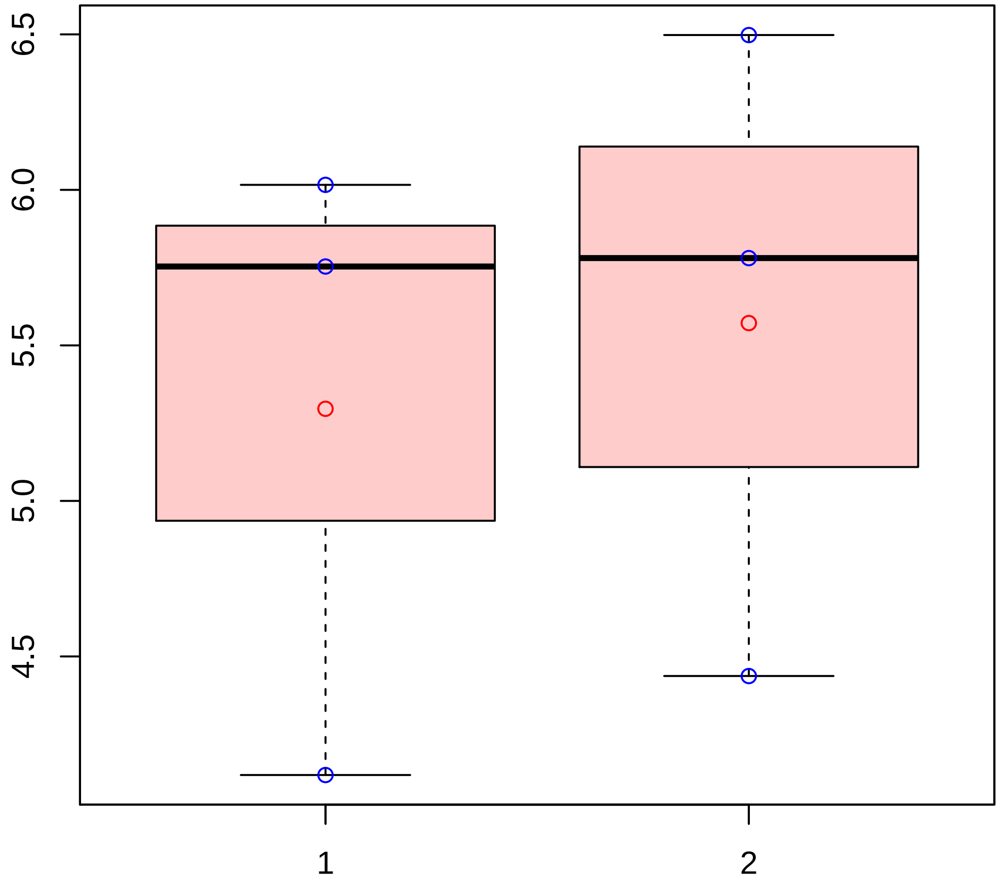


t−Test: p−value = 0.76

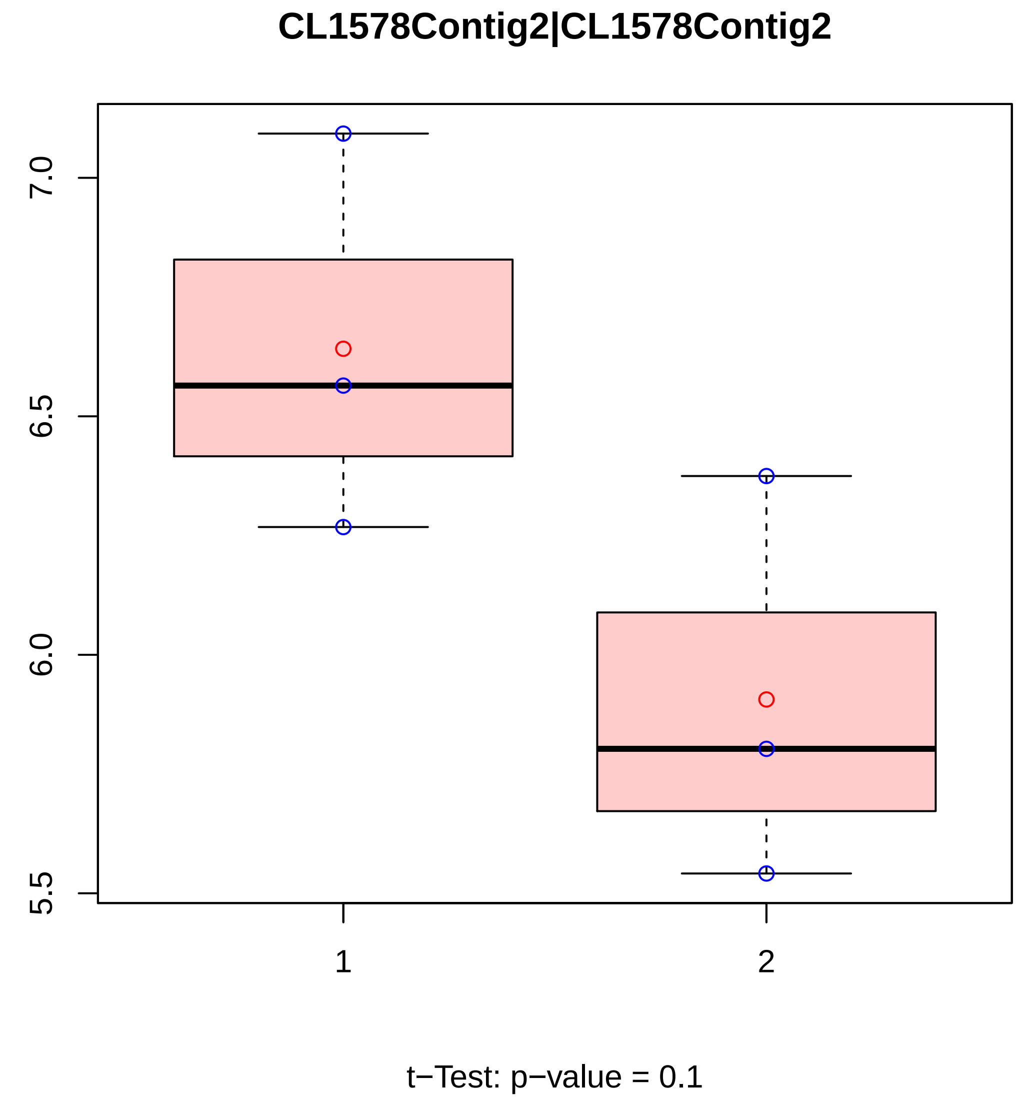
CL1578Contig2|CL1578Contig2
7.0
6.5
6.0
5.5
1
2
t−Test: p−value = 0.1

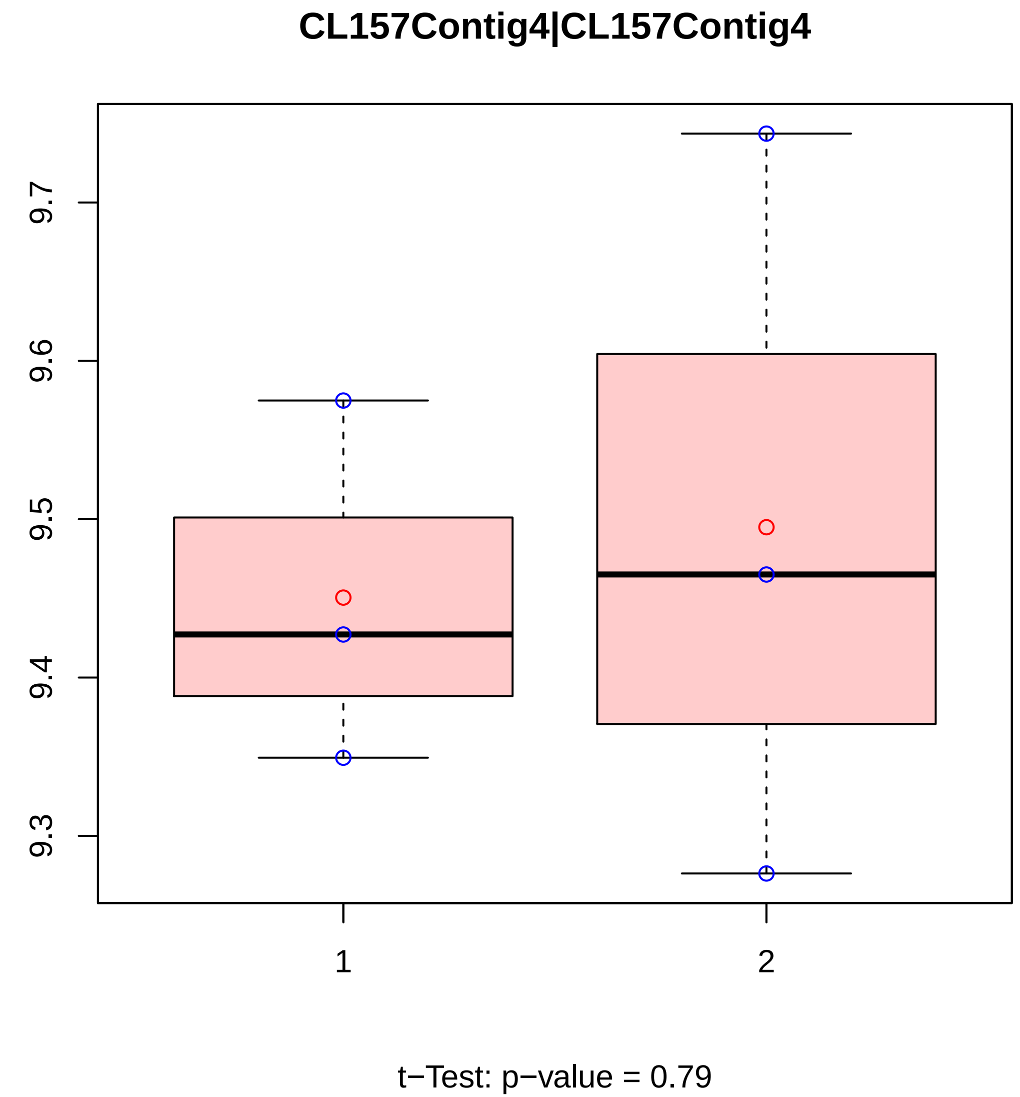
CL157Contig4|CL157Contig4
9.3
9.4
9.5
9.6
9.7
1
2
t−Test: p−value = 0.79

## CL1580Contig2|CL1580Contig2

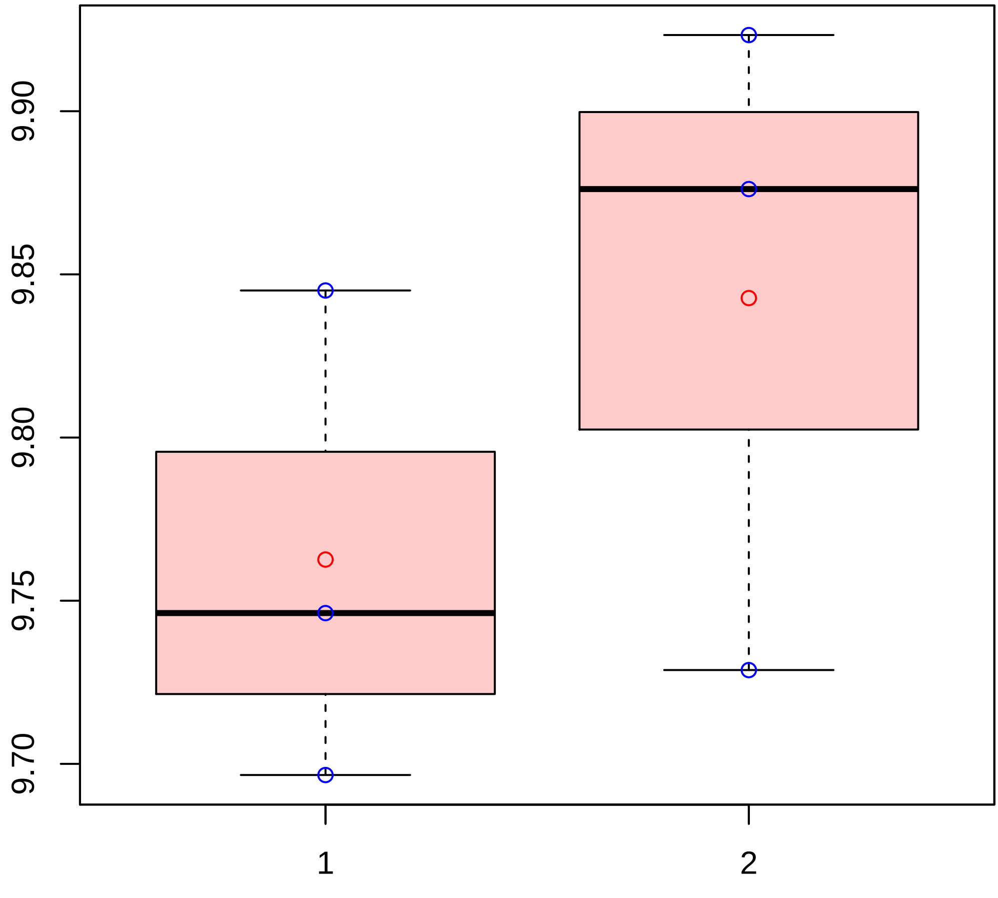


t−Test: p−value = 0.34

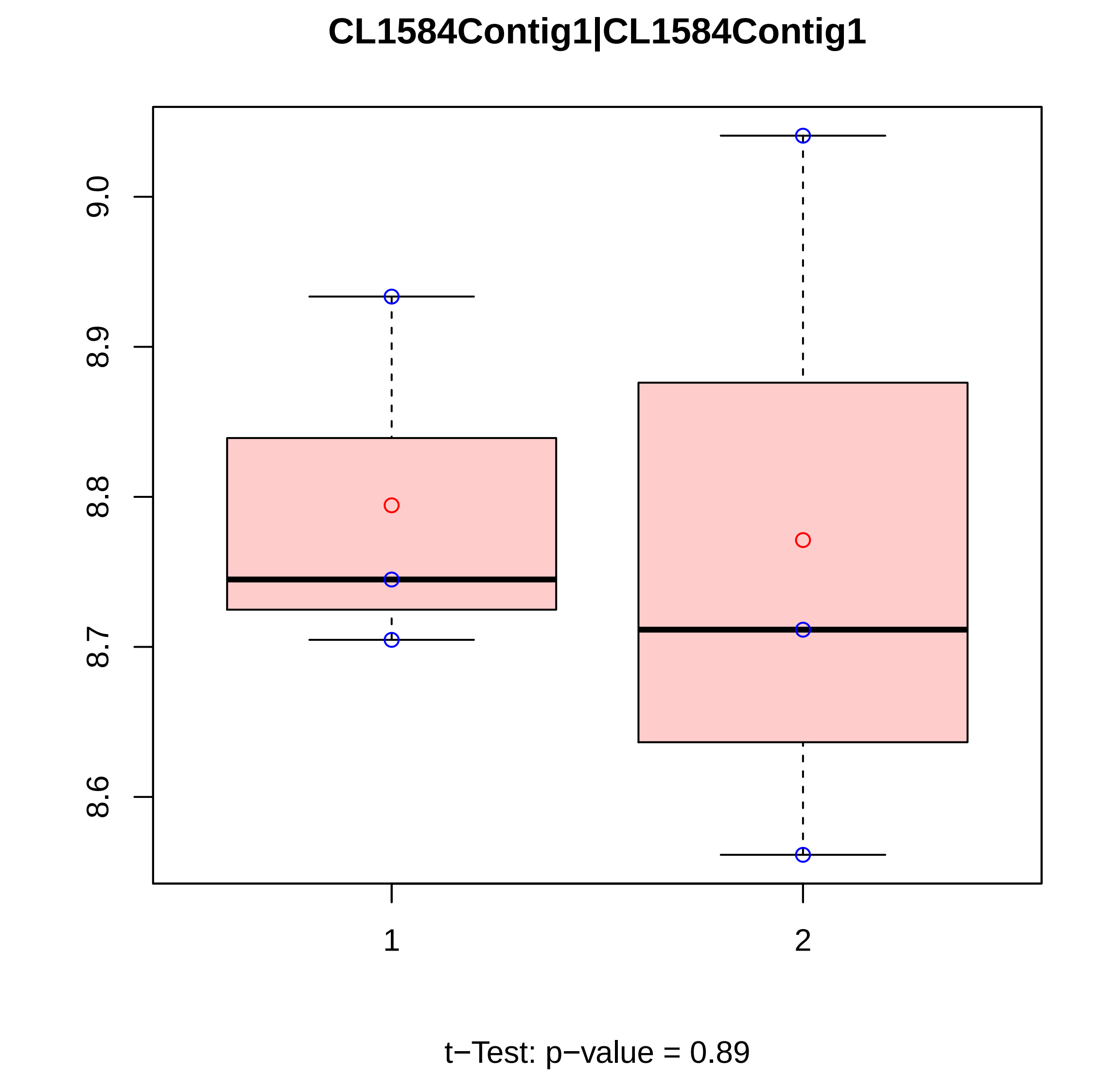
CL1584Contig1|CL1584Contig1
9.0
8.9
8.8
8.7
8.6
1
2
t−Test: p−value = 0.89

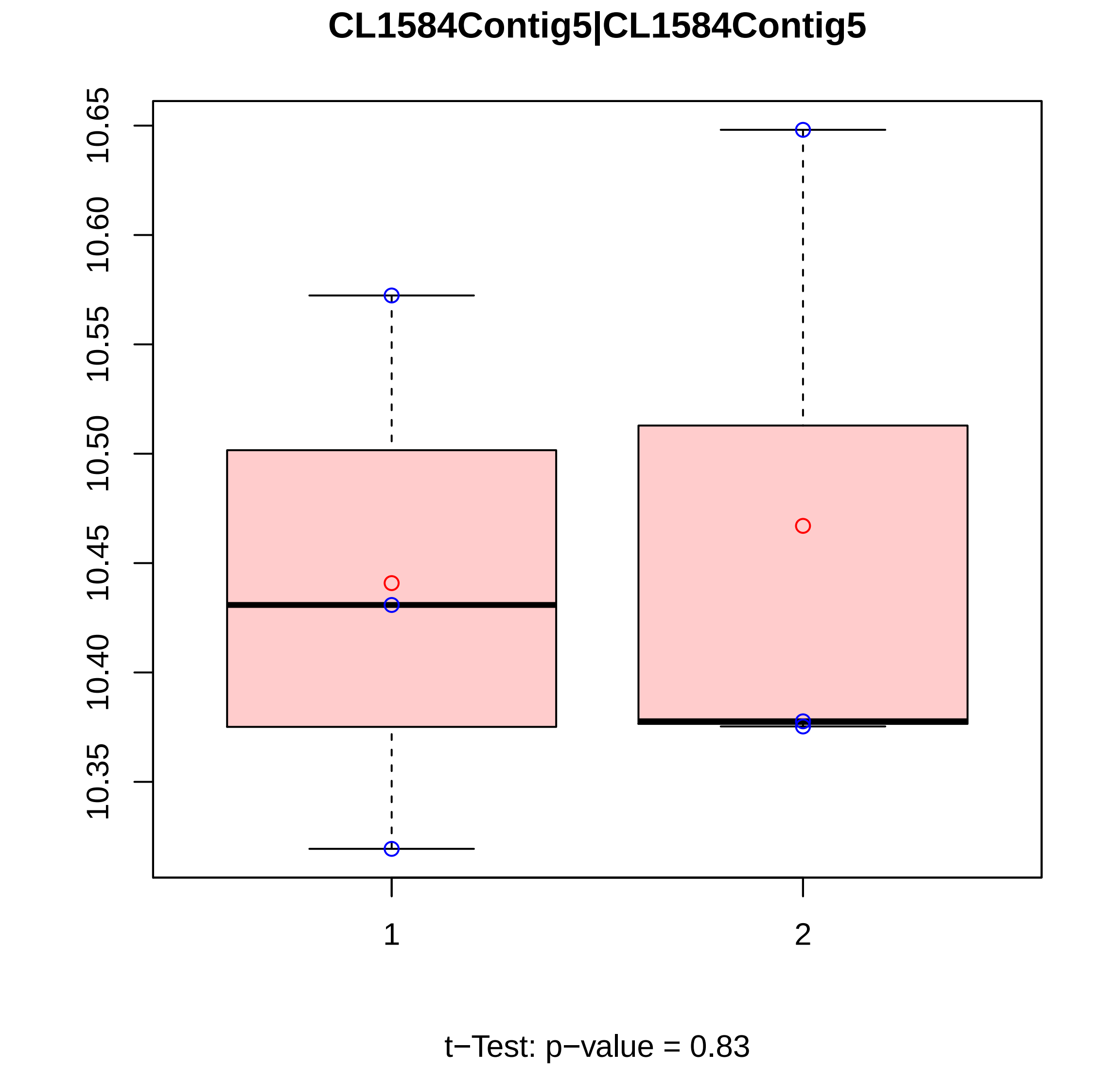
CL1584Contig5|CL1584Contig5
10.35
10.40
10.45
10.50
10.55
10.60
10.65
1
2
t−Test: p−value = 0.83

# CL15873Contig1|CL15873Contig1

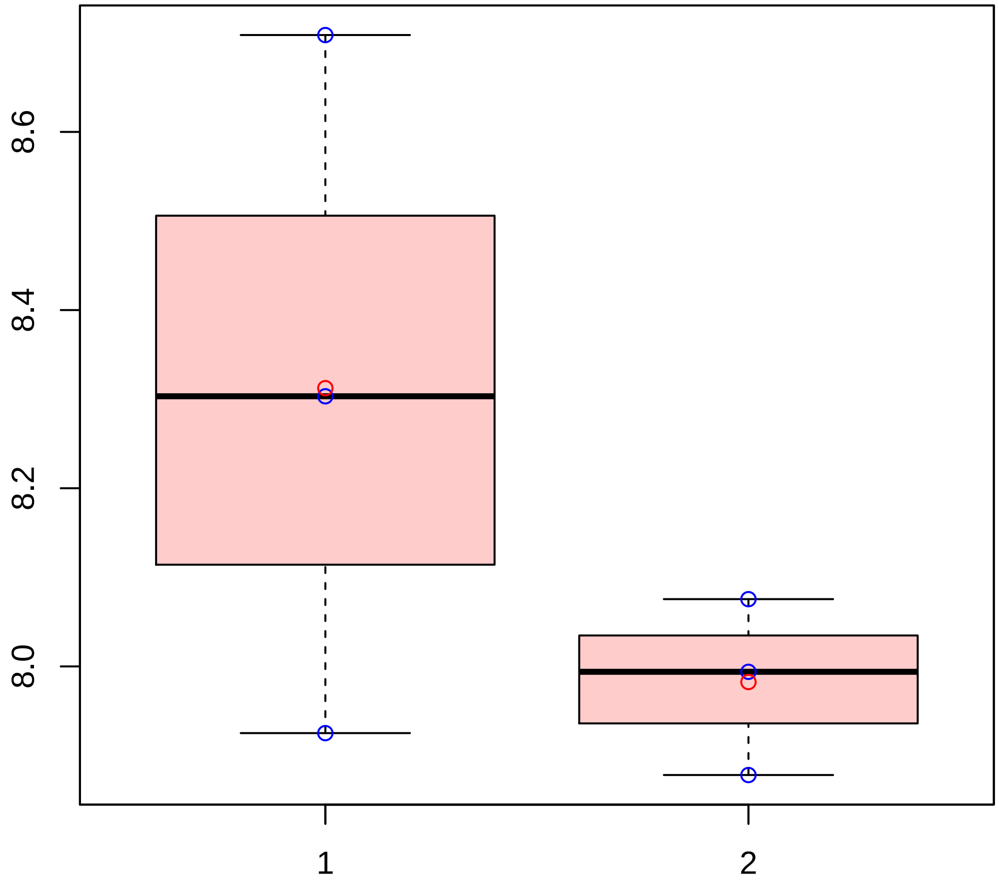


t−Test: p−value = 0.28

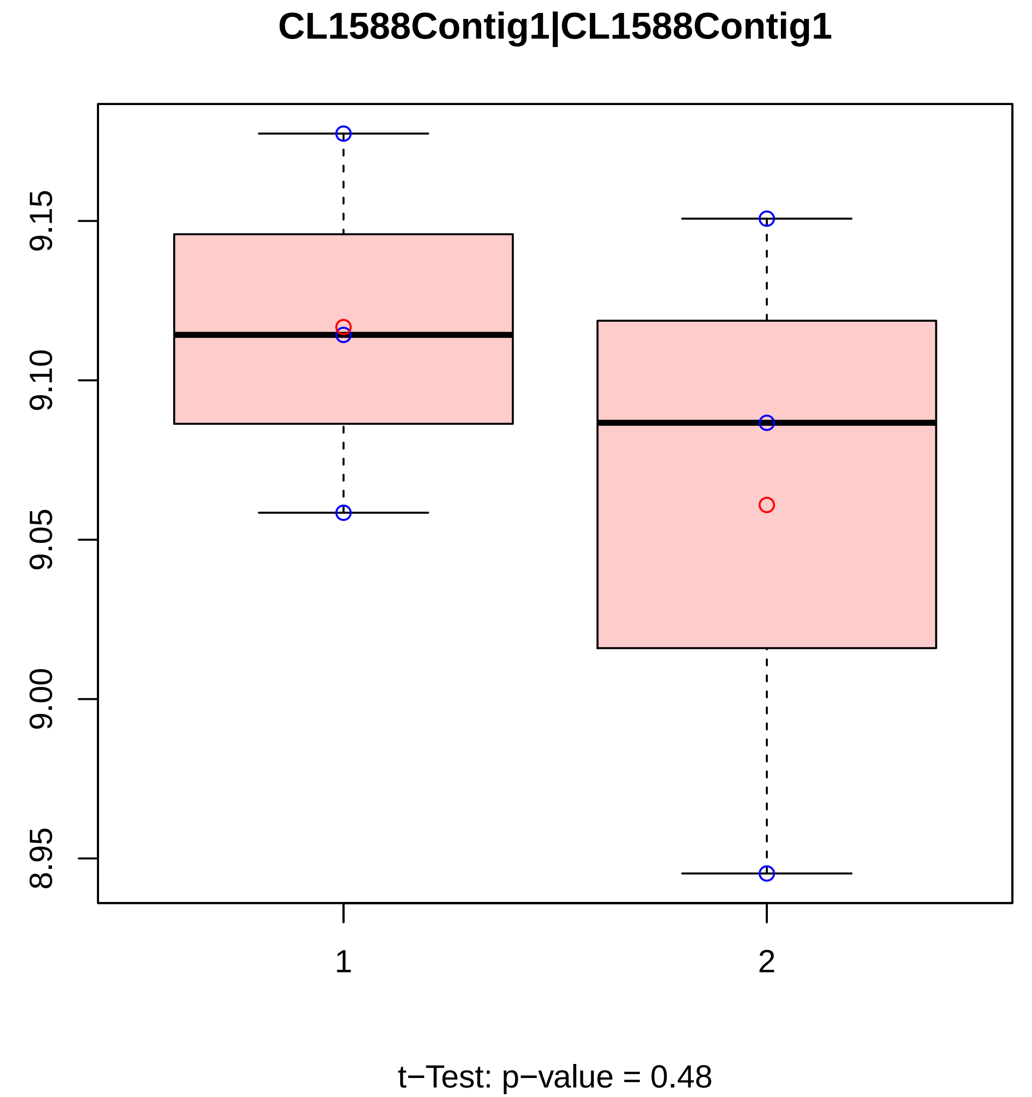
CL1588Contig1|CL1588Contig1
9.15
9.10
9.05
9.00
8.95
1
2
t−Test: p−value = 0.48

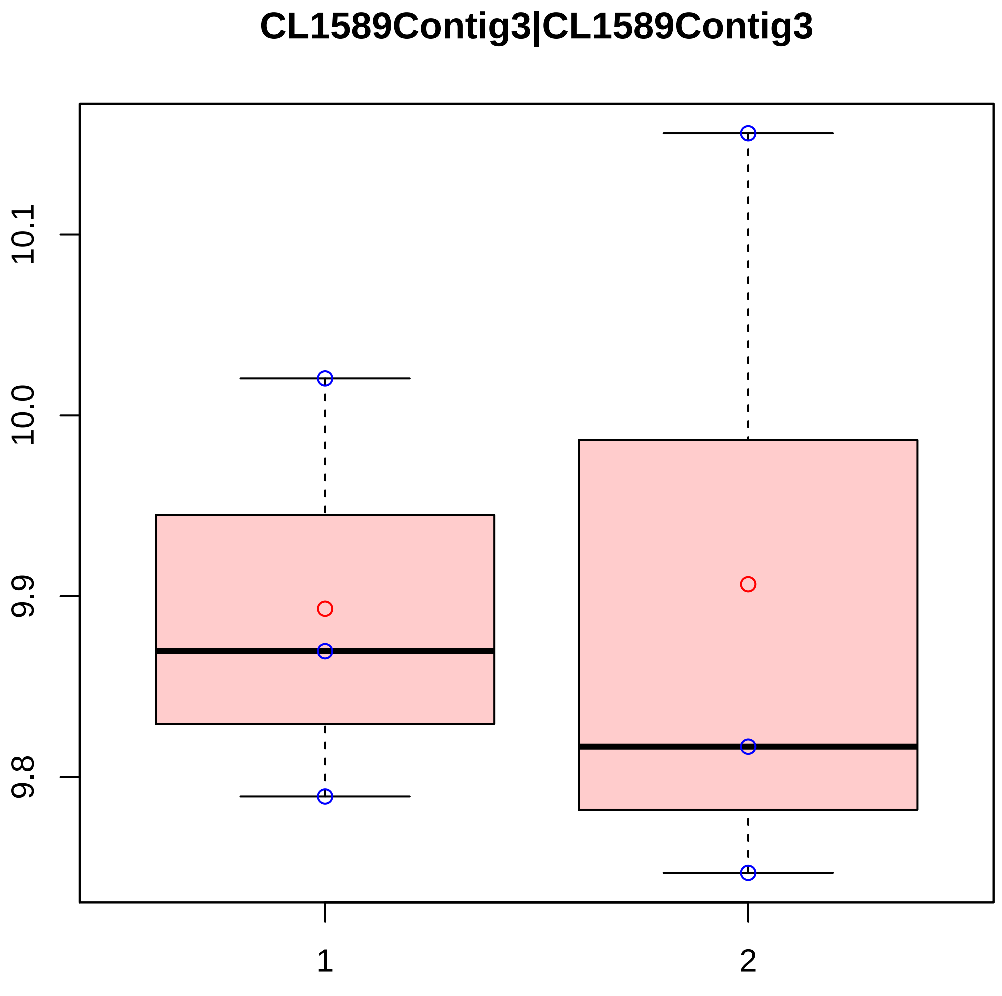


t−Test: p−value = 0.93

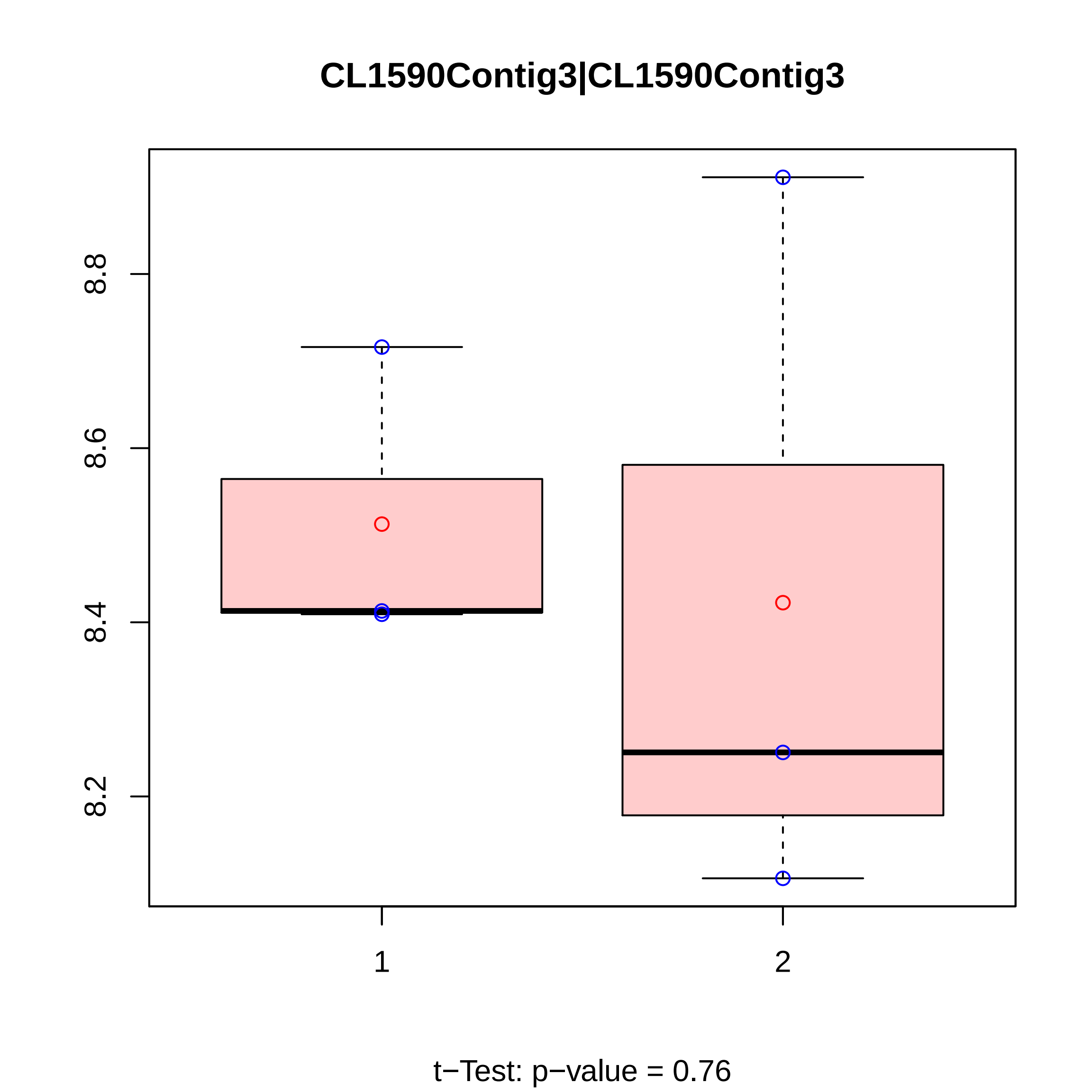

CL1590Contig3|CL1590Contig3
8.2
8.4
8.6
8.8
1
2
t−Test: p−value = 0.76

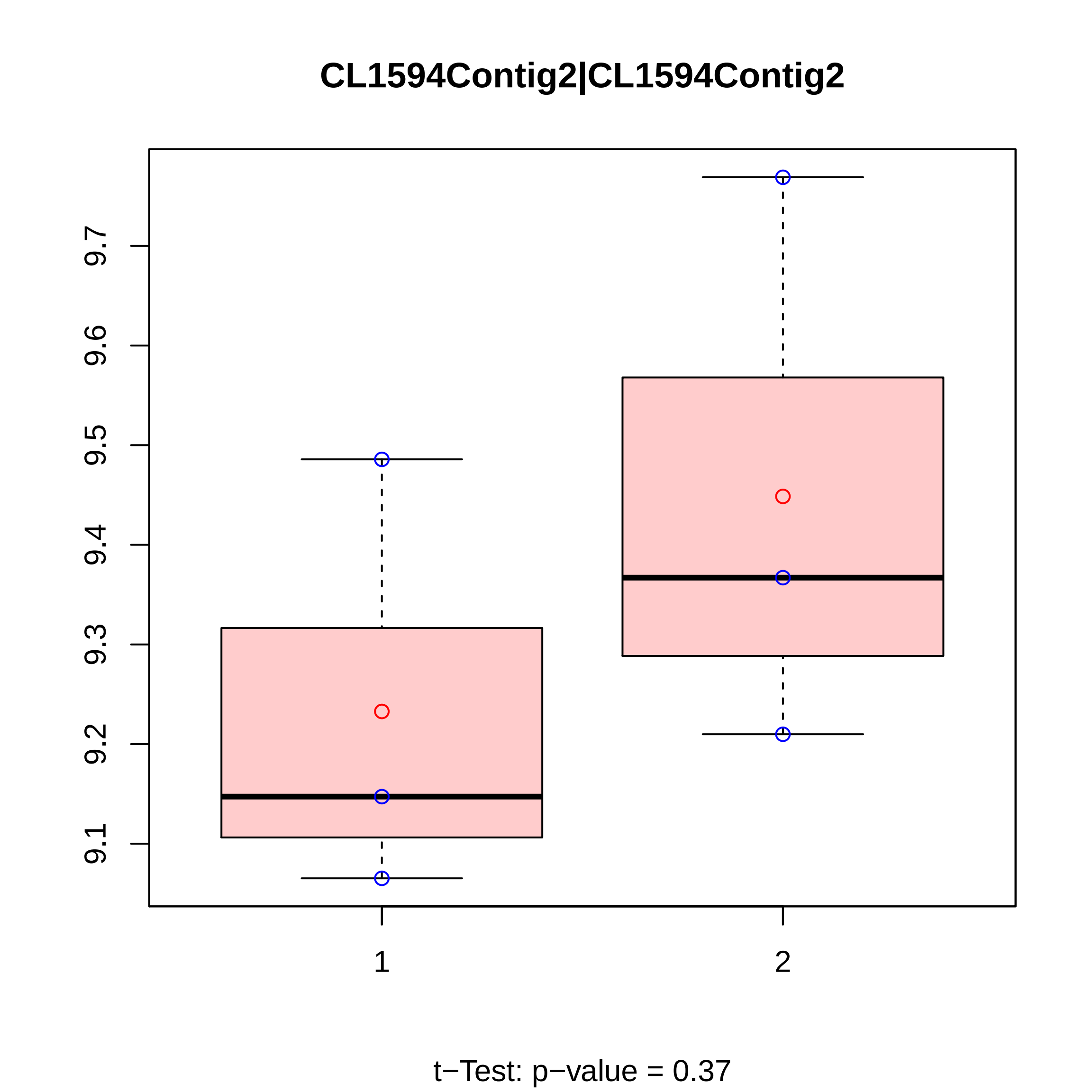

CL1594Contig2|CL1594Contig2
9.1
9.2
9.3
9.4
9.5
9.6
9.7
1
2
t−Test: p−value = 0.37

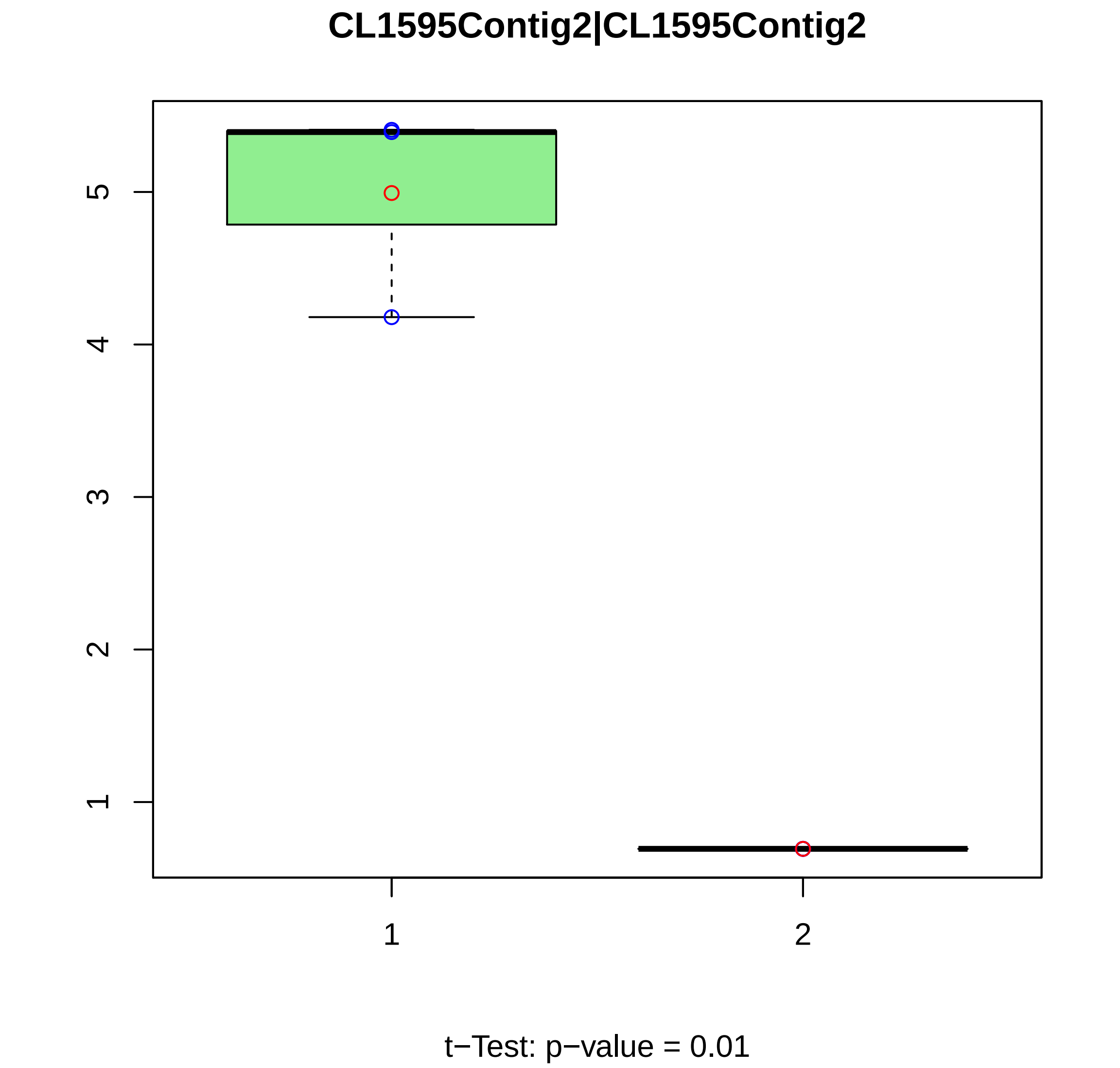

CL1595Contig2|CL1595Contig2
1
2
3
4
5
1
2
t−Test: p−value = 0.01

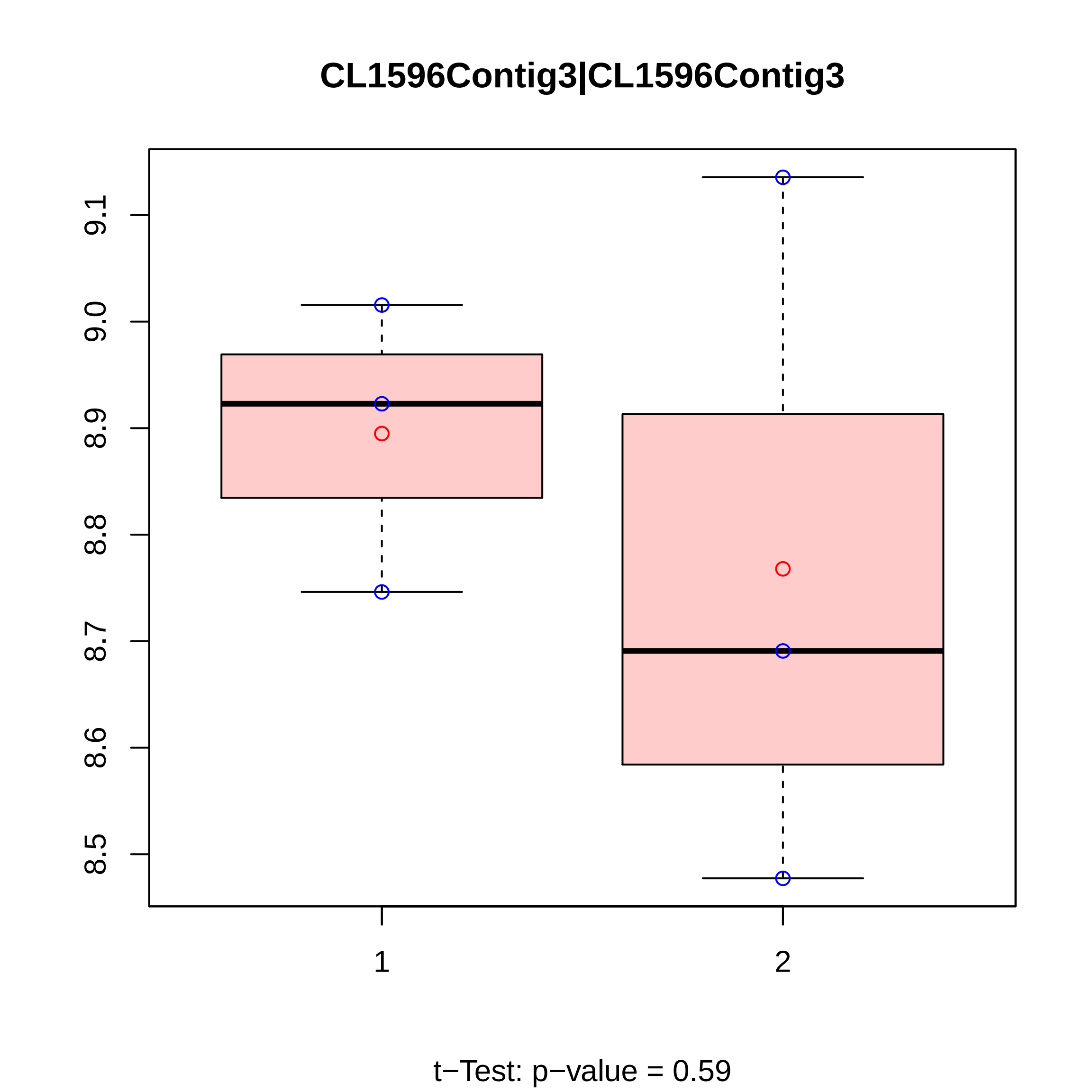
CL1596Contig3|CL1596Contig3
9.1
9.0
8.9
8.8
8.7
8.6
8.5
1
2
t−Test: p−value = 0.59

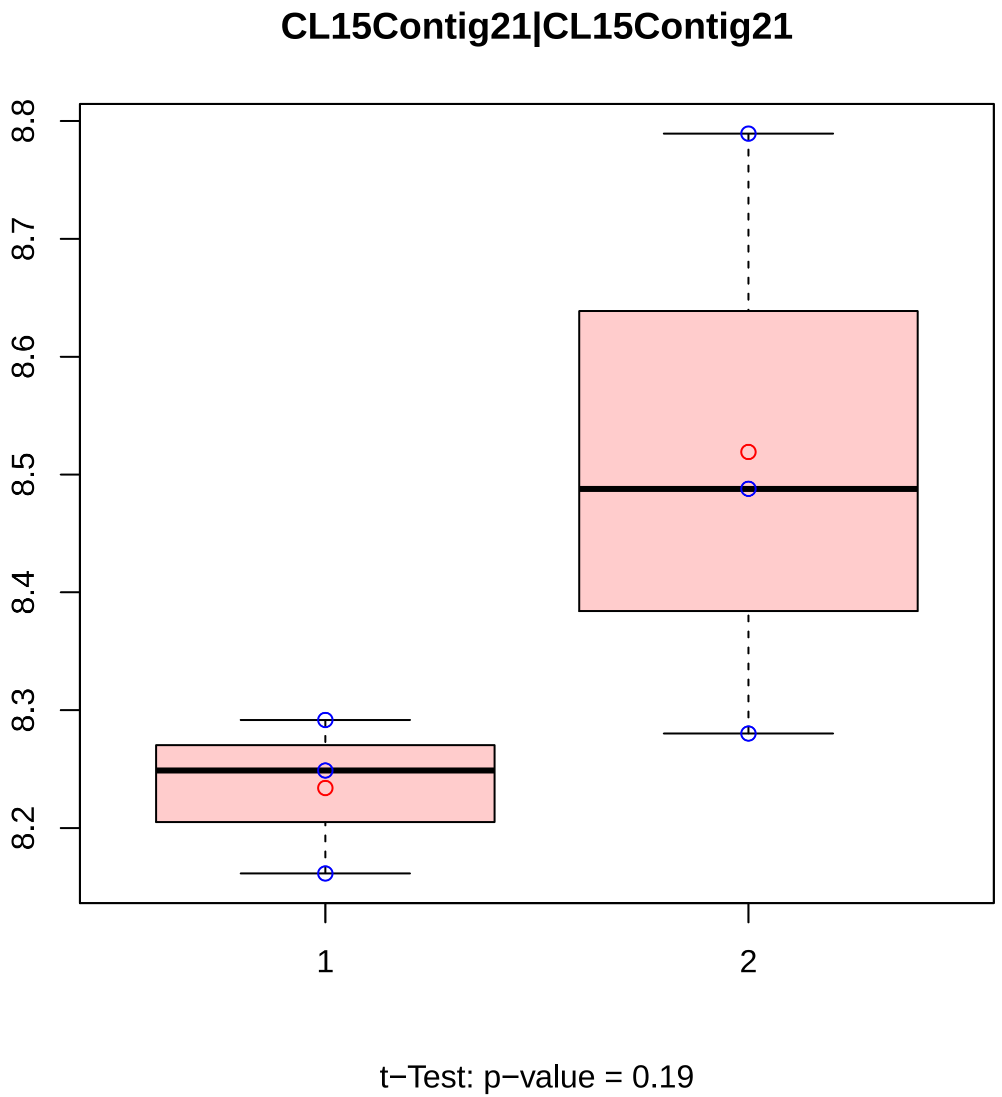
CL15Contig21|CL15Contig21
8.2
8.3
8.4
8.5
8.6
8.7
8.8
1
2
t−Test: p−value = 0.19

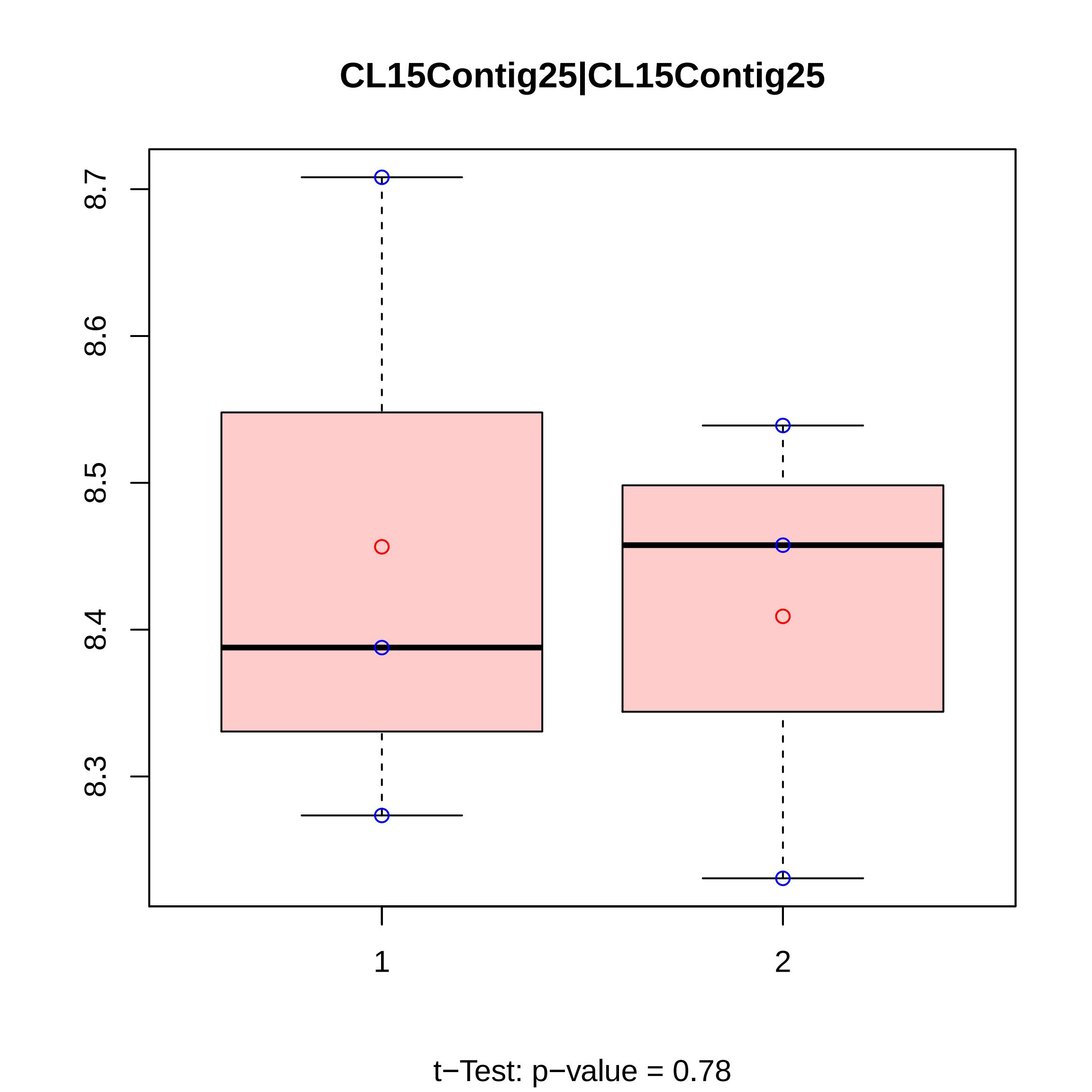
CL15Contig25|CL15Contig25
8.7
8.6
8.5
8.4
8.3
1
2
t−Test: p−value = 0.78

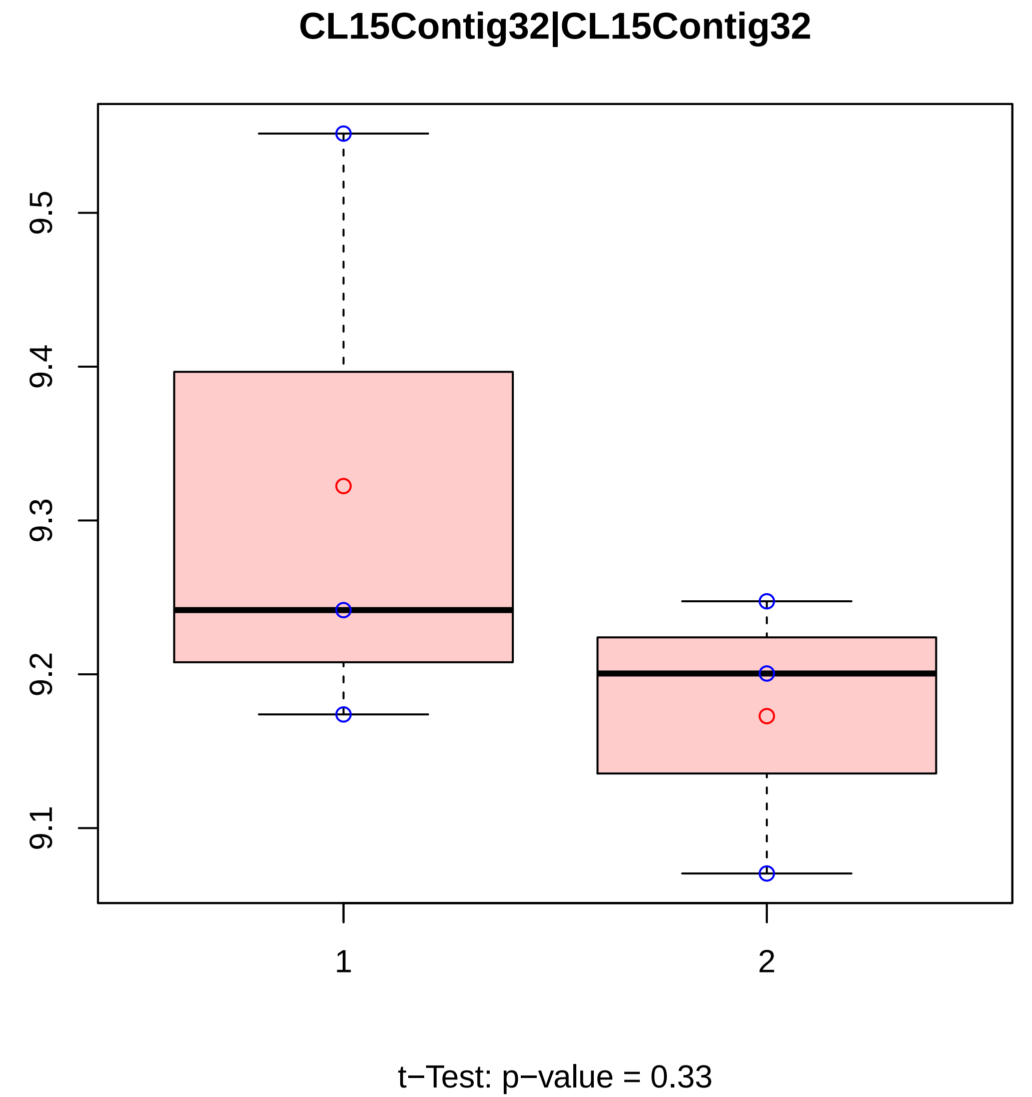

CL15Contig32|CL15Contig32
9.1
9.2
9.3
9.4
9.5
1
2
t−Test: p−value = 0.33

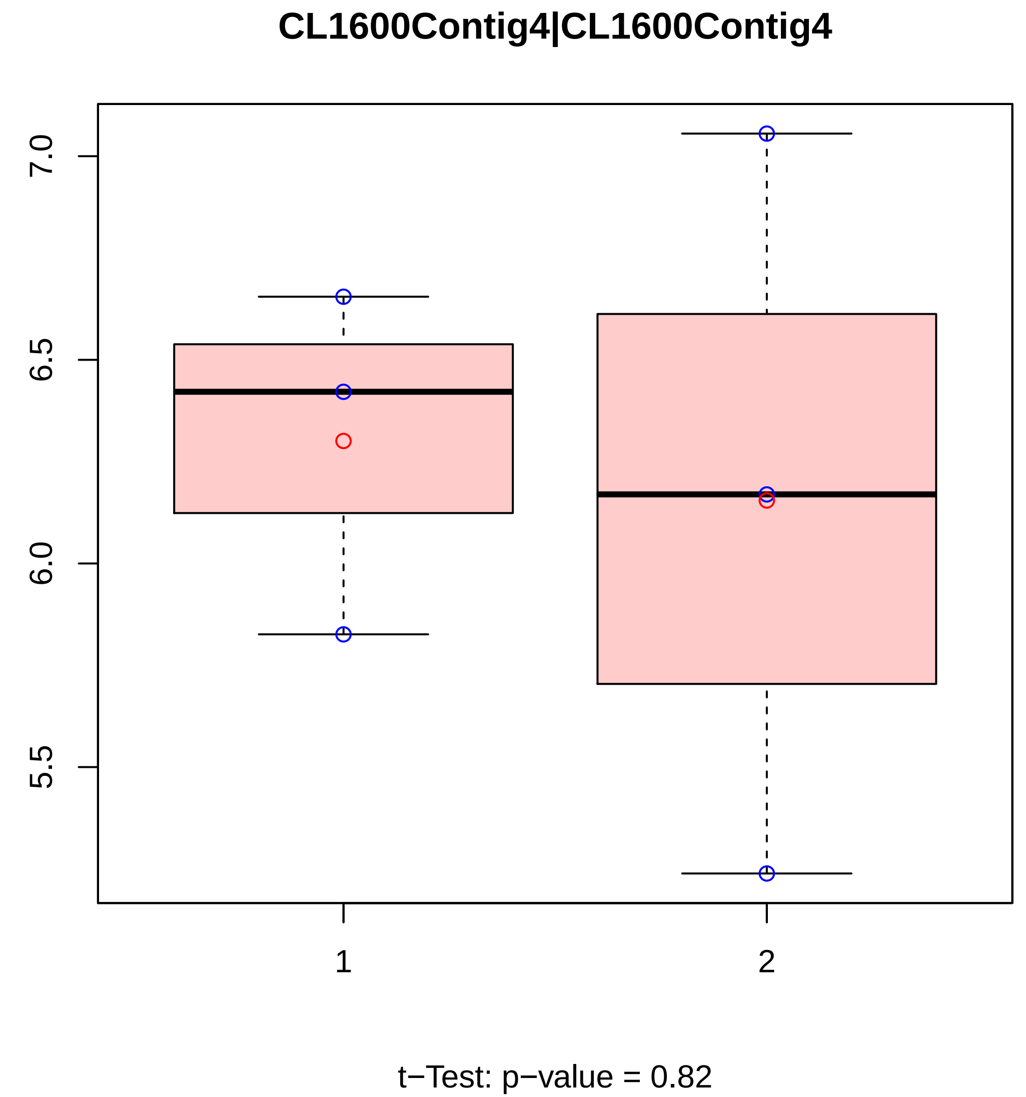
CL1600Contig4|CL1600Contig4
7.0
6.5
6.0
5.5
1
2
t−Test: p−value = 0.82

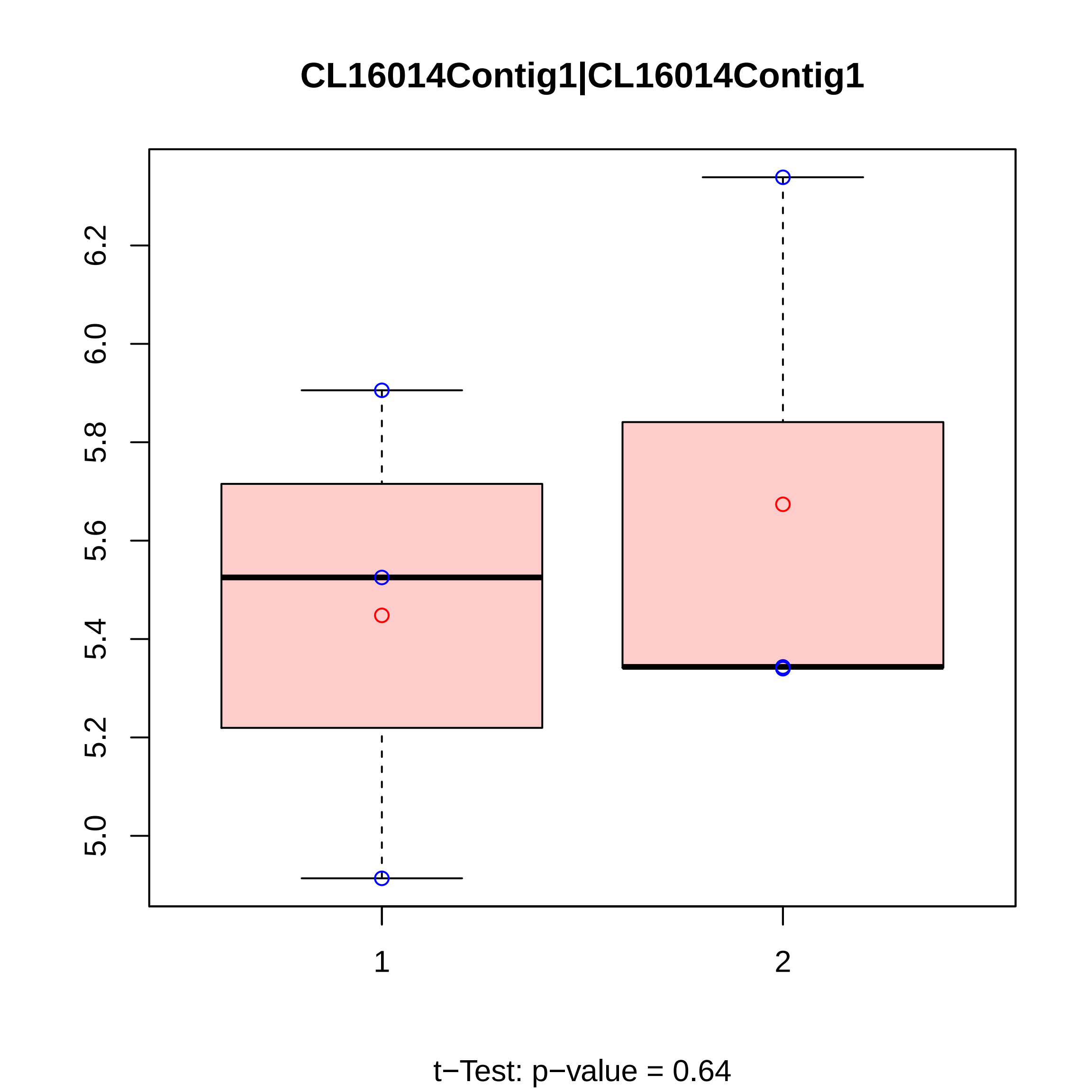

CL16014Contig1|CL16014Contig1
6.2
6.0
5.8
5.6
5.4
5.2
5.0
1
2
t−Test: p−value = 0.64

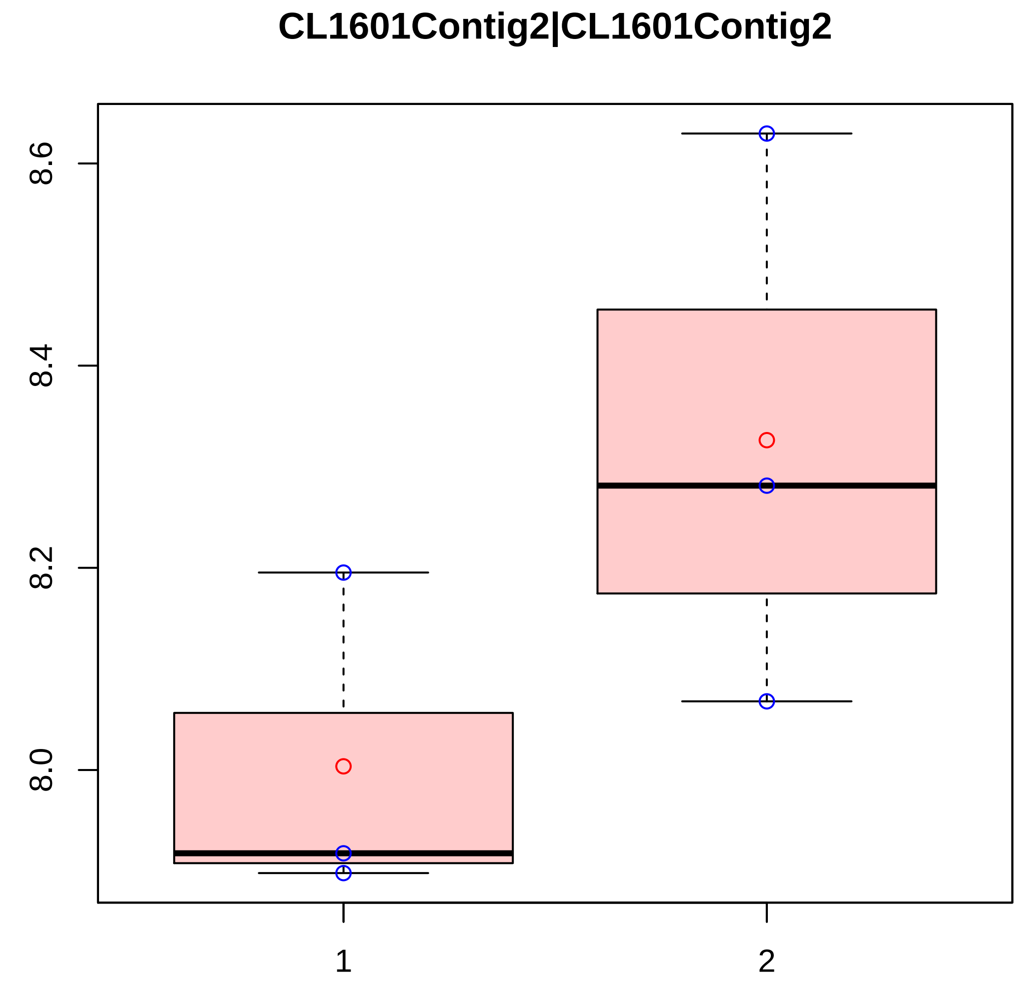


t−Test: p−value = 0.18

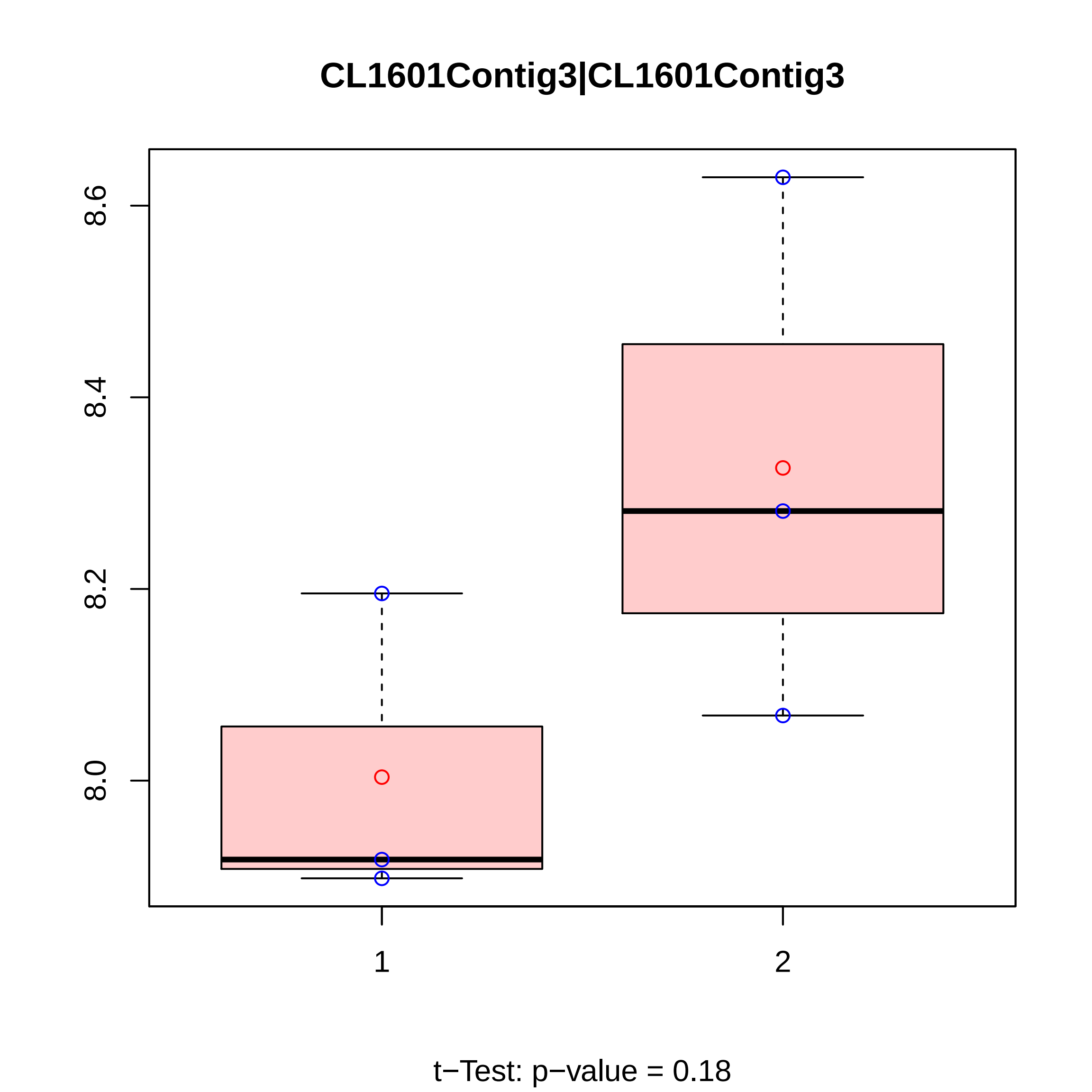

CL1601Contig3|CL1601Contig3
8.0
8.2
8.4
8.6
1
2
t−Test: p−value = 0.18

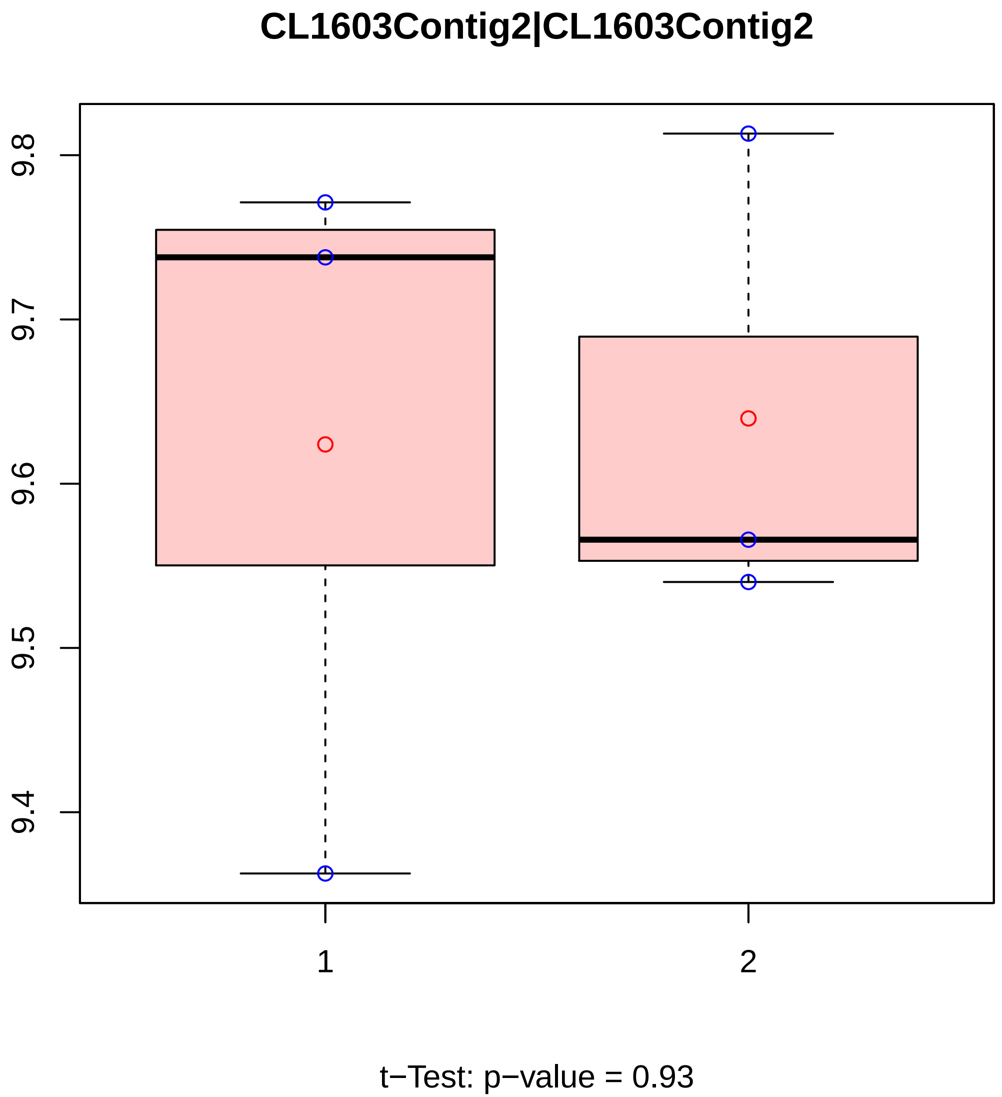

CL1603Contig2|CL1603Contig2
9.4
9.5
9.6
9.7
9.8
1
2
t−Test: p−value = 0.93

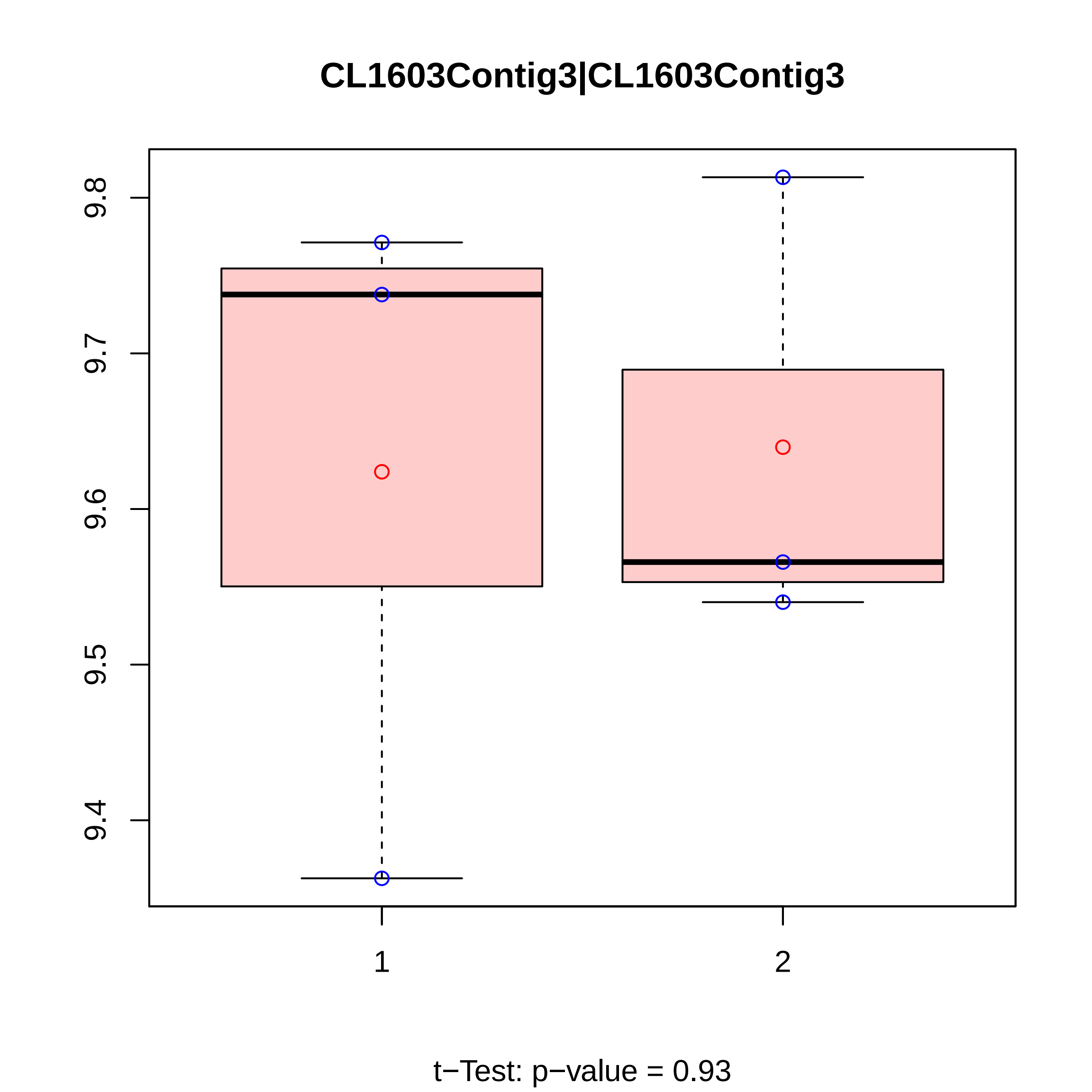
CL1603Contig3|CL1603Contig3
9.4
9.5
9.6
9.7
9.8
1
2
t−Test: p−value = 0.93

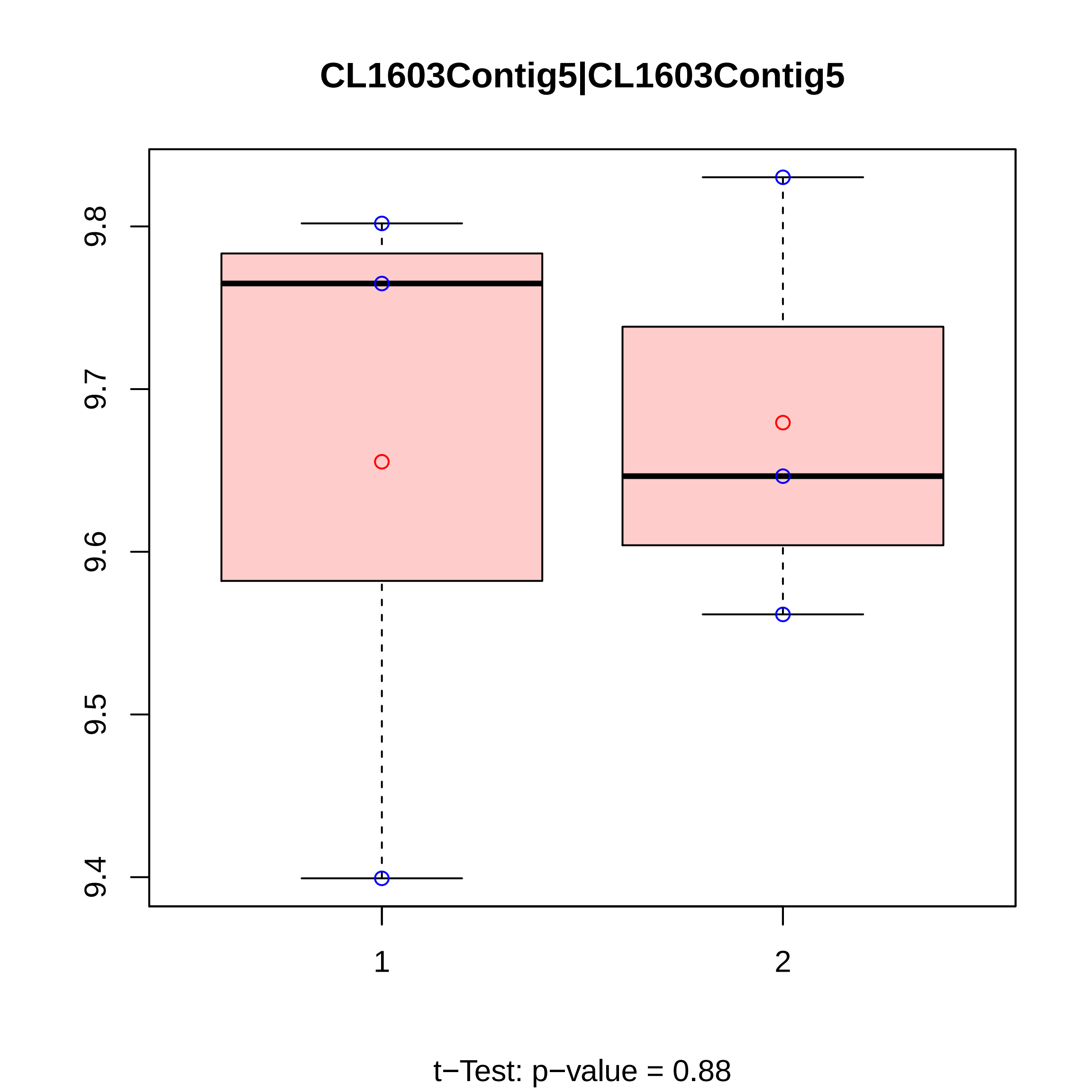
CL1603Contig5|CL1603Contig5
9.4
9.5
9.6
9.7
9.8
1
2
t−Test: p−value = 0.88

# CL1603Contig6|CL1603Contig6

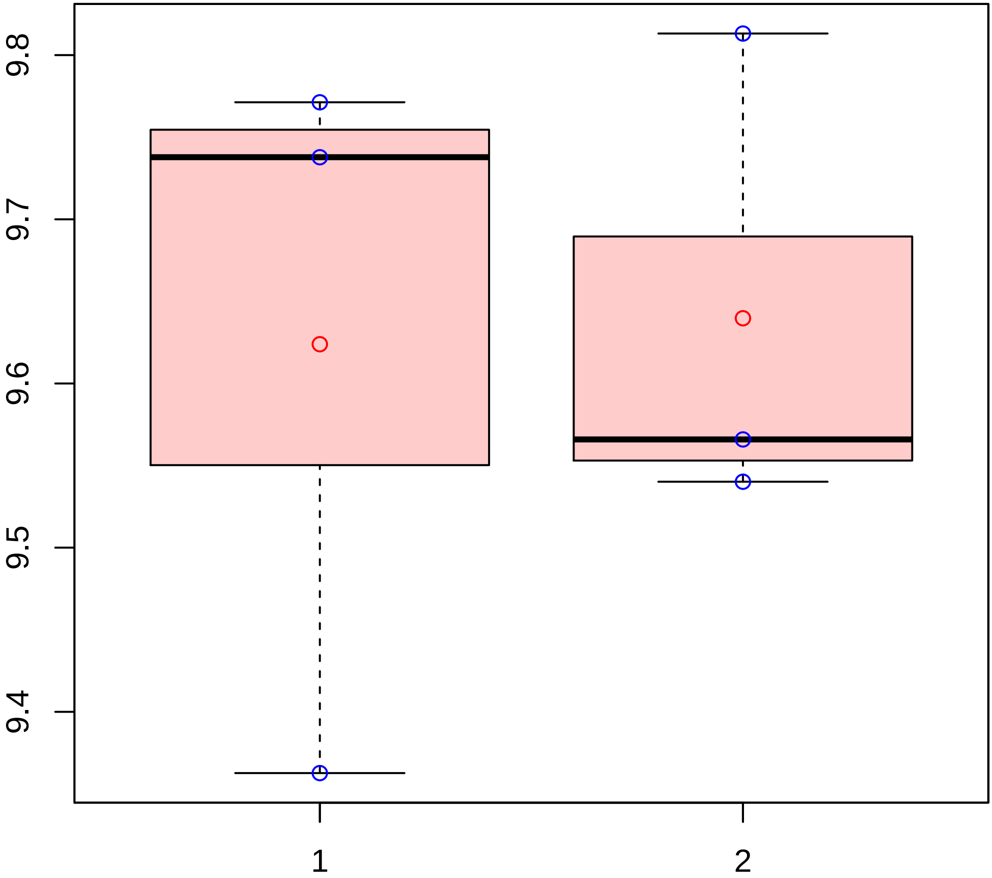


t−Test: p−value = 0.93

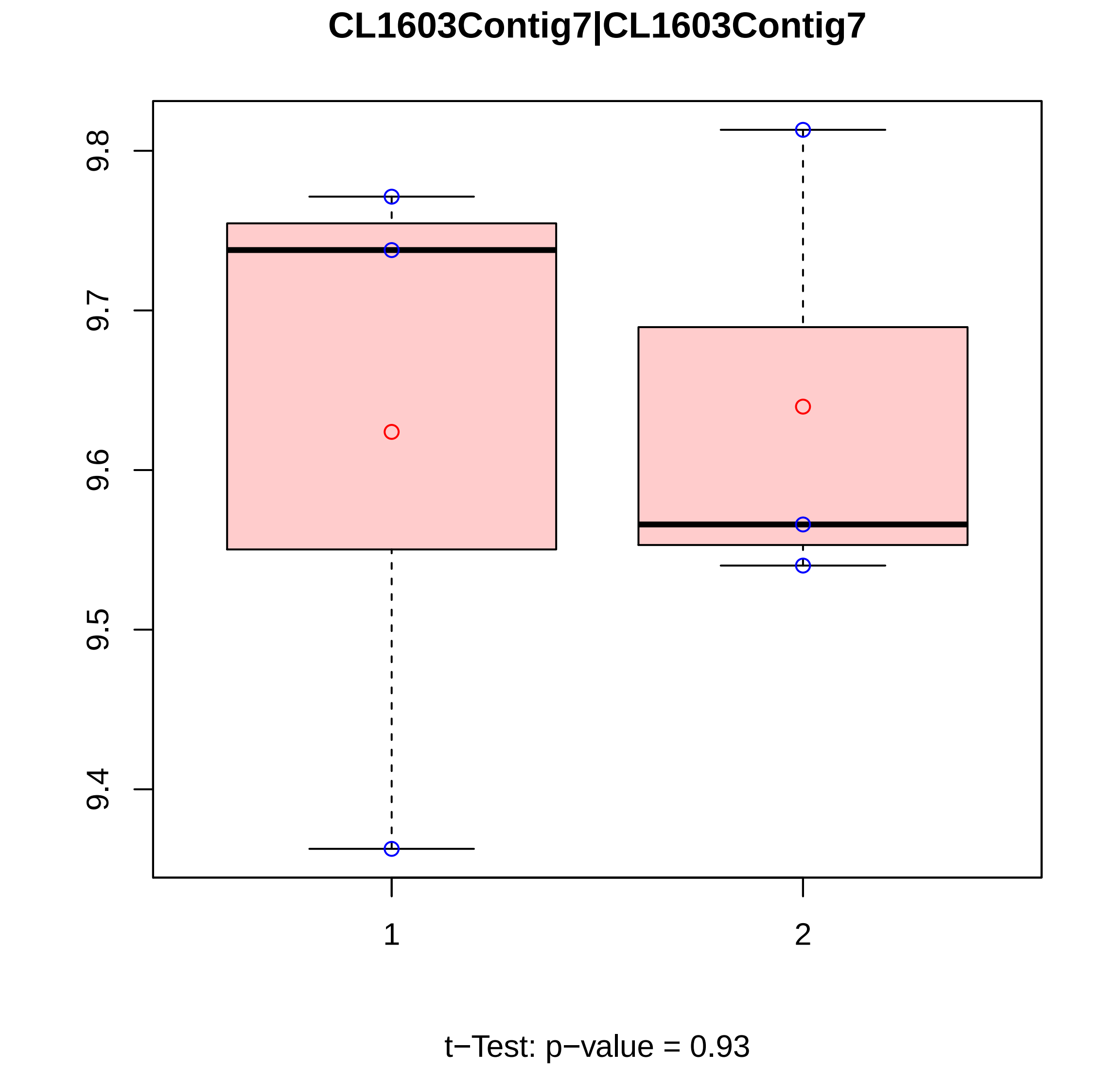

CL1603Contig7|CL1603Contig7
9.4
9.5
9.6
9.7
9.8
1
2
t−Test: p−value = 0.93

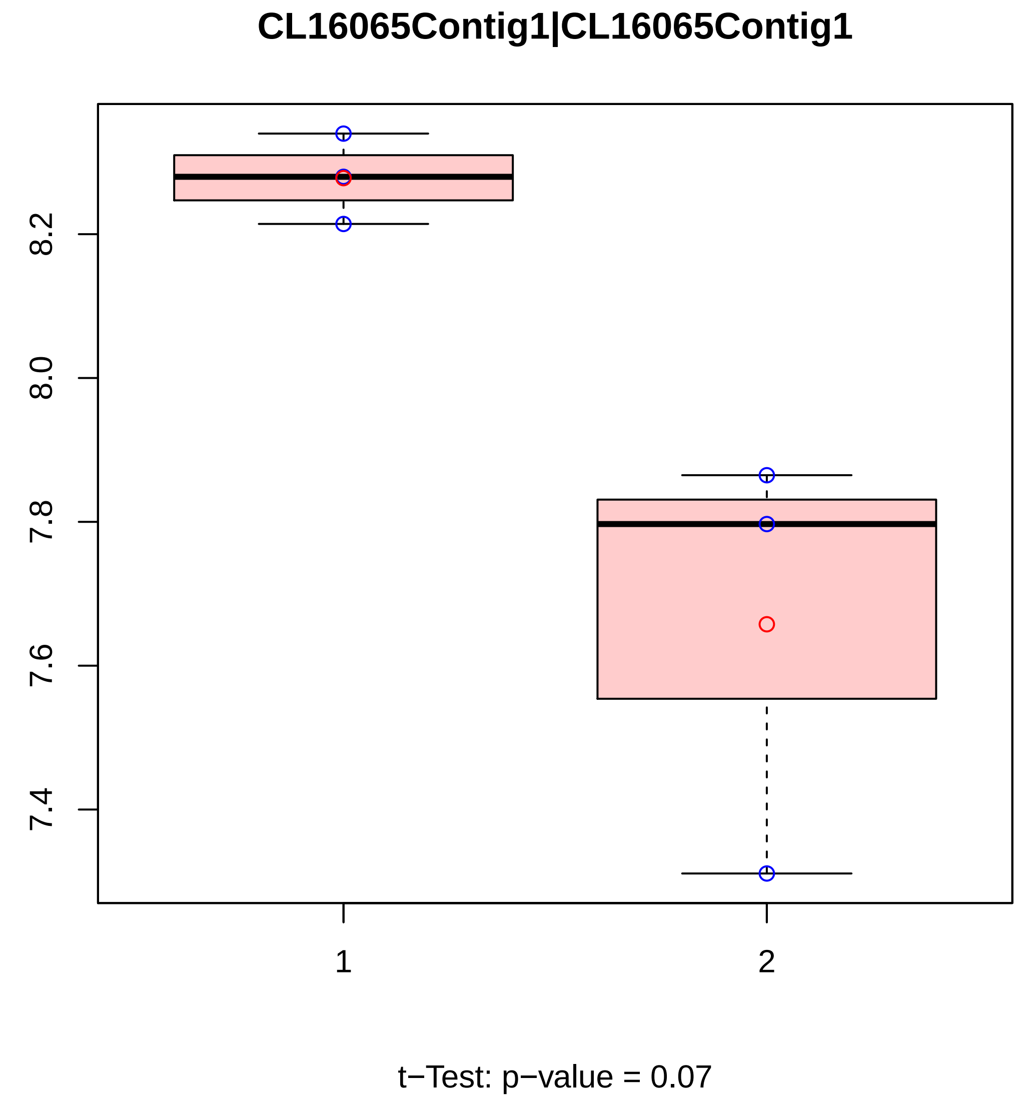
CL16065Contig1|CL16065Contig1
8.2
8.0
7.8
7.6
7.4
1
2
t−Test: p−value = 0.07

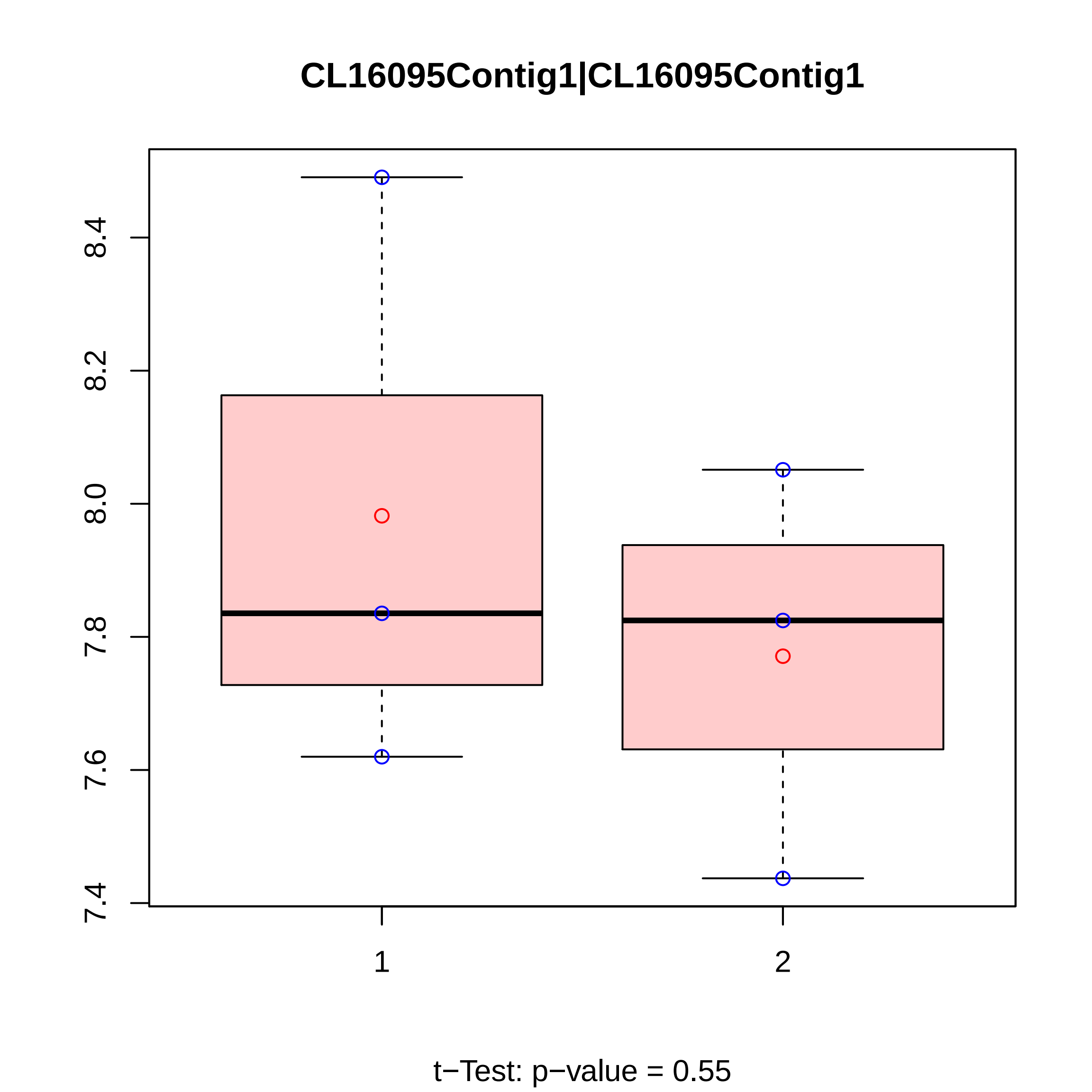

CL16095Contig1|CL16095Contig1
8.4
8.2
8.0
7.8
7.6
7.4
1
2
t−Test: p−value = 0.55

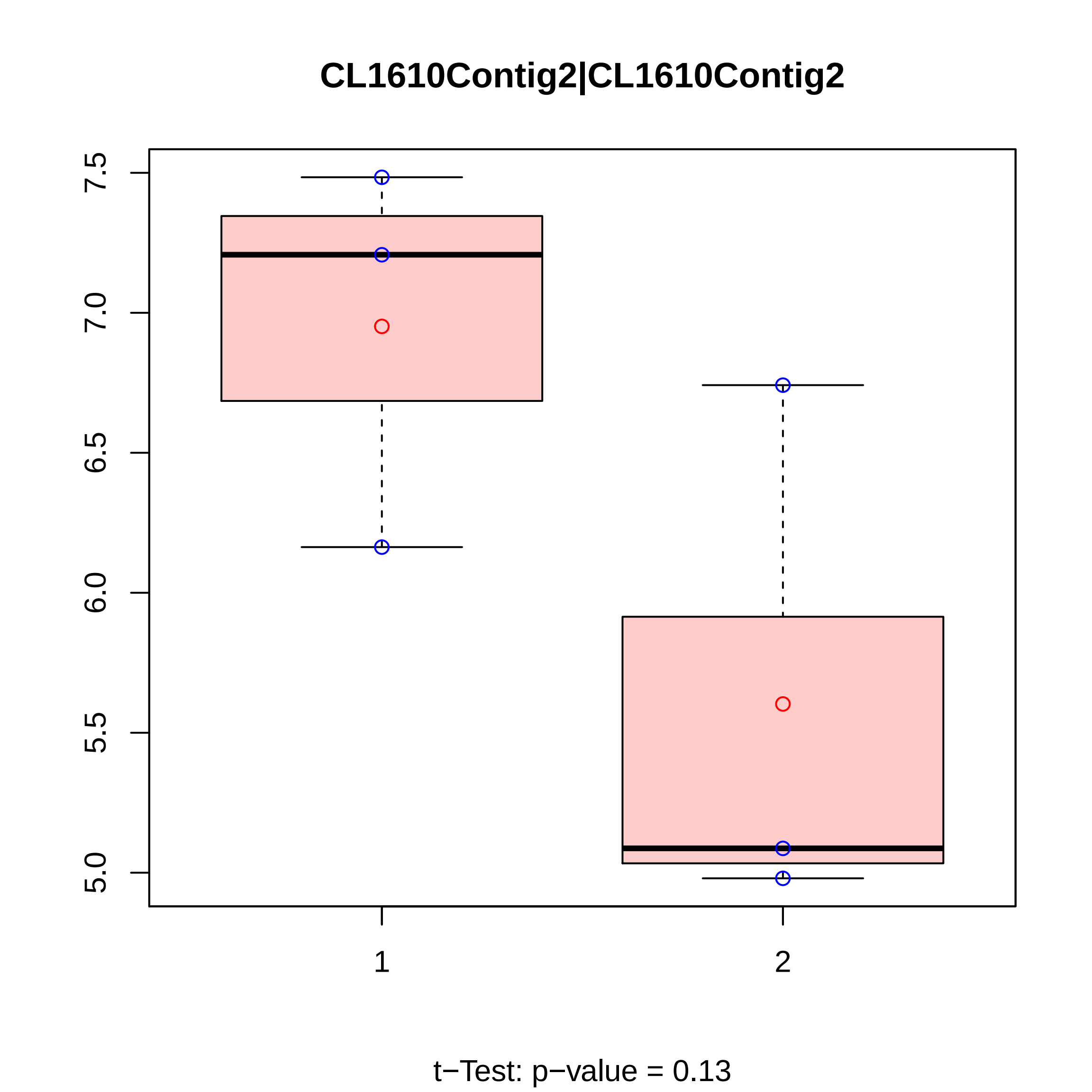
CL1610Contig2|CL1610Contig2
7.5
7.0
6.5
6.0
5.5
5.0
1
2
t−Test: p−value = 0.13

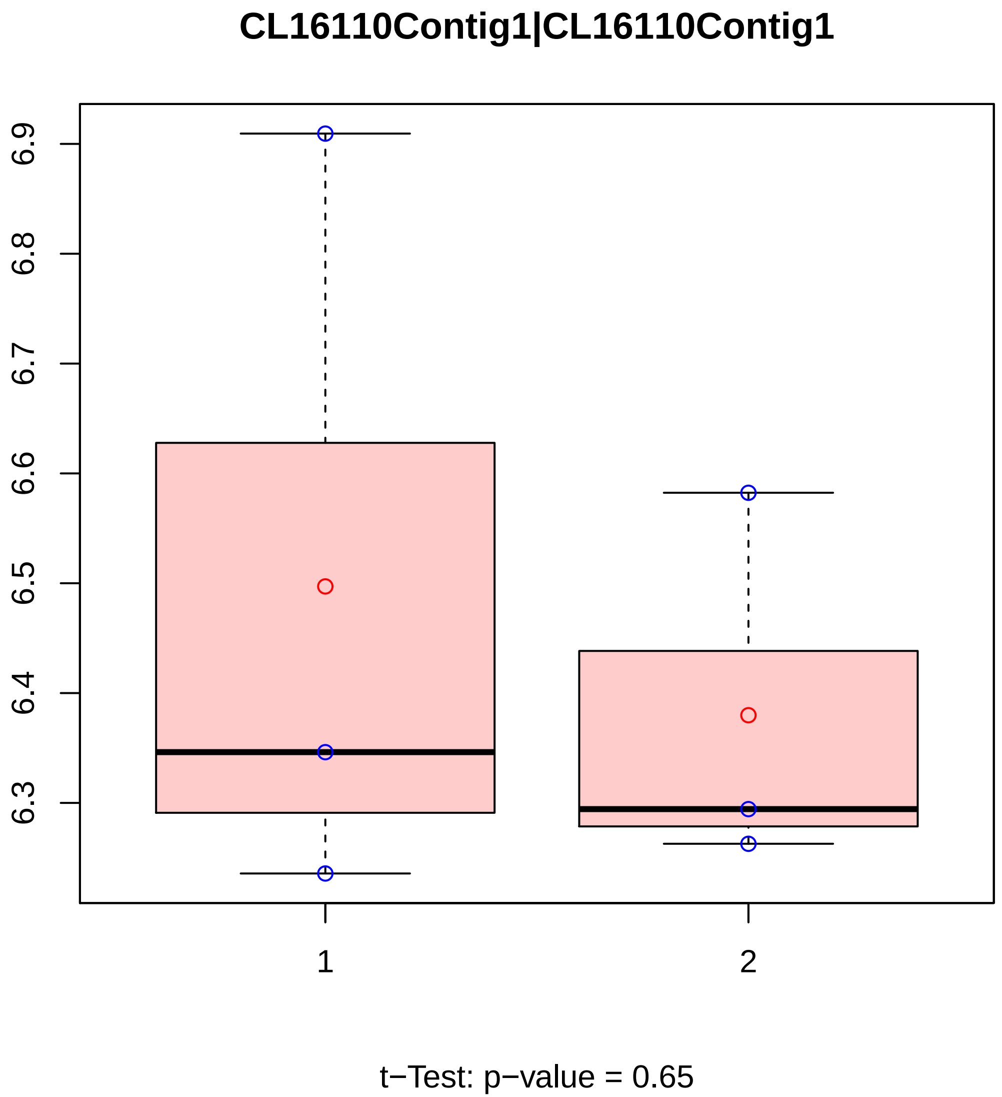
CL16110Contig1|CL16110Contig1
6.3
6.4
6.5
6.6
6.7
6.8
6.9
1
2
t−Test: p−value = 0.65

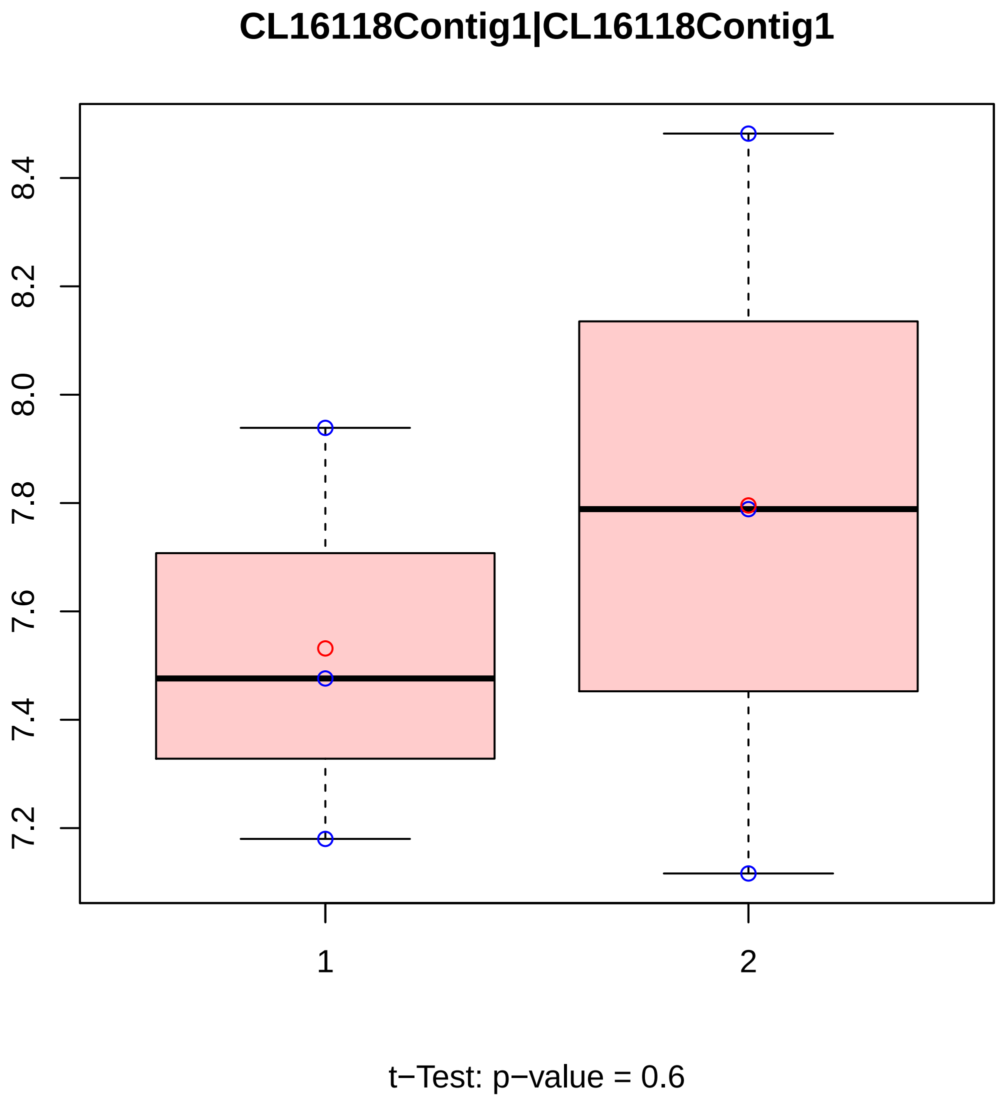
CL16118Contig1|CL16118Contig1
8.4
8.2
8.0
7.8
7.6
7.4
7.2
1
2
t−Test: p−value = 0.6

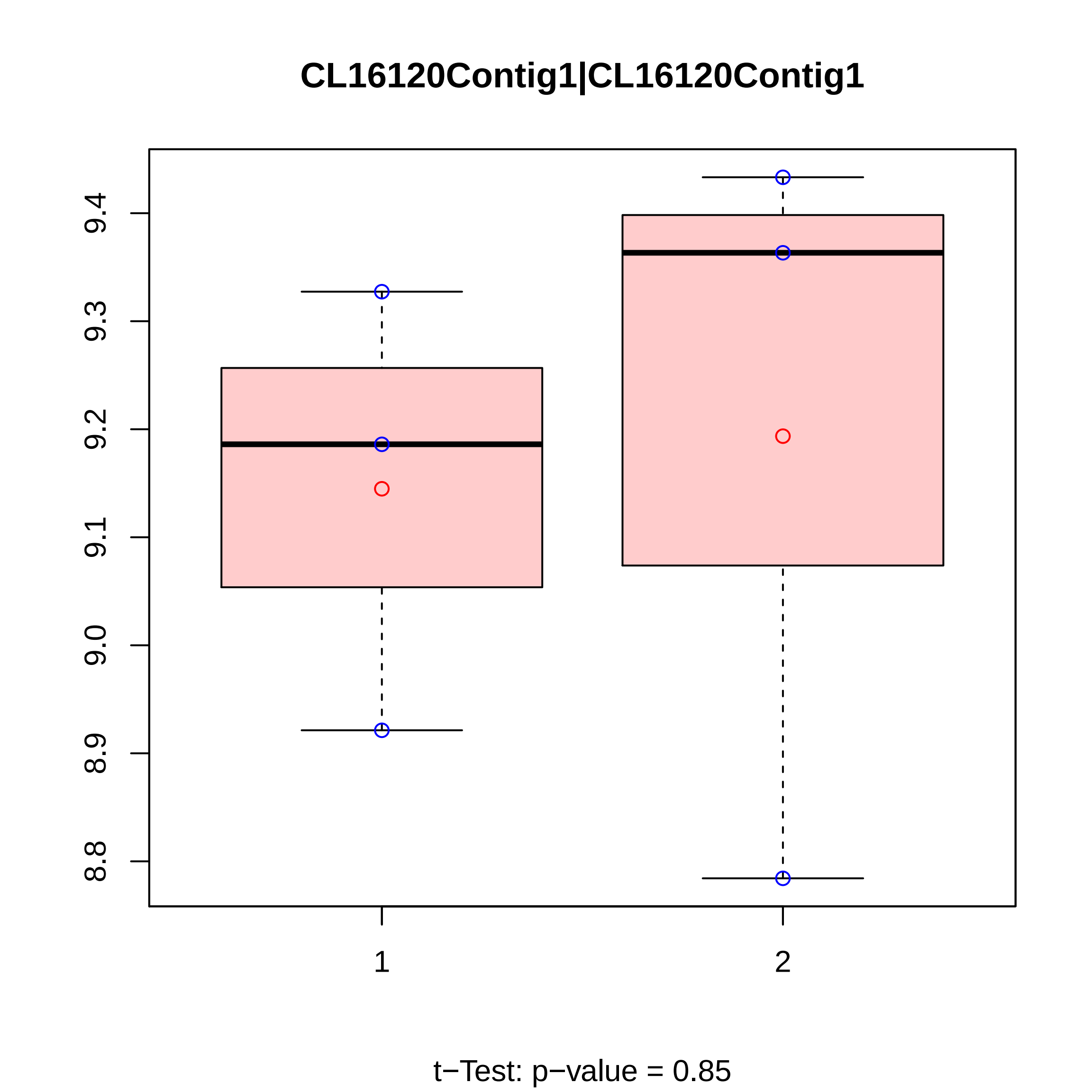

CL16120Contig1|CL16120Contig1
9.4
9.3
9.2
9.1
9.0
8.9
8.8
1
2
t−Test: p−value = 0.85

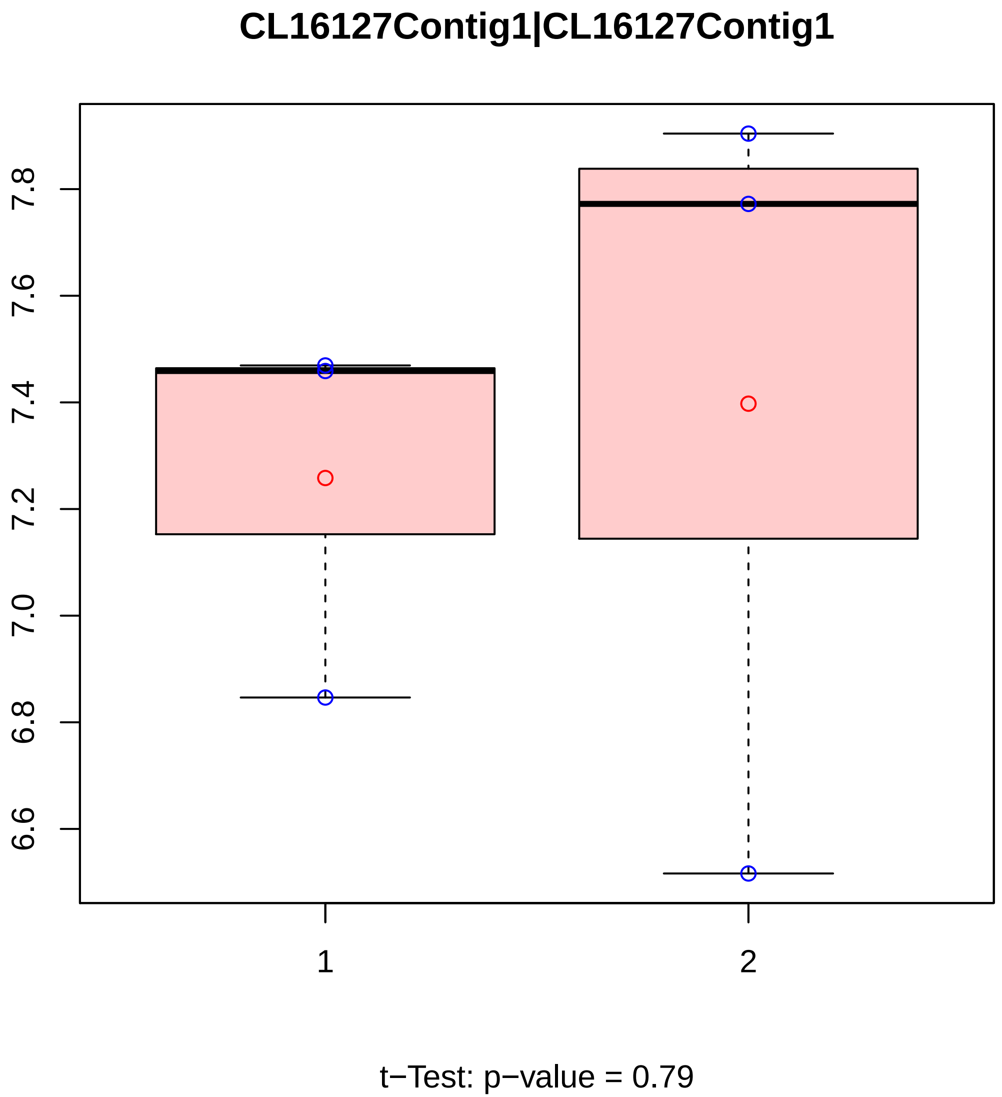
CL16127Contig1|CL16127Contig1
7.8
7.6
7.4
7.2
7.0
6.8
6.6
1
2
t−Test: p−value = 0.79

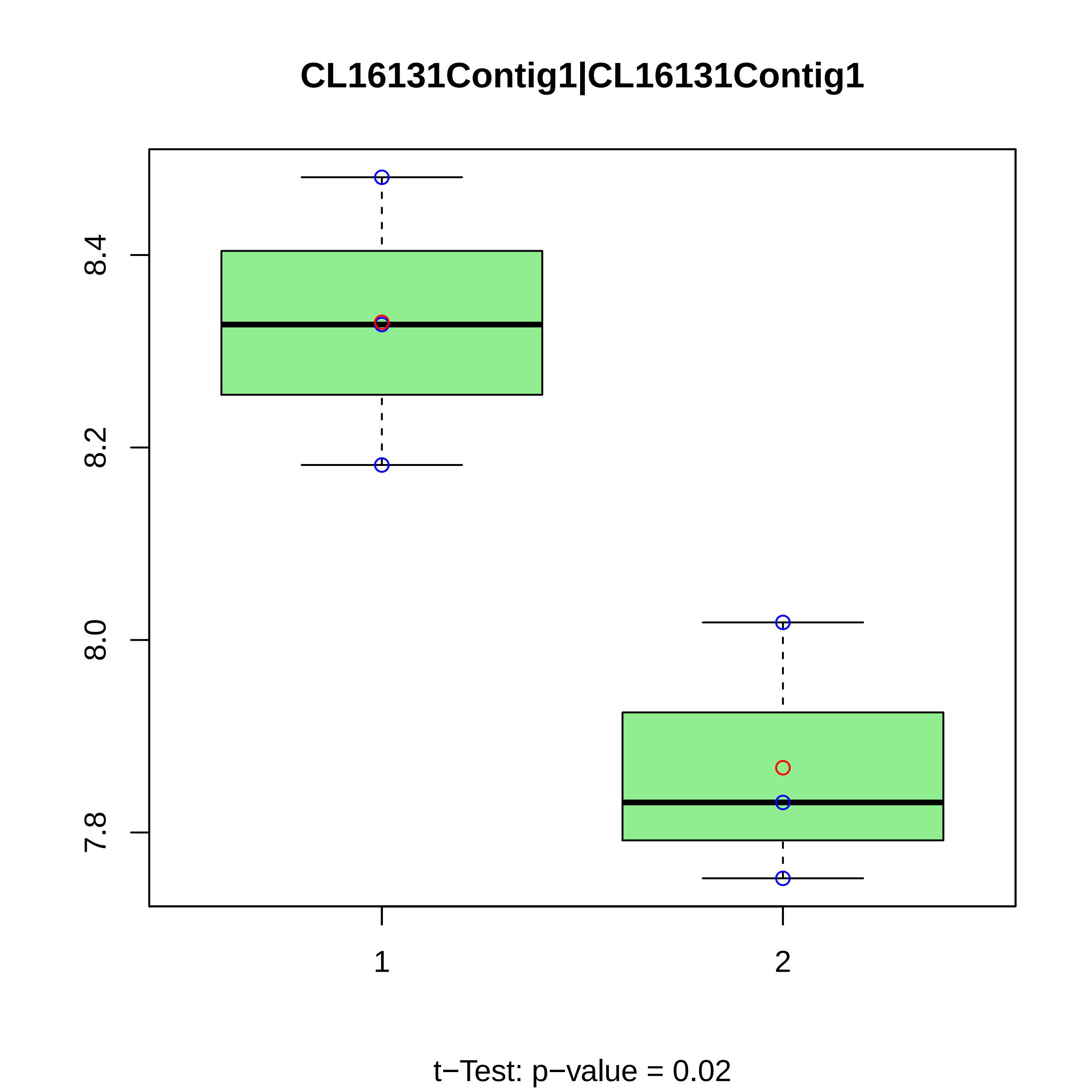
CL16131Contig1|CL16131Contig1
8.4
8.2
8.0
7.8
1
2
t−Test: p−value = 0.02

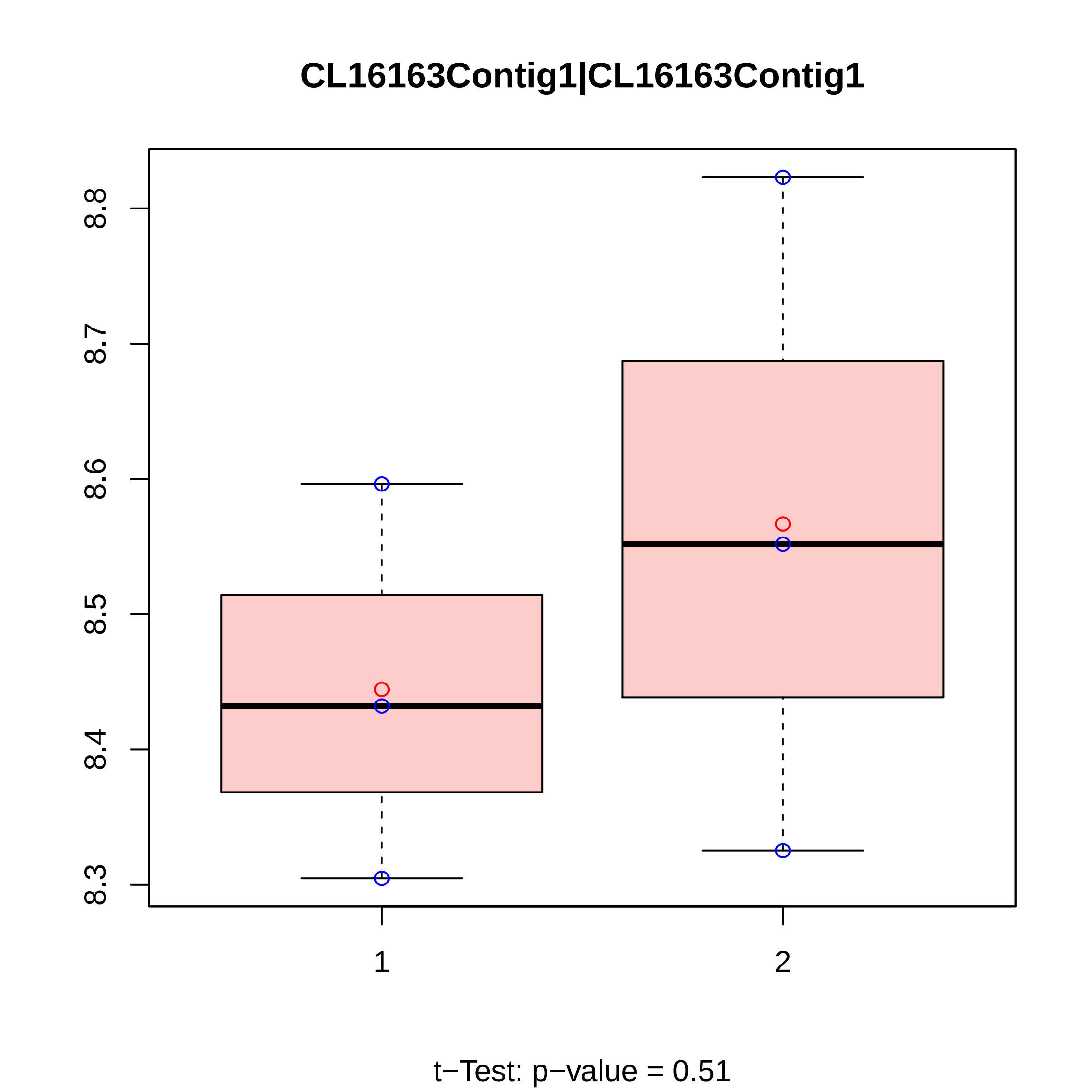
CL16163Contig1|CL16163Contig1
8.3
8.4
8.5
8.6
8.7
8.8
1
2
t−Test: p−value = 0.51

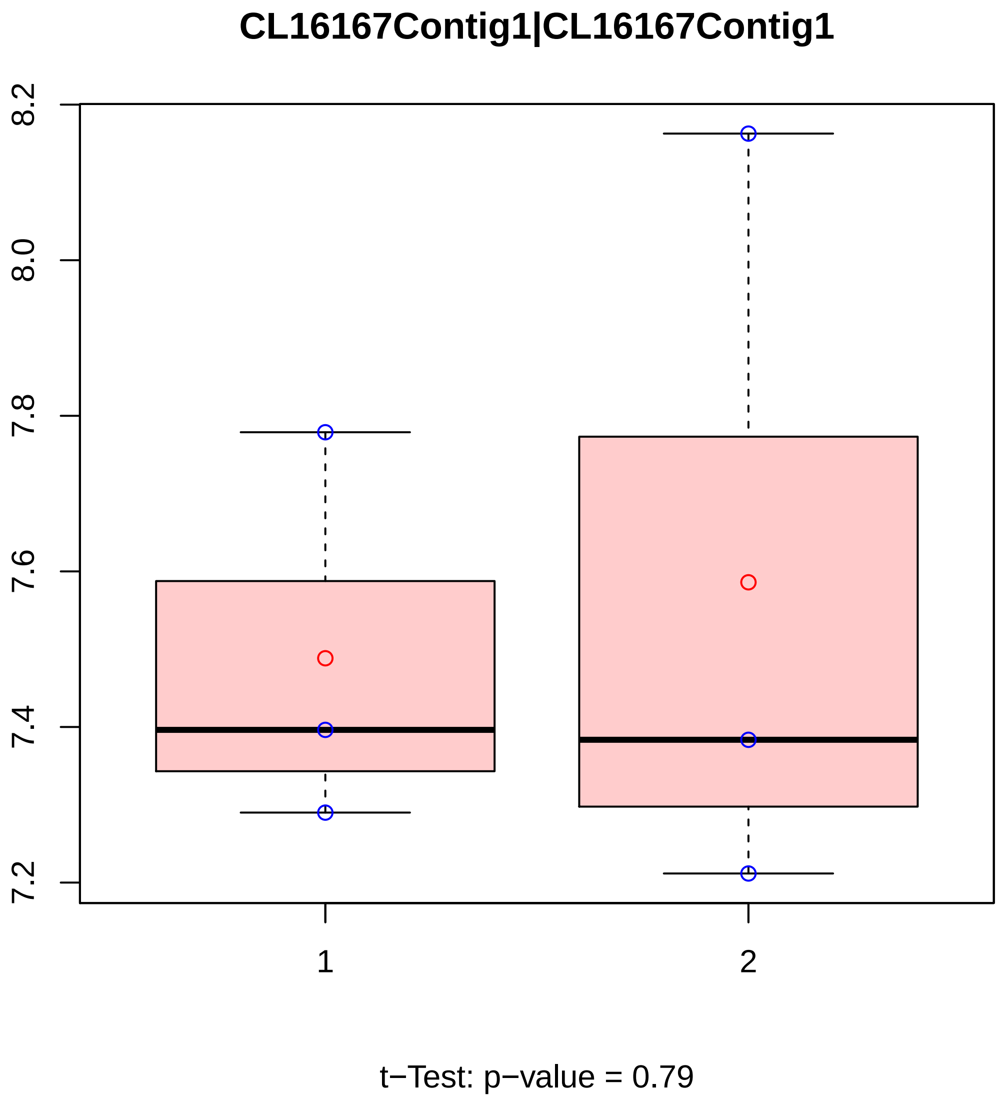

CL16167Contig1|CL16167Contig1
8.2
8.0
7.8
7.6
7.4
7.2
1
2
t−Test: p−value = 0.79

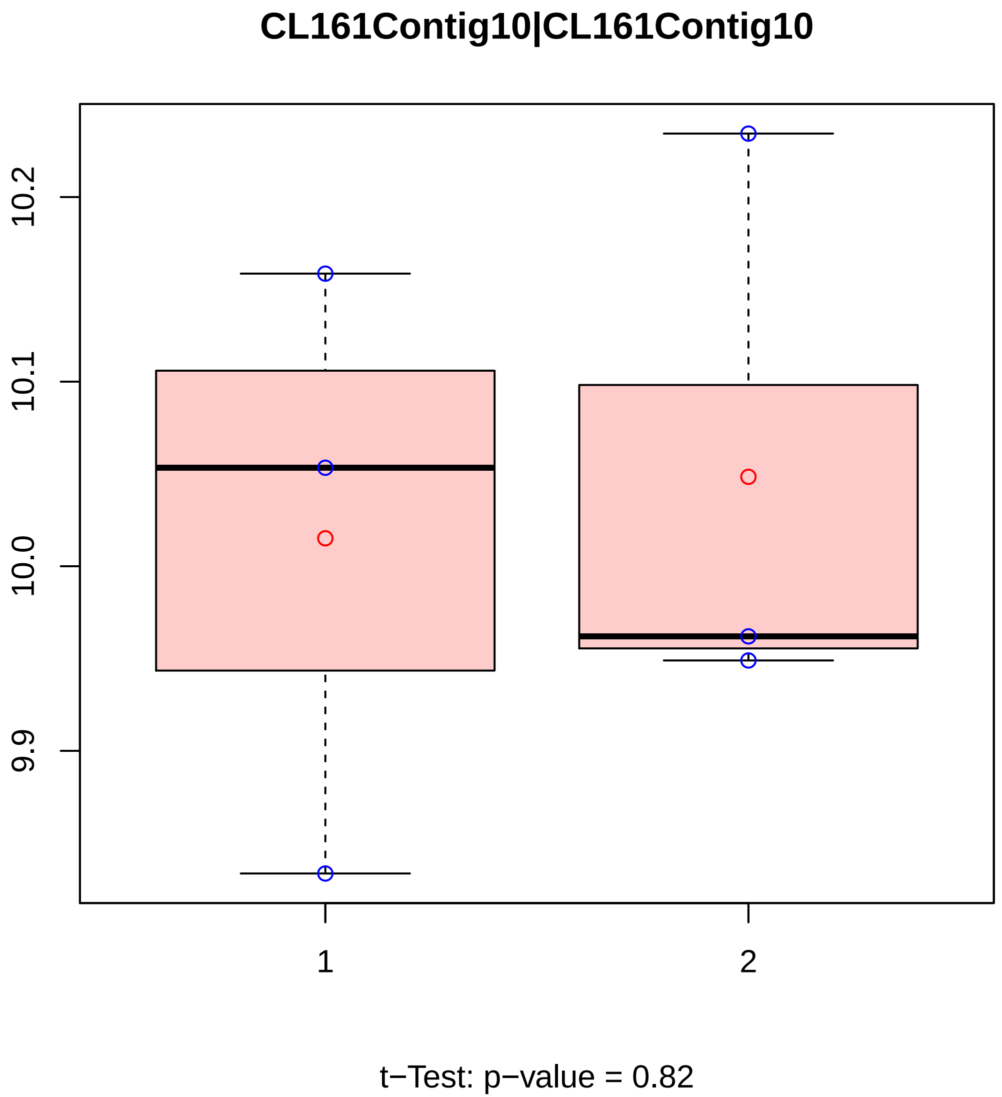
CL161Contig10|CL161Contig10
10.2
10.1
10.0
9.9
1
2
t−Test: p−value = 0.82

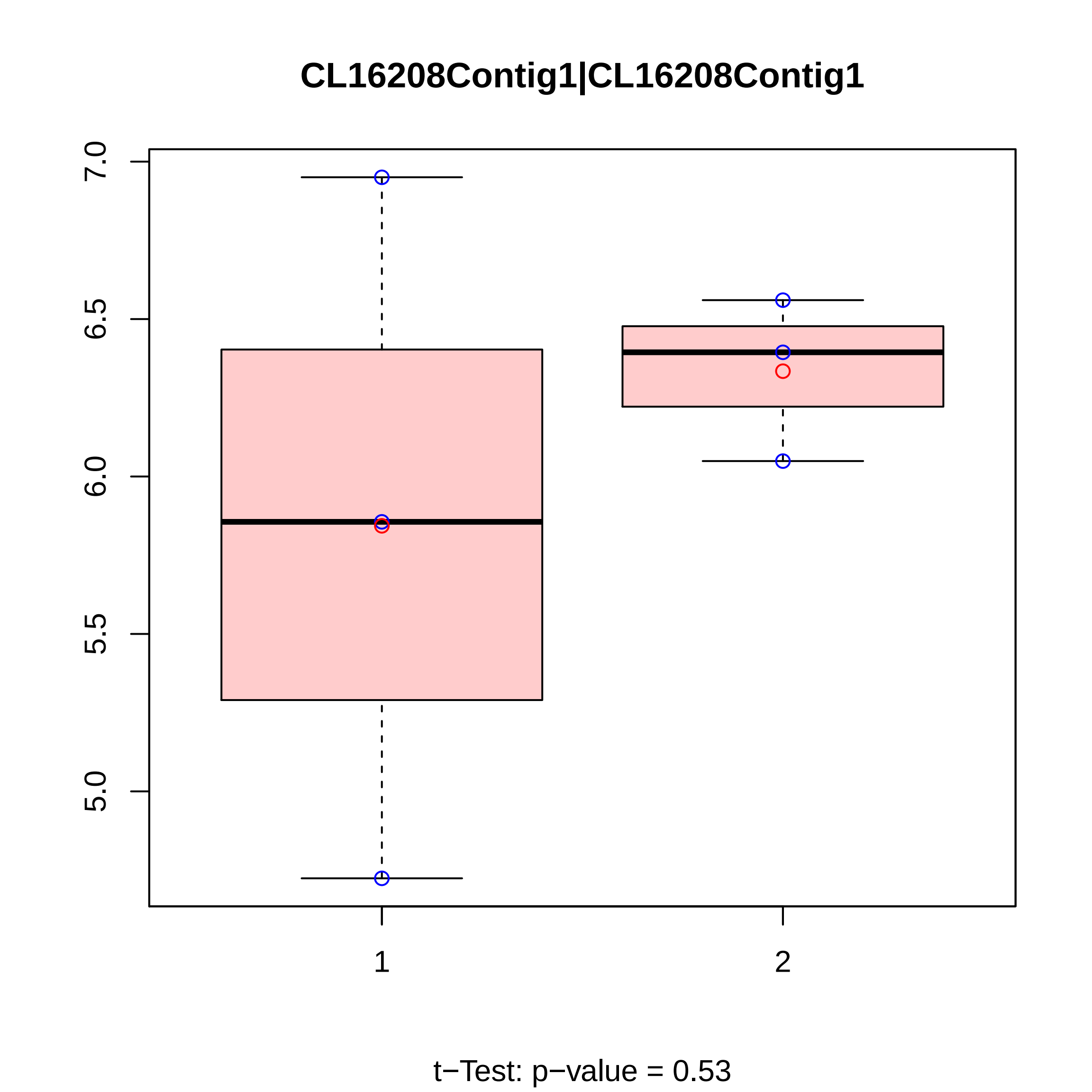
CL16208Contig1|CL16208Contig1
7.0
6.5
6.0
5.5
5.0
1
2
t−Test: p−value = 0.53

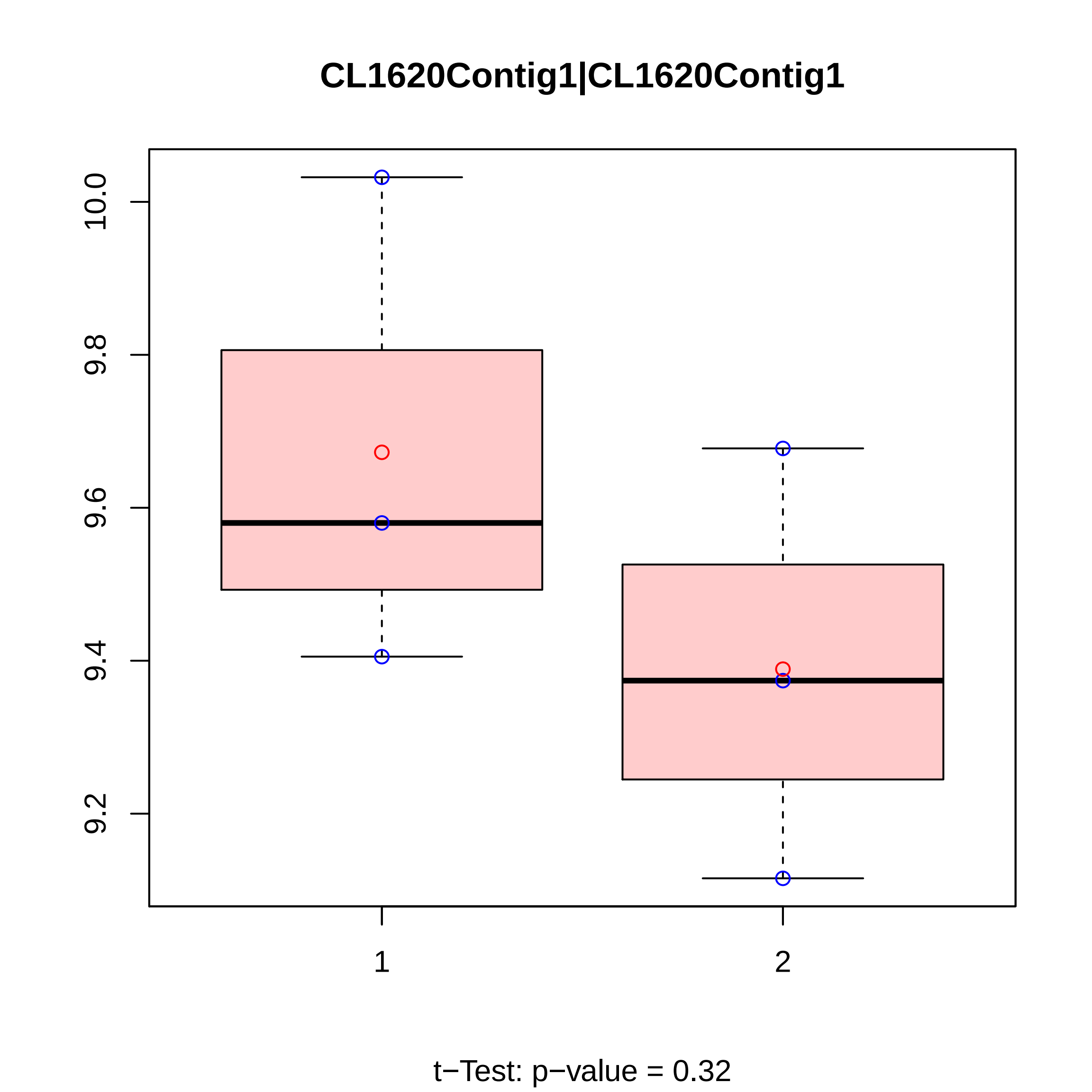
CL1620Contig1|CL1620Contig1
10.0
9.8
9.6
9.4
9.2
1
2
t−Test: p−value = 0.32

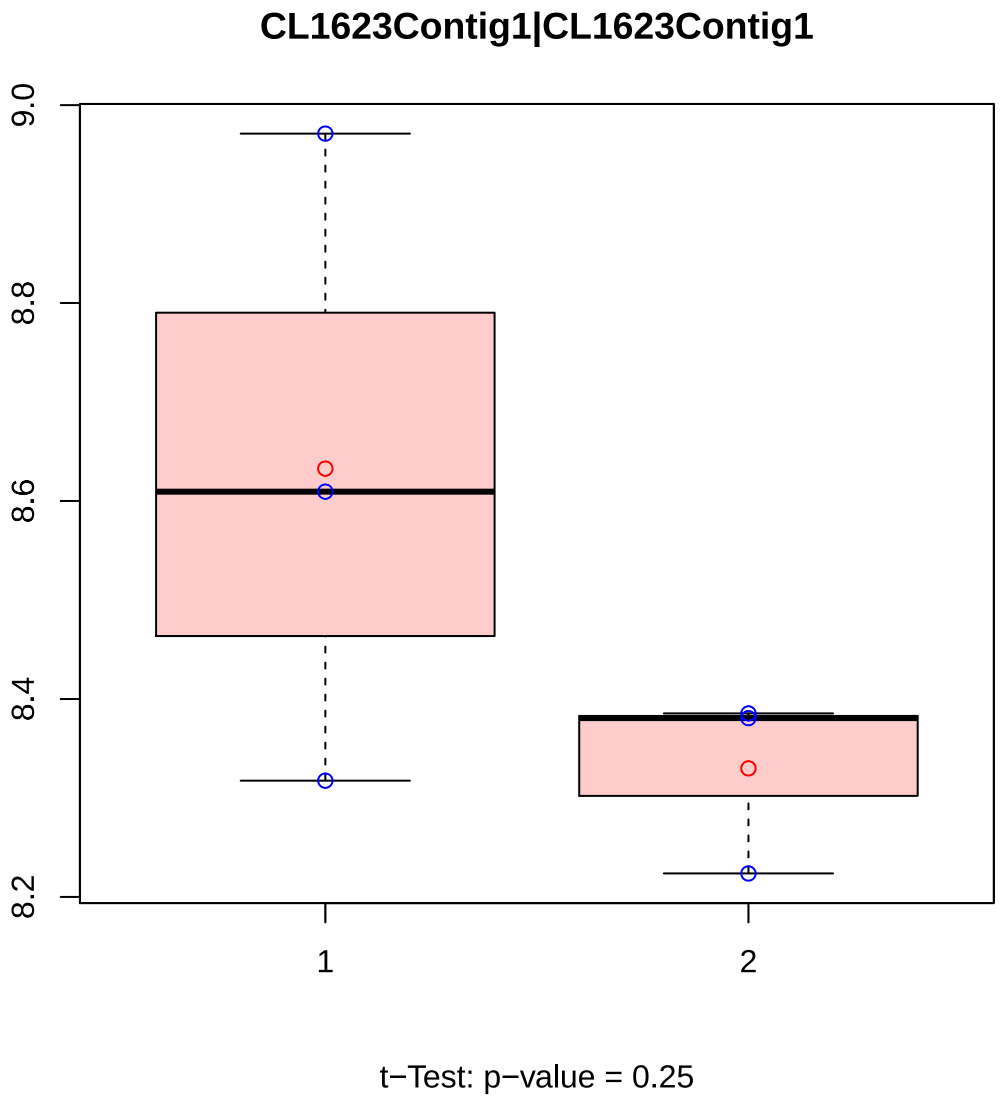
CL1623Contig1|CL1623Contig1
9.0
8.8
8.6
8.4
8.2
1
2
t−Test: p−value = 0.25

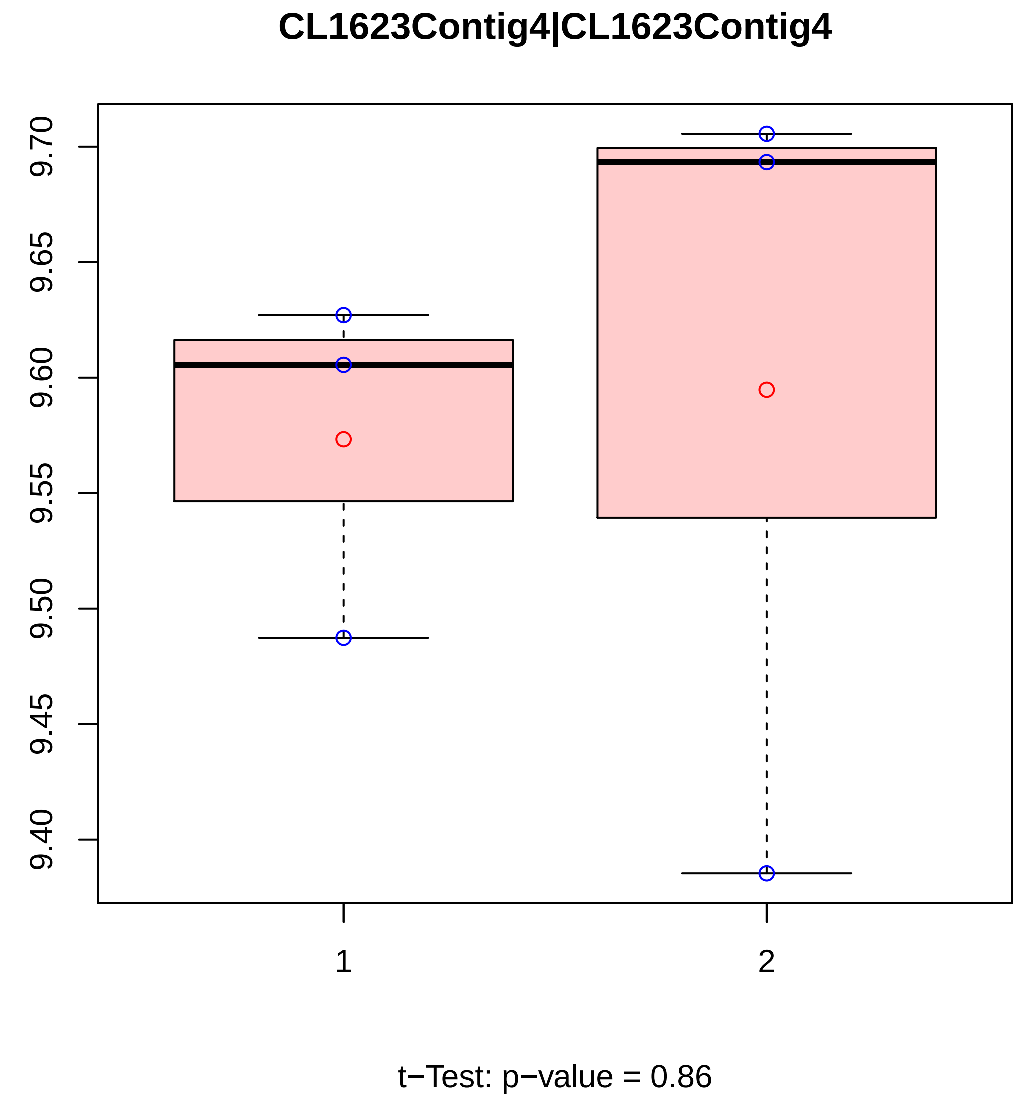

CL1623Contig4|CL1623Contig4
9.70
9.65
9.60
9.55
9.50
9.45
9.40
1
2
t−Test: p−value = 0.86

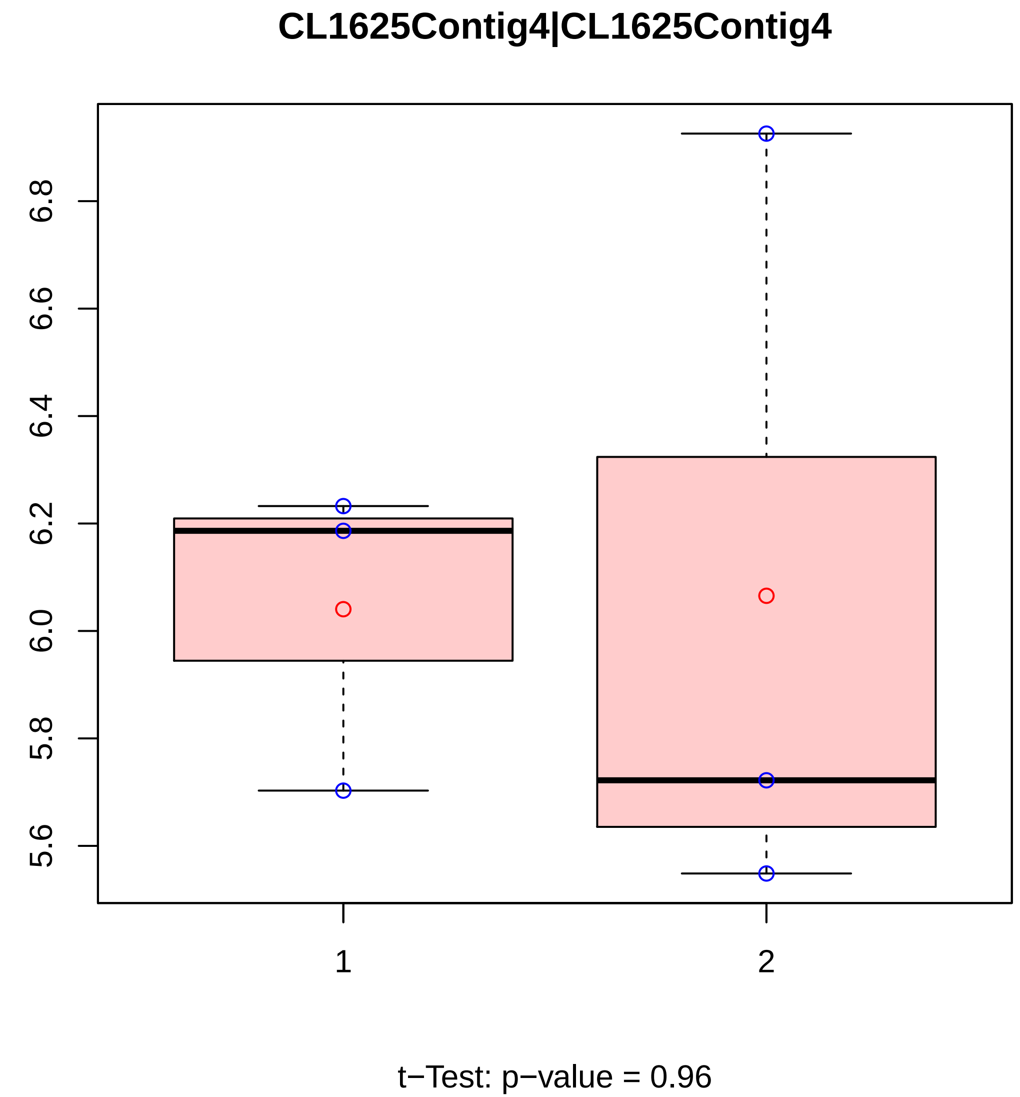
CL1625Contig4|CL1625Contig4
6.8
6.6
6.4
6.2
6.0
5.8
5.6
1
2
t−Test: p−value = 0.96

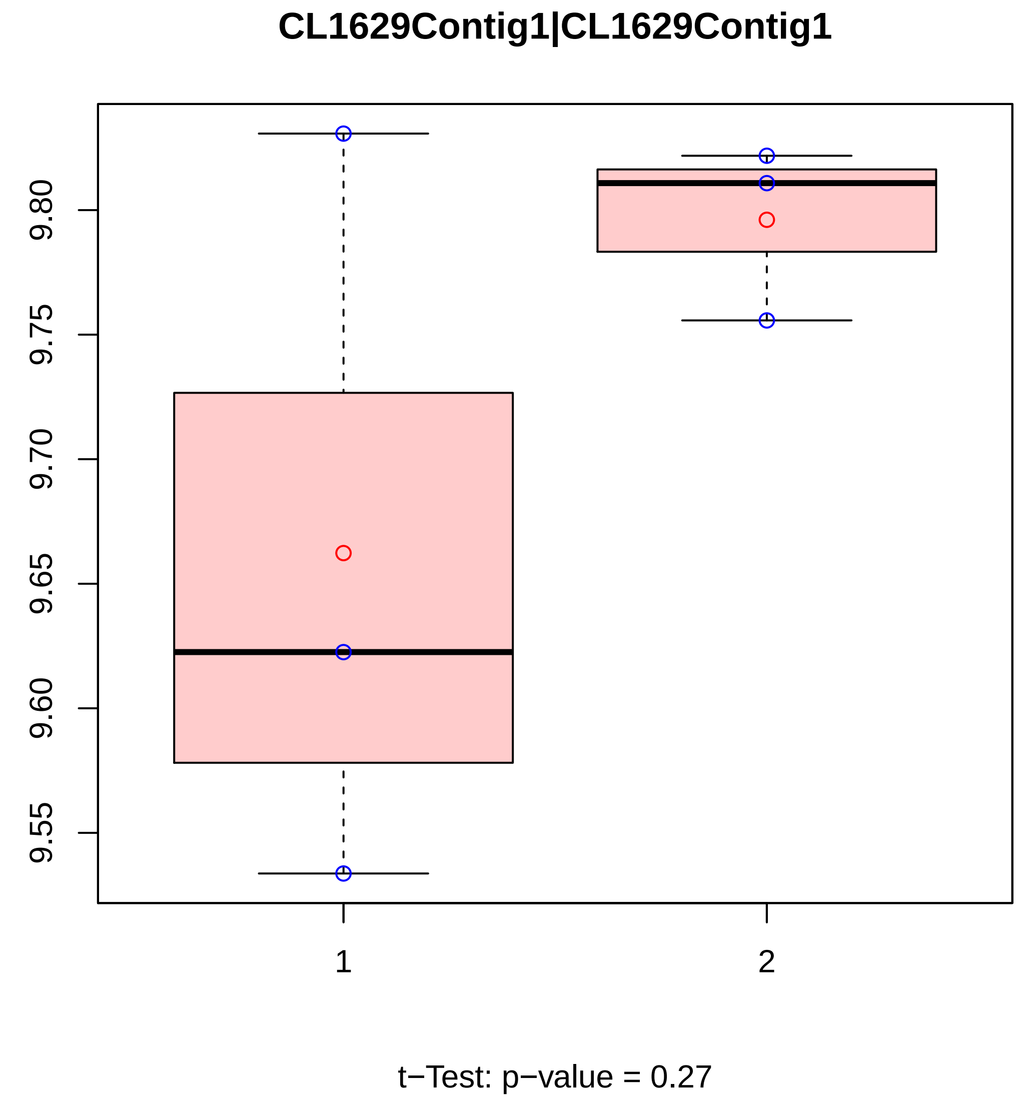
CL1629Contig1|CL1629Contig1
9.55
9.60
9.65
9.70
9.75
9.80
1
2
t−Test: p−value = 0.27

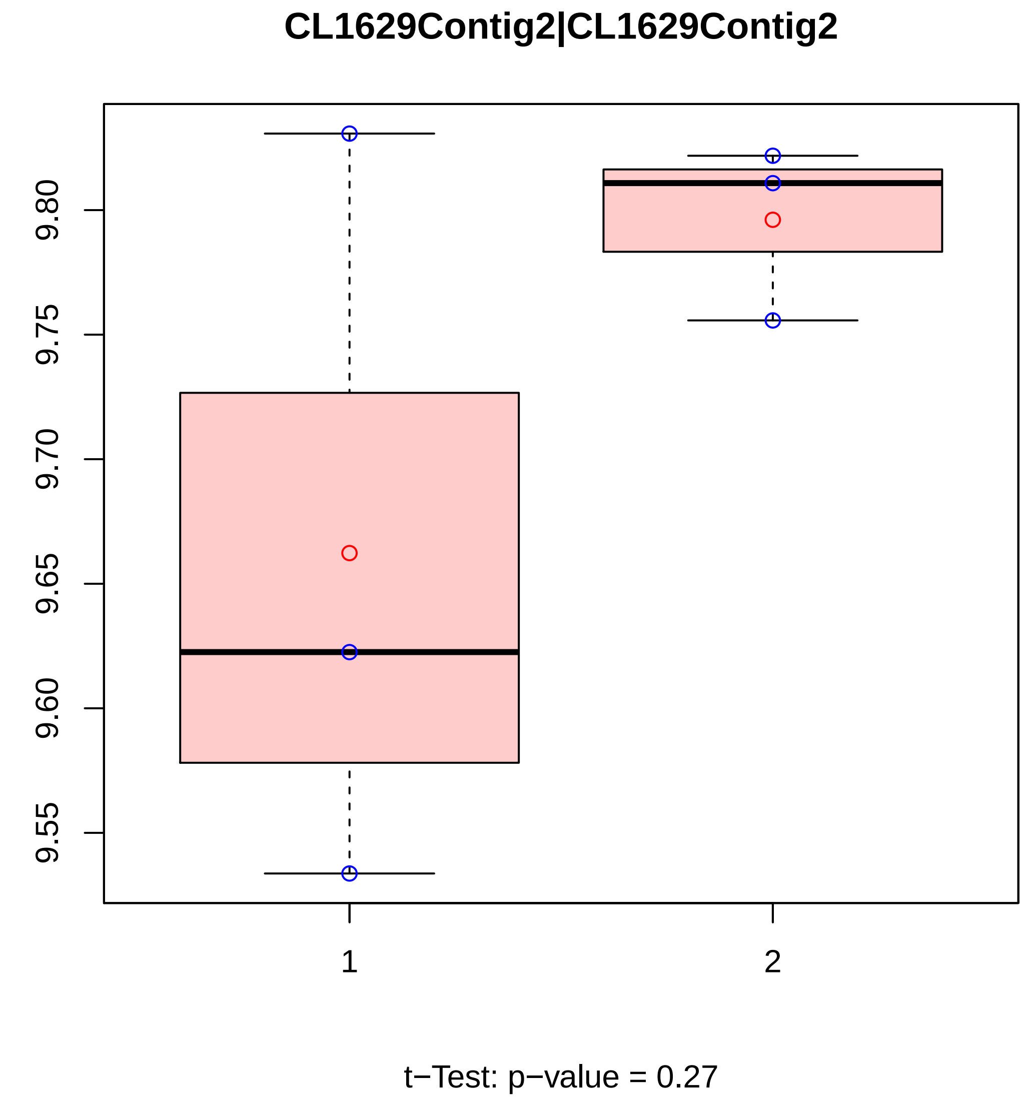
CL1629Contig2|CL1629Contig2
9.80
9.75
9.70
9.65
9.60
9.55
1
2
t−Test: p−value = 0.27

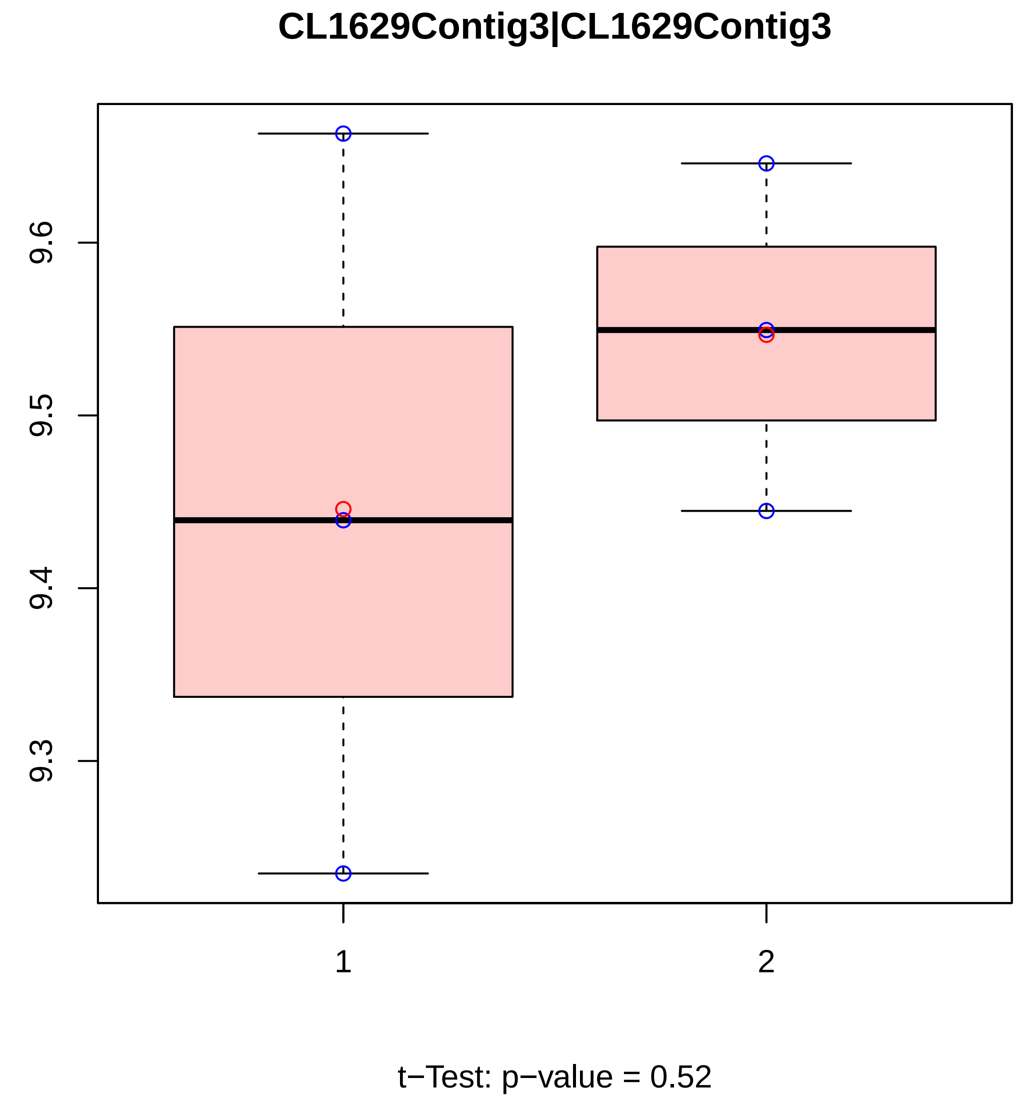
CL1629Contig3|CL1629Contig3
9.3
9.4
9.5
9.6
1
2
t−Test: p−value = 0.52

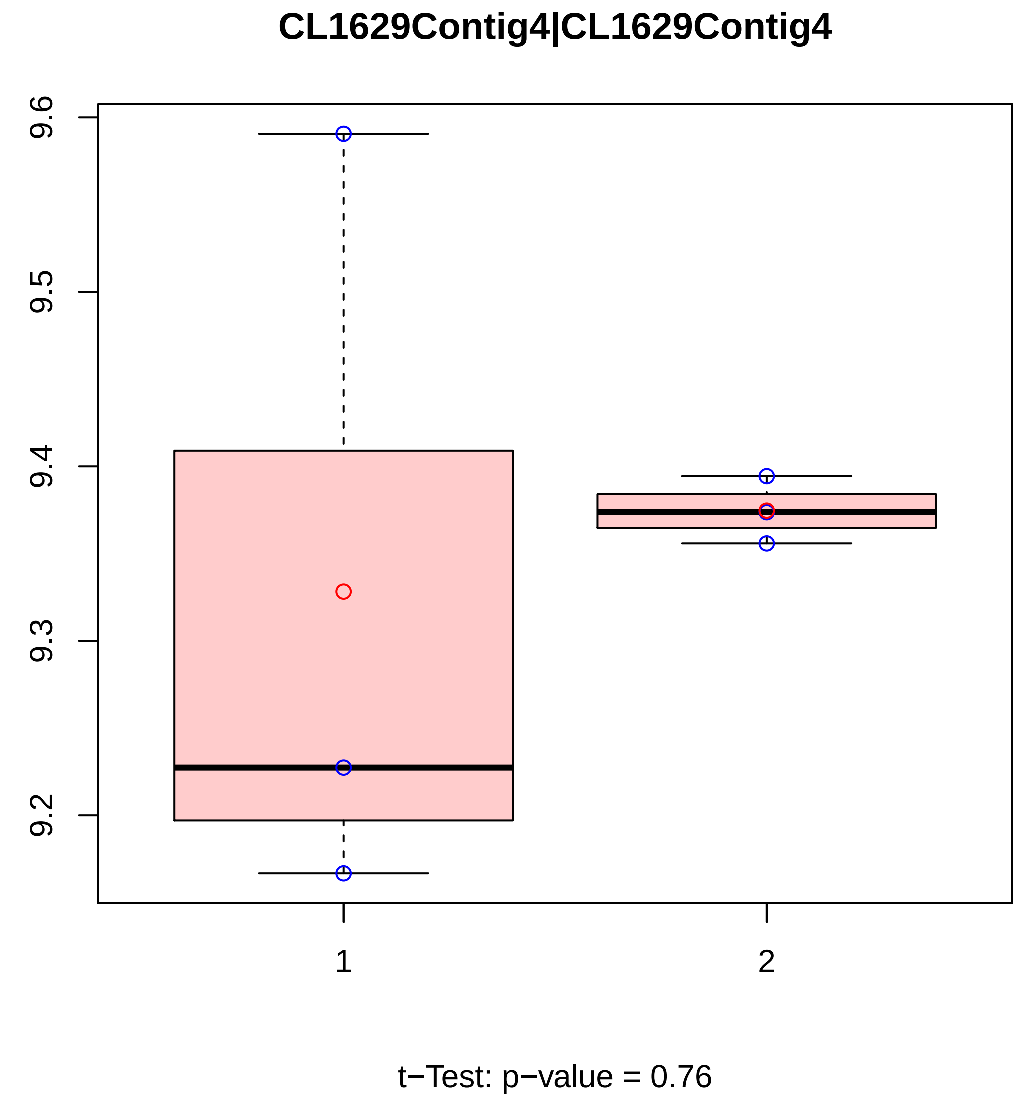
CL1629Contig4|CL1629Contig4
9.2
9.3
9.4
9.5
9.6
1
2
t−Test: p−value = 0.76

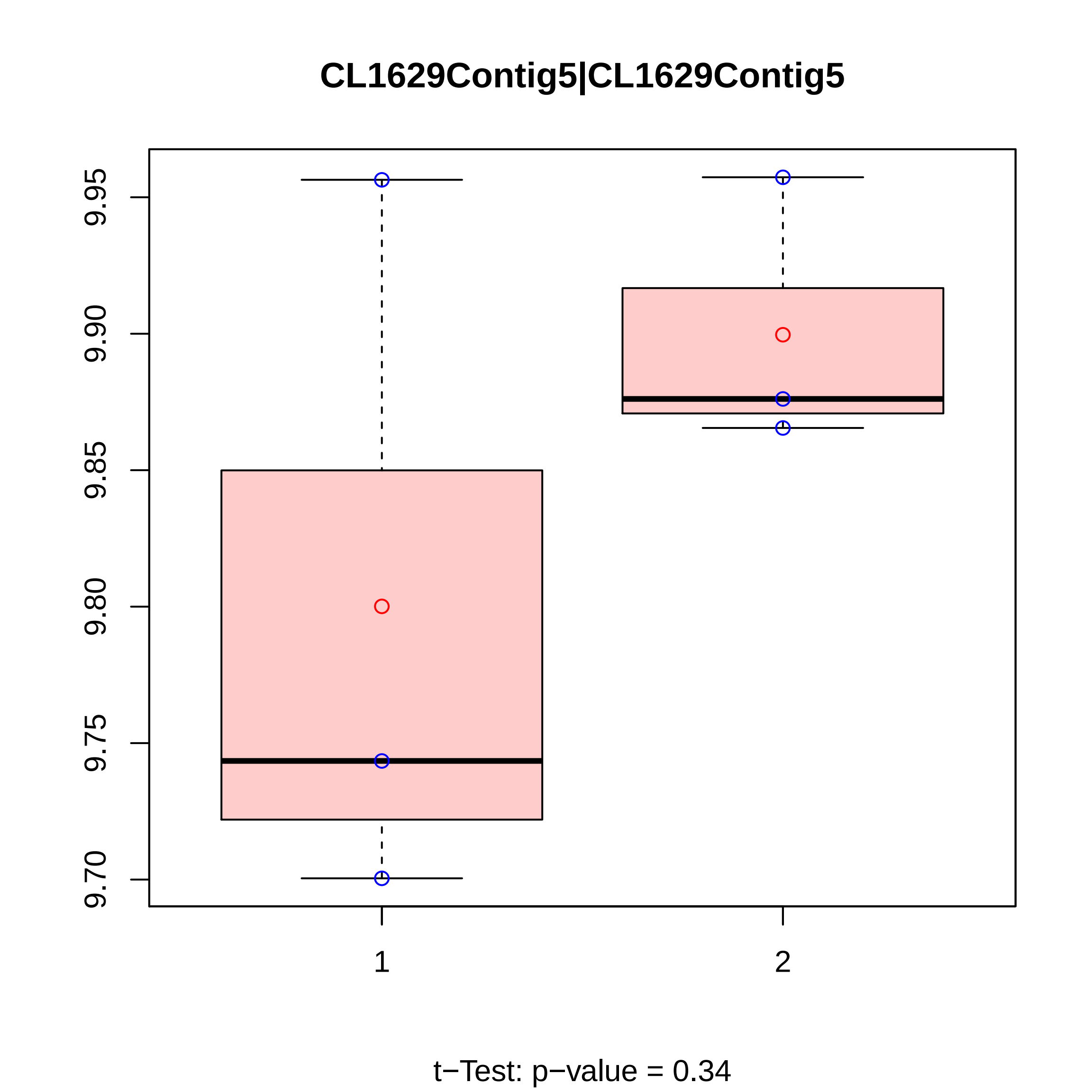
CL1629Contig5|CL1629Contig5
9.70
9.75
9.80
9.85
9.90
9.95
1
2
t−Test: p−value = 0.34

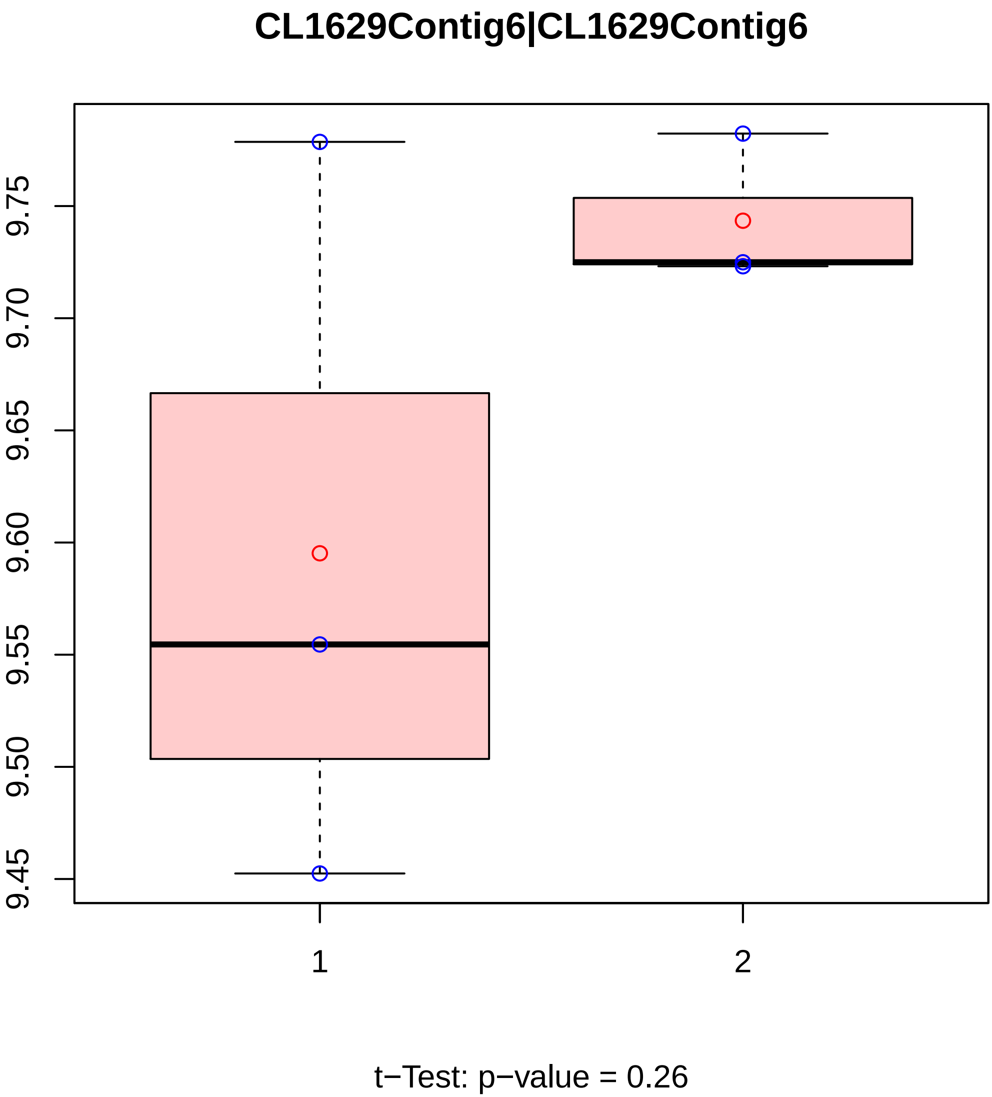
CL1629Contig6|CL1629Contig6
9.45
9.50
9.55
9.60
9.65
9.70
9.75
1
2
t−Test: p−value = 0.26

# CL1629Contig7|CL1629Contig7

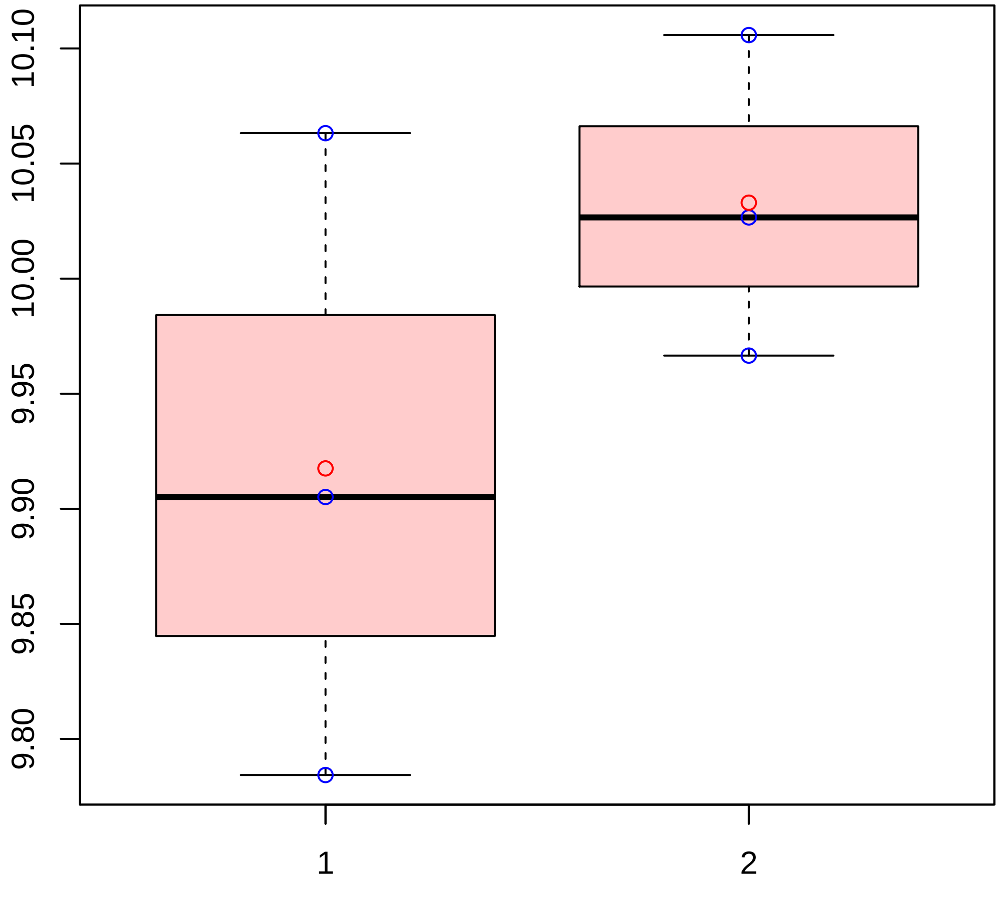


t−Test: p−value = 0.29

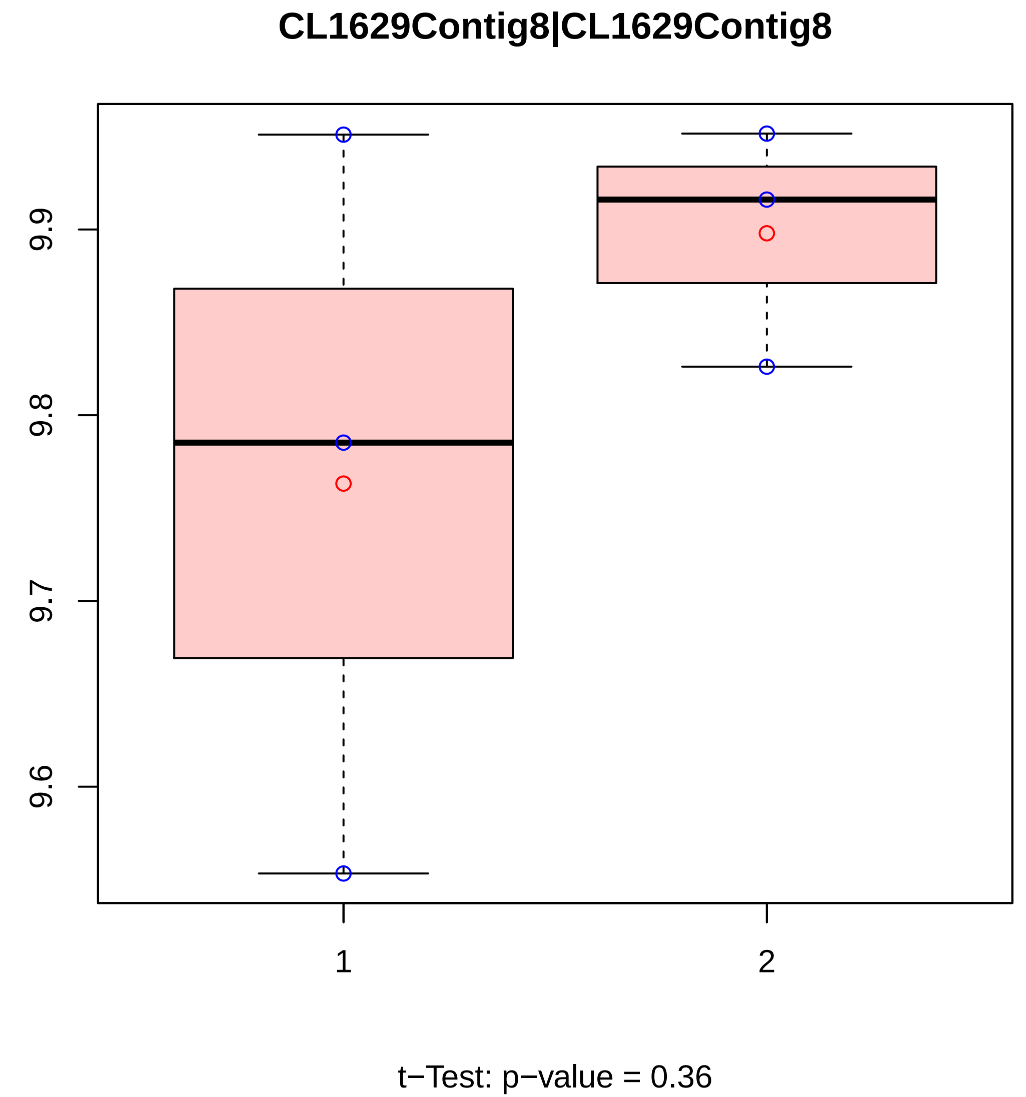
CL1629Contig8|CL1629Contig8
9.6
9.7
9.8
9.9
1
2
t−Test: p−value = 0.36

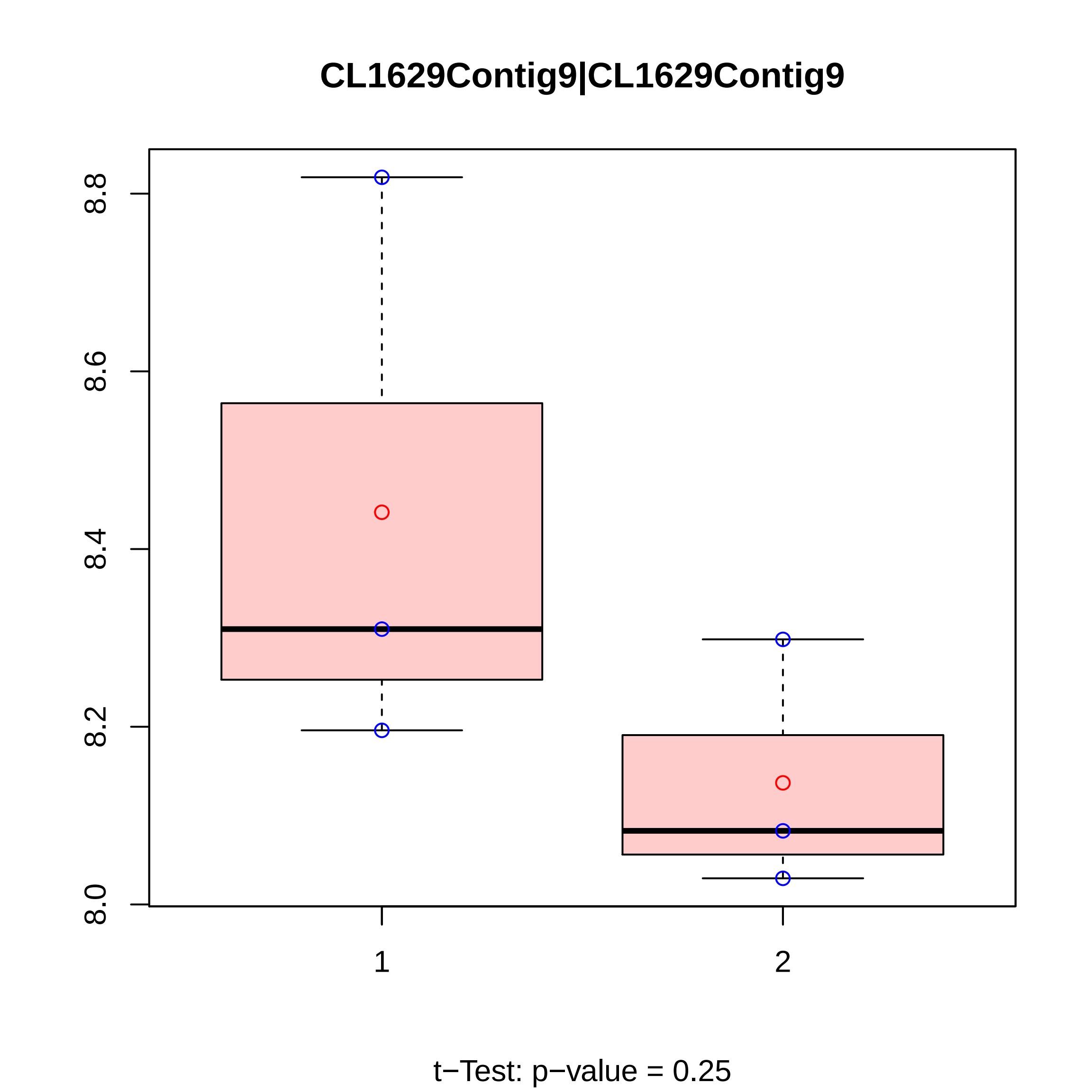
CL1629Contig9|CL1629Contig9
8.0
8.2
8.4
8.6
8.8
1
2
t−Test: p−value = 0.25

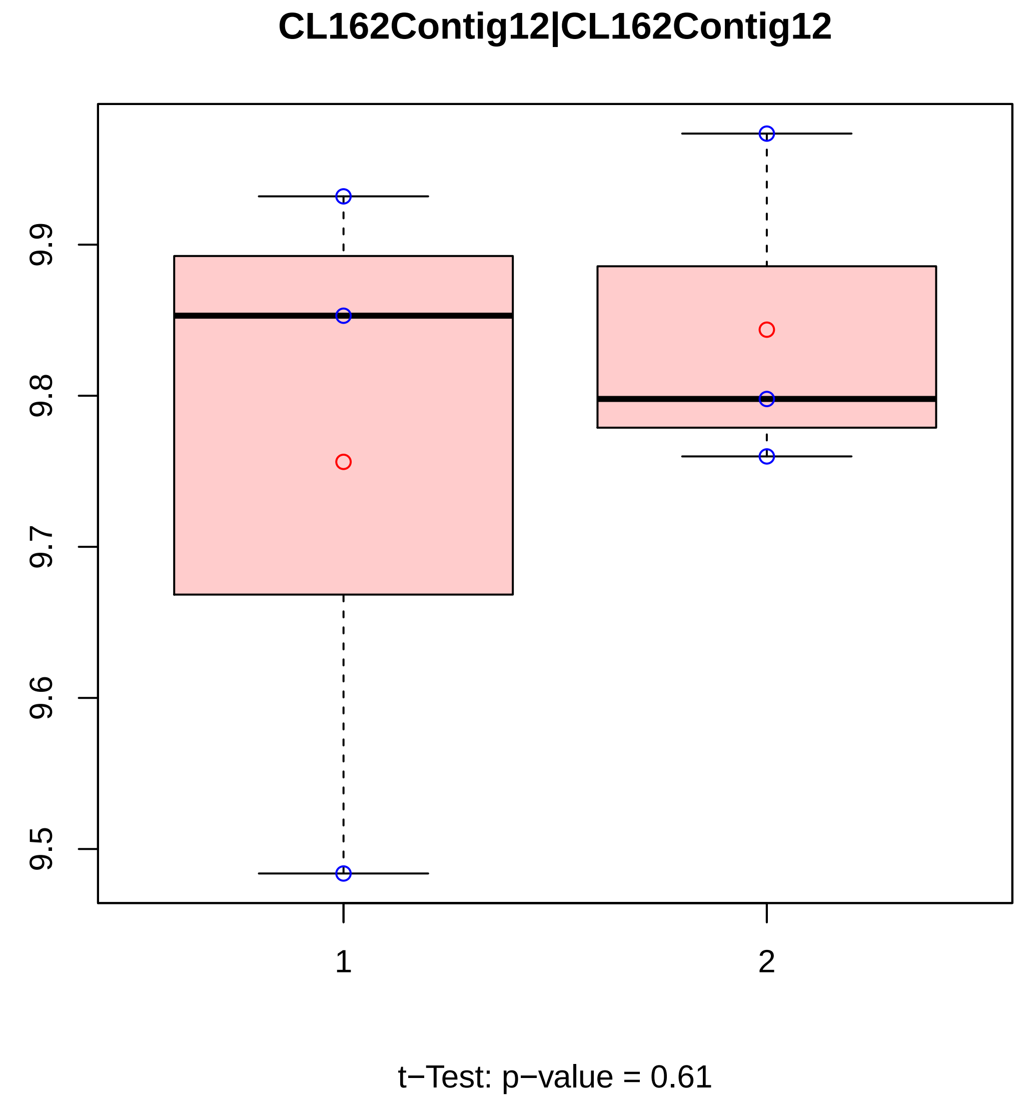
CL162Contig12|CL162Contig12
9.5
9.6
9.7
9.8
9.9
1
2
t−Test: p−value = 0.61

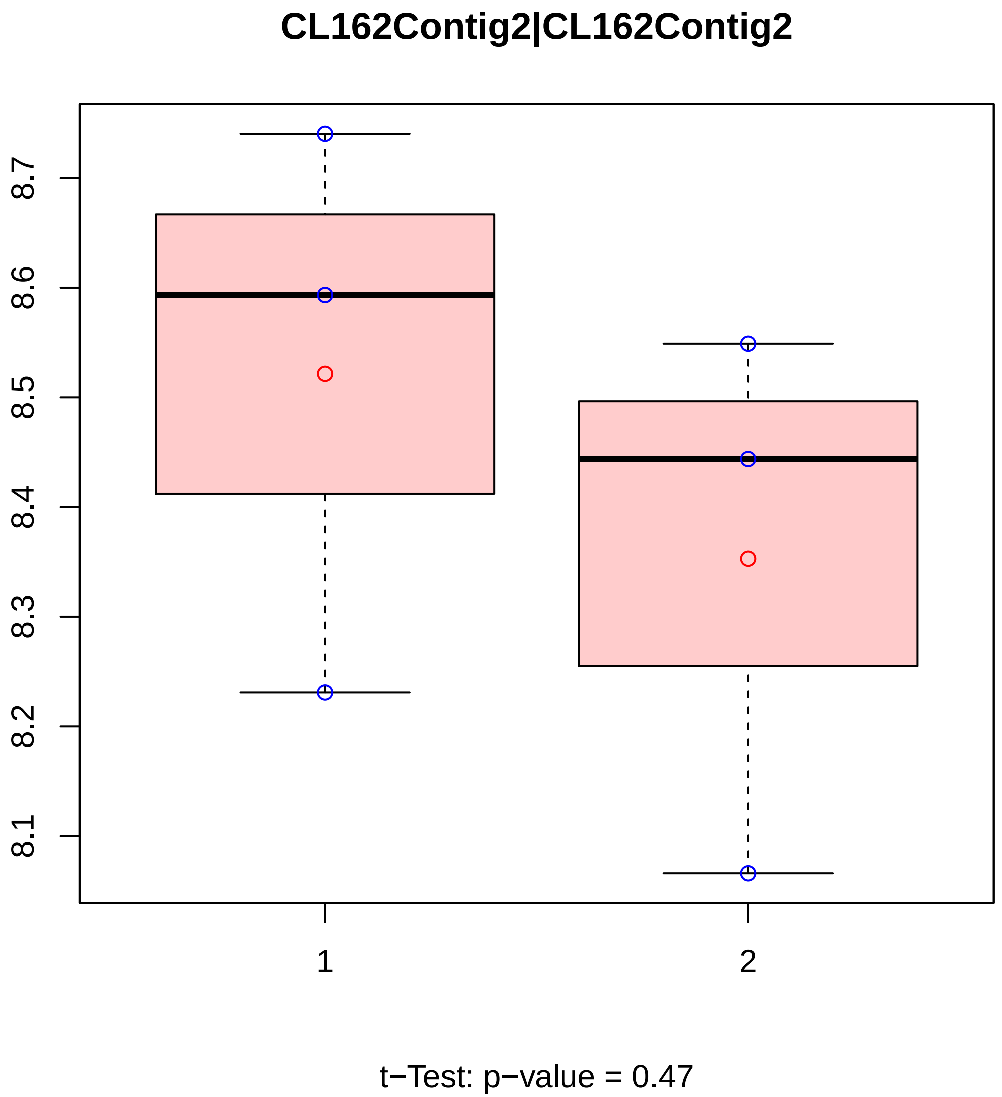
CL162Contig2|CL162Contig2
8.1
8.2
8.3
8.4
8.5
8.6
8.7
1
2
t−Test: p−value = 0.47

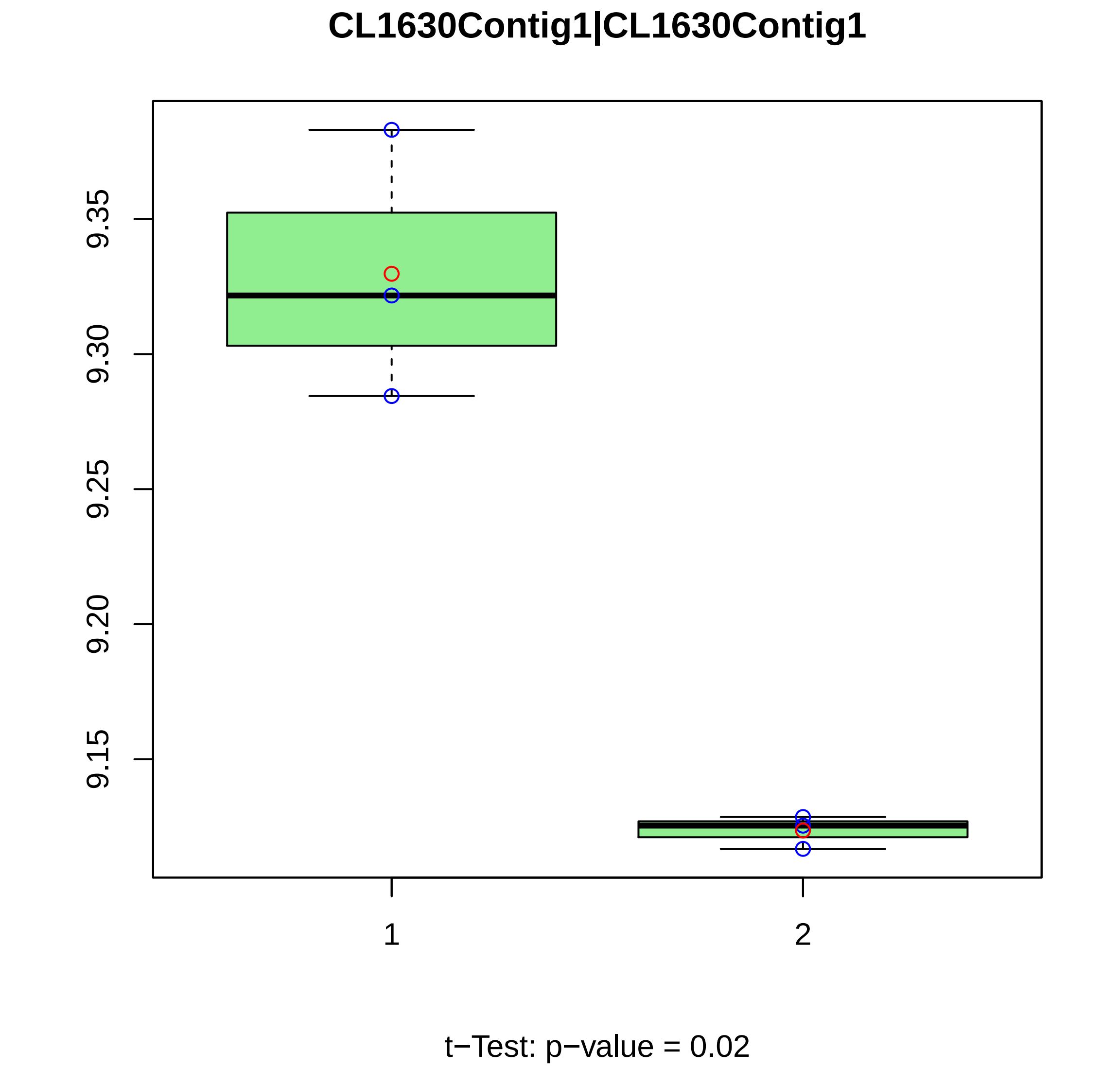
CL1630Contig1|CL1630Contig1
9.35
9.30
9.25
9.20
9.15
1
2
t−Test: p−value = 0.02

# CL1630Contig2|CL1630Contig2

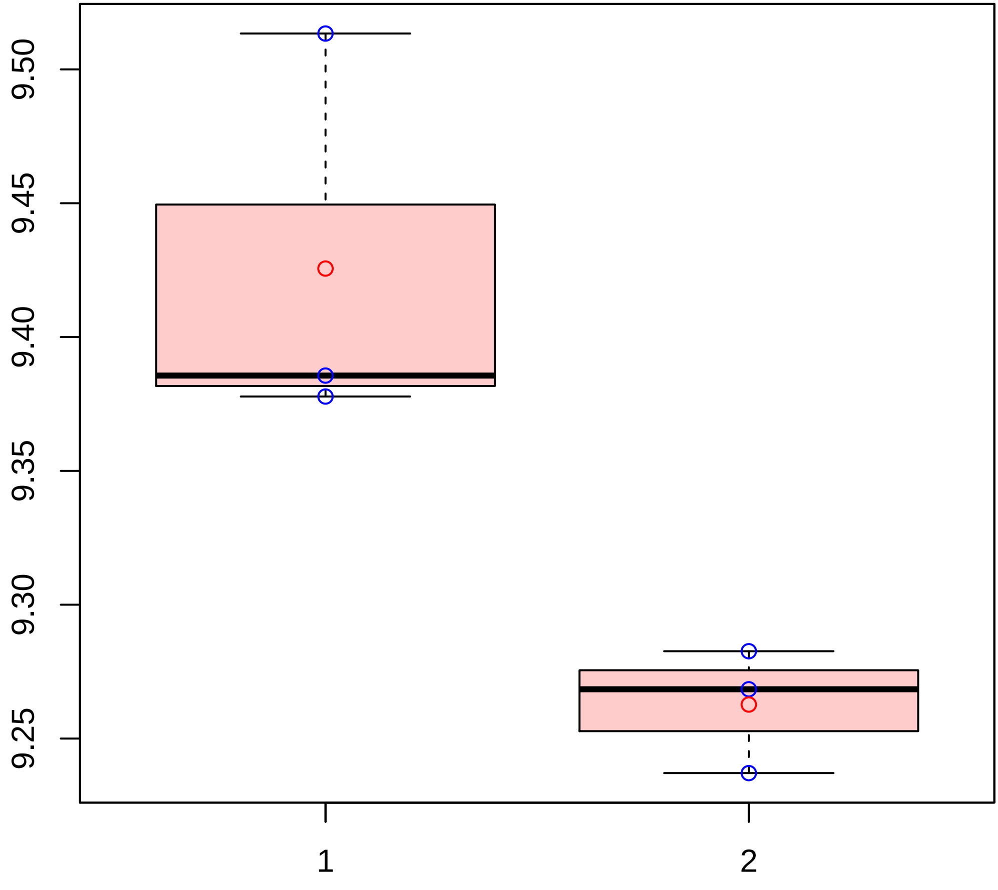


t−Test: p−value = 0.06

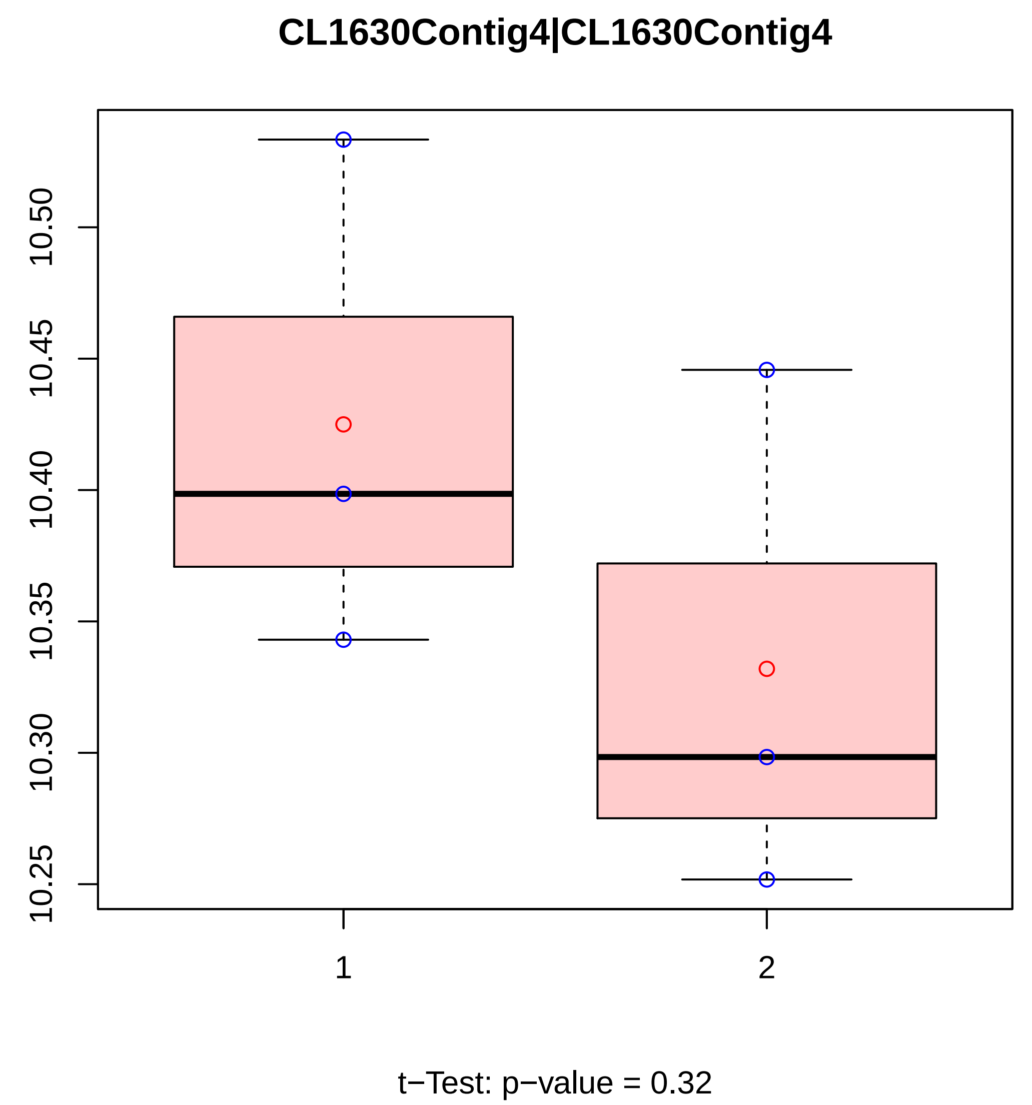
CL1630Contig4|CL1630Contig4
10.25
10.30
10.35
10.40
10.45
10.50
1
2
t−Test: p−value = 0.32

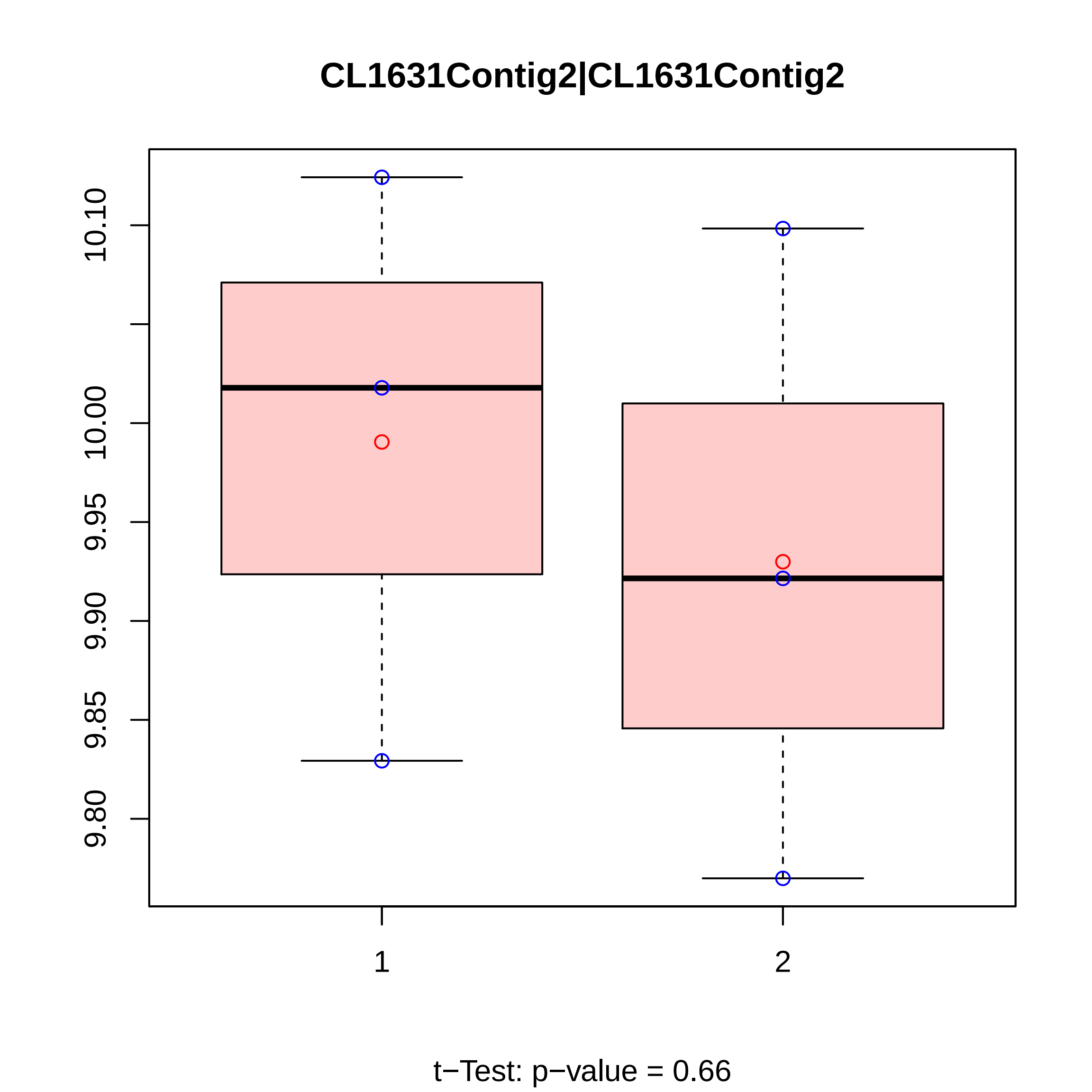
CL1631Contig2|CL1631Contig2
10.10
10.00
9.95
9.90
9.85
9.80
1
2
t−Test: p−value = 0.66

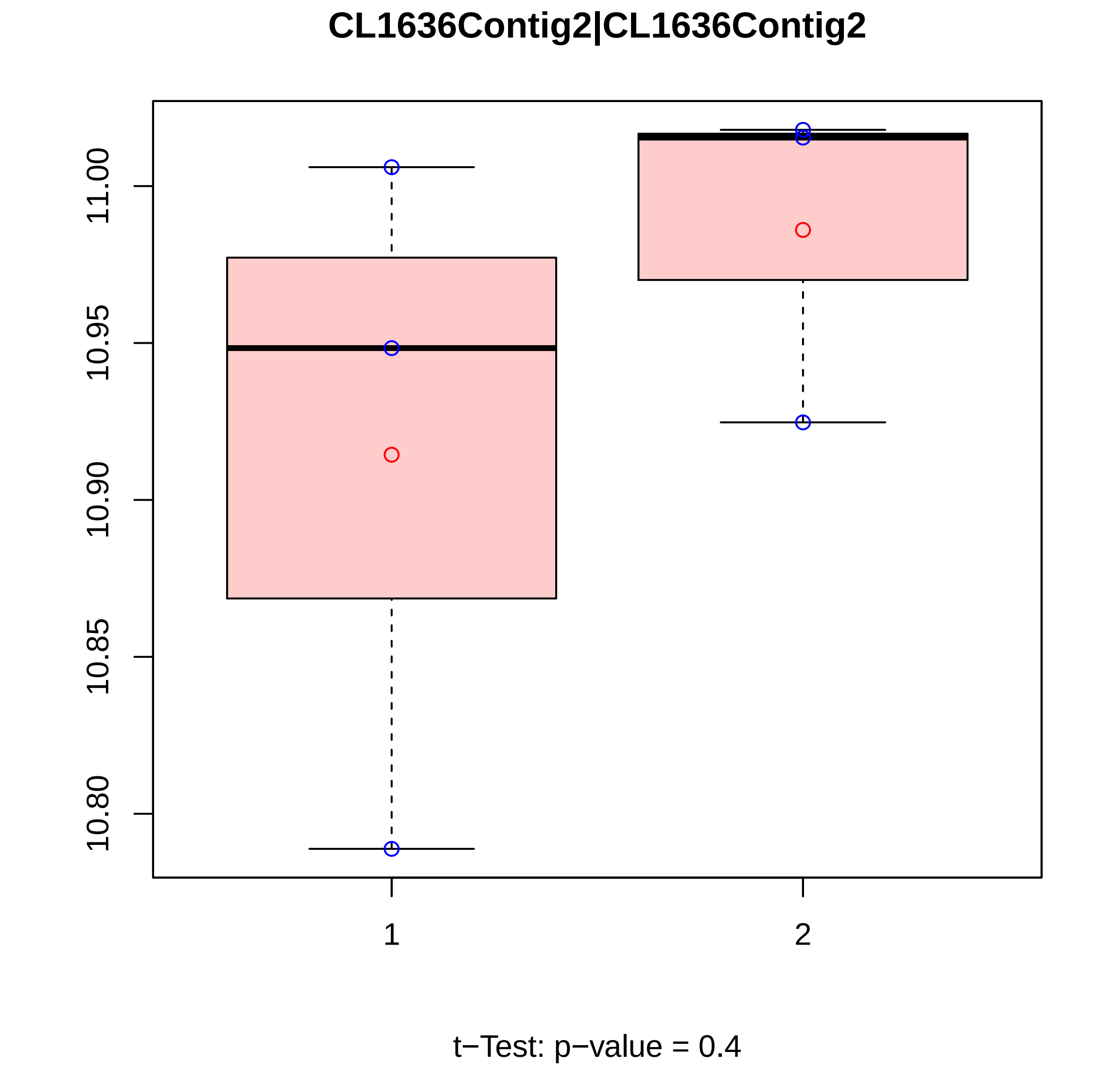
CL1636Contig2|CL1636Contig2
11.00
10.95
10.90
10.85
10.80
1
2
t−Test: p−value = 0.4

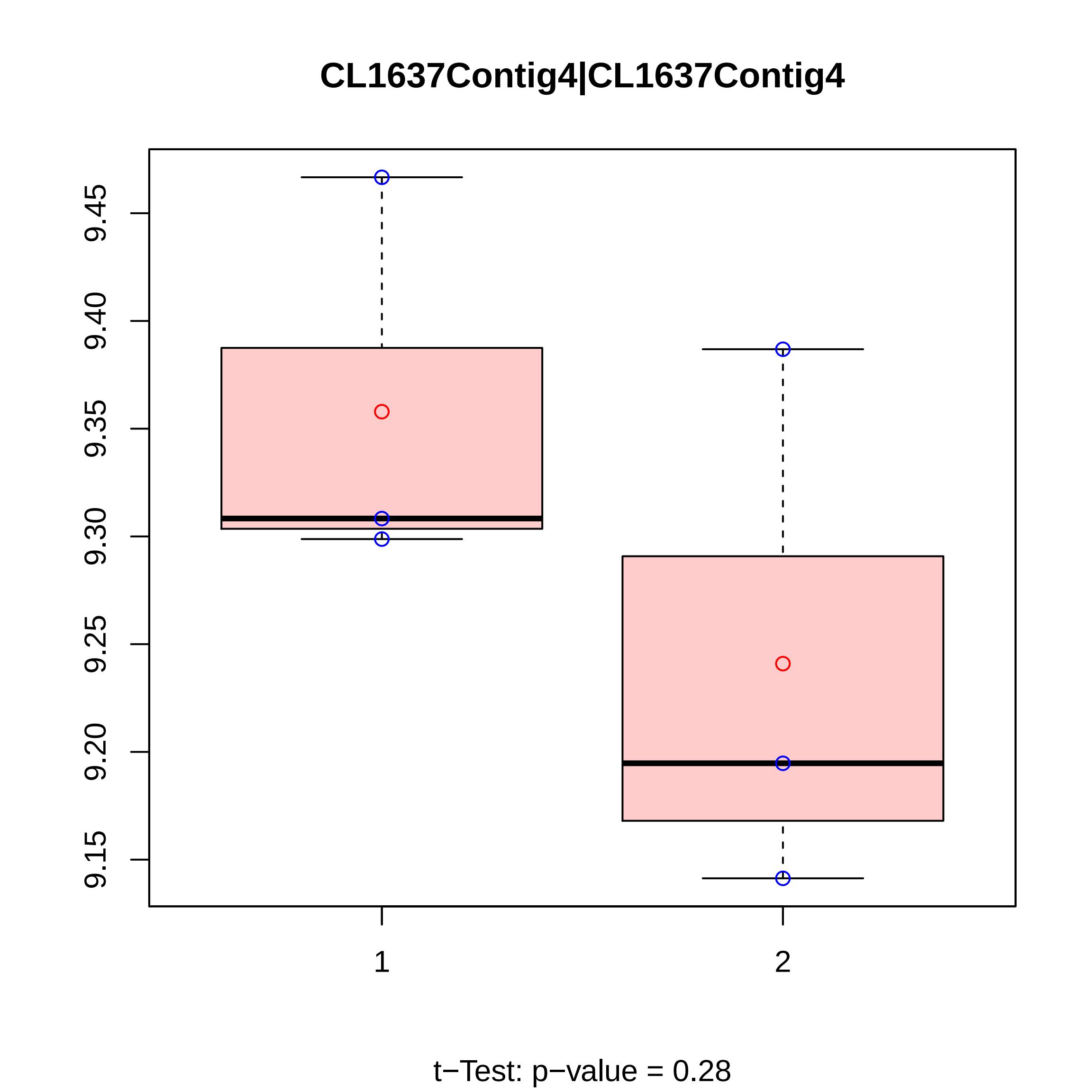
CL1637Contig4|CL1637Contig4
9.15
9.20
9.25
9.30
9.35
9.40
9.45
1
2
t−Test: p−value = 0.28

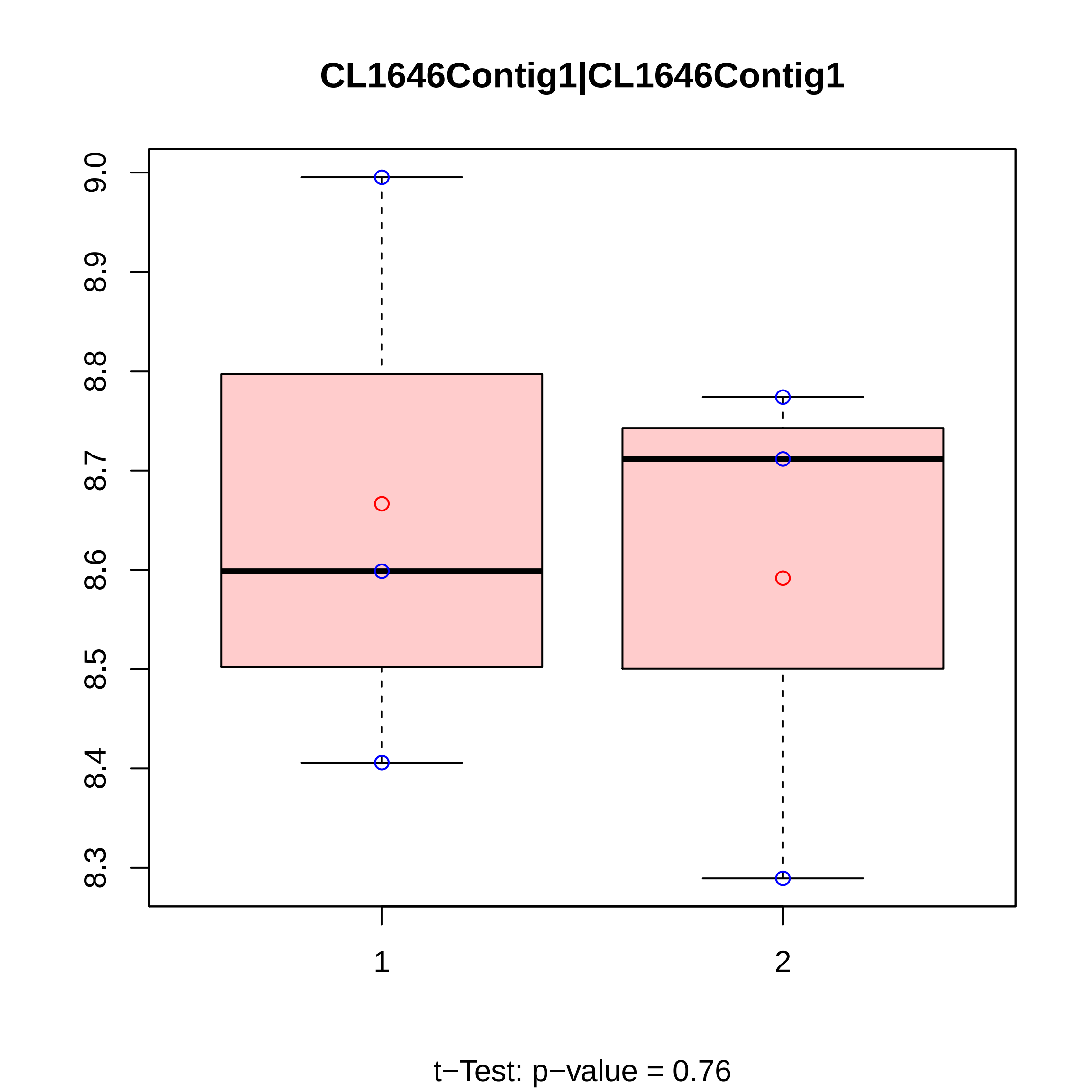
CL1646Contig1|CL1646Contig1
9.0
8.9
8.8
8.7
8.6
8.5
8.4
8.3
1
2
t−Test: p−value = 0.76

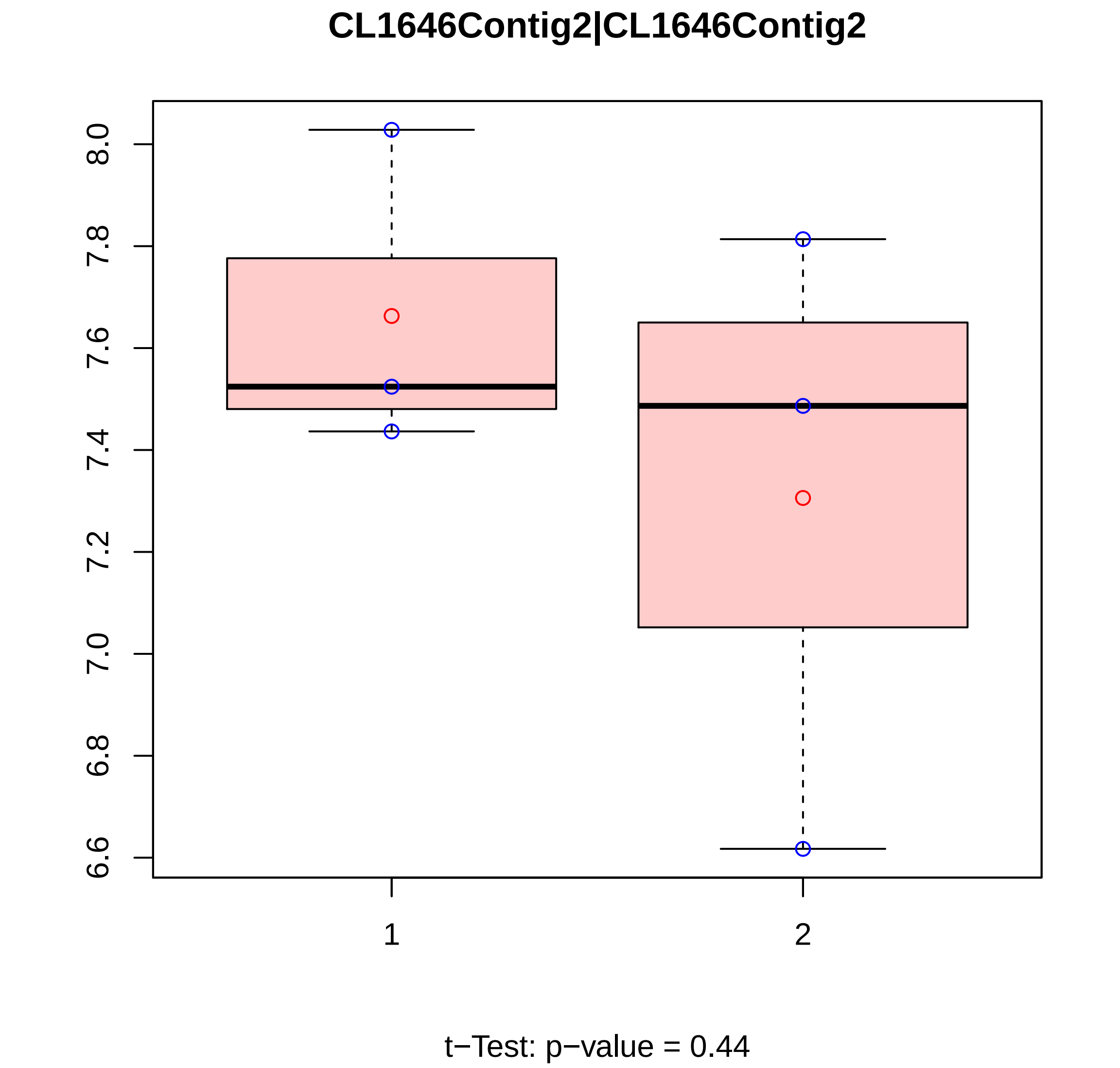
CL1646Contig2|CL1646Contig2
8.0
7.8
7.6
7.4
7.2
7.0
6.8
6.6
1
2
t−Test: p−value = 0.44

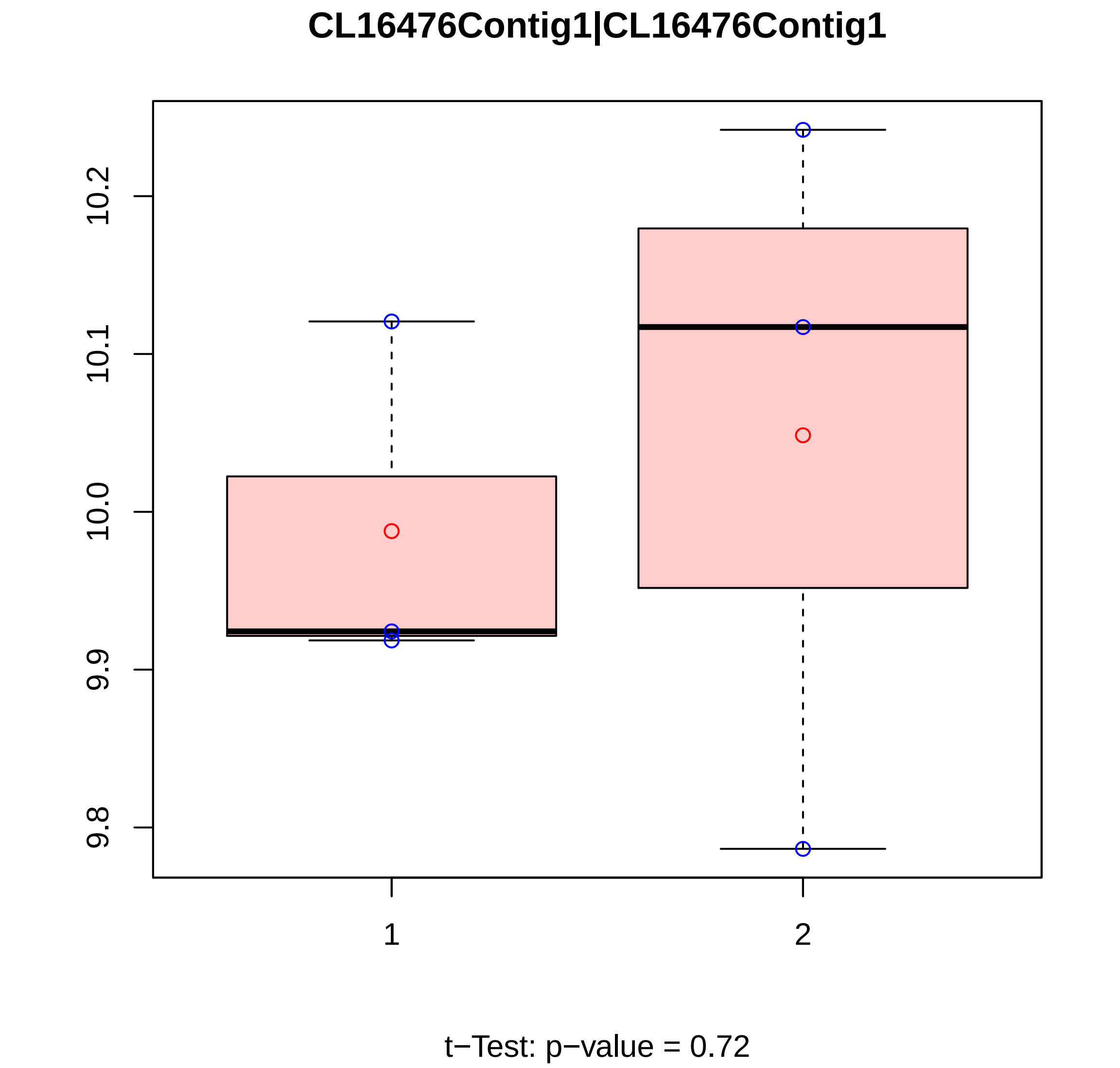
CL16476Contig1|CL16476Contig1
10.2
10.1
10.0
9.9
9.8
1
2
t−Test: p−value = 0.72

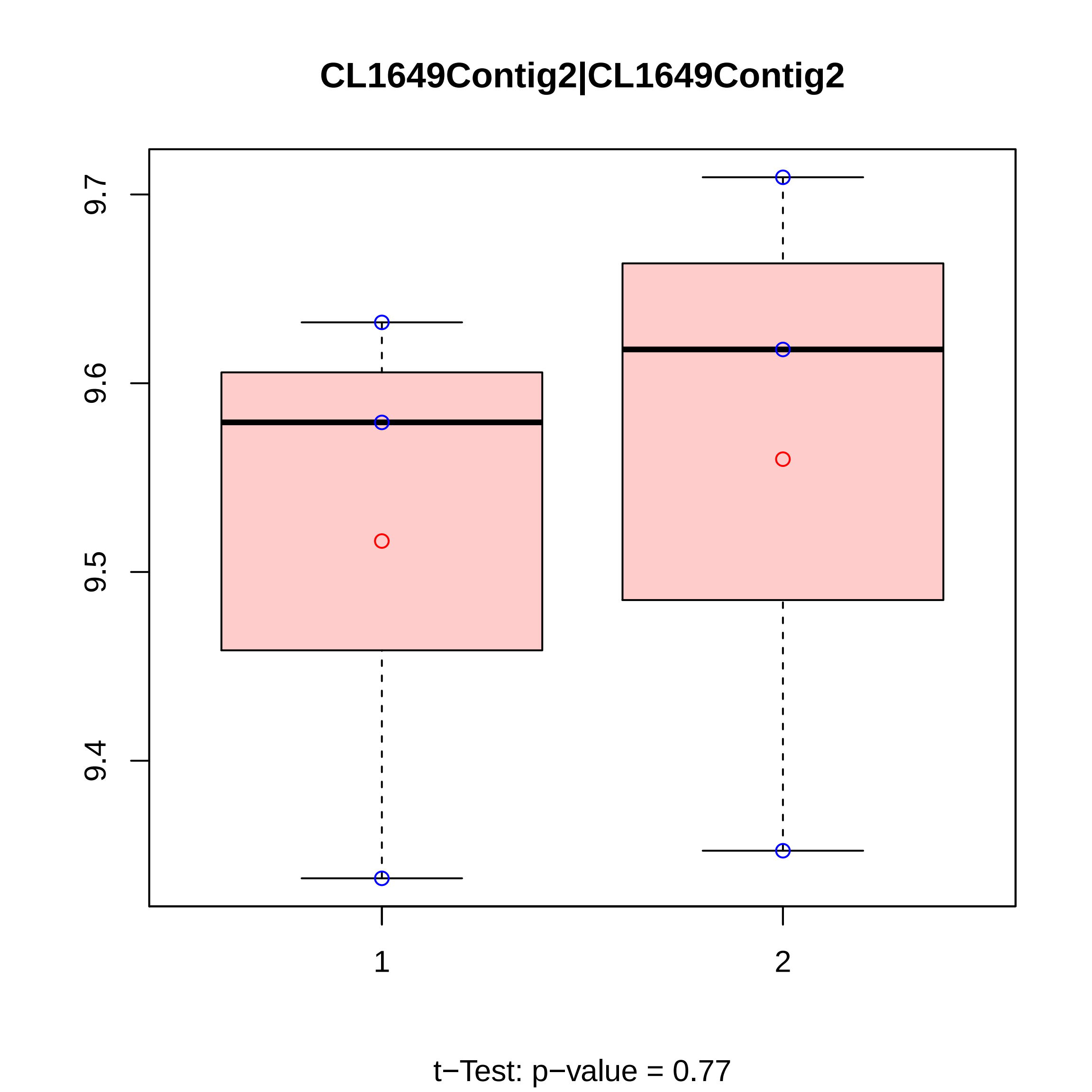
CL1649Contig2|CL1649Contig2
9.4
9.5
9.6
9.7
1
2
t−Test: p−value = 0.77

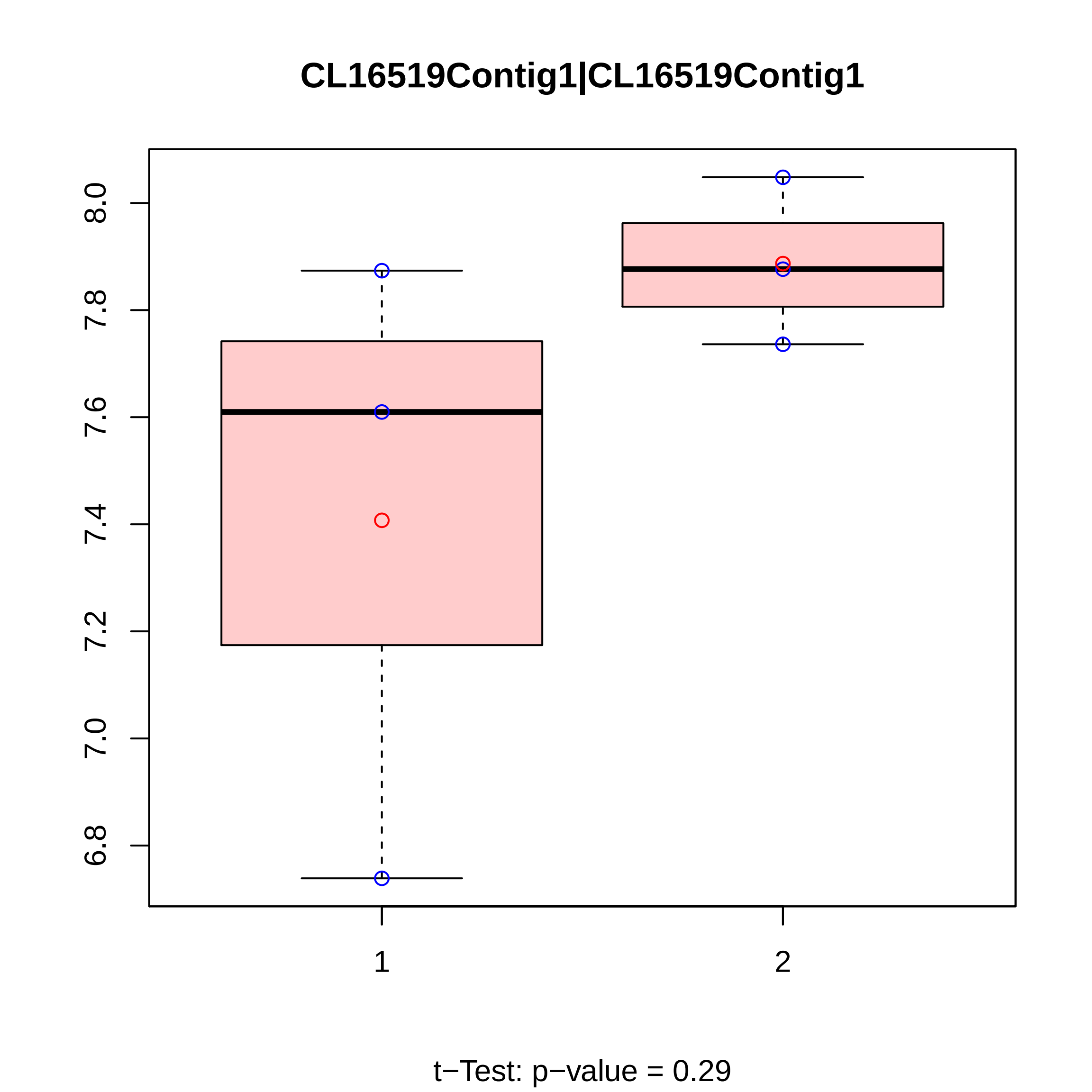
CL16519Contig1|CL16519Contig1
8.0
7.8
7.6
7.4
7.2
7.0
6.8
1
2
t−Test: p−value = 0.29

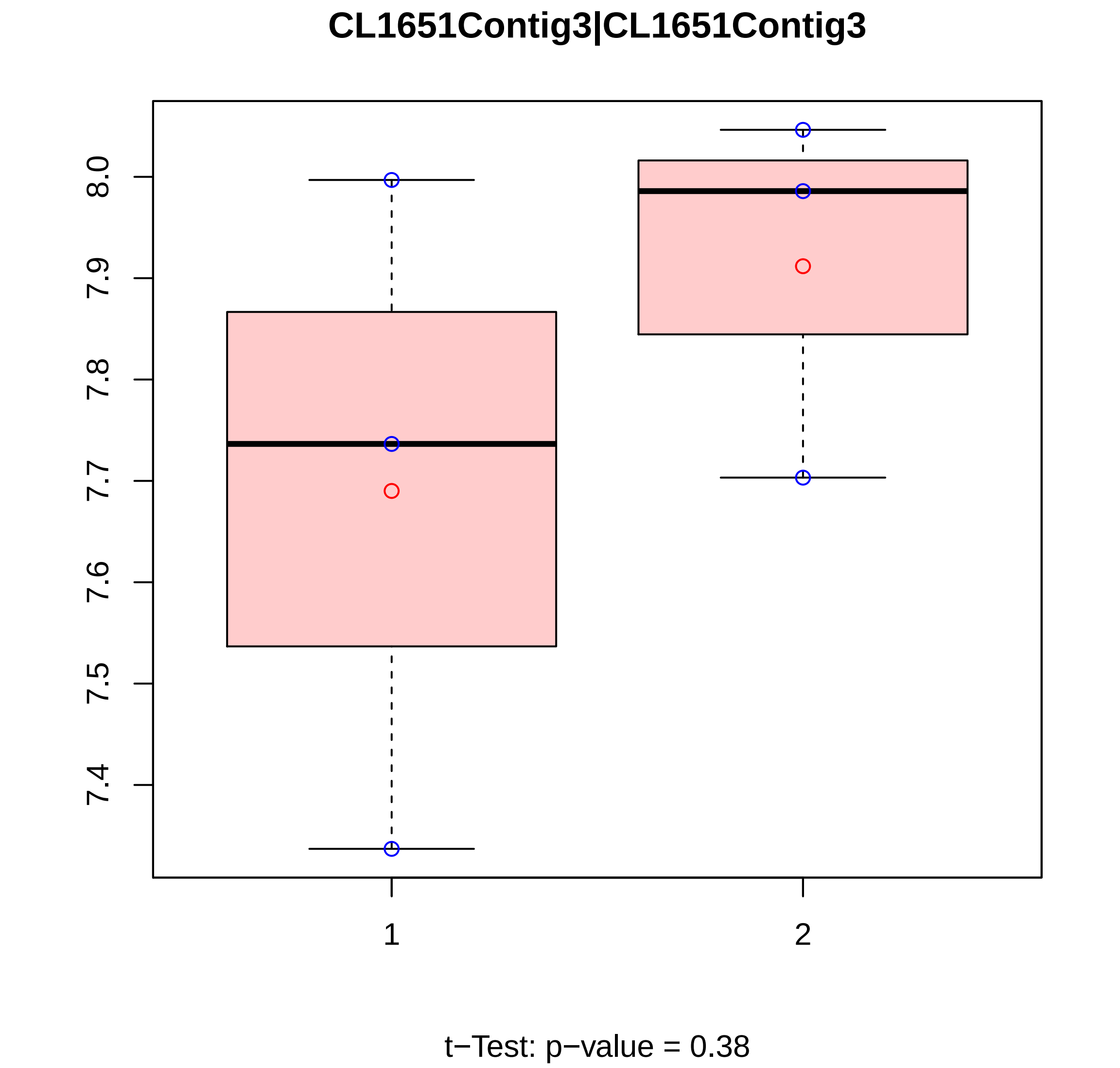

CL1651Contig3|CL1651Contig3
8.0
7.9
7.8
7.7
7.6
7.5
7.4
1
2
t−Test: p−value = 0.38

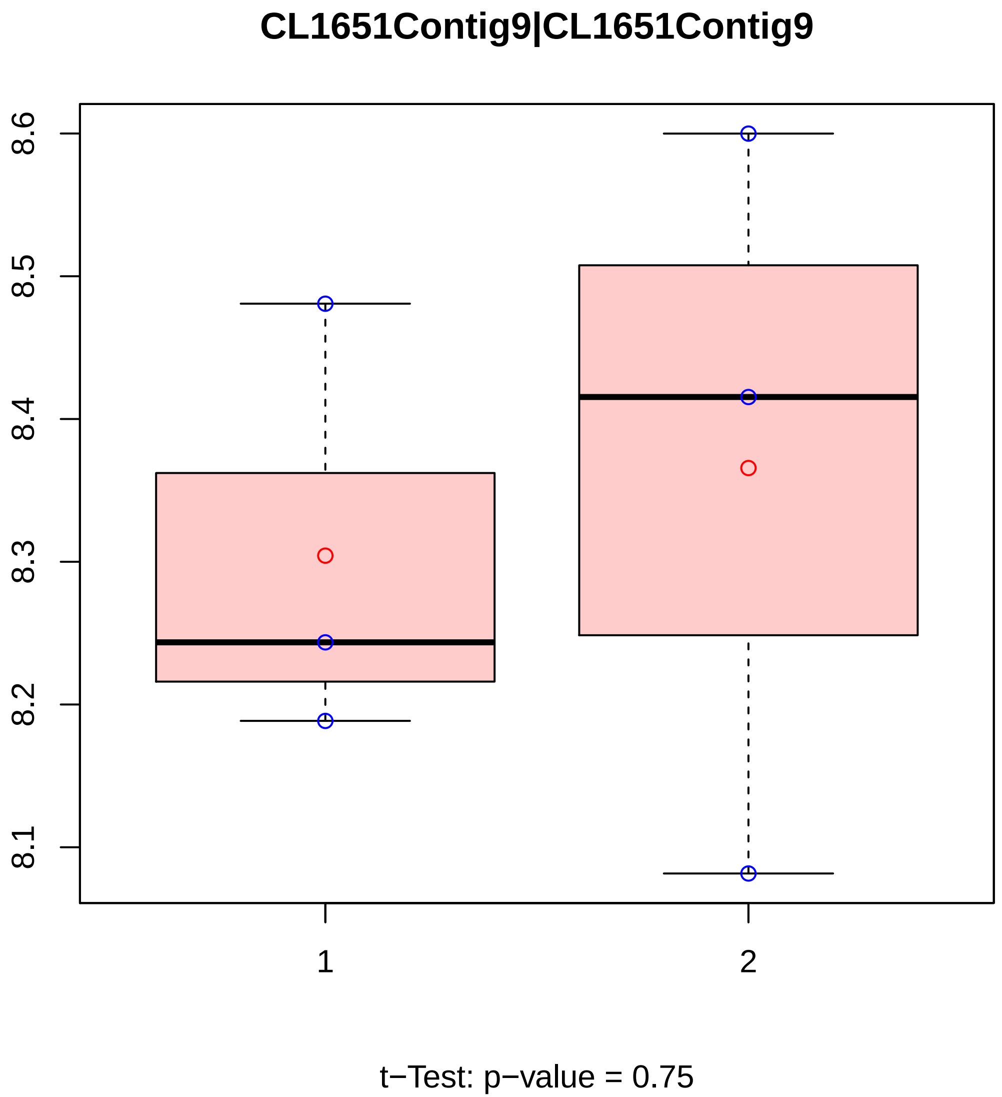

CL1651Contig9|CL1651Contig9
8.1
8.2
8.3
8.4
8.5
8.6
1
2
t−Test: p−value = 0.75

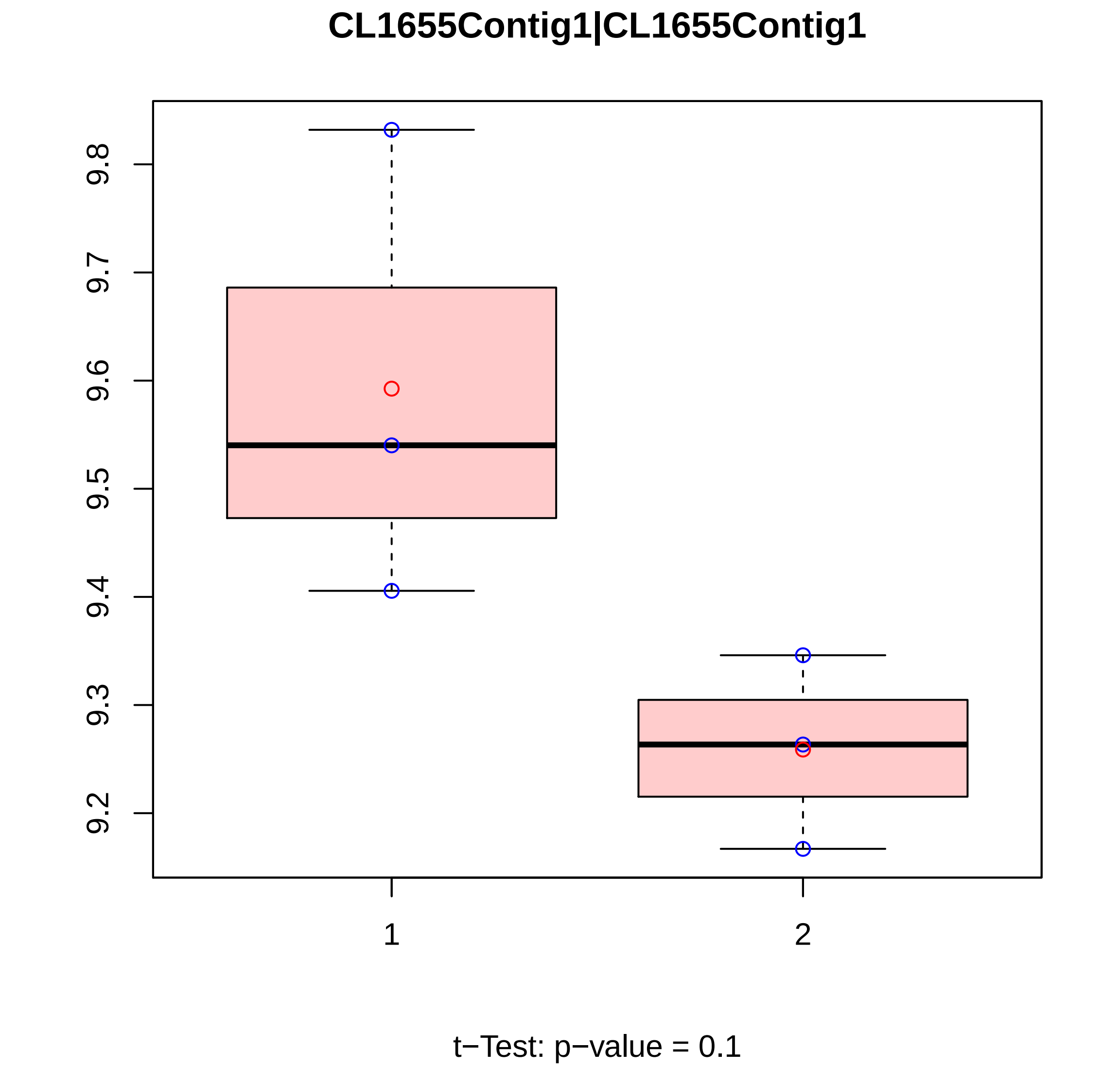
CL1655Contig1|CL1655Contig1
9.2
9.3
9.4
9.5
9.6
9.7
9.8
1
2
t−Test: p−value = 0.1

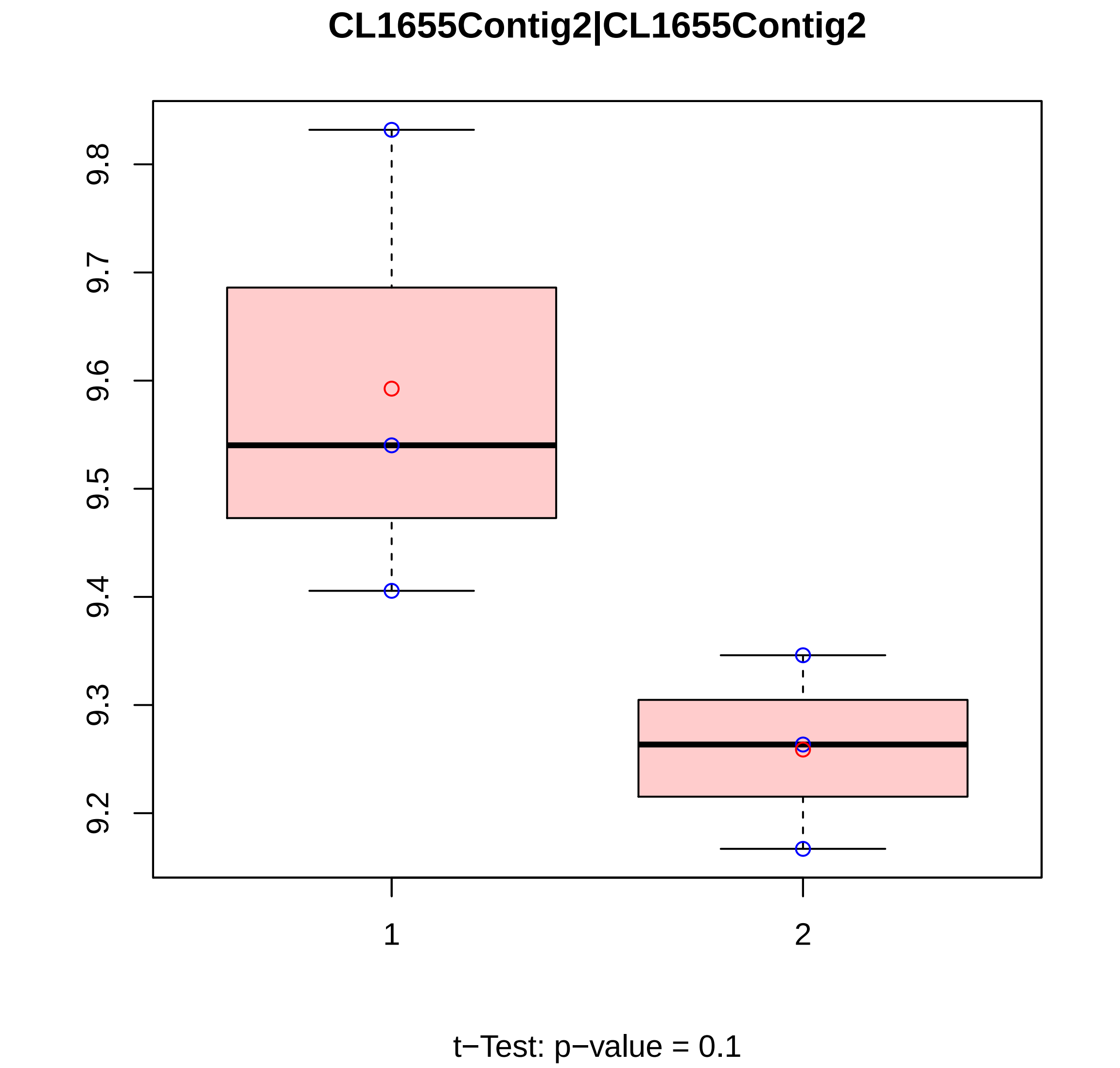
CL1655Contig2|CL1655Contig2
9.2
9.3
9.4
9.5
9.6
9.7
9.8
1
2
t−Test: p−value = 0.1

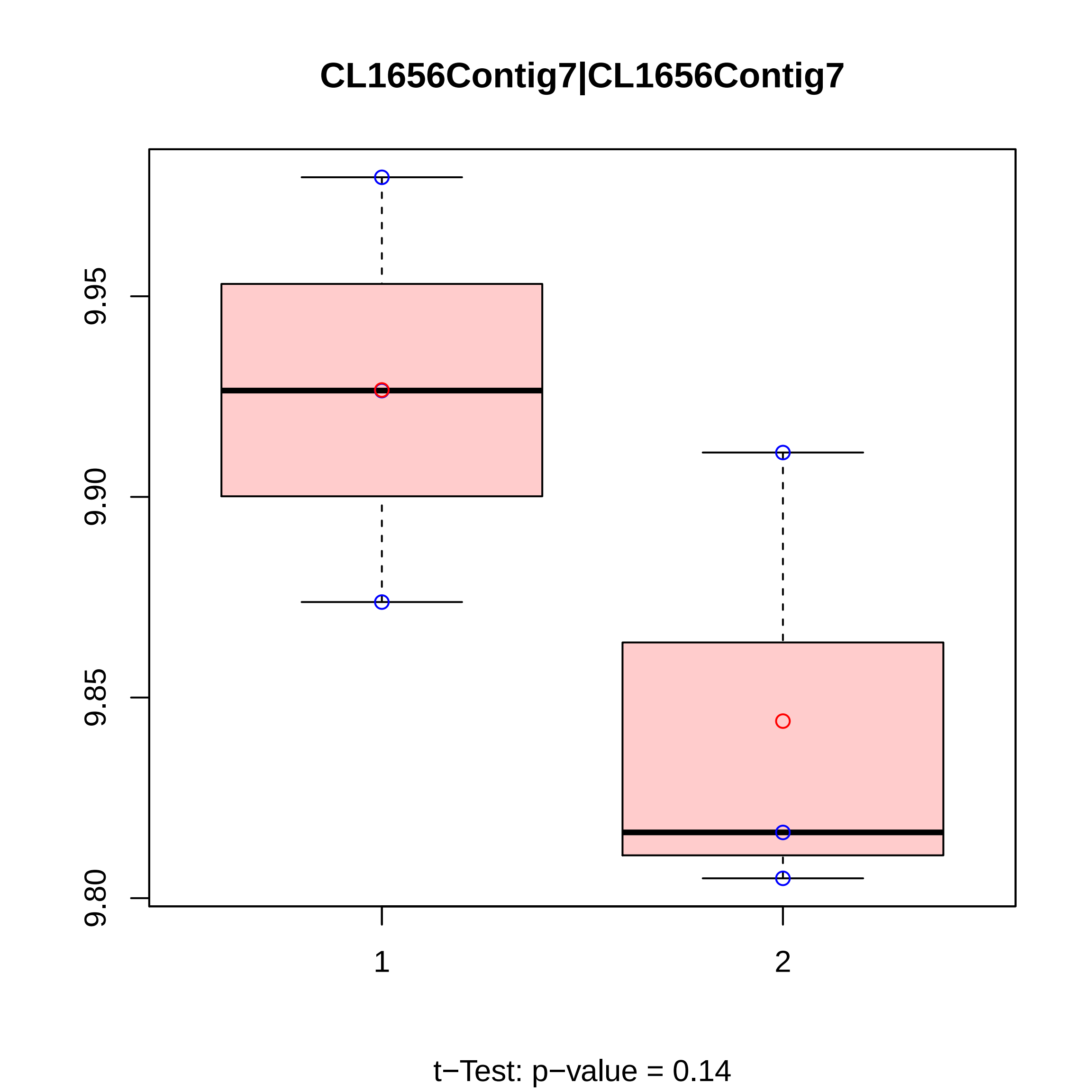
CL1656Contig7|CL1656Contig7
9.80
9.85
9.90
9.95
1
2
t−Test: p−value = 0.14

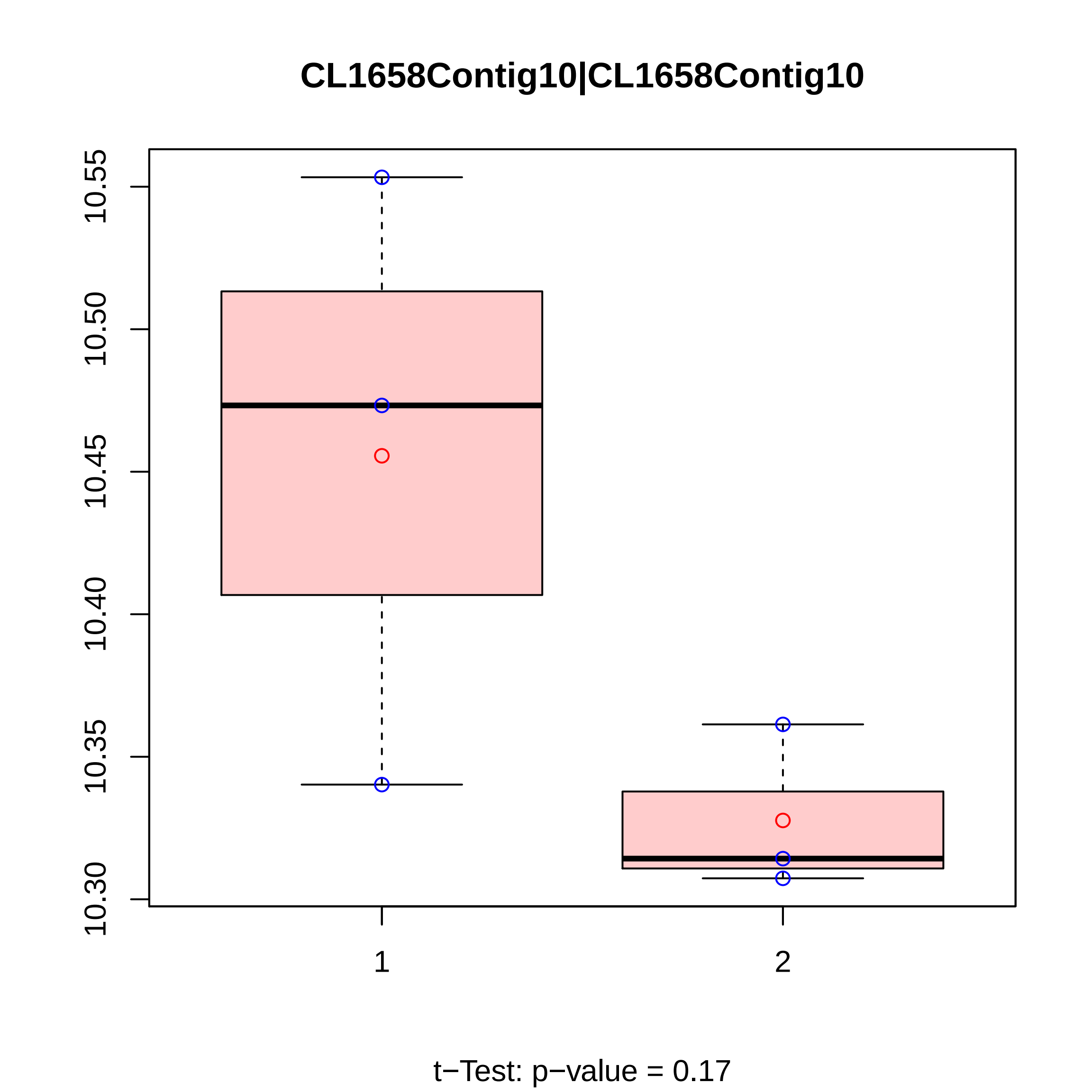
CL1658Contig10|CL1658Contig10
10.30
10.35
10.40
10.45
10.50
10.55
1
2
t−Test: p−value = 0.17

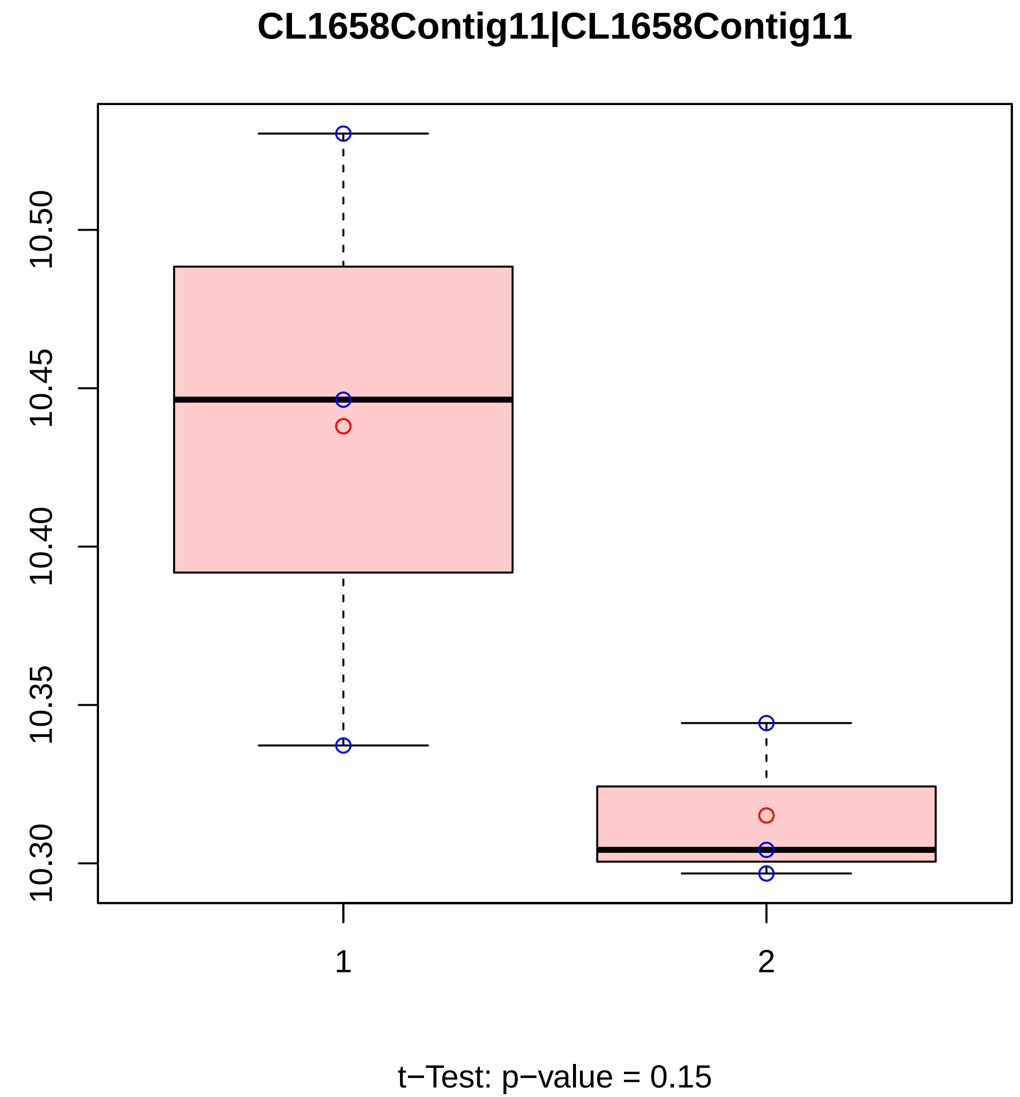
CL1658Contig11|CL1658Contig11
10.30
10.35
10.40
10.45
10.50
1
2
t−Test: p−value = 0.15

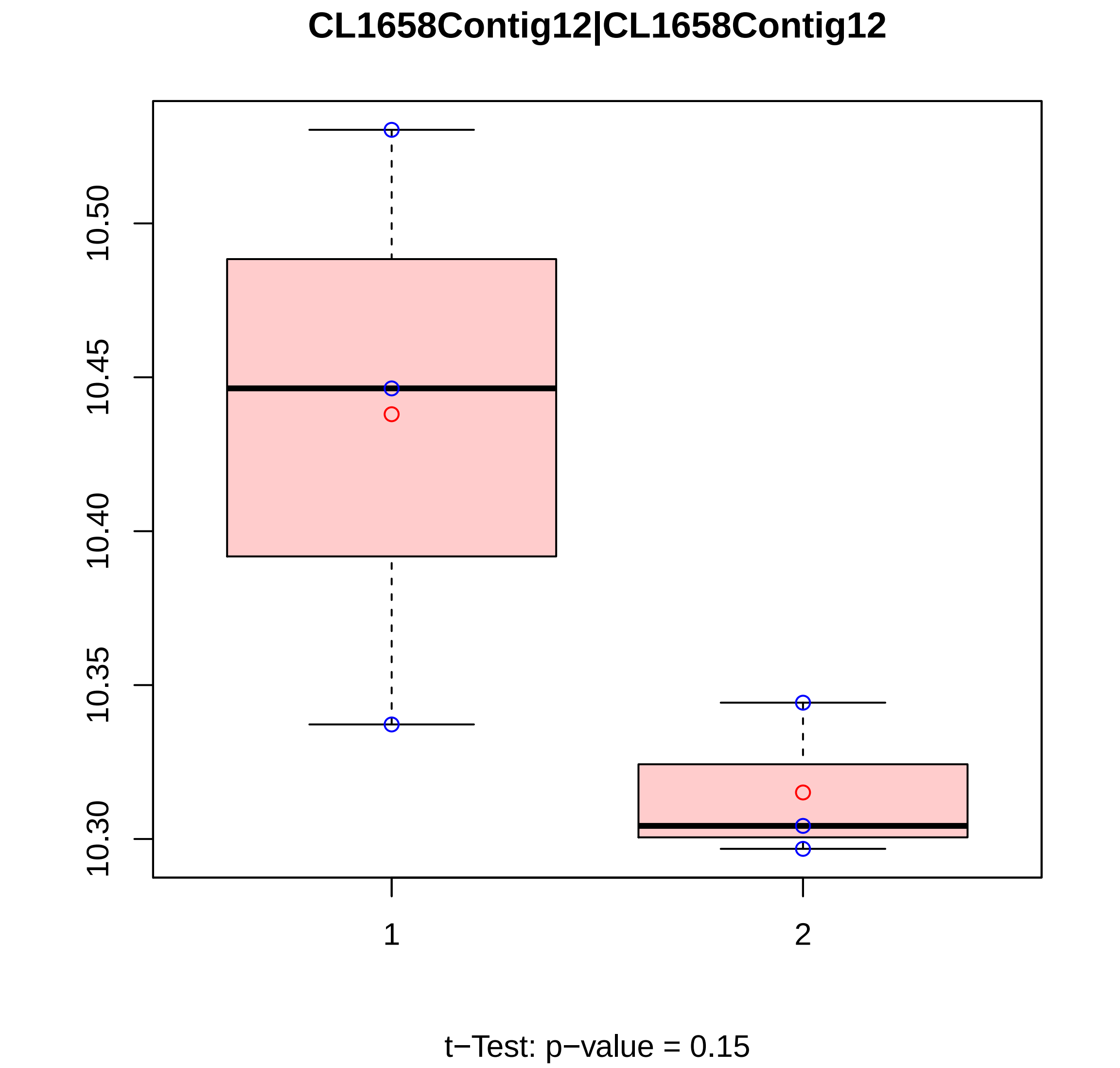

CL1658Contig12|CL1658Contig12
10.30
10.35
10.40
10.45
10.50
1
2
t−Test: p−value = 0.15

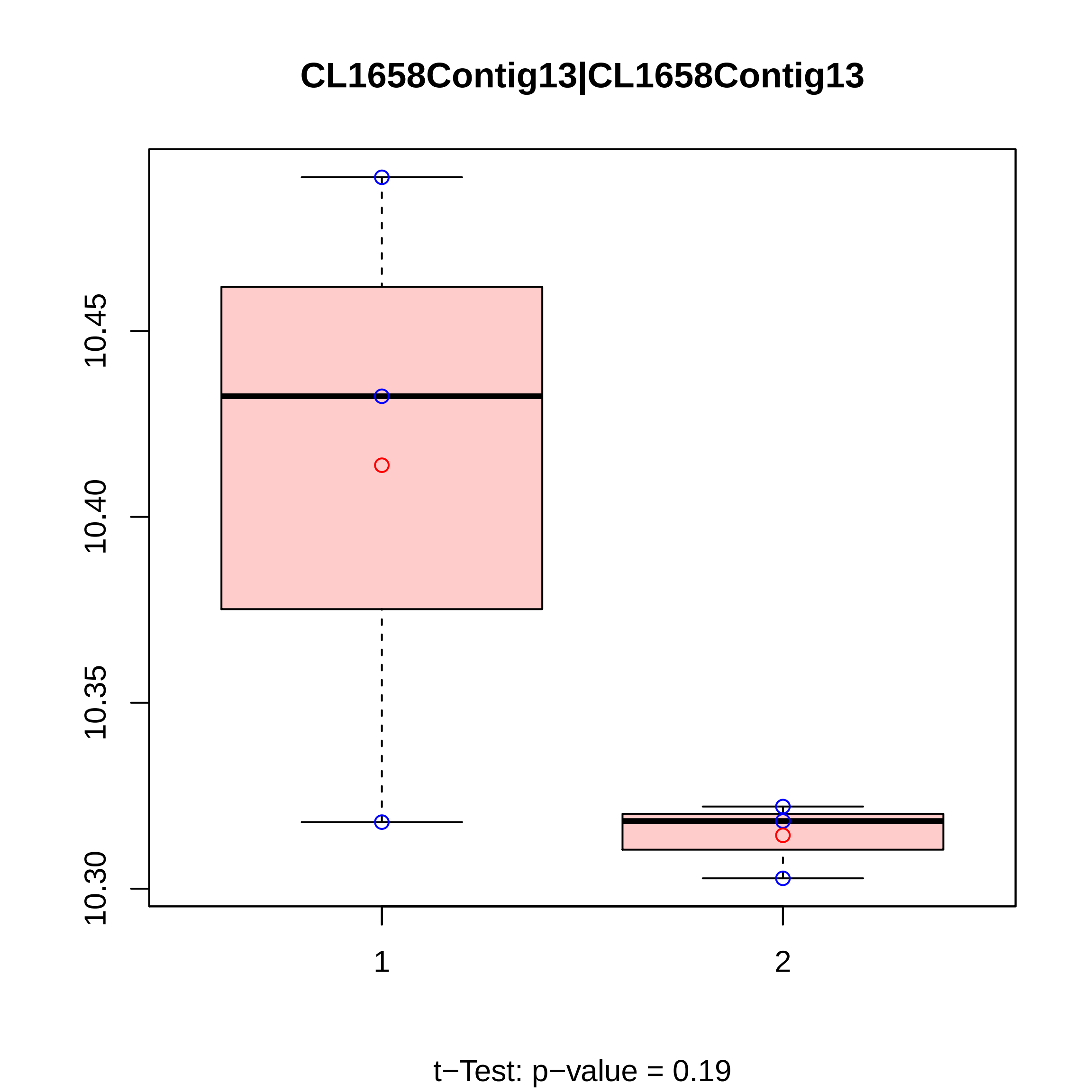

CL1658Contig13|CL1658Contig13
10.30
10.35
10.40
10.45
1
2
t−Test: p−value = 0.19

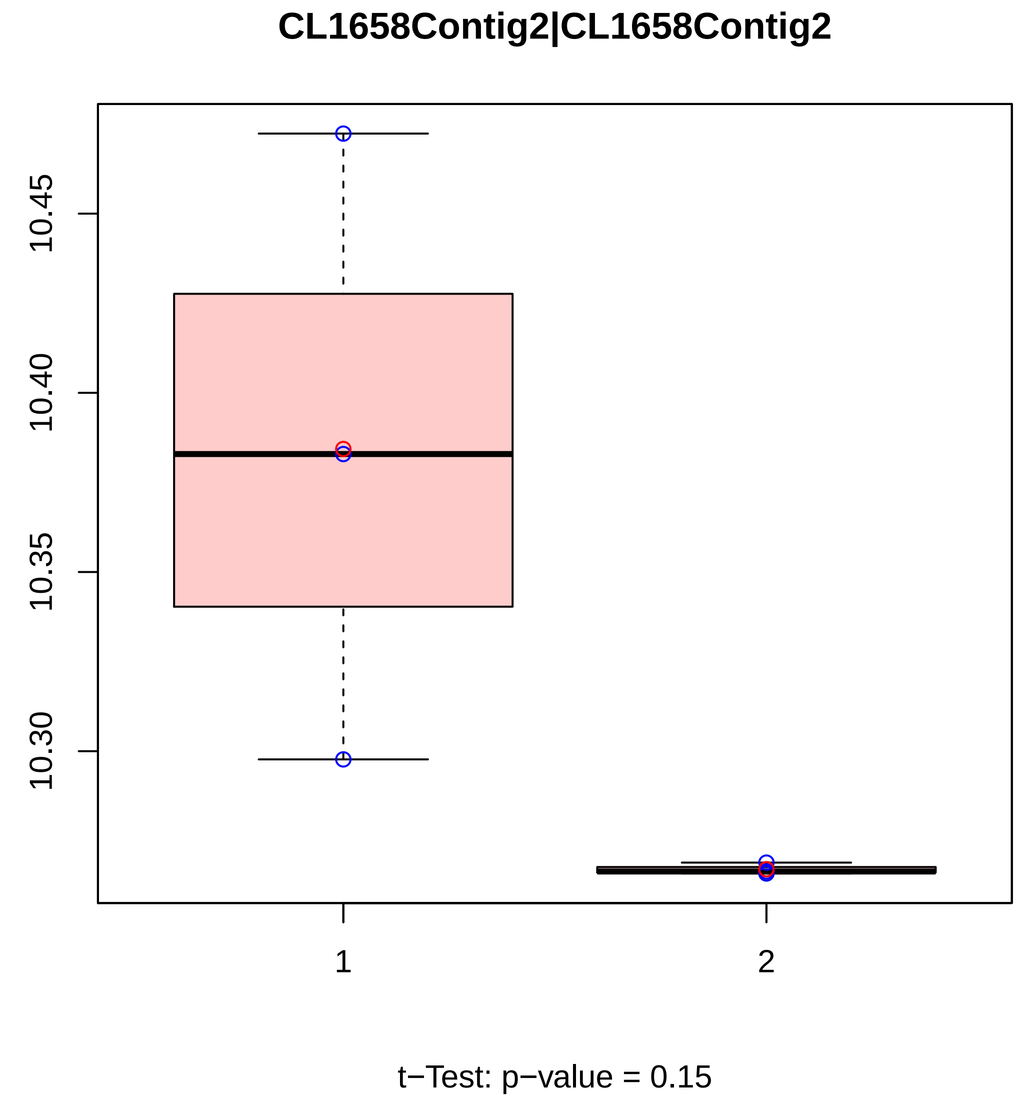

CL1658Contig2|CL1658Contig2
10.30
10.35
10.40
10.45
1
2
t−Test: p−value = 0.15

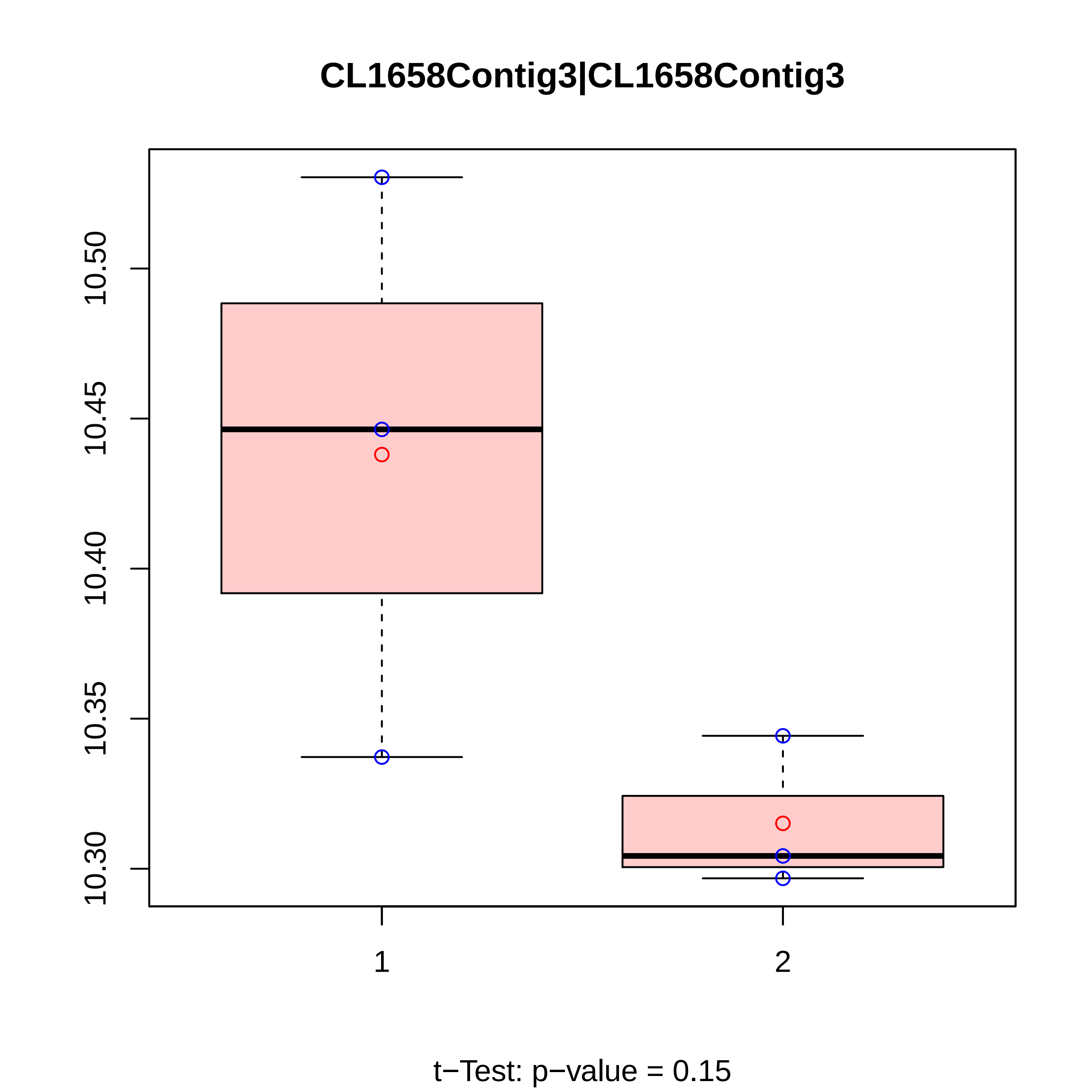

CL1658Contig3|CL1658Contig3
10.30
10.35
10.40
10.45
10.50
1
2
t−Test: p−value = 0.15

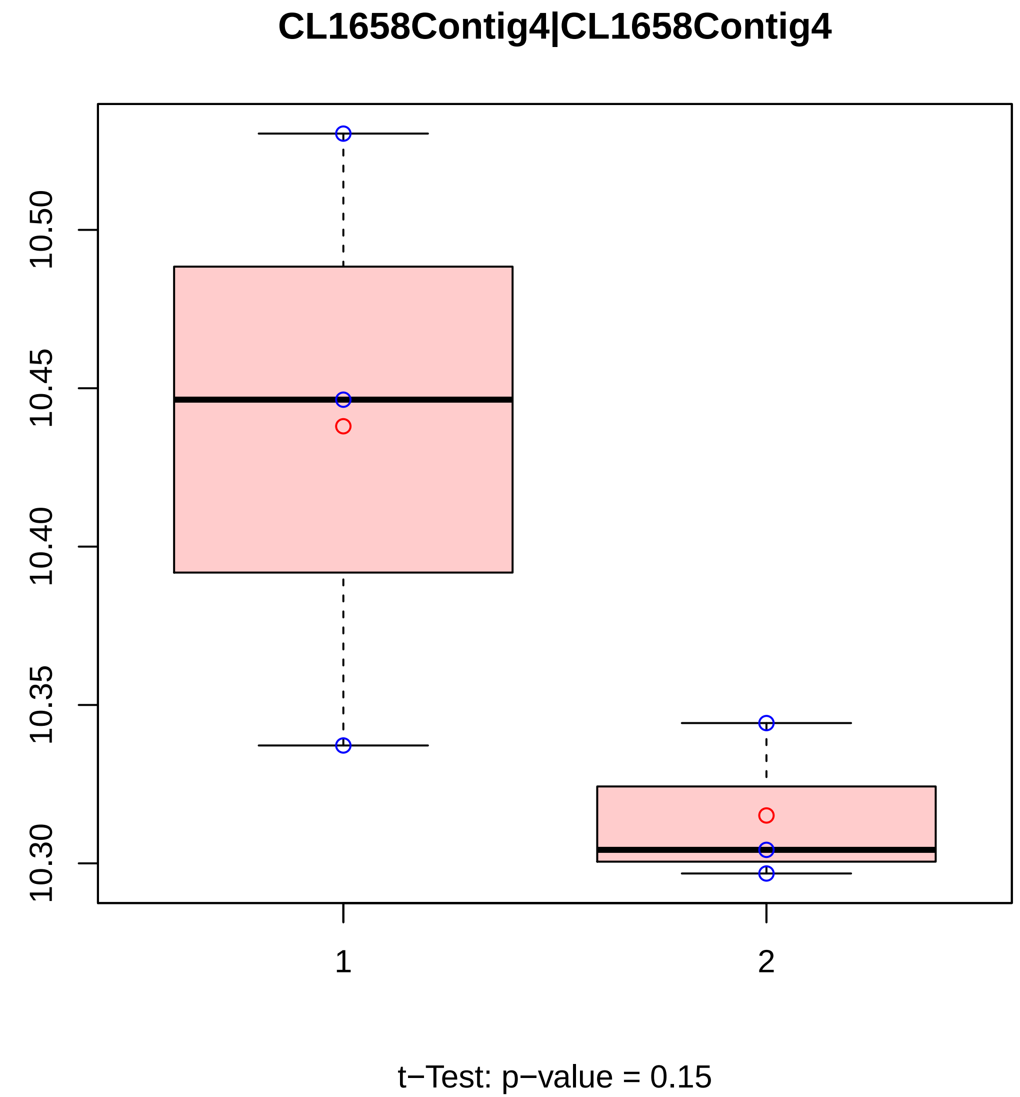

CL1658Contig4|CL1658Contig4
10.30
10.35
10.40
10.45
10.50
1
2
t−Test: p−value = 0.15

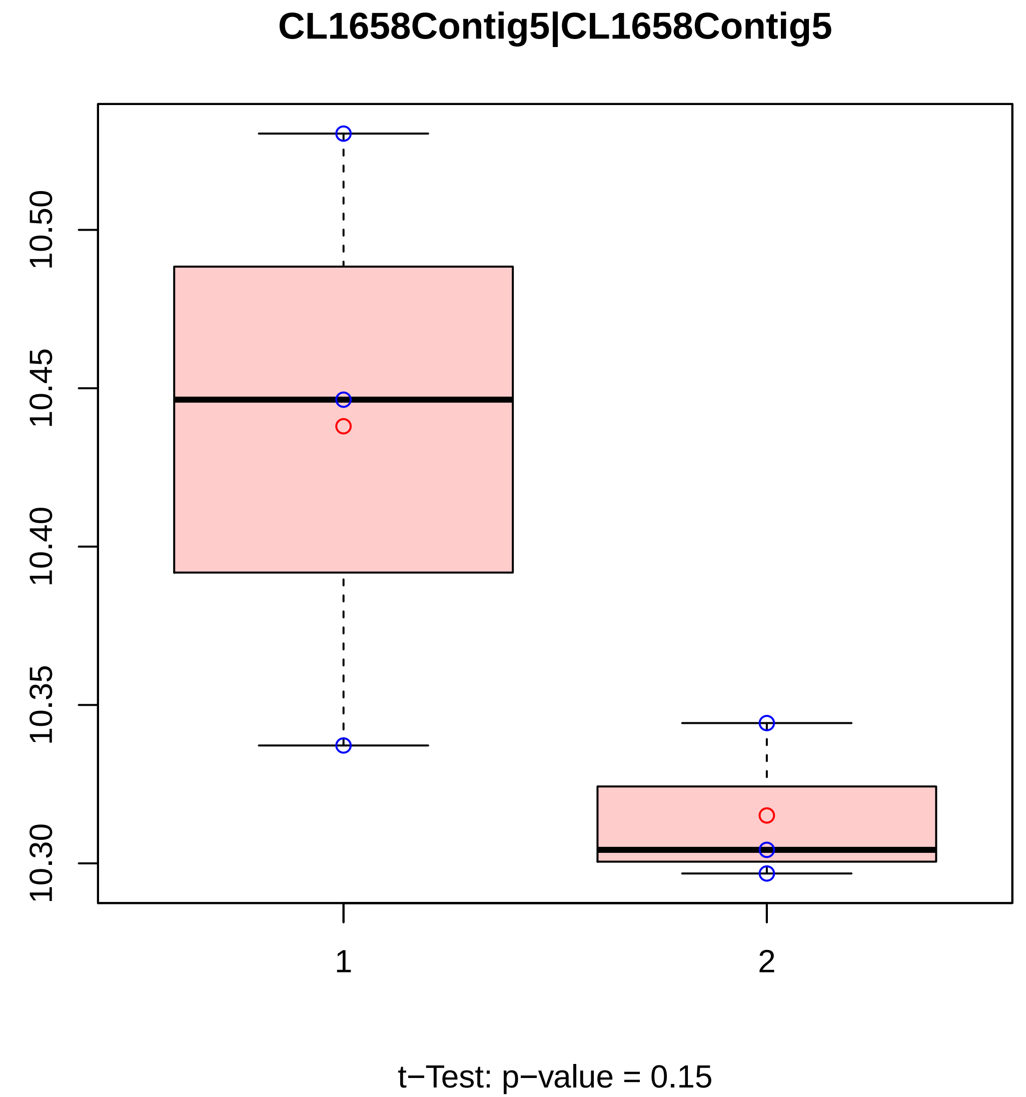
CL1658Contig5|CL1658Contig5
10.30
10.35
10.40
10.45
10.50
1
2
t−Test: p−value = 0.15

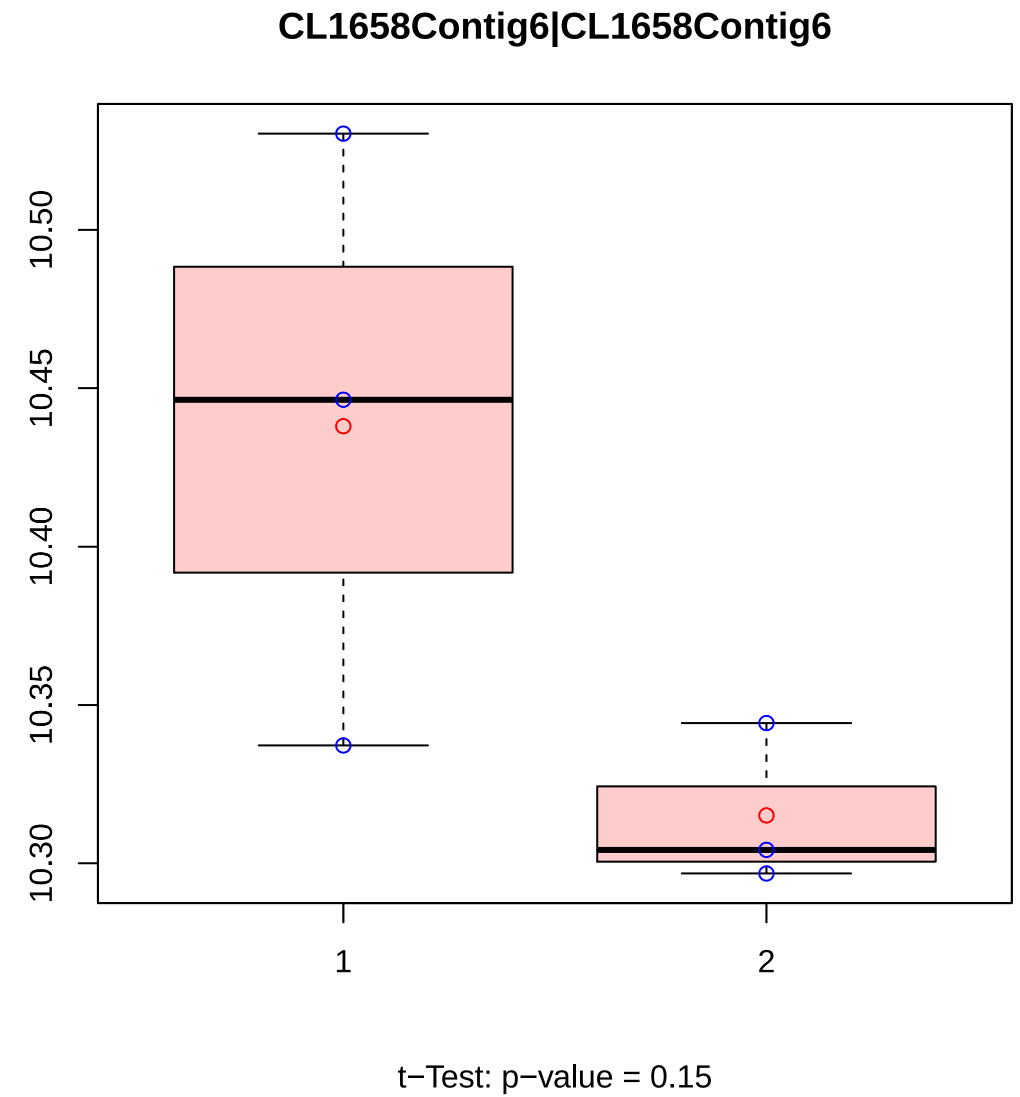
CL1658Contig6|CL1658Contig6
10.30
10.35
10.40
10.45
10.50
1
2
t−Test: p−value = 0.15

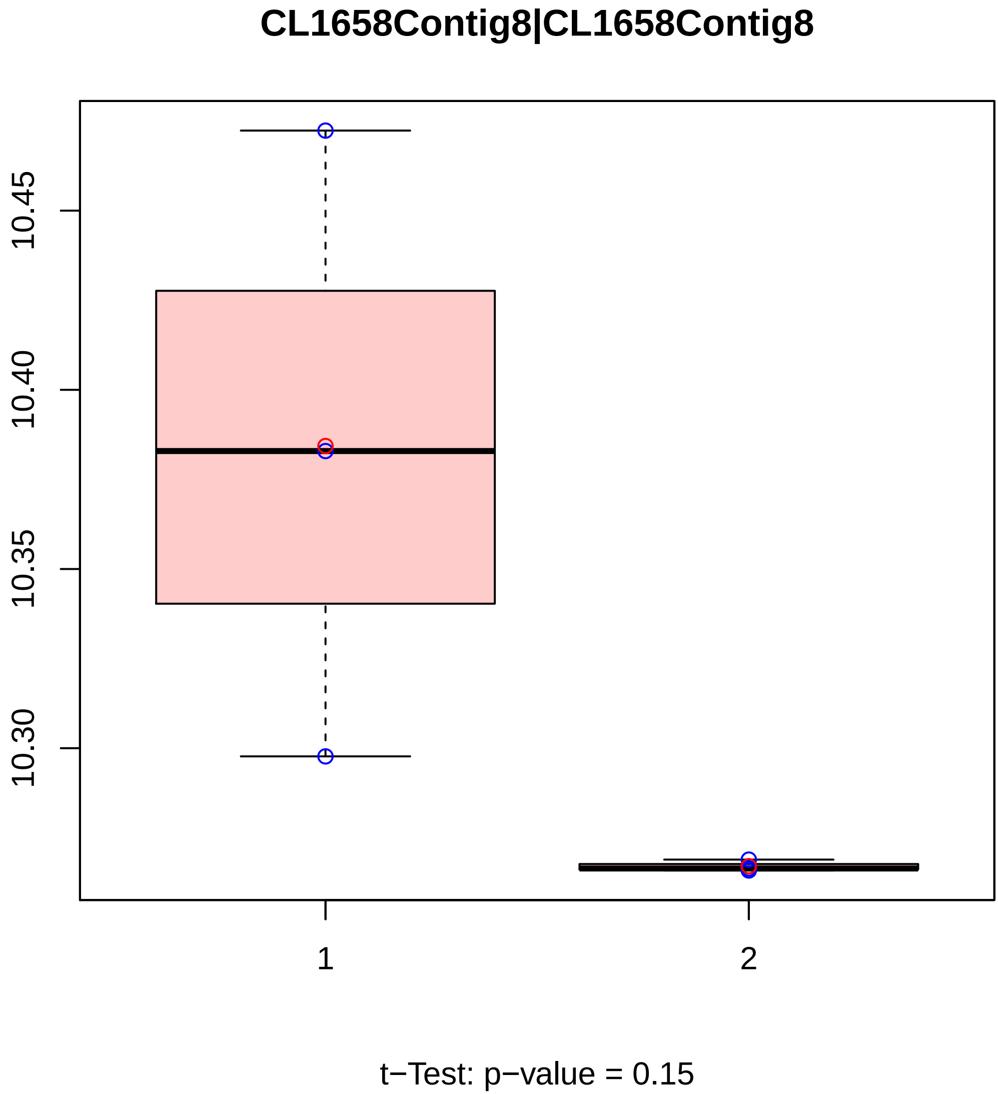

CL1658Contig8|CL1658Contig8
10.30
10.35
10.40
10.45
1
2
t−Test: p−value = 0.15

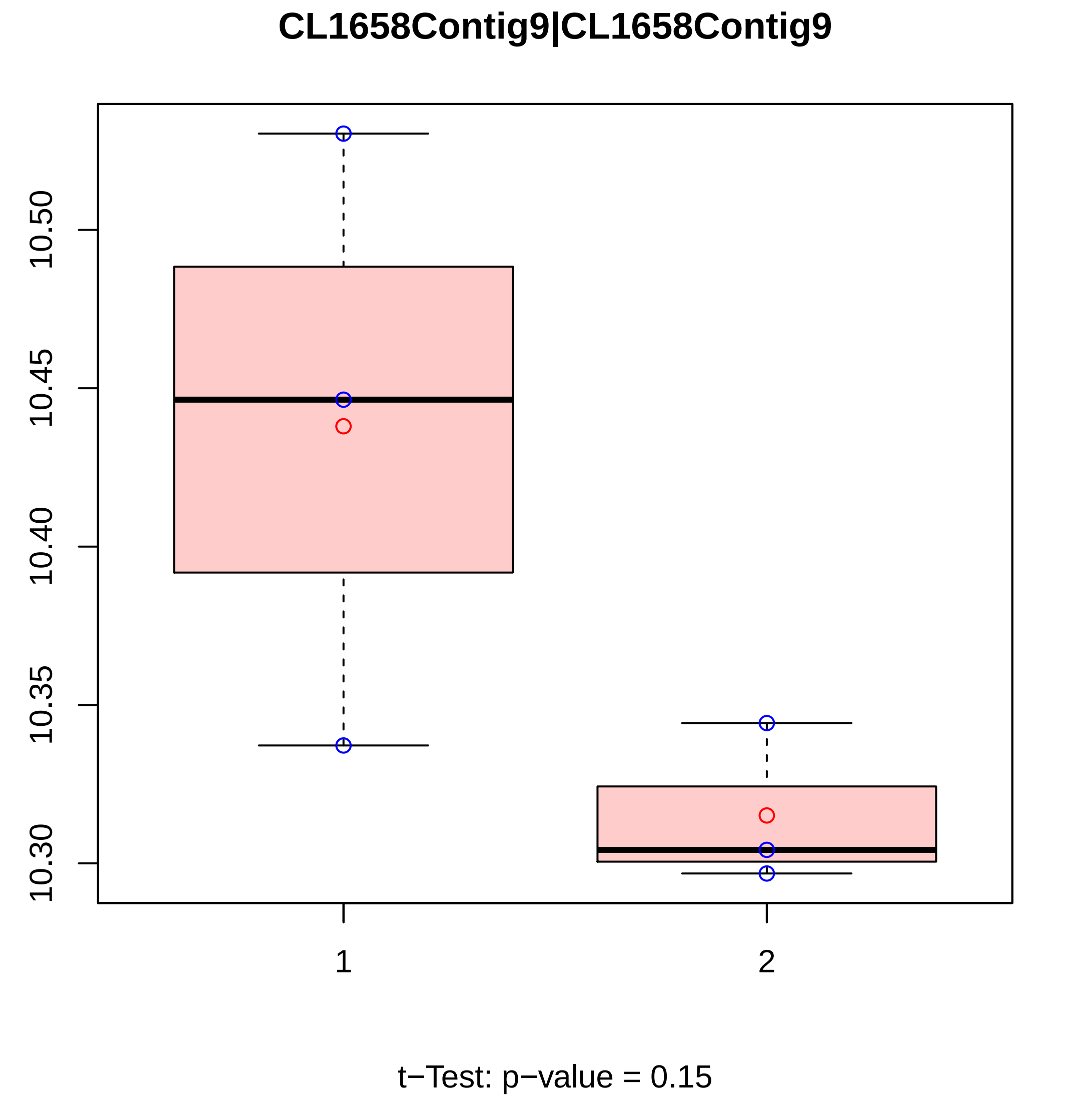
CL1658Contig9|CL1658Contig9
10.30
10.35
10.40
10.45
10.50
1
2
t−Test: p−value = 0.15

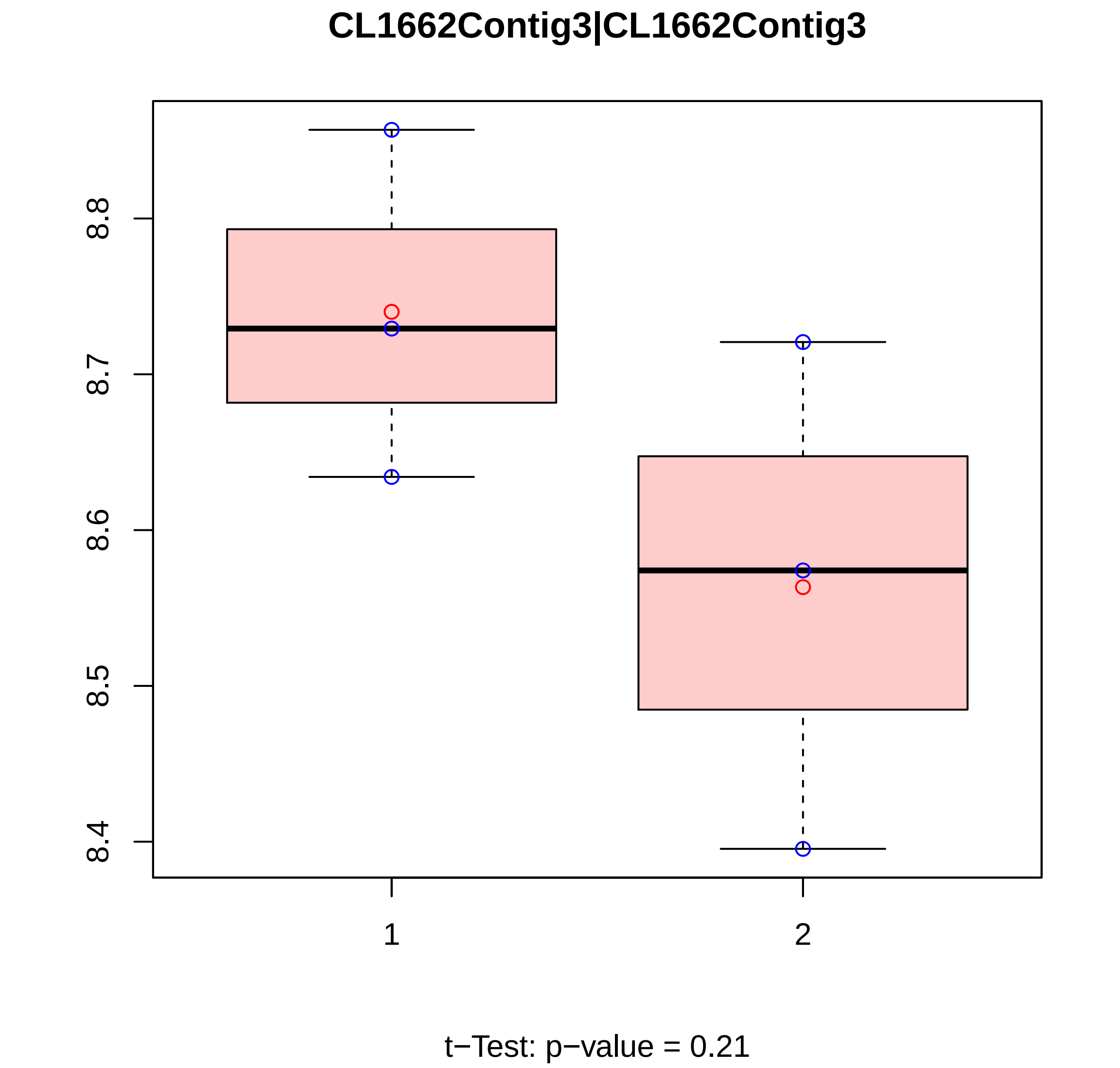
CL1662Contig3|CL1662Contig3
8.4
8.5
8.6
8.7
8.8
1
2
t−Test: p−value = 0.21

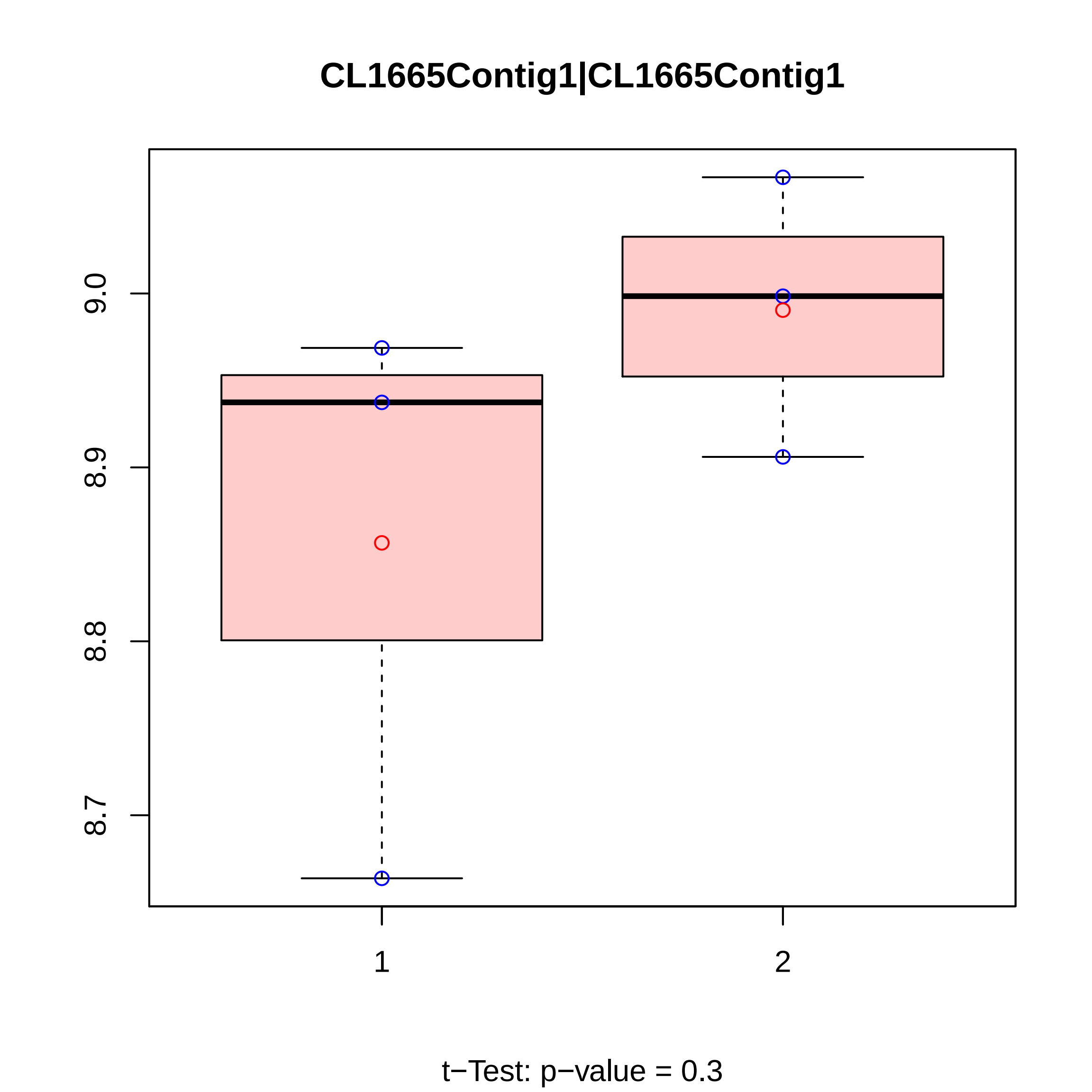
CL1665Contig1|CL1665Contig1
9.0
8.9
8.8
8.7
1
2
t−Test: p−value = 0.3

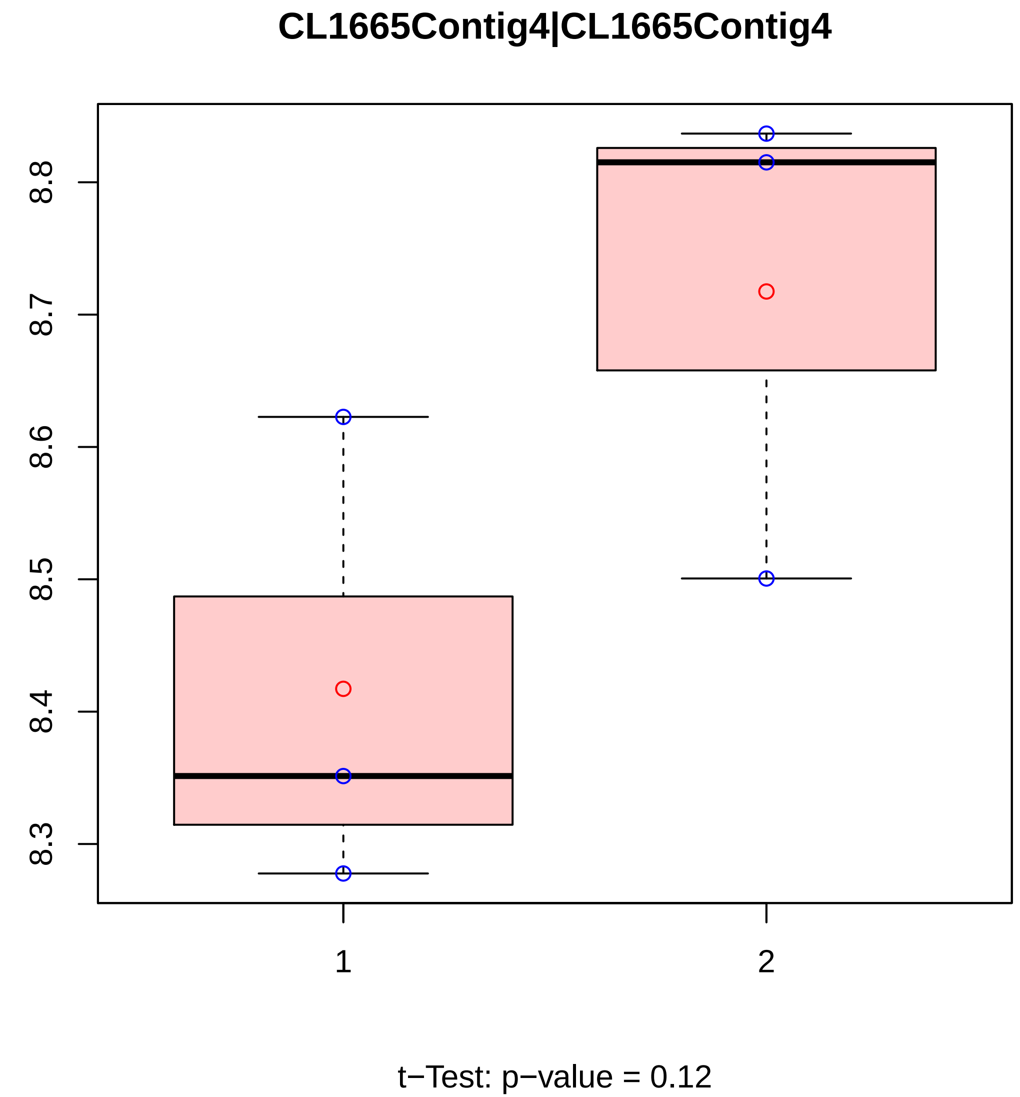

CL1665Contig4|CL1665Contig4
8.3
8.4
8.5
8.6
8.7
8.8
1
2
t−Test: p−value = 0.12

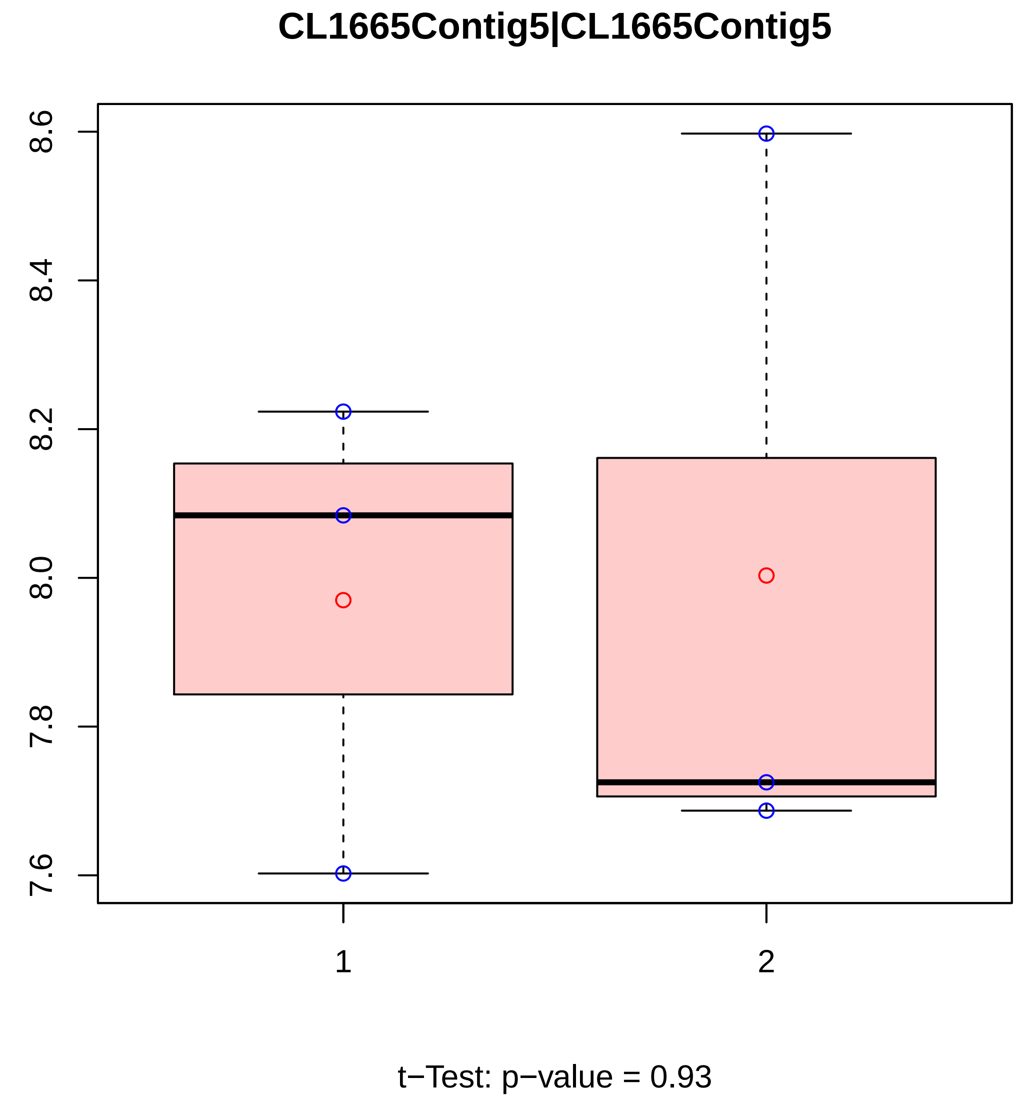
CL1665Contig5|CL1665Contig5
8.6
8.4
8.2
8.0
7.8
7.6
1
2
t−Test: p−value = 0.93

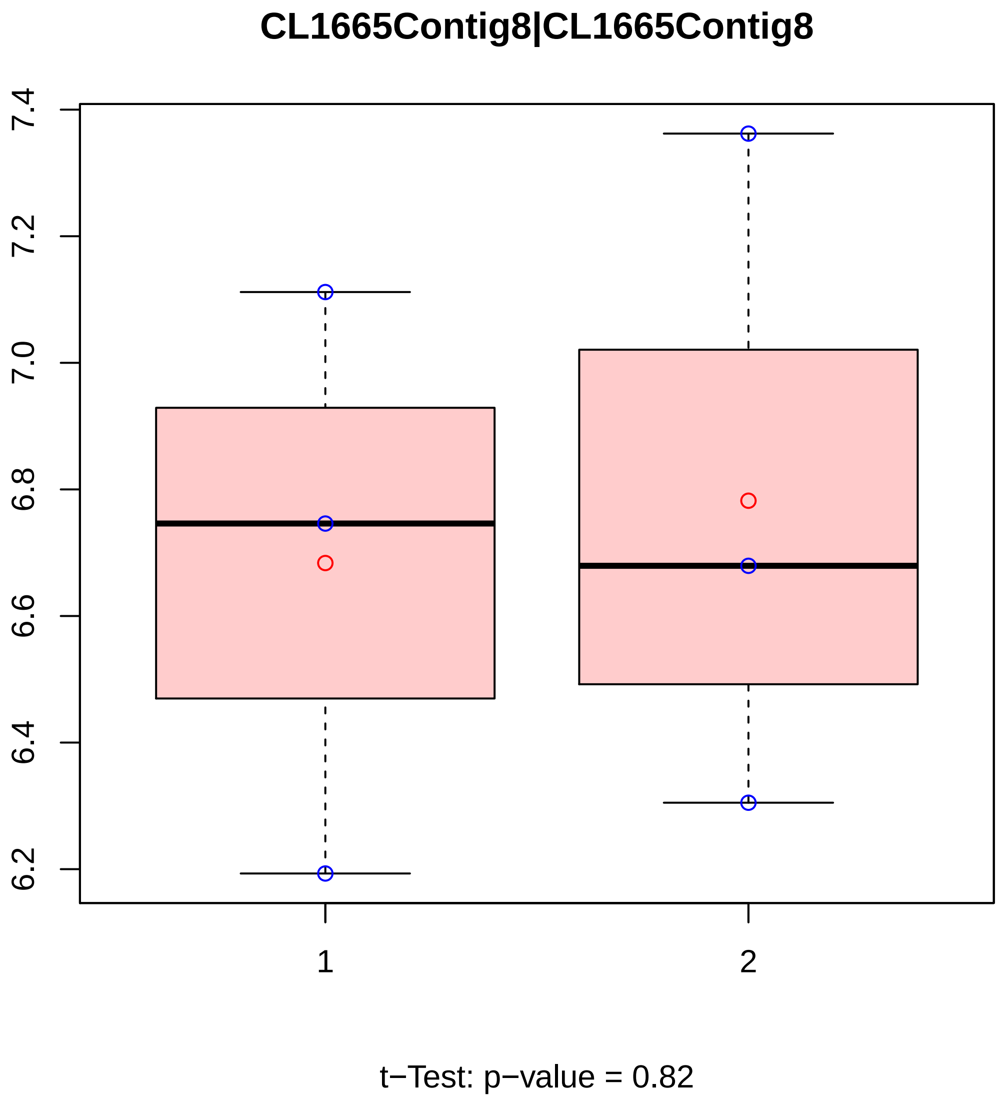
CL1665Contig8|CL1665Contig8
7.4
7.2
7.0
6.8
6.6
6.4
6.2
1
2
t−Test: p−value = 0.82

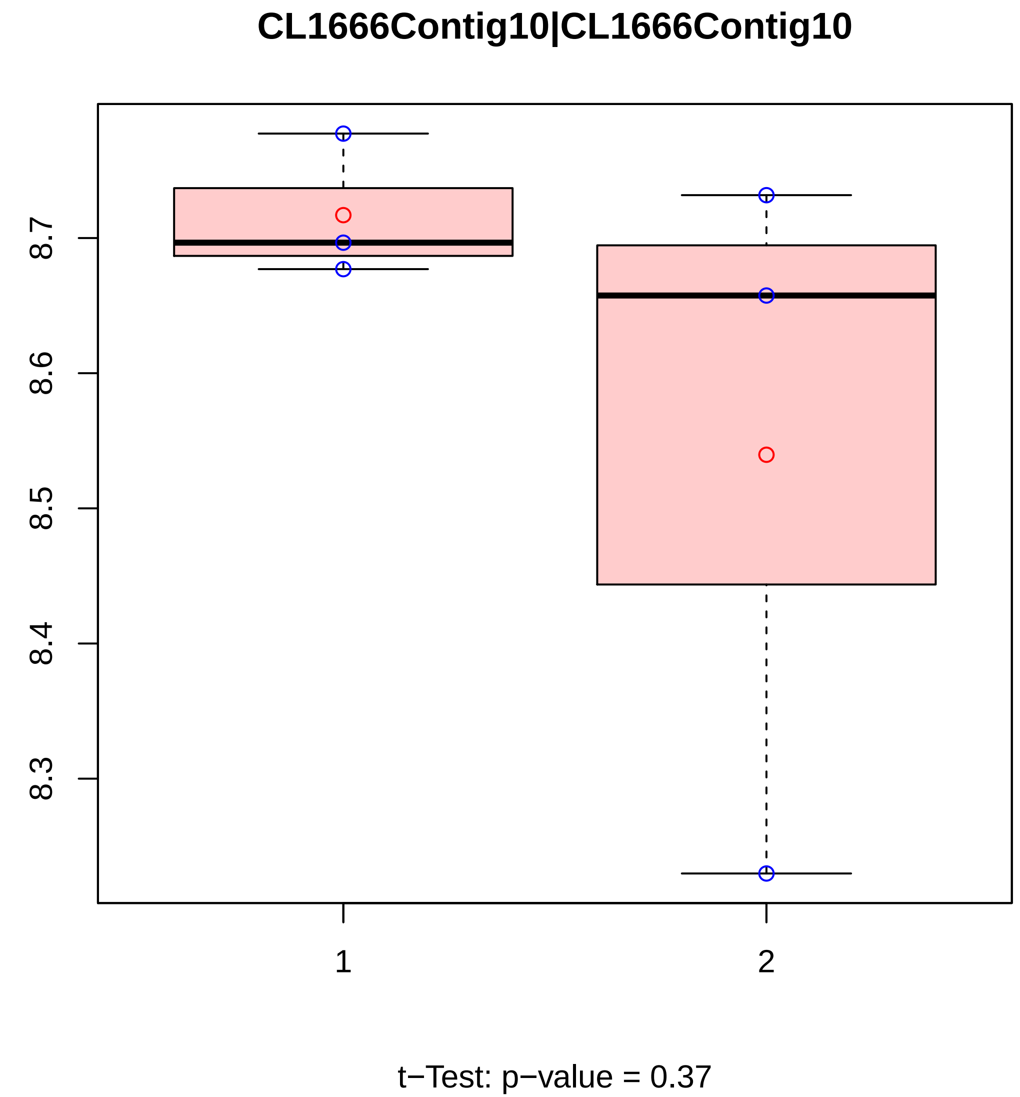
CL1666Contig10|CL1666Contig10
8.3
8.4
8.5
8.6
8.7
1
2
t−Test: p−value = 0.37

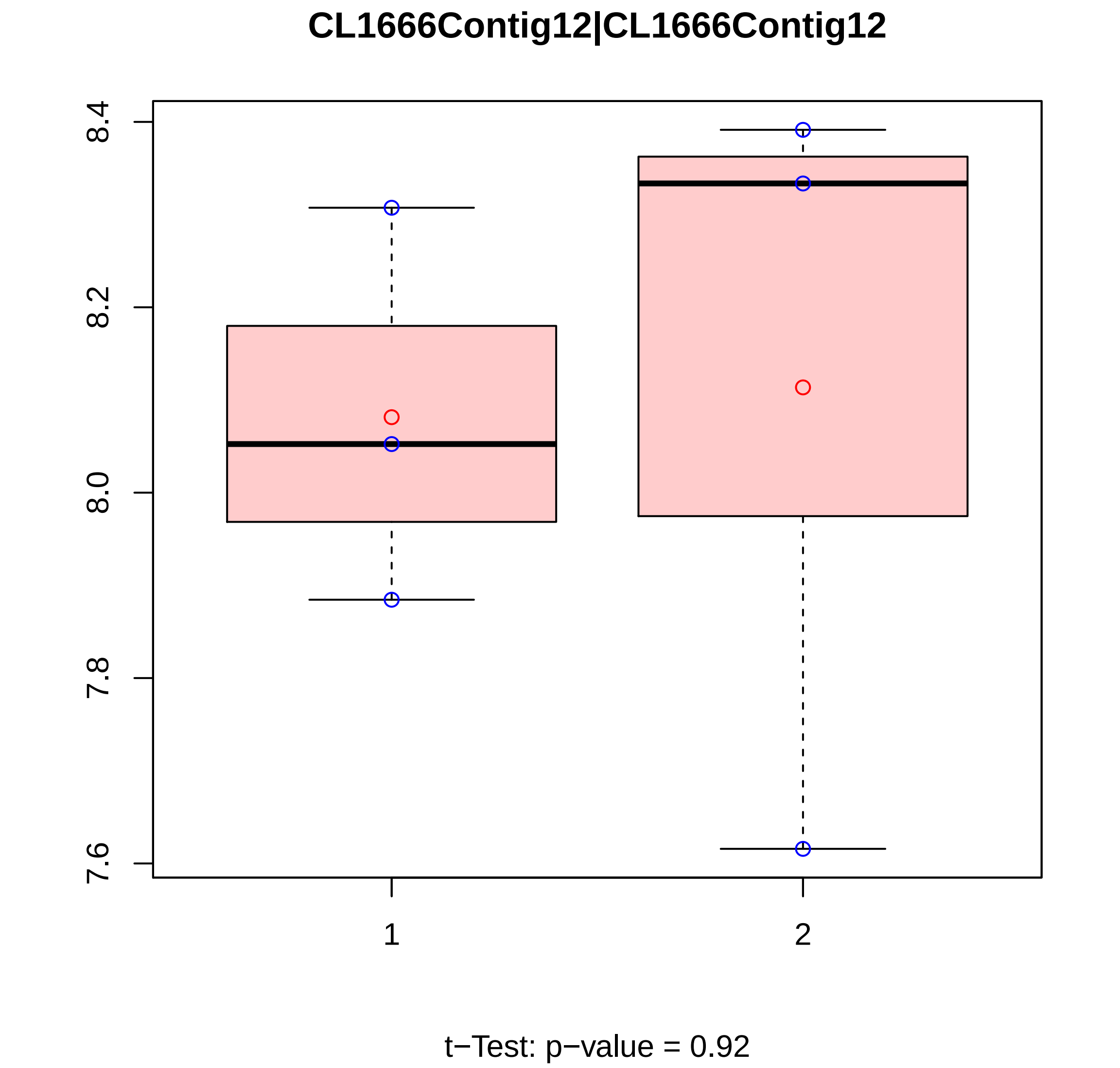
CL1666Contig12|CL1666Contig12
8.4
8.2
8.0
7.8
7.6
1
2
t−Test: p−value = 0.92

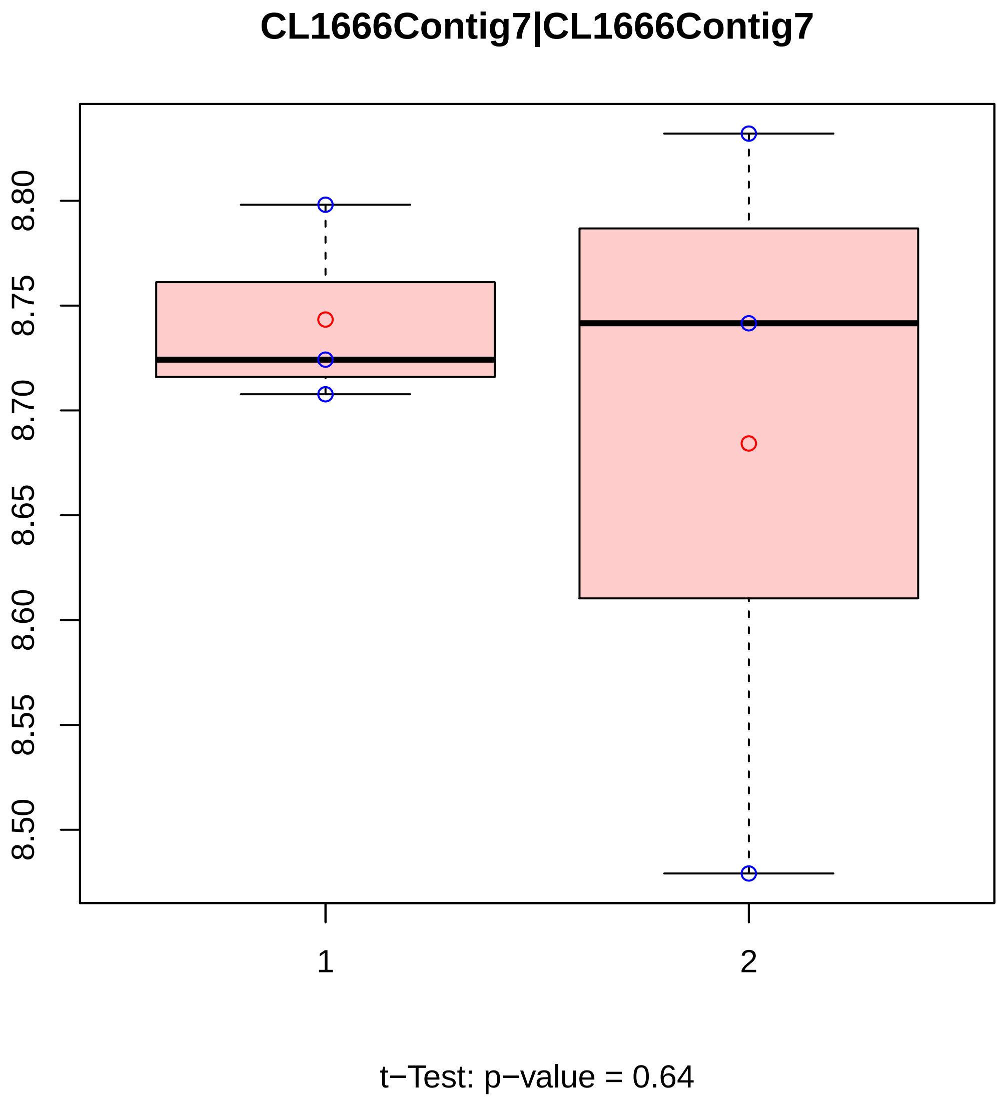
CL1666Contig7|CL1666Contig7
8.50
8.55
8.60
8.65
8.70
8.75
8.80
1
2
t−Test: p−value = 0.64

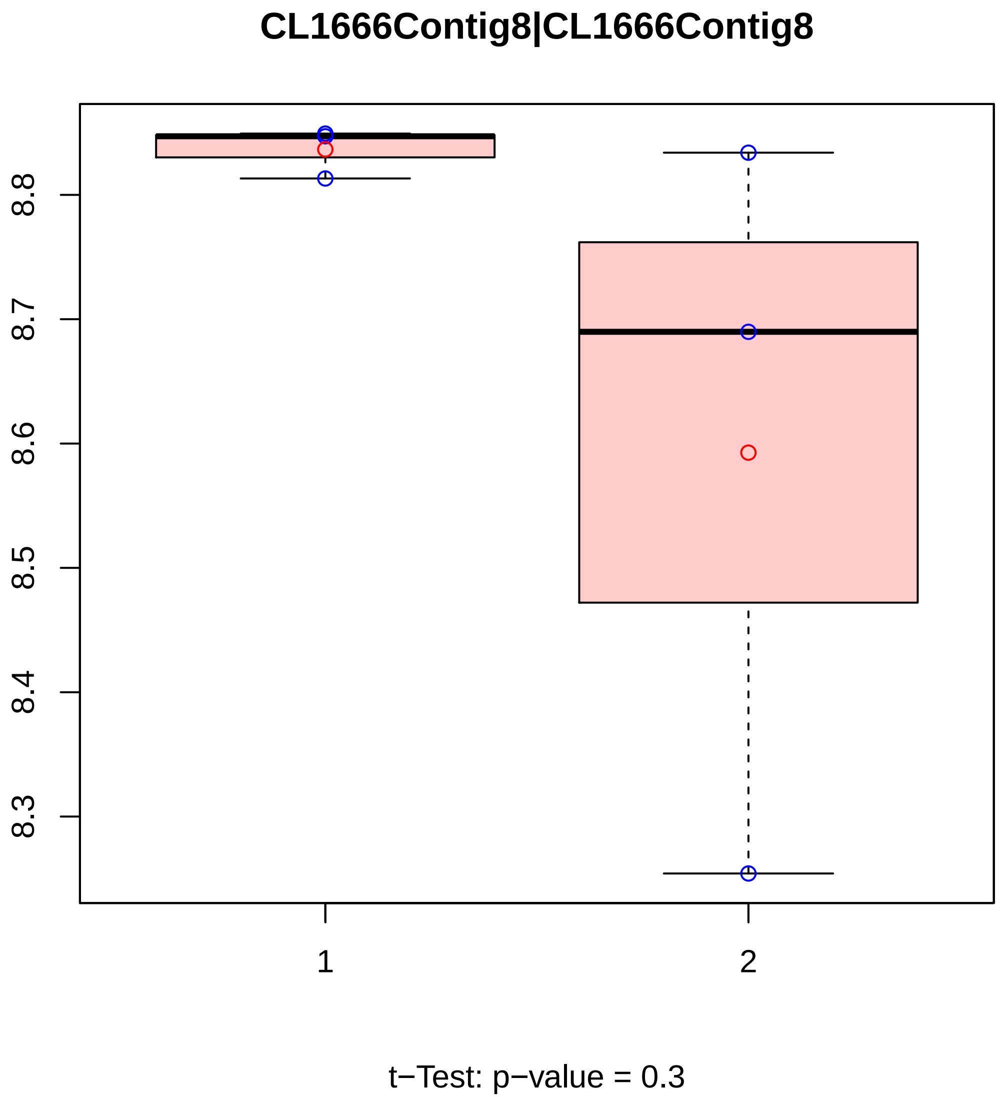
CL1666Contig8|CL1666Contig8
8.3
8.4
8.5
8.6
8.7
8.8
1
2
t−Test: p−value = 0.3

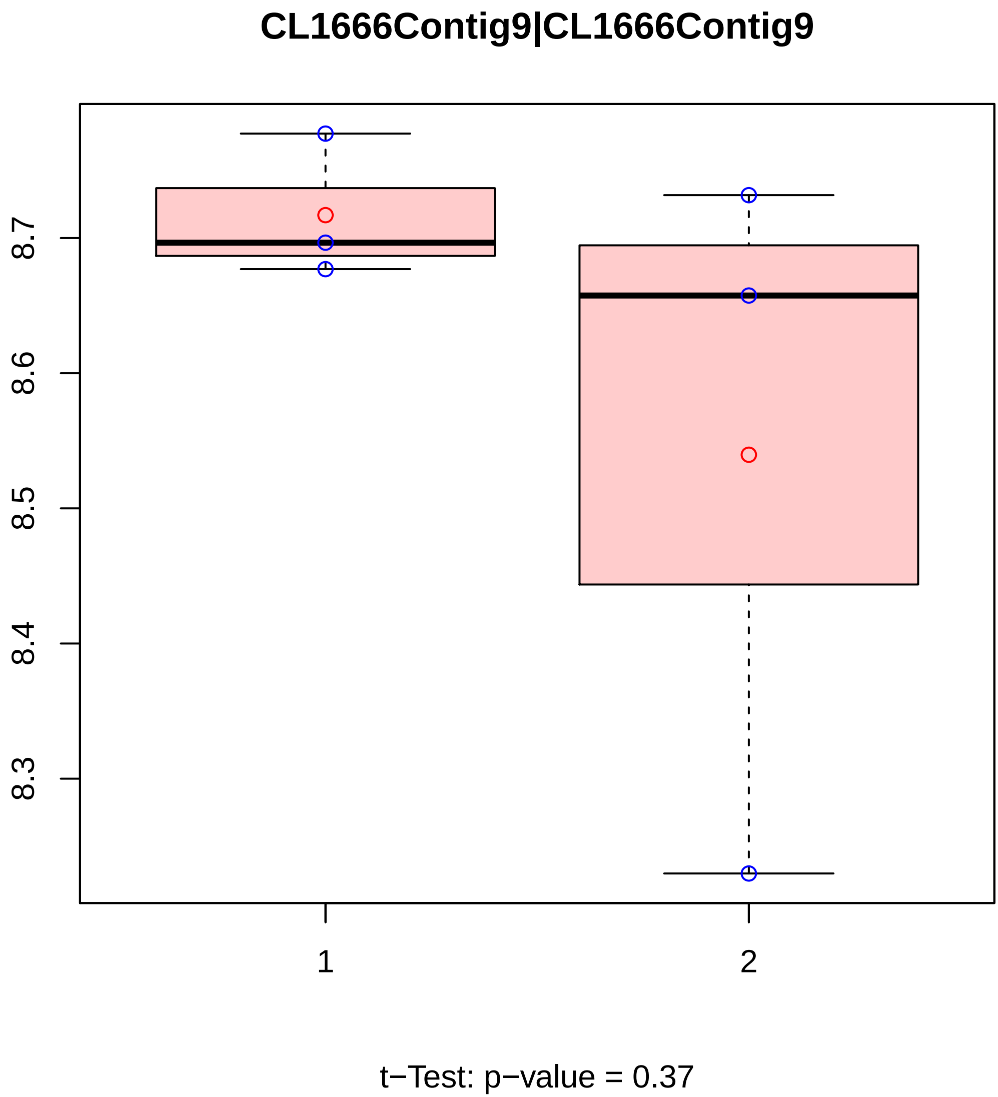
CL1666Contig9|CL1666Contig9
8.3
8.4
8.5
8.6
8.7
1
2
t−Test: p−value = 0.37

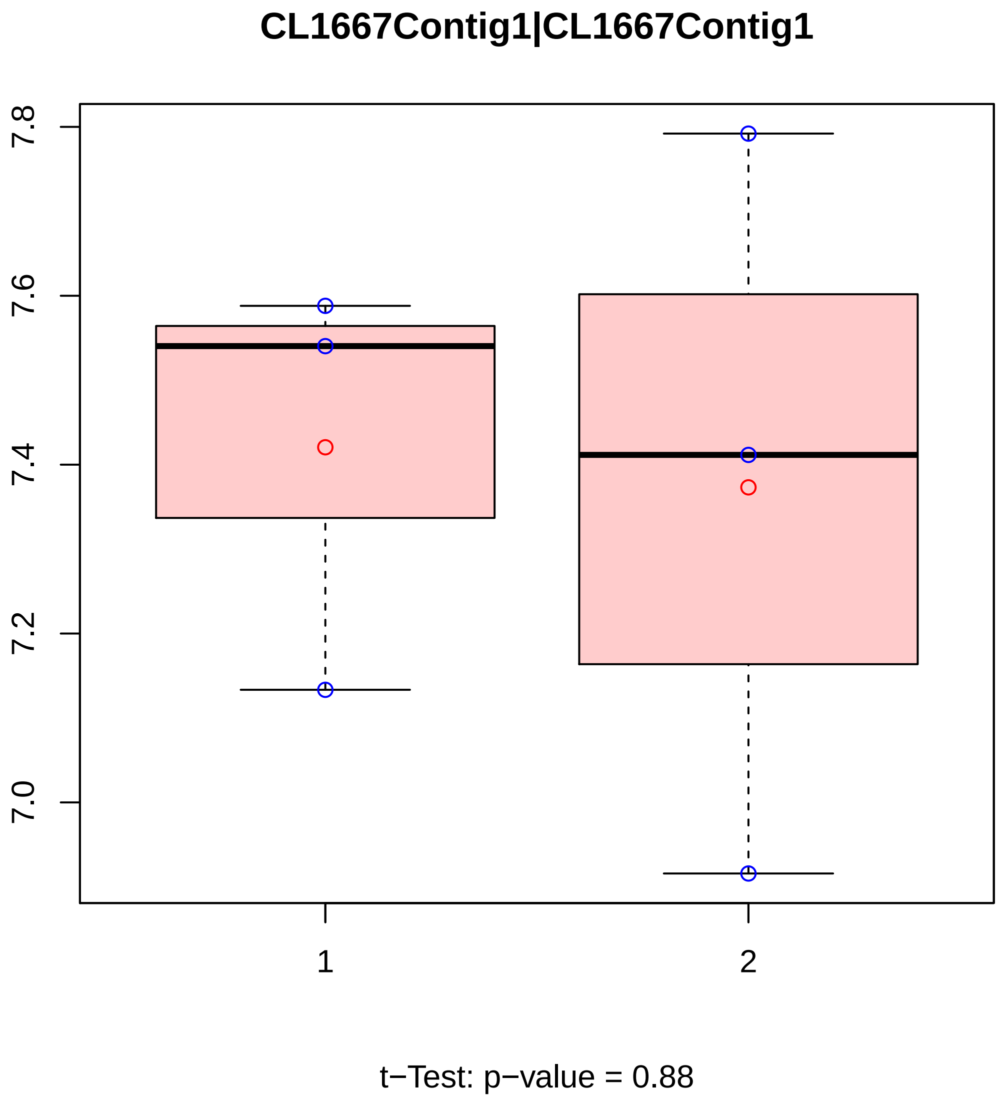
CL1667Contig1|CL1667Contig1
7.8
7.6
7.4
7.2
7.0
1
2
t−Test: p−value = 0.88

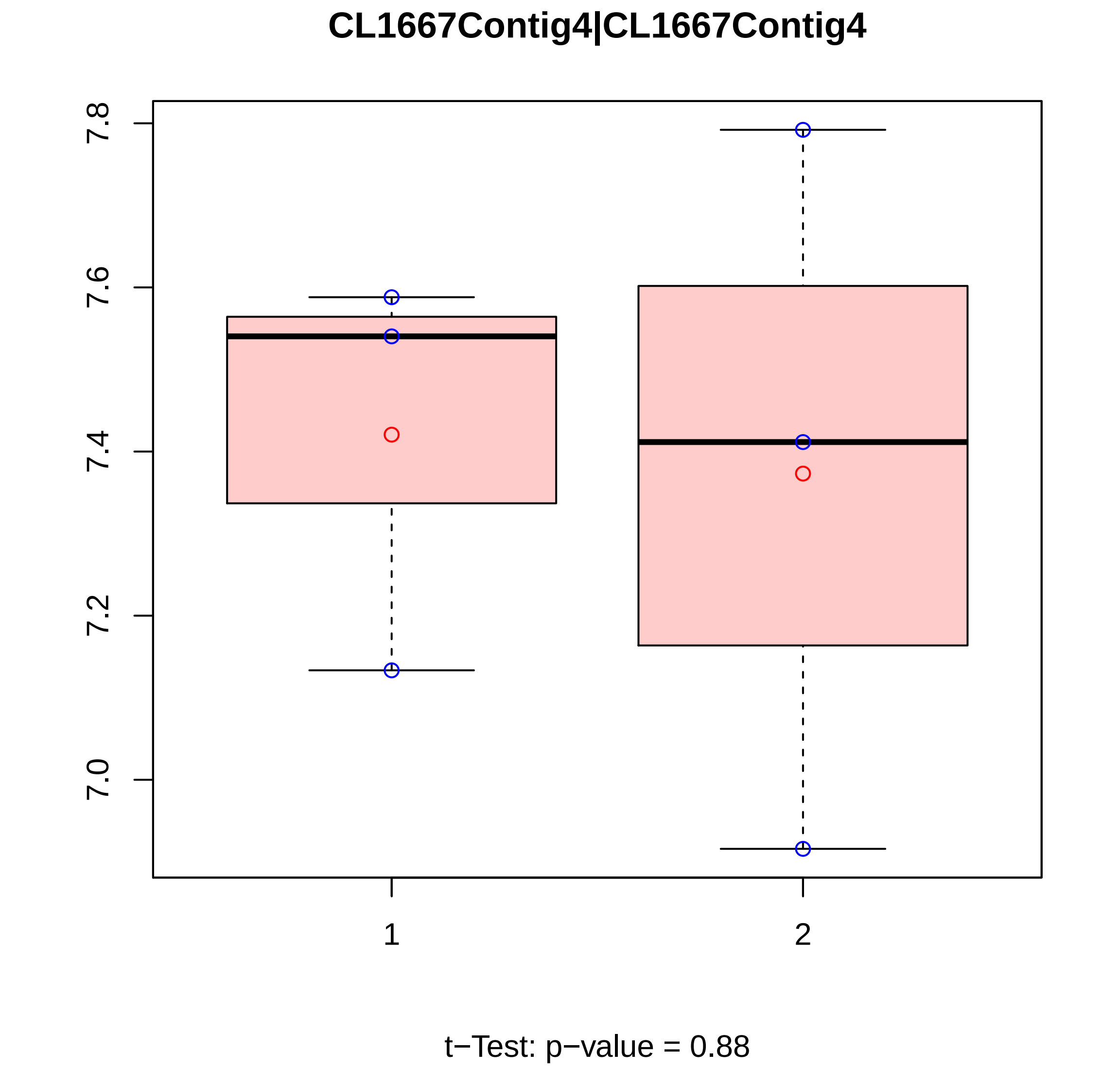
CL1667Contig4|CL1667Contig4
7.0
7.2
7.4
7.6
7.8
1
2
t−Test: p−value = 0.88

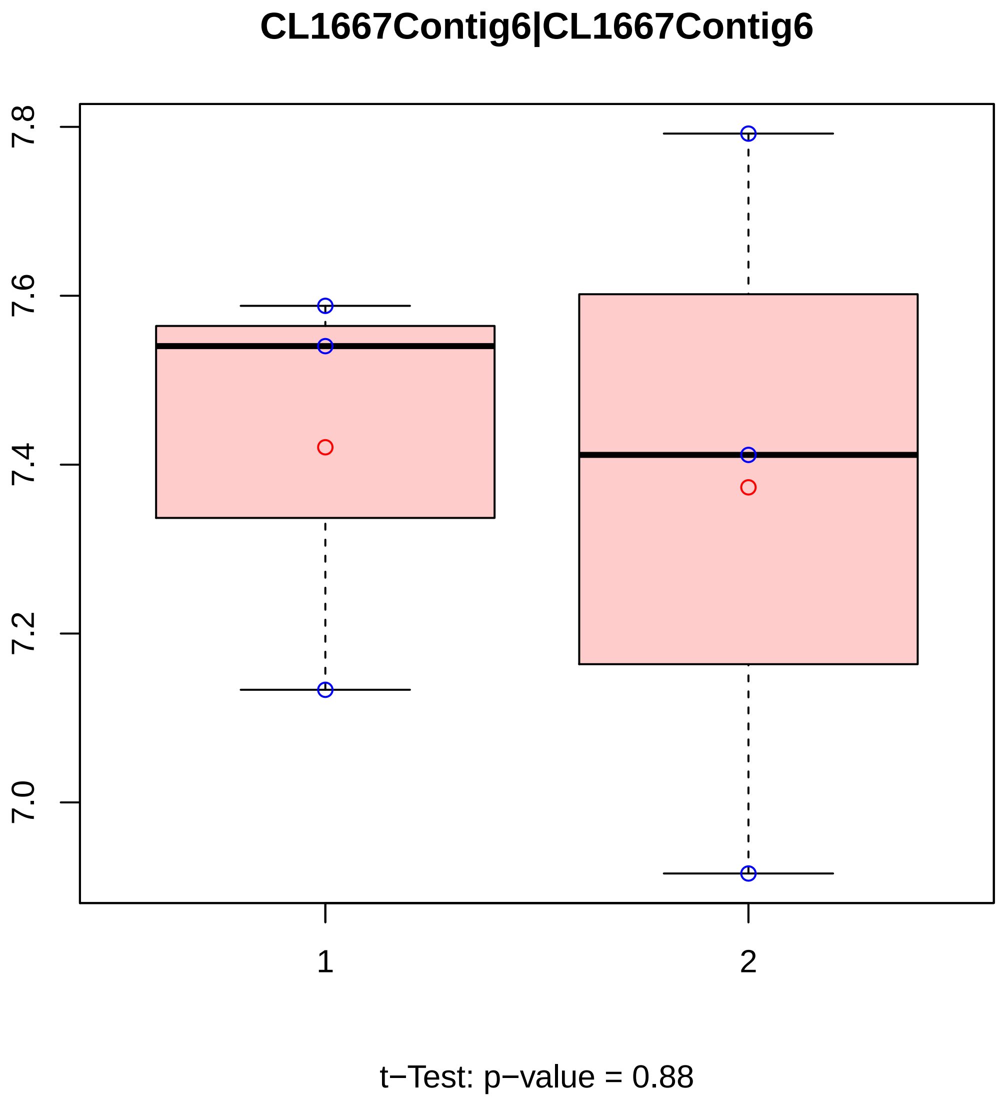

CL1667Contig6|CL1667Contig6
7.0
7.2
7.4
7.6
7.8
1
2
t−Test: p−value = 0.88

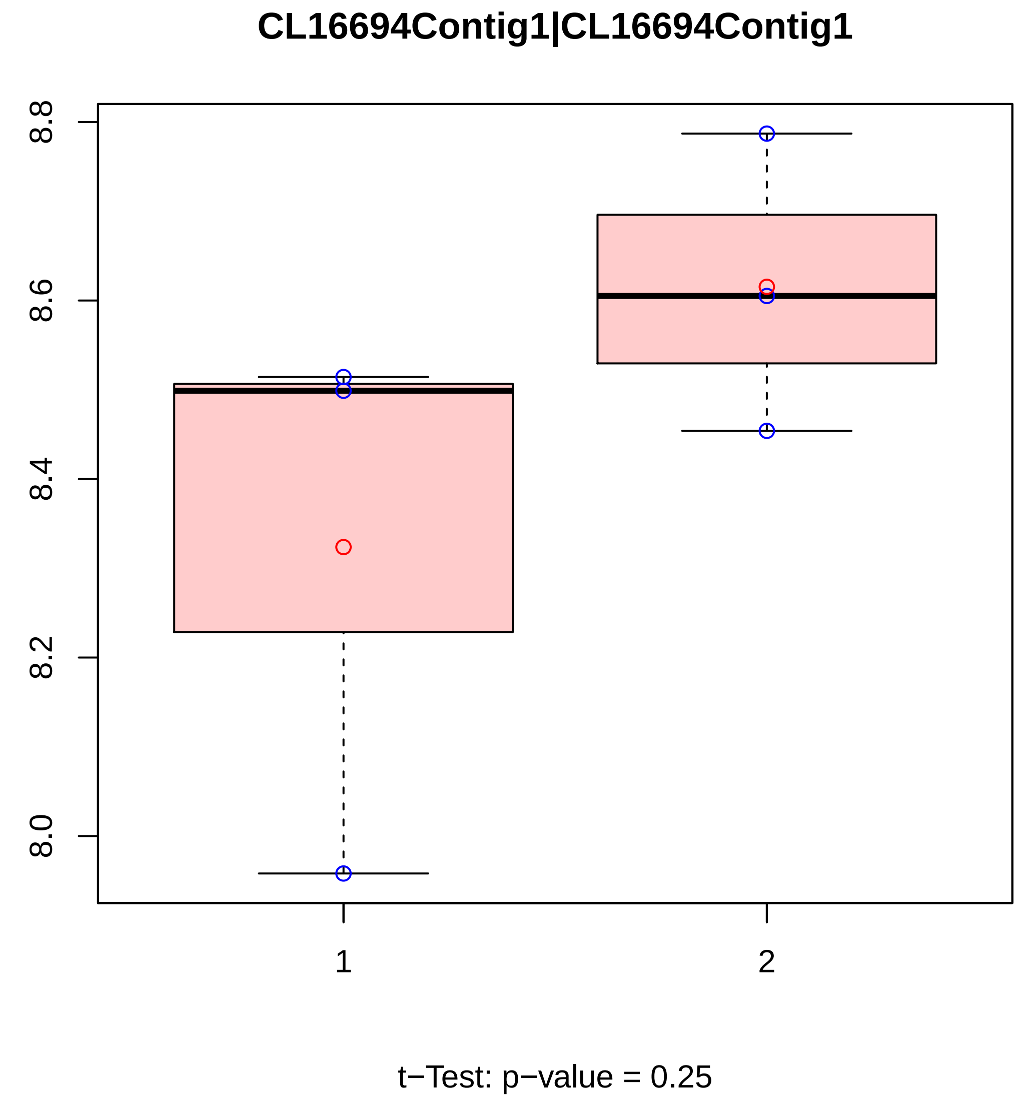

CL16694Contig1|CL16694Contig1
8.0
8.2
8.4
8.6
8.8
1
2
t−Test: p−value = 0.25

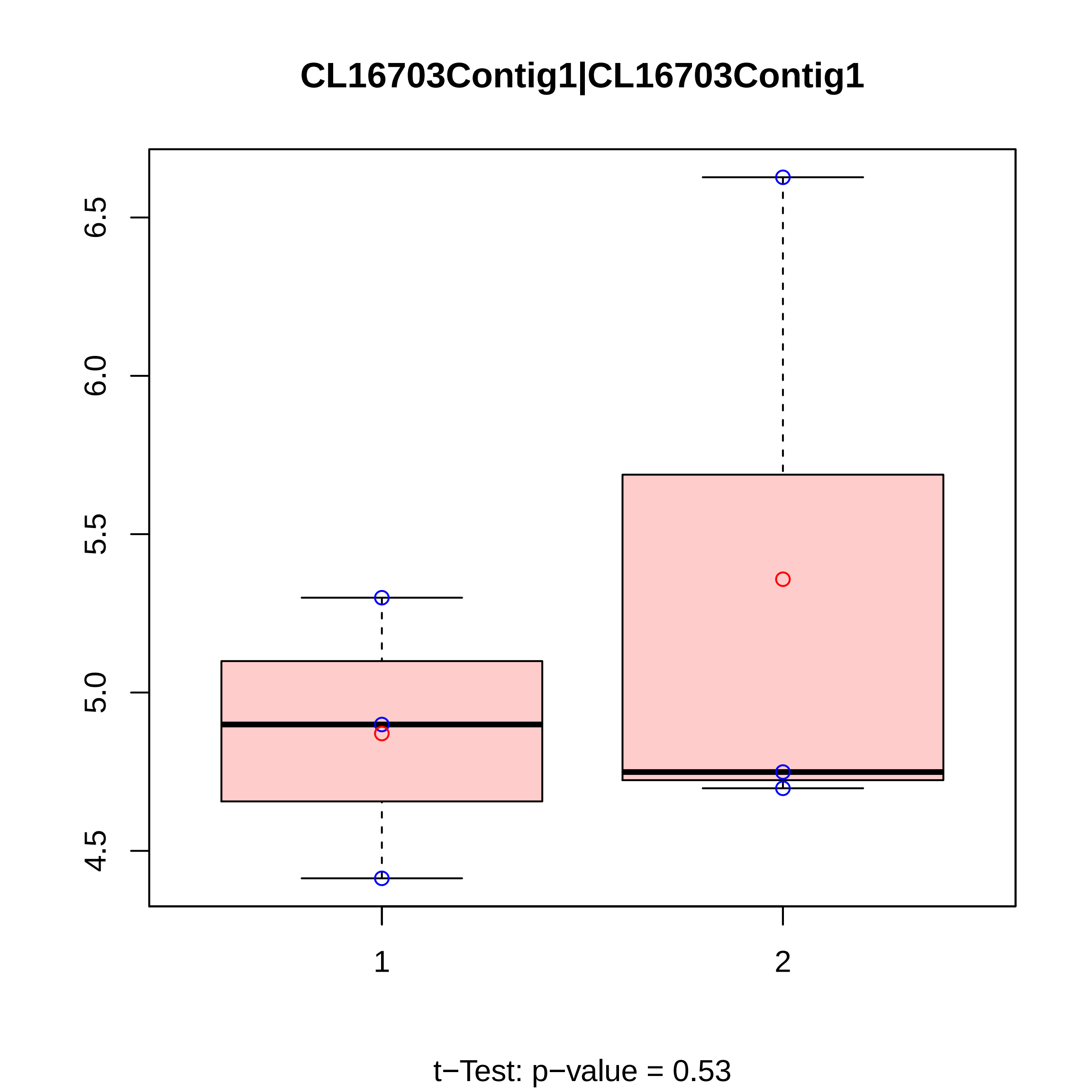

CL16703Contig1|CL16703Contig1
6.5
6.0
5.5
5.0
4.5
1
2
t−Test: p−value = 0.53

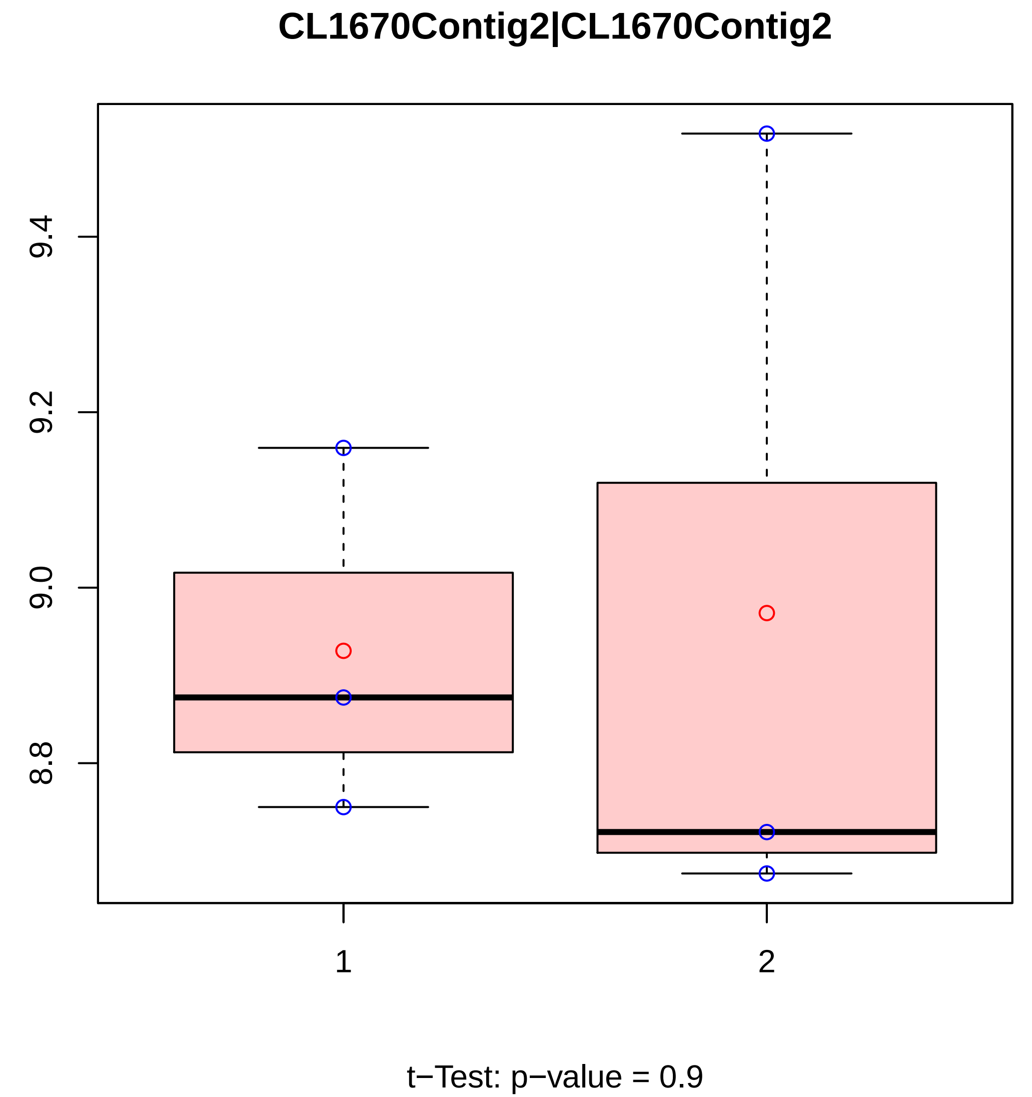
CL1670Contig2|CL1670Contig2
9.4
9.2
9.0
8.8
1
2
t−Test: p−value = 0.9

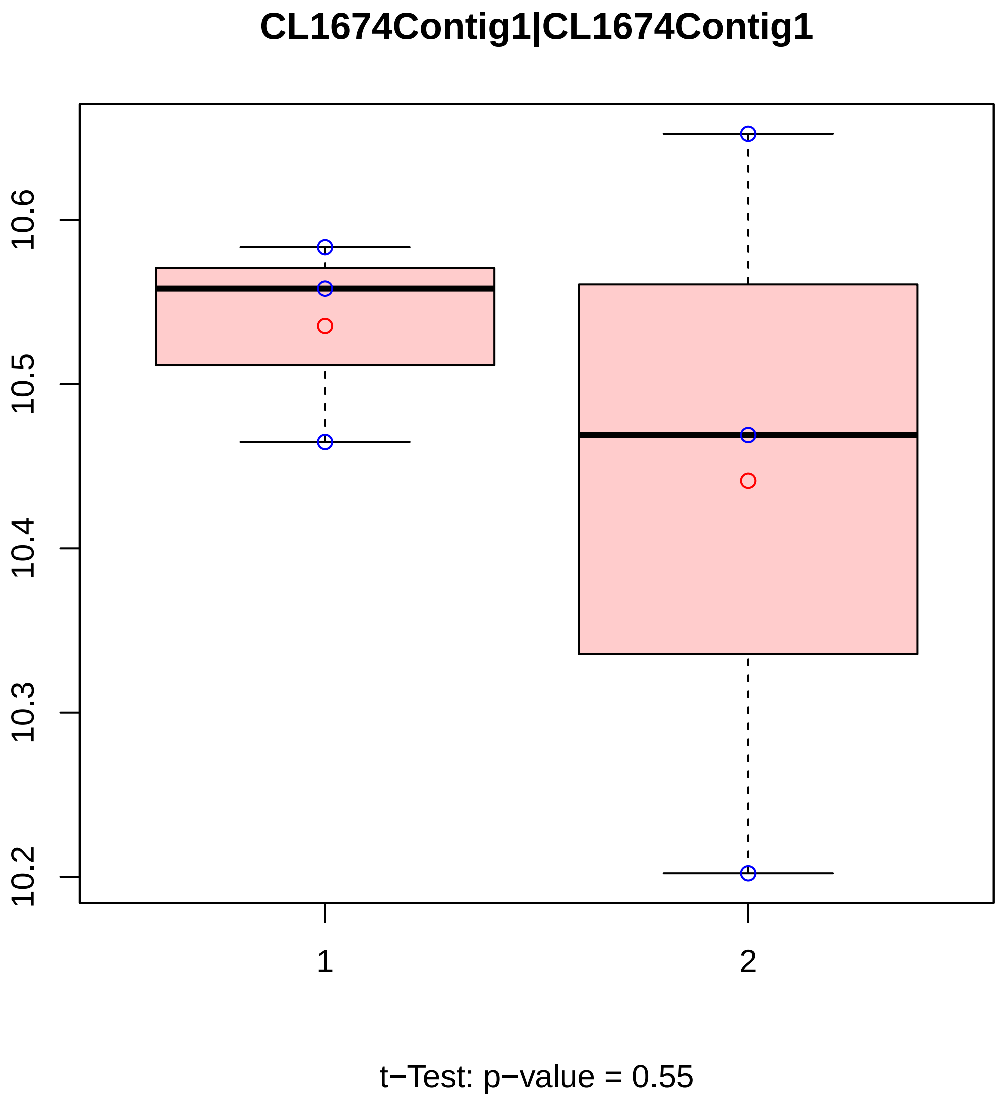
CL1674Contig1|CL1674Contig1
10.2
10.3
10.4
10.5
10.6
1
2
t−Test: p−value = 0.55

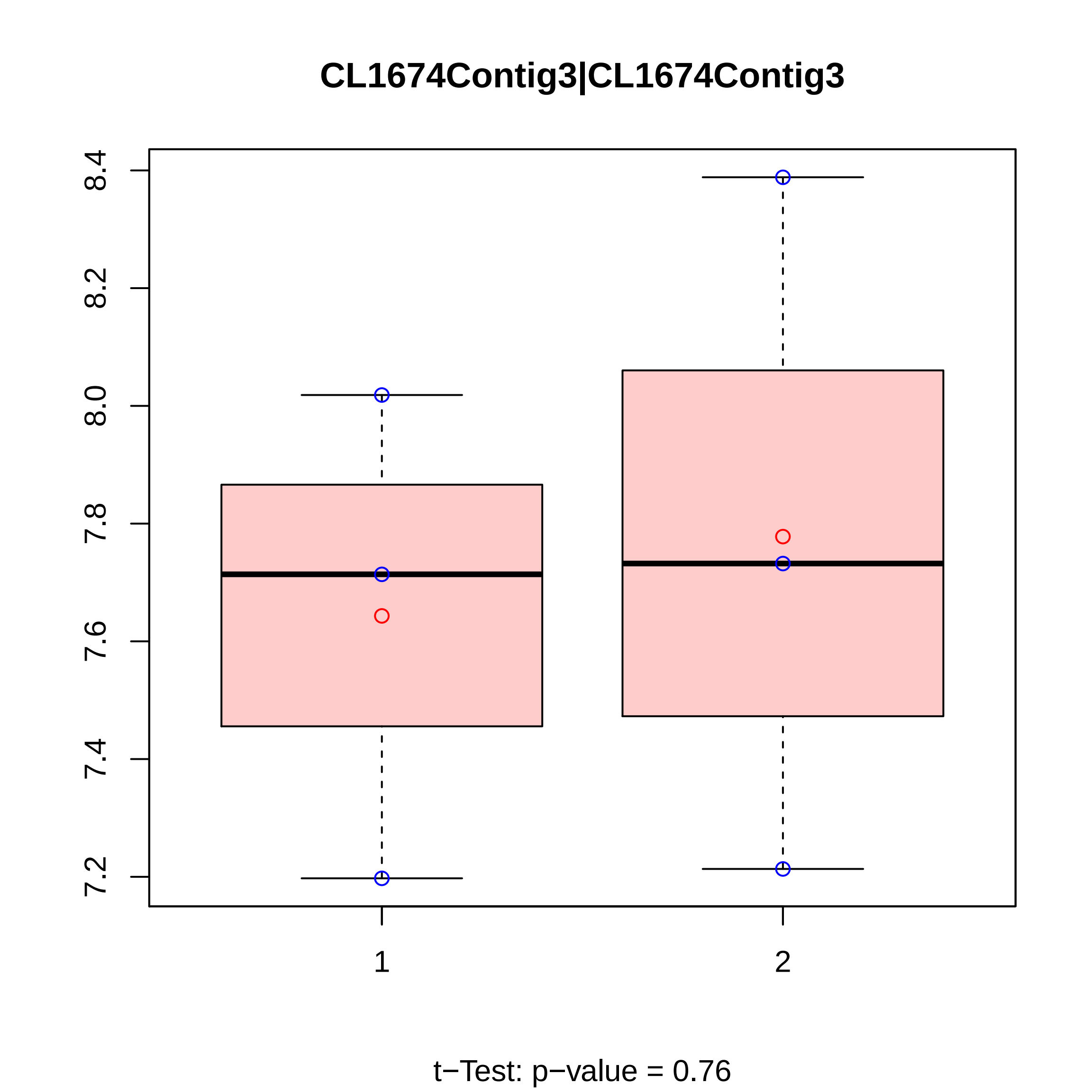

CL1674Contig3|CL1674Contig3
8.4
8.2
8.0
7.8
7.6
7.4
7.2
1
2
t−Test: p−value = 0.76

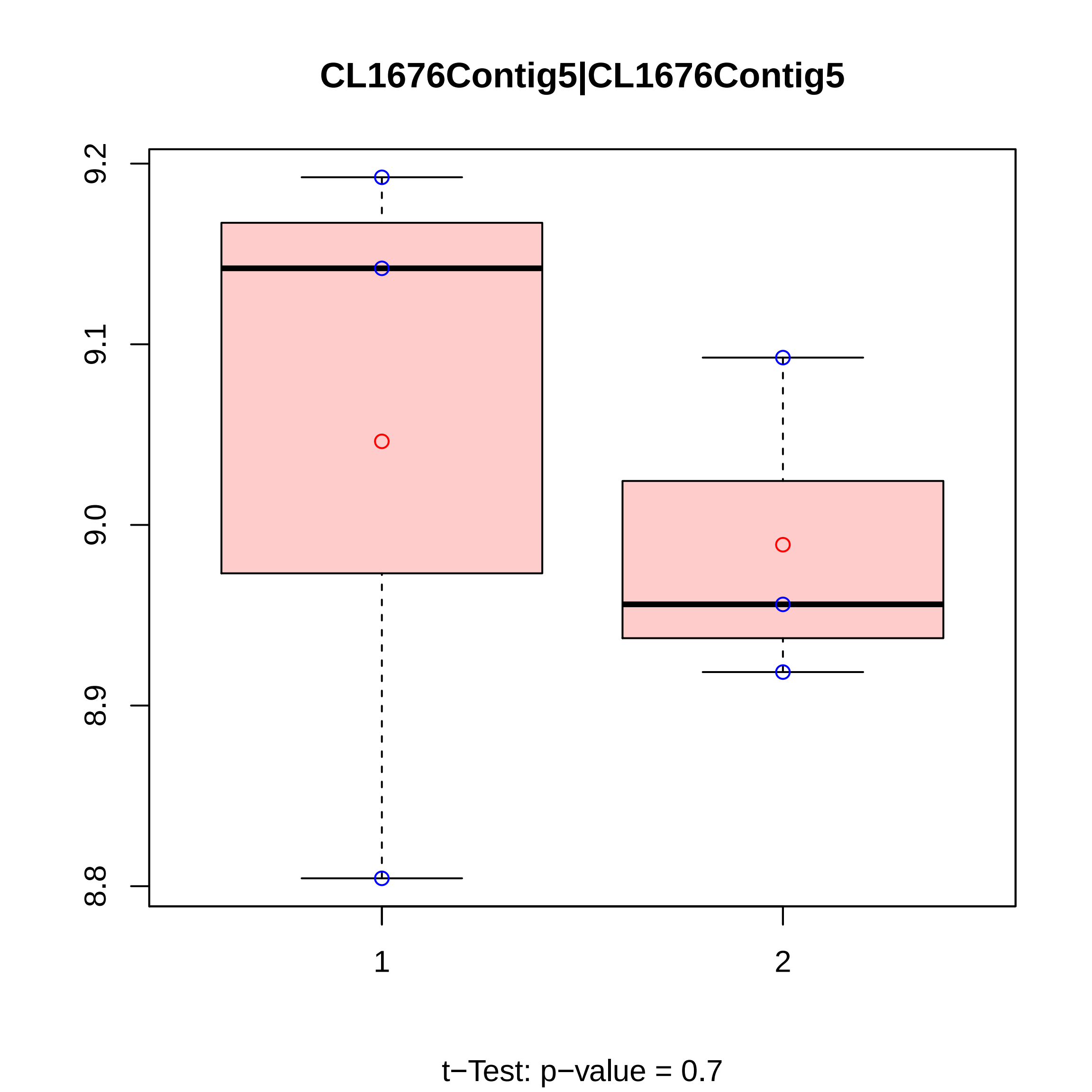
CL1676Contig5|CL1676Contig5
9.2
9.1
9.0
8.9
8.8
1
2
t−Test: p−value = 0.7

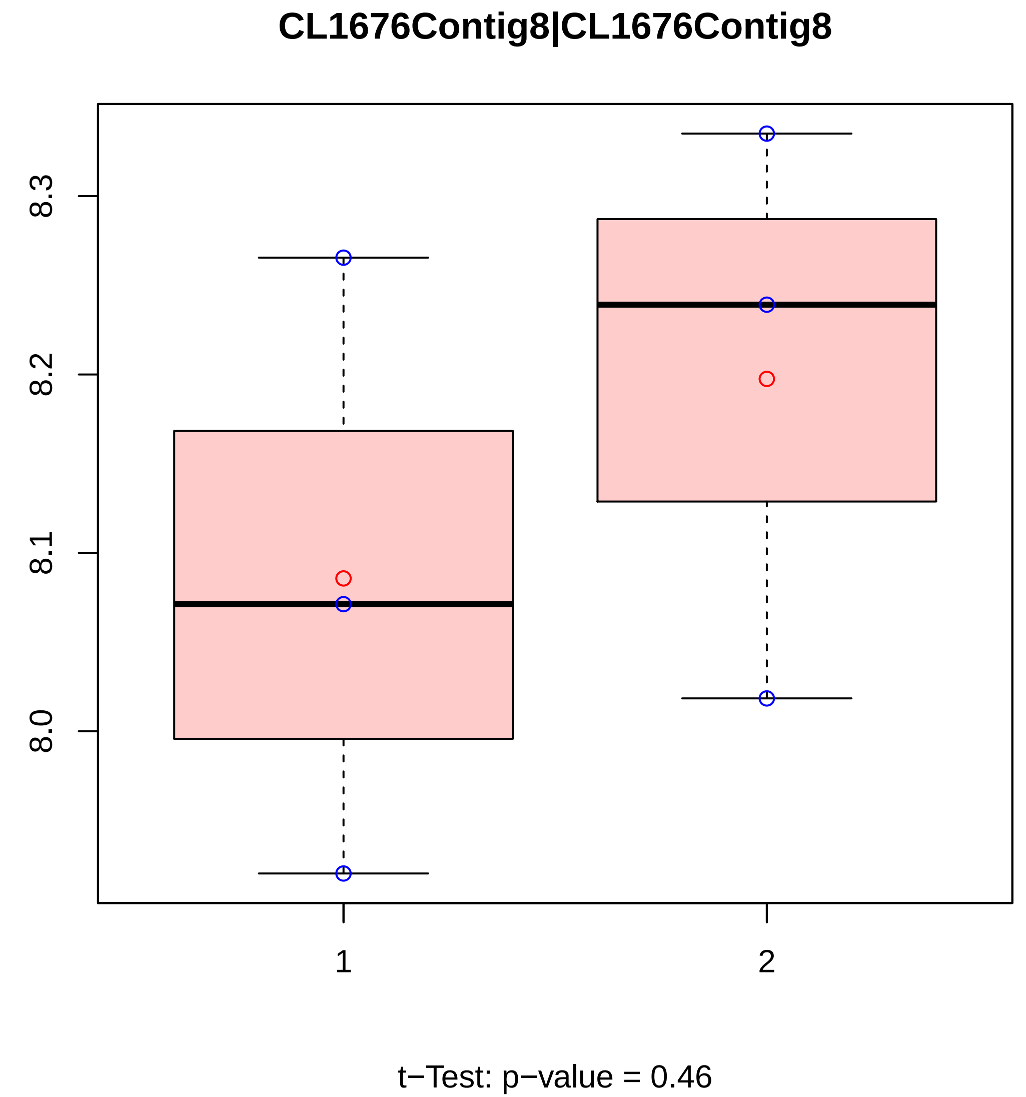
CL1676Contig8|CL1676Contig8
8.0
8.1
8.2
8.3
1
2
t−Test: p−value = 0.46

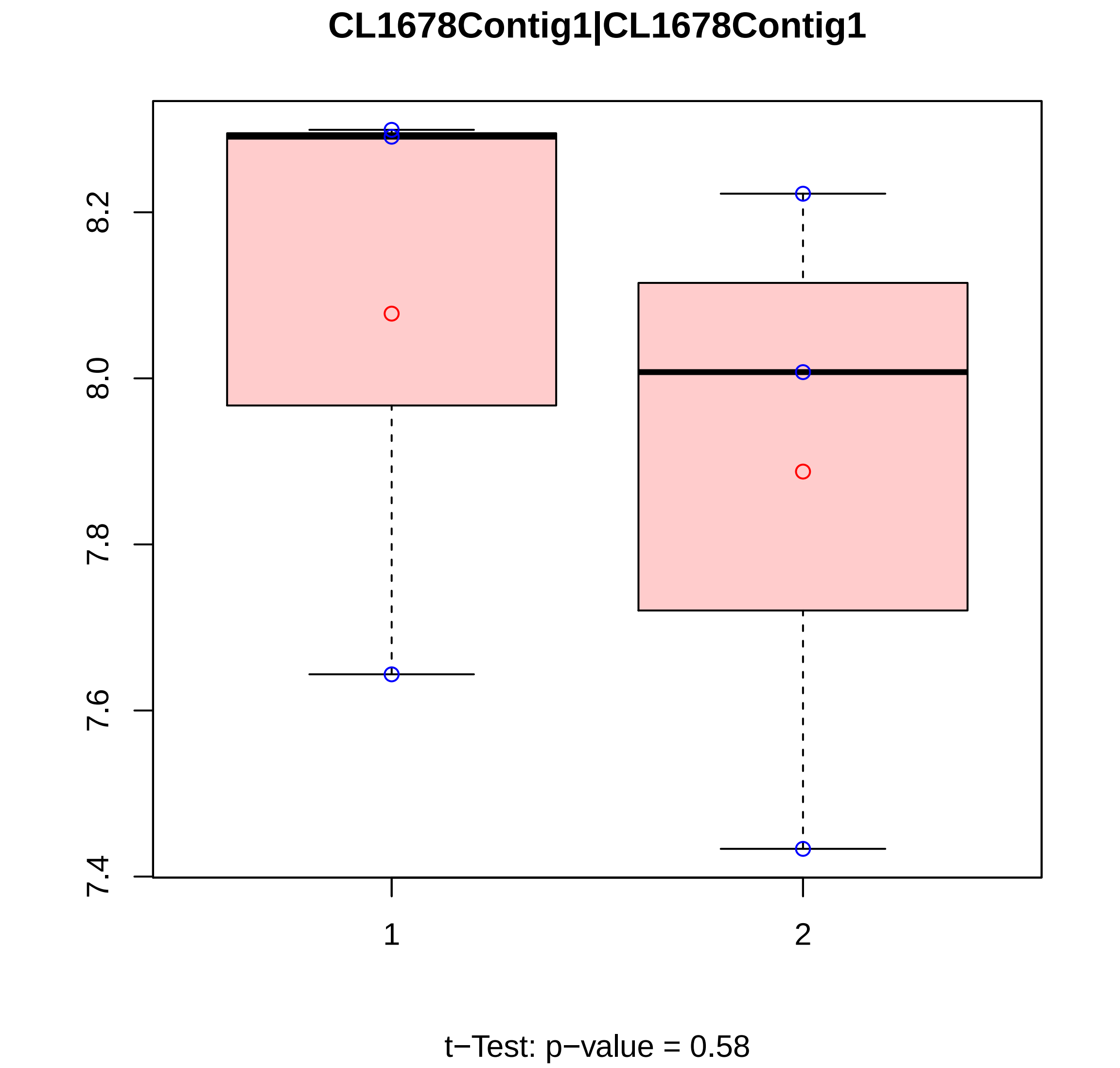

CL1678Contig1|CL1678Contig1
8.2
8.0
7.8
7.6
7.4
1
2
t−Test: p−value = 0.58

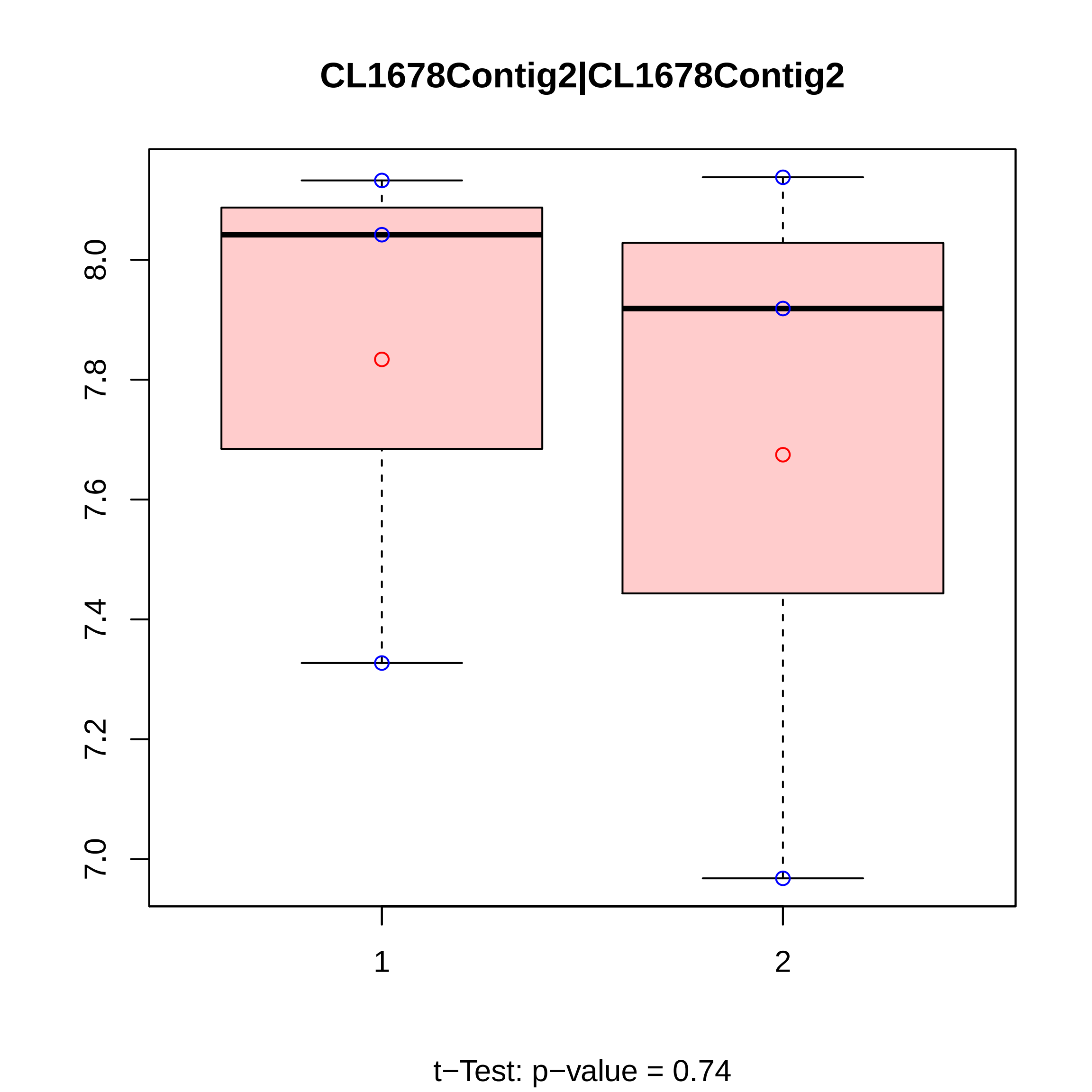
CL1678Contig2|CL1678Contig2
8.0
7.8
7.6
7.4
7.2
7.0
1
2
t−Test: p−value = 0.74

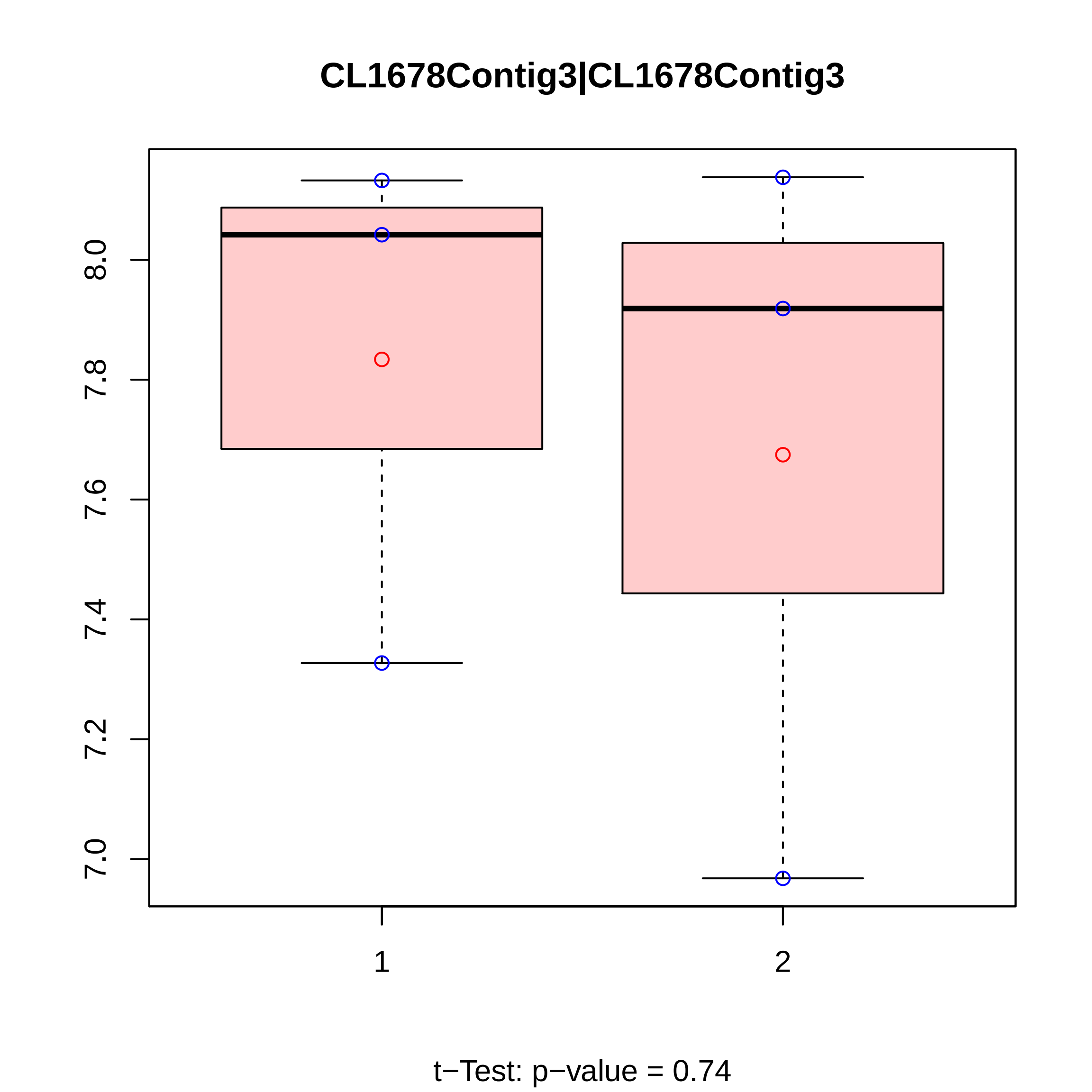
CL1678Contig3|CL1678Contig3
8.0
7.8
7.6
7.4
7.2
7.0
1
2
t−Test: p−value = 0.74

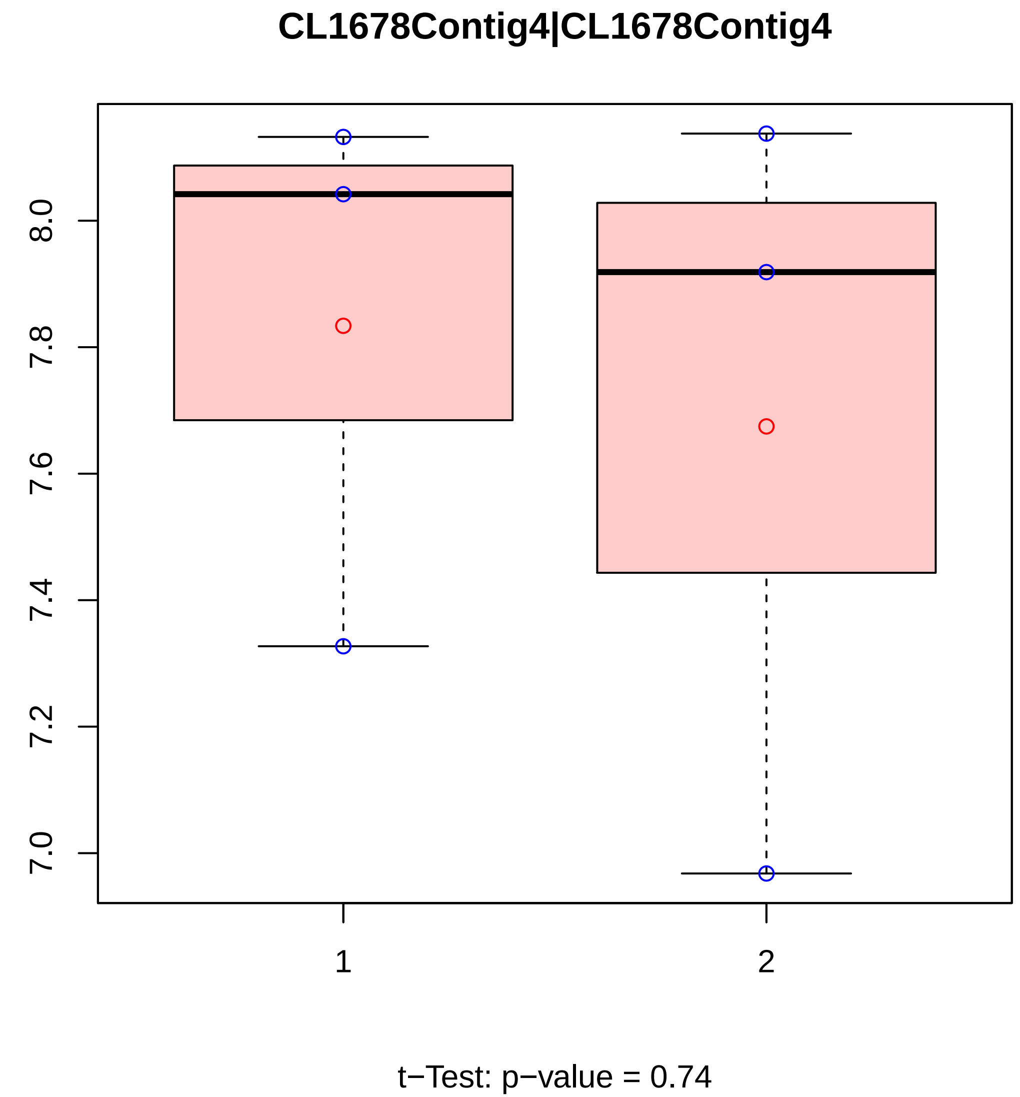
CL1678Contig4|CL1678Contig4
8.0
7.8
7.6
7.4
7.2
7.0
1
2
t−Test: p−value = 0.74

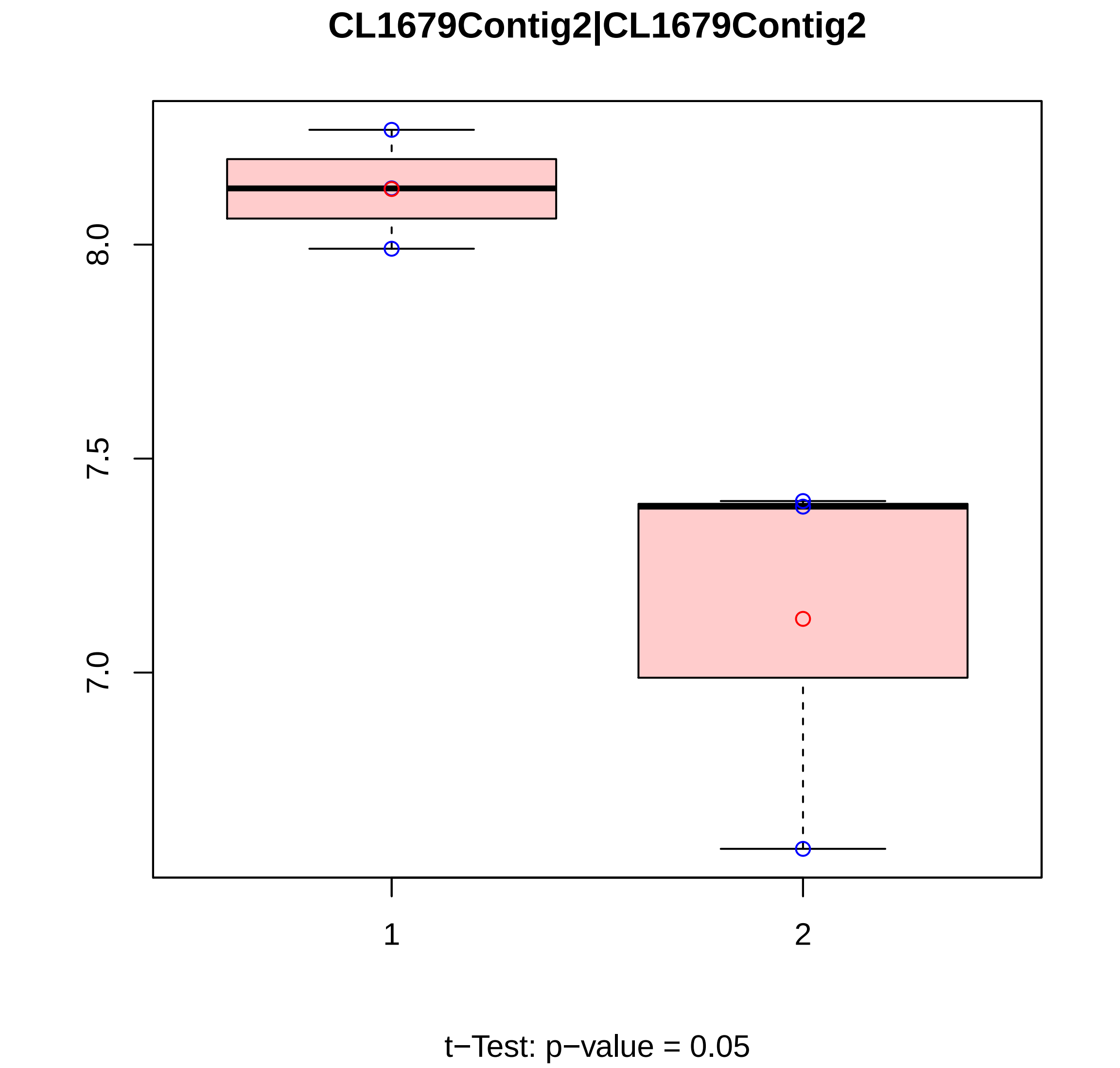
CL1679Contig2|CL1679Contig2
8.0
7.5
7.0
1
2
t−Test: p−value = 0.05

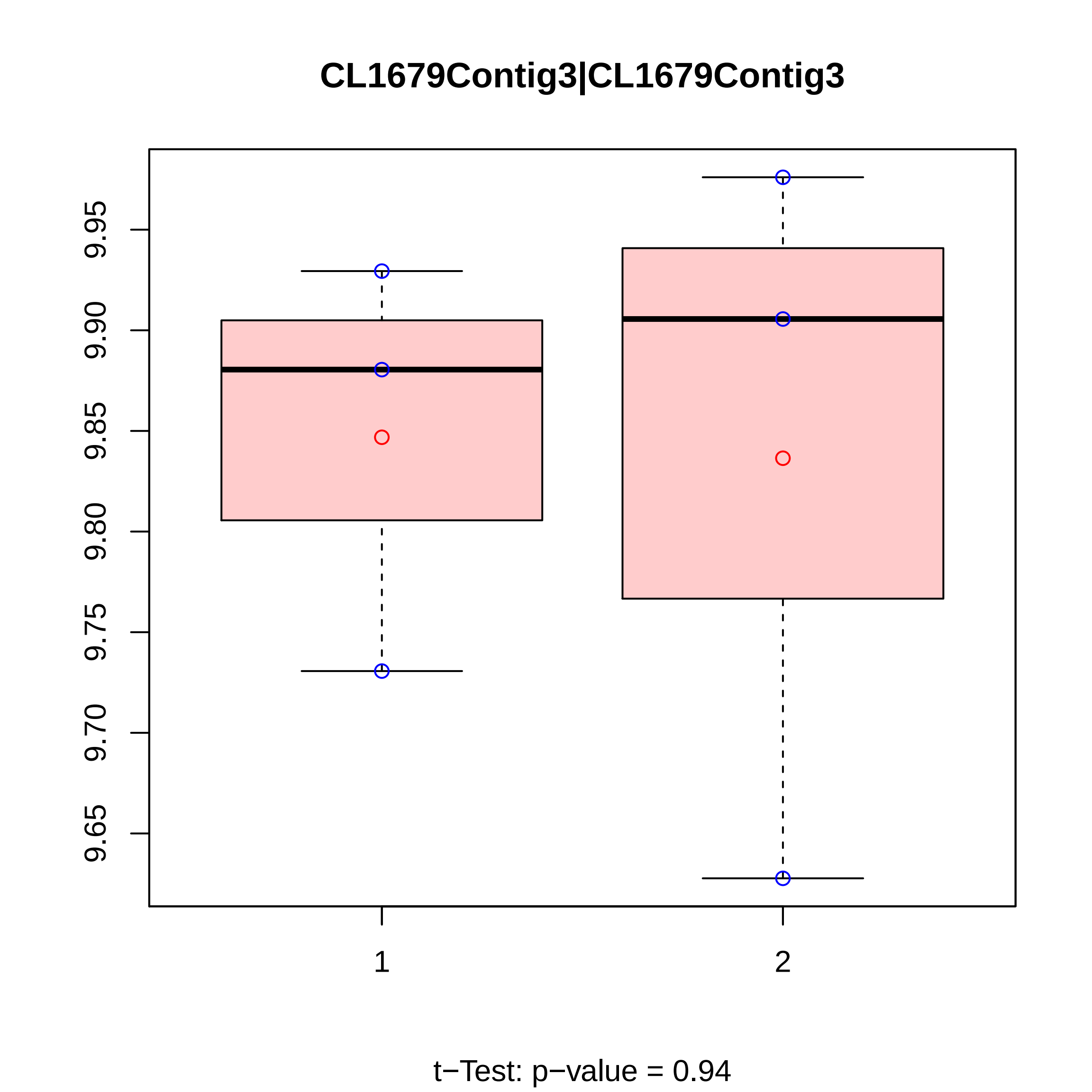
CL1679Contig3|CL1679Contig3
9.65
9.70
9.75
9.80
9.85
9.90
9.95
1
2
t−Test: p−value = 0.94

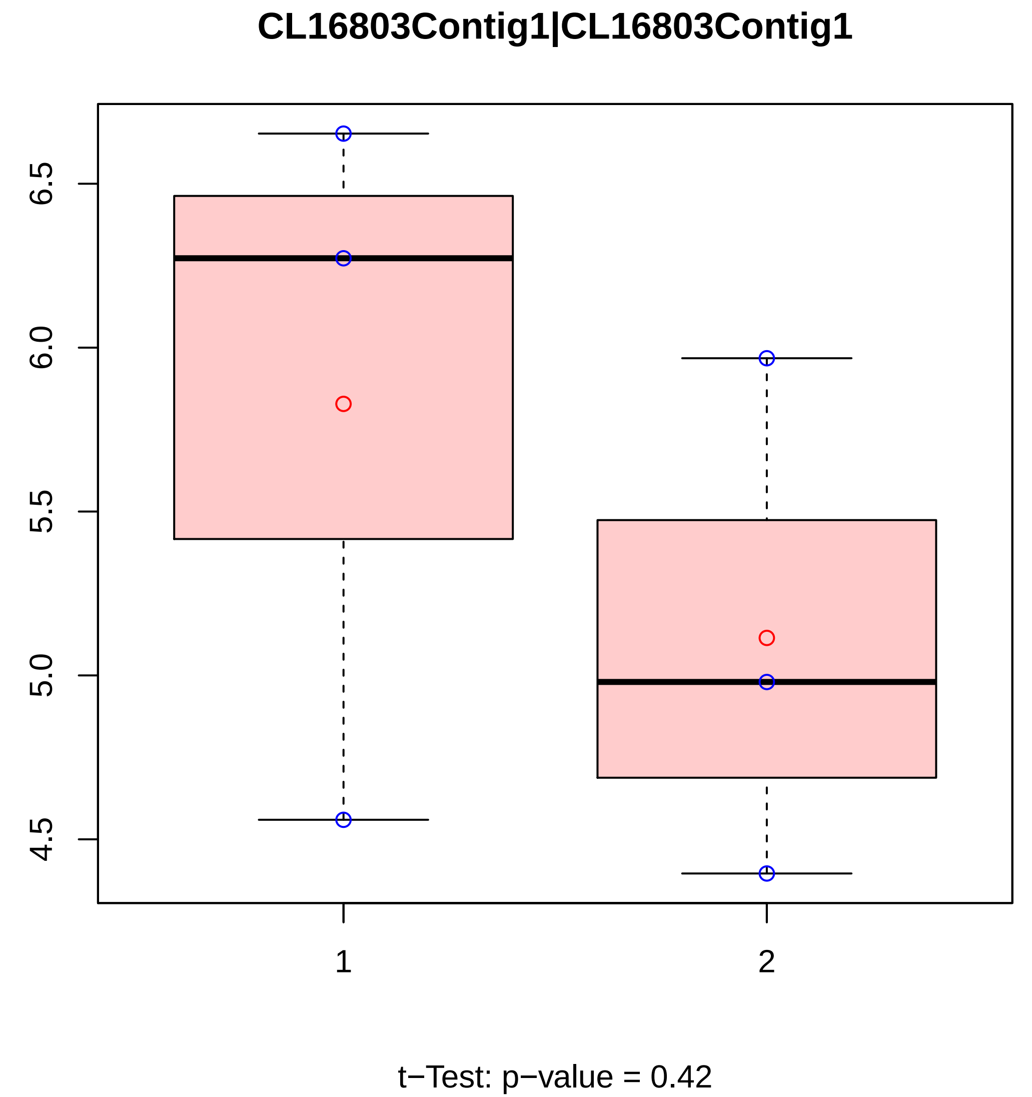

CL16803Contig1|CL16803Contig1
6.5
6.0
5.5
5.0
4.5
1
2
t−Test: p−value = 0.42

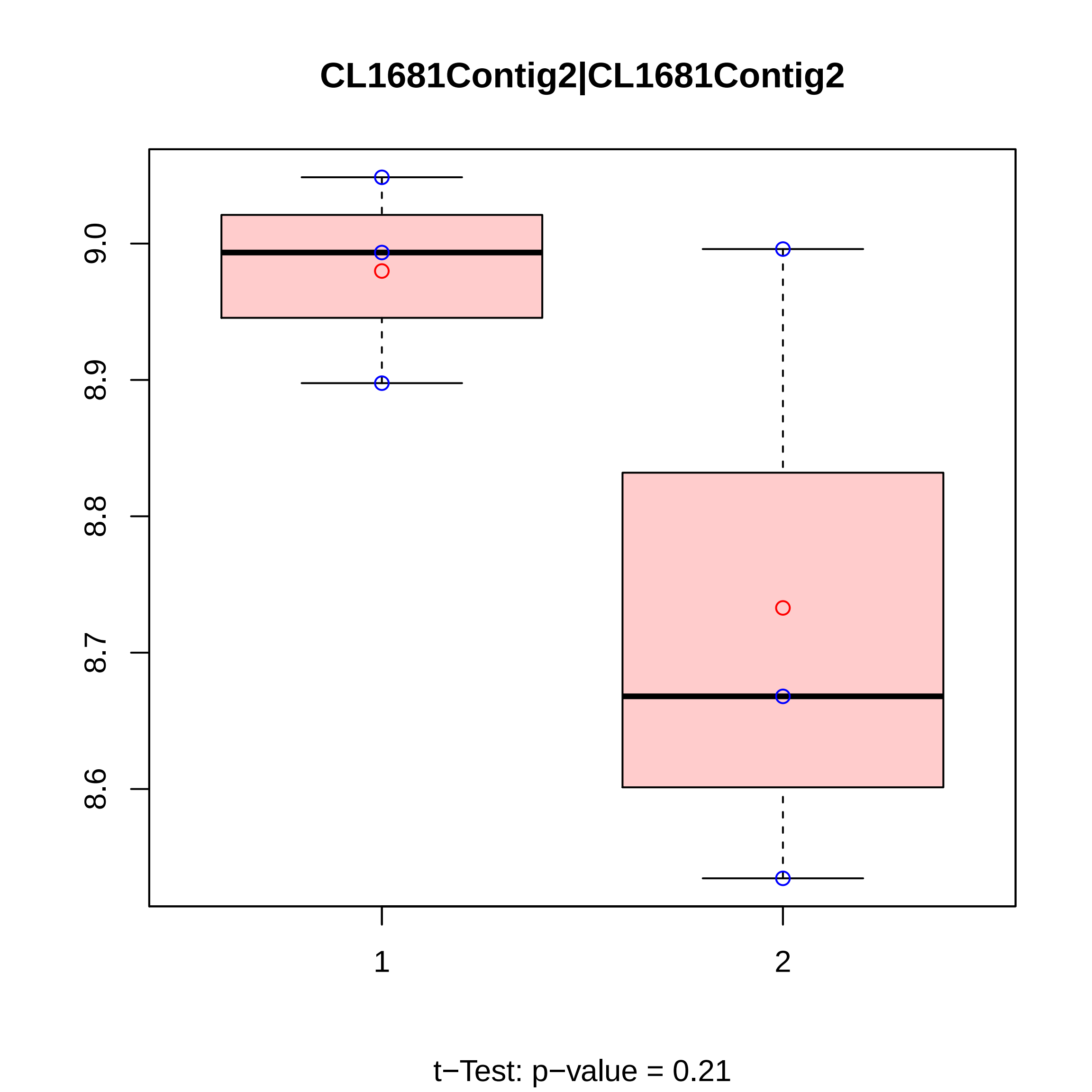
CL1681Contig2|CL1681Contig2
9.0
8.9
8.8
8.7
8.6
1
2
t−Test: p−value = 0.21

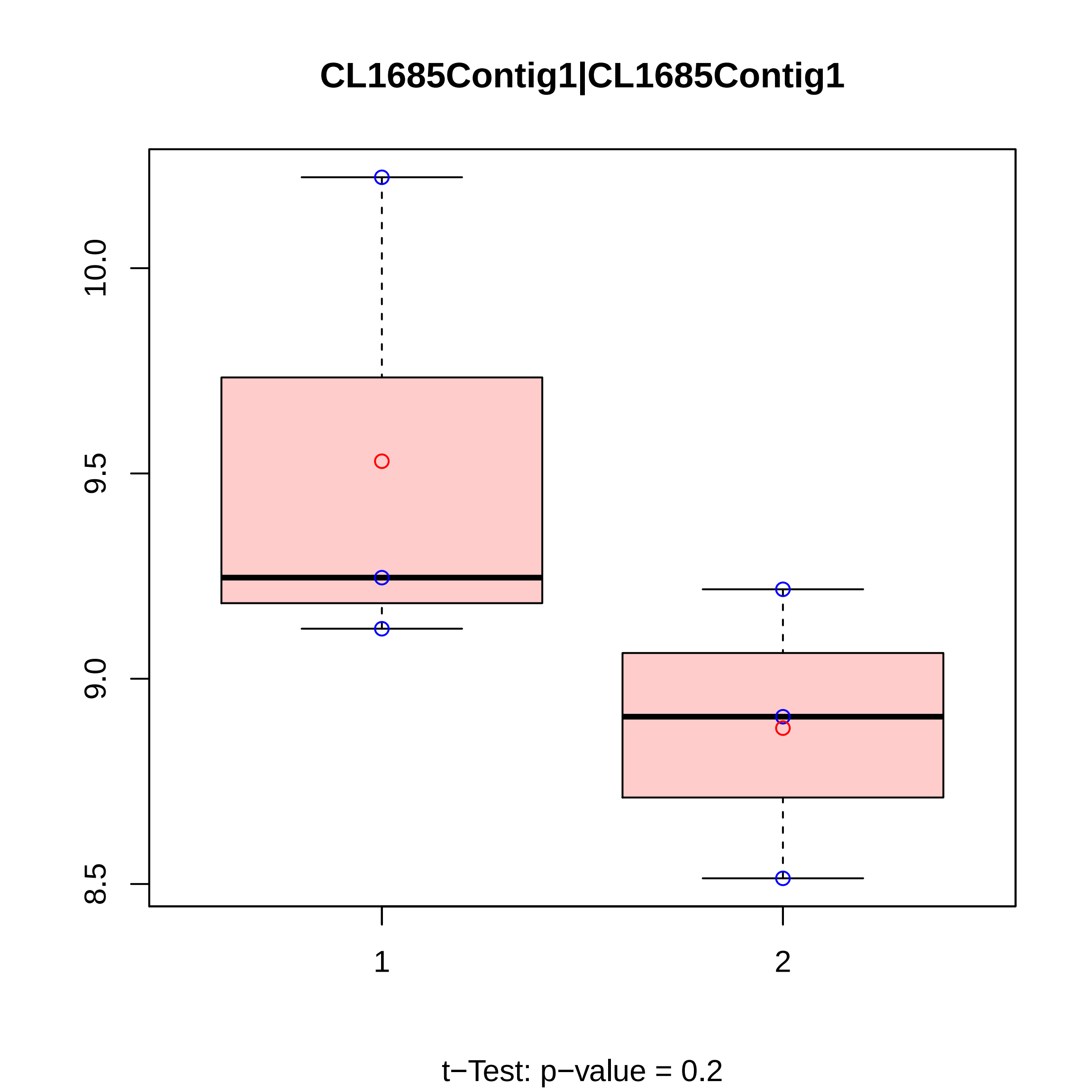
CL1685Contig1|CL1685Contig1
10.0
9.5
9.0
8.5
1
2
t−Test: p−value = 0.2

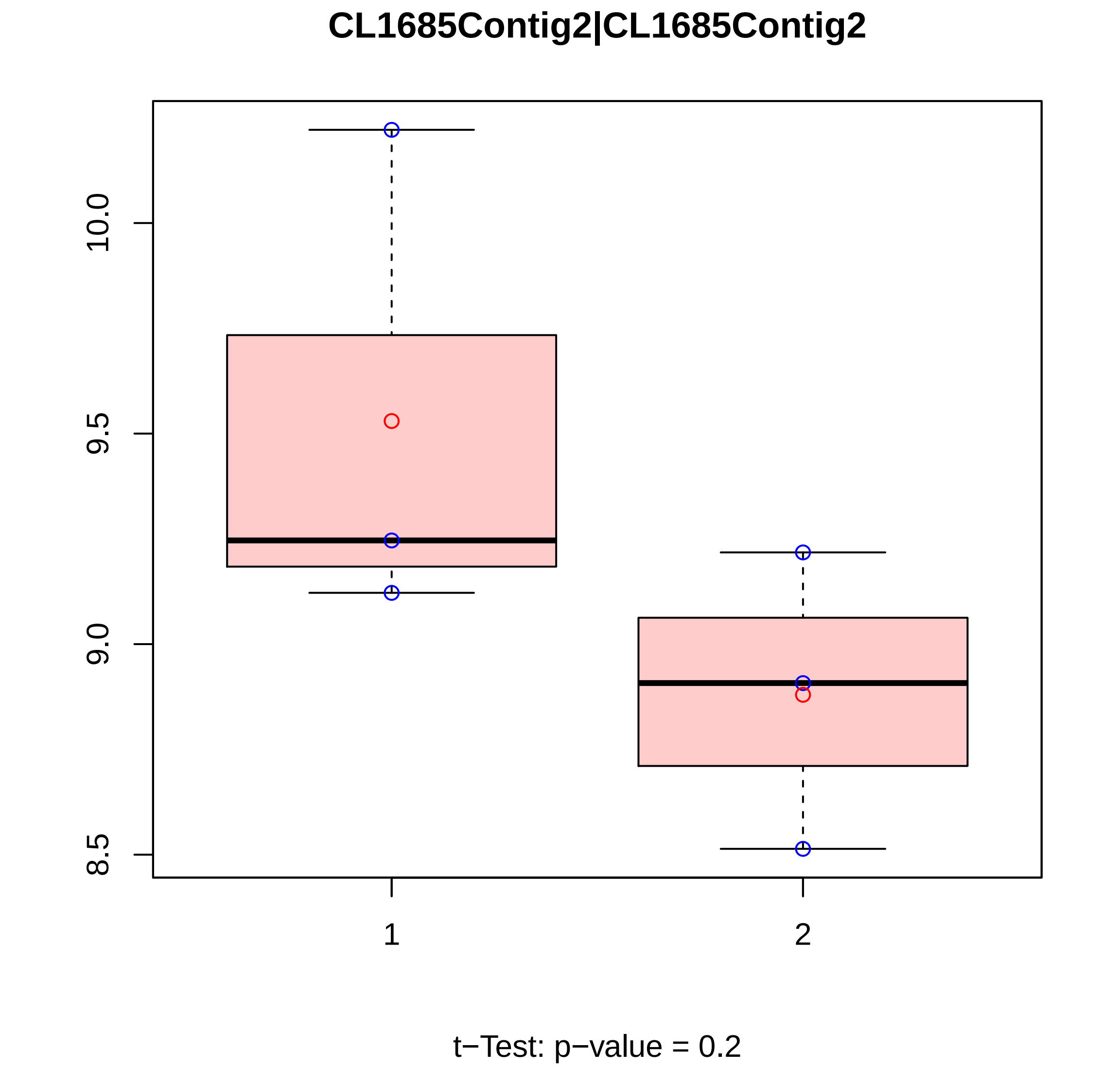
CL1685Contig2|CL1685Contig2
10.0
9.5
9.0
8.5
1
2
t−Test: p−value = 0.2

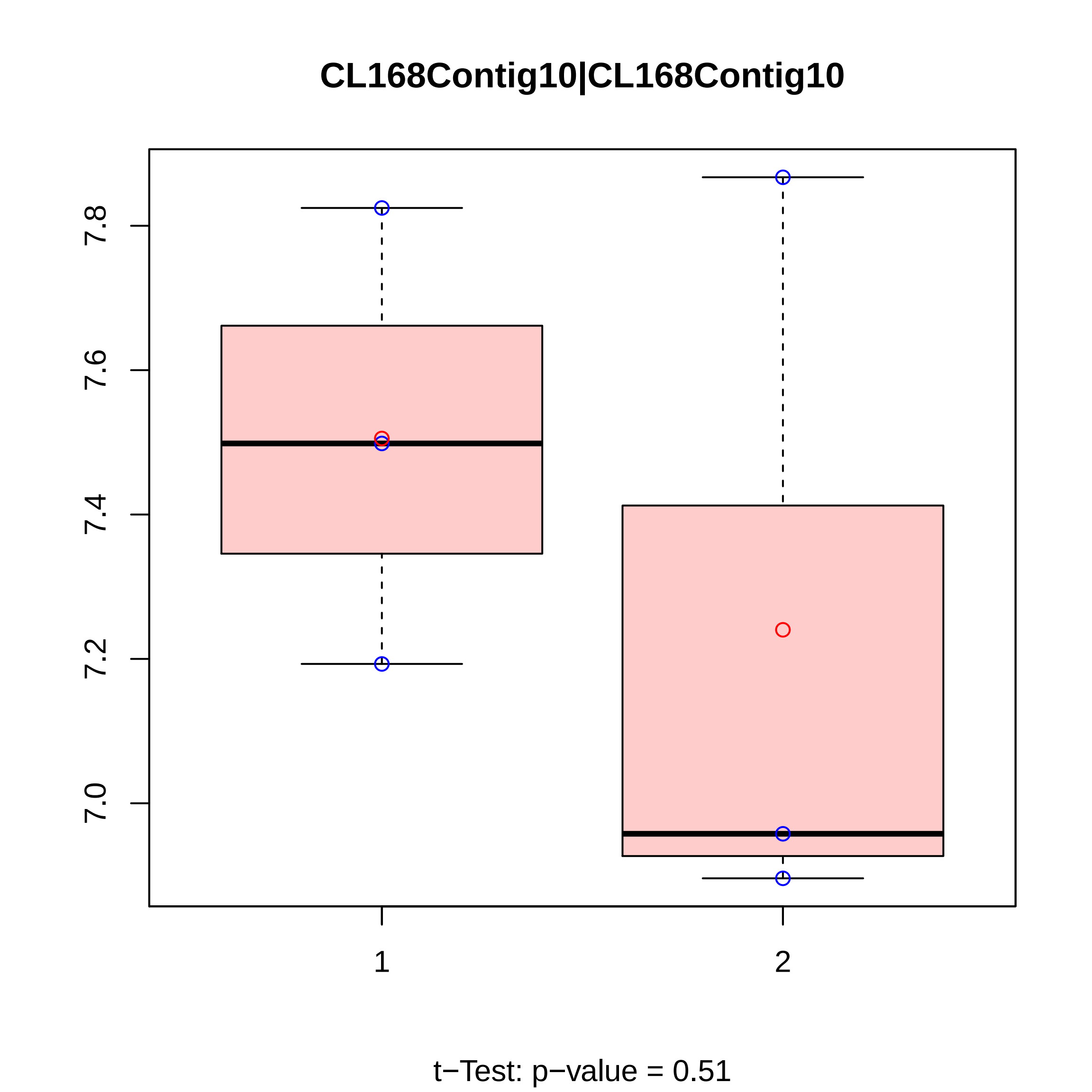
CL168Contig10|CL168Contig10
7.0
7.2
7.4
7.6
7.8
1
2
t−Test: p−value = 0.51

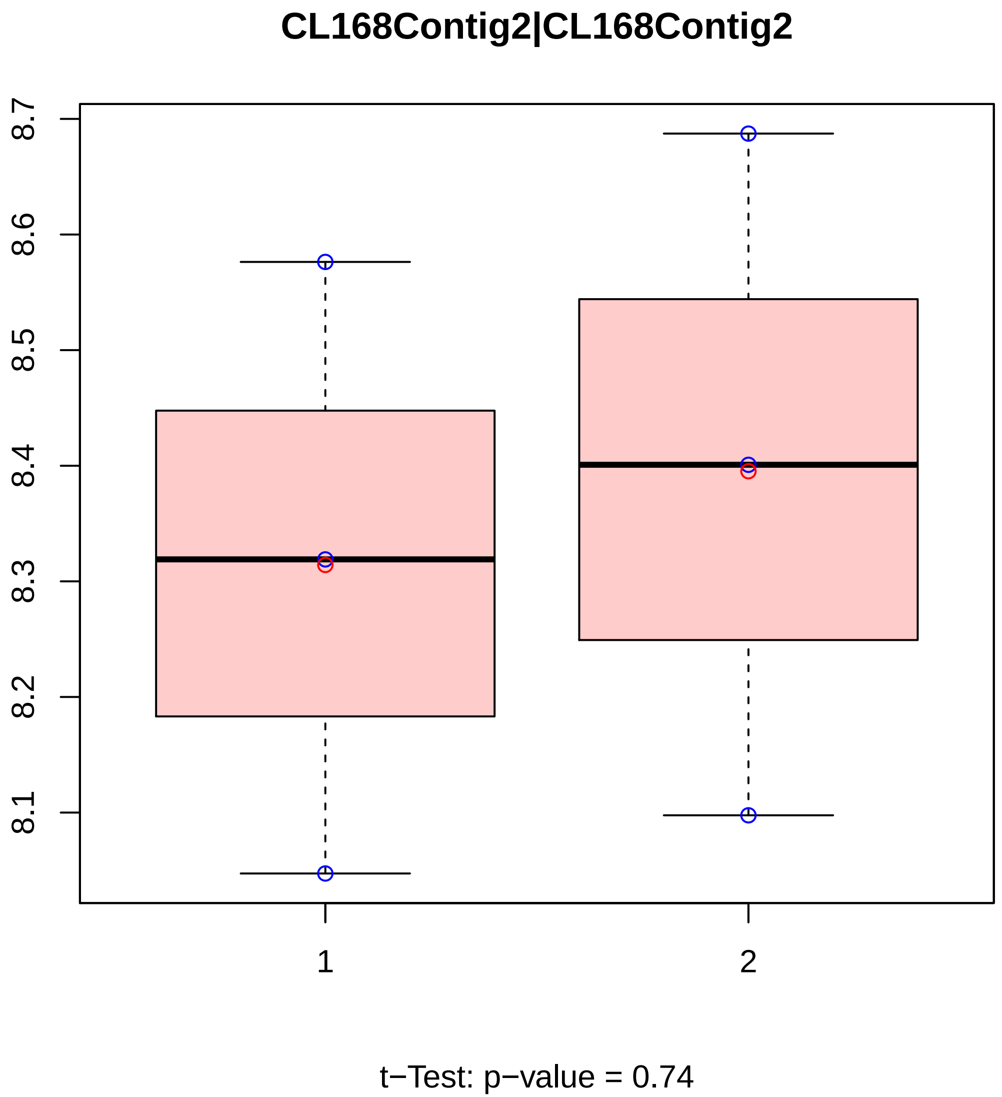
CL168Contig2|CL168Contig2
8.1
8.2
8.3
8.4
8.5
8.6
8.7
1
2
t−Test: p−value = 0.74

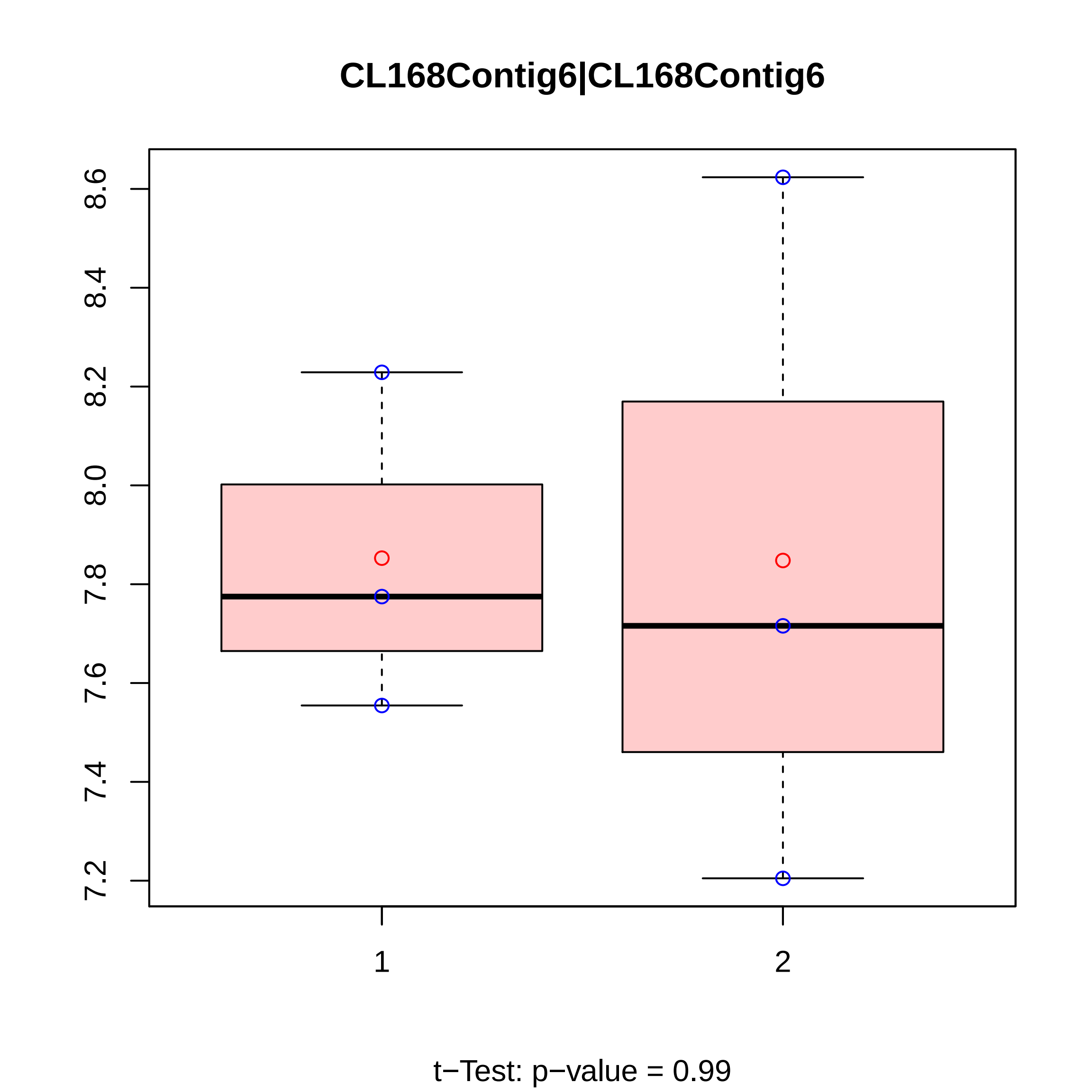
CL168Contig6|CL168Contig6
8.6
8.4
8.2
8.0
7.8
7.6
7.4
7.2
1
2
t−Test: p−value = 0.99

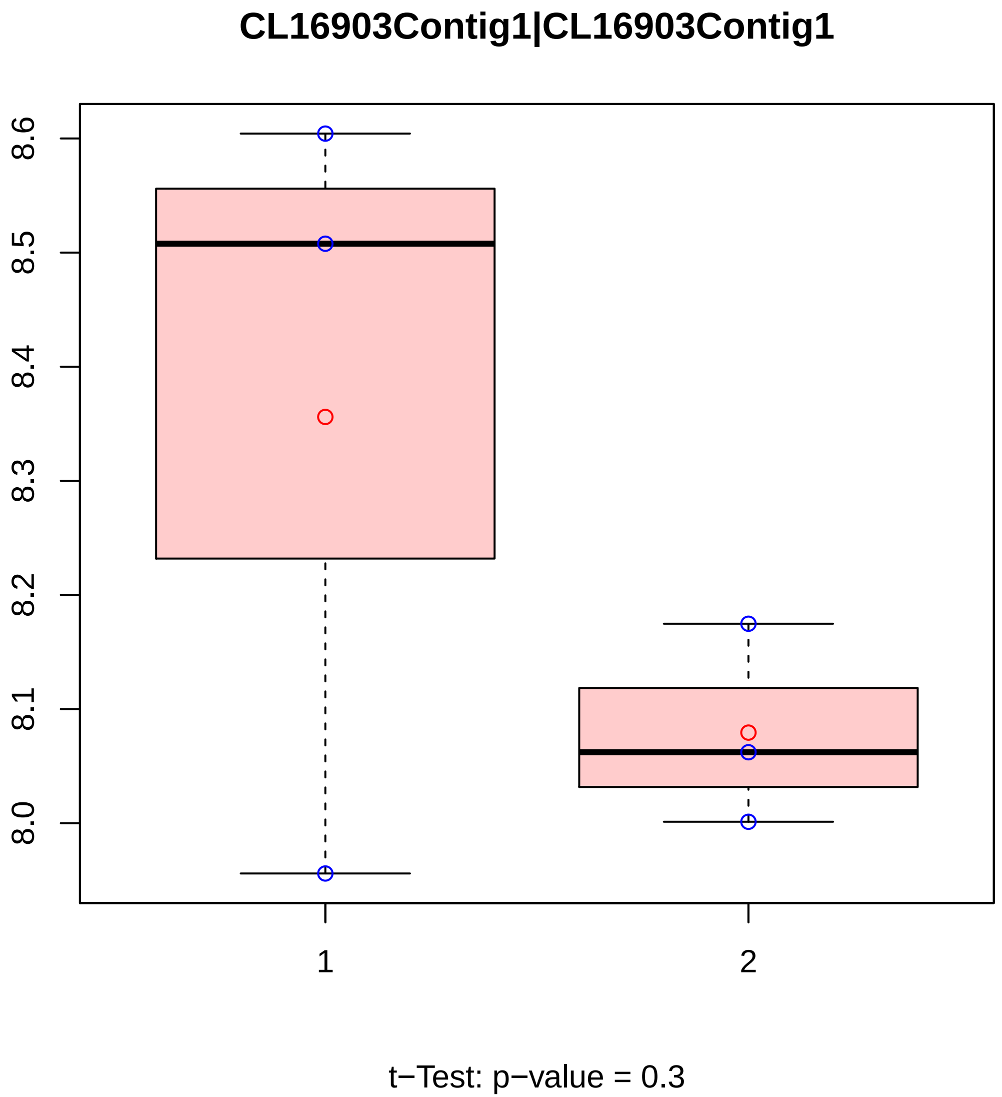
CL16903Contig1|CL16903Contig1
8.0
8.1
8.2
8.3
8.4
8.5
8.6
1
2
t−Test: p−value = 0.3

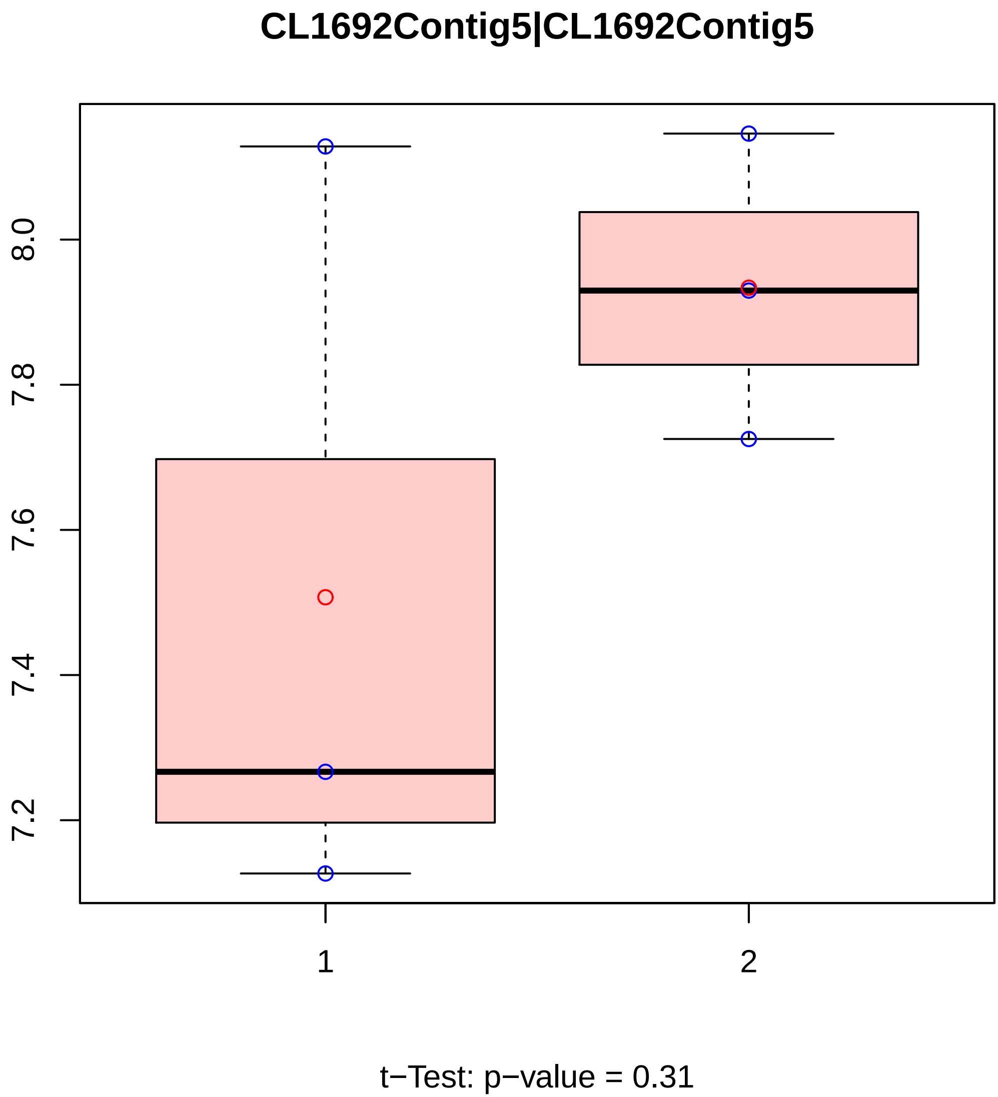
CL1692Contig5|CL1692Contig5
8.0
7.8
7.6
7.4
7.2
1
2
t−Test: p−value = 0.31

CL16961Contig1|CL16961Contig1

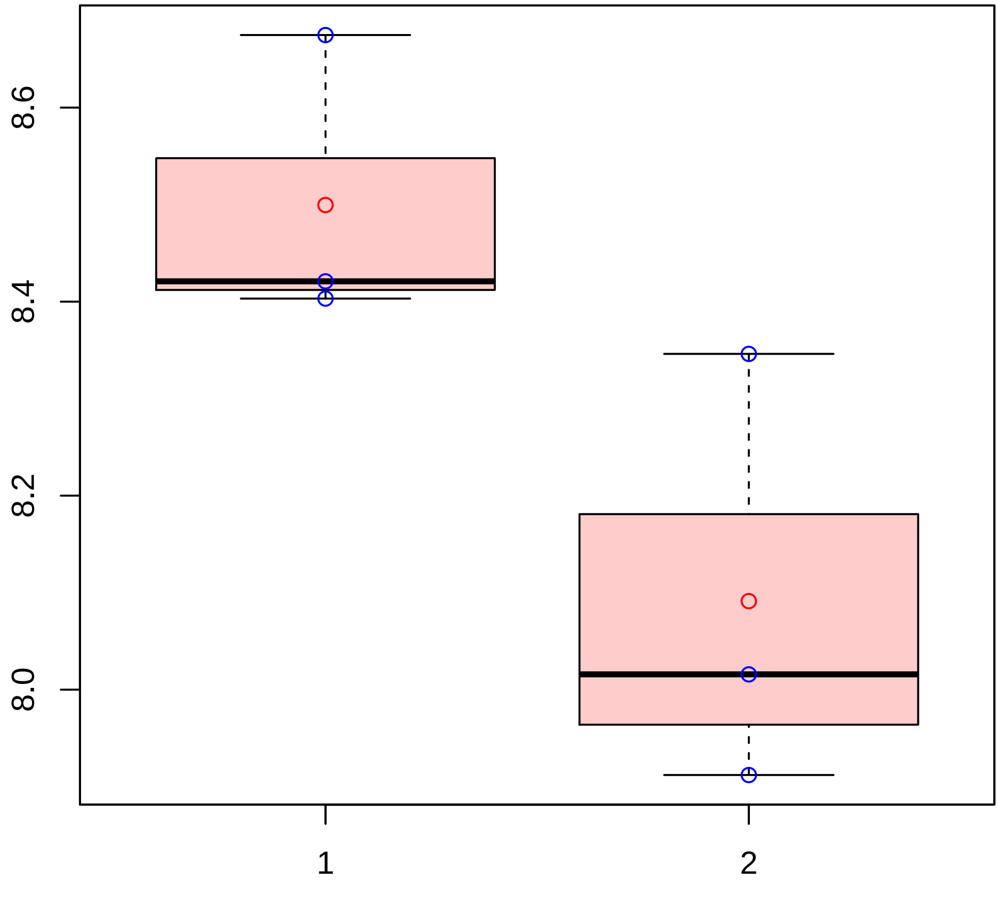


t−Test: p−value = 0.07

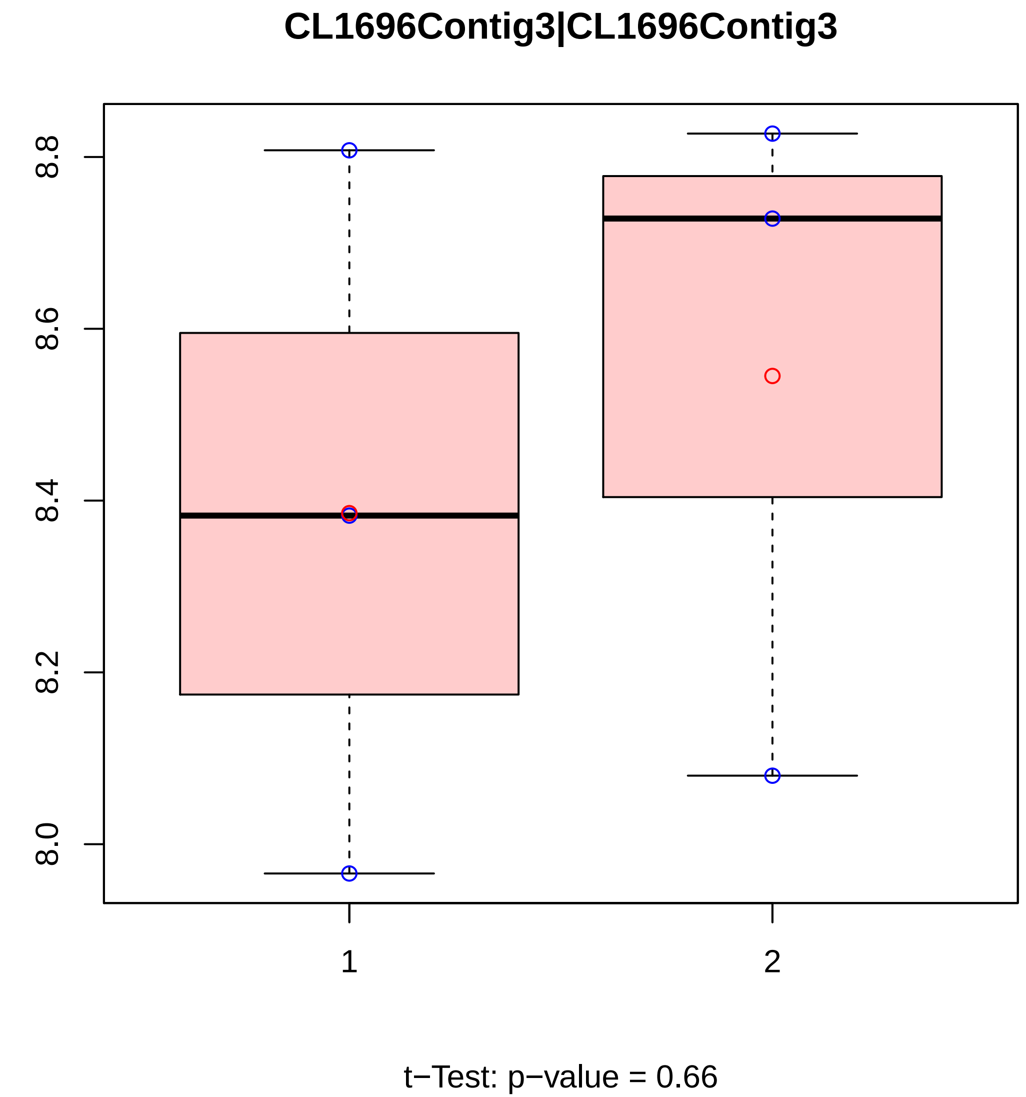
CL1696Contig3|CL1696Contig3
8.0
8.2
8.4
8.6
8.8
1
2
t−Test: p−value = 0.66

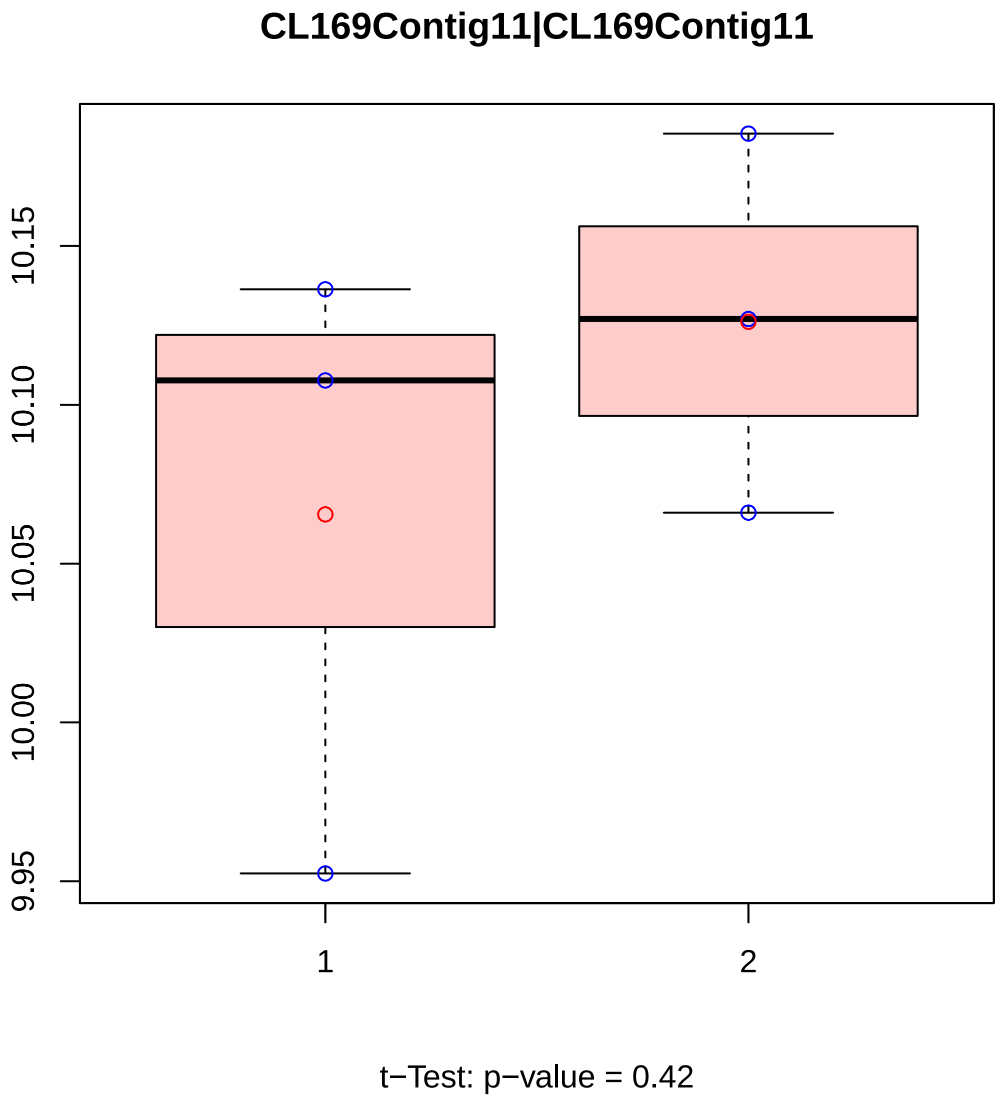
CL169Contig11|CL169Contig11
9.95
10.00
10.05
10.10
10.15
1
2
t−Test: p−value = 0.42

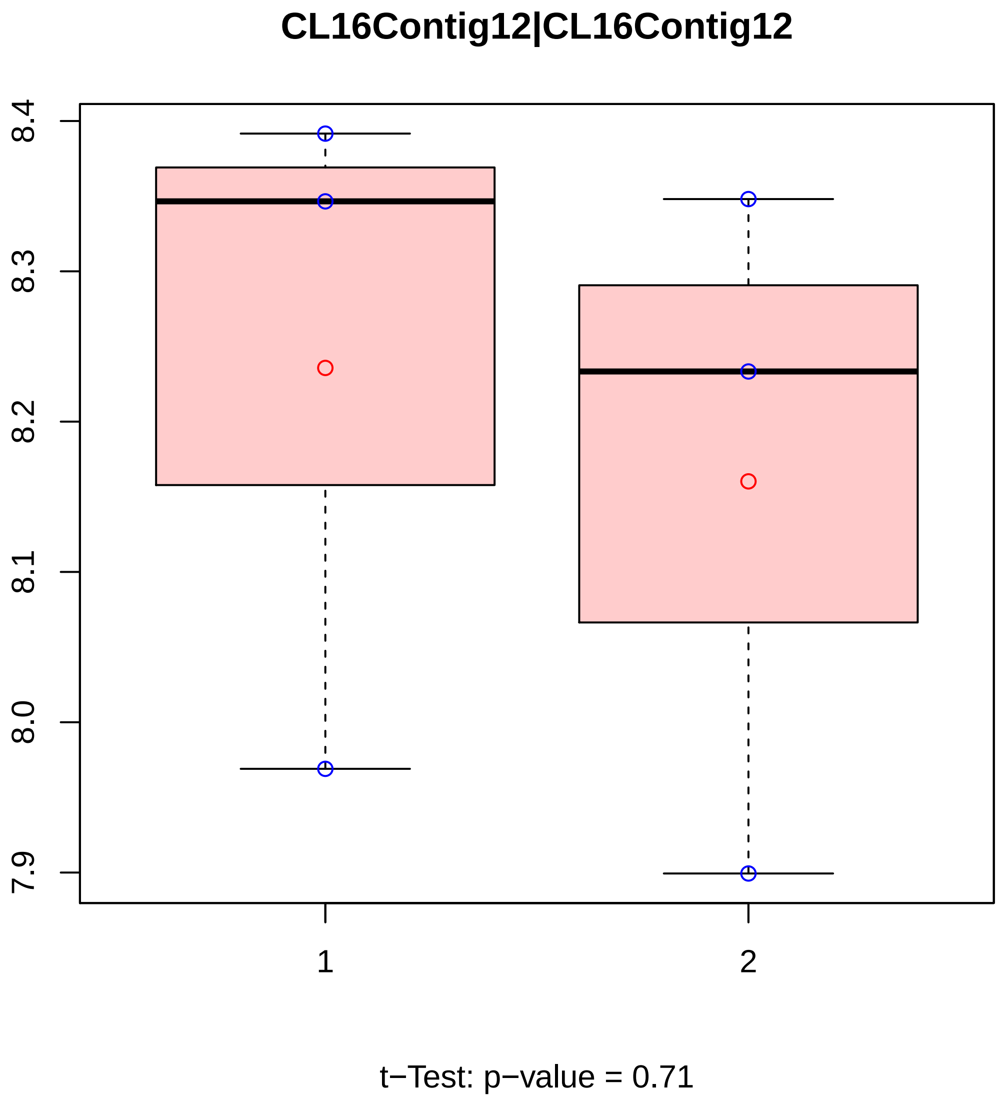
CL16Contig12|CL16Contig12
8.4
8.3
8.2
8.1
8.0
7.9
1
2
t−Test: p−value = 0.71

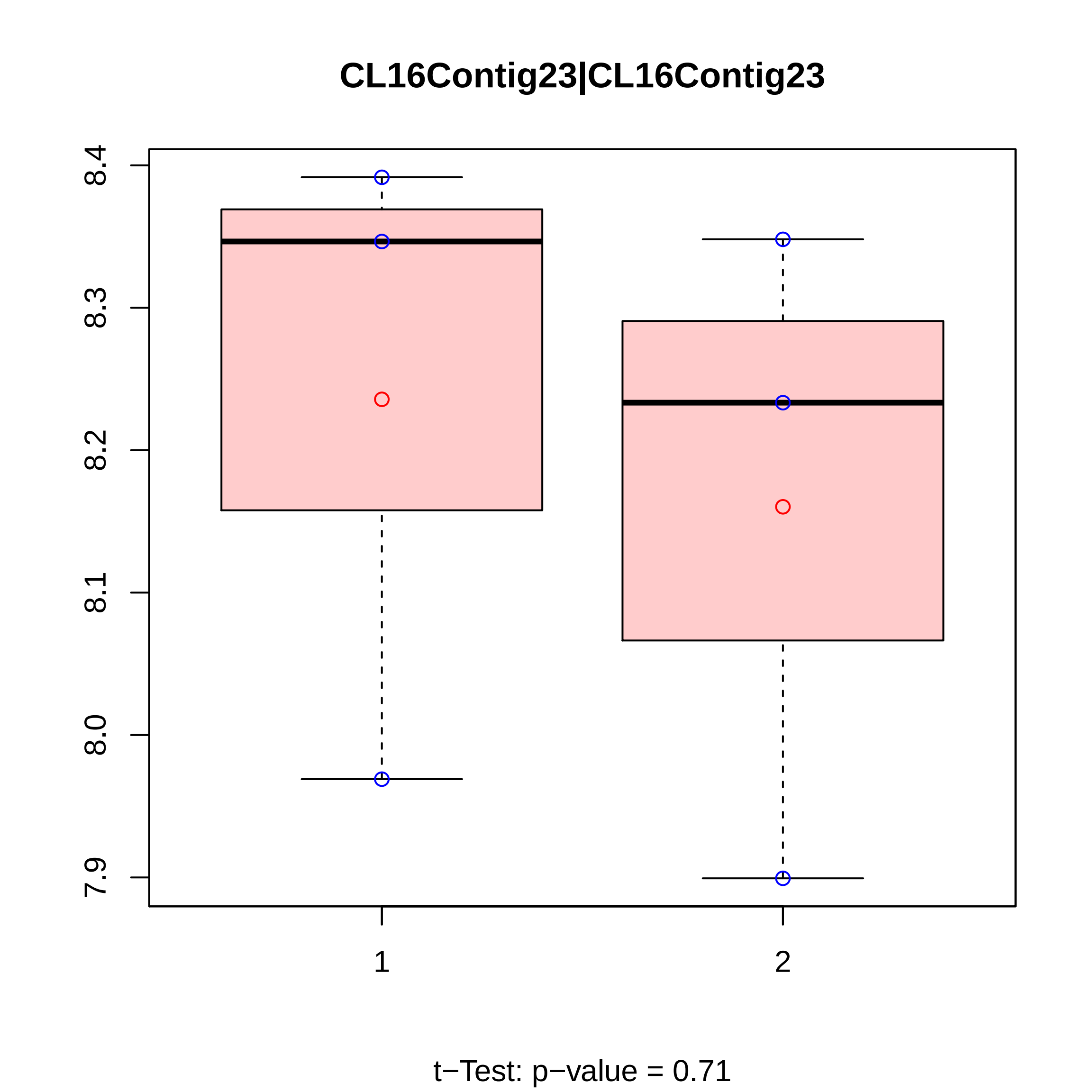
CL16Contig23|CL16Contig23
8.4
8.3
8.2
8.1
8.0
7.9
1
2
t−Test: p−value = 0.71

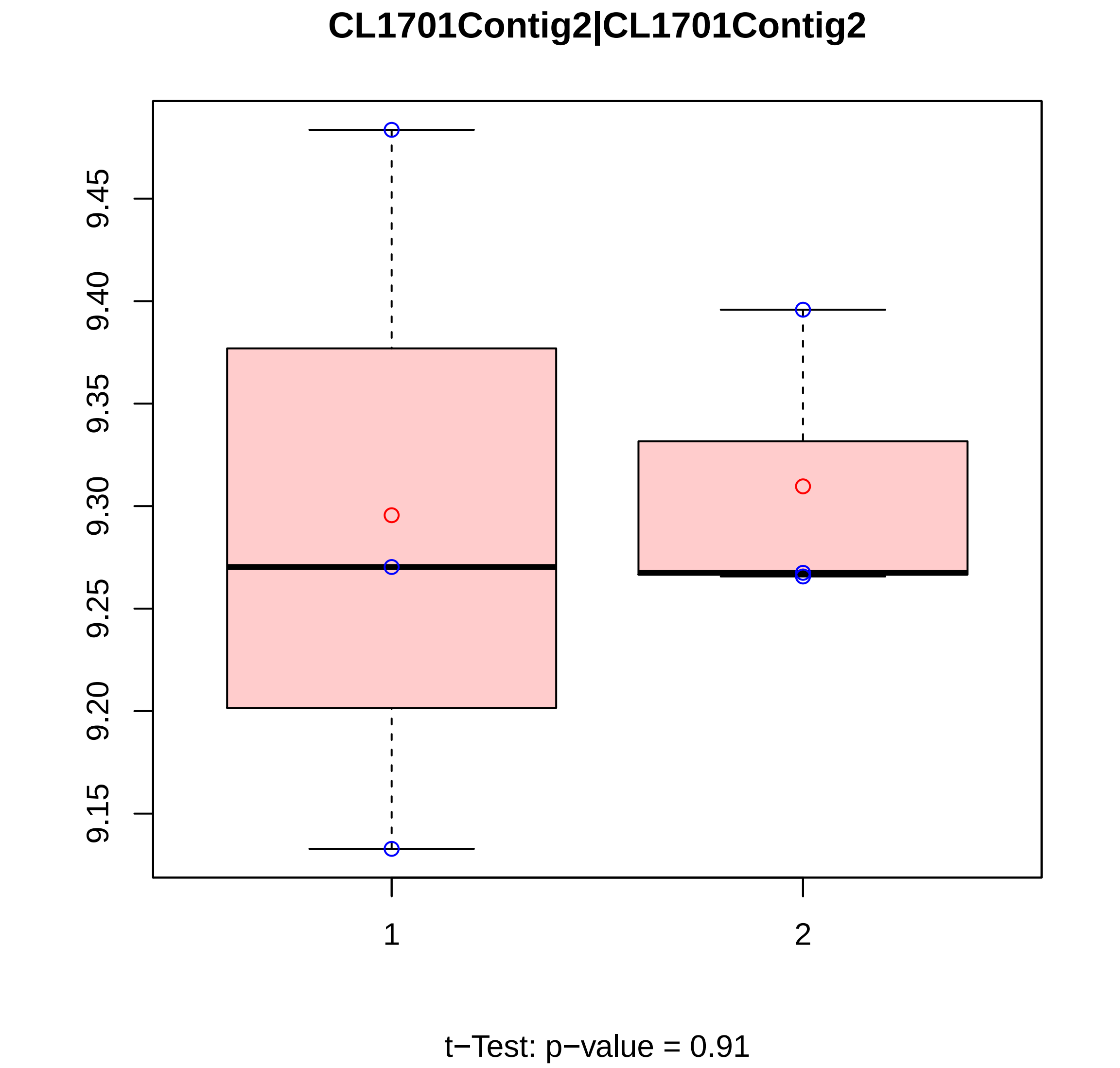
CL1701Contig2|CL1701Contig2
9.15
9.20
9.25
9.30
9.35
9.40
9.45
1
2
t−Test: p−value = 0.91

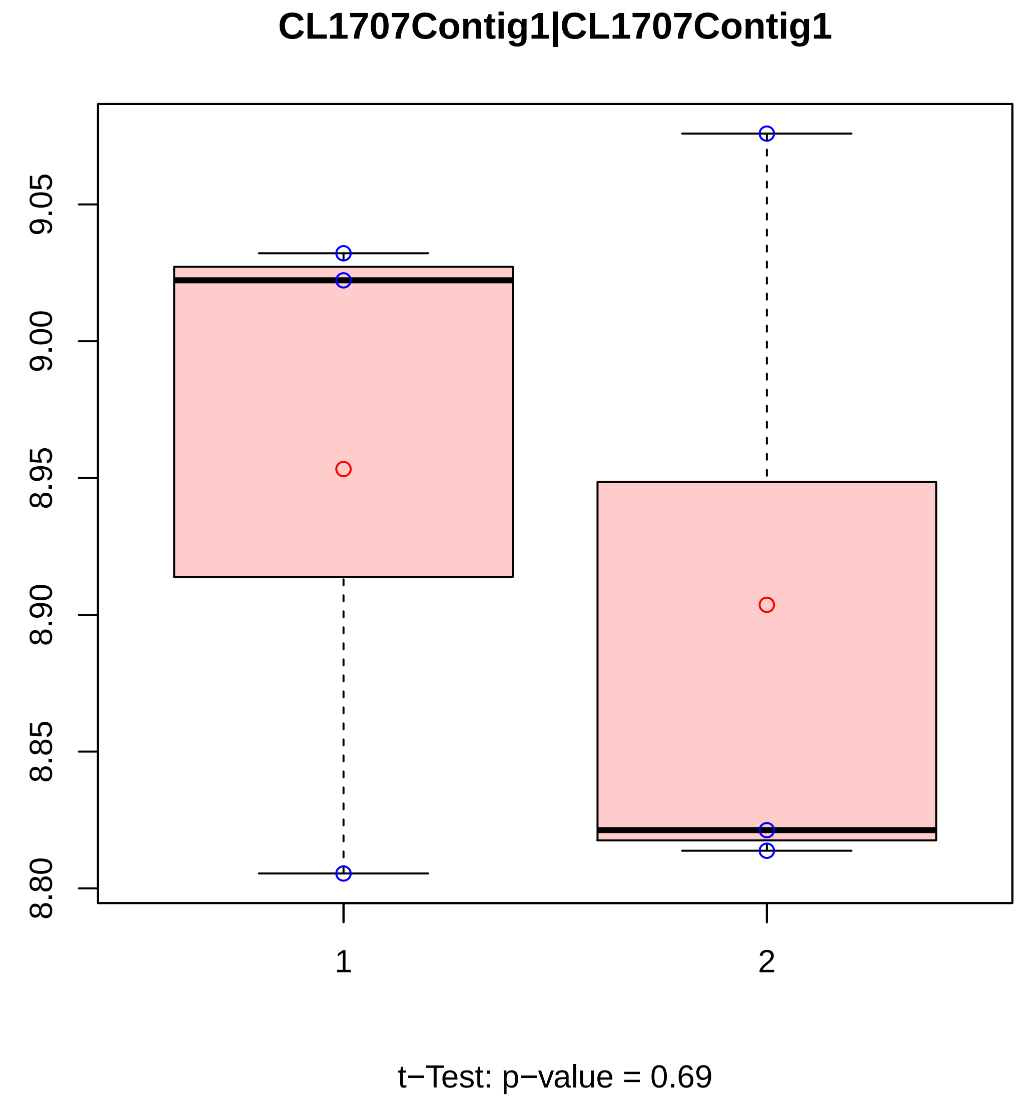

CL1707Contig1|CL1707Contig1
9.05
9.00
8.95
8.90
8.85
8.80
1
2
t−Test: p−value = 0.69

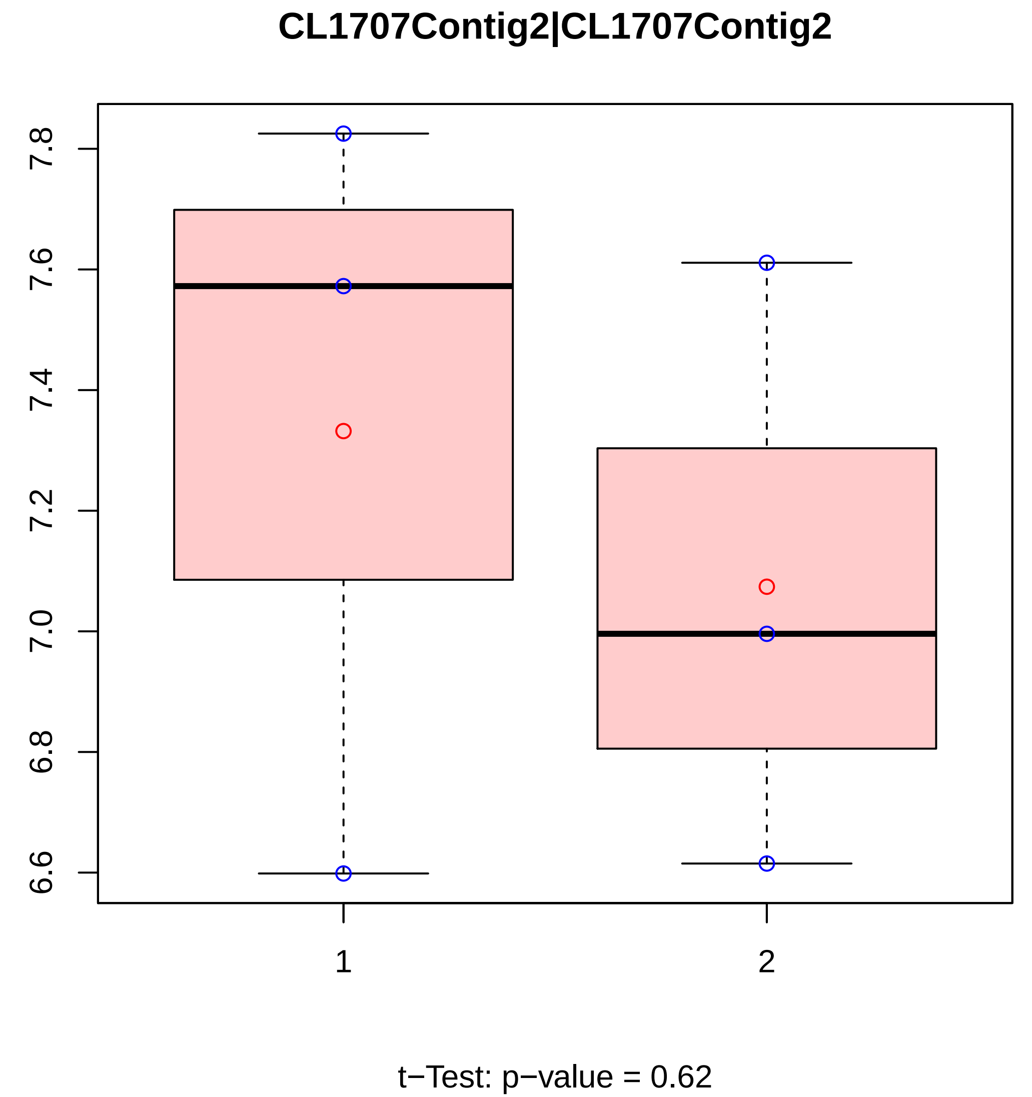

CL1707Contig2|CL1707Contig2
7.8
7.6
7.4
7.2
7.0
6.8
6.6
1
2
t−Test: p−value = 0.62

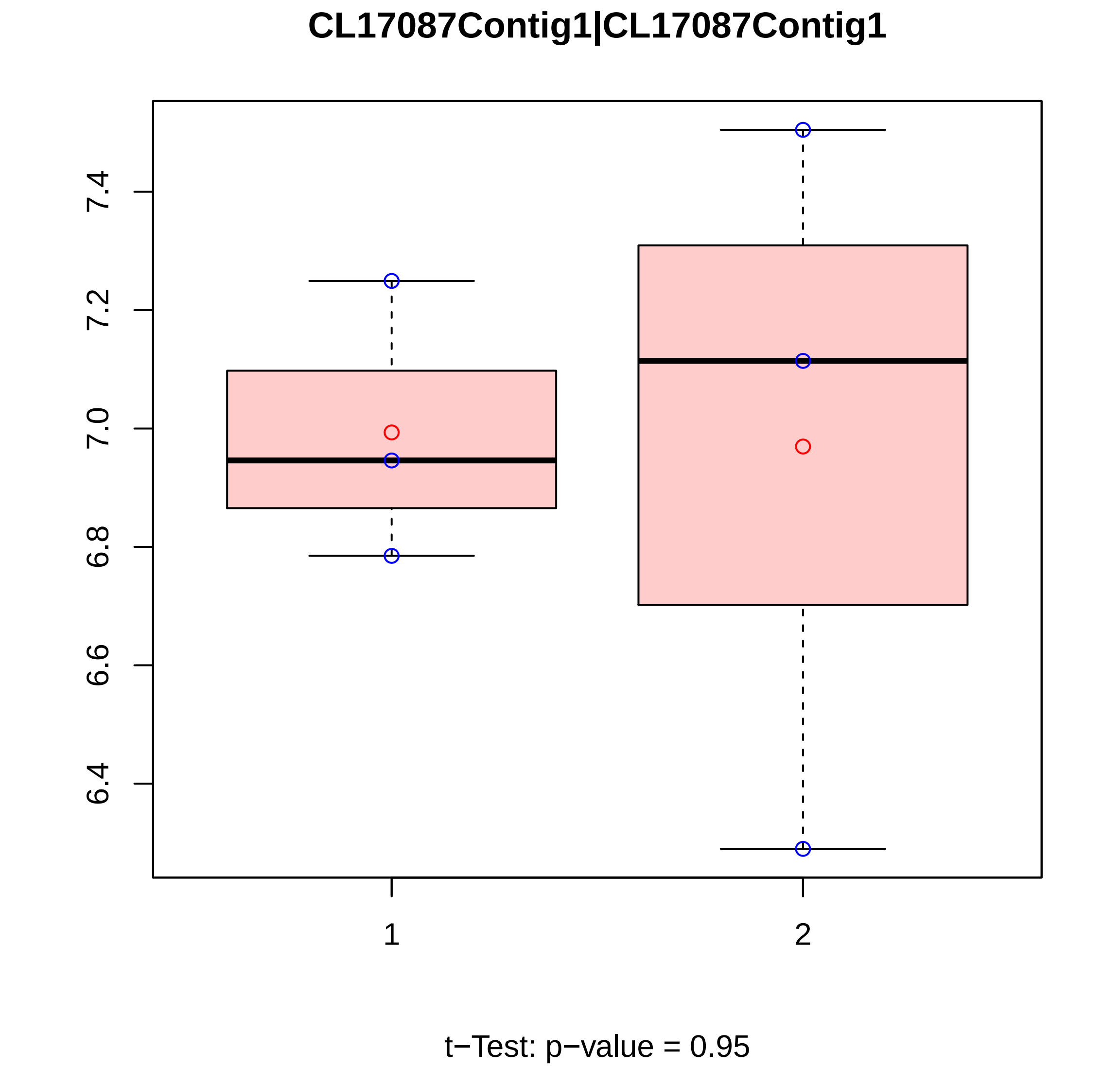
CL17087Contig1|CL17087Contig1
7.4
7.2
7.0
6.8
6.6
6.4
1
2
t−Test: p−value = 0.95

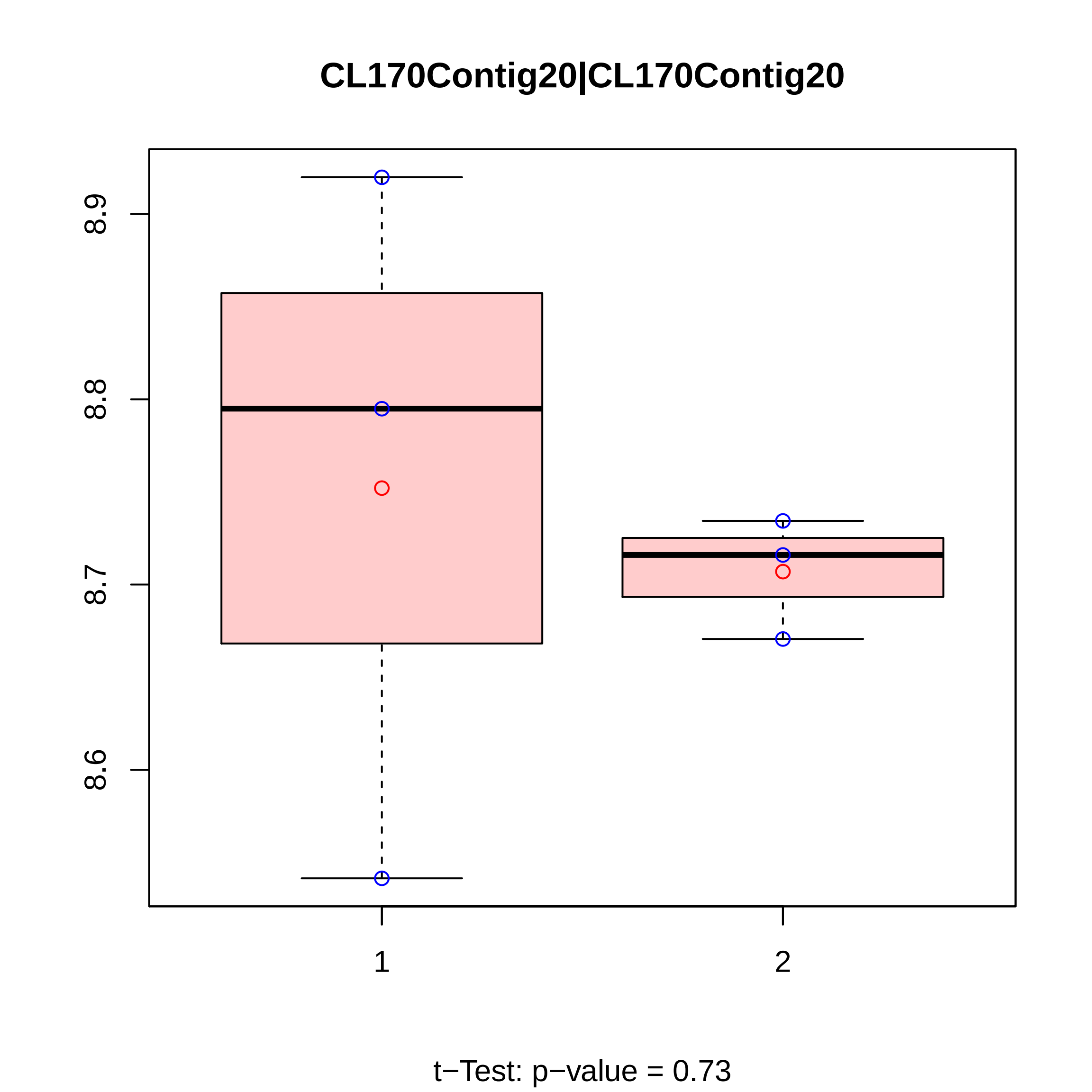
CL170Contig20|CL170Contig20
8.6
8.7
8.8
8.9
1
2
t−Test: p−value = 0.73

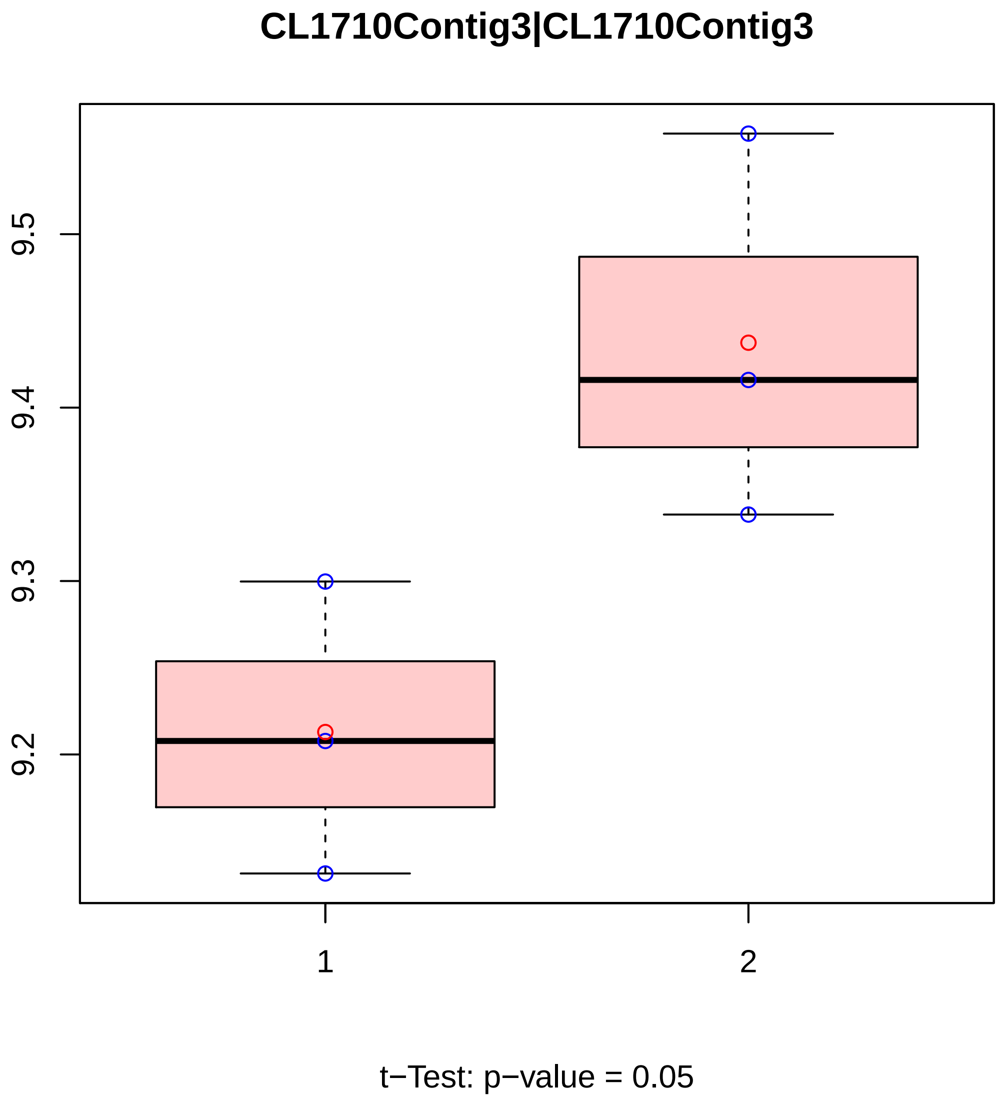

CL1710Contig3|CL1710Contig3
9.2
9.3
9.4
9.5
1
2
t−Test: p−value = 0.05

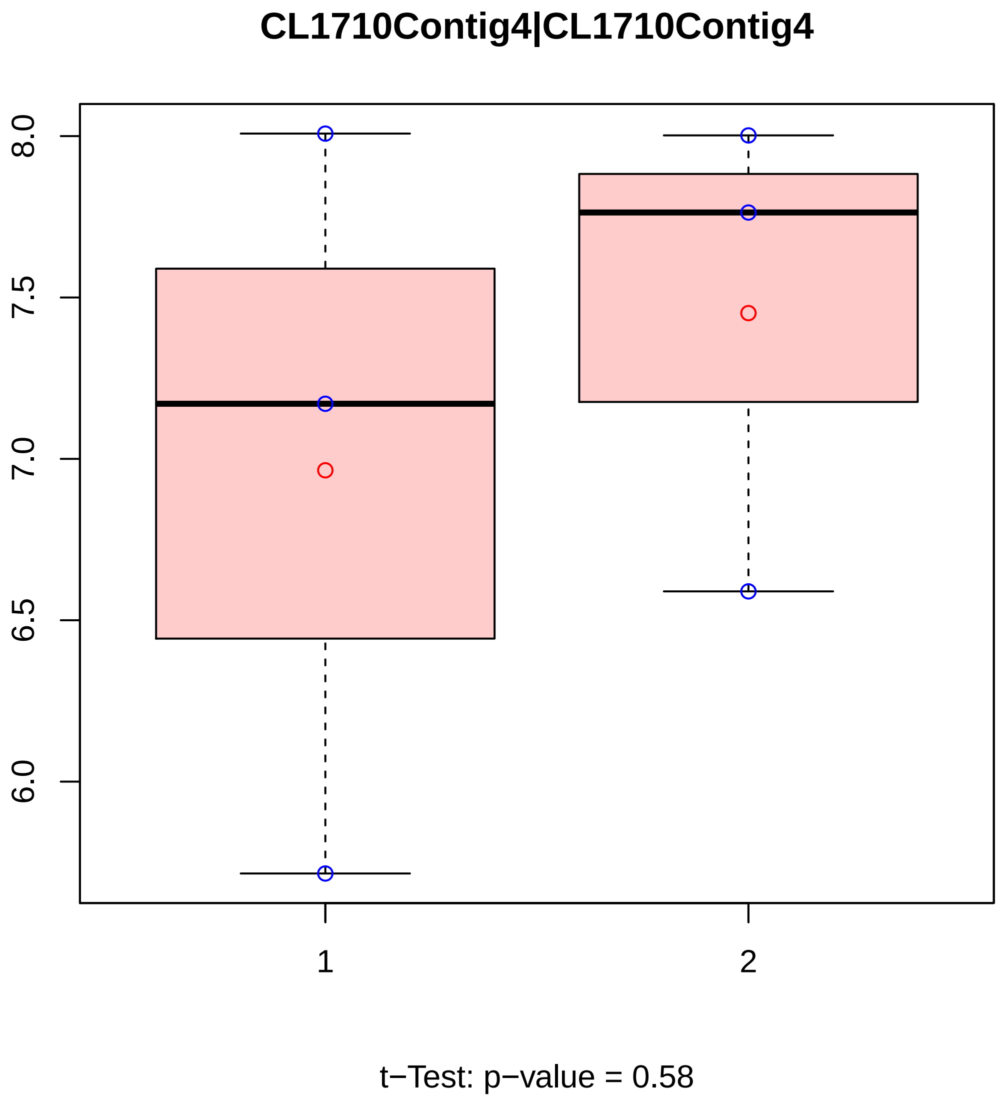
CL1710Contig4|CL1710Contig4
8.0
7.5
7.0
6.5
6.0
1
2
t−Test: p−value = 0.58

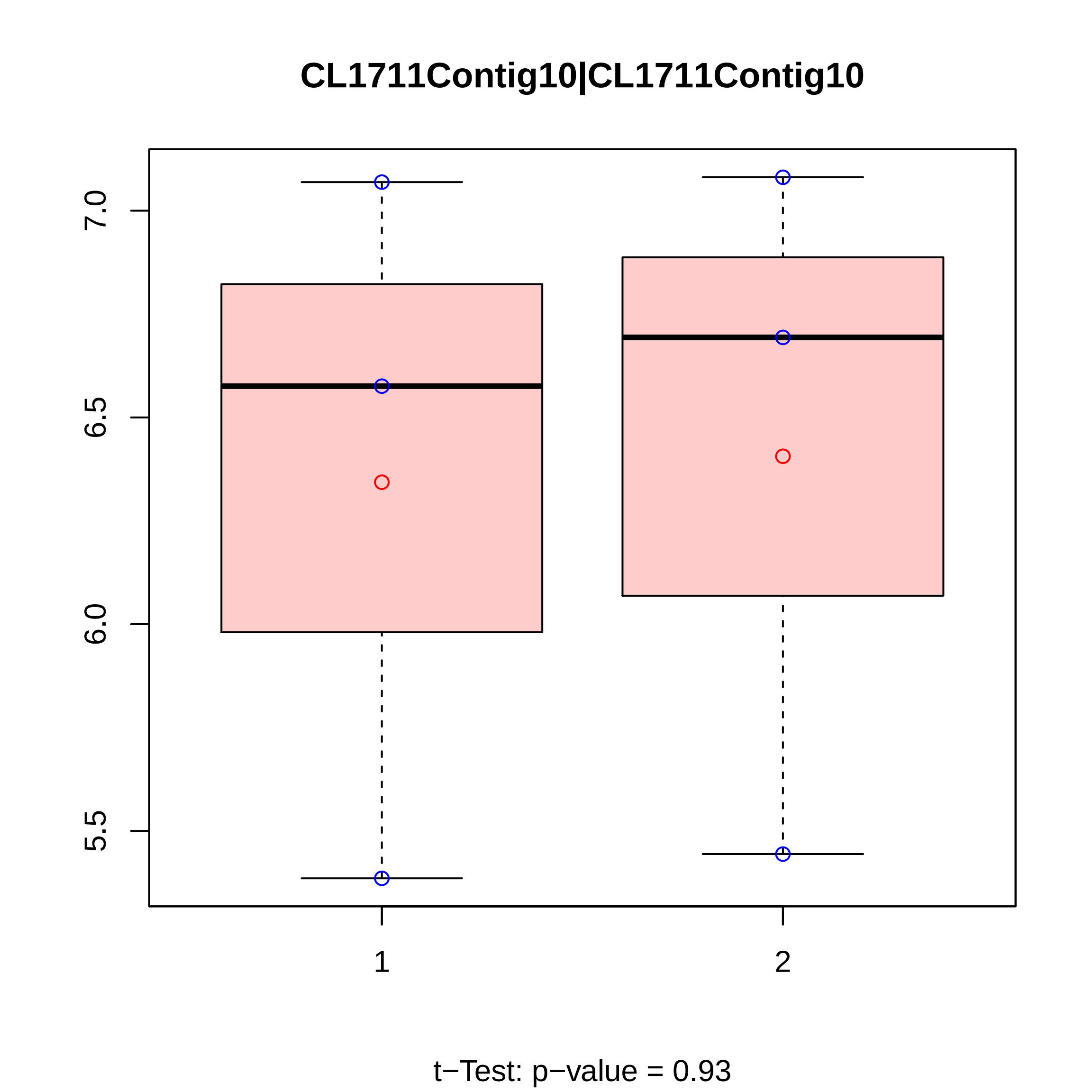
CL1711Contig10|CL1711Contig10
7.0
6.5
6.0
5.5
1
2
t−Test: p−value = 0.93

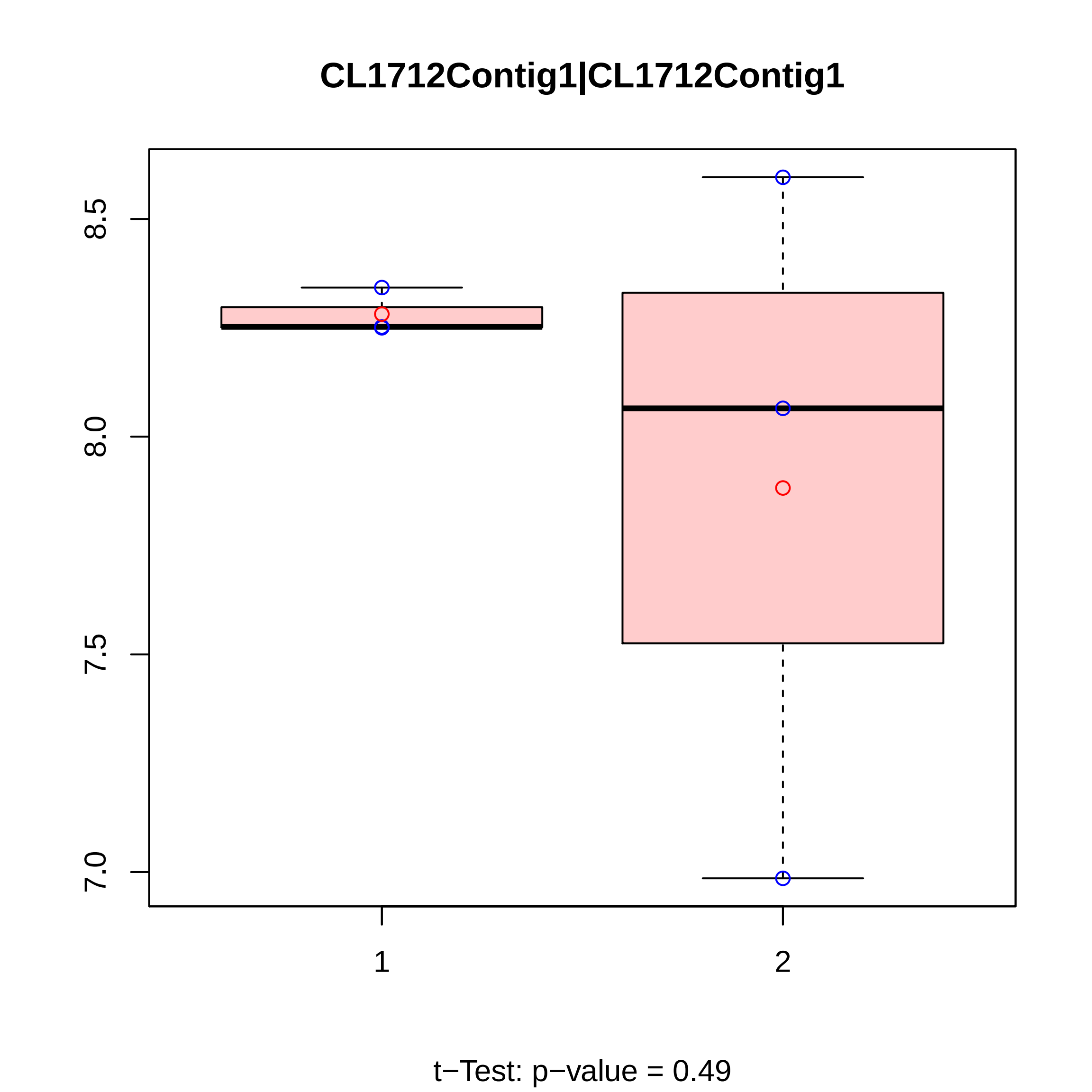
CL1712Contig1|CL1712Contig1
8.5
8.0
7.5
7.0
1
2
t−Test: p−value = 0.49

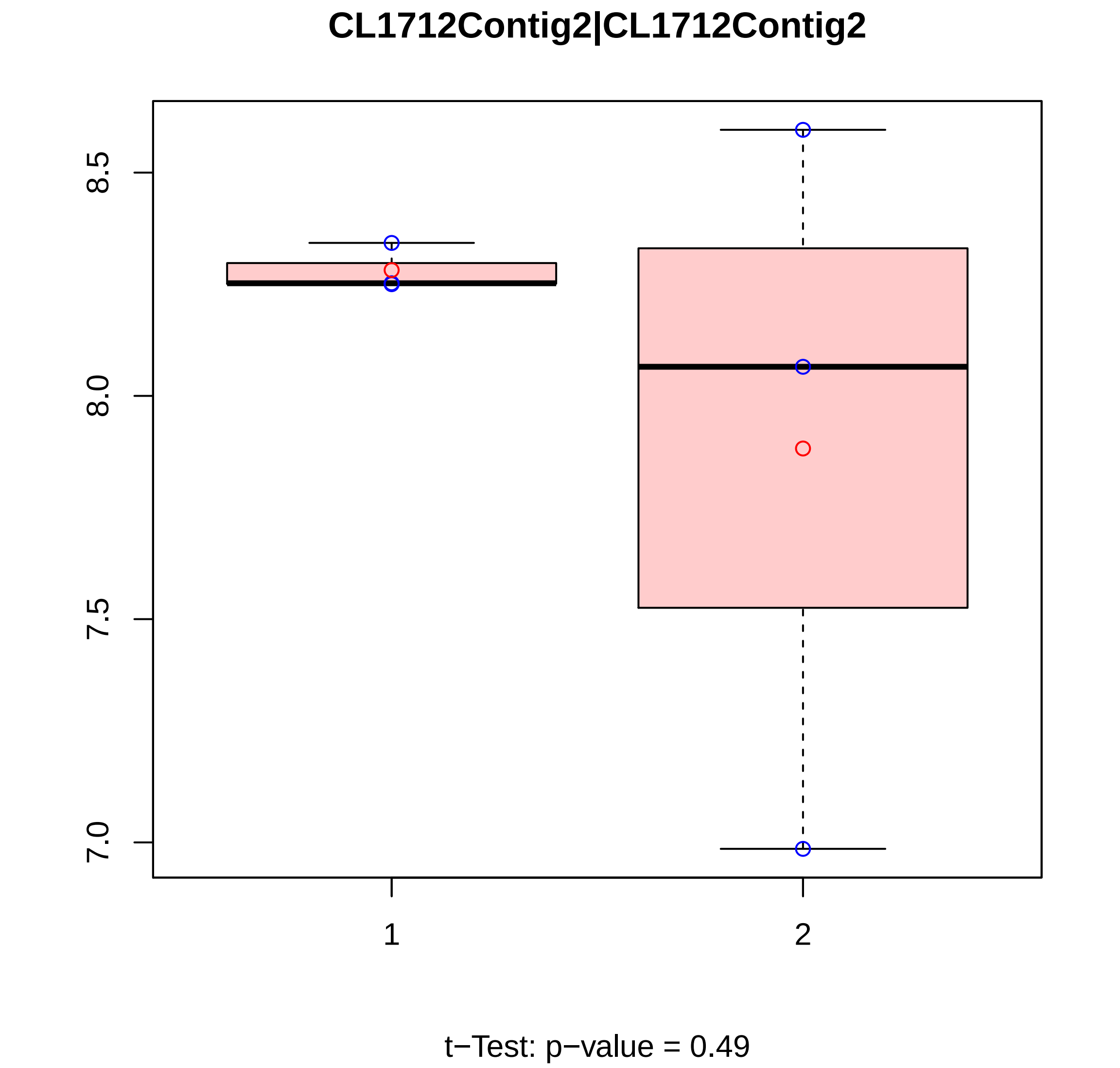
CL1712Contig2|CL1712Contig2
8.5
8.0
7.5
7.0
1
2
t−Test: p−value = 0.49

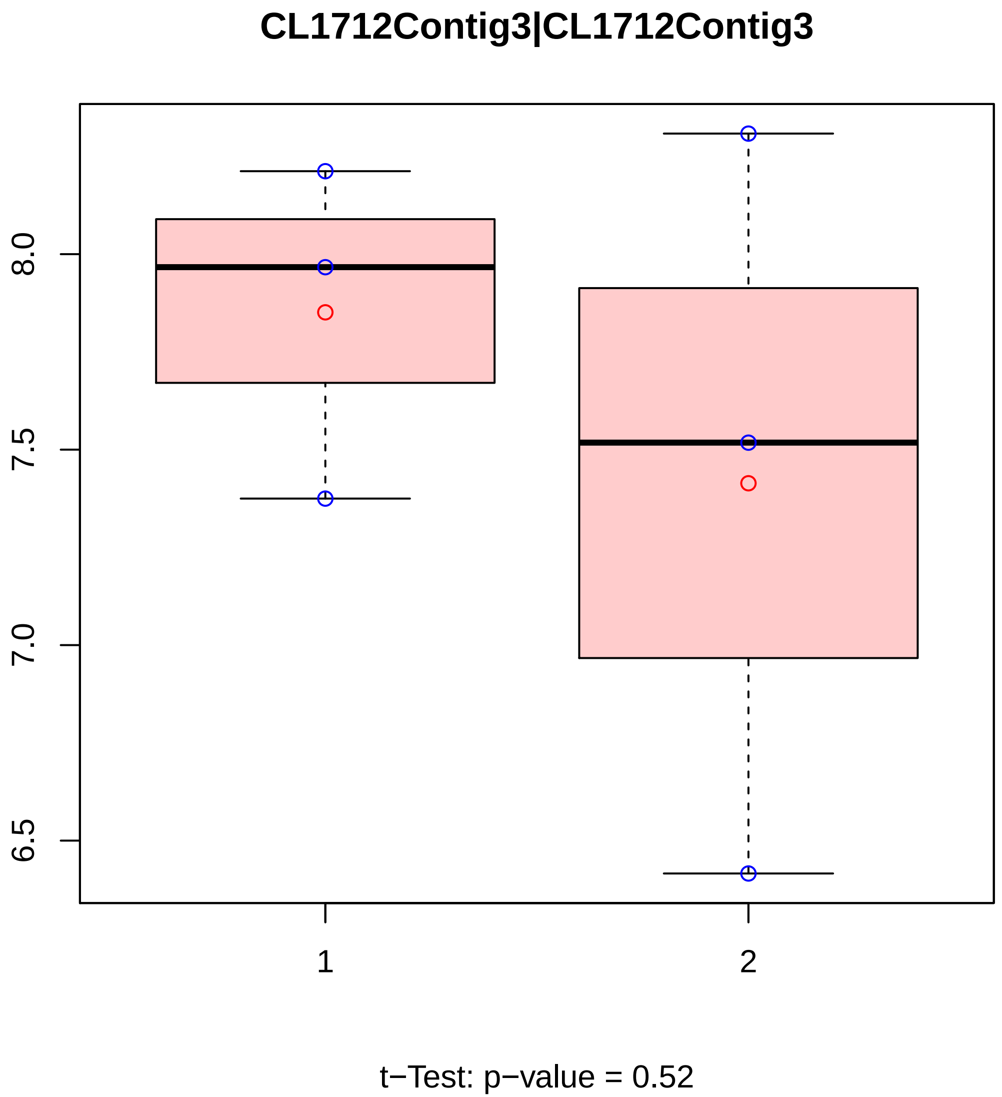
CL1712Contig3|CL1712Contig3
8.0
7.5
7.0
6.5
1
2
t−Test: p−value = 0.52

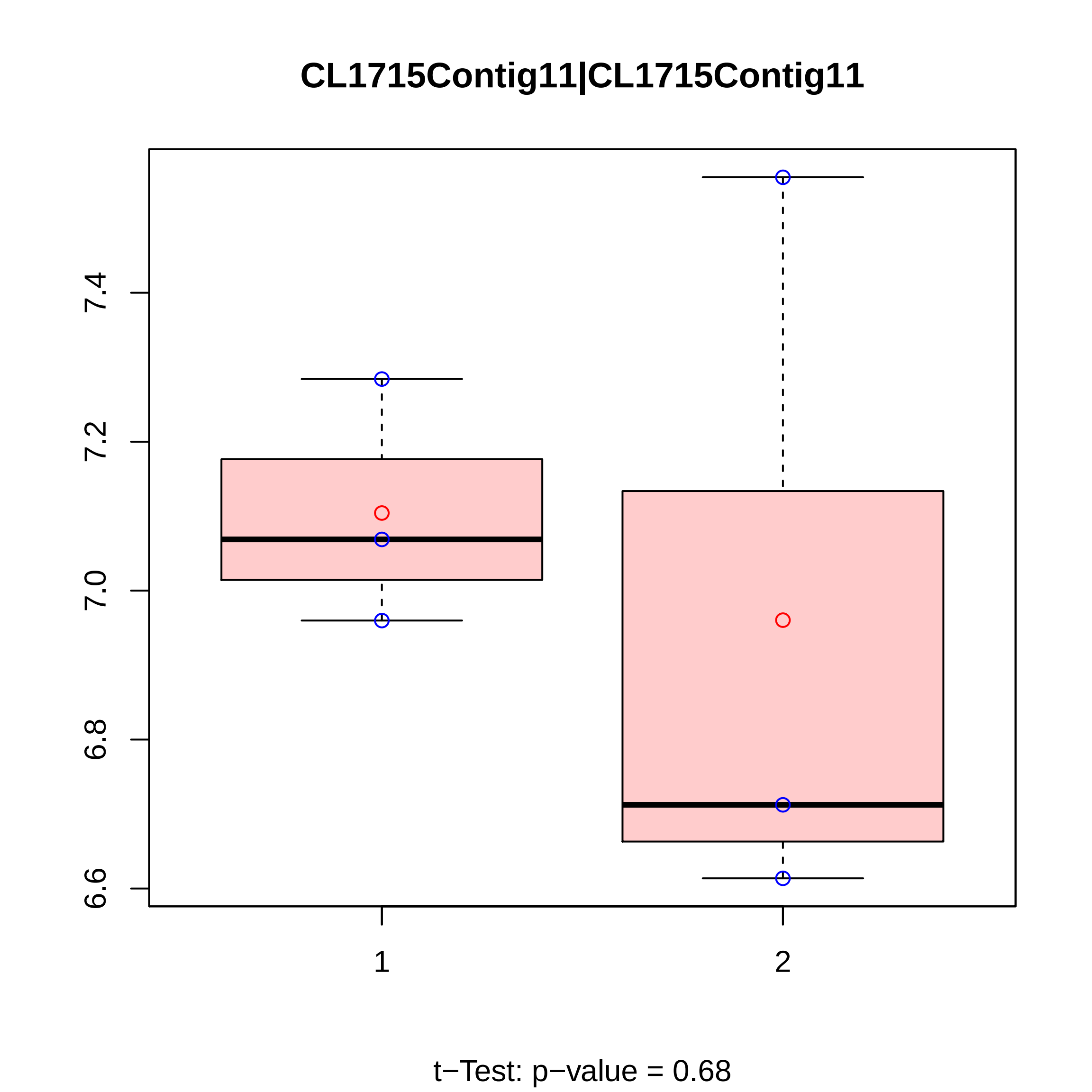
CL1715Contig11|CL1715Contig11
7.4
7.2
7.0
6.8
6.6
1
2
t−Test: p−value = 0.68

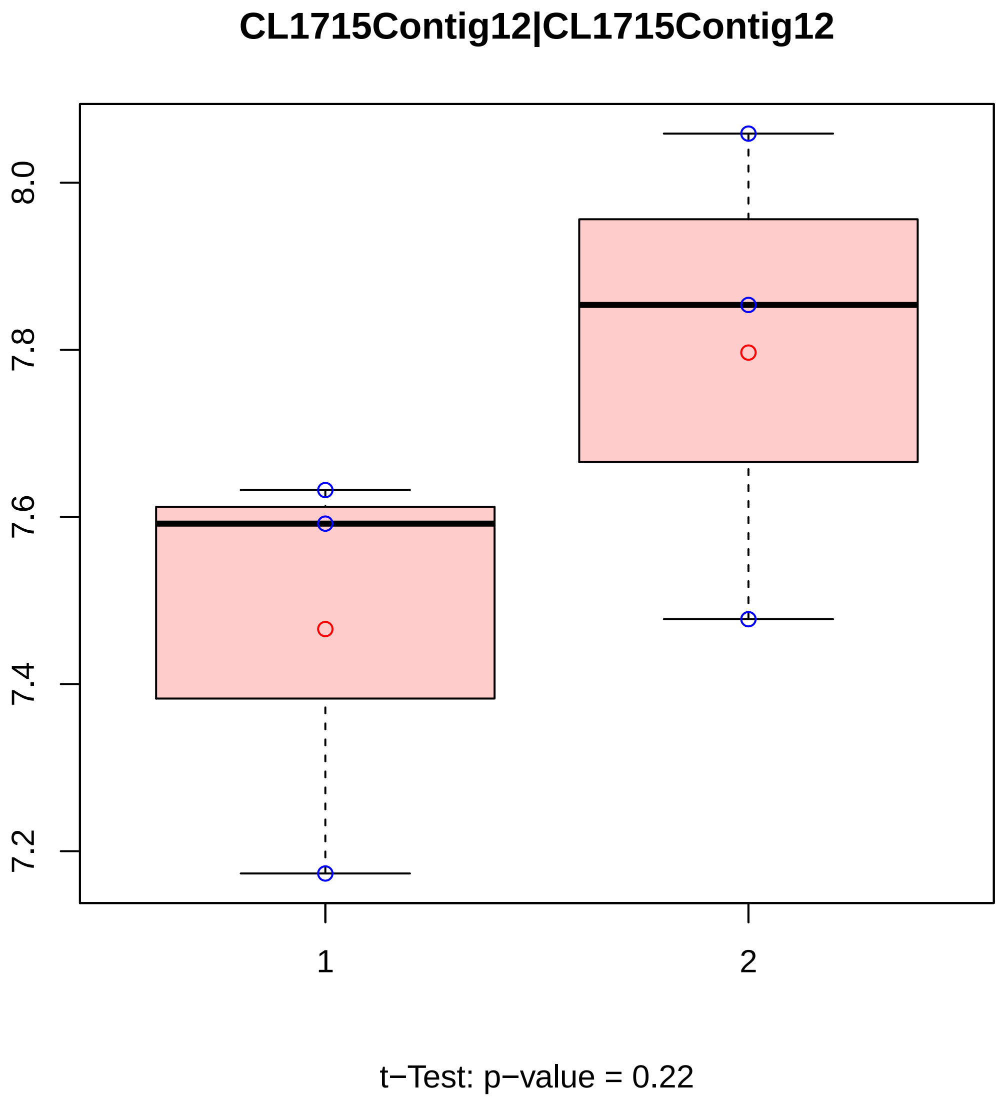

CL1715Contig12|CL1715Contig12
8.0
7.8
7.6
7.4
7.2
1
2
t−Test: p−value = 0.22

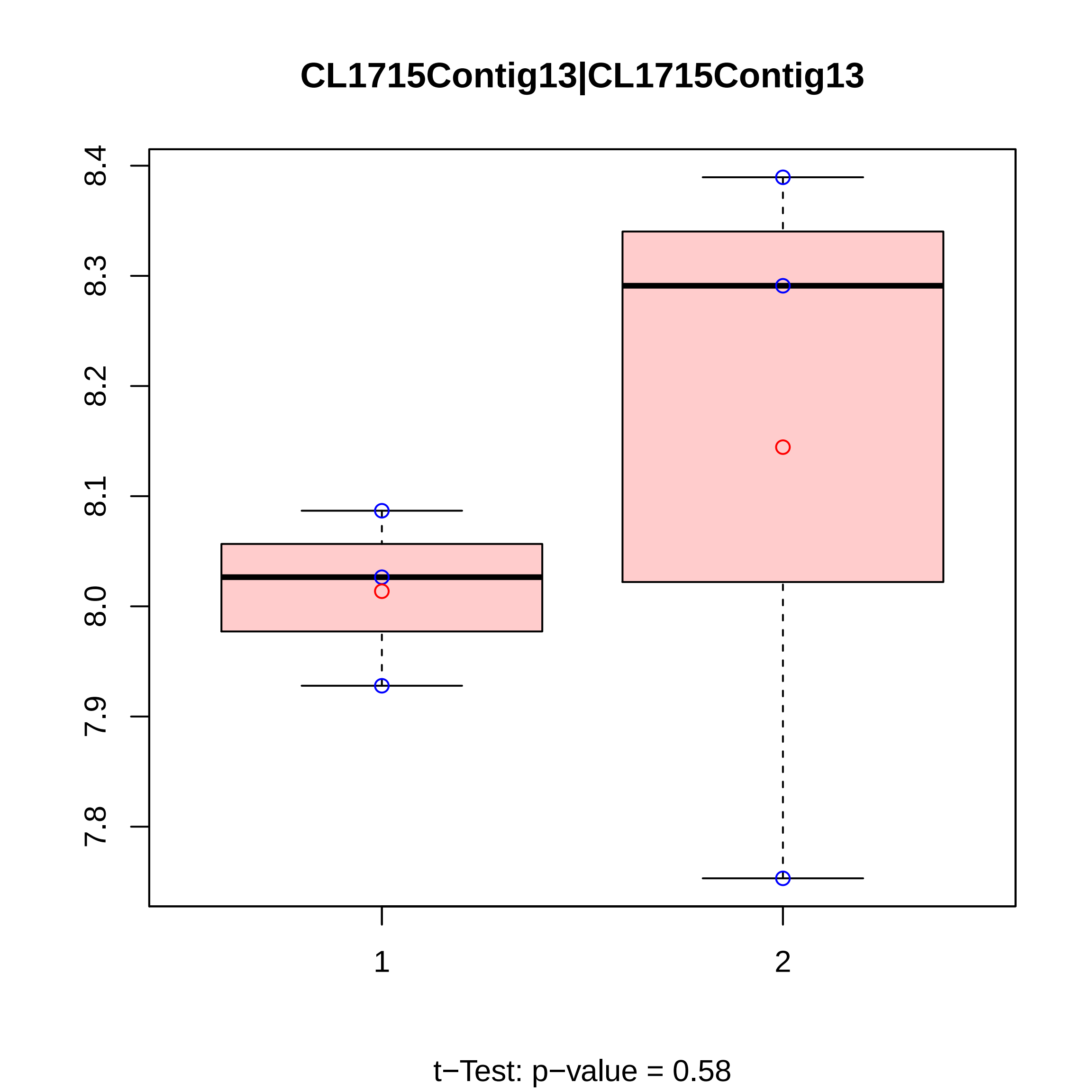
CL1715Contig13|CL1715Contig13
8.4
8.3
8.2
8.1
8.0
7.9
7.8
1
2
t−Test: p−value = 0.58

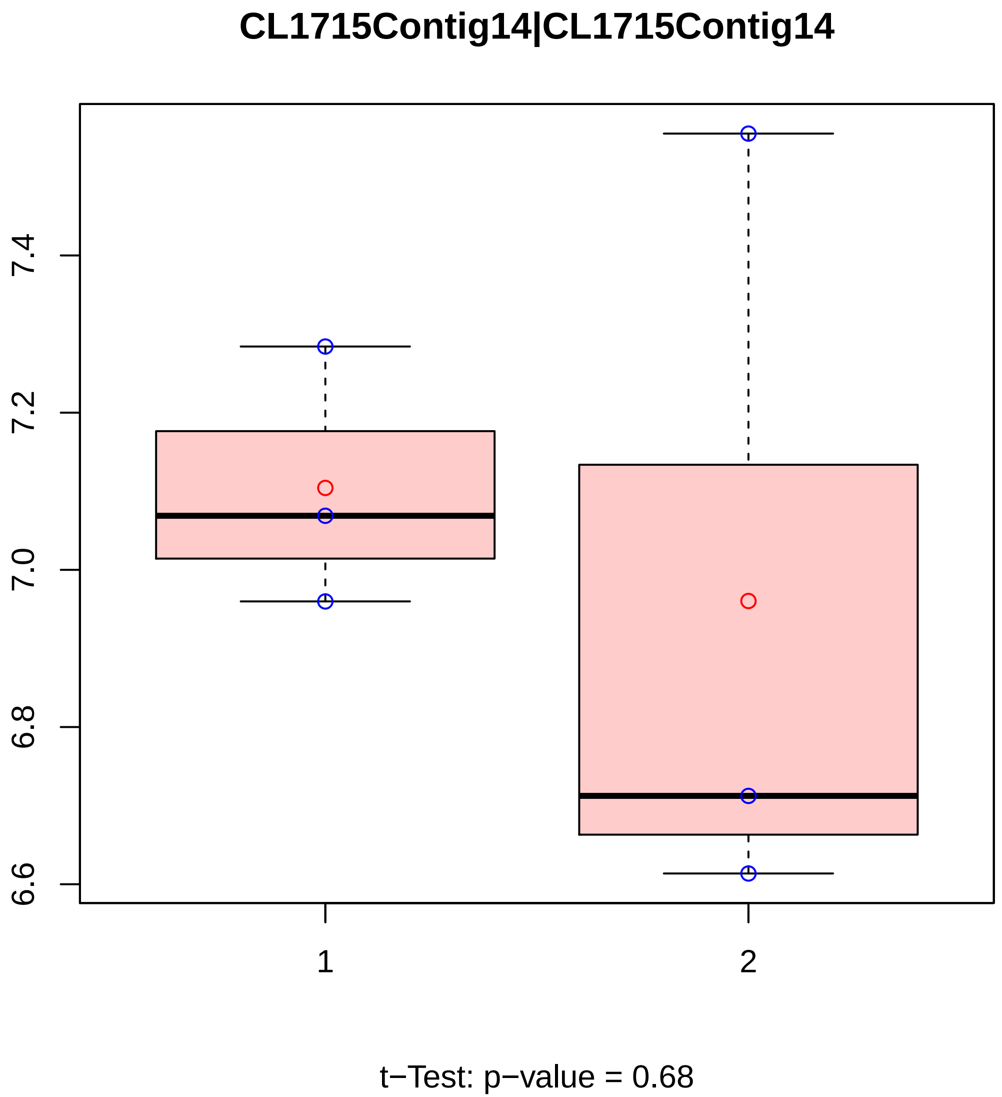
CL1715Contig14|CL1715Contig14
7.4
7.2
7.0
6.8
6.6
1
2
t−Test: p−value = 0.68

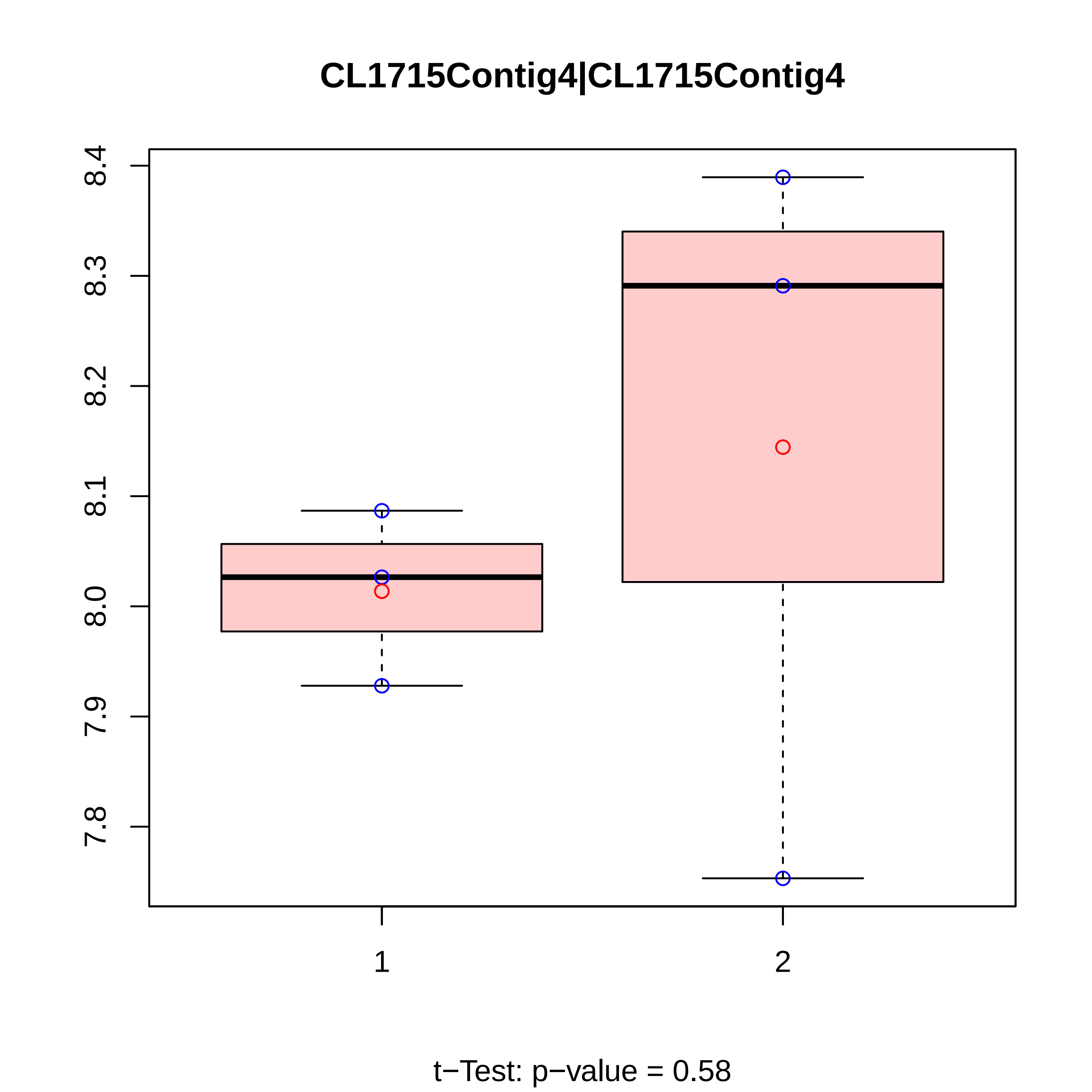

CL1715Contig4|CL1715Contig4
8.4
8.3
8.2
8.1
8.0
7.9
7.8
1
2
t−Test: p−value = 0.58

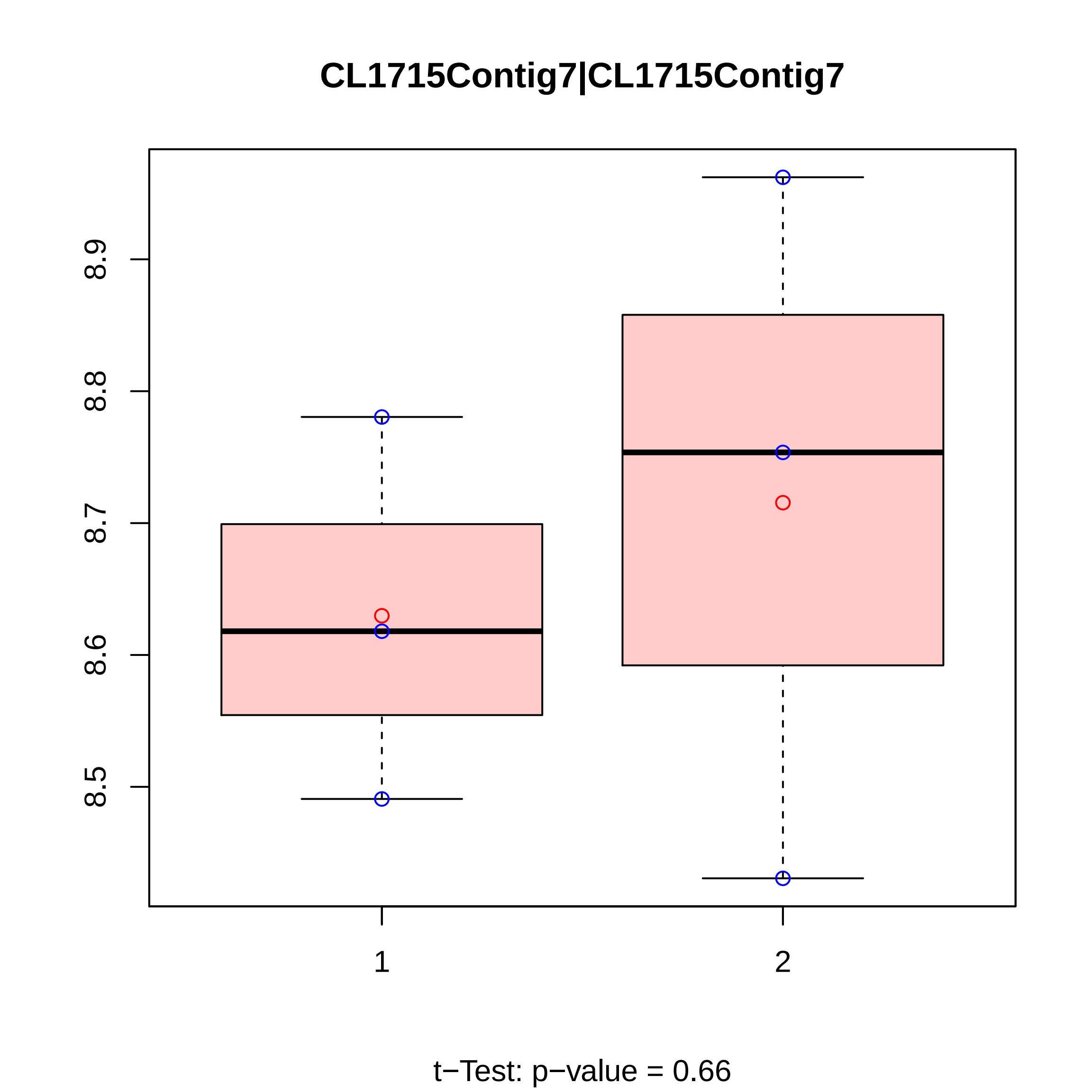
CL1715Contig7|CL1715Contig7
8.5
8.6
8.7
8.8
8.9
1
2
t−Test: p−value = 0.66

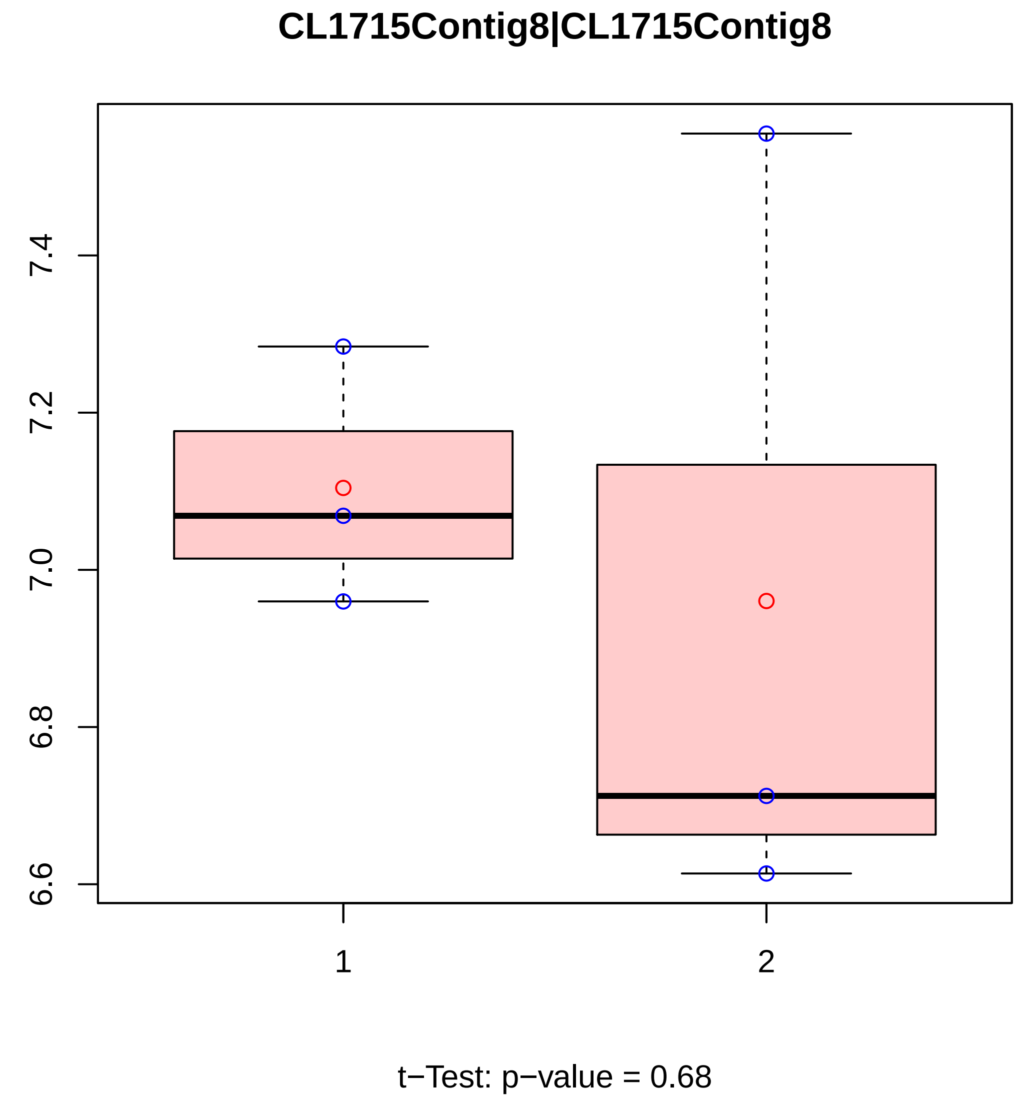
CL1715Contig8|CL1715Contig8
7.4
7.2
7.0
6.8
6.6
1
2
t−Test: p−value = 0.68

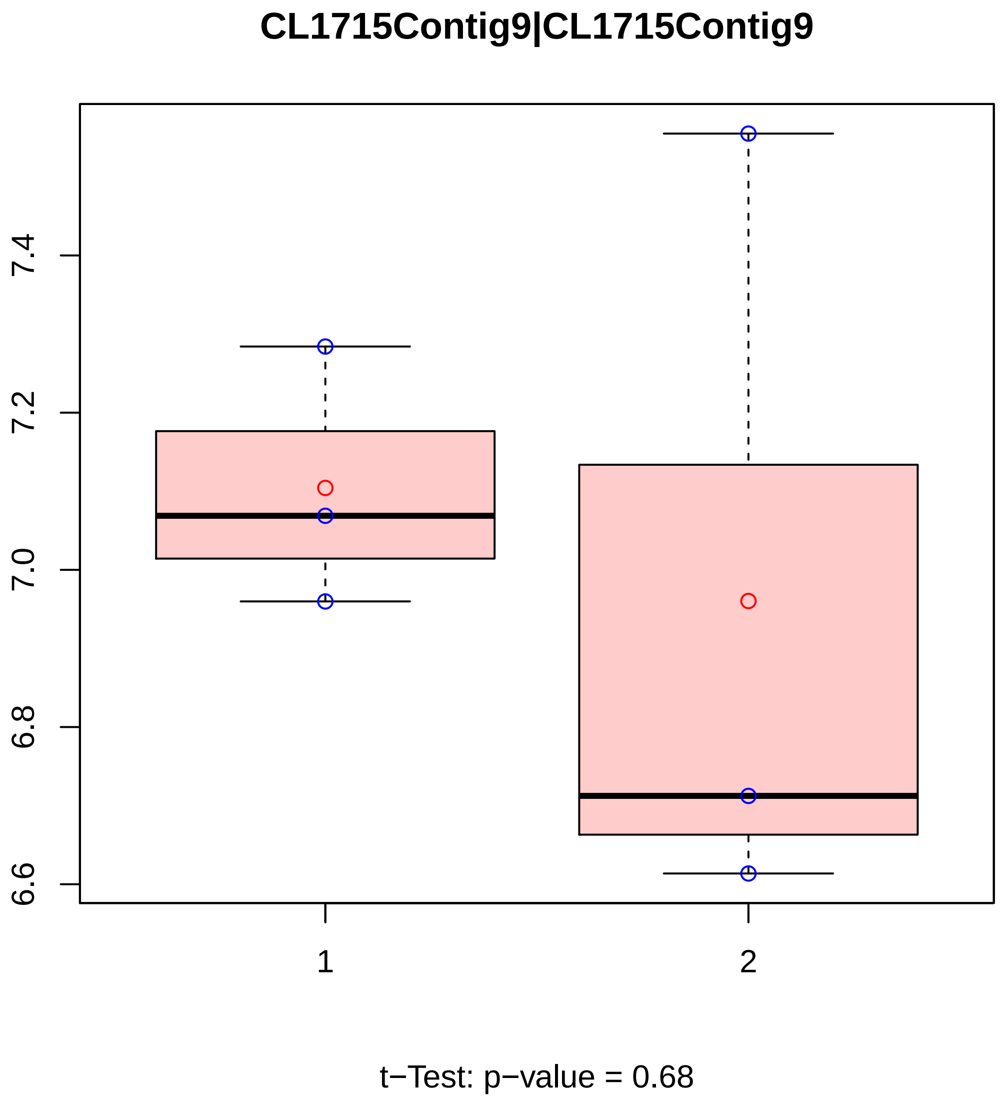
CL1715Contig9|CL1715Contig9
7.4
7.2
7.0
6.8
6.6
1
2
t−Test: p−value = 0.68

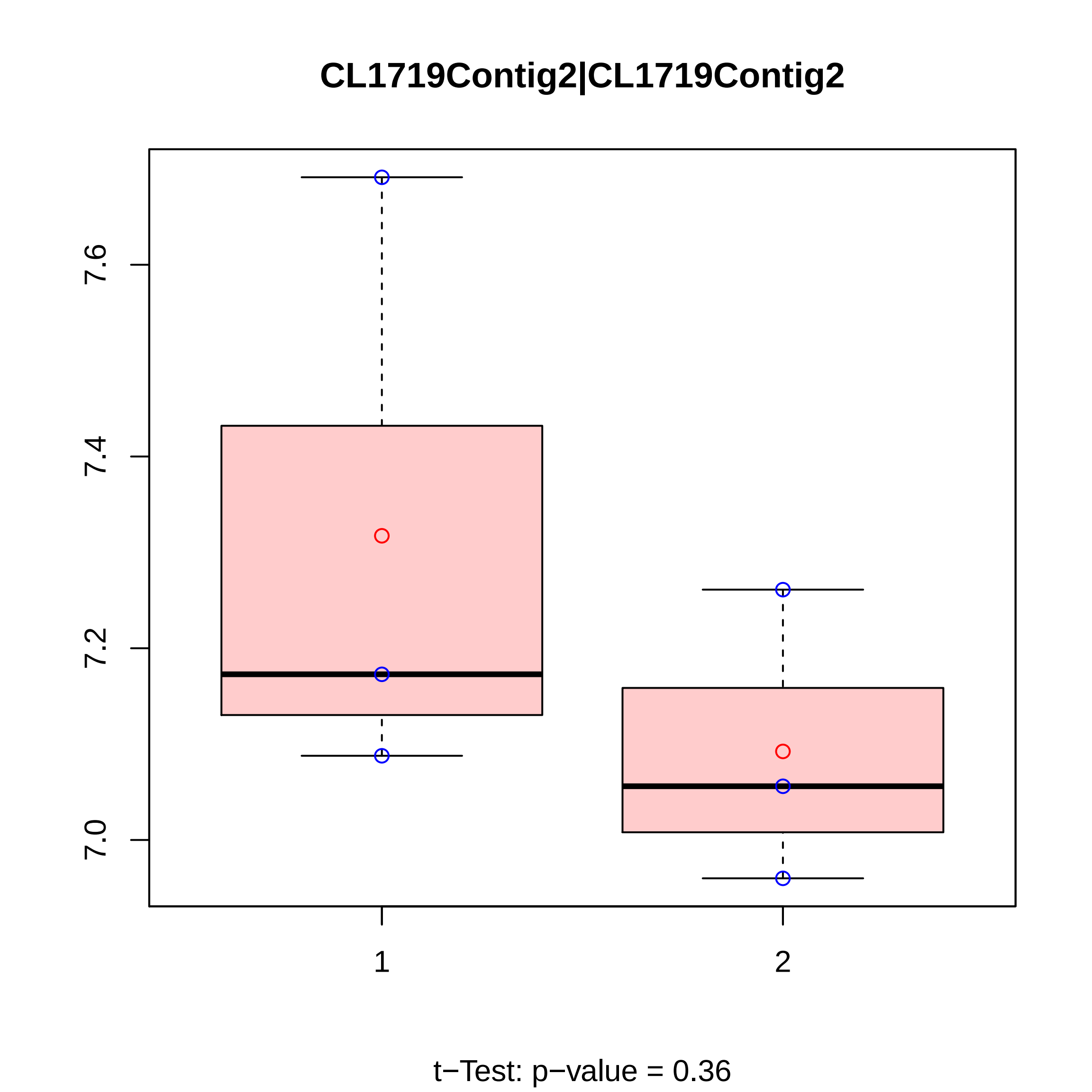
CL1719Contig2|CL1719Contig2
7.0
7.2
7.4
7.6
1
2
t−Test: p−value = 0.36

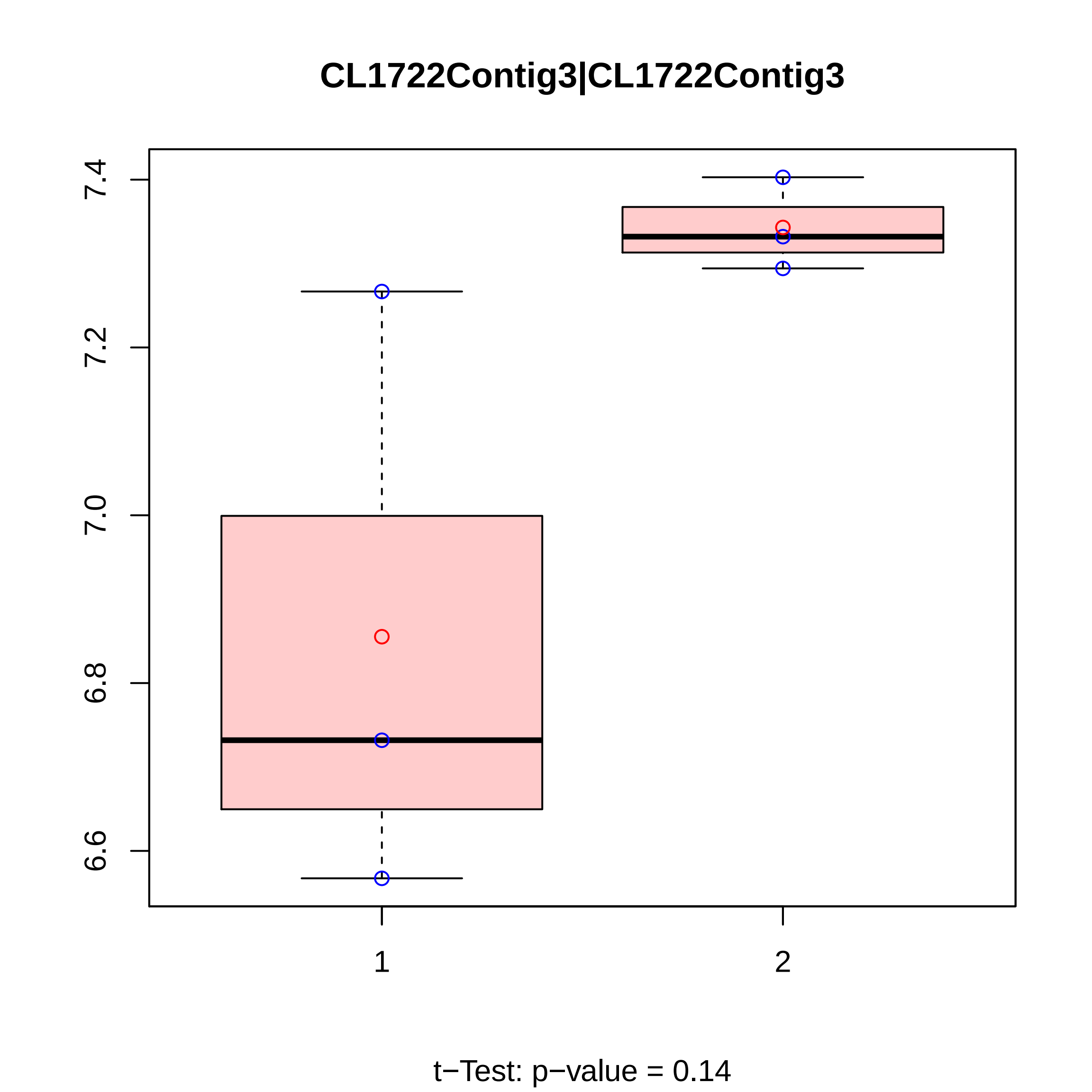
CL1722Contig3|CL1722Contig3
7.4
7.2
7.0
6.8
6.6
1
2
t−Test: p−value = 0.14

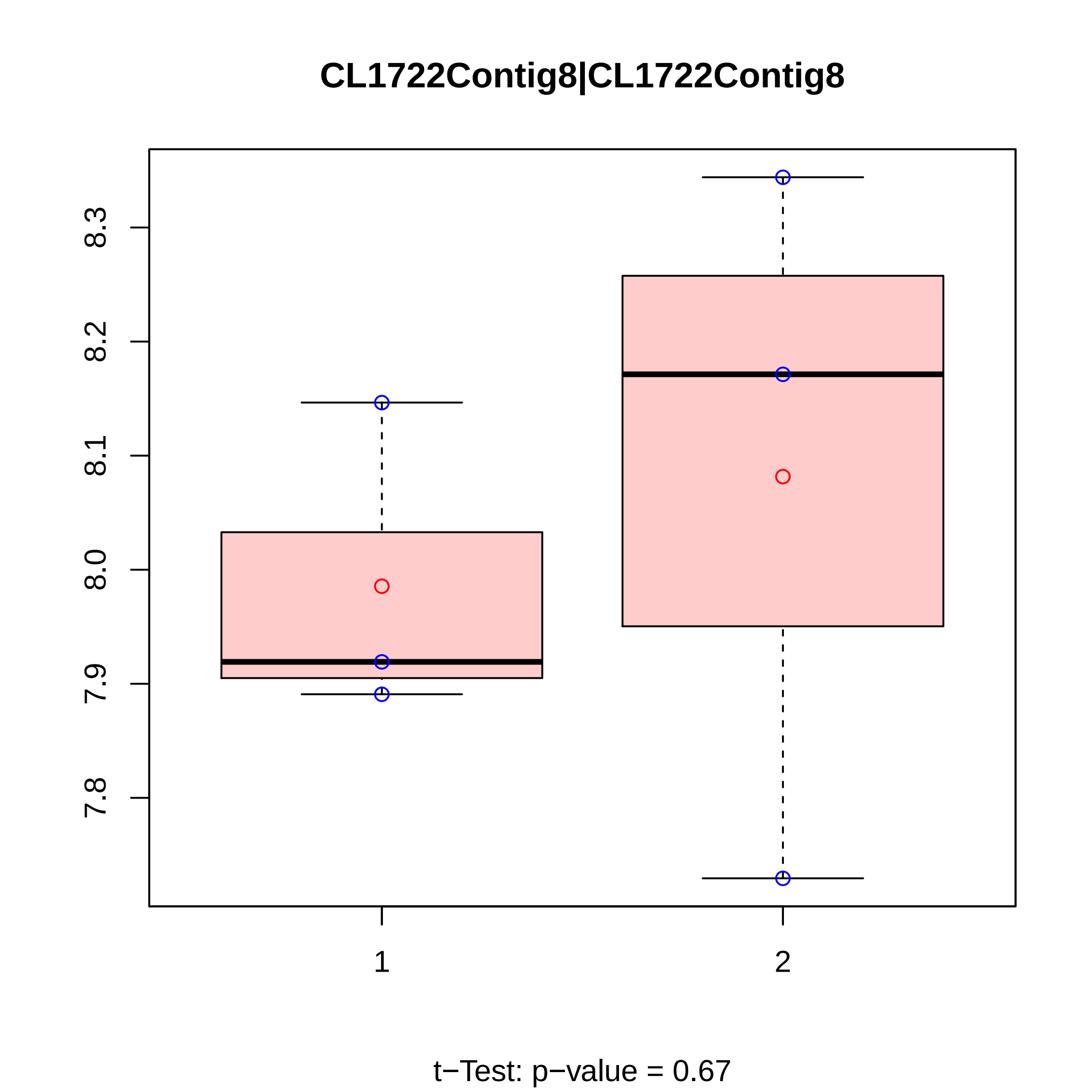
CL1722Contig8|CL1722Contig8
8.3
8.2
8.1
8.0
7.9
7.8
1
2
t−Test: p−value = 0.67

# CL1723Contig10|CL1723Contig10

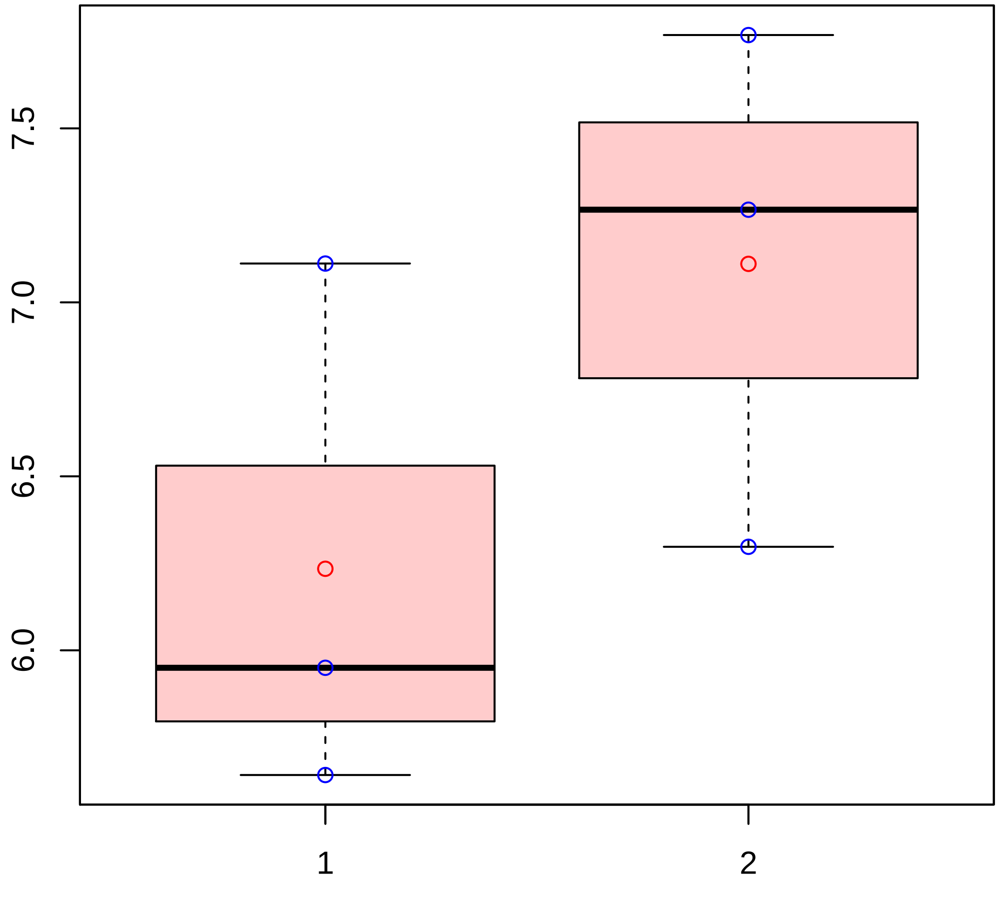


t−Test: p−value = 0.23

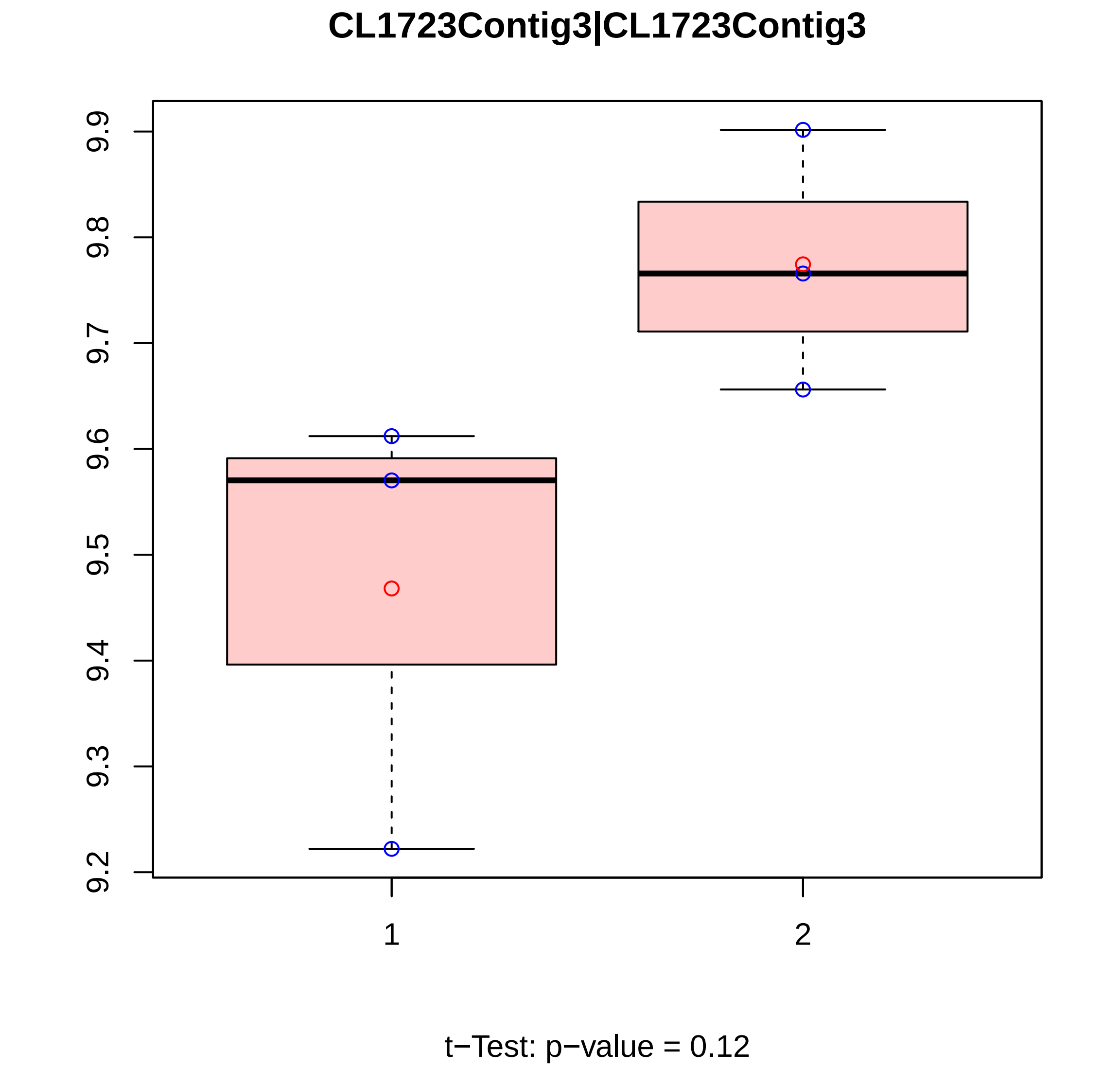
CL1723Contig3|CL1723Contig3
9.2
9.3
9.4
9.5
9.6
9.7
9.8
9.9
1
2
t−Test: p−value = 0.12

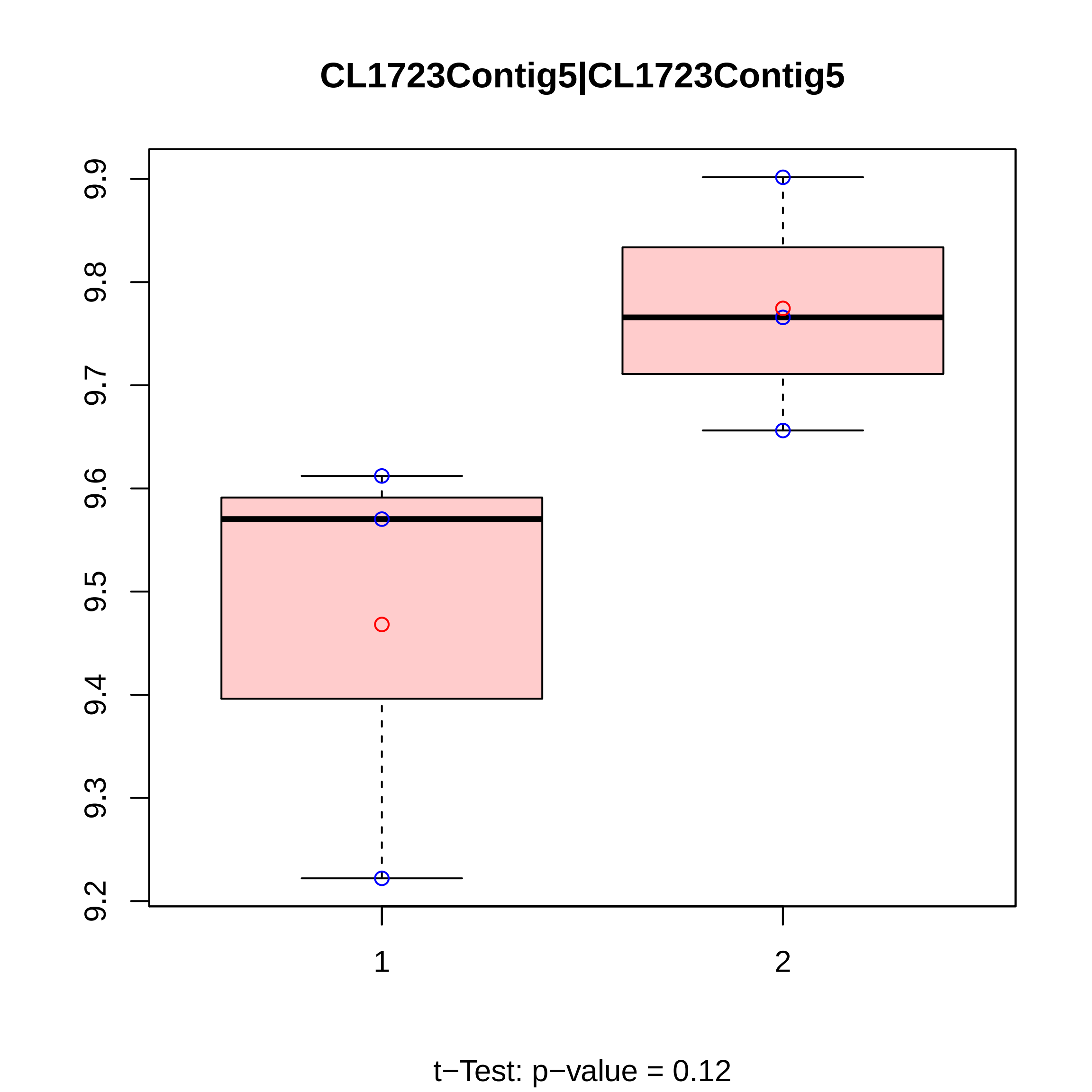

CL1723Contig5|CL1723Contig5
9.2
9.3
9.4
9.5
9.6
9.7
9.8
9.9
1
2
t−Test: p−value = 0.12

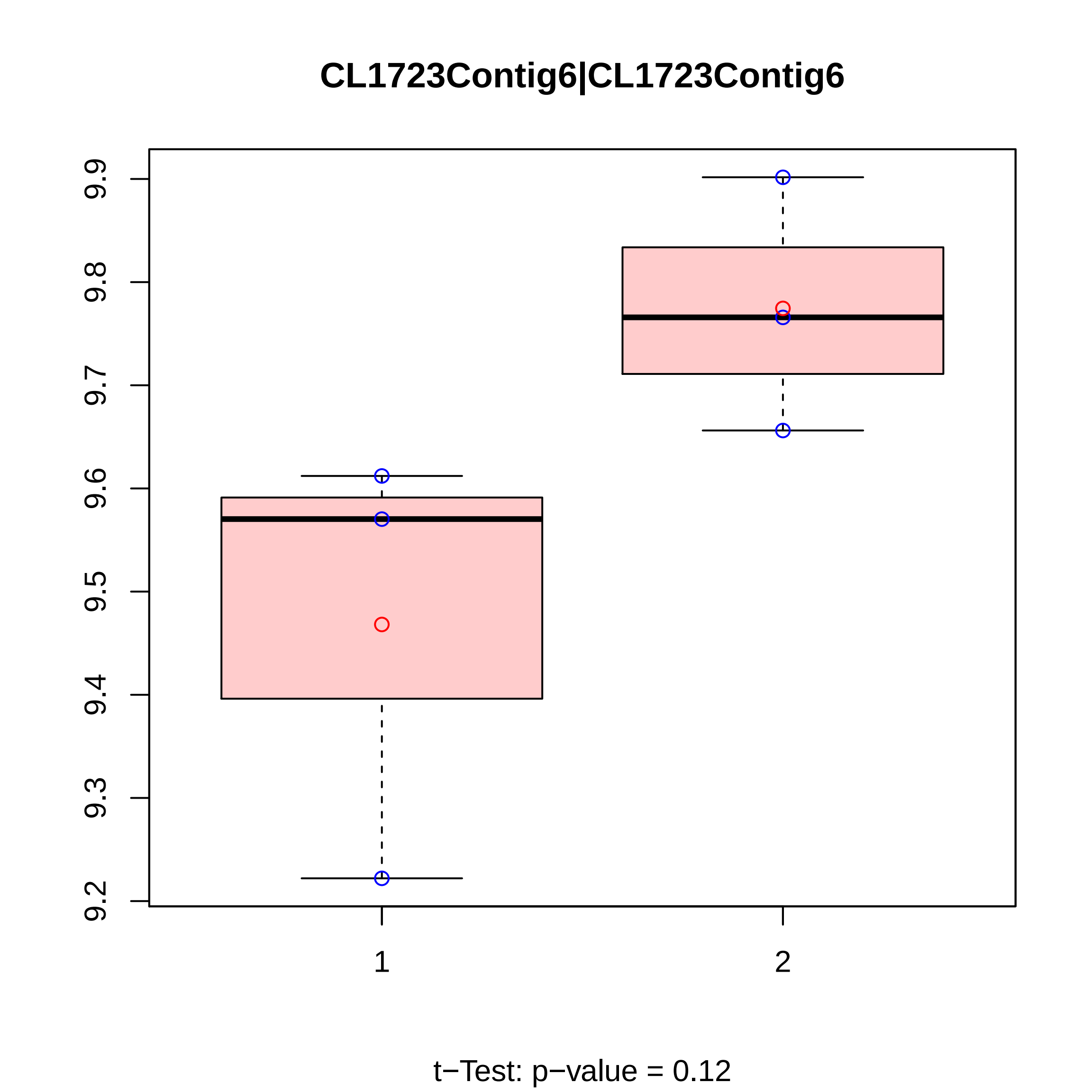

CL1723Contig6|CL1723Contig6
9.2
9.3
9.4
9.5
9.6
9.7
9.8
9.9
1
2
t−Test: p−value = 0.12

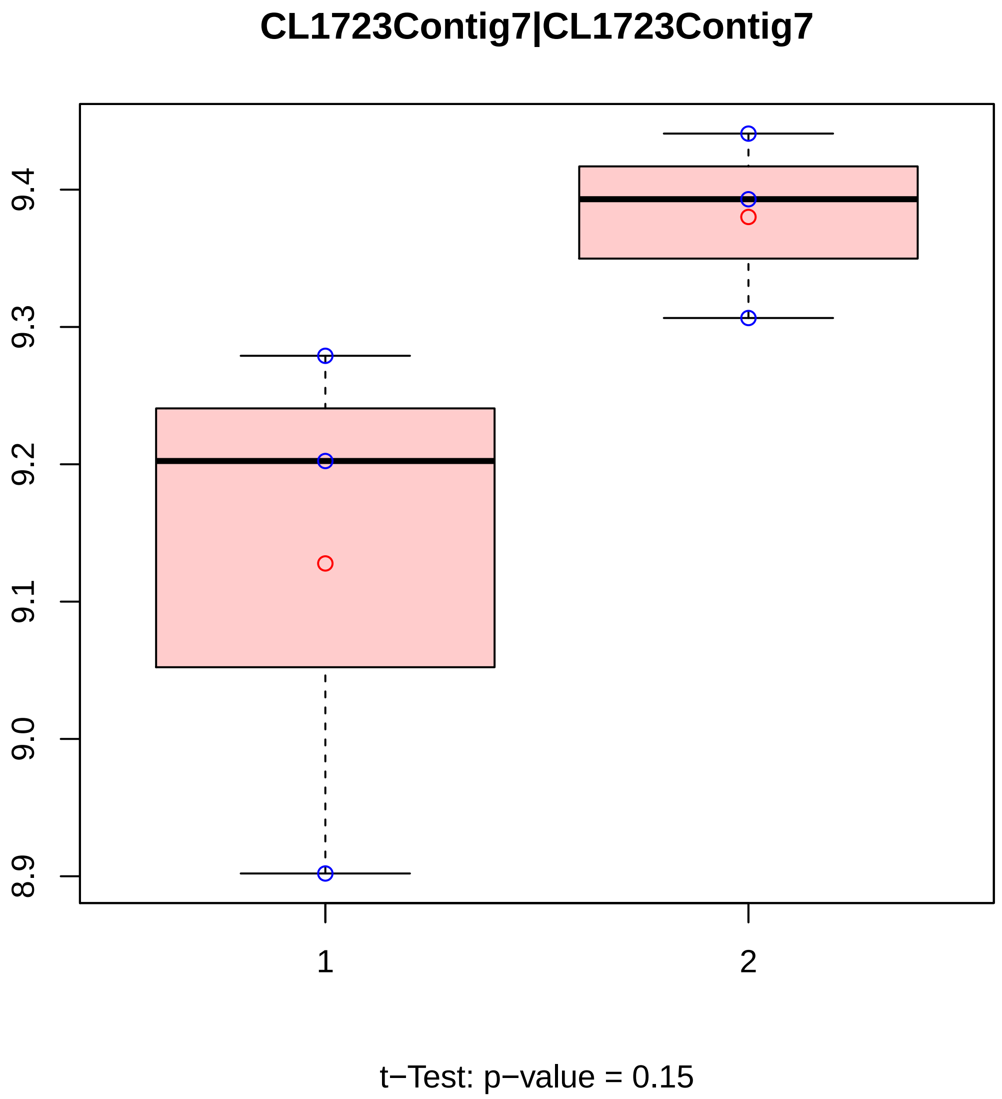

CL1723Contig7|CL1723Contig7
9.4
9.3
9.2
9.1
9.0
8.9
1
2
t−Test: p−value = 0.15

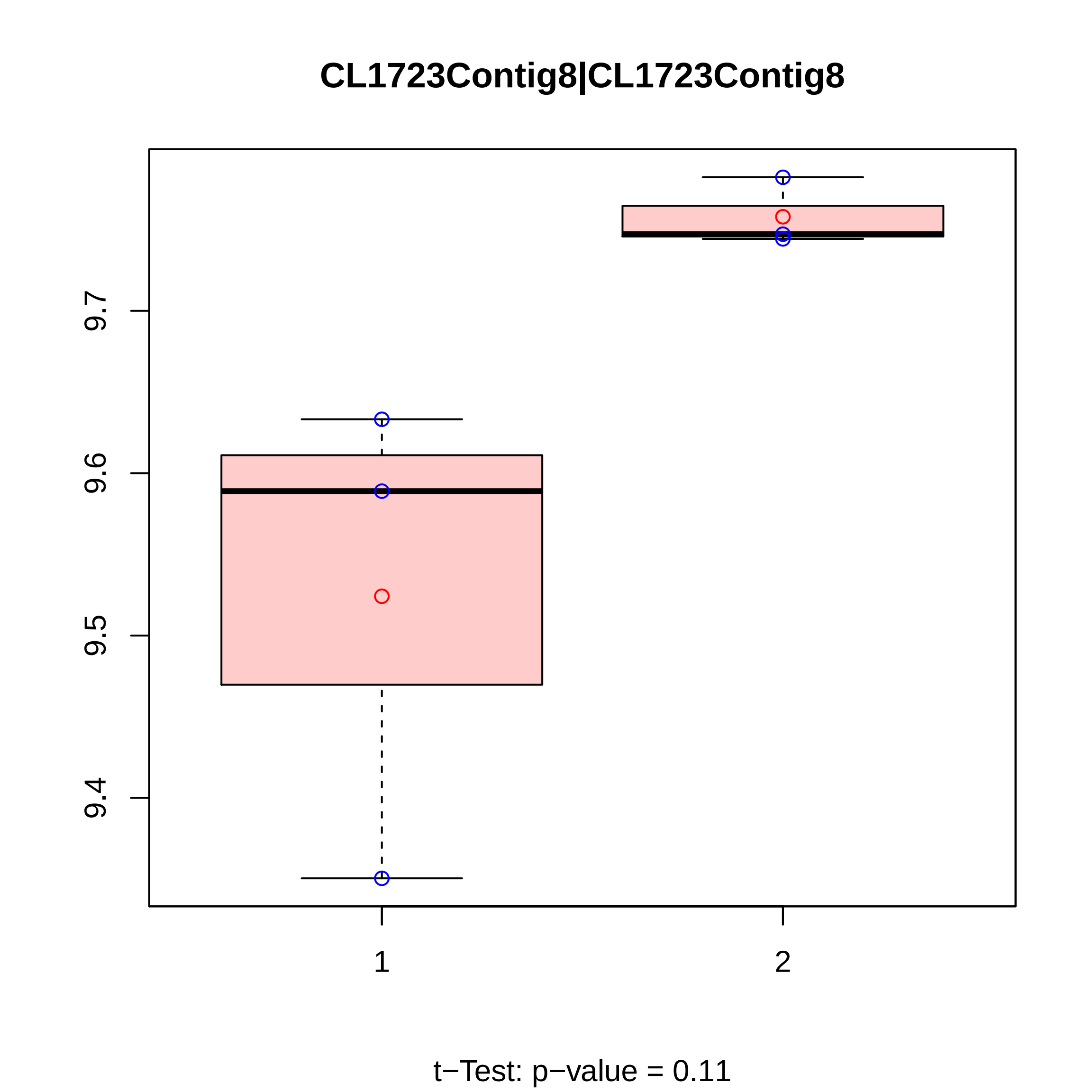

CL1723Contig8|CL1723Contig8
9.4
9.5
9.6
9.7
1
2
t−Test: p−value = 0.11

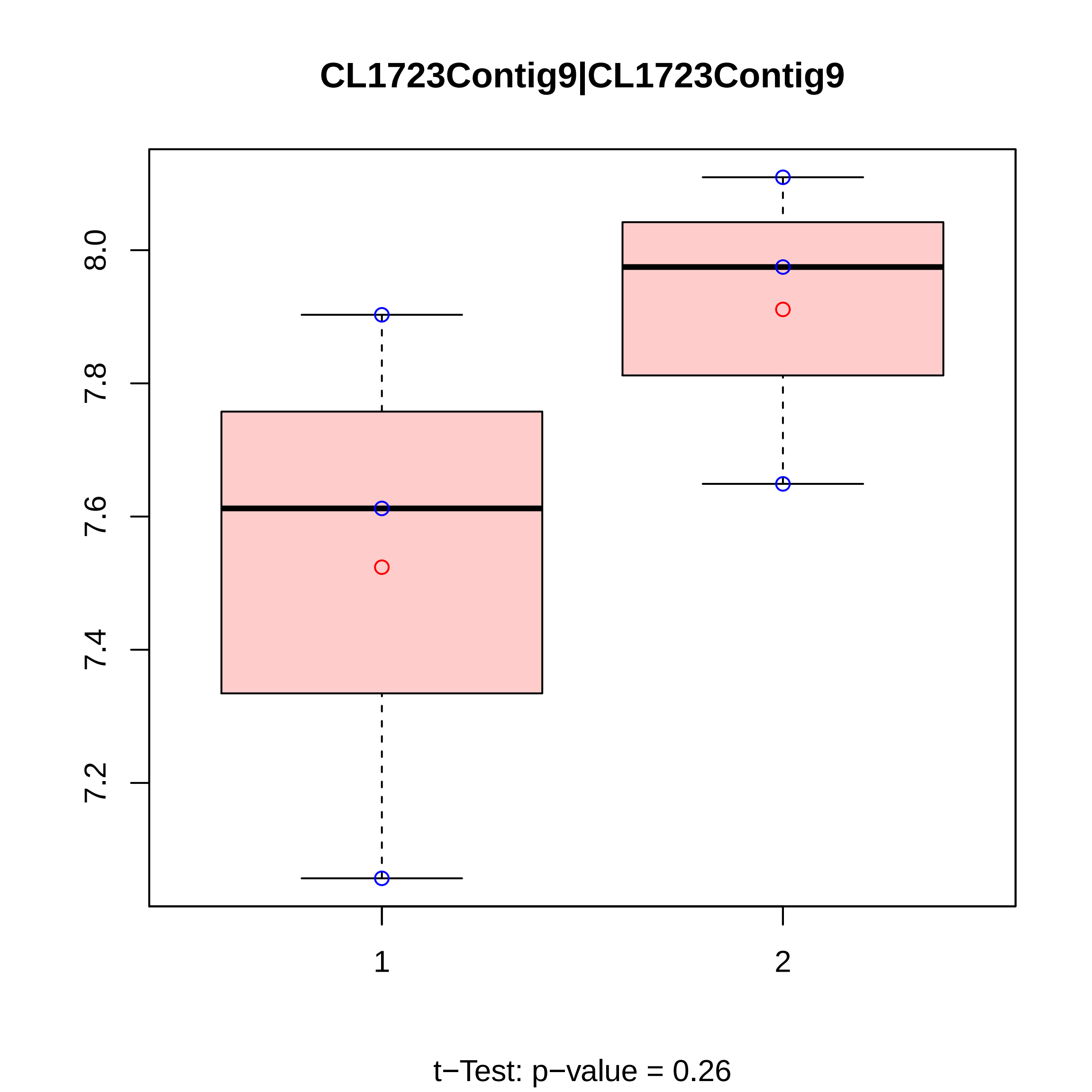
CL1723Contig9|CL1723Contig9
8.0
7.8
7.6
7.4
7.2
1
2
t−Test: p−value = 0.26

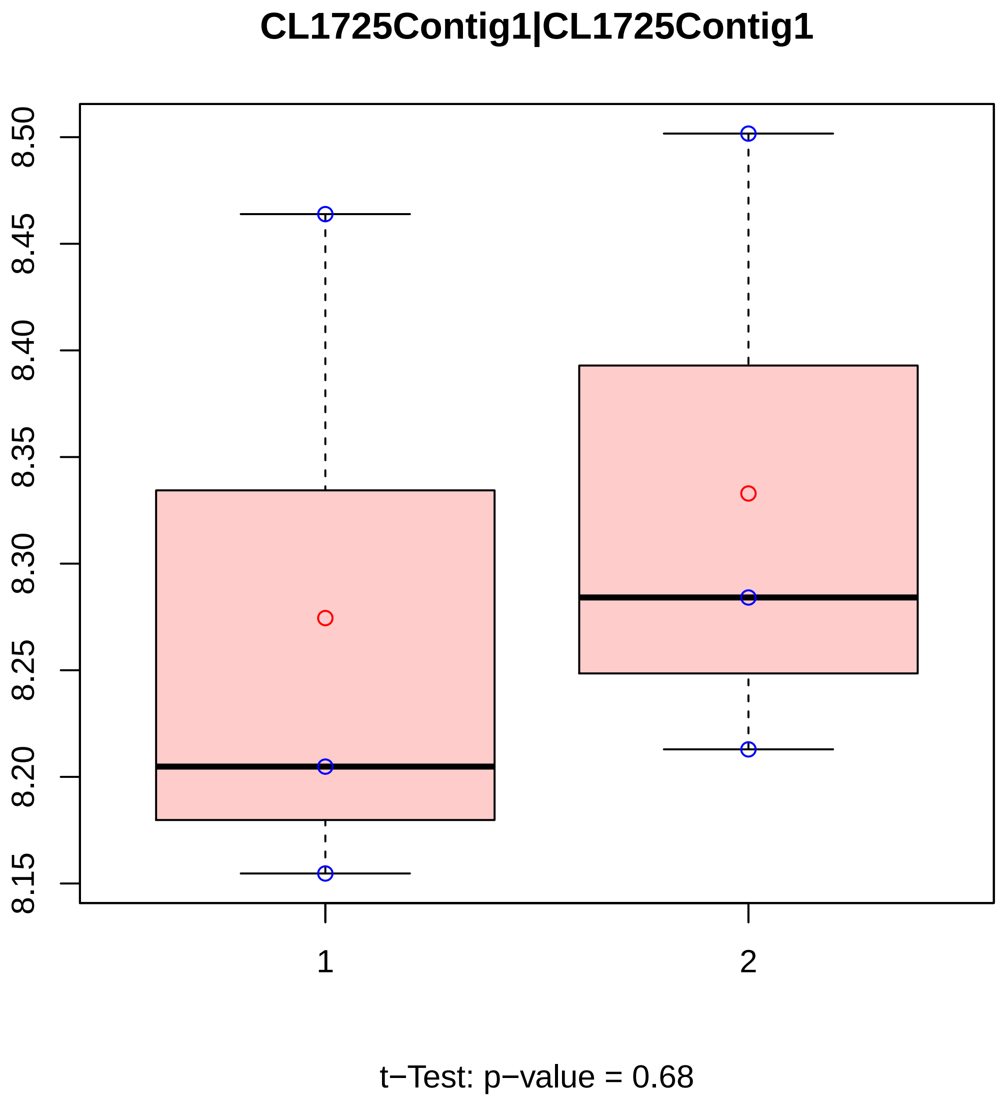

CL1725Contig1|CL1725Contig1
8.50
8.45
8.40
8.35
8.30
8.25
8.20
8.15
1
2
t−Test: p−value = 0.68

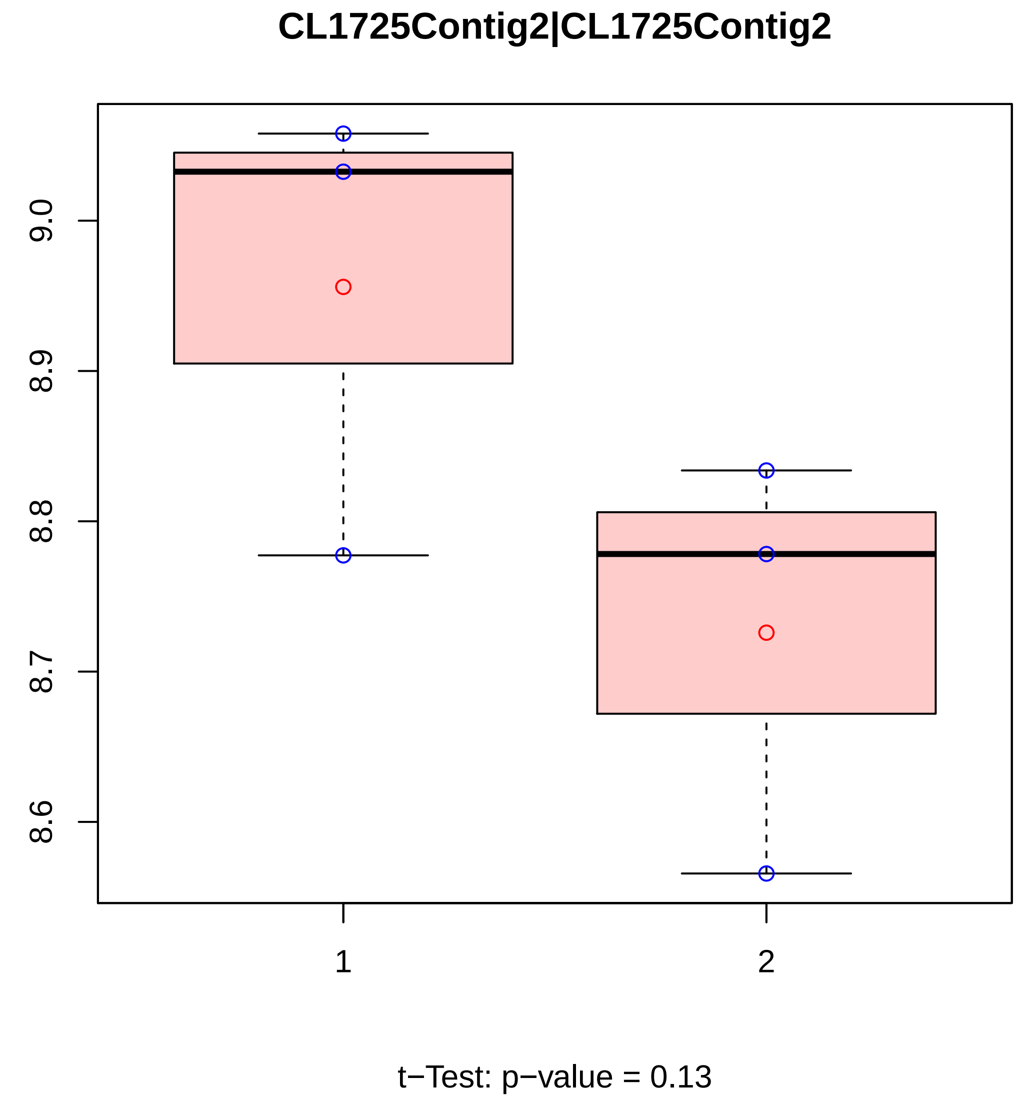
CL1725Contig2|CL1725Contig2
9.0
8.9
8.8
8.7
8.6
1
2
t−Test: p−value = 0.13

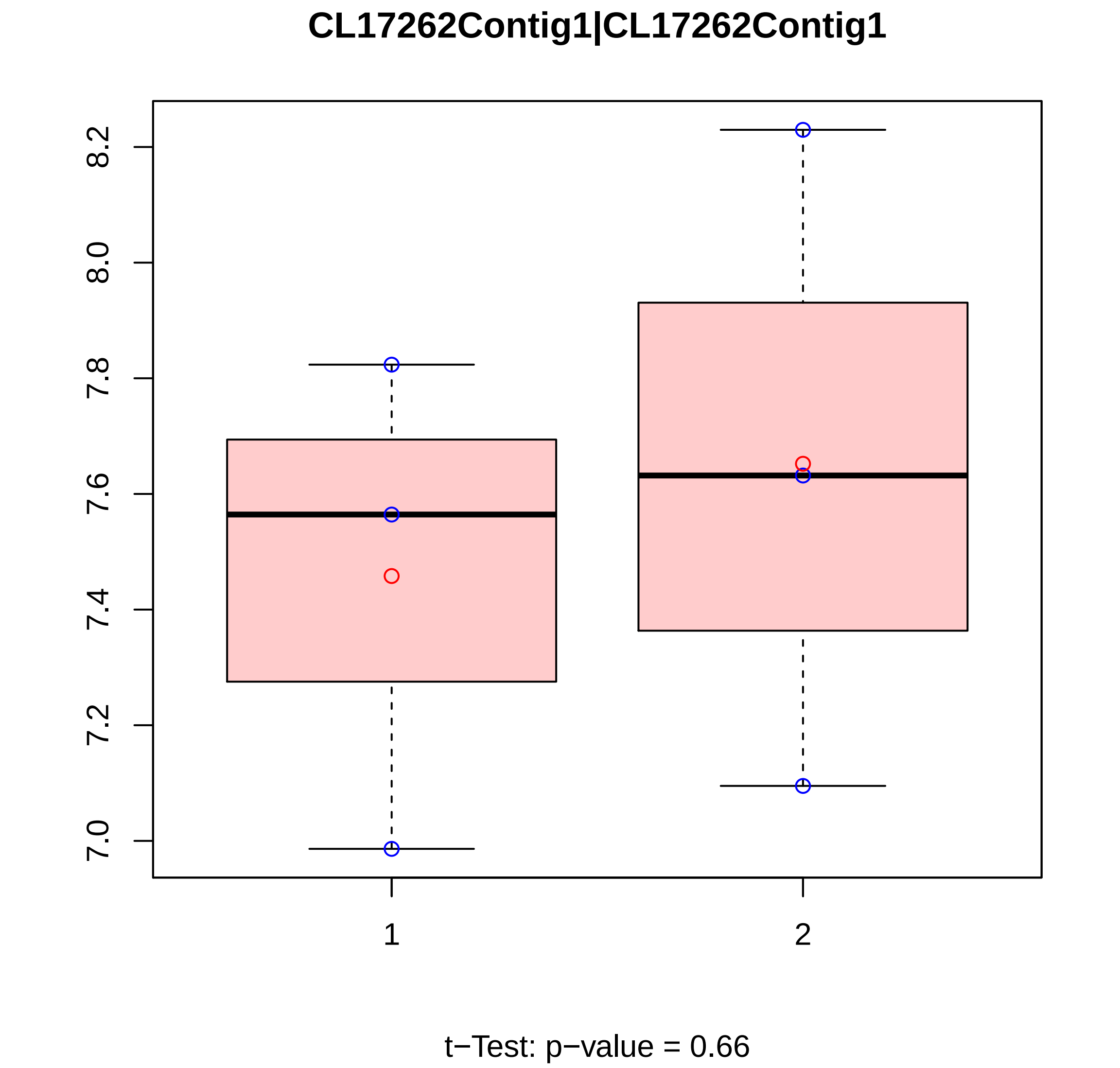

CL17262Contig1|CL17262Contig1
8.2
8.0
7.8
7.6
7.4
7.2
7.0
1
2
t−Test: p−value = 0.66

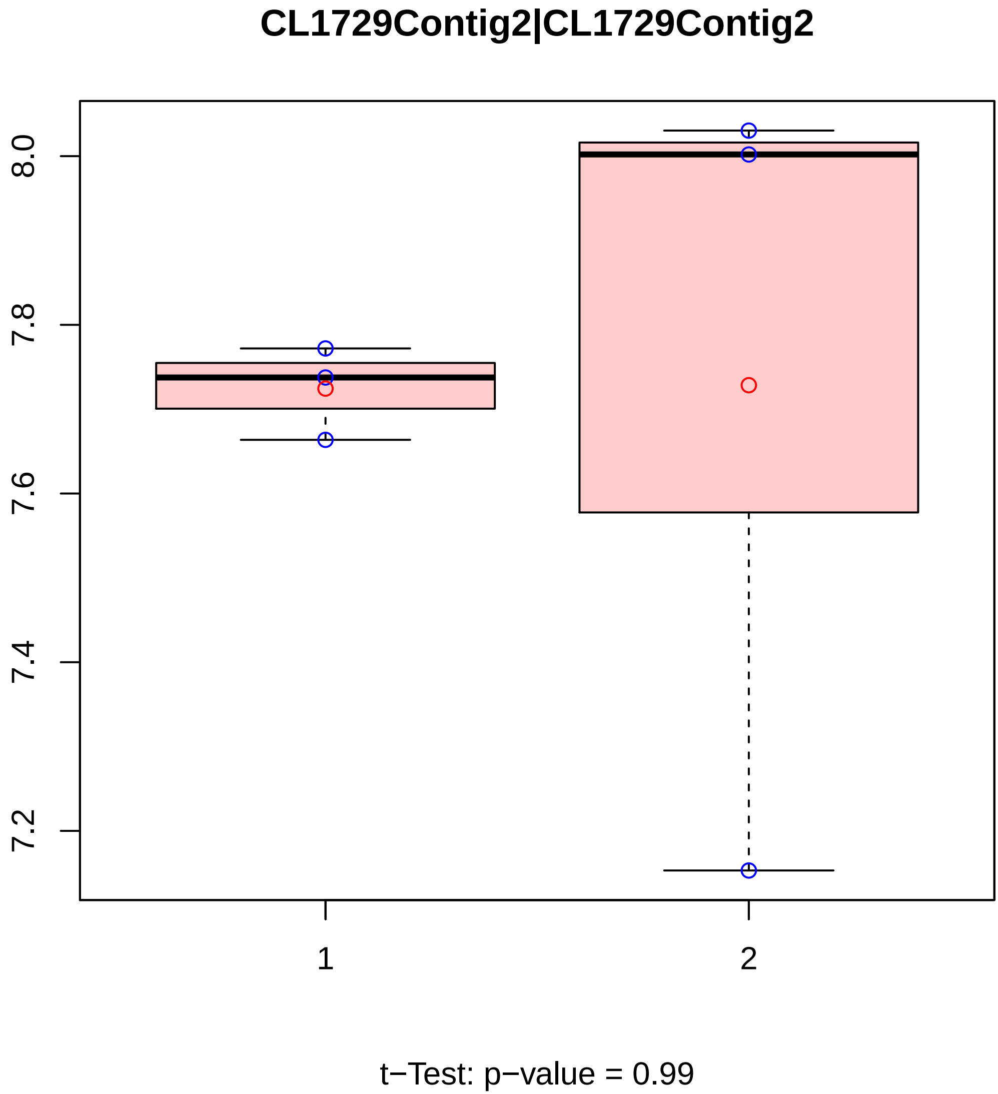

CL1729Contig2|CL1729Contig2
8.0
7.8
7.6
7.4
7.2
1
2
t−Test: p−value = 0.99

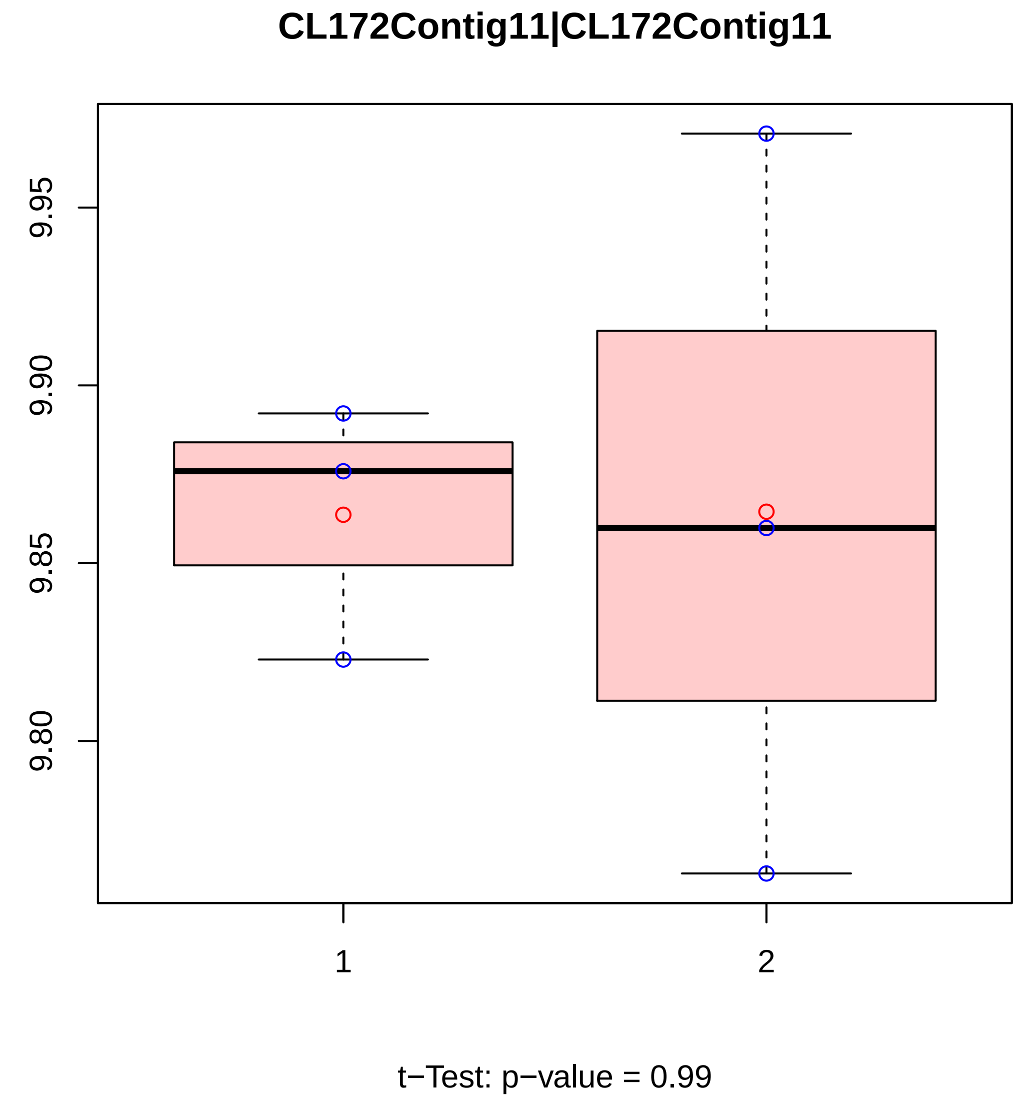

CL172Contig11|CL172Contig11
9.80
9.85
9.90
9.95
1
2
t−Test: p−value = 0.99

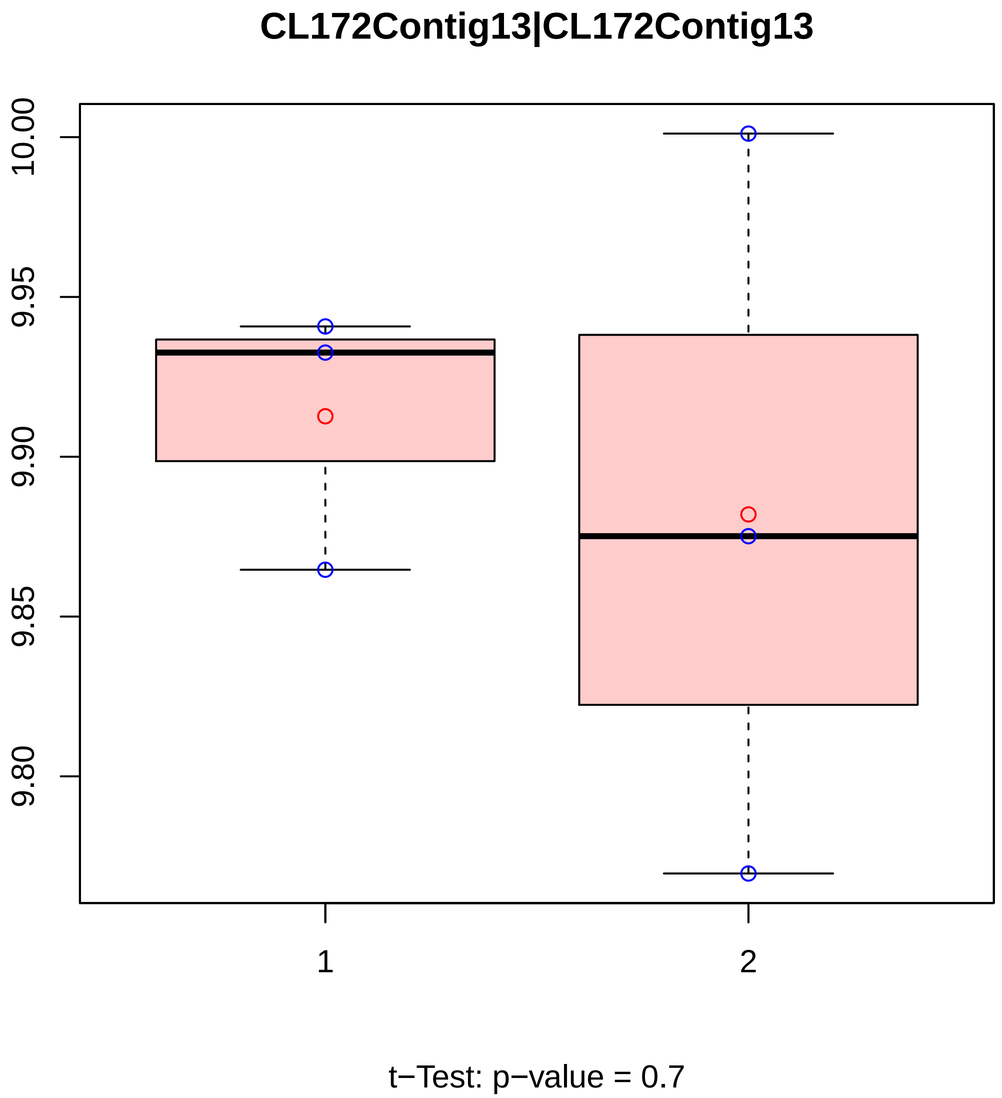

CL172Contig13|CL172Contig13
10.00
9.95
9.90
9.85
9.80
1
2
t−Test: p−value = 0.7

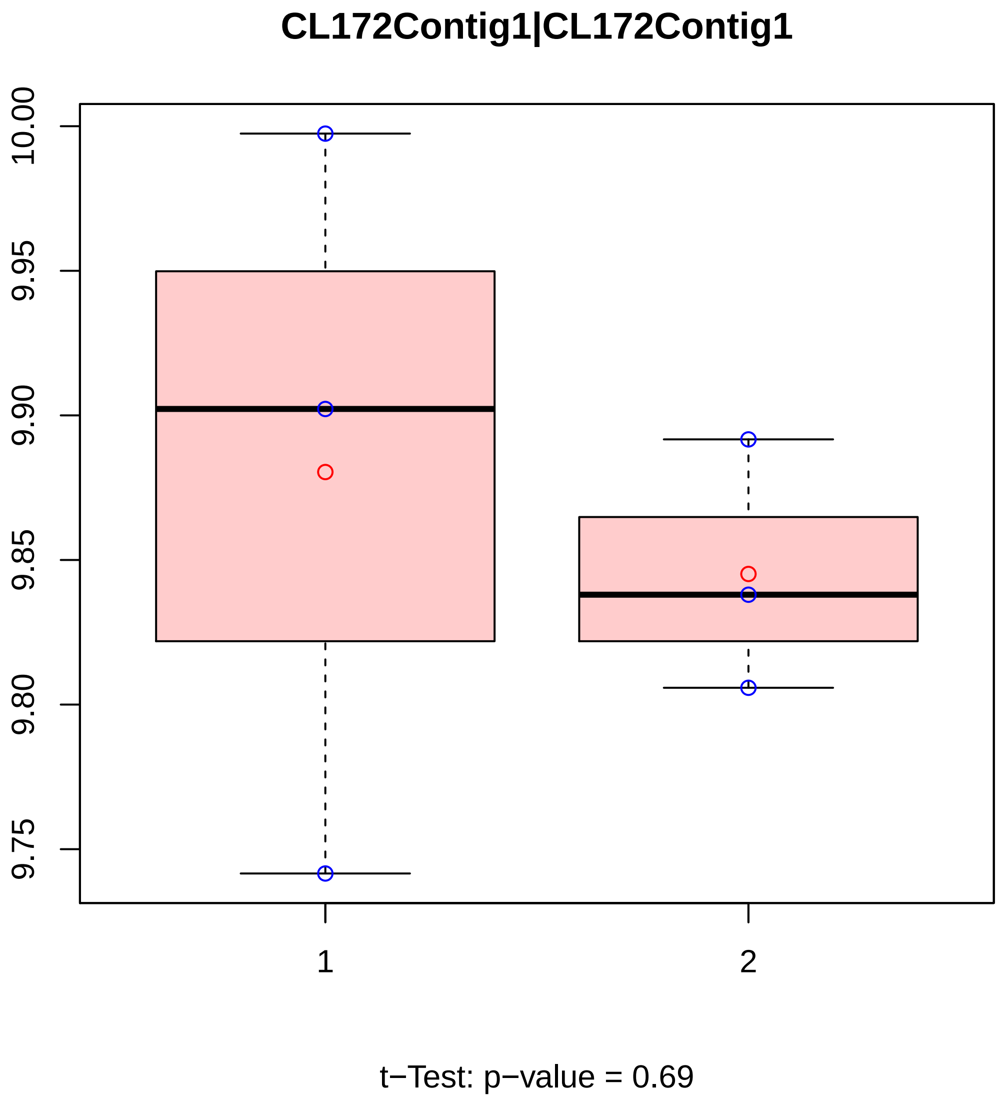
CL172Contig1|CL172Contig1
10.00
9.95
9.90
9.85
9.80
9.75
1
2
t−Test: p−value = 0.69

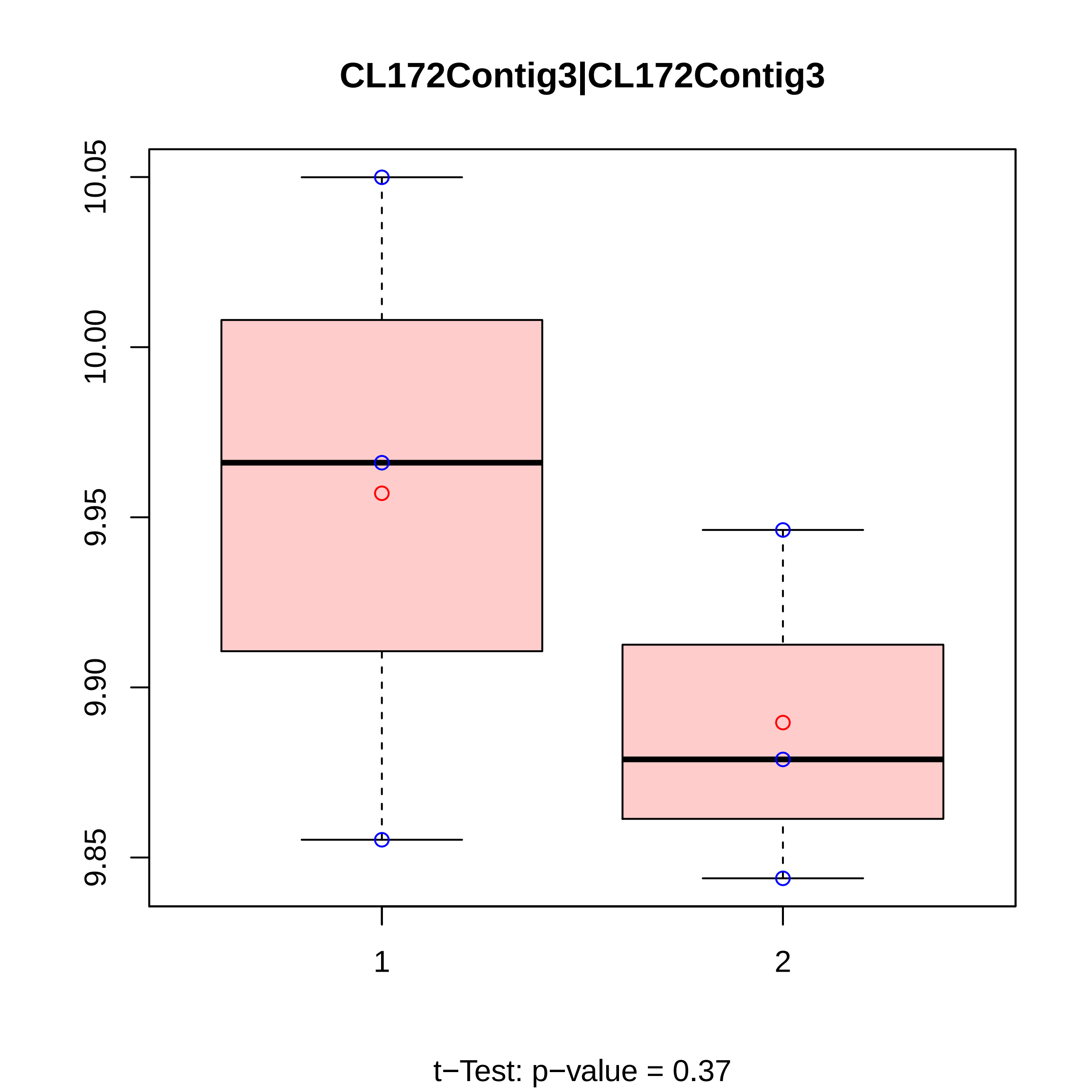
CL172Contig3|CL172Contig3
10.05
10.00
9.95
9.90
9.85
1
2
t−Test: p−value = 0.37

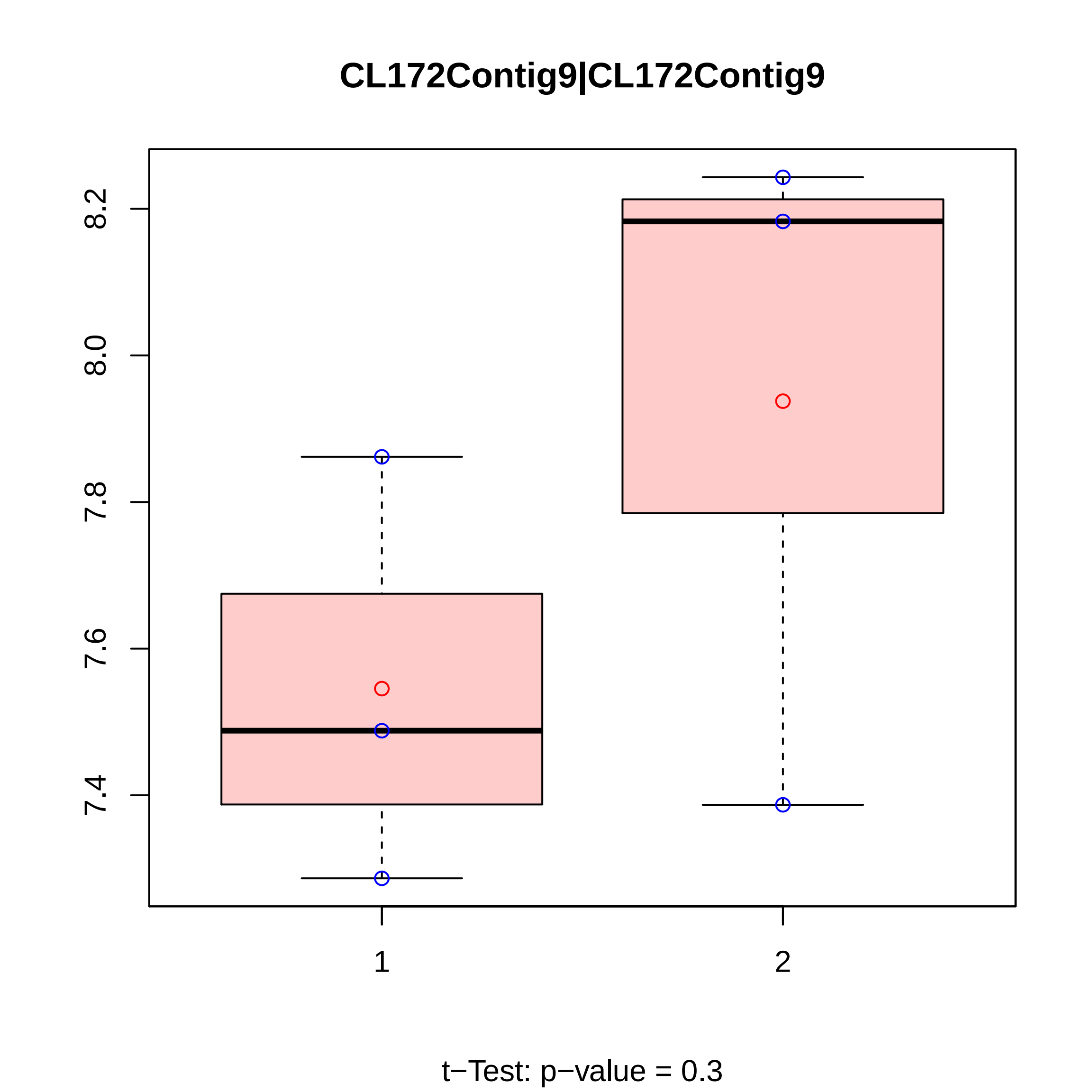
CL172Contig9|CL172Contig9
8.2
8.0
7.8
7.6
7.4
1
2
t−Test: p−value = 0.3

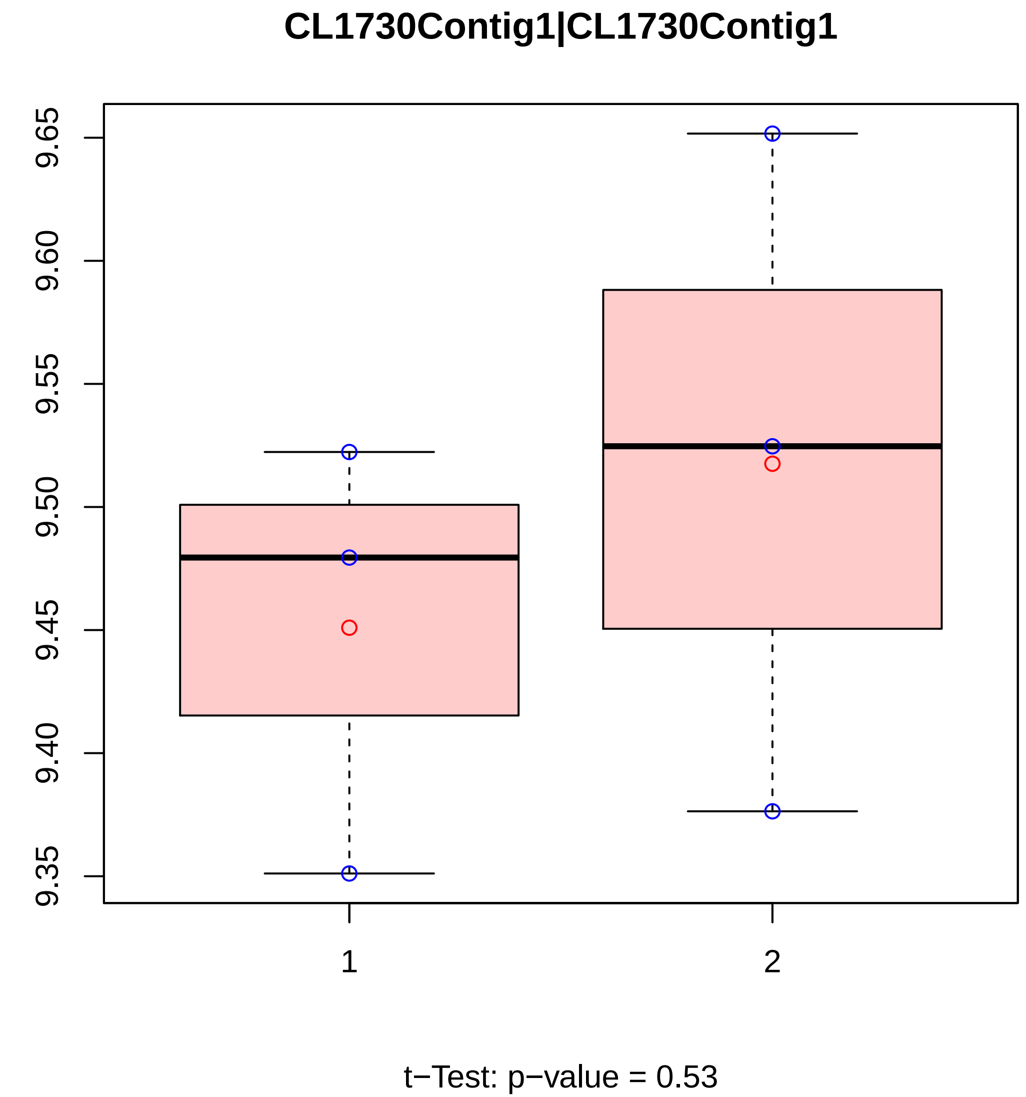
CL1730Contig1|CL1730Contig1
9.35
9.40
9.45
9.50
9.55
9.60
9.65
1
2
t−Test: p−value = 0.53

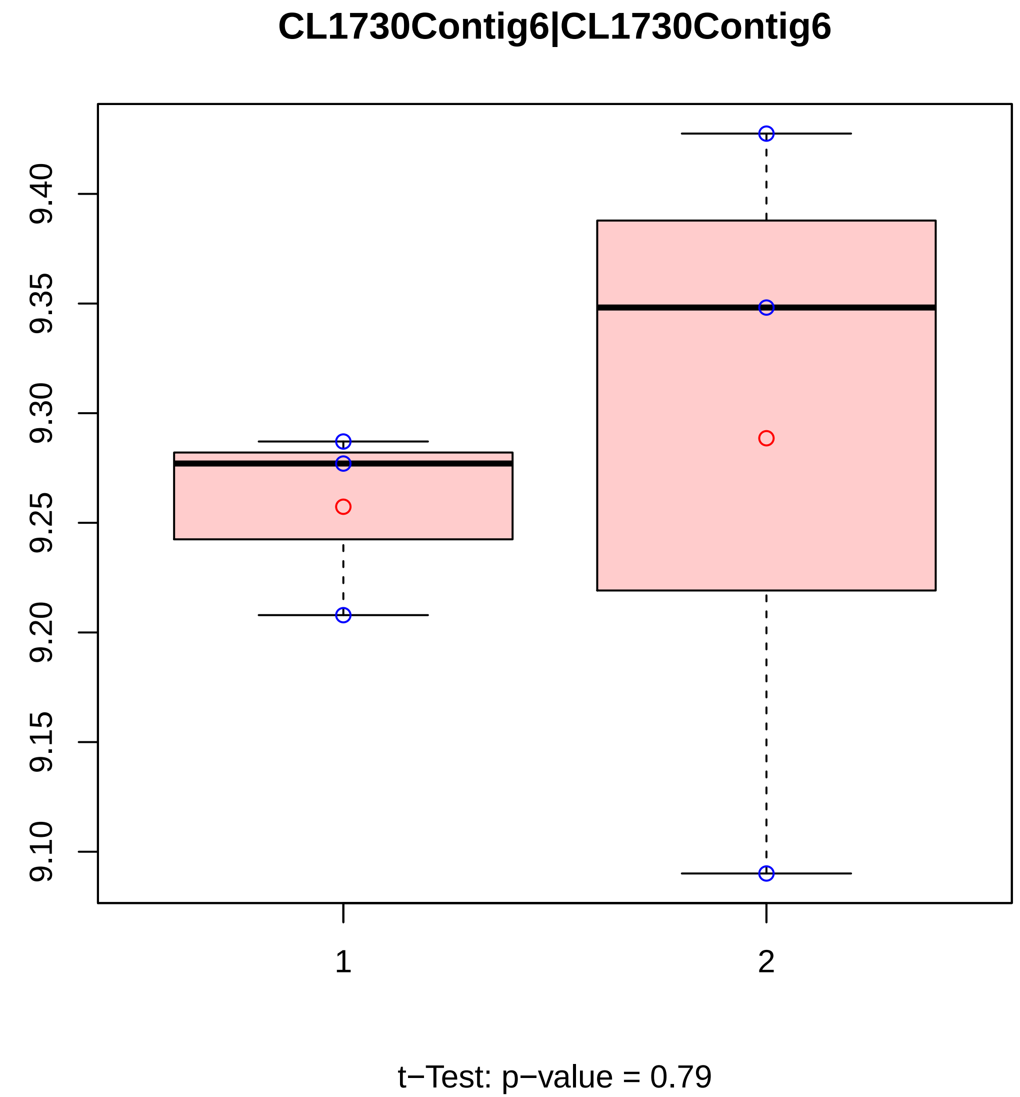
CL1730Contig6|CL1730Contig6
9.10
9.15
9.20
9.25
9.30
9.35
9.40
1
2
t−Test: p−value = 0.79

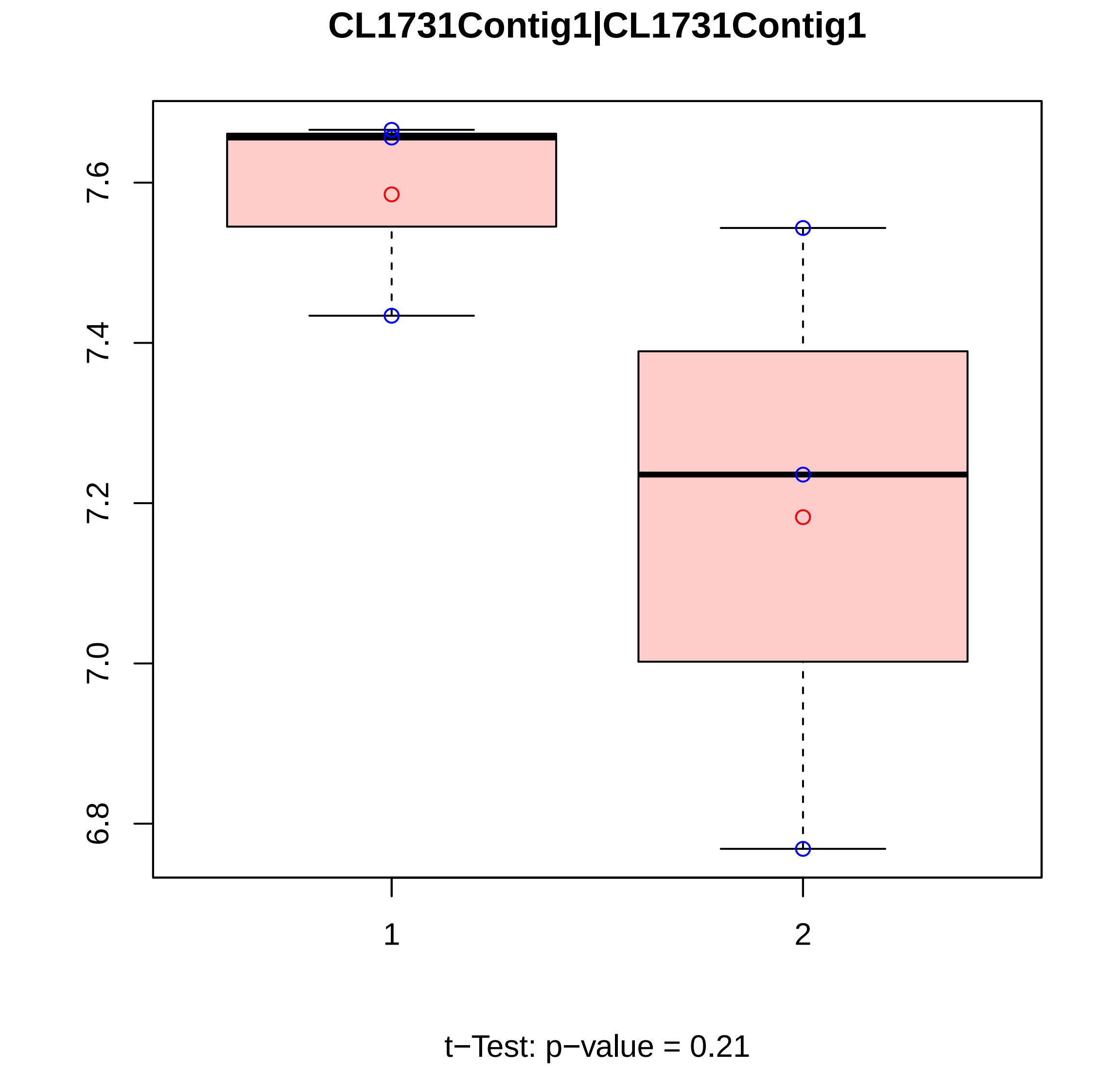
CL1731Contig1|CL1731Contig1
7.6
7.4
7.2
7.0
6.8
1
2
t−Test: p−value = 0.21

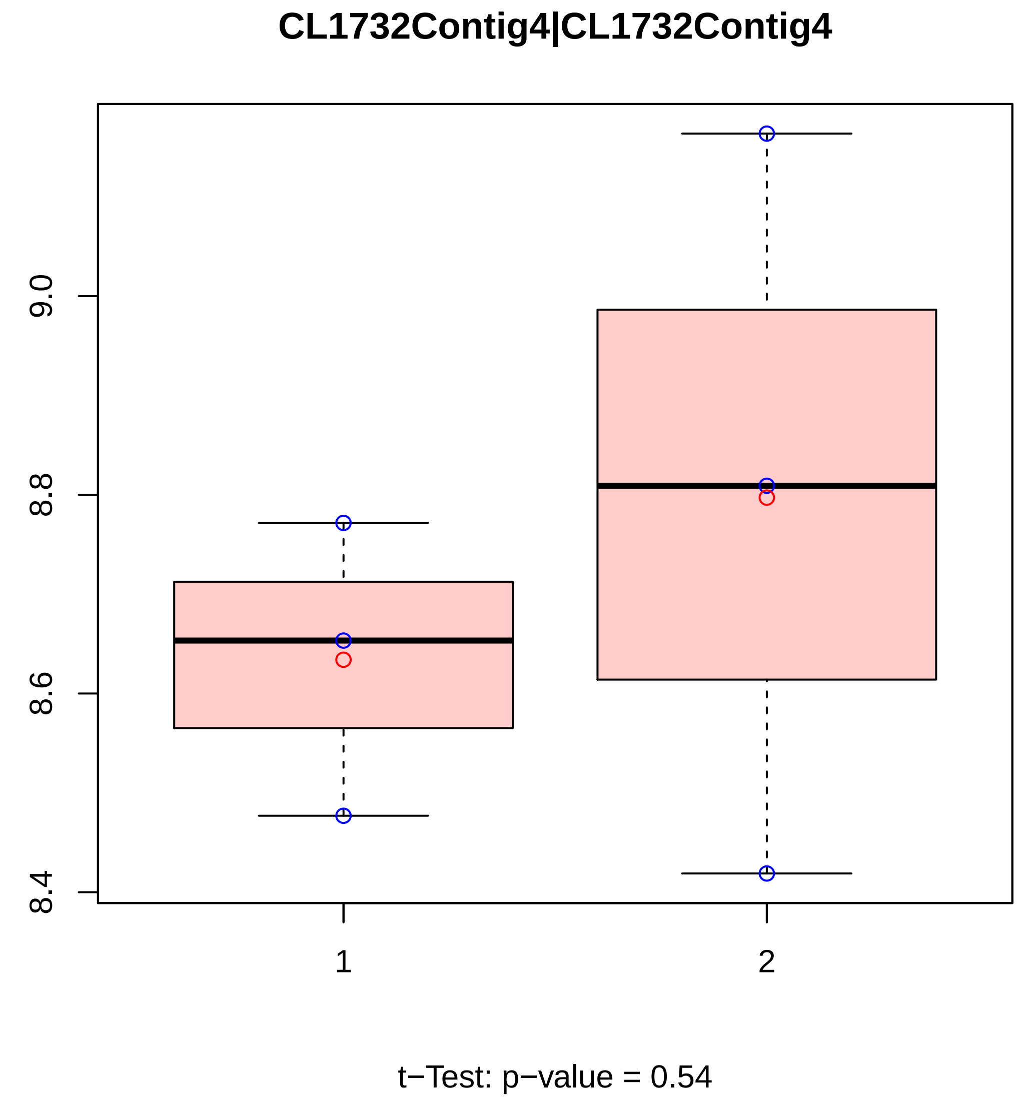
CL1732Contig4|CL1732Contig4
9.0
8.8
8.6
8.4
1
2
t−Test: p−value = 0.54

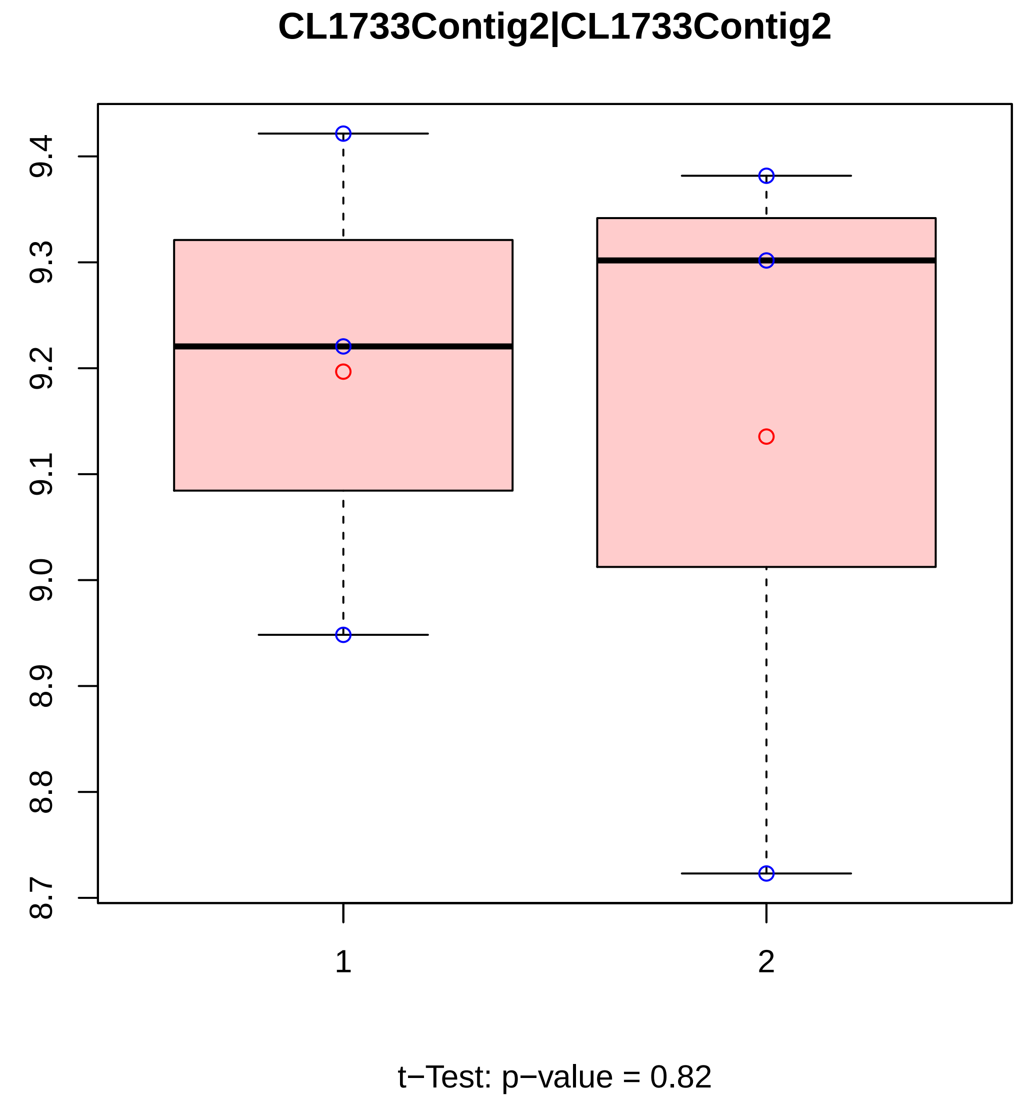
CL1733Contig2|CL1733Contig2
9.4
9.3
9.2
9.1
9.0
8.9
8.8
8.7
1
2
t−Test: p−value = 0.82

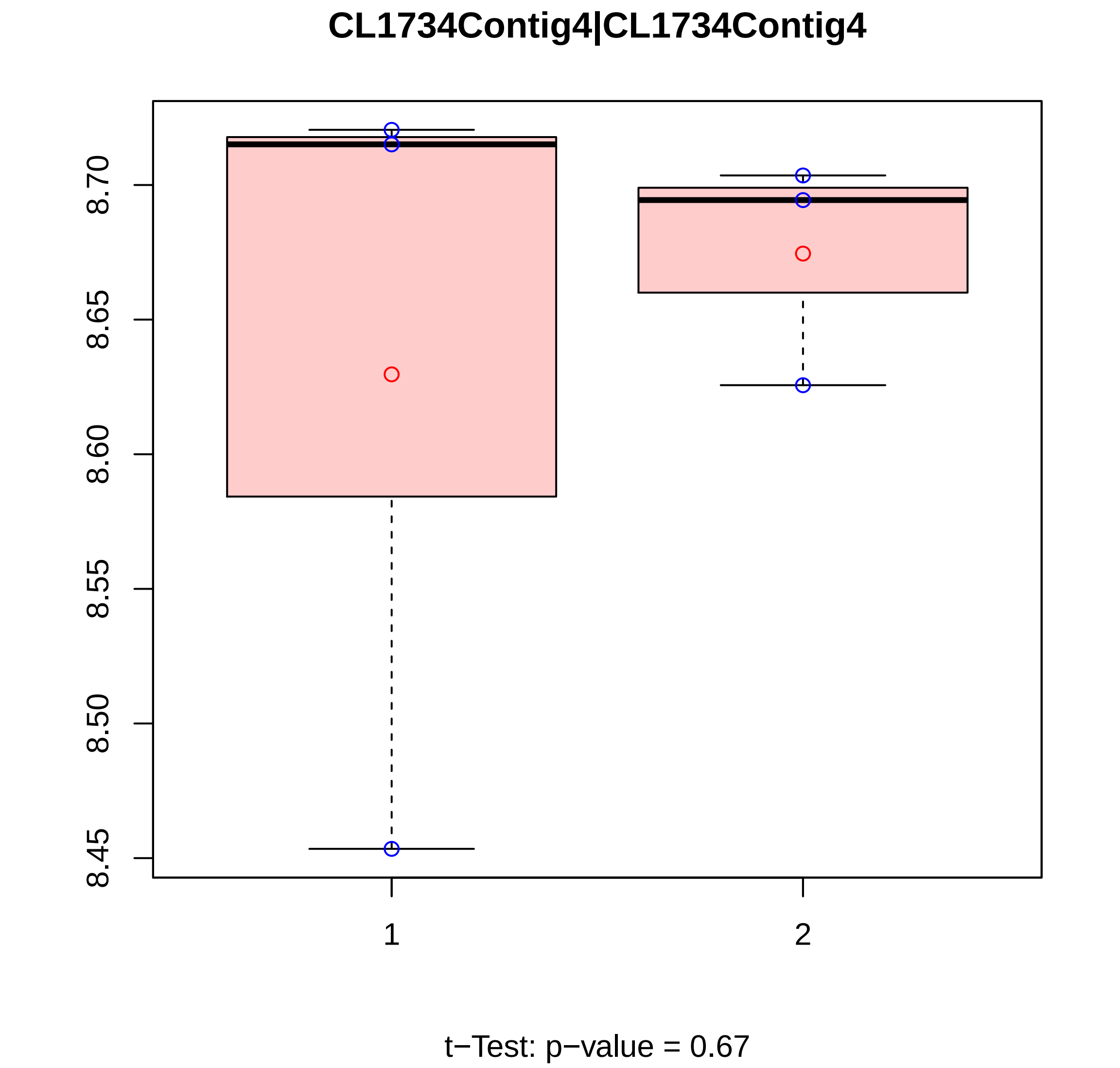
CL1734Contig4|CL1734Contig4
8.45
8.50
8.55
8.60
8.65
8.70
1
2
t−Test: p−value = 0.67

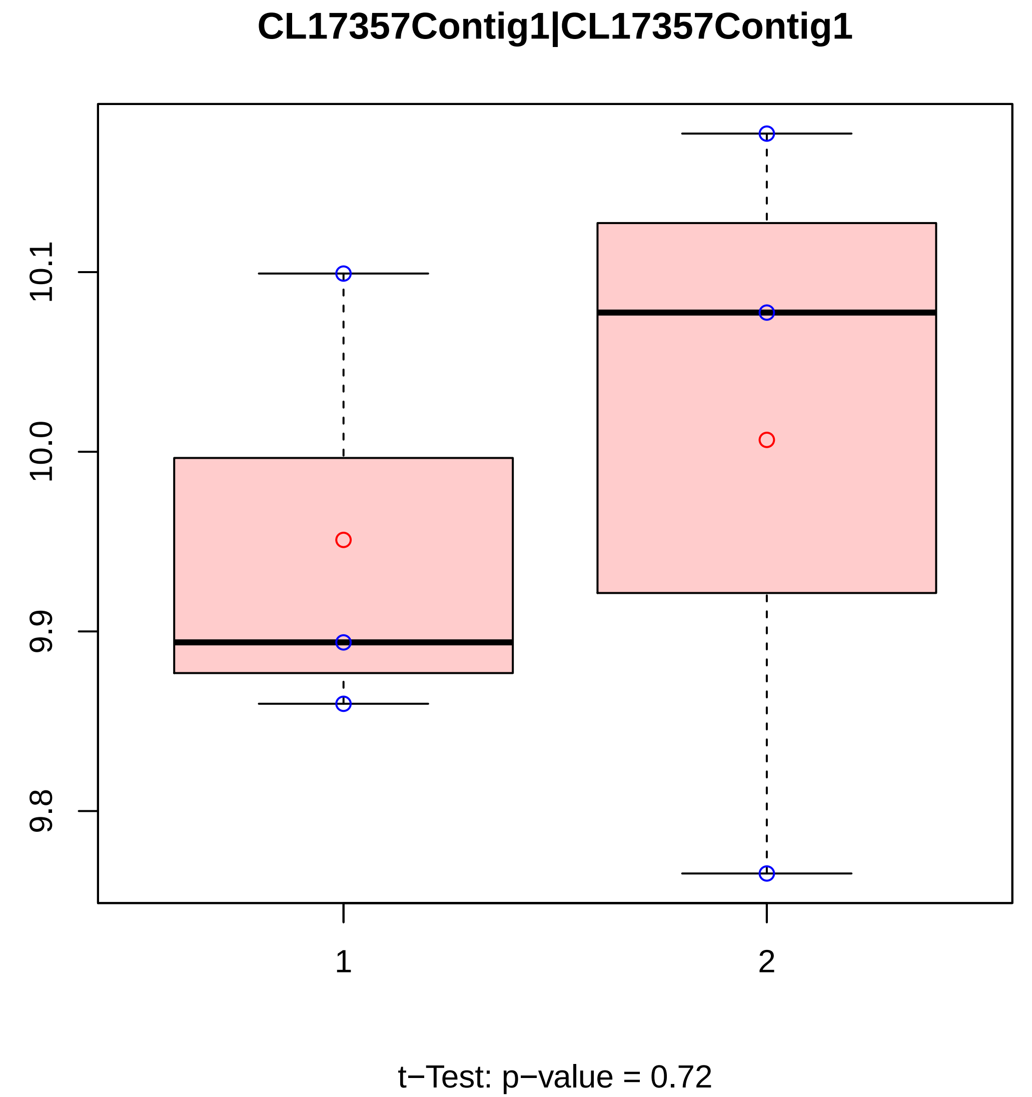
CL17357Contig1|CL17357Contig1
10.1
10.0
9.9
9.8
1
2
t−Test: p−value = 0.72

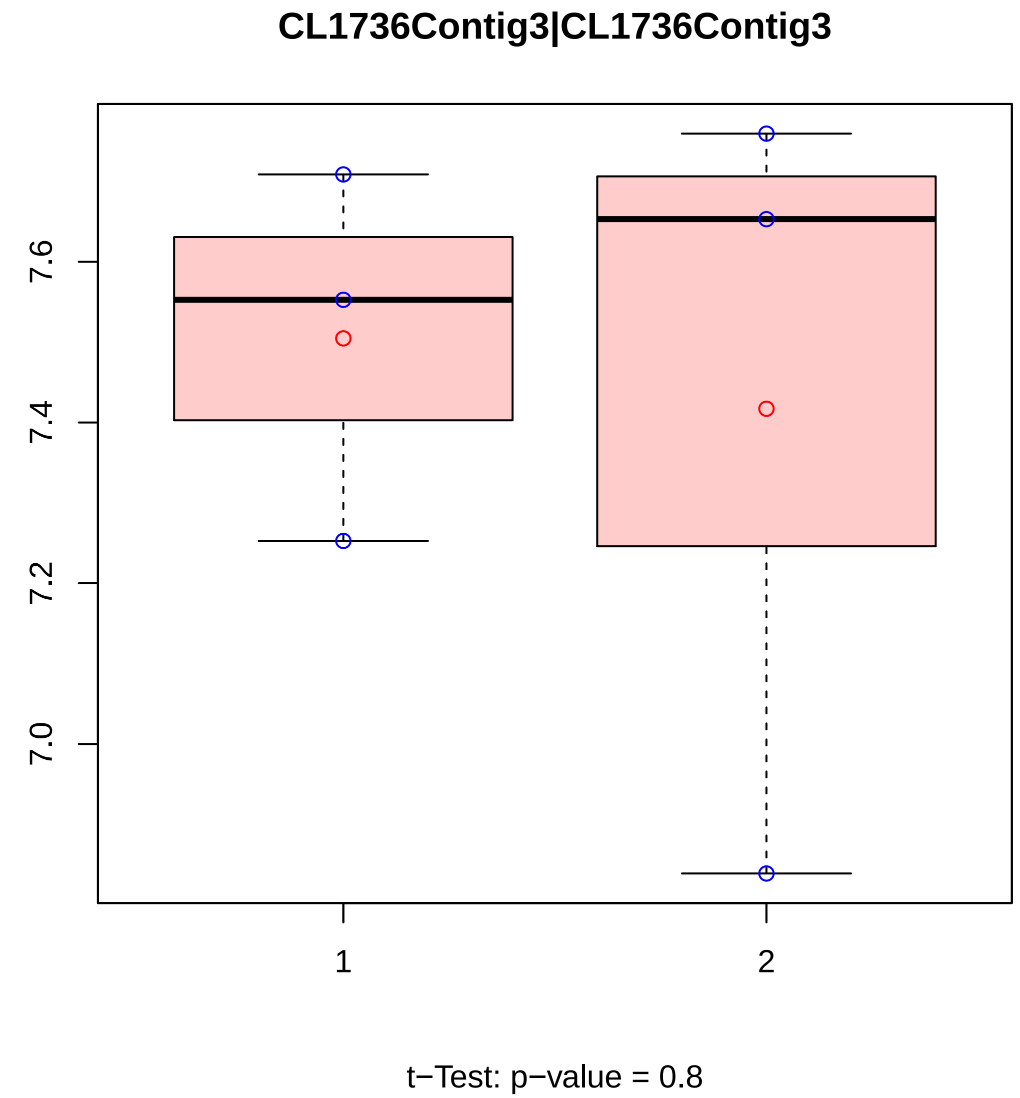

CL1736Contig3|CL1736Contig3
7.0
7.2
7.4
7.6
1
2
t−Test: p−value = 0.8

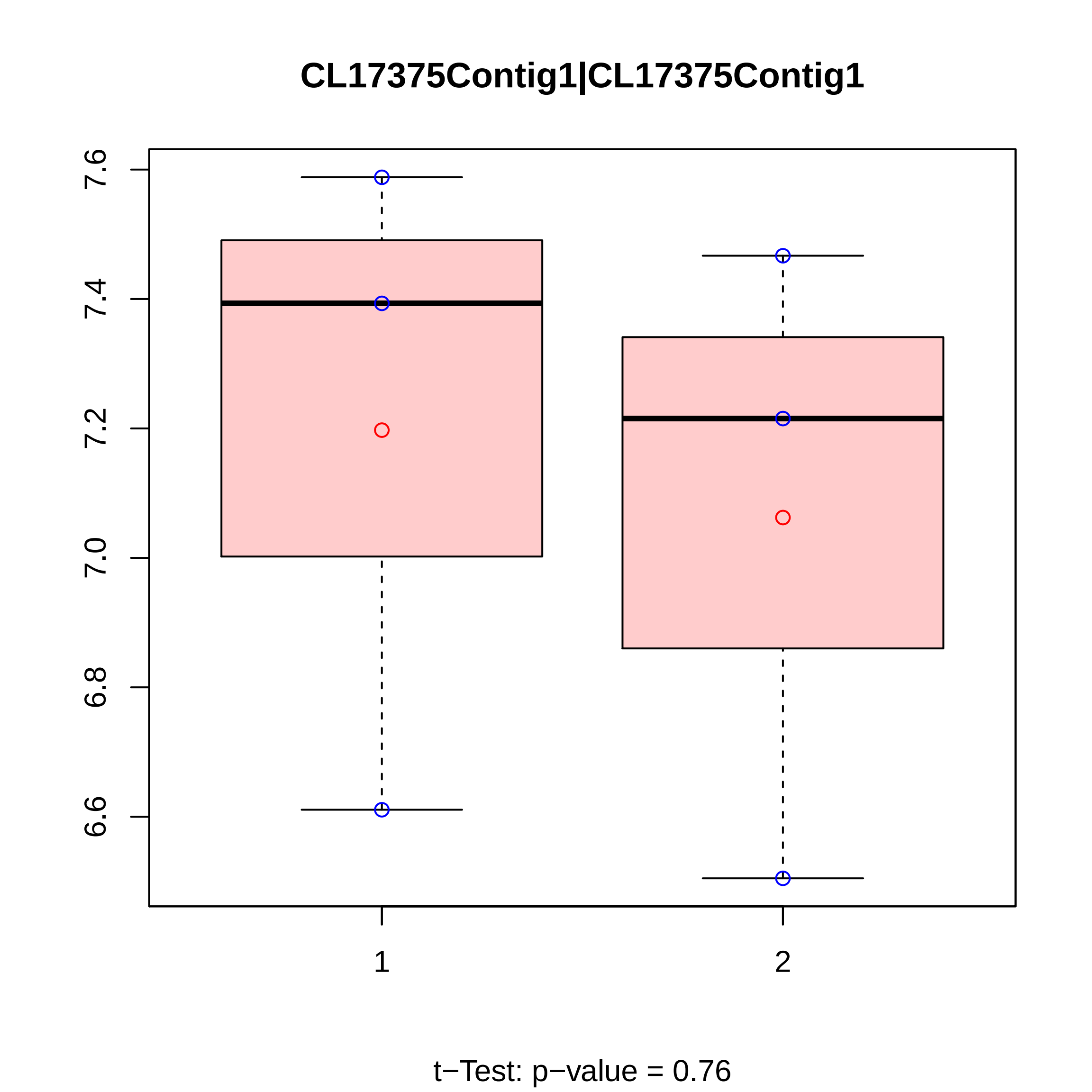
CL17375Contig1|CL17375Contig1
7.6
7.4
7.2
7.0
6.8
6.6
1
2
t−Test: p−value = 0.76

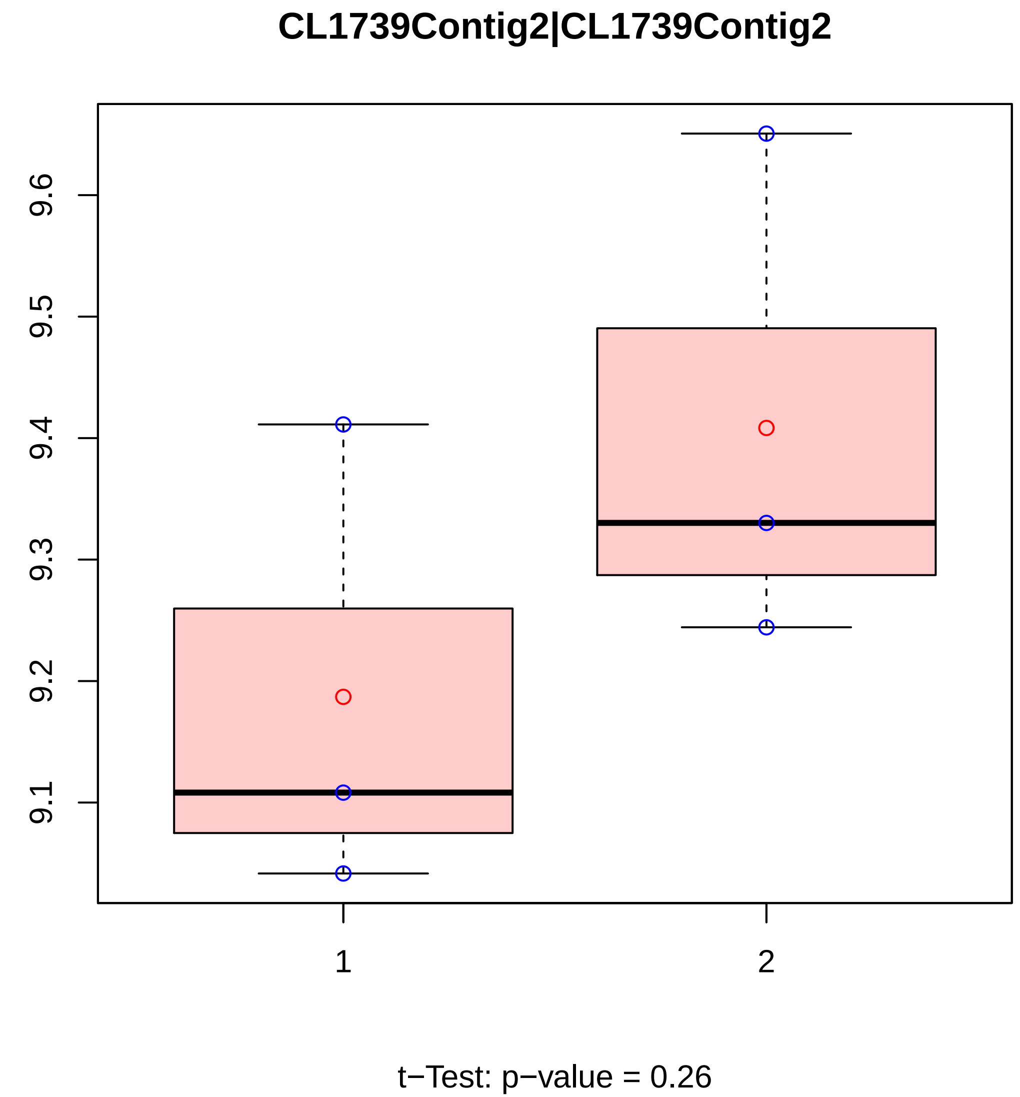
CL1739Contig2|CL1739Contig2
9.1
9.2
9.3
9.4
9.5
9.6
1
2
t−Test: p−value = 0.26

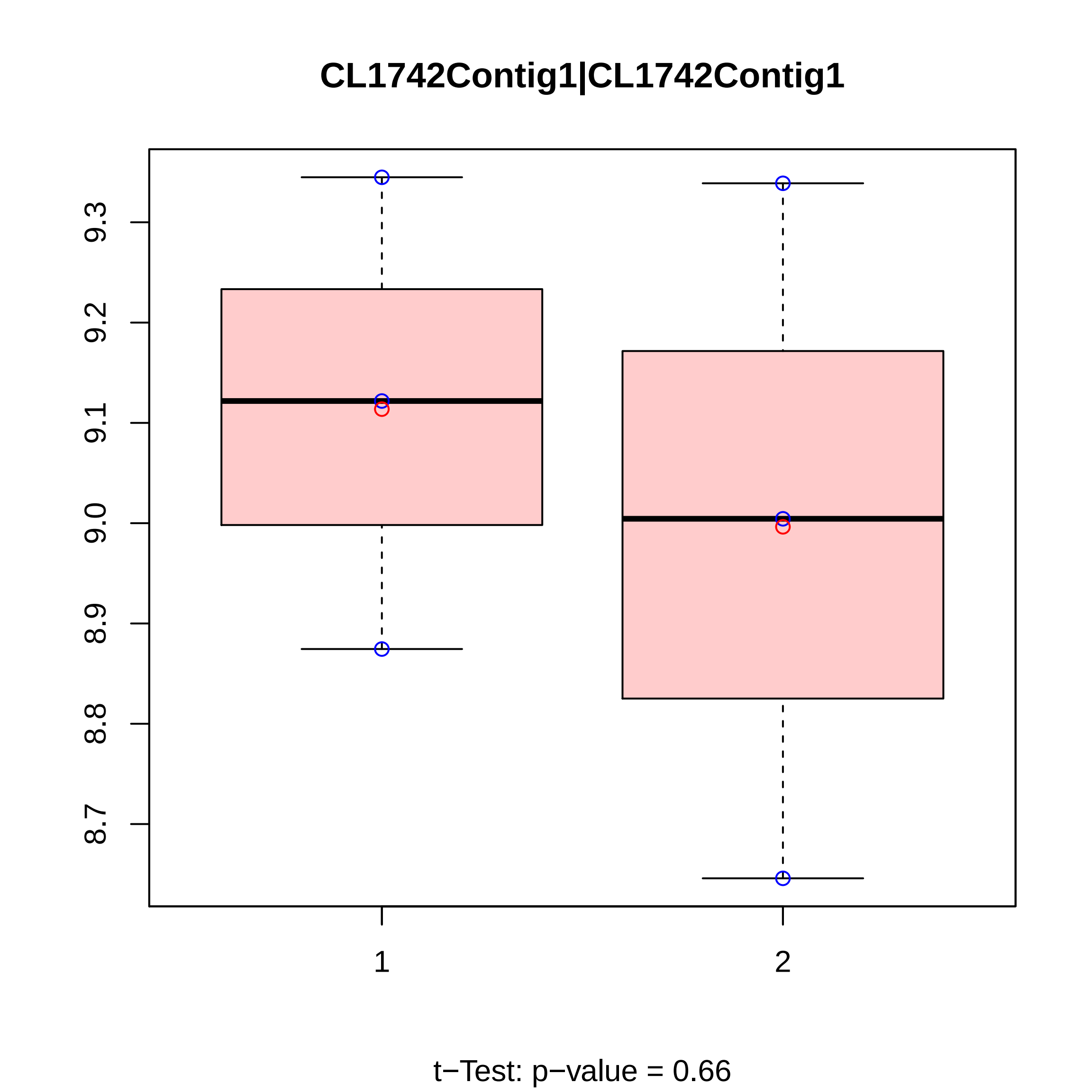

CL1742Contig1|CL1742Contig1
9.3
9.2
9.1
9.0
8.9
8.8
8.7
1
2
t−Test: p−value = 0.66

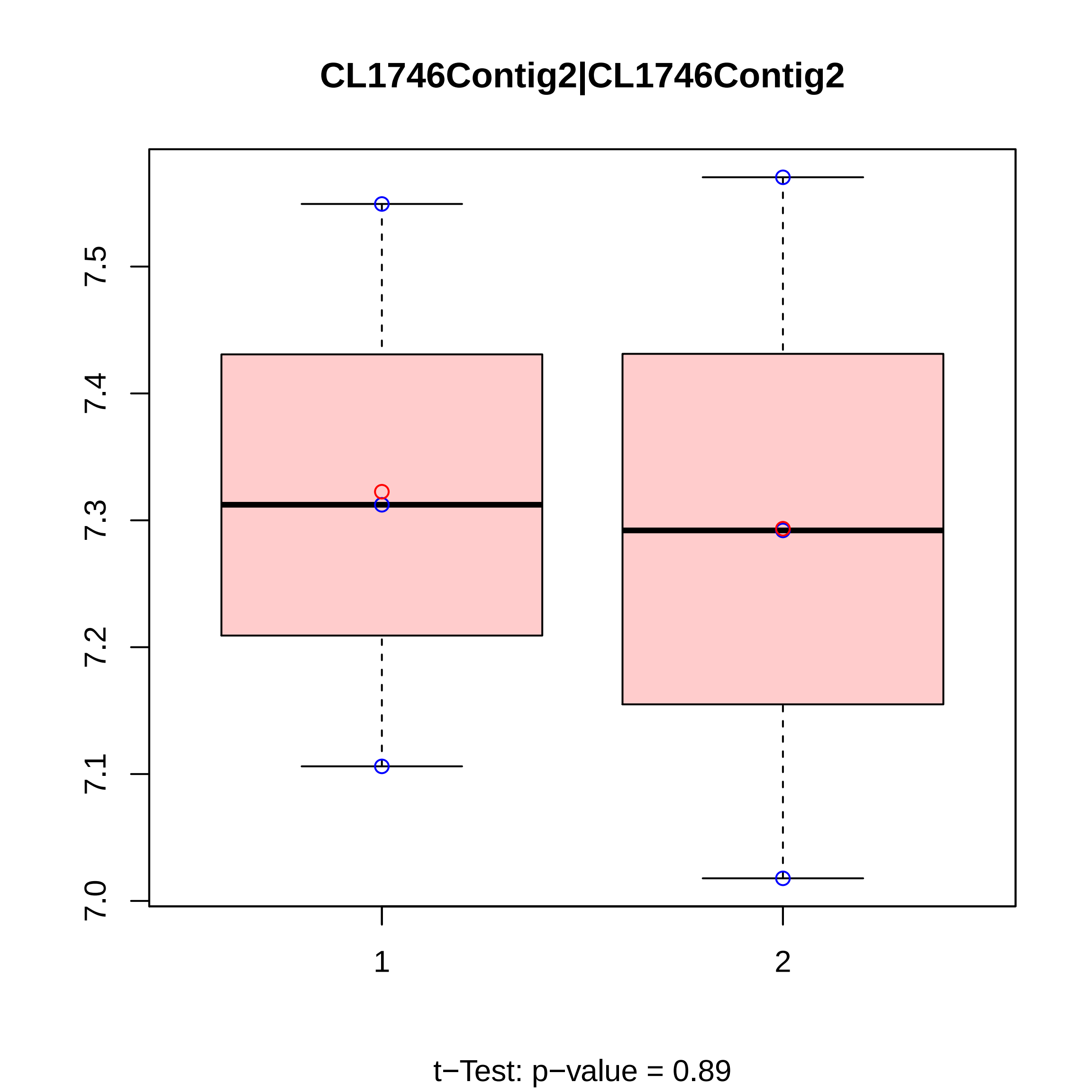
CL1746Contig2|CL1746Contig2
7.0
7.1
7.2
7.3
7.4
7.5
1
2
t−Test: p−value = 0.89

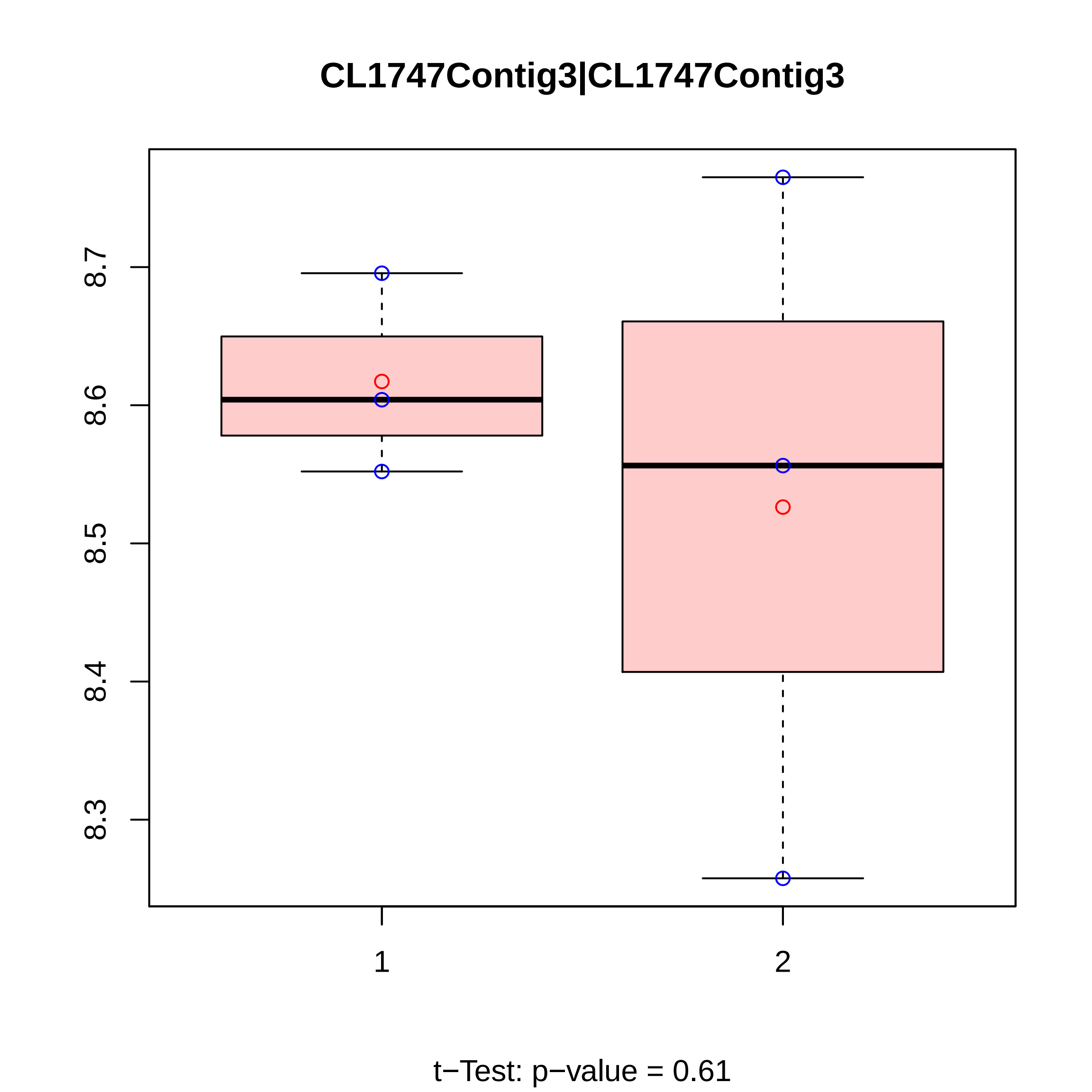

CL1747Contig3|CL1747Contig3
8.7
8.6
8.5
8.4
8.3
1
2
t−Test: p−value = 0.61

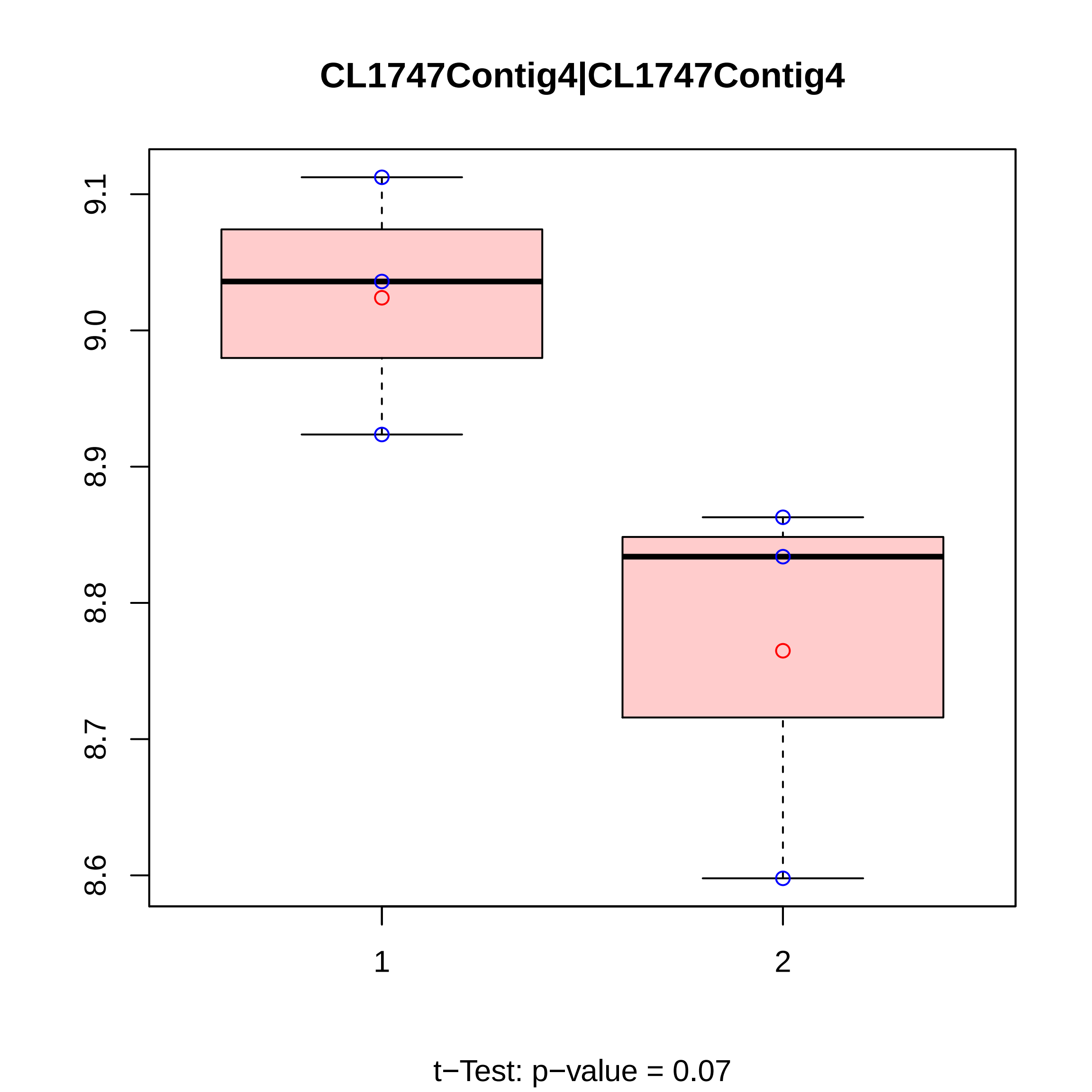

CL1747Contig4|CL1747Contig4
9.1
9.0
8.9
8.8
8.7
8.6
1
2
t−Test: p−value = 0.07

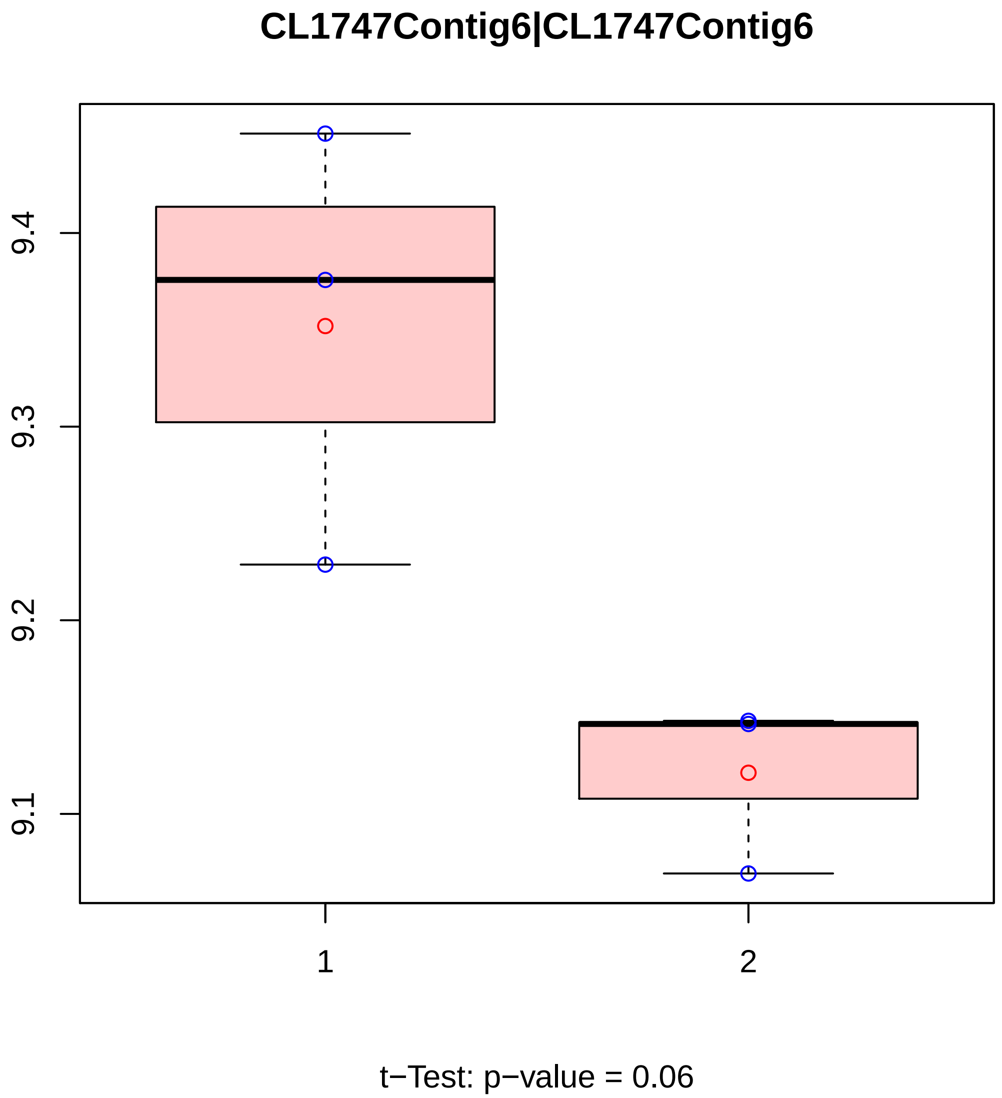
CL1747Contig6|CL1747Contig6
9.4
9.3
9.2
9.1
1
2
t−Test: p−value = 0.06

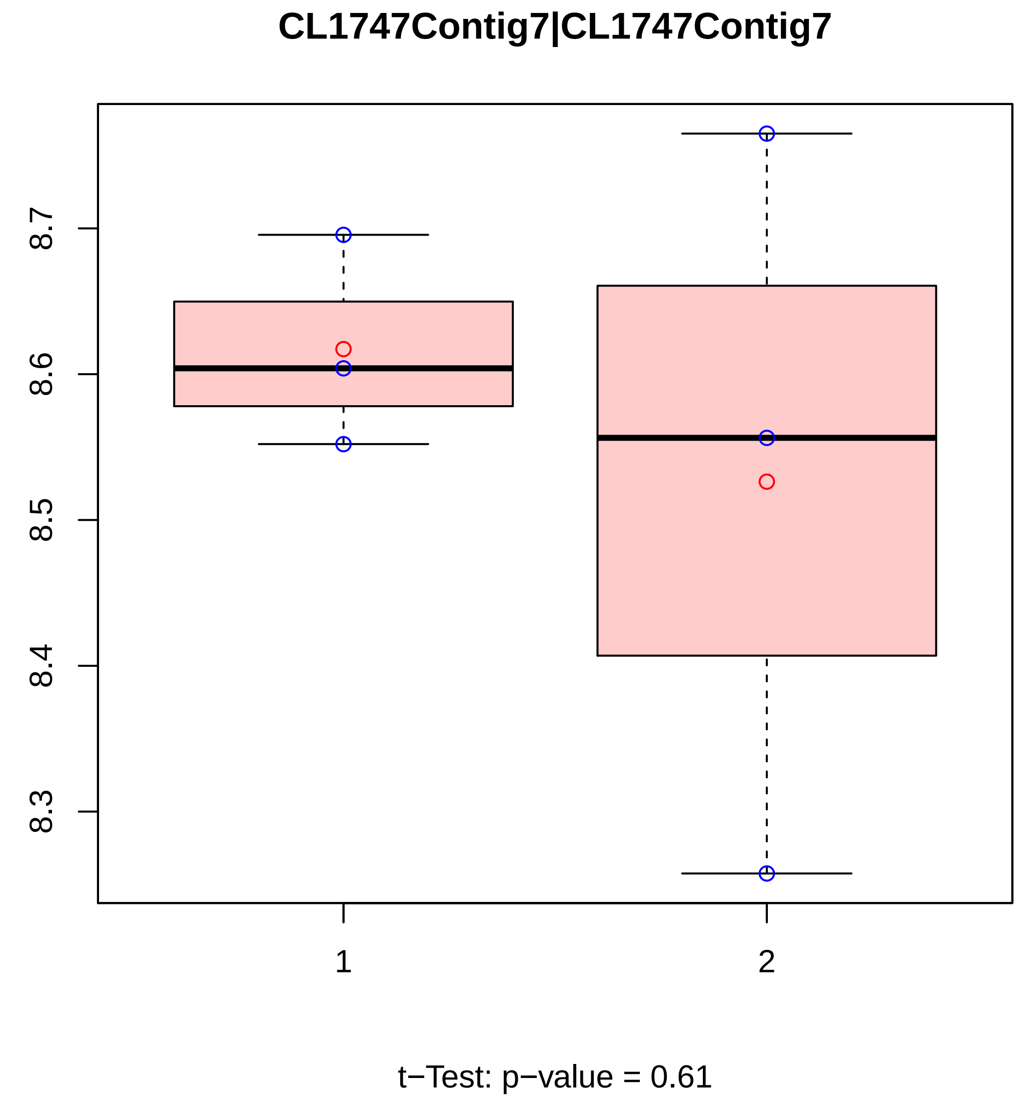

CL1747Contig7|CL1747Contig7
8.3
8.4
8.5
8.6
8.7
1
2
t−Test: p−value = 0.61

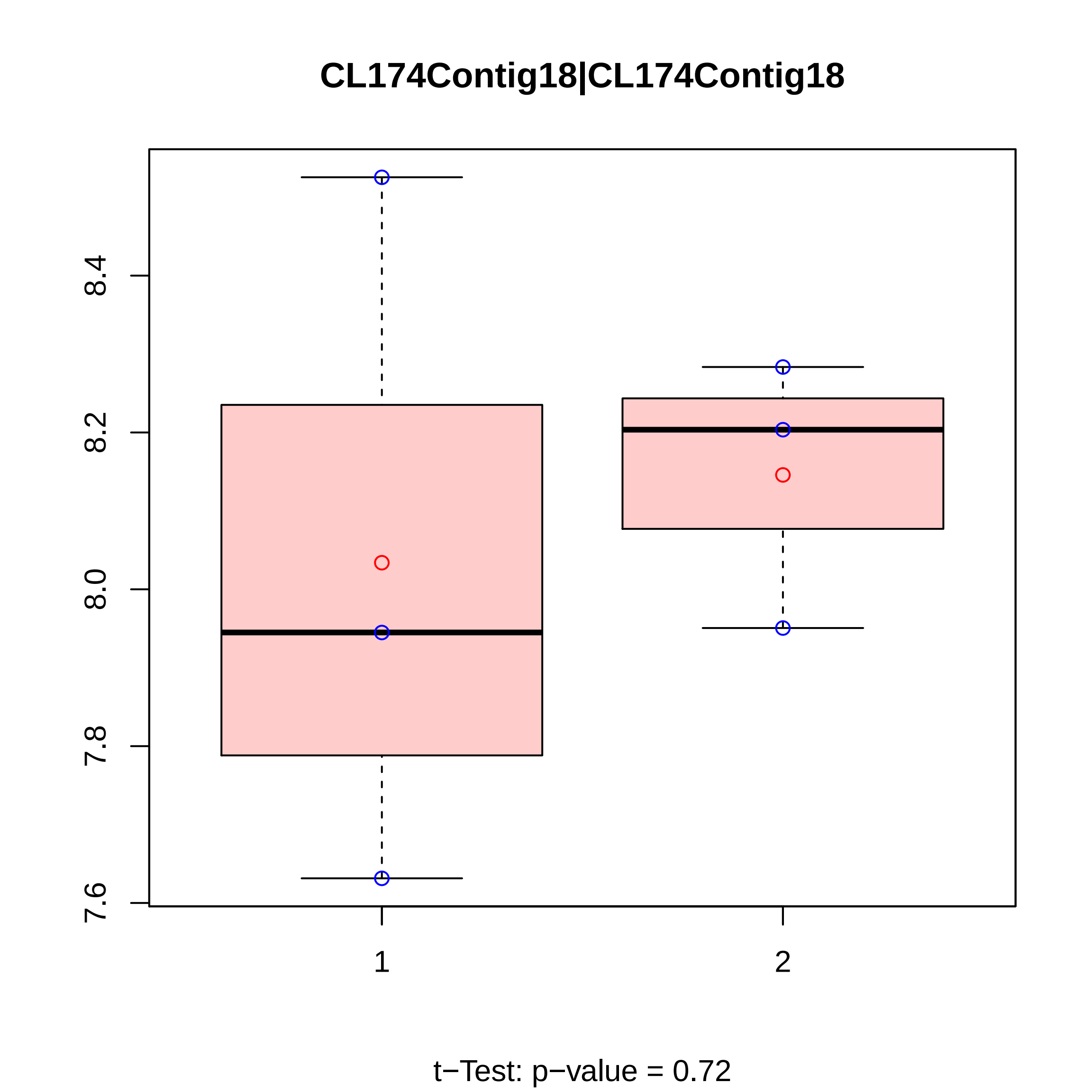
CL174Contig18|CL174Contig18
8.4
8.2
8.0
7.8
7.6
1
2
t−Test: p−value = 0.72

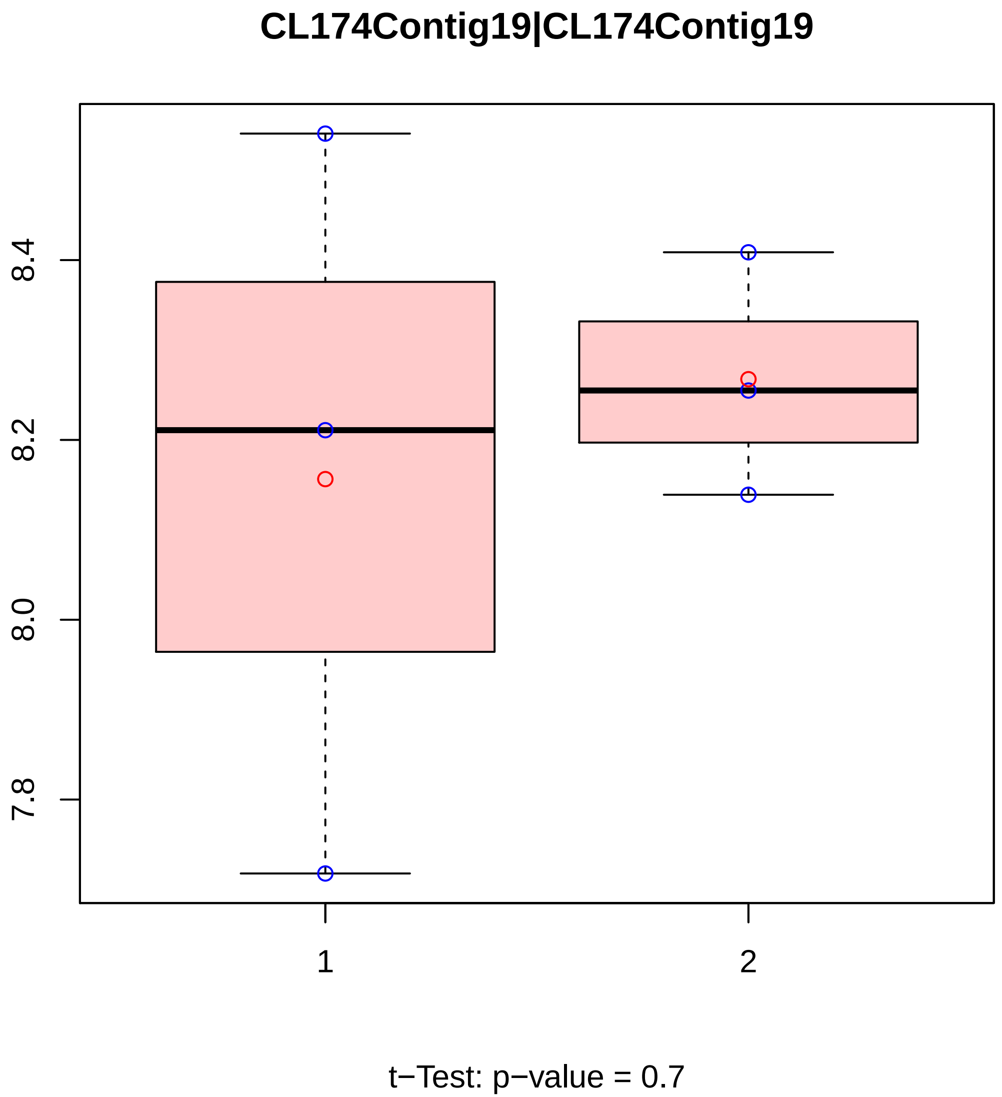

CL174Contig19|CL174Contig19
8.4
8.2
8.0
7.8
1
2
t−Test: p−value = 0.7

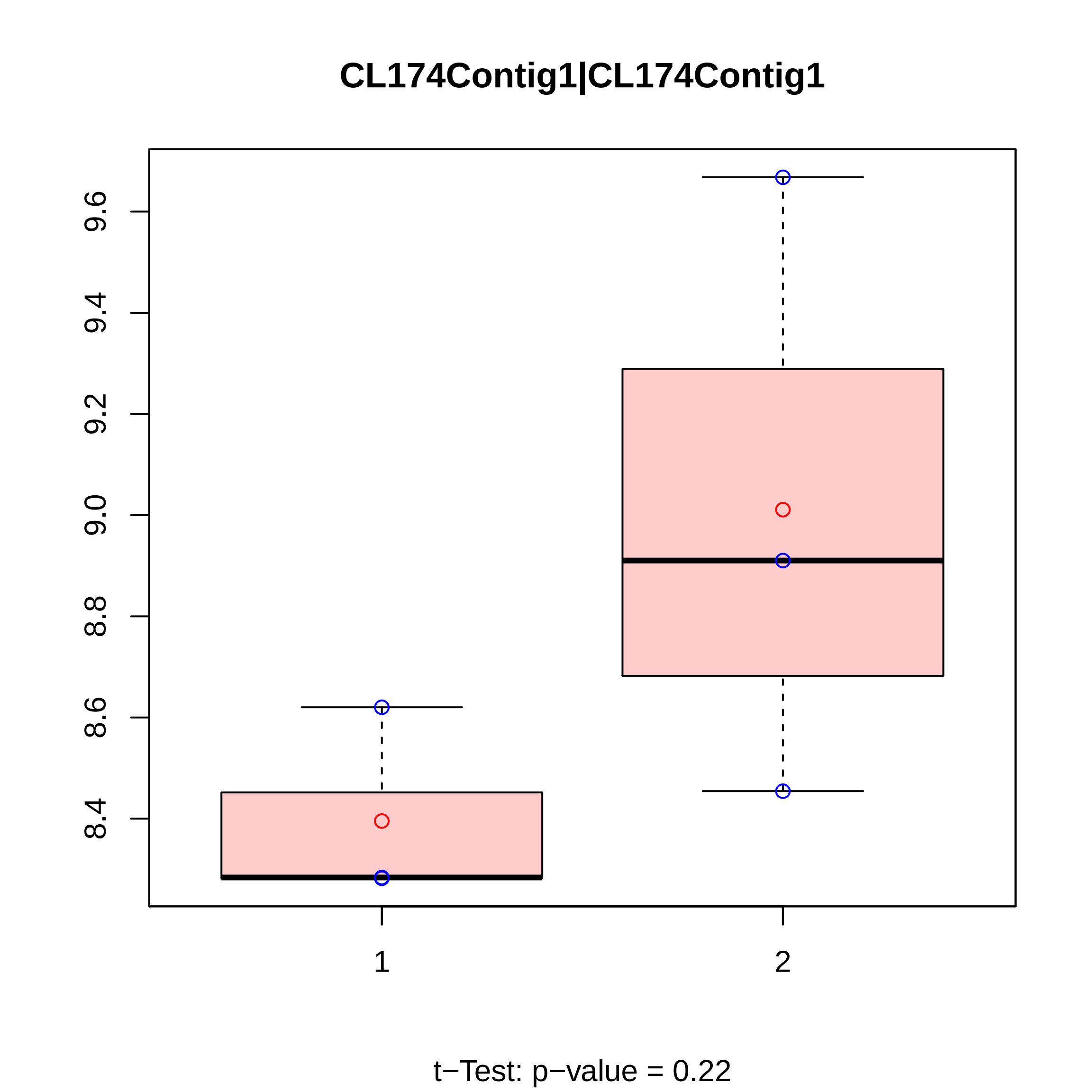

CL174Contig1|CL174Contig1
9.6
9.4
9.2
9.0
8.8
8.6
8.4
1
2
t−Test: p−value = 0.22

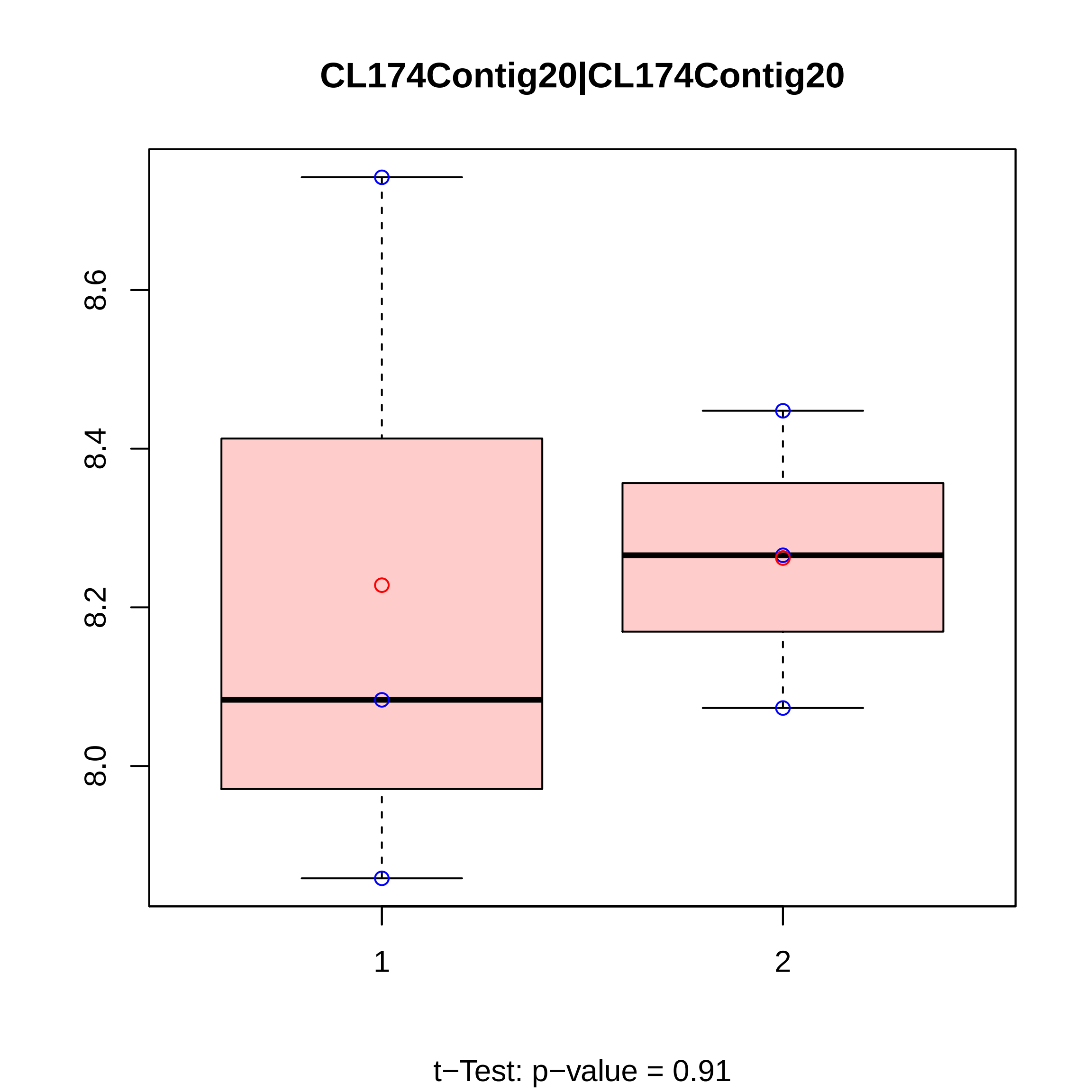
CL174Contig20|CL174Contig20
8.0
8.2
8.4
8.6
1
2
t−Test: p−value = 0.91

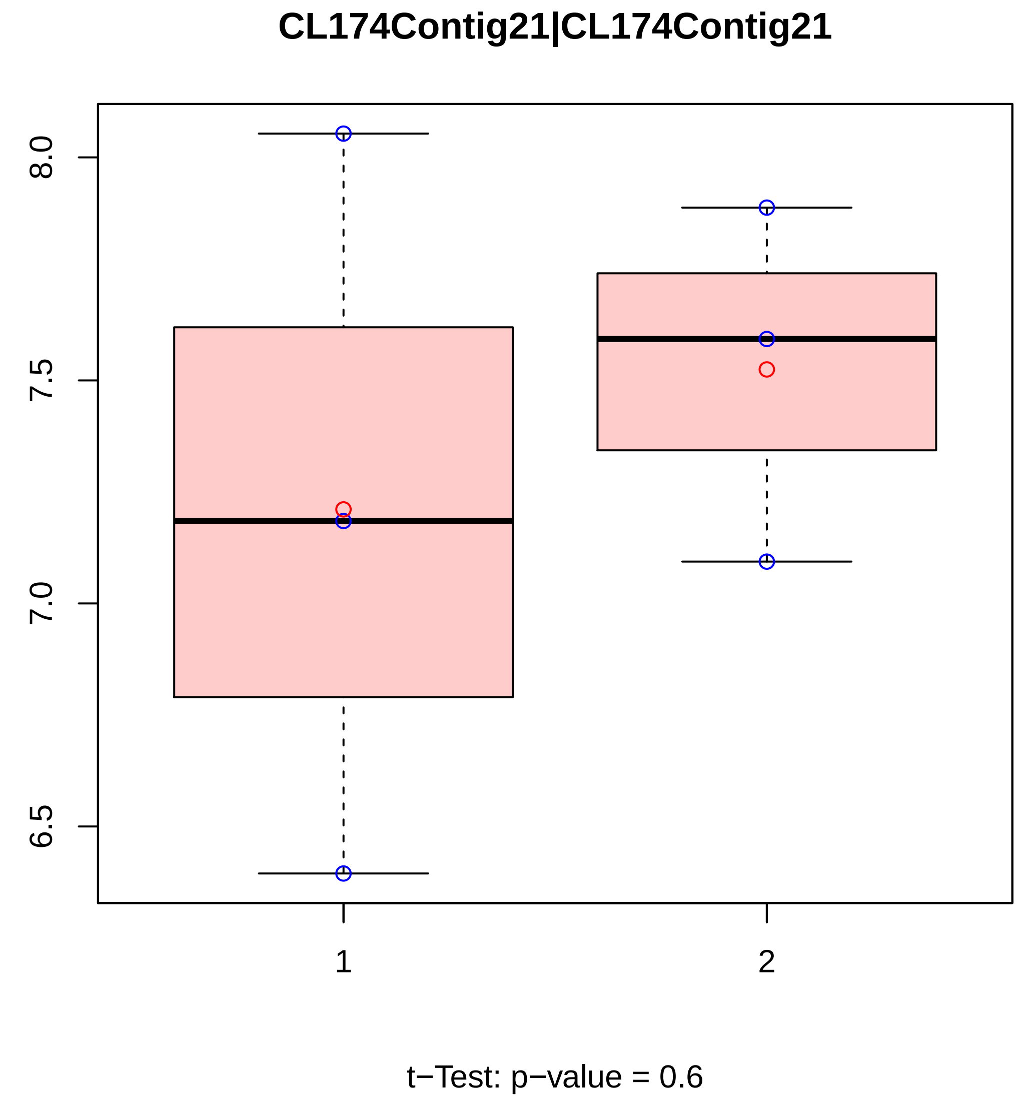

CL174Contig21|CL174Contig21
8.0
7.5
7.0
6.5
1
2
t−Test: p−value = 0.6

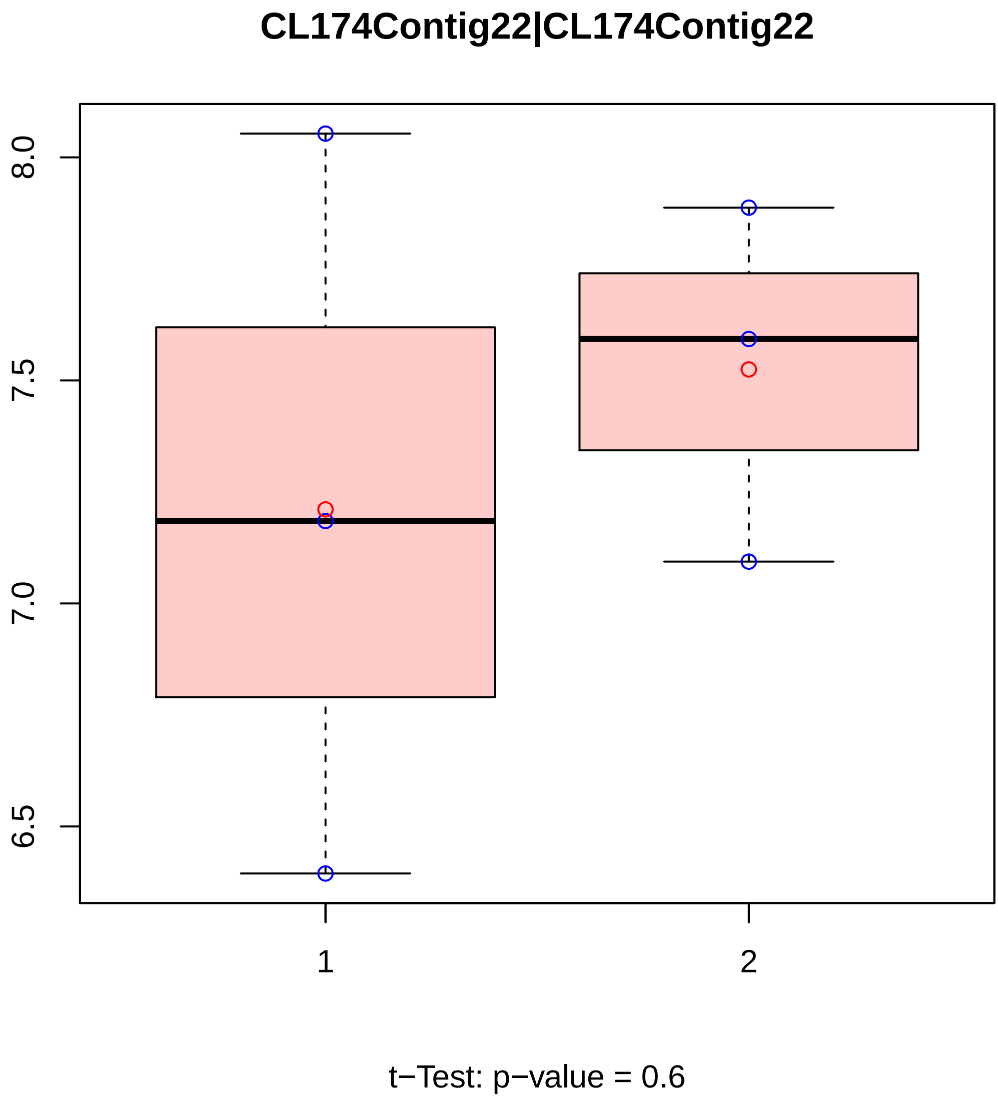
CL174Contig22|CL174Contig22
8.0
7.5
7.0
6.5
1
2
t−Test: p−value = 0.6

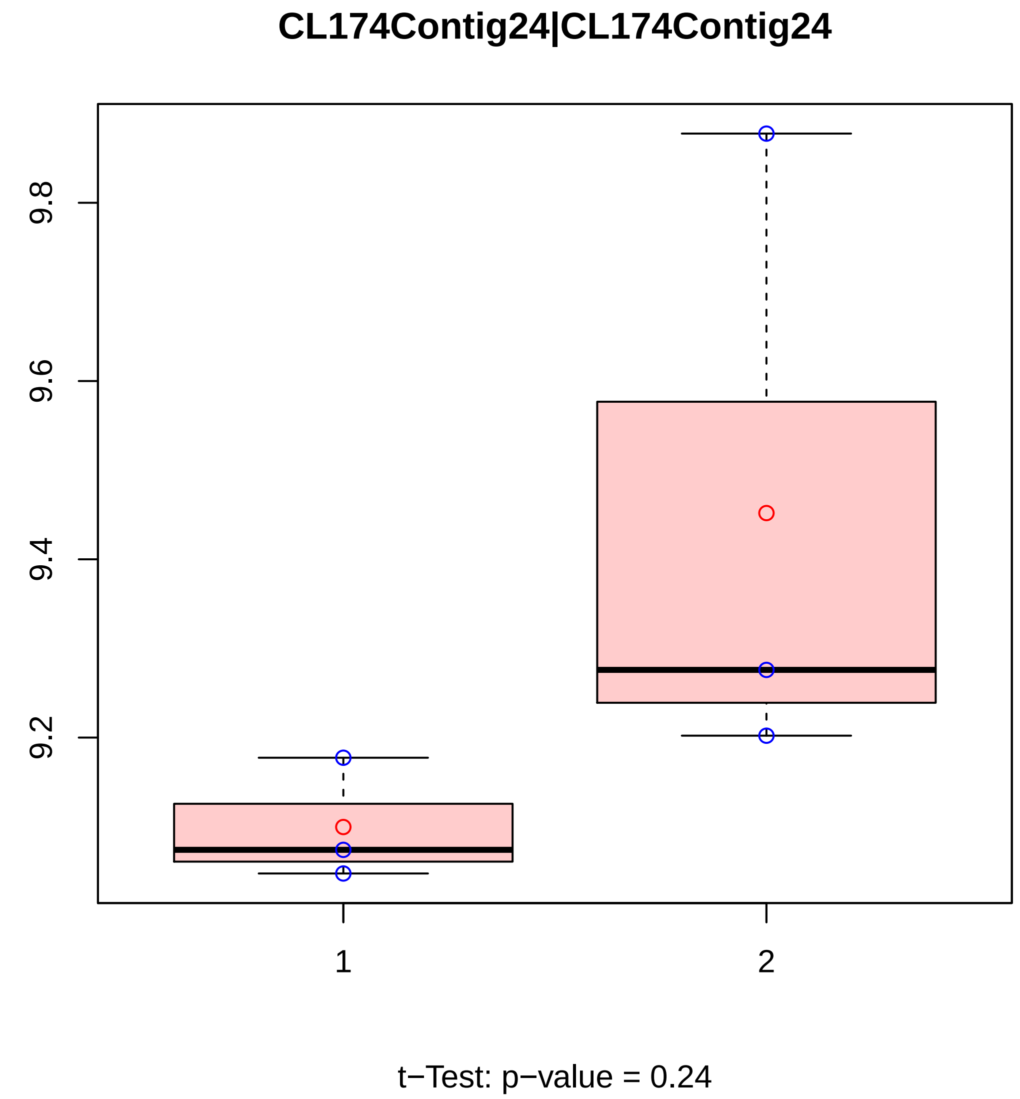
CL174Contig24|CL174Contig24
9.2
9.4
9.6
9.8
1
2
t−Test: p−value = 0.24

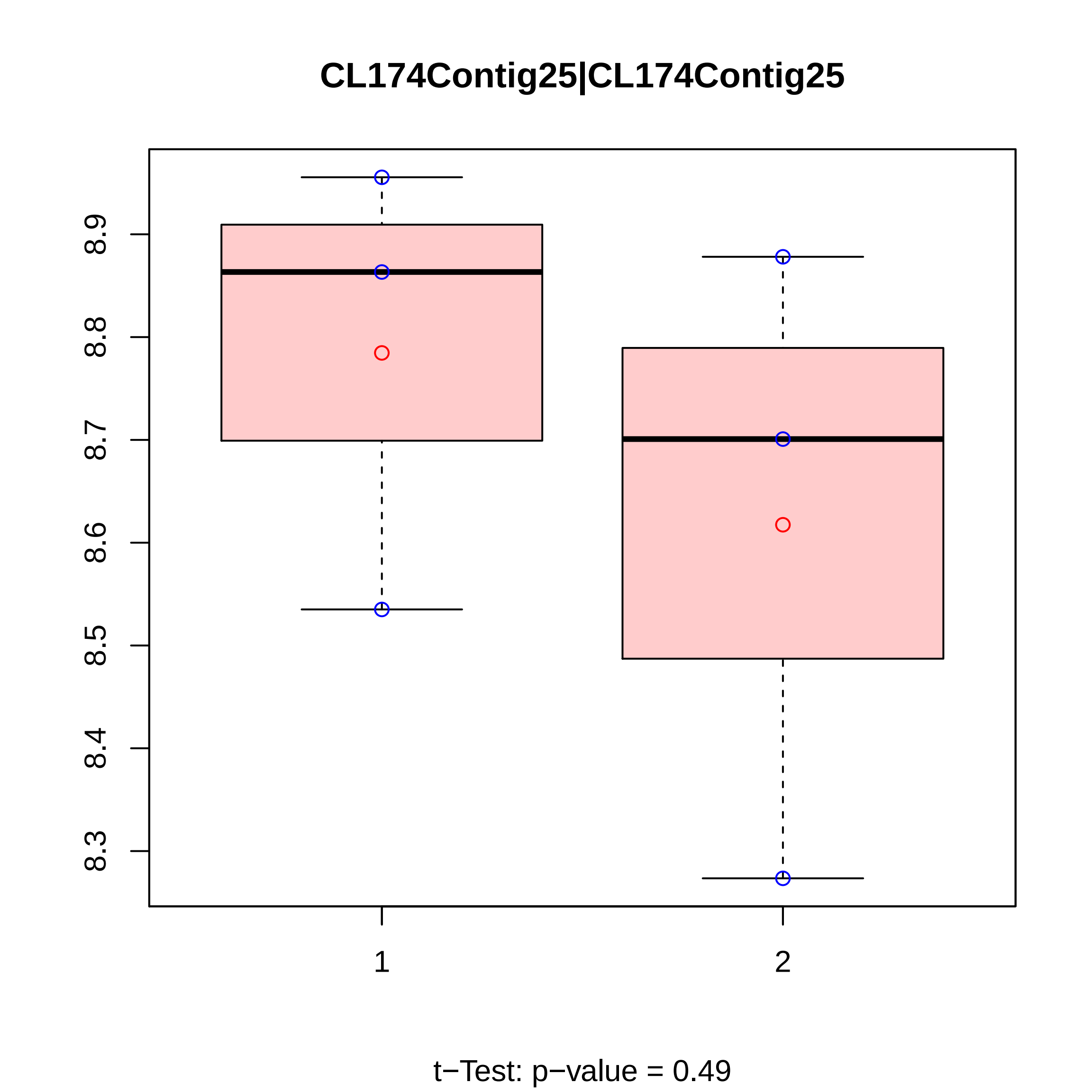
CL174Contig25|CL174Contig25
8.3
8.4
8.5
8.6
8.7
8.8
8.9
1
2
t−Test: p−value = 0.49

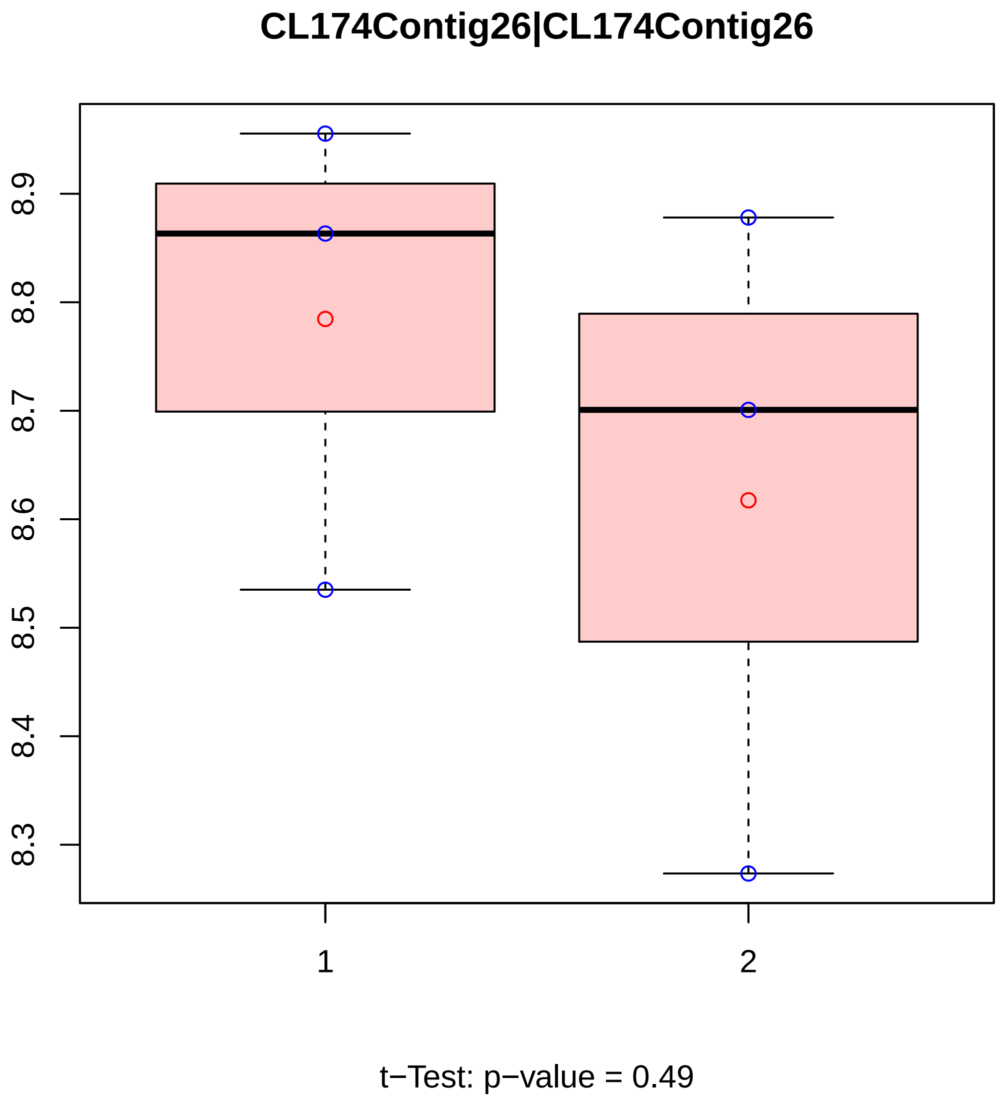
CL174Contig26|CL174Contig26
8.3
8.4
8.5
8.6
8.7
8.8
8.9
1
2
t−Test: p−value = 0.49

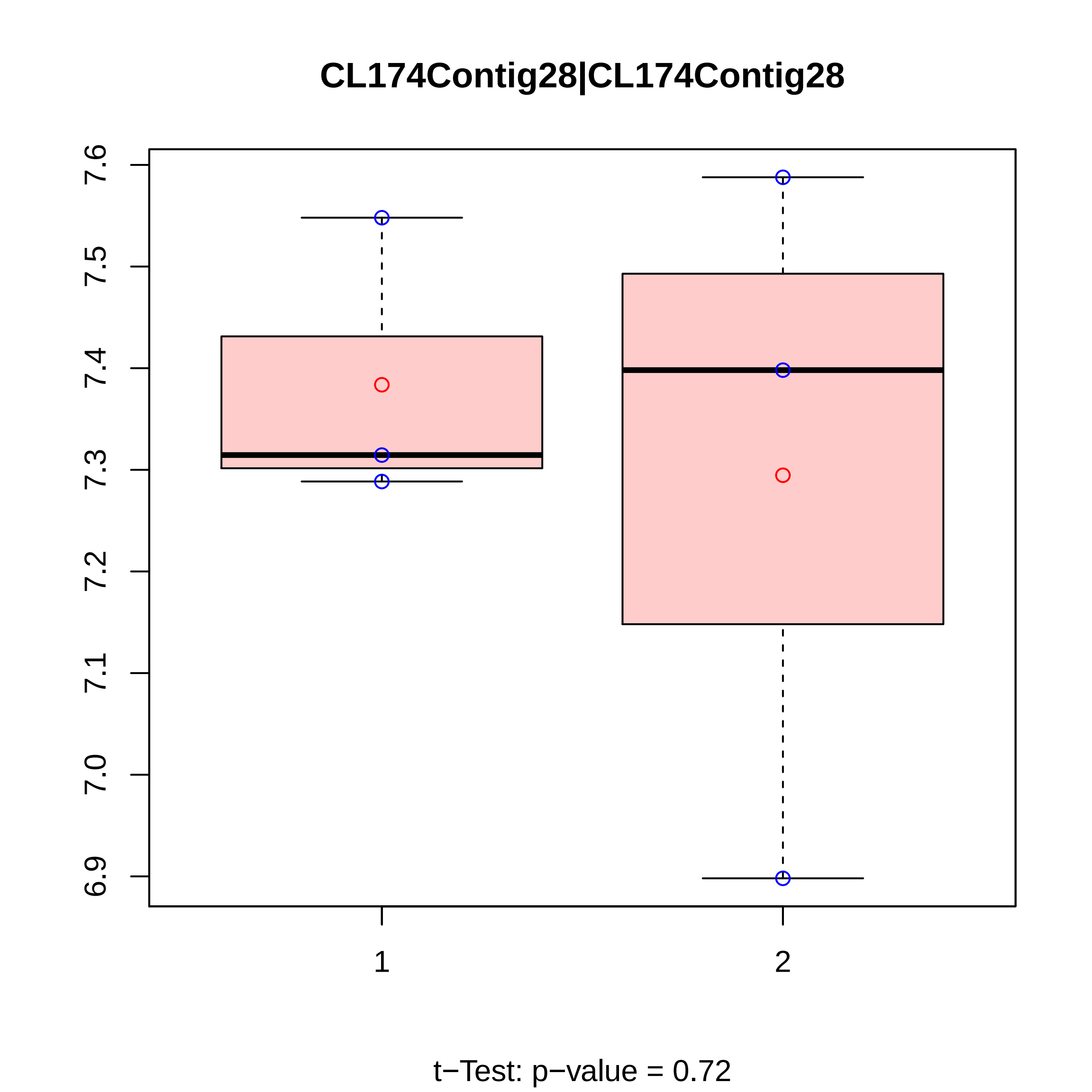

CL174Contig28|CL174Contig28
7.6
7.5
7.4
7.3
7.2
7.1
7.0
6.9
1
2
t−Test: p−value = 0.72

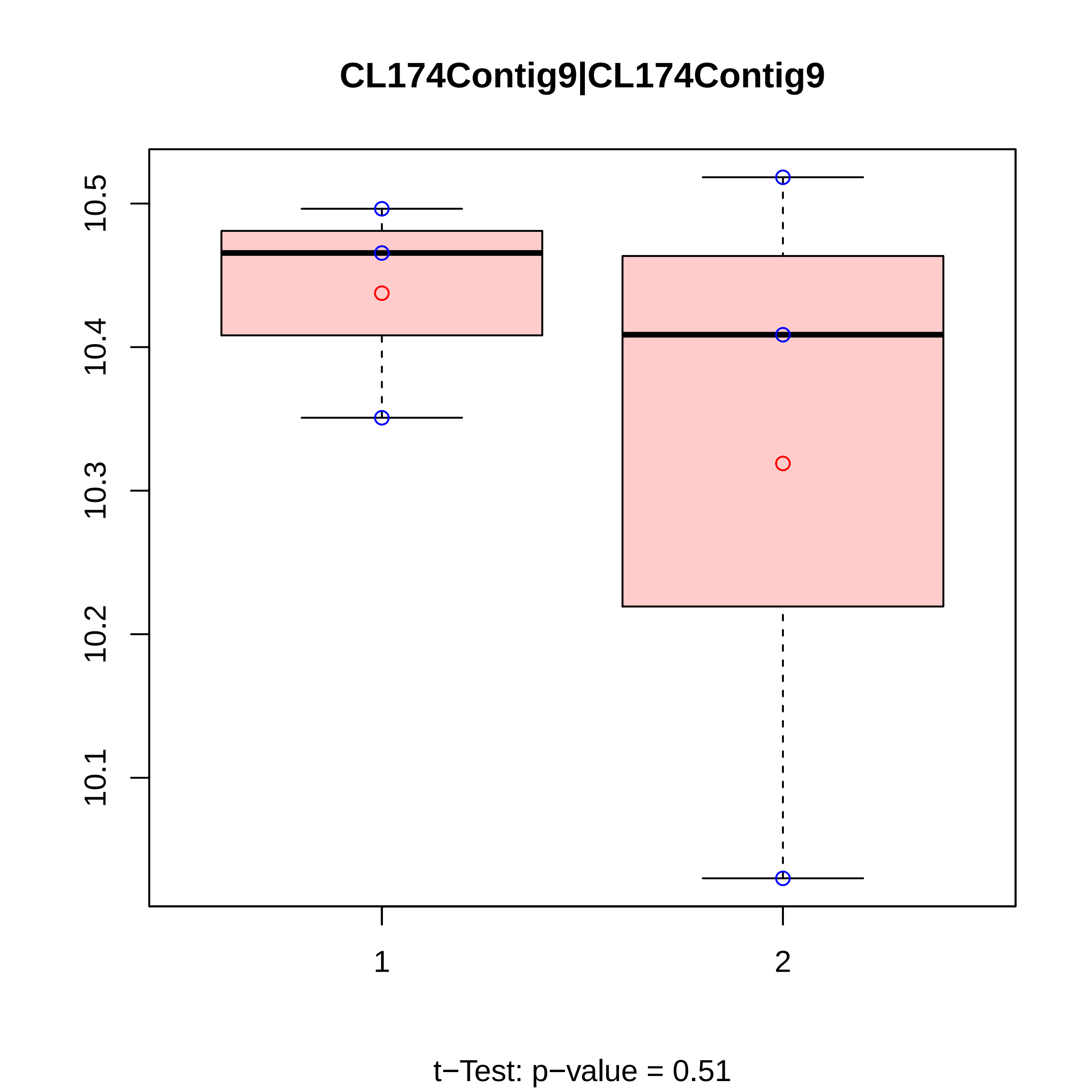

CL174Contig9|CL174Contig9
10.5
10.4
10.3
10.2
10.1
1
2
t−Test: p−value = 0.51

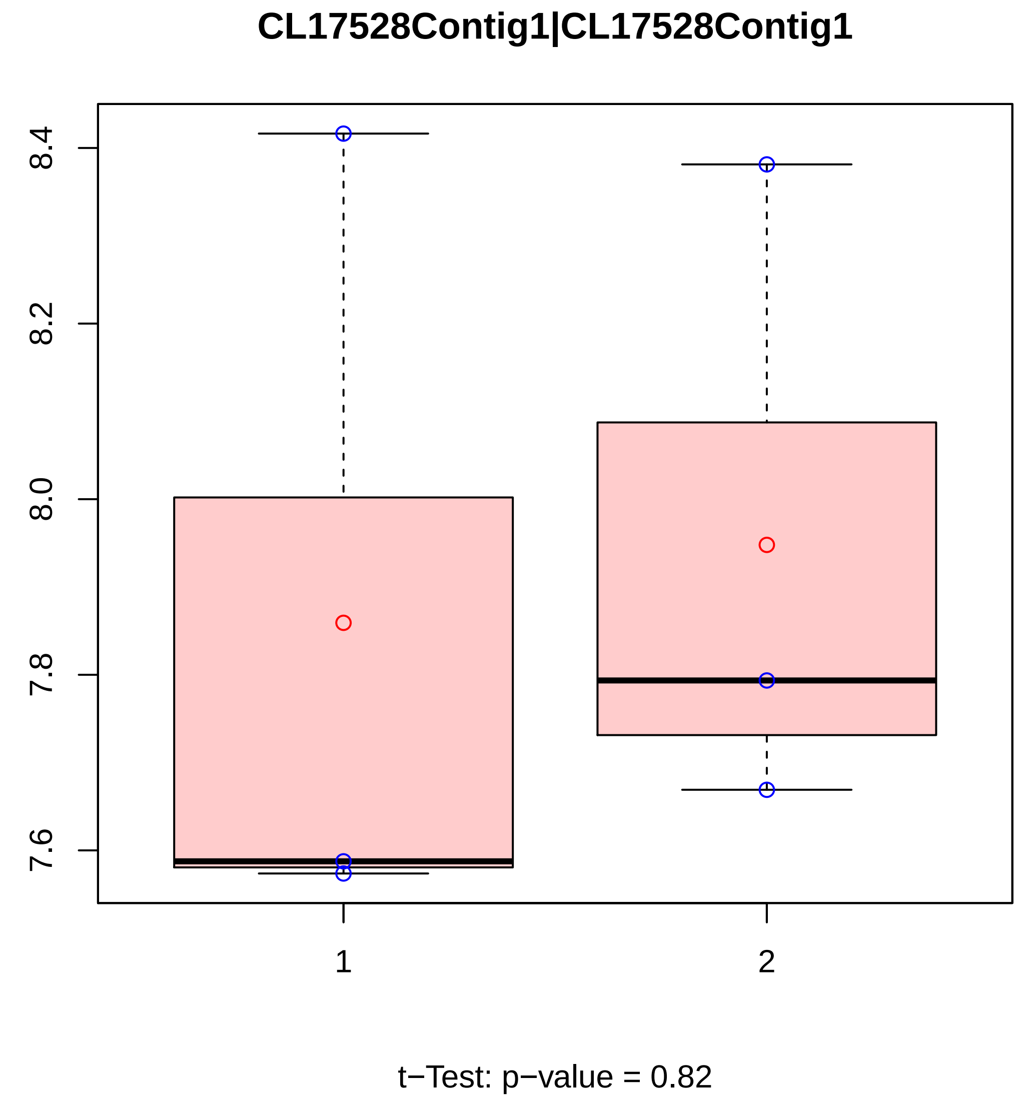

CL17528Contig1|CL17528Contig1
8.4
8.2
8.0
7.8
7.6
1
2
t−Test: p−value = 0.82

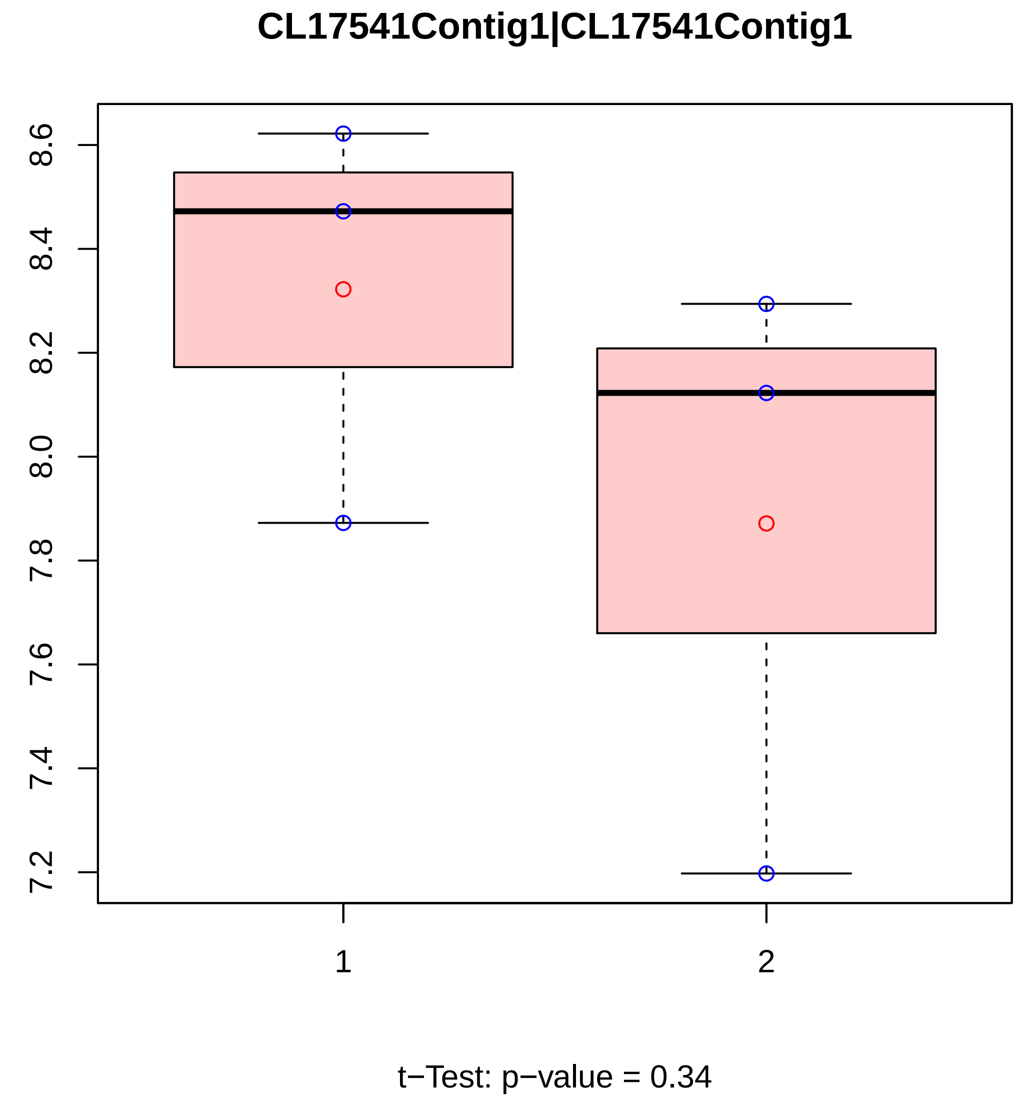
CL17541Contig1|CL17541Contig1
8.6
8.4
8.2
8.0
7.8
7.6
7.4
7.2
1
2
t−Test: p−value = 0.34

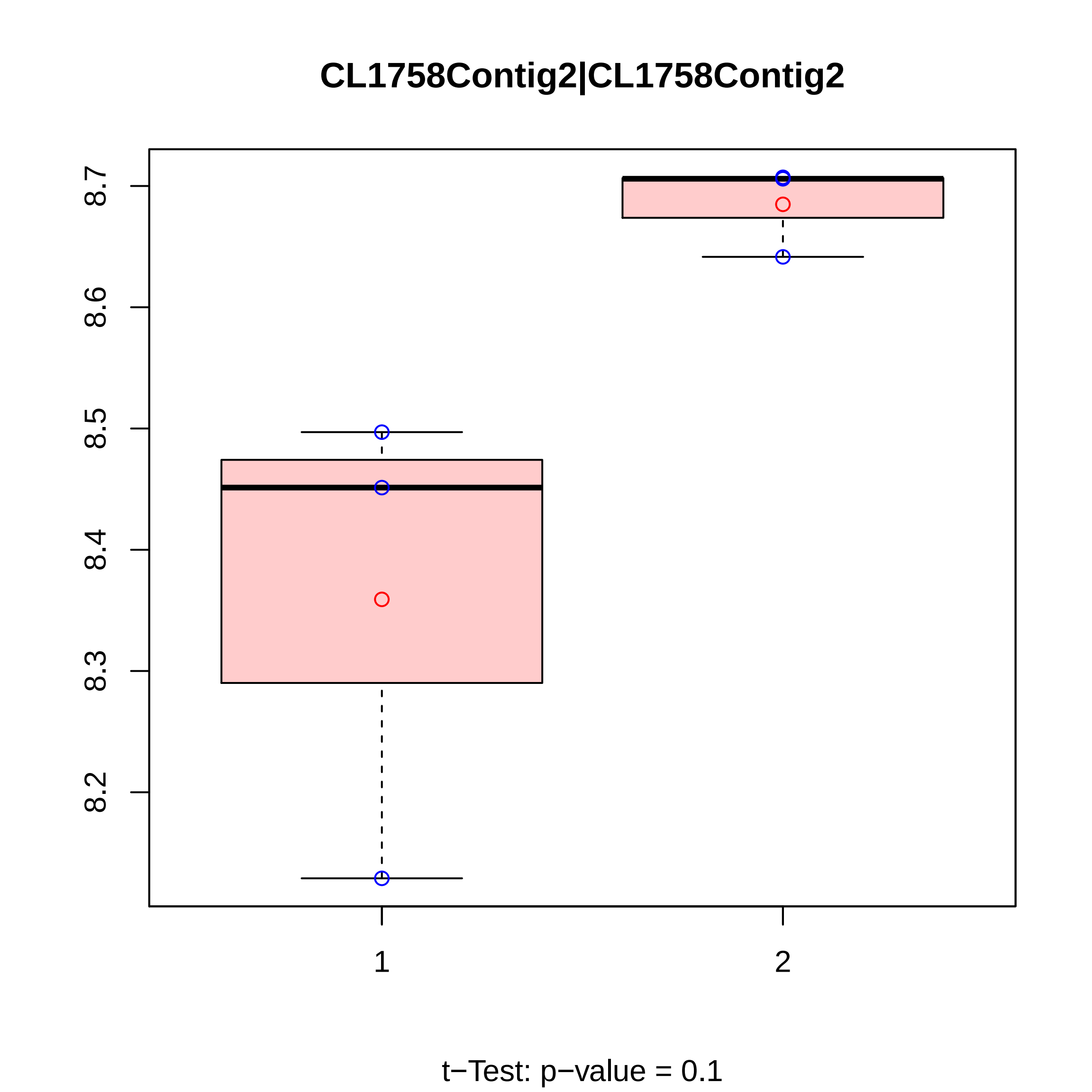
CL1758Contig2|CL1758Contig2
8.2
8.3
8.4
8.5
8.6
8.7
1
2
t−Test: p−value = 0.1

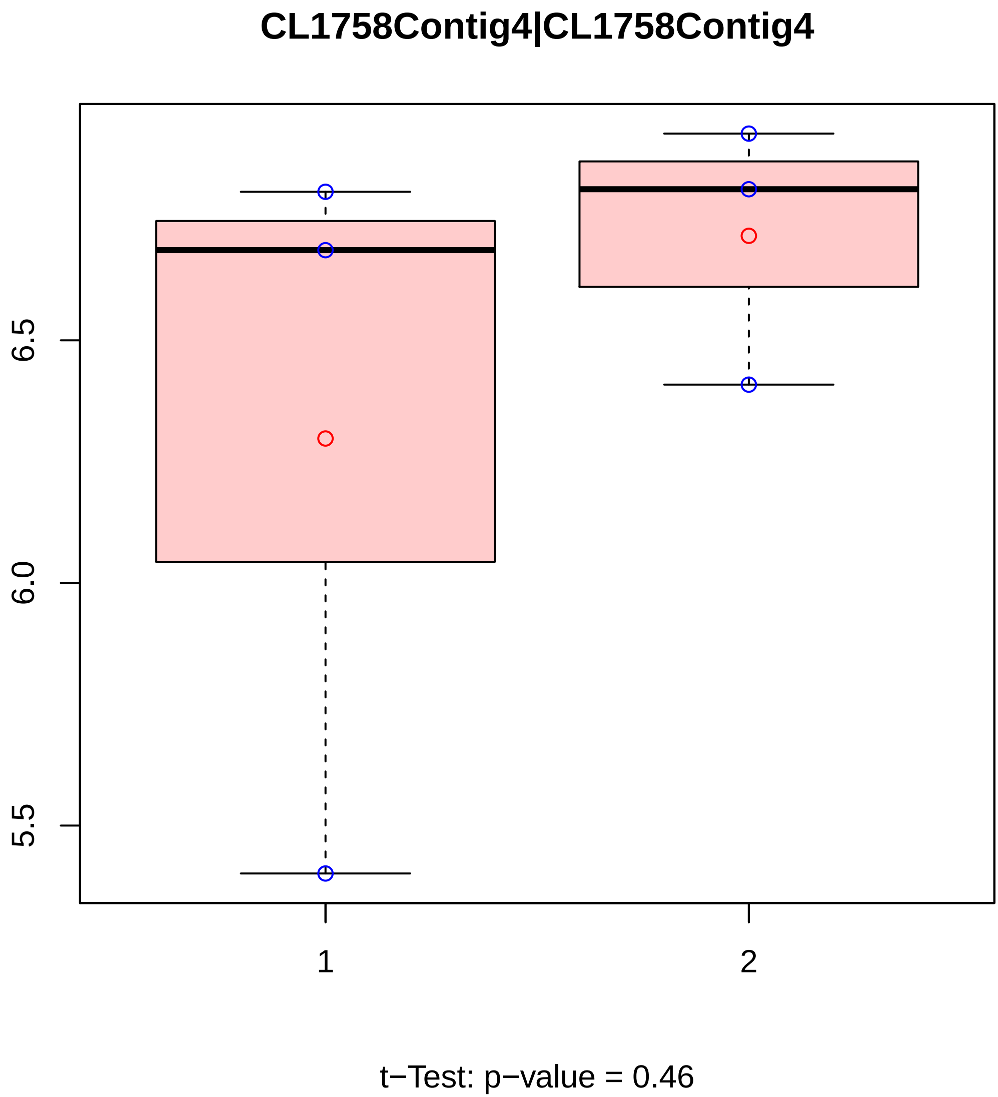
CL1758Contig4|CL1758Contig4
6.5
6.0
5.5
1
2
t−Test: p−value = 0.46

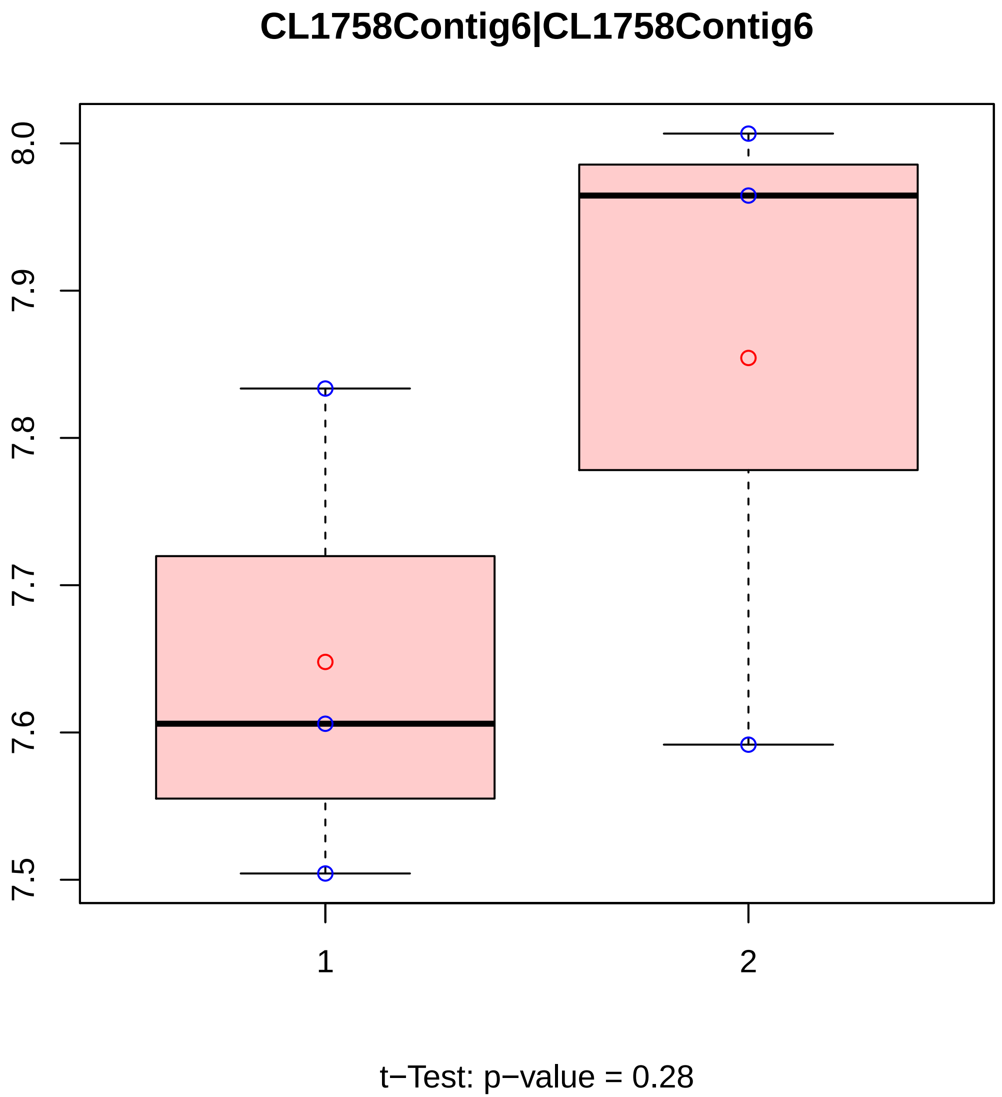
CL1758Contig6|CL1758Contig6
8.0
7.9
7.8
7.7
7.6
7.5
1
2
t−Test: p−value = 0.28

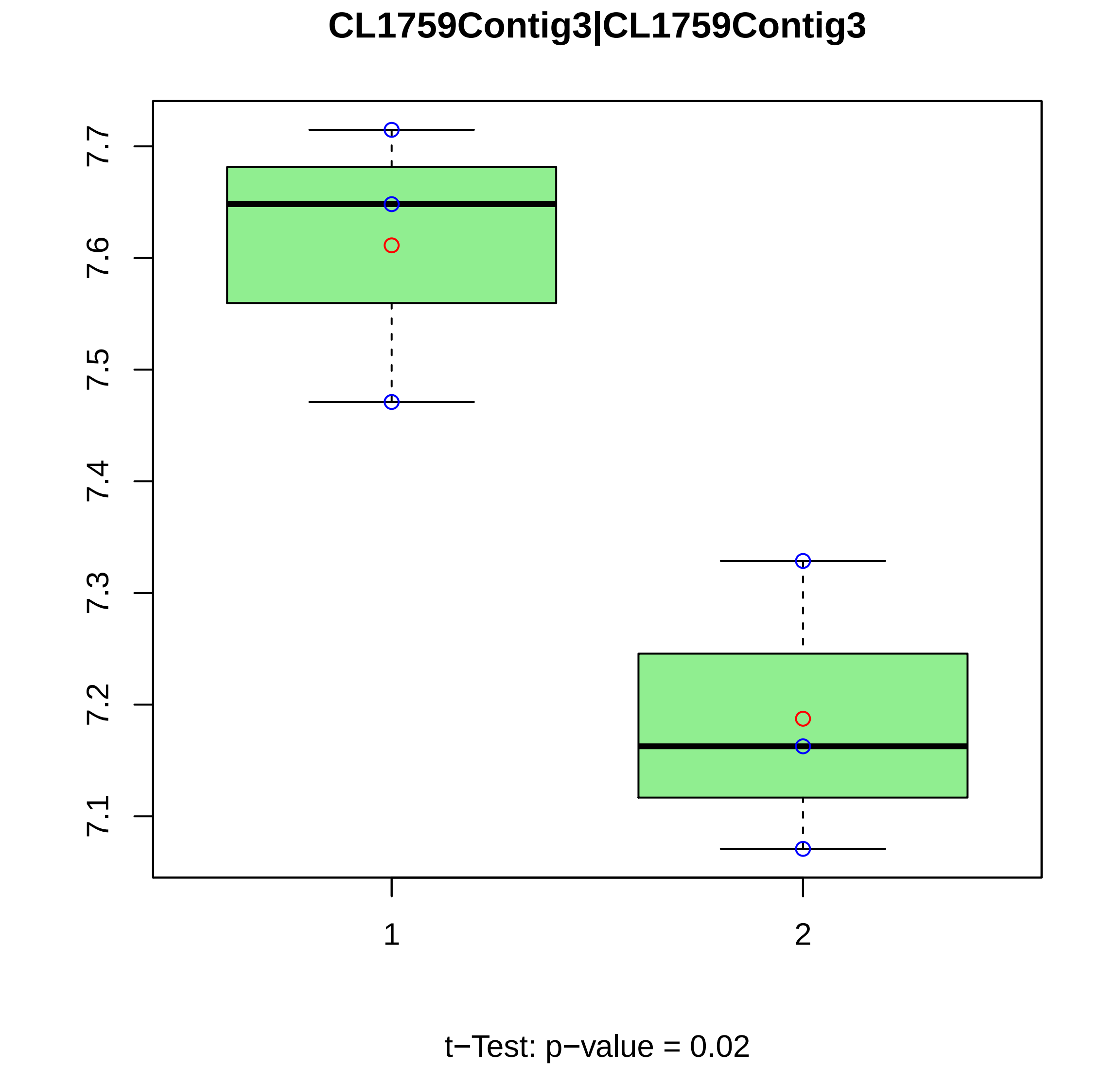
CL1759Contig3|CL1759Contig3
7.7
7.6
7.5
7.4
7.3
7.2
7.1
1
2
t−Test: p−value = 0.02

# CL175Contig13|CL175Contig13

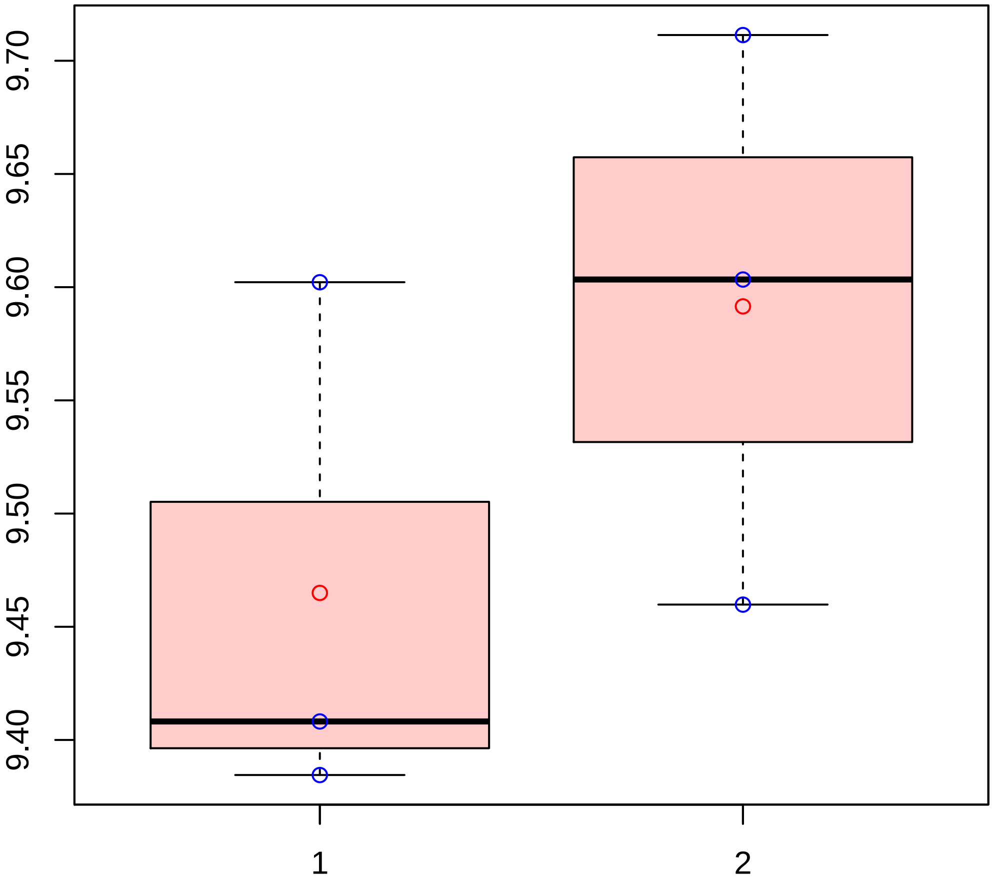


t−Test: p−value = 0.28

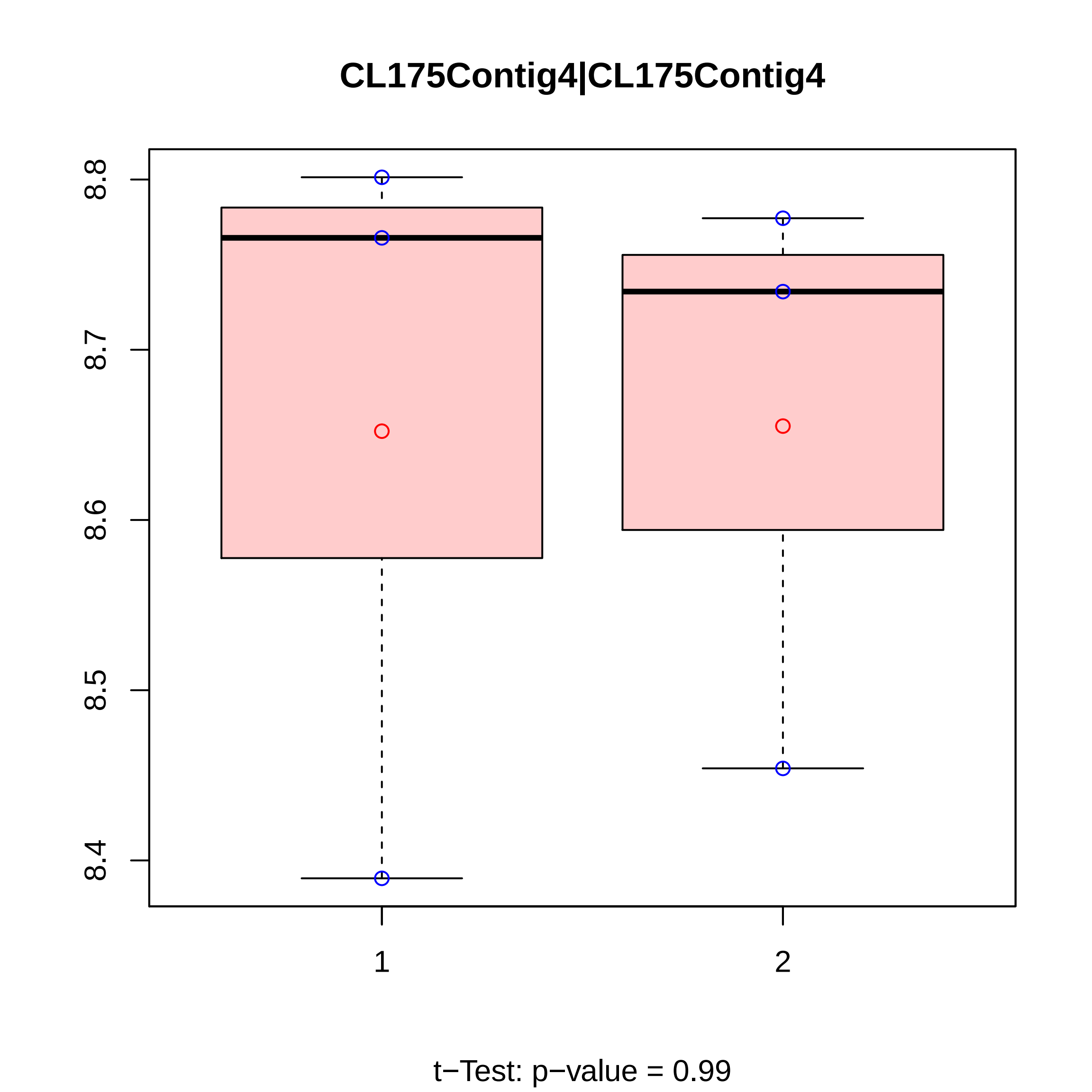

CL175Contig4|CL175Contig4
8.8
8.7
8.6
8.5
8.4
1
2
t−Test: p−value = 0.99

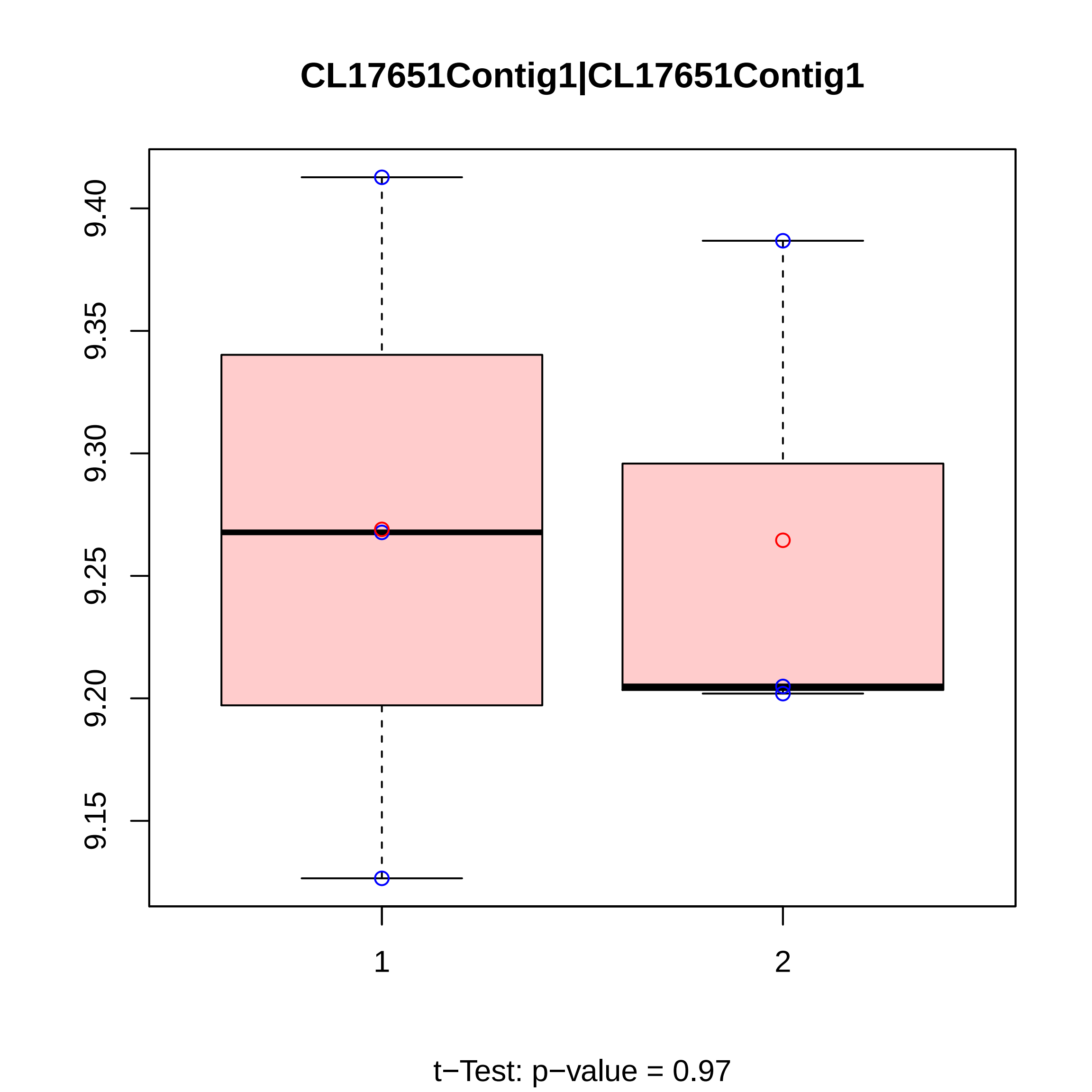

CL17651Contig1|CL17651Contig1
9.40
9.35
9.30
9.25
9.20
9.15
1
2
t−Test: p−value = 0.97

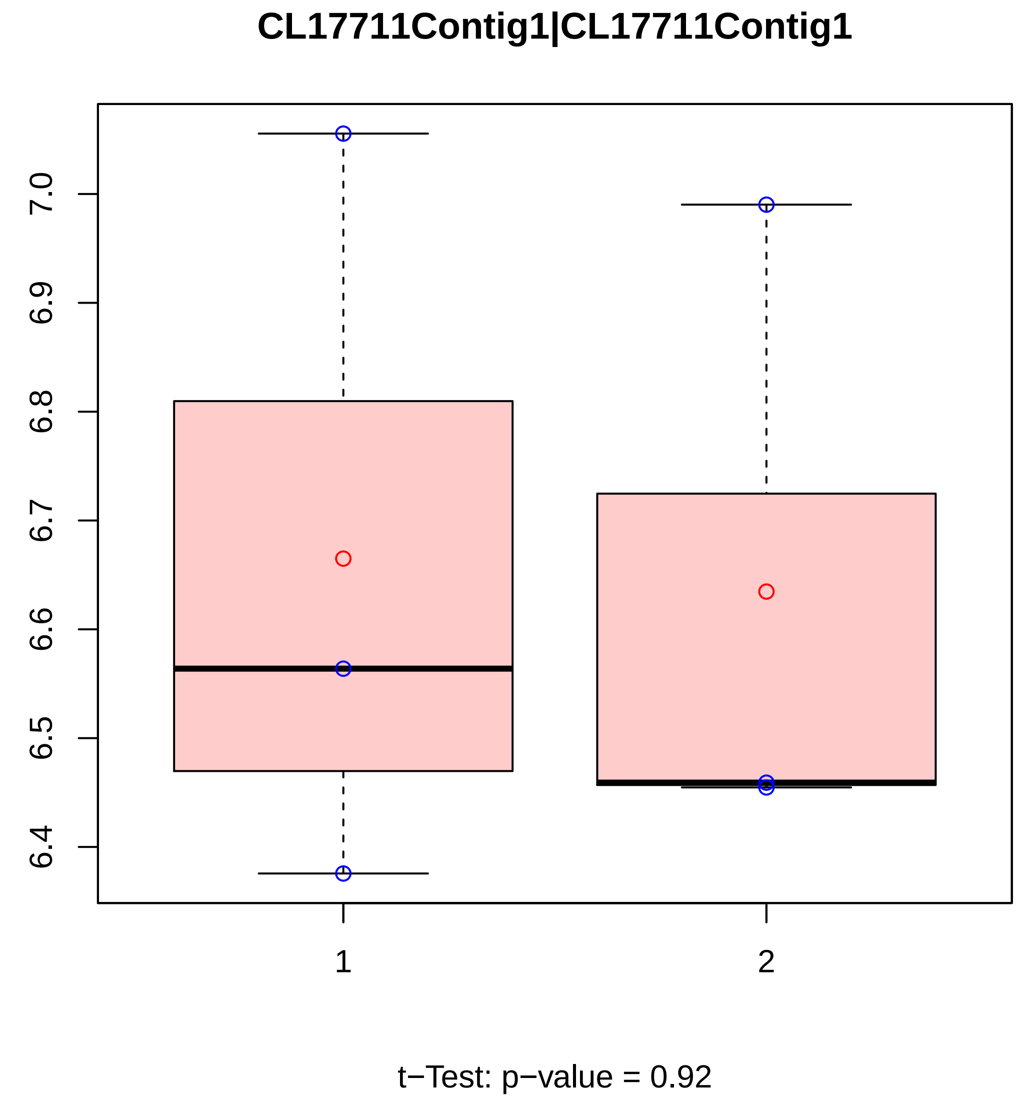
CL17711Contig1|CL17711Contig1
7.0
6.9
6.8
6.7
6.6
6.5
6.4
1
2
t−Test: p−value = 0.92

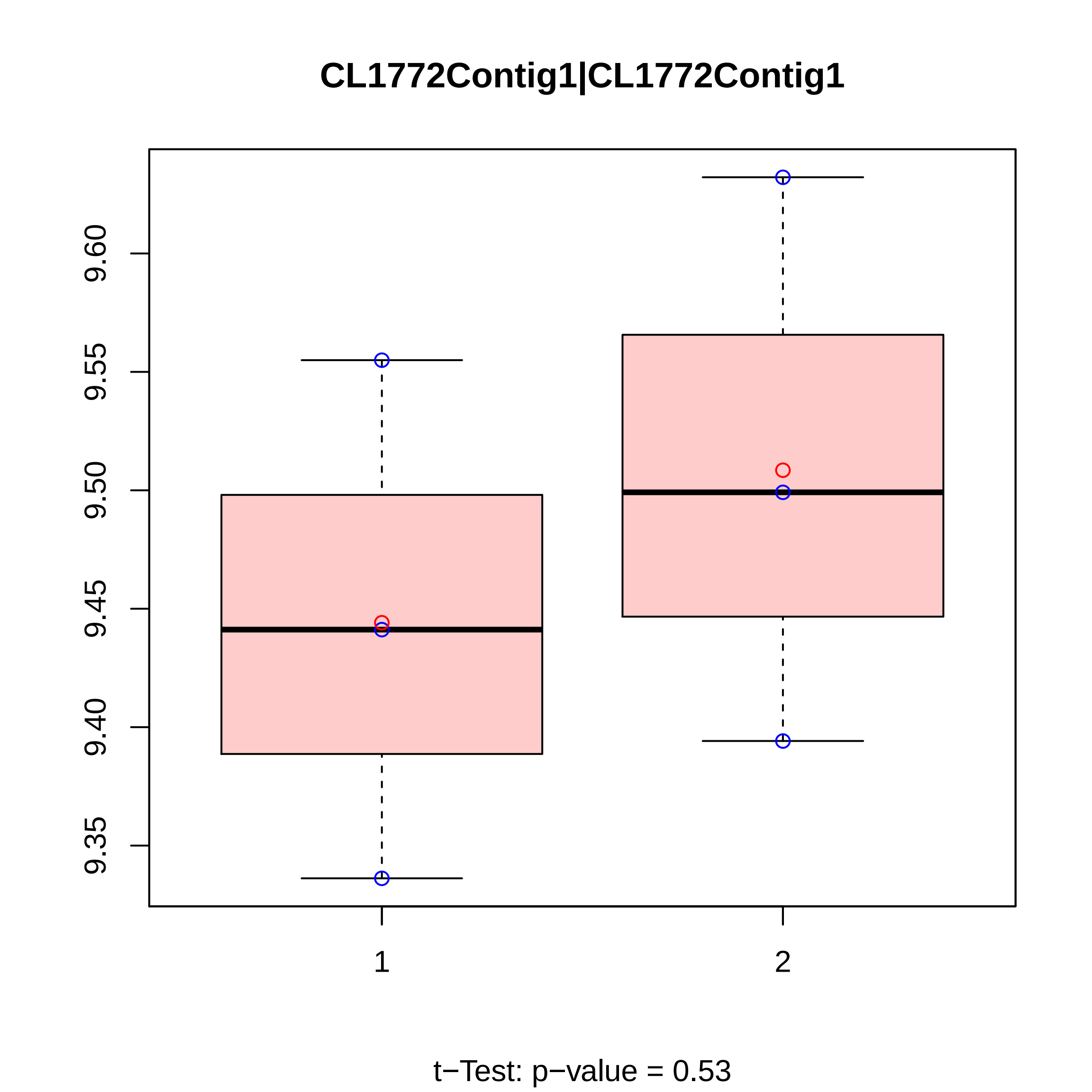
CL1772Contig1|CL1772Contig1
9.35
9.40
9.45
9.50
9.55
9.60
1
2
t−Test: p−value = 0.53

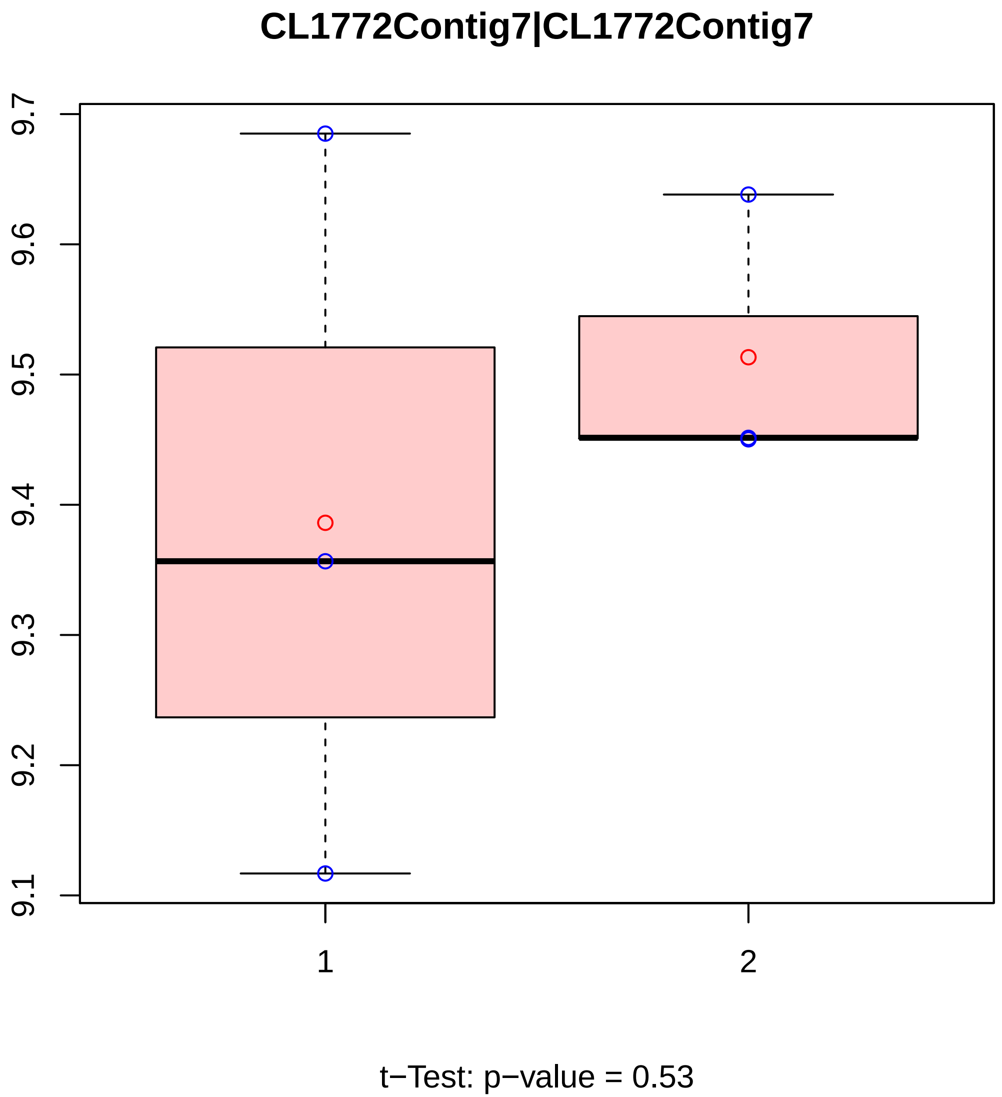

CL1772Contig7|CL1772Contig7
9.1
9.2
9.3
9.4
9.5
9.6
9.7
1
2
t−Test: p−value = 0.53

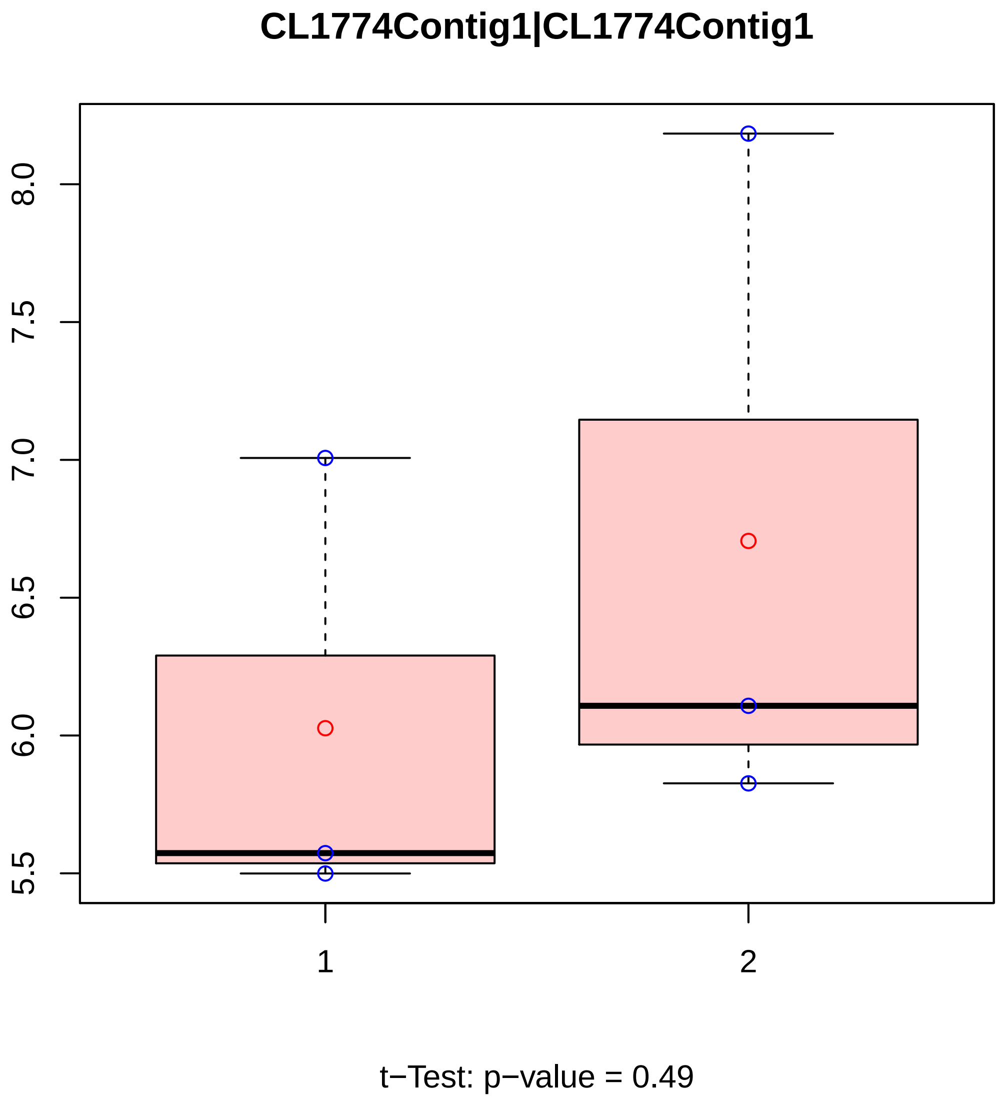

CL1774Contig1|CL1774Contig1
8.0
7.5
7.0
6.5
6.0
5.5
1
2
t−Test: p−value = 0.49

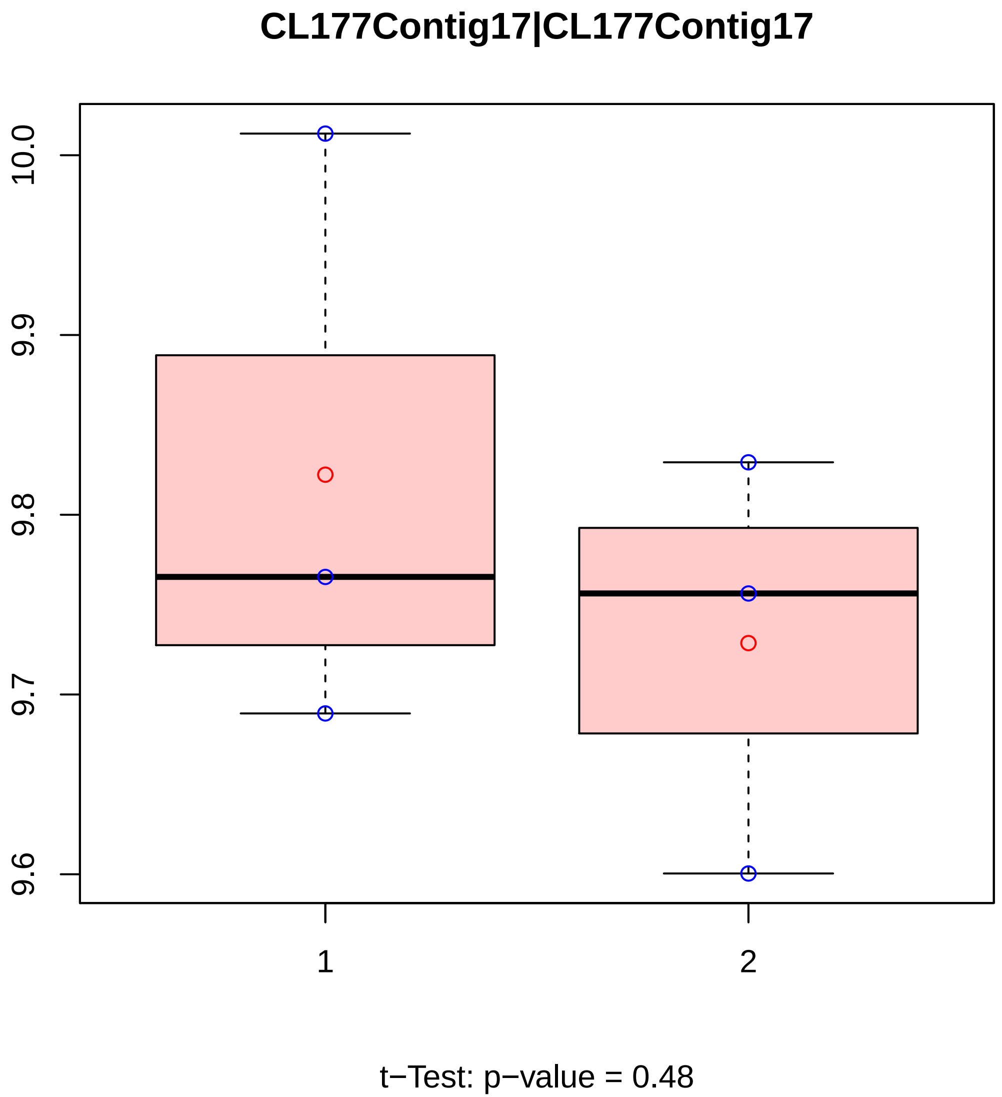
CL177Contig17|CL177Contig17
10.0
9.9
9.8
9.7
9.6
1
2
t−Test: p−value = 0.48

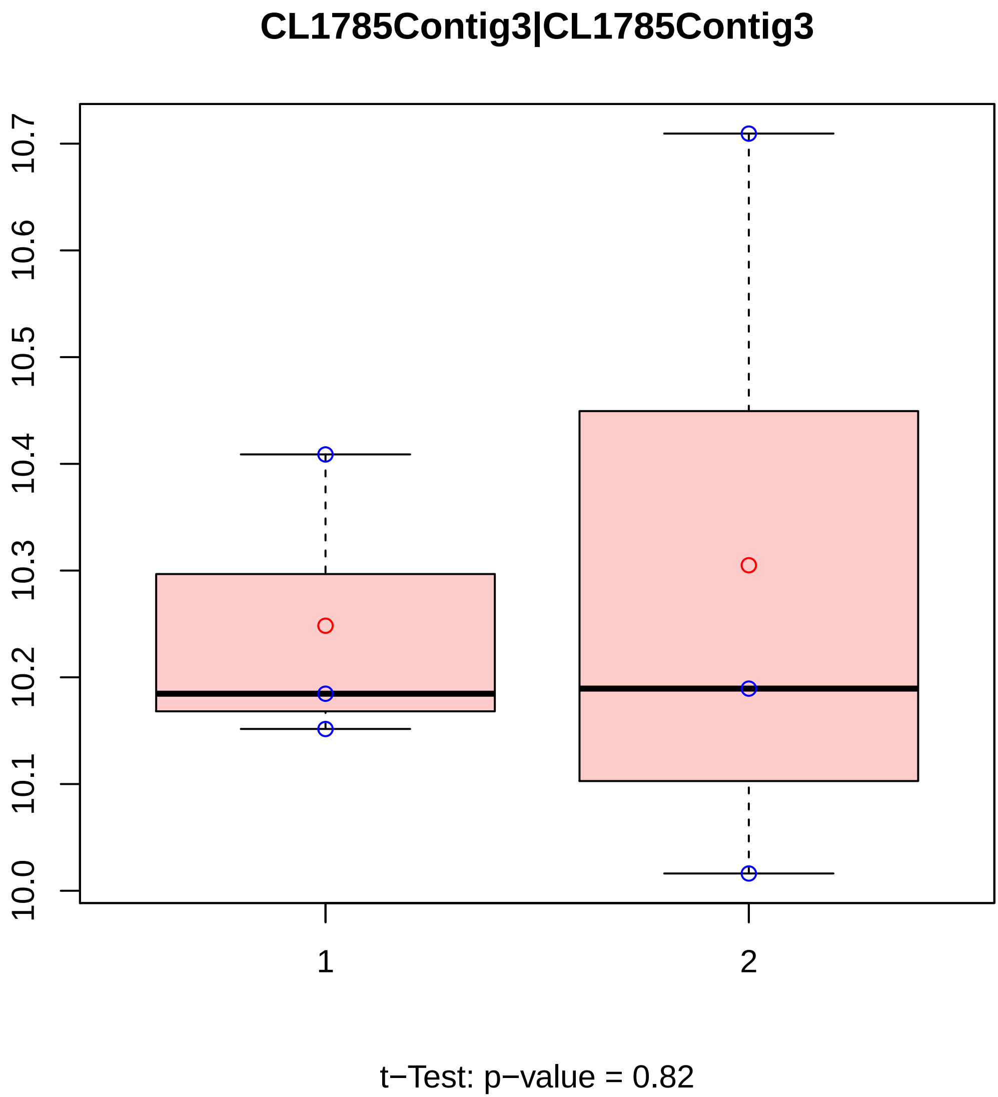
CL1785Contig3|CL1785Contig3
10.0
10.1
10.2
10.3
10.4
10.5
10.6
10.7
1
2
t−Test: p−value = 0.82

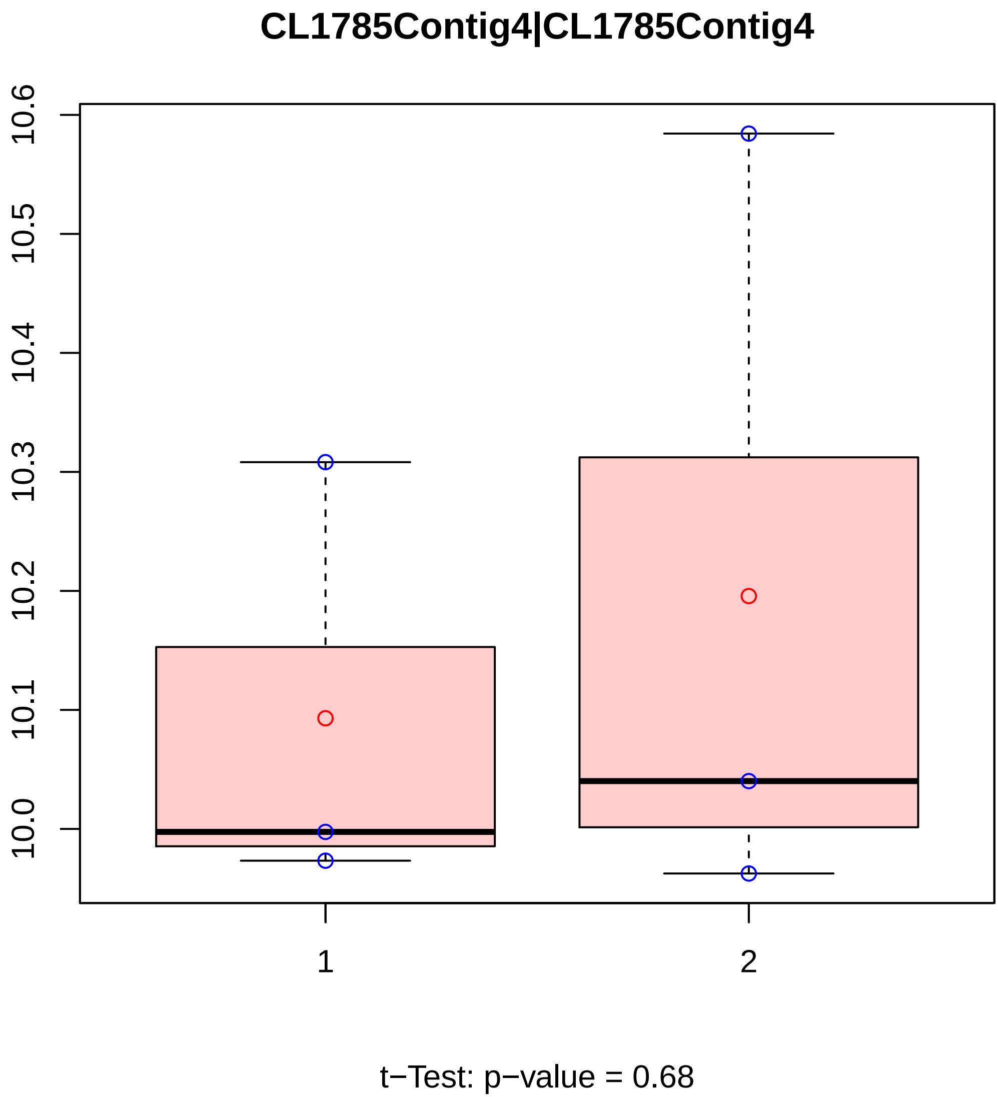
CL1785Contig4|CL1785Contig4
10.0
10.1
10.2
10.3
10.4
10.5
10.6
1
2
t−Test: p−value = 0.68

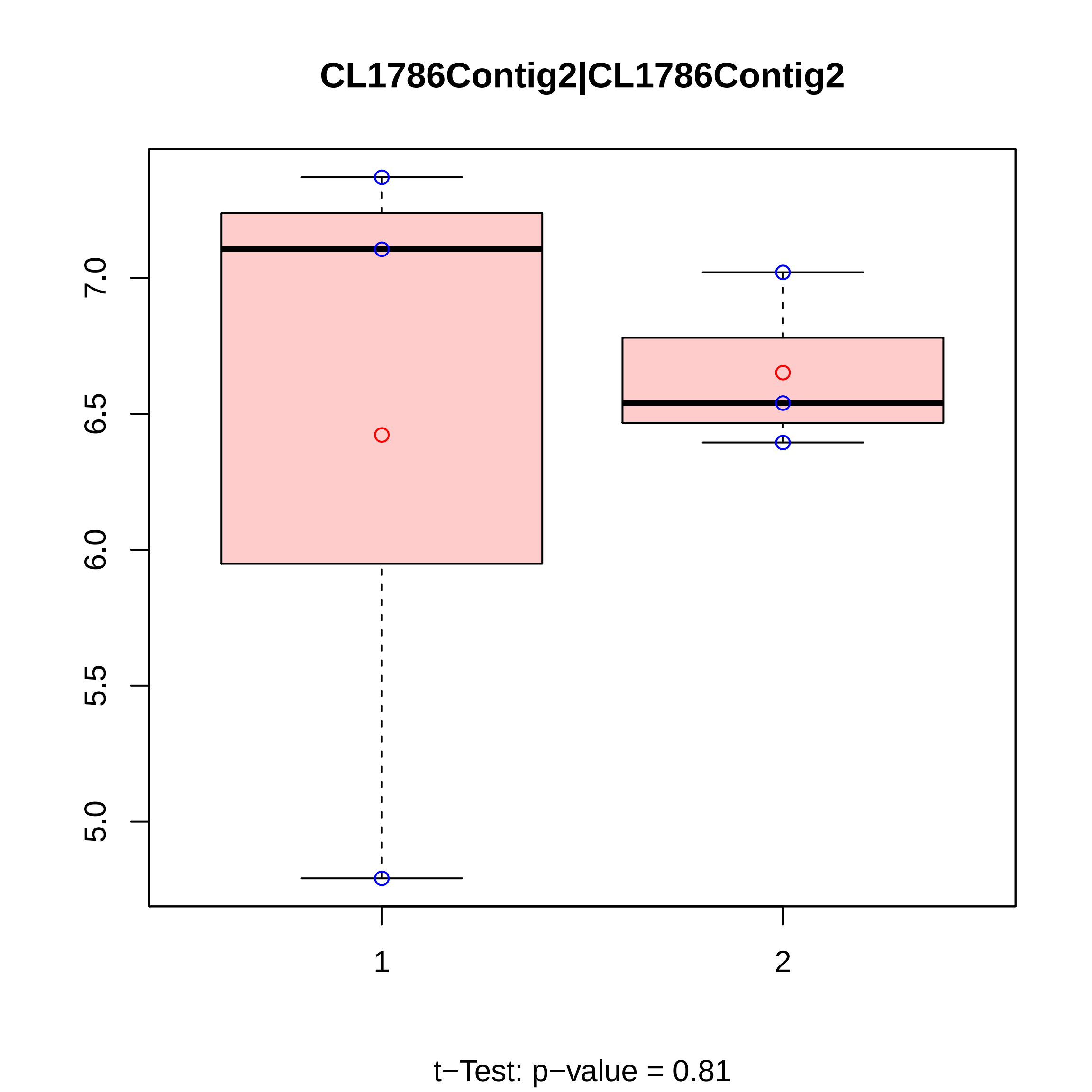

CL1786Contig2|CL1786Contig2
7.0
6.5
6.0
5.5
5.0
1
2
t−Test: p−value = 0.81

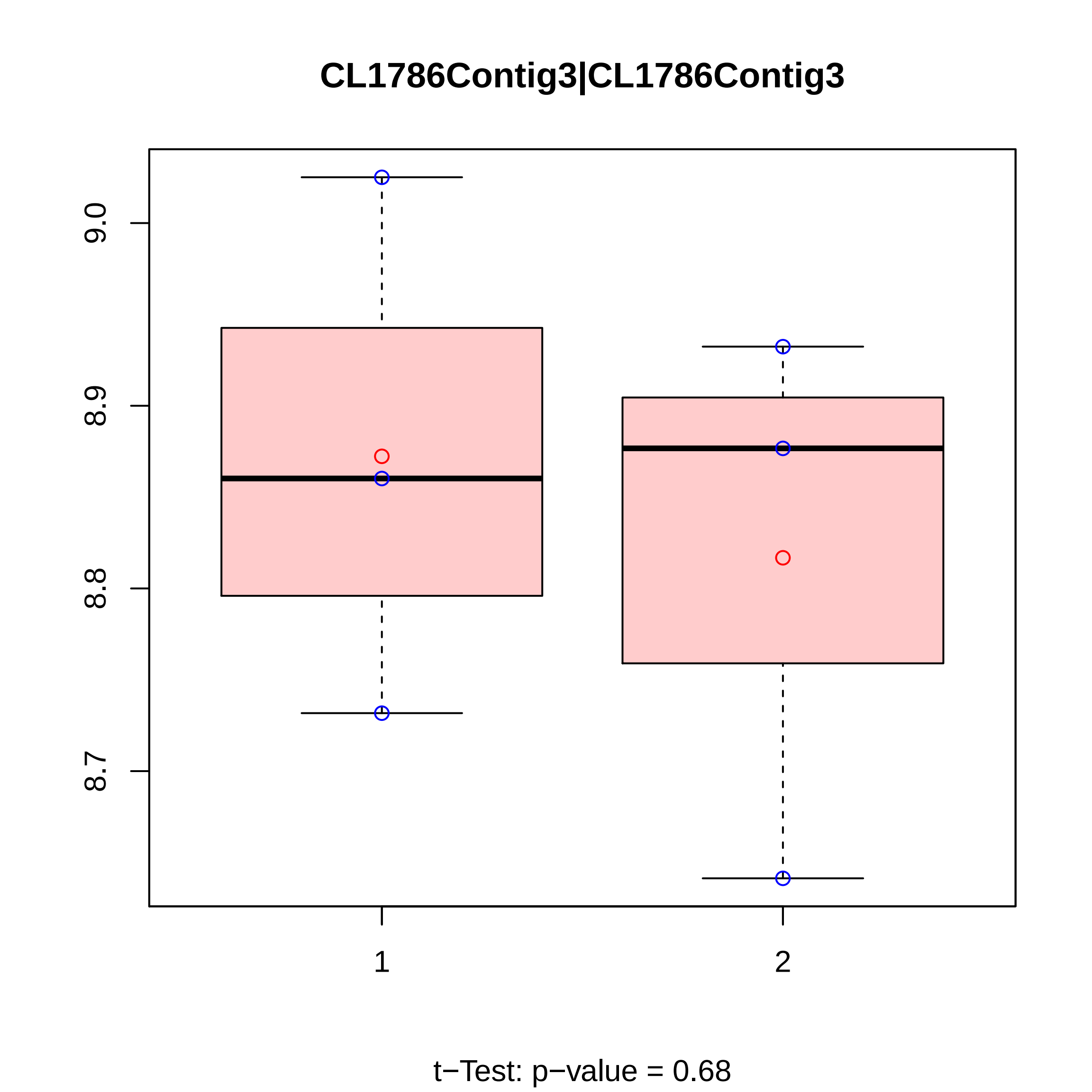
CL1786Contig3|CL1786Contig3
9.0
8.9
8.8
8.7
1
2
t−Test: p−value = 0.68

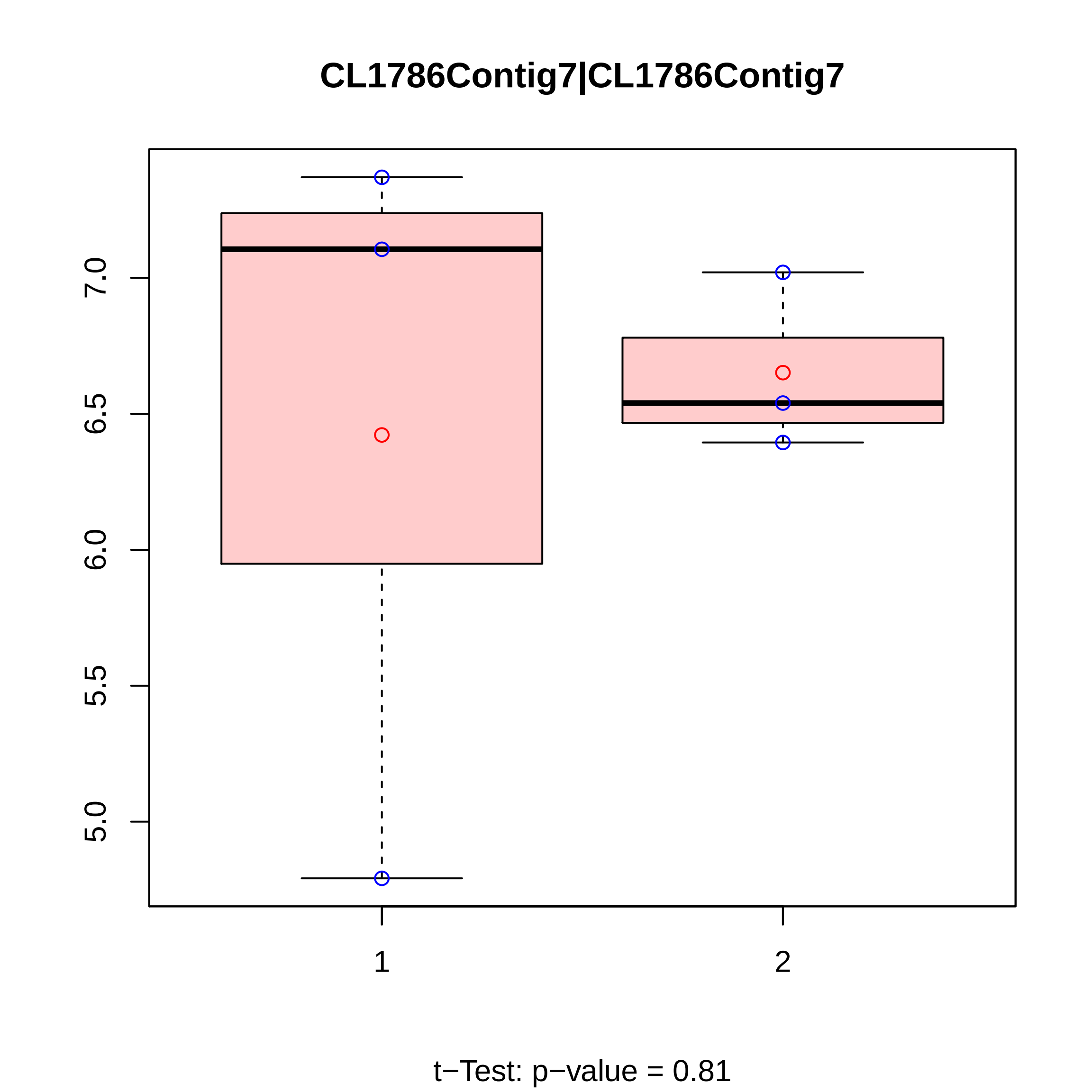
CL1786Contig7|CL1786Contig7
7.0
6.5
6.0
5.5
5.0
1
2
t−Test: p−value = 0.81

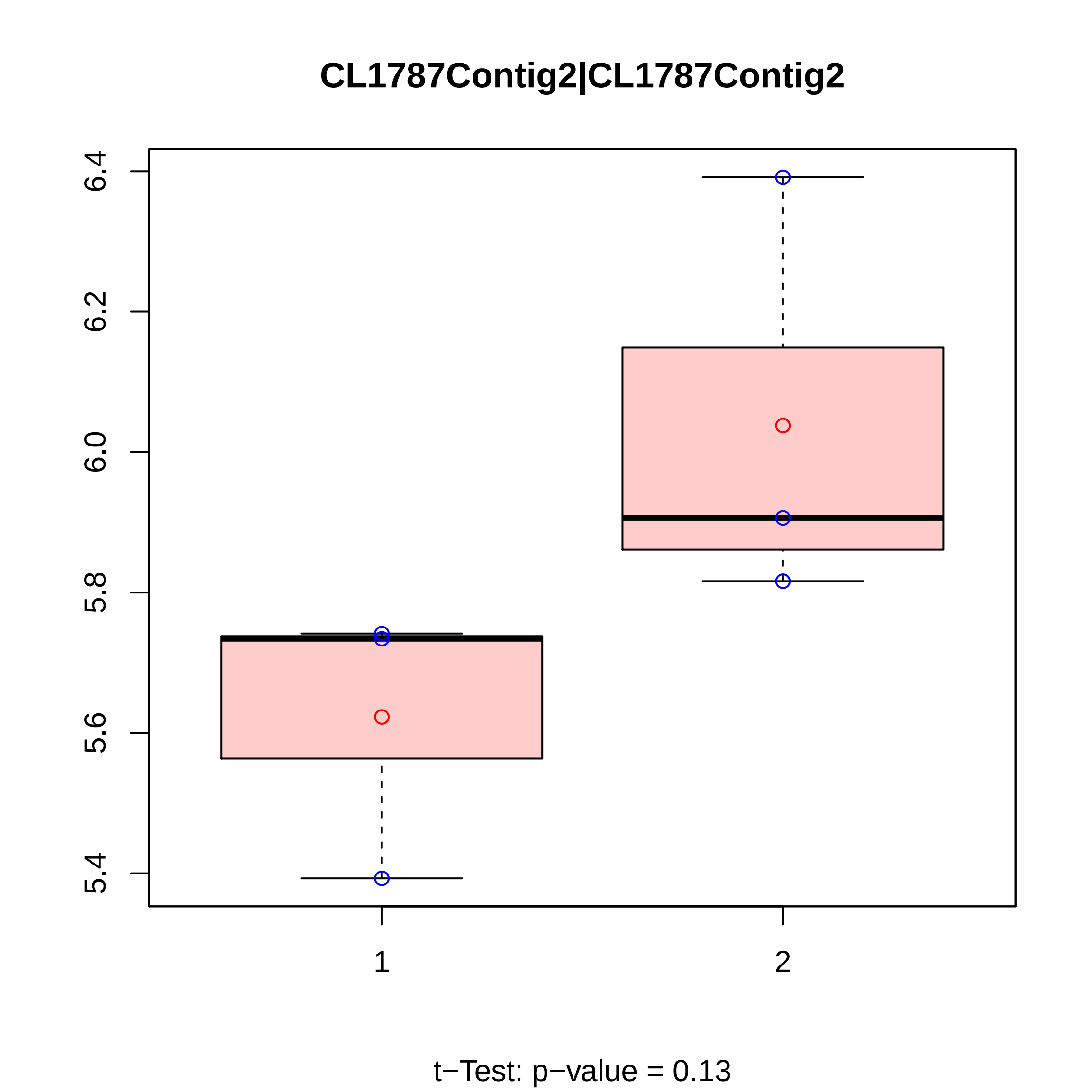

CL1787Contig2|CL1787Contig2
6.4
6.2
6.0
5.8
5.6
5.4
1
2
t−Test: p−value = 0.13

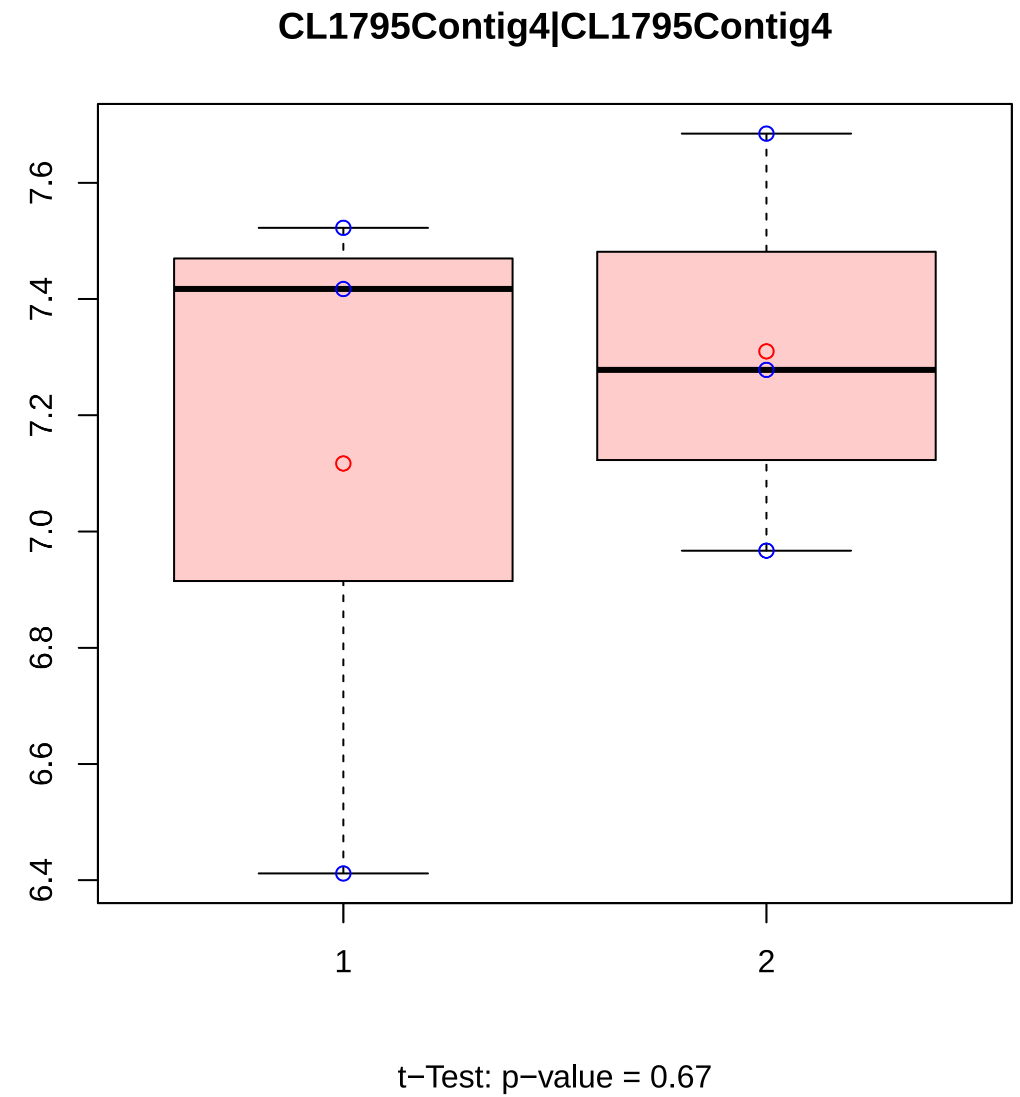
CL1795Contig4|CL1795Contig4
7.6
7.4
7.2
7.0
6.8
6.6
6.4
1
2
t−Test: p−value = 0.67

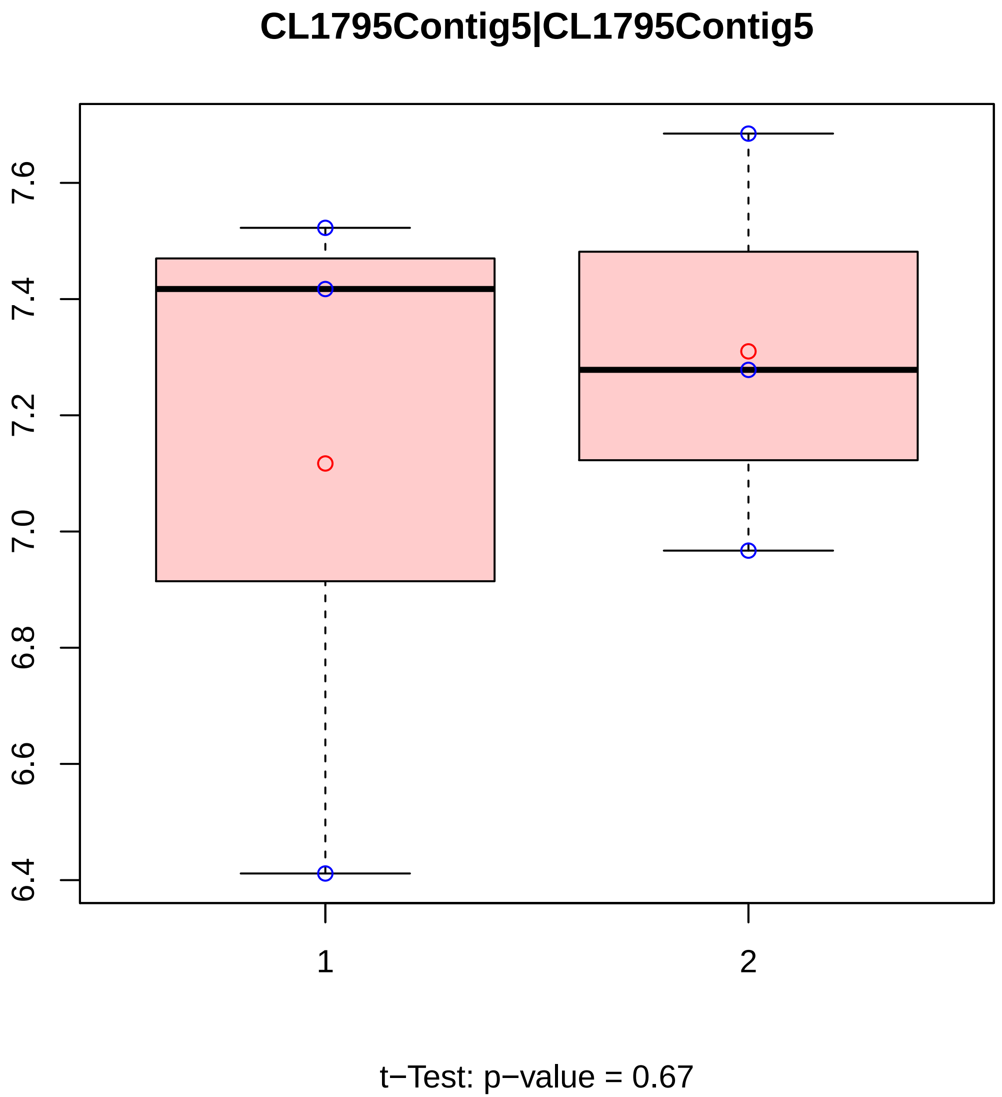
CL1795Contig5|CL1795Contig5
7.6
7.4
7.2
7.0
6.8
6.6
6.4
1
2
t−Test: p−value = 0.67

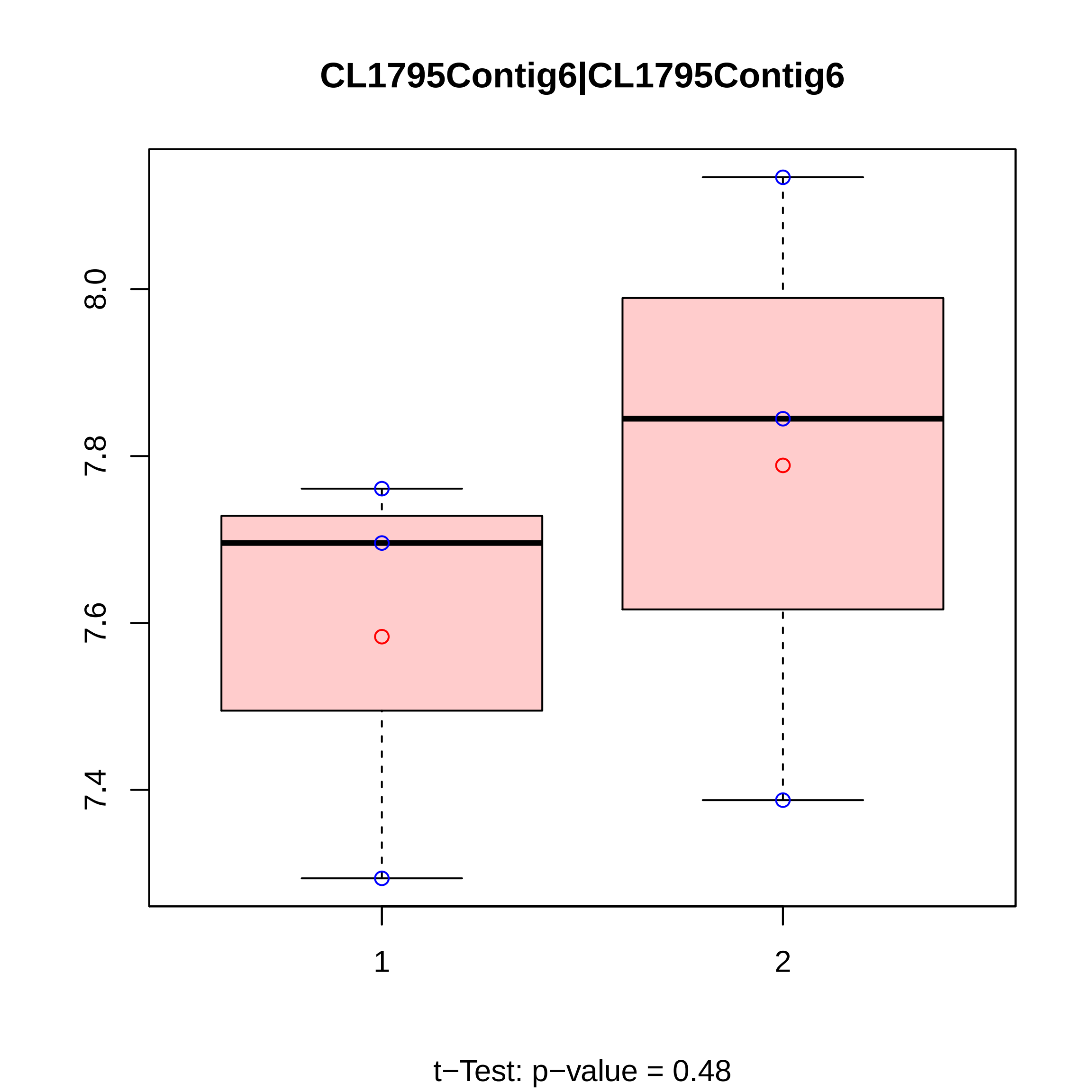

CL1795Contig6|CL1795Contig6
8.0
7.8
7.6
7.4
1
2
t−Test: p−value = 0.48

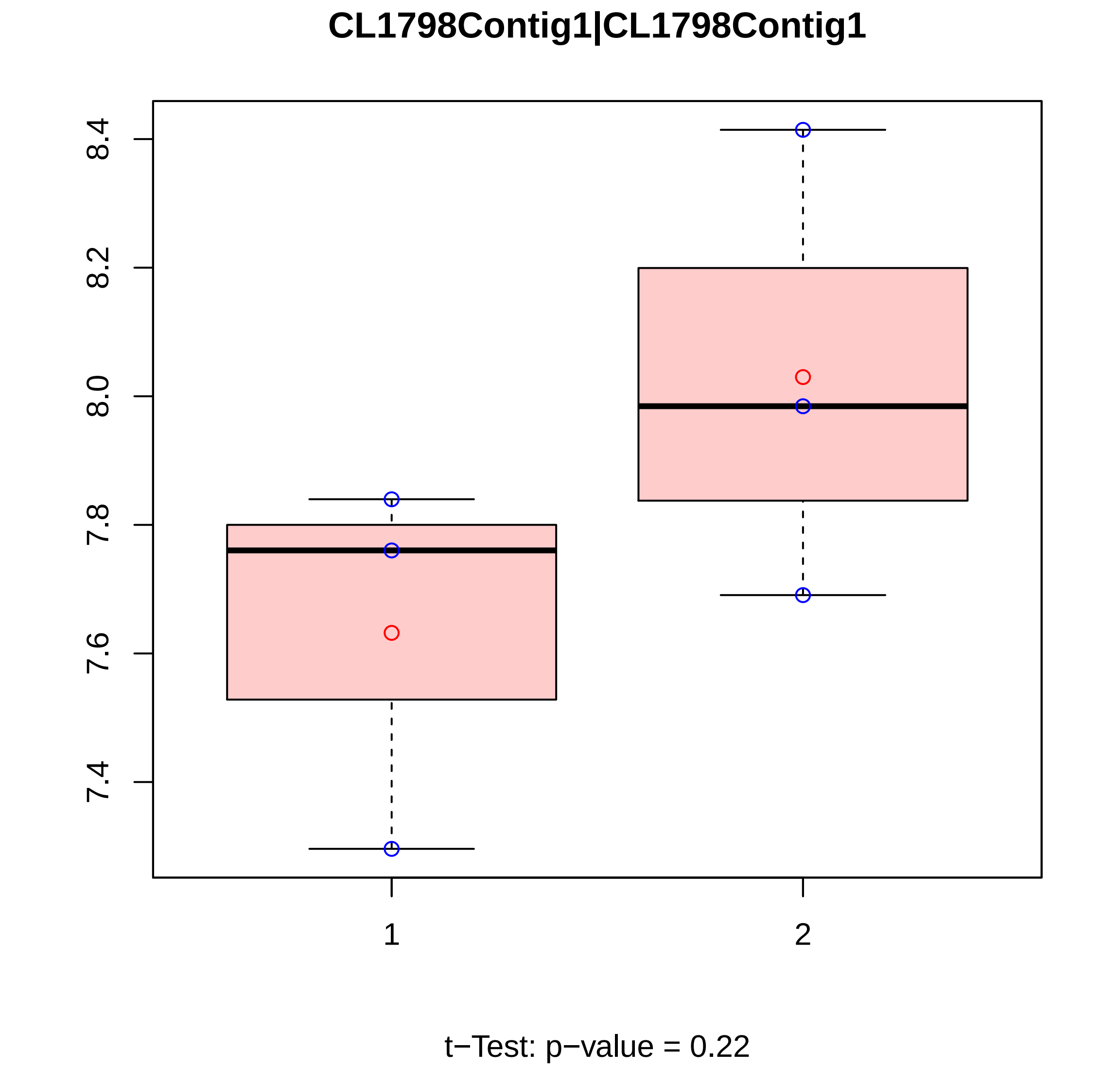
CL1798Contig1|CL1798Contig1
8.4
8.2
8.0
7.8
7.6
7.4
1
2
t−Test: p−value = 0.22

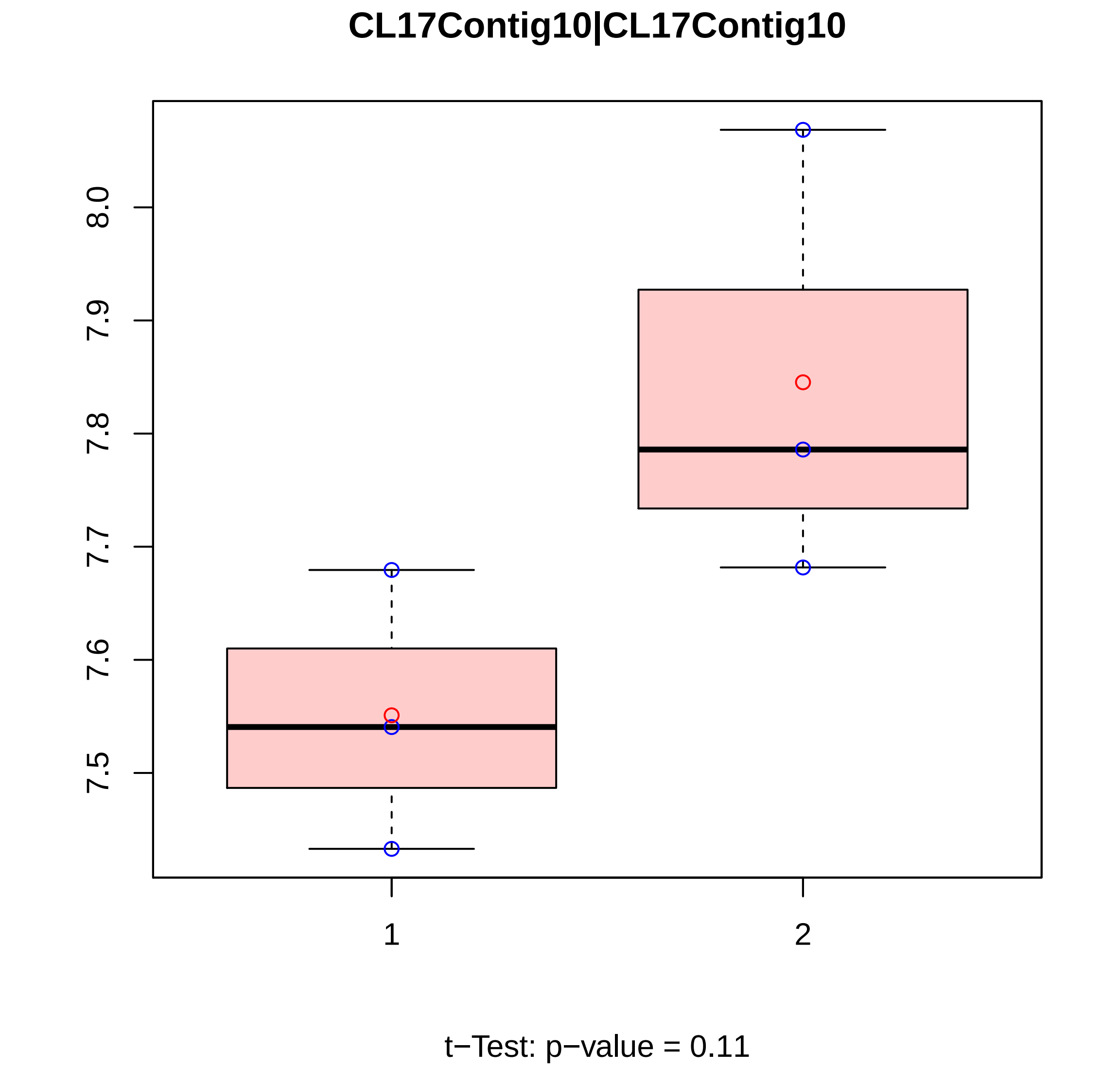

CL17Contig10|CL17Contig10
8.0
7.9
7.8
7.7
7.6
7.5
1
2
t−Test: p−value = 0.11

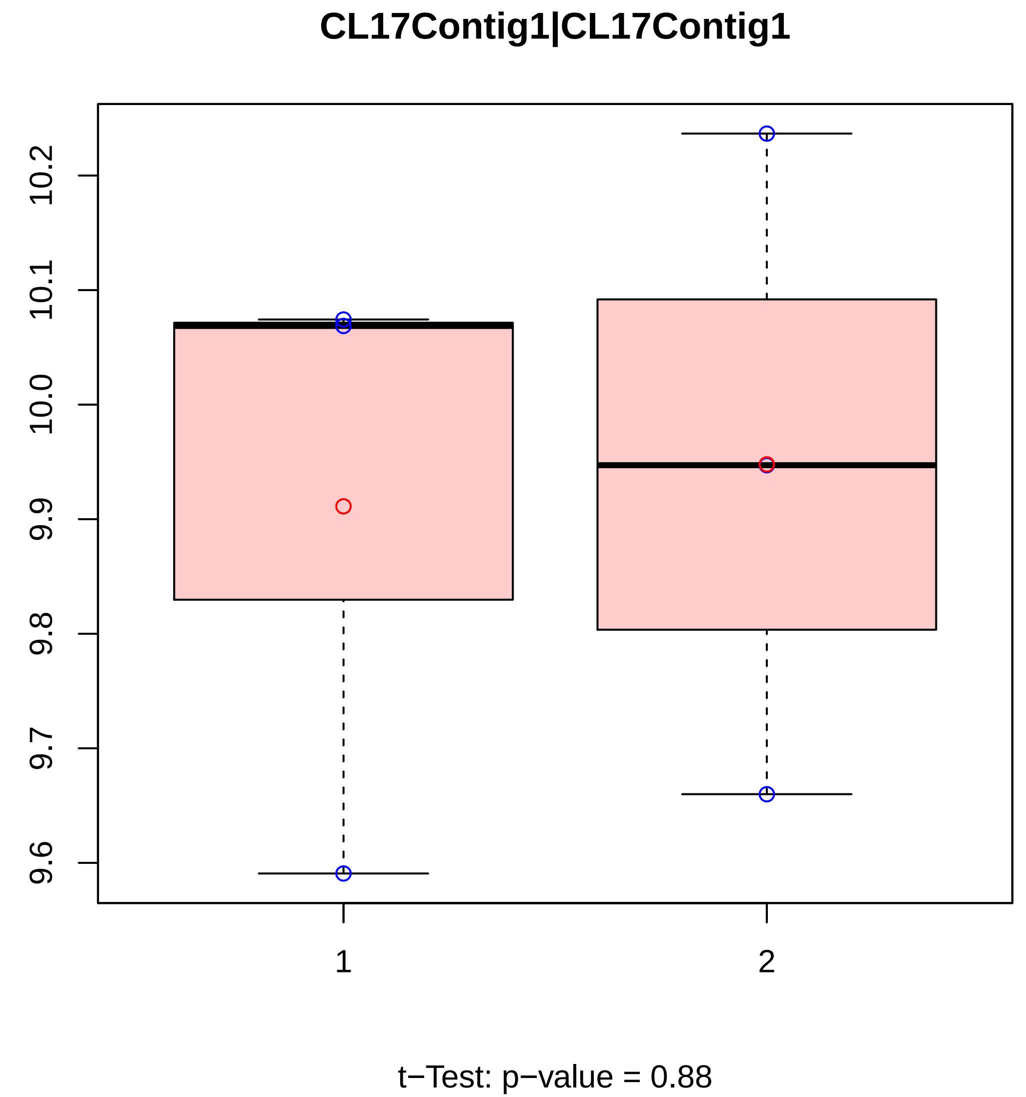
CL17Contig1|CL17Contig1
10.2
10.1
10.0
9.9
9.8
9.7
9.6
1
2
t−Test: p−value = 0.88

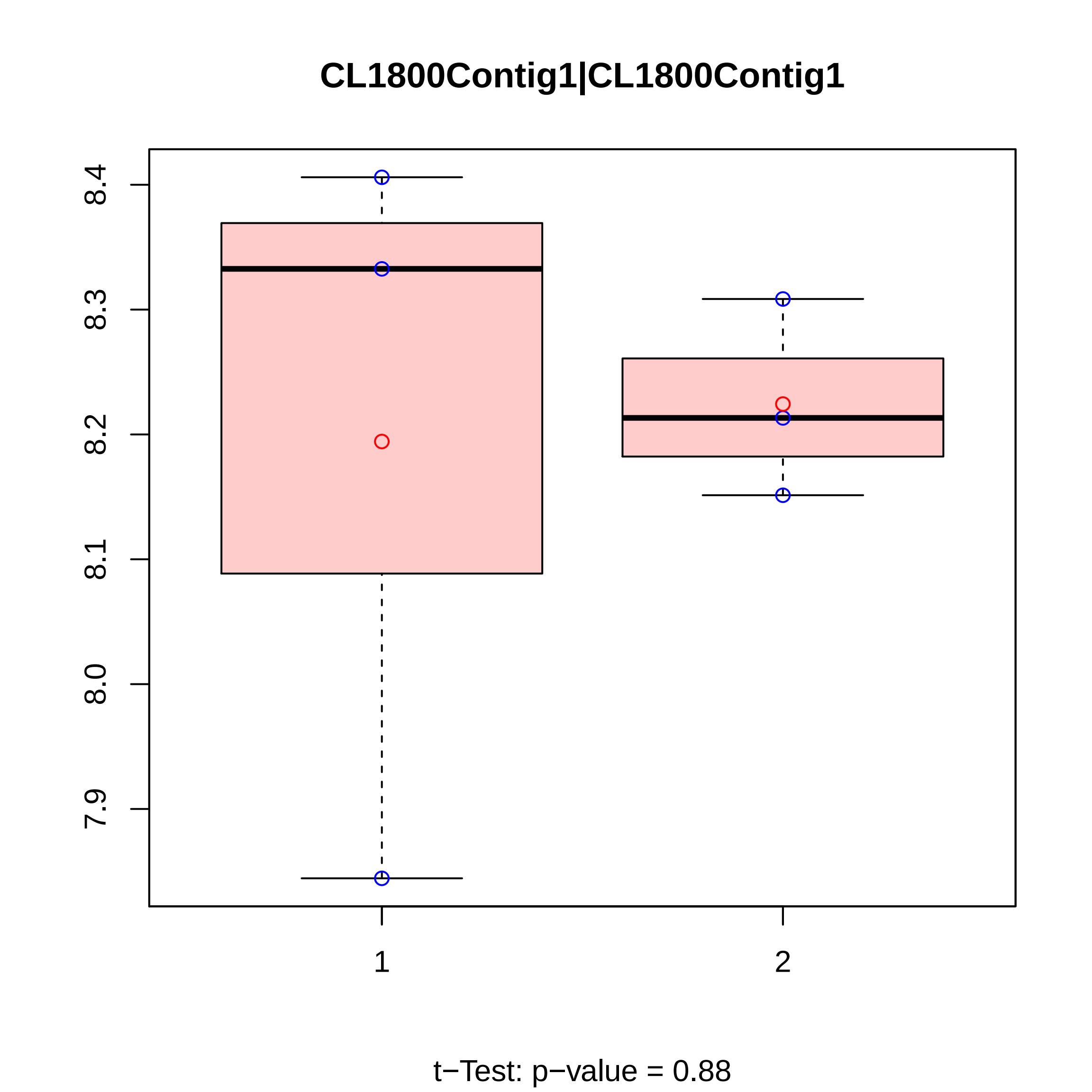
CL1800Contig1|CL1800Contig1
8.4
8.3
8.2
8.1
8.0
7.9
1
2
t−Test: p−value = 0.88

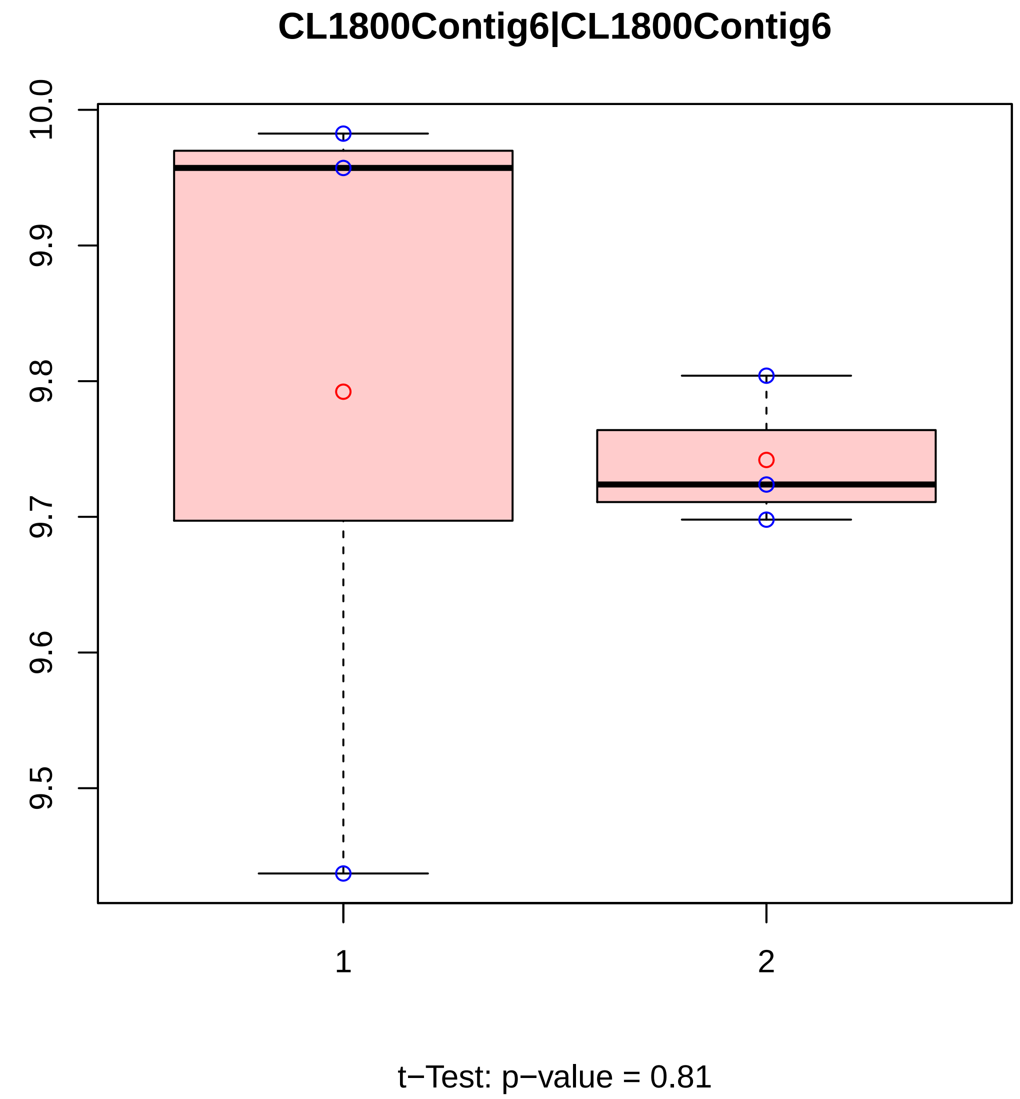
CL1800Contig6|CL1800Contig6
10.0
9.9
9.8
9.7
9.6
9.5
1
2
t−Test: p−value = 0.81

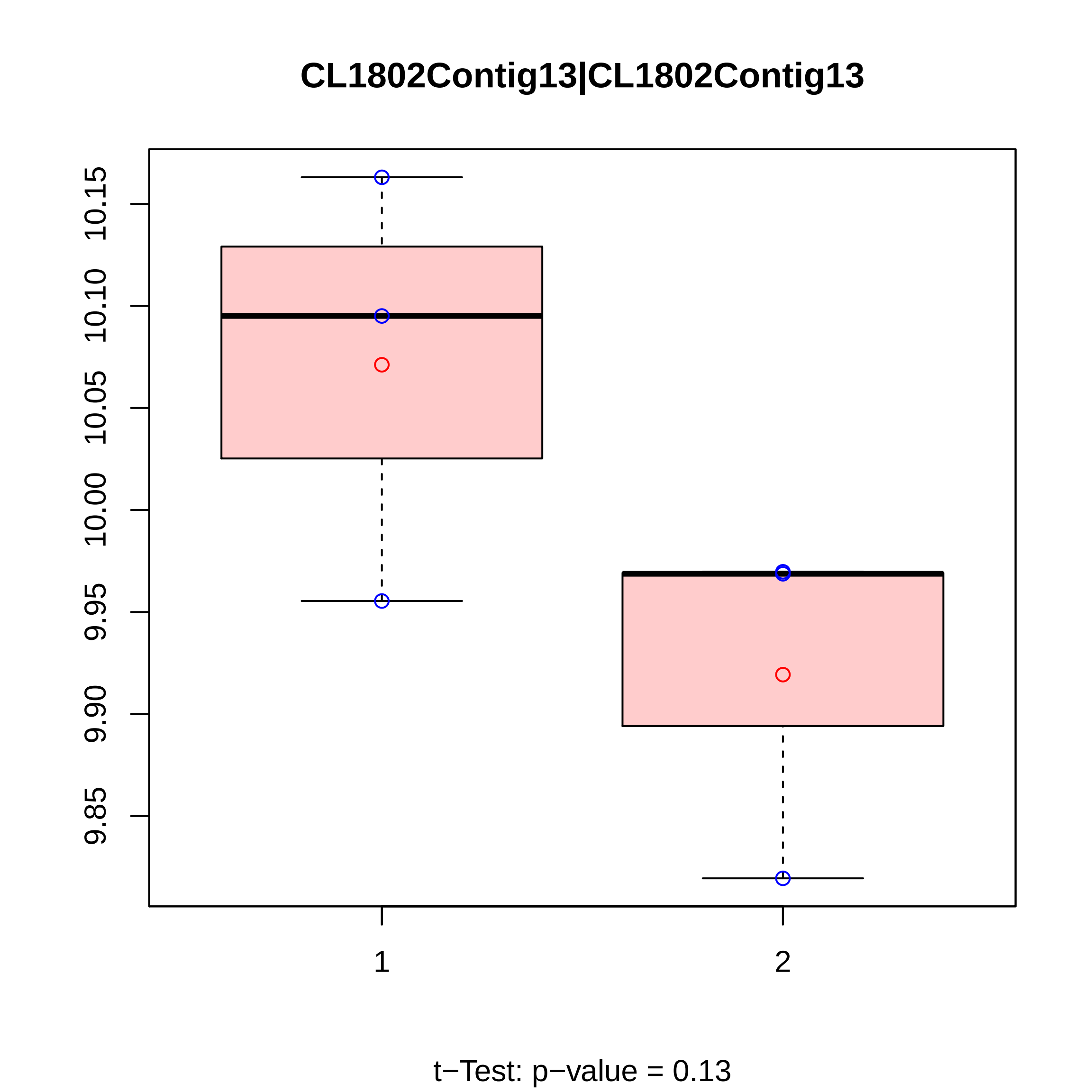
CL1802Contig13|CL1802Contig13
10.15
10.10
10.05
10.00
9.95
9.90
9.85
1
2
t−Test: p−value = 0.13

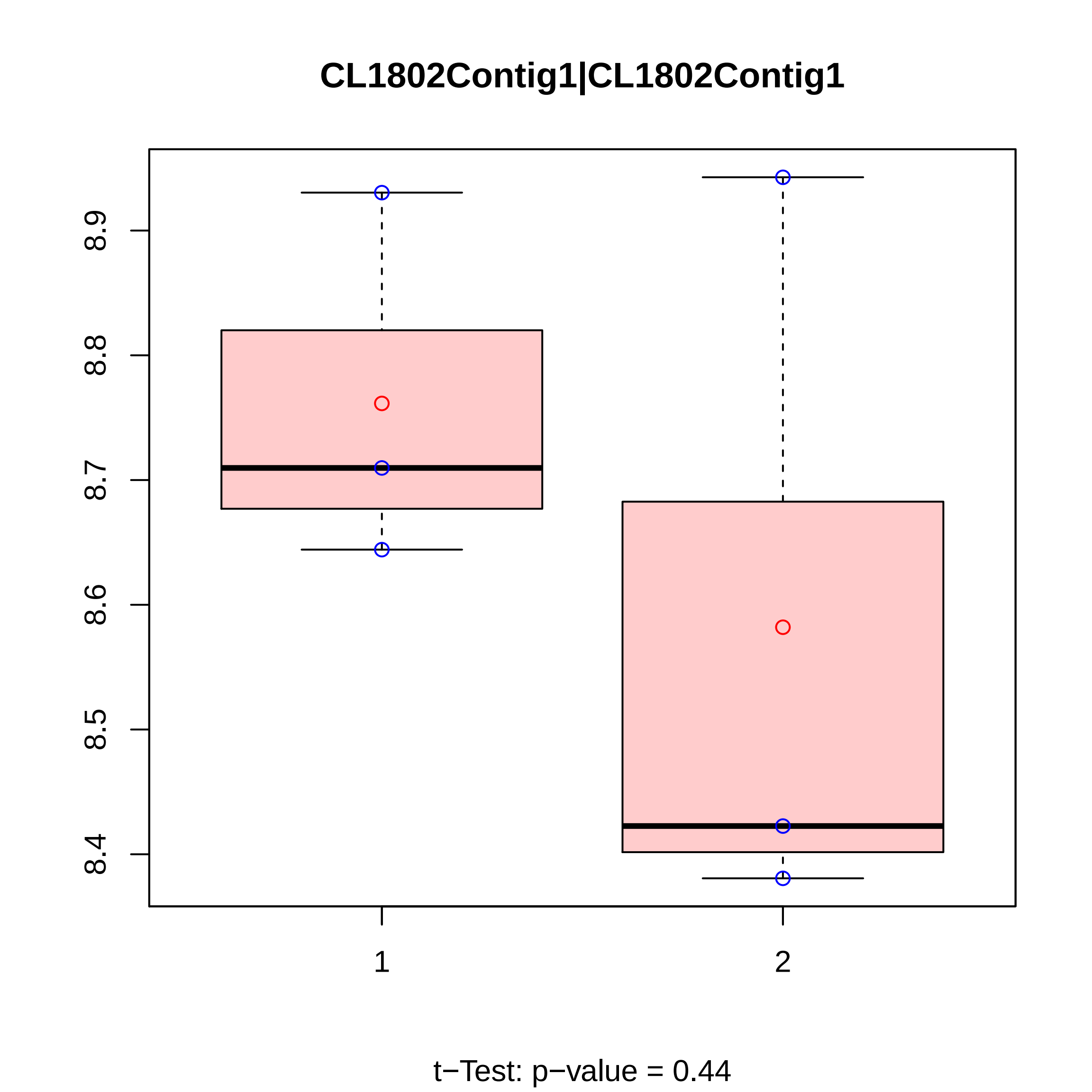

CL1802Contig1|CL1802Contig1
8.4
8.5
8.6
8.7
8.8
8.9
1
2
t−Test: p−value = 0.44

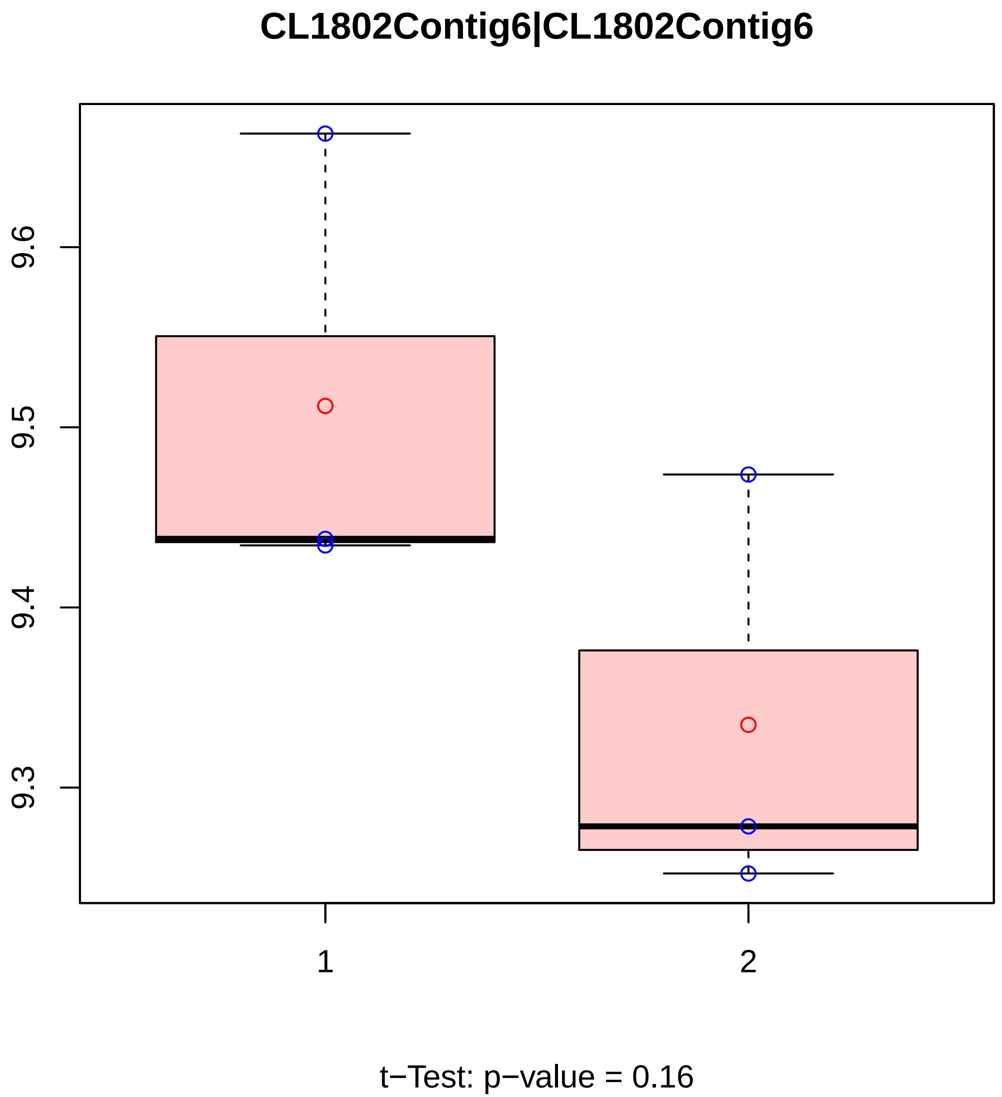
CL1802Contig6|CL1802Contig6
9.3
9.4
9.5
9.6
1
2
t−Test: p−value = 0.16

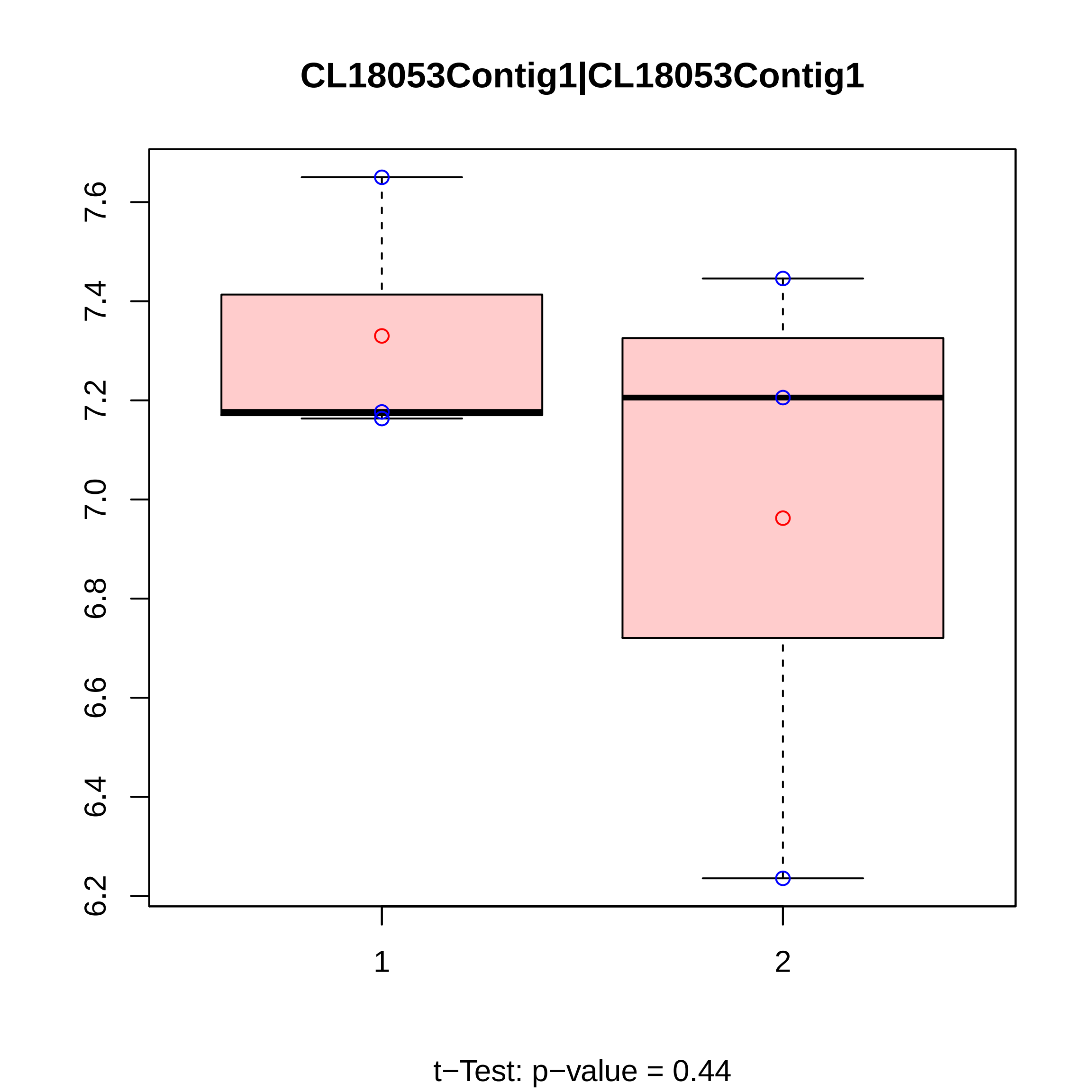

CL18053Contig1|CL18053Contig1
7.6
7.4
7.2
7.0
6.8
6.6
6.4
6.2
1
2
t−Test: p−value = 0.44

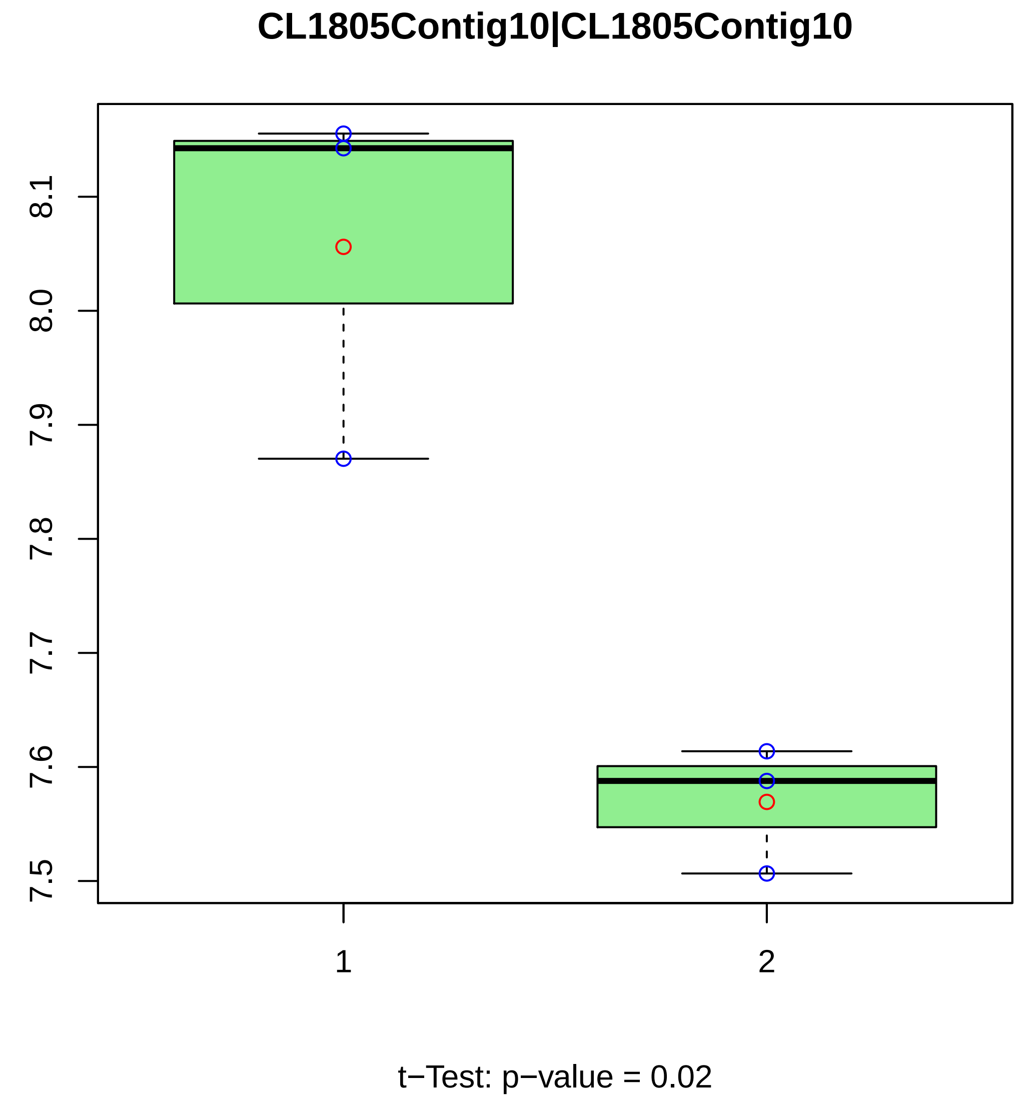
CL1805Contig10|CL1805Contig10
8.1
8.0
7.9
7.8
7.7
7.6
7.5
1
2
t−Test: p−value = 0.02

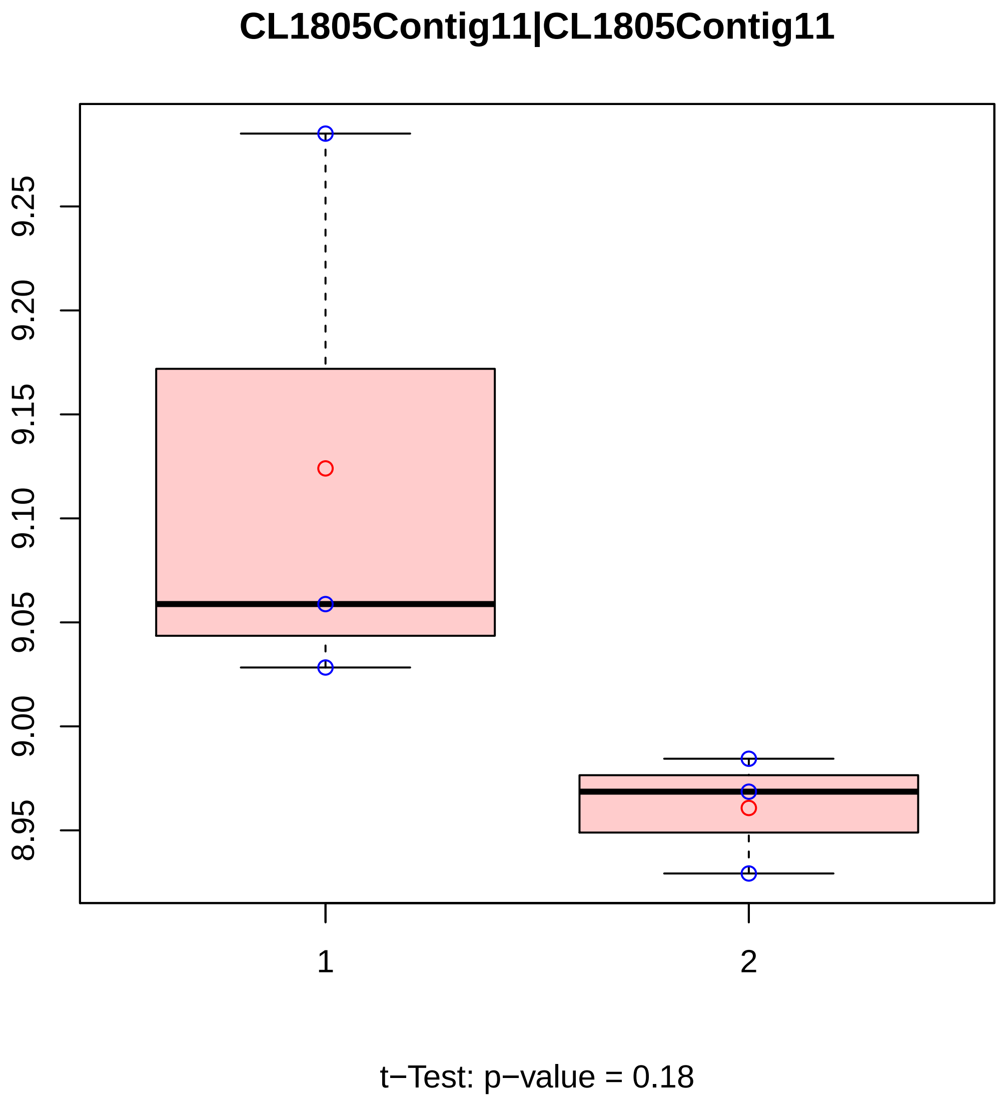
CL1805Contig11|CL1805Contig11
9.25
9.20
9.15
9.10
9.05
9.00
8.95
1
2
t−Test: p−value = 0.18

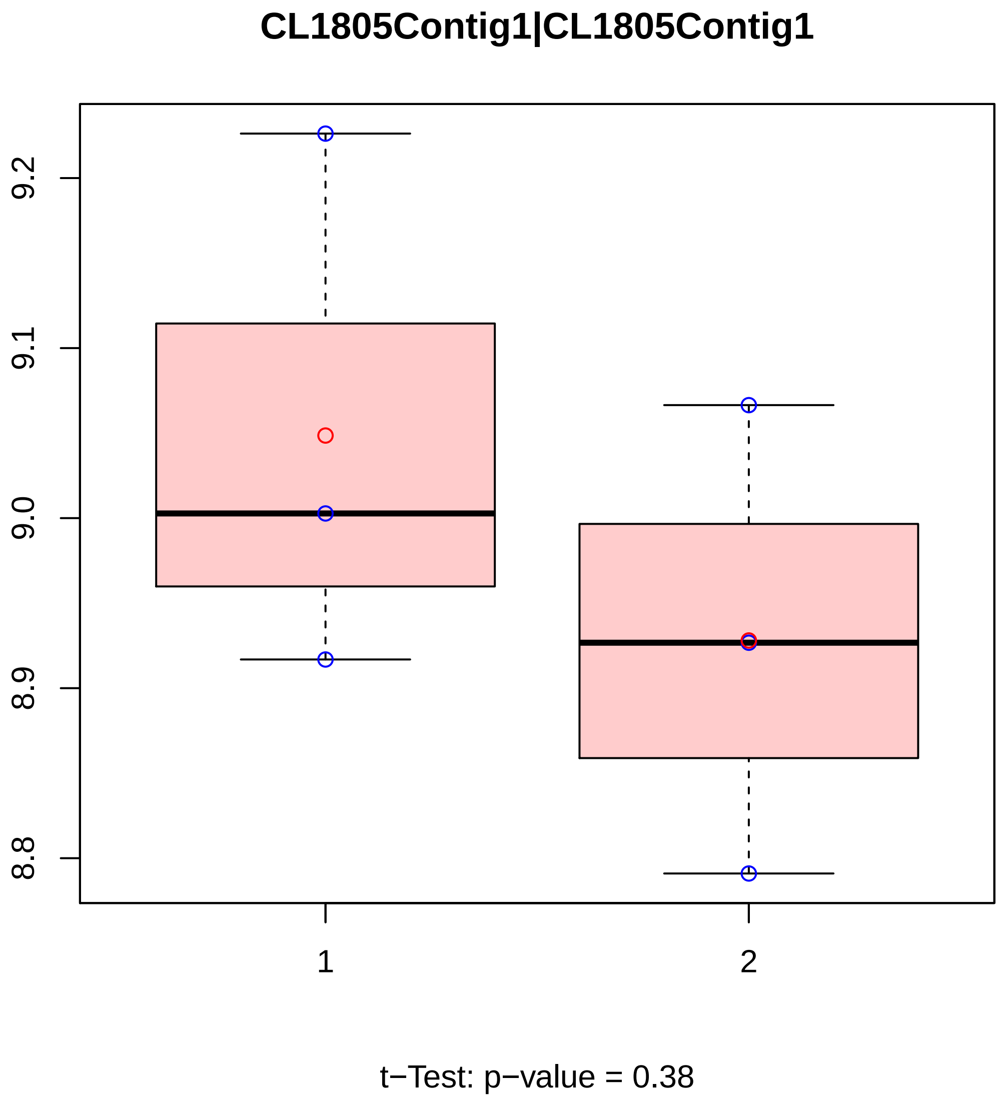

CL1805Contig1|CL1805Contig1
9.2
9.1
9.0
8.9
8.8
1
2
t−Test: p−value = 0.38

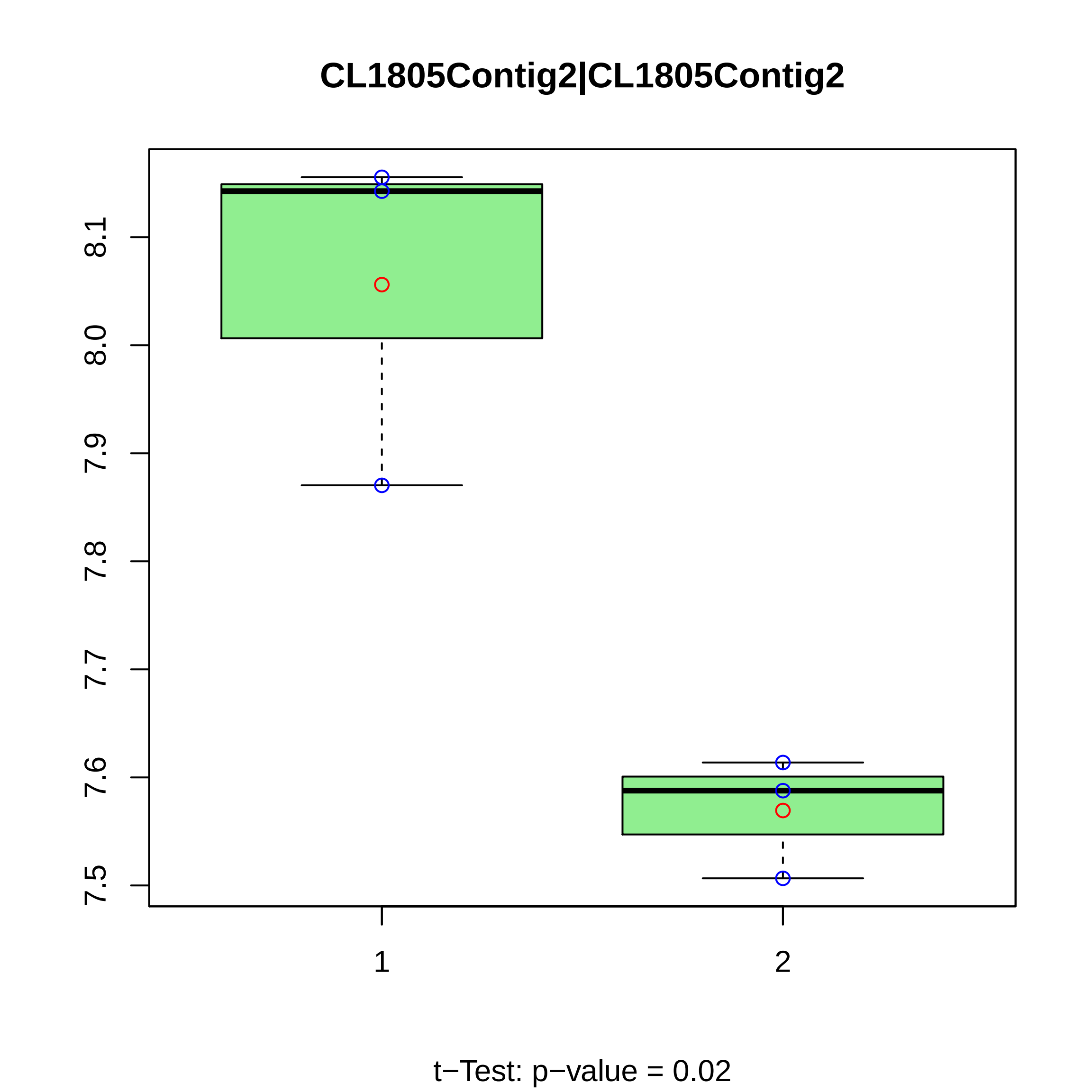
CL1805Contig2|CL1805Contig2
8.1
8.0
7.9
7.8
7.7
7.6
7.5
1
2
t−Test: p−value = 0.02

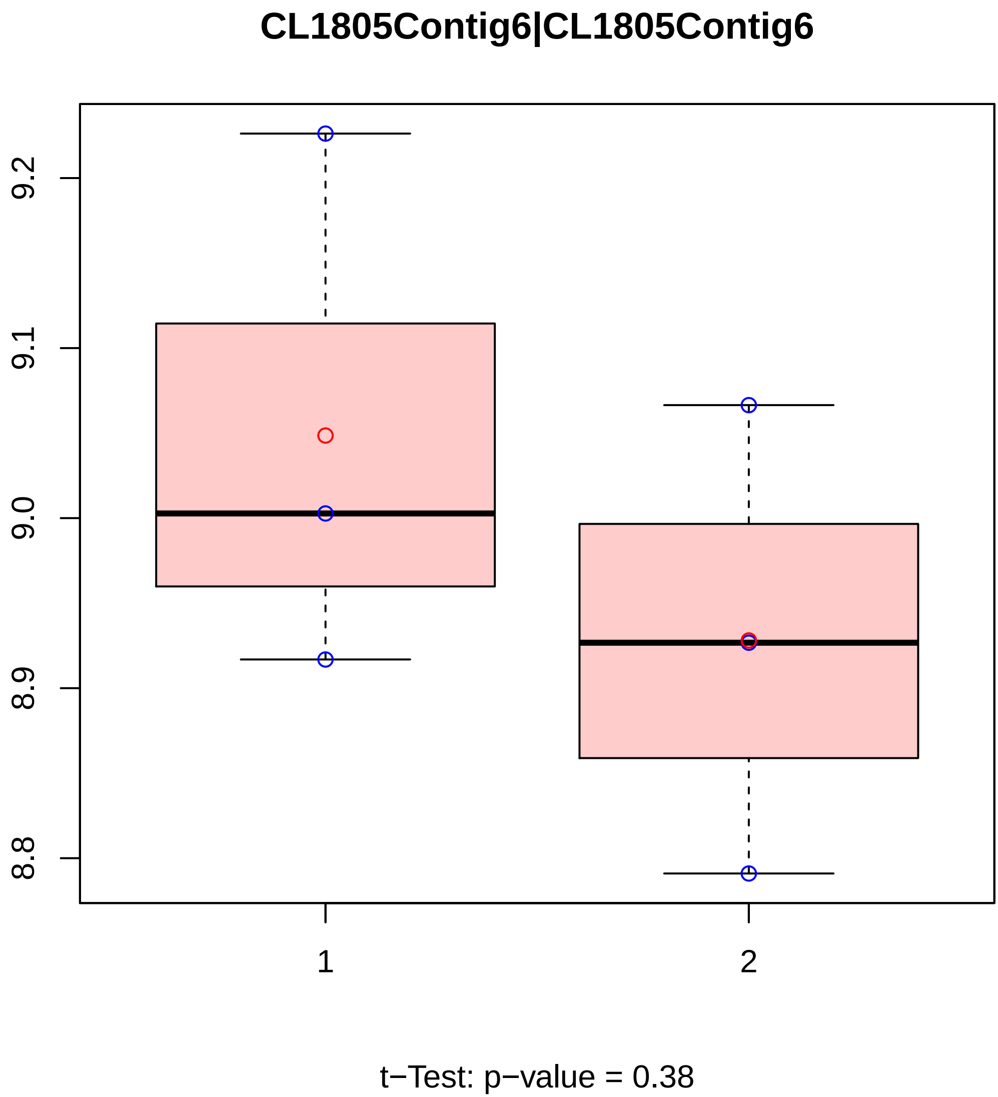

CL1805Contig6|CL1805Contig6
9.2
9.1
9.0
8.9
8.8
1
2
t−Test: p−value = 0.38

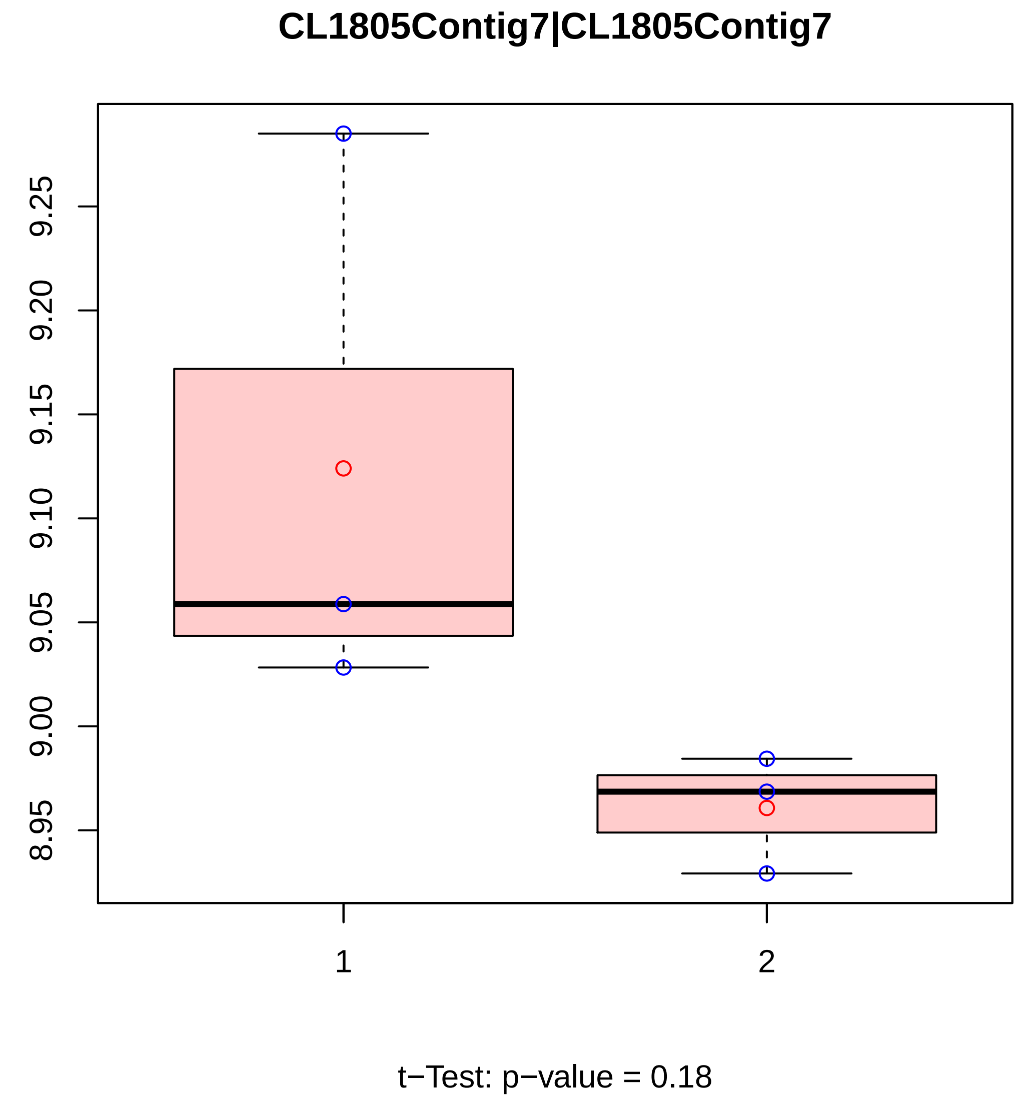
CL1805Contig7|CL1805Contig7
9.25
9.20
9.15
9.10
9.05
9.00
8.95
1
2
t−Test: p−value = 0.18

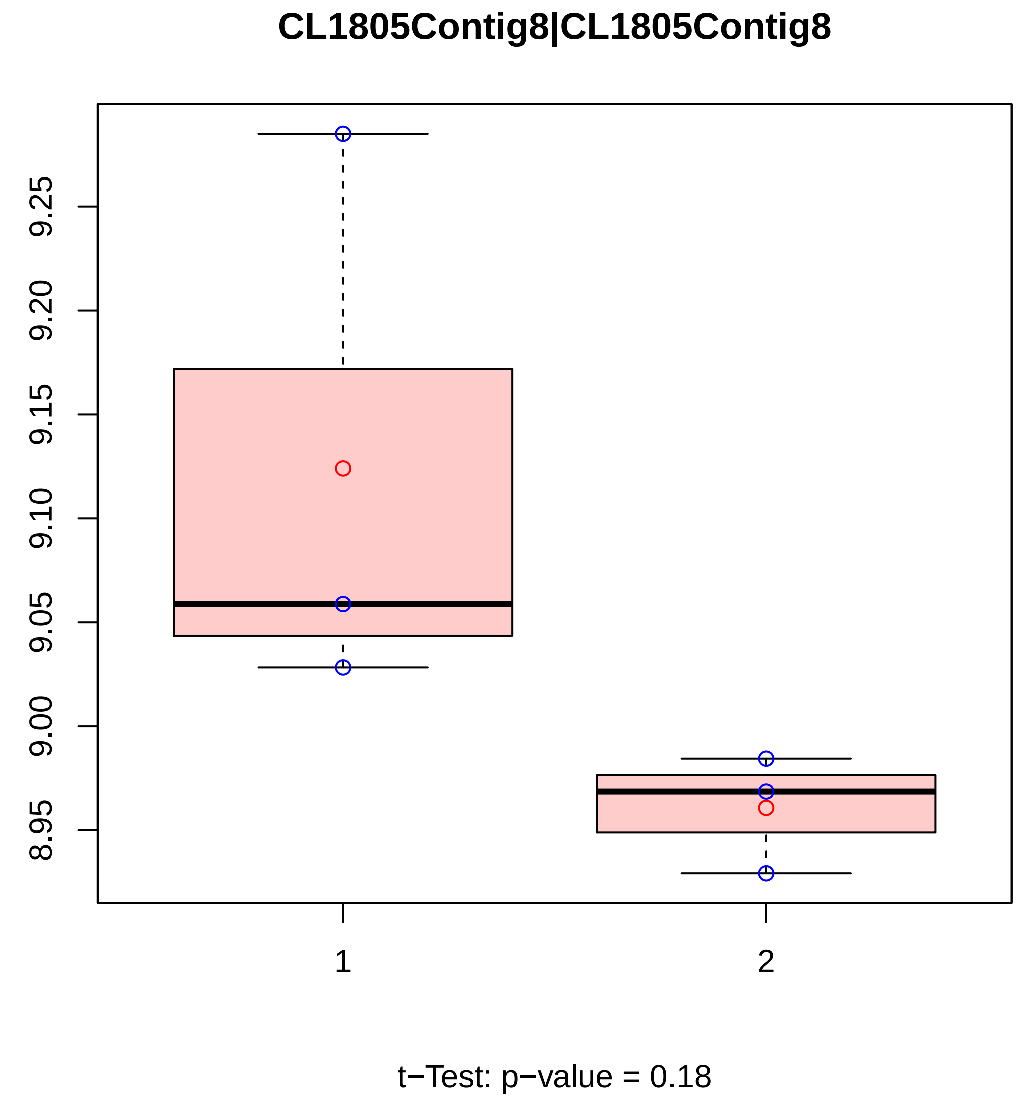

CL1805Contig8|CL1805Contig8
9.25
9.20
9.15
9.10
9.05
9.00
8.95
1
2
t−Test: p−value = 0.18

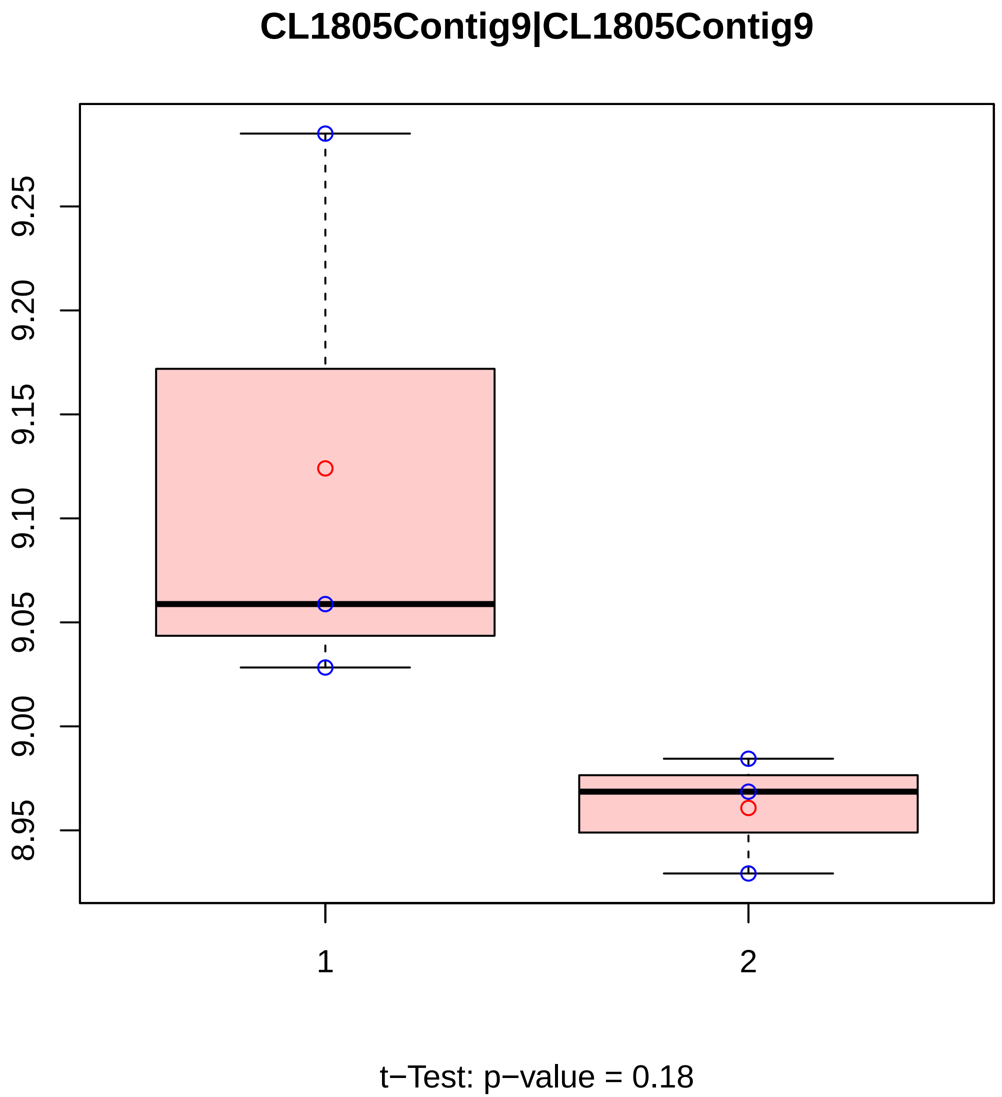

CL1805Contig9|CL1805Contig9
9.25
9.20
9.15
9.10
9.05
9.00
8.95
1
2
t−Test: p−value = 0.18

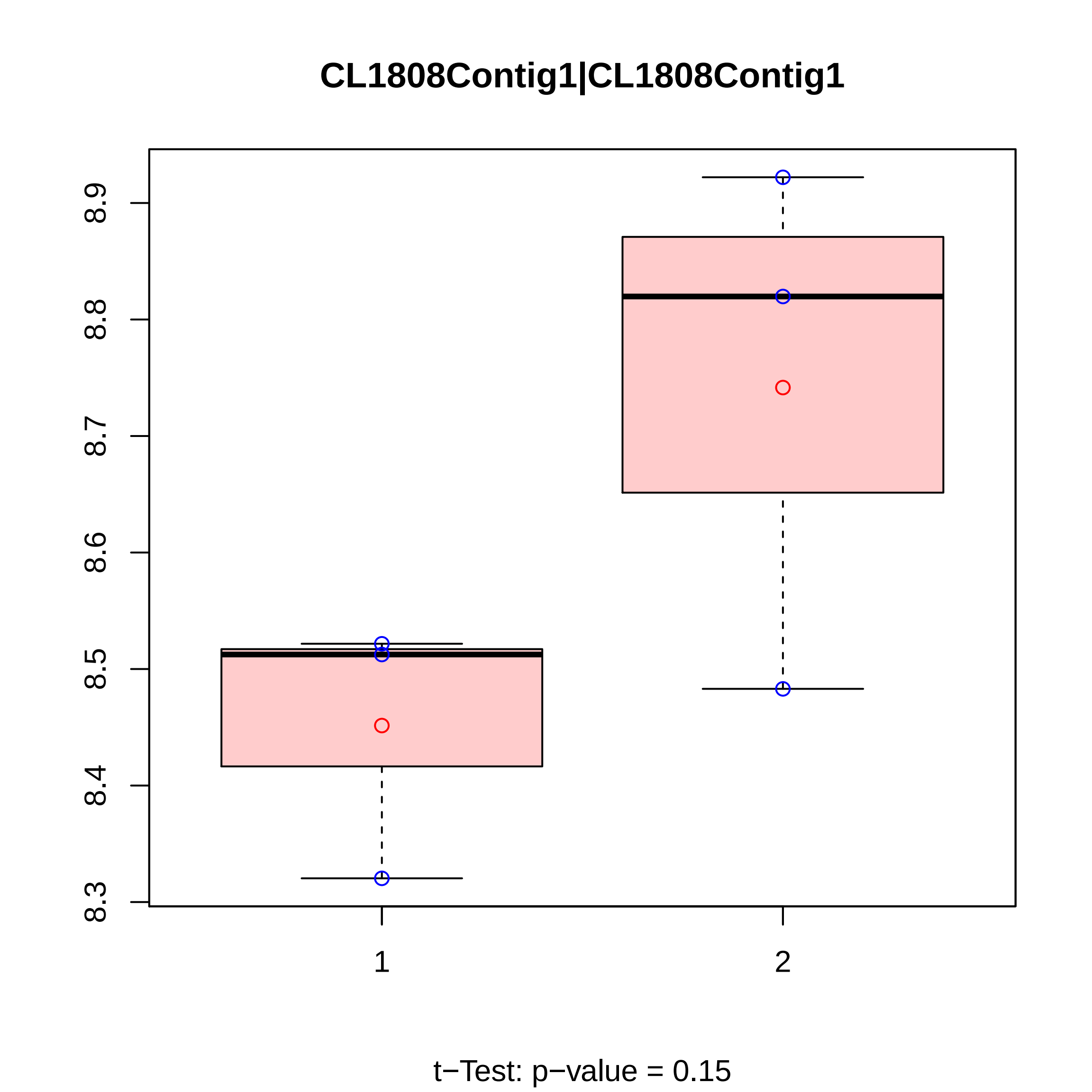

CL1808Contig1|CL1808Contig1
8.3
8.4
8.5
8.6
8.7
8.8
8.9
1
2
t−Test: p−value = 0.15

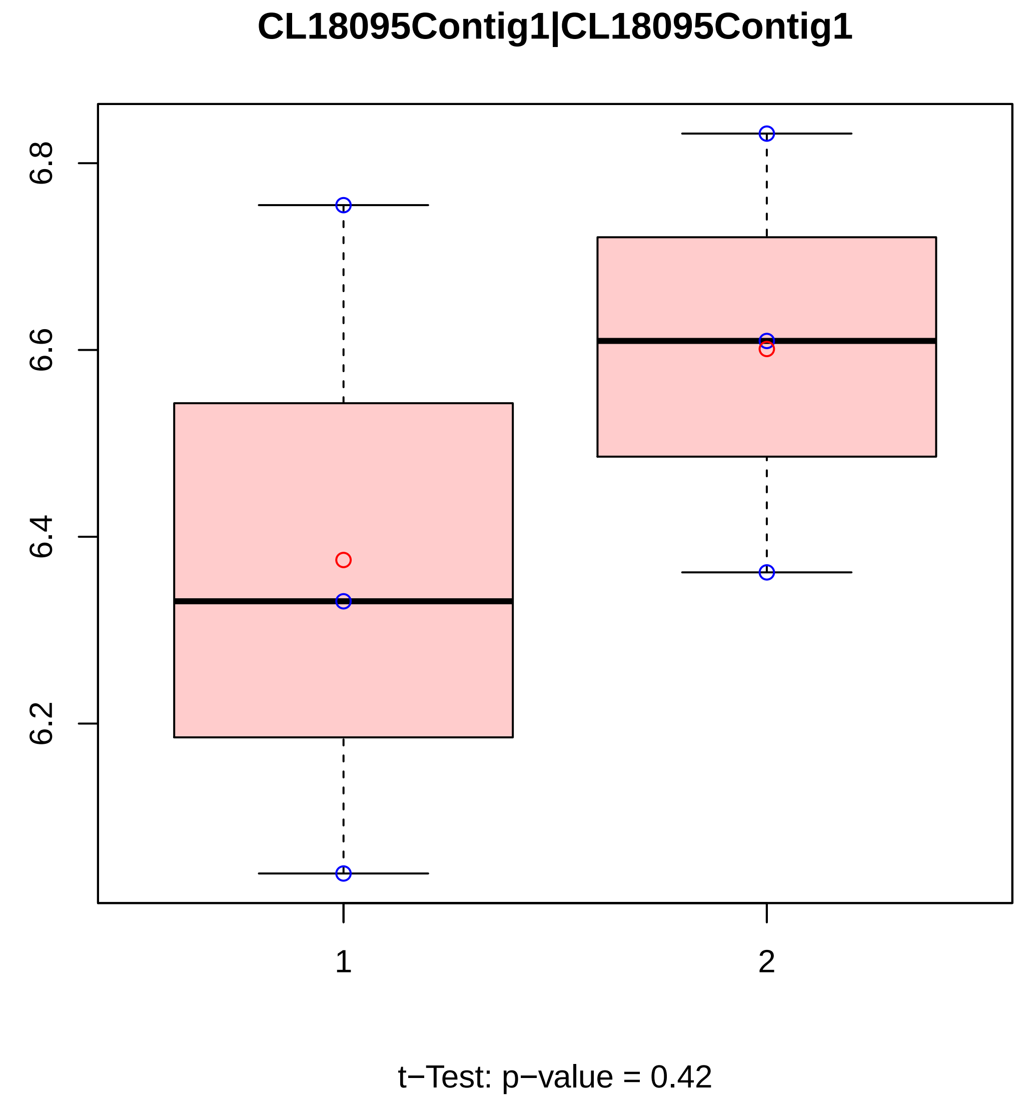

CL18095Contig1|CL18095Contig1
6.2
6.4
6.6
6.8
1
2
t−Test: p−value = 0.42

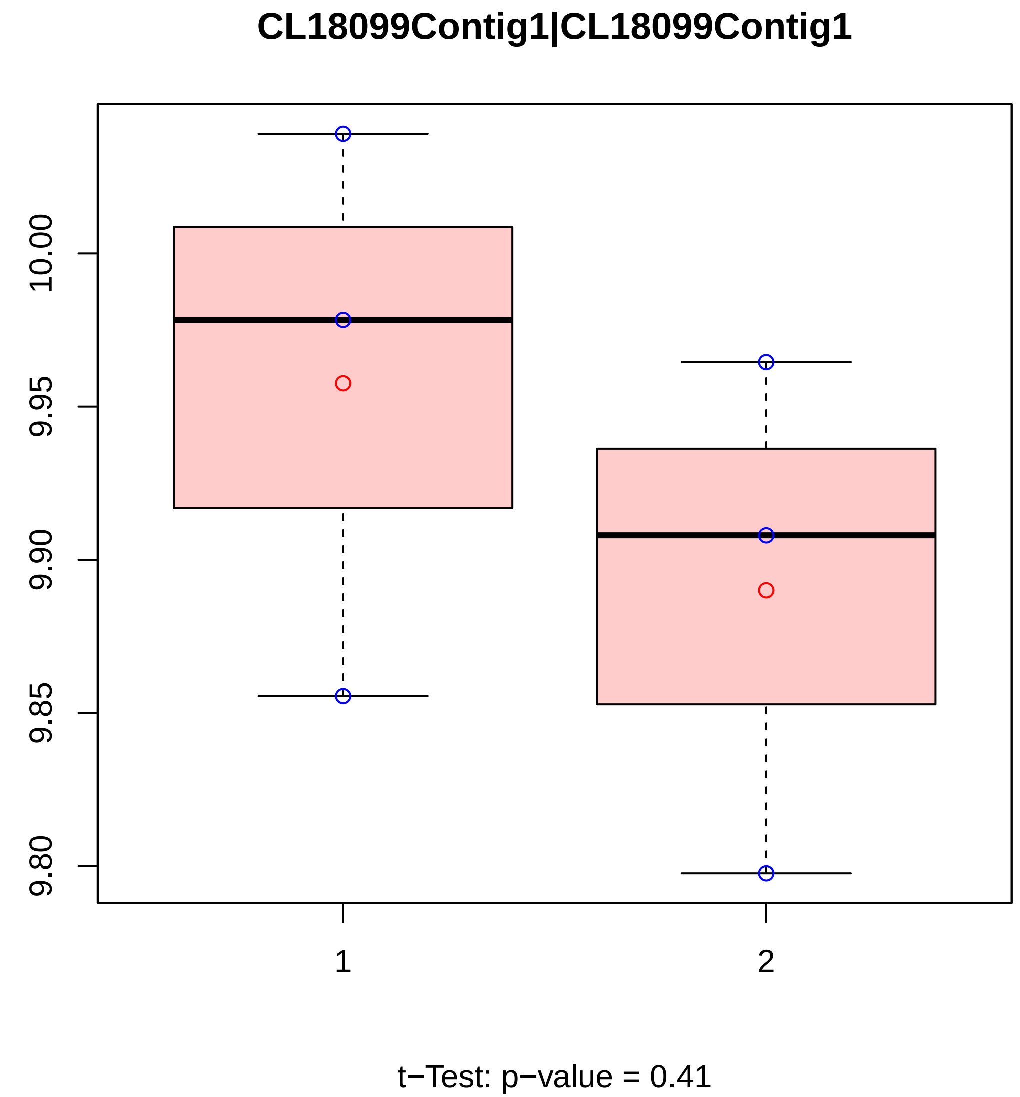
CL18099Contig1|CL18099Contig1
10.00
9.95
9.90
9.85
9.80
1
2
t−Test: p−value = 0.41

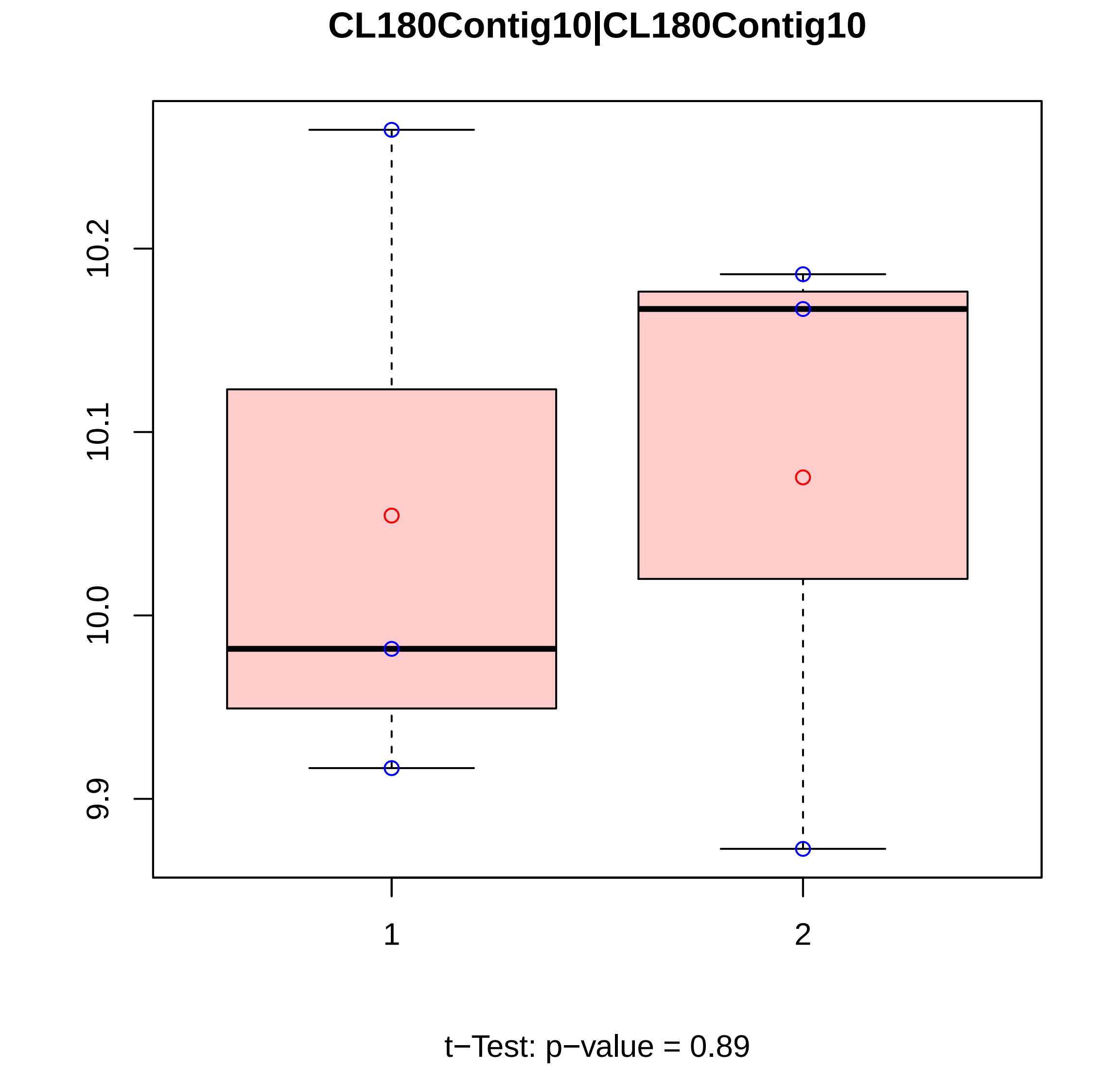
CL180Contig10|CL180Contig10
10.2
10.1
10.0
9.9
1
2
t−Test: p−value = 0.89

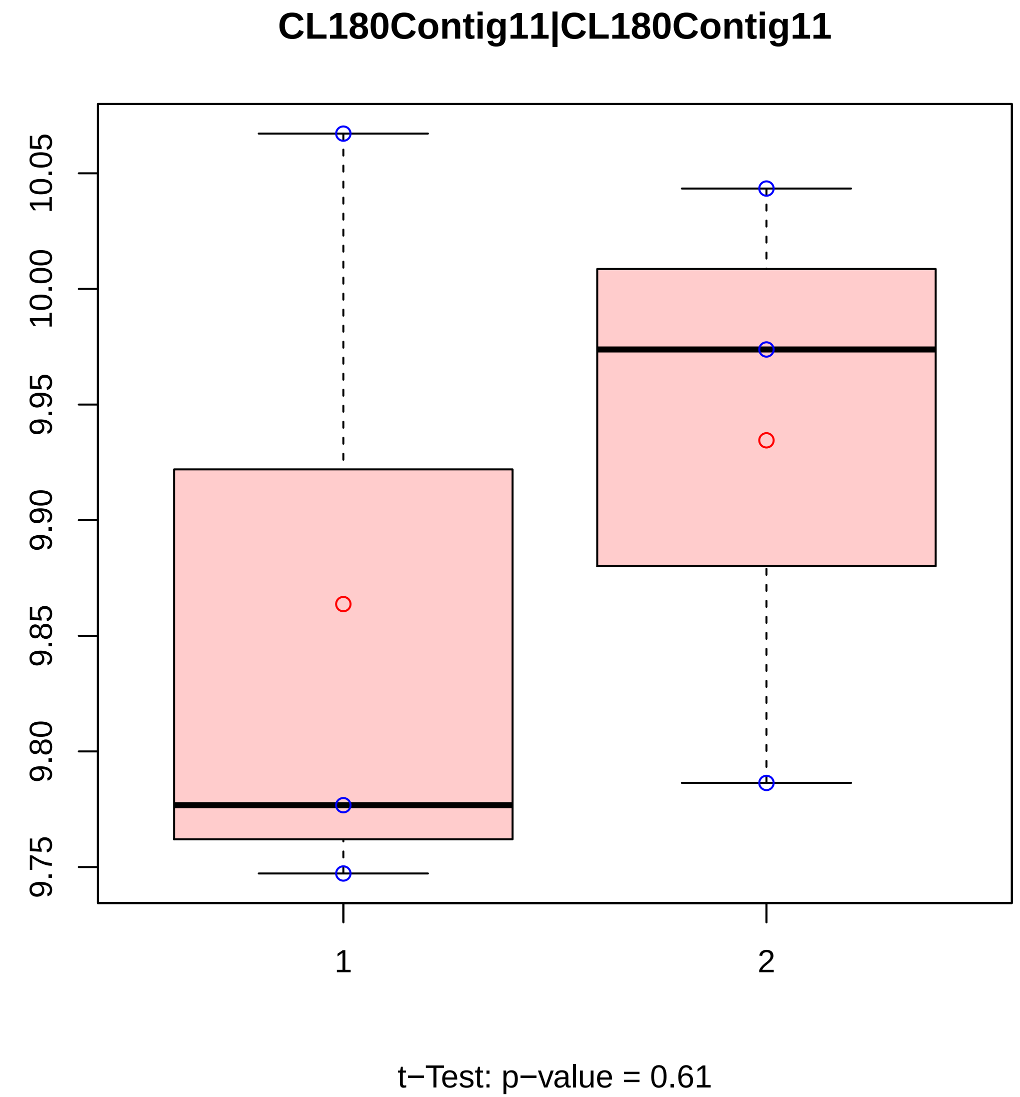

CL180Contig11|CL180Contig11
10.05
10.00
9.95
9.90
9.85
9.80
9.75
1
2
t−Test: p−value = 0.61

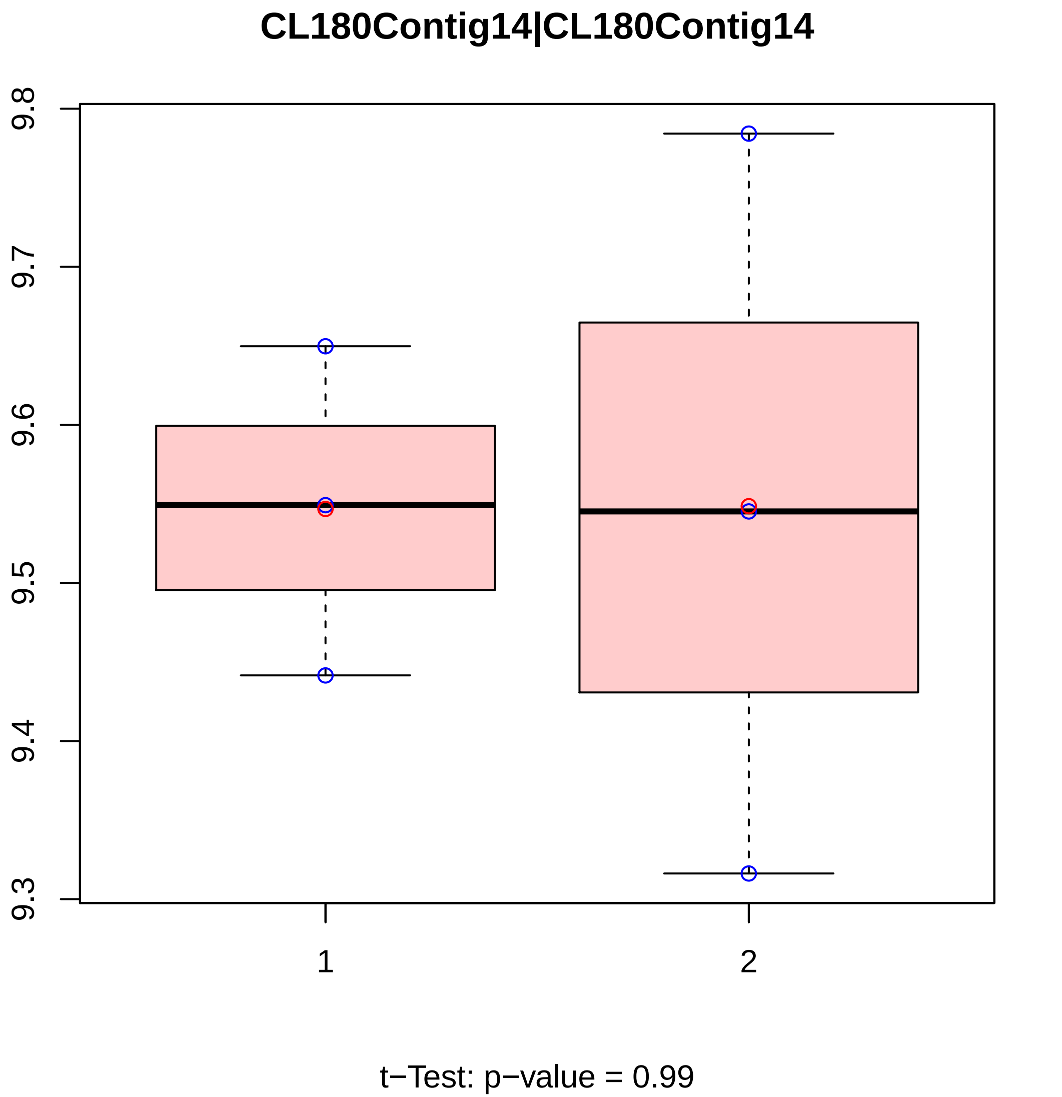
CL180Contig14|CL180Contig14
9.8
9.7
9.6
9.5
9.4
9.3
1
2
t−Test: p−value = 0.99

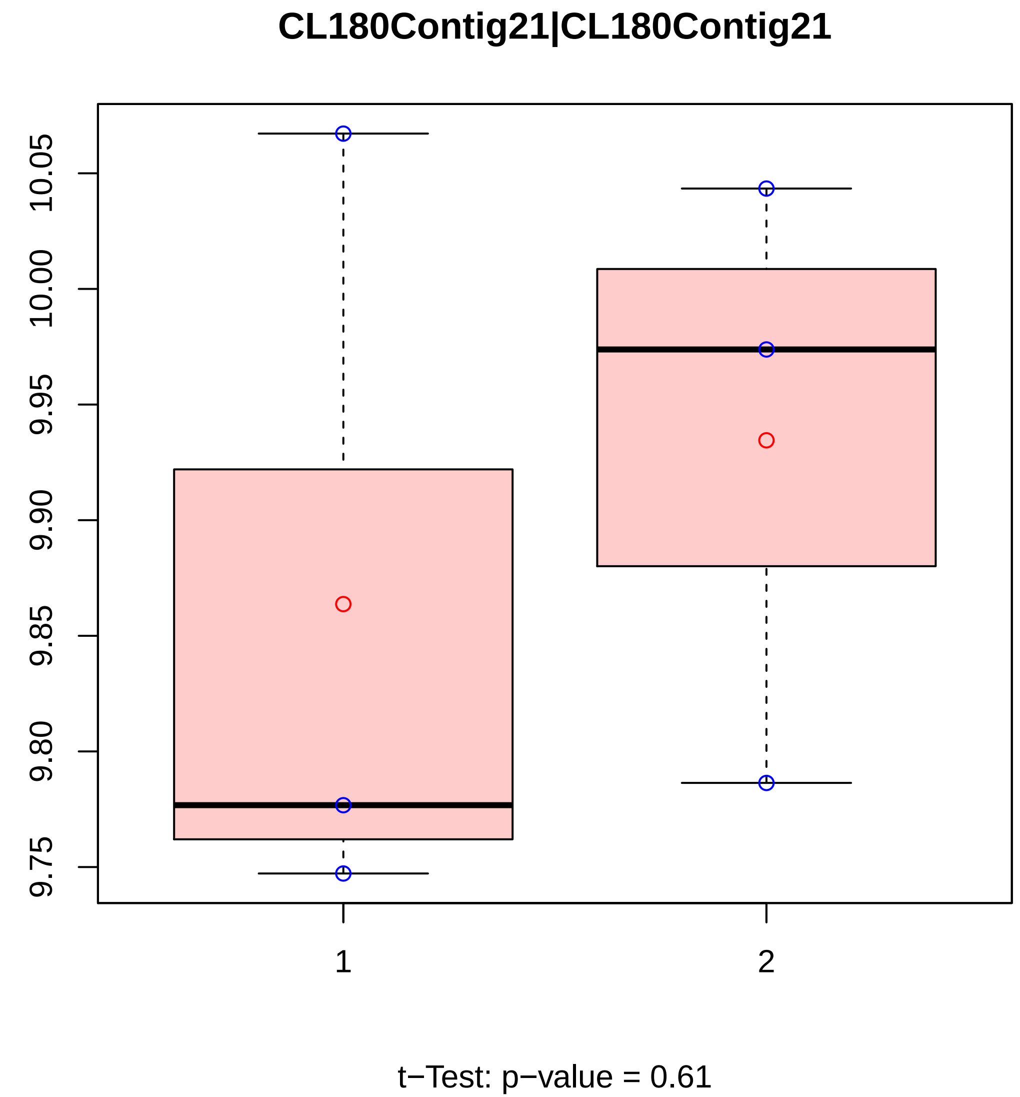
CL180Contig21|CL180Contig21
10.05
10.00
9.95
9.90
9.85
9.80
9.75
1
2
t−Test: p−value = 0.61

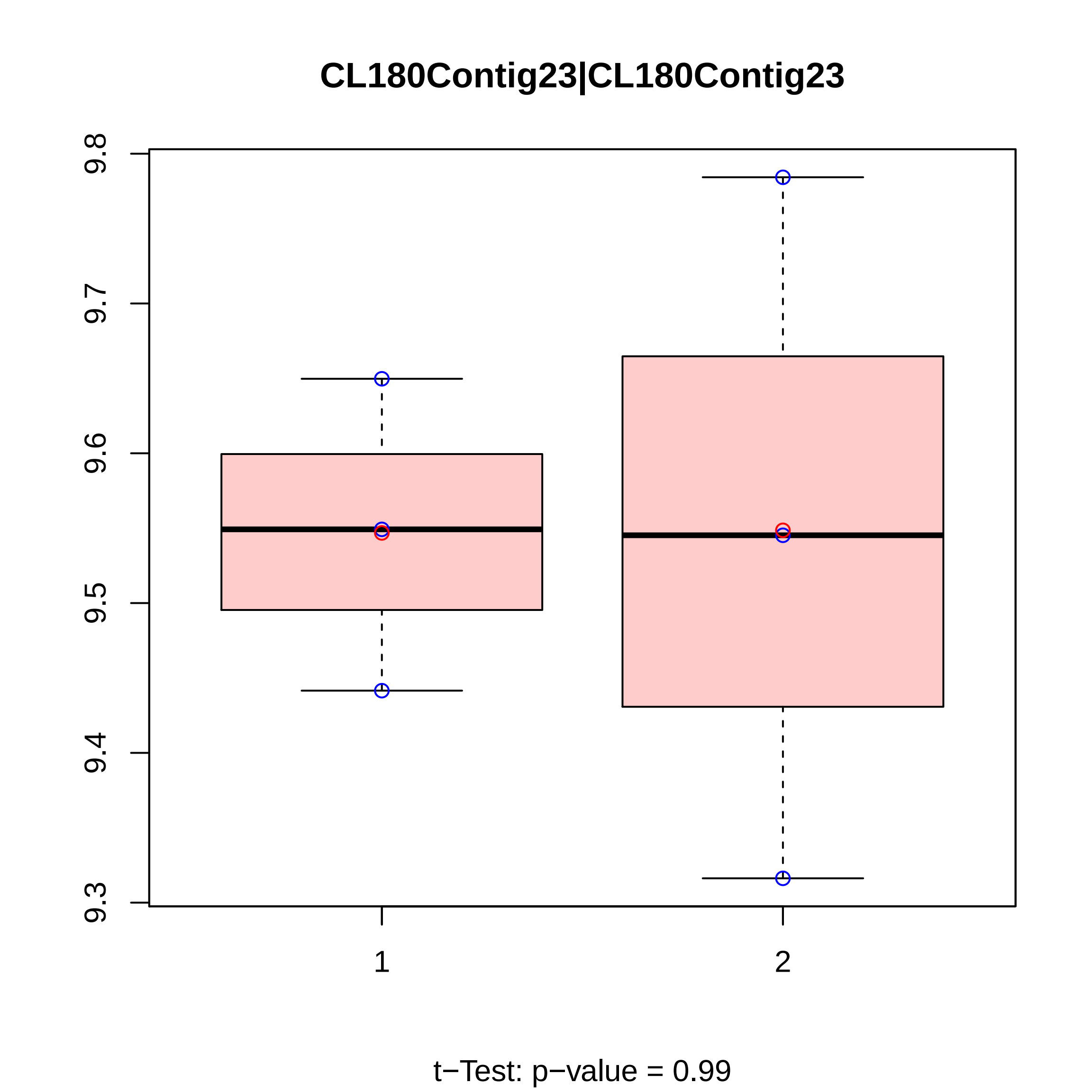

CL180Contig23|CL180Contig23
9.3
9.4
9.5
9.6
9.7
9.8
1
2
t−Test: p−value = 0.99

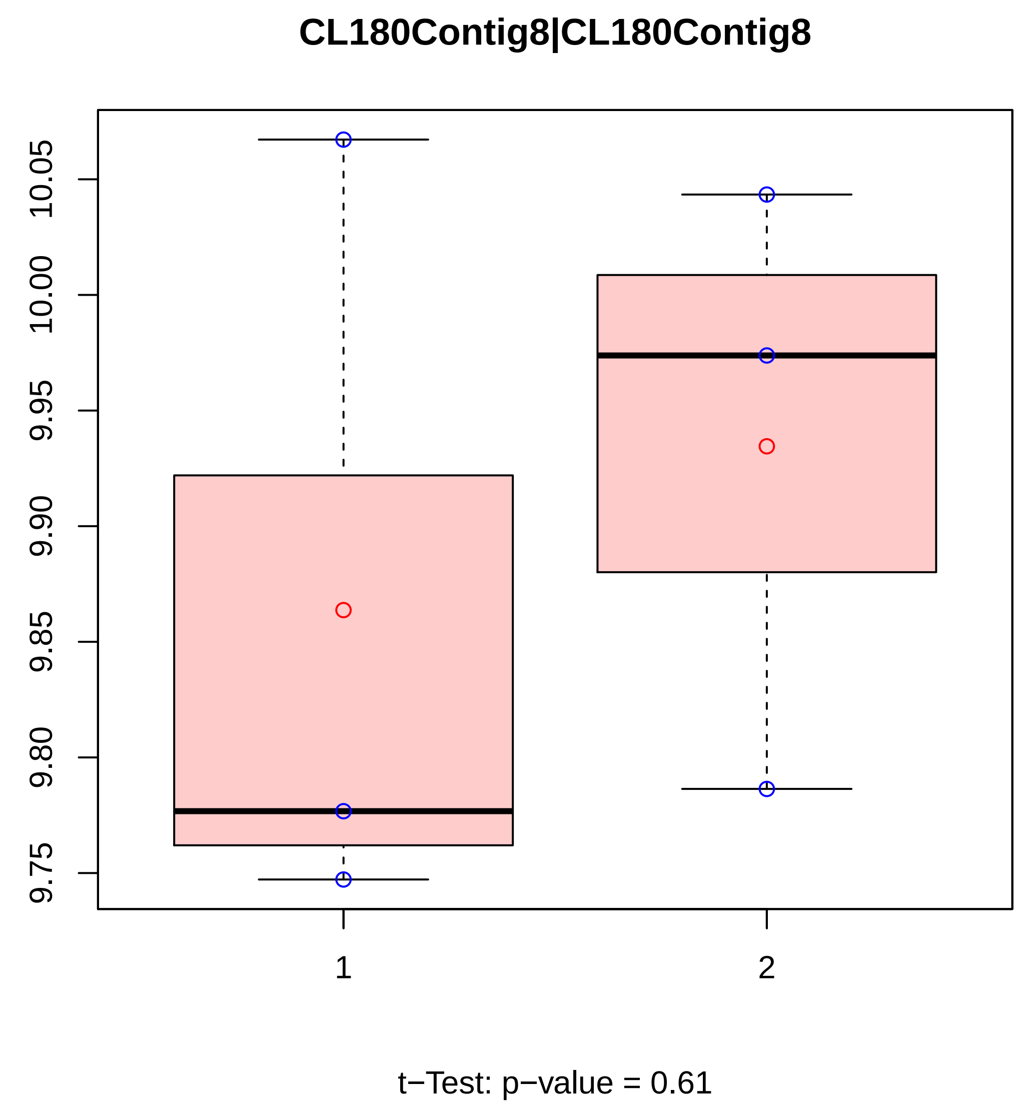
CL180Contig8|CL180Contig8
10.05
10.00
9.95
9.90
9.85
9.80
9.75
1
2
t−Test: p−value = 0.61

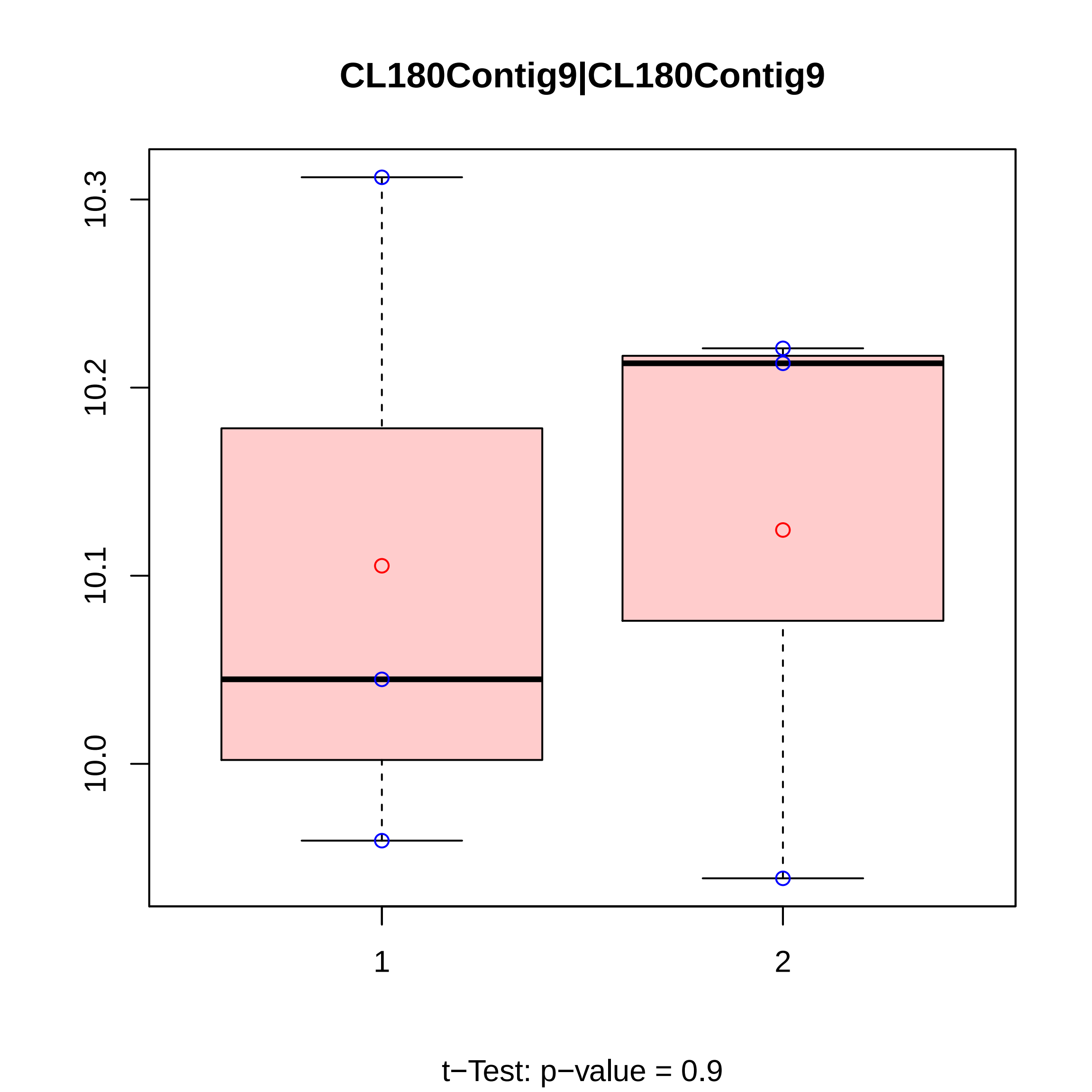
CL180Contig9|CL180Contig9
10.3
10.2
10.1
10.0
1
2
t−Test: p−value = 0.9

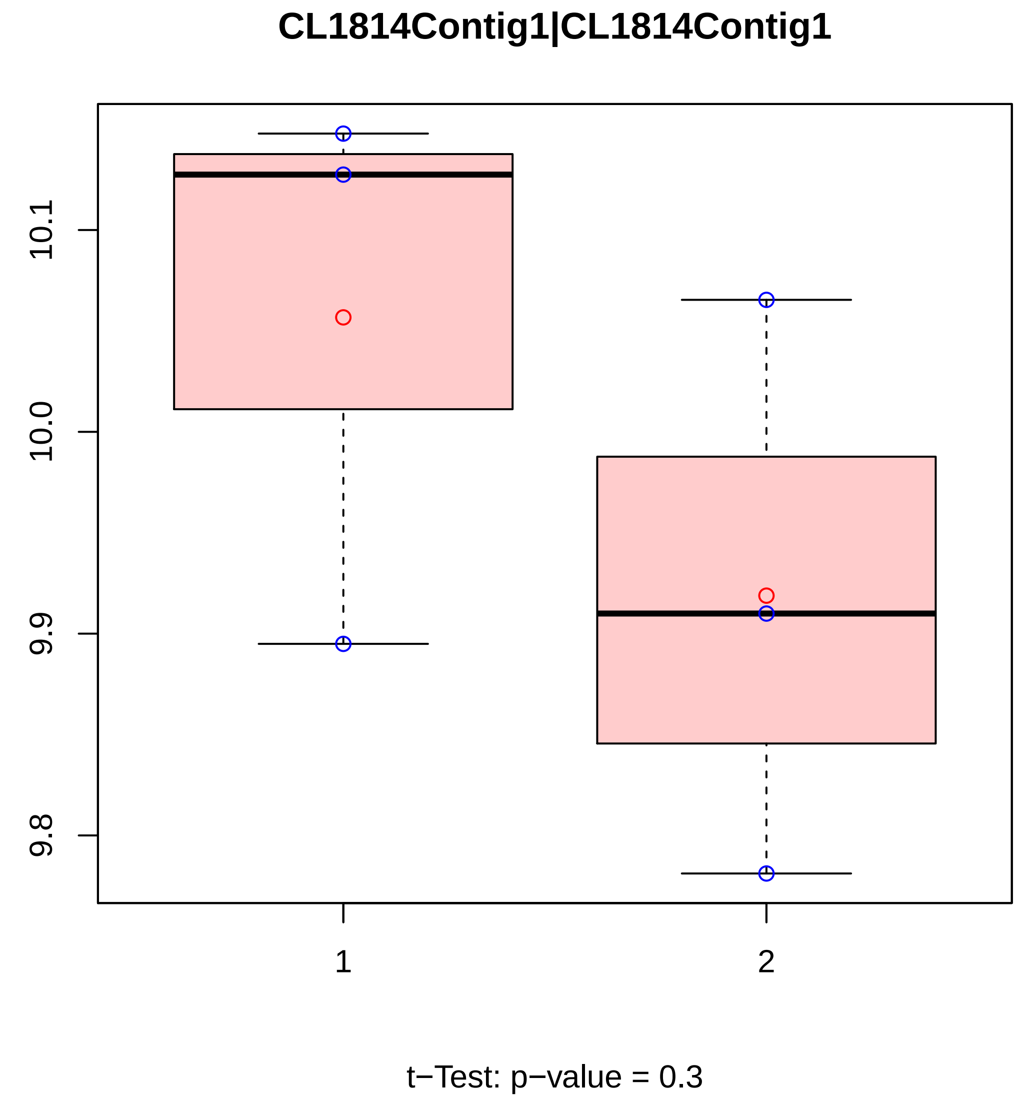

CL1814Contig1|CL1814Contig1
10.1
10.0
9.9
9.8
1
2
t−Test: p−value = 0.3

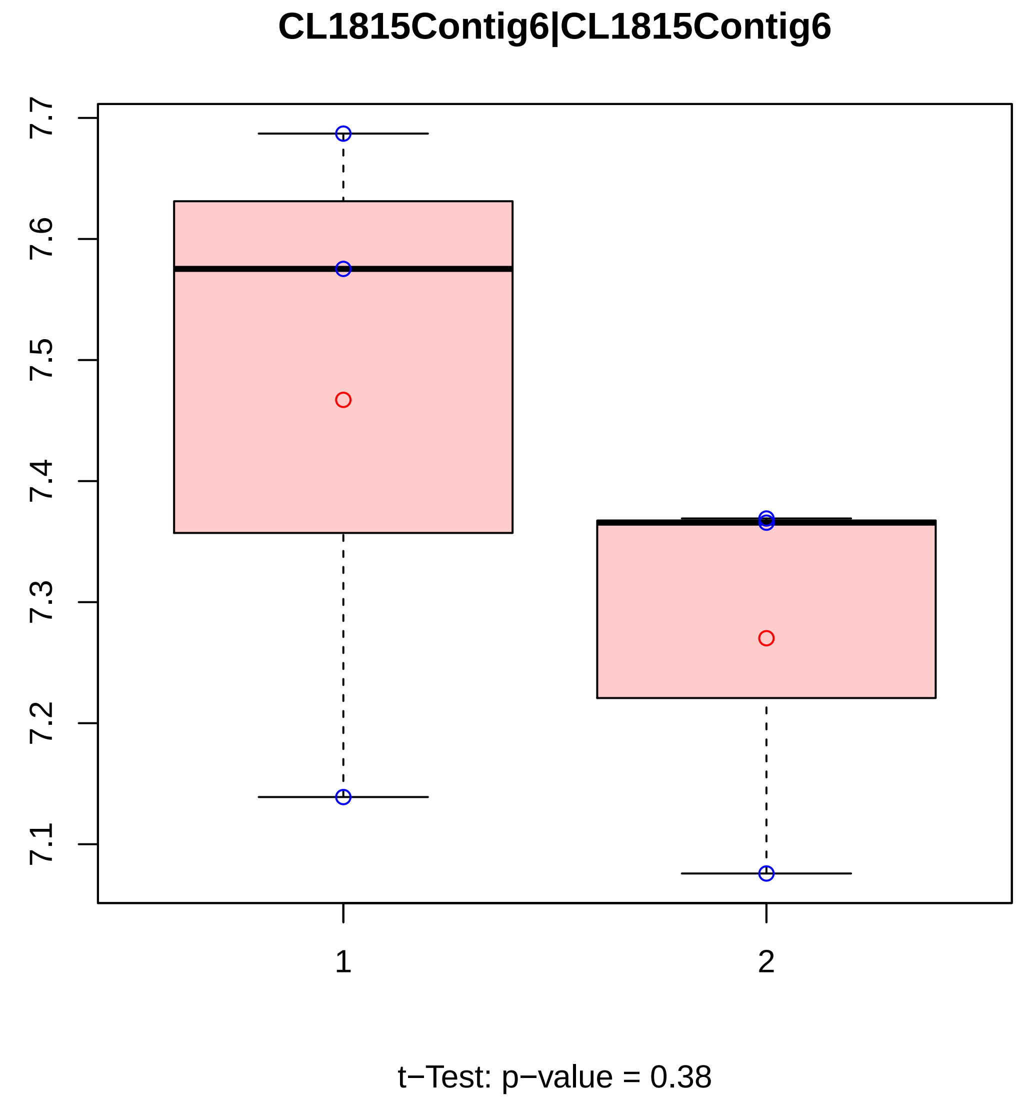

CL1815Contig6|CL1815Contig6
7.1
7.2
7.3
7.4
7.5
7.6
7.7
1
2
t−Test: p−value = 0.38

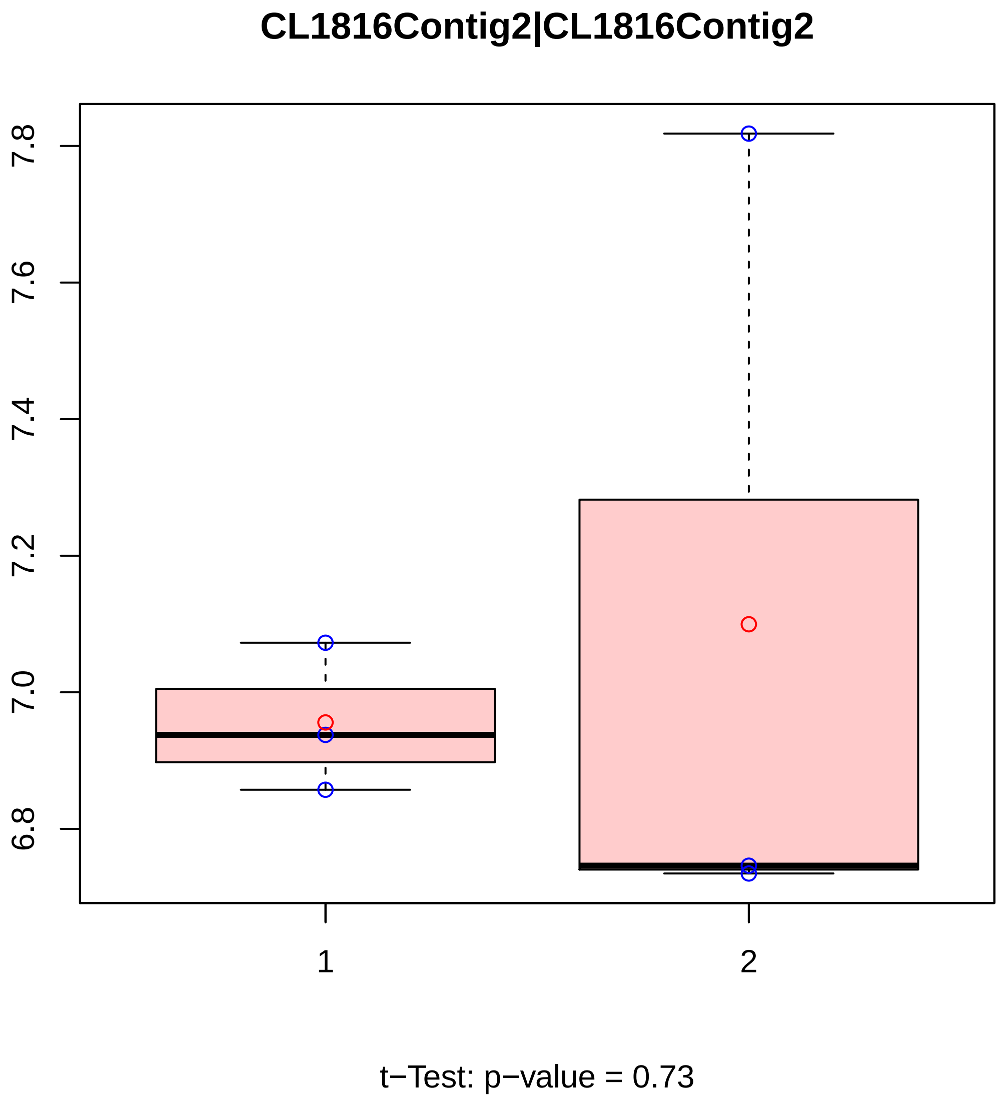
CL1816Contig2|CL1816Contig2
7.8
7.6
7.4
7.2
7.0
6.8
1
2
t−Test: p−value = 0.73

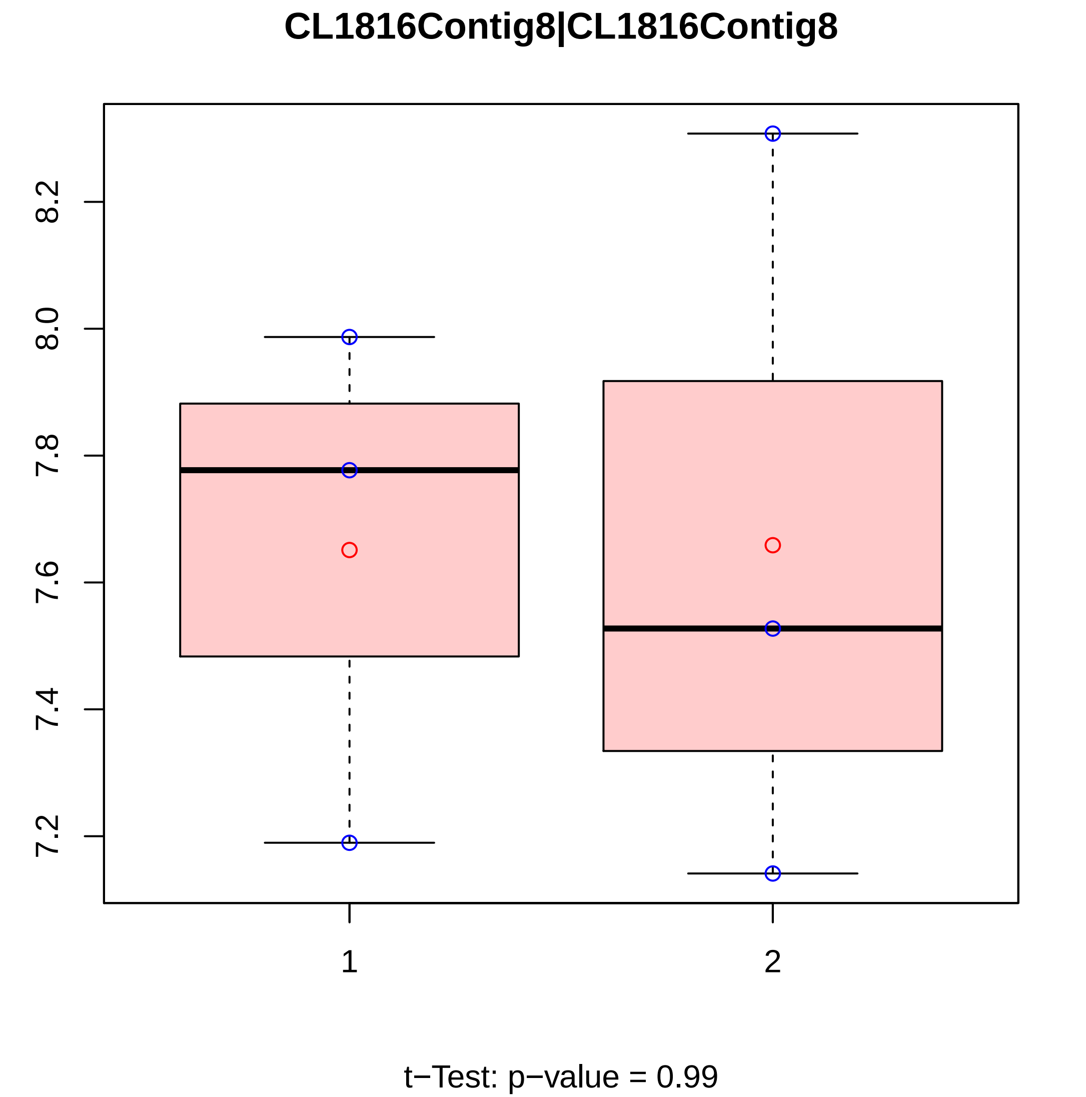
CL1816Contig8|CL1816Contig8
8.2
8.0
7.8
7.6
7.4
7.2
1
2
t−Test: p−value = 0.99

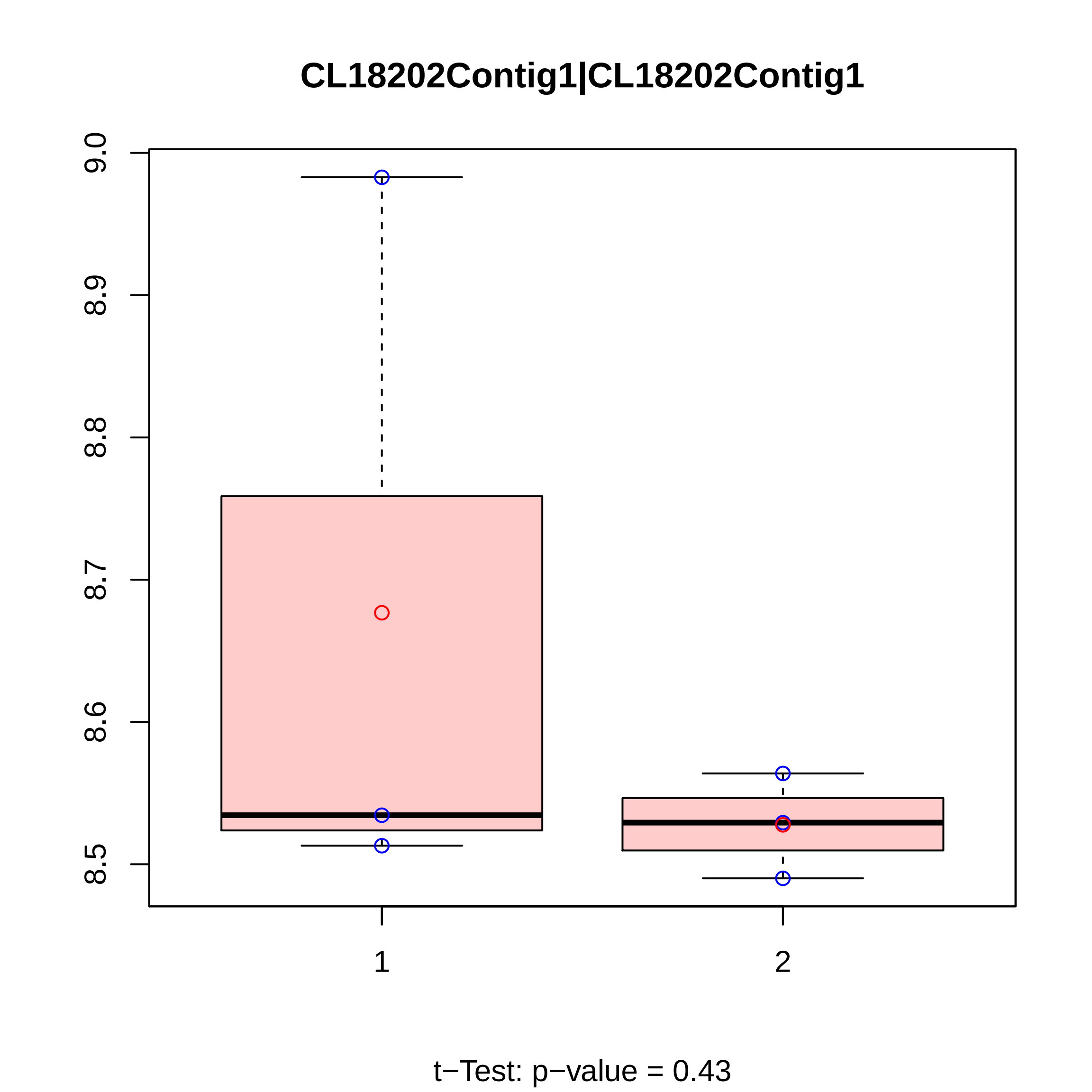
CL18202Contig1|CL18202Contig1
9.0
8.9
8.8
8.7
8.6
8.5
1
2
t−Test: p−value = 0.43

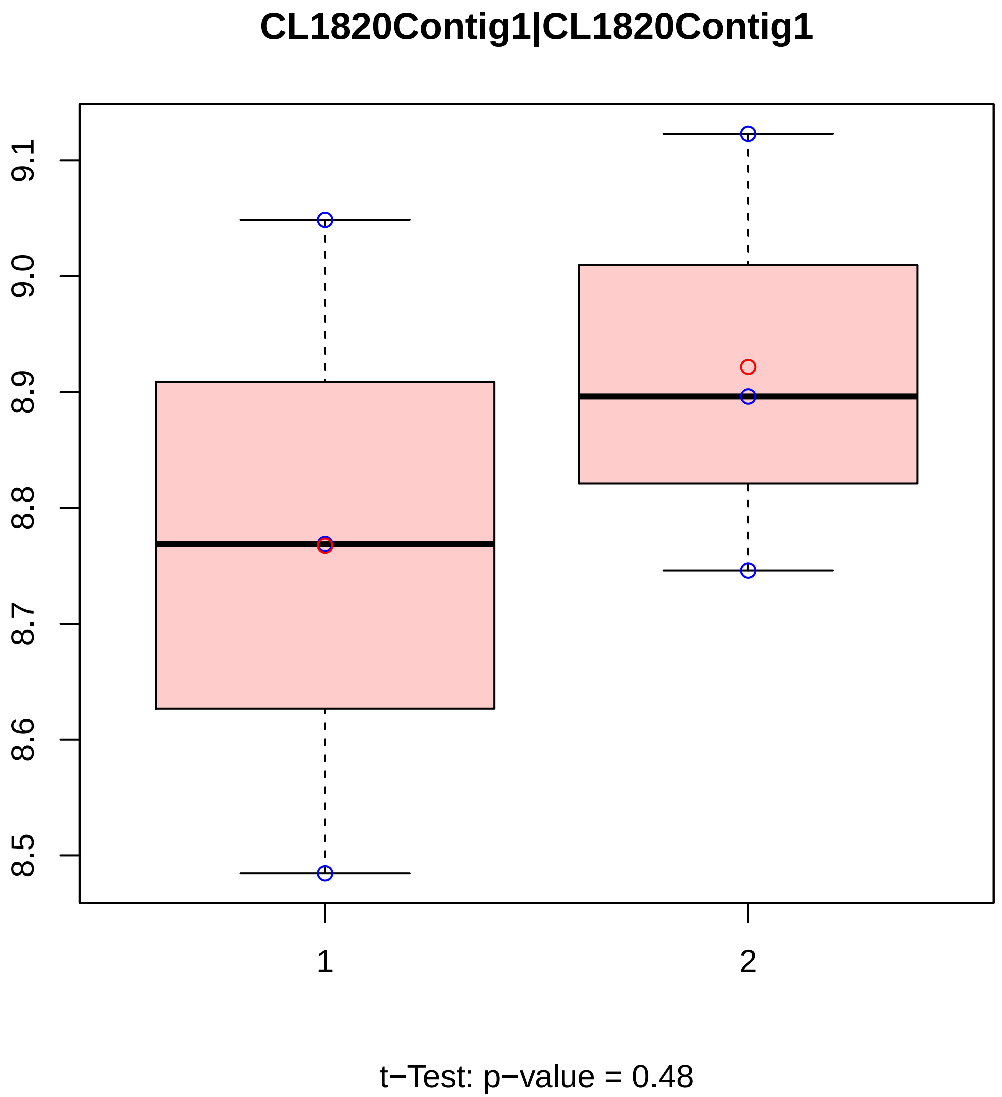
CL1820Contig1|CL1820Contig1
9.1
9.0
8.9
8.8
8.7
8.6
8.5
1
2
t−Test: p−value = 0.48

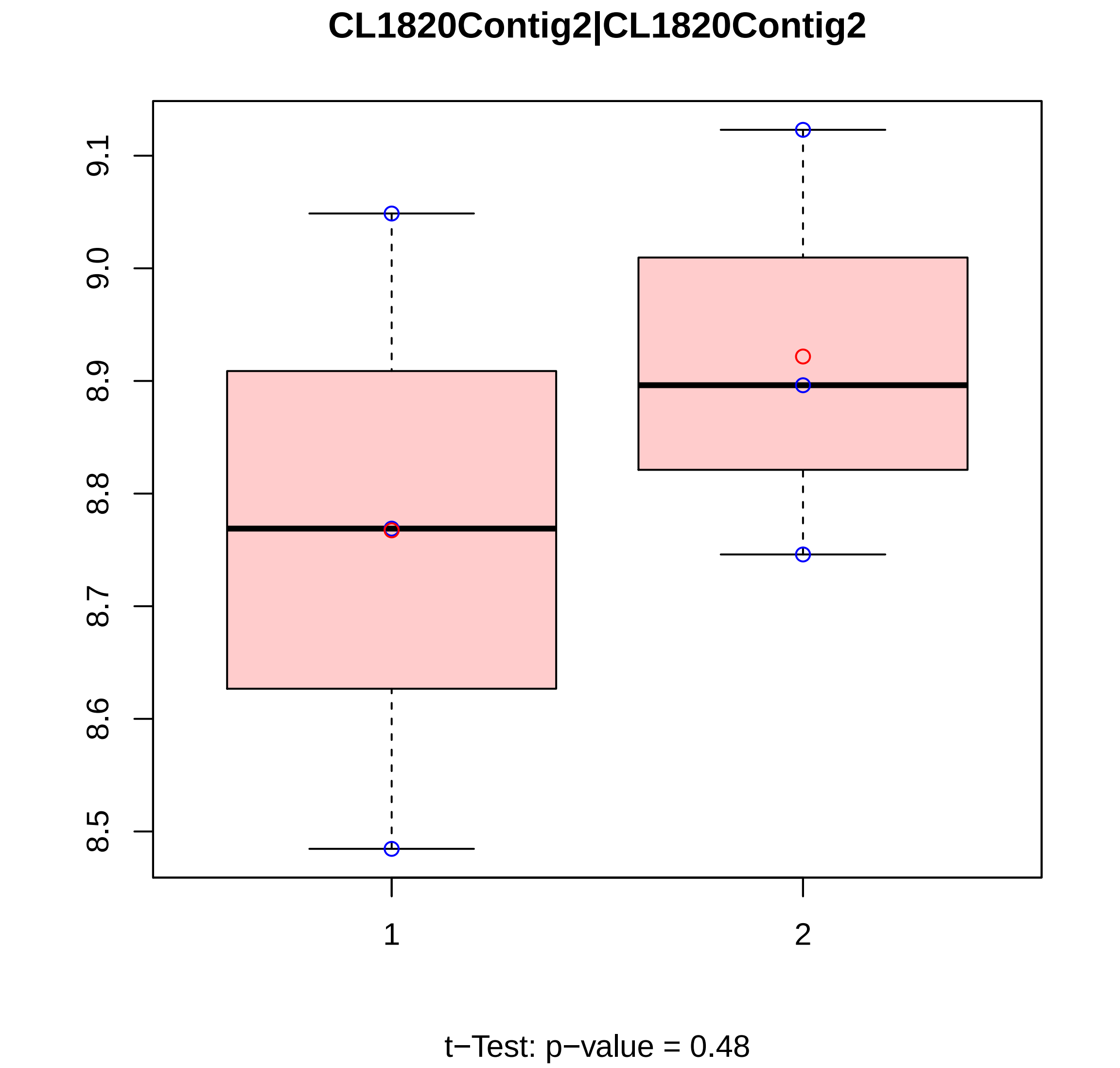

CL1820Contig2|CL1820Contig2
9.1
9.0
8.9
8.8
8.7
8.6
8.5
1
2
t−Test: p−value = 0.48

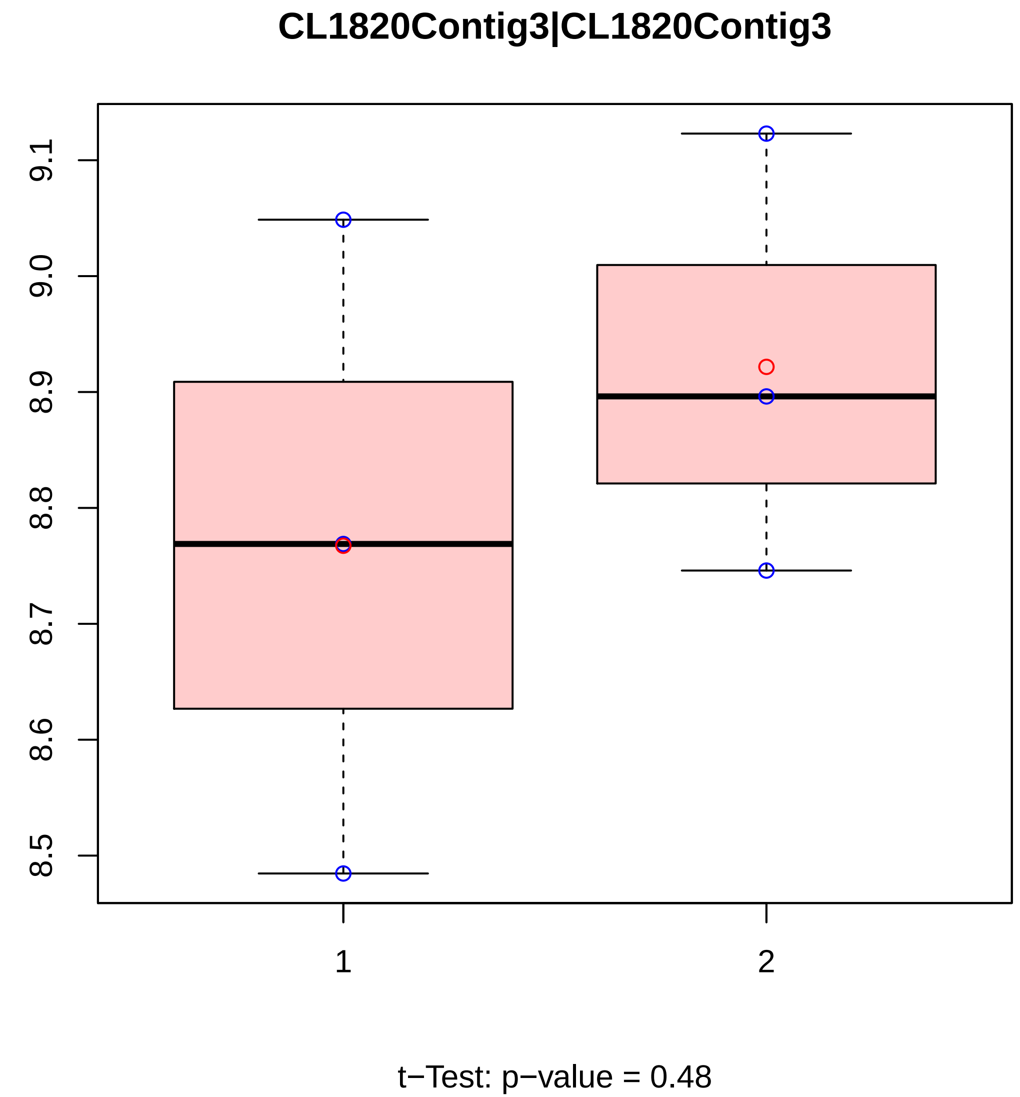

CL1820Contig3|CL1820Contig3
9.1
9.0
8.9
8.8
8.7
8.6
8.5
1
2
t−Test: p−value = 0.48

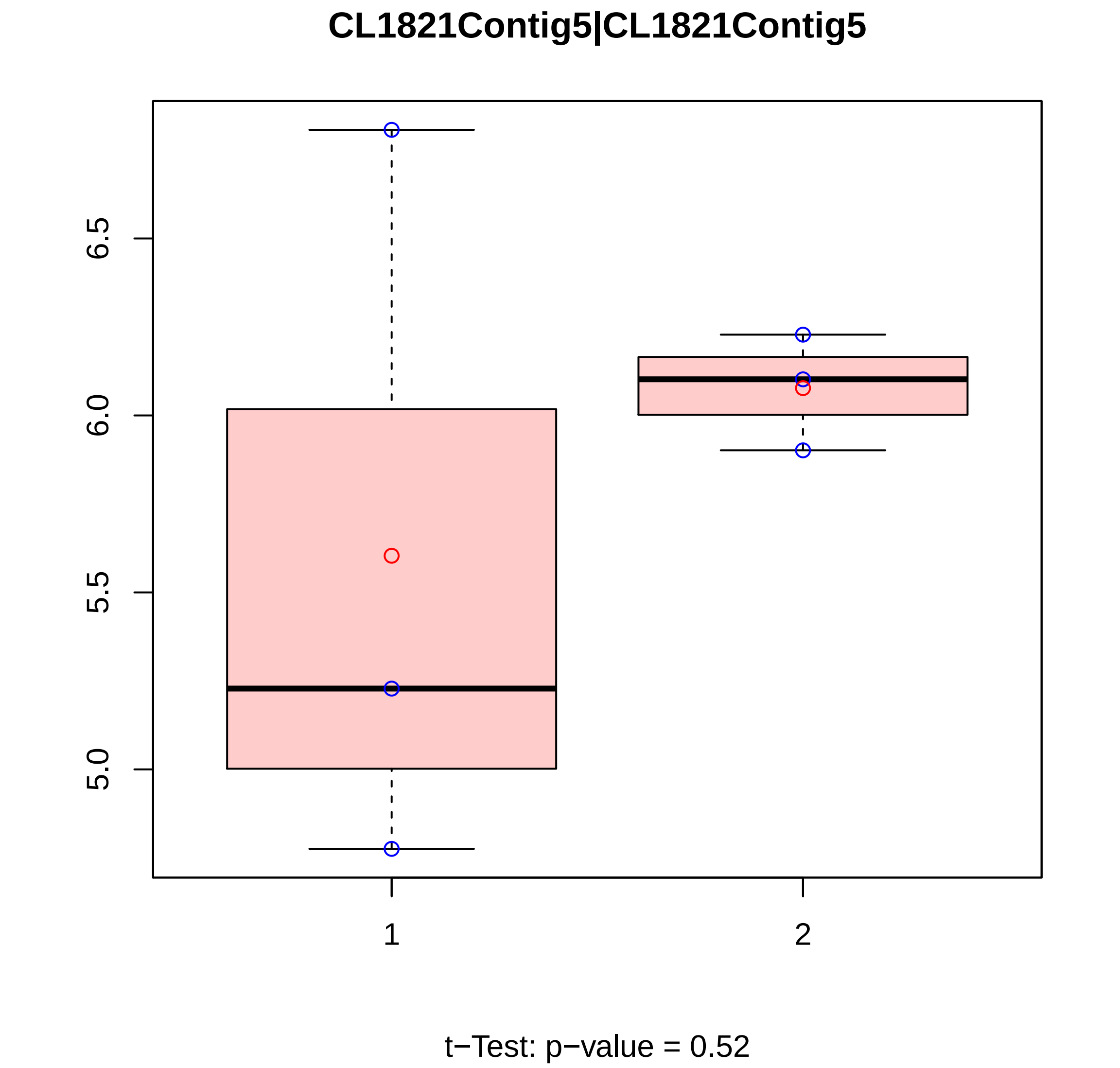
CL1821Contig5|CL1821Contig5
6.5
6.0
5.5
5.0
1
2
t−Test: p−value = 0.52

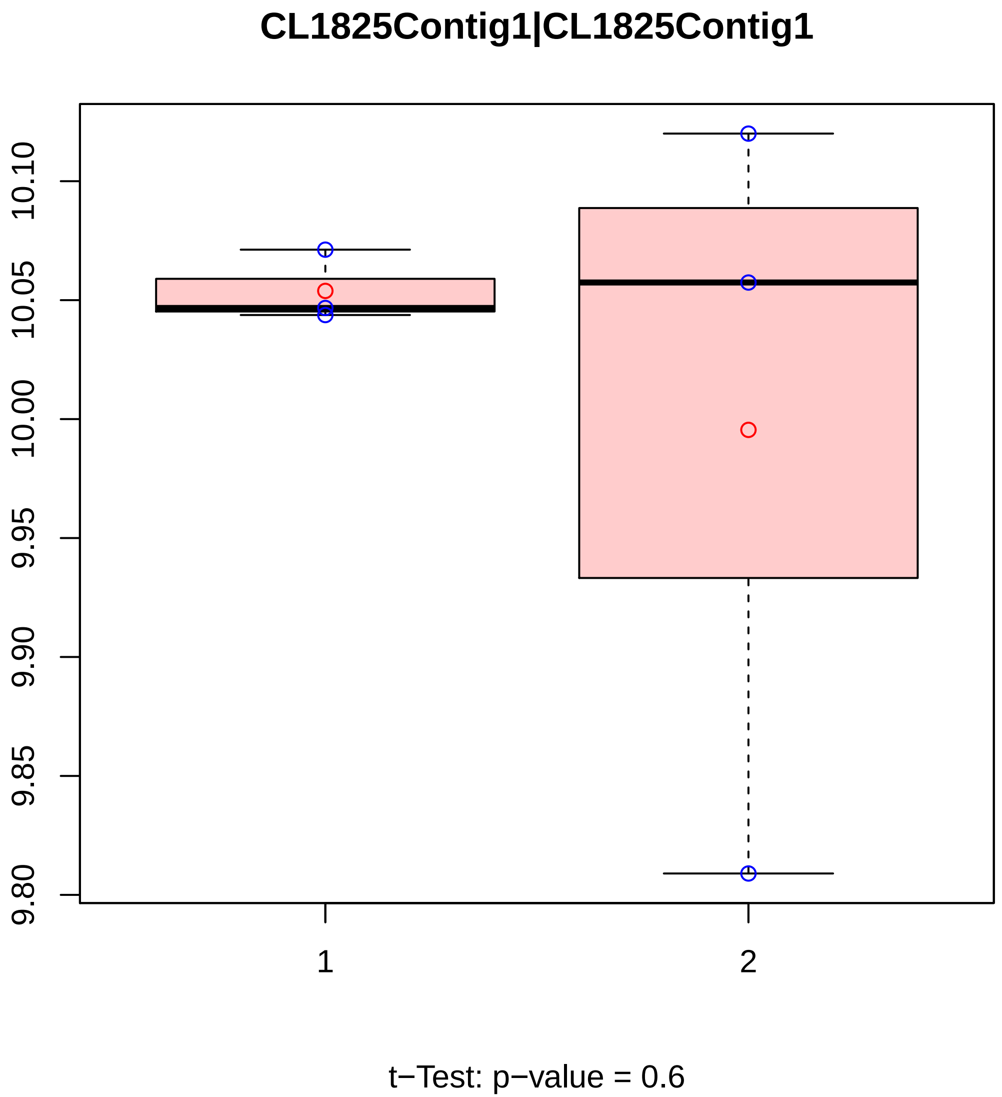
CL1825Contig1|CL1825Contig1
10.10
10.05
10.00
9.95
9.90
9.85
9.80
1
2
t−Test: p−value = 0.6

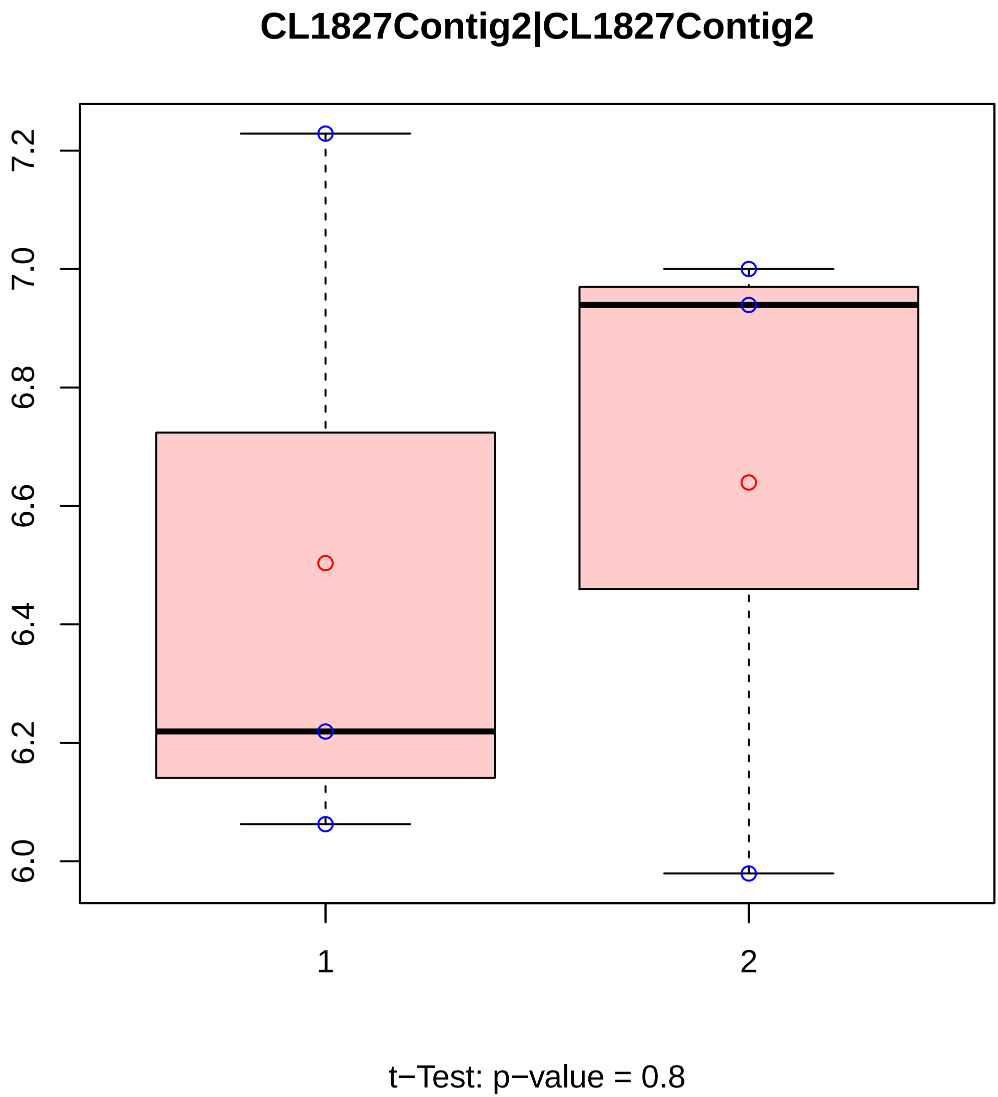

CL1827Contig2|CL1827Contig2
7.2
7.0
6.8
6.6
6.4
6.2
6.0
1
2
t−Test: p−value = 0.8

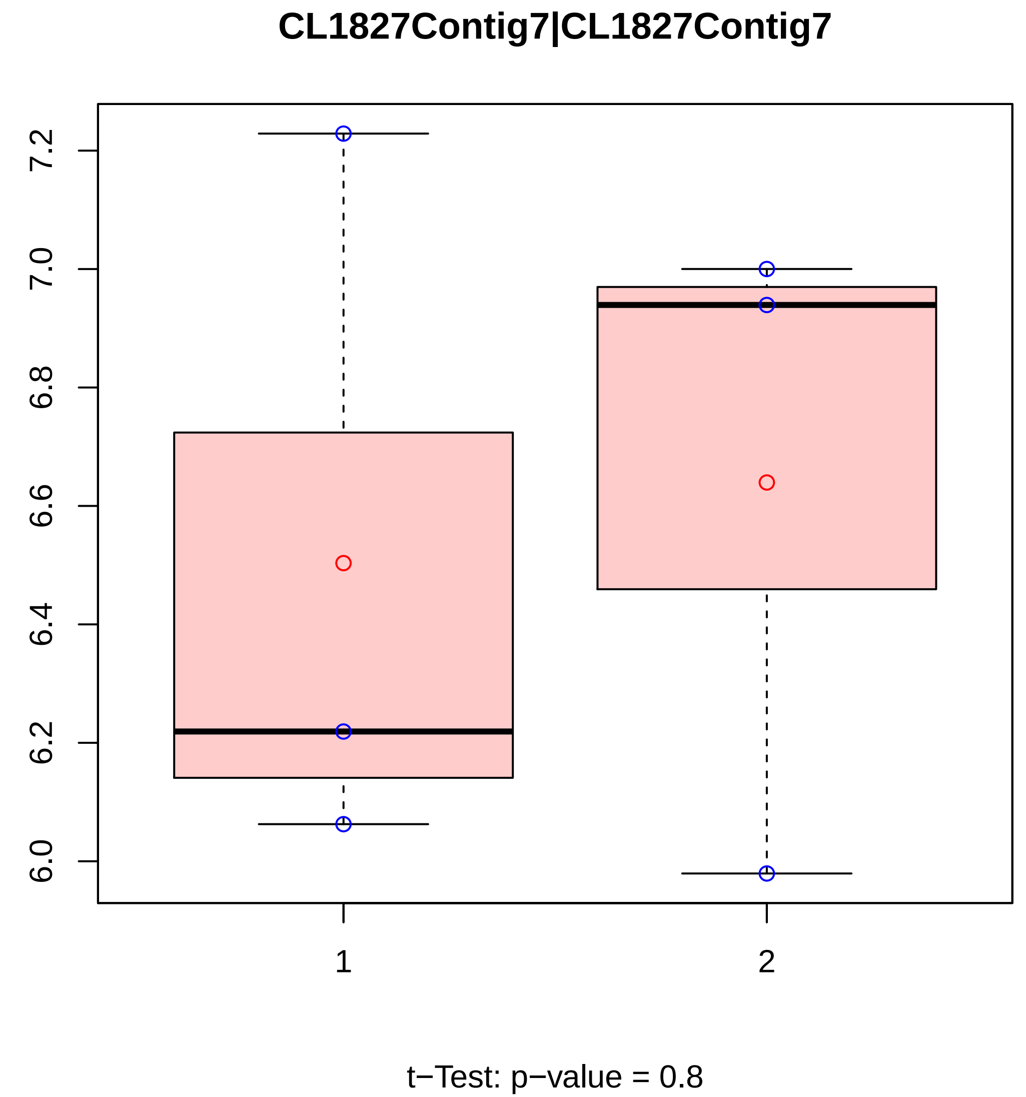
CL1827Contig7|CL1827Contig7
7.2
7.0
6.8
6.6
6.4
6.2
6.0
1
2
t−Test: p−value = 0.8

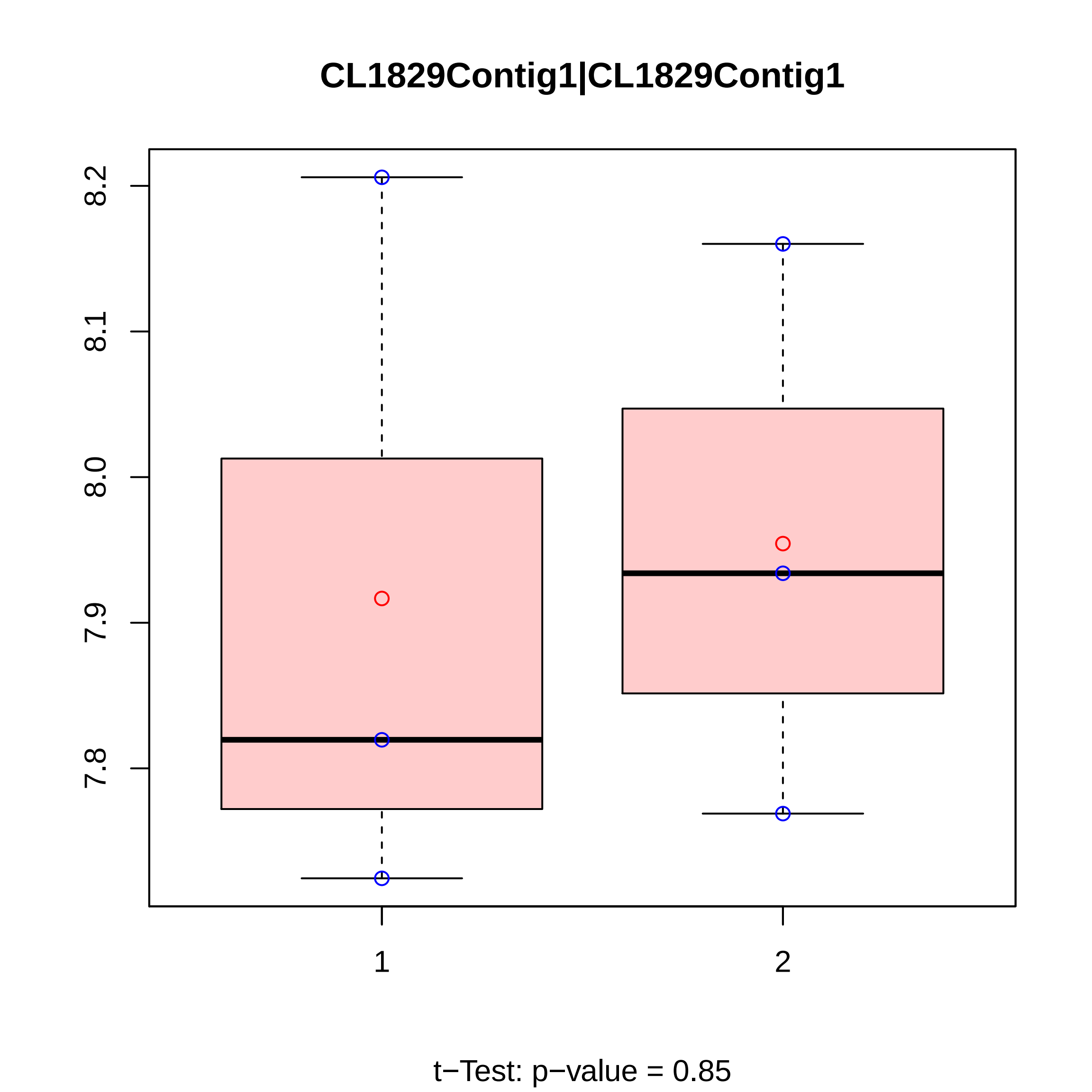

CL1829Contig1|CL1829Contig1
8.2
8.1
8.0
7.9
7.8
1
2
t−Test: p−value = 0.85

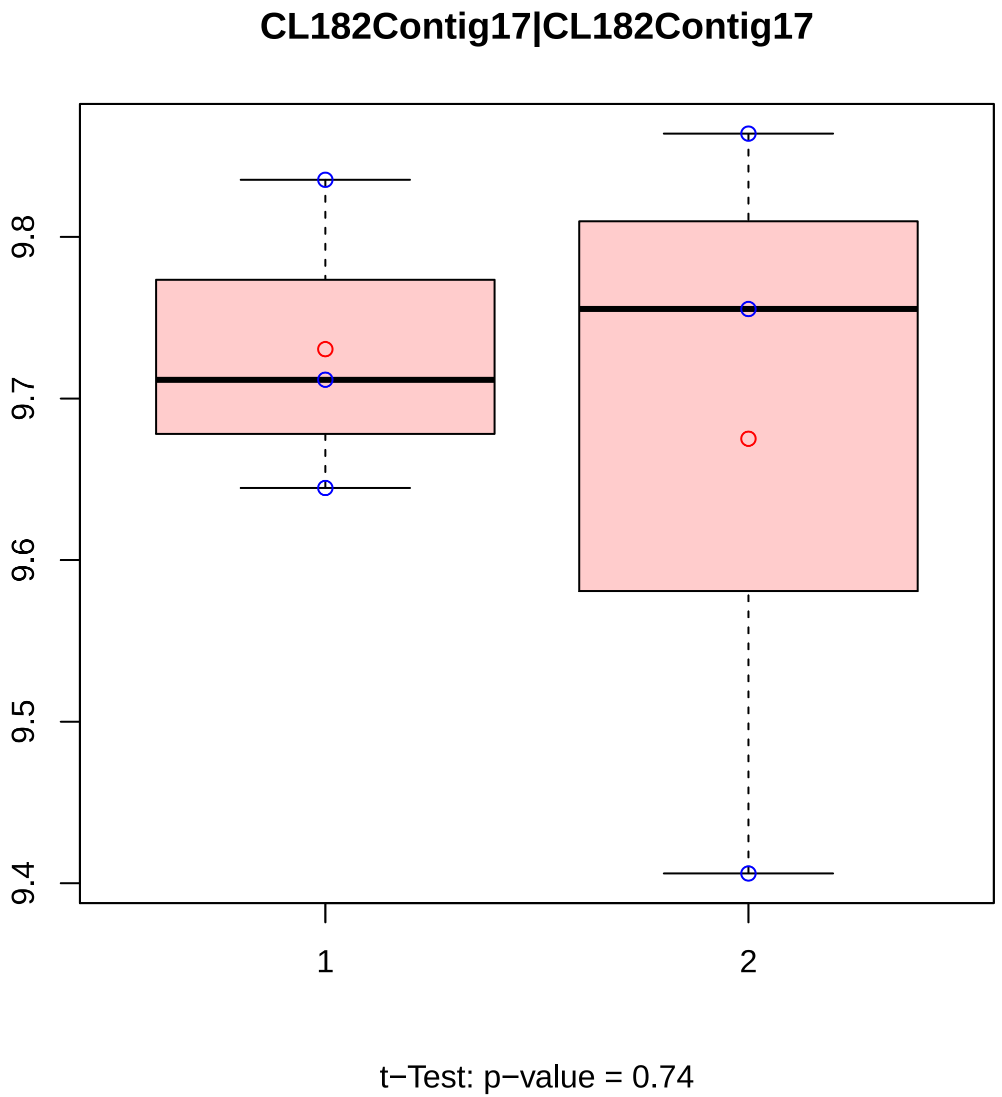
CL182Contig17|CL182Contig17
9.4
9.5
9.6
9.7
9.8
1
2
t−Test: p−value = 0.74

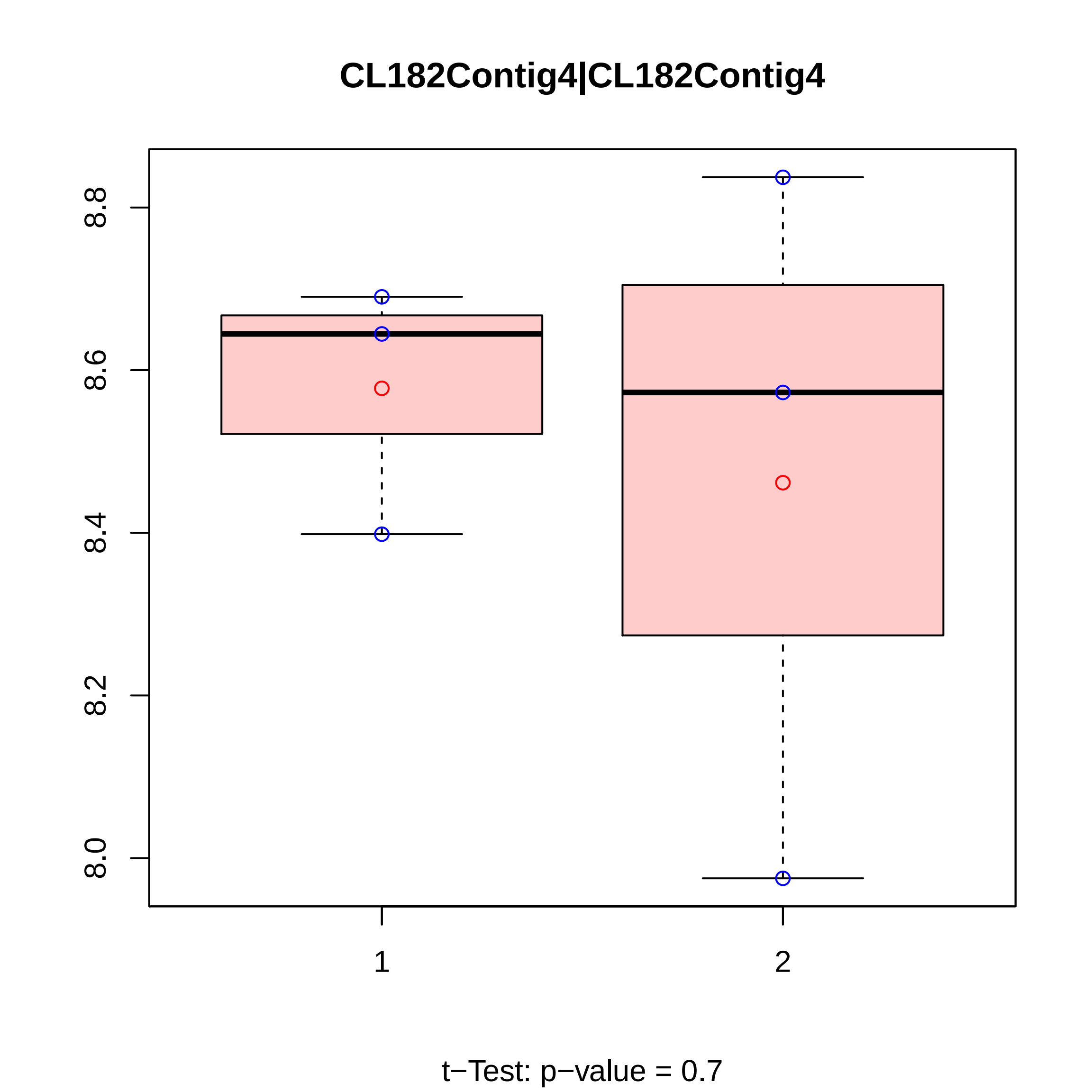
CL182Contig4|CL182Contig4
8.0
8.2
8.4
8.6
8.8
1
2
t−Test: p−value = 0.7

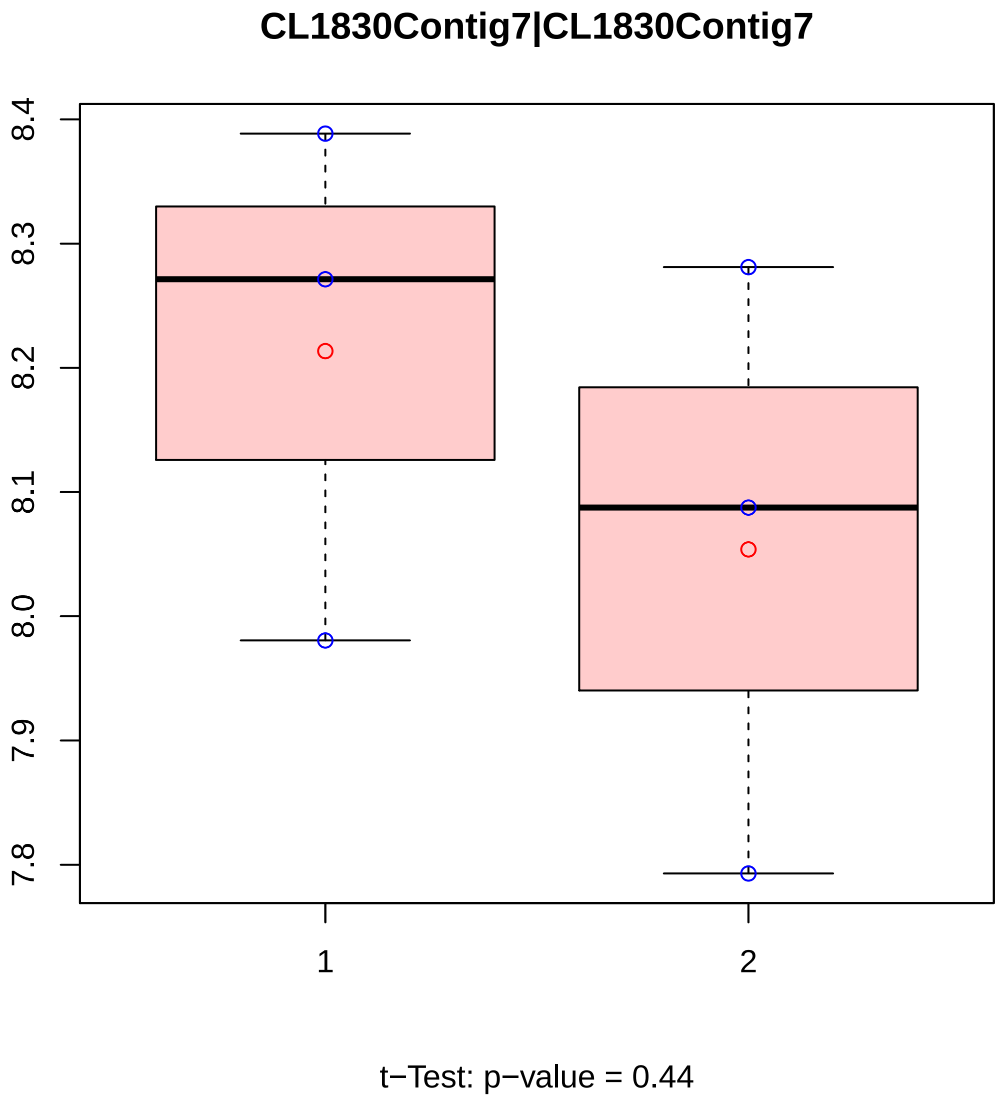
CL1830Contig7|CL1830Contig7
8.4
8.3
8.2
8.1
8.0
7.9
7.8
1
2
t−Test: p−value = 0.44

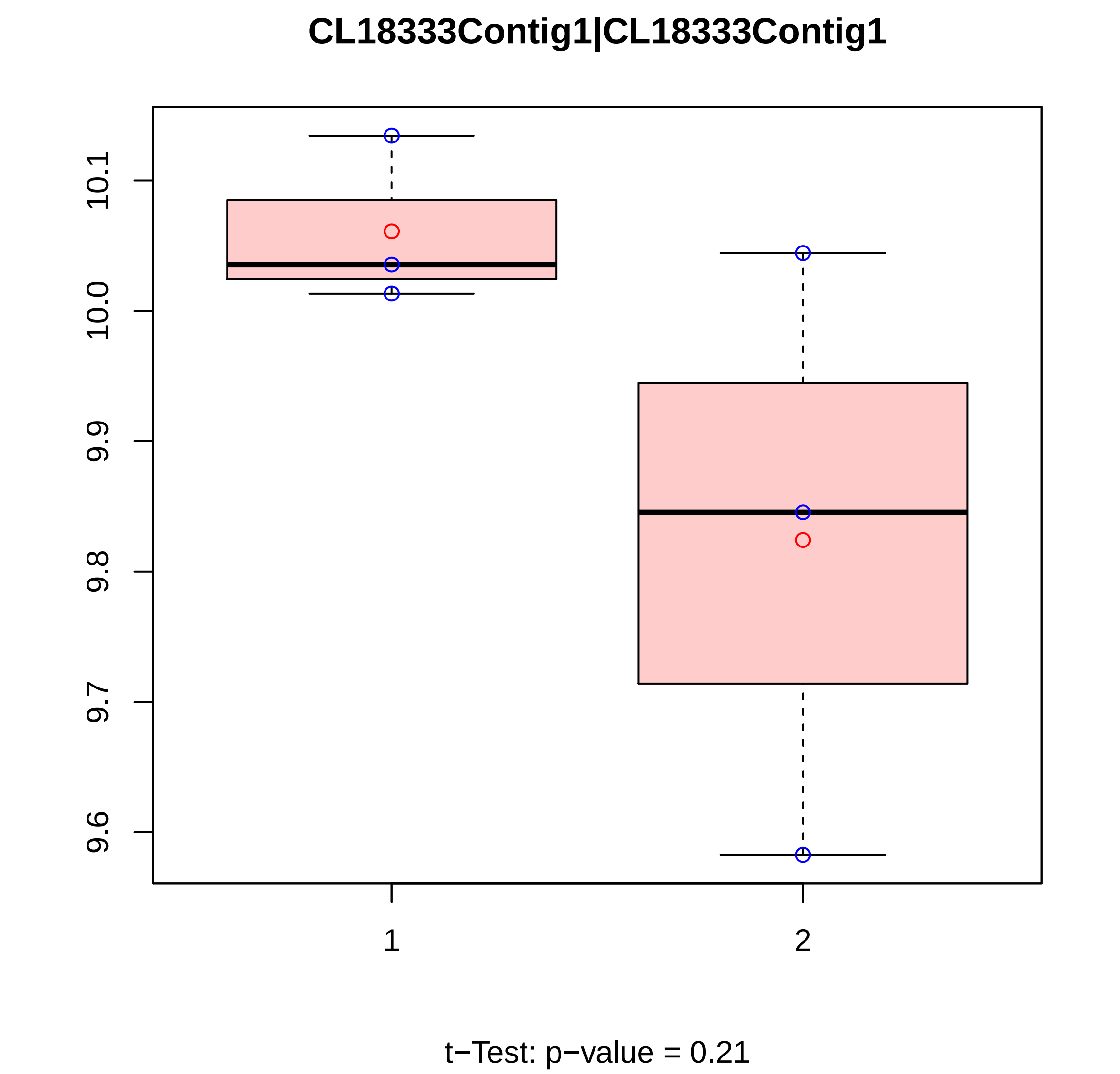
CL18333Contig1|CL18333Contig1
10.1
10.0
9.9
9.8
9.7
9.6
1
2
t−Test: p−value = 0.21

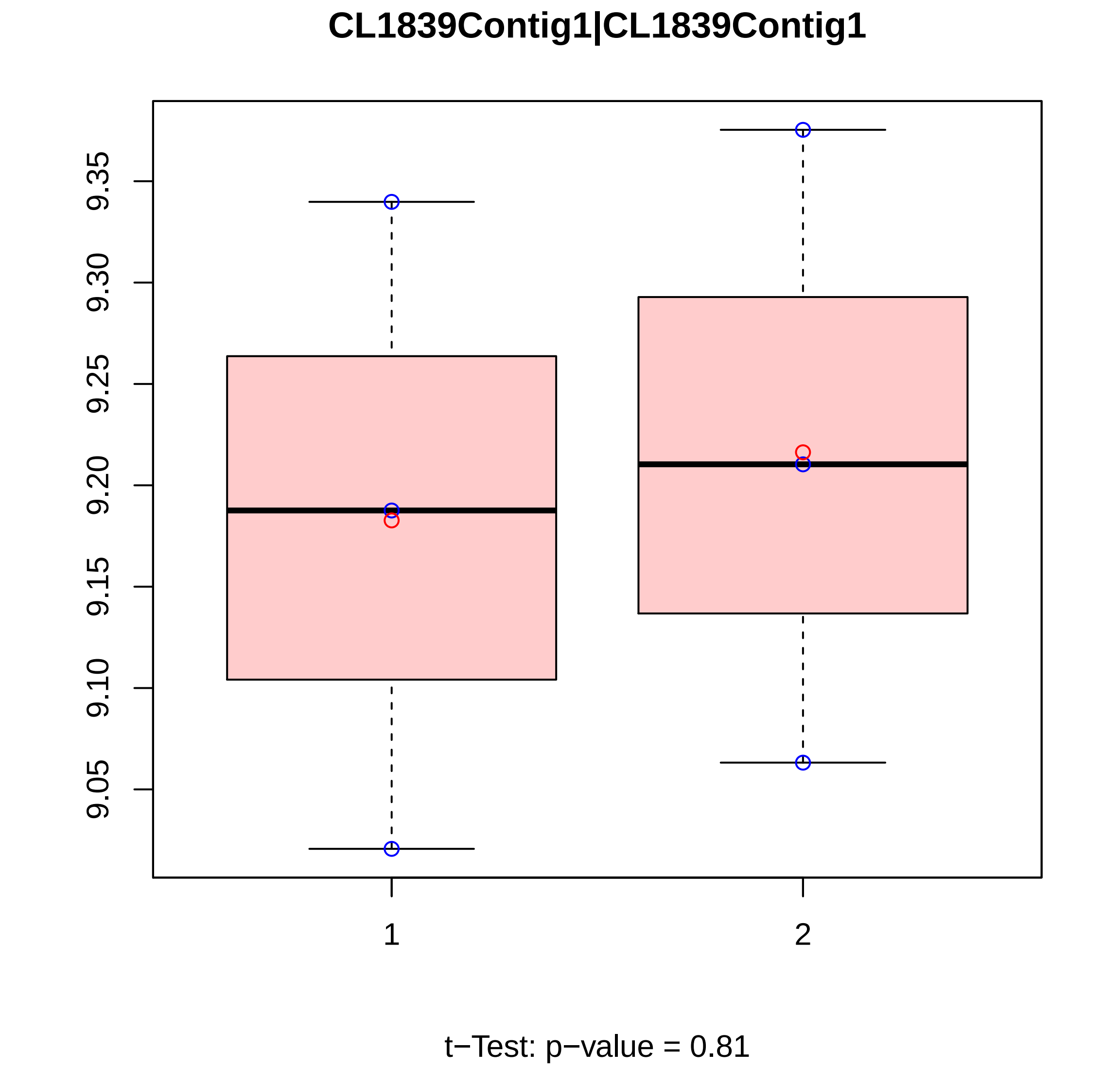

CL1839Contig1|CL1839Contig1
9.05
9.10
9.15
9.20
9.25
9.30
9.35
1
2
t−Test: p−value = 0.81

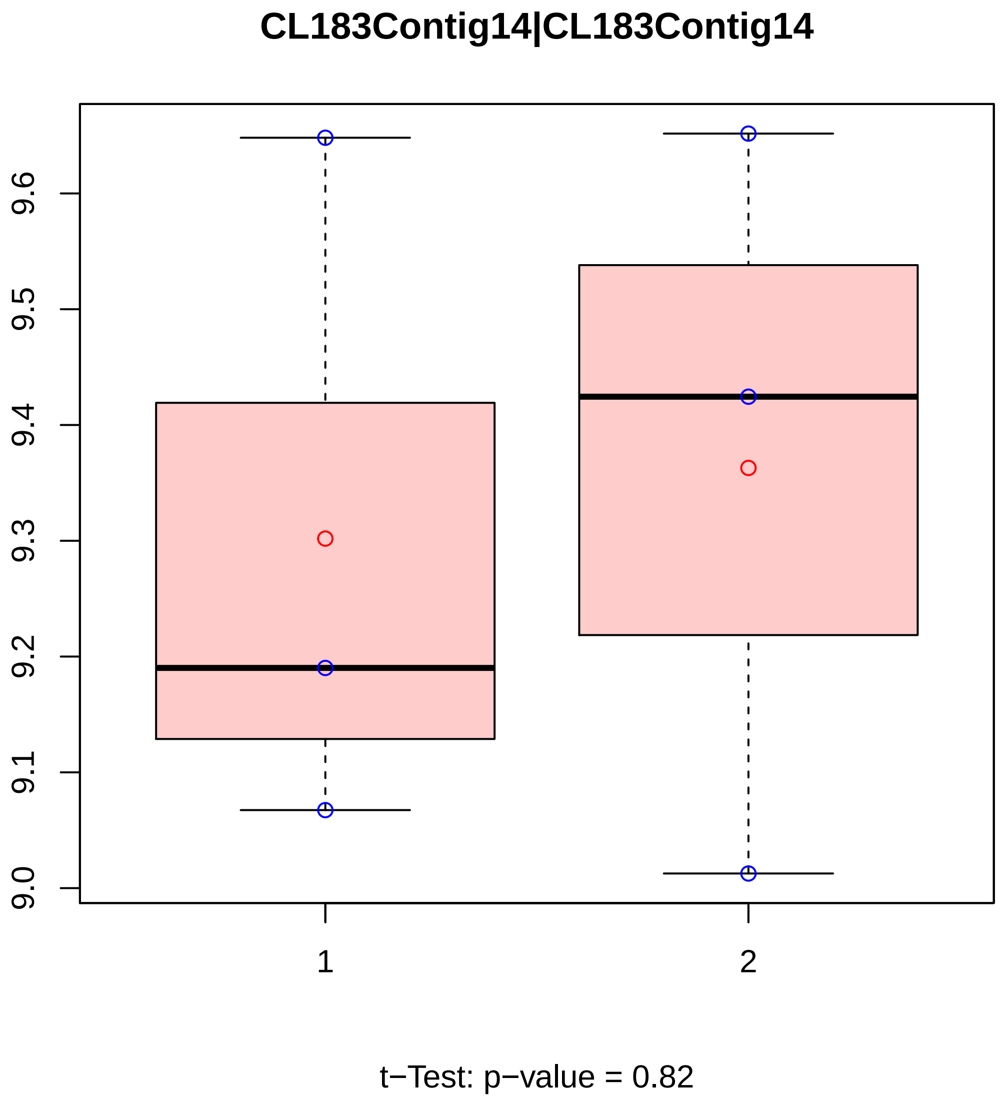
CL183Contig14|CL183Contig14
9.0
9.1
9.2
9.3
9.4
9.5
9.6
1
2
t−Test: p−value = 0.82

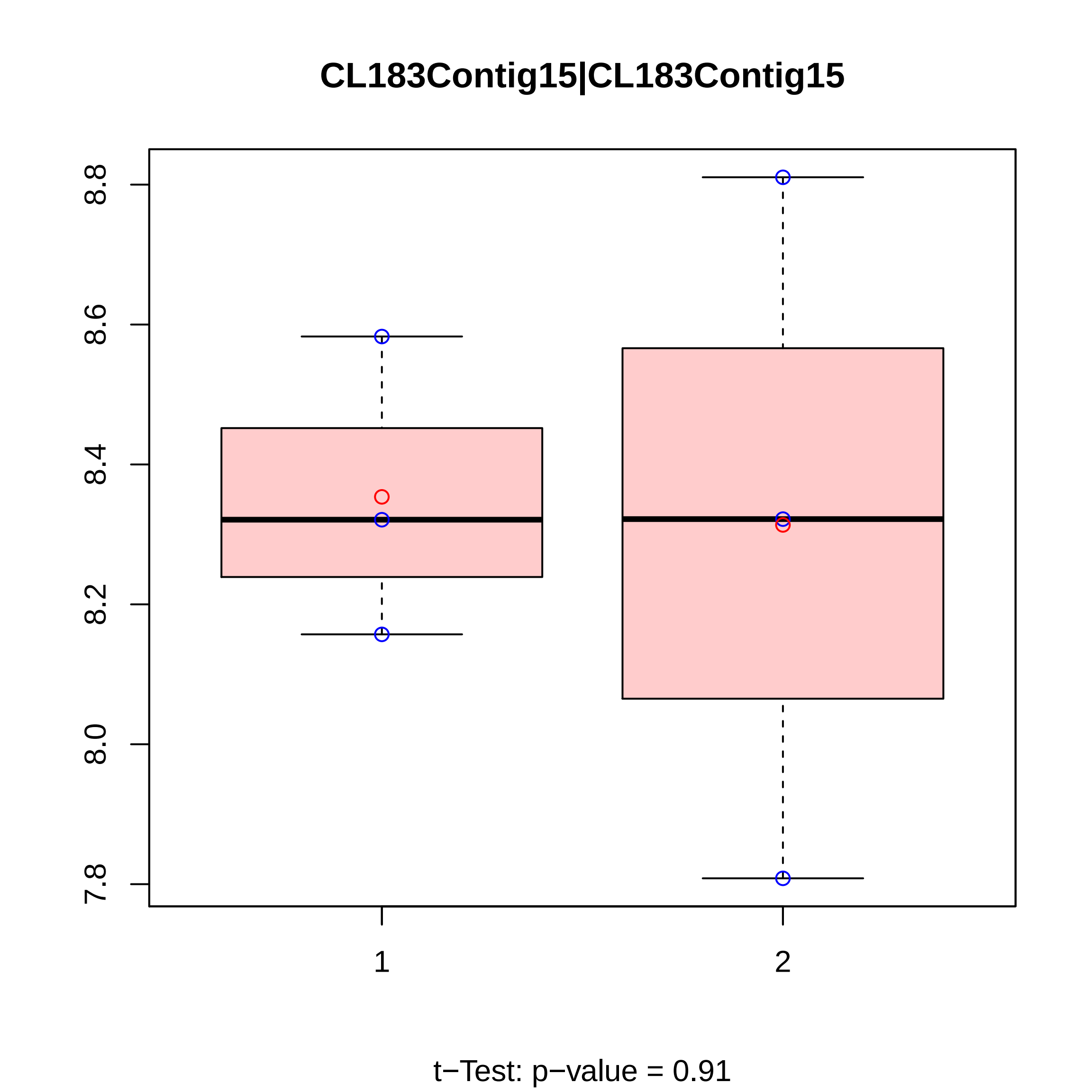

CL183Contig15|CL183Contig15
8.8
8.6
8.4
8.2
8.0
7.8
1
2
t−Test: p−value = 0.91

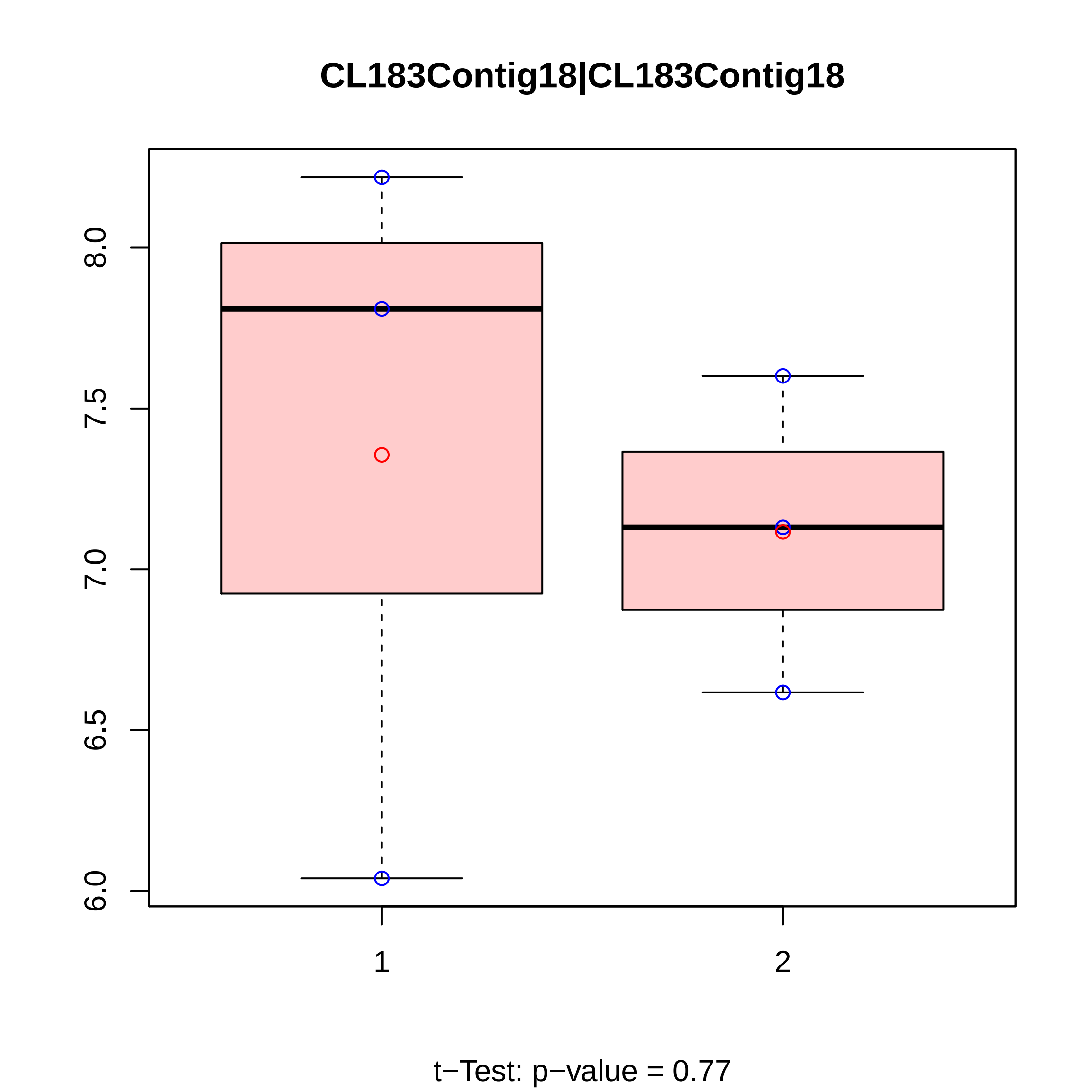
CL183Contig18|CL183Contig18
8.0
7.5
7.0
6.5
6.0
1
2
t−Test: p−value = 0.77

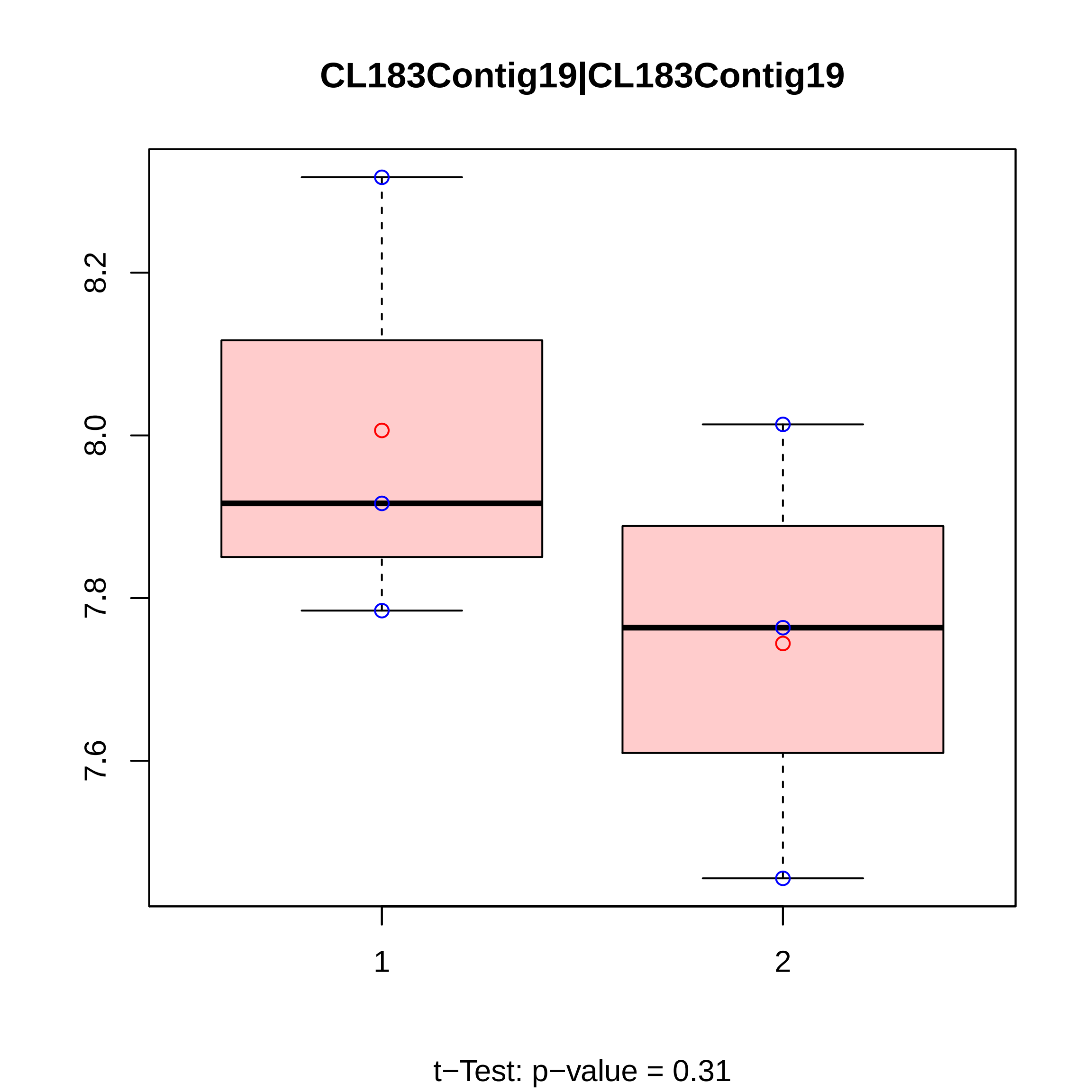

CL183Contig19|CL183Contig19
8.2
8.0
7.8
7.6
1
2
t−Test: p−value = 0.31

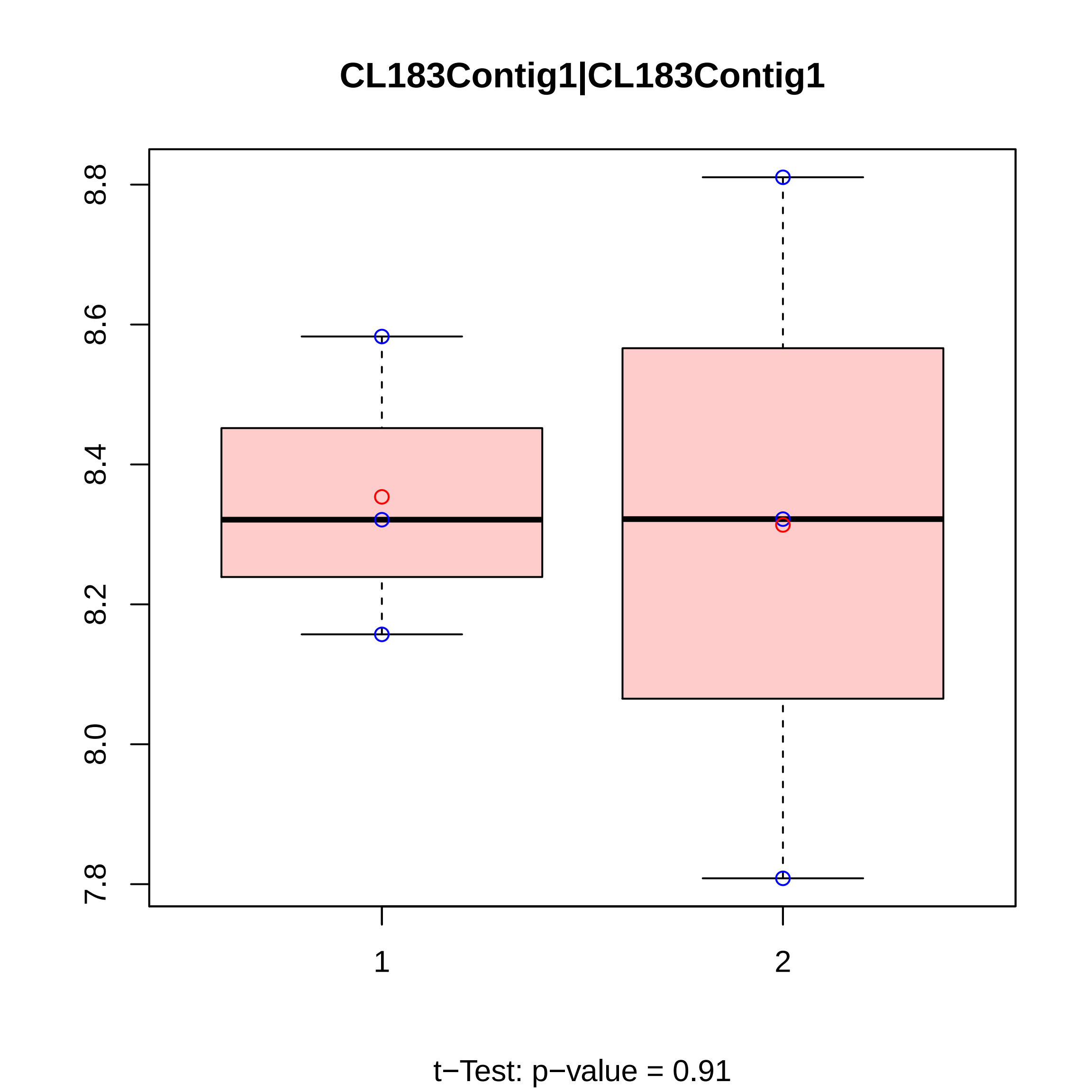

CL183Contig1|CL183Contig1
8.8
8.6
8.4
8.2
8.0
7.8
1
2
t−Test: p−value = 0.91

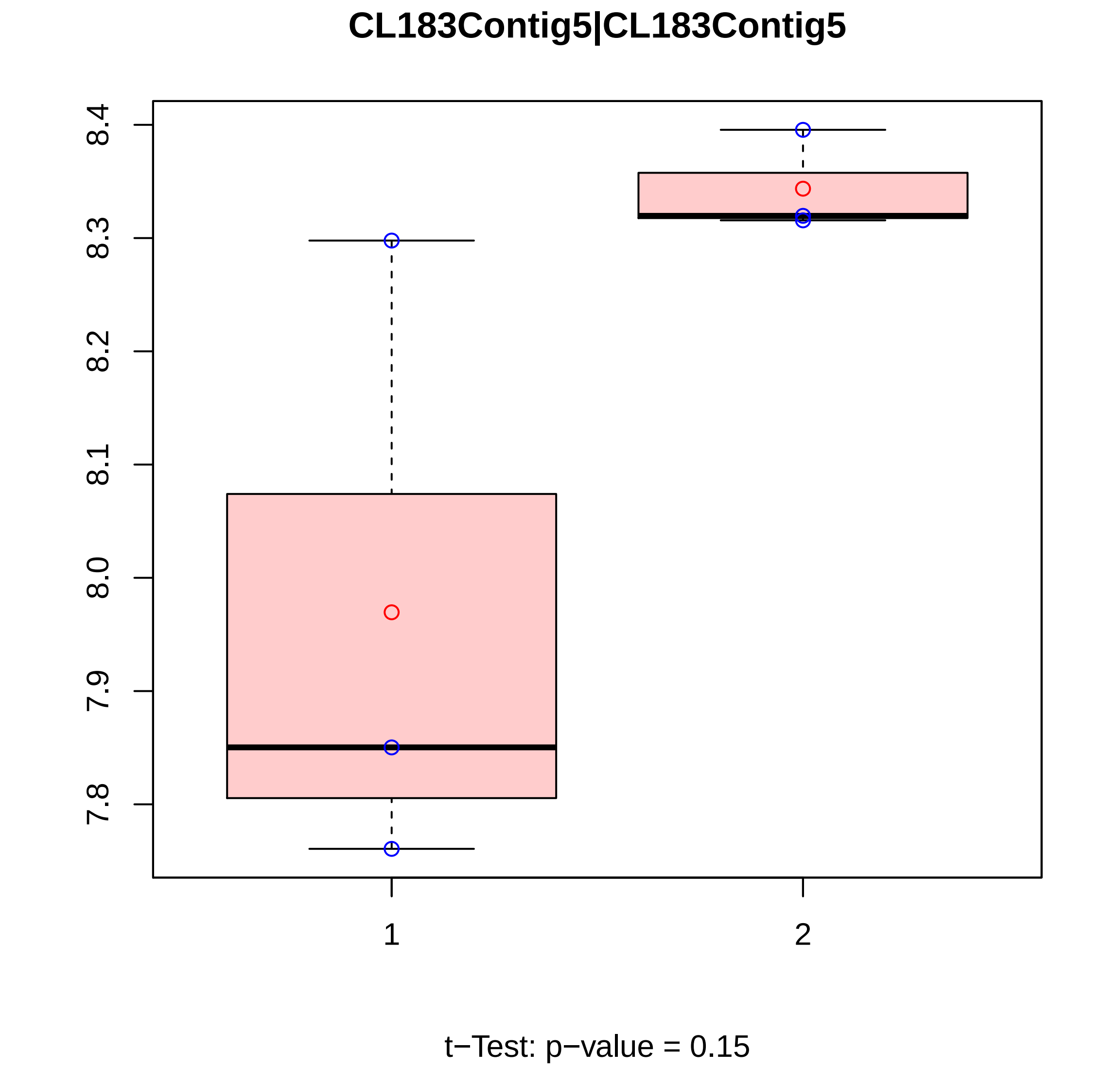

CL183Contig5|CL183Contig5
8.4
8.3
8.2
8.1
8.0
7.9
7.8
1
2
t−Test: p−value = 0.15

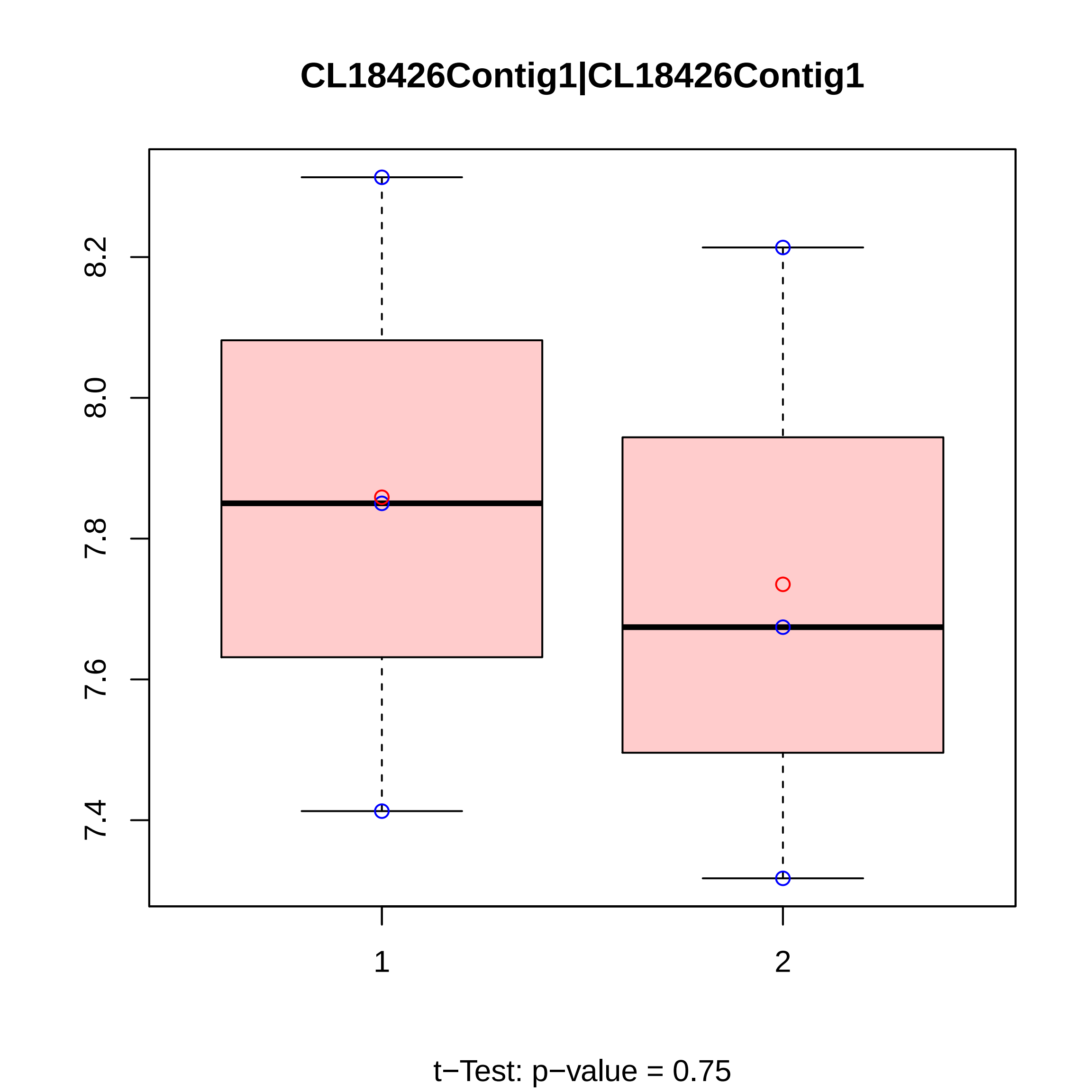
CL18426Contig1|CL18426Contig1
8.2
8.0
7.8
7.6
7.4
1
2
t−Test: p−value = 0.75

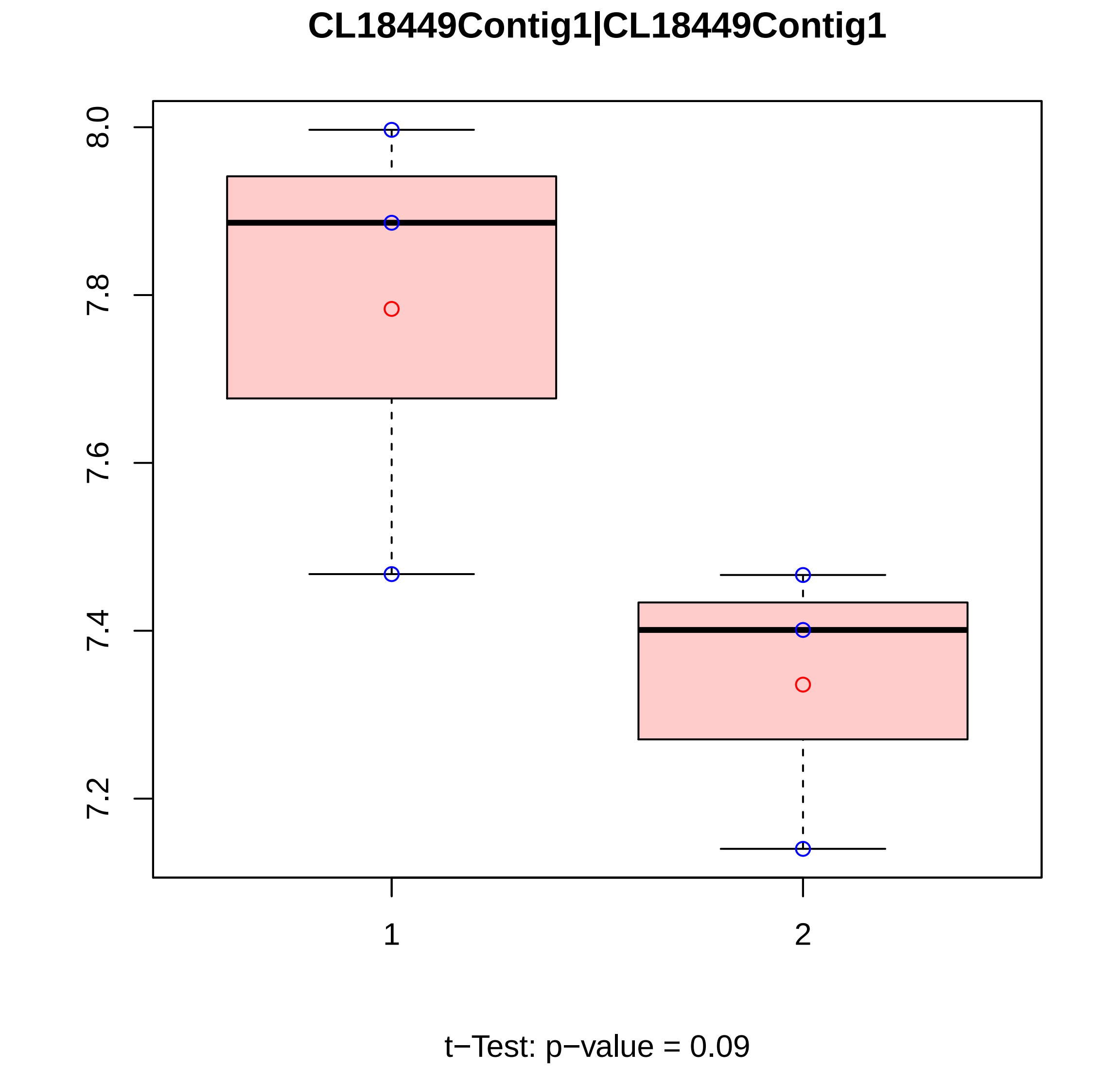
CL18449Contig1|CL18449Contig1
8.0
7.8
7.6
7.4
7.2
1
2
t−Test: p−value = 0.09

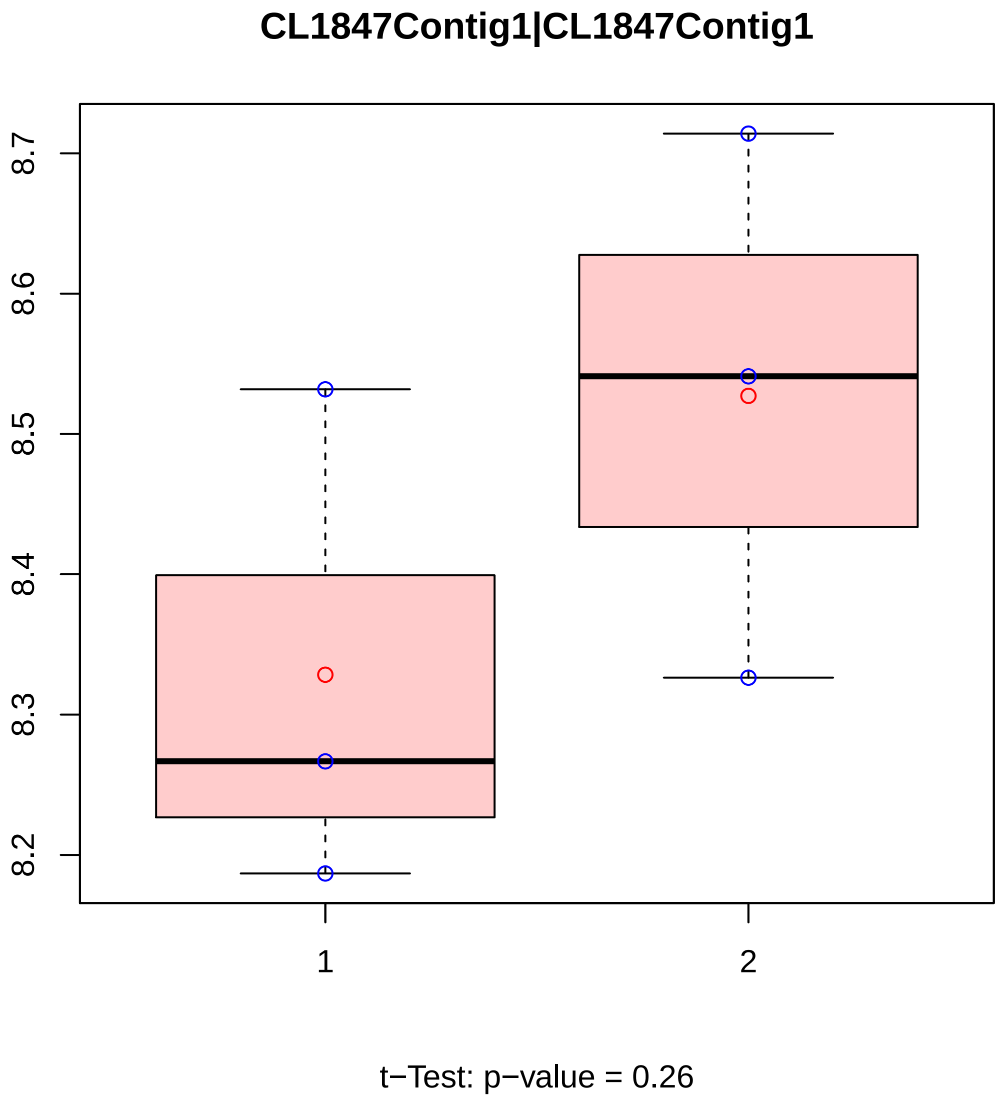

CL1847Contig1|CL1847Contig1
8.2
8.3
8.4
8.5
8.6
8.7
1
2
t−Test: p−value = 0.26

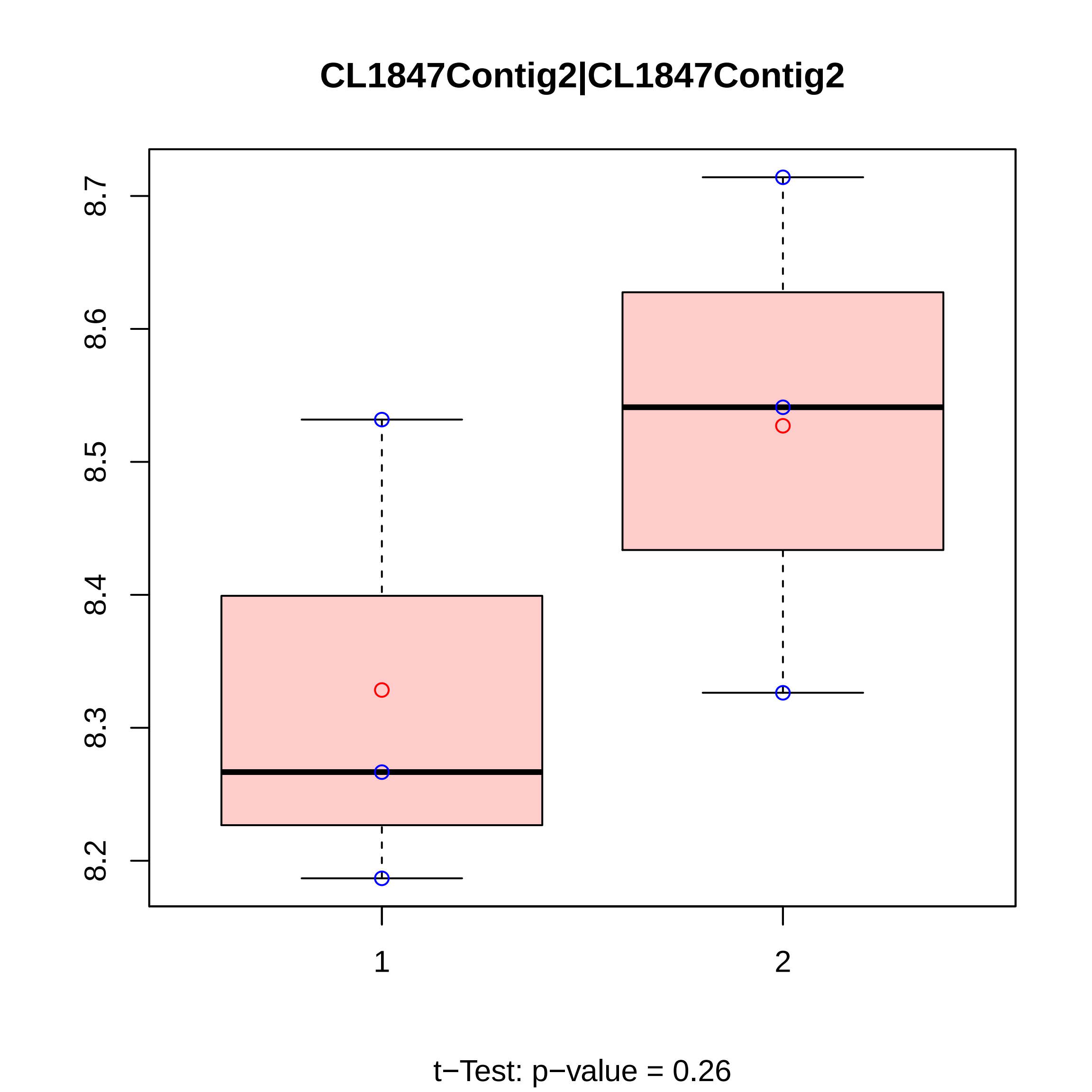
CL1847Contig2|CL1847Contig2
8.2
8.3
8.4
8.5
8.6
8.7
1
2
t−Test: p−value = 0.26

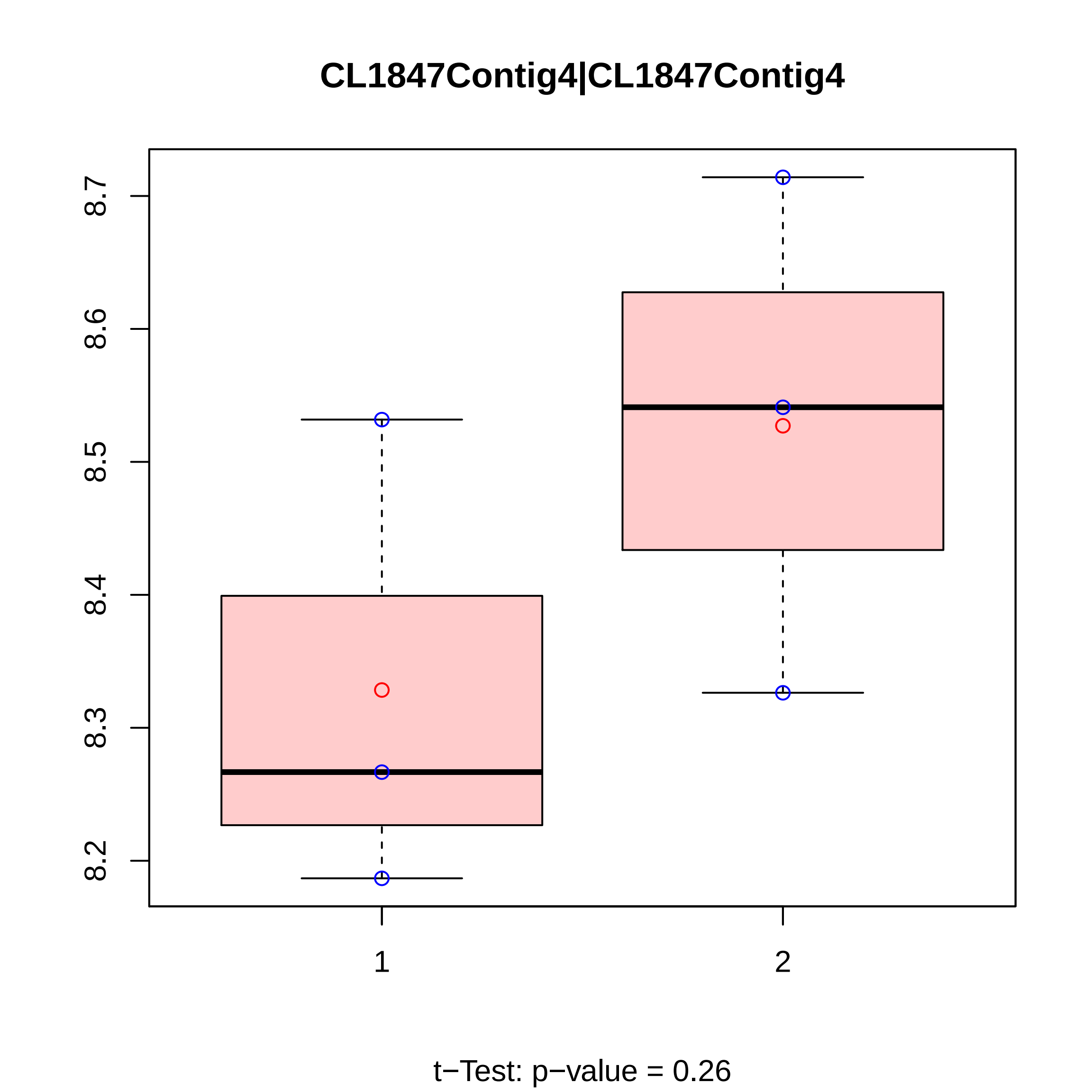
CL1847Contig4|CL1847Contig4
8.2
8.3
8.4
8.5
8.6
8.7
1
2
t−Test: p−value = 0.26

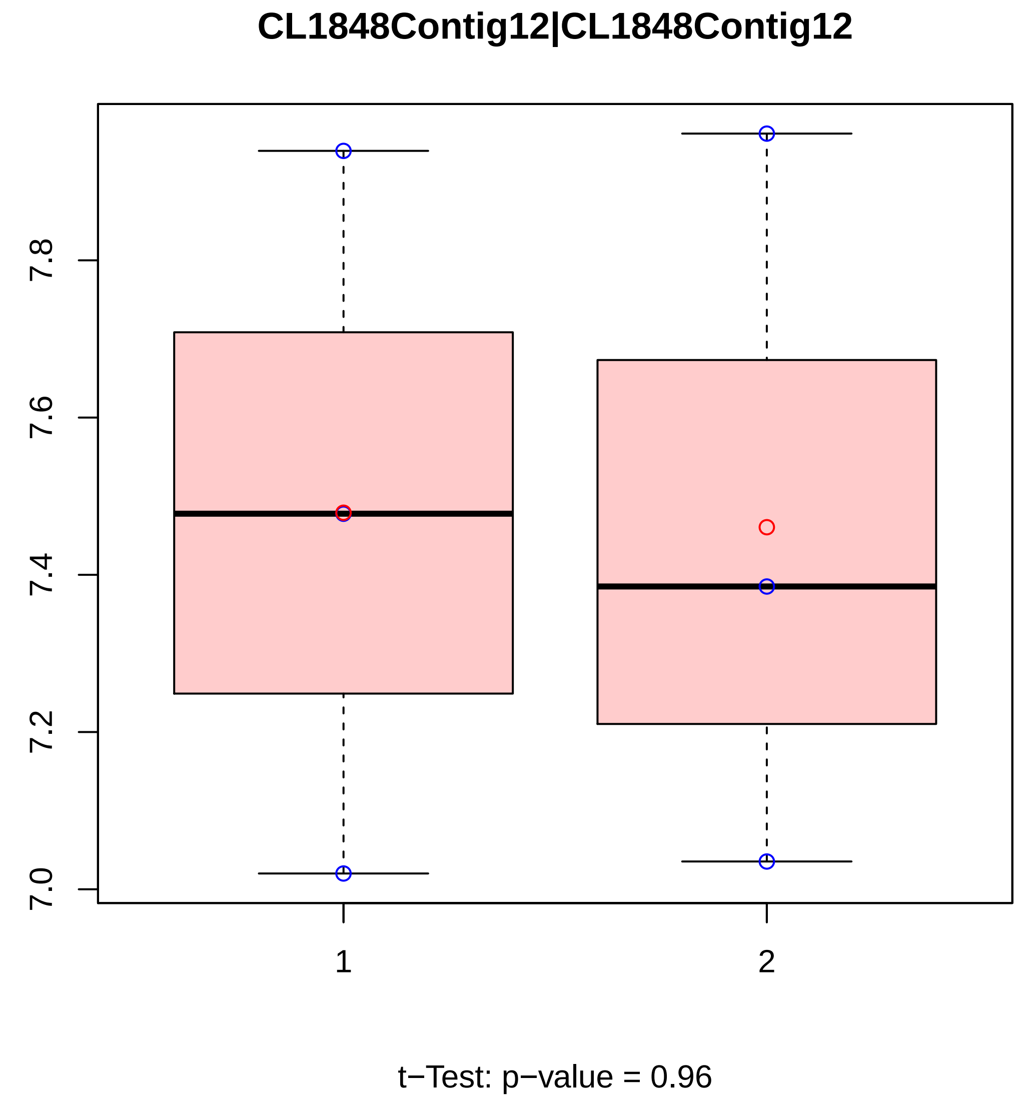
CL1848Contig12|CL1848Contig12
7.0
7.2
7.4
7.6
7.8
1
2
t−Test: p−value = 0.96

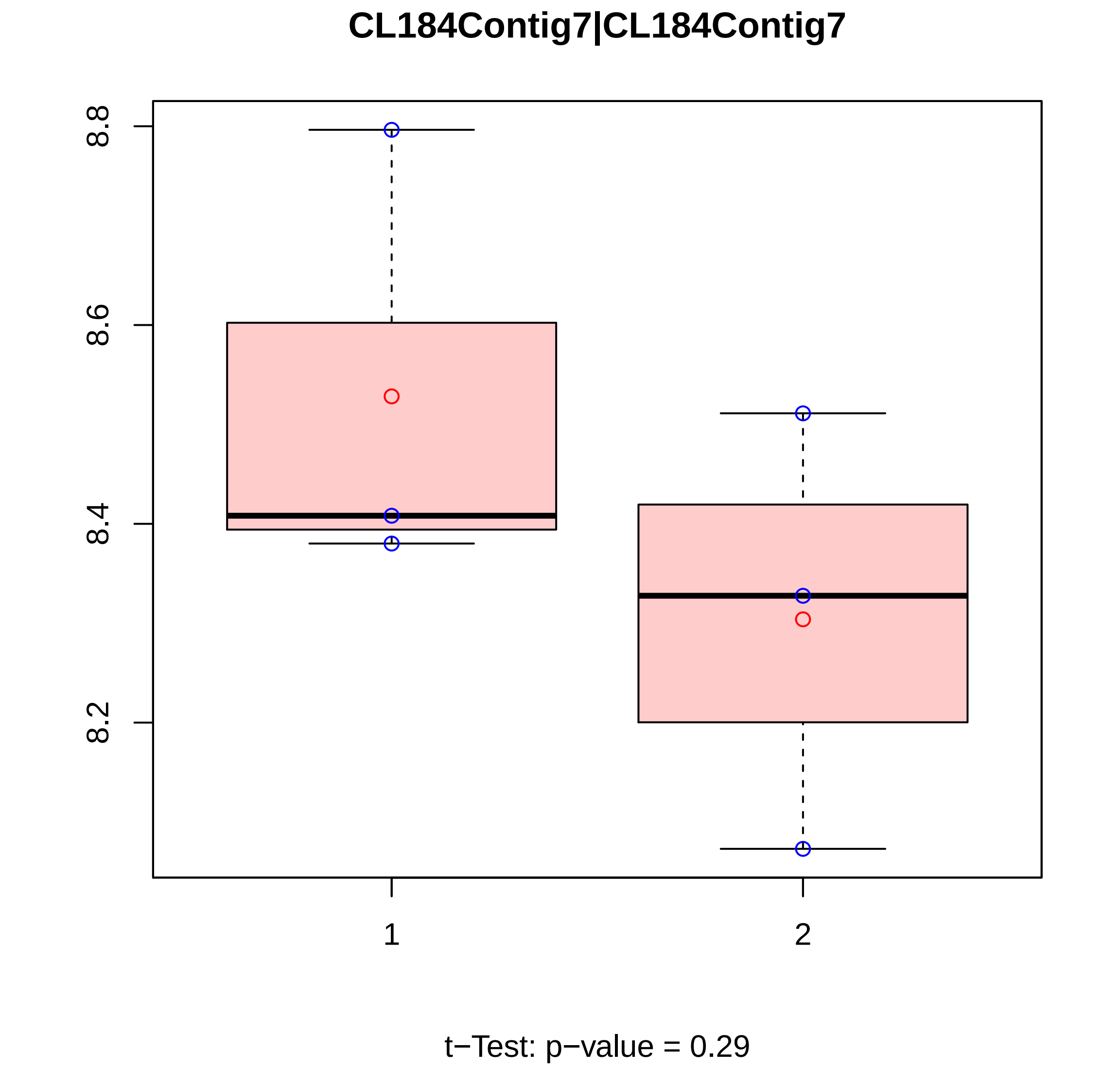
CL184Contig7|CL184Contig7
8.8
8.6
8.4
8.2
1
2
t−Test: p−value = 0.29

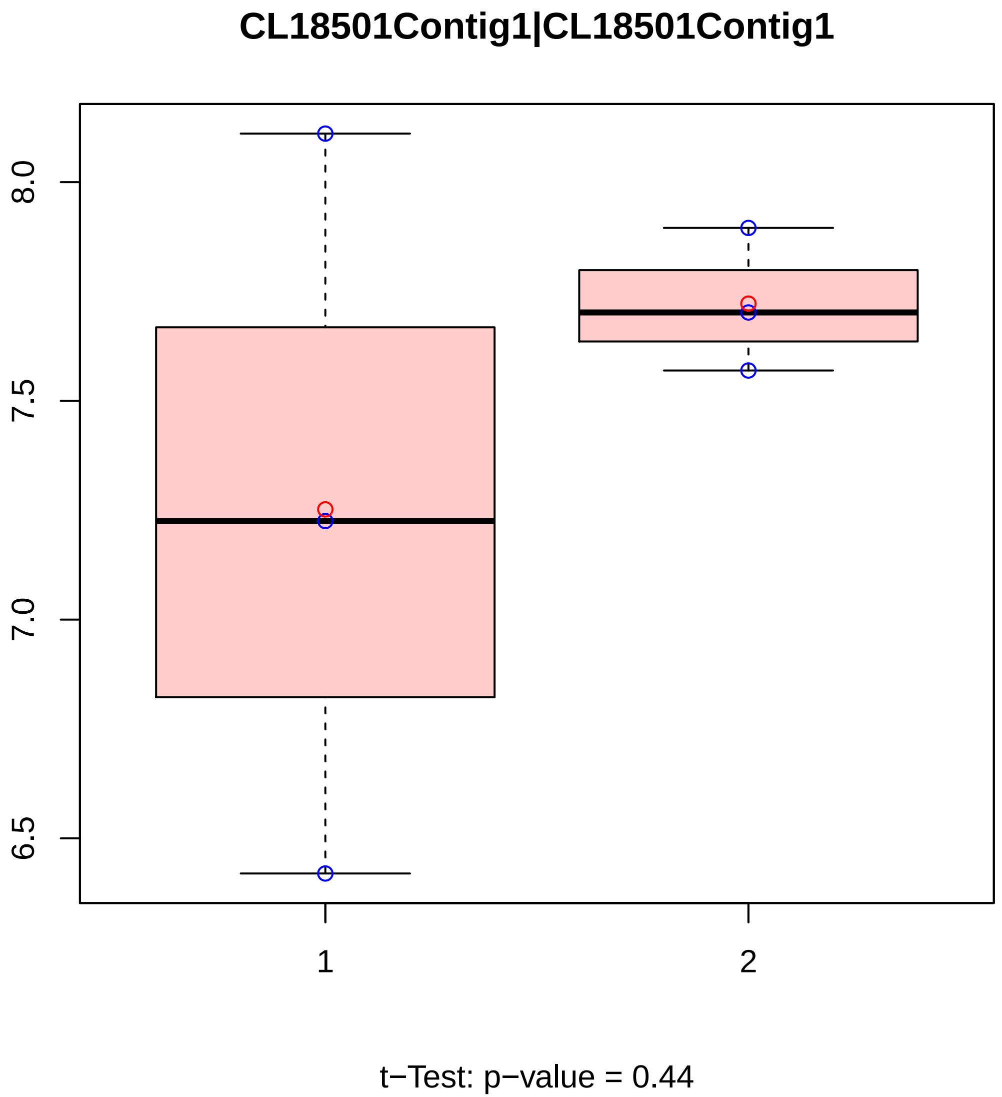
CL18501Contig1|CL18501Contig1
8.0
7.5
7.0
6.5
1
2
t−Test: p−value = 0.44

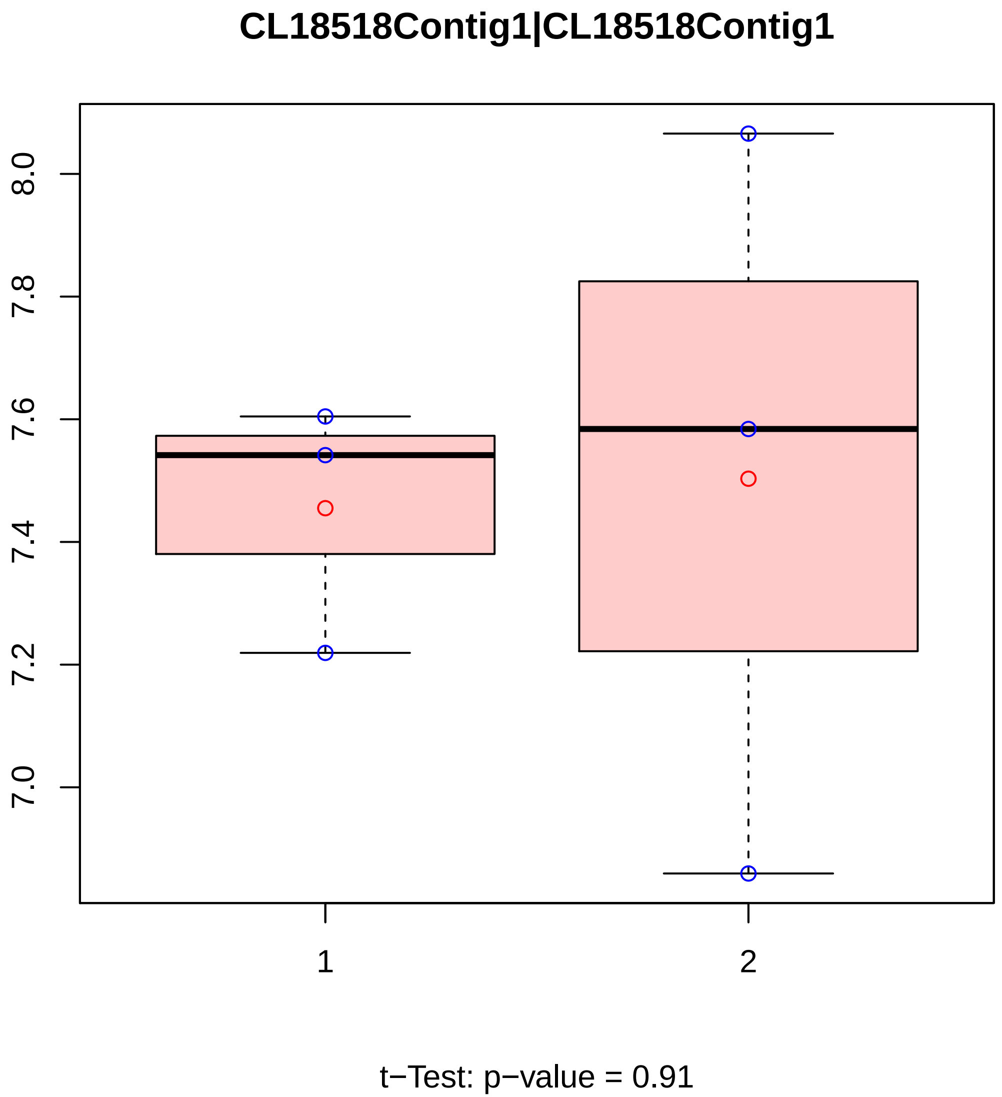
CL18518Contig1|CL18518Contig1
8.0
7.8
7.6
7.4
7.2
7.0
1
2
t−Test: p−value = 0.91

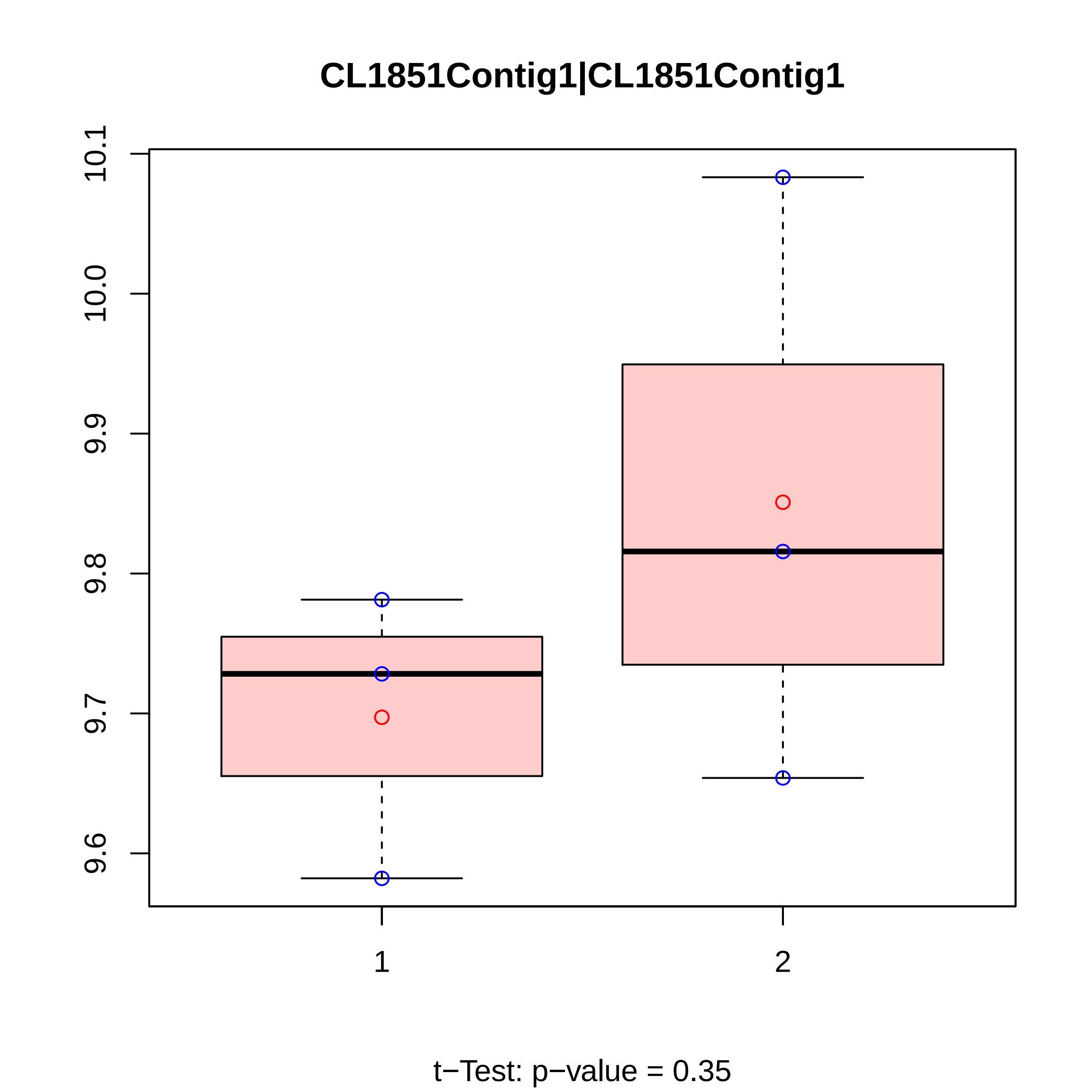
CL1851Contig1|CL1851Contig1
10.1
10.0
9.9
9.8
9.7
9.6
1
2
t−Test: p−value = 0.35

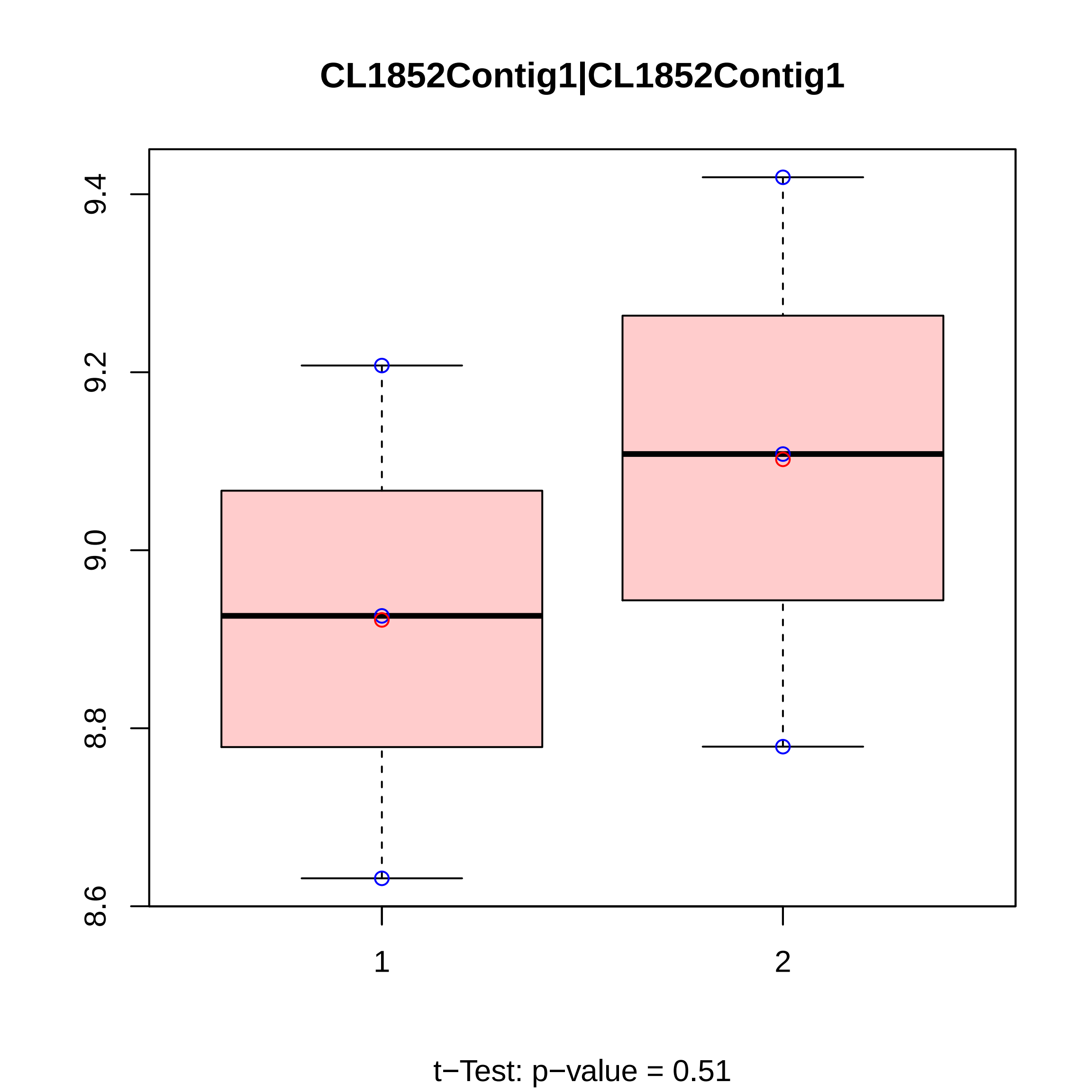

CL1852Contig1|CL1852Contig1
9.4
9.2
9.0
8.8
8.6
1
2
t−Test: p−value = 0.51

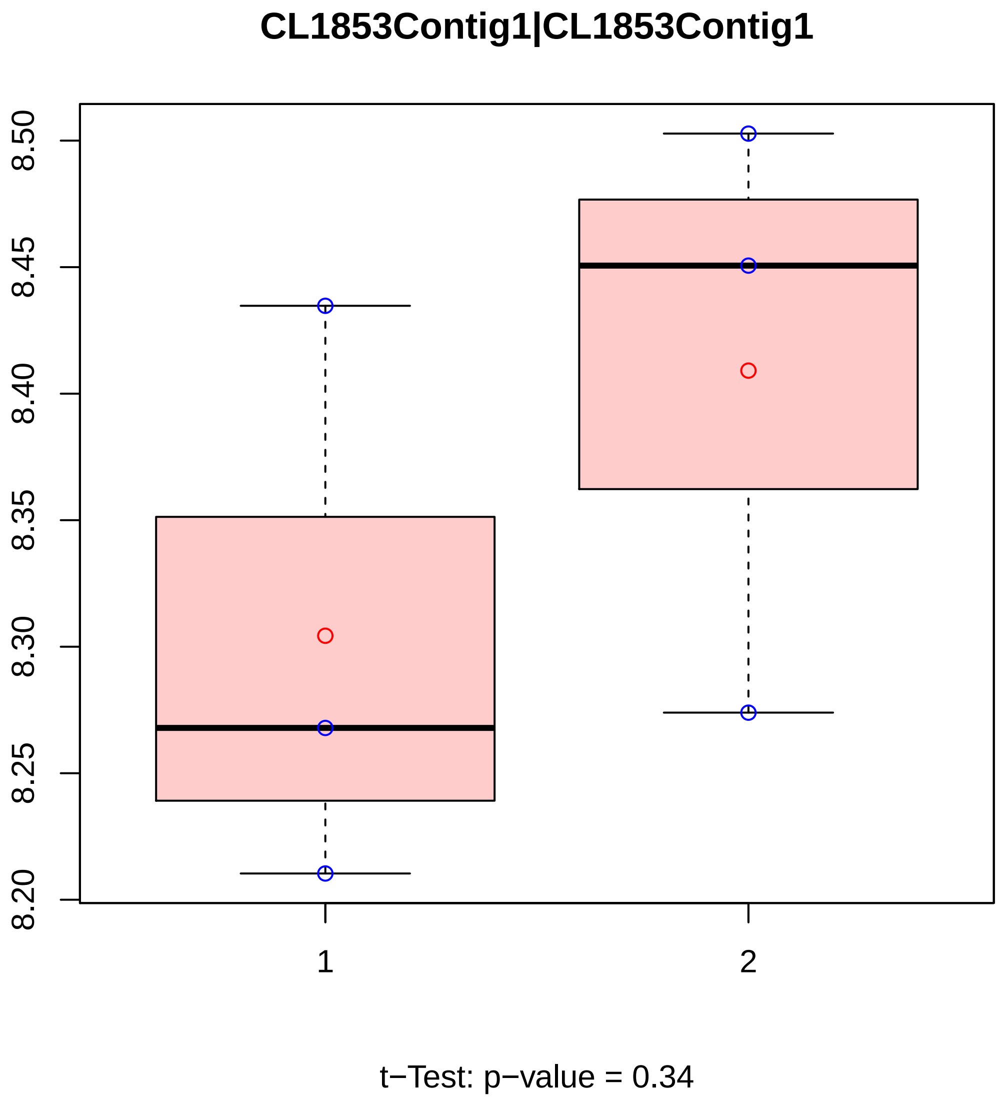

CL1853Contig1|CL1853Contig1
8.20
8.25
8.30
8.35
8.40
8.45
8.50
1
2
t−Test: p−value = 0.34

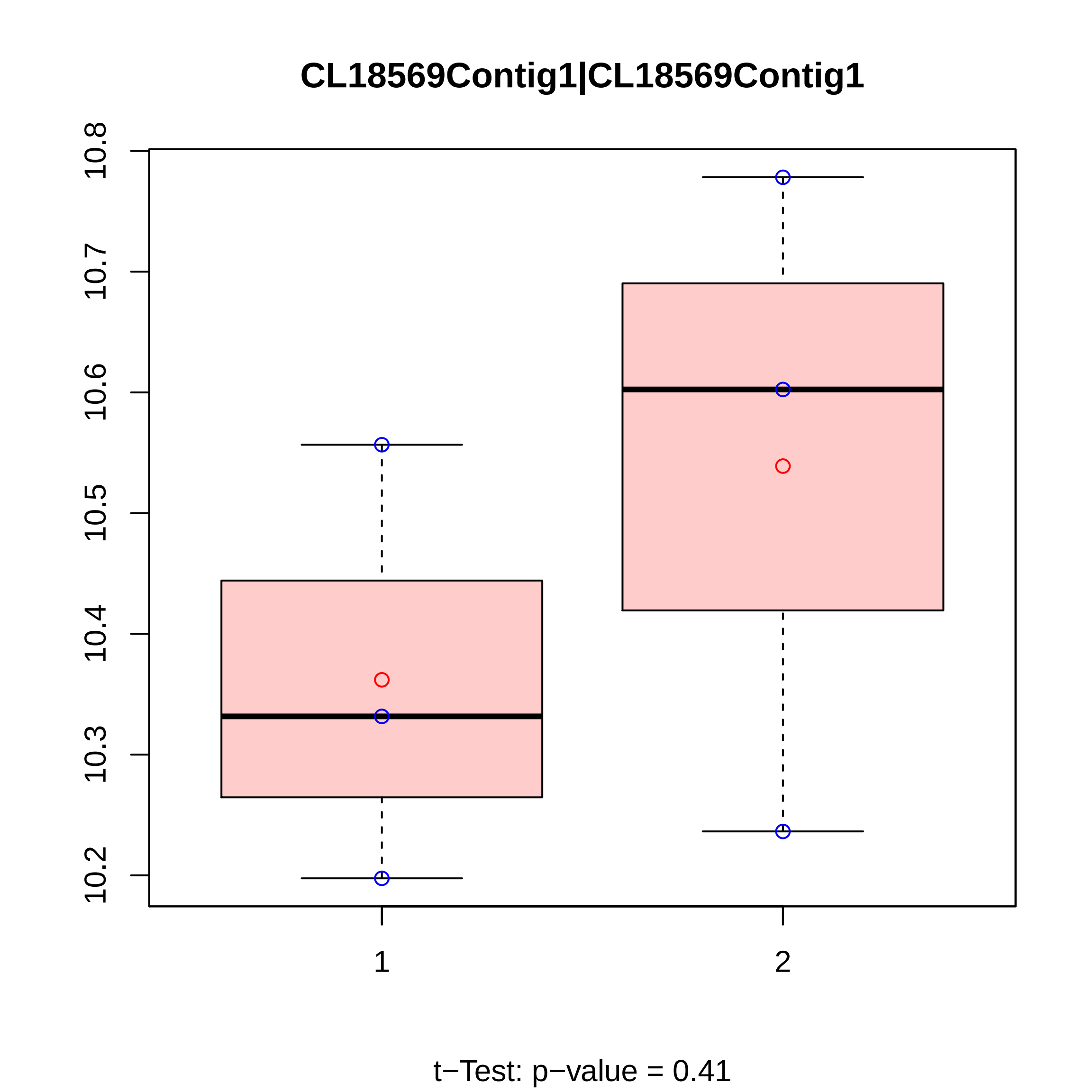

CL18569Contig1|CL18569Contig1
10.2
10.3
10.4
10.5
10.6
10.7
10.8
1
2
t−Test: p−value = 0.41

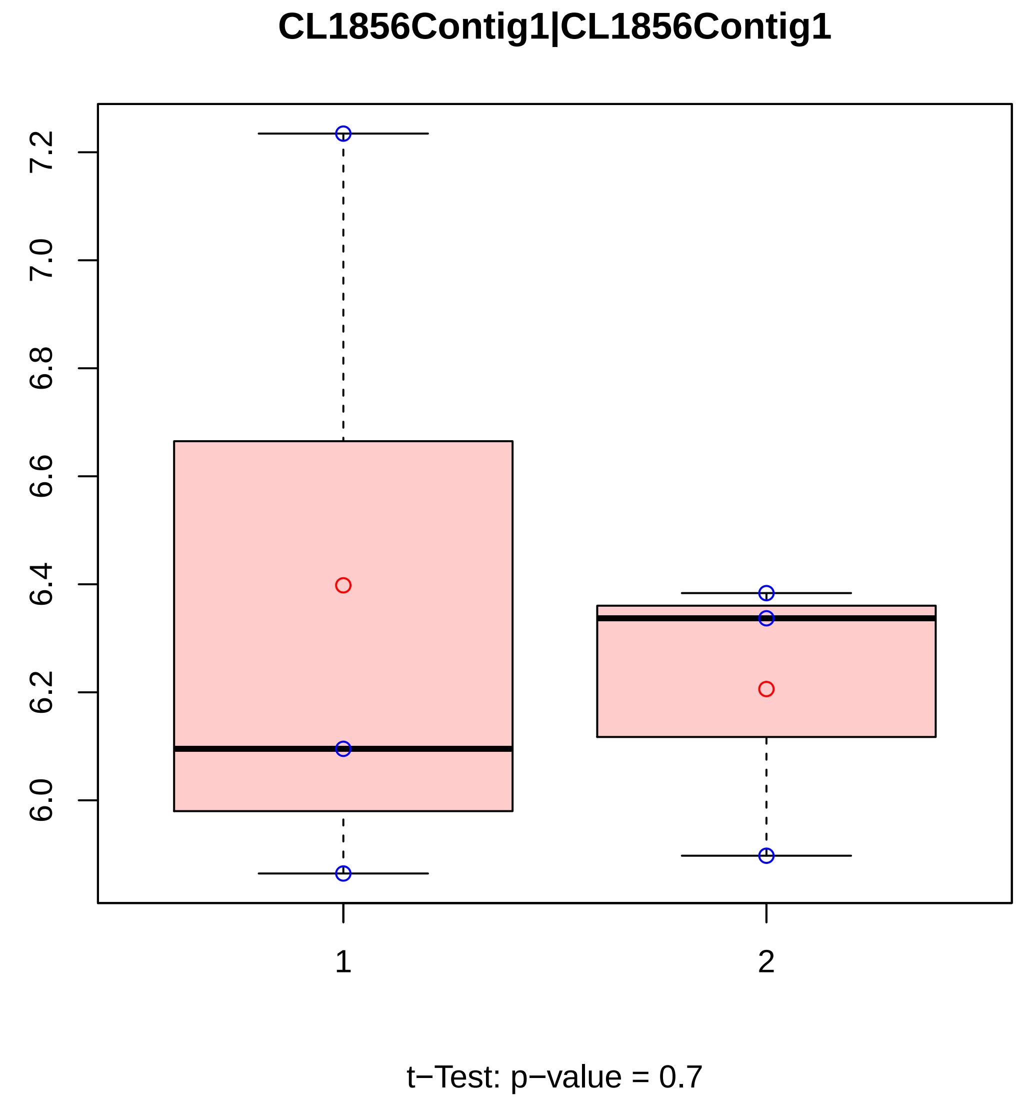
CL1856Contig1|CL1856Contig1
7.2
7.0
6.8
6.6
6.4
6.2
6.0
1
2
t−Test: p−value = 0.7

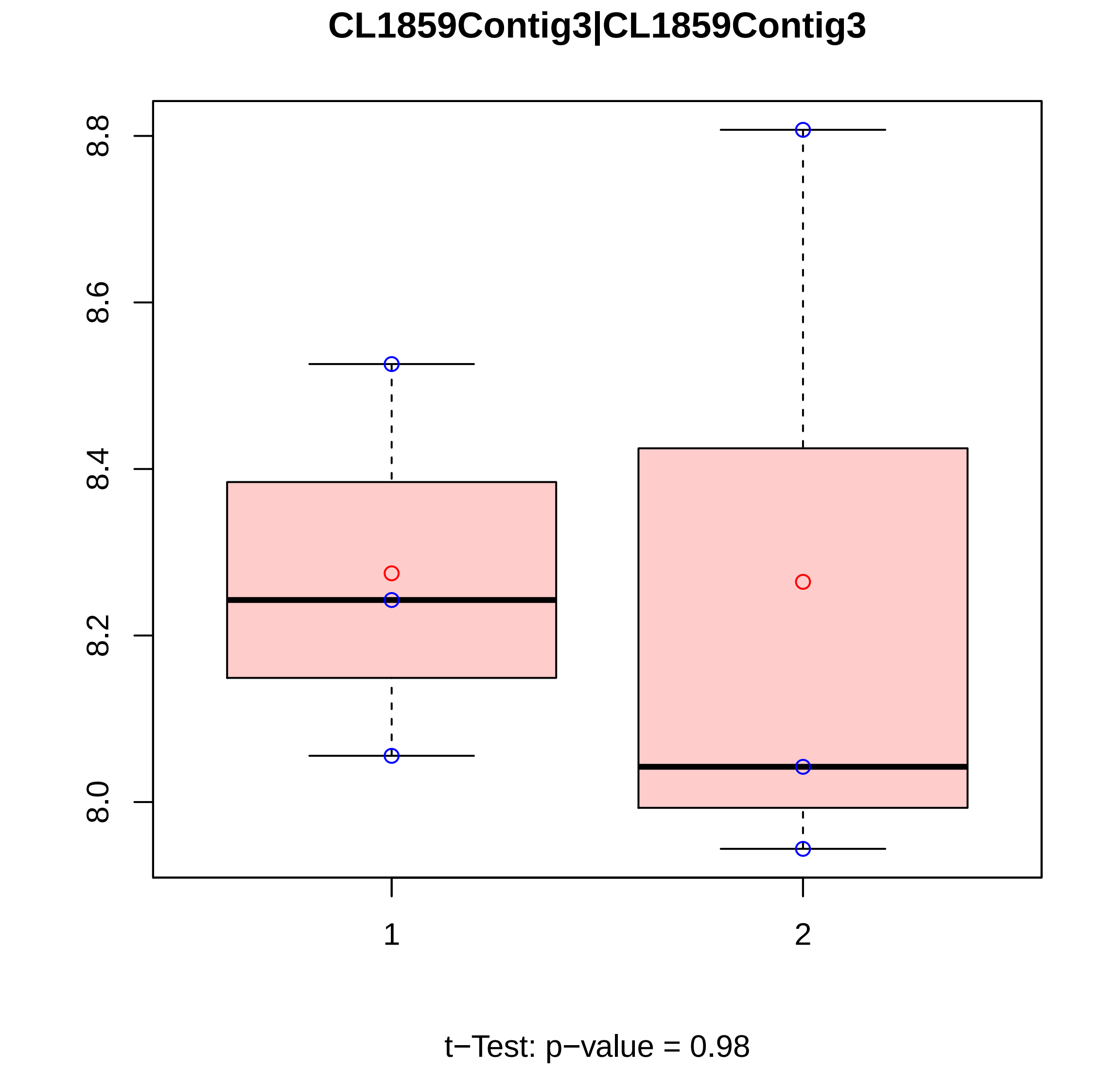
CL1859Contig3|CL1859Contig3
8.0
8.2
8.4
8.6
8.8
1
2
t−Test: p−value = 0.98

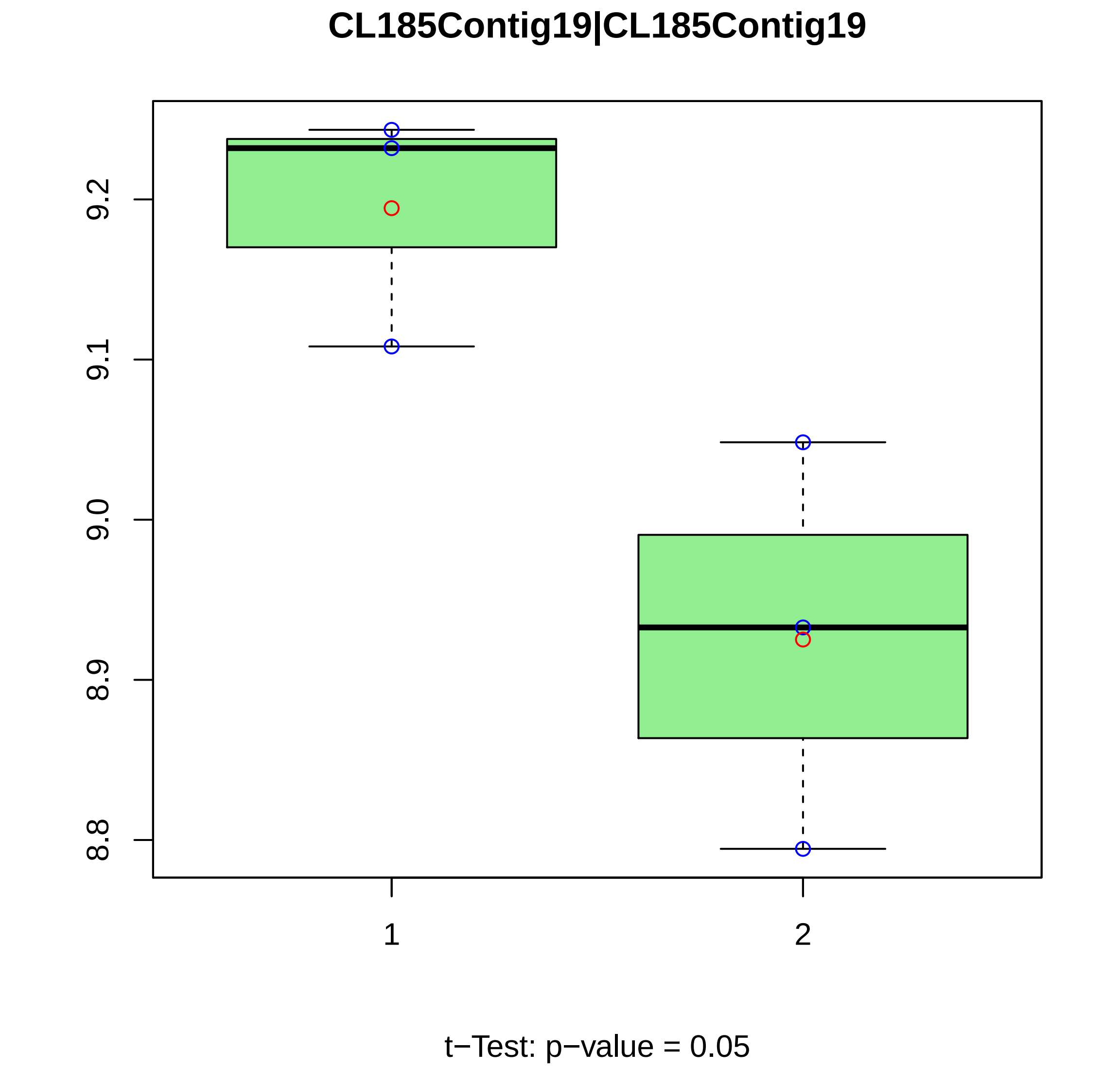
CL185Contig19|CL185Contig19
9.2
9.1
9.0
8.9
8.8
1
2
t−Test: p−value = 0.05

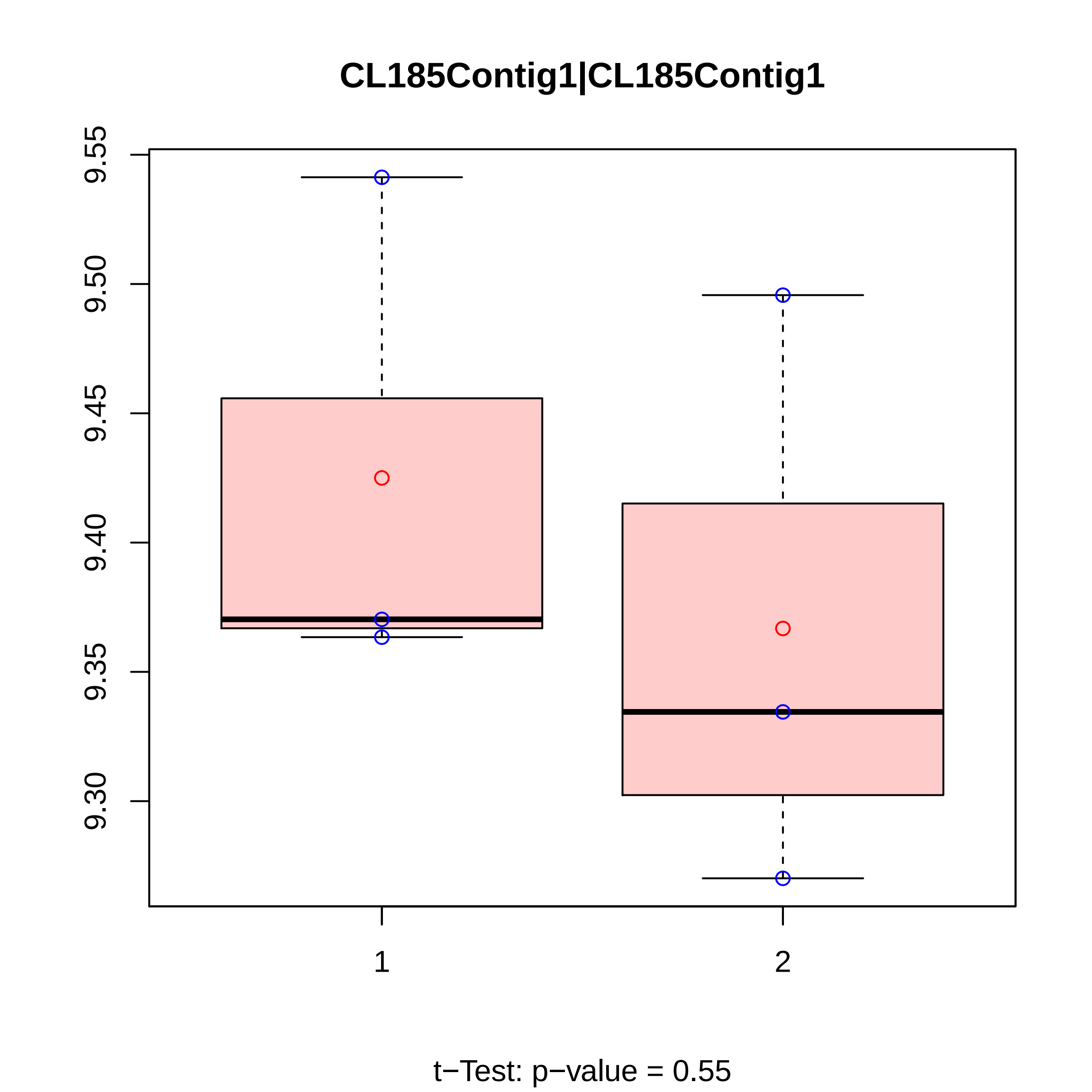

CL185Contig1|CL185Contig1
9.30
9.35
9.40
9.45
9.50
9.55
1
2
t−Test: p−value = 0.55

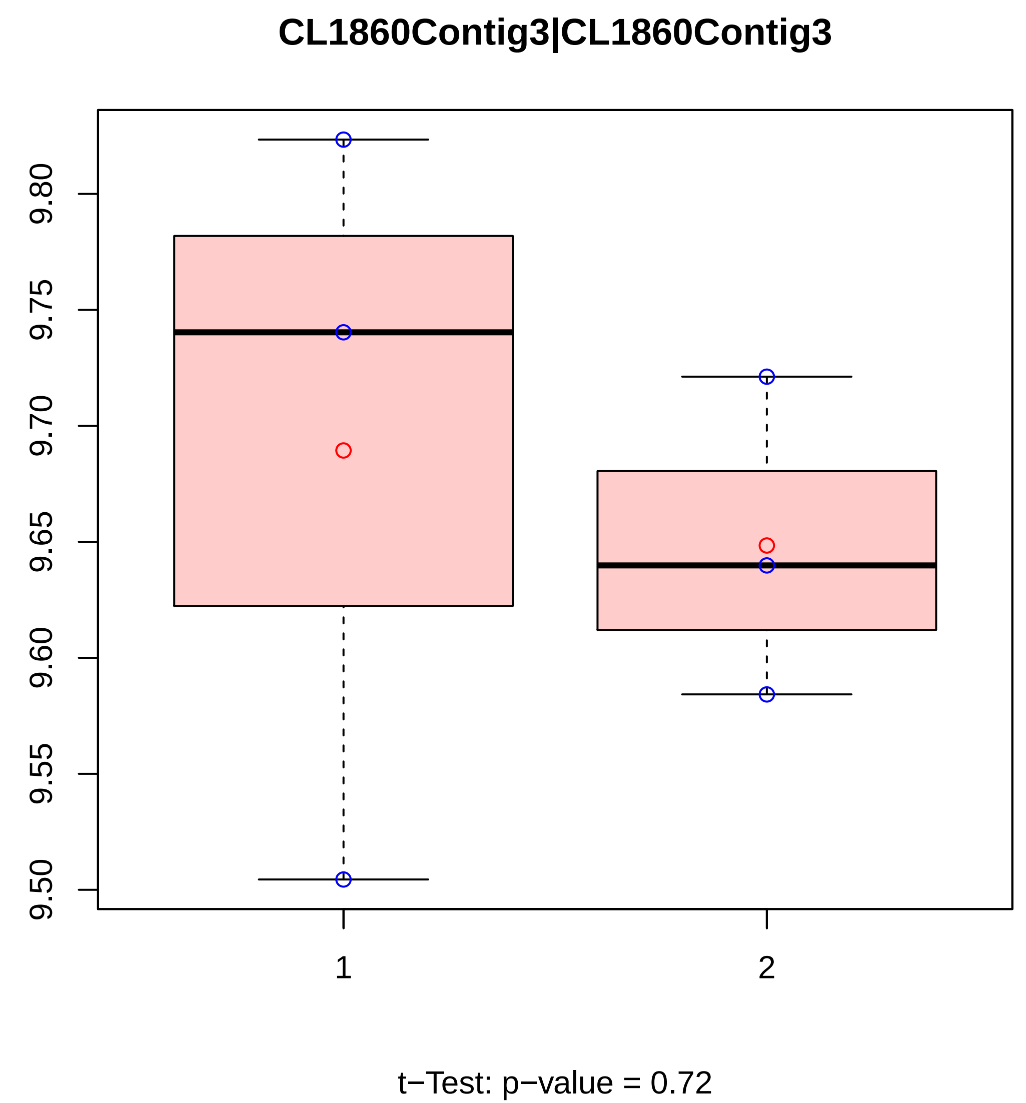

CL1860Contig3|CL1860Contig3
9.50
9.55
9.60
9.65
9.70
9.75
9.80
1
2
t−Test: p−value = 0.72

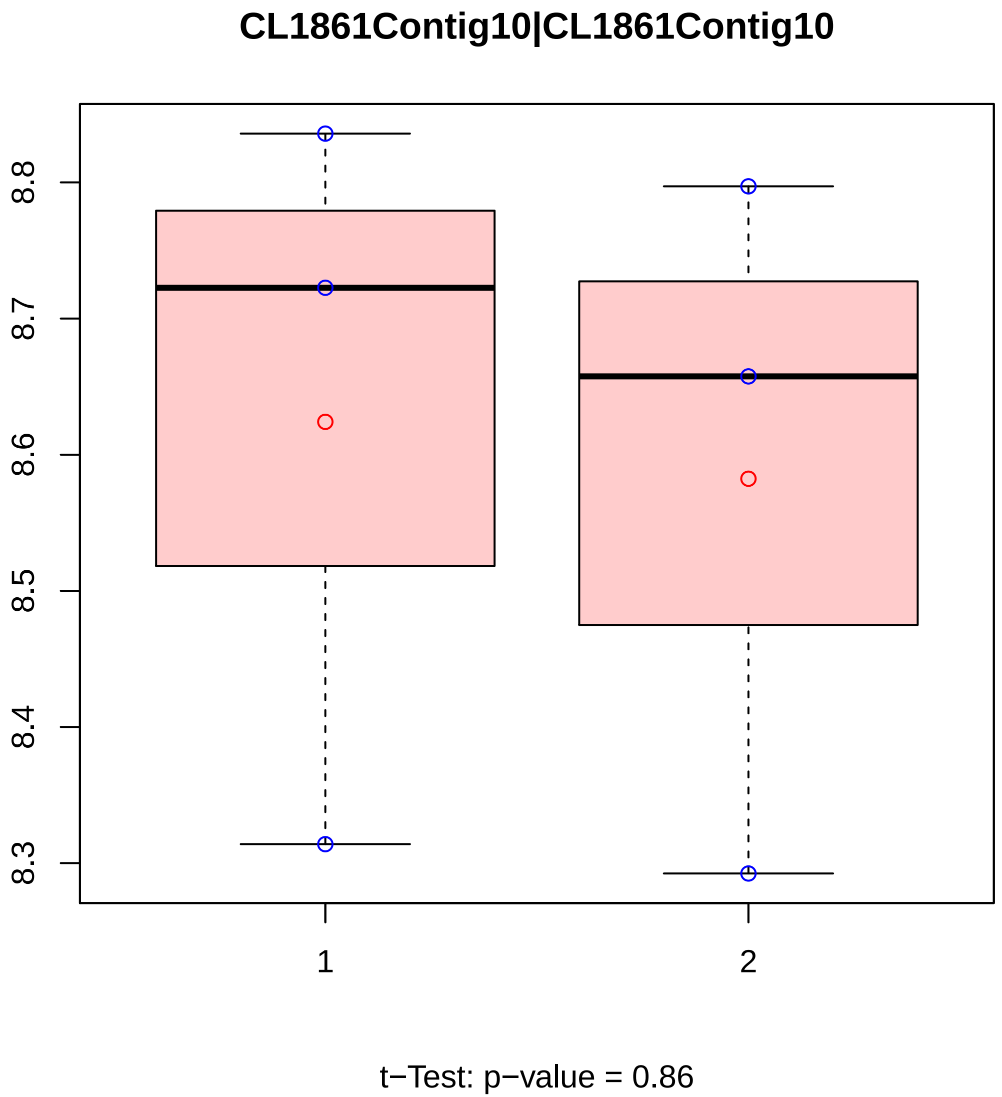
CL1861Contig10|CL1861Contig10
8.3
8.4
8.5
8.6
8.7
8.8
1
2
t−Test: p−value = 0.86

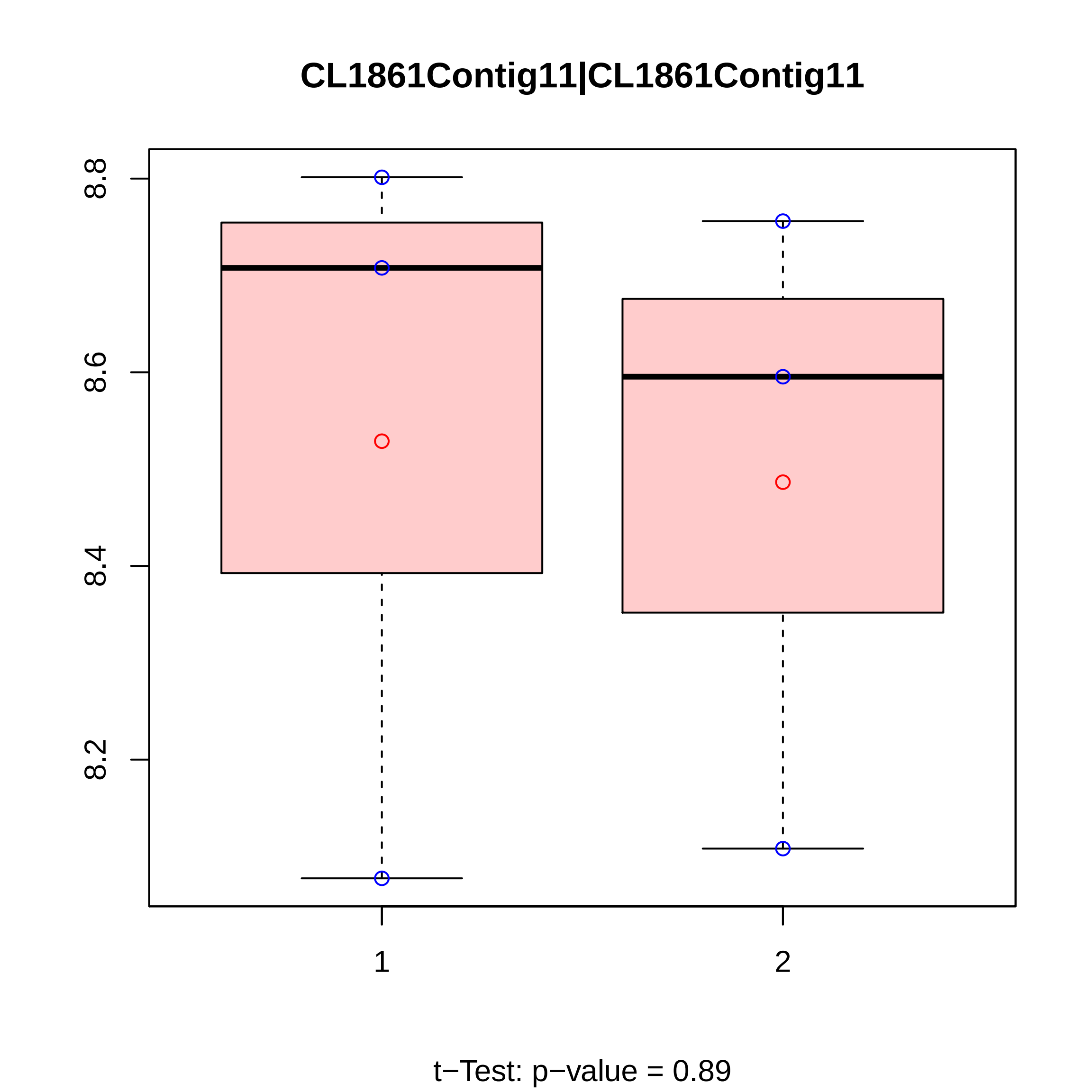

CL1861Contig11|CL1861Contig11
8.8
8.6
8.4
8.2
1
2
t−Test: p−value = 0.89

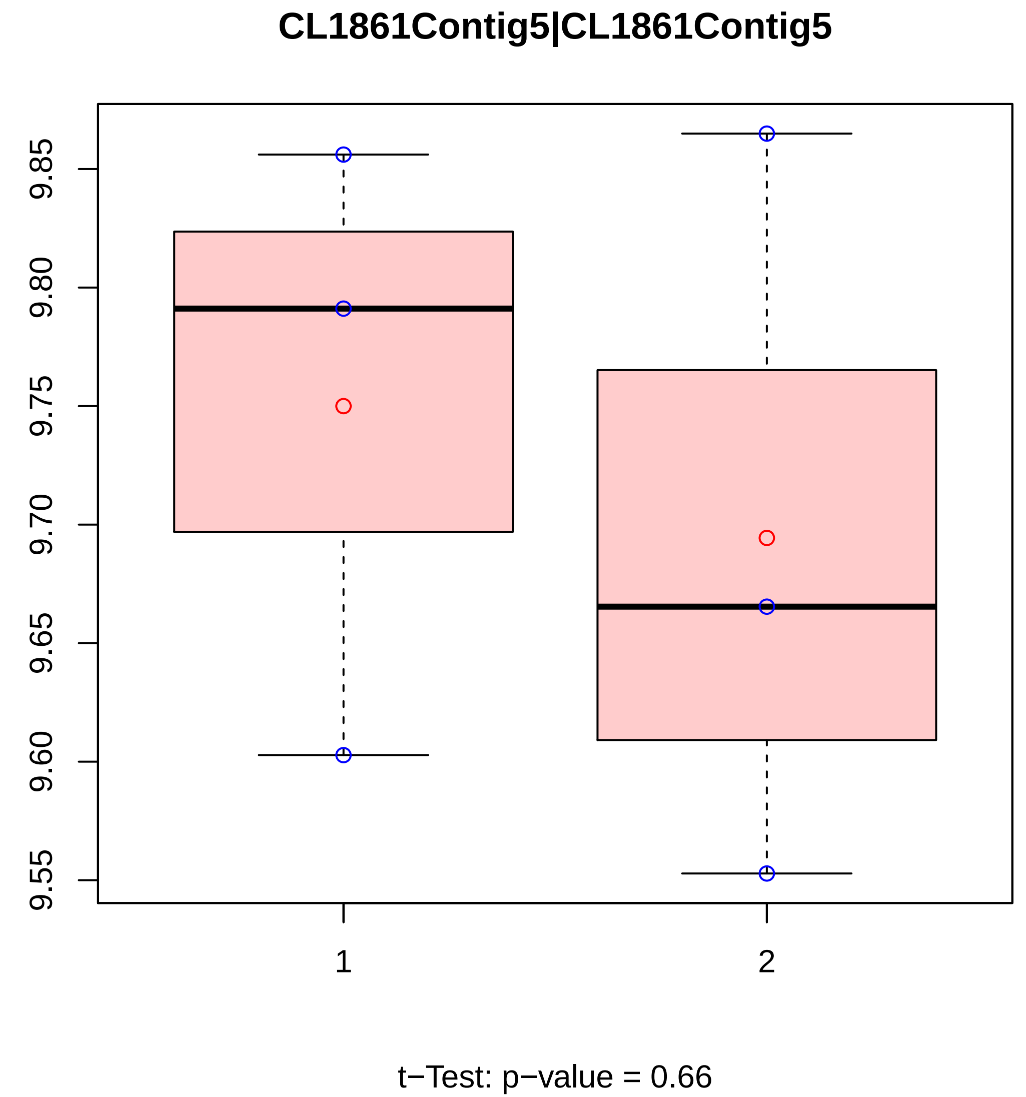
CL1861Contig5|CL1861Contig5
9.55
9.60
9.65
9.70
9.75
9.80
9.85
1
2
t−Test: p−value = 0.66

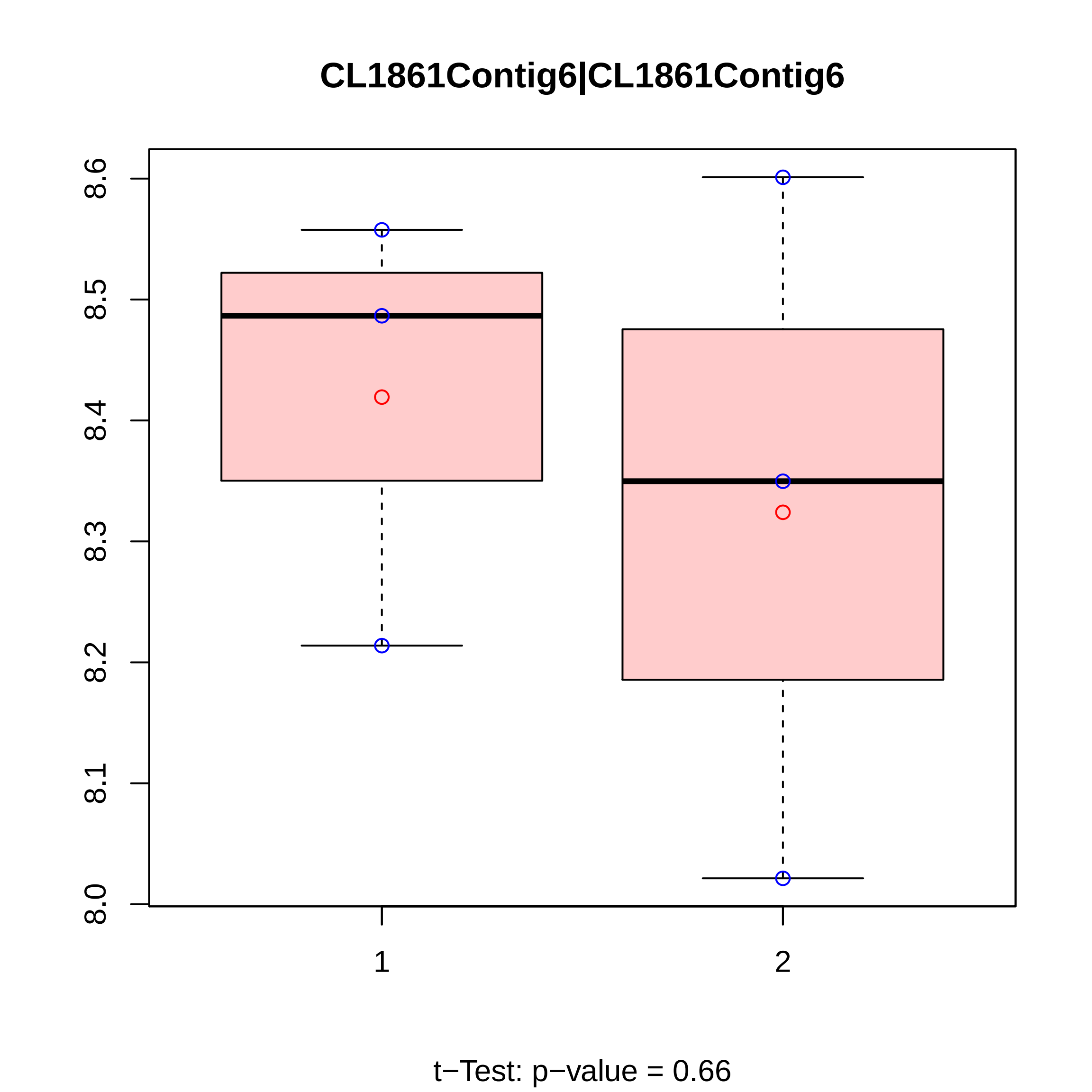

CL1861Contig6|CL1861Contig6
8.0
8.1
8.2
8.3
8.4
8.5
8.6
1
2
t−Test: p−value = 0.66

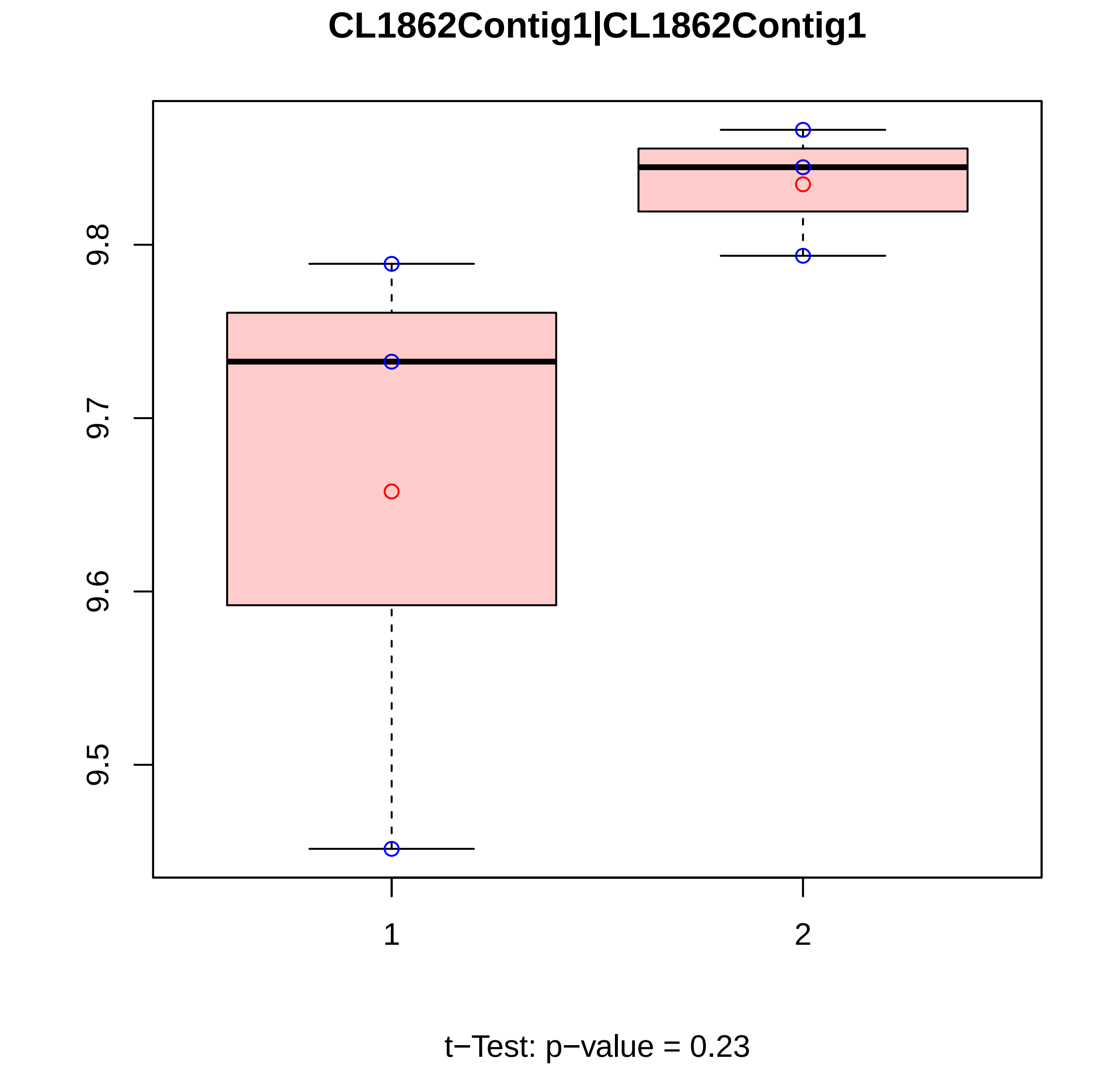
CL1862Contig1|CL1862Contig1
9.5
9.6
9.7
9.8
1
2
t−Test: p−value = 0.23

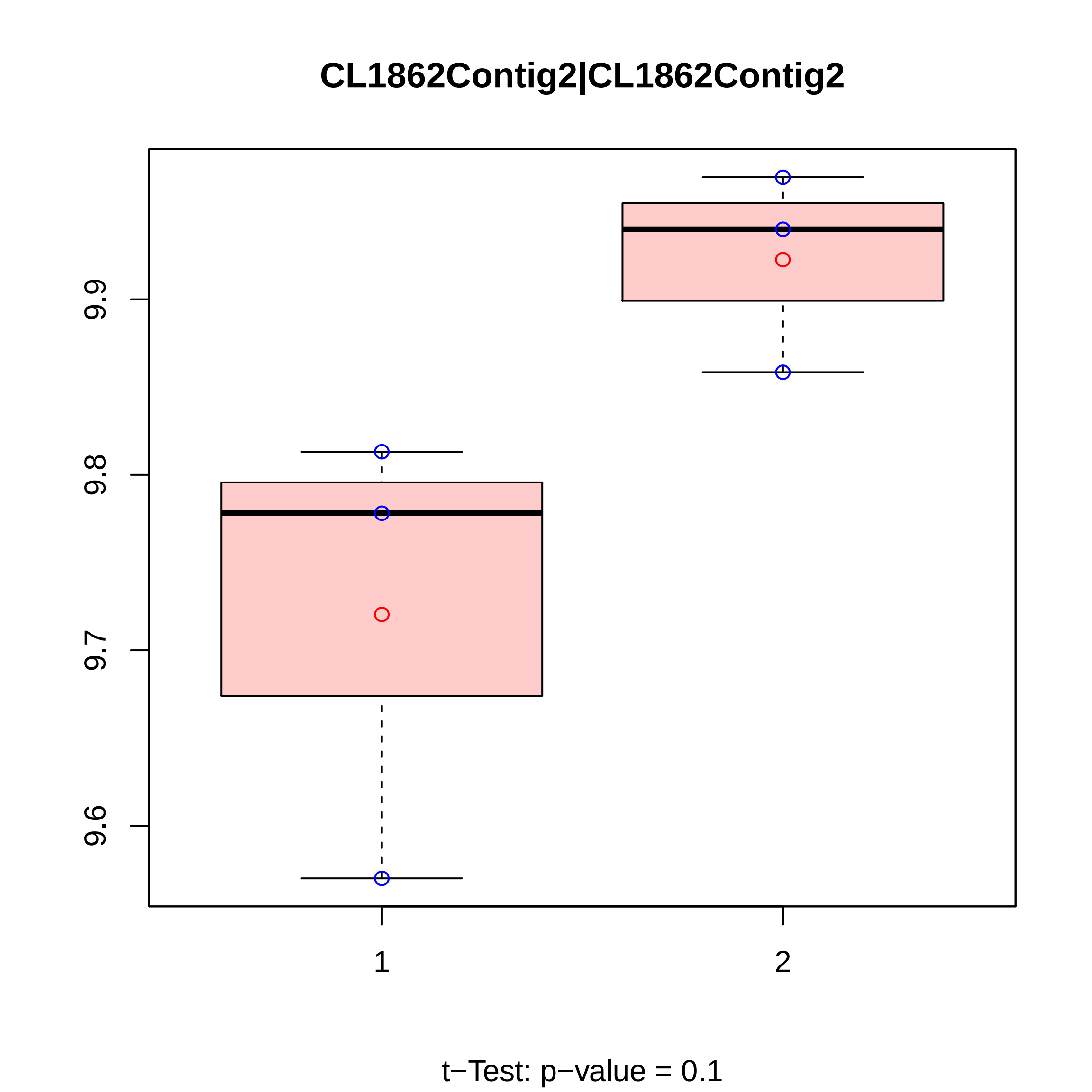
CL1862Contig2|CL1862Contig2
9.6
9.7
9.8
9.9
1
2
t−Test: p−value = 0.1

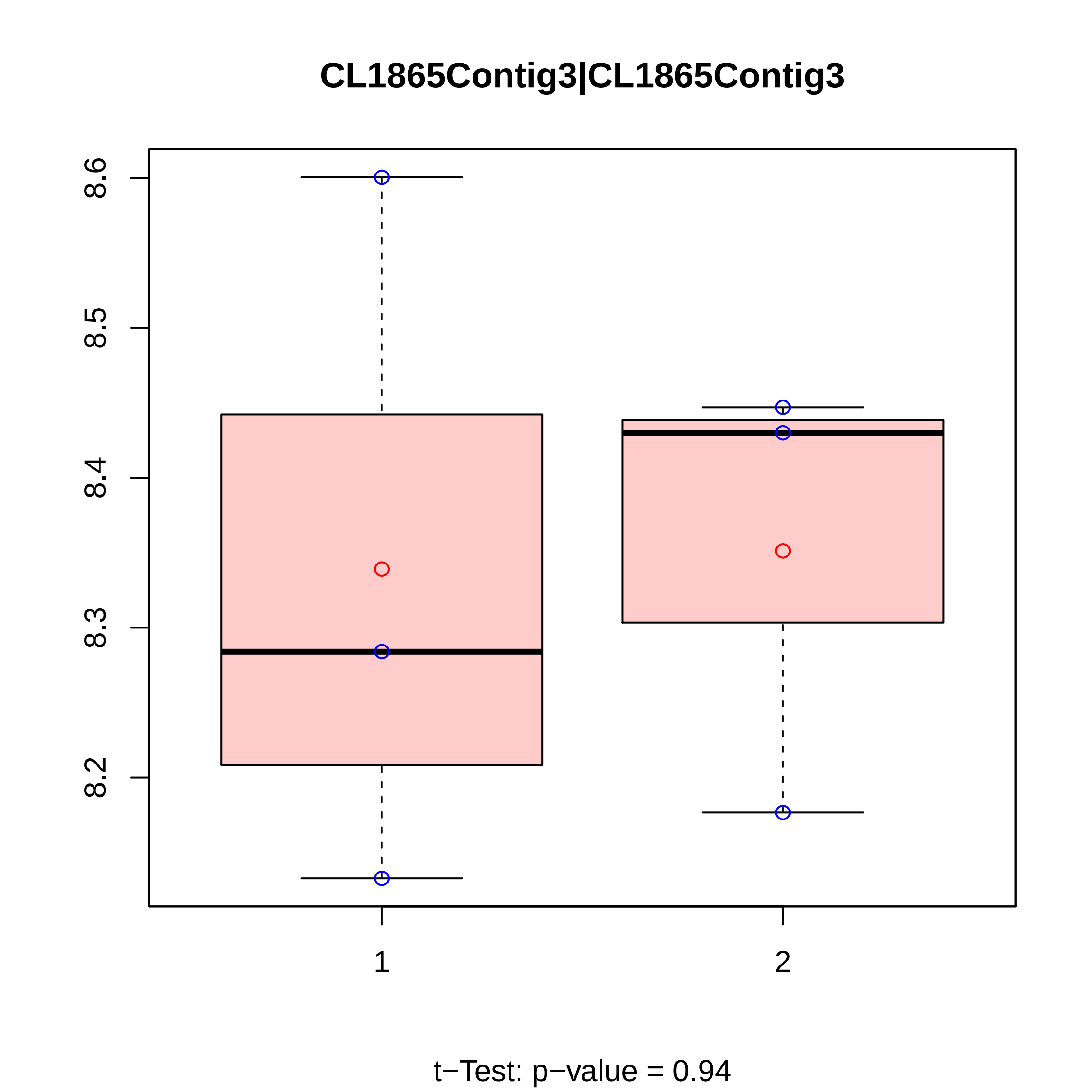

CL1865Contig3|CL1865Contig3
8.6
8.5
8.4
8.3
8.2
1
2
t−Test: p−value = 0.94

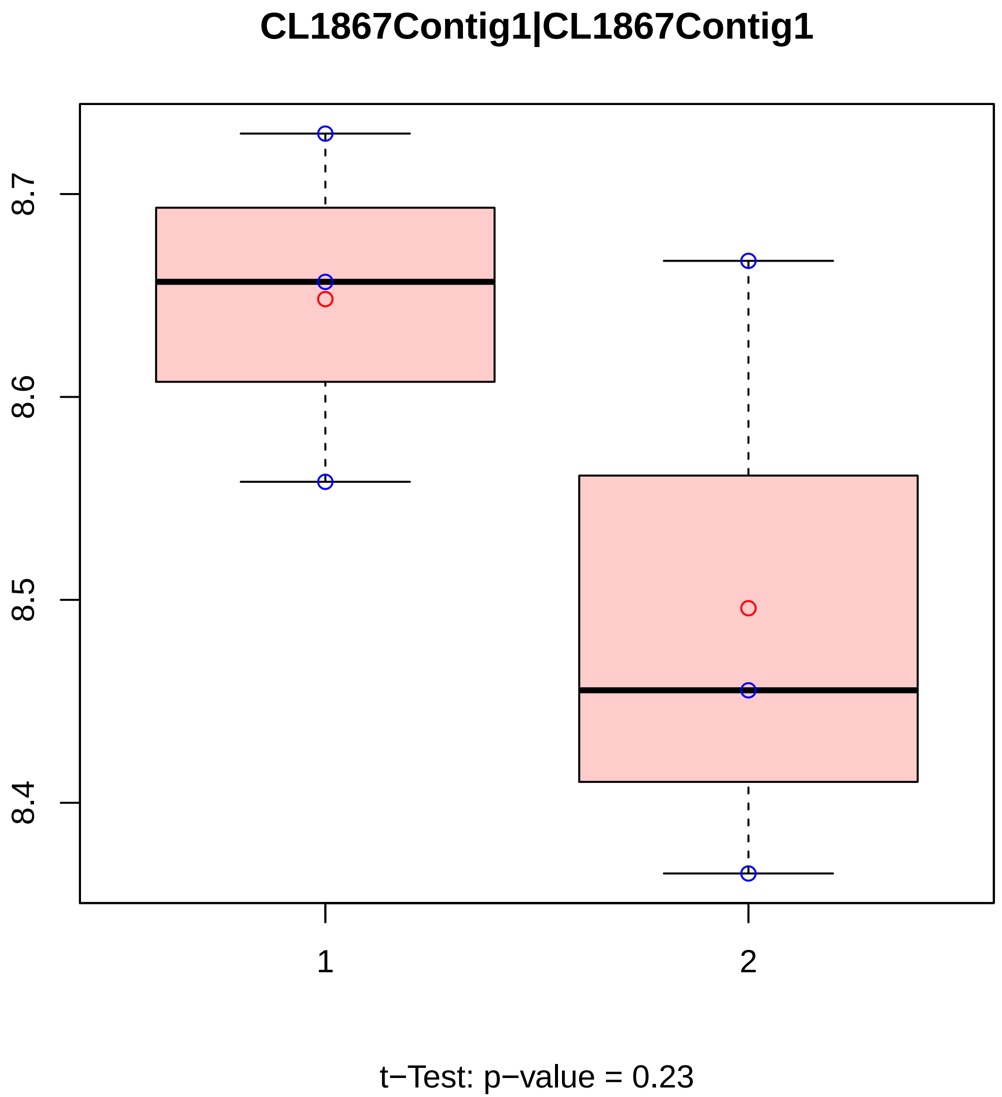

CL1867Contig1|CL1867Contig1
8.4
8.5
8.6
8.7
1
2
t−Test: p−value = 0.23

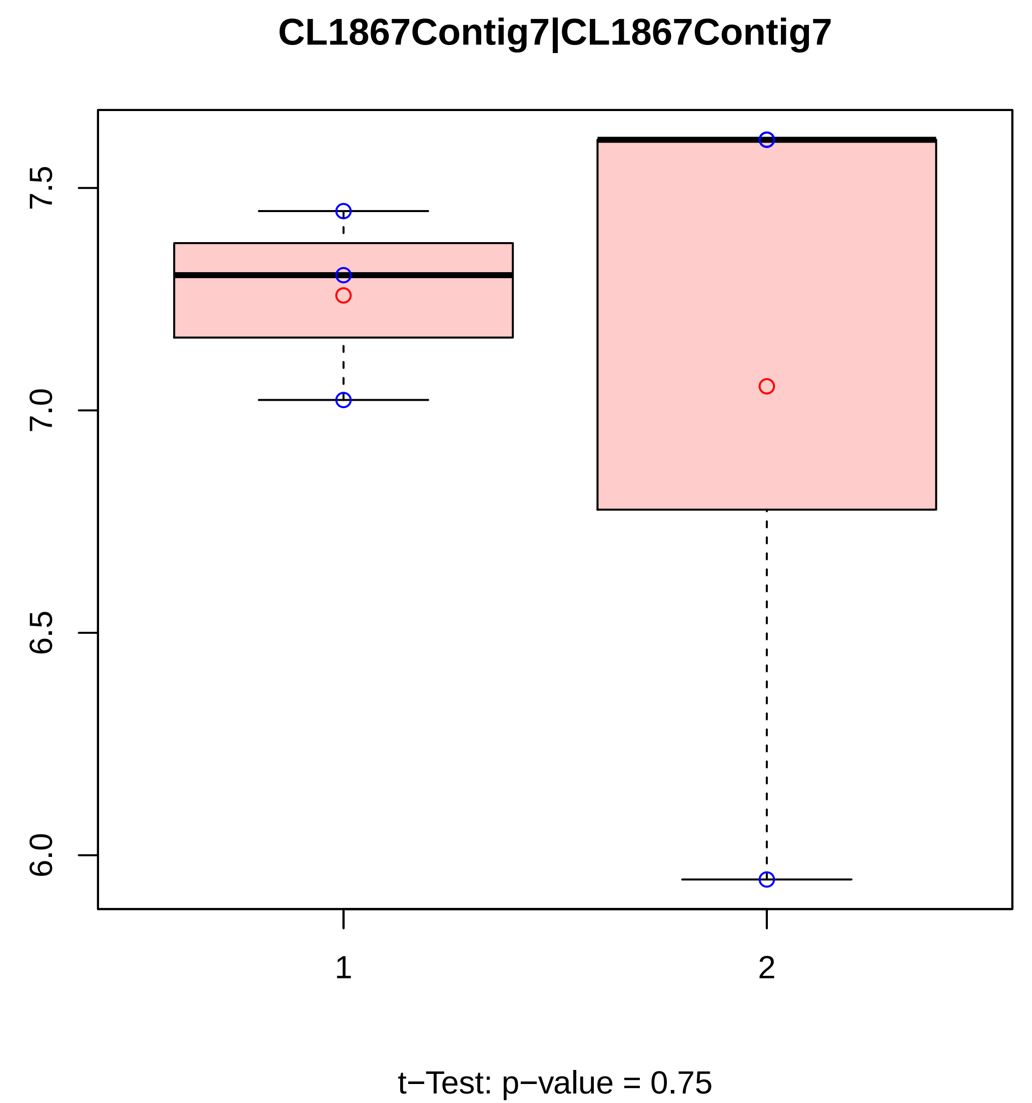

CL1867Contig7|CL1867Contig7
7.5
7.0
6.5
6.0
1
2
t−Test: p−value = 0.75

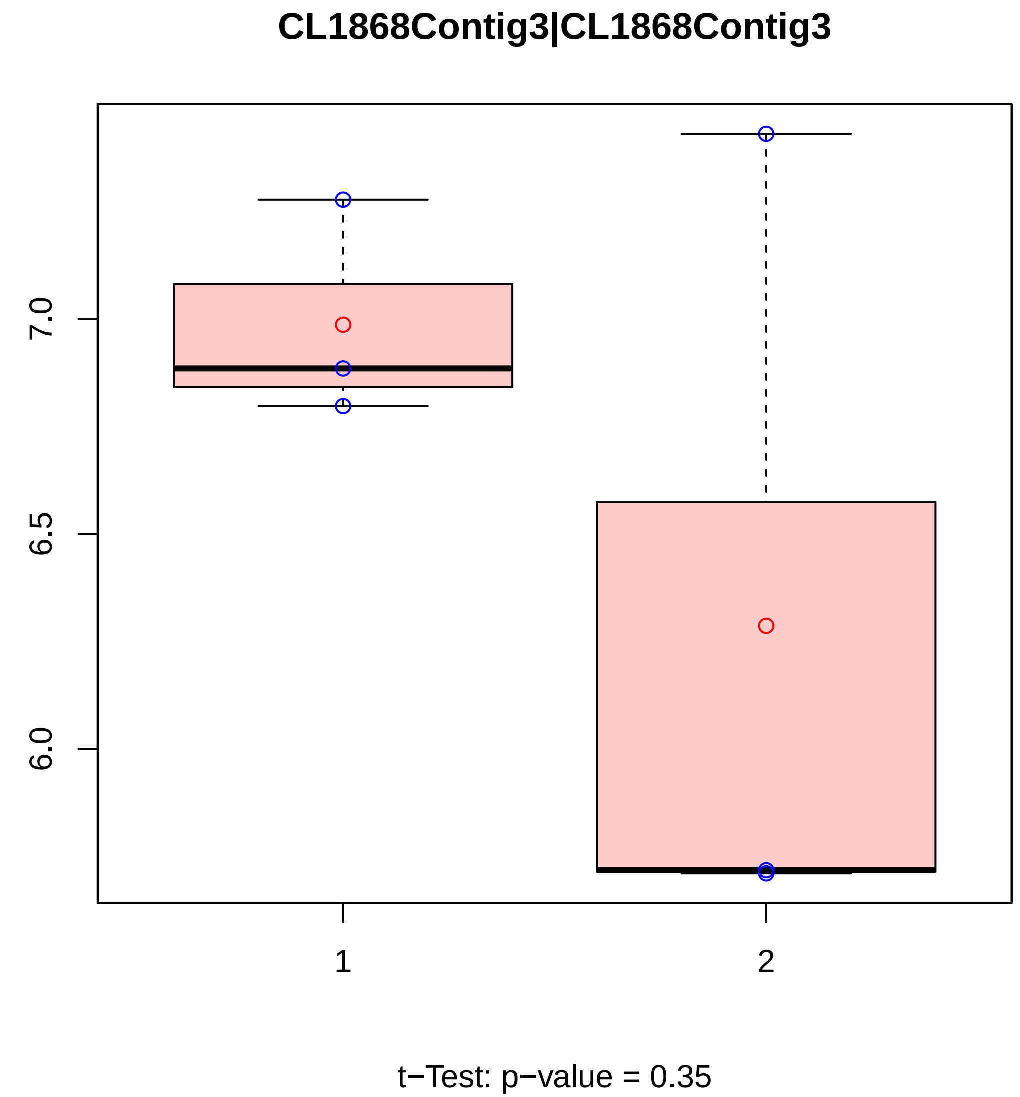
CL1868Contig3|CL1868Contig3
7.0
6.5
6.0
1
2
t−Test: p−value = 0.35

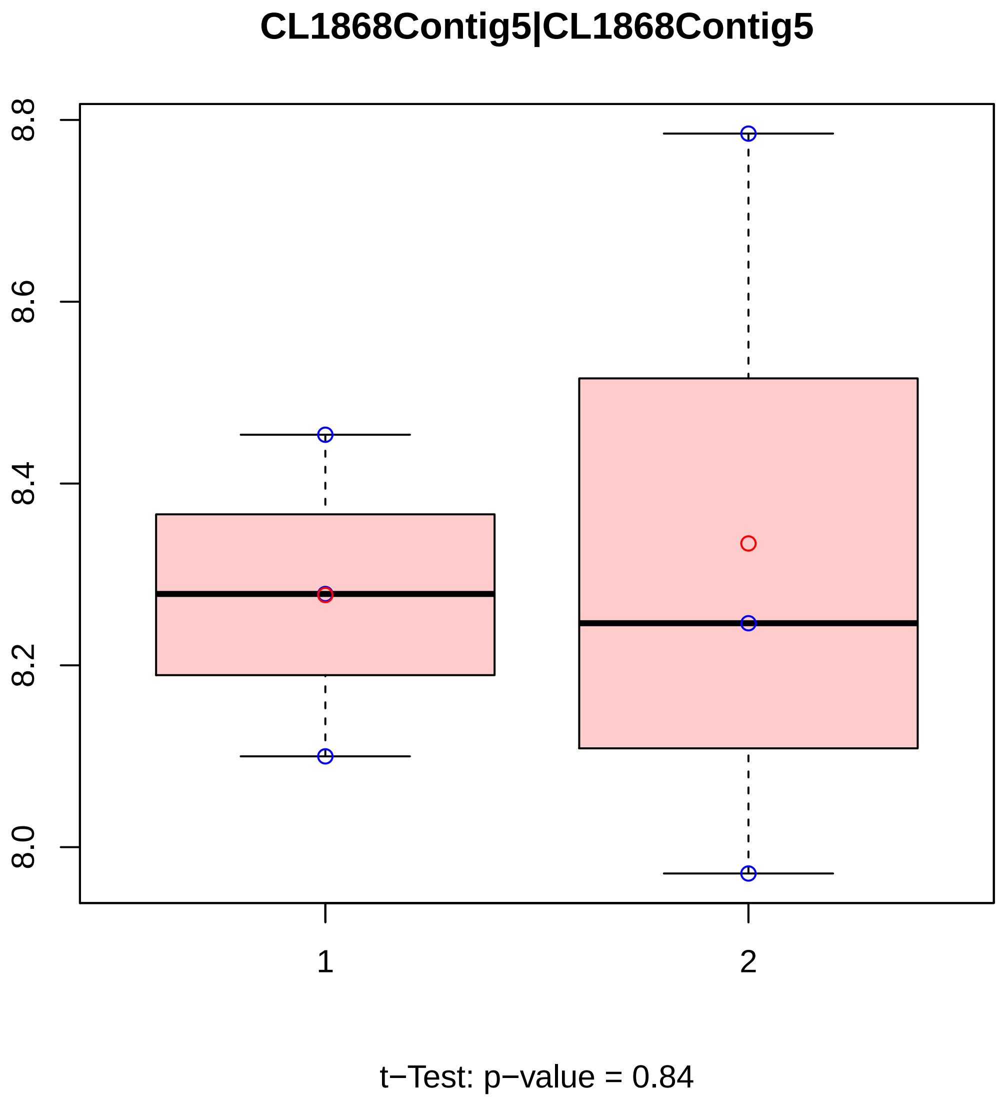
CL1868Contig5|CL1868Contig5
8.0
8.2
8.4
8.6
8.8
1
2
t−Test: p−value = 0.84

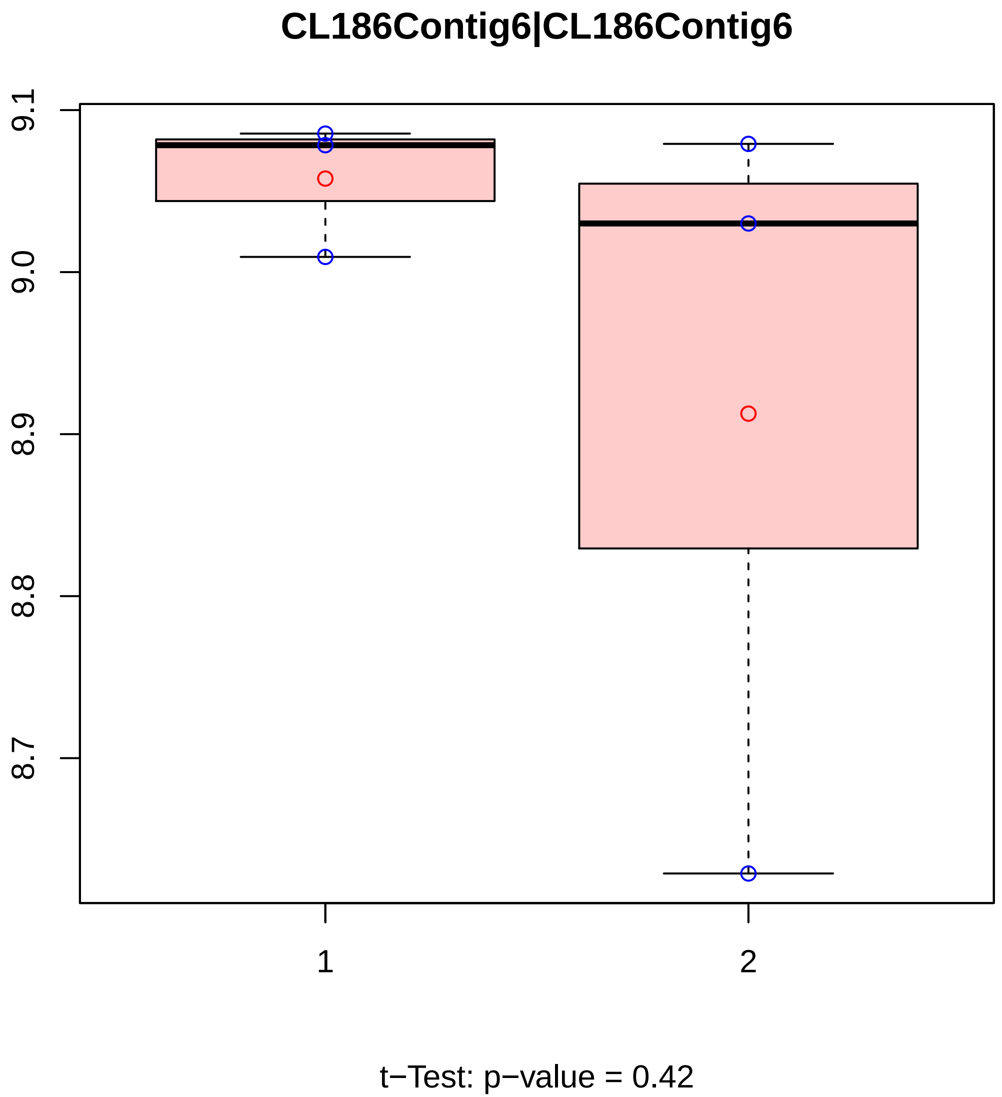
CL186Contig6|CL186Contig6
9.1
9.0
8.9
8.8
8.7
1
2
t−Test: p−value = 0.42

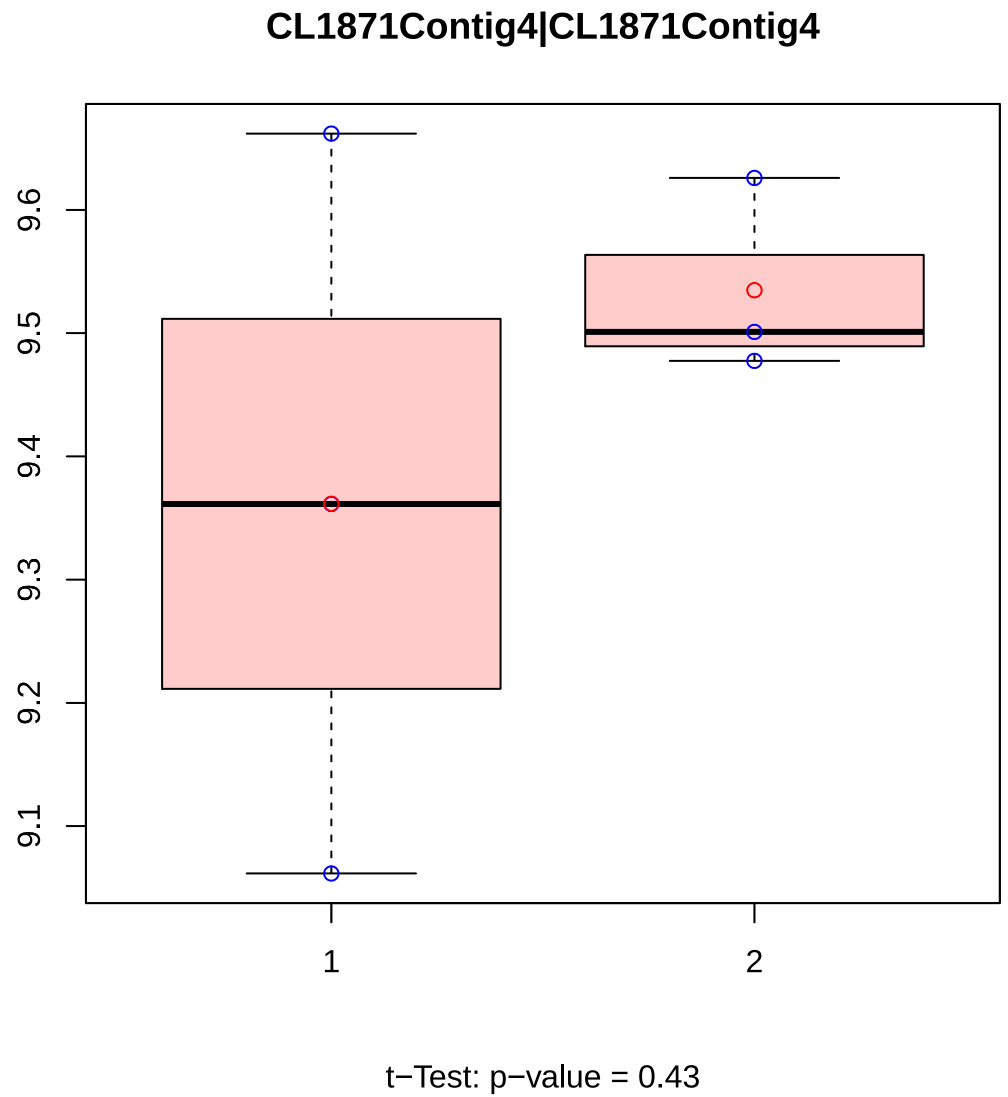

CL1871Contig4|CL1871Contig4
9.1
9.2
9.3
9.4
9.5
9.6
1
2
t−Test: p−value = 0.43

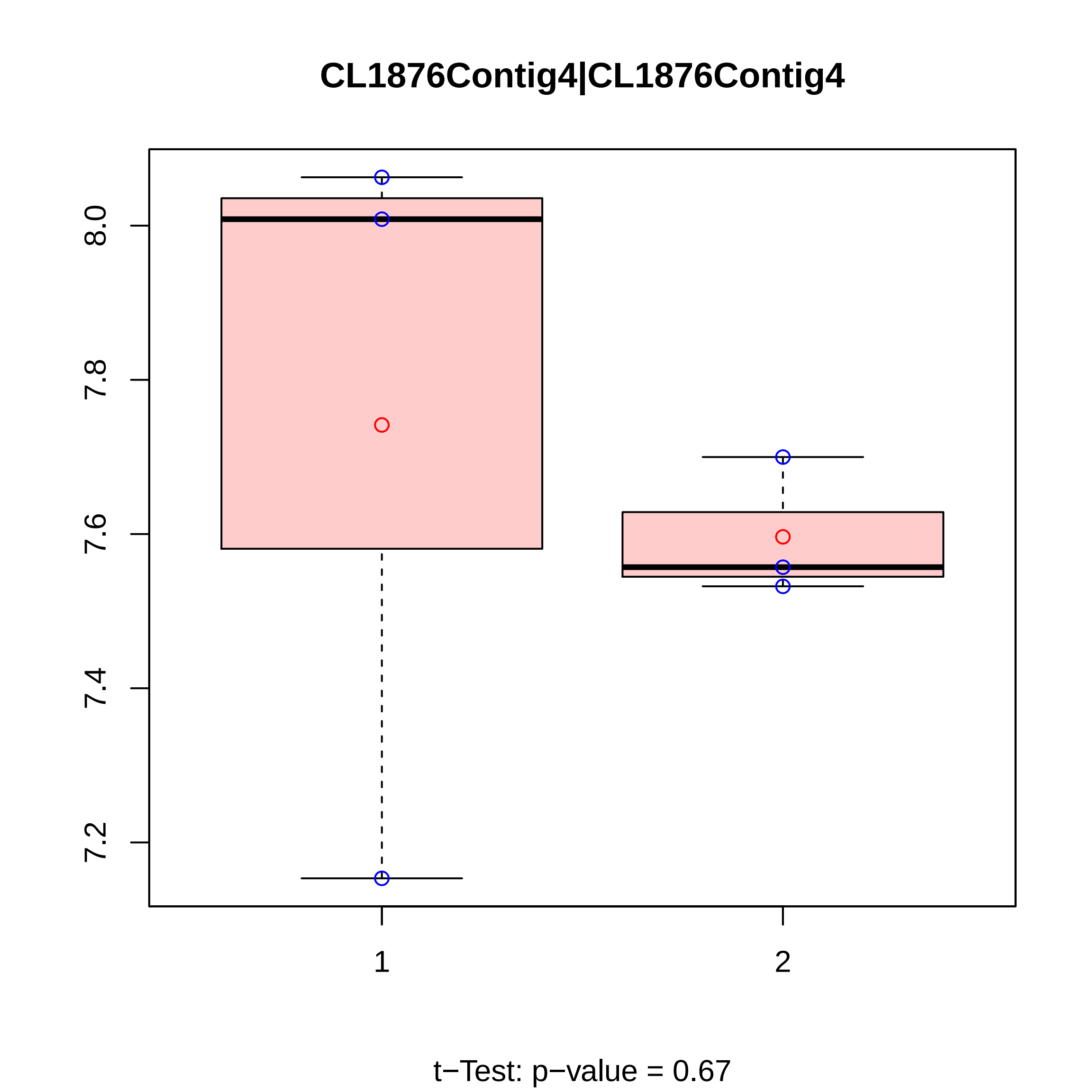

CL1876Contig4|CL1876Contig4
8.0
7.8
7.6
7.4
7.2
1
2
t−Test: p−value = 0.67

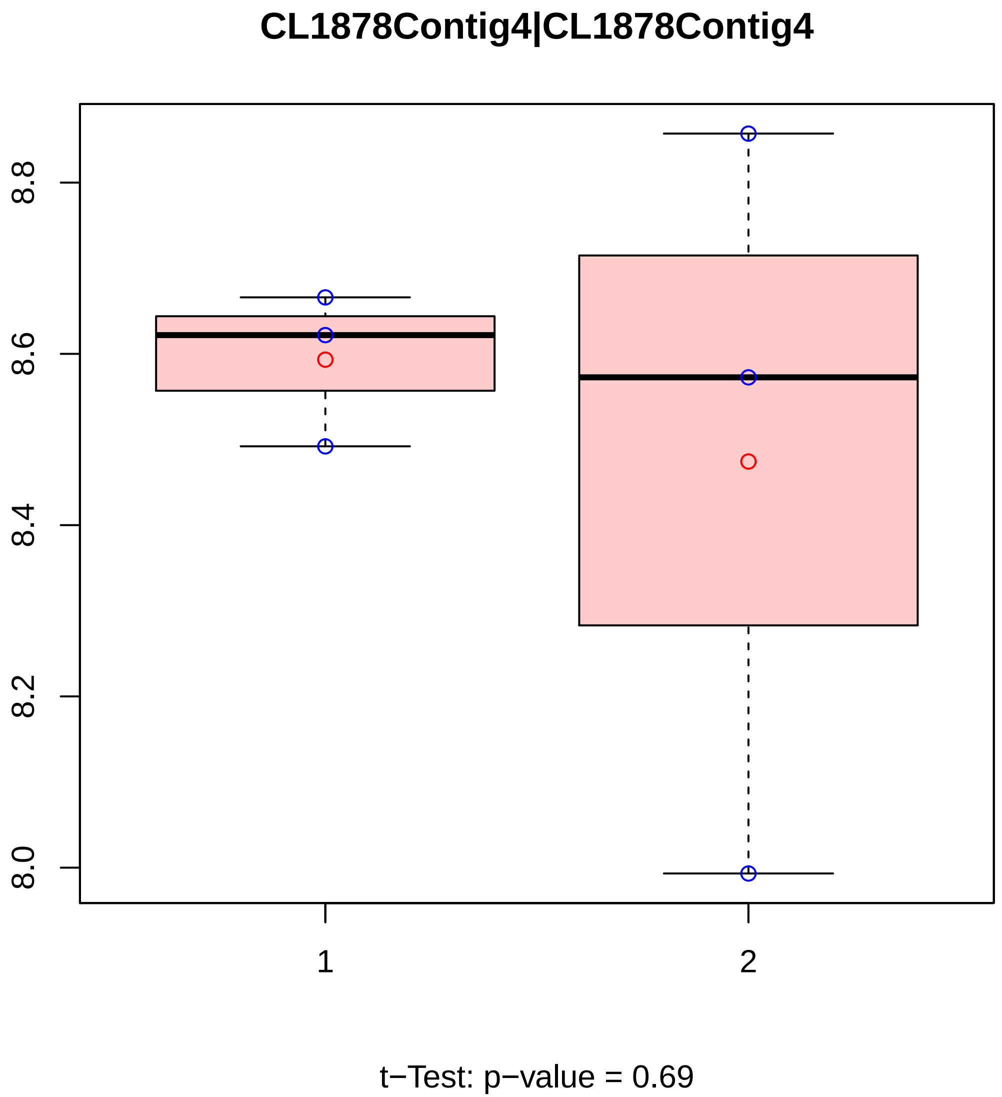

CL1878Contig4|CL1878Contig4
8.0
8.2
8.4
8.6
8.8
1
2
t−Test: p−value = 0.69

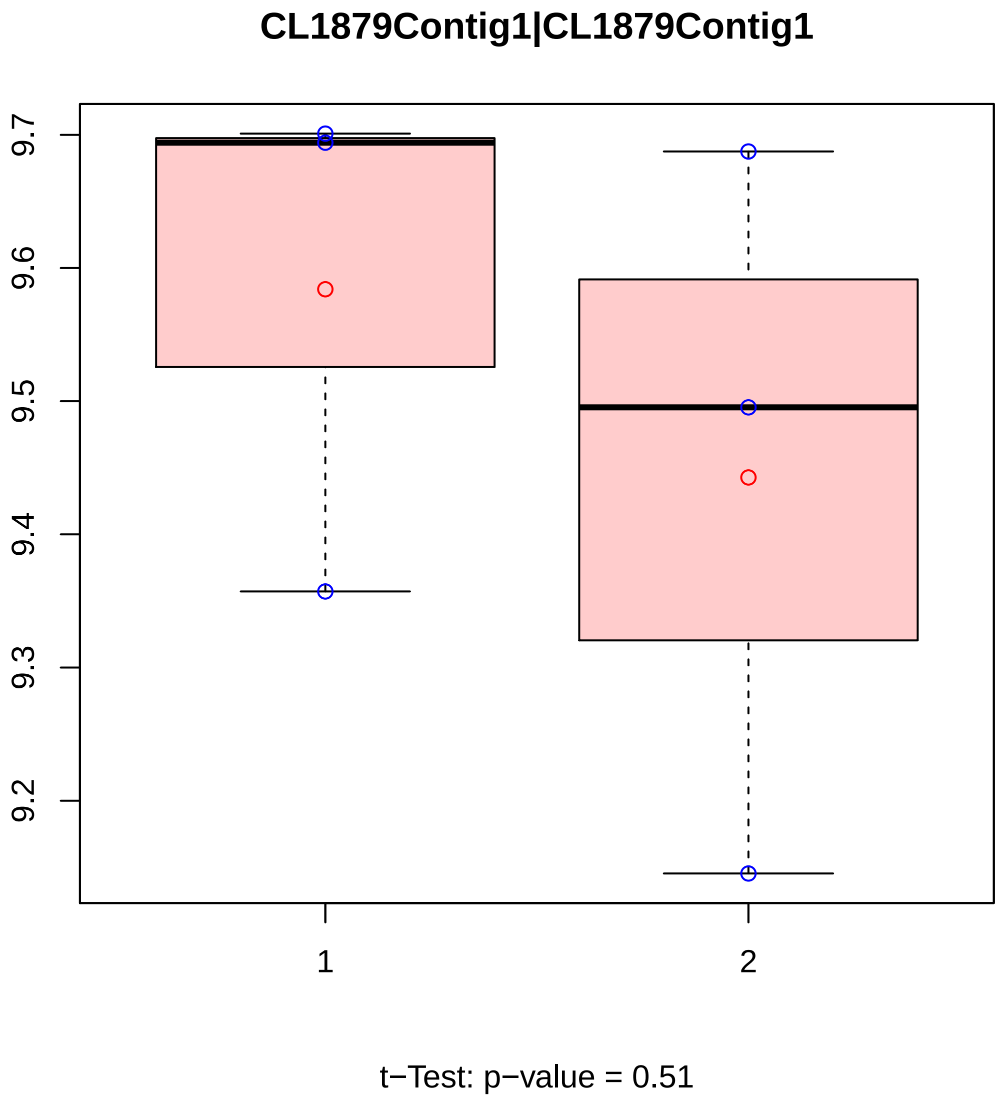
CL1879Contig1|CL1879Contig1
9.7
9.6
9.5
9.4
9.3
9.2
1
2
t−Test: p−value = 0.51

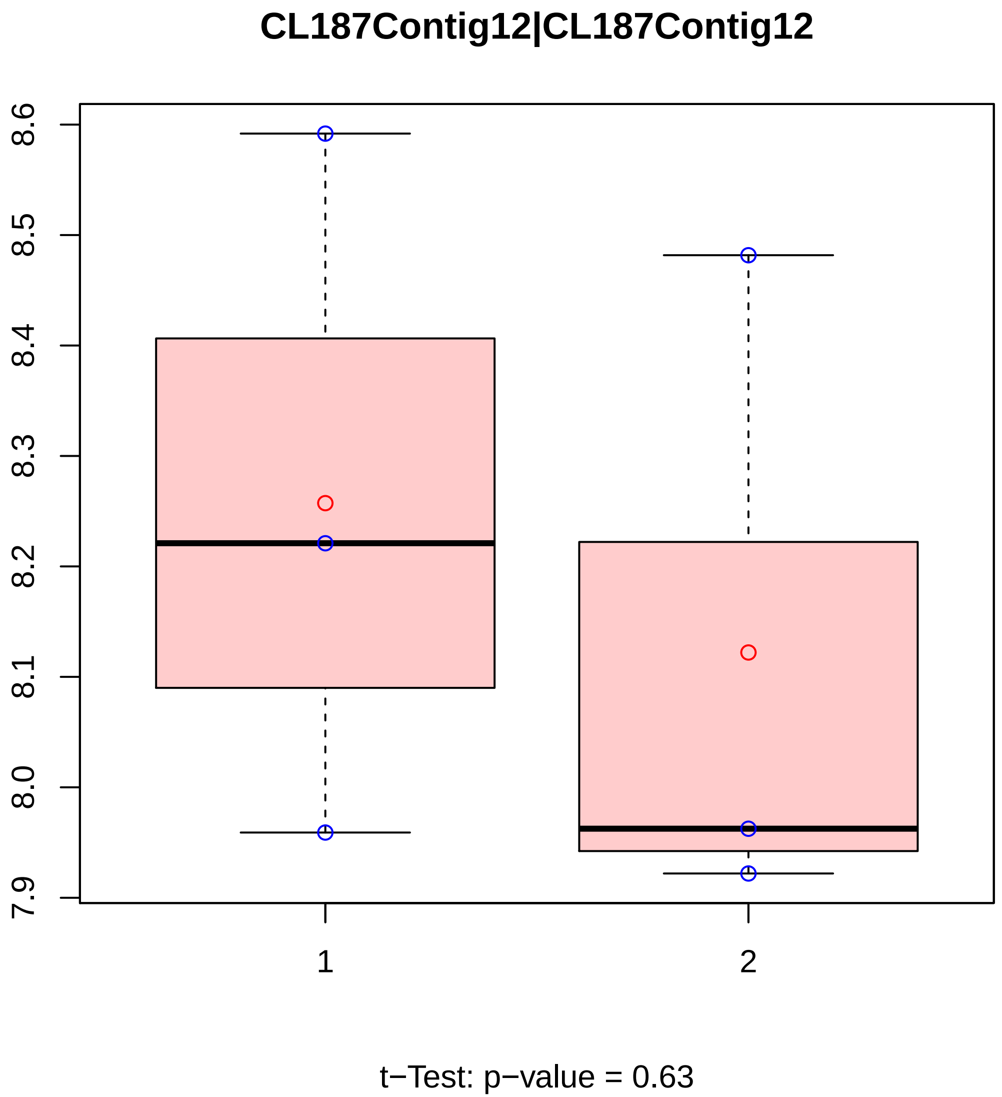
CL187Contig12|CL187Contig12
8.6
8.5
8.4
8.3
8.2
8.1
8.0
7.9
1
2
t−Test: p−value = 0.63

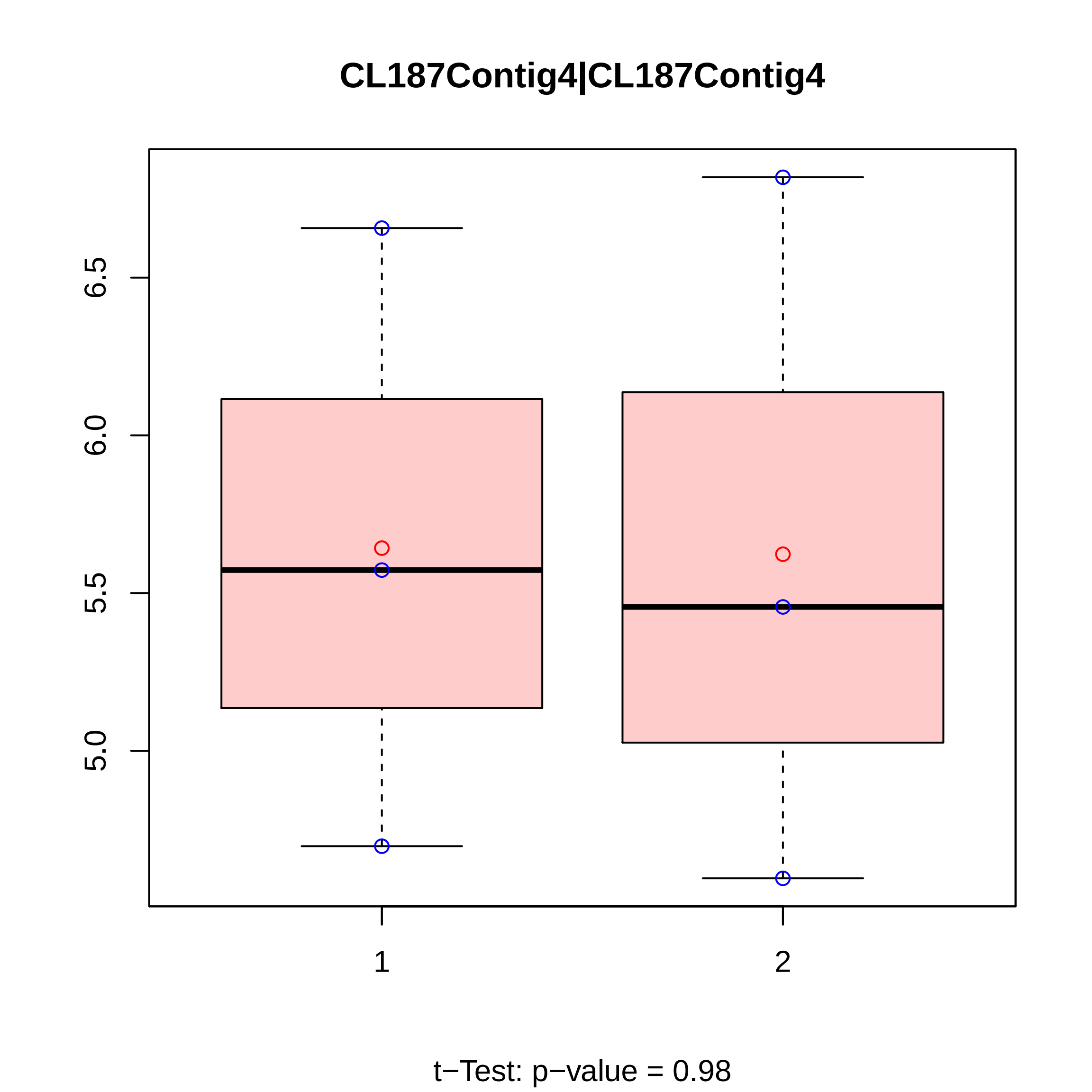

CL187Contig4|CL187Contig4
6.5
6.0
5.5
5.0
1
2
t−Test: p−value = 0.98

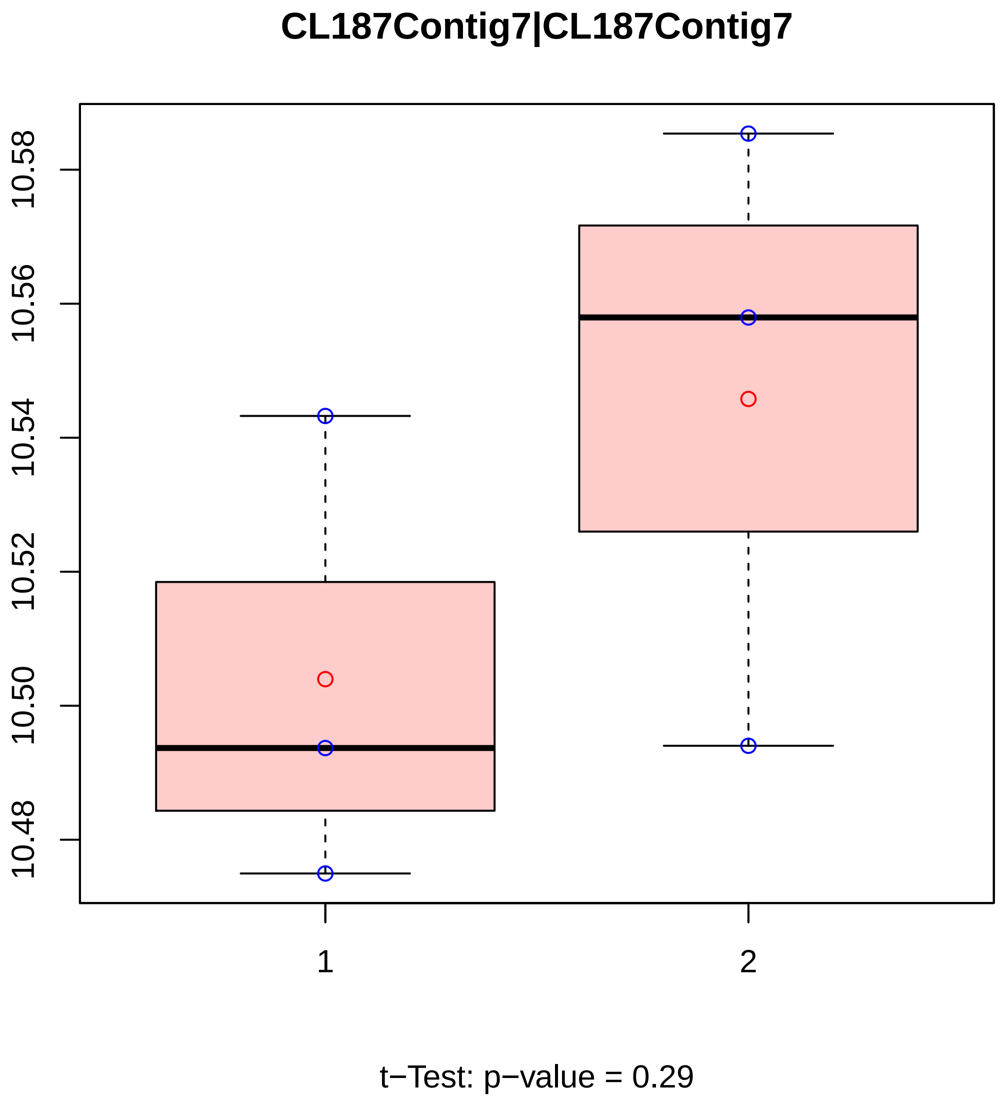
CL187Contig7|CL187Contig7
10.48
10.50
10.52
10.54
10.56
10.58
1
2
t−Test: p−value = 0.29

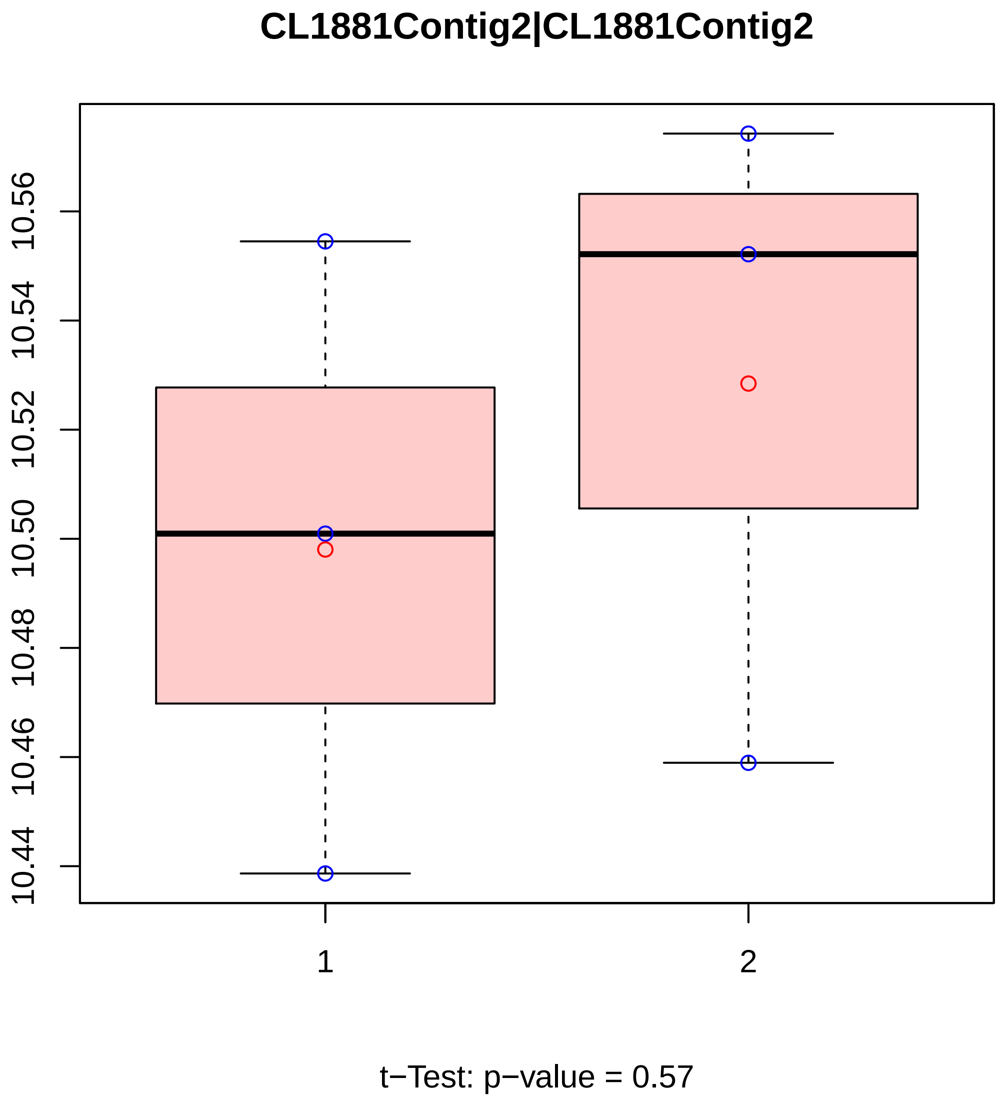

CL1881Contig2|CL1881Contig2
10.44
10.46
10.48
10.50
10.52
10.54
10.56
1
2
t−Test: p−value = 0.57

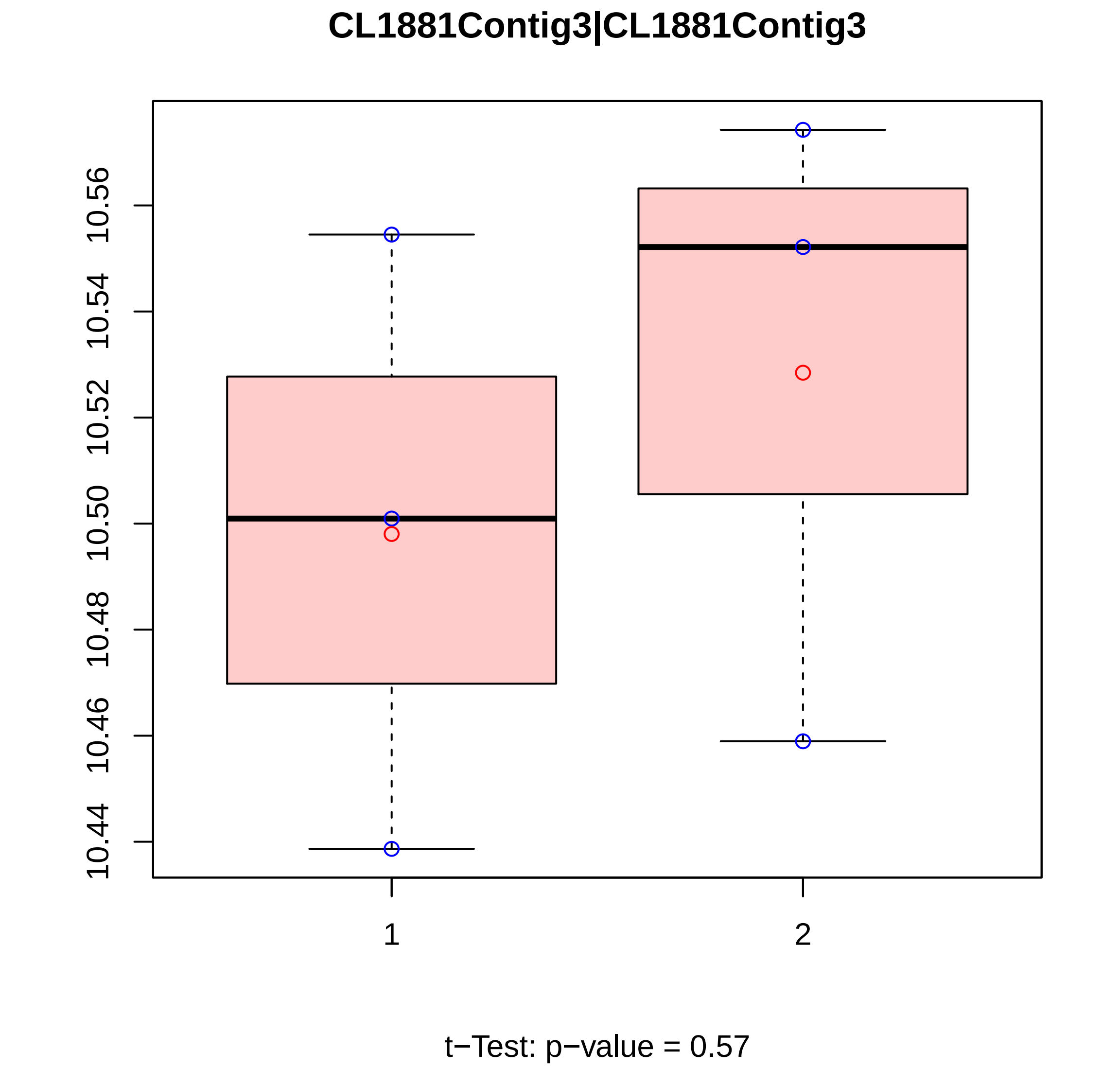

CL1881Contig3|CL1881Contig3
10.44
10.46
10.48
10.50
10.52
10.54
10.56
1
2
t−Test: p−value = 0.57

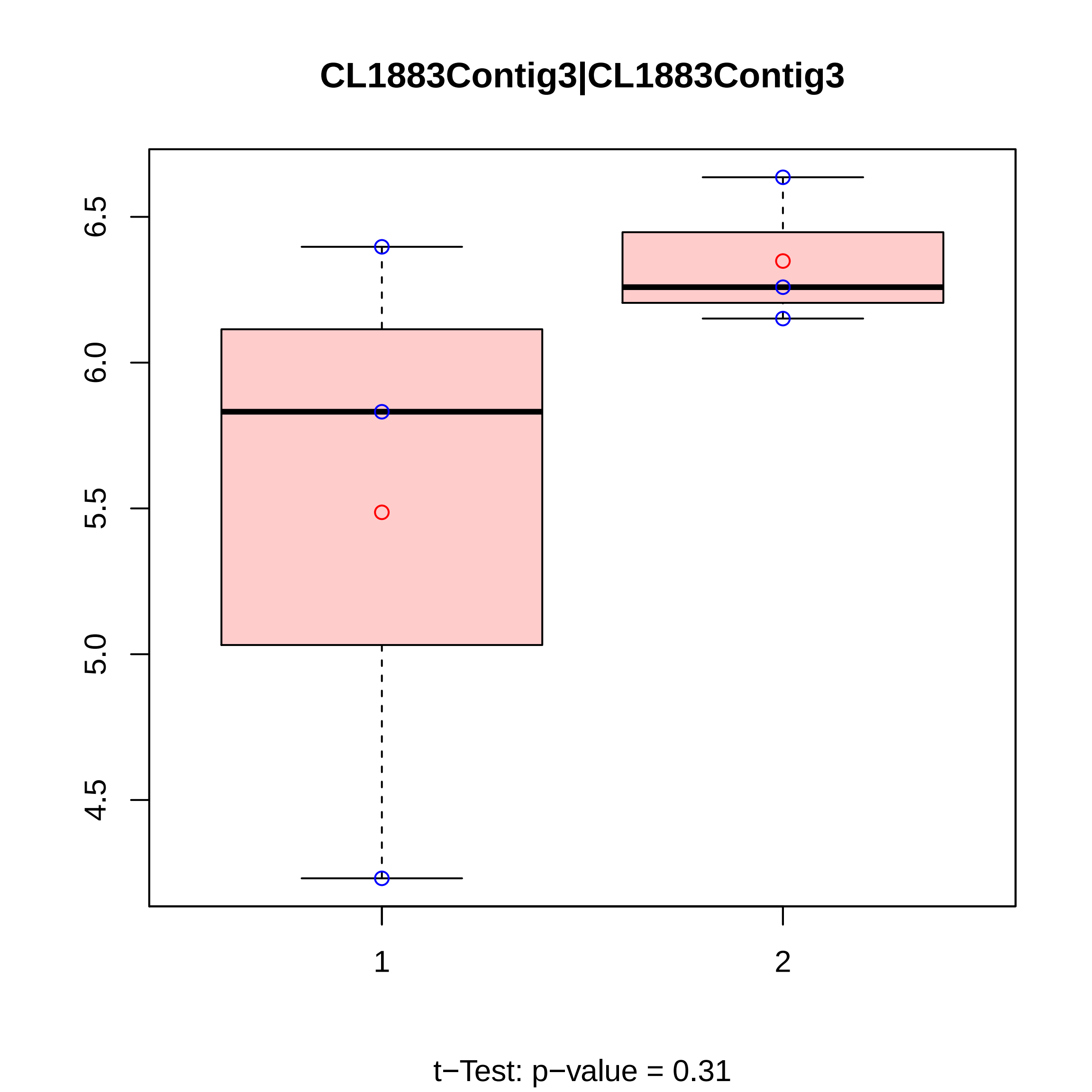

CL1883Contig3|CL1883Contig3
6.5
6.0
5.5
5.0
4.5
1
2
t−Test: p−value = 0.31

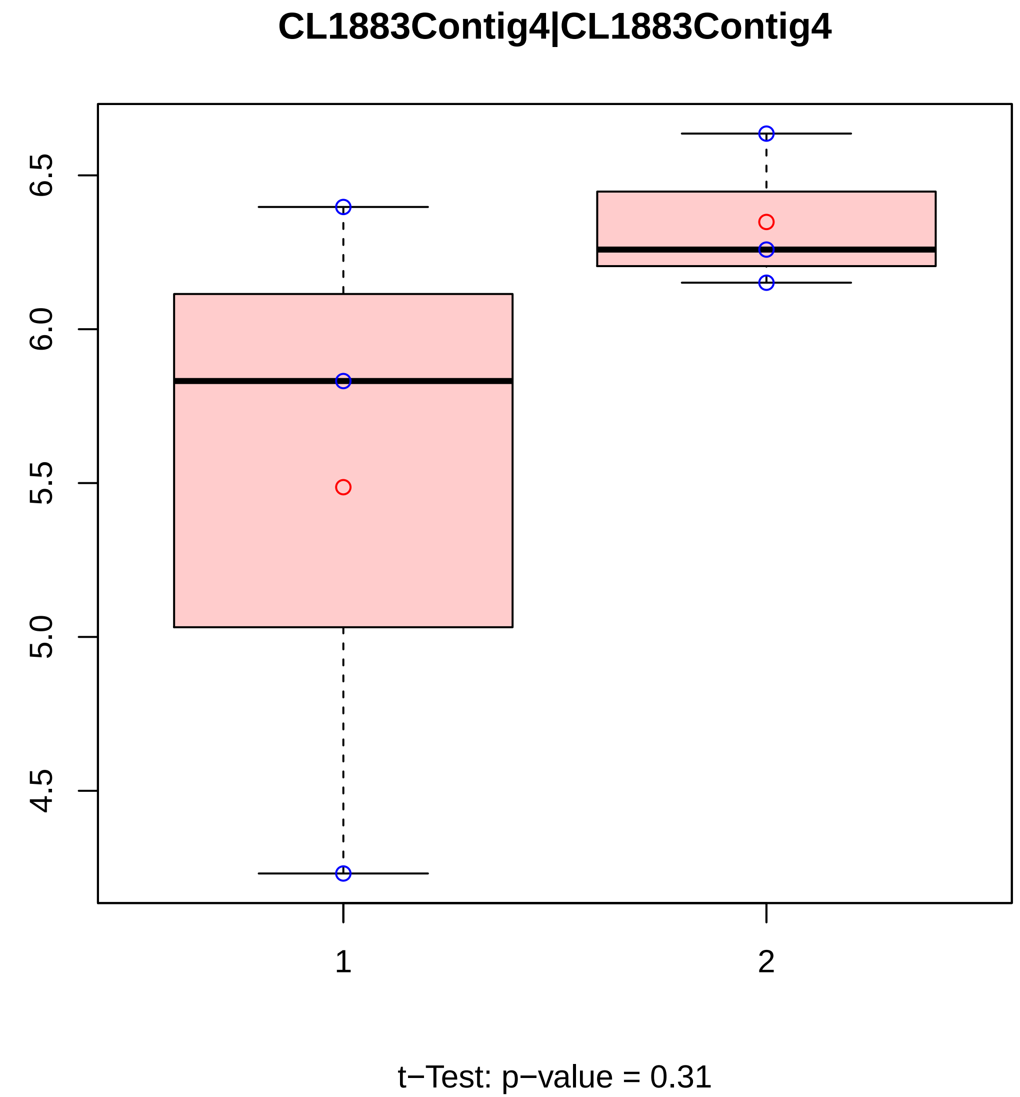
CL1883Contig4|CL1883Contig4
6.5
6.0
5.5
5.0
4.5
1
2
t−Test: p−value = 0.31

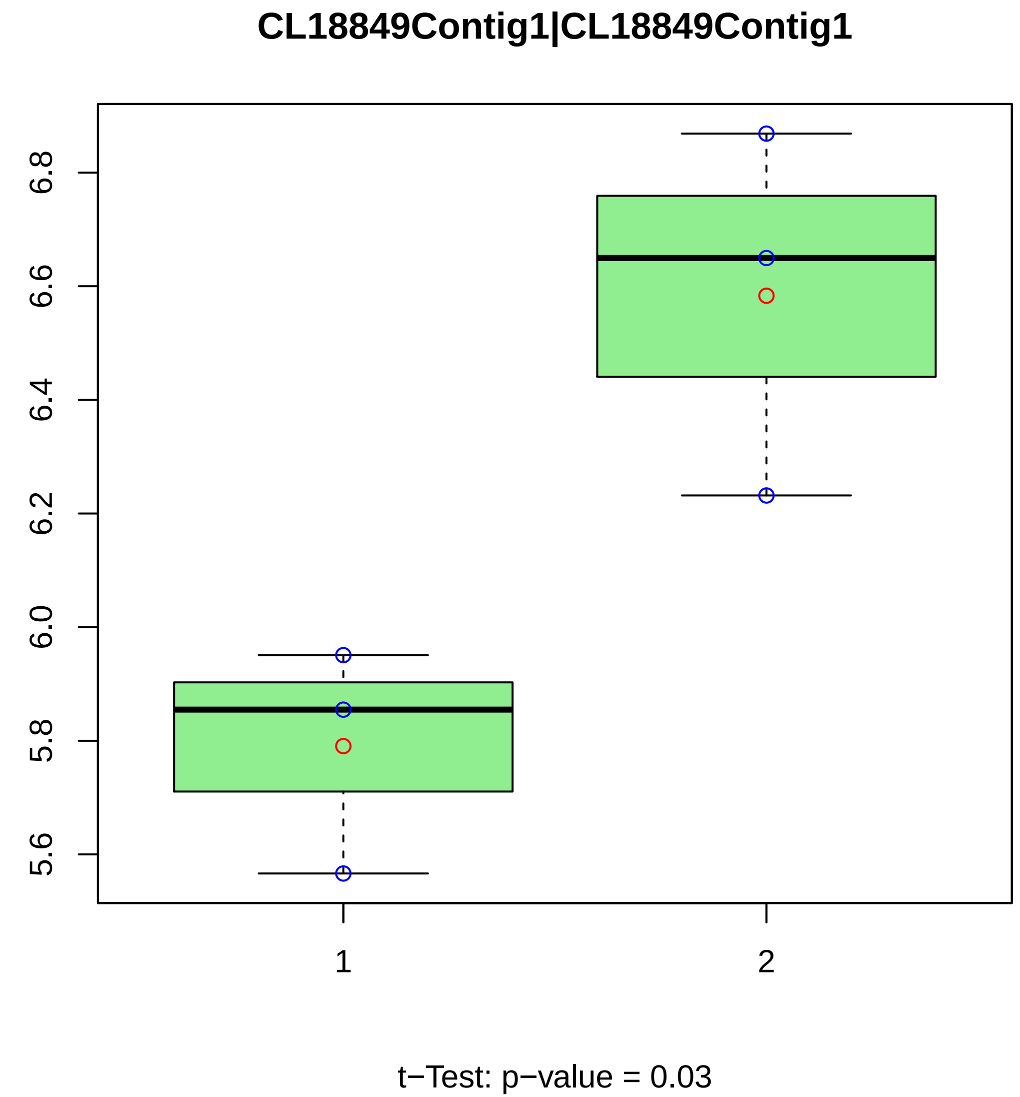
CL18849Contig1|CL18849Contig1
6.8
6.6
6.4
6.2
6.0
5.8
5.6
1
2
t−Test: p−value = 0.03

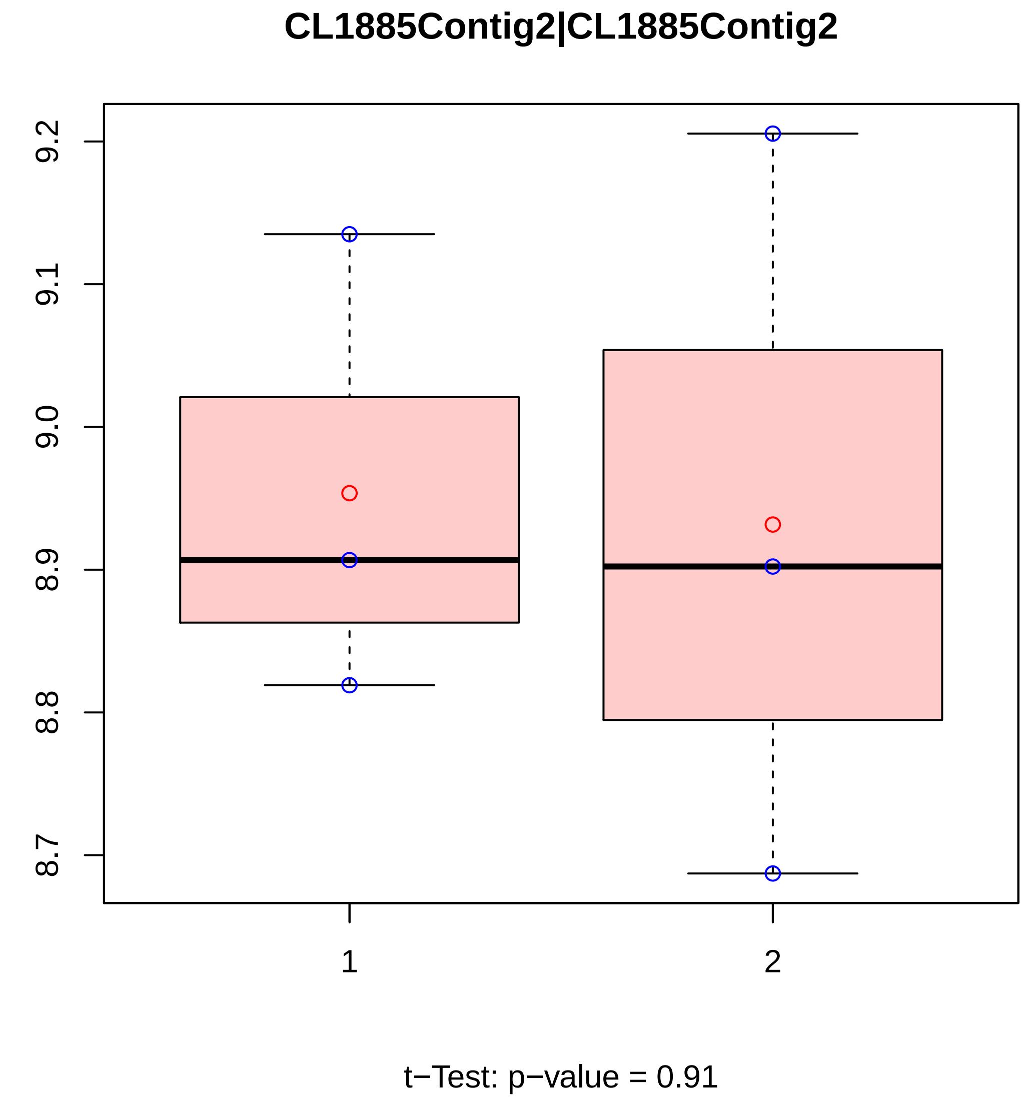
CL1885Contig2|CL1885Contig2
9.2
9.1
9.0
8.9
8.8
8.7
1
2
t−Test: p−value = 0.91

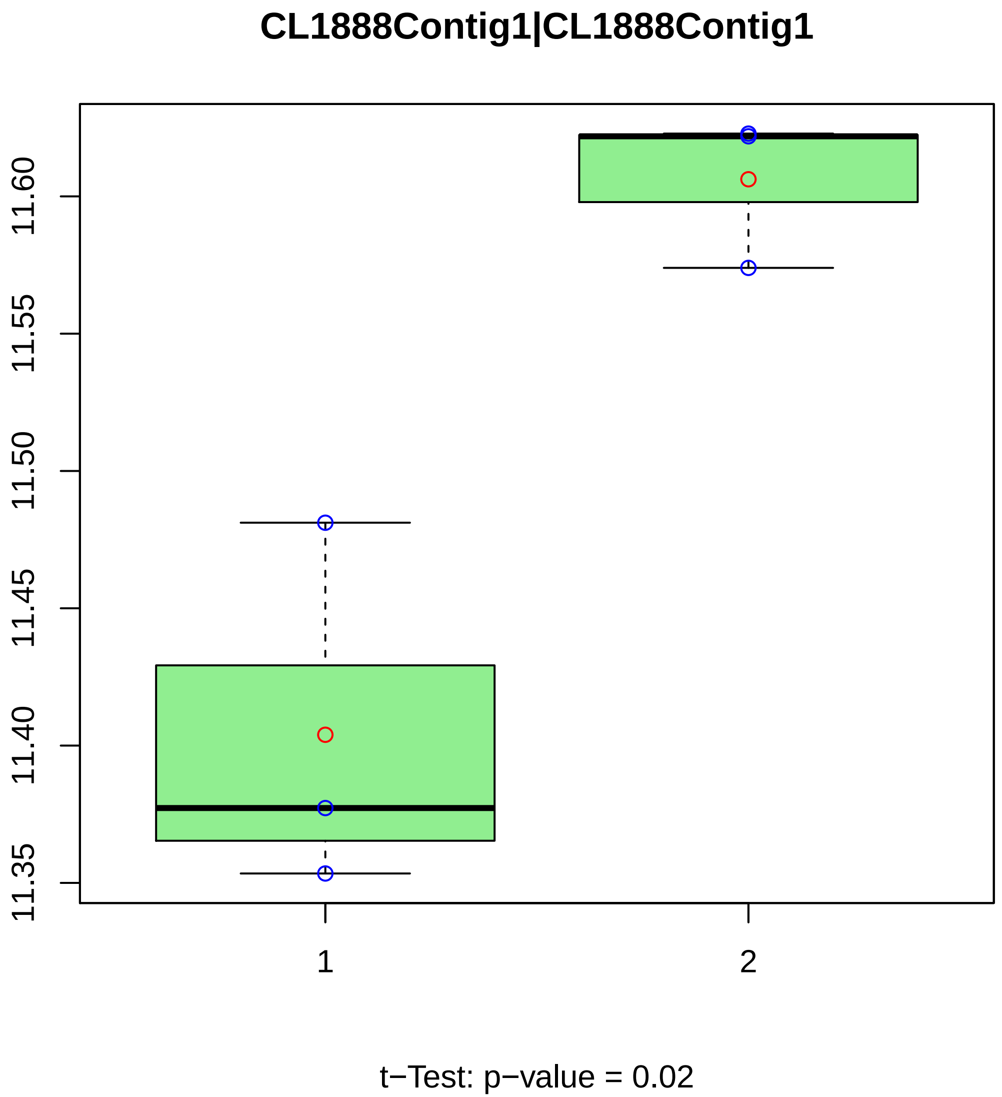
CL1888Contig1|CL1888Contig1
11.35
11.40
11.45
11.50
11.55
11.60
1
2
t−Test: p−value = 0.02

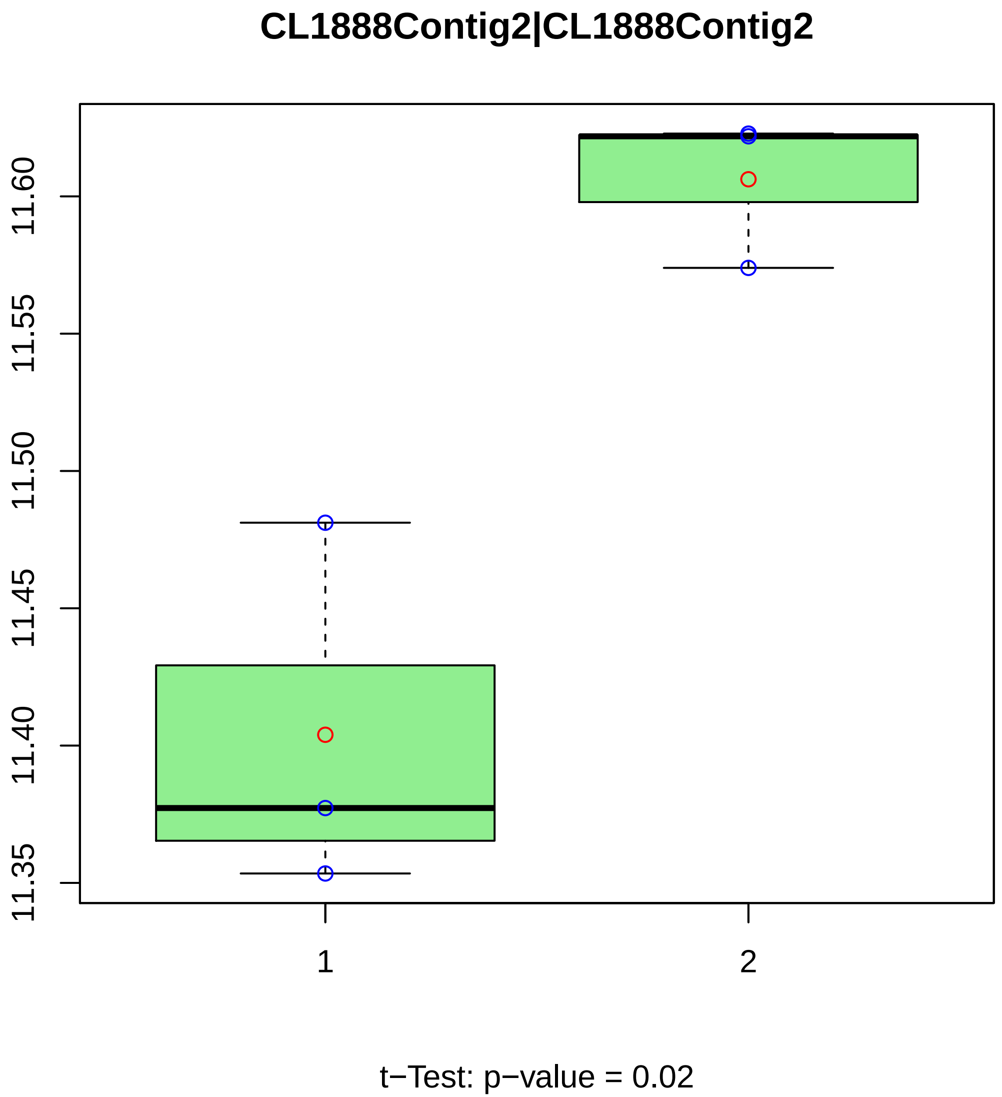

CL1888Contig2|CL1888Contig2
11.35
11.40
11.45
11.50
11.55
11.60
1
2
t−Test: p−value = 0.02

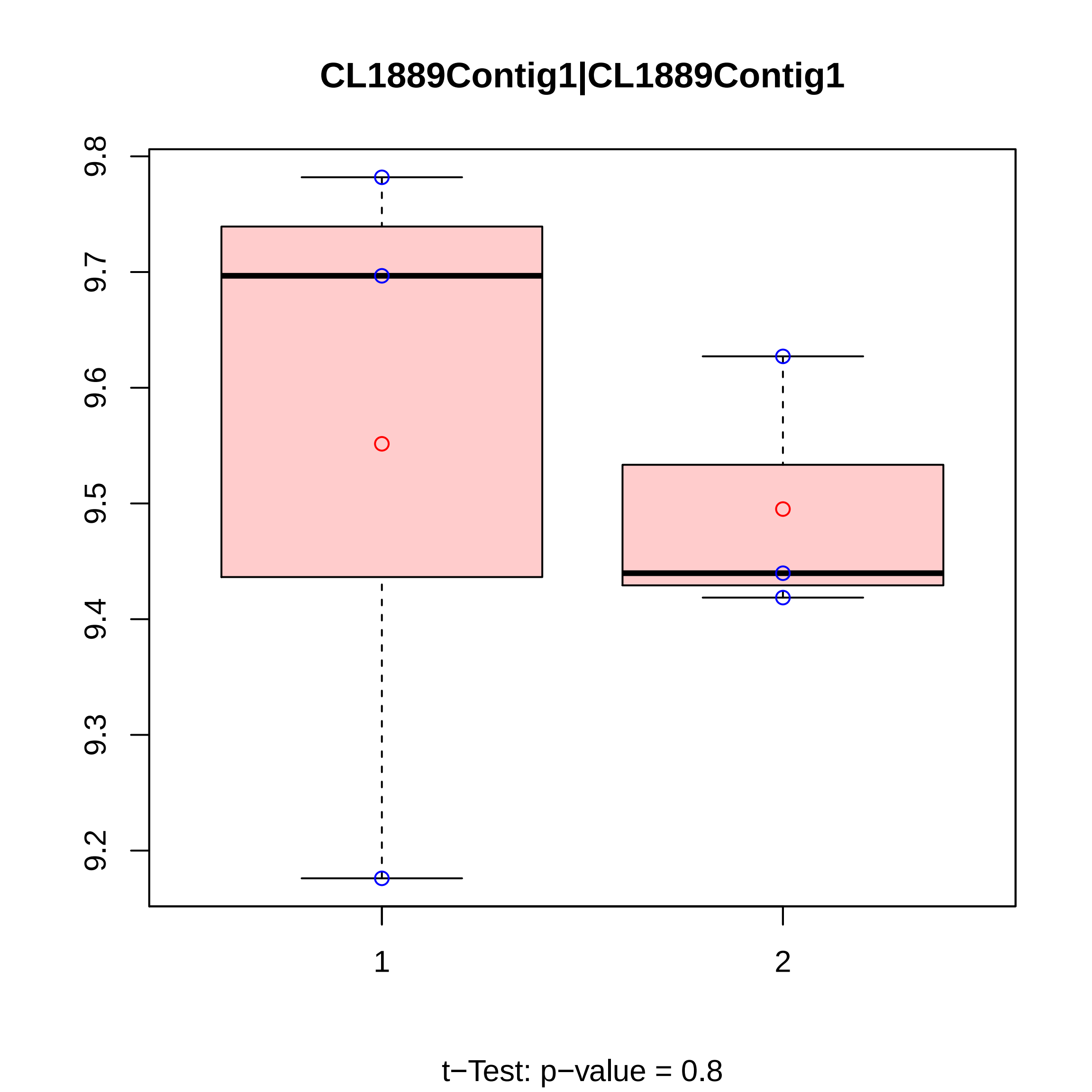

CL1889Contig1|CL1889Contig1
9.2
9.3
9.4
9.5
9.6
9.7
9.8
1
2
t−Test: p−value = 0.8

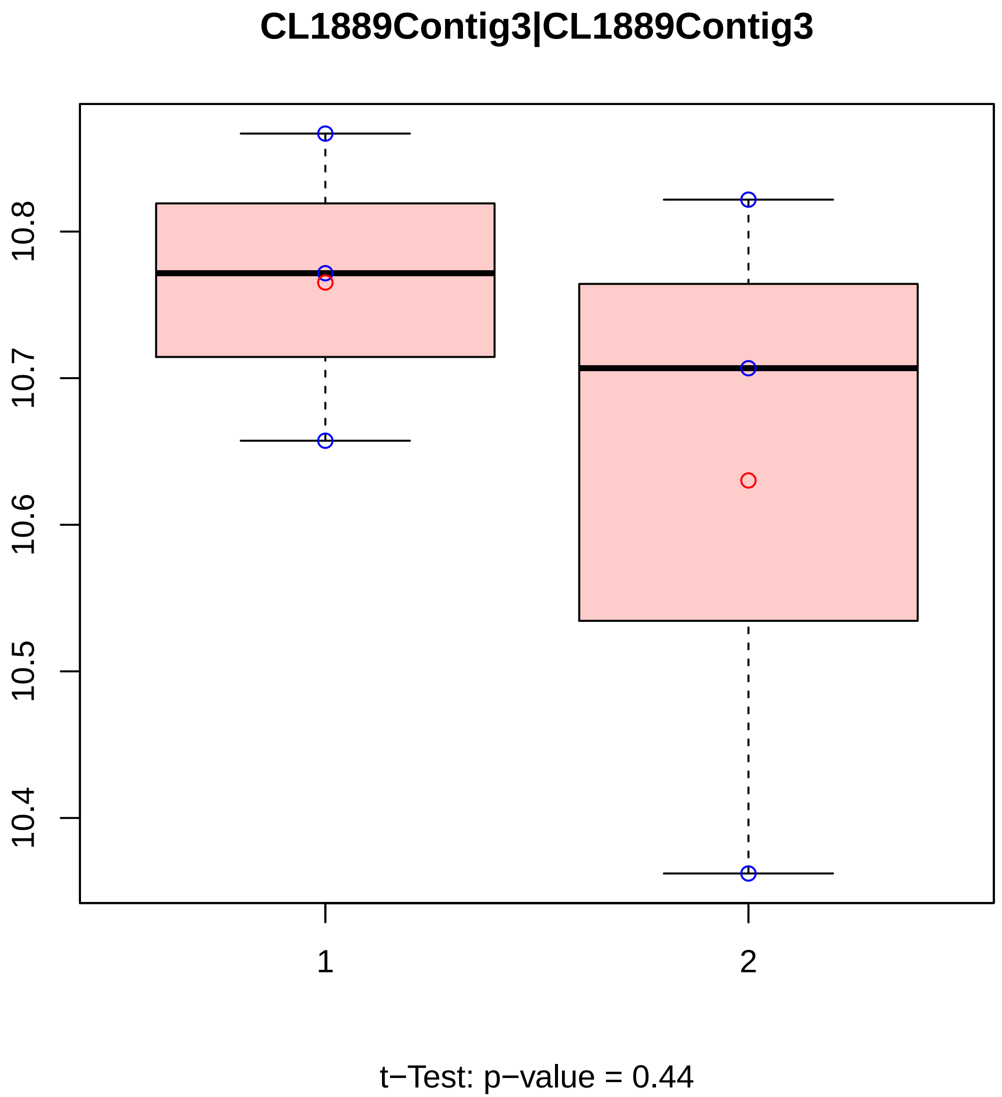

CL1889Contig3|CL1889Contig3
10.4
10.5
10.6
10.7
10.8
1
2
t−Test: p−value = 0.44

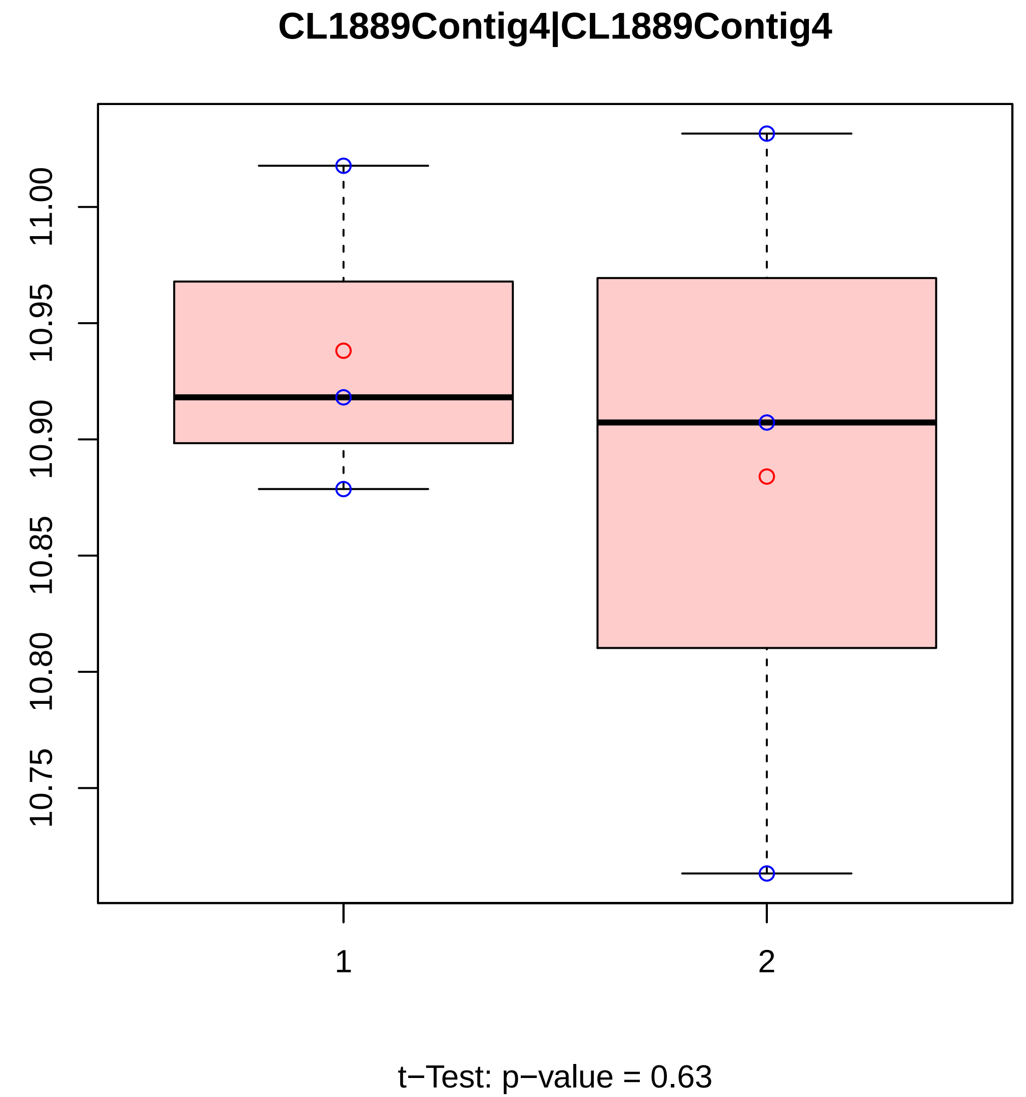
CL1889Contig4|CL1889Contig4
11.00
10.95
10.90
10.85
10.80
10.75
1
2
t−Test: p−value = 0.63

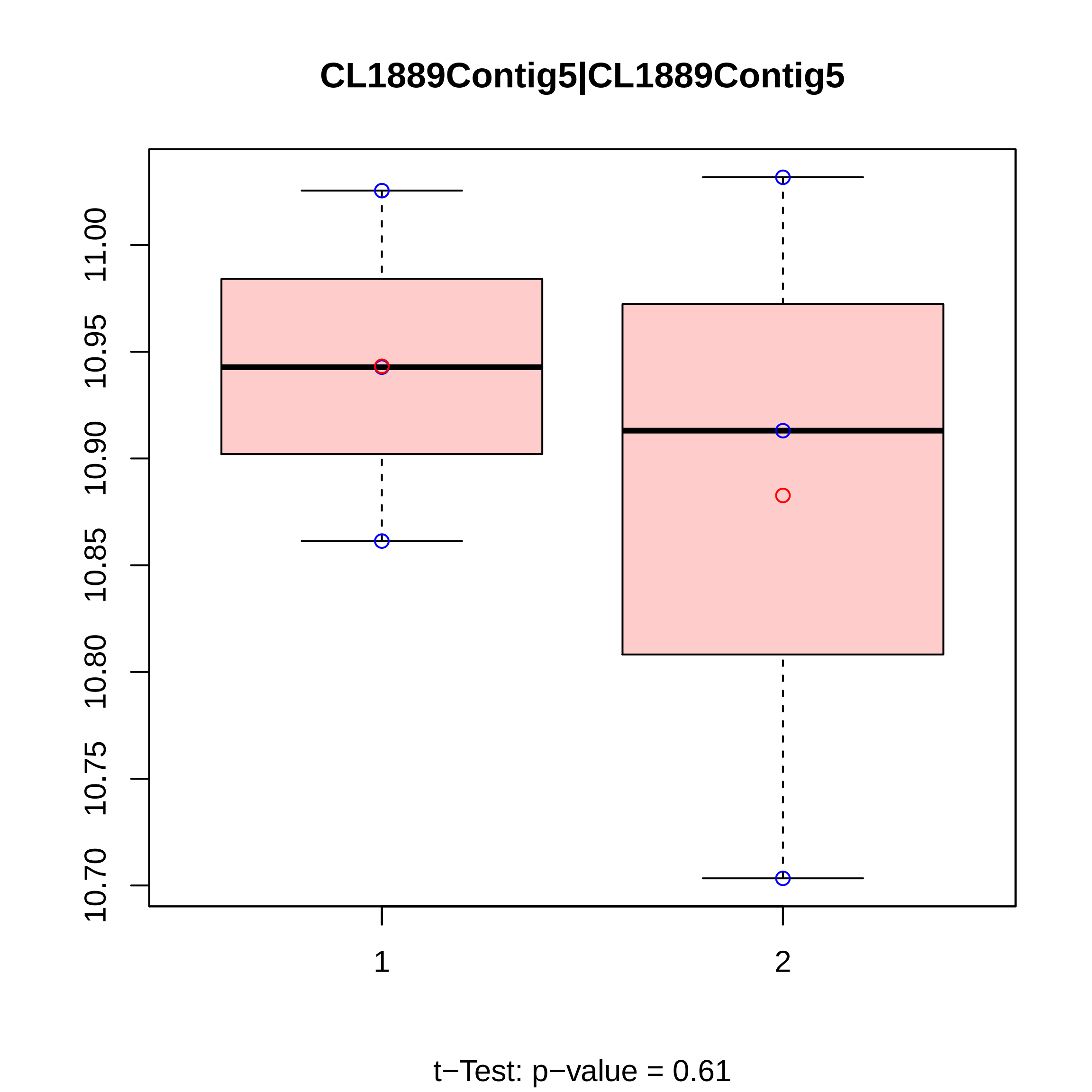

CL1889Contig5|CL1889Contig5
11.00
10.95
10.90
10.85
10.80
10.75
10.70
1
2
t−Test: p−value = 0.61

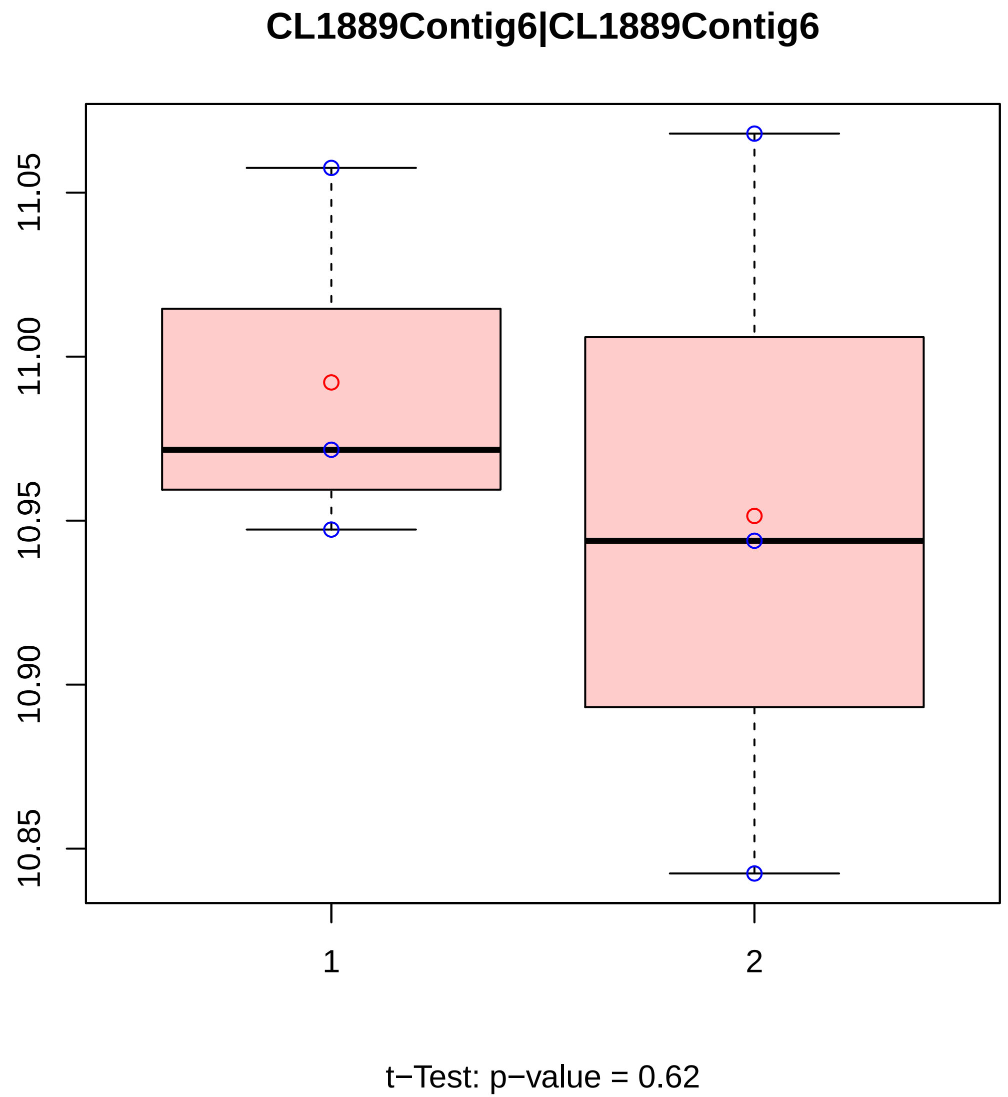

CL1889Contig6|CL1889Contig6
11.05
11.00
10.95
10.90
10.85
1
2
t−Test: p−value = 0.62

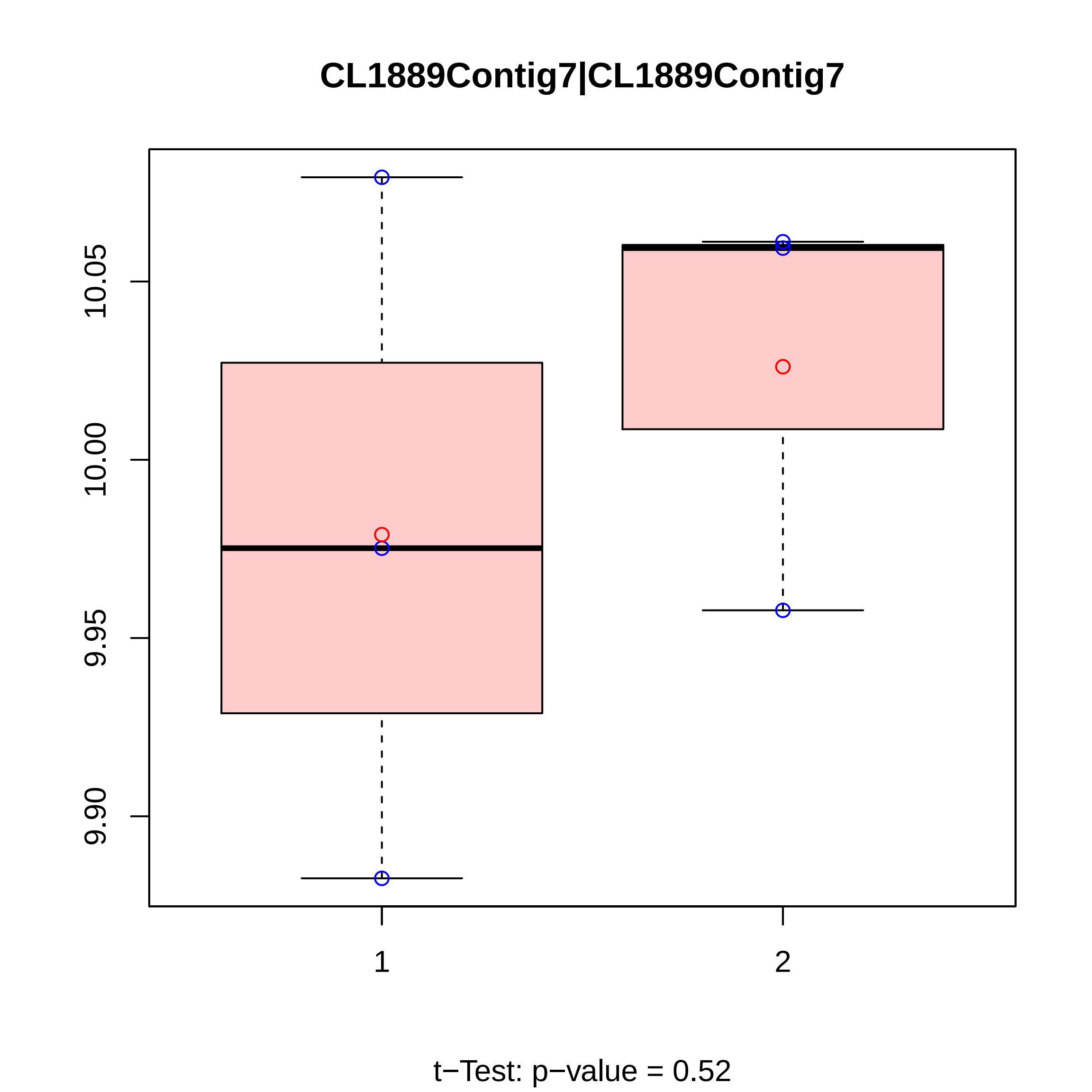
CL1889Contig7|CL1889Contig7
10.05
10.00
9.95
9.90
1
2
t−Test: p−value = 0.52

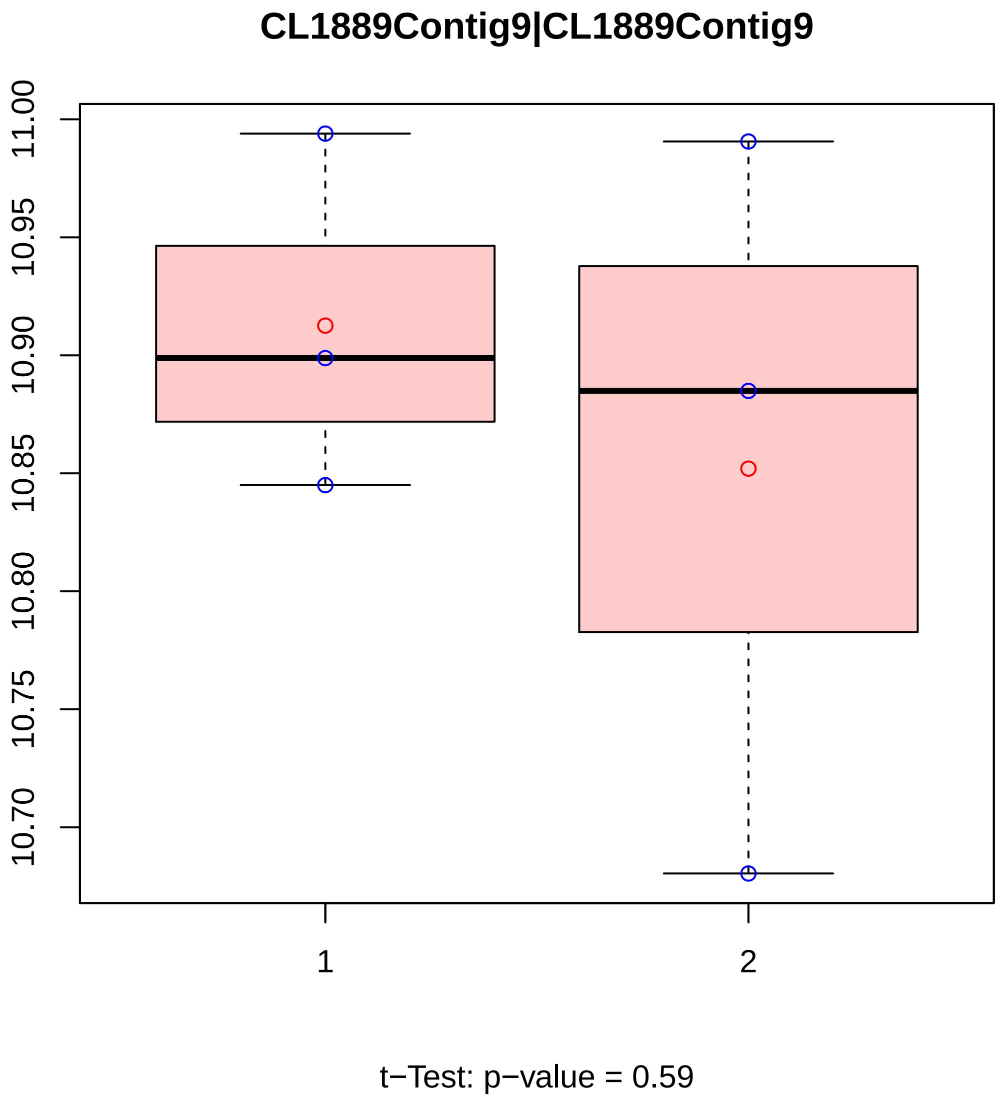
CL1889Contig9|CL1889Contig9
11.00
10.95
10.90
10.85
10.80
10.75
10.70
1
2
t−Test: p−value = 0.59

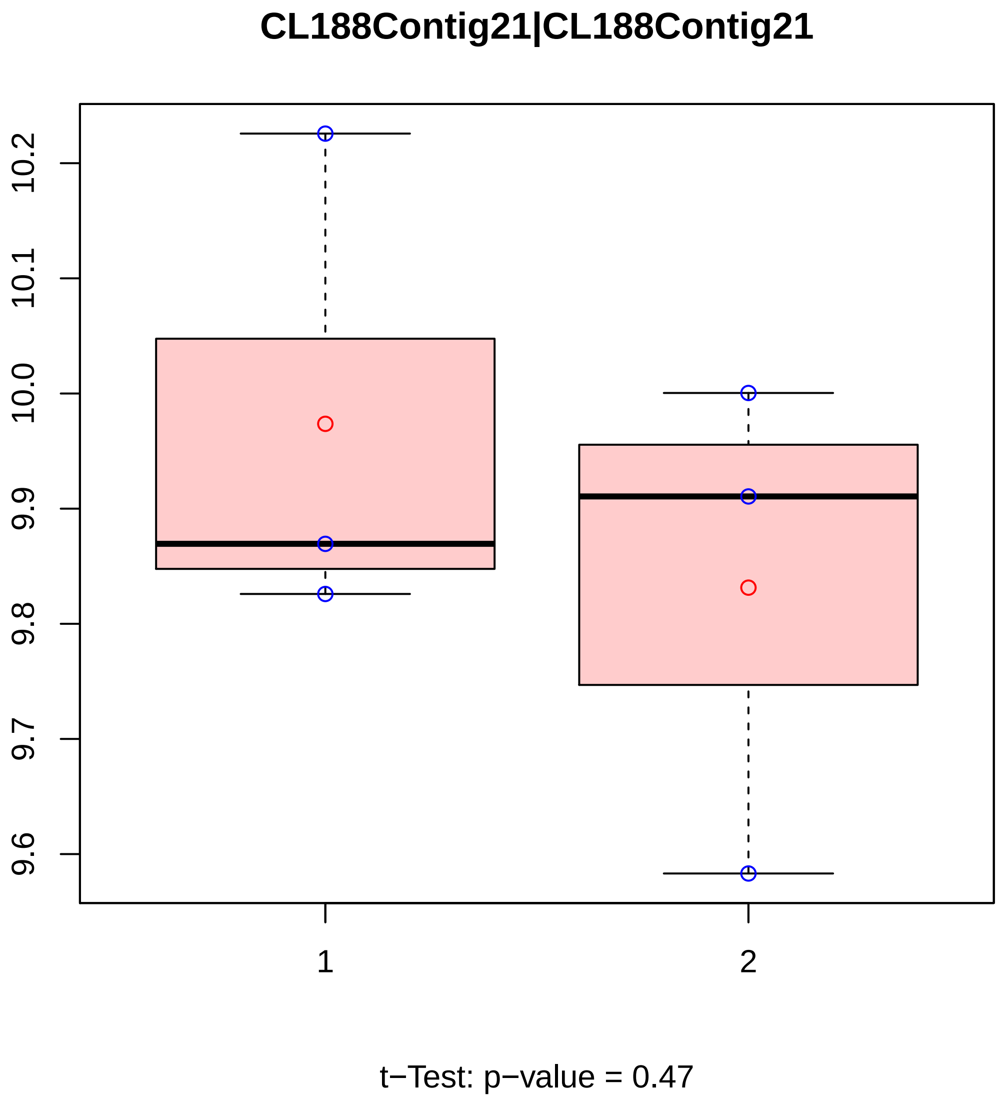
CL188Contig21|CL188Contig21
10.2
10.1
10.0
9.9
9.8
9.7
9.6
1
2
t−Test: p−value = 0.47

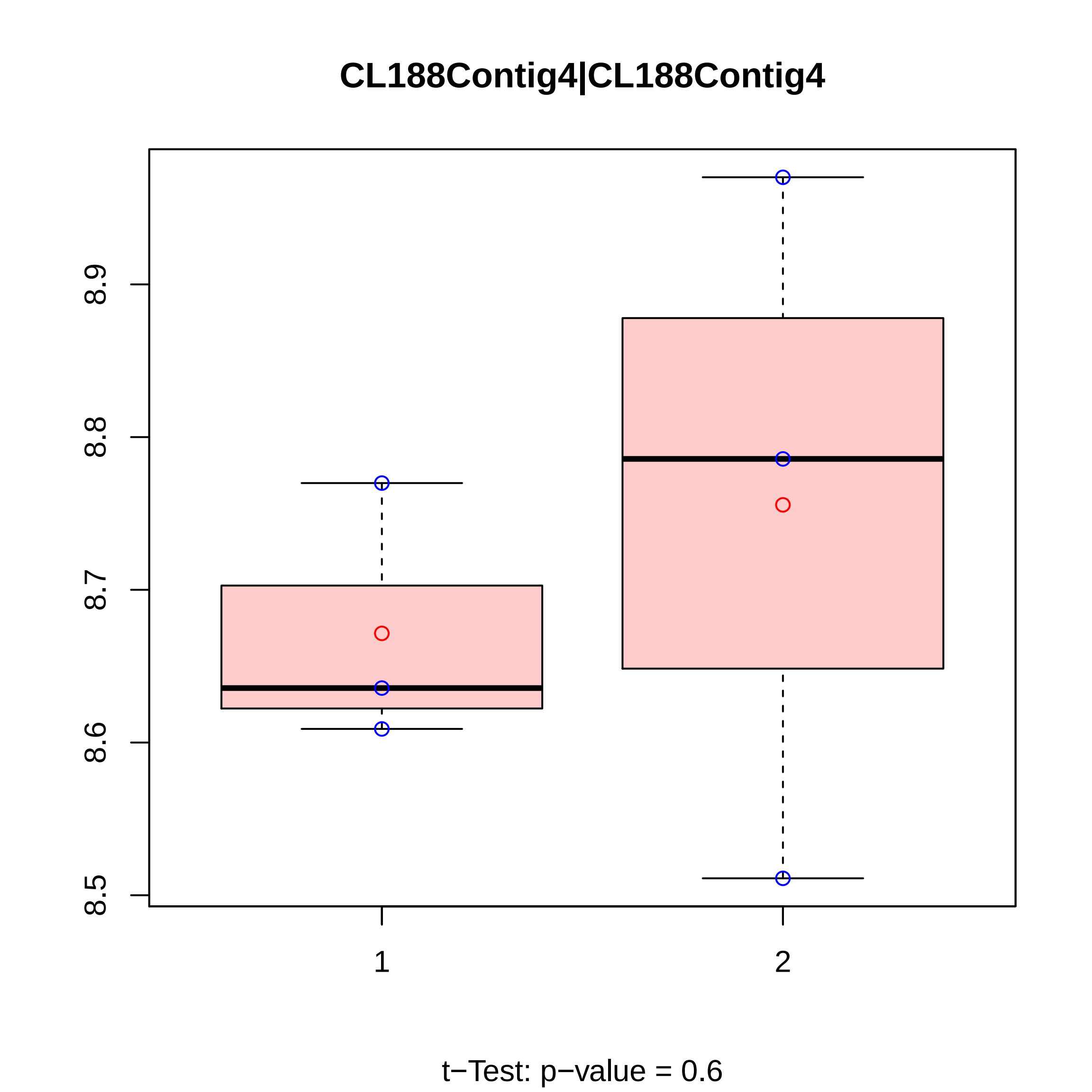
CL188Contig4|CL188Contig4
8.5
8.6
8.7
8.8
8.9
1
2
t−Test: p−value = 0.6

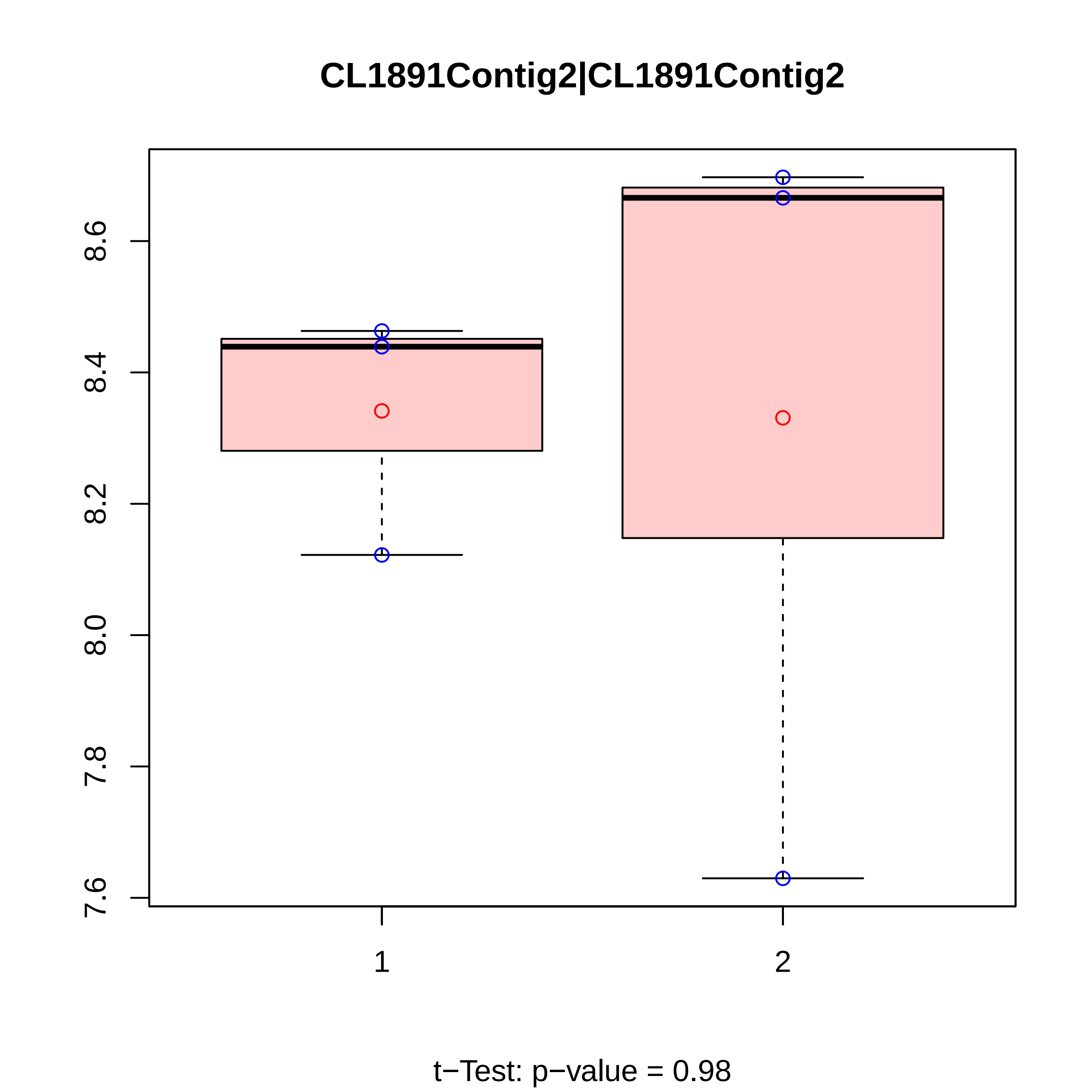

CL1891Contig2|CL1891Contig2
8.6
8.4
8.2
8.0
7.8
7.6
1
2
t−Test: p−value = 0.98

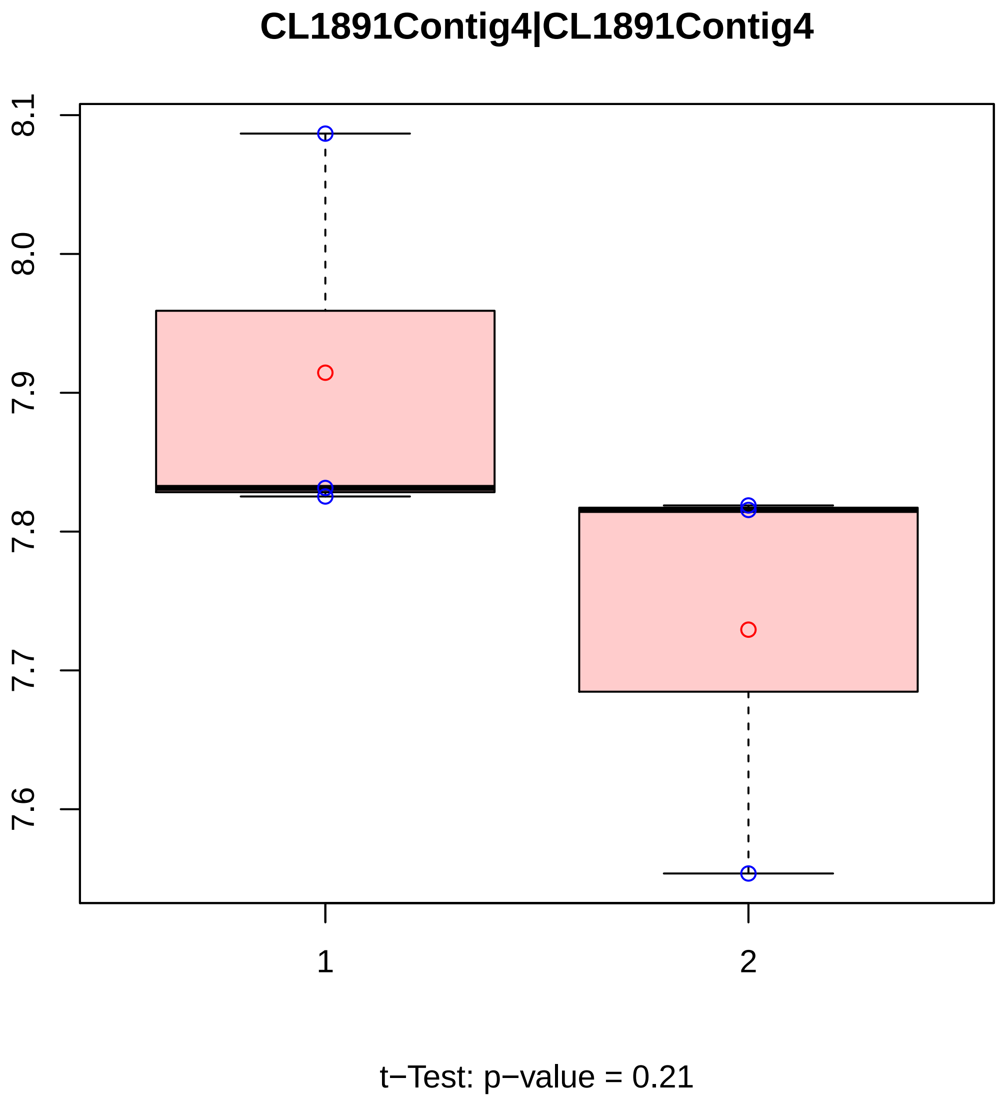

CL1891Contig4|CL1891Contig4
8.1
8.0
7.9
7.8
7.7
7.6
1
2
t−Test: p−value = 0.21

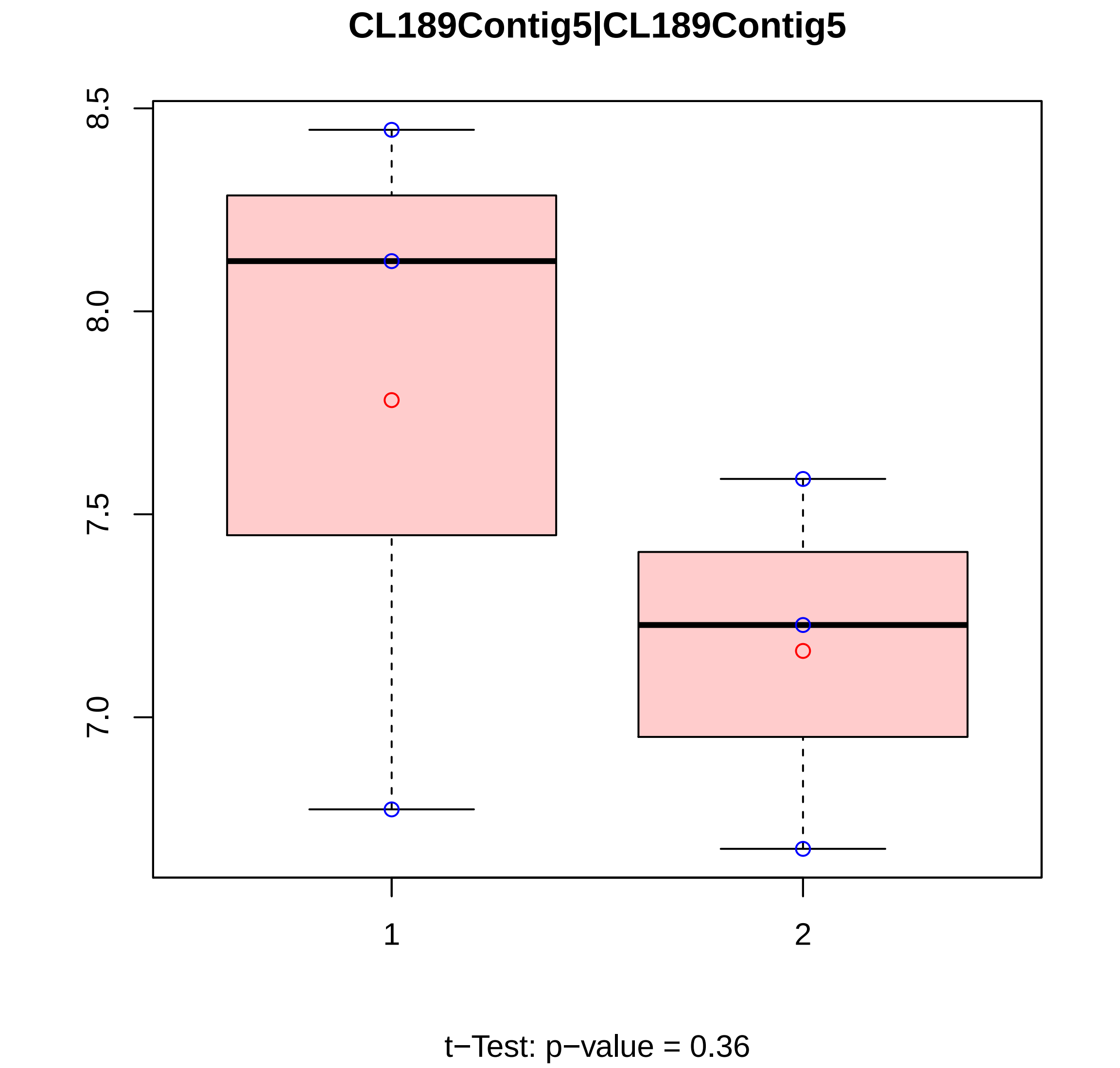
CL189Contig5|CL189Contig5
8.5
8.0
7.5
7.0
1
2
t−Test: p−value = 0.36

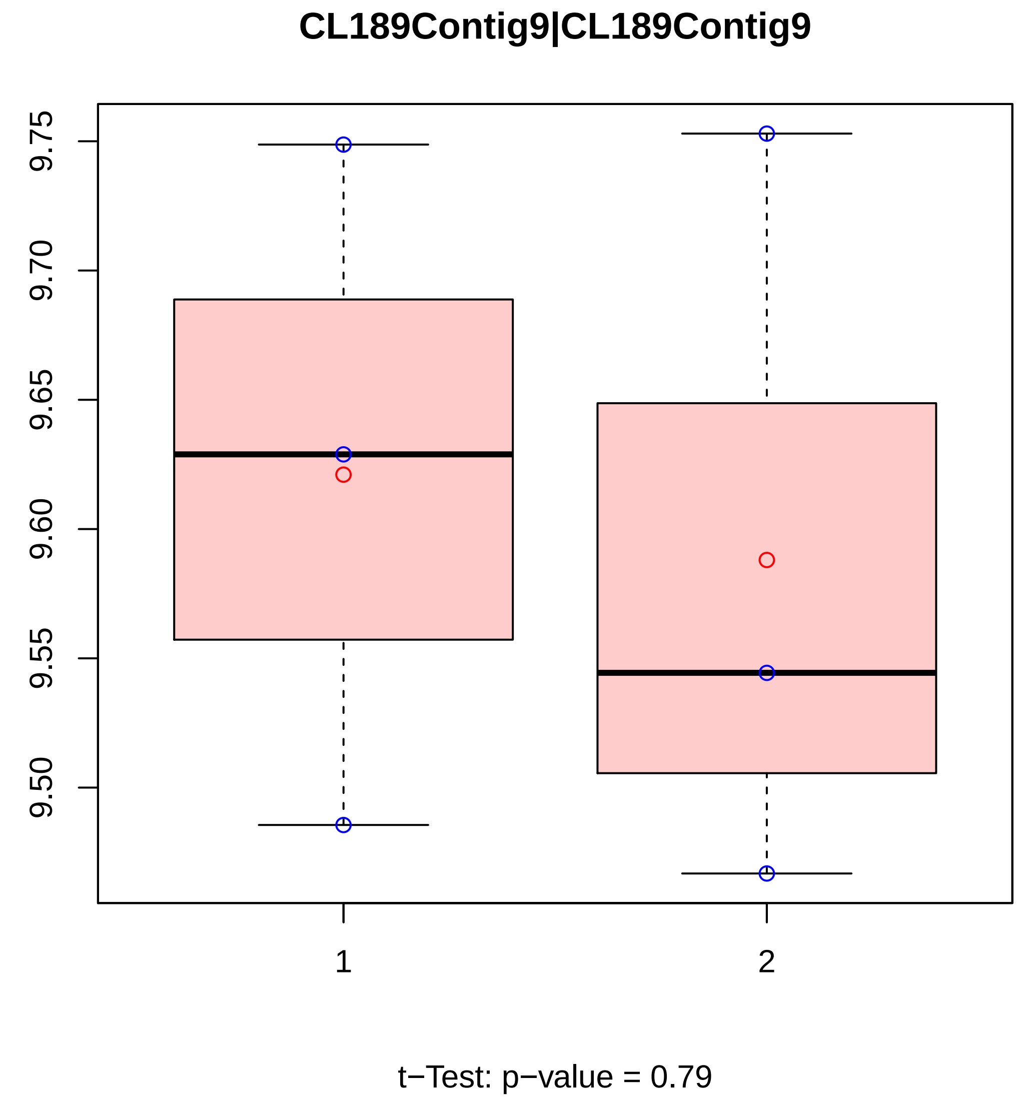

CL189Contig9|CL189Contig9
9.50
9.55
9.60
9.65
9.70
9.75
1
2
t−Test: p−value = 0.79

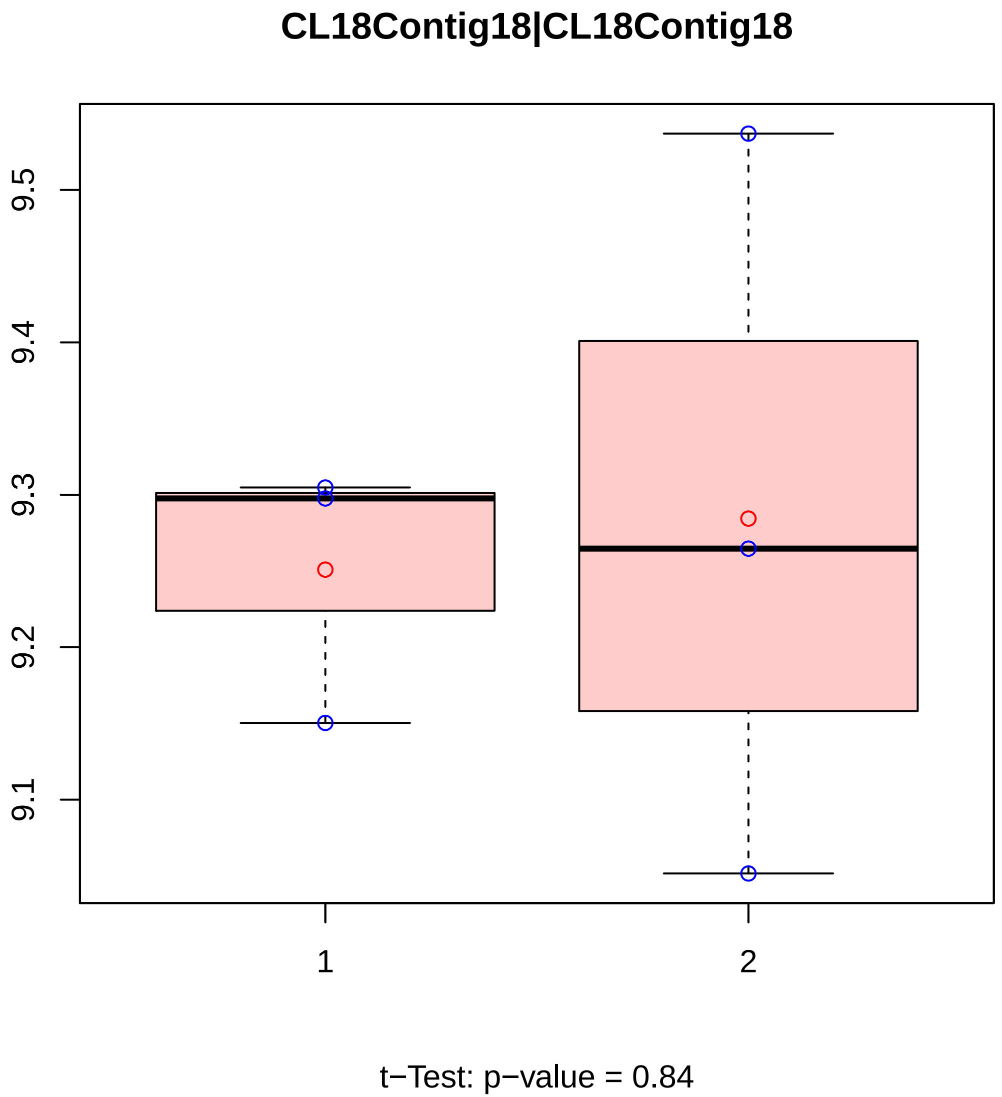

CL18Contig18|CL18Contig18
9.1
9.2
9.3
9.4
9.5
1
2
t−Test: p−value = 0.84

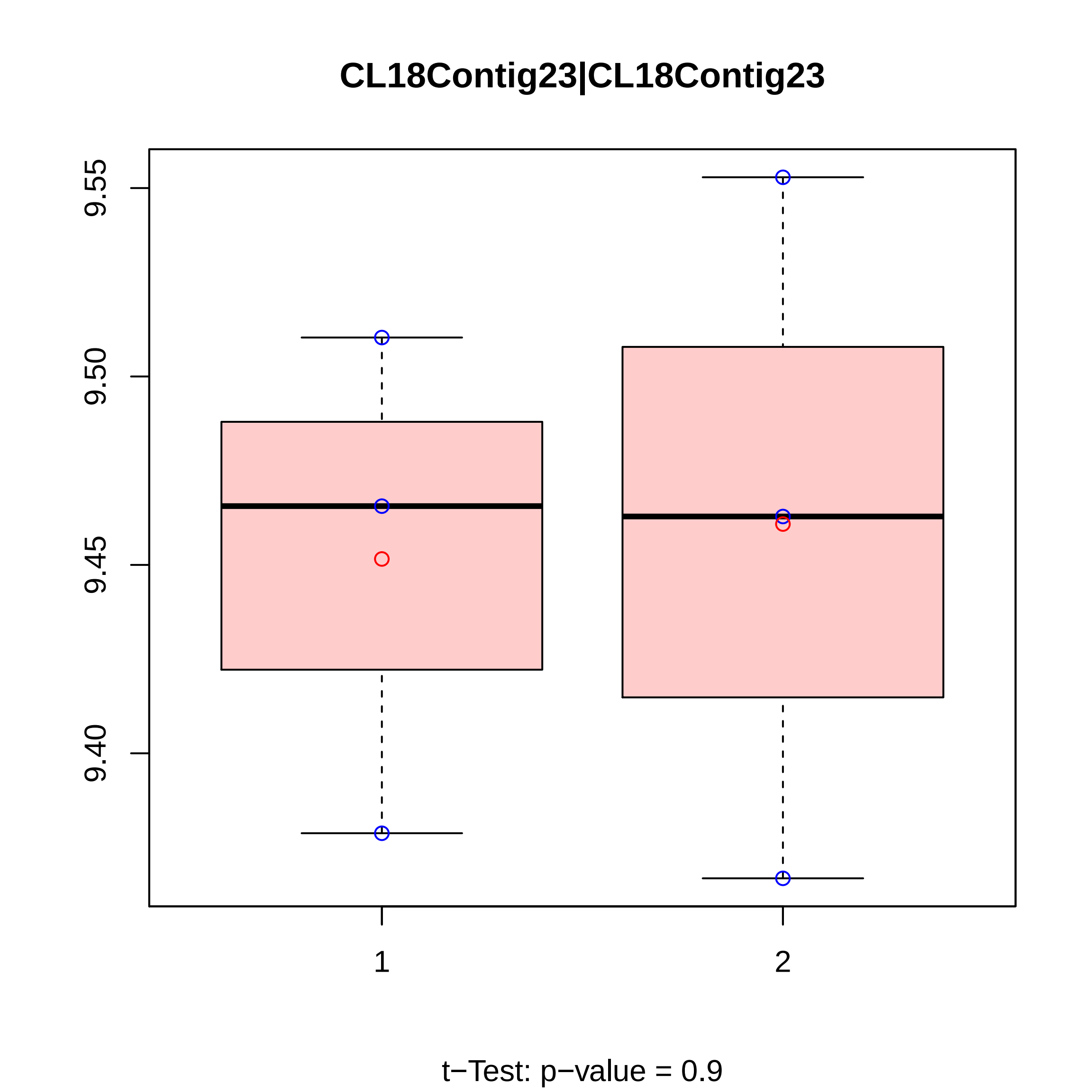
CL18Contig23|CL18Contig23
9.55
9.50
9.45
9.40
1
2
t−Test: p−value = 0.9

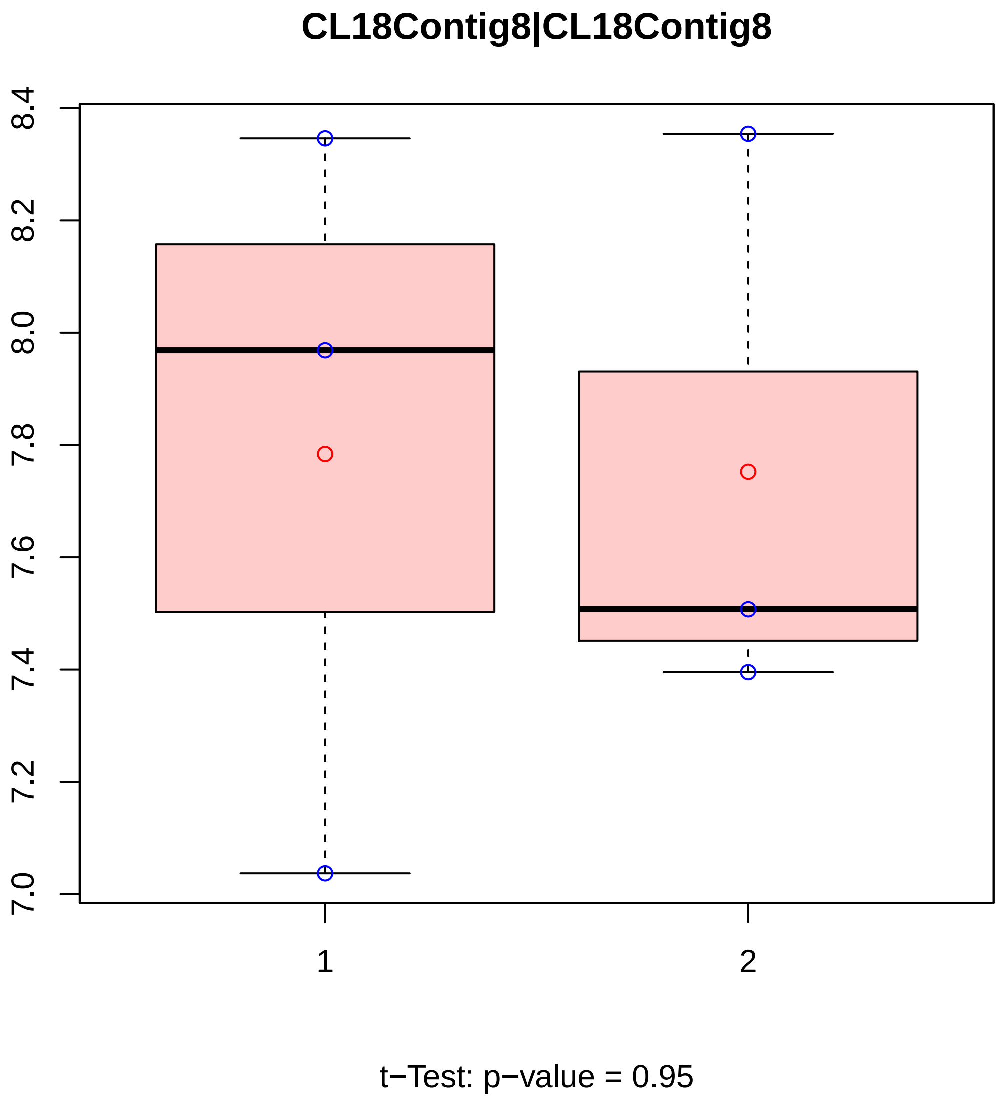

CL18Contig8|CL18Contig8
8.4
8.2
8.0
7.8
7.6
7.4
7.2
7.0
1
2
t−Test: p−value = 0.95

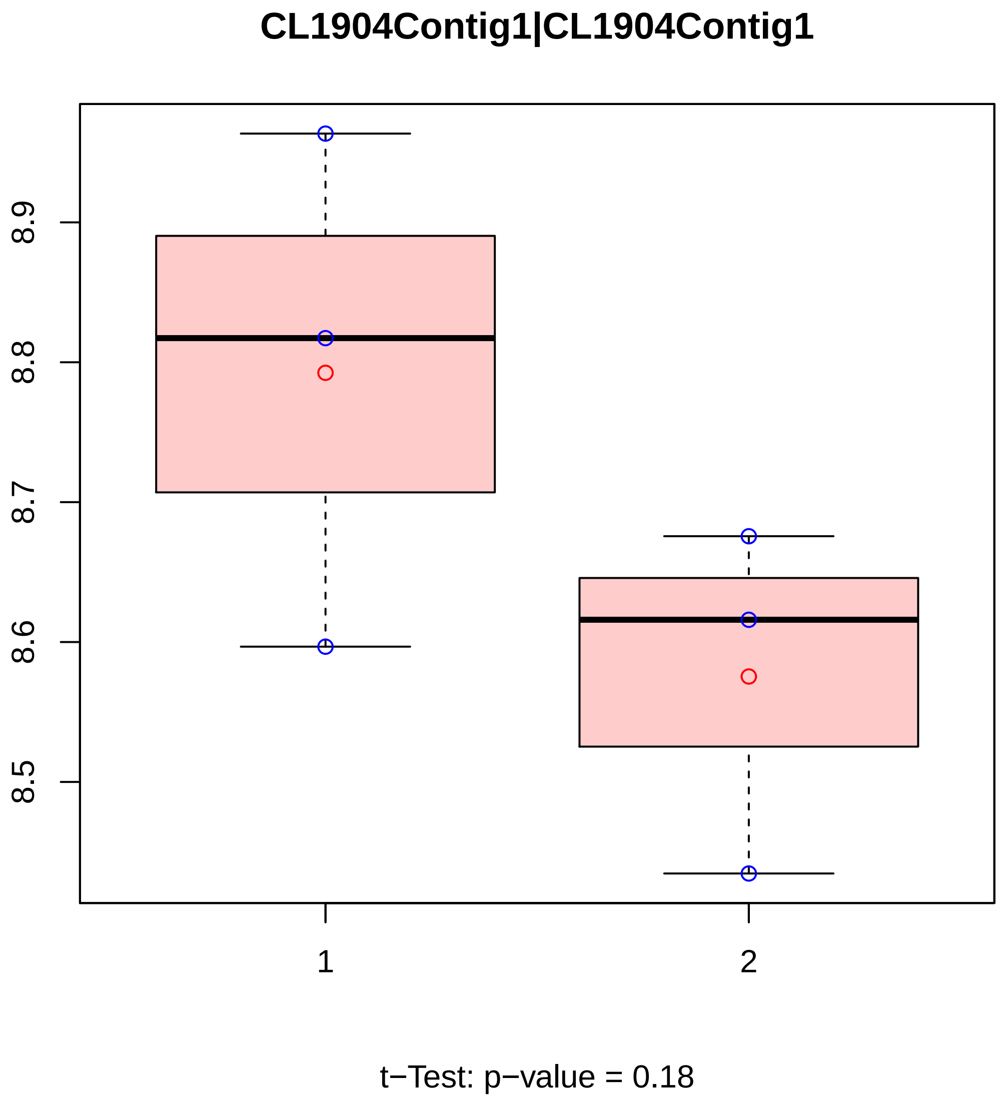
CL1904Contig1|CL1904Contig1
8.5
8.6
8.7
8.8
8.9
1
2
t−Test: p−value = 0.18

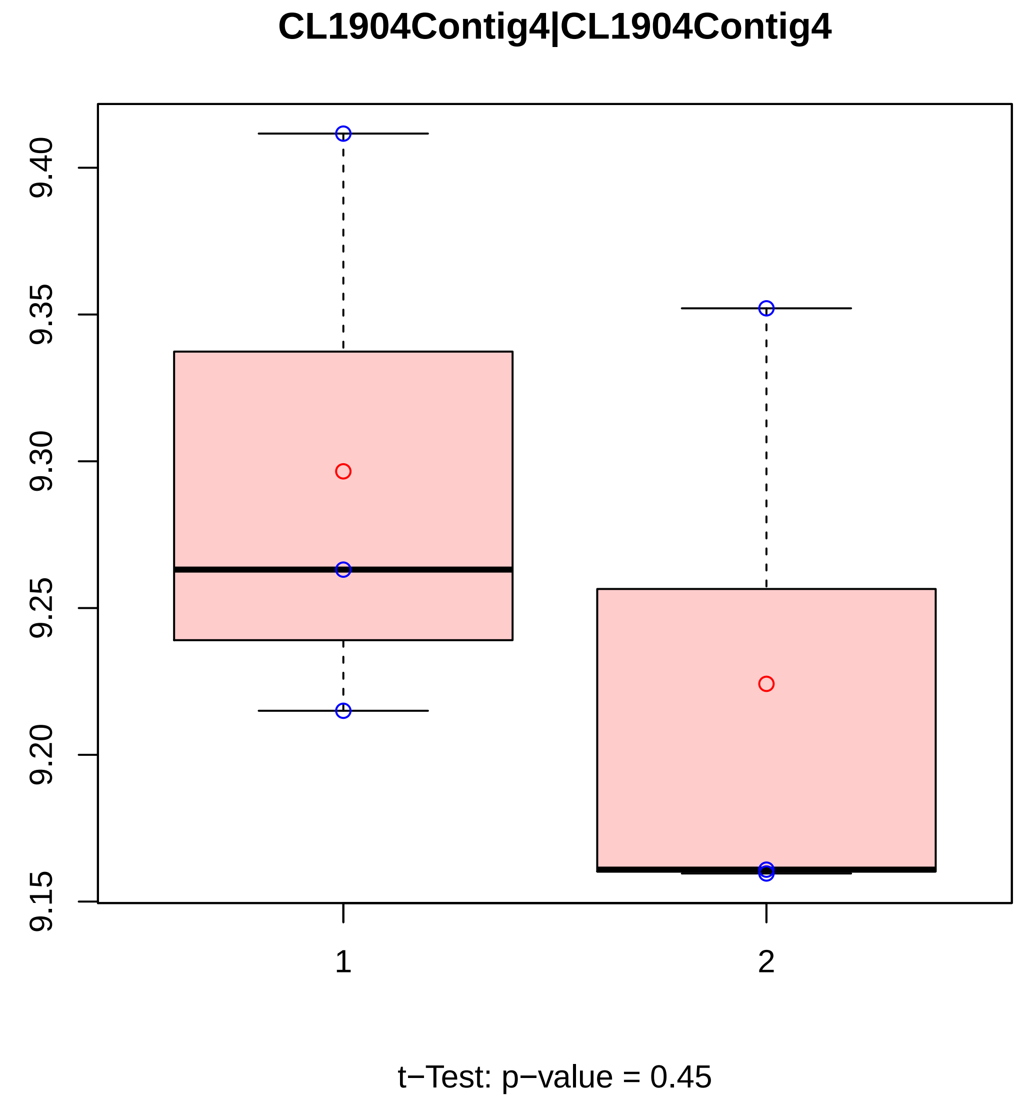
CL1904Contig4|CL1904Contig4
9.40
9.35
9.30
9.25
9.20
9.15
1
2
t−Test: p−value = 0.45

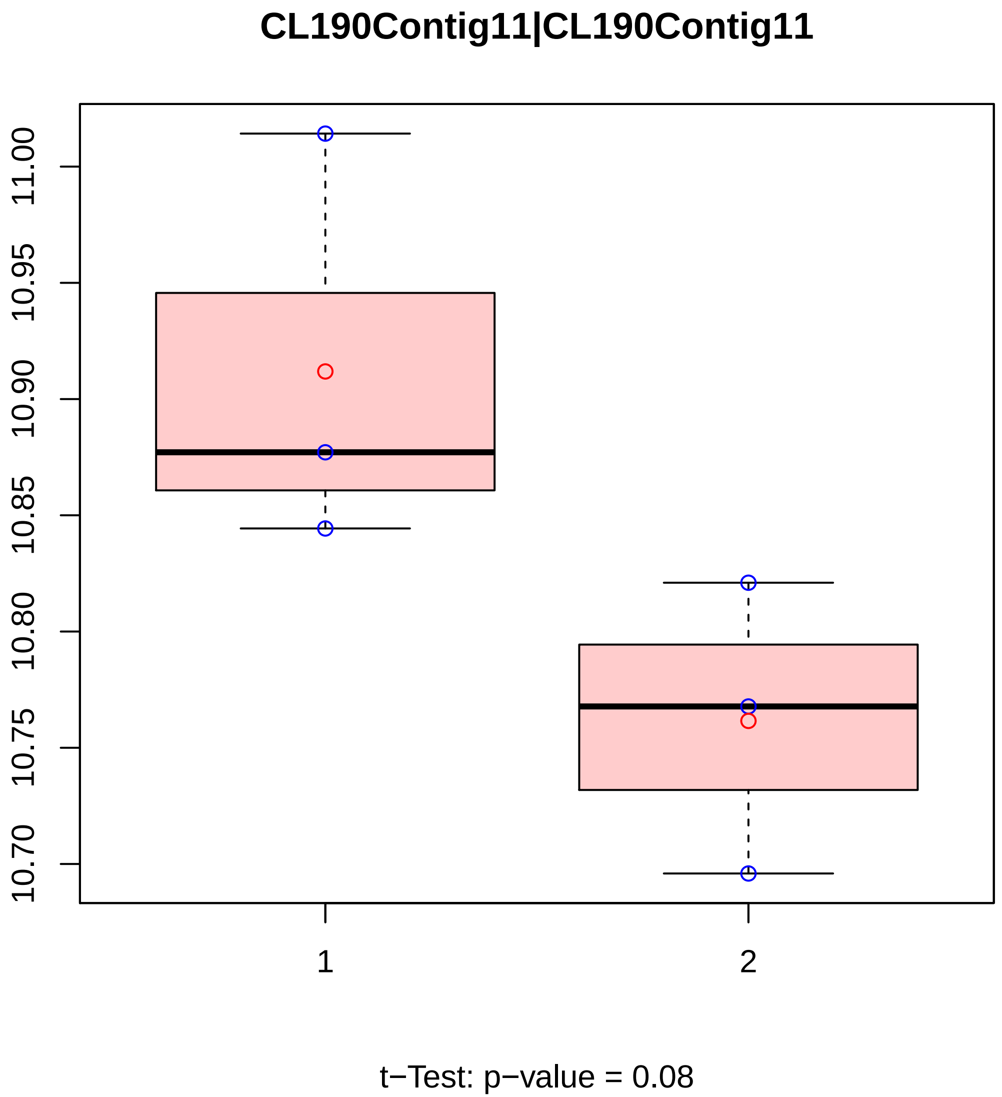
CL190Contig11|CL190Contig11
11.00
10.95
10.90
10.85
10.80
10.75
10.70
1
2
t−Test: p−value = 0.08

# CL190Contig18|CL190Contig18

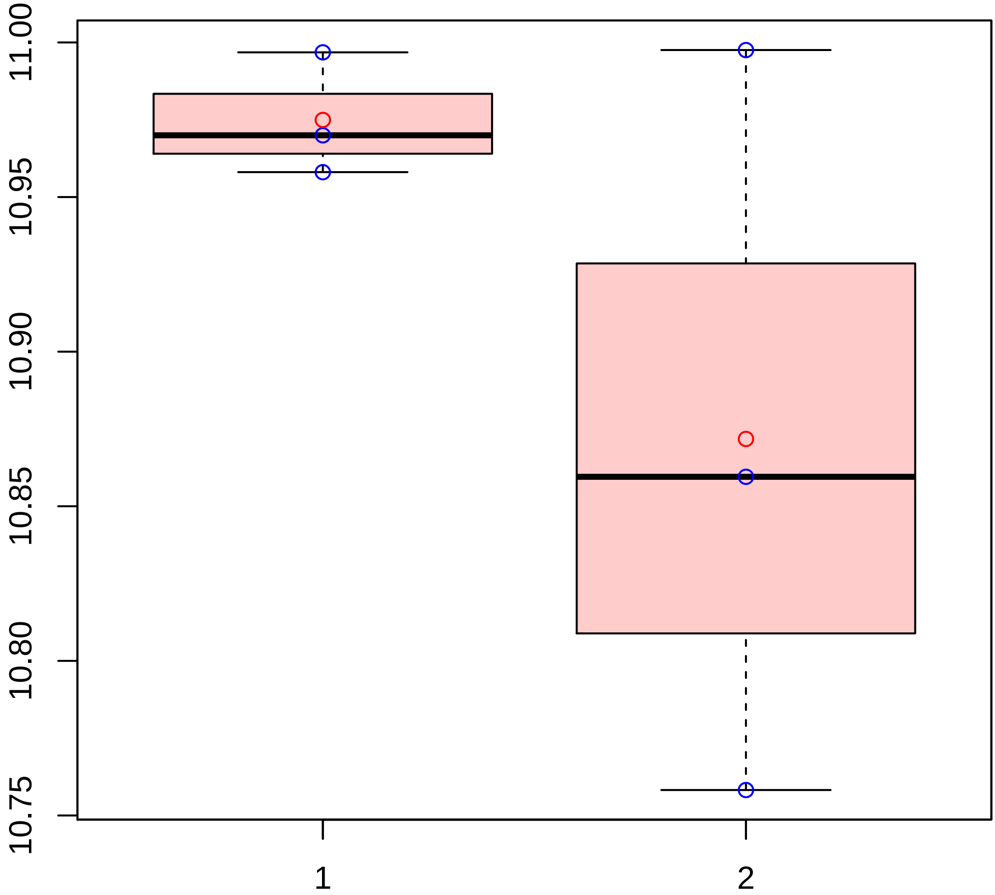


t−Test: p−value = 0.27

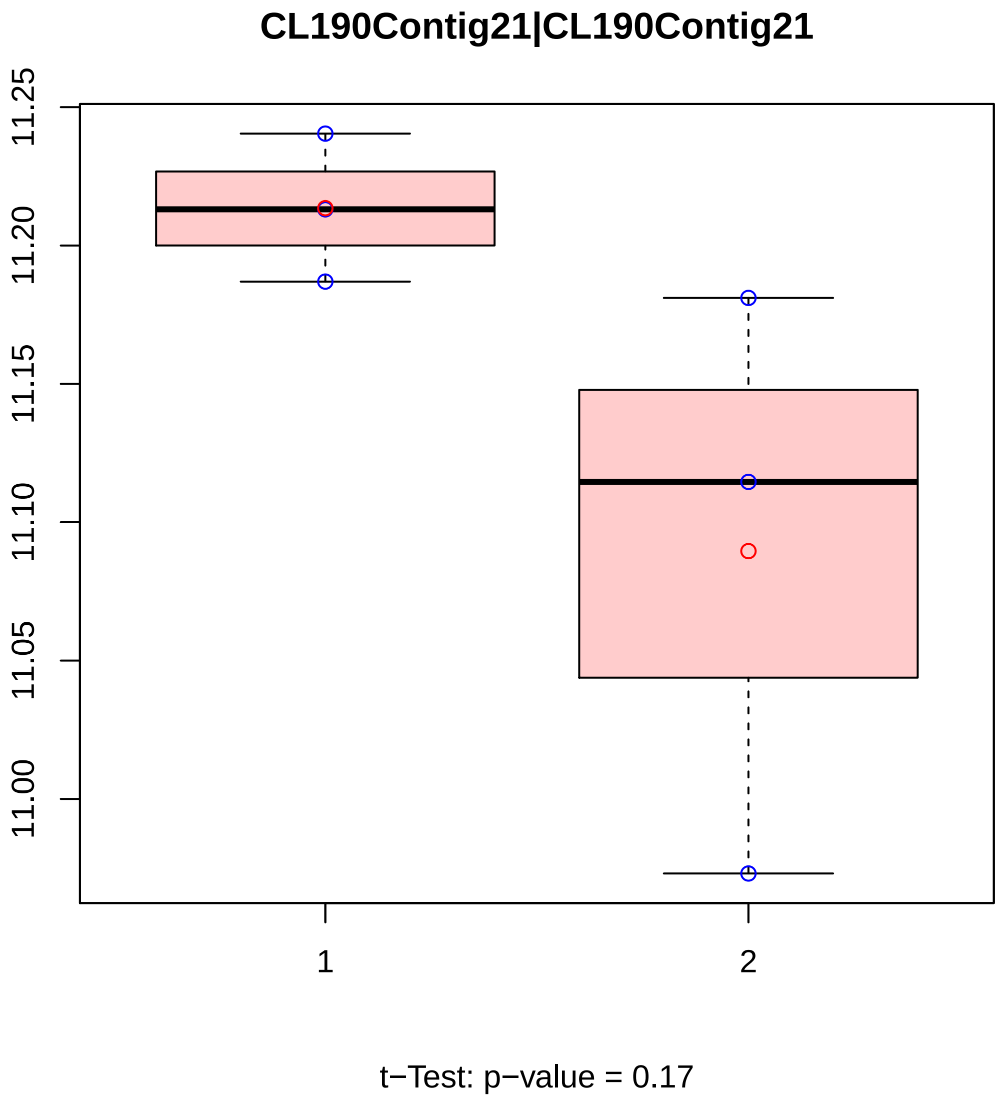

CL190Contig21|CL190Contig21
11.25
11.20
11.15
11.10
11.05
11.00
1
2
t−Test: p−value = 0.17

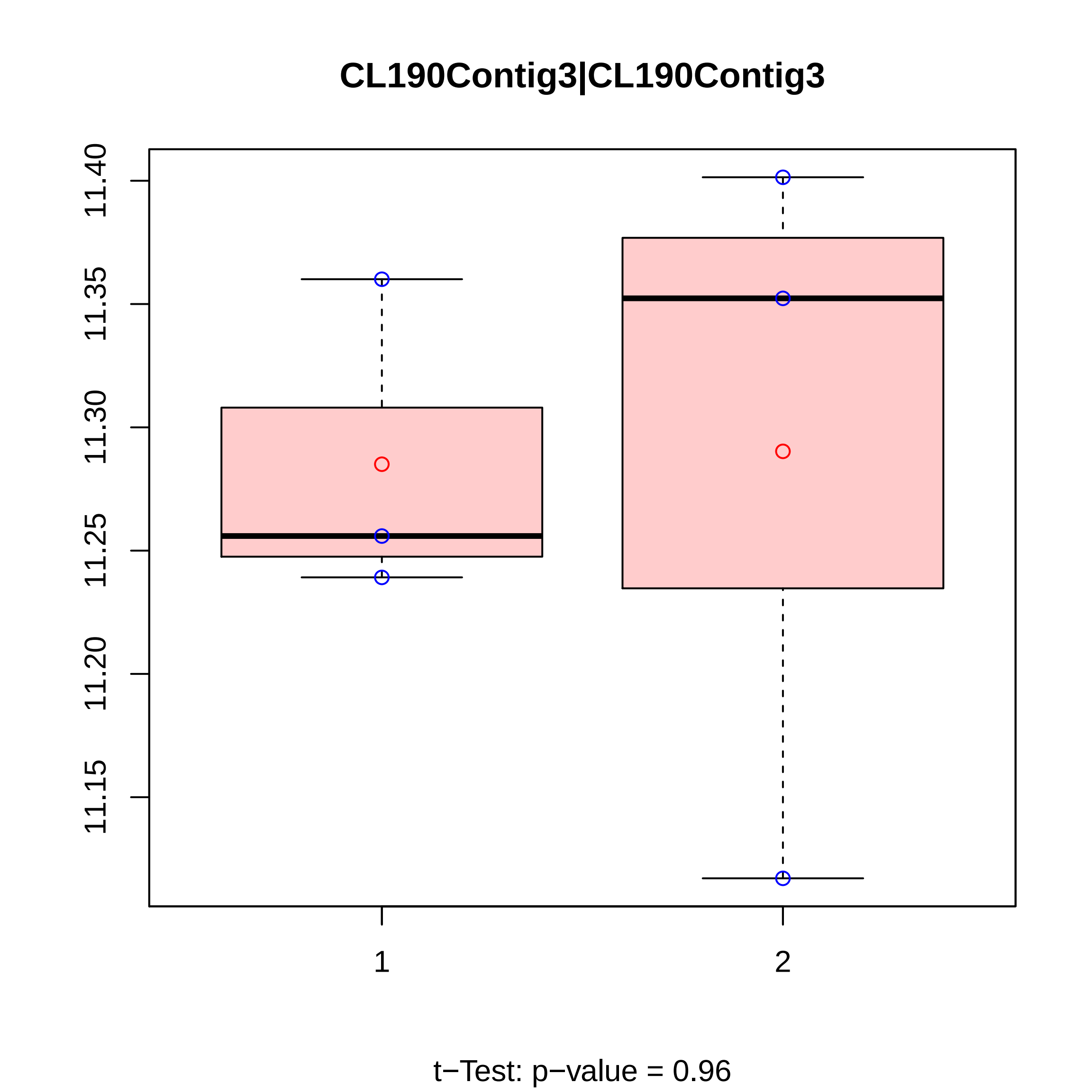

CL190Contig3|CL190Contig3
11.40
11.35
11.30
11.25
11.20
11.15
1
2
t−Test: p−value = 0.96

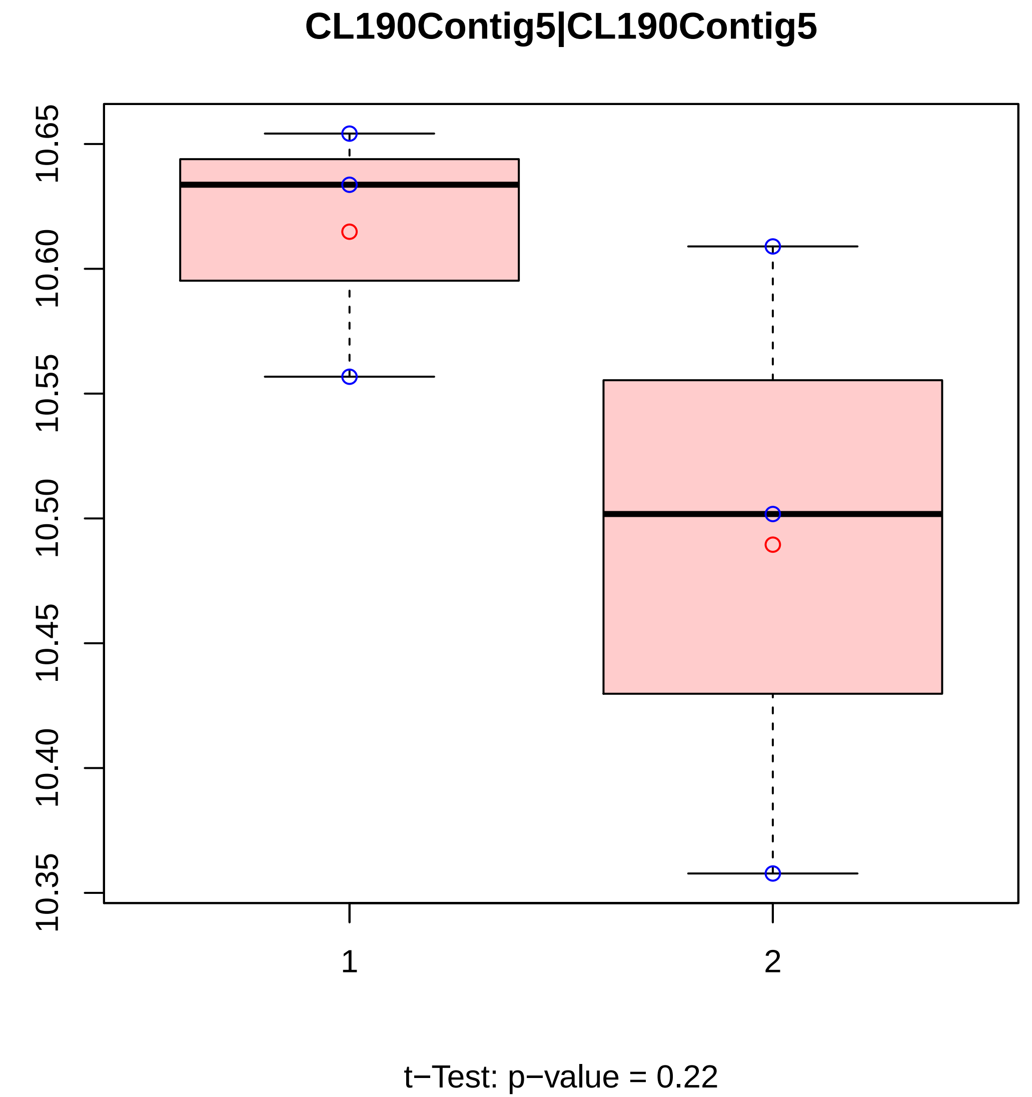
CL190Contig5|CL190Contig5
10.35
10.40
10.45
10.50
10.55
10.60
10.65
1
2
t−Test: p−value = 0.22

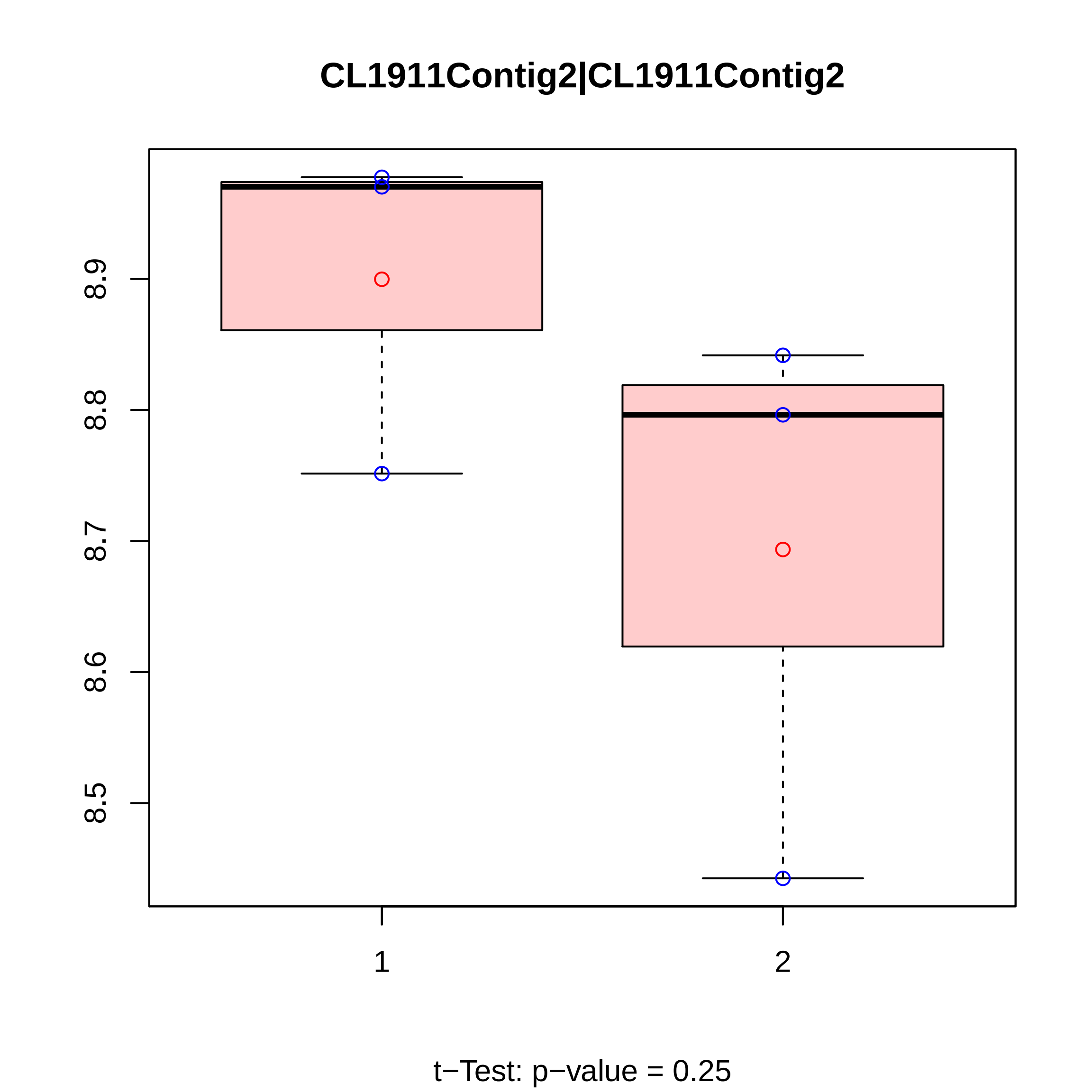
CL1911Contig2|CL1911Contig2
8.5
8.6
8.7
8.8
8.9
1
2
t−Test: p−value = 0.25

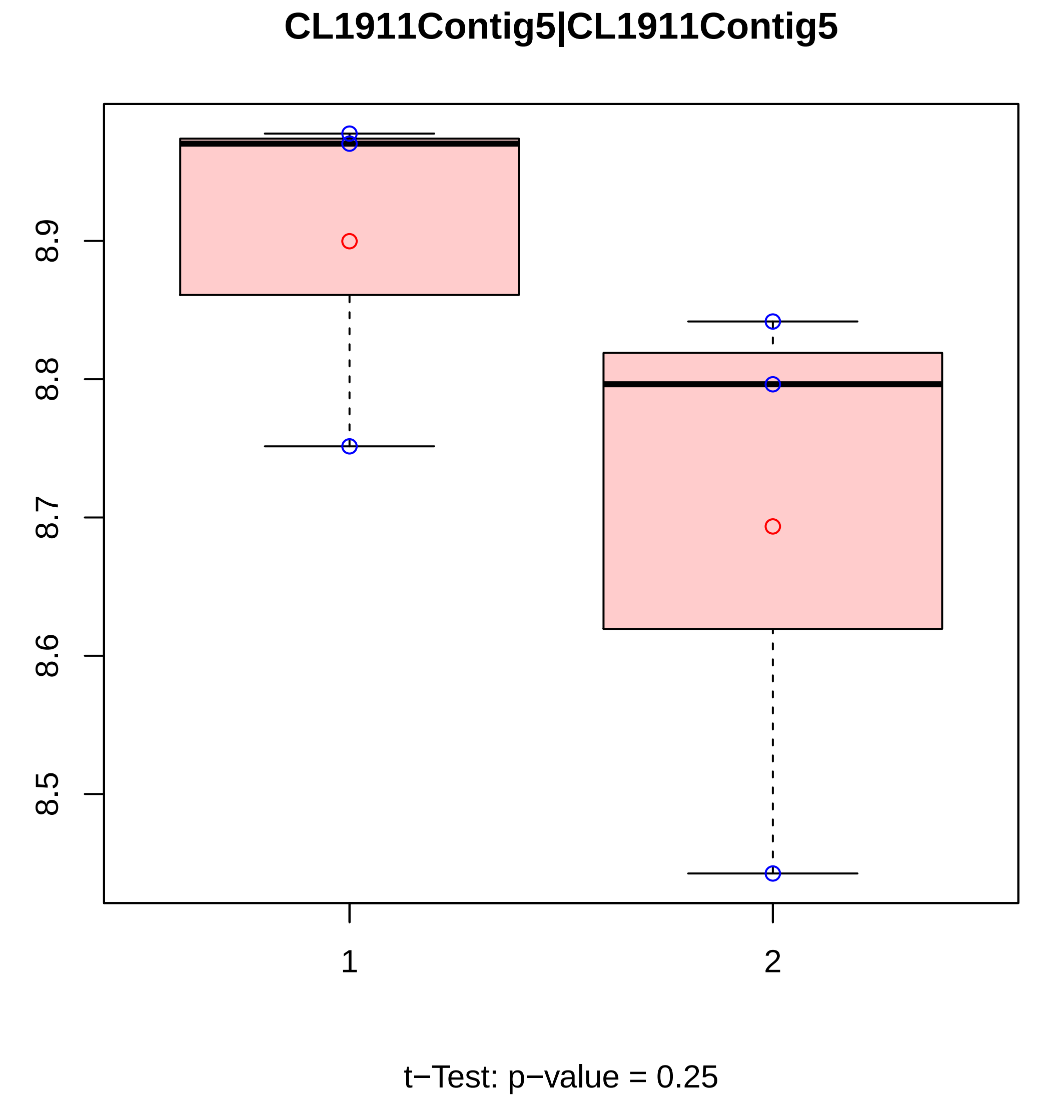

CL1911Contig5|CL1911Contig5
8.5
8.6
8.7
8.8
8.9
1
2
t−Test: p−value = 0.25

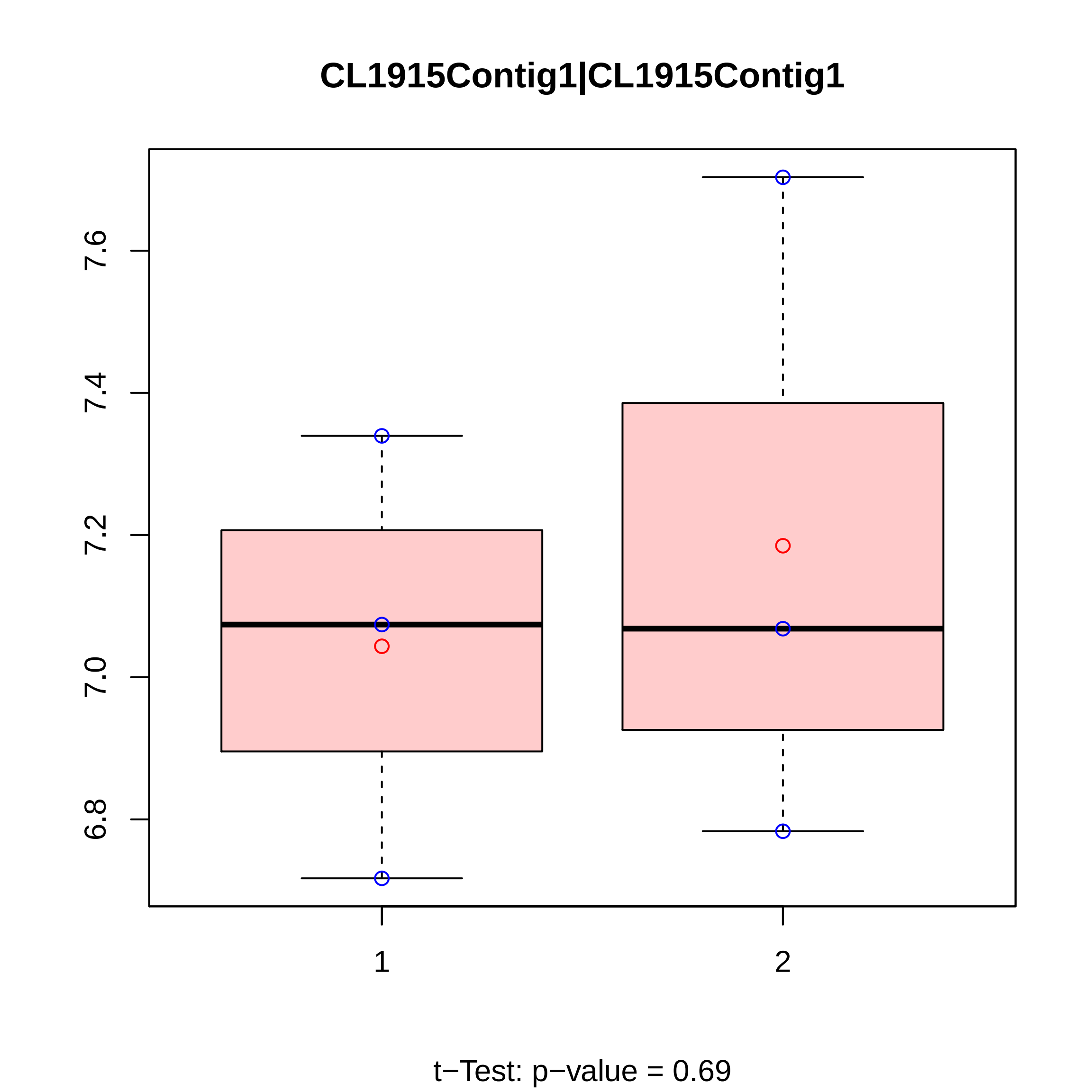
CL1915Contig1|CL1915Contig1
7.6
7.4
7.2
7.0
6.8
1
2
t−Test: p−value = 0.69

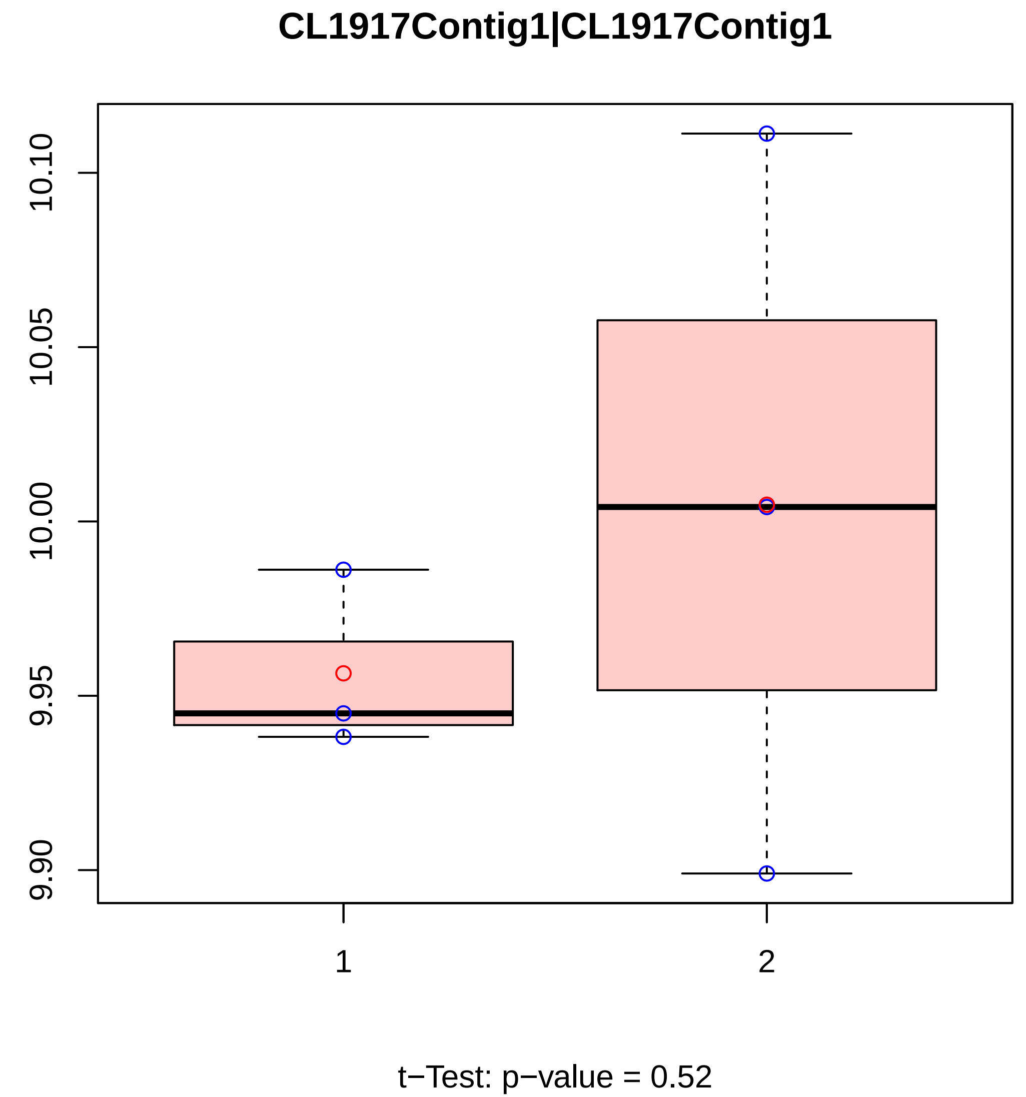
CL1917Contig1|CL1917Contig1
10.10
10.05
10.00
9.95
9.90
1
2
t−Test: p−value = 0.52

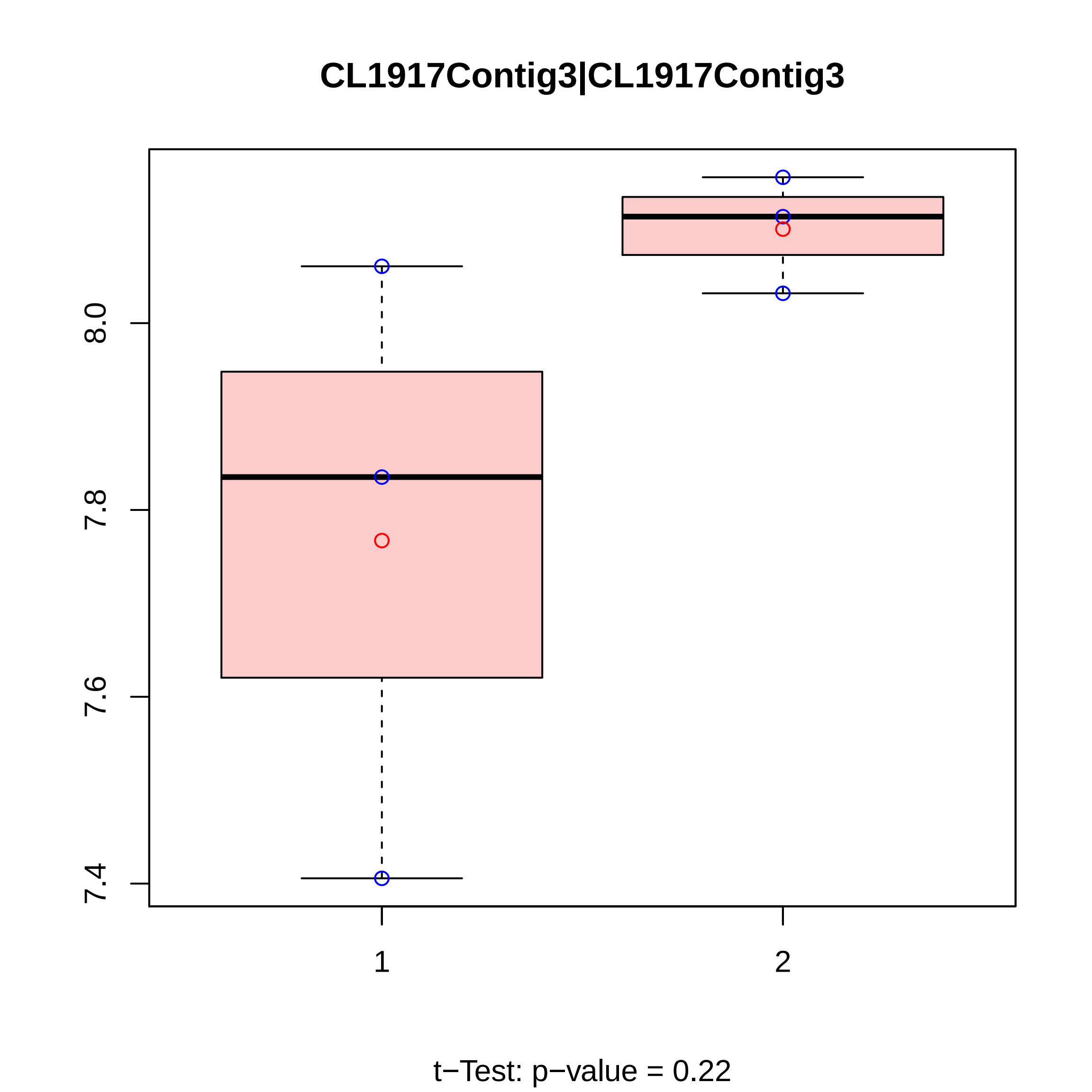
CL1917Contig3|CL1917Contig3
8.0
7.8
7.6
7.4
1
2
t−Test: p−value = 0.22

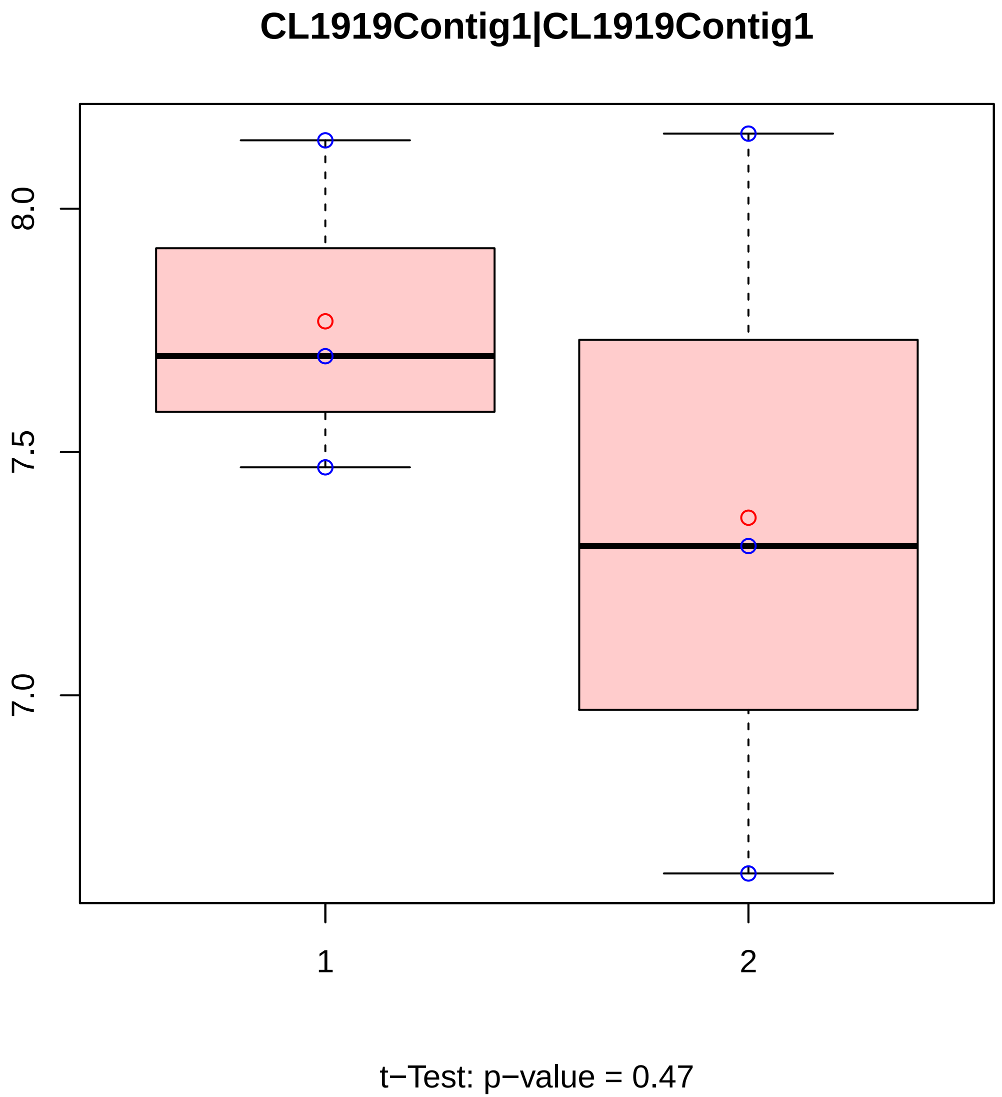
CL1919Contig1|CL1919Contig1
8.0
7.5
7.0
1
2
t−Test: p−value = 0.47

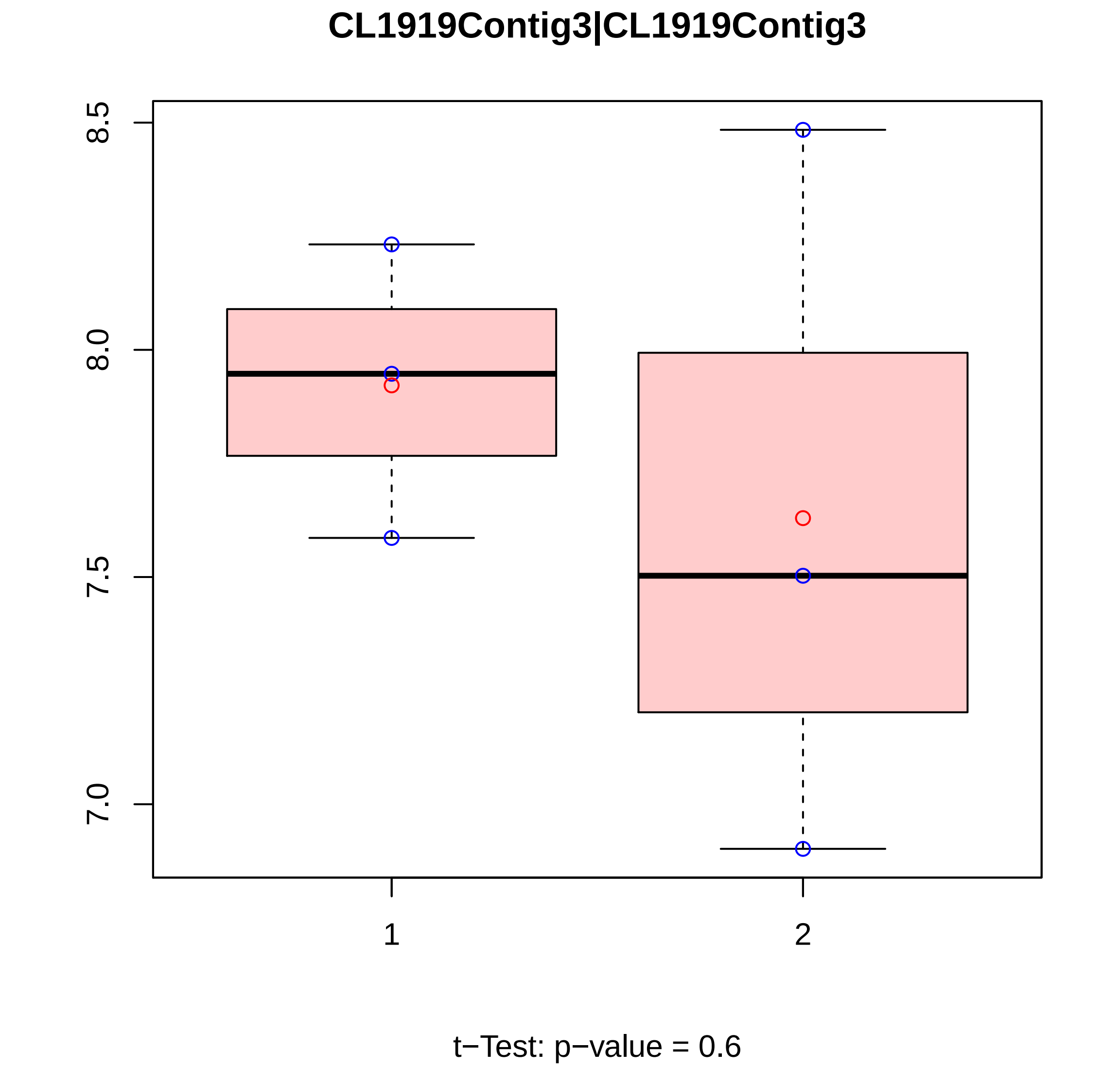
CL1919Contig3|CL1919Contig3
8.5
8.0
7.5
7.0
1
2
t−Test: p−value = 0.6

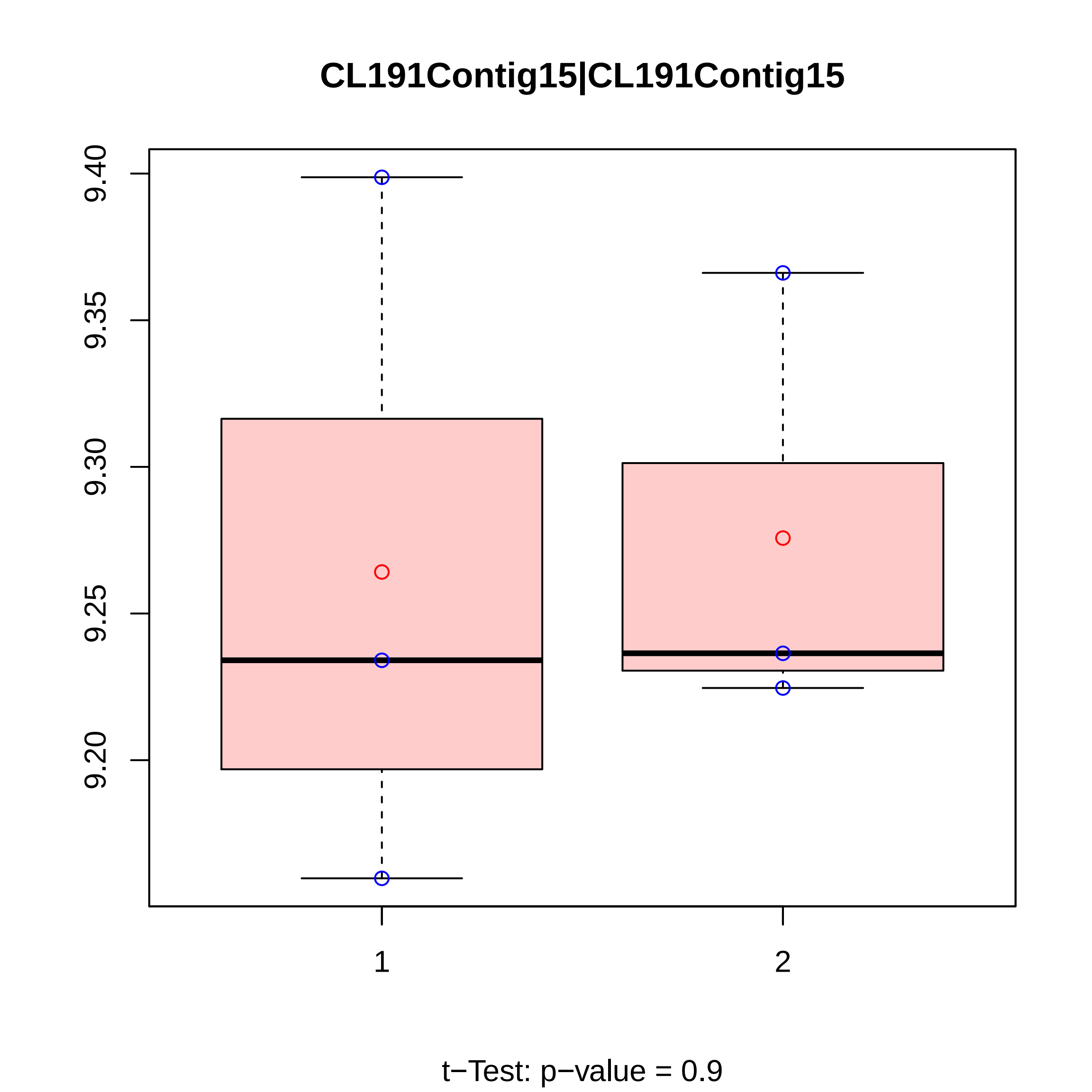
CL191Contig15|CL191Contig15
9.40
9.35
9.30
9.25
9.20
1
2
t−Test: p−value = 0.9

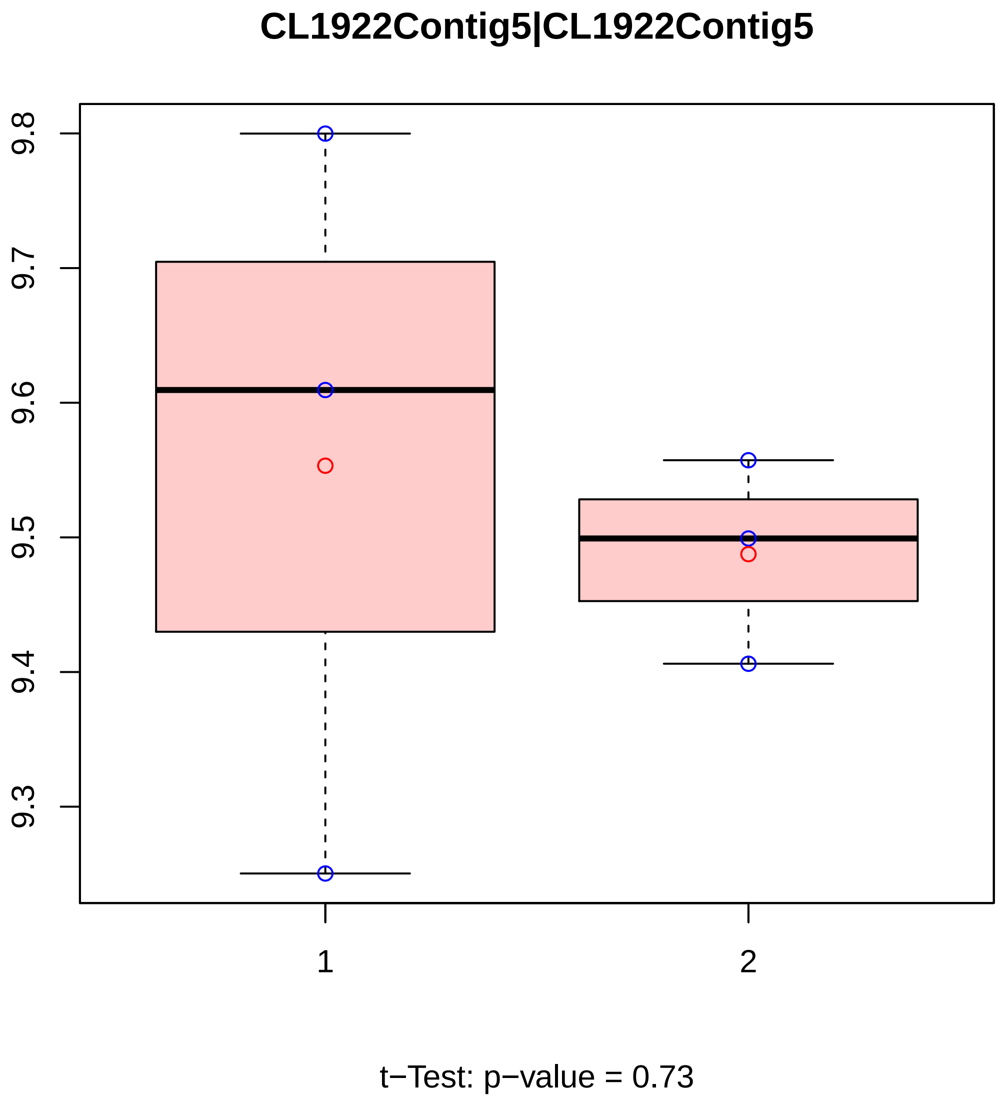

CL1922Contig5|CL1922Contig5
9.8
9.7
9.6
9.5
9.4
9.3
1
2
t−Test: p−value = 0.73

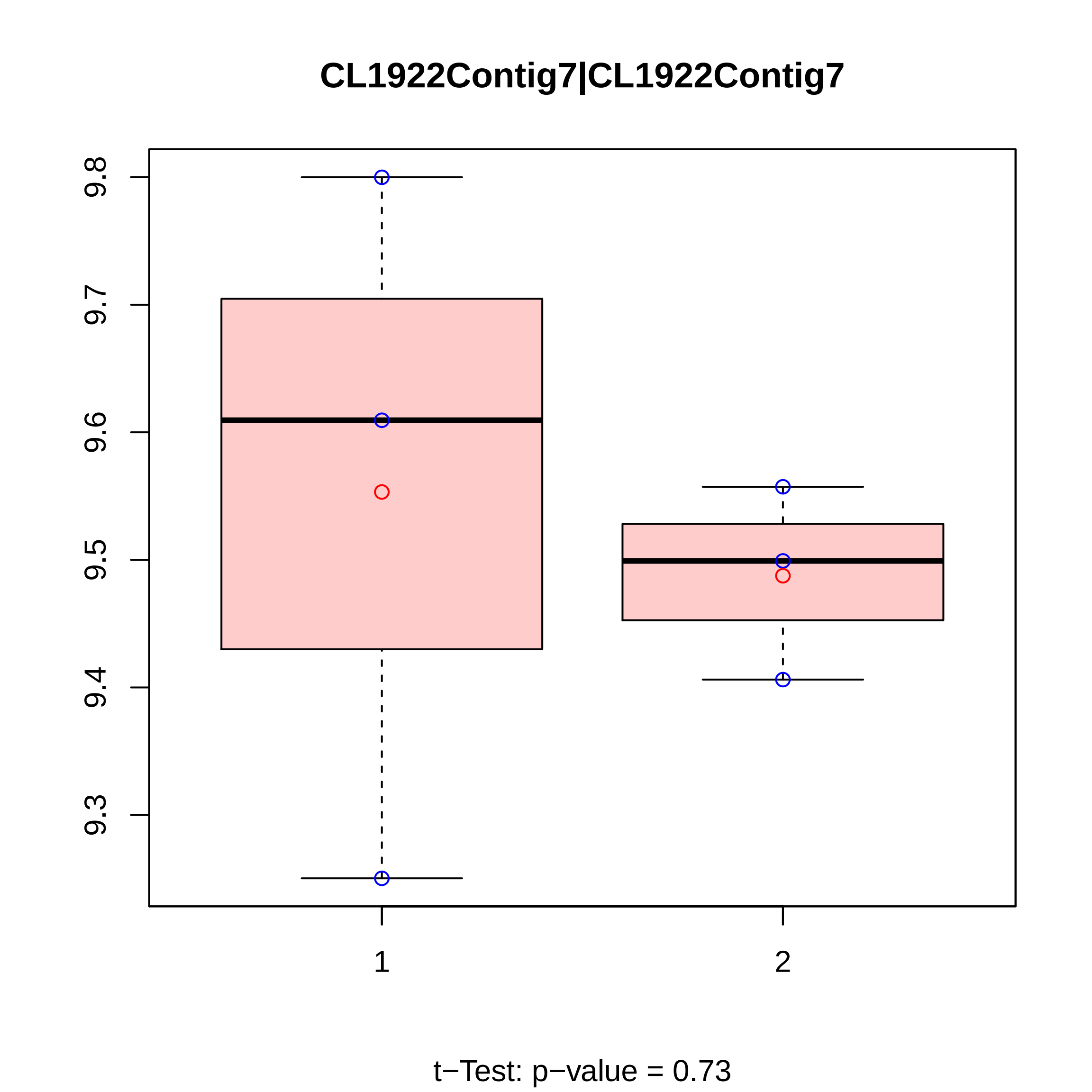

CL1922Contig7|CL1922Contig7
9.8
9.7
9.6
9.5
9.4
9.3
1
2
t−Test: p−value = 0.73

## CL1923Contig1|CL1923Contig1

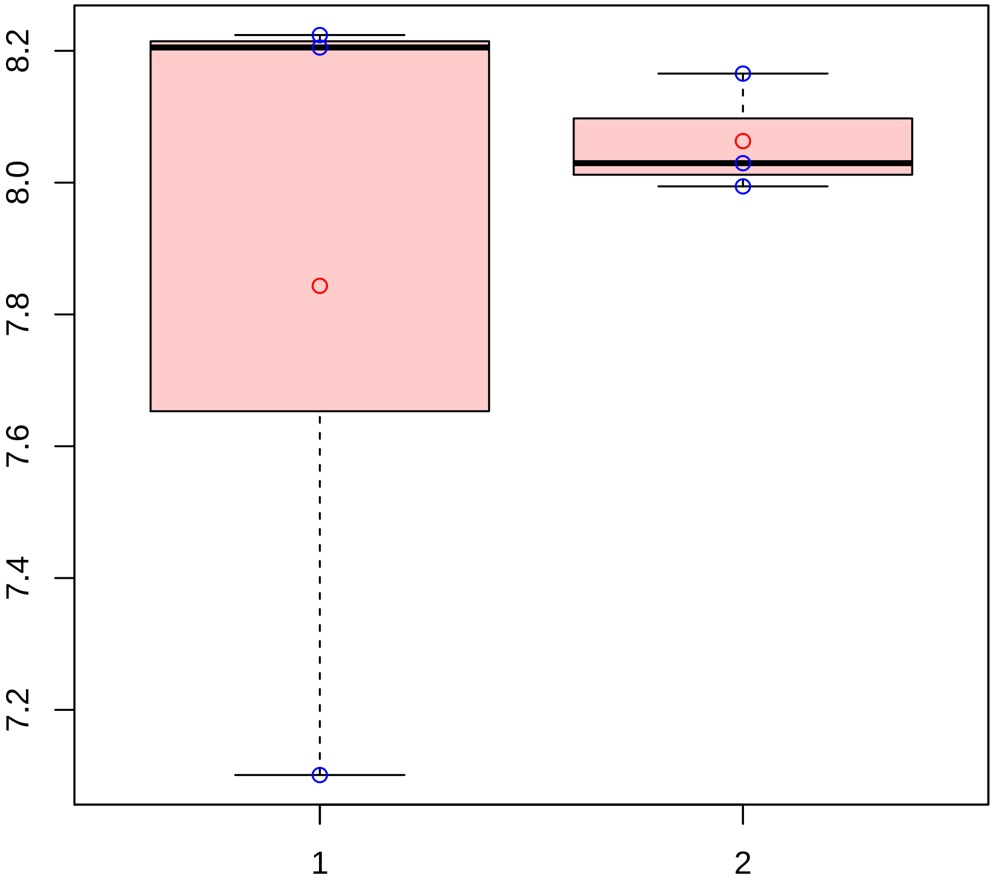


t−Test: p−value = 0.62

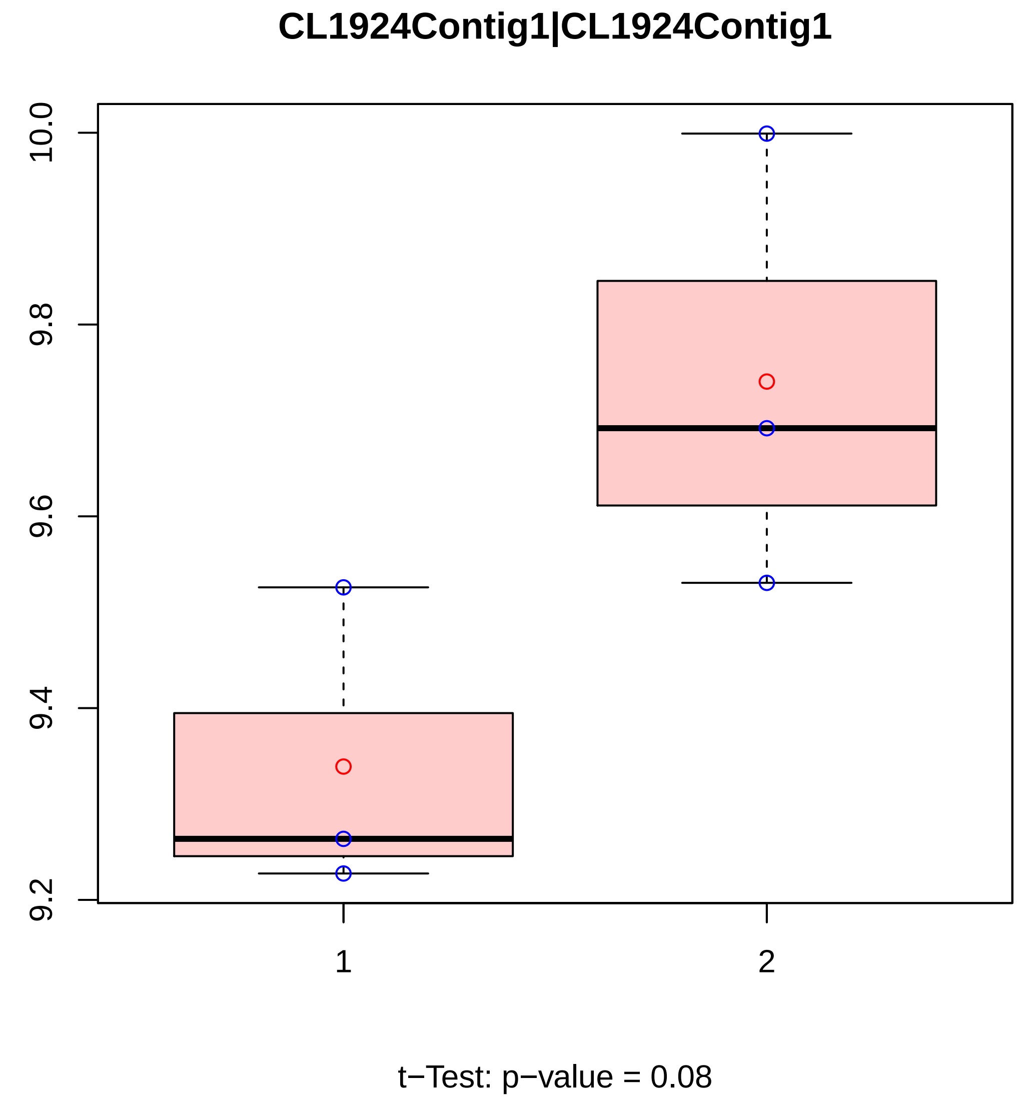
CL1924Contig1|CL1924Contig1
10.0
9.8
9.6
9.4
9.2
1
2
t−Test: p−value = 0.08

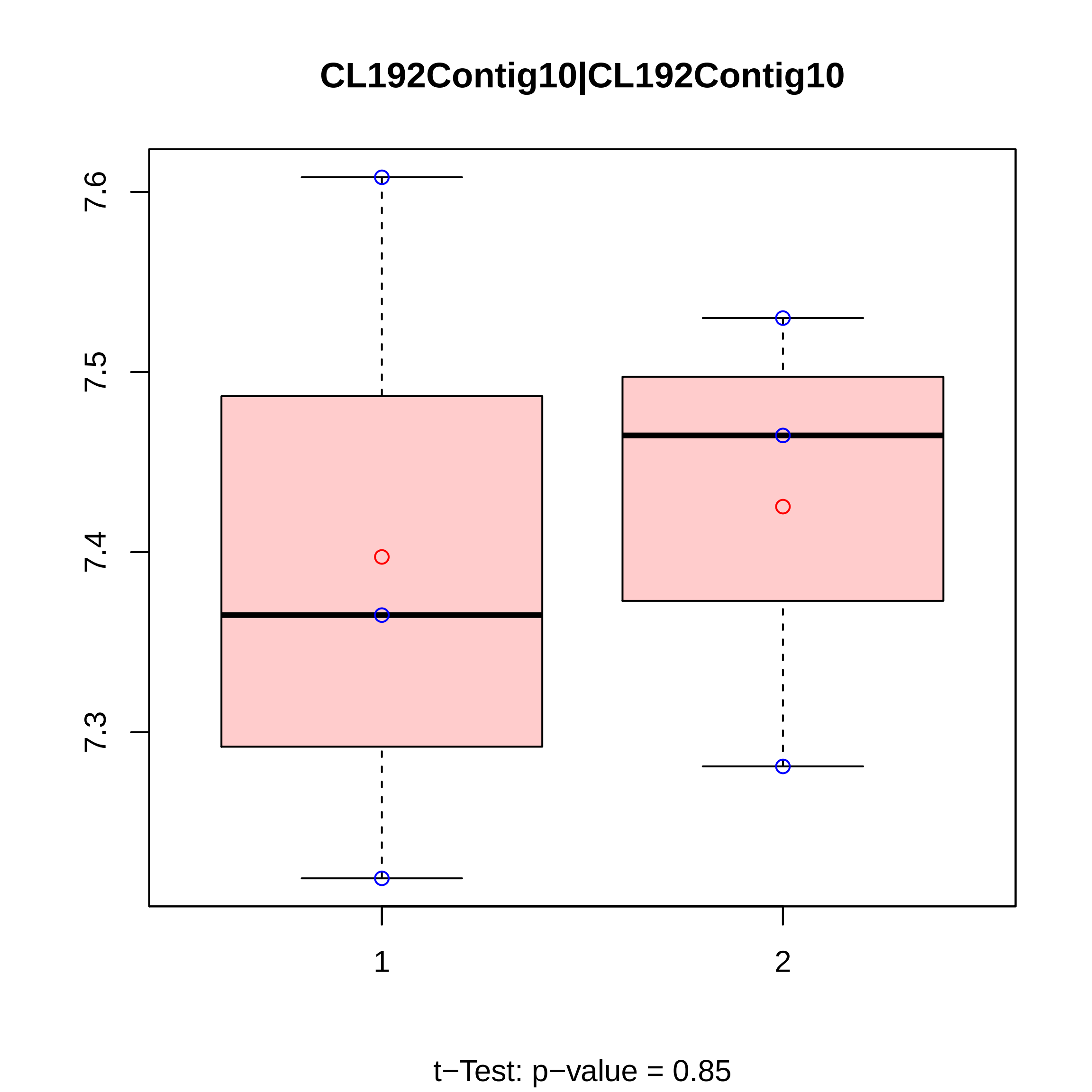
CL192Contig10|CL192Contig10
7.3
7.4
7.5
7.6
1
2
t−Test: p−value = 0.85

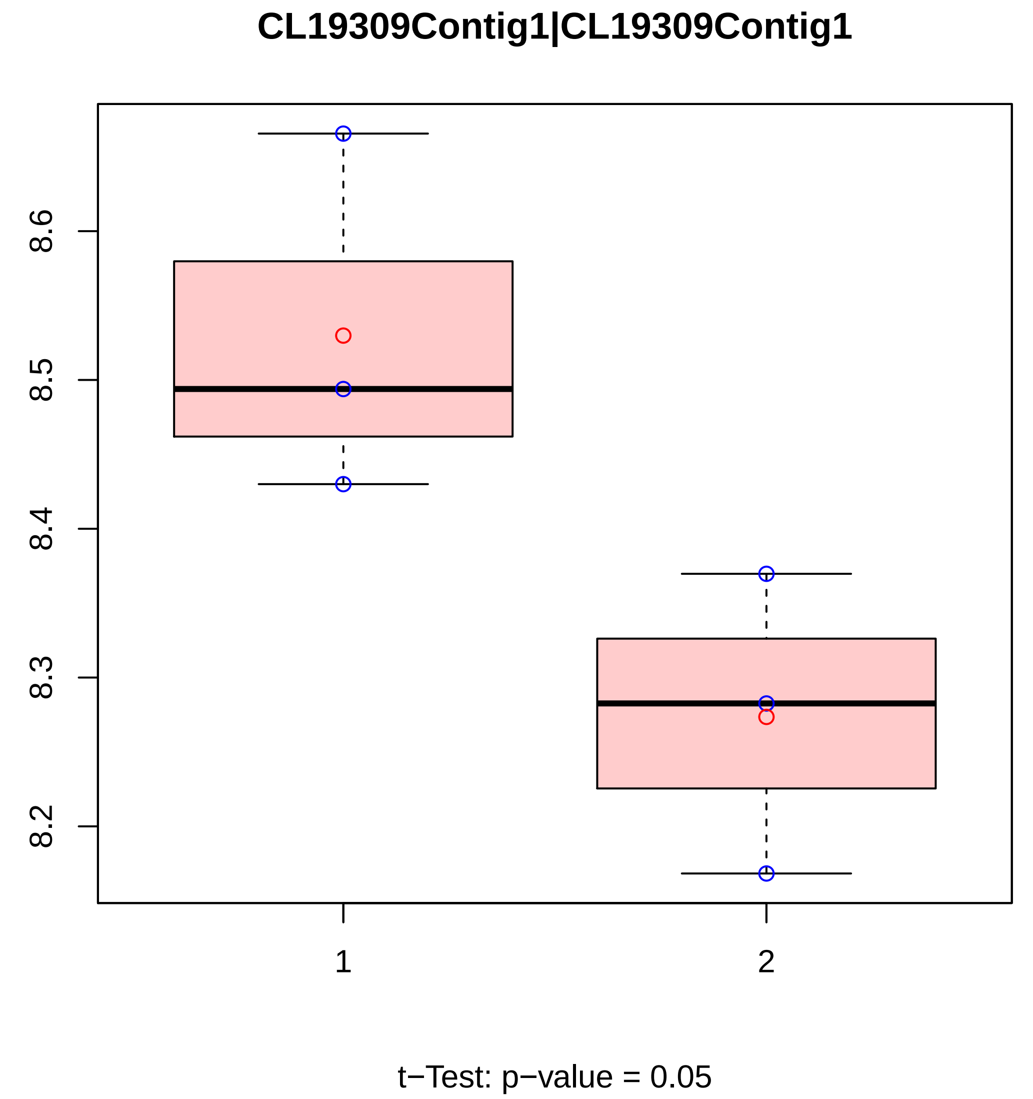
CL19309Contig1|CL19309Contig1
8.2
8.3
8.4
8.5
8.6
1
2
t−Test: p−value = 0.05

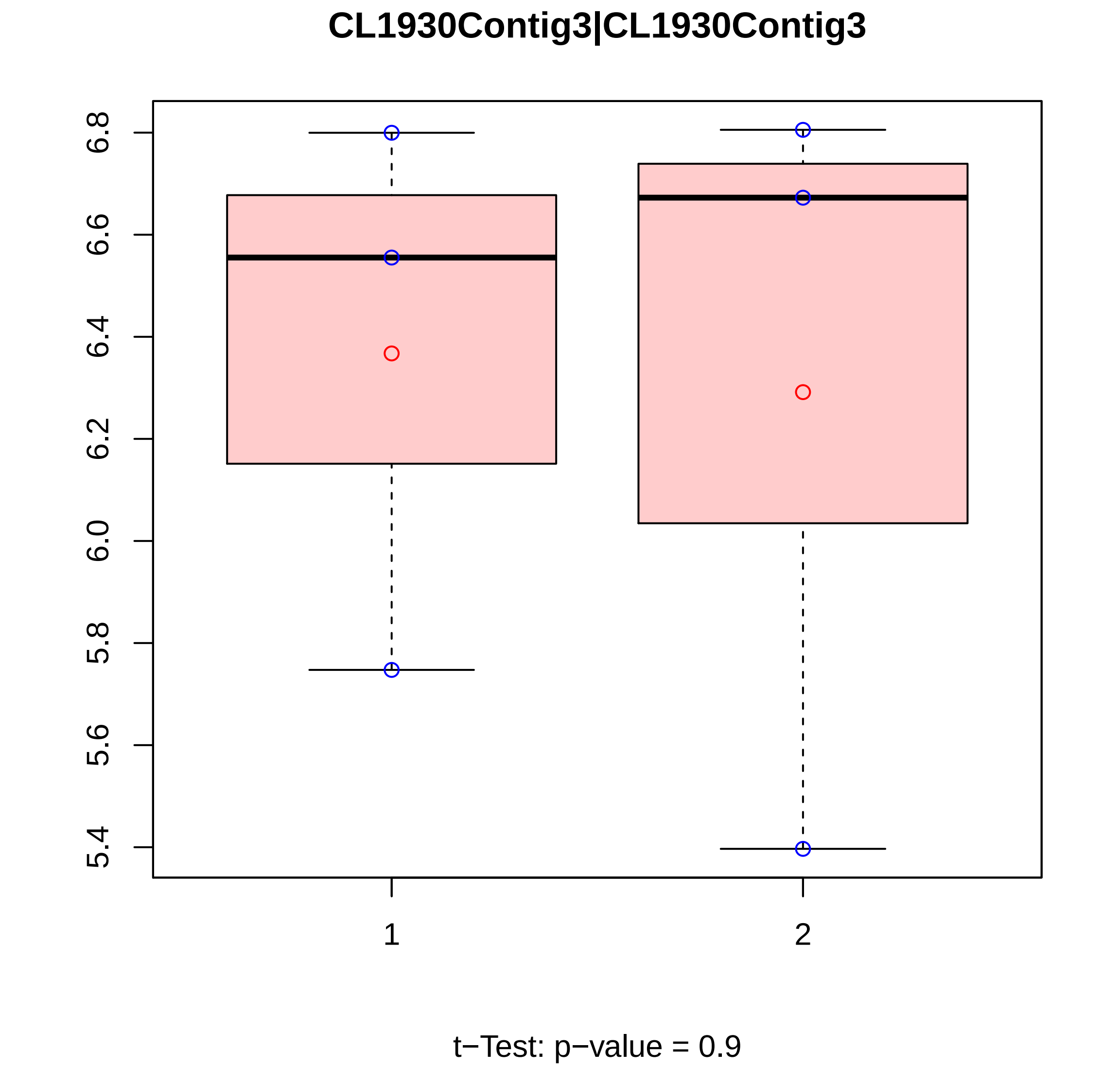
CL1930Contig3|CL1930Contig3
6.8
6.6
6.4
6.2
6.0
5.8
5.6
5.4
1
2
t−Test: p−value = 0.9

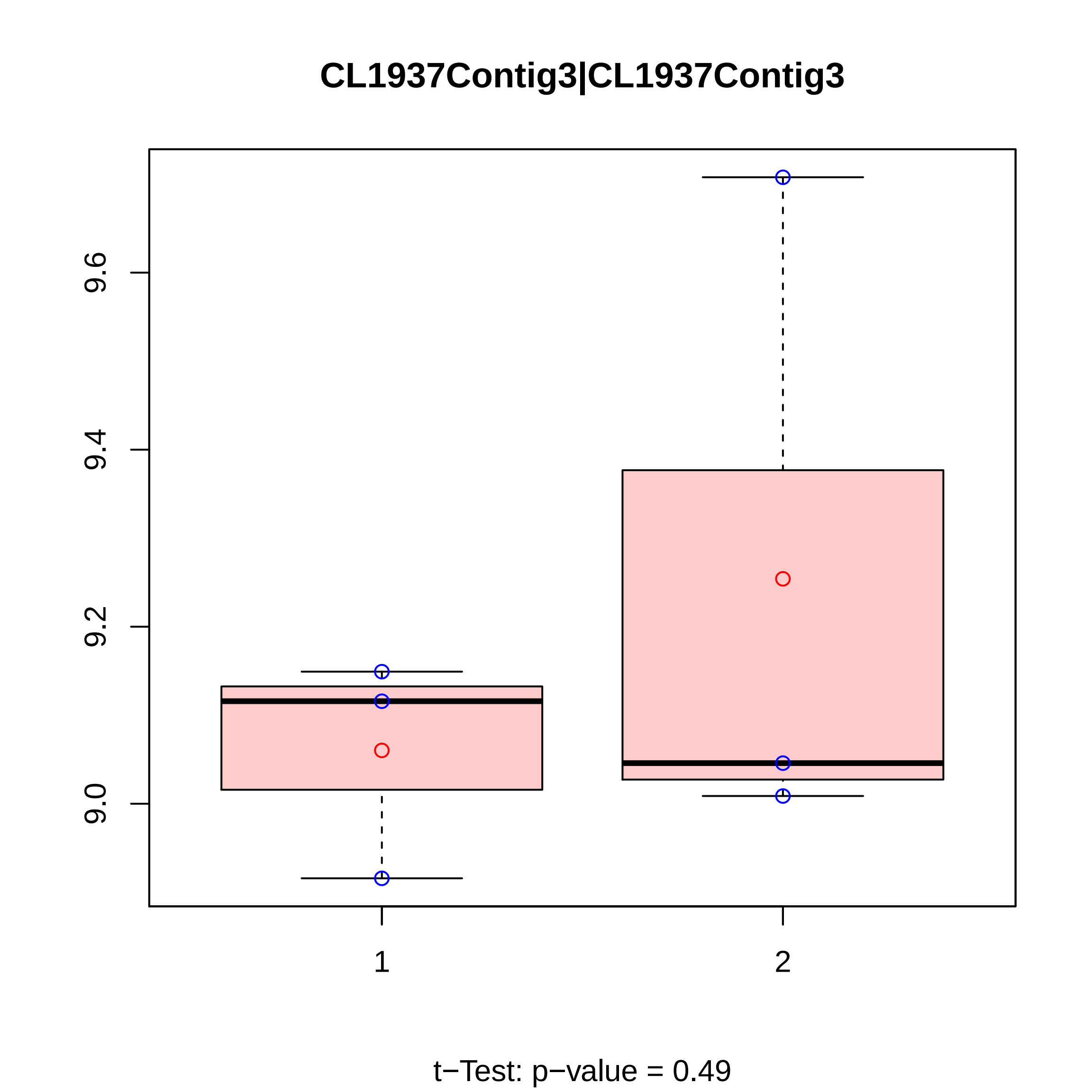
CL1937Contig3|CL1937Contig3
9.0
9.2
9.4
9.6
1
2
t−Test: p−value = 0.49

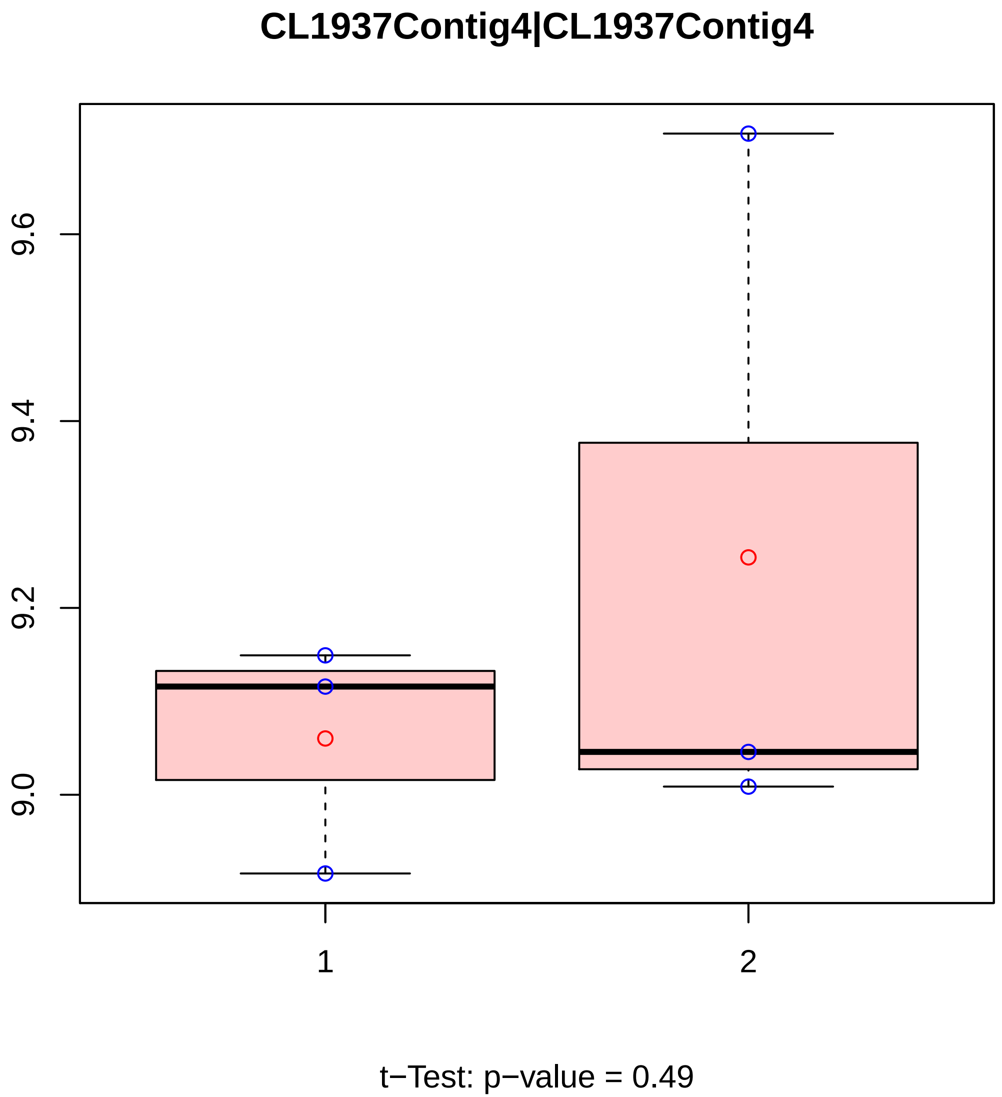
CL1937Contig4|CL1937Contig4
9.0
9.2
9.4
9.6
1
2
t−Test: p−value = 0.49

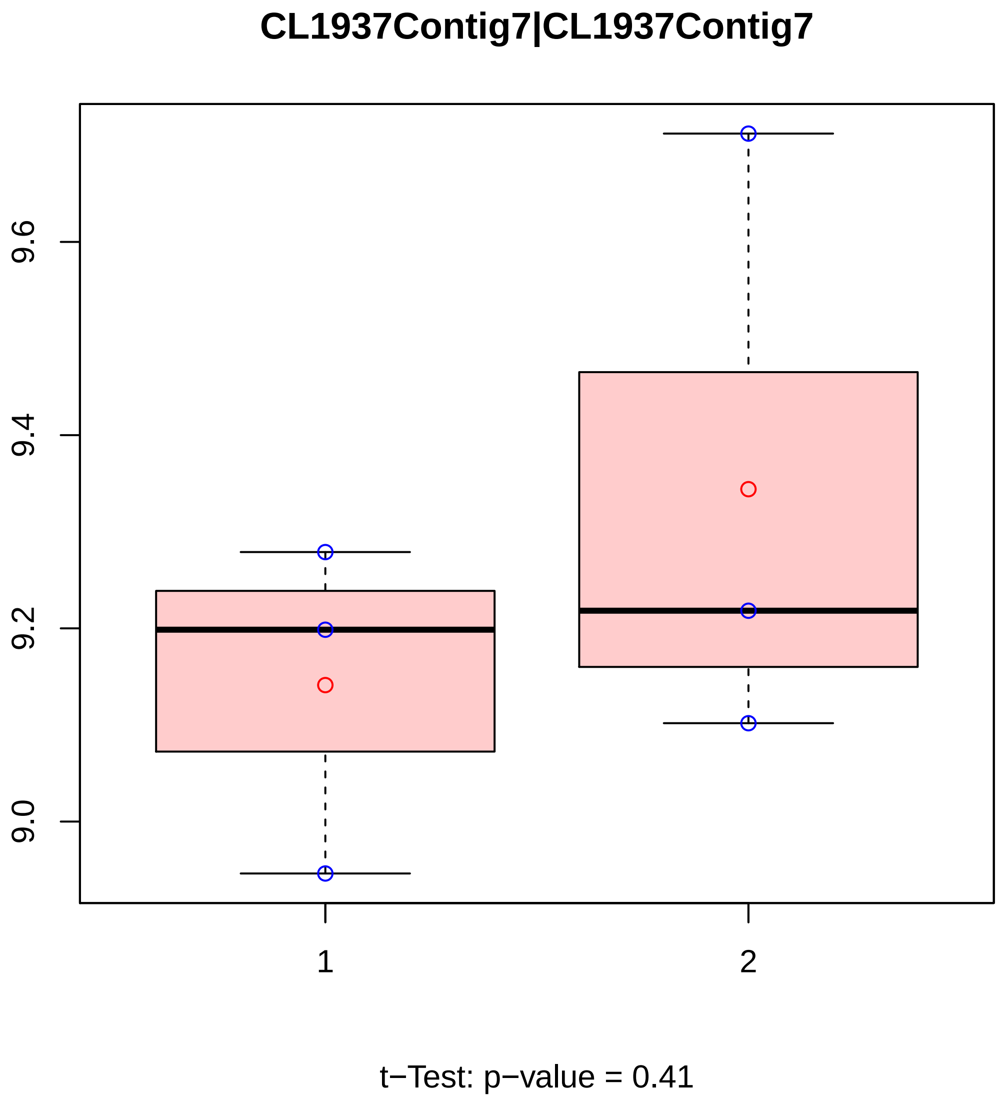

CL1937Contig7|CL1937Contig7
9.0
9.2
9.4
9.6
1
2
t−Test: p−value = 0.41

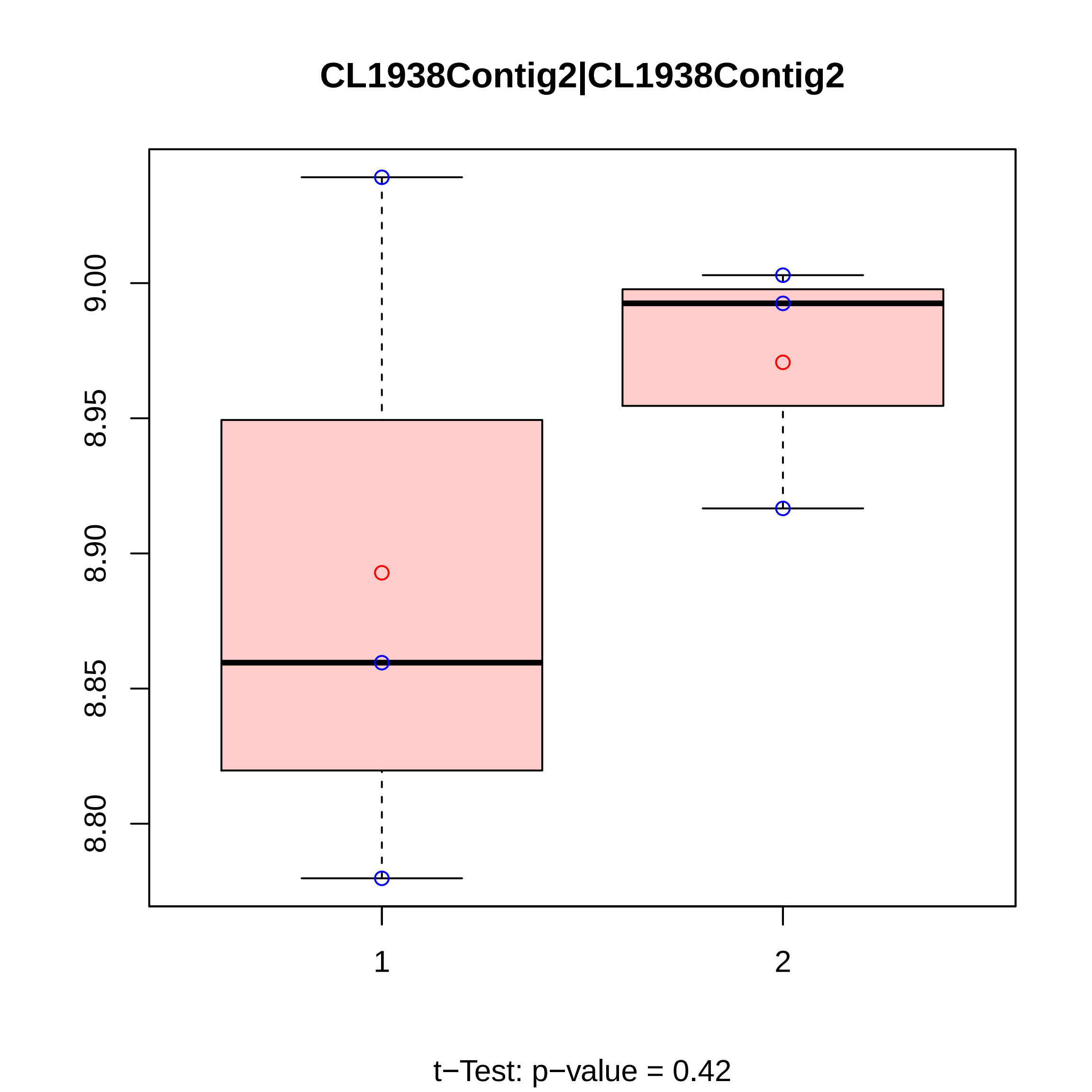
CL1938Contig2|CL1938Contig2
9.00
8.95
8.90
8.85
8.80
1
2
t−Test: p−value = 0.42

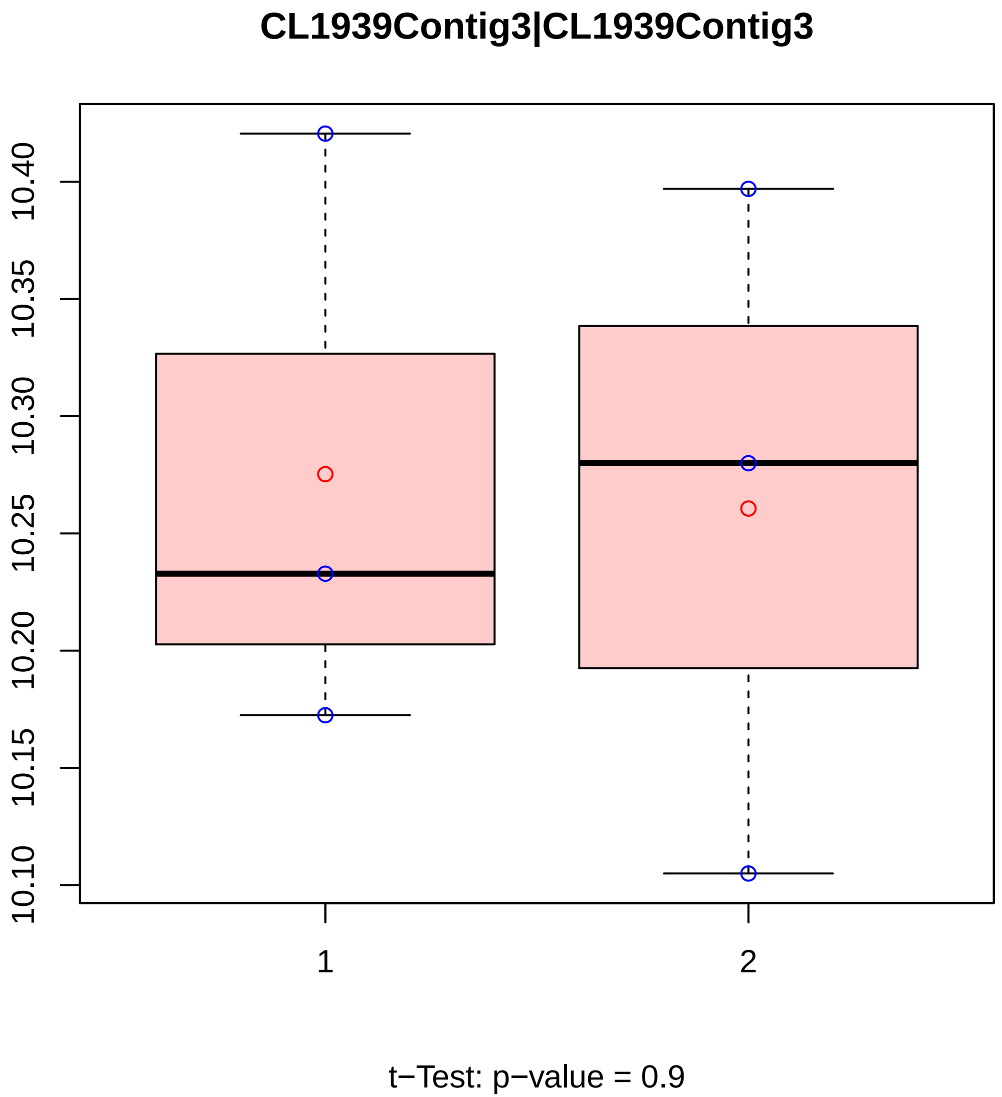

CL1939Contig3|CL1939Contig3
10.10
10.15
10.20
10.25
10.30
10.35
10.40
1
2
t−Test: p−value = 0.9

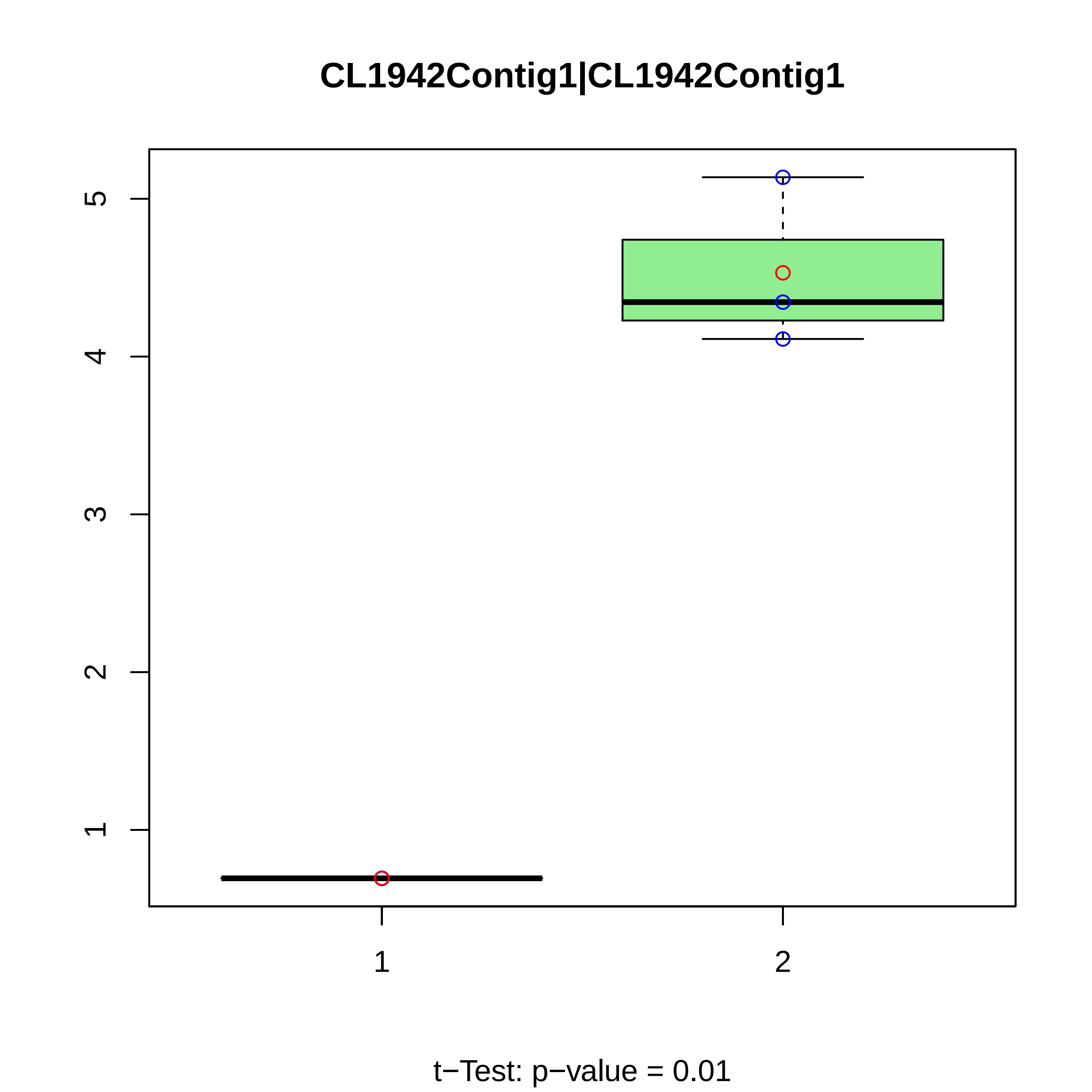
CL1942Contig1|CL1942Contig1
5
4
3
2
1
1
2
t−Test: p−value = 0.01

## CL1945Contig11|CL1945Contig11

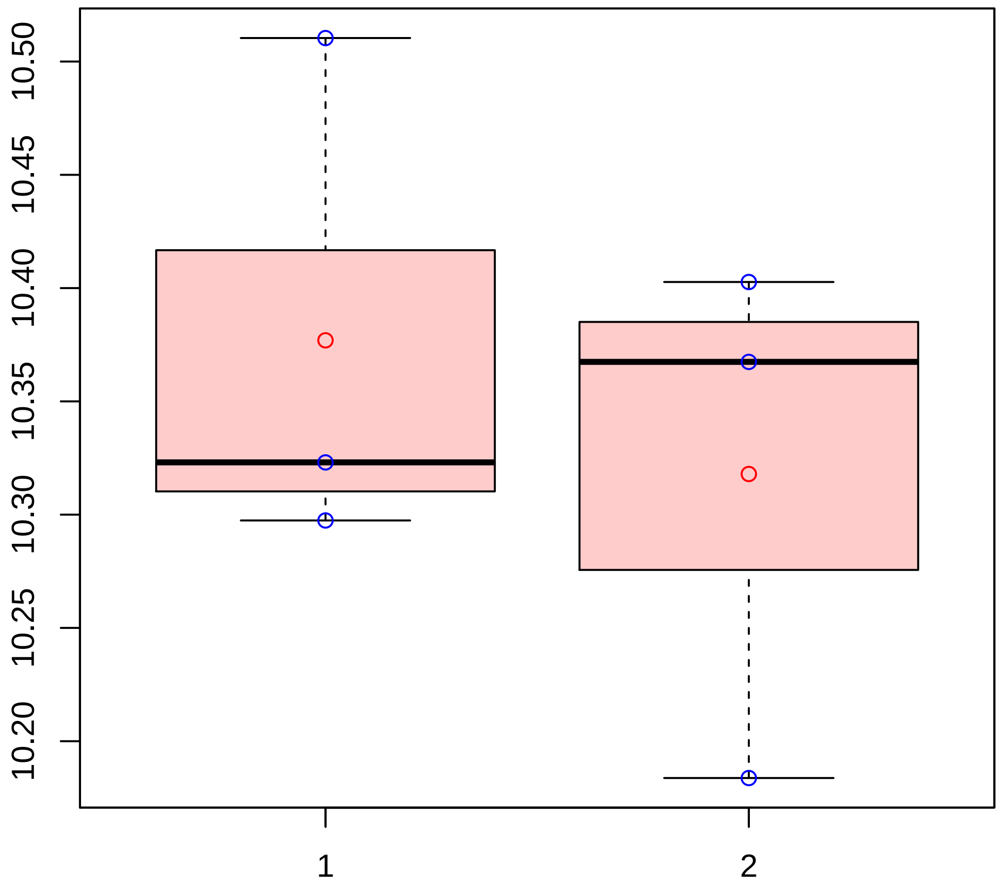


t−Test: p−value = 0.57

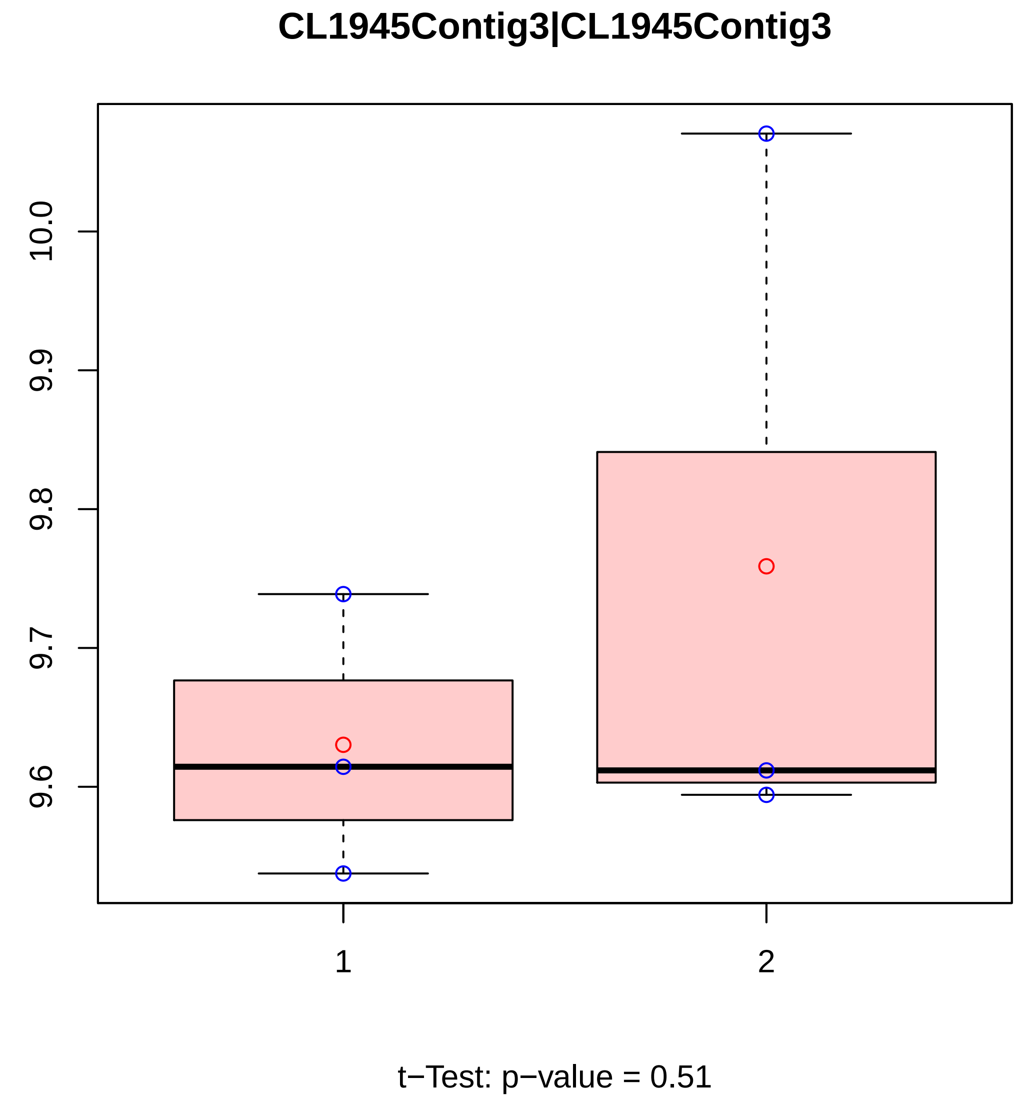
CL1945Contig3|CL1945Contig3
10.0
9.9
9.8
9.7
9.6
1
2
t−Test: p−value = 0.51

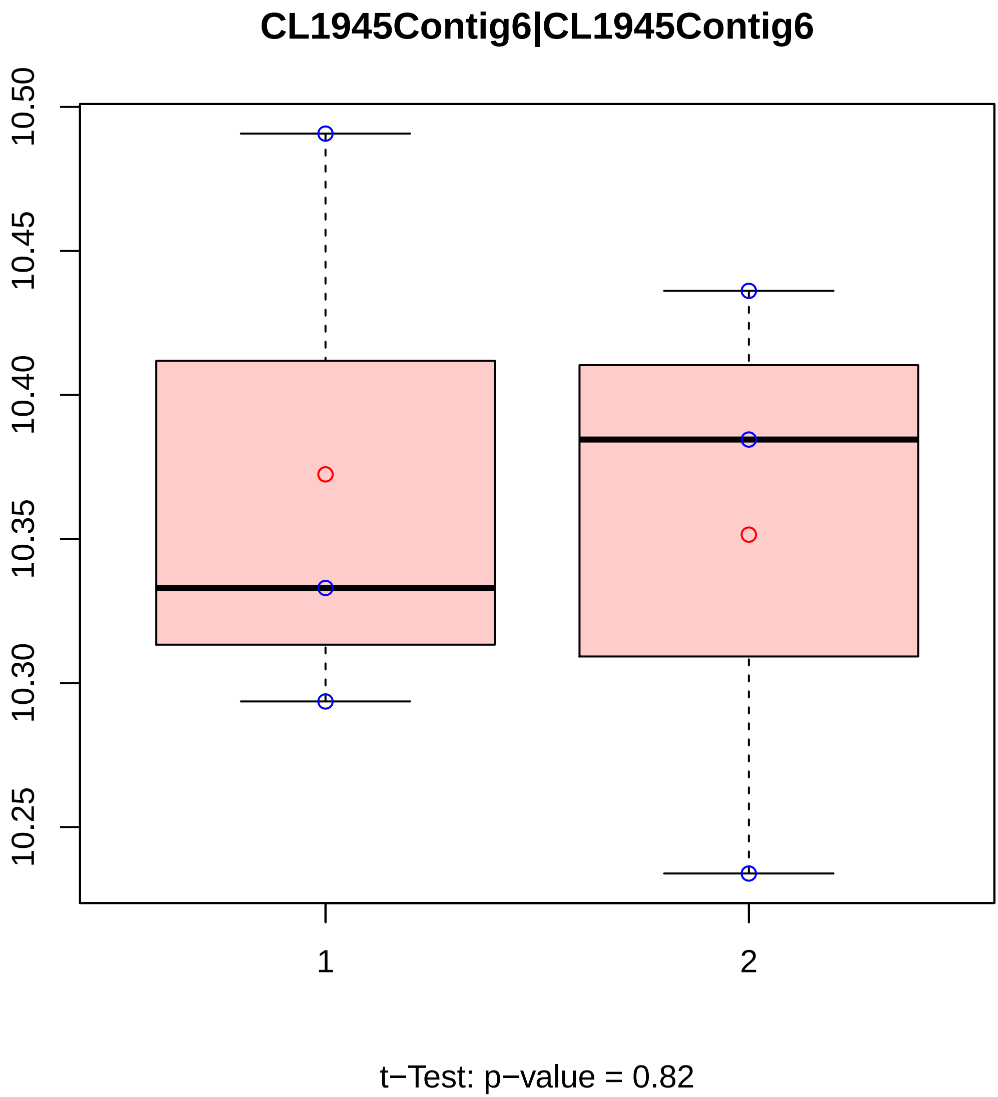
CL1945Contig6|CL1945Contig6
10.50
10.45
10.40
10.35
10.30
10.25
1
2
t−Test: p−value = 0.82

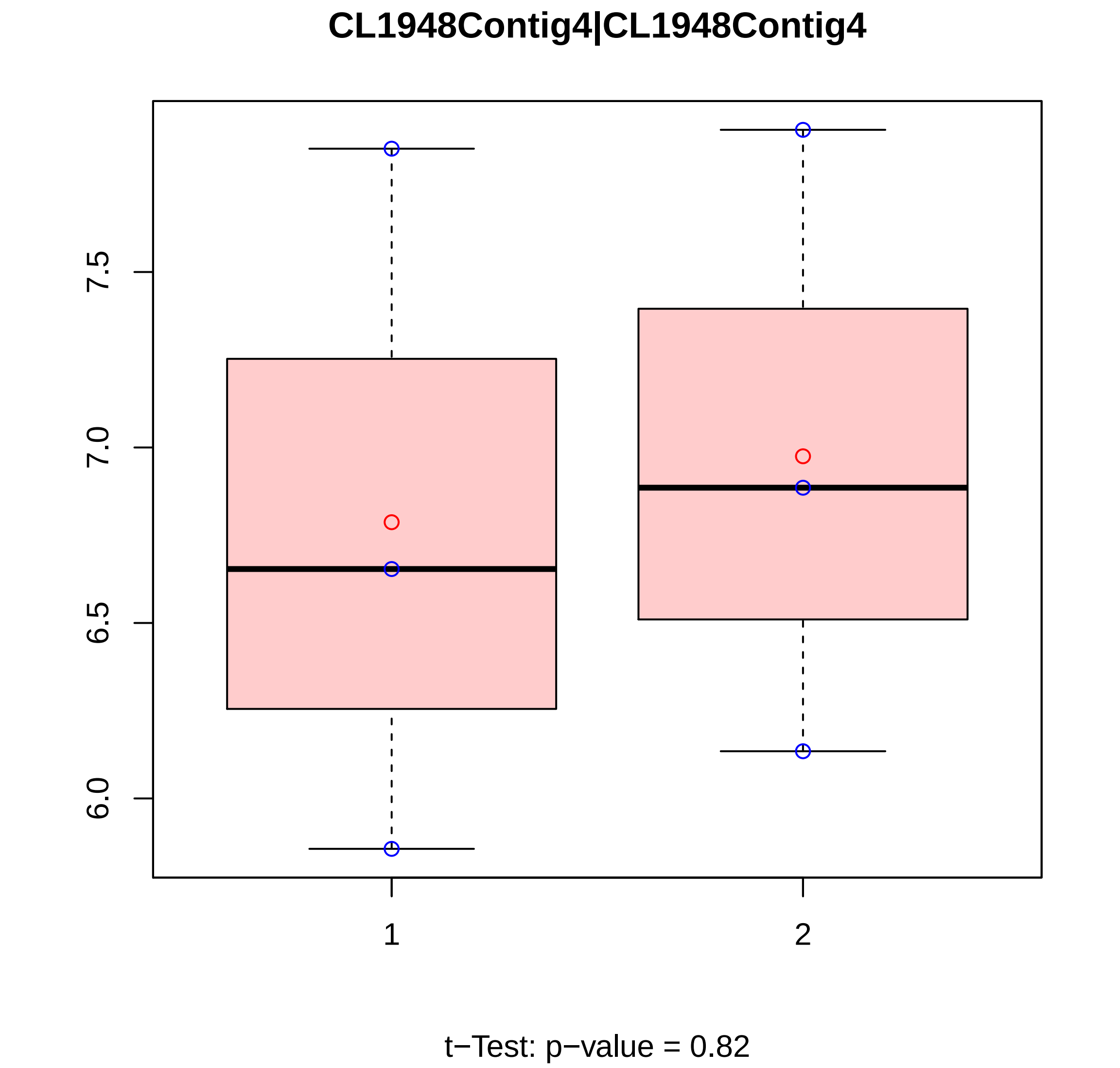

CL1948Contig4|CL1948Contig4
7.5
7.0
6.5
6.0
1
2
t−Test: p−value = 0.82

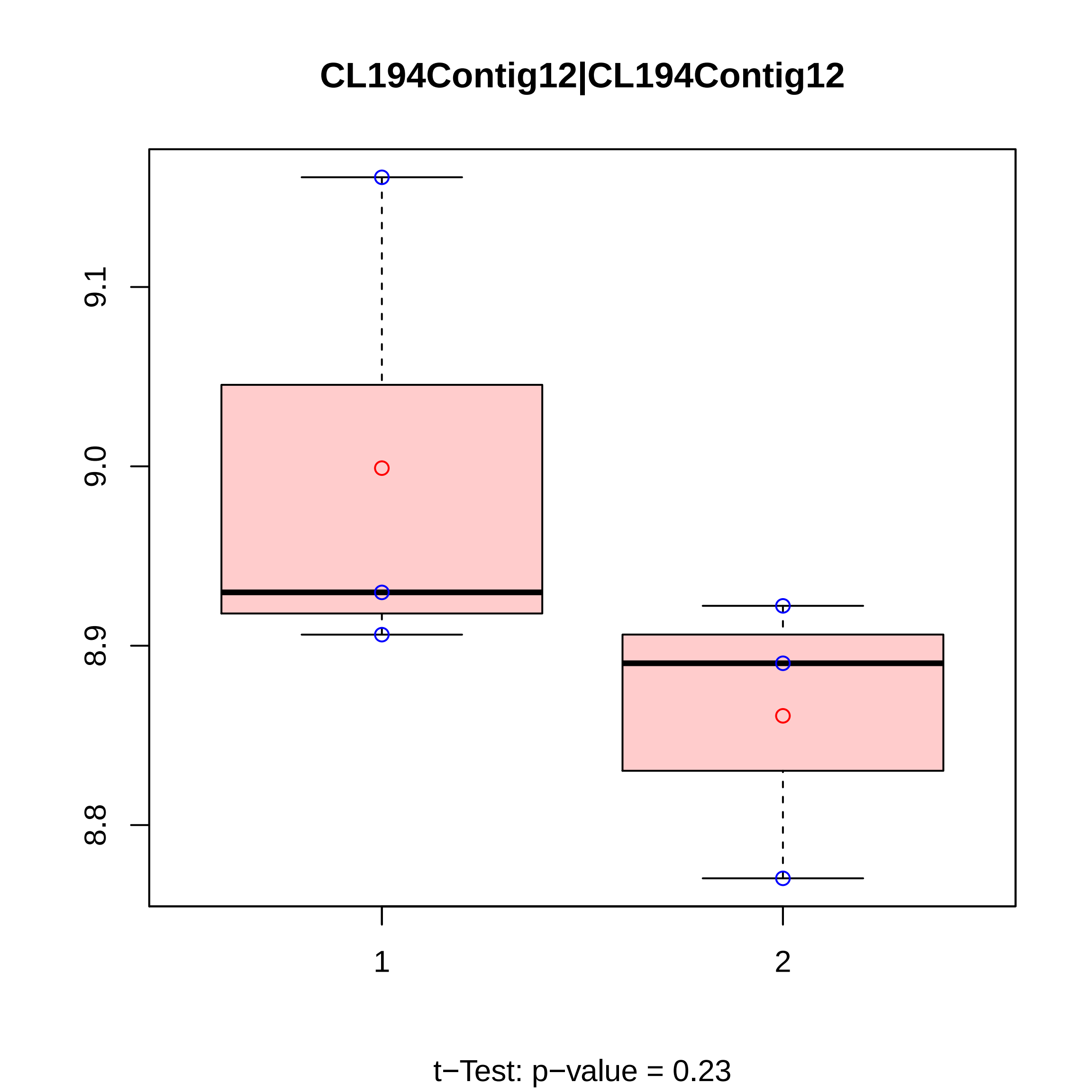
CL194Contig12|CL194Contig12
9.1
9.0
8.9
8.8
1
2
t−Test: p−value = 0.23

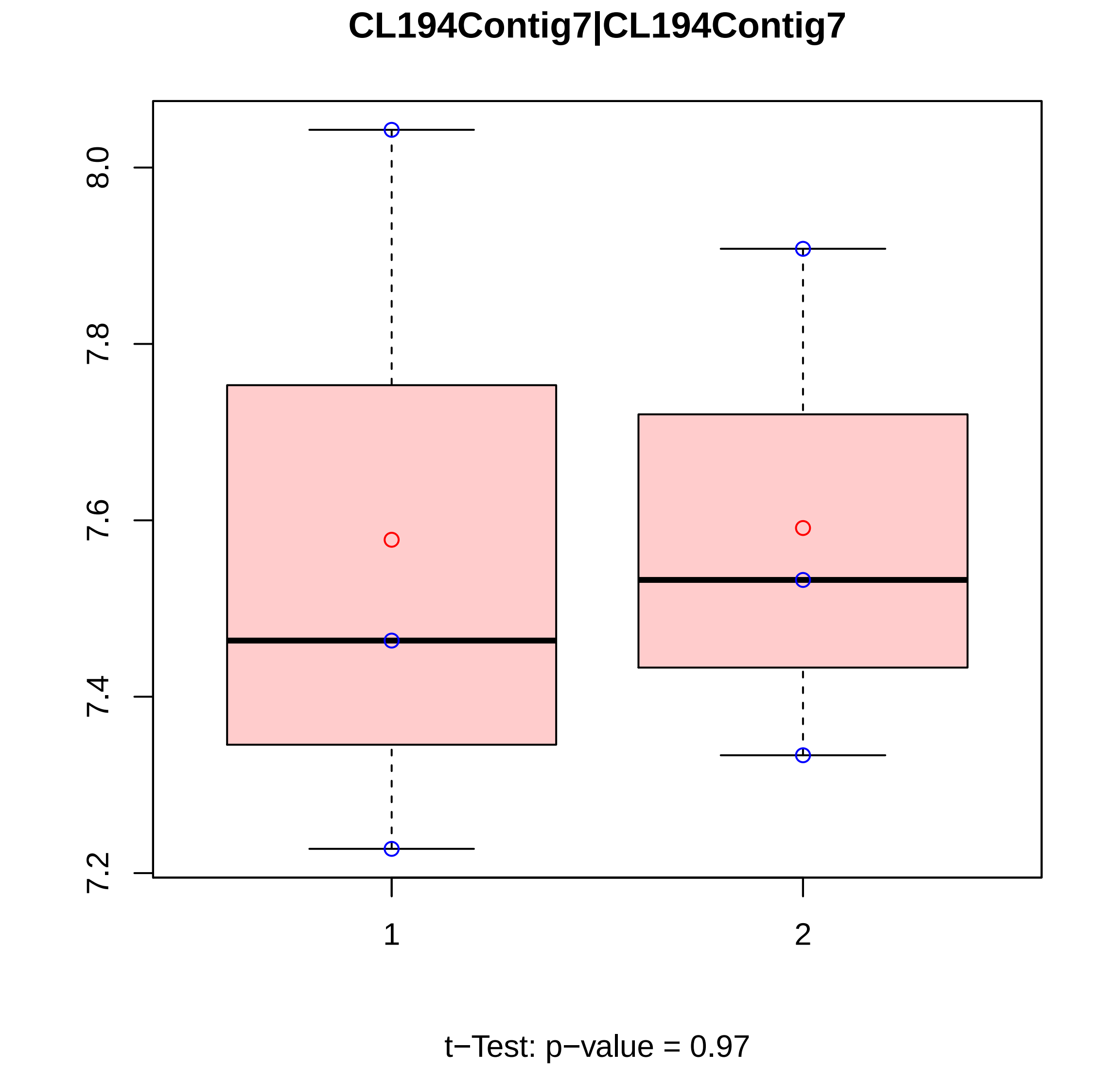
CL194Contig7|CL194Contig7
8.0
7.8
7.6
7.4
7.2
1
2
t−Test: p−value = 0.97

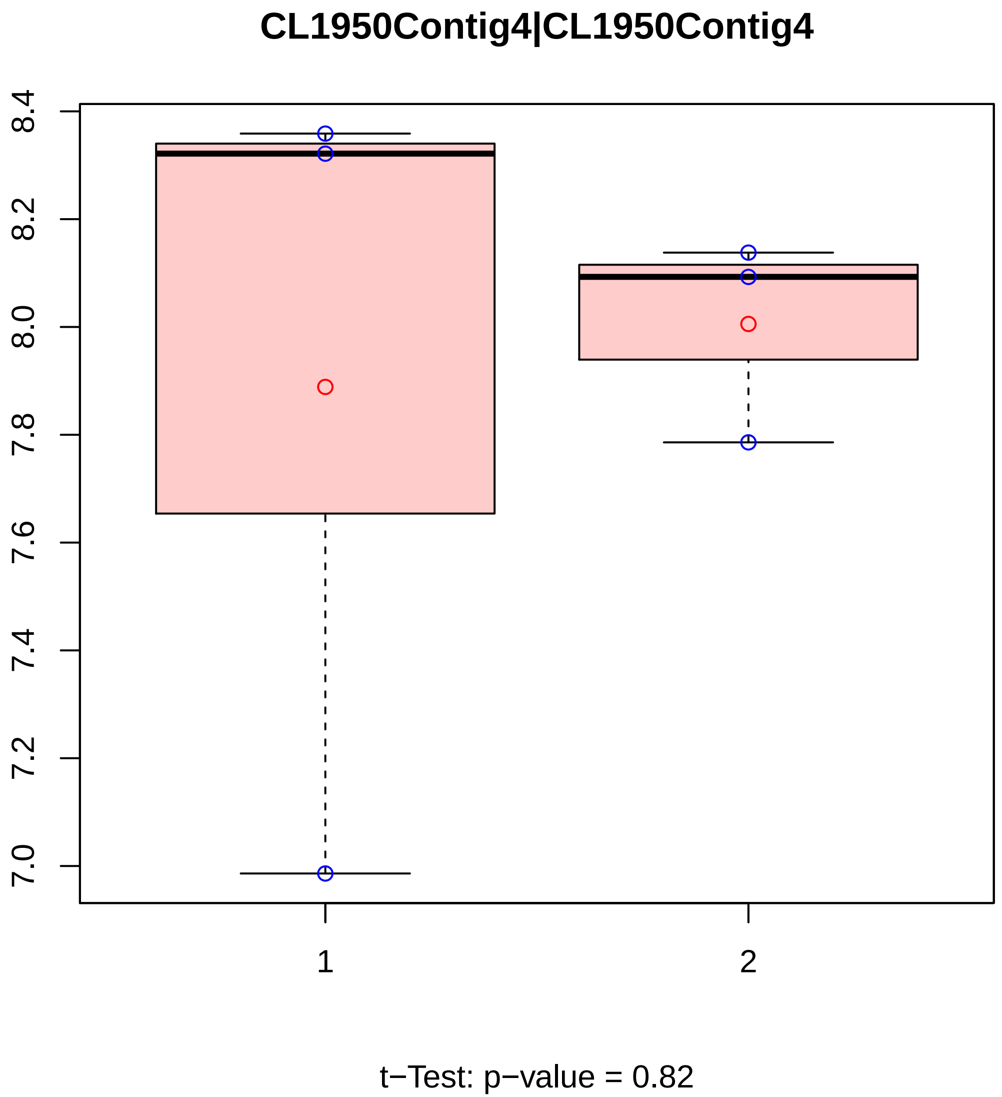

CL1950Contig4|CL1950Contig4
8.4
8.2
8.0
7.8
7.6
7.4
7.2
7.0
1
2
t−Test: p−value = 0.82

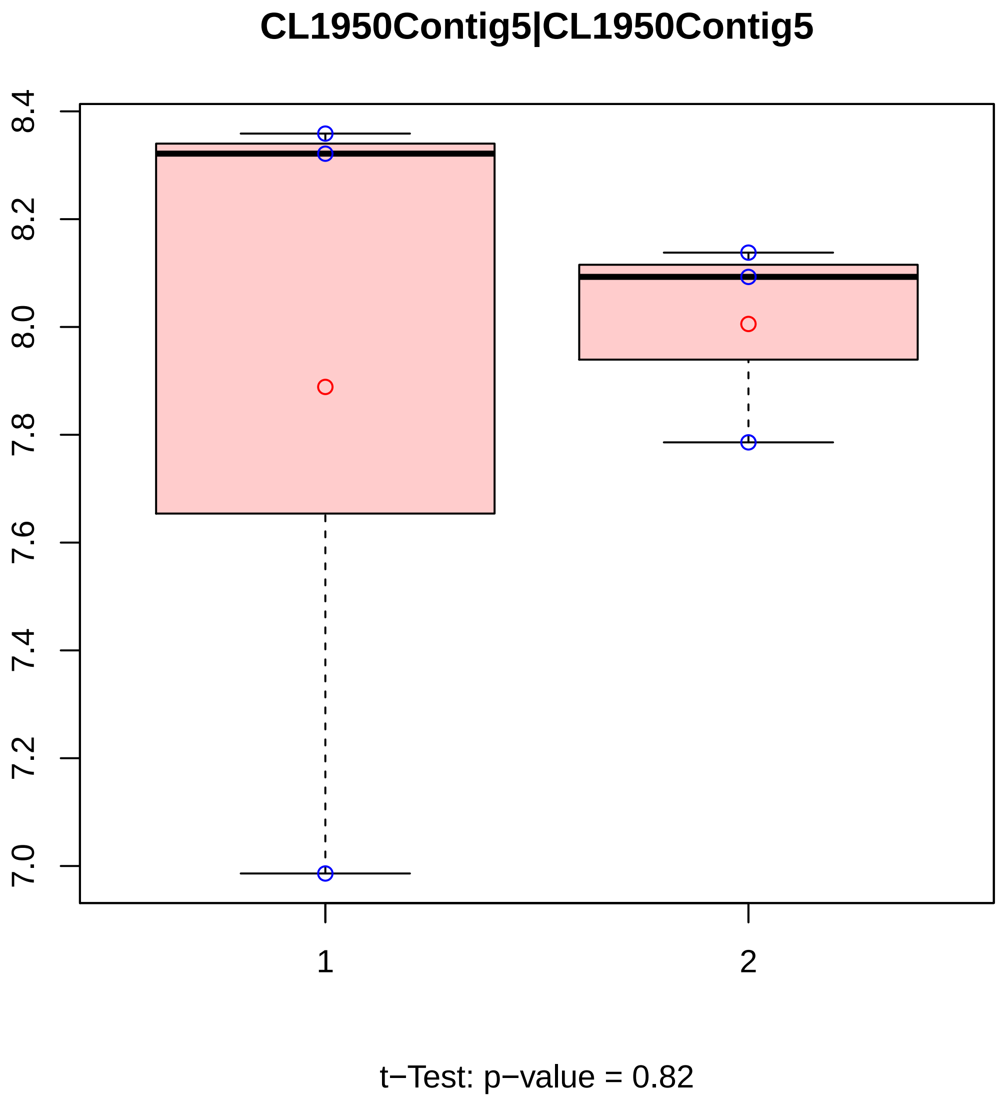

CL1950Contig5|CL1950Contig5
8.4
8.2
8.0
7.8
7.6
7.4
7.2
7.0
1
2
t−Test: p−value = 0.82

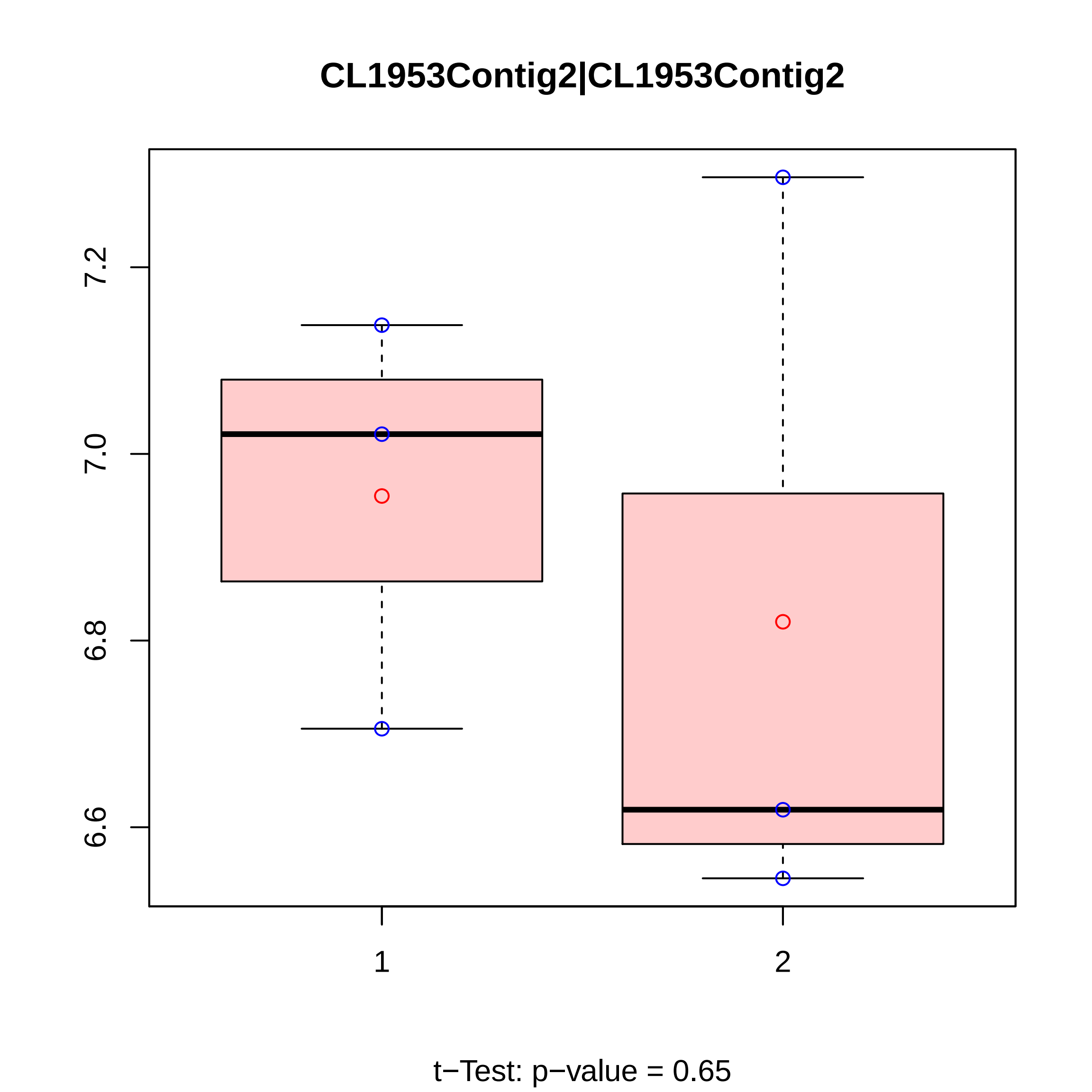

CL1953Contig2|CL1953Contig2
7.2
7.0
6.8
6.6
1
2
t−Test: p−value = 0.65

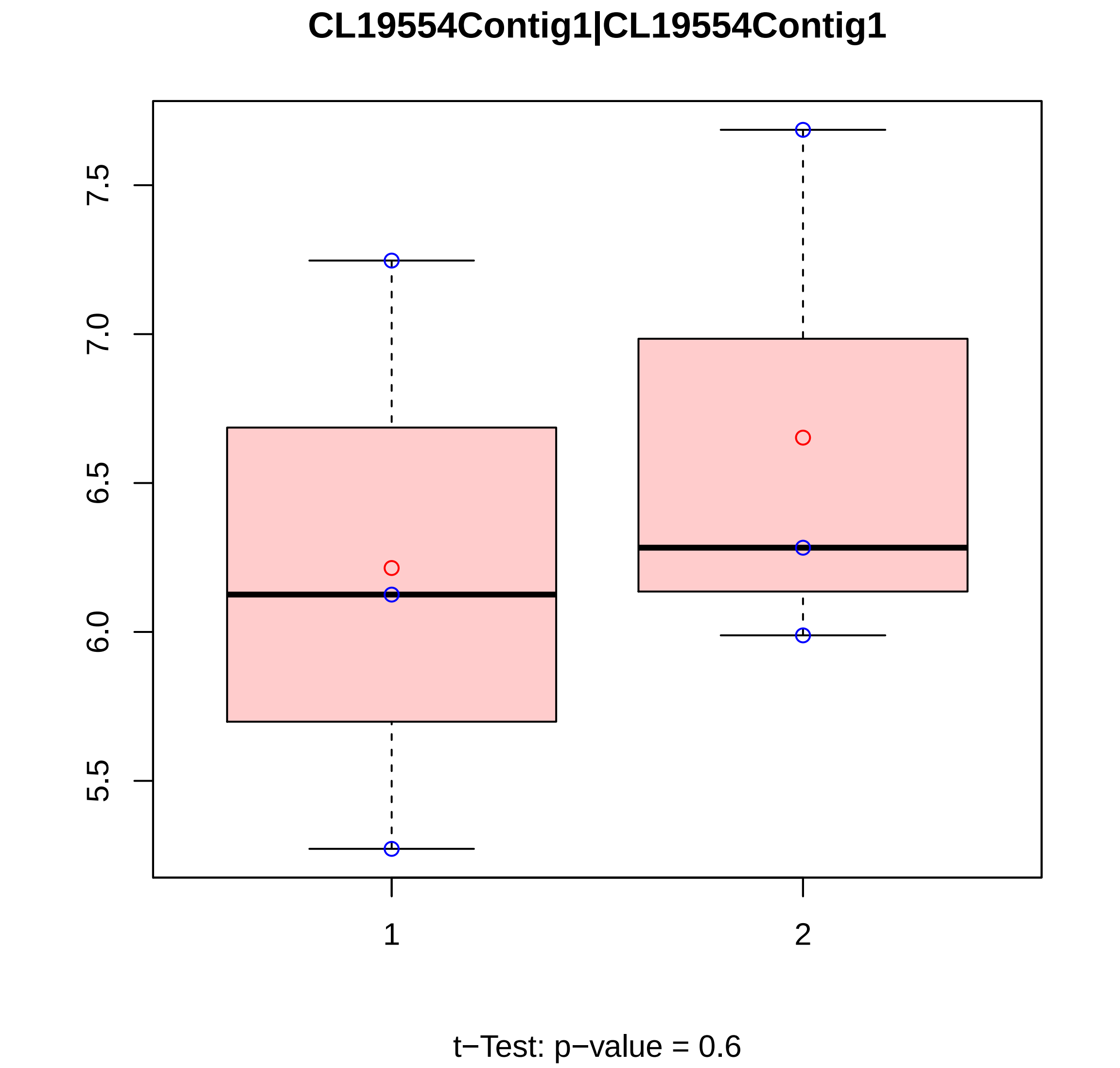
CL19554Contig1|CL19554Contig1
7.5
7.0
6.5
6.0
5.5
1
2
t−Test: p−value = 0.6

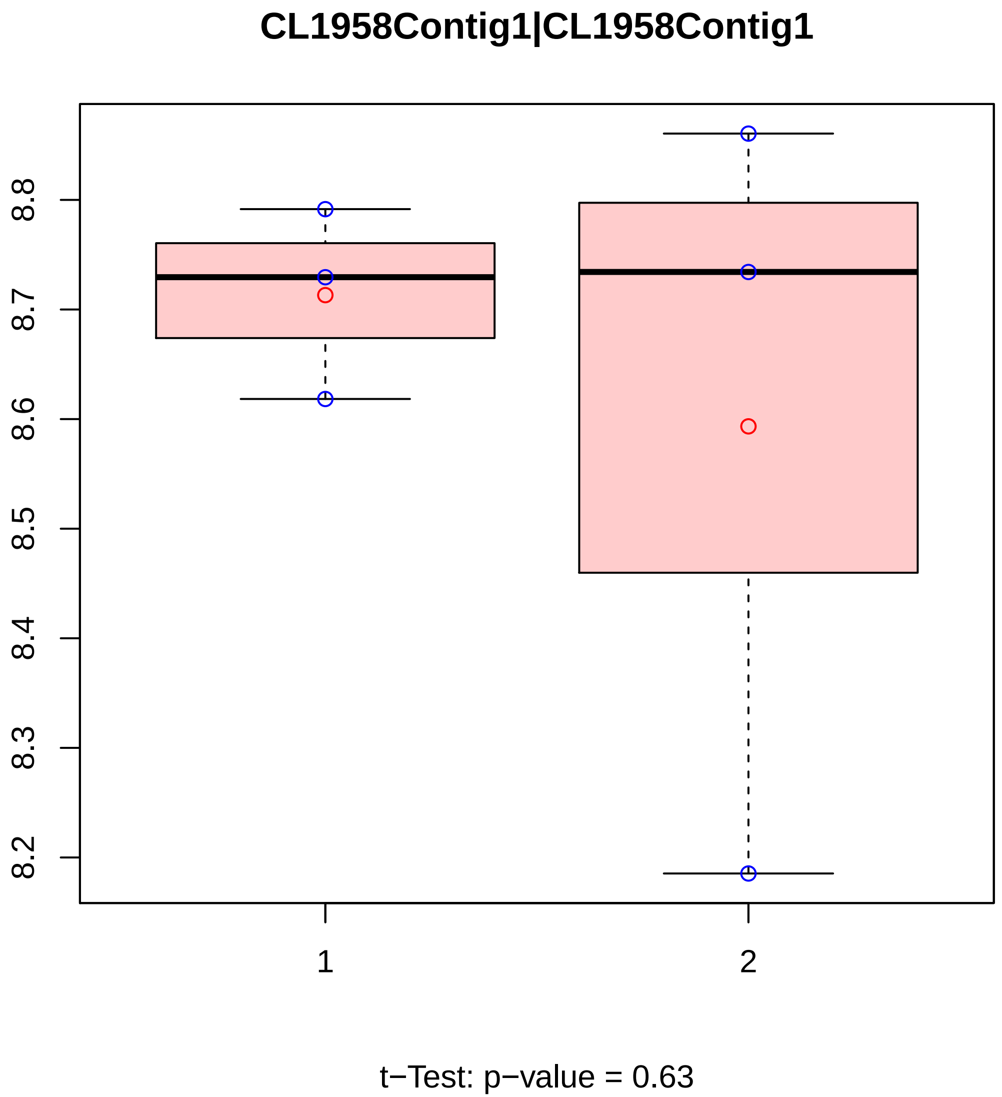

CL1958Contig1|CL1958Contig1
8.2
8.3
8.4
8.5
8.6
8.7
8.8
1
2
t−Test: p−value = 0.63

CL1960Contig3|CL1960Contig3

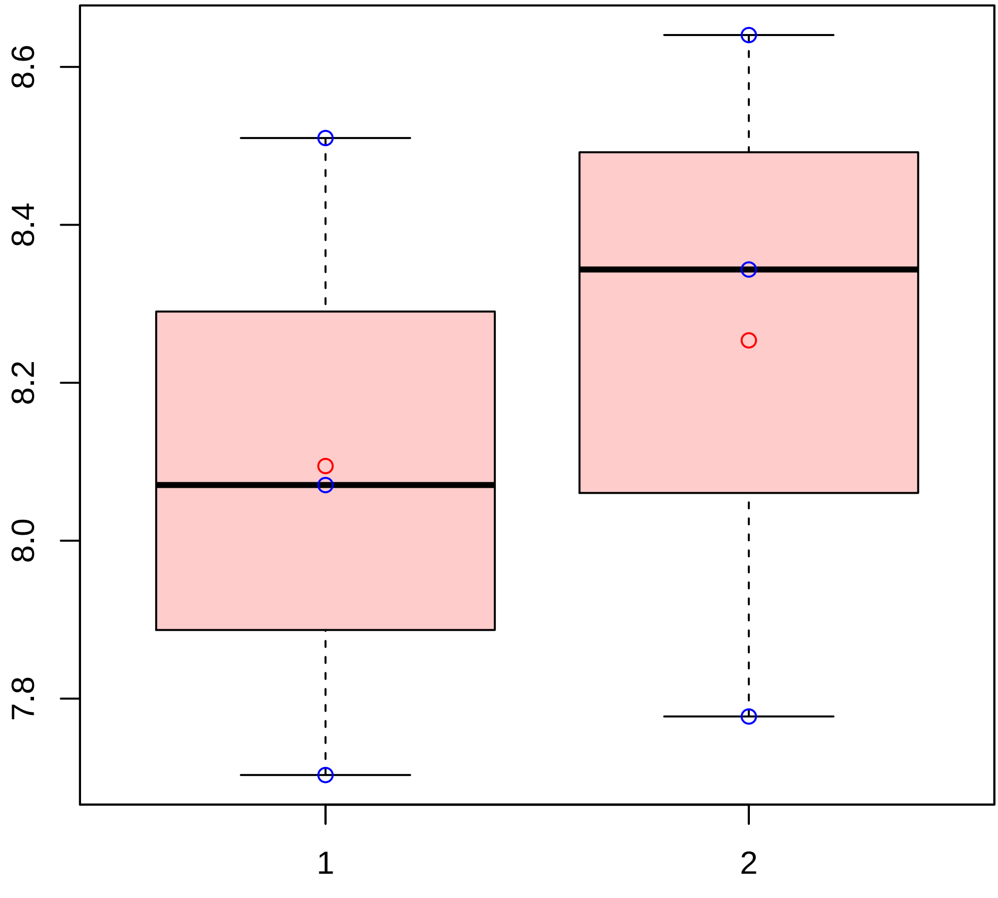


t−Test: p−value = 0.67

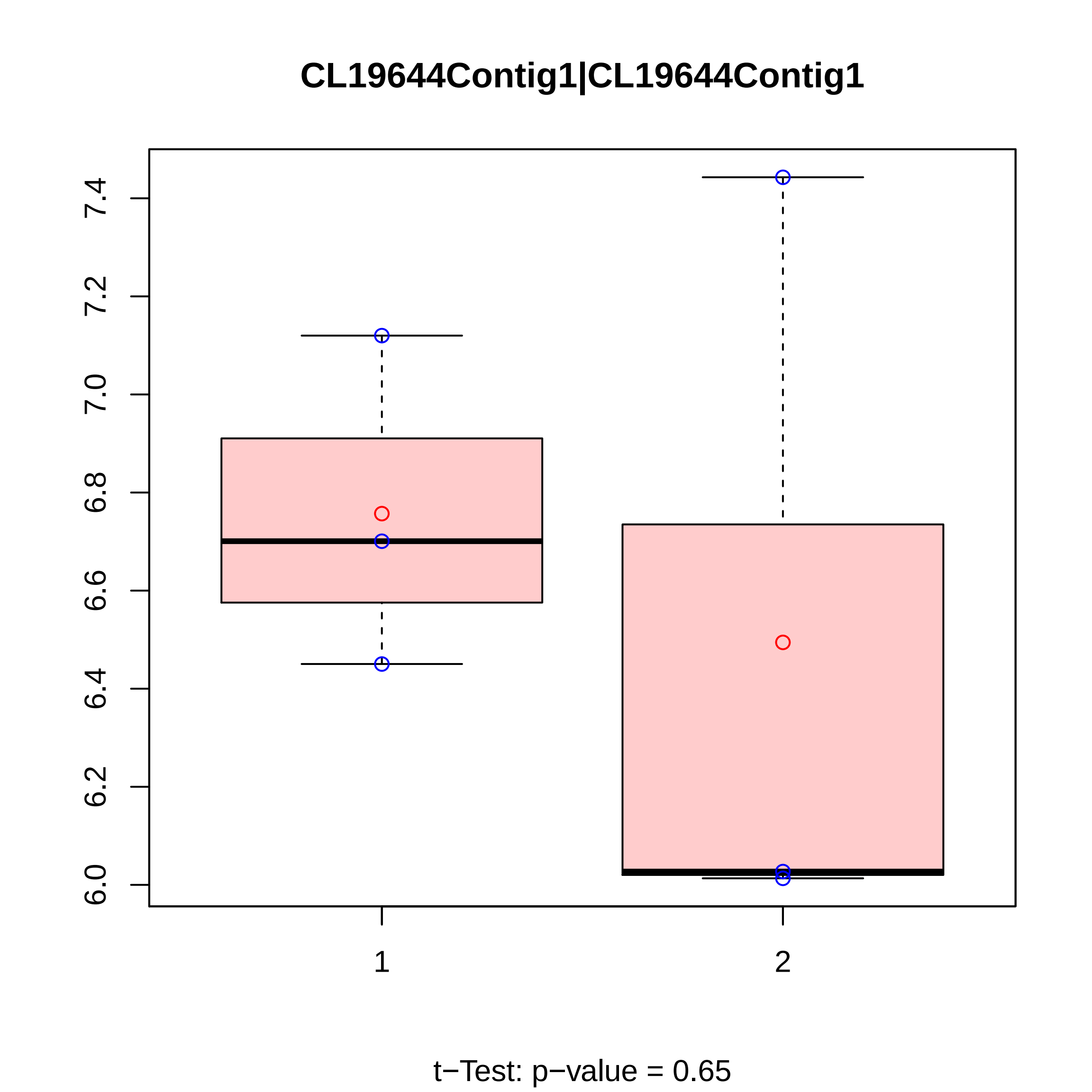
CL19644Contig1|CL19644Contig1
7.4
7.2
7.0
6.8
6.6
6.4
6.2
6.0
1
2
t−Test: p−value = 0.65

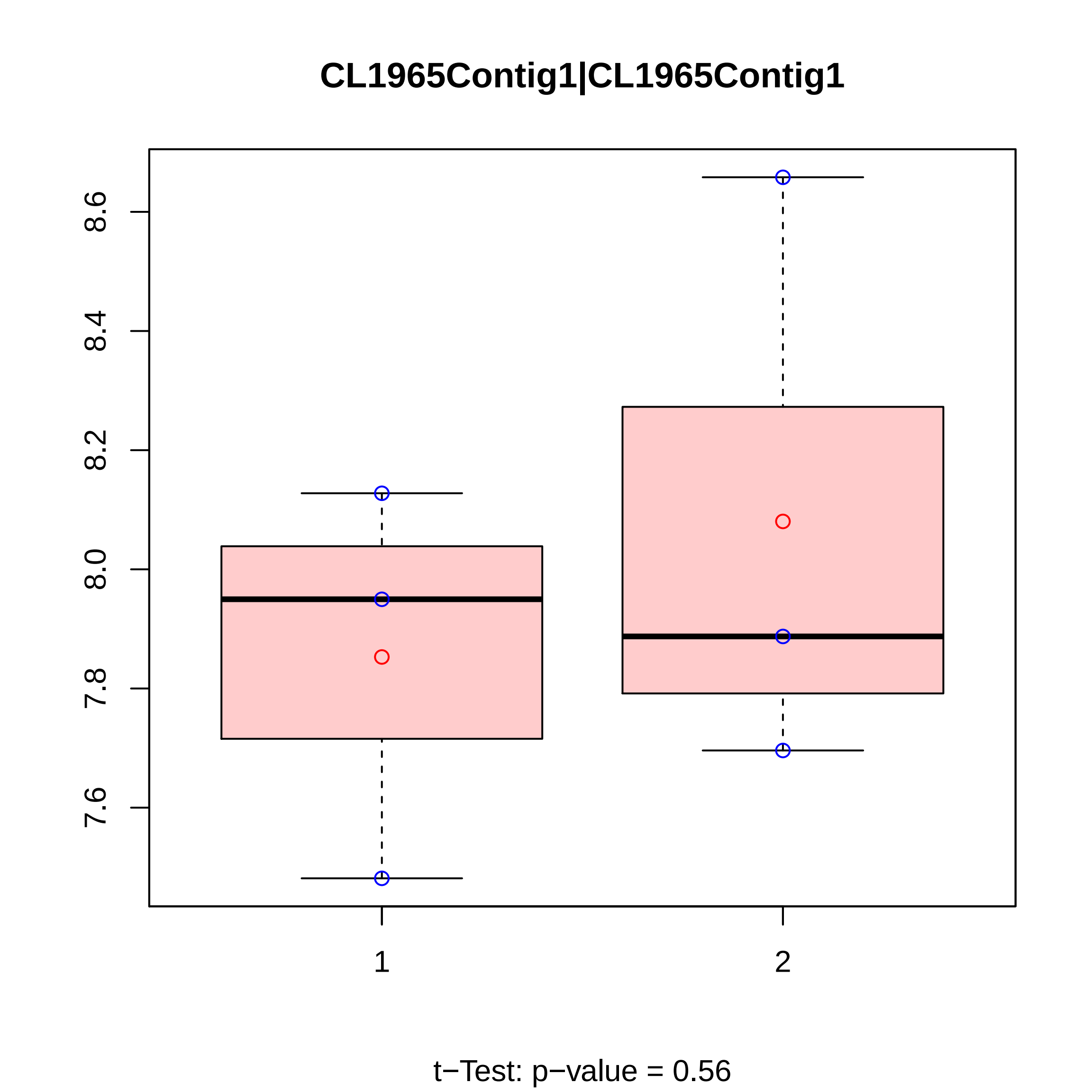
CL1965Contig1|CL1965Contig1
8.6
8.4
8.2
8.0
7.8
7.6
1
2
t−Test: p−value = 0.56

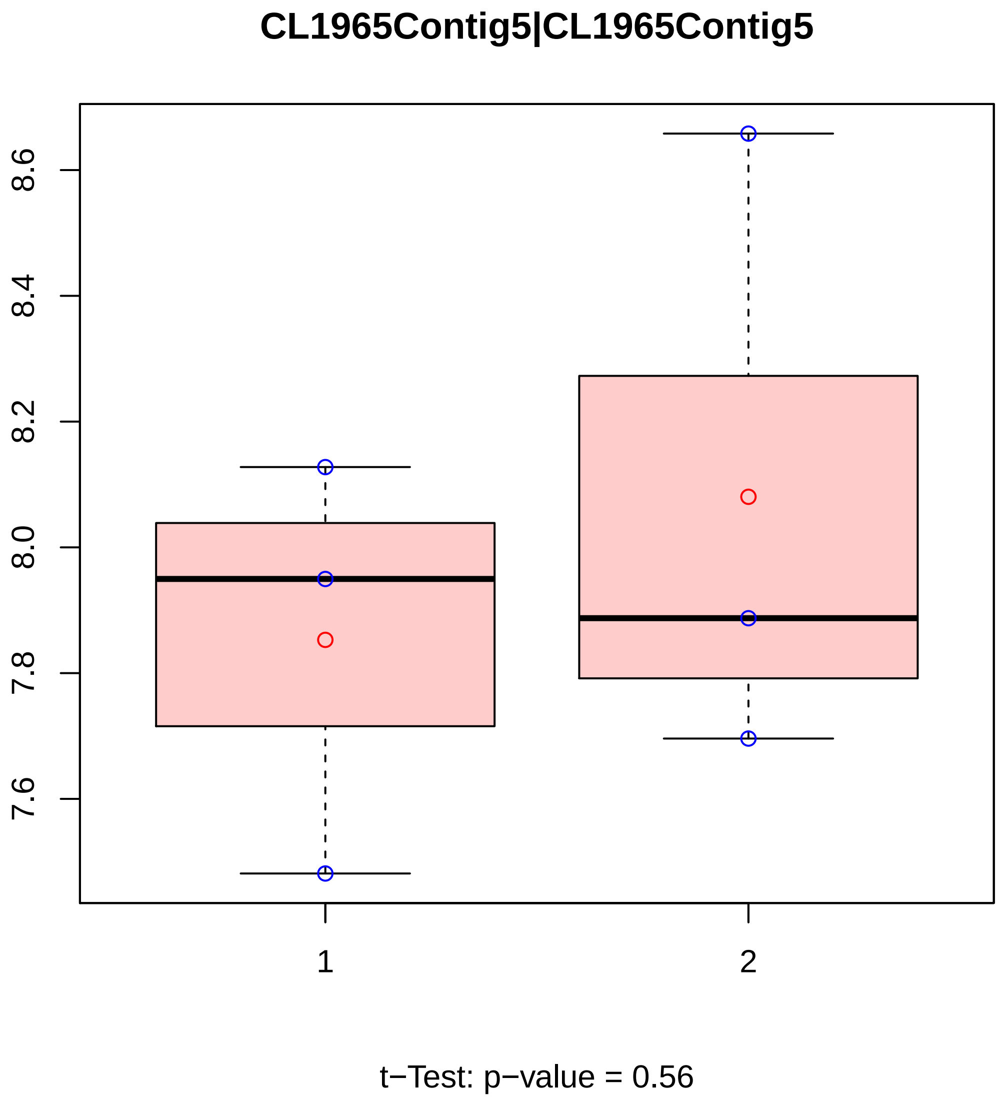

CL1965Contig5|CL1965Contig5
8.6
8.4
8.2
8.0
7.8
7.6
1
2
t−Test: p−value = 0.56

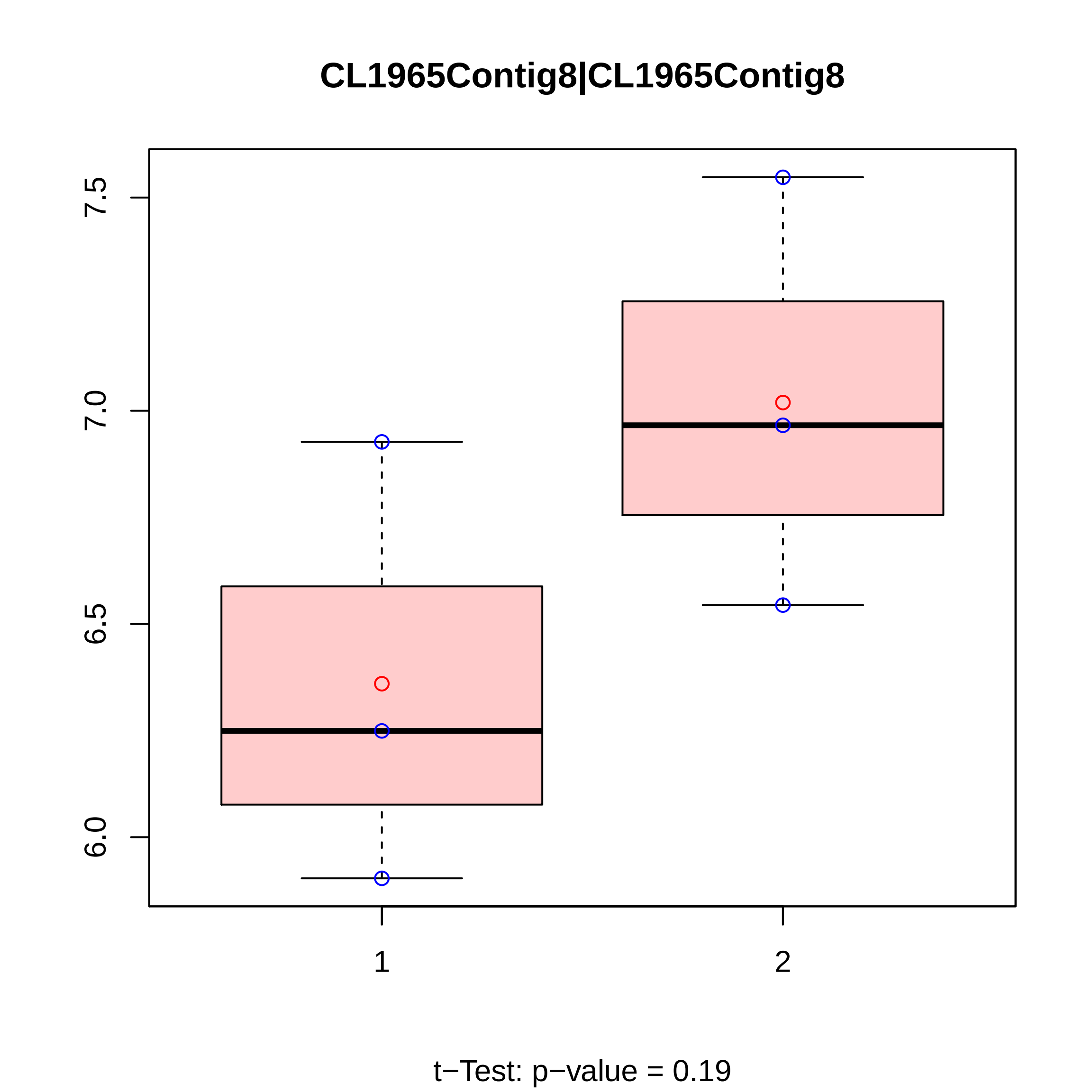
CL1965Contig8|CL1965Contig8
7.5
7.0
6.5
6.0
1
2
t−Test: p−value = 0.19

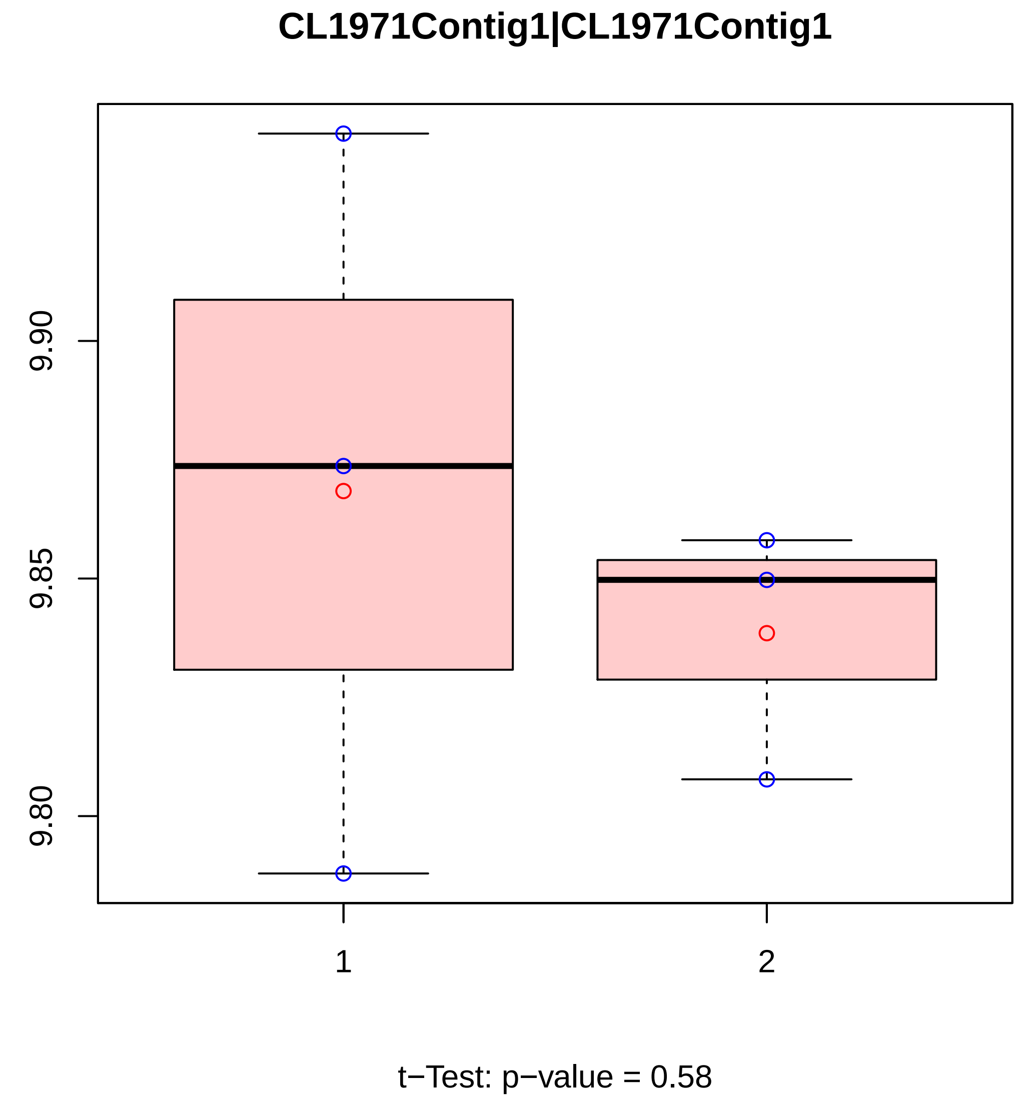
CL1971Contig1|CL1971Contig1
9.80
9.85
9.90
1
2
t−Test: p−value = 0.58

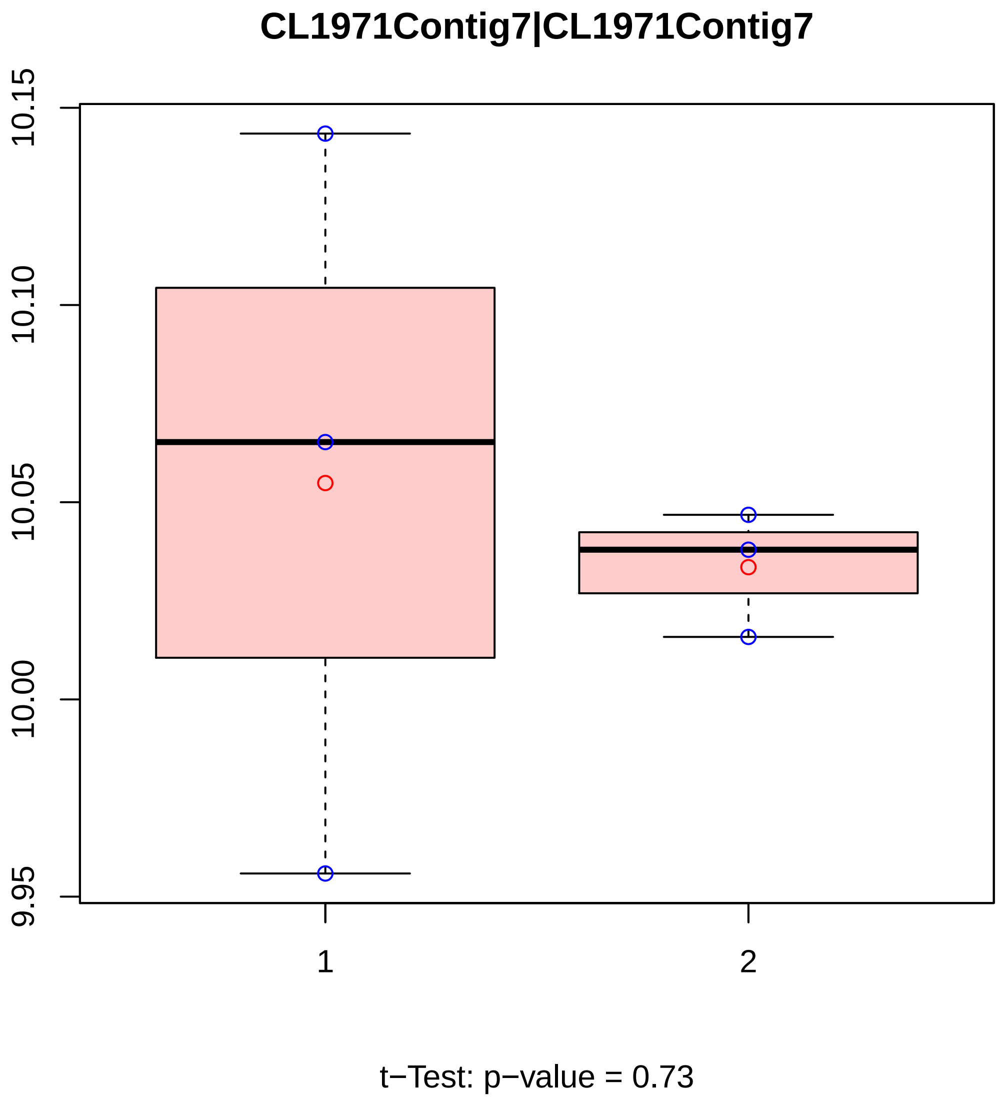

CL1971Contig7|CL1971Contig7
10.15
10.10
10.05
10.00
9.95
1
2
t−Test: p−value = 0.73

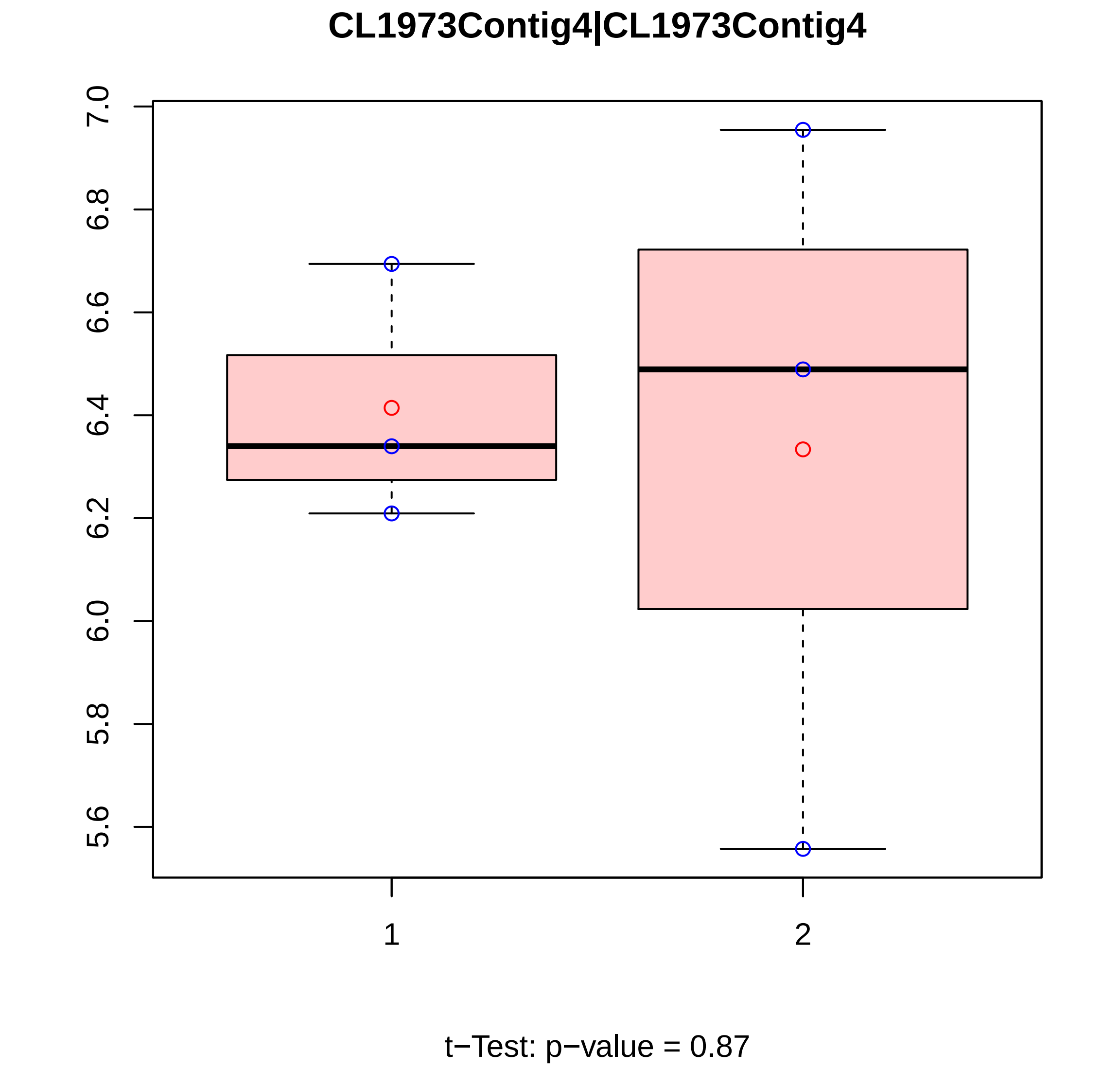
CL1973Contig4|CL1973Contig4
7.0
6.8
6.6
6.4
6.2
6.0
5.8
5.6
1
2
t−Test: p−value = 0.87

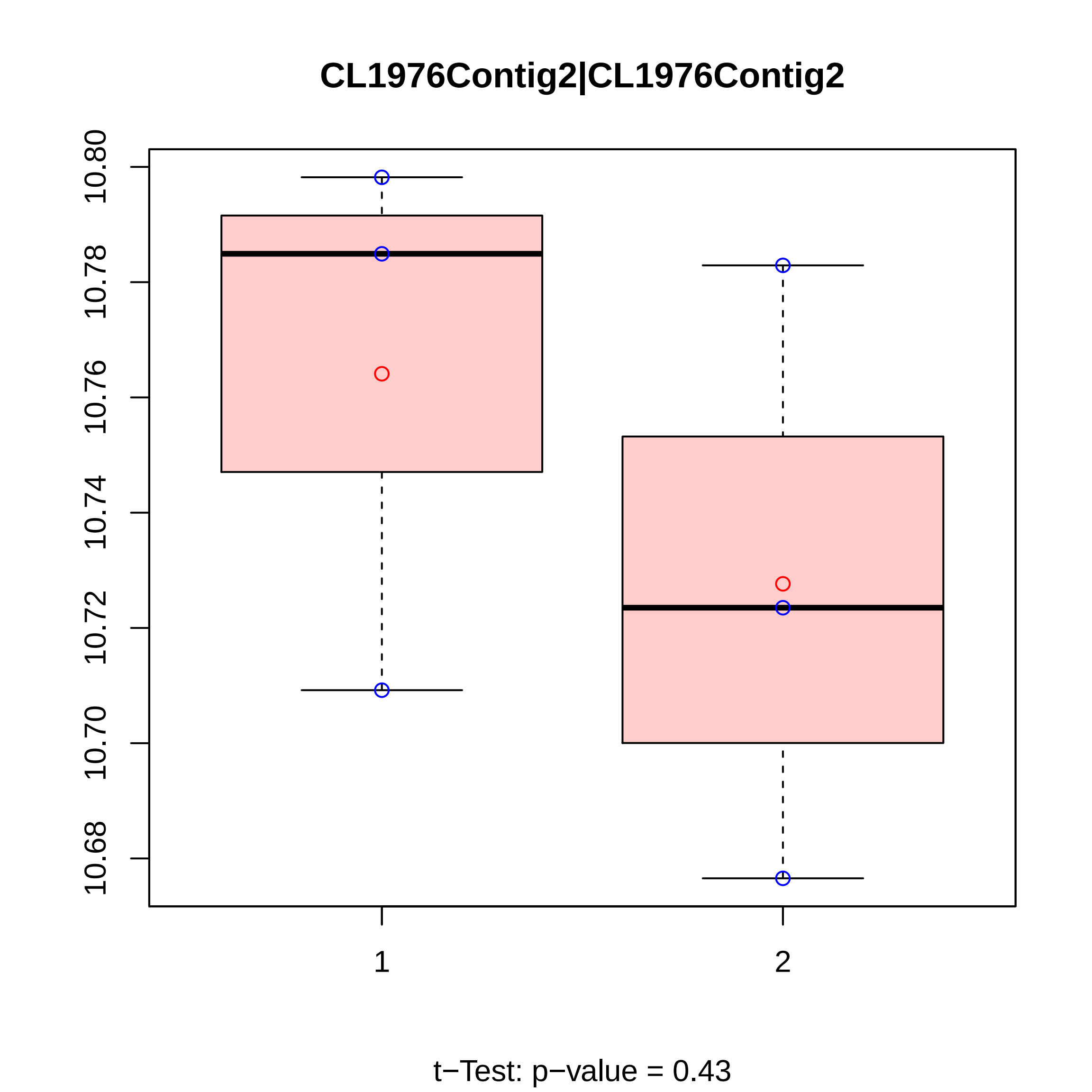

CL1976Contig2|CL1976Contig2
10.68
10.70
10.72
10.74
10.76
10.78
10.80
1
2
t−Test: p−value = 0.43

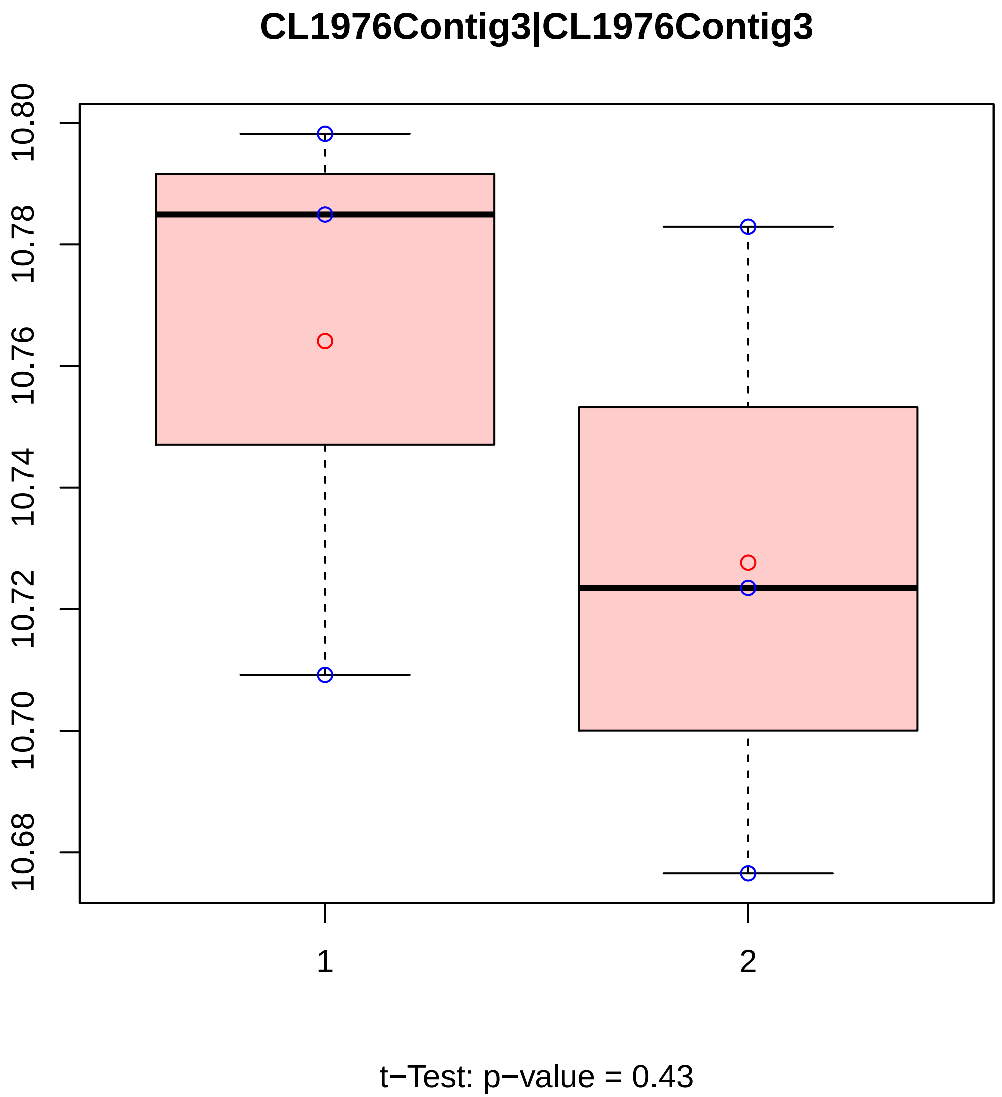

CL1976Contig3|CL1976Contig3
10.80
10.78
10.76
10.74
10.72
10.70
10.68
1
2
t−Test: p−value = 0.43

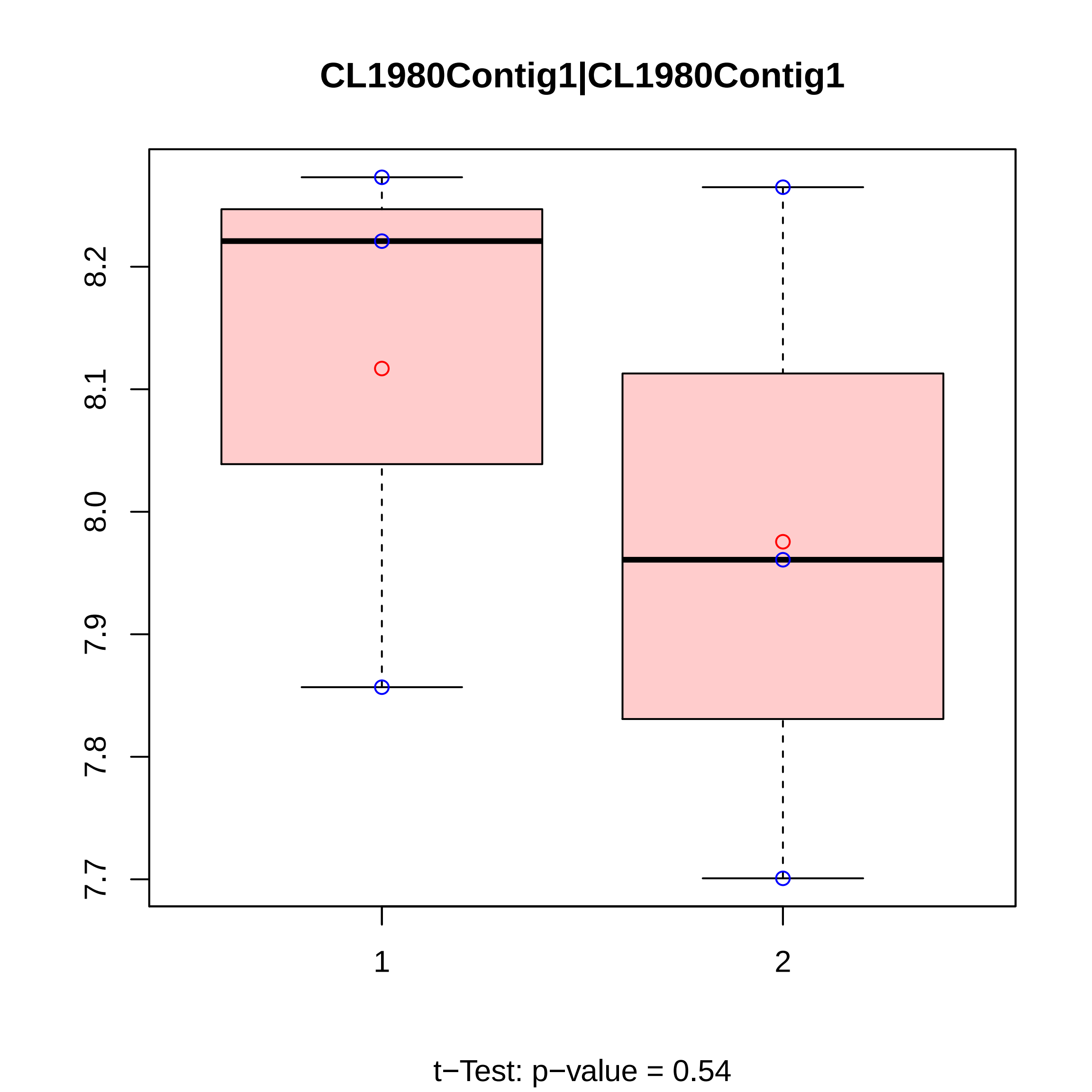
CL1980Contig1|CL1980Contig1
8.2
8.1
8.0
7.9
7.8
7.7
1
2
t−Test: p−value = 0.54

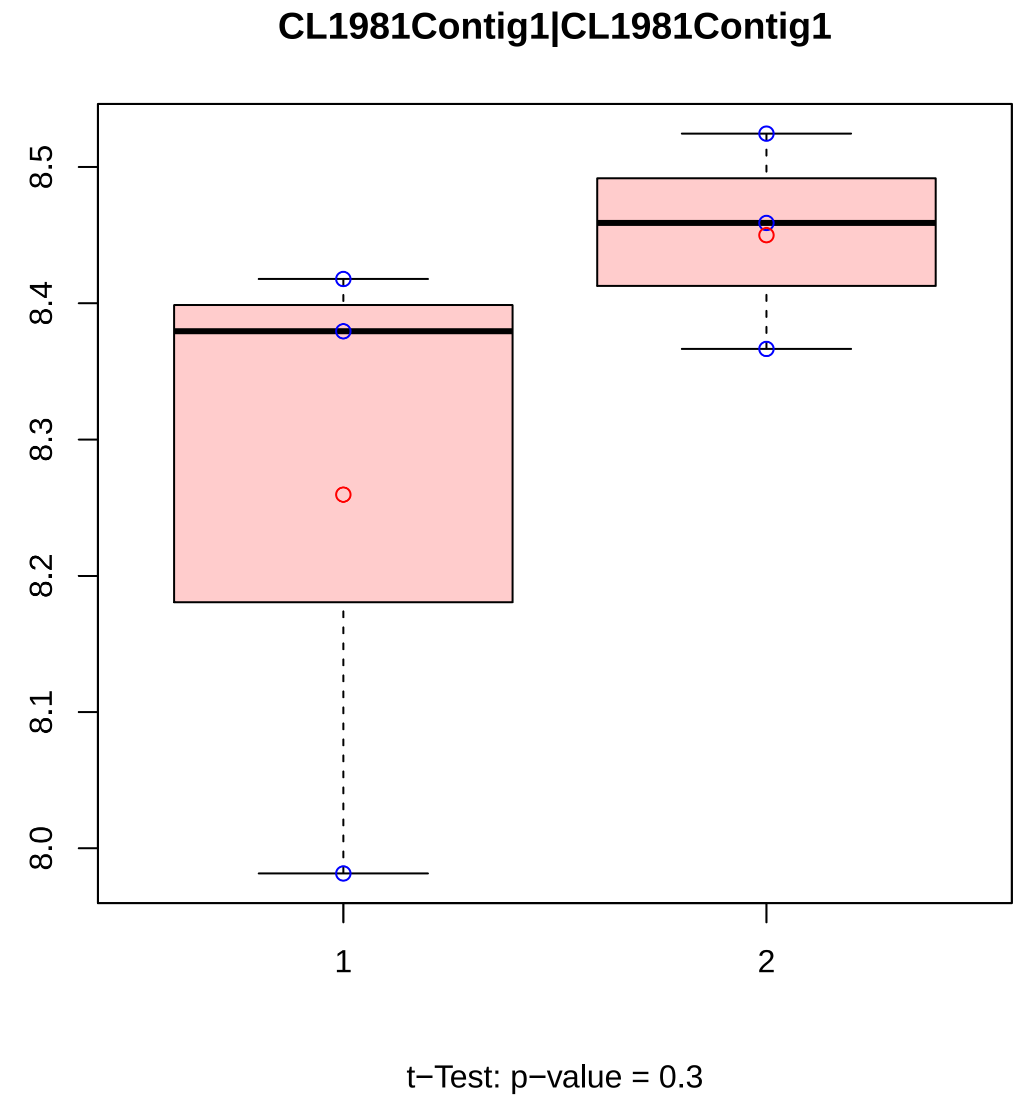

CL1981Contig1|CL1981Contig1
8.0
8.1
8.2
8.3
8.4
8.5
1
2
t−Test: p−value = 0.3

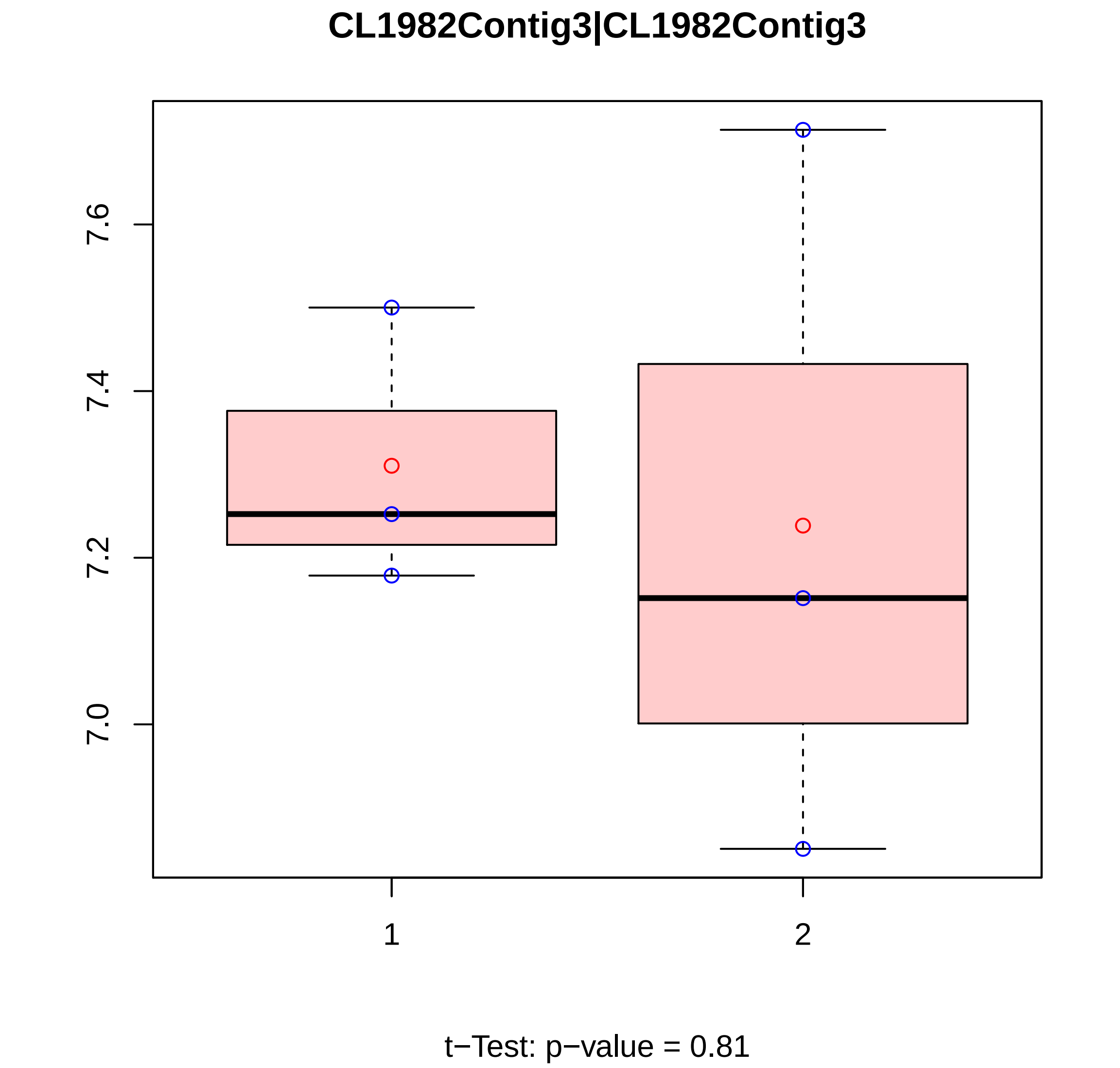
CL1982Contig3|CL1982Contig3
7.0
7.2
7.4
7.6
1
2
t−Test: p−value = 0.81

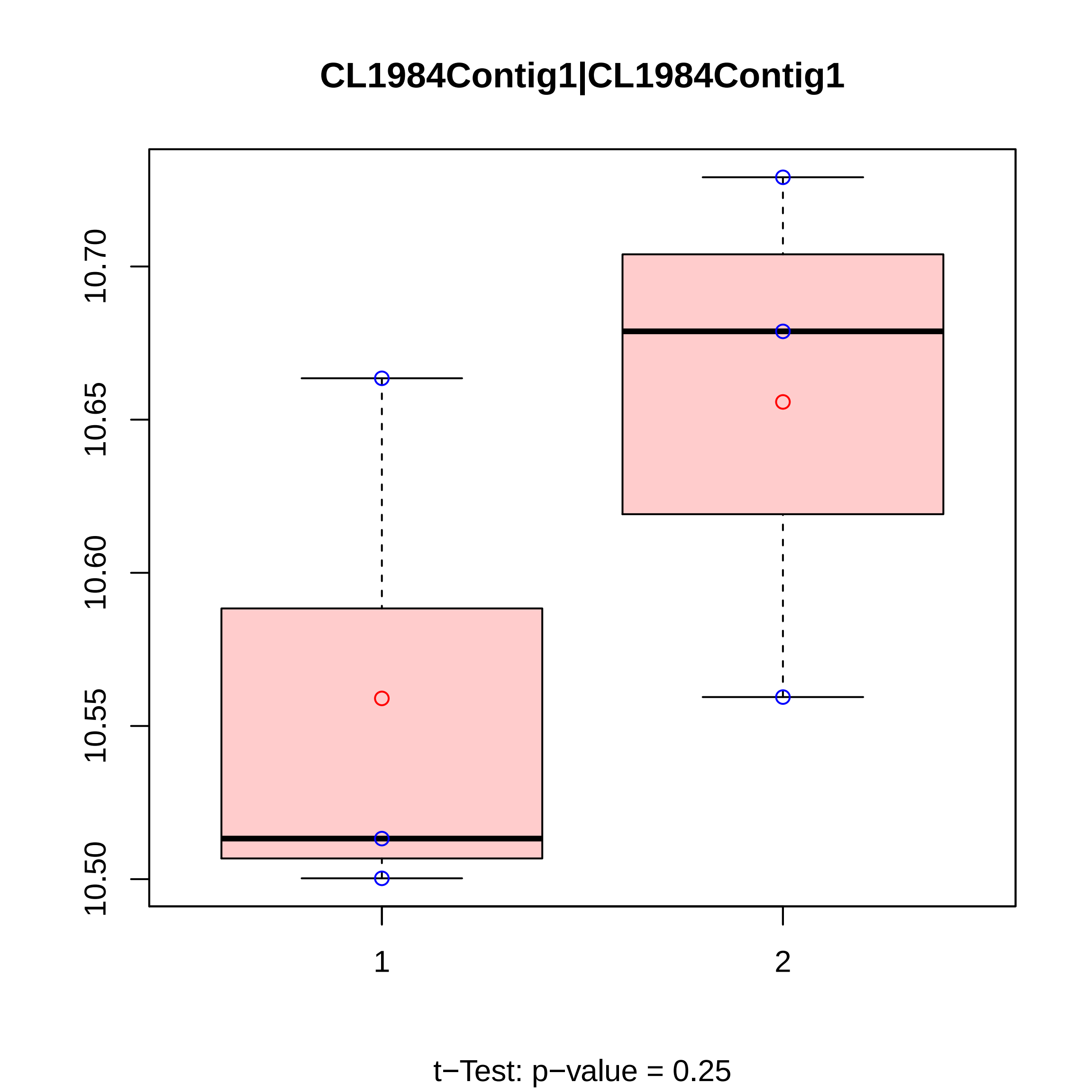

CL1984Contig1|CL1984Contig1
10.50
10.55
10.60
10.65
10.70
1
2
t−Test: p−value = 0.25

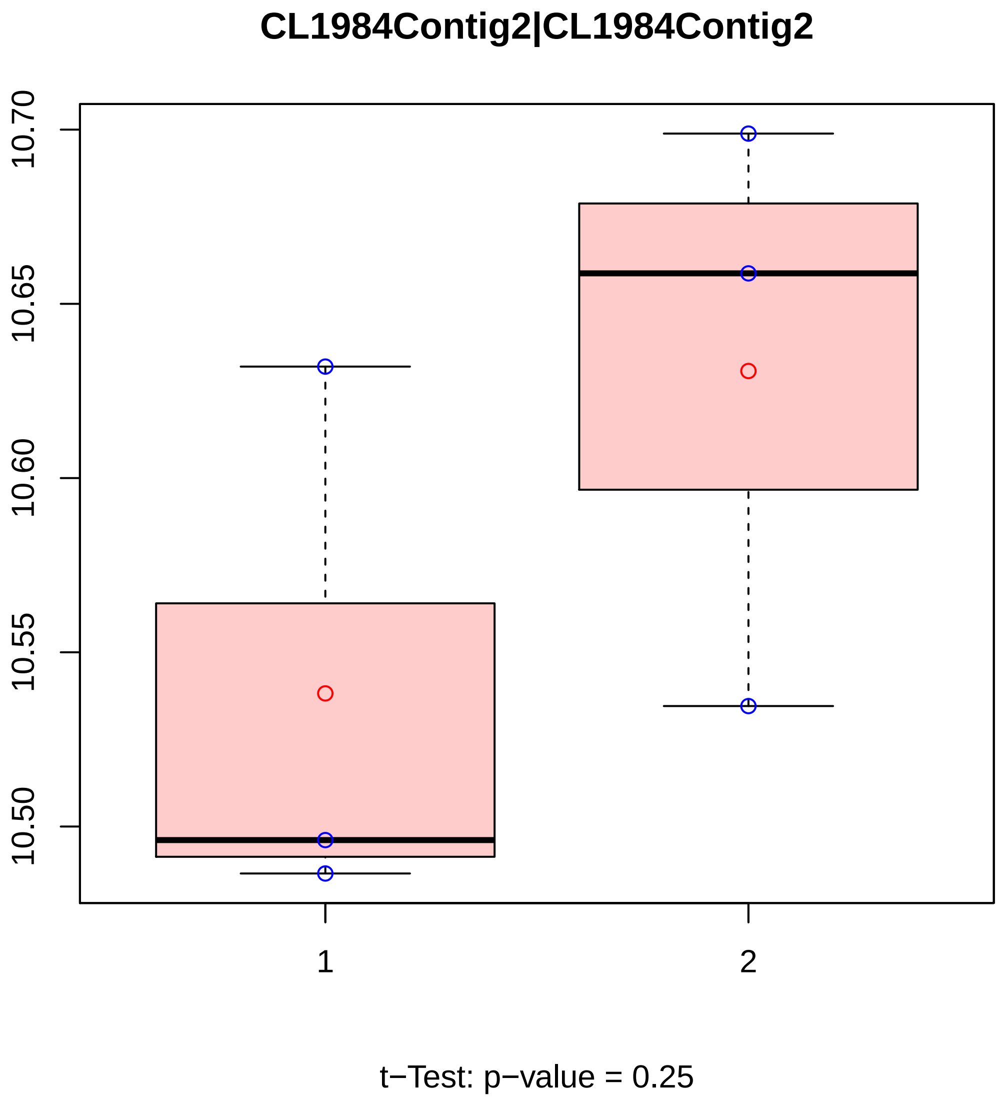

CL1984Contig2|CL1984Contig2
10.70
10.65
10.60
10.55
10.50
1
2
t−Test: p−value = 0.25

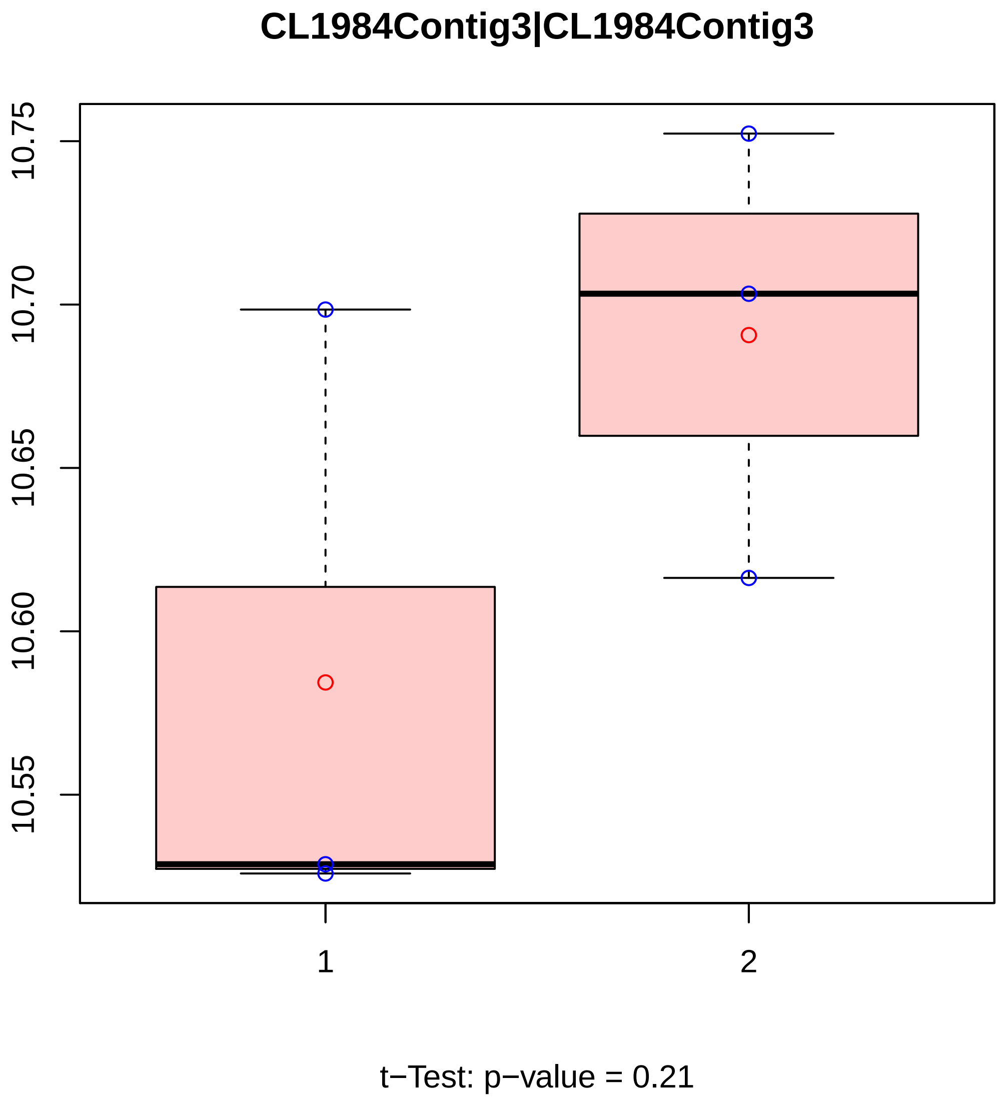

CL1984Contig3|CL1984Contig3
10.55
10.60
10.65
10.70
10.75
1
2
t−Test: p−value = 0.21

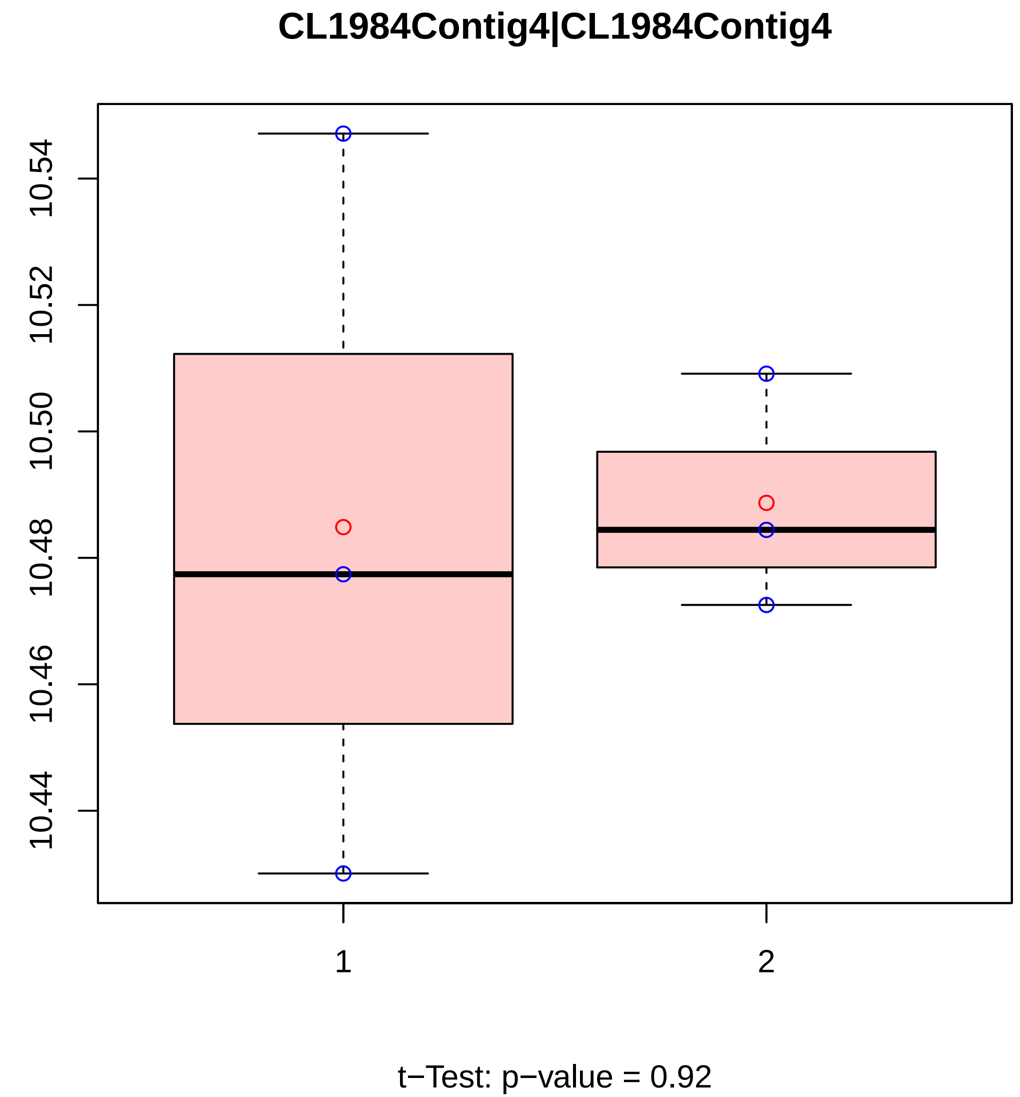
CL1984Contig4|CL1984Contig4
10.44
10.46
10.48
10.50
10.52
10.54
1
2
t−Test: p−value = 0.92

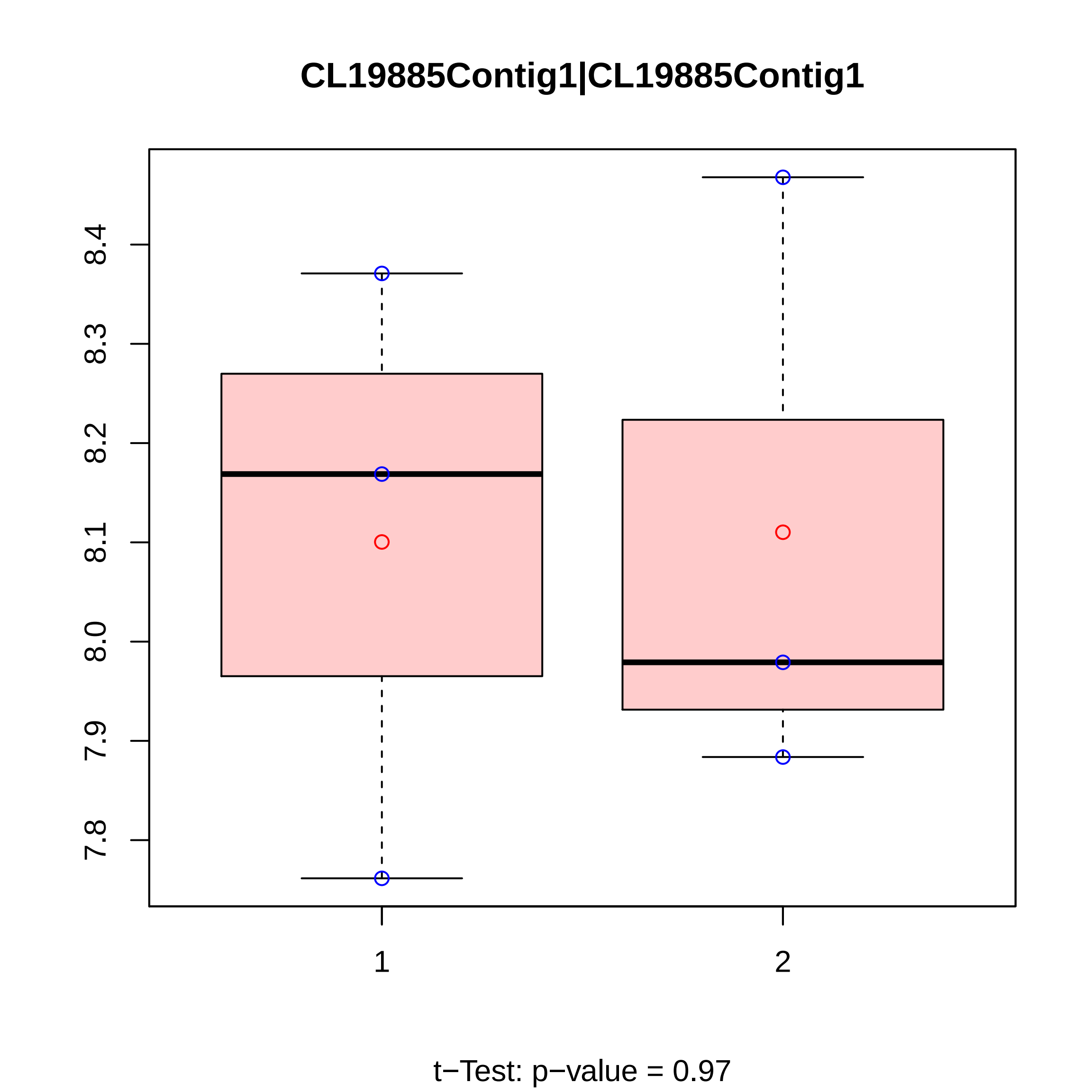
CL19885Contig1|CL19885Contig1
8.4
8.3
8.2
8.1
8.0
7.9
7.8
1
2
t−Test: p−value = 0.97

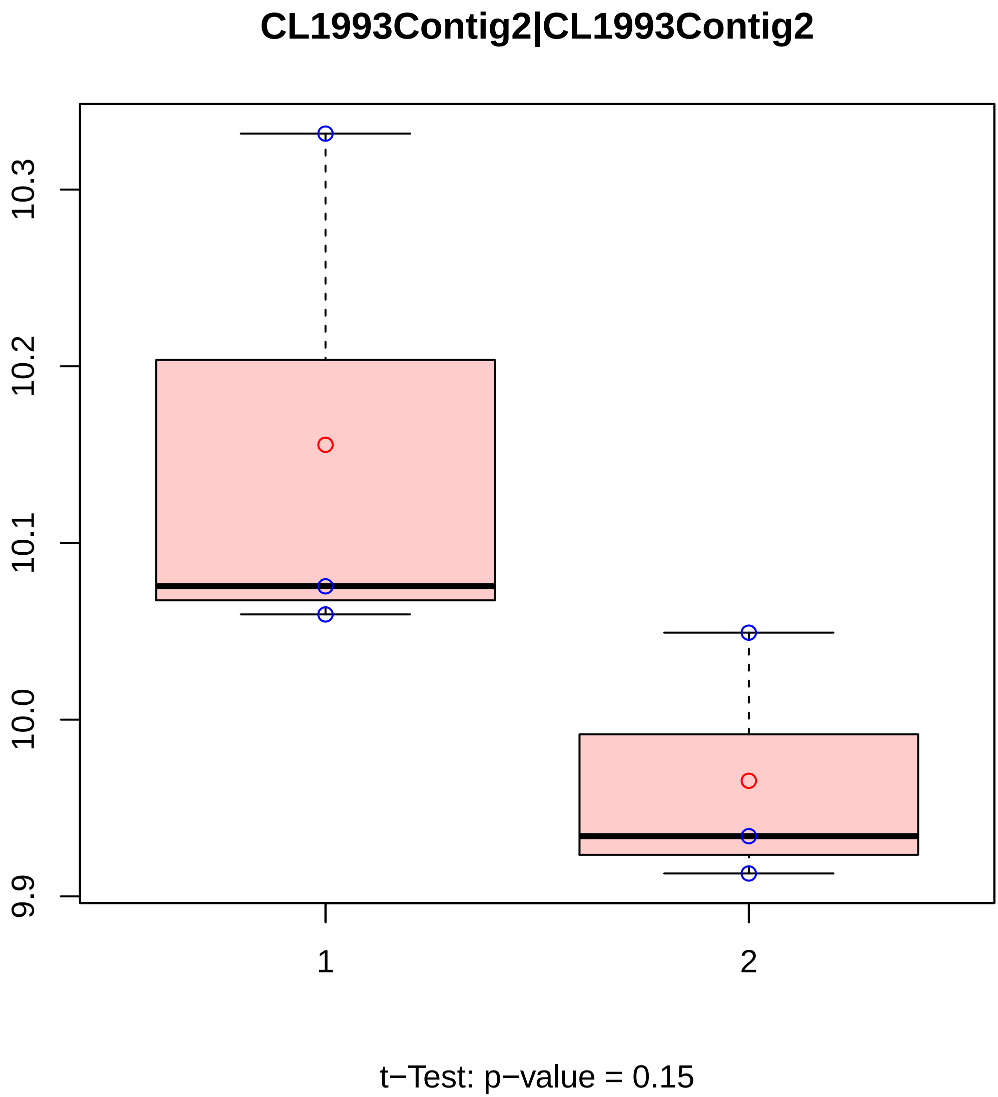
CL1993Contig2|CL1993Contig2
10.3
10.2
10.1
10.0
9.9
1
2
t−Test: p−value = 0.15

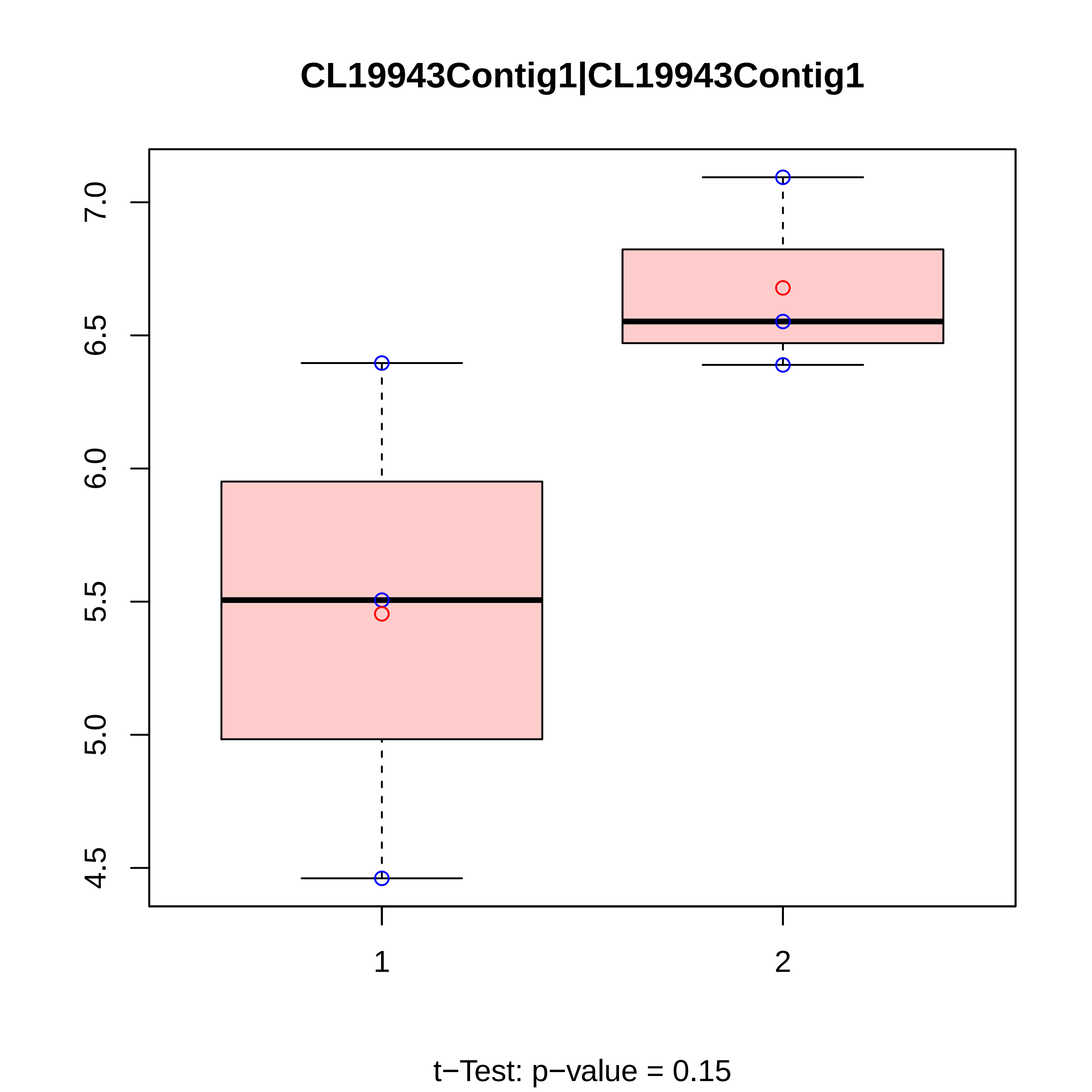

CL19943Contig1|CL19943Contig1
7.0
6.5
6.0
5.5
5.0
4.5
1
2
t−Test: p−value = 0.15

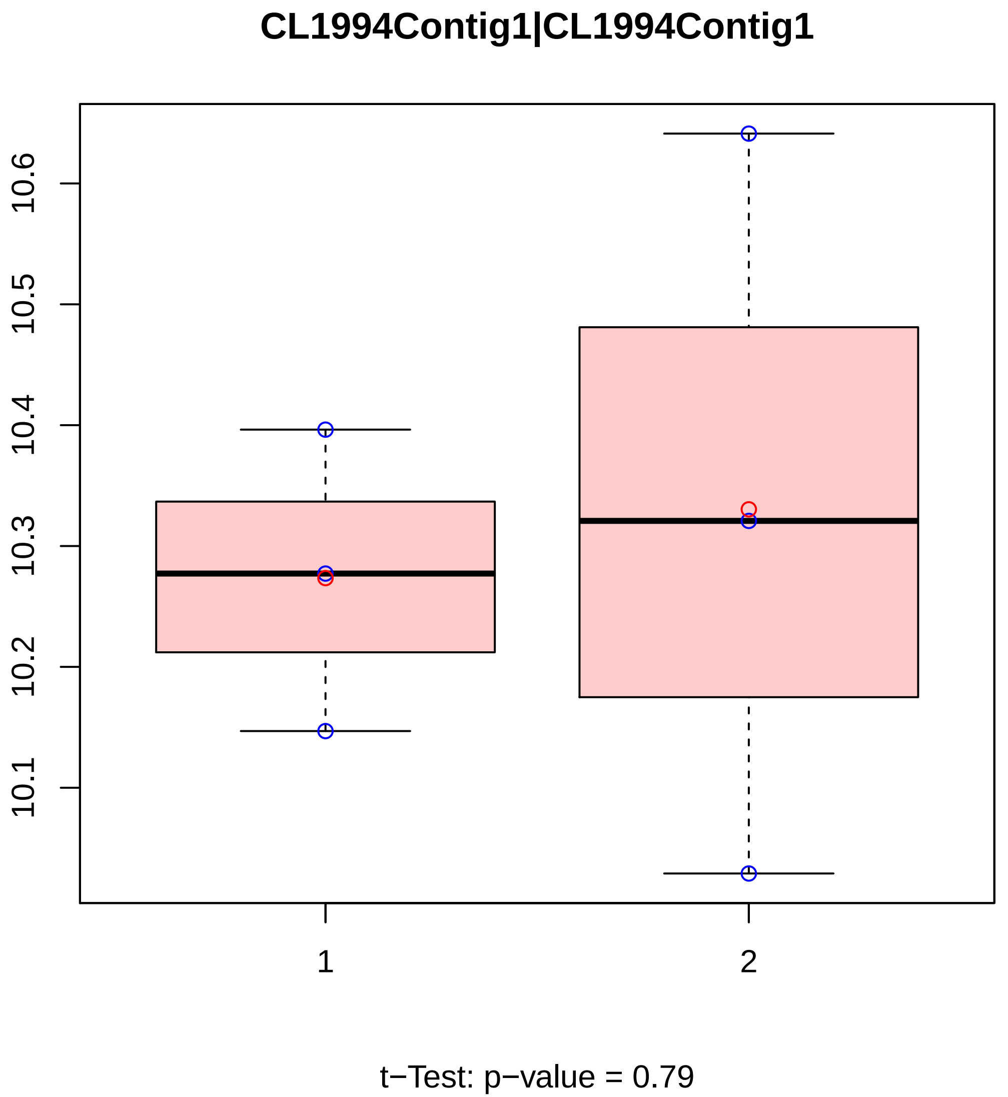

CL1994Contig1|CL1994Contig1
10.1
10.2
10.3
10.4
10.5
10.6
1
2
t−Test: p−value = 0.79

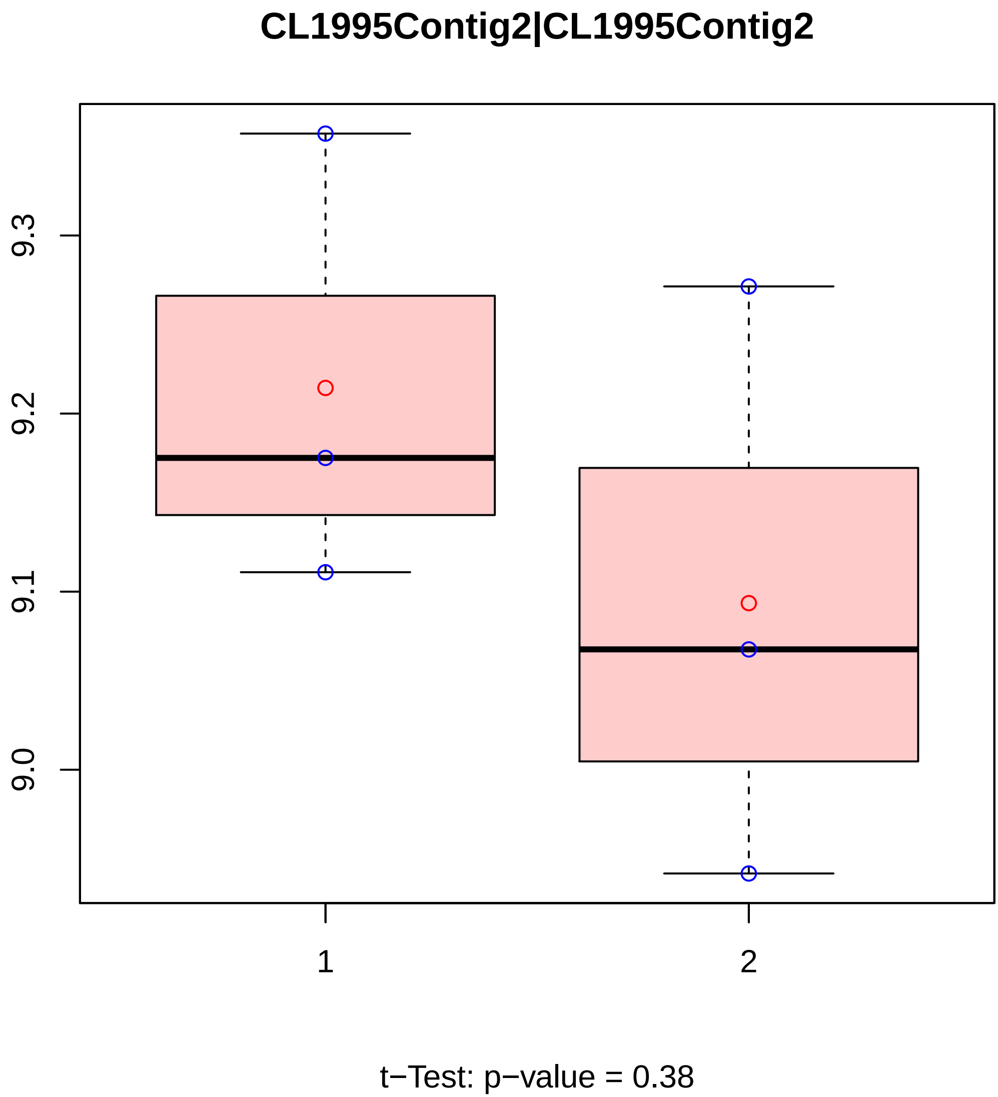
CL1995Contig2|CL1995Contig2
9.0
9.1
9.2
9.3
1
2
t−Test: p−value = 0.38

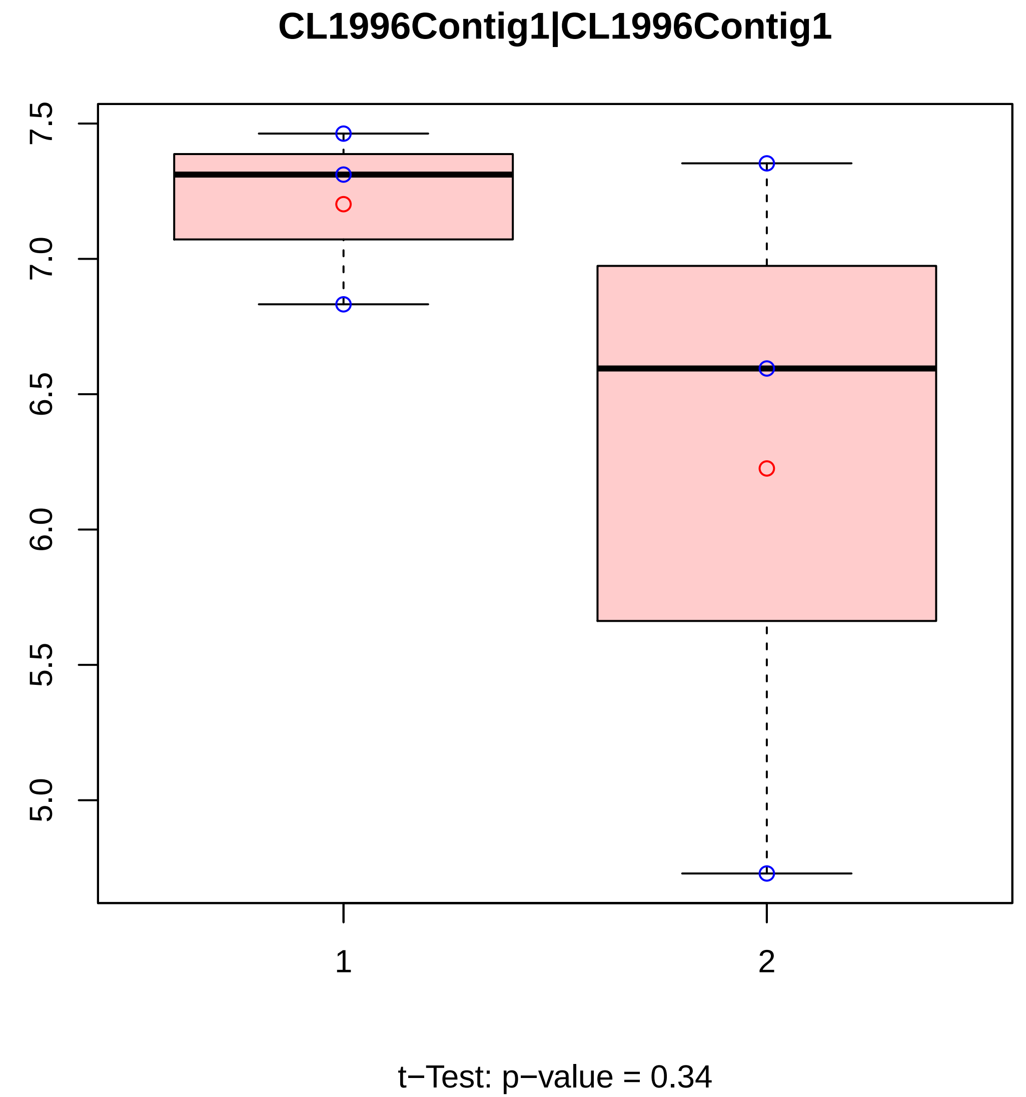

CL1996Contig1|CL1996Contig1
7.5
7.0
6.5
6.0
5.5
5.0
1
2
t−Test: p−value = 0.34

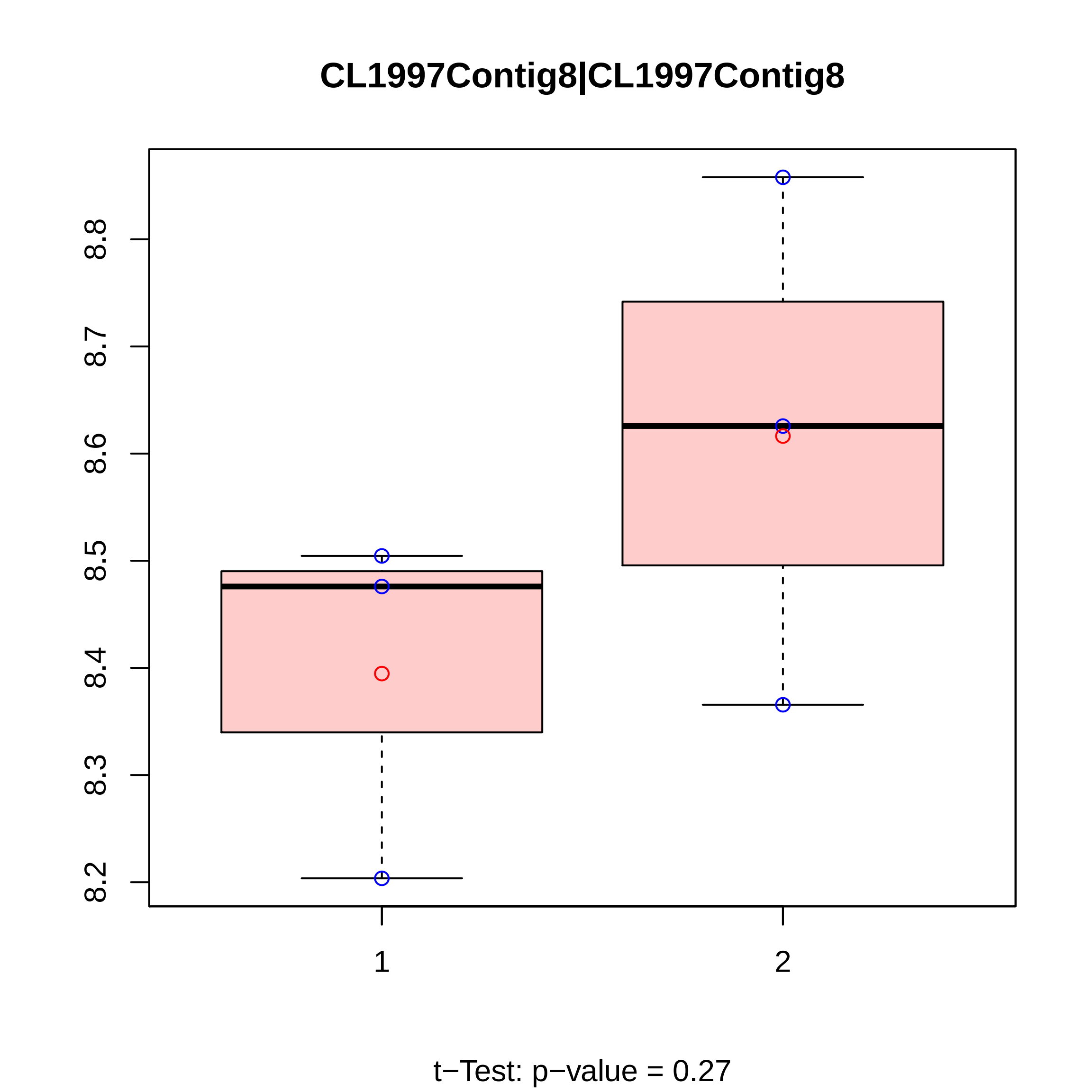
CL1997Contig8|CL1997Contig8
8.2
8.3
8.4
8.5
8.6
8.7
8.8
1
2
t−Test: p−value = 0.27

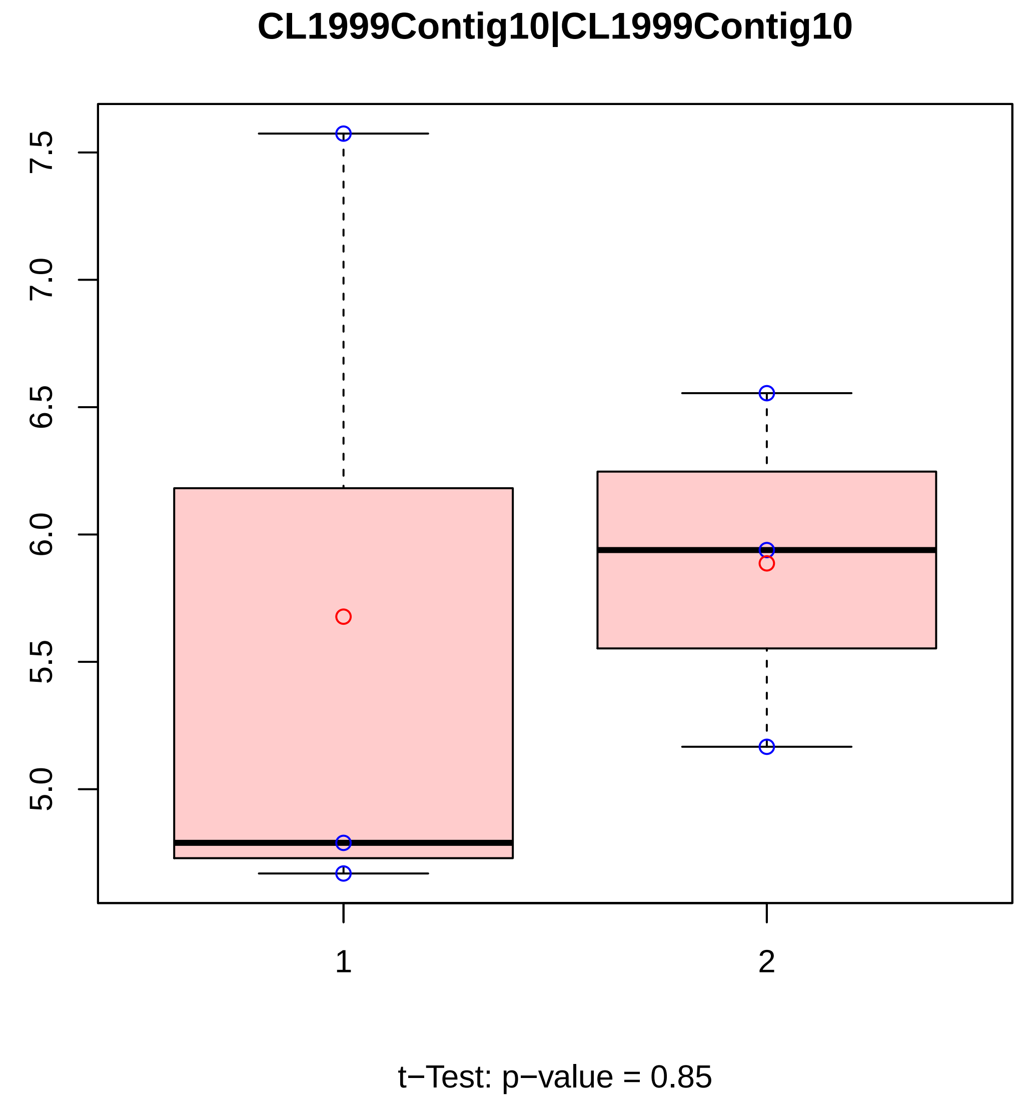
CL1999Contig10|CL1999Contig10
7.5
7.0
6.5
6.0
5.5
5.0
1
2
t−Test: p−value = 0.85

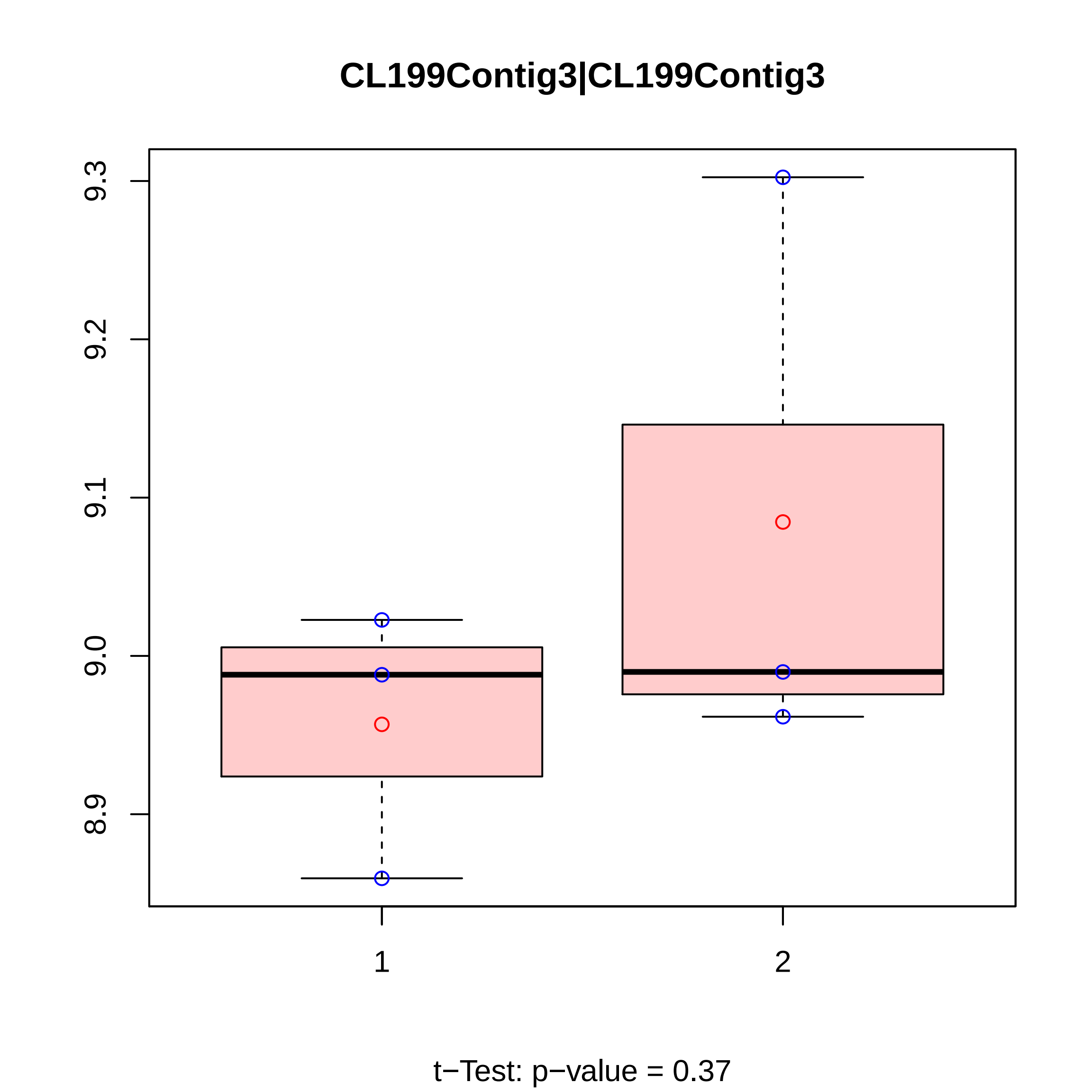
CL199Contig3|CL199Contig3
9.3
9.2
9.1
9.0
8.9
1
2
t−Test: p−value = 0.37

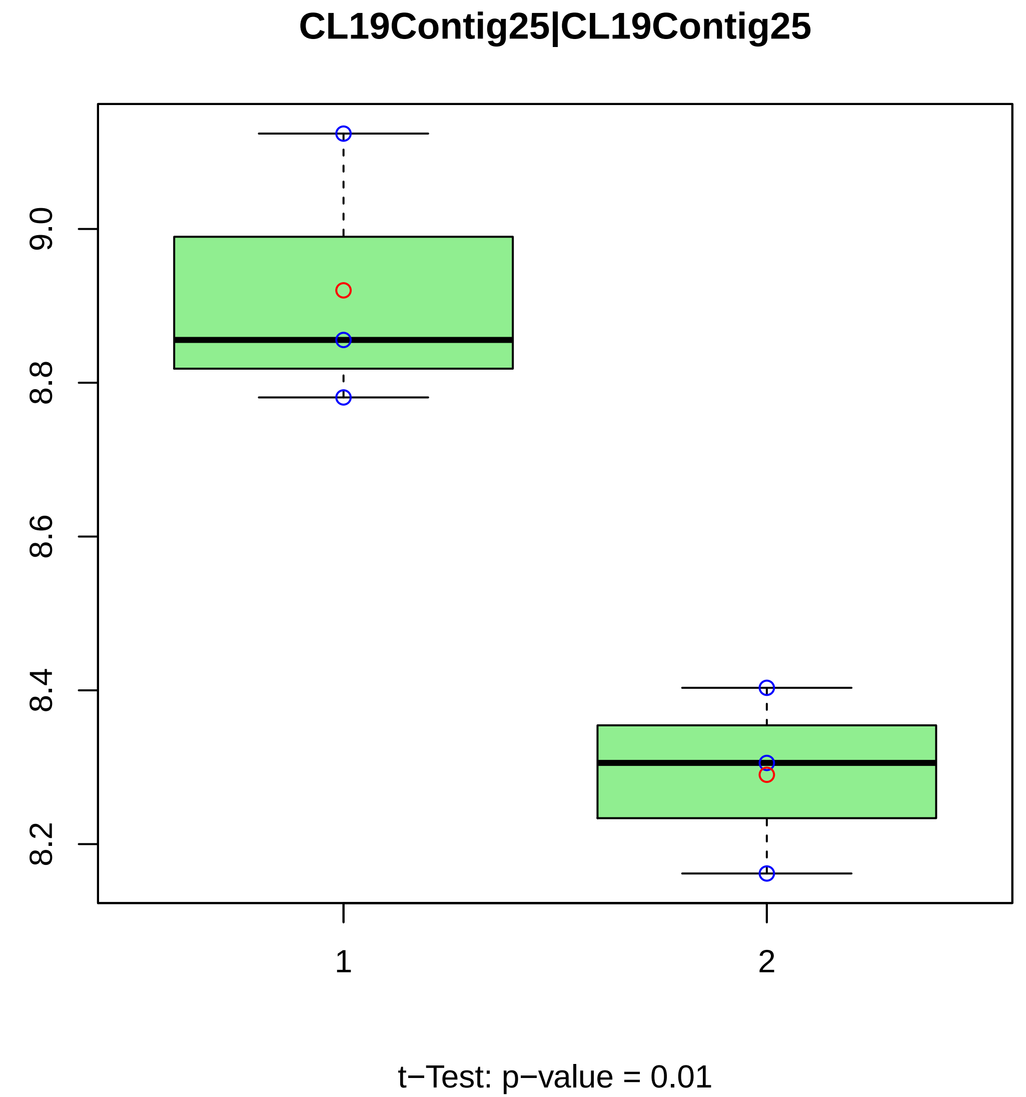

CL19Contig25|CL19Contig25
9.0
8.8
8.6
8.4
8.2
1
2
t−Test: p−value = 0.01

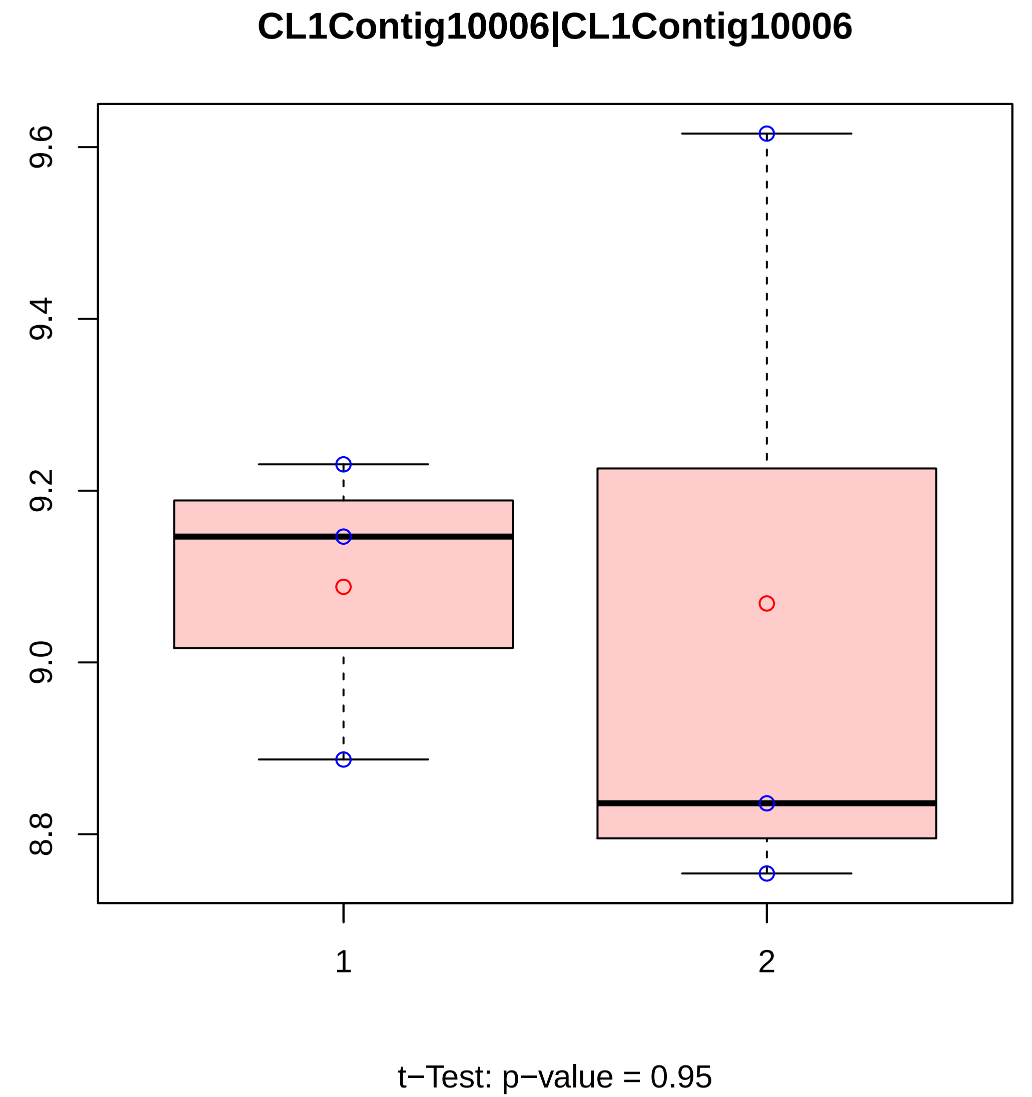
CL1Contig10006|CL1Contig10006
9.6
9.4
9.2
9.0
8.8
1
2
t−Test: p−value = 0.95

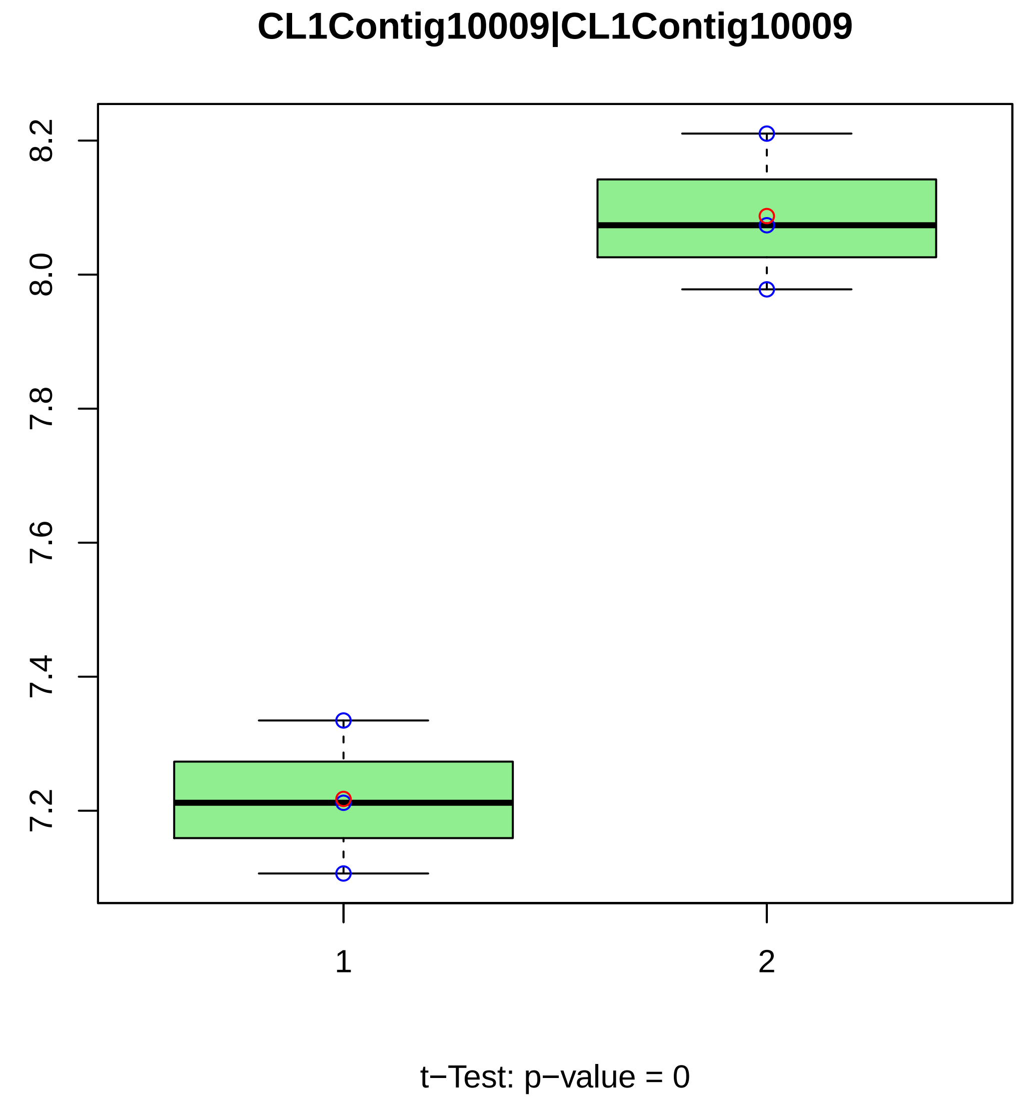

CL1Contig10009|CL1Contig10009
8.2
8.0
7.8
7.6
7.4
7.2
1
2
t−Test: p−value = 0

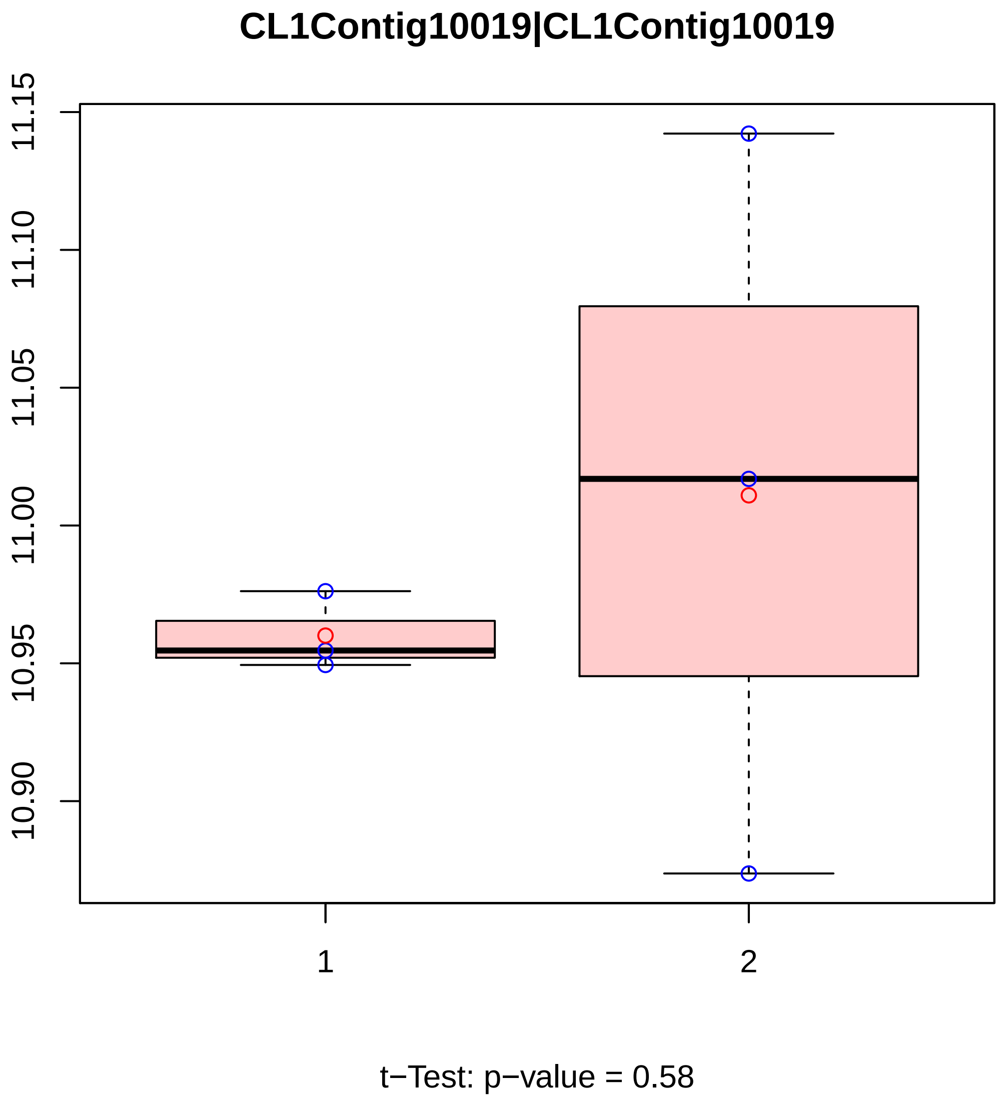

CL1Contig10019|CL1Contig10019
11.15
11.10
11.05
11.00
10.95
10.90
1
2
t−Test: p−value = 0.58

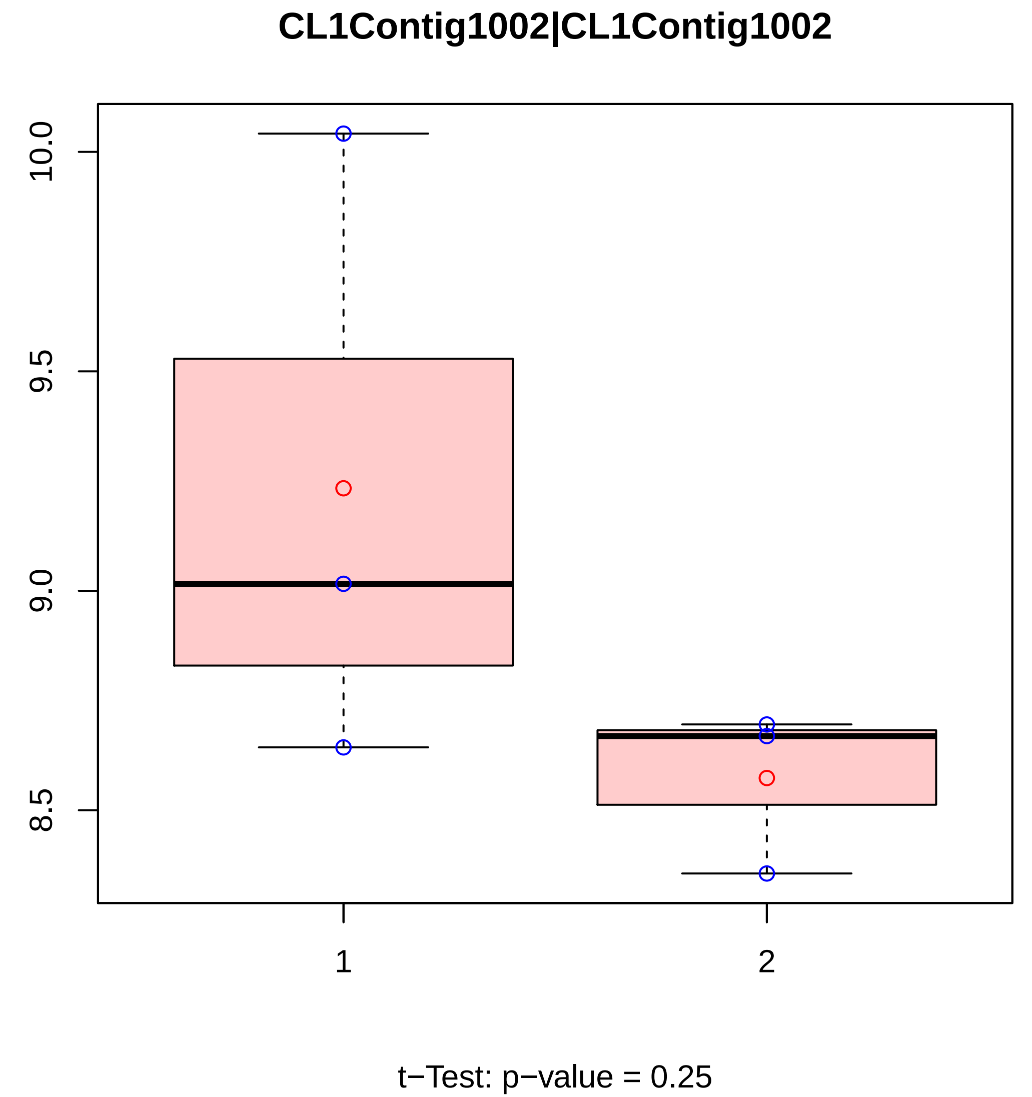
CL1Contig1002|CL1Contig1002
10.0
9.5
9.0
8.5
1
2
t−Test: p−value = 0.25

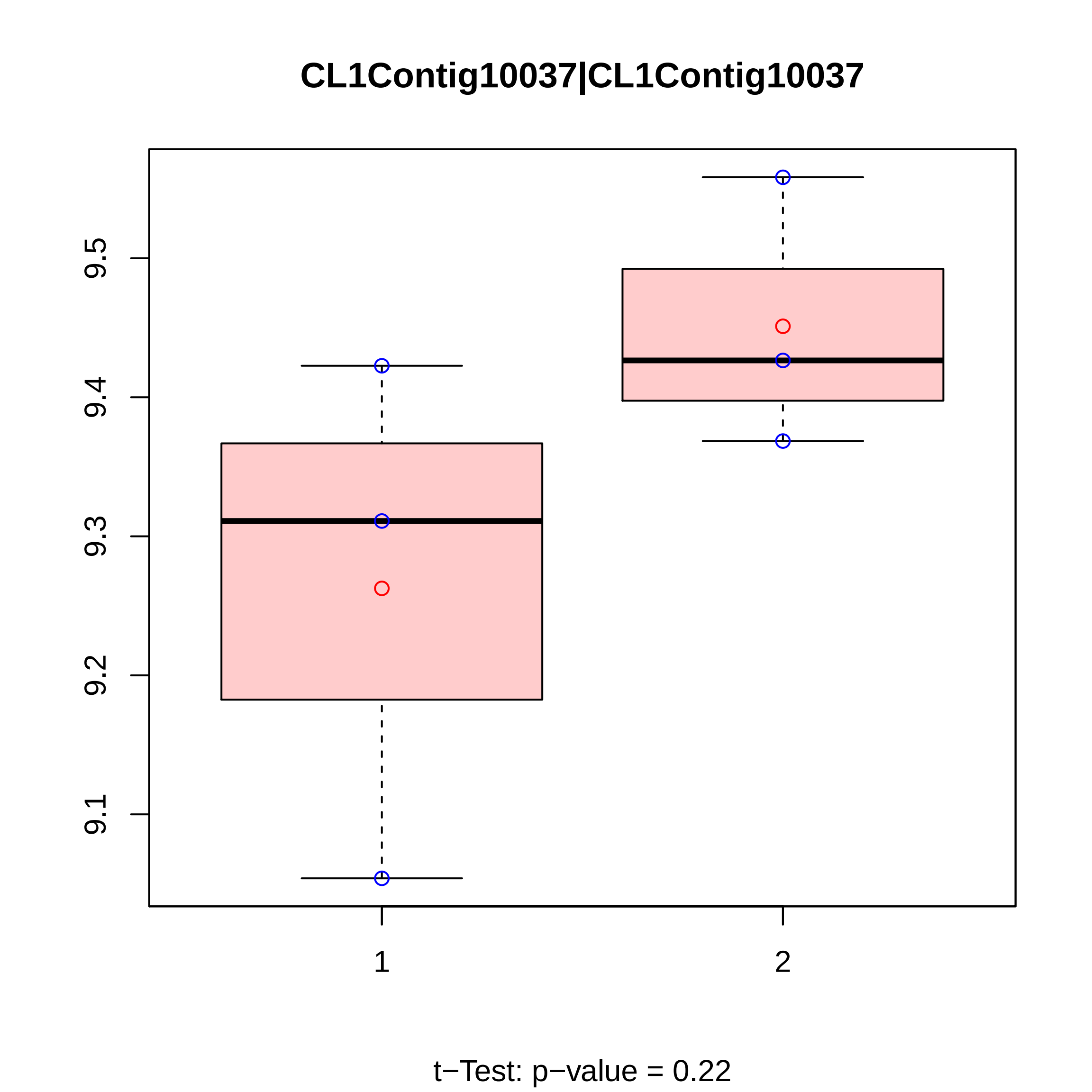
CL1Contig10037|CL1Contig10037
9.1
9.2
9.3
9.4
9.5
1
2
t−Test: p−value = 0.22

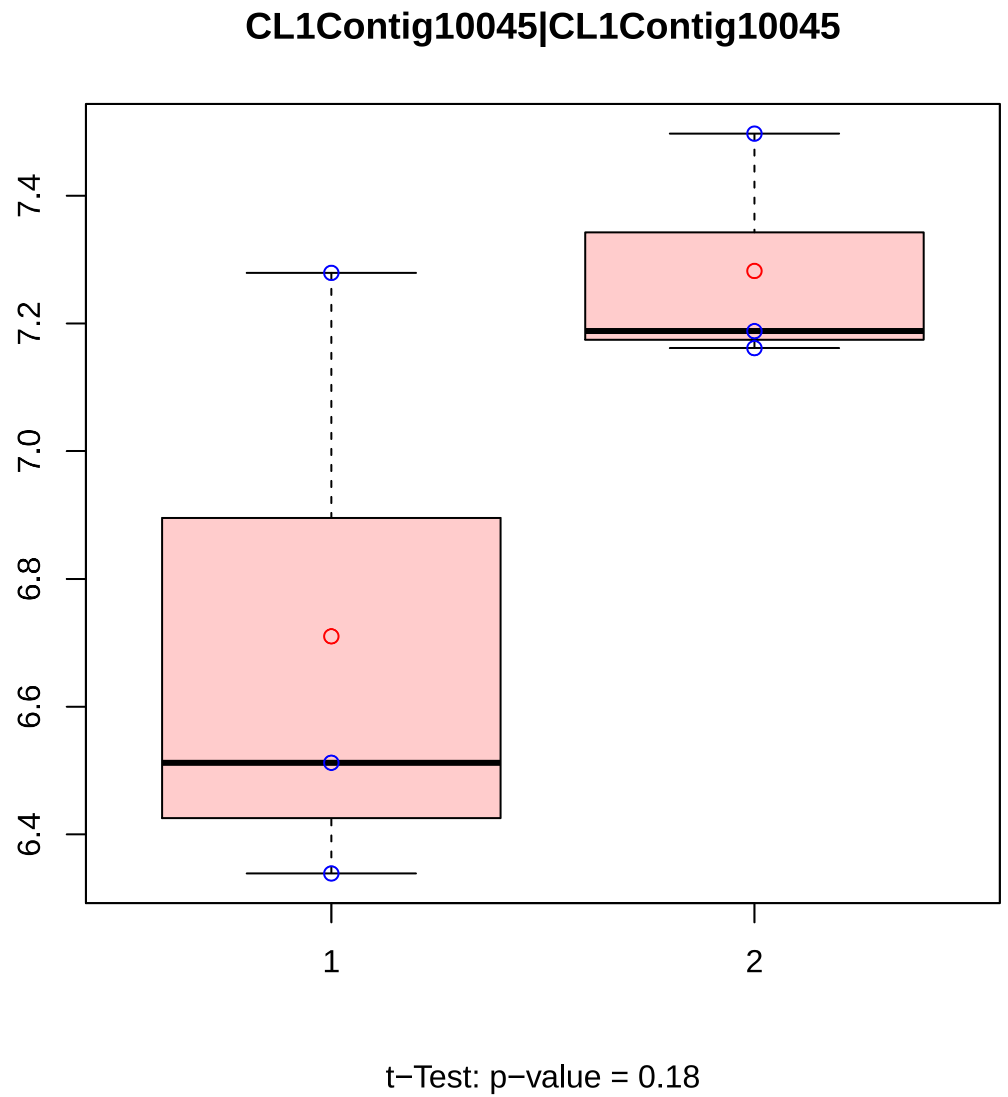

CL1Contig10045|CL1Contig10045
7.4
7.2
7.0
6.8
6.6
6.4
1
2
t−Test: p−value = 0.18

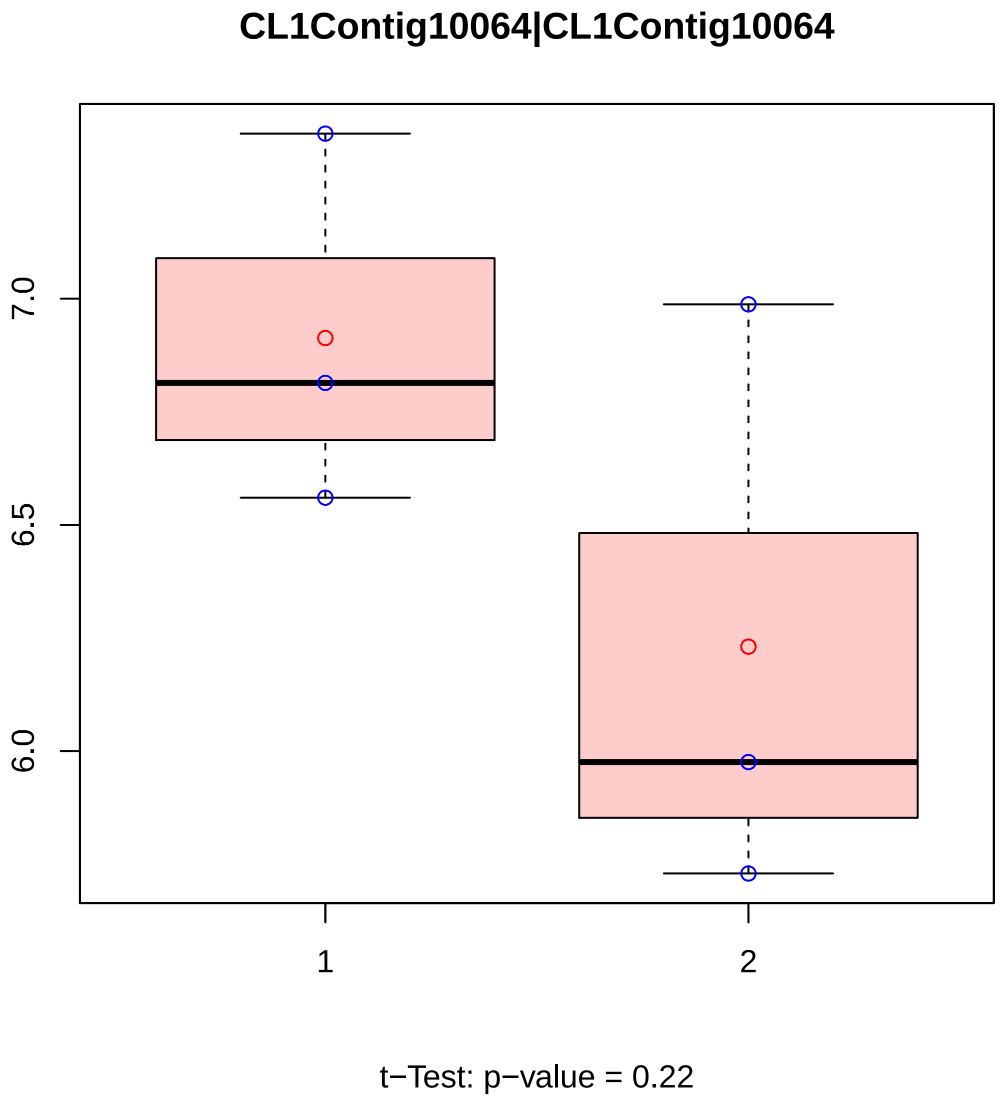
CL1Contig10064|CL1Contig10064
7.0
6.5
6.0
1
2
t−Test: p−value = 0.22

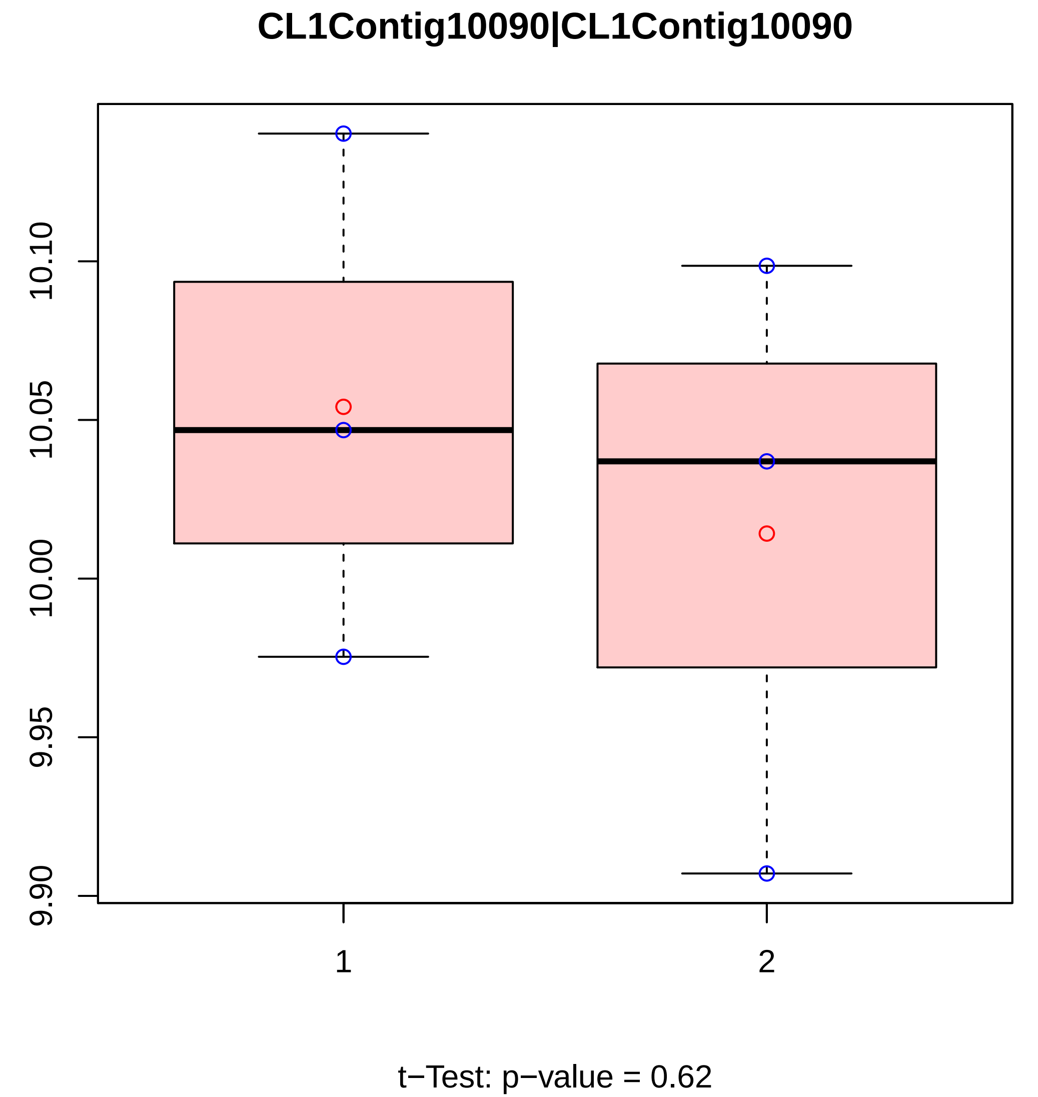
CL1Contig10090|CL1Contig10090
10.10
10.05
10.00
9.95
9.90
1
2
t−Test: p−value = 0.62

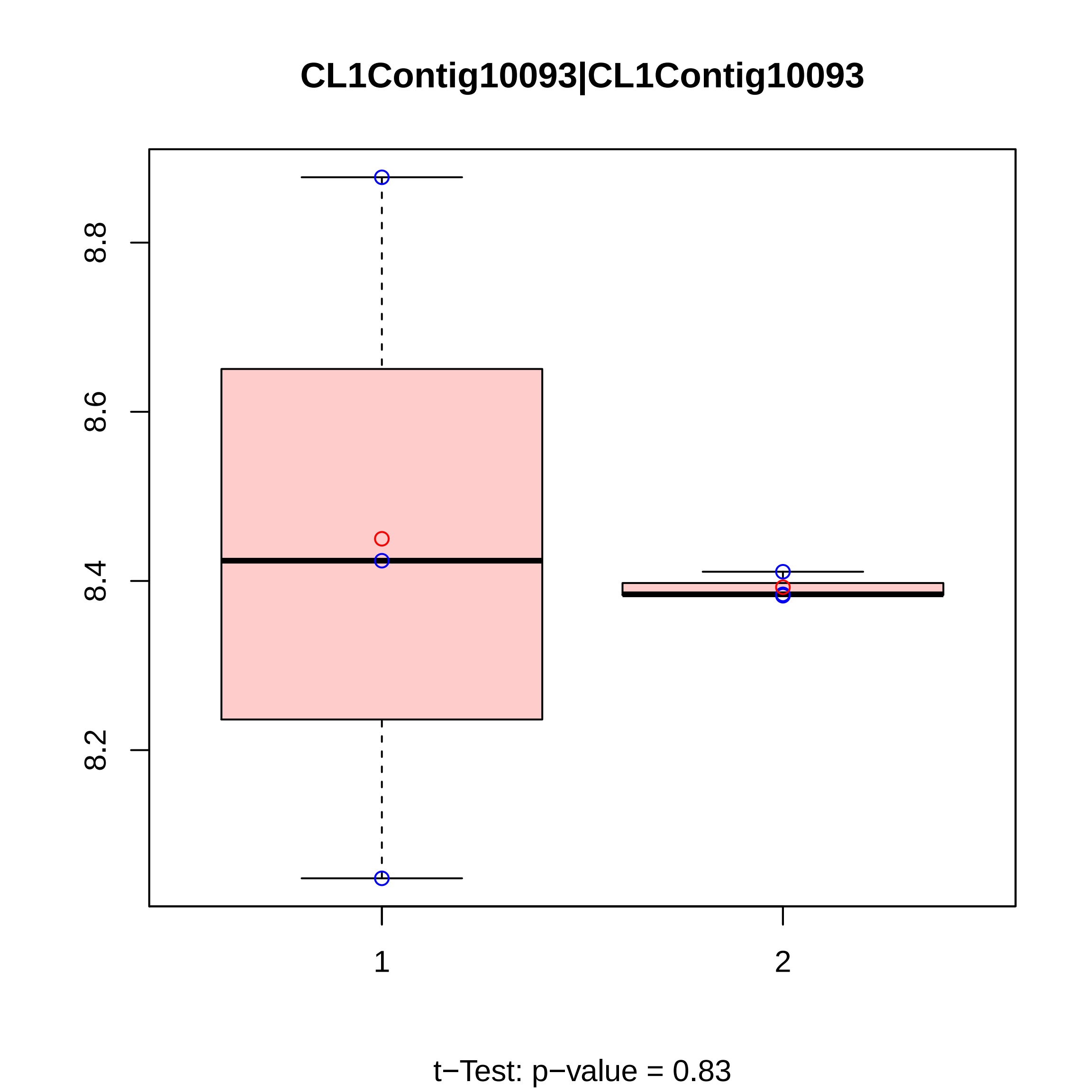

CL1Contig10093|CL1Contig10093
8.2
8.4
8.6
8.8
1
2
t−Test: p−value = 0.83

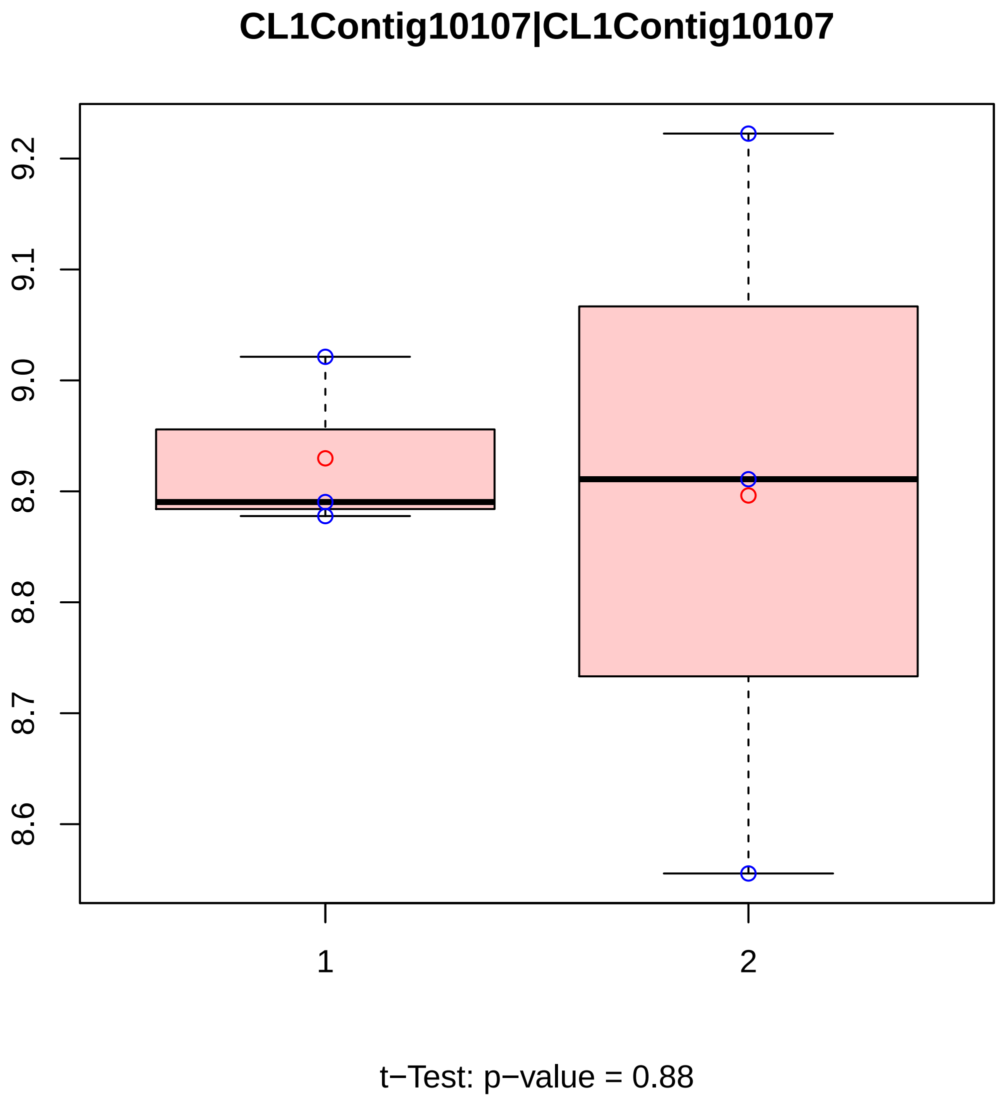
CL1Contig10107|CL1Contig10107
9.2
9.1
9.0
8.9
8.8
8.7
8.6
1
2
t−Test: p−value = 0.88

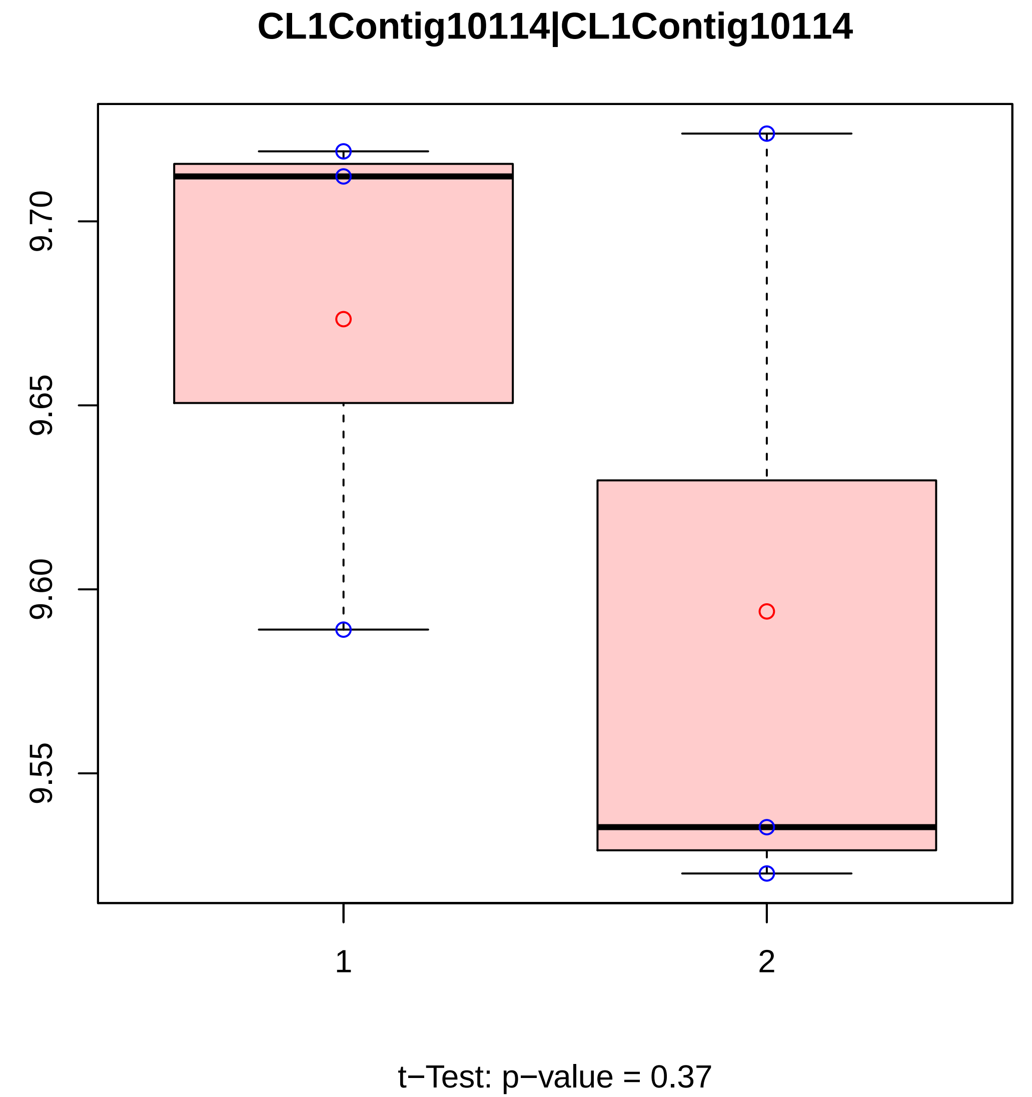
CL1Contig10114|CL1Contig10114
9.70
9.65
9.60
9.55
1
2
t−Test: p−value = 0.37

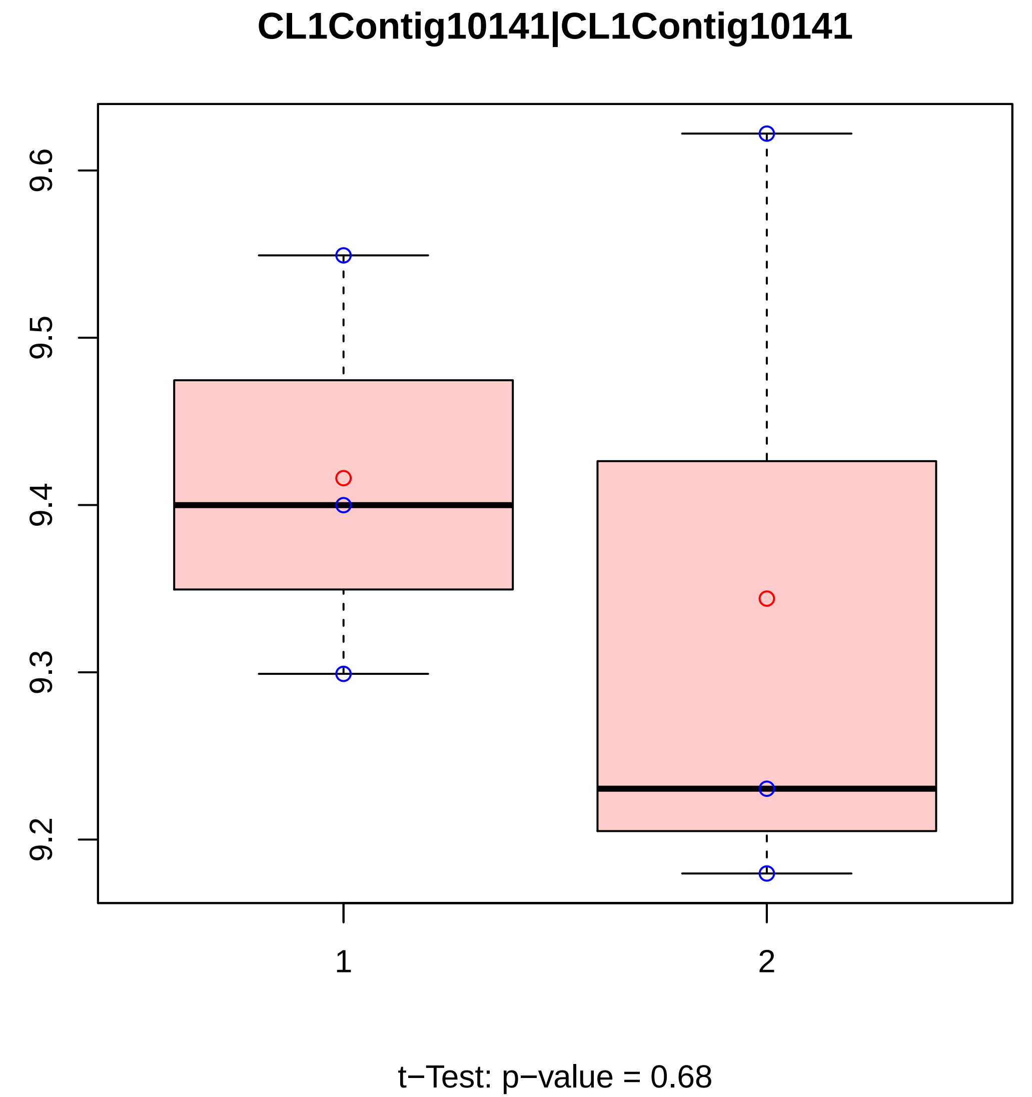
CL1Contig10141|CL1Contig10141
9.2
9.3
9.4
9.5
9.6
1
2
t−Test: p−value = 0.68

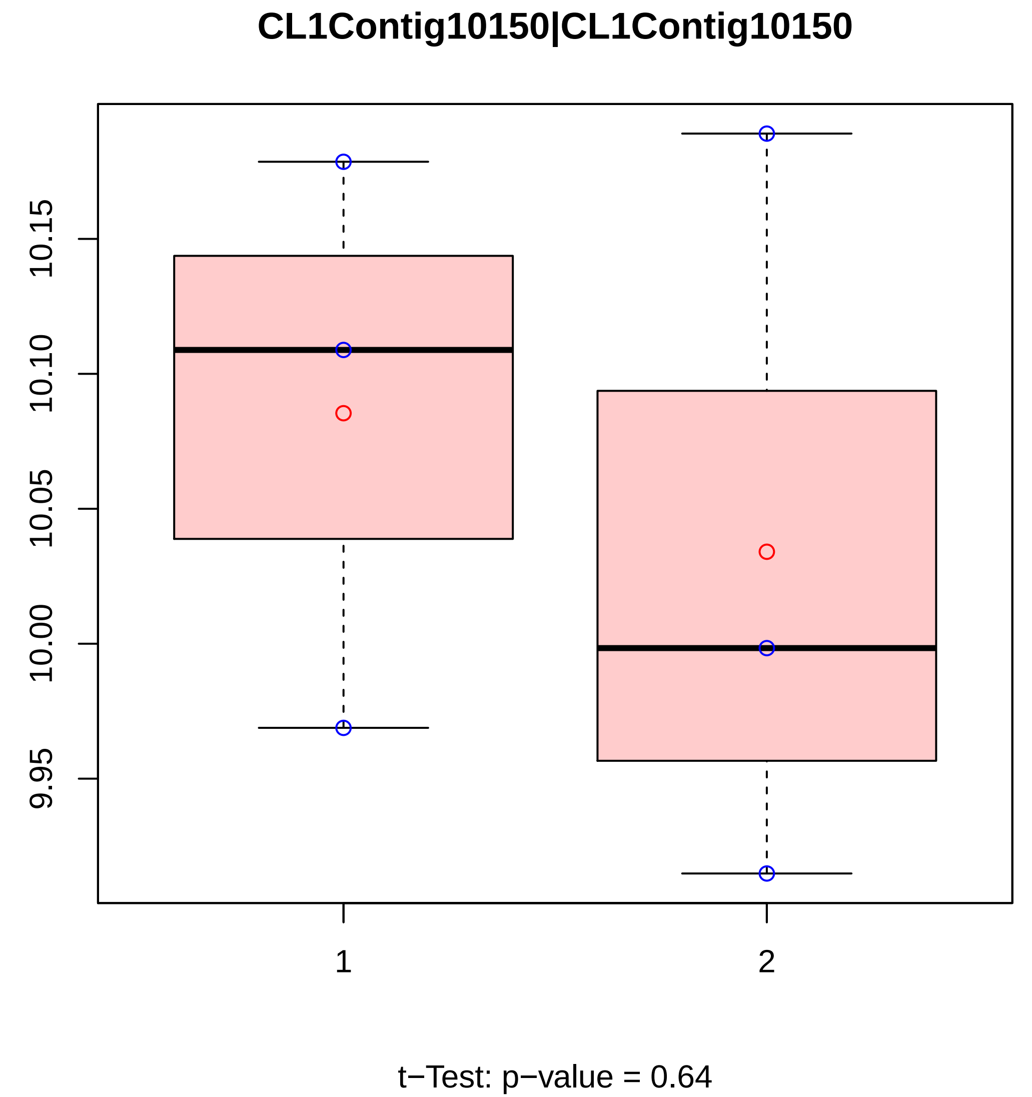
CL1Contig10150|CL1Contig10150
10.15
10.10
10.05
10.00
9.95
1
2
t−Test: p−value = 0.64

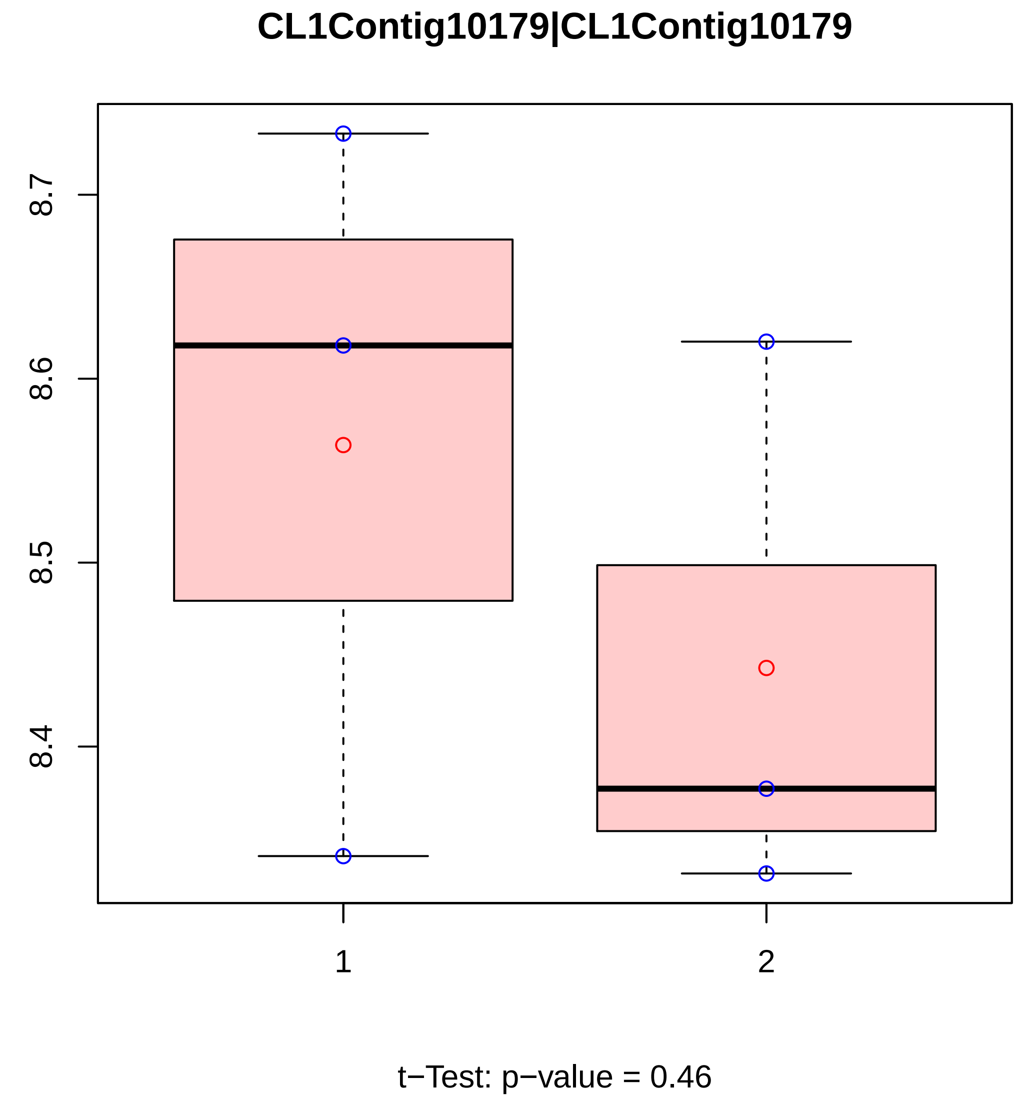
CL1Contig10179|CL1Contig10179
8.4
8.5
8.6
8.7
1
2
t−Test: p−value = 0.46

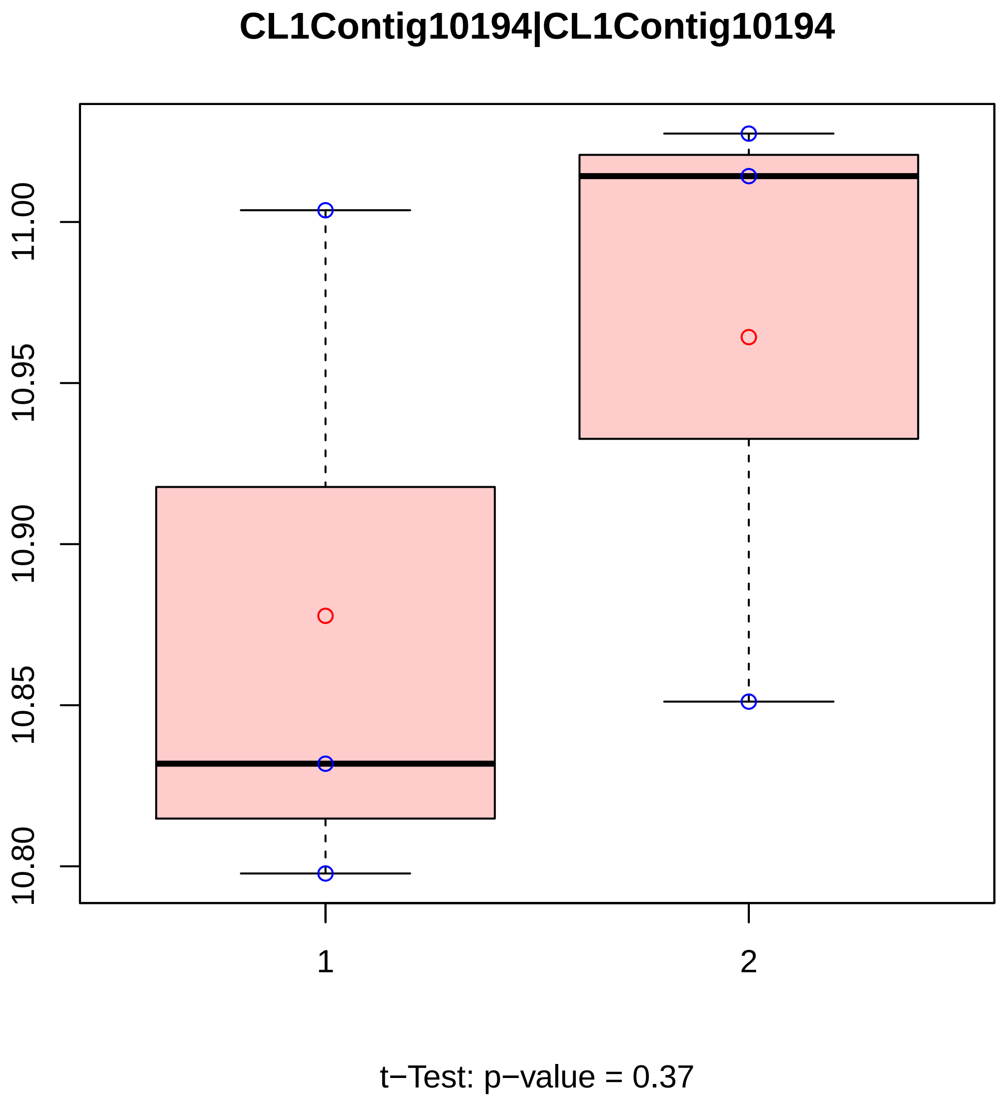

CL1Contig10194|CL1Contig10194
11.00
10.95
10.90
10.85
10.80
1
2
t−Test: p−value = 0.37

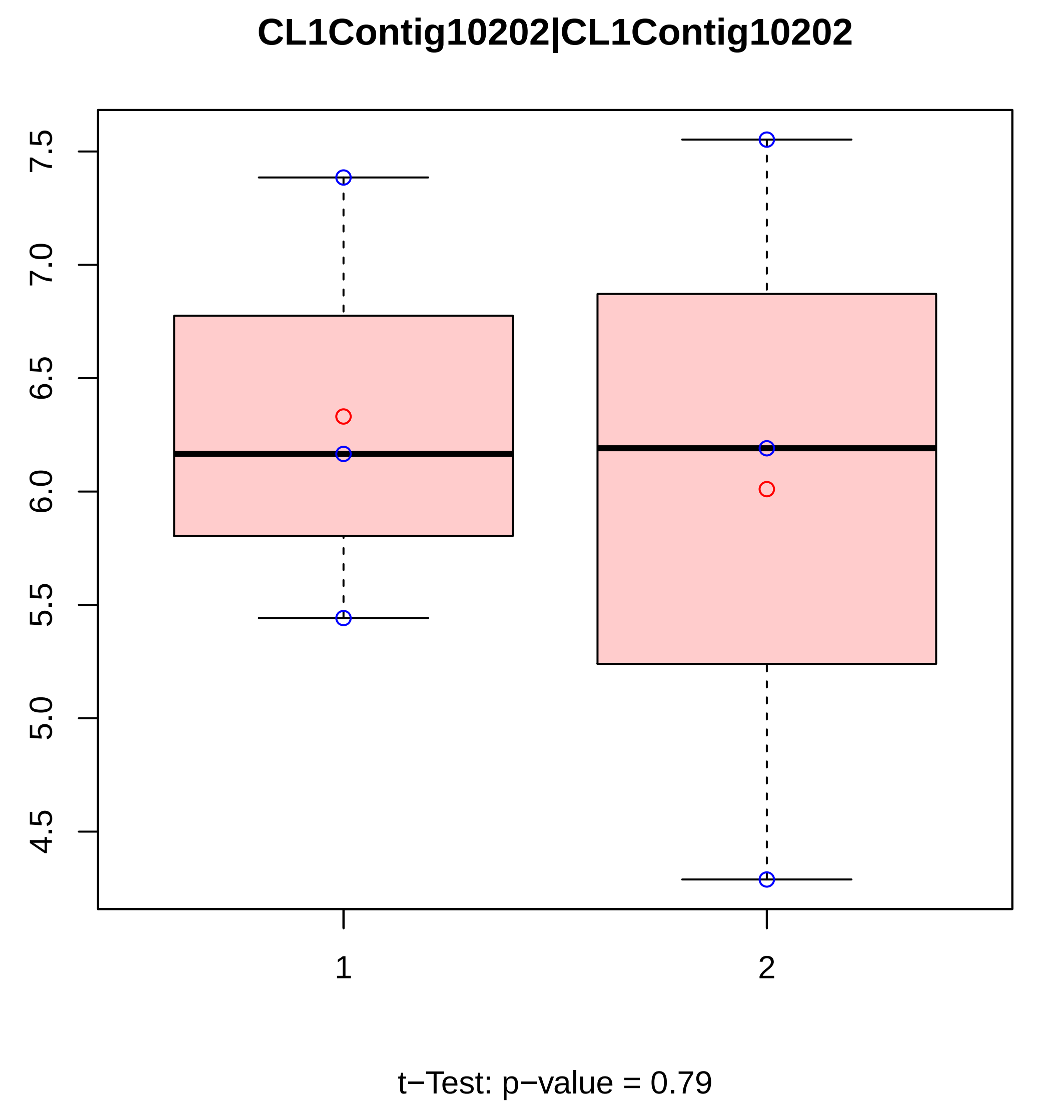

CL1Contig10202|CL1Contig10202
7.5
7.0
6.5
6.0
5.5
5.0
4.5
1
2
t−Test: p−value = 0.79

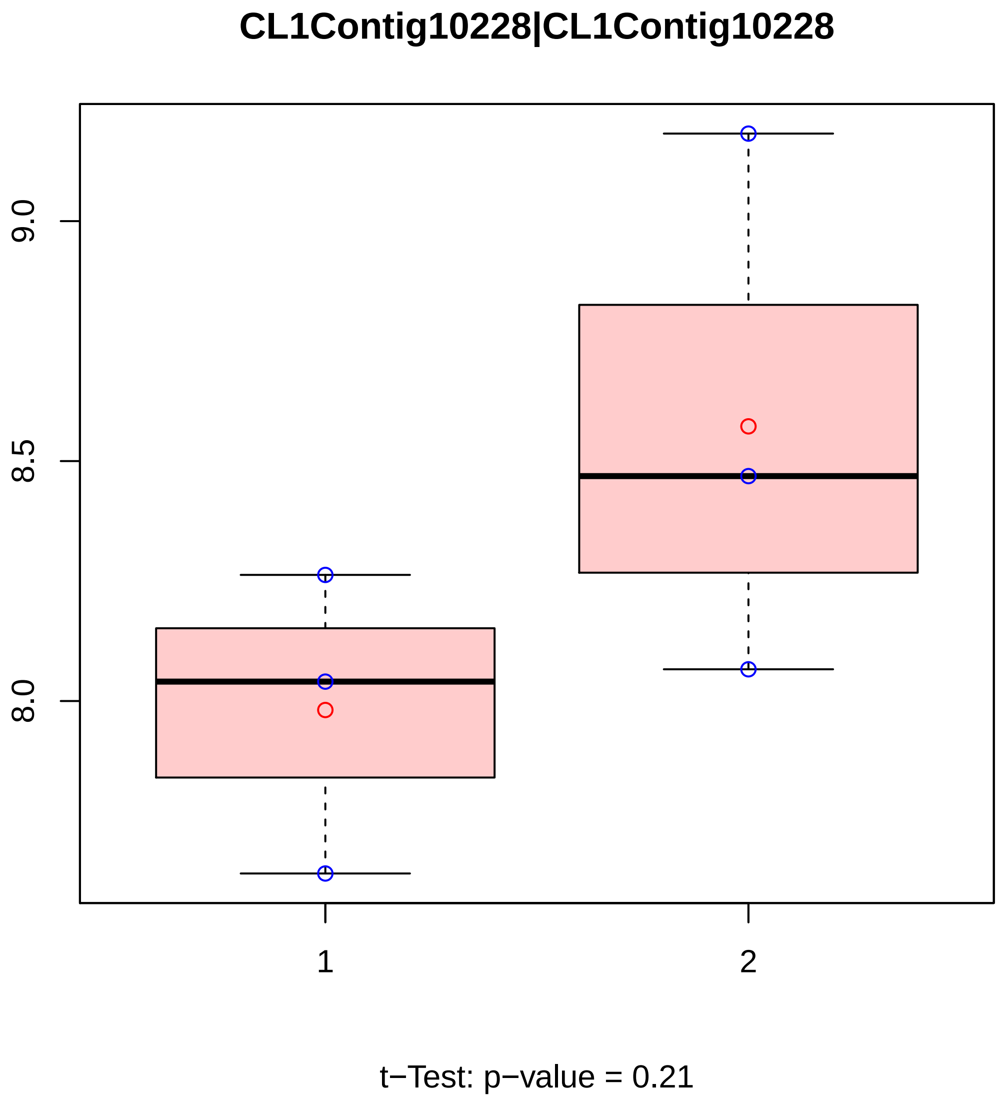
CL1Contig10228|CL1Contig10228
9.0
8.5
8.0
1
2
t−Test: p−value = 0.21

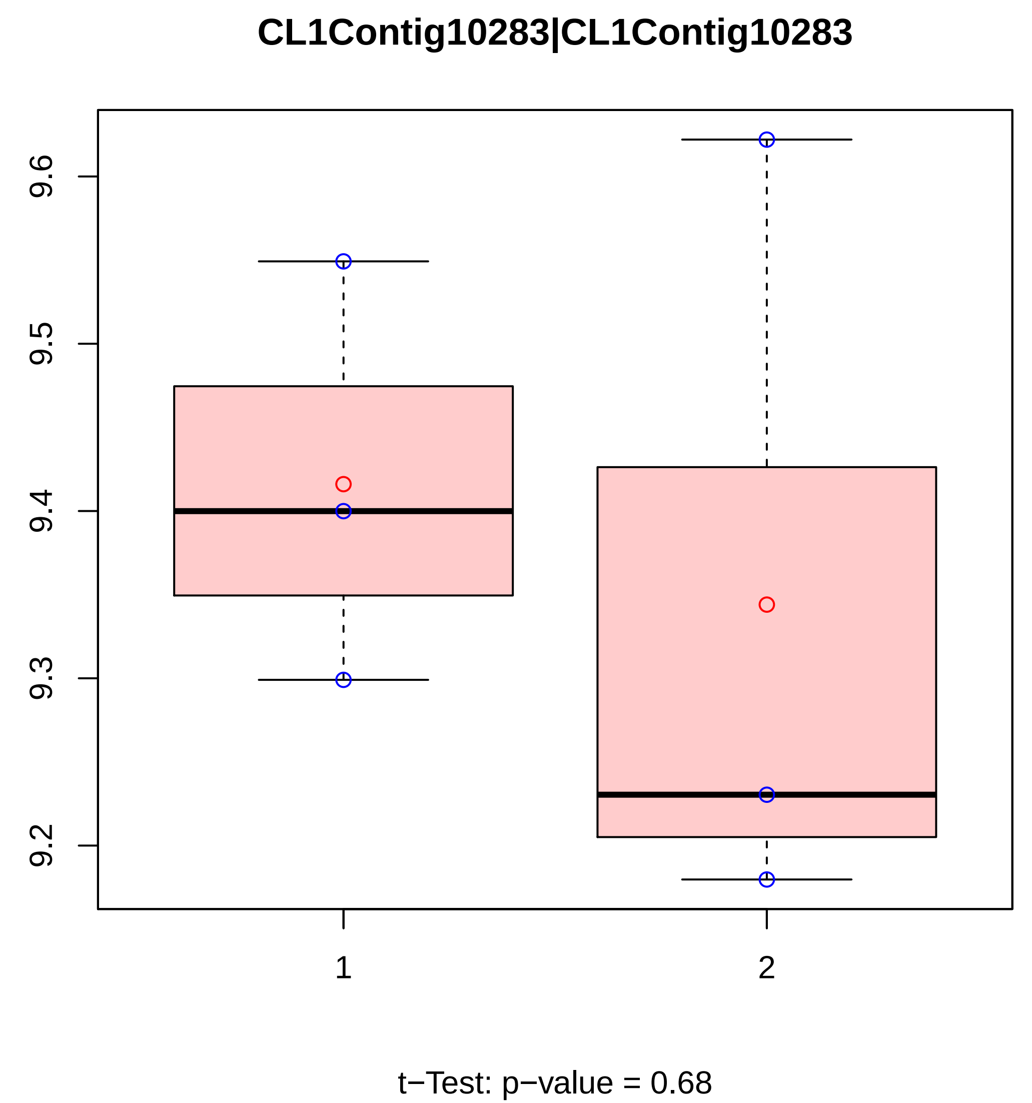
CL1Contig10283|CL1Contig10283
9.2
9.3
9.4
9.5
9.6
1
2
t−Test: p−value = 0.68

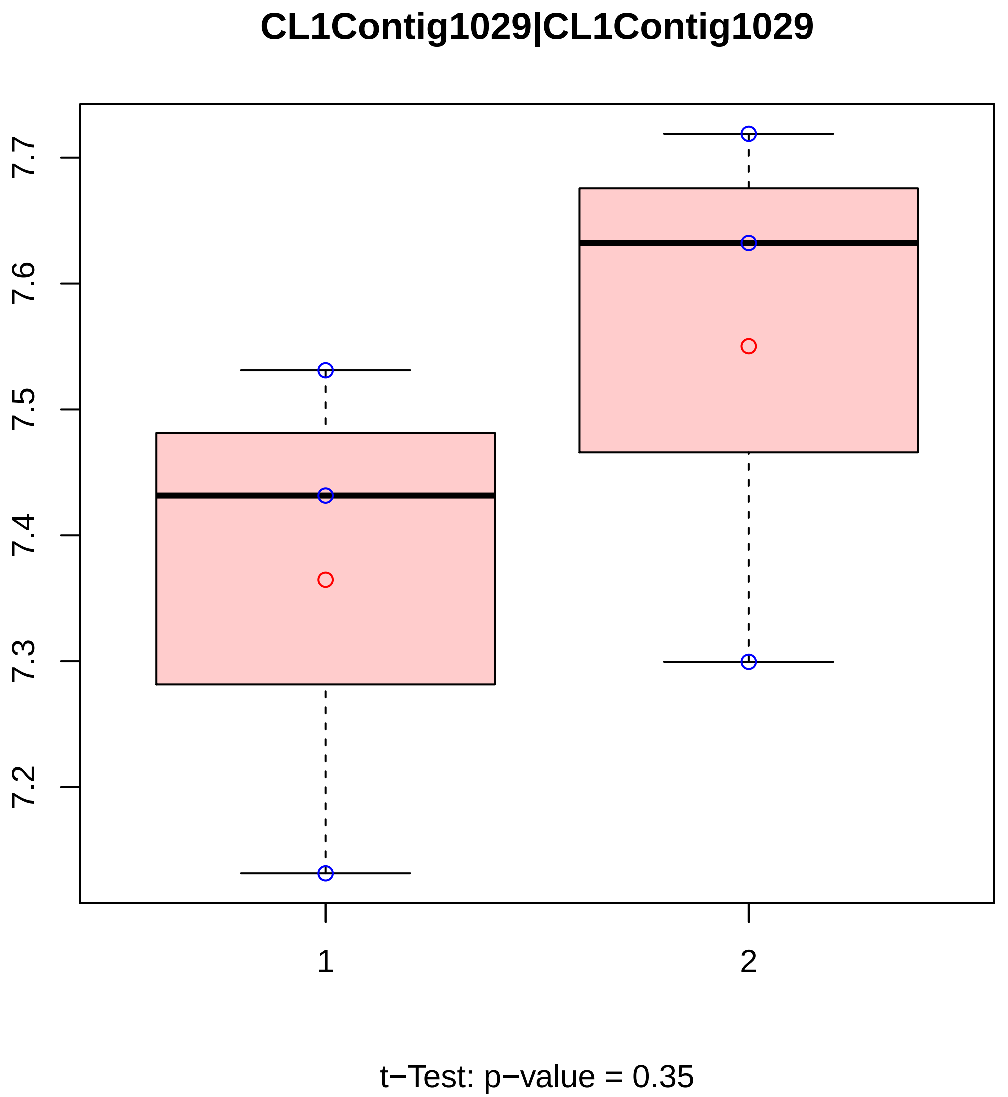
CL1Contig1029|CL1Contig1029
7.2
7.3
7.4
7.5
7.6
7.7
1
2
t−Test: p−value = 0.35

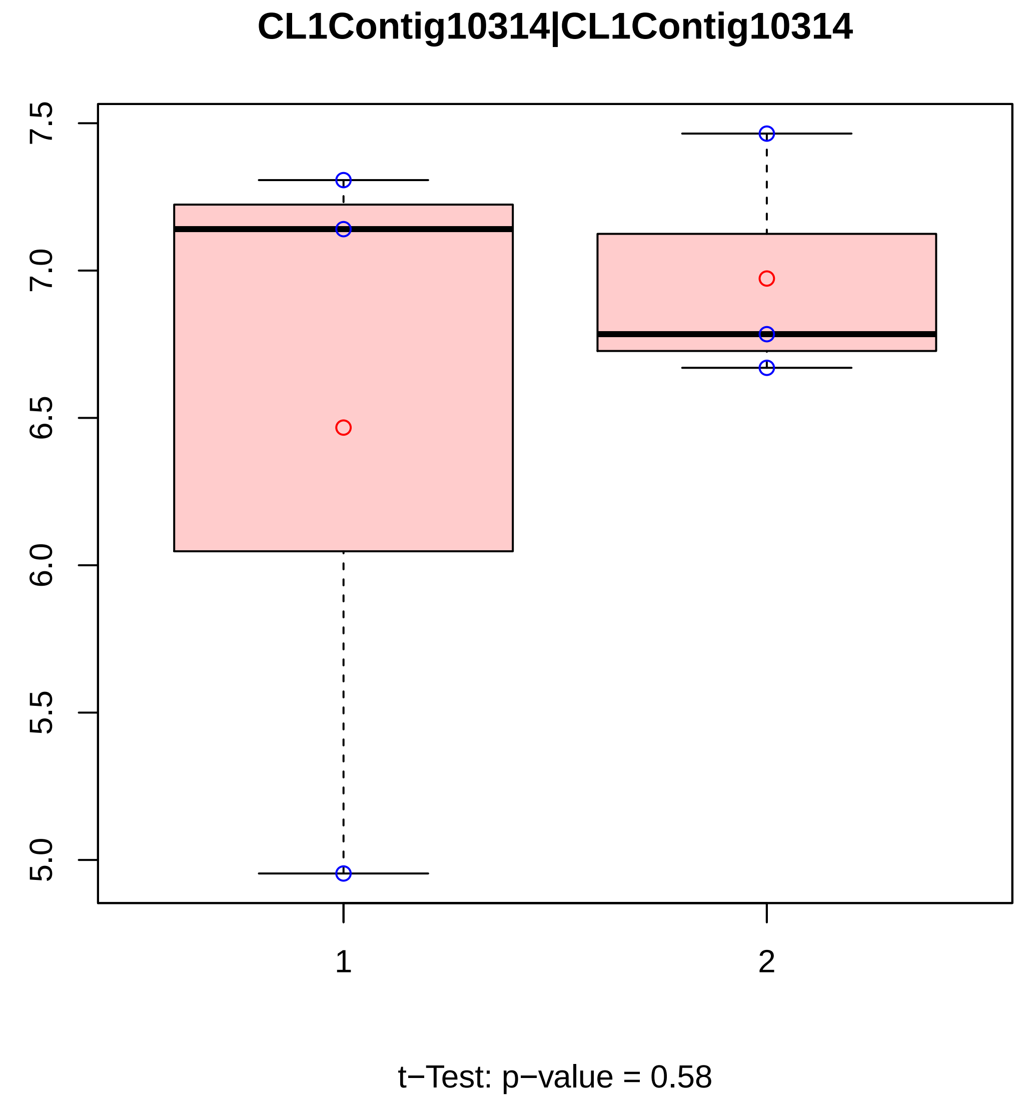
CL1Contig10314|CL1Contig10314
7.5
7.0
6.5
6.0
5.5
5.0
1
2
t−Test: p−value = 0.58

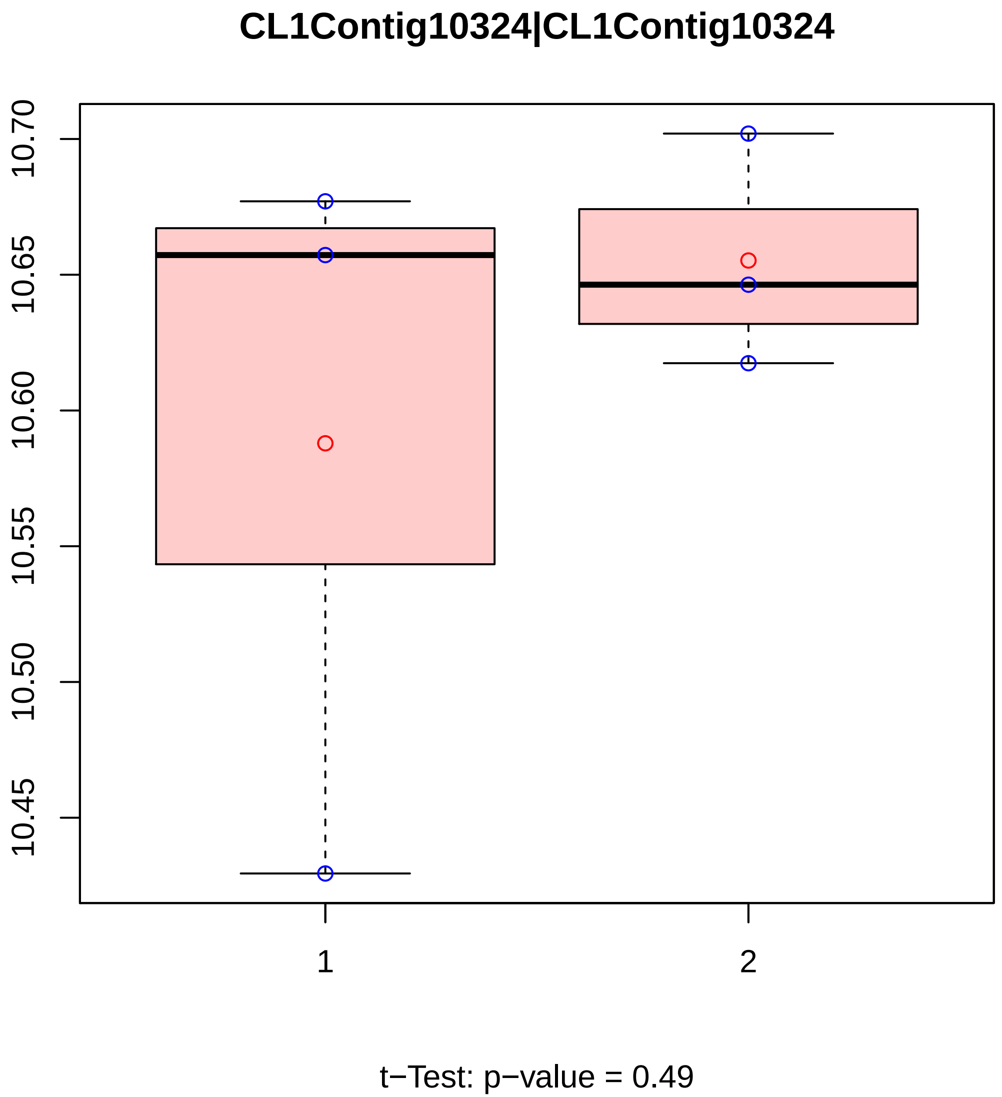
CL1Contig10324|CL1Contig10324
10.70
10.65
10.60
10.55
10.50
10.45
1
2
t−Test: p−value = 0.49

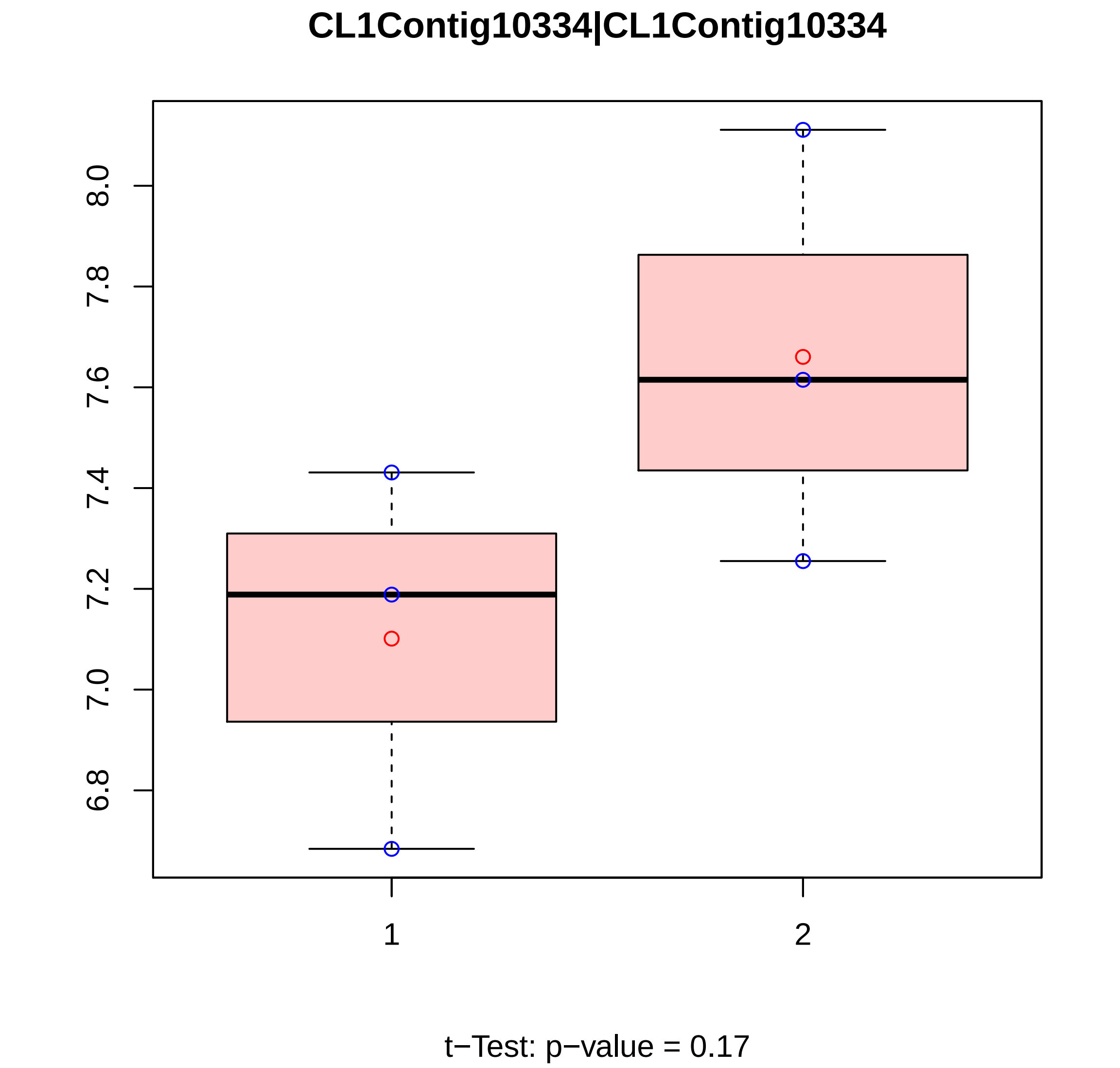

CL1Contig10334|CL1Contig10334
8.0
7.8
7.6
7.4
7.2
7.0
6.8
1
2
t−Test: p−value = 0.17

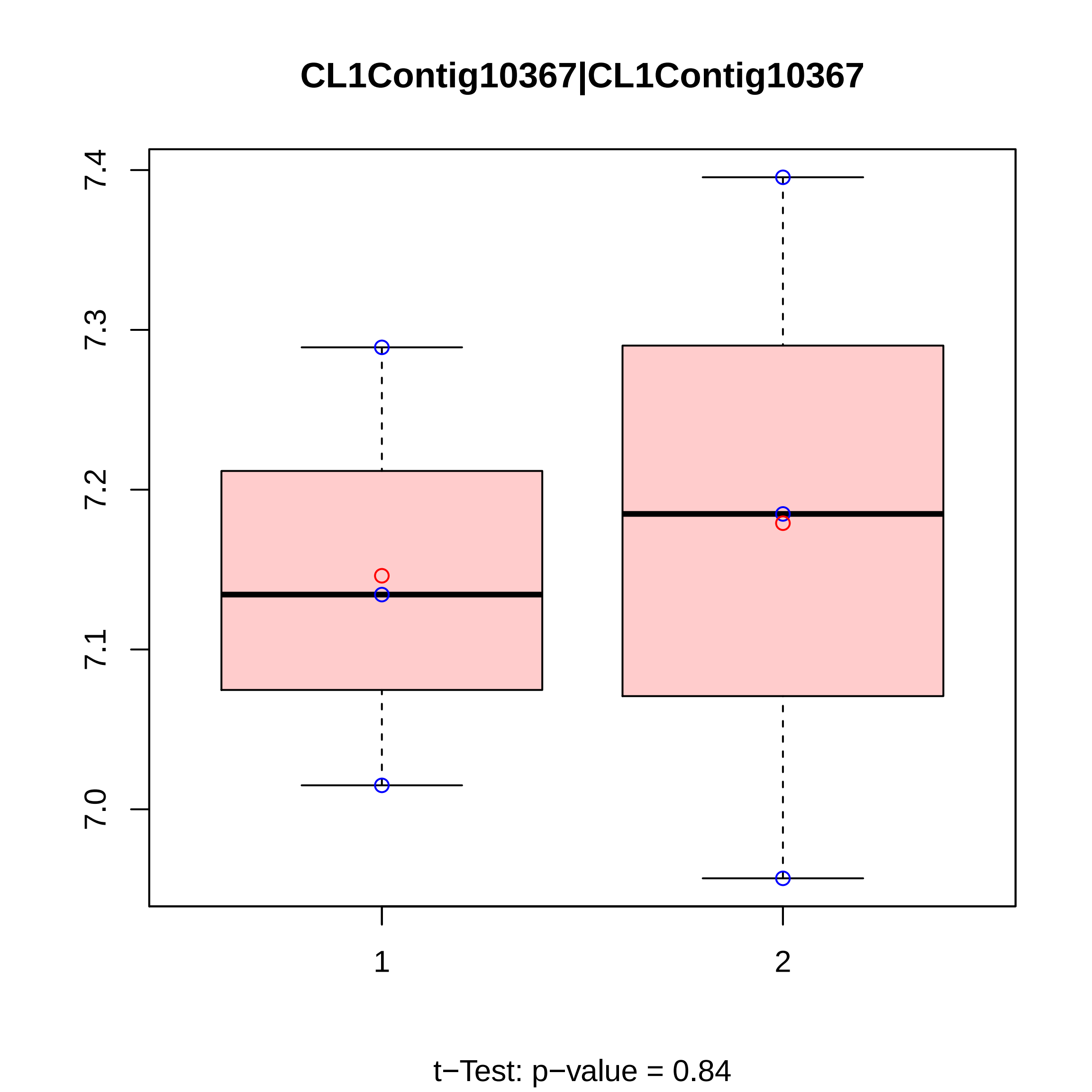
CL1Contig10367|CL1Contig10367
7.4
7.3
7.2
7.1
7.0
1
2
t−Test: p−value = 0.84

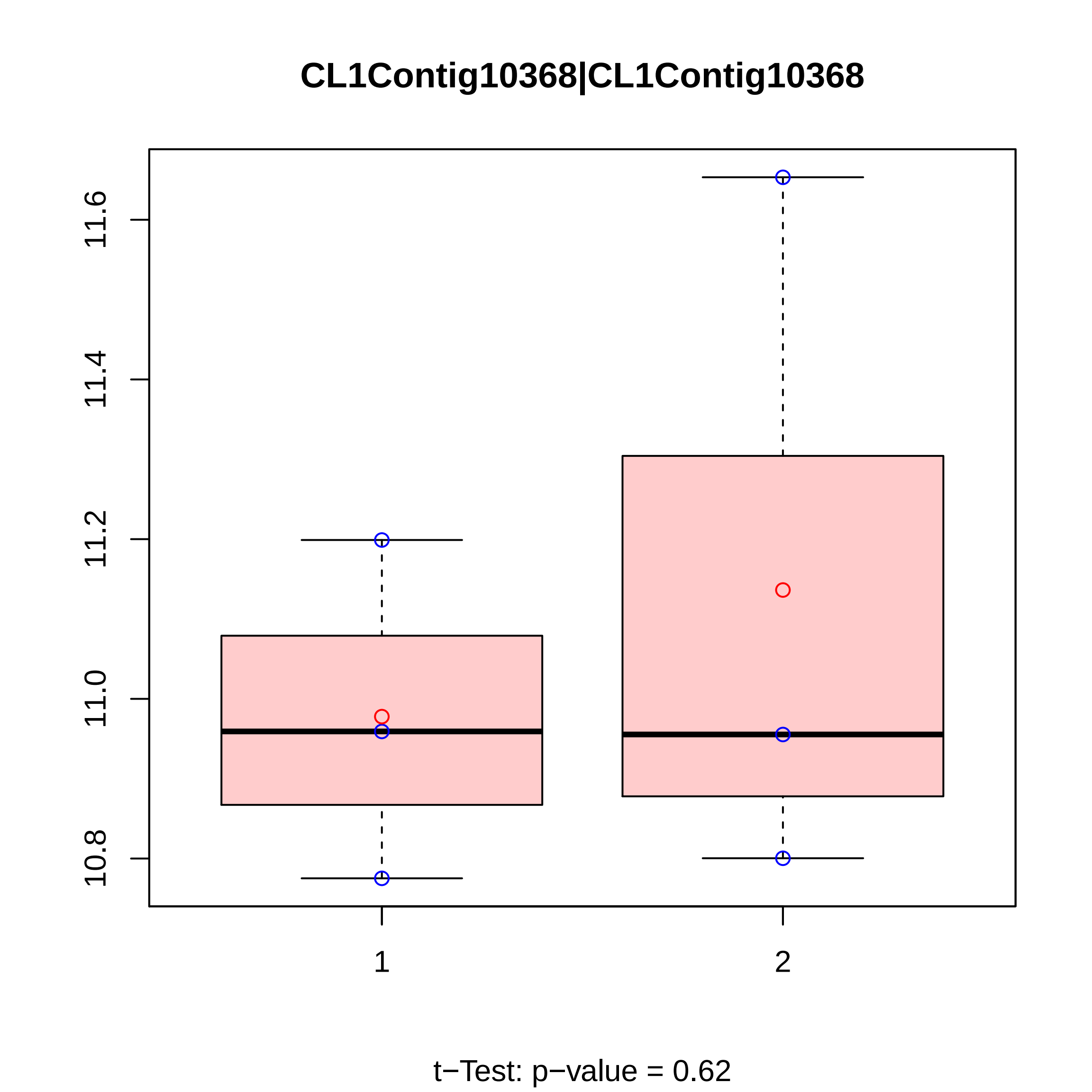
CL1Contig10368|CL1Contig10368
11.6
11.4
11.2
11.0
10.8
1
2
t−Test: p−value = 0.62

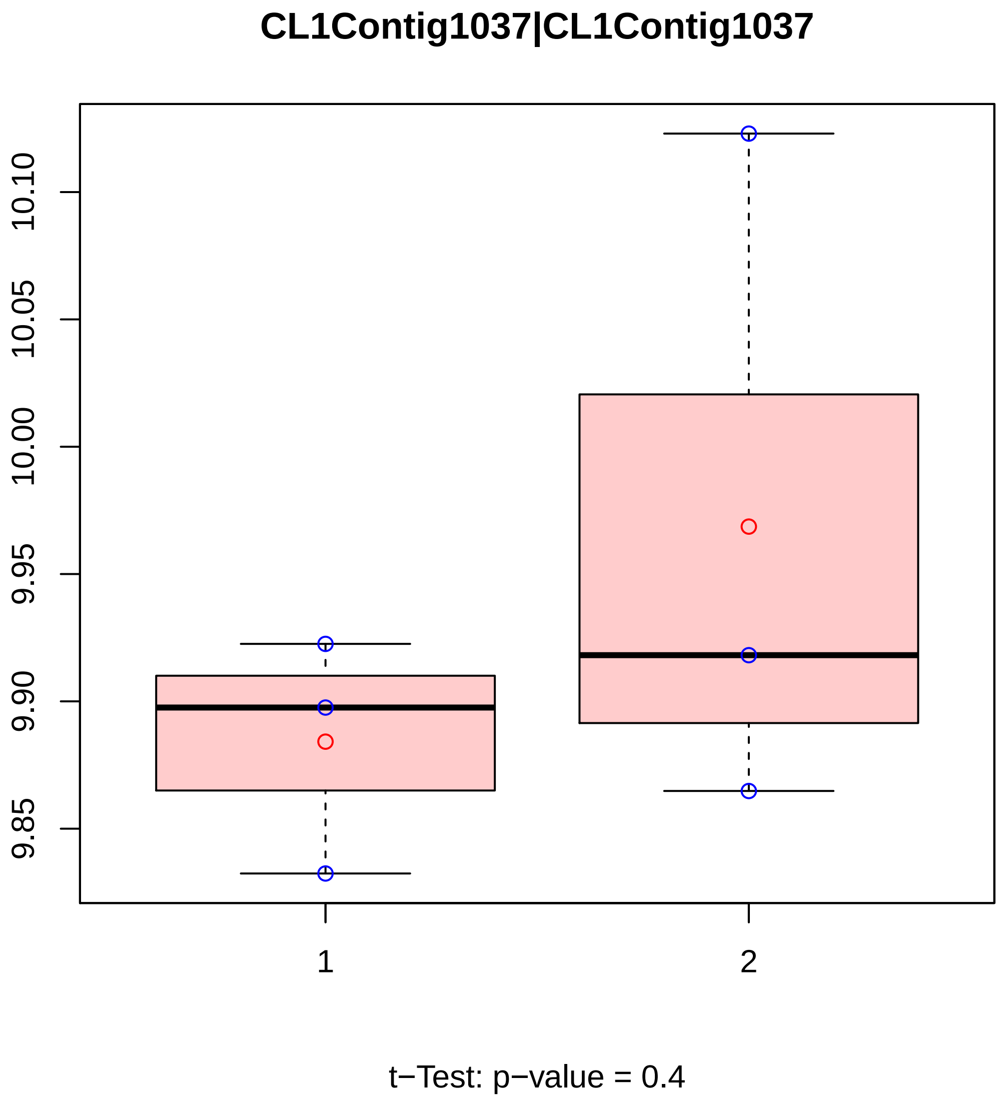
CL1Contig1037|CL1Contig1037
10.10
10.05
10.00
9.95
9.90
9.85
1
2
t−Test: p−value = 0.4

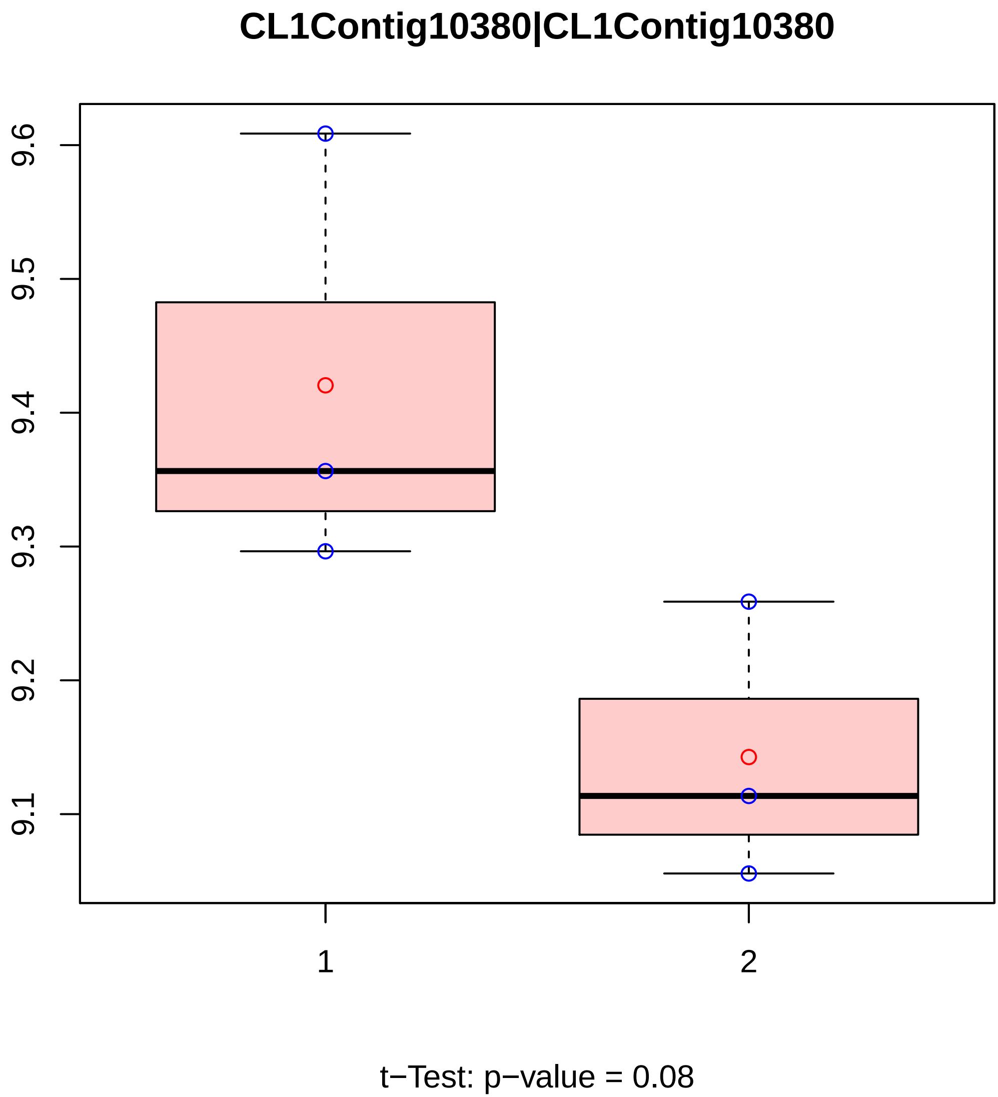
CL1Contig10380|CL1Contig10380
9.6
9.5
9.4
9.3
9.2
9.1
1
2
t−Test: p−value = 0.08

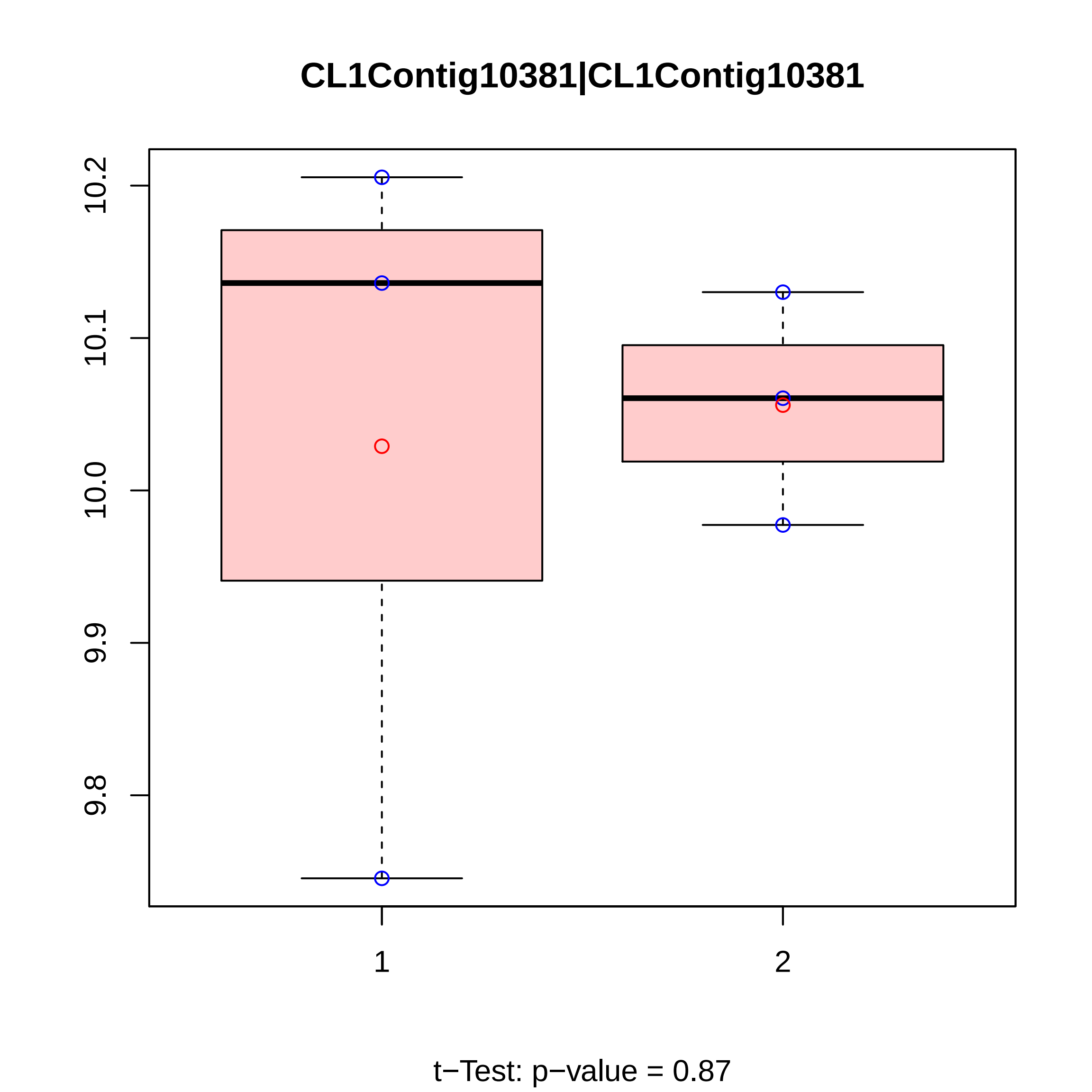

CL1Contig10381|CL1Contig10381
10.2
10.1
10.0
9.9
9.8
1
2
t−Test: p−value = 0.87

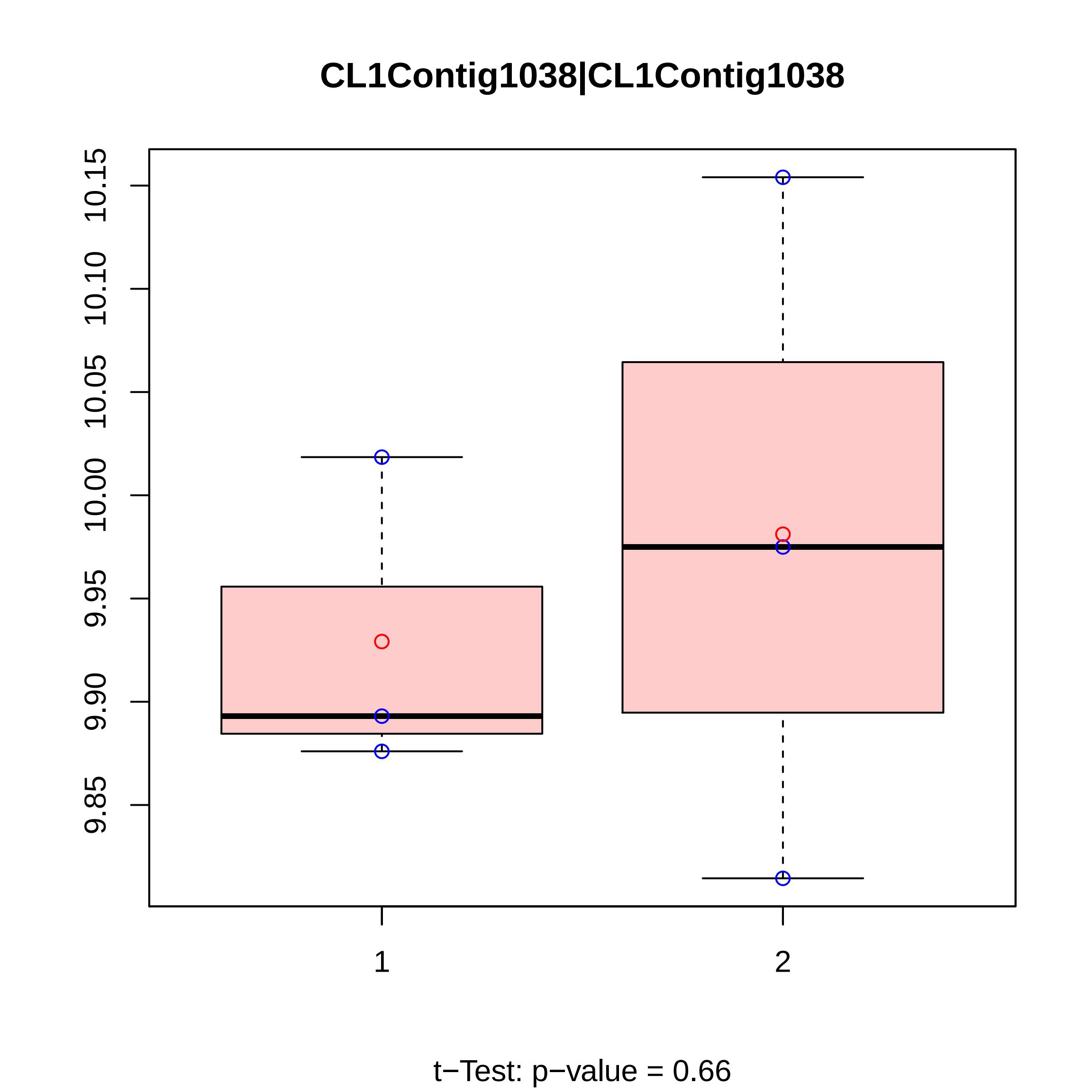
CL1Contig1038|CL1Contig1038
9.85
9.90
9.95
10.00
10.05
10.10
10.15
1
2
t−Test: p−value = 0.66

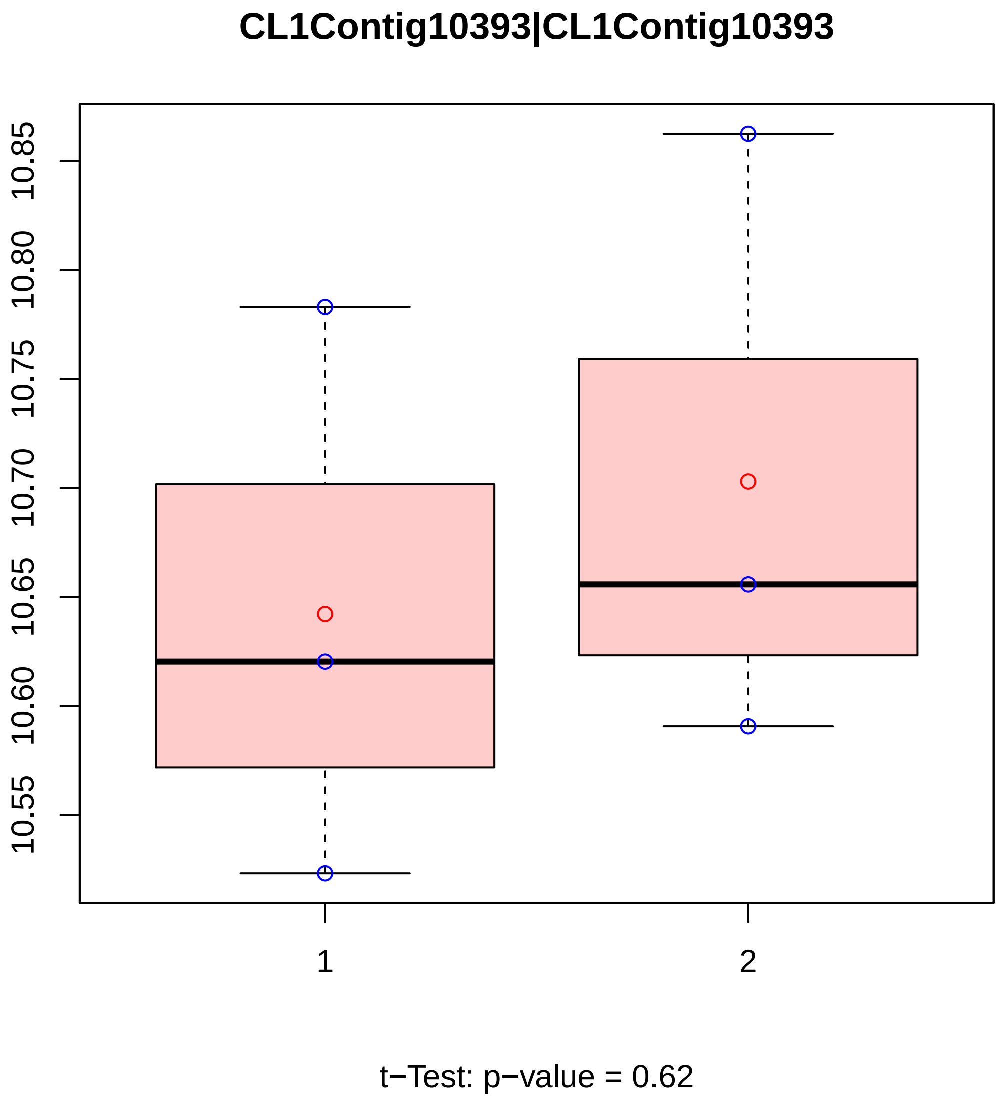

CL1Contig10393|CL1Contig10393
10.55
10.60
10.65
10.70
10.75
10.80
10.85
1
2
t−Test: p−value = 0.62

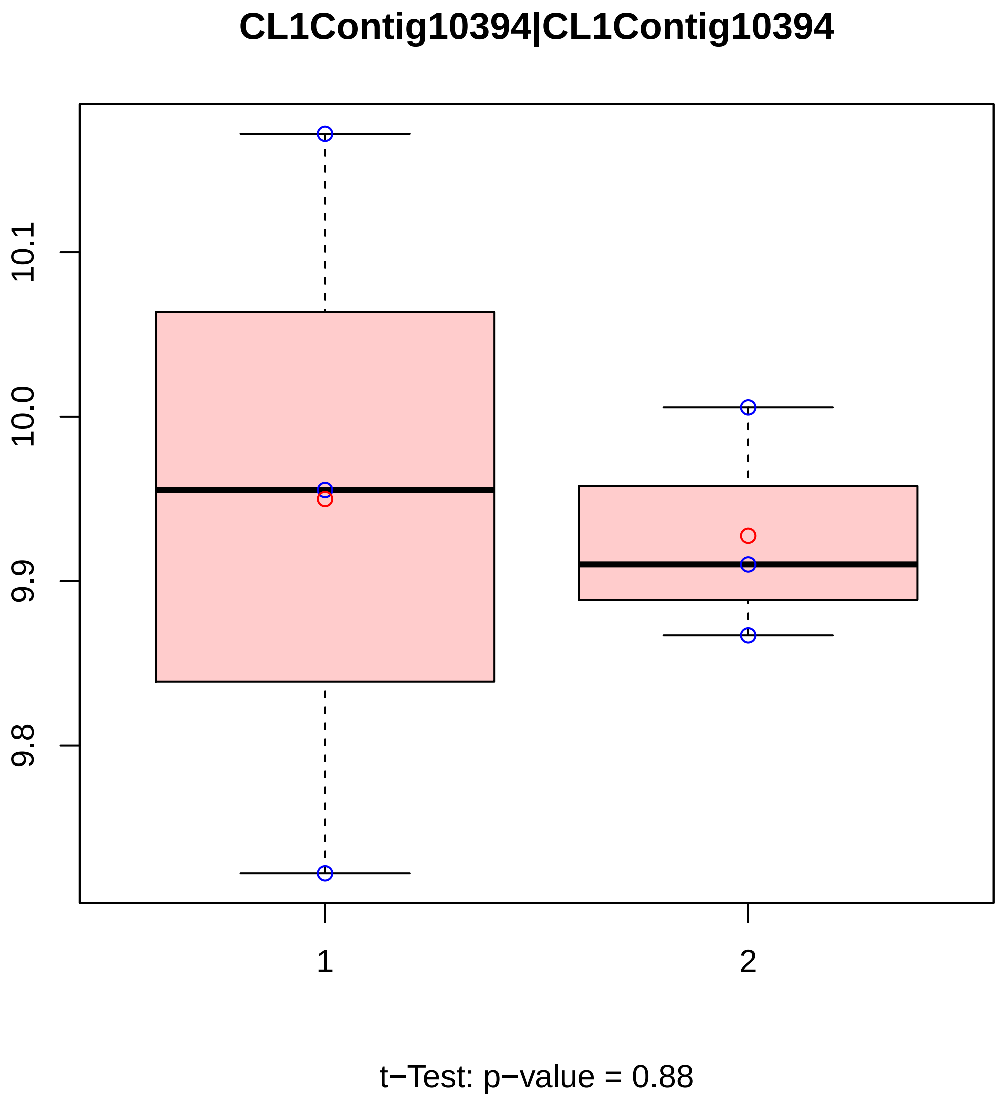
CL1Contig10394|CL1Contig10394
10.1
10.0
9.9
9.8
1
2
t−Test: p−value = 0.88

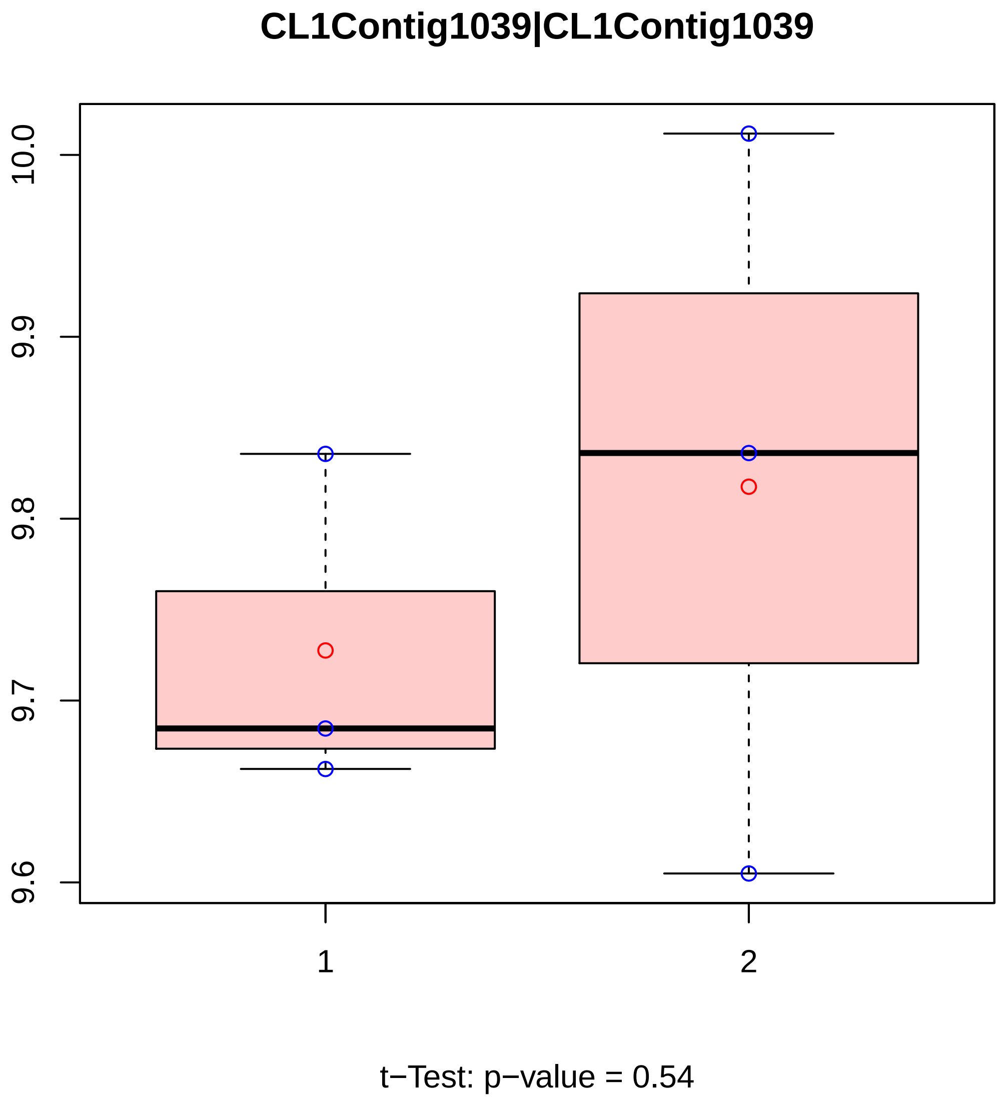

CL1Contig1039|CL1Contig1039
10.0
9.9
9.8
9.7
9.6
1
2
t−Test: p−value = 0.54

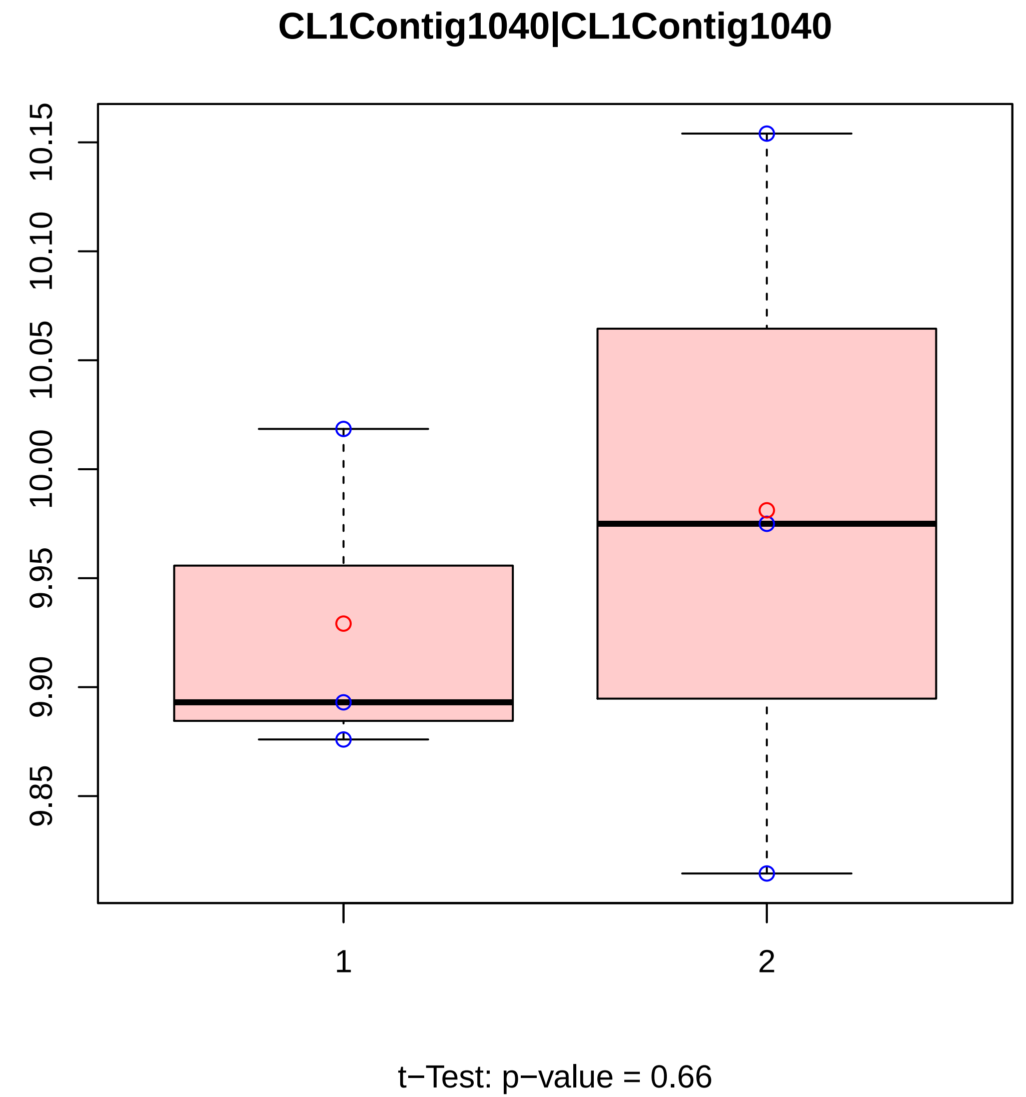
CL1Contig1040|CL1Contig1040
10.15
10.10
10.05
10.00
9.95
9.90
9.85
1
2
t−Test: p−value = 0.66

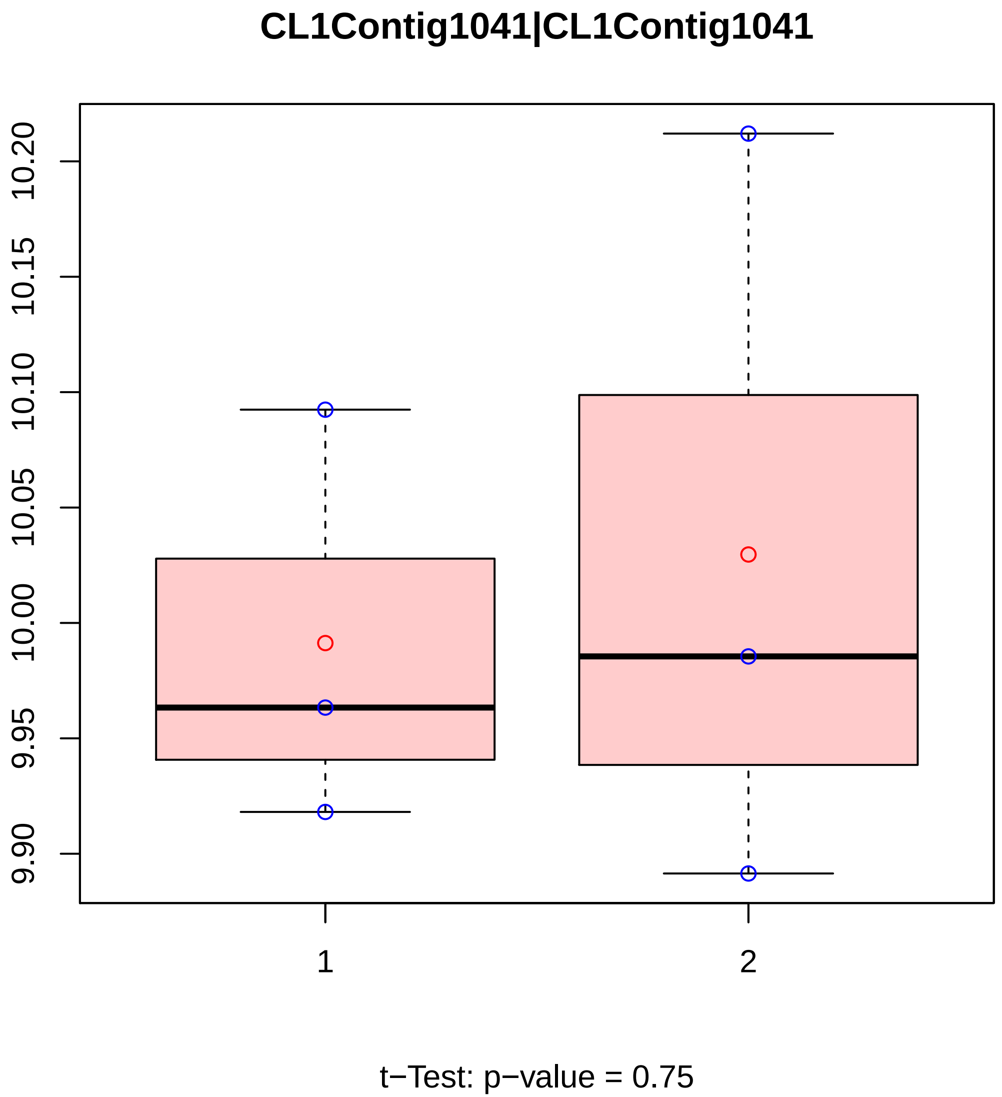
CL1Contig1041|CL1Contig1041
10.20
10.15
10.10
10.05
10.00
9.95
9.90
1
2
t−Test: p−value = 0.75

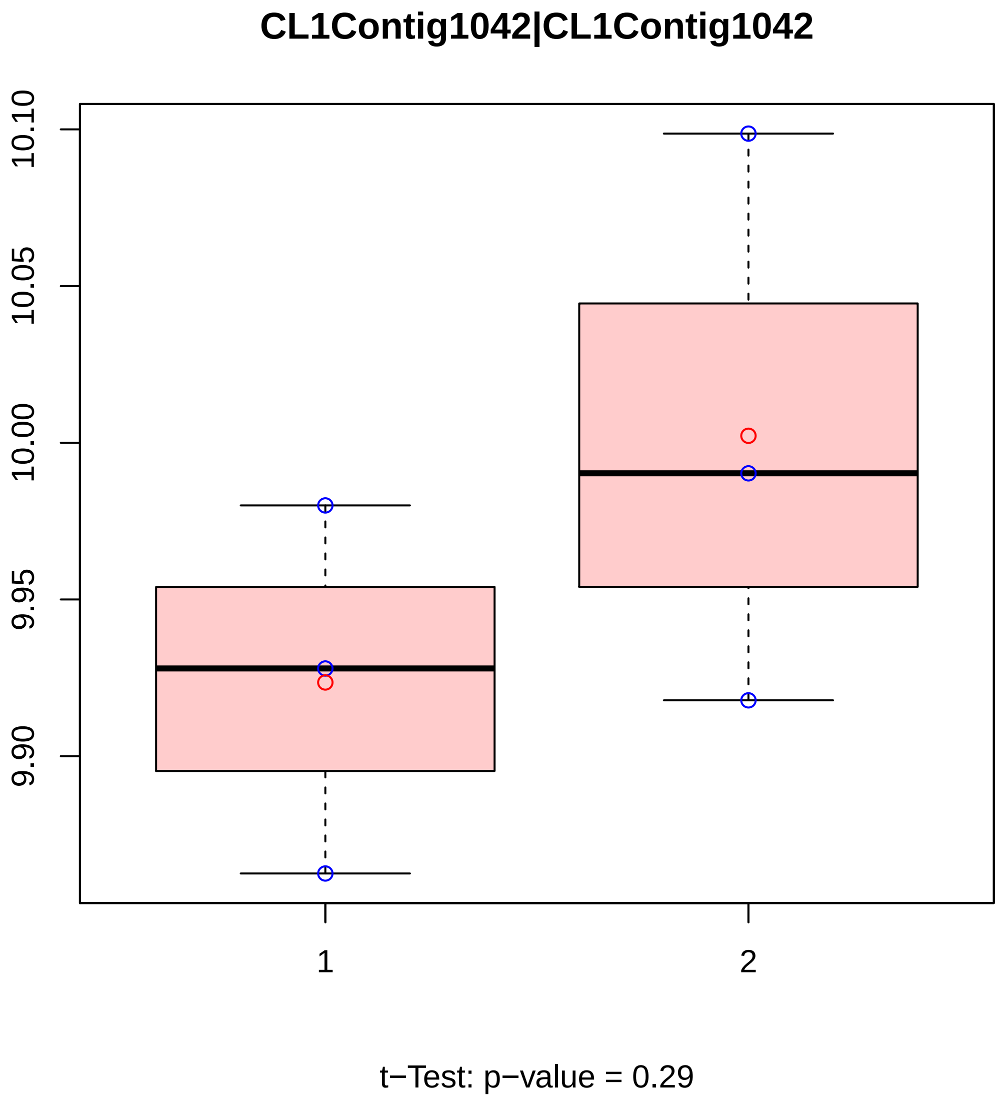

CL1Contig1042|CL1Contig1042
10.10
10.05
10.00
9.95
9.90
1
2
t−Test: p−value = 0.29

## CL1Contig10445|CL1Contig10445

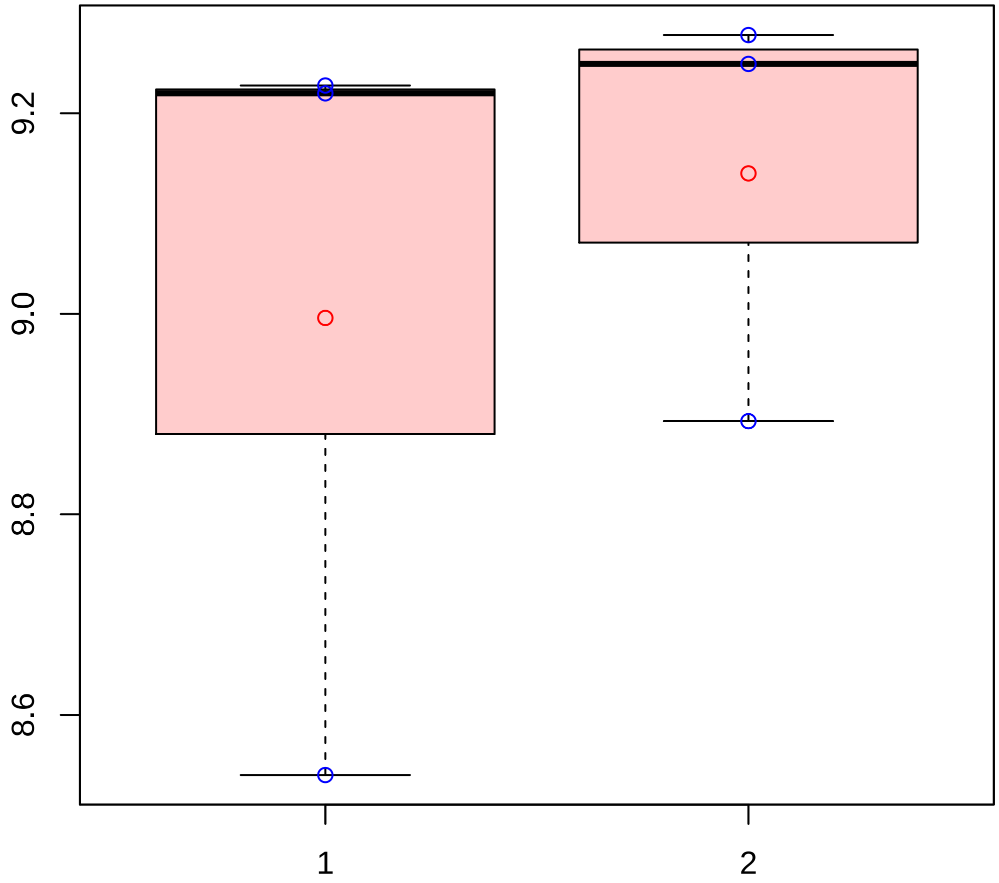


t−Test: p−value = 0.62

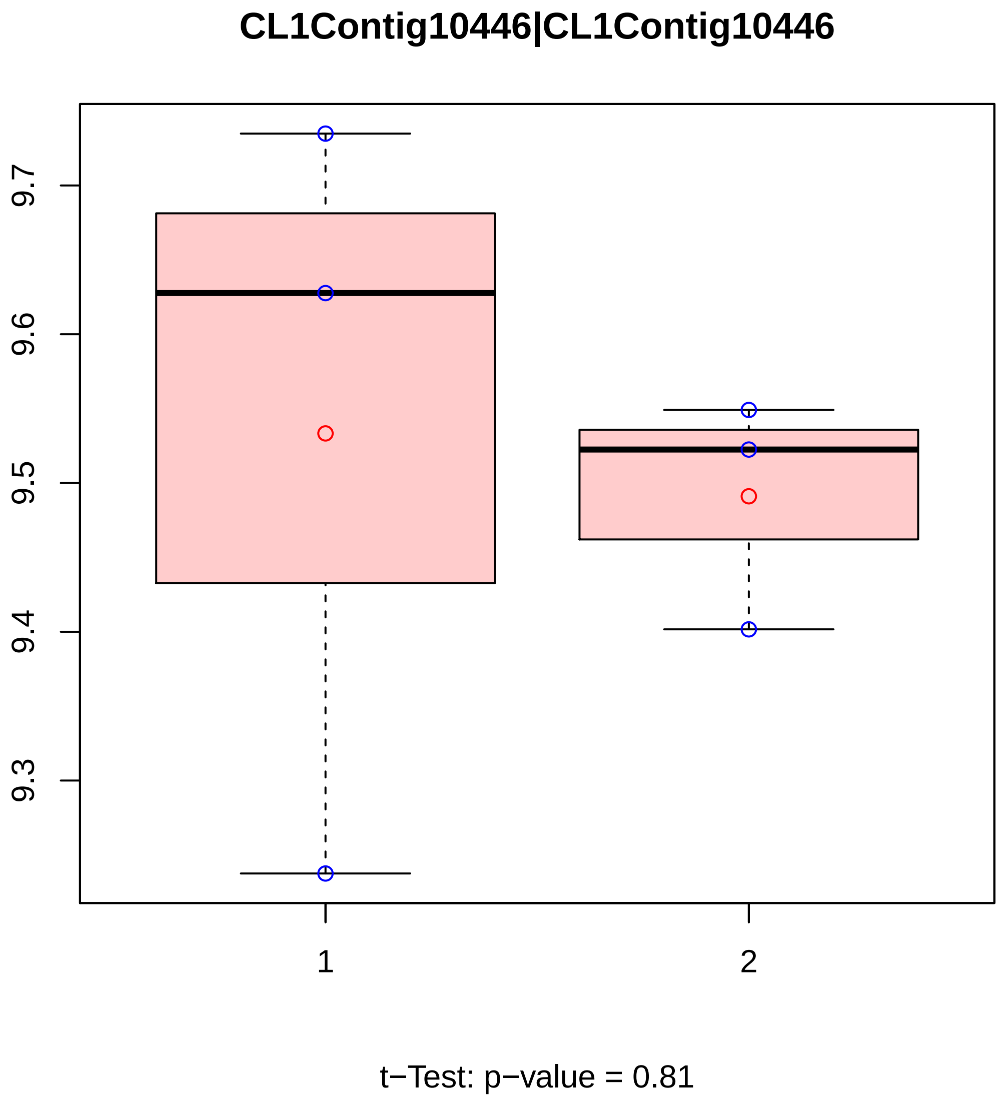

CL1Contig10446|CL1Contig10446
9.3
9.4
9.5
9.6
9.7
1
2
t−Test: p−value = 0.81

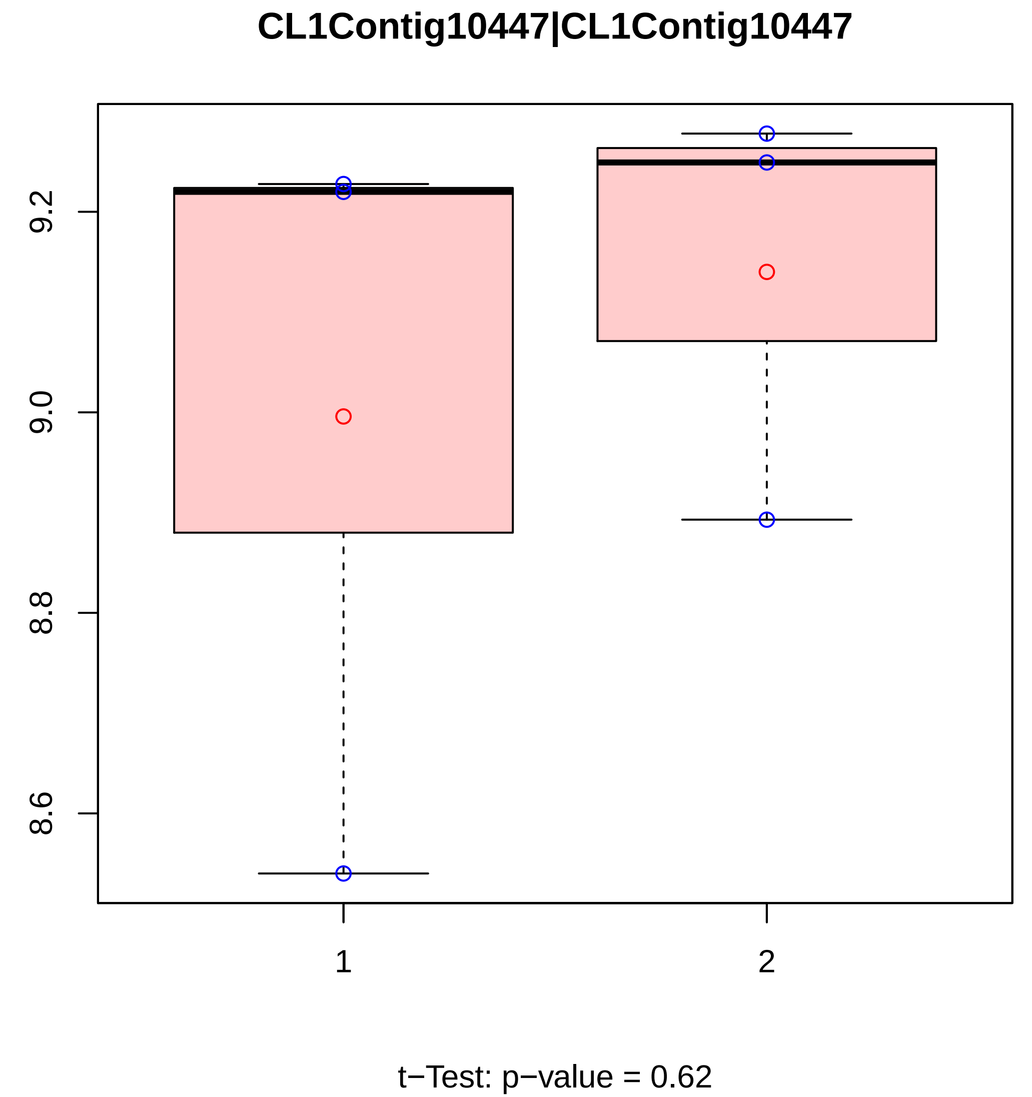
CL1Contig10447|CL1Contig10447
9.2
9.0
8.8
8.6
1
2
t−Test: p−value = 0.62

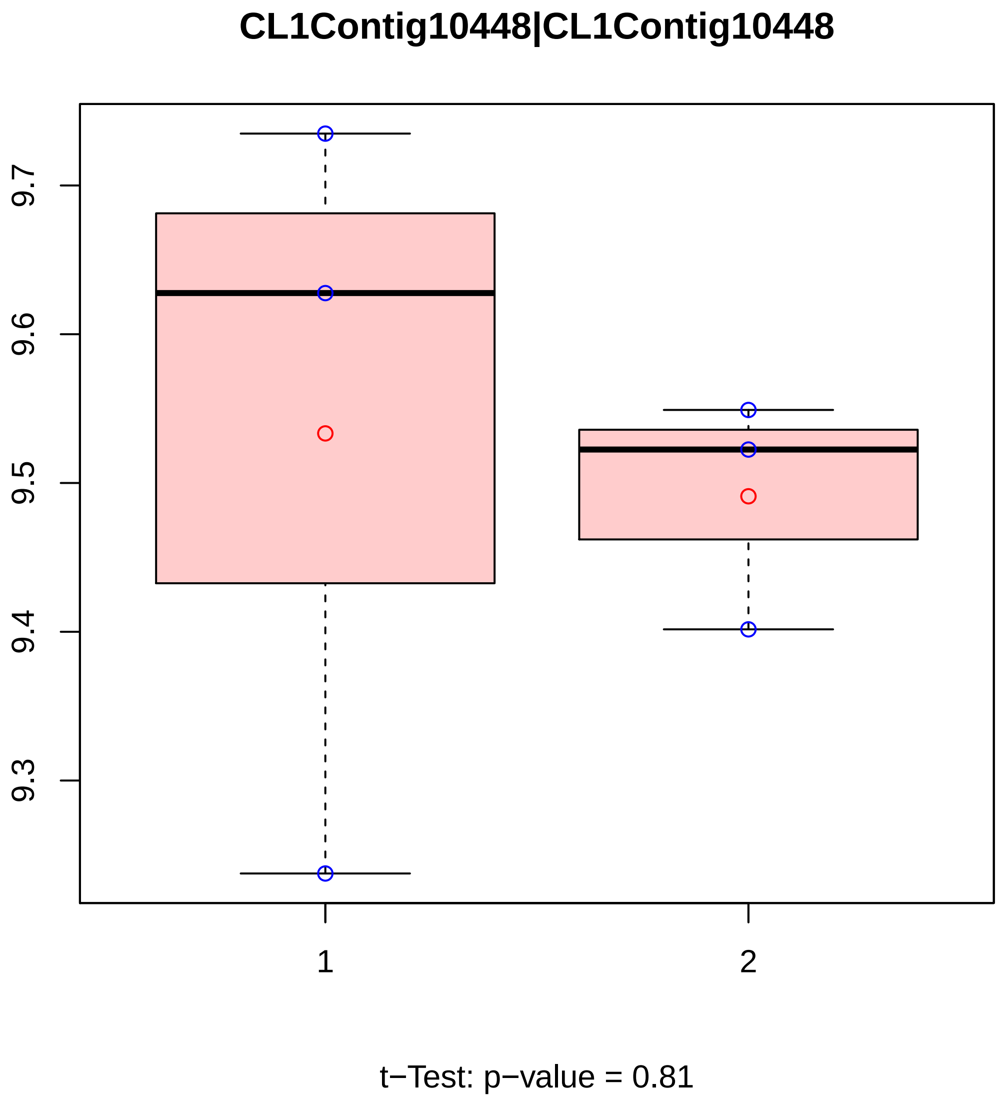

CL1Contig10448|CL1Contig10448
9.7
9.6
9.5
9.4
9.3
1
2
t−Test: p−value = 0.81

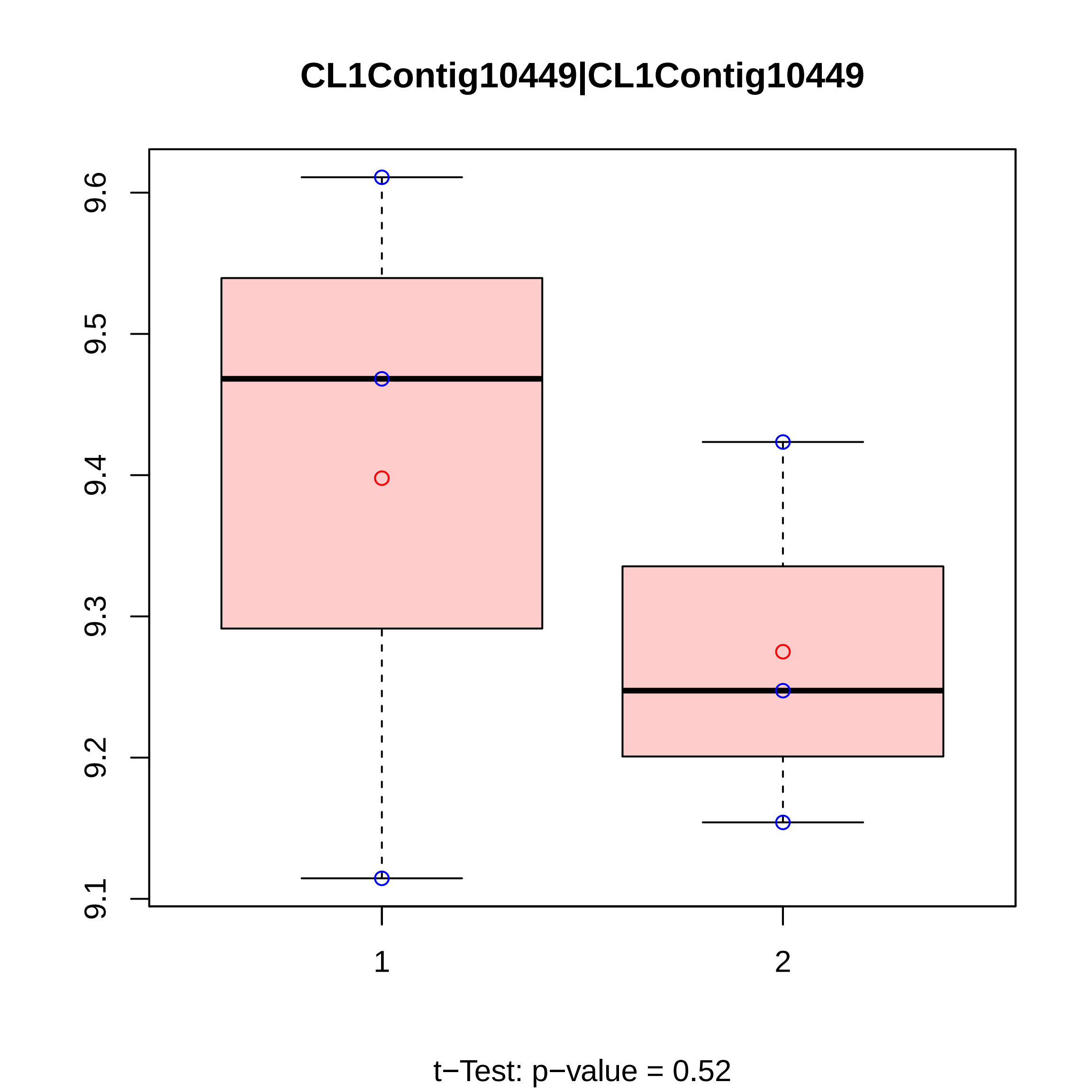
CL1Contig10449|CL1Contig10449
9.1
9.2
9.3
9.4
9.5
9.6
1
2
t−Test: p−value = 0.52

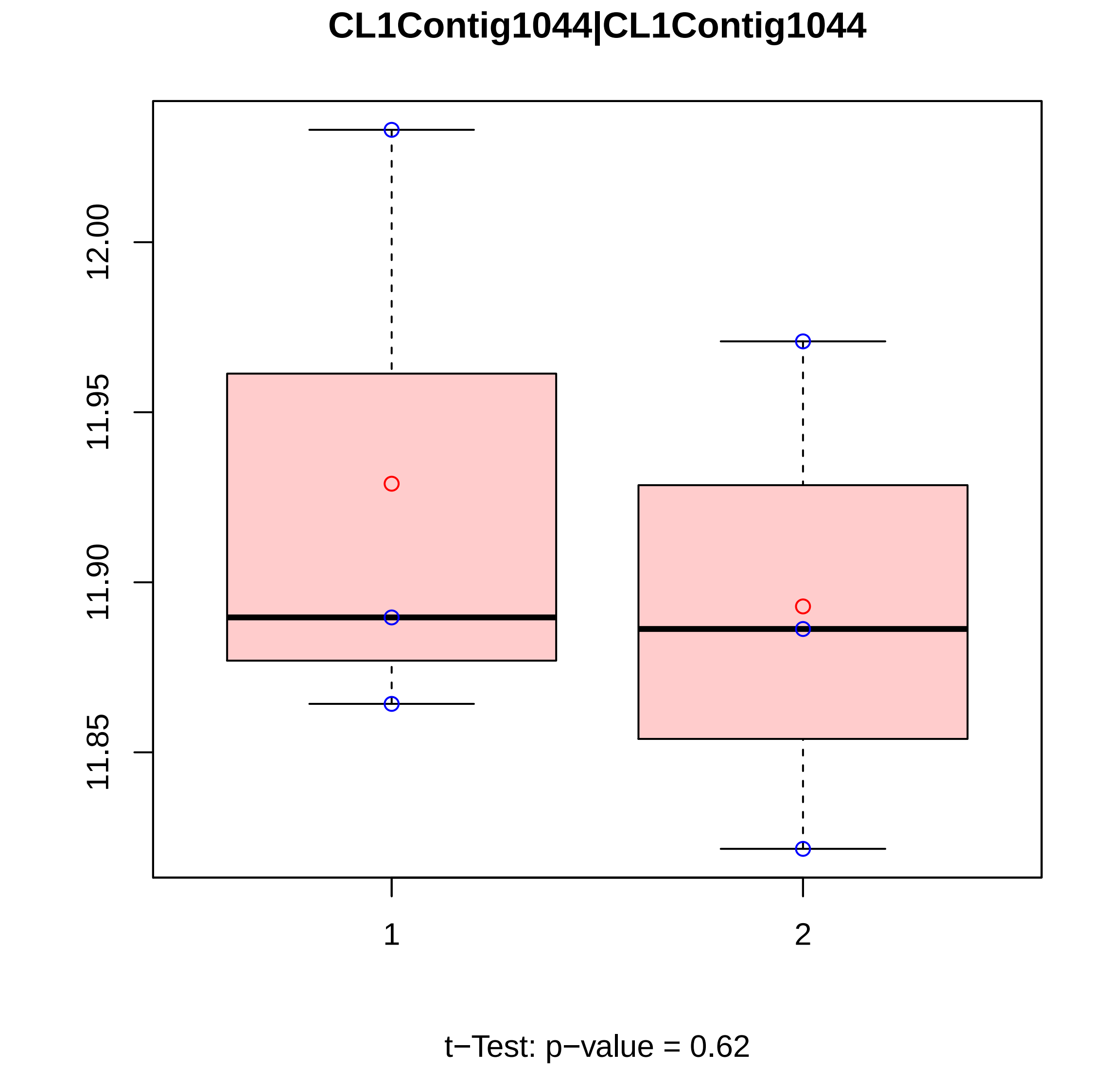

CL1Contig1044|CL1Contig1044
12.00
11.95
11.90
11.85
1
2
t−Test: p−value = 0.62

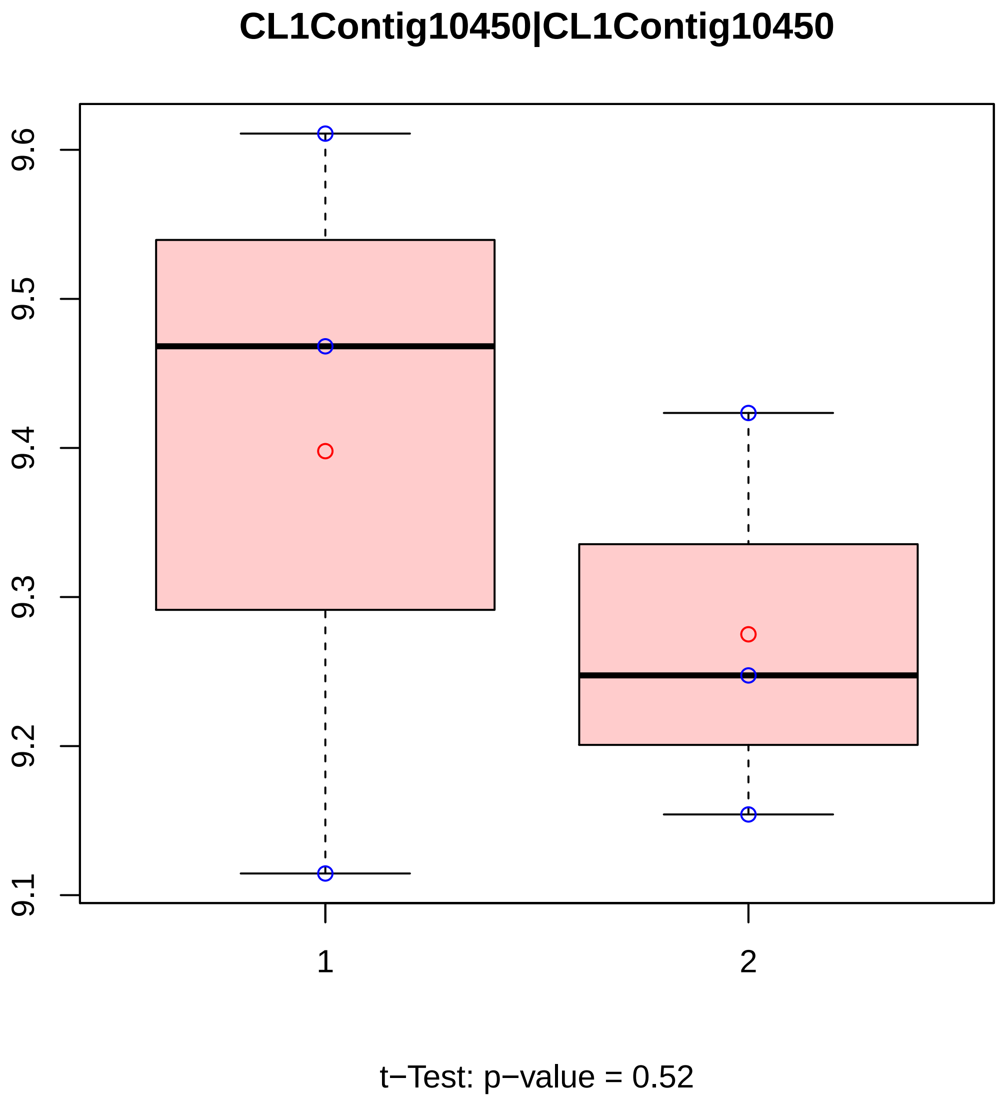
CL1Contig10450|CL1Contig10450
9.1
9.2
9.3
9.4
9.5
9.6
1
2
t−Test: p−value = 0.52

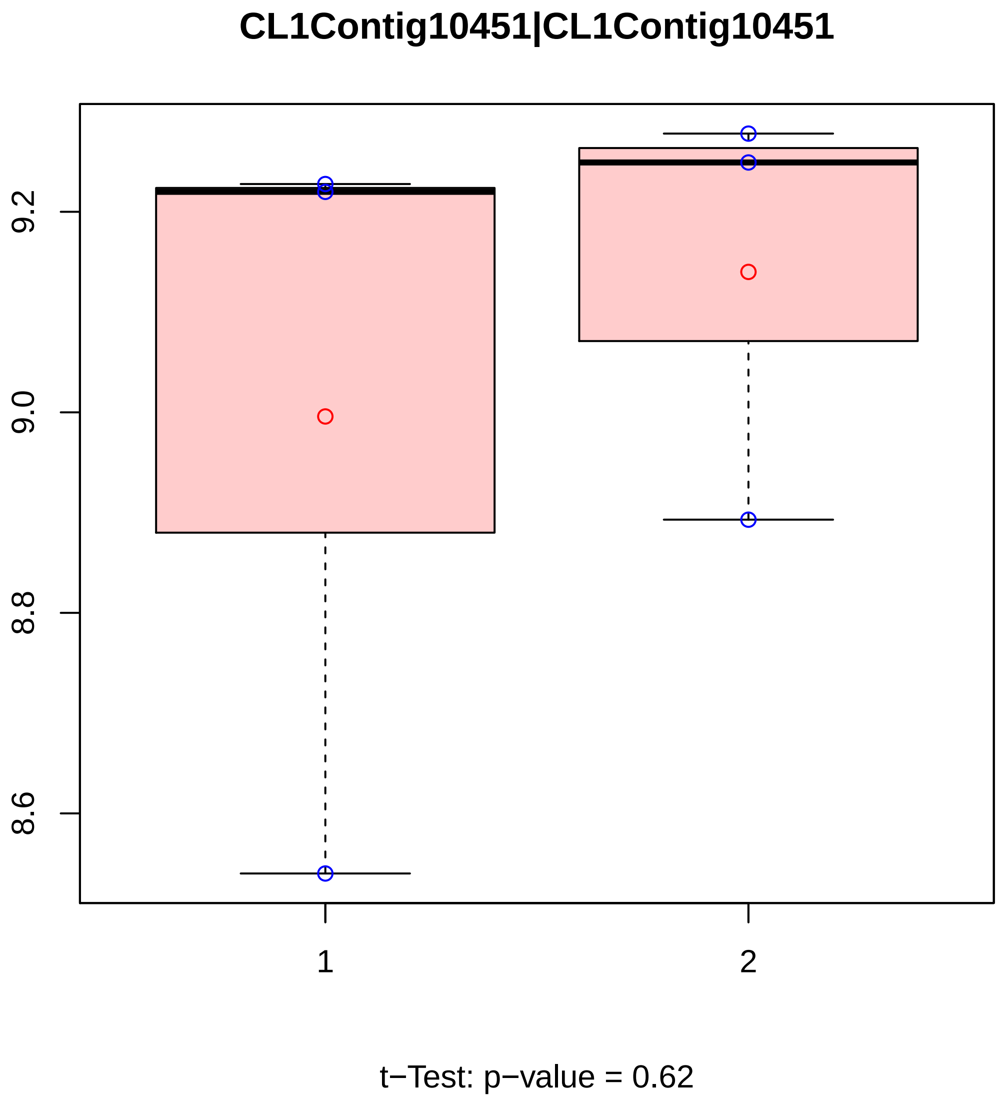
CL1Contig10451|CL1Contig10451
9.2
9.0
8.8
8.6
1
2
t−Test: p−value = 0.62

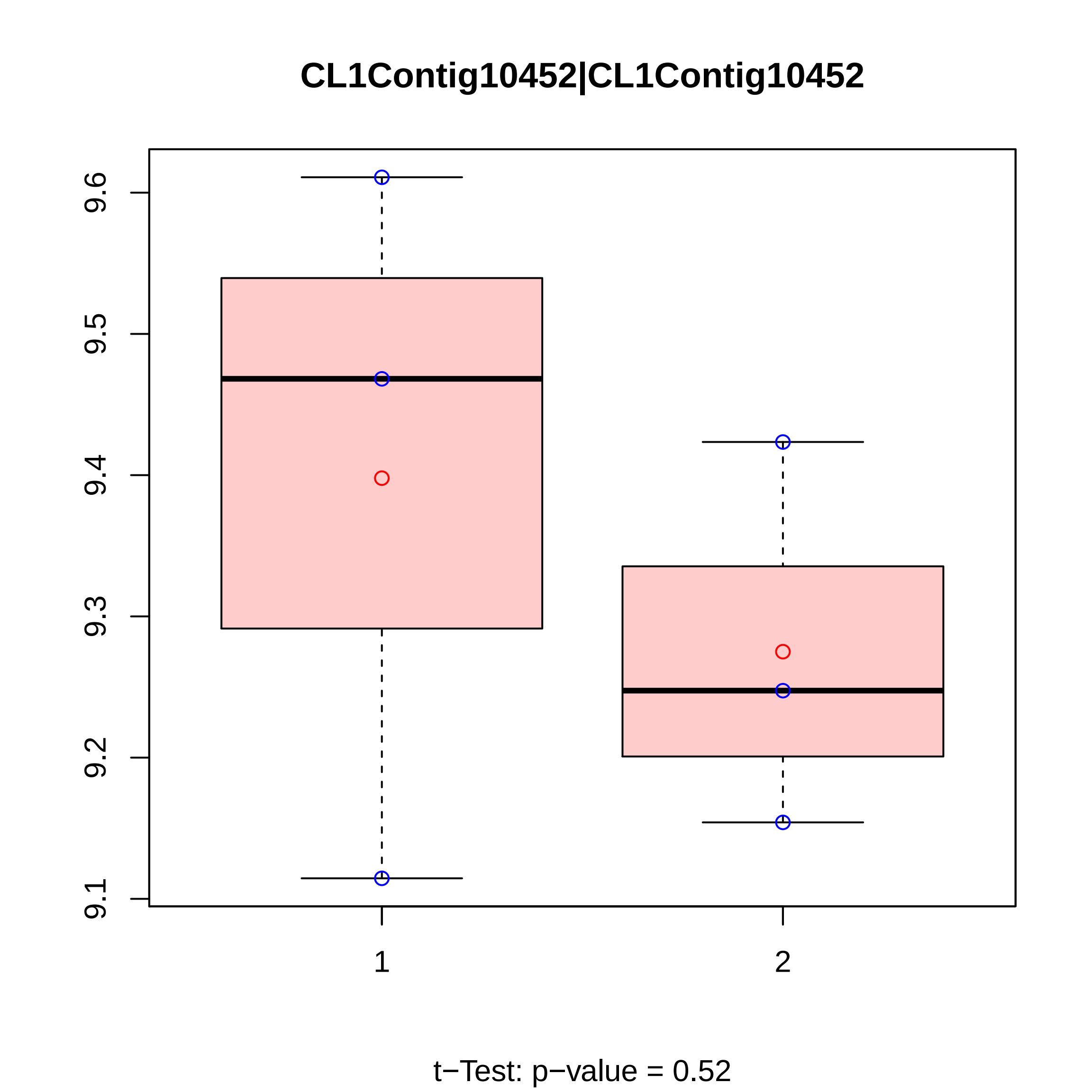
CL1Contig10452|CL1Contig10452
9.1
9.2
9.3
9.4
9.5
9.6
1
2
t−Test: p−value = 0.52

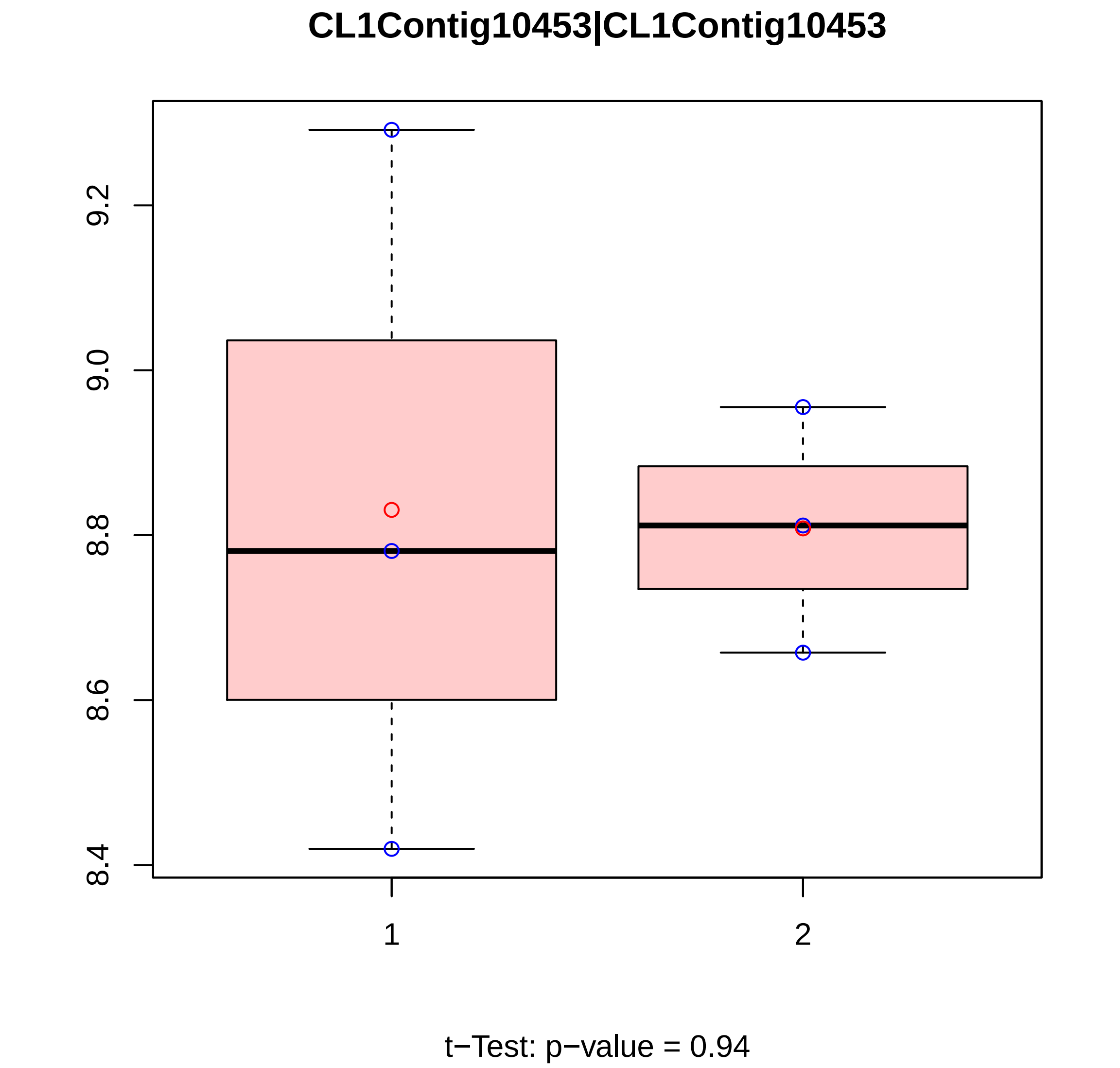
CL1Contig10453|CL1Contig10453
9.2
9.0
8.8
8.6
8.4
1
2
t−Test: p−value = 0.94

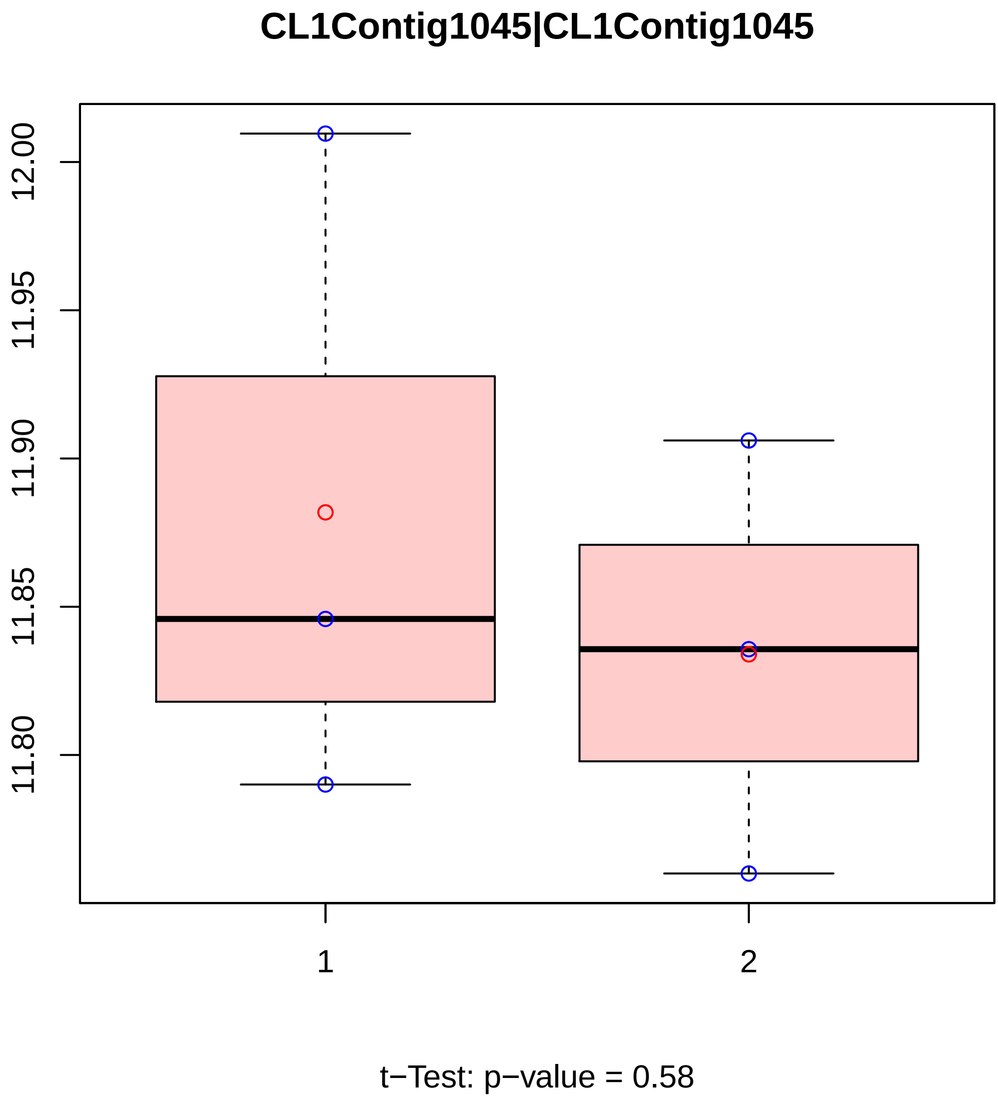
CL1Contig1045|CL1Contig1045
12.00
11.95
11.90
11.85
11.80
1
2
t−Test: p−value = 0.58

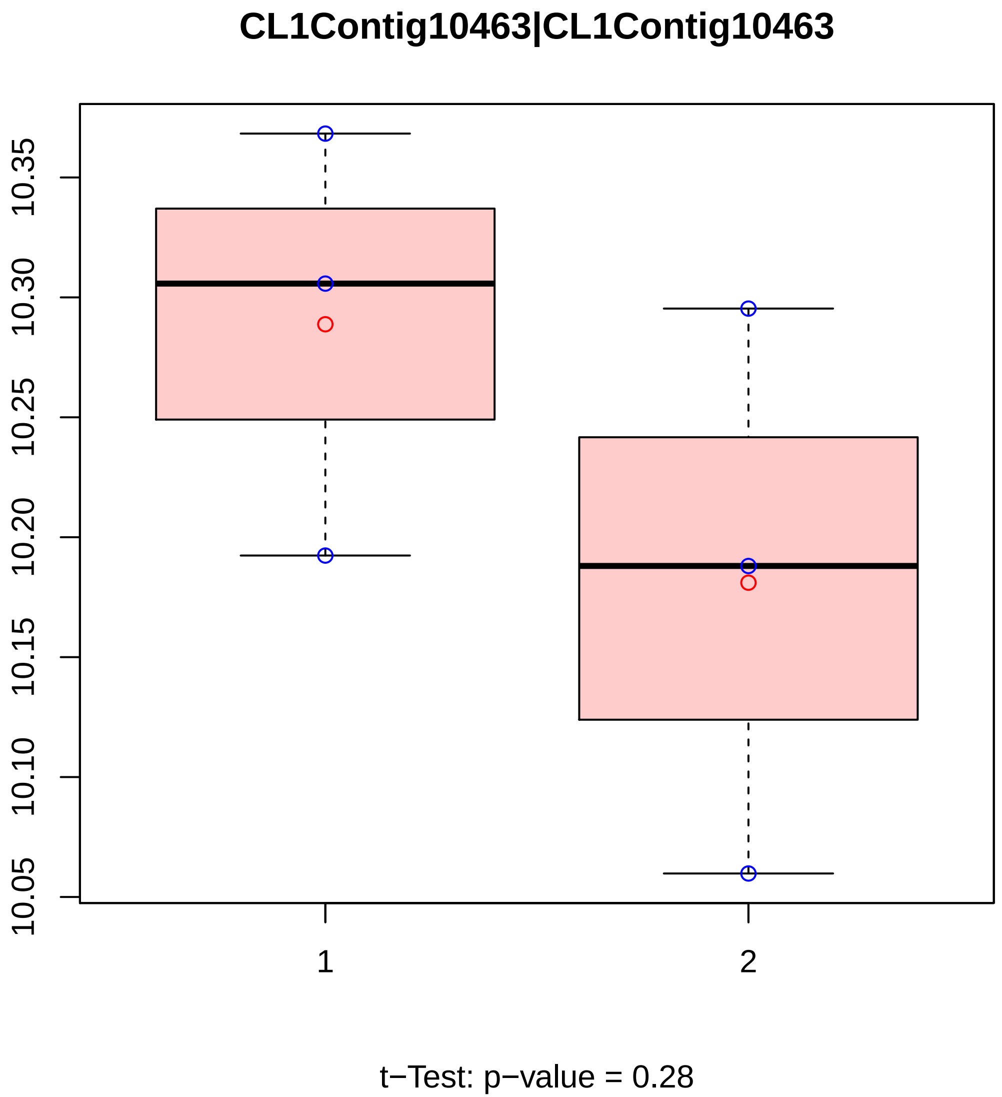

CL1Contig10463|CL1Contig10463
10.05
10.10
10.15
10.20
10.25
10.30
10.35
1
2
t−Test: p−value = 0.28

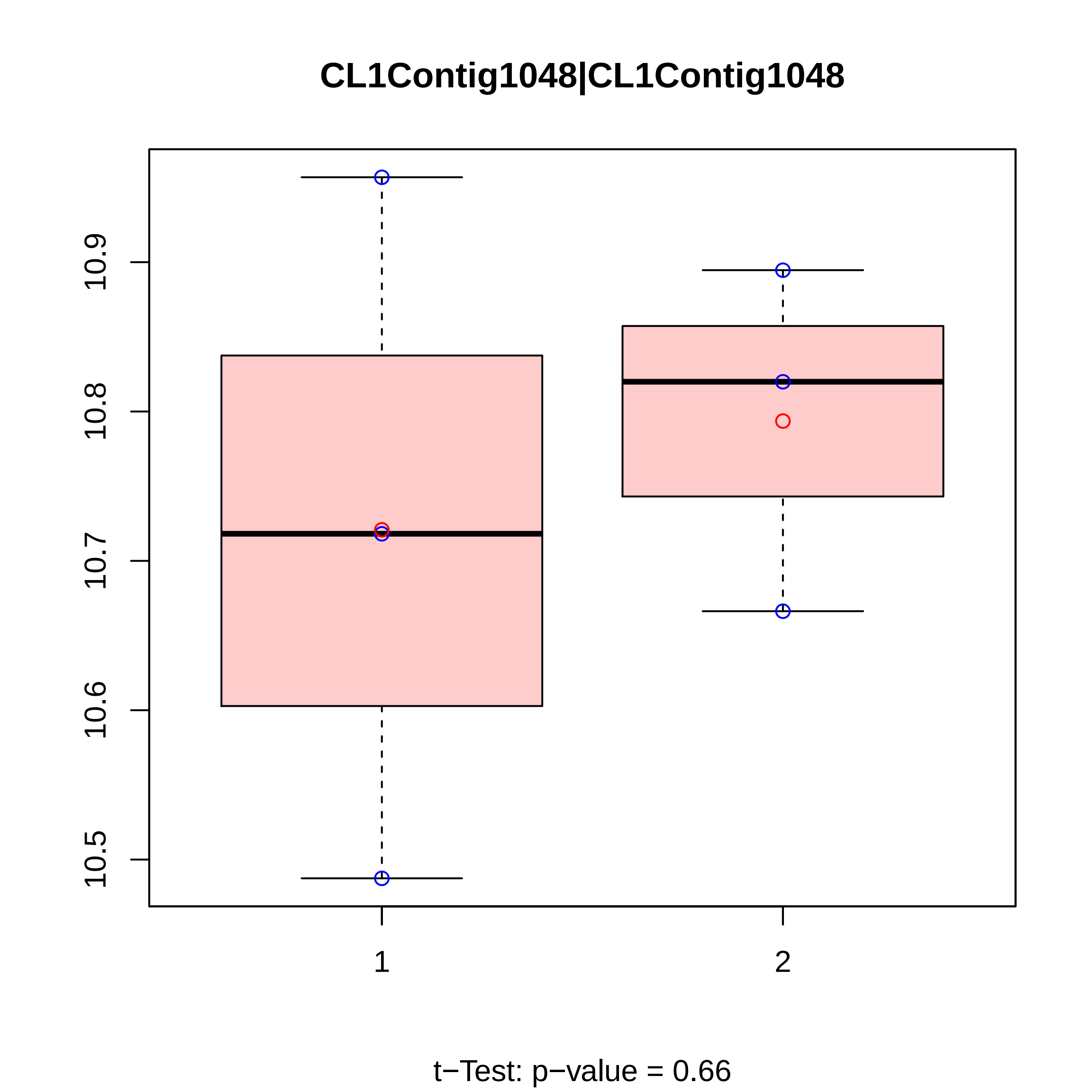
CL1Contig1048|CL1Contig1048
10.5
10.6
10.7
10.8
10.9
1
2
t−Test: p−value = 0.66

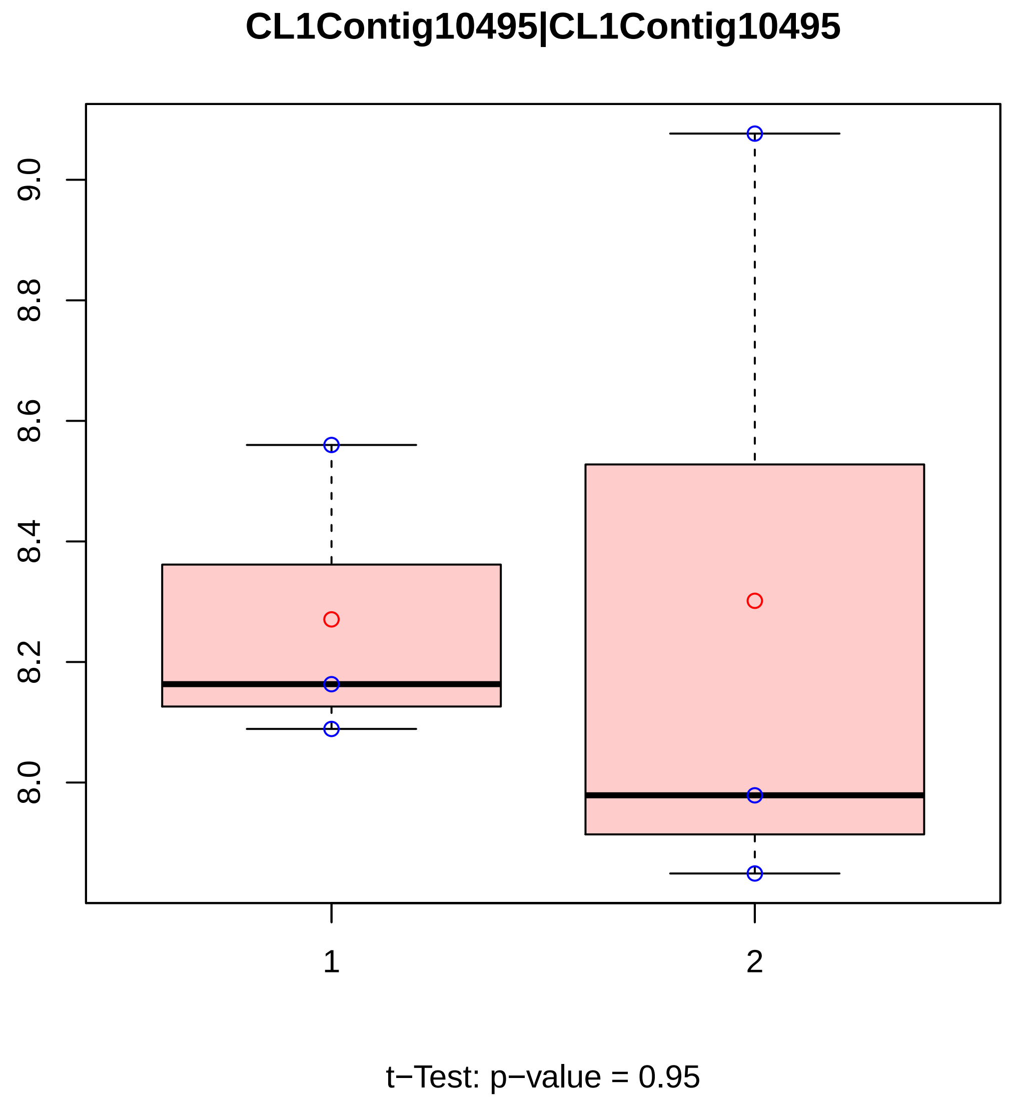

CL1Contig10495|CL1Contig10495
9.0
8.8
8.6
8.4
8.2
8.0
1
2
t−Test: p−value = 0.95

# CL1Contig10513|CL1Contig10513

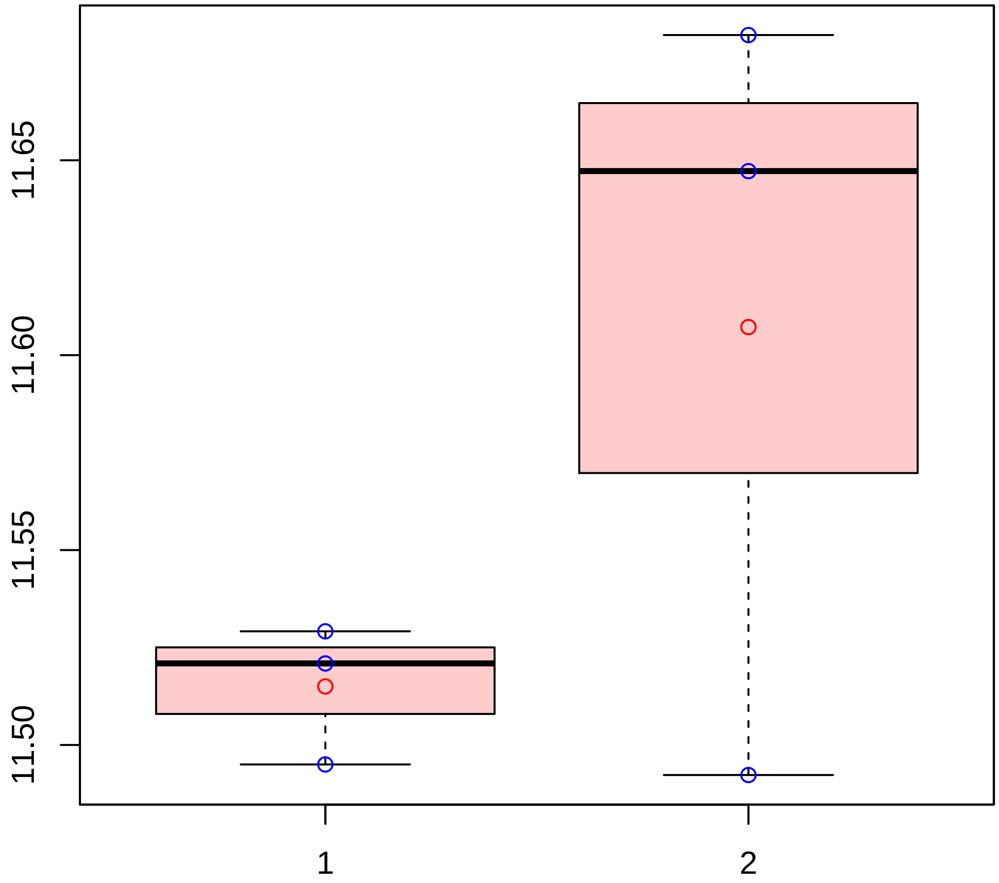


t−Test: p−value = 0.25

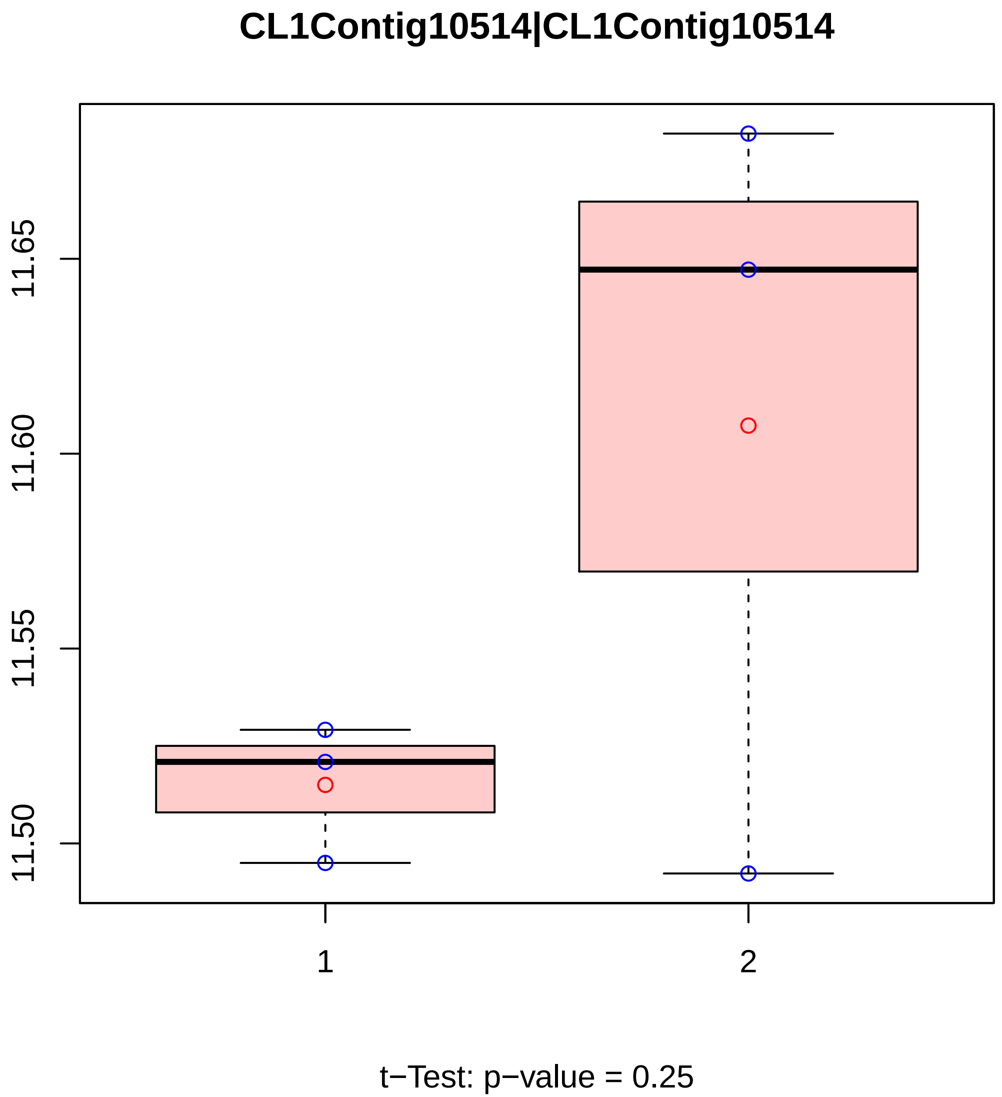
CL1Contig10514|CL1Contig10514
11.65
11.60
11.55
11.50
1
2
t−Test: p−value = 0.25

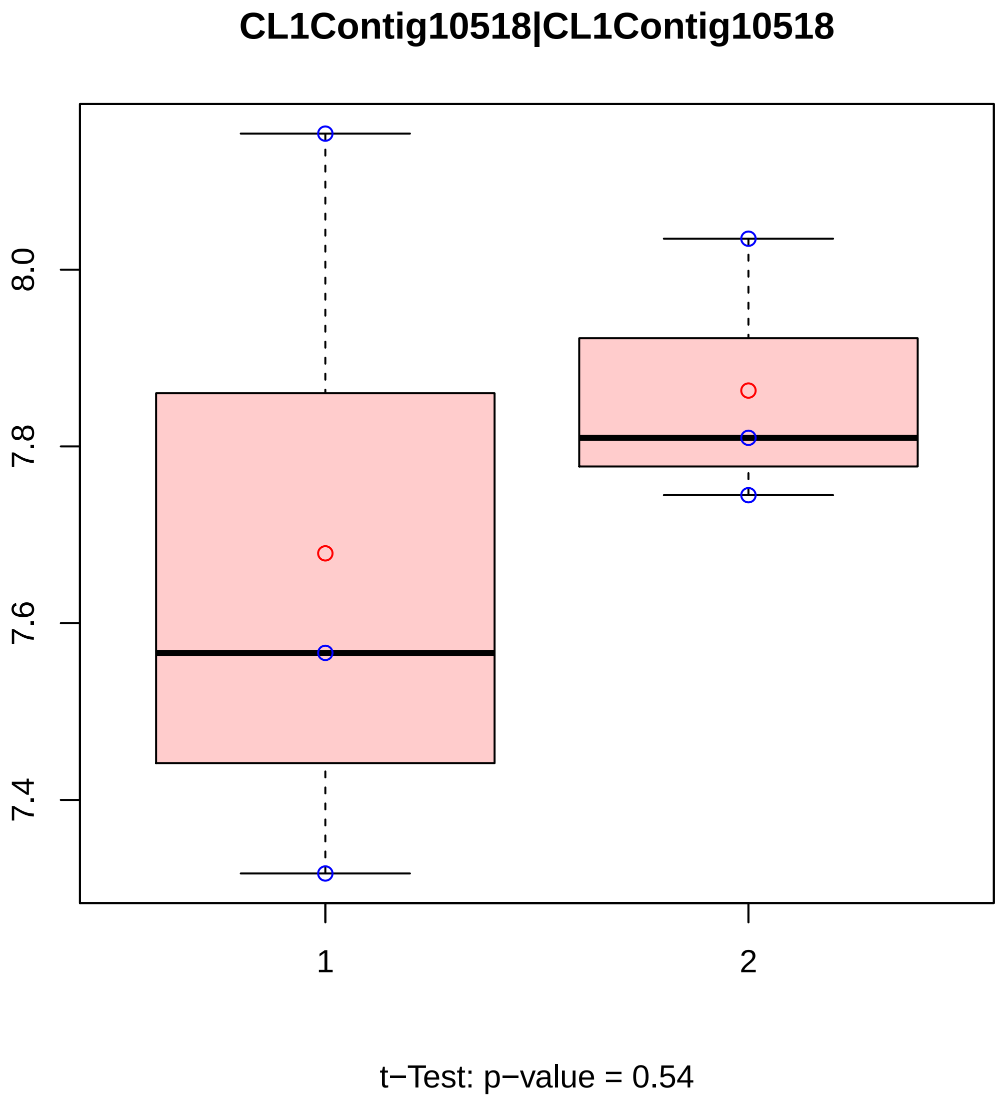
CL1Contig10518|CL1Contig10518
8.0
7.8
7.6
7.4
1
2
t−Test: p−value = 0.54

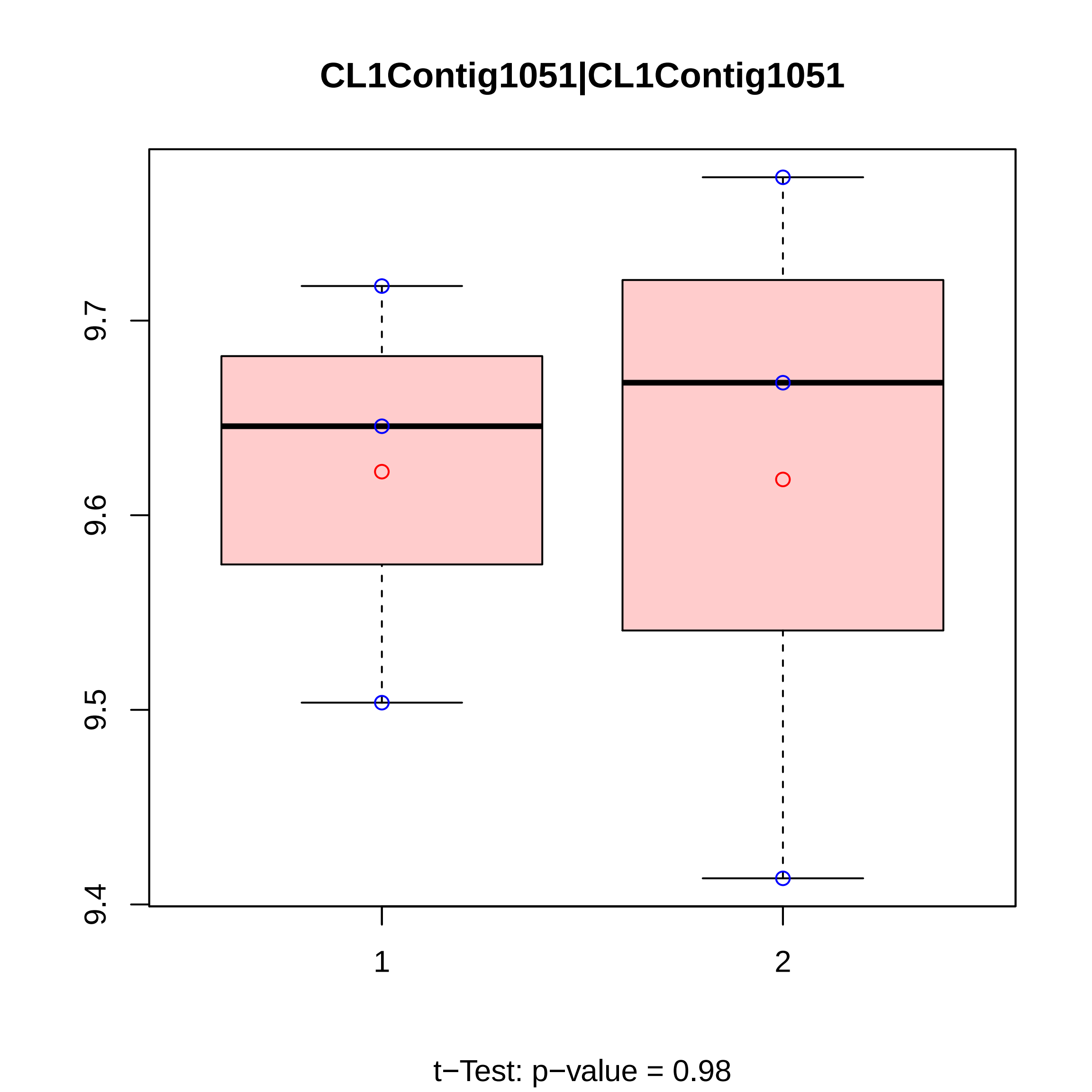

CL1Contig1051|CL1Contig1051
9.4
9.5
9.6
9.7
1
2
t−Test: p−value = 0.98

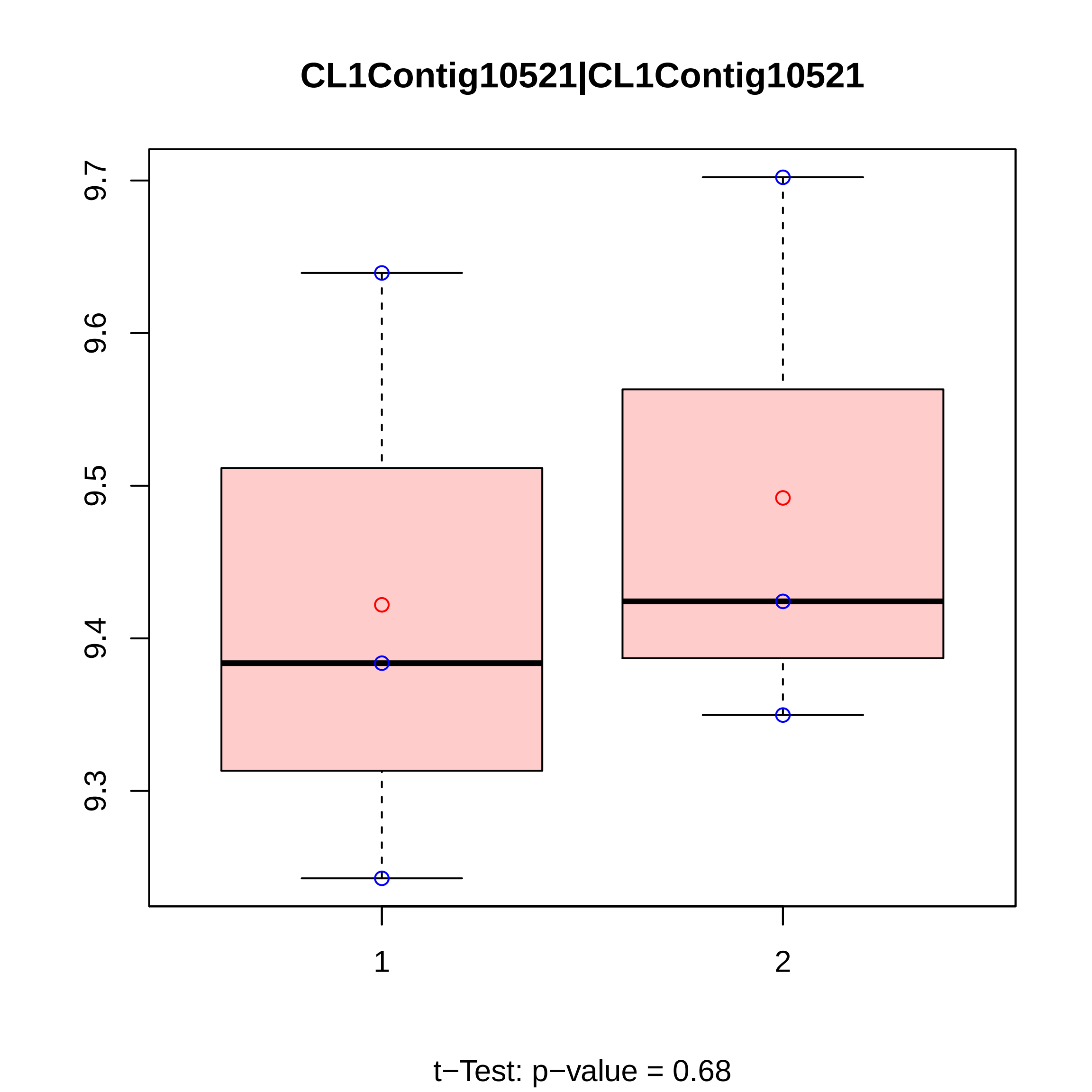
CL1Contig10521|CL1Contig10521
9.3
9.4
9.5
9.6
9.7
1
2
t−Test: p−value = 0.68

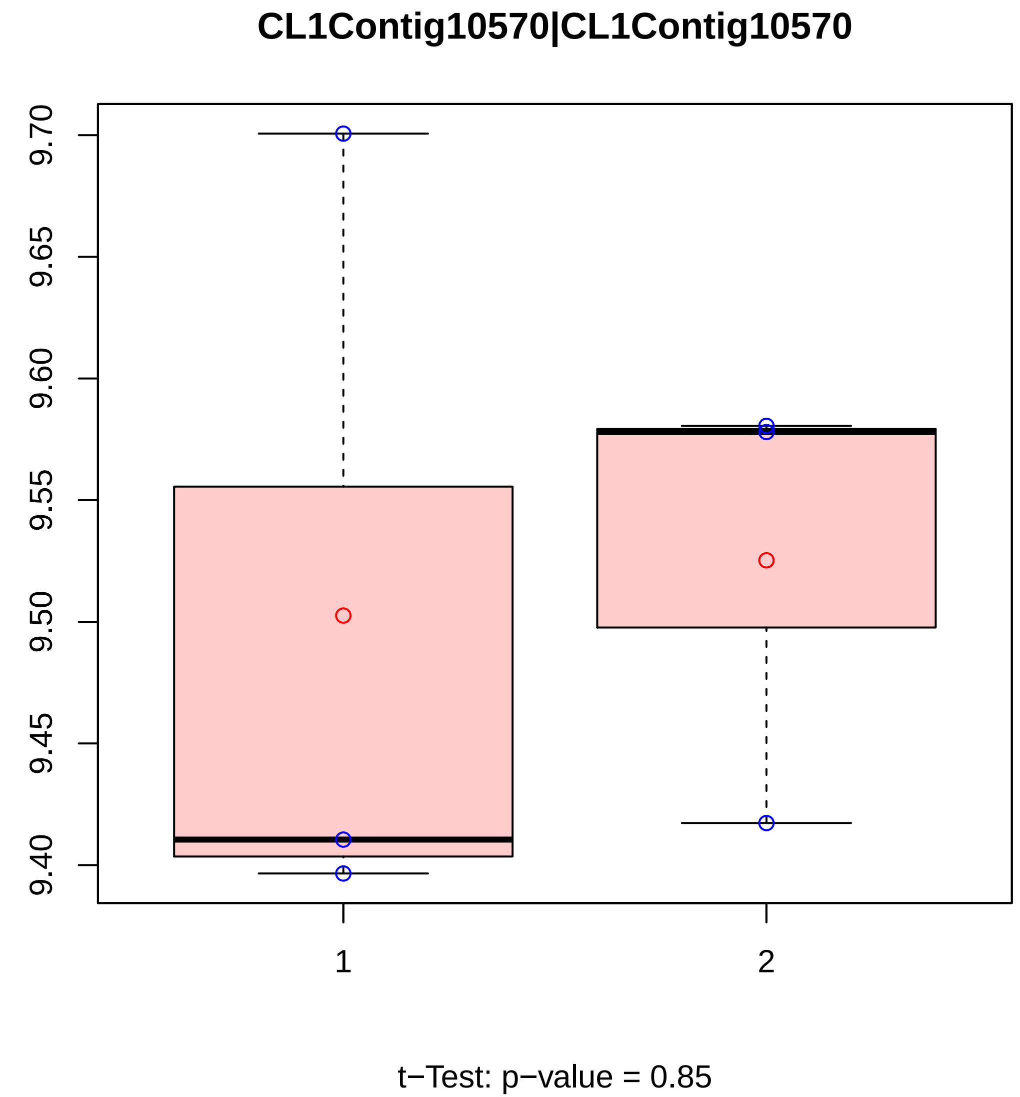
CL1Contig10570|CL1Contig10570
9.70
9.65
9.60
9.55
9.50
9.45
9.40
1
2
t−Test: p−value = 0.85

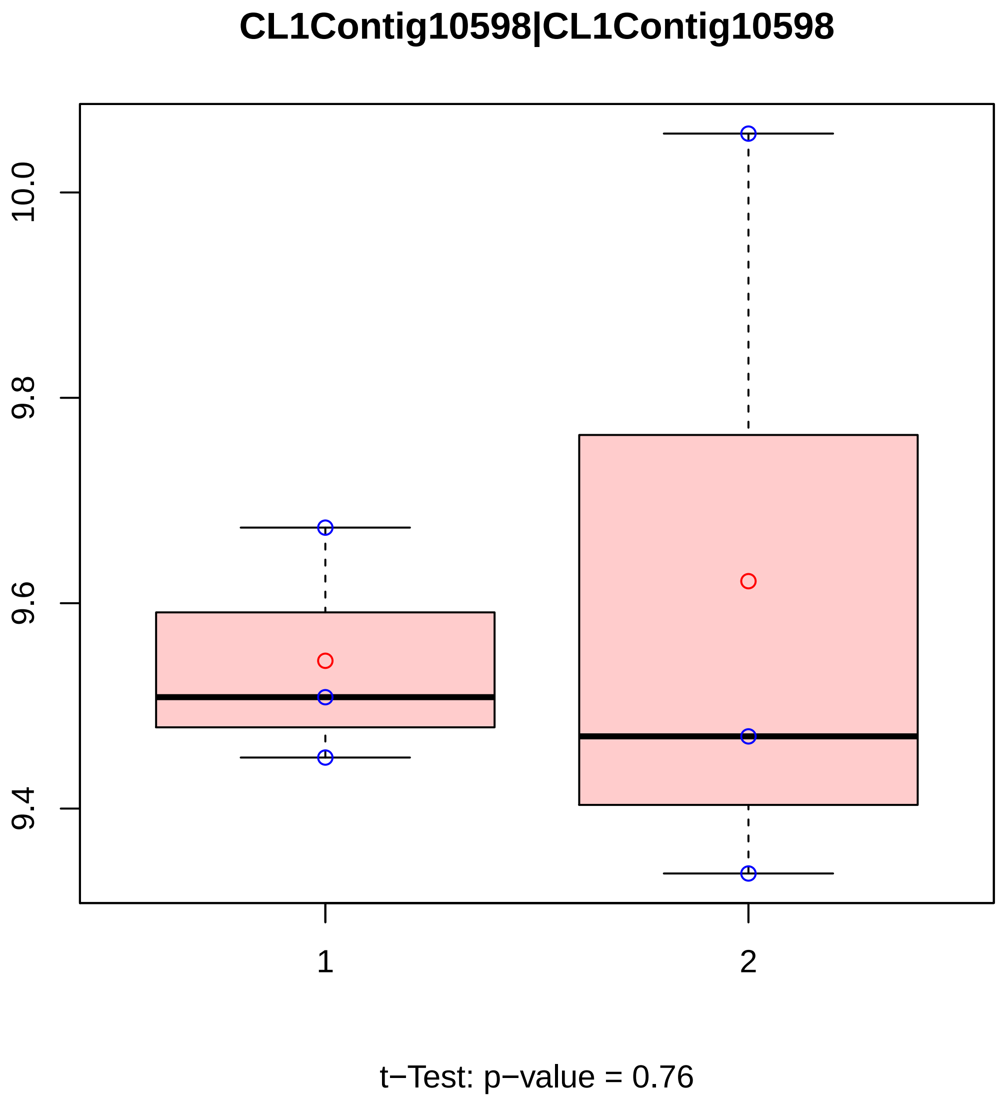
CL1Contig10598|CL1Contig10598
10.0
9.8
9.6
9.4
1
2
t−Test: p−value = 0.76

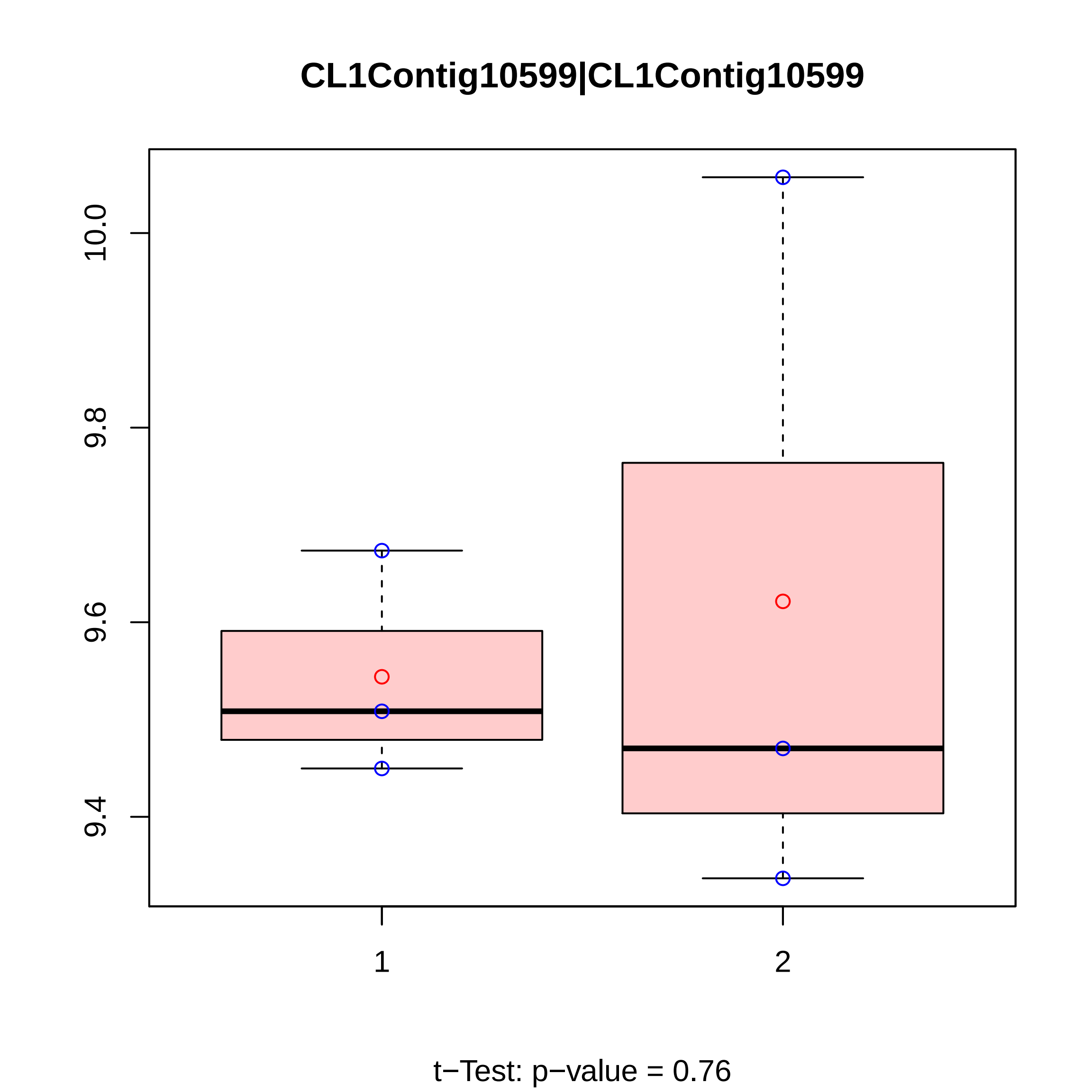
CL1Contig10599|CL1Contig10599
10.0
9.8
9.6
9.4
1
2
t−Test: p−value = 0.76

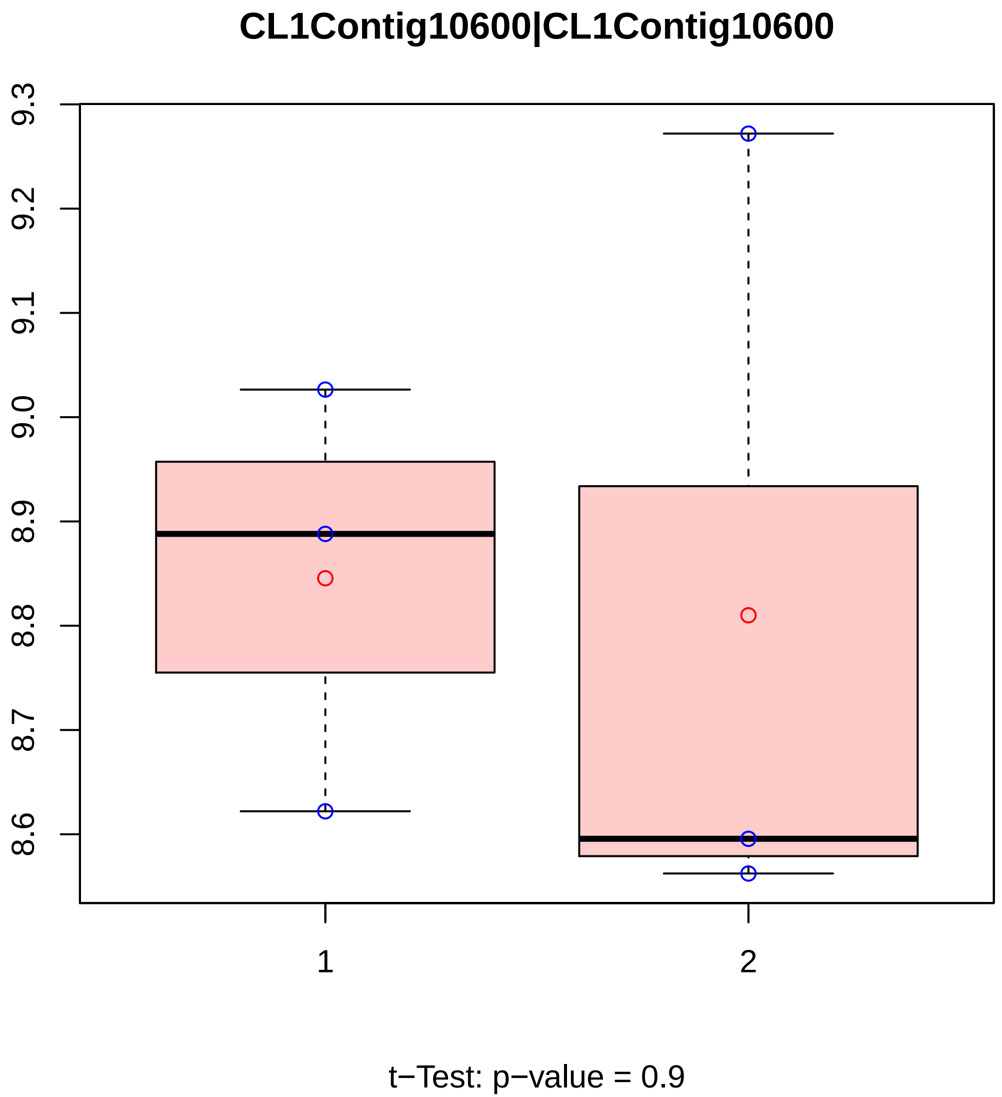

CL1Contig10600|CL1Contig10600
9.3
9.2
9.1
9.0
8.9
8.8
8.7
8.6
1
2
t−Test: p−value = 0.9

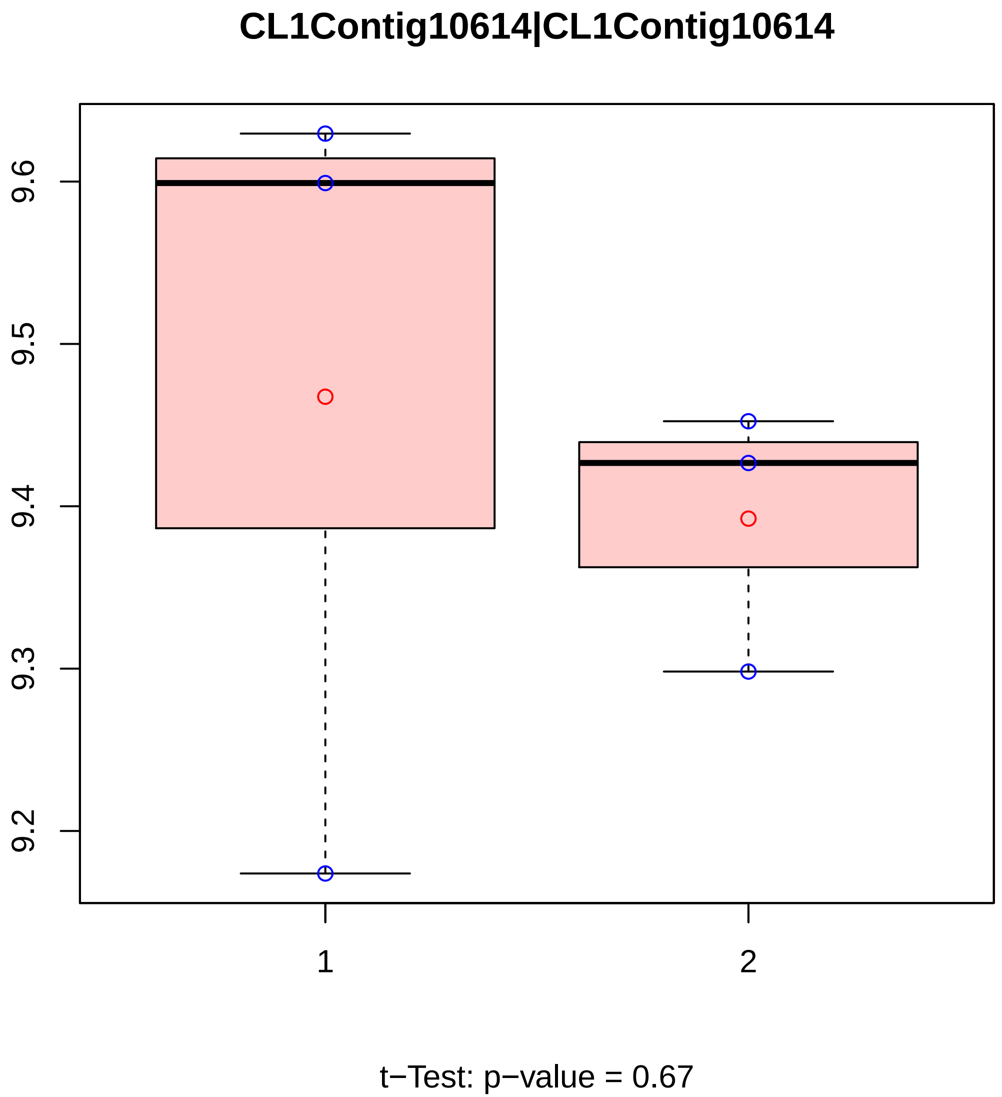
CL1Contig10614|CL1Contig10614
9.2
9.3
9.4
9.5
9.6
1
2
t−Test: p−value = 0.67

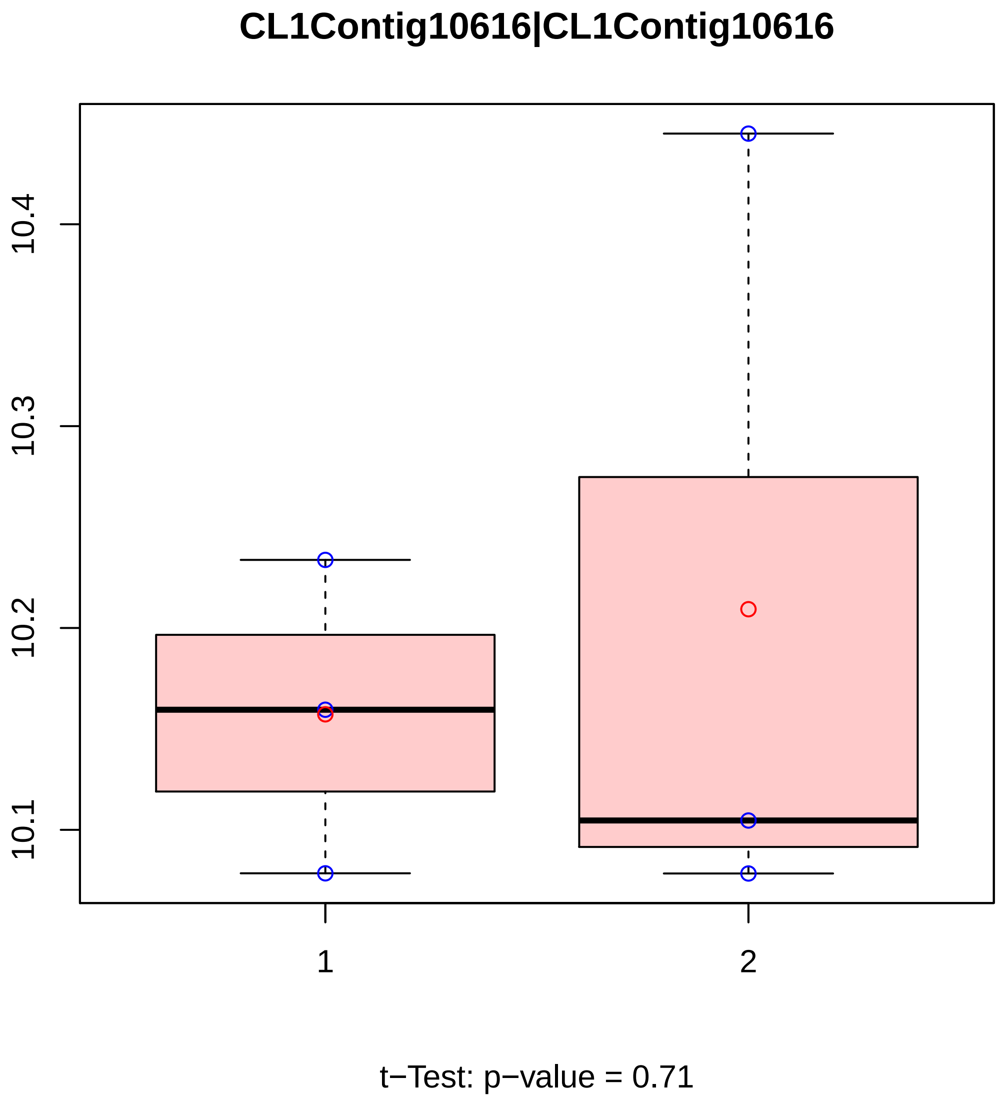
CL1Contig10616|CL1Contig10616
10.4
10.3
10.2
10.1
1
2
t−Test: p−value = 0.71

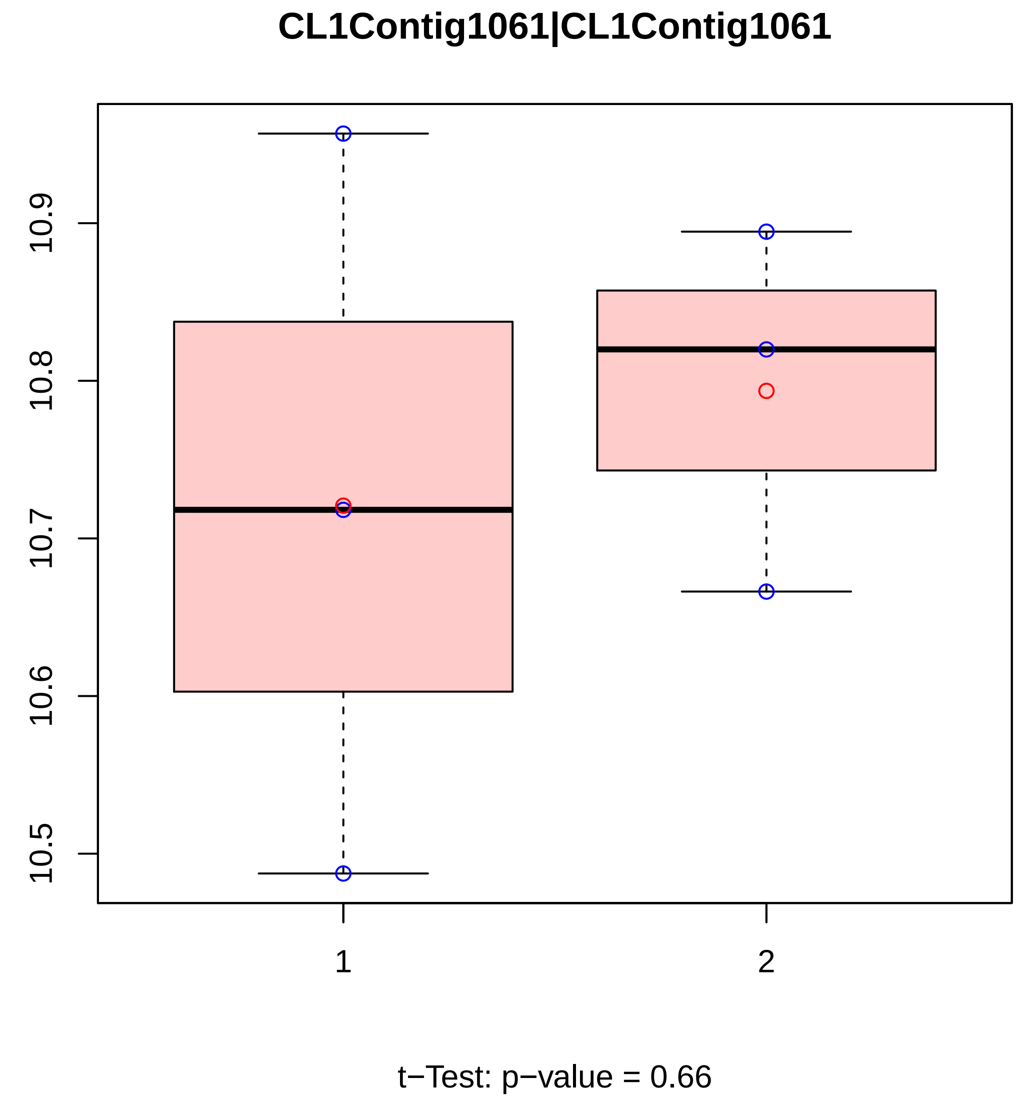
CL1Contig1061|CL1Contig1061
10.5
10.6
10.7
10.8
10.9
1
2
t−Test: p−value = 0.66

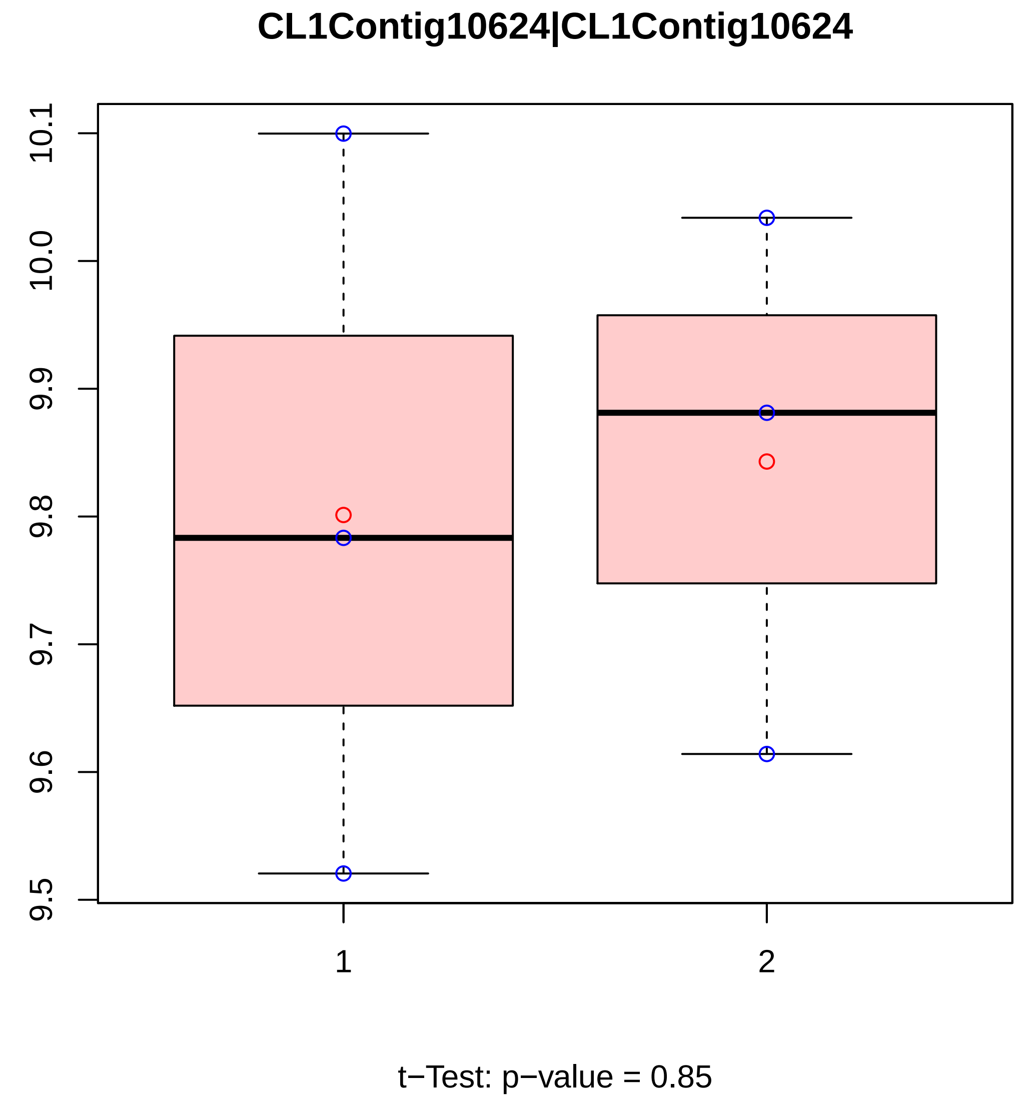
CL1Contig10624|CL1Contig10624
10.1
10.0
9.9
9.8
9.7
9.6
9.5
1
2
t−Test: p−value = 0.85

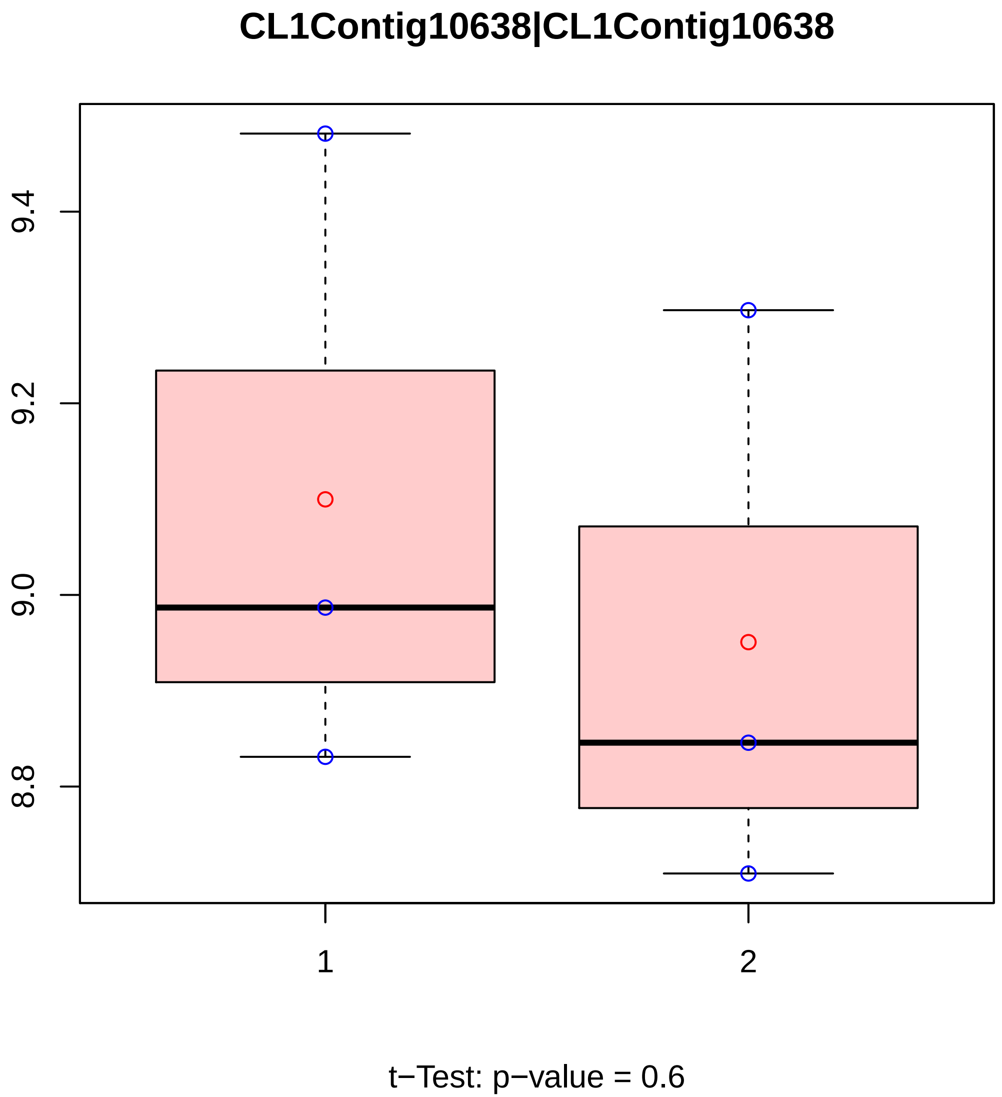
CL1Contig10638|CL1Contig10638
9.4
9.2
9.0
8.8
1
2
t−Test: p−value = 0.6

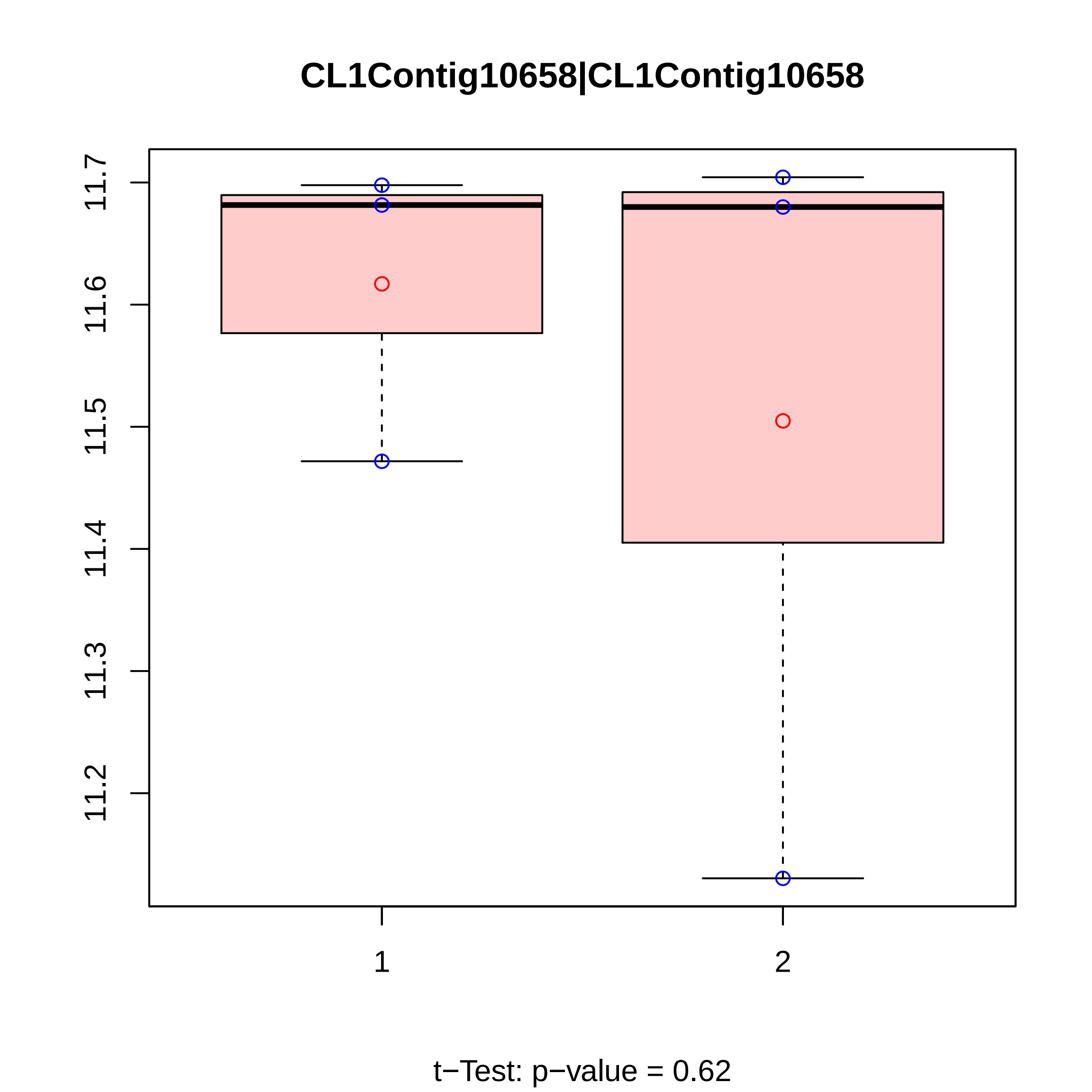

CL1Contig10658|CL1Contig10658
11.2
11.3
11.4
11.5
11.6
11.7
1
2
t−Test: p−value = 0.62

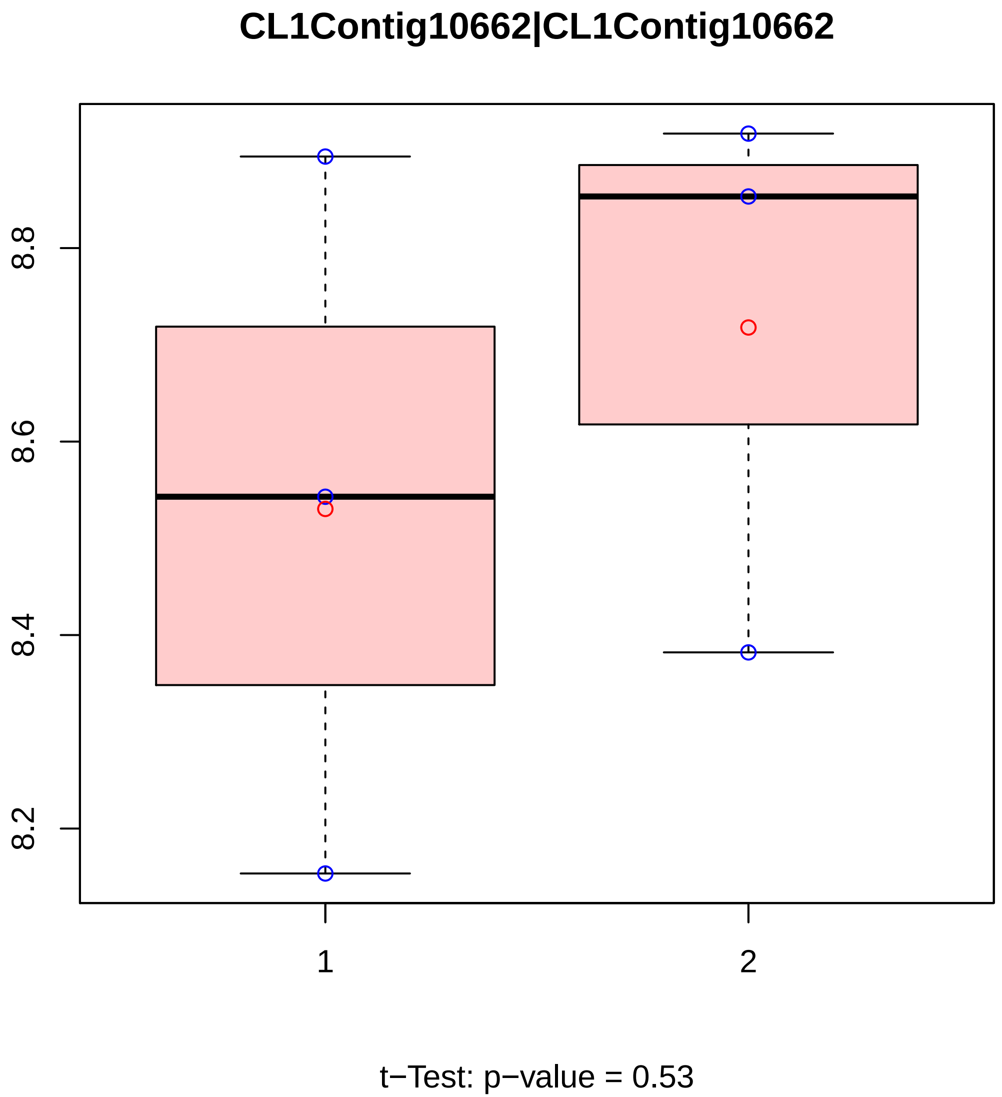

CL1Contig10662|CL1Contig10662
8.2
8.4
8.6
8.8
1
2
t−Test: p−value = 0.53

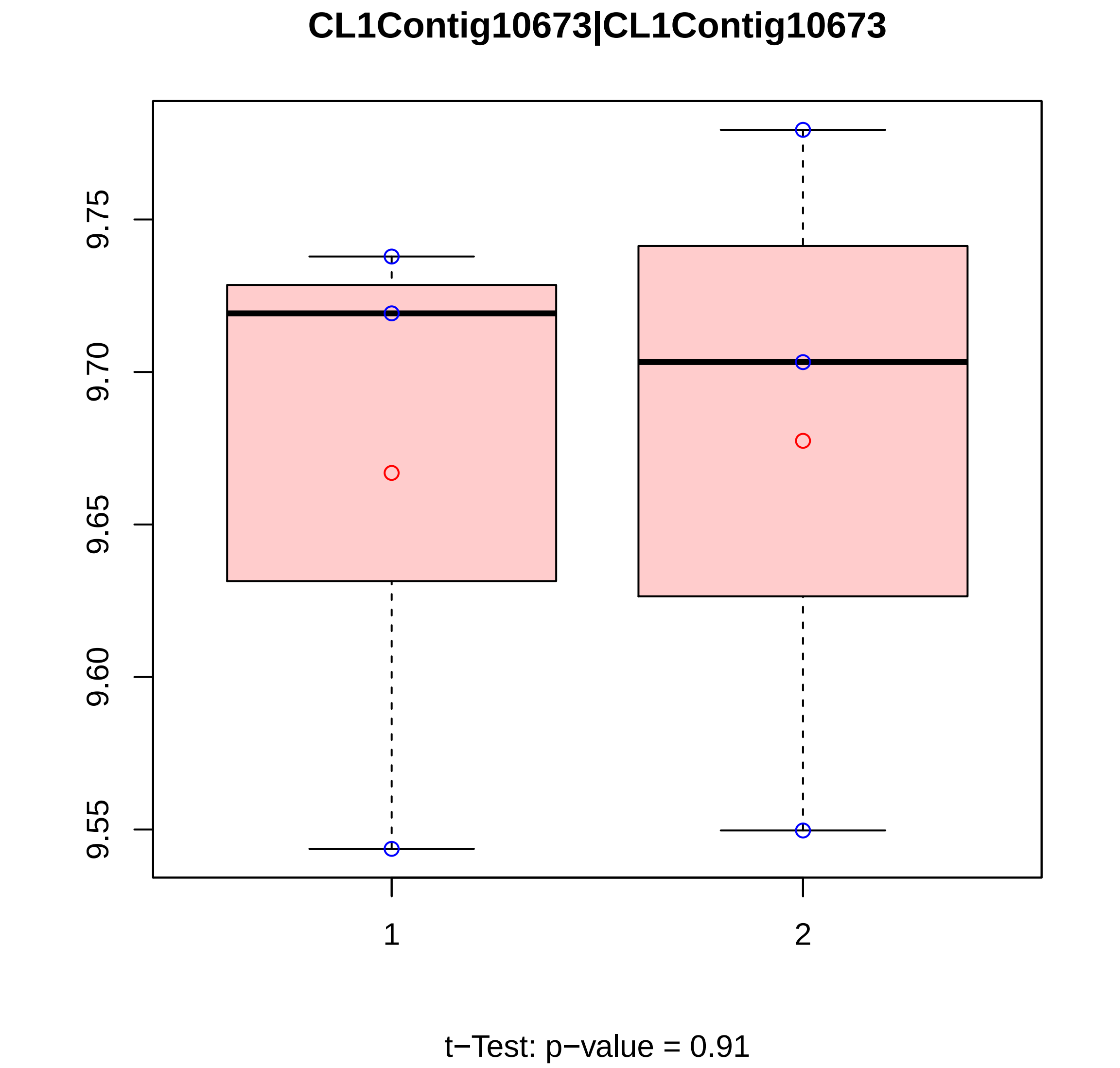
CL1Contig10673|CL1Contig10673
9.55
9.60
9.65
9.70
9.75
1
2
t−Test: p−value = 0.91

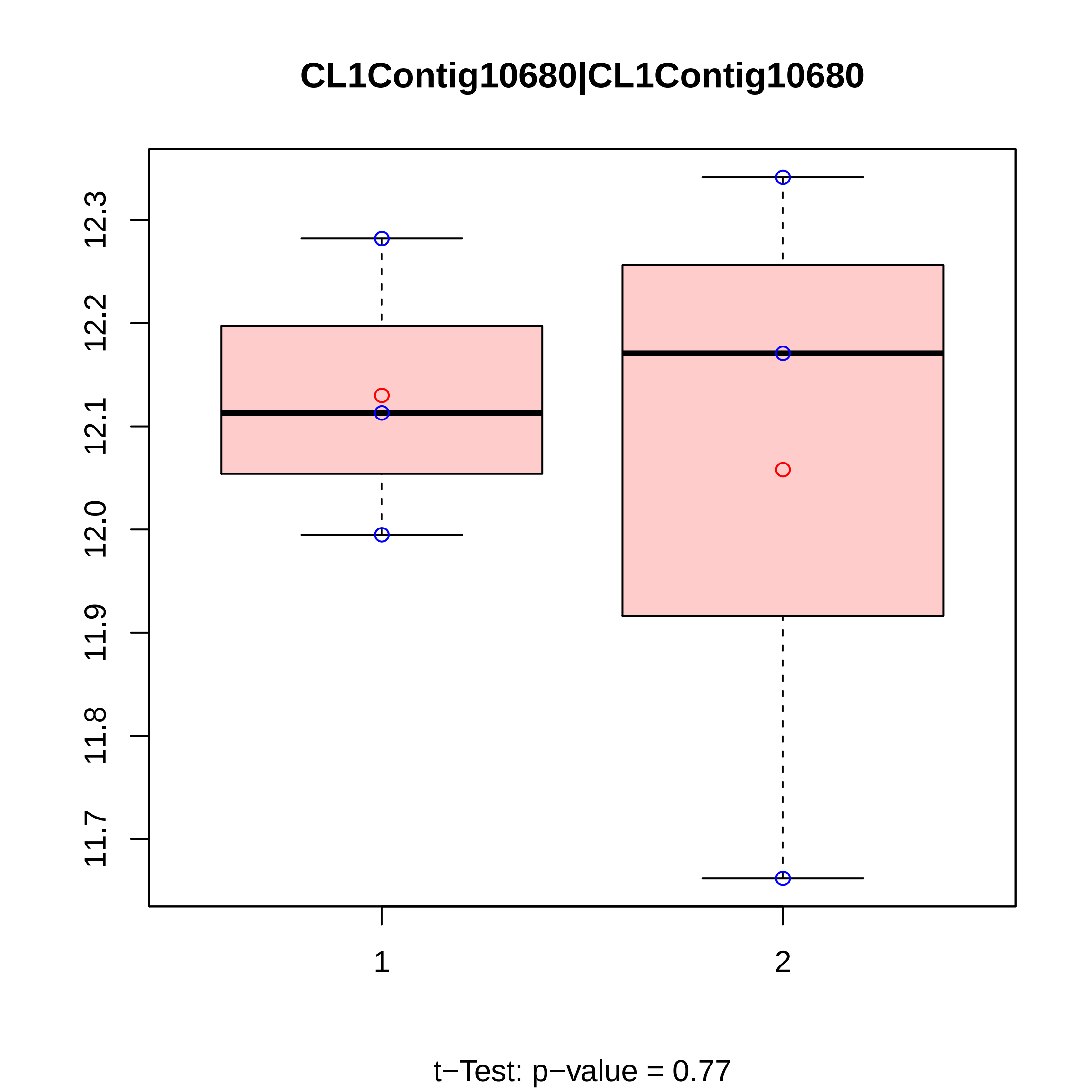
CL1Contig10680|CL1Contig10680
12.3
12.2
12.1
12.0
11.9
11.8
11.7
1
2
t−Test: p−value = 0.77

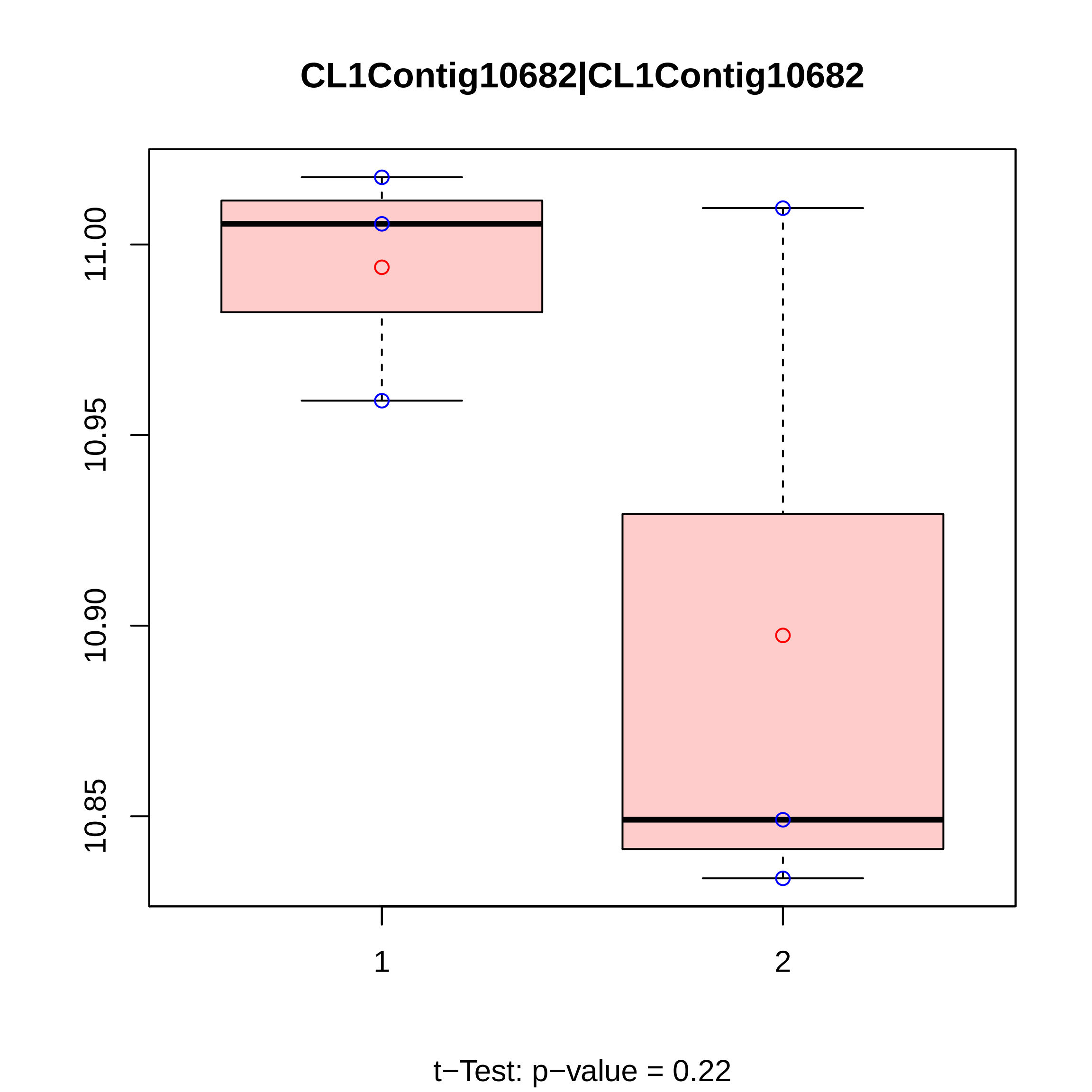

CL1Contig10682|CL1Contig10682
11.00
10.95
10.90
10.85
1
2
t−Test: p−value = 0.22

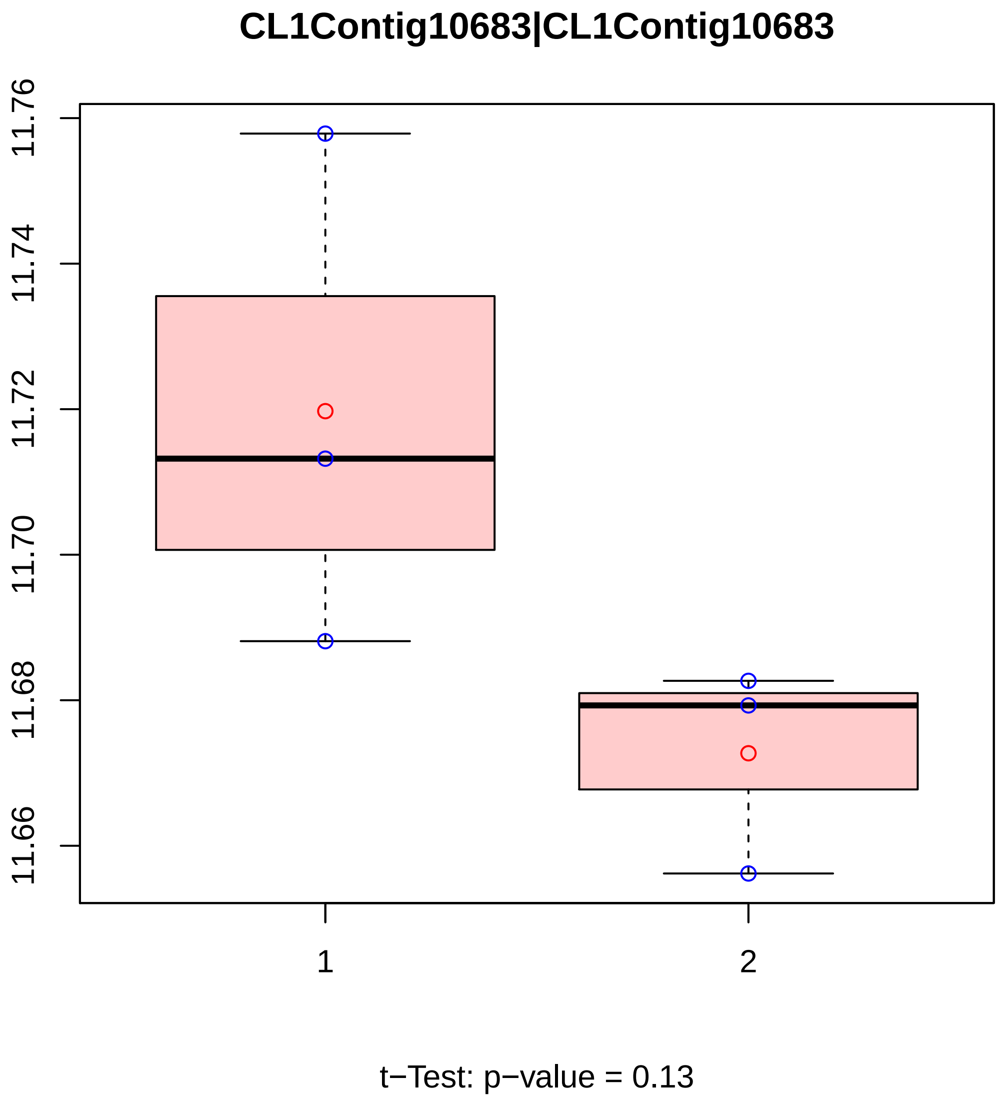
CL1Contig10683|CL1Contig10683
11.76
11.74
11.72
11.70
11.68
11.66
1
2
t−Test: p−value = 0.13

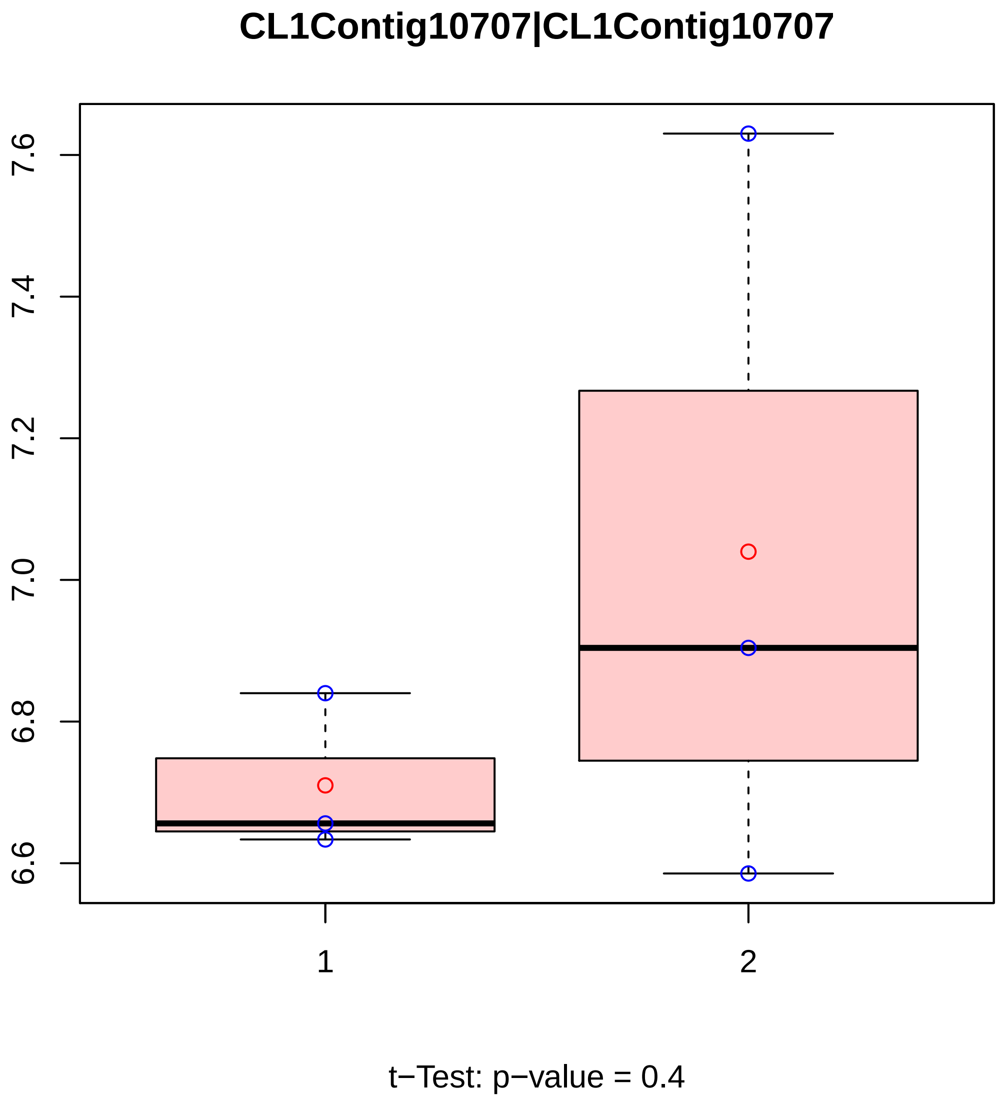

CL1Contig10707|CL1Contig10707
7.6
7.4
7.2
7.0
6.8
6.6
1
2
t−Test: p−value = 0.4

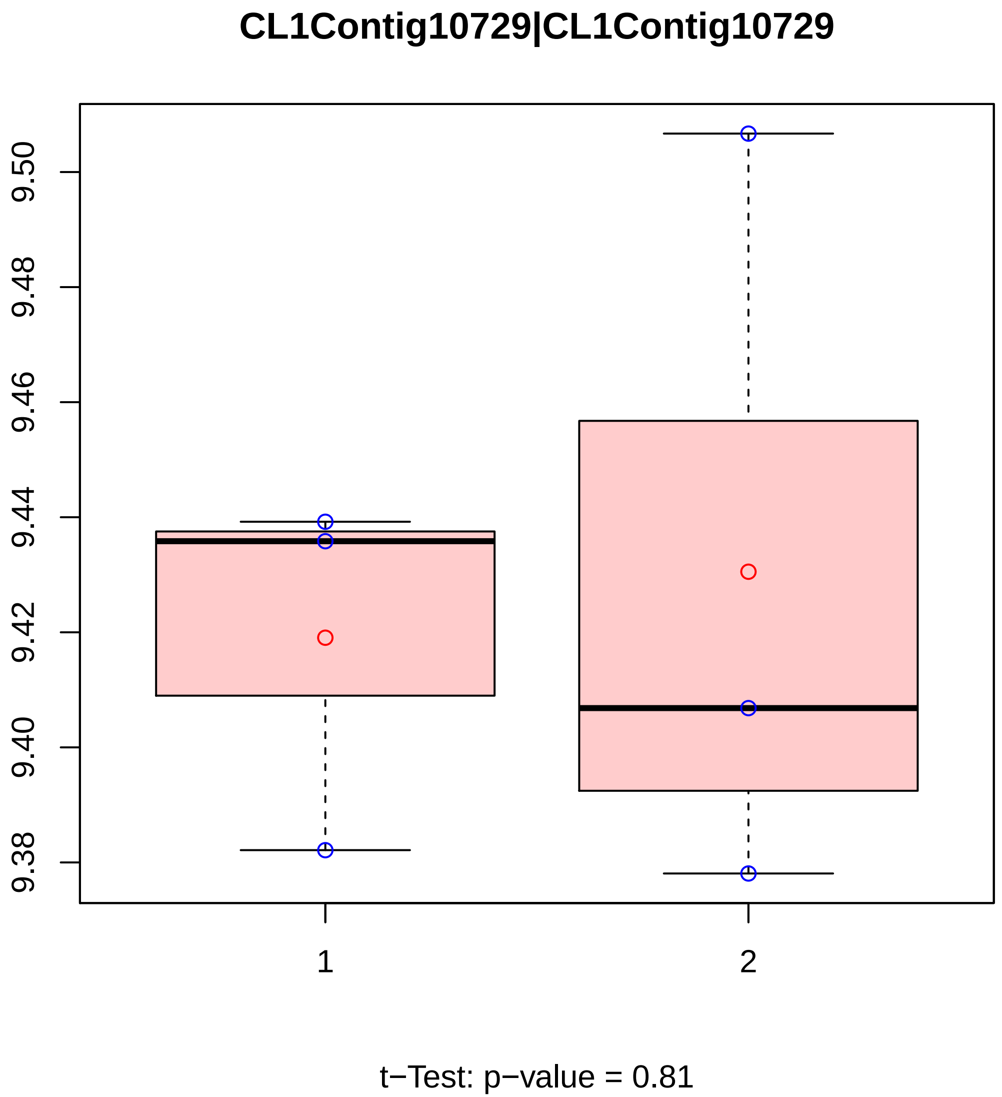
CL1Contig10729|CL1Contig10729
9.38
9.40
9.42
9.44
9.46
9.48
9.50
1
2
t−Test: p−value = 0.81

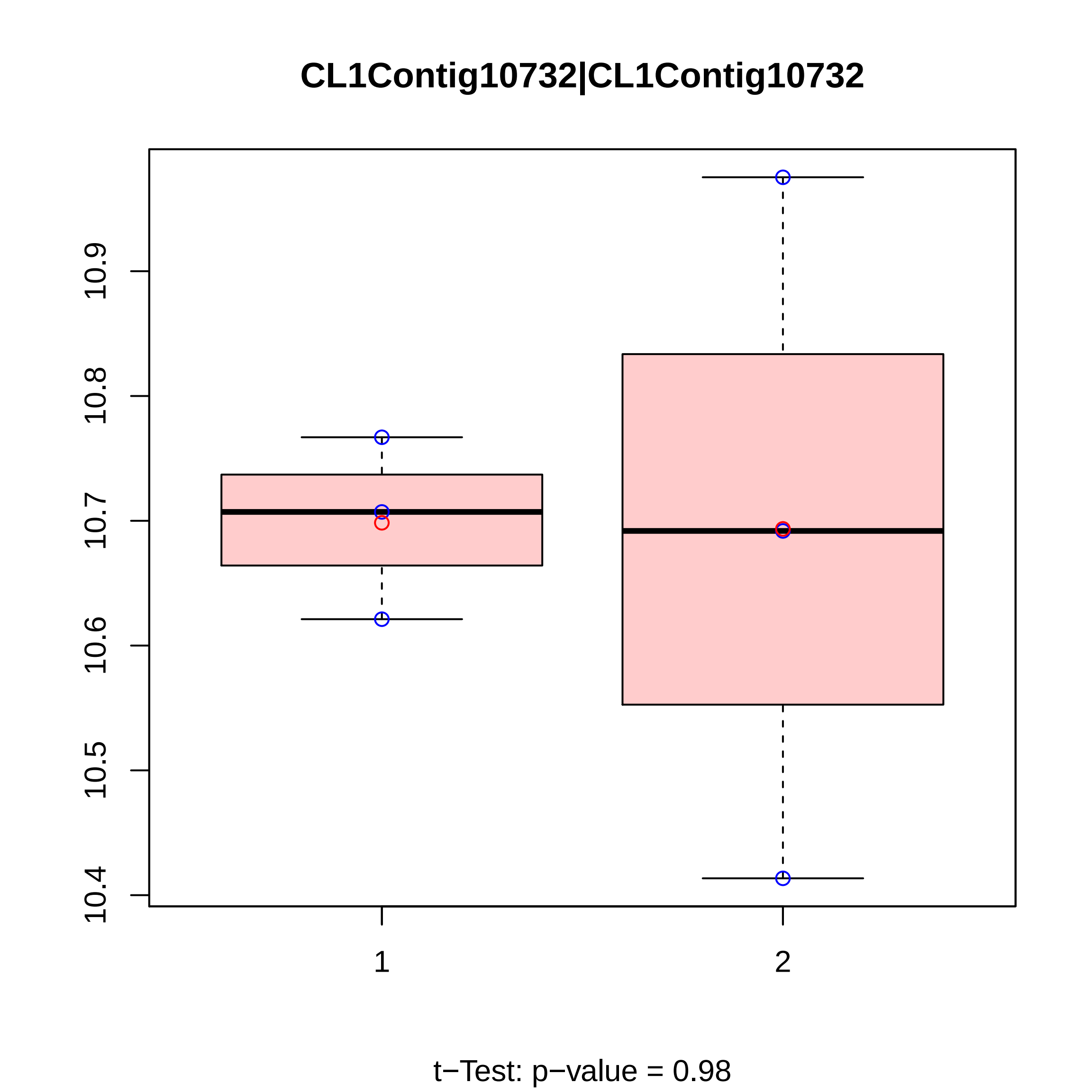
CL1Contig10732|CL1Contig10732
10.4
10.5
10.6
10.7
10.8
10.9
1
2
t−Test: p−value = 0.98

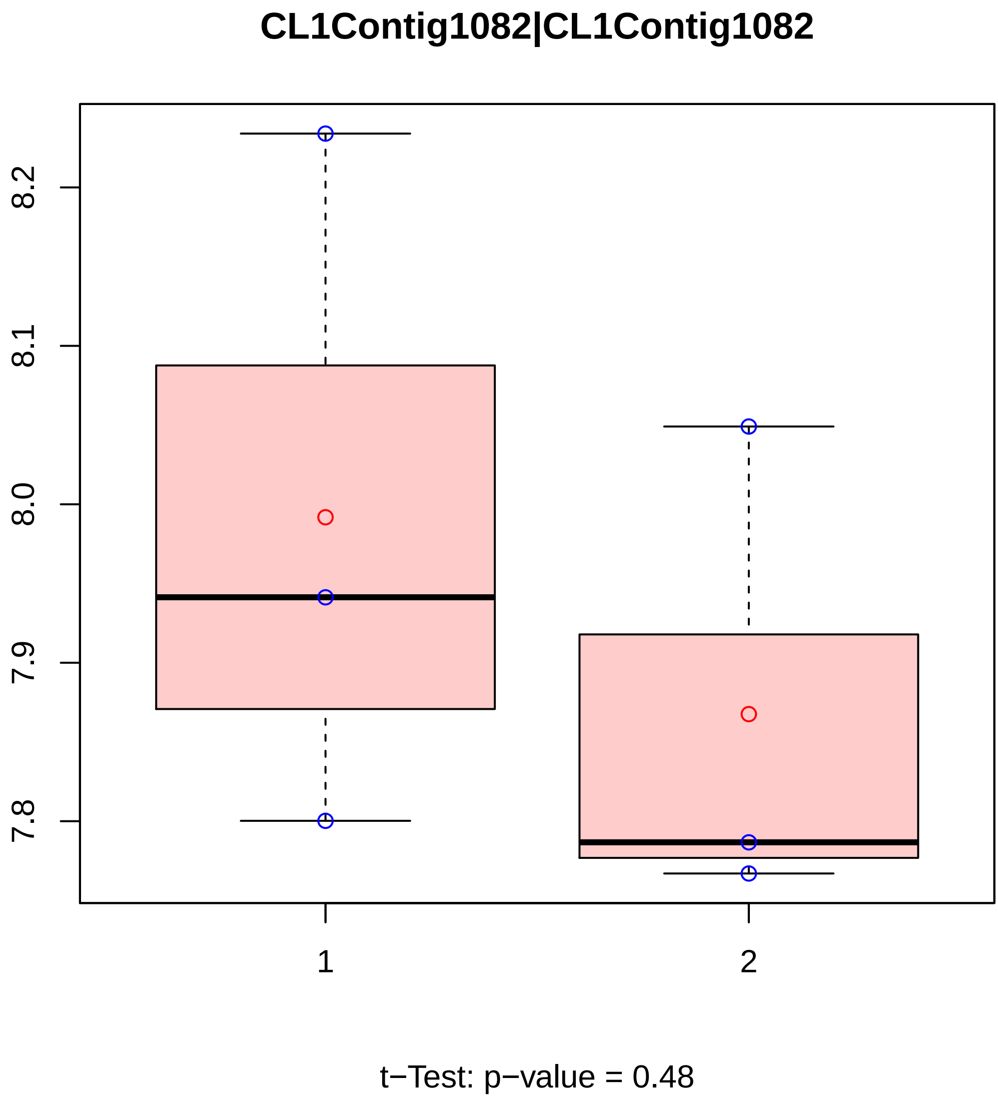

CL1Contig1082|CL1Contig1082
8.2
8.1
8.0
7.9
7.8
1
2
t−Test: p−value = 0.48

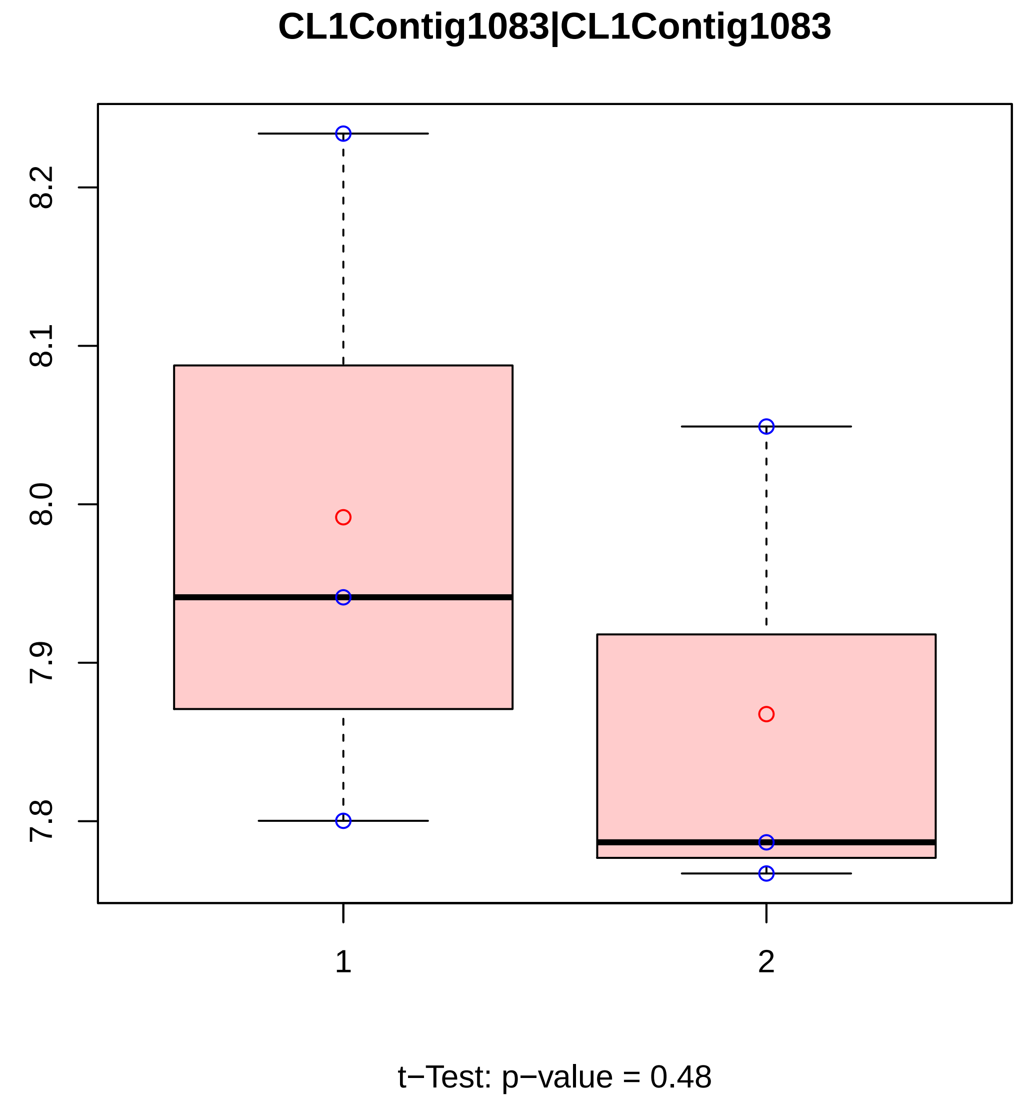
CL1Contig1083|CL1Contig1083
8.2
8.1
8.0
7.9
7.8
1
2
t−Test: p−value = 0.48

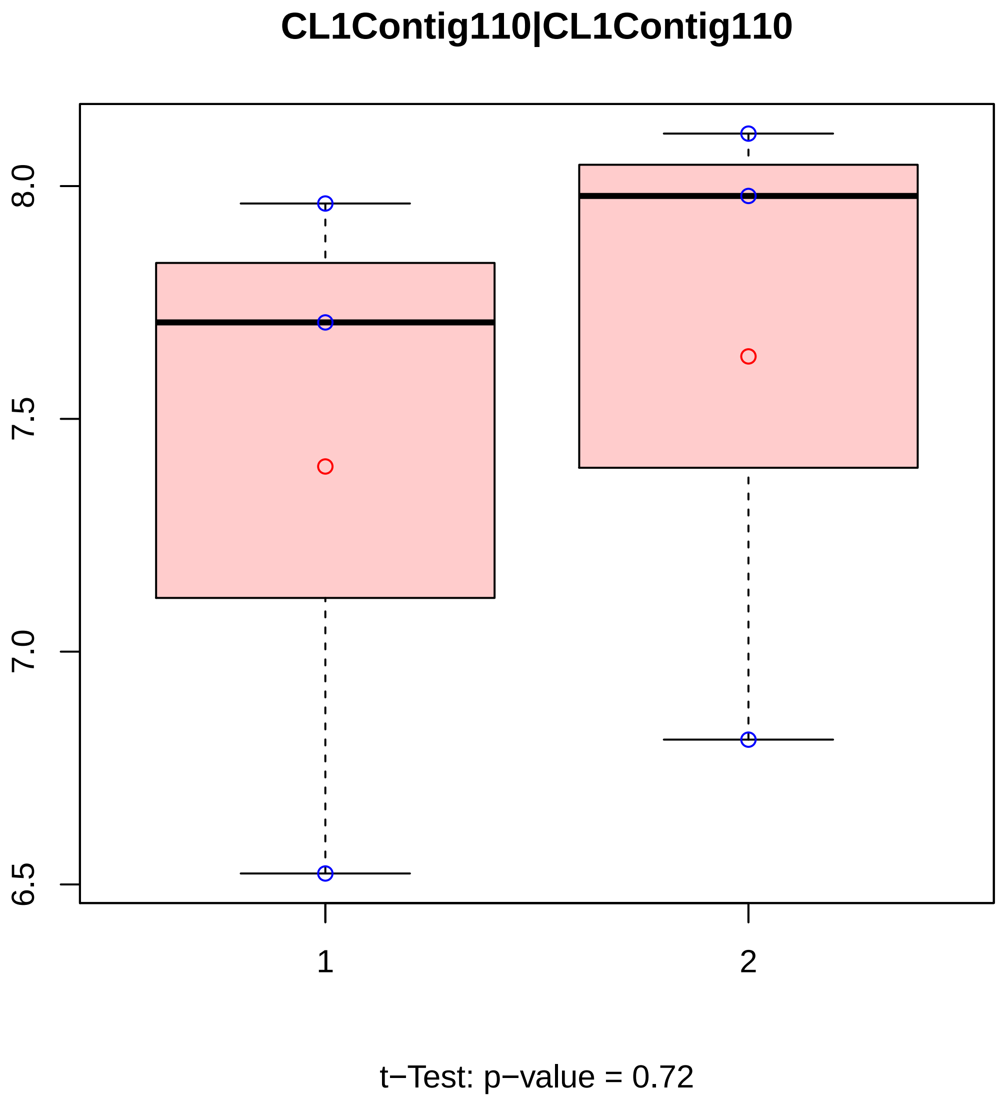

CL1Contig110|CL1Contig110
8.0
7.5
7.0
6.5
1
2
t−Test: p−value = 0.72

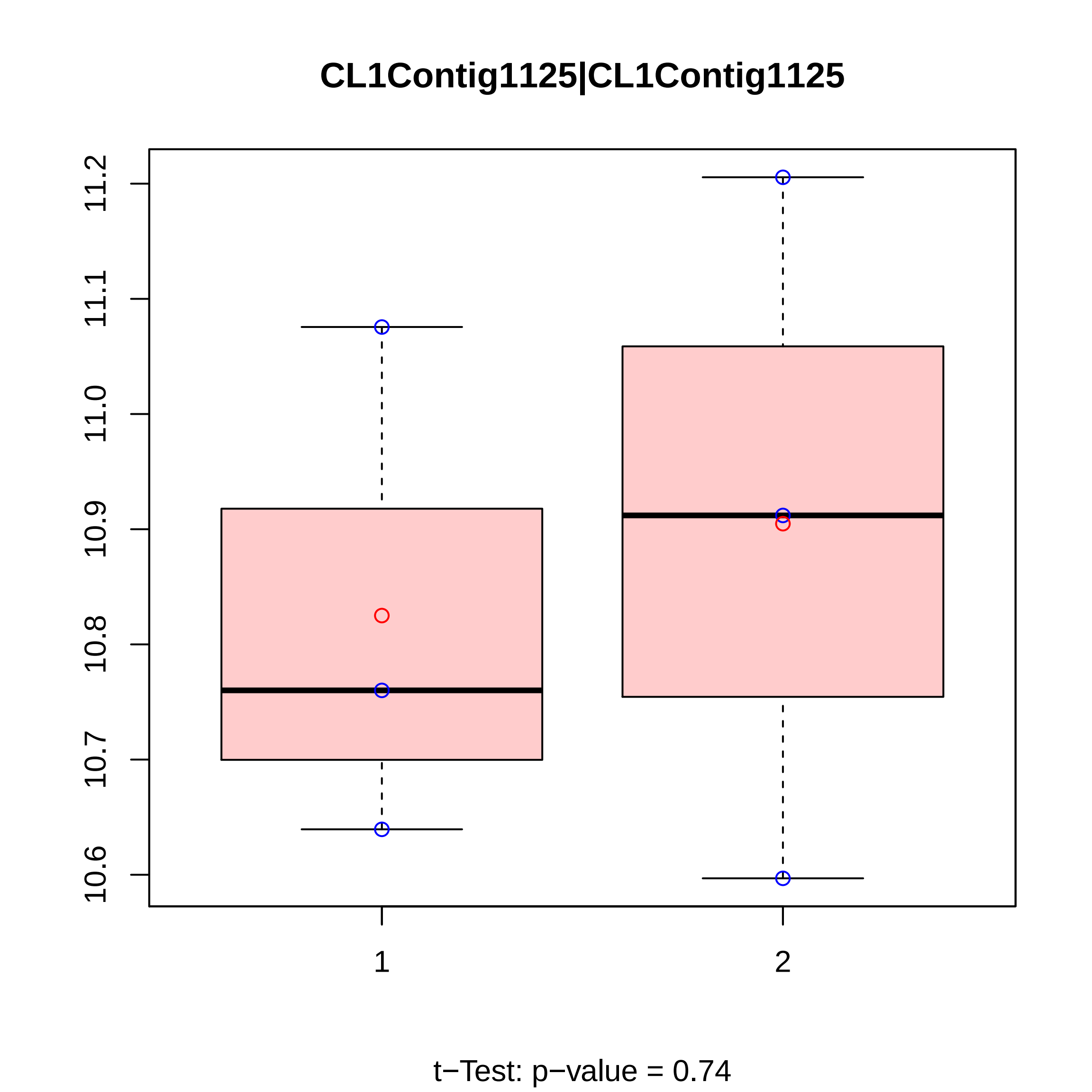
CL1Contig1125|CL1Contig1125
11.2
11.1
11.0
10.9
10.8
10.7
10.6
1
2
t−Test: p−value = 0.74

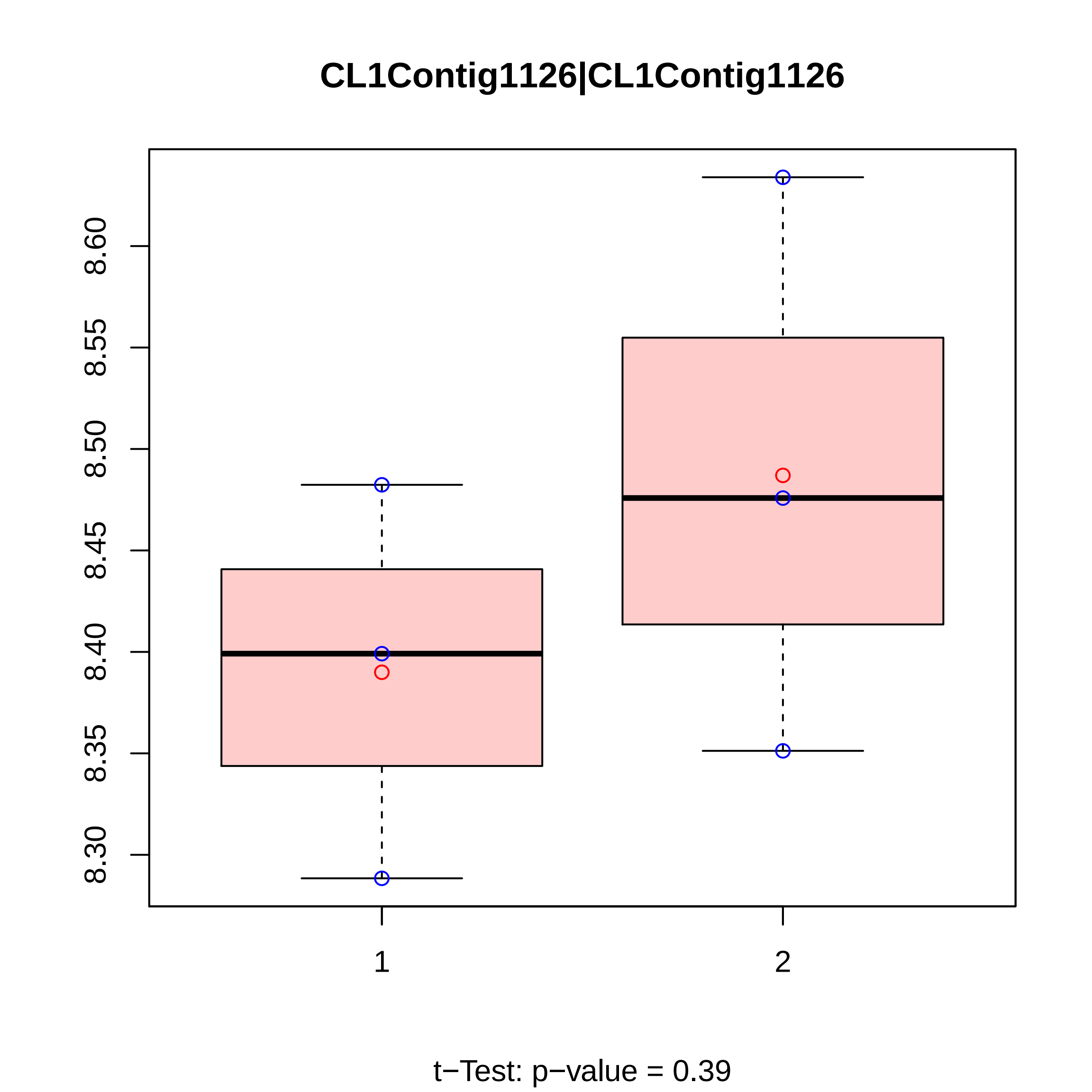
CL1Contig1126|CL1Contig1126
8.30
8.35
8.40
8.45
8.50
8.55
8.60
1
2
t−Test: p−value = 0.39

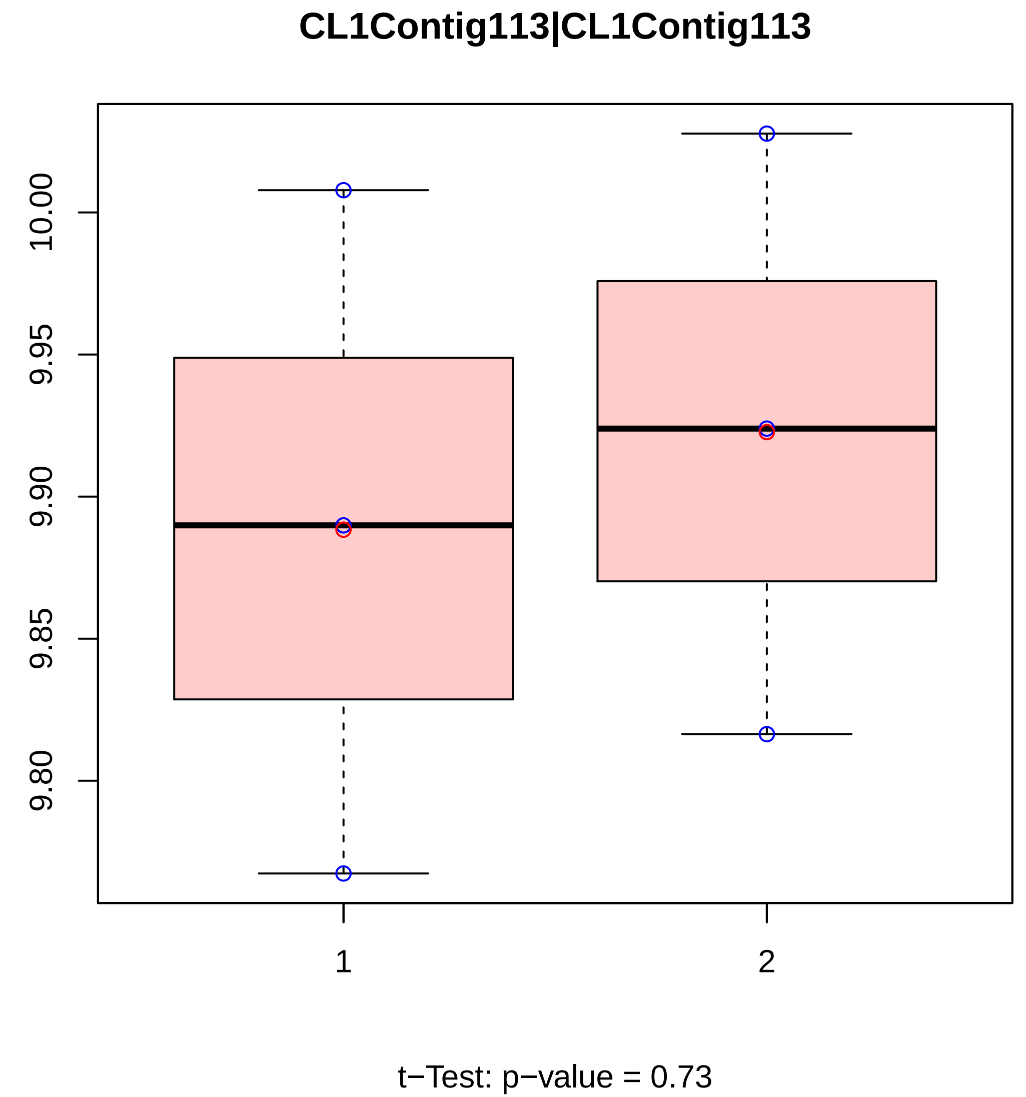

CL1Contig113|CL1Contig113
10.00
9.95
9.90
9.85
9.80
1
2
t−Test: p−value = 0.73

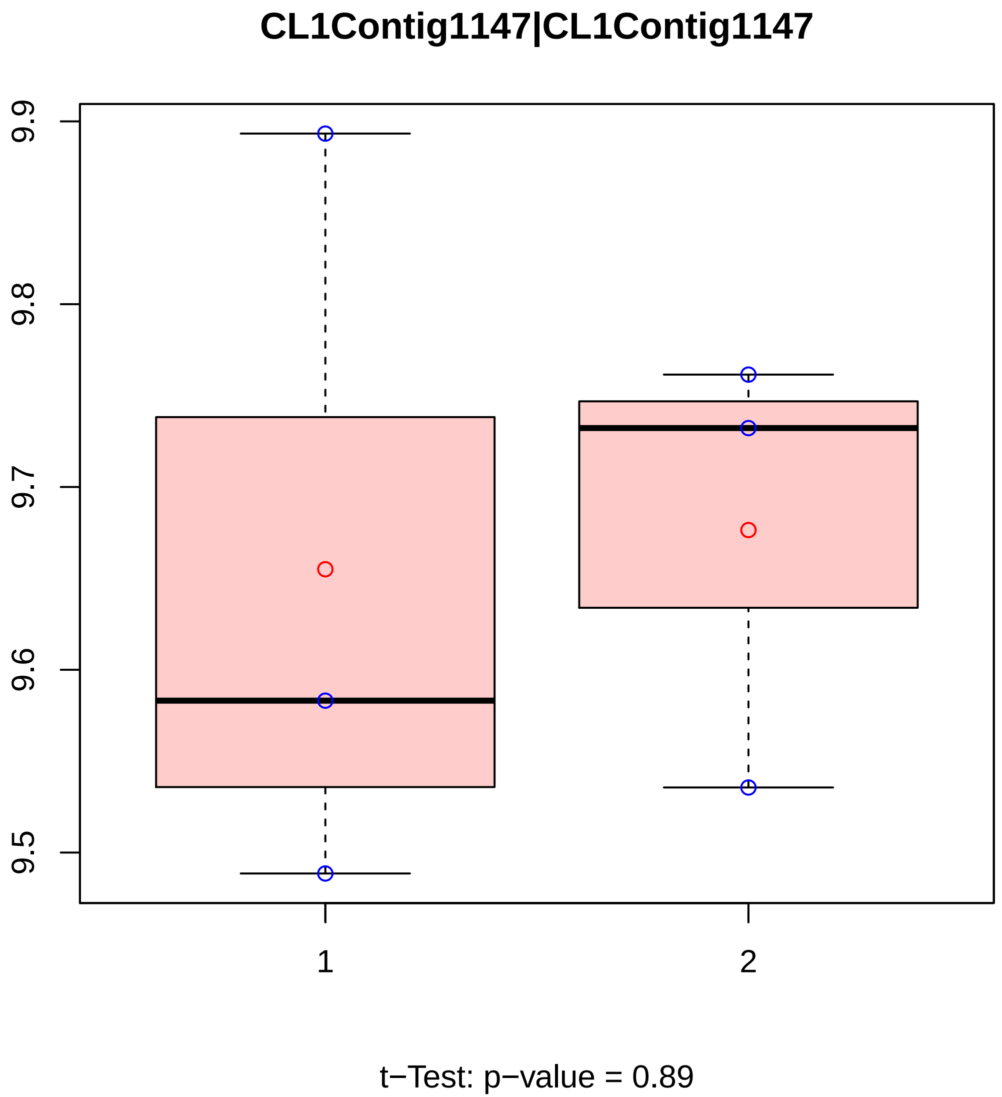

CL1Contig1147|CL1Contig1147
9.5
9.6
9.7
9.8
9.9
1
2
t−Test: p−value = 0.89

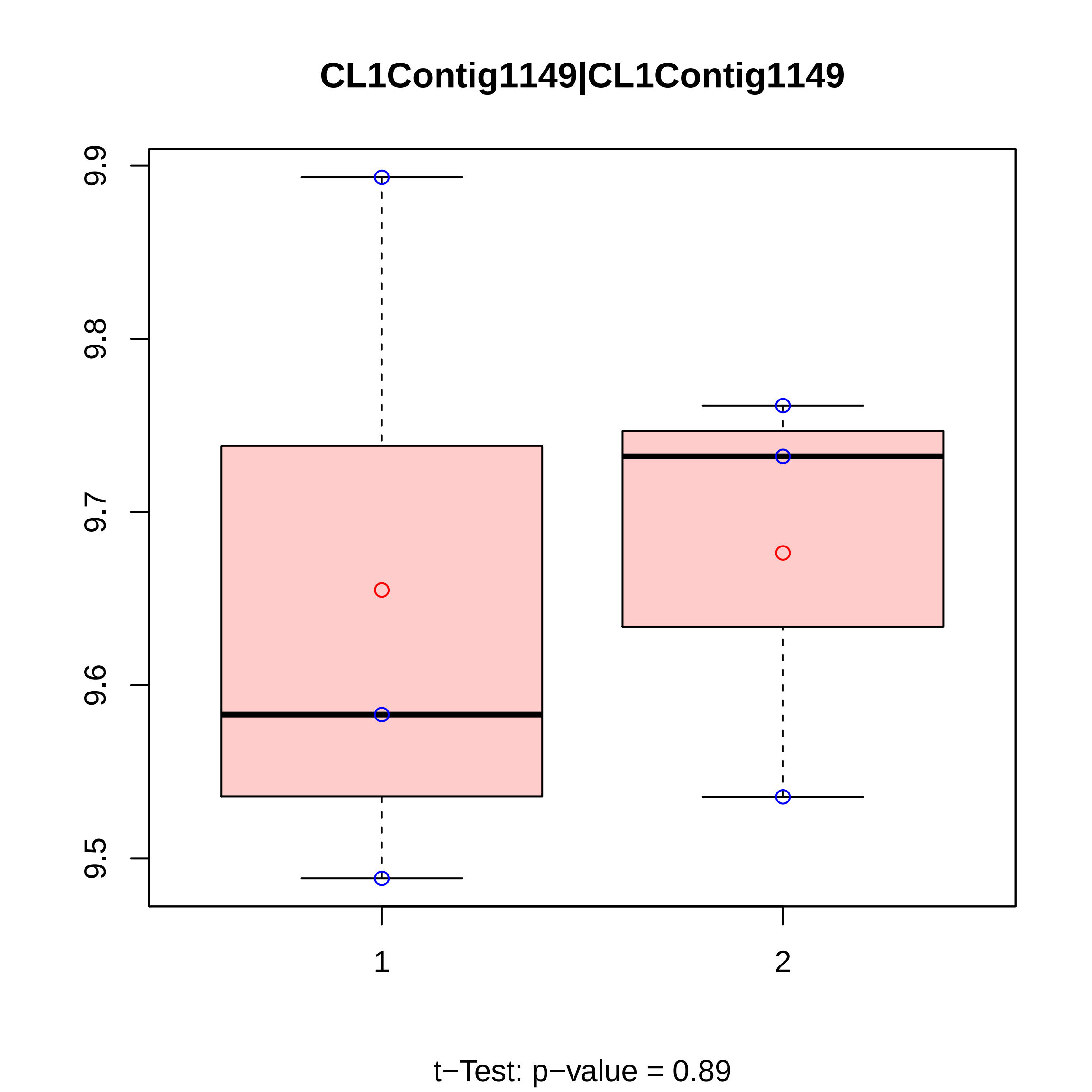

CL1Contig1149|CL1Contig1149
9.5
9.6
9.7
9.8
9.9
1
2
t−Test: p−value = 0.89

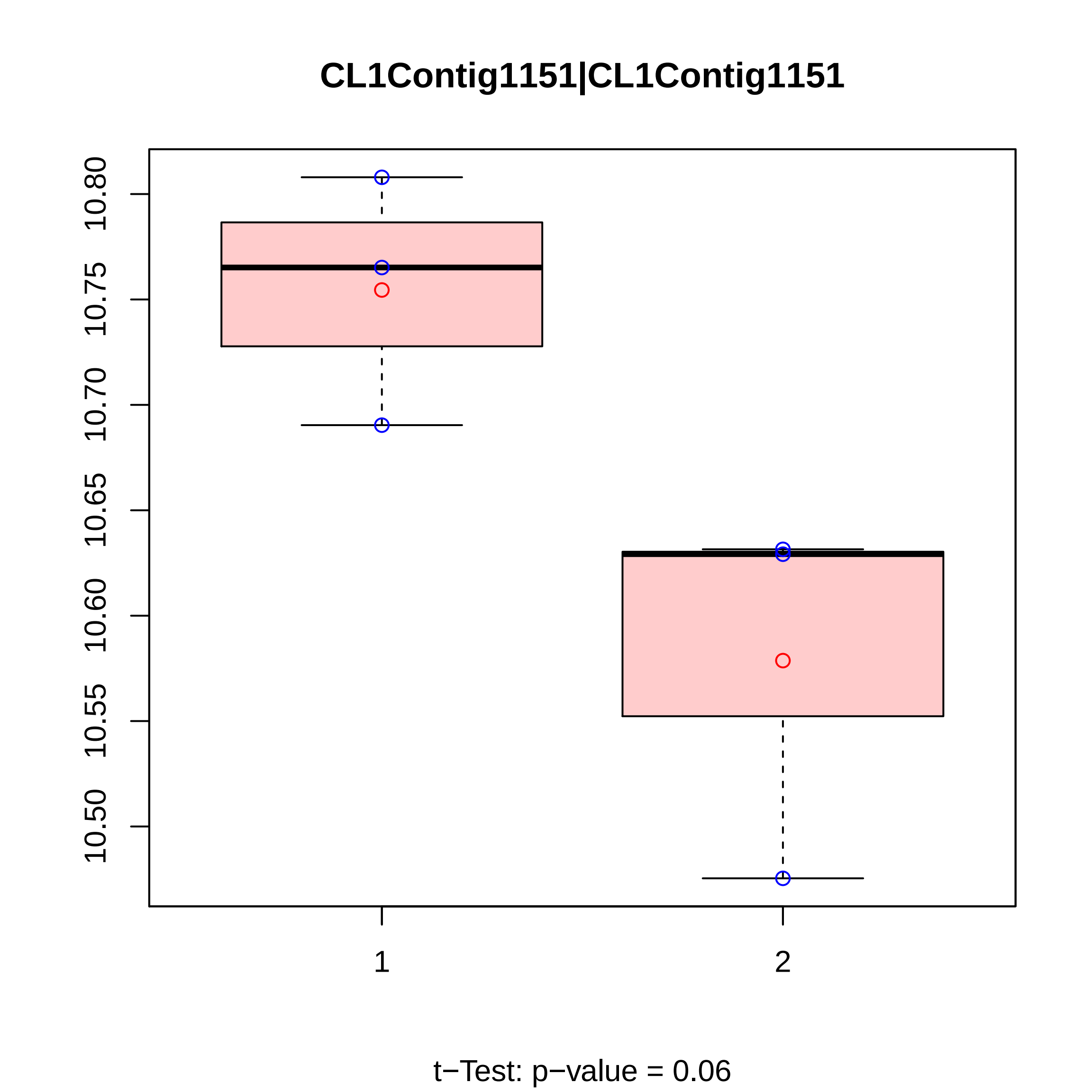
CL1Contig1151|CL1Contig1151
10.50
10.55
10.60
10.65
10.70
10.75
10.80
1
2
t−Test: p−value = 0.06

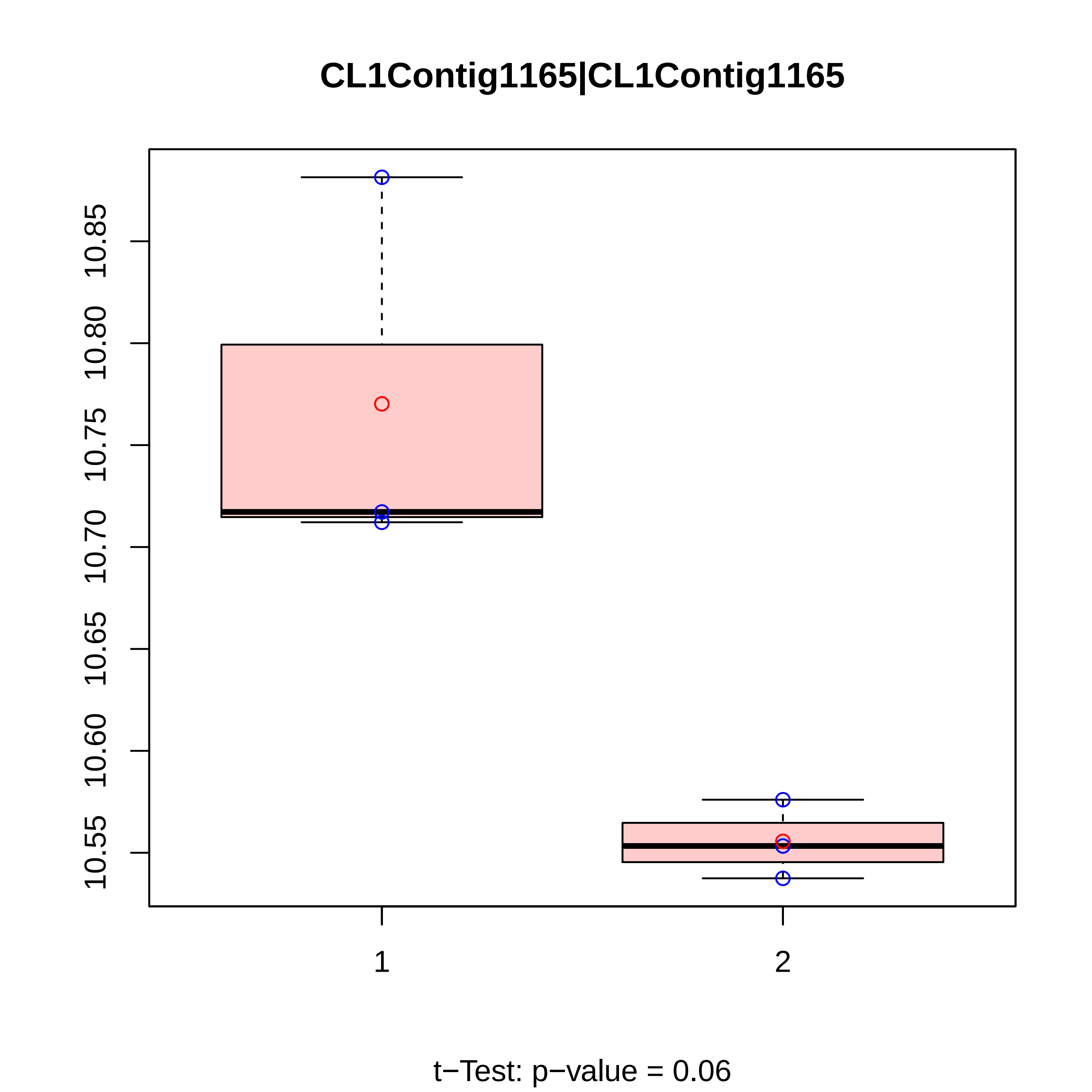
CL1Contig1165|CL1Contig1165
10.55
10.60
10.65
10.70
10.75
10.80
10.85
1
2
t−Test: p−value = 0.06

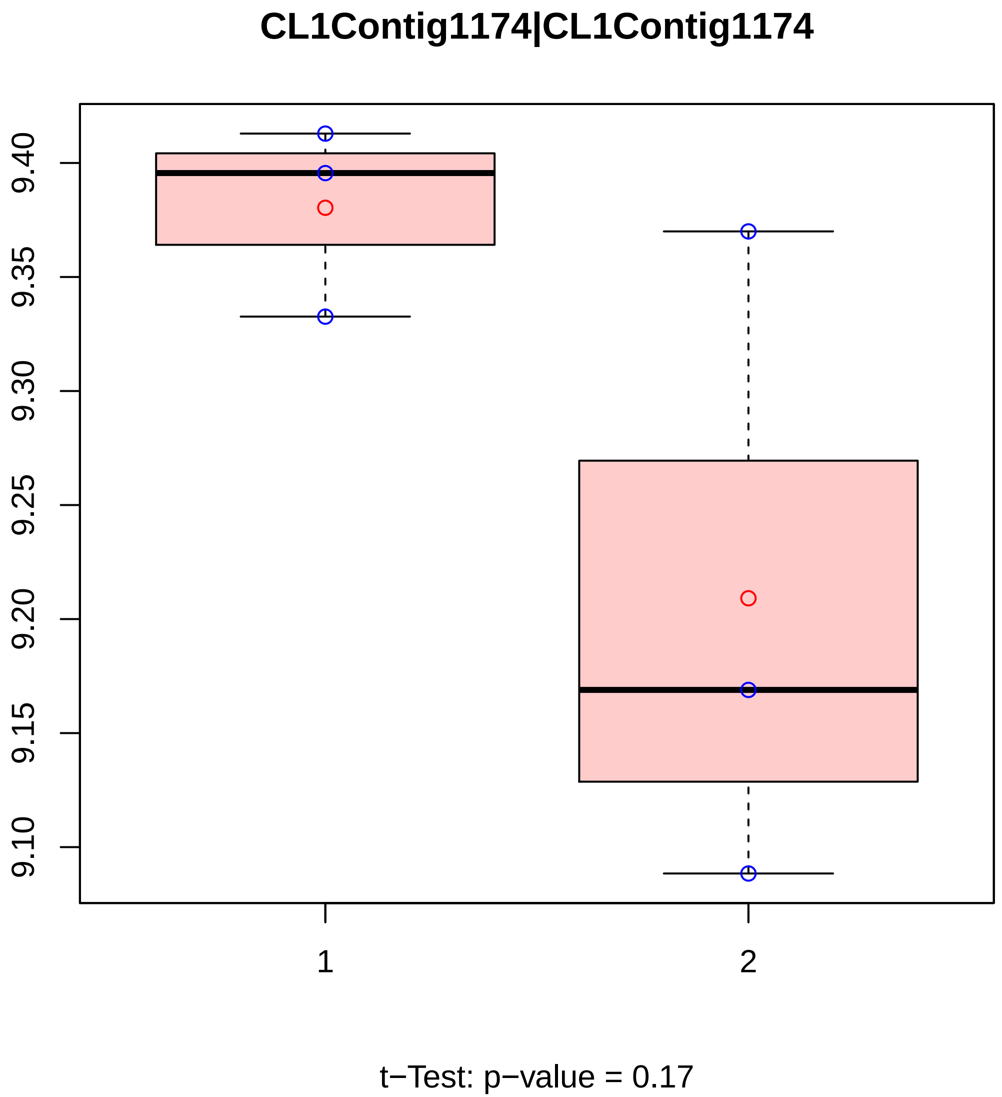
CL1Contig1174|CL1Contig1174
9.40
9.35
9.30
9.25
9.20
9.15
9.10
1
2
t−Test: p−value = 0.17

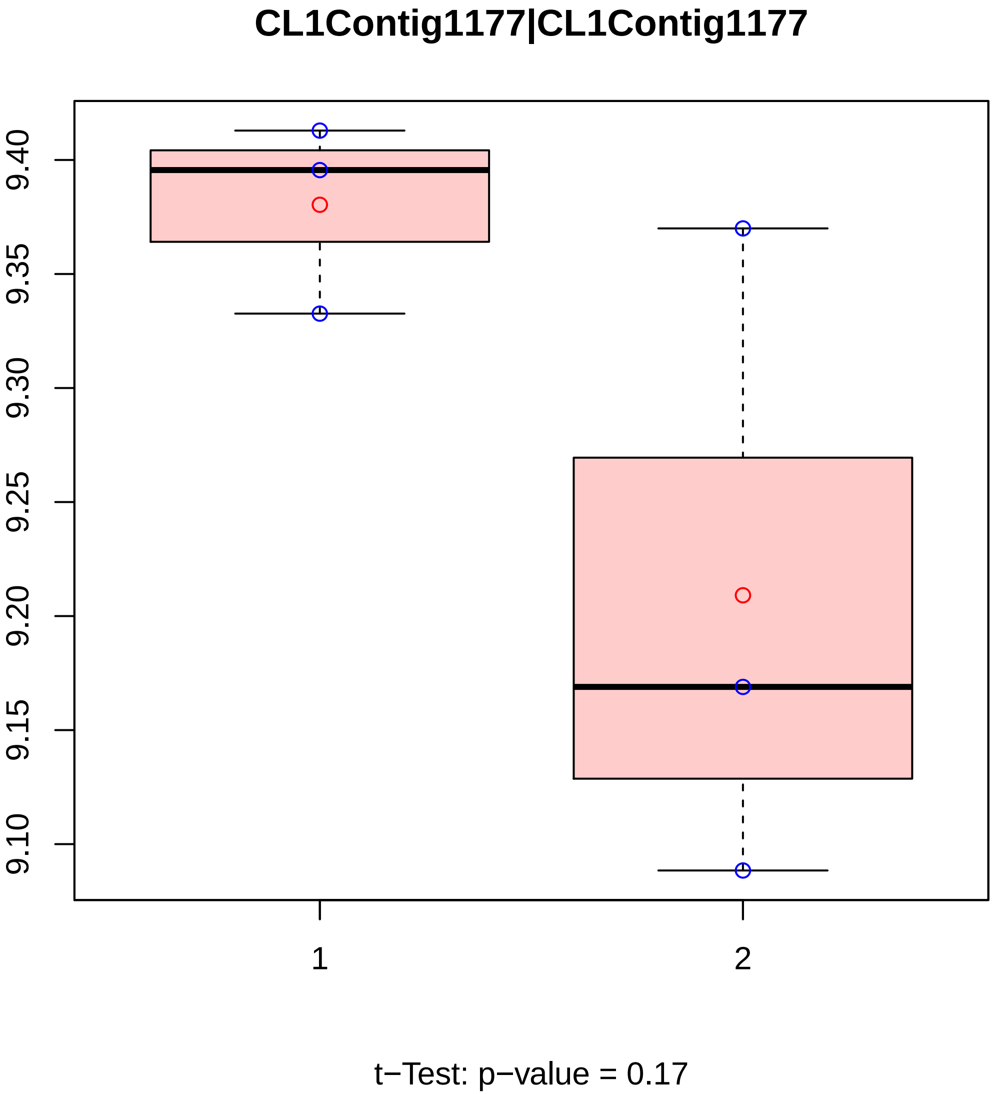

CL1Contig1177|CL1Contig1177
9.10
9.15
9.20
9.25
9.30
9.35
9.40
1
2
t−Test: p−value = 0.17

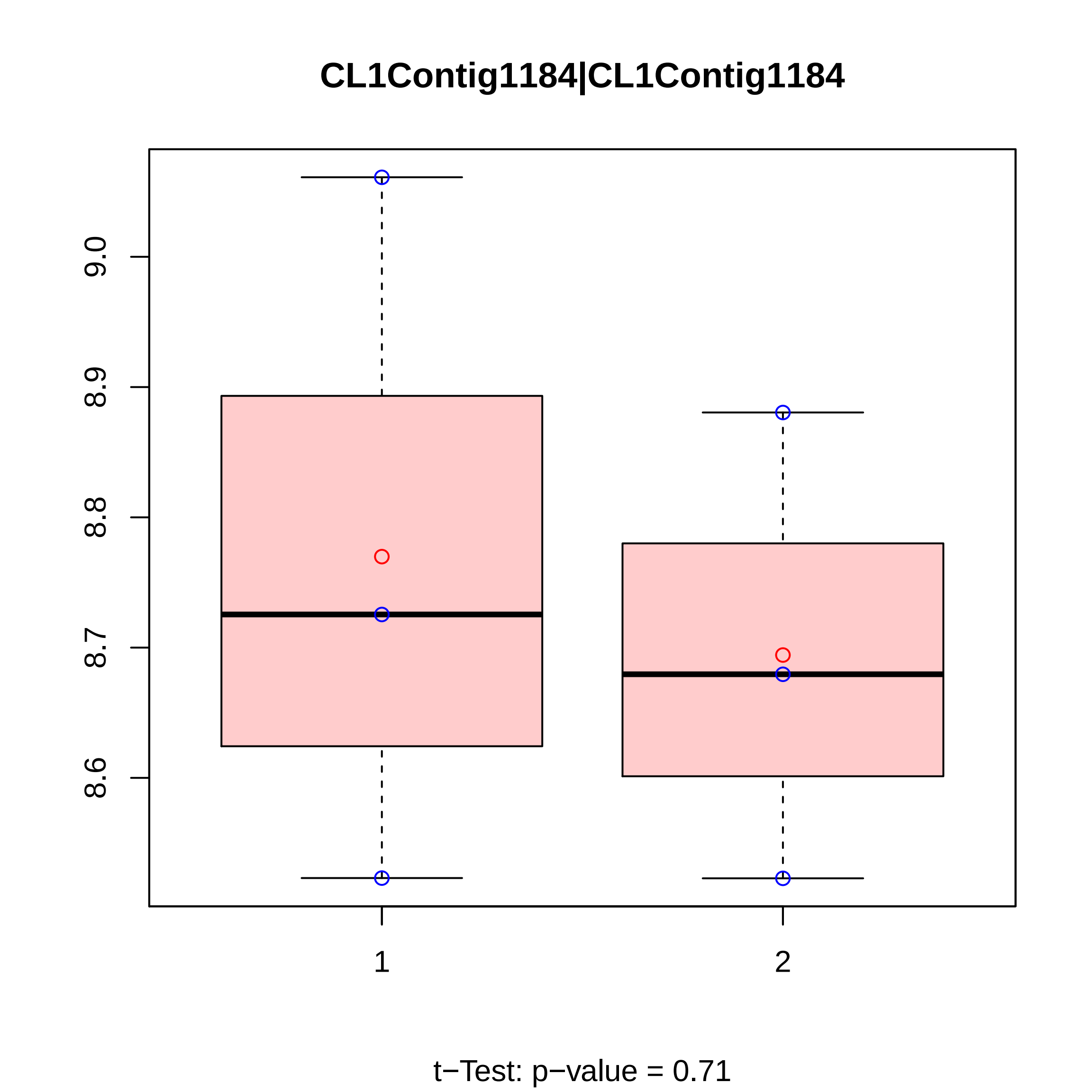
CL1Contig1184|CL1Contig1184
9.0
8.9
8.8
8.7
8.6
1
2
t−Test: p−value = 0.71

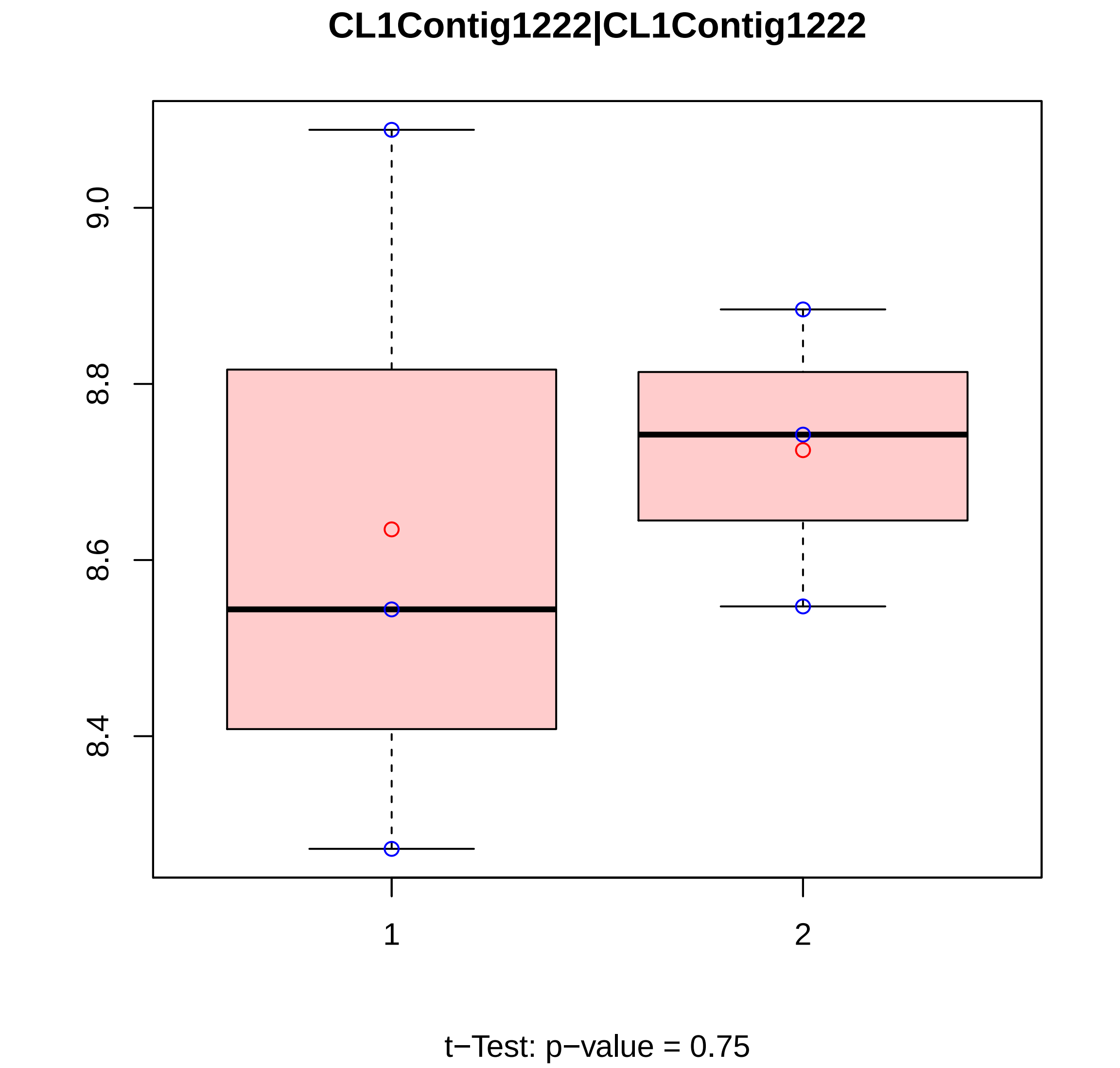

CL1Contig1222|CL1Contig1222
9.0
8.8
8.6
8.4
1
2
t−Test: p−value = 0.75

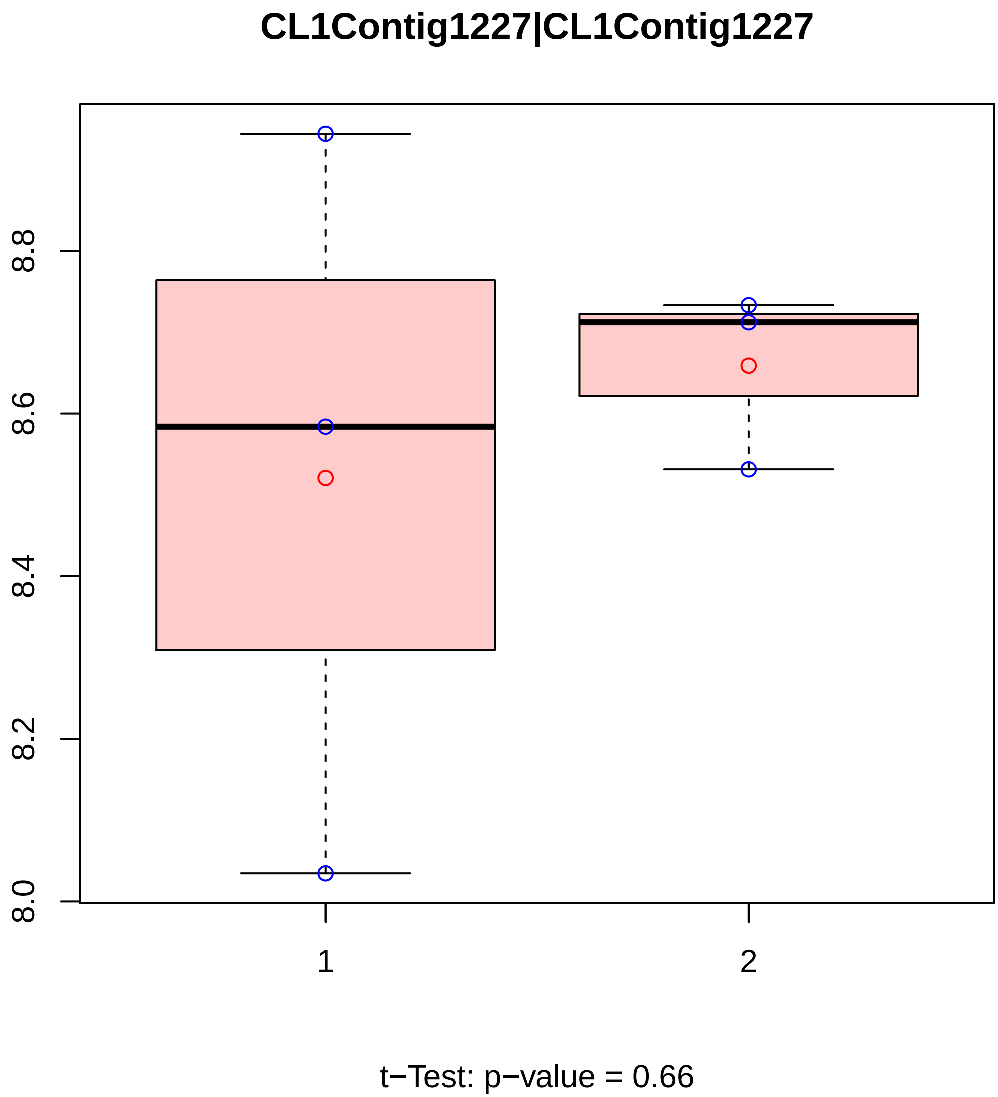

CL1Contig1227|CL1Contig1227
8.0
8.2
8.4
8.6
8.8
1
2
t−Test: p−value = 0.66

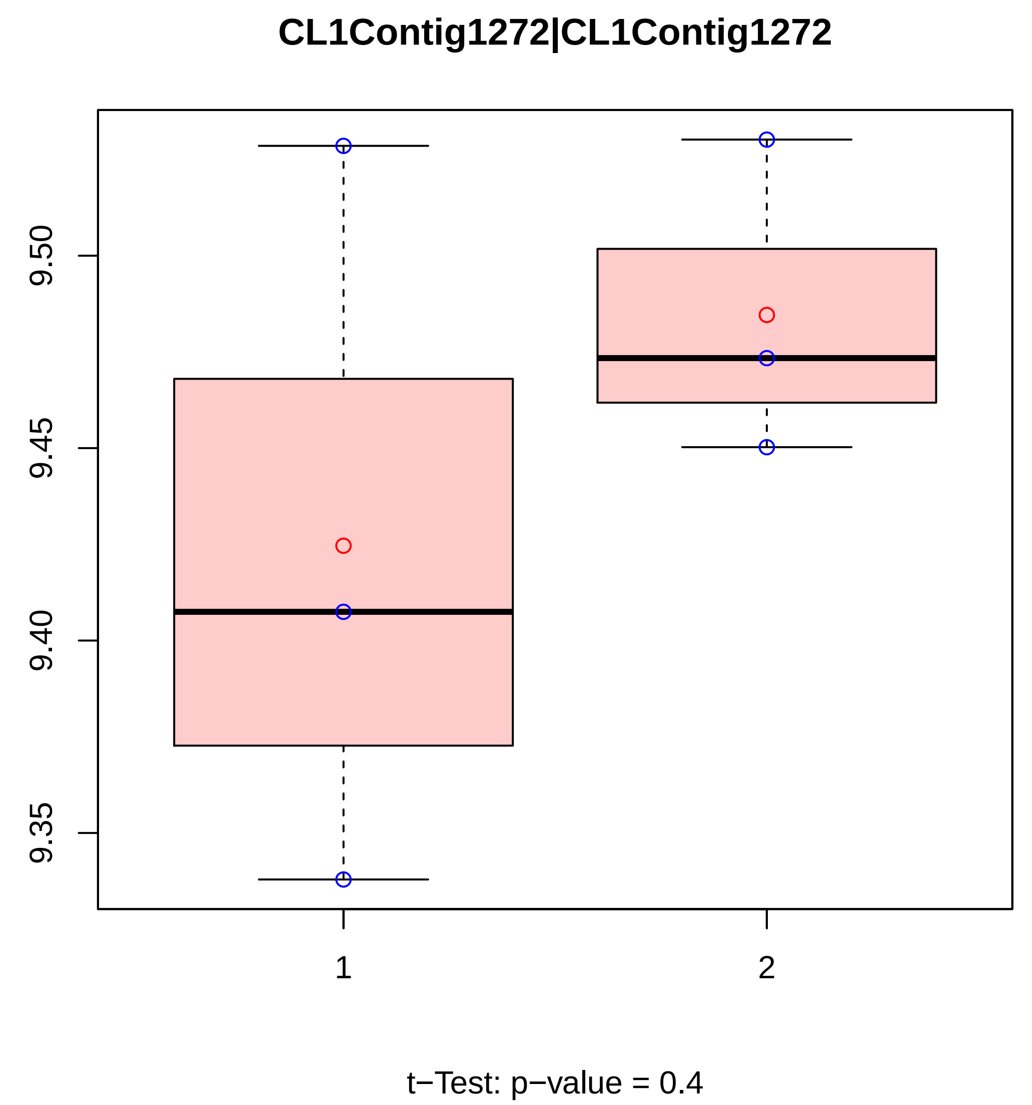
CL1Contig1272|CL1Contig1272
9.35
9.40
9.45
9.50
1
2
t−Test: p−value = 0.4

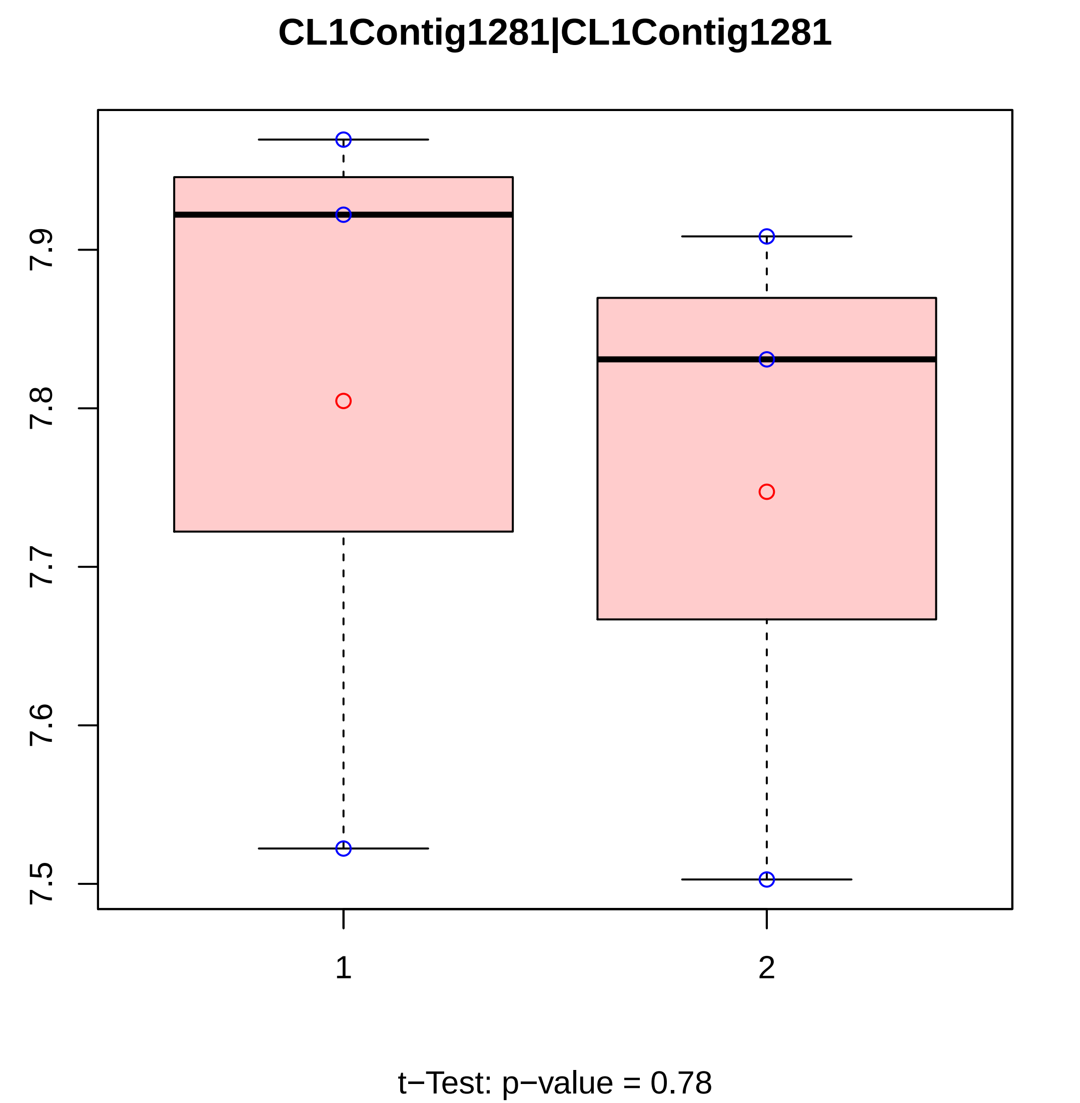
CL1Contig1281|CL1Contig1281
7.5
7.6
7.7
7.8
7.9
1
2
t−Test: p−value = 0.78

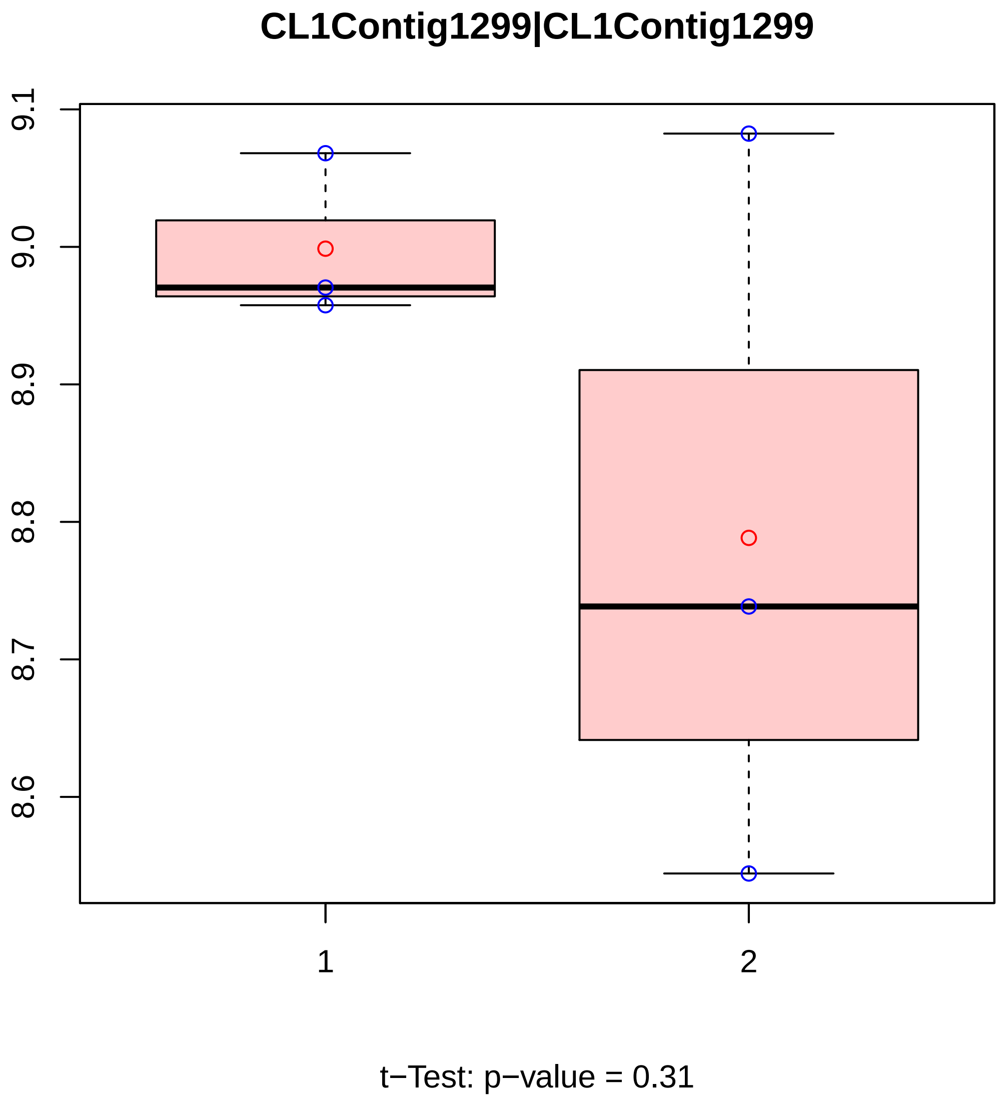

CL1Contig1299|CL1Contig1299
9.1
9.0
8.9
8.8
8.7
8.6
1
2
t−Test: p−value = 0.31

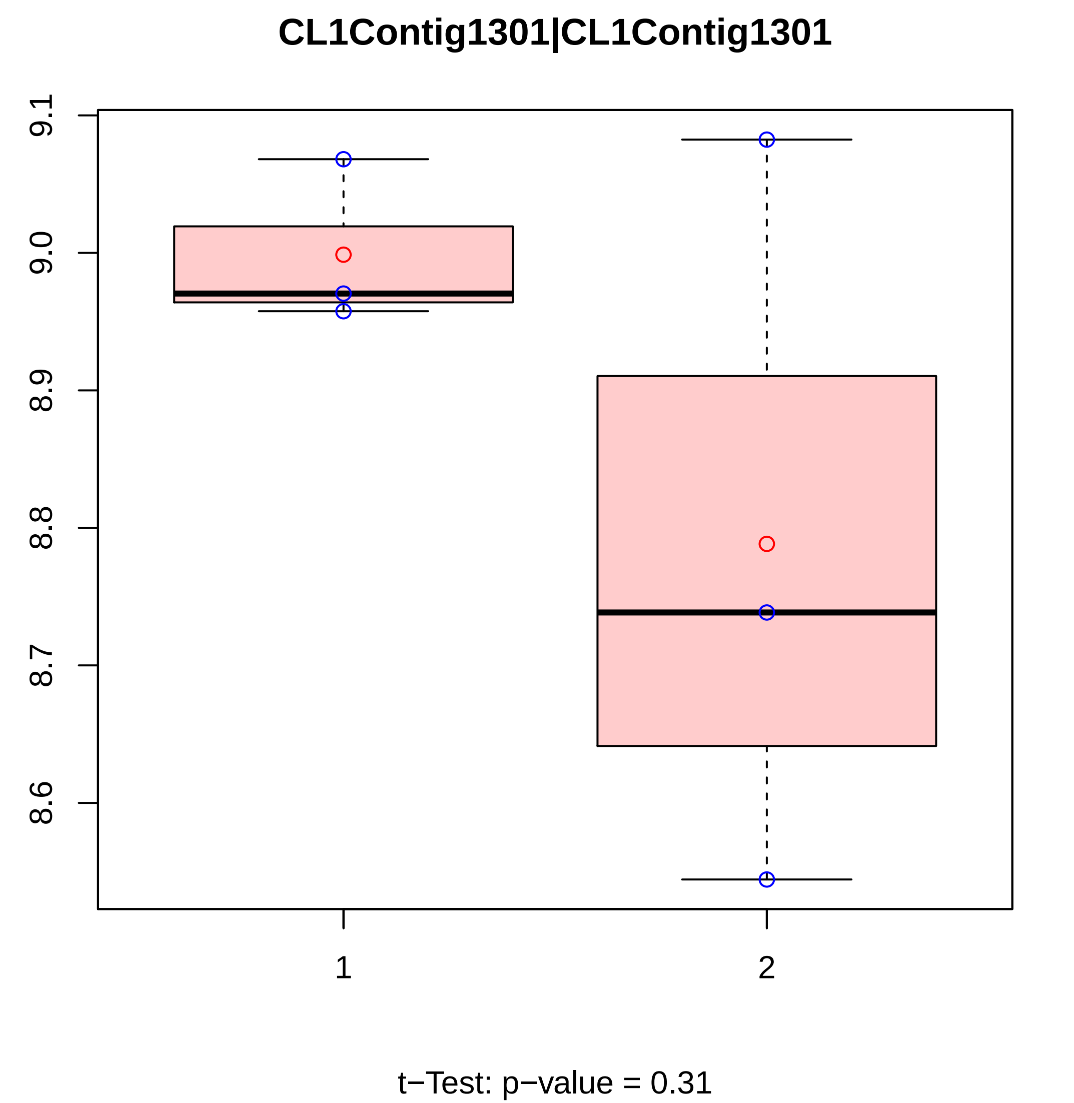

CL1Contig1301|CL1Contig1301
9.1
9.0
8.9
8.8
8.7
8.6
1
2
t−Test: p−value = 0.31

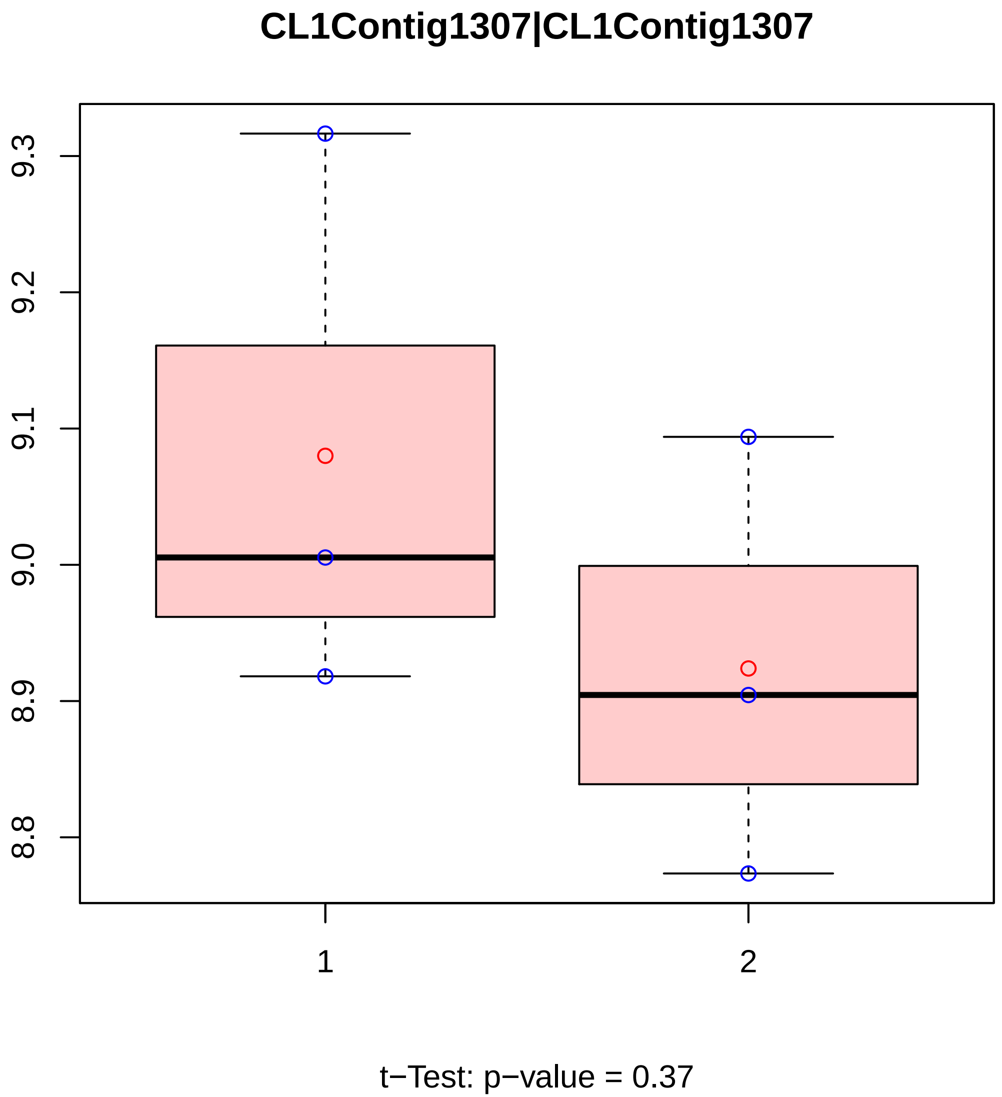
CL1Contig1307|CL1Contig1307
9.3
9.2
9.1
9.0
8.9
8.8
1
2
t−Test: p−value = 0.37

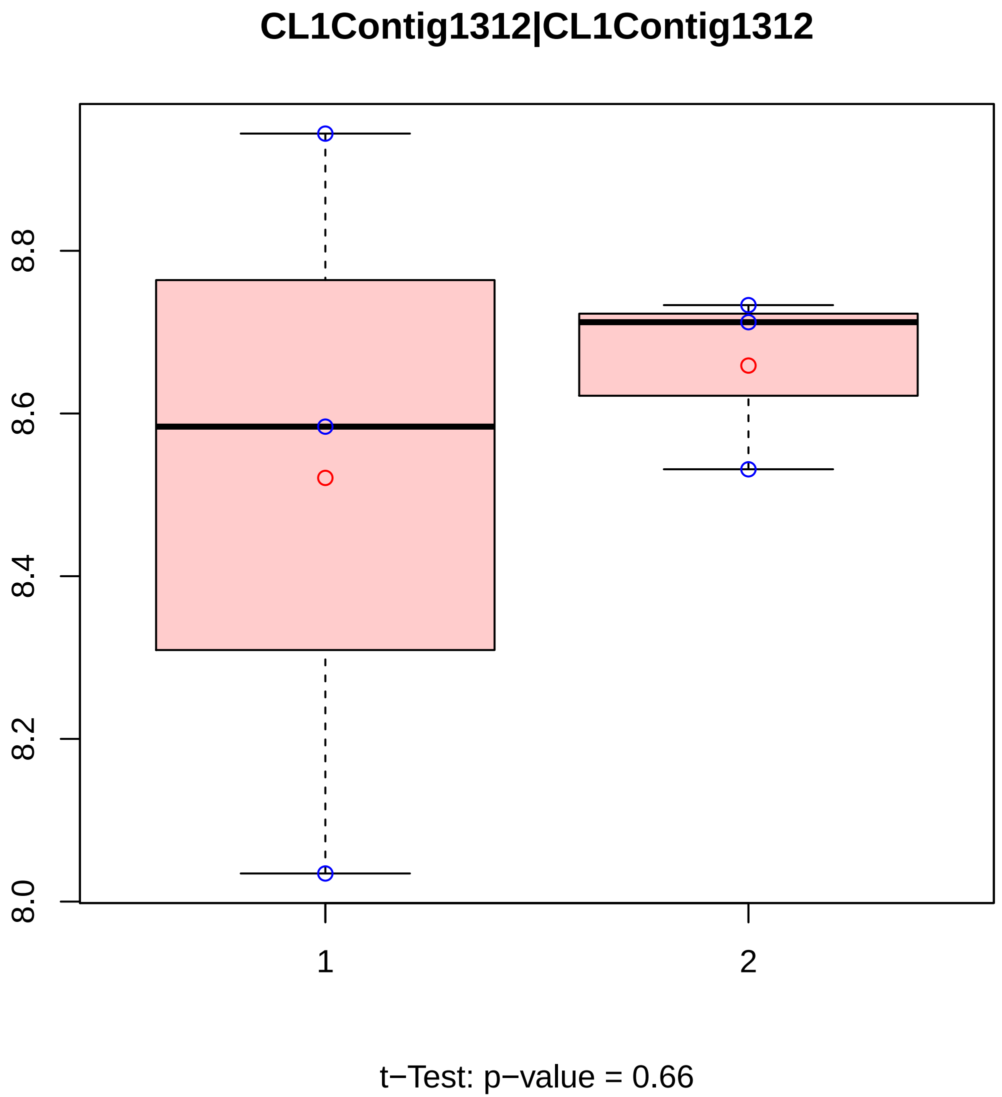

CL1Contig1312|CL1Contig1312
8.0
8.2
8.4
8.6
8.8
1
2
t−Test: p−value = 0.66

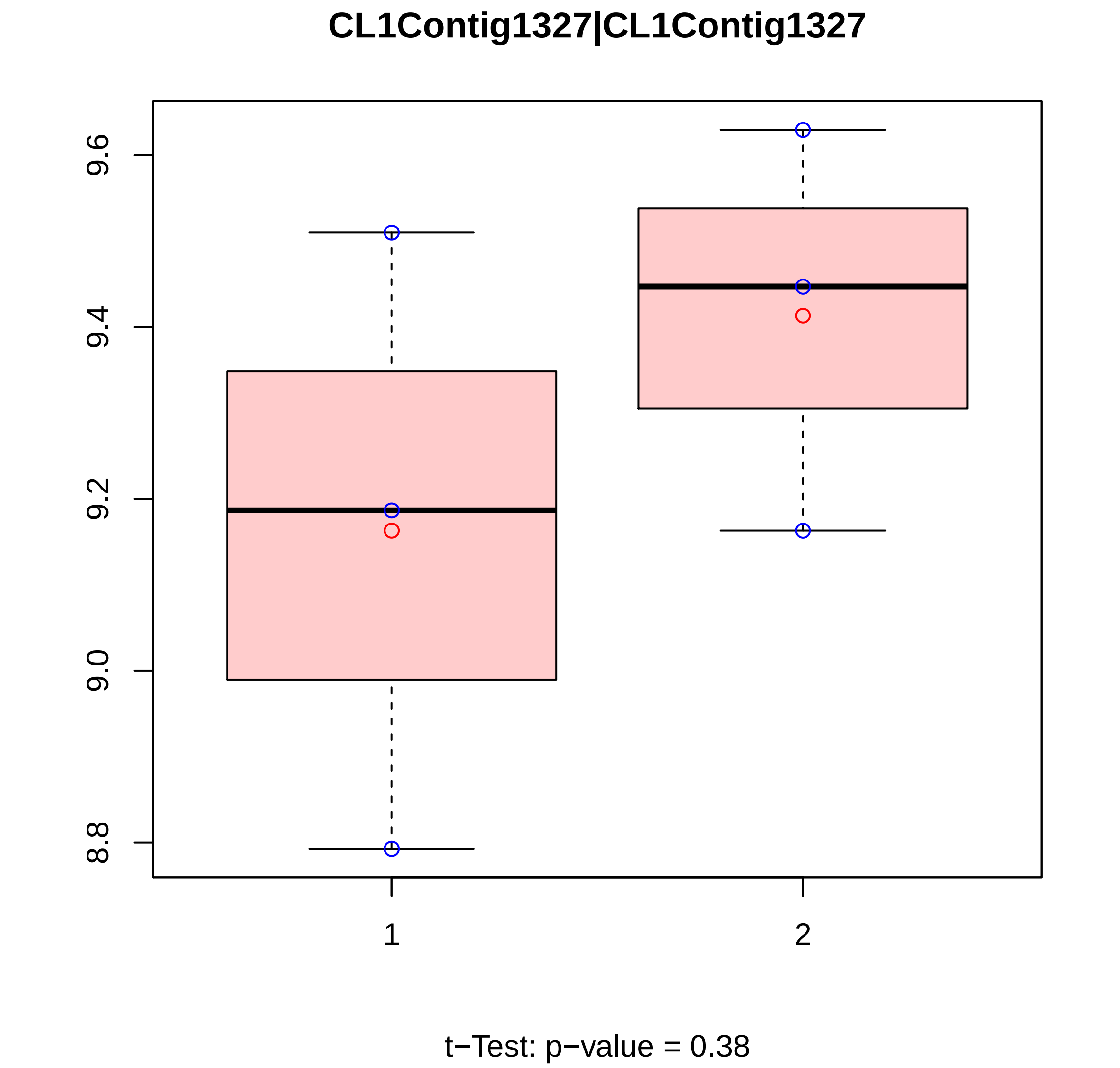

CL1Contig1327|CL1Contig1327
9.6
9.4
9.2
9.0
8.8
1
2
t−Test: p−value = 0.38

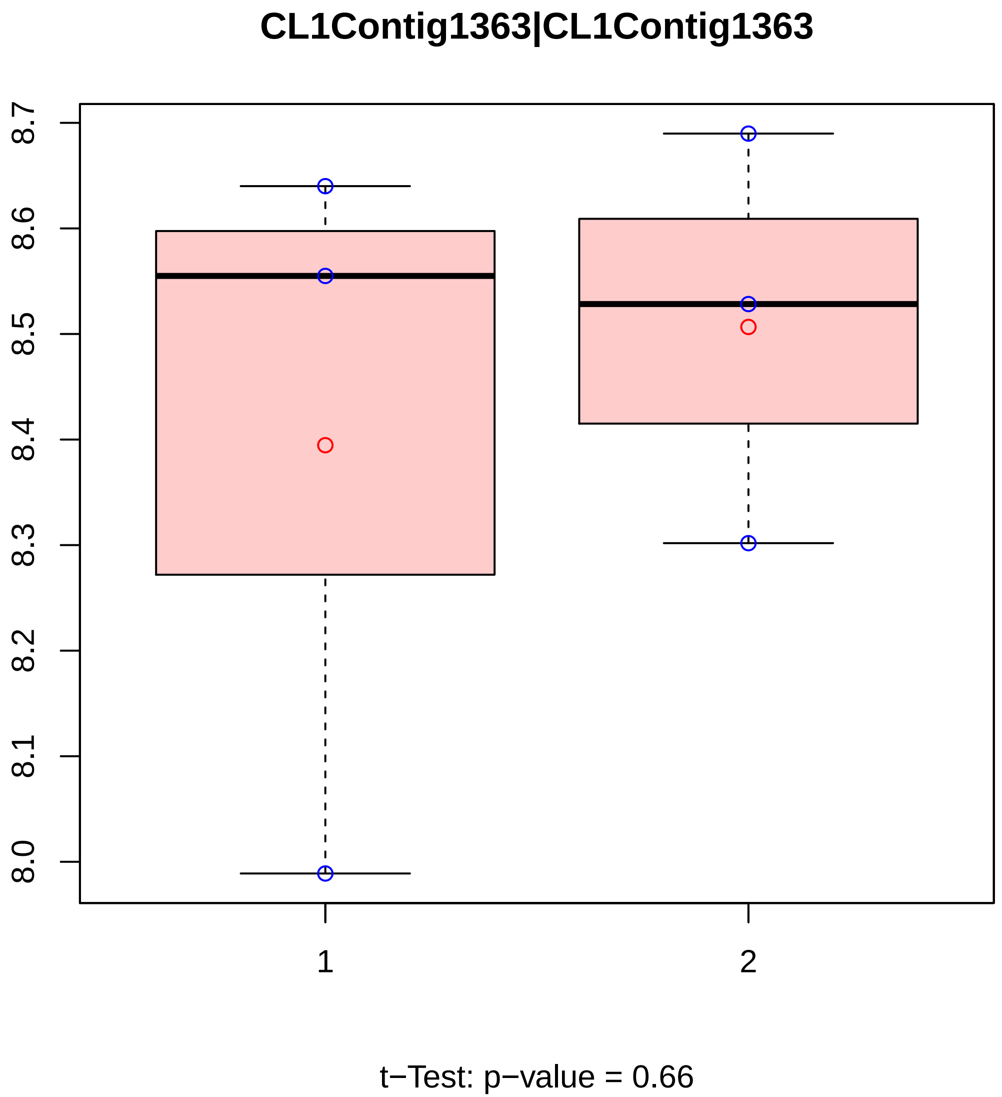
CL1Contig1363|CL1Contig1363
8.0
8.1
8.2
8.3
8.4
8.5
8.6
8.7
1
2
t−Test: p−value = 0.66

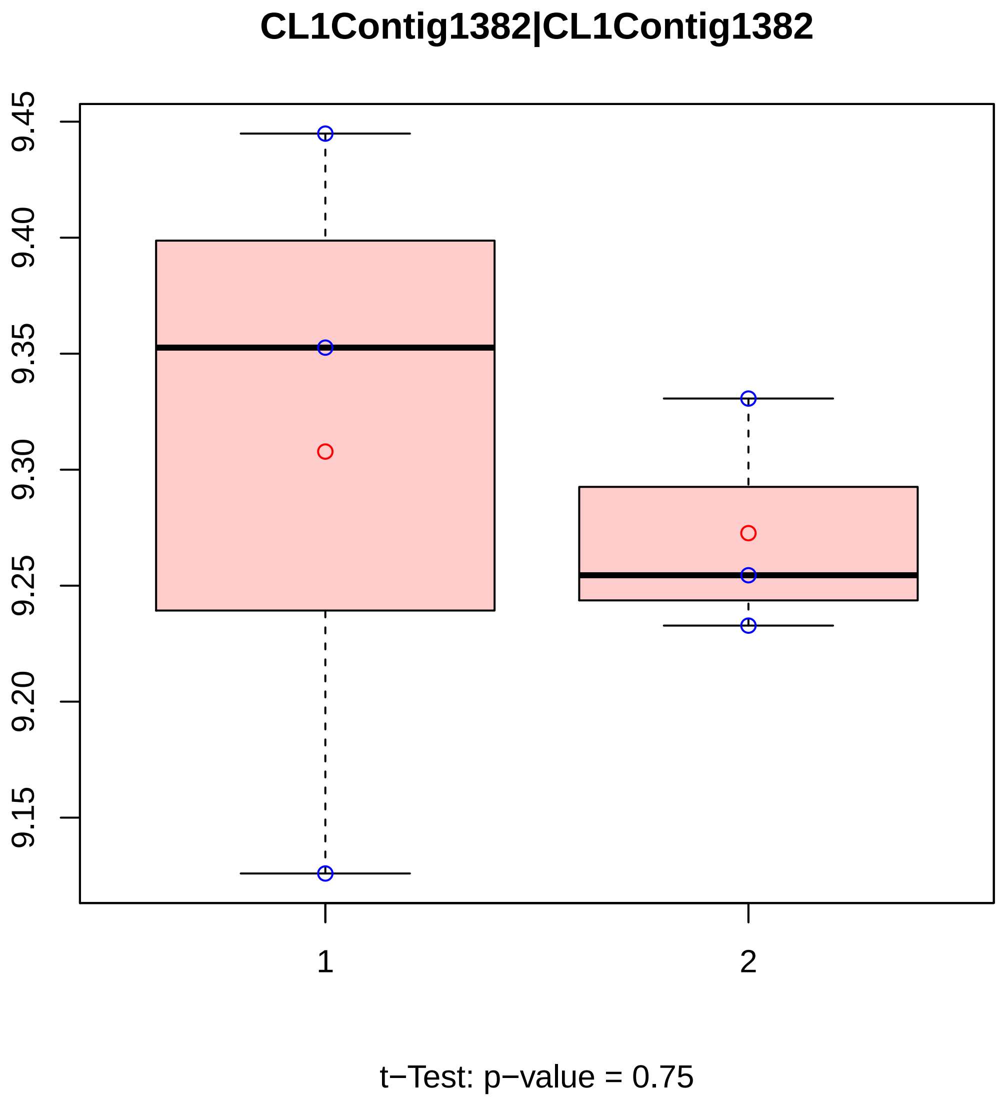
CL1Contig1382|CL1Contig1382
9.15
9.20
9.25
9.30
9.35
9.40
9.45
1
2
t−Test: p−value = 0.75

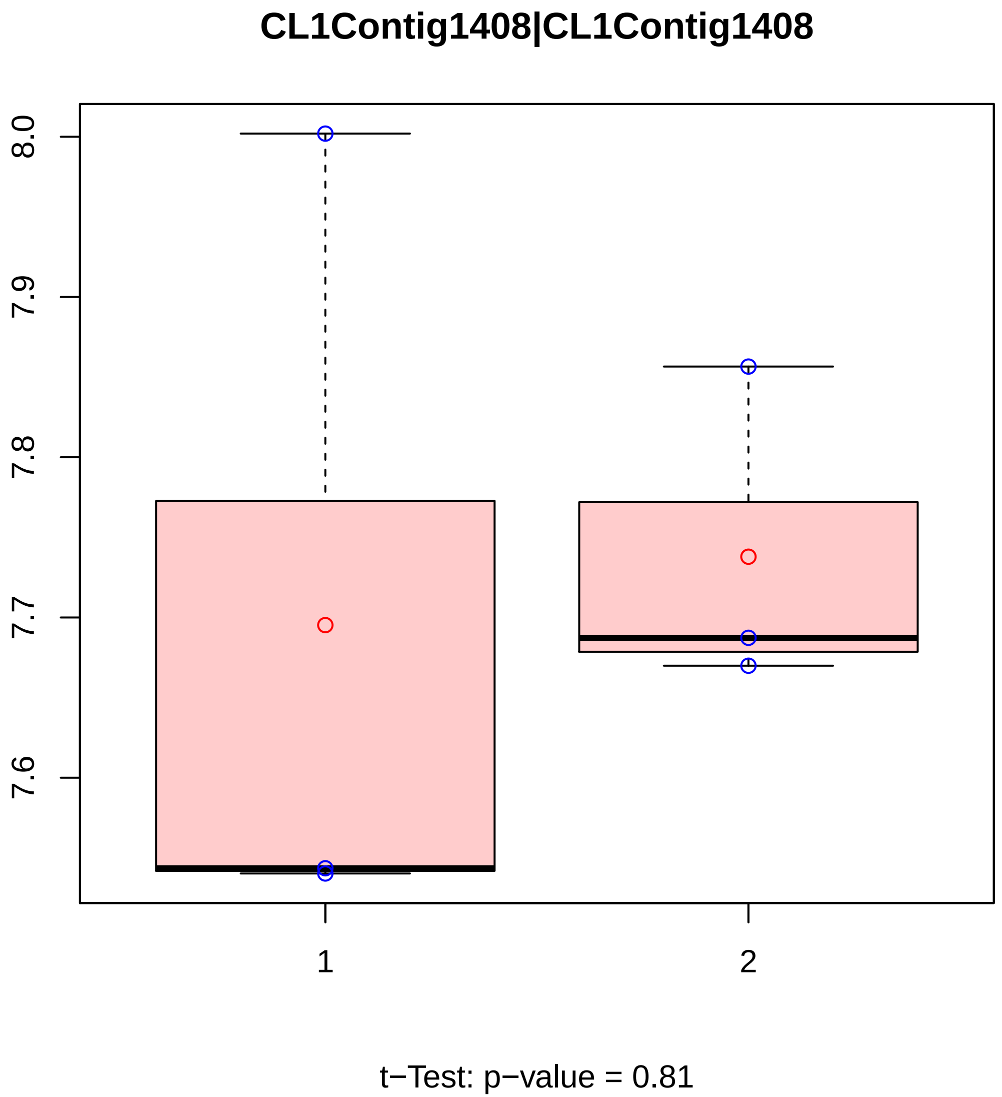

CL1Contig1408|CL1Contig1408
8.0
7.9
7.8
7.7
7.6
1
2
t−Test: p−value = 0.81

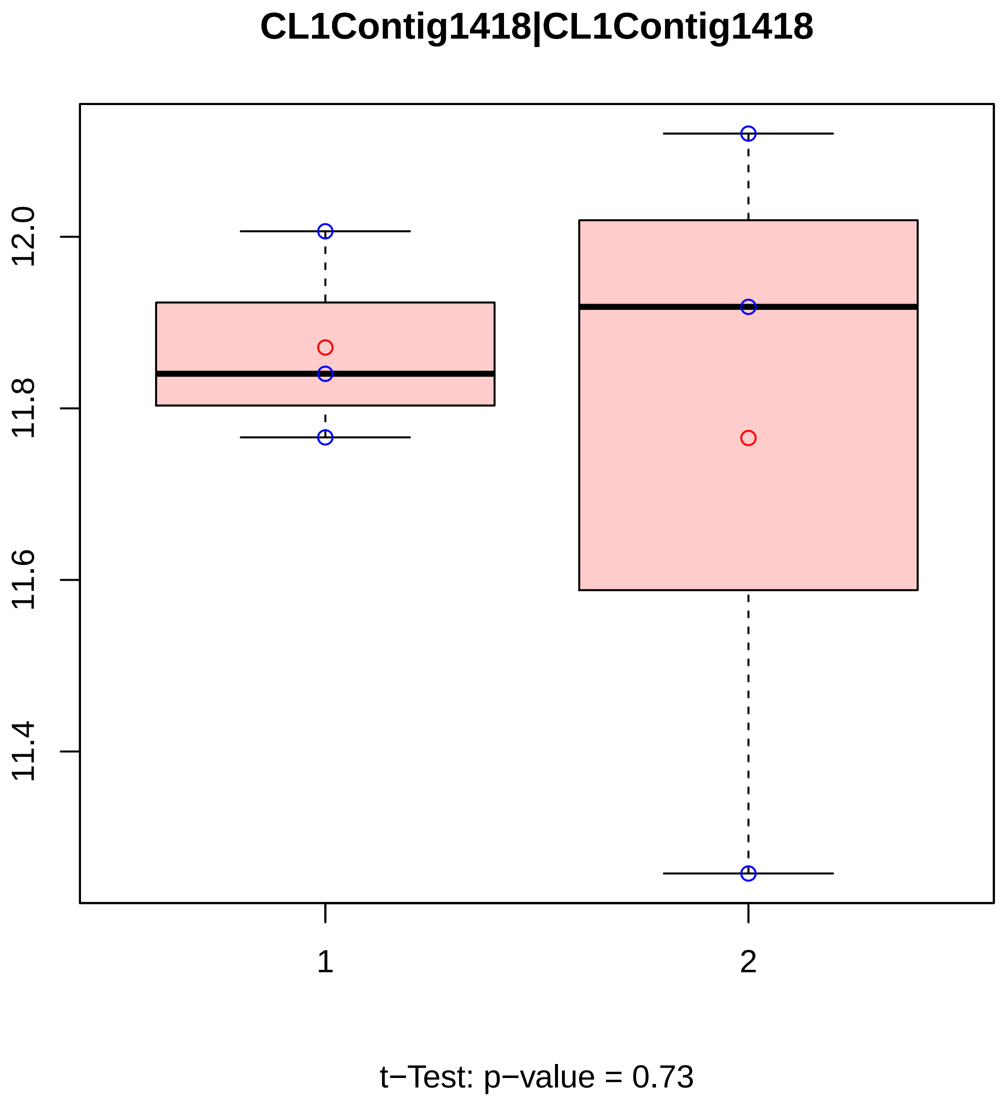
CL1Contig1418|CL1Contig1418
12.0
11.8
11.6
11.4
1
2
t−Test: p−value = 0.73

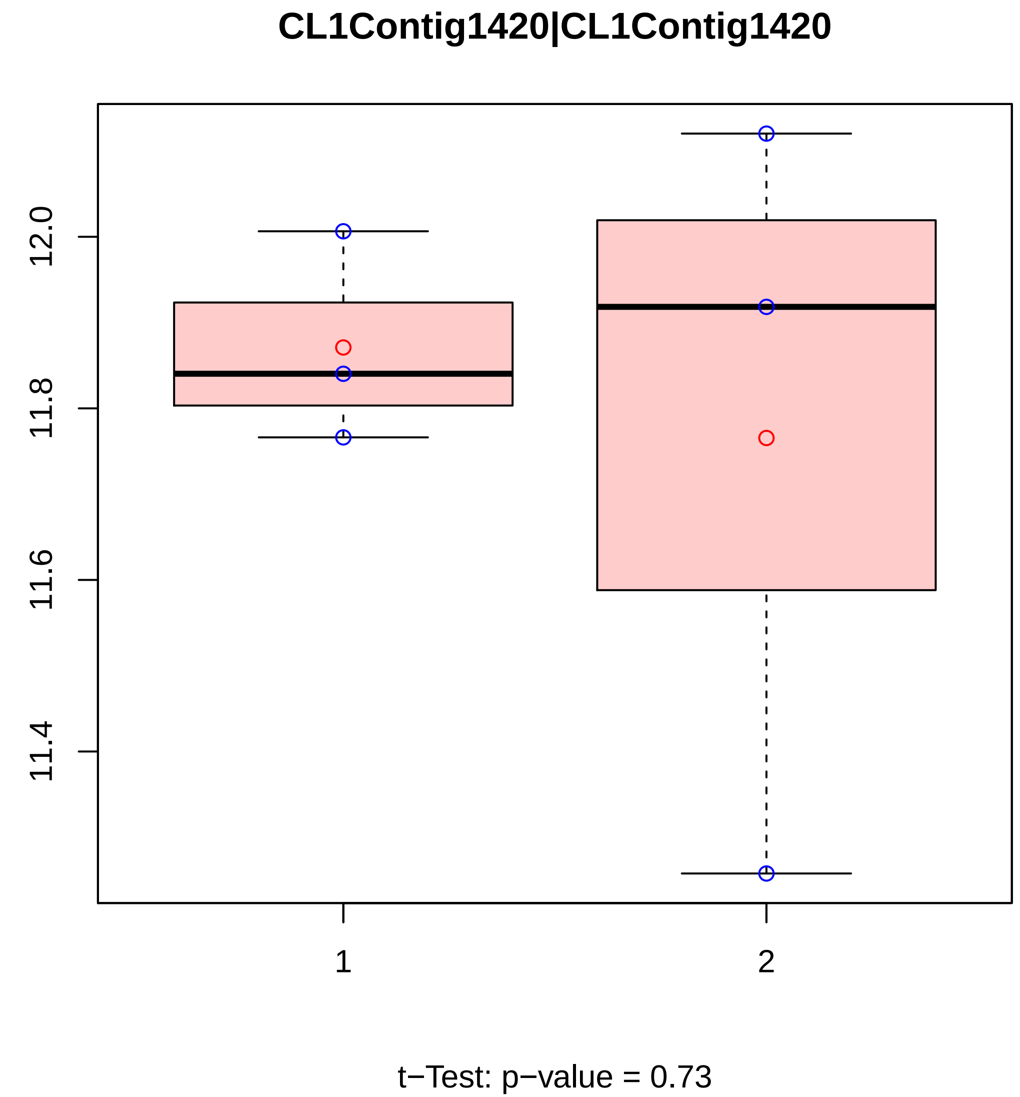
CL1Contig1420|CL1Contig1420
12.0
11.8
11.6
11.4
1
2
t−Test: p−value = 0.73

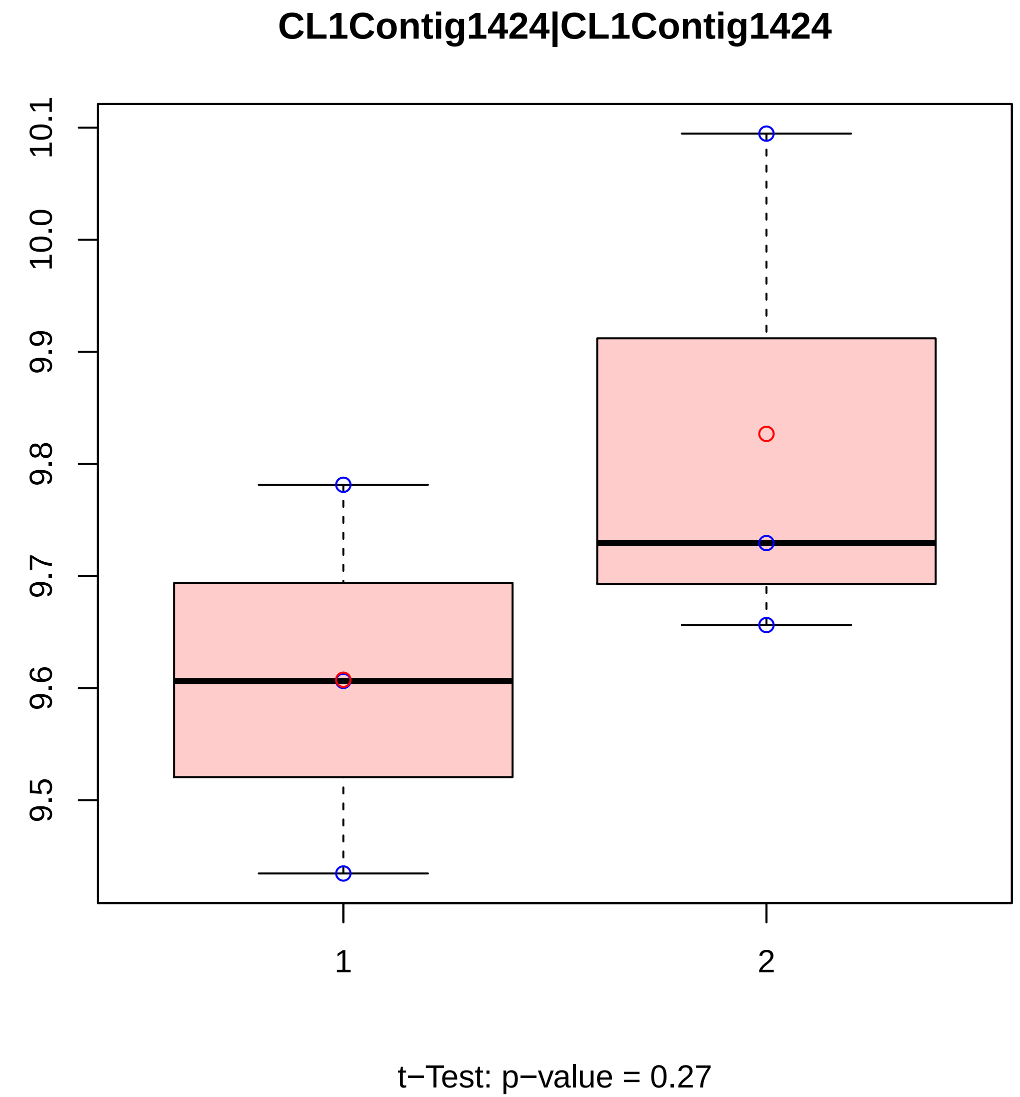

CL1Contig1424|CL1Contig1424
10.1
10.0
9.9
9.8
9.7
9.6
9.5
1
2
t−Test: p−value = 0.27

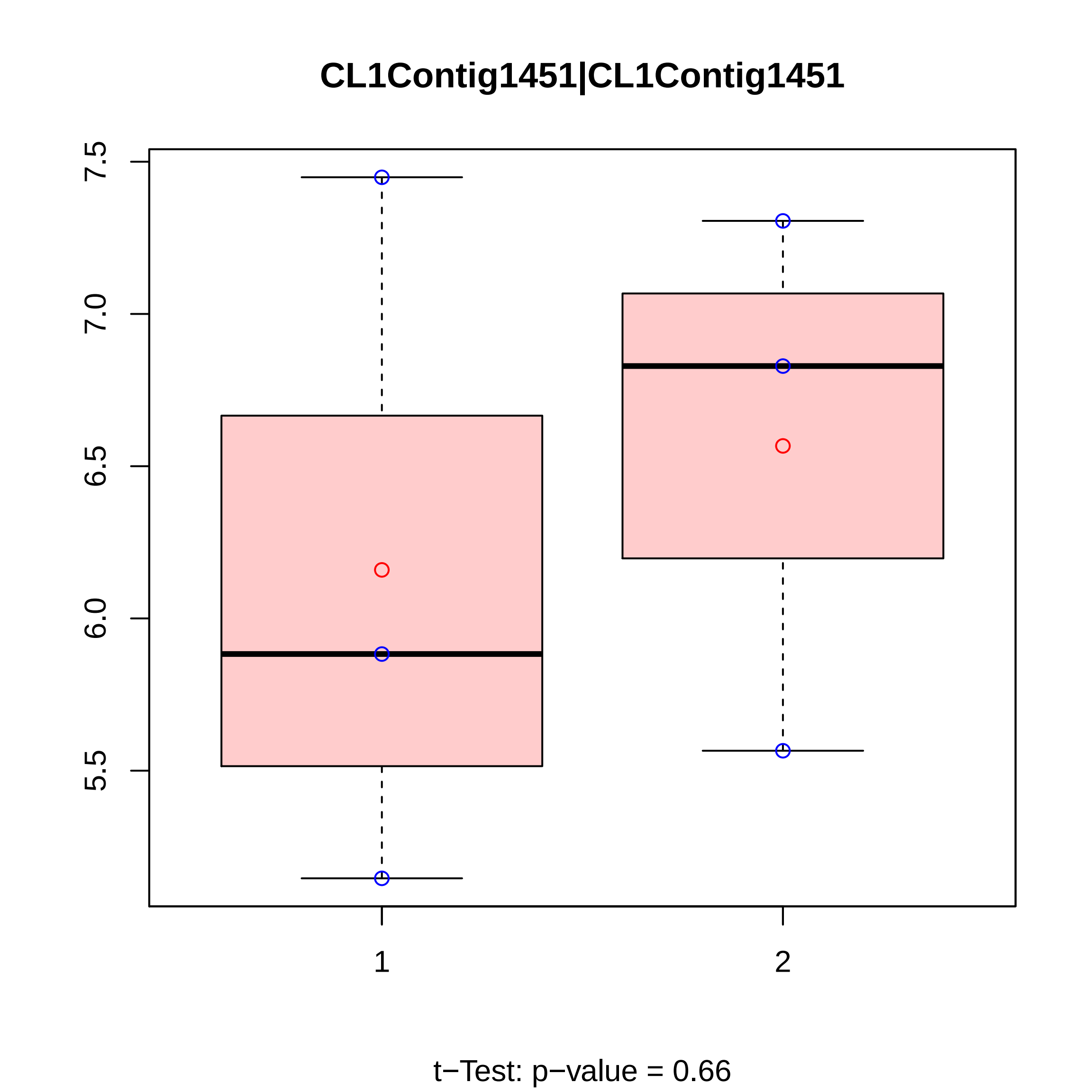
CL1Contig1451|CL1Contig1451
7.5
7.0
6.5
6.0
5.5
1
2
t−Test: p−value = 0.66

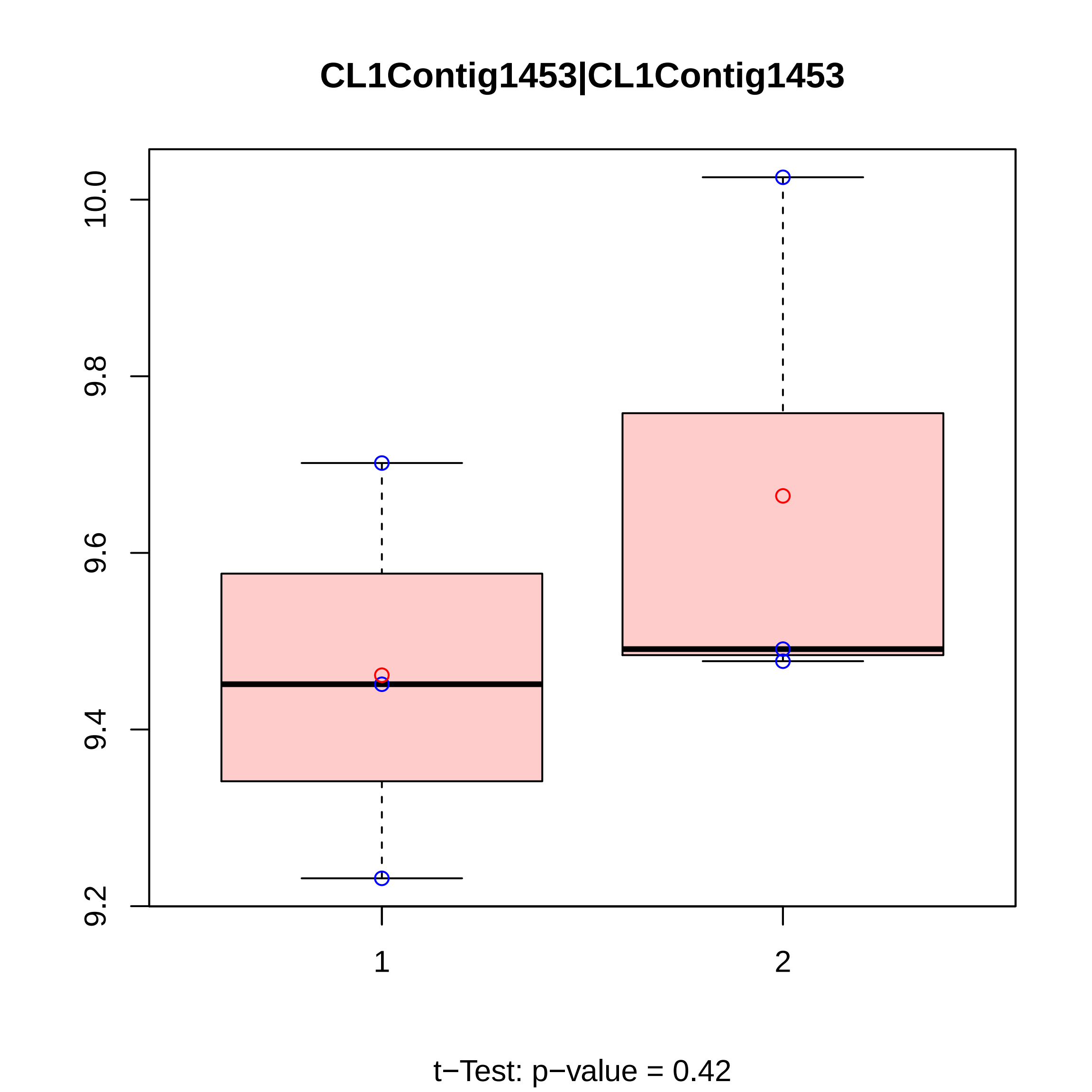

CL1Contig1453|CL1Contig1453
10.0
9.8
9.6
9.4
9.2
1
2
t−Test: p−value = 0.42

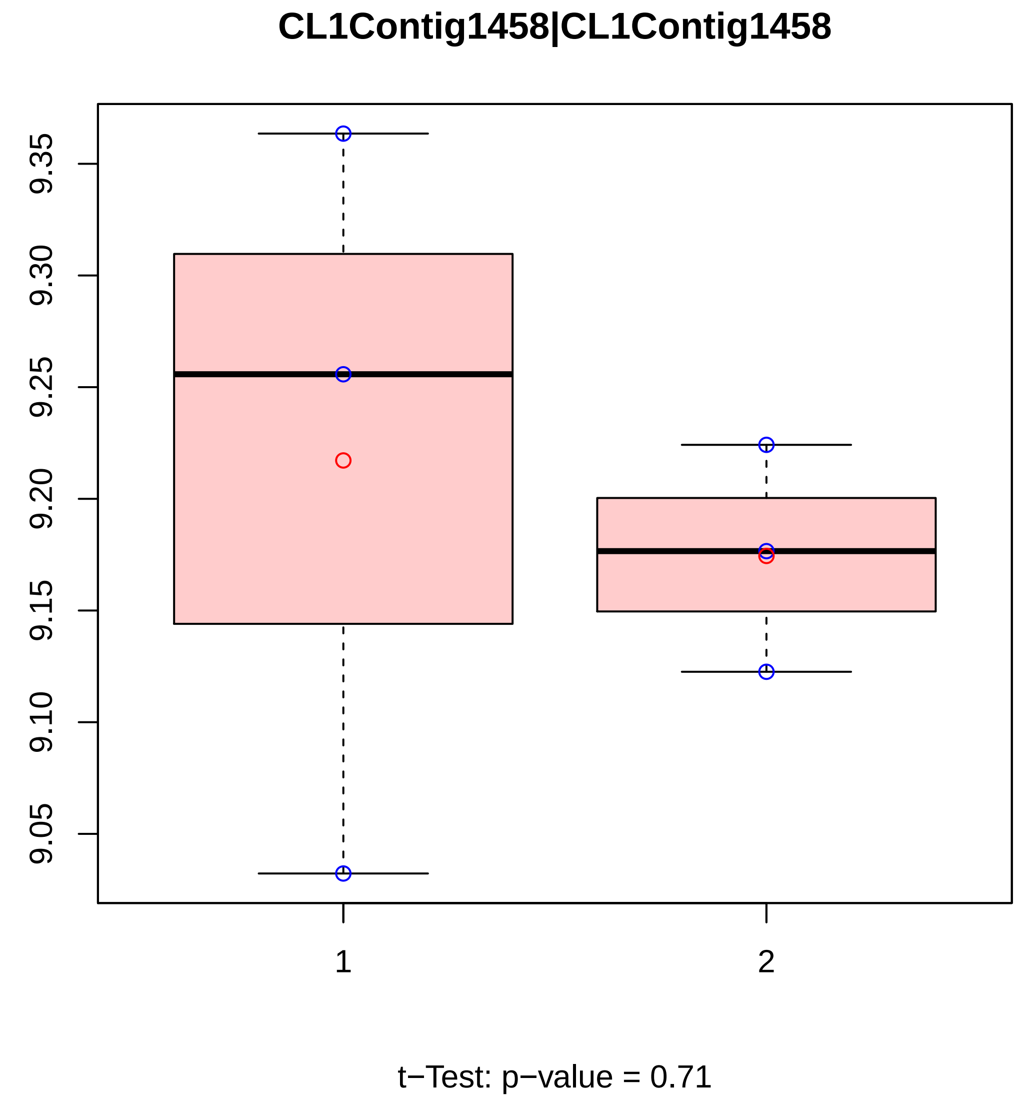
CL1Contig1458|CL1Contig1458
9.35
9.30
9.25
9.20
9.15
9.10
9.05
1
2
t−Test: p−value = 0.71

# CL1Contig149|CL1Contig149

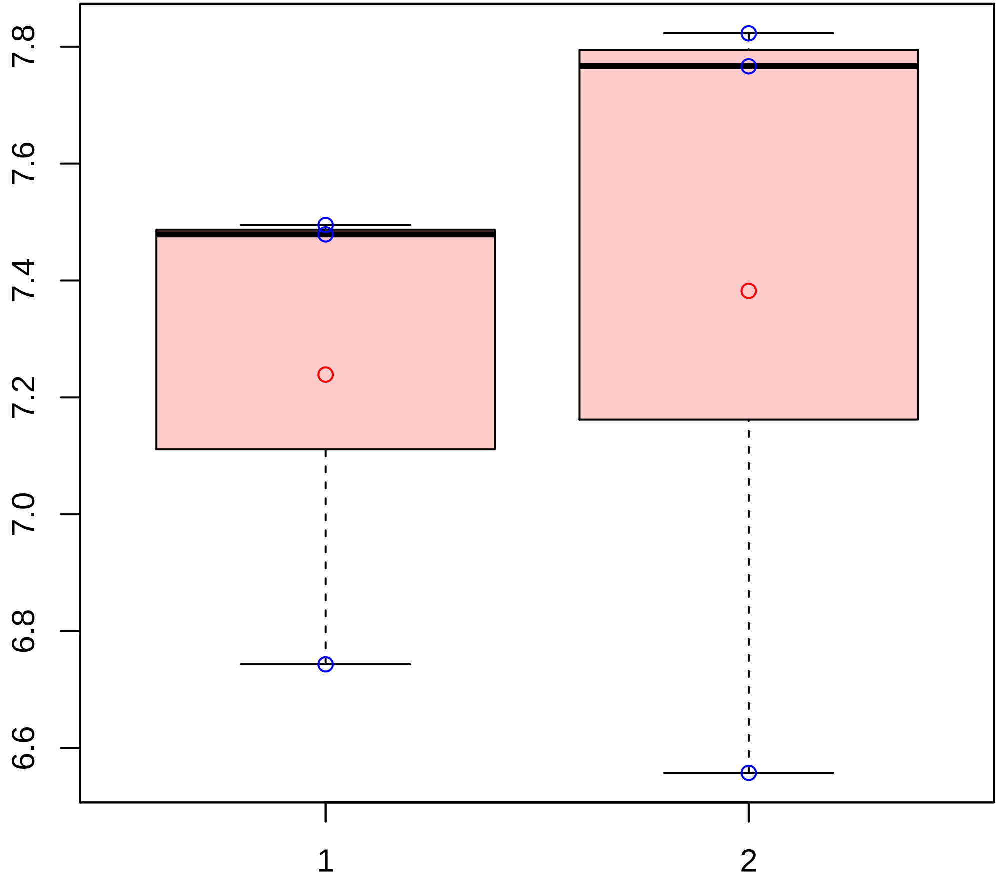


t−Test: p−value = 0.78

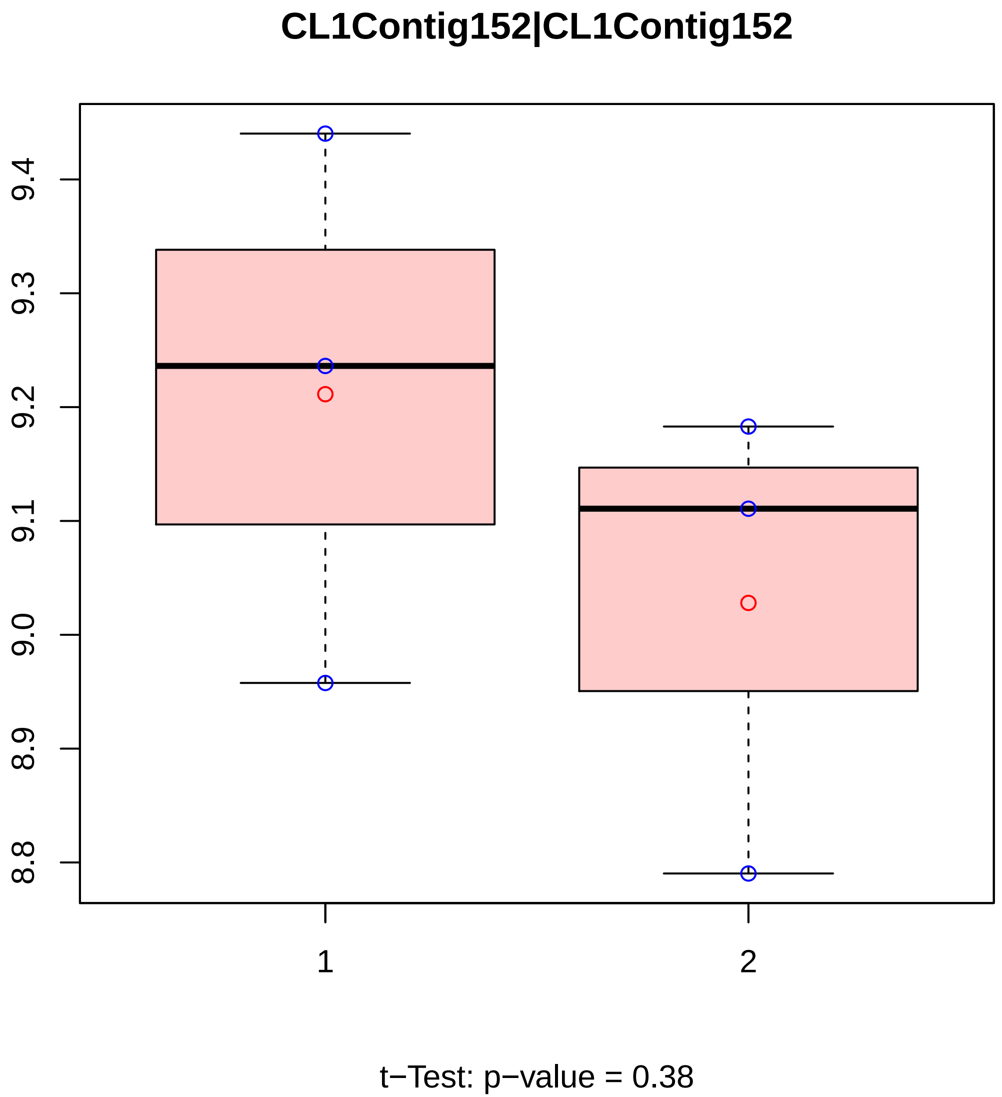
CL1Contig152|CL1Contig152
9.4
9.3
9.2
9.1
9.0
8.9
8.8
1
2
t−Test: p−value = 0.38

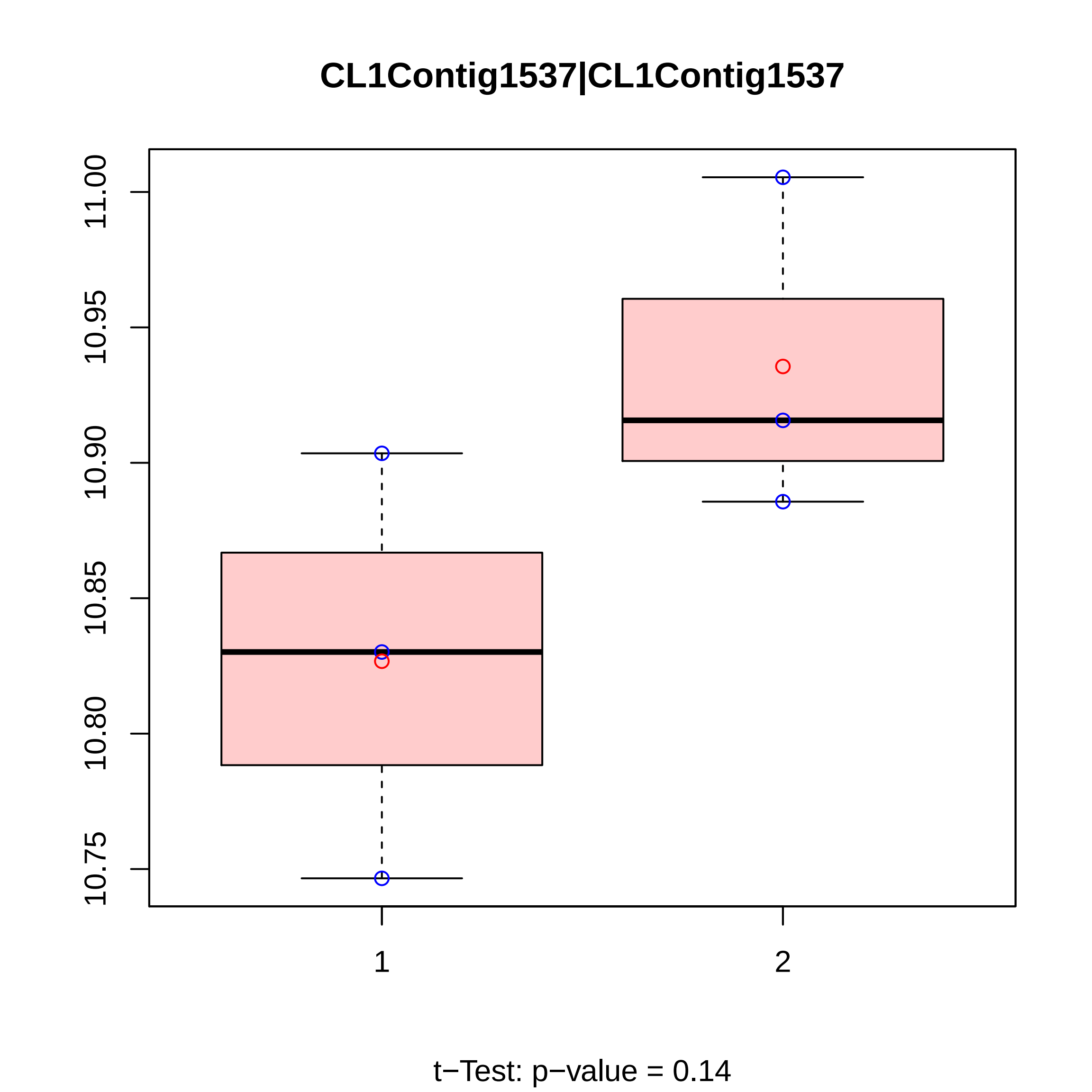

CL1Contig1537|CL1Contig1537
11.00
10.95
10.90
10.85
10.80
10.75
1
2
t−Test: p−value = 0.14

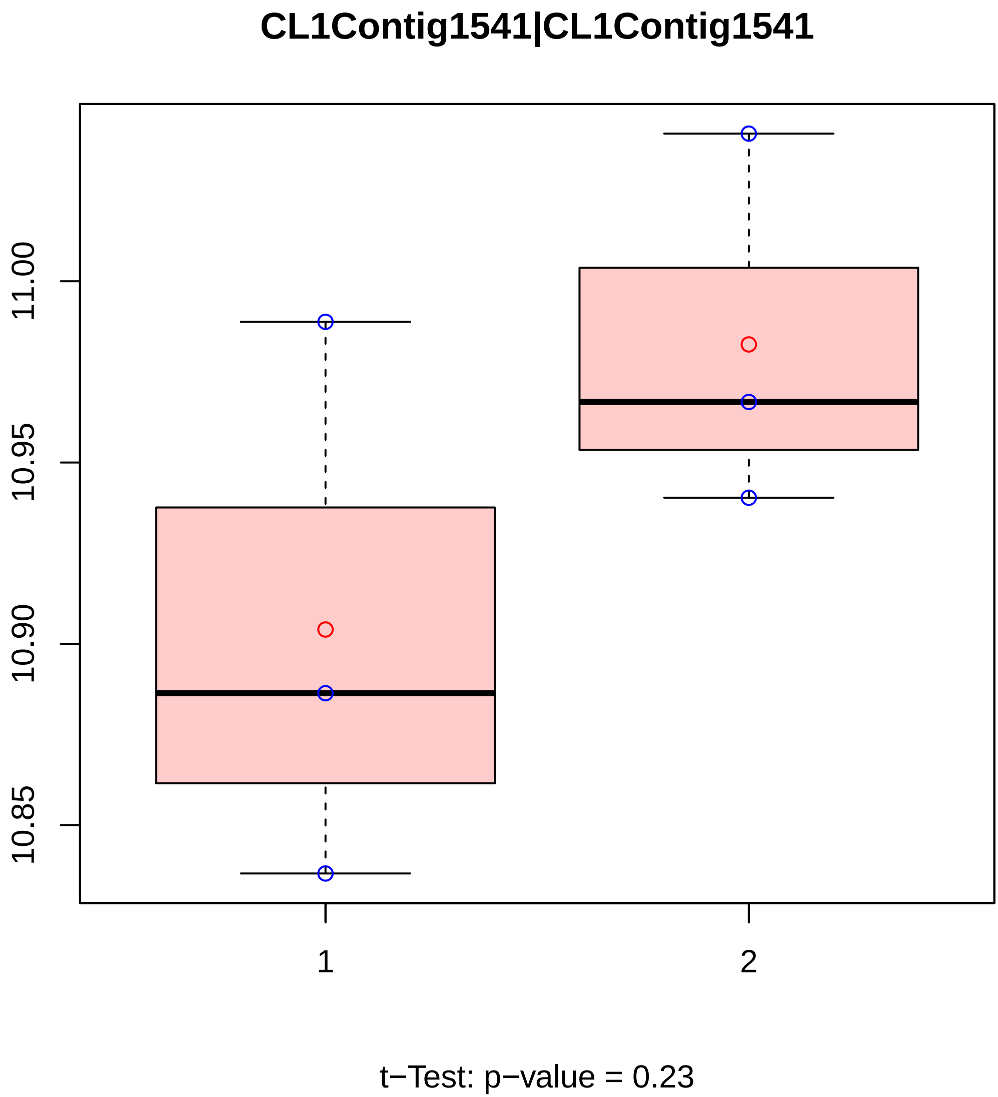

CL1Contig1541|CL1Contig1541
11.00
10.95
10.90
10.85
1
2
t−Test: p−value = 0.23

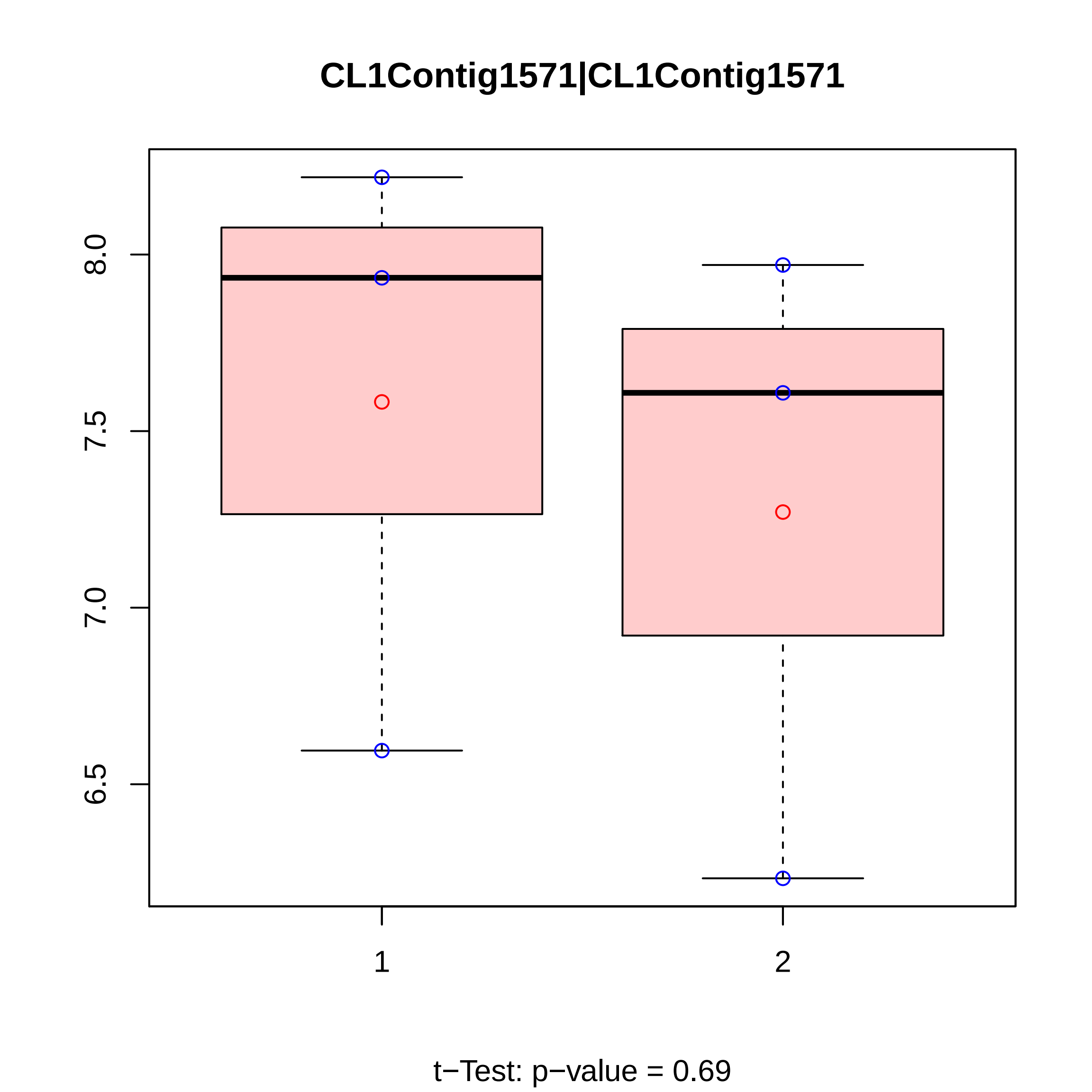
CL1Contig1571|CL1Contig1571
8.0
7.5
7.0
6.5
1
2
t−Test: p−value = 0.69

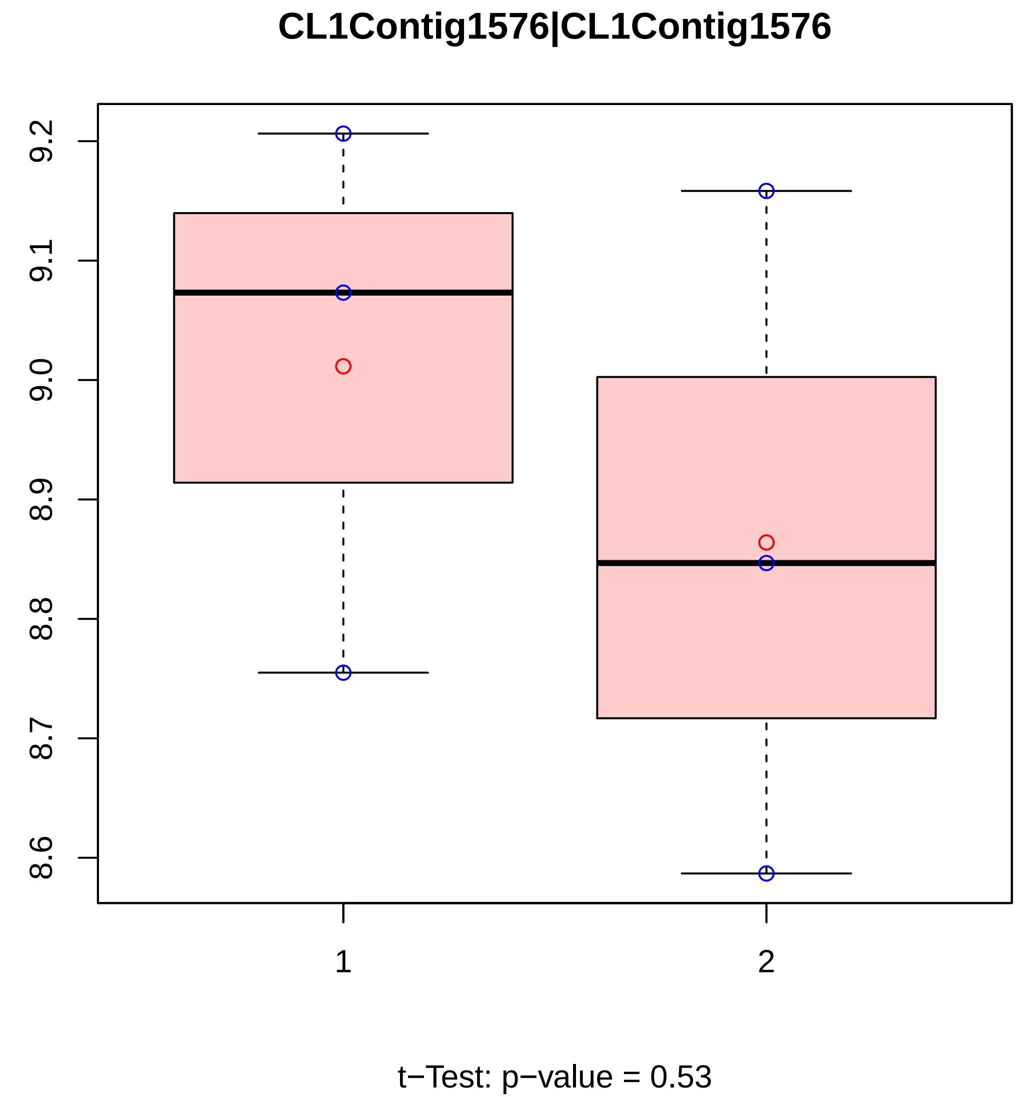

CL1Contig1576|CL1Contig1576
9.2
9.1
9.0
8.9
8.8
8.7
8.6
1
2
t−Test: p−value = 0.53

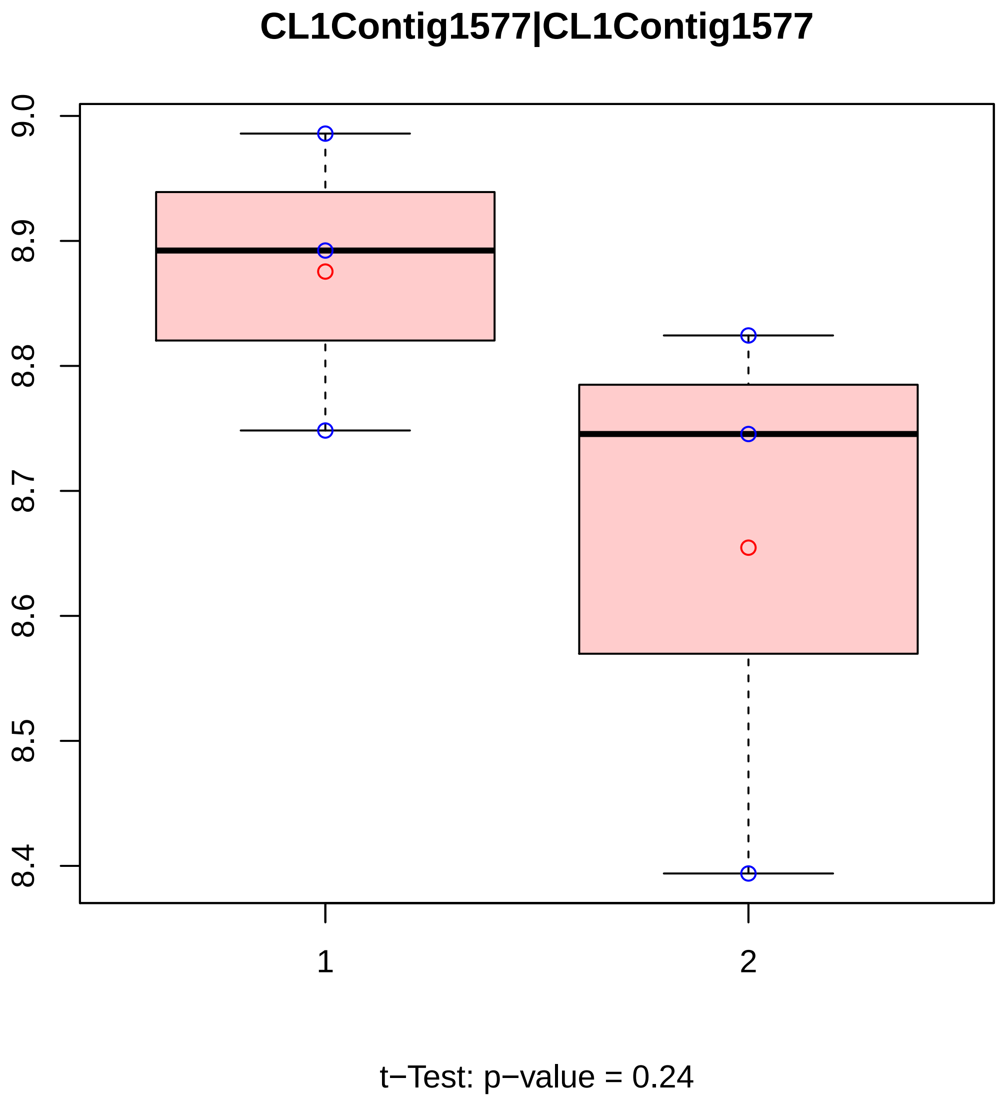
CL1Contig1577|CL1Contig1577
9.0
8.9
8.8
8.7
8.6
8.5
8.4
1
2
t−Test: p−value = 0.24

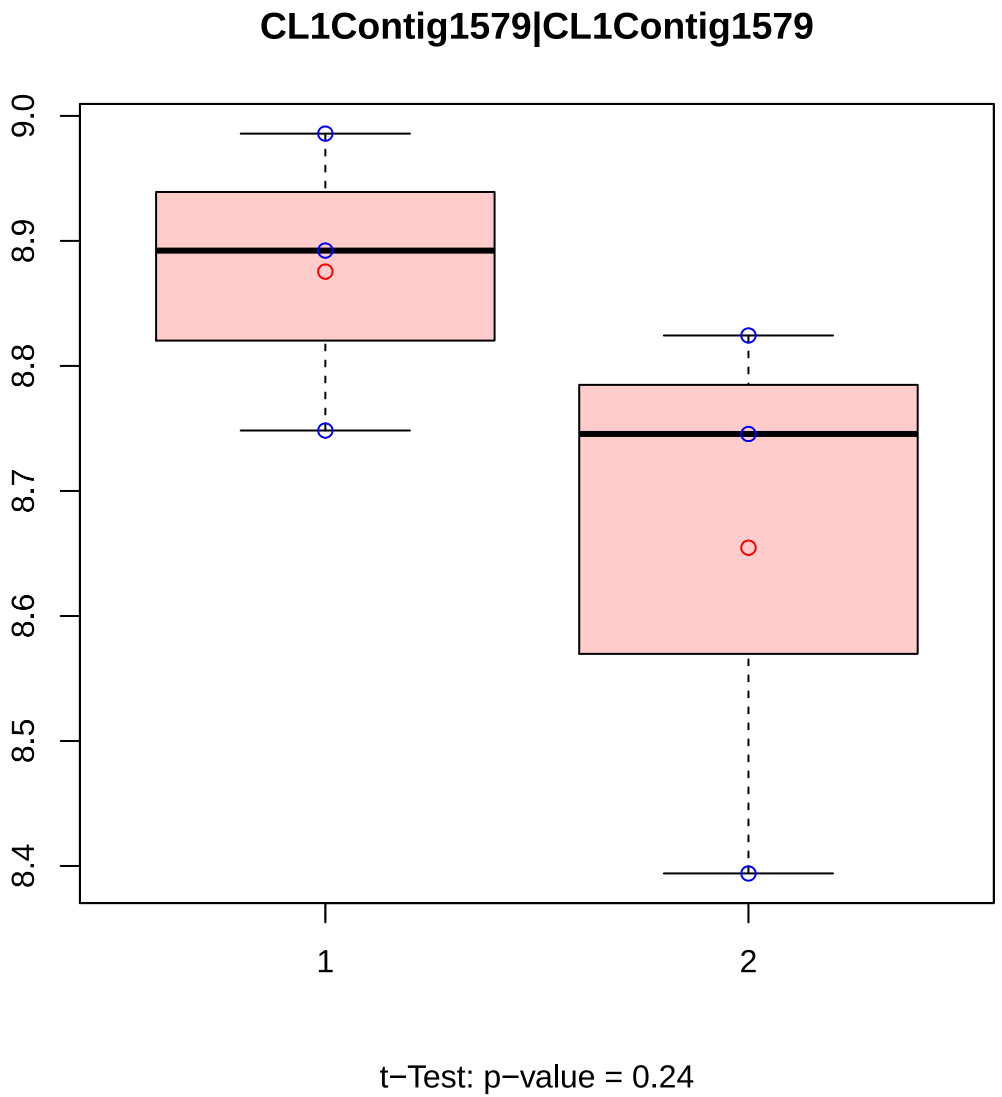
CL1Contig1579|CL1Contig1579
9.0
8.9
8.8
8.7
8.6
8.5
8.4
1
2
t−Test: p−value = 0.24

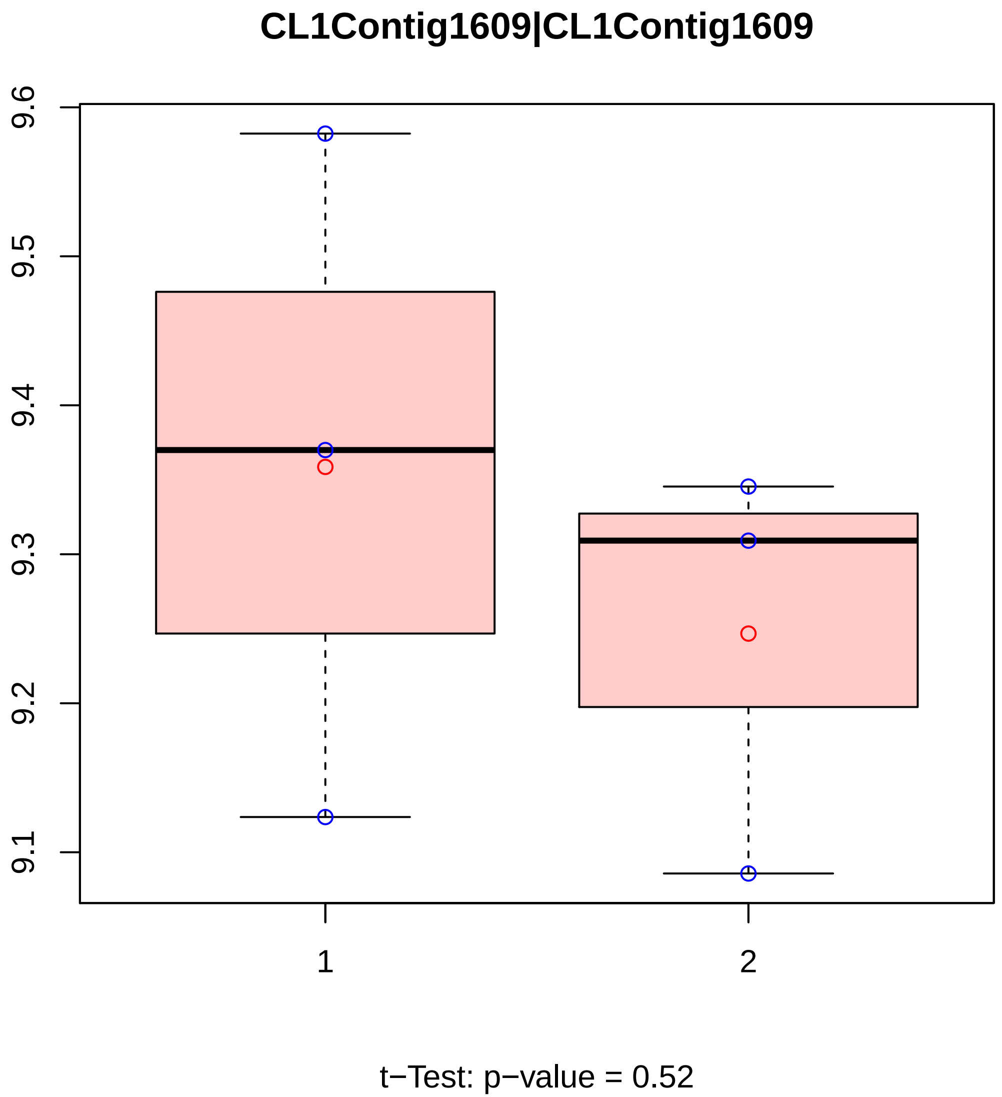

CL1Contig1609|CL1Contig1609
9.6
9.5
9.4
9.3
9.2
9.1
1
2
t−Test: p−value = 0.52

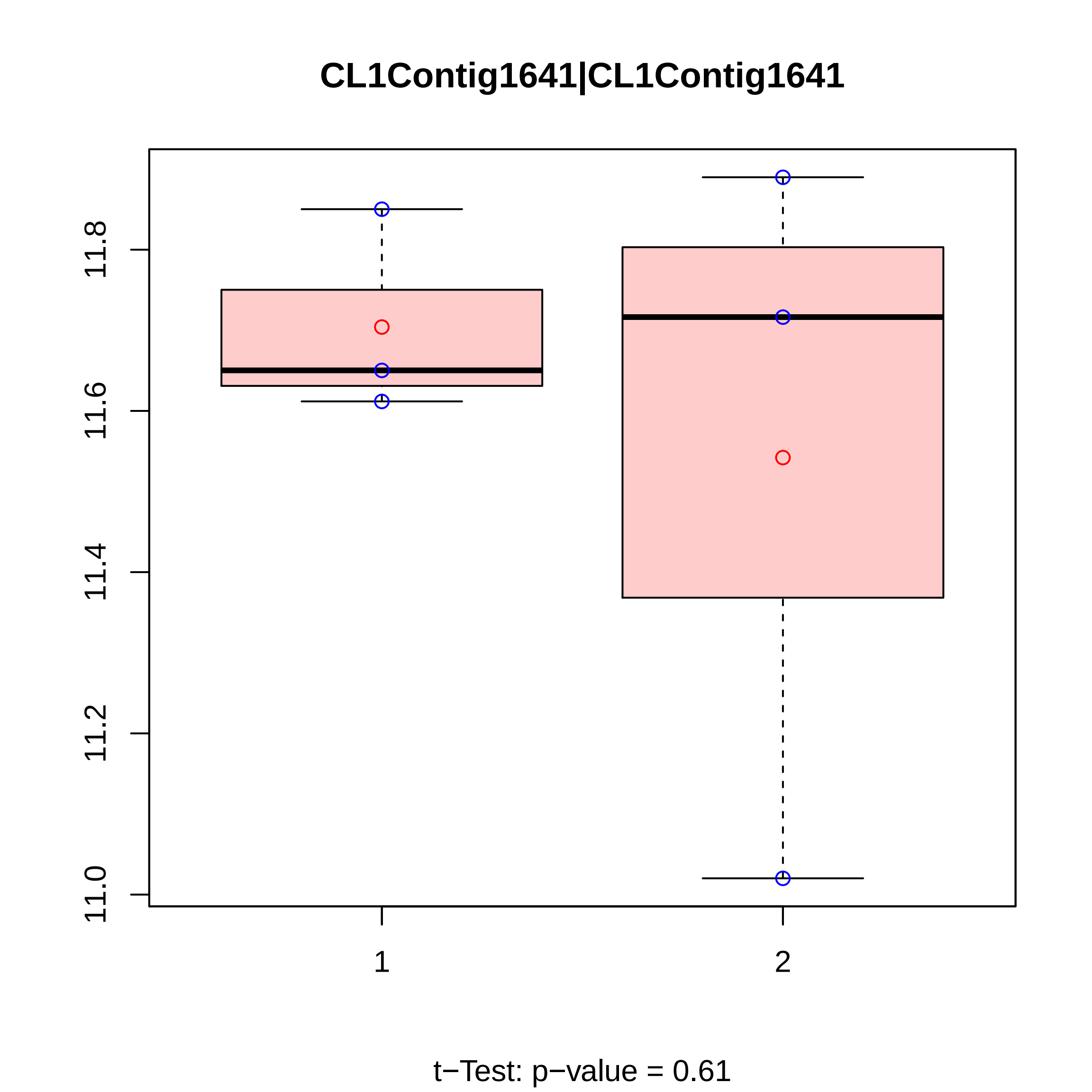

CL1Contig1641|CL1Contig1641
11.0
11.2
11.4
11.6
11.8
1
2
t−Test: p−value = 0.61

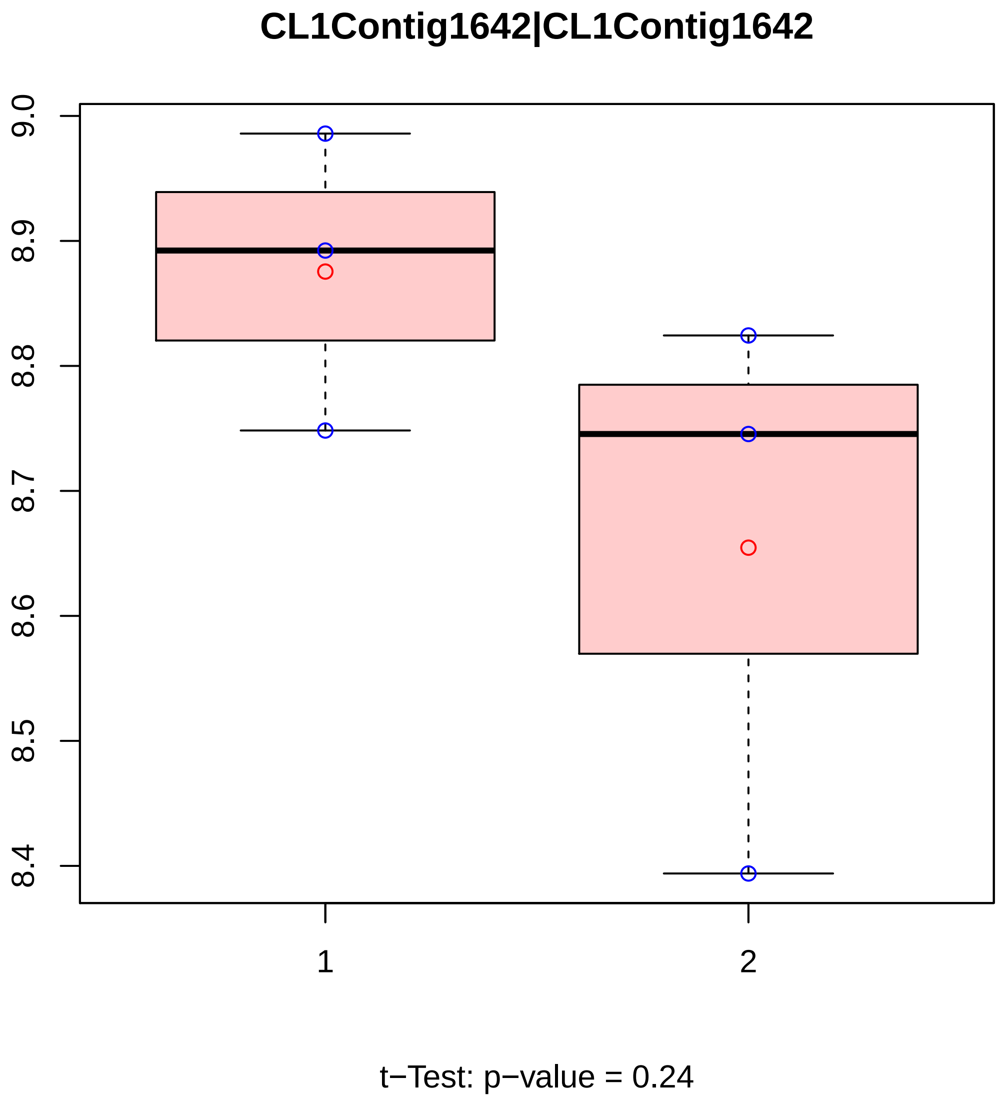
CL1Contig1642|CL1Contig1642
9.0
8.9
8.8
8.7
8.6
8.5
8.4
1
2
t−Test: p−value = 0.24

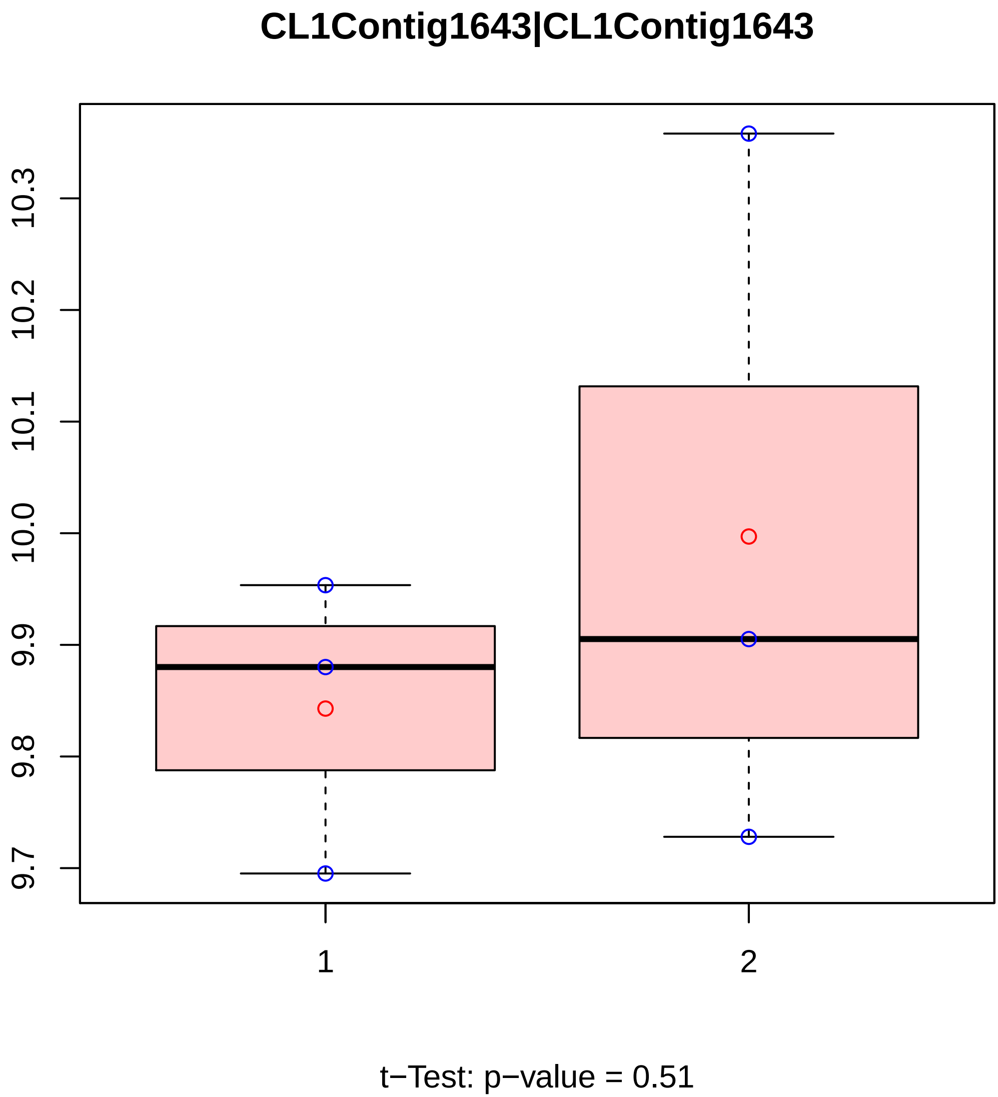

CL1Contig1643|CL1Contig1643
10.3
10.2
10.1
10.0
9.9
9.8
9.7
1
2
t−Test: p−value = 0.51

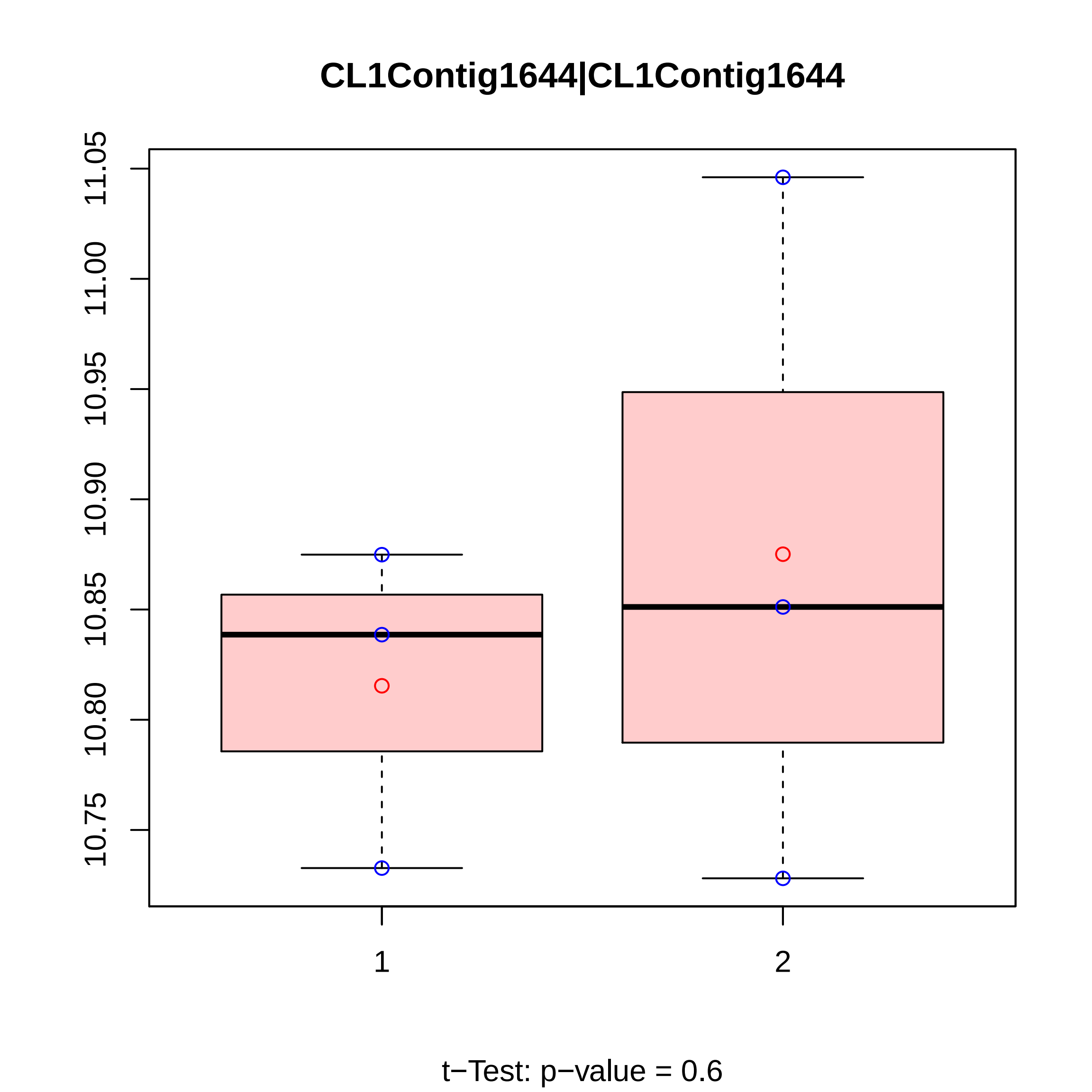

CL1Contig1644|CL1Contig1644
11.05
11.00
10.95
10.90
10.85
10.80
10.75
1
2
t−Test: p−value = 0.6

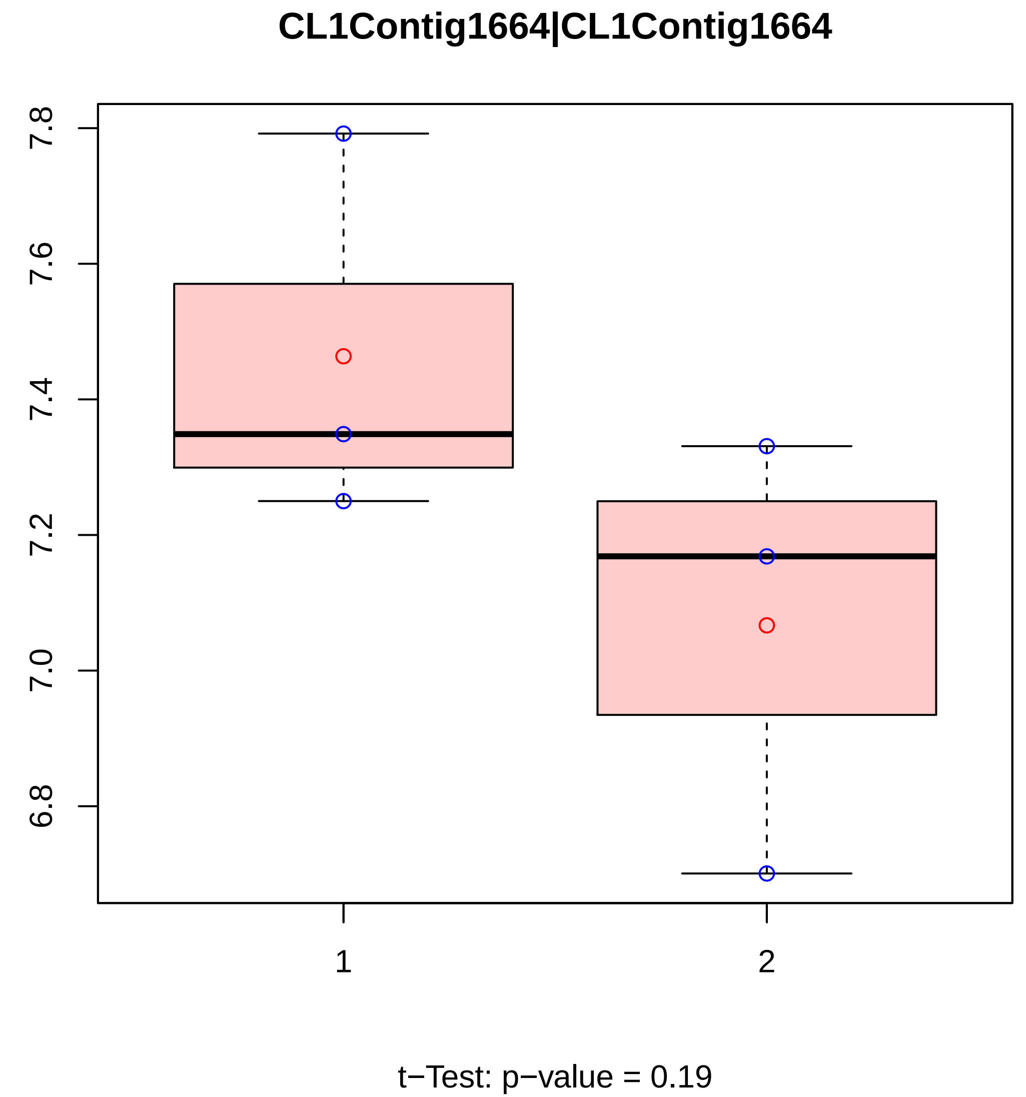
CL1Contig1664|CL1Contig1664
7.8
7.6
7.4
7.2
7.0
6.8
1
2
t−Test: p−value = 0.19

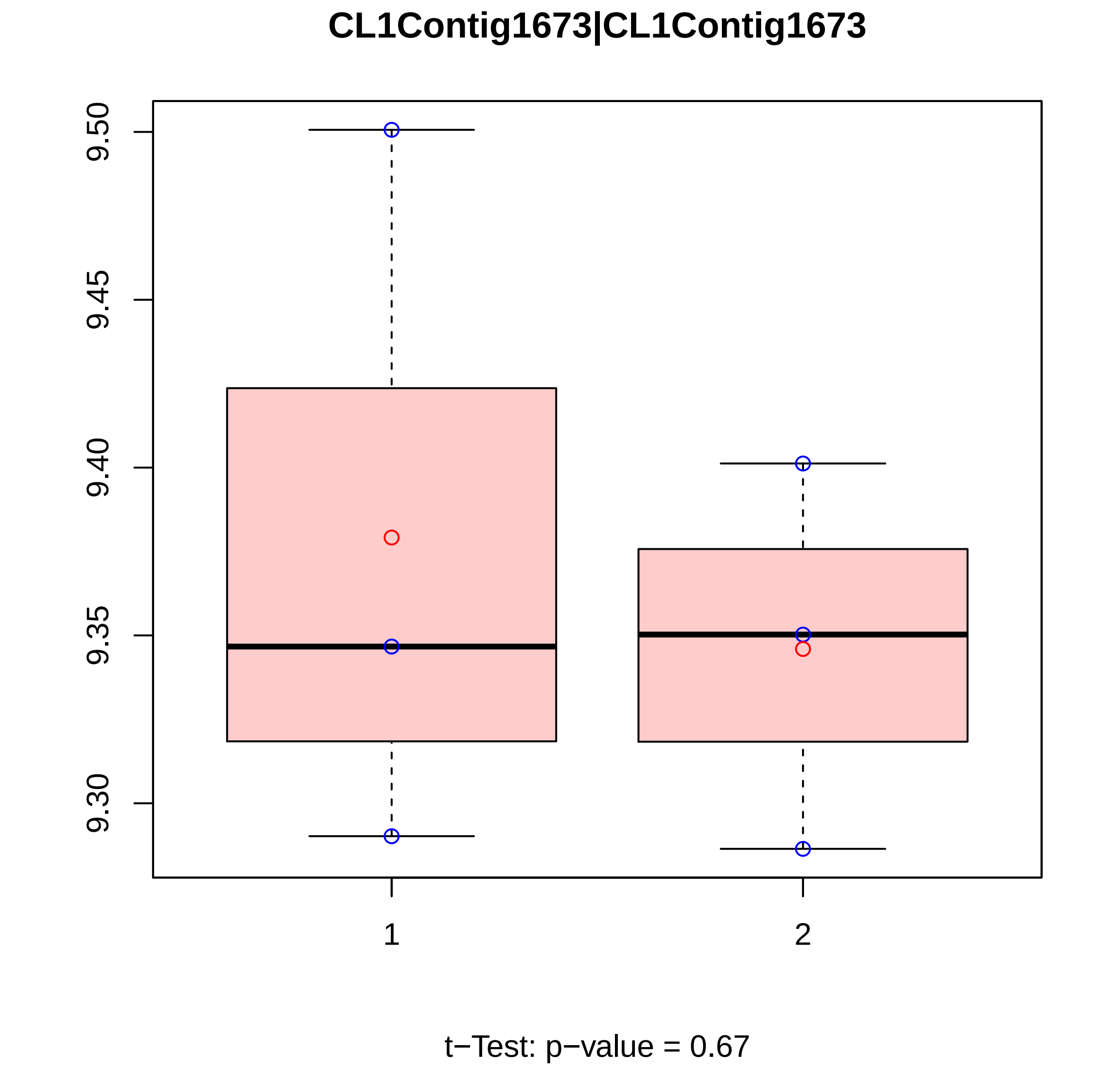

CL1Contig1673|CL1Contig1673
9.50
9.45
9.40
9.35
9.30
1
2
t−Test: p−value = 0.67

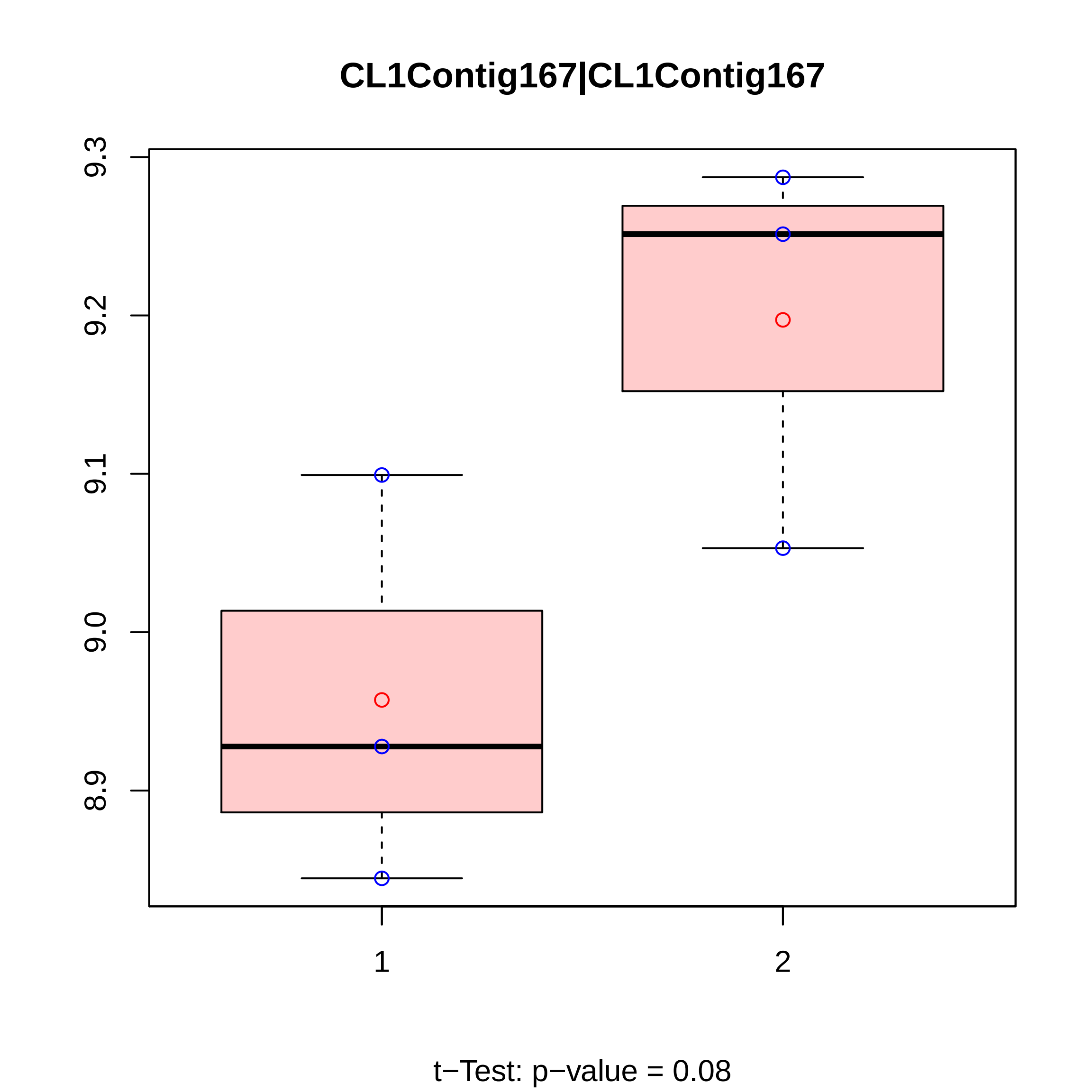

CL1Contig167|CL1Contig167
9.3
9.2
9.1
9.0
8.9
1
2
t−Test: p−value = 0.08

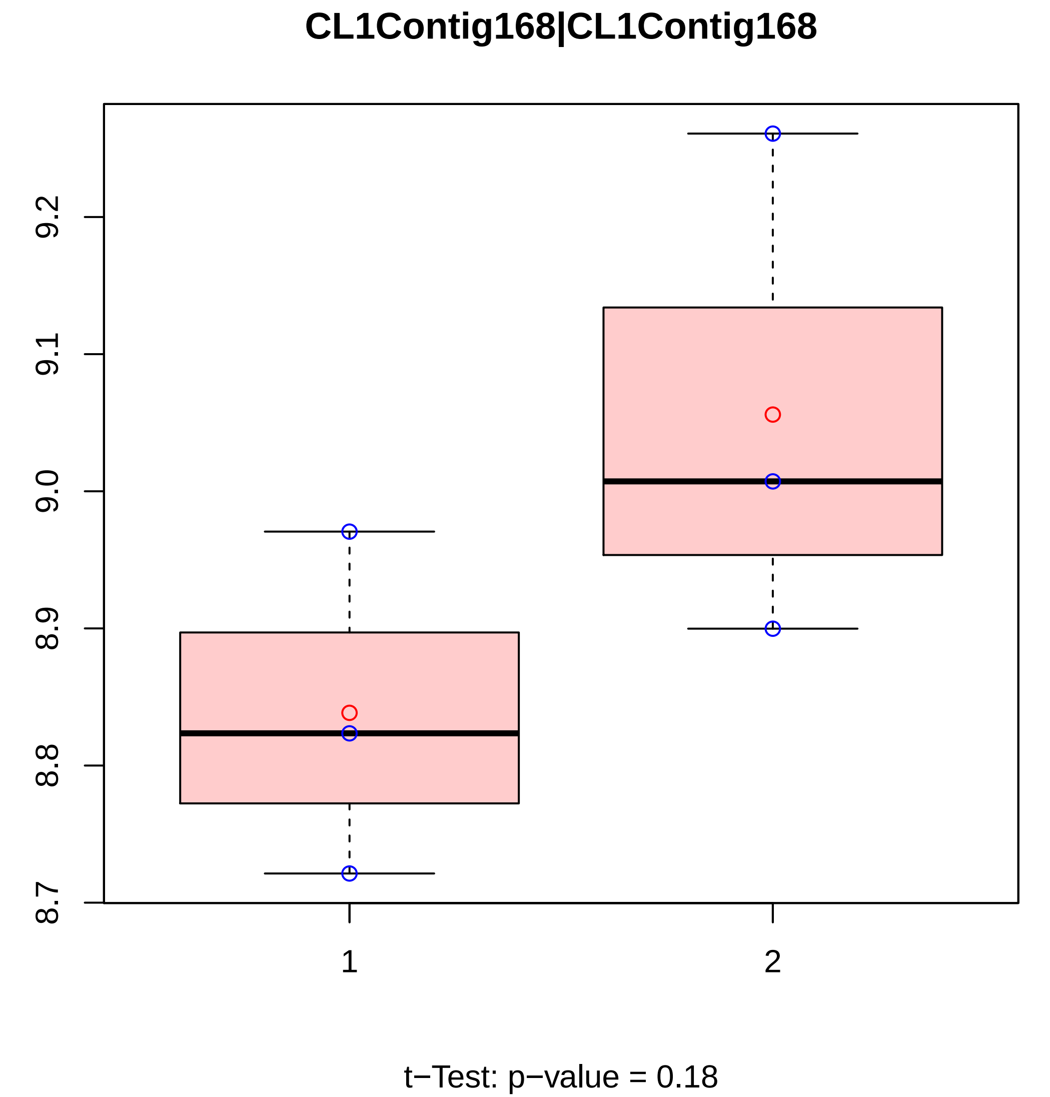

CL1Contig168|CL1Contig168
9.2
9.1
9.0
8.9
8.8
8.7
1
2
t−Test: p−value = 0.18

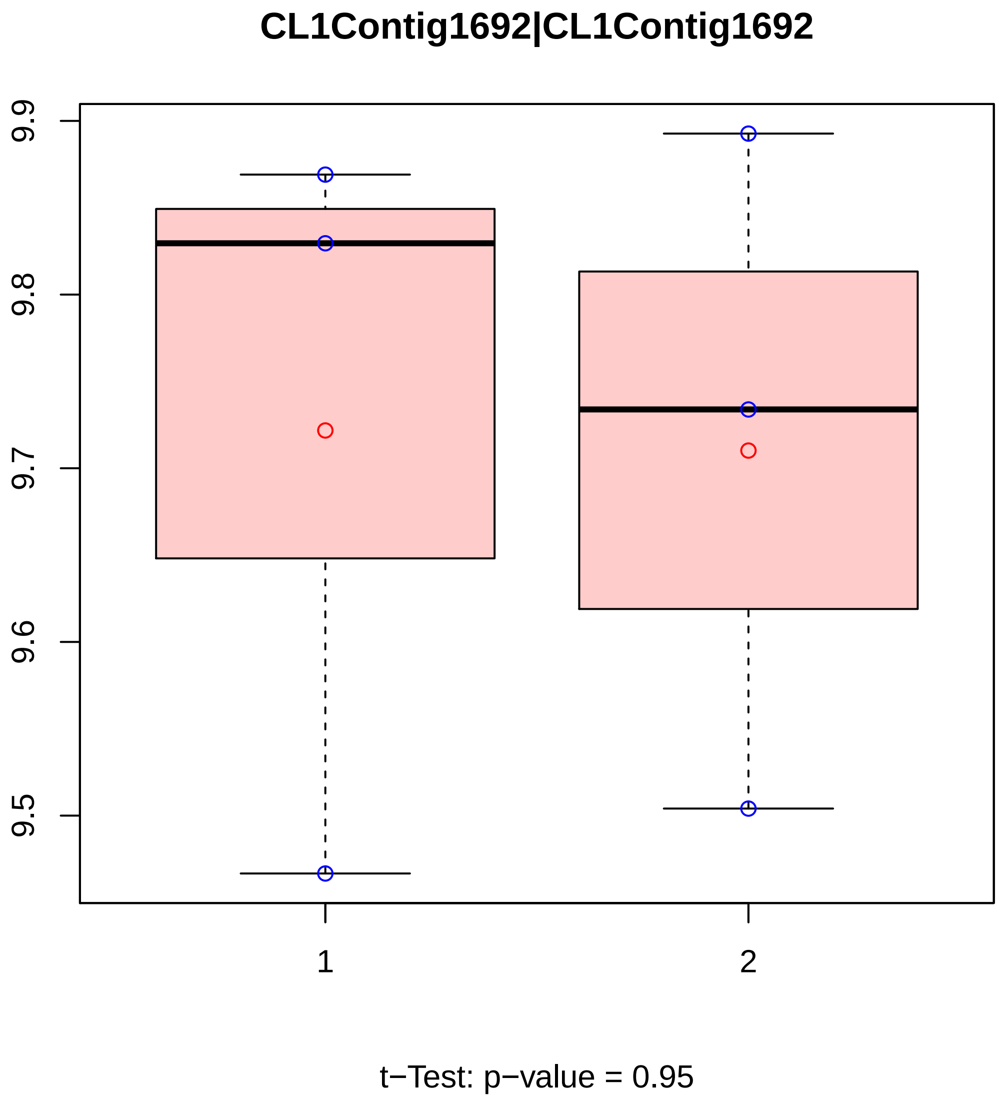

CL1Contig1692|CL1Contig1692
9.9
9.8
9.7
9.6
9.5
1
2
t−Test: p−value = 0.95

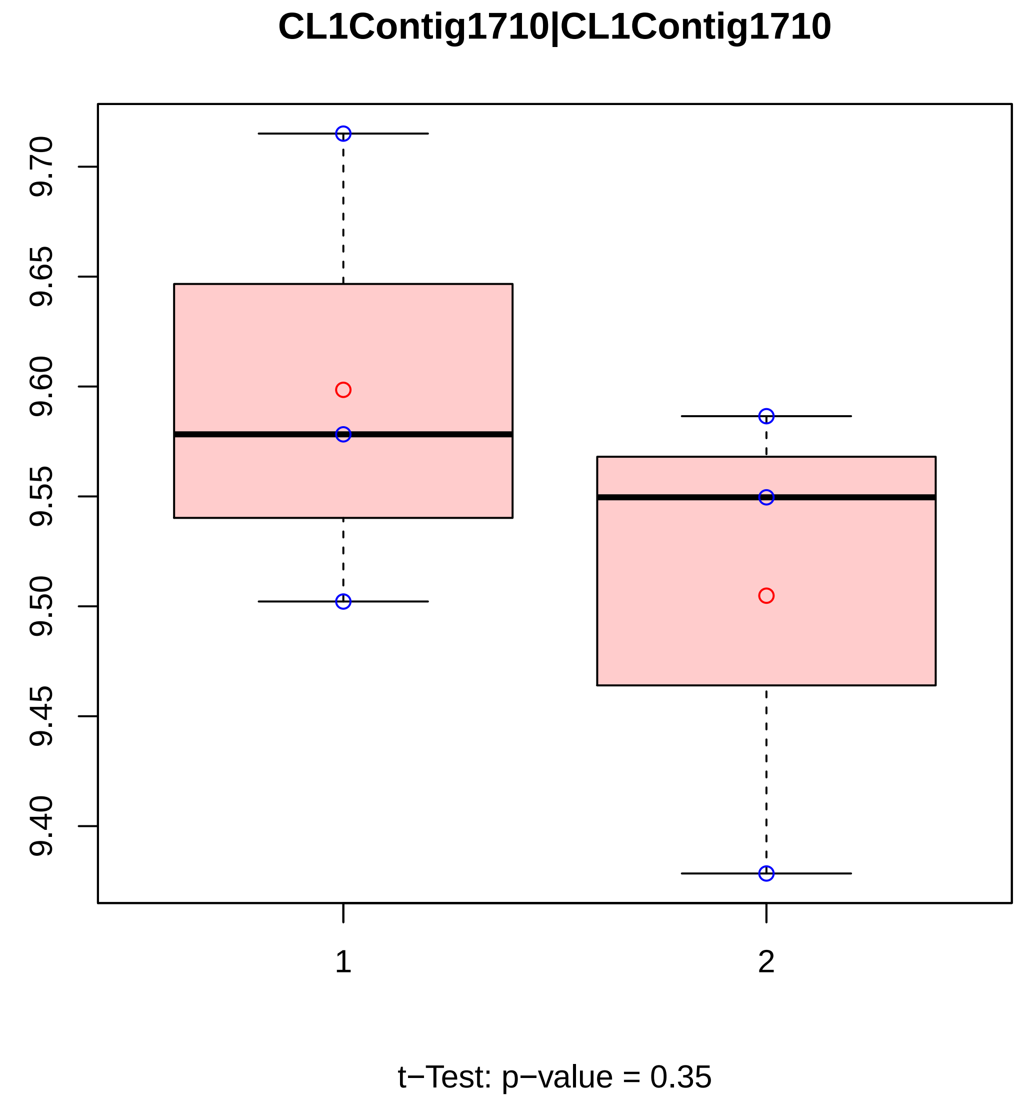
CL1Contig1710|CL1Contig1710
9.70
9.65
9.60
9.55
9.50
9.45
9.40
1
2
t−Test: p−value = 0.35

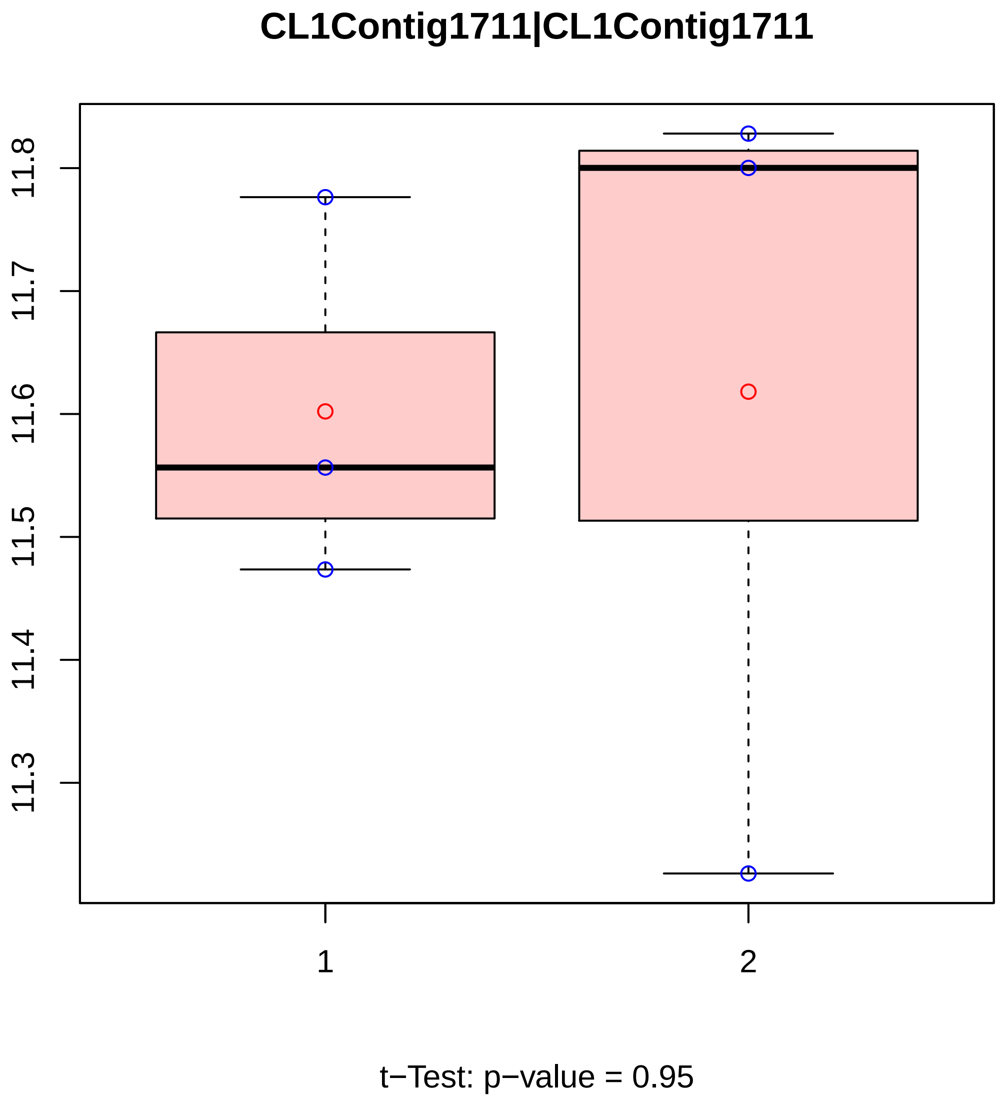

CL1Contig1711|CL1Contig1711
11.3
11.4
11.5
11.6
11.7
11.8
1
2
t−Test: p−value = 0.95

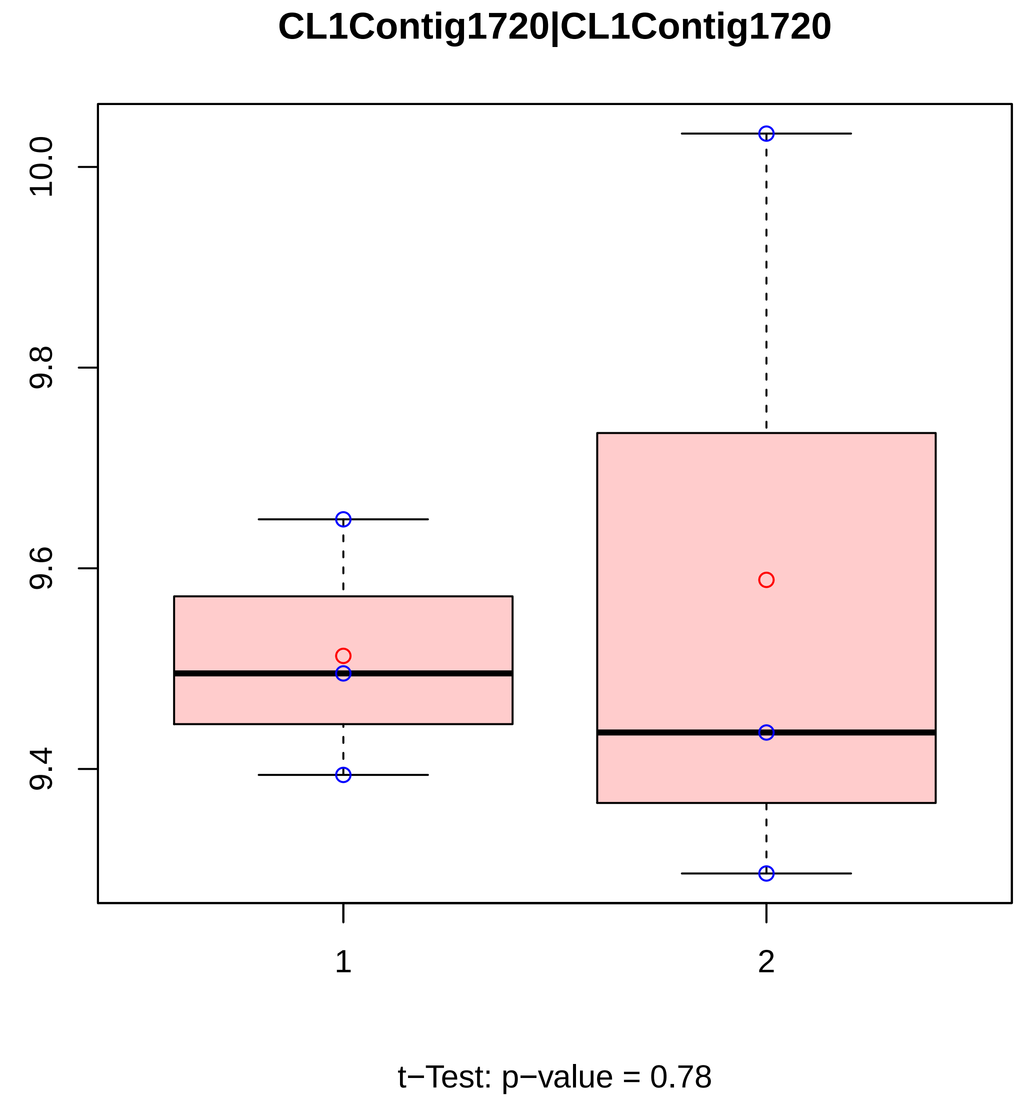

CL1Contig1720|CL1Contig1720
10.0
9.8
9.6
9.4
1
2
t−Test: p−value = 0.78

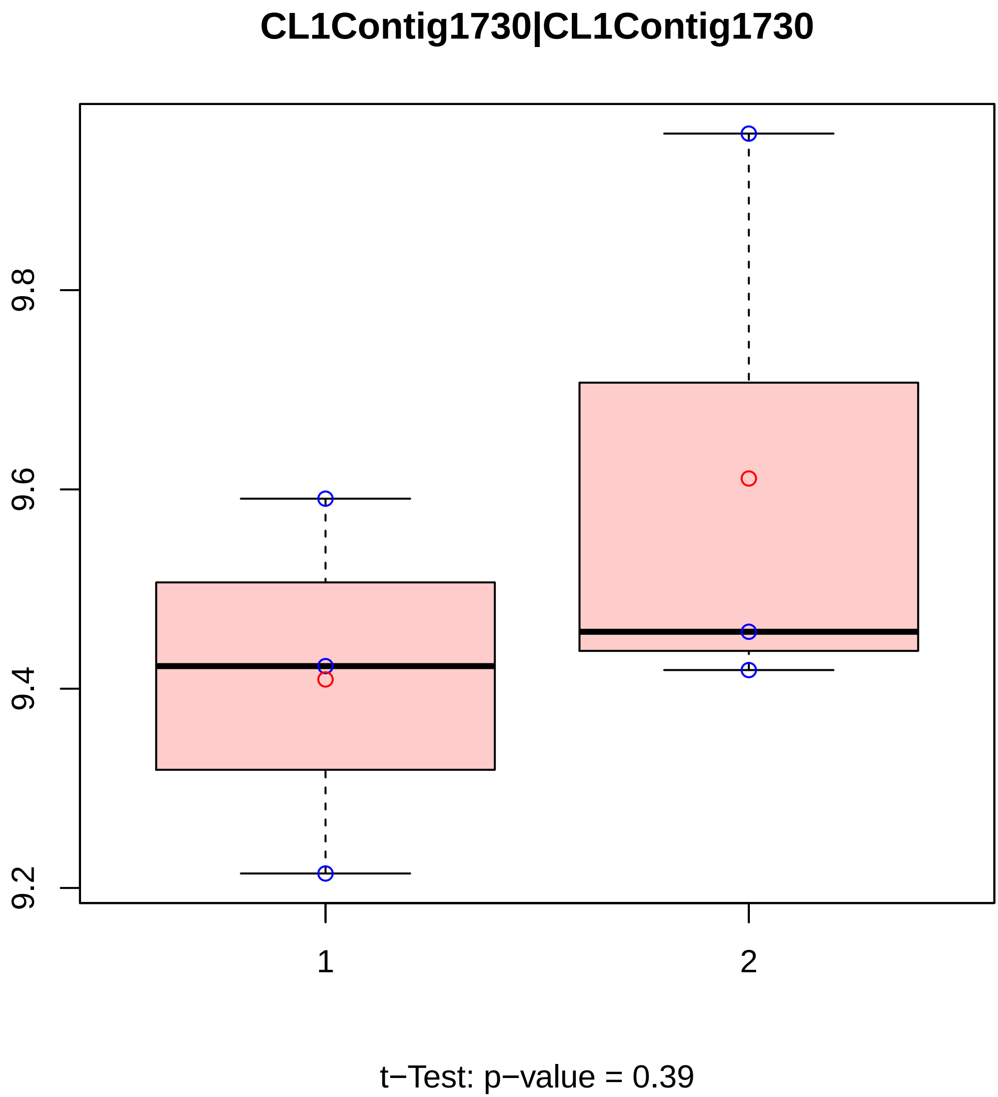
CL1Contig1730|CL1Contig1730
9.2
9.4
9.6
9.8
1
2
t−Test: p−value = 0.39

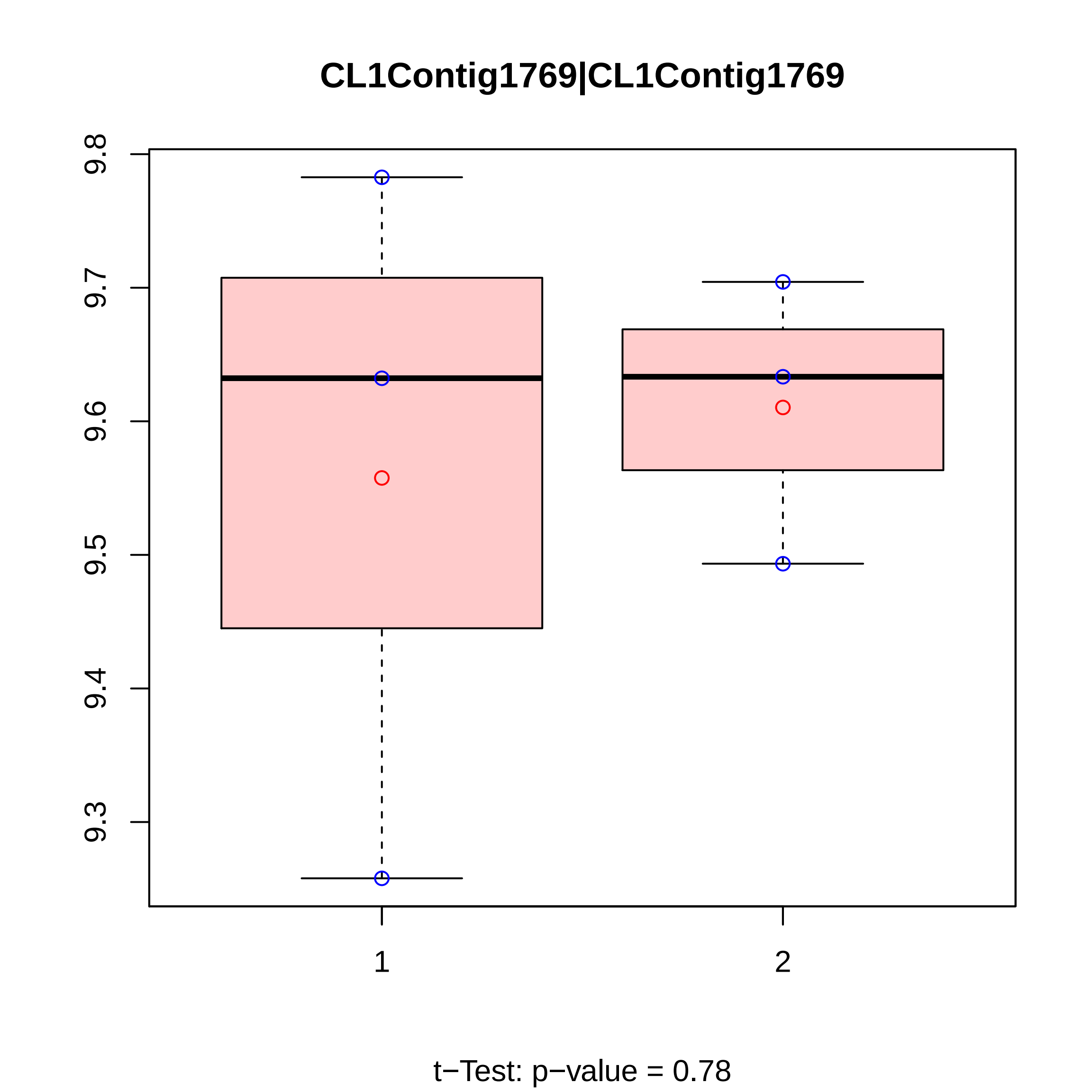

CL1Contig1769|CL1Contig1769
9.8
9.7
9.6
9.5
9.4
9.3
1
2
t−Test: p−value = 0.78

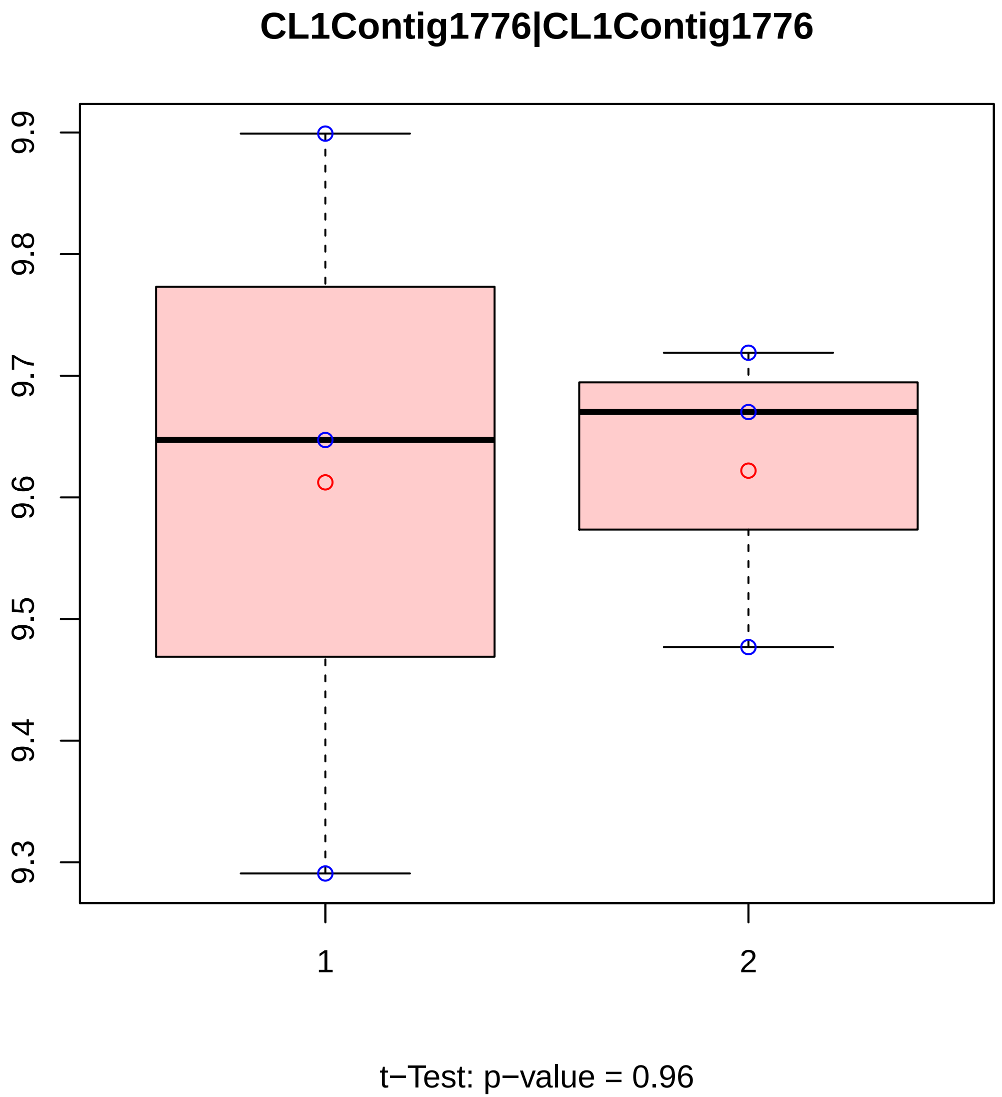
CL1Contig1776|CL1Contig1776
9.3
9.4
9.5
9.6
9.7
9.8
9.9
1
2
t−Test: p−value = 0.96

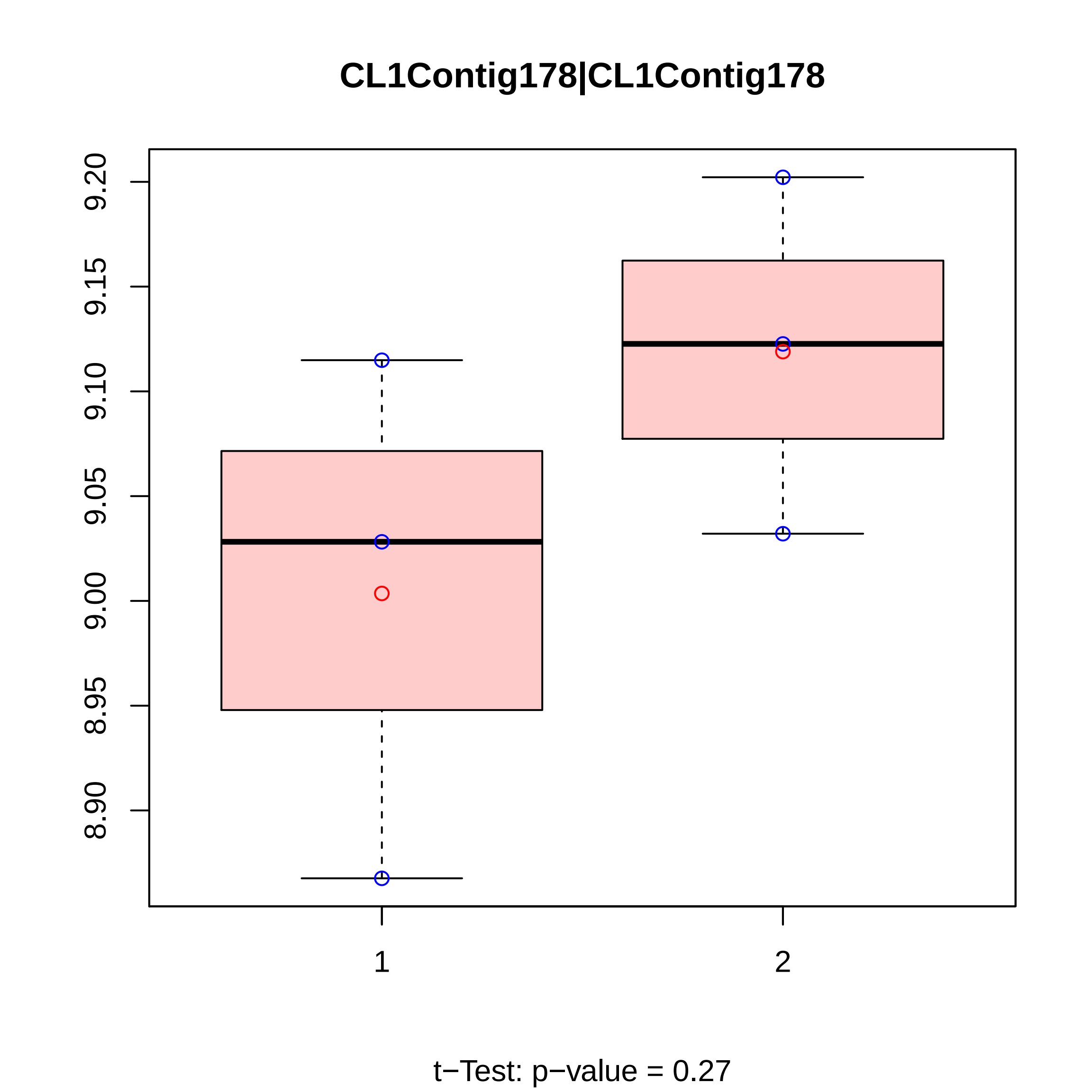

CL1Contig178|CL1Contig178
9.20
9.15
9.10
9.05
9.00
8.95
8.90
1
2
t−Test: p−value = 0.27

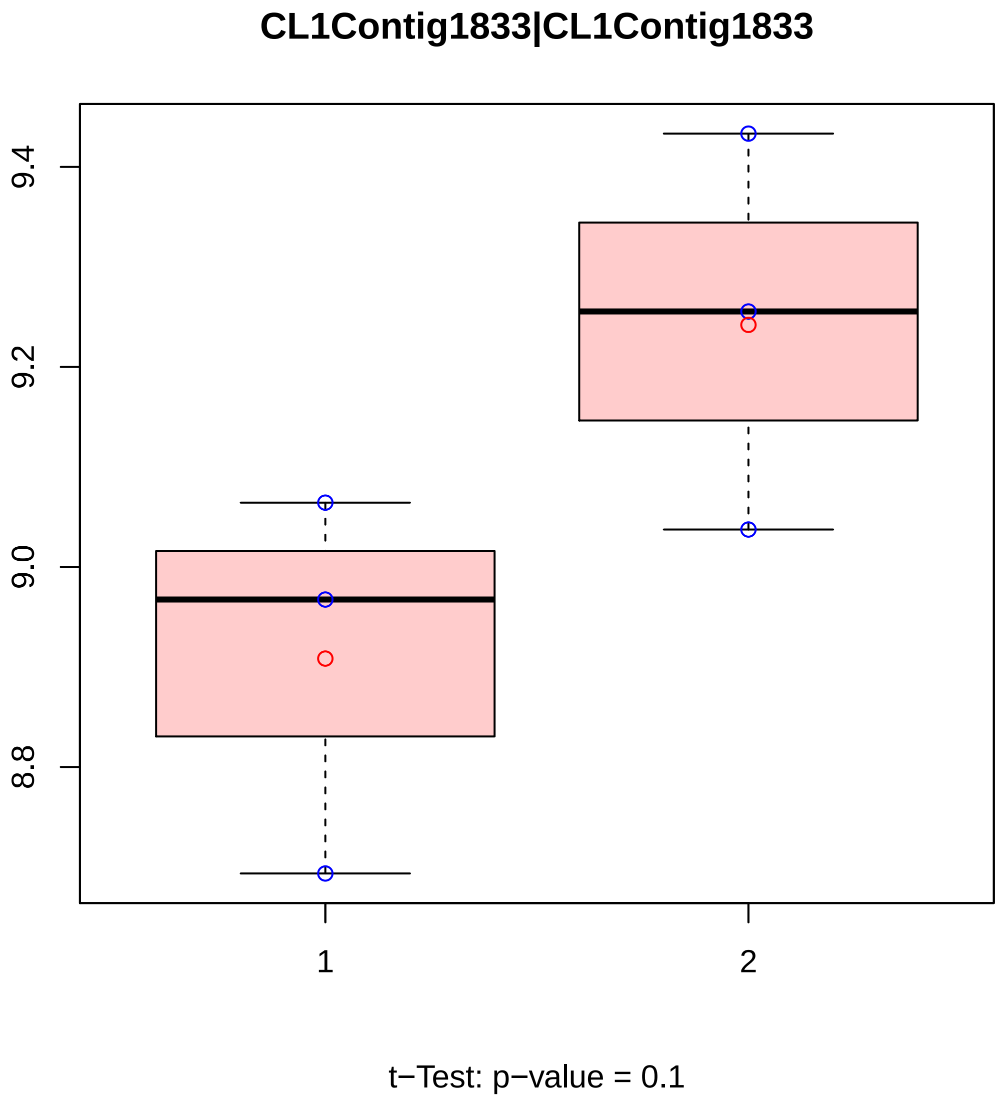
CL1Contig1833|CL1Contig1833
9.4
9.2
9.0
8.8
1
2
t−Test: p−value = 0.1

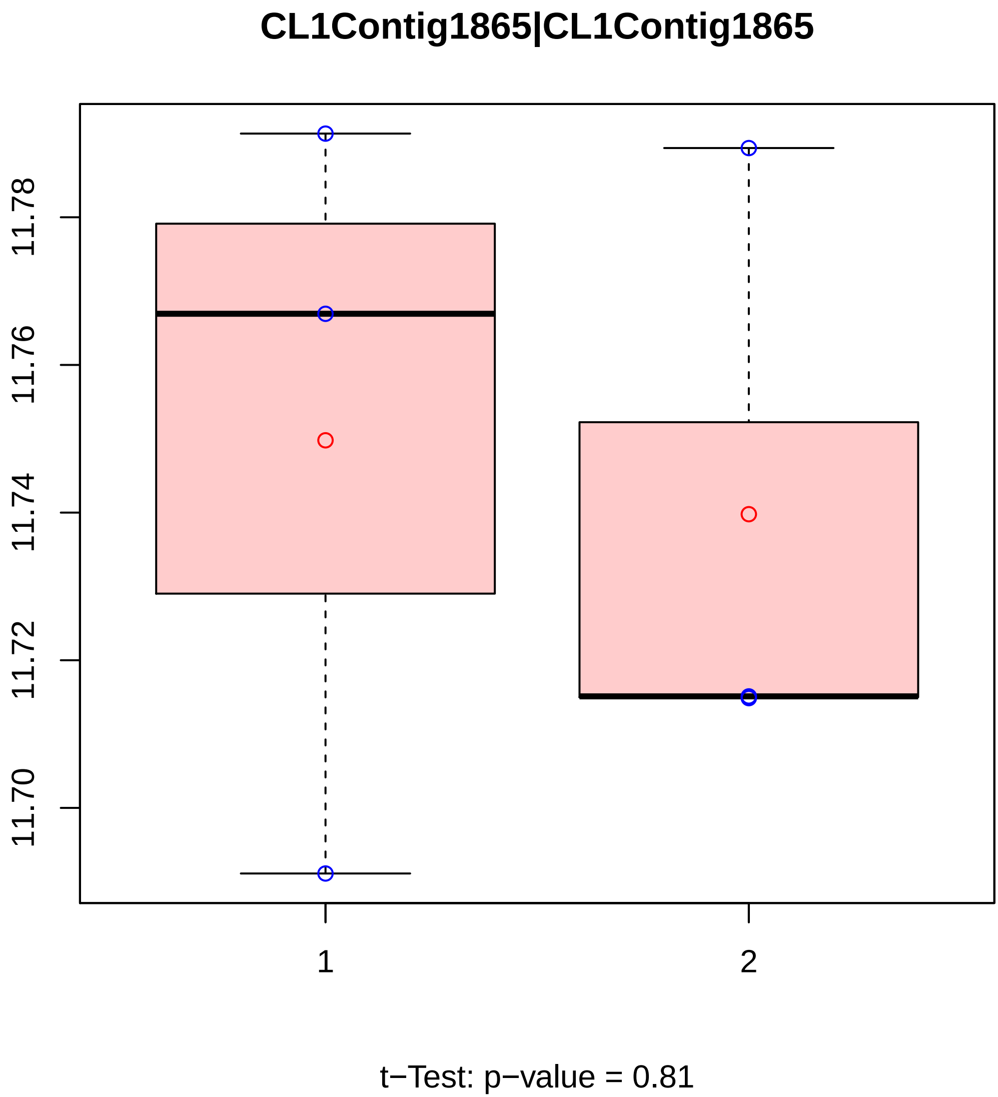

CL1Contig1865|CL1Contig1865
11.70
11.72
11.74
11.76
11.78
1
2
t−Test: p−value = 0.81

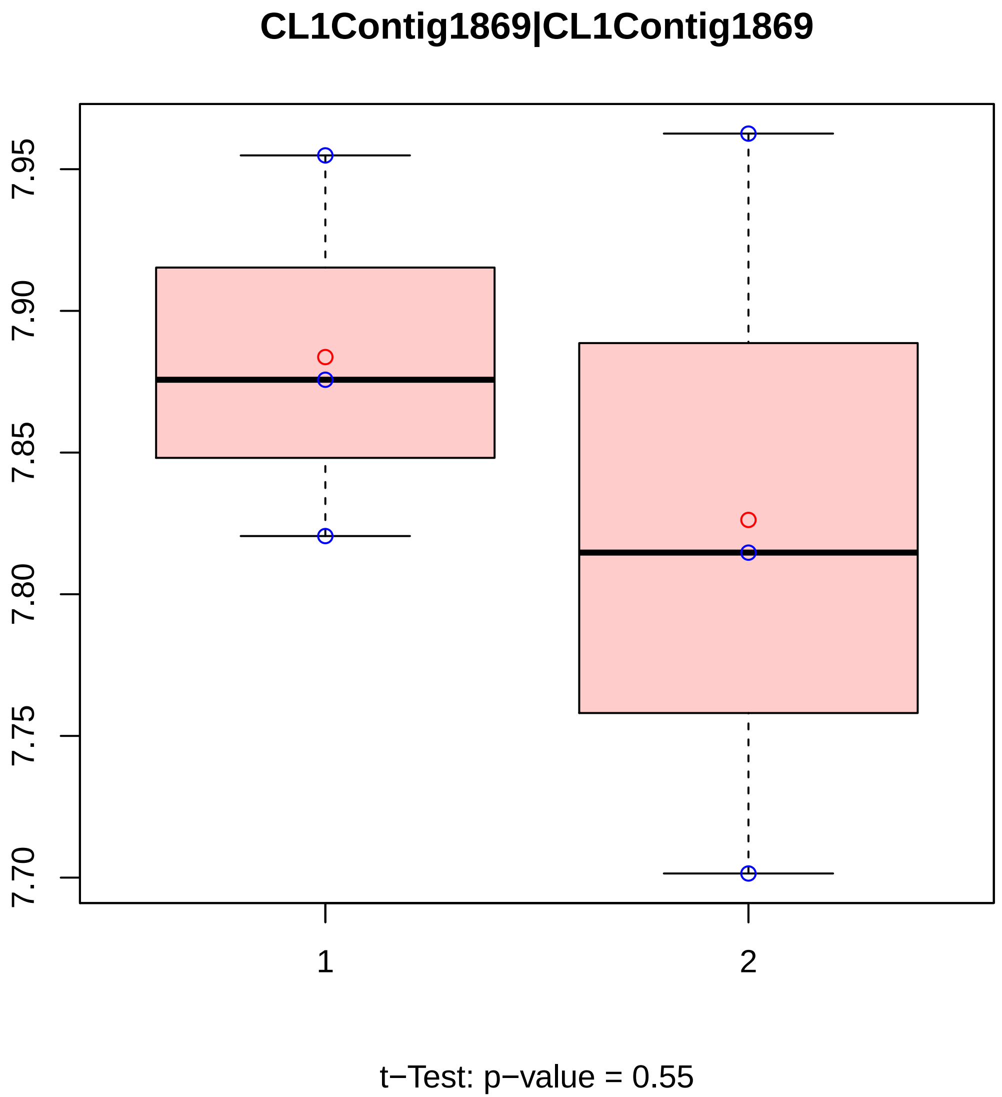
CL1Contig1869|CL1Contig1869
7.70
7.75
7.80
7.85
7.90
7.95
1
2
t−Test: p−value = 0.55

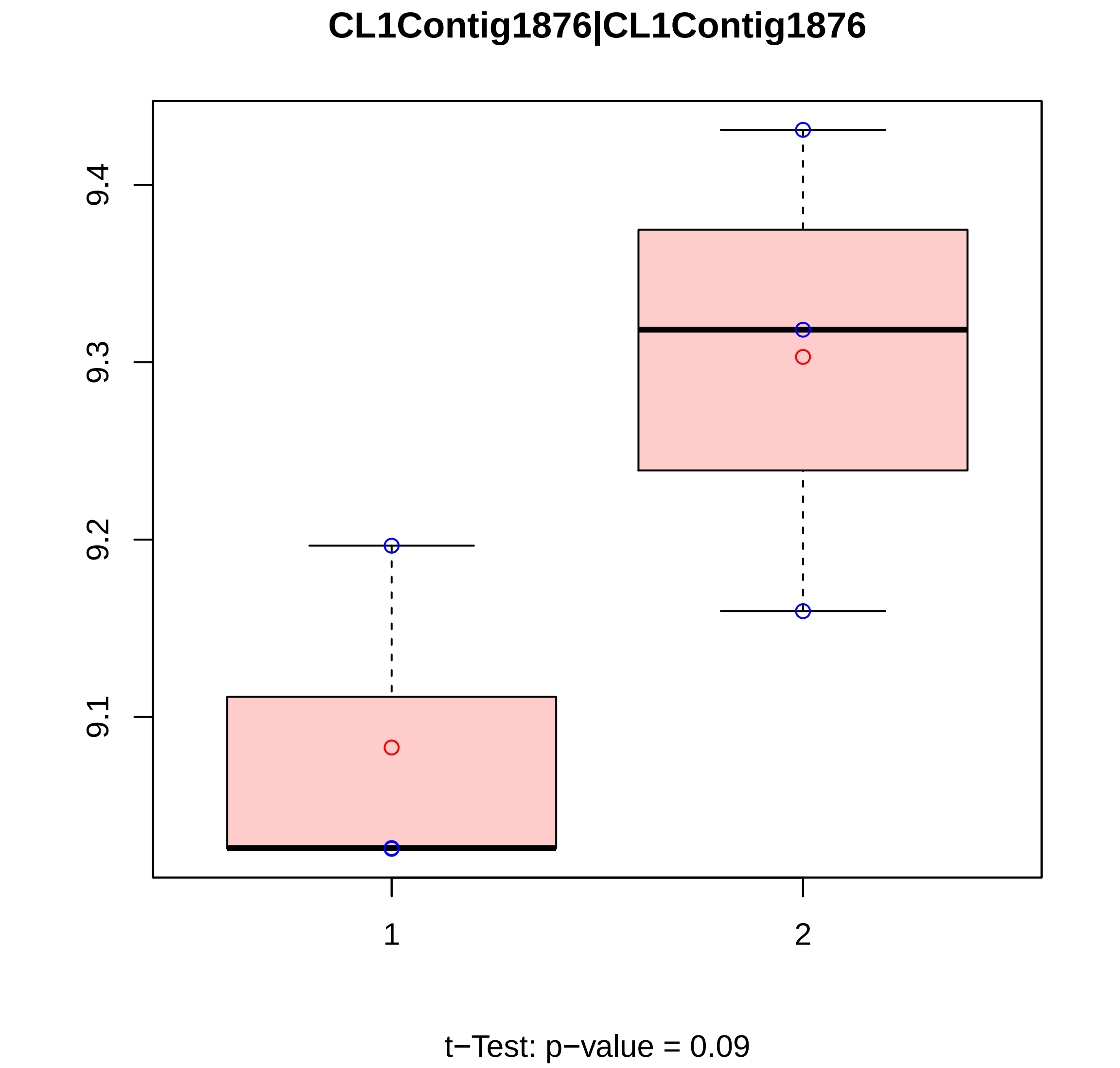

CL1Contig1876|CL1Contig1876
9.1
9.2
9.3
9.4
1
2
t−Test: p−value = 0.09

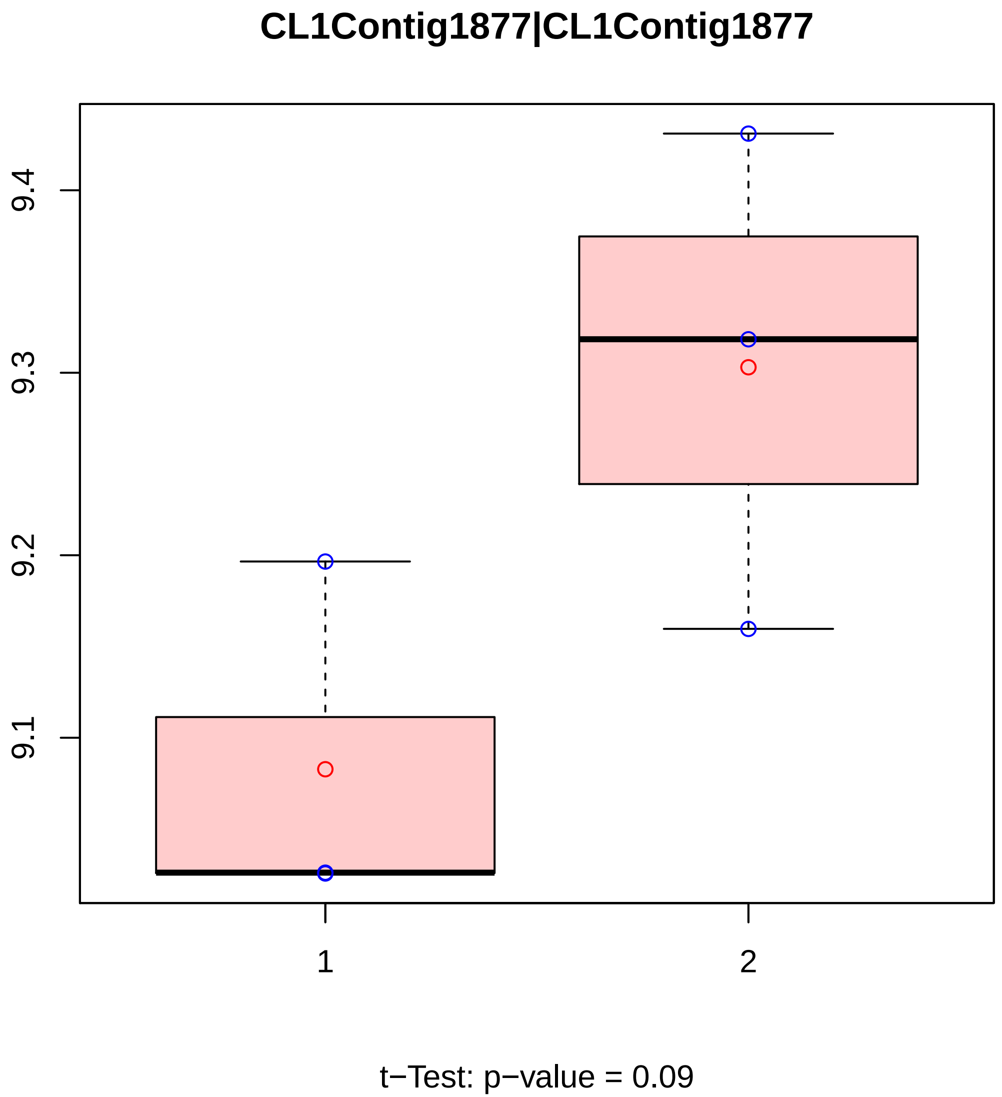
CL1Contig1877|CL1Contig1877
9.1
9.2
9.3
9.4
1
2
t−Test: p−value = 0.09

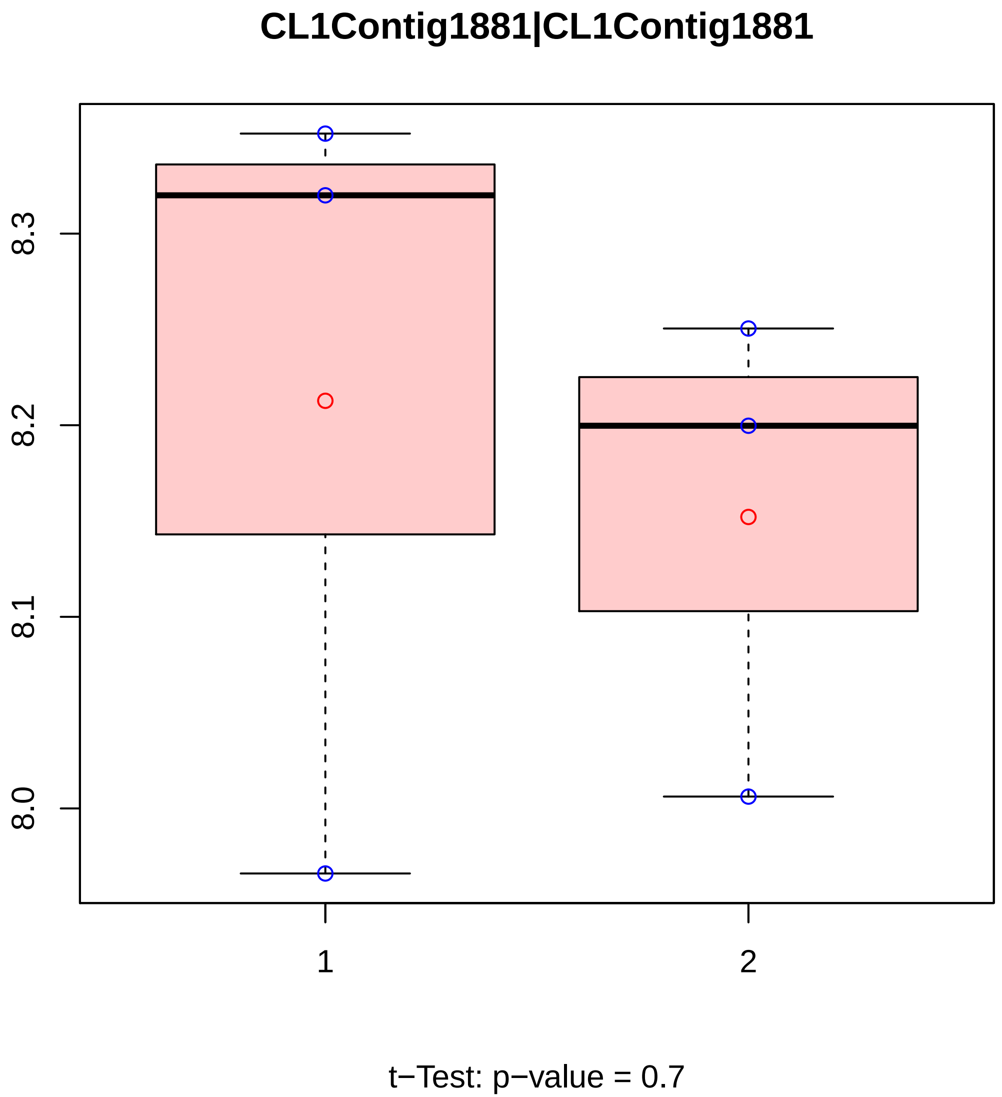
CL1Contig1881|CL1Contig1881
8.0
8.1
8.2
8.3
1
2
t−Test: p−value = 0.7

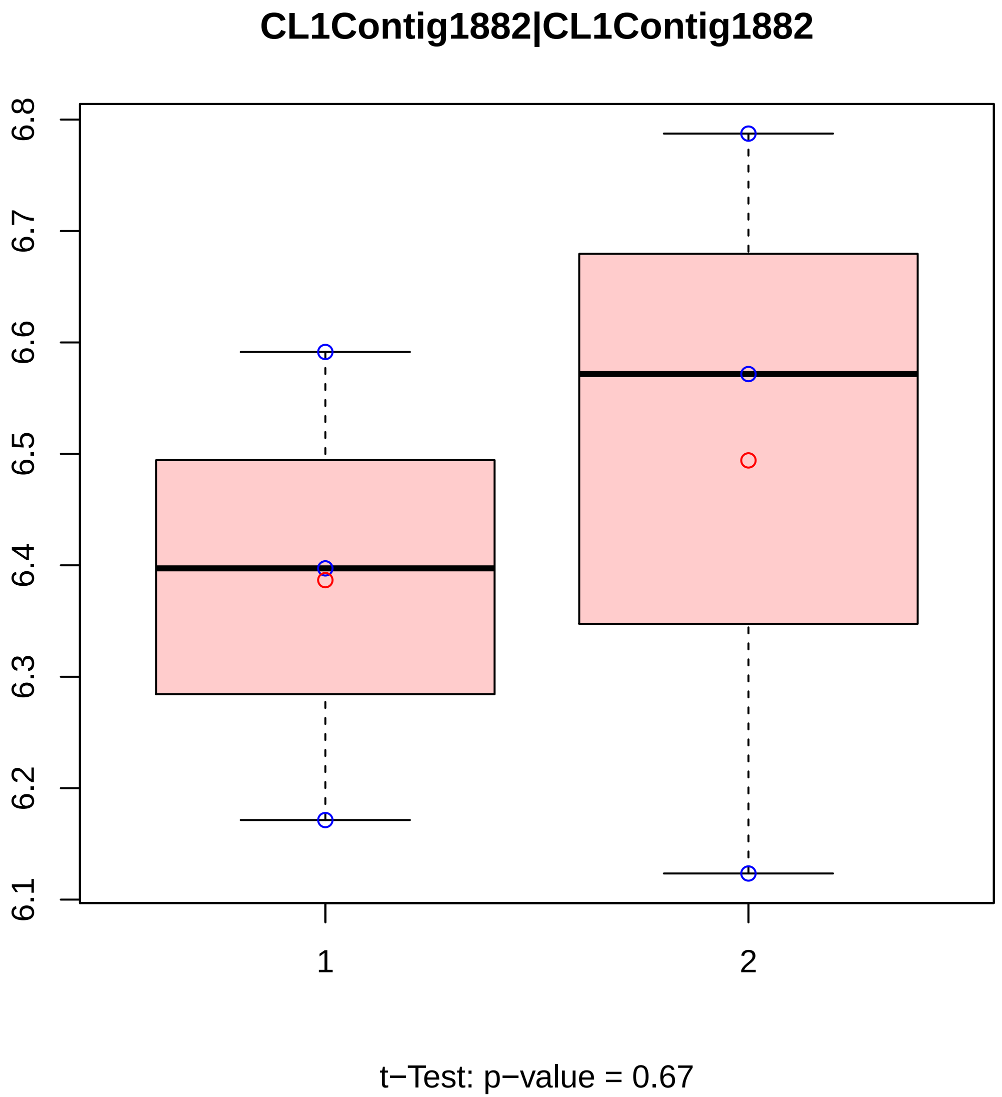

CL1Contig1882|CL1Contig1882
6.1
6.2
6.3
6.4
6.5
6.6
6.7
6.8
1
2
t−Test: p−value = 0.67

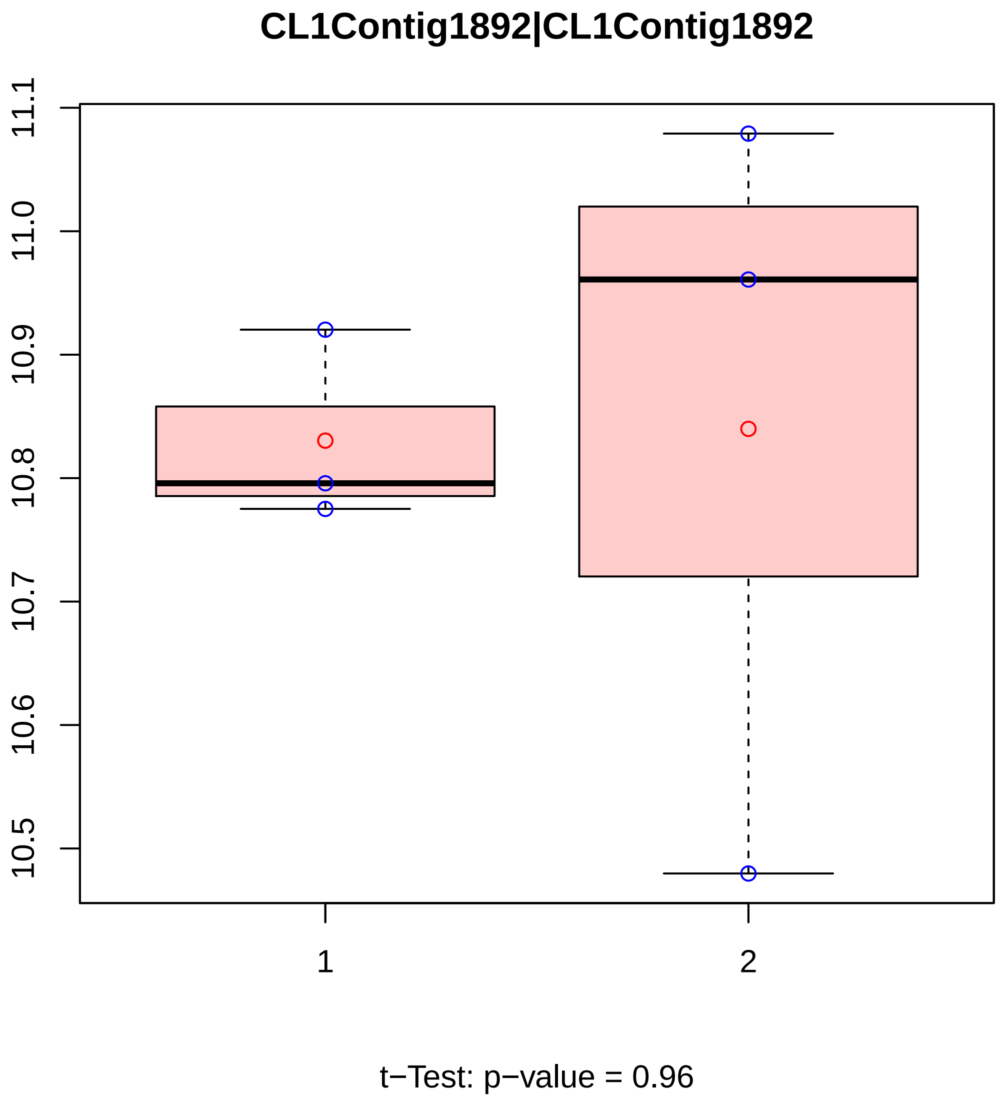
CL1Contig1892|CL1Contig1892
11.1
11.0
10.9
10.8
10.7
10.6
10.5
1
2
t−Test: p−value = 0.96

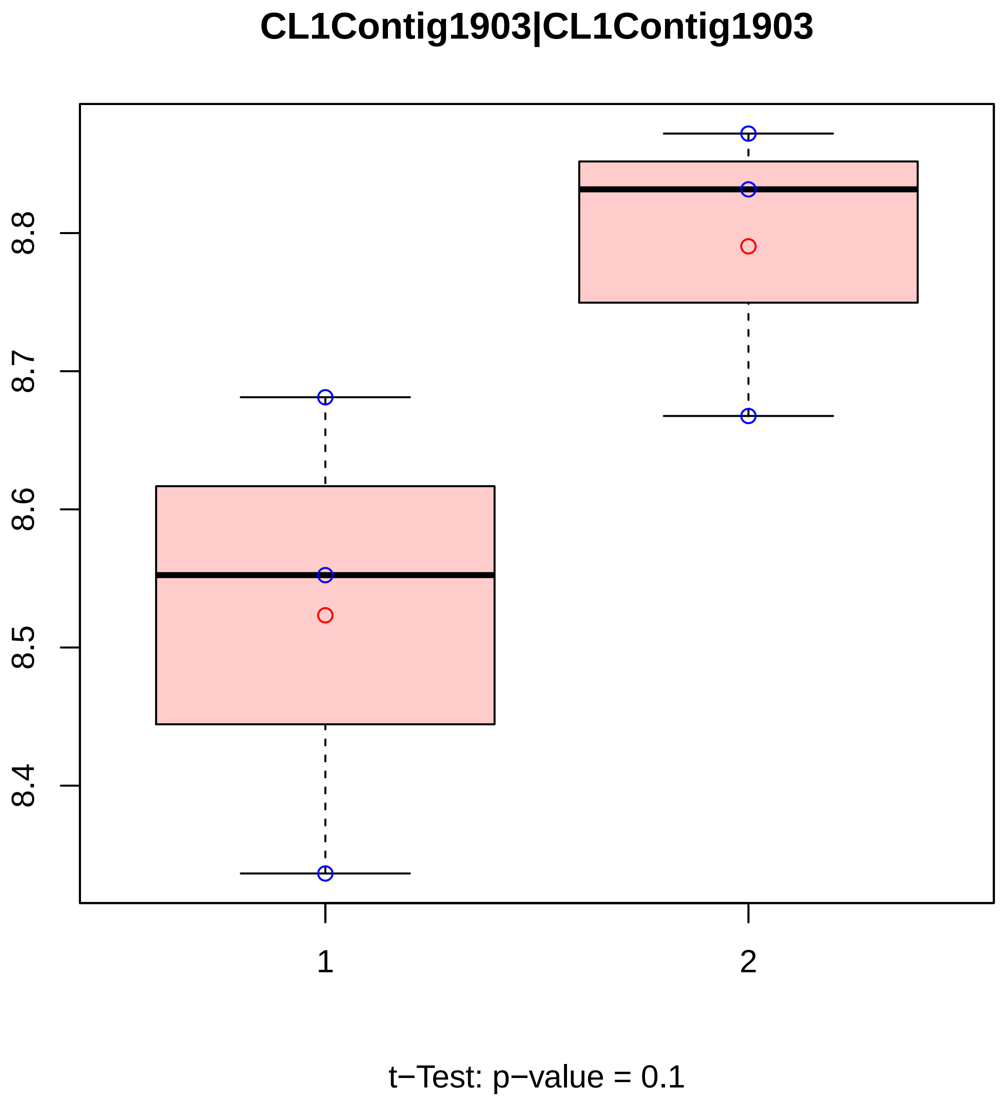

CL1Contig1903|CL1Contig1903
8.4
8.5
8.6
8.7
8.8
1
2
t−Test: p−value = 0.1

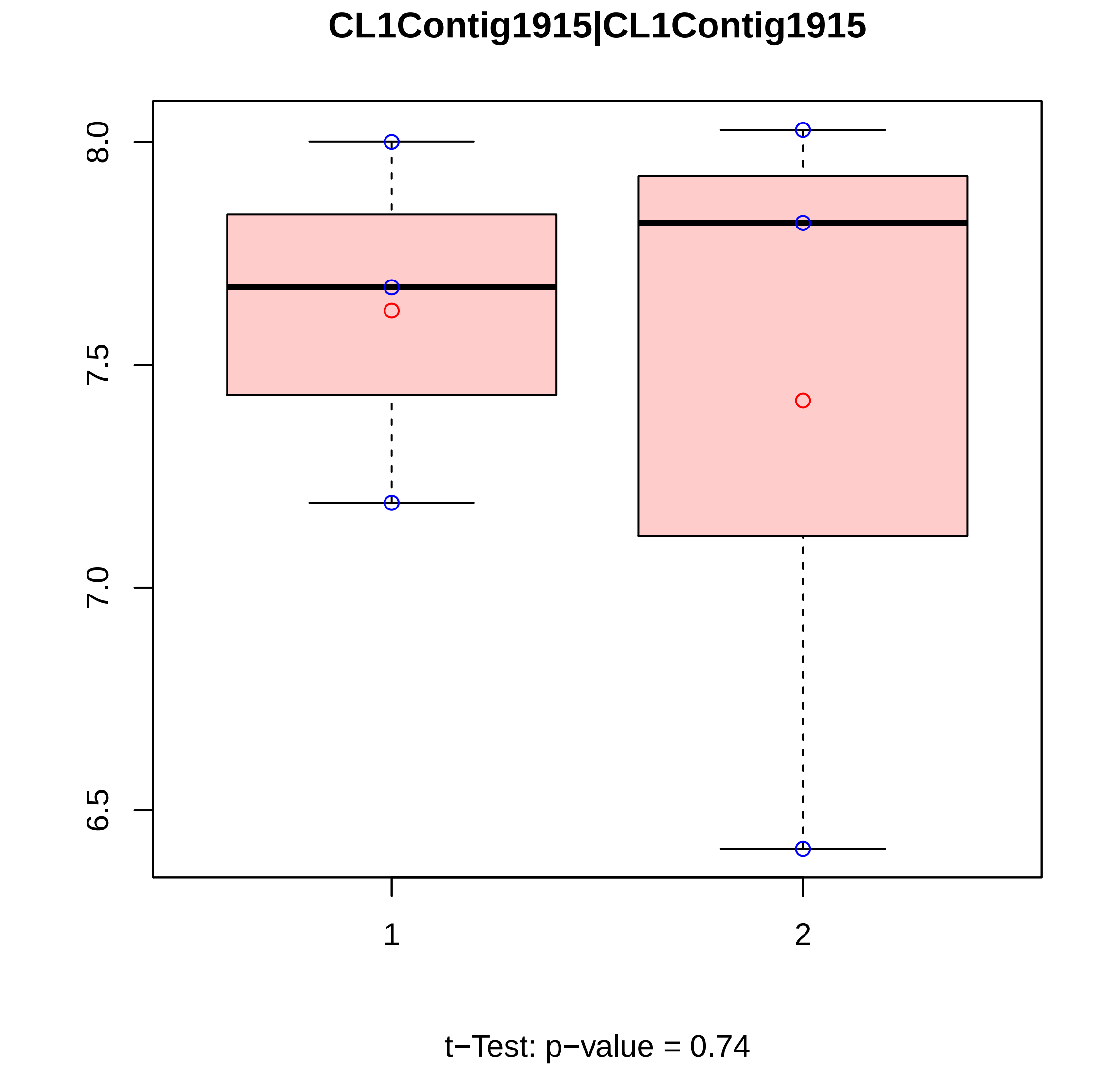
CL1Contig1915|CL1Contig1915
8.0
7.5
7.0
6.5
1
2
t−Test: p−value = 0.74

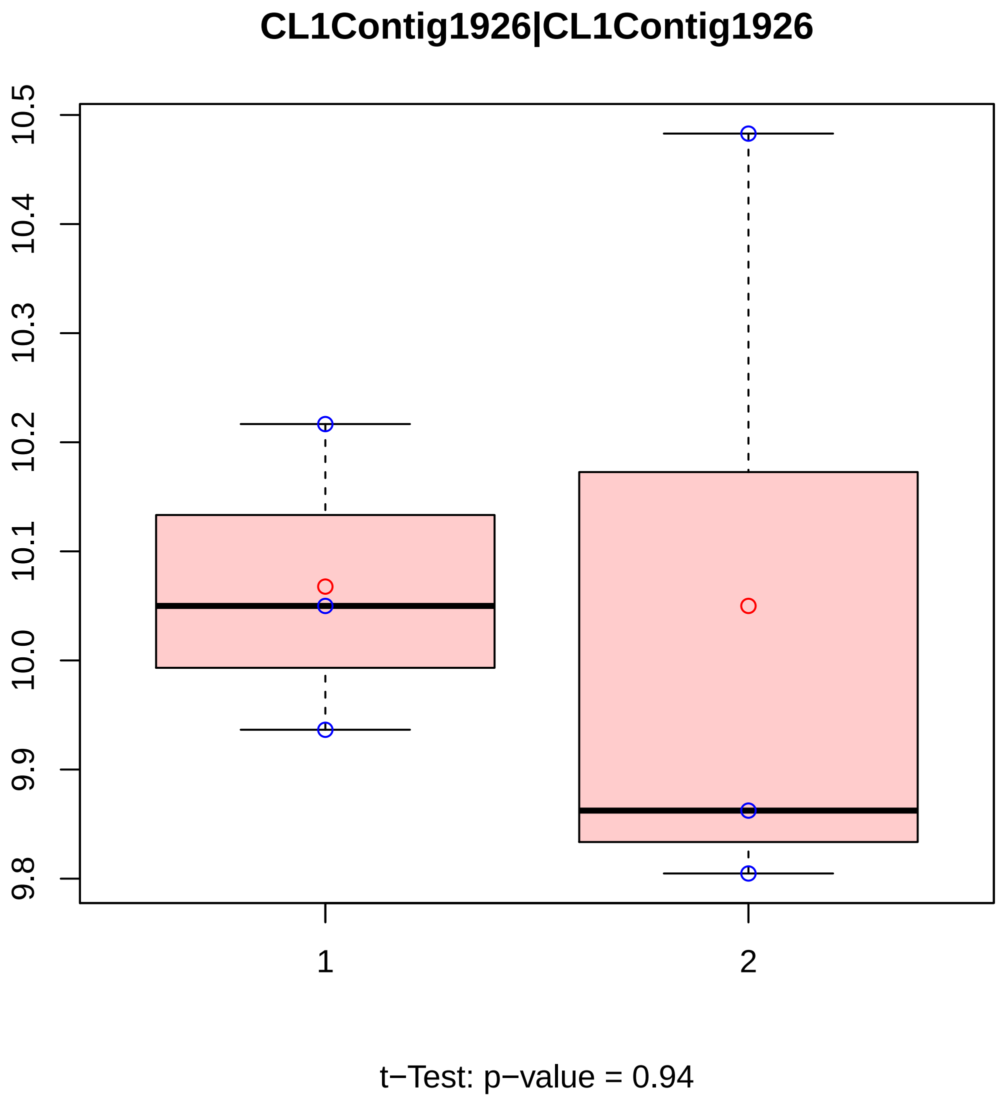

CL1Contig1926|CL1Contig1926
10.5
10.4
10.3
10.2
10.1
10.0
9.9
9.8
1
2
t−Test: p−value = 0.94

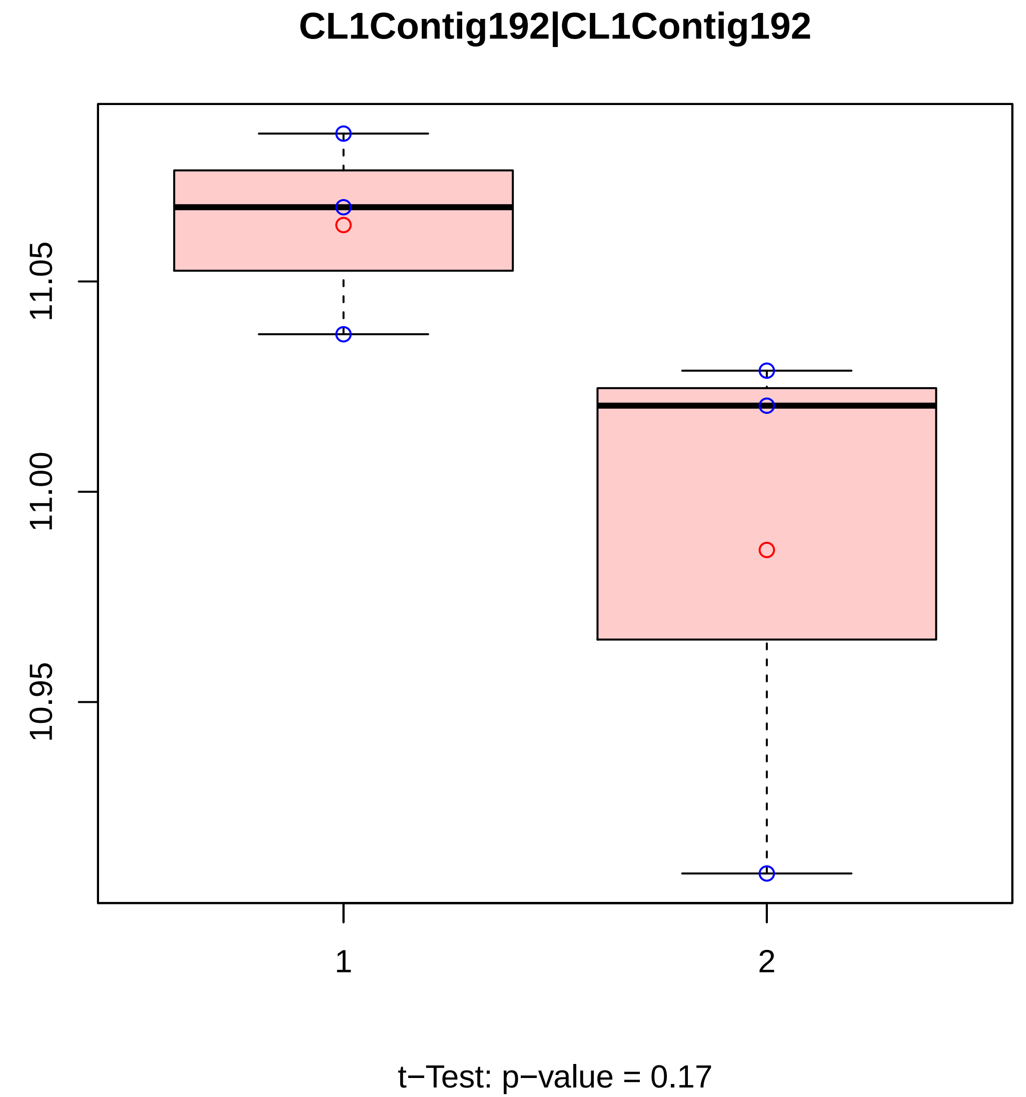
CL1Contig192|CL1Contig192
11.05
11.00
10.95
1
2
t−Test: p−value = 0.17

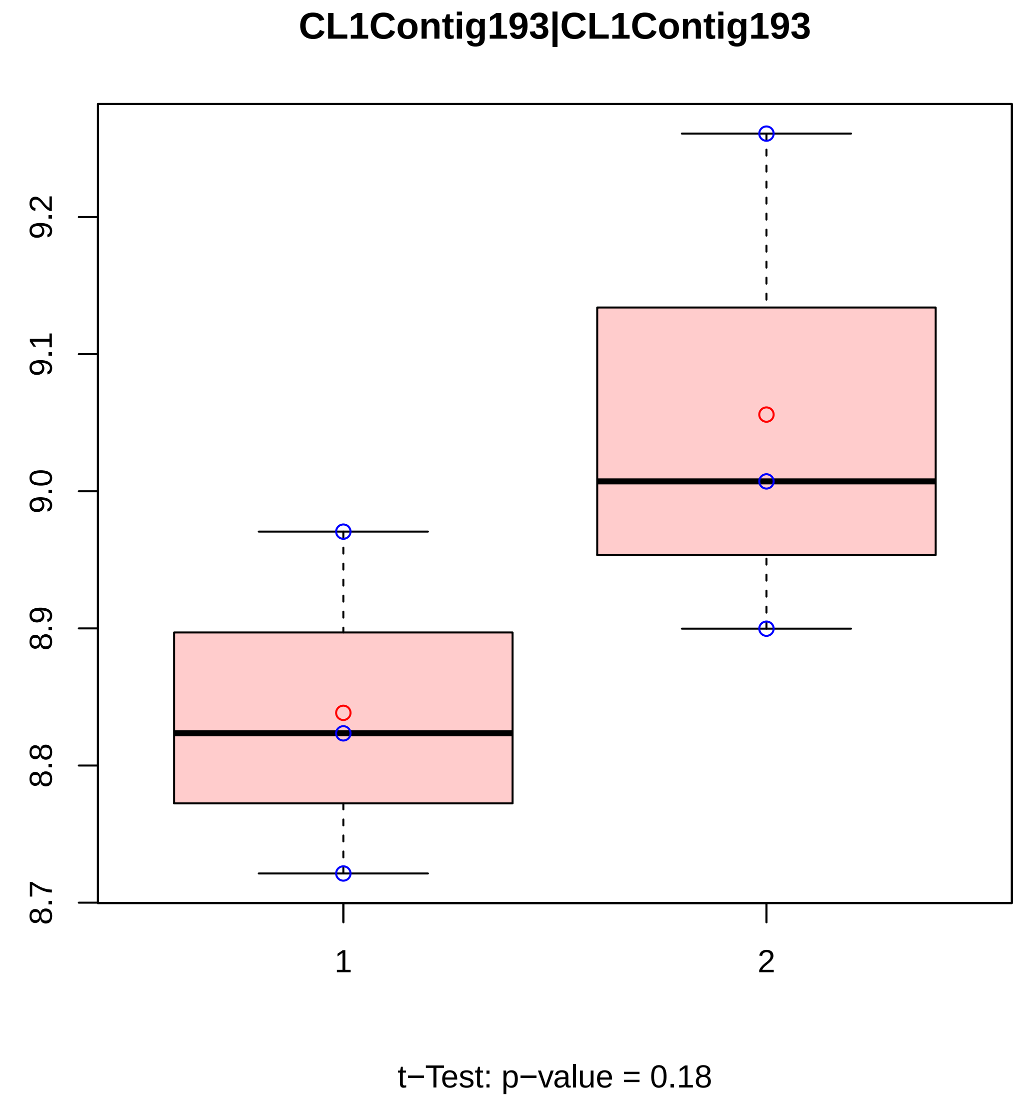
CL1Contig193|CL1Contig193
9.2
9.1
9.0
8.9
8.8
8.7
1
2
t−Test: p−value = 0.18

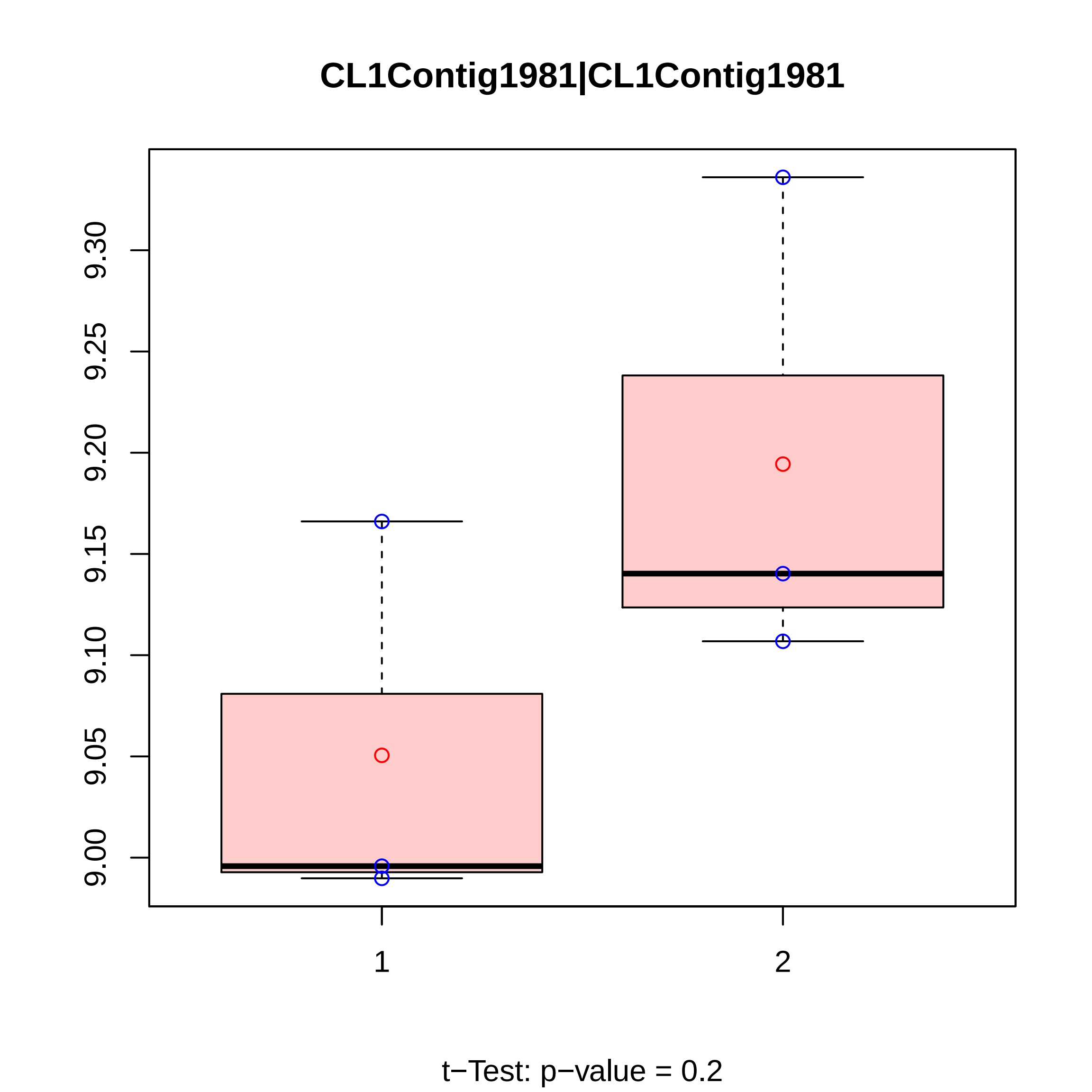
CL1Contig1981|CL1Contig1981
9.00
9.05
9.10
9.15
9.20
9.25
9.30
1
2
t−Test: p−value = 0.2

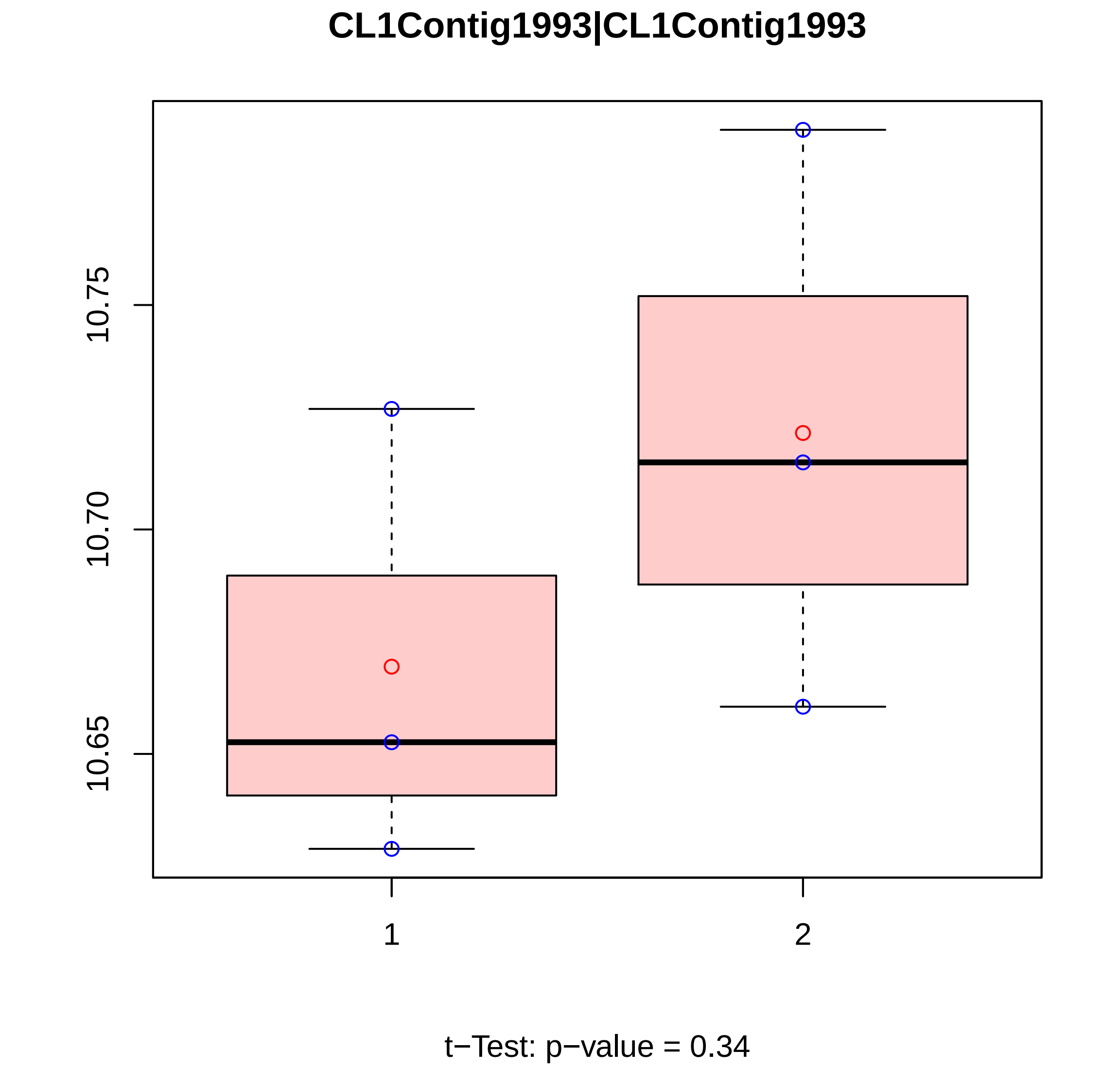
CL1Contig1993|CL1Contig1993
10.65
10.70
10.75
1
2
t−Test: p−value = 0.34

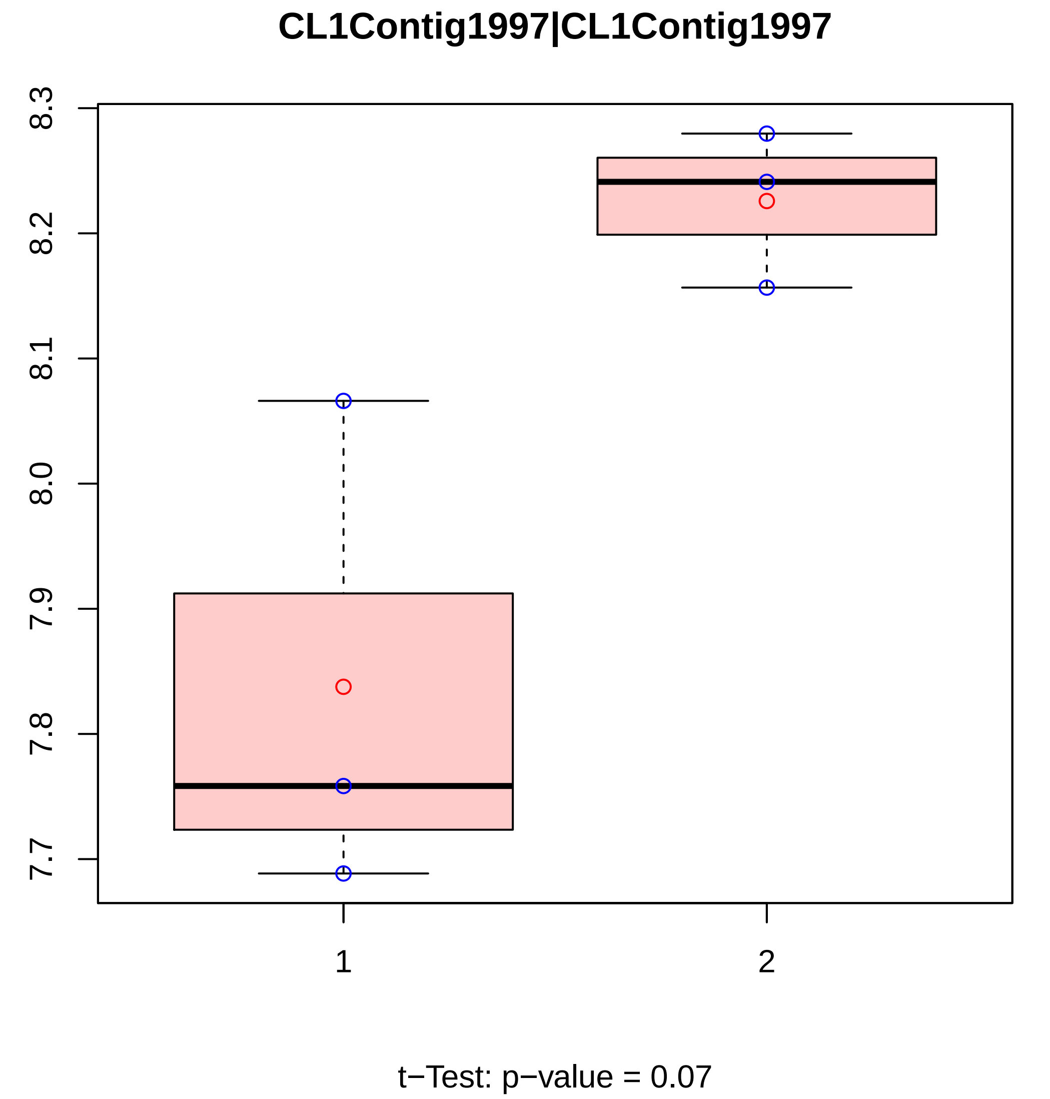

CL1Contig1997|CL1Contig1997
8.3
8.2
8.1
8.0
7.9
7.8
7.7
1
2
t−Test: p−value = 0.07

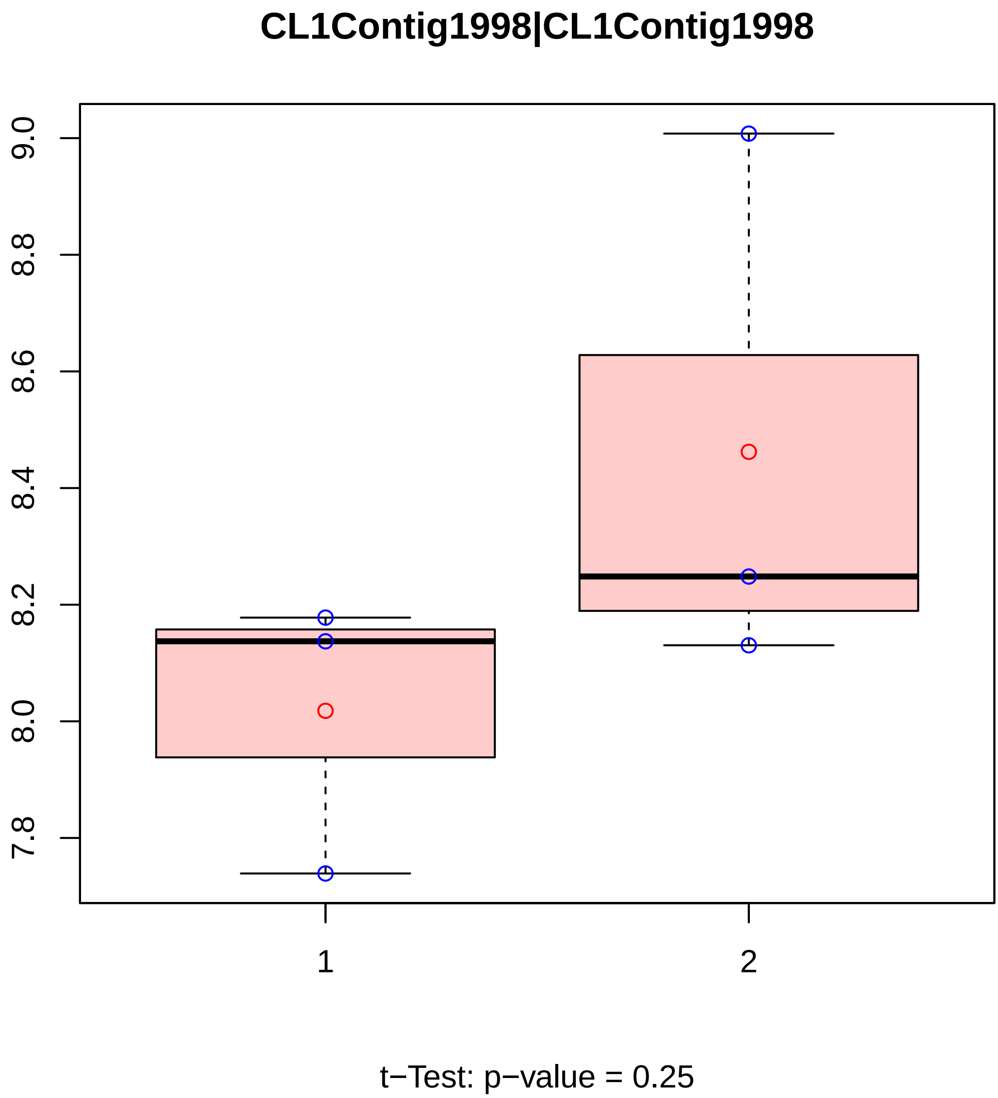
CL1Contig1998|CL1Contig1998
9.0
8.8
8.6
8.4
8.2
8.0
7.8
1
2
t−Test: p−value = 0.25

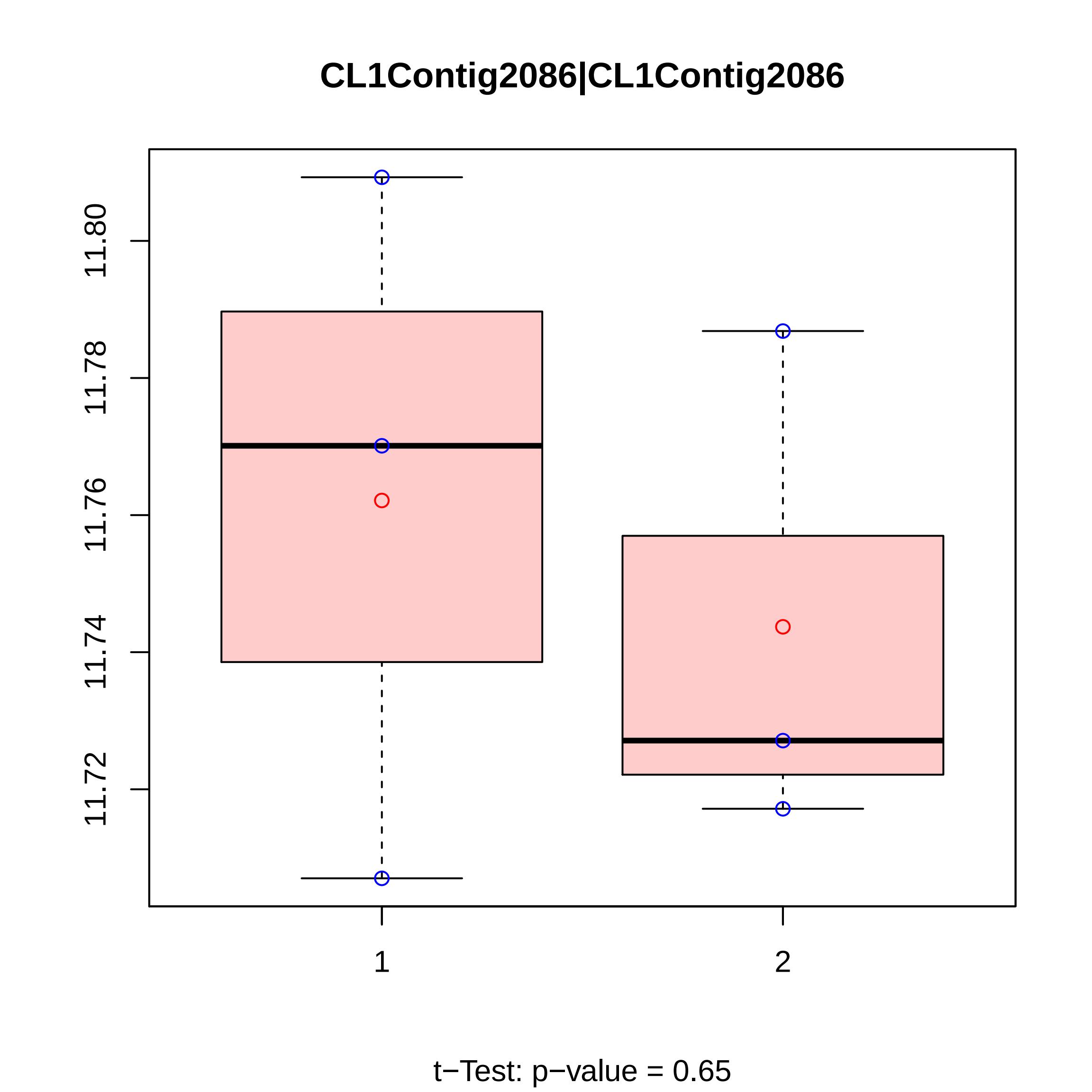

CL1Contig2086|CL1Contig2086
11.72
11.74
11.76
11.78
11.80
1
2
t−Test: p−value = 0.65

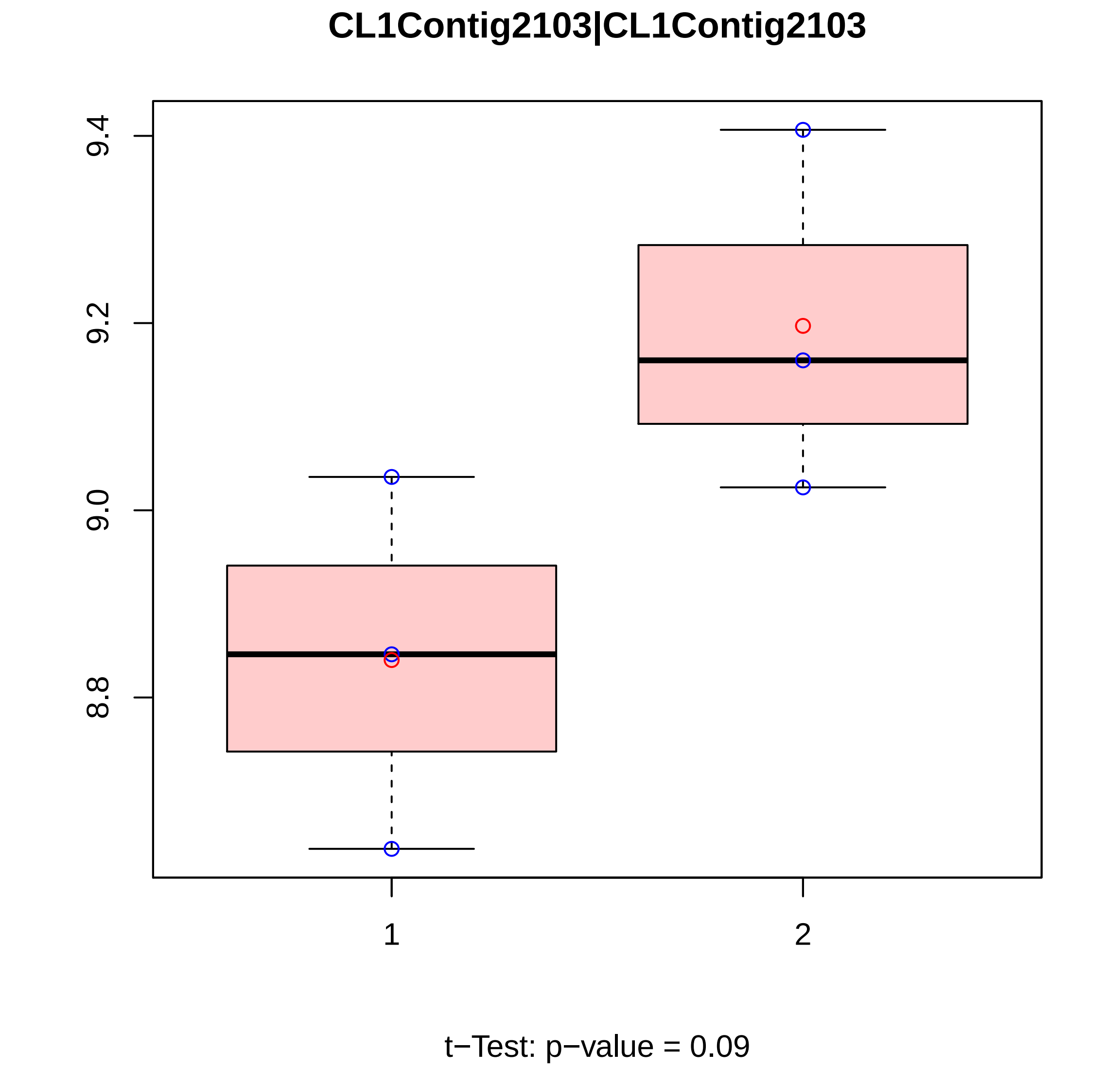
CL1Contig2103|CL1Contig2103
9.4
9.2
9.0
8.8
1
2
t−Test: p−value = 0.09

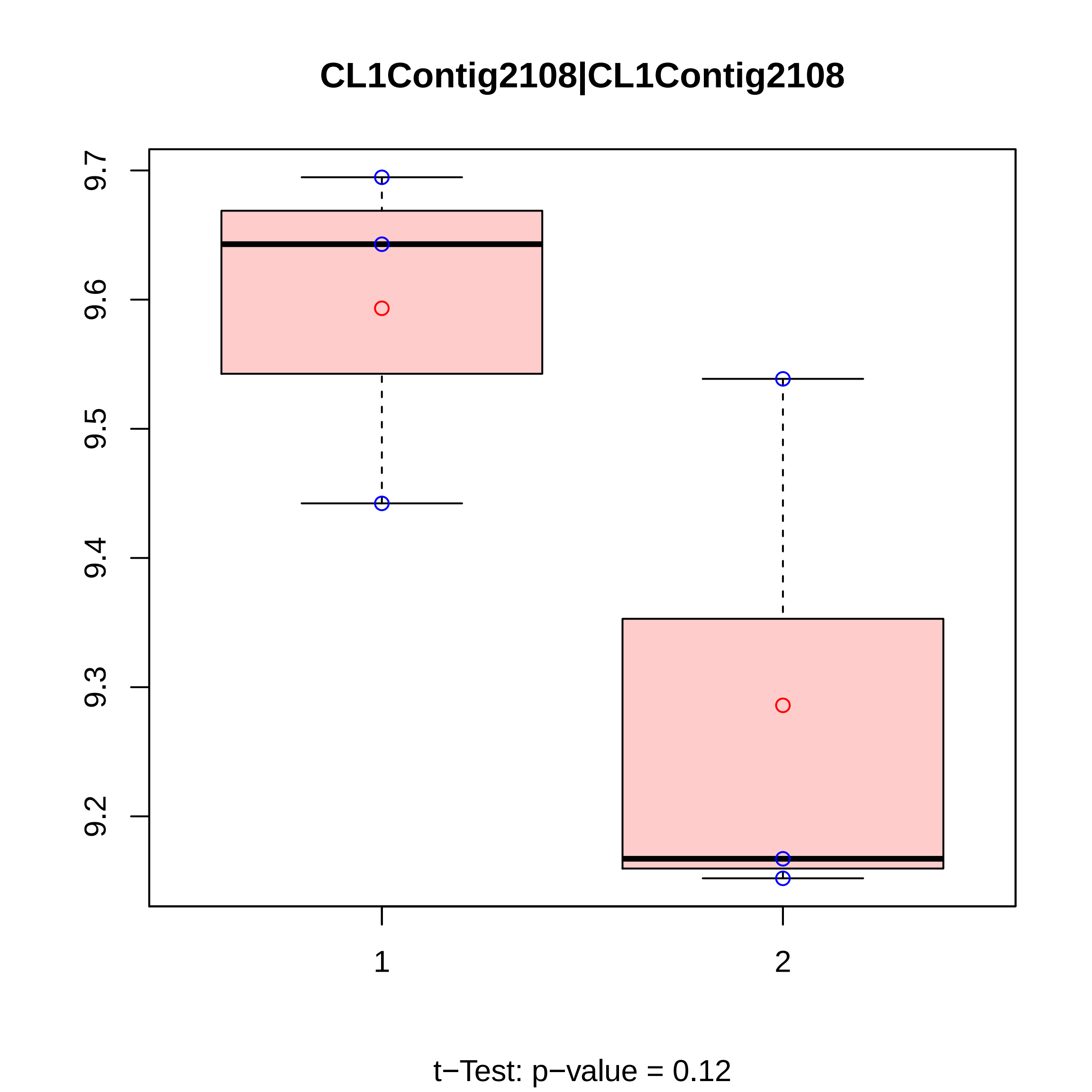

CL1Contig2108|CL1Contig2108
9.7
9.6
9.5
9.4
9.3
9.2
1
2
t−Test: p−value = 0.12

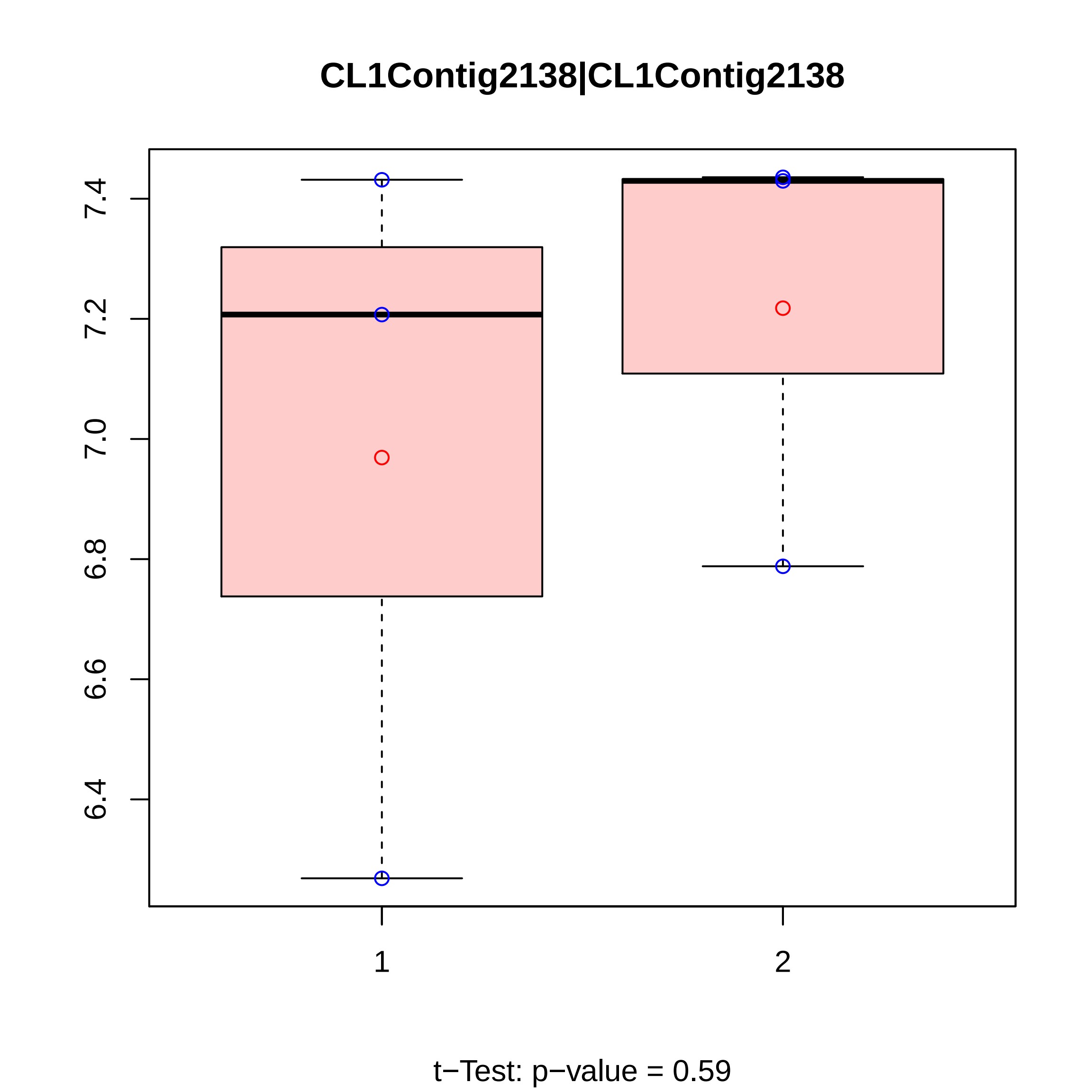
CL1Contig2138|CL1Contig2138
7.4
7.2
7.0
6.8
6.6
6.4
1
2
t−Test: p−value = 0.59

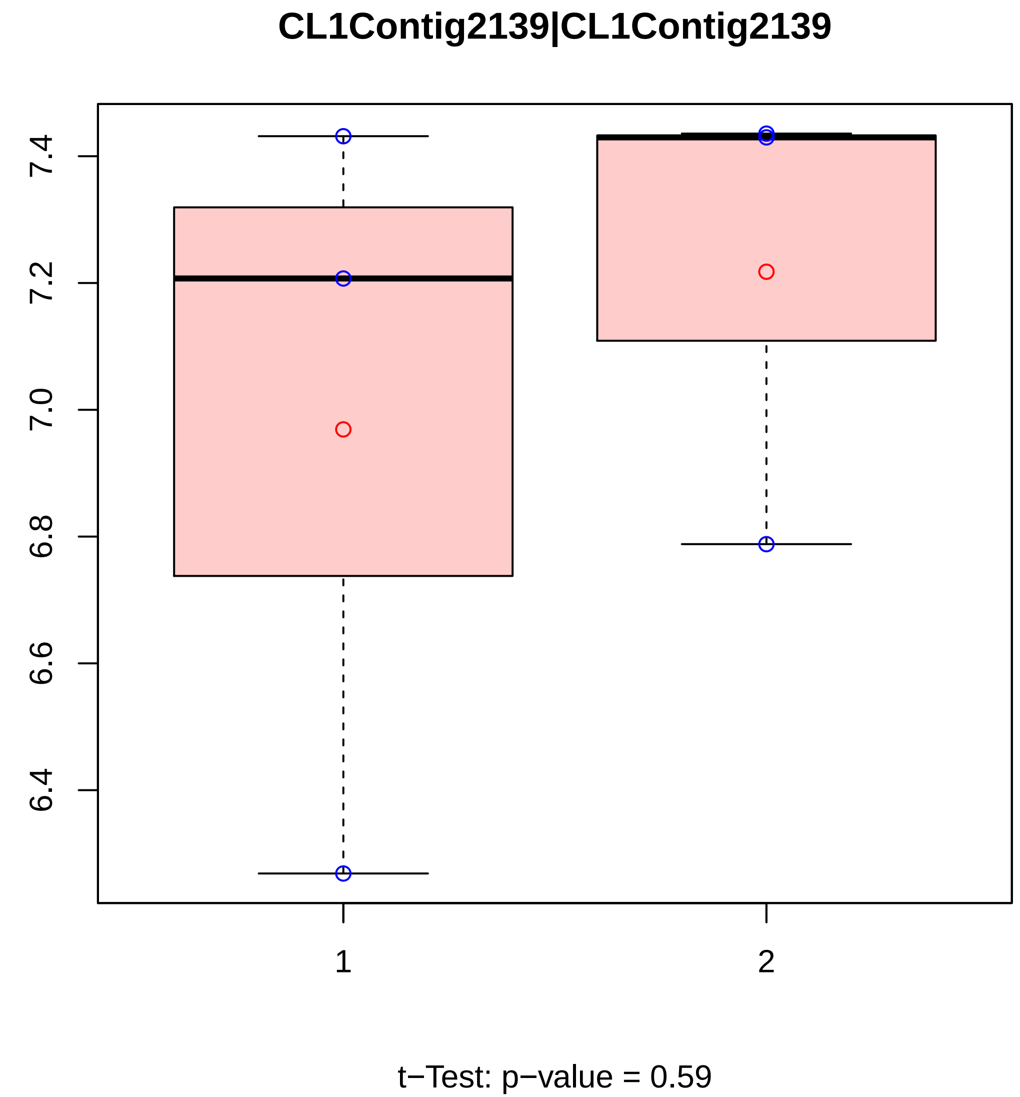
CL1Contig2139|CL1Contig2139
7.4
7.2
7.0
6.8
6.6
6.4
1
2
t−Test: p−value = 0.59

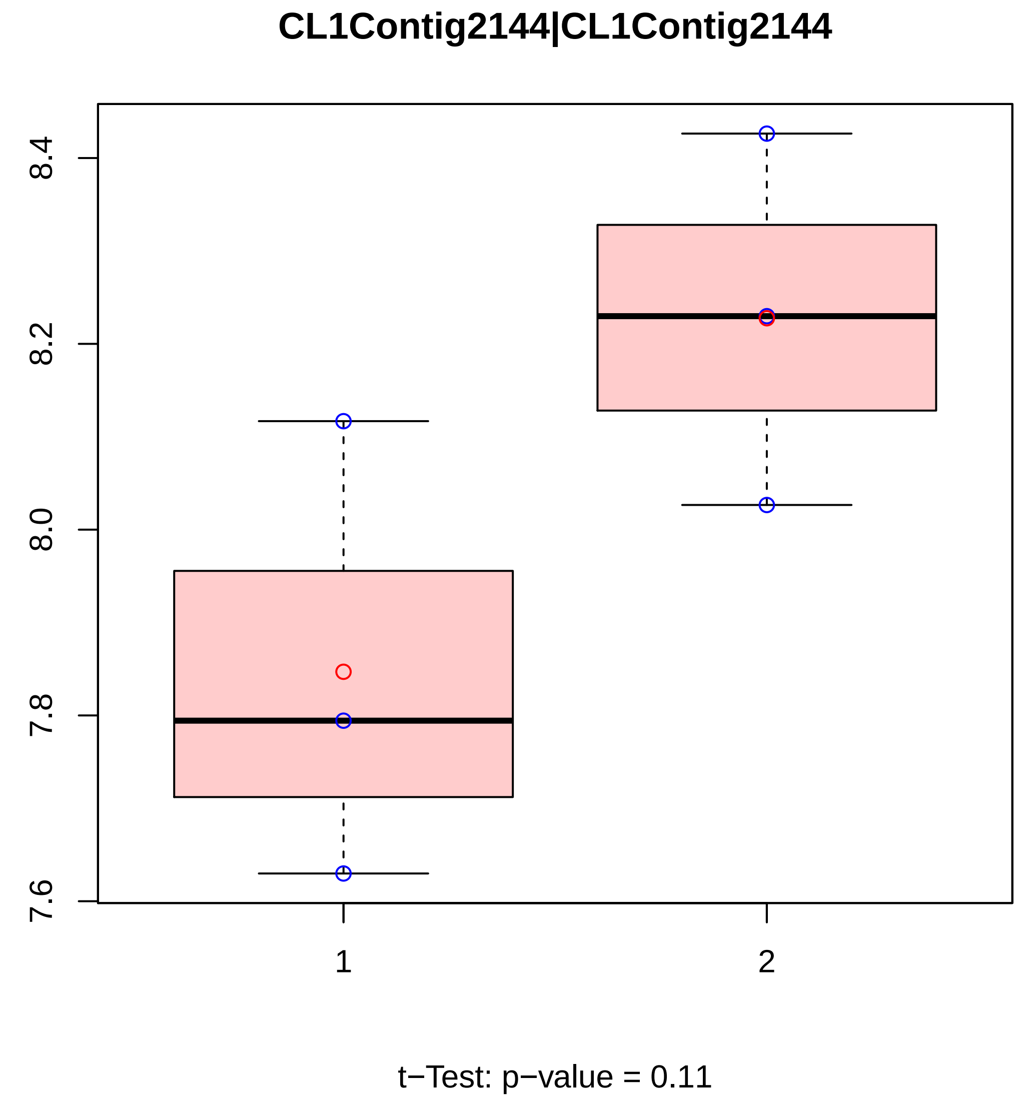
CL1Contig2144|CL1Contig2144
8.4
8.2
8.0
7.8
7.6
1
2
t−Test: p−value = 0.11

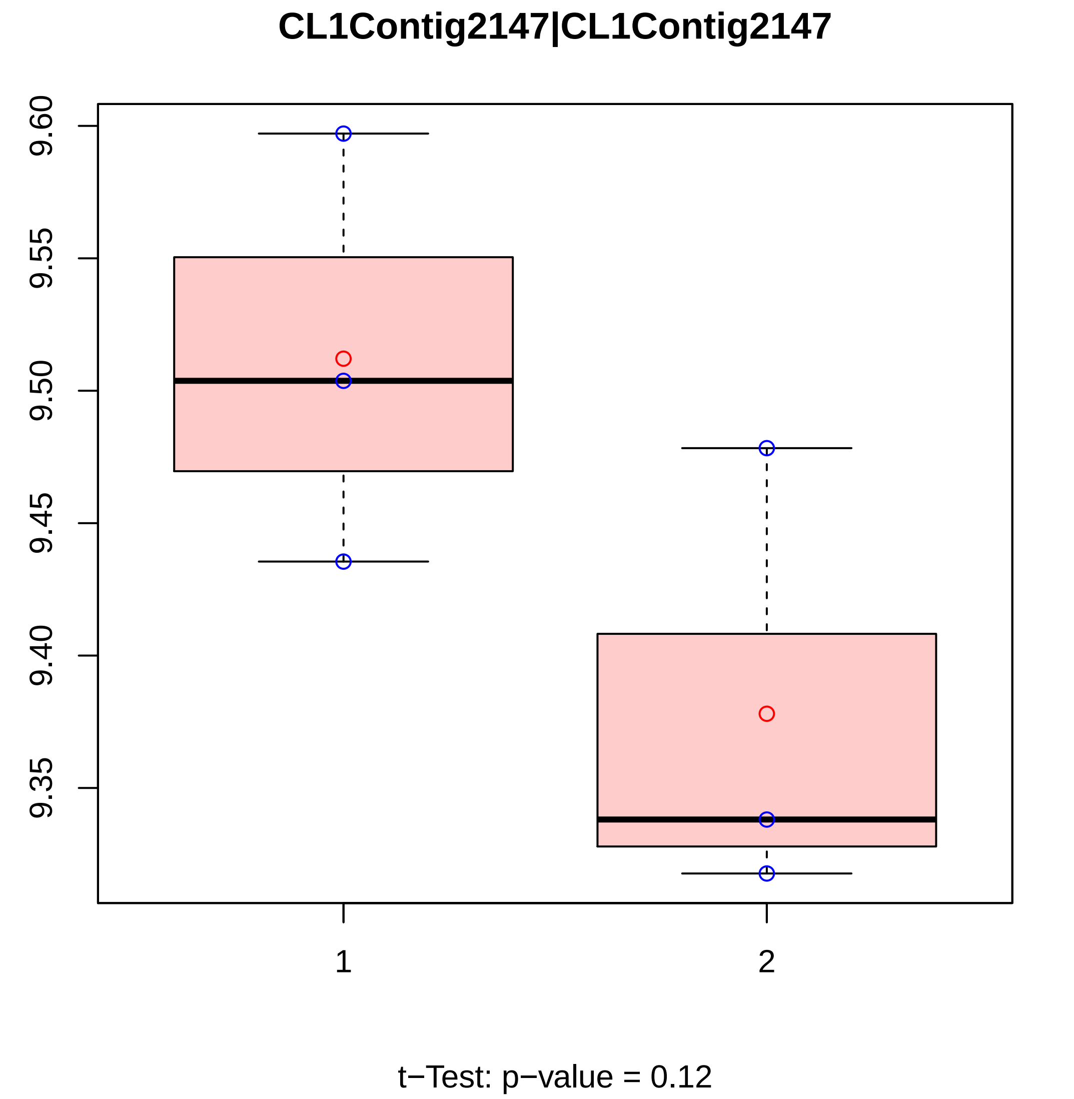
CL1Contig2147|CL1Contig2147
9.60
9.55
9.50
9.45
9.40
9.35
1
2
t−Test: p−value = 0.12

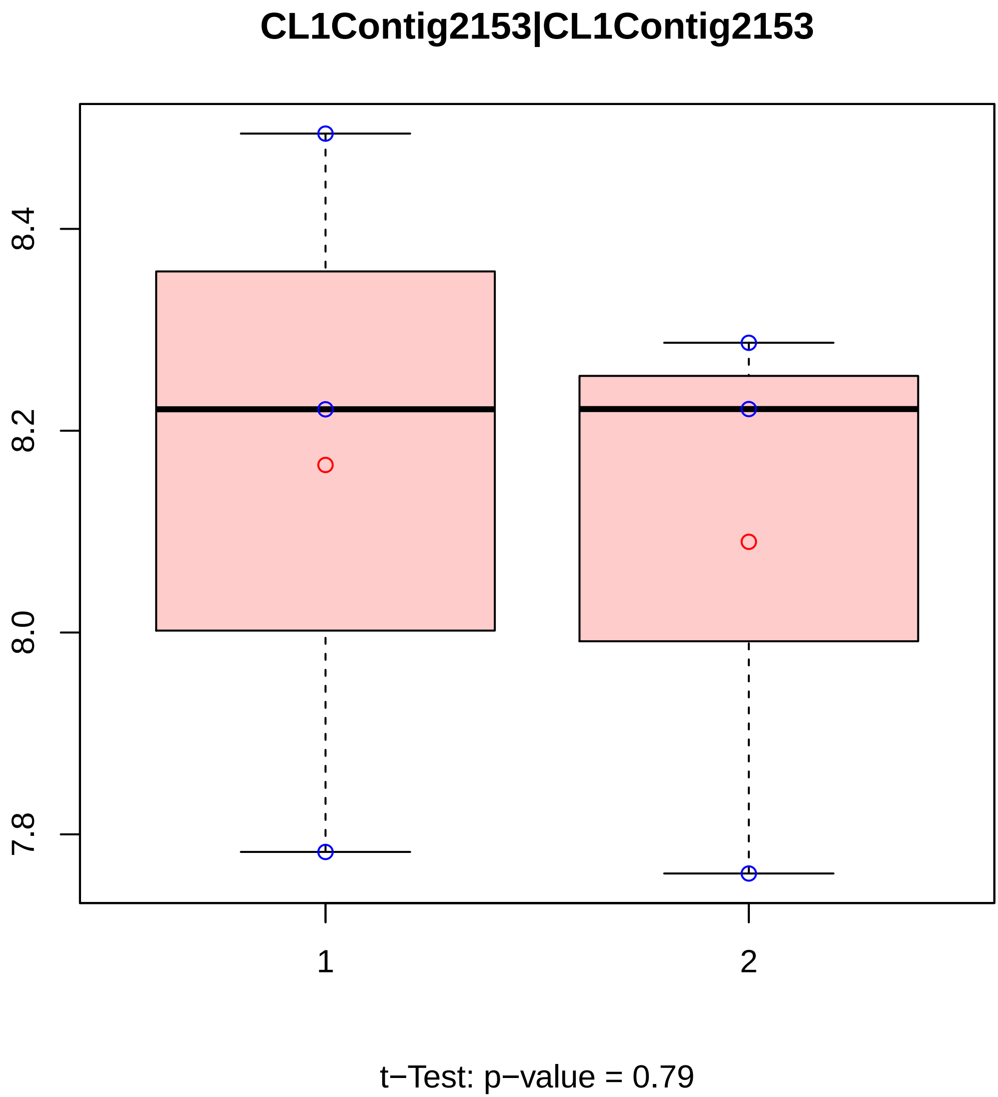
CL1Contig2153|CL1Contig2153
7.8
8.0
8.2
8.4
1
2
t−Test: p−value = 0.79

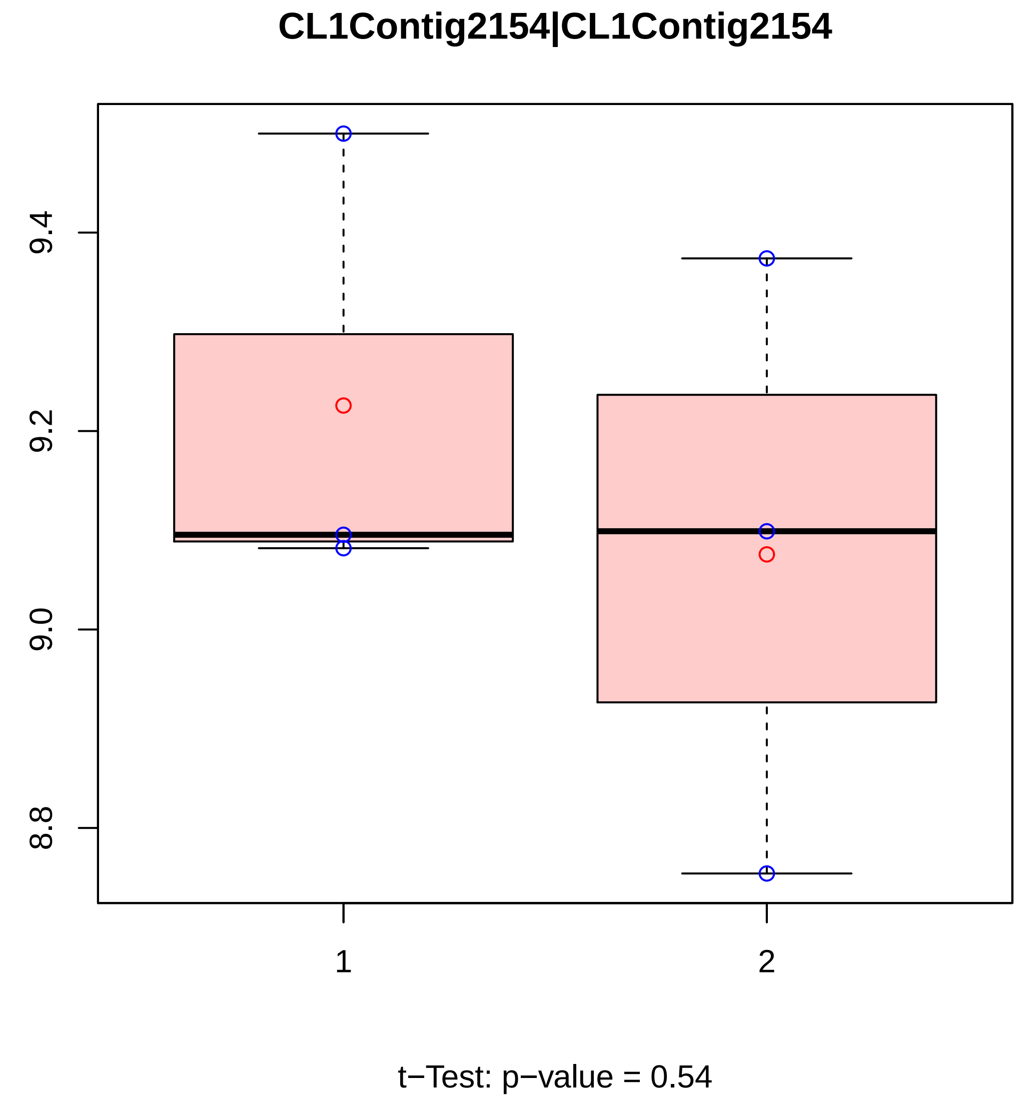

CL1Contig2154|CL1Contig2154
9.4
9.2
9.0
8.8
1
2
t−Test: p−value = 0.54

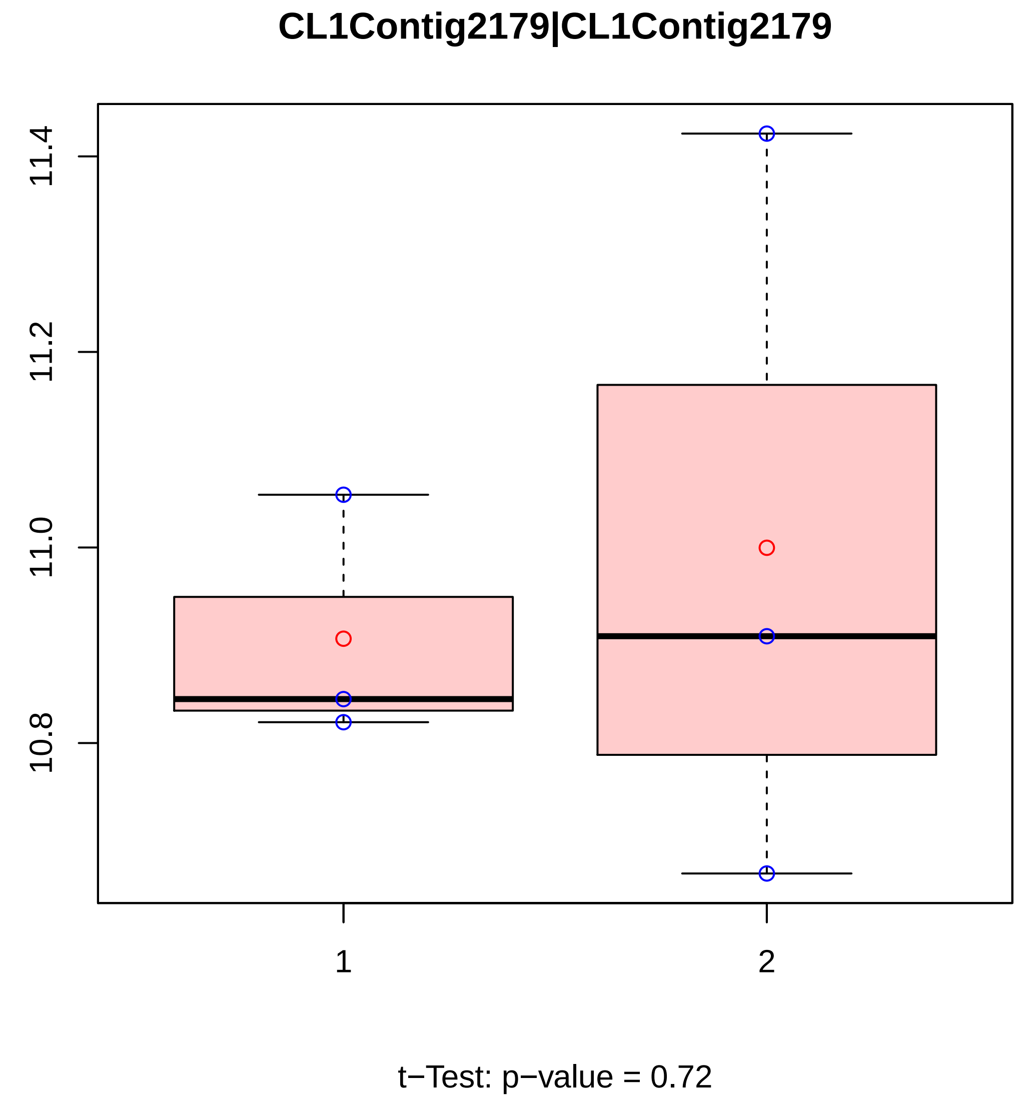
CL1Contig2179|CL1Contig2179
11.4
11.2
11.0
10.8
1
2
t−Test: p−value = 0.72

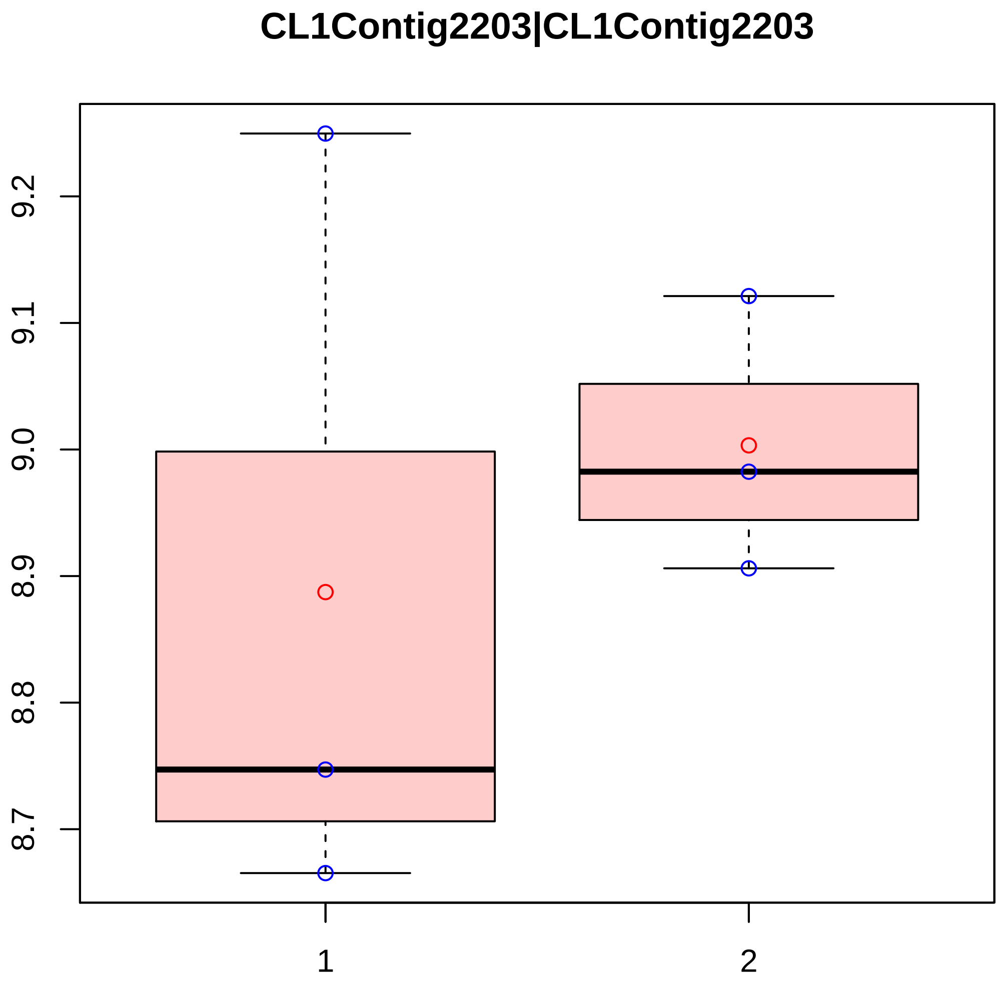


t−Test: p−value = 0.6

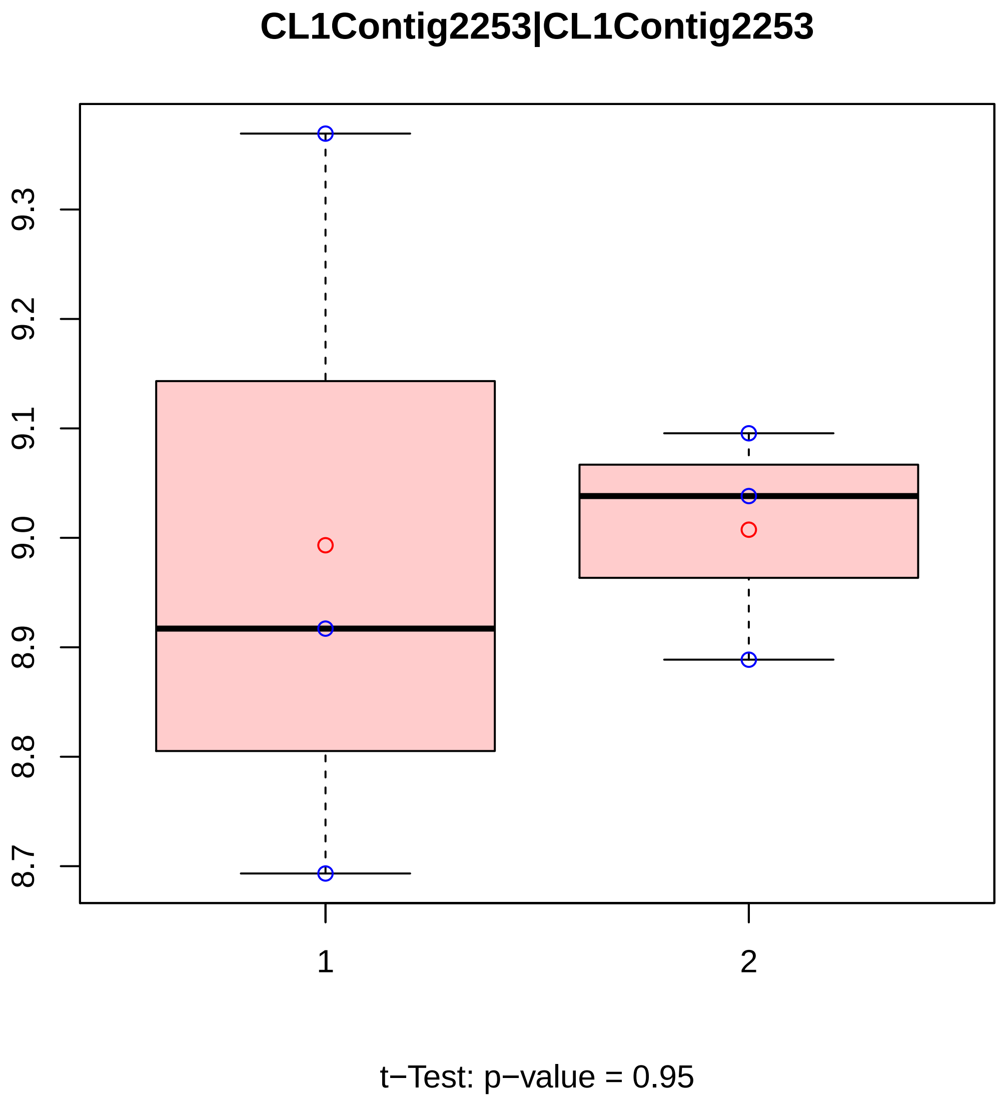
CL1Contig2253|CL1Contig2253
9.3
9.2
9.1
9.0
8.9
8.8
8.7
1
2
t−Test: p−value = 0.95

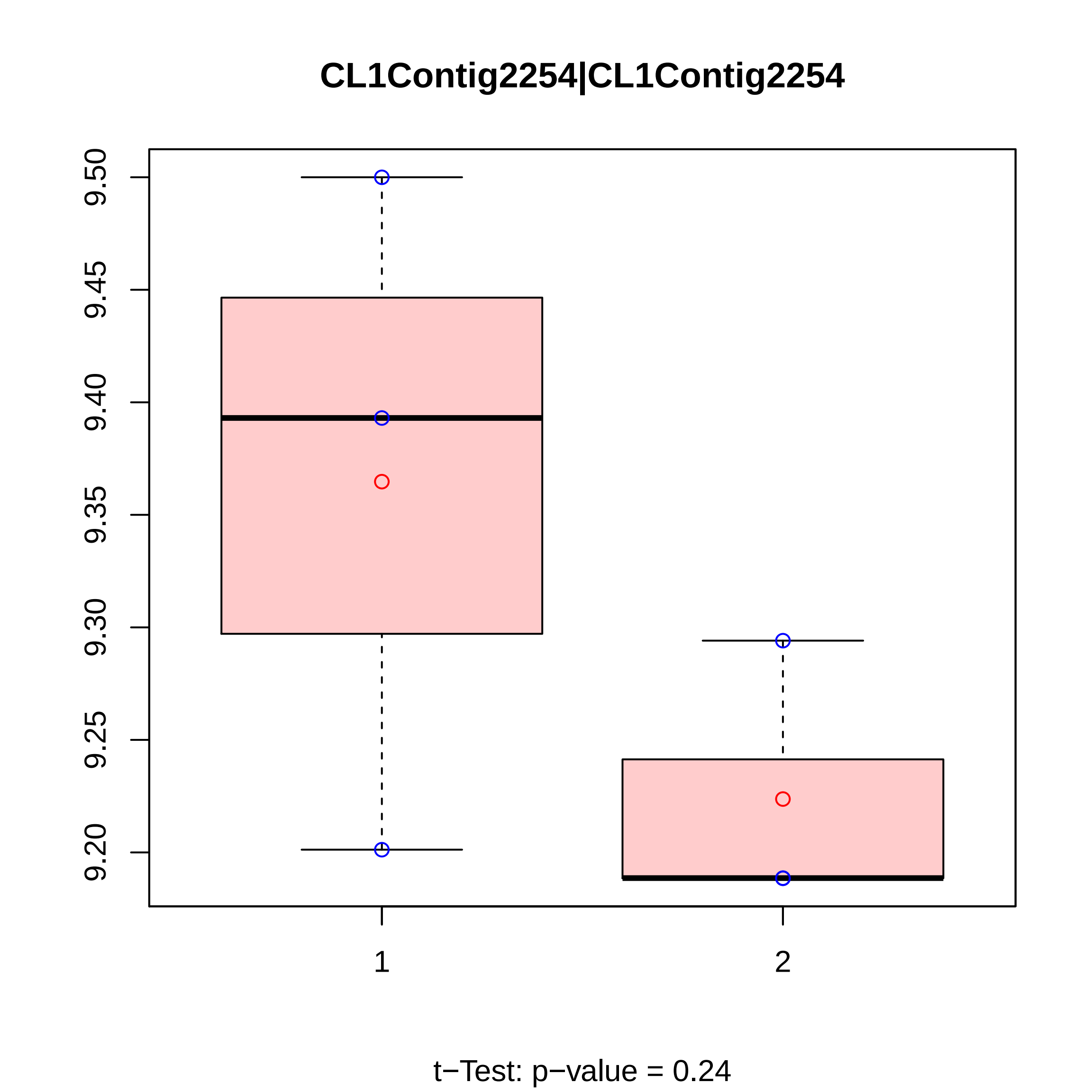
CL1Contig2254|CL1Contig2254
9.50
9.45
9.40
9.35
9.30
9.25
9.20
1
2
t−Test: p−value = 0.24

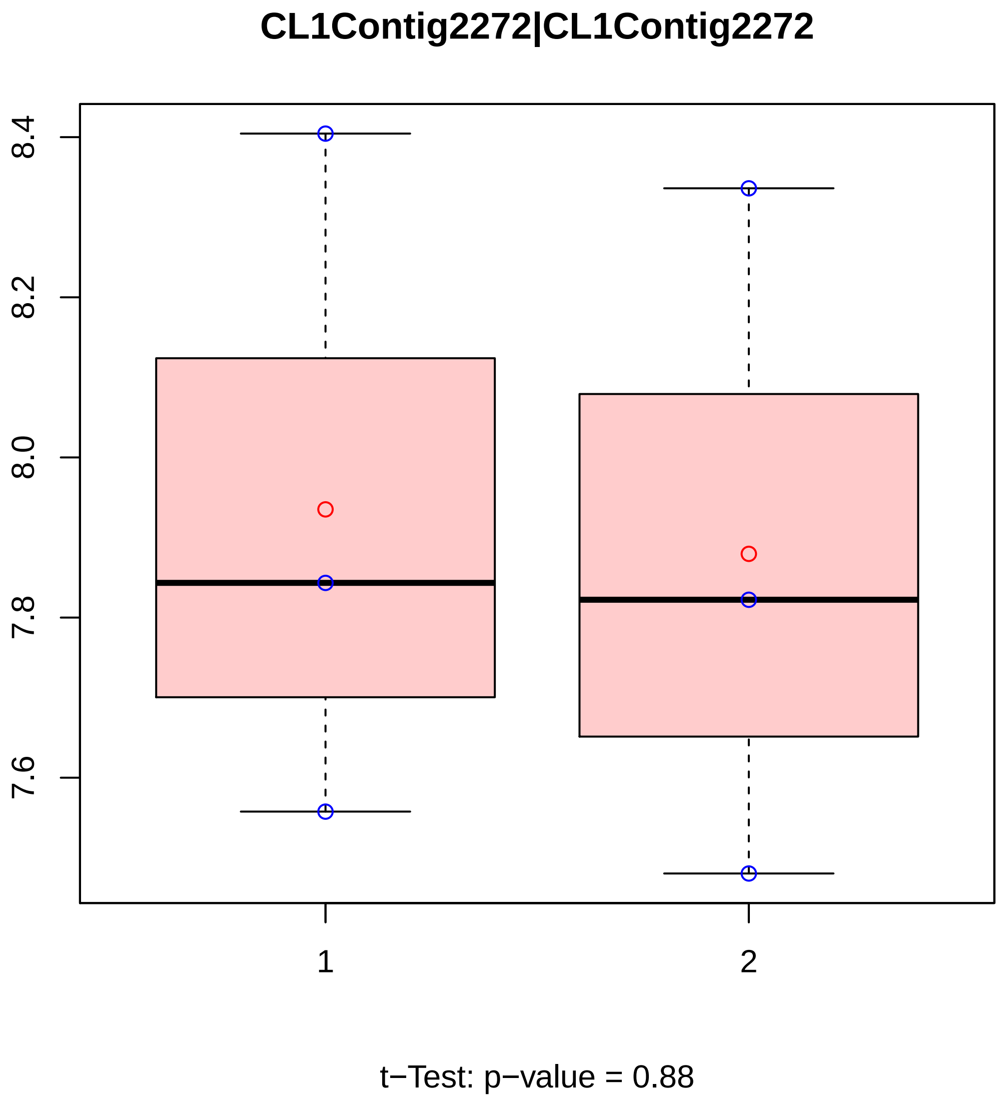

CL1Contig2272|CL1Contig2272
8.4
8.2
8.0
7.8
7.6
1
2
t−Test: p−value = 0.88

## CL1Contig2280|CL1Contig2280

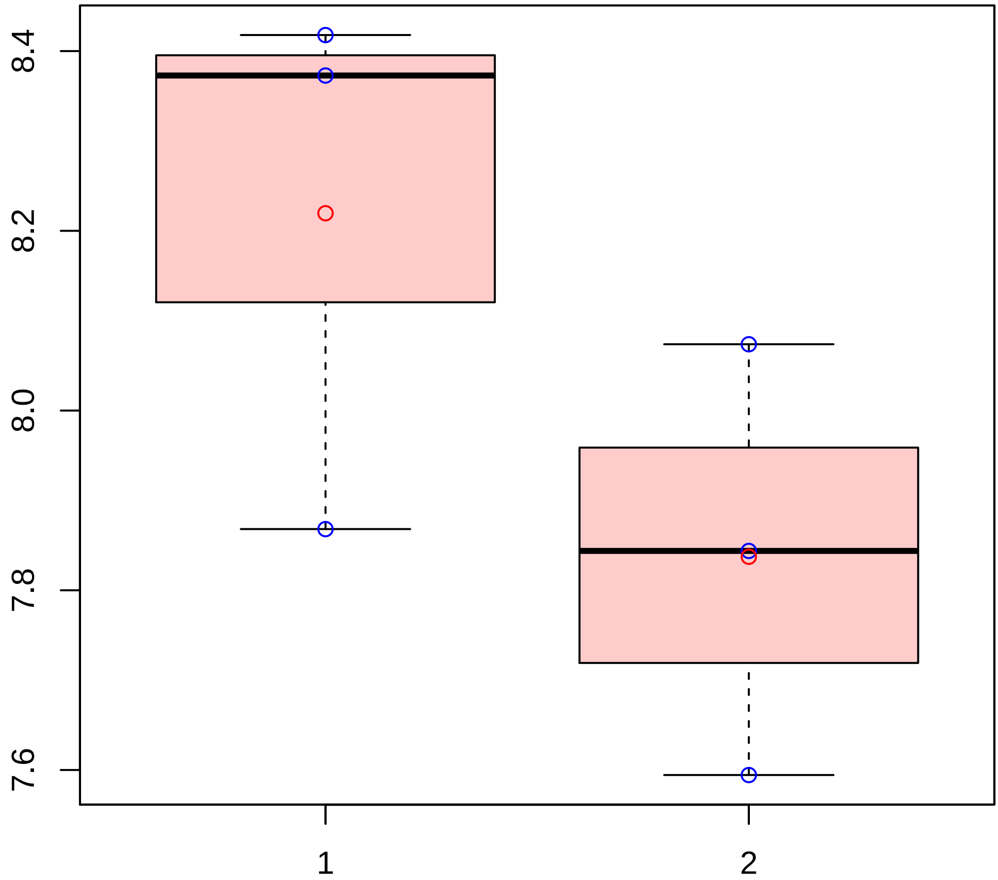


t−Test: p−value = 0.17

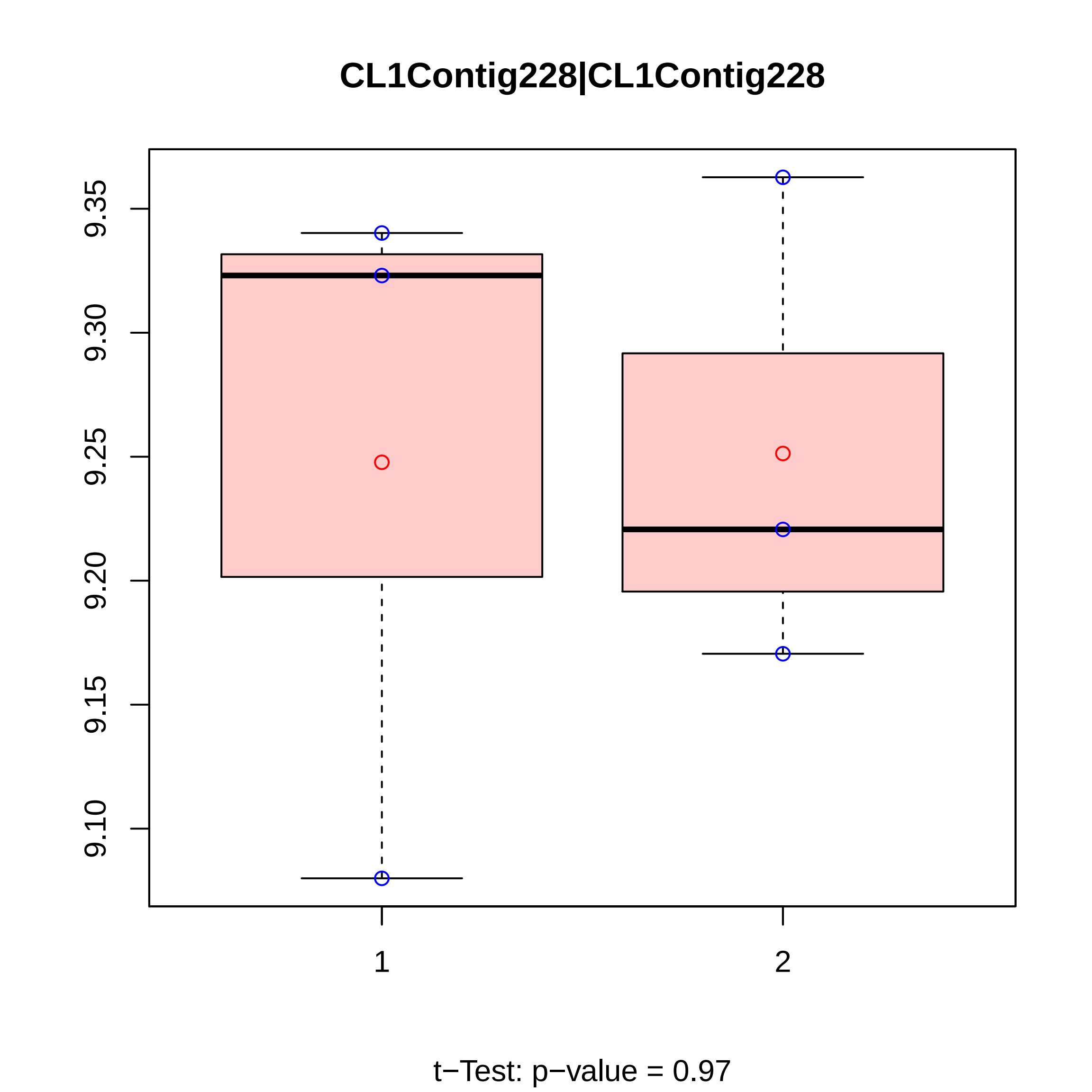

CL1Contig228|CL1Contig228
9.35
9.30
9.25
9.20
9.15
9.10
1
2
t−Test: p−value = 0.97

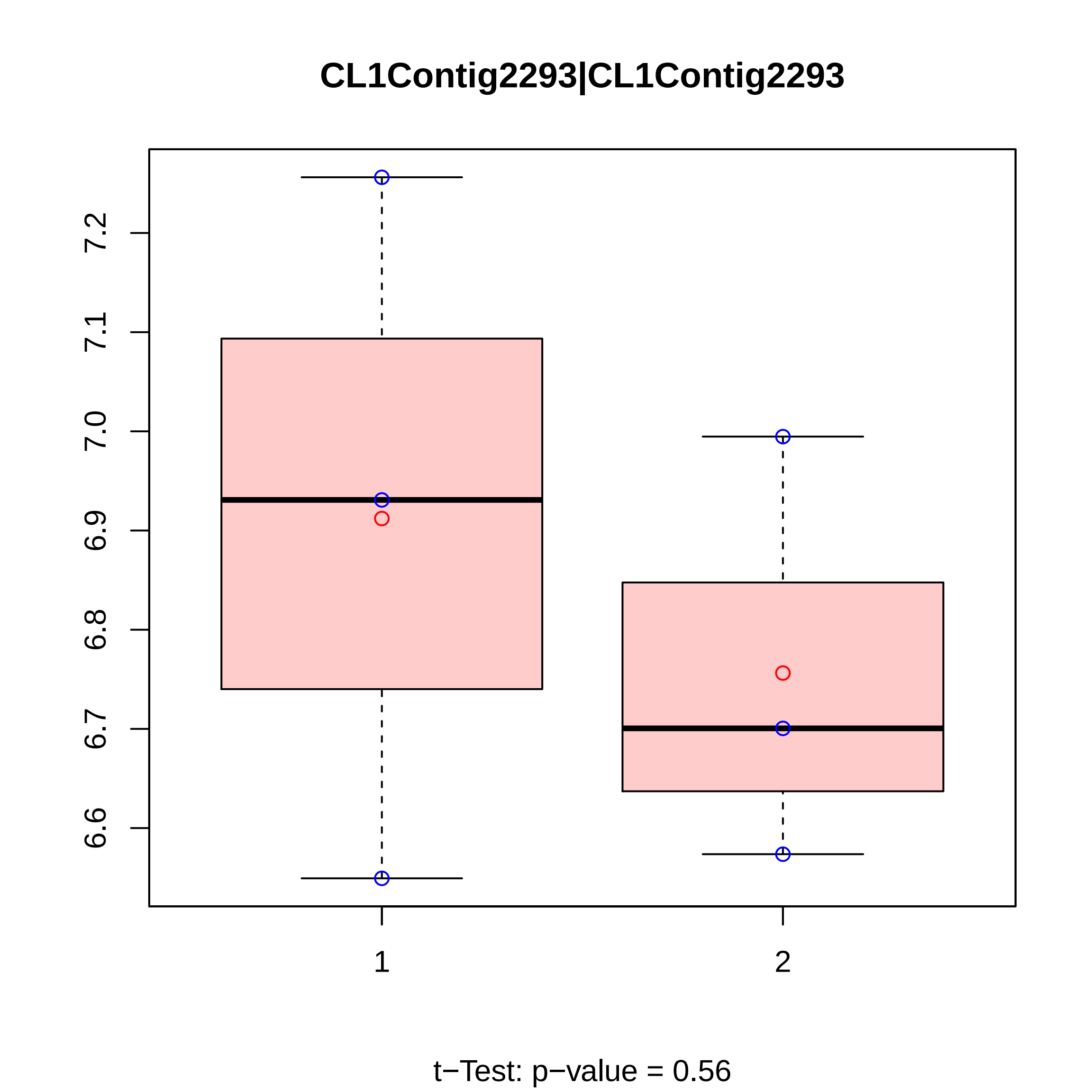
CL1Contig2293|CL1Contig2293
7.2
7.1
7.0
6.9
6.8
6.7
6.6
1
2
t−Test: p−value = 0.56

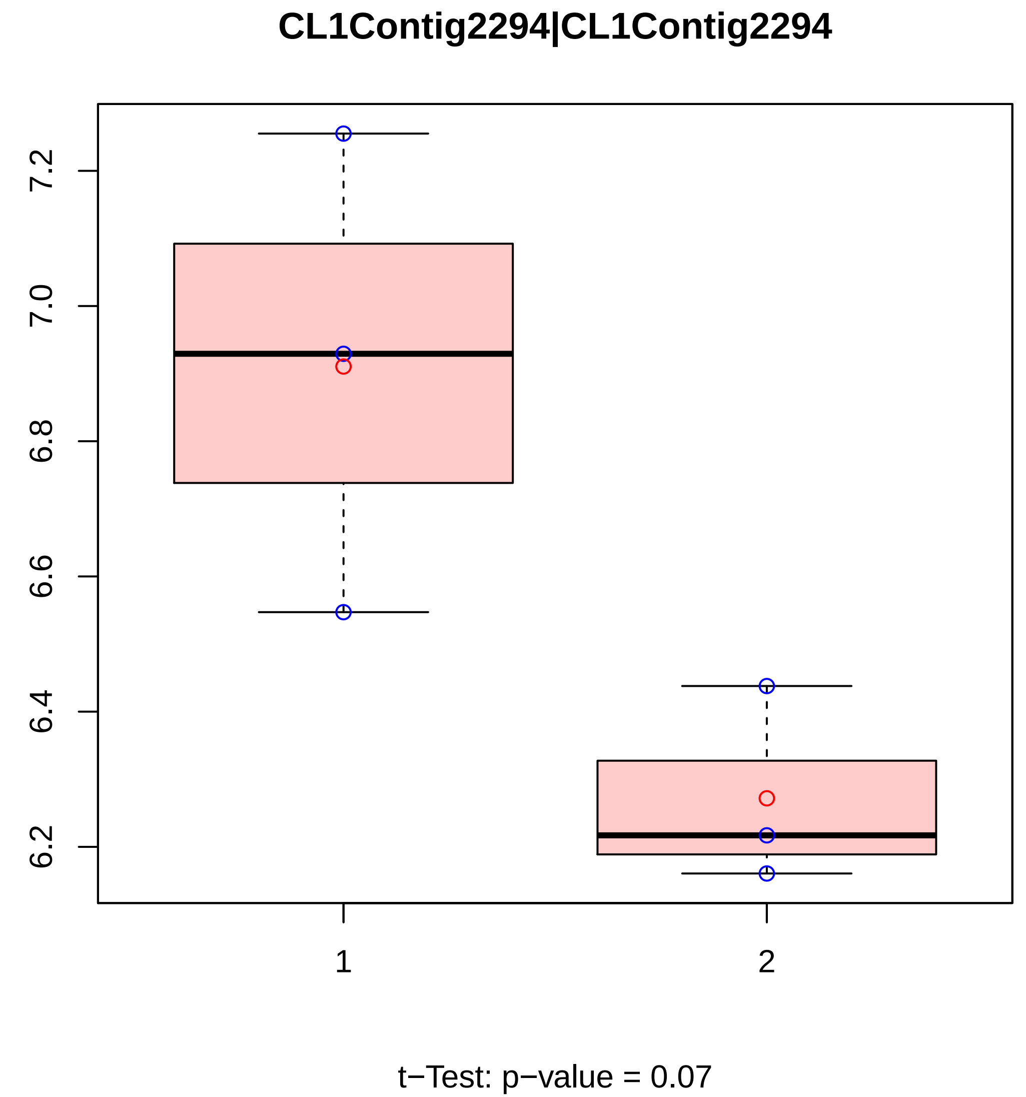
CL1Contig2294|CL1Contig2294
7.2
7.0
6.8
6.6
6.4
6.2
1
2
t−Test: p−value = 0.07

# CL1Contig229|CL1Contig229

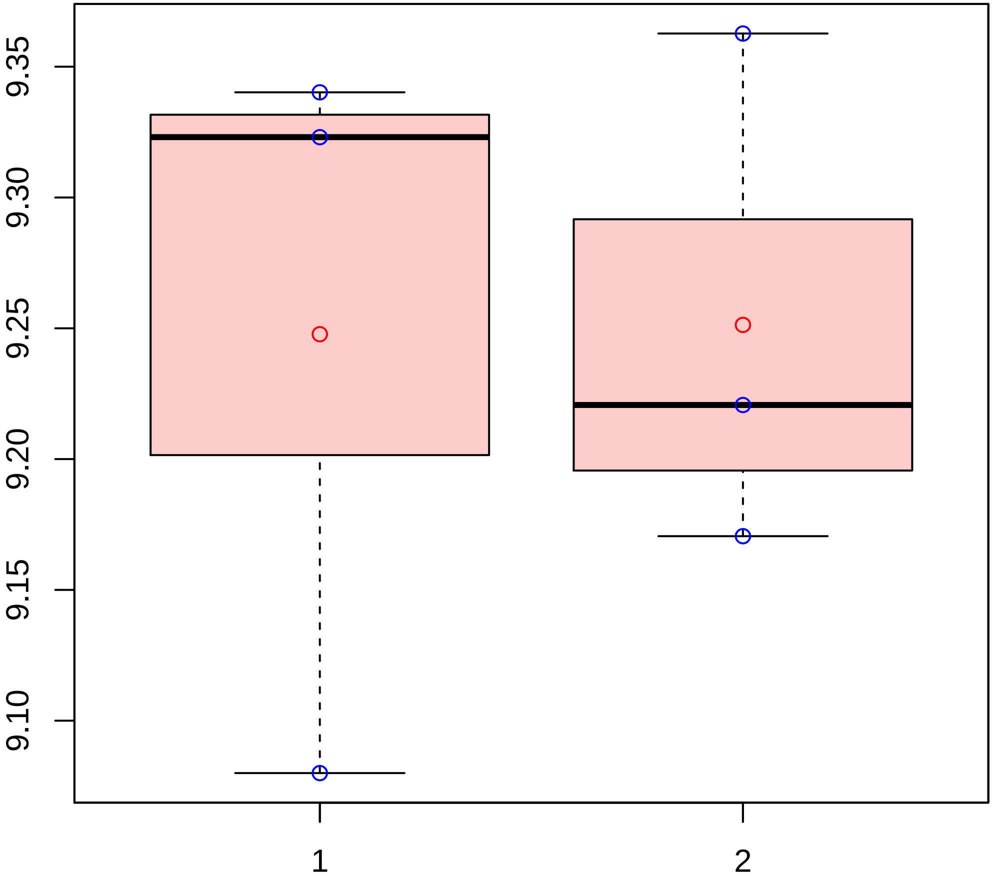


t−Test: p−value = 0.97

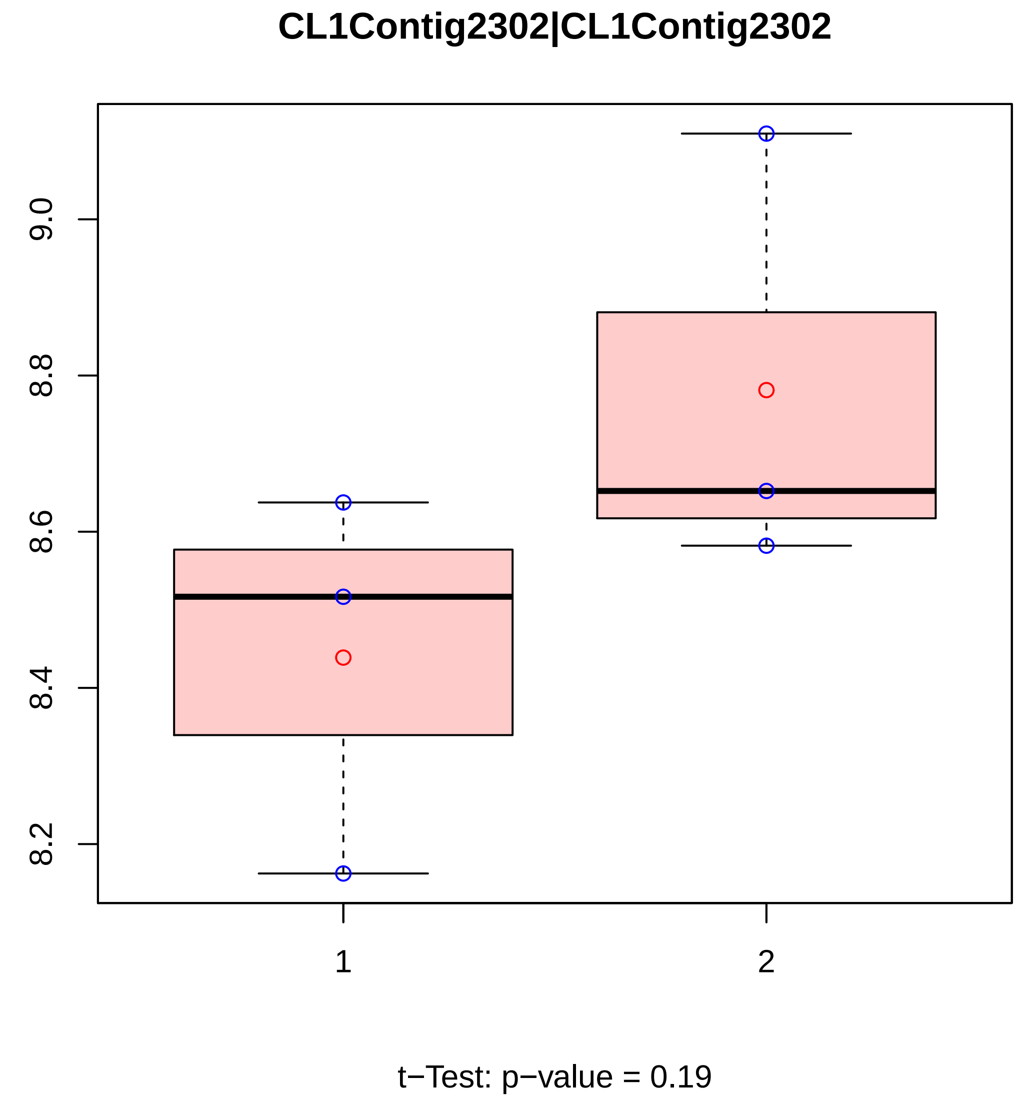
CL1Contig2302|CL1Contig2302
9.0
8.8
8.6
8.4
8.2
1
2
t−Test: p−value = 0.19

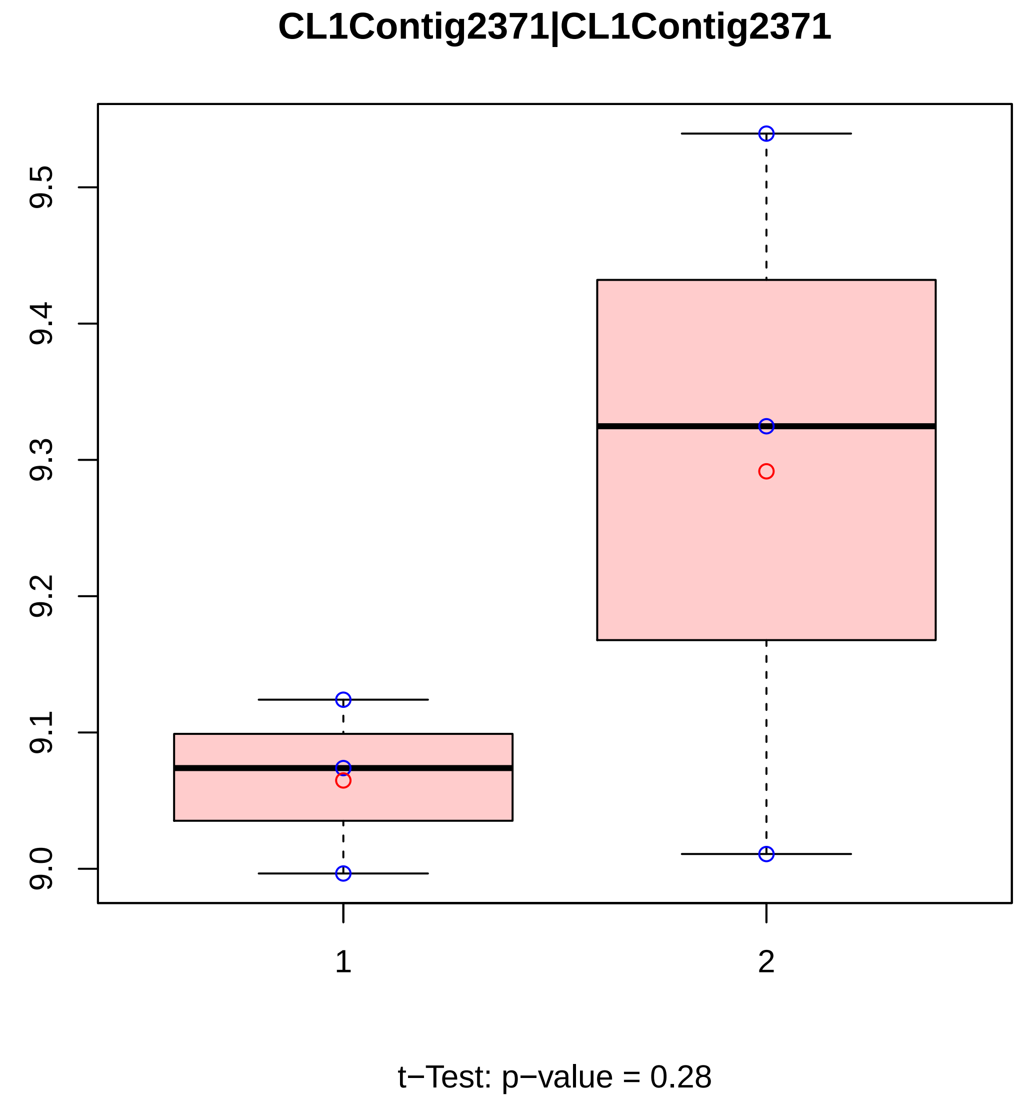

CL1Contig2371|CL1Contig2371
9.0
9.1
9.2
9.3
9.4
9.5
1
2
t−Test: p−value = 0.28

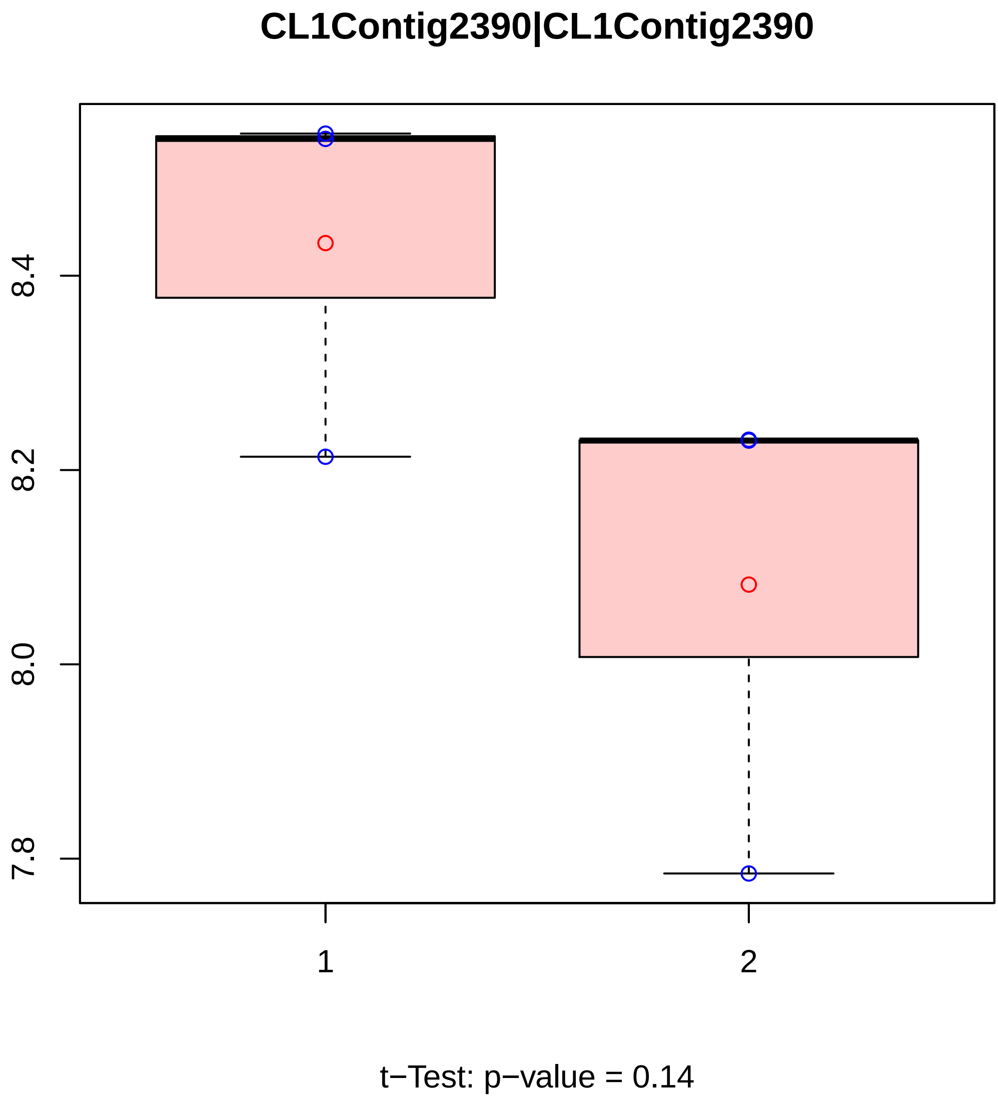
CL1Contig2390|CL1Contig2390
8.4
8.2
8.0
7.8
1
2
t−Test: p−value = 0.14

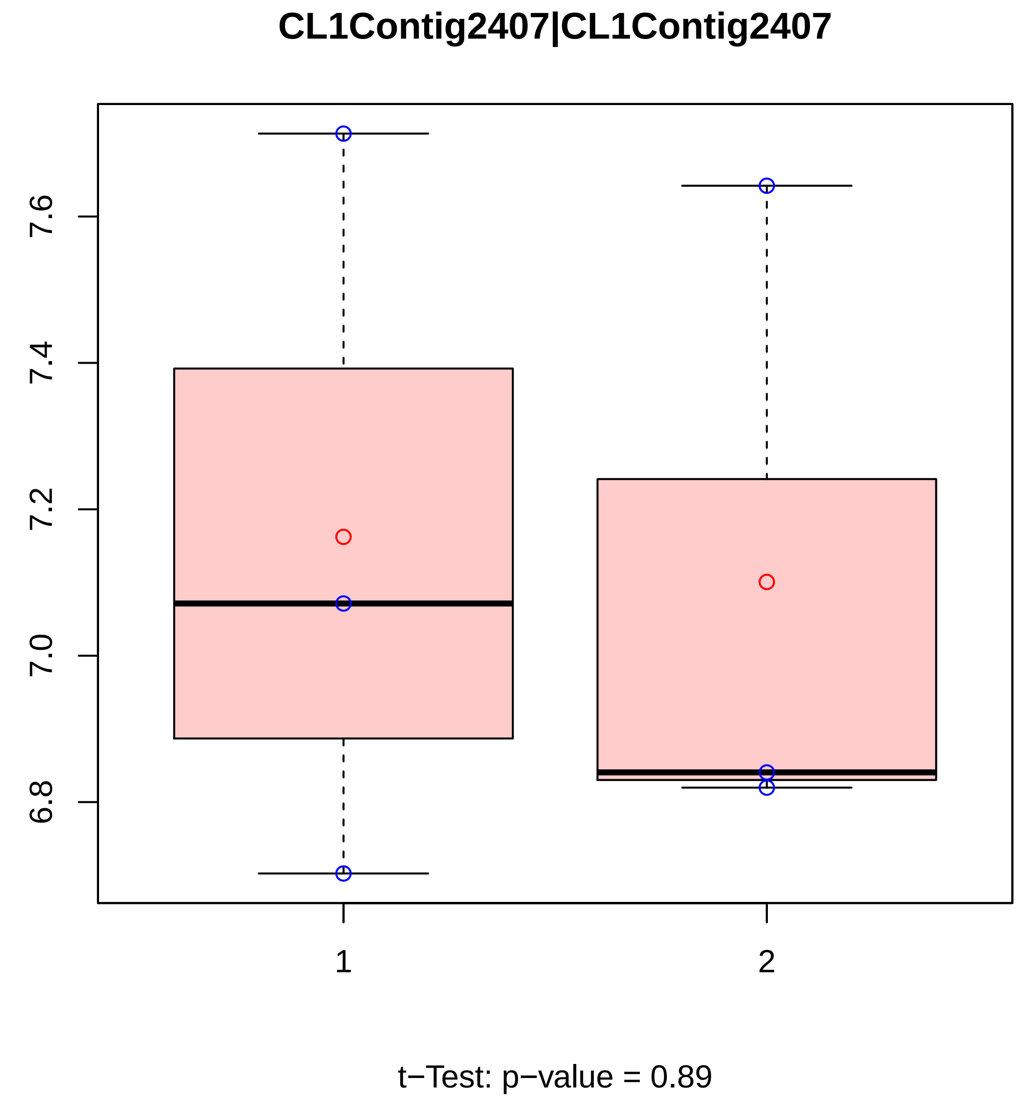
CL1Contig2407|CL1Contig2407
7.6
7.4
7.2
7.0
6.8
1
2
t−Test: p−value = 0.89

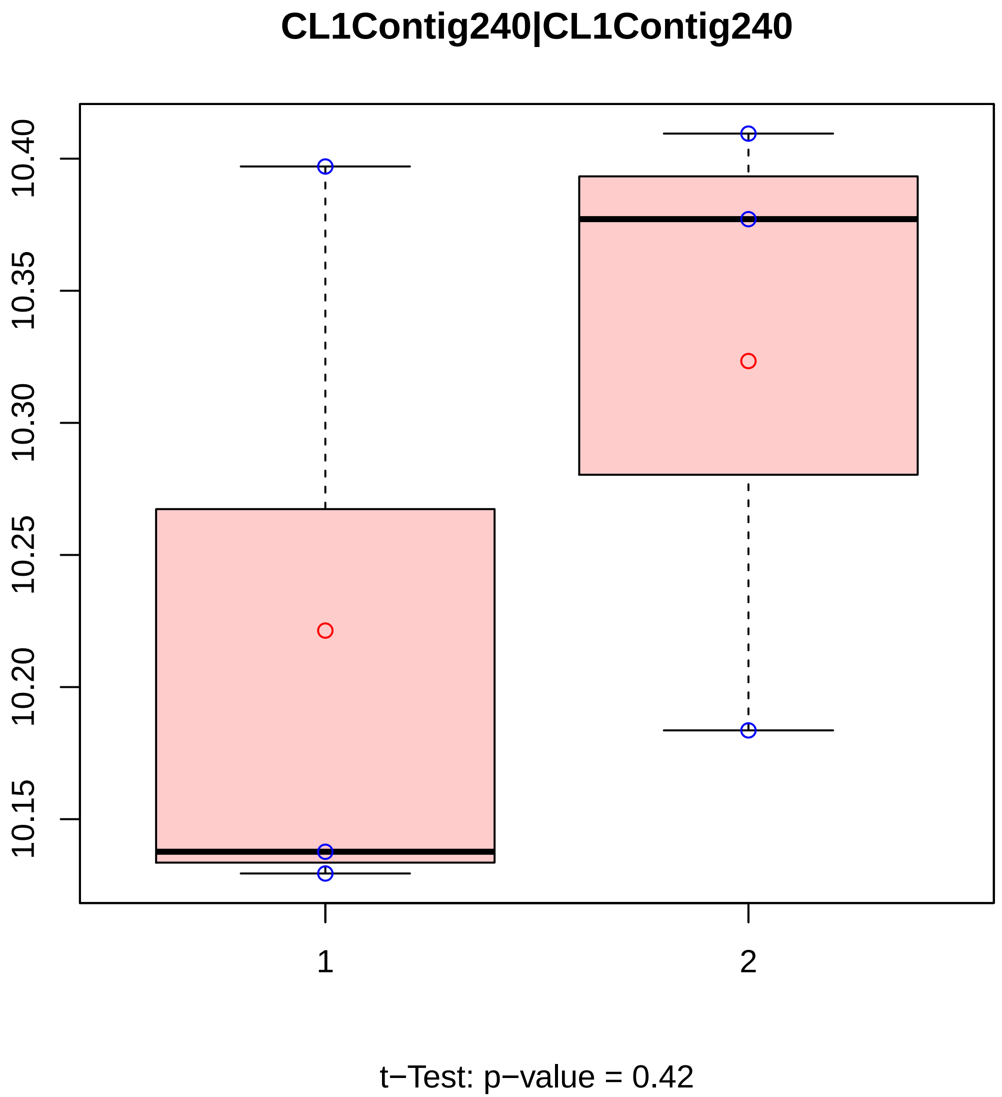

CL1Contig240|CL1Contig240
10.15
10.20
10.25
10.30
10.35
10.40
1
2
t−Test: p−value = 0.42

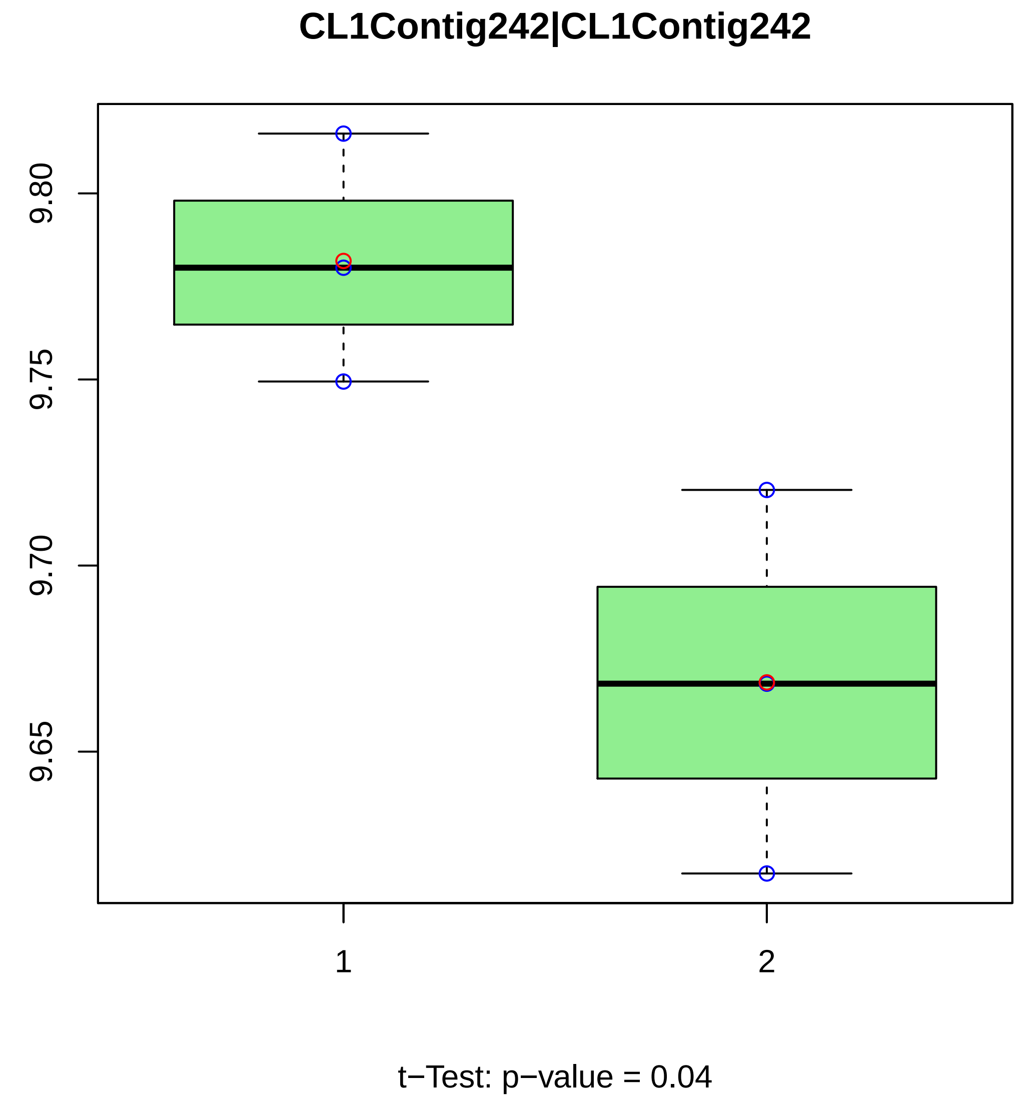
CL1Contig242|CL1Contig242
9.80
9.75
9.70
9.65
1
2
t−Test: p−value = 0.04

CL1Contig2436|CL1Contig2436

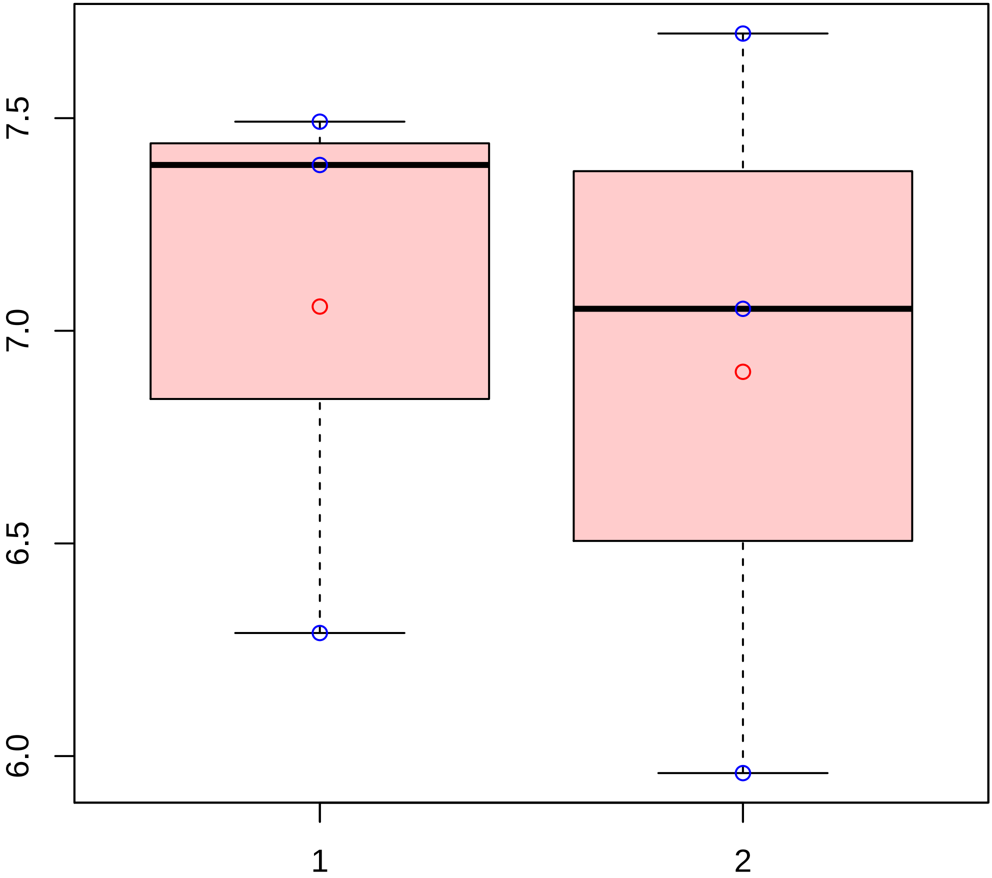


t−Test: p−value = 0.82

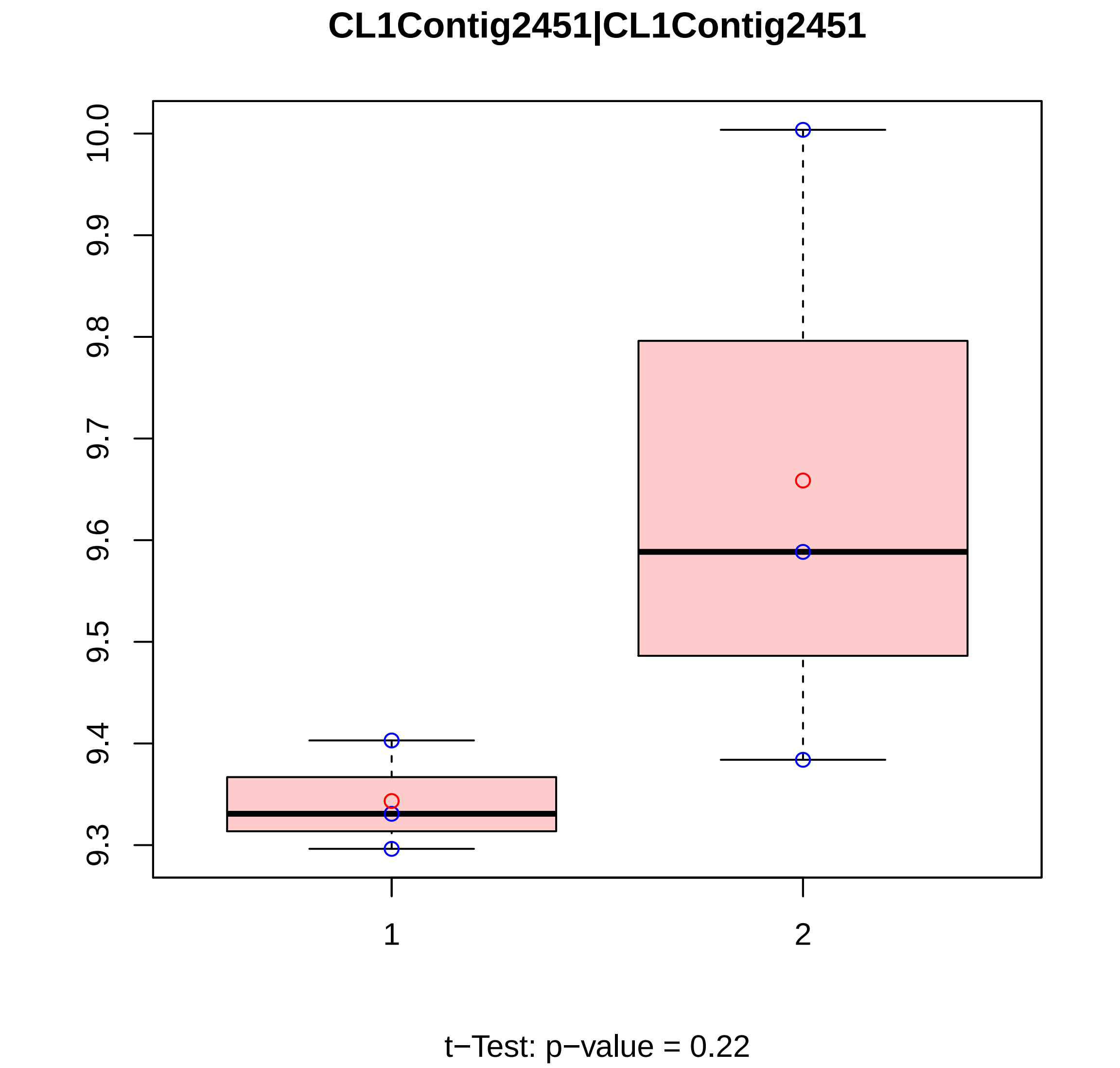
CL1Contig2451|CL1Contig2451
10.0
9.9
9.8
9.7
9.6
9.5
9.4
9.3
1
2
t−Test: p−value = 0.22

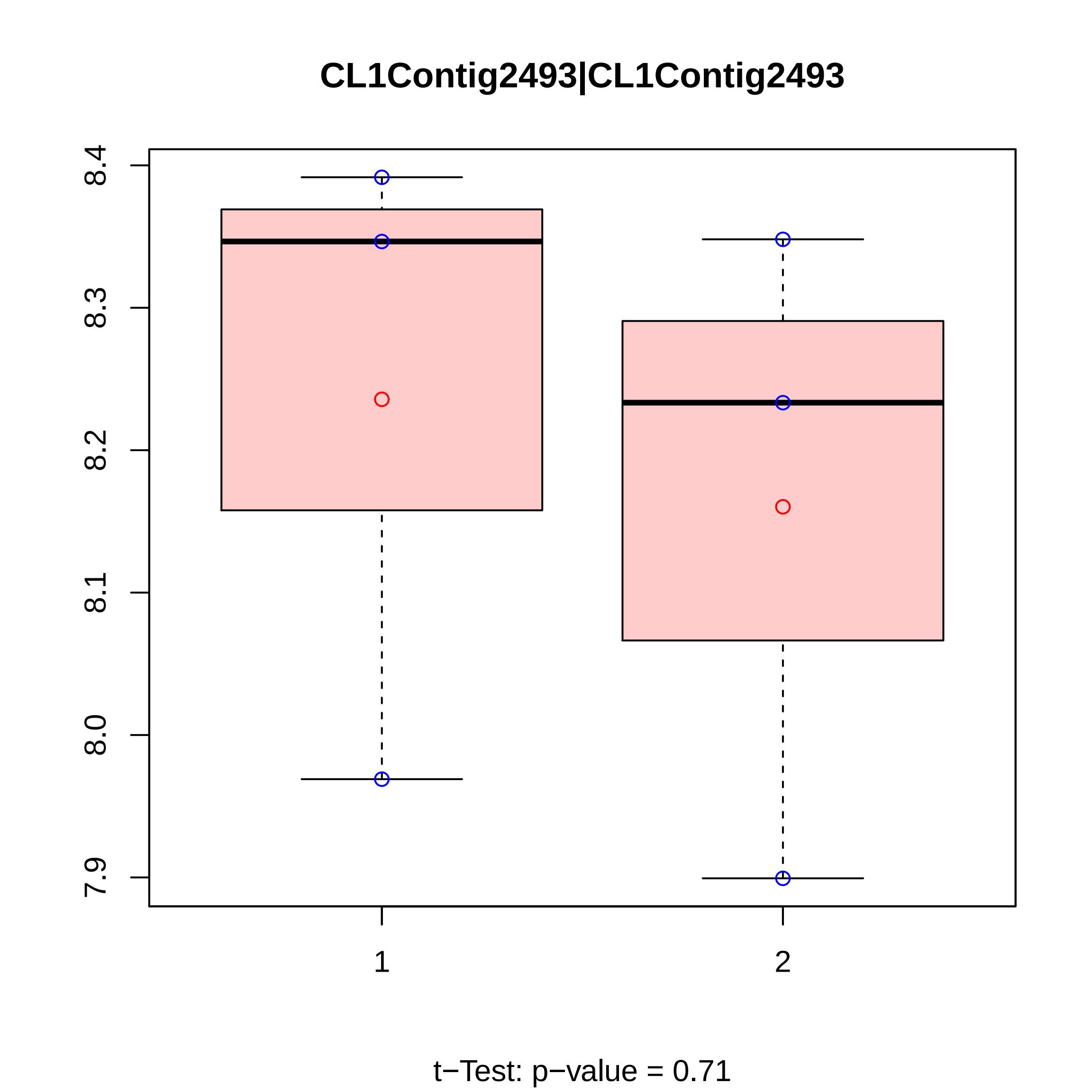

CL1Contig2493|CL1Contig2493
8.4
8.3
8.2
8.1
8.0
7.9
1
2
t−Test: p−value = 0.71

# CL1Contig249|CL1Contig249

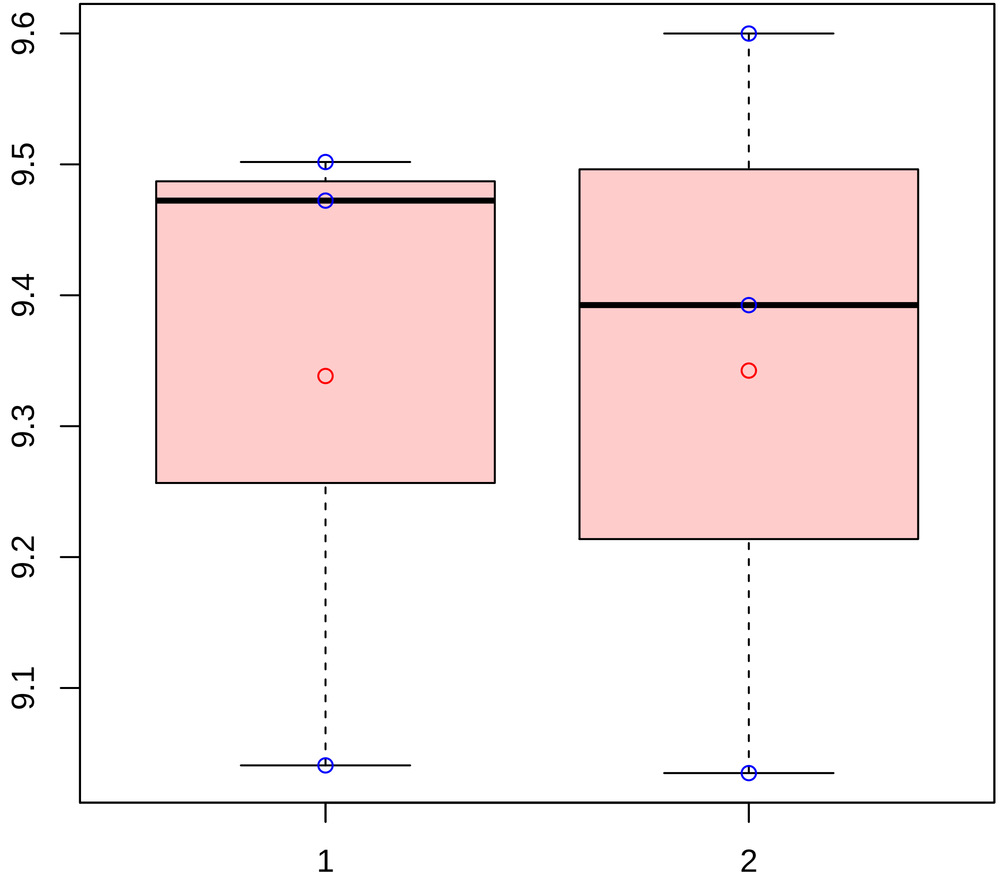


t−Test: p−value = 0.99

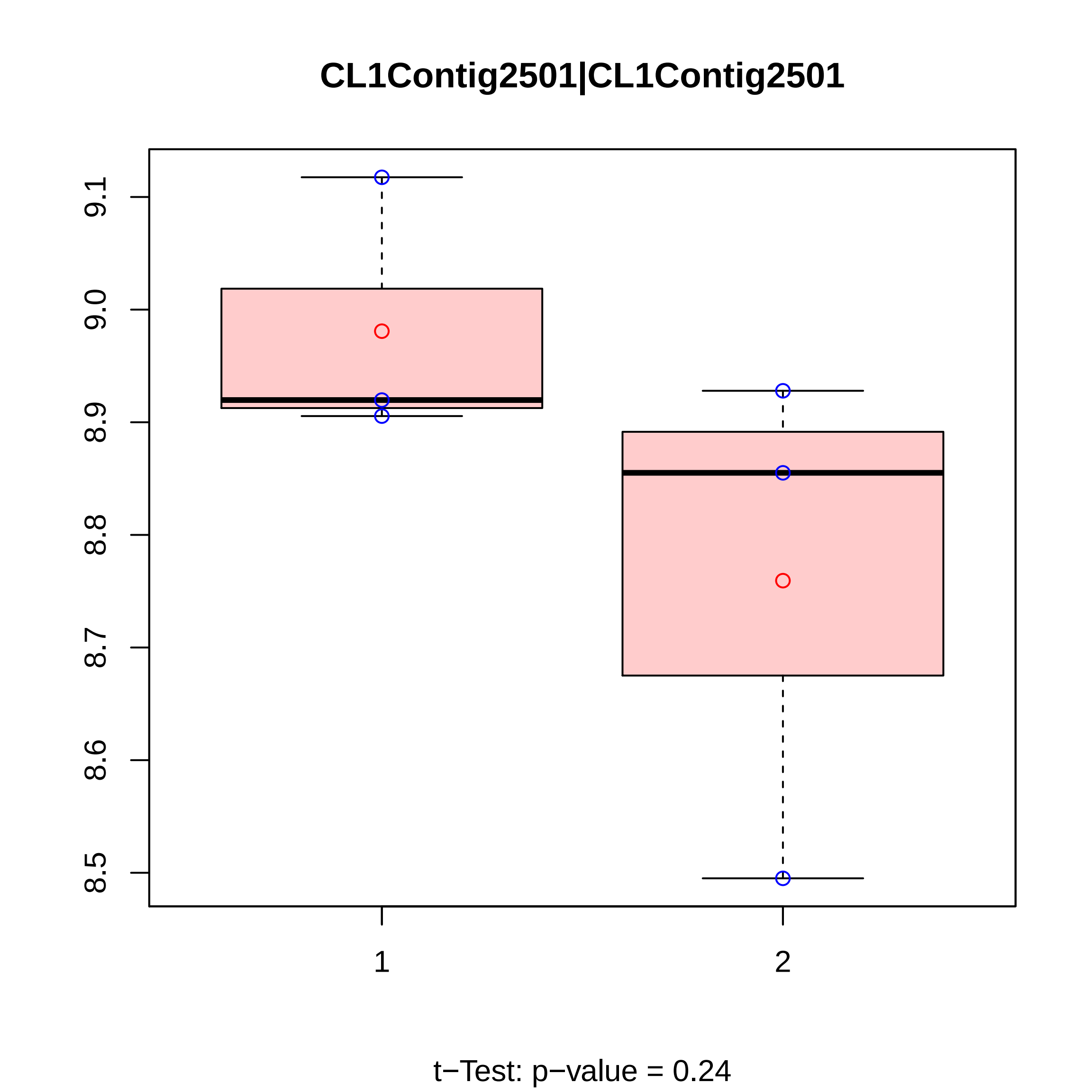
CL1Contig2501|CL1Contig2501
9.1
9.0
8.9
8.8
8.7
8.6
8.5
1
2
t−Test: p−value = 0.24

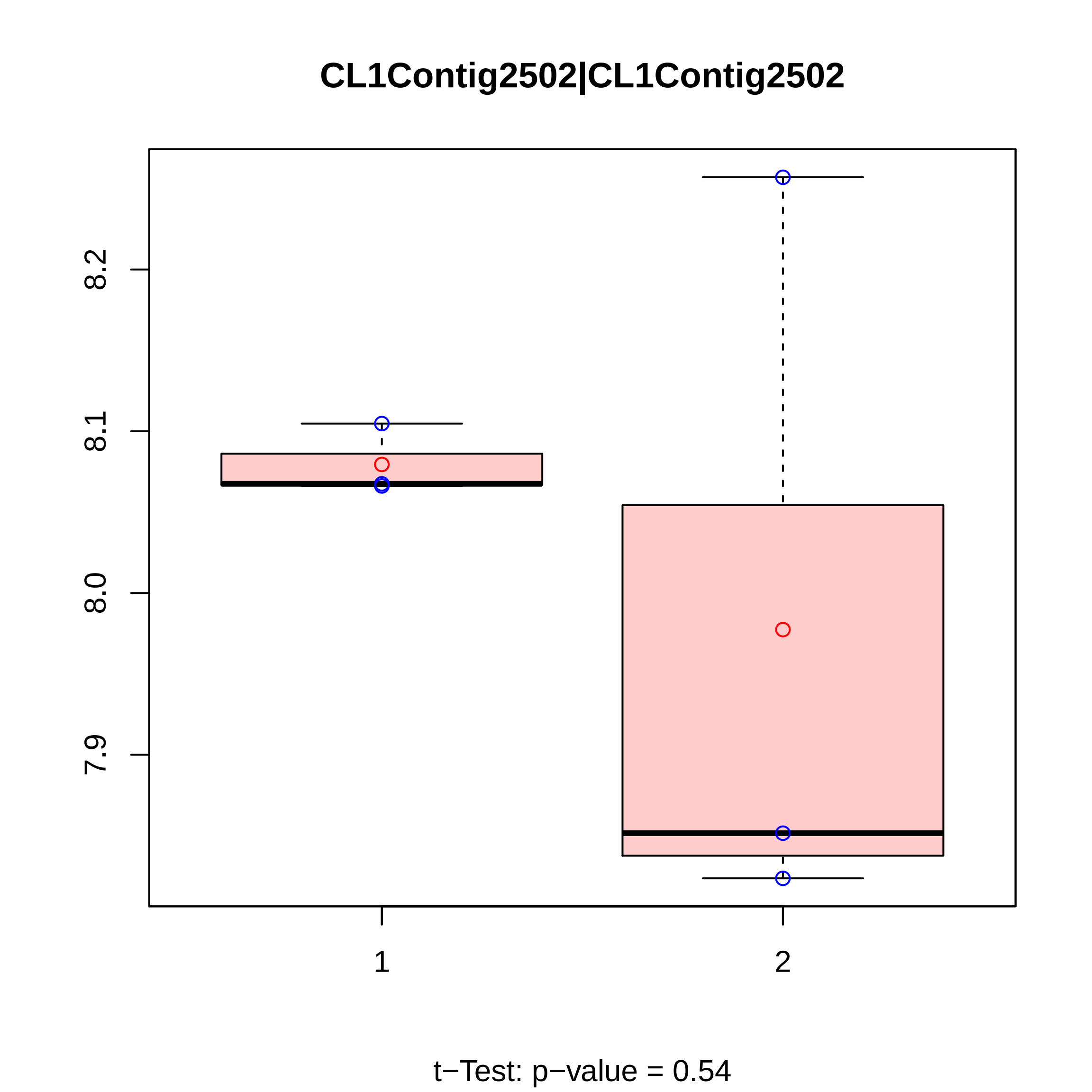

CL1Contig2502|CL1Contig2502
8.2
8.1
8.0
7.9
1
2
t−Test: p−value = 0.54

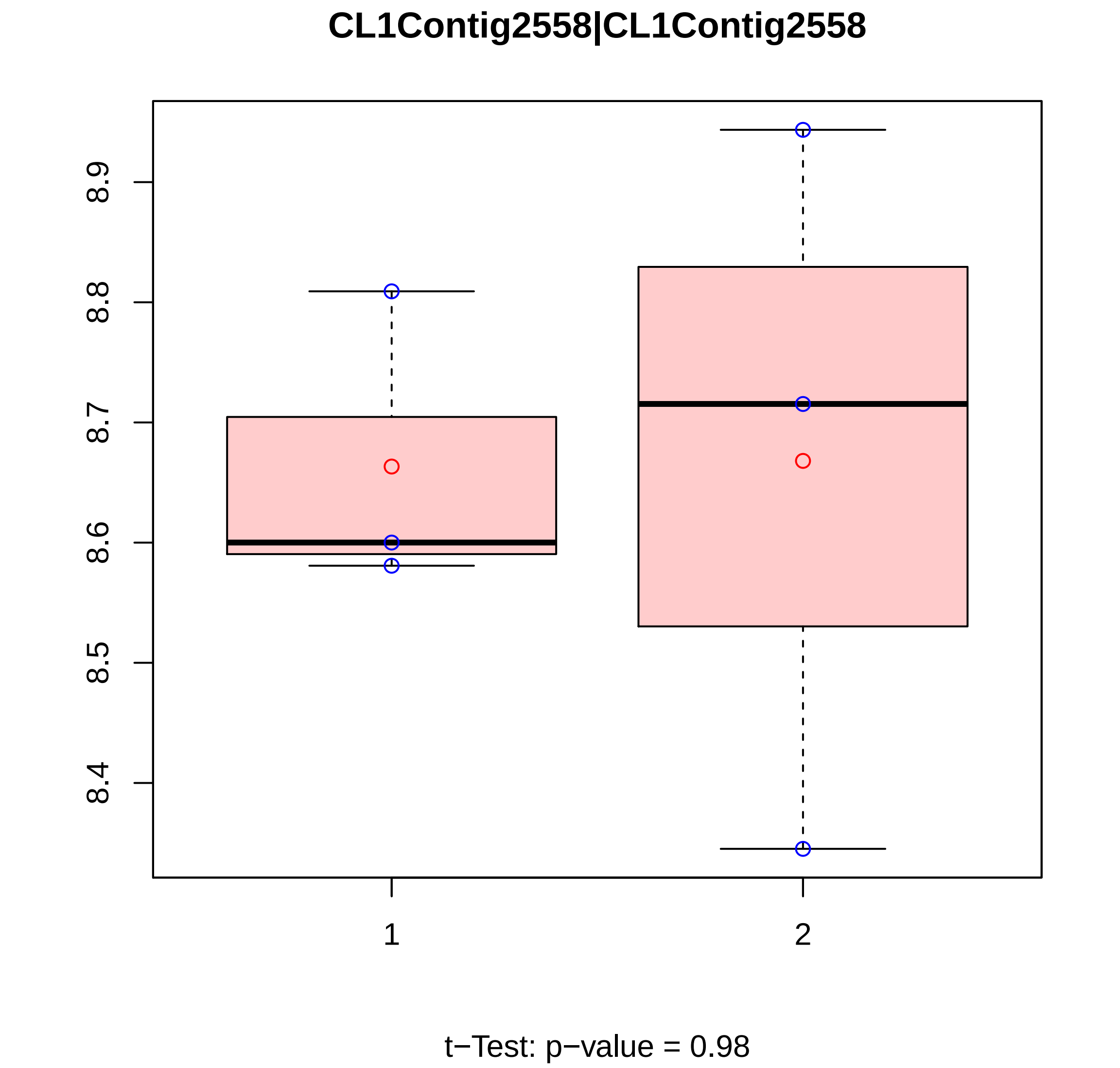

CL1Contig2558|CL1Contig2558
8.4
8.5
8.6
8.7
8.8
8.9
1
2
t−Test: p−value = 0.98

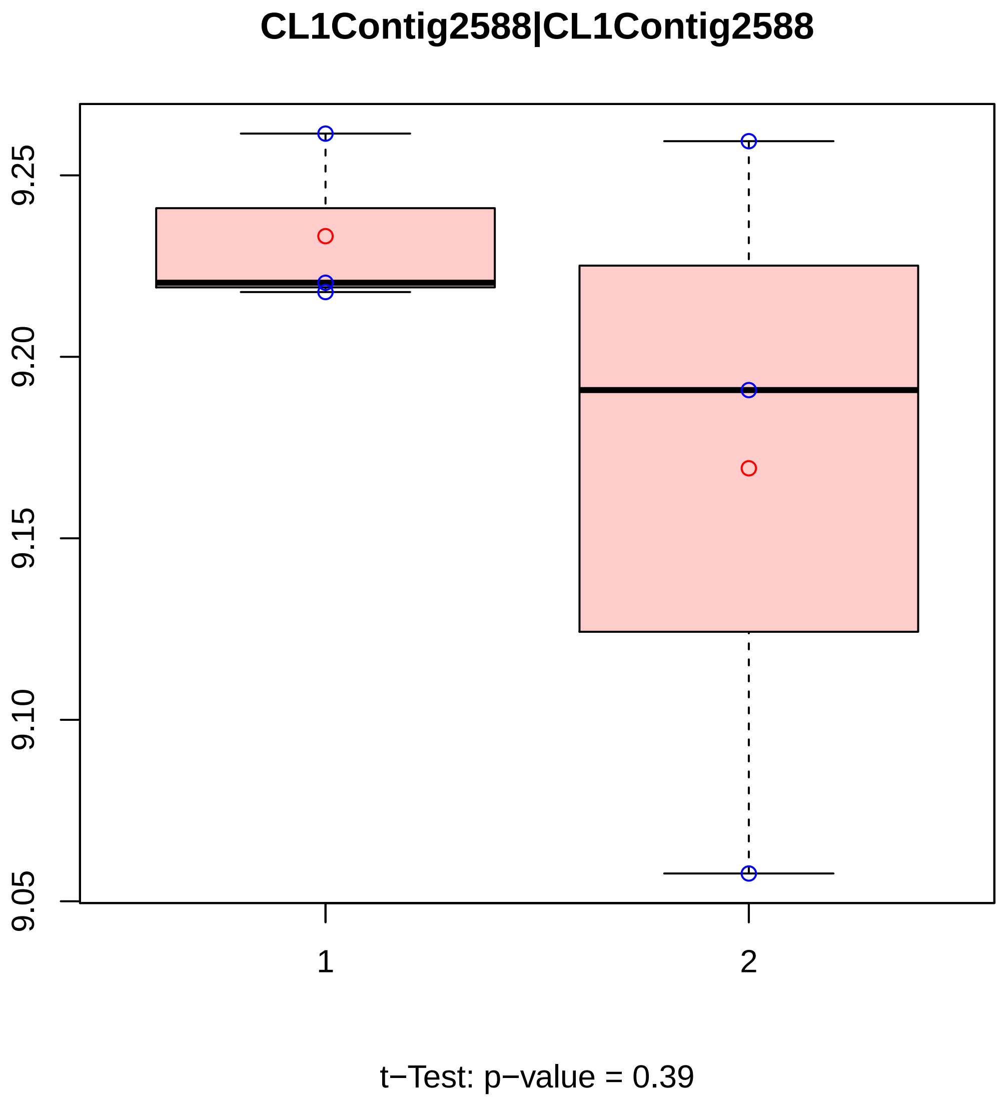

CL1Contig2588|CL1Contig2588
9.25
9.20
9.15
9.10
9.05
1
2
t−Test: p−value = 0.39

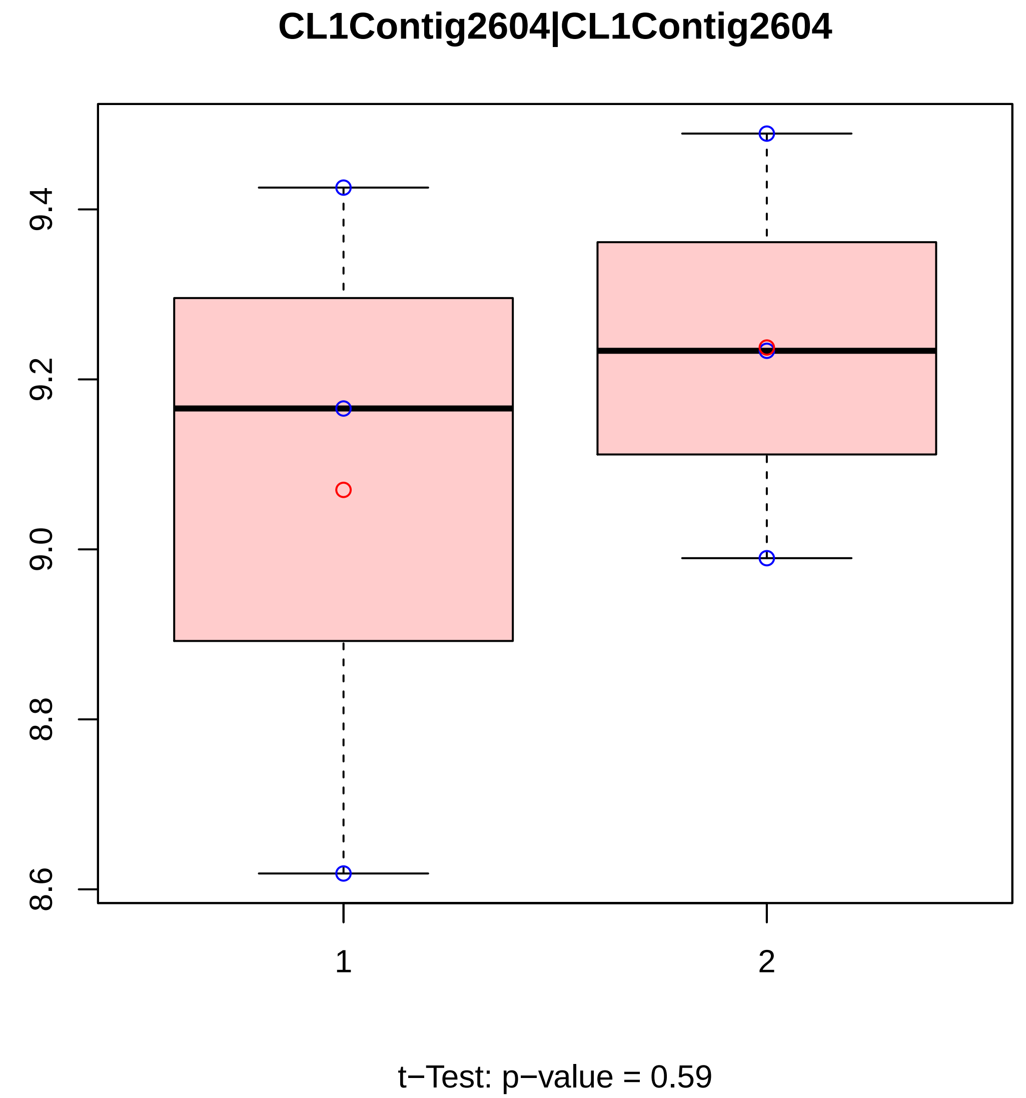
CL1Contig2604|CL1Contig2604
9.4
9.2
9.0
8.8
8.6
1
2
t−Test: p−value = 0.59

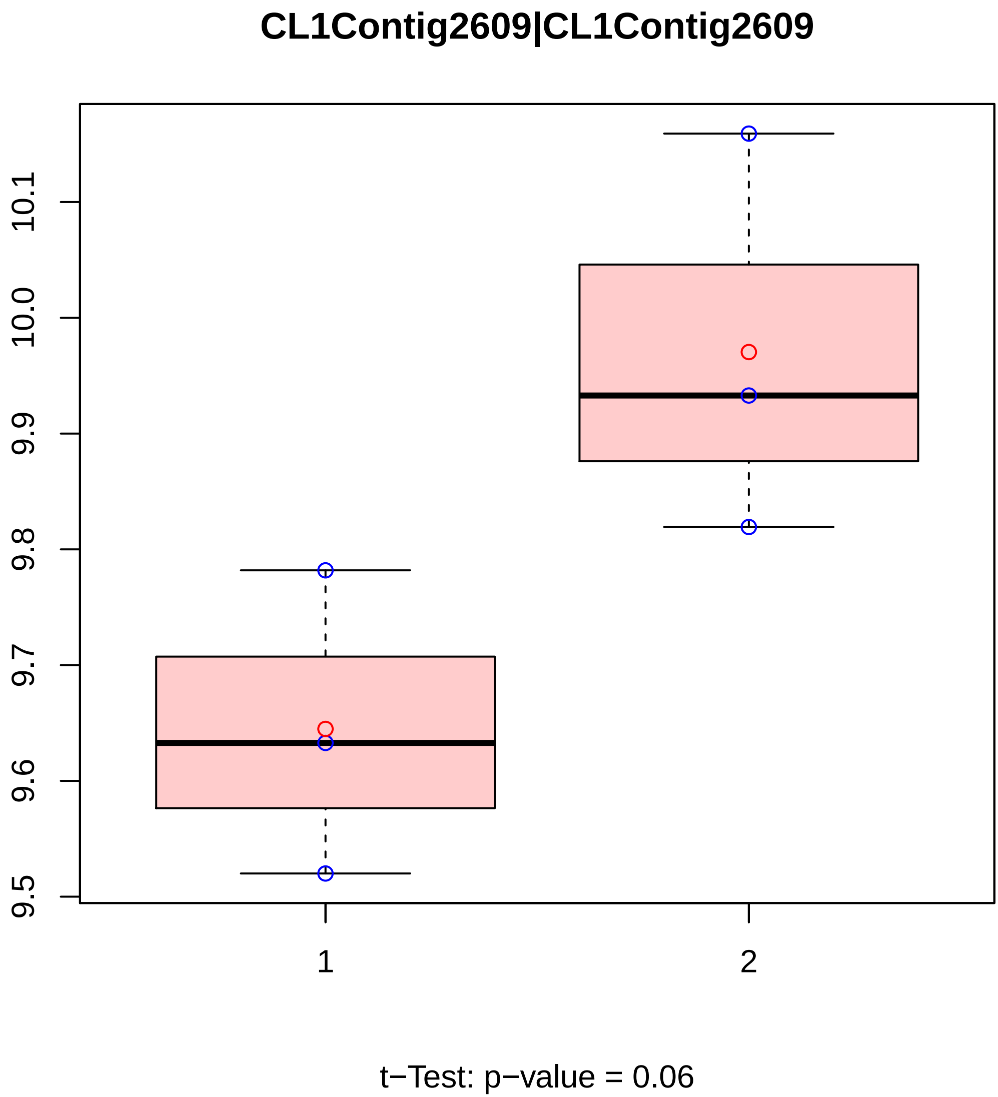
CL1Contig2609|CL1Contig2609
10.1
10.0
9.9
9.8
9.7
9.6
9.5
1
2
t−Test: p−value = 0.06

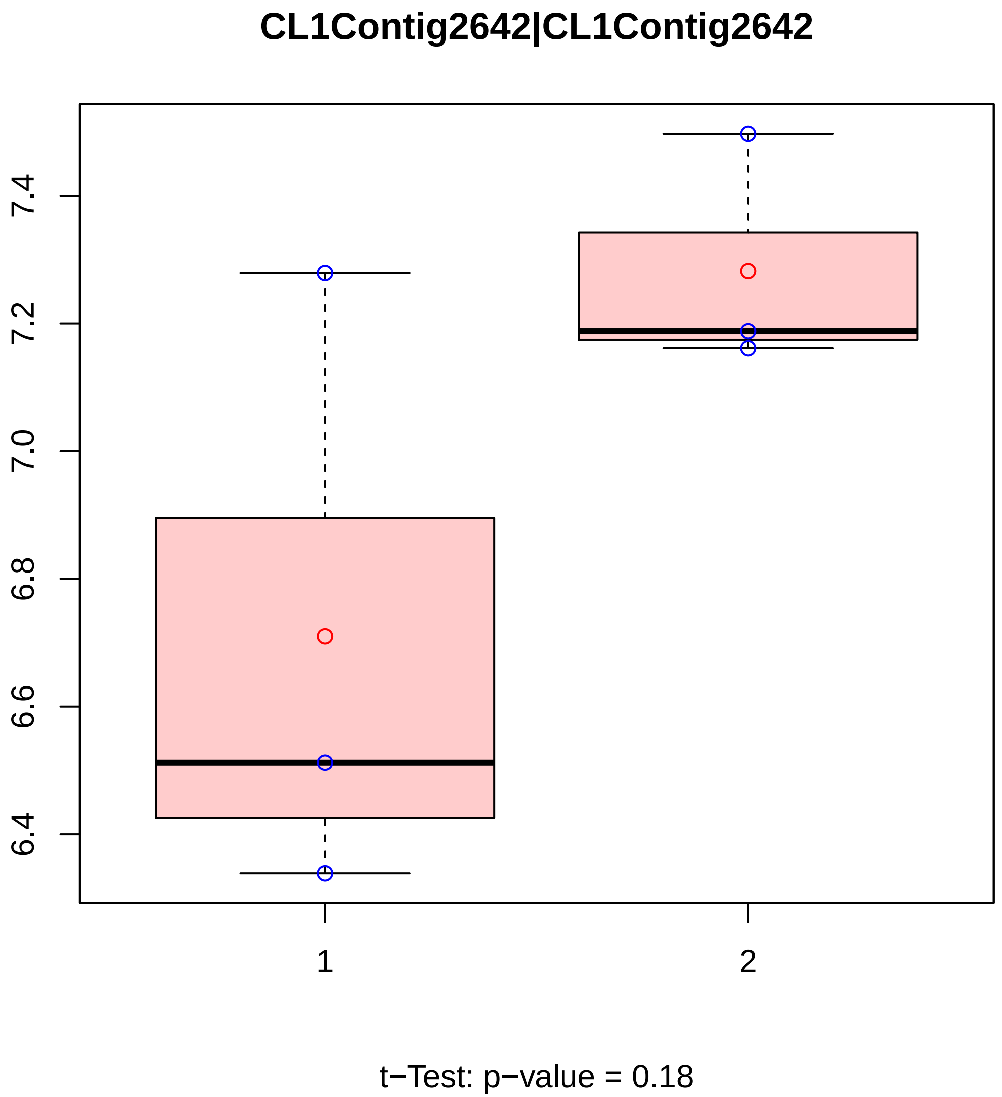
CL1Contig2642|CL1Contig2642
7.4
7.2
7.0
6.8
6.6
6.4
1
2
t−Test: p−value = 0.18

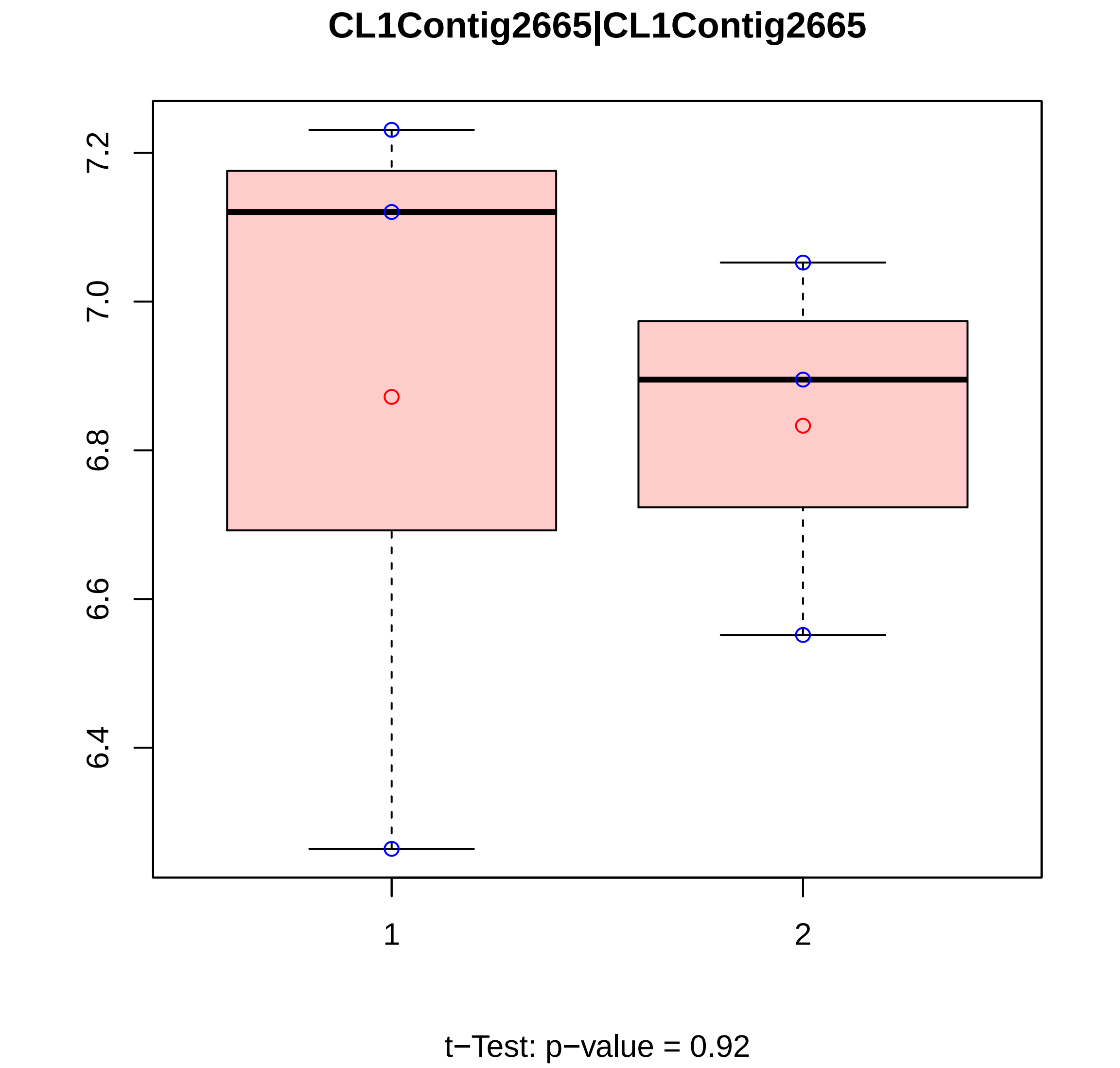
CL1Contig2665|CL1Contig2665
7.2
7.0
6.8
6.6
6.4
1
2
t−Test: p−value = 0.92

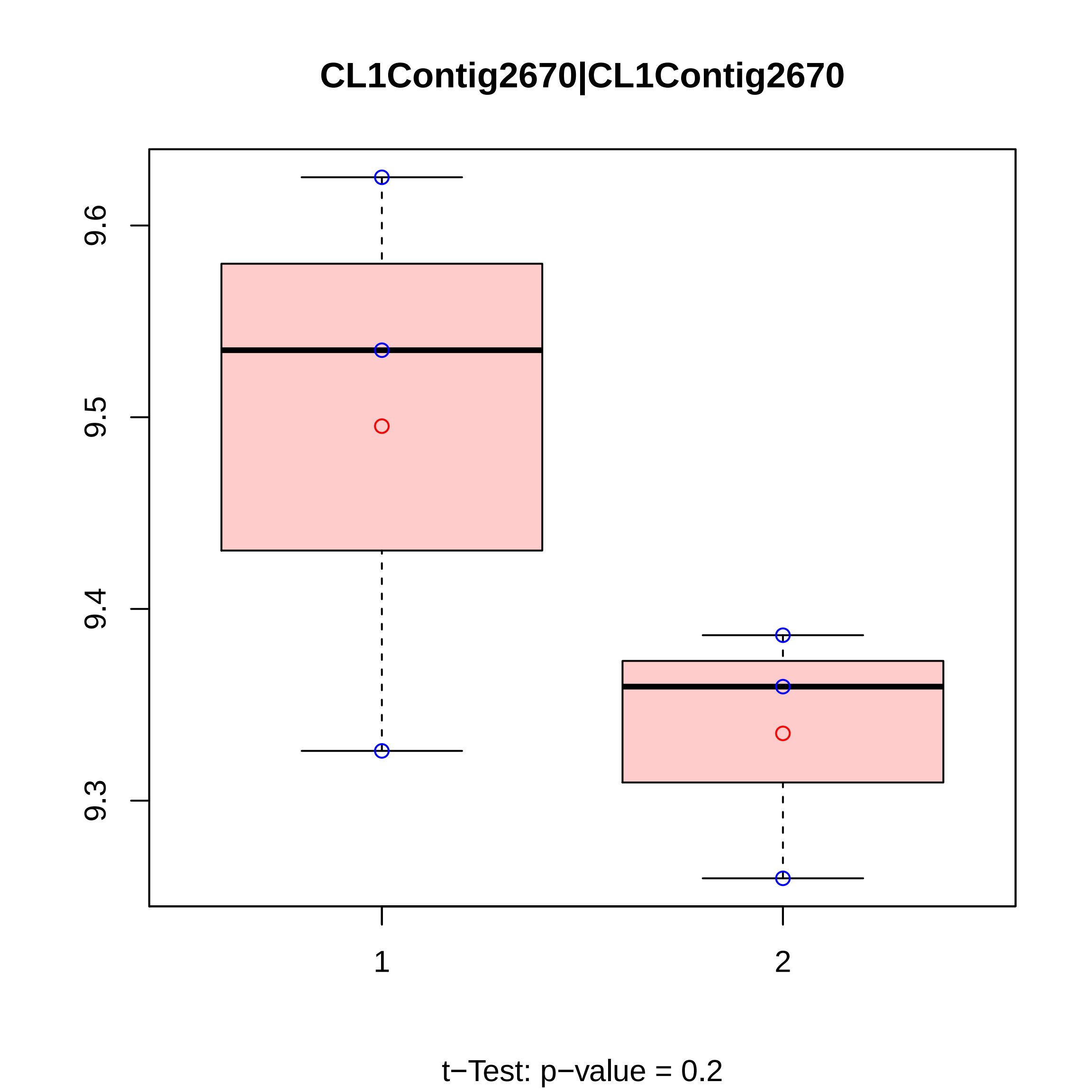
CL1Contig2670|CL1Contig2670
9.6
9.5
9.4
9.3
1
2
t−Test: p−value = 0.2

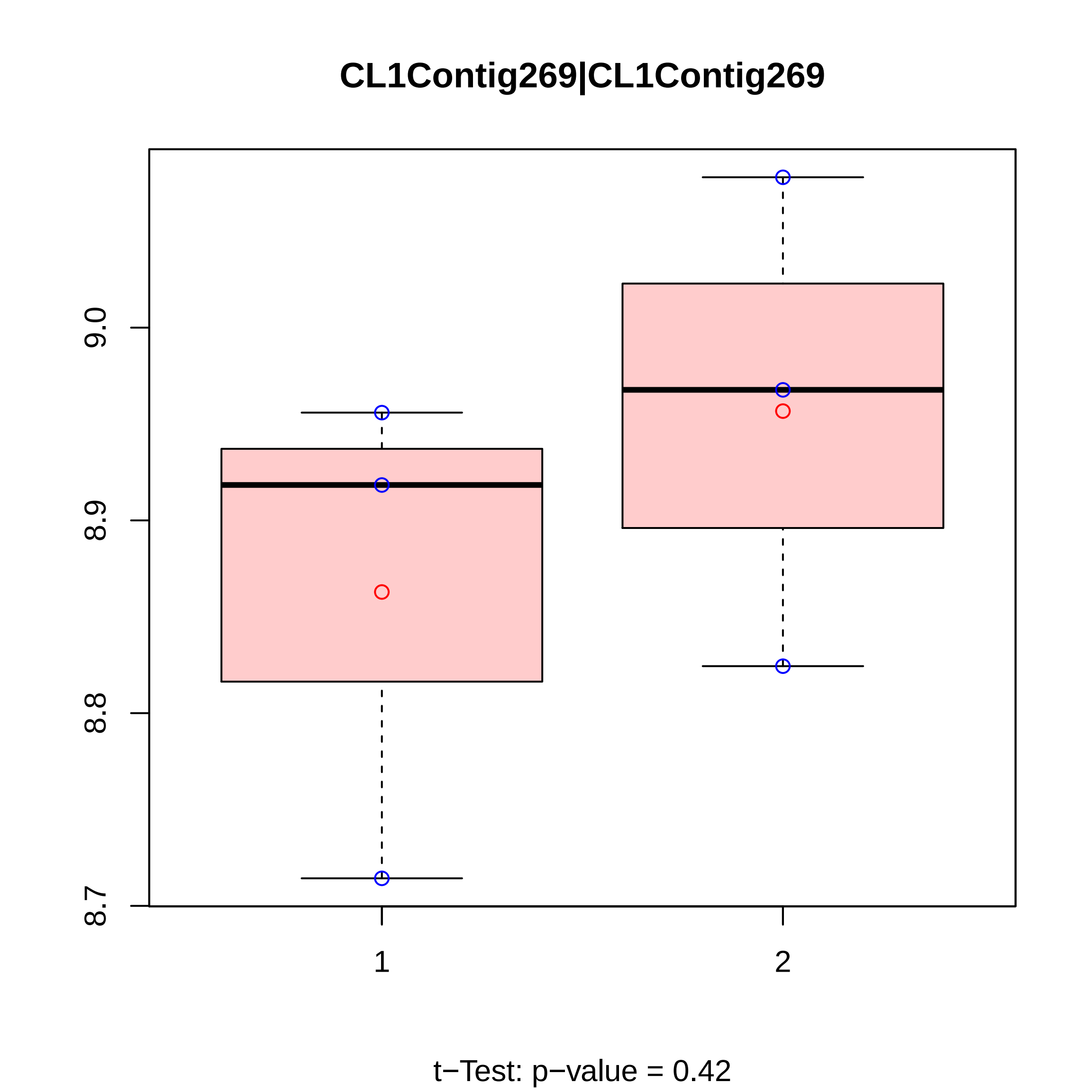

CL1Contig269|CL1Contig269
9.0
8.9
8.8
8.7
1
2
t−Test: p−value = 0.42

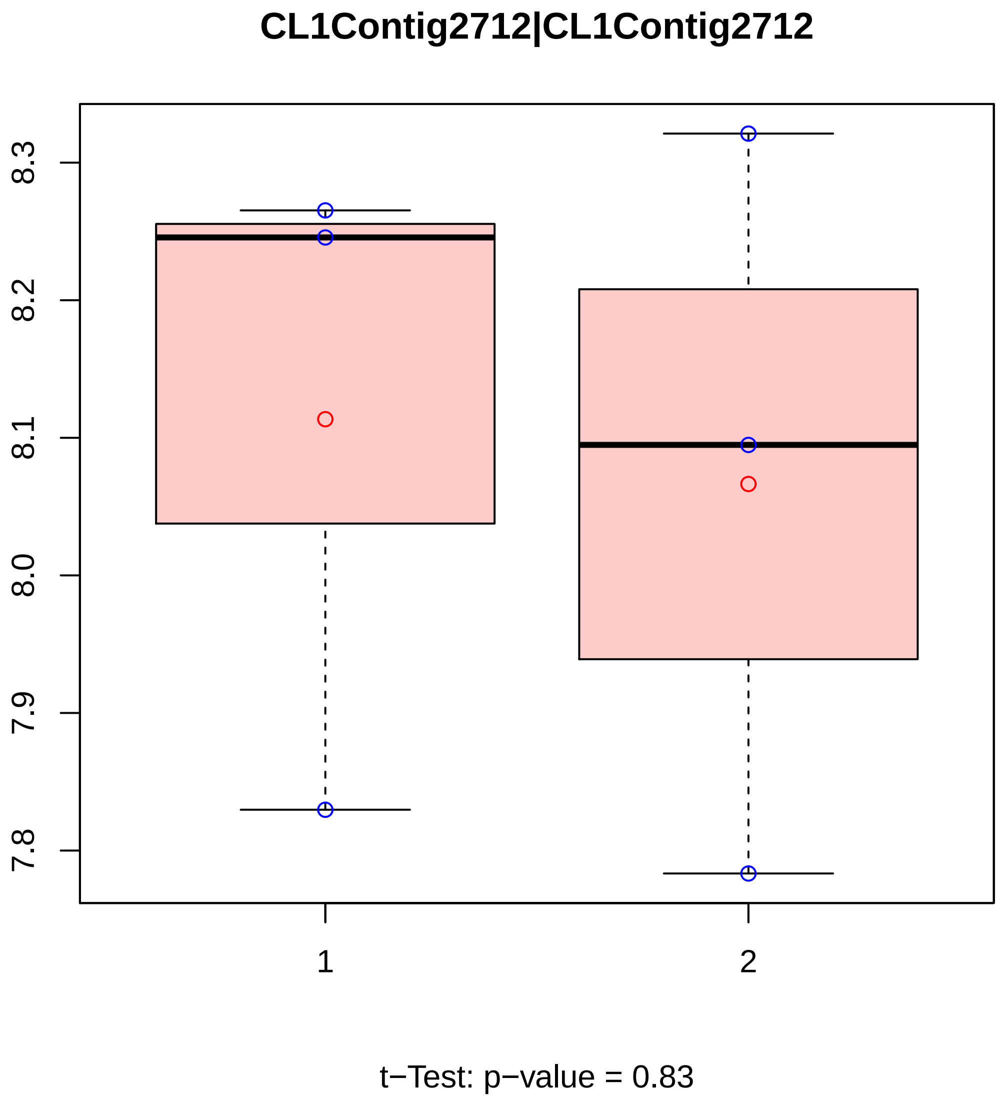

CL1Contig2712|CL1Contig2712
8.3
8.2
8.1
8.0
7.9
7.8
1
2
t−Test: p−value = 0.83

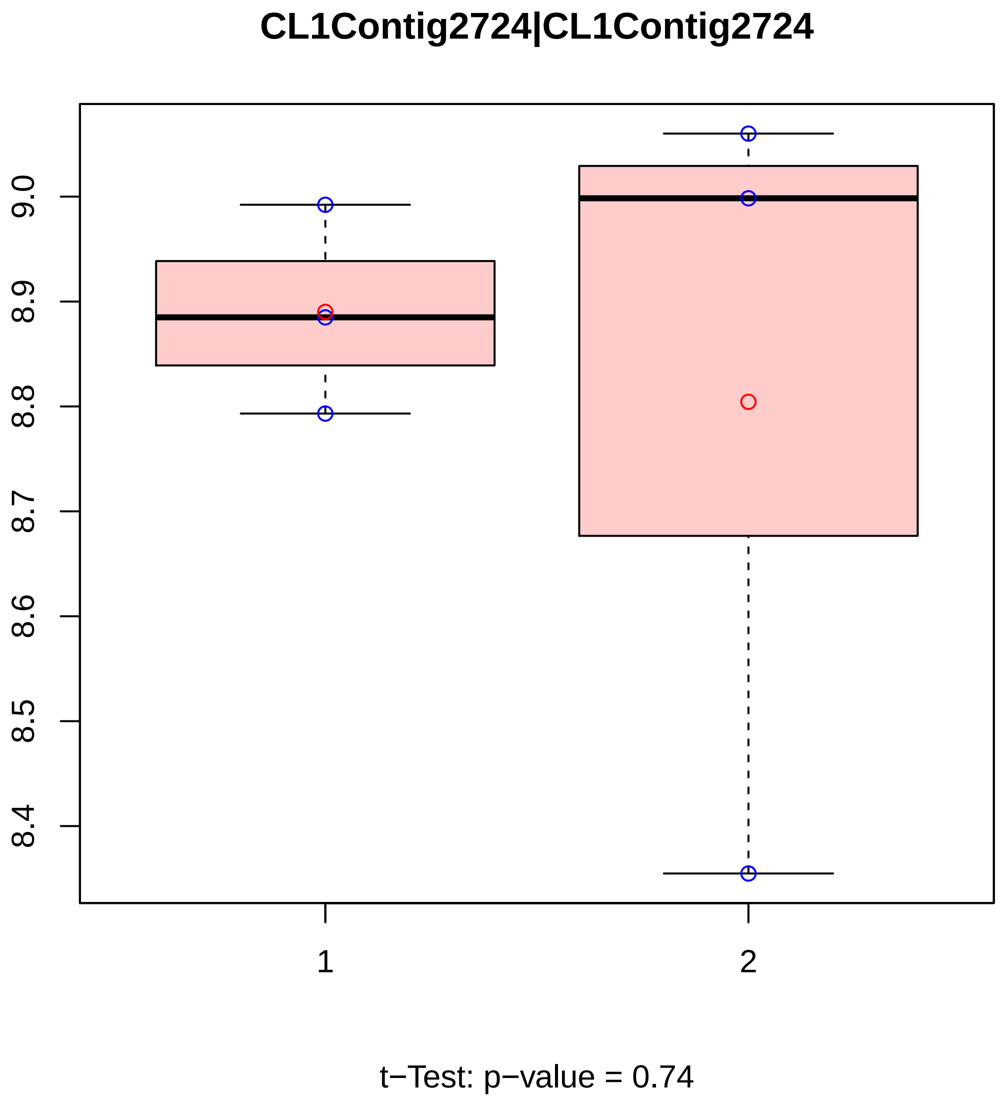
CL1Contig2724|CL1Contig2724
9.0
8.9
8.8
8.7
8.6
8.5
8.4
1
2
t−Test: p−value = 0.74

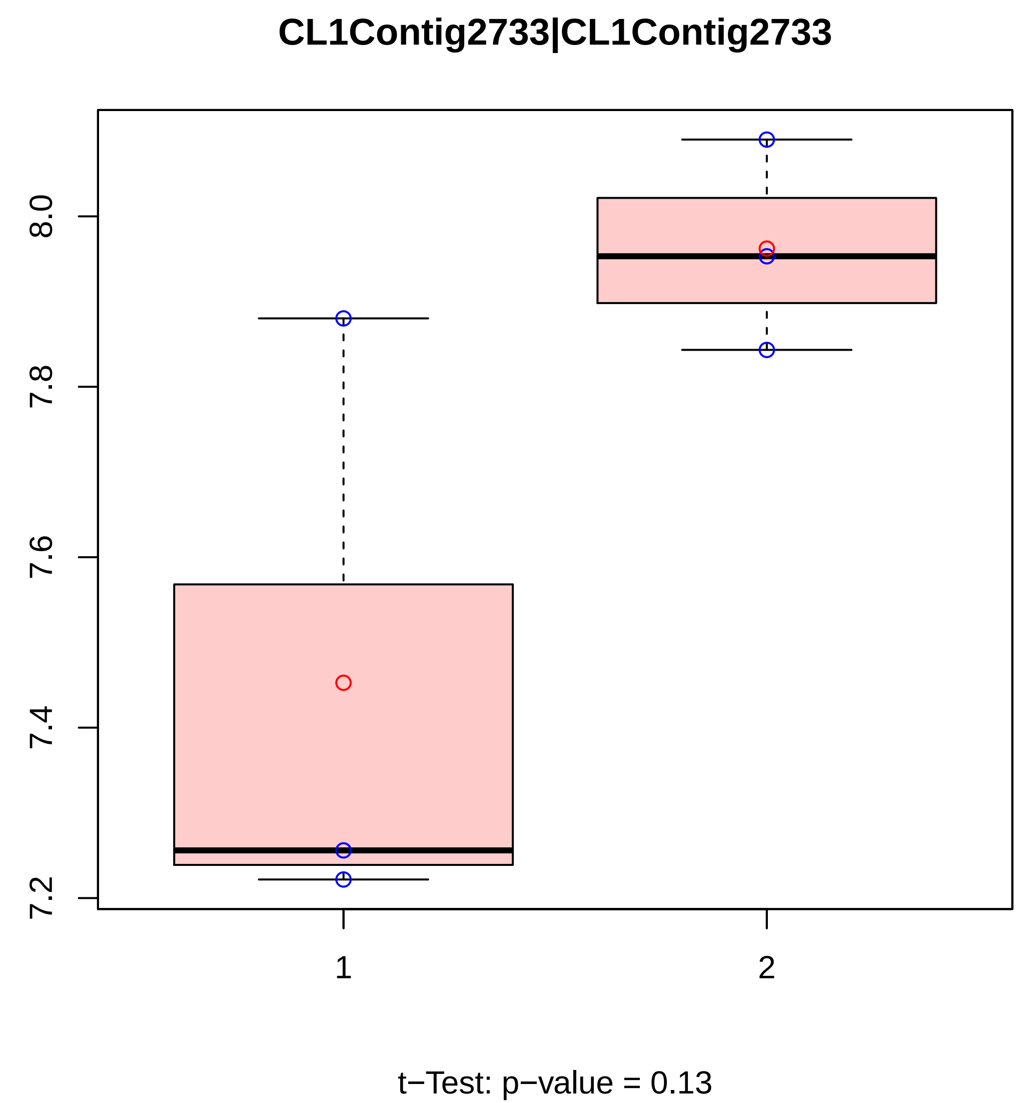

CL1Contig2733|CL1Contig2733
7.2
7.4
7.6
7.8
8.0
1
2
t−Test: p−value = 0.13

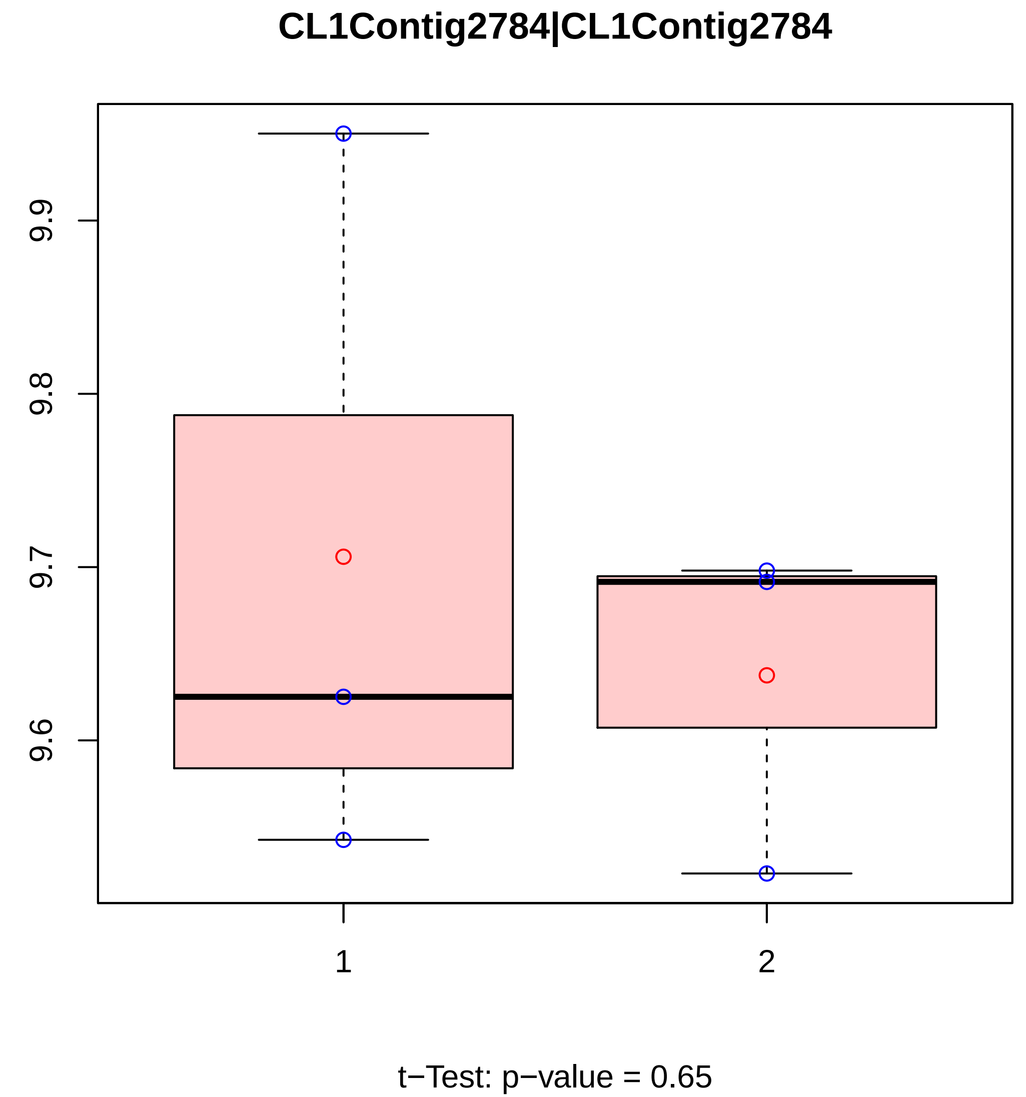
CL1Contig2784|CL1Contig2784
9.6
9.7
9.8
9.9
1
2
t−Test: p−value = 0.65

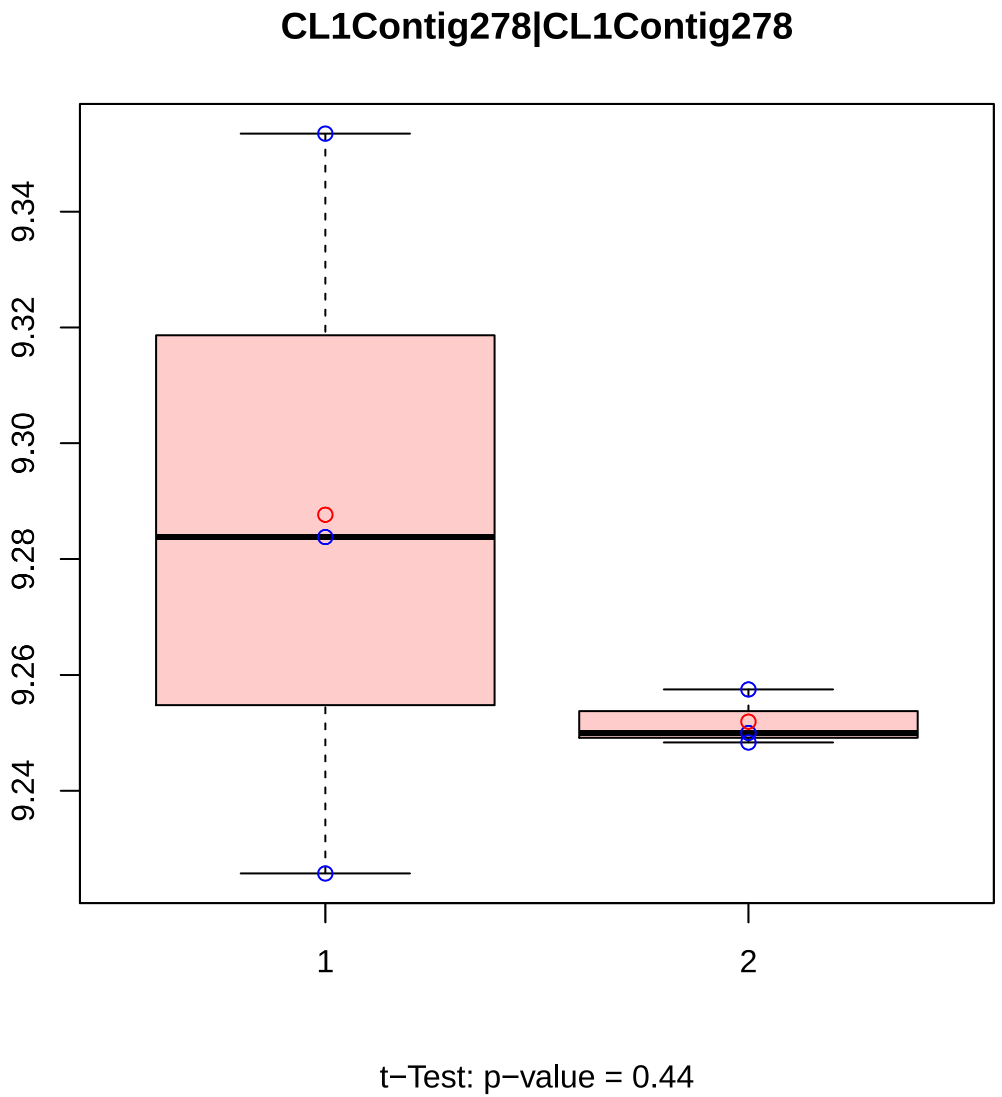
CL1Contig278|CL1Contig278
9.24
9.26
9.28
9.30
9.32
9.34
1
2
t−Test: p−value = 0.44

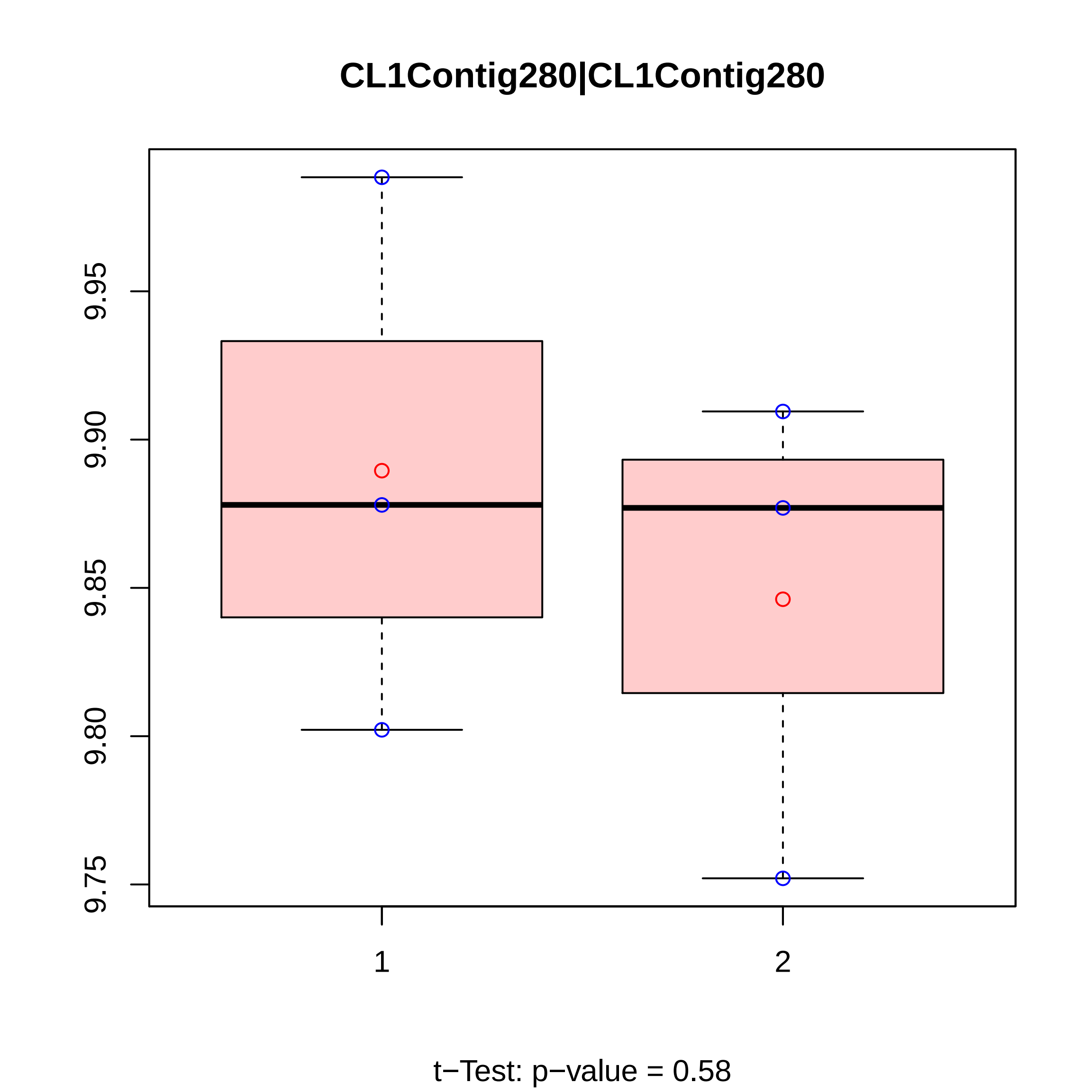

CL1Contig280|CL1Contig280
9.75
9.80
9.85
9.90
9.95
1
2
t−Test: p−value = 0.58

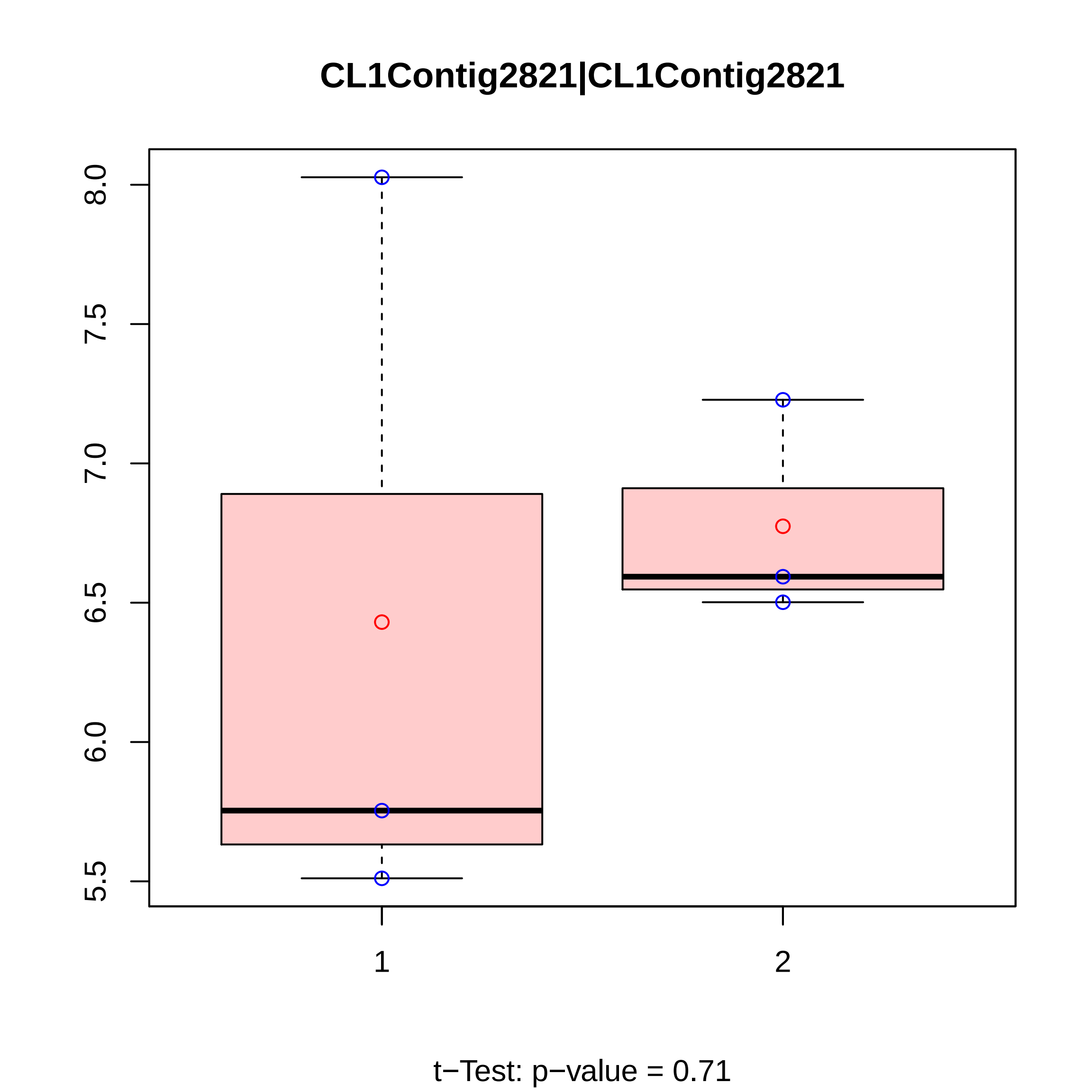
CL1Contig2821|CL1Contig2821
8.0
7.5
7.0
6.5
6.0
5.5
1
2
t−Test: p−value = 0.71

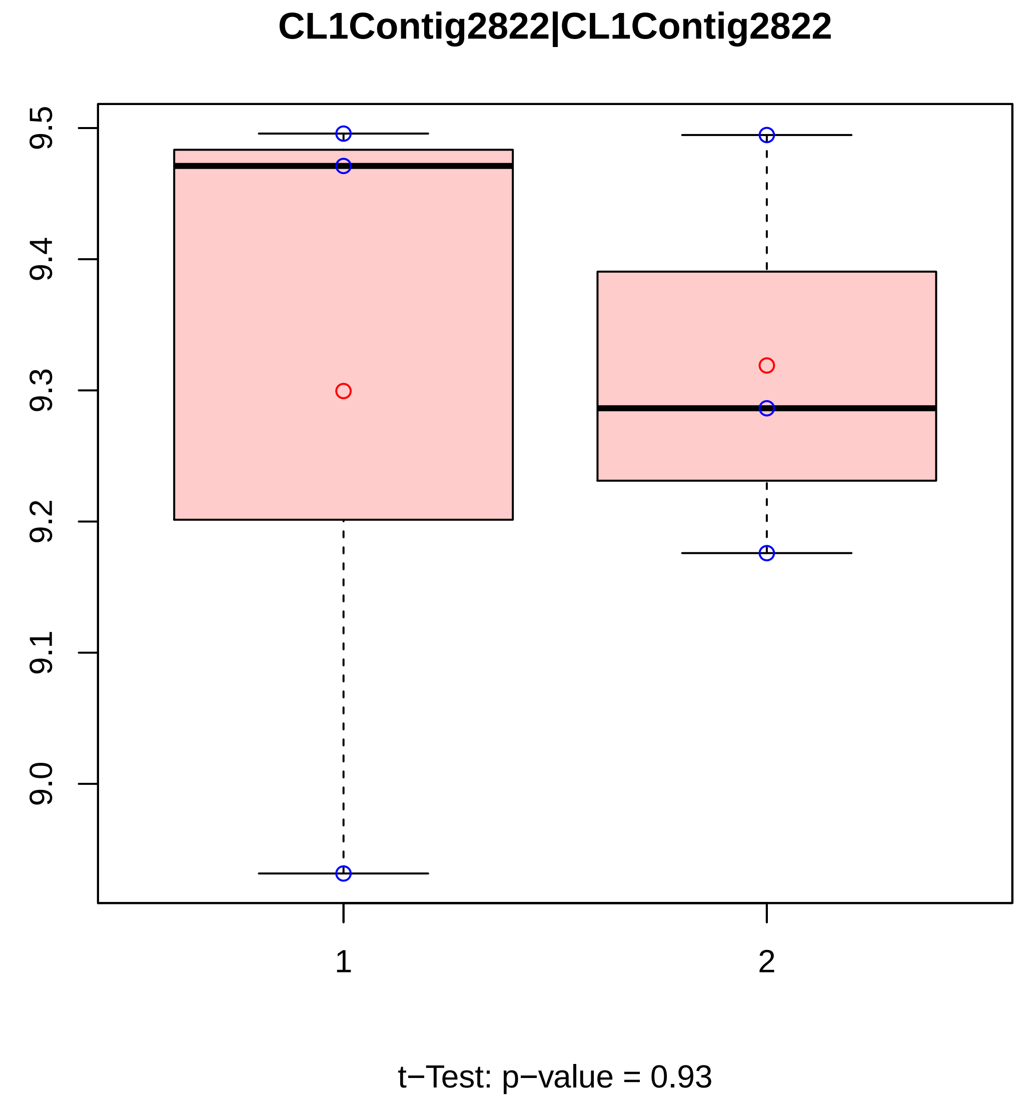
CL1Contig2822|CL1Contig2822
9.5
9.4
9.3
9.2
9.1
9.0
1
2
t−Test: p−value = 0.93

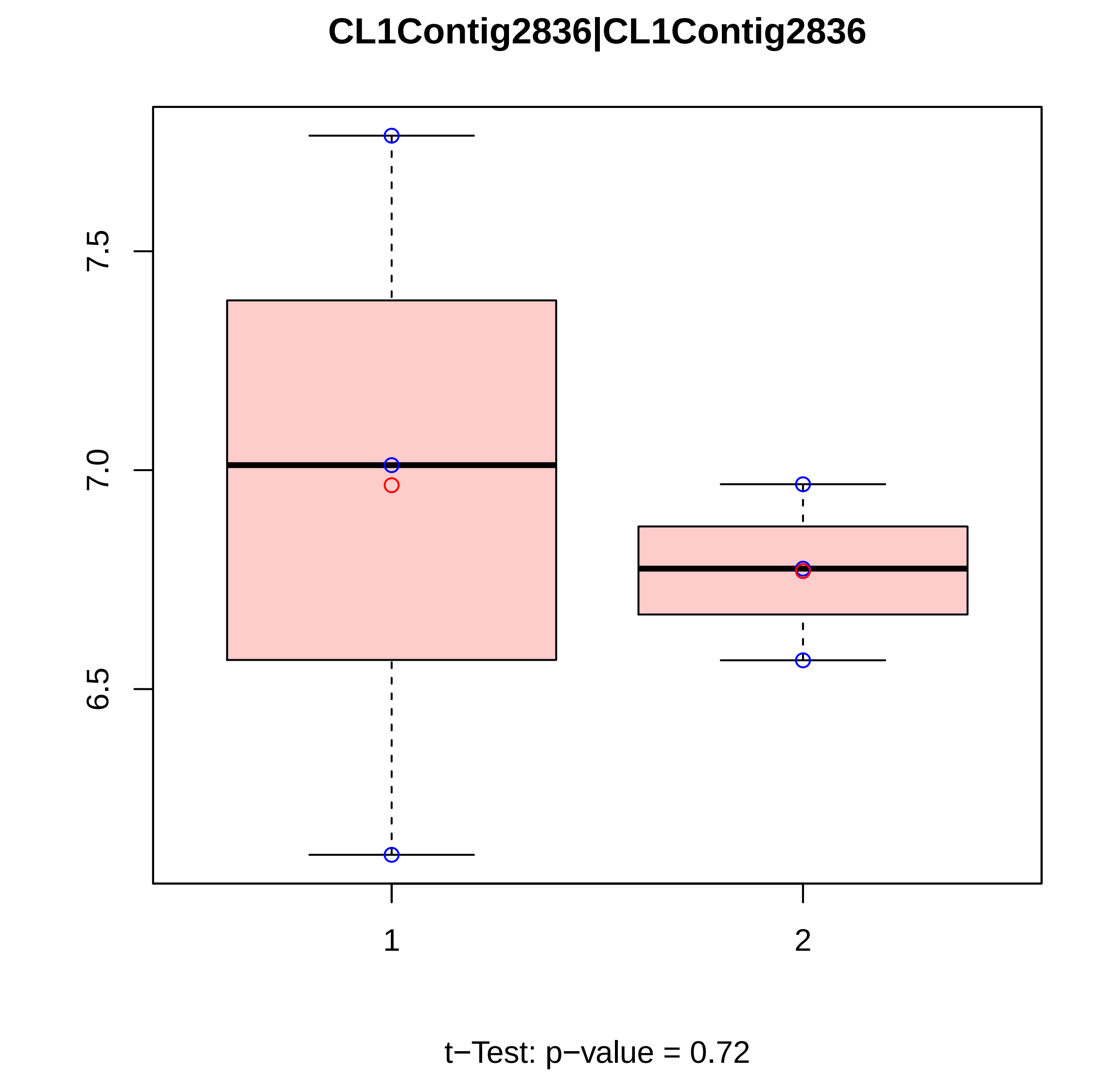
CL1Contig2836|CL1Contig2836
7.5
7.0
6.5
1
2
t−Test: p−value = 0.72

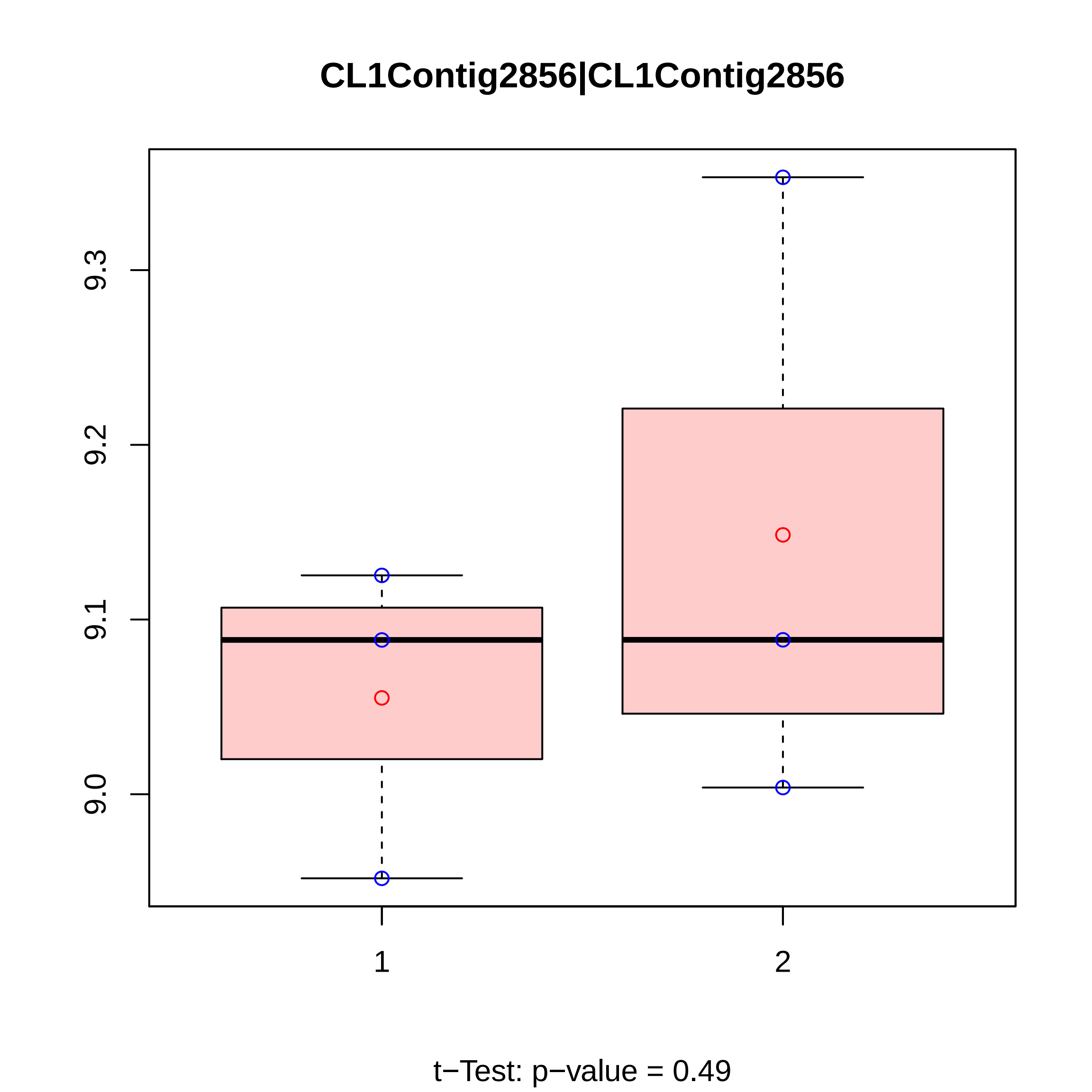
CL1Contig2856|CL1Contig2856
9.0
9.1
9.2
9.3
1
2
t−Test: p−value = 0.49

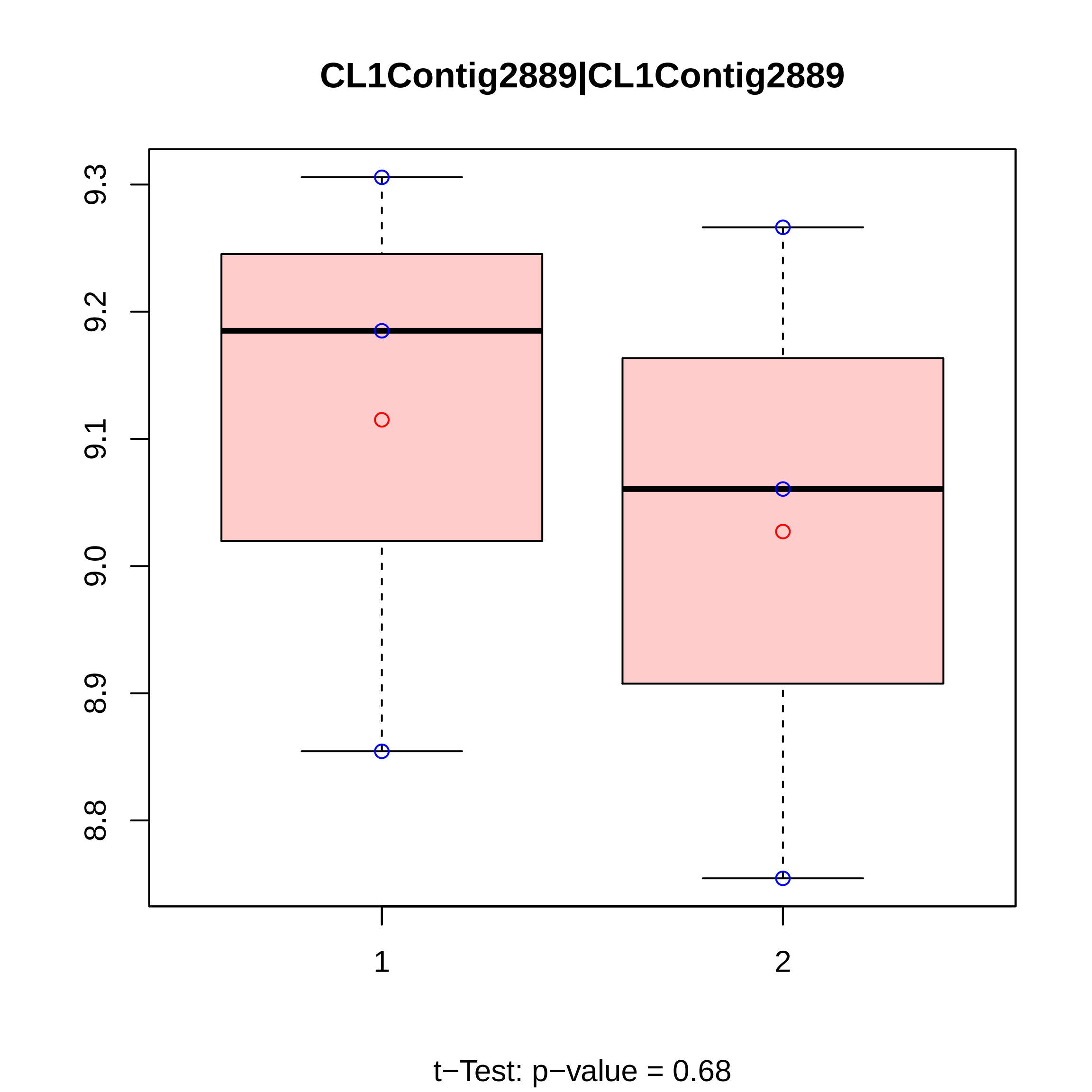

CL1Contig2889|CL1Contig2889
9.3
9.2
9.1
9.0
8.9
8.8
1
2
t−Test: p−value = 0.68

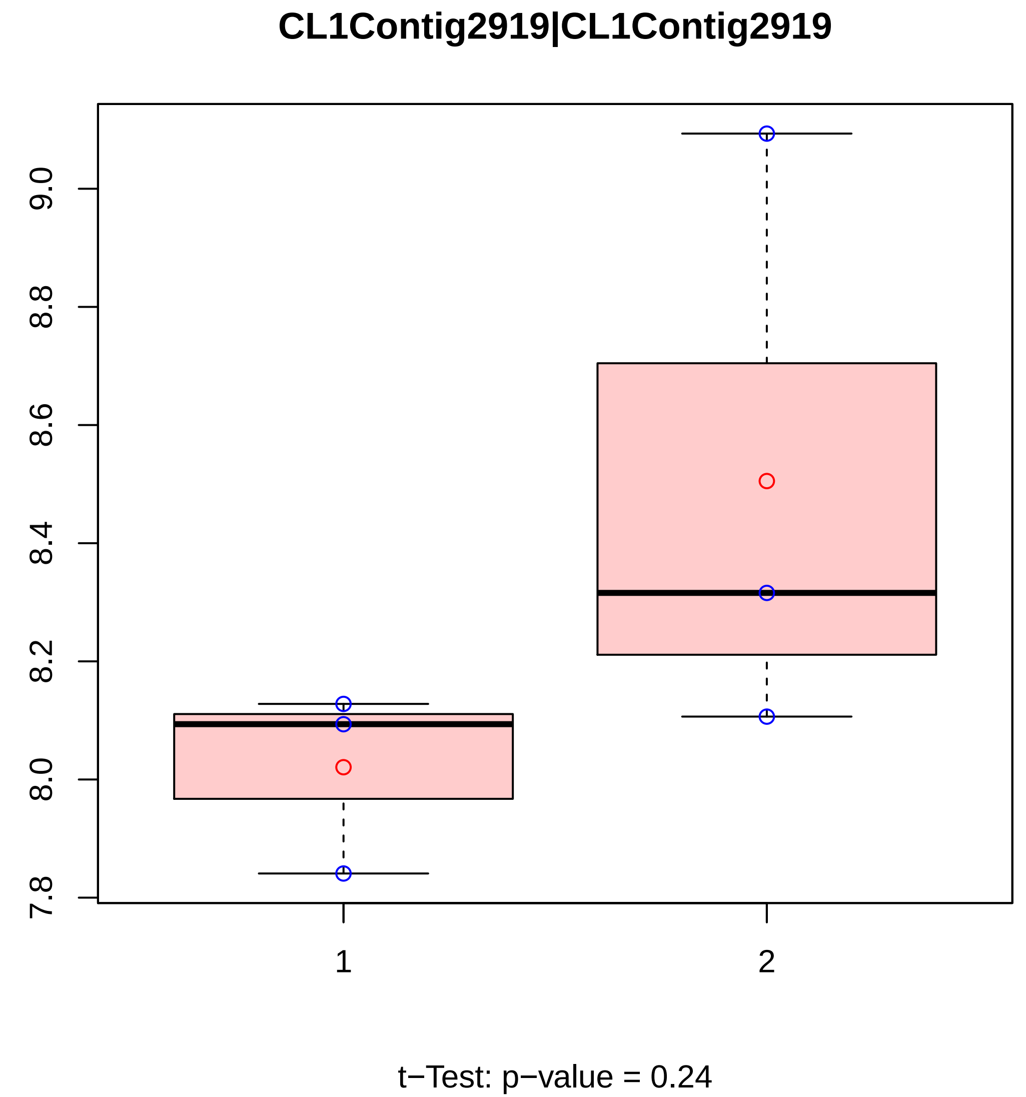

CL1Contig2919|CL1Contig2919
9.0
8.8
8.6
8.4
8.2
8.0
7.8
1
2
t−Test: p−value = 0.24

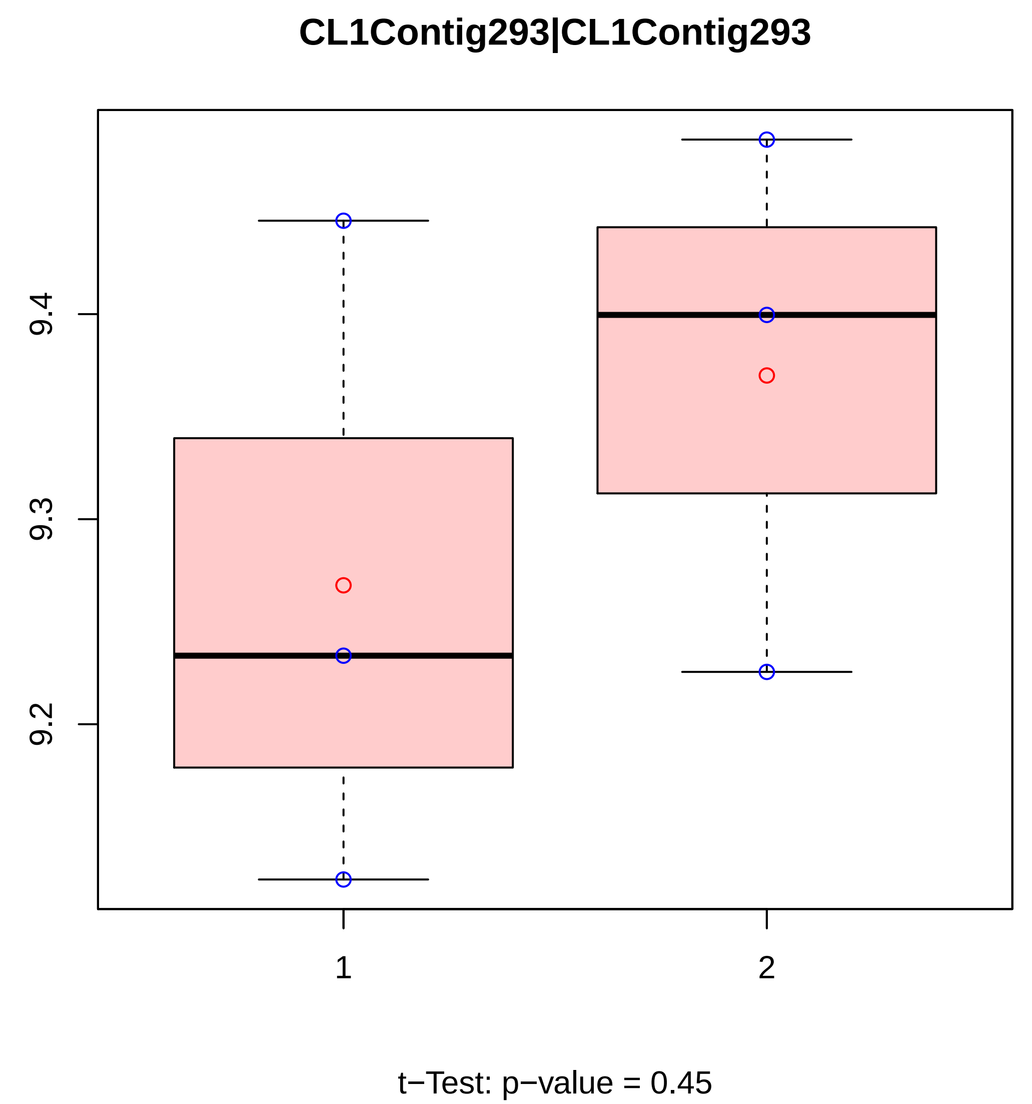
CL1Contig293|CL1Contig293
9.2
9.3
9.4
1
2
t−Test: p−value = 0.45

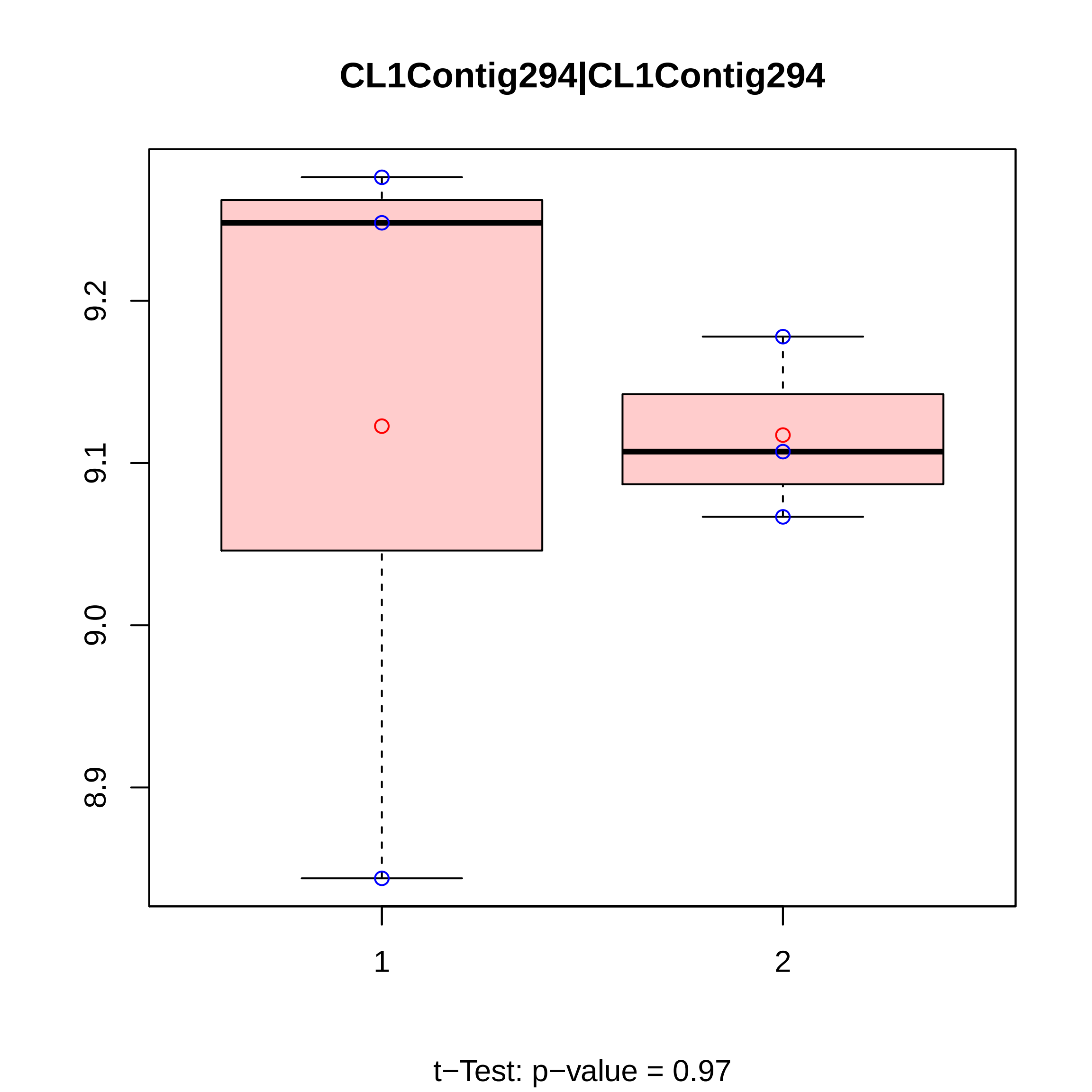
CL1Contig294|CL1Contig294
9.2
9.1
9.0
8.9
1
2
t−Test: p−value = 0.97

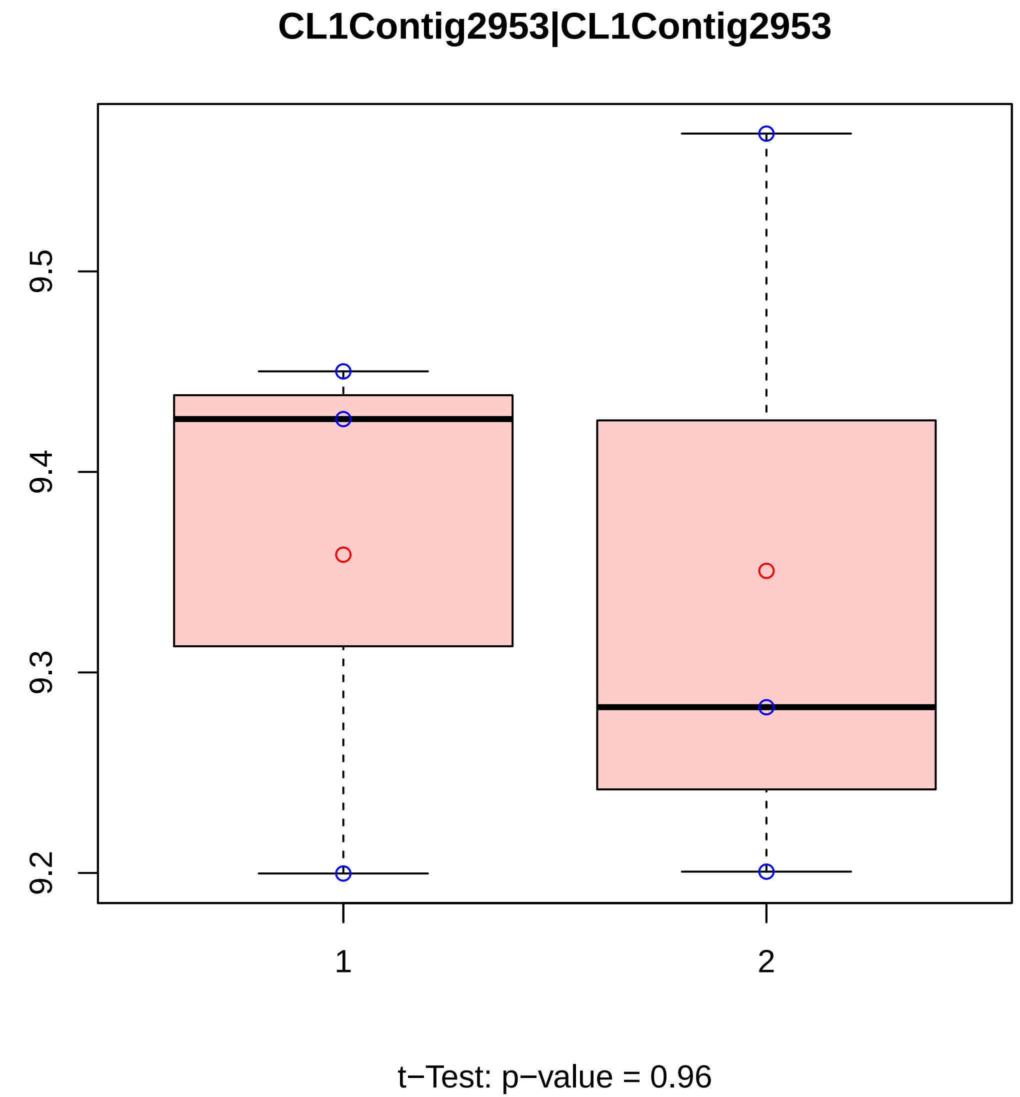
CL1Contig2953|CL1Contig2953
9.2
9.3
9.4
9.5
1
2
t−Test: p−value = 0.96

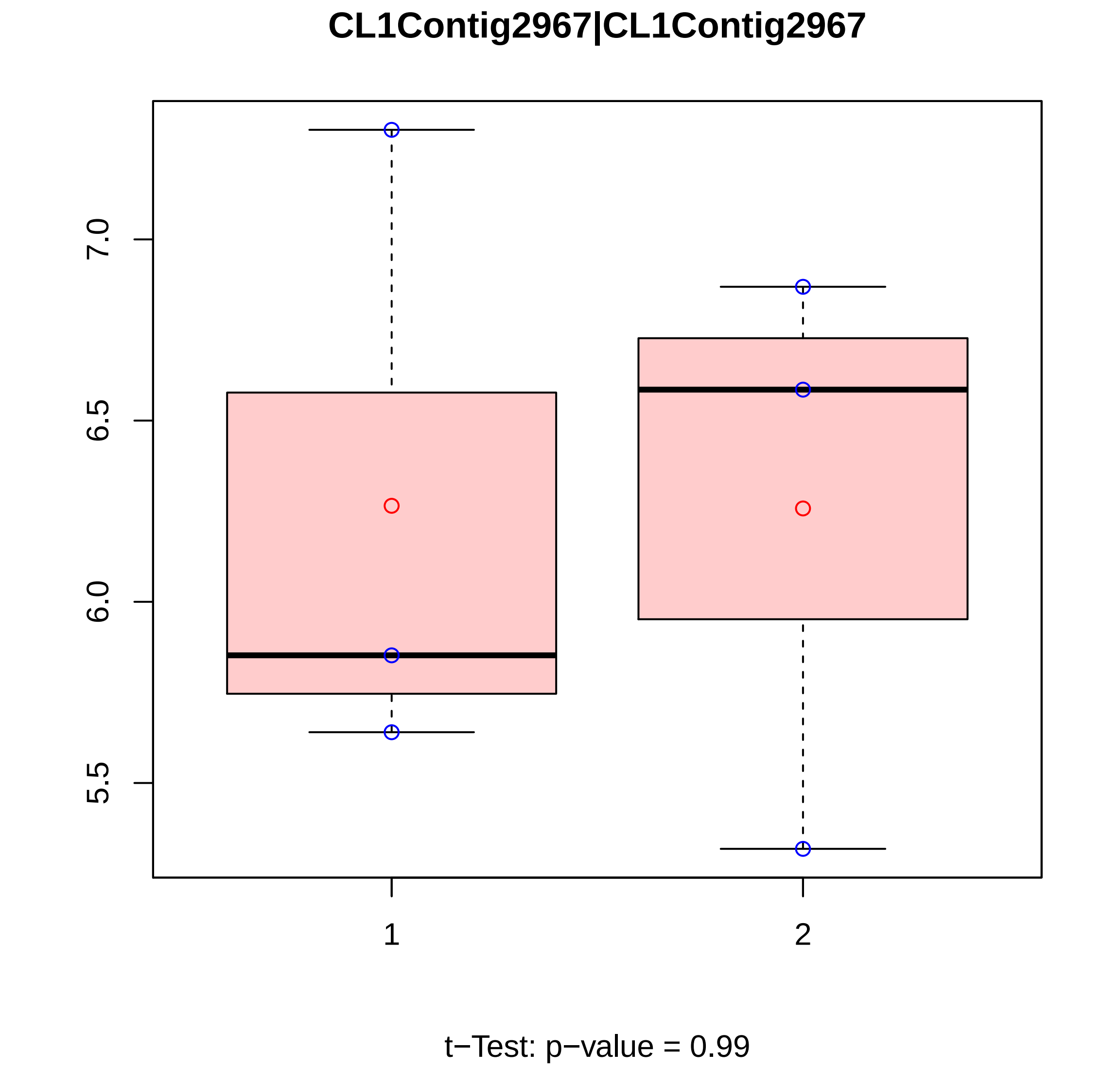
CL1Contig2967|CL1Contig2967
7.0
6.5
6.0
5.5
1
2
t−Test: p−value = 0.99

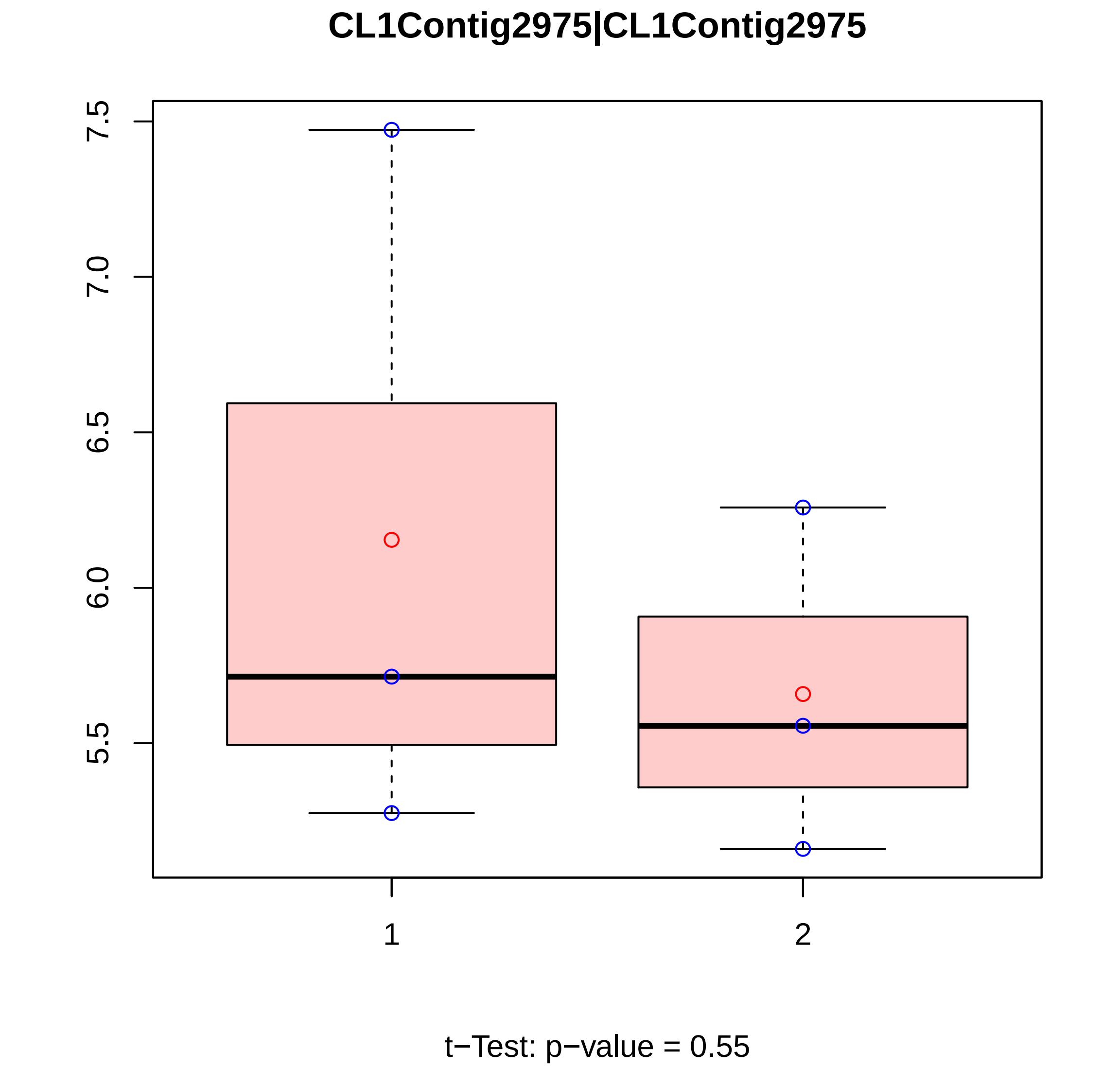

CL1Contig2975|CL1Contig2975
7.5
7.0
6.5
6.0
5.5
1
2
t−Test: p−value = 0.55

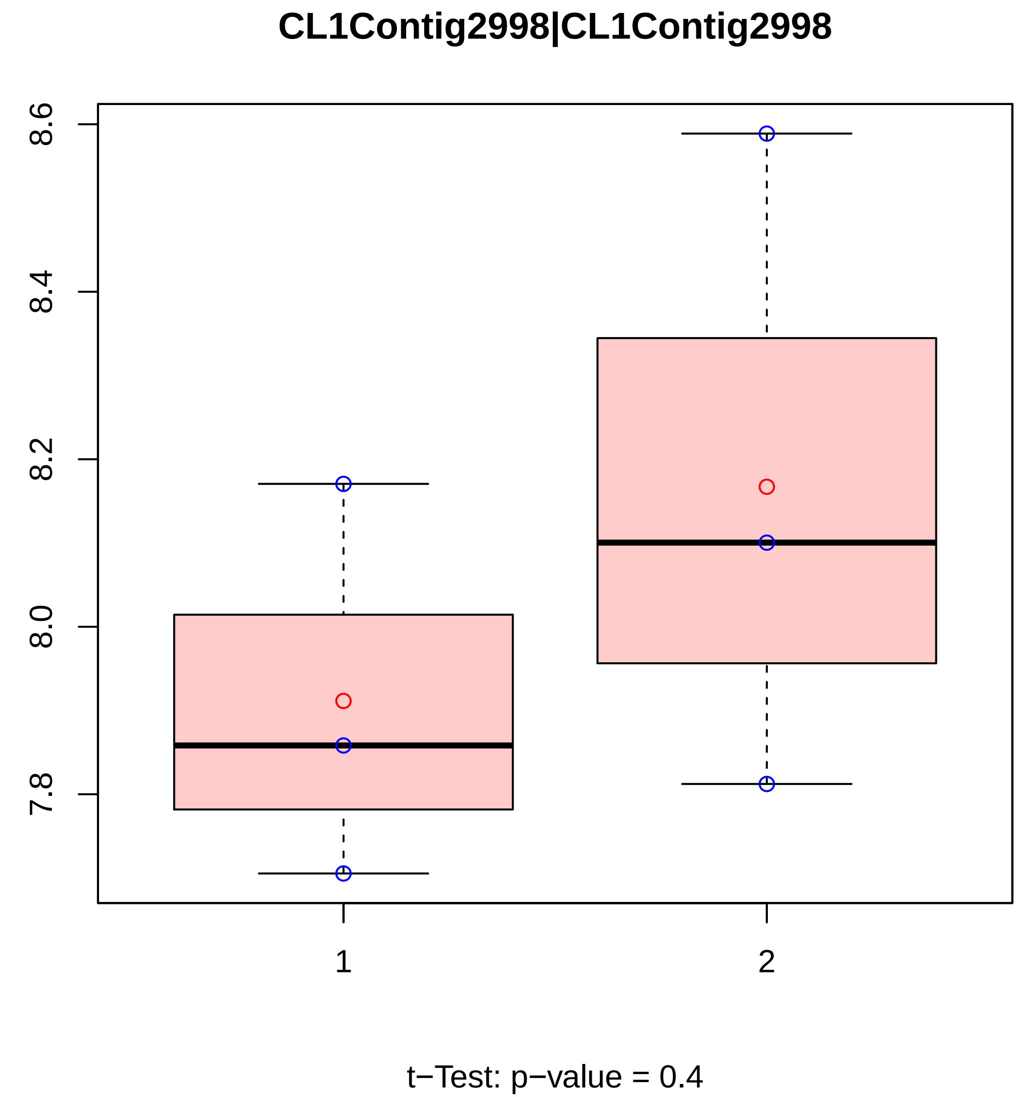
CL1Contig2998|CL1Contig2998
8.6
8.4
8.2
8.0
7.8
1
2
t−Test: p−value = 0.4

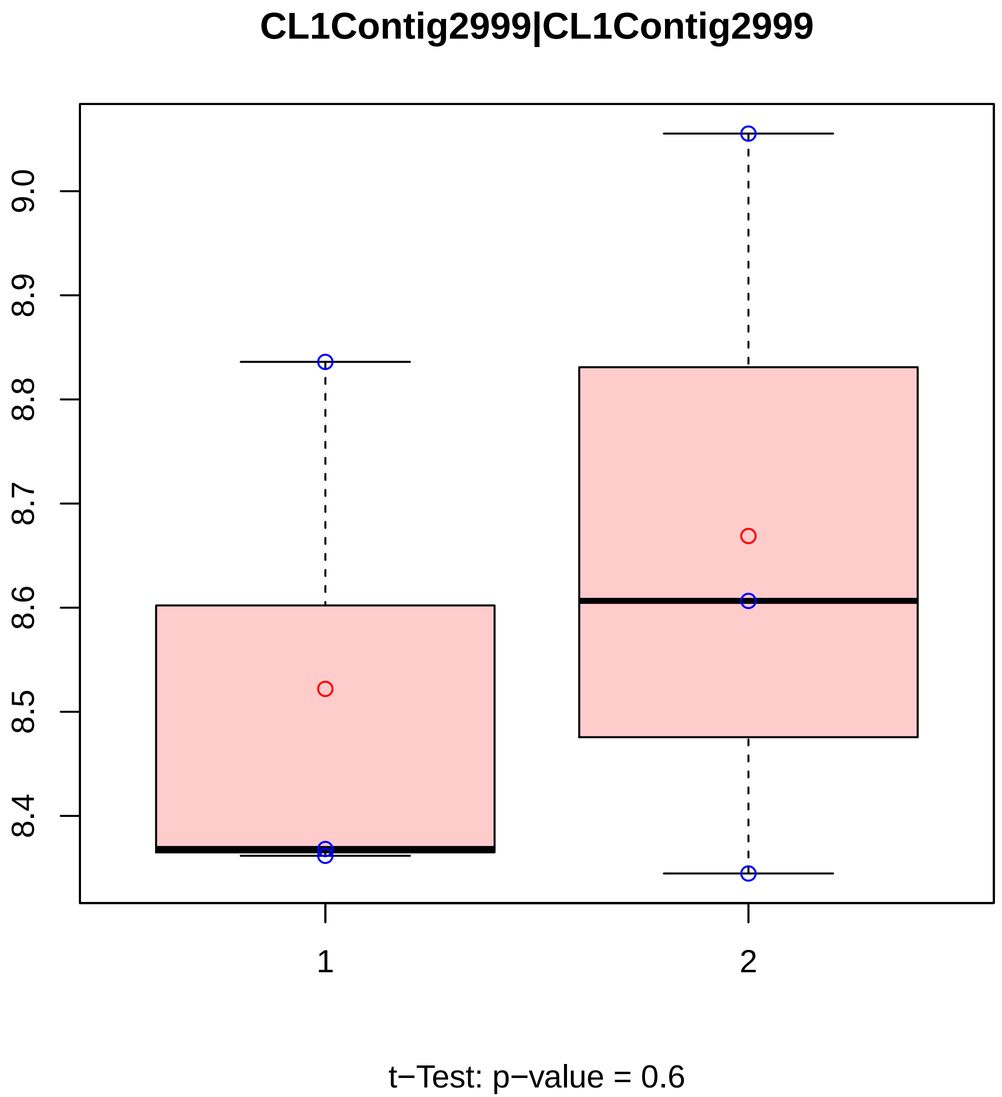
CL1Contig2999|CL1Contig2999
9.0
8.9
8.8
8.7
8.6
8.5
8.4
1
2
t−Test: p−value = 0.6

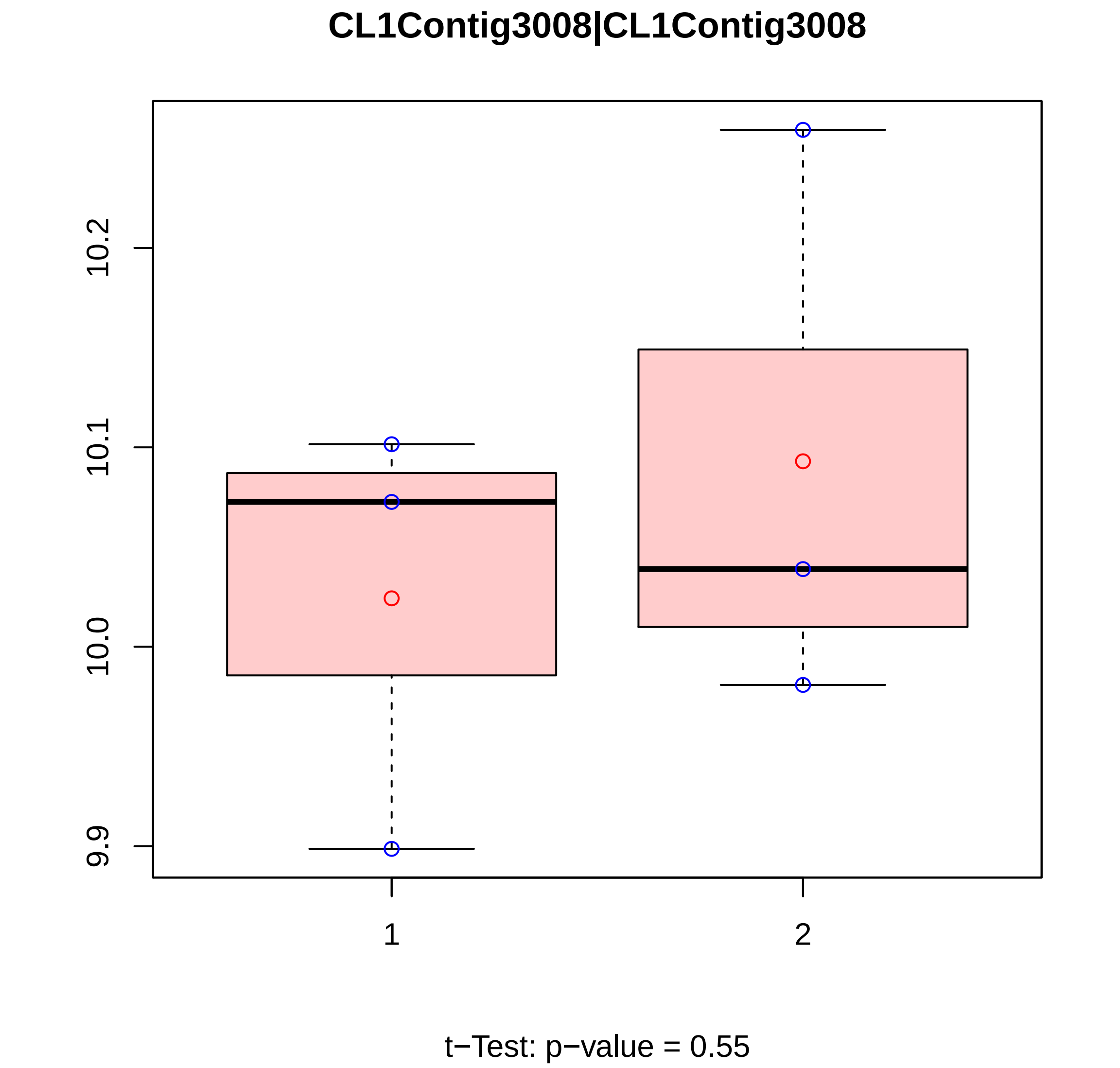

CL1Contig3008|CL1Contig3008
10.2
10.1
10.0
9.9
1
2
t−Test: p−value = 0.55

**CL1Contig3030|CL1Contig3030**

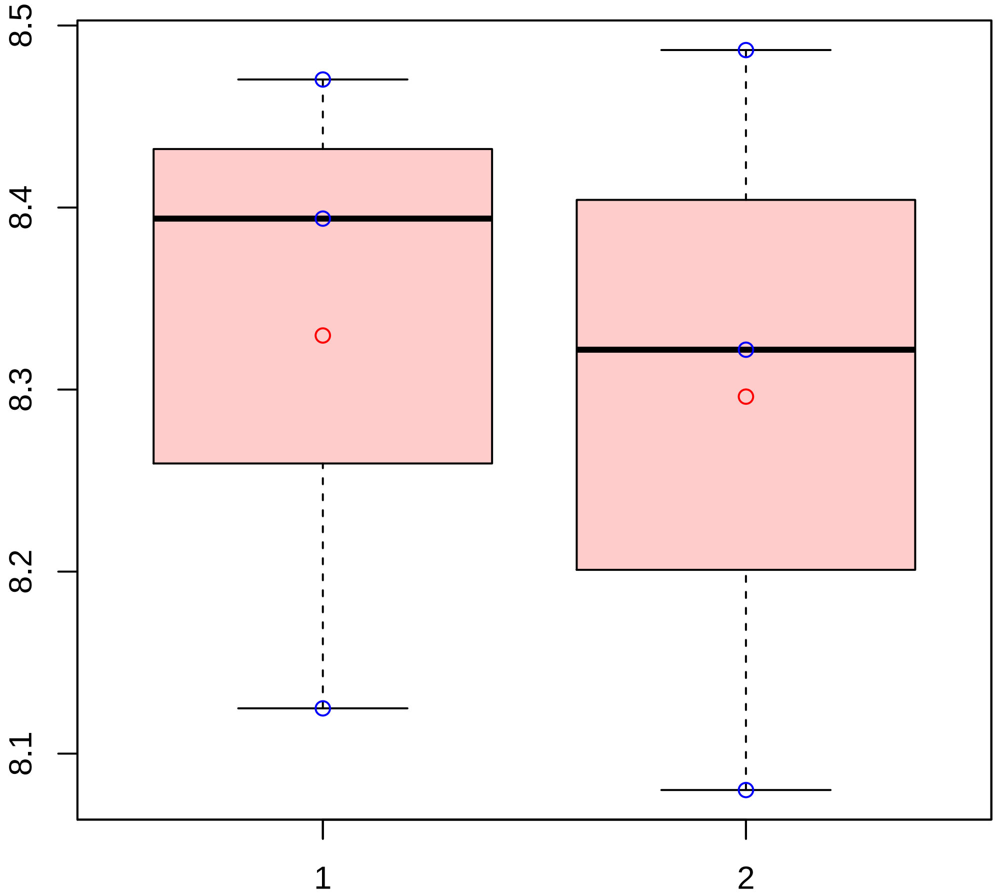


t−Test: p−value = 0.84

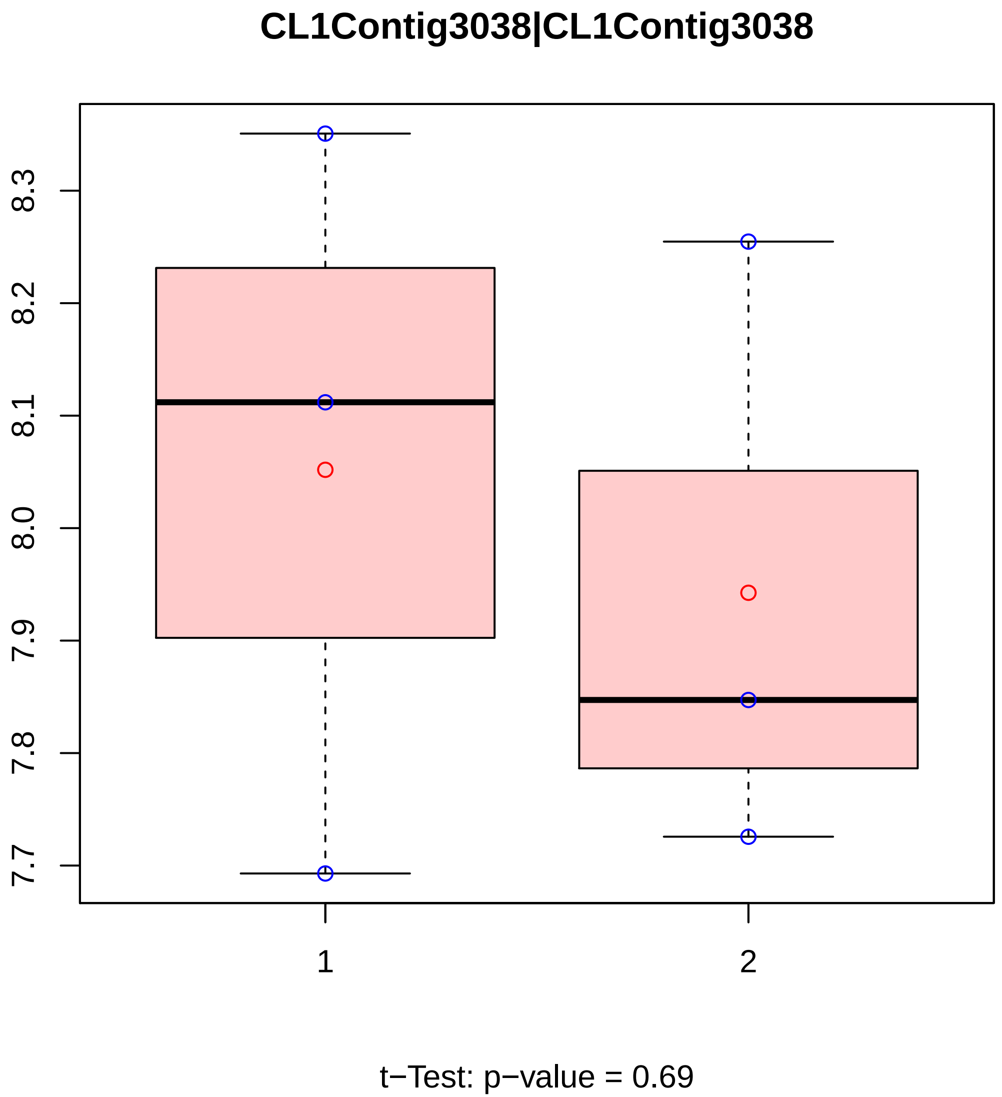
CL1Contig3038|CL1Contig3038
8.3
8.2
8.1
8.0
7.9
7.8
7.7
1
2
t−Test: p−value = 0.69

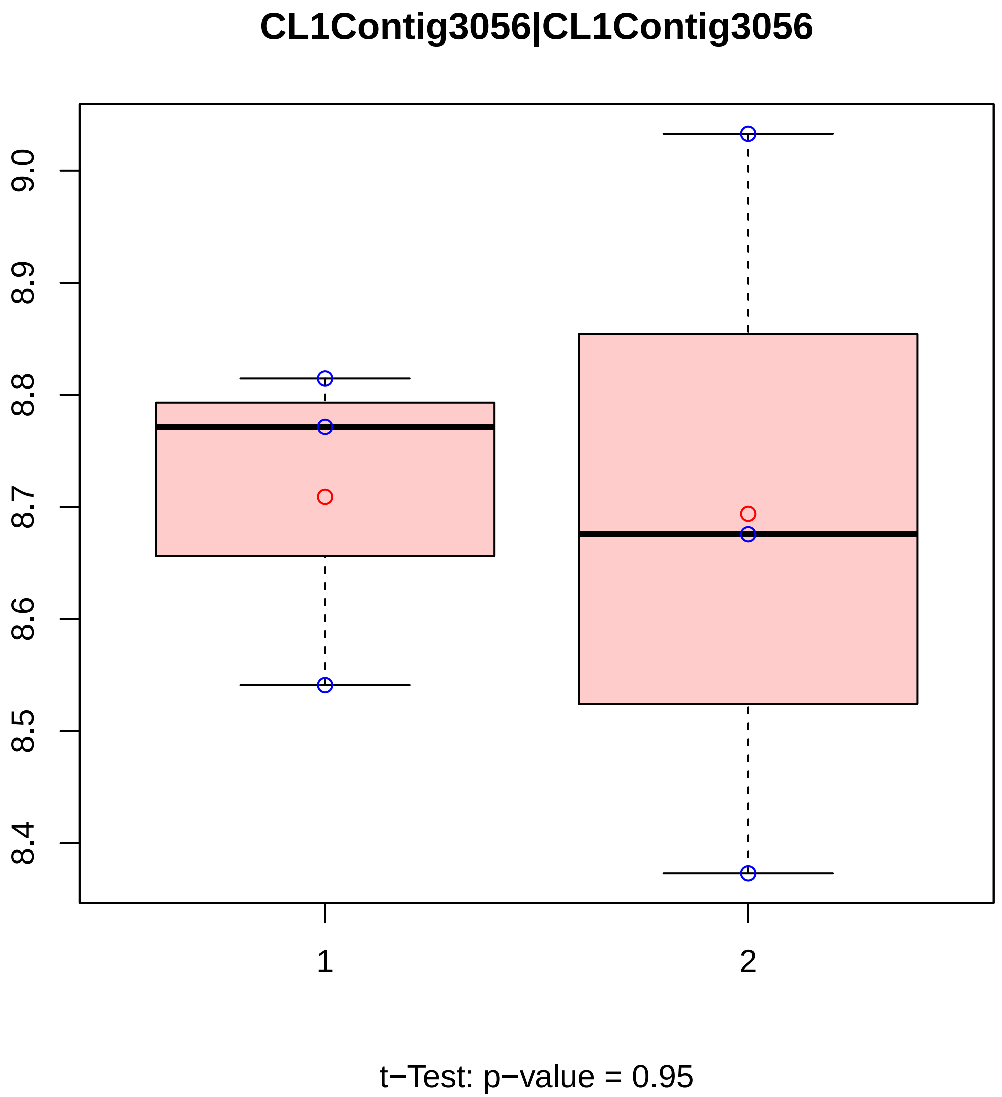
CL1Contig3056|CL1Contig3056
9.0
8.9
8.8
8.7
8.6
8.5
8.4
1
2
t−Test: p−value = 0.95

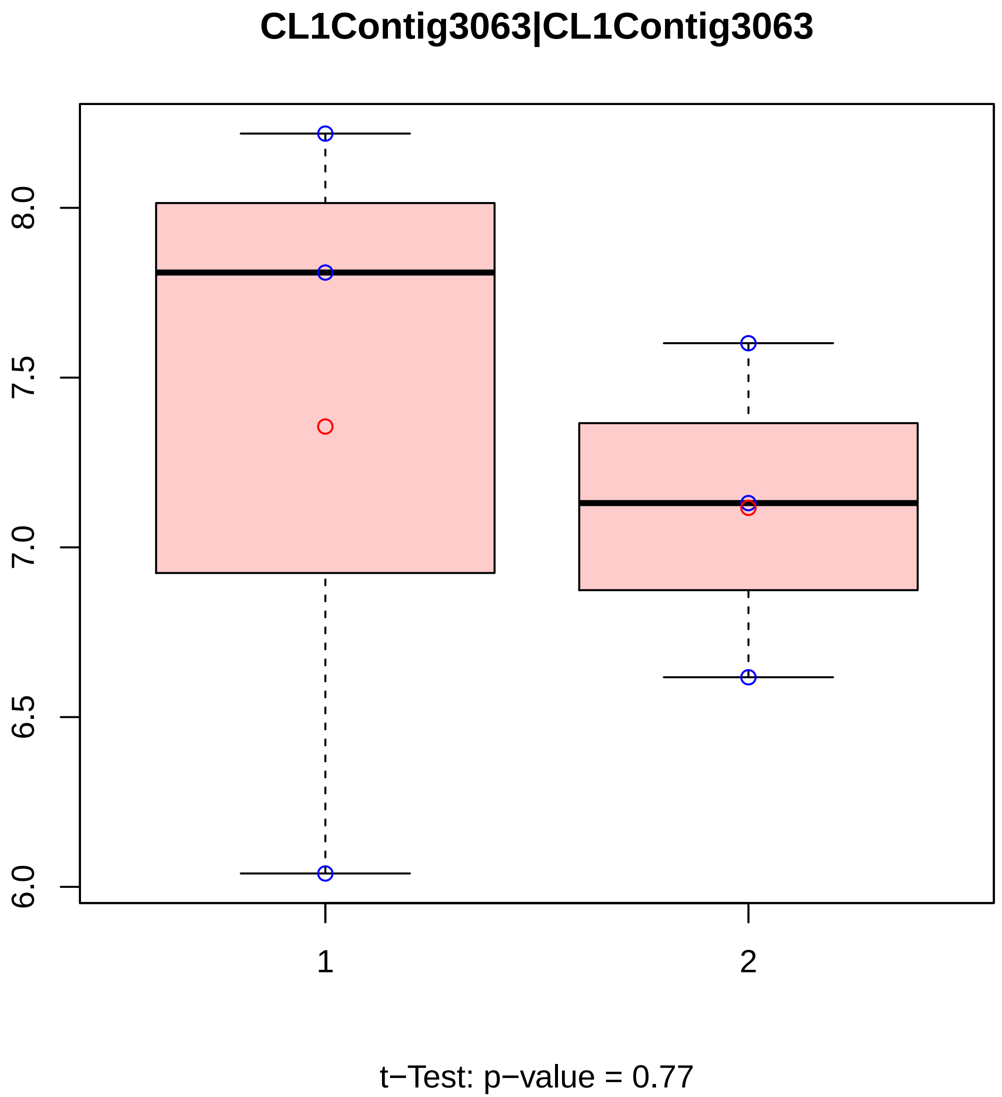
CL1Contig3063|CL1Contig3063
8.0
7.5
7.0
6.5
6.0
1
2
t−Test: p−value = 0.77

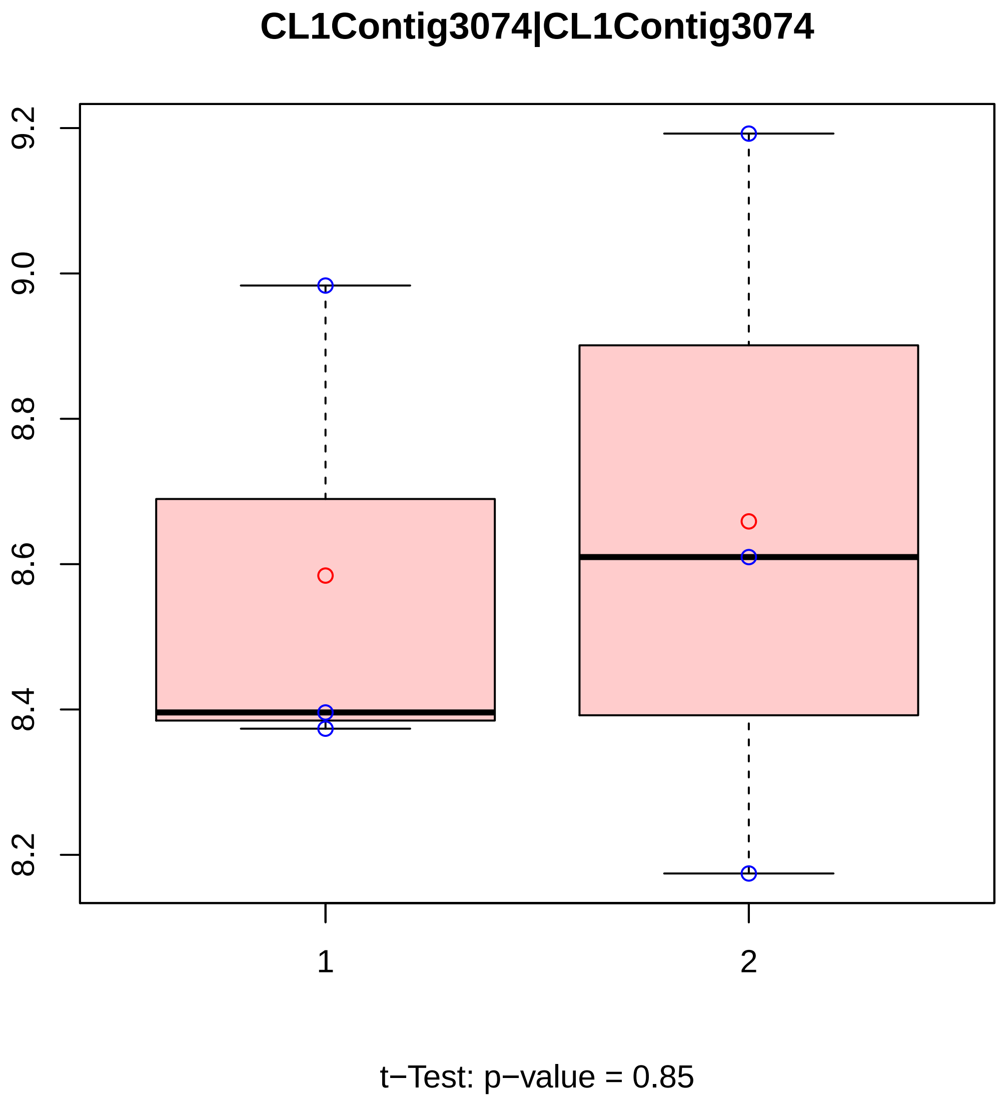
CL1Contig3074|CL1Contig3074
9.2
9.0
8.8
8.6
8.4
8.2
1
2
t−Test: p−value = 0.85

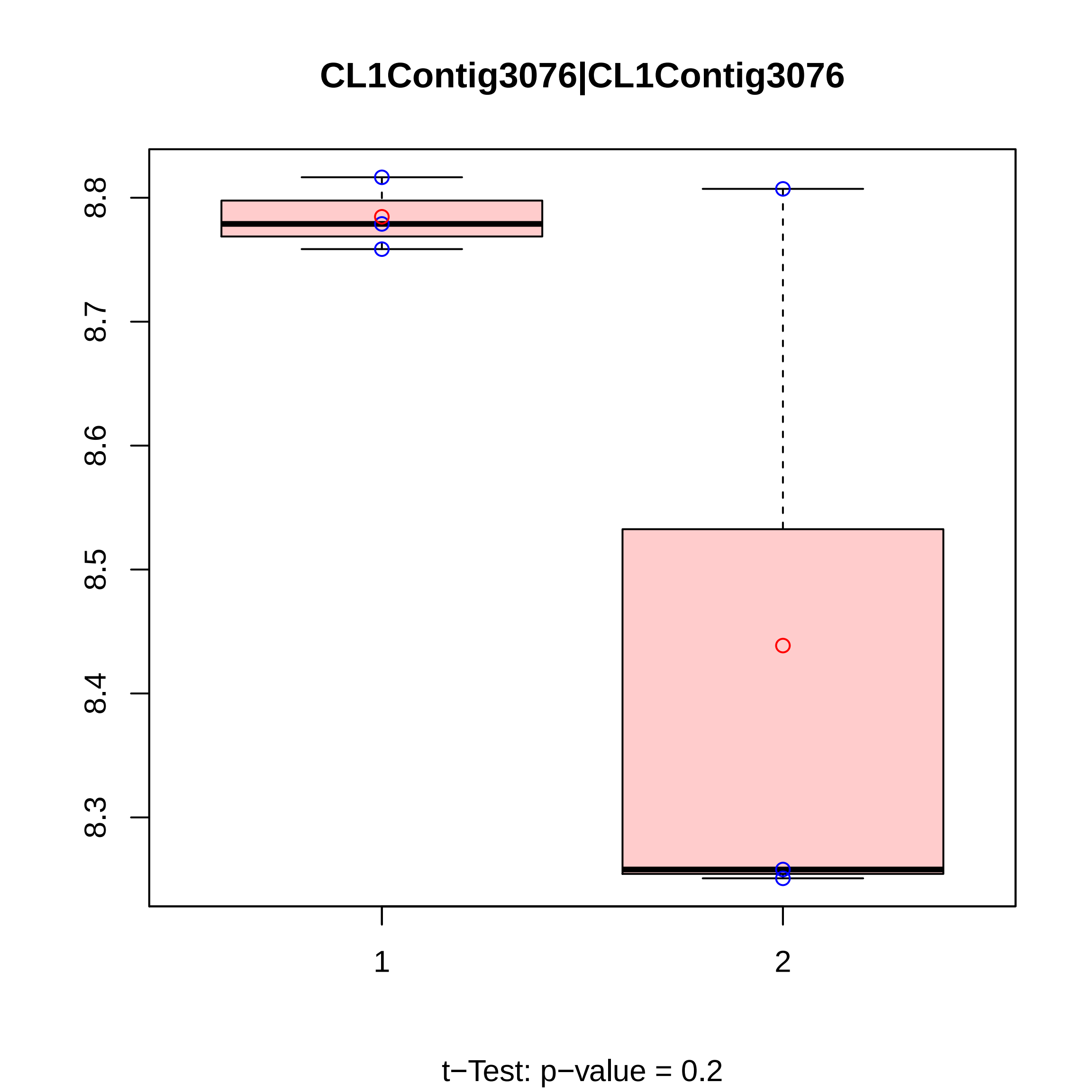
CL1Contig3076|CL1Contig3076
8.3
8.4
8.5
8.6
8.7
8.8
1
2
t−Test: p−value = 0.2

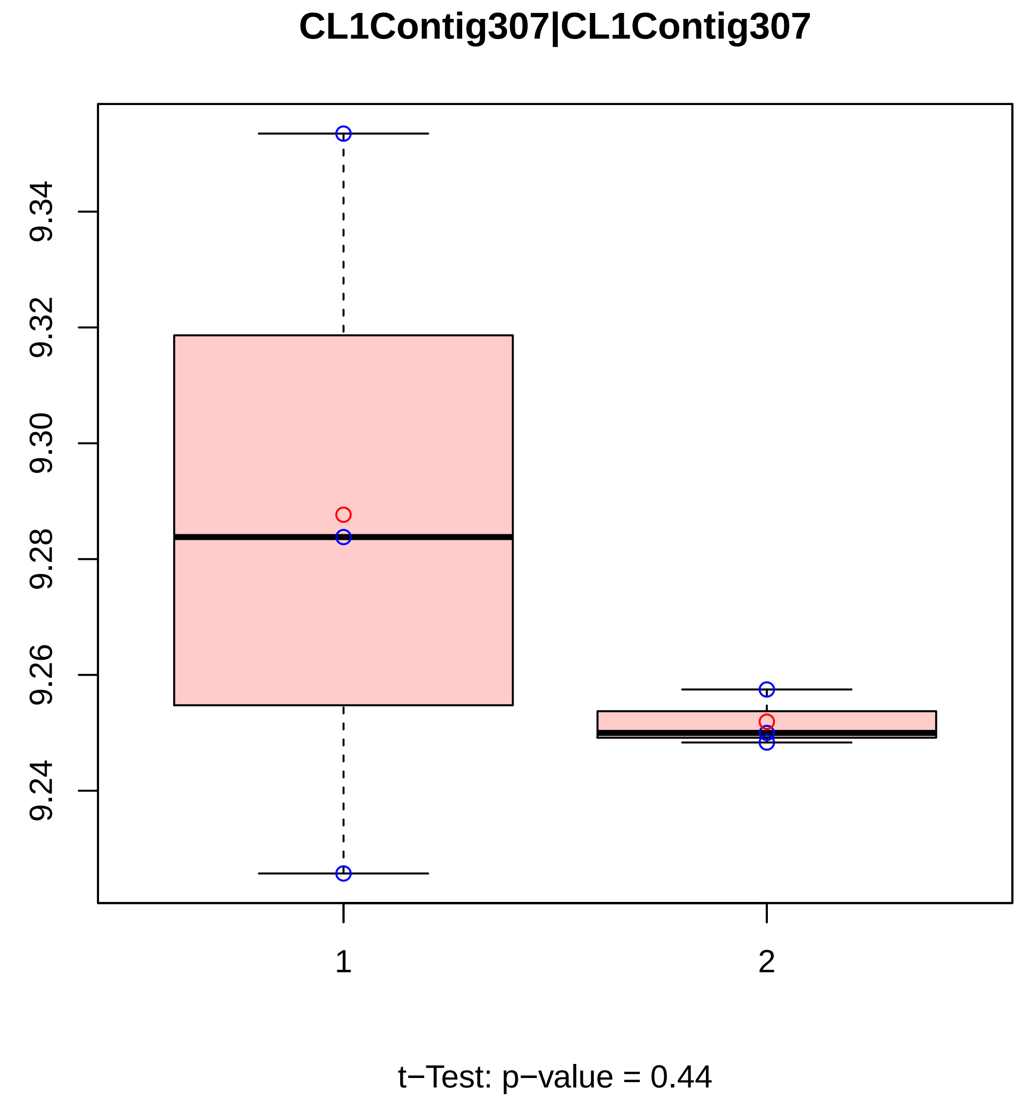

CL1Contig307|CL1Contig307
9.24
9.26
9.28
9.30
9.32
9.34
1
2
t−Test: p−value = 0.44

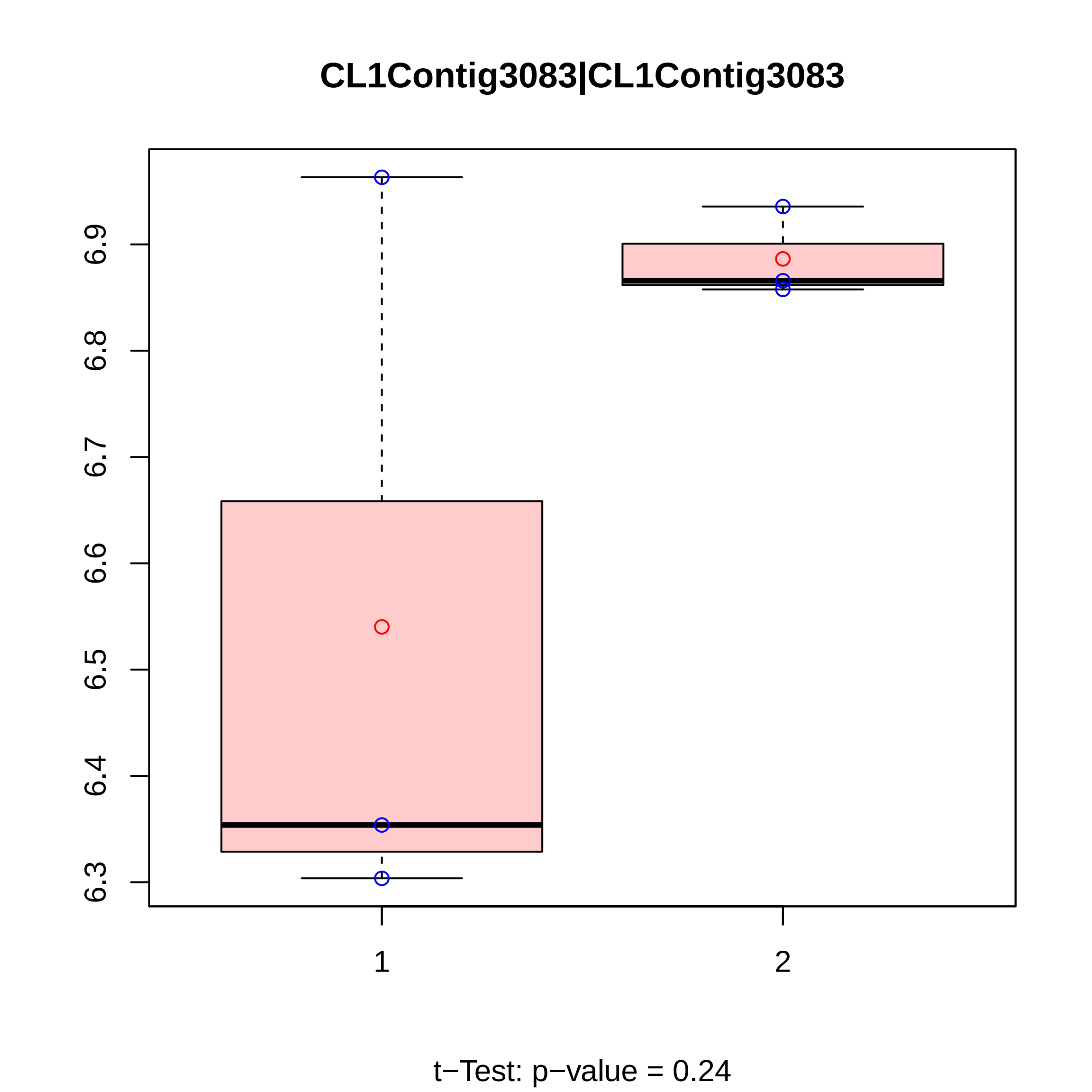

CL1Contig3083|CL1Contig3083
6.3
6.4
6.5
6.6
6.7
6.8
6.9
1
2
t−Test: p−value = 0.24

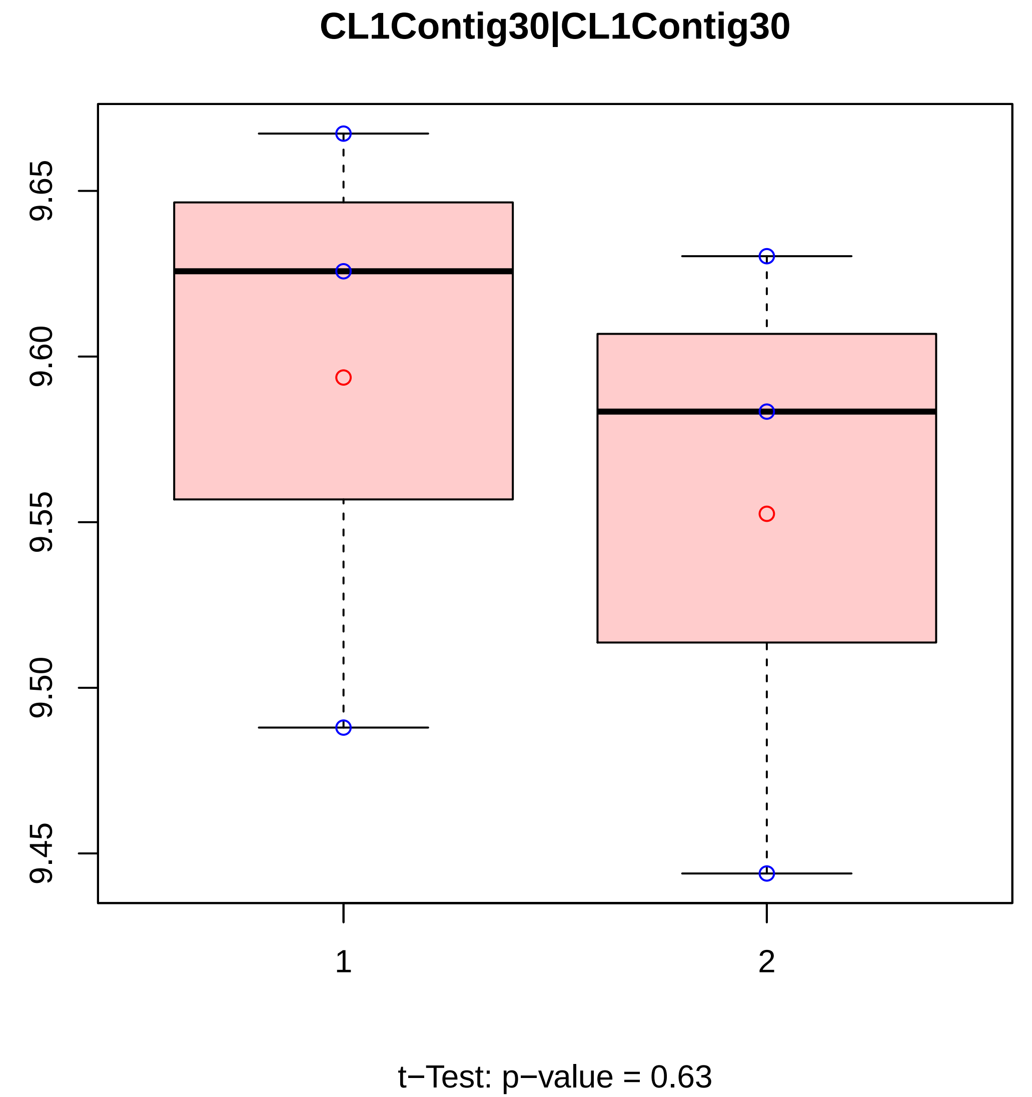
CL1Contig30|CL1Contig30
9.45
9.50
9.55
9.60
9.65
1
2
t−Test: p−value = 0.63

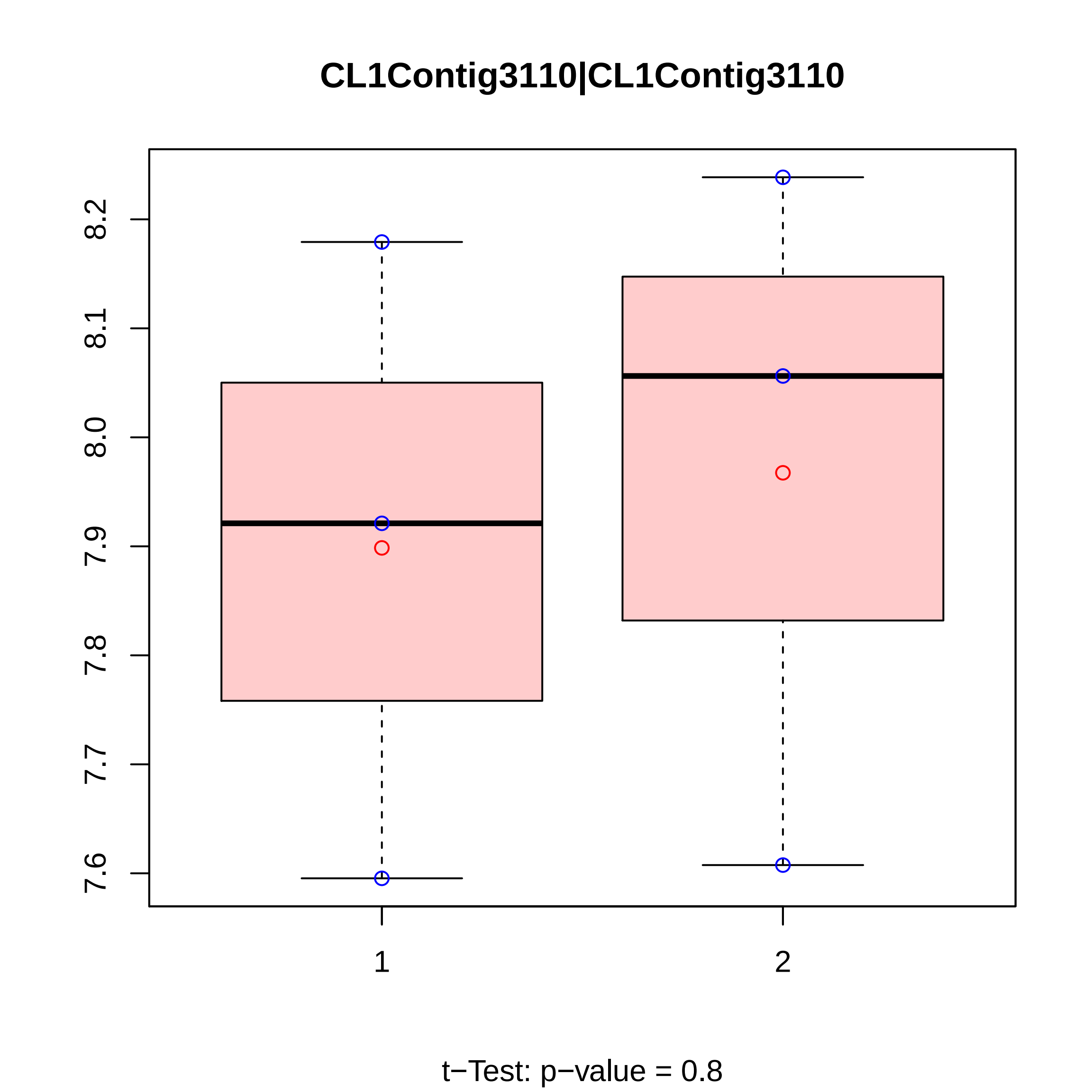
CL1Contig3110|CL1Contig3110
8.2
8.1
8.0
7.9
7.8
7.7
7.6
1
2
t−Test: p−value = 0.8

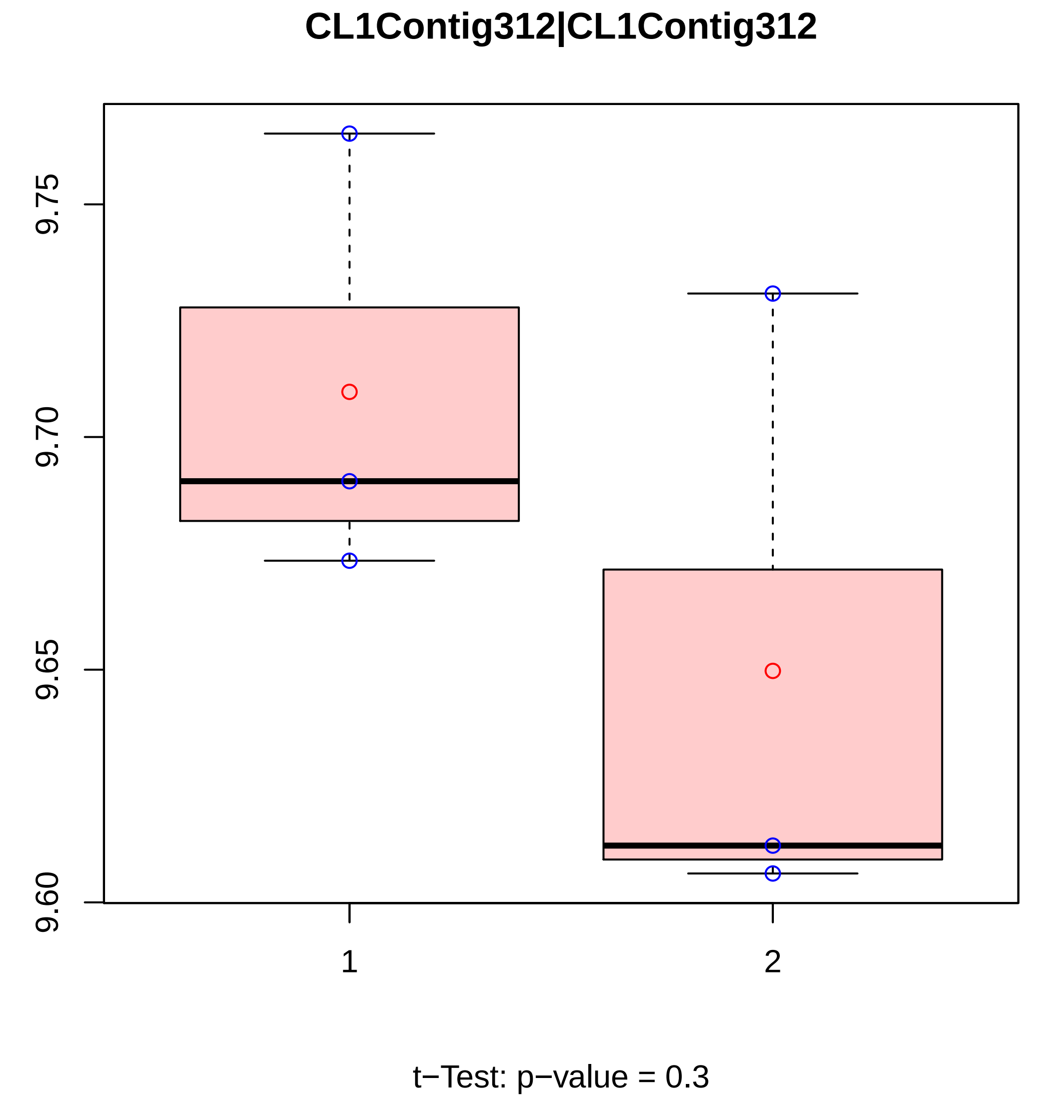
CL1Contig312|CL1Contig312
9.60
9.65
9.70
9.75
1
2
t−Test: p−value = 0.3

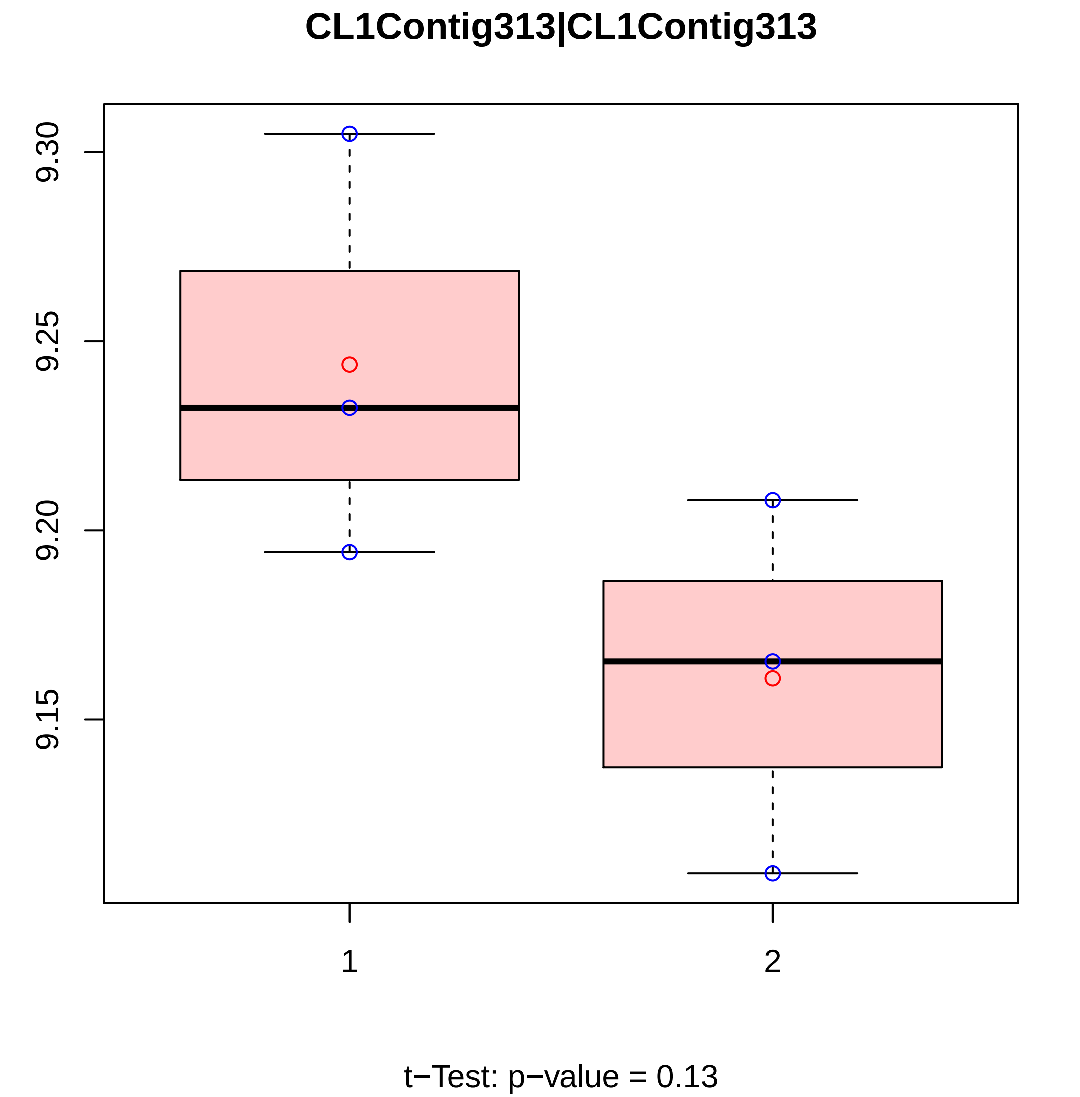
CL1Contig313|CL1Contig313
9.30
9.25
9.20
9.15
1
2
t−Test: p−value = 0.13

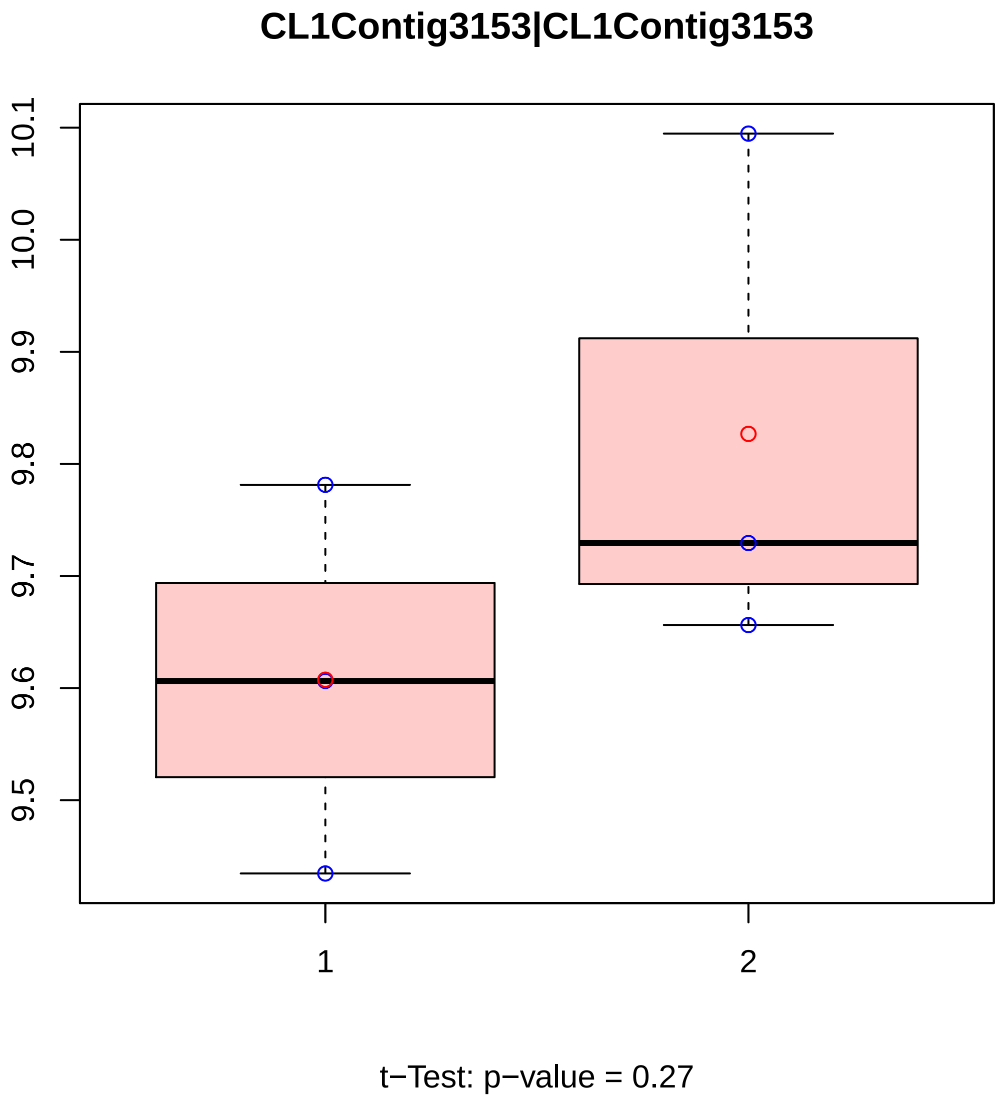
CL1Contig3153|CL1Contig3153
10.1
10.0
9.9
9.8
9.7
9.6
9.5
1
2
t−Test: p−value = 0.27

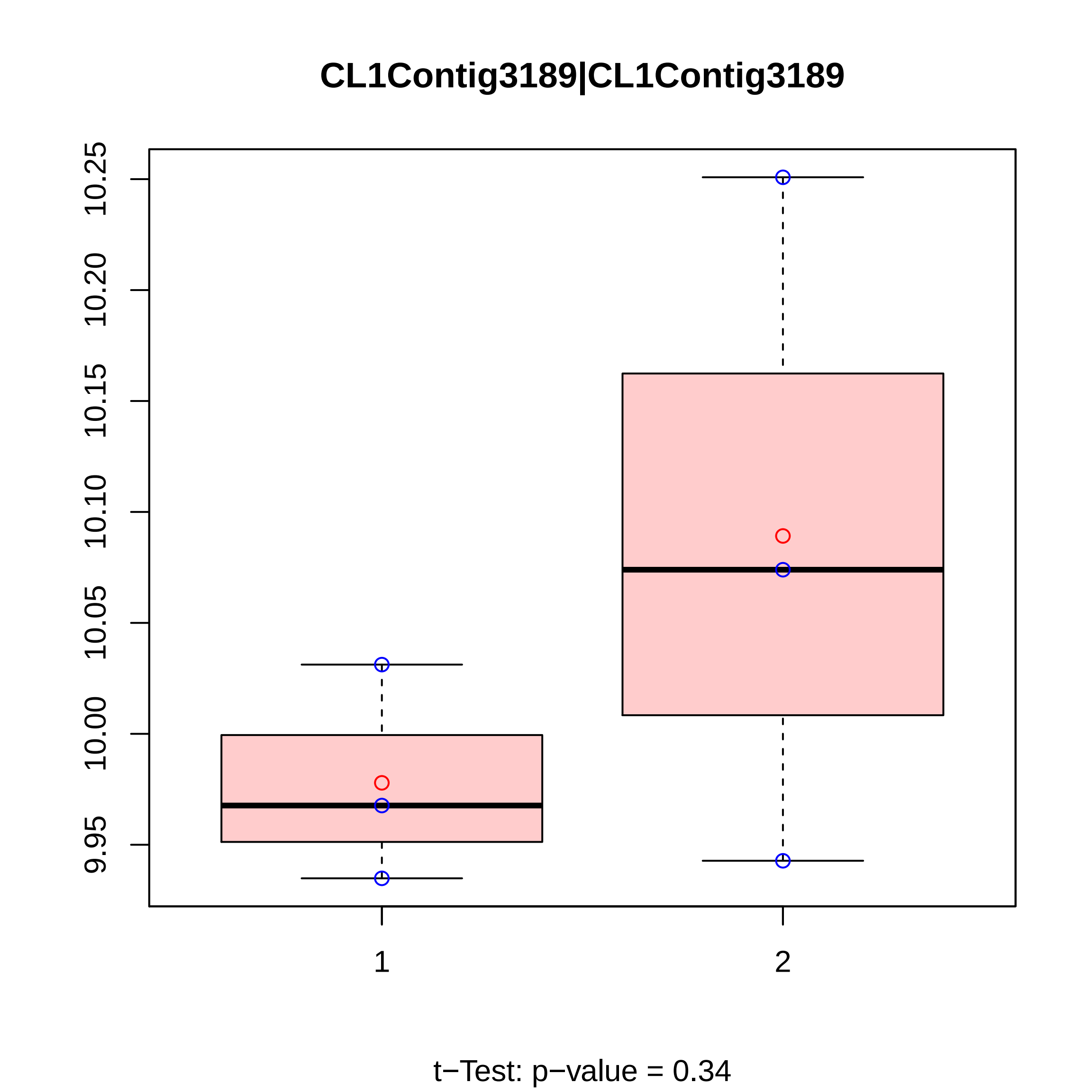
CL1Contig3189|CL1Contig3189
10.25
10.20
10.15
10.10
10.05
10.00
9.95
1
2
t−Test: p−value = 0.34

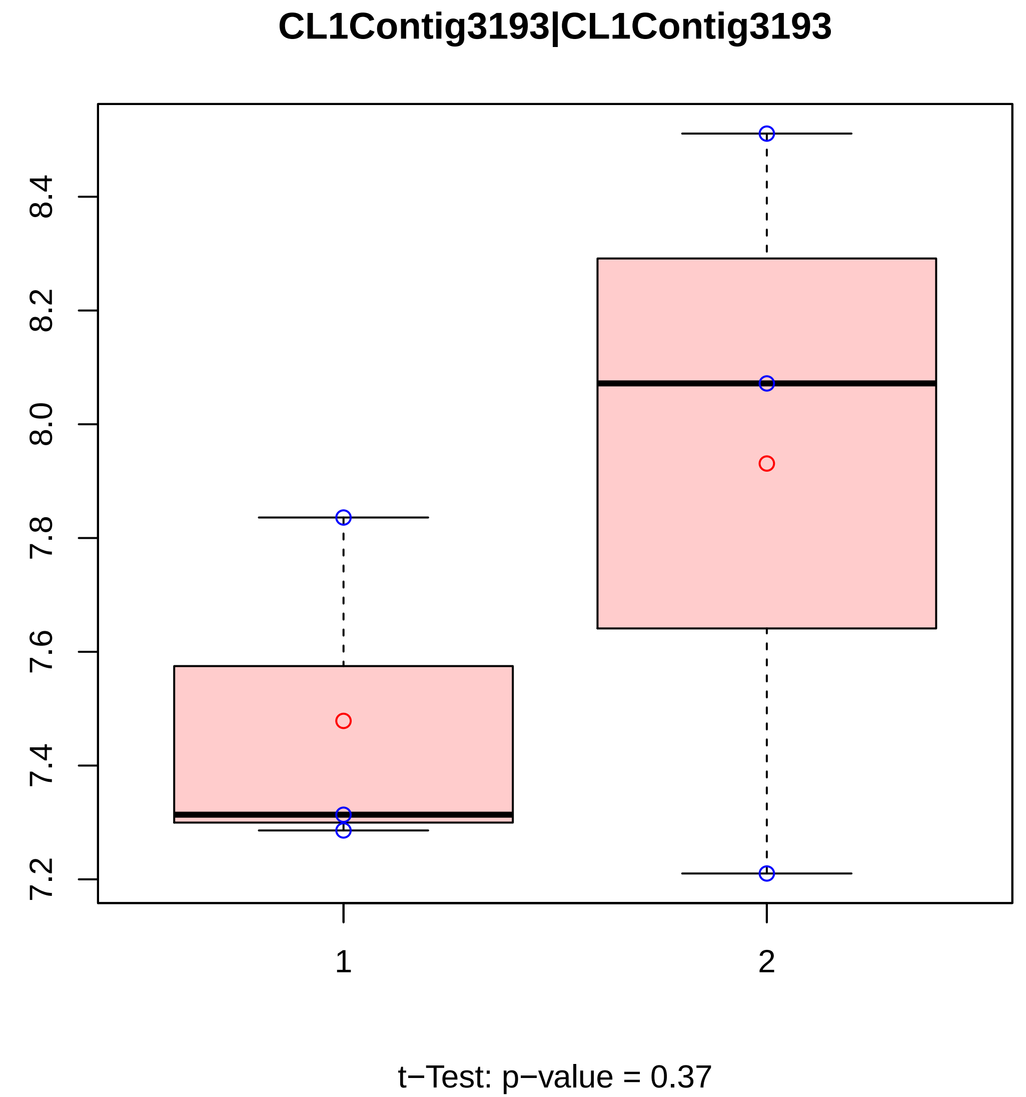

CL1Contig3193|CL1Contig3193
8.4
8.2
8.0
7.8
7.6
7.4
7.2
1
2
t−Test: p−value = 0.37

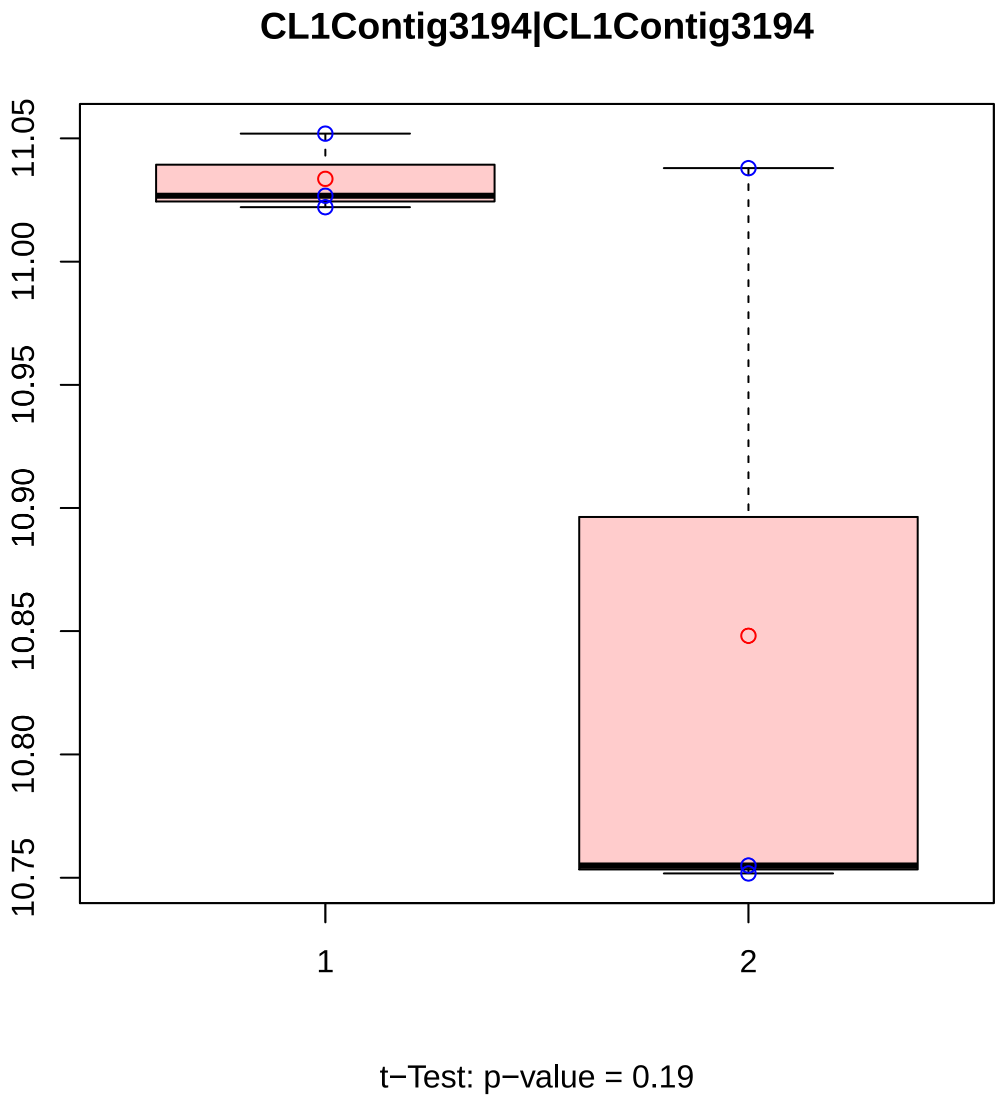
CL1Contig3194|CL1Contig3194
11.05
11.00
10.95
10.90
10.85
10.80
10.75
1
2
t−Test: p−value = 0.19

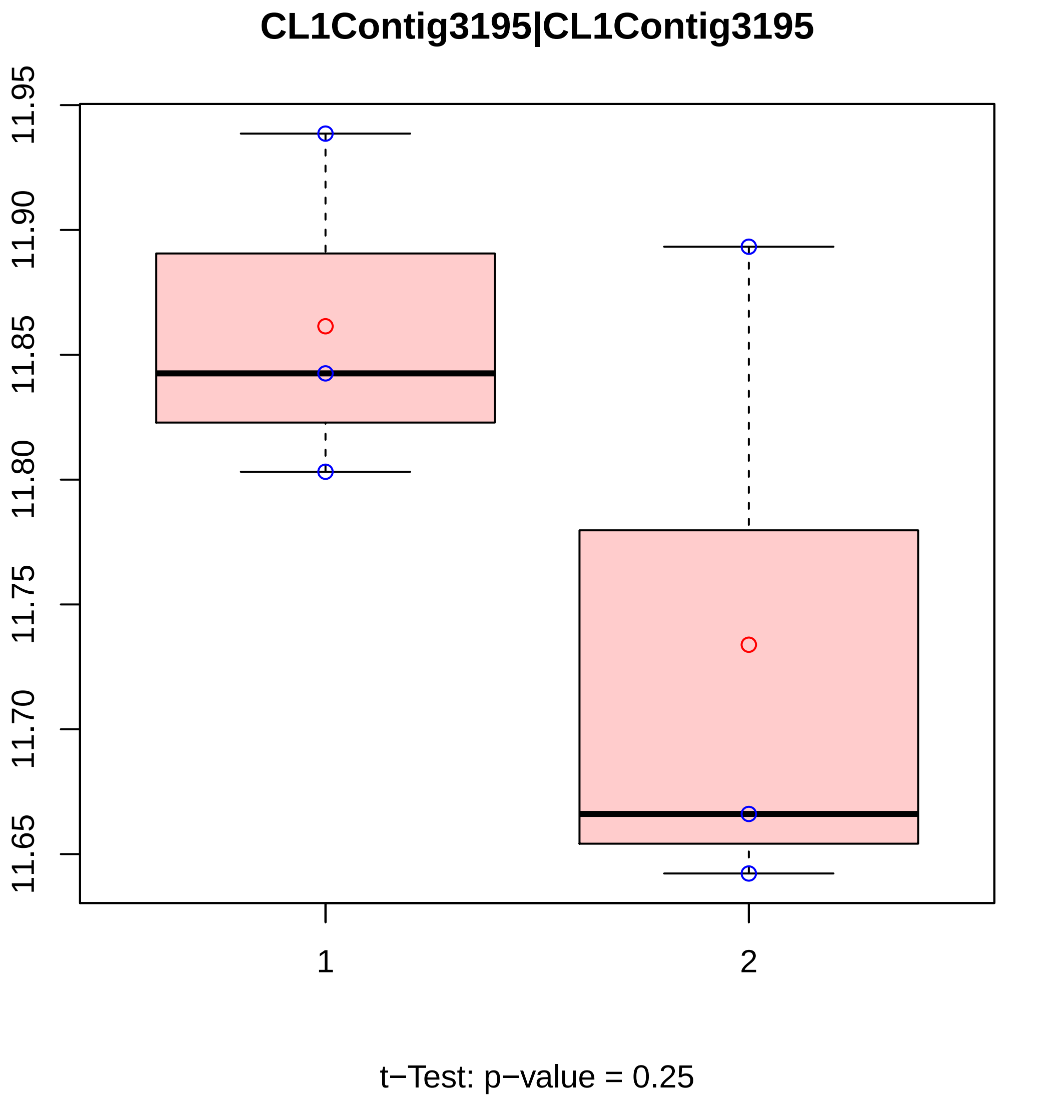

CL1Contig3195|CL1Contig3195
11.95
11.90
11.85
11.80
11.75
11.70
11.65
1
2
t−Test: p−value = 0.25

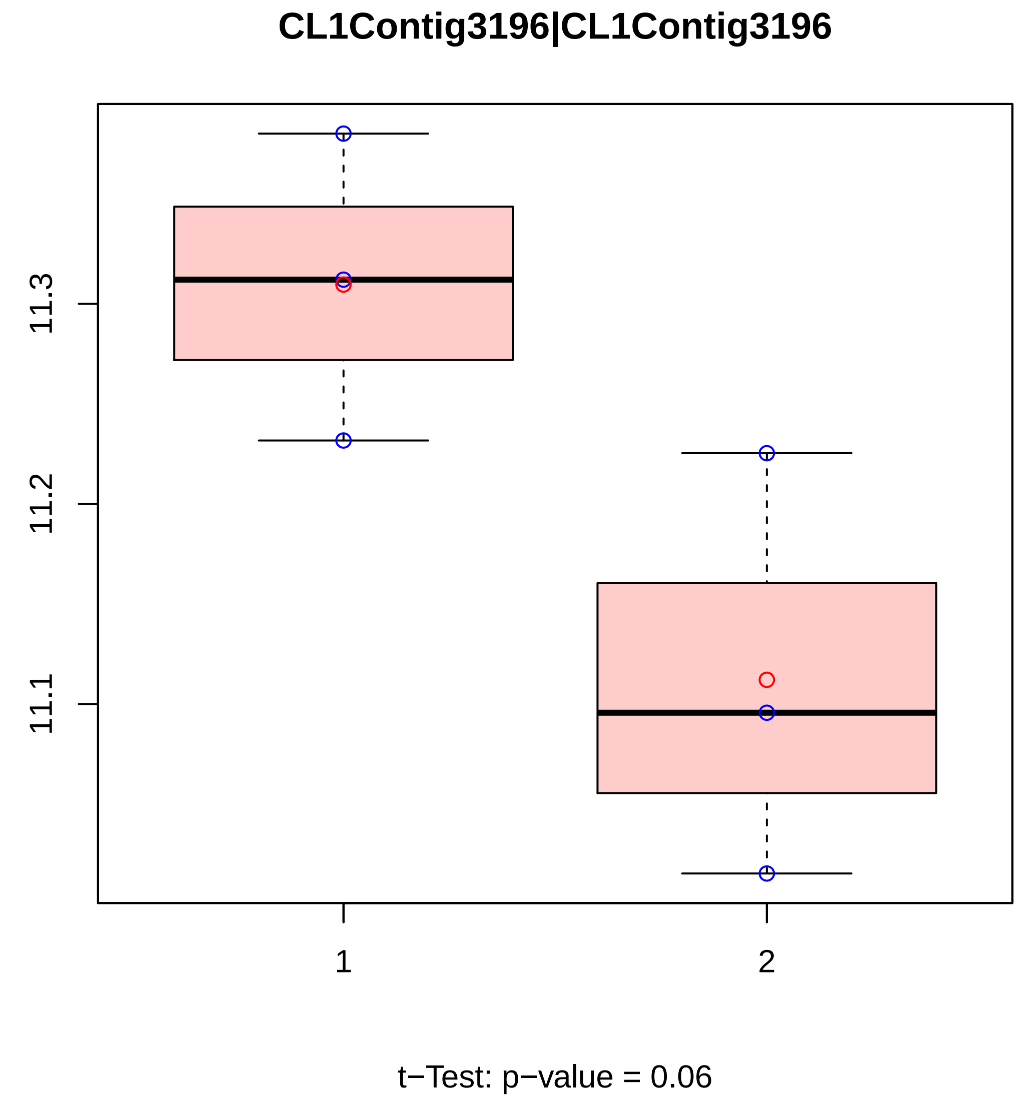

CL1Contig3196|CL1Contig3196
11.1
11.2
11.3
1
2
t−Test: p−value = 0.06

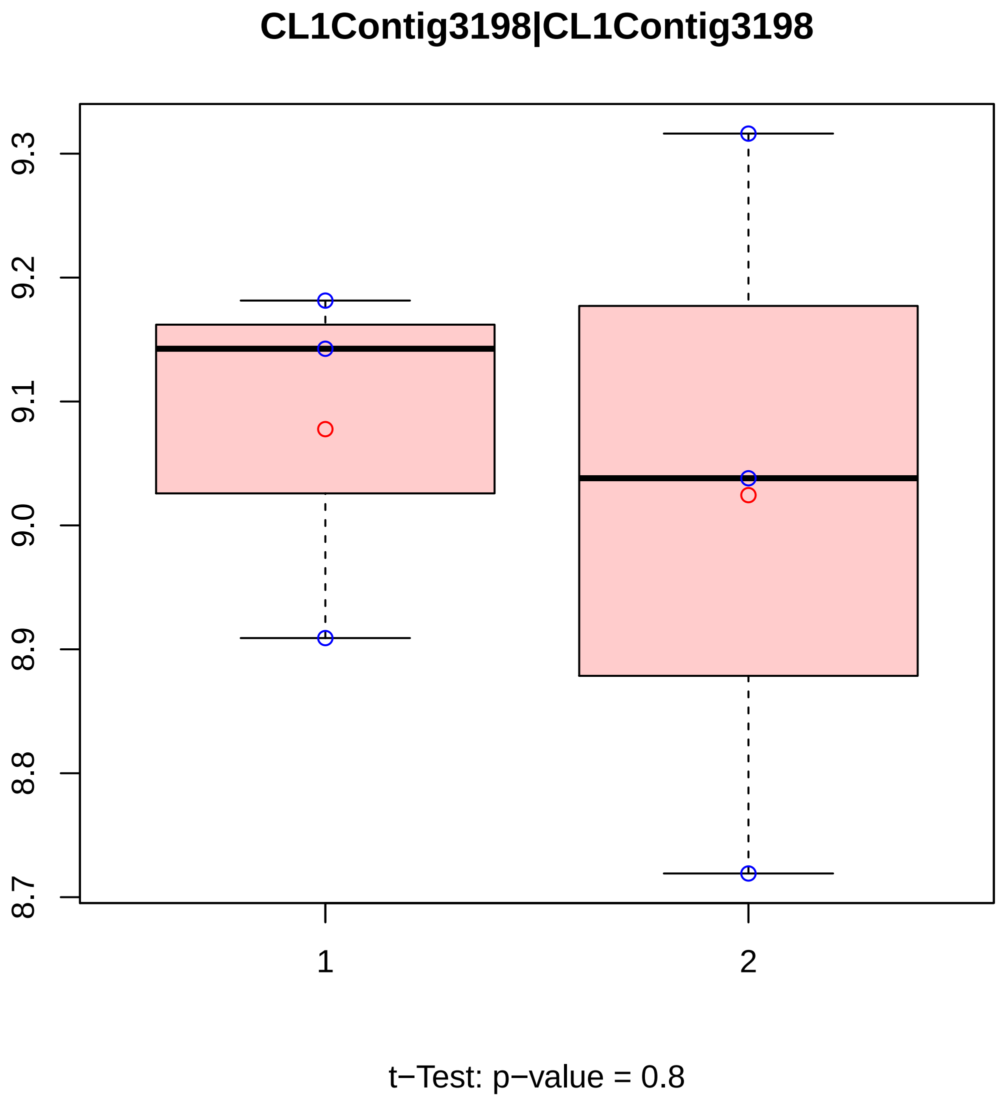

CL1Contig3198|CL1Contig3198
9.3
9.2
9.1
9.0
8.9
8.8
8.7
1
2
t−Test: p−value = 0.8

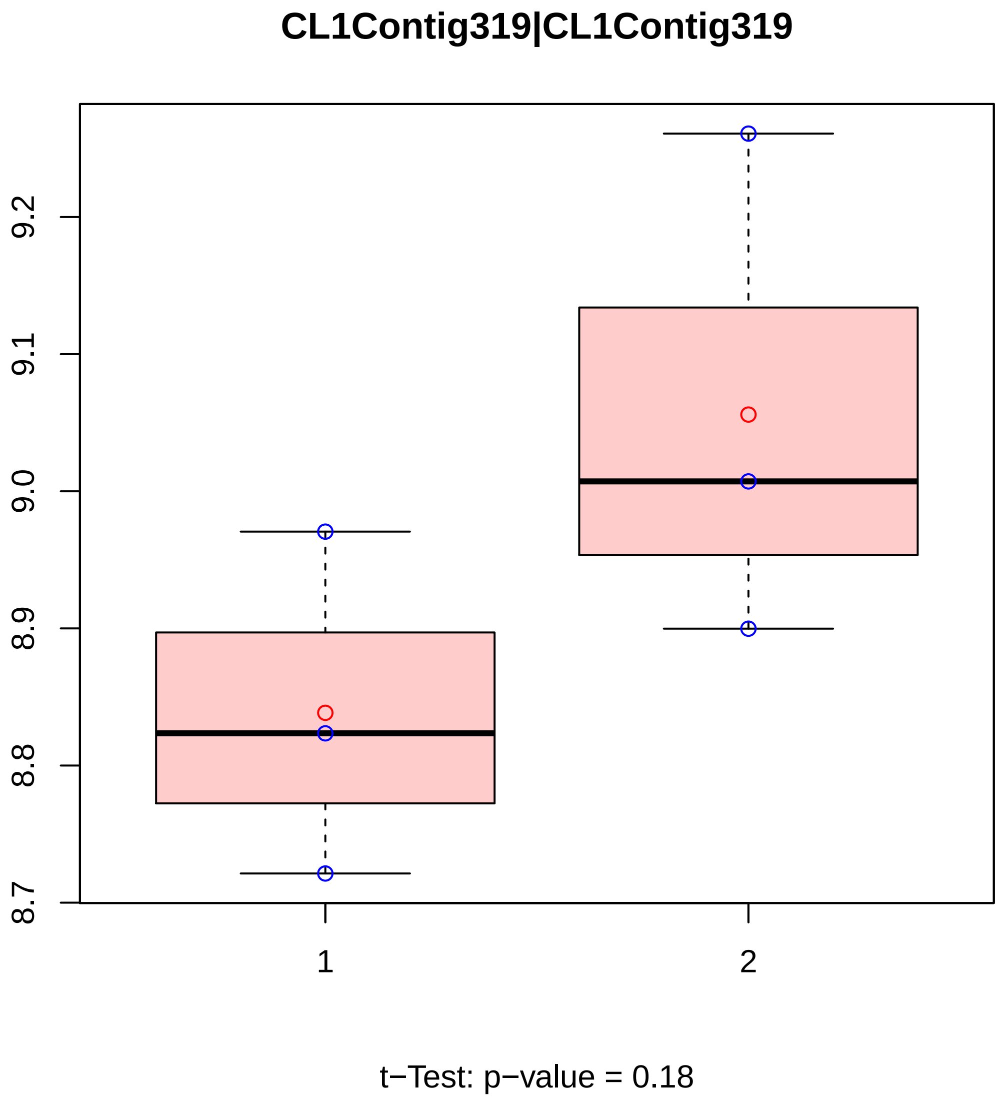
CL1Contig319|CL1Contig319
9.2
9.1
9.0
8.9
8.8
8.7
1
2
t−Test: p−value = 0.18

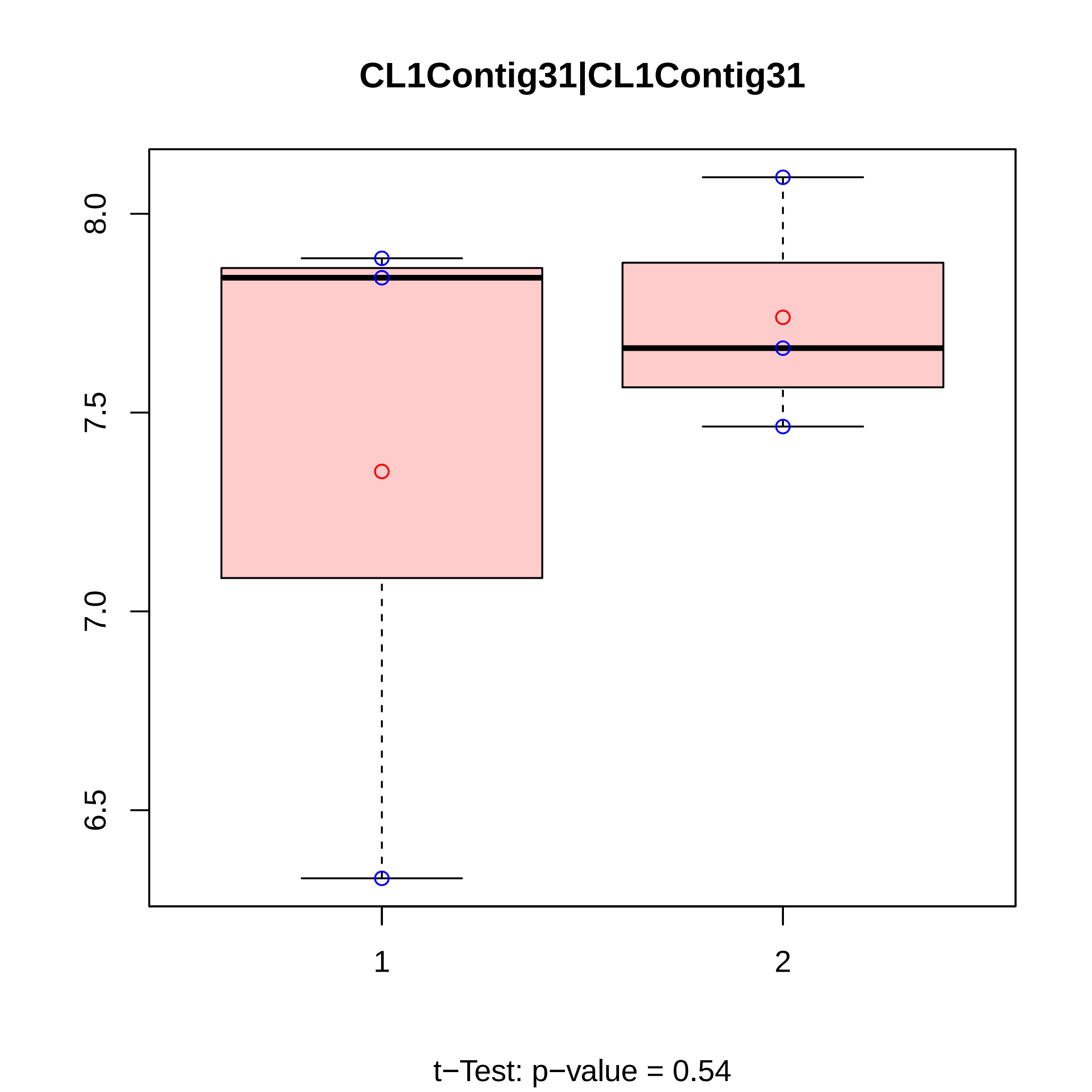

CL1Contig31|CL1Contig31
8.0
7.5
7.0
6.5
1
2
t−Test: p−value = 0.54

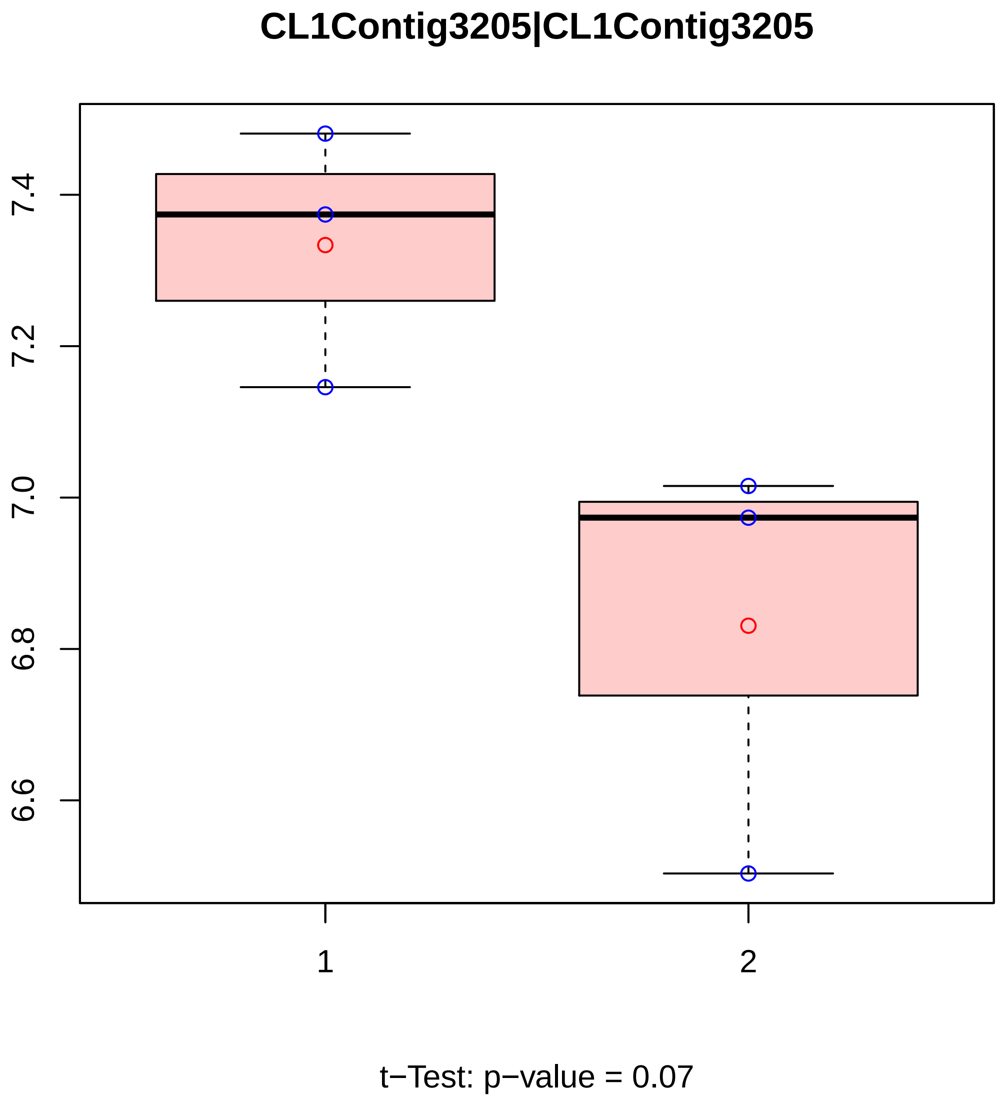
CL1Contig3205|CL1Contig3205
7.4
7.2
7.0
6.8
6.6
1
2
t−Test: p−value = 0.07

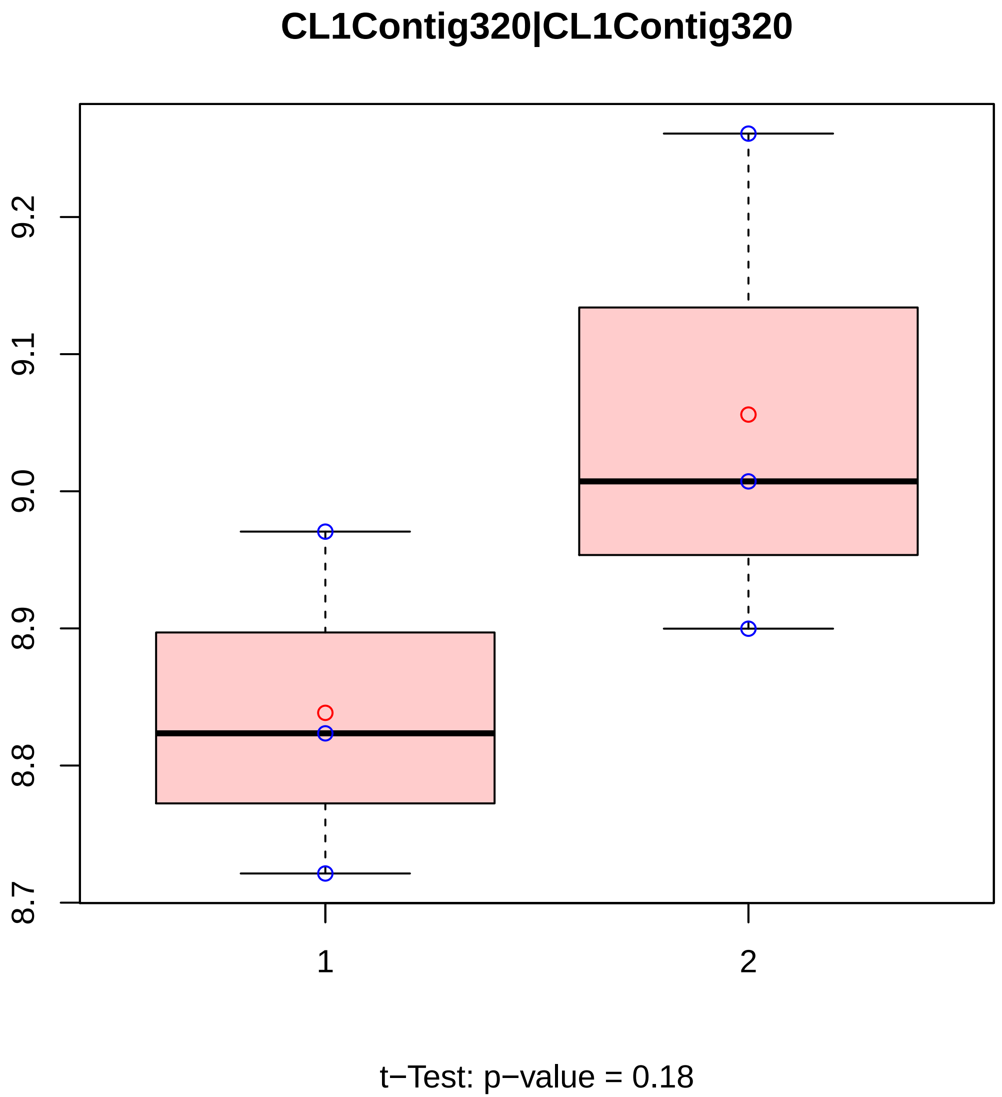

CL1Contig320|CL1Contig320
9.2
9.1
9.0
8.9
8.8
8.7
1
2
t−Test: p−value = 0.18

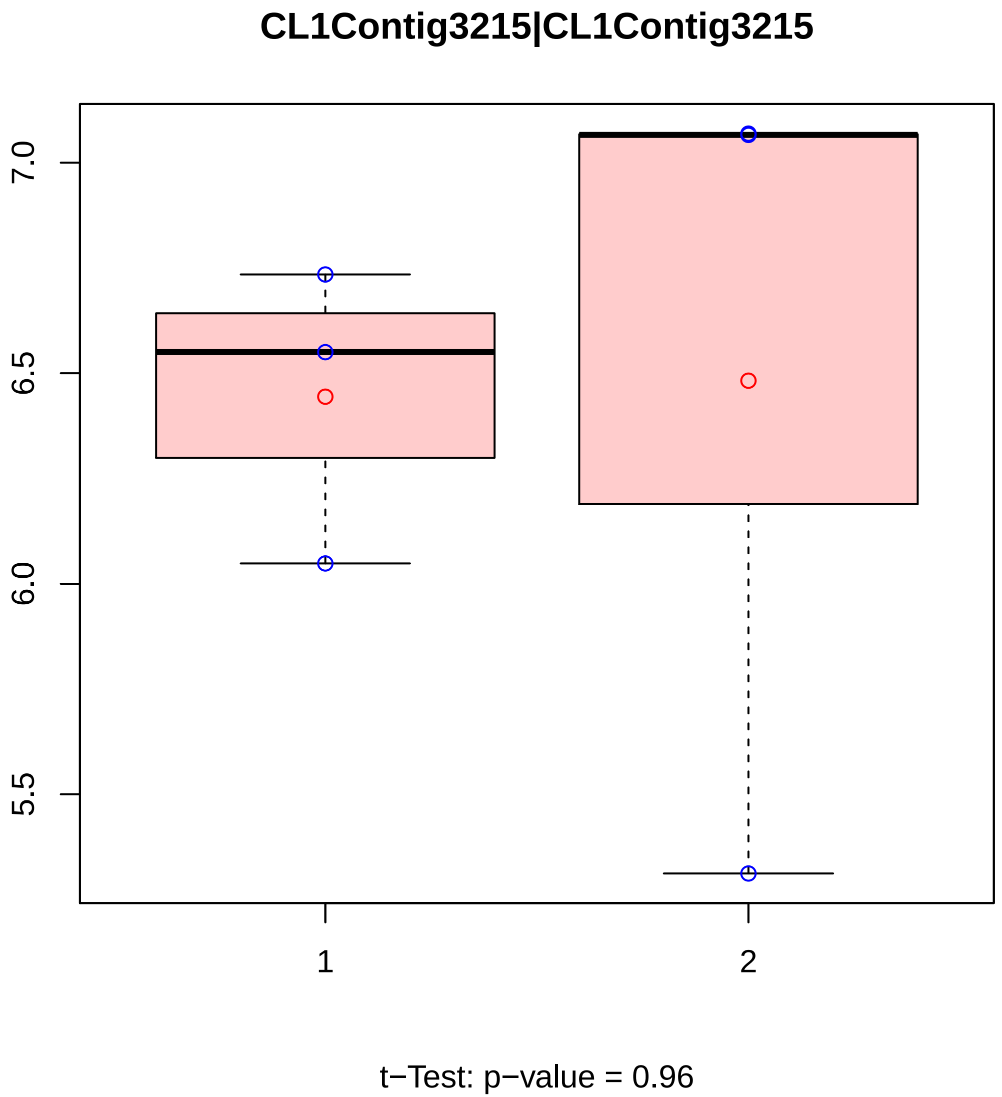

CL1Contig3215|CL1Contig3215
7.0
6.5
6.0
5.5
1
2
t−Test: p−value = 0.96

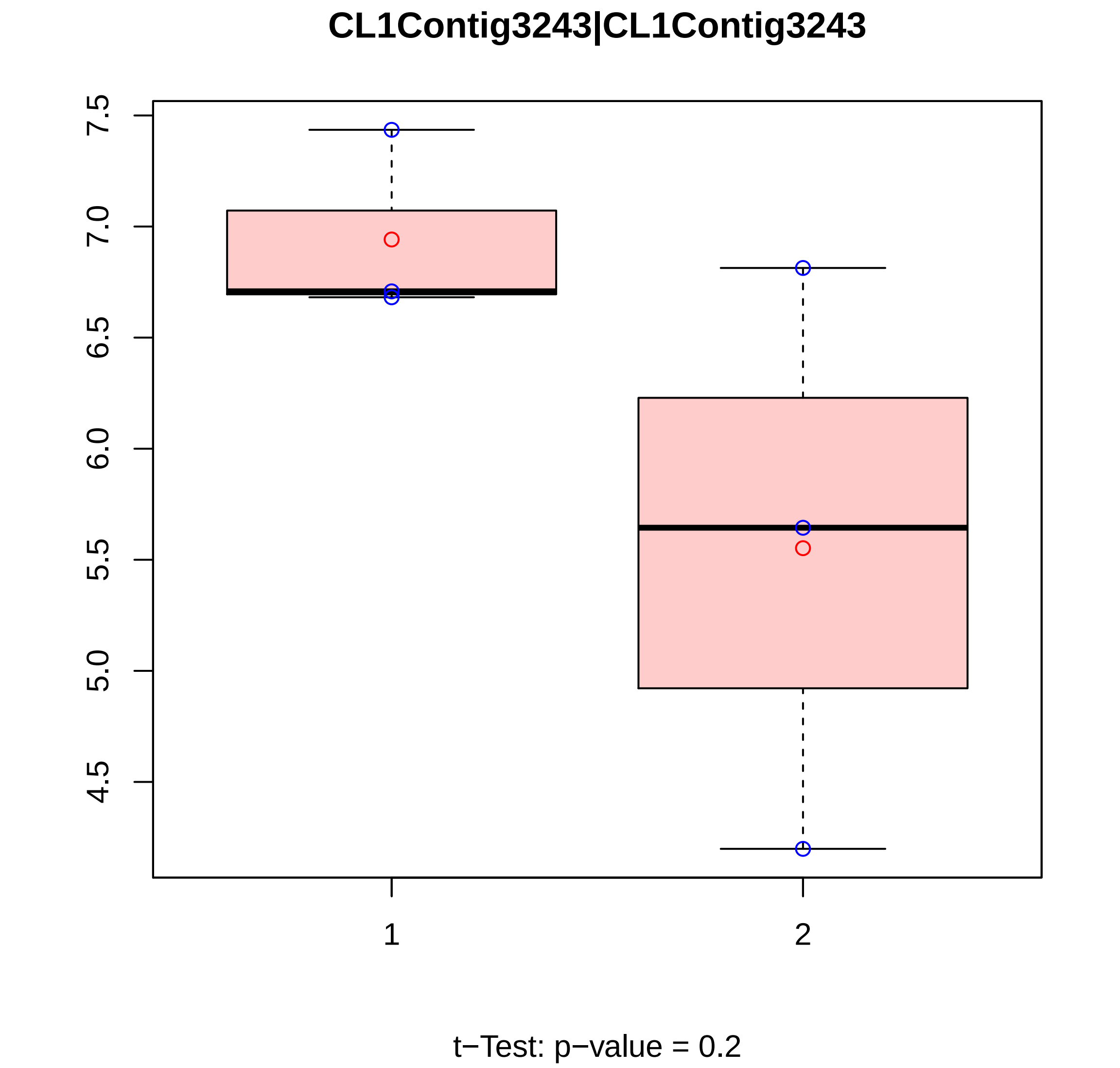

CL1Contig3243|CL1Contig3243
7.5
7.0
6.5
6.0
5.5
5.0
4.5
1
2
t−Test: p−value = 0.2

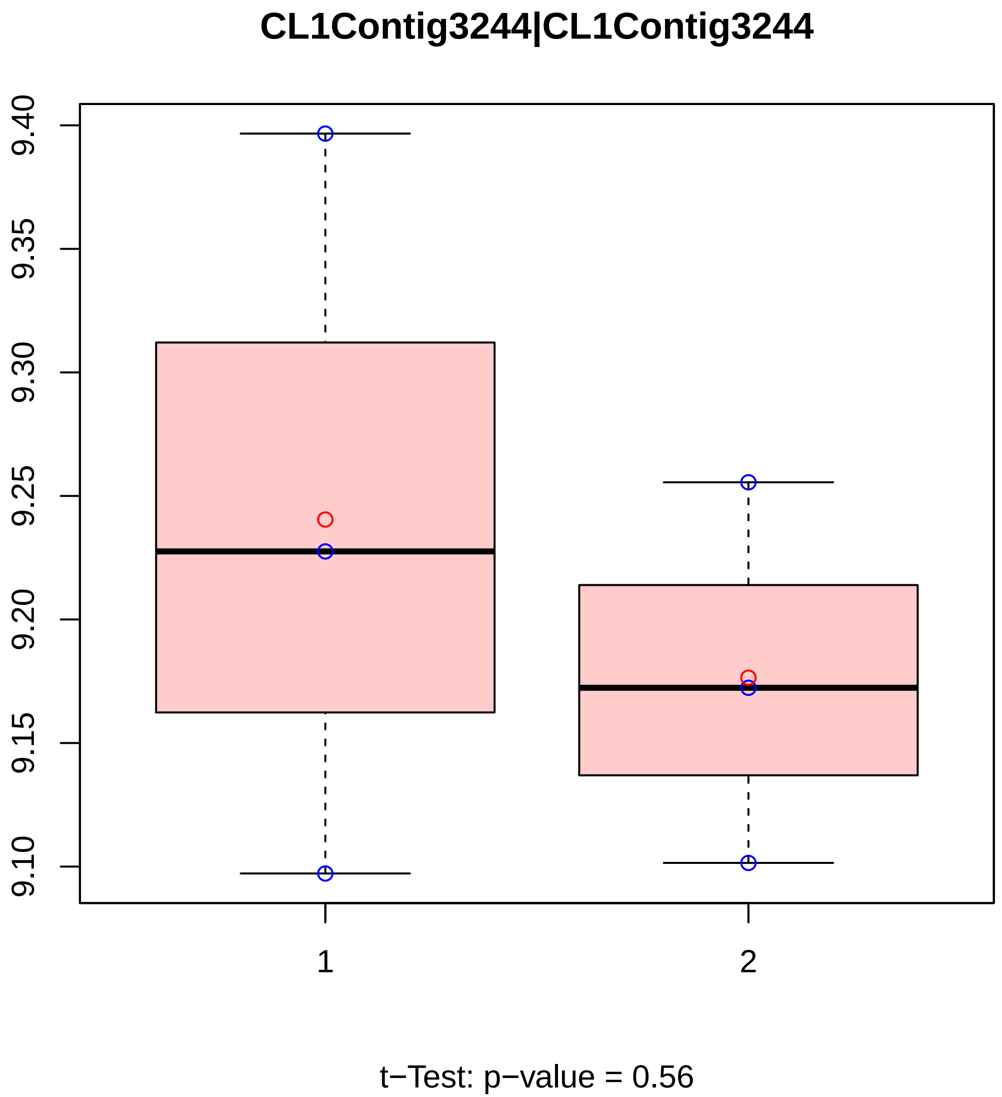
CL1Contig3244|CL1Contig3244
9.40
9.35
9.30
9.25
9.20
9.15
9.10
1
2
t−Test: p−value = 0.56

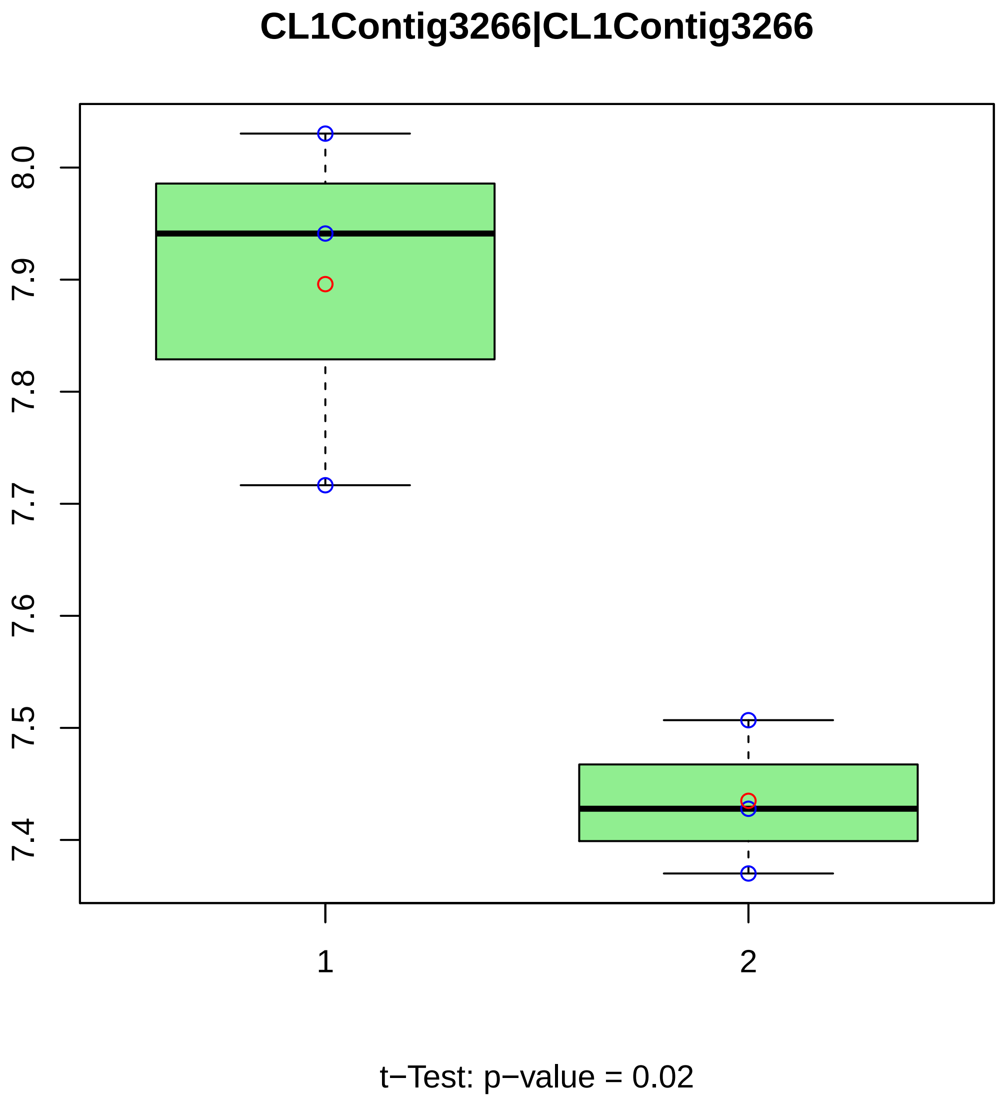
CL1Contig3266|CL1Contig3266
8.0
7.9
7.8
7.7
7.6
7.5
7.4
1
2
t−Test: p−value = 0.02

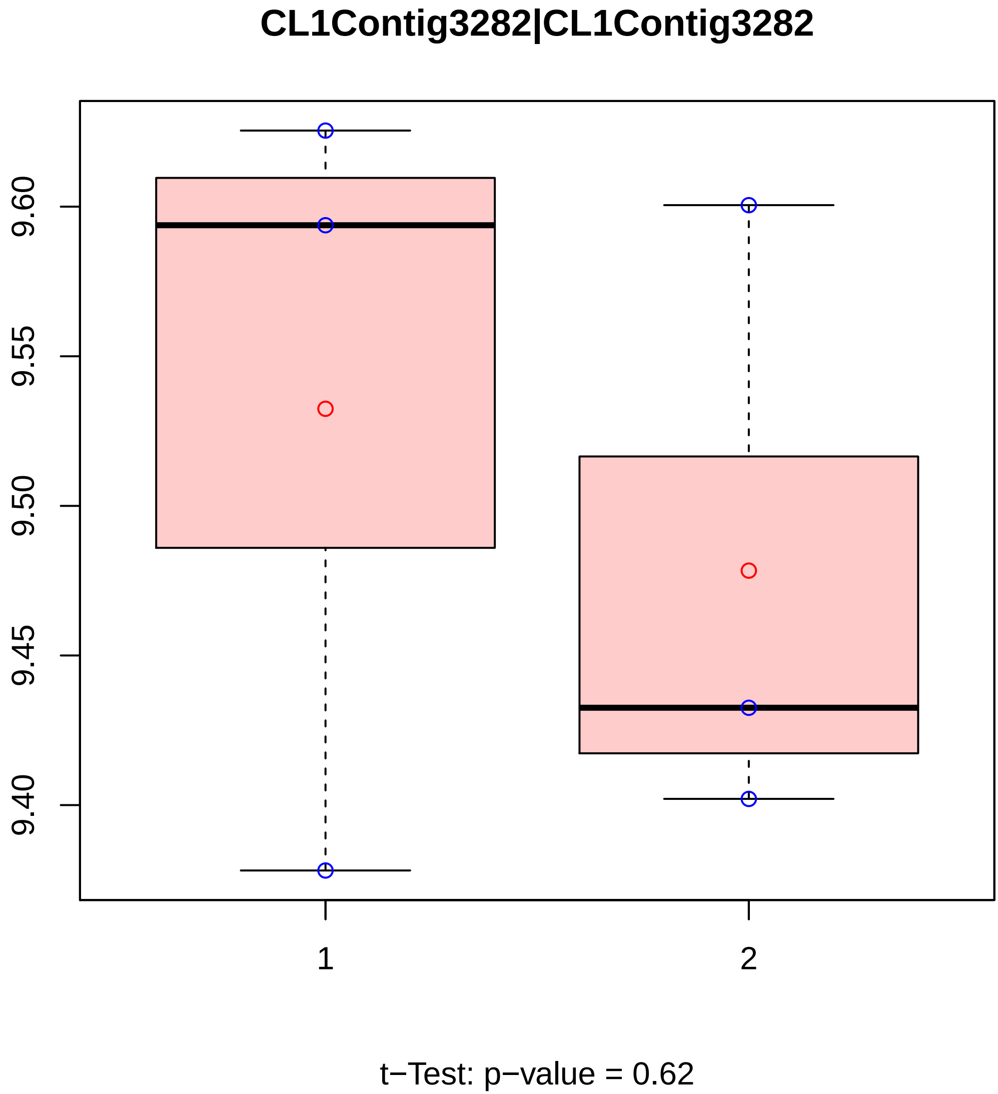

CL1Contig3282|CL1Contig3282
9.40
9.45
9.50
9.55
9.60
1
2
t−Test: p−value = 0.62

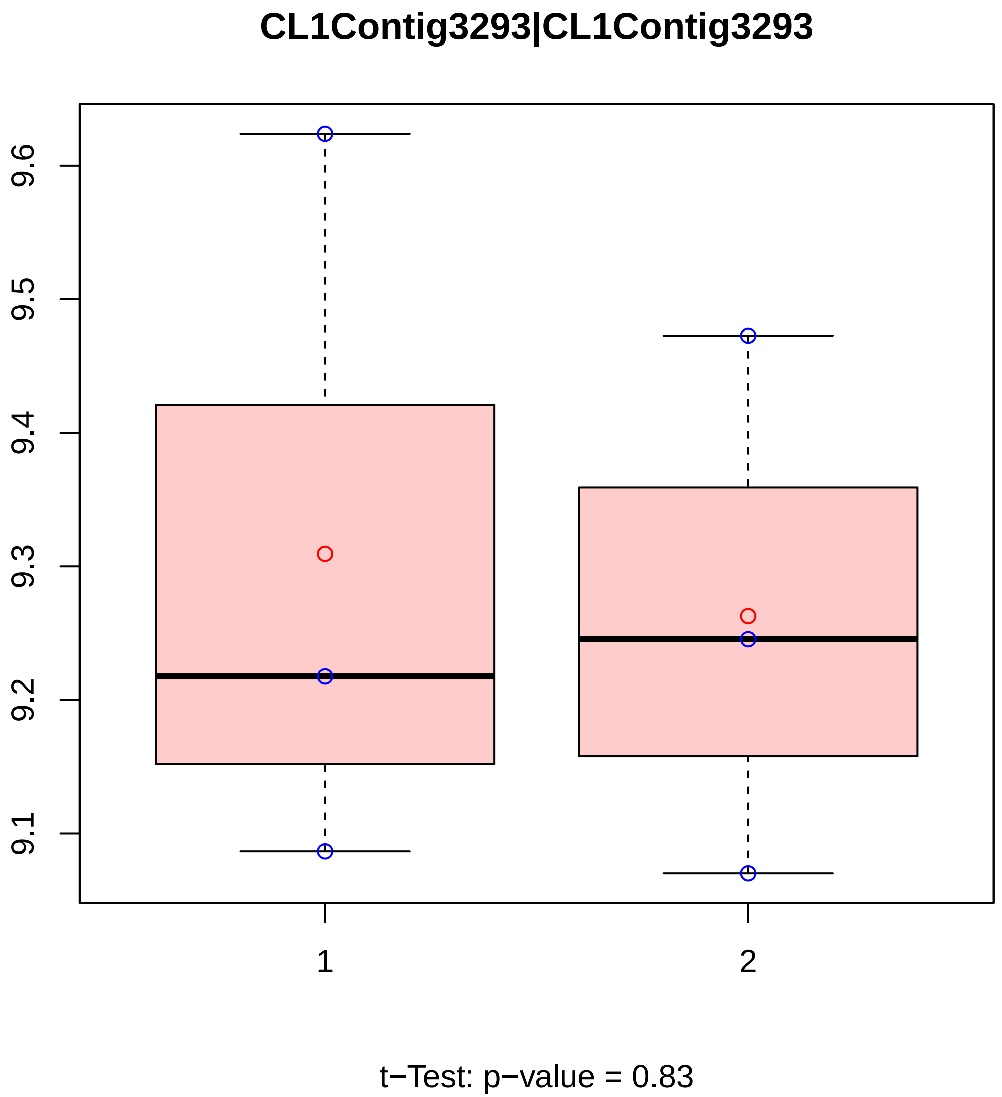
CL1Contig3293|CL1Contig3293
9.1
9.2
9.3
9.4
9.5
9.6
1
2
t−Test: p−value = 0.83

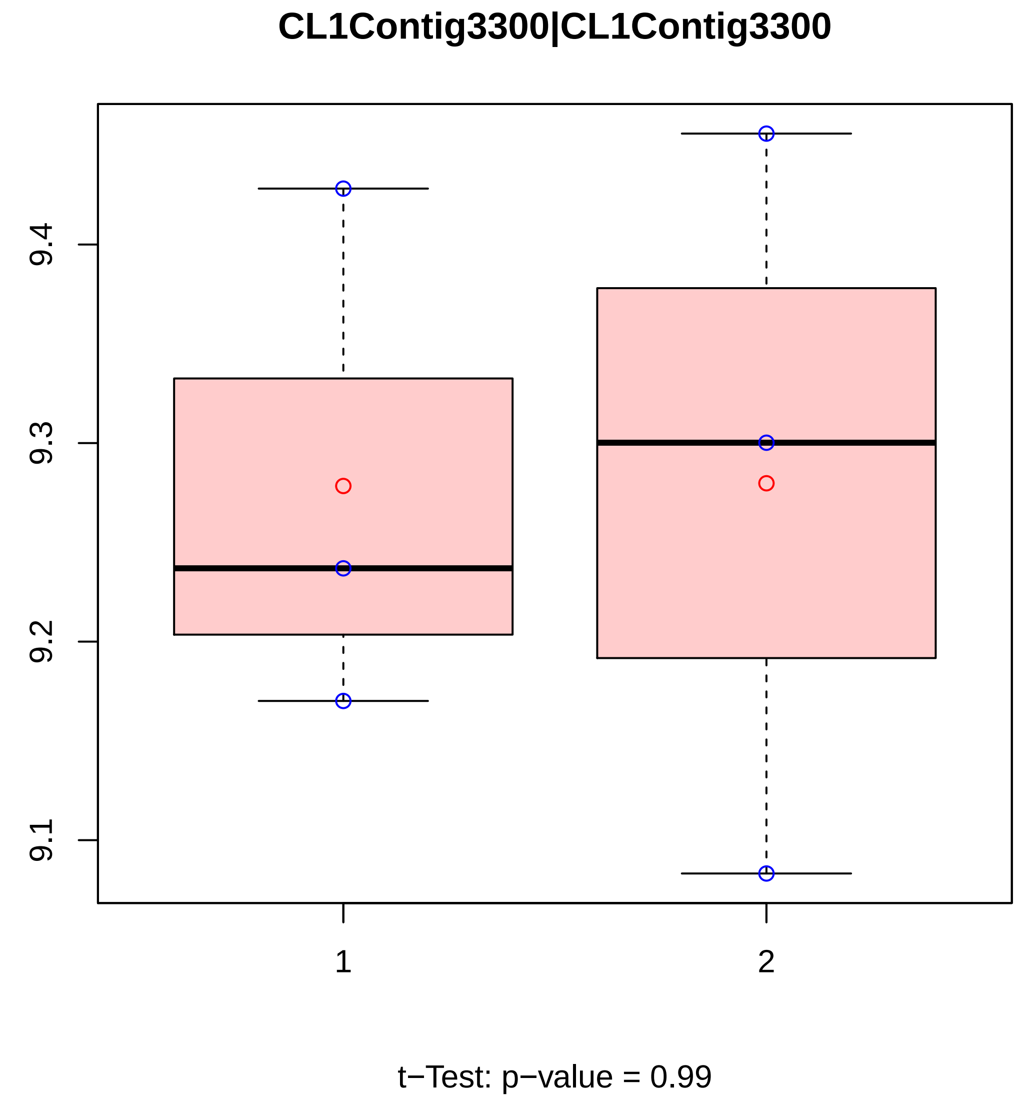
CL1Contig3300|CL1Contig3300
9.1
9.2
9.3
9.4
1
2
t−Test: p−value = 0.99

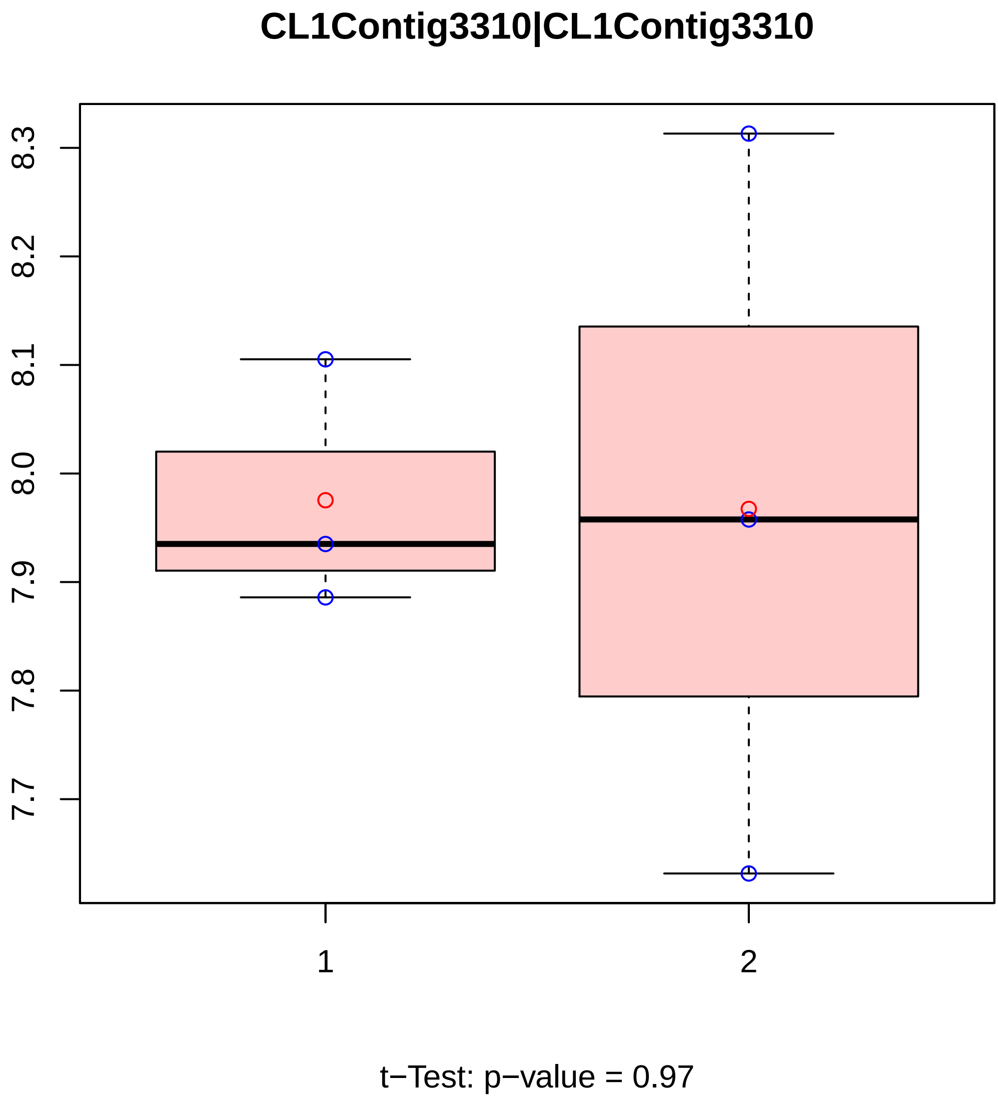

CL1Contig3310|CL1Contig3310
8.3
8.2
8.1
8.0
7.9
7.8
7.7
1
2
t−Test: p−value = 0.97

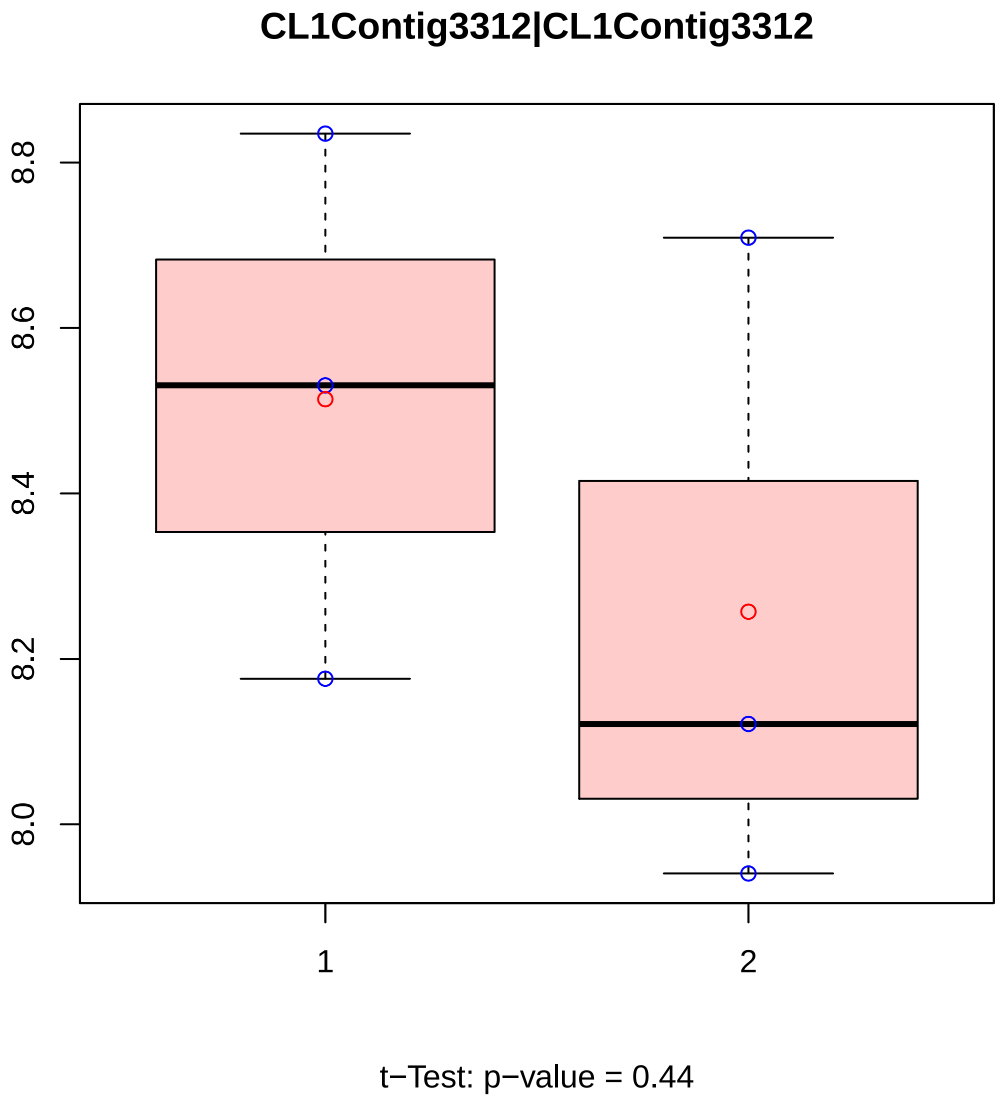
CL1Contig3312|CL1Contig3312
8.0
8.2
8.4
8.6
8.8
1
2
t−Test: p−value = 0.44

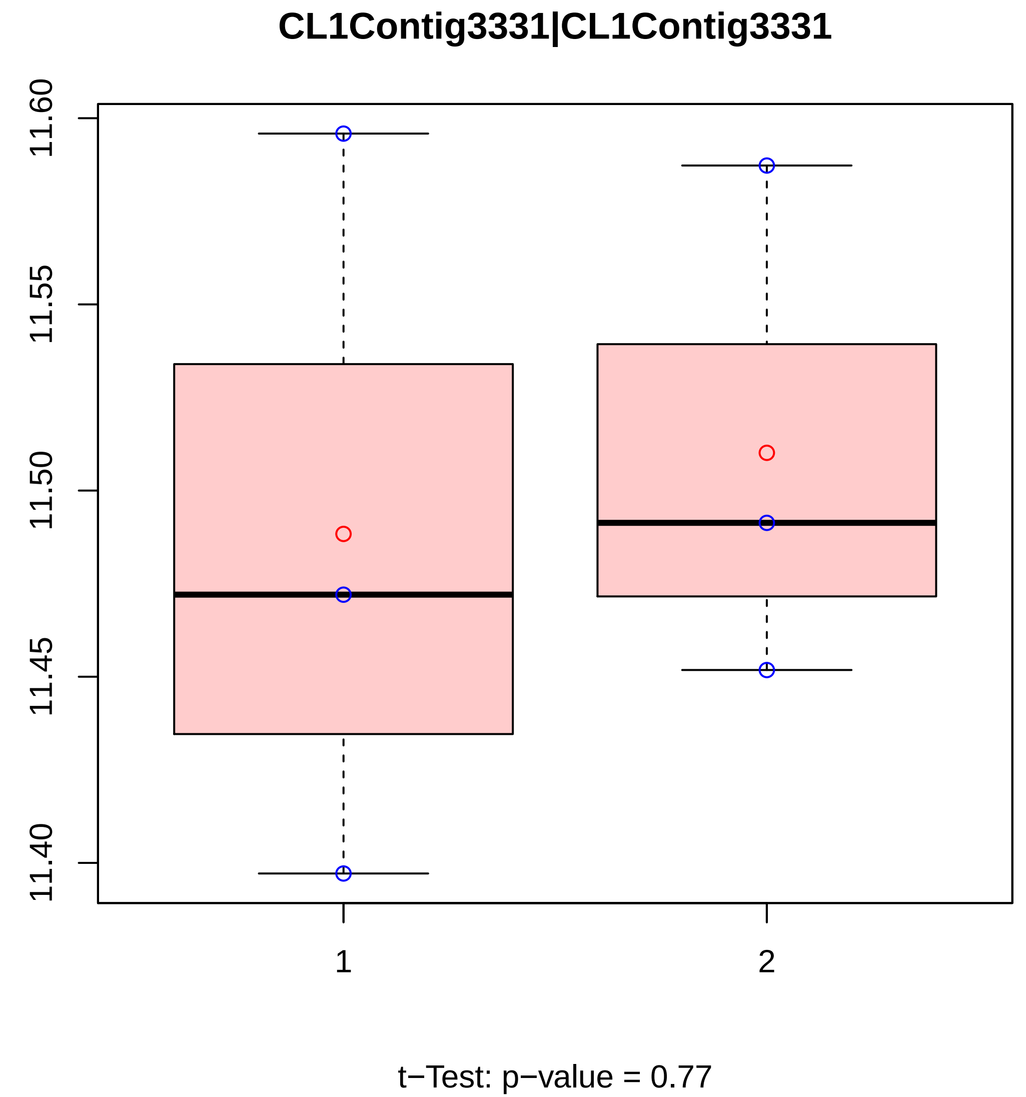
CL1Contig3331|CL1Contig3331
11.40
11.45
11.50
11.55
11.60
1
2
t−Test: p−value = 0.77

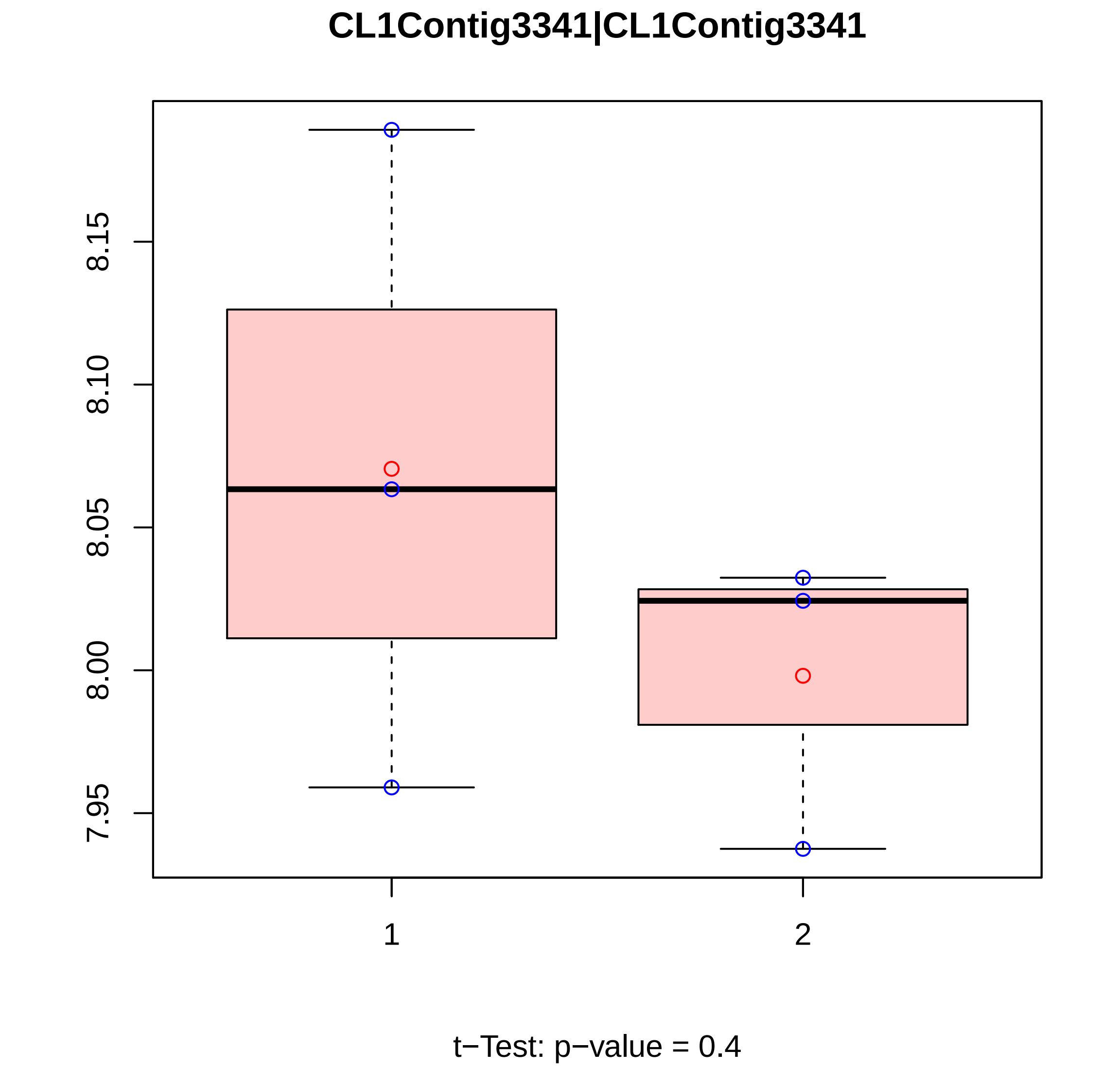
CL1Contig3341|CL1Contig3341
8.15
8.10
8.05
8.00
7.95
1
2
t−Test: p−value = 0.4

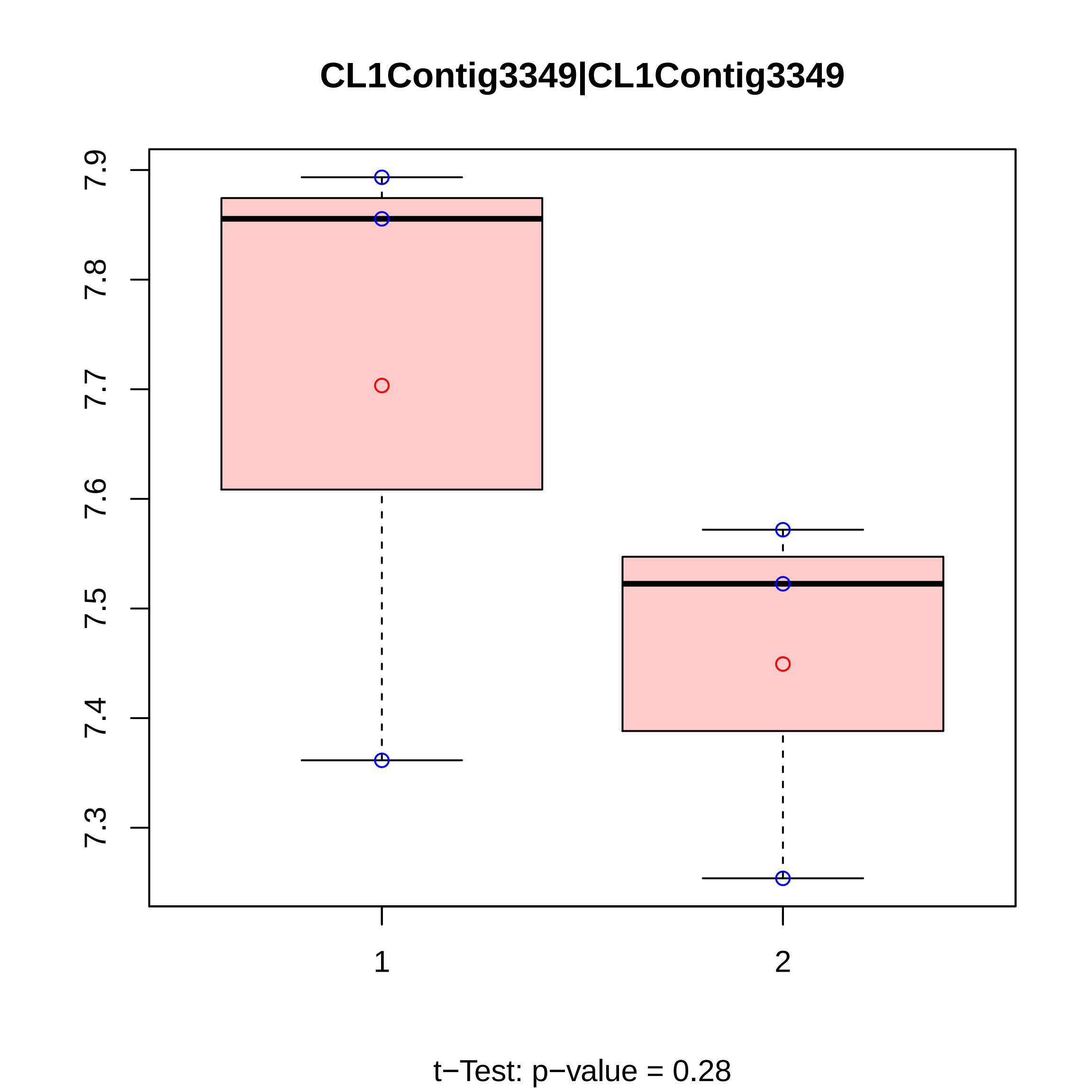

CL1Contig3349|CL1Contig3349
7.3
7.4
7.5
7.6
7.7
7.8
7.9
1
2
t−Test: p−value = 0.28

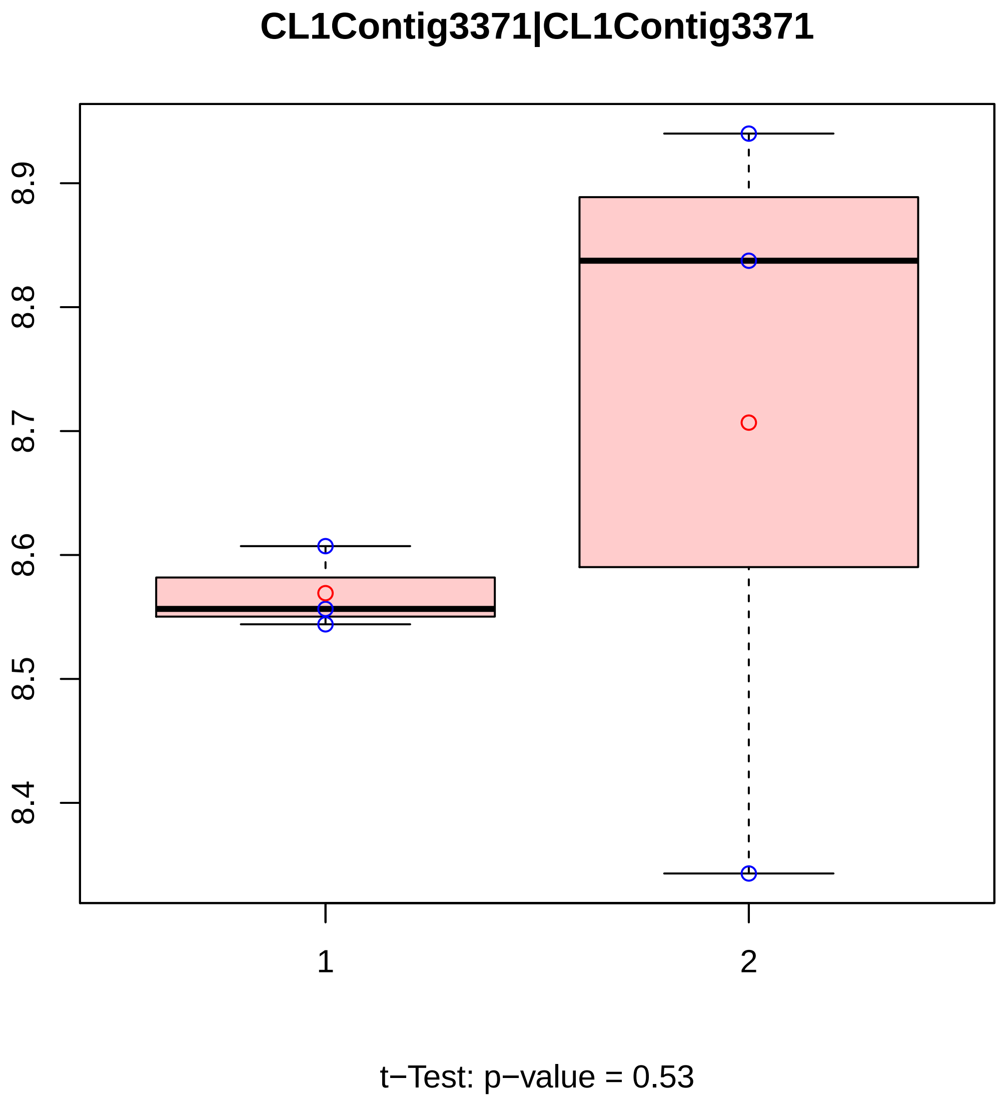
CL1Contig3371|CL1Contig3371
8.4
8.5
8.6
8.7
8.8
8.9
1
2
t−Test: p−value = 0.53

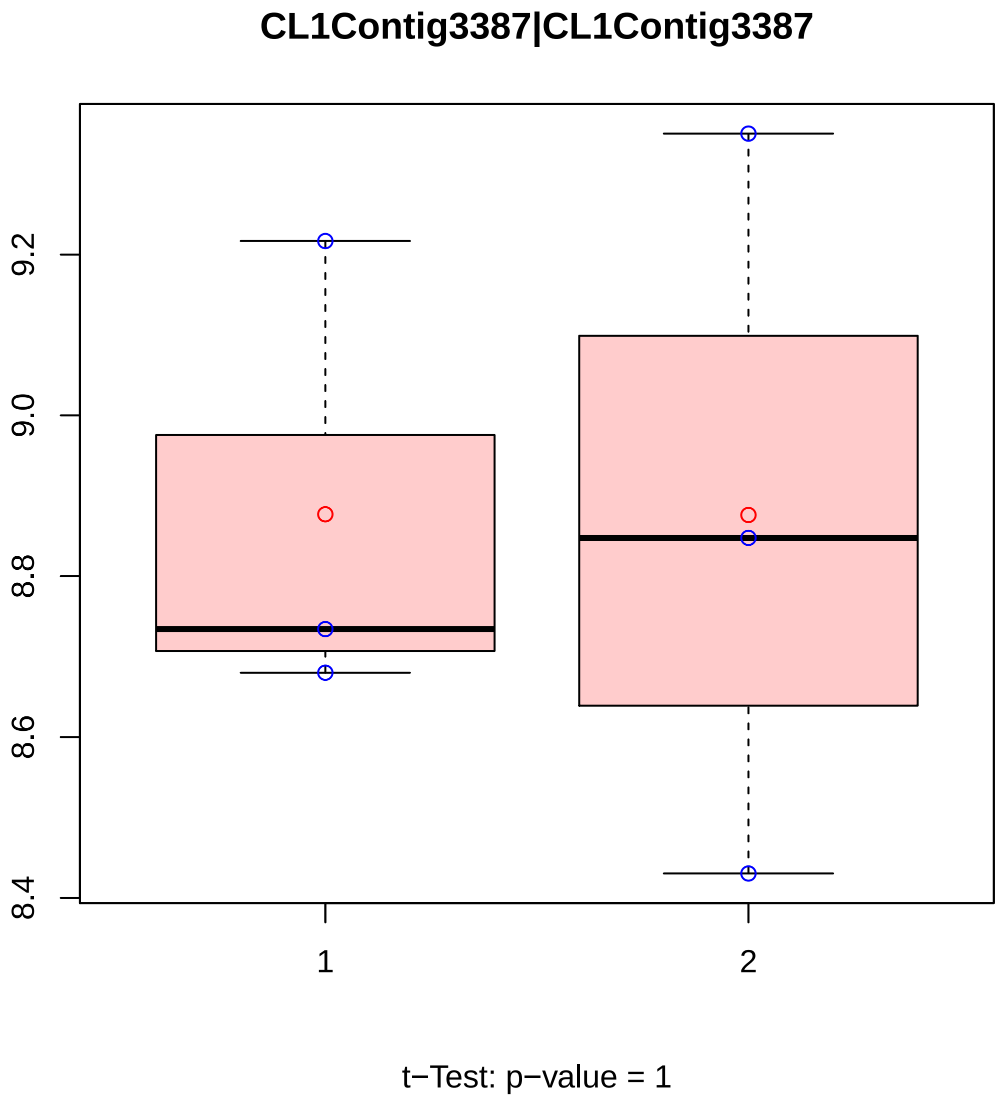
CL1Contig3387|CL1Contig3387
9.2
9.0
8.8
8.6
8.4
1
2
t−Test: p−value = 1

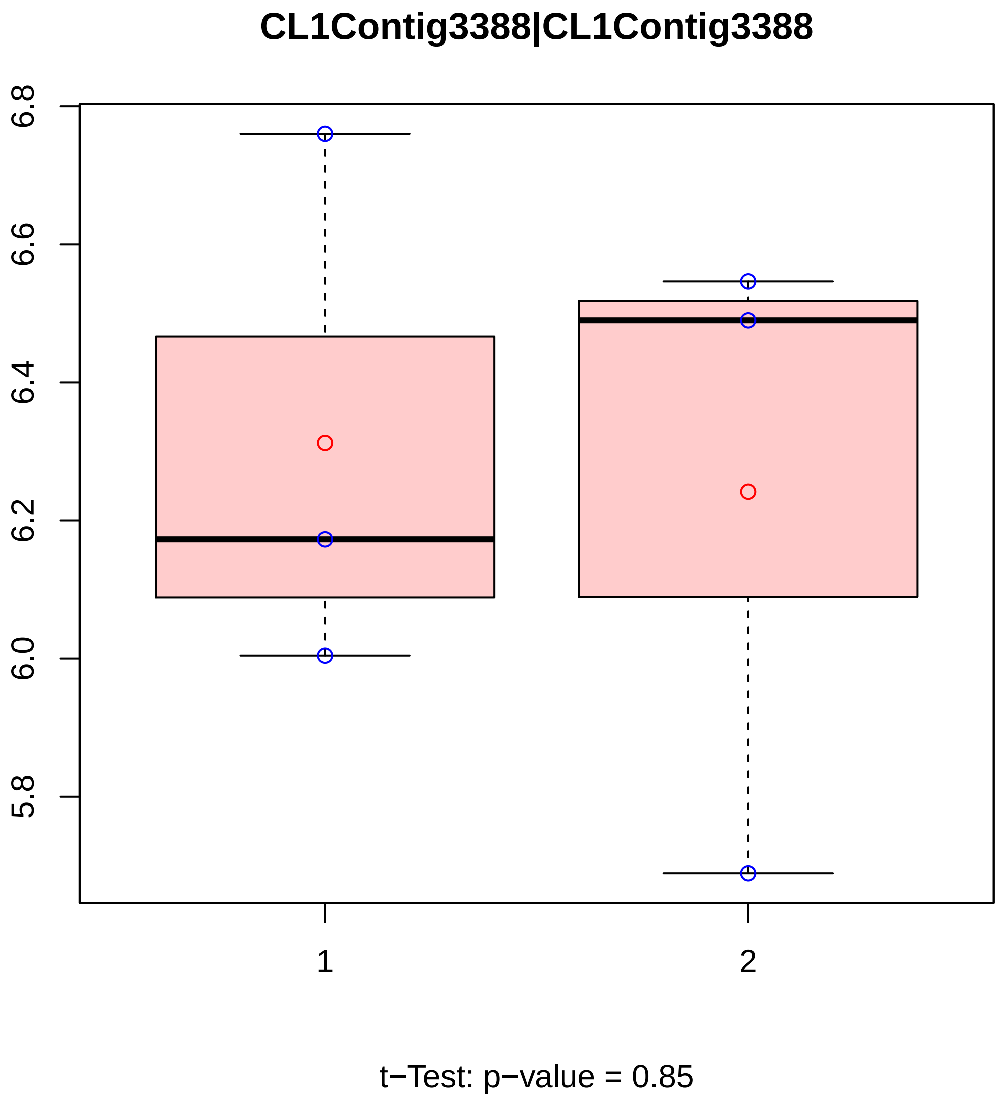

CL1Contig3388|CL1Contig3388
6.8
6.6
6.4
6.2
6.0
5.8
1
2
t−Test: p−value = 0.85

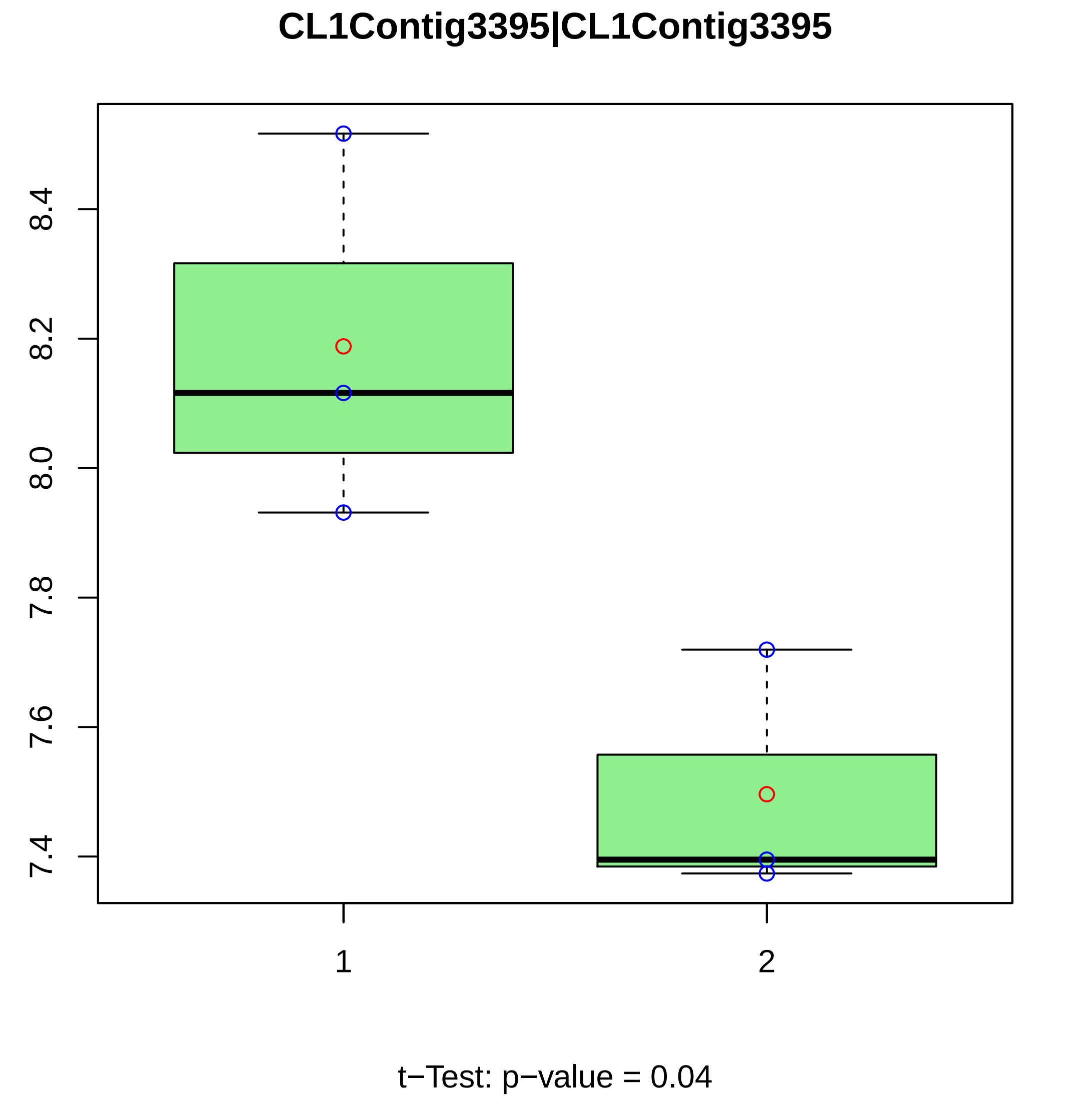
CL1Contig3395|CL1Contig3395
8.4
8.2
8.0
7.8
7.6
7.4
1
2
t−Test: p−value = 0.04

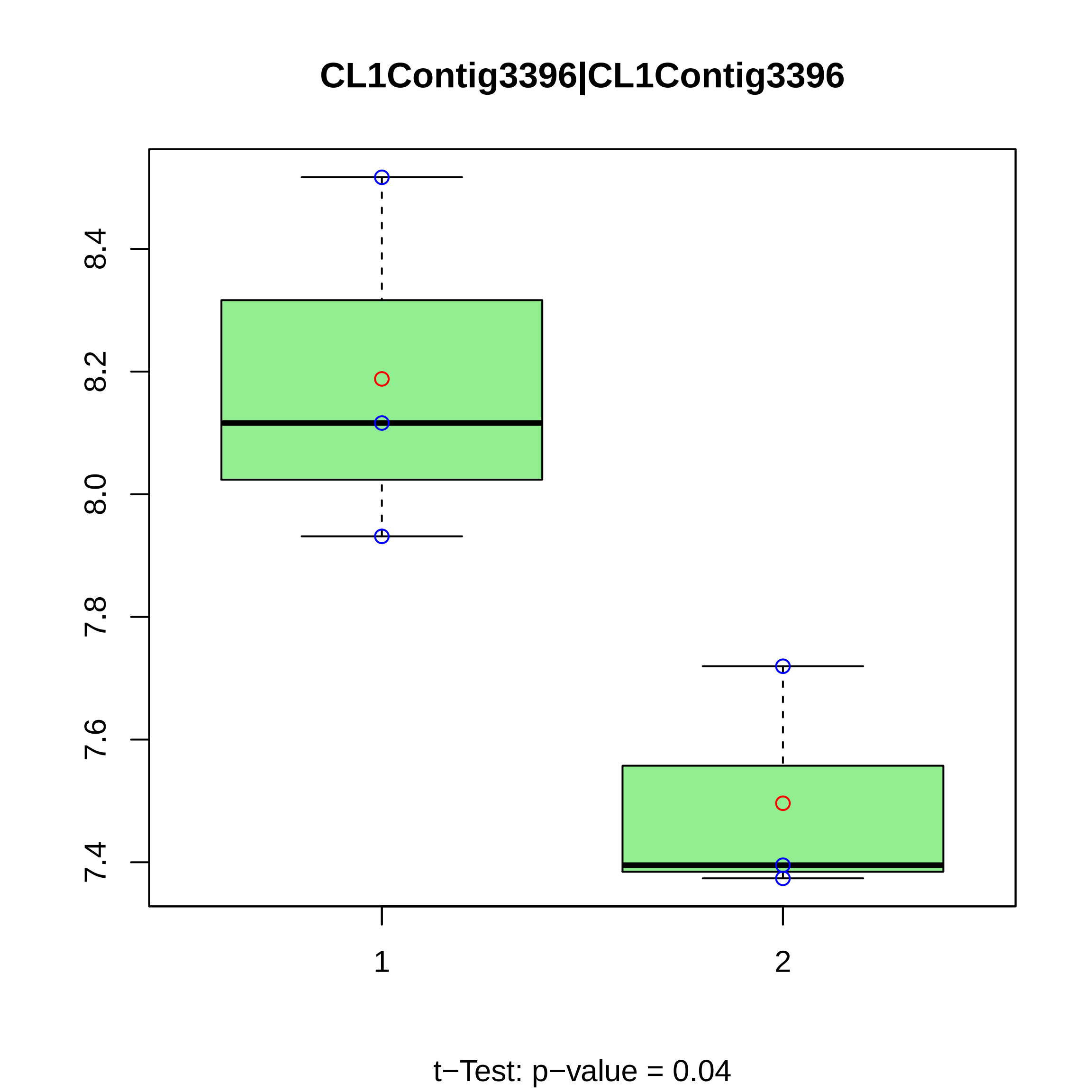
CL1Contig3396|CL1Contig3396
8.4
8.2
8.0
7.8
7.6
7.4
1
2
t−Test: p−value = 0.04

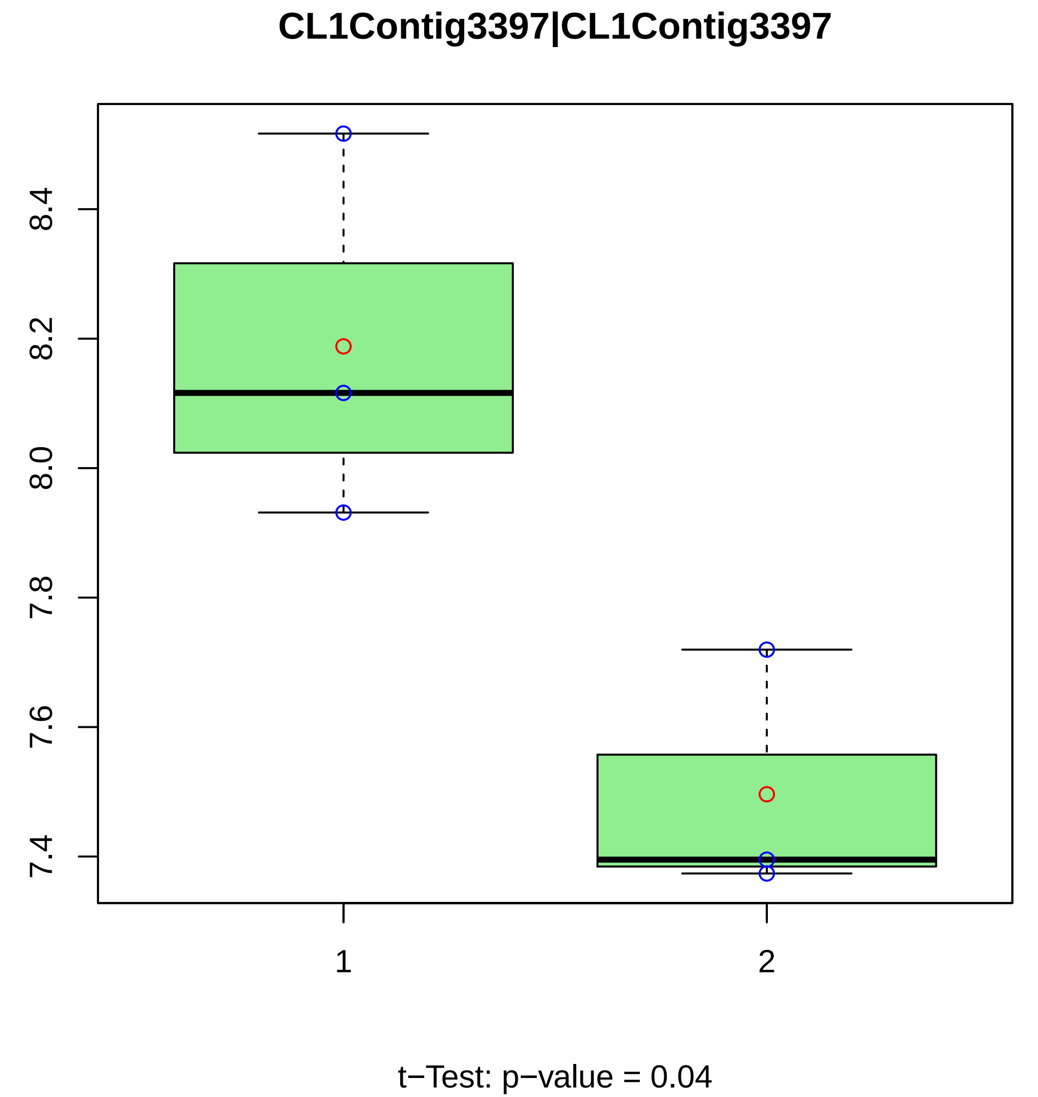
CL1Contig3397|CL1Contig3397
8.4
8.2
8.0
7.8
7.6
7.4
1
2
t−Test: p−value = 0.04

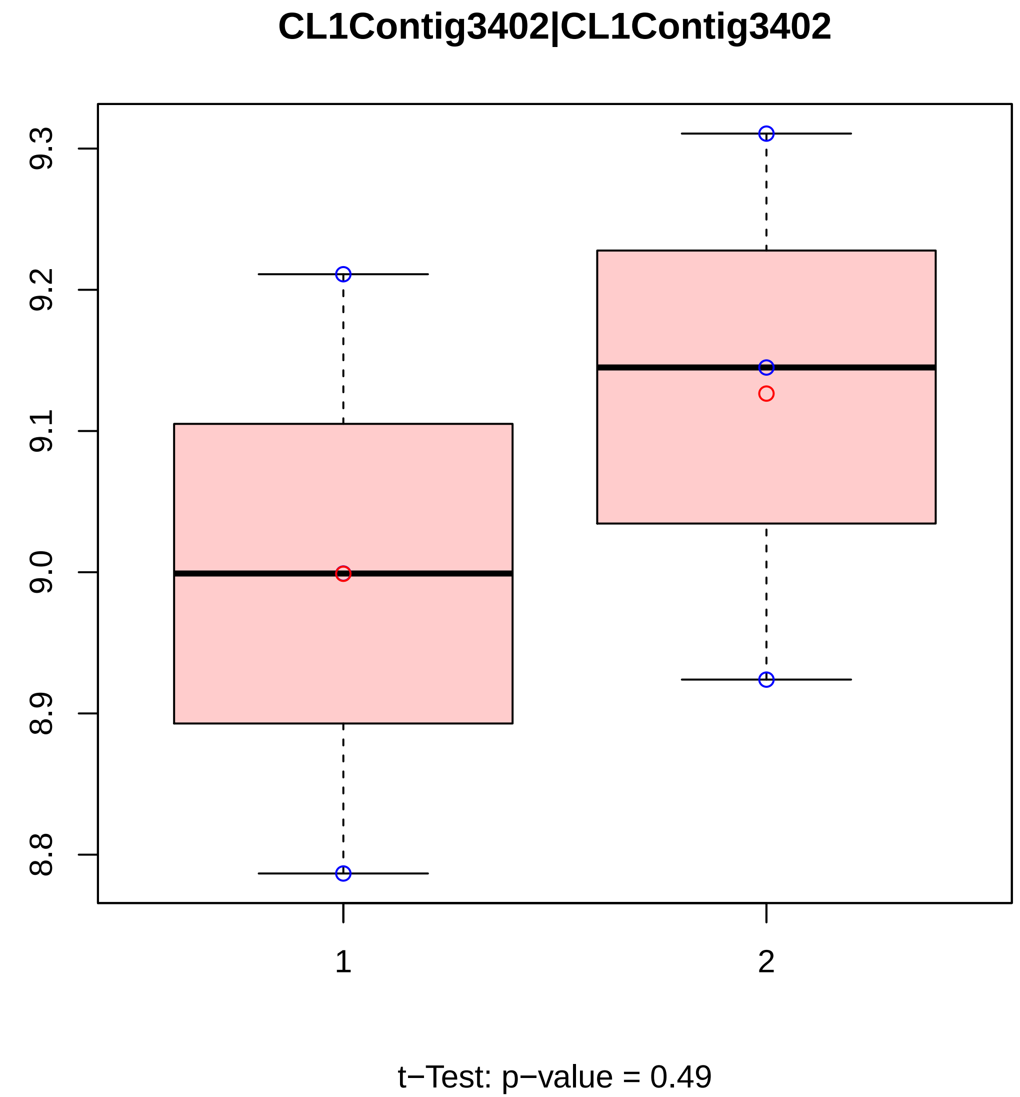

CL1Contig3402|CL1Contig3402
9.3
9.2
9.1
9.0
8.9
8.8
1
2
t−Test: p−value = 0.49

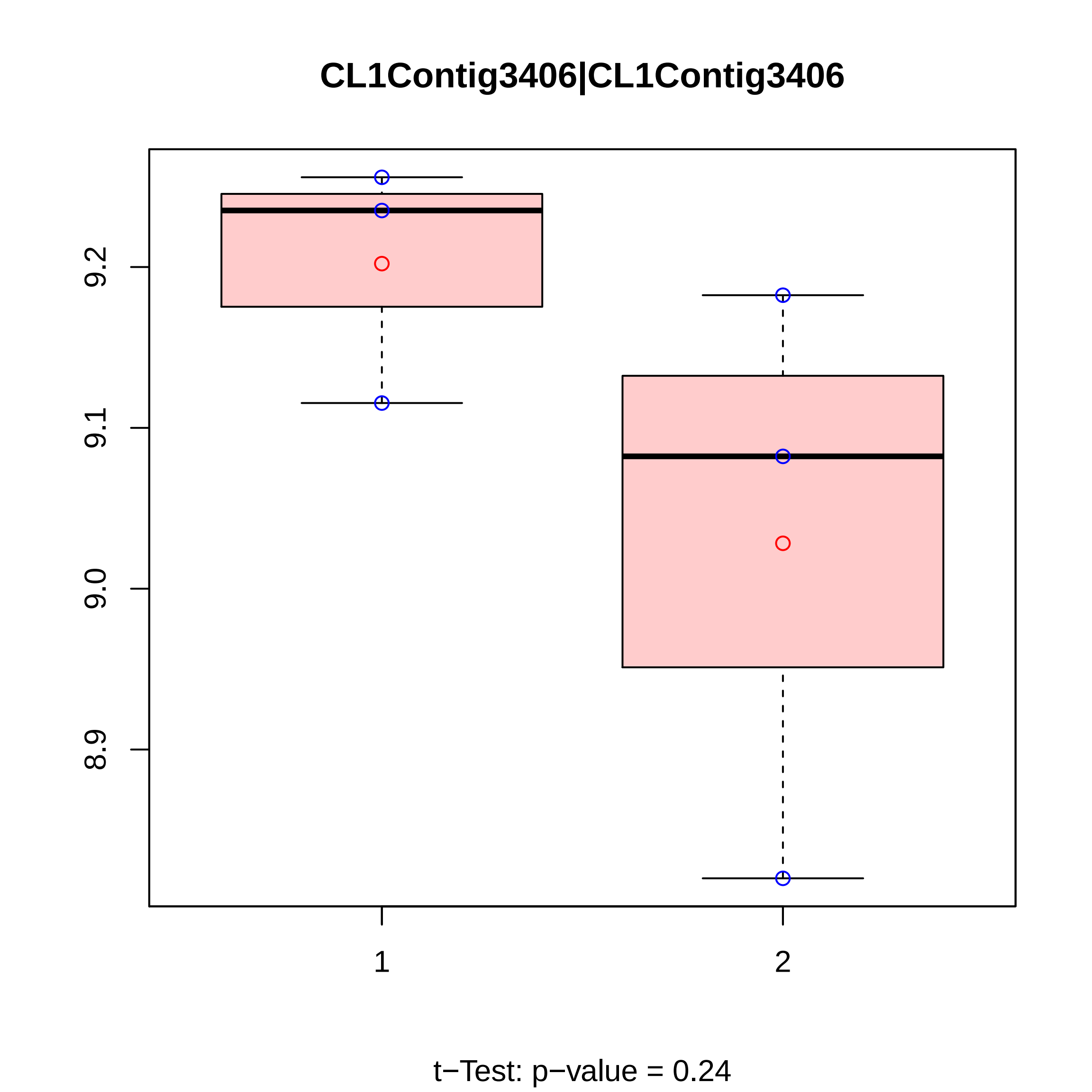
CL1Contig3406|CL1Contig3406
9.2
9.1
9.0
8.9
1
2
t−Test: p−value = 0.24

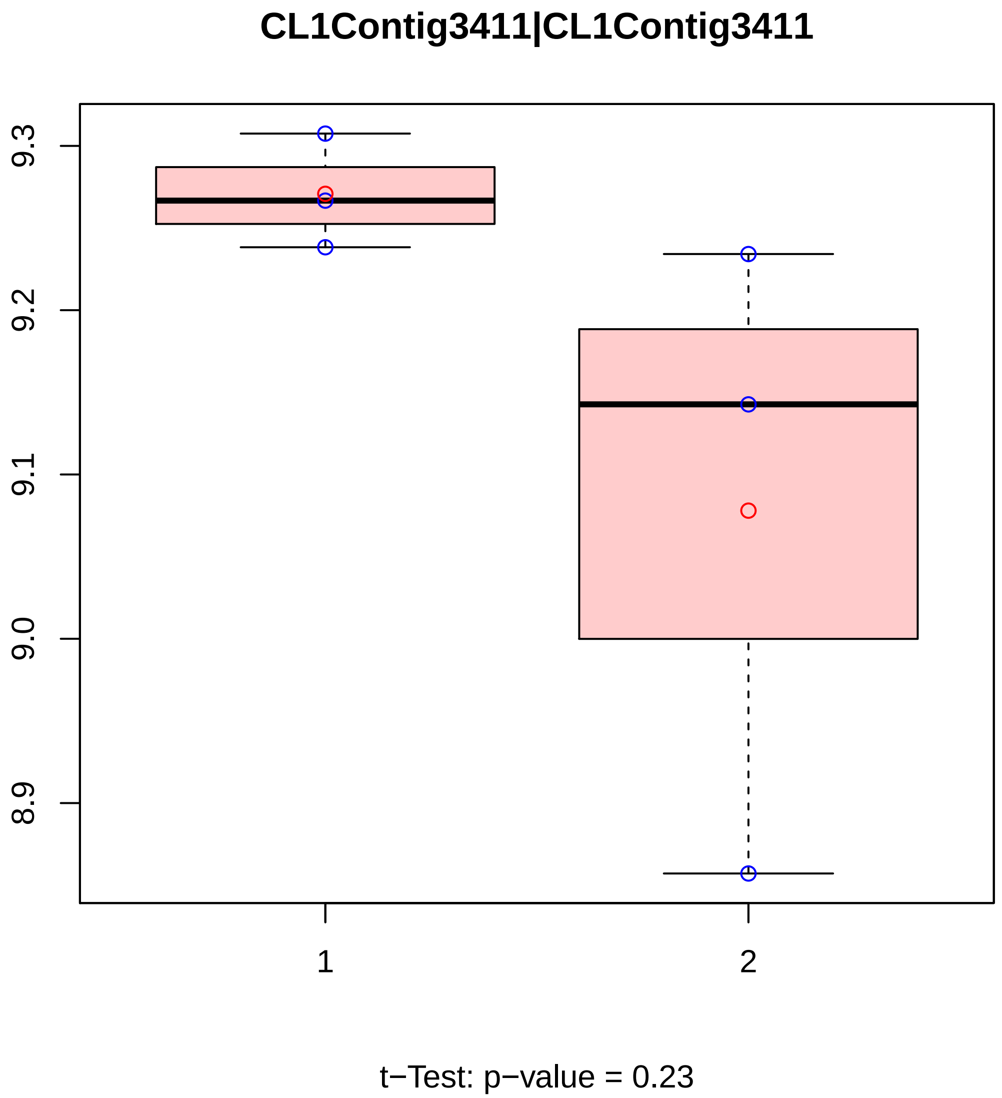

CL1Contig3411|CL1Contig3411
9.3
9.2
9.1
9.0
8.9
1
2
t−Test: p−value = 0.23

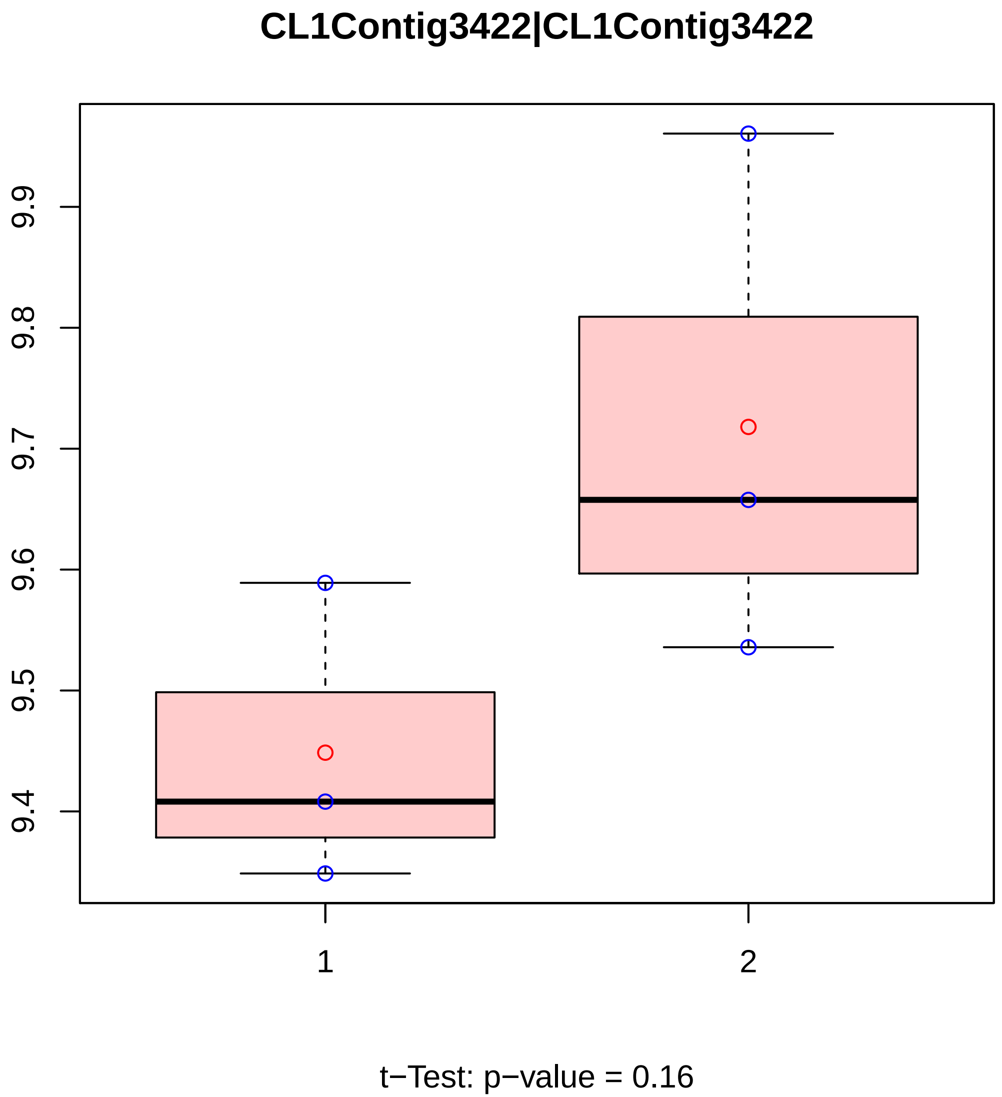
CL1Contig3422|CL1Contig3422
9.4
9.5
9.6
9.7
9.8
9.9
1
2
t−Test: p−value = 0.16

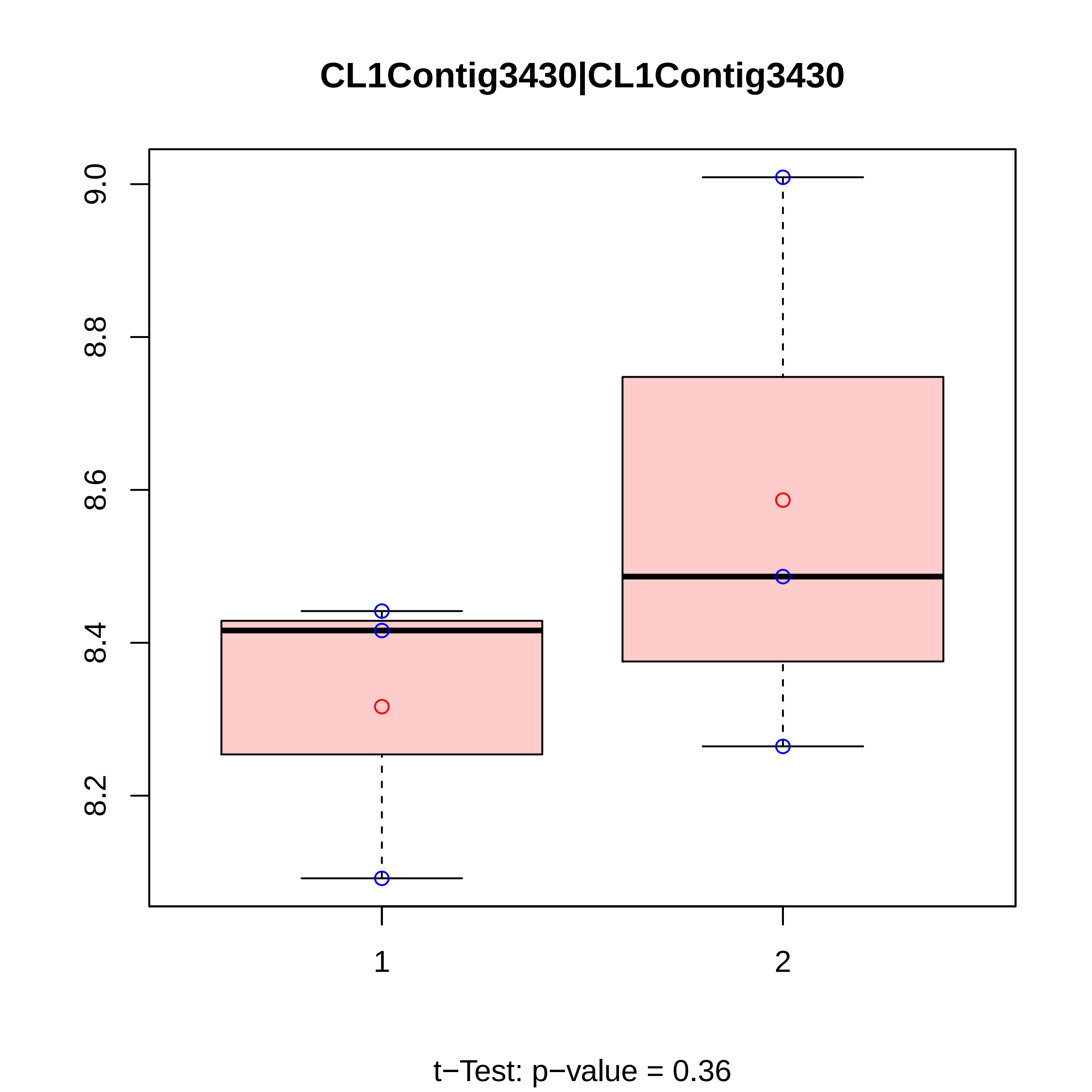
CL1Contig3430|CL1Contig3430
9.0
8.8
8.6
8.4
8.2
1
2
t−Test: p−value = 0.36

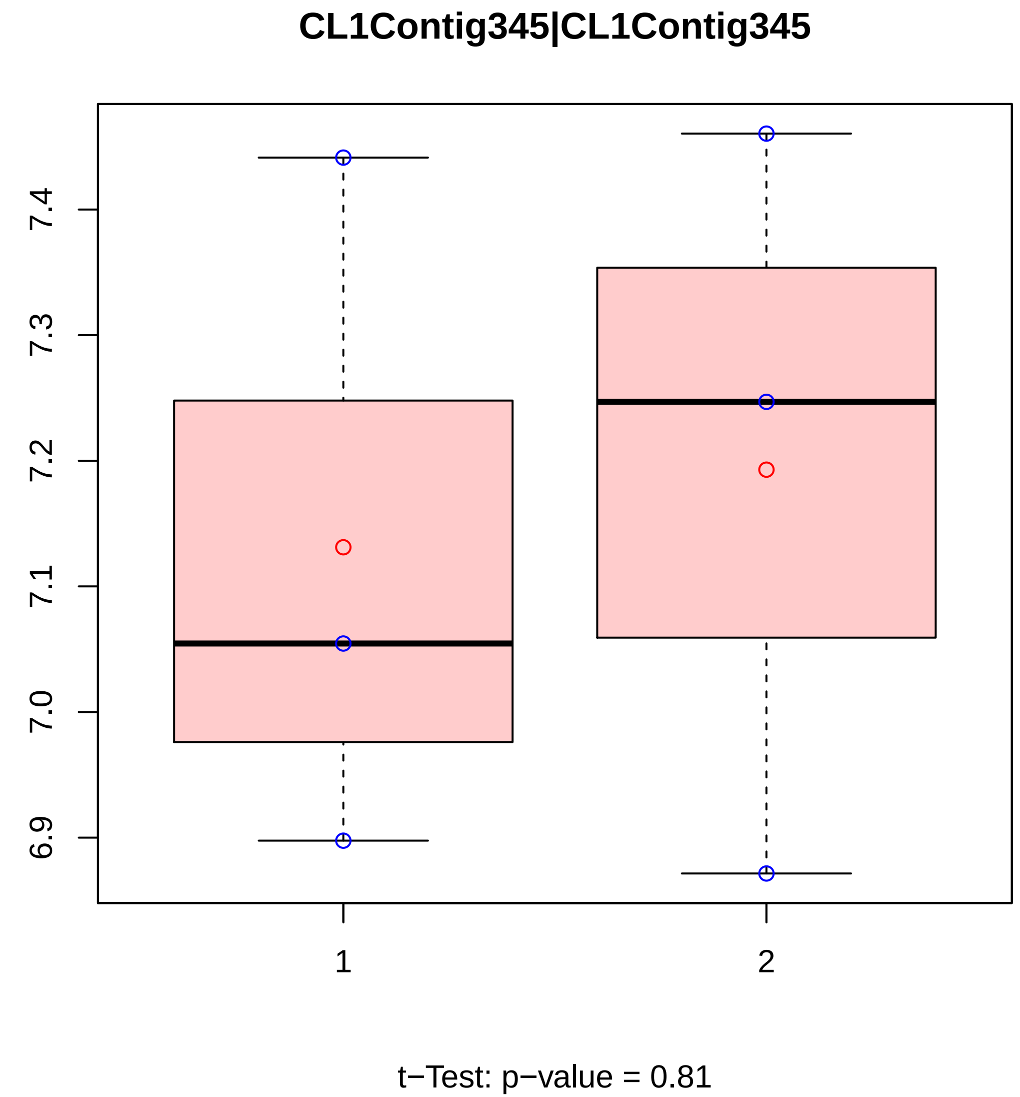

CL1Contig345|CL1Contig345
7.4
7.3
7.2
7.1
7.0
6.9
1
2
t−Test: p−value = 0.81

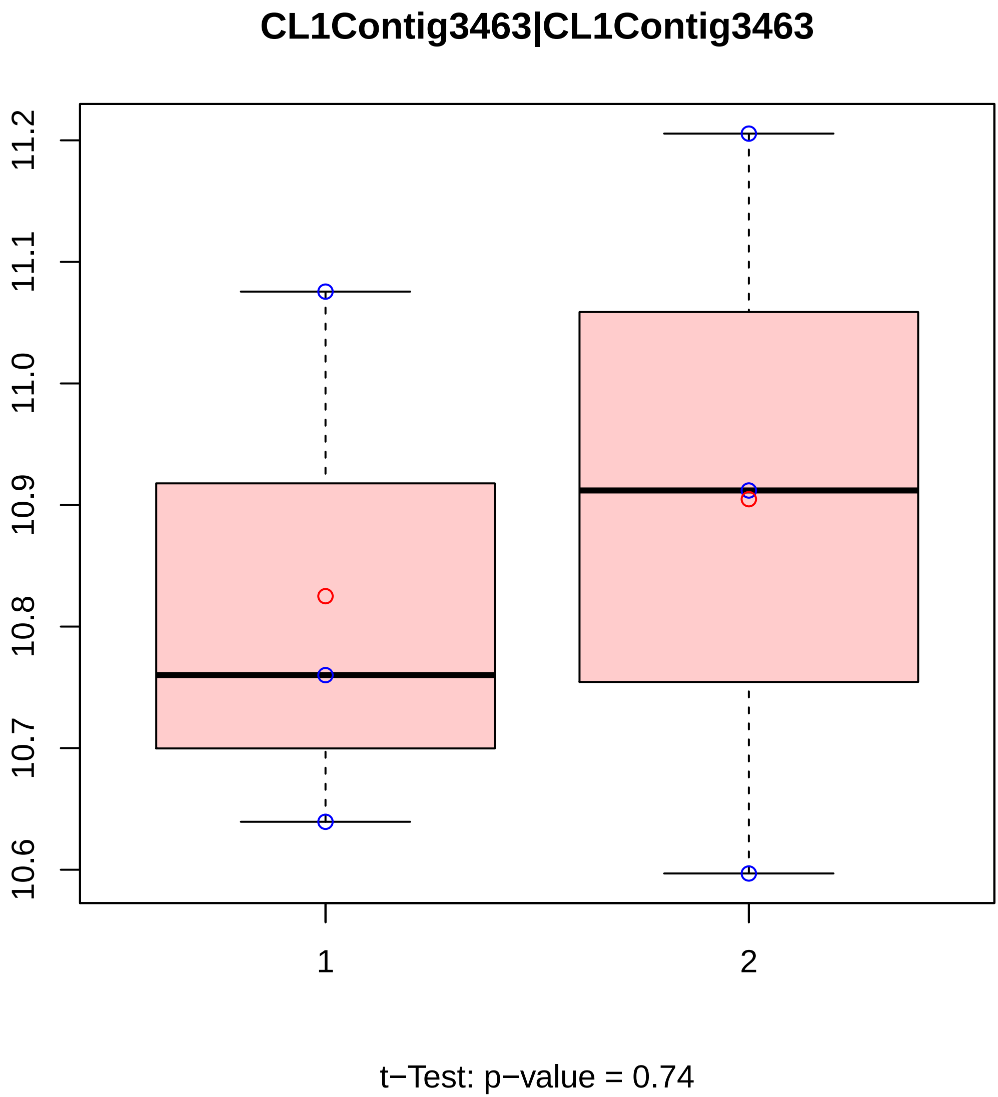
CL1Contig3463|CL1Contig3463
11.2
11.1
11.0
10.9
10.8
10.7
10.6
1
2
t−Test: p−value = 0.74

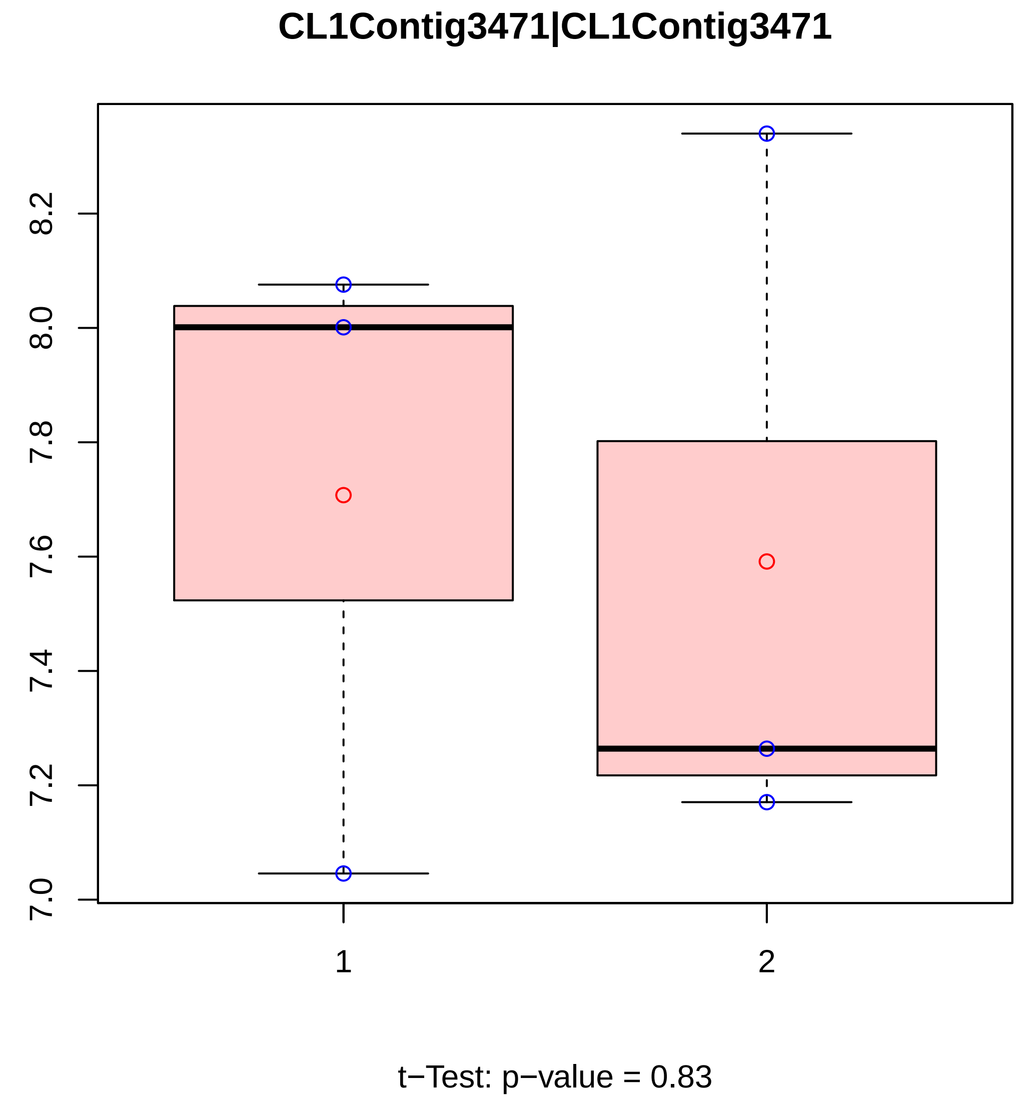
CL1Contig3471|CL1Contig3471
8.2
8.0
7.8
7.6
7.4
7.2
7.0
1
2
t−Test: p−value = 0.83

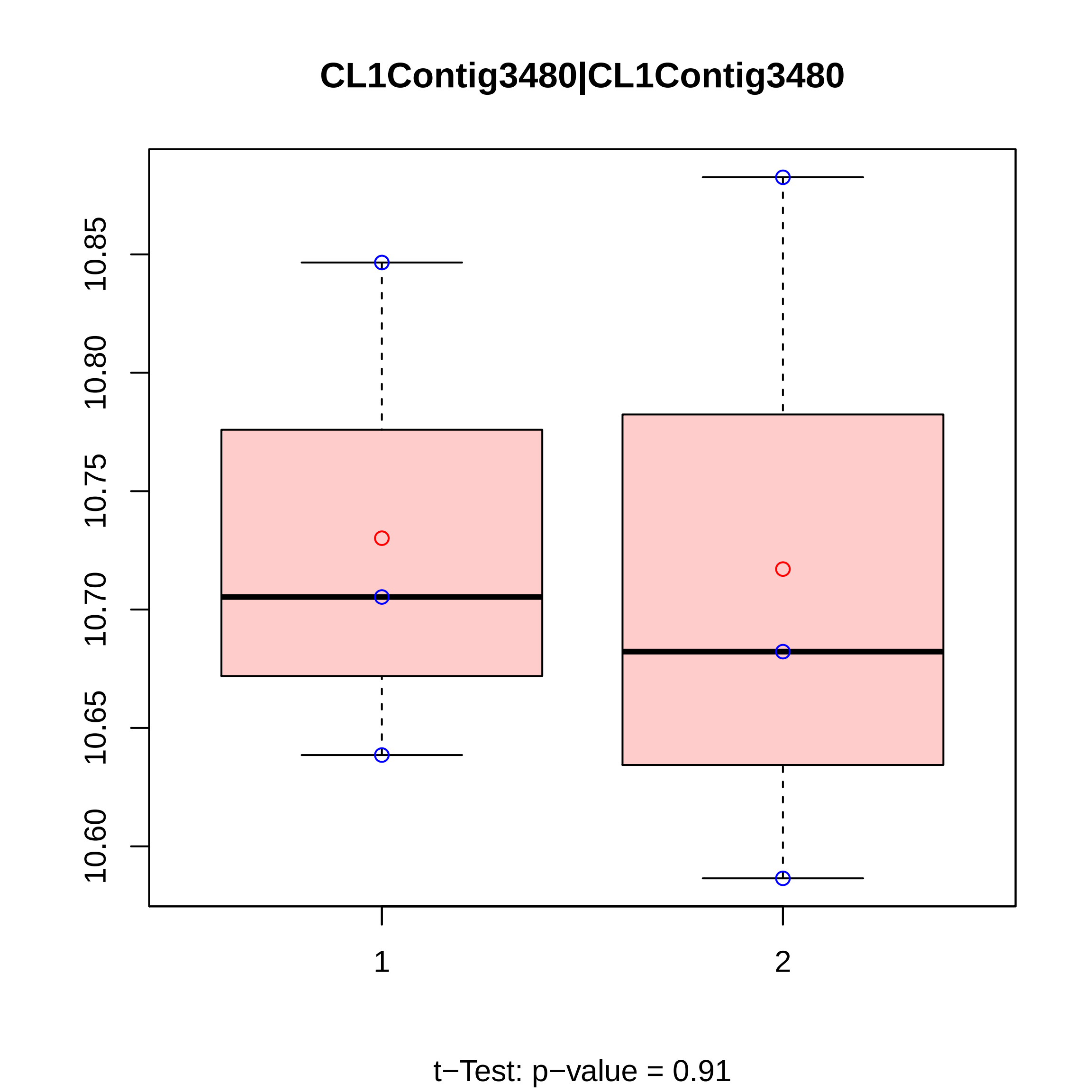
CL1Contig3480|CL1Contig3480
10.60
10.65
10.70
10.75
10.80
10.85
1
2
t−Test: p−value = 0.91

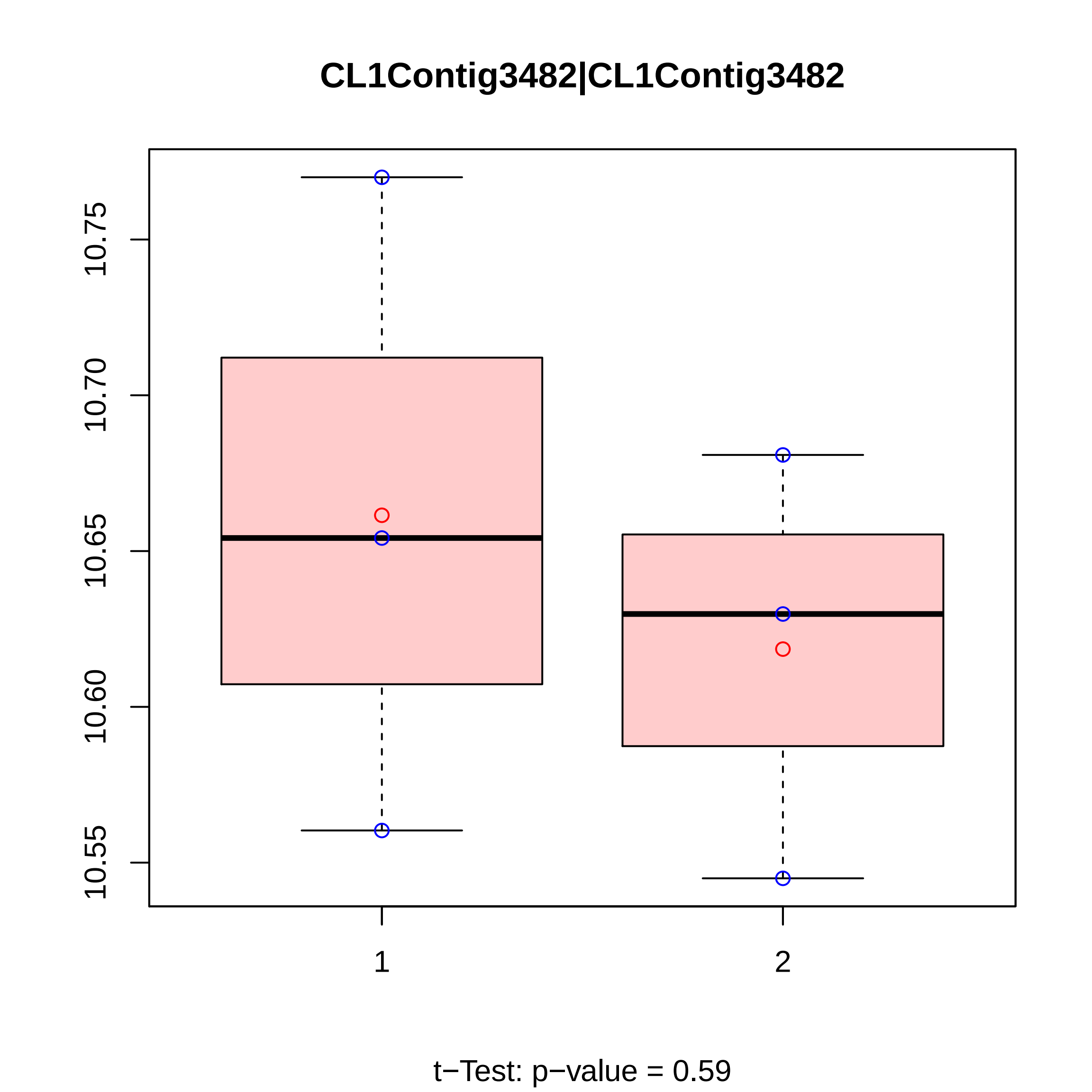
CL1Contig3482|CL1Contig3482
10.55
10.60
10.65
10.70
10.75
1
2
t−Test: p−value = 0.59

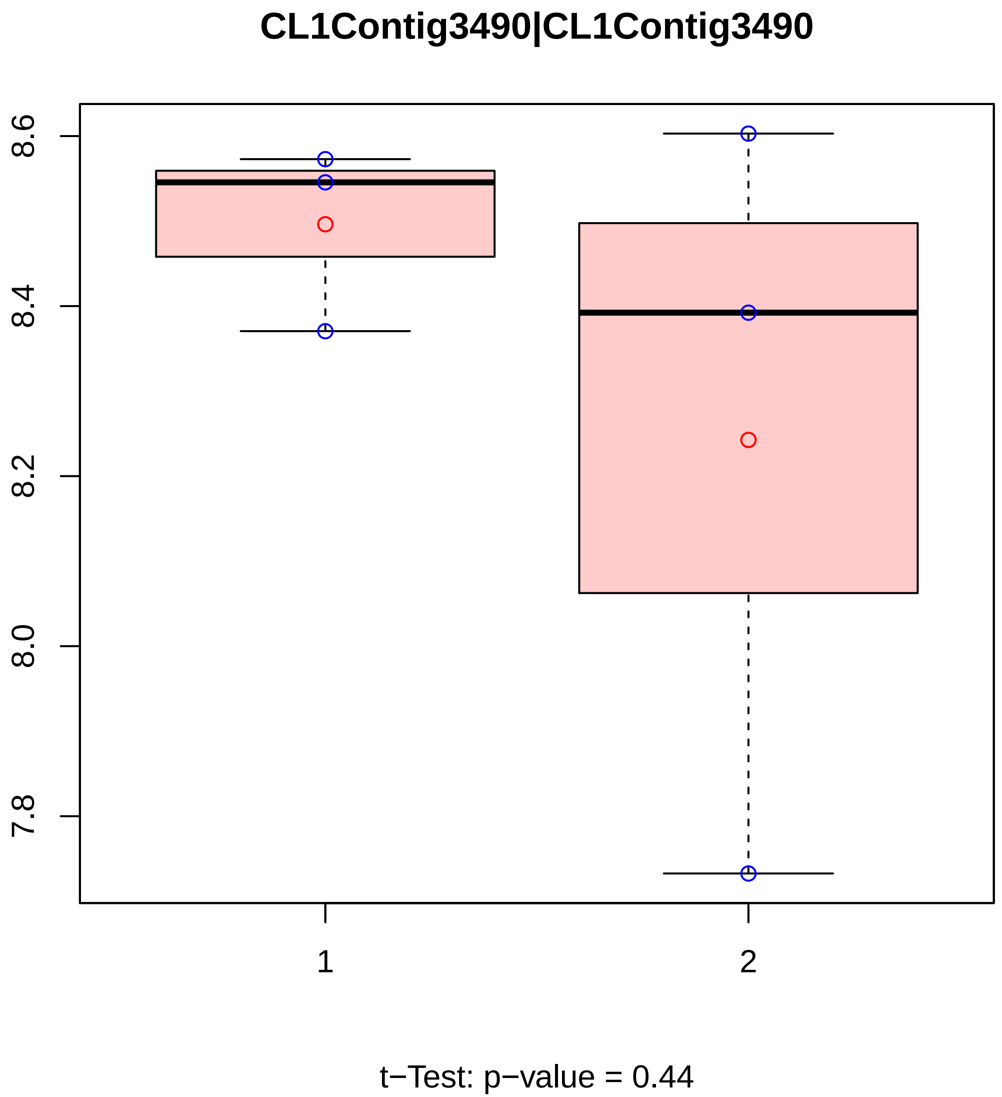
CL1Contig3490|CL1Contig3490
8.6
8.4
8.2
8.0
7.8
1
2
t−Test: p−value = 0.44

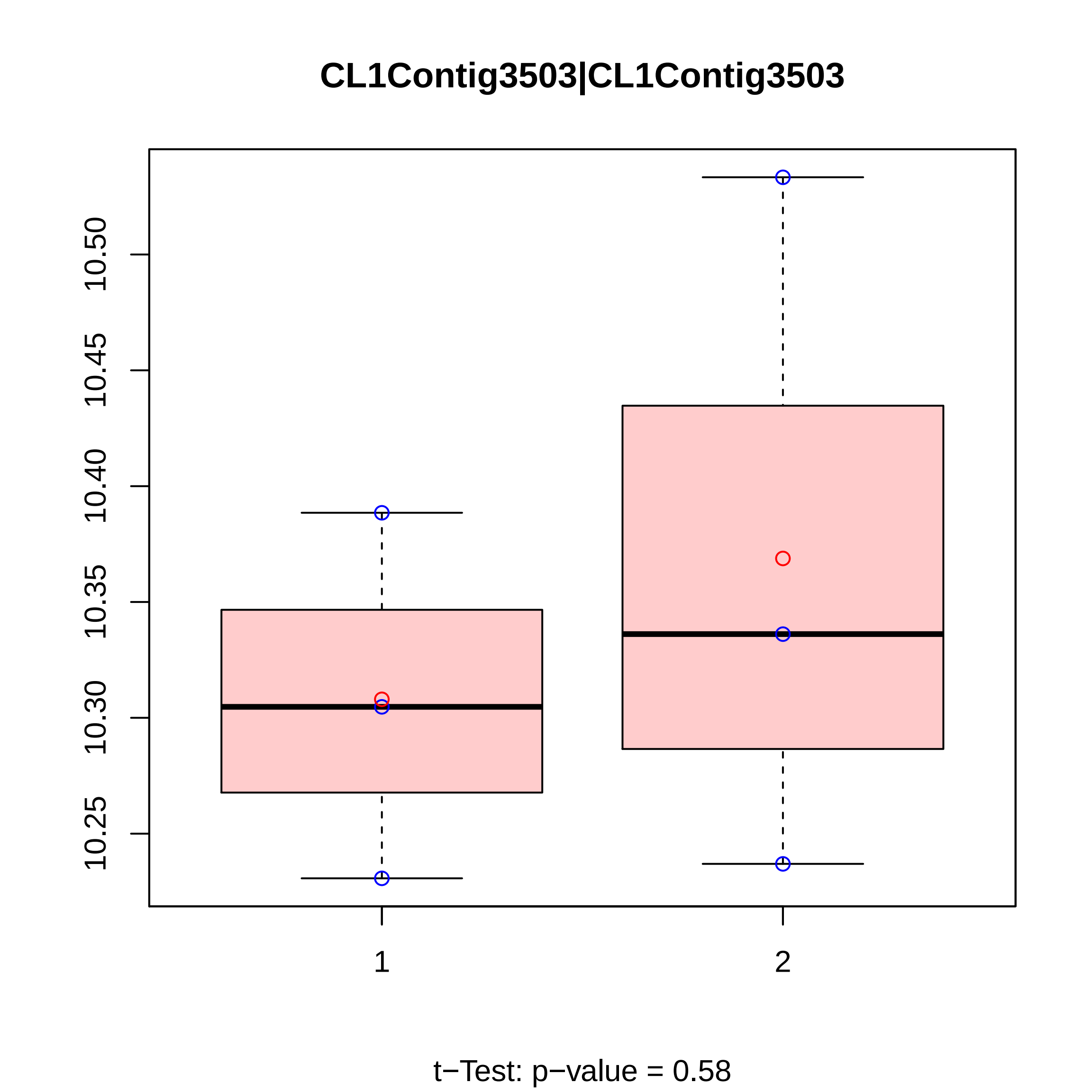

CL1Contig3503|CL1Contig3503
10.25
10.30
10.35
10.40
10.45
10.50
1
2
t−Test: p−value = 0.58

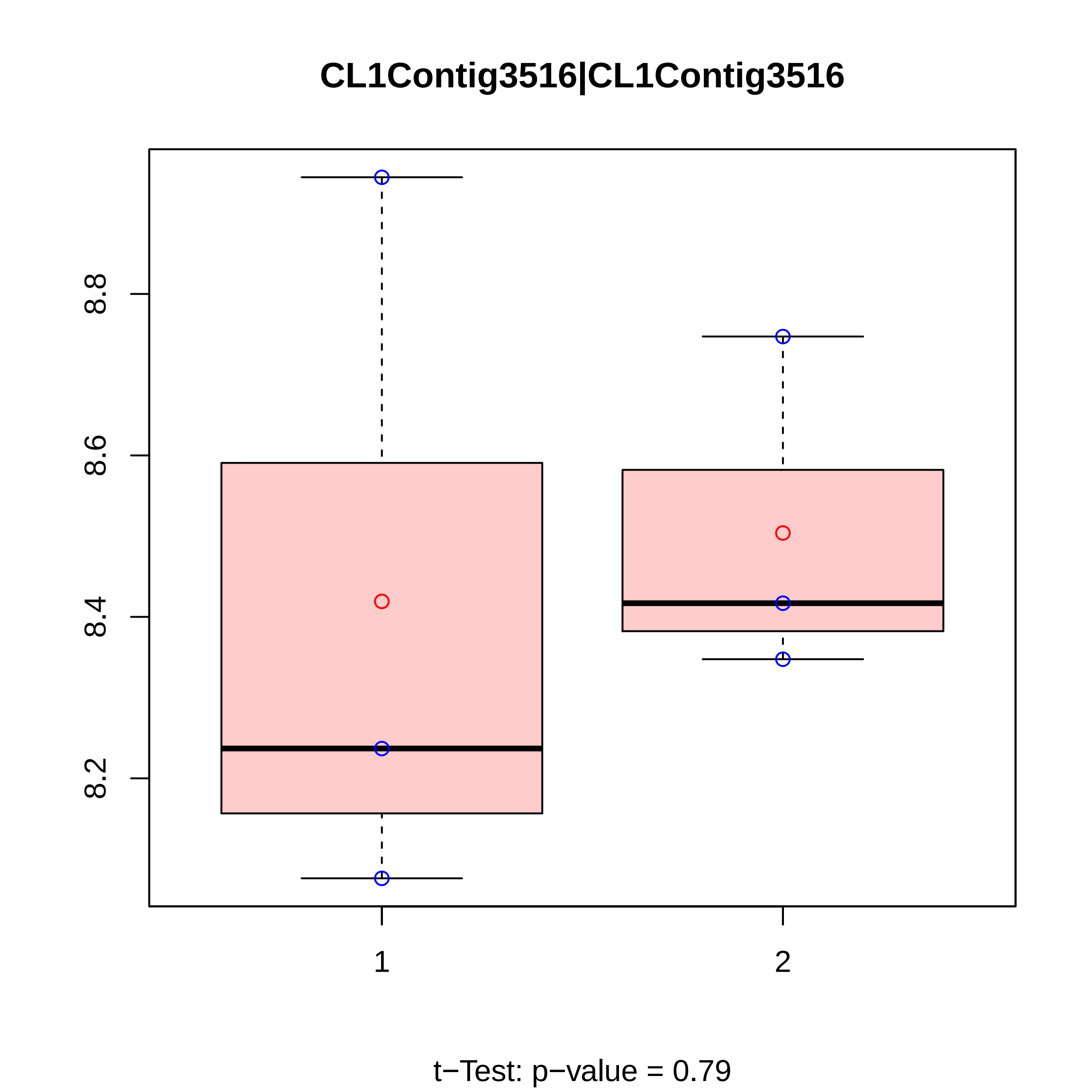
CL1Contig3516|CL1Contig3516
8.2
8.4
8.6
8.8
1
2
t−Test: p−value = 0.79

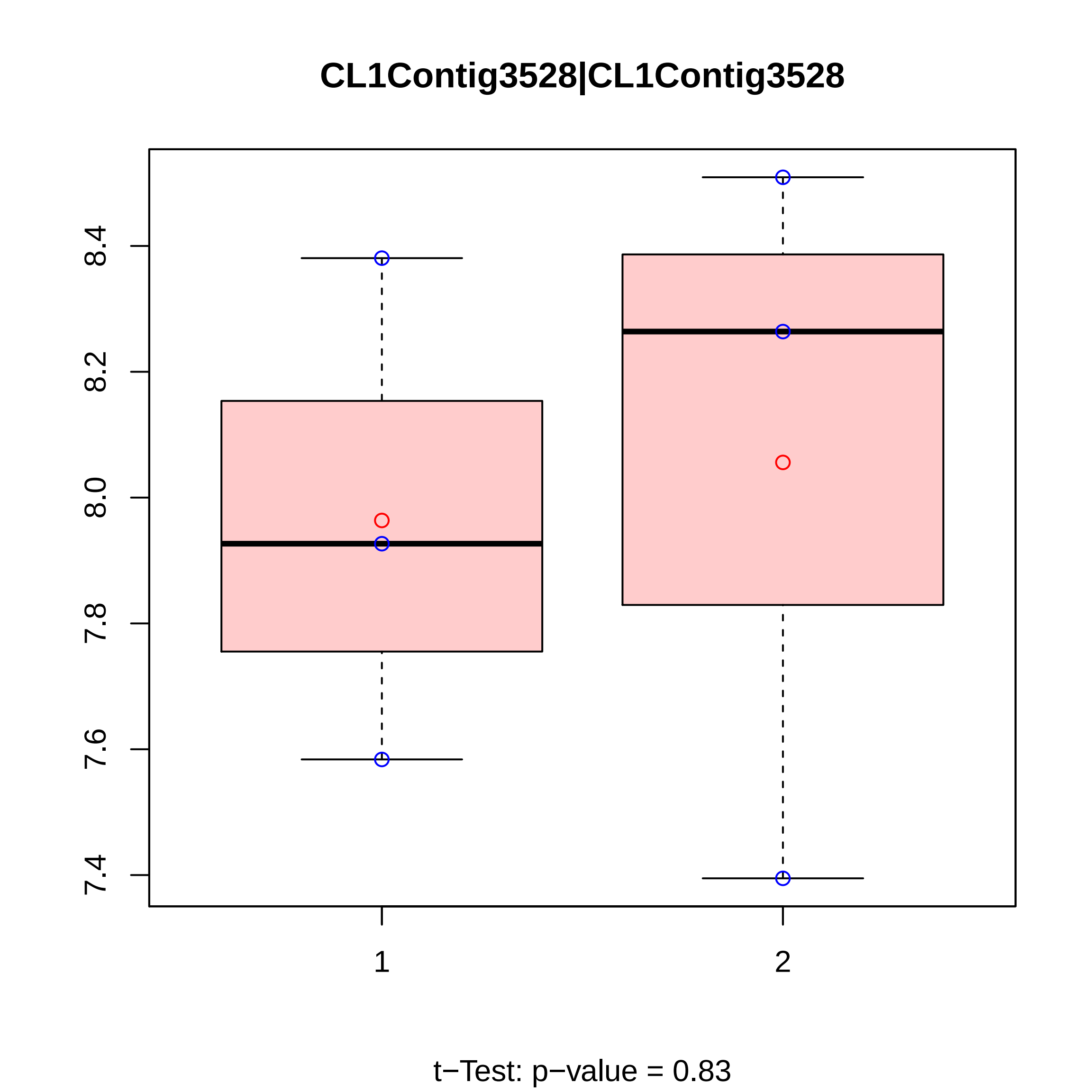
CL1Contig3528|CL1Contig3528
8.4
8.2
8.0
7.8
7.6
7.4
1
2
t−Test: p−value = 0.83

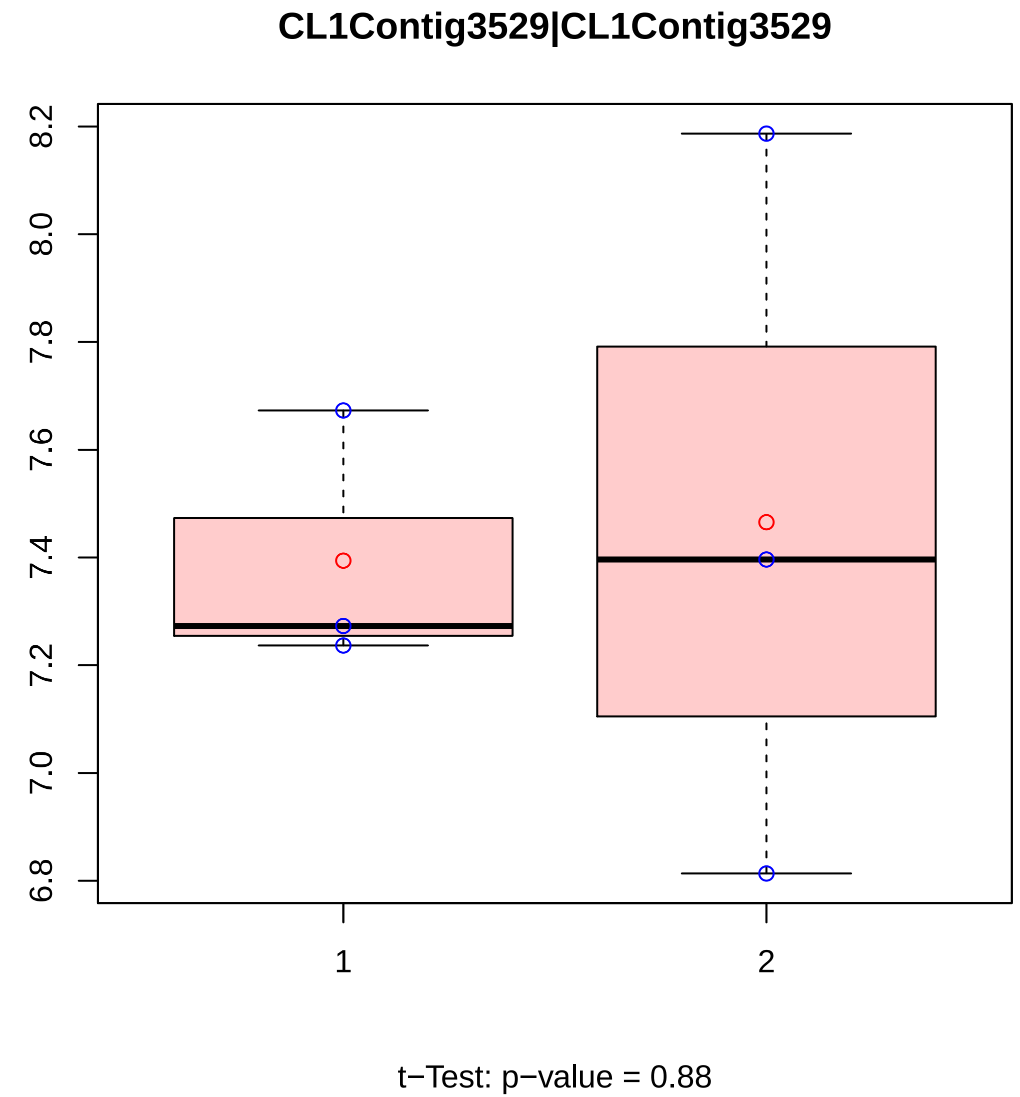
CL1Contig3529|CL1Contig3529
8.2
8.0
7.8
7.6
7.4
7.2
7.0
6.8
1
2
t−Test: p−value = 0.88

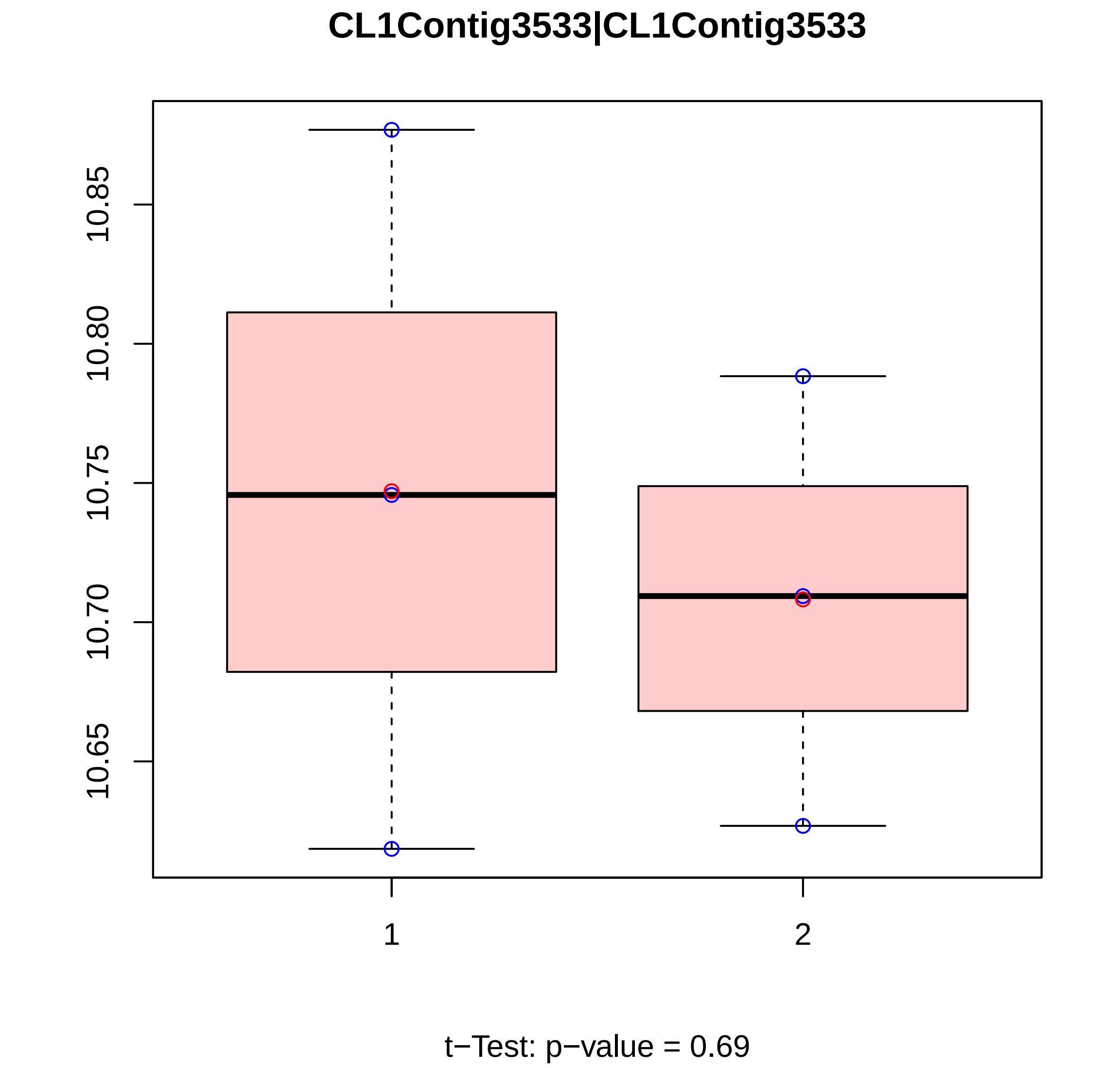
CL1Contig3533|CL1Contig3533
10.65
10.70
10.75
10.80
10.85
1
2
t−Test: p−value = 0.69

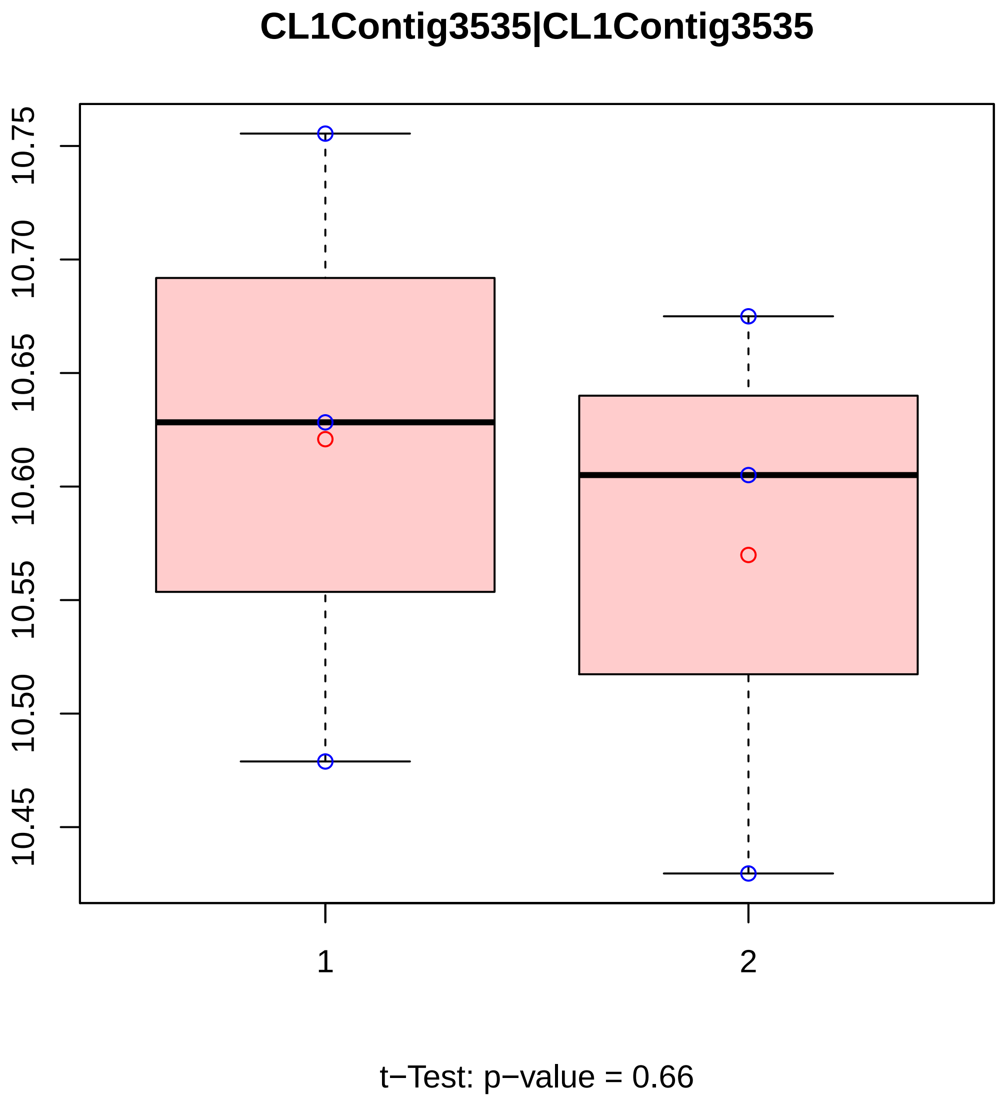

CL1Contig3535|CL1Contig3535
10.45
10.50
10.55
10.60
10.65
10.70
10.75
1
2
t−Test: p−value = 0.66

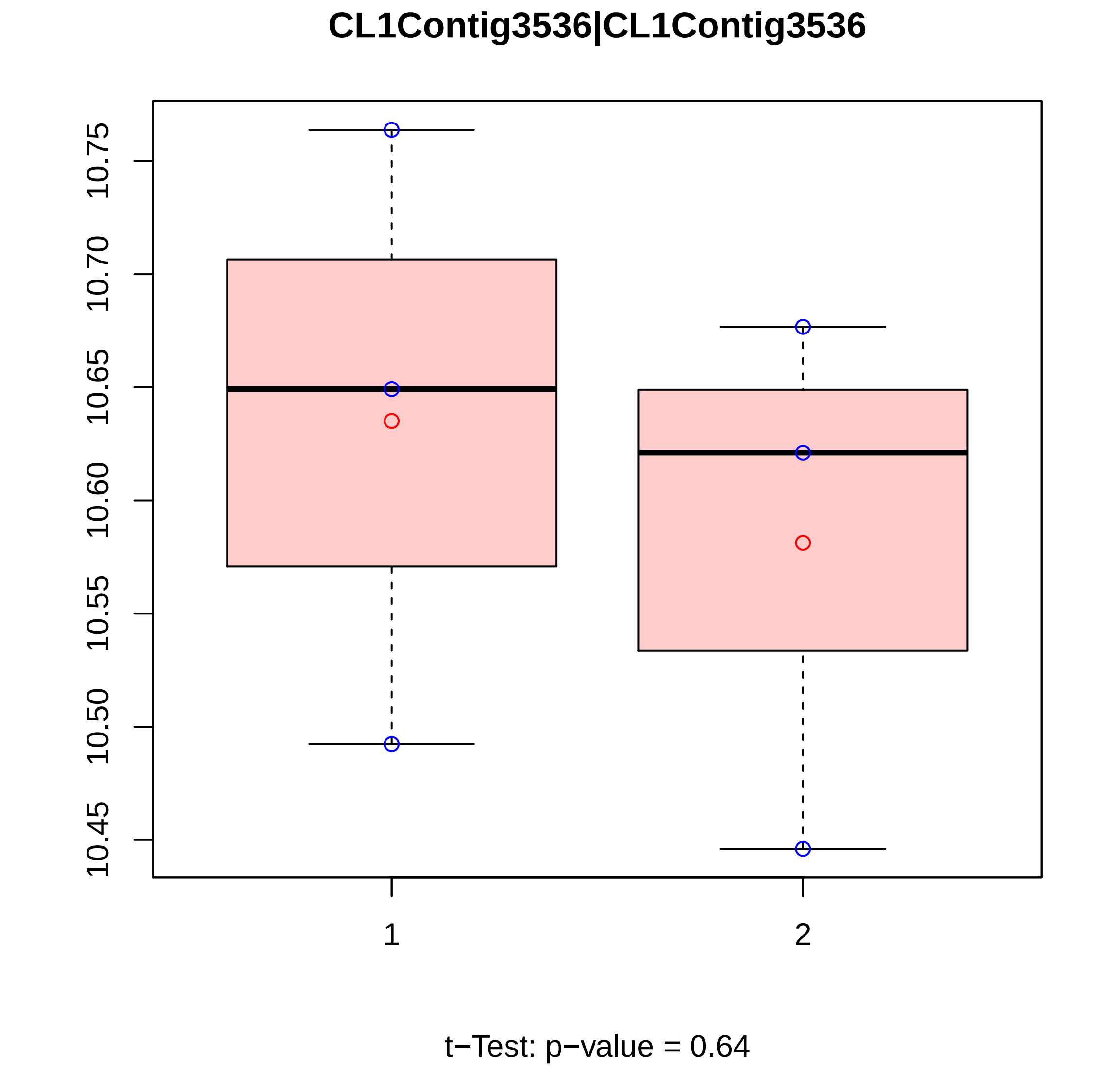

CL1Contig3536|CL1Contig3536
10.45
10.50
10.55
10.60
10.65
10.70
10.75
1
2
t−Test: p−value = 0.64

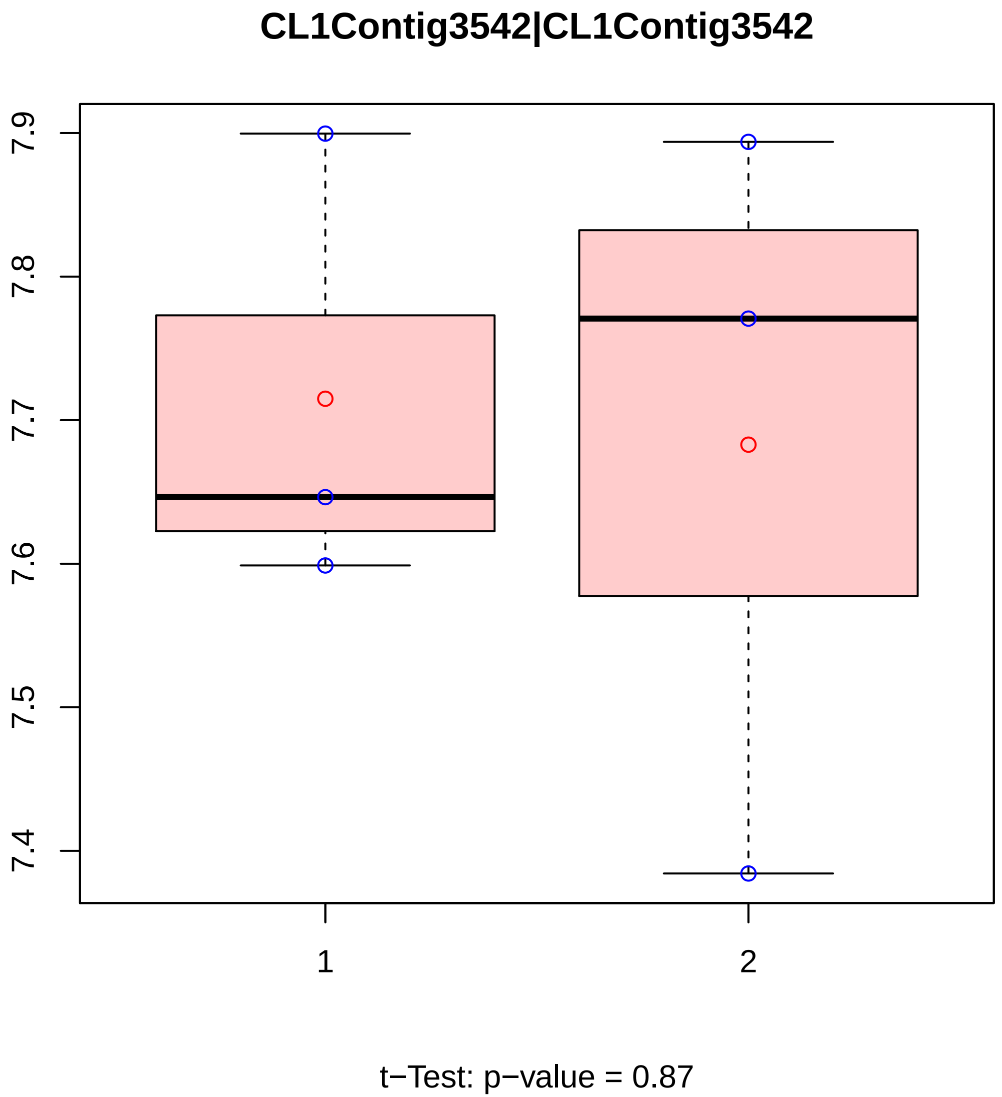

CL1Contig3542|CL1Contig3542
7.9
7.8
7.7
7.6
7.5
7.4
1
2
t−Test: p−value = 0.87

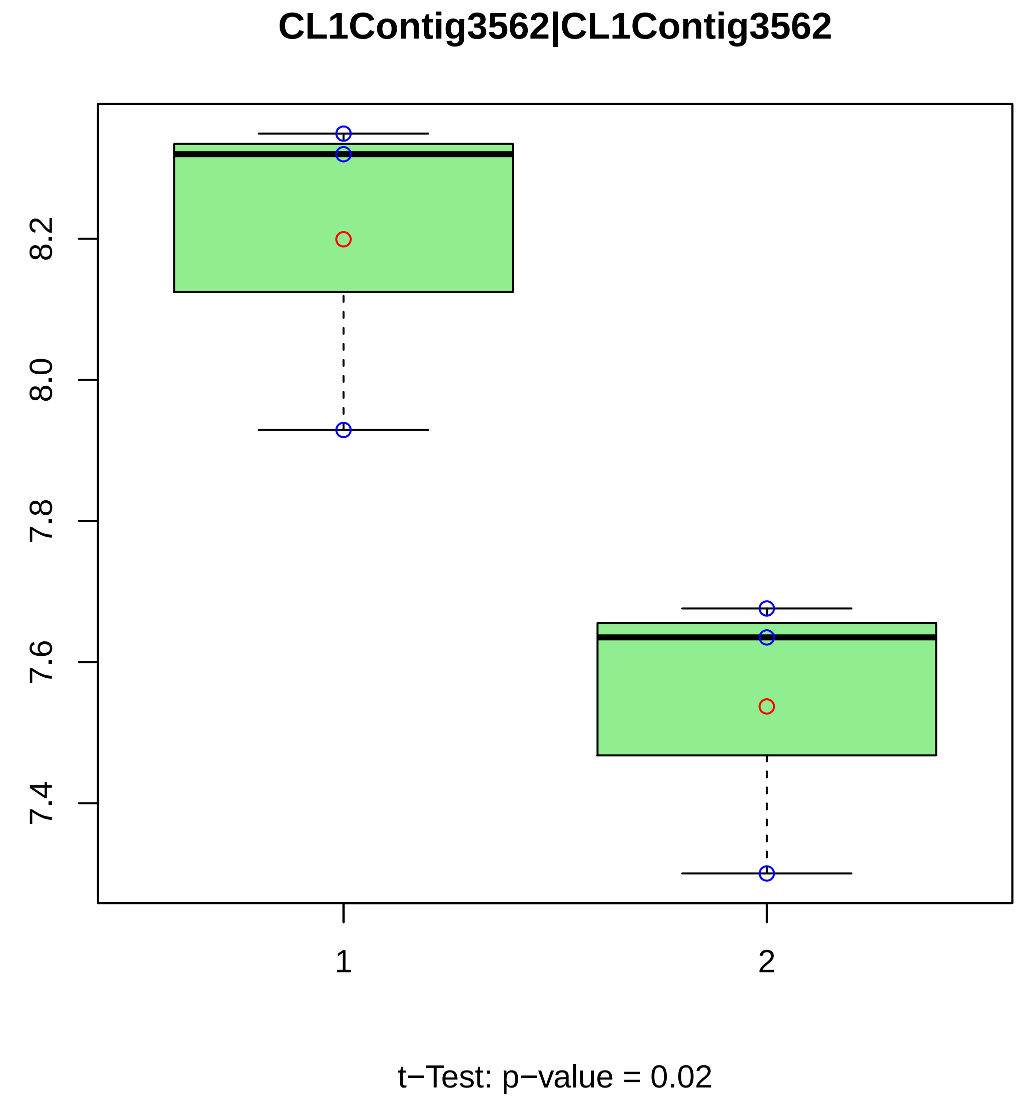

CL1Contig3562|CL1Contig3562
8.2
8.0
7.8
7.6
7.4
1
2
t−Test: p−value = 0.02

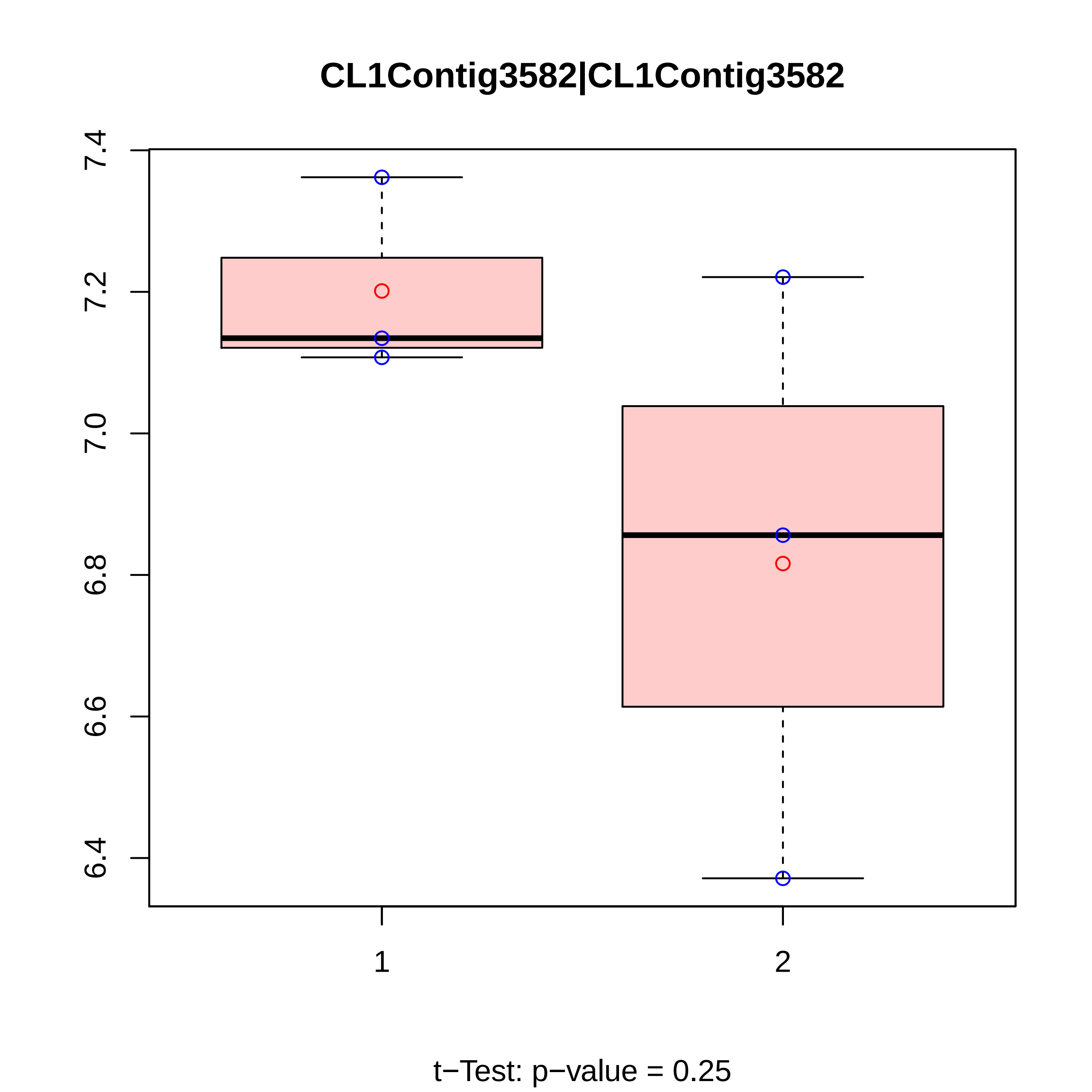

CL1Contig3582|CL1Contig3582
7.4
7.2
7.0
6.8
6.6
6.4
1
2
t−Test: p−value = 0.25

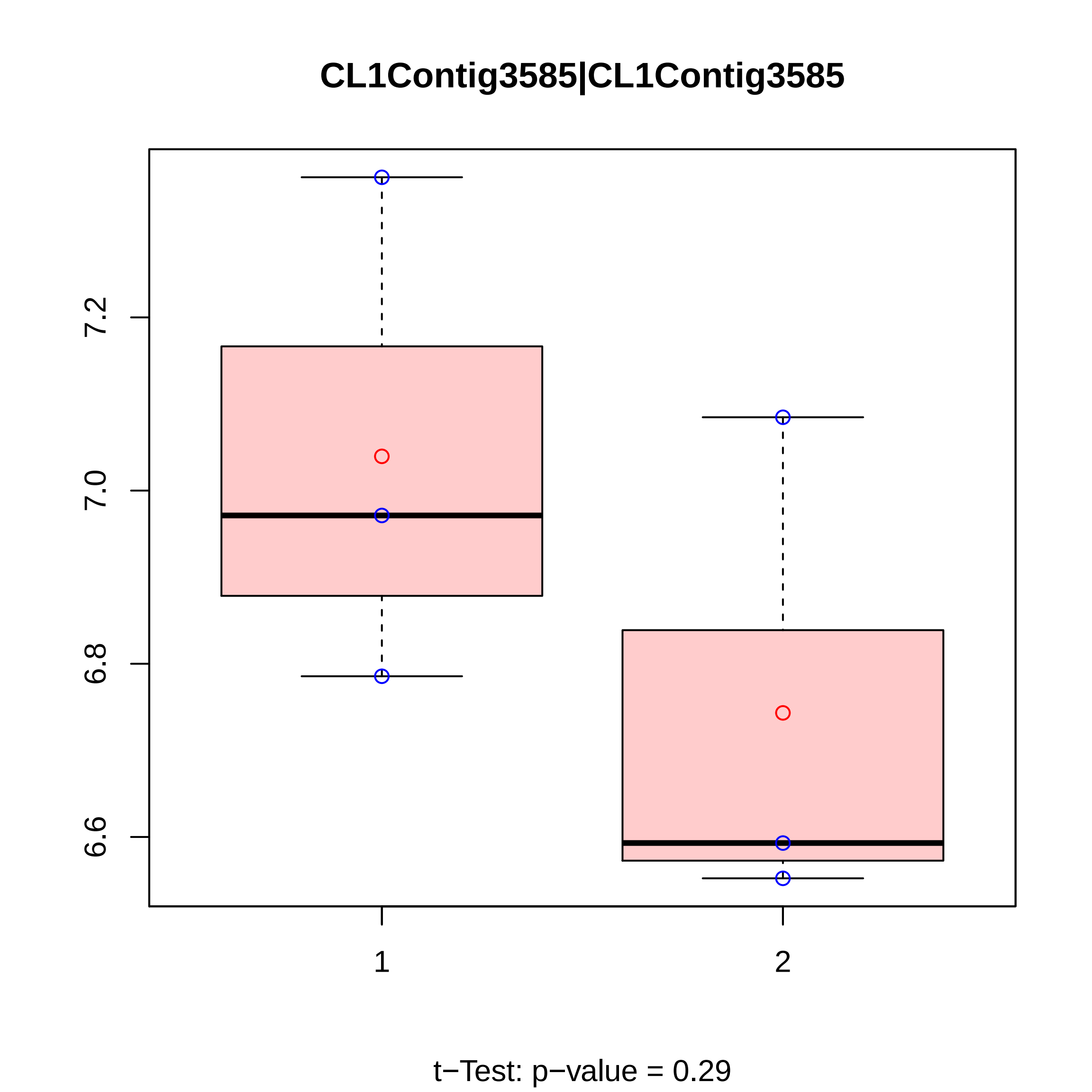
CL1Contig3585|CL1Contig3585
7.2
7.0
6.8
6.6
1
2
t−Test: p−value = 0.29

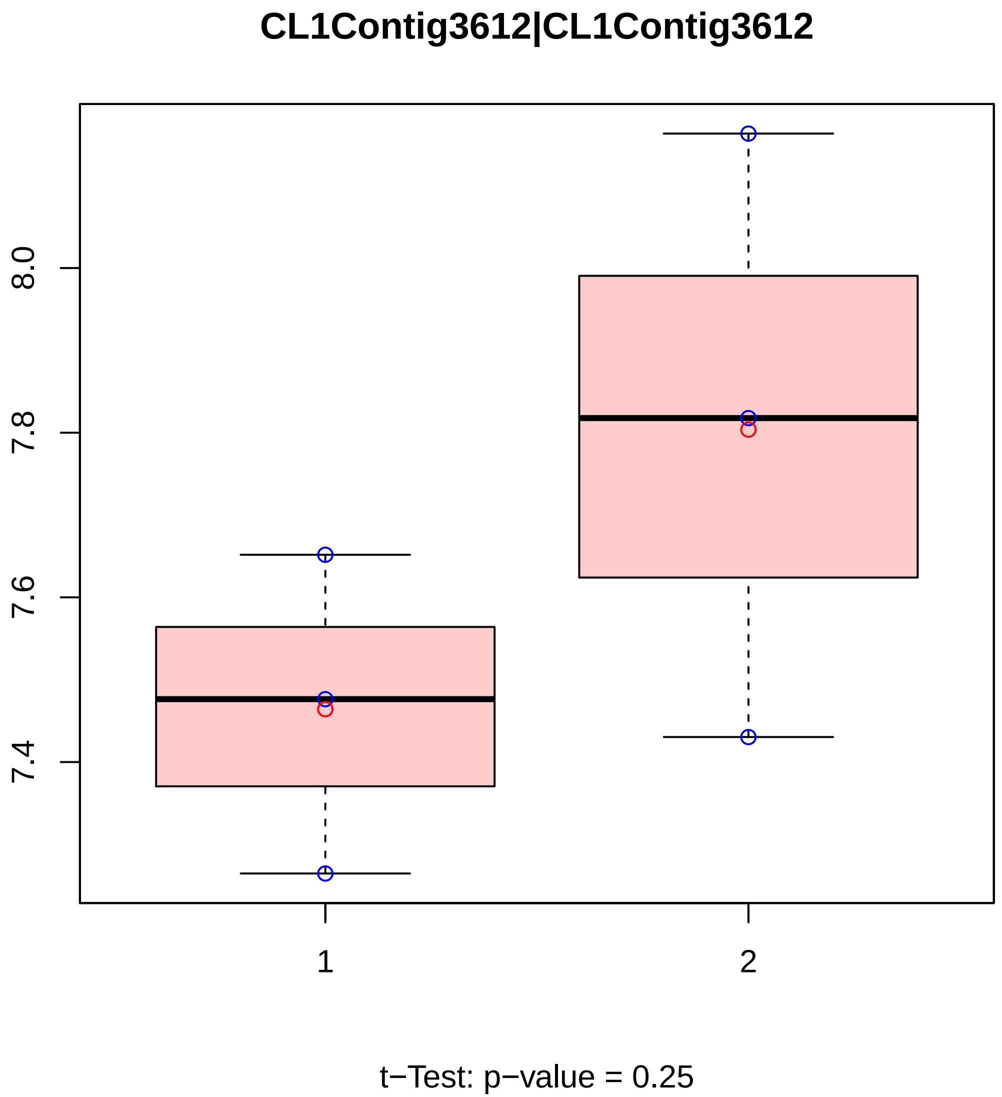
CL1Contig3612|CL1Contig3612
8.0
7.8
7.6
7.4
1
2
t−Test: p−value = 0.25

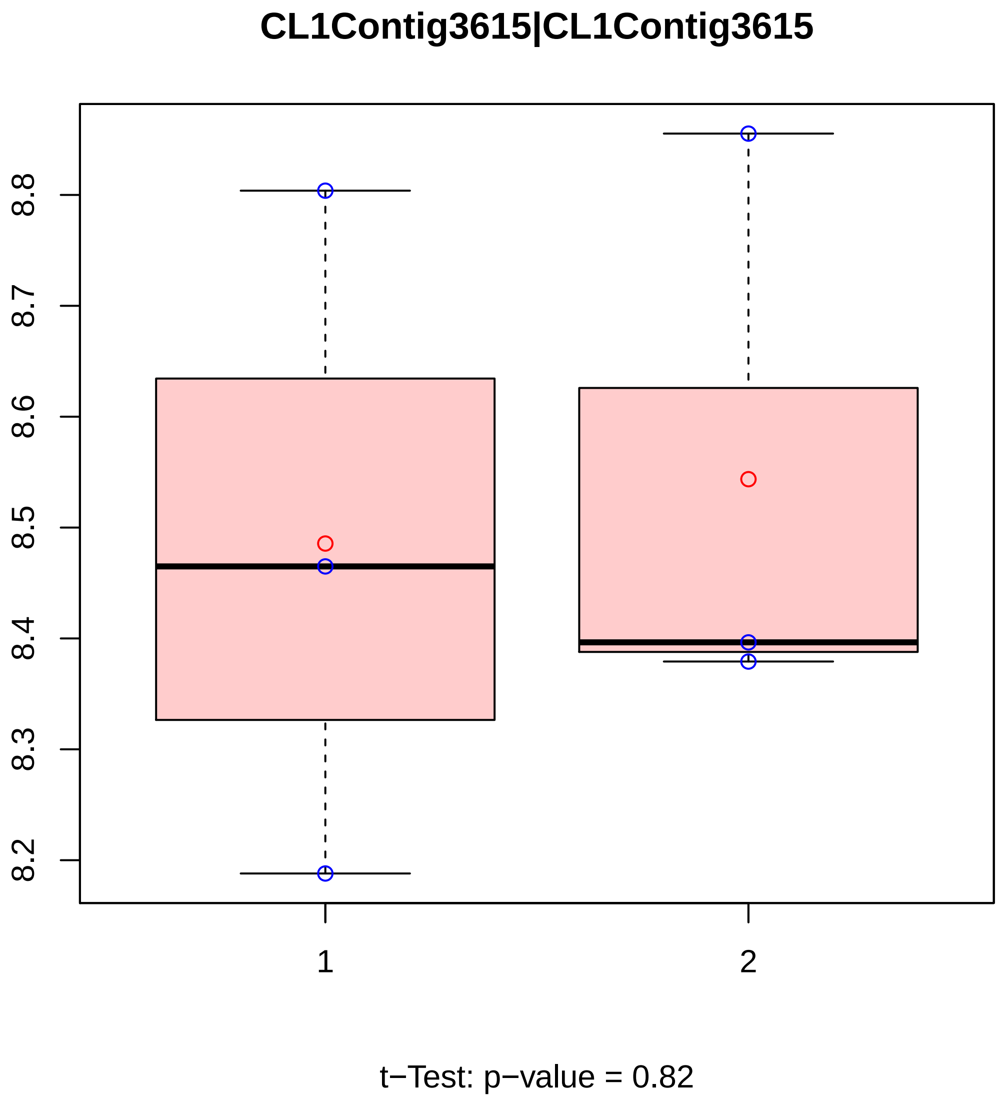

CL1Contig3615|CL1Contig3615
8.2
8.3
8.4
8.5
8.6
8.7
8.8
1
2
t−Test: p−value = 0.82

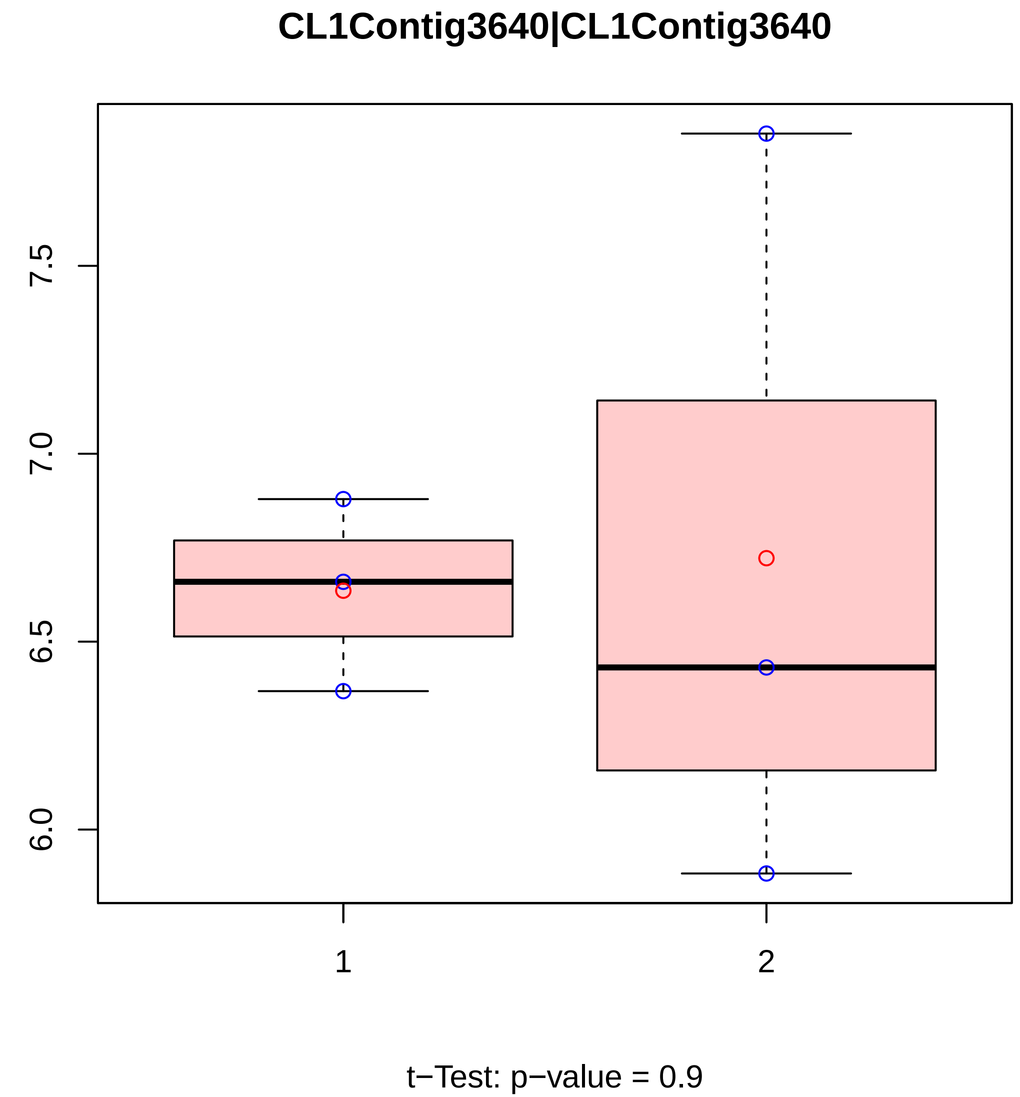
CL1Contig3640|CL1Contig3640
7.5
7.0
6.5
6.0
1
2
t−Test: p−value = 0.9

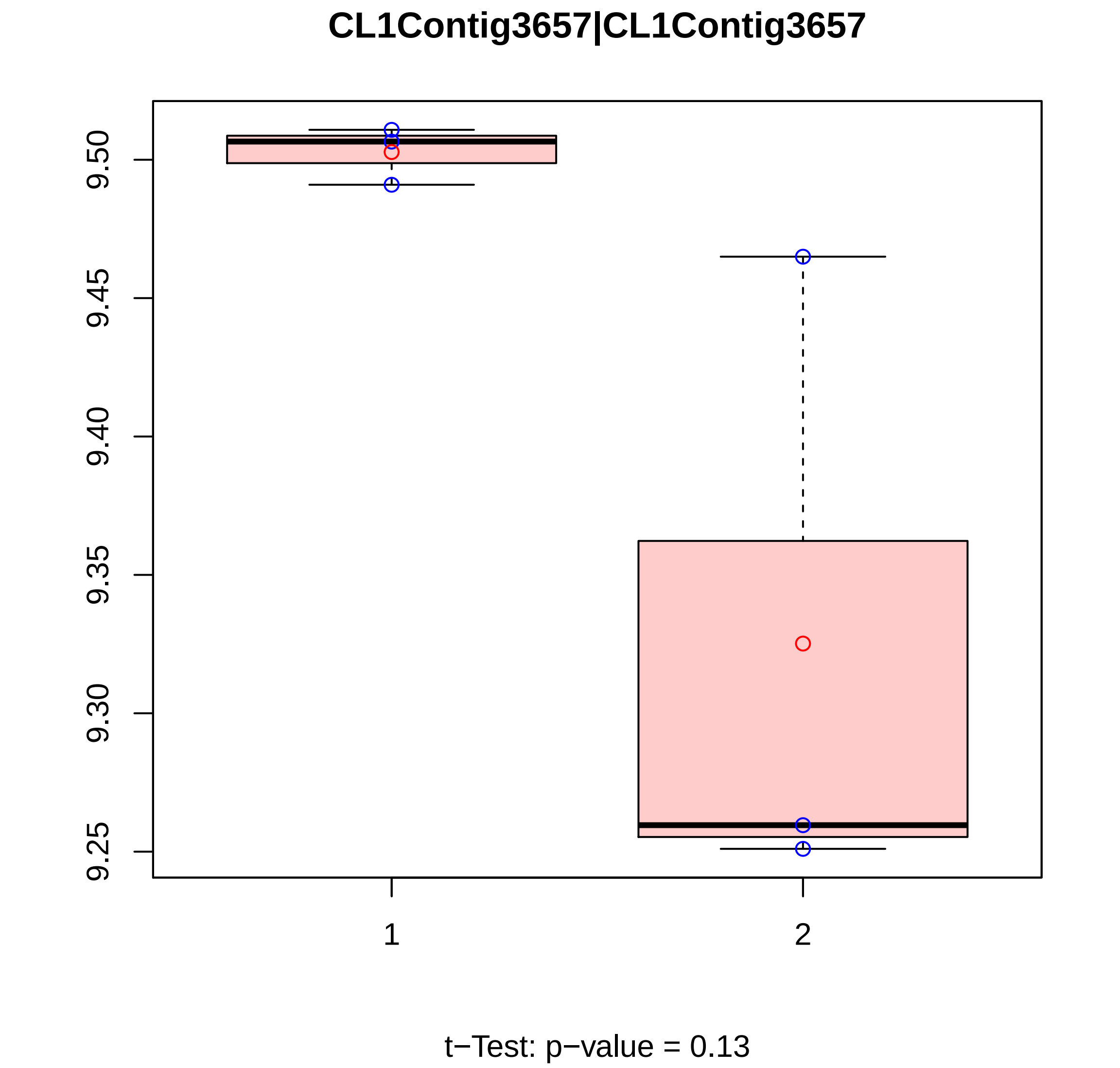
CL1Contig3657|CL1Contig3657
9.50
9.45
9.40
9.35
9.30
9.25
1
2
t−Test: p−value = 0.13

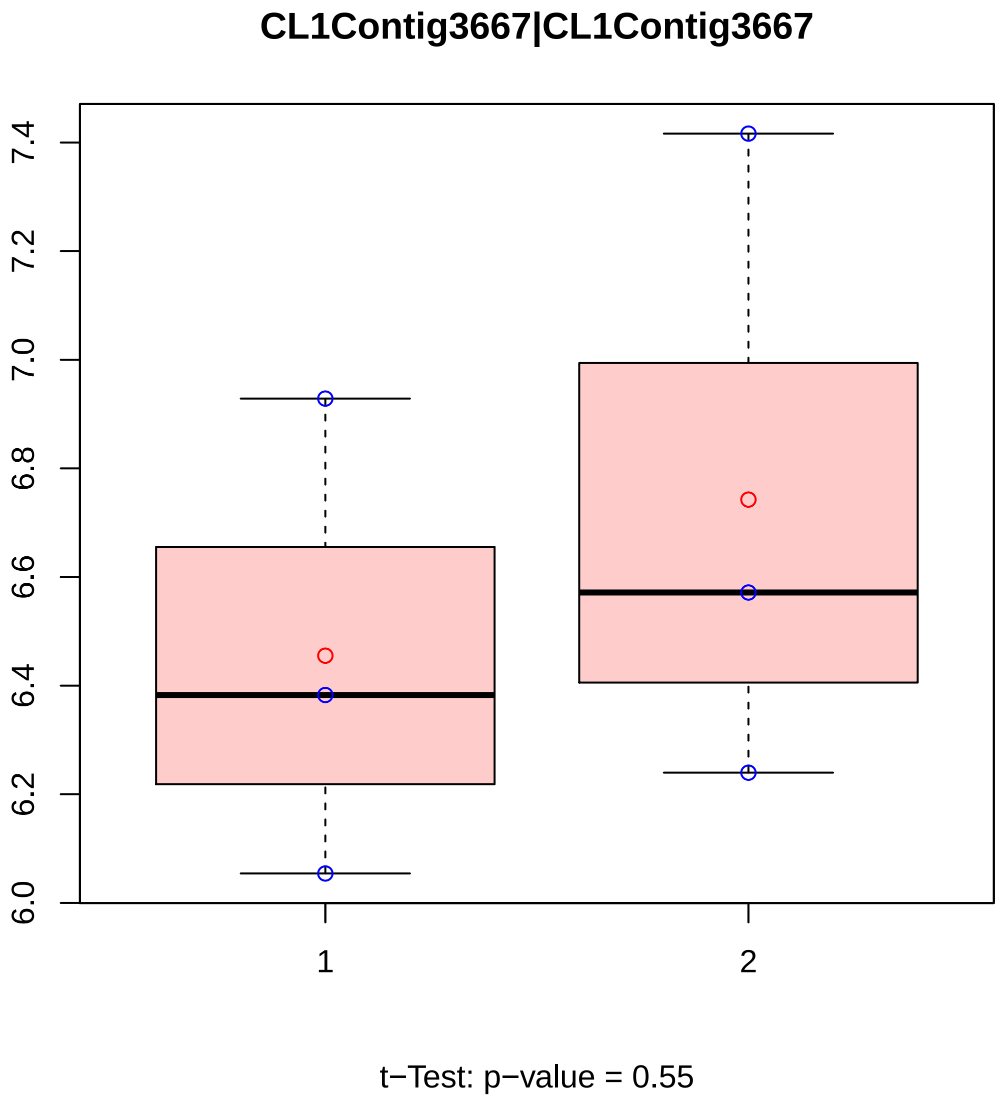
CL1Contig3667|CL1Contig3667
6.0
6.2
6.4
6.6
6.8
7.0
7.2
7.4
1
2
t−Test: p−value = 0.55

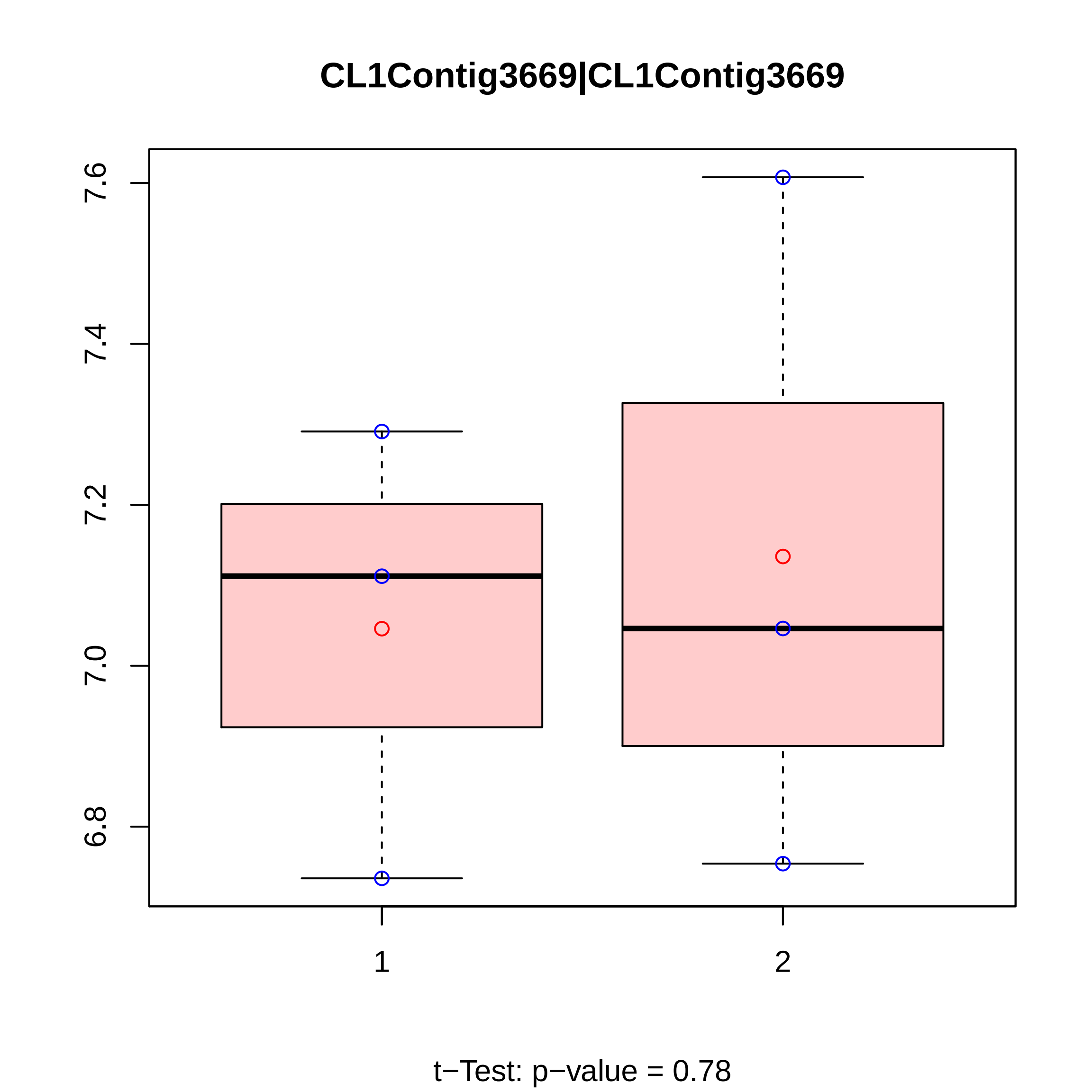
CL1Contig3669|CL1Contig3669
7.6
7.4
7.2
7.0
6.8
1
2
t−Test: p−value = 0.78

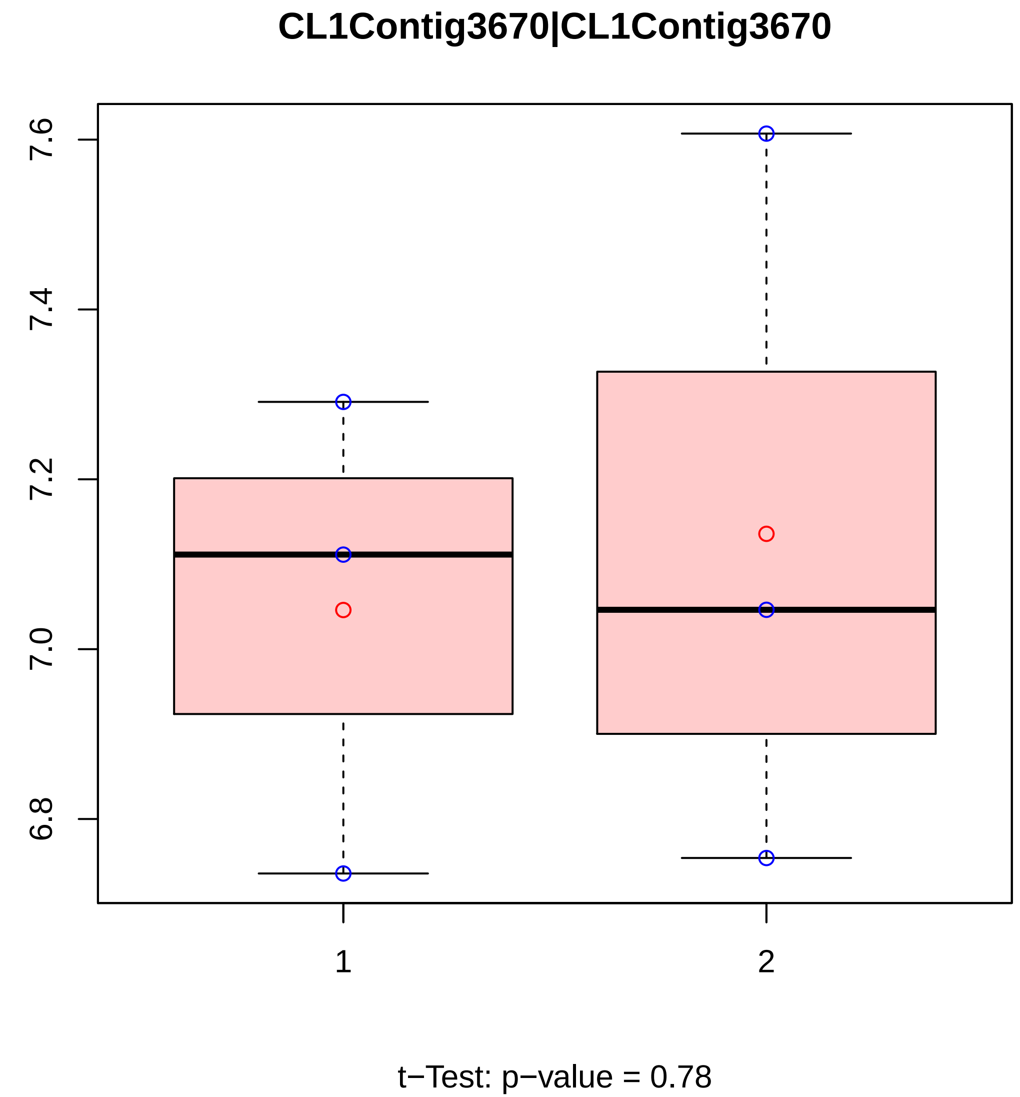
CL1Contig3670|CL1Contig3670
7.6
7.4
7.2
7.0
6.8
1
2
t−Test: p−value = 0.78

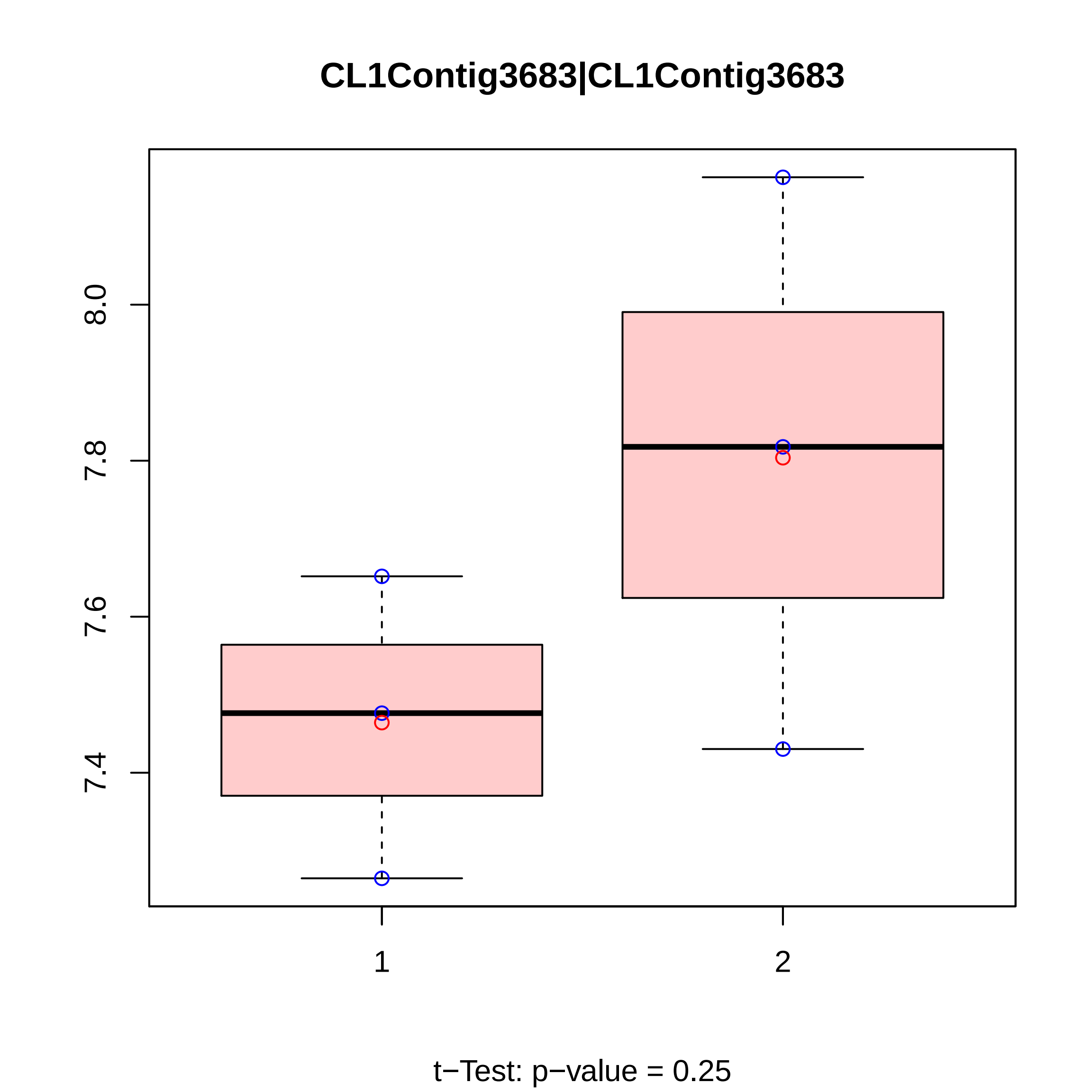
CL1Contig3683|CL1Contig3683
8.0
7.8
7.6
7.4
1
2
t−Test: p−value = 0.25

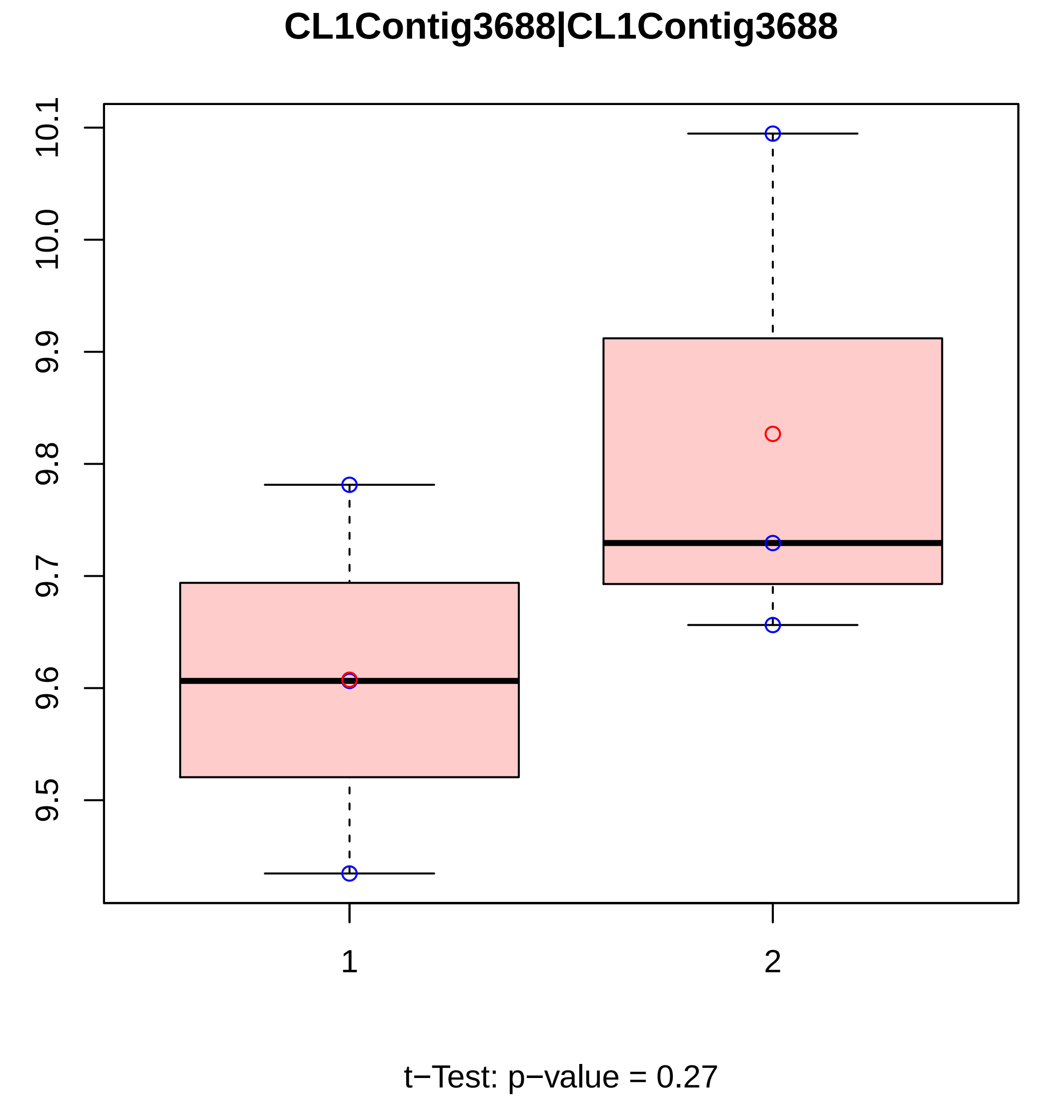
CL1Contig3688|CL1Contig3688
10.1
10.0
9.9
9.8
9.7
9.6
9.5
1
2
t−Test: p−value = 0.27

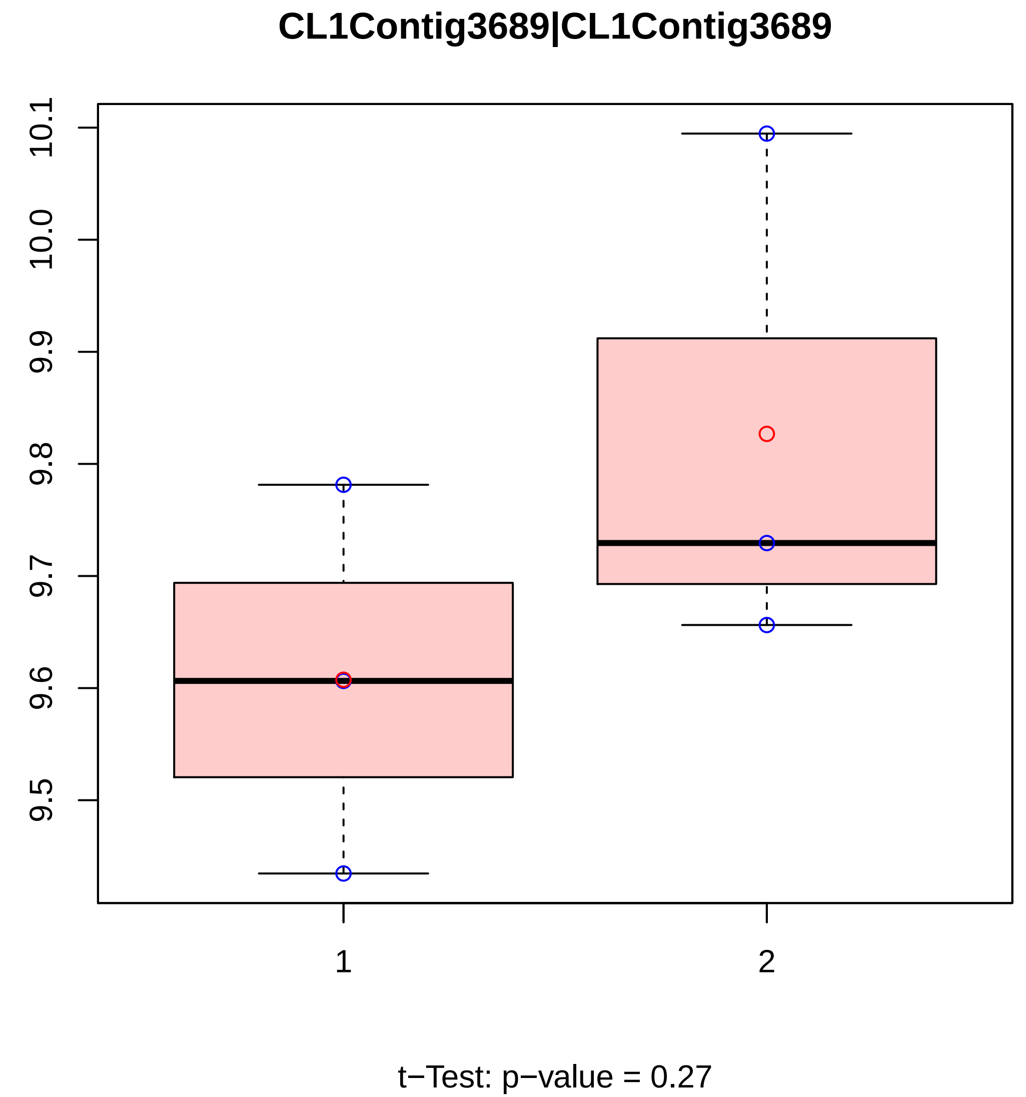

CL1Contig3689|CL1Contig3689
10.1
10.0
9.9
9.8
9.7
9.6
9.5
1
2
t−Test: p−value = 0.27

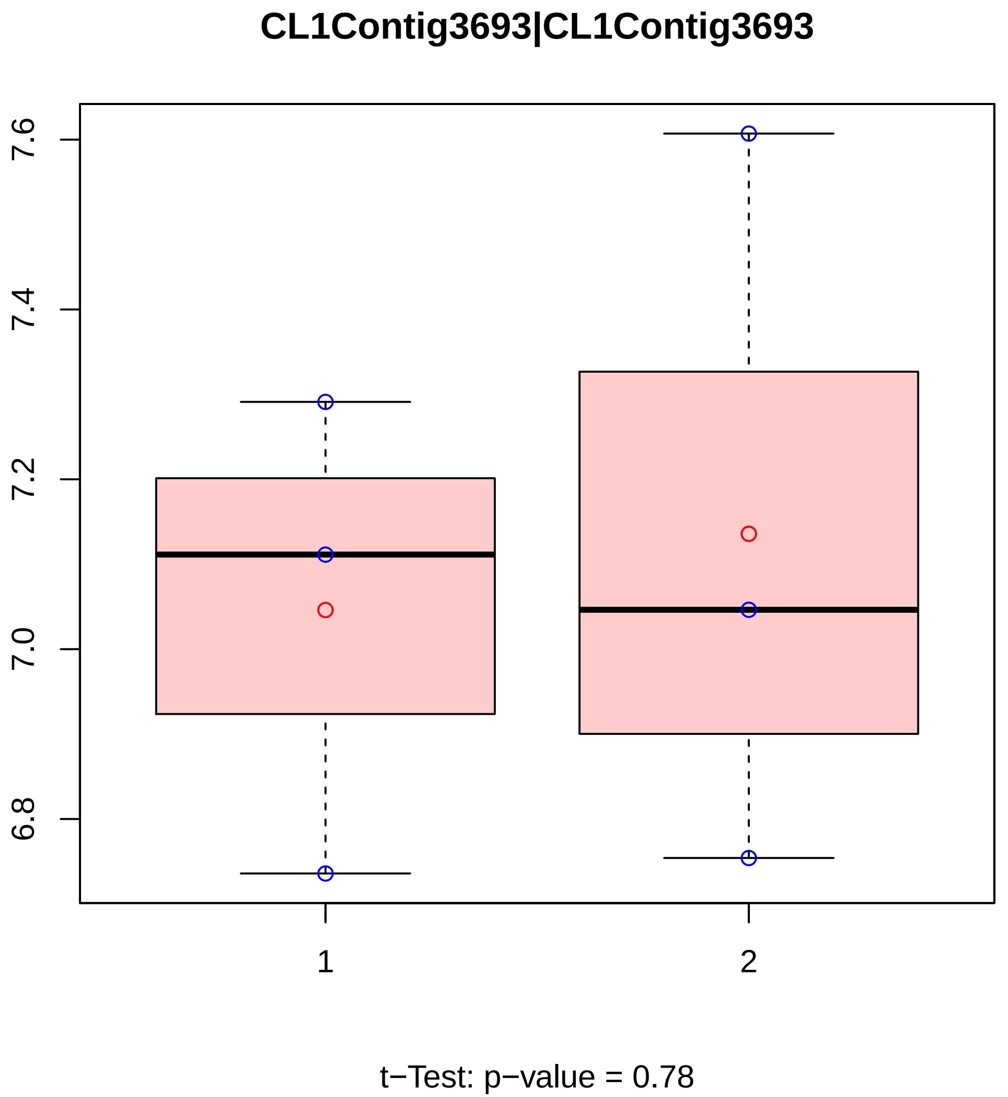
CL1Contig3693|CL1Contig3693
7.6
7.4
7.2
7.0
6.8
1
2
t−Test: p−value = 0.78

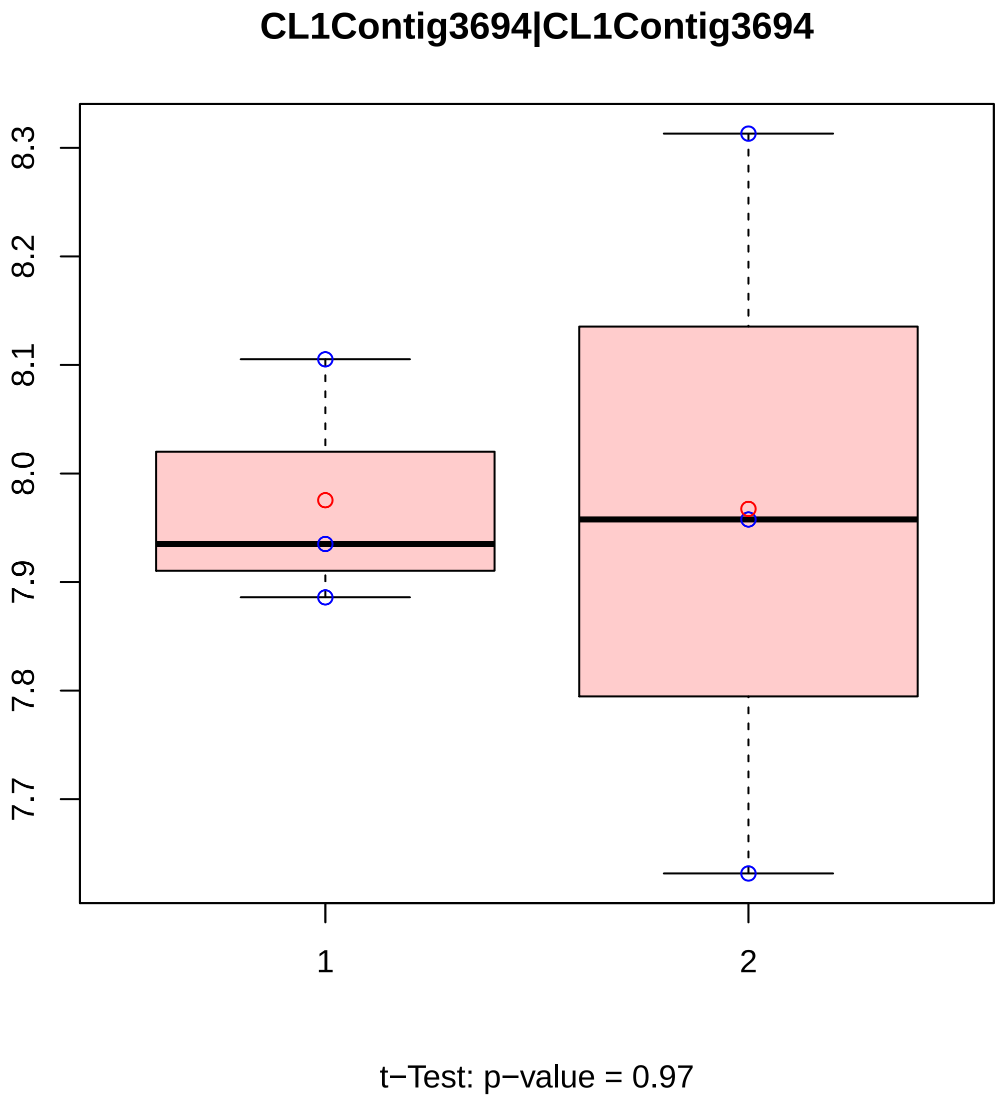

CL1Contig3694|CL1Contig3694
8.3
8.2
8.1
8.0
7.9
7.8
7.7
1
2
t−Test: p−value = 0.97

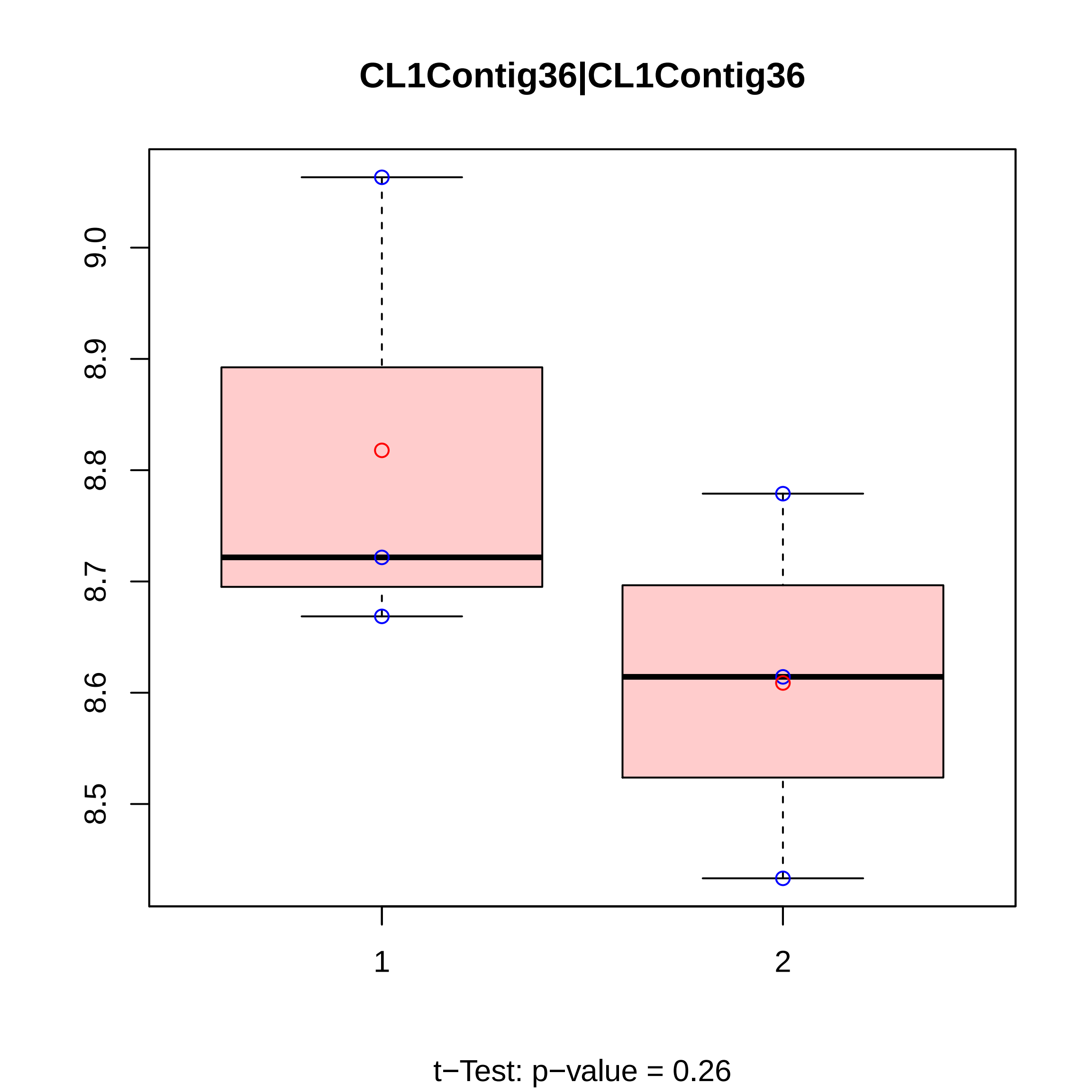

CL1Contig36|CL1Contig36
9.0
8.9
8.8
8.7
8.6
8.5
1
2
t−Test: p−value = 0.26

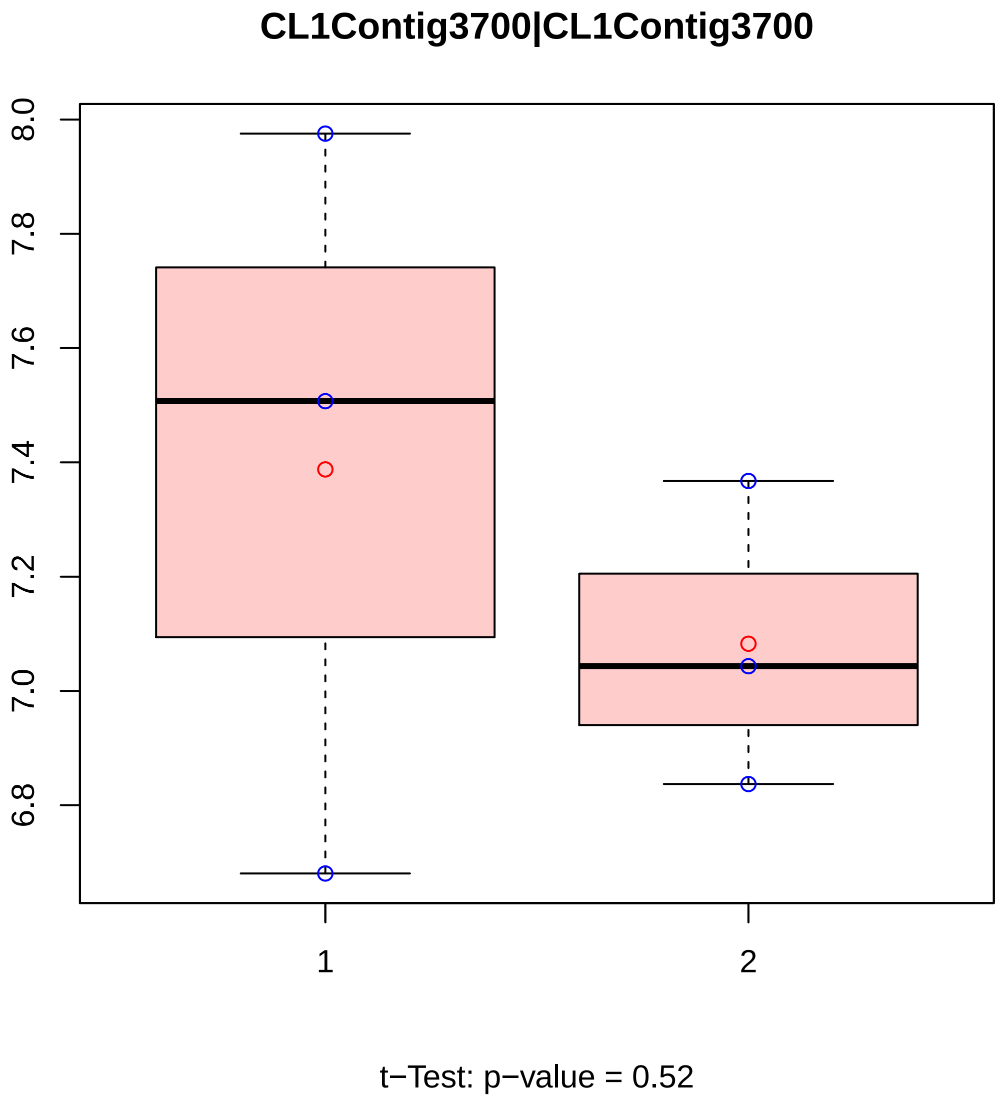
CL1Contig3700|CL1Contig3700
8.0
7.8
7.6
7.4
7.2
7.0
6.8
1
2
t−Test: p−value = 0.52

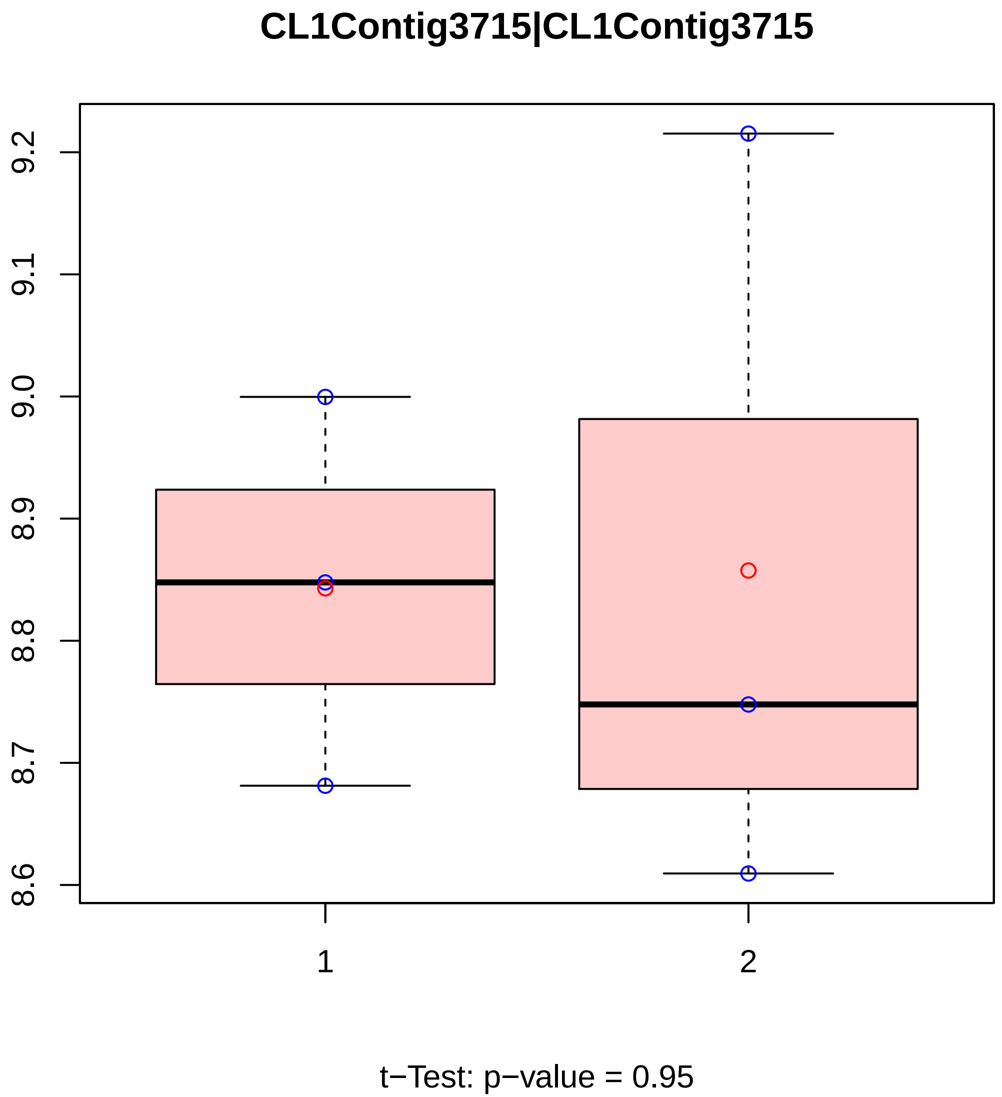
CL1Contig3715|CL1Contig3715
9.2
9.1
9.0
8.9
8.8
8.7
8.6
1
2
t−Test: p−value = 0.95

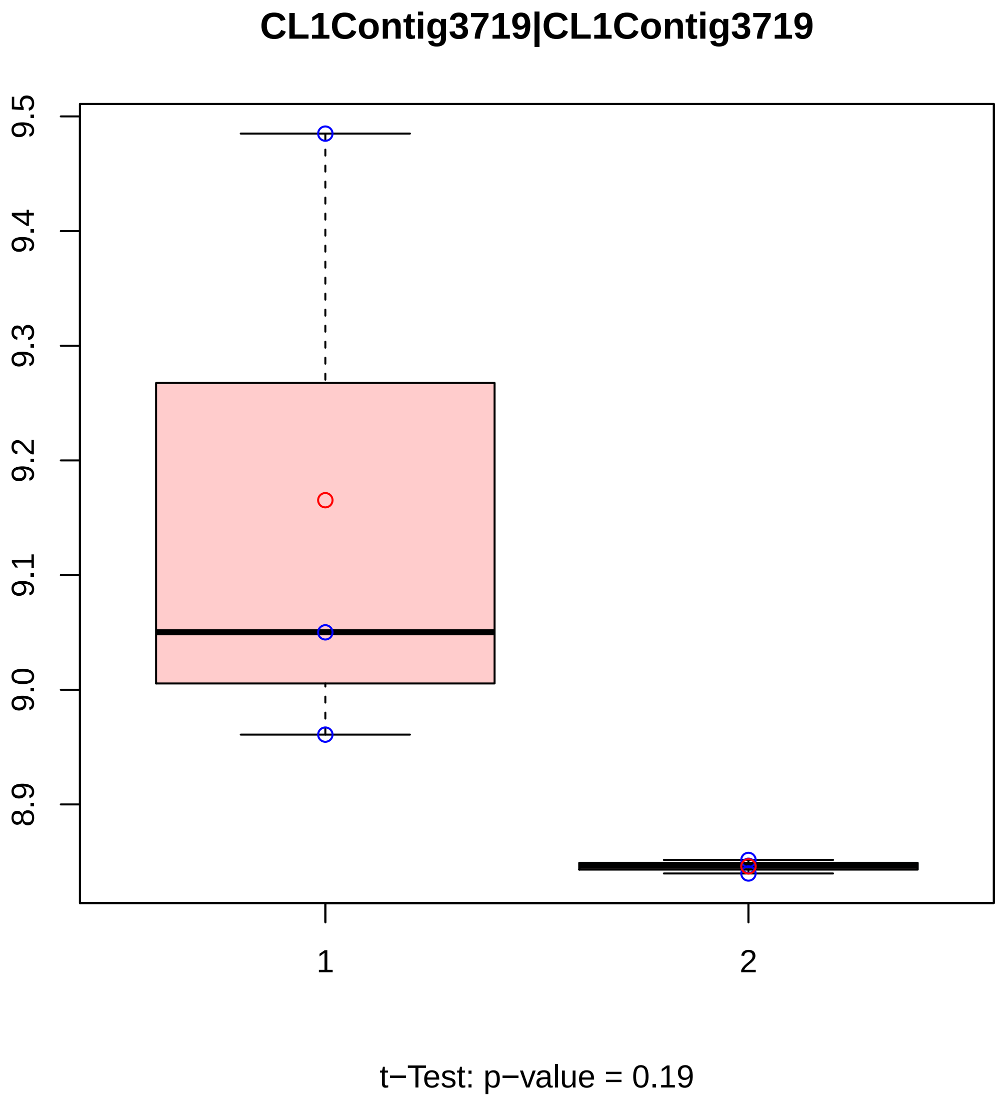
CL1Contig3719|CL1Contig3719
9.5
9.4
9.3
9.2
9.1
9.0
8.9
1
2
t−Test: p−value = 0.19

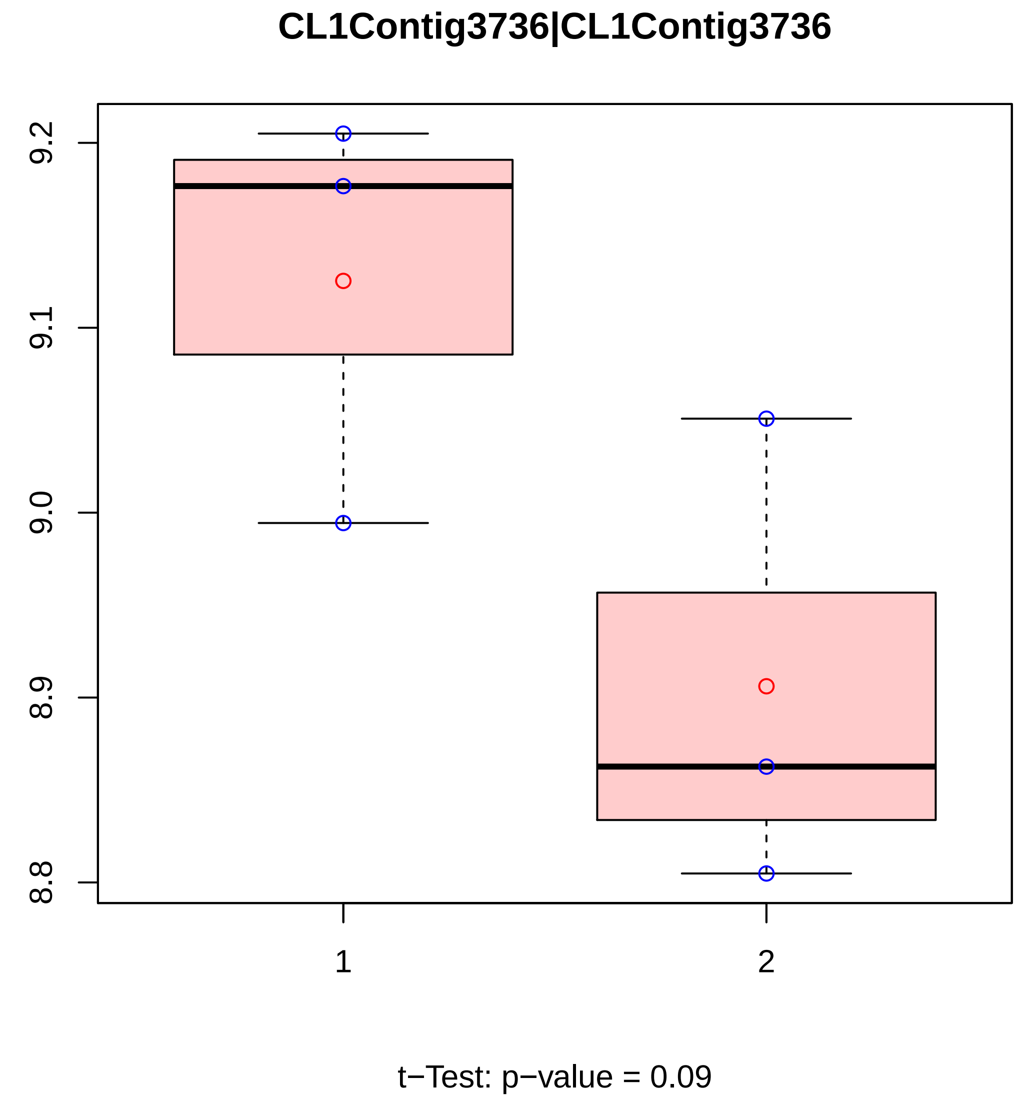
CL1Contig3736|CL1Contig3736
9.2
9.1
9.0
8.9
8.8
1
2
t−Test: p−value = 0.09

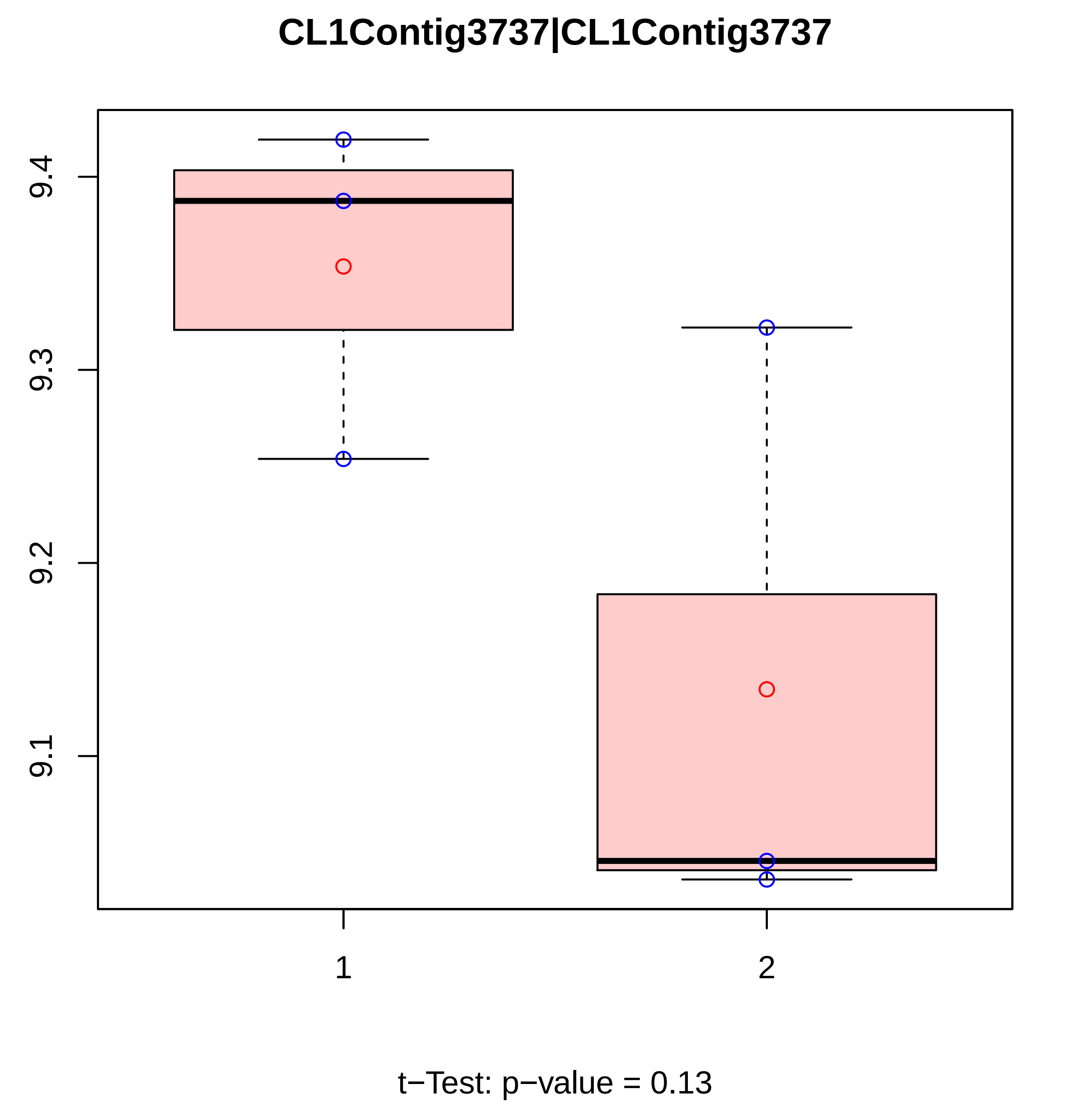
CL1Contig3737|CL1Contig3737
9.4
9.3
9.2
9.1
1
2
t−Test: p−value = 0.13

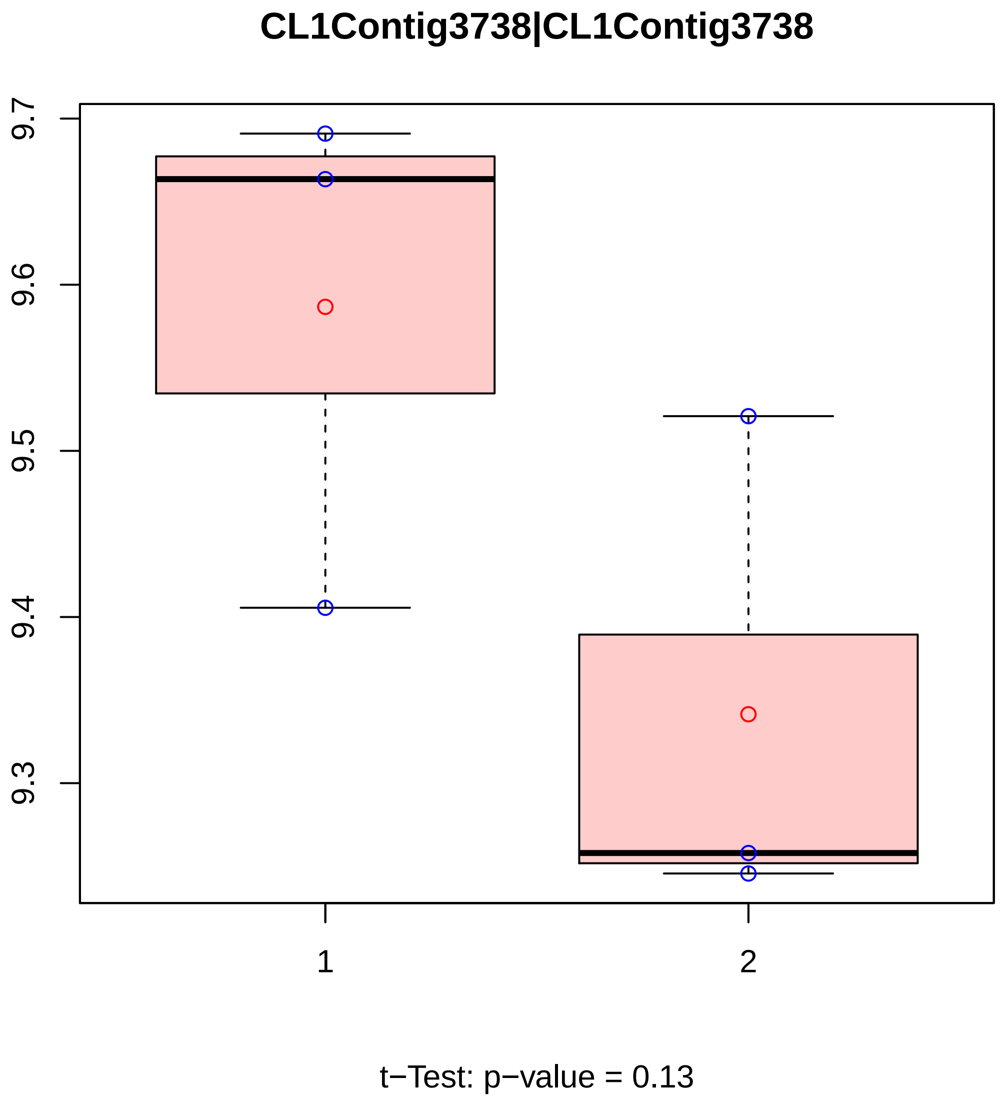
CL1Contig3738|CL1Contig3738
9.7
9.6
9.5
9.4
9.3
1
2
t−Test: p−value = 0.13

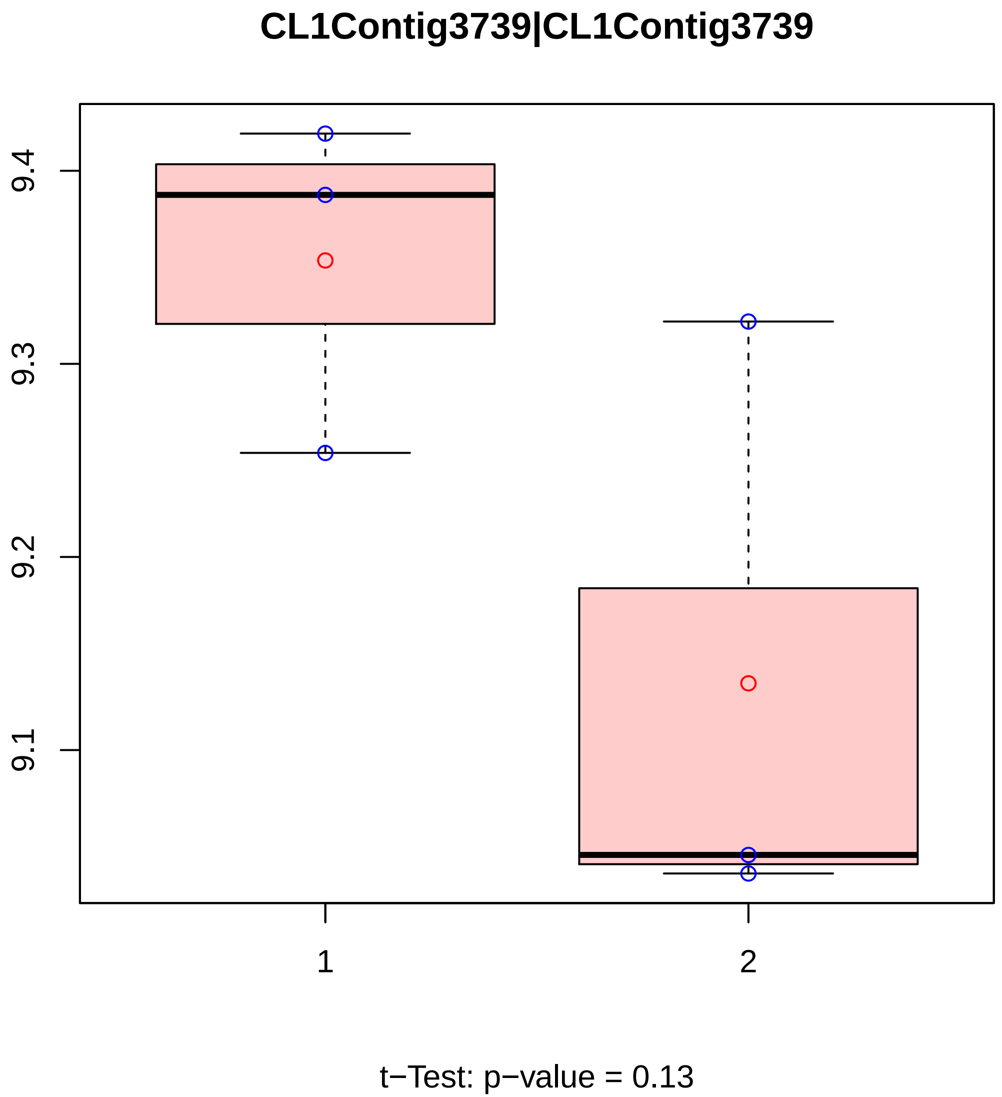

CL1Contig3739|CL1Contig3739
9.4
9.3
9.2
9.1
1
2
t−Test: p−value = 0.13

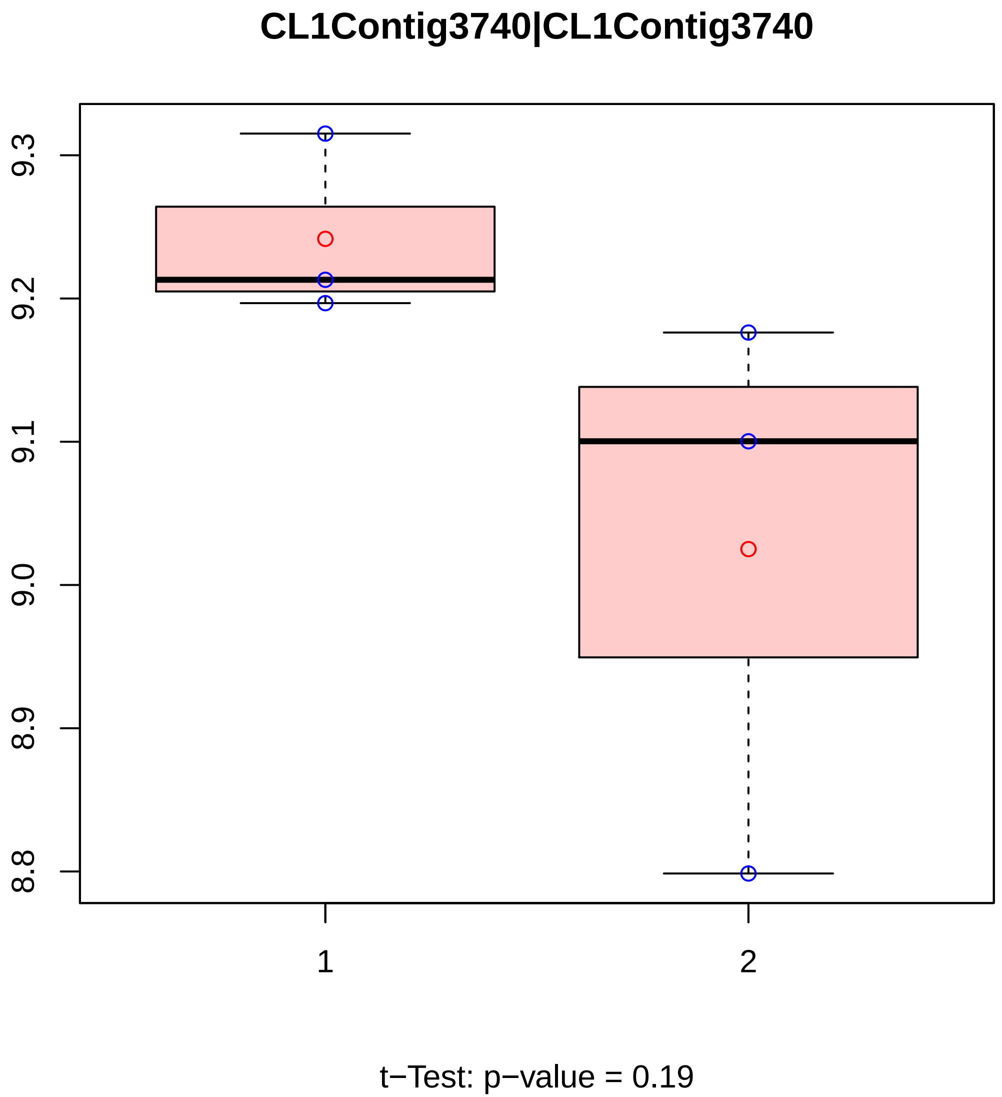

CL1Contig3740|CL1Contig3740
9.3
9.2
9.1
9.0
8.9
8.8
1
2
t−Test: p−value = 0.19

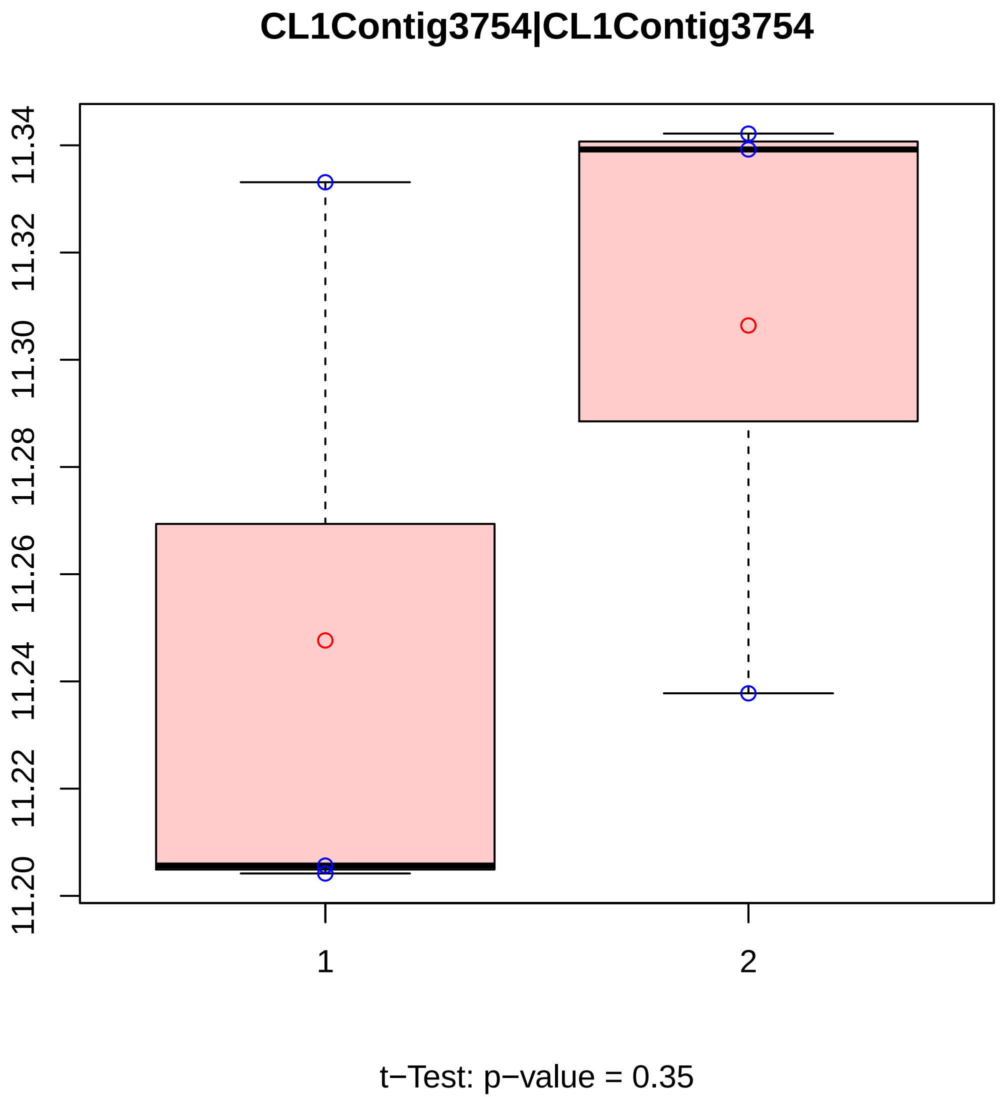
CL1Contig3754|CL1Contig3754
11.20
11.22
11.24
11.26
11.28
11.30
11.32
11.34
1
2
t−Test: p−value = 0.35

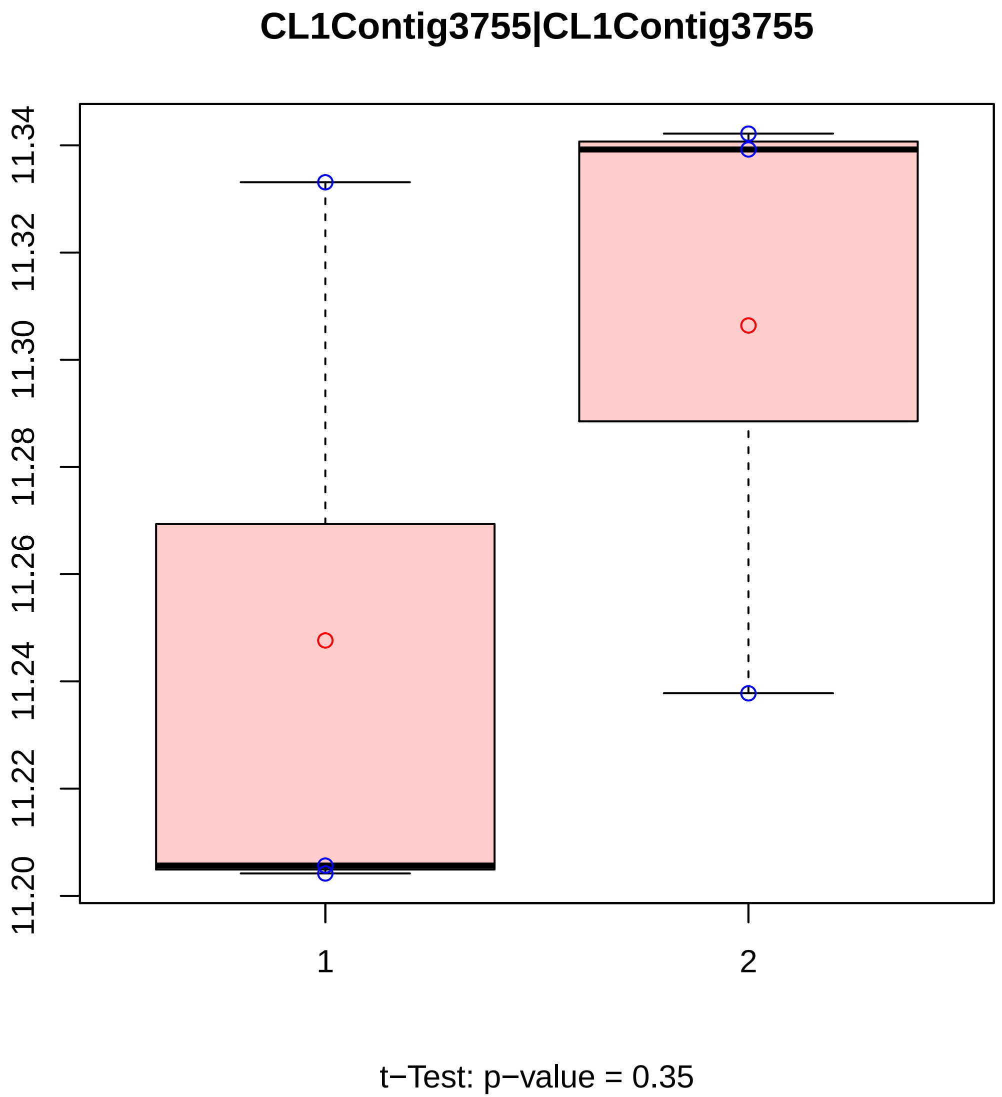

CL1Contig3755|CL1Contig3755
11.20
11.22
11.24
11.26
11.28
11.30
11.32
11.34
1
2
t−Test: p−value = 0.35

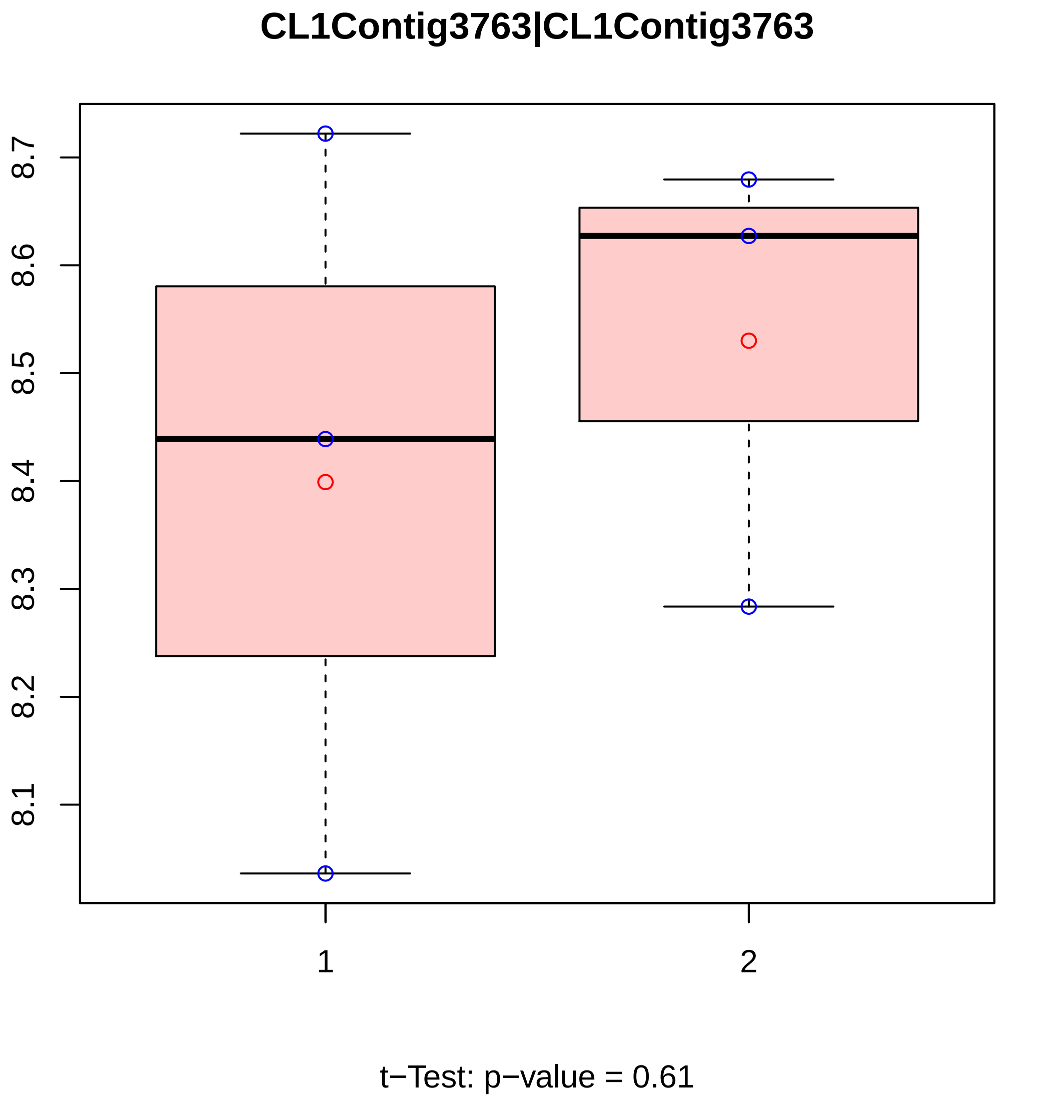
CL1Contig3763|CL1Contig3763
8.1
8.2
8.3
8.4
8.5
8.6
8.7
1
2
t−Test: p−value = 0.61

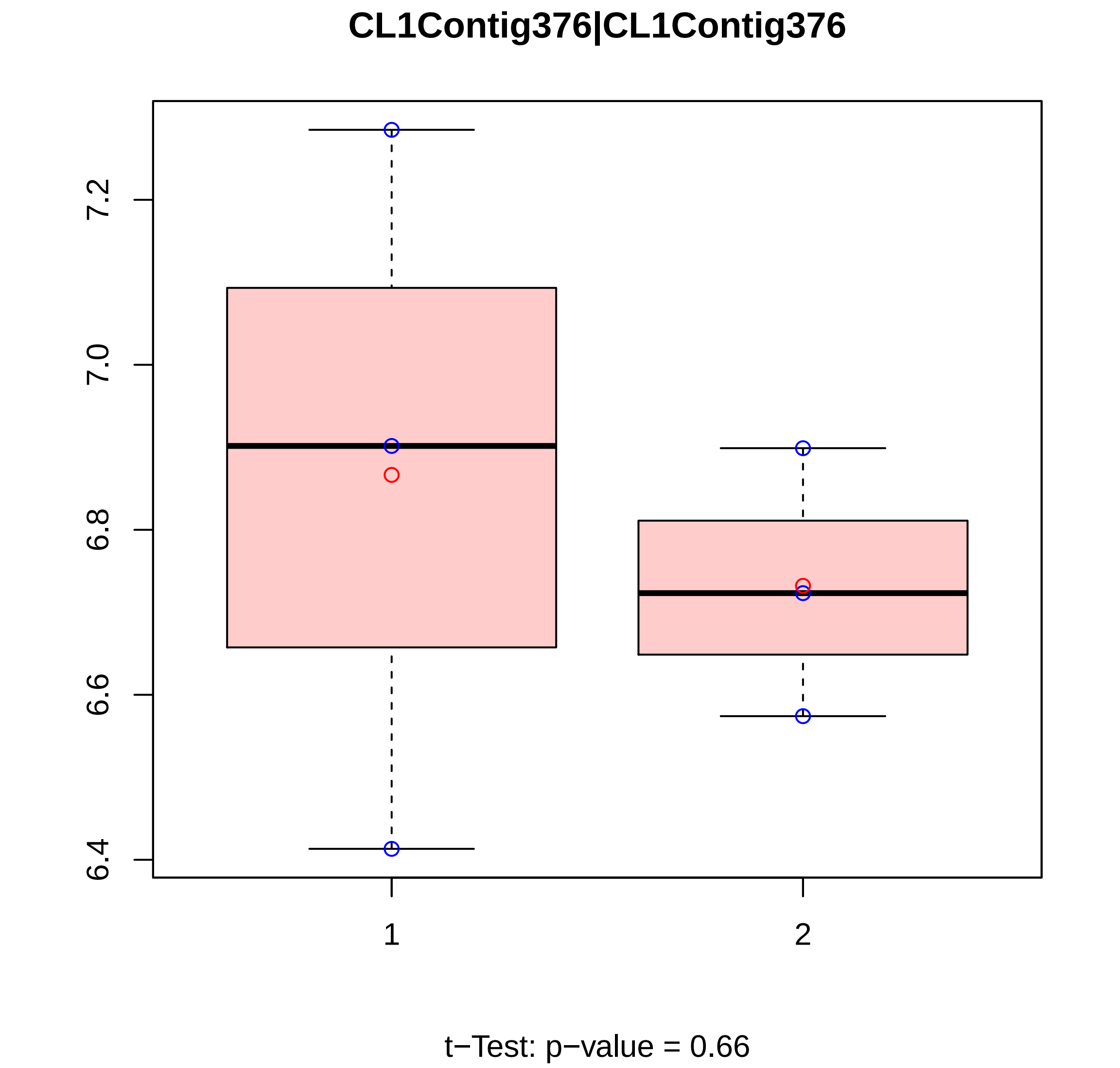

CL1Contig376|CL1Contig376
7.2
7.0
6.8
6.6
6.4
1
2
t−Test: p−value = 0.66

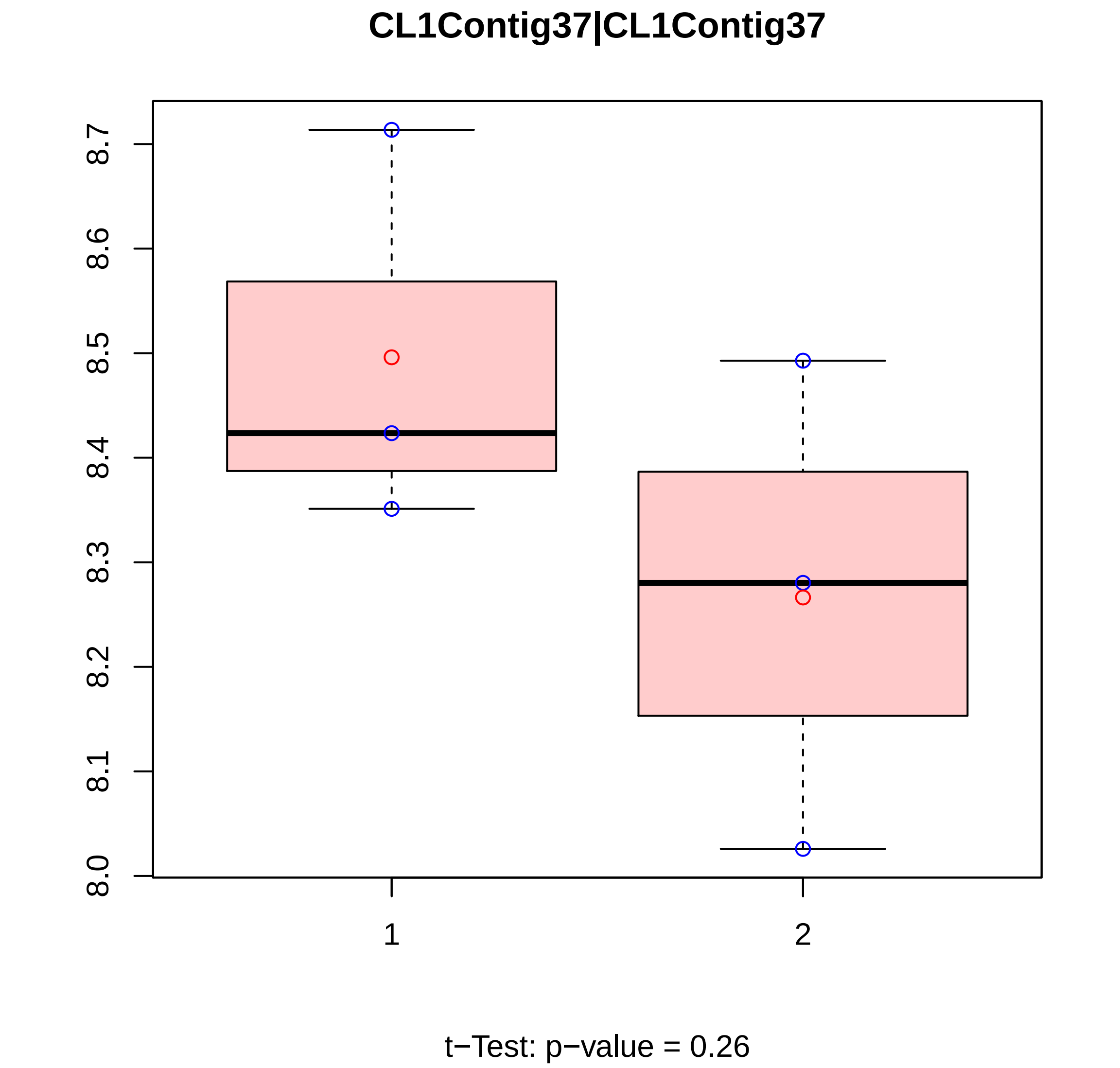
CL1Contig37|CL1Contig37
8.0
8.1
8.2
8.3
8.4
8.5
8.6
8.7
1
2
t−Test: p−value = 0.26

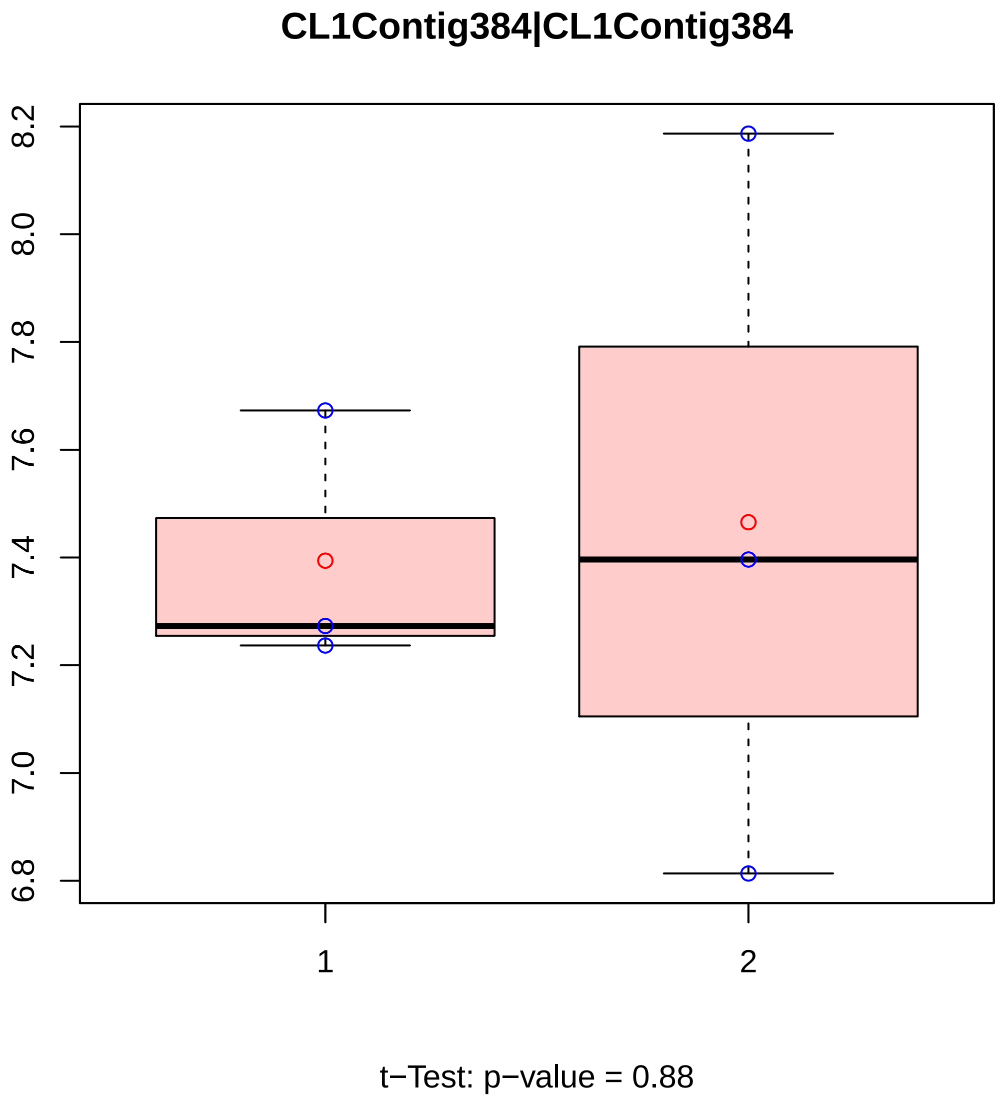

CL1Contig384|CL1Contig384
8.2
8.0
7.8
7.6
7.4
7.2
7.0
6.8
1
2
t−Test: p−value = 0.88

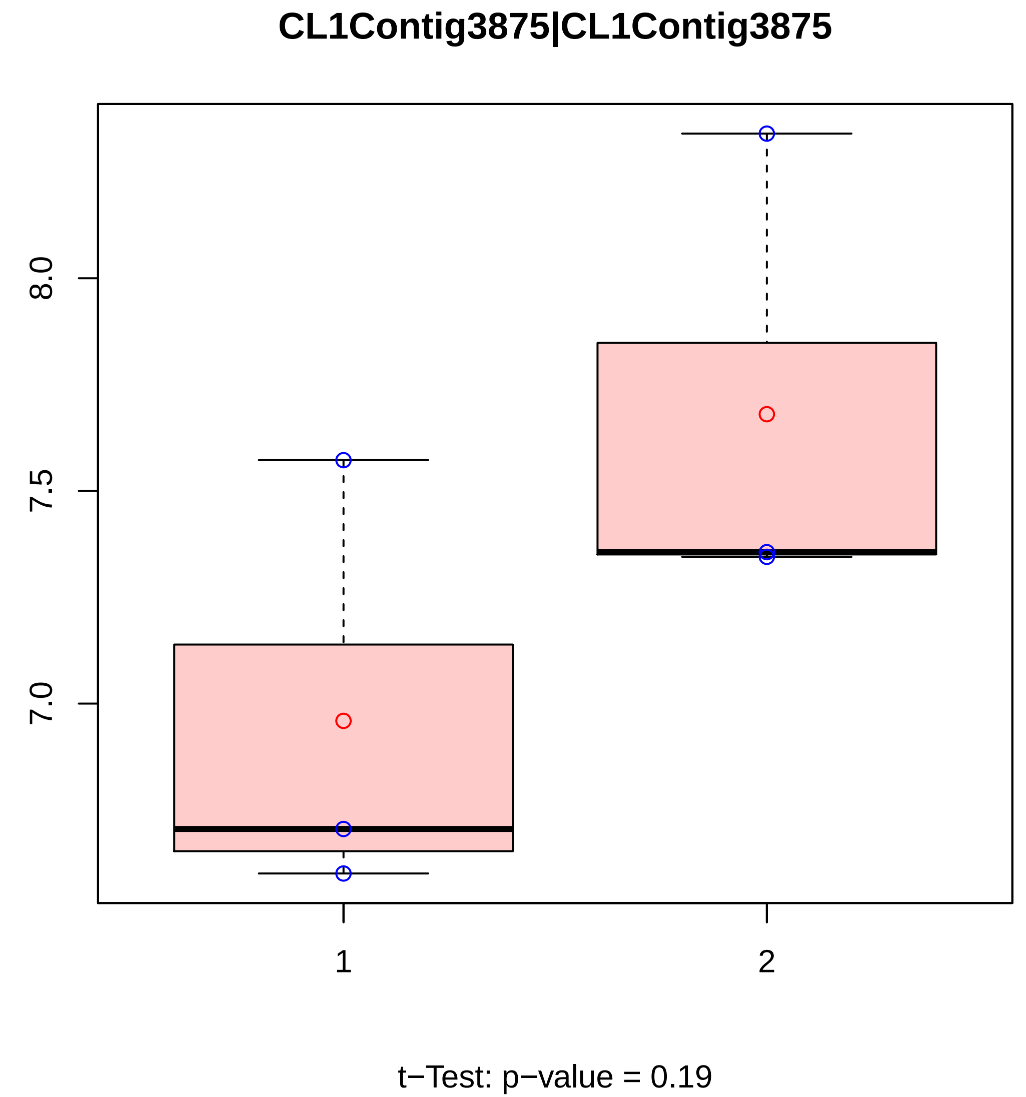
CL1Contig3875|CL1Contig3875
8.0
7.5
7.0
1
2
t−Test: p−value = 0.19

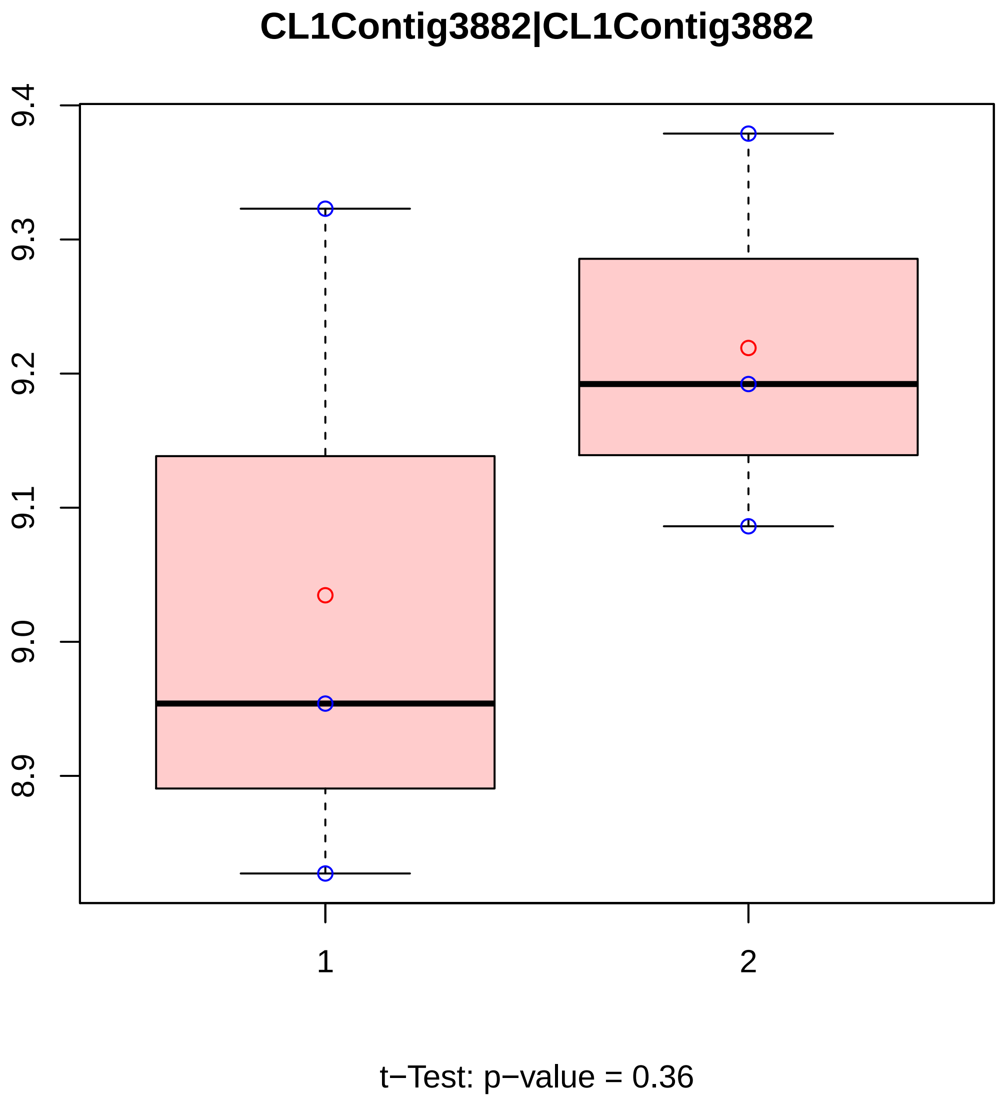
CL1Contig3882|CL1Contig3882
9.4
9.3
9.2
9.1
9.0
8.9
1
2
t−Test: p−value = 0.36

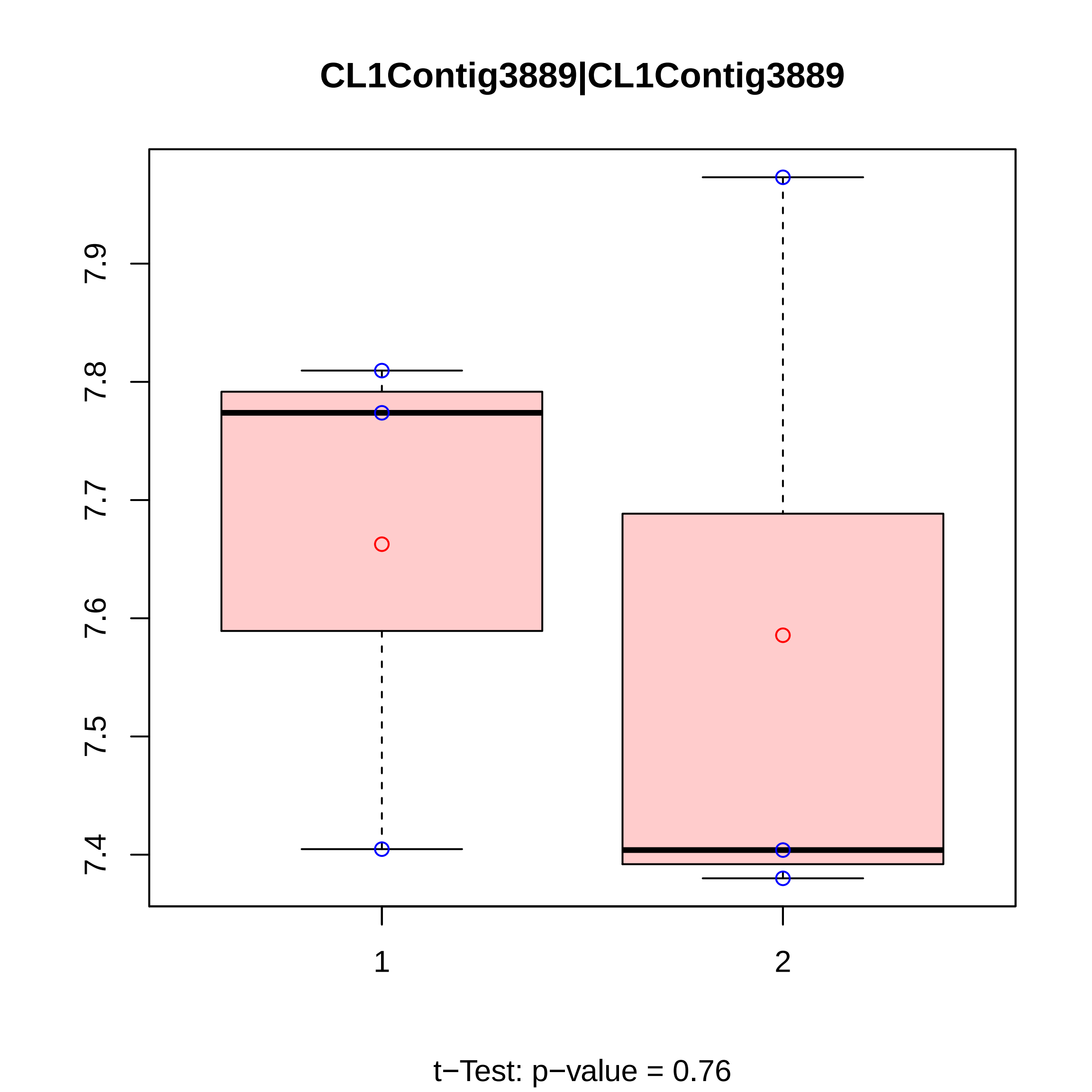

CL1Contig3889|CL1Contig3889
7.4
7.5
7.6
7.7
7.8
7.9
1
2
t−Test: p−value = 0.76

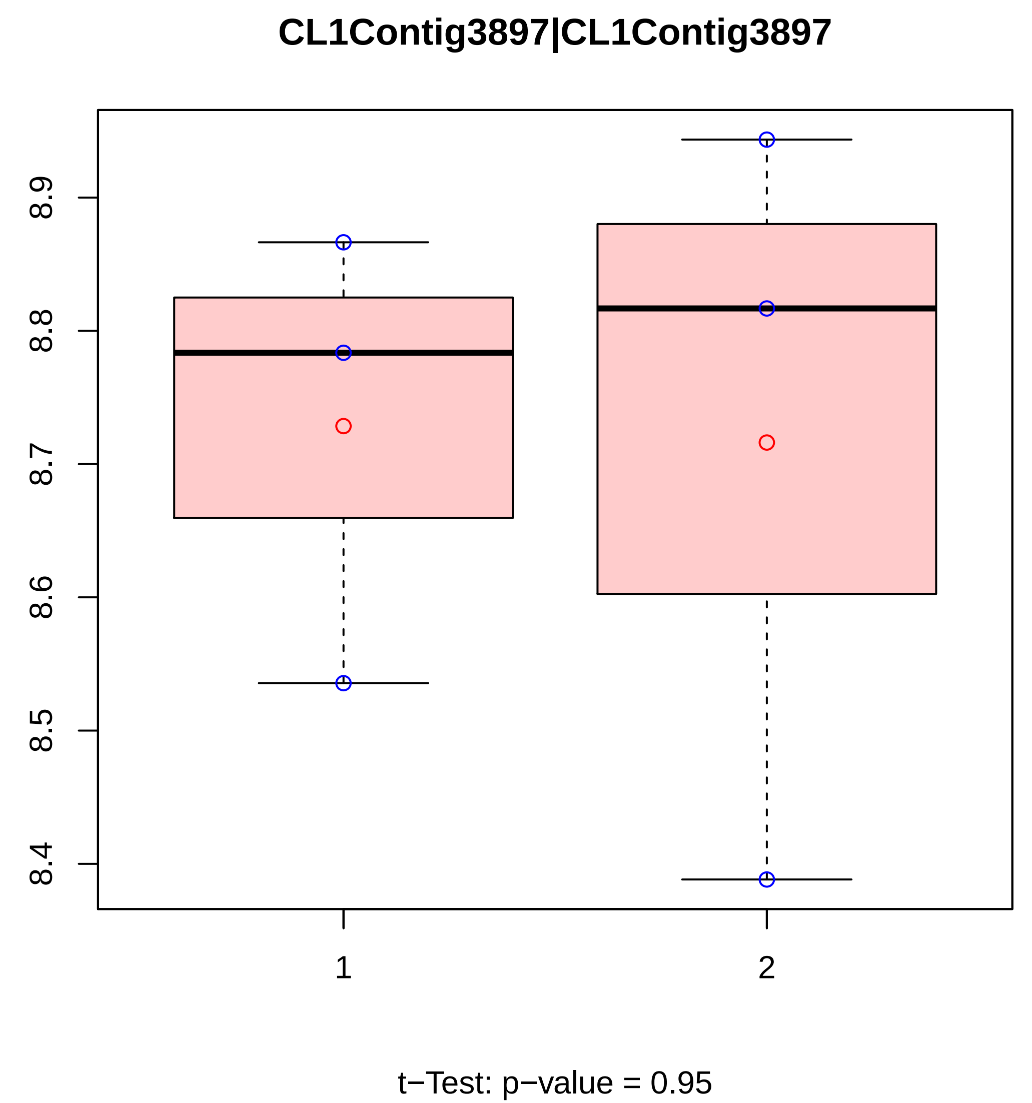
CL1Contig3897|CL1Contig3897
8.4
8.5
8.6
8.7
8.8
8.9
1
2
t−Test: p−value = 0.95

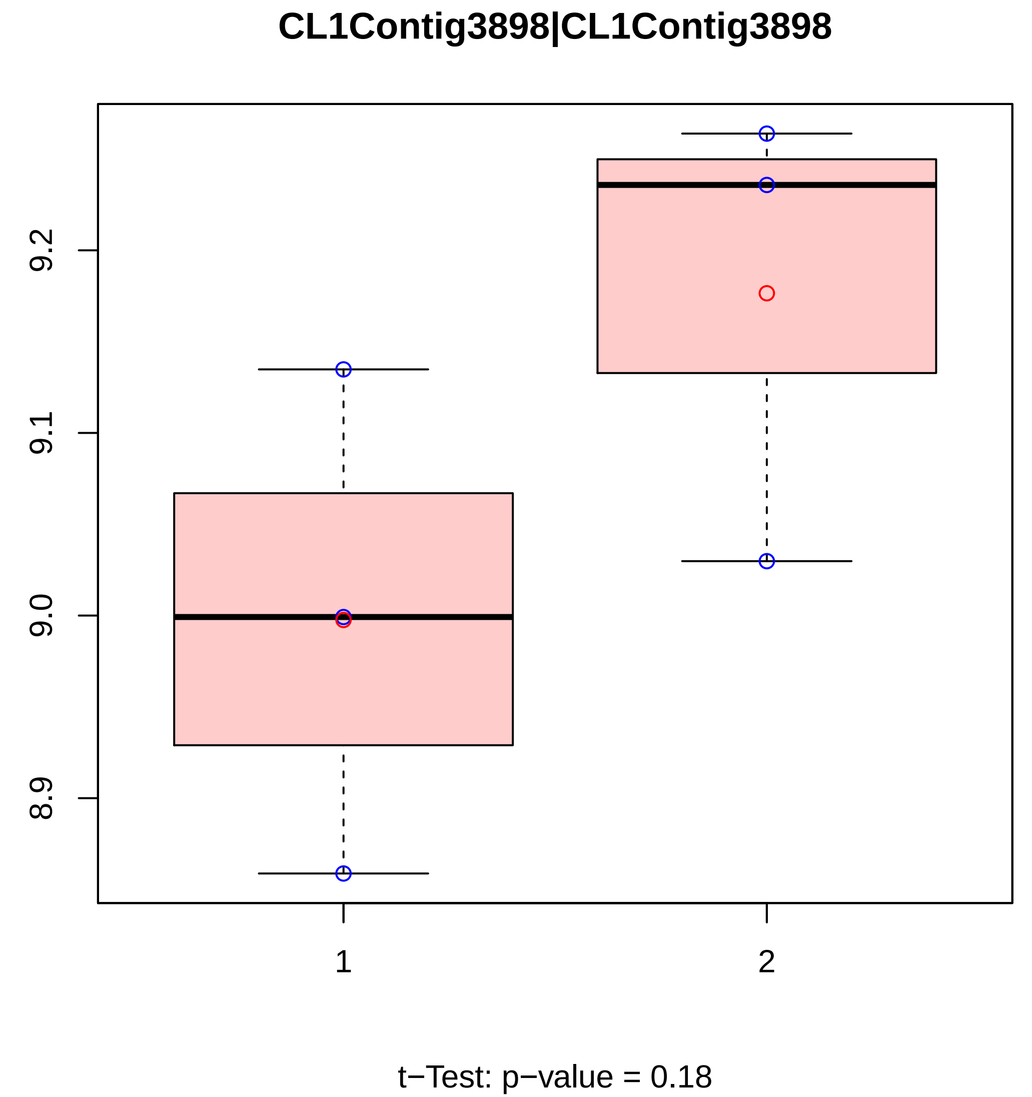
CL1Contig3898|CL1Contig3898
9.2
9.1
9.0
8.9
1
2
t−Test: p−value = 0.18

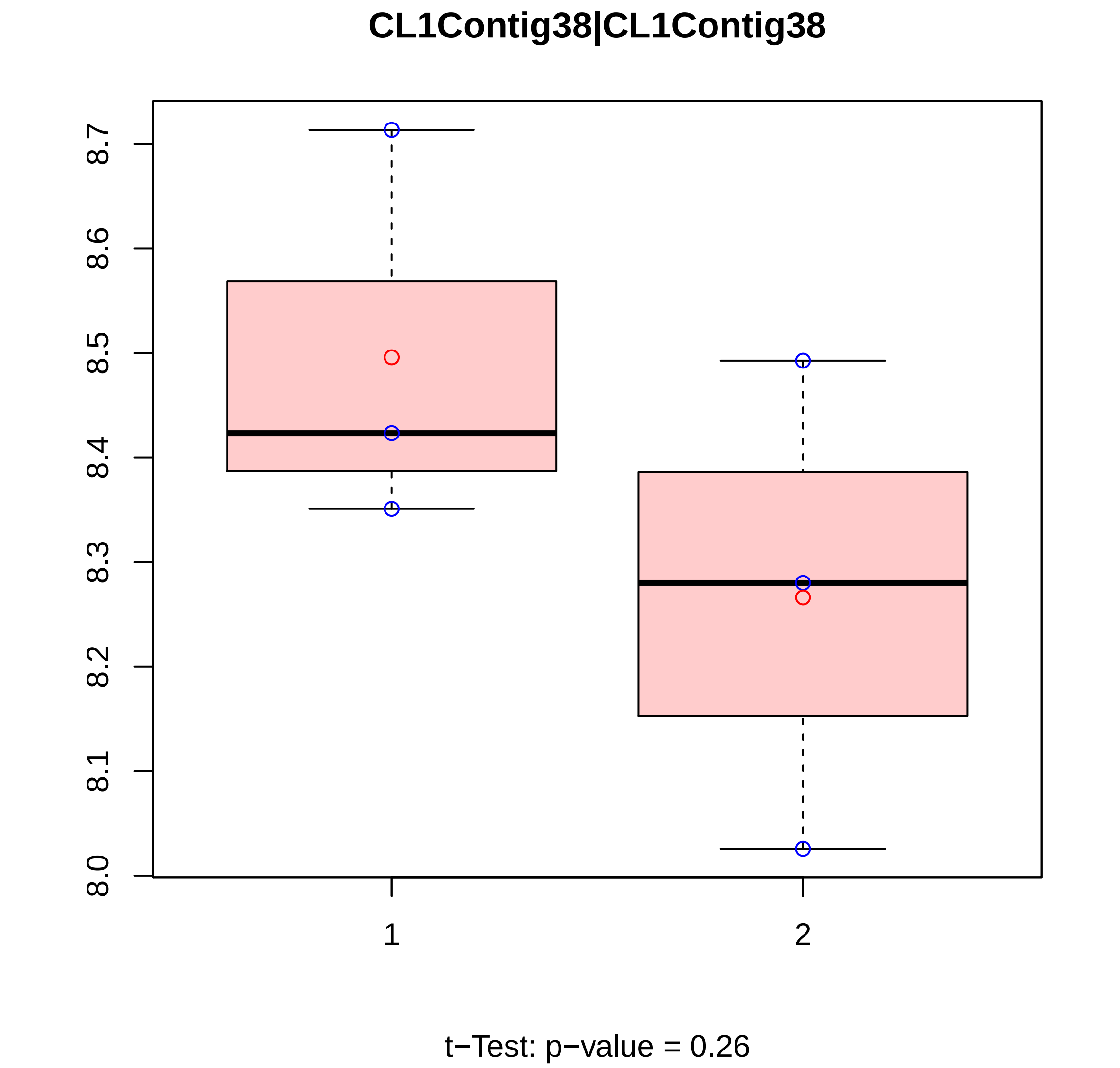

CL1Contig38|CL1Contig38
8.0
8.1
8.2
8.3
8.4
8.5
8.6
8.7
1
2
t−Test: p−value = 0.26

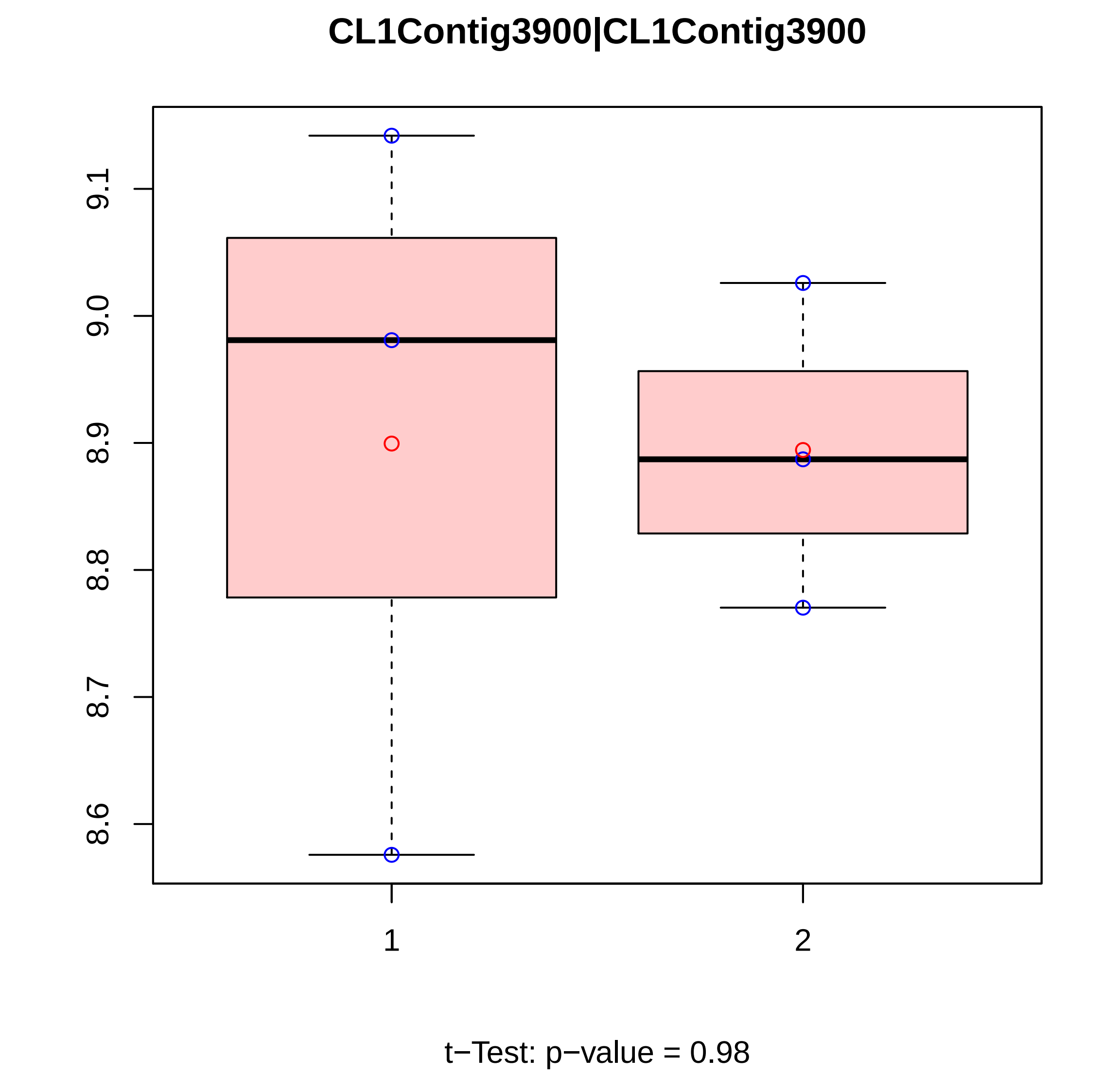

CL1Contig3900|CL1Contig3900
9.1
9.0
8.9
8.8
8.7
8.6
1
2
t−Test: p−value = 0.98

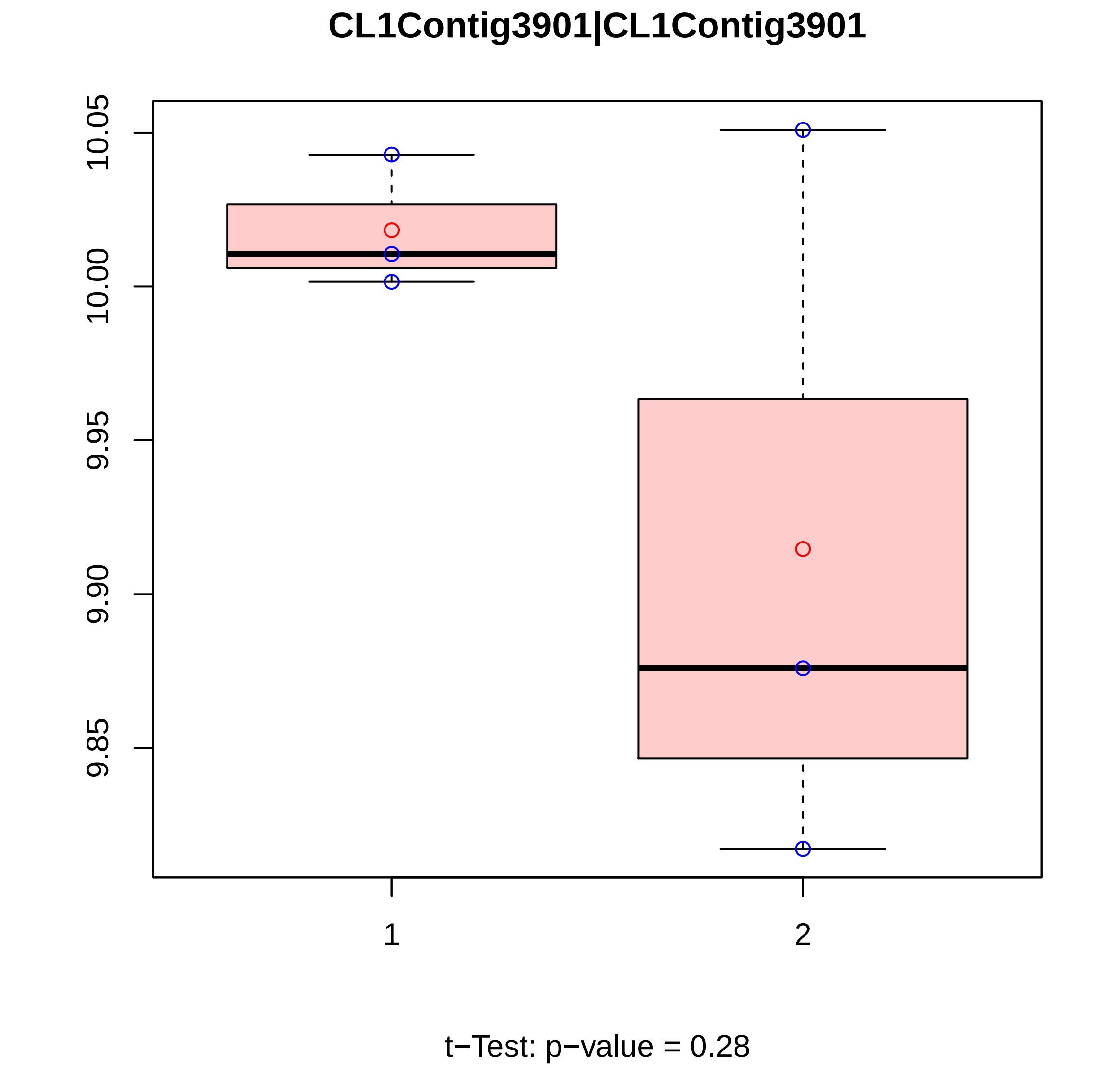
CL1Contig3901|CL1Contig3901
10.05
10.00
9.95
9.90
9.85
1
2
t−Test: p−value = 0.28

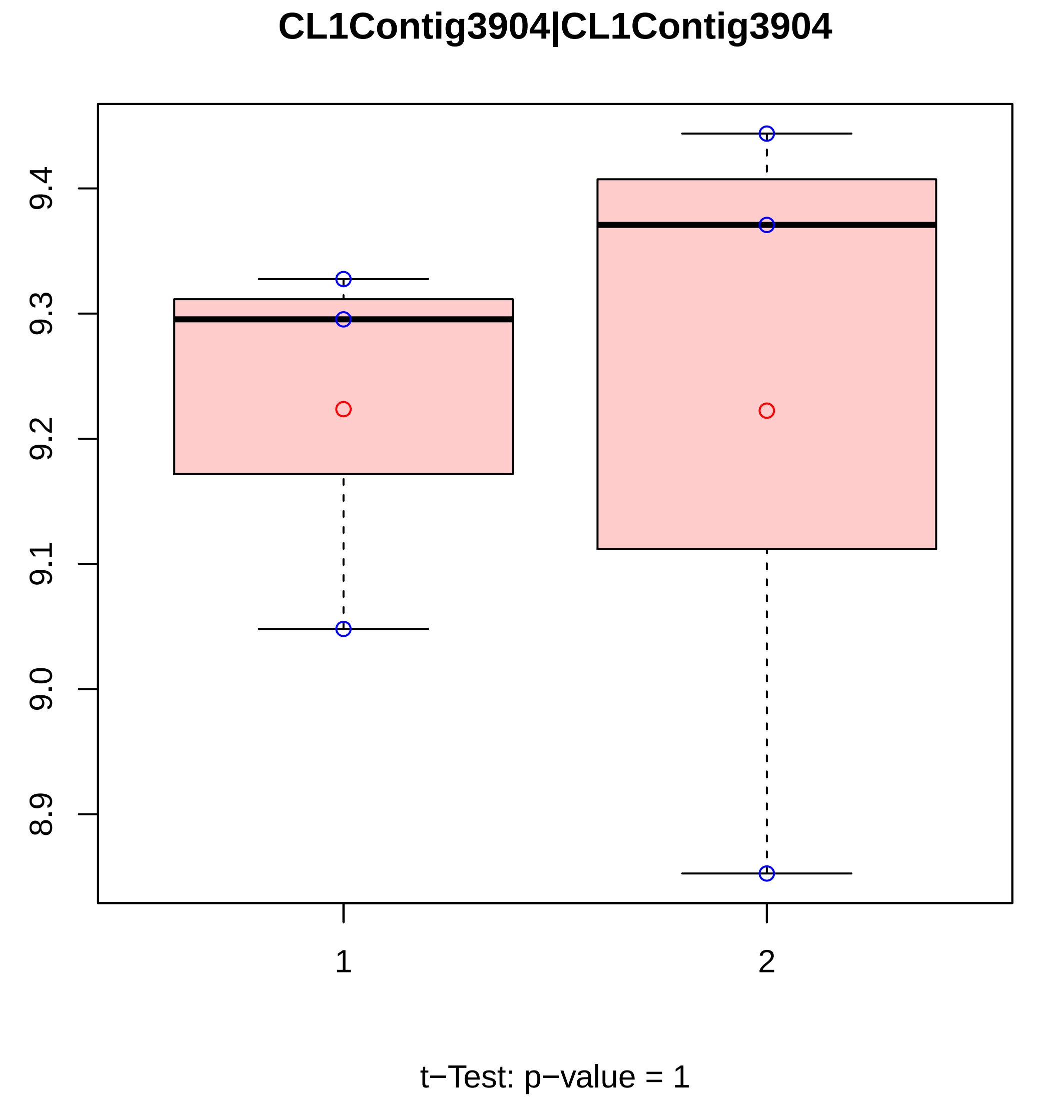

CL1Contig3904|CL1Contig3904
9.4
9.3
9.2
9.1
9.0
8.9
1
2
t−Test: p−value = 1

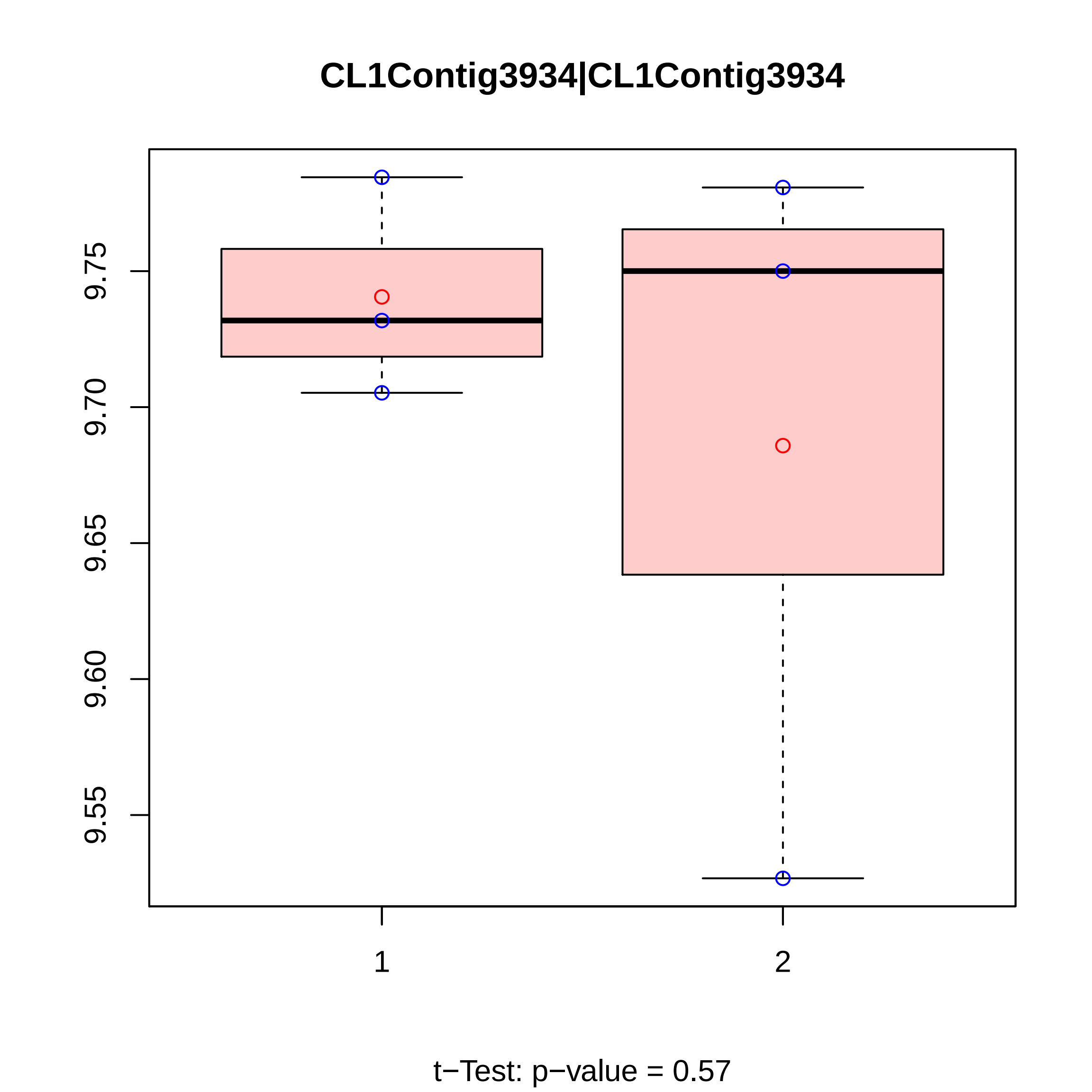
CL1Contig3934|CL1Contig3934
9.55
9.60
9.65
9.70
9.75
1
2
t−Test: p−value = 0.57

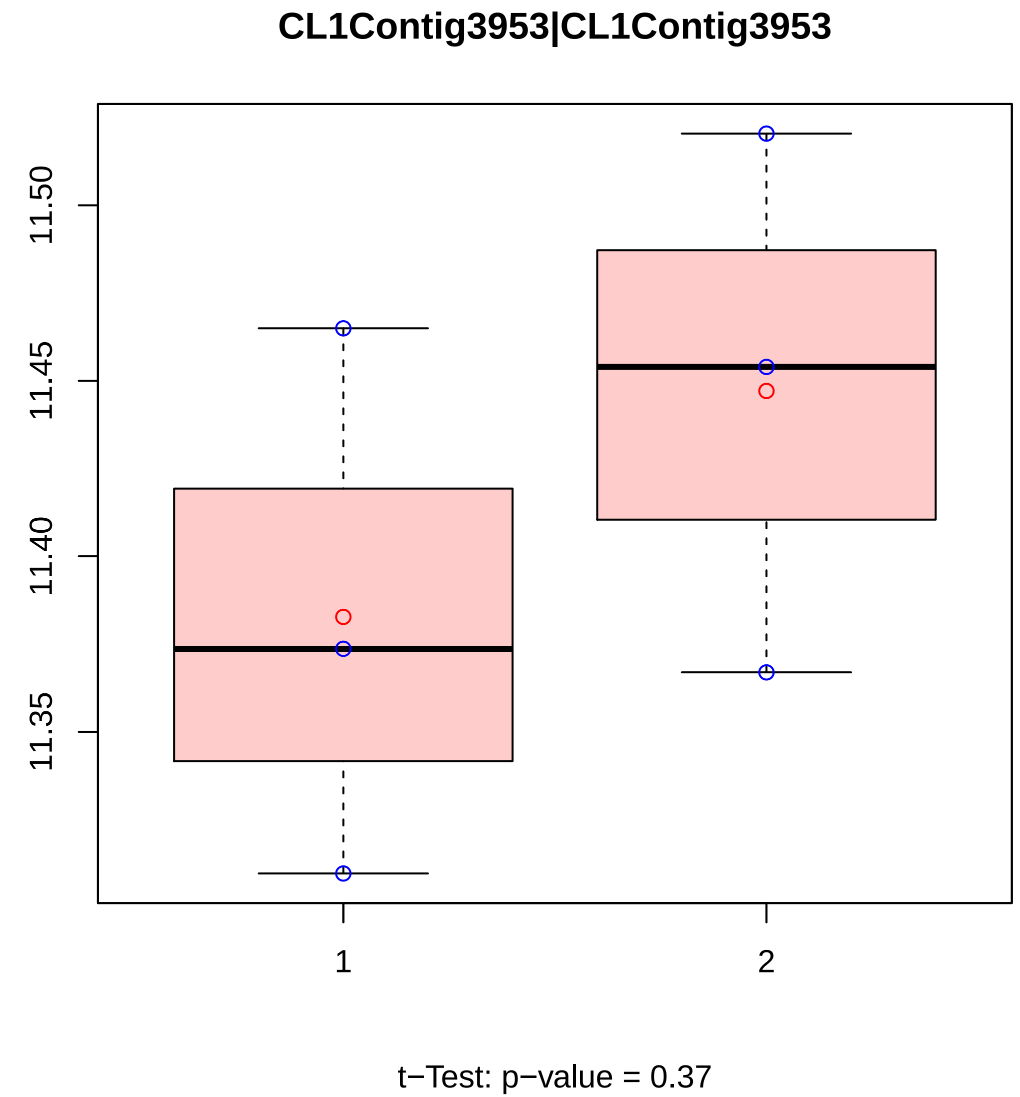
CL1Contig3953|CL1Contig3953
11.35
11.40
11.45
11.50
1
2
t−Test: p−value = 0.37

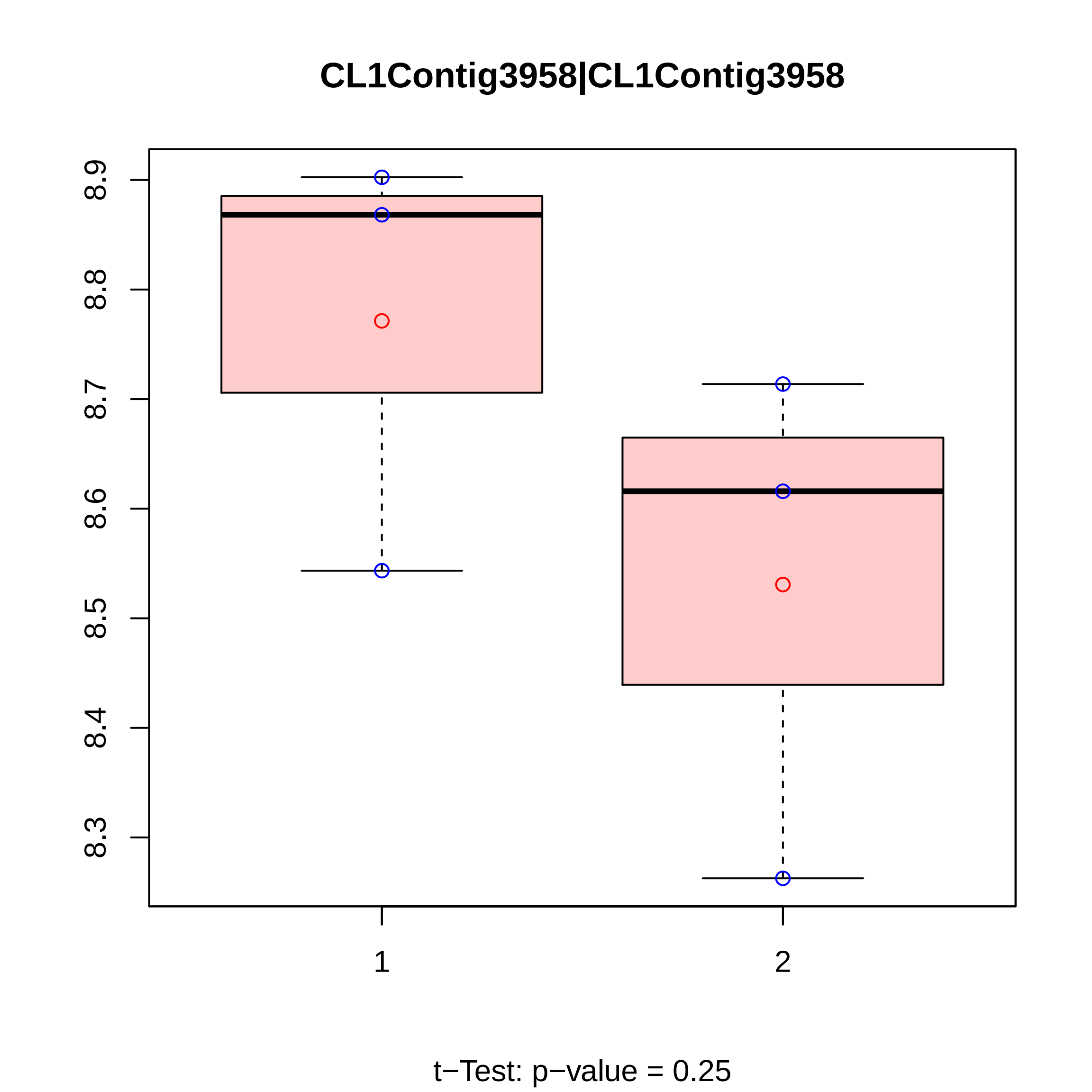
CL1Contig3958|CL1Contig3958
8.3
8.4
8.5
8.6
8.7
8.8
8.9
1
2
t−Test: p−value = 0.25

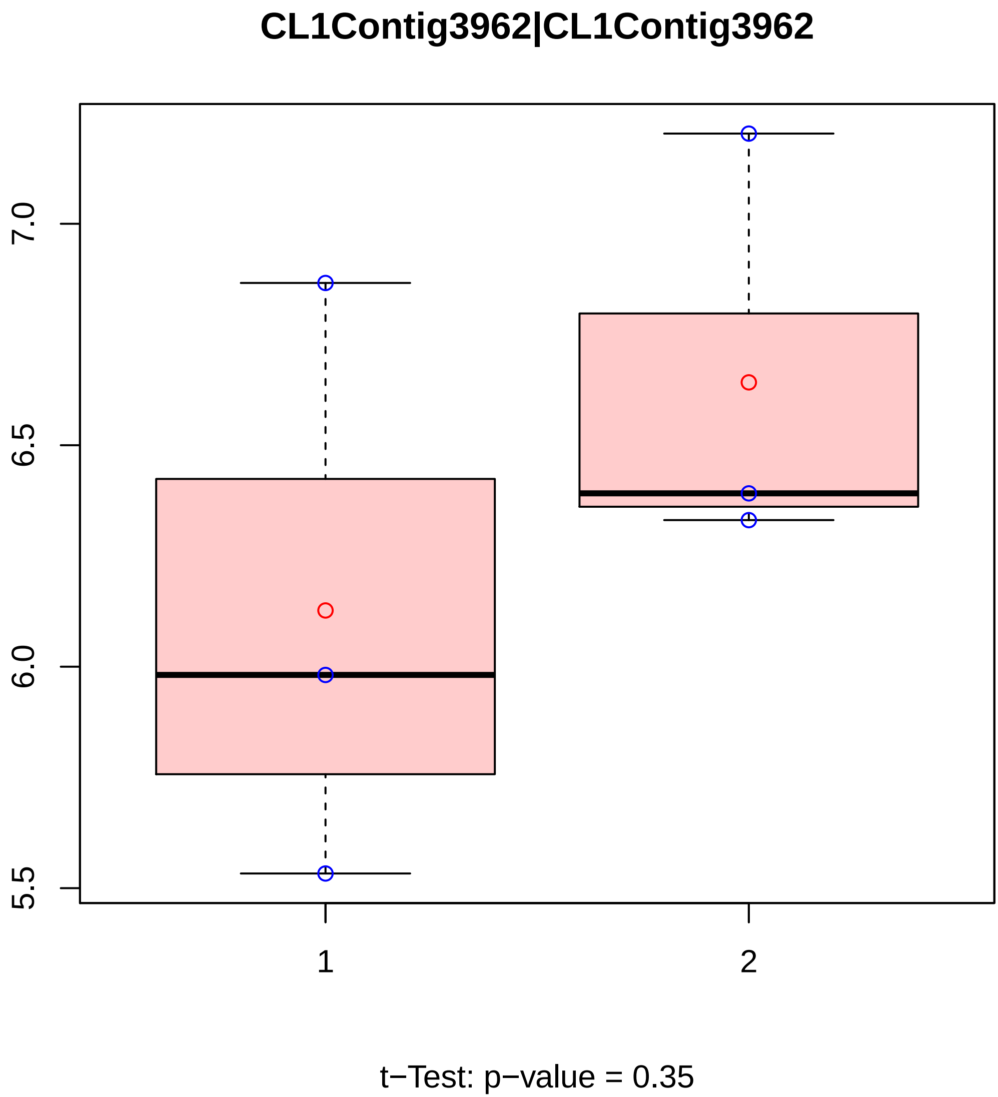

CL1Contig3962|CL1Contig3962
7.0
6.5
6.0
5.5
1
2
t−Test: p−value = 0.35

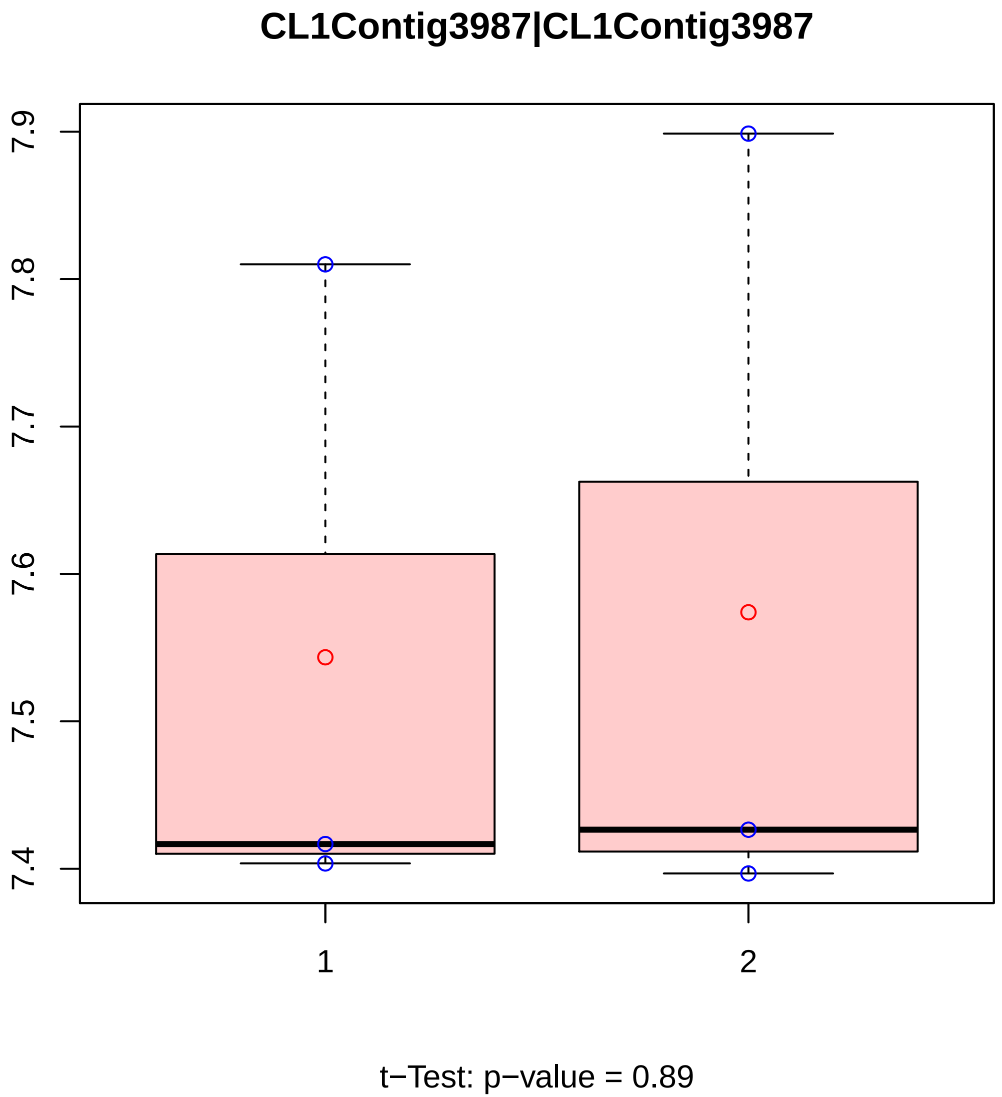

CL1Contig3987|CL1Contig3987
7.9
7.8
7.7
7.6
7.5
7.4
1
2
t−Test: p−value = 0.89

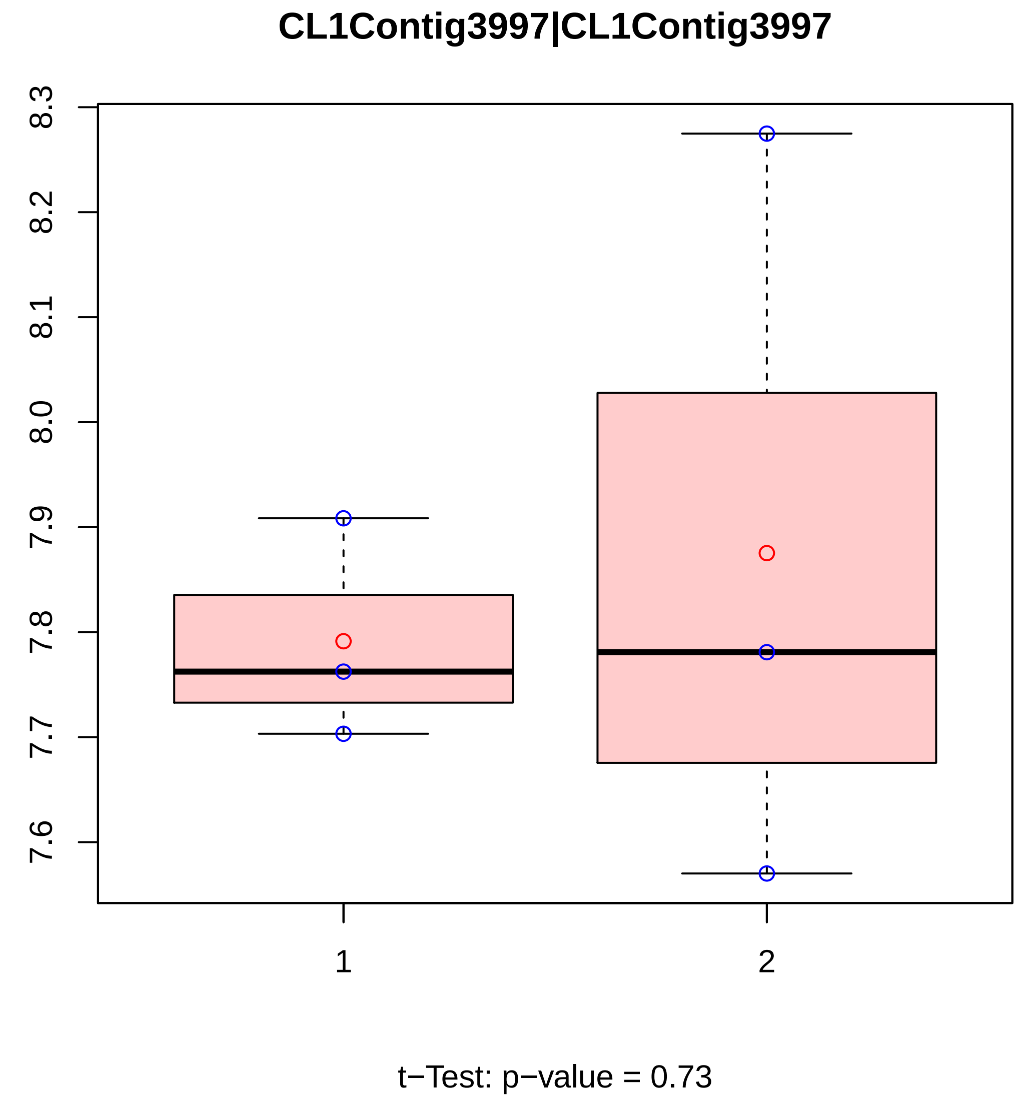
CL1Contig3997|CL1Contig3997
8.3
8.2
8.1
8.0
7.9
7.8
7.7
7.6
1
2
t−Test: p−value = 0.73

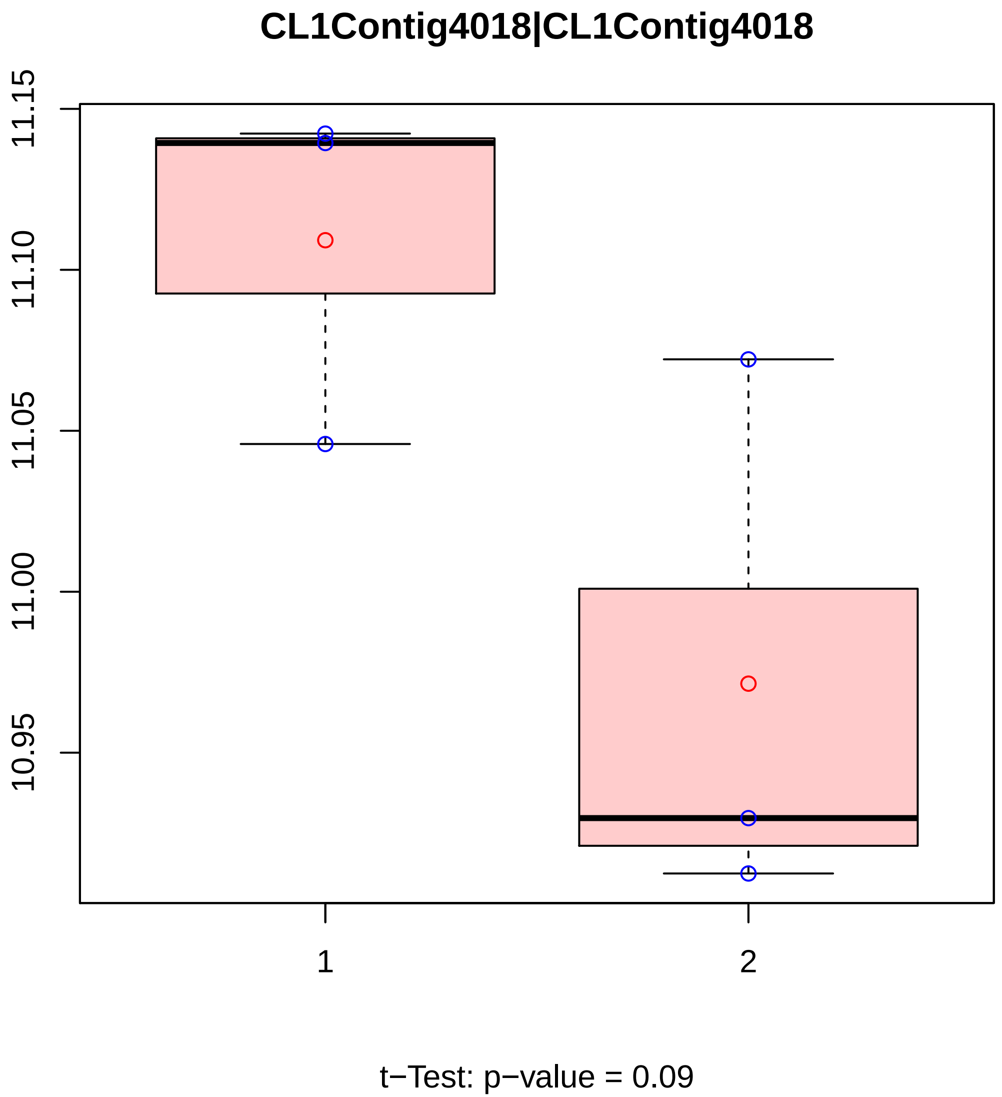

CL1Contig4018|CL1Contig4018
11.15
11.10
11.05
11.00
10.95
1
2
t−Test: p−value = 0.09

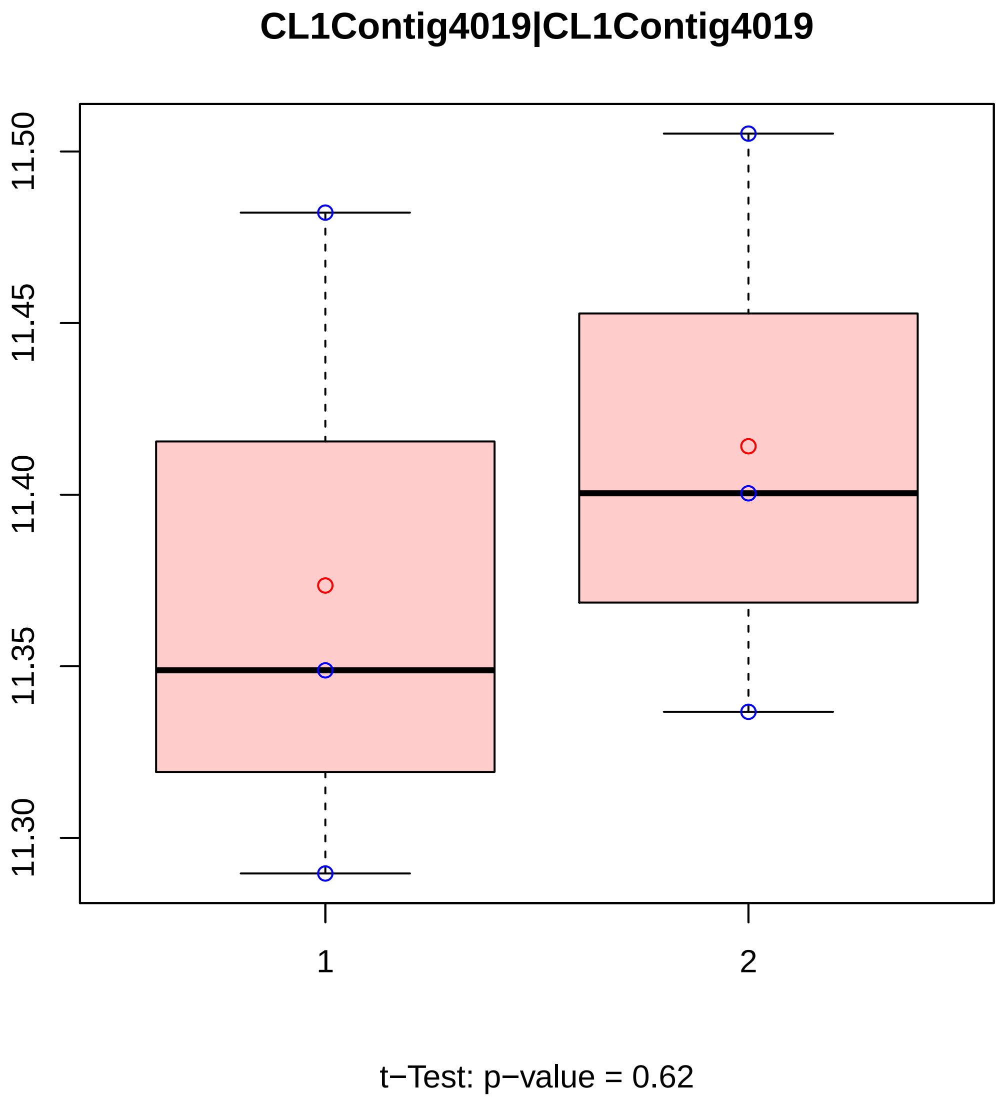
CL1Contig4019|CL1Contig4019
11.30
11.35
11.40
11.45
11.50
1
2
t−Test: p−value = 0.62

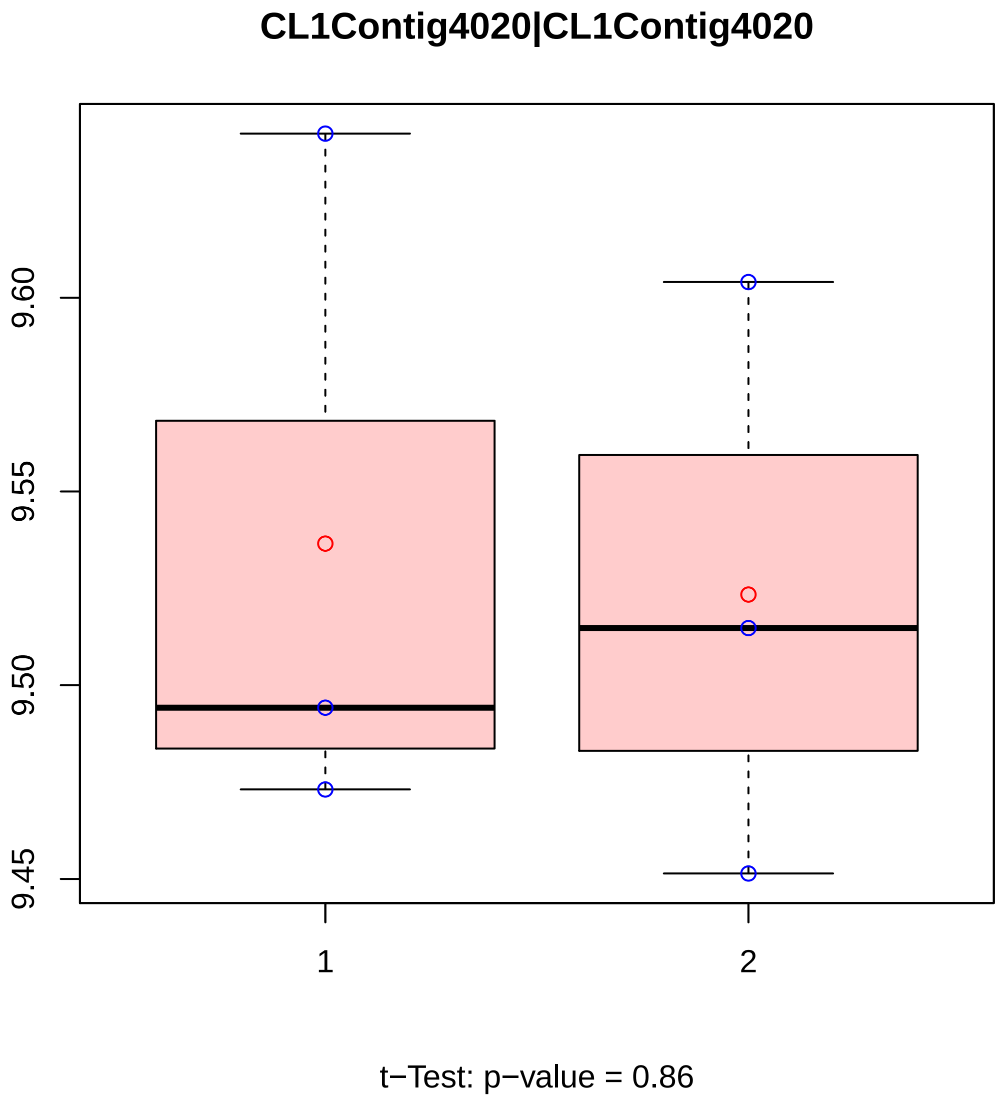

CL1Contig4020|CL1Contig4020
9.45
9.50
9.55
9.60
1
2
t−Test: p−value = 0.86

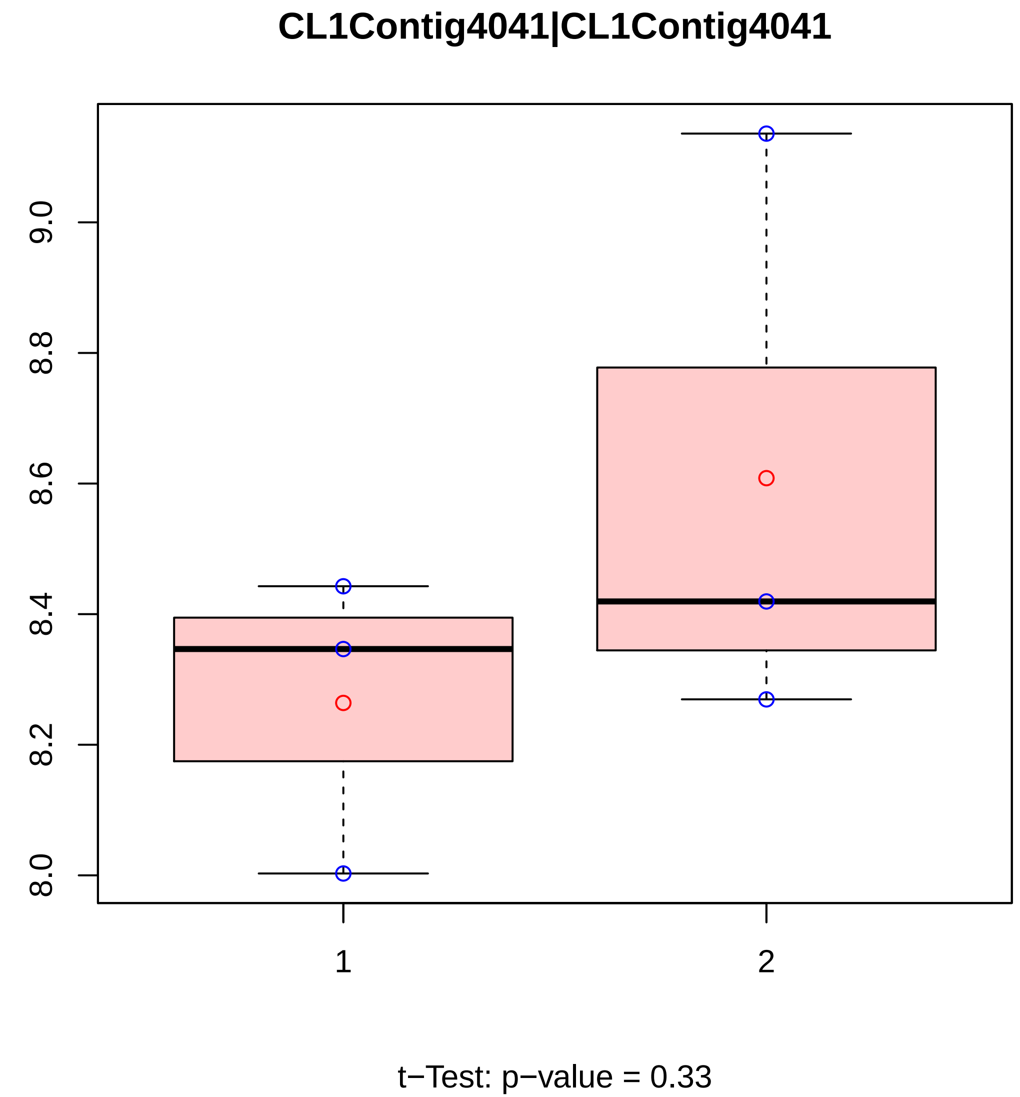

CL1Contig4041|CL1Contig4041
8.0
8.2
8.4
8.6
8.8
9.0
1
2
t−Test: p−value = 0.33

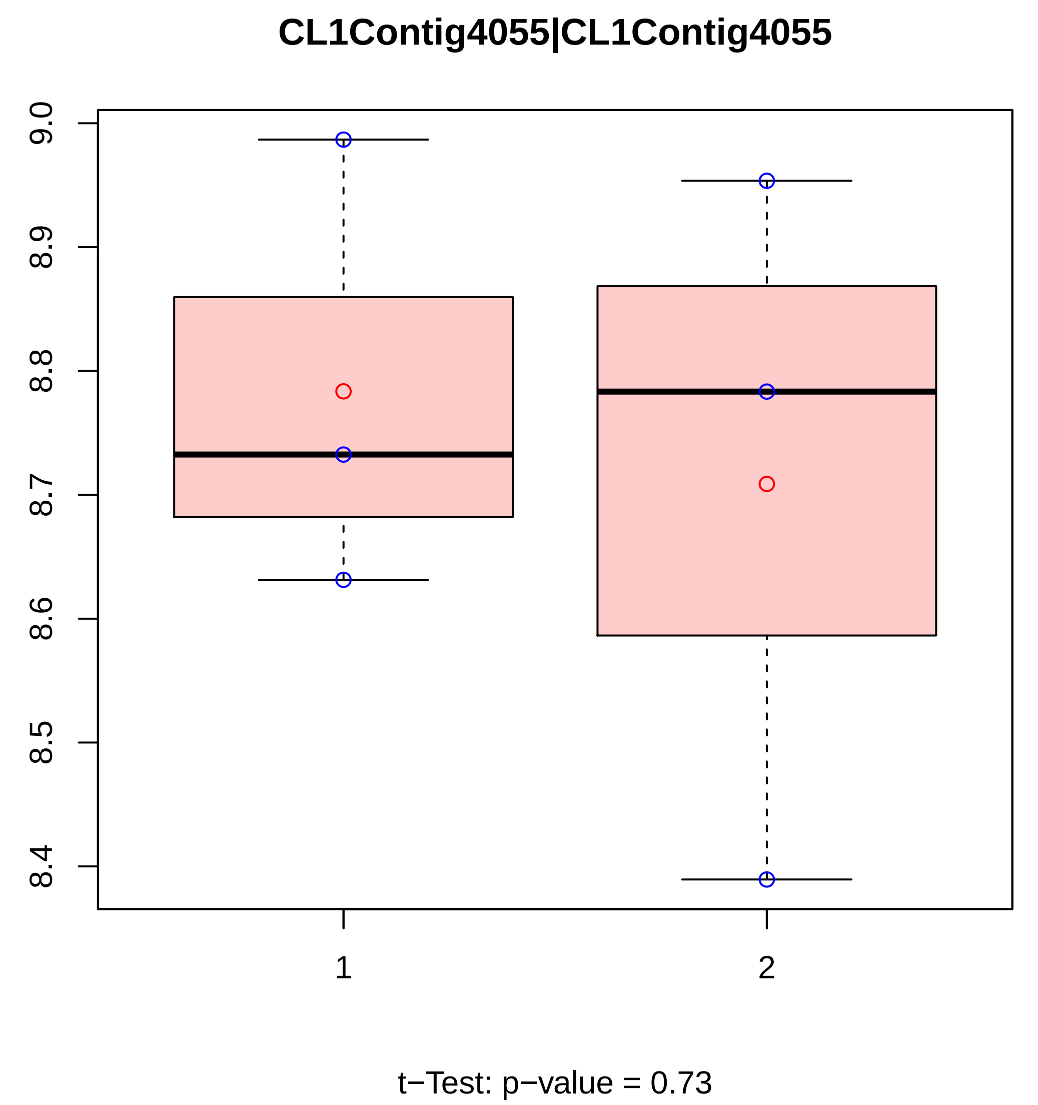

CL1Contig4055|CL1Contig4055
9.0
8.9
8.8
8.7
8.6
8.5
8.4
1
2
t−Test: p−value = 0.73

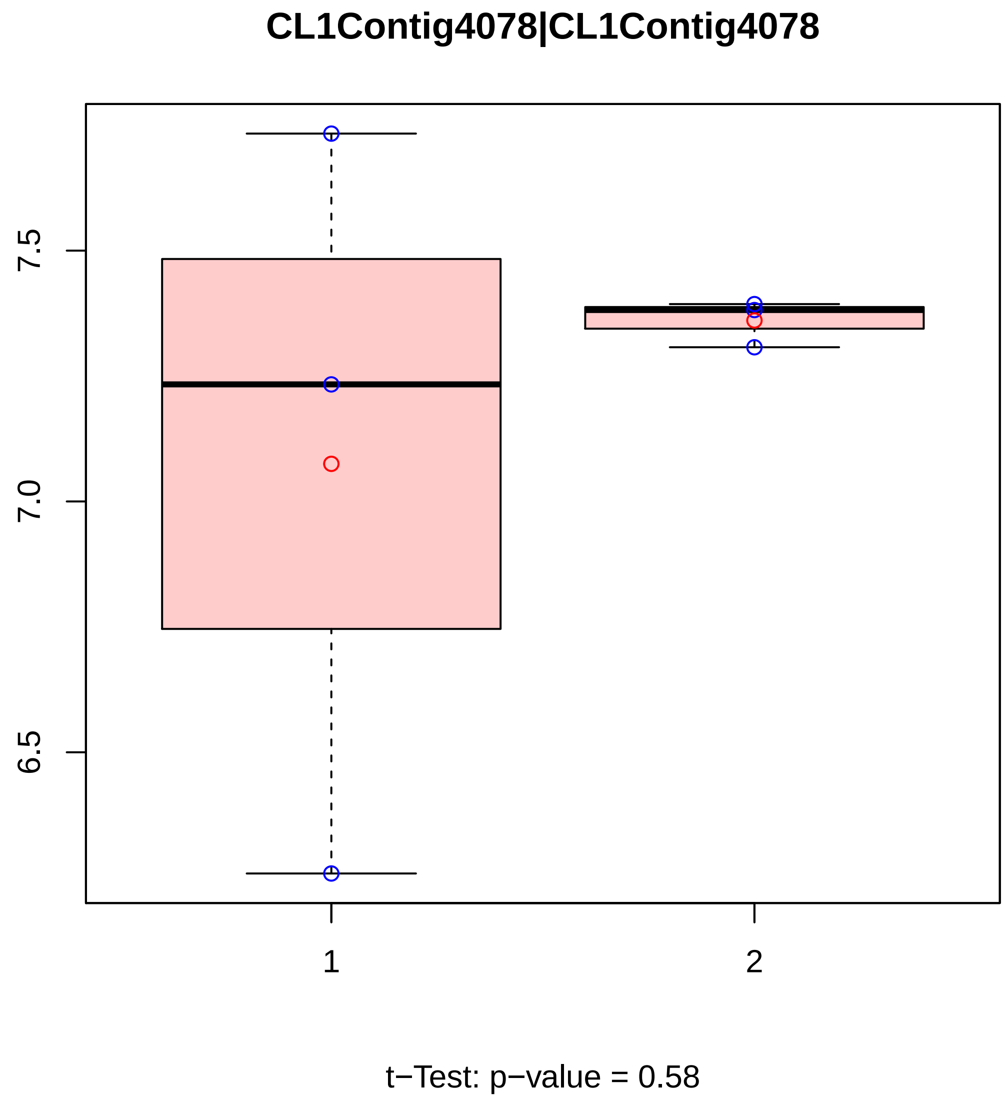
CL1Contig4078|CL1Contig4078
7.5
7.0
6.5
1
2
t−Test: p−value = 0.58

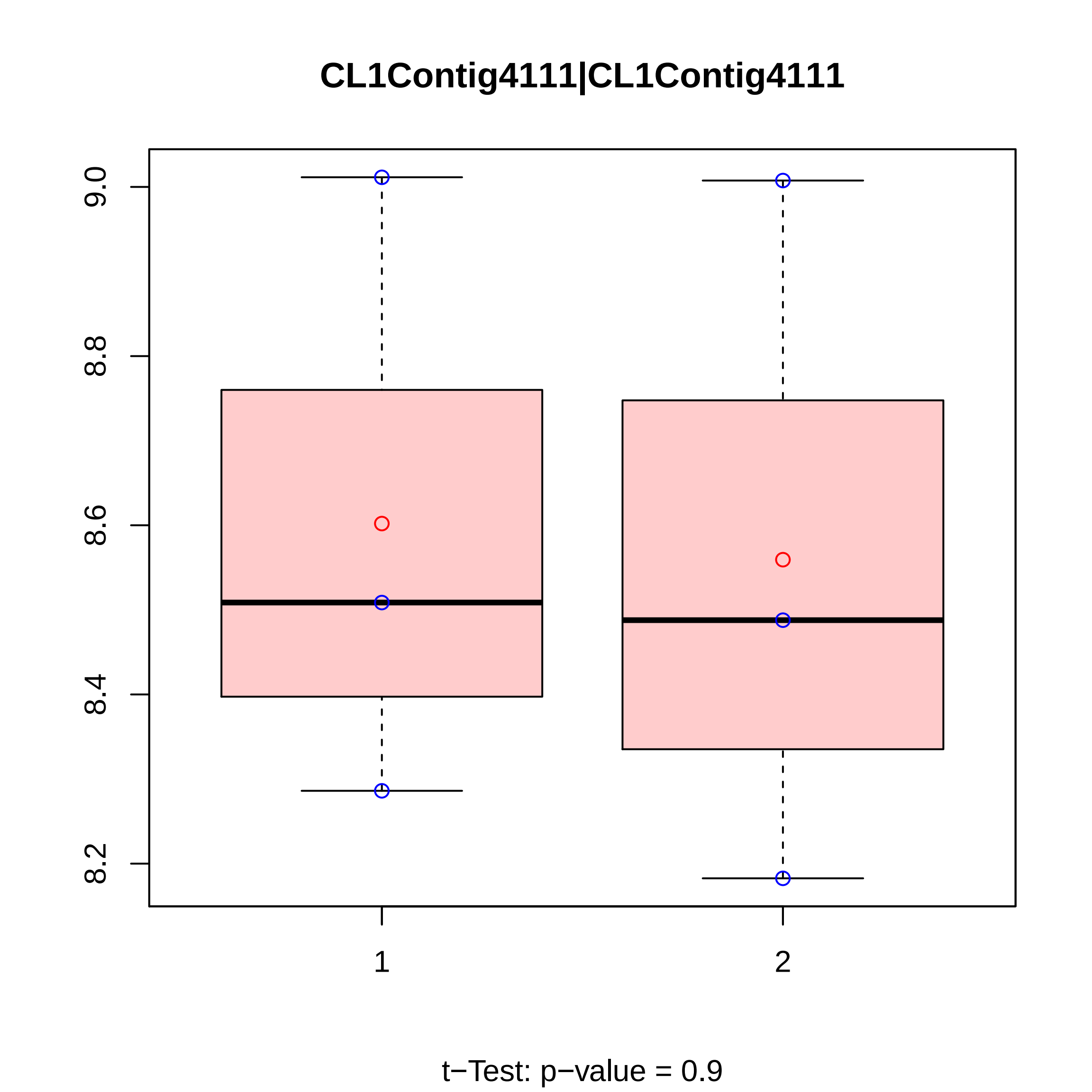
CL1Contig4111|CL1Contig4111
9.0
8.8
8.6
8.4
8.2
1
2
t−Test: p−value = 0.9

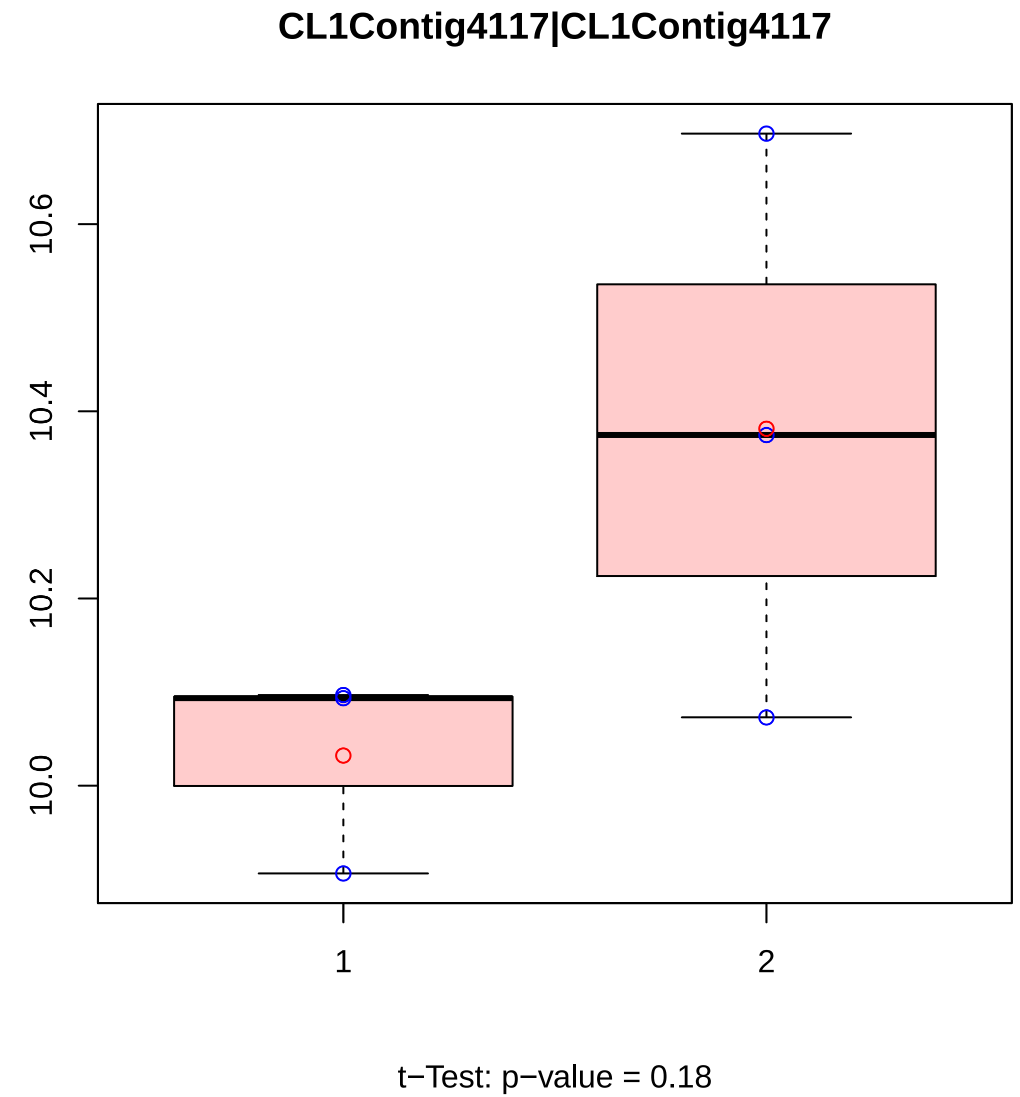

CL1Contig4117|CL1Contig4117
10.6
10.4
10.2
10.0
1
2
t−Test: p−value = 0.18

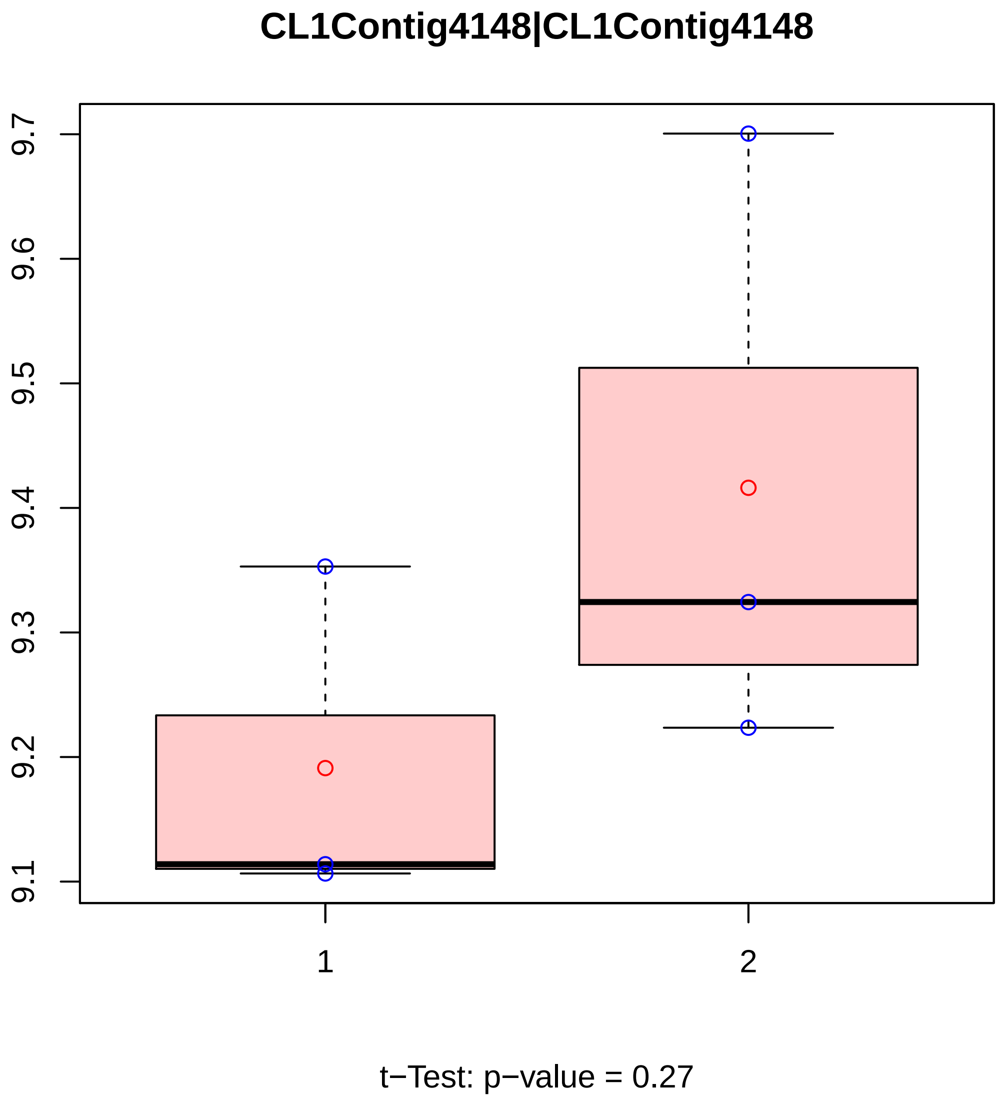
CL1Contig4148|CL1Contig4148
9.1
9.2
9.3
9.4
9.5
9.6
9.7
1
2
t−Test: p−value = 0.27

# CL1Contig414|CL1Contig414

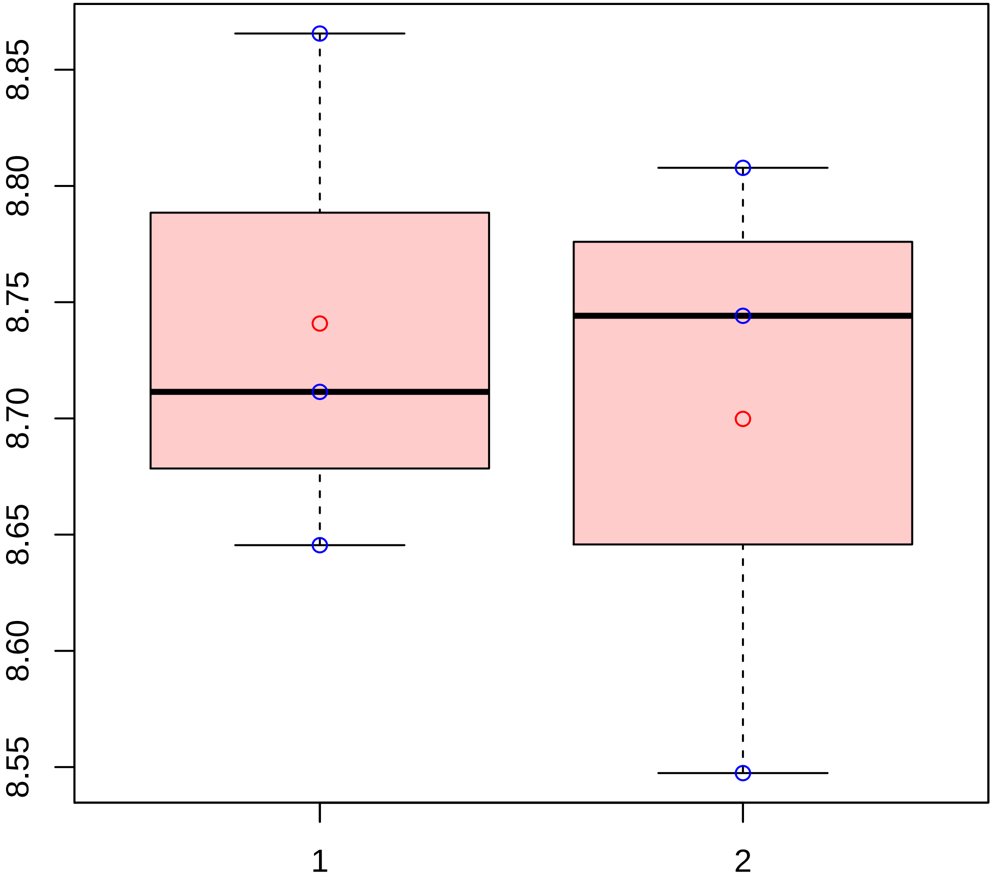


t−Test: p−value = 0.71

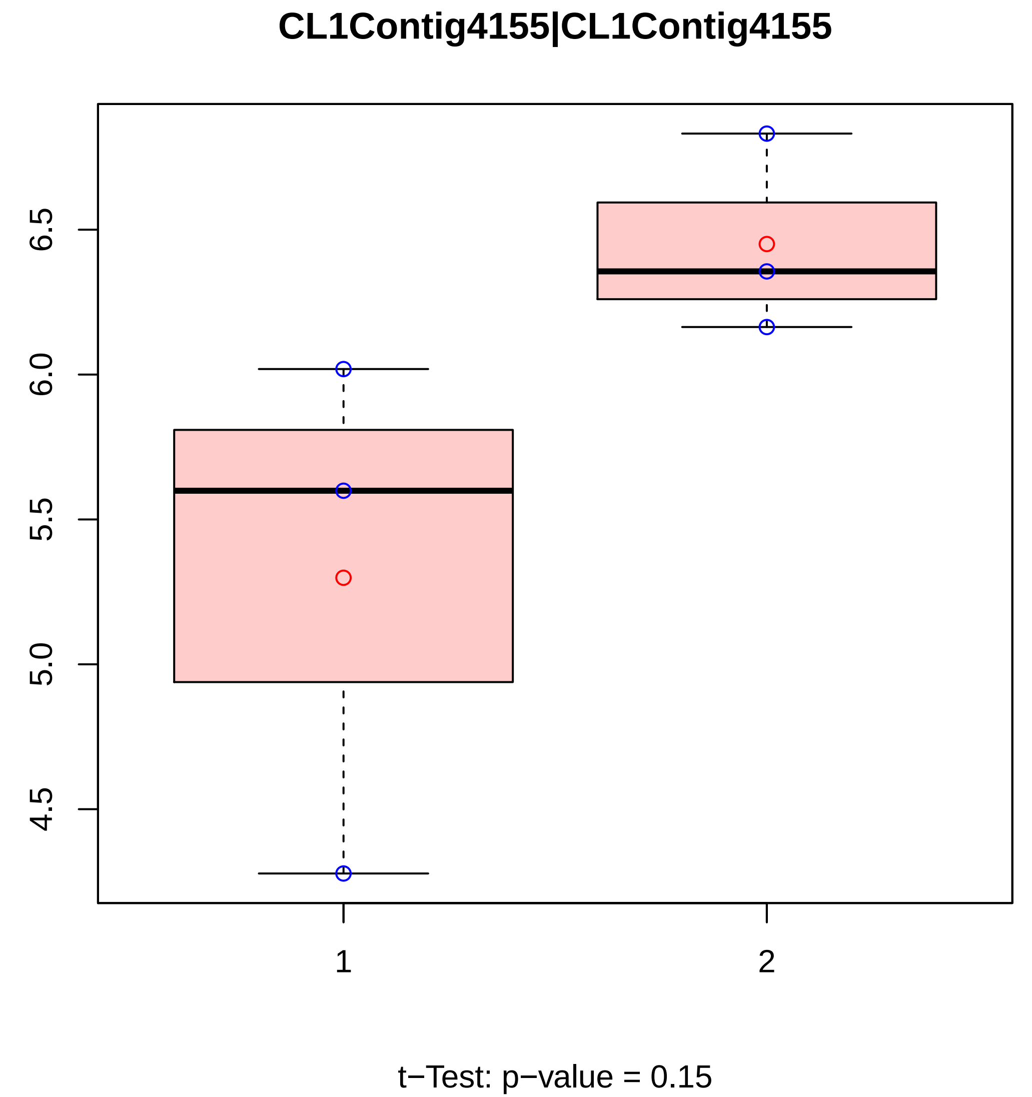

CL1Contig4155|CL1Contig4155
6.5
6.0
5.5
5.0
4.5
1
2
t−Test: p−value = 0.15

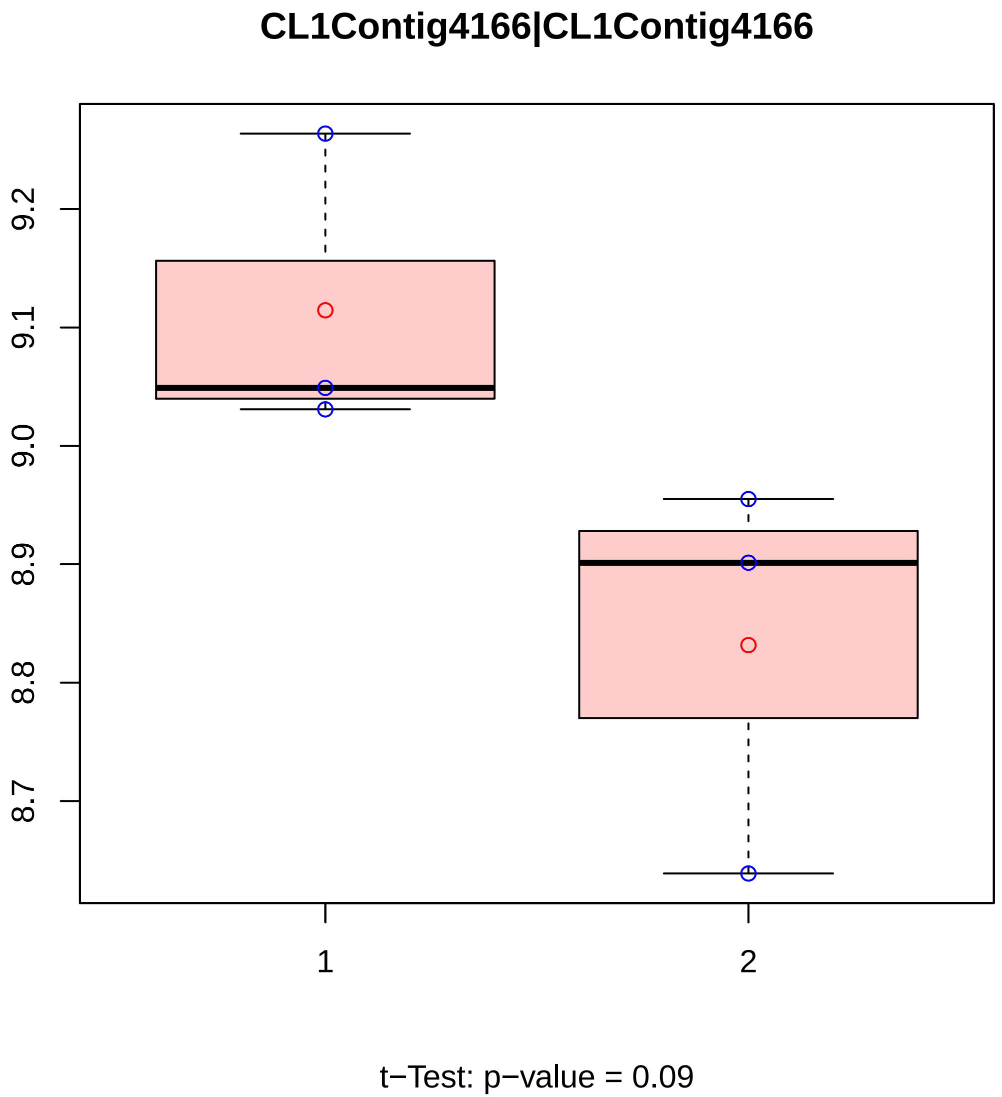
CL1Contig4166|CL1Contig4166
9.2
9.1
9.0
8.9
8.8
8.7
1
2
t−Test: p−value = 0.09

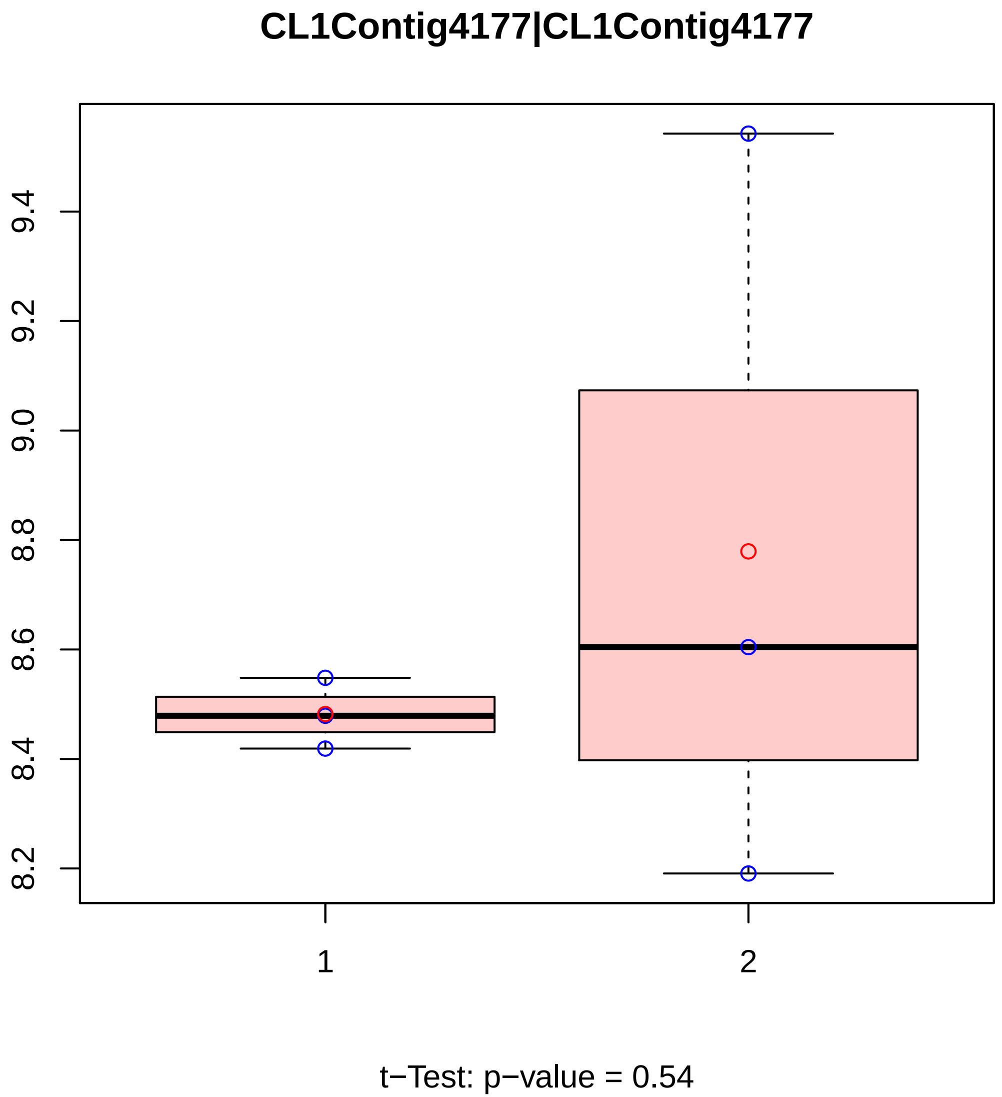
CL1Contig4177|CL1Contig4177
9.4
9.2
9.0
8.8
8.6
8.4
8.2
1
2
t−Test: p−value = 0.54

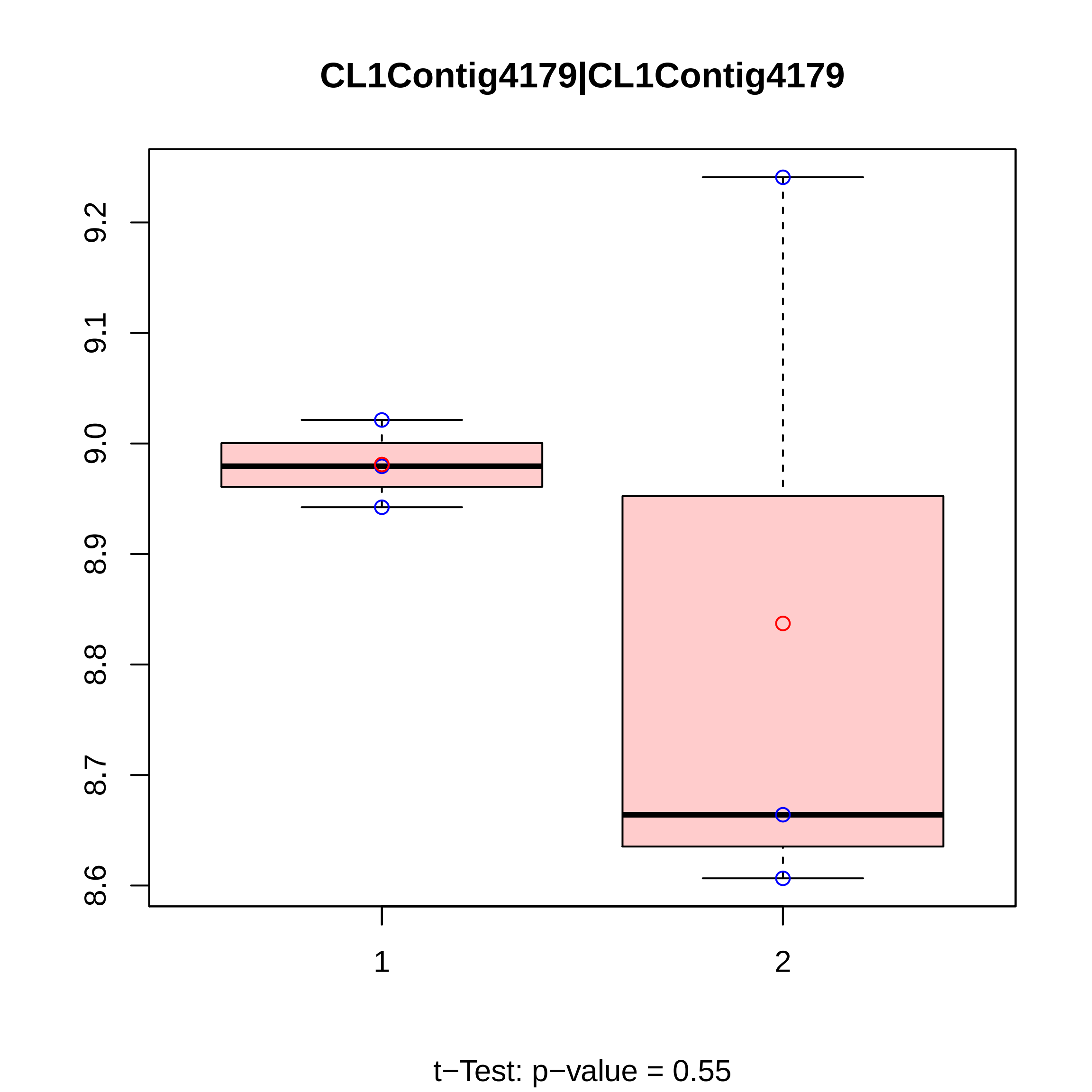

CL1Contig4179|CL1Contig4179
9.2
9.1
9.0
8.9
8.8
8.7
8.6
1
2
t−Test: p−value = 0.55

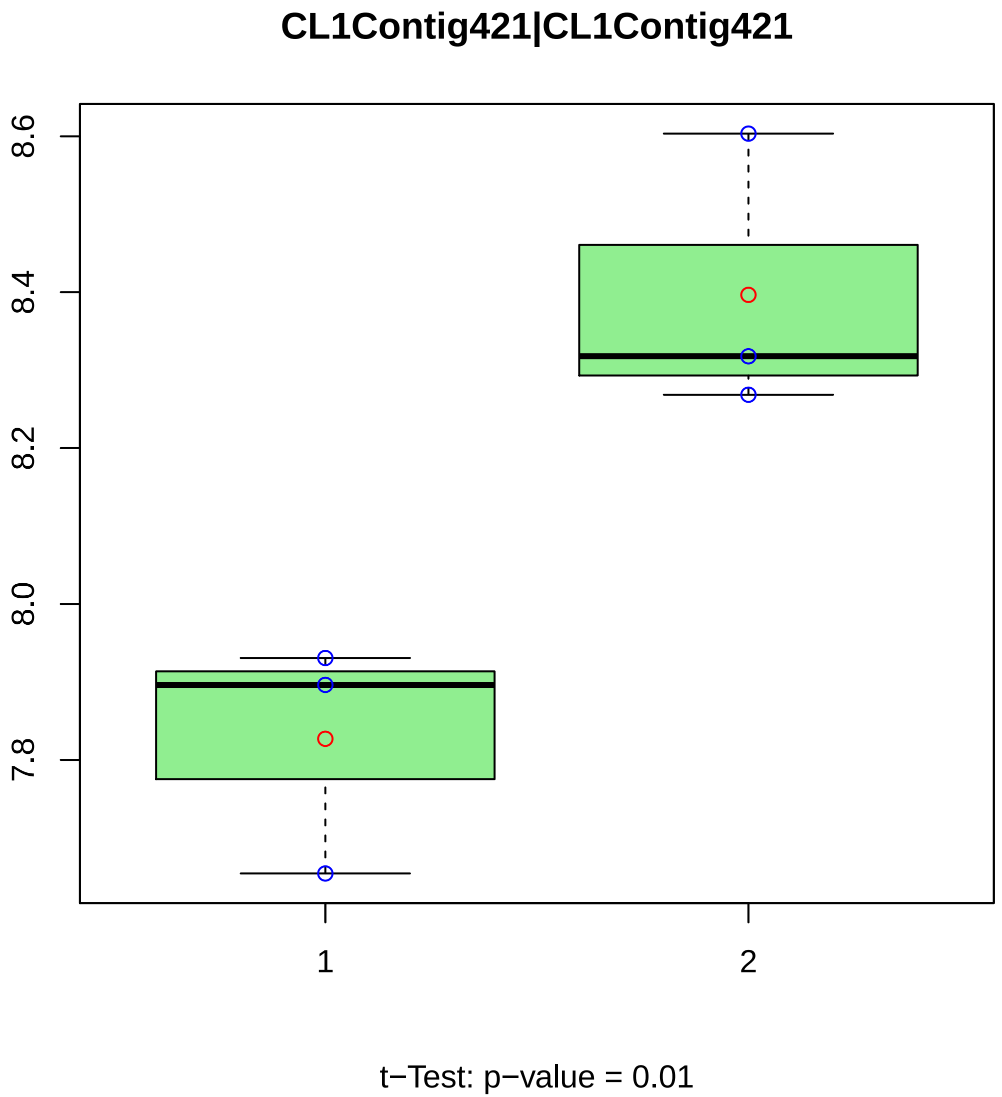
CL1Contig421|CL1Contig421
8.6
8.4
8.2
8.0
7.8
1
2
t−Test: p−value = 0.01

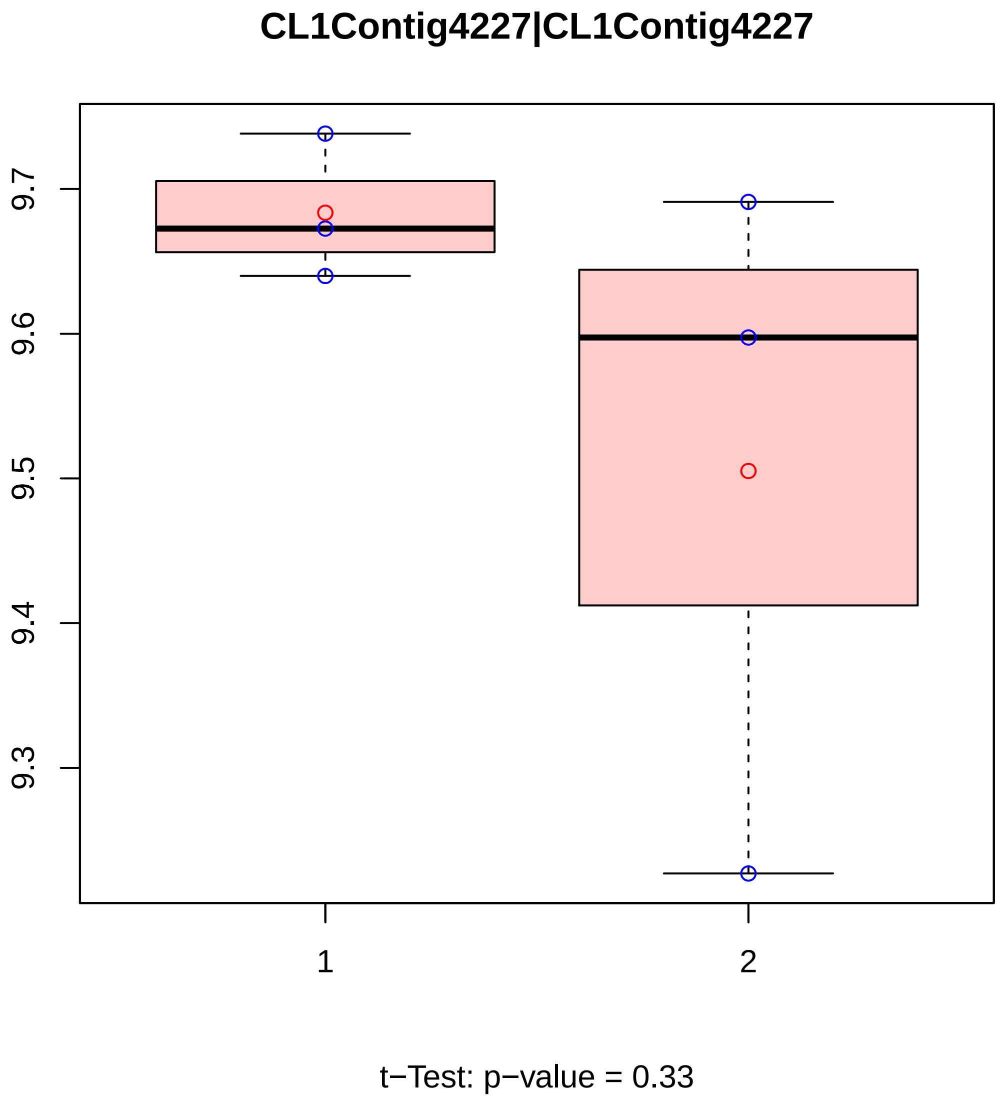
CL1Contig4227|CL1Contig4227
9.3
9.4
9.5
9.6
9.7
1
2
t−Test: p−value = 0.33

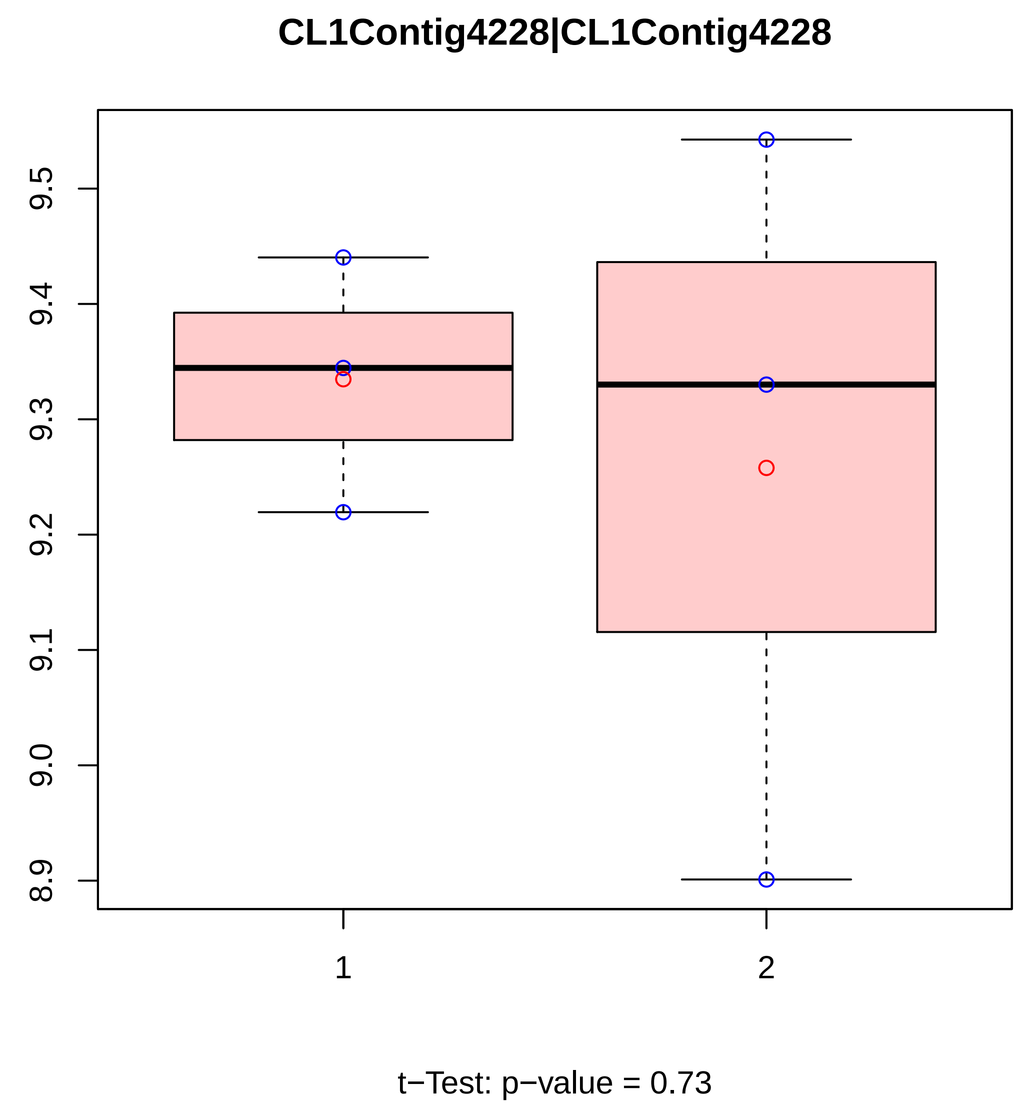
CL1Contig4228|CL1Contig4228
9.5
9.4
9.3
9.2
9.1
9.0
8.9
1
2
t−Test: p−value = 0.73

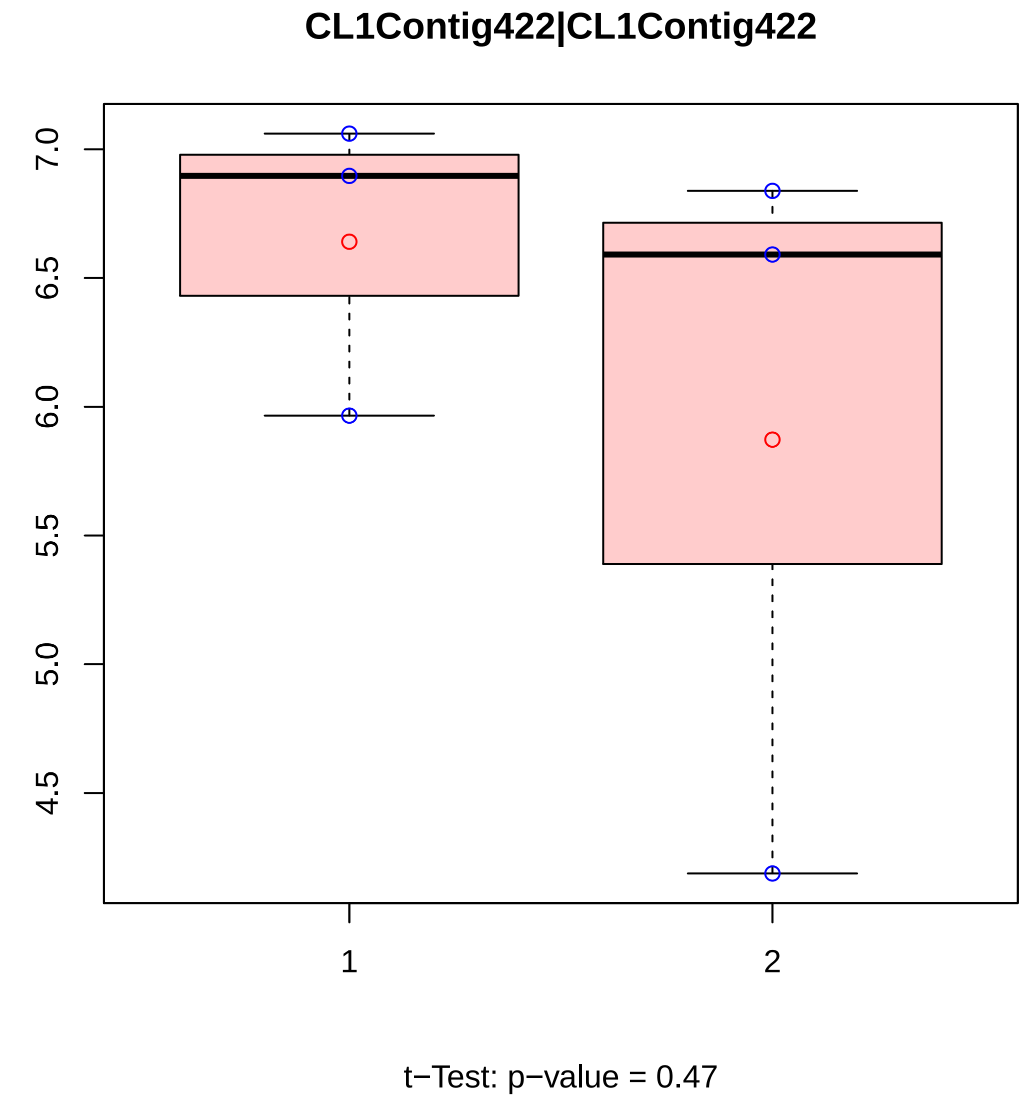

CL1Contig422|CL1Contig422
7.0
6.5
6.0
5.5
5.0
4.5
1
2
t−Test: p−value = 0.47

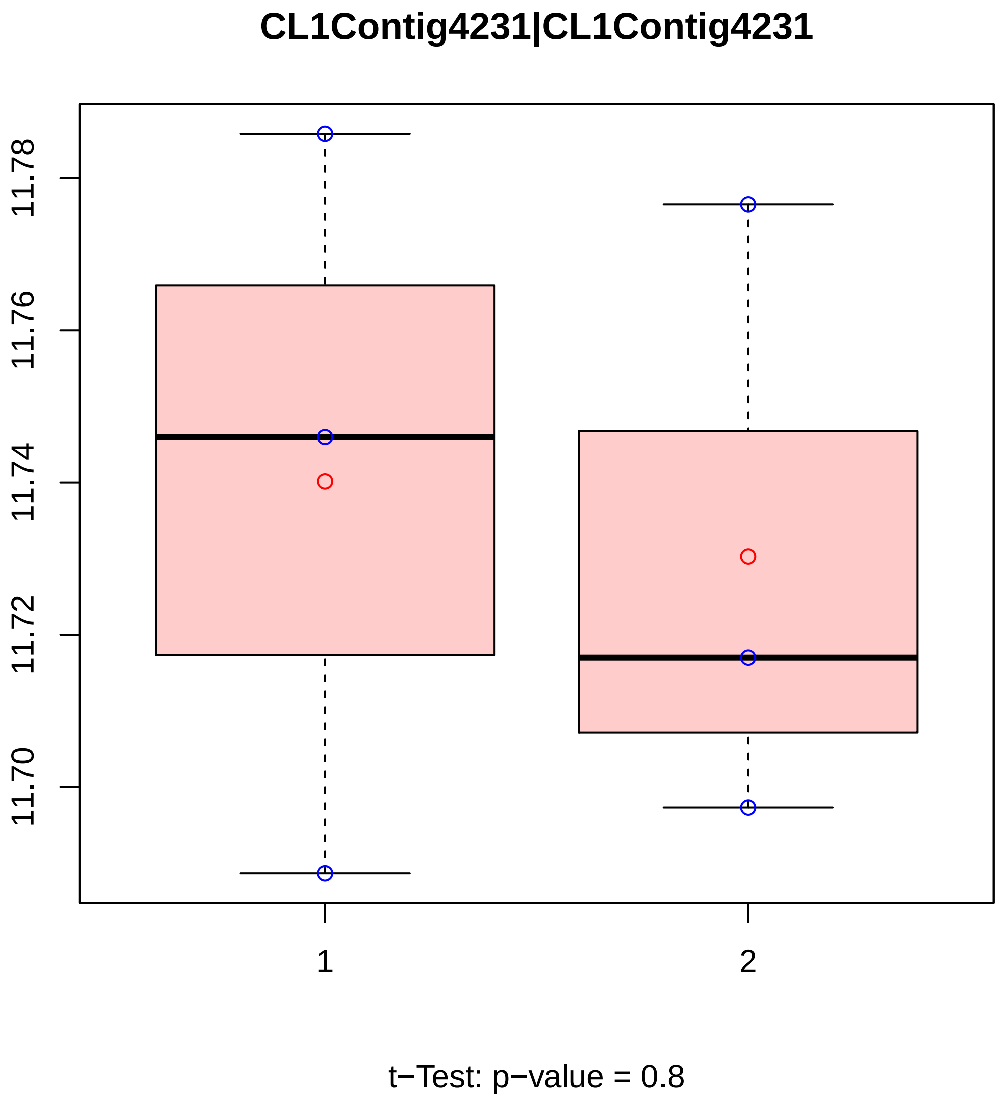

CL1Contig4231|CL1Contig4231
11.70
11.72
11.74
11.76
11.78
1
2
t−Test: p−value = 0.8

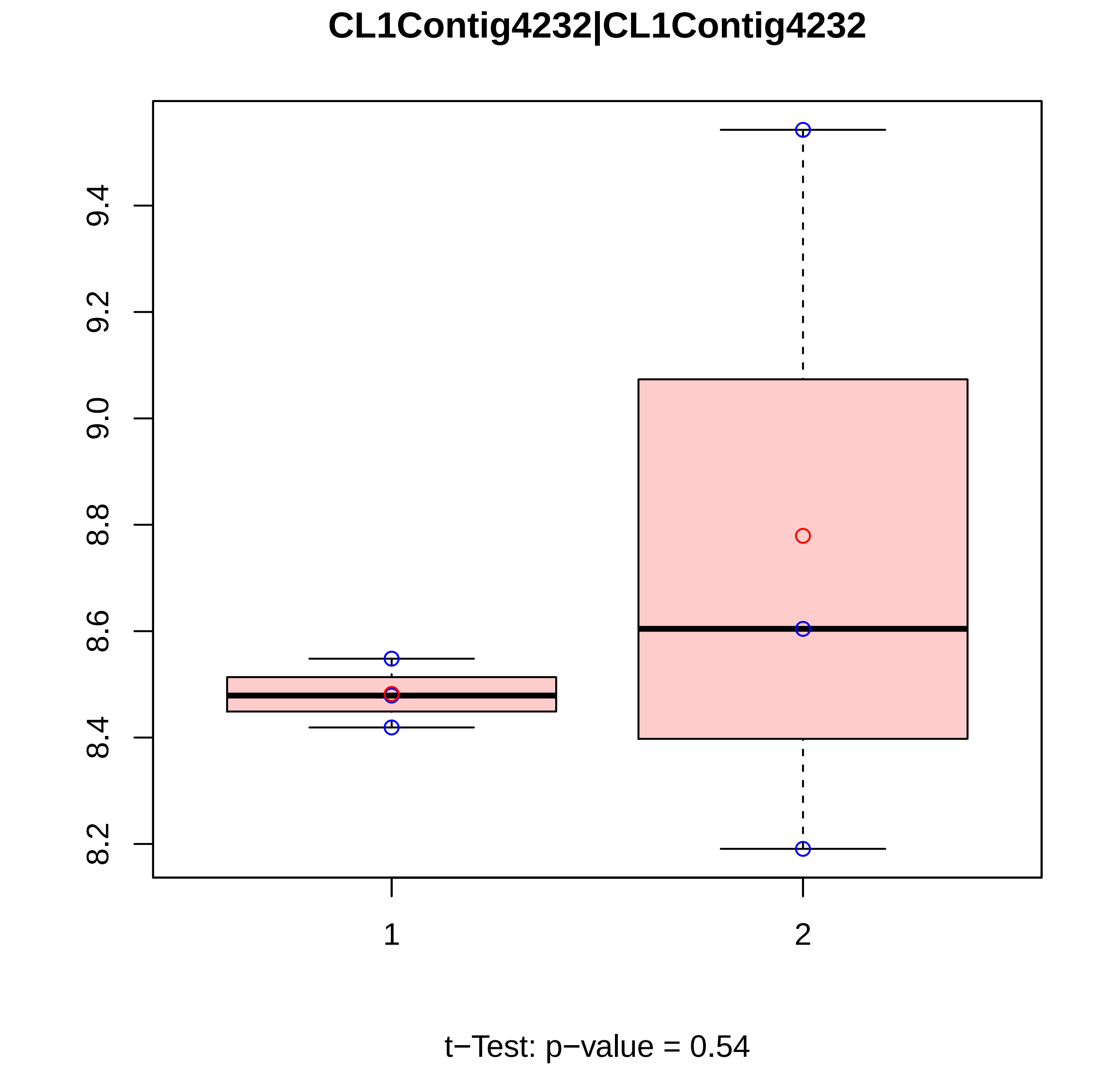
CL1Contig4232|CL1Contig4232
9.4
9.2
9.0
8.8
8.6
8.4
8.2
1
2
t−Test: p−value = 0.54

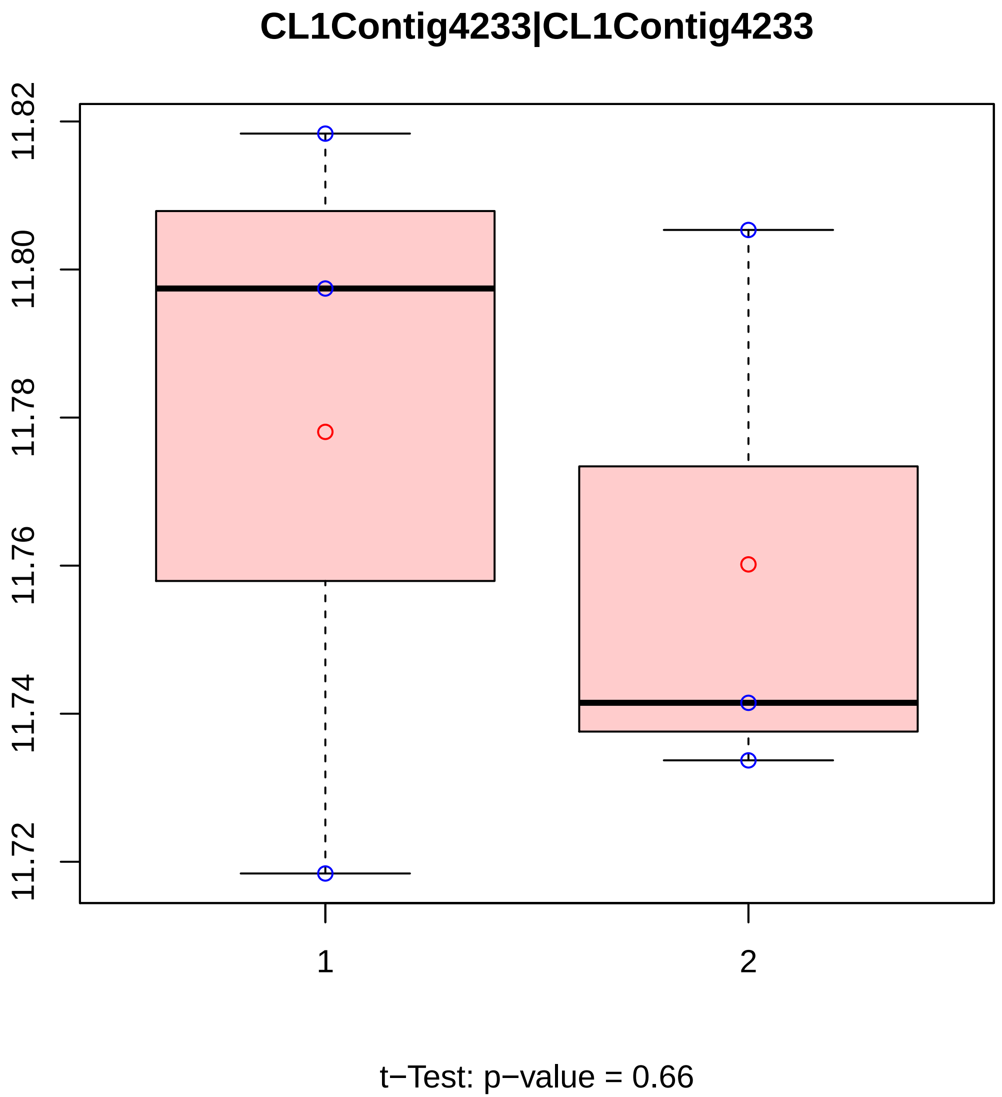

CL1Contig4233|CL1Contig4233
11.82
11.80
11.78
11.76
11.74
11.72
1
2
t−Test: p−value = 0.66

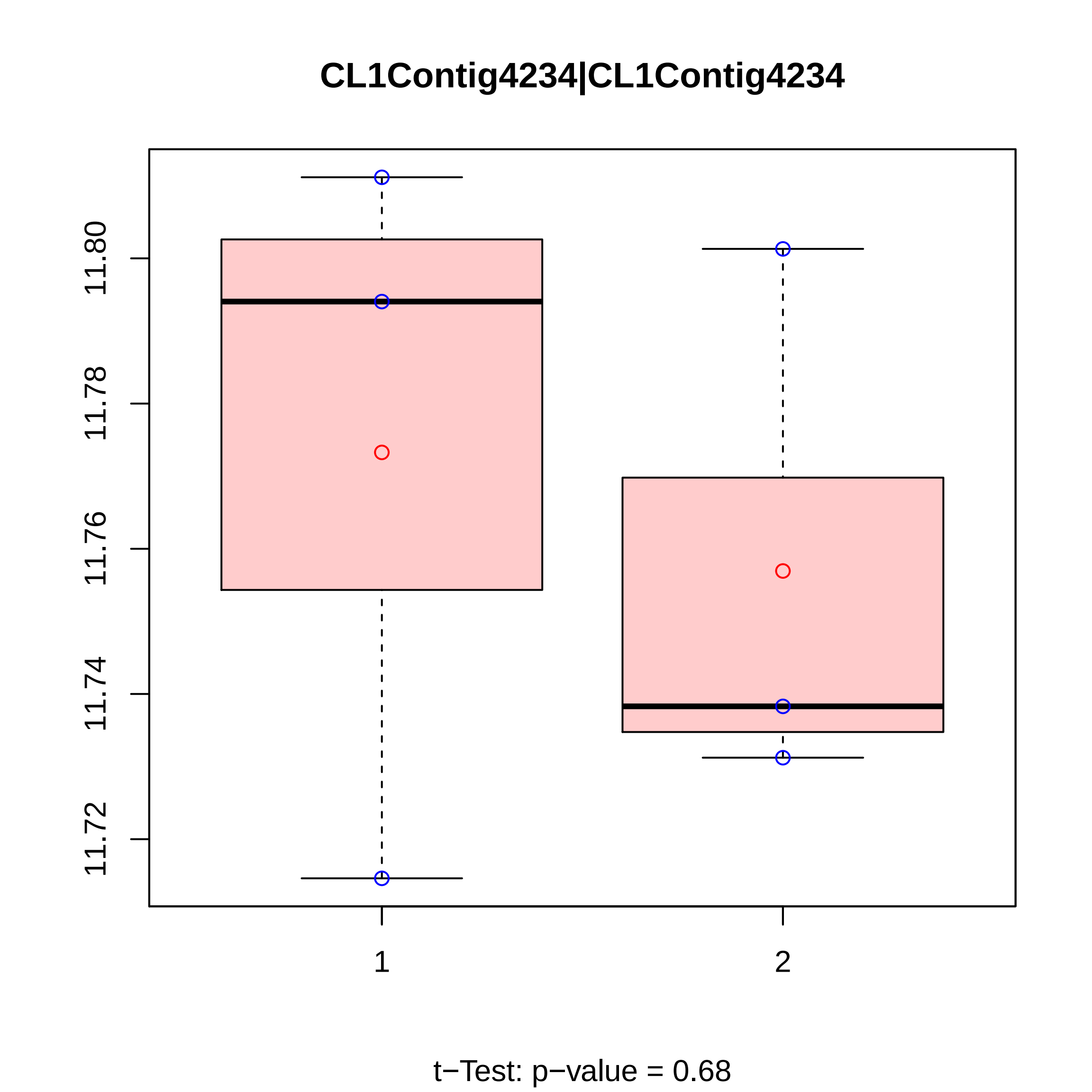
CL1Contig4234|CL1Contig4234
11.72
11.74
11.76
11.78
11.80
1
2
t−Test: p−value = 0.68

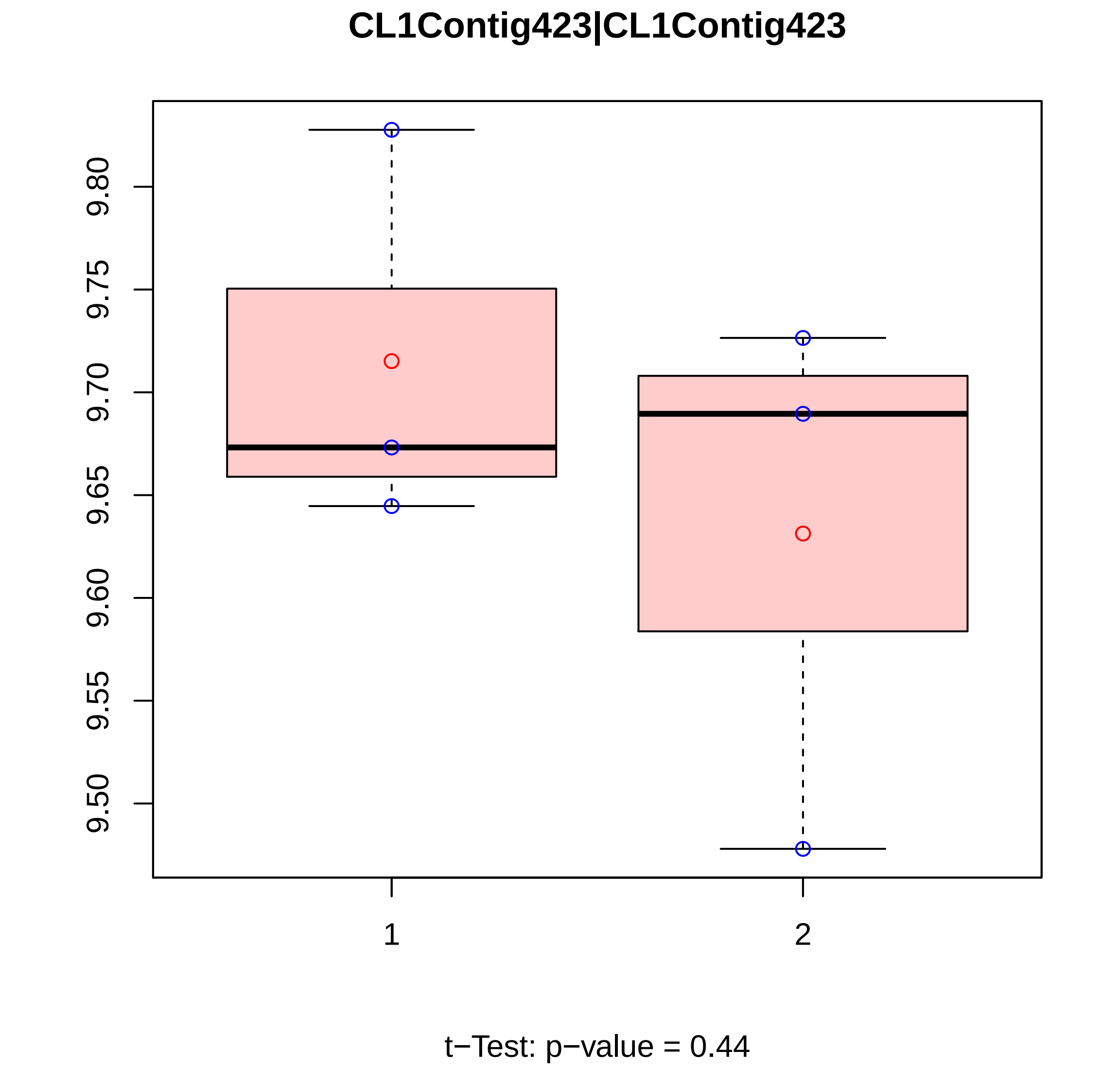
CL1Contig423|CL1Contig423
9.50
9.55
9.60
9.65
9.70
9.75
9.80
1
2
t−Test: p−value = 0.44

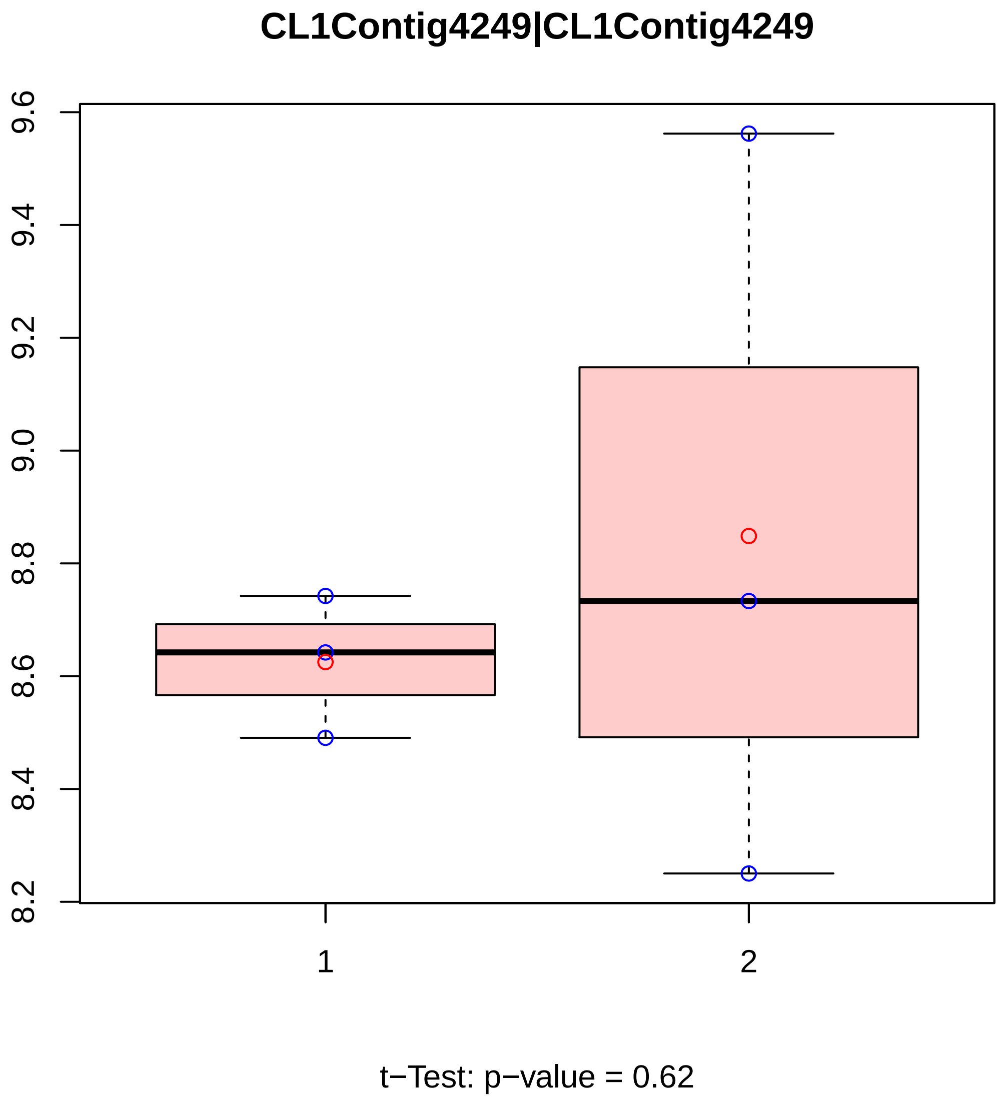
CL1Contig4249|CL1Contig4249
9.6
9.4
9.2
9.0
8.8
8.6
8.4
8.2
1
2
t−Test: p−value = 0.62

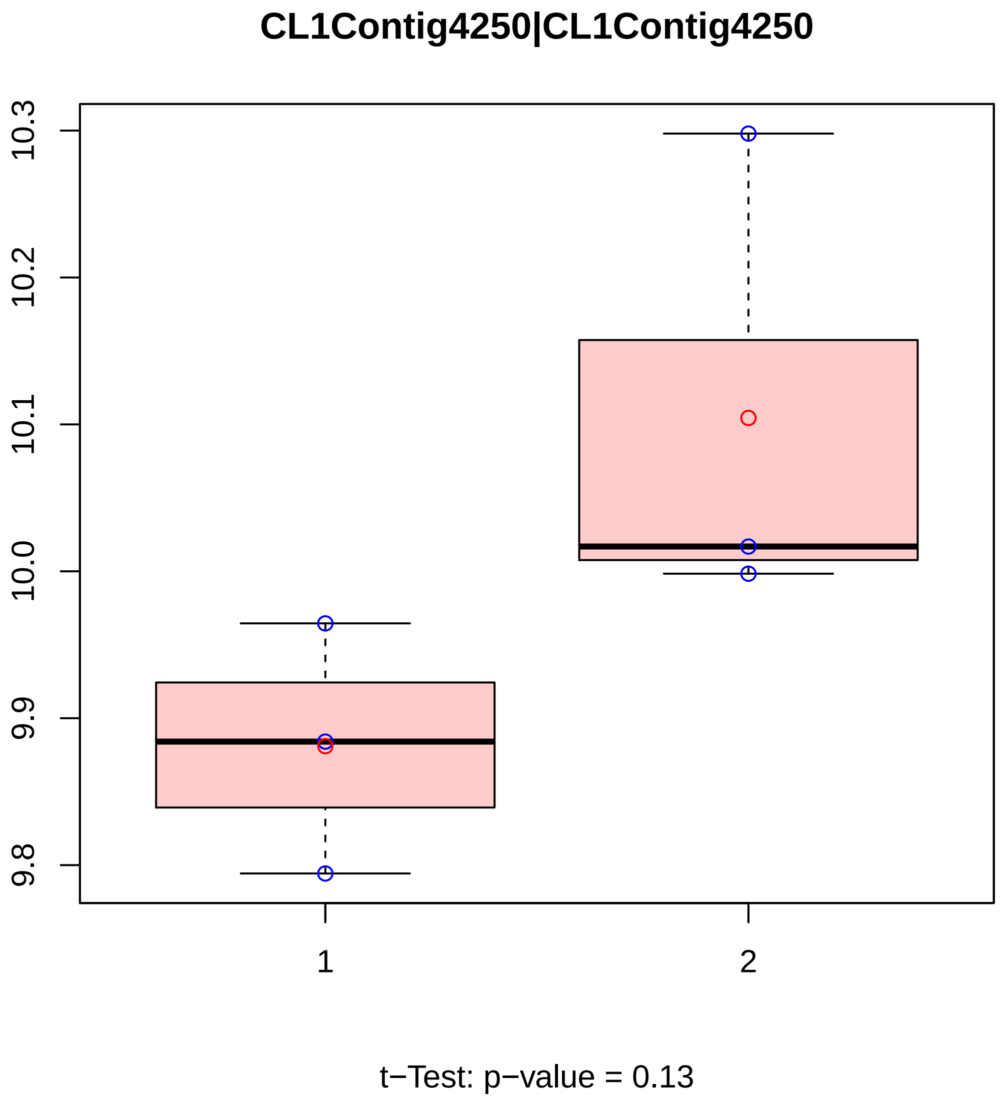
CL1Contig4250|CL1Contig4250
10.3
10.2
10.1
10.0
9.9
9.8
1
2
t−Test: p−value = 0.13

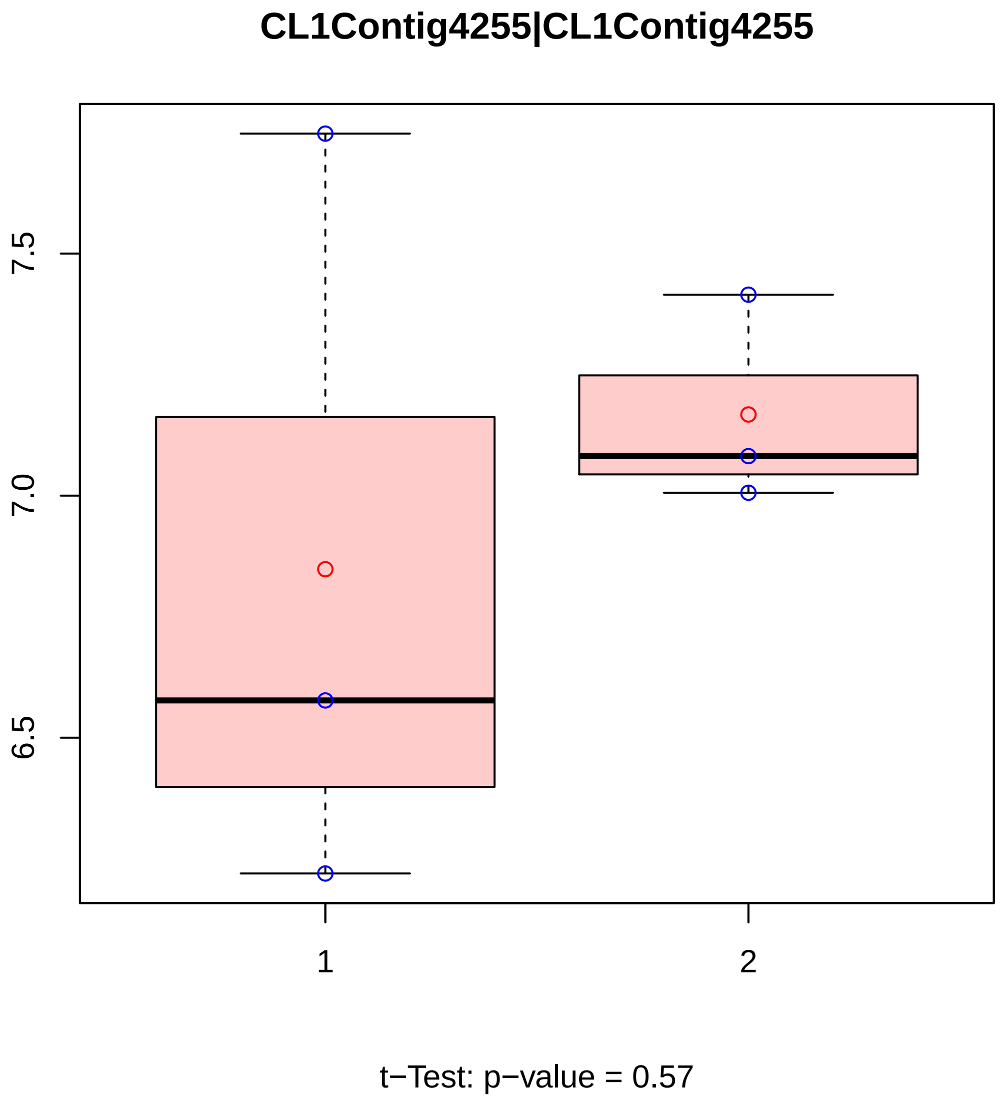

CL1Contig4255|CL1Contig4255
7.5
7.0
6.5
1
2
t−Test: p−value = 0.57

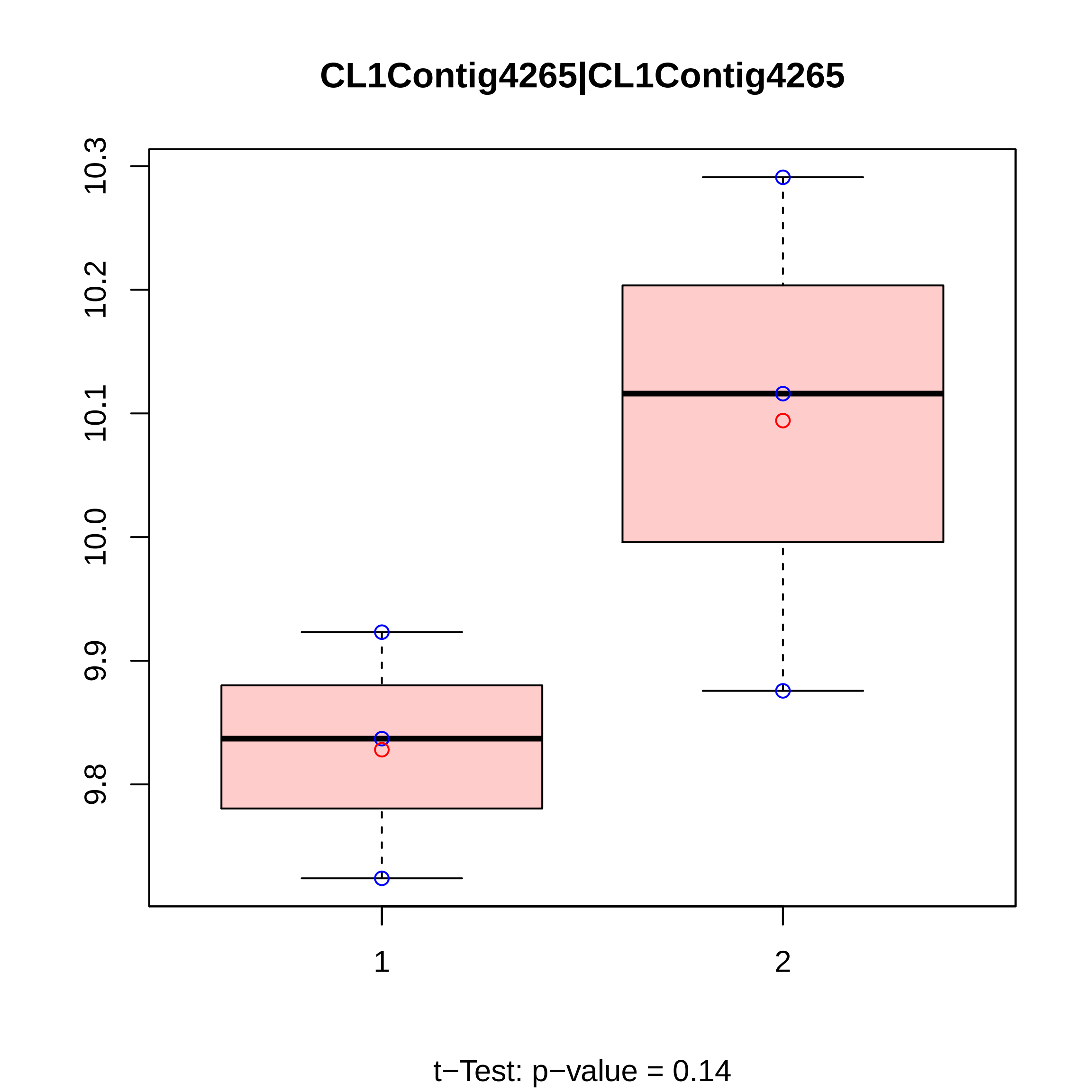

CL1Contig4265|CL1Contig4265
10.3
10.2
10.1
10.0
9.9
9.8
1
2
t−Test: p−value = 0.14

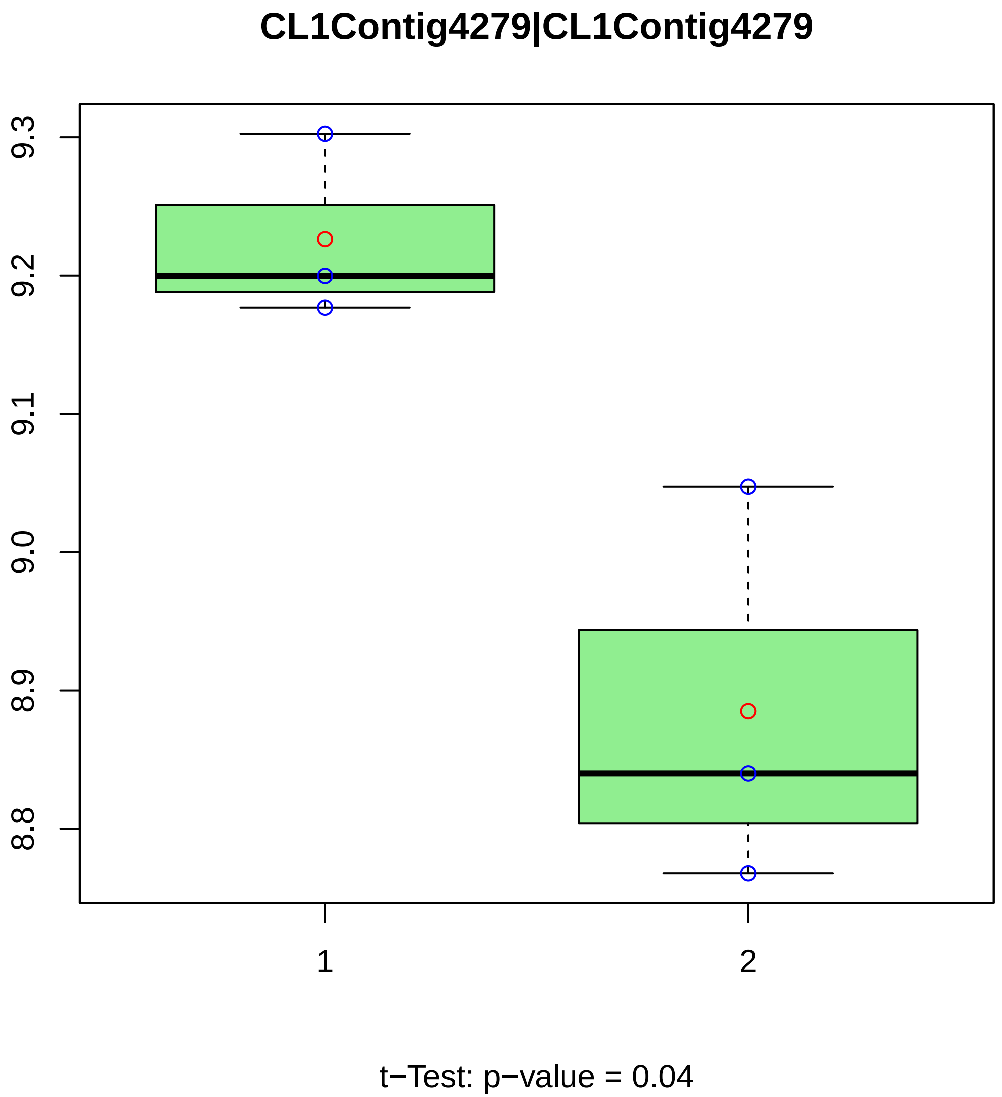

CL1Contig4279|CL1Contig4279
9.3
9.2
9.1
9.0
8.9
8.8
1
2
t−Test: p−value = 0.04

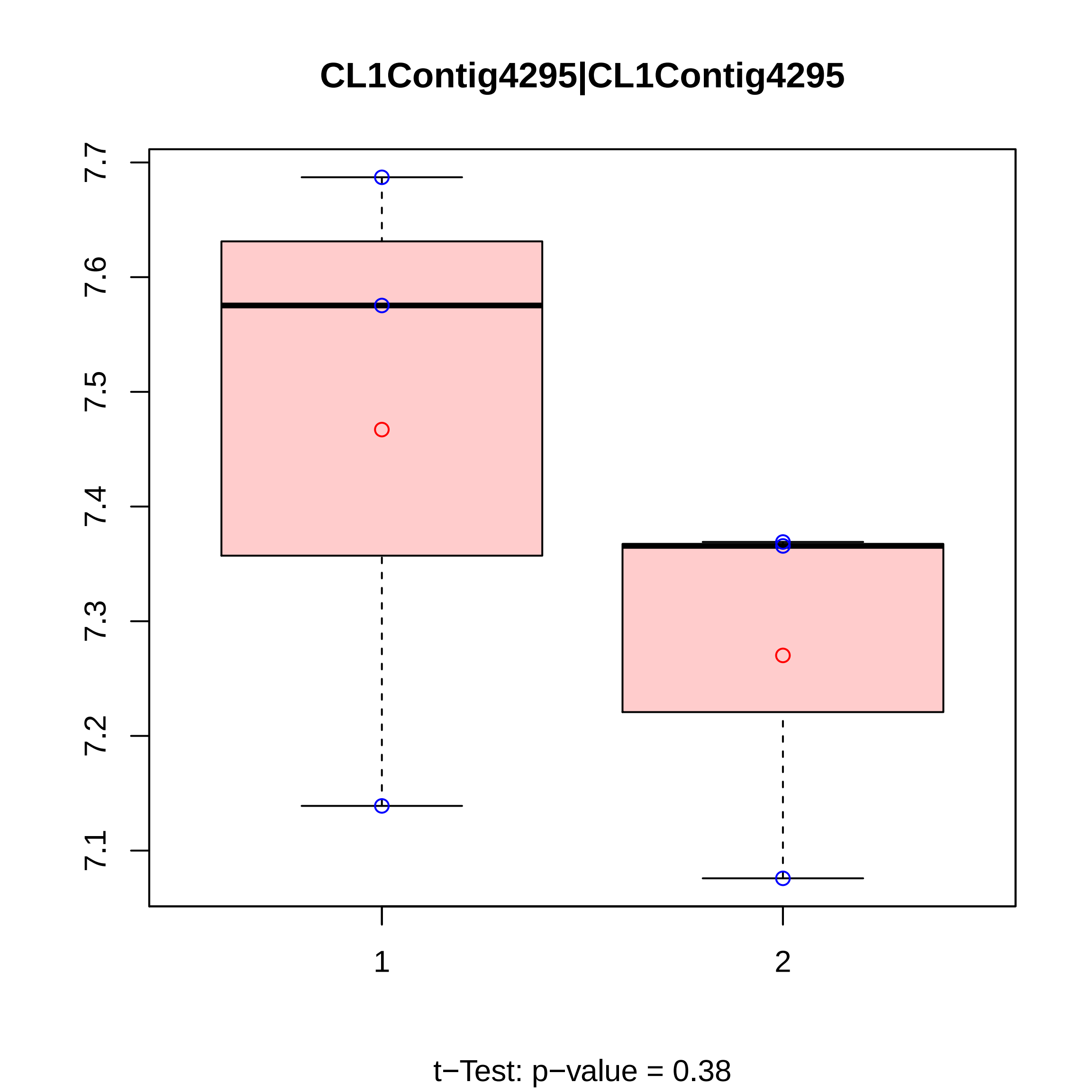
CL1Contig4295|CL1Contig4295
7.7
7.6
7.5
7.4
7.3
7.2
7.1
1
2
t−Test: p−value = 0.38

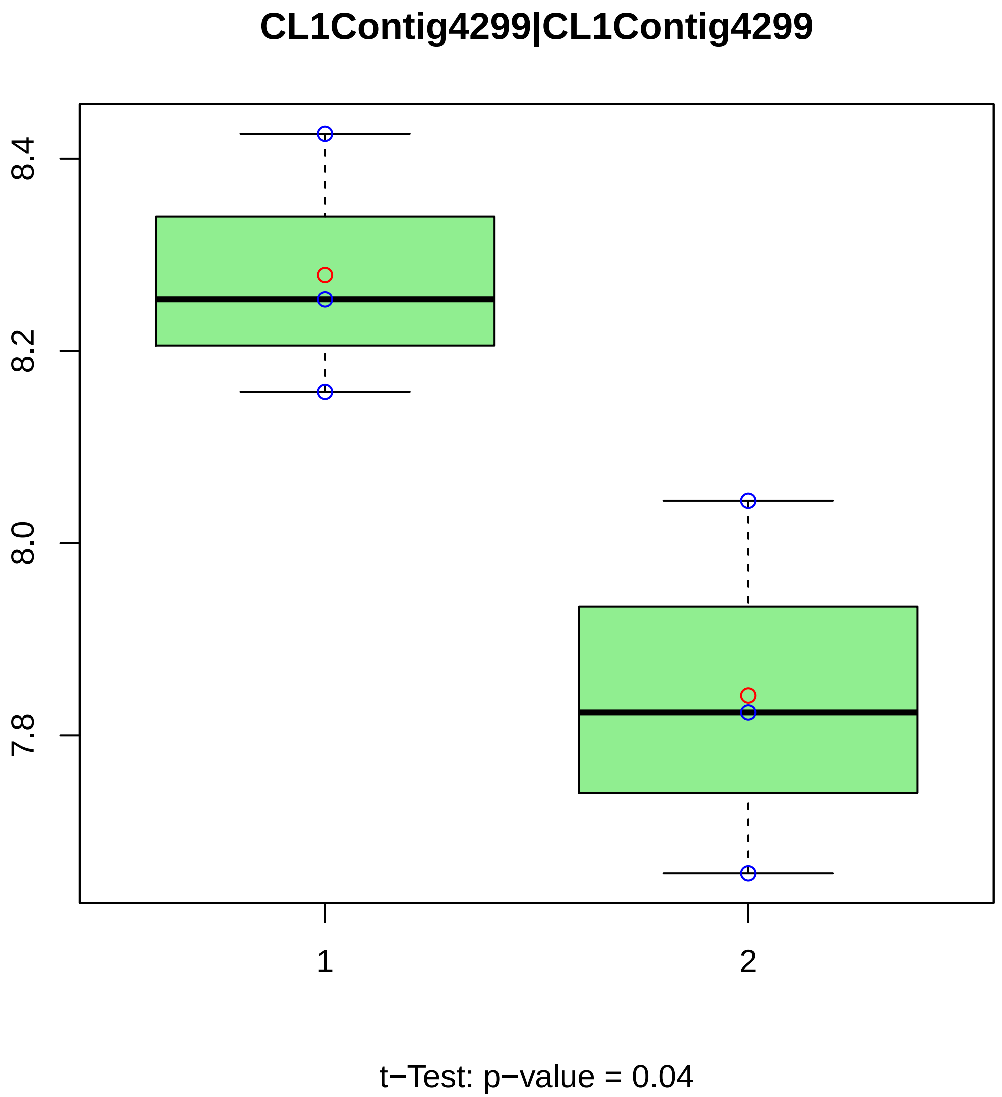
CL1Contig4299|CL1Contig4299
8.4
8.2
8.0
7.8
1
2
t−Test: p−value = 0.04

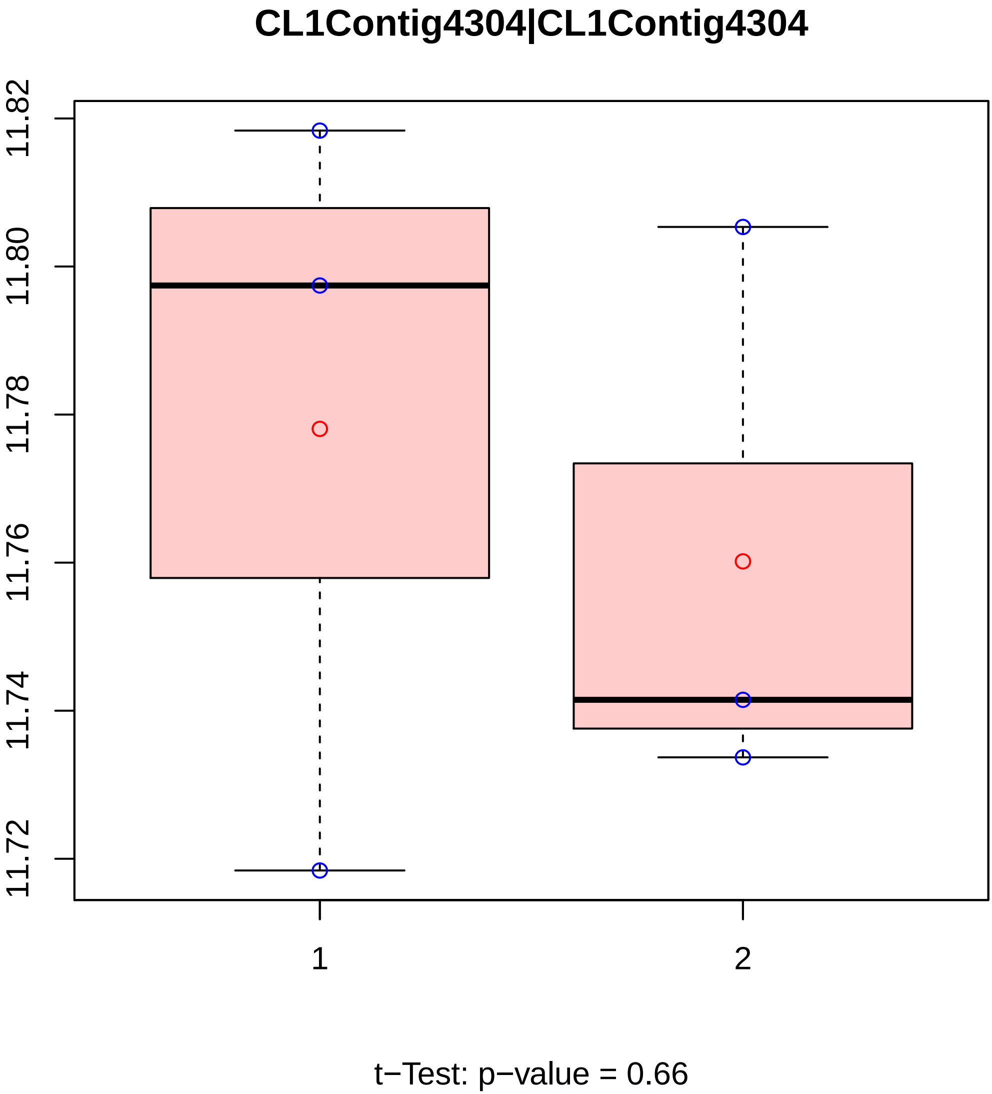

CL1Contig4304|CL1Contig4304
11.82
11.80
11.78
11.76
11.74
11.72
1
2
t−Test: p−value = 0.66

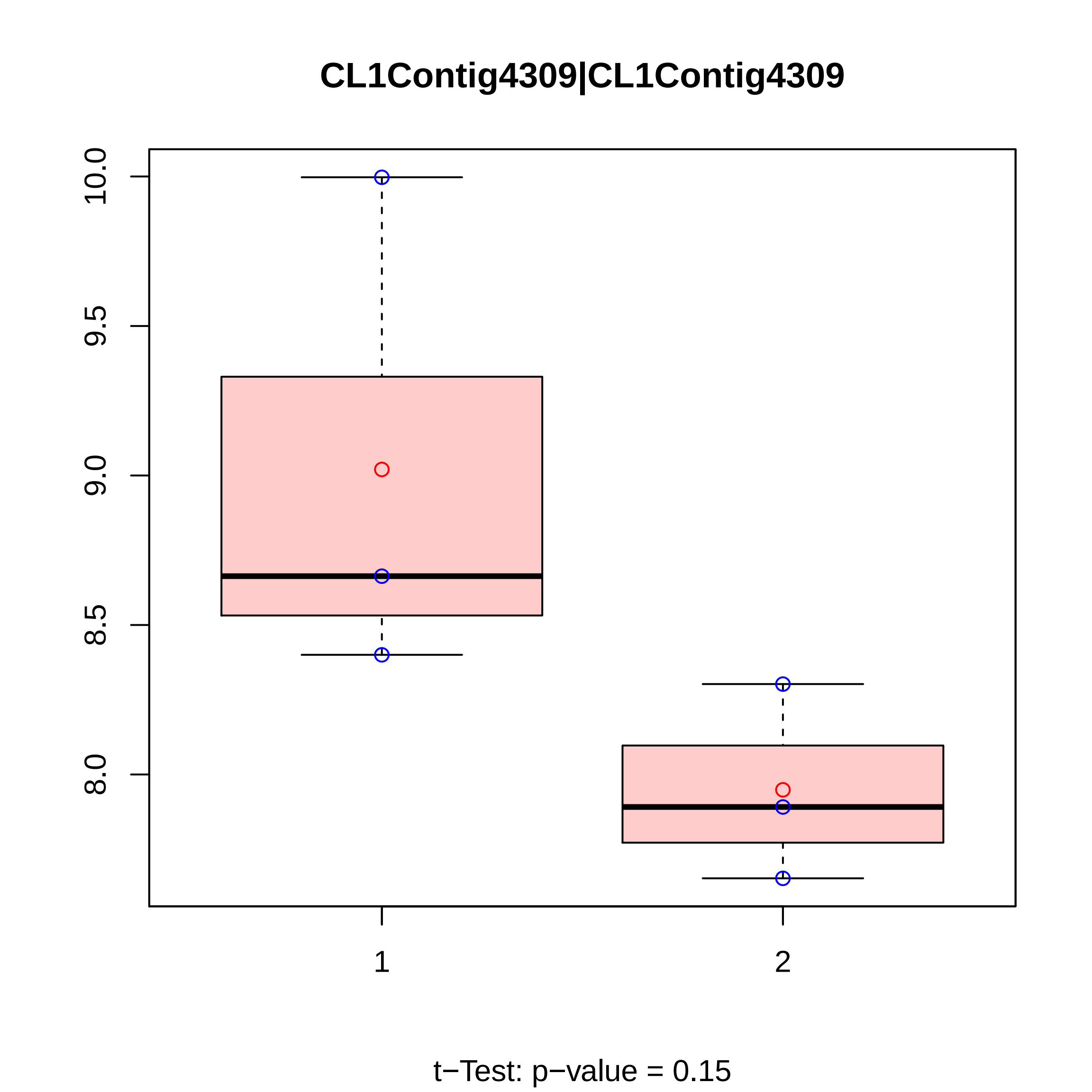

CL1Contig4309|CL1Contig4309
10.0
9.5
9.0
8.5
8.0
1
2
t−Test: p−value = 0.15

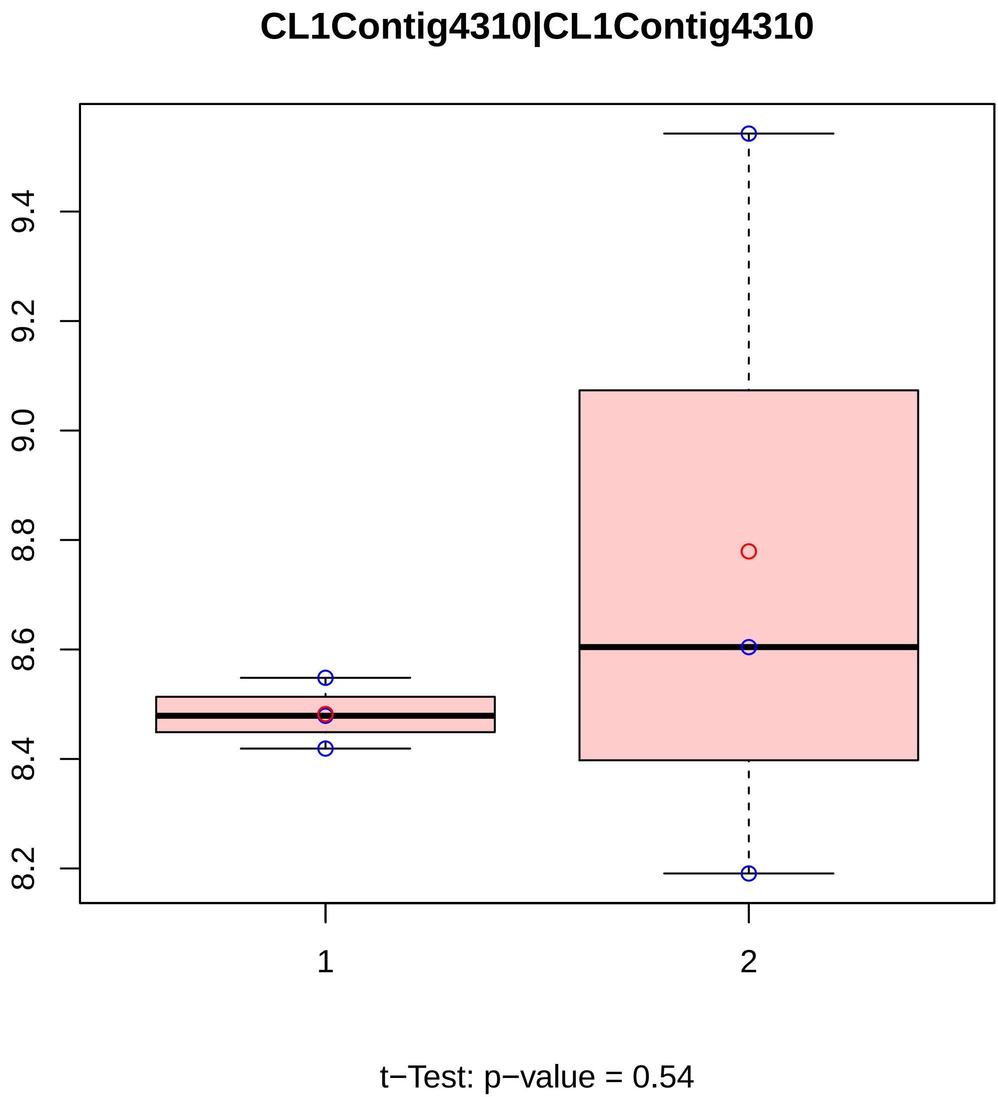
CL1Contig4310|CL1Contig4310
9.4
9.2
9.0
8.8
8.6
8.4
8.2
1
2
t−Test: p−value = 0.54

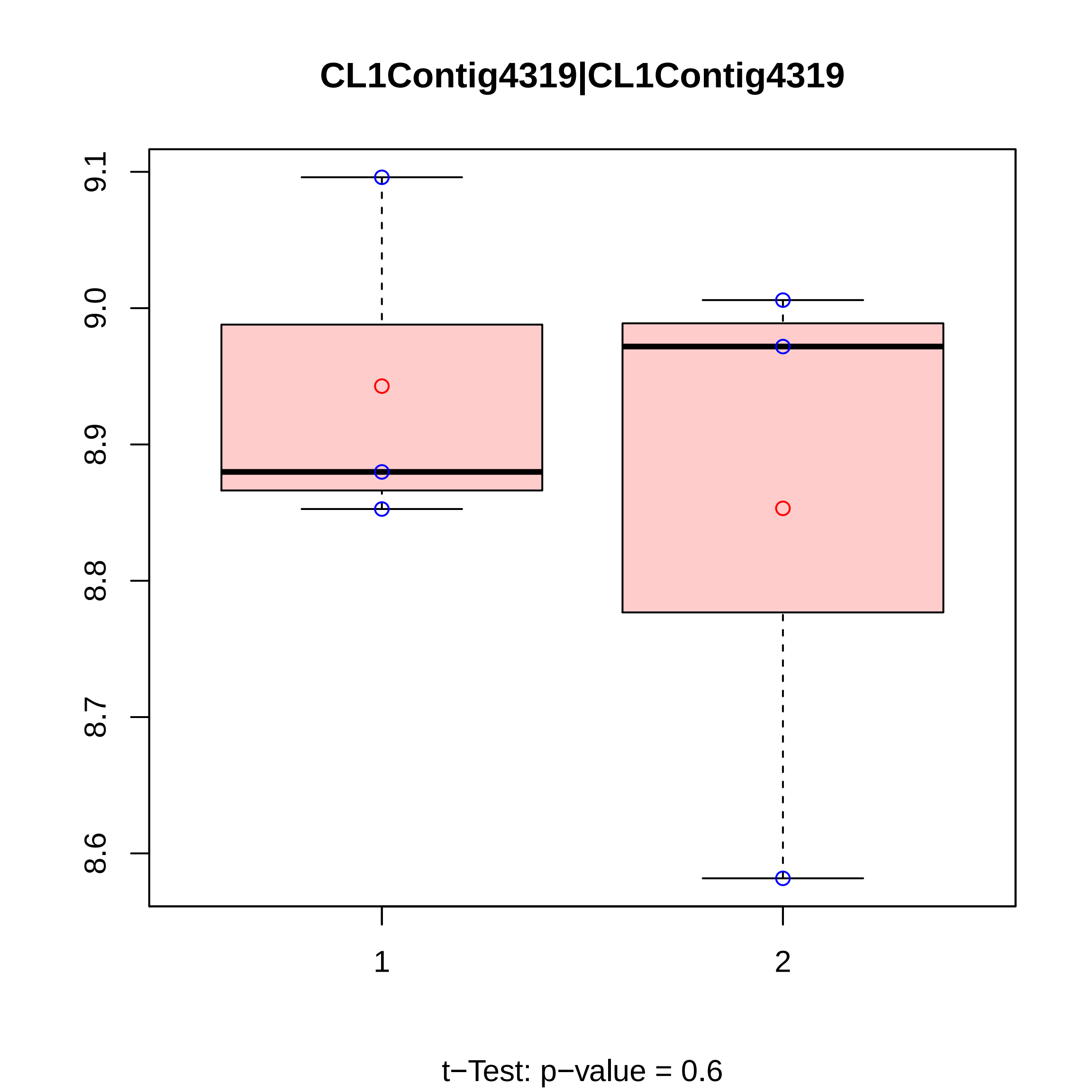
CL1Contig4319|CL1Contig4319
9.1
9.0
8.9
8.8
8.7
8.6
1
2
t−Test: p−value = 0.6

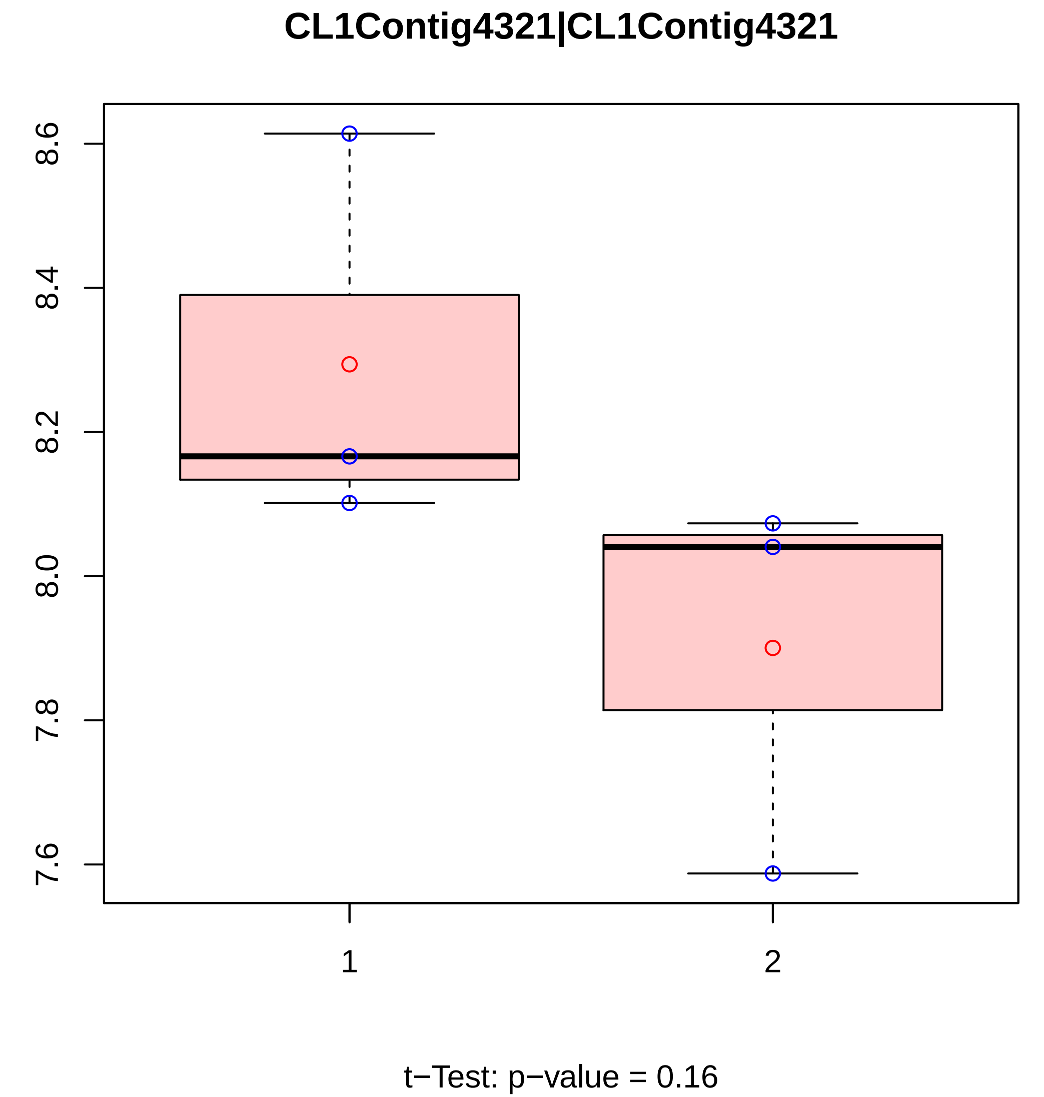
CL1Contig4321|CL1Contig4321
8.6
8.4
8.2
8.0
7.8
7.6
1
2
t−Test: p−value = 0.16

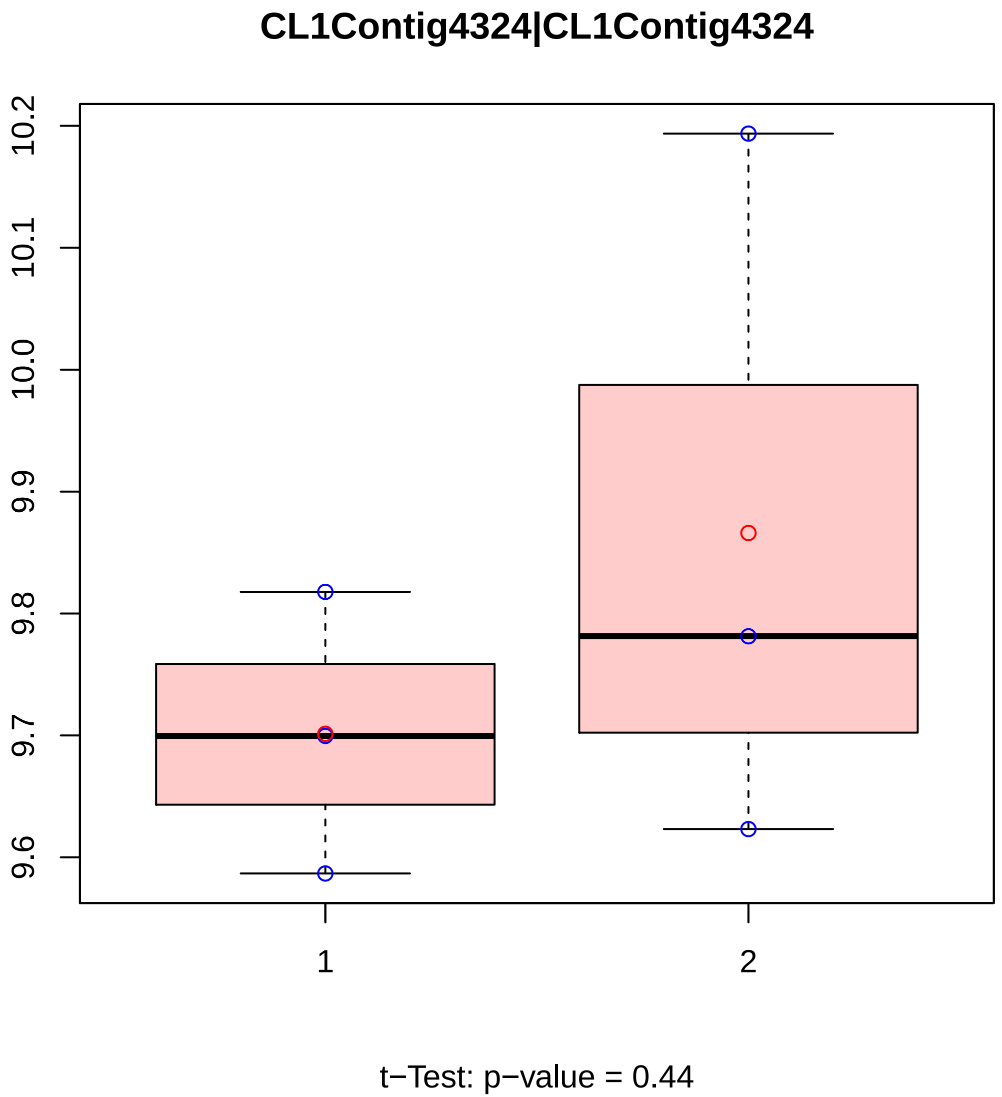
CL1Contig4324|CL1Contig4324
10.2
10.1
10.0
9.9
9.8
9.7
9.6
1
2
t−Test: p−value = 0.44

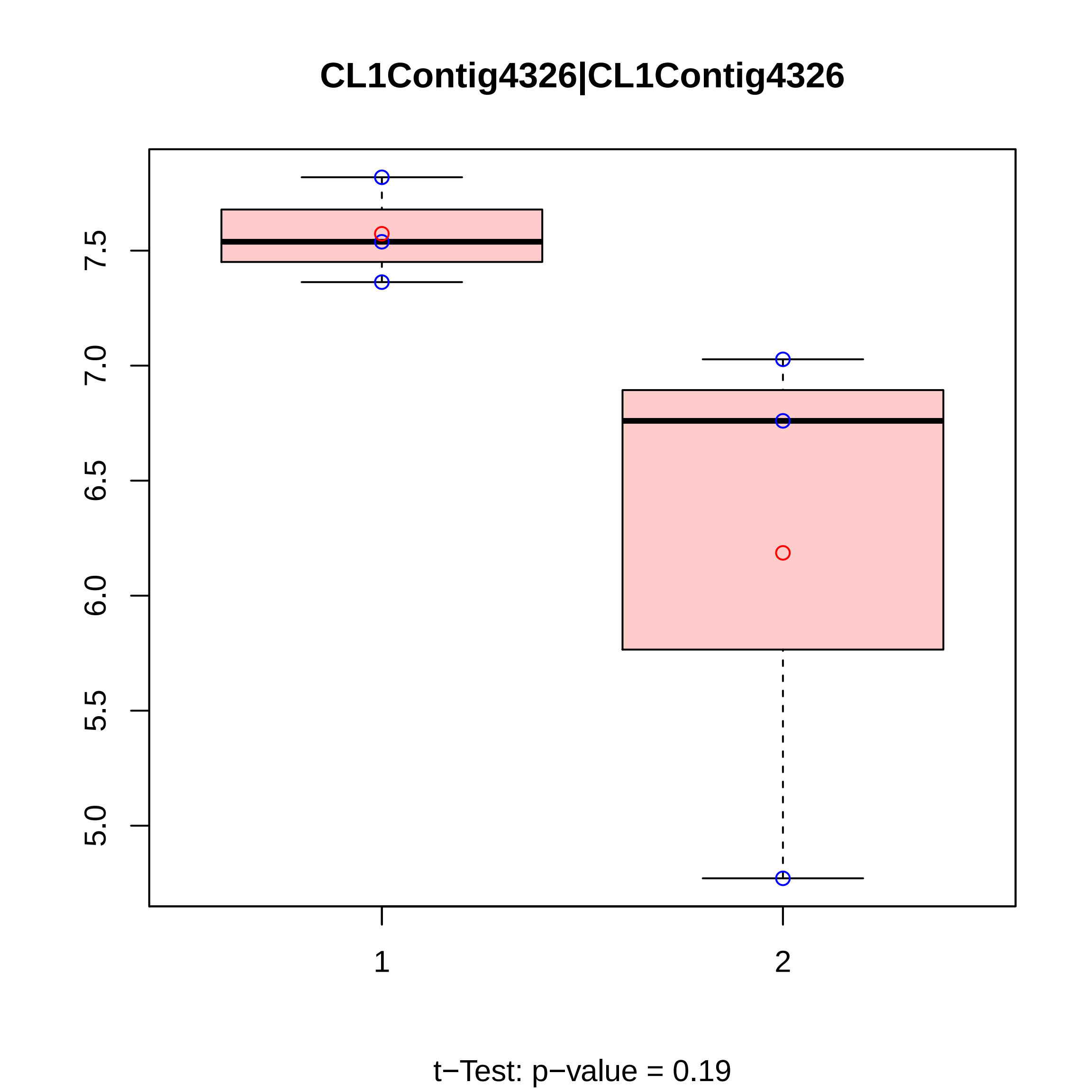

CL1Contig4326|CL1Contig4326
7.5
7.0
6.5
6.0
5.5
5.0
1
2
t−Test: p−value = 0.19

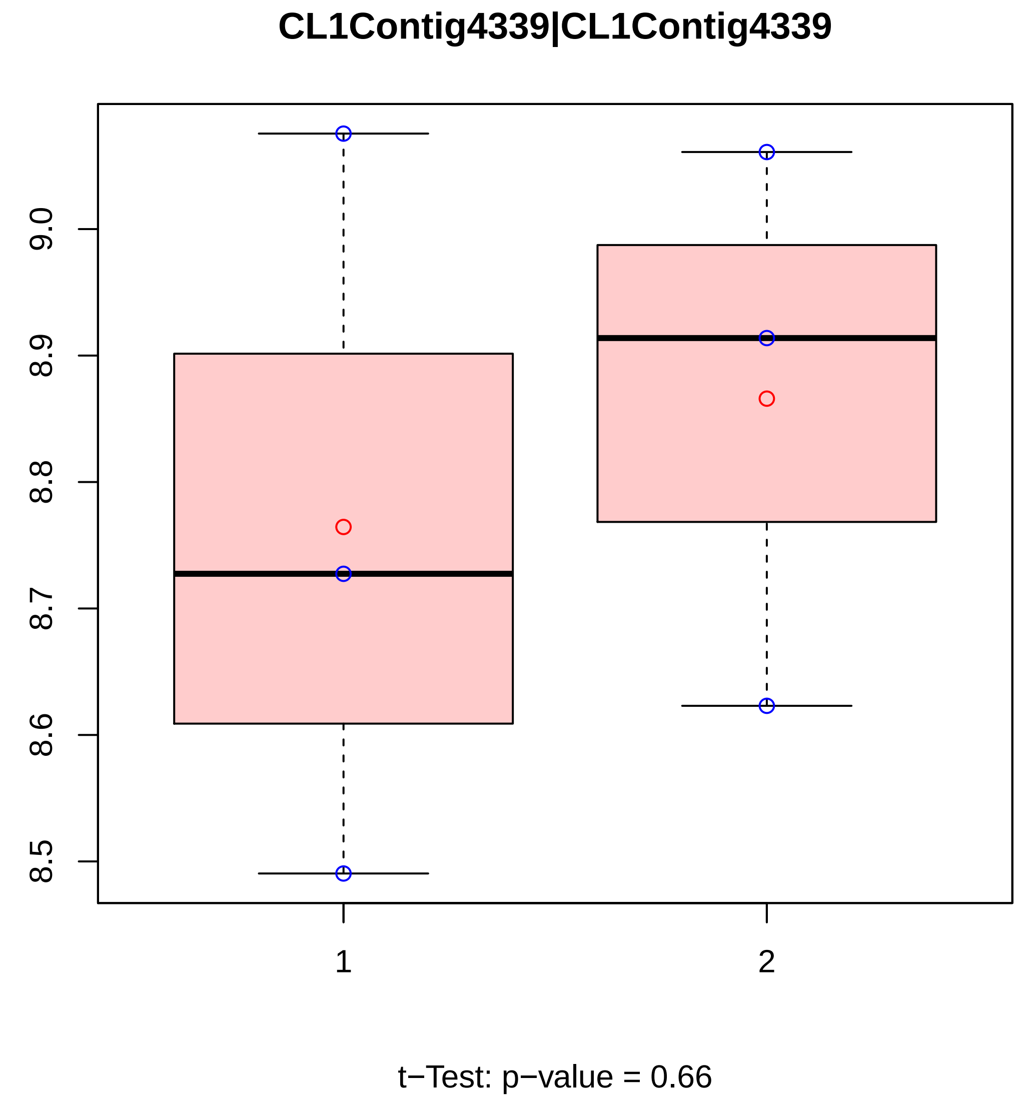
CL1Contig4339|CL1Contig4339
9.0
8.9
8.8
8.7
8.6
8.5
1
2
t−Test: p−value = 0.66

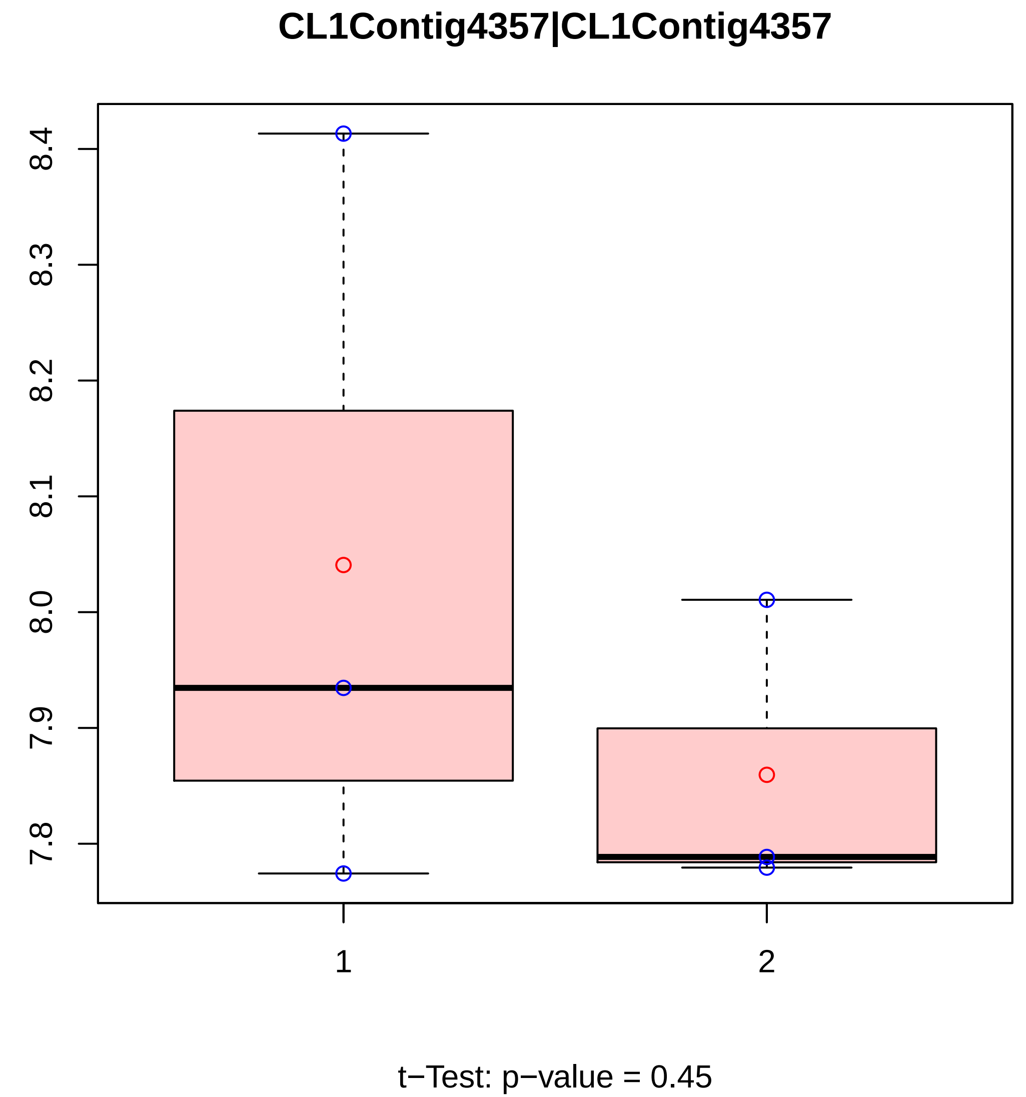
CL1Contig4357|CL1Contig4357
8.4
8.3
8.2
8.1
8.0
7.9
7.8
1
2
t−Test: p−value = 0.45

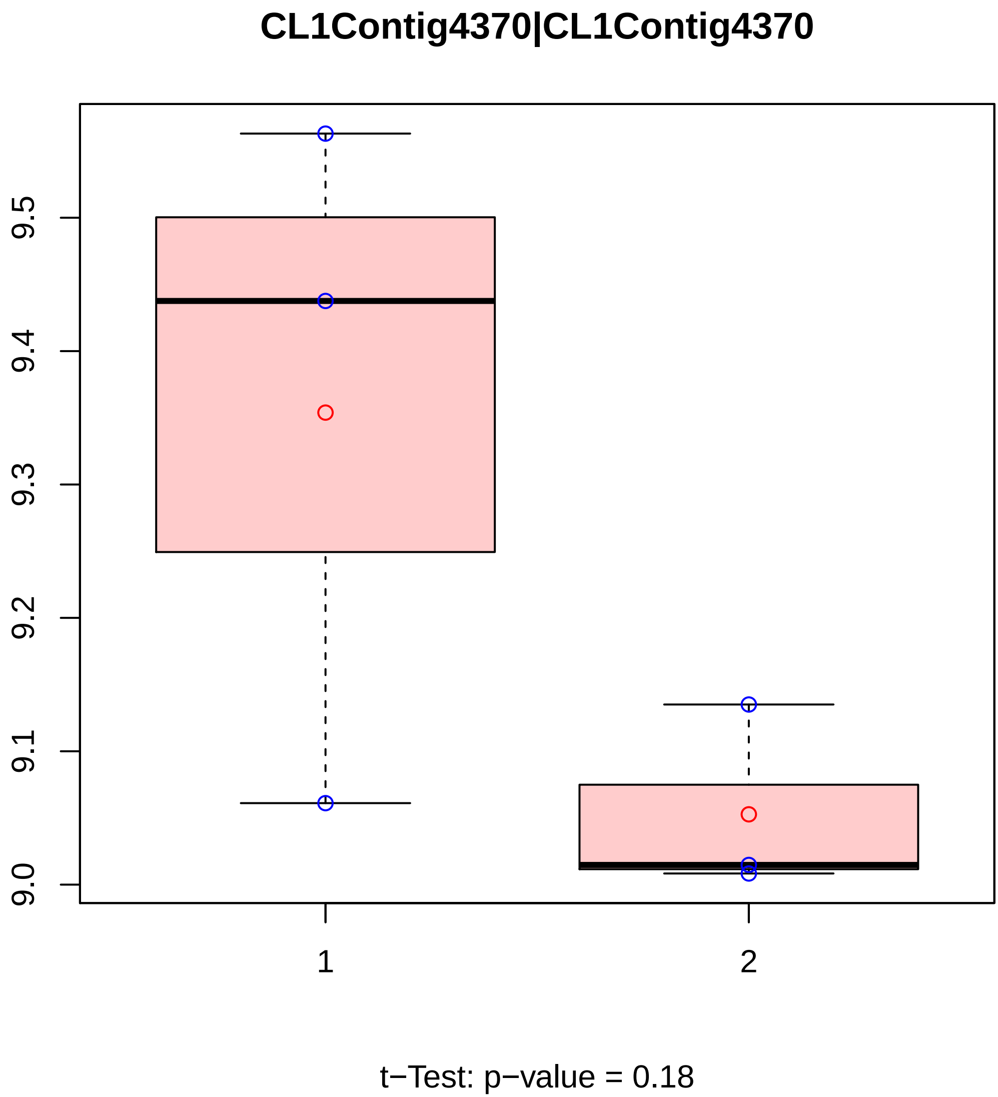

CL1Contig4370|CL1Contig4370
9.0
9.1
9.2
9.3
9.4
9.5
1
2
t−Test: p−value = 0.18

# CL1Contig4379|CL1Contig4379

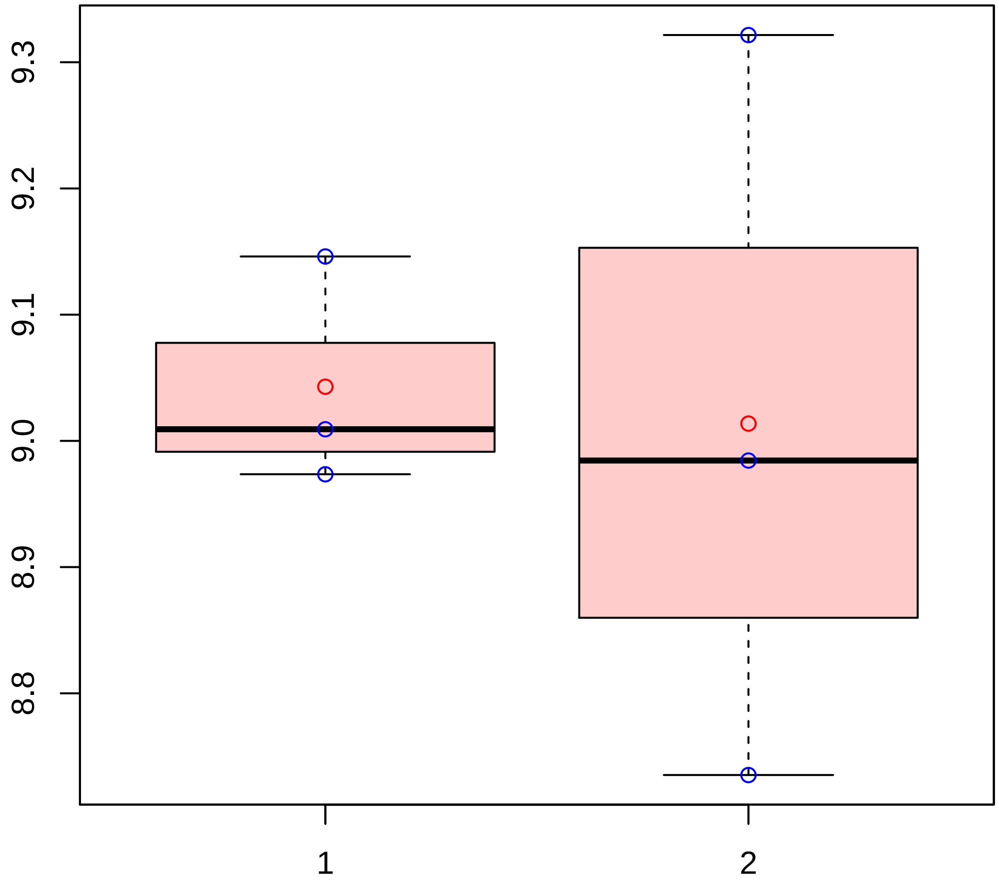


t−Test: p−value = 0.88

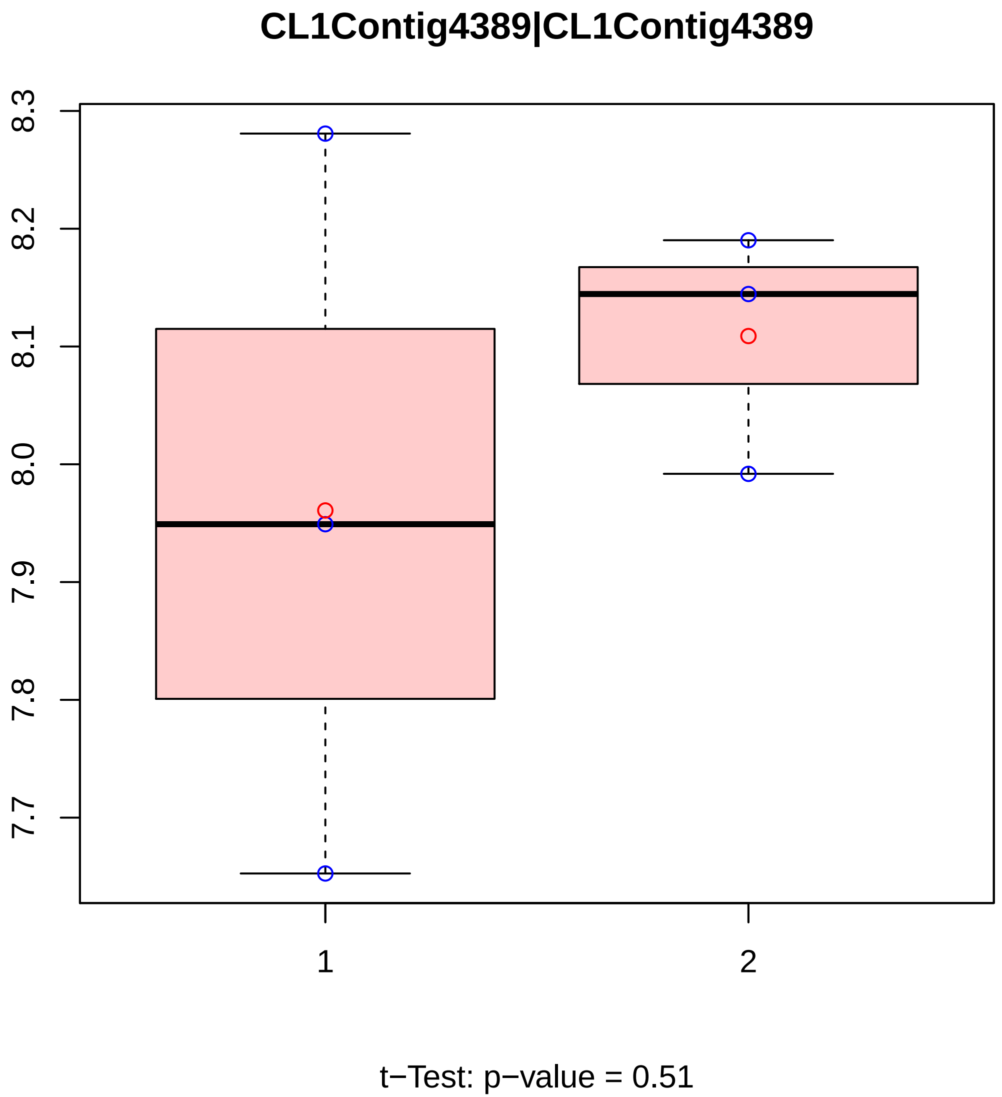
CL1Contig4389|CL1Contig4389
8.3
8.2
8.1
8.0
7.9
7.8
7.7
1
2
t−Test: p−value = 0.51

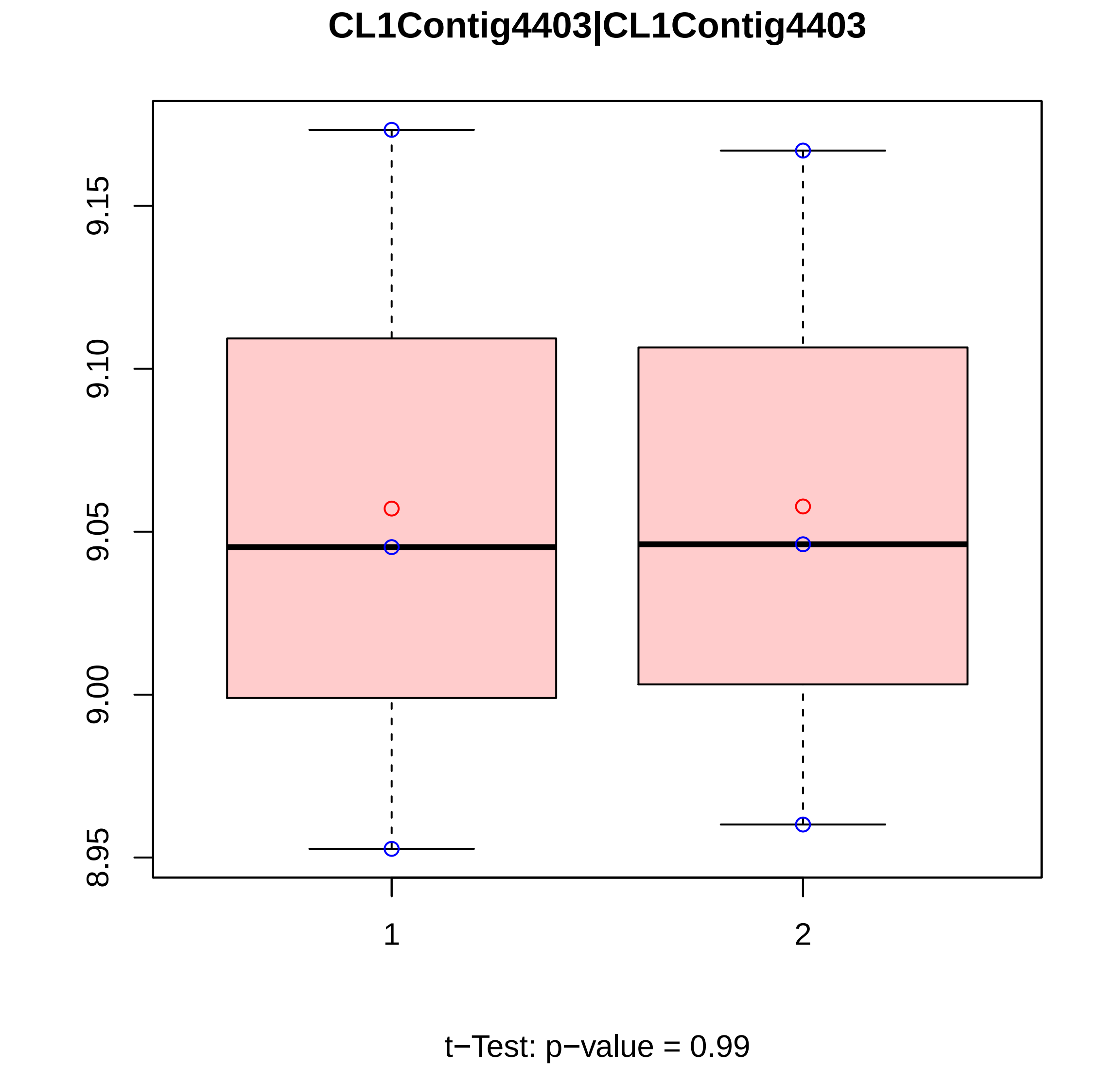
CL1Contig4403|CL1Contig4403
9.15
9.10
9.05
9.00
8.95
1
2
t−Test: p−value = 0.99

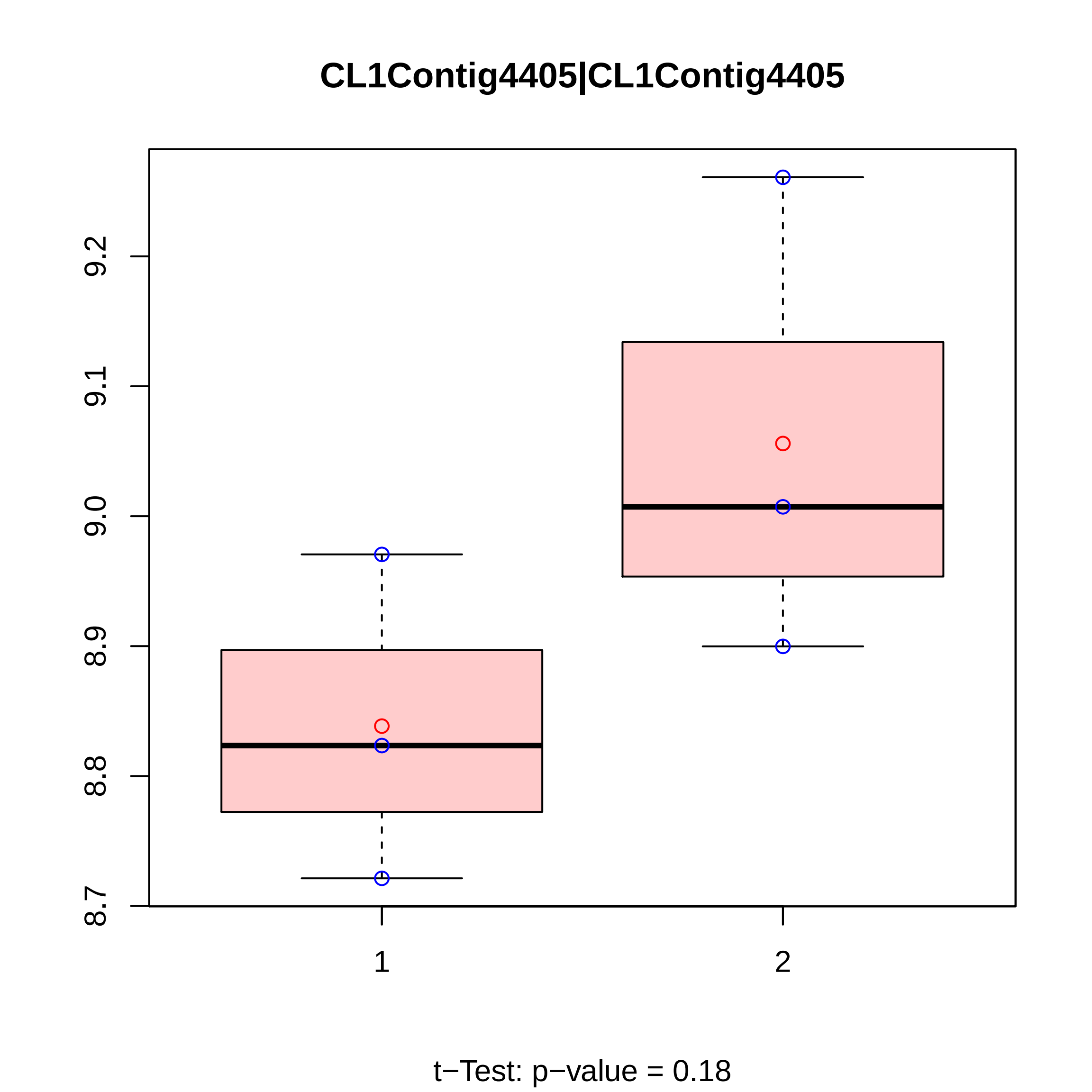
CL1Contig4405|CL1Contig4405
9.2
9.1
9.0
8.9
8.8
8.7
1
2
t−Test: p−value = 0.18

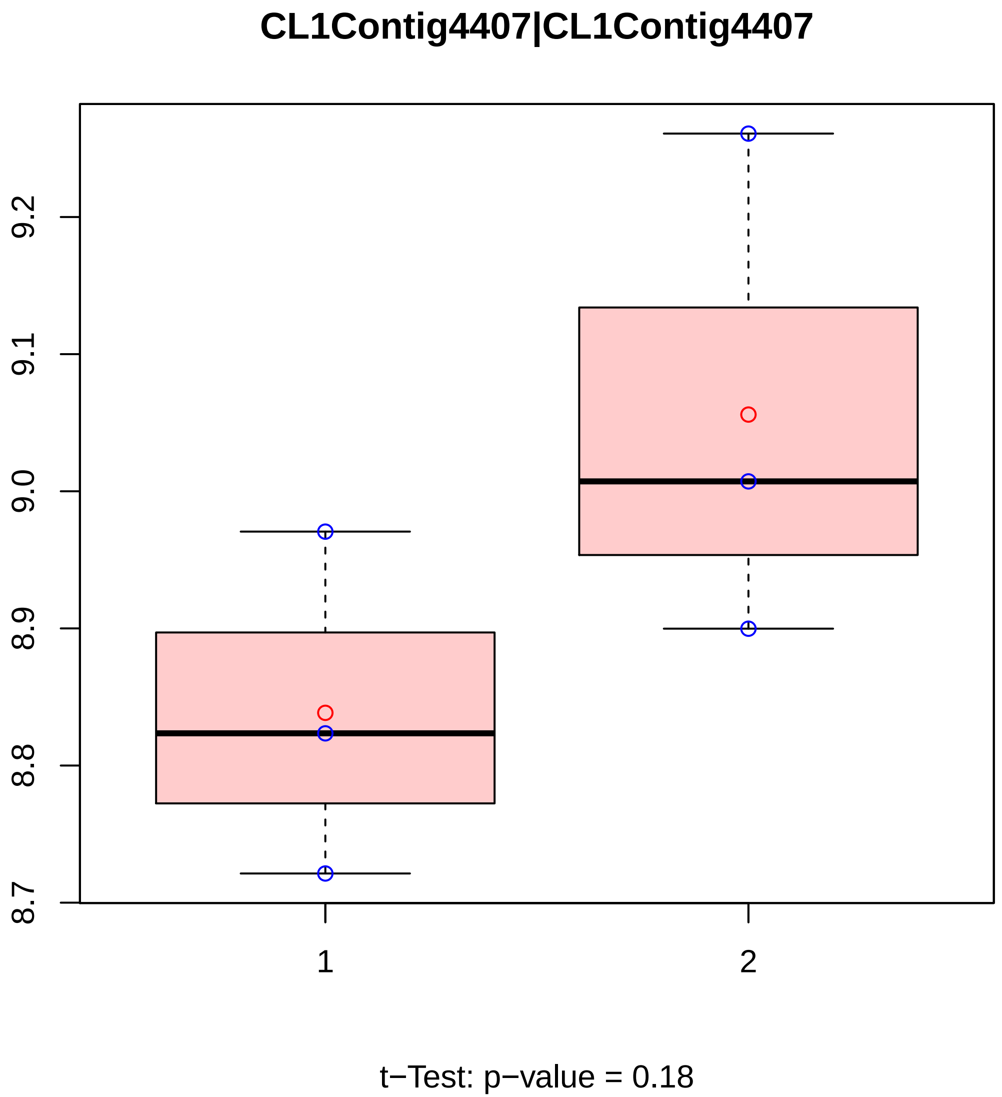
CL1Contig4407|CL1Contig4407
9.2
9.1
9.0
8.9
8.8
8.7
1
2
t−Test: p−value = 0.18

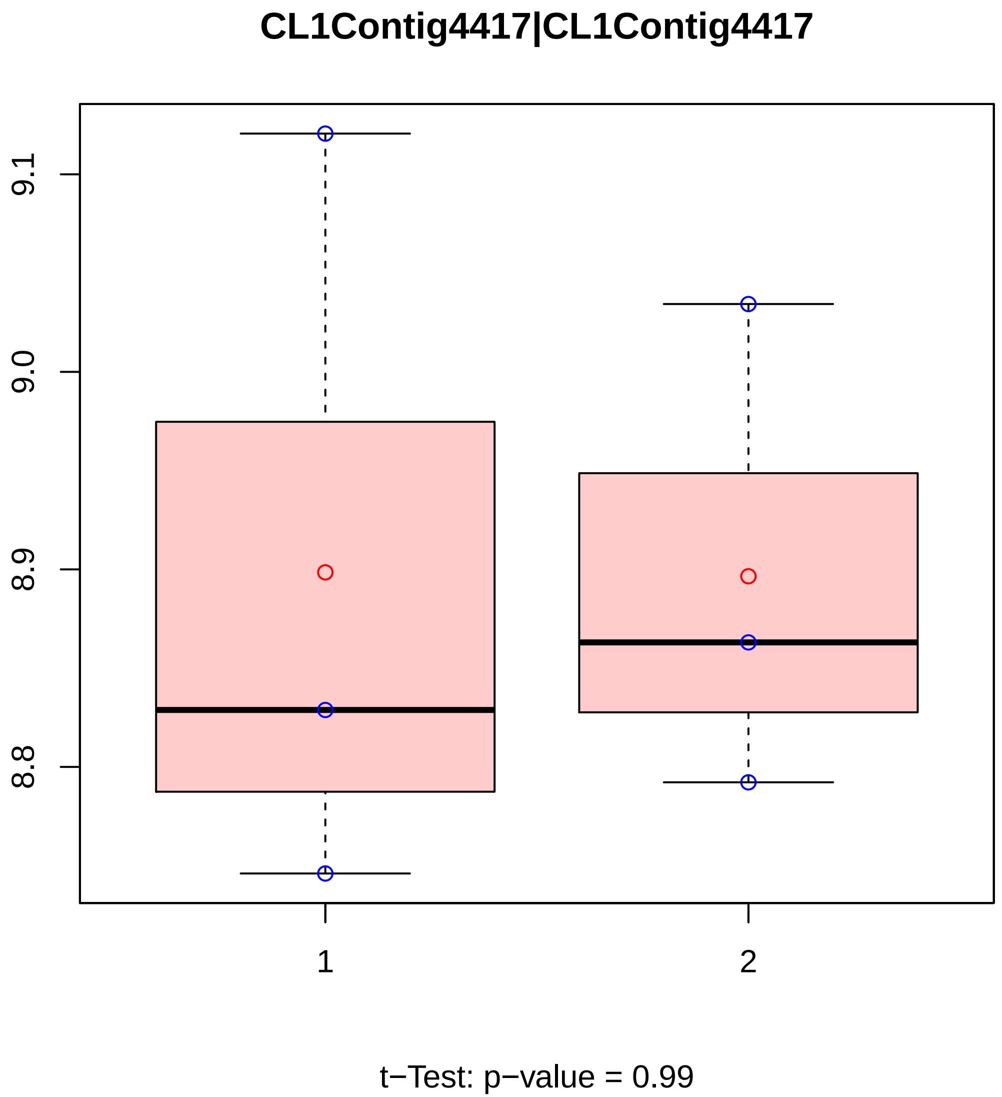
CL1Contig4417|CL1Contig4417
9.1
9.0
8.9
8.8
1
2
t−Test: p−value = 0.99

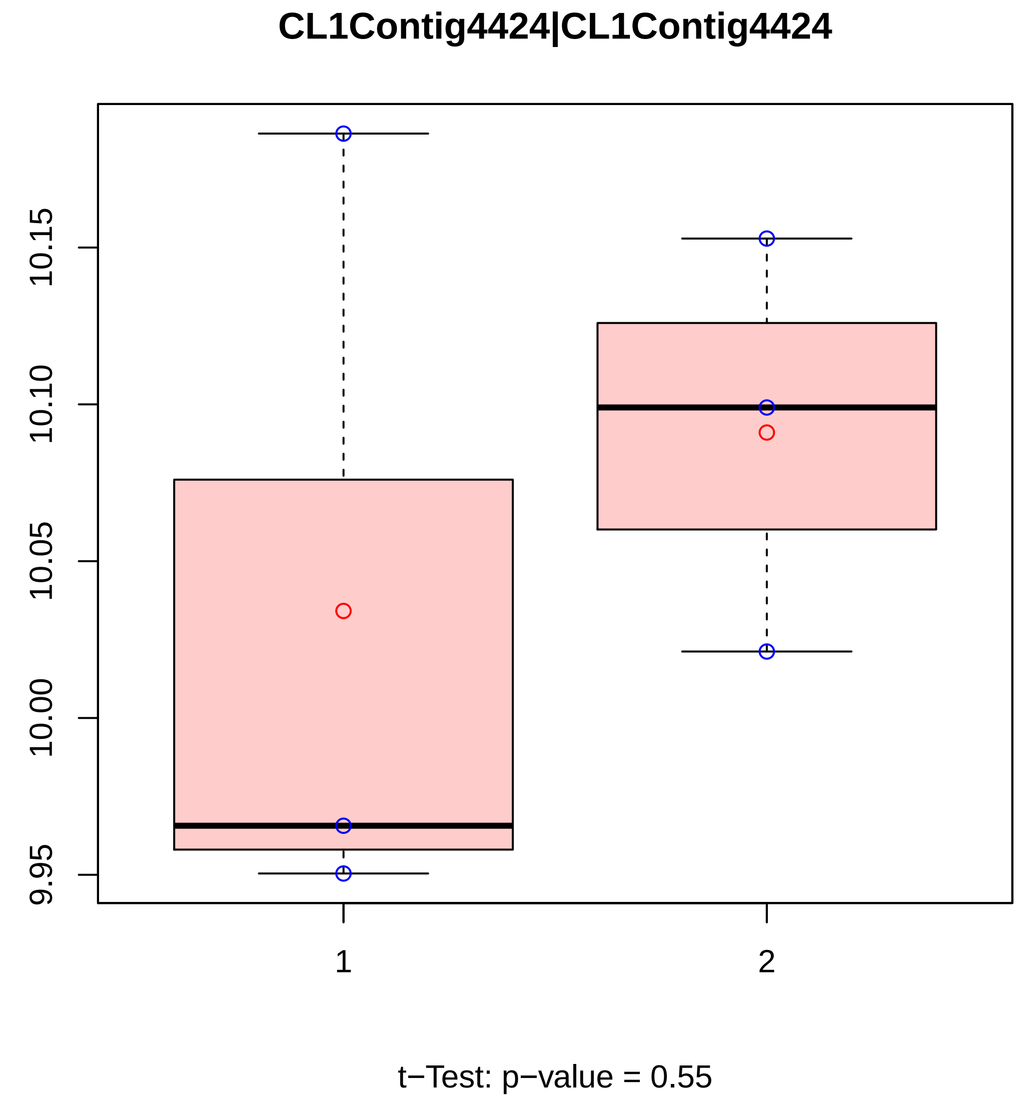

CL1Contig4424|CL1Contig4424
10.15
10.10
10.05
10.00
9.95
1
2
t−Test: p−value = 0.55

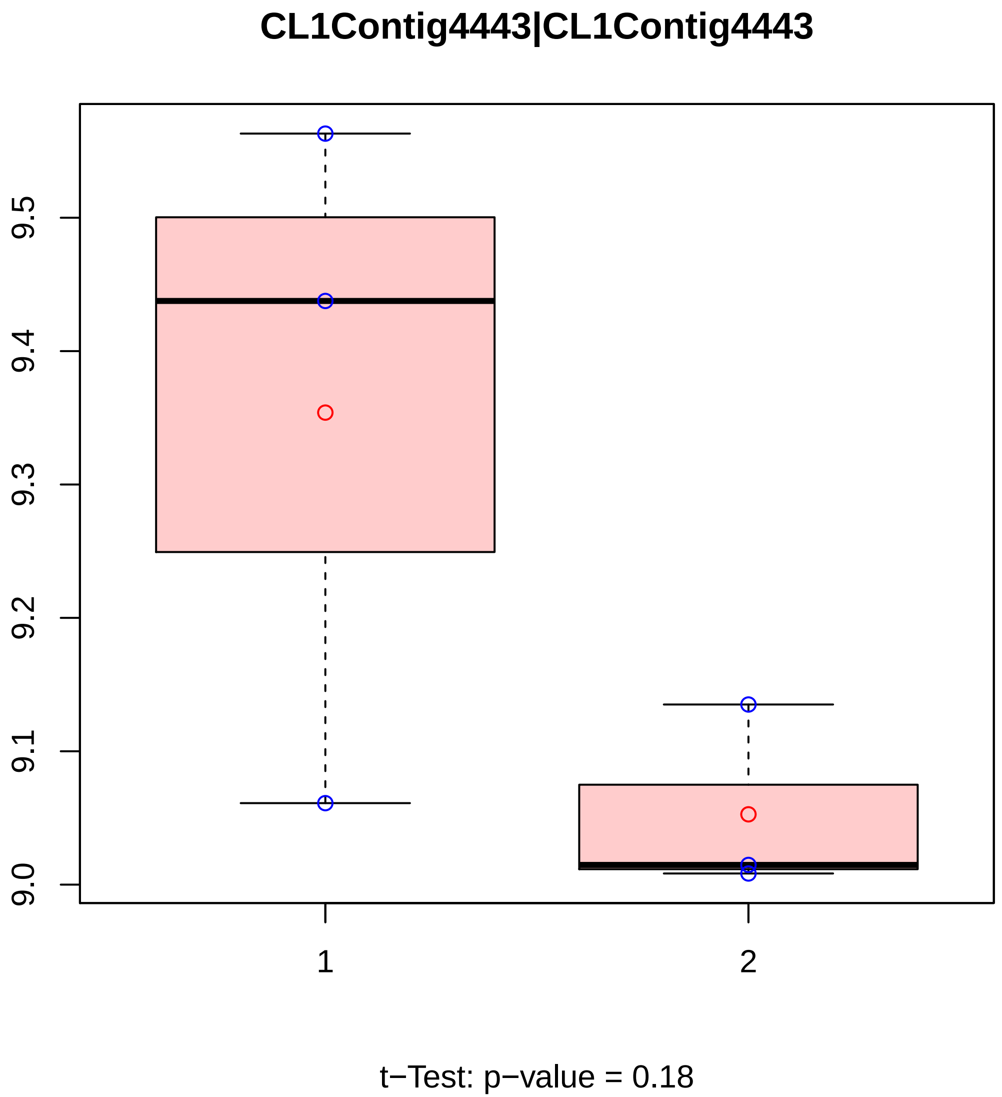

CL1Contig4443|CL1Contig4443
9.0
9.1
9.2
9.3
9.4
9.5
1
2
t−Test: p−value = 0.18

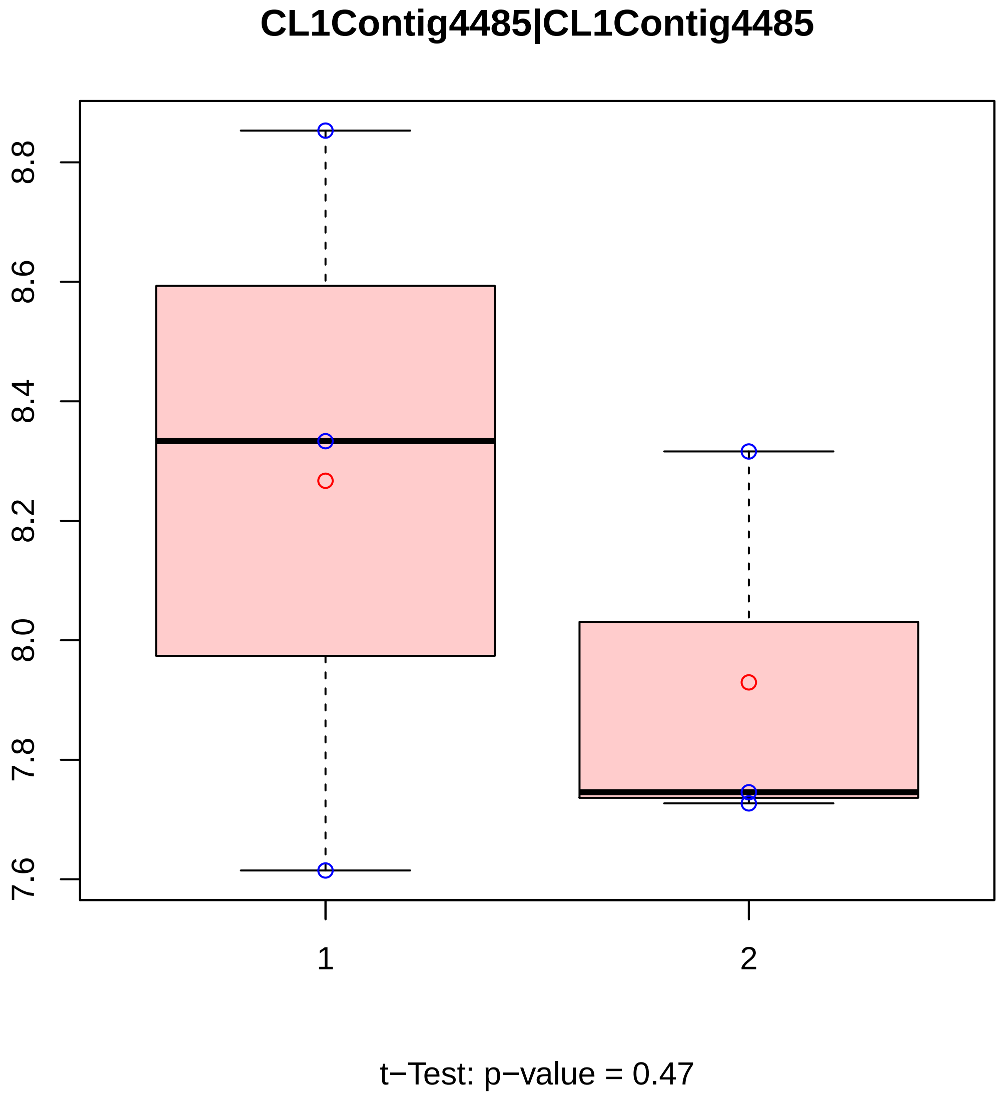
CL1Contig4485|CL1Contig4485
8.8
8.6
8.4
8.2
8.0
7.8
7.6
1
2
t−Test: p−value = 0.47

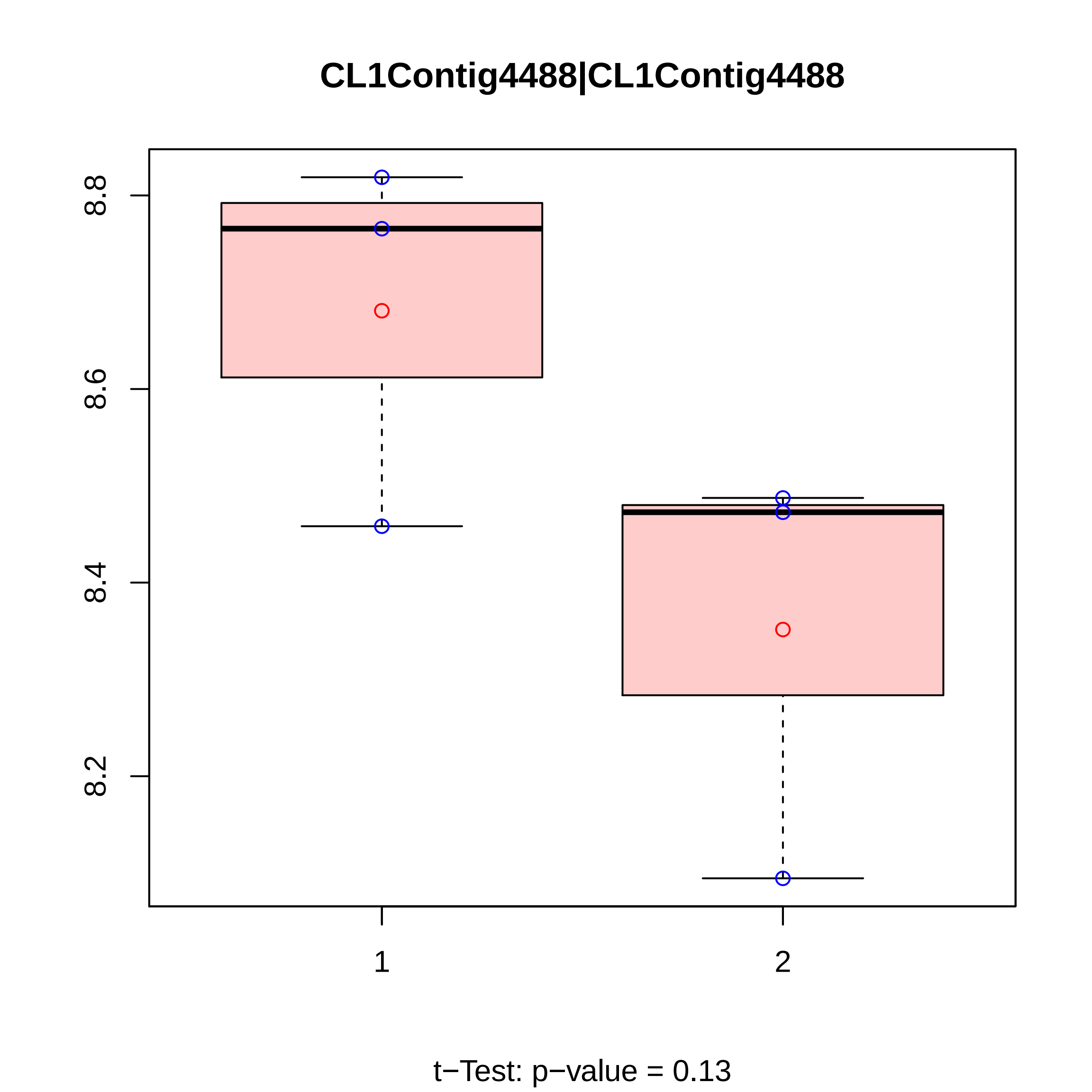
CL1Contig4488|CL1Contig4488
8.2
8.4
8.6
8.8
1
2
t−Test: p−value = 0.13

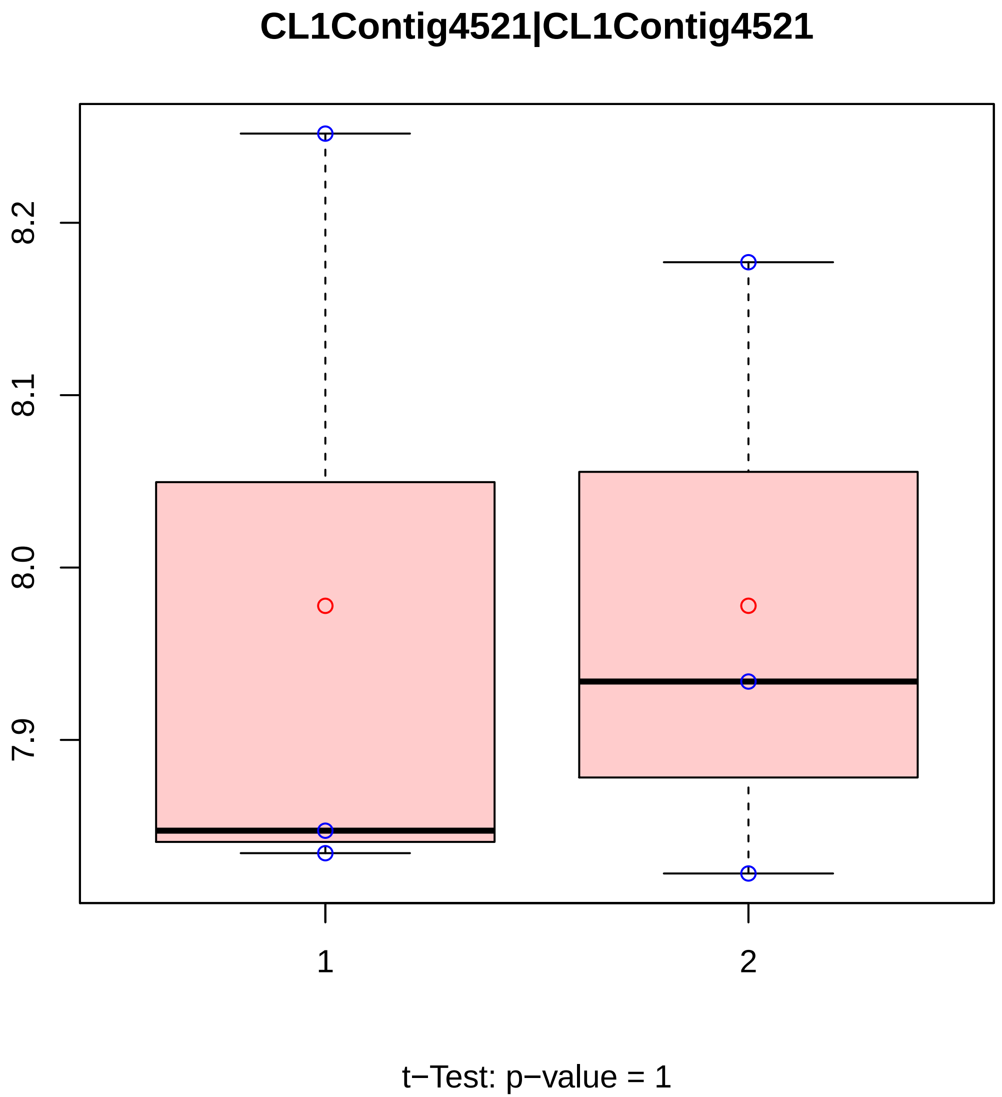
CL1Contig4521|CL1Contig4521
8.2
8.1
8.0
7.9
1
2
t−Test: p−value = 1

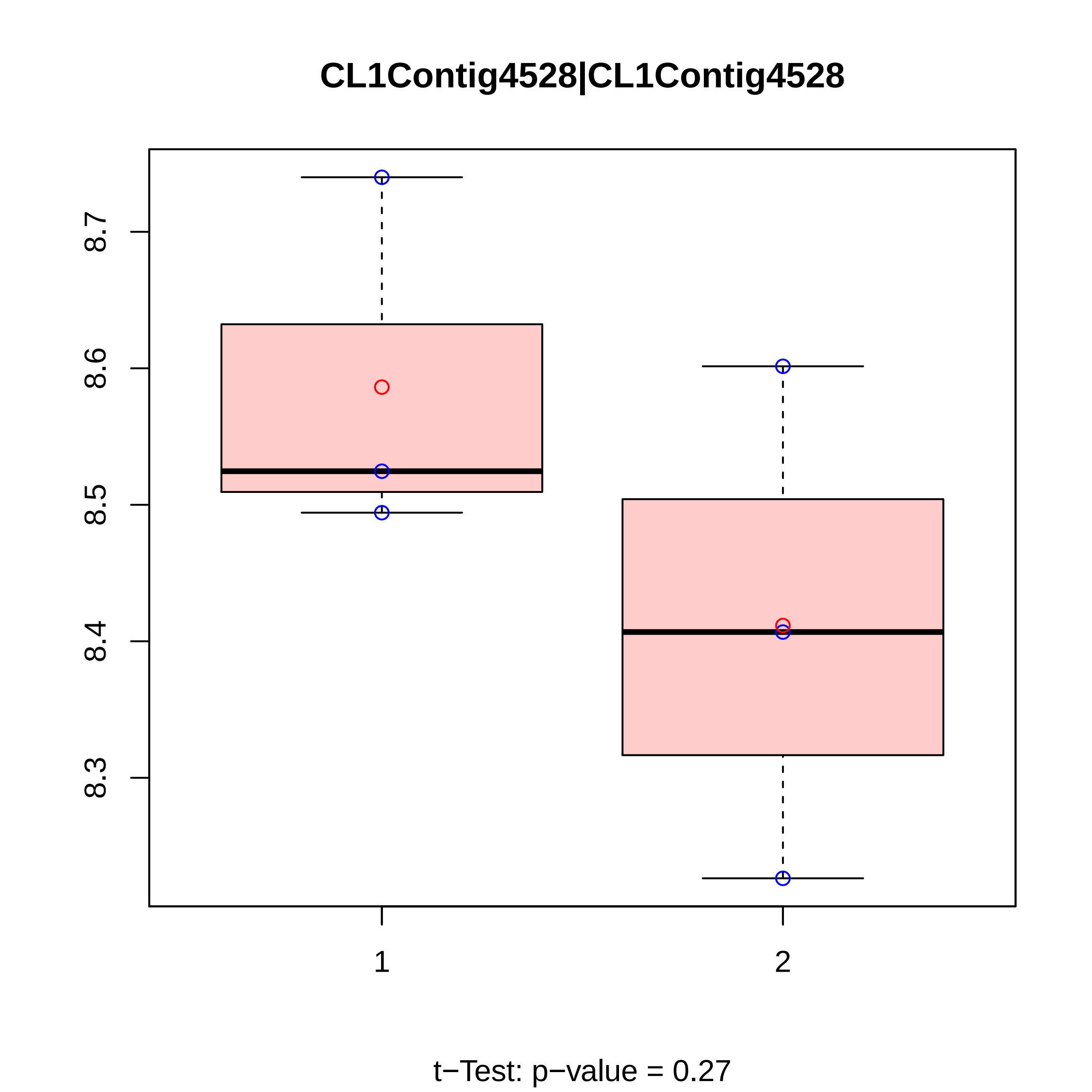
CL1Contig4528|CL1Contig4528
8.3
8.4
8.5
8.6
8.7
1
2
t−Test: p−value = 0.27

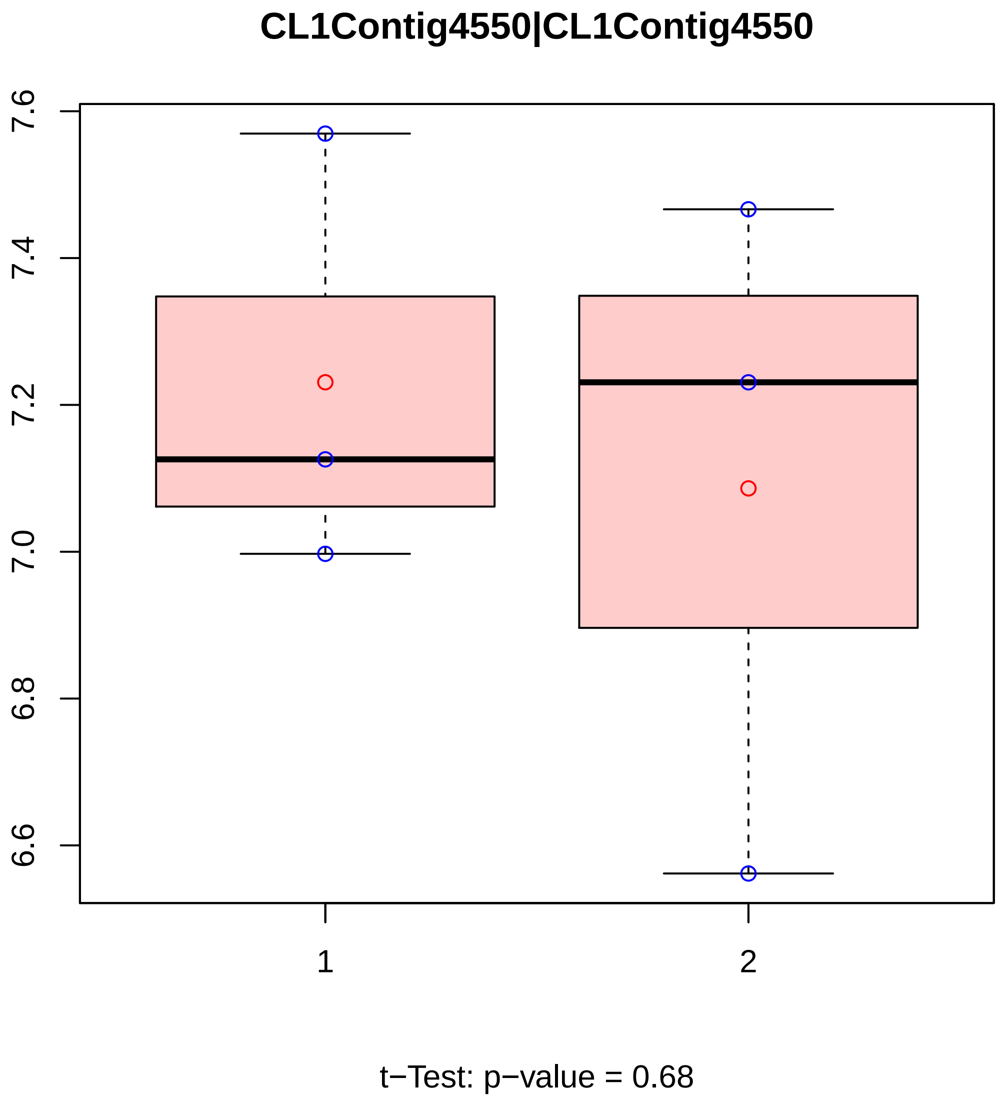

CL1Contig4550|CL1Contig4550
7.6
7.4
7.2
7.0
6.8
6.6
1
2
t−Test: p−value = 0.68

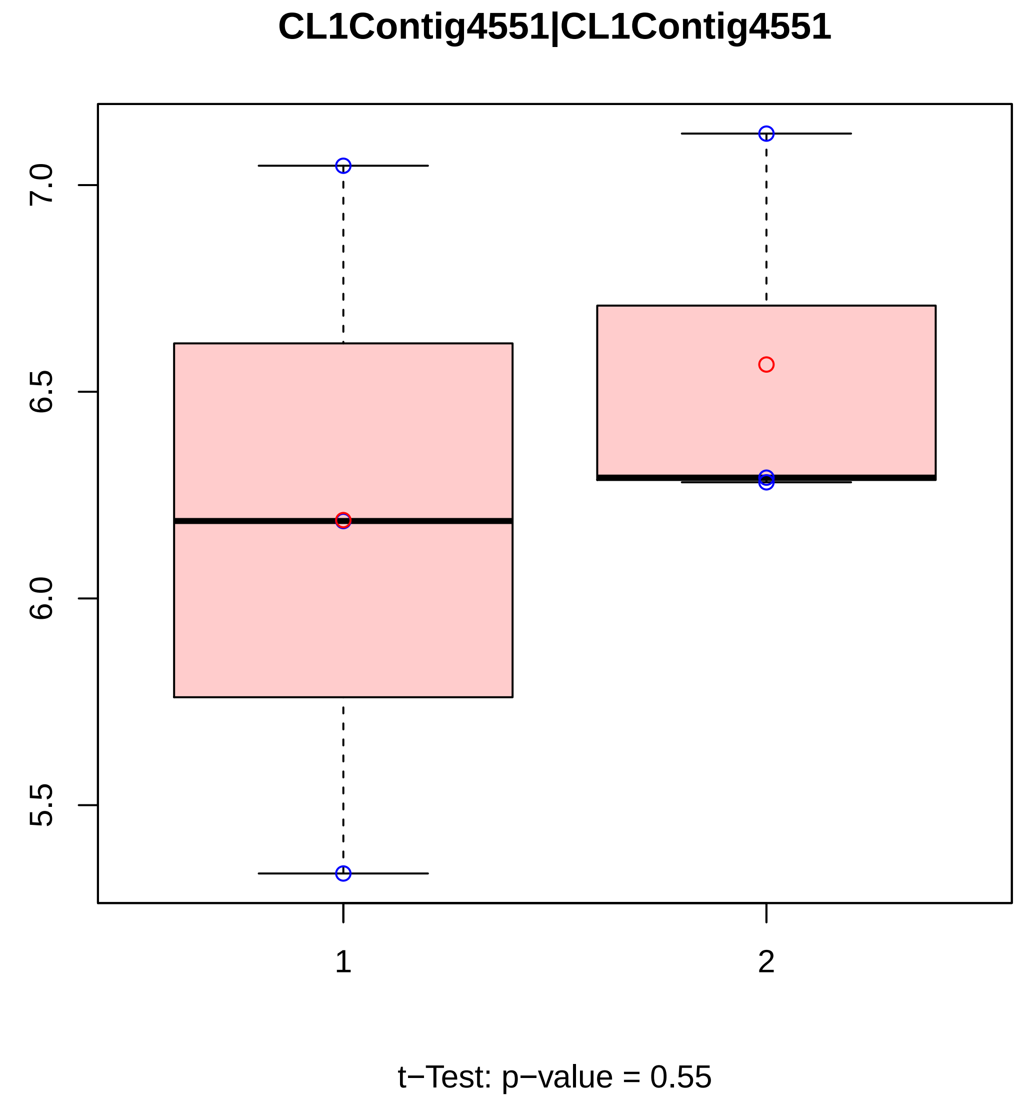

CL1Contig4551|CL1Contig4551
7.0
6.5
6.0
5.5
1
2
t−Test: p−value = 0.55

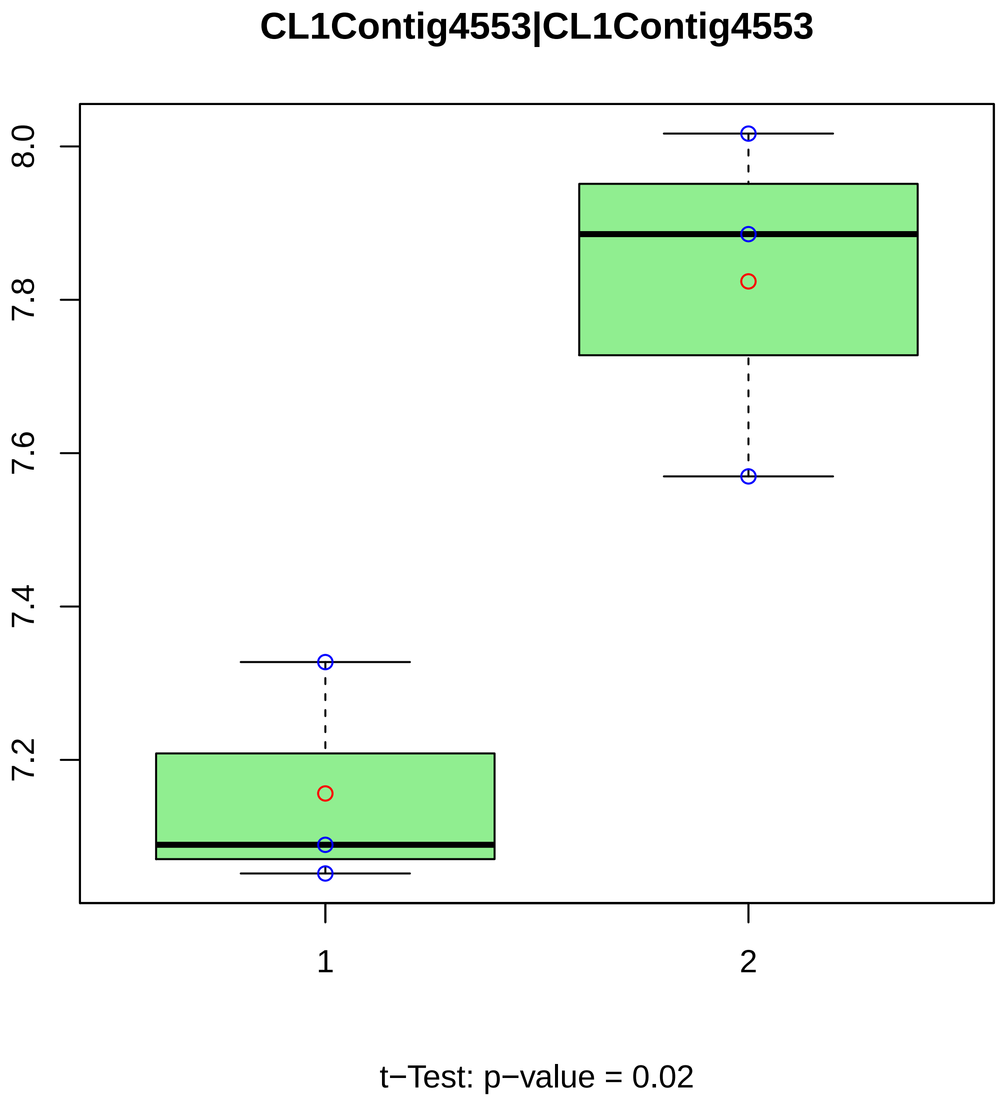

CL1Contig4553|CL1Contig4553
8.0
7.8
7.6
7.4
7.2
1
2
t−Test: p−value = 0.02

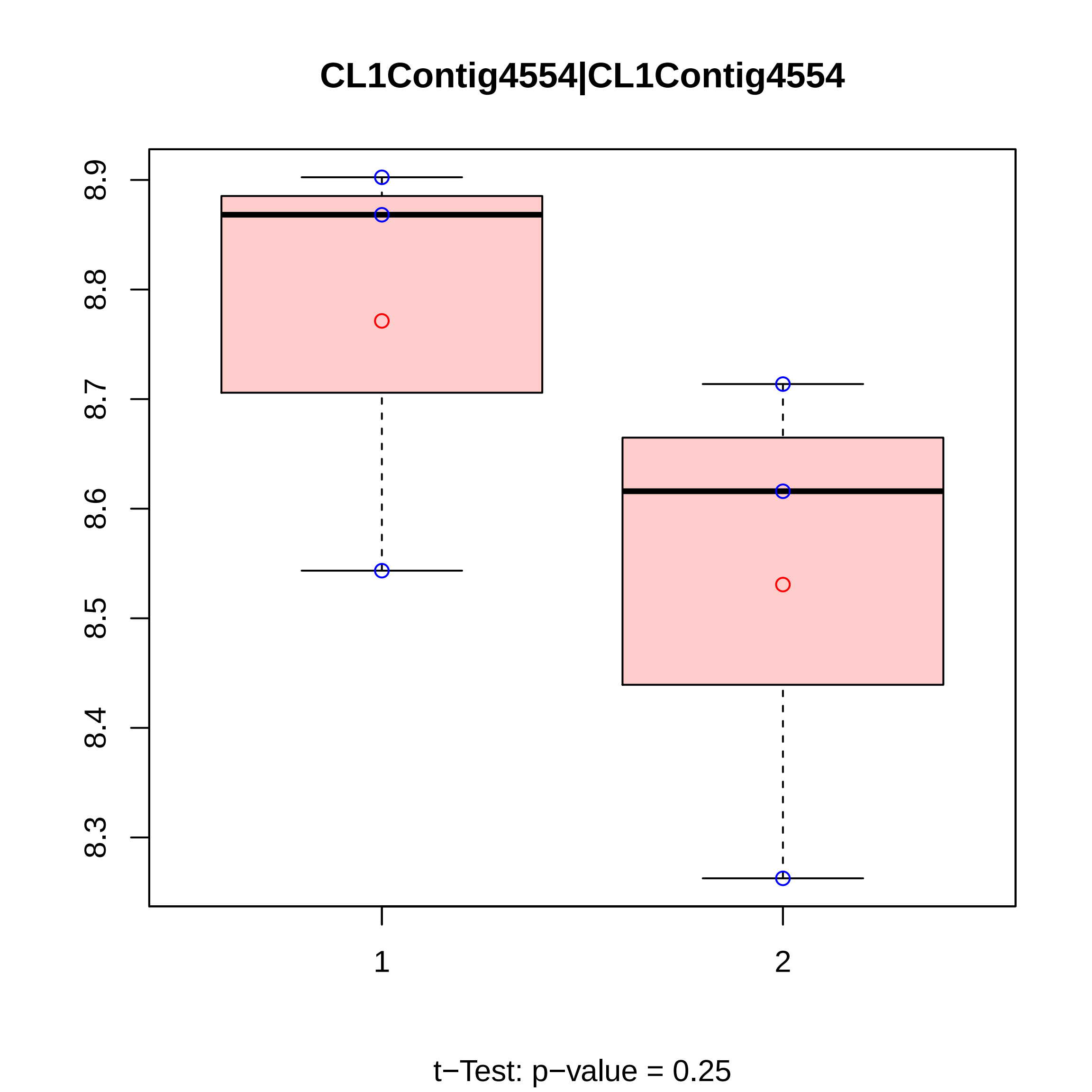
CL1Contig4554|CL1Contig4554
8.3
8.4
8.5
8.6
8.7
8.8
8.9
1
2
t−Test: p−value = 0.25

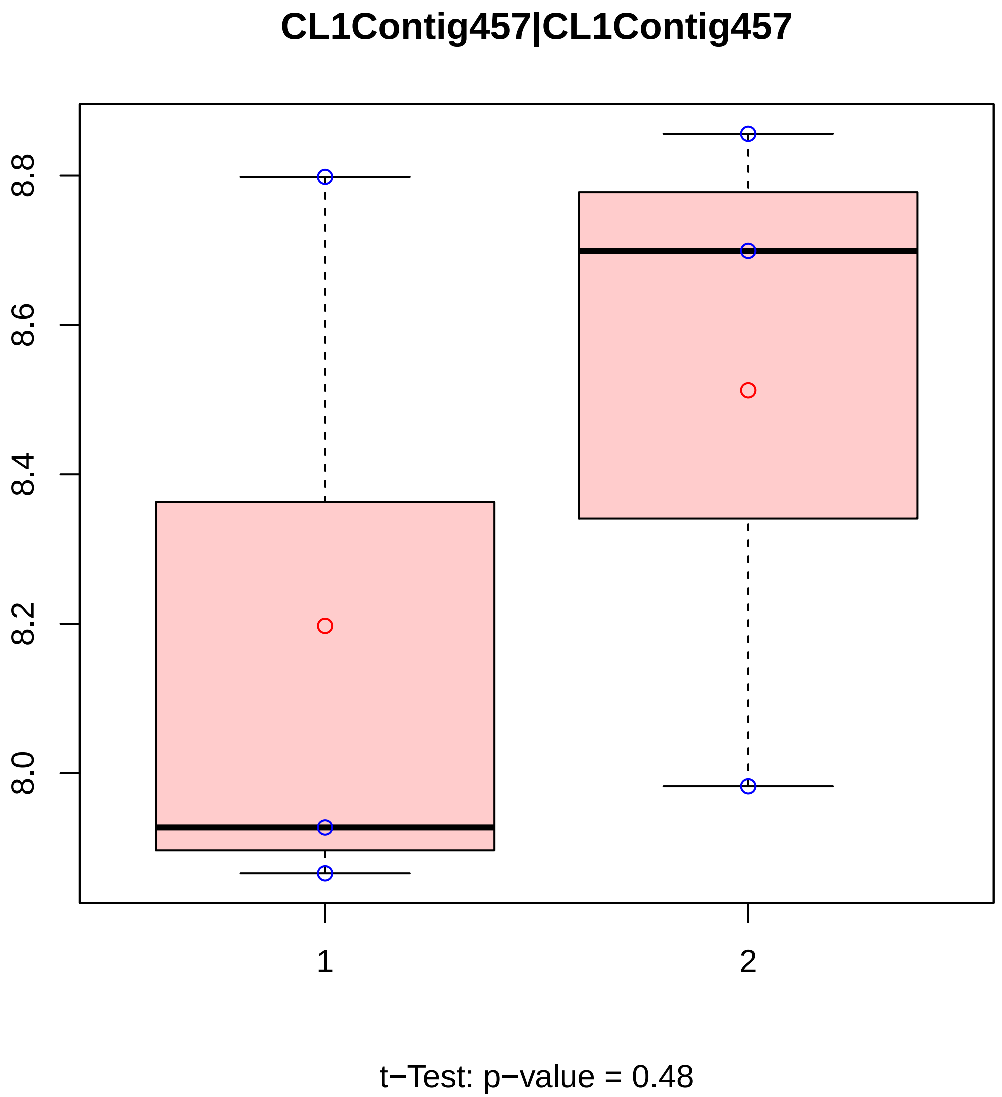
CL1Contig457|CL1Contig457
8.0
8.2
8.4
8.6
8.8
1
2
t−Test: p−value = 0.48

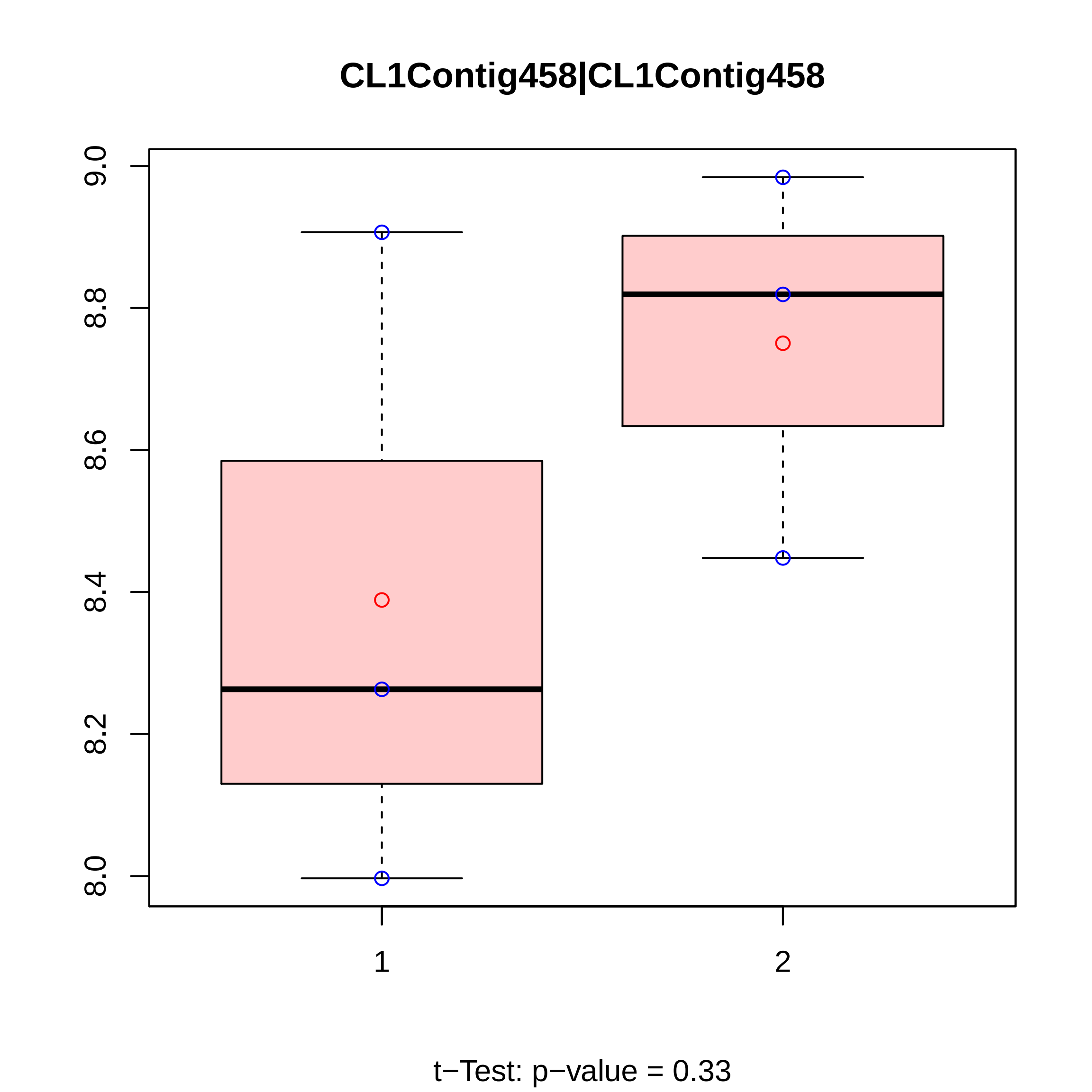

CL1Contig458|CL1Contig458
9.0
8.8
8.6
8.4
8.2
8.0
1
2
t−Test: p−value = 0.33

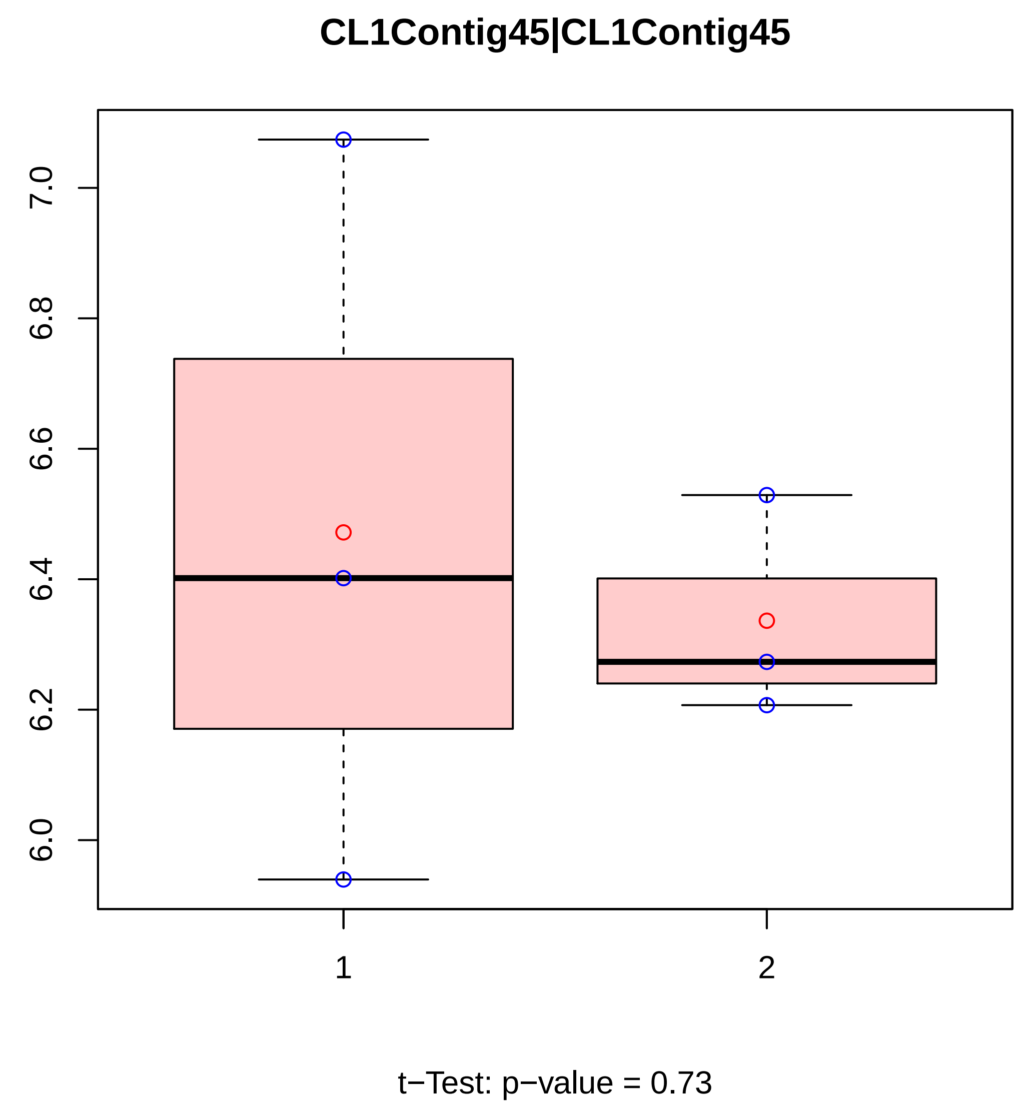

CL1Contig45|CL1Contig45
7.0
6.8
6.6
6.4
6.2
6.0
1
2
t−Test: p−value = 0.73

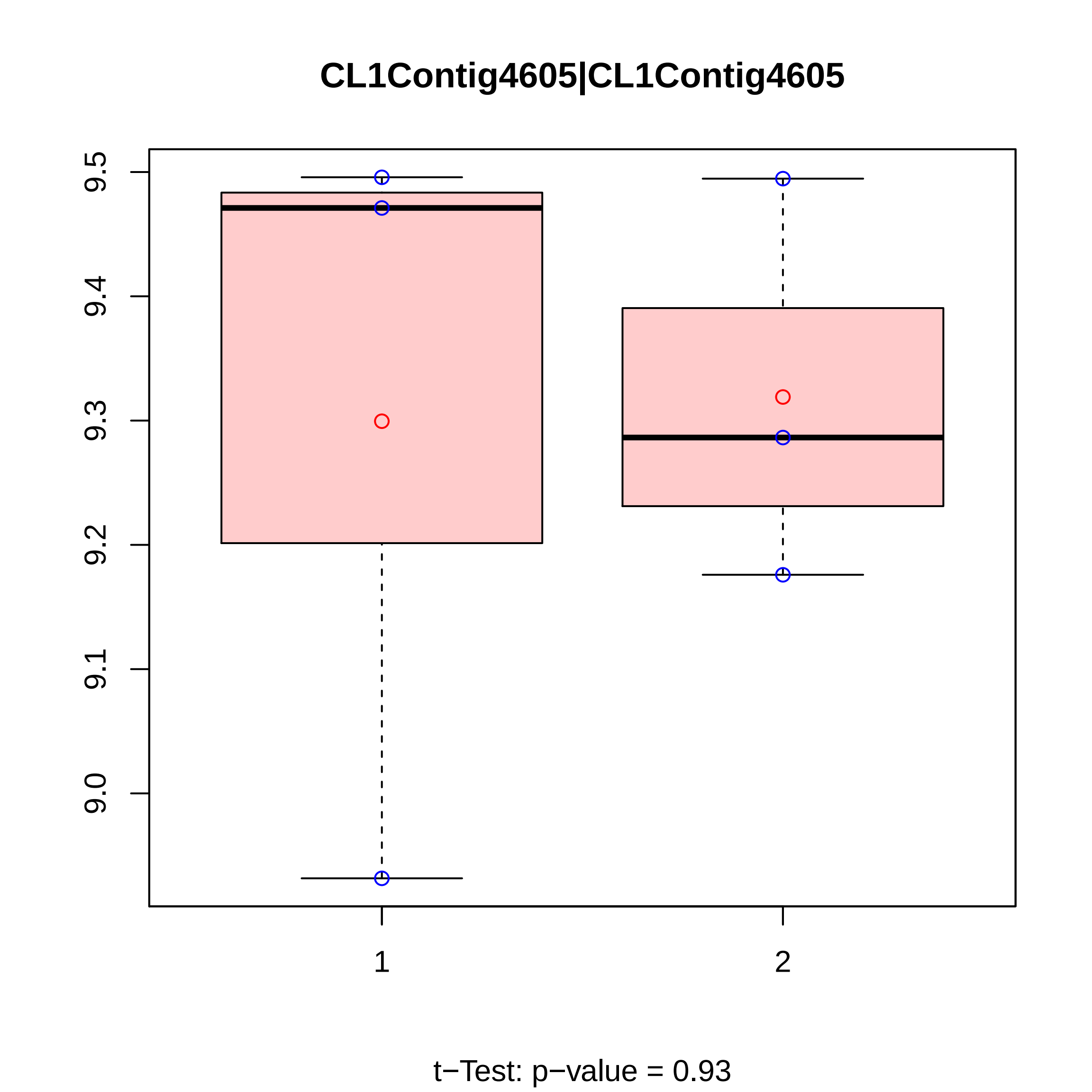
CL1Contig4605|CL1Contig4605
9.5
9.4
9.3
9.2
9.1
9.0
1
2
t−Test: p−value = 0.93

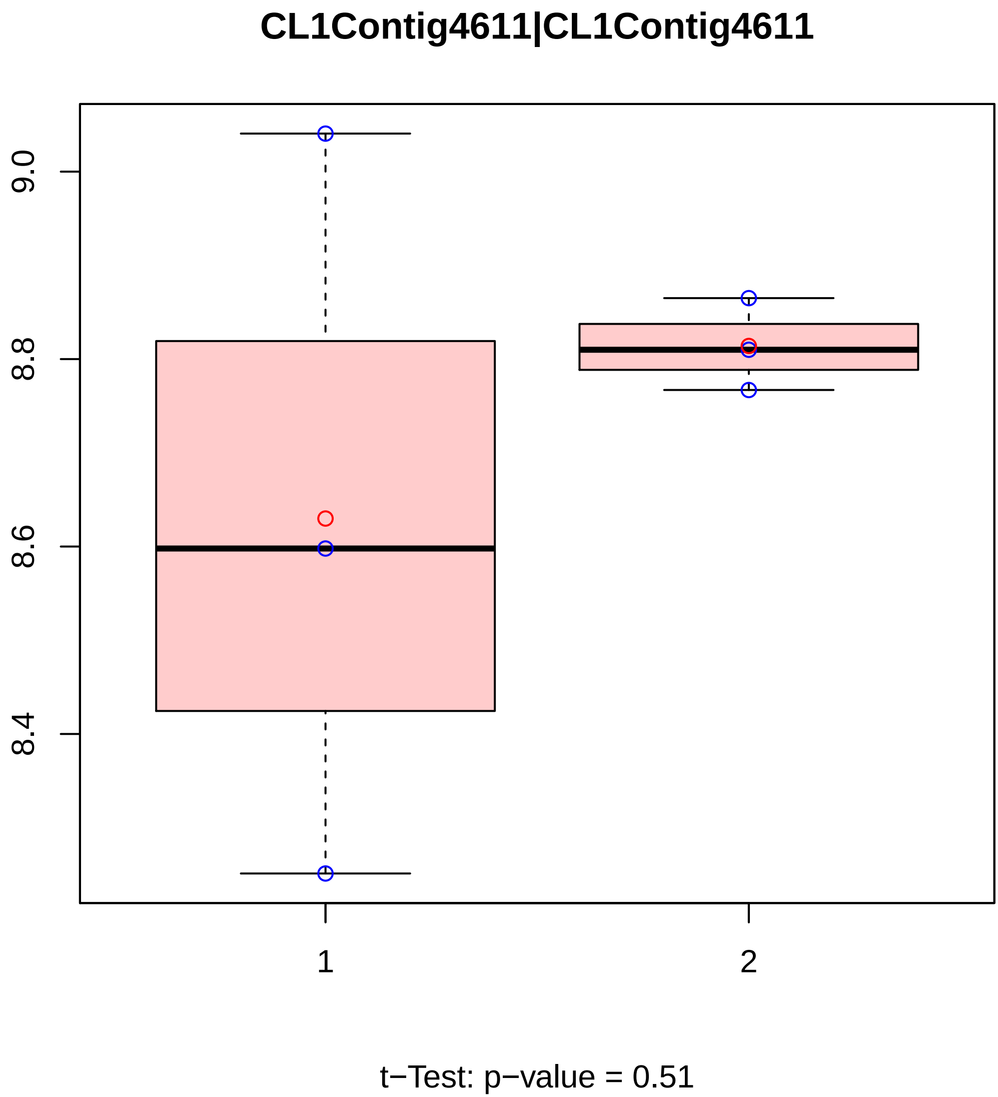
CL1Contig4611|CL1Contig4611
9.0
8.8
8.6
8.4
1
2
t−Test: p−value = 0.51

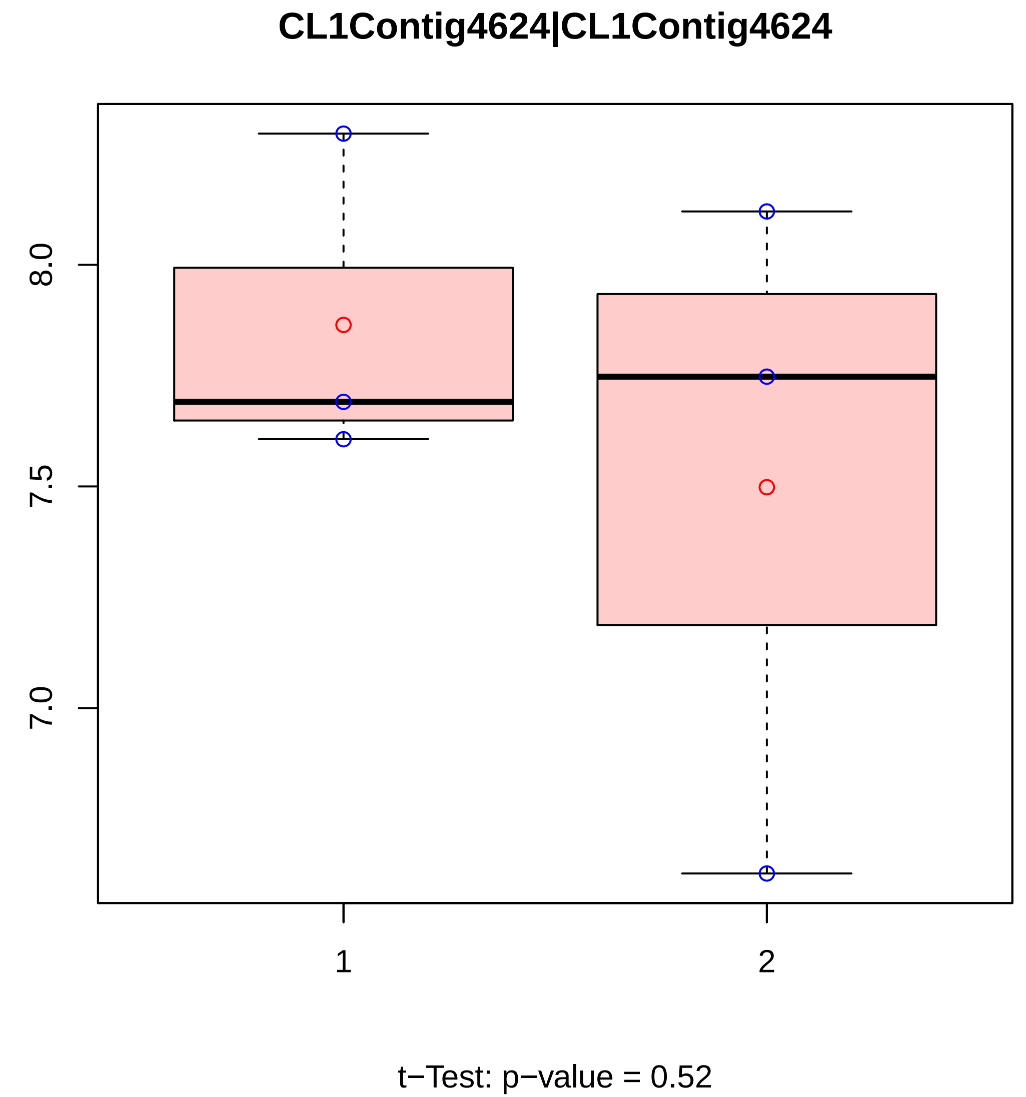

CL1Contig4624|CL1Contig4624
8.0
7.5
7.0
1
2
t−Test: p−value = 0.52

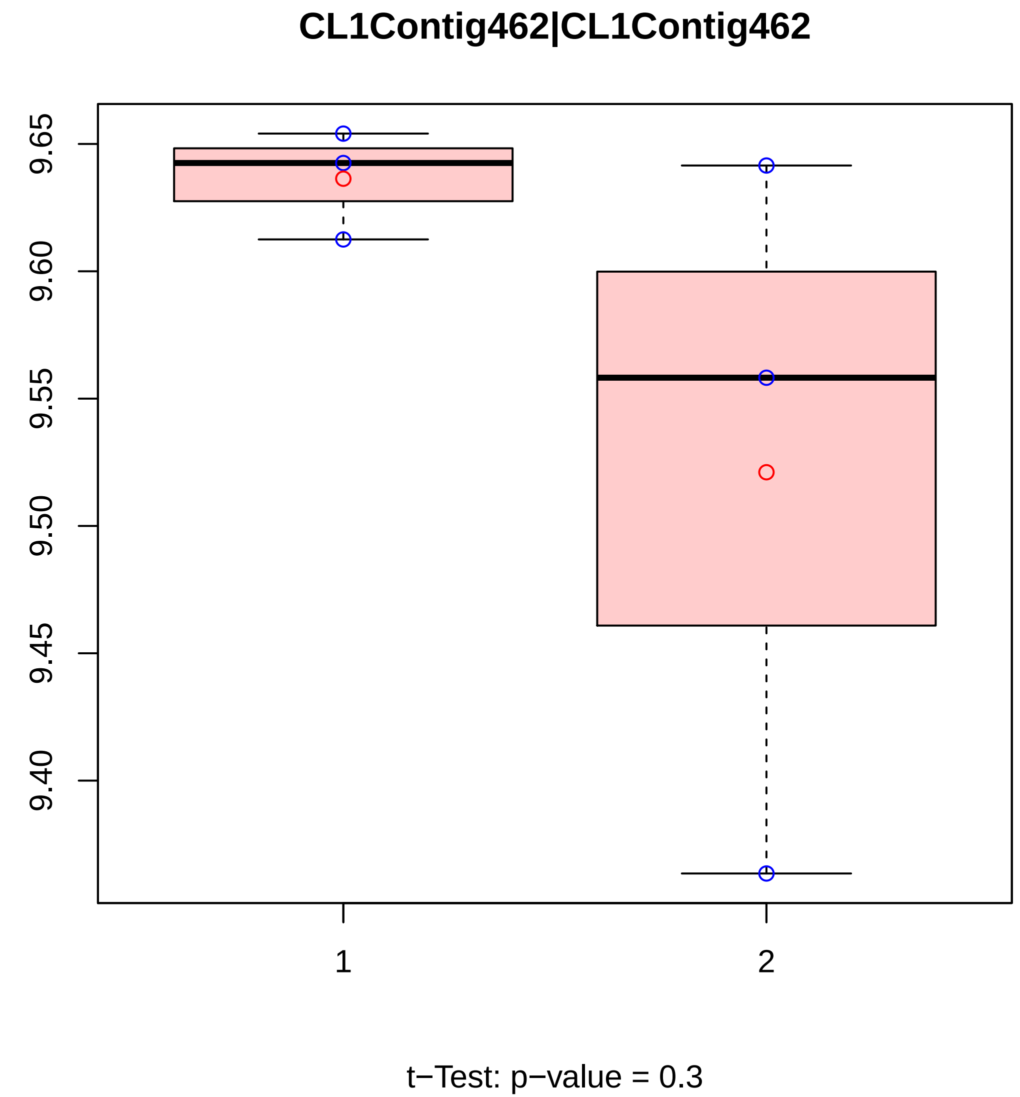
CL1Contig462|CL1Contig462
9.40
9.45
9.50
9.55
9.60
9.65
1
2
t−Test: p−value = 0.3

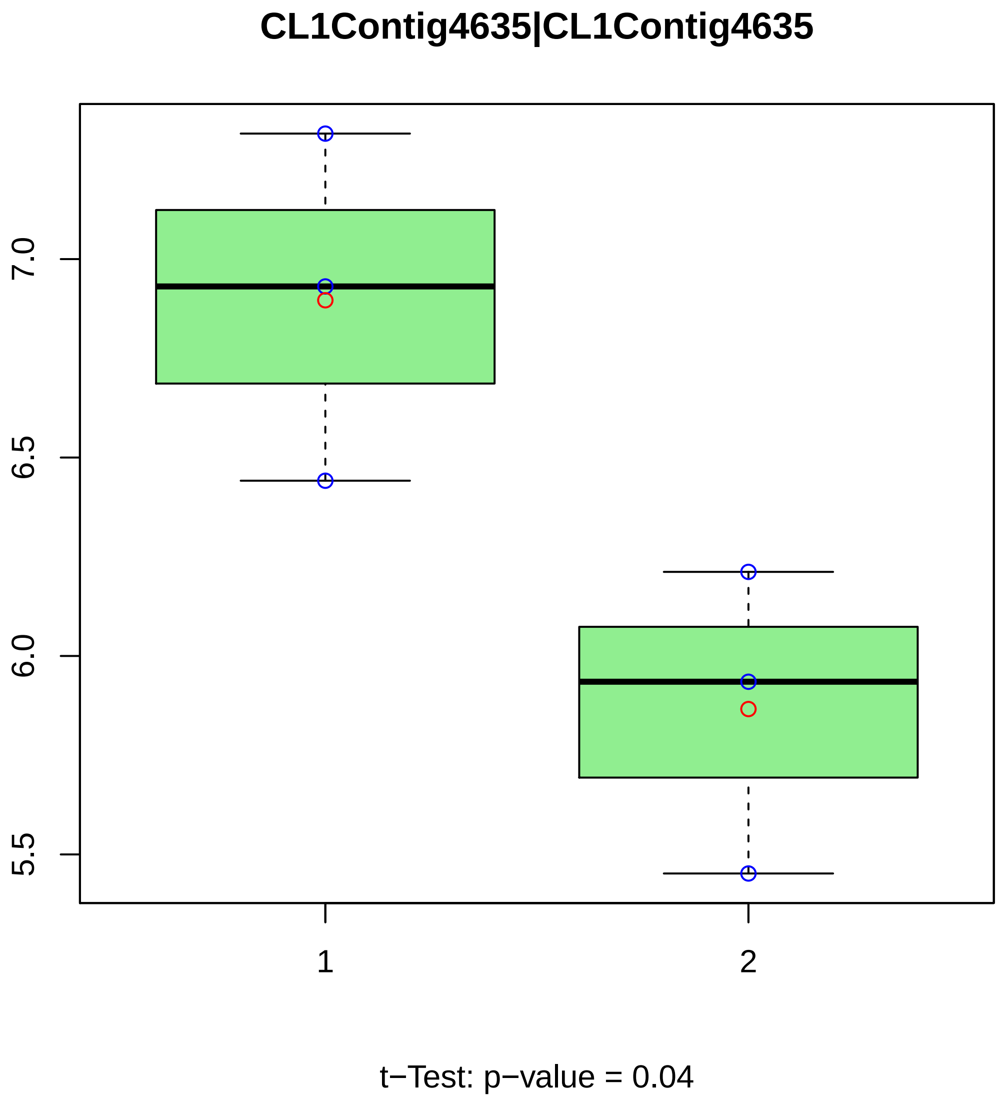
CL1Contig4635|CL1Contig4635
7.0
6.5
6.0
5.5
1
2
t−Test: p−value = 0.04

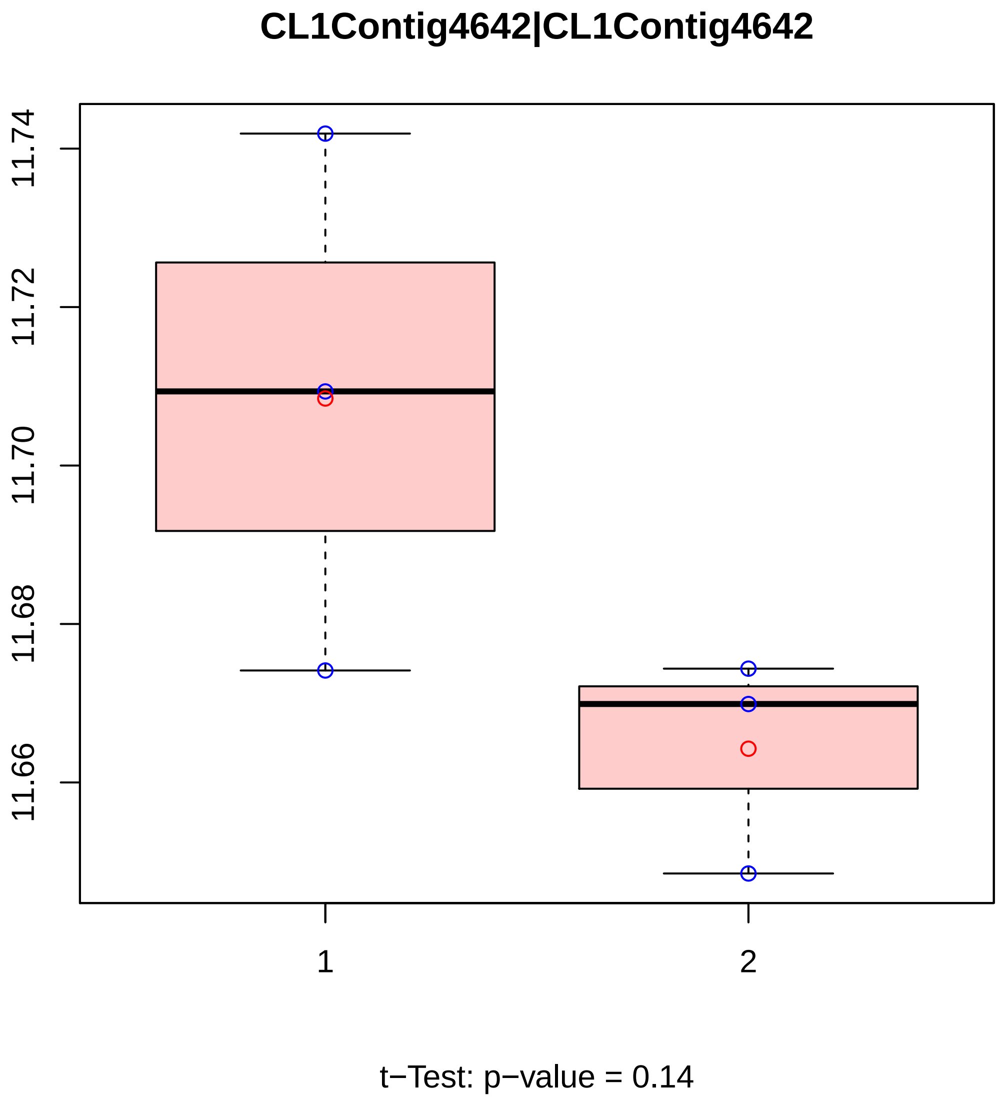

CL1Contig4642|CL1Contig4642
11.74
11.72
11.70
11.68
11.66
1
2
t−Test: p−value = 0.14

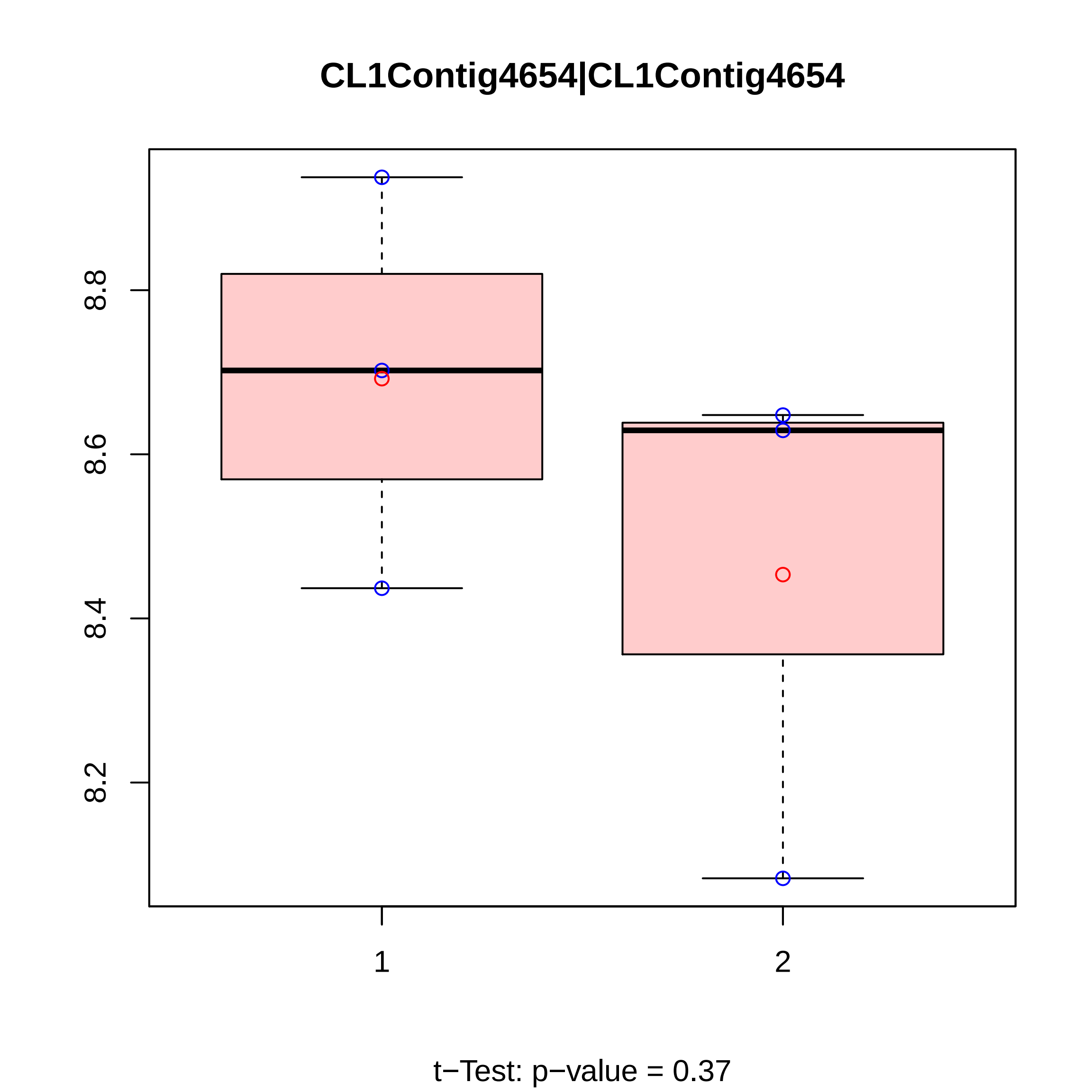
CL1Contig4654|CL1Contig4654
8.2
8.4
8.6
8.8
1
2
t−Test: p−value = 0.37

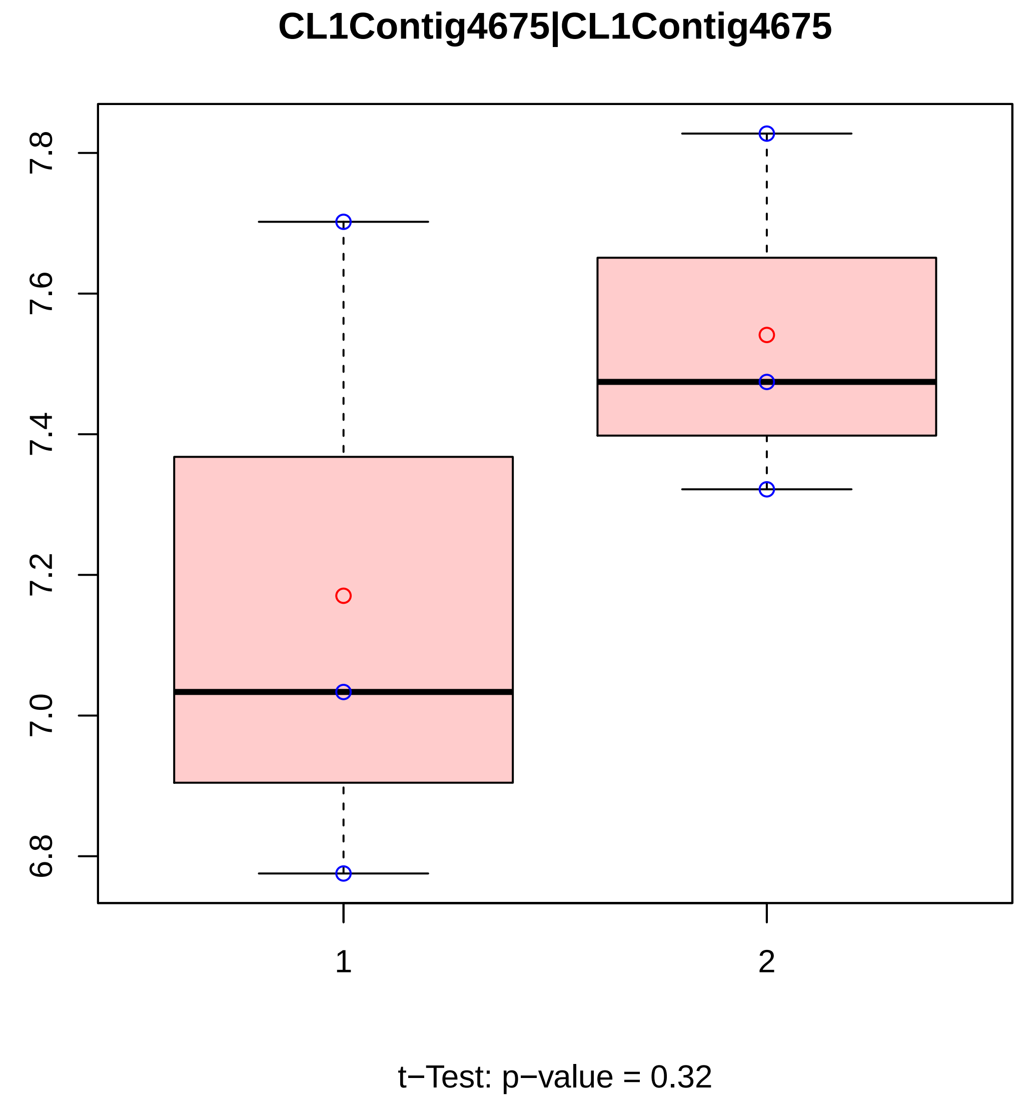
CL1Contig4675|CL1Contig4675
7.8
7.6
7.4
7.2
7.0
6.8
1
2
t−Test: p−value = 0.32

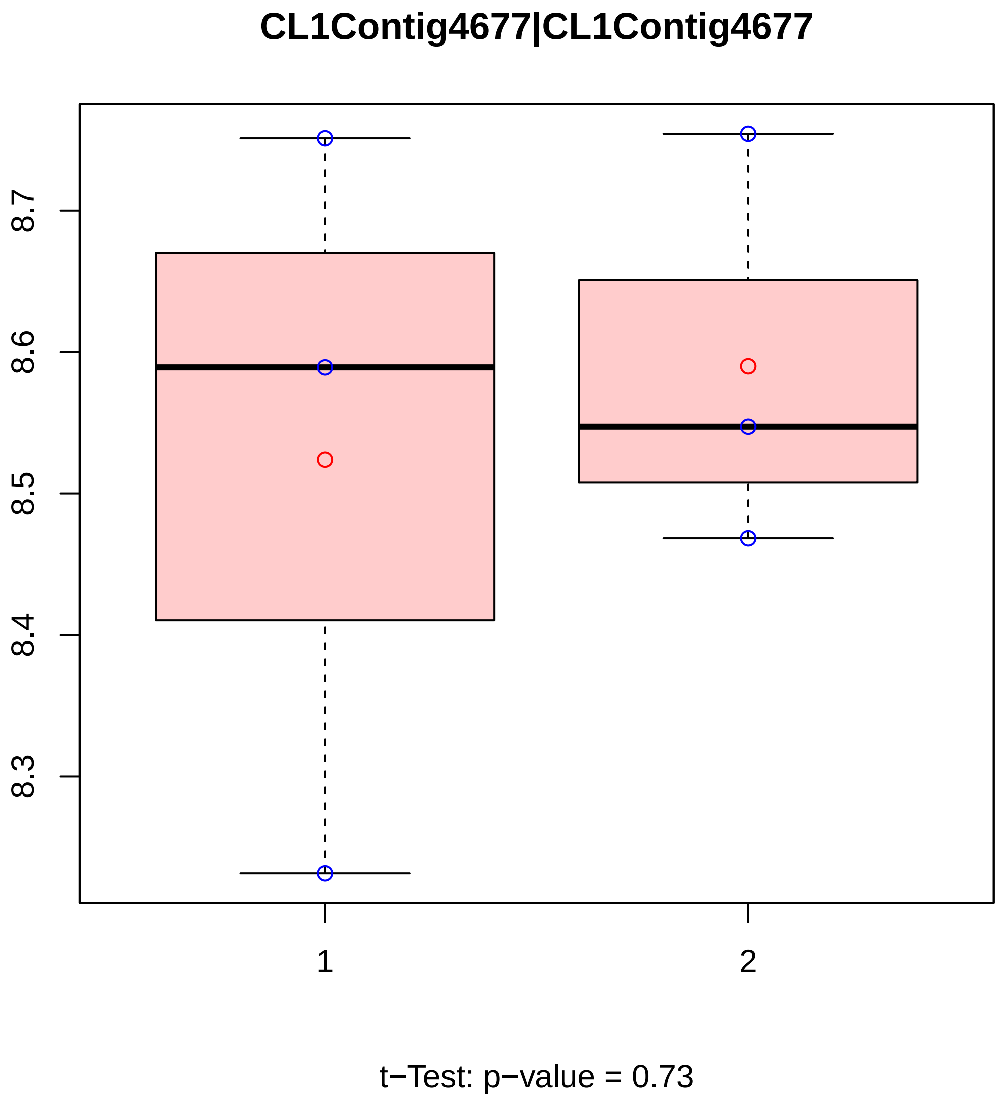

CL1Contig4677|CL1Contig4677
8.3
8.4
8.5
8.6
8.7
1
2
t−Test: p−value = 0.73

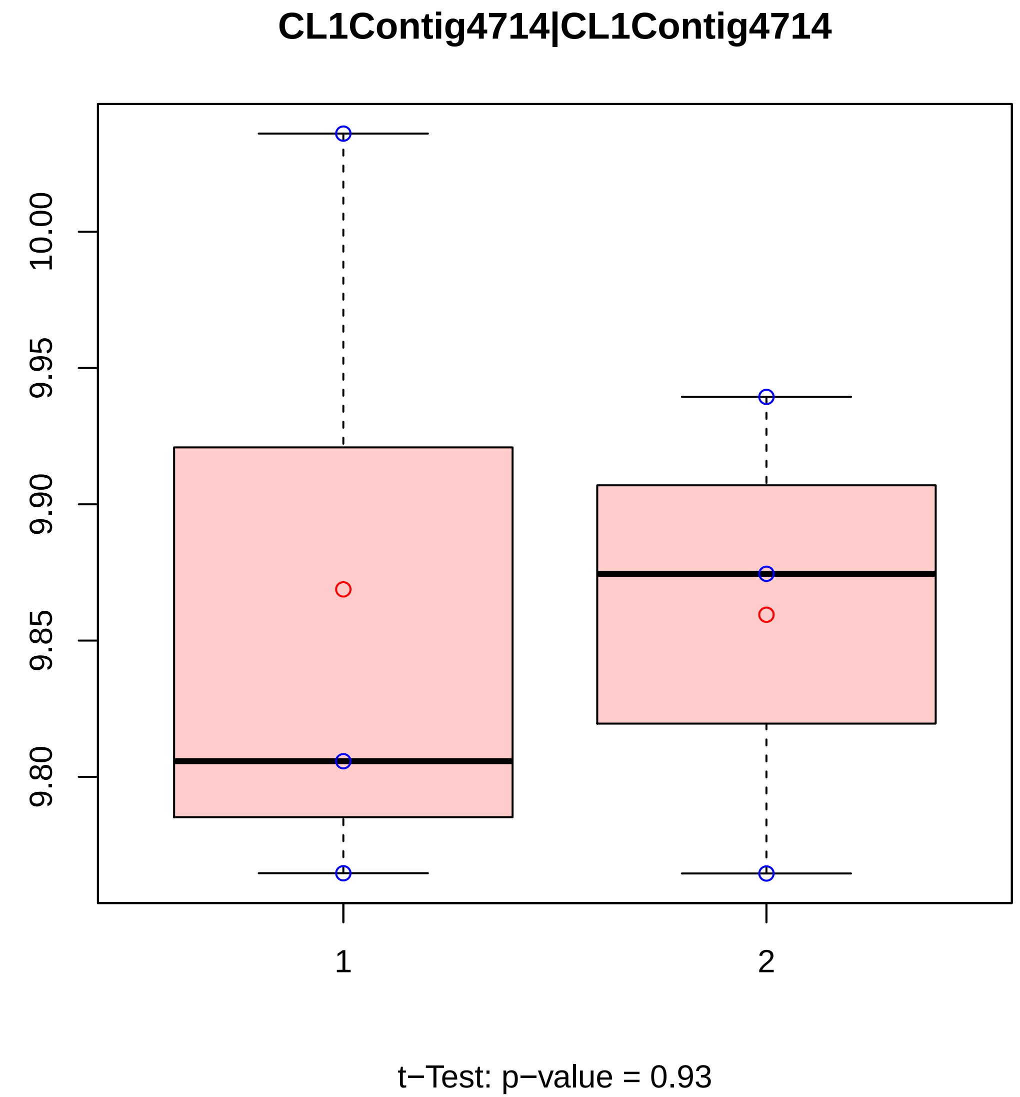
CL1Contig4714|CL1Contig4714
10.00
9.95
9.90
9.85
9.80
1
2
t−Test: p−value = 0.93

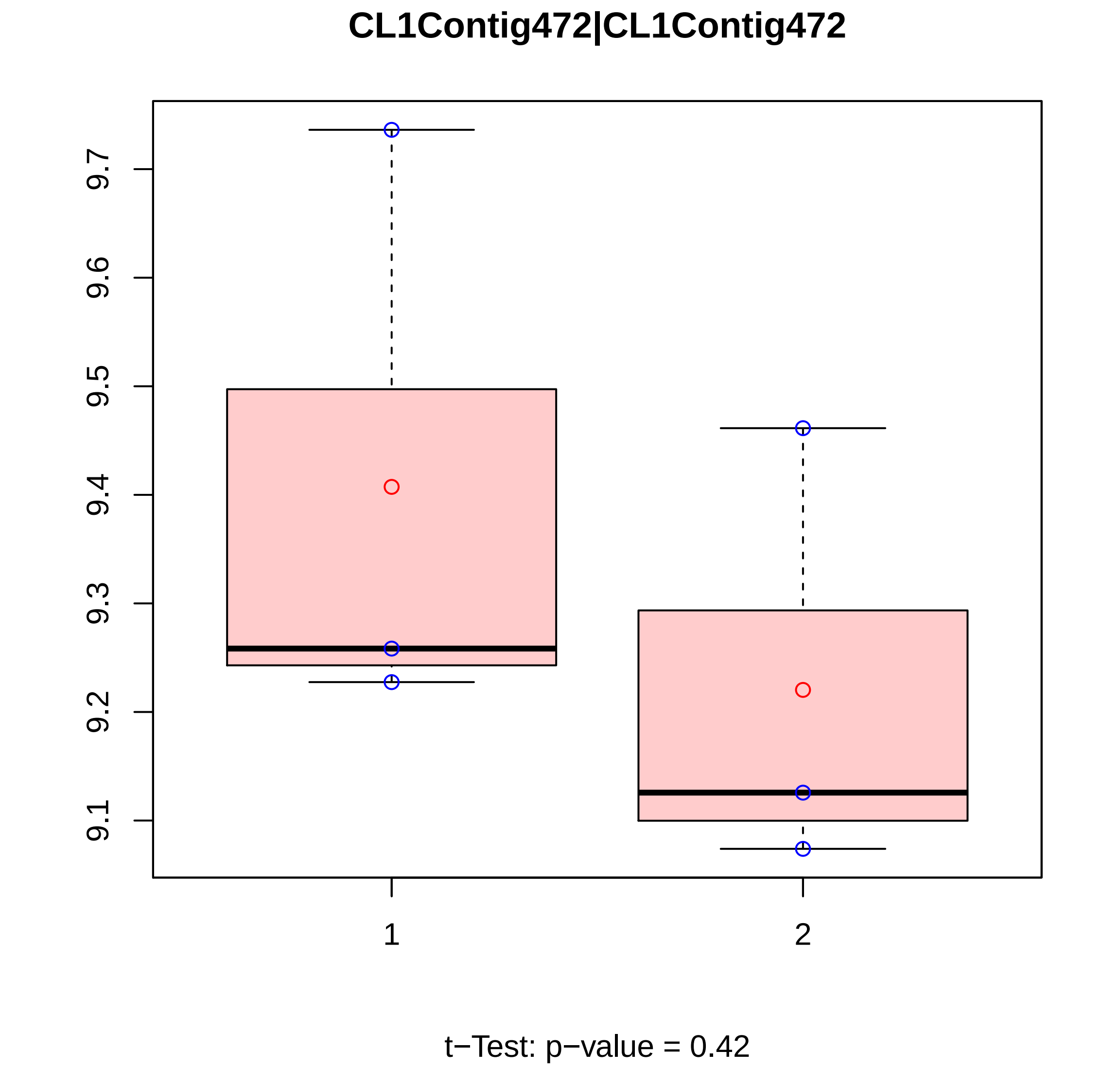
CL1Contig472|CL1Contig472
9.1
9.2
9.3
9.4
9.5
9.6
9.7
1
2
t−Test: p−value = 0.42

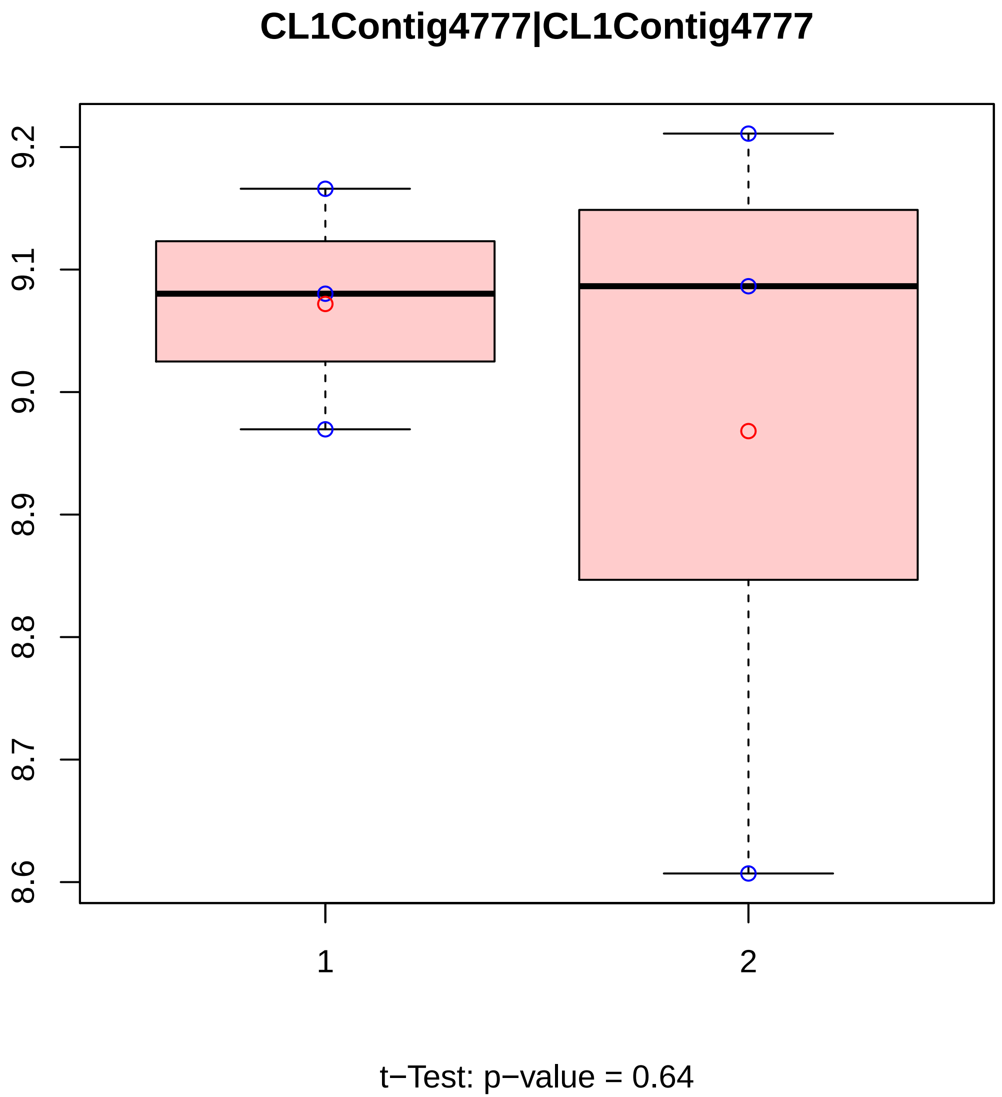
CL1Contig4777|CL1Contig4777
9.2
9.1
9.0
8.9
8.8
8.7
8.6
1
2
t−Test: p−value = 0.64

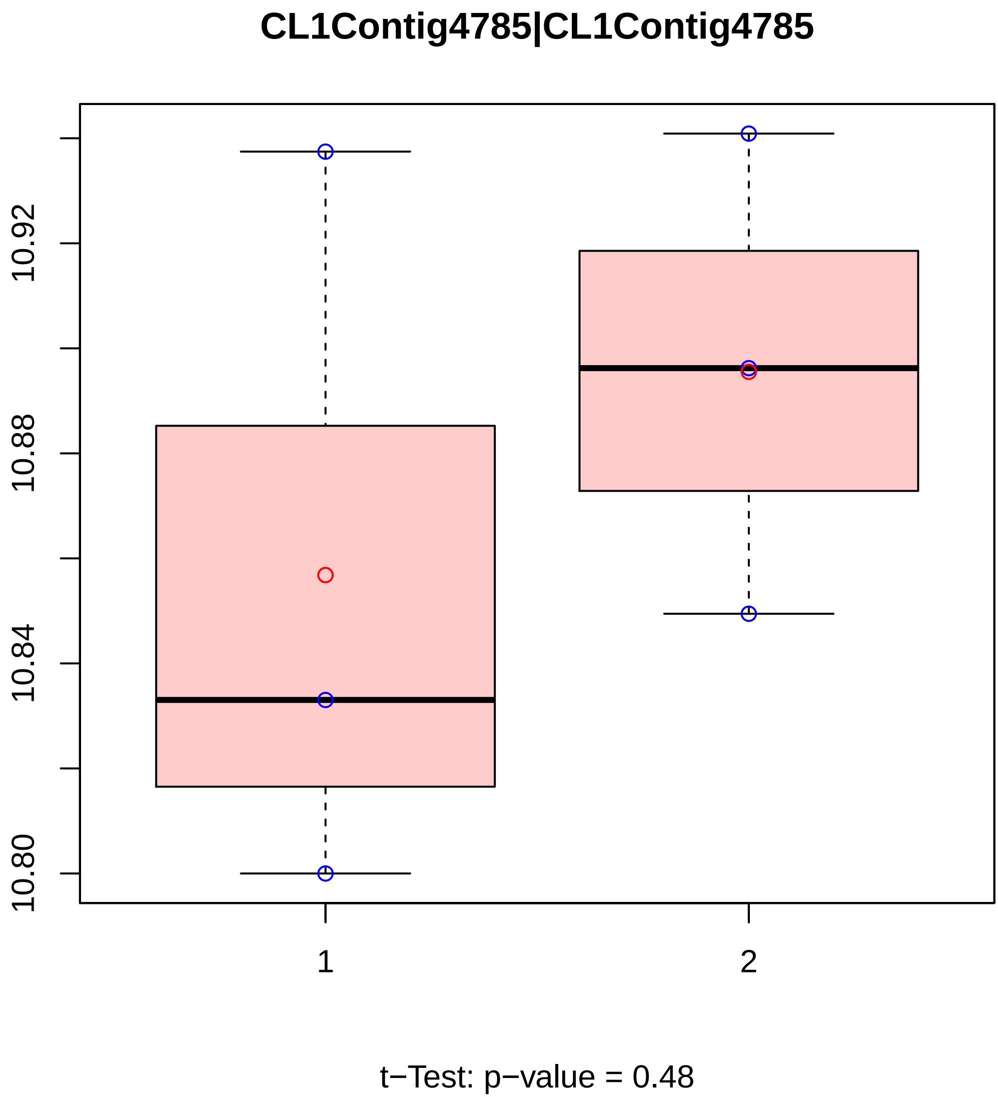
CL1Contig4785|CL1Contig4785
10.80
10.84
10.88
10.92
1
2
t−Test: p−value = 0.48

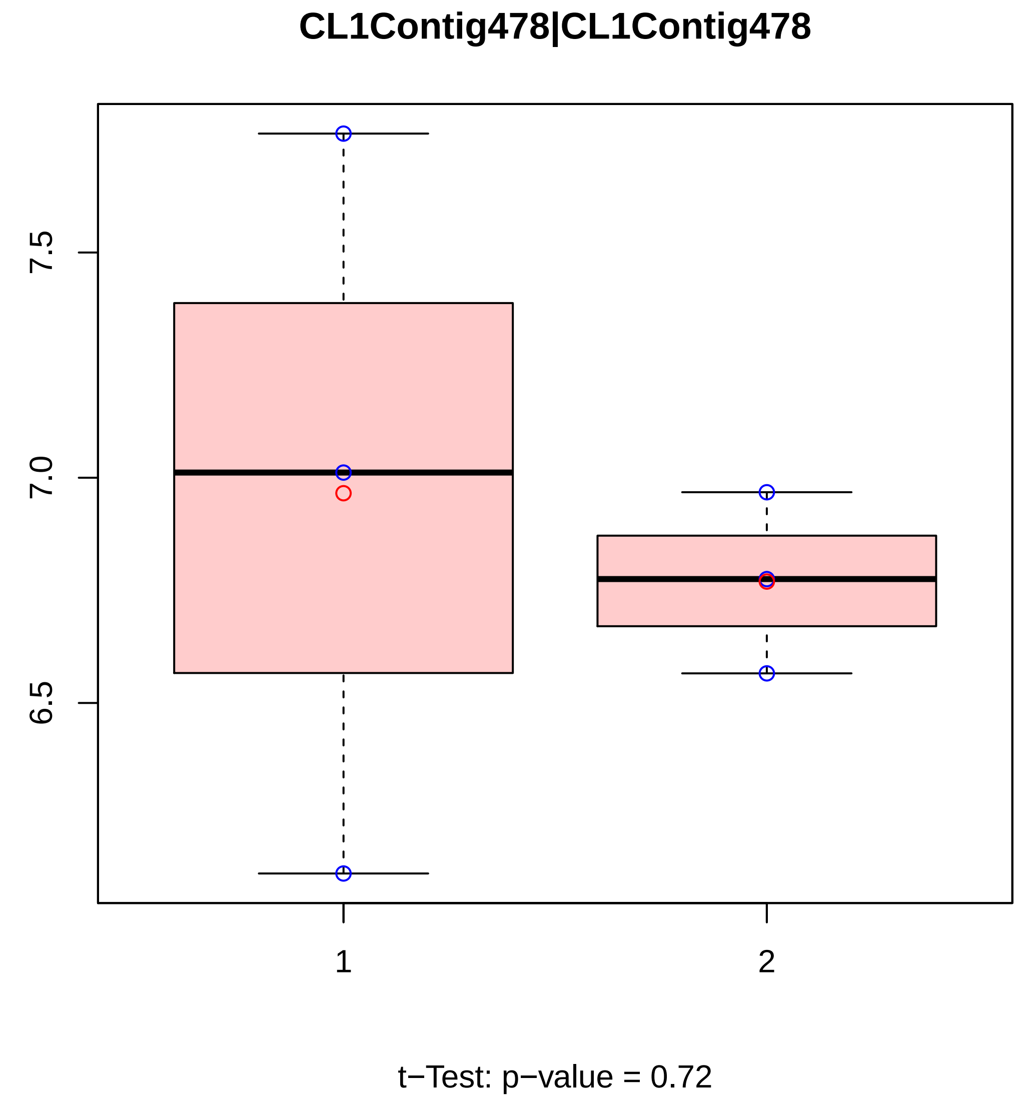
CL1Contig478|CL1Contig478
7.5
7.0
6.5
1
2
t−Test: p−value = 0.72

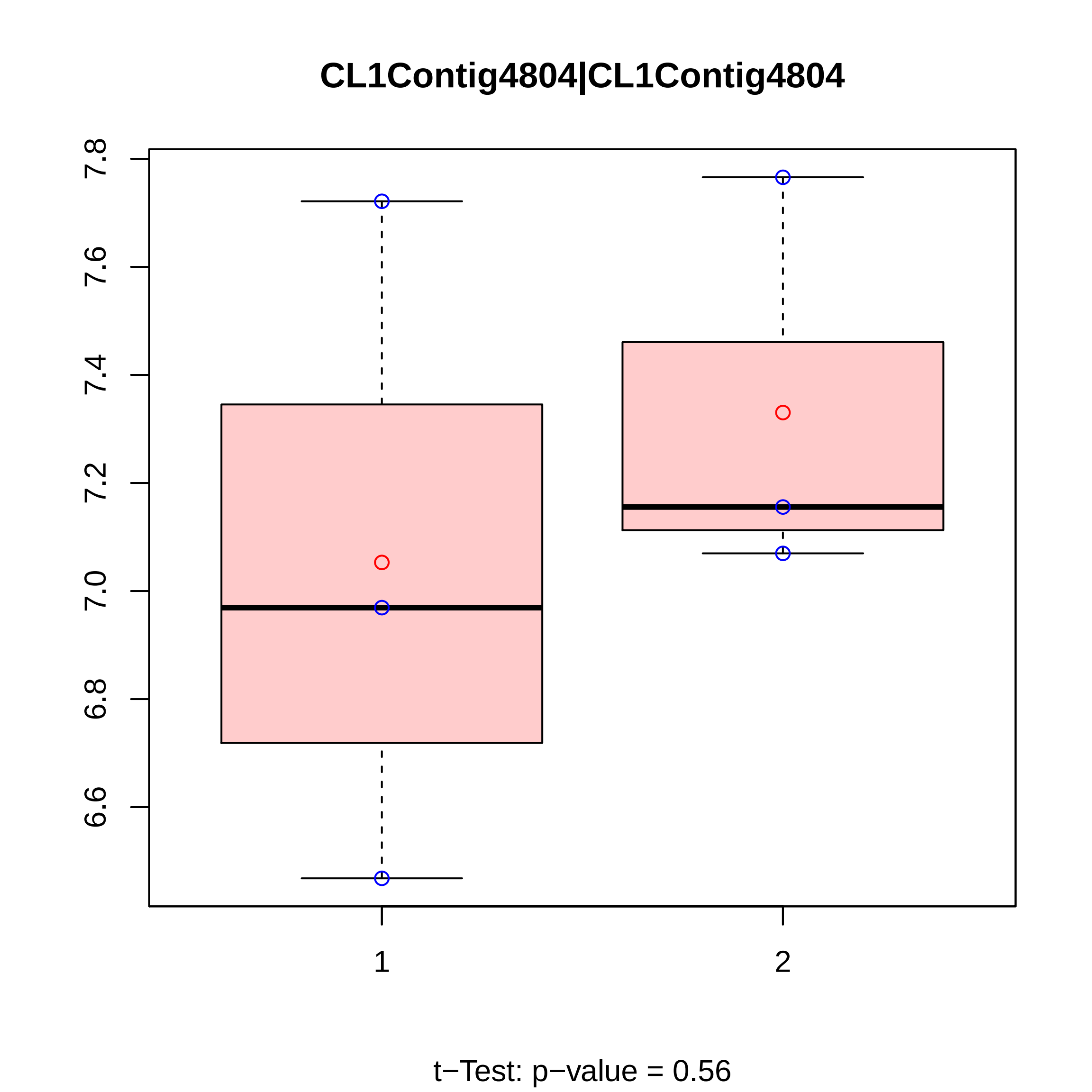
CL1Contig4804|CL1Contig4804
7.8
7.6
7.4
7.2
7.0
6.8
6.6
1
2
t−Test: p−value = 0.56

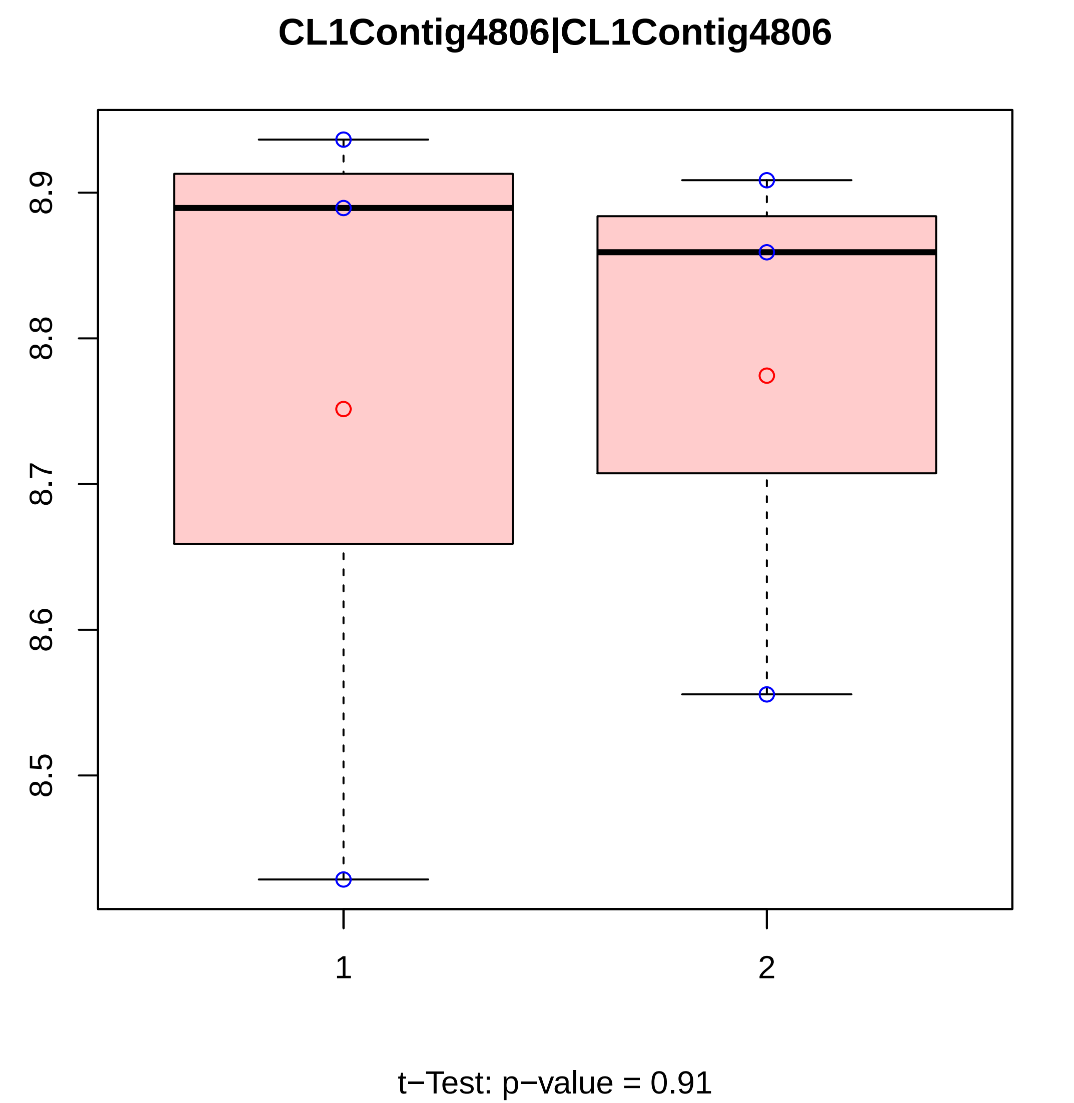
CL1Contig4806|CL1Contig4806
8.9
8.8
8.7
8.6
8.5
1
2
t−Test: p−value = 0.91

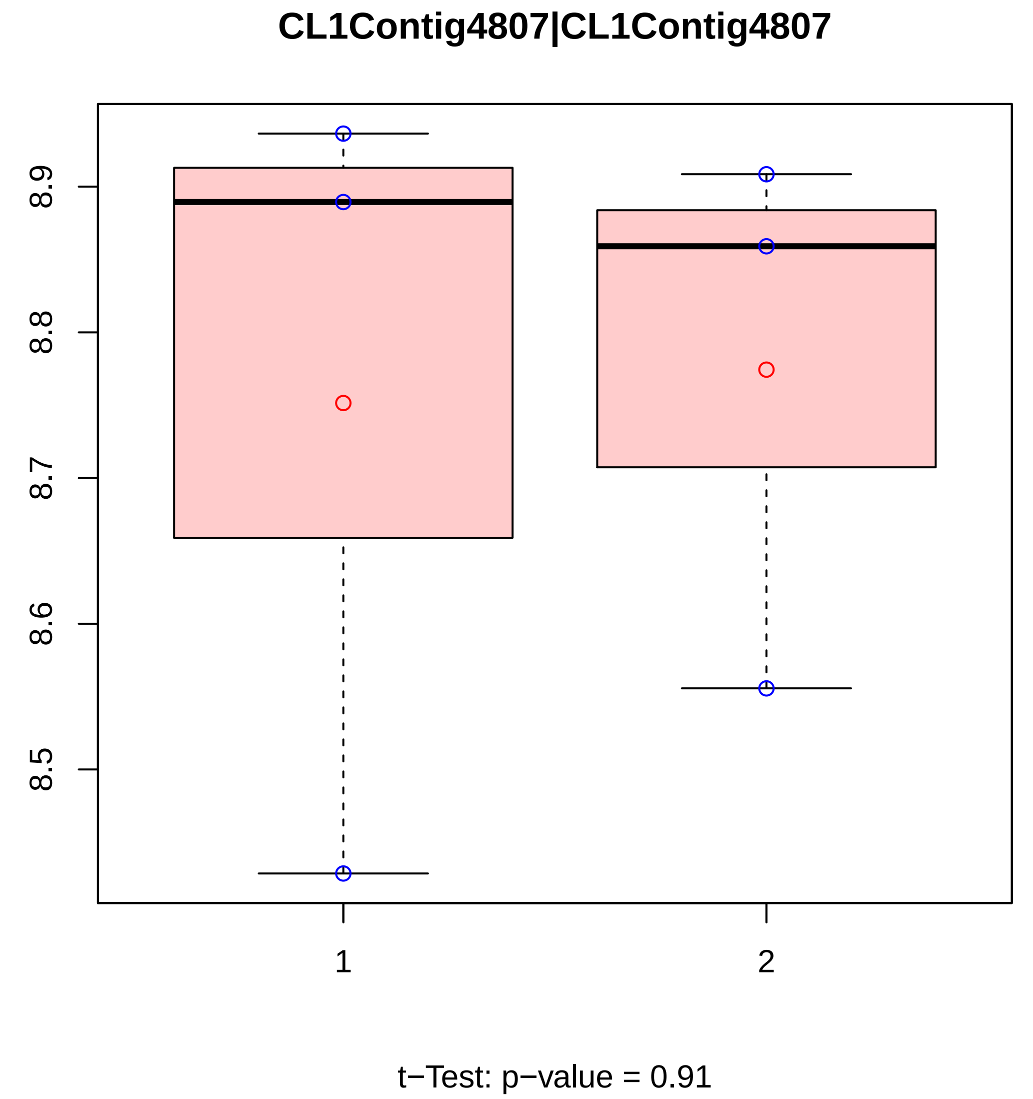

CL1Contig4807|CL1Contig4807
8.5
8.6
8.7
8.8
8.9
1
2
t−Test: p−value = 0.91

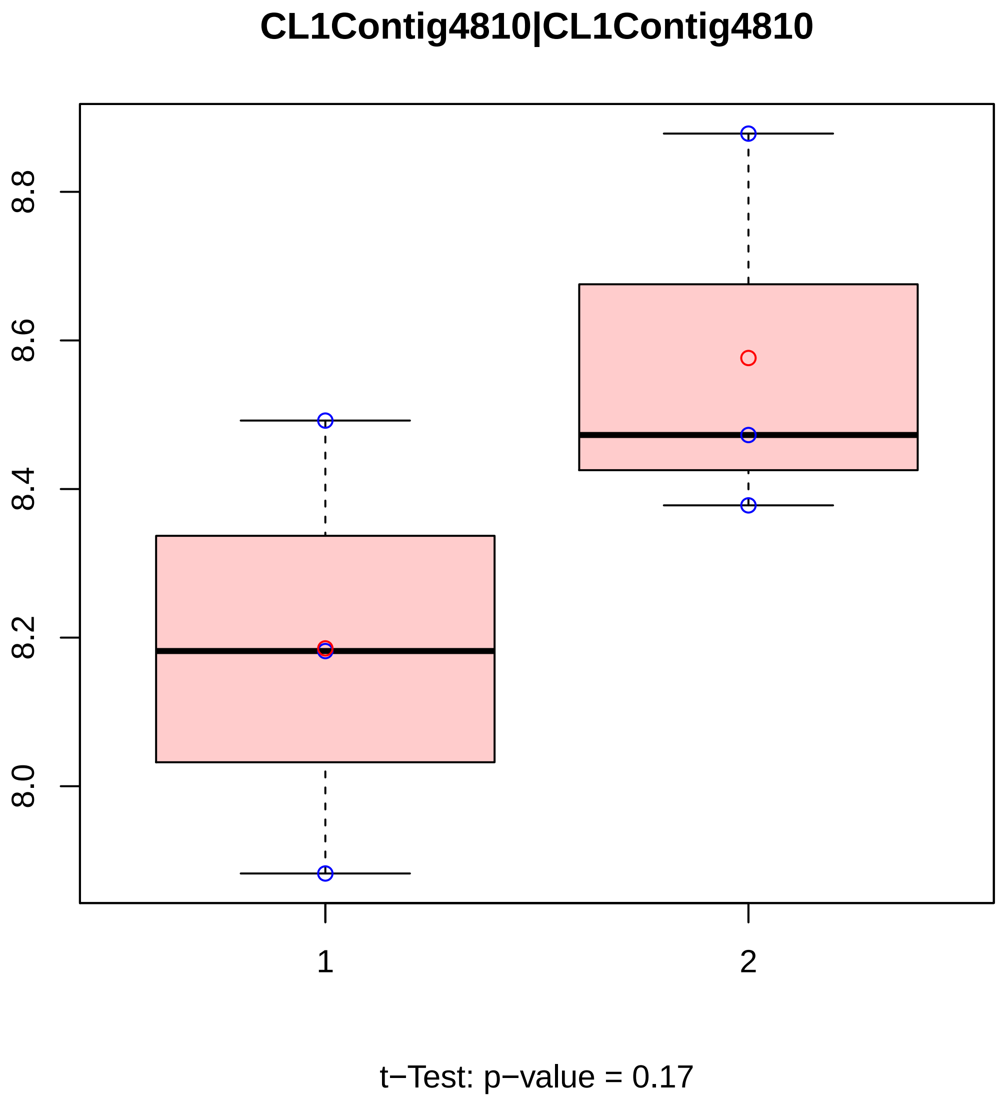
CL1Contig4810|CL1Contig4810
8.0
8.2
8.4
8.6
8.8
1
2
t−Test: p−value = 0.17

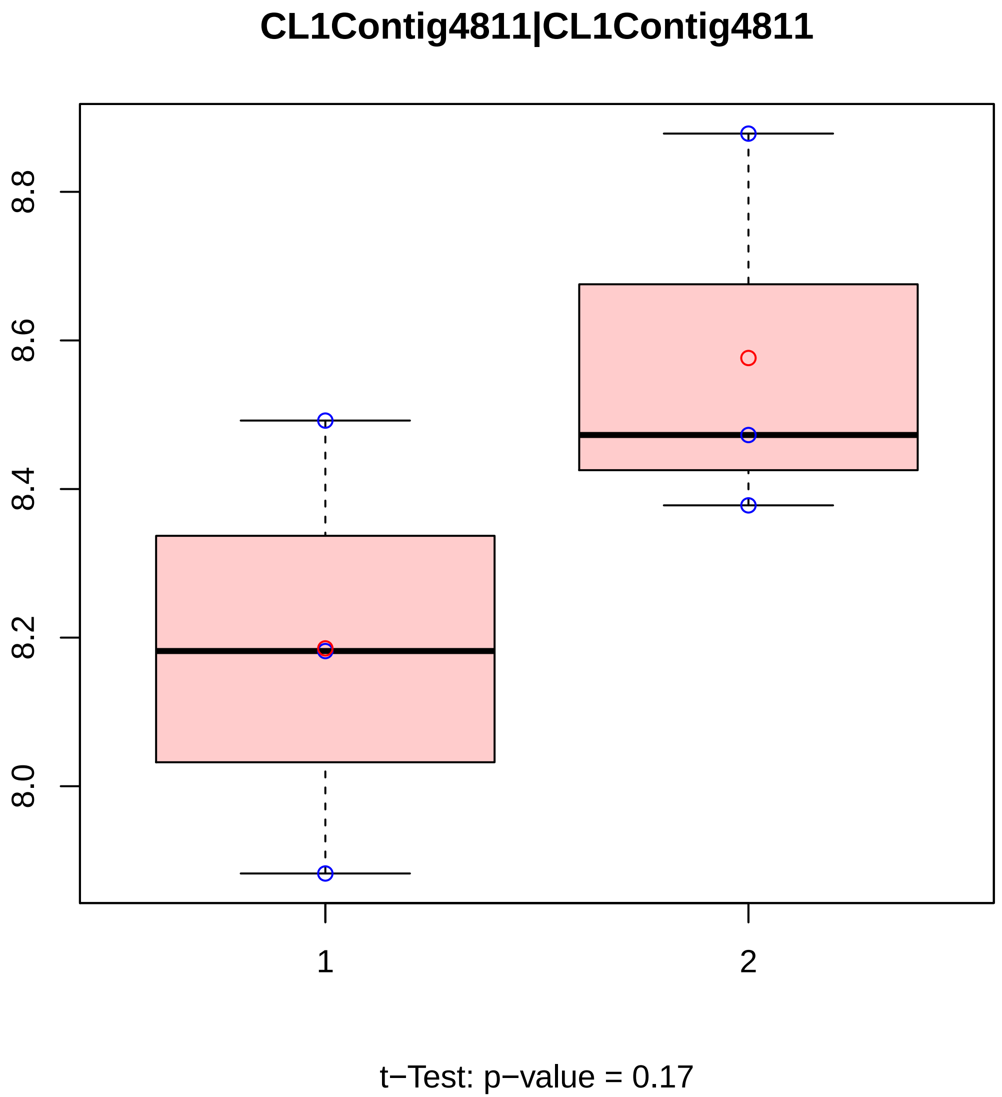
CL1Contig4811|CL1Contig4811
8.0
8.2
8.4
8.6
8.8
1
2
t−Test: p−value = 0.17

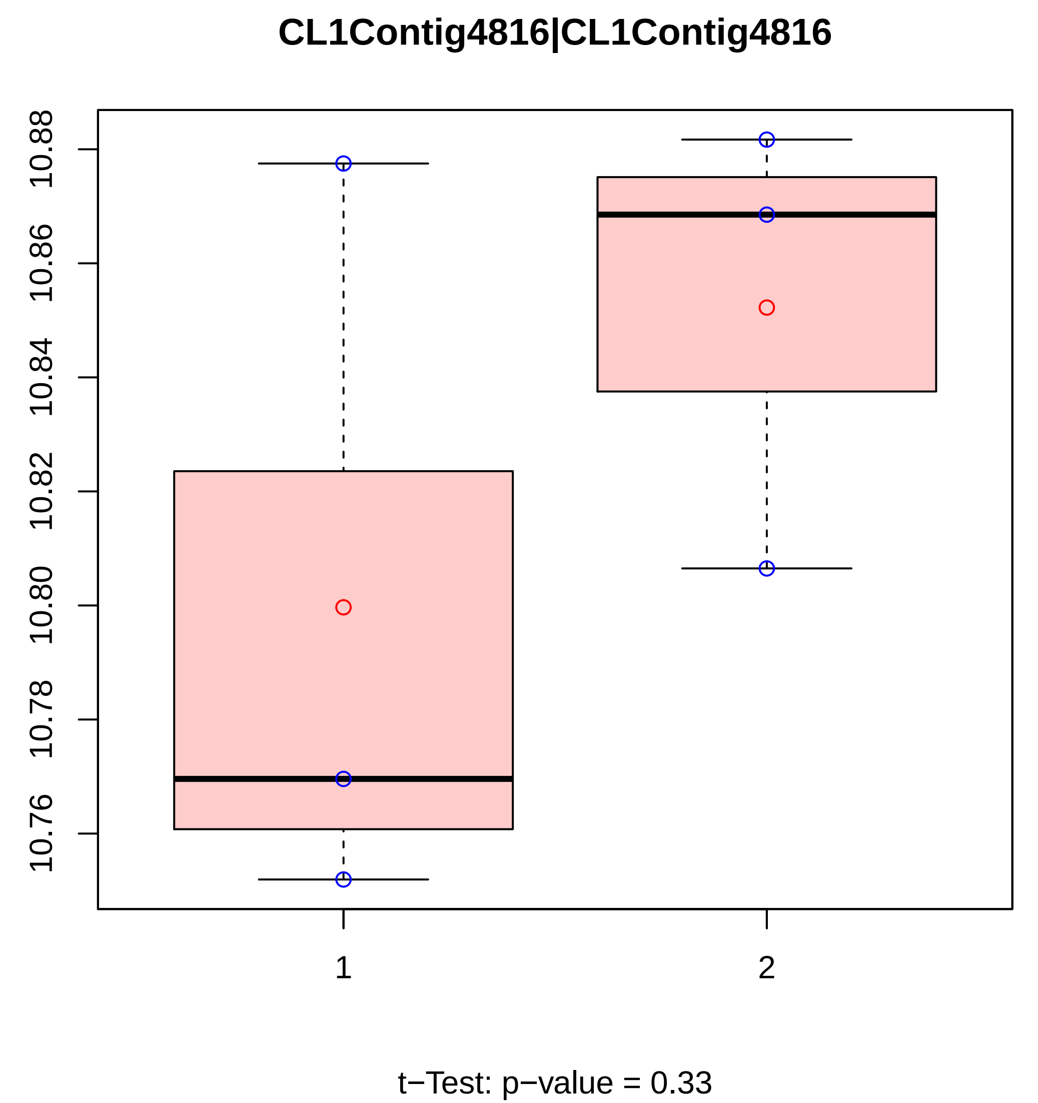
CL1Contig4816|CL1Contig4816
10.76
10.78
10.80
10.82
10.84
10.86
10.88
1
2
t−Test: p−value = 0.33

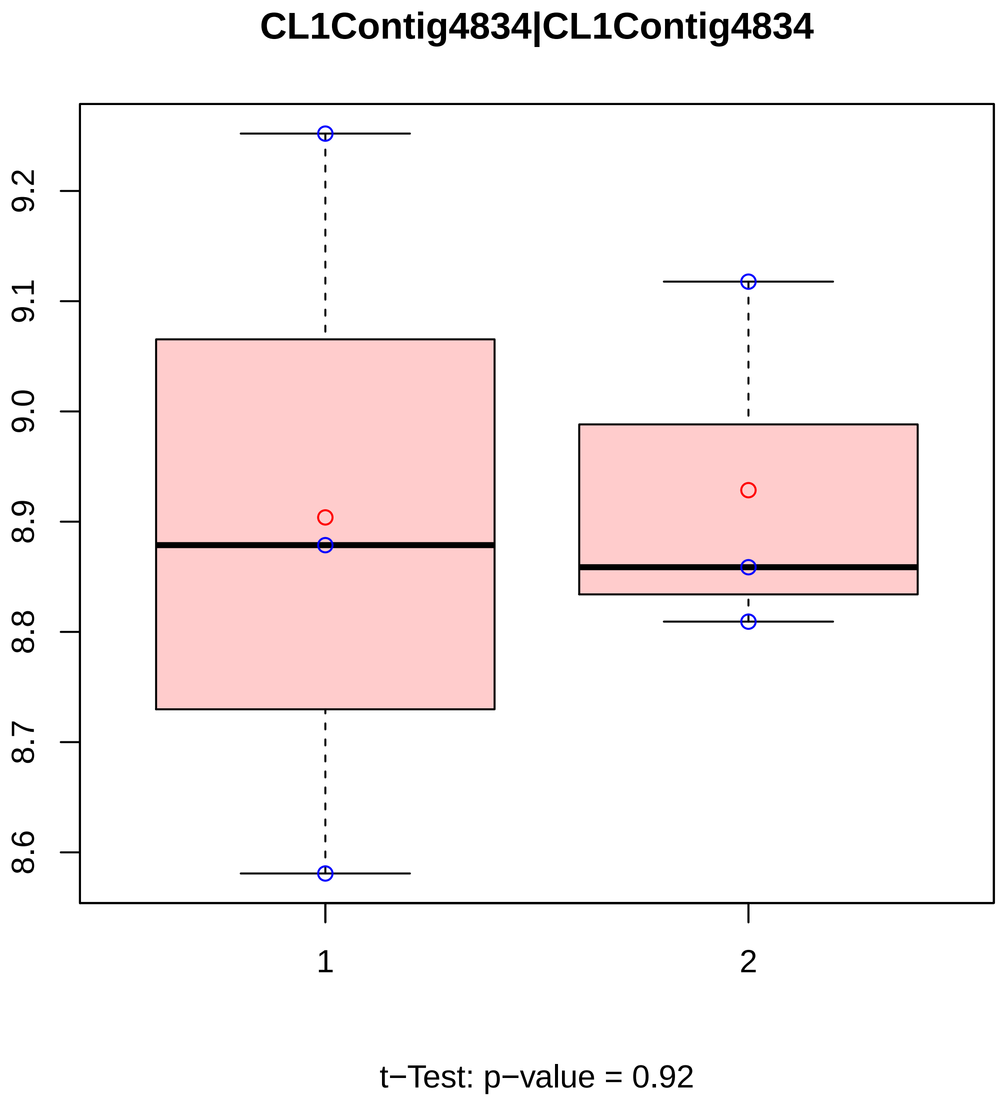
CL1Contig4834|CL1Contig4834
9.2
9.1
9.0
8.9
8.8
8.7
8.6
1
2
t−Test: p−value = 0.92

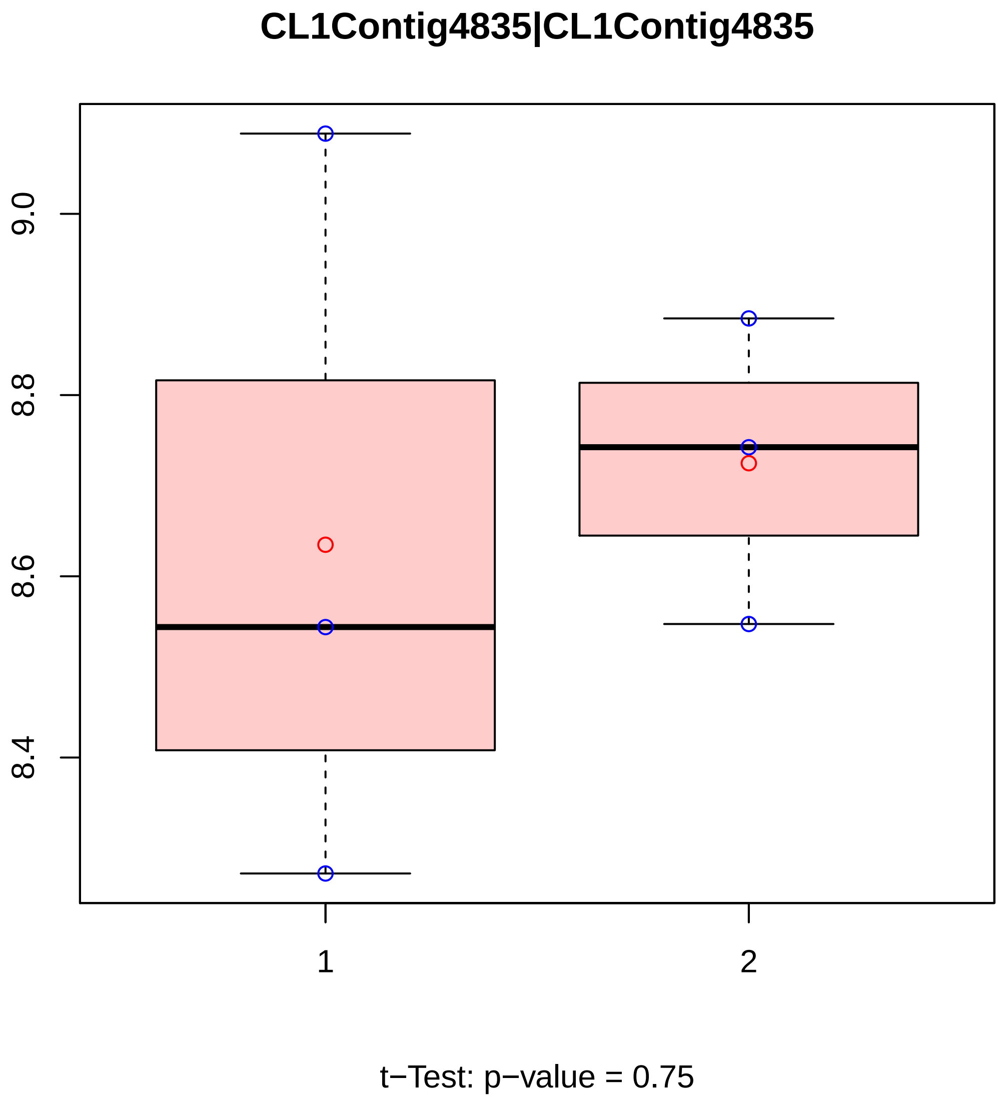
CL1Contig4835|CL1Contig4835
9.0
8.8
8.6
8.4
1
2
t−Test: p−value = 0.75

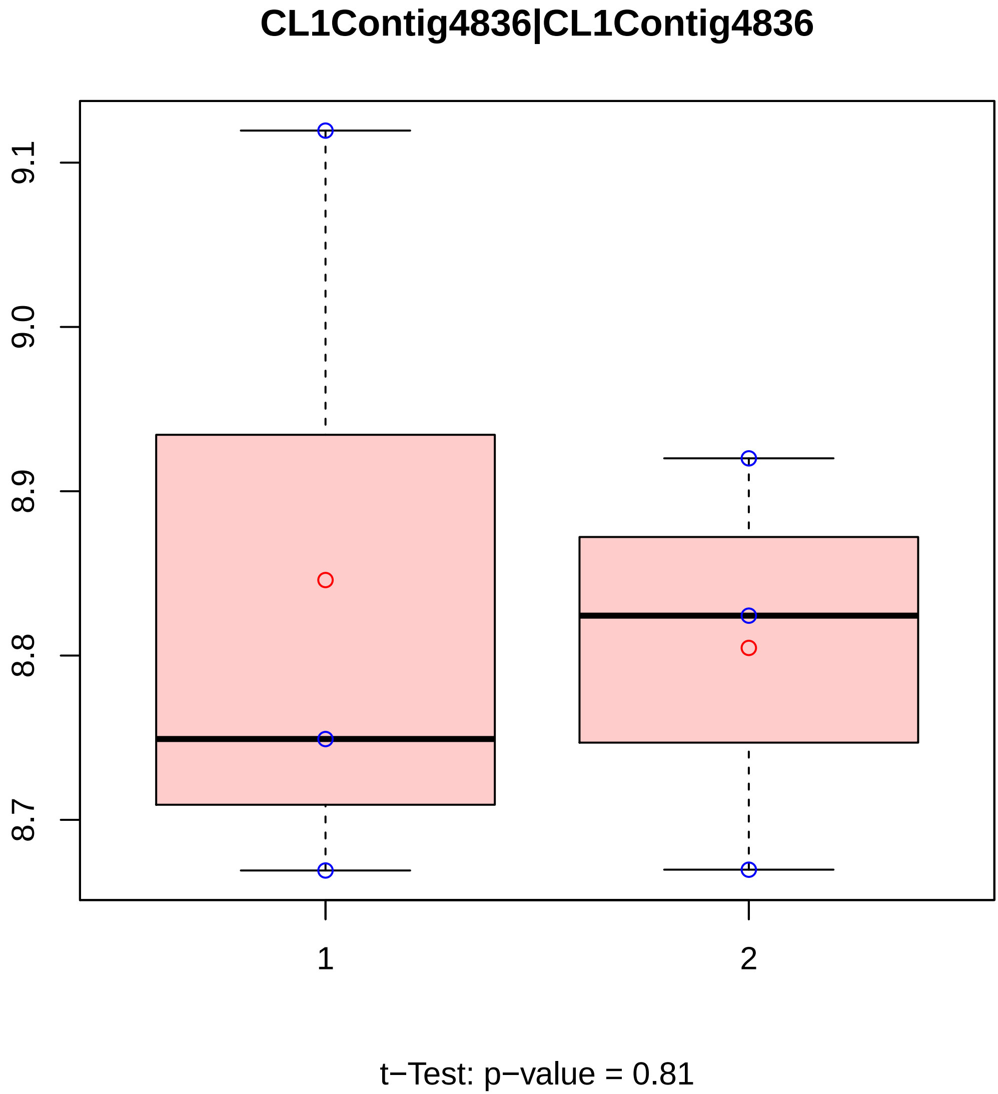

CL1Contig4836|CL1Contig4836
9.1
9.0
8.9
8.8
8.7
1
2
t−Test: p−value = 0.81

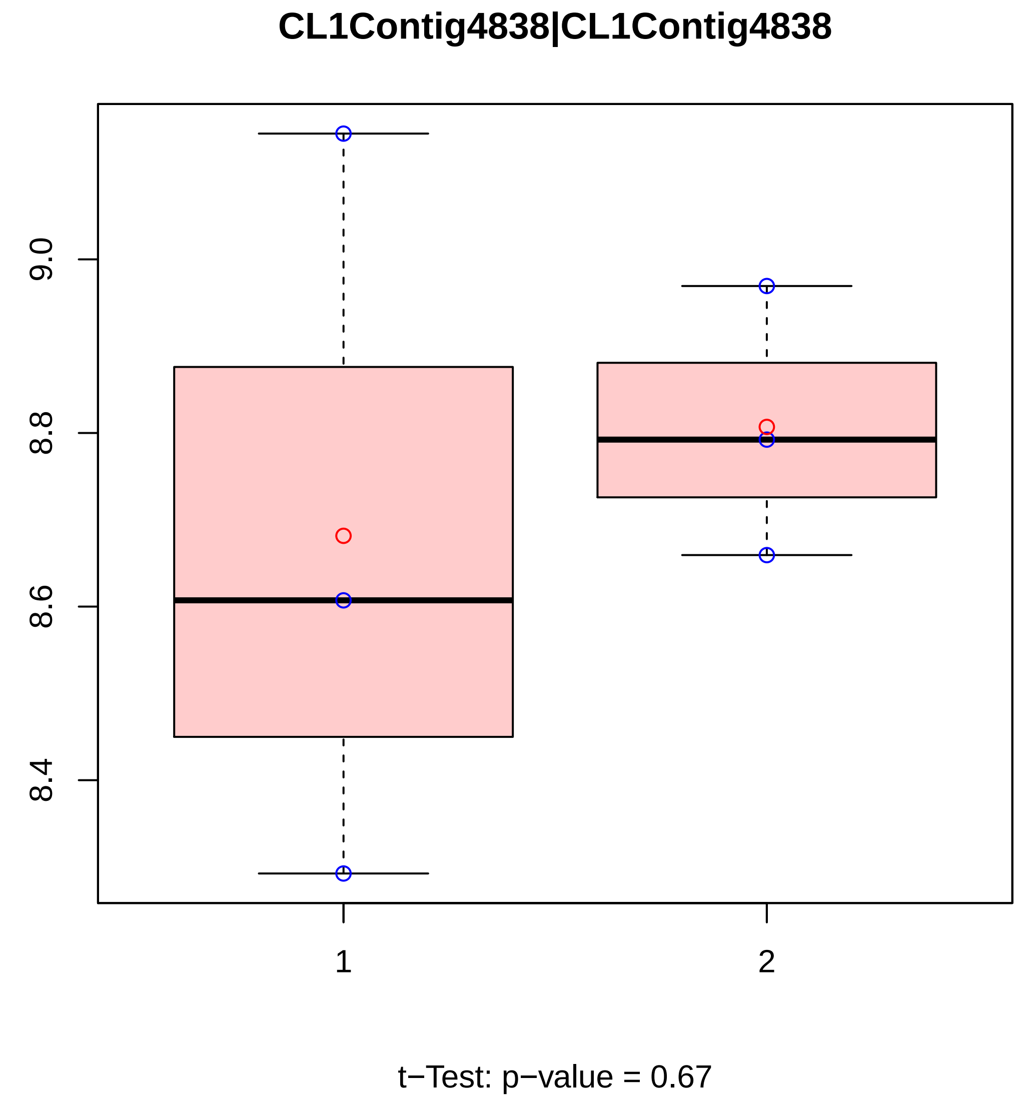
CL1Contig4838|CL1Contig4838
9.0
8.8
8.6
8.4
1
2
t−Test: p−value = 0.67

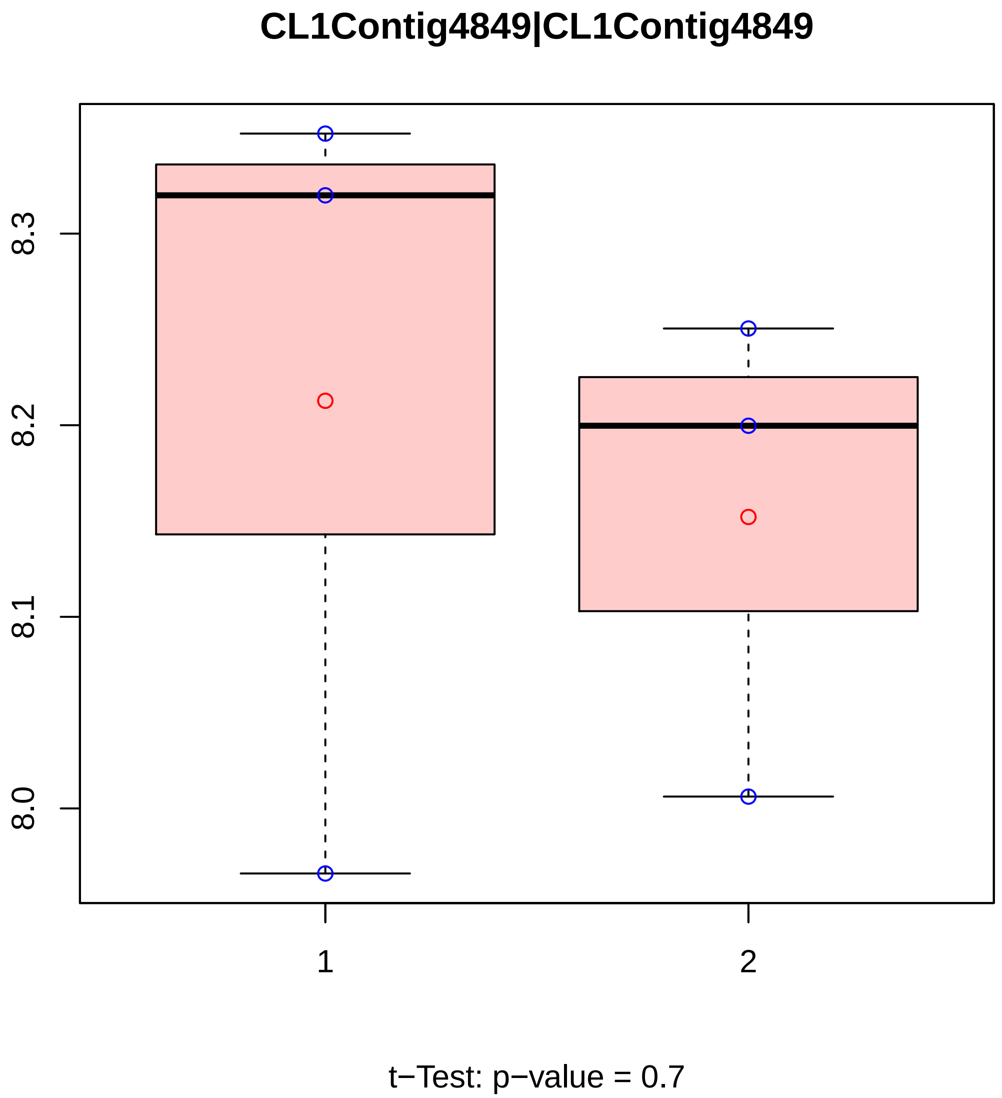
CL1Contig4849|CL1Contig4849
8.0
8.1
8.2
8.3
1
2
t−Test: p−value = 0.7

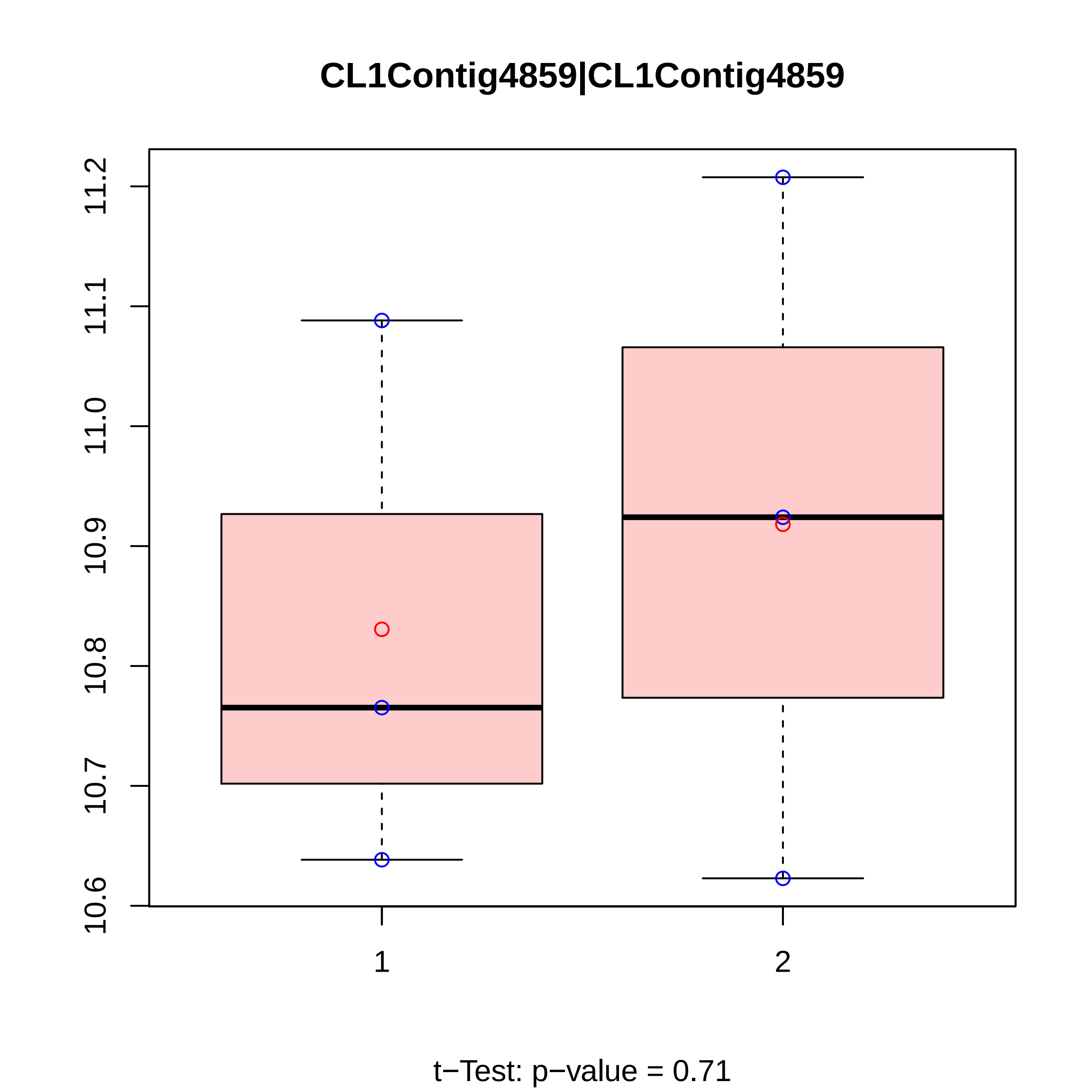

CL1Contig4859|CL1Contig4859
11.2
11.1
11.0
10.9
10.8
10.7
10.6
1
2
t−Test: p−value = 0.71

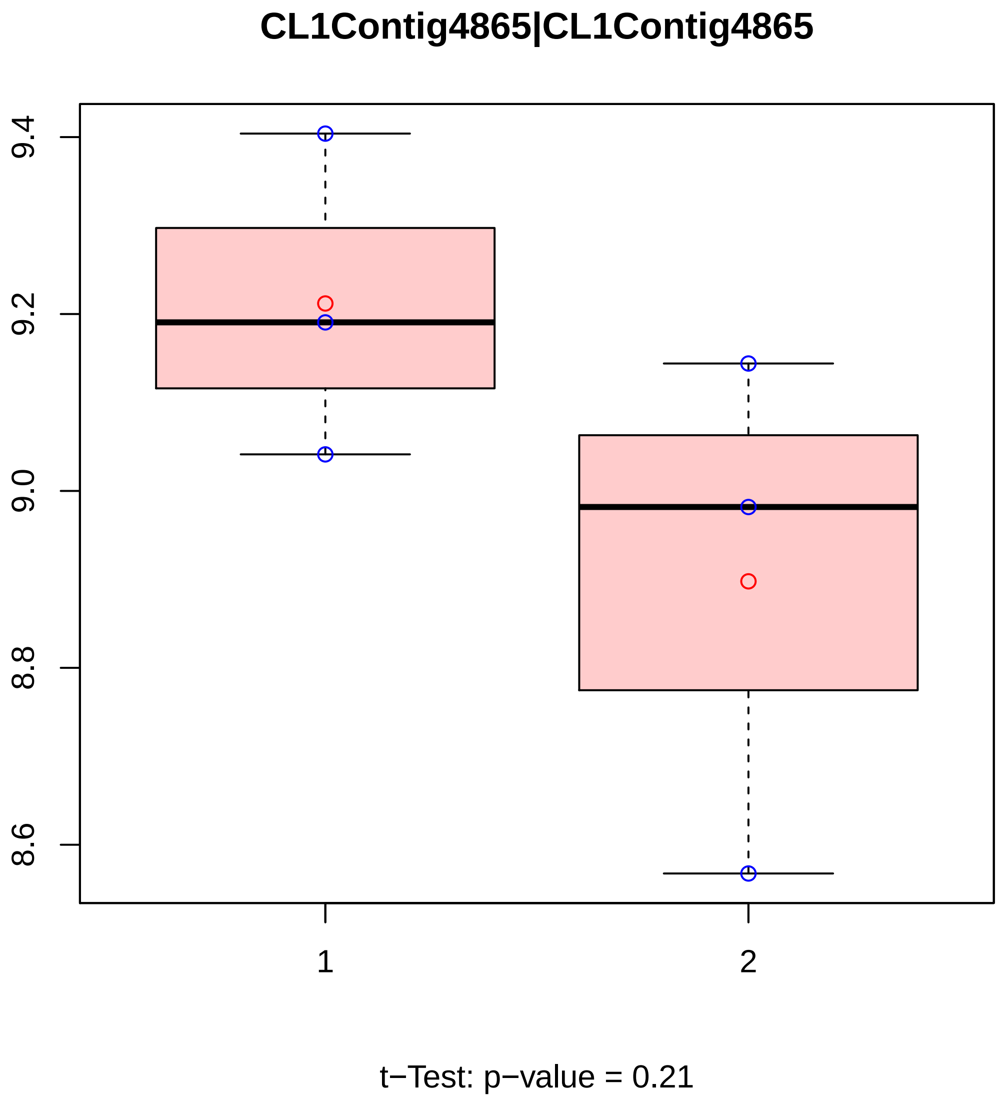
CL1Contig4865|CL1Contig4865
9.4
9.2
9.0
8.8
8.6
1
2
t−Test: p−value = 0.21

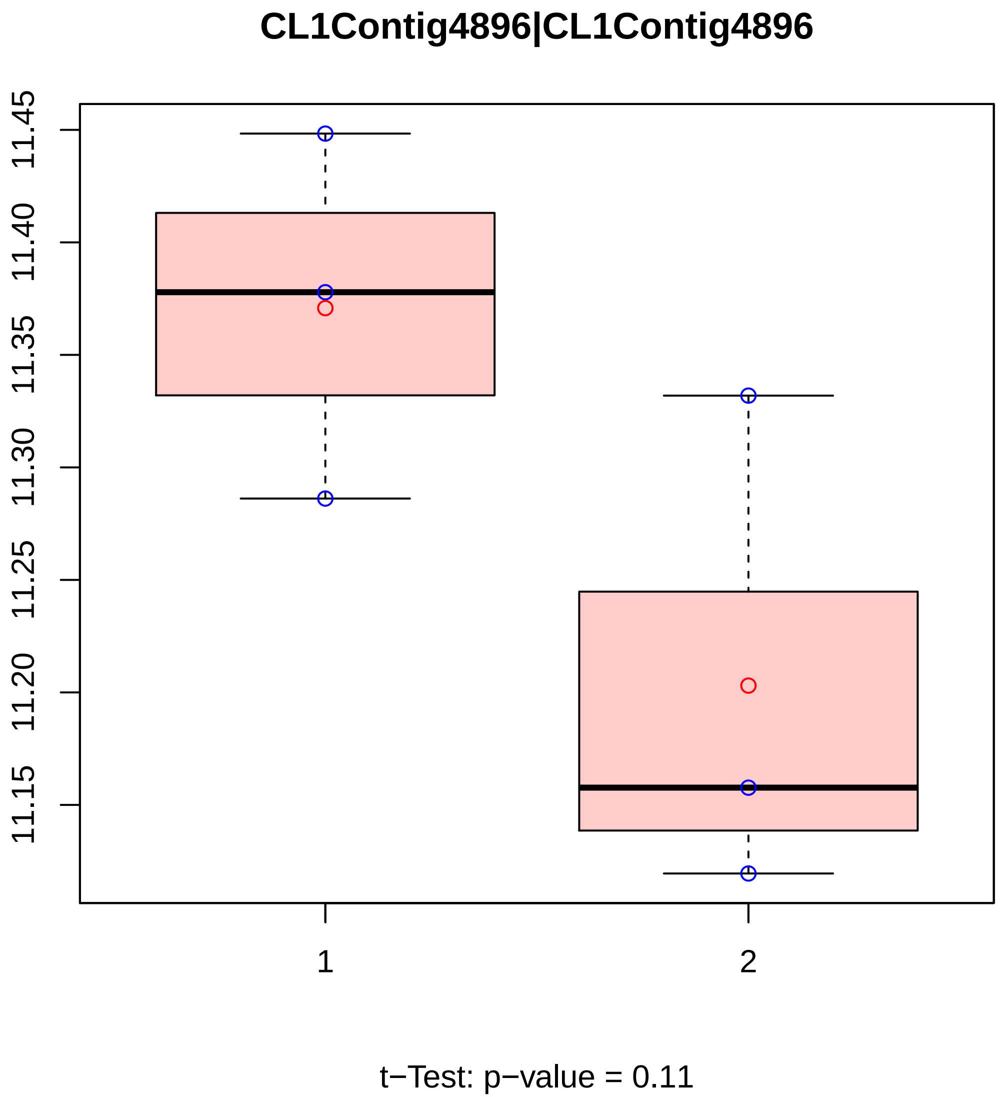

CL1Contig4896|CL1Contig4896
11.15
11.20
11.25
11.30
11.35
11.40
11.45
1
2
t−Test: p−value = 0.11

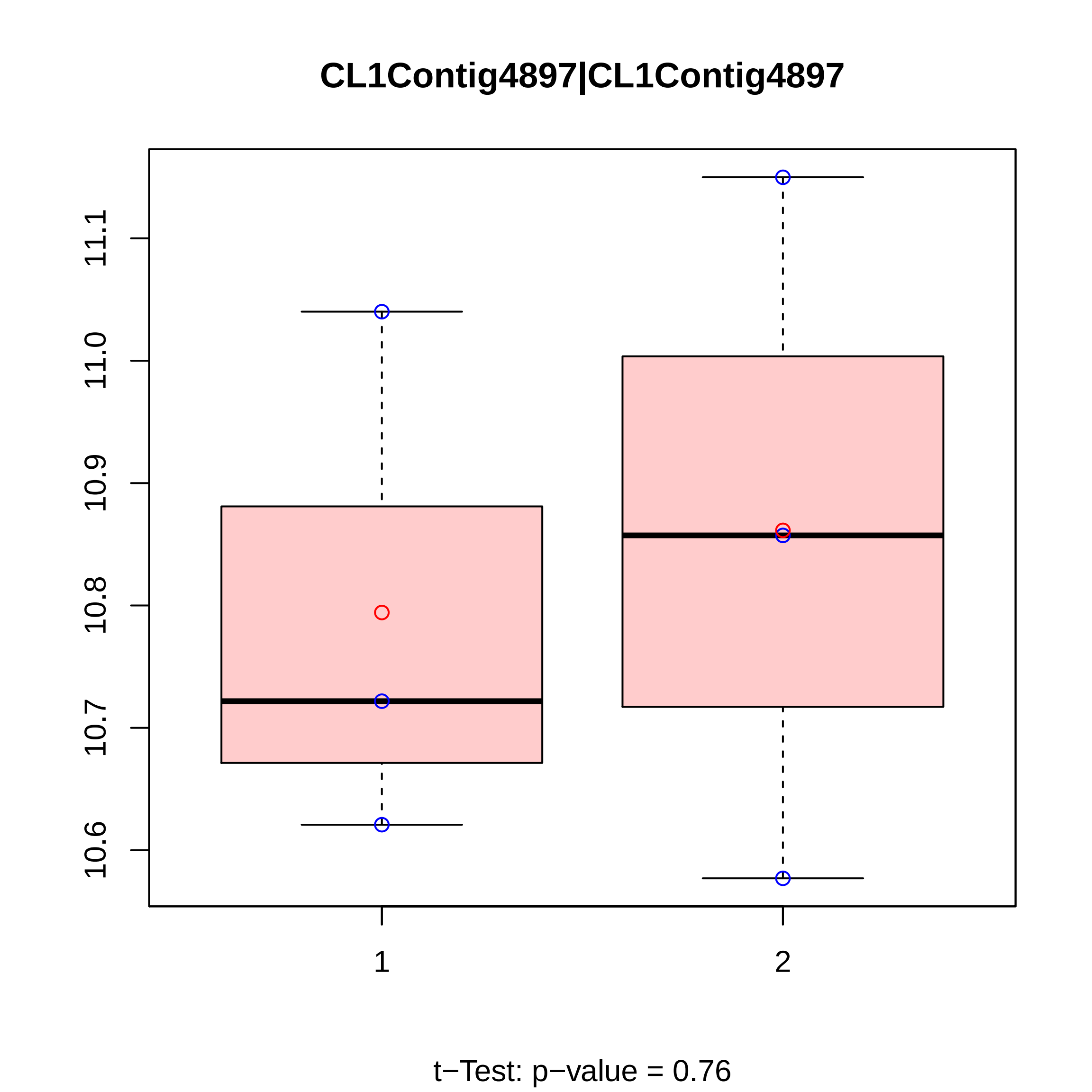
CL1Contig4897|CL1Contig4897
11.1
11.0
10.9
10.8
10.7
10.6
1
2
t−Test: p−value = 0.76

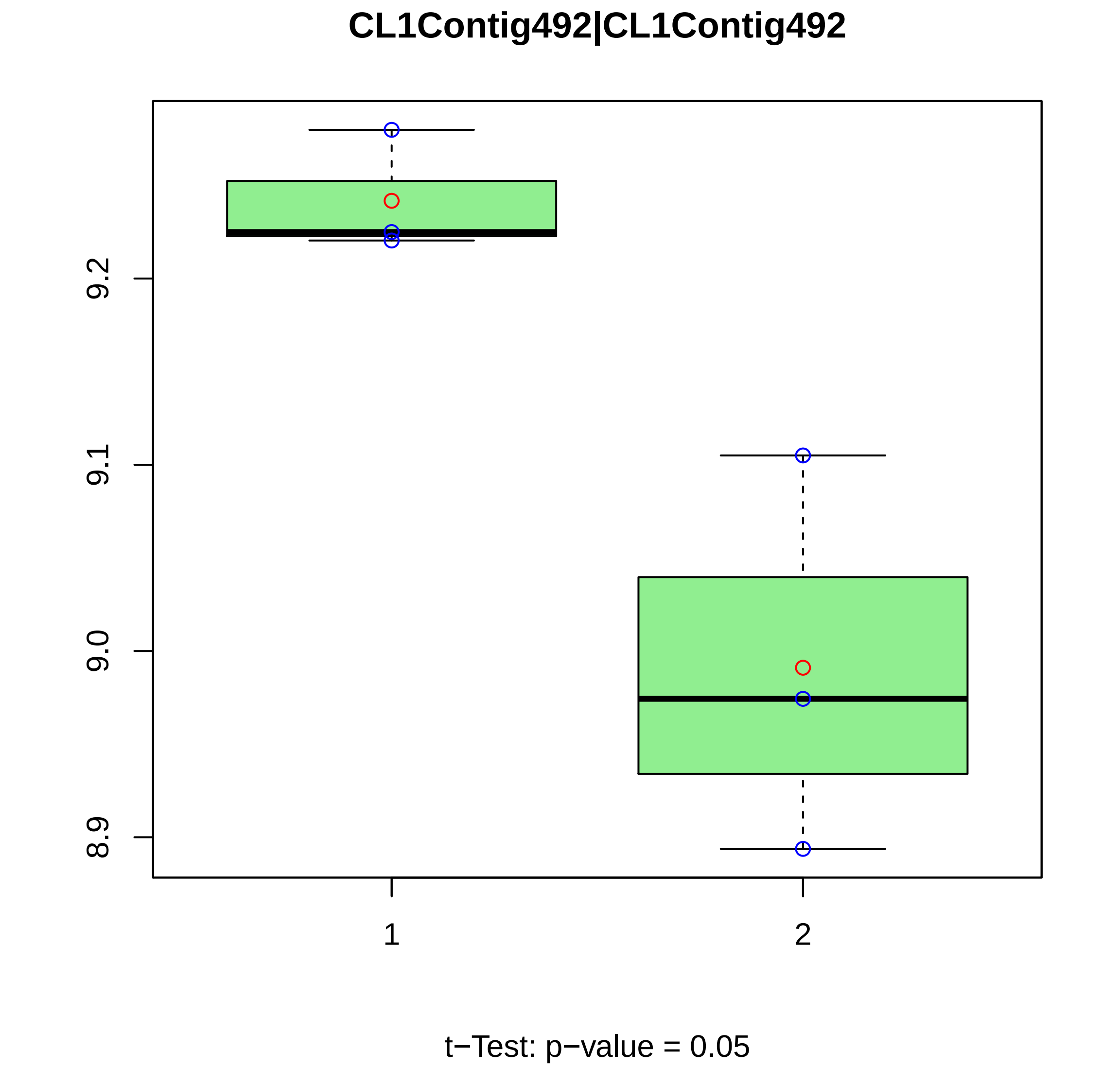

CL1Contig492|CL1Contig492
9.2
9.1
9.0
8.9
1
2
t−Test: p−value = 0.05

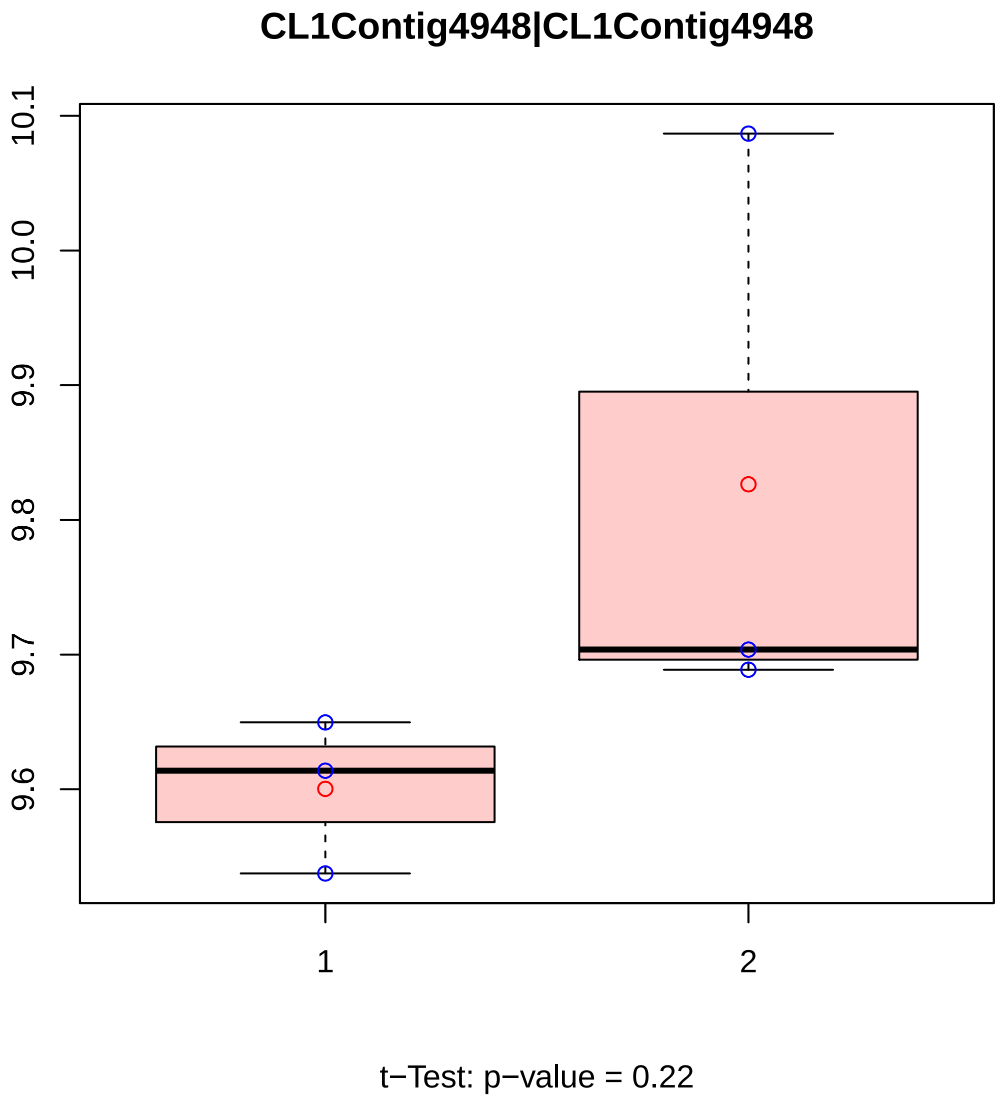
CL1Contig4948|CL1Contig4948
10.1
10.0
9.9
9.8
9.7
9.6
1
2
t−Test: p−value = 0.22

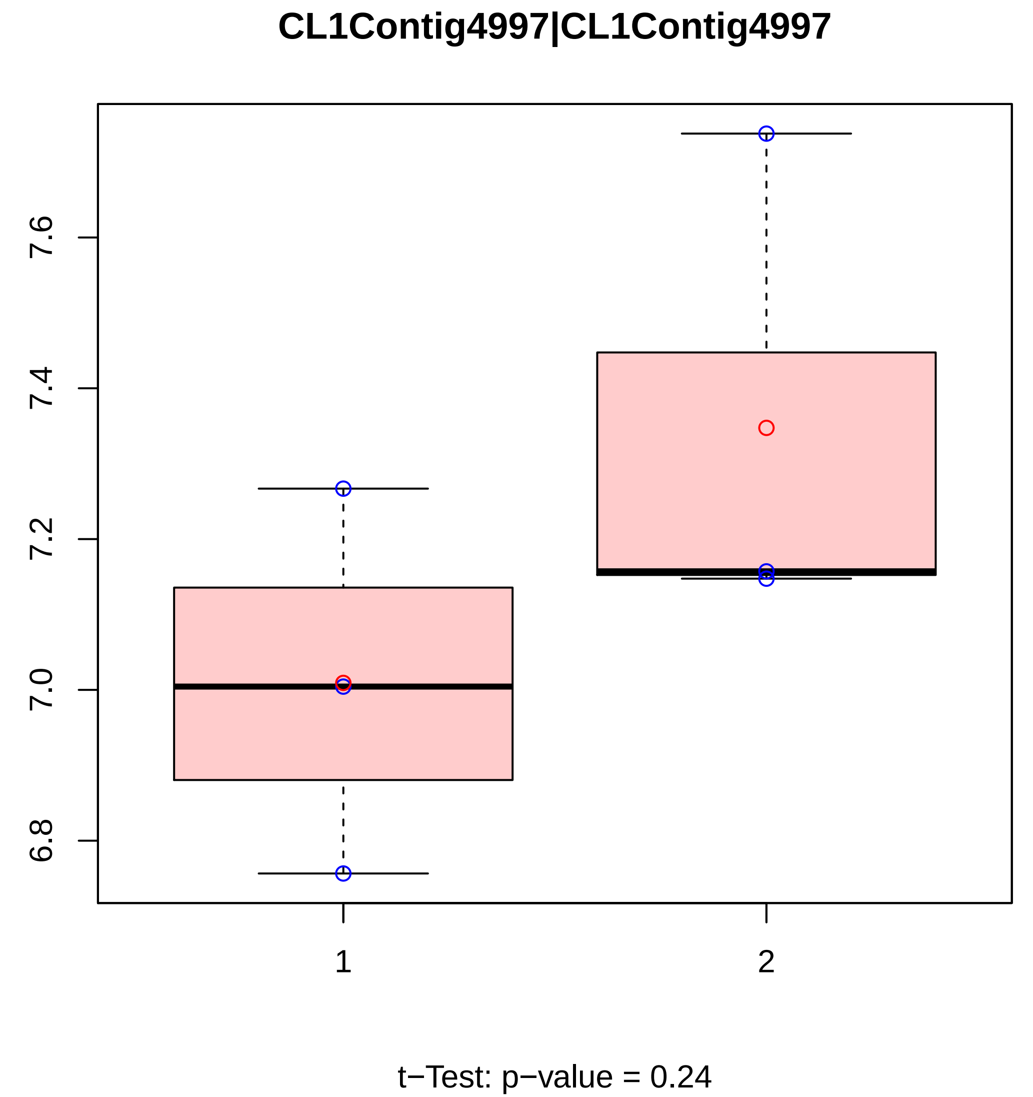
CL1Contig4997|CL1Contig4997
7.6
7.4
7.2
7.0
6.8
1
2
t−Test: p−value = 0.24

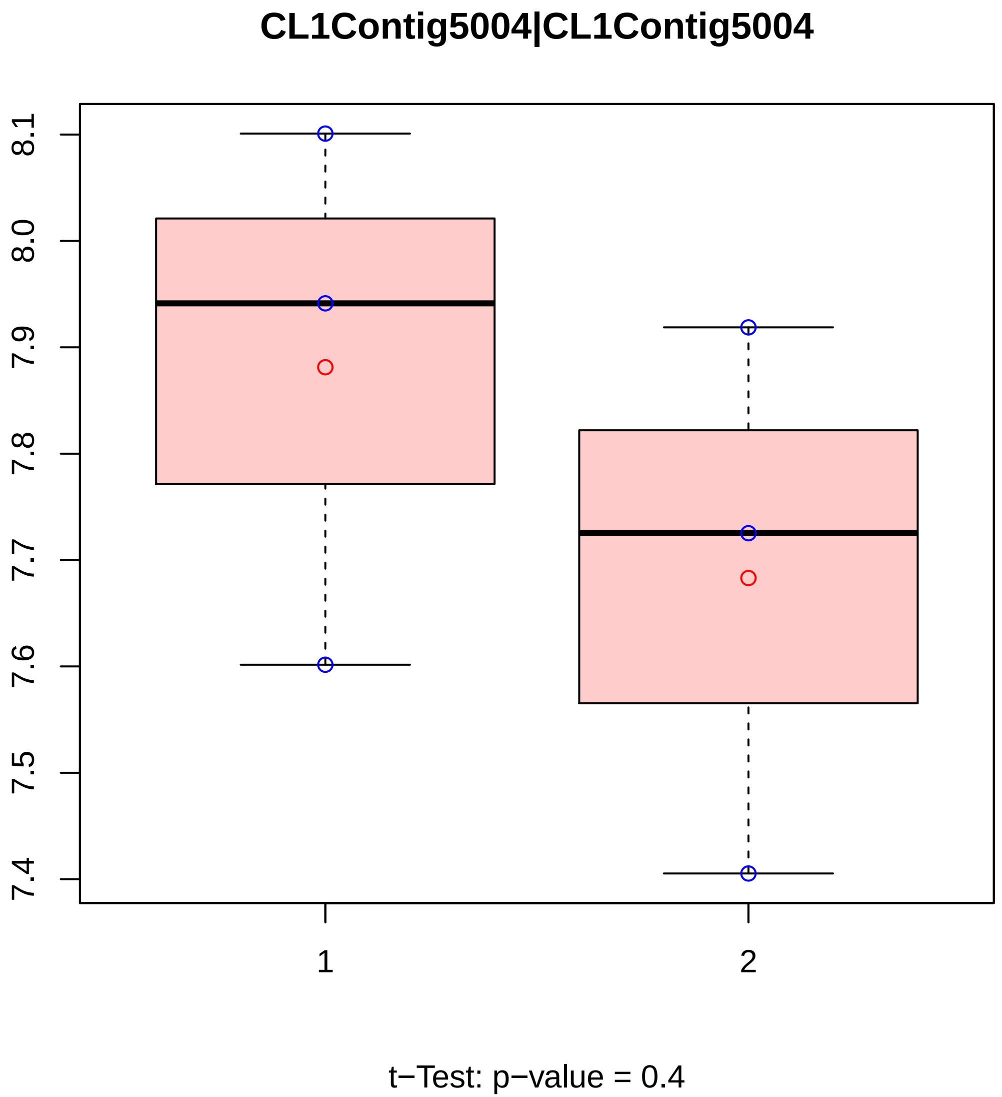
CL1Contig5004|CL1Contig5004
8.1
8.0
7.9
7.8
7.7
7.6
7.5
7.4
1
2
t−Test: p−value = 0.4

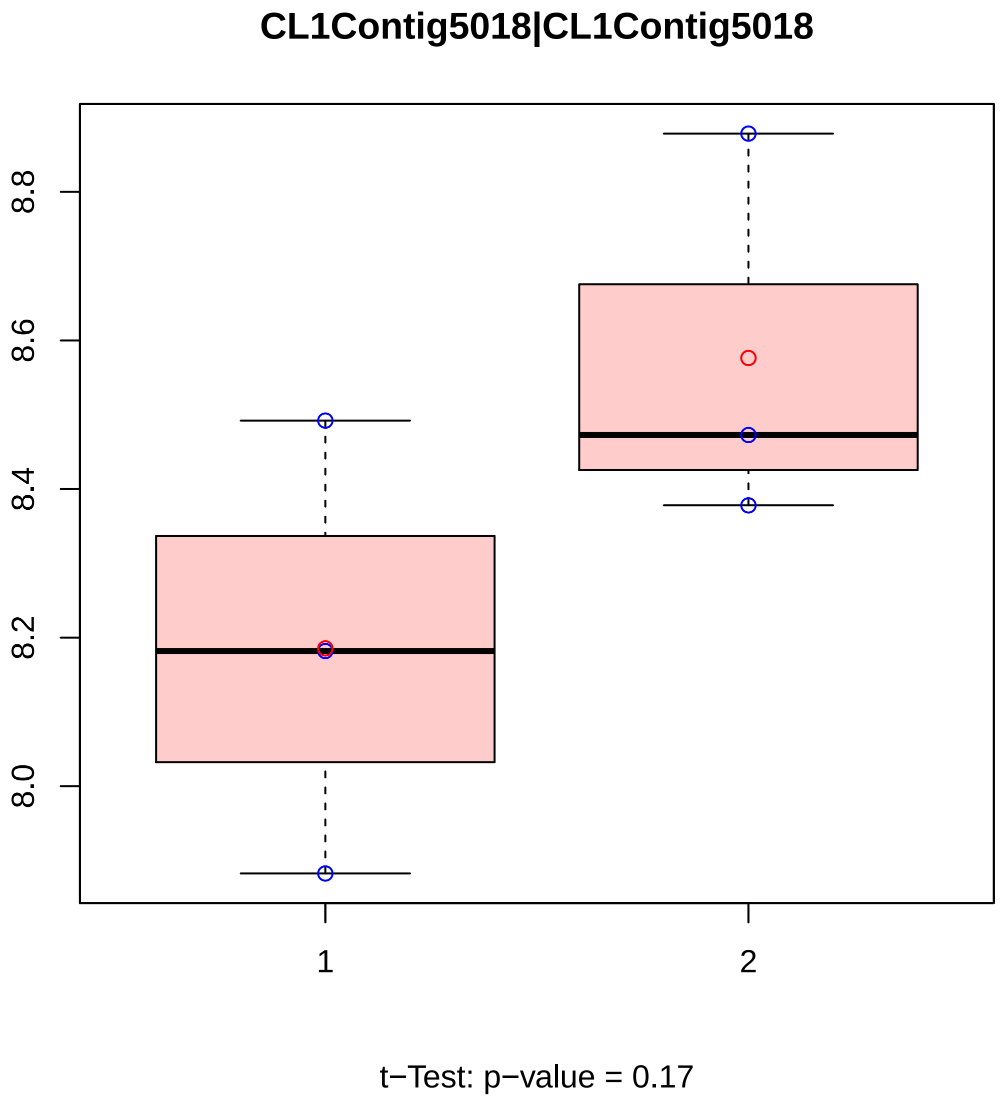

CL1Contig5018|CL1Contig5018
8.0
8.2
8.4
8.6
8.8
1
2
t−Test: p−value = 0.17

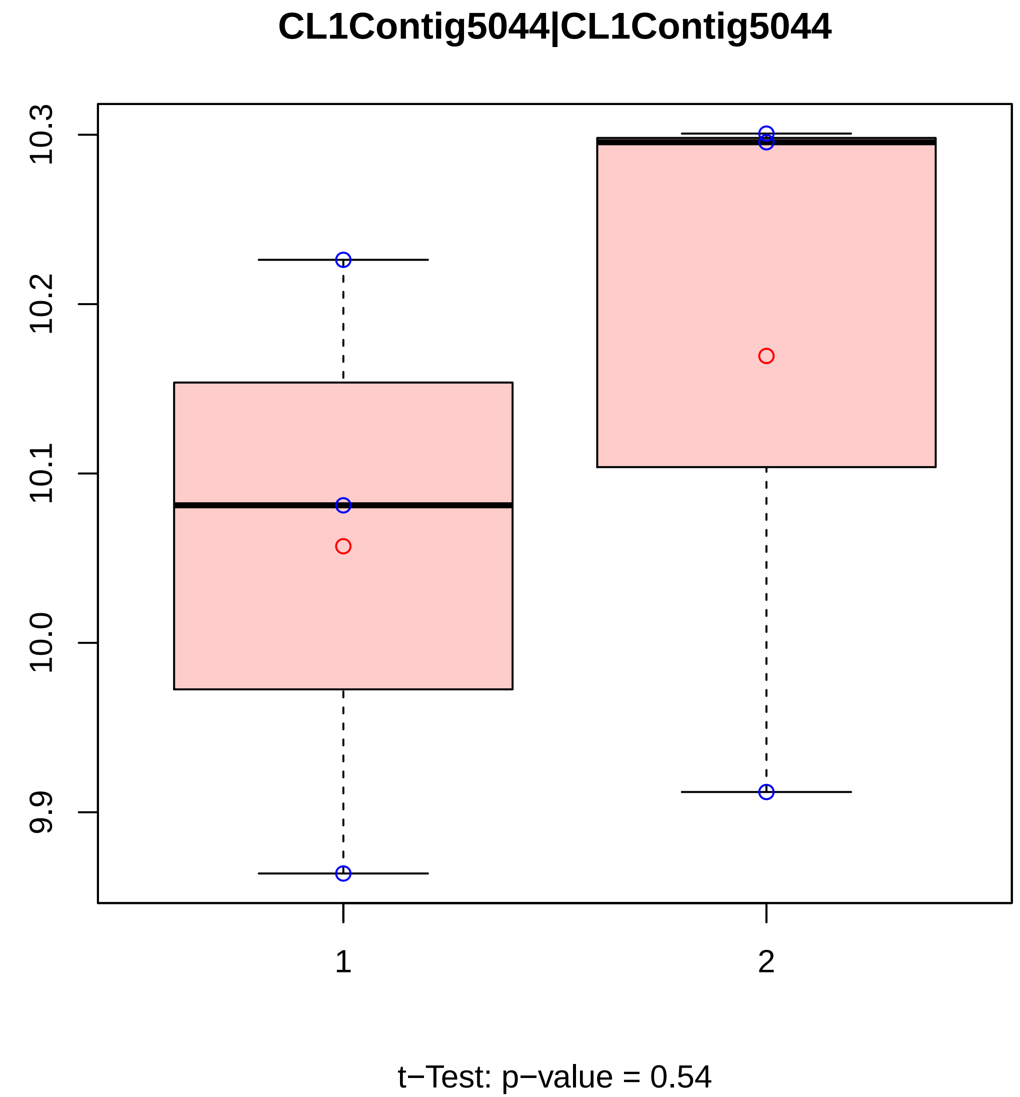
CL1Contig5044|CL1Contig5044
10.3
10.2
10.1
10.0
9.9
1
2
t−Test: p−value = 0.54

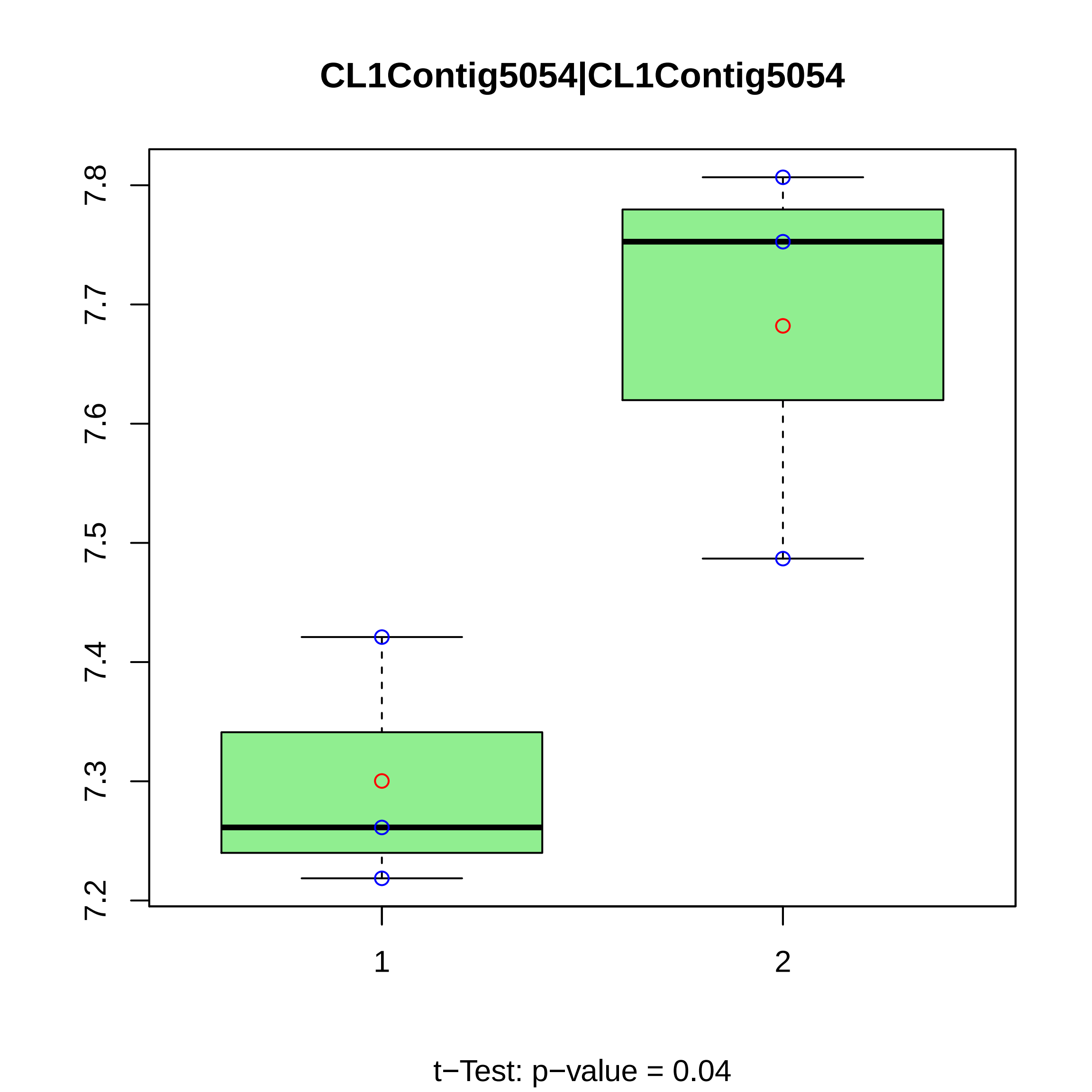
CL1Contig5054|CL1Contig5054
7.2
7.3
7.4
7.5
7.6
7.7
7.8
1
2
t−Test: p−value = 0.04

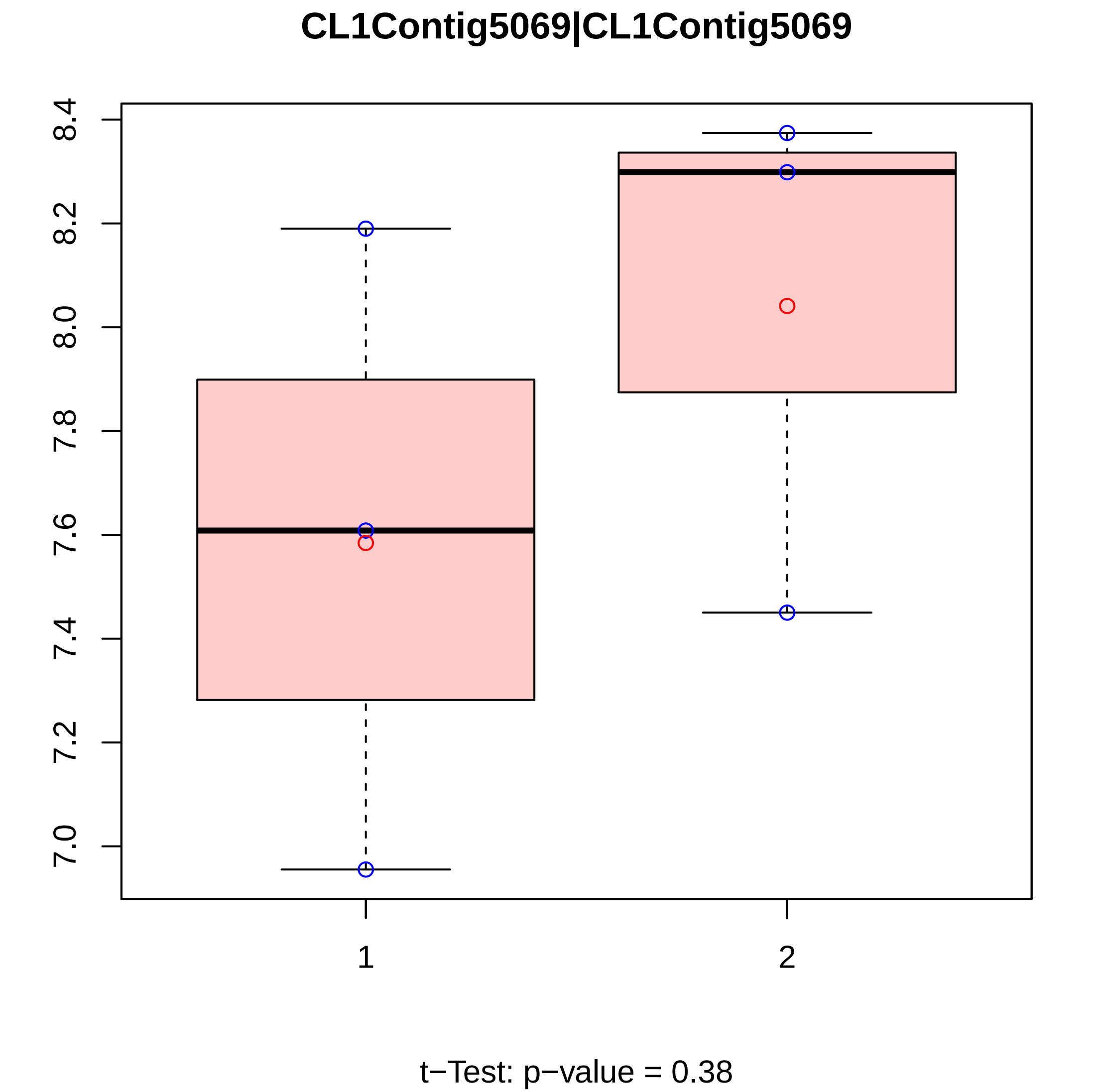

CL1Contig5069|CL1Contig5069
8.4
8.2
8.0
7.8
7.6
7.4
7.2
7.0
1
2
t−Test: p−value = 0.38

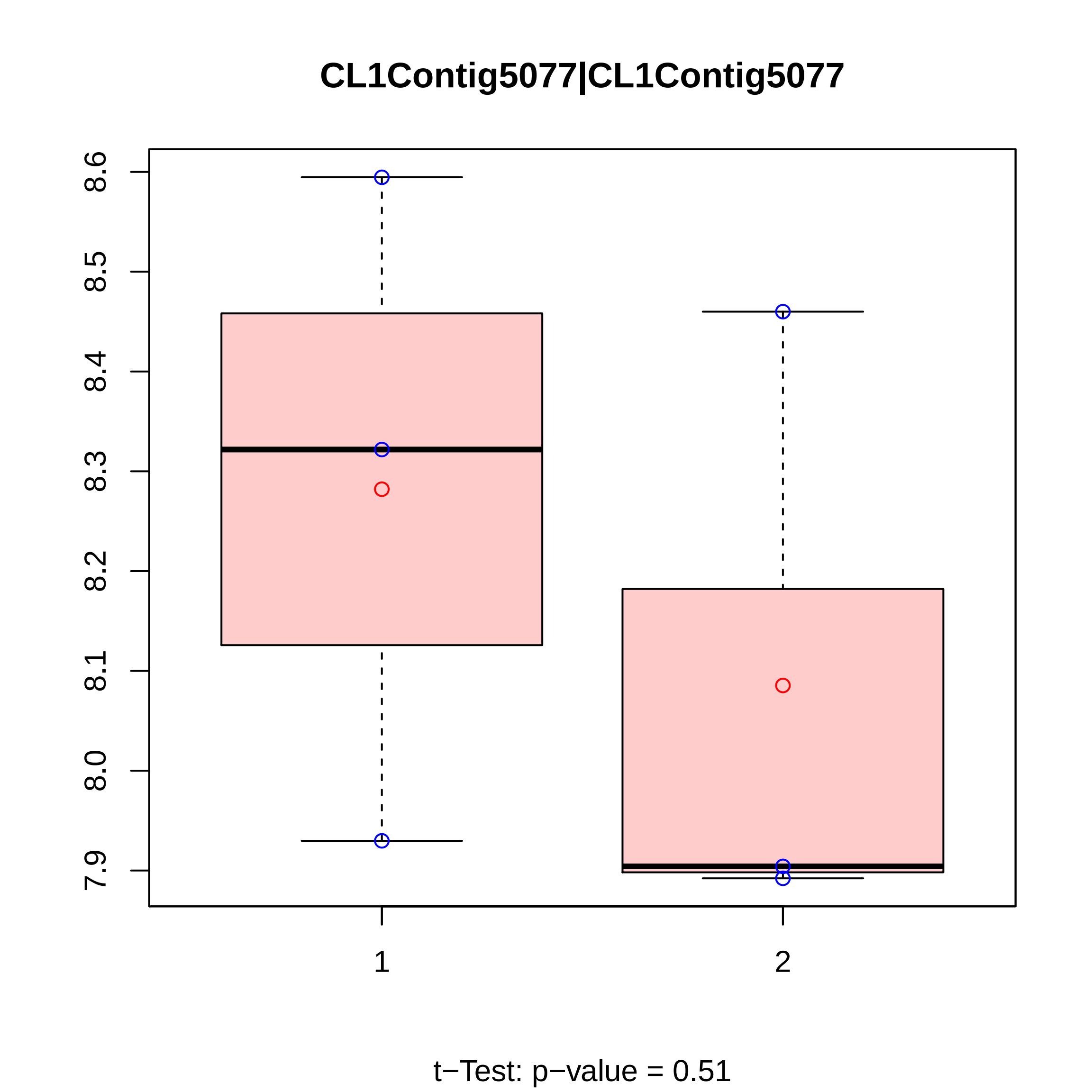
CL1Contig5077|CL1Contig5077
8.6
8.5
8.4
8.3
8.2
8.1
8.0
7.9
1
2
t−Test: p−value = 0.51

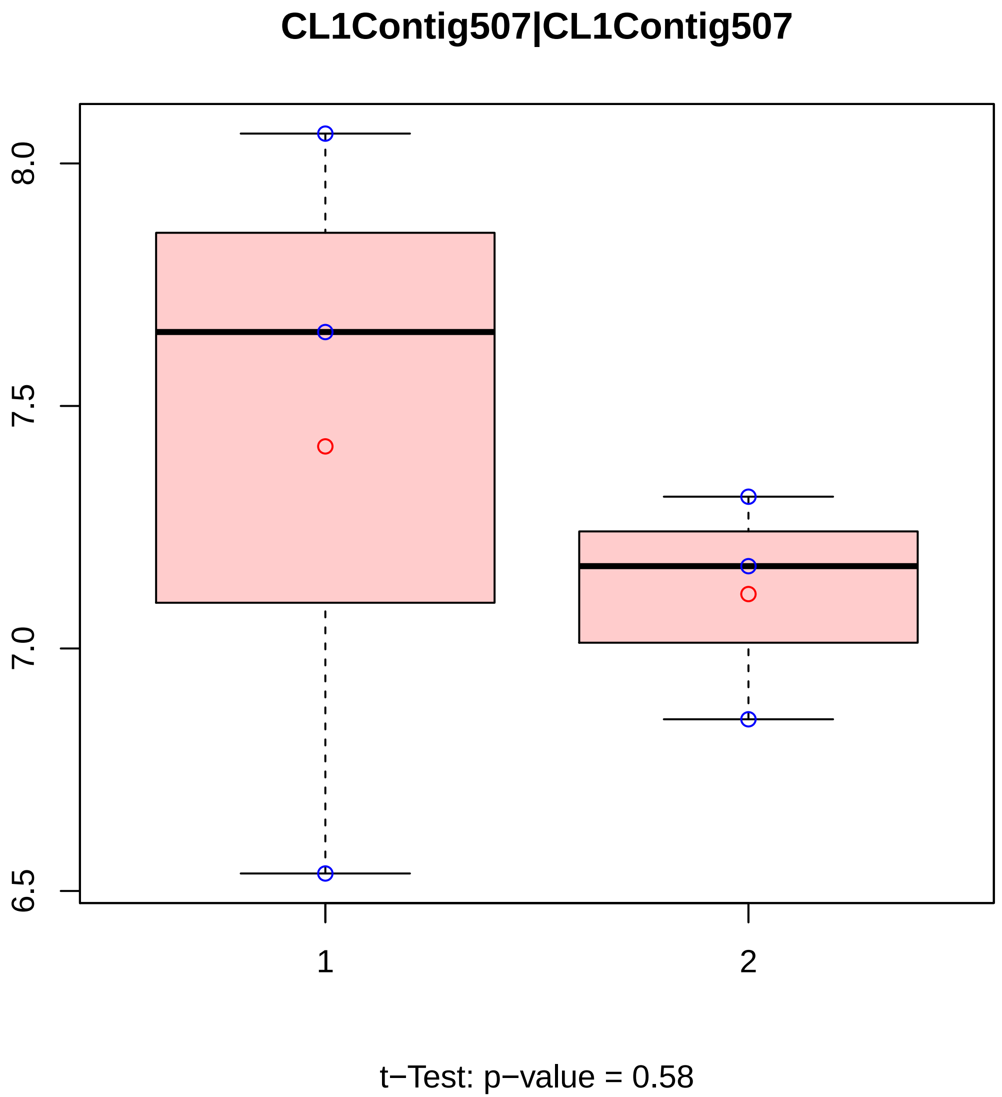
CL1Contig507|CL1Contig507
8.0
7.5
7.0
6.5
1
2
t−Test: p−value = 0.58

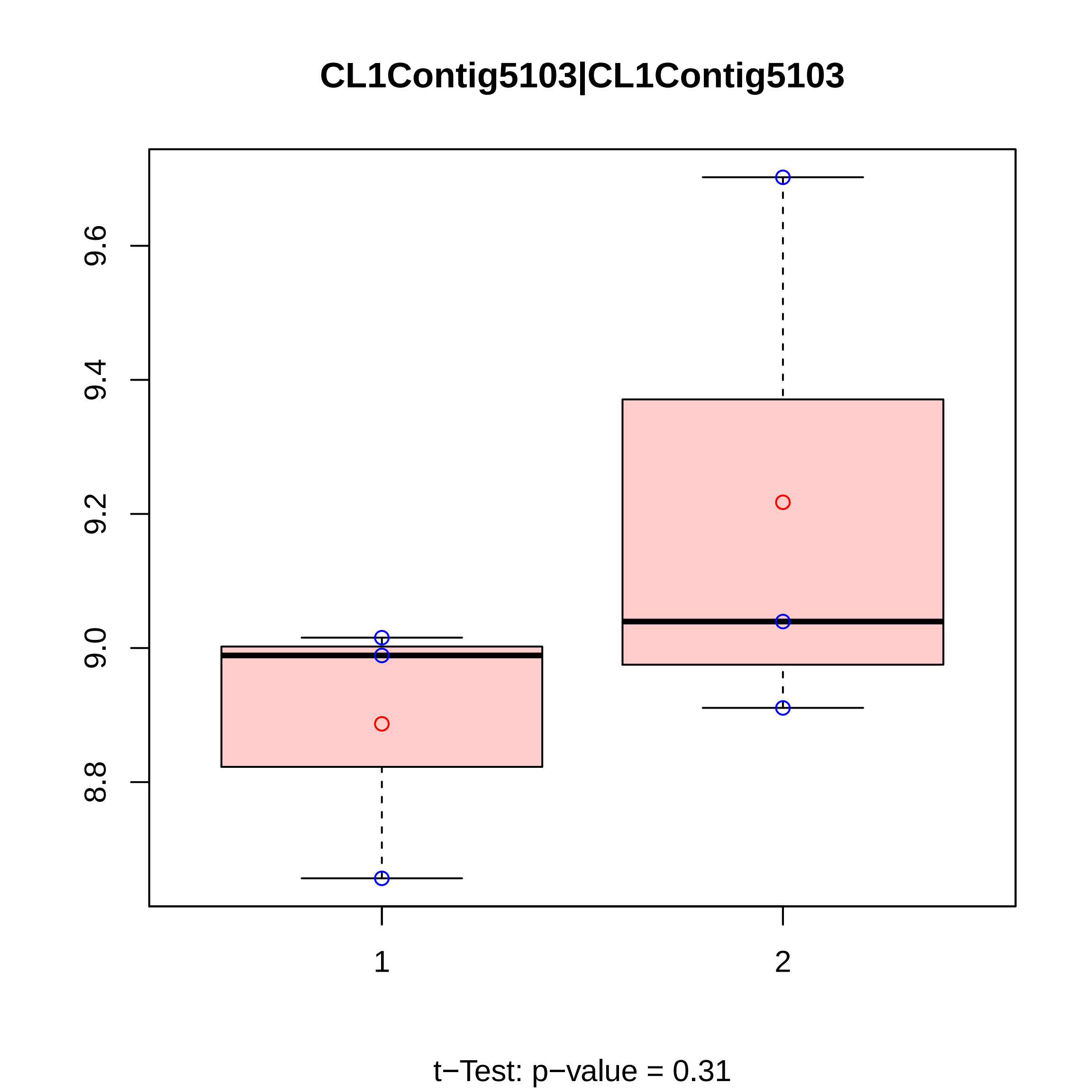
CL1Contig5103|CL1Contig5103
9.6
9.4
9.2
9.0
8.8
1
2
t−Test: p−value = 0.31

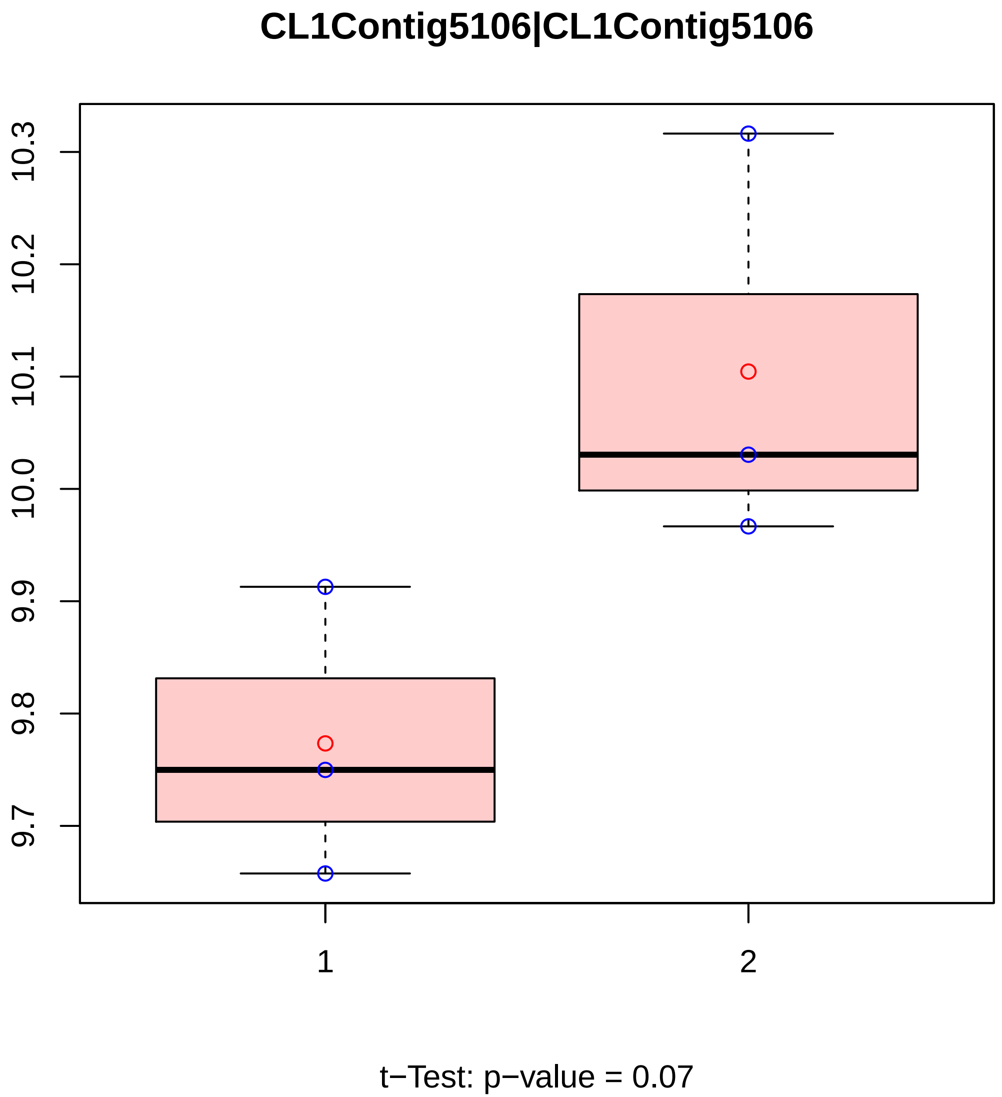
CL1Contig5106|CL1Contig5106
10.3
10.2
10.1
10.0
9.9
9.8
9.7
1
2
t−Test: p−value = 0.07

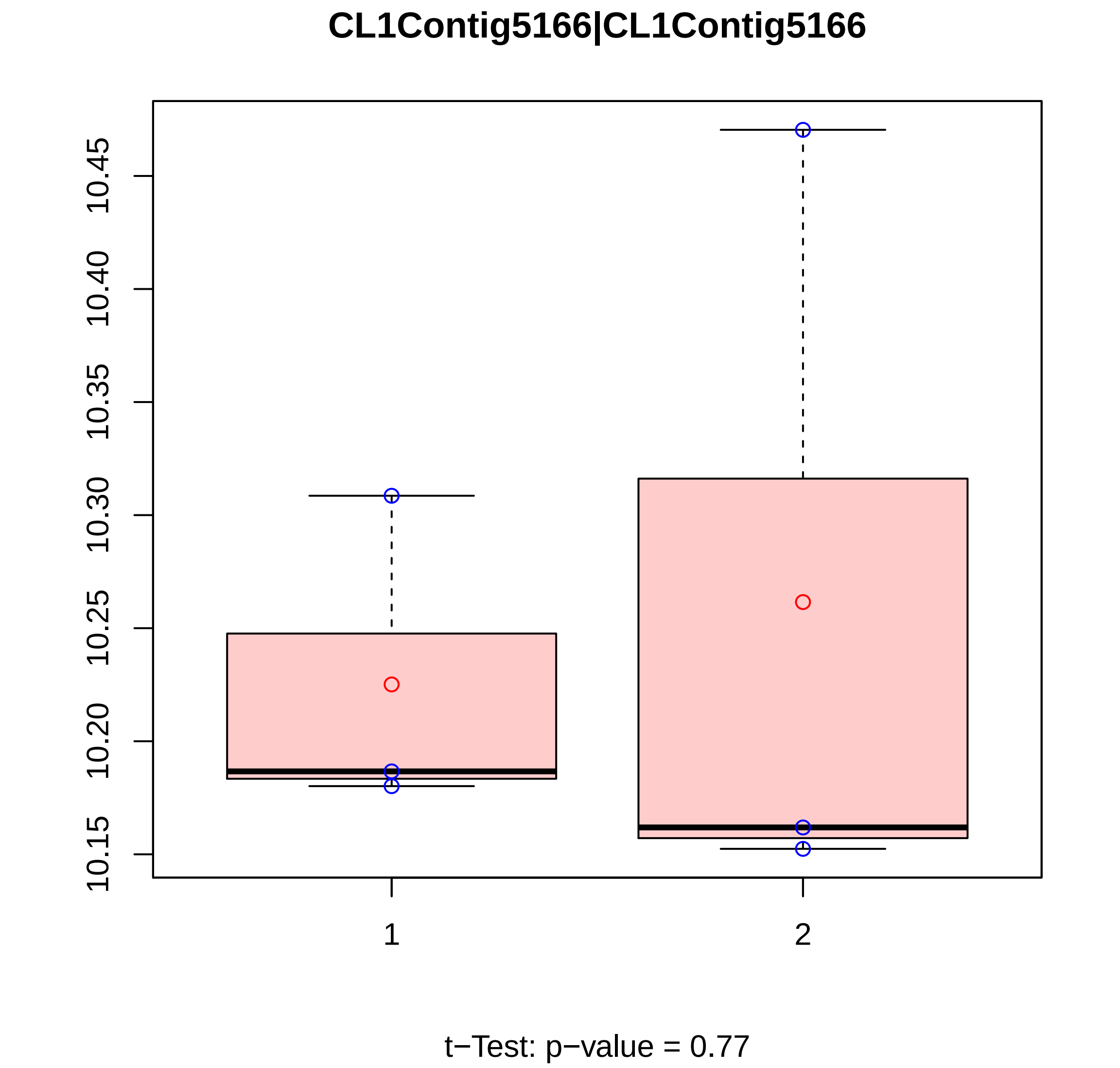
CL1Contig5166|CL1Contig5166
10.15
10.20
10.25
10.30
10.35
10.40
10.45
1
2
t−Test: p−value = 0.77

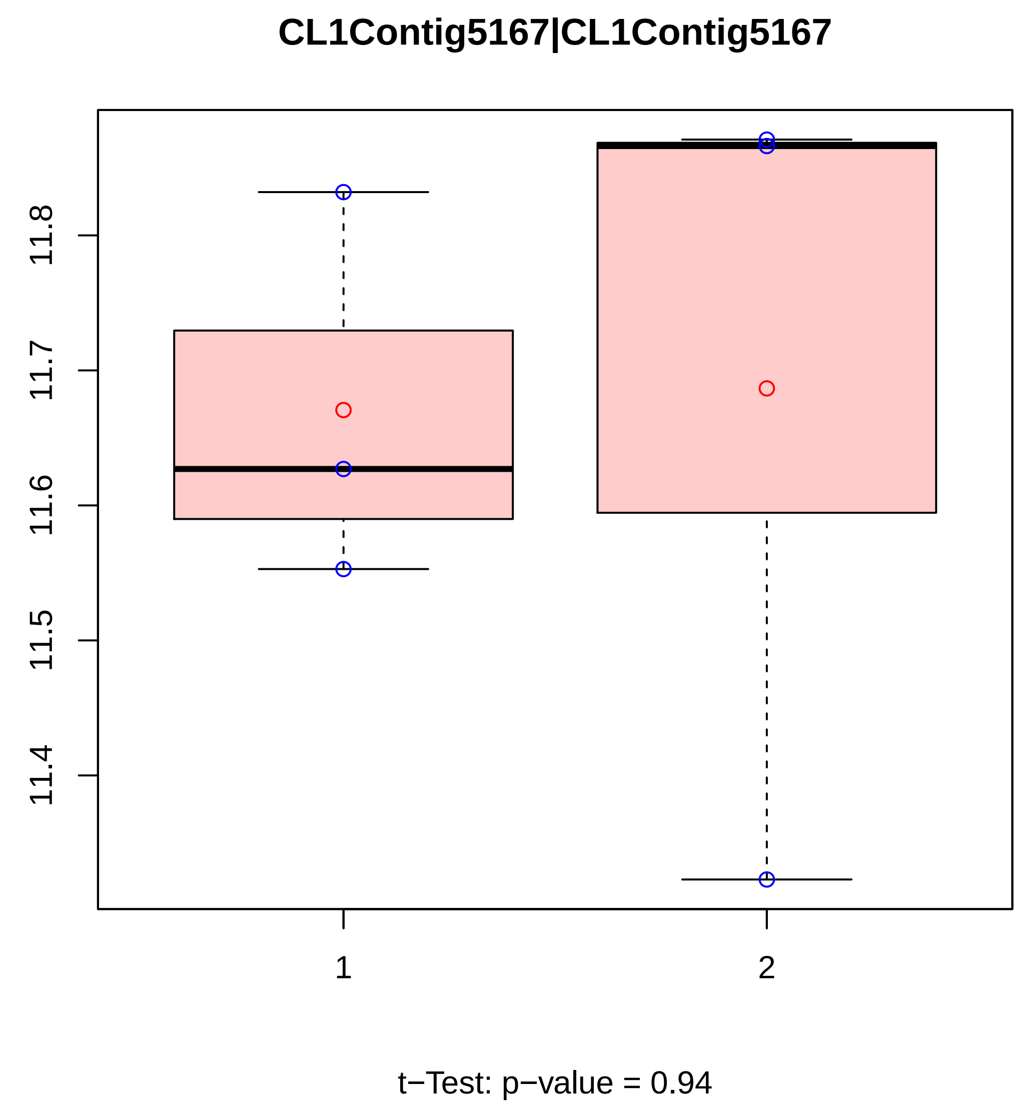
CL1Contig5167|CL1Contig5167
11.4
11.5
11.6
11.7
11.8
1
2
t−Test: p−value = 0.94

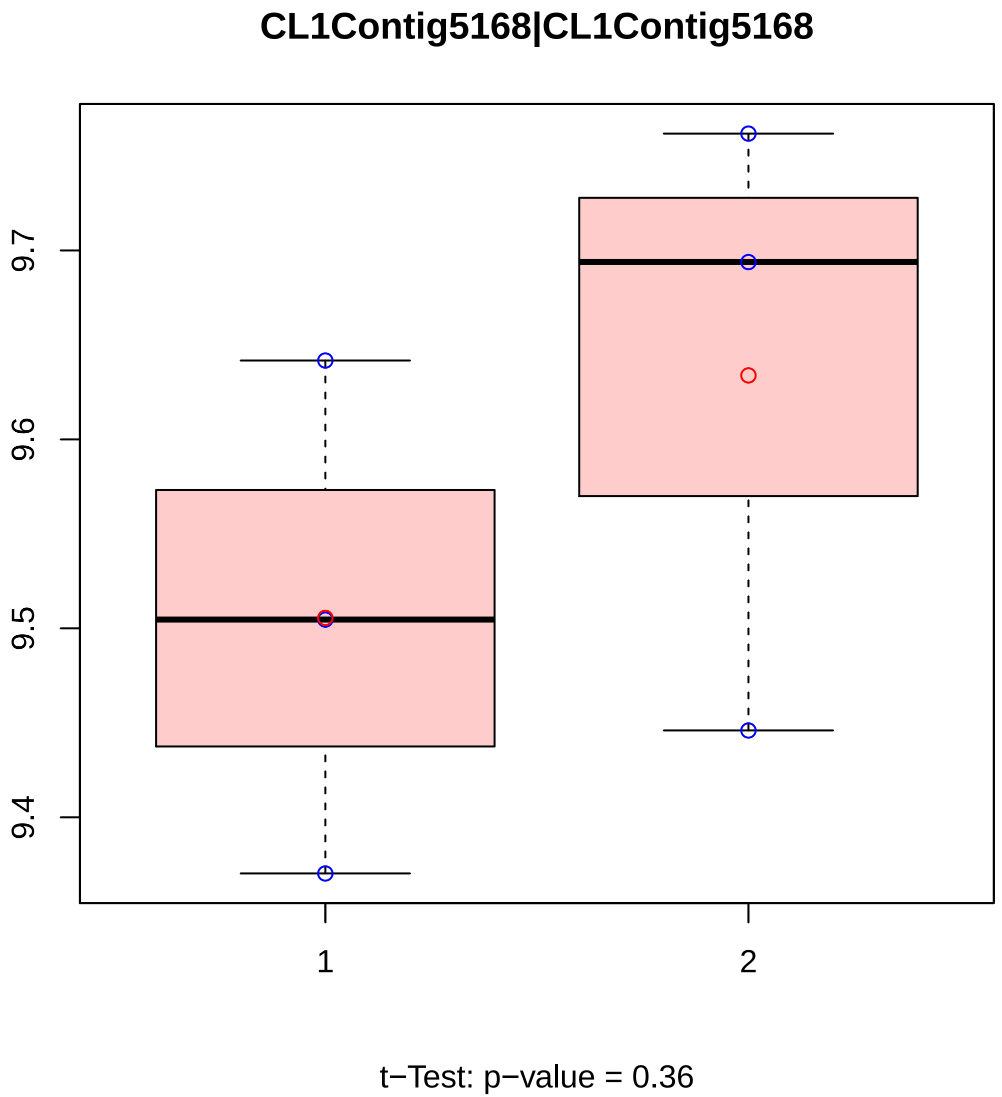
CL1Contig5168|CL1Contig5168
9.4
9.5
9.6
9.7
1
2
t−Test: p−value = 0.36

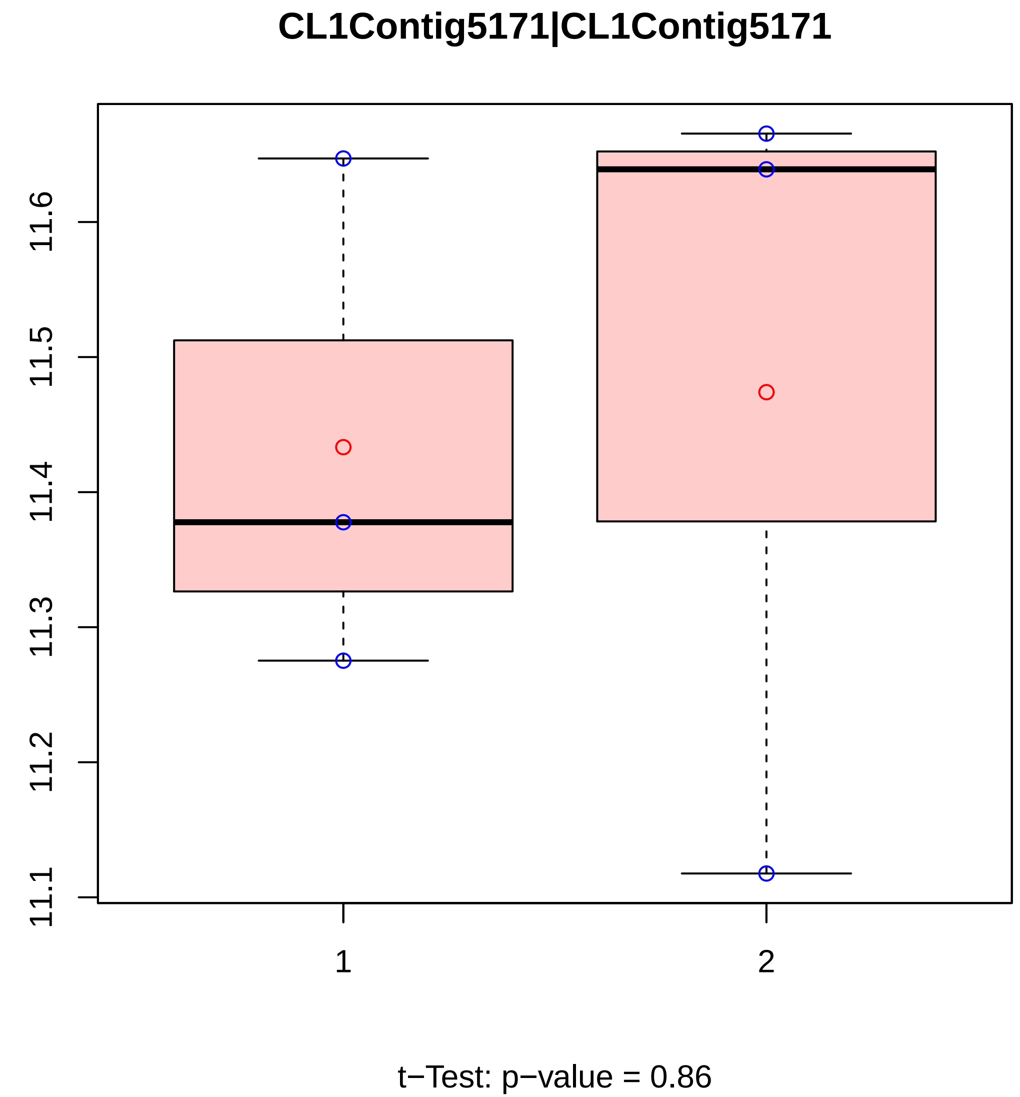
CL1Contig5171|CL1Contig5171
11.1
11.2
11.3
11.4
11.5
11.6
1
2
t−Test: p−value = 0.86

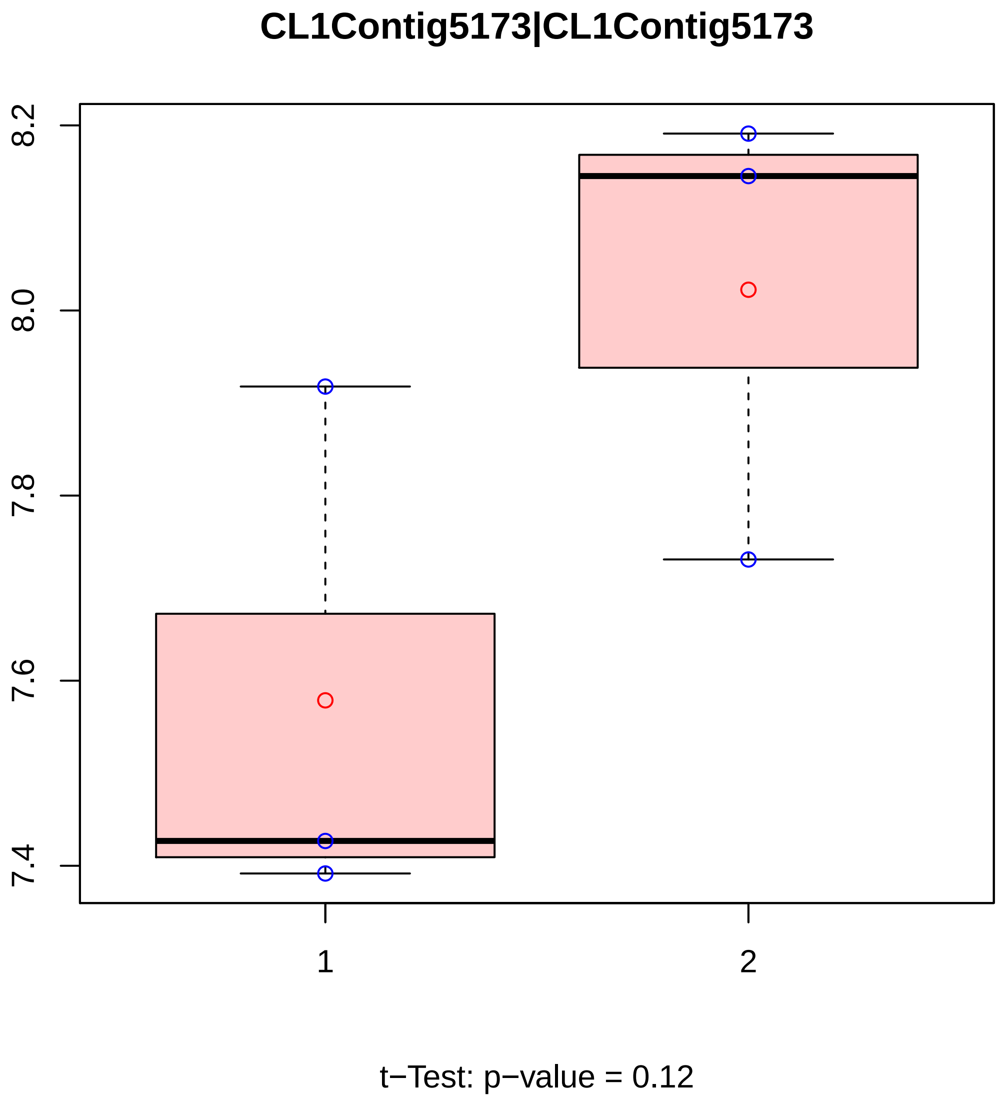
CL1Contig5173|CL1Contig5173
8.2
8.0
7.8
7.6
7.4
1
2
t−Test: p−value = 0.12

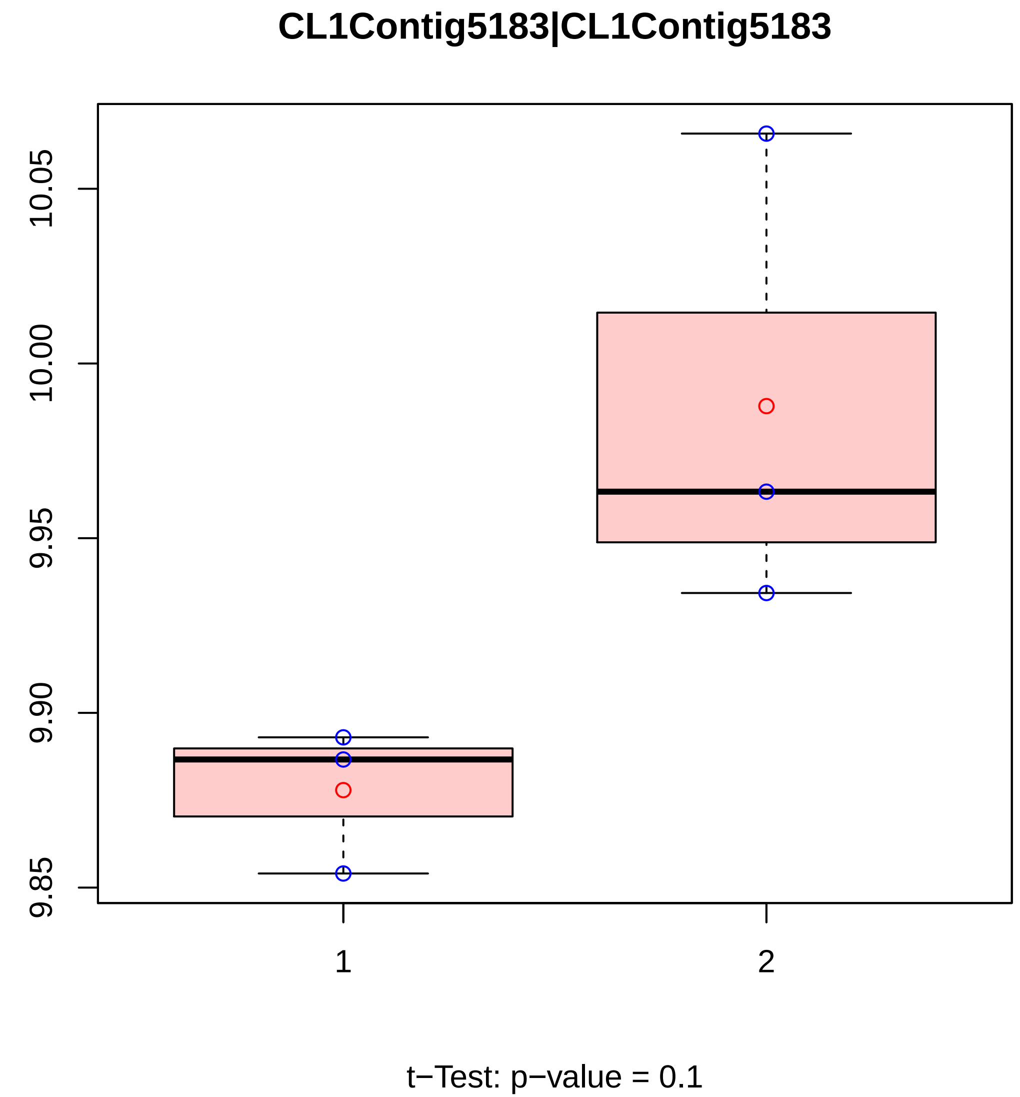

CL1Contig5183|CL1Contig5183
10.05
10.00
9.95
9.90
9.85
1
2
t−Test: p−value = 0.1

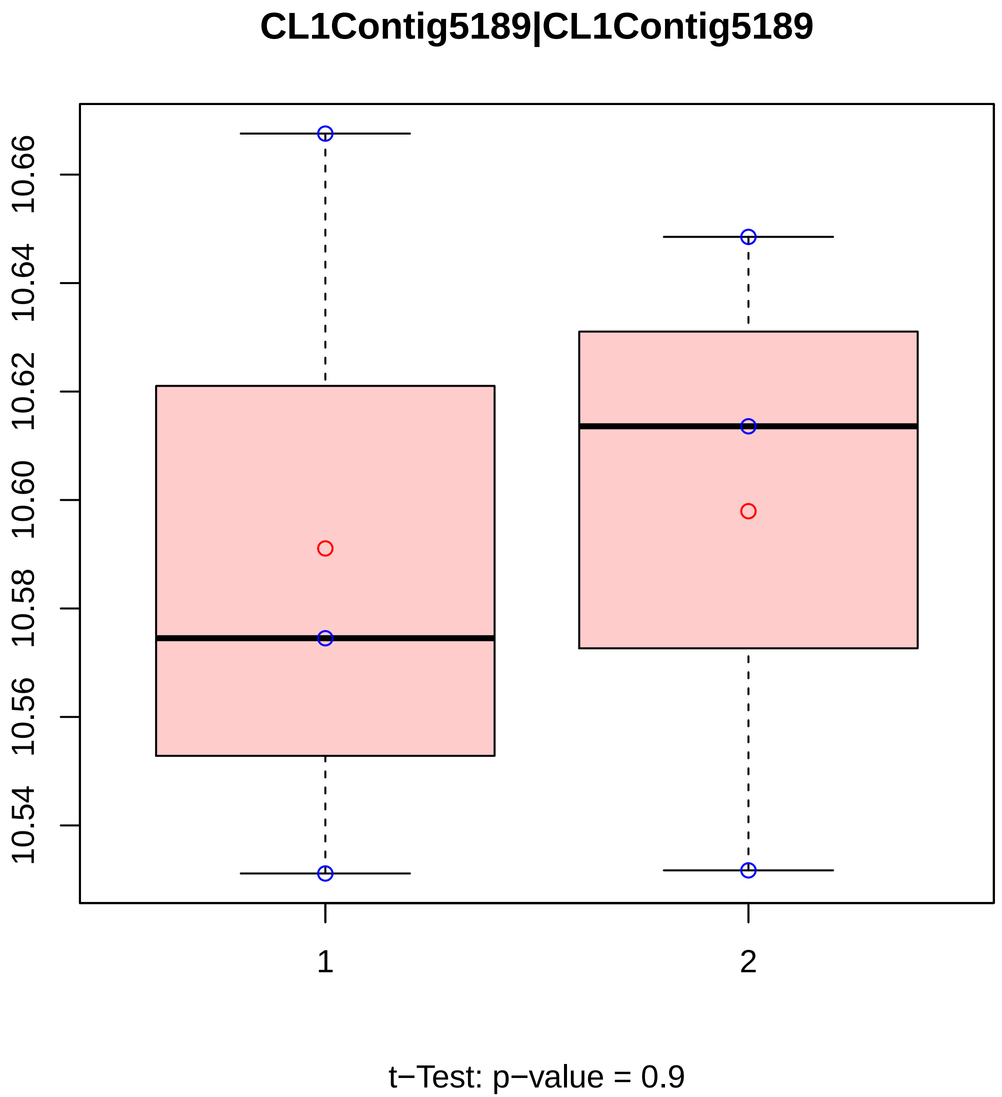
CL1Contig5189|CL1Contig5189
10.54
10.56
10.58
10.60
10.62
10.64
10.66
1
2
t−Test: p−value = 0.9

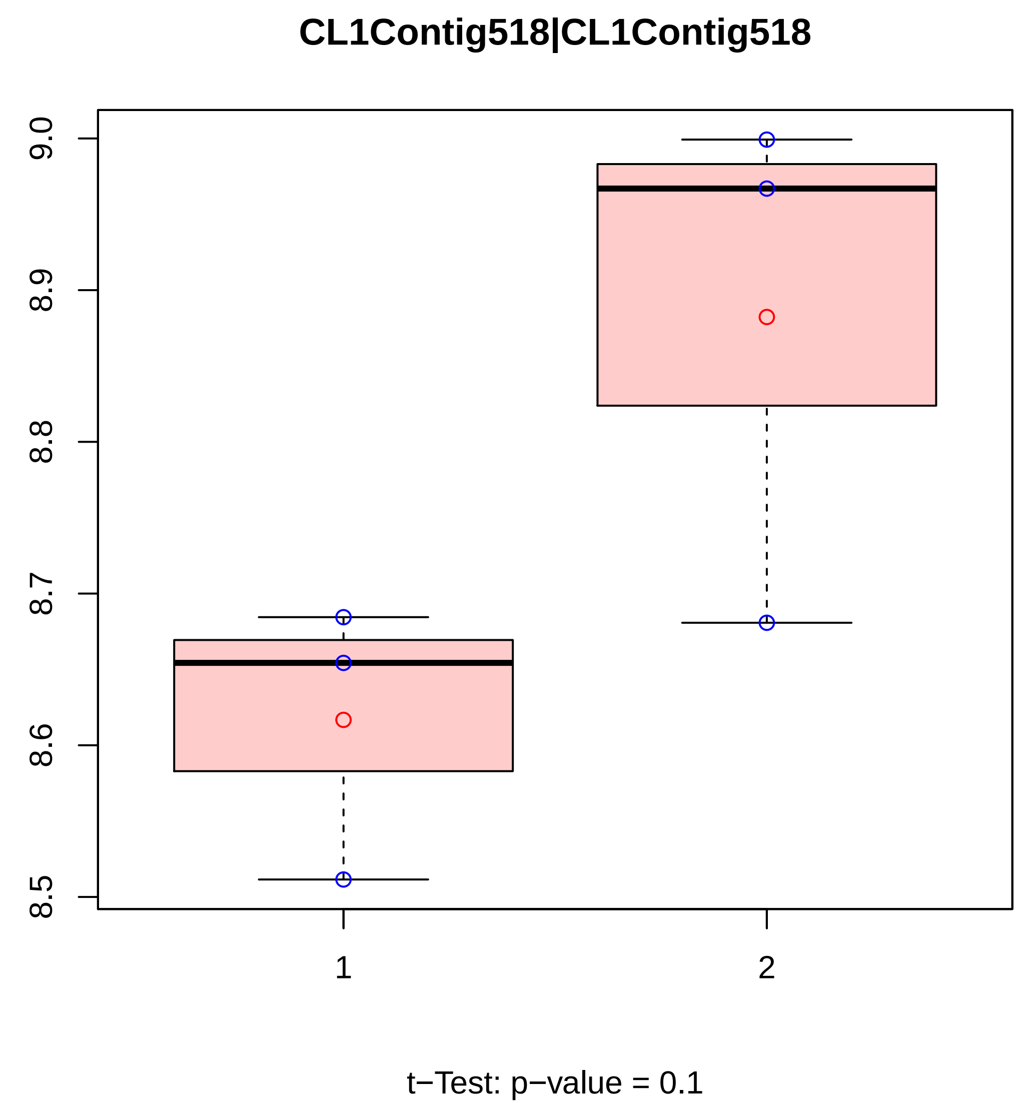
CL1Contig518|CL1Contig518
9.0
8.9
8.8
8.7
8.6
8.5
1
2
t−Test: p−value = 0.1

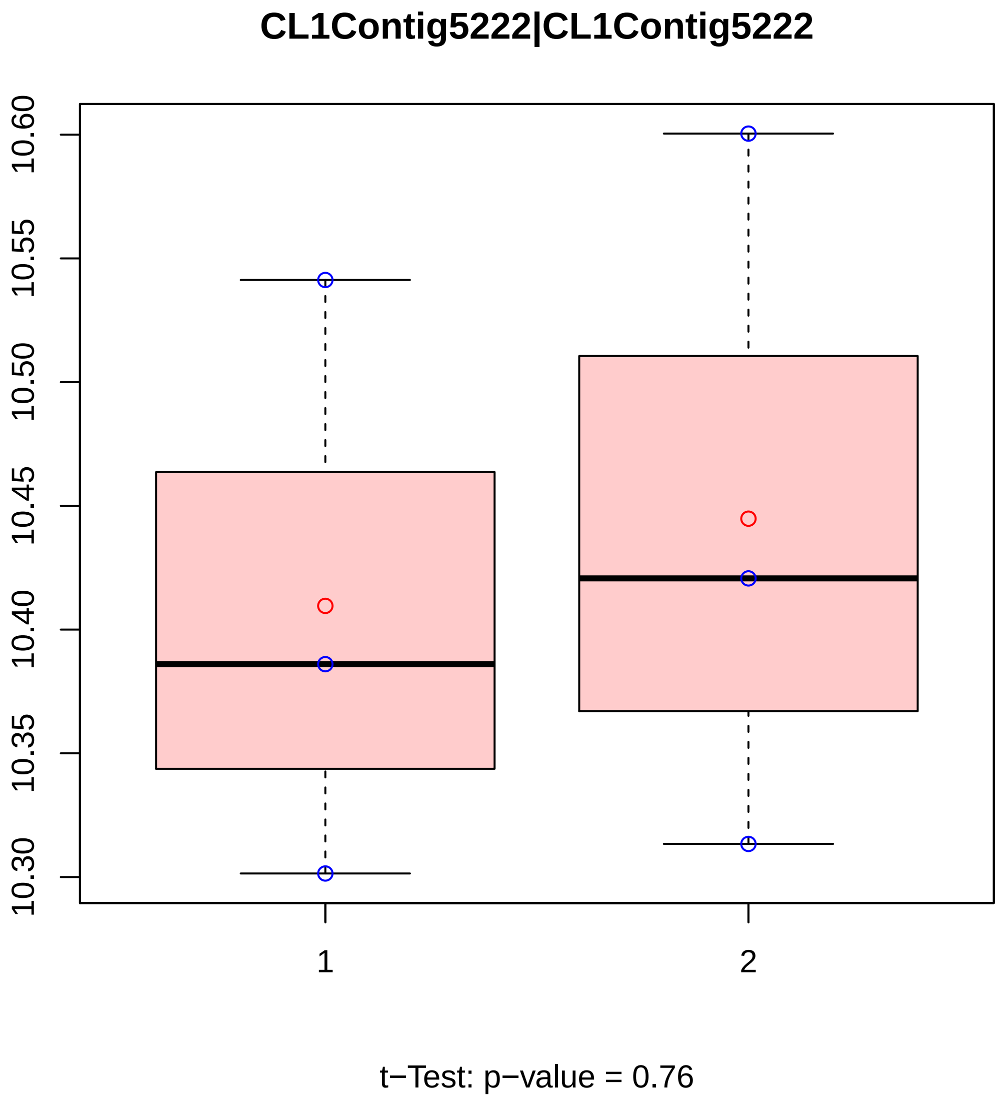

CL1Contig5222|CL1Contig5222
10.30
10.35
10.40
10.45
10.50
10.55
10.60
1
2
t−Test: p−value = 0.76

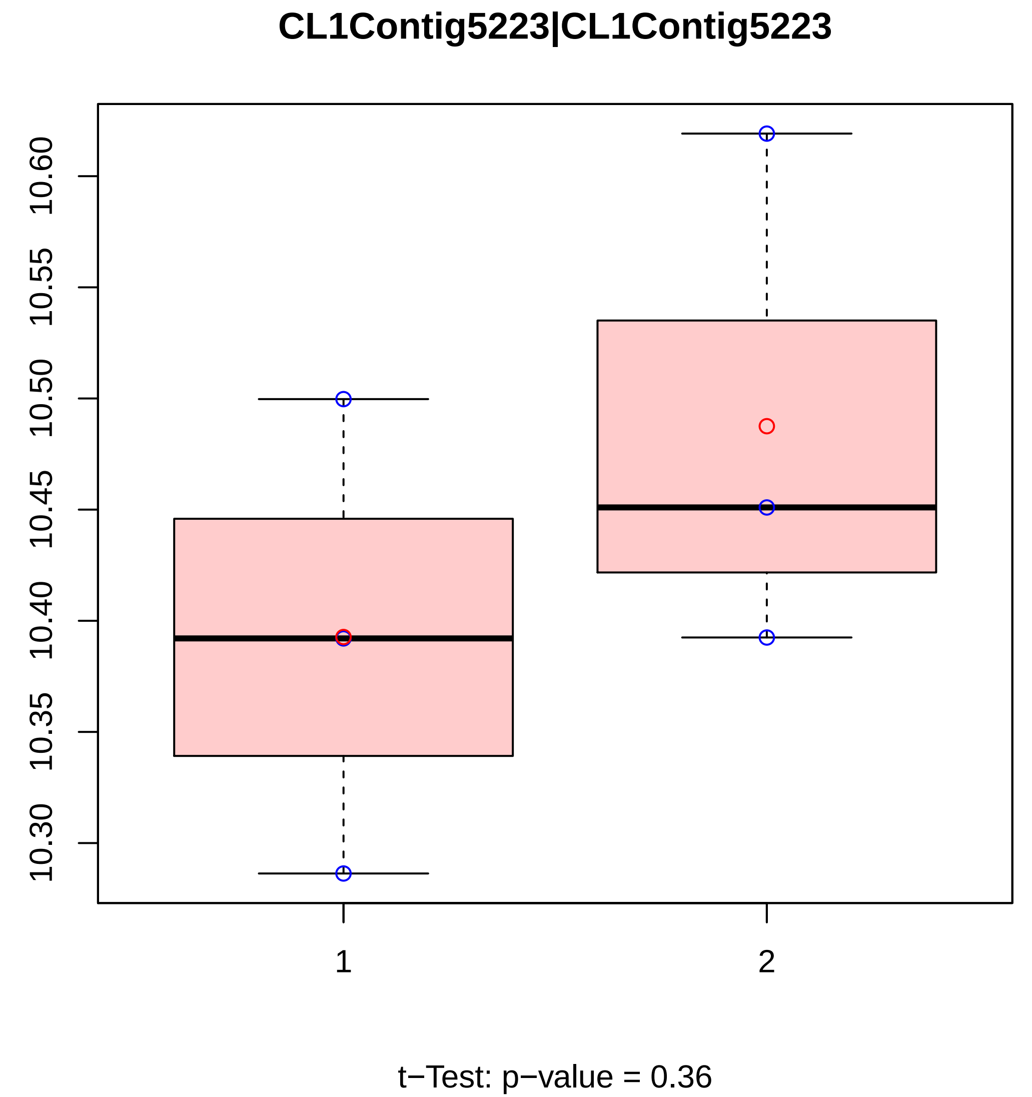
CL1Contig5223|CL1Contig5223
10.30
10.35
10.40
10.45
10.50
10.55
10.60
1
2
t−Test: p−value = 0.36

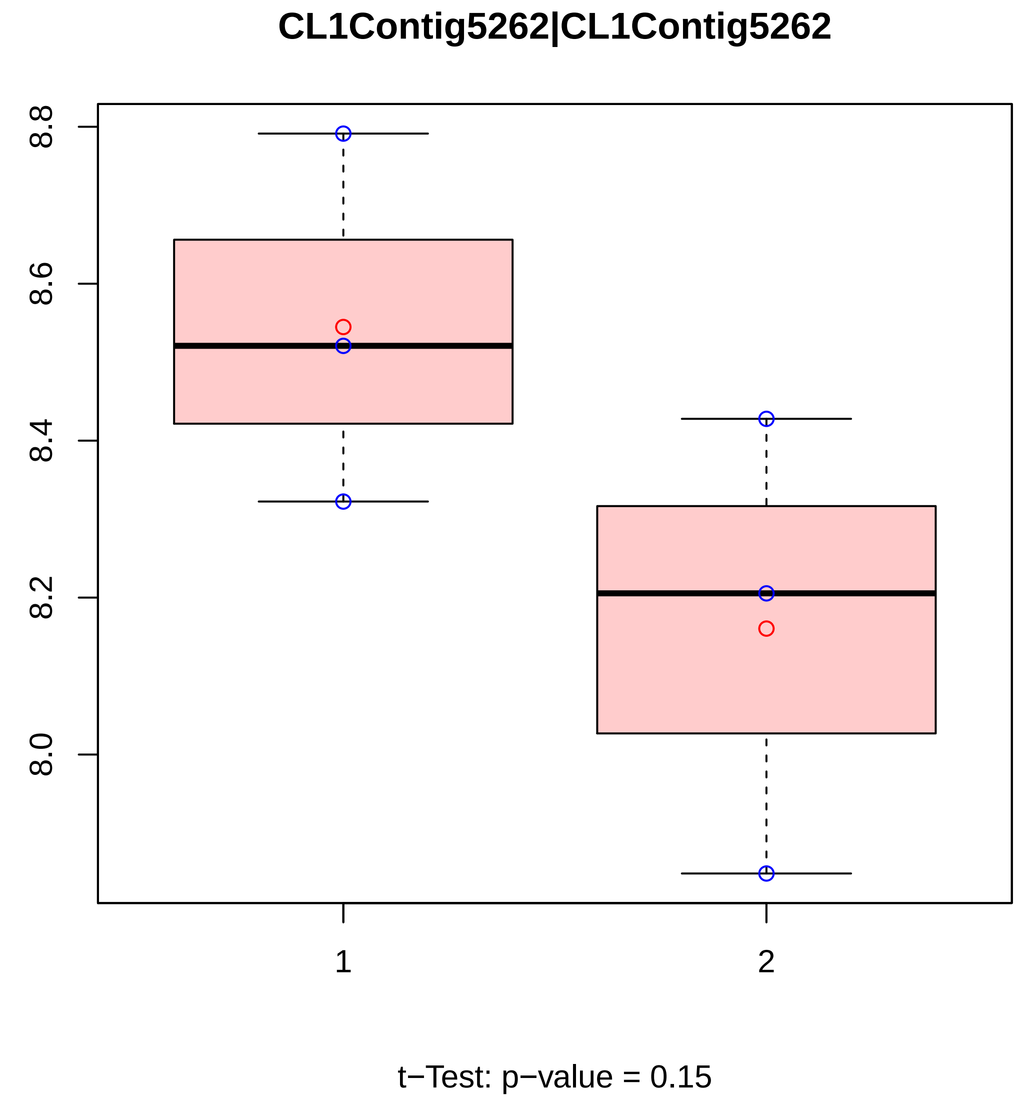

CL1Contig5262|CL1Contig5262
8.0
8.2
8.4
8.6
8.8
1
2
t−Test: p−value = 0.15

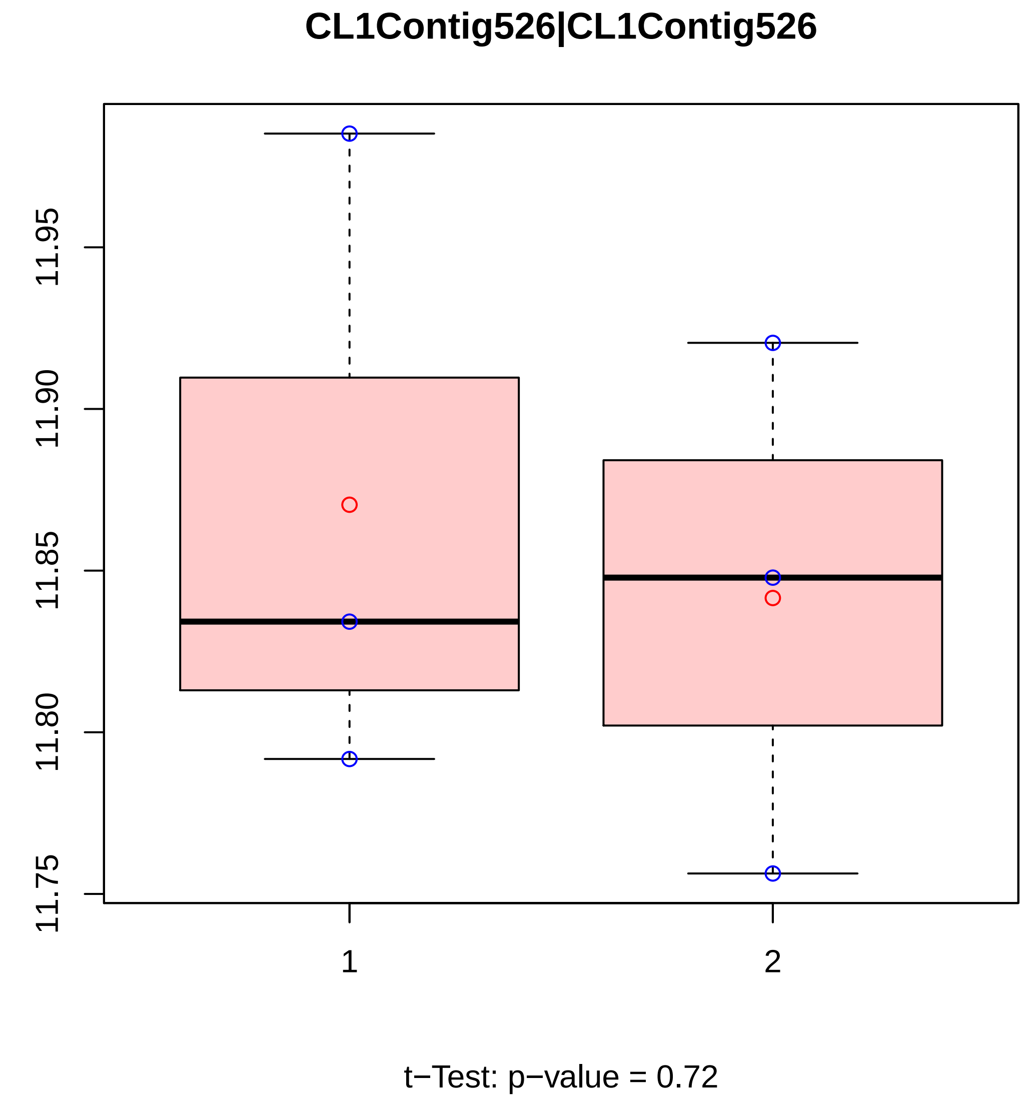

CL1Contig526|CL1Contig526
11.75
11.80
11.85
11.90
11.95
1
2
t−Test: p−value = 0.72

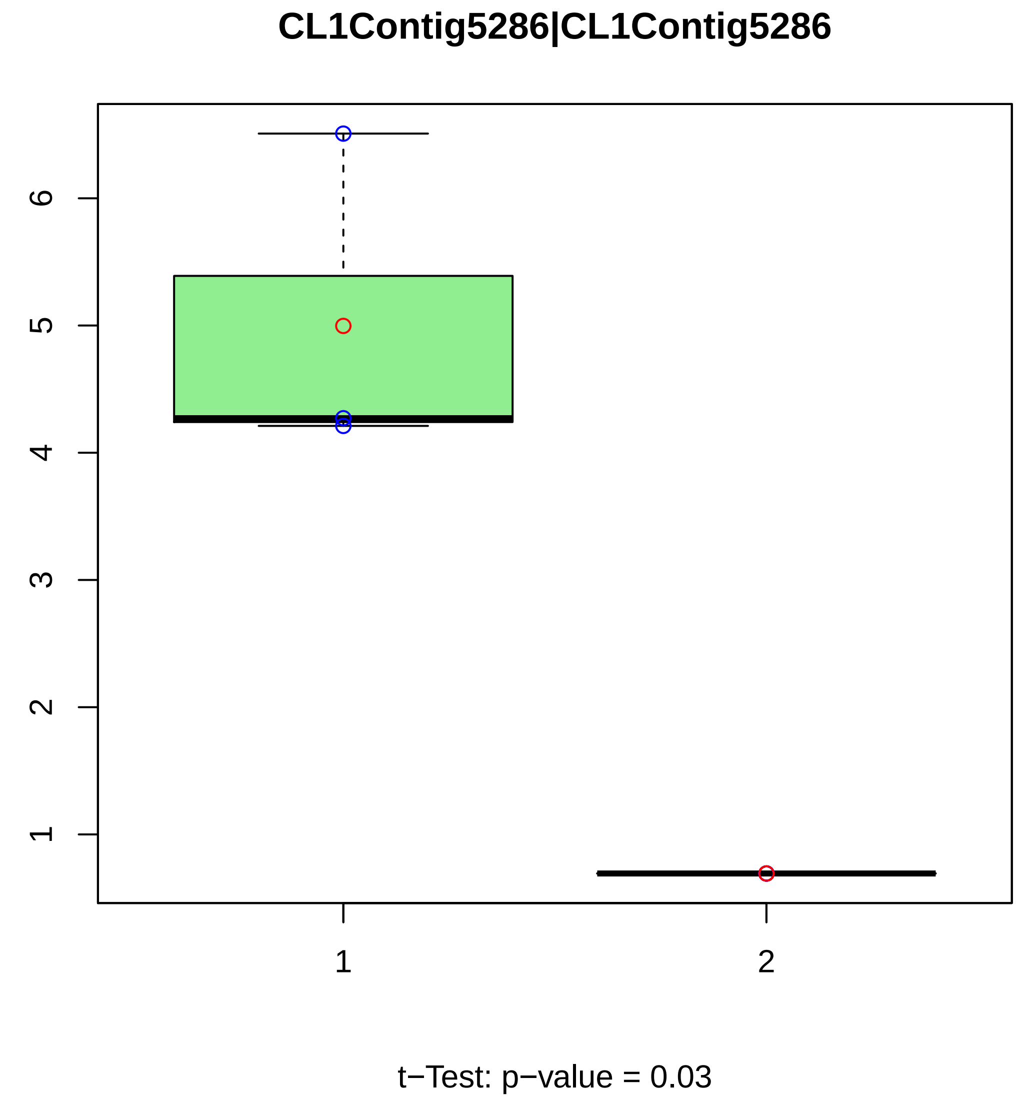

CL1Contig5286|CL1Contig5286
1
2
3
4
5
6
1
2
t−Test: p−value = 0.03

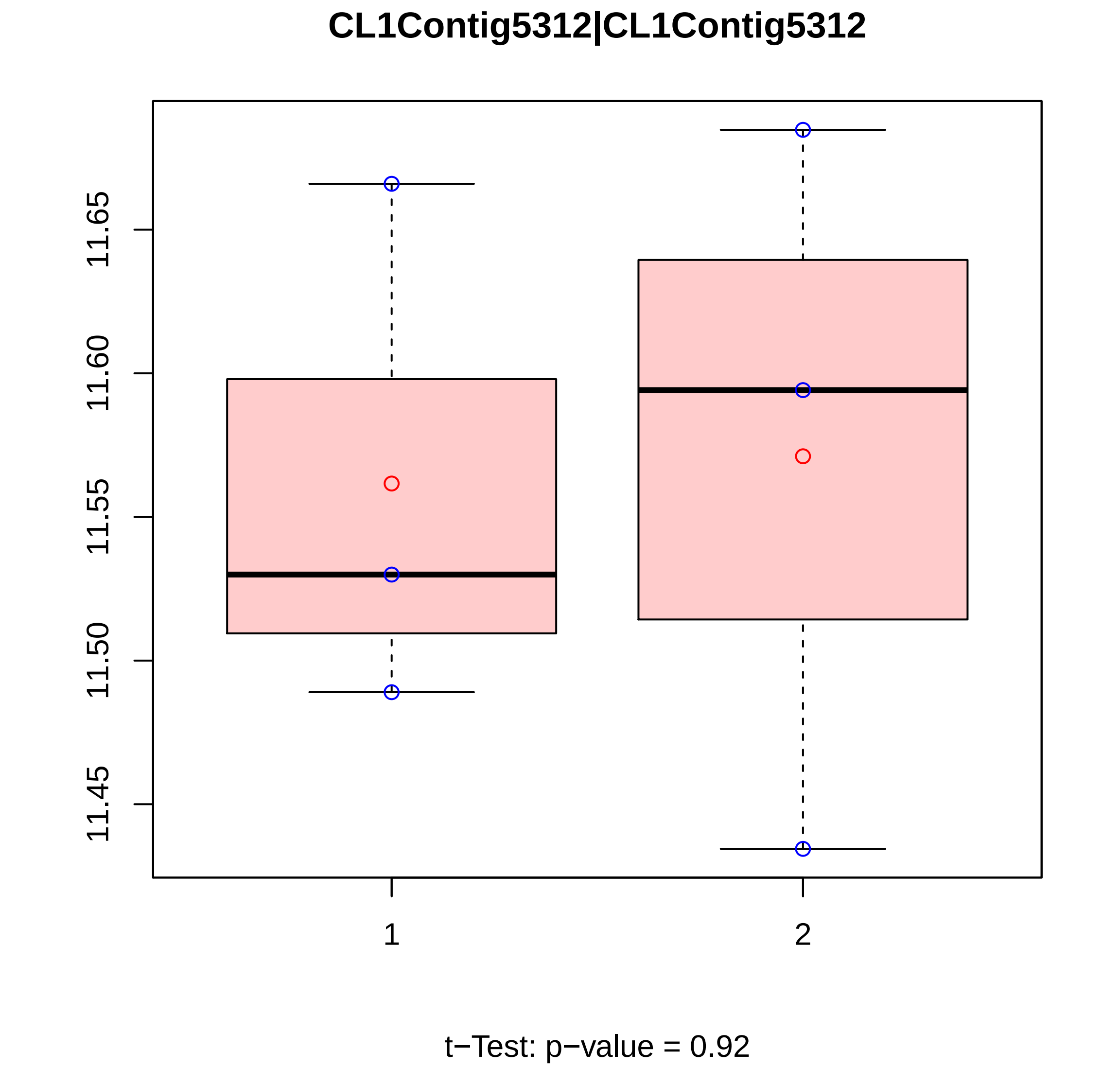

CL1Contig5312|CL1Contig5312
11.45
11.50
11.55
11.60
11.65
1
2
t−Test: p−value = 0.92

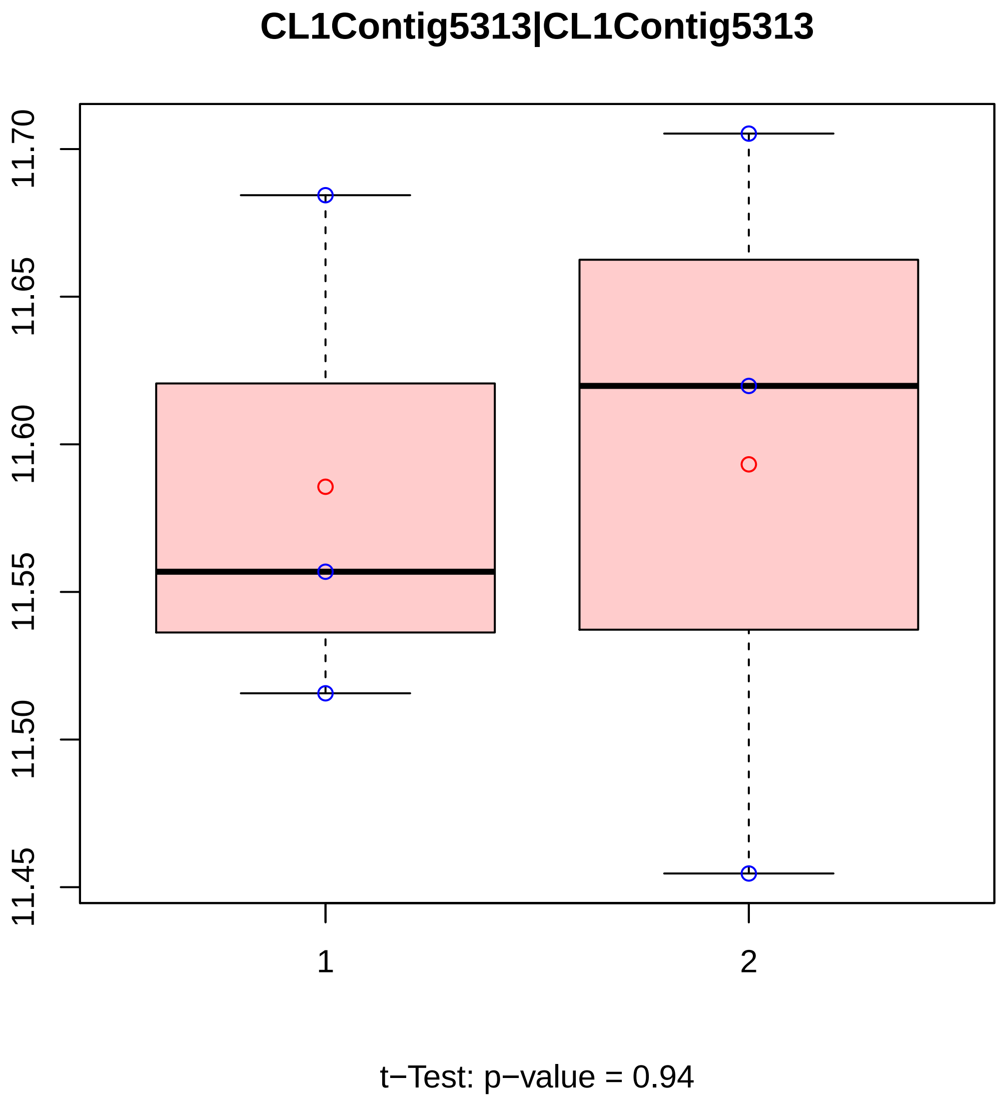

CL1Contig5313|CL1Contig5313
11.70
11.65
11.60
11.55
11.50
11.45
1
2
t−Test: p−value = 0.94

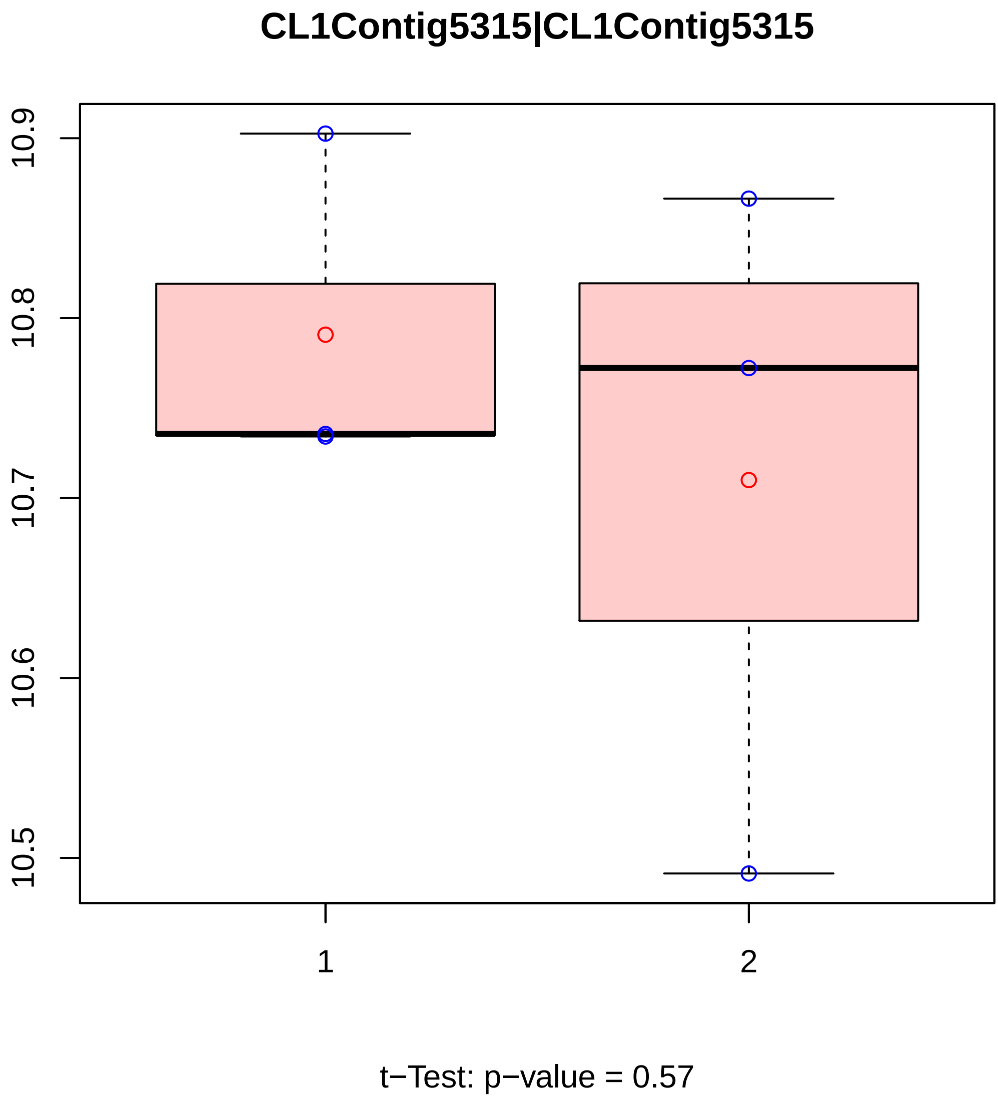

CL1Contig5315|CL1Contig5315
10.9
10.8
10.7
10.6
10.5
1
2
t−Test: p−value = 0.57

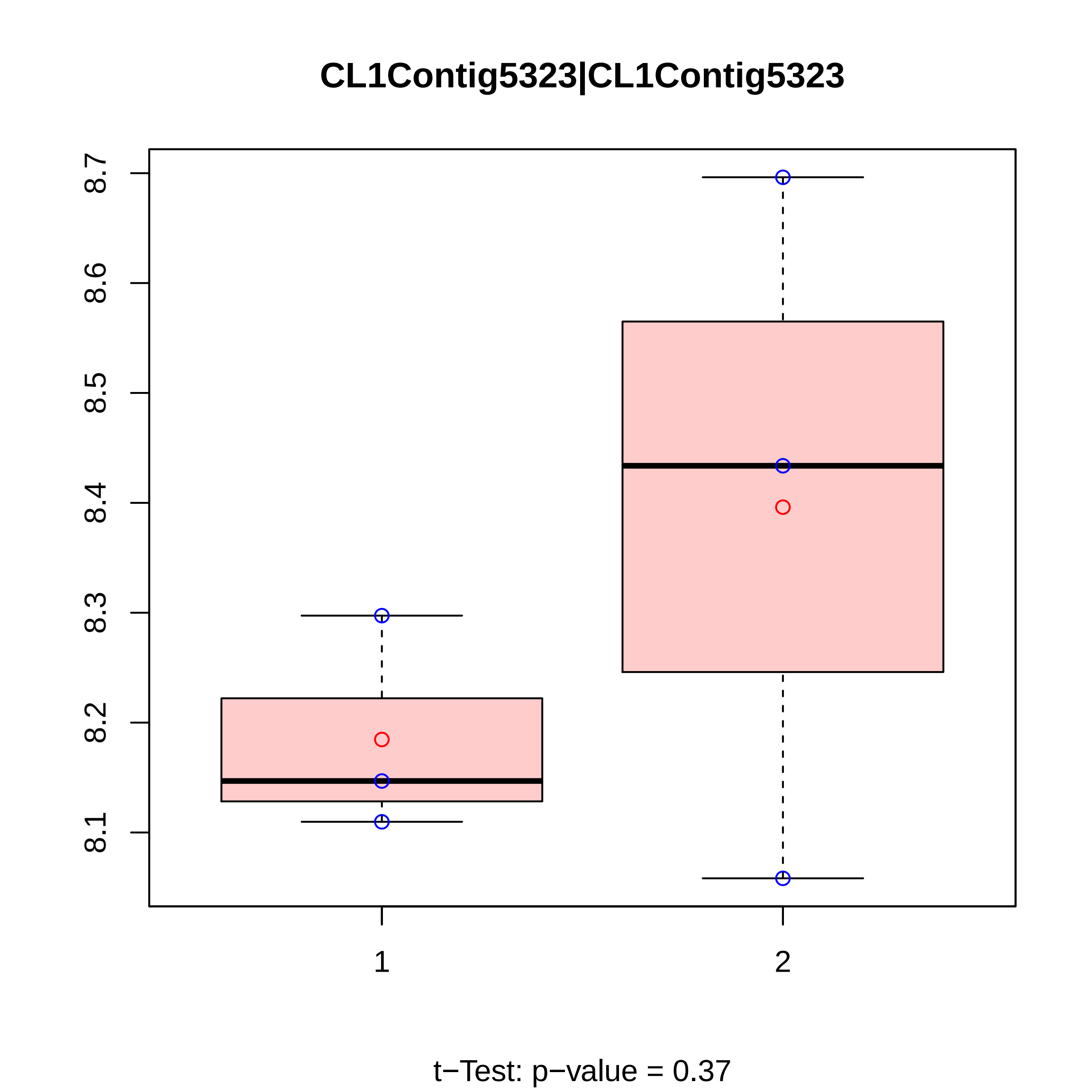
CL1Contig5323|CL1Contig5323
8.1
8.2
8.3
8.4
8.5
8.6
8.7
1
2
t−Test: p−value = 0.37

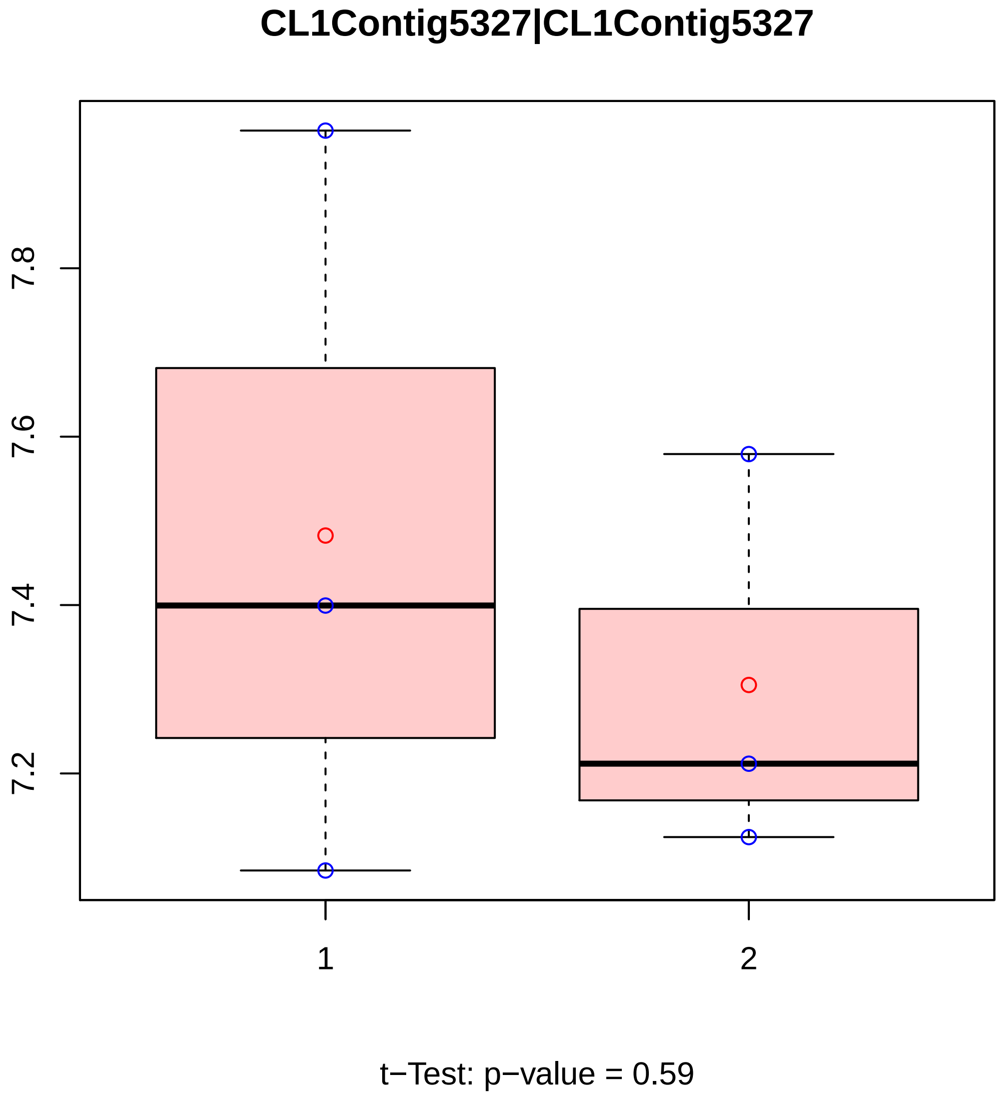

CL1Contig5327|CL1Contig5327
7.2
7.4
7.6
7.8
1
2
t−Test: p−value = 0.59

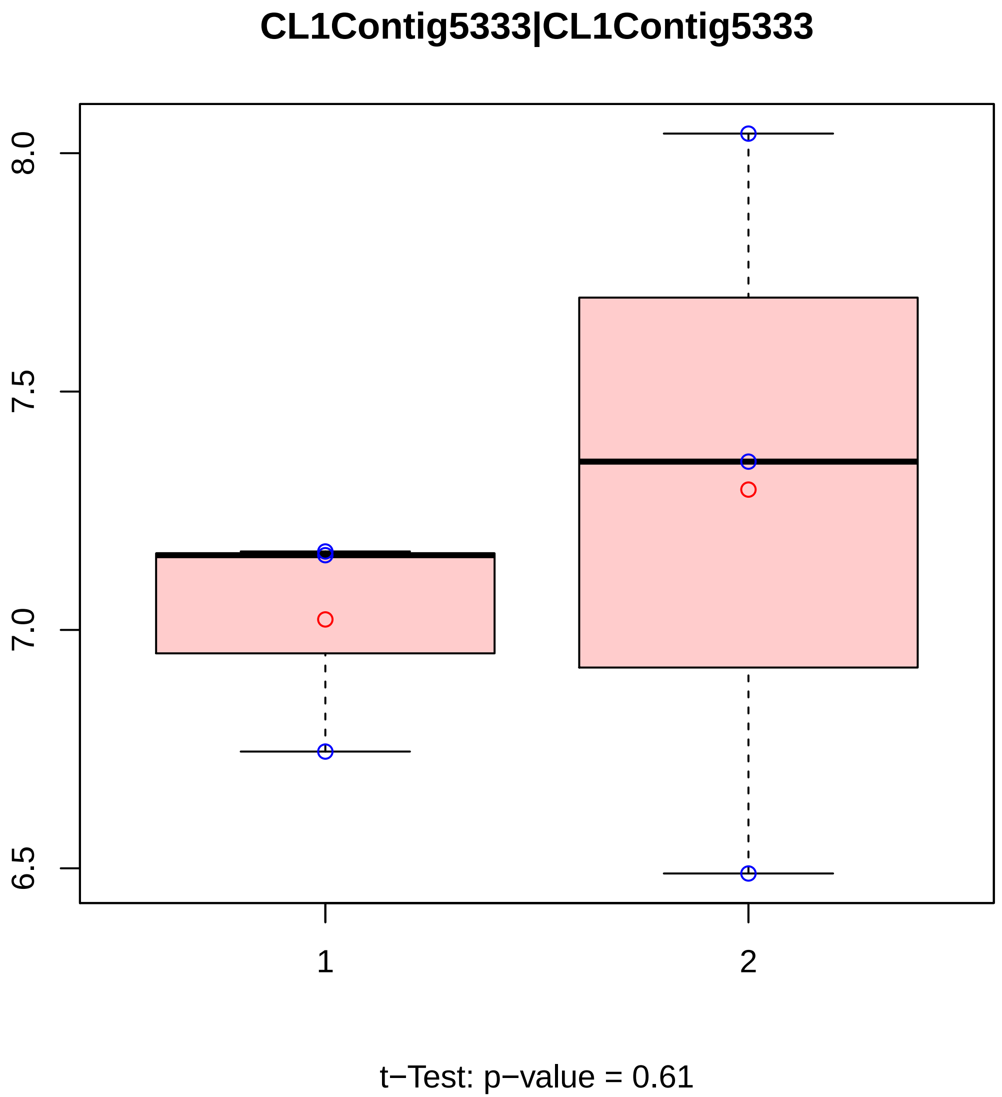
CL1Contig5333|CL1Contig5333
8.0
7.5
7.0
6.5
1
2
t−Test: p−value = 0.61

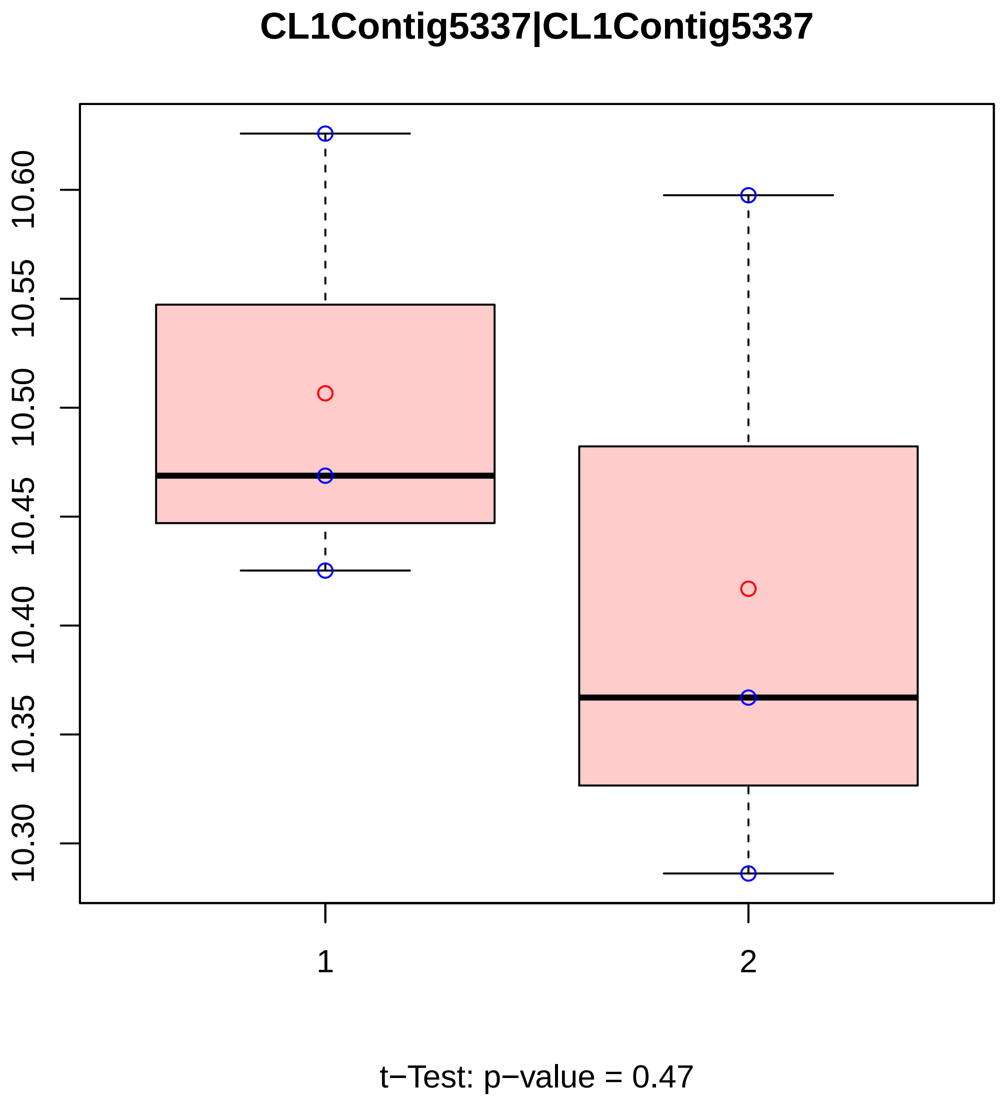

CL1Contig5337|CL1Contig5337
10.30
10.35
10.40
10.45
10.50
10.55
10.60
1
2
t−Test: p−value = 0.47

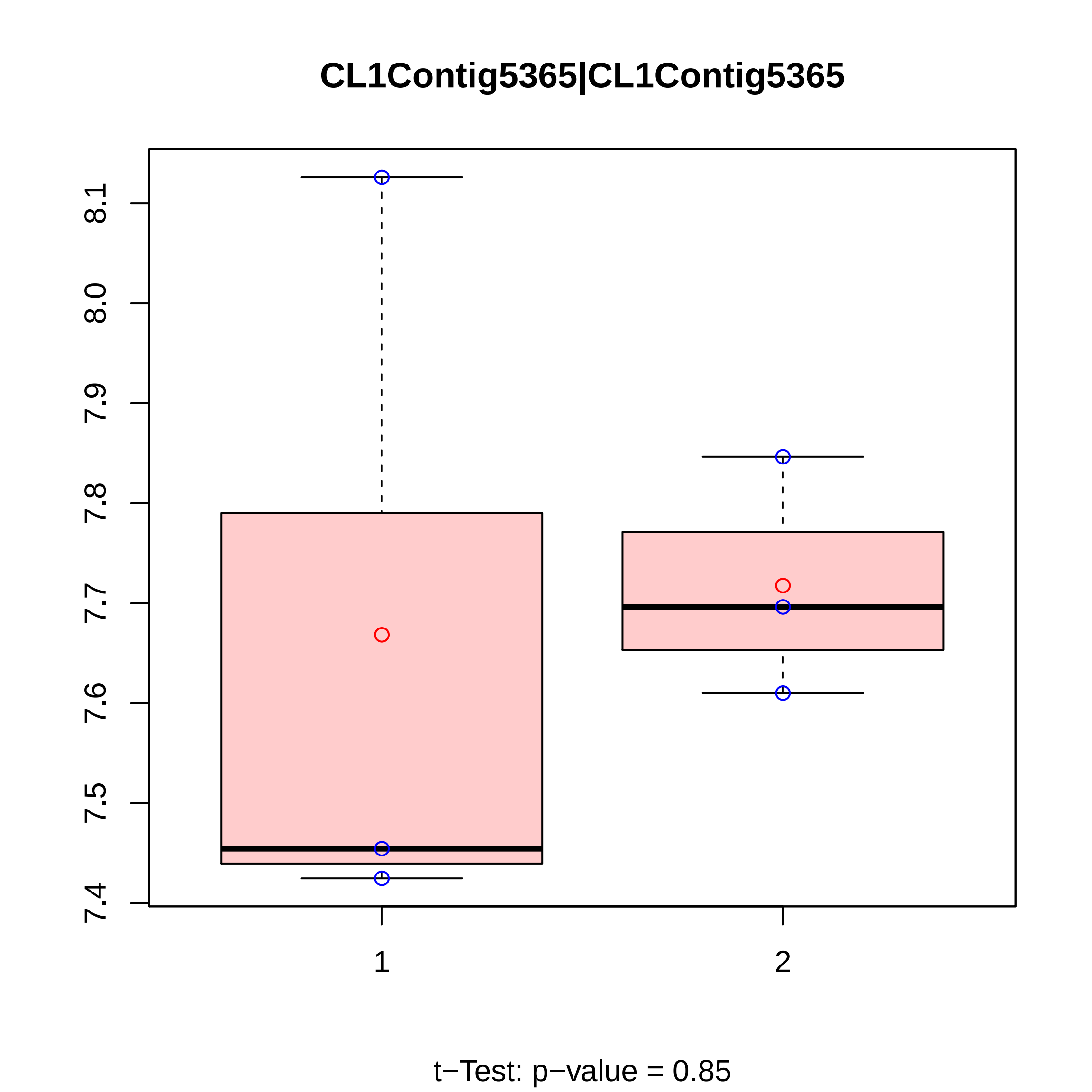

CL1Contig5365|CL1Contig5365
8.1
8.0
7.9
7.8
7.7
7.6
7.5
7.4
1
2
t−Test: p−value = 0.85

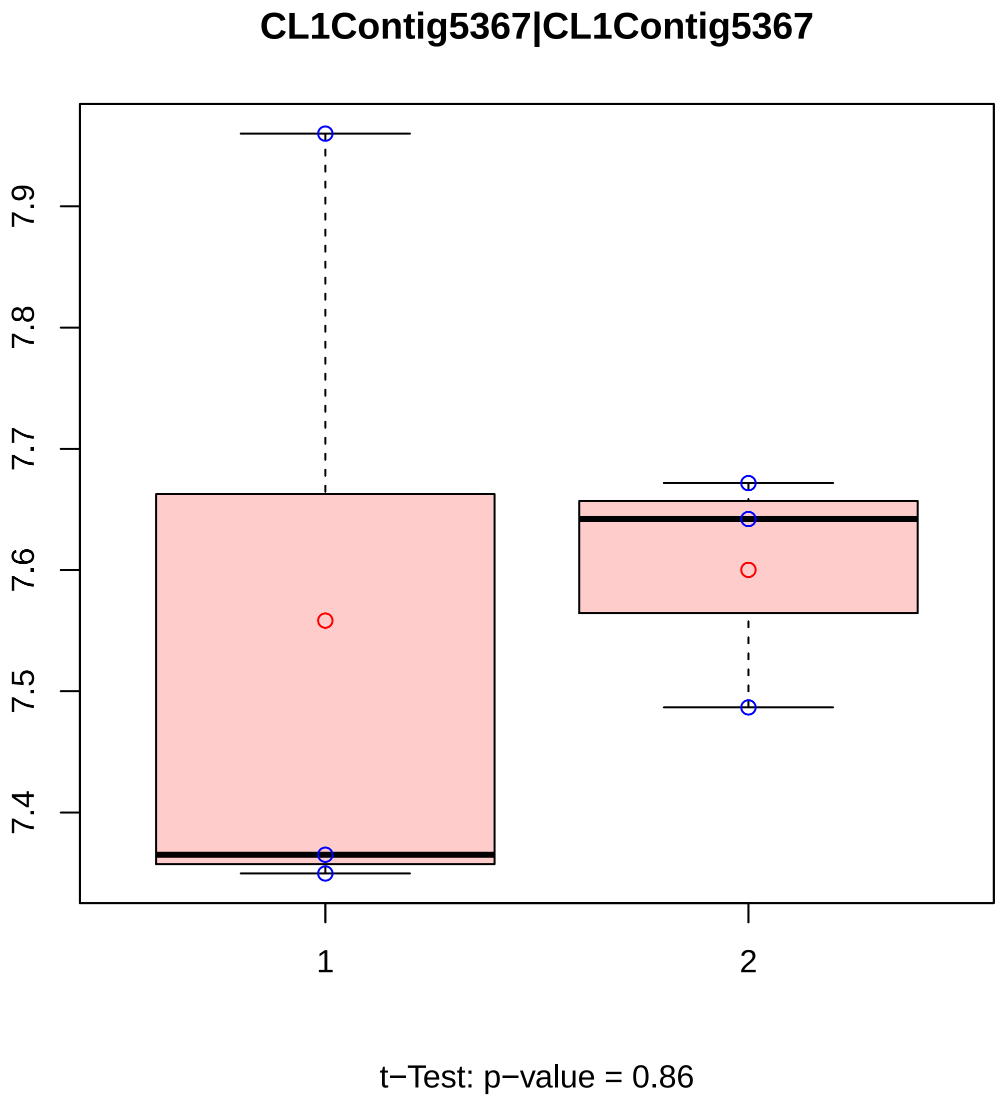

CL1Contig5367|CL1Contig5367
7.4
7.5
7.6
7.7
7.8
7.9
1
2
t−Test: p−value = 0.86

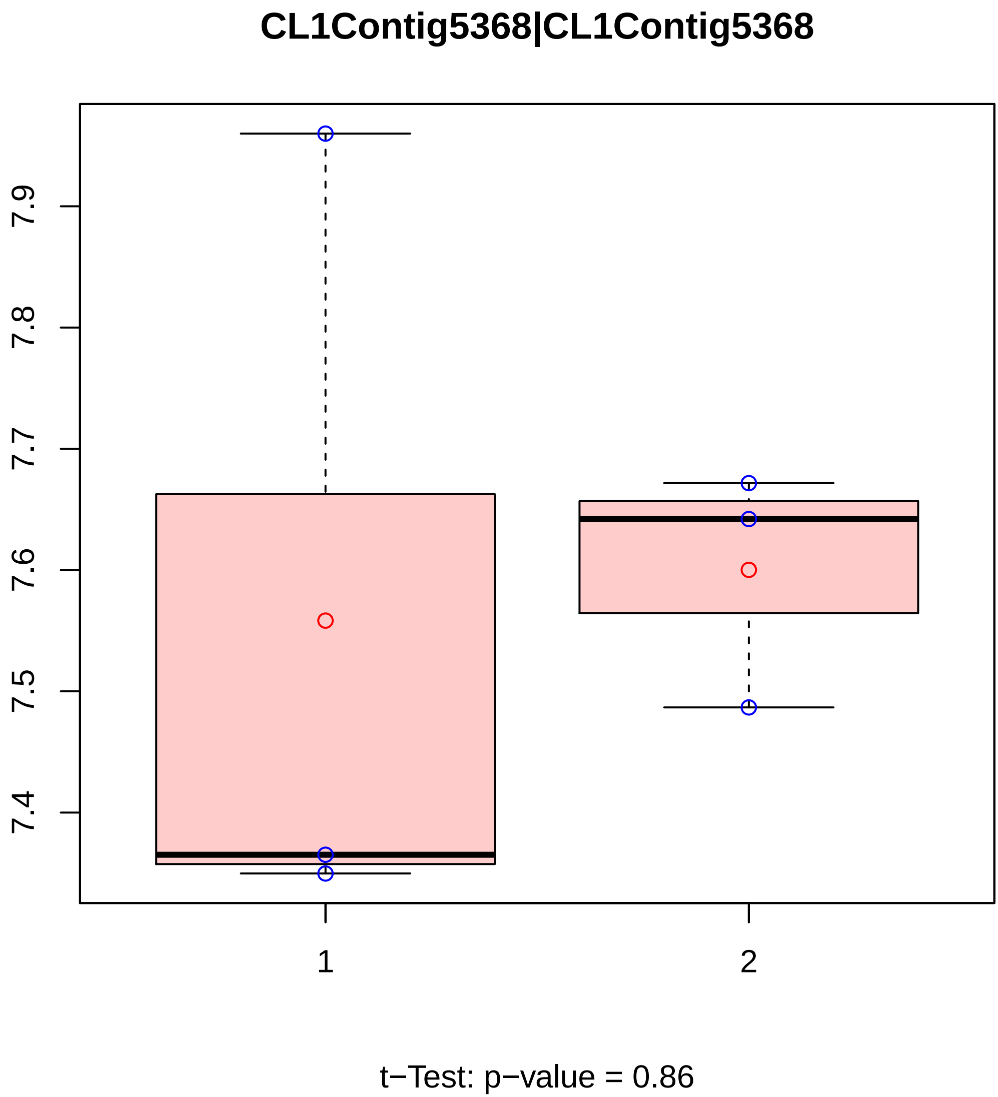
CL1Contig5368|CL1Contig5368
7.4
7.5
7.6
7.7
7.8
7.9
1
2
t−Test: p−value = 0.86

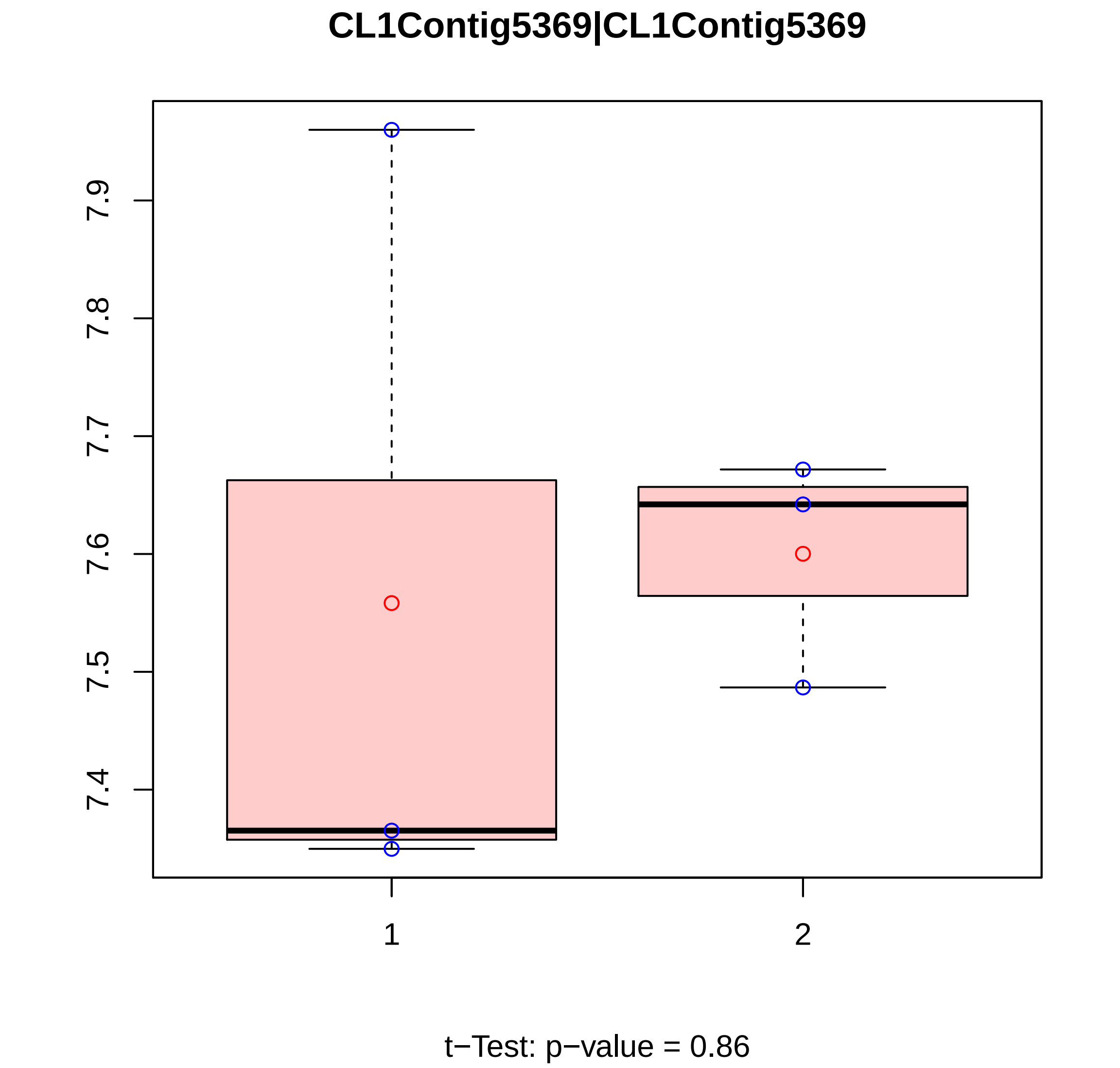
CL1Contig5369|CL1Contig5369
7.4
7.5
7.6
7.7
7.8
7.9
1
2
t−Test: p−value = 0.86

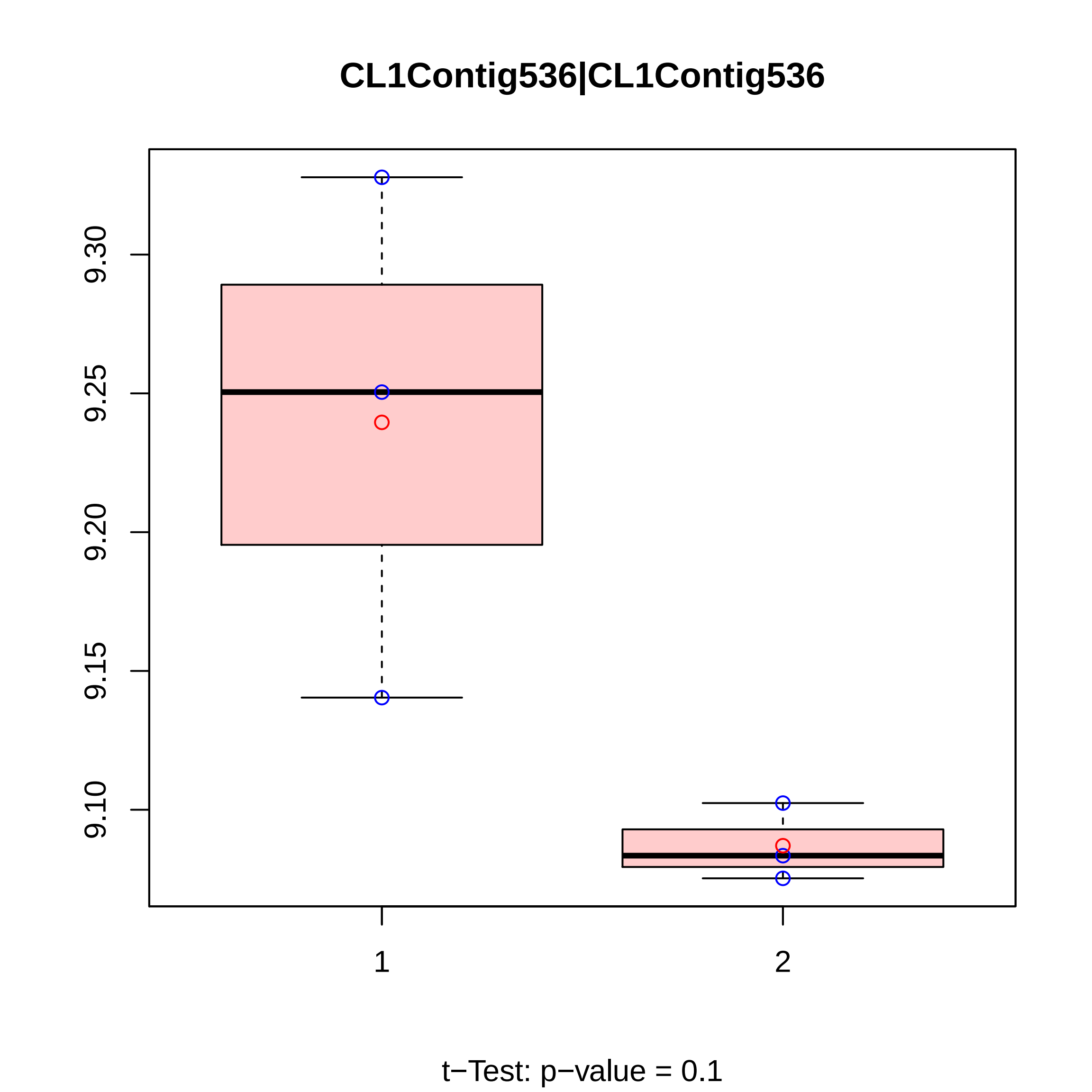

CL1Contig536|CL1Contig536
9.30
9.25
9.20
9.15
9.10
1
2
t−Test: p−value = 0.1

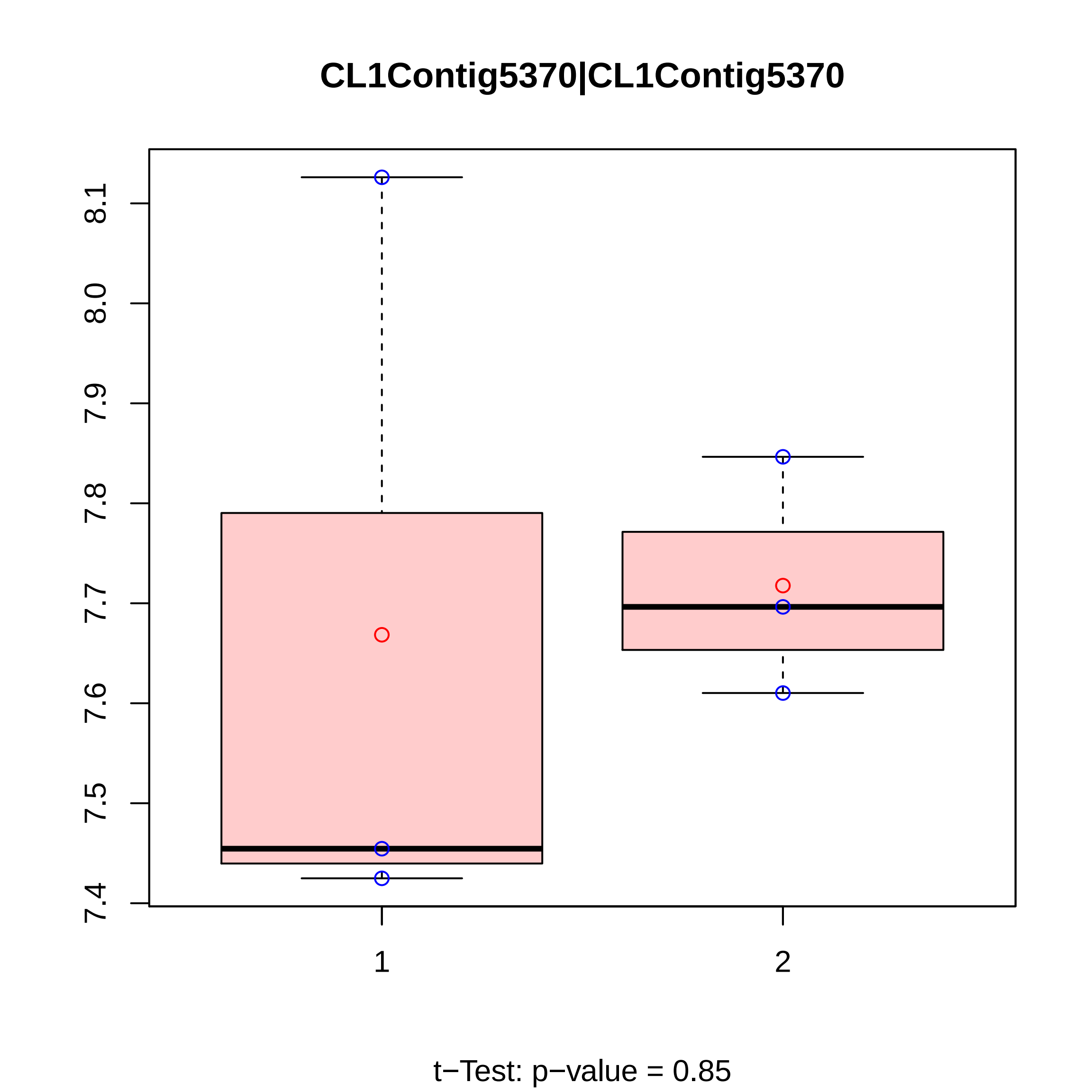
CL1Contig5370|CL1Contig5370
8.1
8.0
7.9
7.8
7.7
7.6
7.5
7.4
1
2
t−Test: p−value = 0.85

# CL1Contig5371|CL1Contig5371

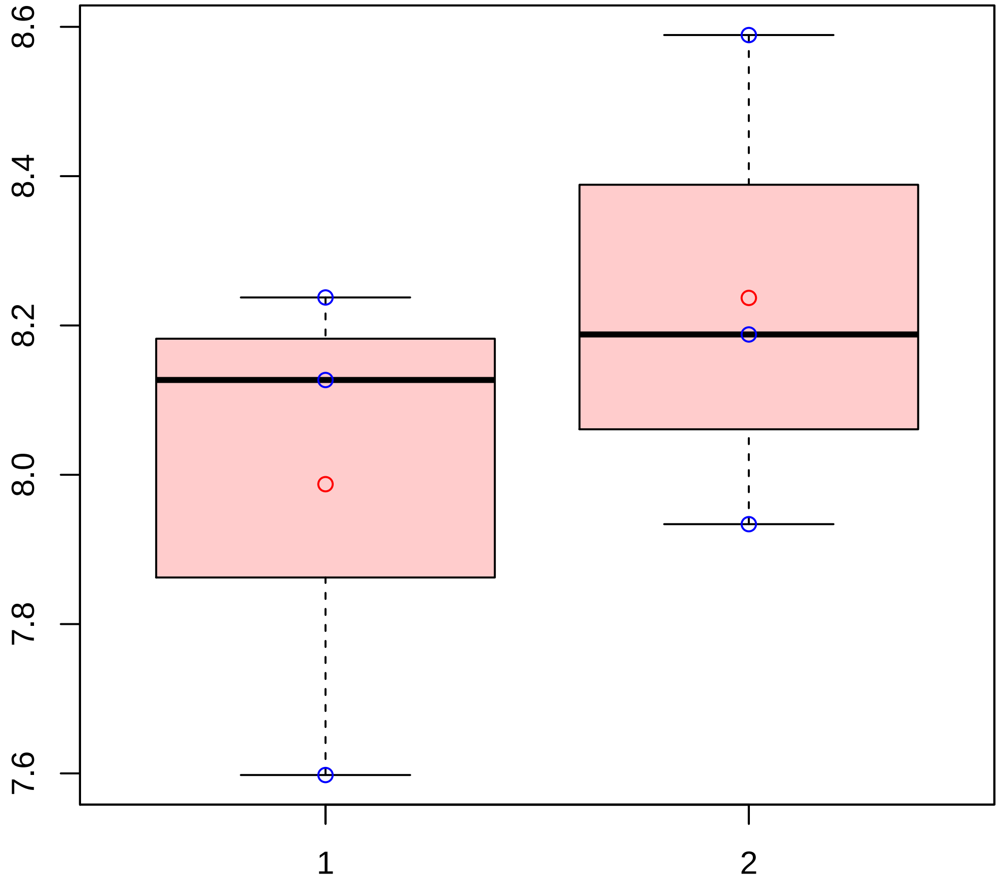


t−Test: p−value = 0.41

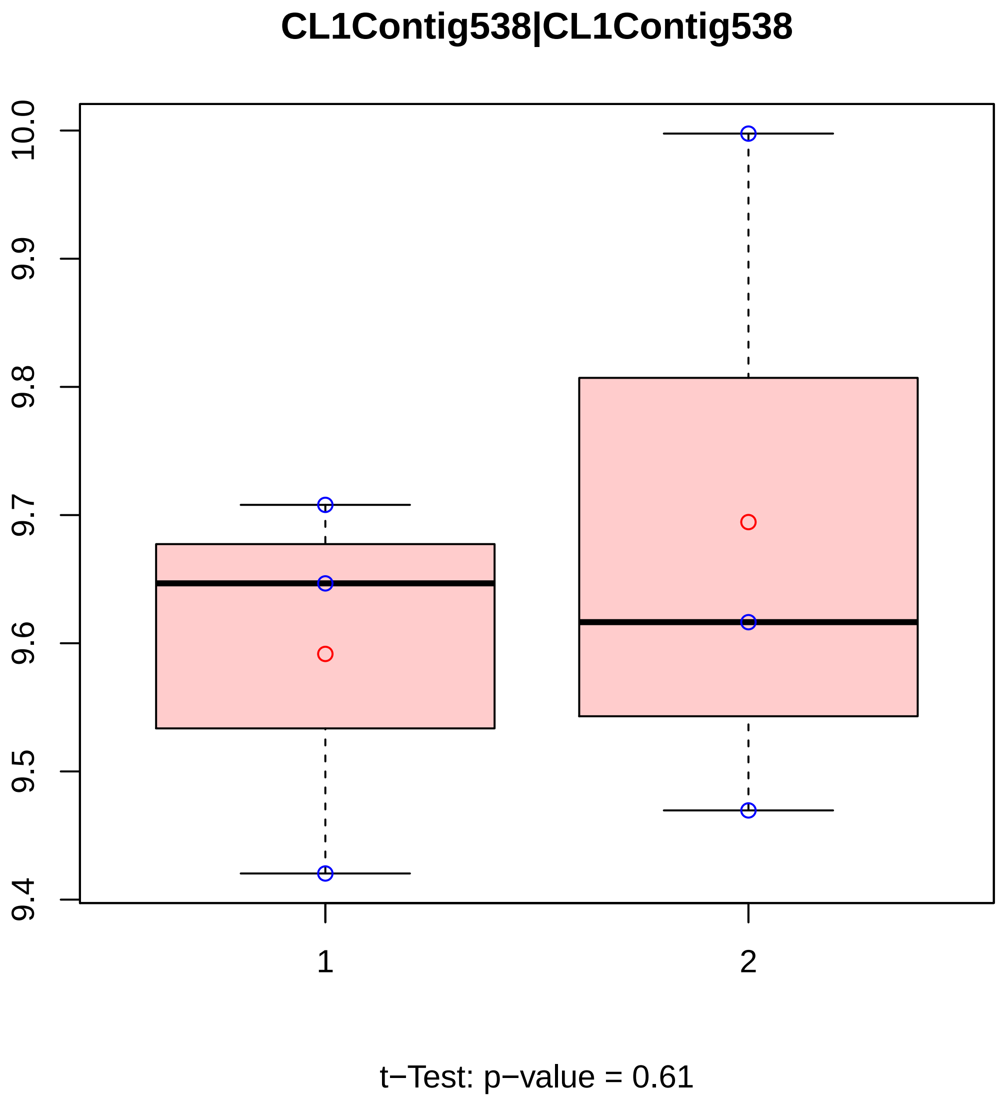

CL1Contig538|CL1Contig538
10.0
9.9
9.8
9.7
9.6
9.5
9.4
1
2
t−Test: p−value = 0.61

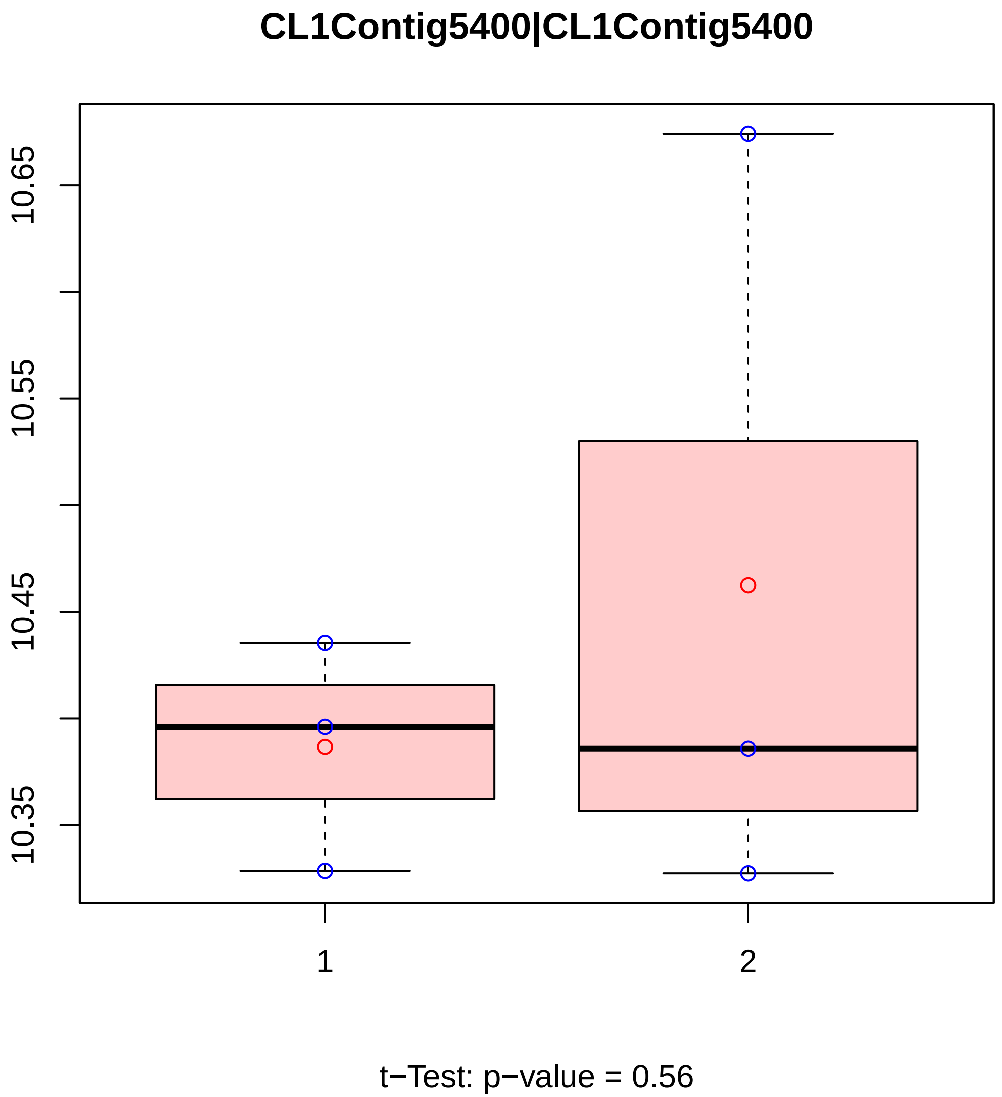
CL1Contig5400|CL1Contig5400
10.35
10.45
10.55
10.65
1
2
t−Test: p−value = 0.56

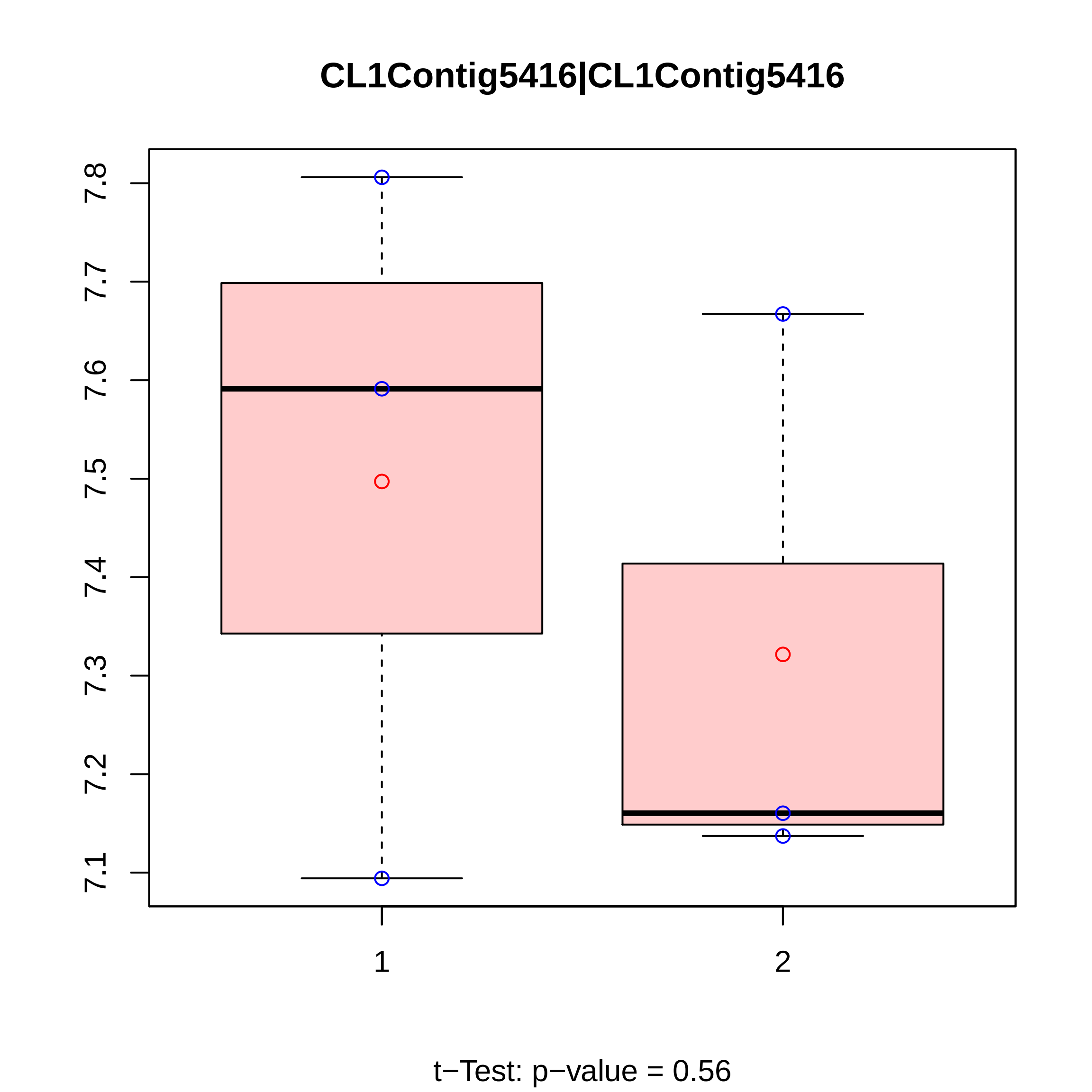

CL1Contig5416|CL1Contig5416
7.1
7.2
7.3
7.4
7.5
7.6
7.7
7.8
1
2
t−Test: p−value = 0.56

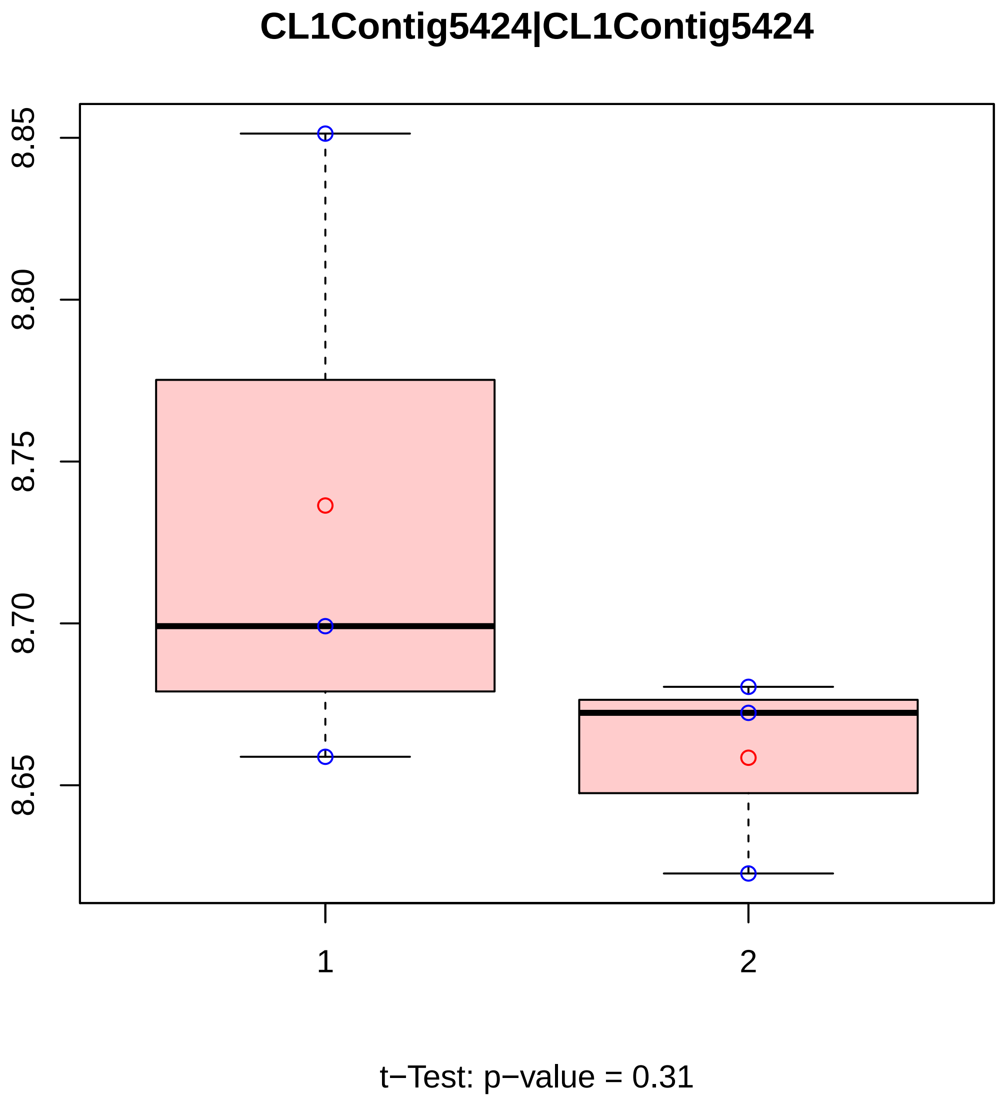
CL1Contig5424|CL1Contig5424
8.85
8.80
8.75
8.70
8.65
1
2
t−Test: p−value = 0.31

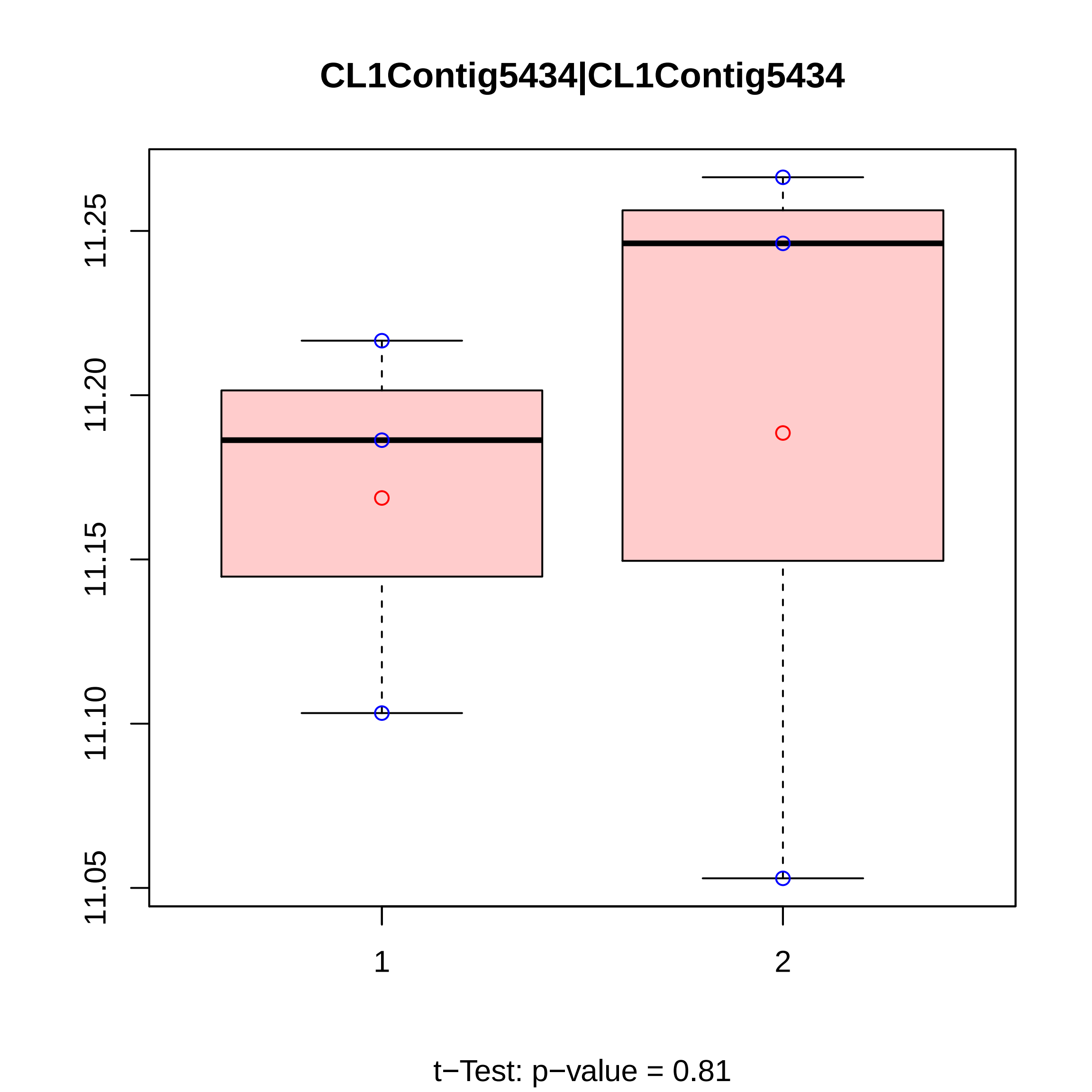
CL1Contig5434|CL1Contig5434
11.05
11.10
11.15
11.20
11.25
1
2
t−Test: p−value = 0.81

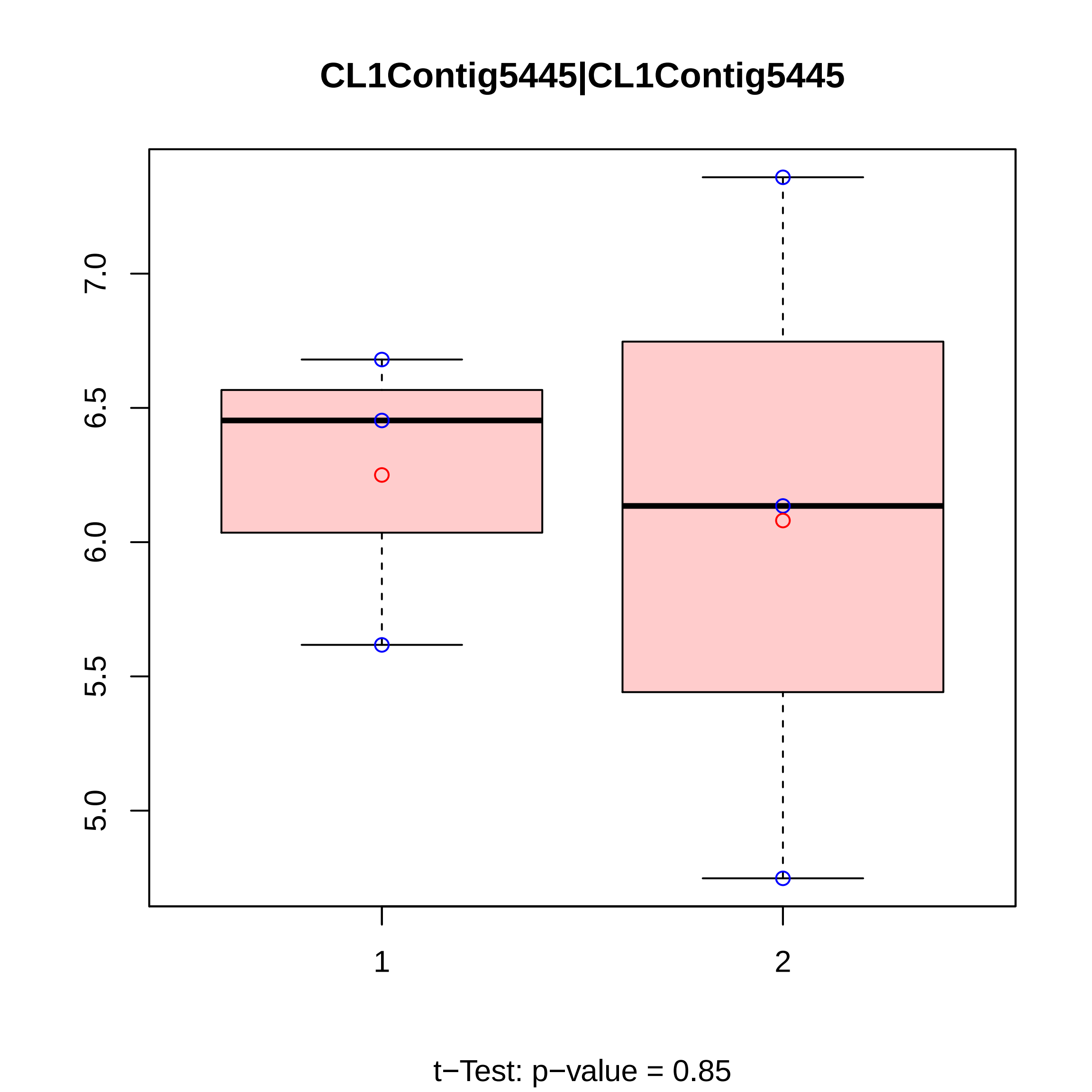

CL1Contig5445|CL1Contig5445
7.0
6.5
6.0
5.5
5.0
1
2
t−Test: p−value = 0.85

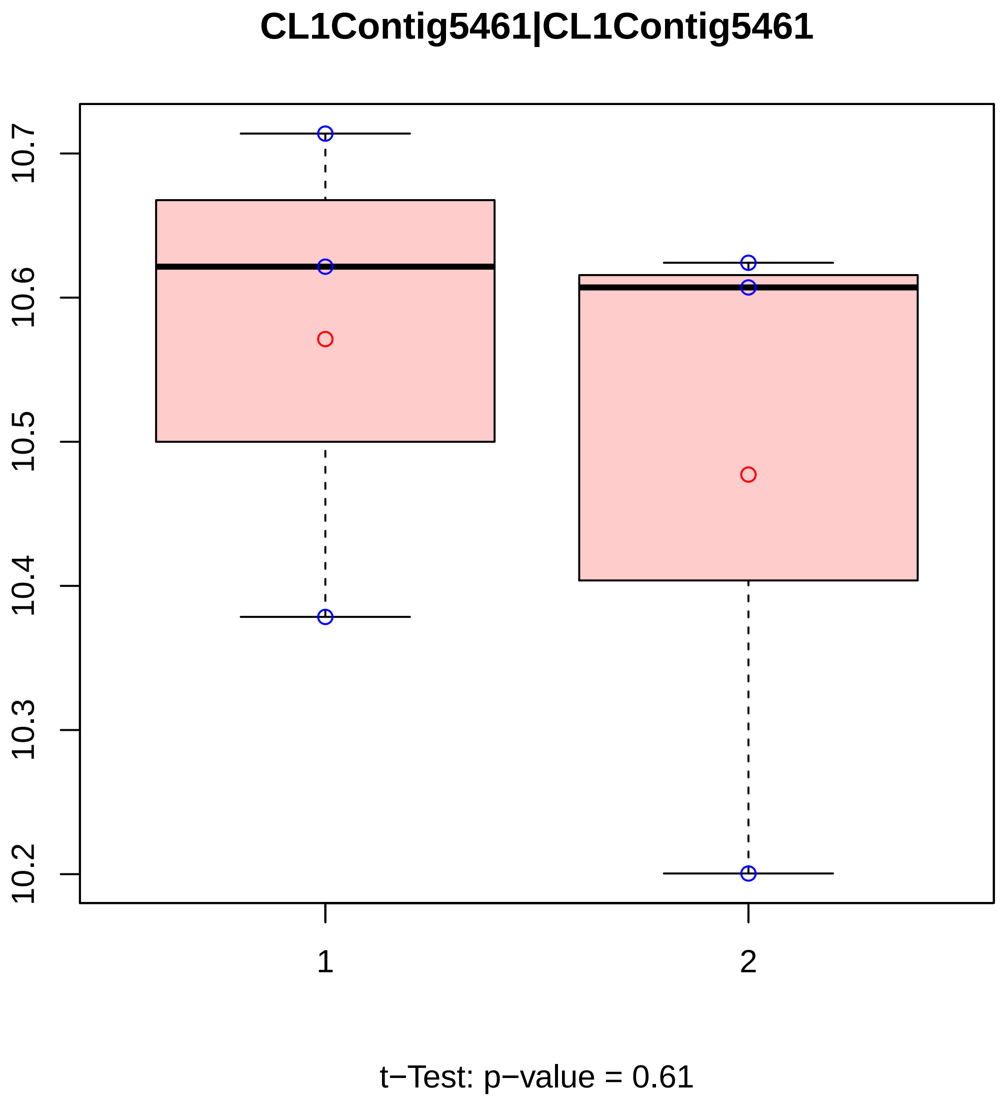
CL1Contig5461|CL1Contig5461
10.7
10.6
10.5
10.4
10.3
10.2
1
2
t−Test: p−value = 0.61

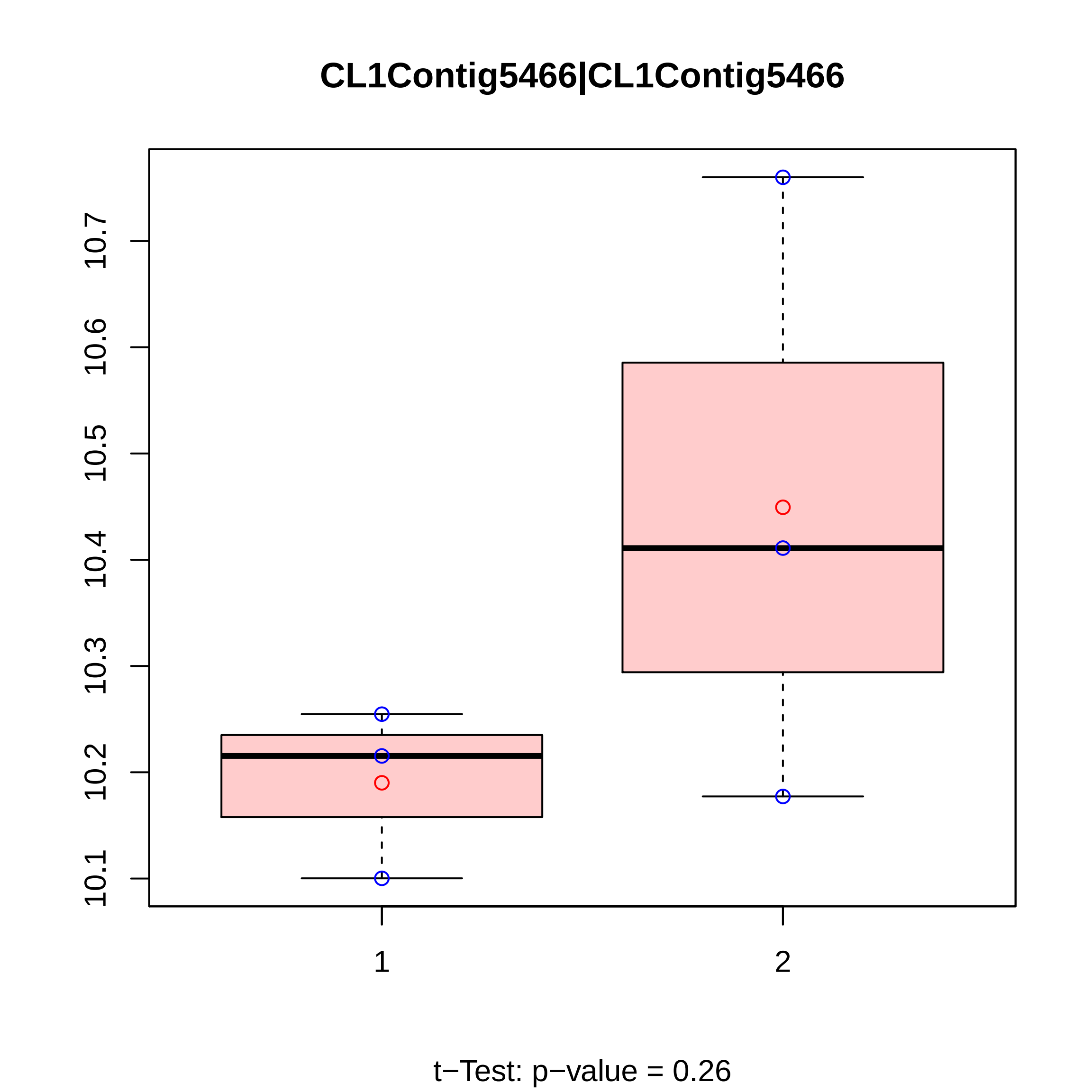
CL1Contig5466|CL1Contig5466
10.1
10.2
10.3
10.4
10.5
10.6
10.7
1
2
t−Test: p−value = 0.26

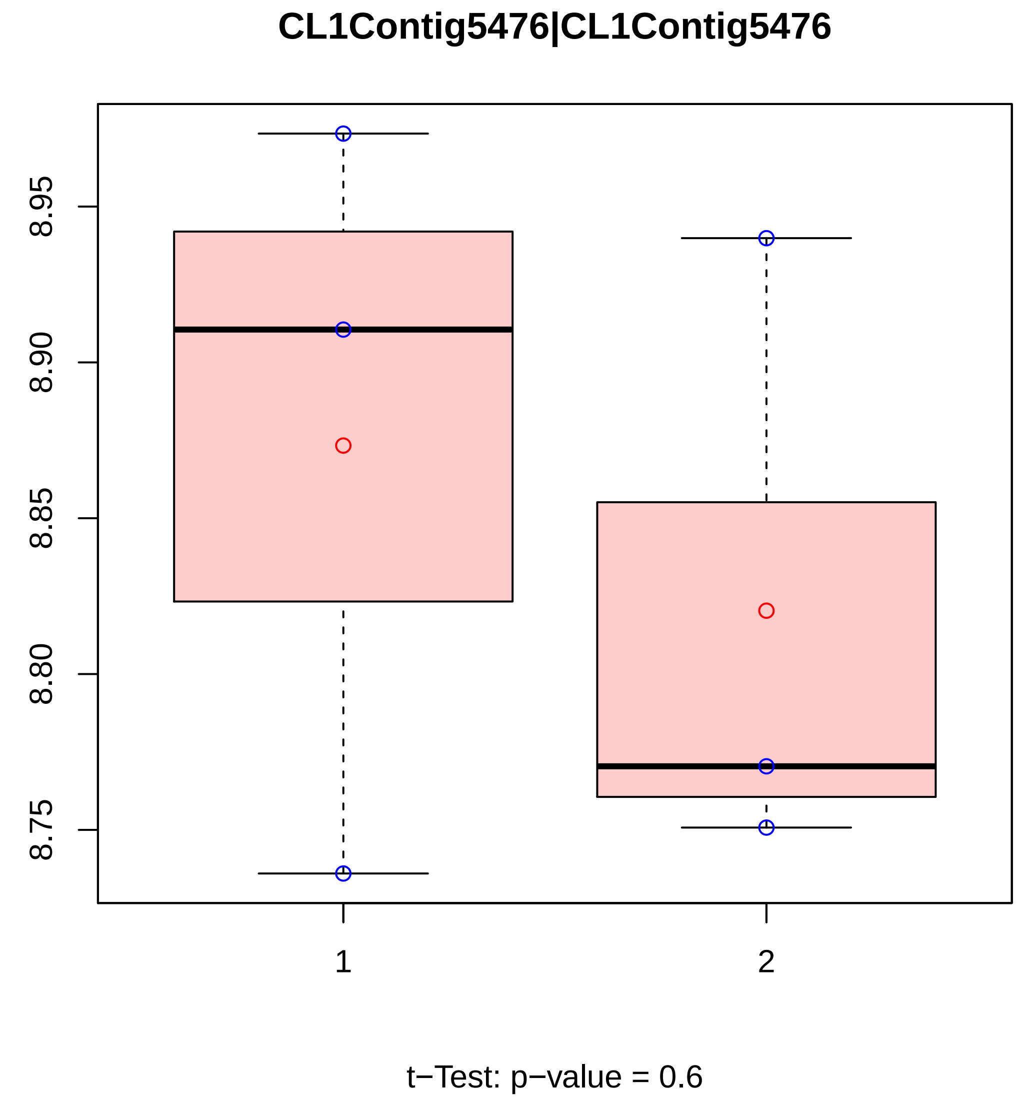
CL1Contig5476|CL1Contig5476
8.75
8.80
8.85
8.90
8.95
1
2
t−Test: p−value = 0.6

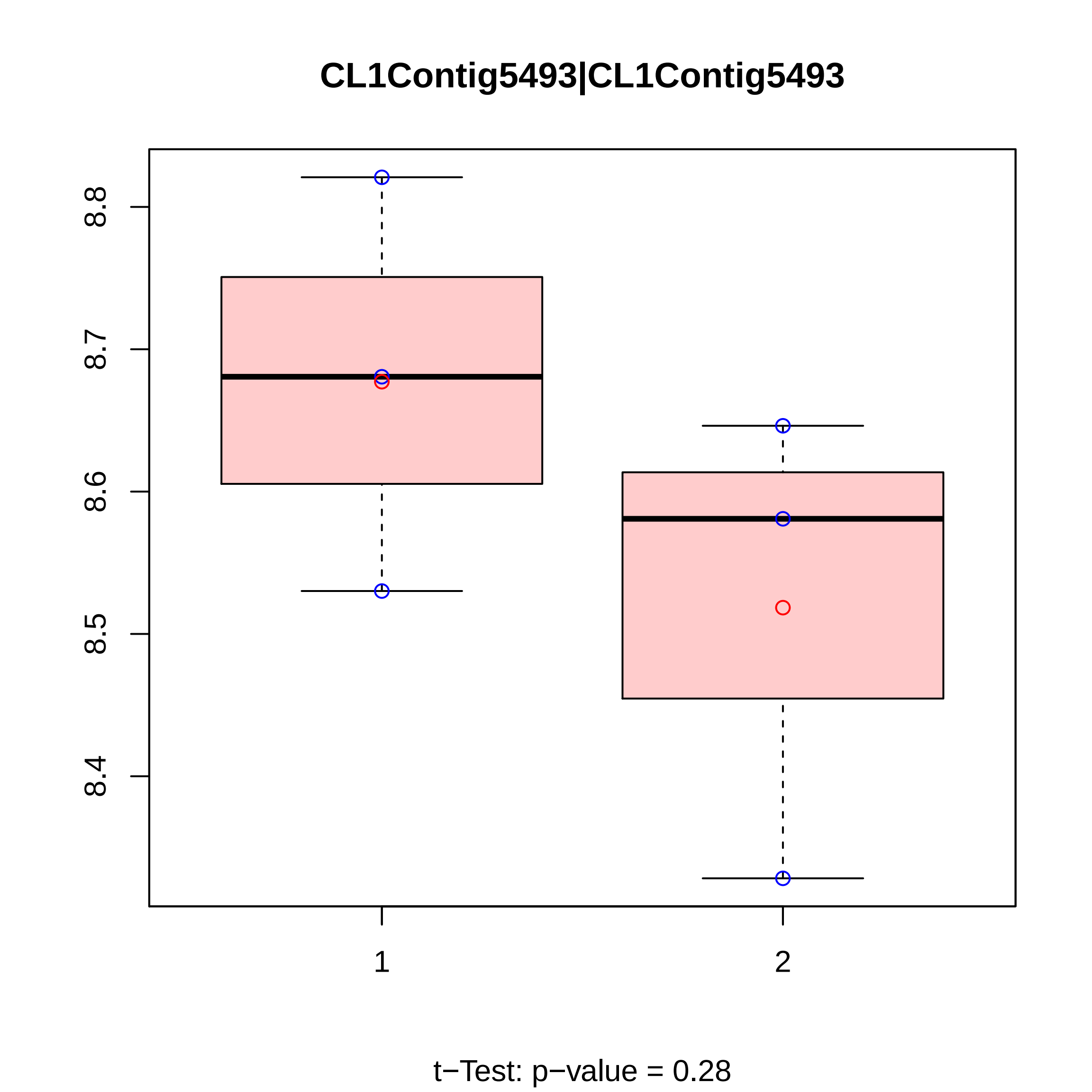

CL1Contig5493|CL1Contig5493
8.4
8.5
8.6
8.7
8.8
1
2
t−Test: p−value = 0.28

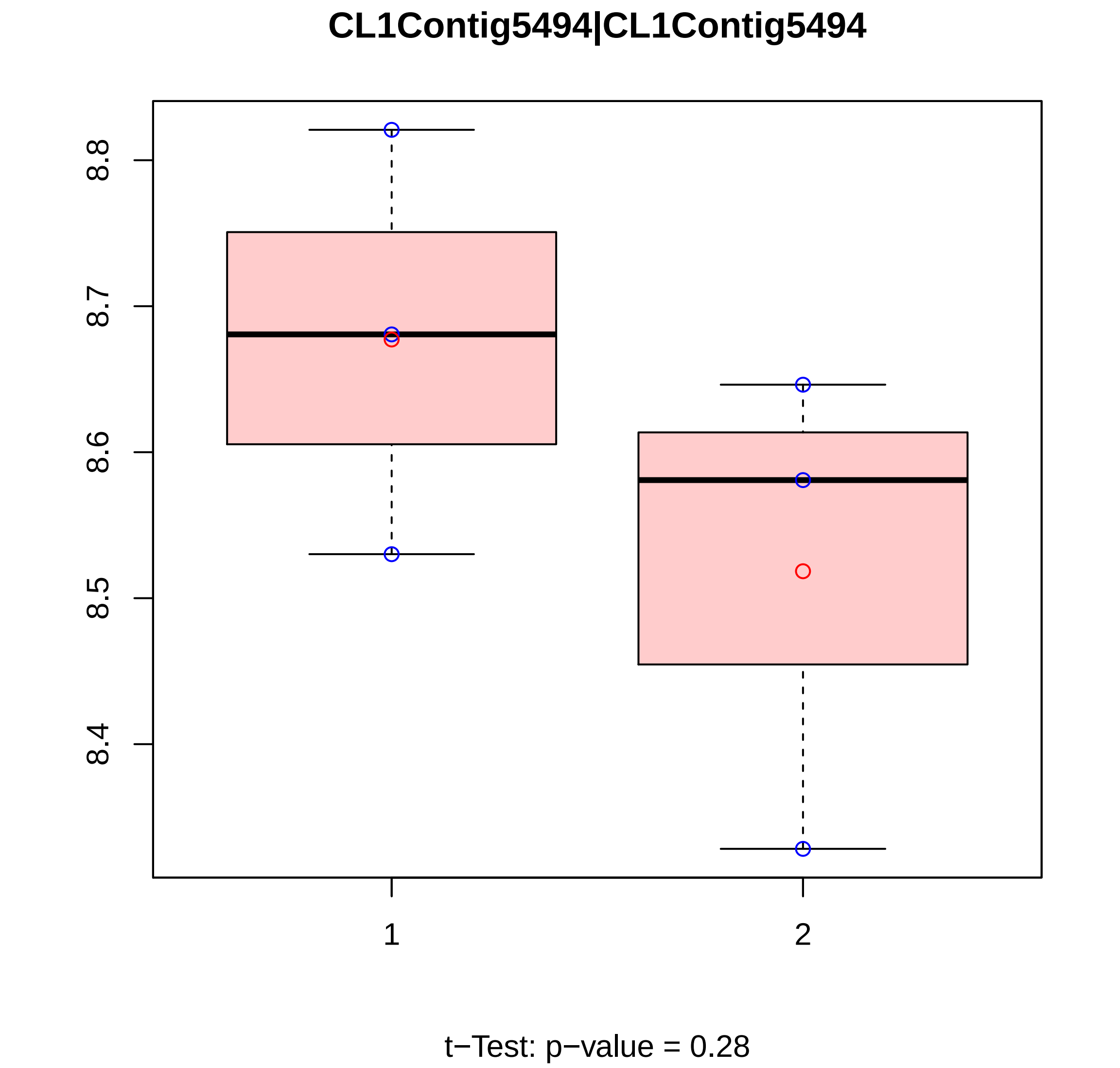
CL1Contig5494|CL1Contig5494
8.4
8.5
8.6
8.7
8.8
1
2
t−Test: p−value = 0.28

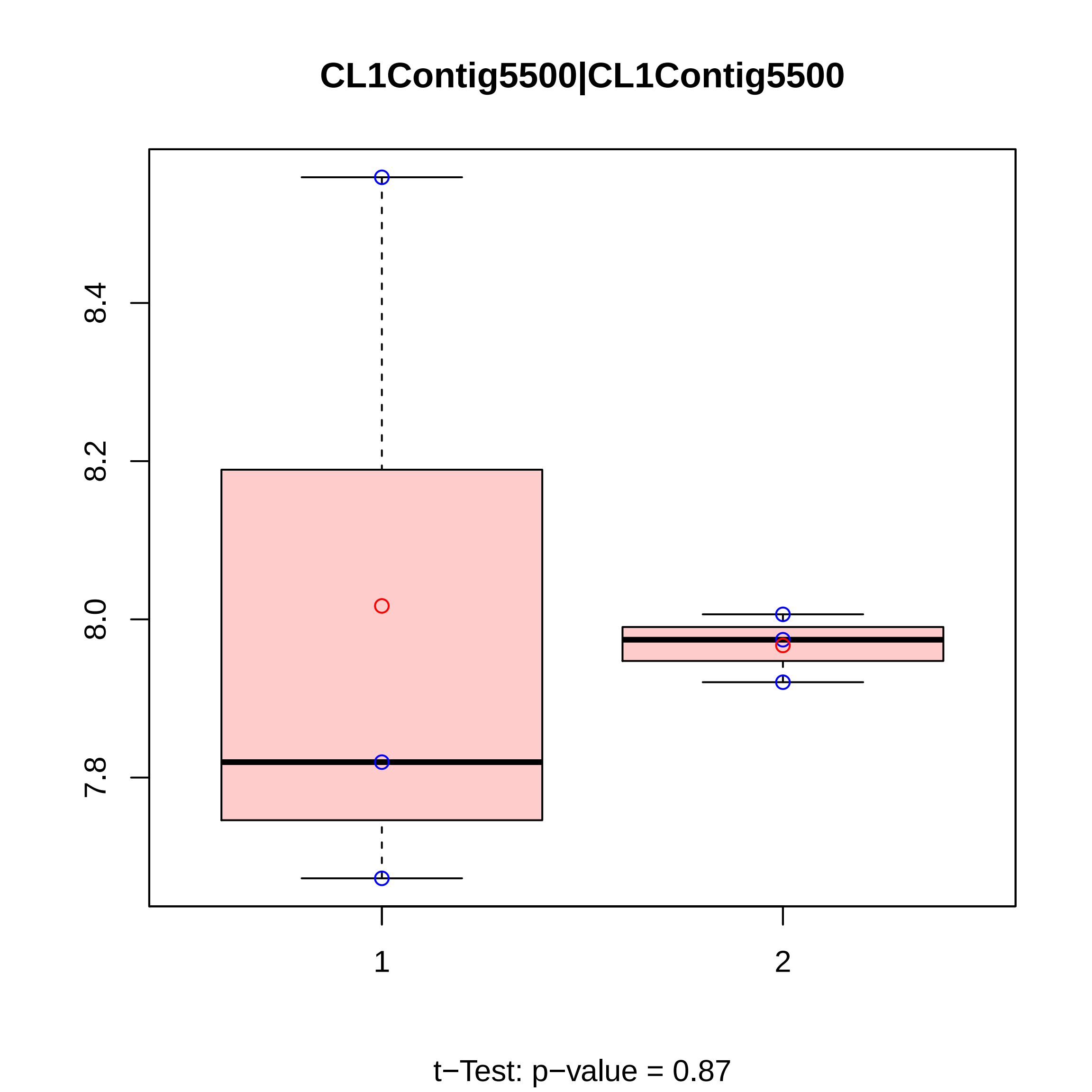

CL1Contig5500|CL1Contig5500
8.4
8.2
8.0
7.8
1
2
t−Test: p−value = 0.87

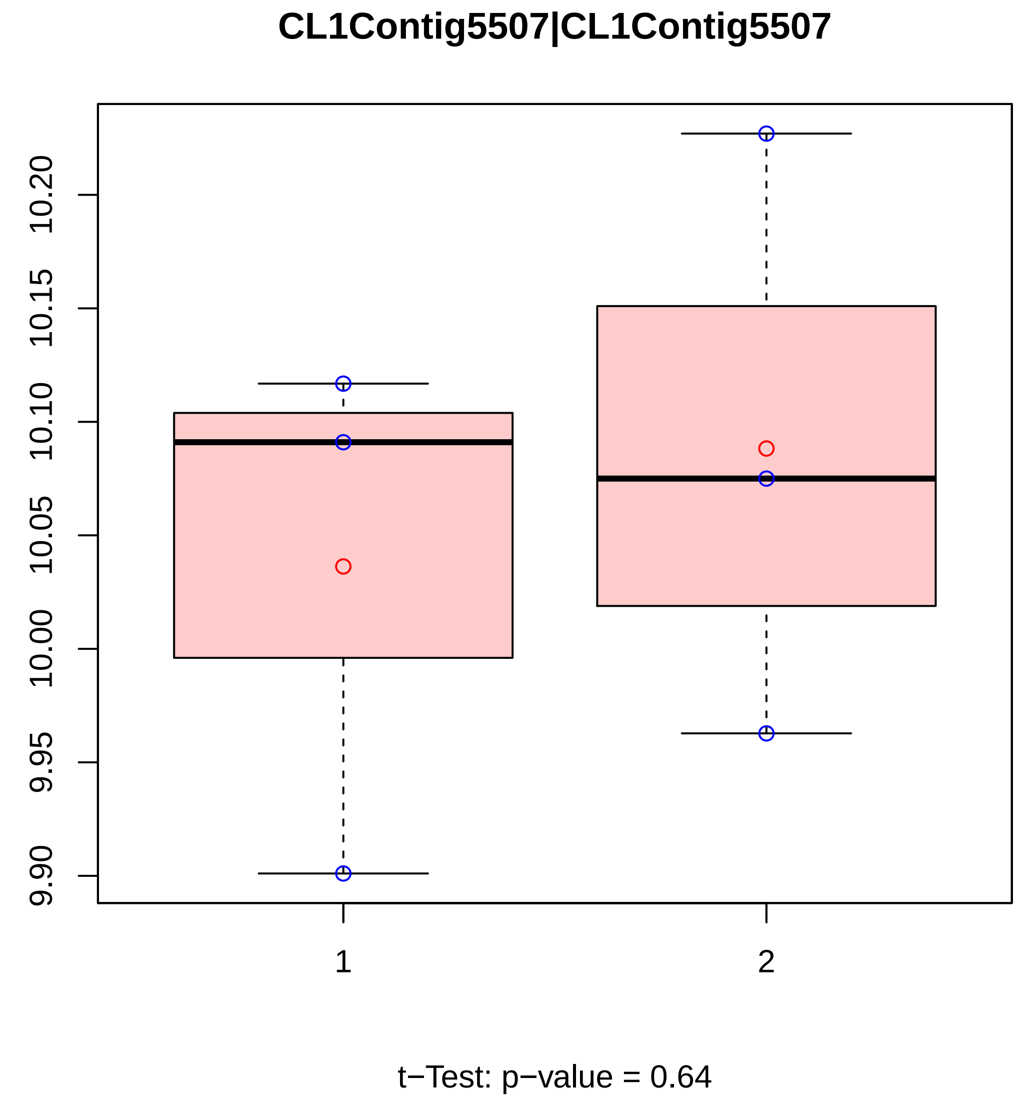

CL1Contig5507|CL1Contig5507
9.90
9.95
10.00
10.05
10.10
10.15
10.20
1
2
t−Test: p−value = 0.64

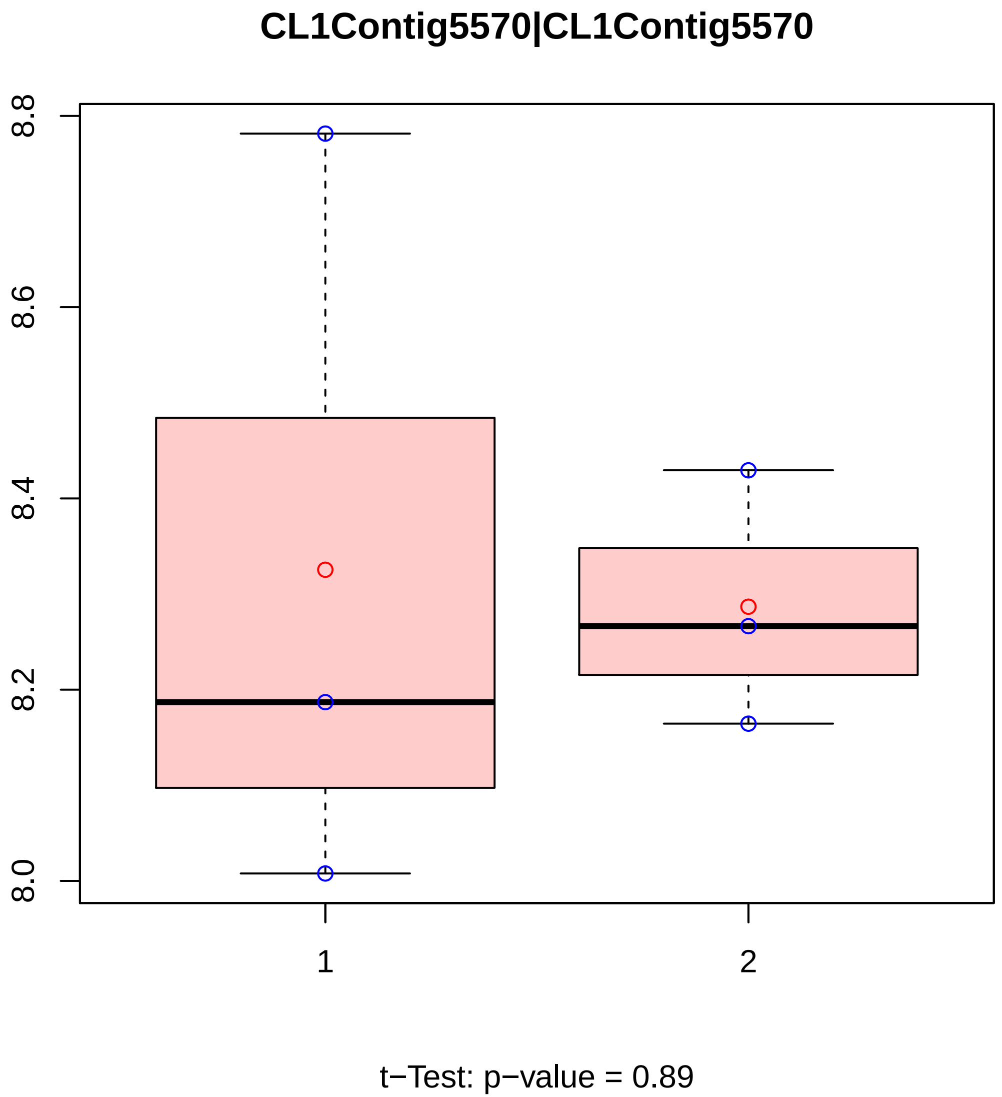
CL1Contig5570|CL1Contig5570
8.0
8.2
8.4
8.6
8.8
1
2
t−Test: p−value = 0.89

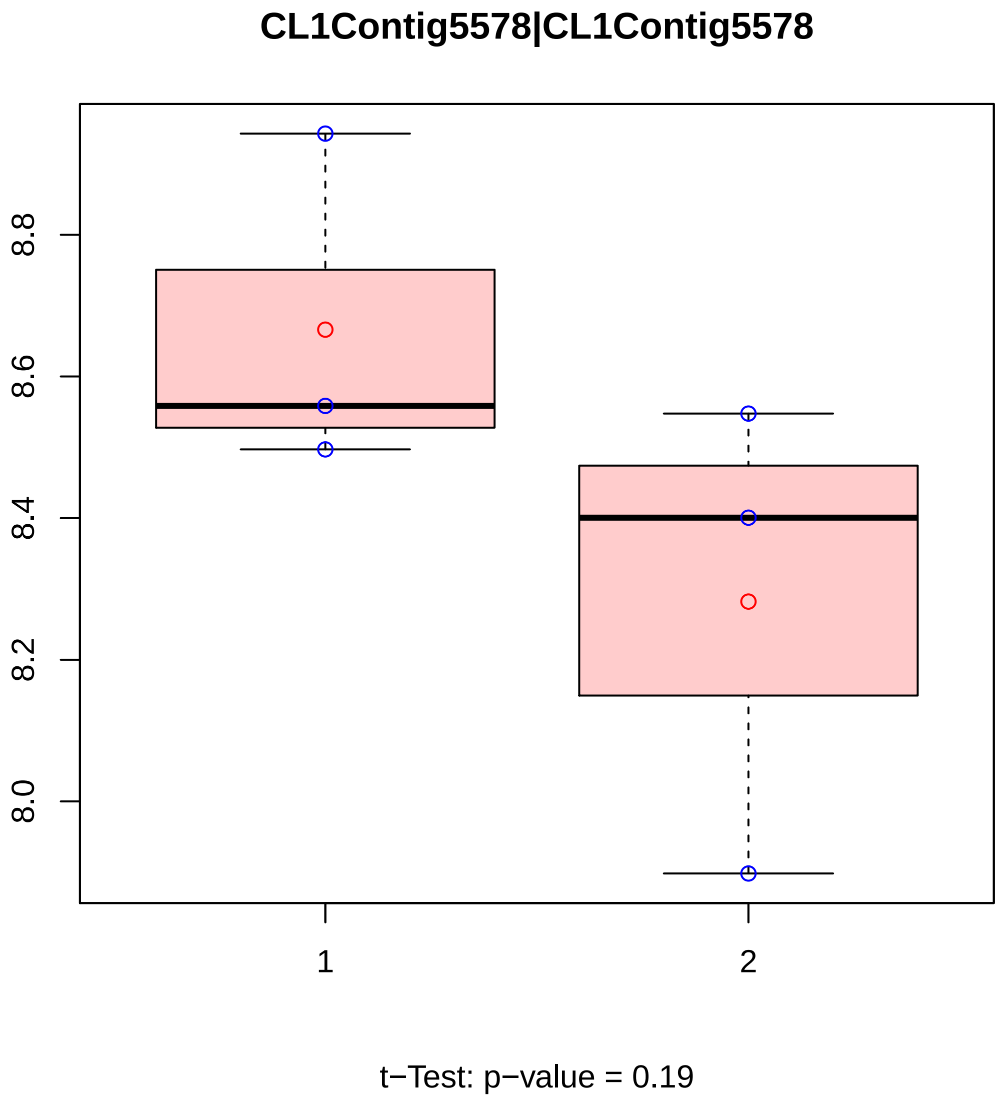
CL1Contig5578|CL1Contig5578
8.0
8.2
8.4
8.6
8.8
1
2
t−Test: p−value = 0.19

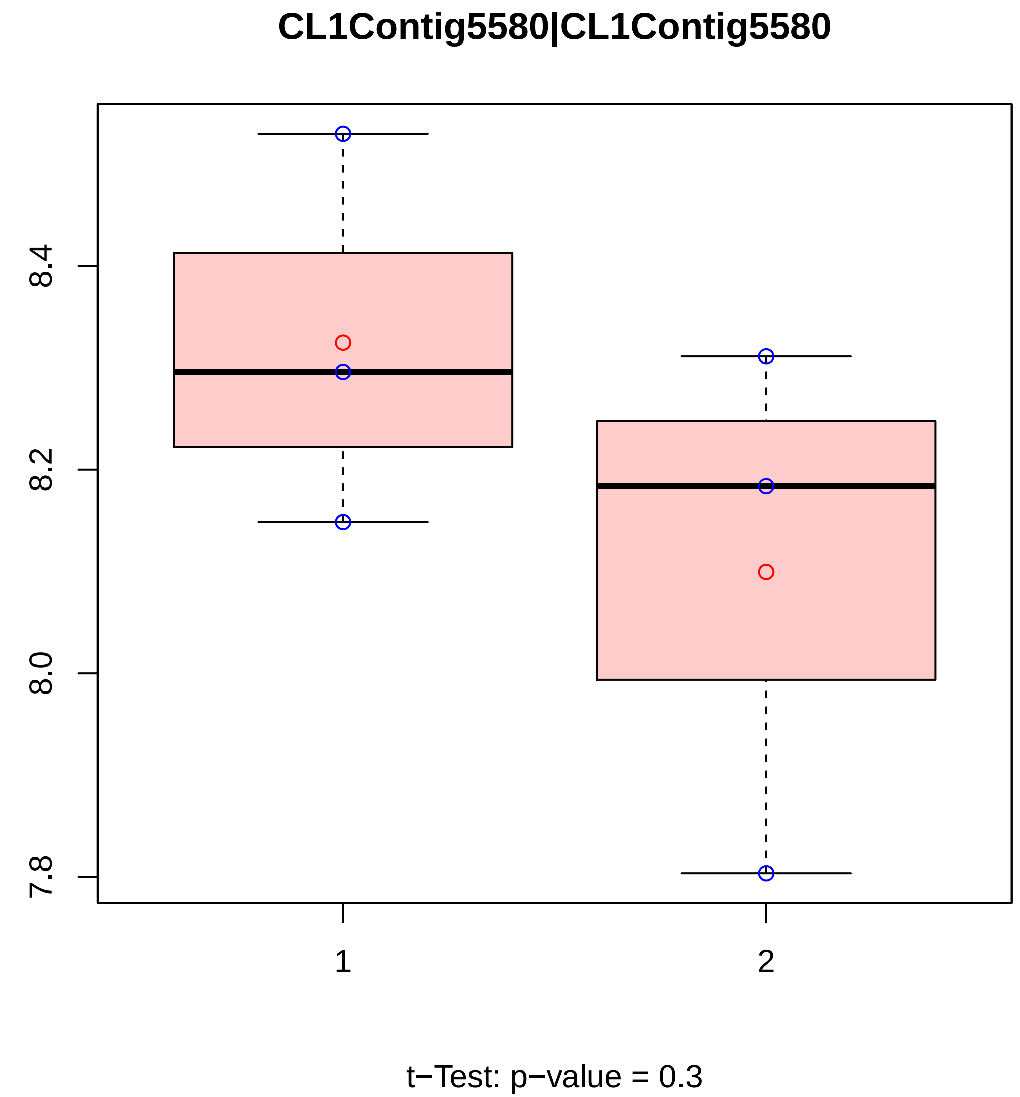

CL1Contig5580|CL1Contig5580
8.4
8.2
8.0
7.8
1
2
t−Test: p−value = 0.3

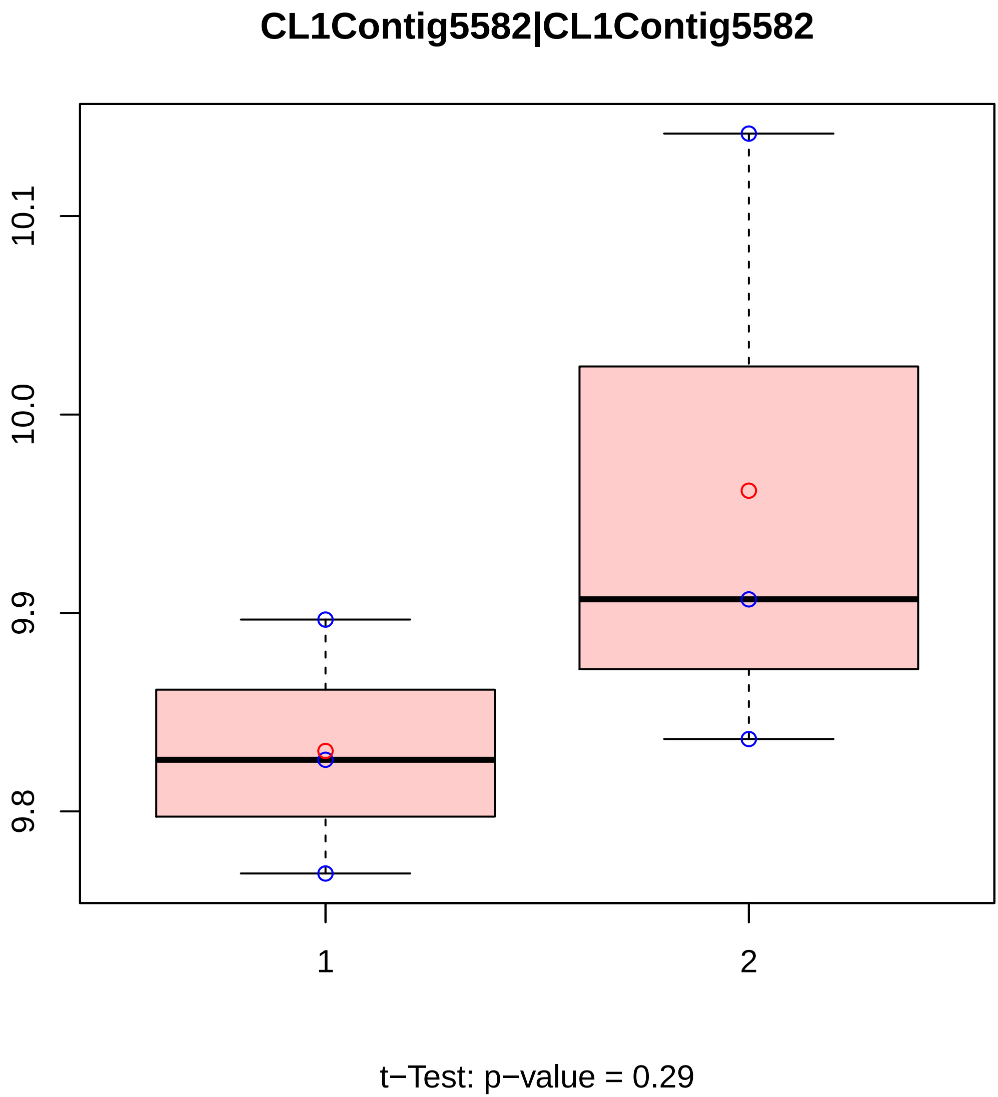
CL1Contig5582|CL1Contig5582
10.1
10.0
9.9
9.8
1
2
t−Test: p−value = 0.29

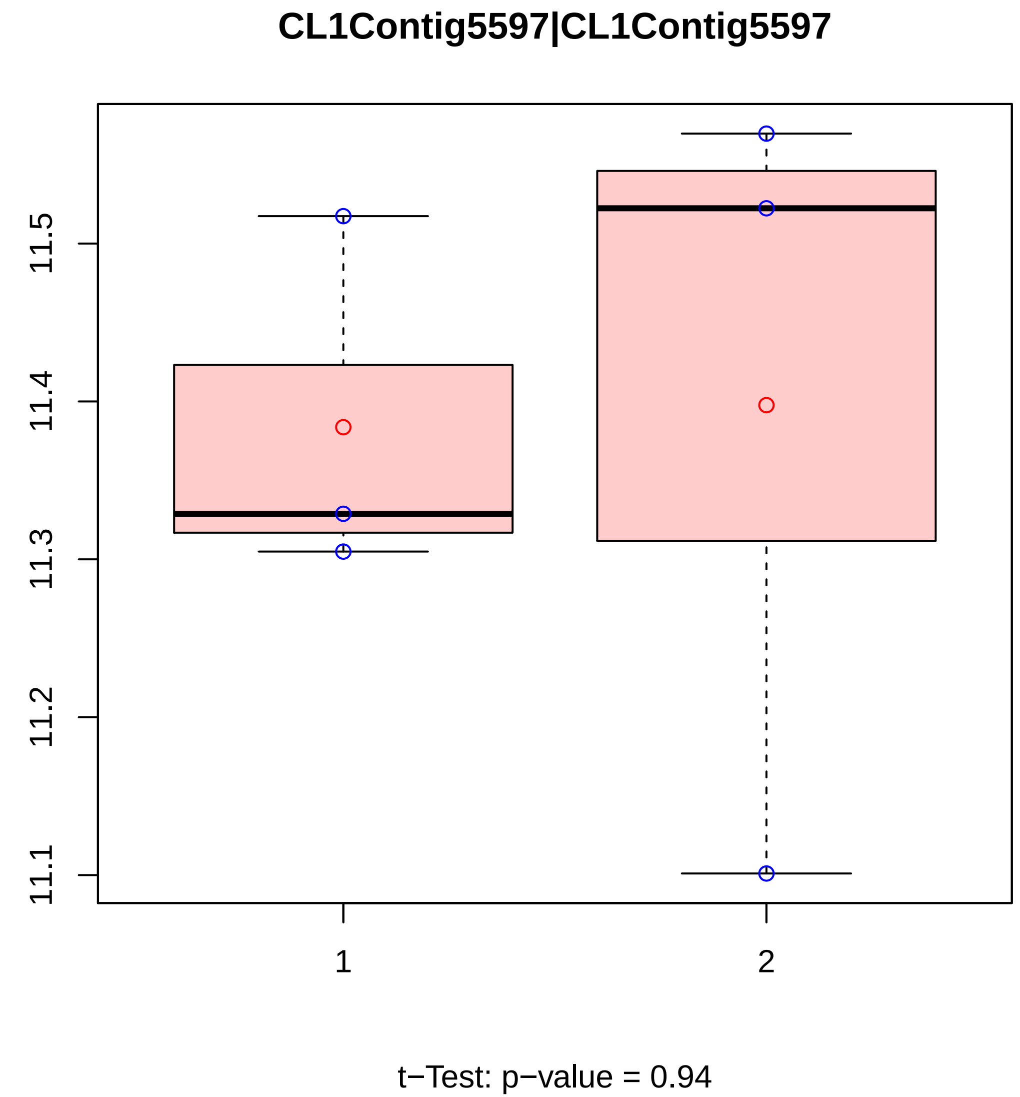

CL1Contig5597|CL1Contig5597
11.1
11.2
11.3
11.4
11.5
1
2
t−Test: p−value = 0.94

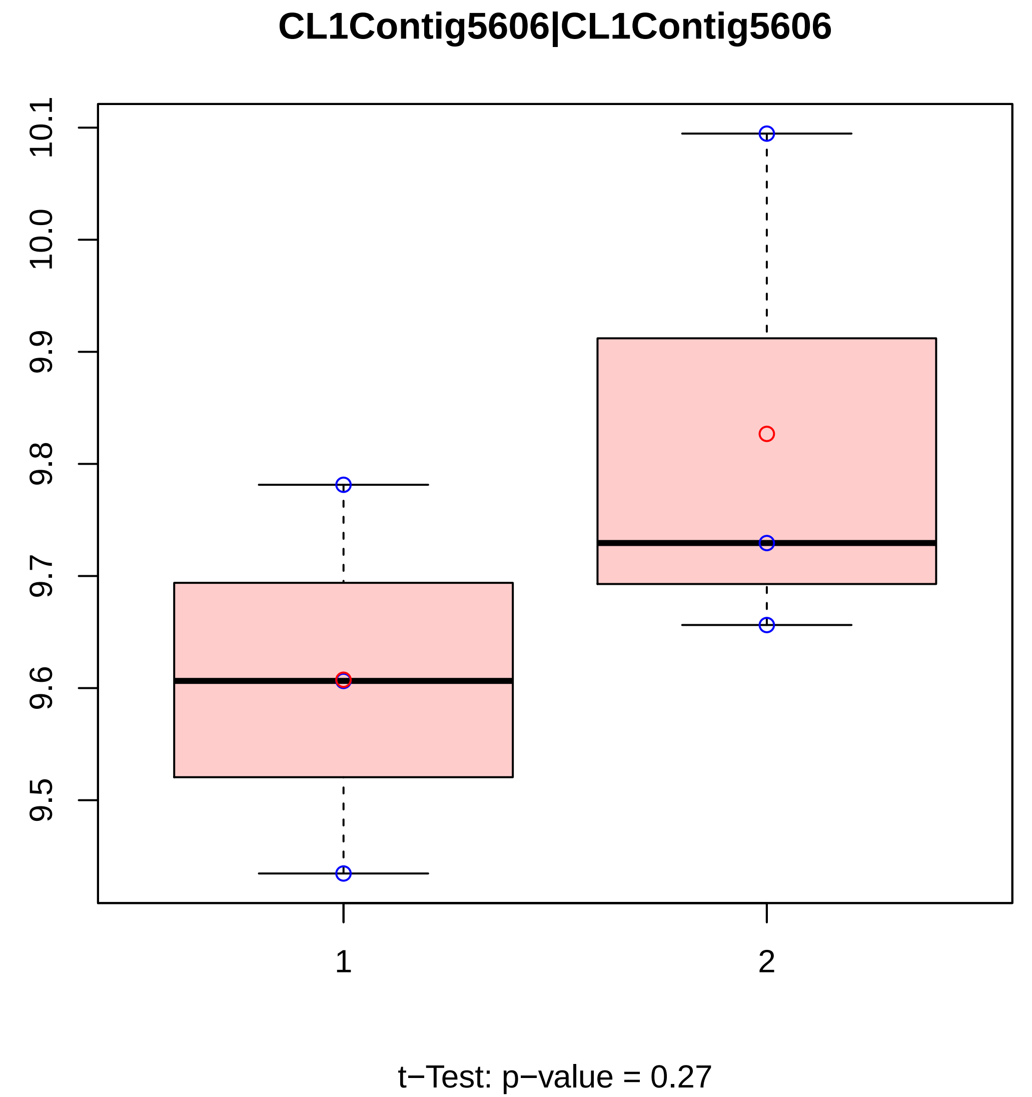

CL1Contig5606|CL1Contig5606
10.1
10.0
9.9
9.8
9.7
9.6
9.5
1
2
t−Test: p−value = 0.27

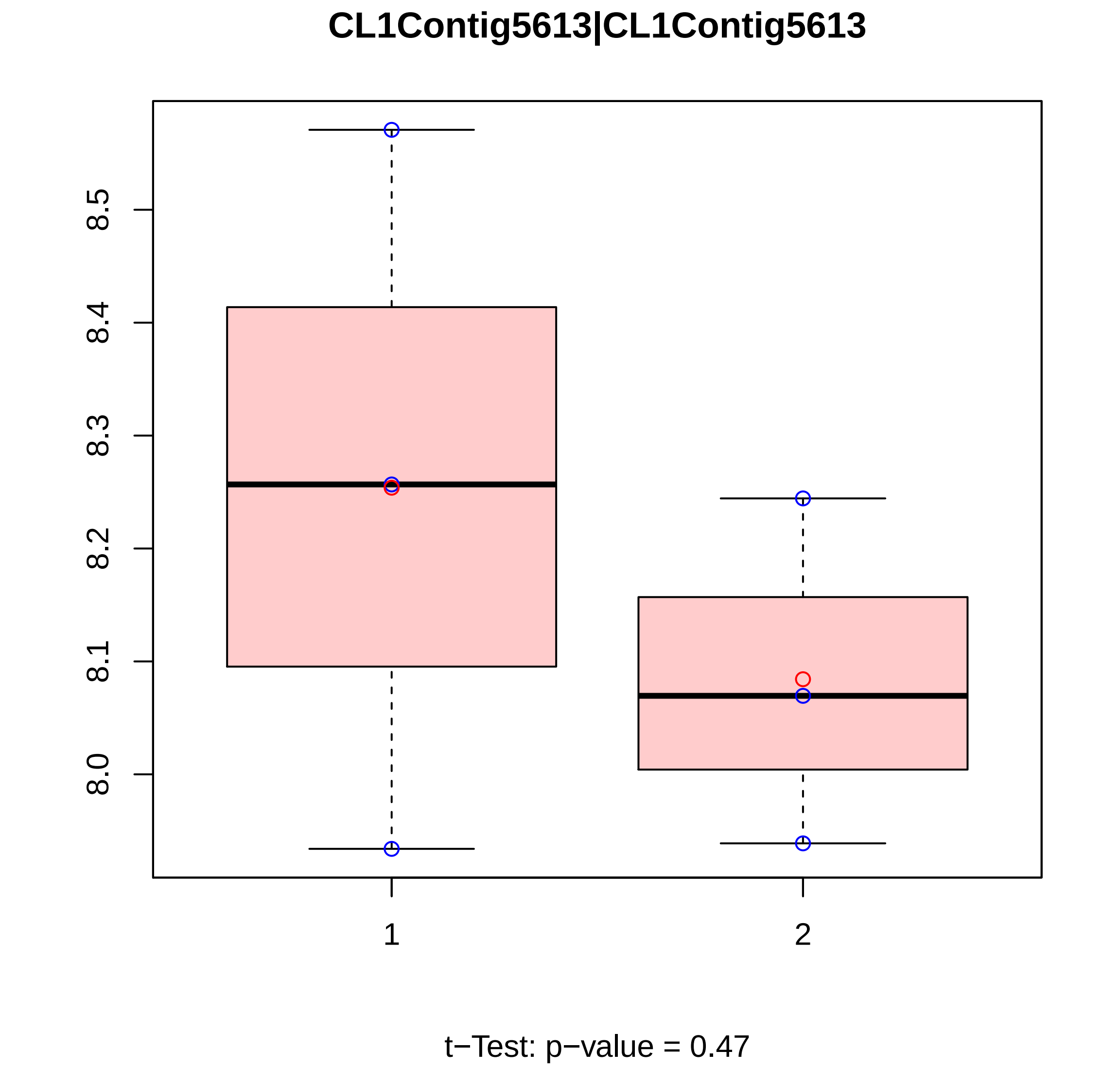
CL1Contig5613|CL1Contig5613
8.0
8.1
8.2
8.3
8.4
8.5
1
2
t−Test: p−value = 0.47

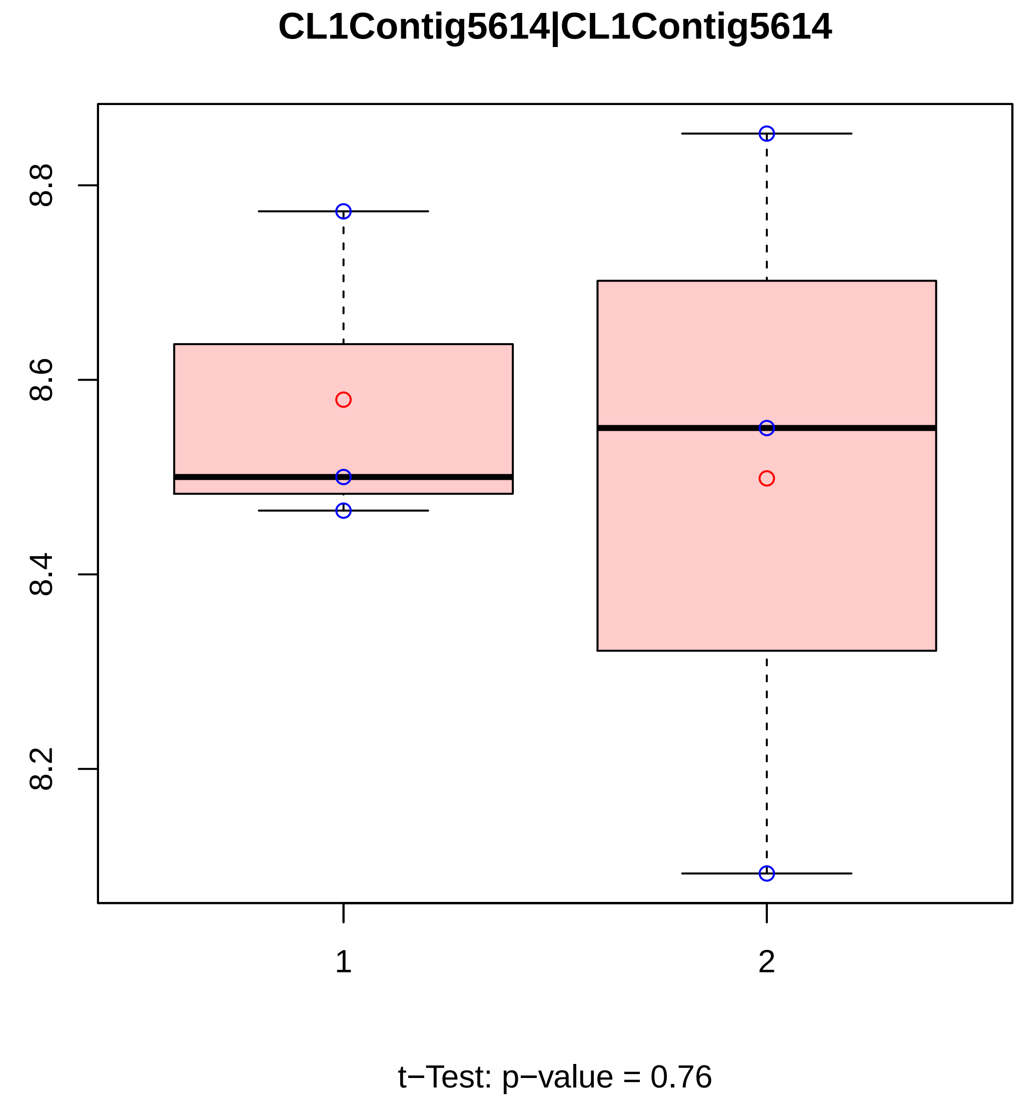
CL1Contig5614|CL1Contig5614
8.2
8.4
8.6
8.8
1
2
t−Test: p−value = 0.76

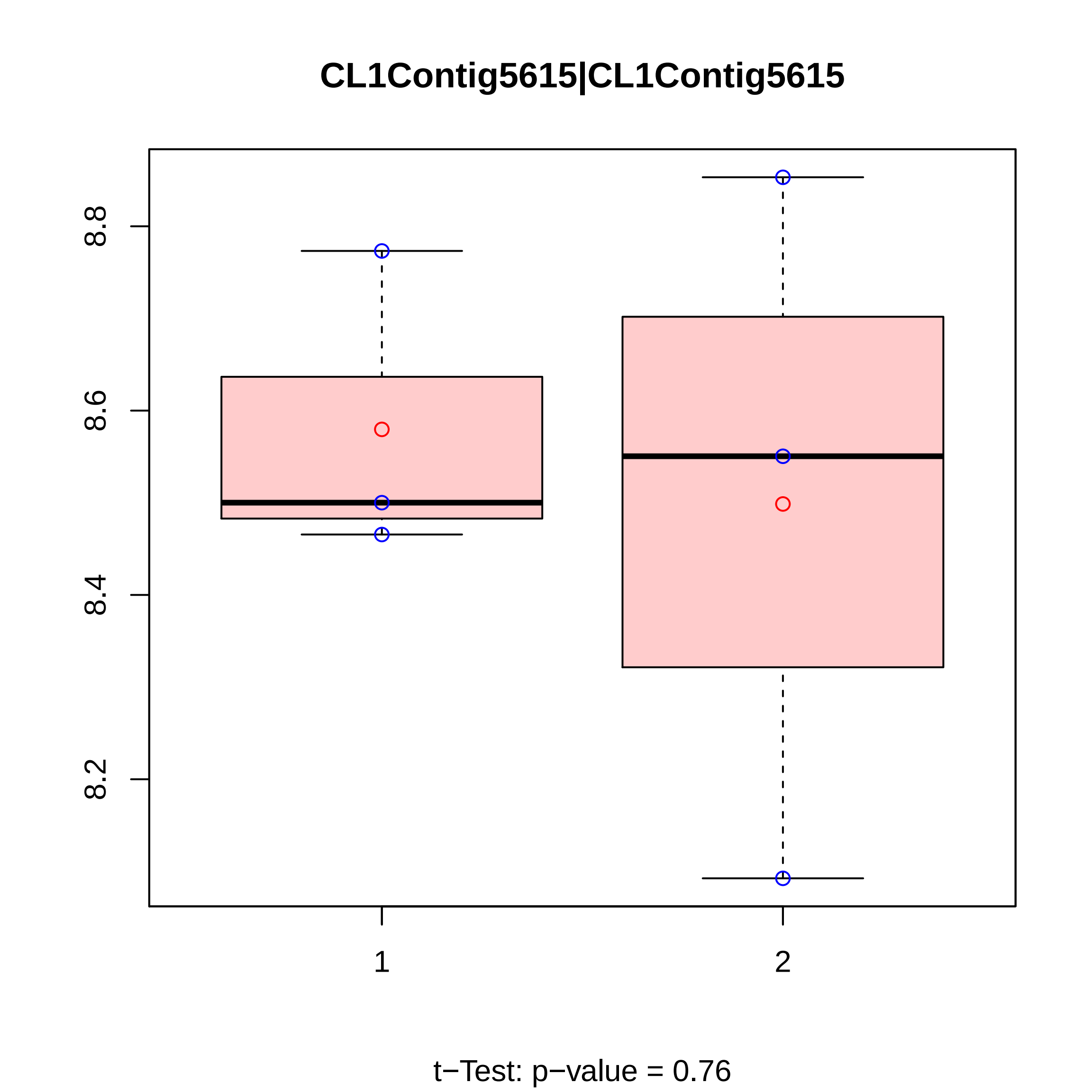
CL1Contig5615|CL1Contig5615
8.2
8.4
8.6
8.8
1
2
t−Test: p−value = 0.76

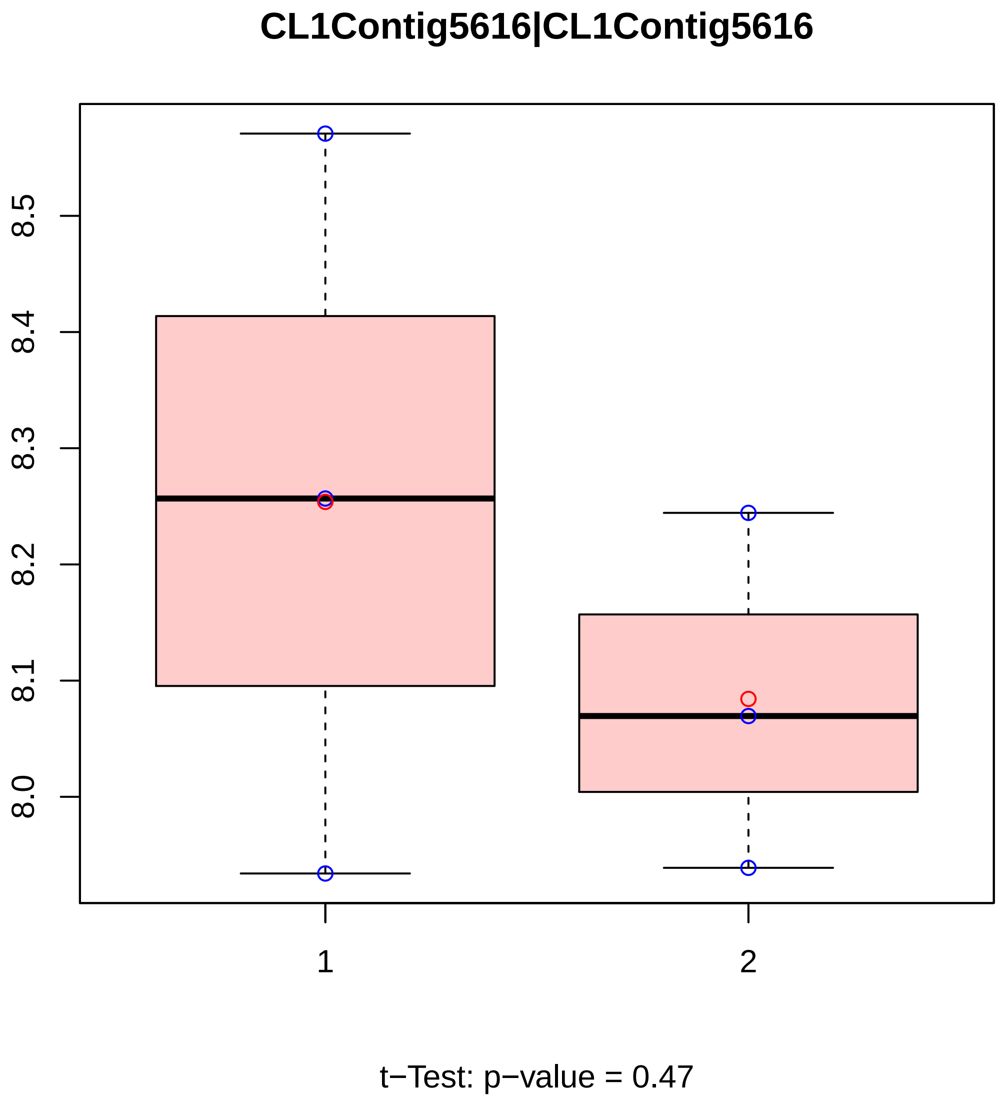
CL1Contig5616|CL1Contig5616
8.0
8.1
8.2
8.3
8.4
8.5
1
2
t−Test: p−value = 0.47

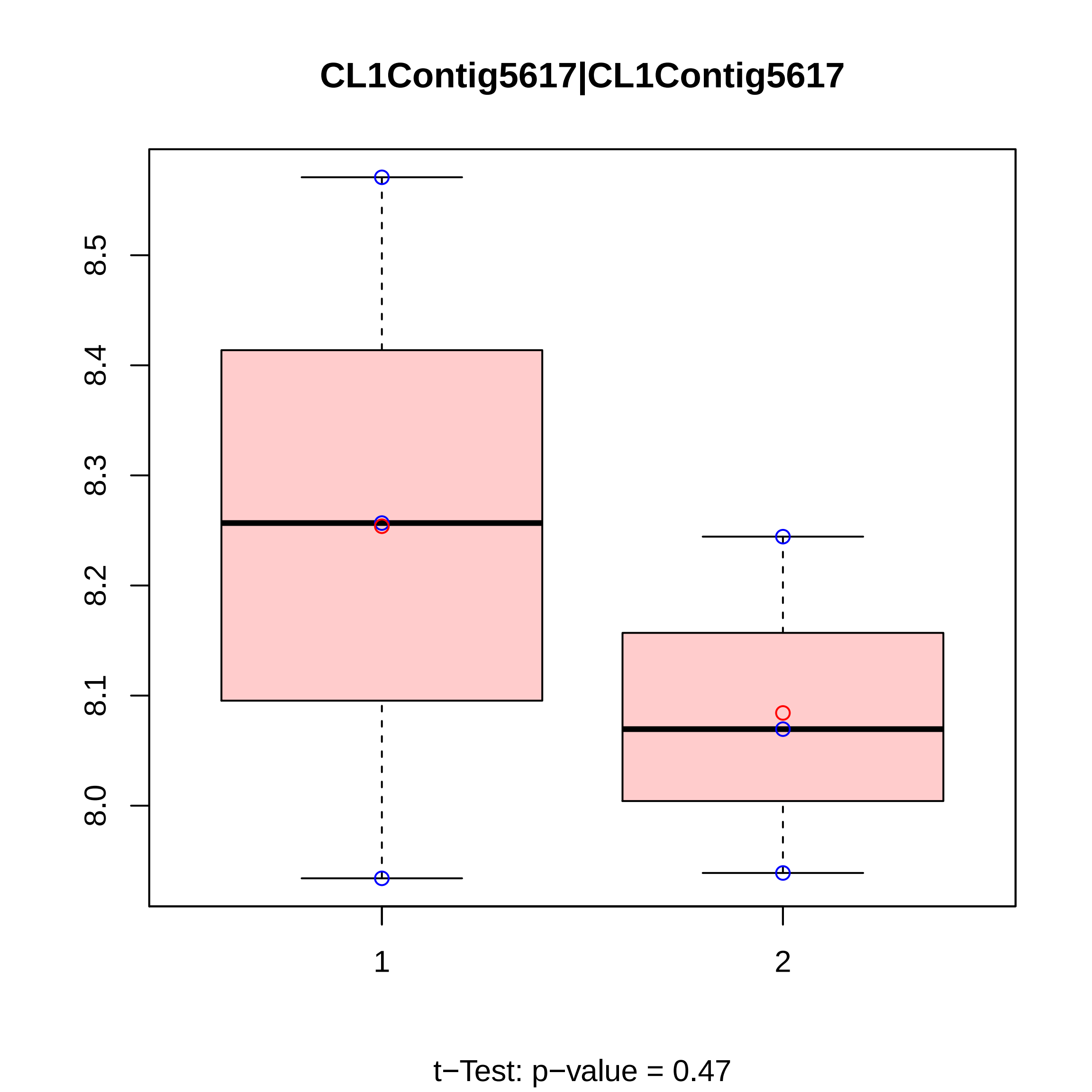
CL1Contig5617|CL1Contig5617
8.0
8.1
8.2
8.3
8.4
8.5
1
2
t−Test: p−value = 0.47

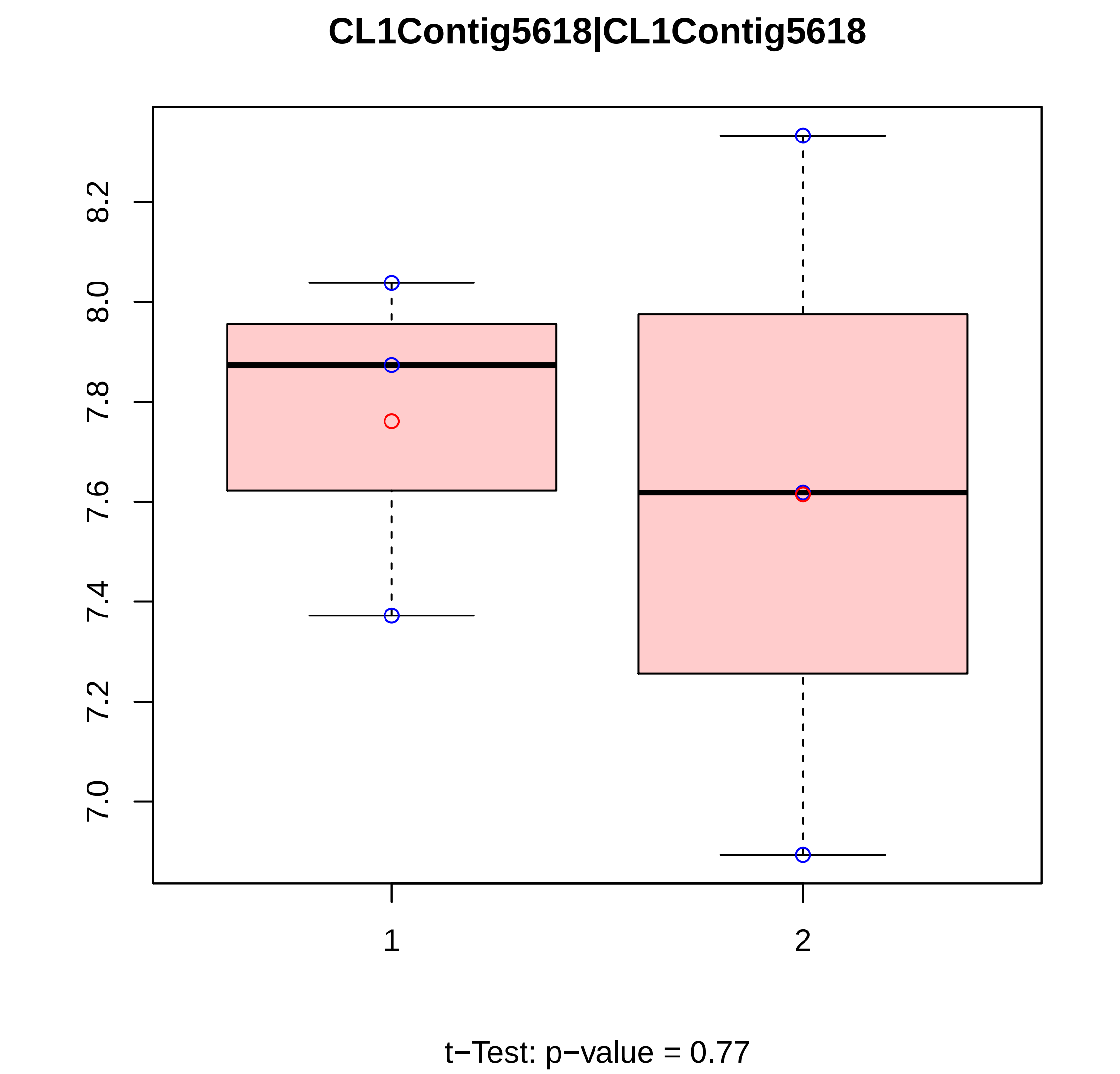

CL1Contig5618|CL1Contig5618
8.2
8.0
7.8
7.6
7.4
7.2
7.0
1
2
t−Test: p−value = 0.77

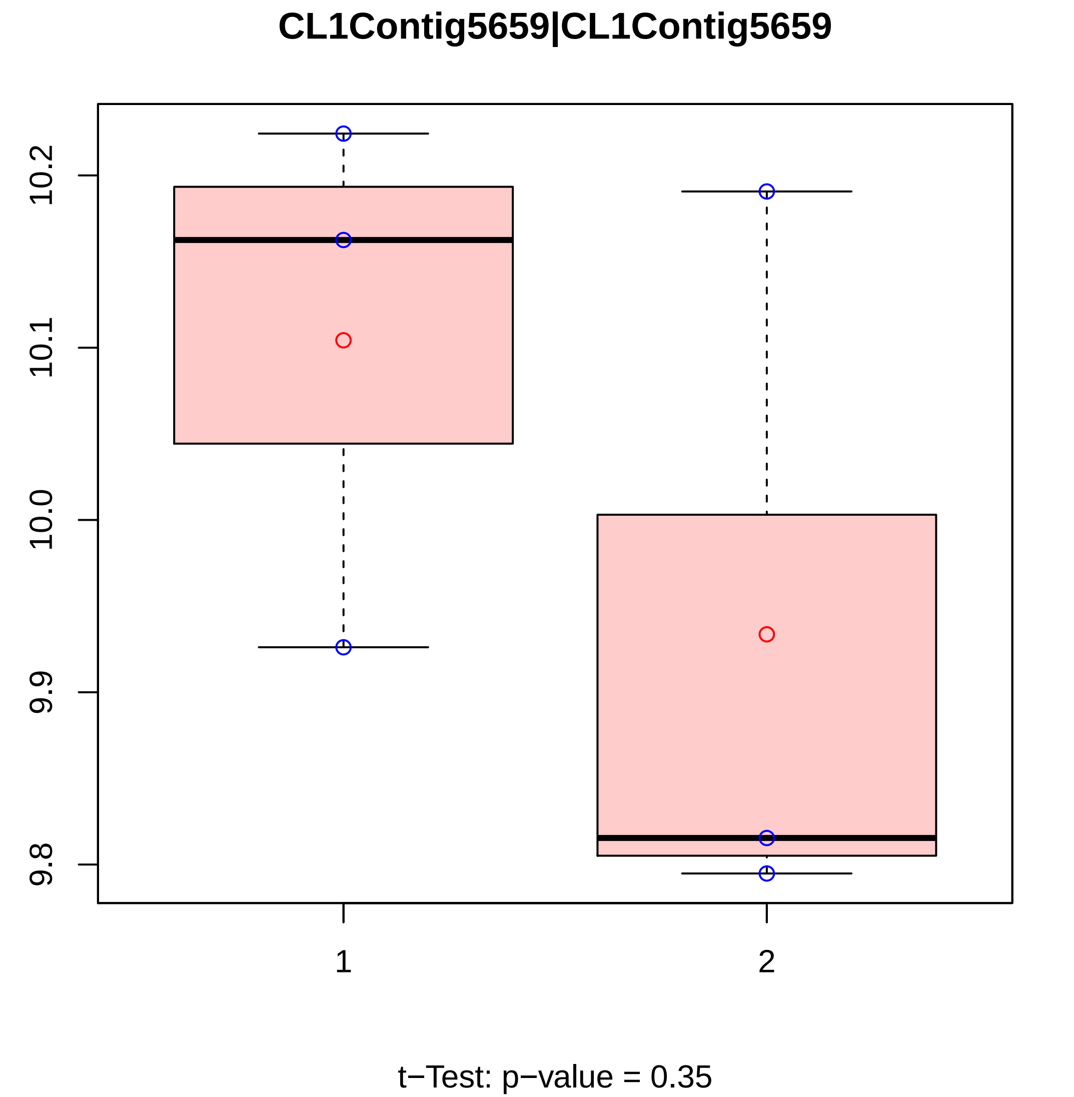
CL1Contig5659|CL1Contig5659
10.2
10.1
10.0
9.9
9.8
1
2
t−Test: p−value = 0.35

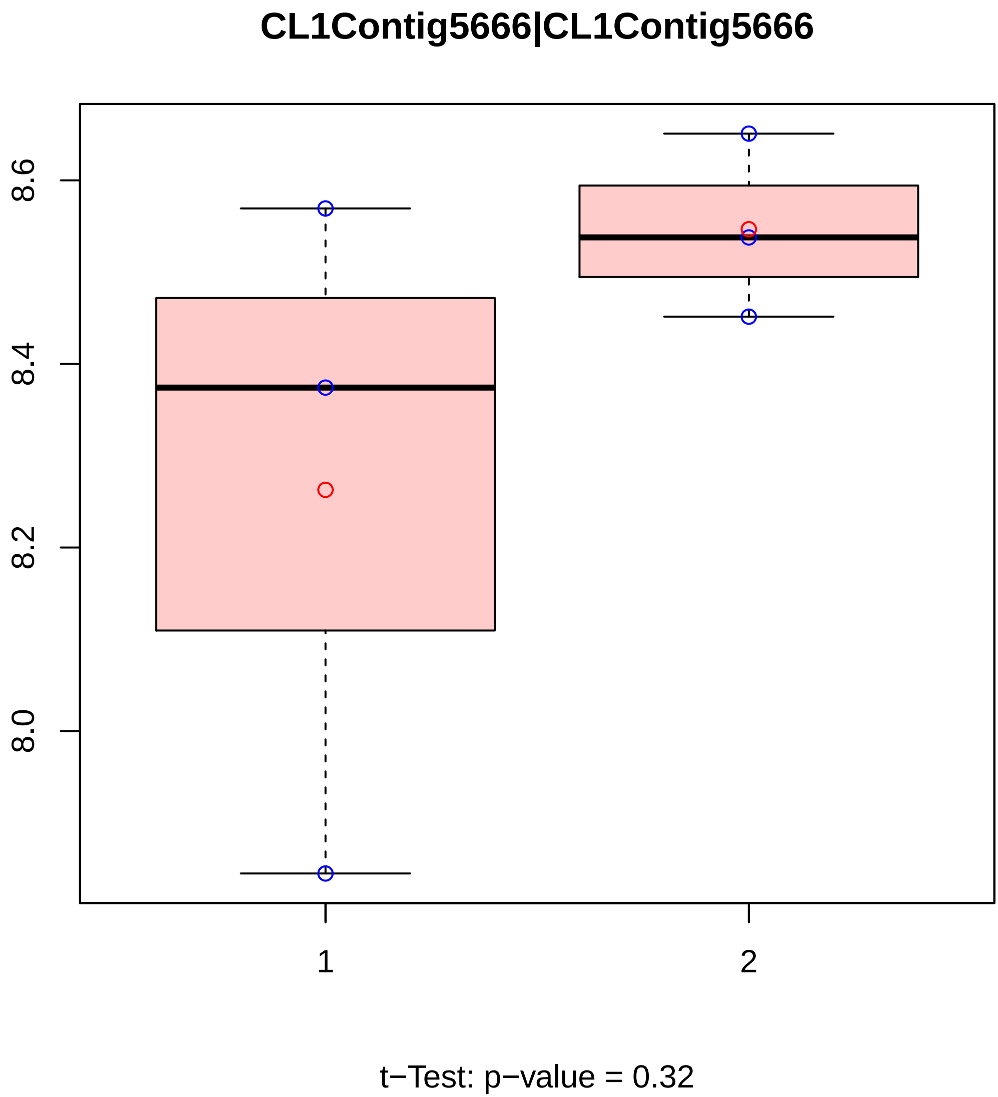
CL1Contig5666|CL1Contig5666
8.0
8.2
8.4
8.6
1
2
t−Test: p−value = 0.32

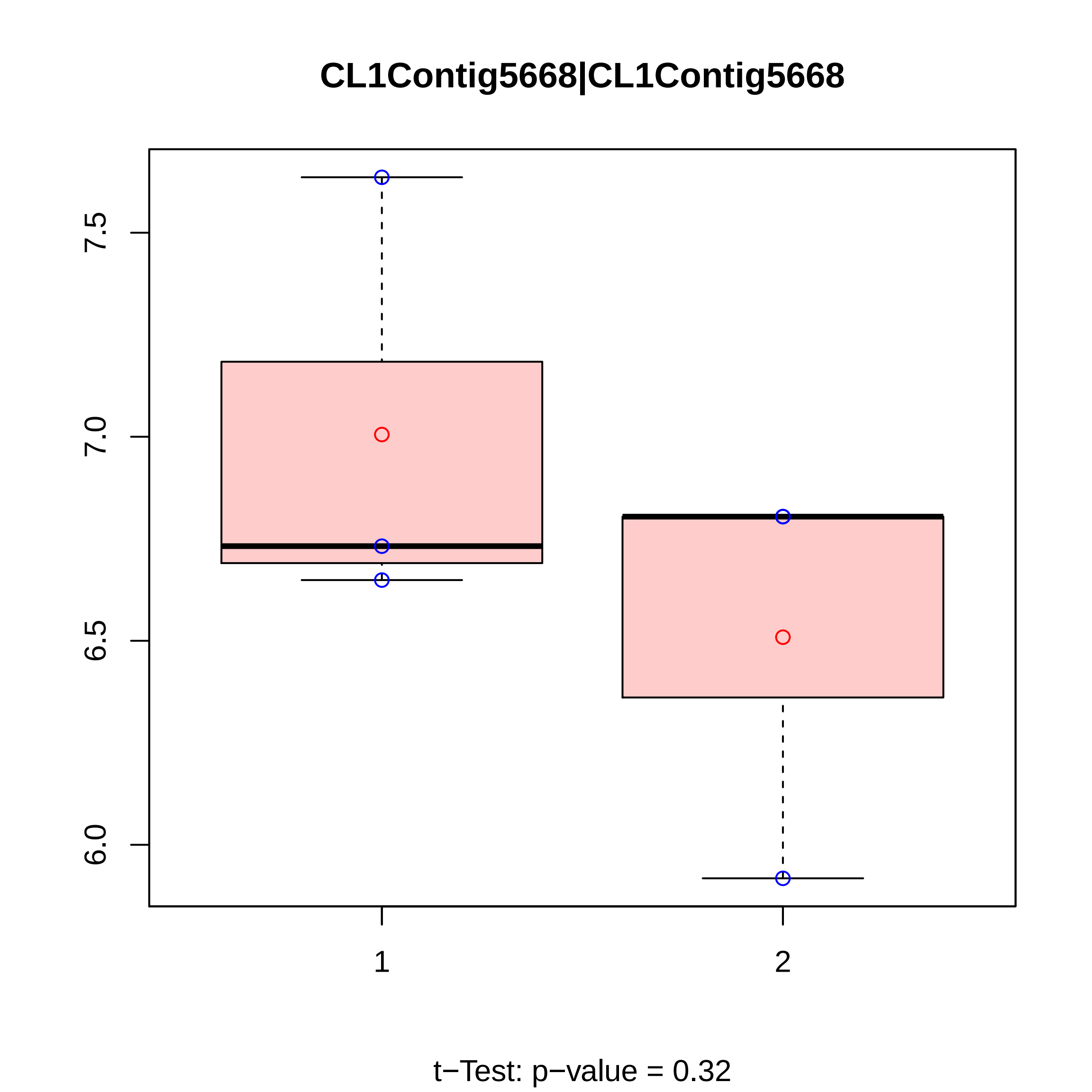

CL1Contig5668|CL1Contig5668
7.5
7.0
6.5
6.0
1
2
t−Test: p−value = 0.32

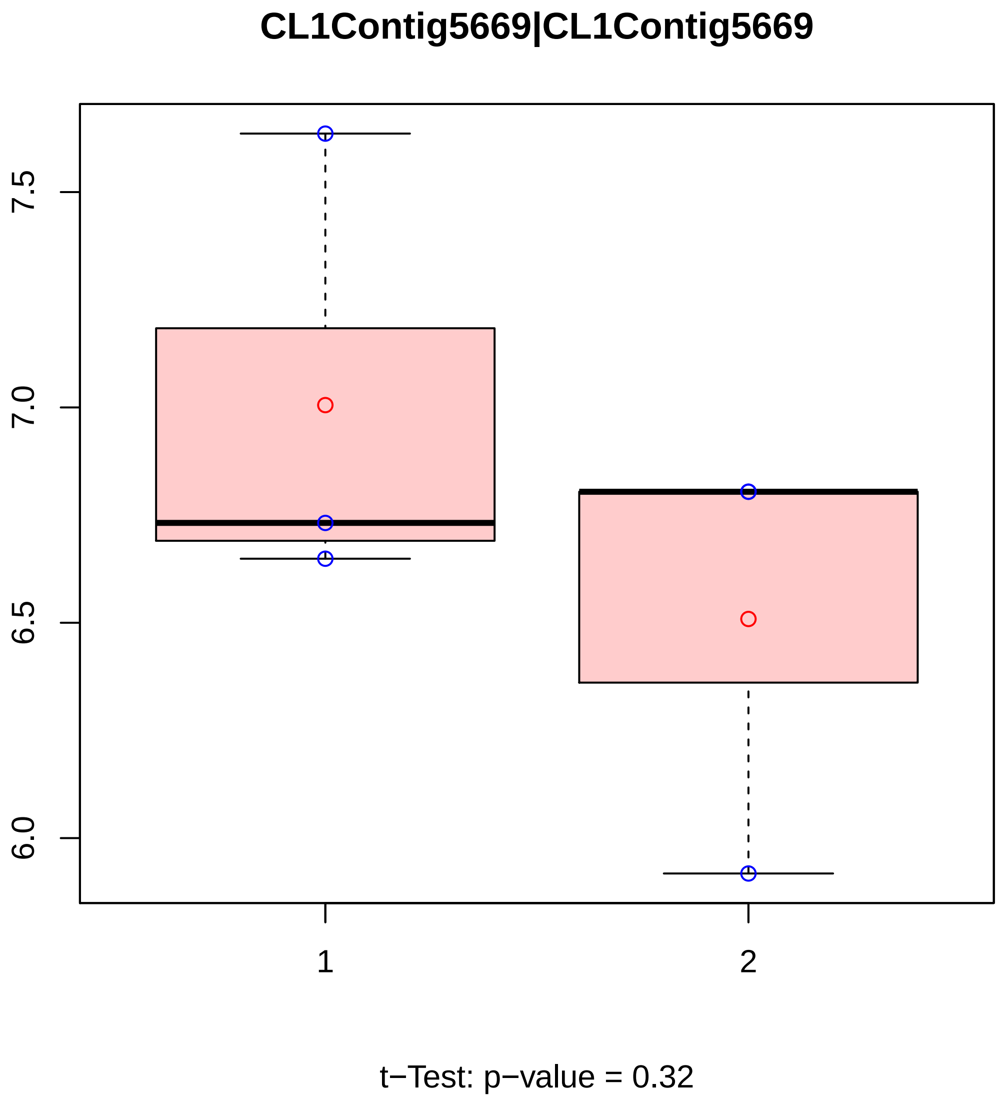
CL1Contig5669|CL1Contig5669
7.5
7.0
6.5
6.0
1
2
t−Test: p−value = 0.32

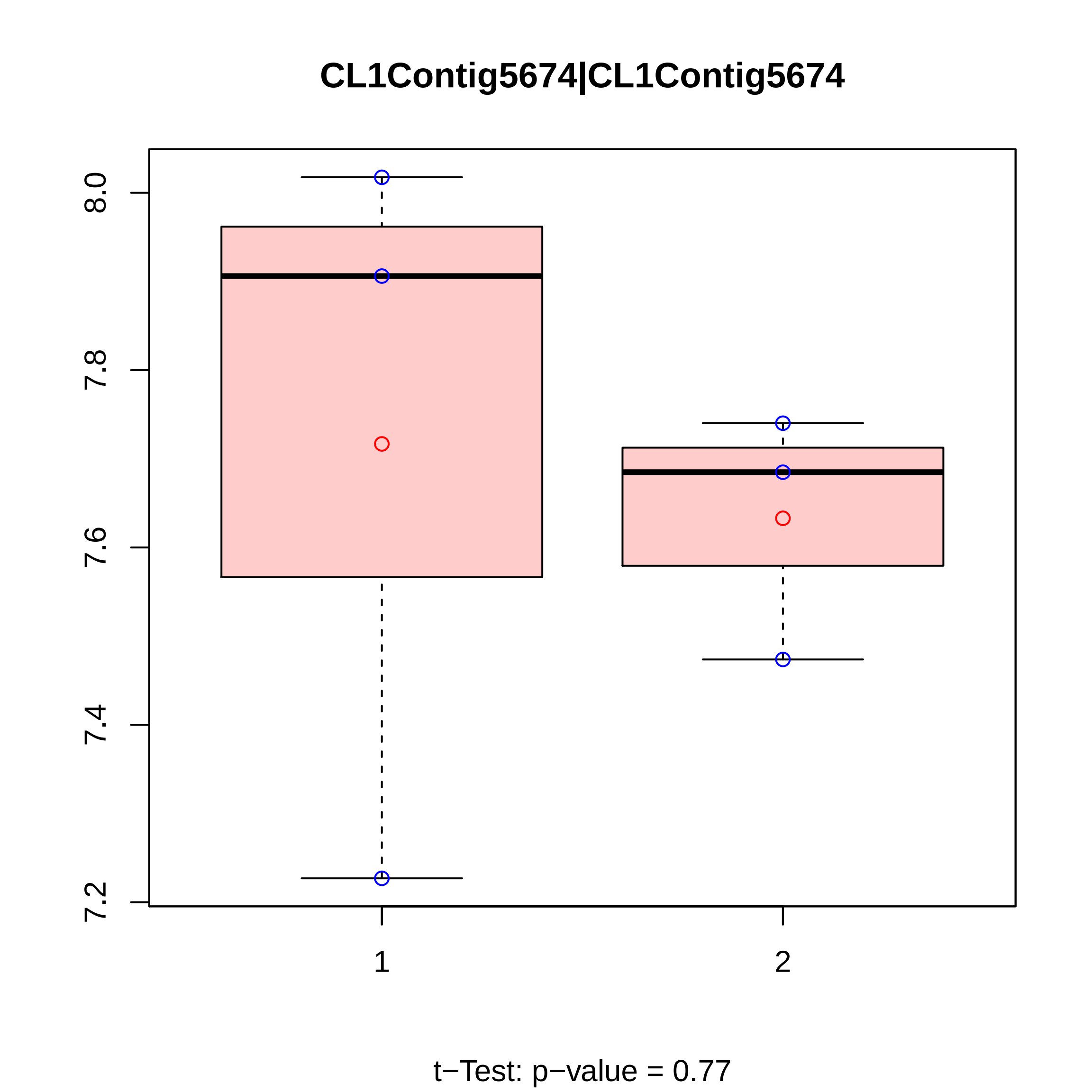

CL1Contig5674|CL1Contig5674
8.0
7.8
7.6
7.4
7.2
1
2
t−Test: p−value = 0.77

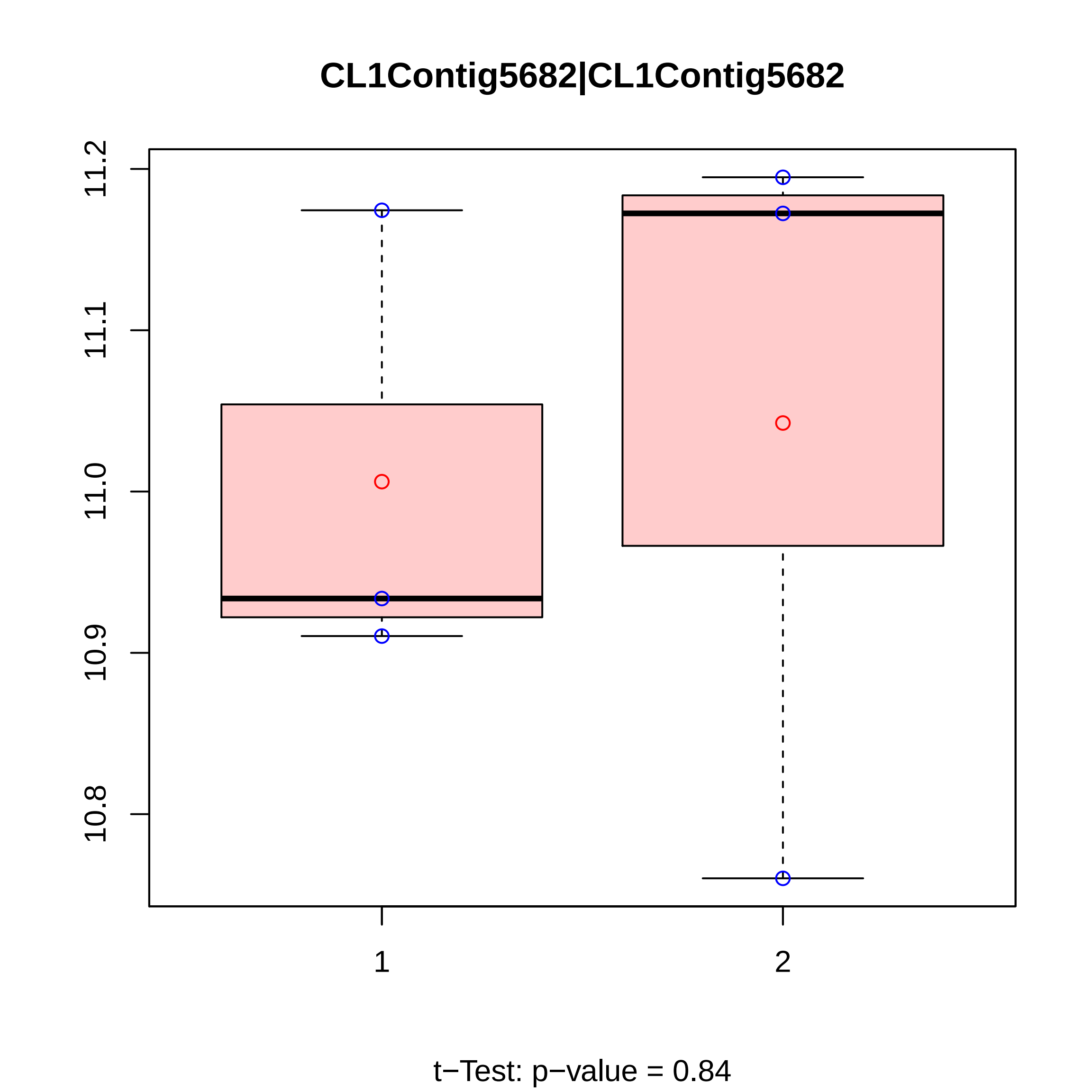

CL1Contig5682|CL1Contig5682
11.2
11.1
11.0
10.9
10.8
1
2
t−Test: p−value = 0.84

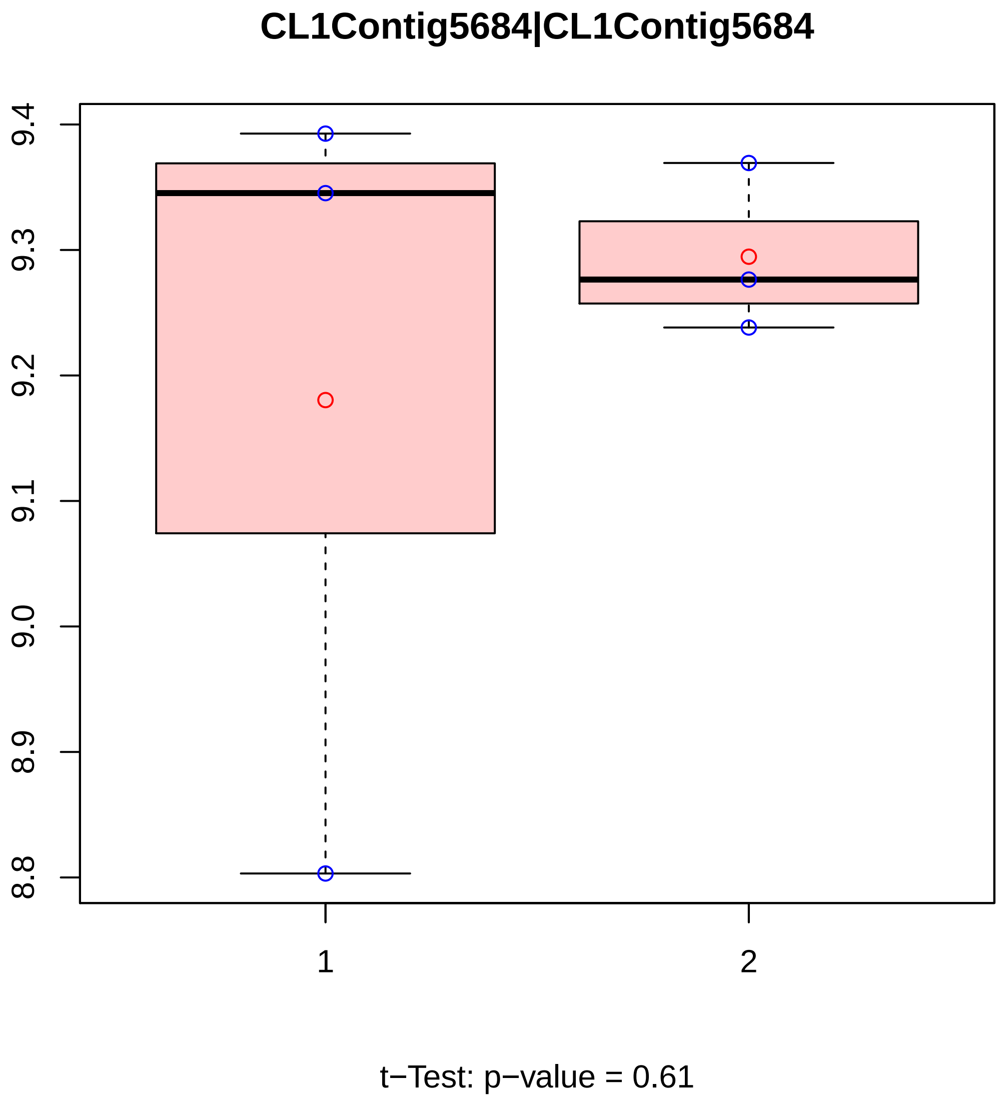

CL1Contig5684|CL1Contig5684
9.4
9.3
9.2
9.1
9.0
8.9
8.8
1
2
t−Test: p−value = 0.61

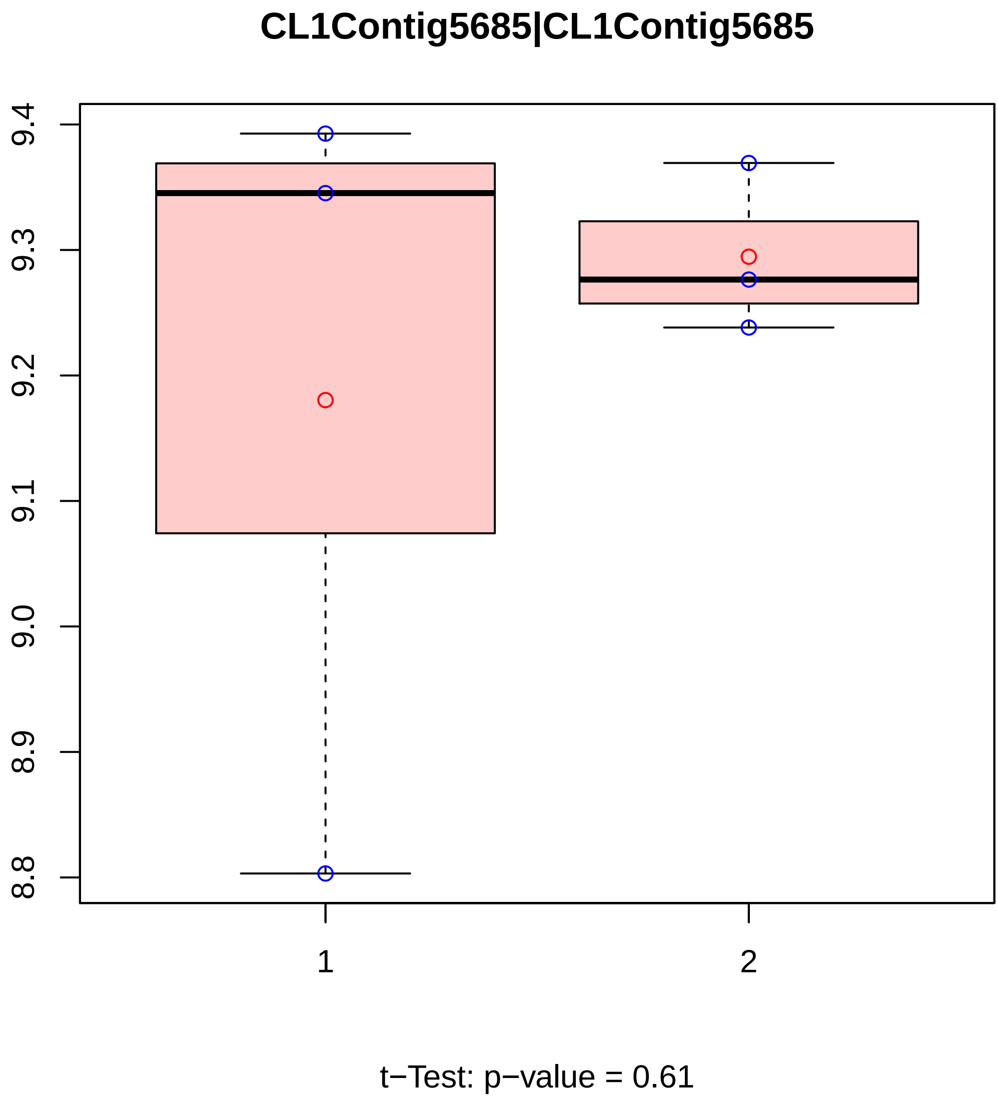

CL1Contig5685|CL1Contig5685
9.4
9.3
9.2
9.1
9.0
8.9
8.8
1
2
t−Test: p−value = 0.61

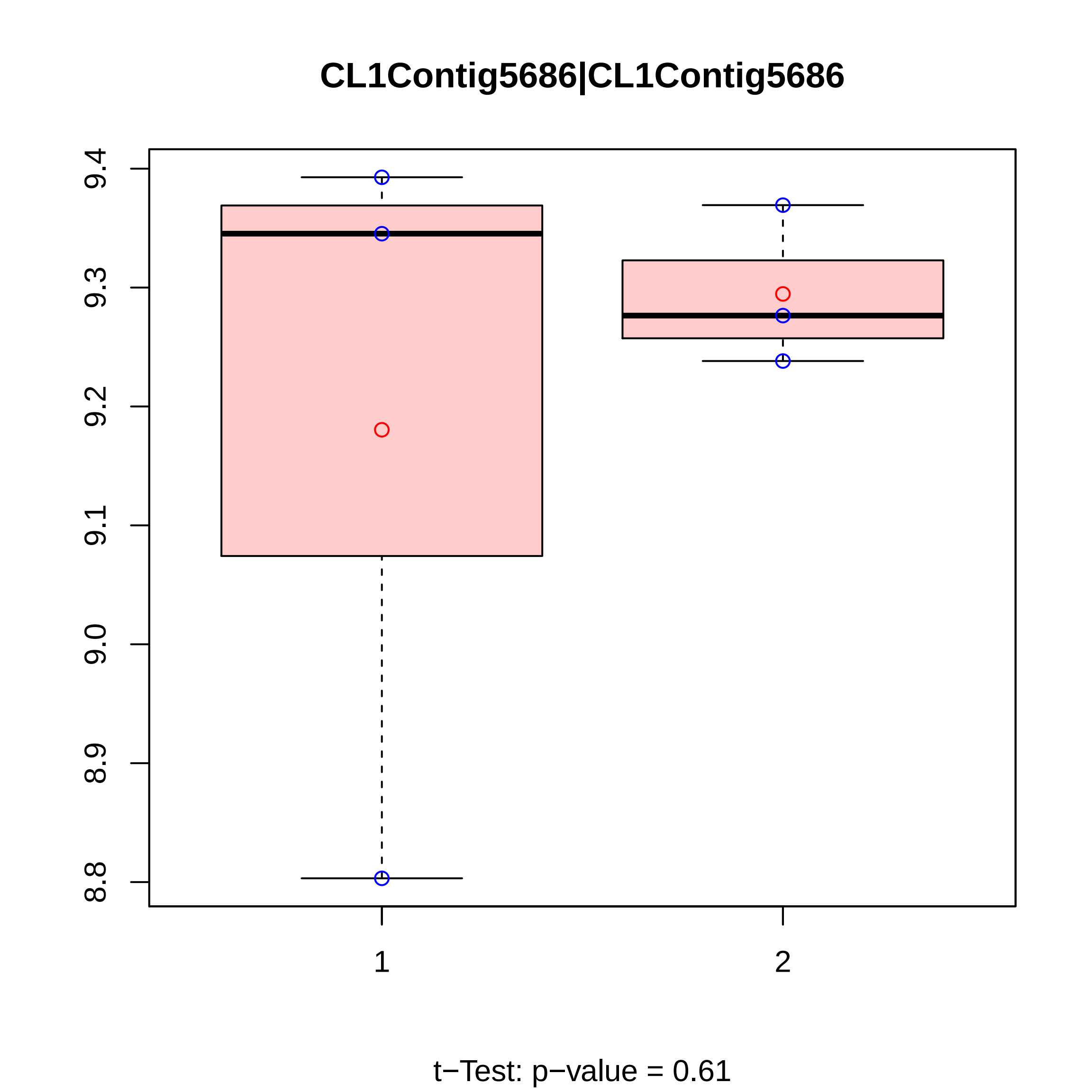

CL1Contig5686|CL1Contig5686
9.4
9.3
9.2
9.1
9.0
8.9
8.8
1
2
t−Test: p−value = 0.61

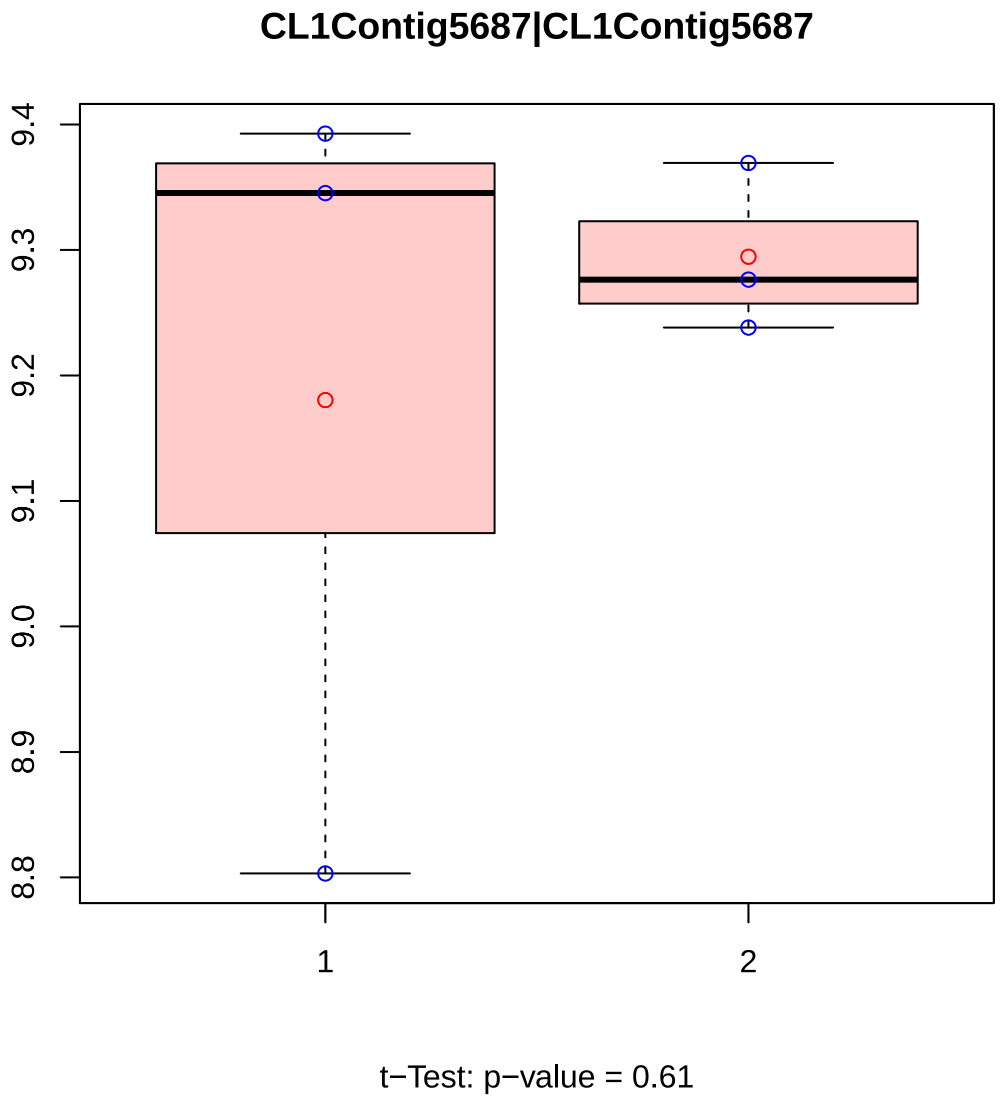

CL1Contig5687|CL1Contig5687
9.4
9.3
9.2
9.1
9.0
8.9
8.8
1
2
t−Test: p−value = 0.61

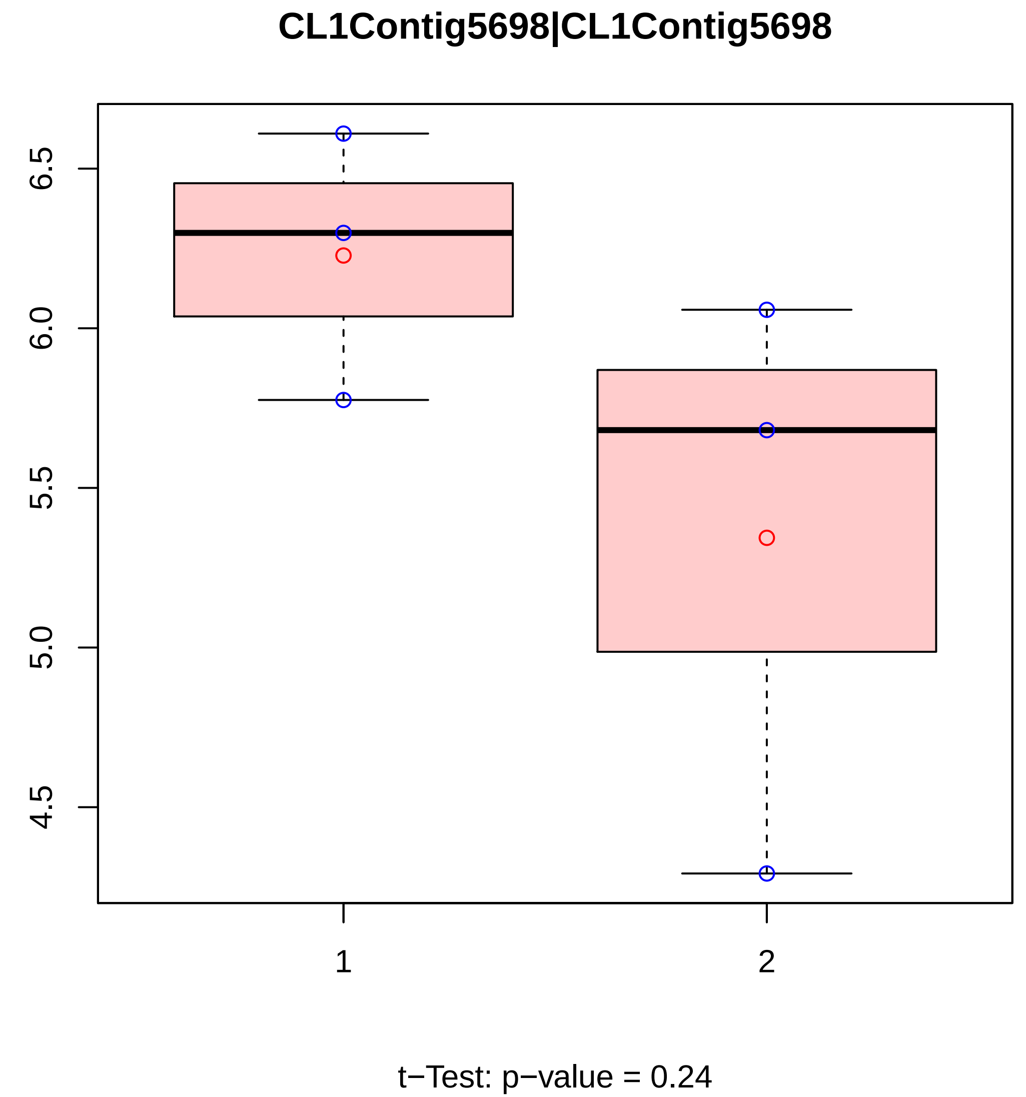

CL1Contig5698|CL1Contig5698
6.5
6.0
5.5
5.0
4.5
1
2
t−Test: p−value = 0.24

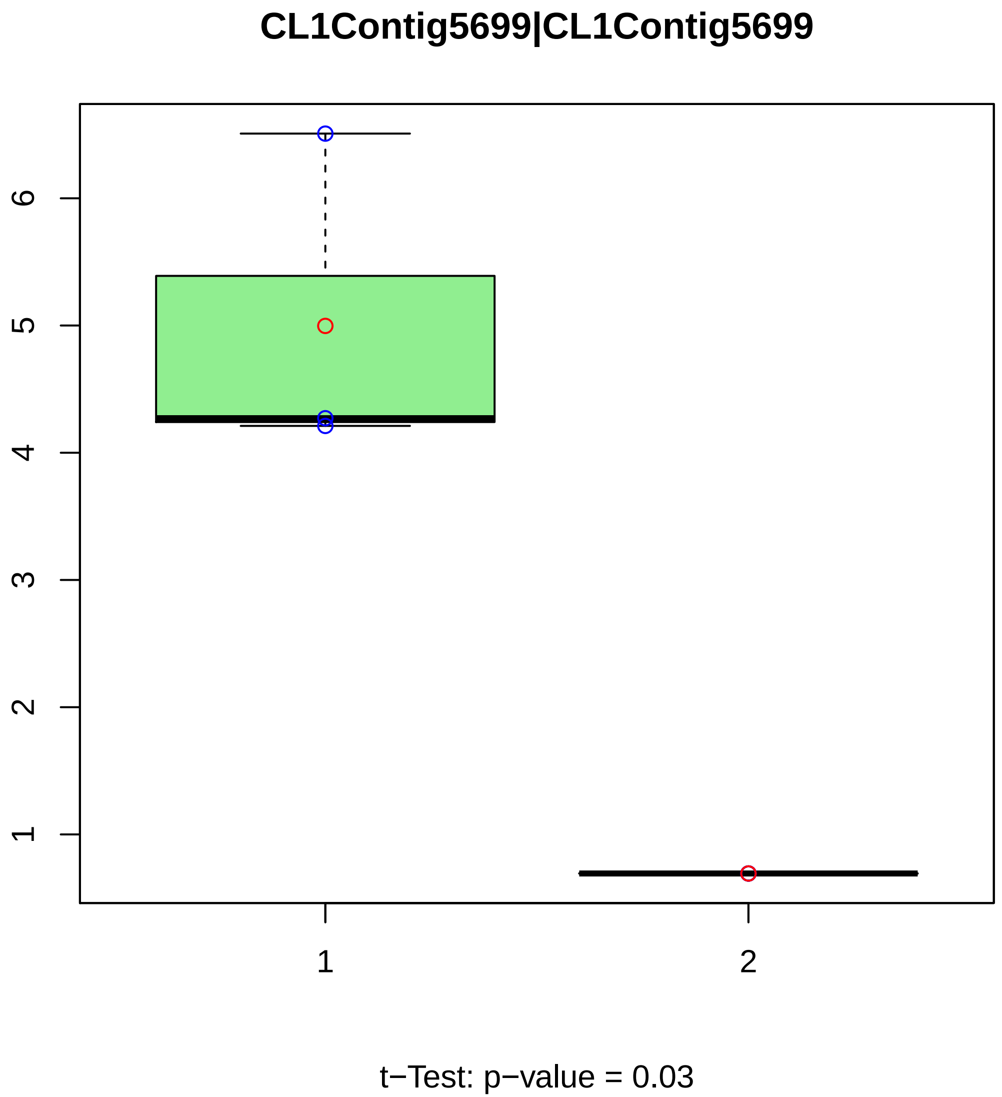
CL1Contig5699|CL1Contig5699
1
2
3
4
5
6
1
2
t−Test: p−value = 0.03

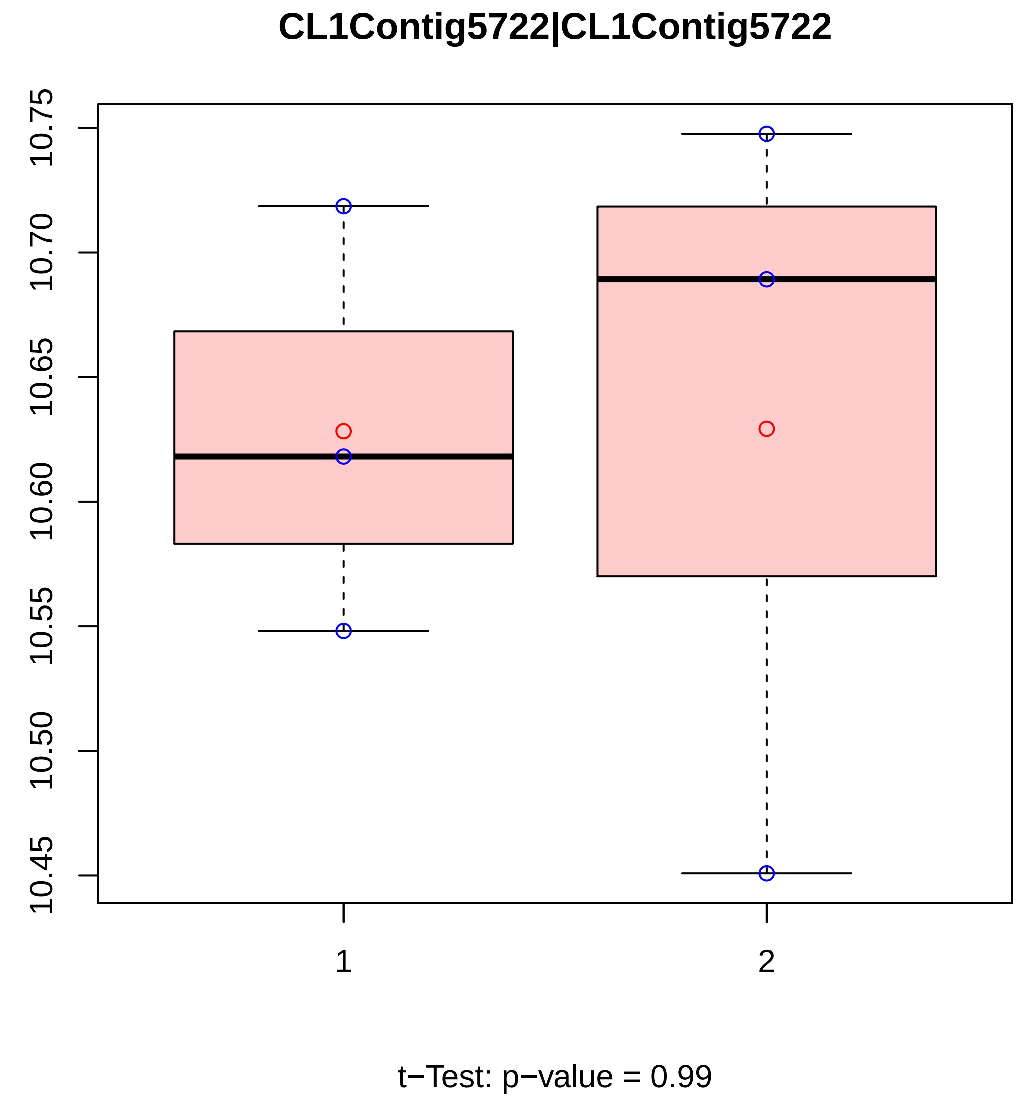

CL1Contig5722|CL1Contig5722
10.45
10.50
10.55
10.60
10.65
10.70
10.75
1
2
t−Test: p−value = 0.99

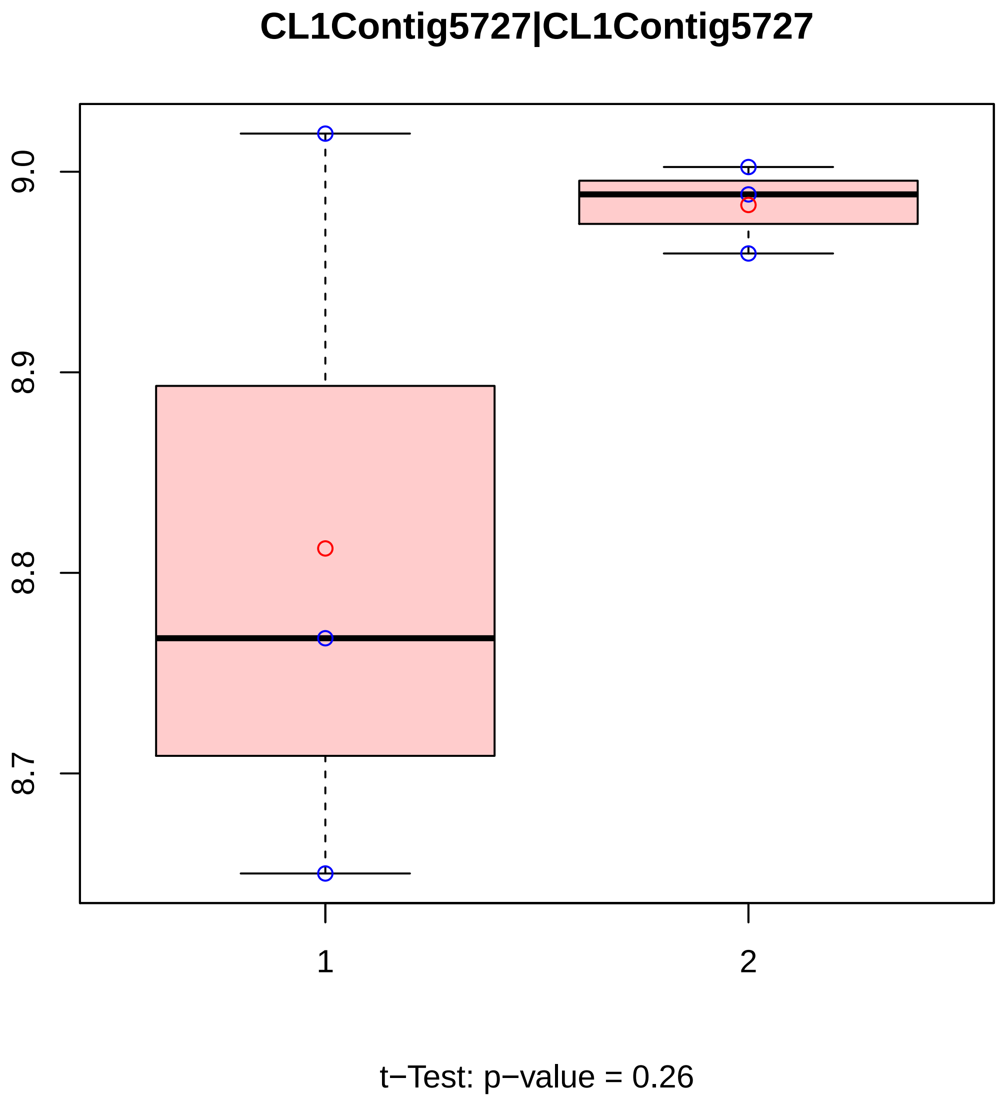
CL1Contig5727|CL1Contig5727
9.0
8.9
8.8
8.7
1
2
t−Test: p−value = 0.26

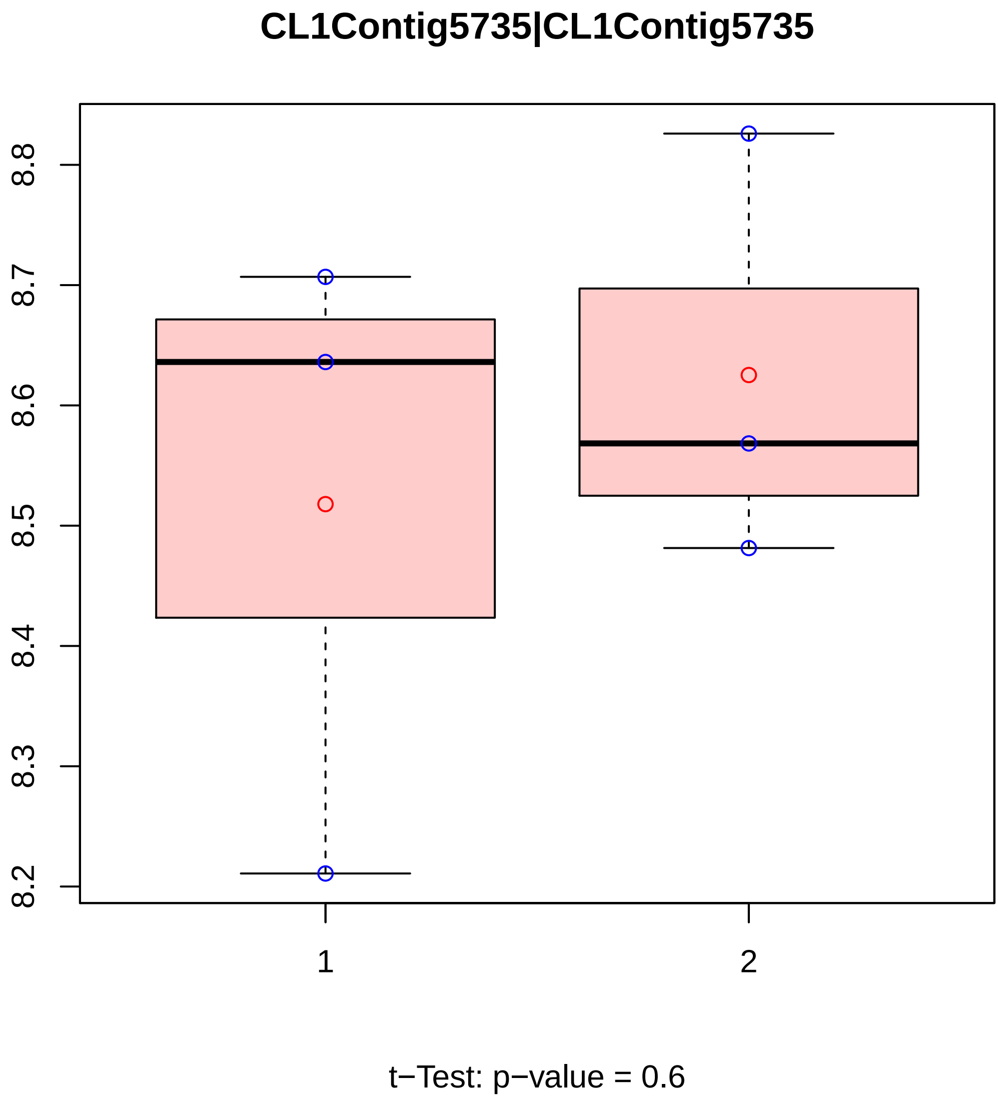
CL1Contig5735|CL1Contig5735
8.2
8.3
8.4
8.5
8.6
8.7
8.8
1
2
t−Test: p−value = 0.6

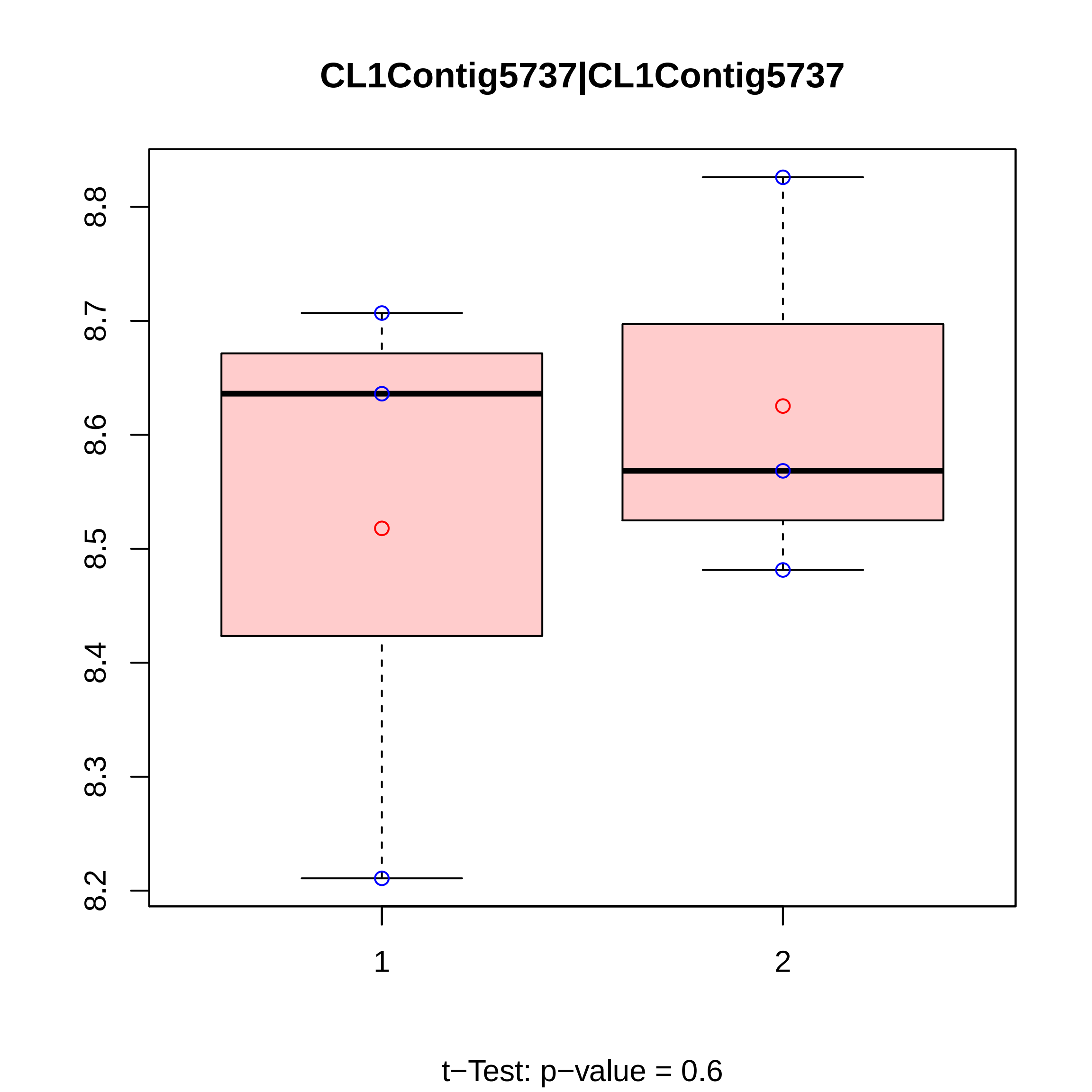

CL1Contig5737|CL1Contig5737
8.2
8.3
8.4
8.5
8.6
8.7
8.8
1
2
t−Test: p−value = 0.6

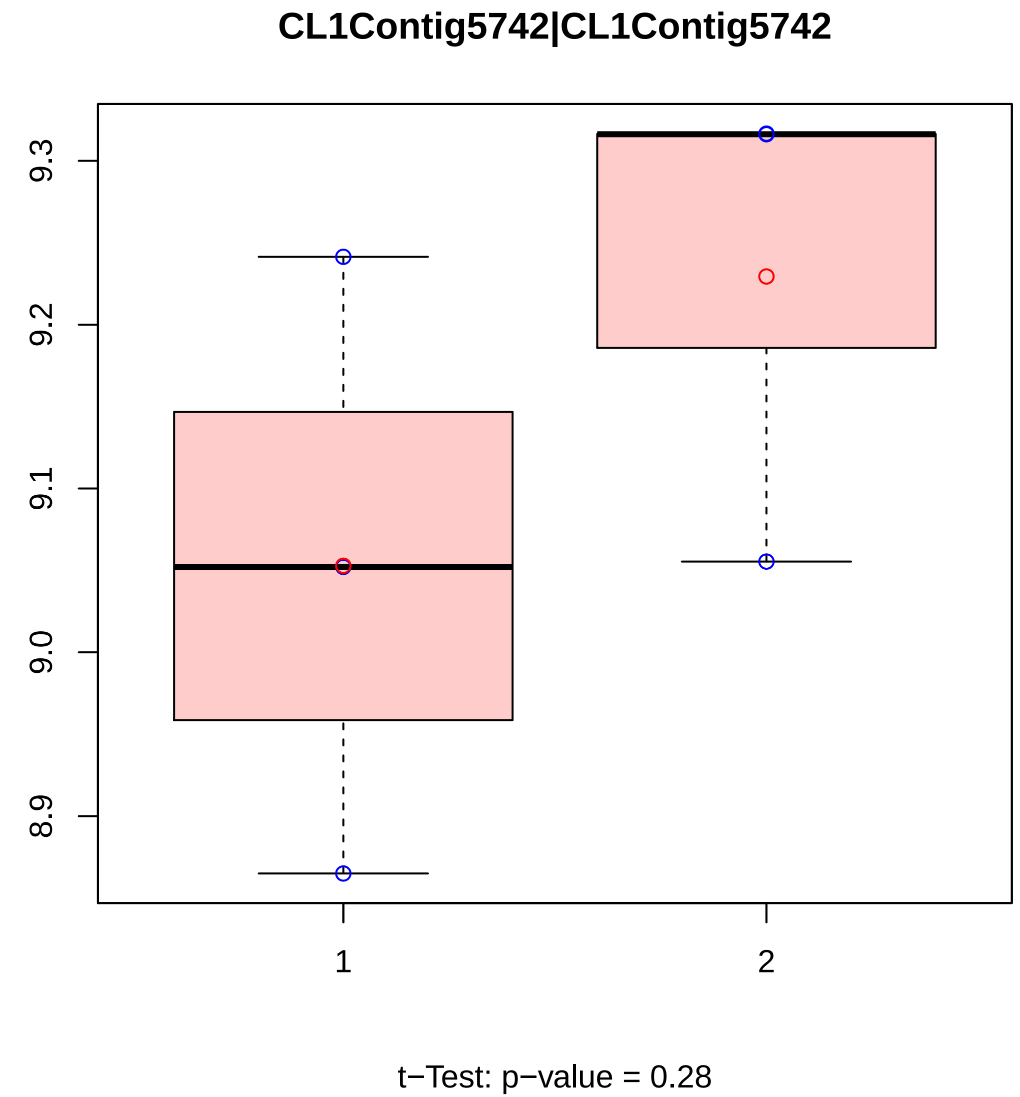
CL1Contig5742|CL1Contig5742
9.3
9.2
9.1
9.0
8.9
1
2
t−Test: p−value = 0.28

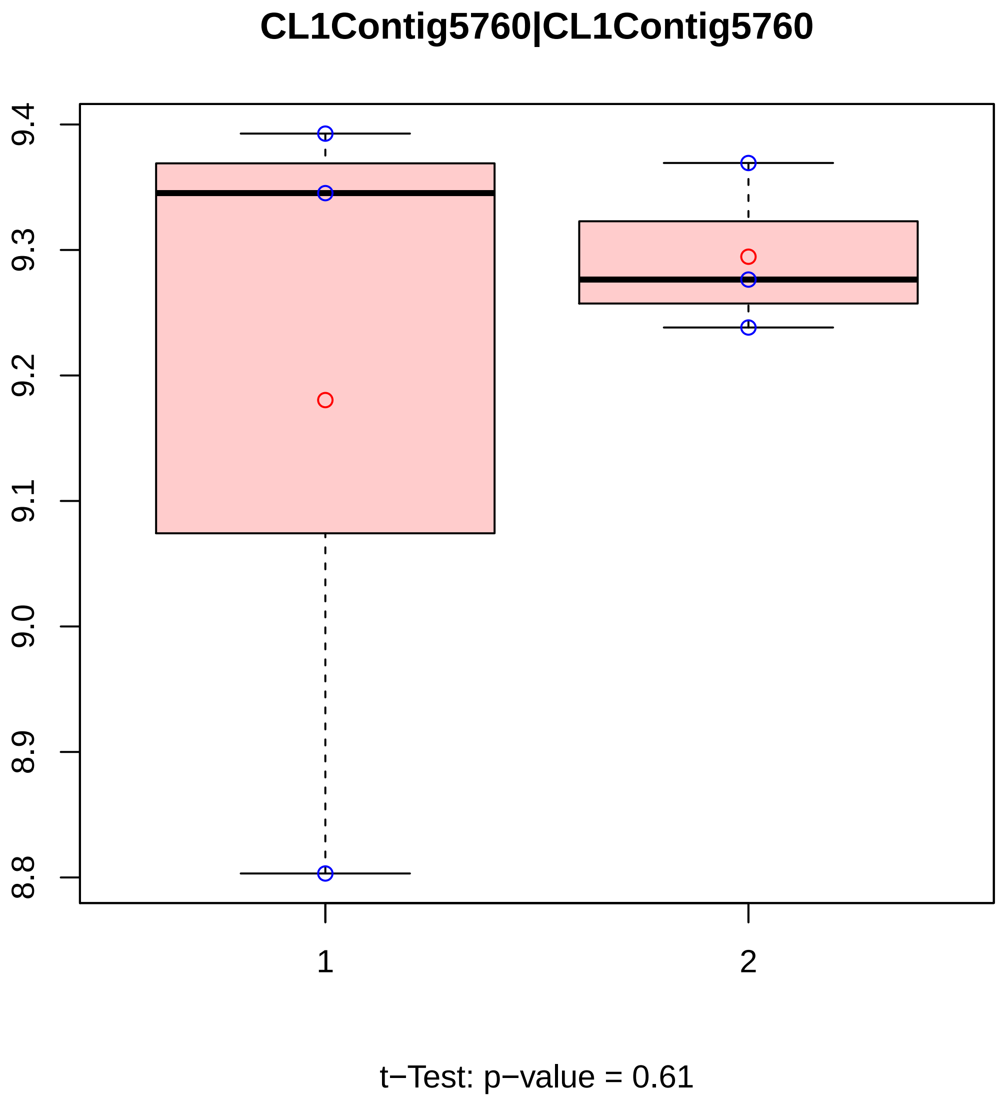

CL1Contig5760|CL1Contig5760
9.4
9.3
9.2
9.1
9.0
8.9
8.8
1
2
t−Test: p−value = 0.61

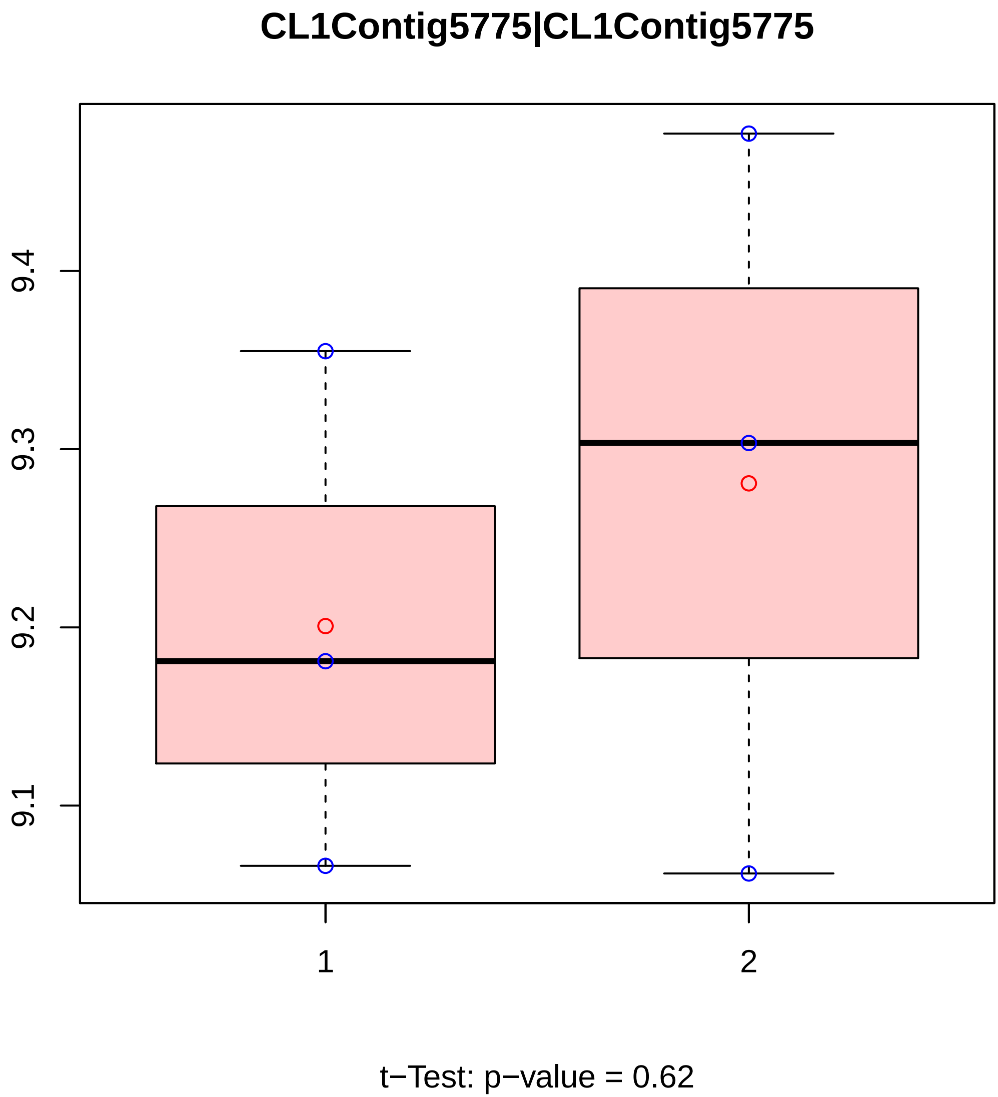

CL1Contig5775|CL1Contig5775
9.1
9.2
9.3
9.4
1
2
t−Test: p−value = 0.62

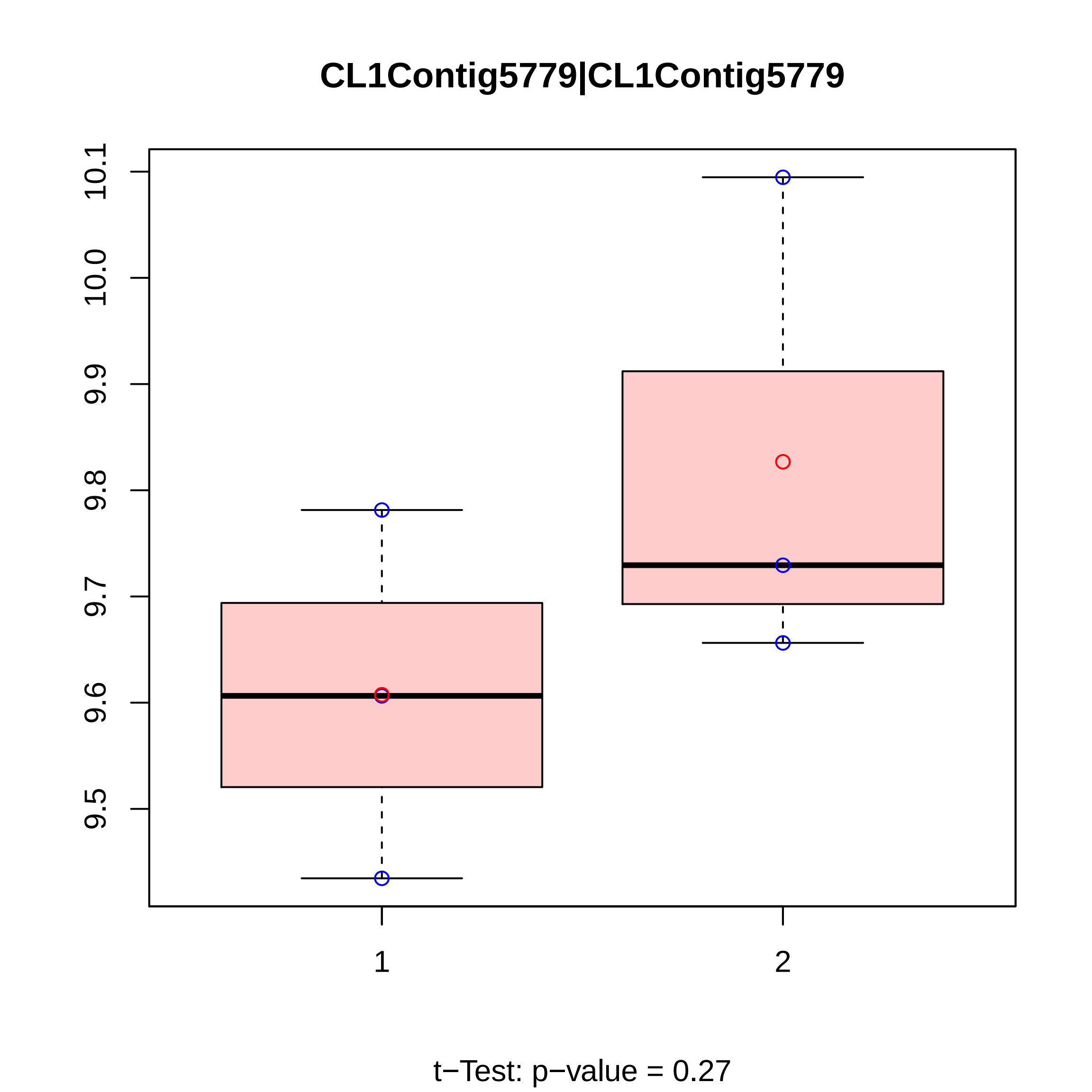

CL1Contig5779|CL1Contig5779
10.1
10.0
9.9
9.8
9.7
9.6
9.5
1
2
t−Test: p−value = 0.27

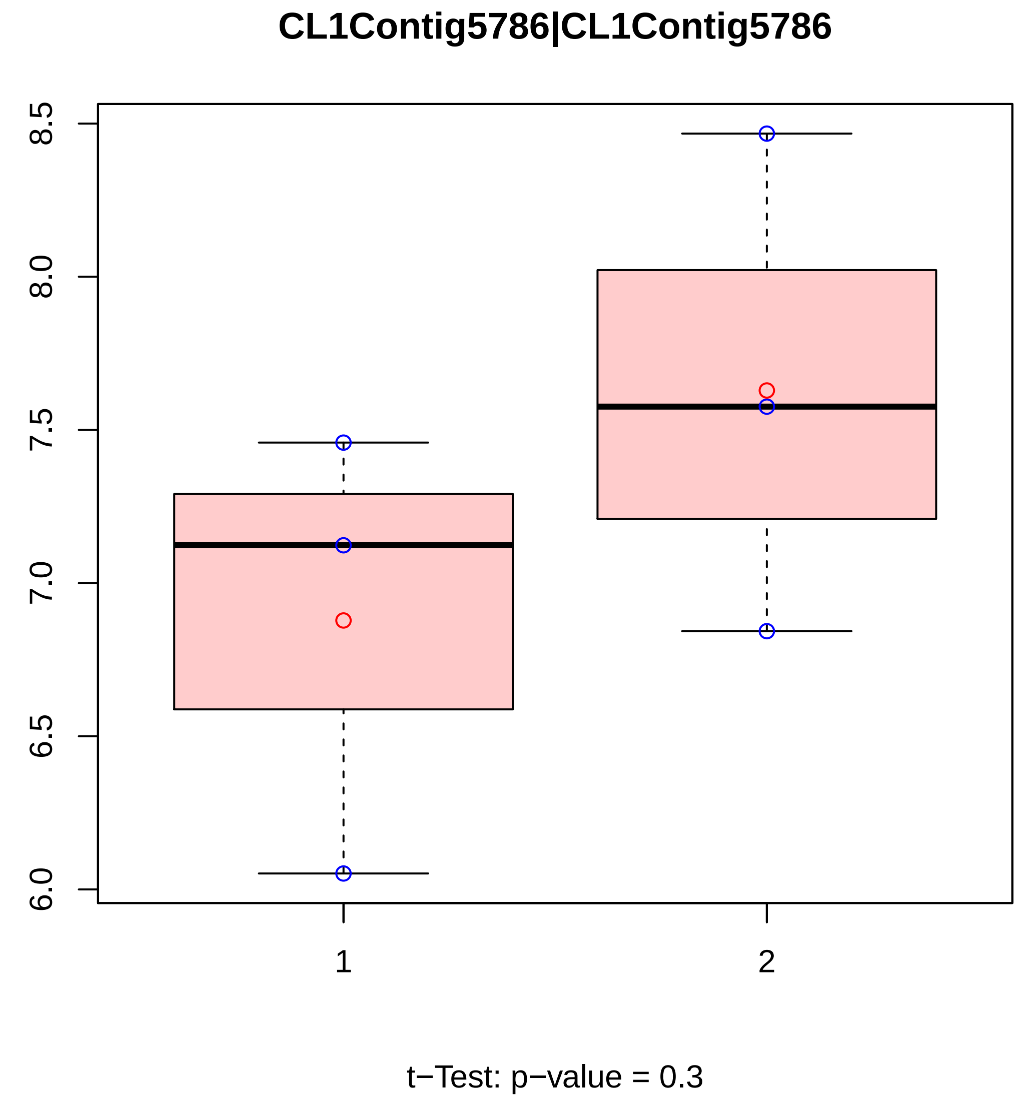

CL1Contig5786|CL1Contig5786
8.5
8.0
7.5
7.0
6.5
6.0
1
2
t−Test: p−value = 0.3

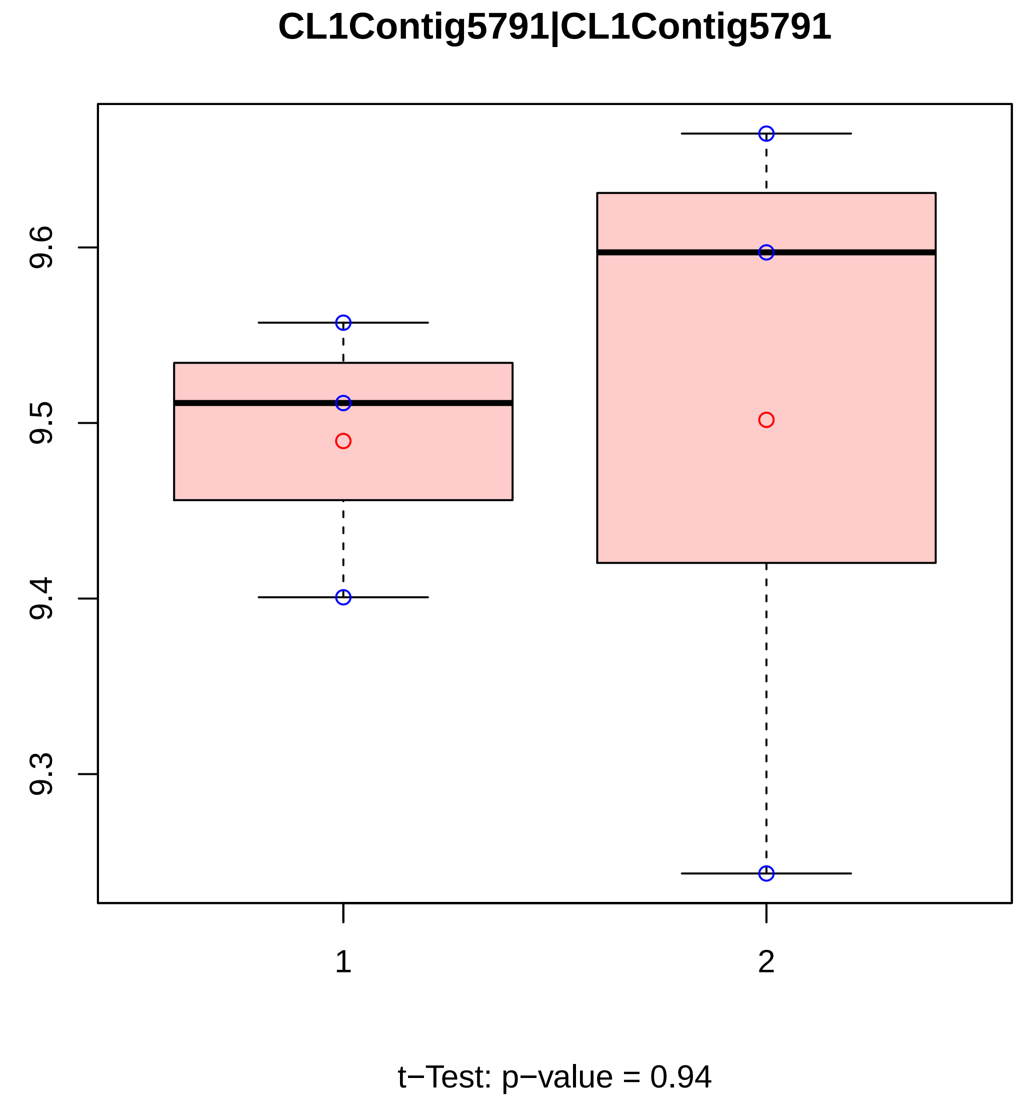

CL1Contig5791|CL1Contig5791
9.3
9.4
9.5
9.6
1
2
t−Test: p−value = 0.94

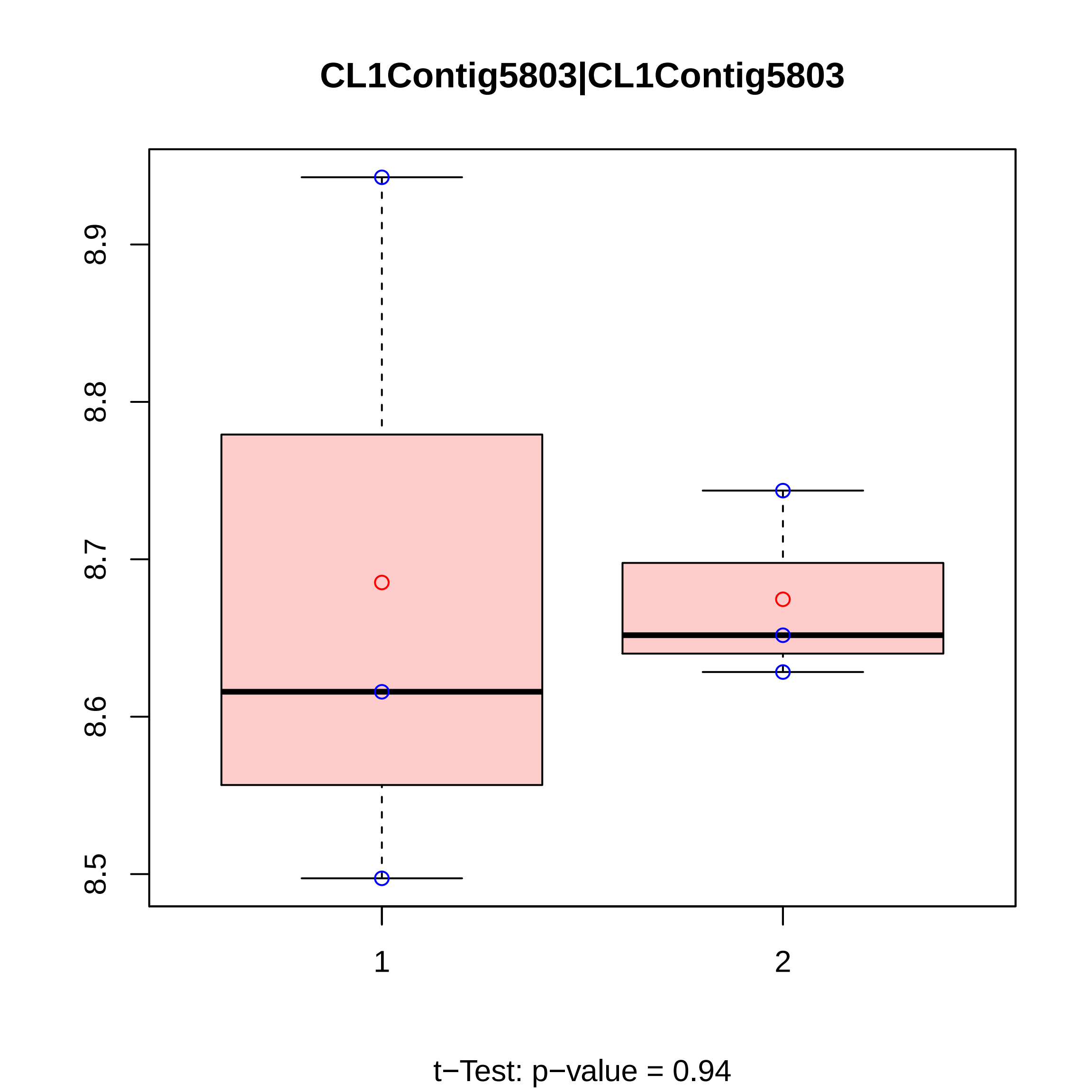
CL1Contig5803|CL1Contig5803
8.5
8.6
8.7
8.8
8.9
1
2
t−Test: p−value = 0.94

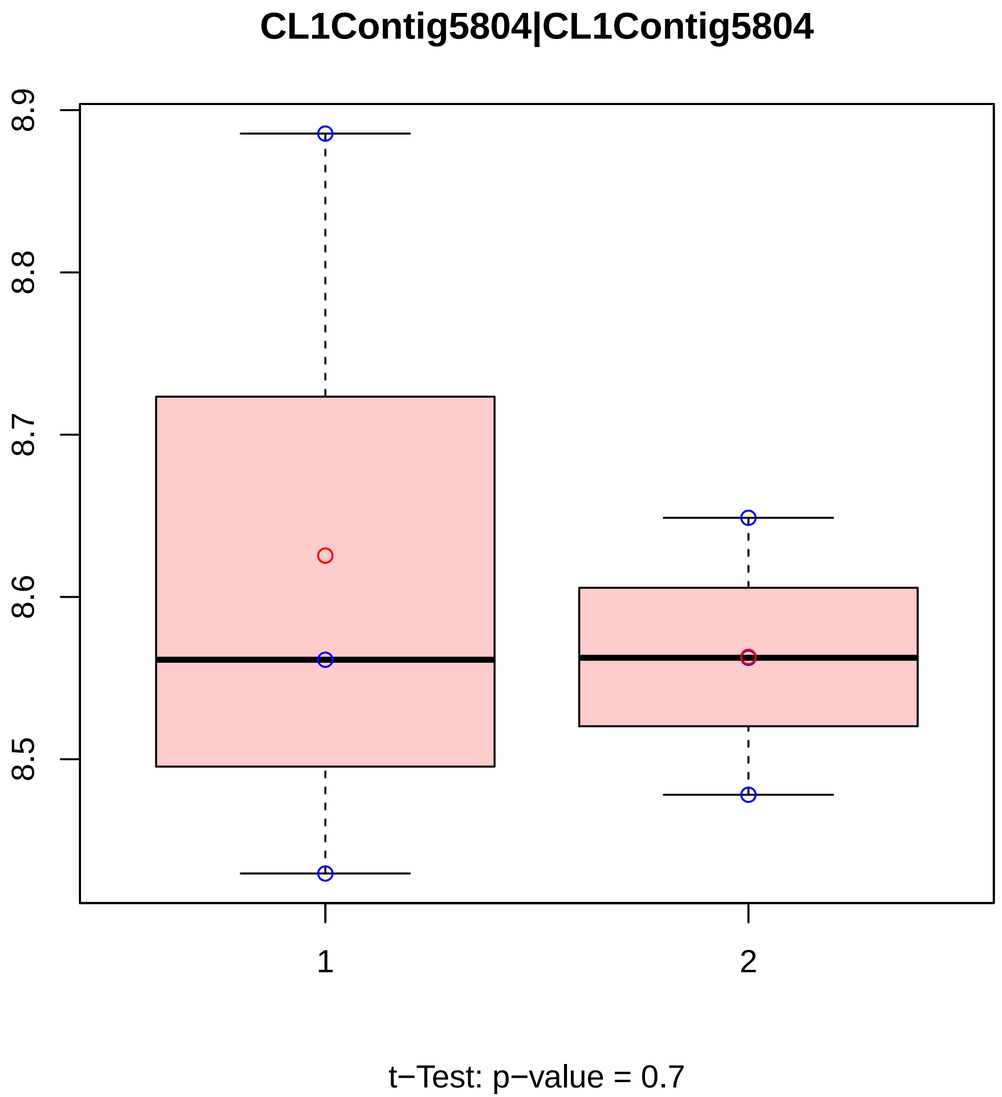
CL1Contig5804|CL1Contig5804
8.5
8.6
8.7
8.8
8.9
1
2
t−Test: p−value = 0.7

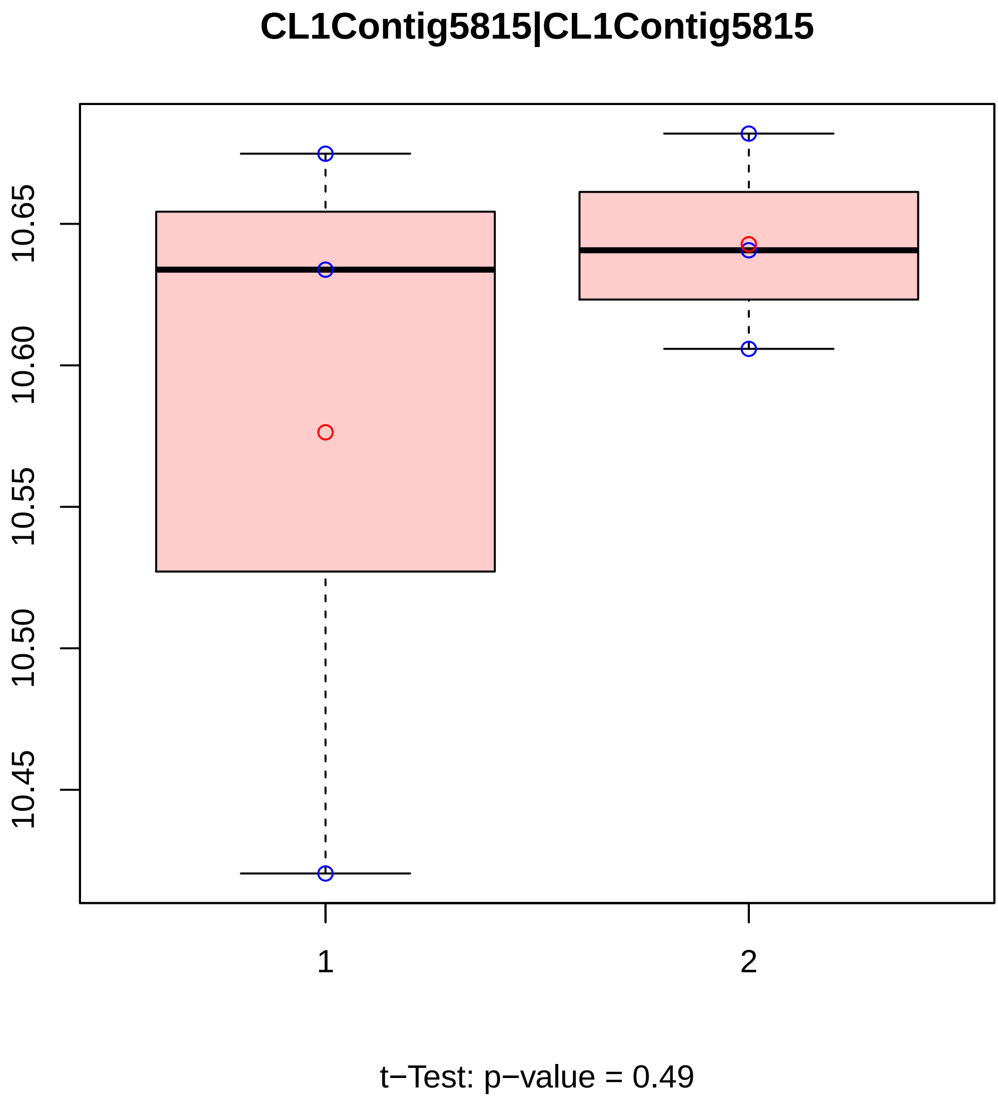
CL1Contig5815|CL1Contig5815
10.45
10.50
10.55
10.60
10.65
1
2
t−Test: p−value = 0.49

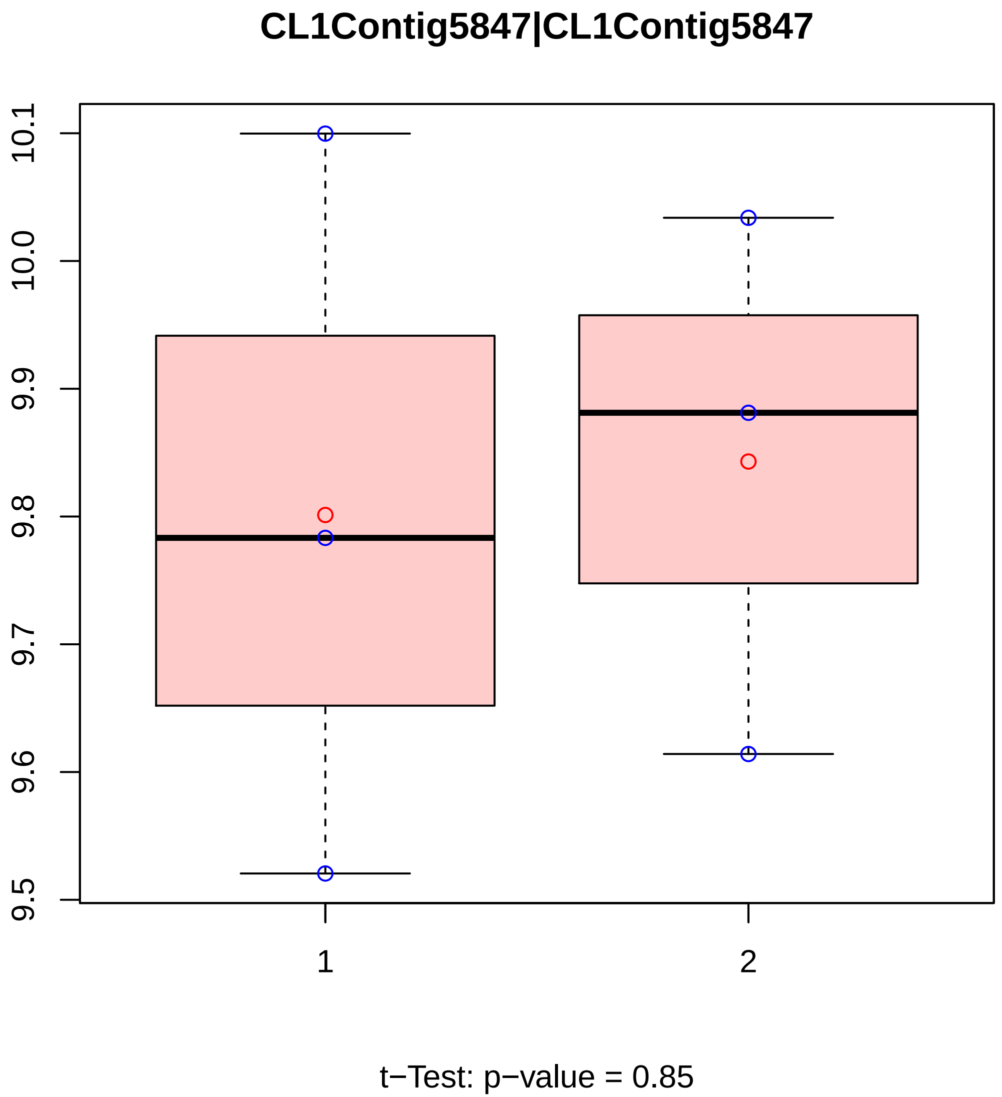

CL1Contig5847|CL1Contig5847
10.1
10.0
9.9
9.8
9.7
9.6
9.5
1
2
t−Test: p−value = 0.85

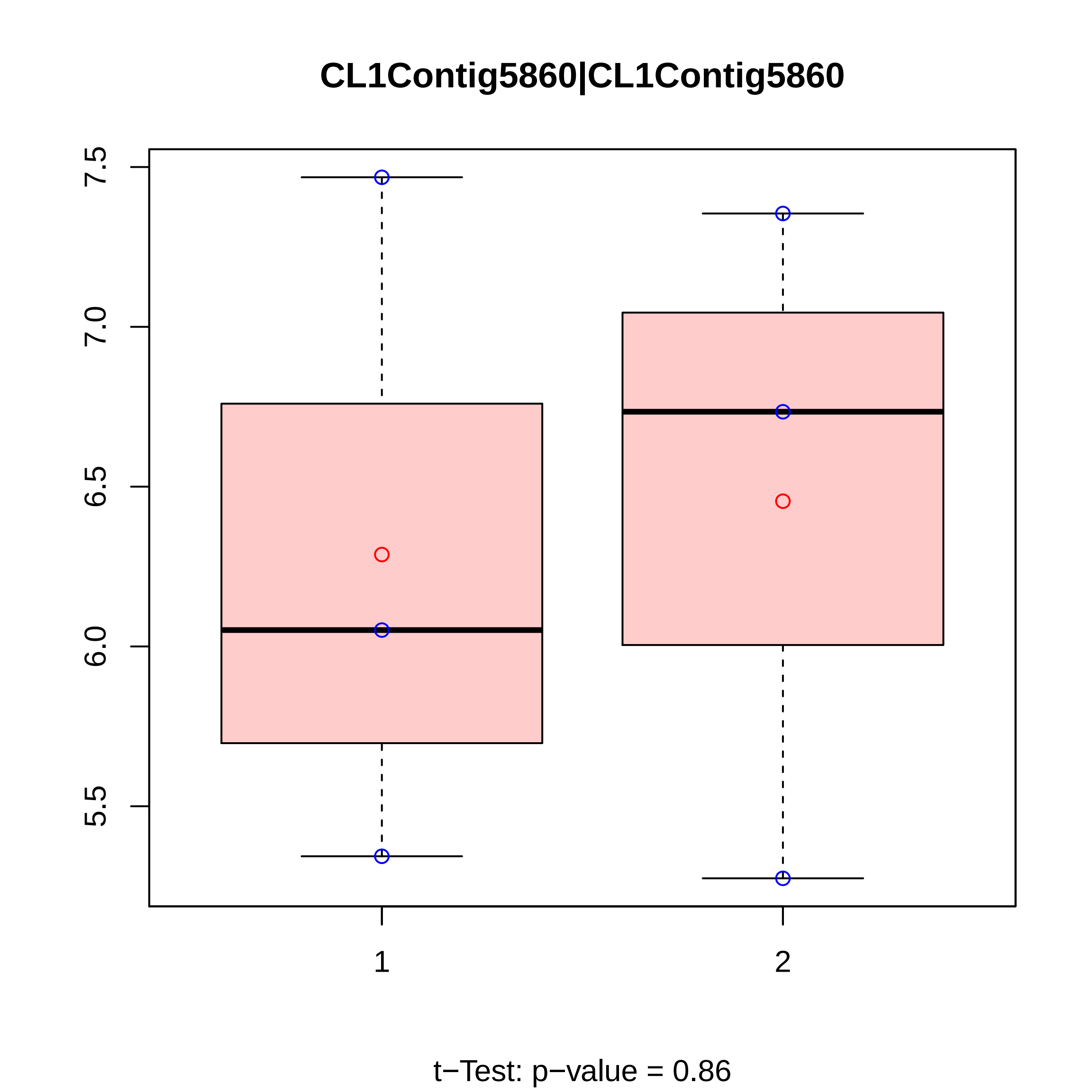

CL1Contig5860|CL1Contig5860
7.5
7.0
6.5
6.0
5.5
1
2
t−Test: p−value = 0.86

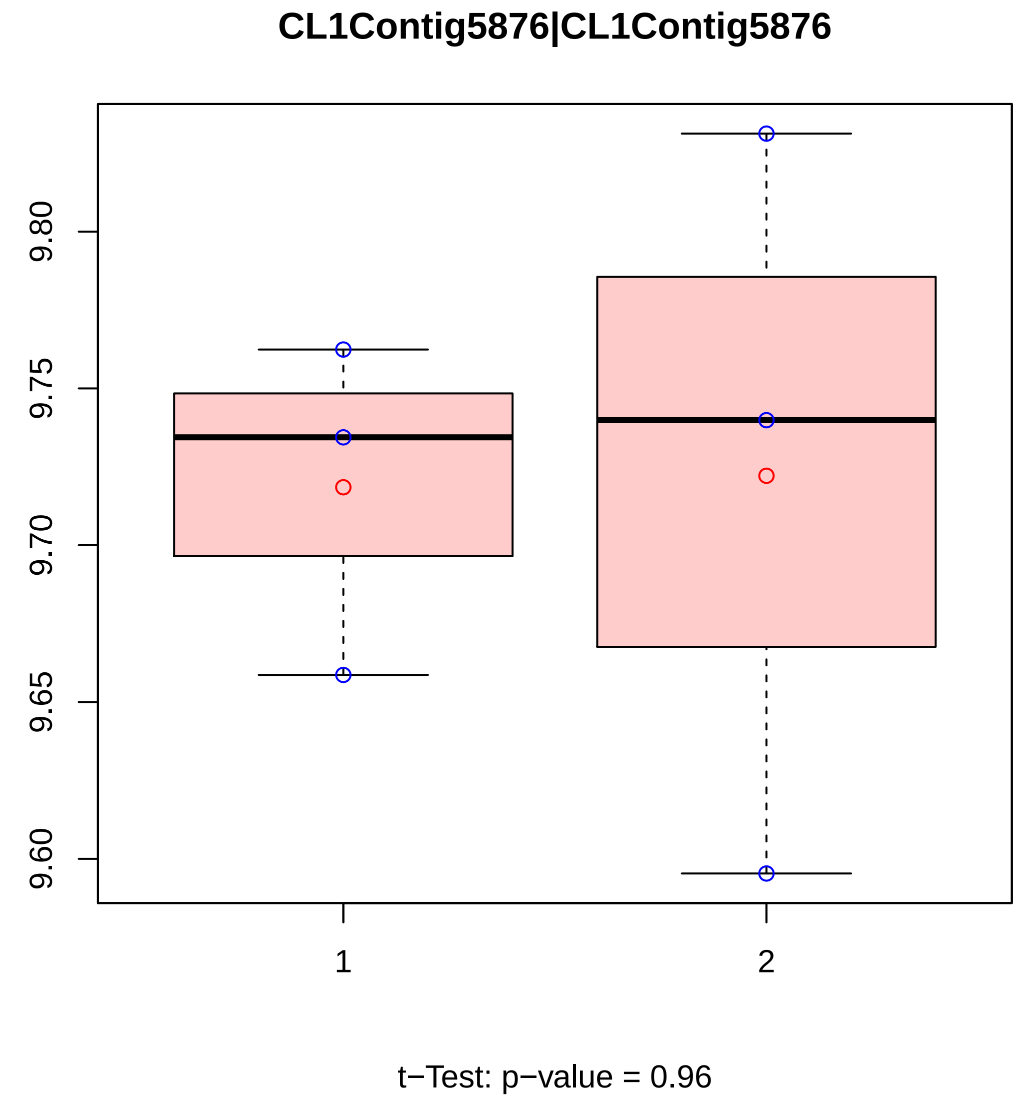
CL1Contig5876|CL1Contig5876
9.60
9.65
9.70
9.75
9.80
1
2
t−Test: p−value = 0.96

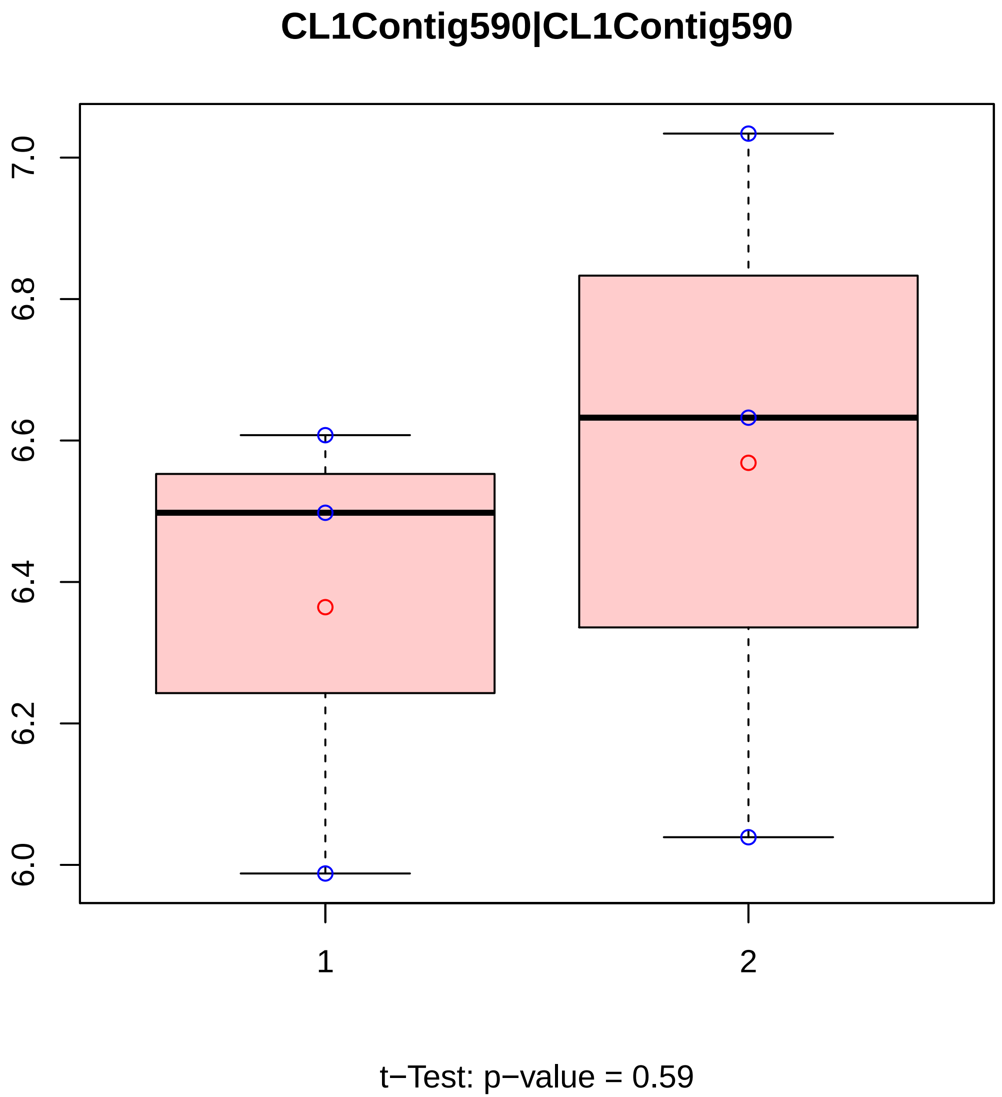

CL1Contig590|CL1Contig590
7.0
6.8
6.6
6.4
6.2
6.0
1
2
t−Test: p−value = 0.59

## CL1Contig5923|CL1Contig5923

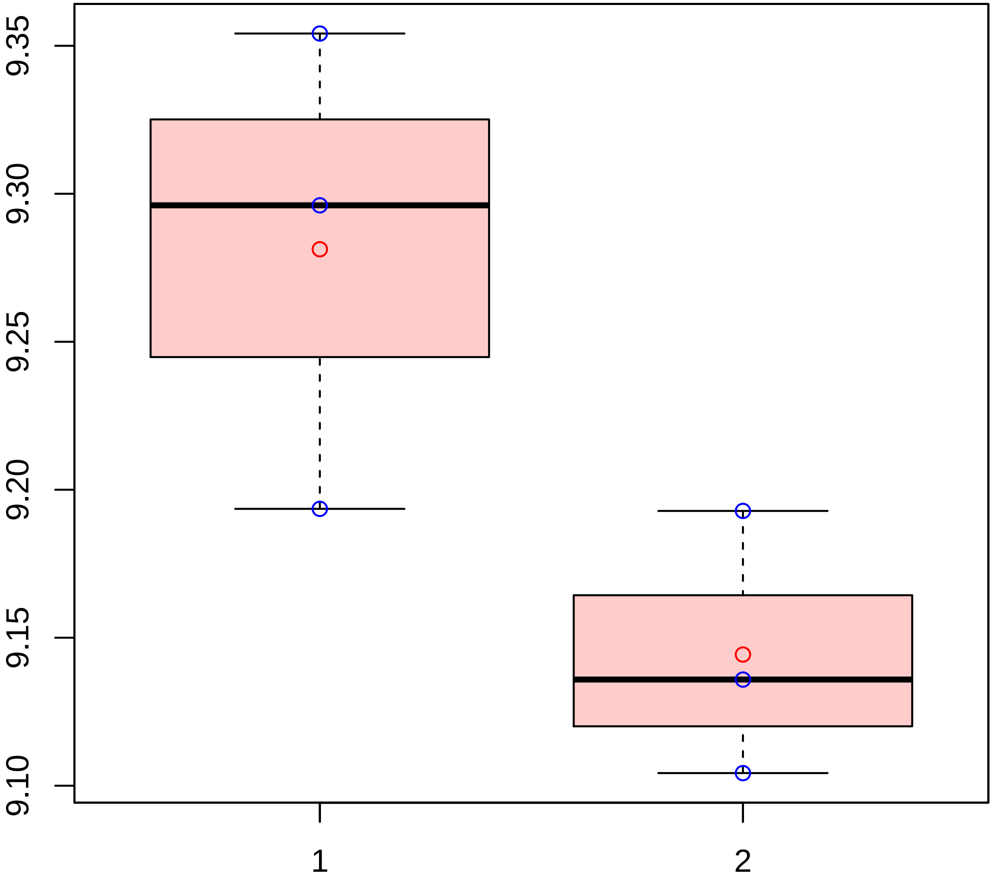


t−Test: p−value = 0.08

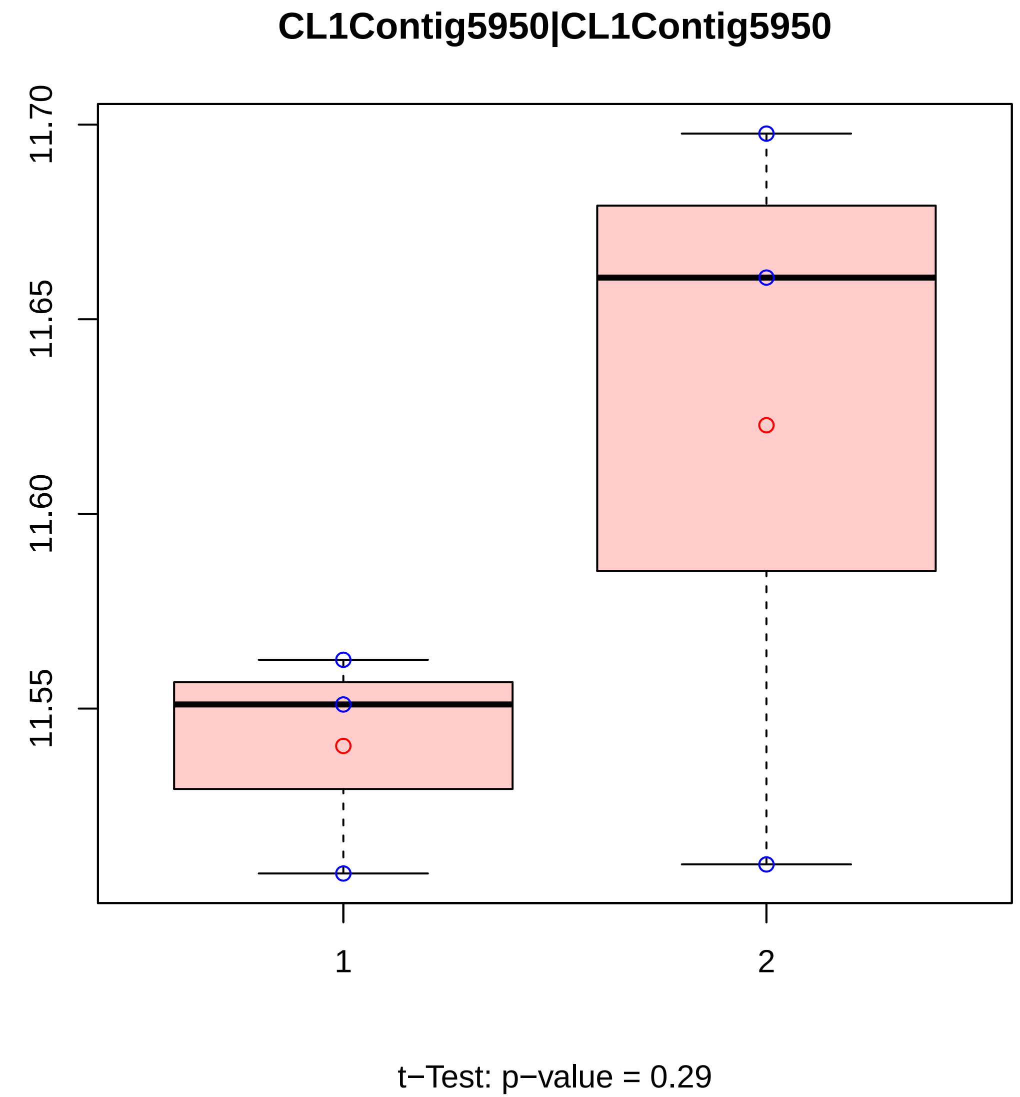
CL1Contig5950|CL1Contig5950
11.70
11.65
11.60
11.55
1
2
t−Test: p−value = 0.29

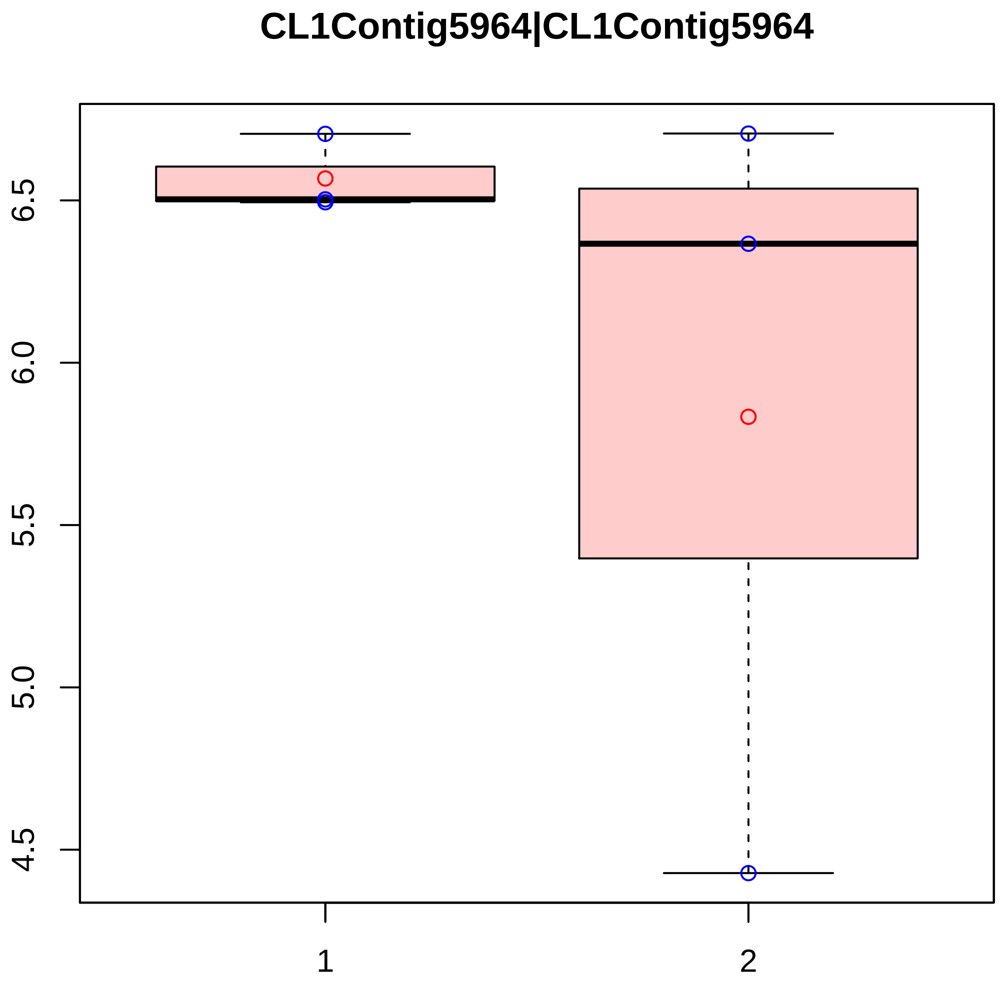

t−Test: p−value = 0.41

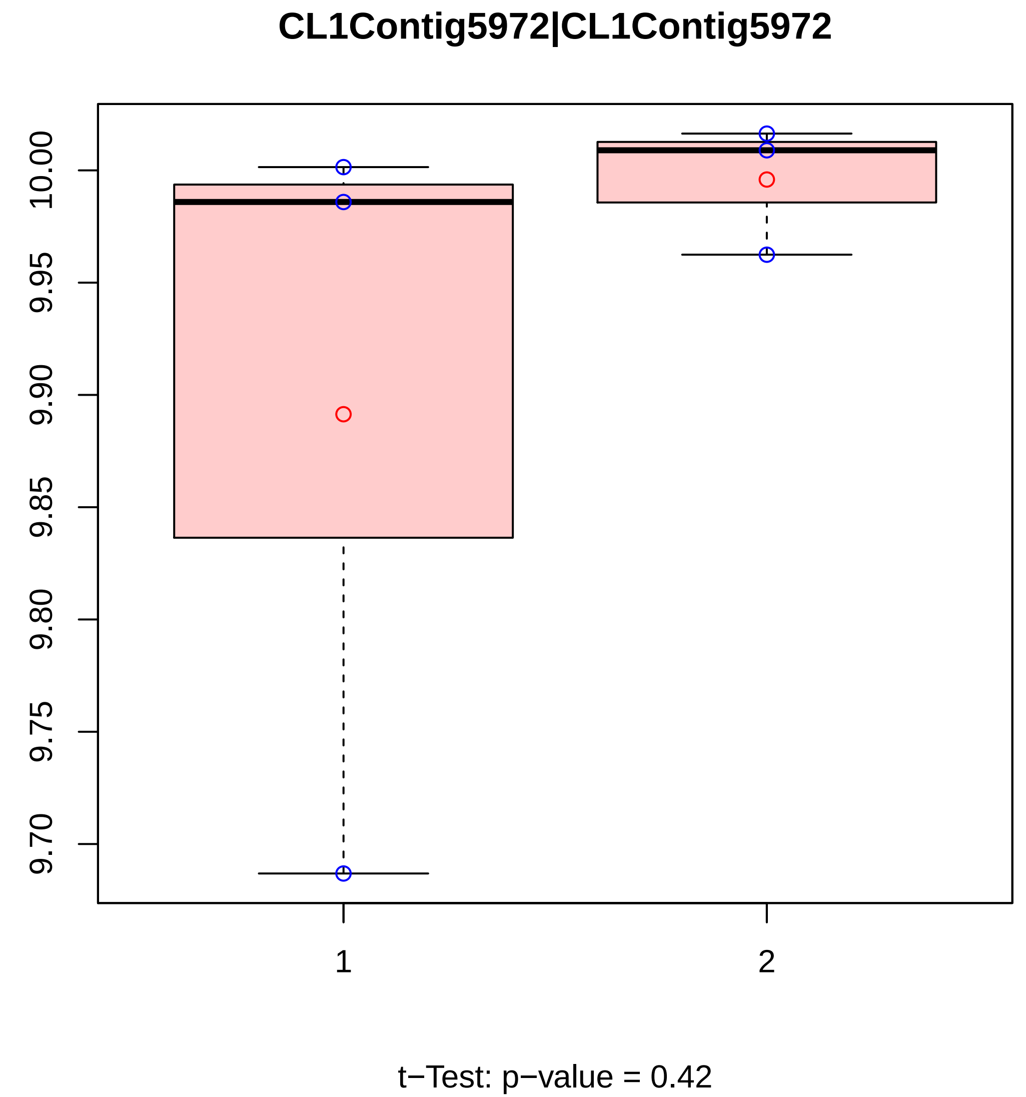

CL1Contig5972|CL1Contig5972
10.00
9.95
9.90
9.85
9.80
9.75
9.70
1
2
t−Test: p−value = 0.42

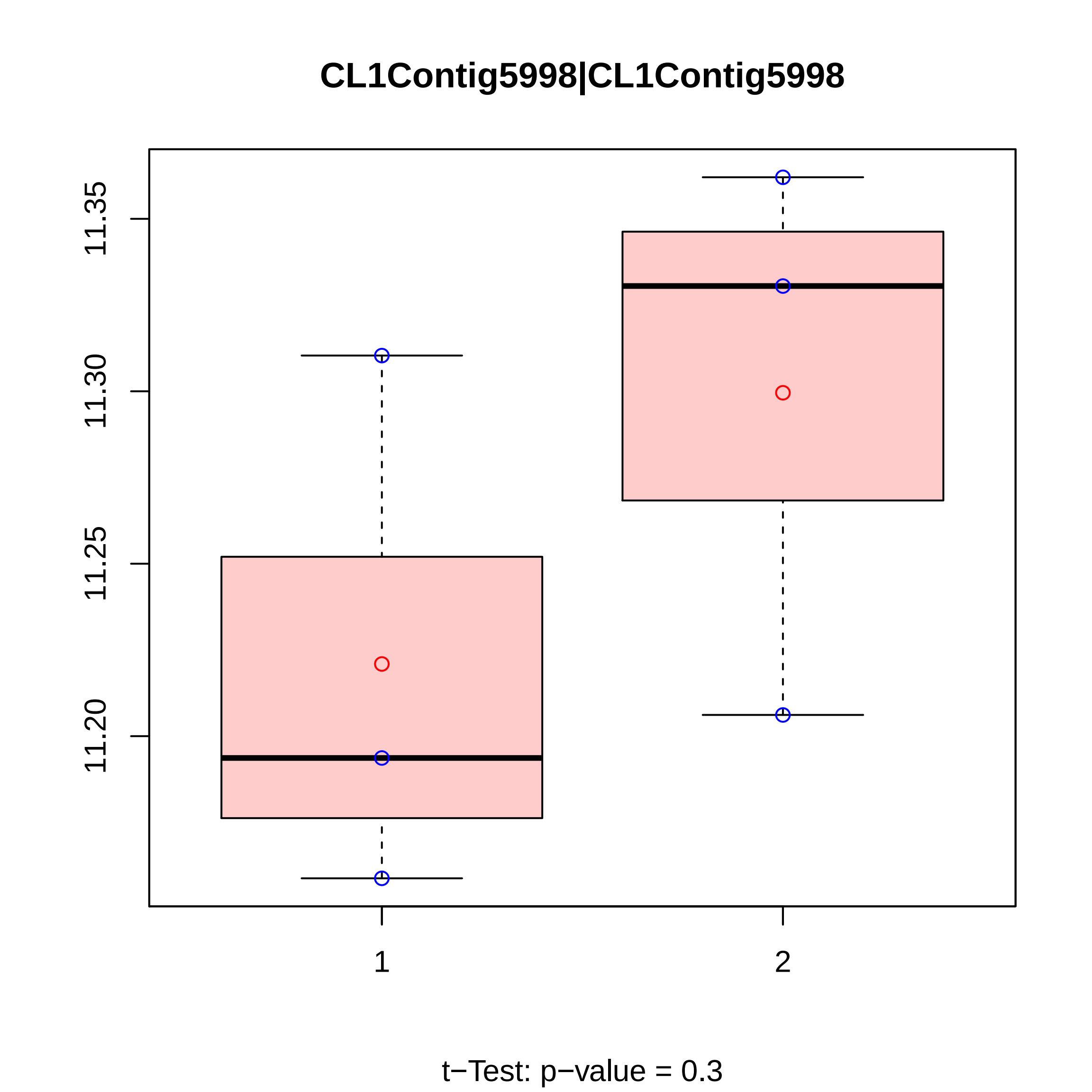

CL1Contig5998|CL1Contig5998
11.20
11.25
11.30
11.35
1
2
t−Test: p−value = 0.3

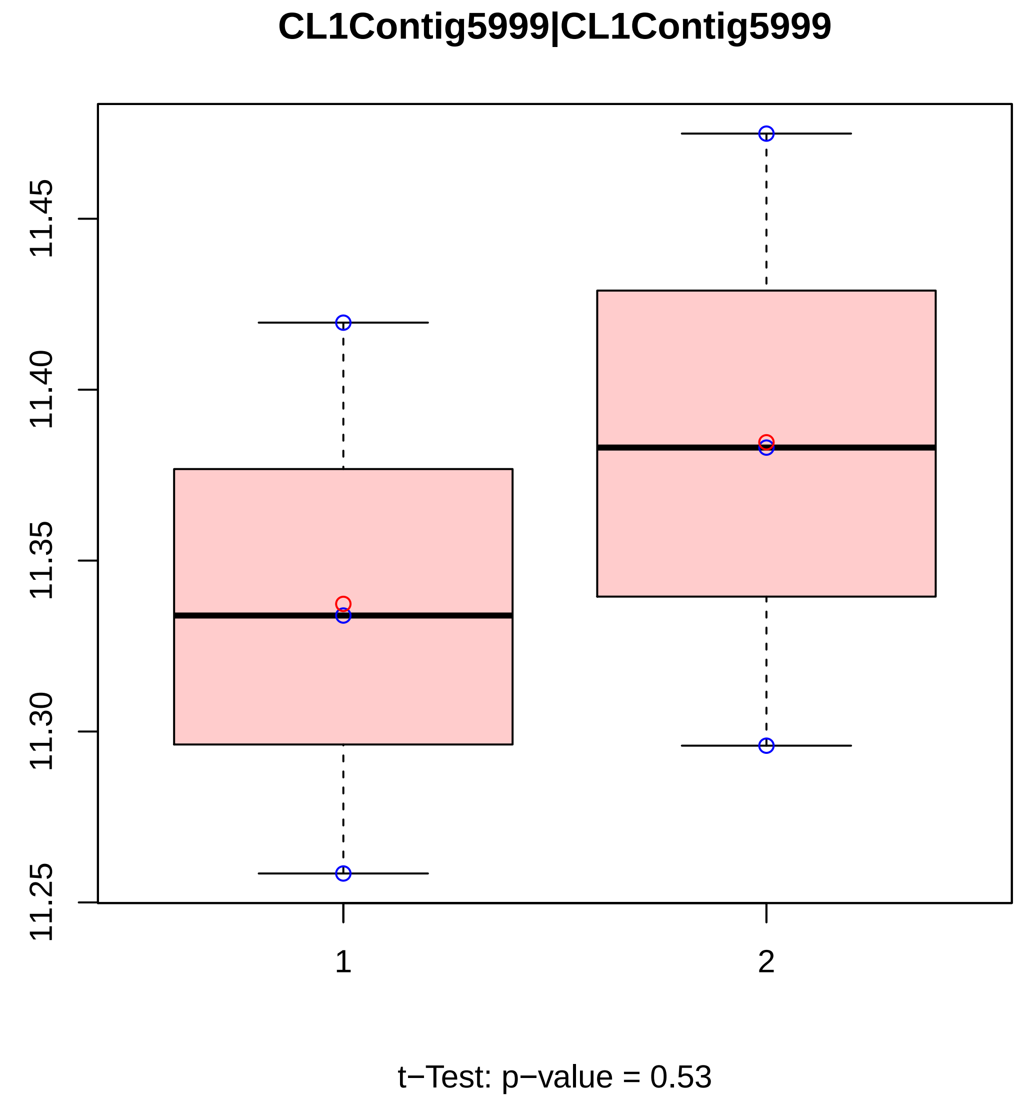
CL1Contig5999|CL1Contig5999
11.25
11.30
11.35
11.40
11.45
1
2
t−Test: p−value = 0.53

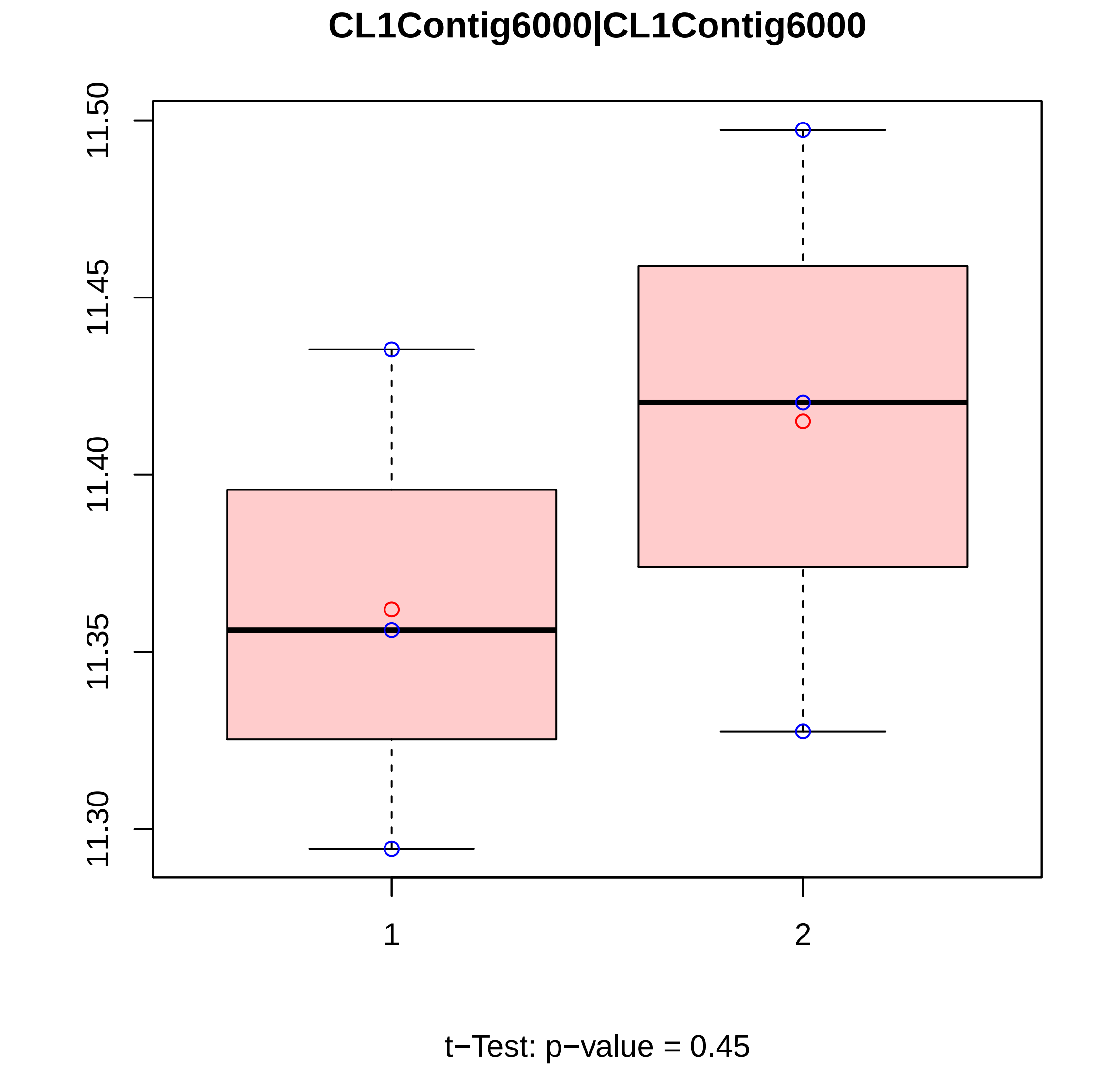
CL1Contig6000|CL1Contig6000
11.30
11.35
11.40
11.45
11.50
1
2
t−Test: p−value = 0.45

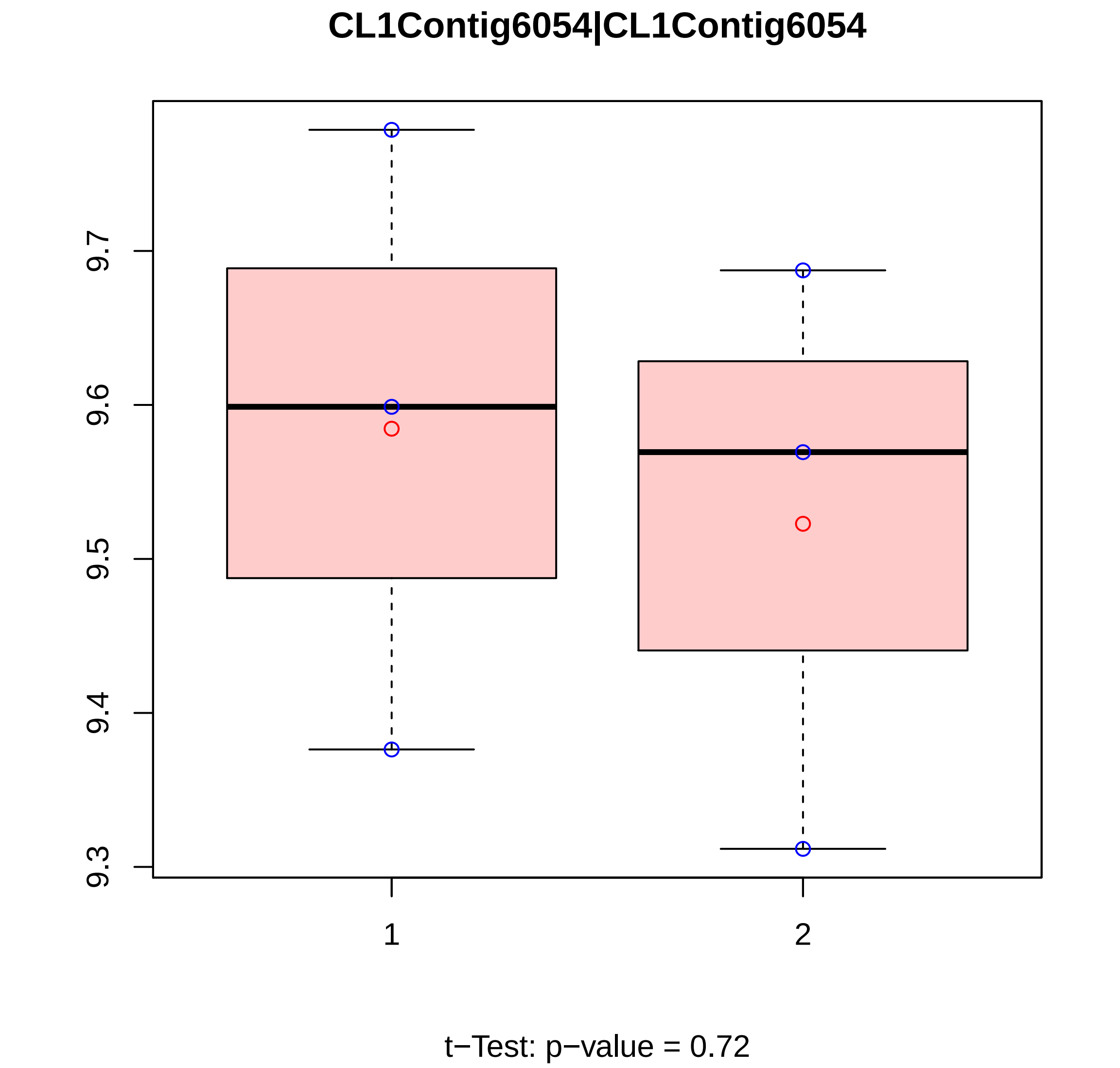

CL1Contig6054|CL1Contig6054
9.3
9.4
9.5
9.6
9.7
1
2
t−Test: p−value = 0.72

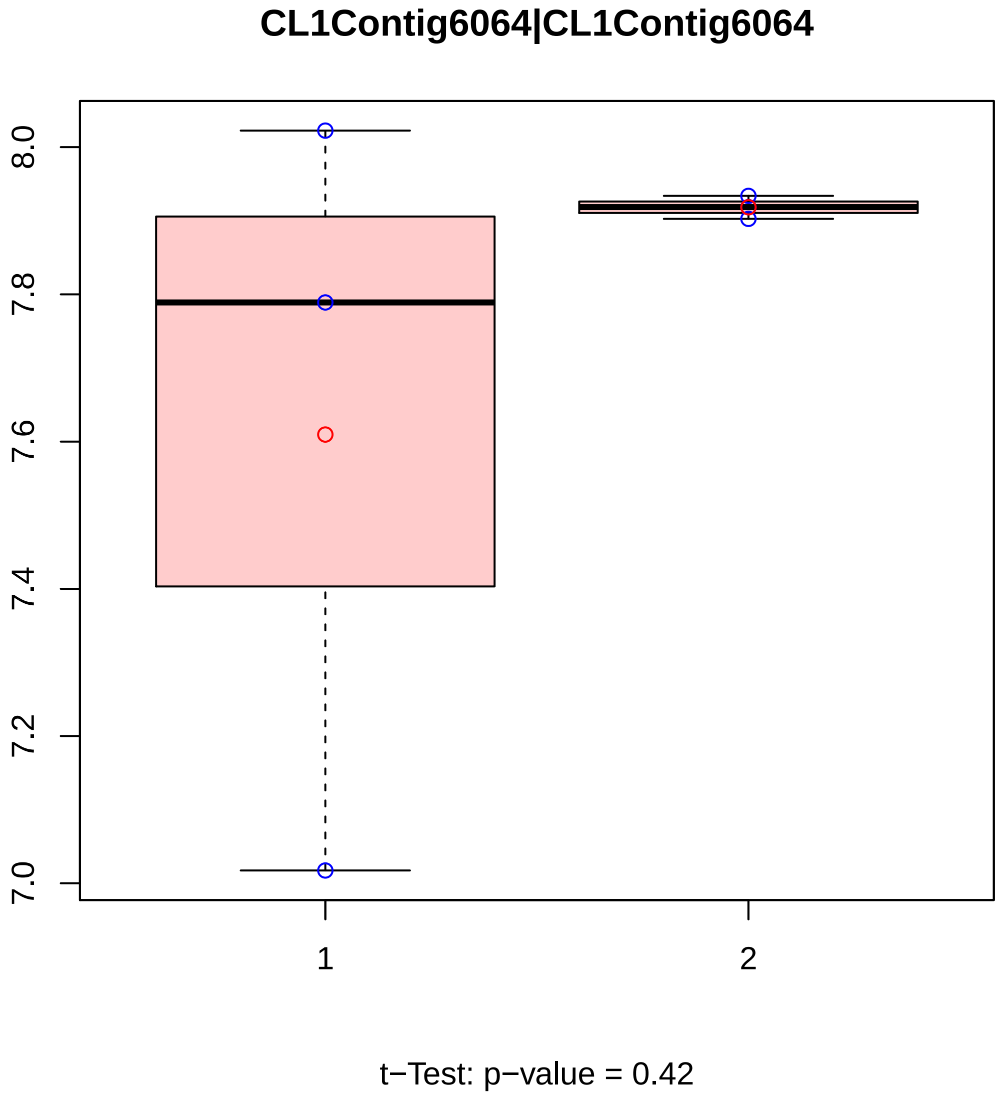

CL1Contig6064|CL1Contig6064
8.0
7.8
7.6
7.4
7.2
7.0
1
2
t−Test: p−value = 0.42

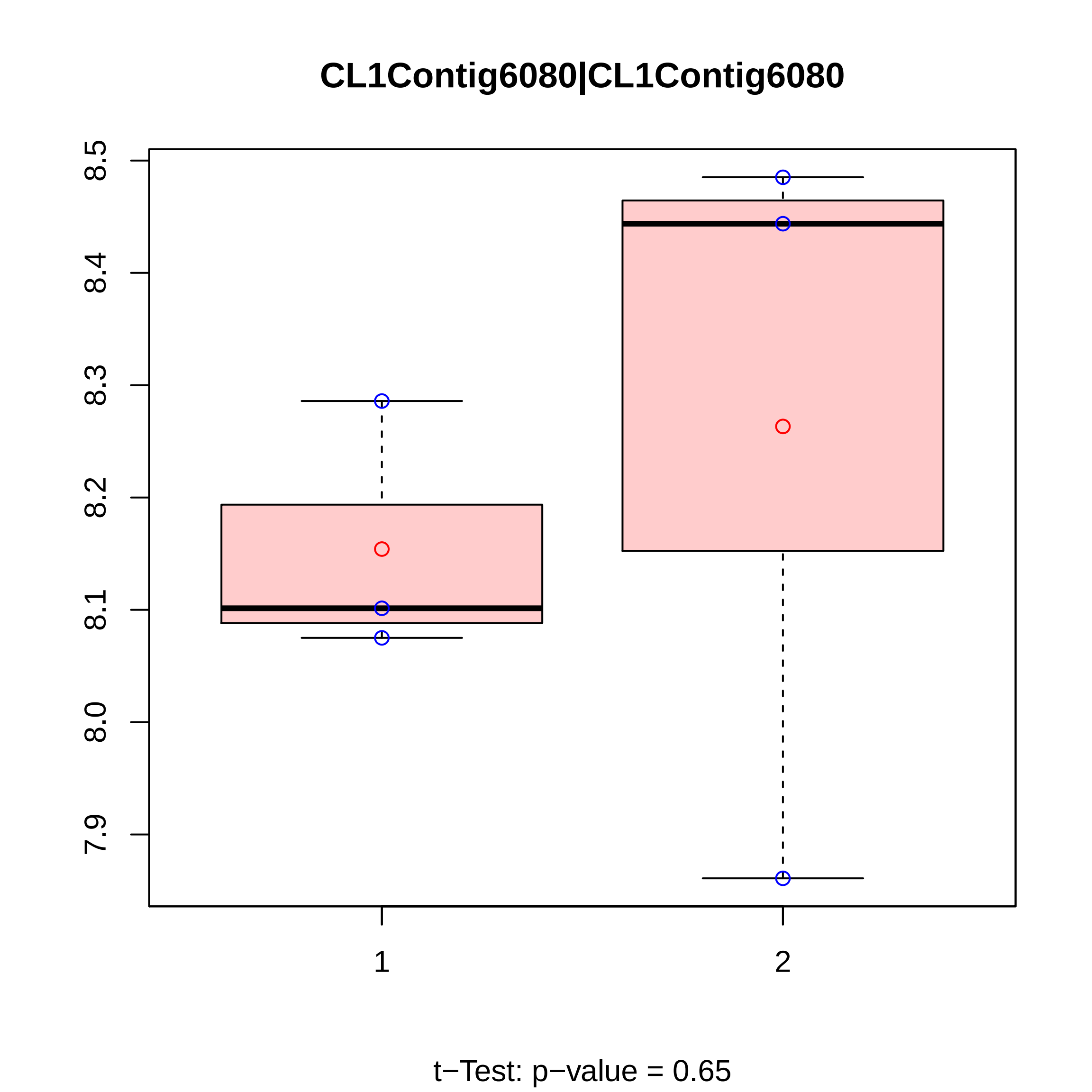
CL1Contig6080|CL1Contig6080
8.5
8.4
8.3
8.2
8.1
8.0
7.9
1
2
t−Test: p−value = 0.65

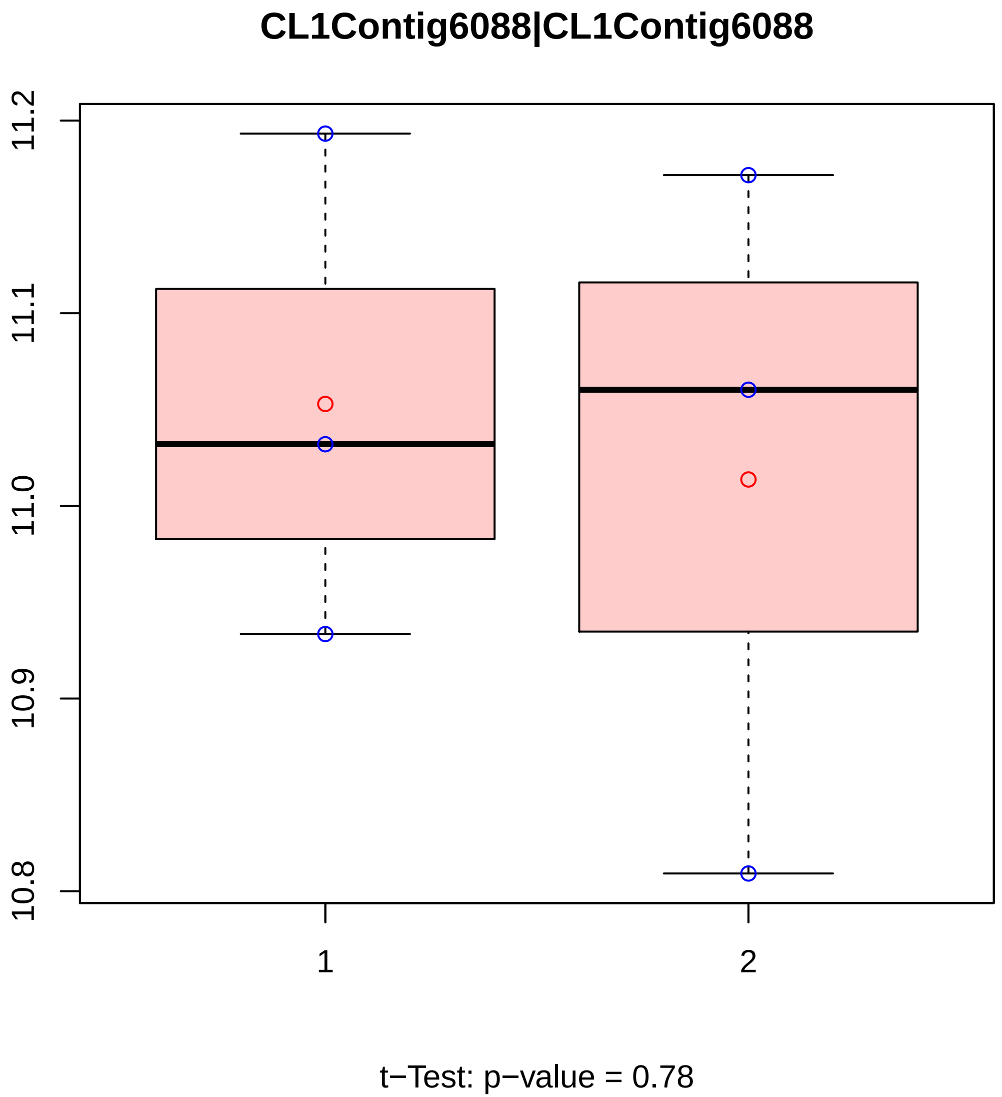
CL1Contig6088|CL1Contig6088
11.2
11.1
11.0
10.9
10.8
1
2
t−Test: p−value = 0.78

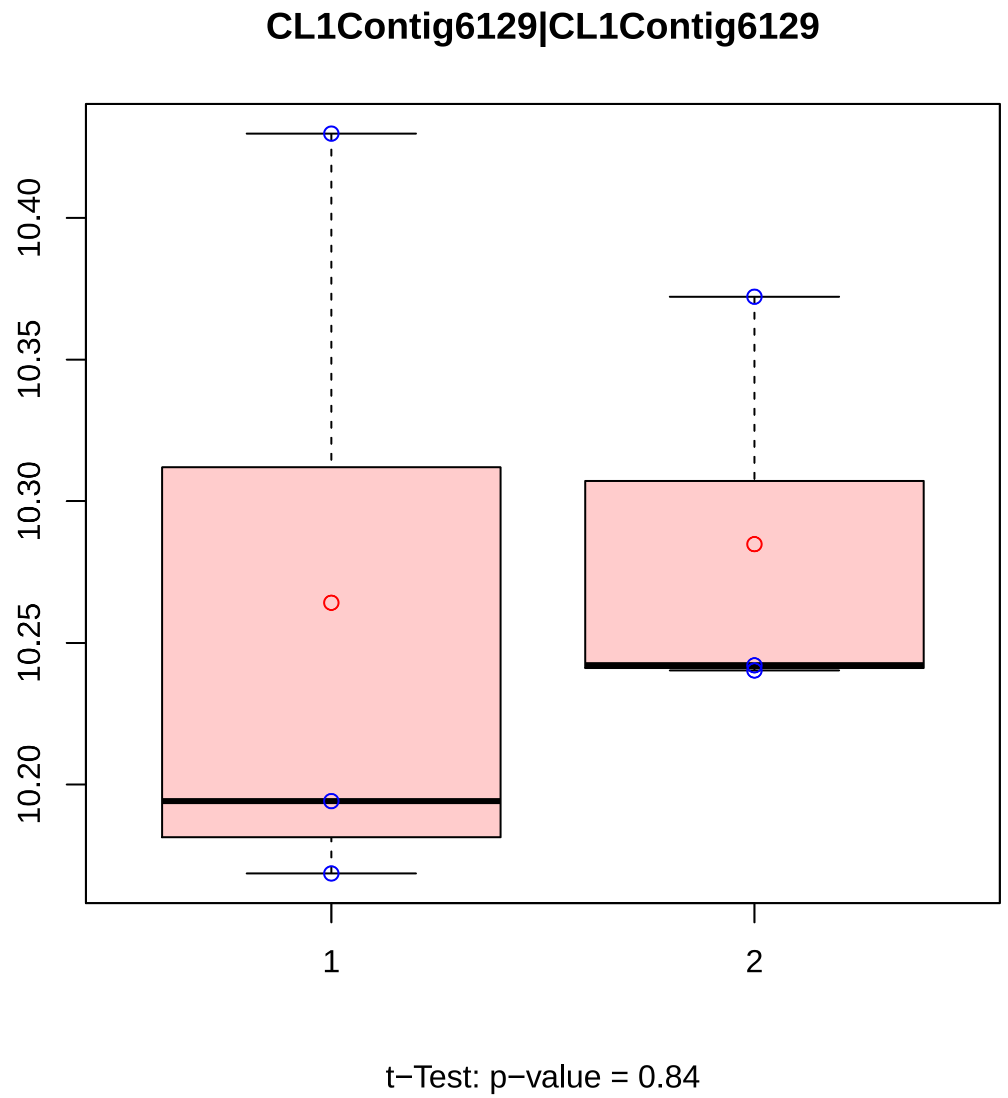

CL1Contig6129|CL1Contig6129
10.20
10.25
10.30
10.35
10.40
1
2
t−Test: p−value = 0.84

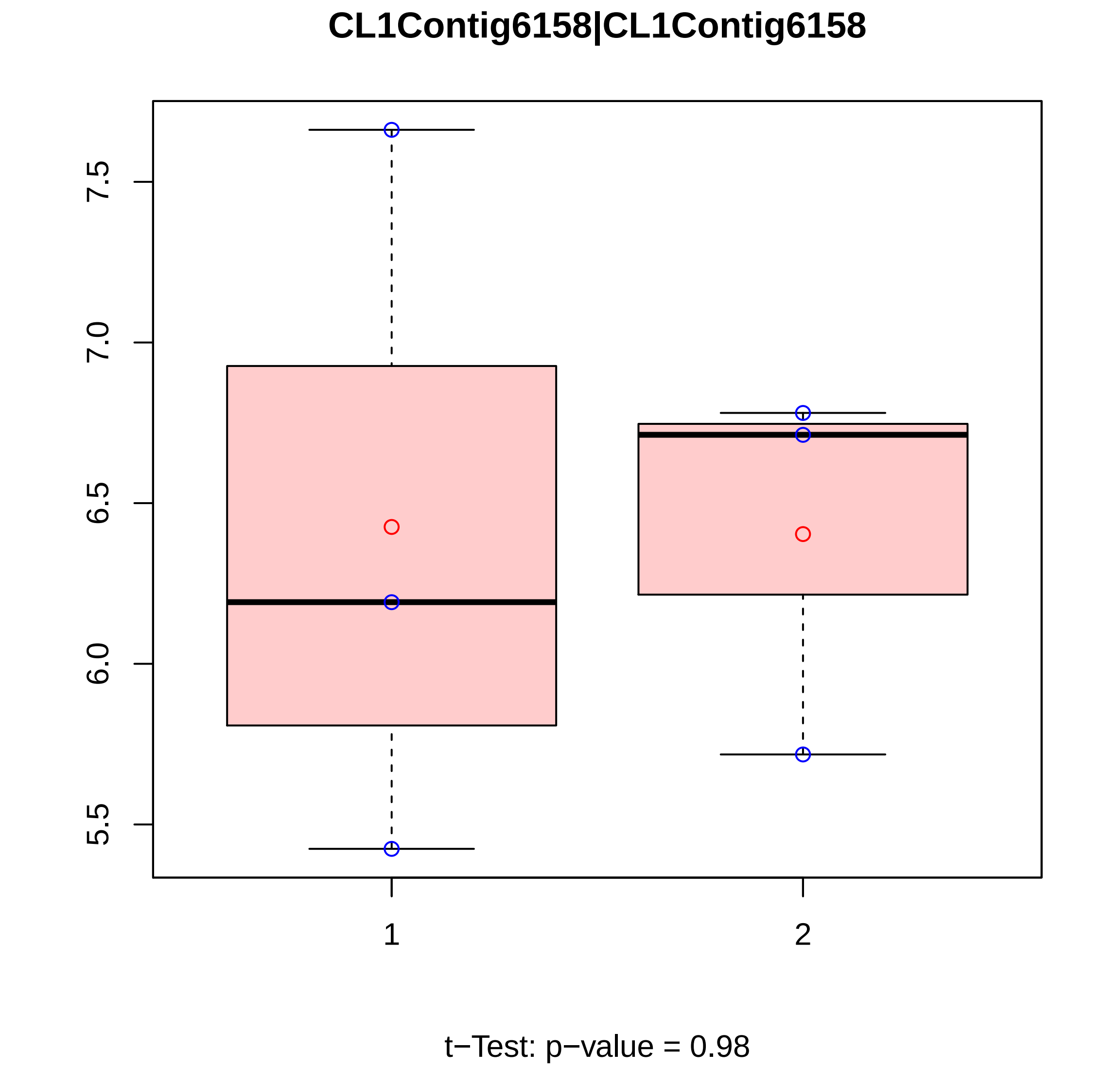
CL1Contig6158|CL1Contig6158
7.5
7.0
6.5
6.0
5.5
1
2
t−Test: p−value = 0.98

# CL1Contig6172|CL1Contig6172

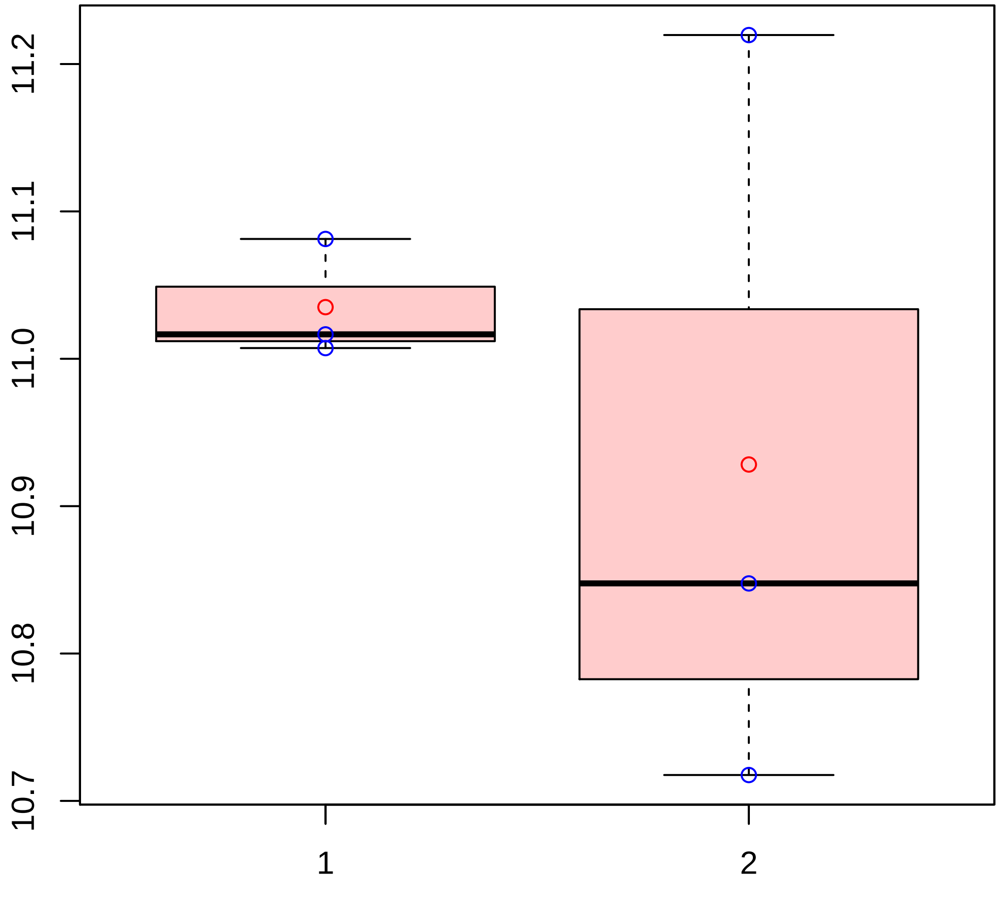


t−Test: p−value = 0.55

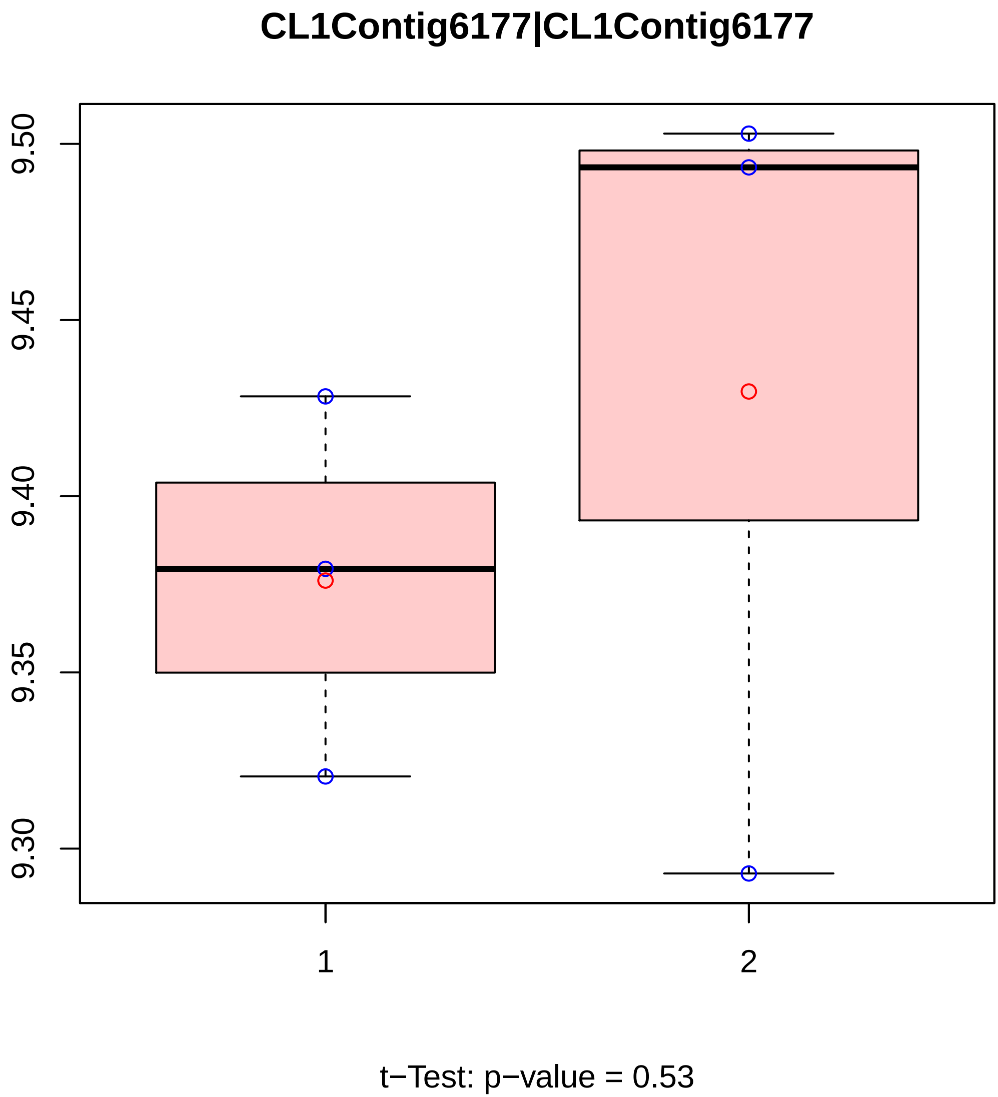
CL1Contig6177|CL1Contig6177
9.50
9.45
9.40
9.35
9.30
1
2
t−Test: p−value = 0.53

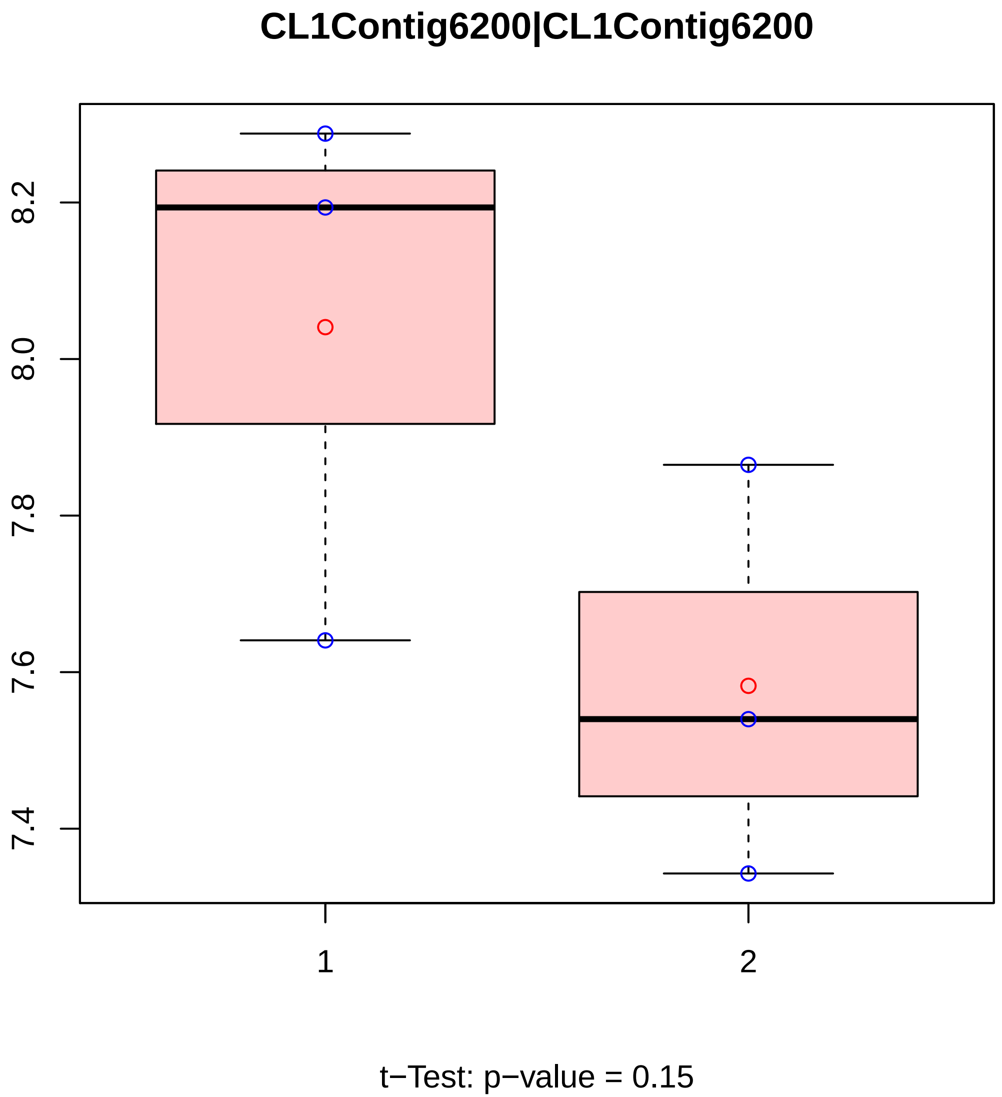

CL1Contig6200|CL1Contig6200
8.2
8.0
7.8
7.6
7.4
1
2
t−Test: p−value = 0.15

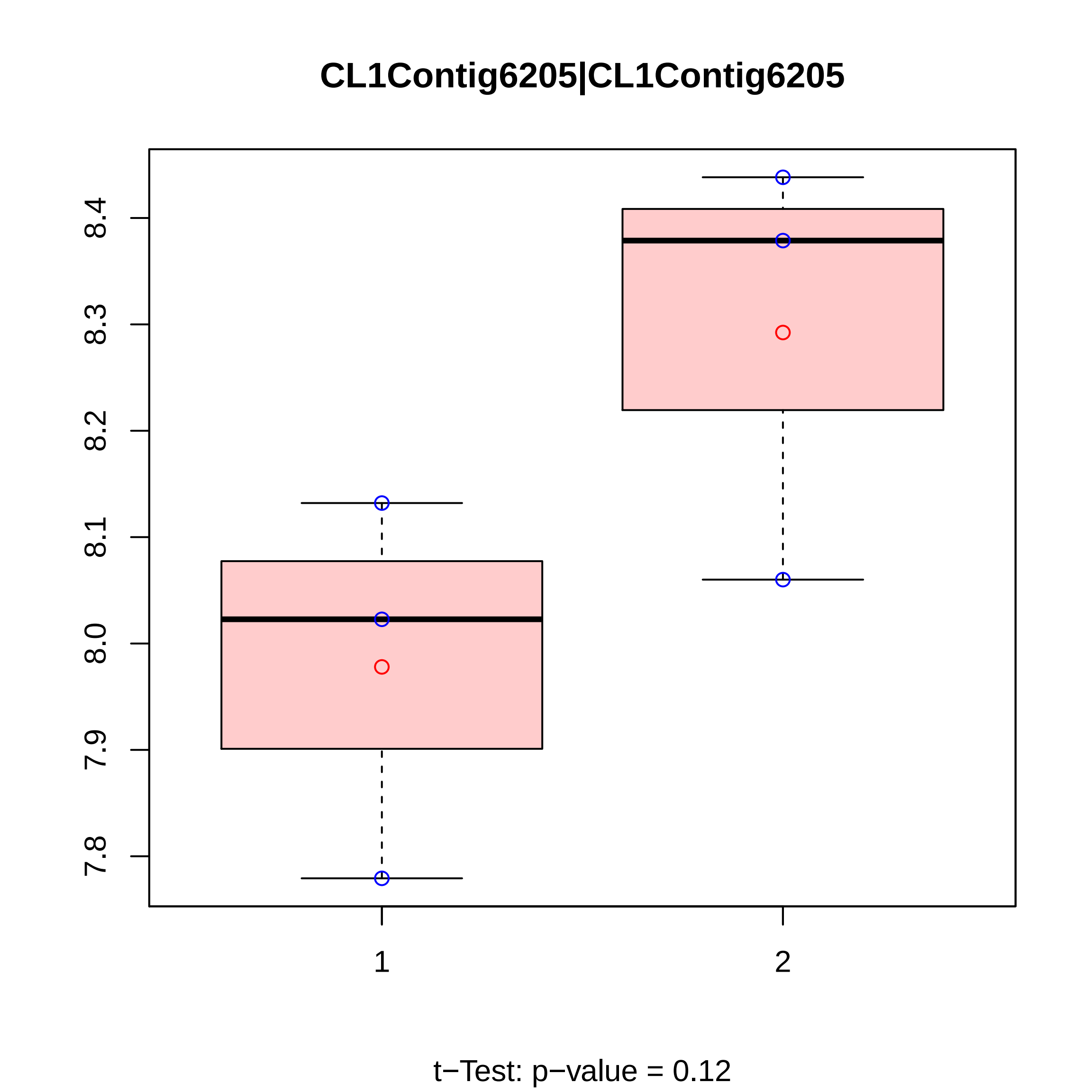
CL1Contig6205|CL1Contig6205
8.4
8.3
8.2
8.1
8.0
7.9
7.8
1
2
t−Test: p−value = 0.12

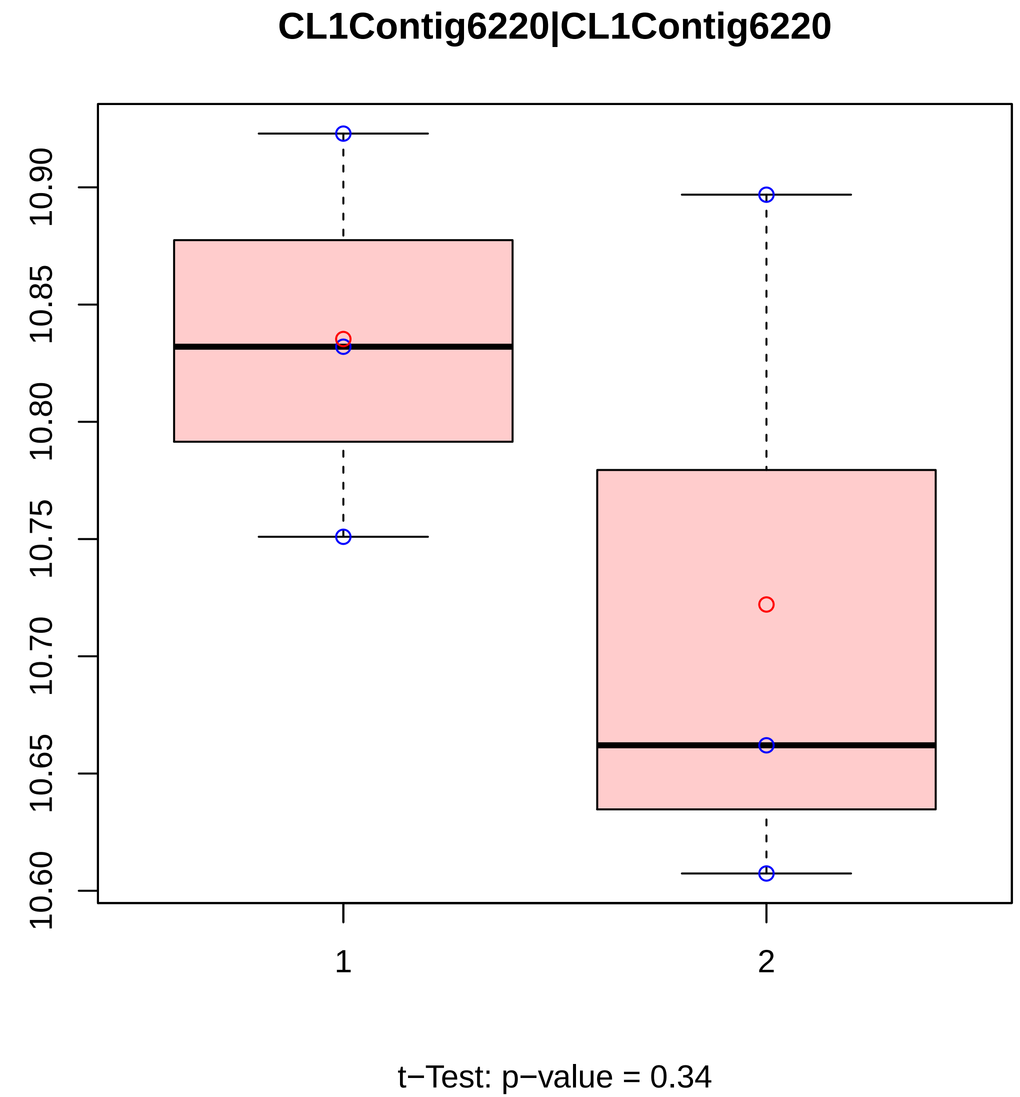
CL1Contig6220|CL1Contig6220
10.60
10.65
10.70
10.75
10.80
10.85
10.90
1
2
t−Test: p−value = 0.34

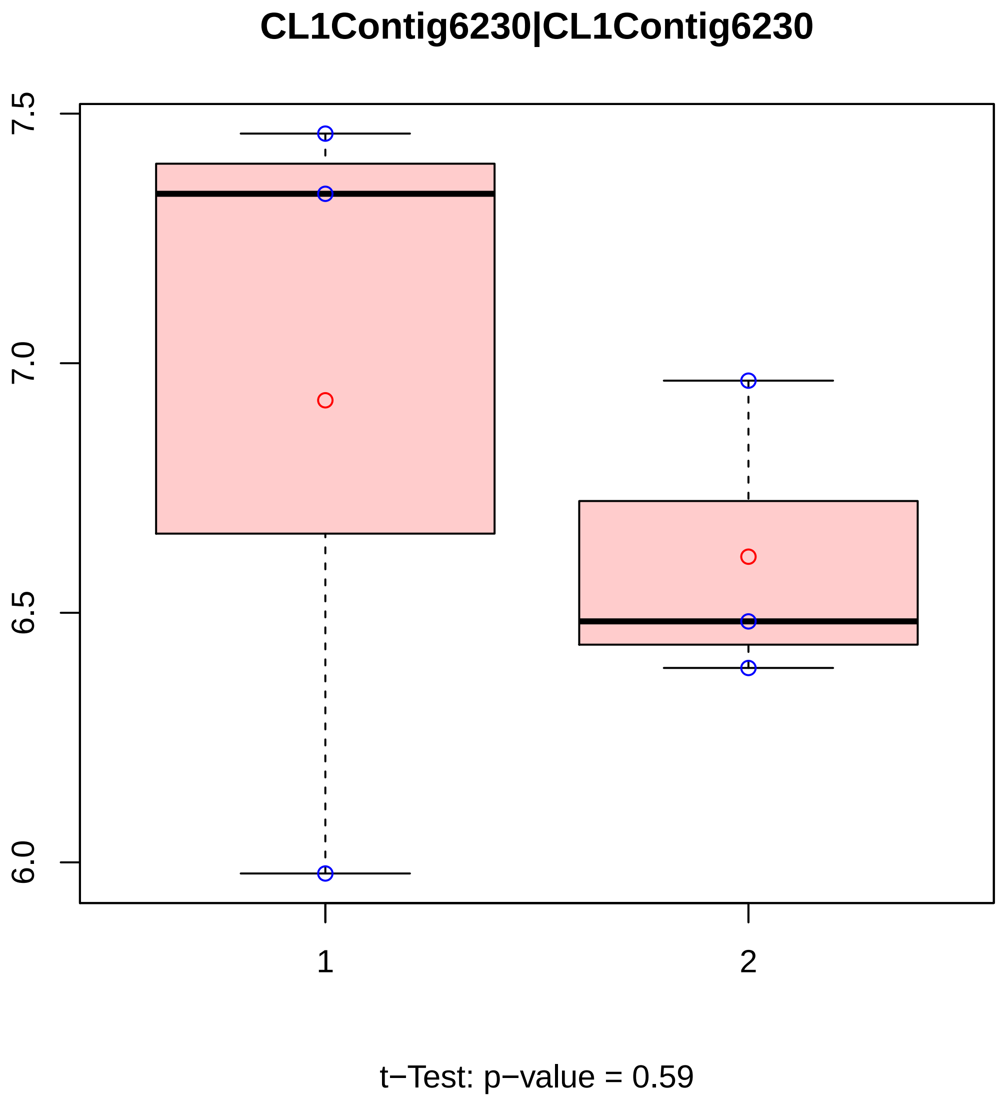
CL1Contig6230|CL1Contig6230
7.5
7.0
6.5
6.0
1
2
t−Test: p−value = 0.59

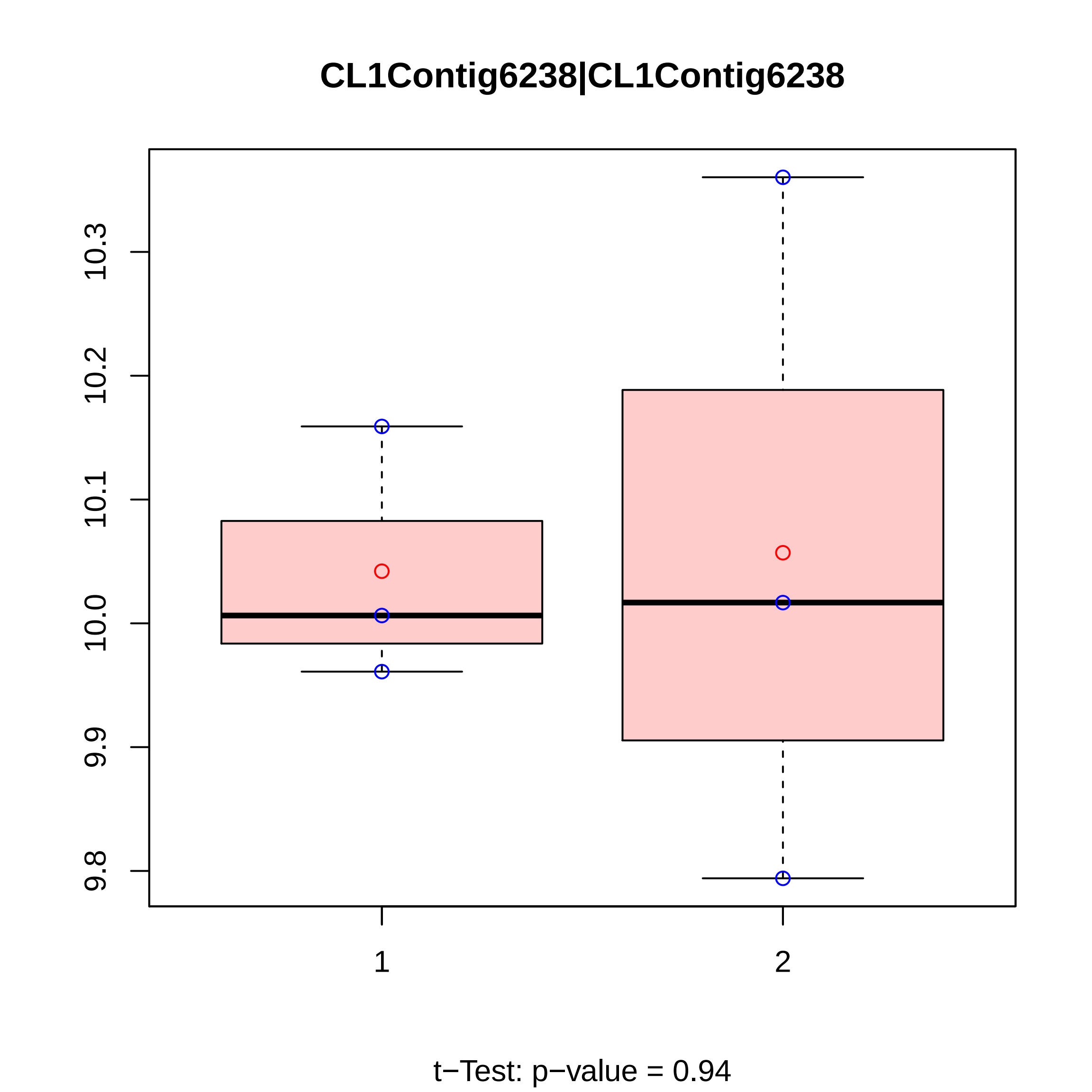
CL1Contig6238|CL1Contig6238
10.3
10.2
10.1
10.0
9.9
9.8
1
2
t−Test: p−value = 0.94

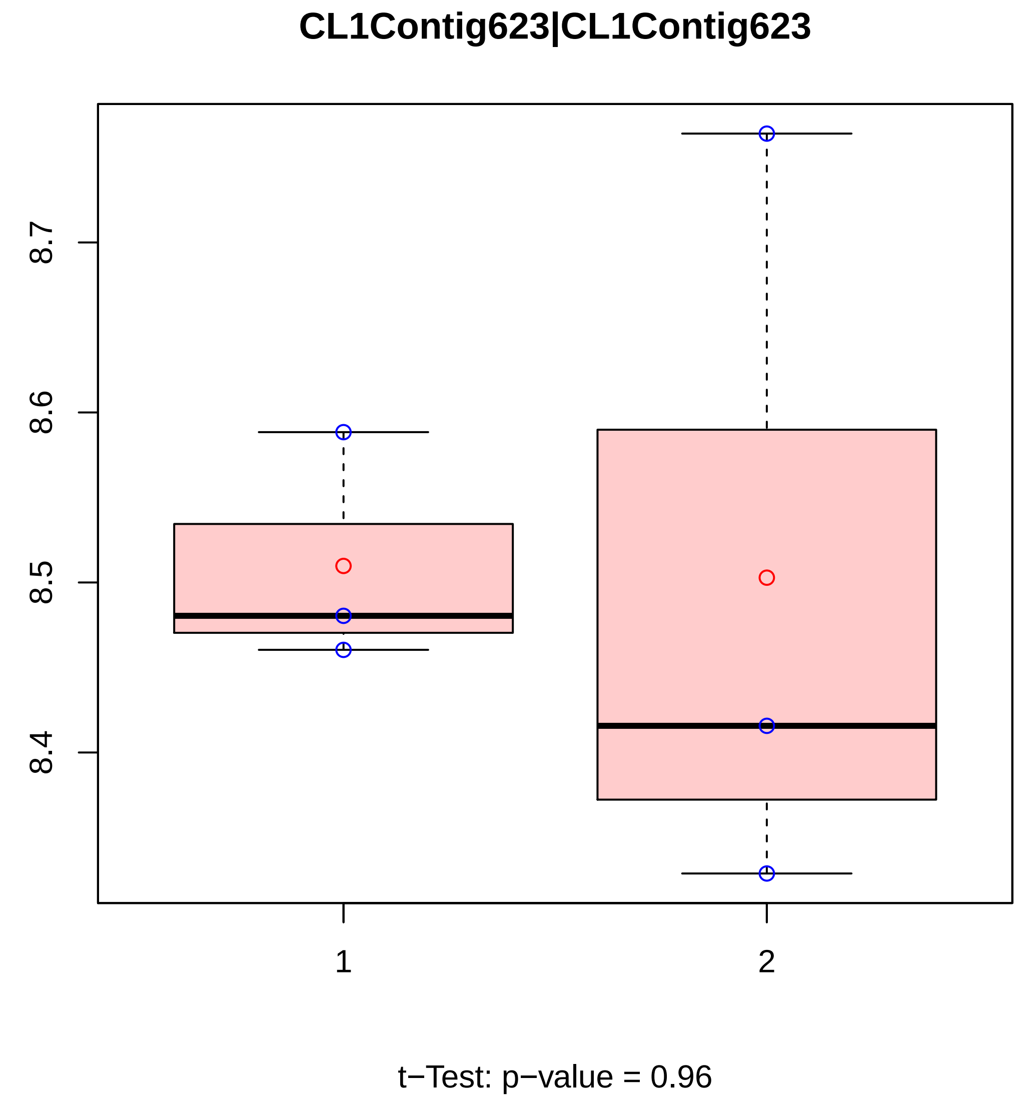
CL1Contig623|CL1Contig623
8.4
8.5
8.6
8.7
1
2
t−Test: p−value = 0.96

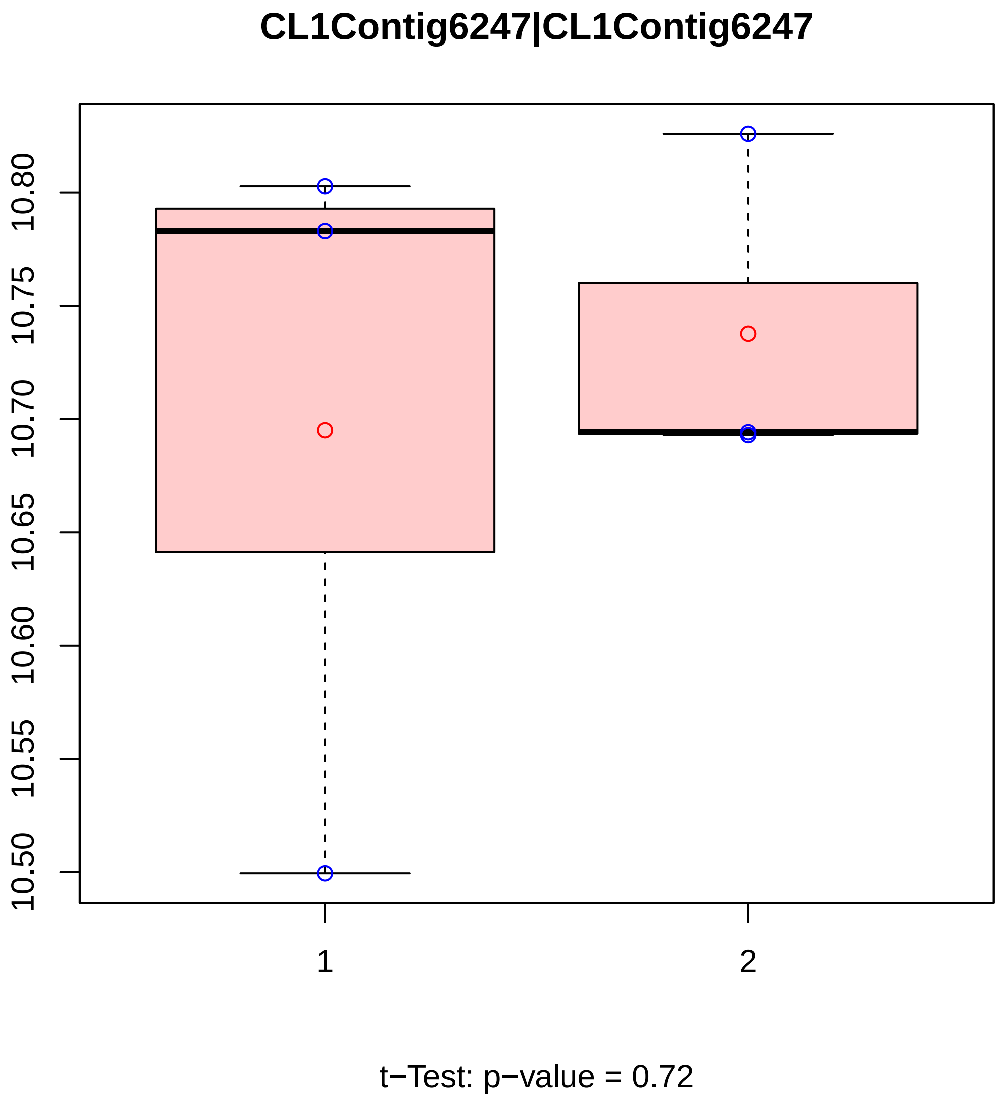

CL1Contig6247|CL1Contig6247
10.50
10.55
10.60
10.65
10.70
10.75
10.80
1
2
t−Test: p−value = 0.72

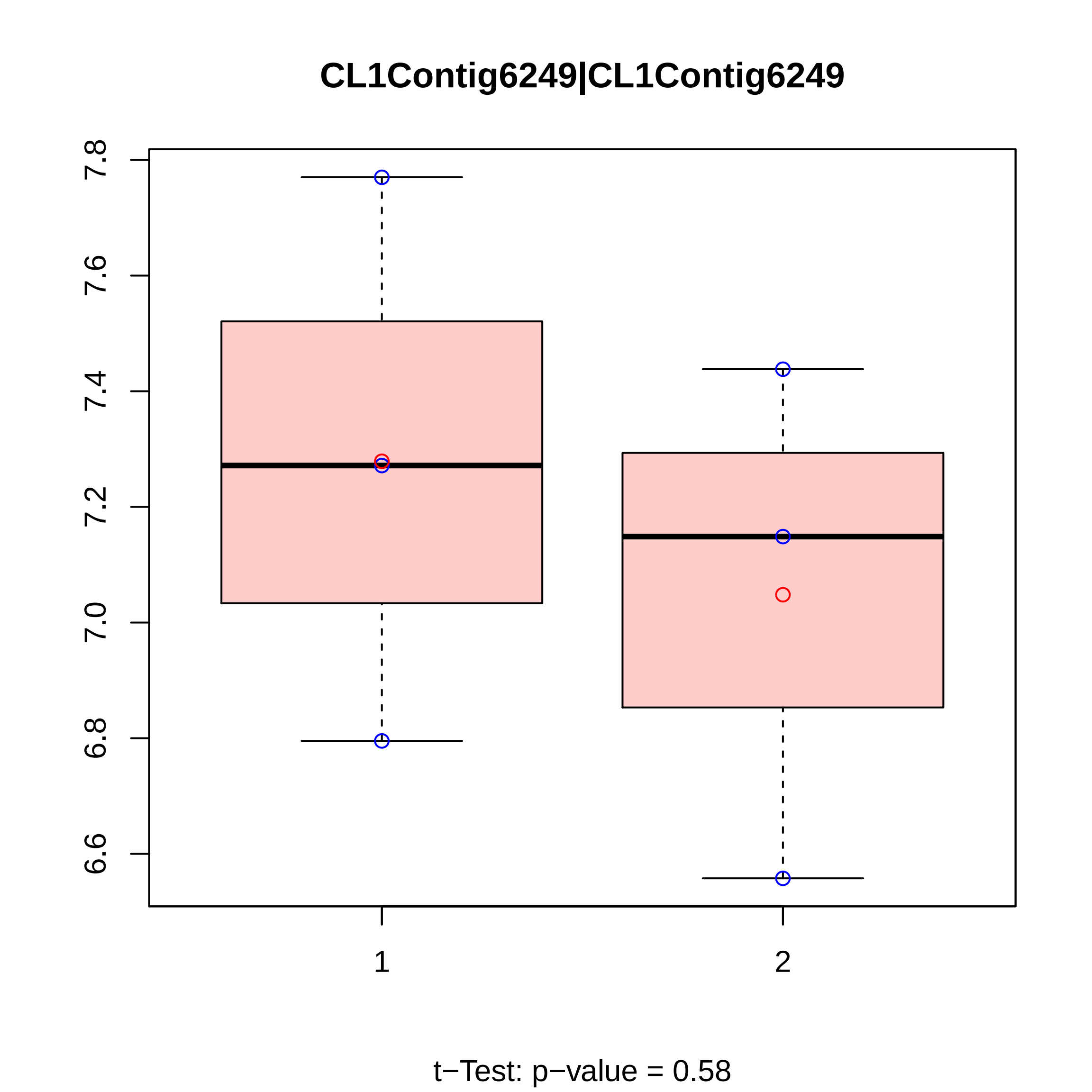
CL1Contig6249|CL1Contig6249
7.8
7.6
7.4
7.2
7.0
6.8
6.6
1
2
t−Test: p−value = 0.58

# CL1Contig6250|CL1Contig6250

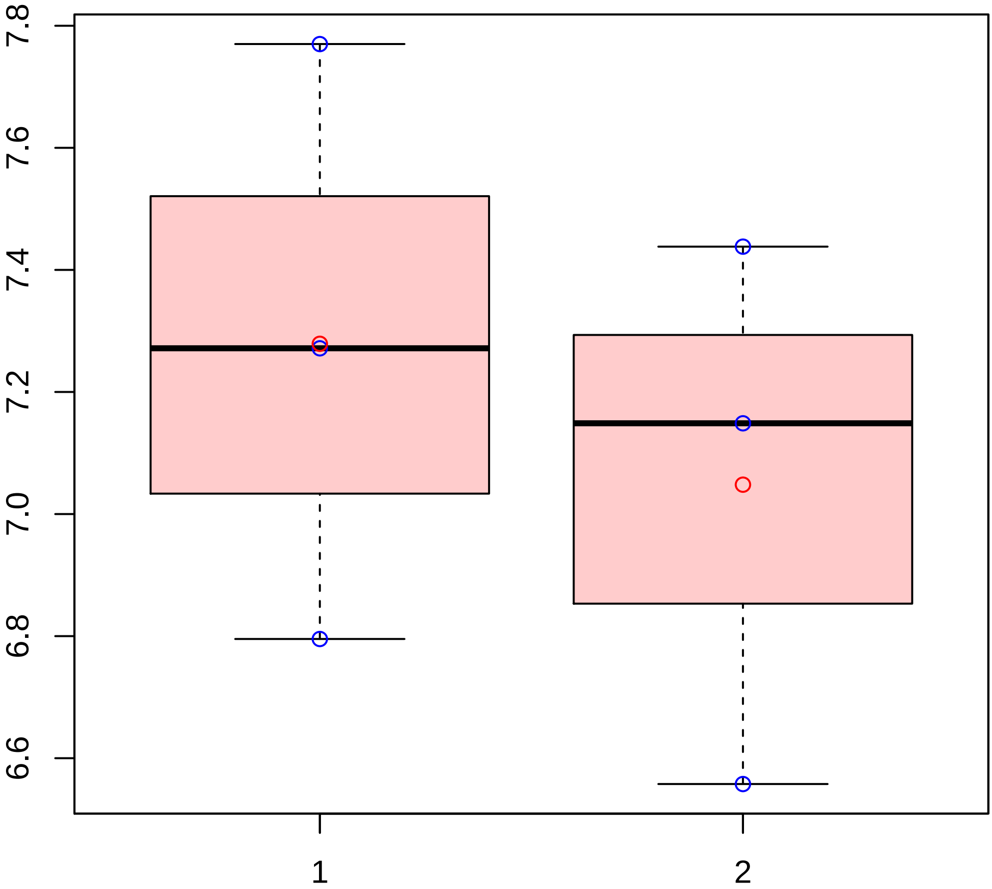


t−Test: p−value = 0.58

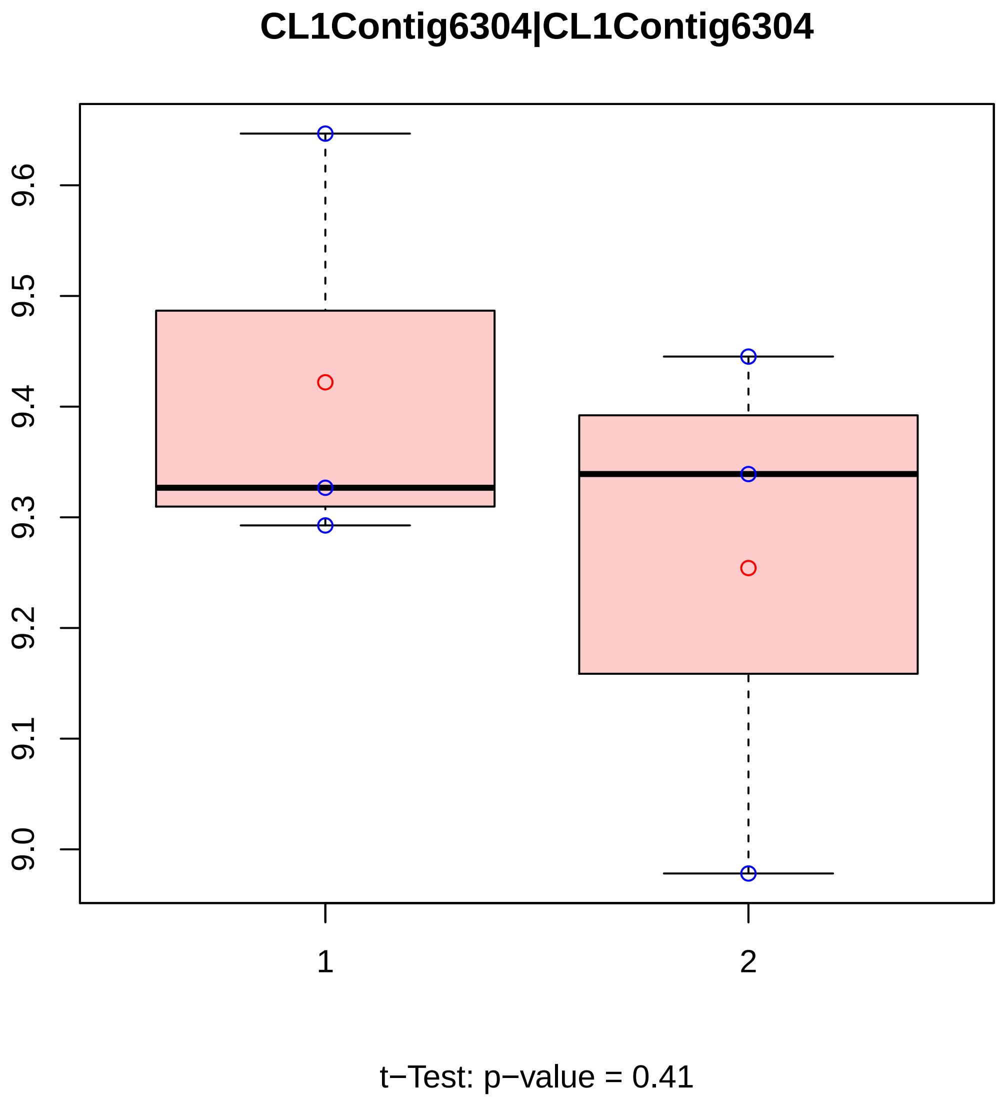

CL1Contig6304|CL1Contig6304
9.0
9.1
9.2
9.3
9.4
9.5
9.6
1
2
t−Test: p−value = 0.41

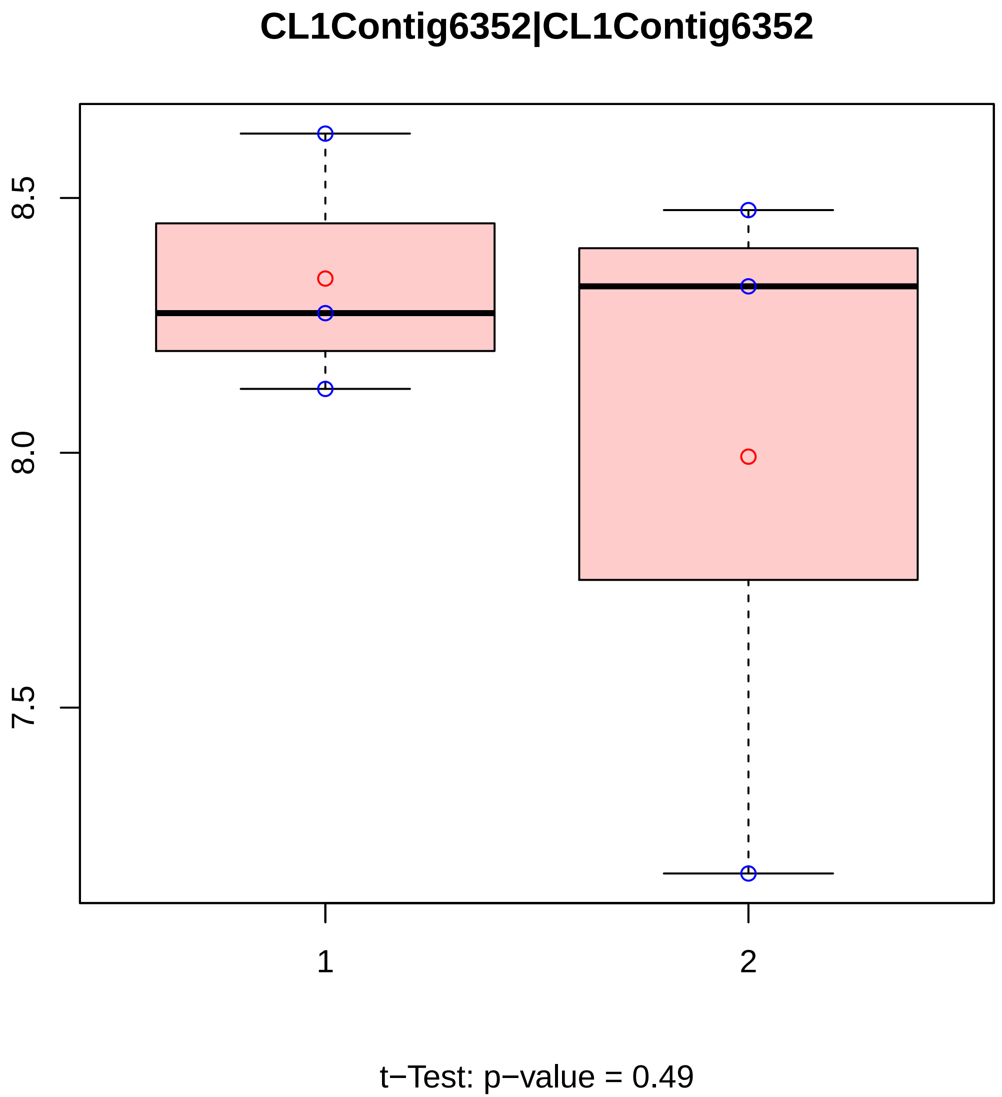
CL1Contig6352|CL1Contig6352
8.5
8.0
7.5
1
2
t−Test: p−value = 0.49

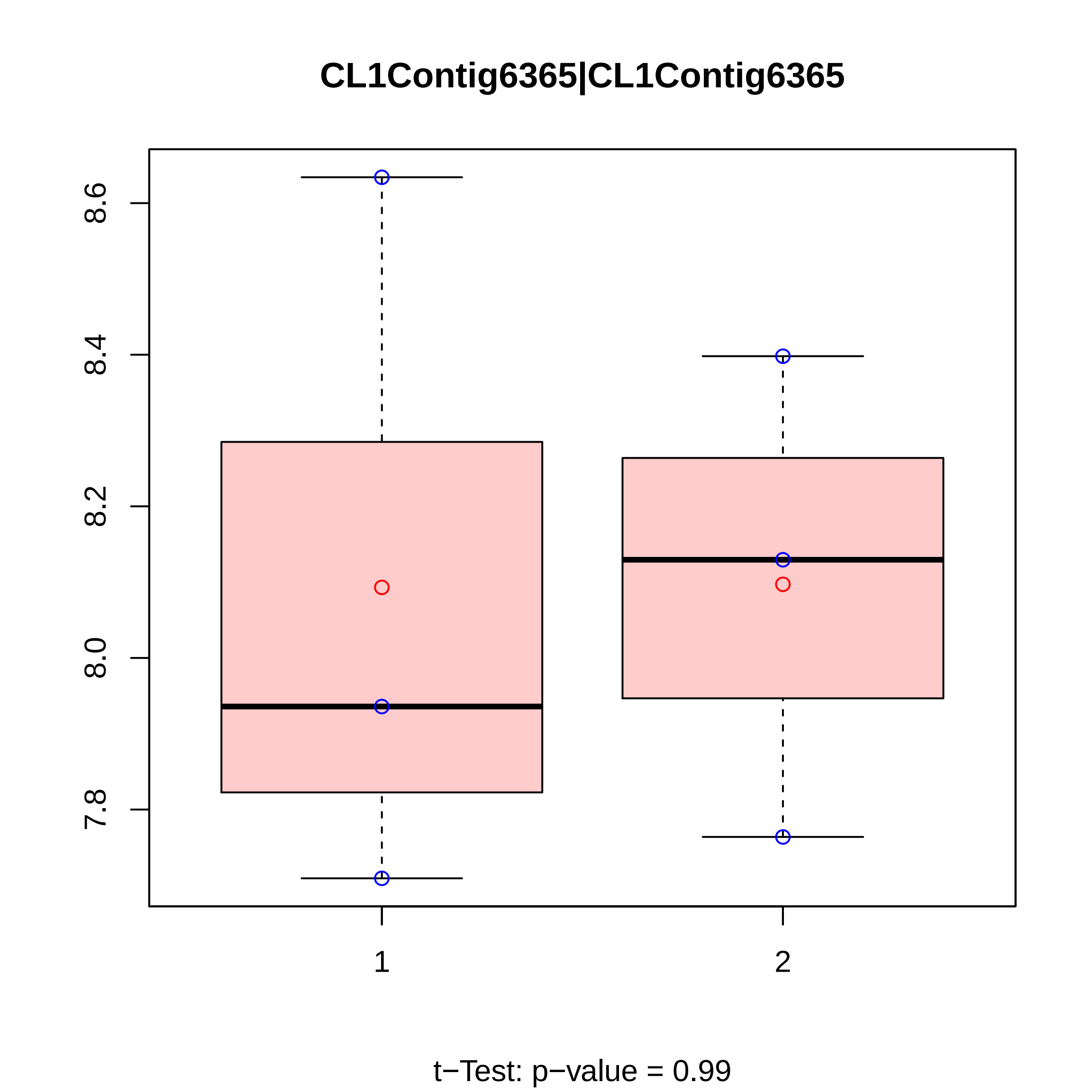
CL1Contig6365|CL1Contig6365
8.6
8.4
8.2
8.0
7.8
1
2
t−Test: p−value = 0.99

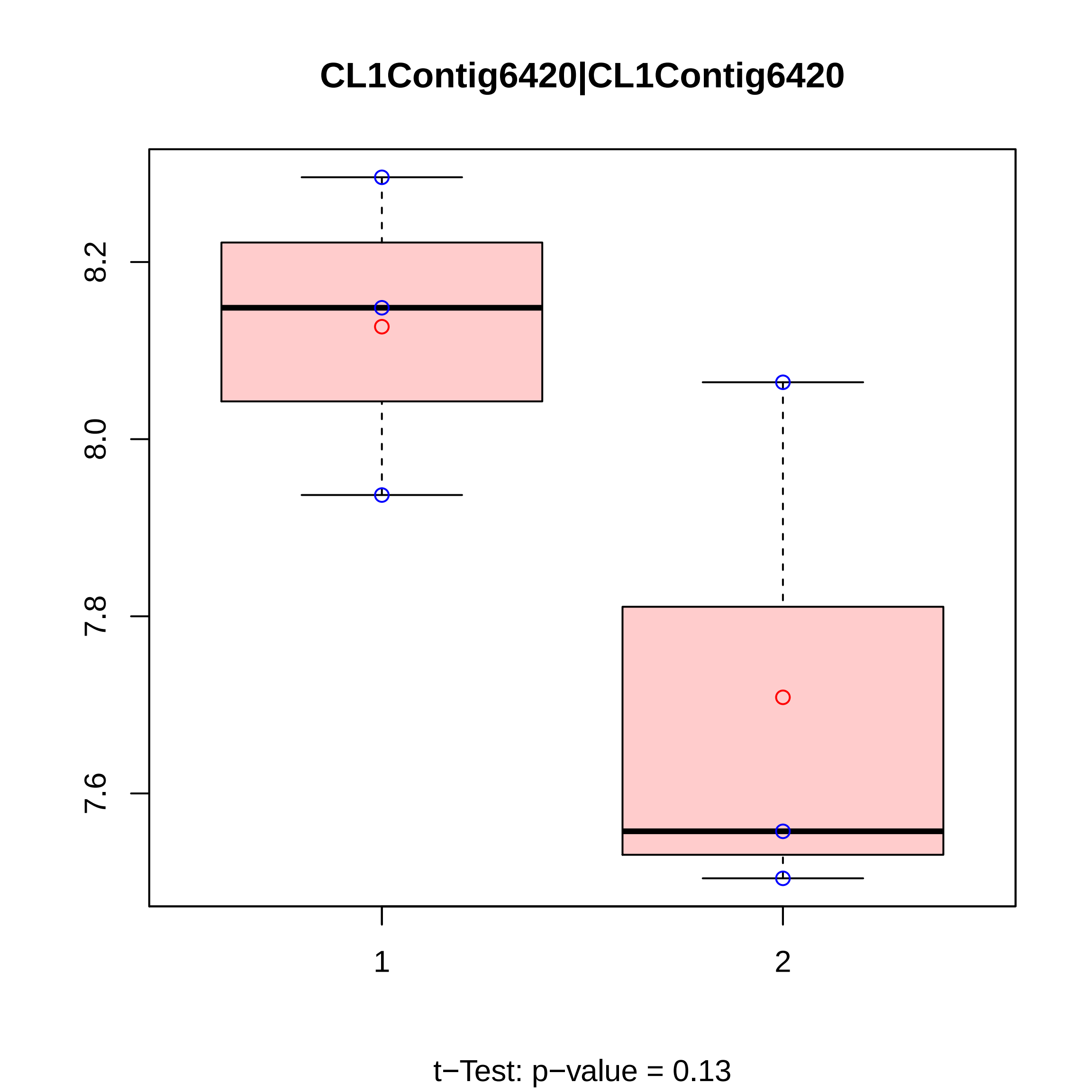
CL1Contig6420|CL1Contig6420
8.2
8.0
7.8
7.6
1
2
t−Test: p−value = 0.13

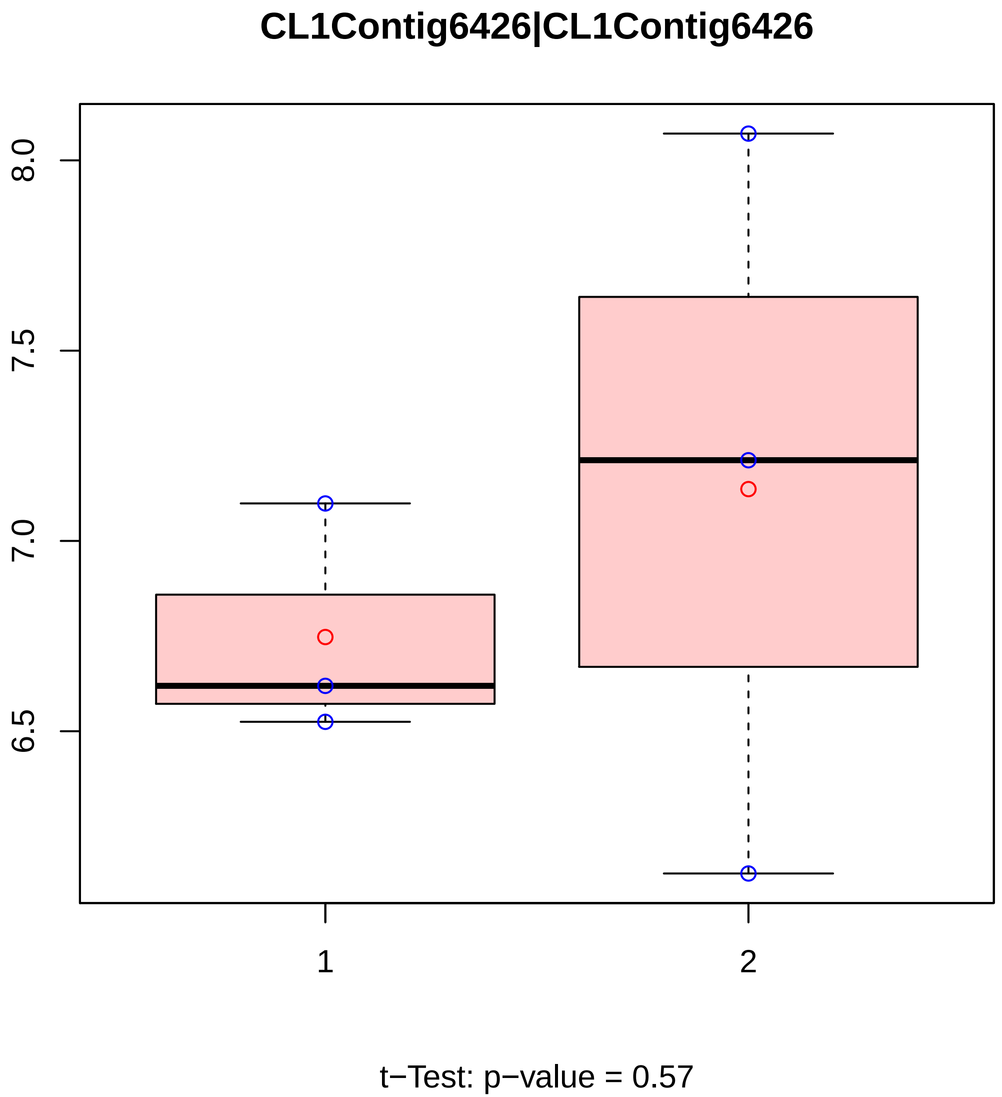

CL1Contig6426|CL1Contig6426
8.0
7.5
7.0
6.5
1
2
t−Test: p−value = 0.57

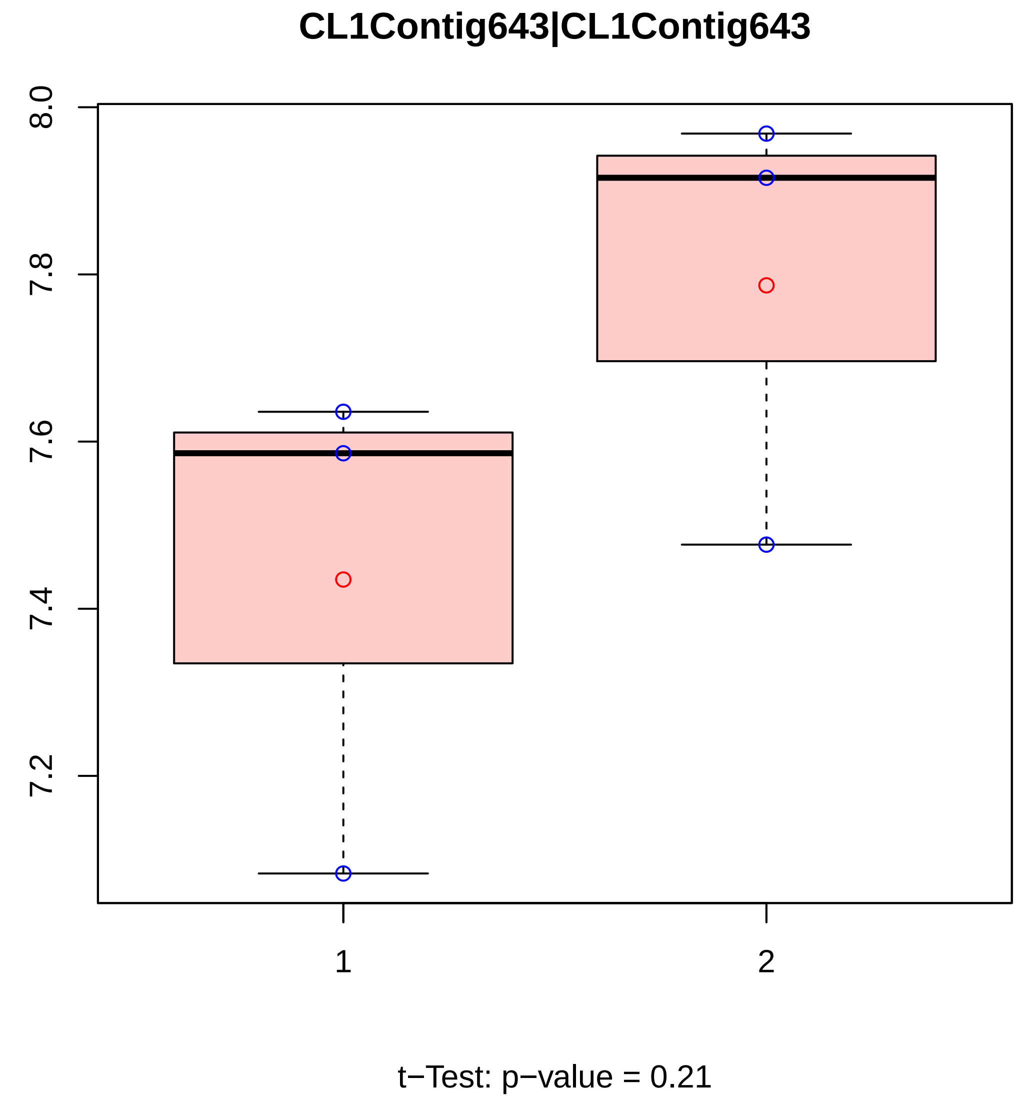
CL1Contig643|CL1Contig643
8.0
7.8
7.6
7.4
7.2
1
2
t−Test: p−value = 0.21

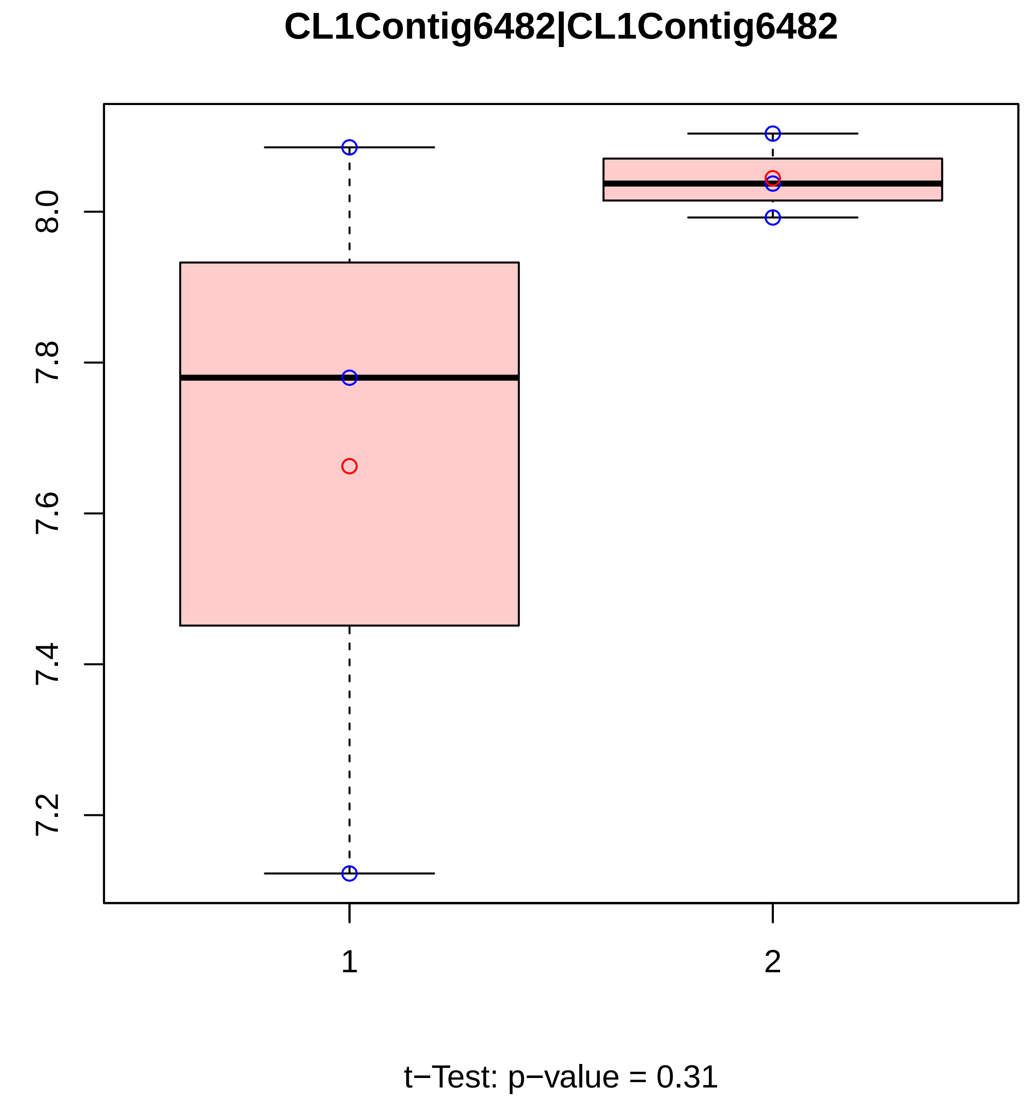

CL1Contig6482|CL1Contig6482
8.0
7.8
7.6
7.4
7.2
1
2
t−Test: p−value = 0.31

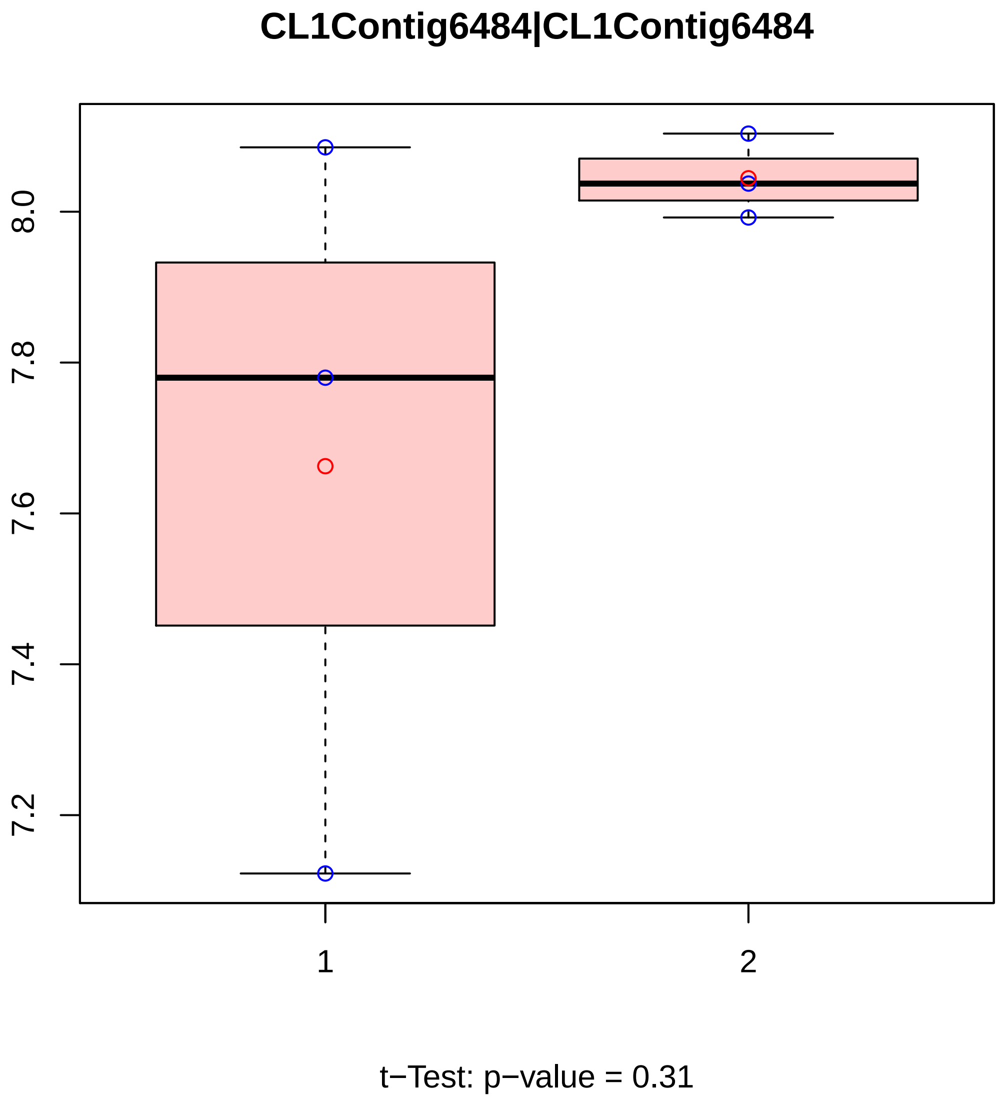
CL1Contig6484|CL1Contig6484
8.0
7.8
7.6
7.4
7.2
1
2
t−Test: p−value = 0.31

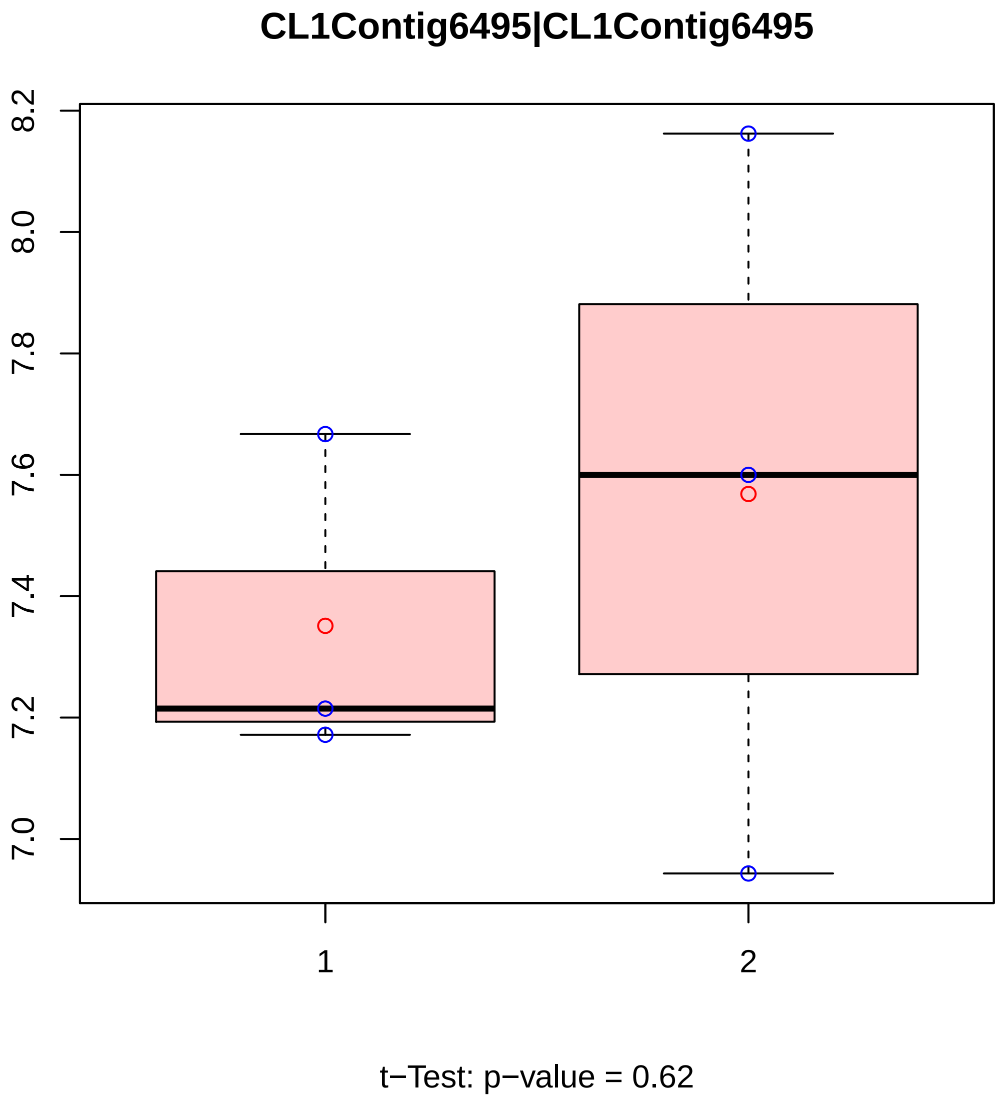
CL1Contig6495|CL1Contig6495
8.2
8.0
7.8
7.6
7.4
7.2
7.0
1
2
t−Test: p−value = 0.62

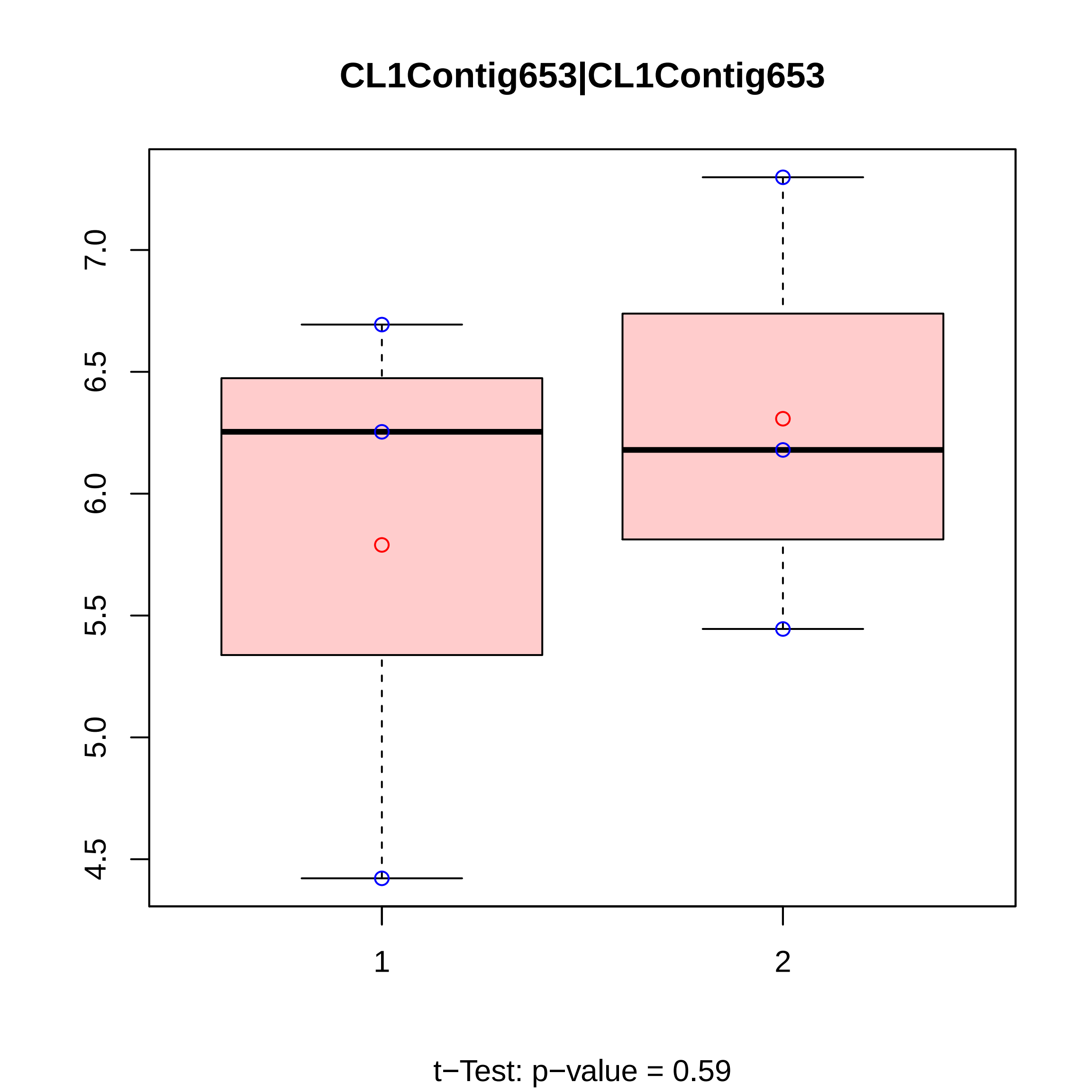

CL1Contig653|CL1Contig653
7.0
6.5
6.0
5.5
5.0
4.5
1
2
t−Test: p−value = 0.59

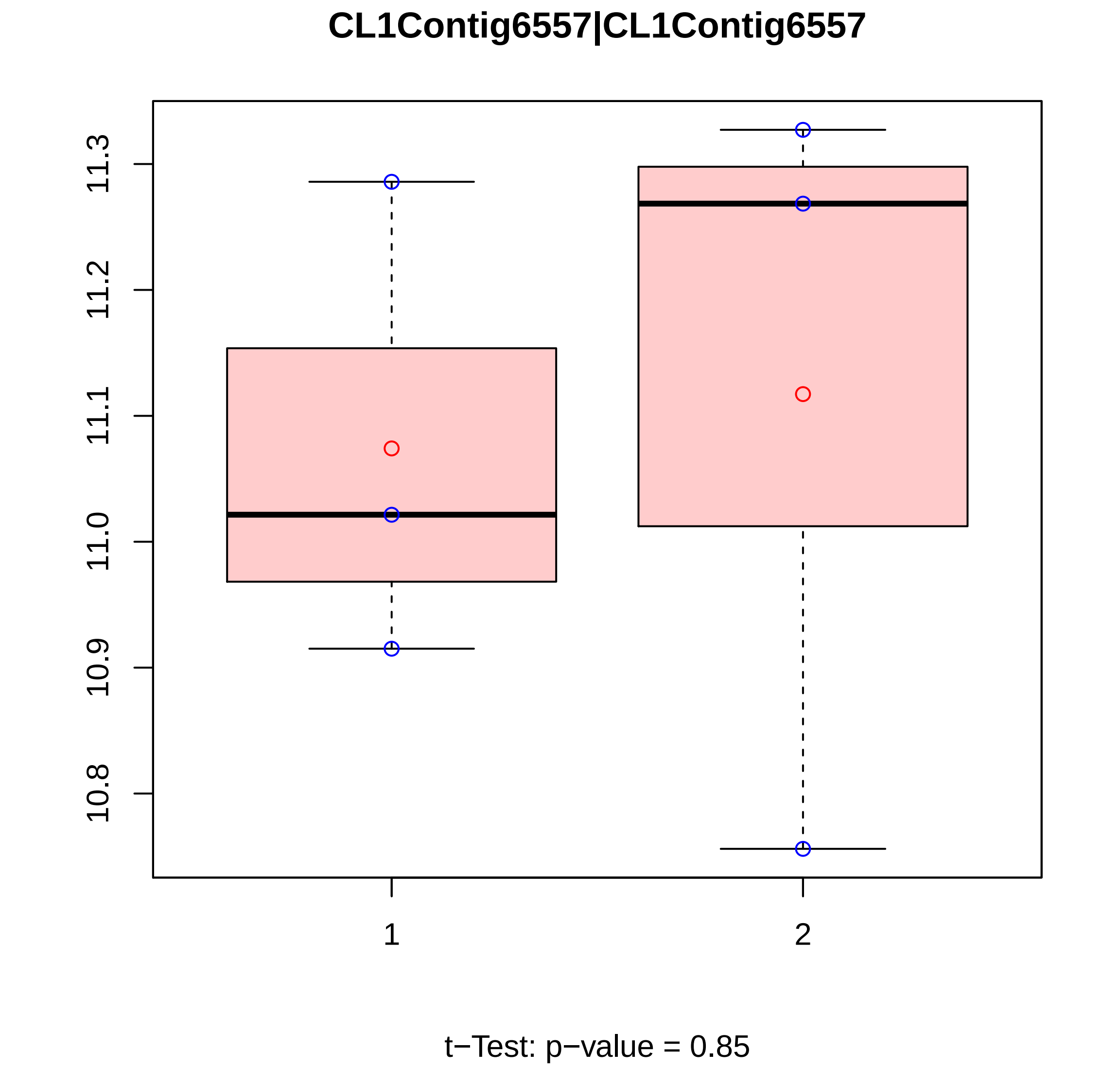
CL1Contig6557|CL1Contig6557
11.3
11.2
11.1
11.0
10.9
10.8
1
2
t−Test: p−value = 0.85

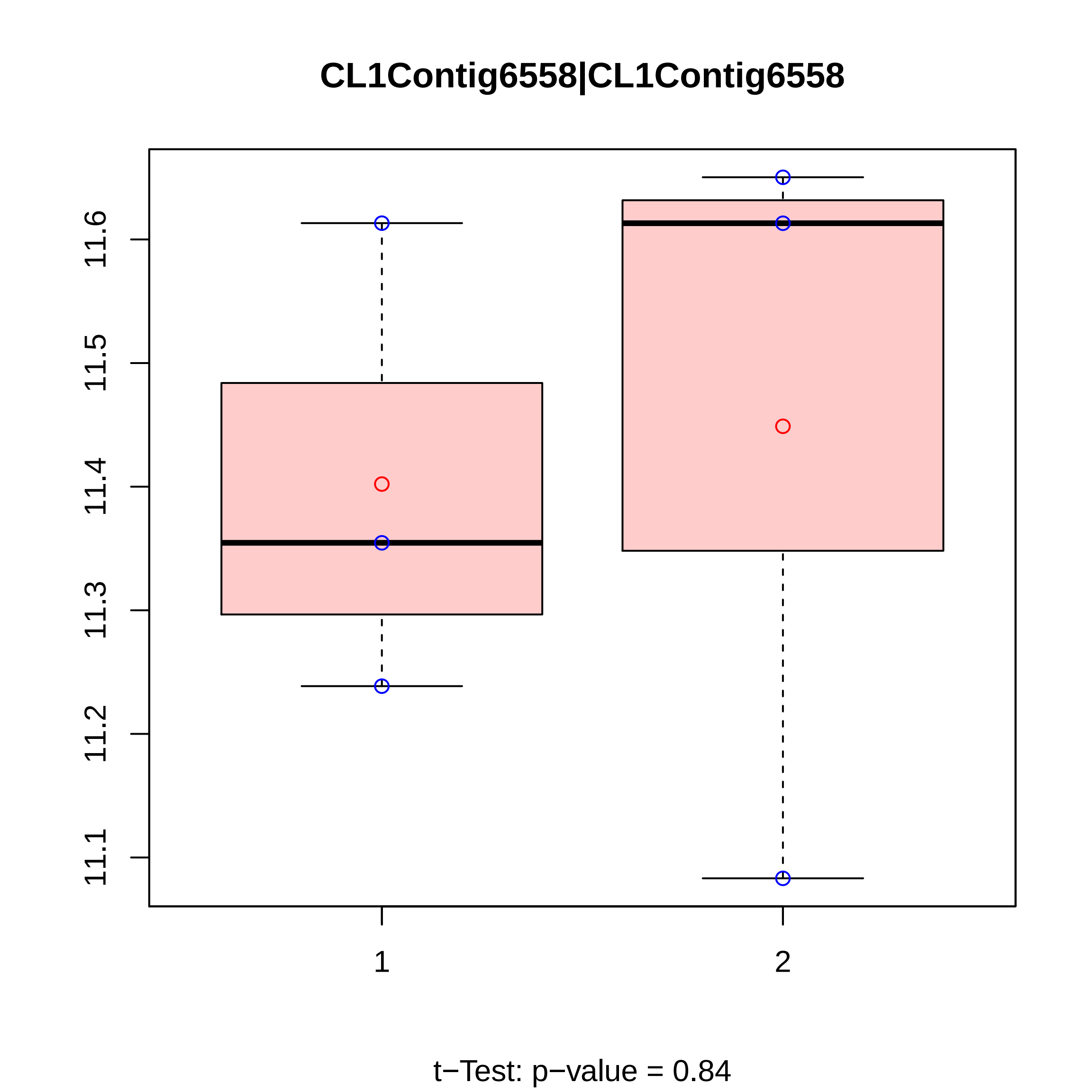
CL1Contig6558|CL1Contig6558
11.1
11.2
11.3
11.4
11.5
11.6
1
2
t−Test: p−value = 0.84

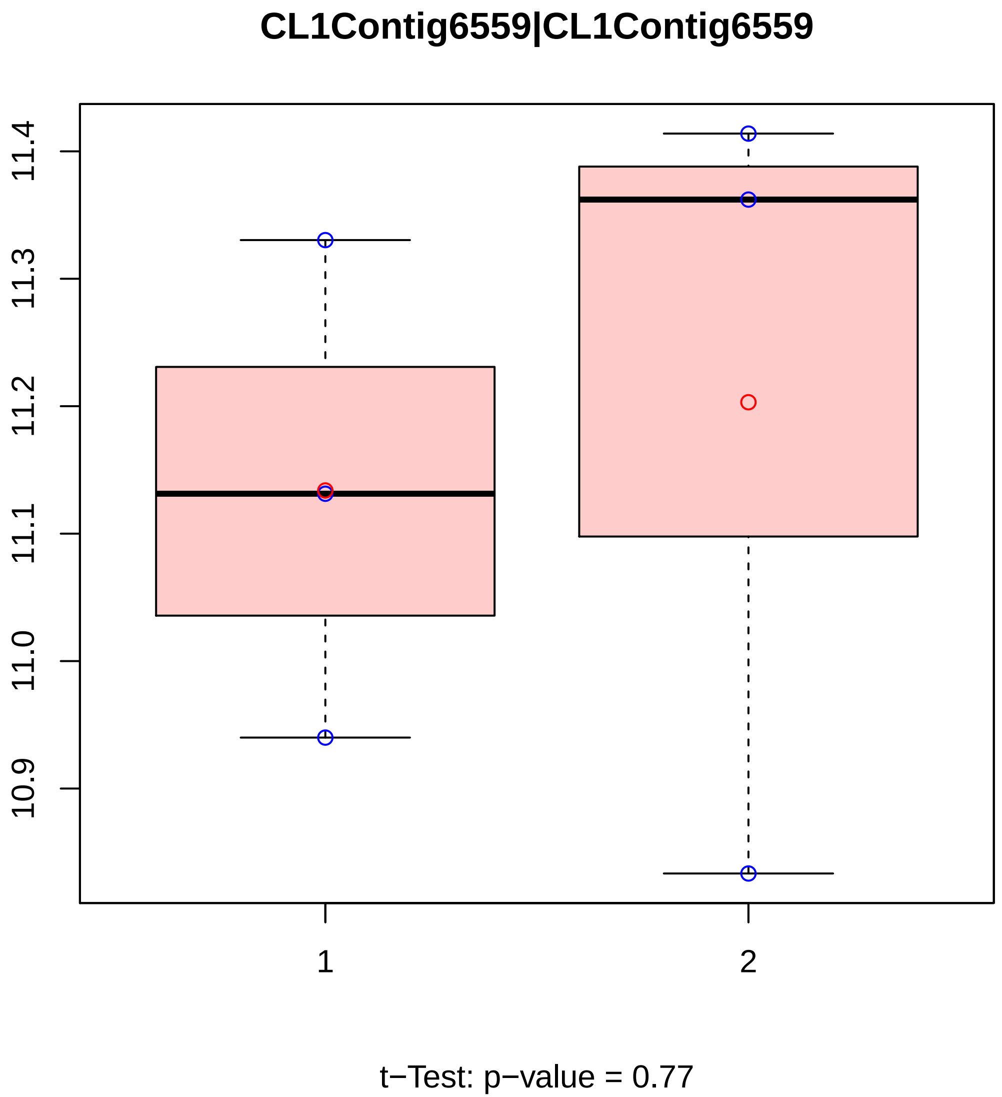
CL1Contig6559|CL1Contig6559
11.4
11.3
11.2
11.1
11.0
10.9
1
2
t−Test: p−value = 0.77

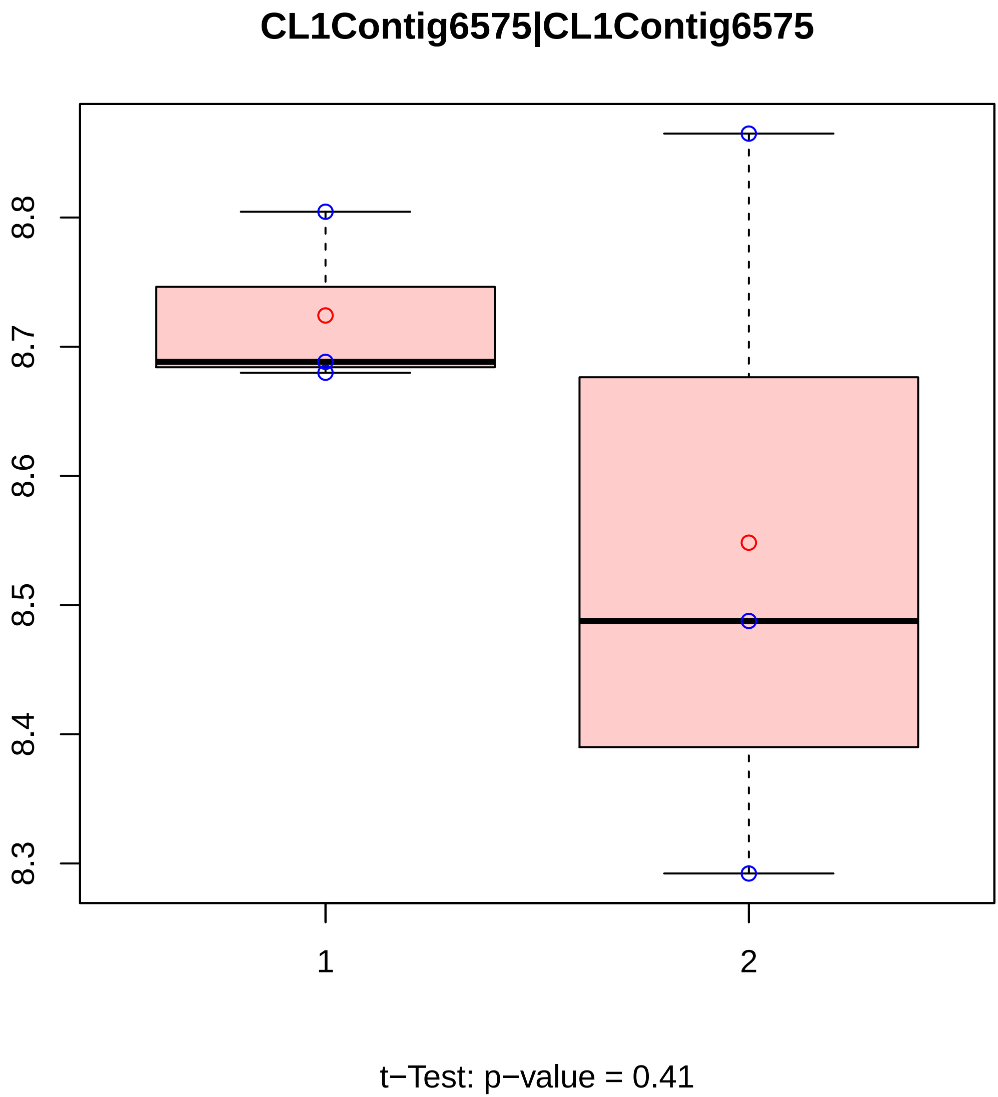
CL1Contig6575|CL1Contig6575
8.3
8.4
8.5
8.6
8.7
8.8
1
2
t−Test: p−value = 0.41

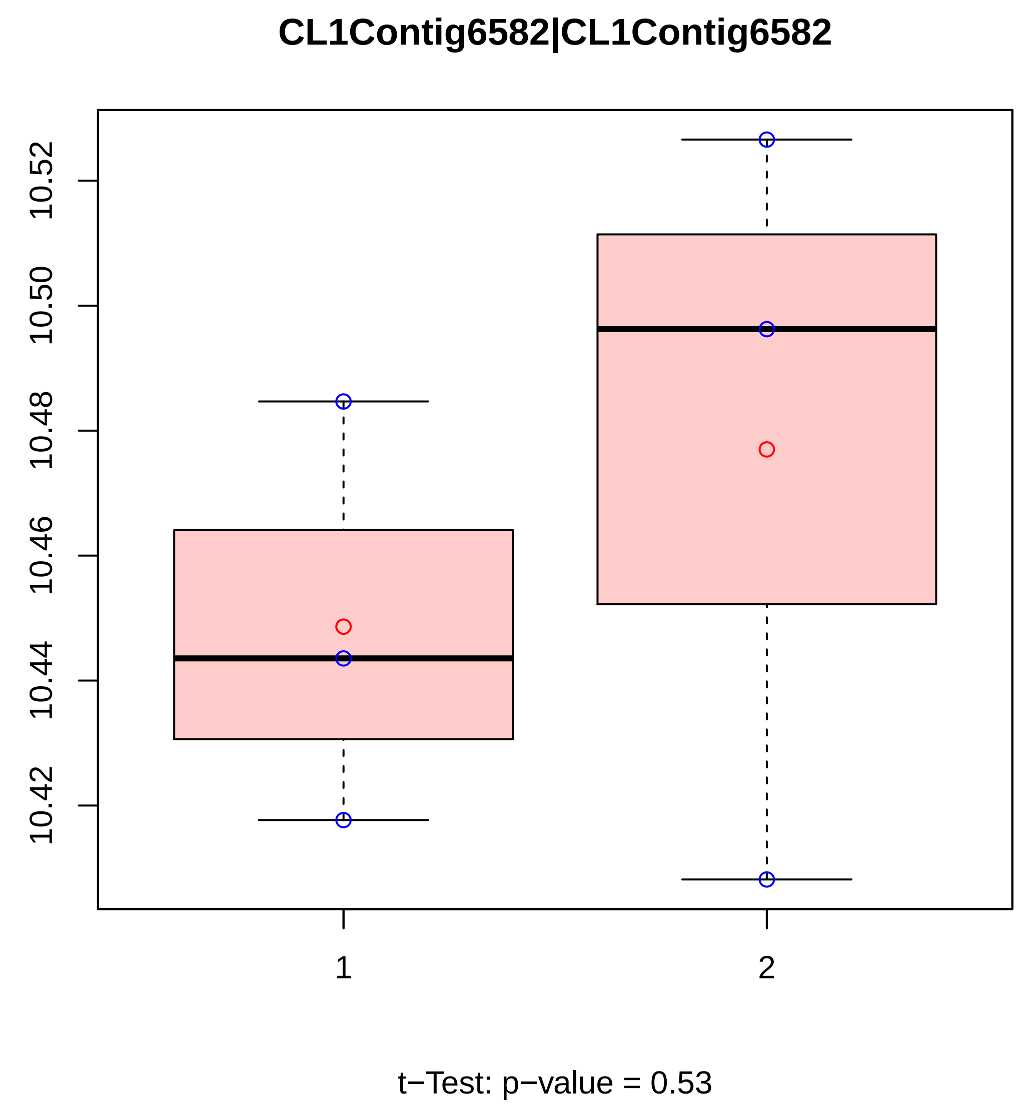
CL1Contig6582|CL1Contig6582
10.42
10.44
10.46
10.48
10.50
10.52
1
2
t−Test: p−value = 0.53

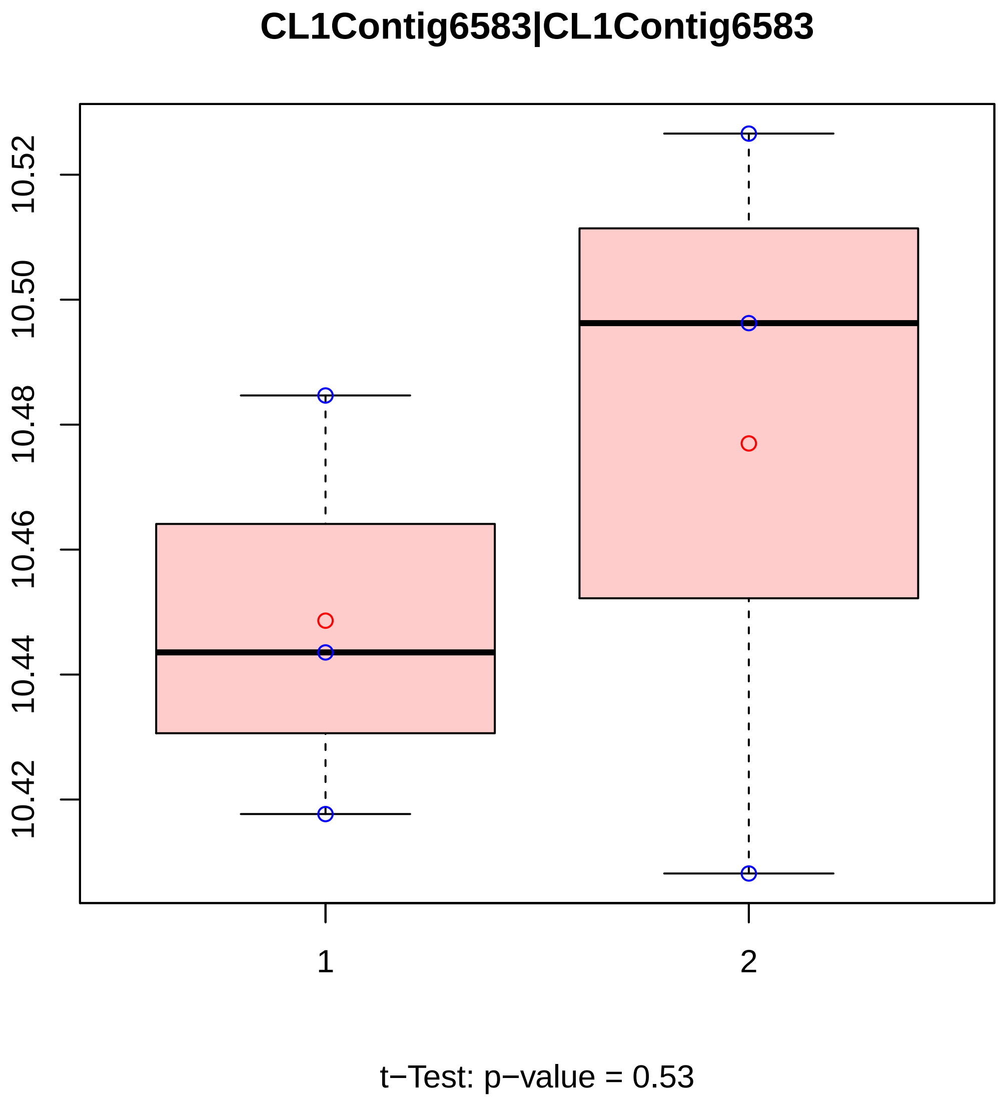
CL1Contig6583|CL1Contig6583
10.42
10.44
10.46
10.48
10.50
10.52
1
2
t−Test: p−value = 0.53

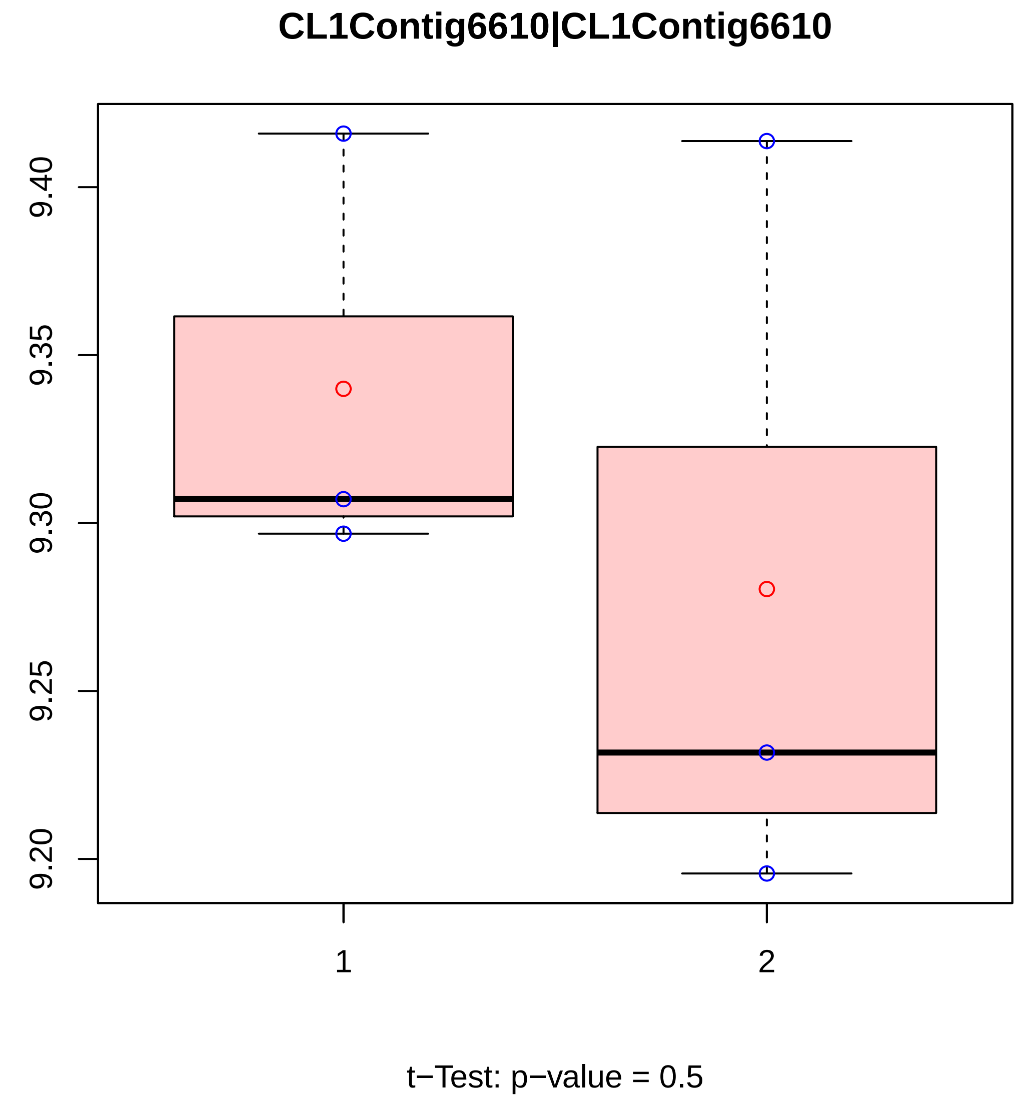
CL1Contig6610|CL1Contig6610
9.40
9.35
9.30
9.25
9.20
1
2
t−Test: p−value = 0.5

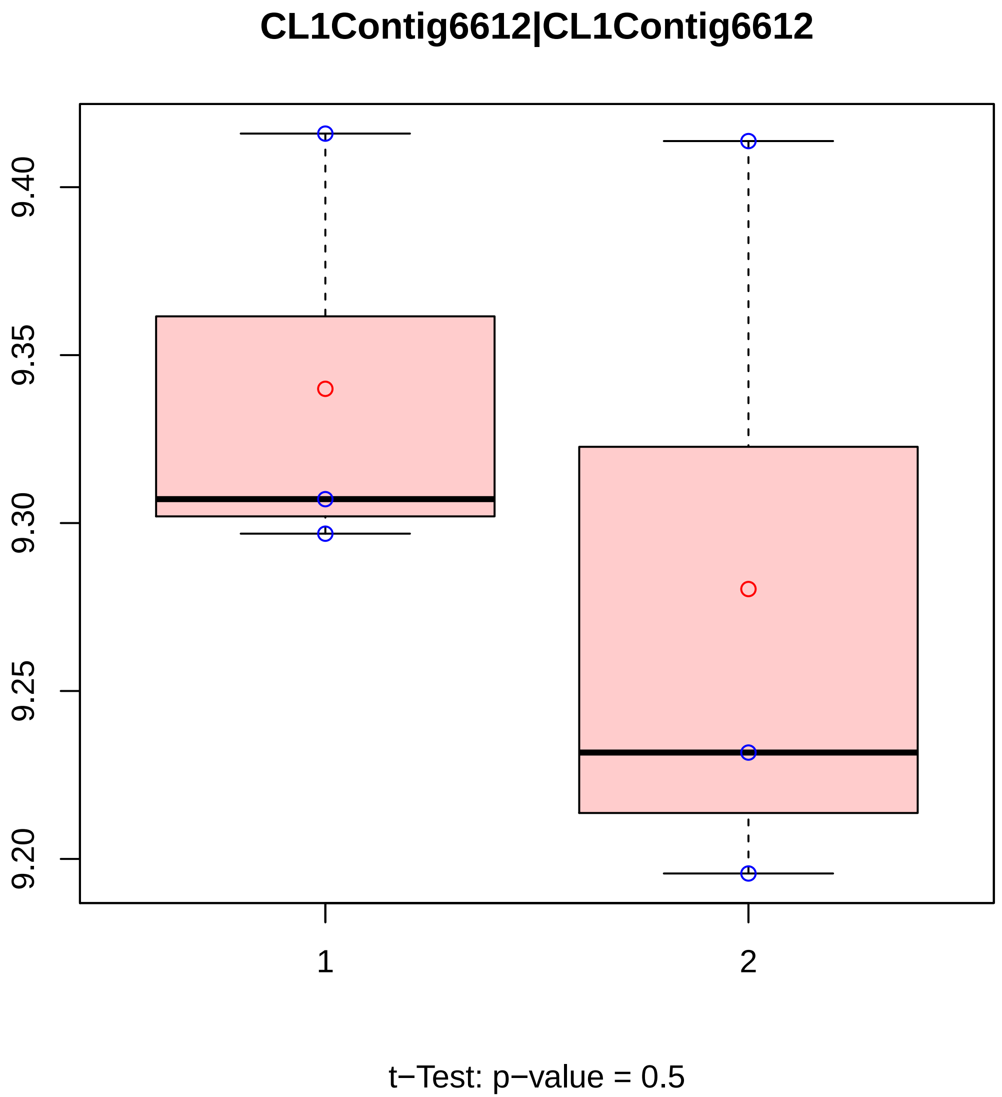

CL1Contig6612|CL1Contig6612
9.40
9.35
9.30
9.25
9.20
1
2
t−Test: p−value = 0.5

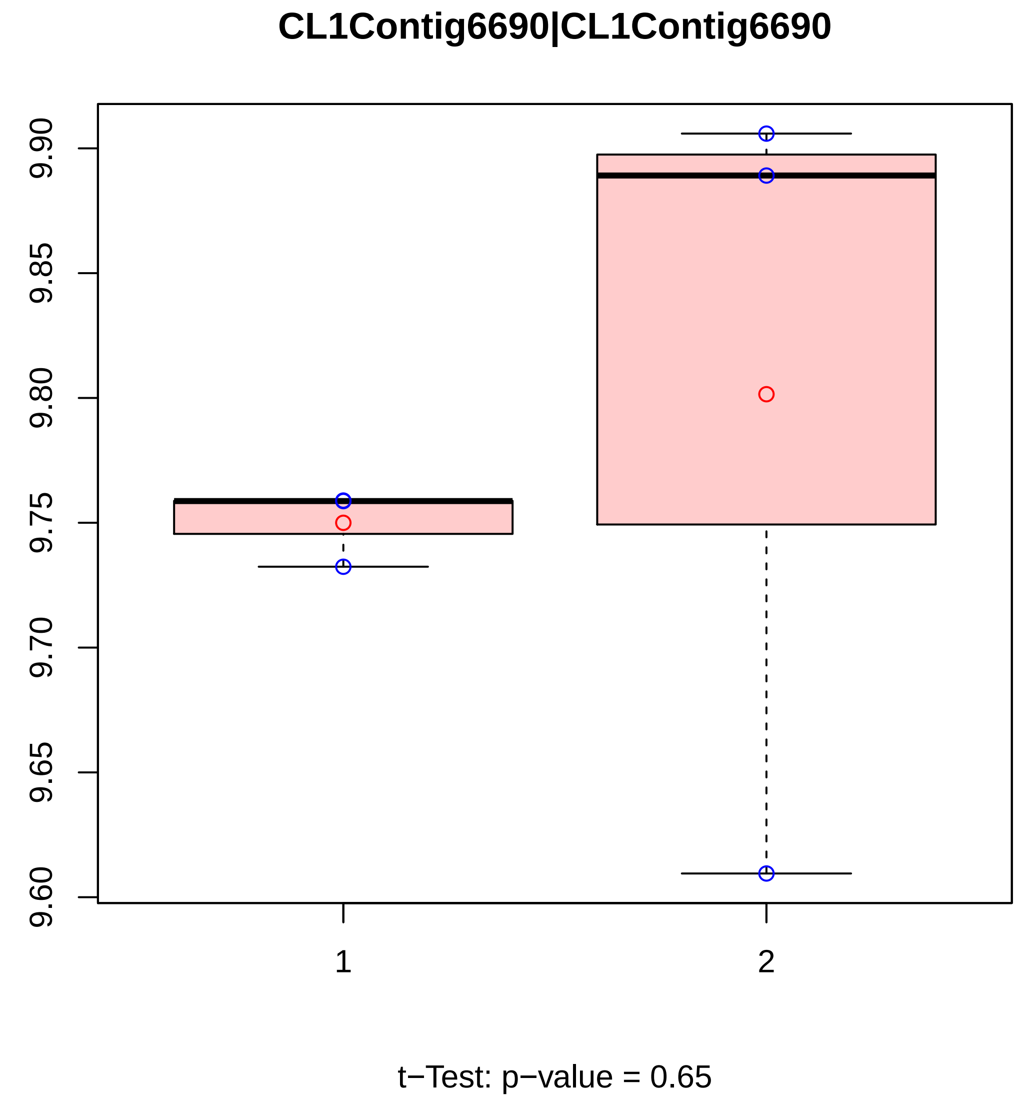
CL1Contig6690|CL1Contig6690
9.60
9.65
9.70
9.75
9.80
9.85
9.90
1
2
t−Test: p−value = 0.65

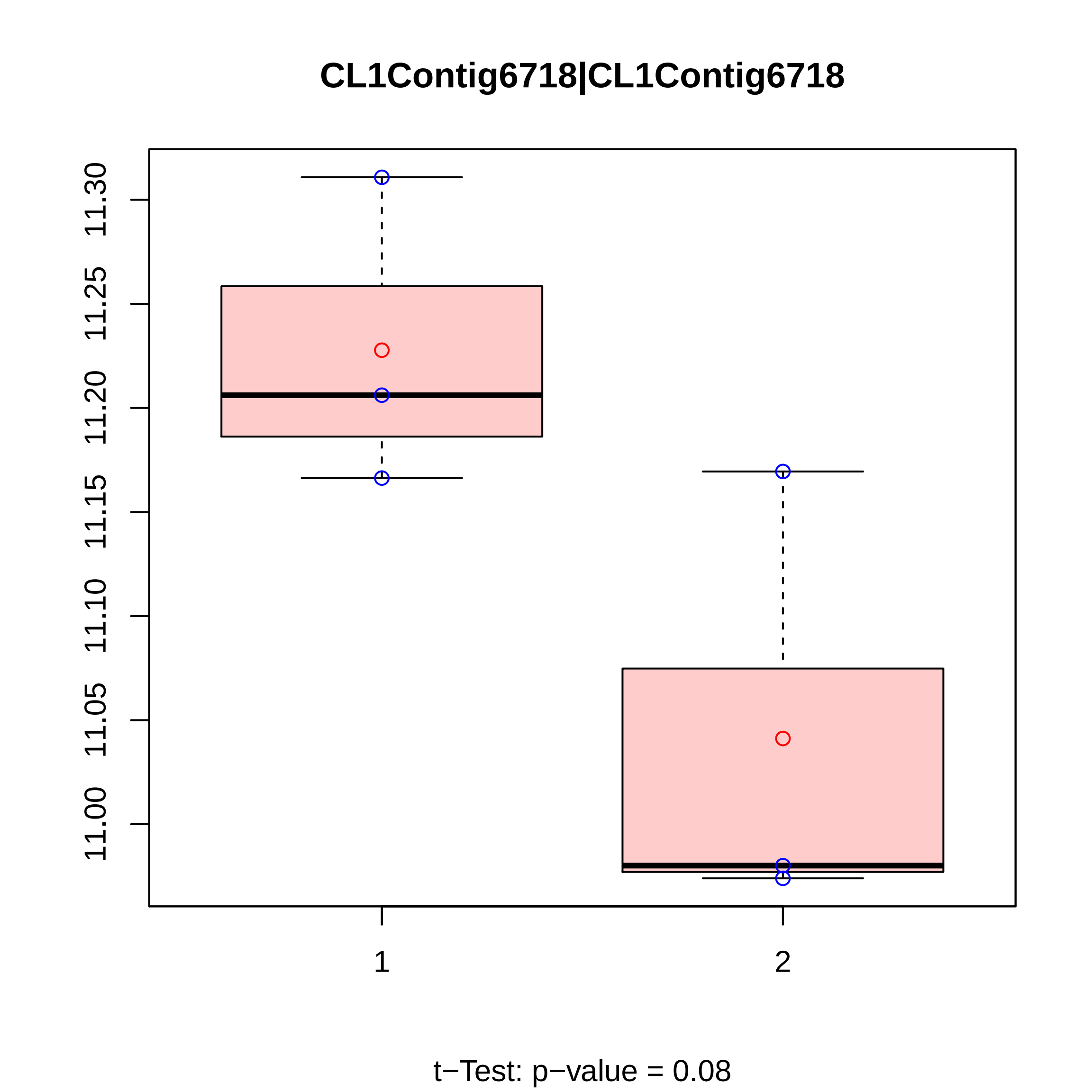
CL1Contig6718|CL1Contig6718
11.00
11.05
11.10
11.15
11.20
11.25
11.30
1
2
t−Test: p−value = 0.08

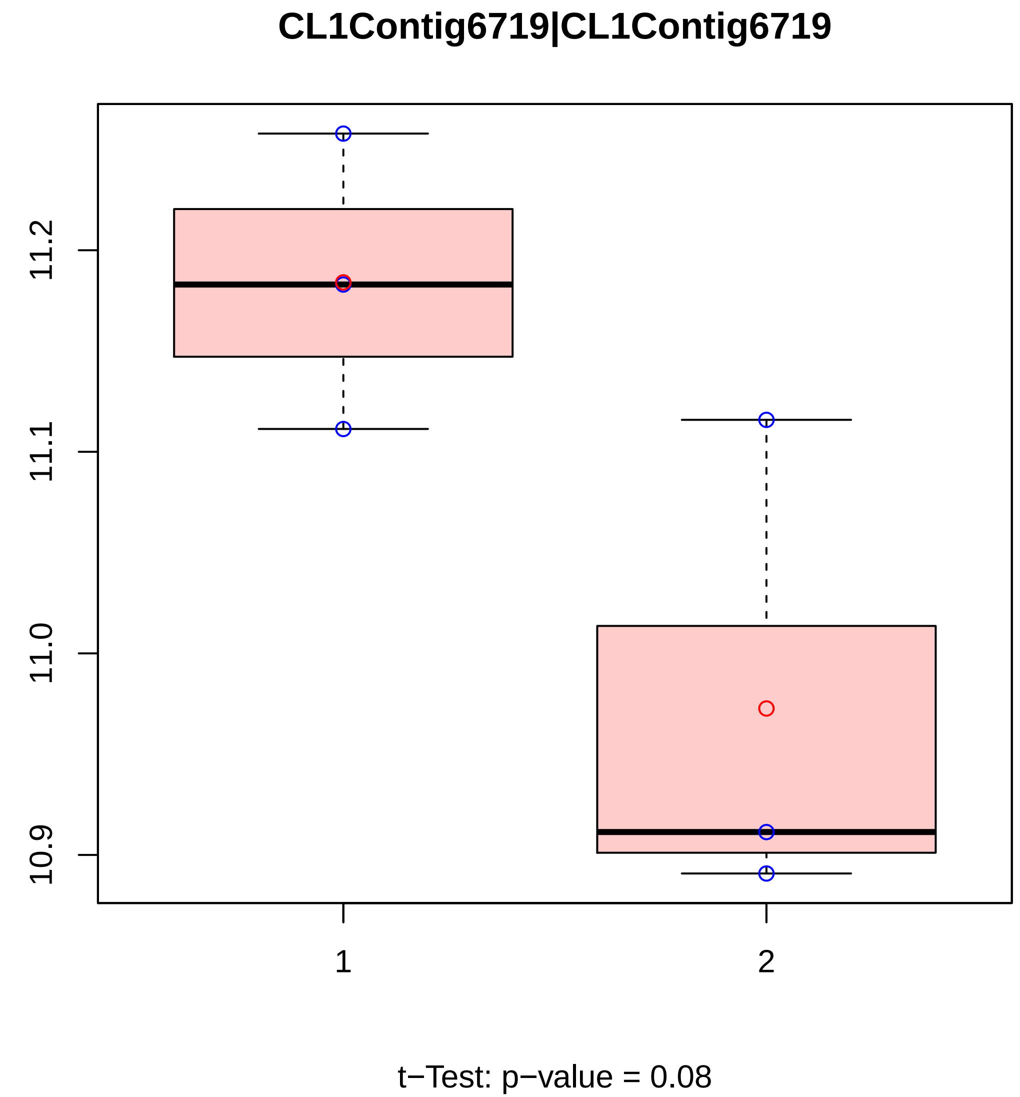
CL1Contig6719|CL1Contig6719
11.2
11.1
11.0
10.9
1
2
t−Test: p−value = 0.08

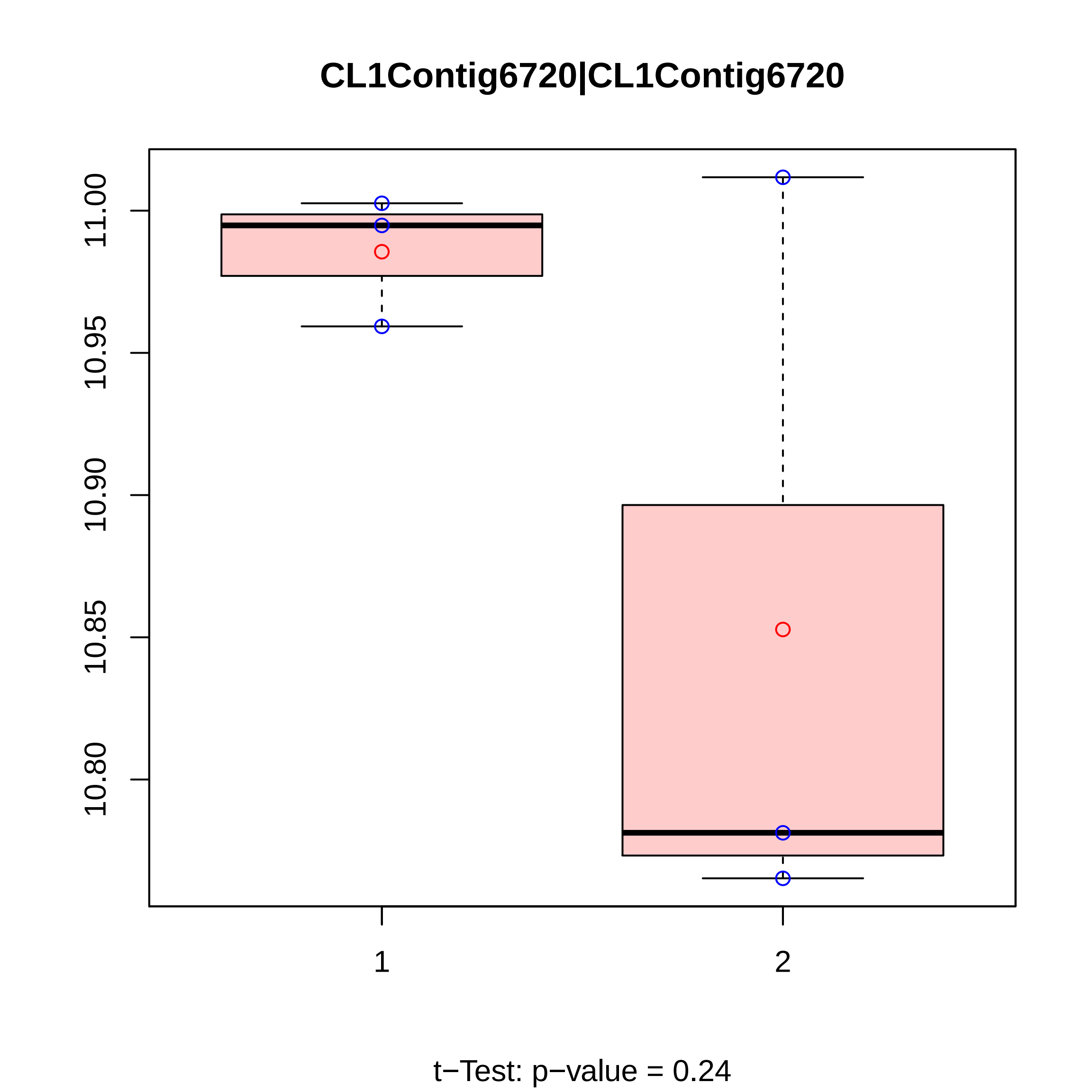
CL1Contig6720|CL1Contig6720
11.00
10.95
10.90
10.85
10.80
1
2
t−Test: p−value = 0.24

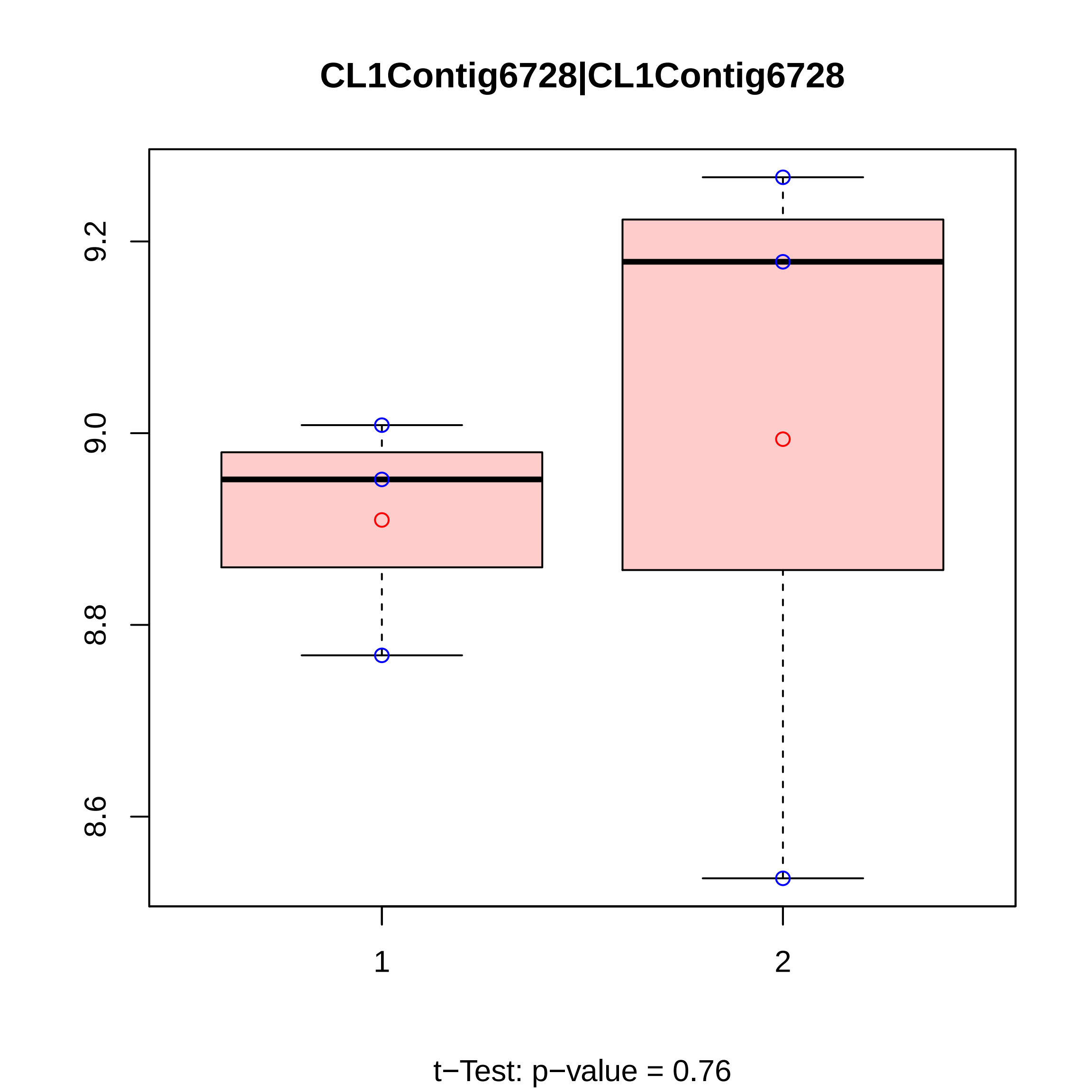
CL1Contig6728|CL1Contig6728
9.2
9.0
8.8
8.6
1
2
t−Test: p−value = 0.76

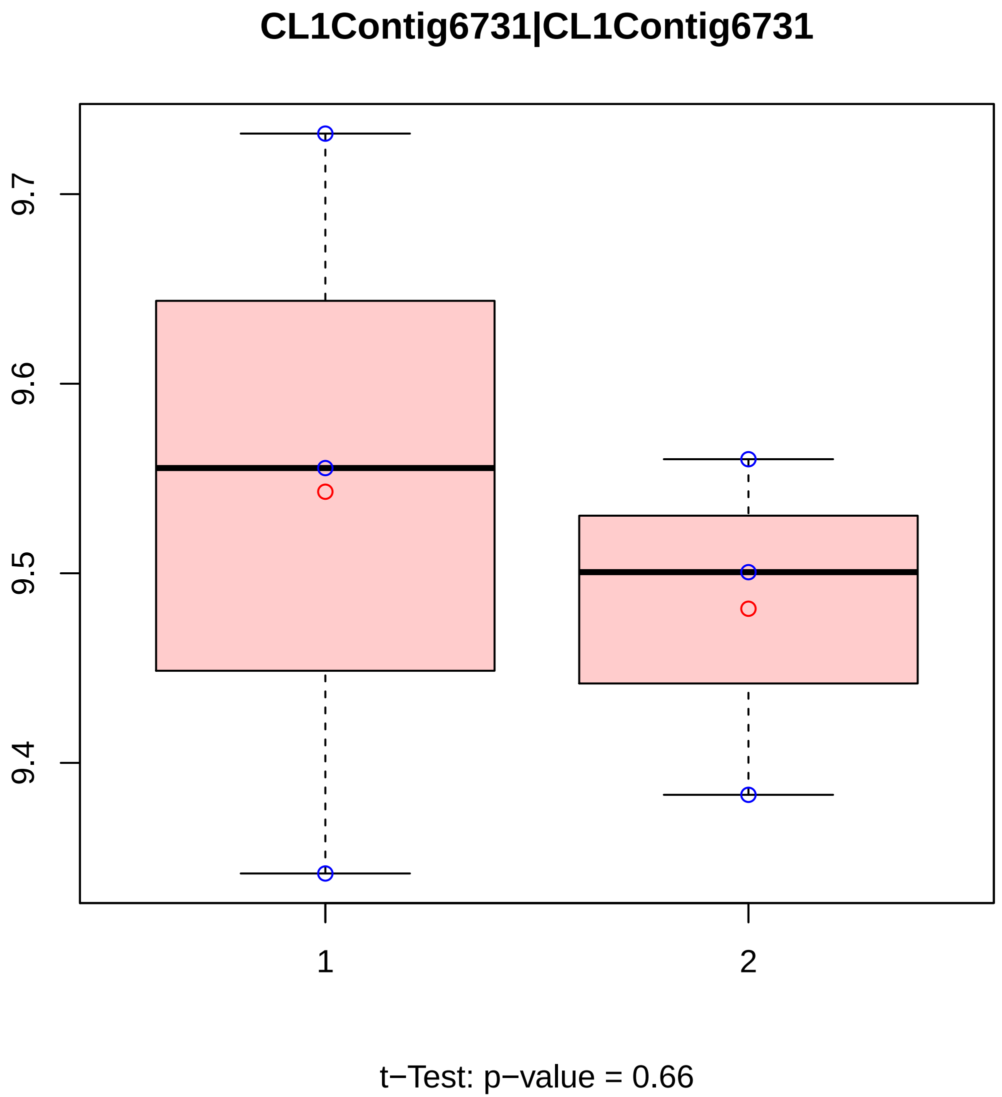

CL1Contig6731|CL1Contig6731
9.4
9.5
9.6
9.7
1
2
t−Test: p−value = 0.66

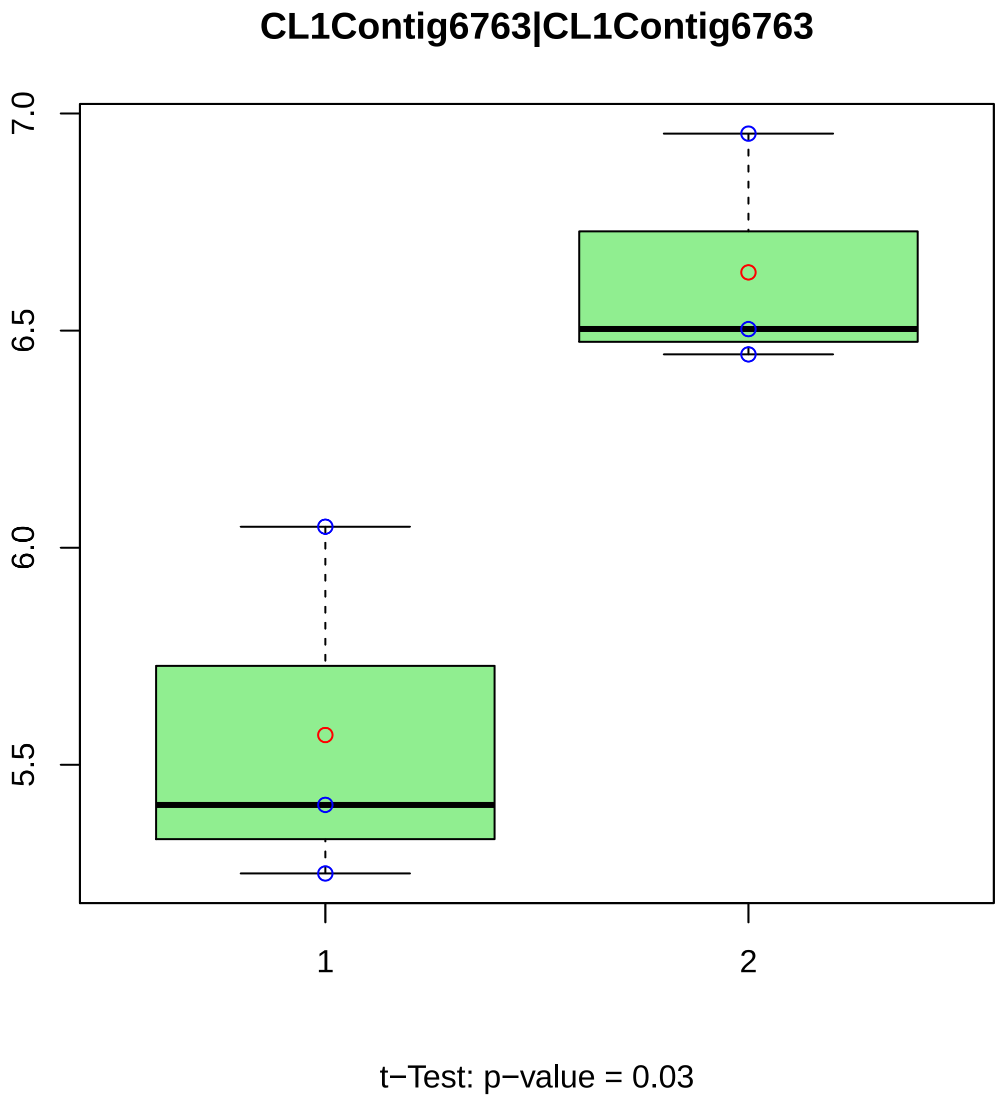
CL1Contig6763|CL1Contig6763
7.0
6.5
6.0
5.5
1
2
t−Test: p−value = 0.03

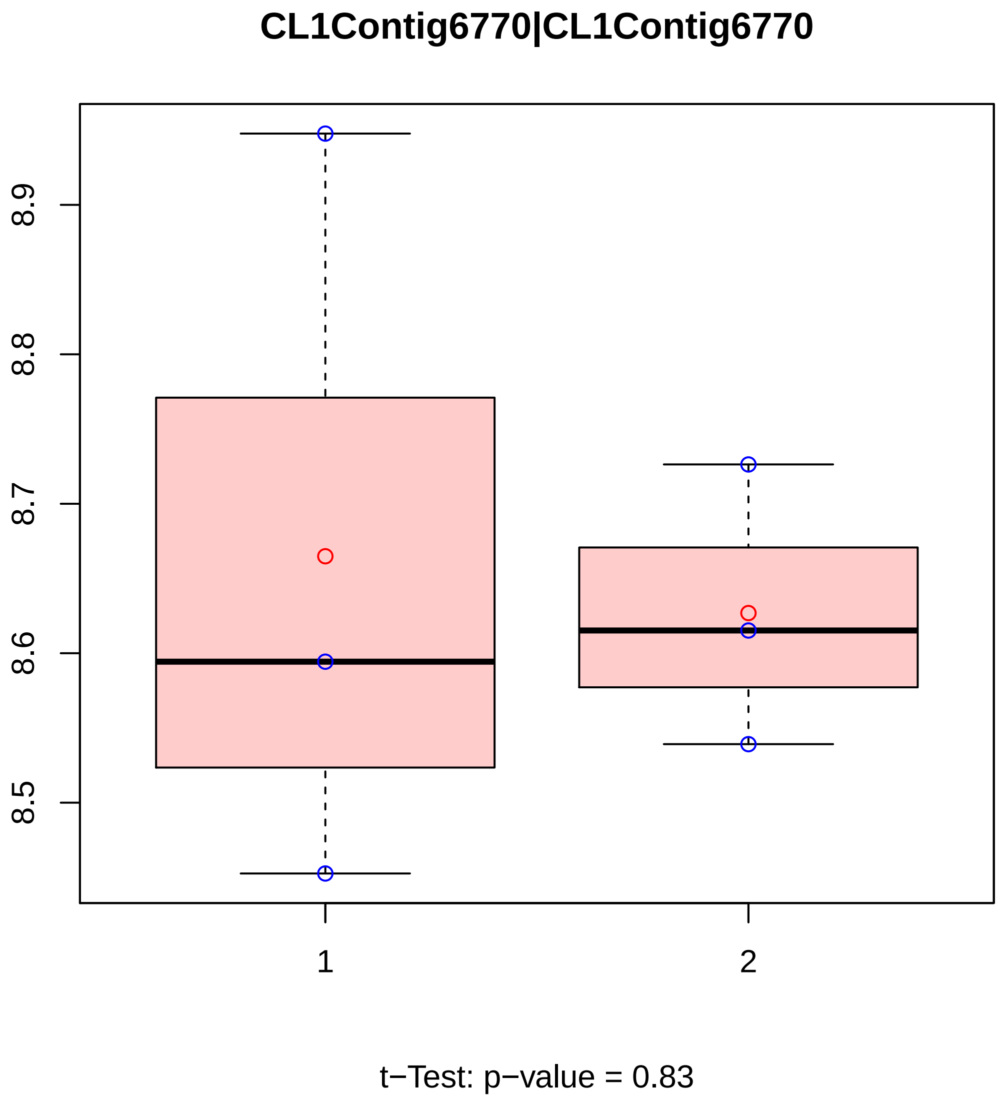
CL1Contig6770|CL1Contig6770
8.5
8.6
8.7
8.8
8.9
1
2
t−Test: p−value = 0.83

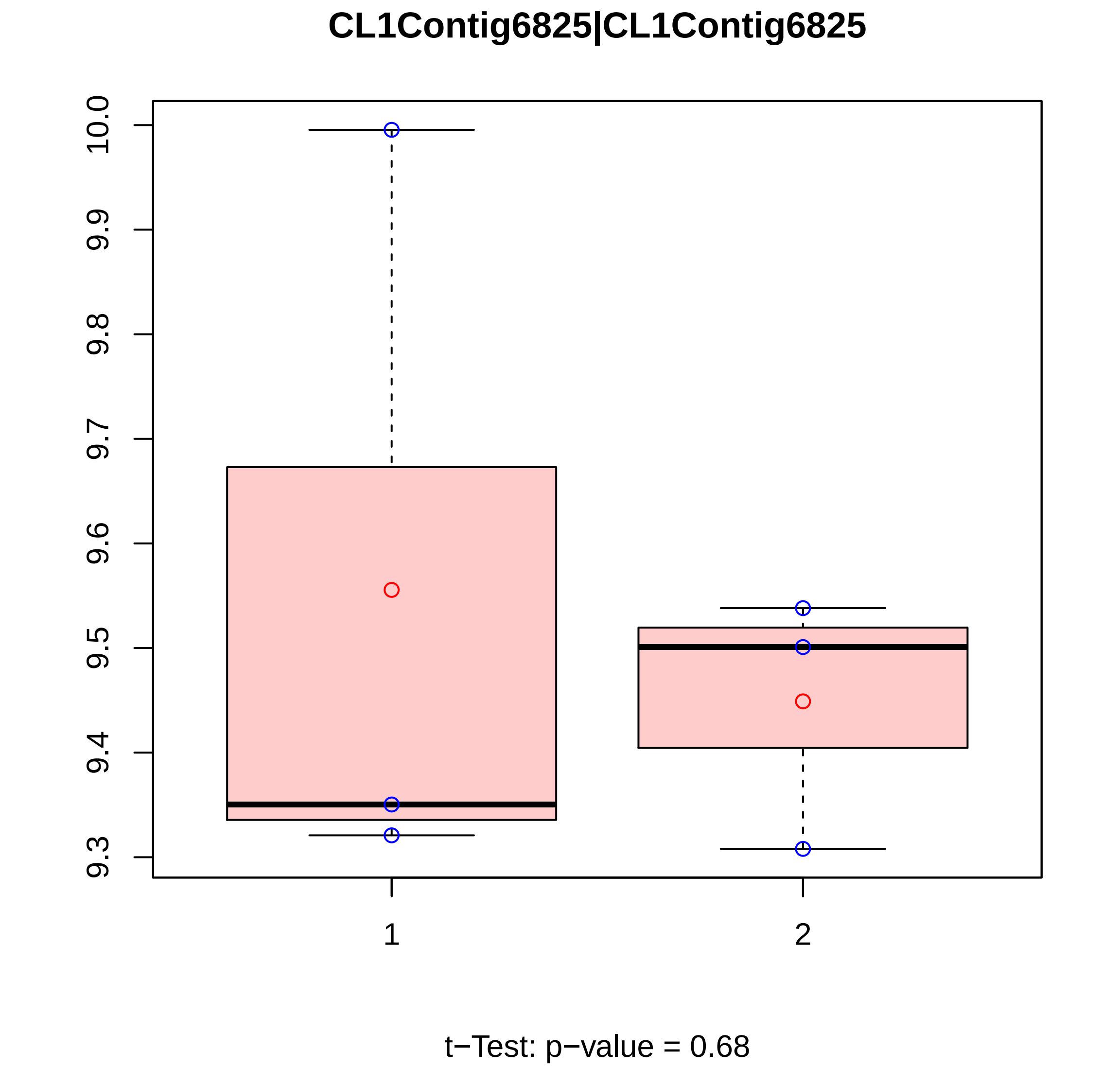
CL1Contig6825|CL1Contig6825
10.0
9.9
9.8
9.7
9.6
9.5
9.4
9.3
1
2
t−Test: p−value = 0.68

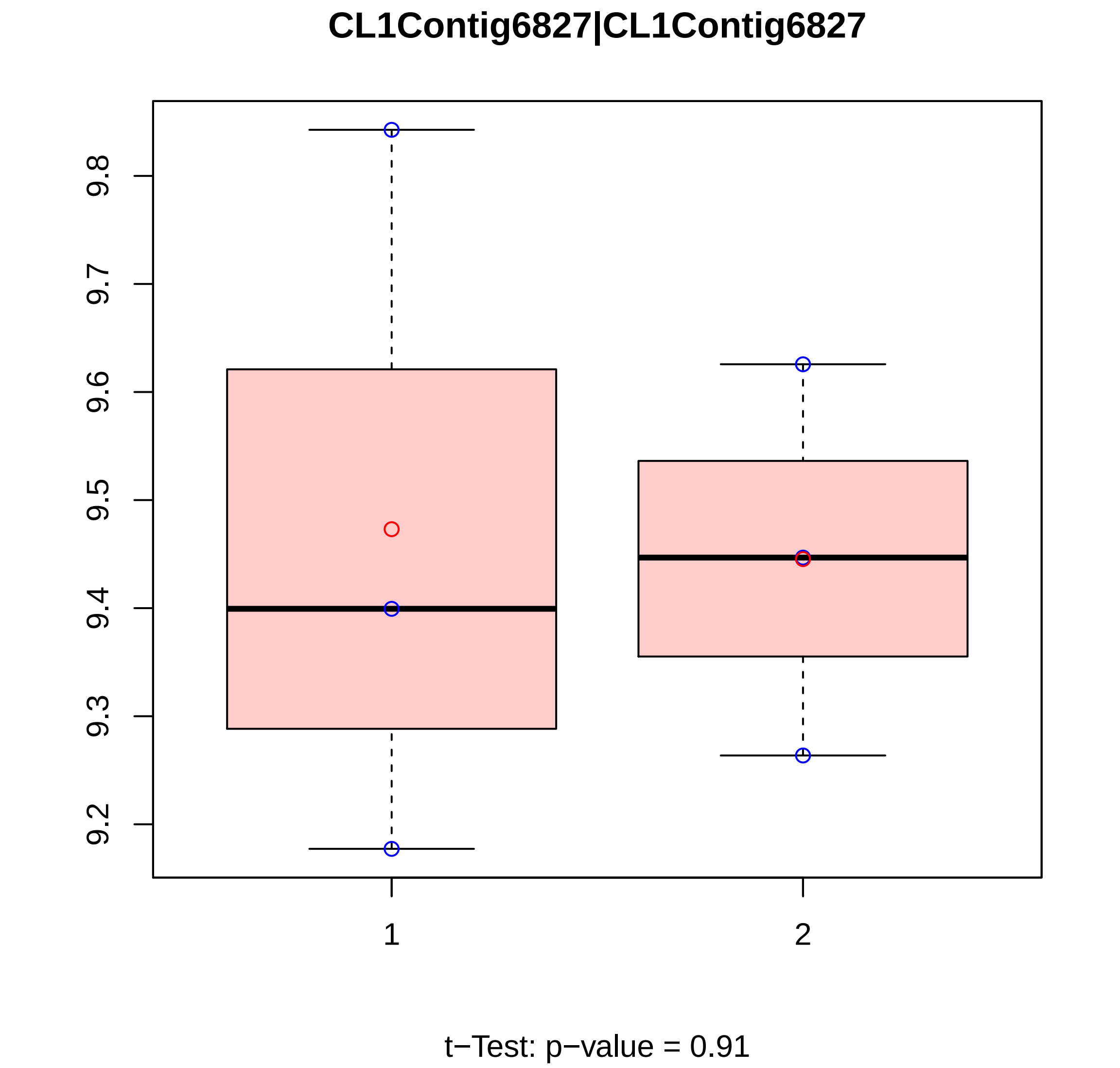
CL1Contig6827|CL1Contig6827
9.2
9.3
9.4
9.5
9.6
9.7
9.8
1
2
t−Test: p−value = 0.91

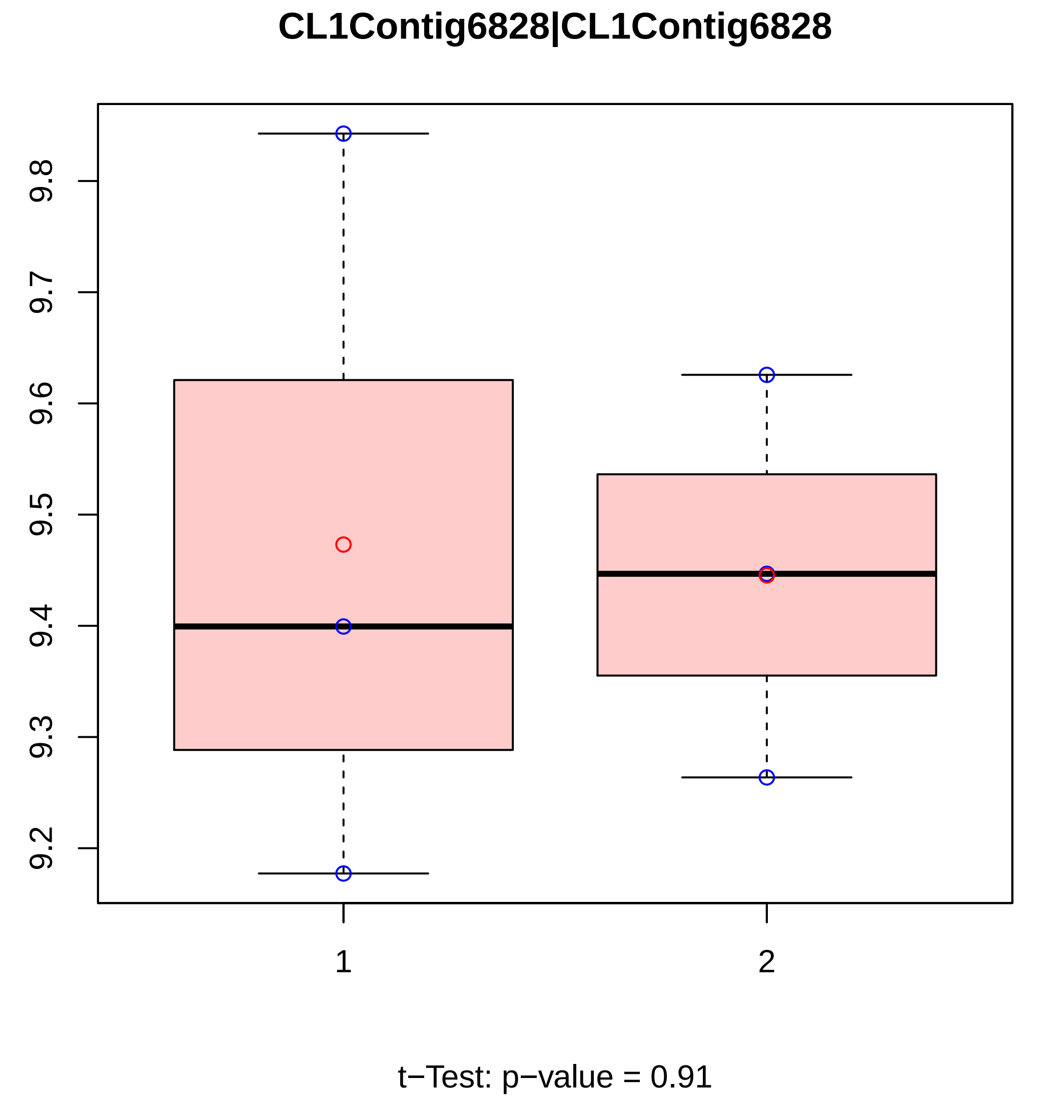
CL1Contig6828|CL1Contig6828
9.2
9.3
9.4
9.5
9.6
9.7
9.8
1
2
t−Test: p−value = 0.91

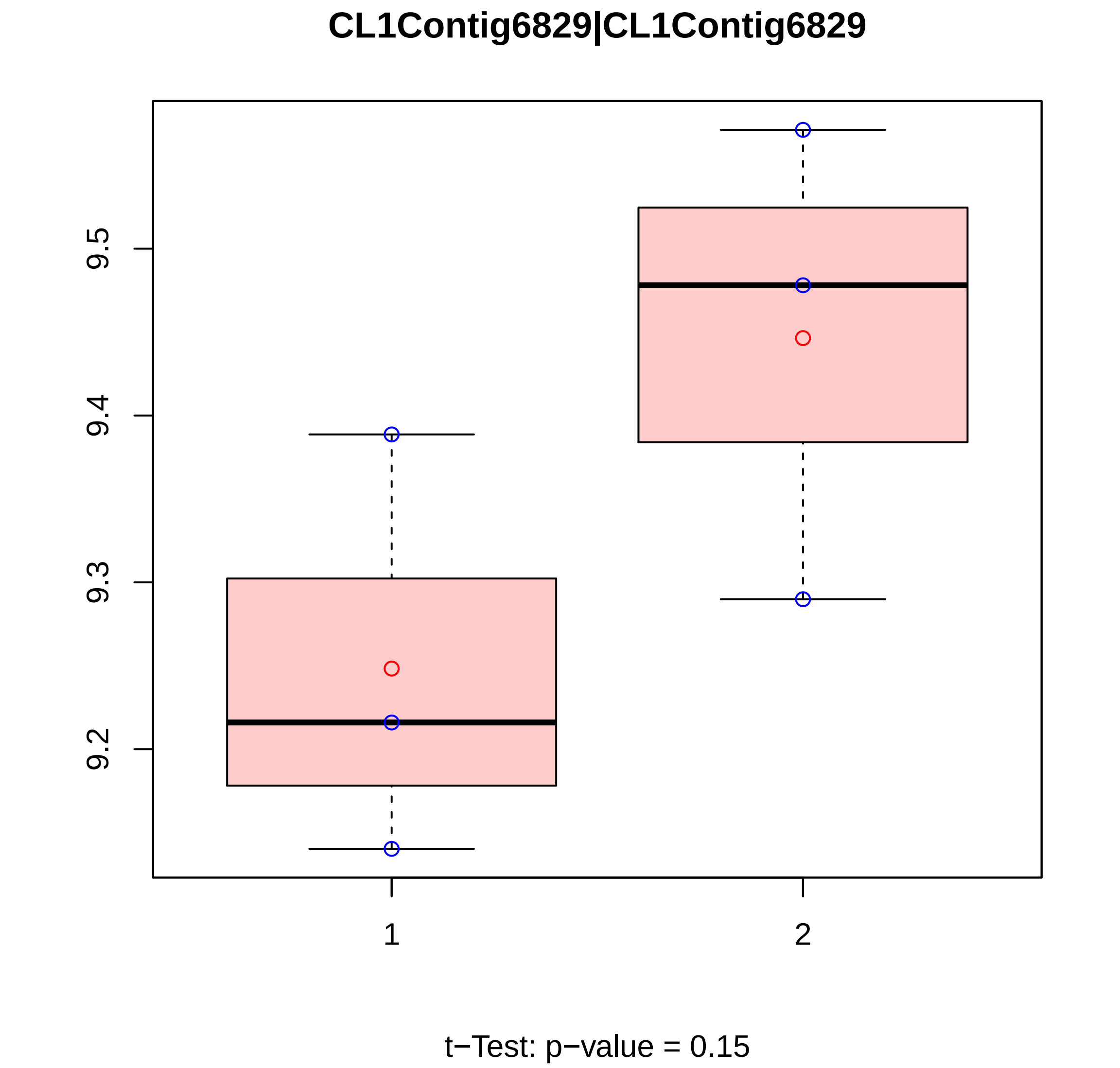
CL1Contig6829|CL1Contig6829
9.2
9.3
9.4
9.5
1
2
t−Test: p−value = 0.15

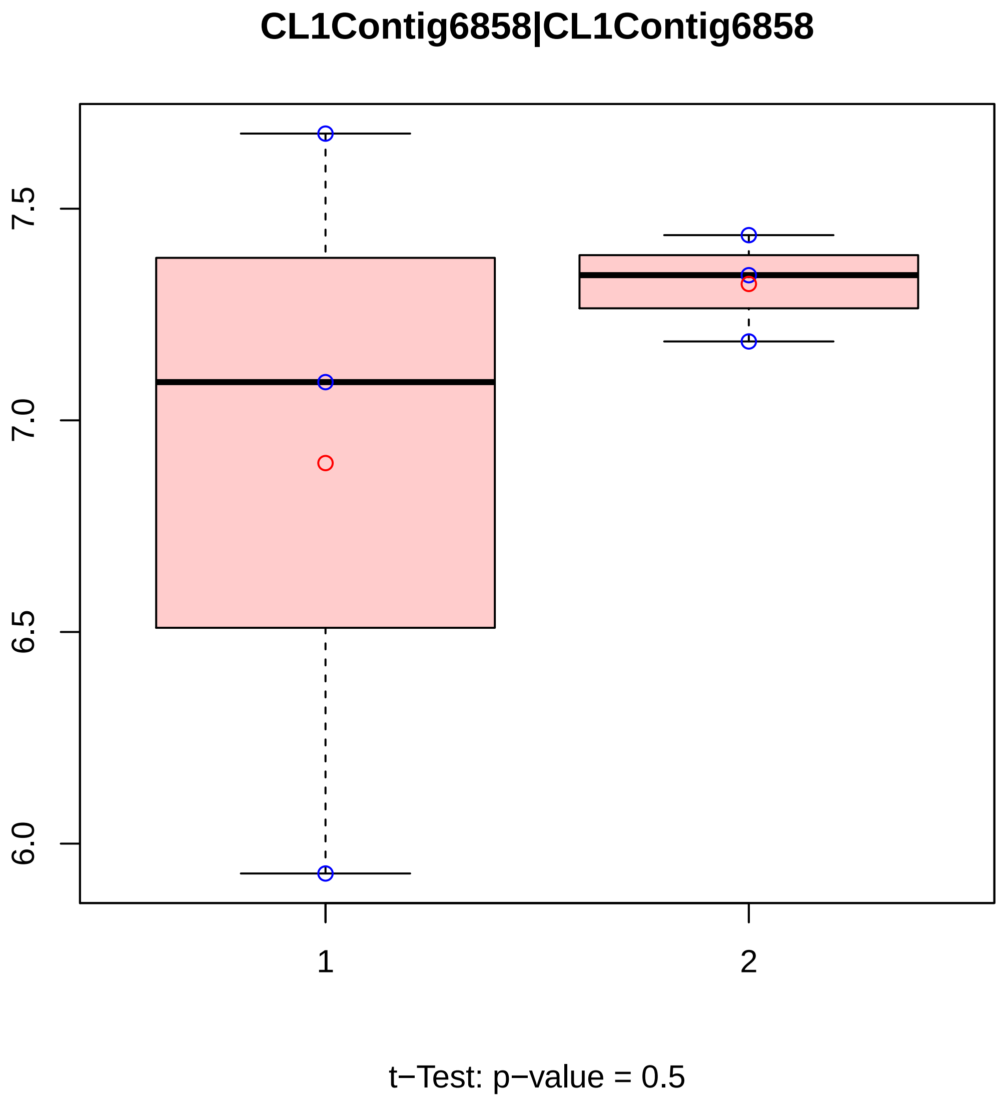

CL1Contig6858|CL1Contig6858
7.5
7.0
6.5
6.0
1
2
t−Test: p−value = 0.5

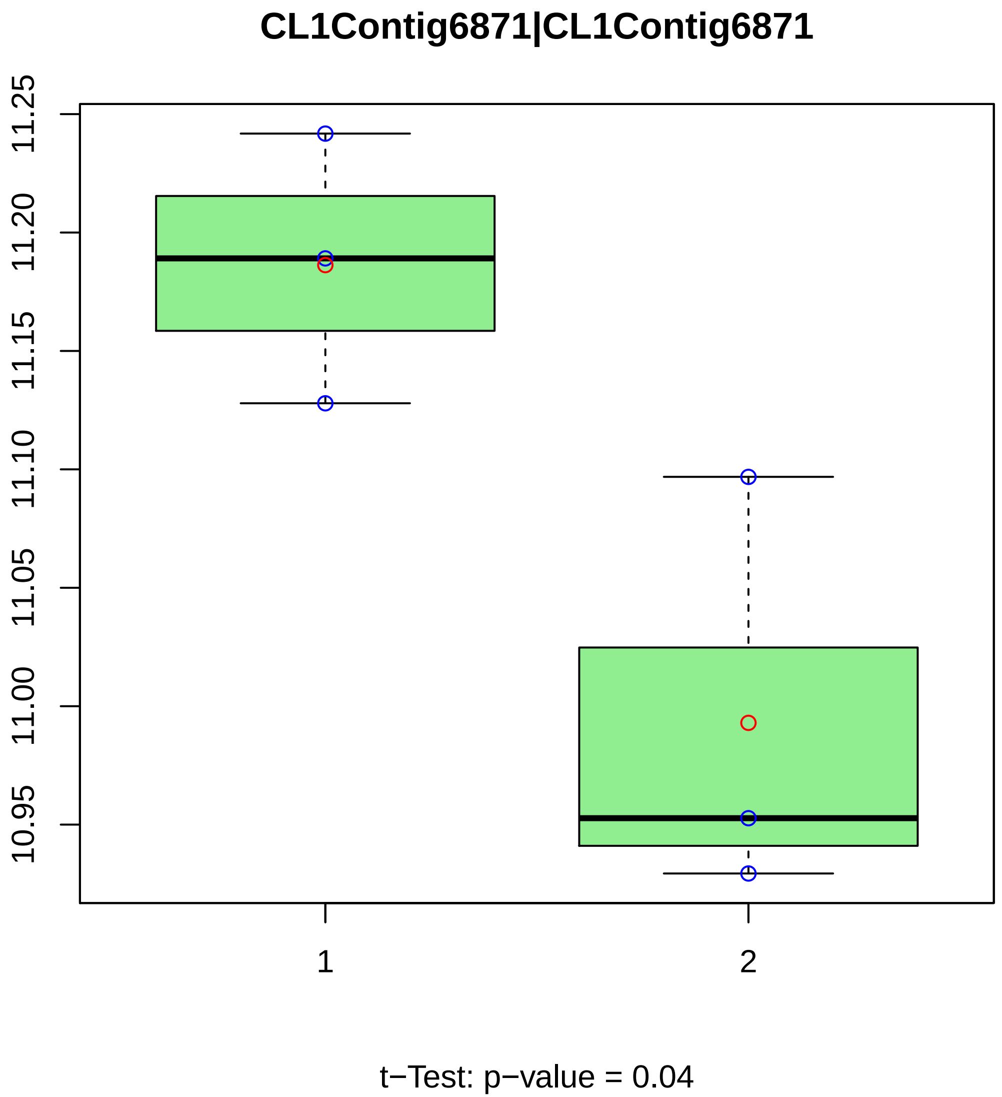

CL1Contig6871|CL1Contig6871
11.25
11.20
11.15
11.10
11.05
11.00
10.95
1
2
t−Test: p−value = 0.04

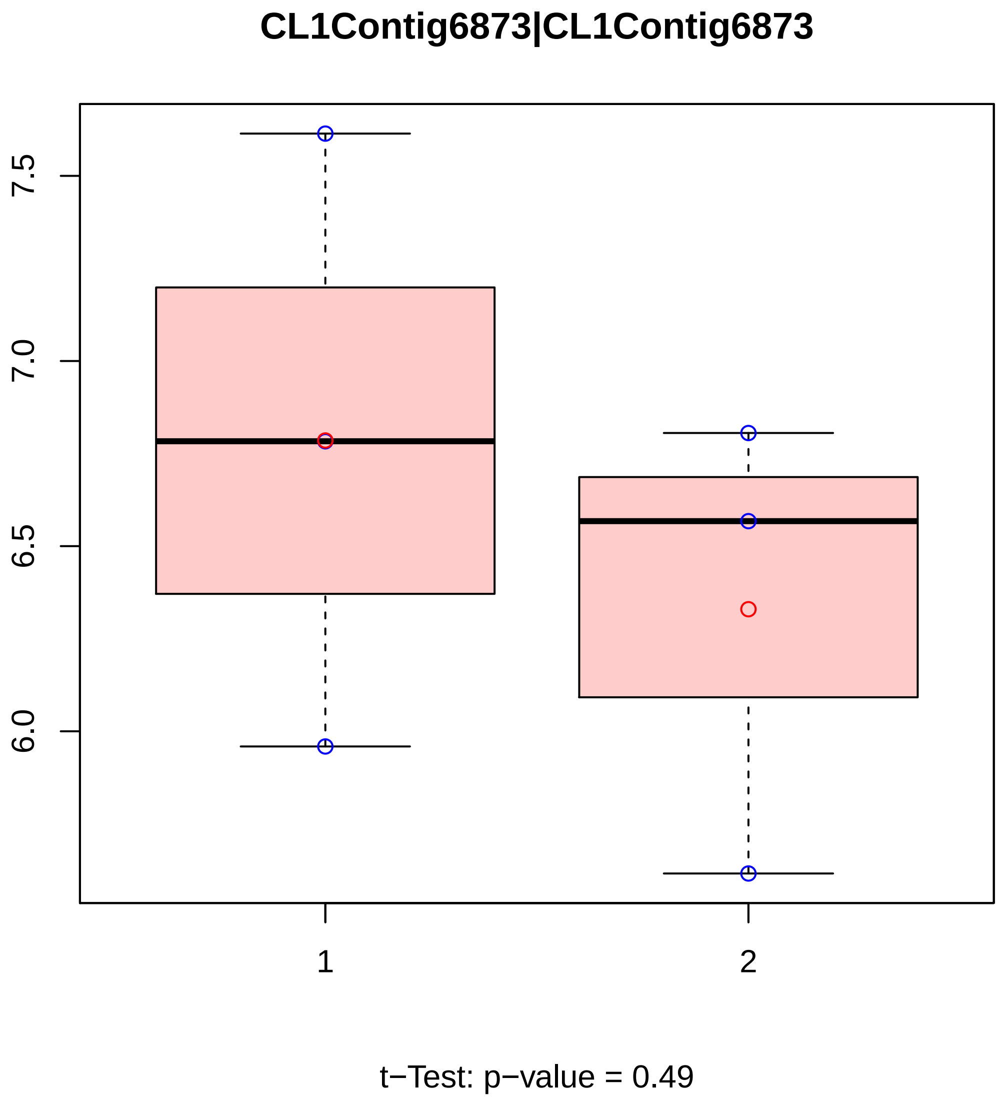
CL1Contig6873|CL1Contig6873
7.5
7.0
6.5
6.0
1
2
t−Test: p−value = 0.49

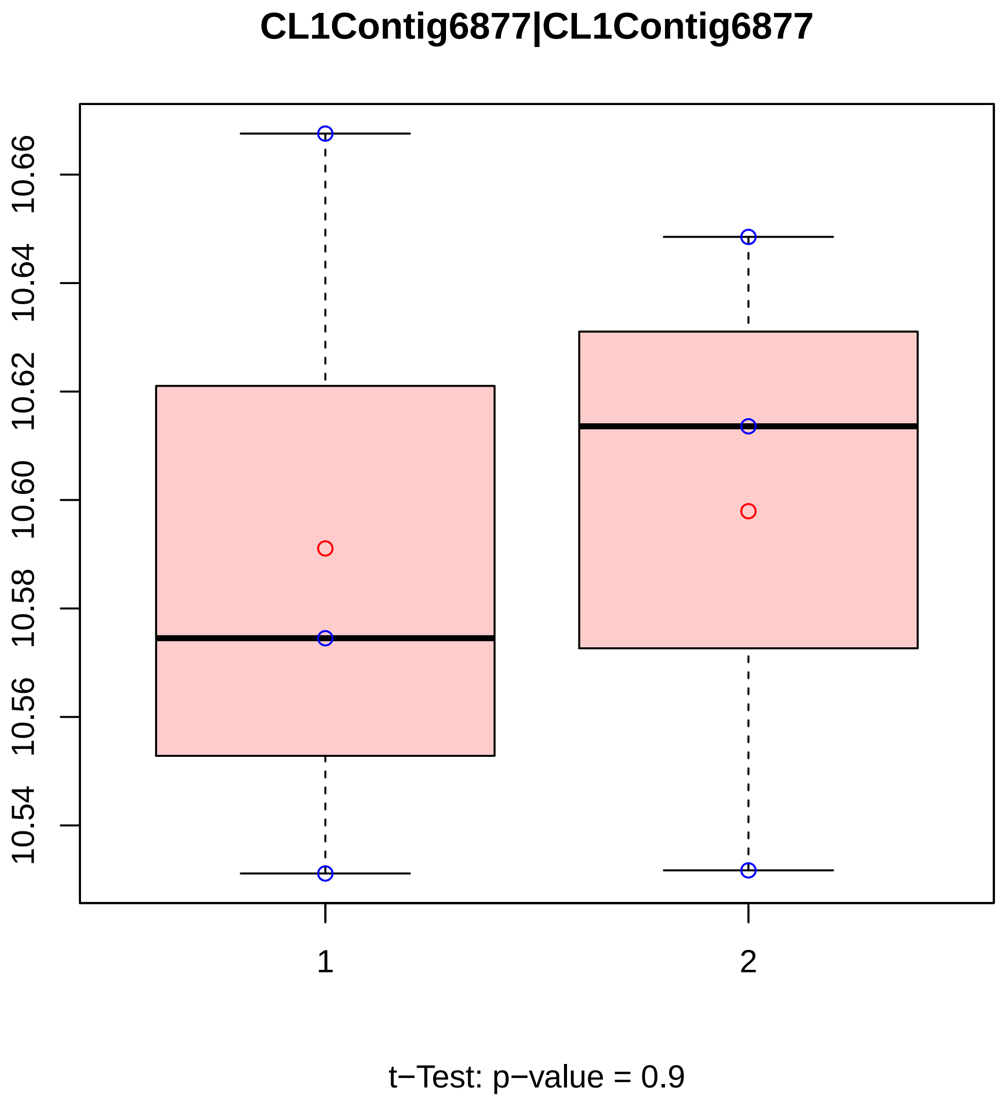

CL1Contig6877|CL1Contig6877
10.54
10.56
10.58
10.60
10.62
10.64
10.66
1
2
t−Test: p−value = 0.9

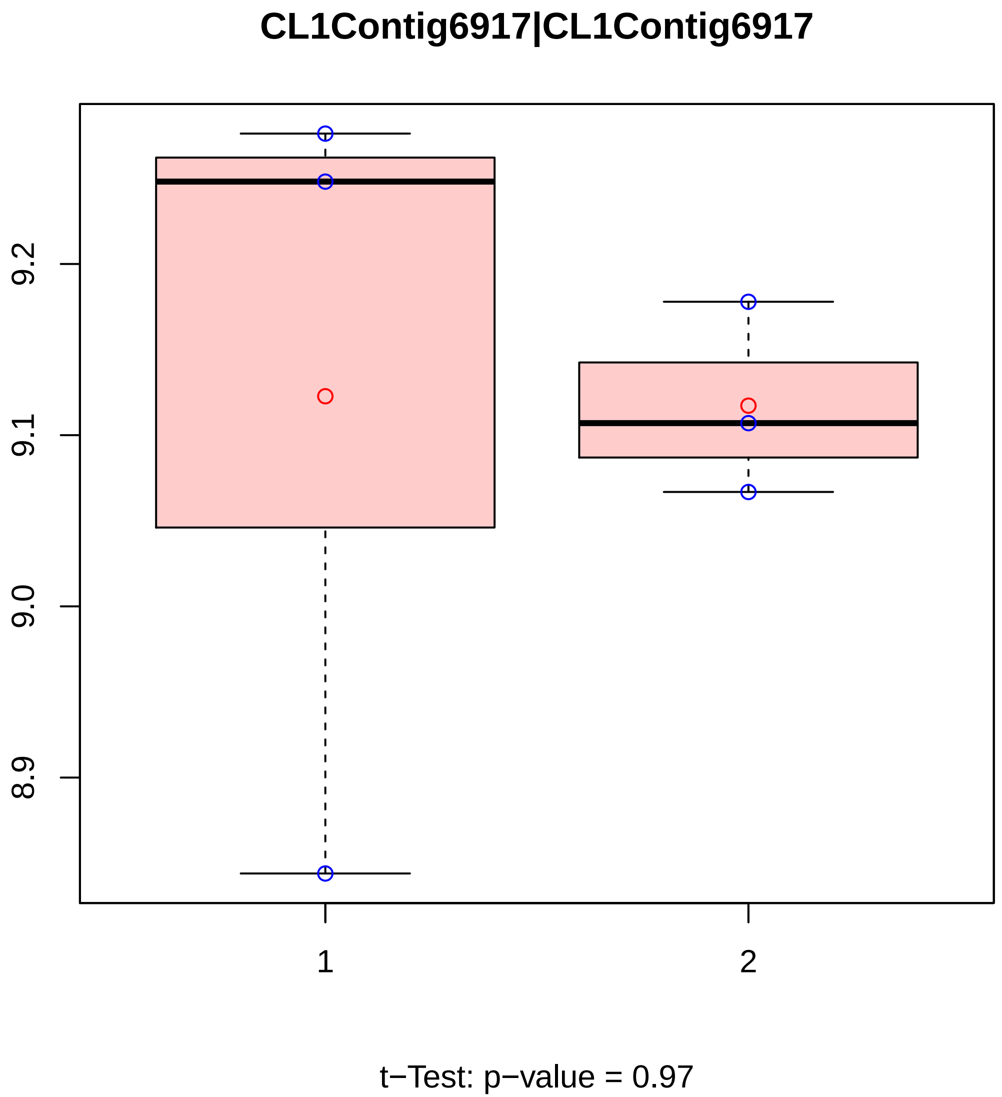

CL1Contig6917|CL1Contig6917
9.2
9.1
9.0
8.9
1
2
t−Test: p−value = 0.97

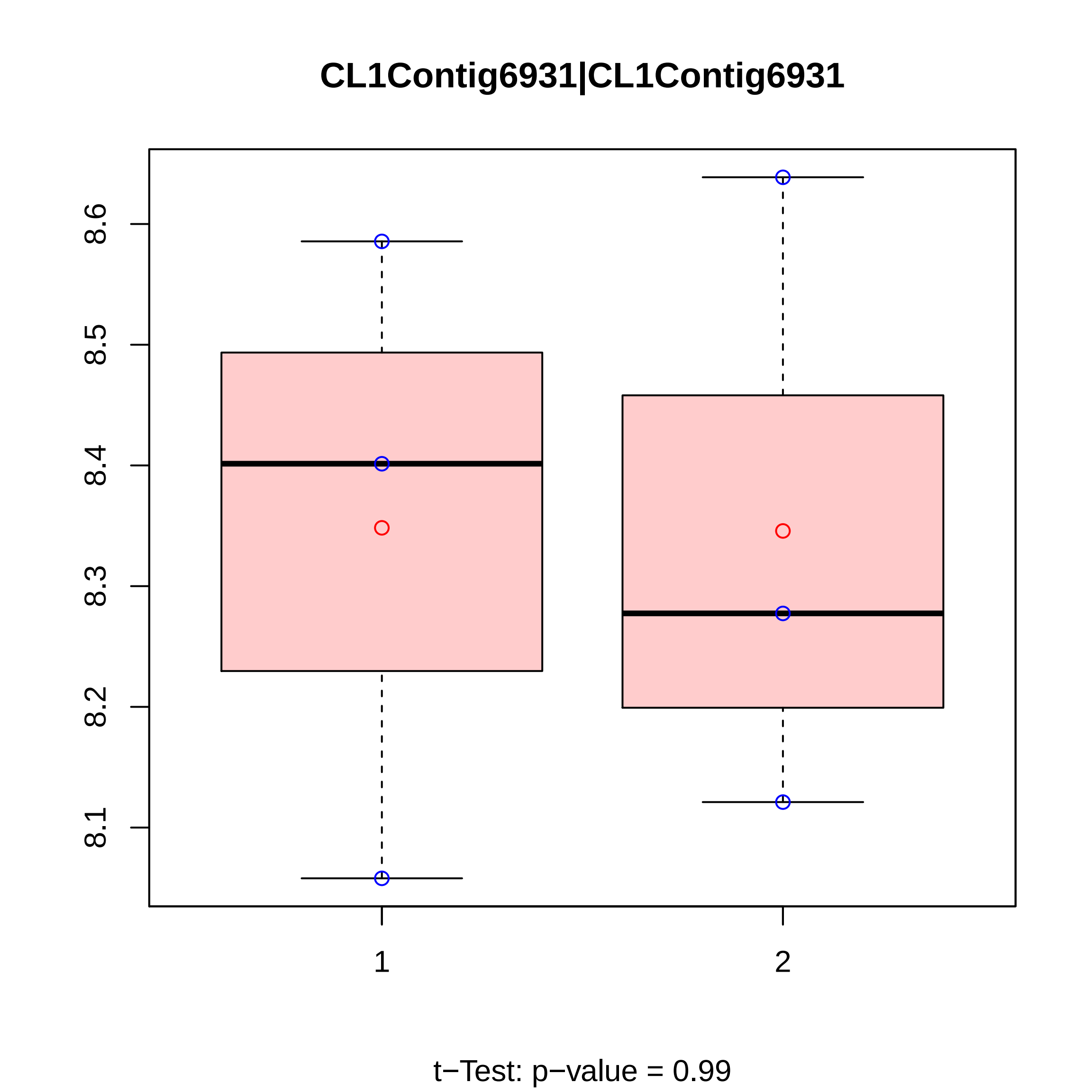
CL1Contig6931|CL1Contig6931
8.1
8.2
8.3
8.4
8.5
8.6
1
2
t−Test: p−value = 0.99

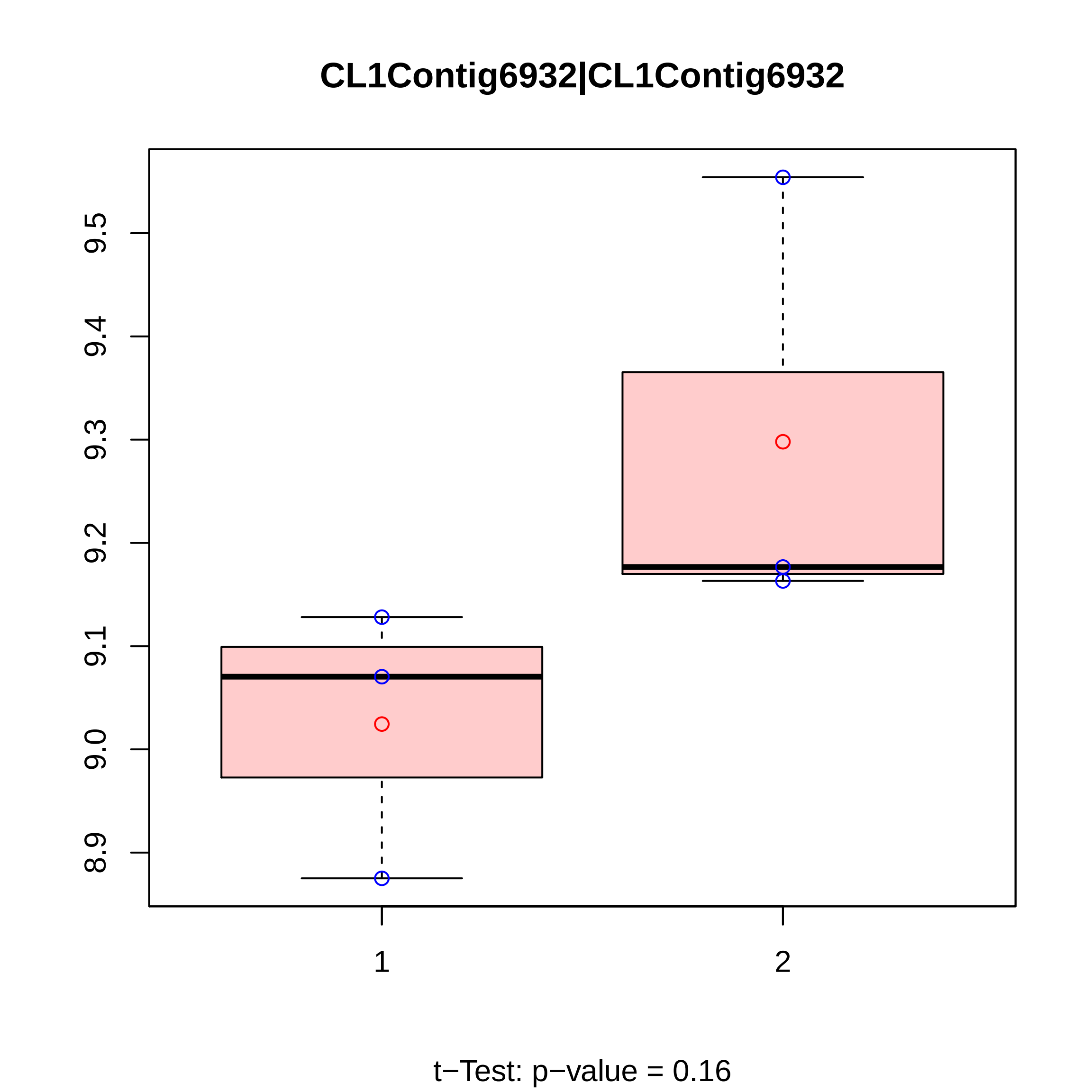

CL1Contig6932|CL1Contig6932
9.5
9.4
9.3
9.2
9.1
9.0
8.9
1
2
t−Test: p−value = 0.16

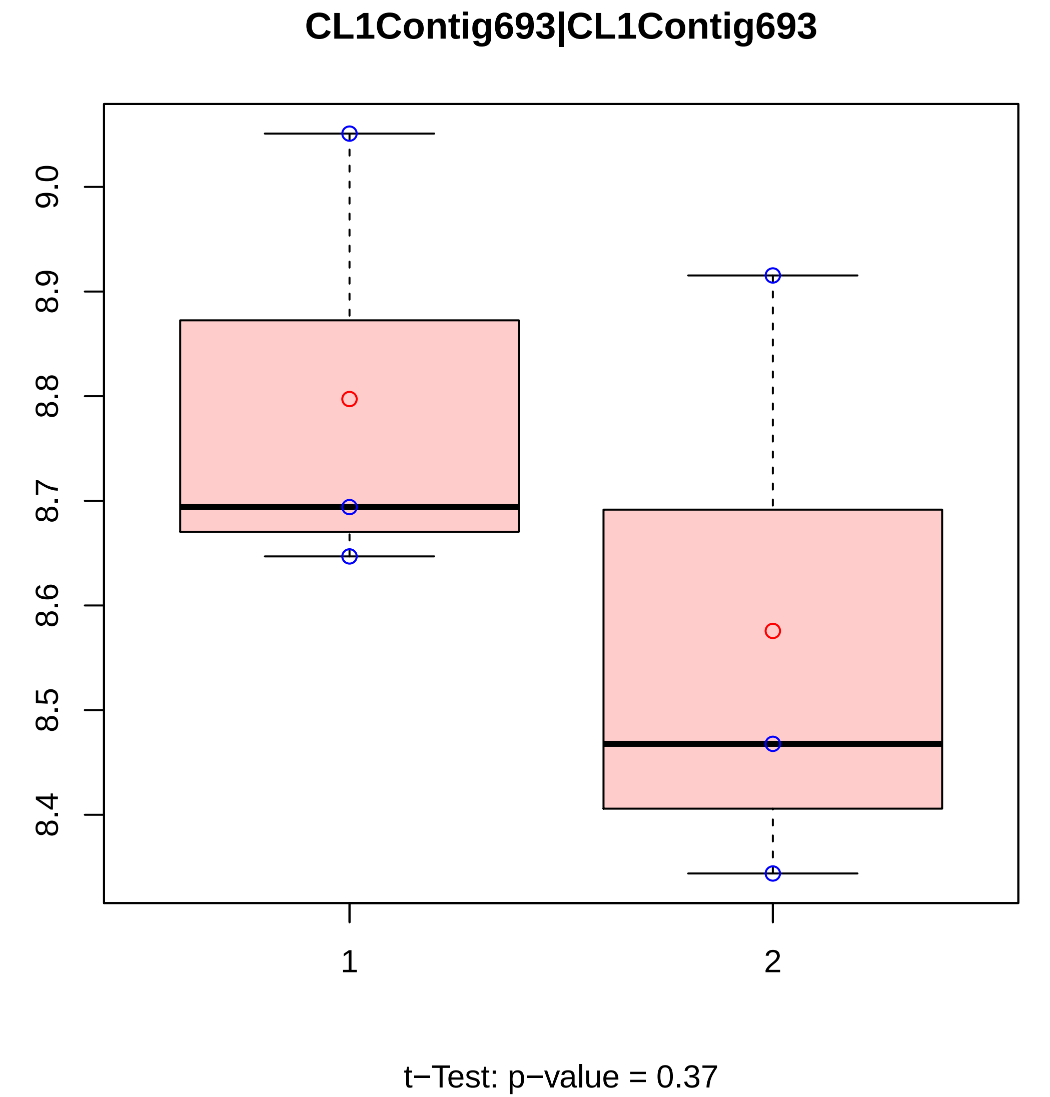
CL1Contig693|CL1Contig693
9.0
8.9
8.8
8.7
8.6
8.5
8.4
1
2
t−Test: p−value = 0.37

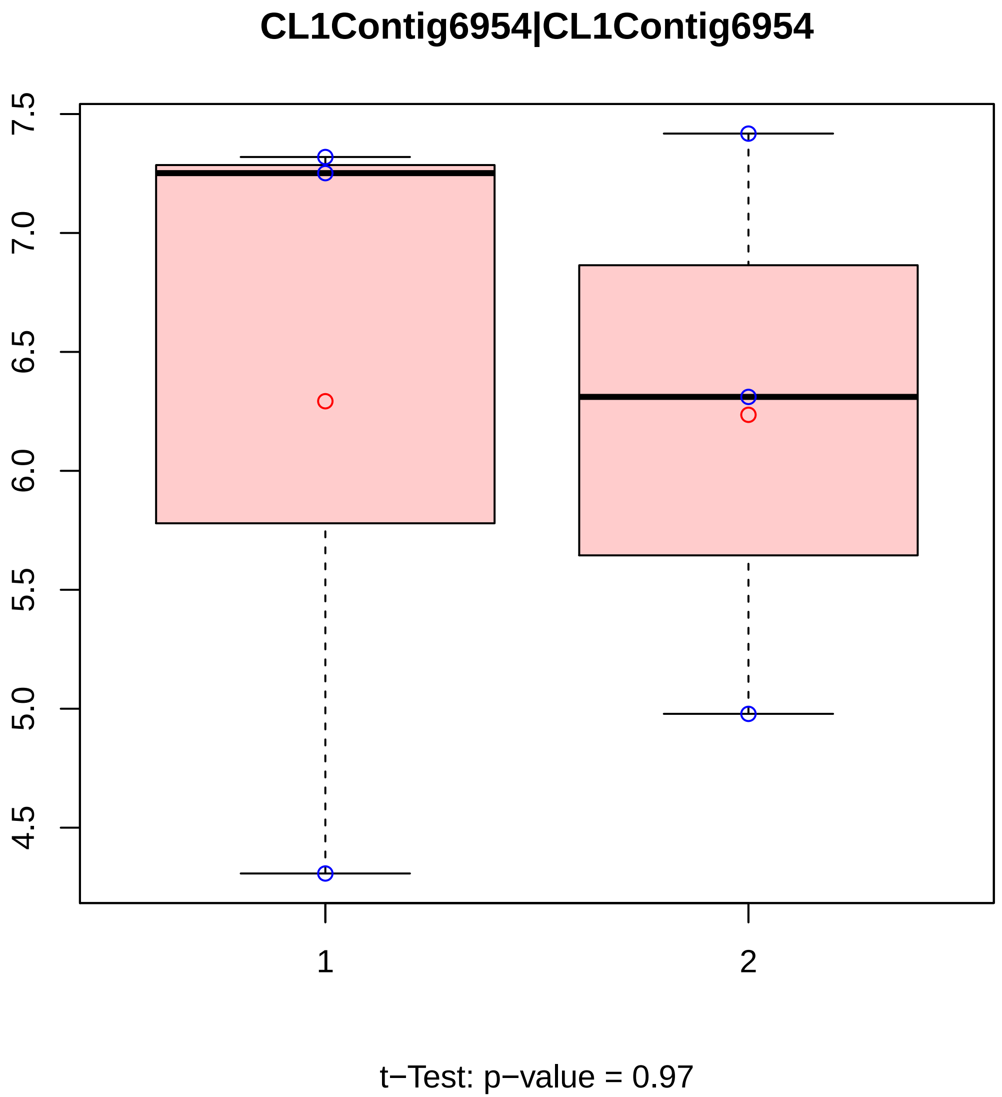
CL1Contig6954|CL1Contig6954
7.5
7.0
6.5
6.0
5.5
5.0
4.5
1
2
t−Test: p−value = 0.97

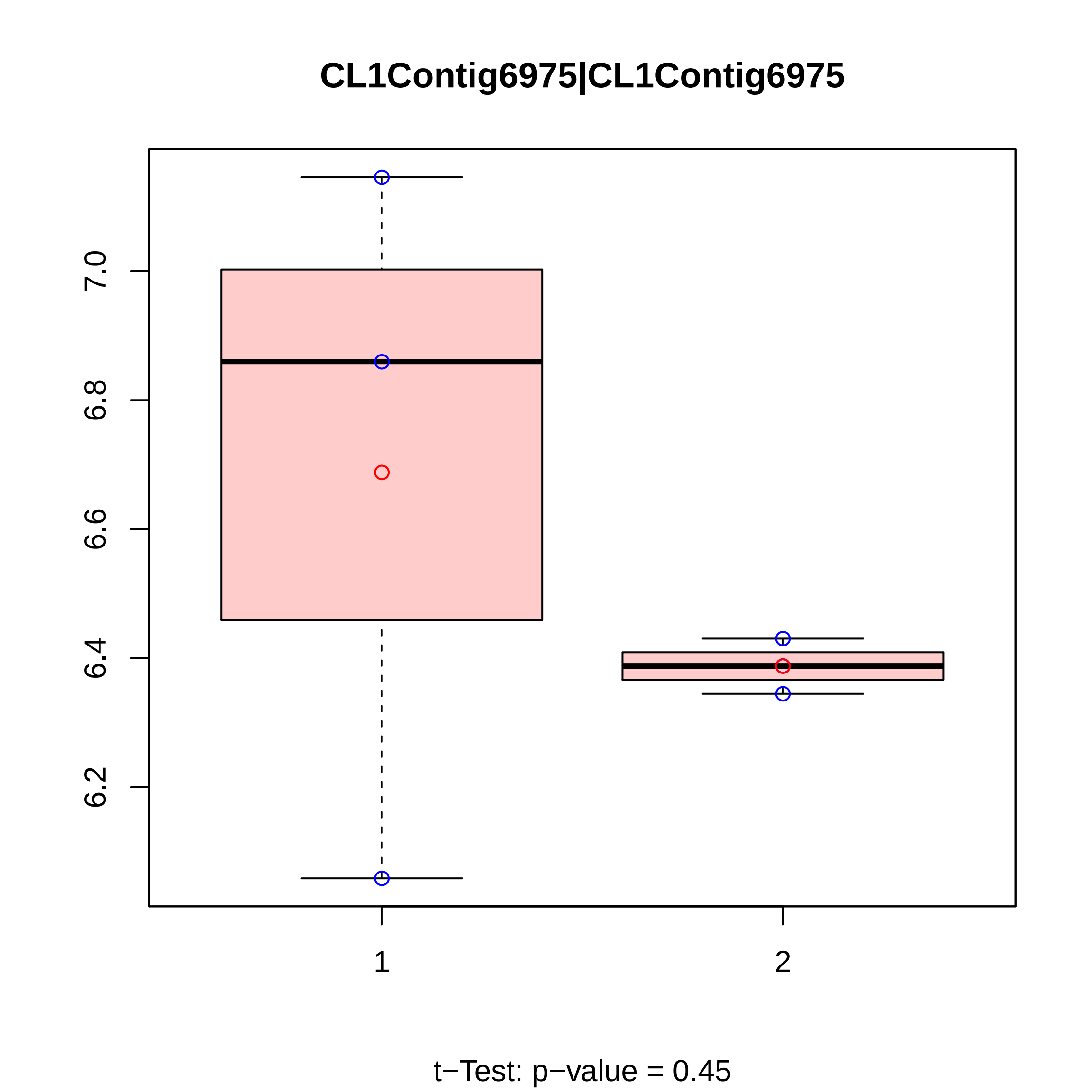
CL1Contig6975|CL1Contig6975
7.0
6.8
6.6
6.4
6.2
1
2
t−Test: p−value = 0.45

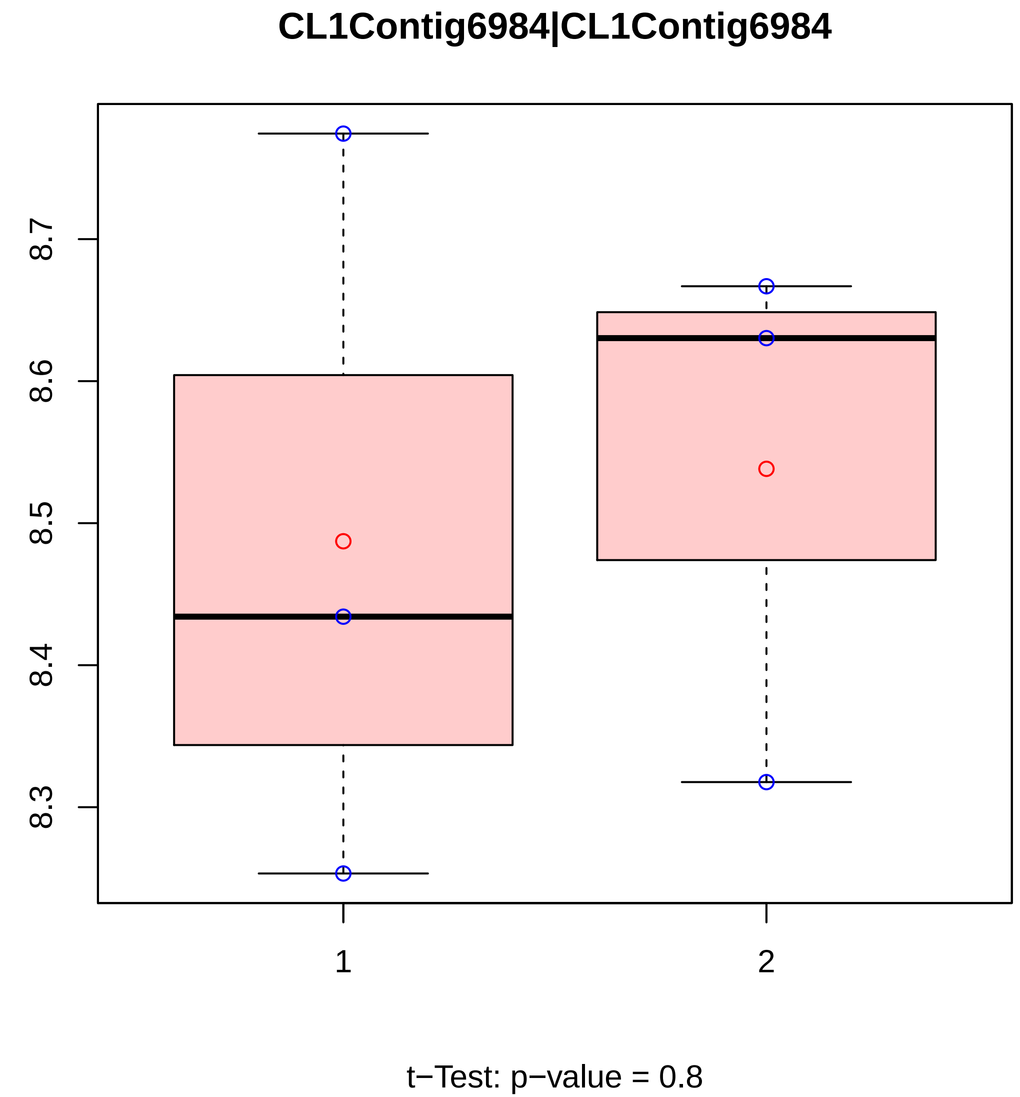
CL1Contig6984|CL1Contig6984
8.3
8.4
8.5
8.6
8.7
1
2
t−Test: p−value = 0.8

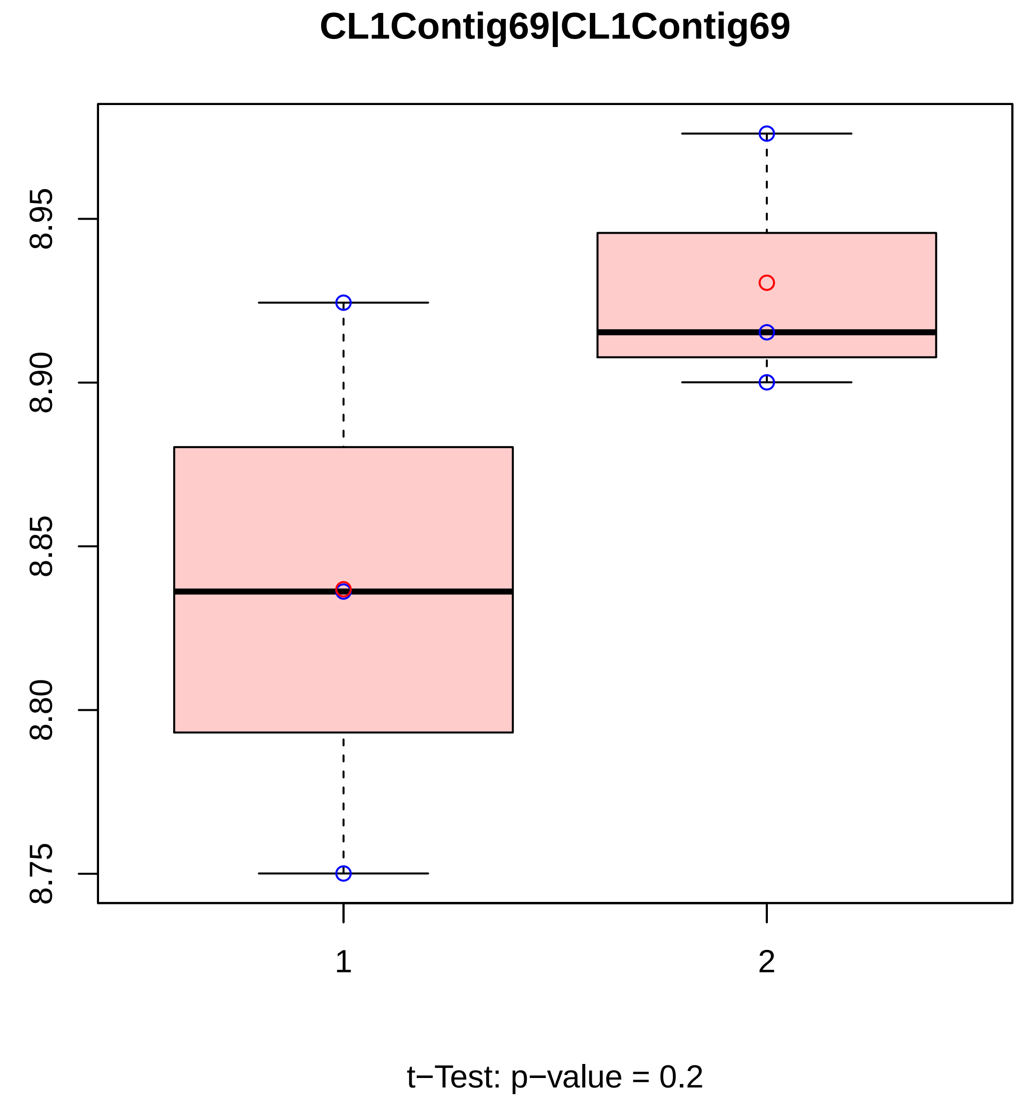
CL1Contig69|CL1Contig69
8.75
8.80
8.85
8.90
8.95
1
2
t−Test: p−value = 0.2

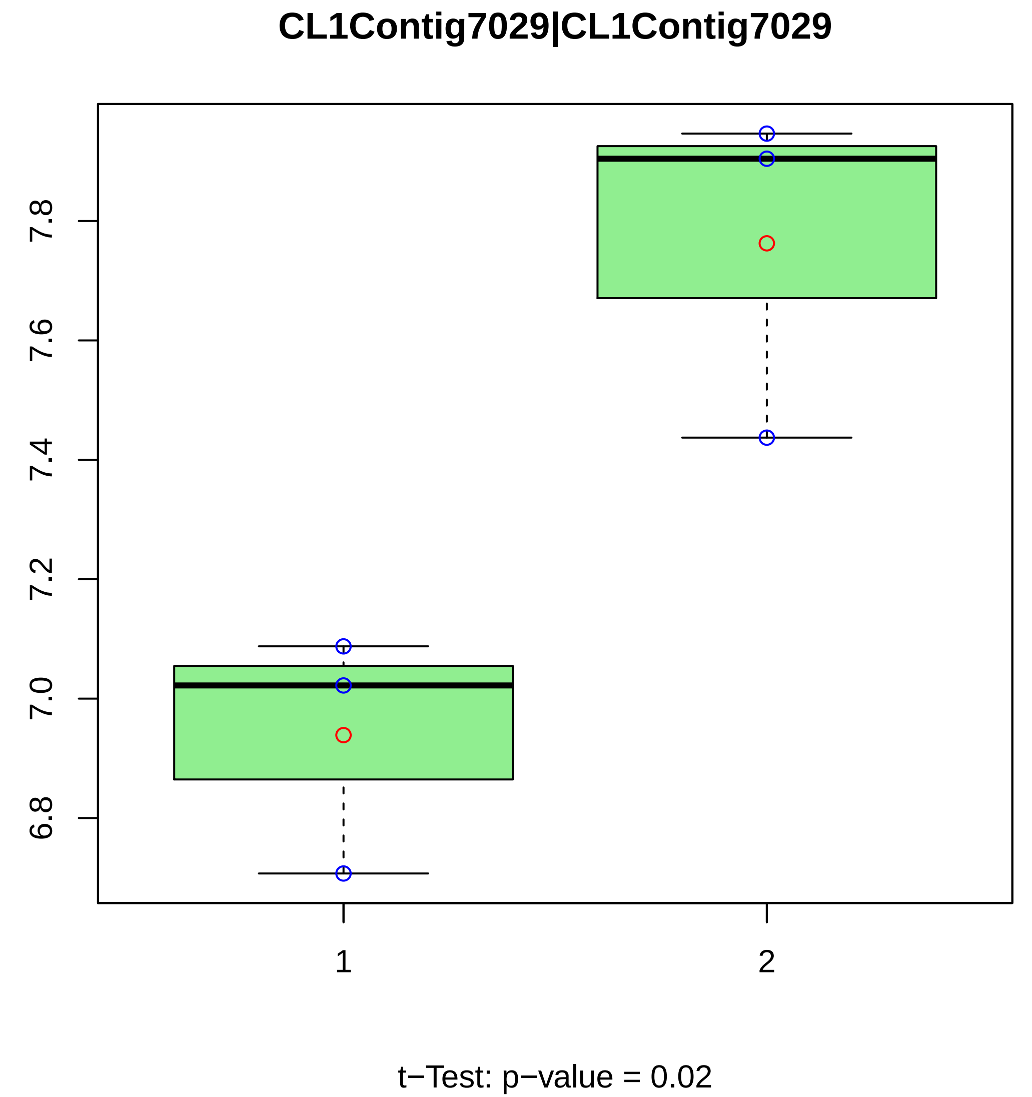
CL1Contig7029|CL1Contig7029
7.8
7.6
7.4
7.2
7.0
6.8
1
2
t−Test: p−value = 0.02

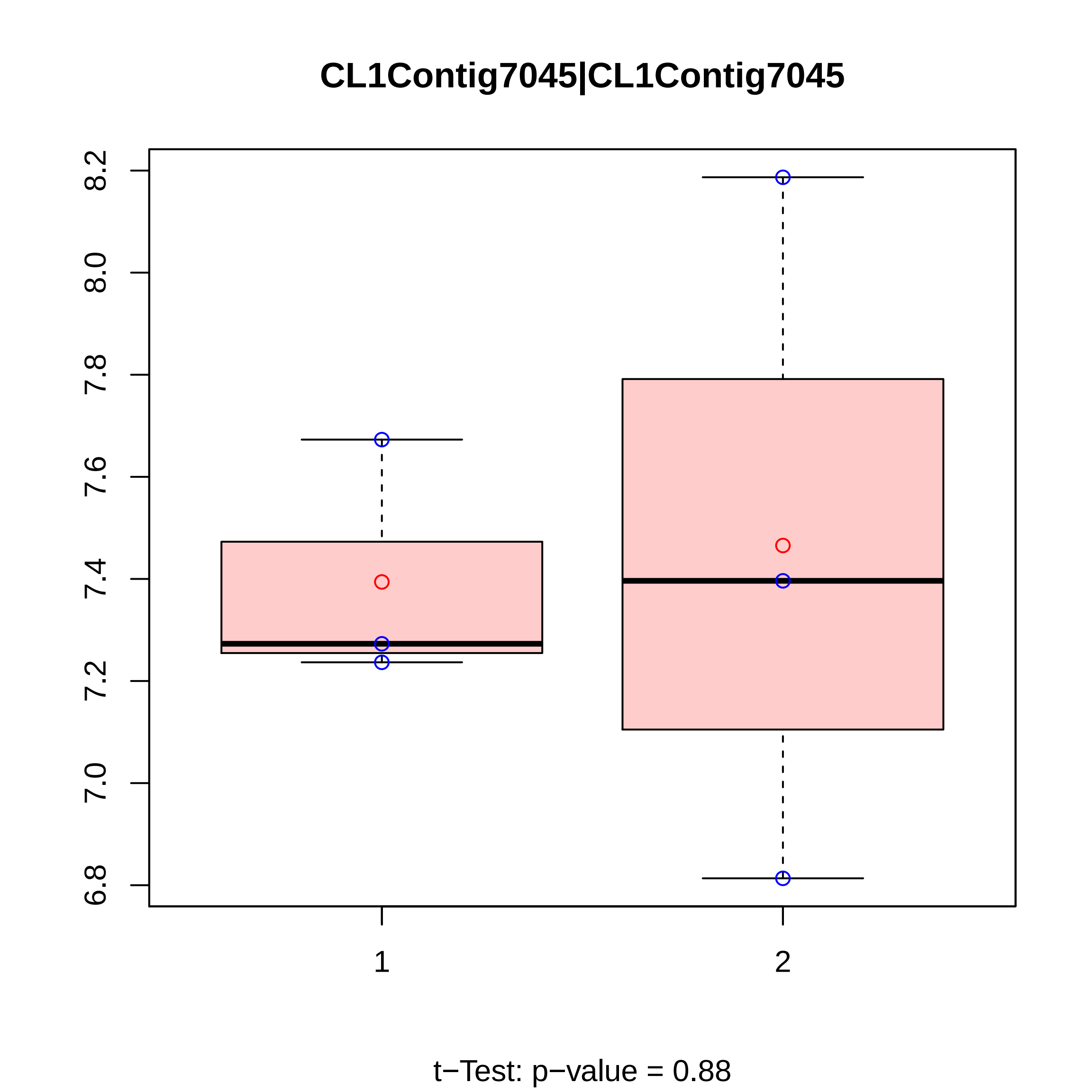
CL1Contig7045|CL1Contig7045
8.2
8.0
7.8
7.6
7.4
7.2
7.0
6.8
1
2
t−Test: p−value = 0.88

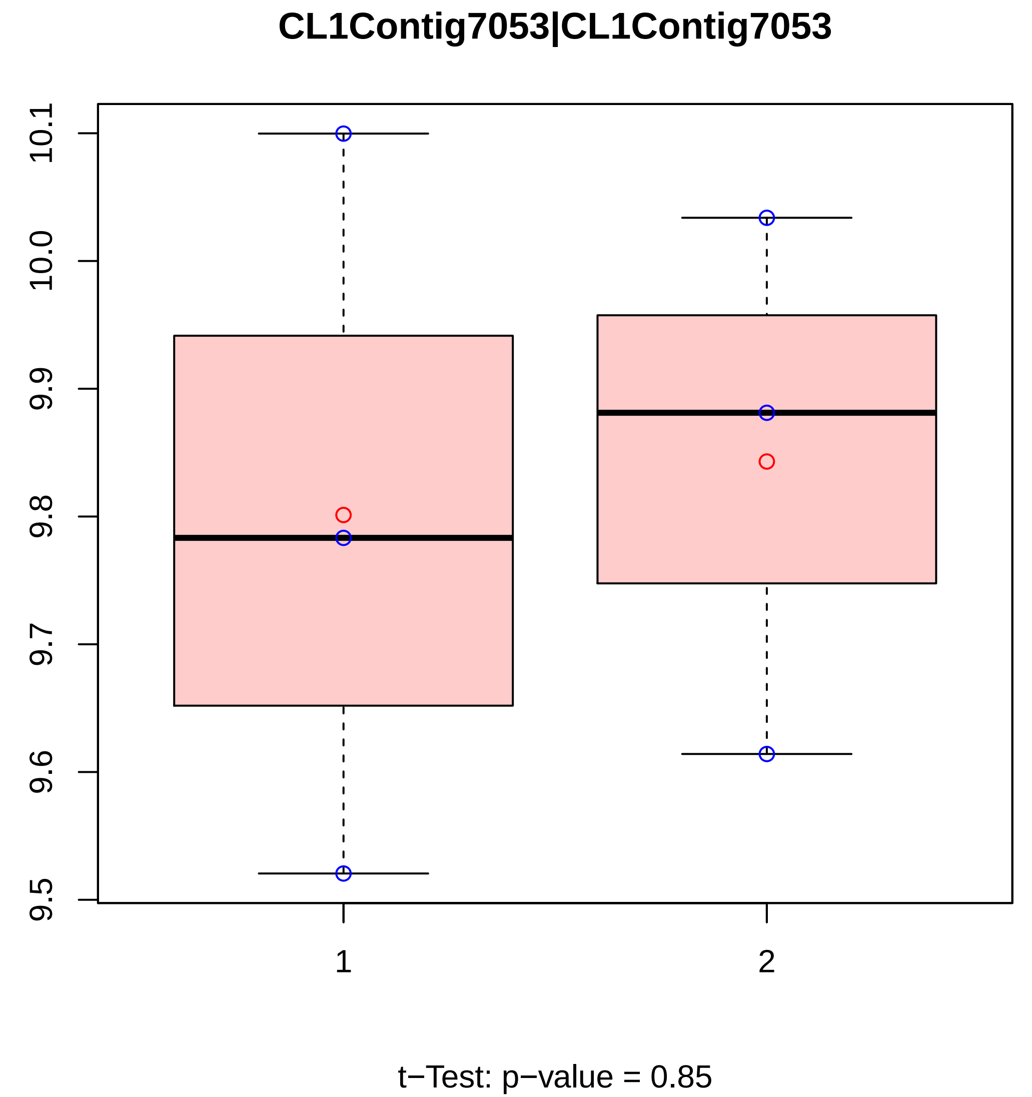

CL1Contig7053|CL1Contig7053
10.1
10.0
9.9
9.8
9.7
9.6
9.5
1
2
t−Test: p−value = 0.85

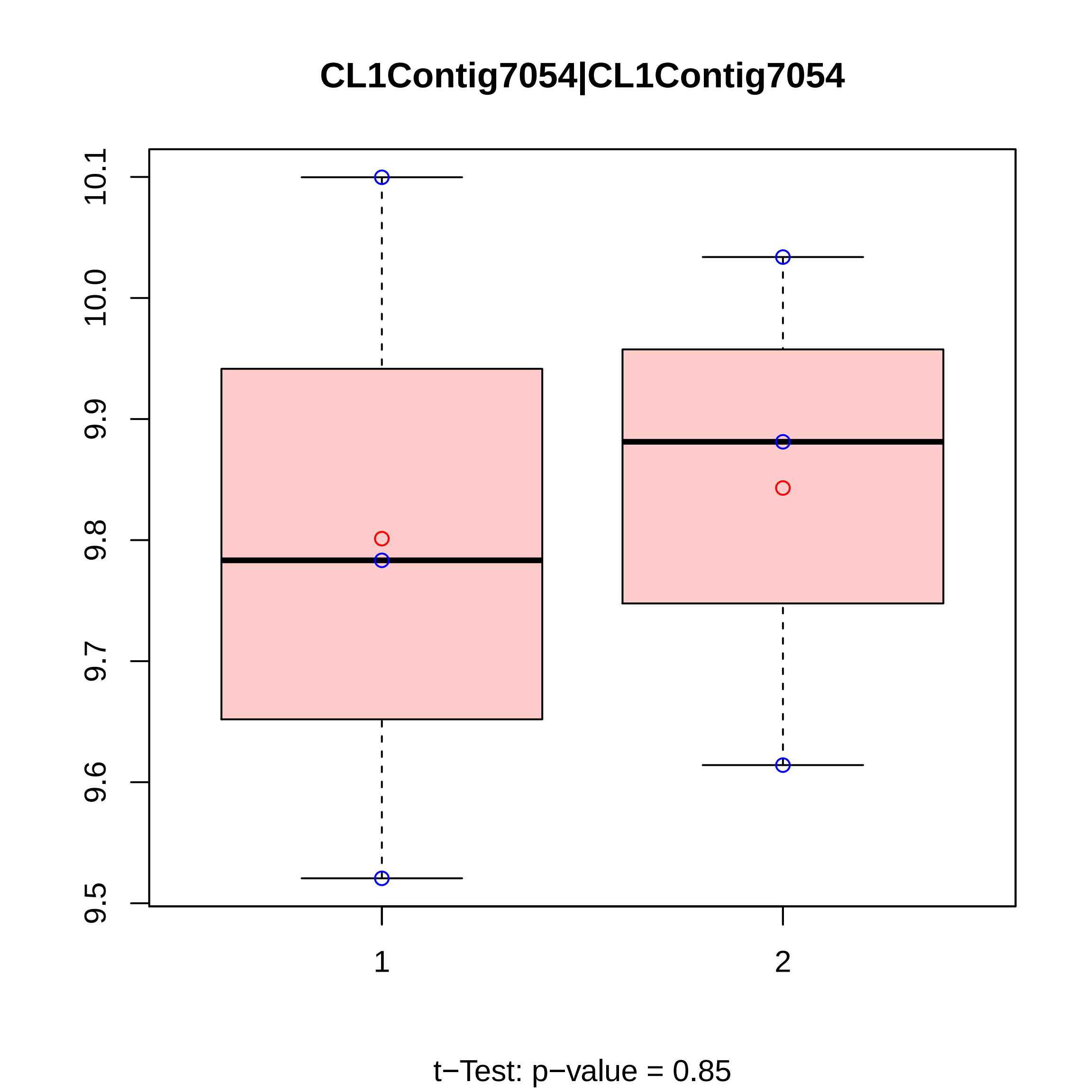

CL1Contig7054|CL1Contig7054
10.1
10.0
9.9
9.8
9.7
9.6
9.5
1
2
t−Test: p−value = 0.85

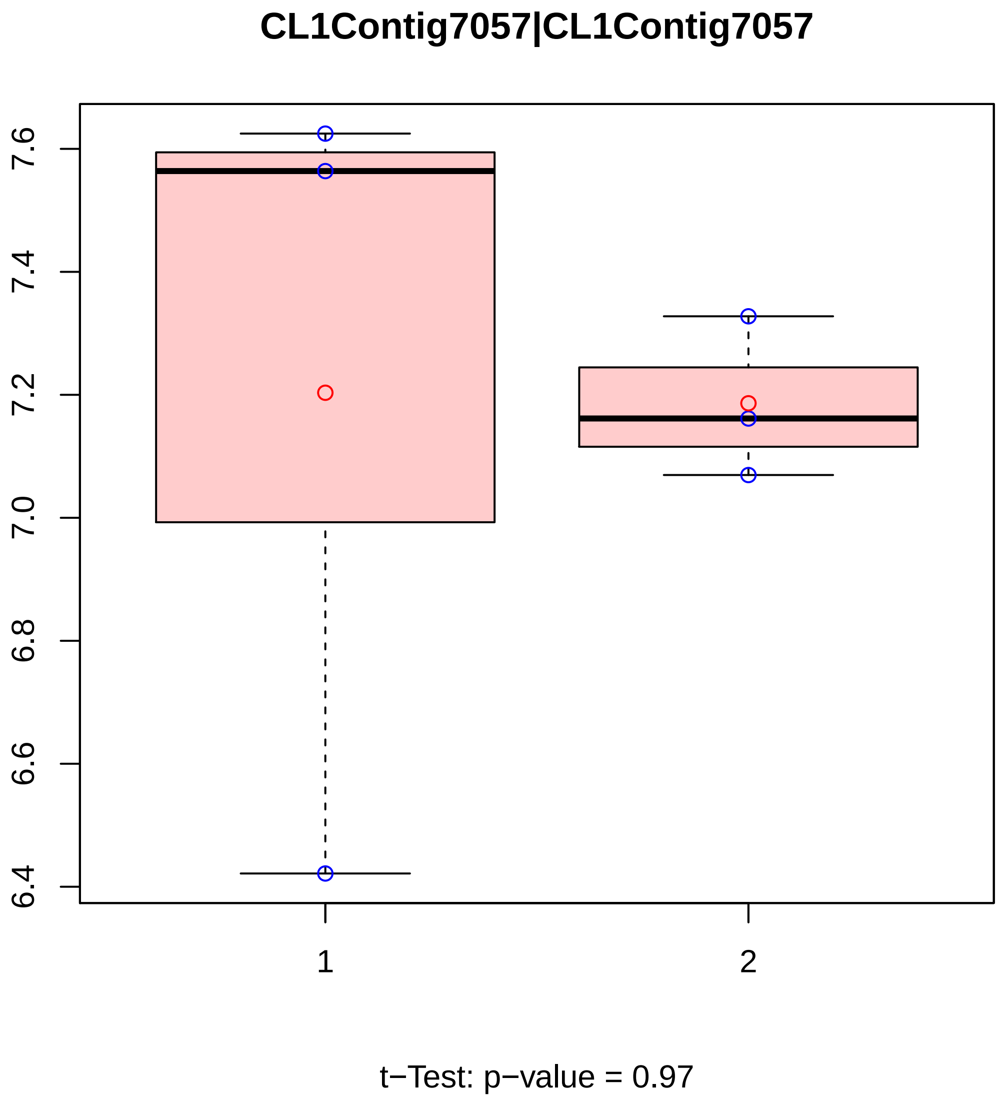
CL1Contig7057|CL1Contig7057
7.6
7.4
7.2
7.0
6.8
6.6
6.4
1
2
t−Test: p−value = 0.97

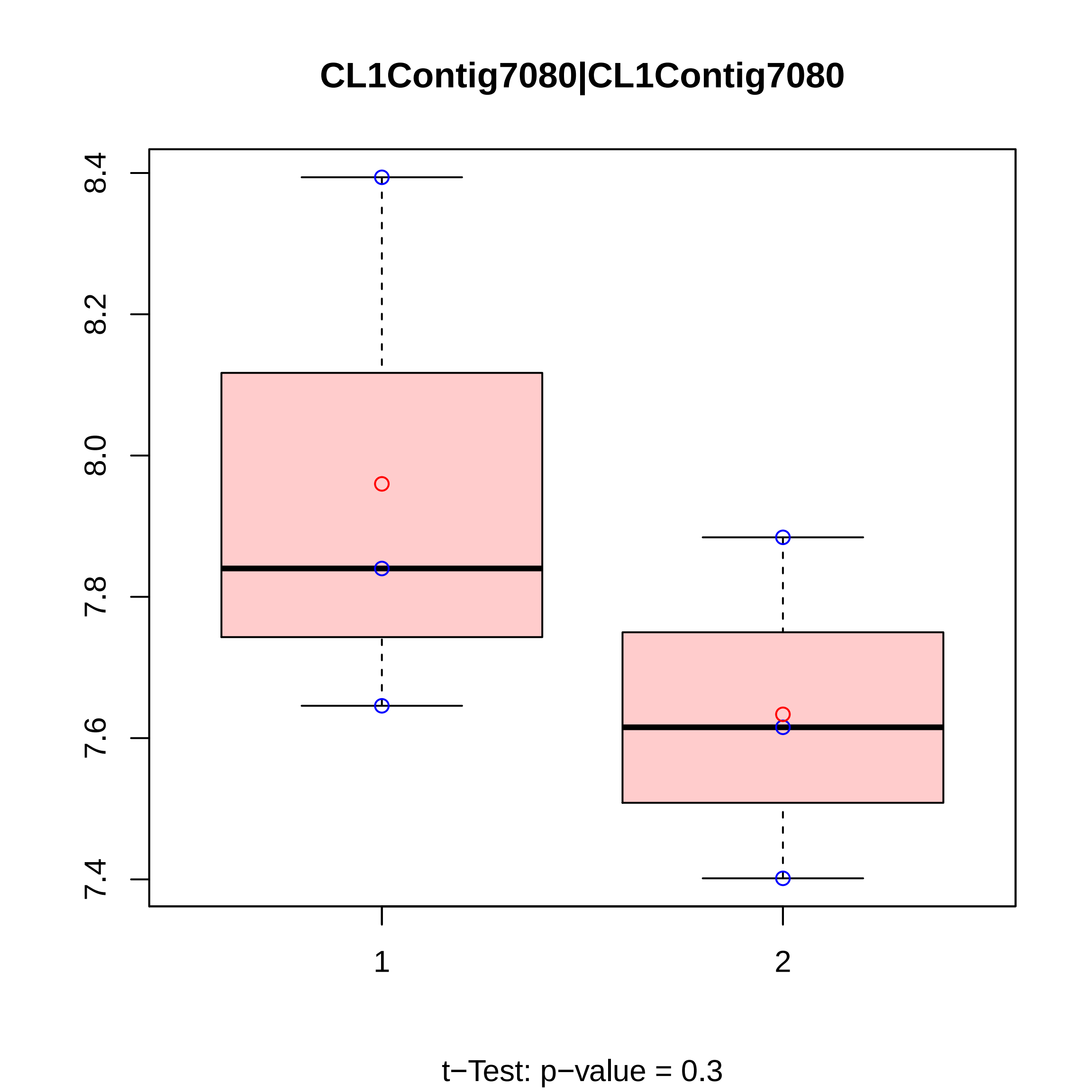
CL1Contig7080|CL1Contig7080
8.4
8.2
8.0
7.8
7.6
7.4
1
2
t−Test: p−value = 0.3

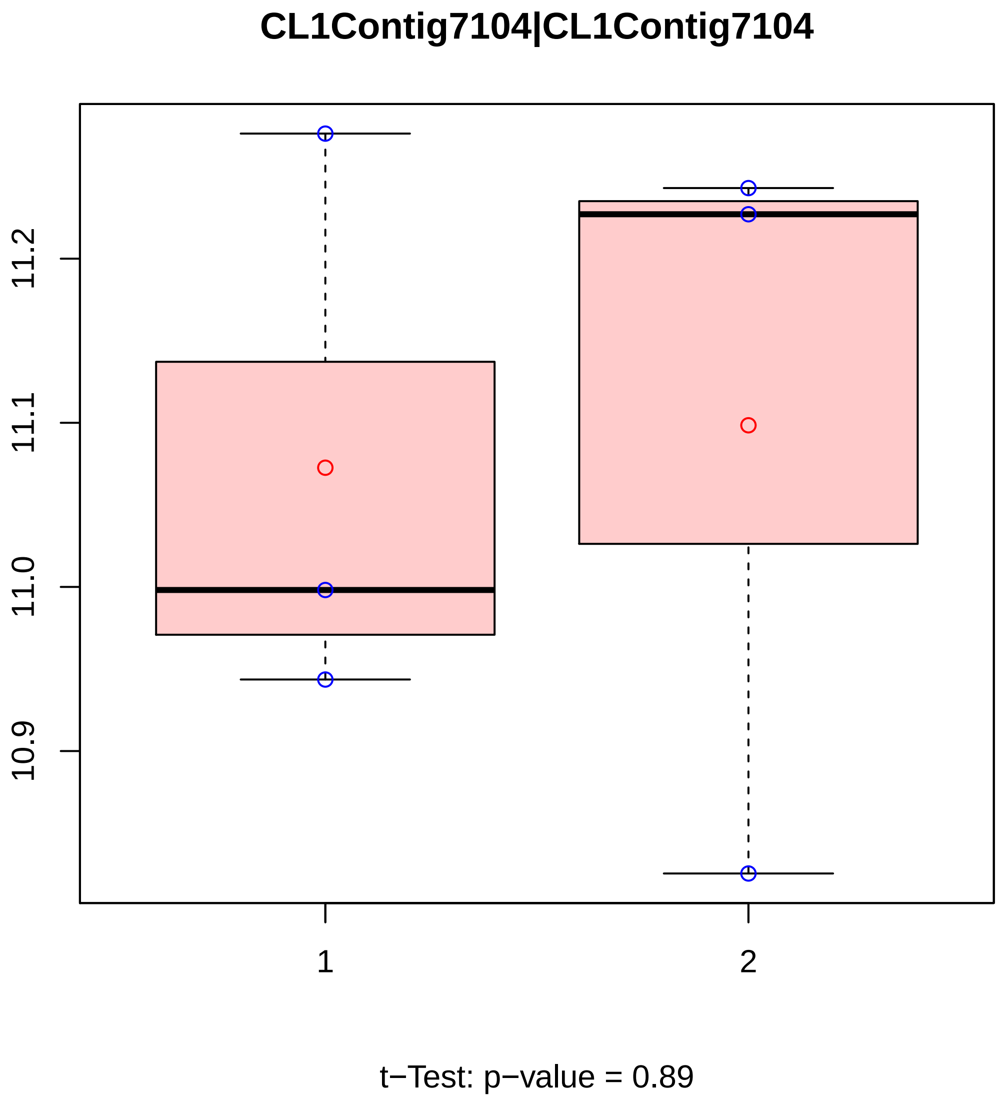
CL1Contig7104|CL1Contig7104
11.2
11.1
11.0
10.9
1
2
t−Test: p−value = 0.89

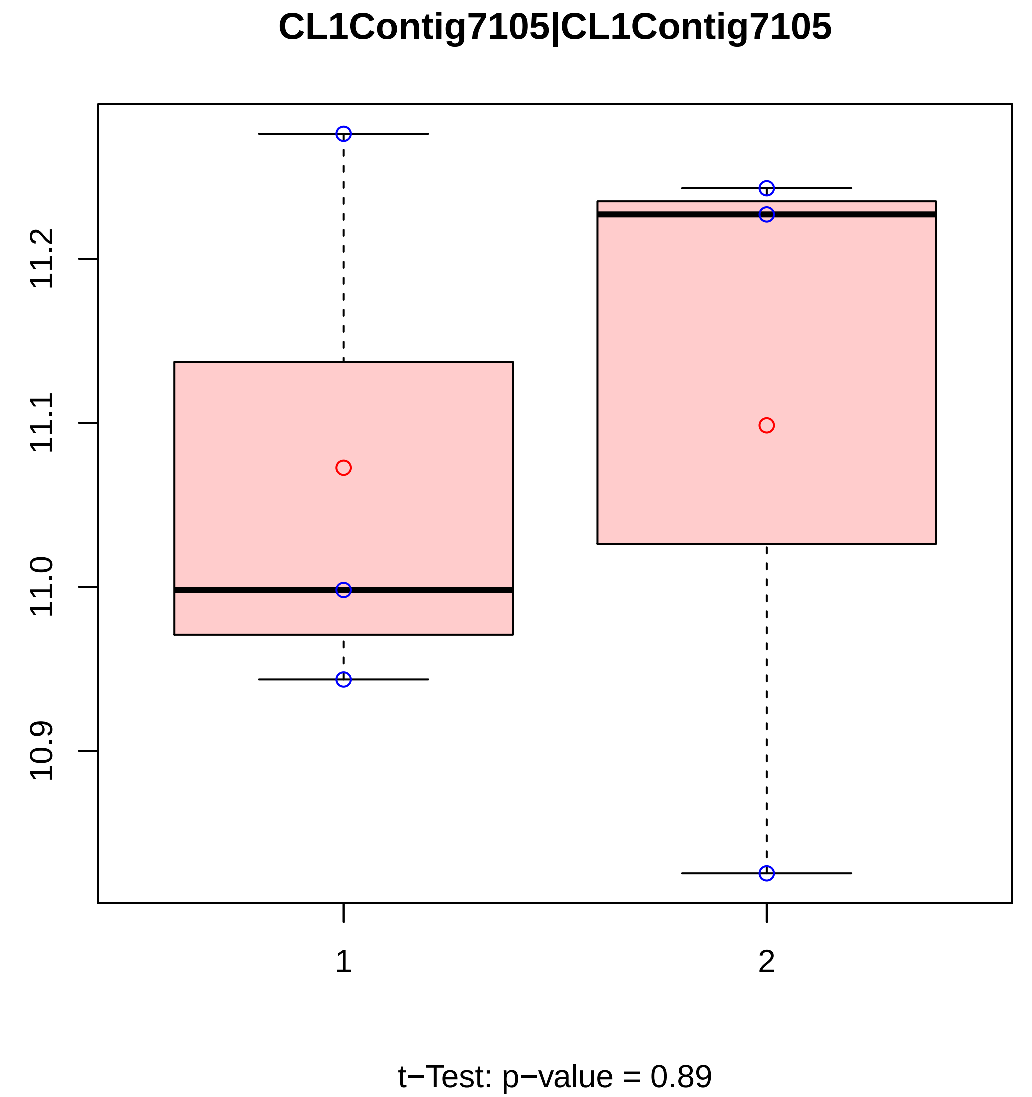

CL1Contig7105|CL1Contig7105
11.2
11.1
11.0
10.9
1
2
t−Test: p−value = 0.89

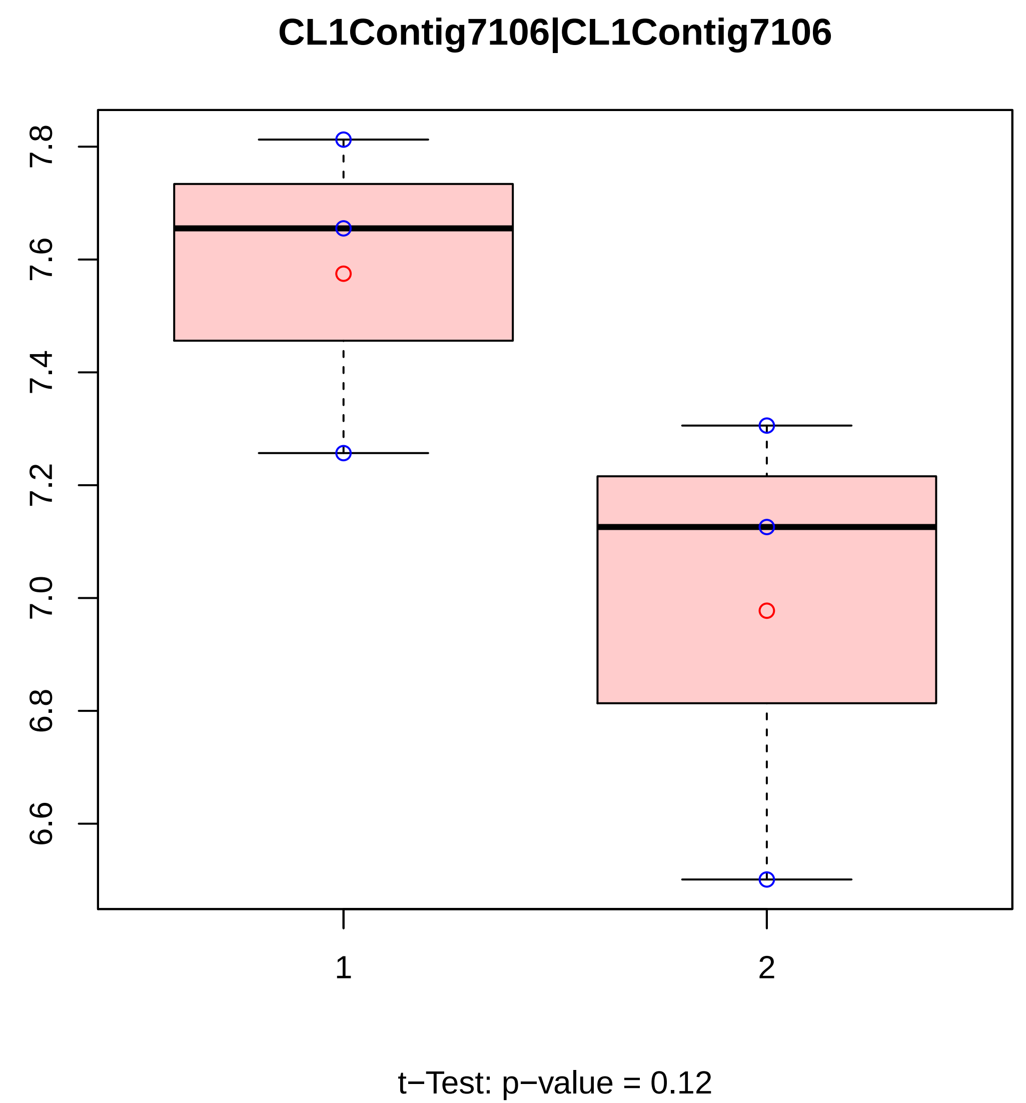

CL1Contig7106|CL1Contig7106
7.8
7.6
7.4
7.2
7.0
6.8
6.6
1
2
t−Test: p−value = 0.12

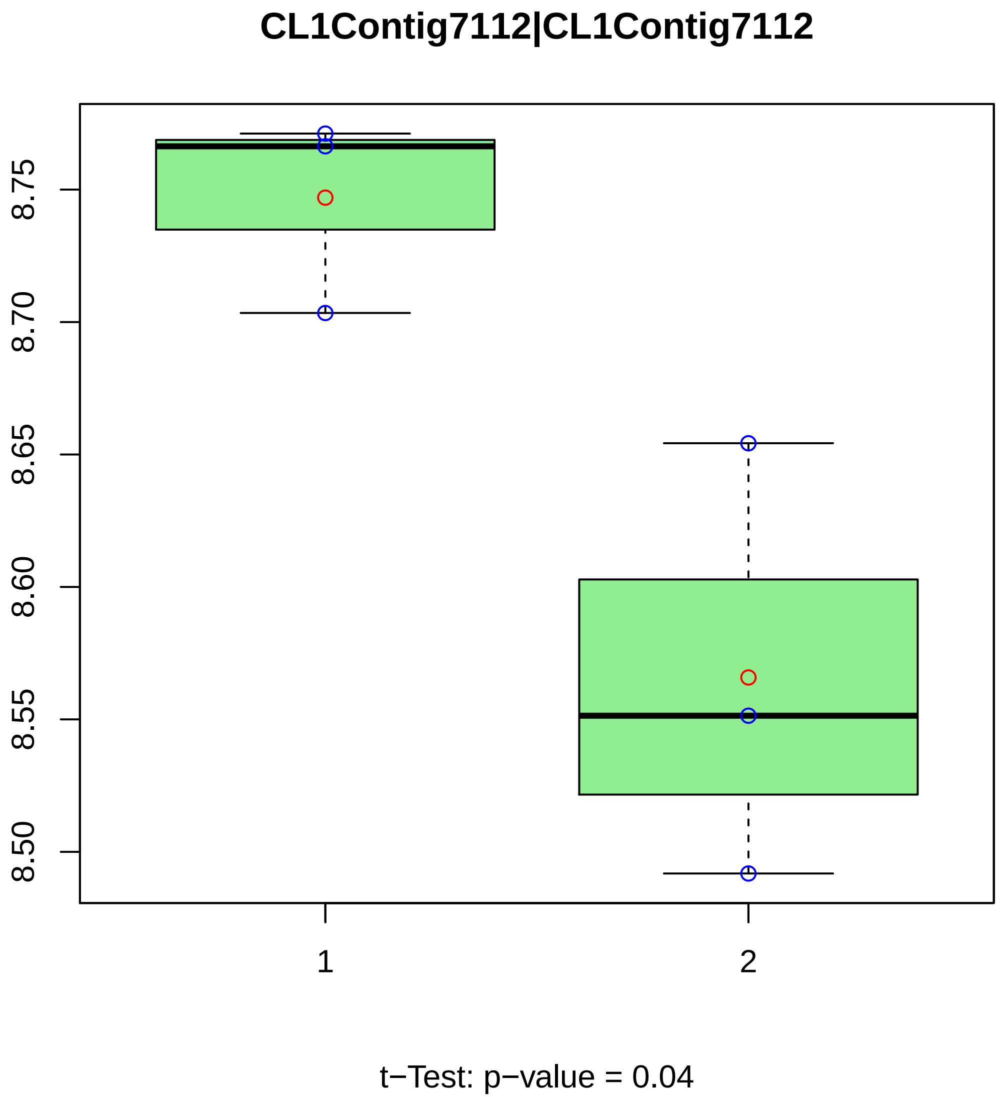
CL1Contig7112|CL1Contig7112
8.50
8.55
8.60
8.65
8.70
8.75
1
2
t−Test: p−value = 0.04

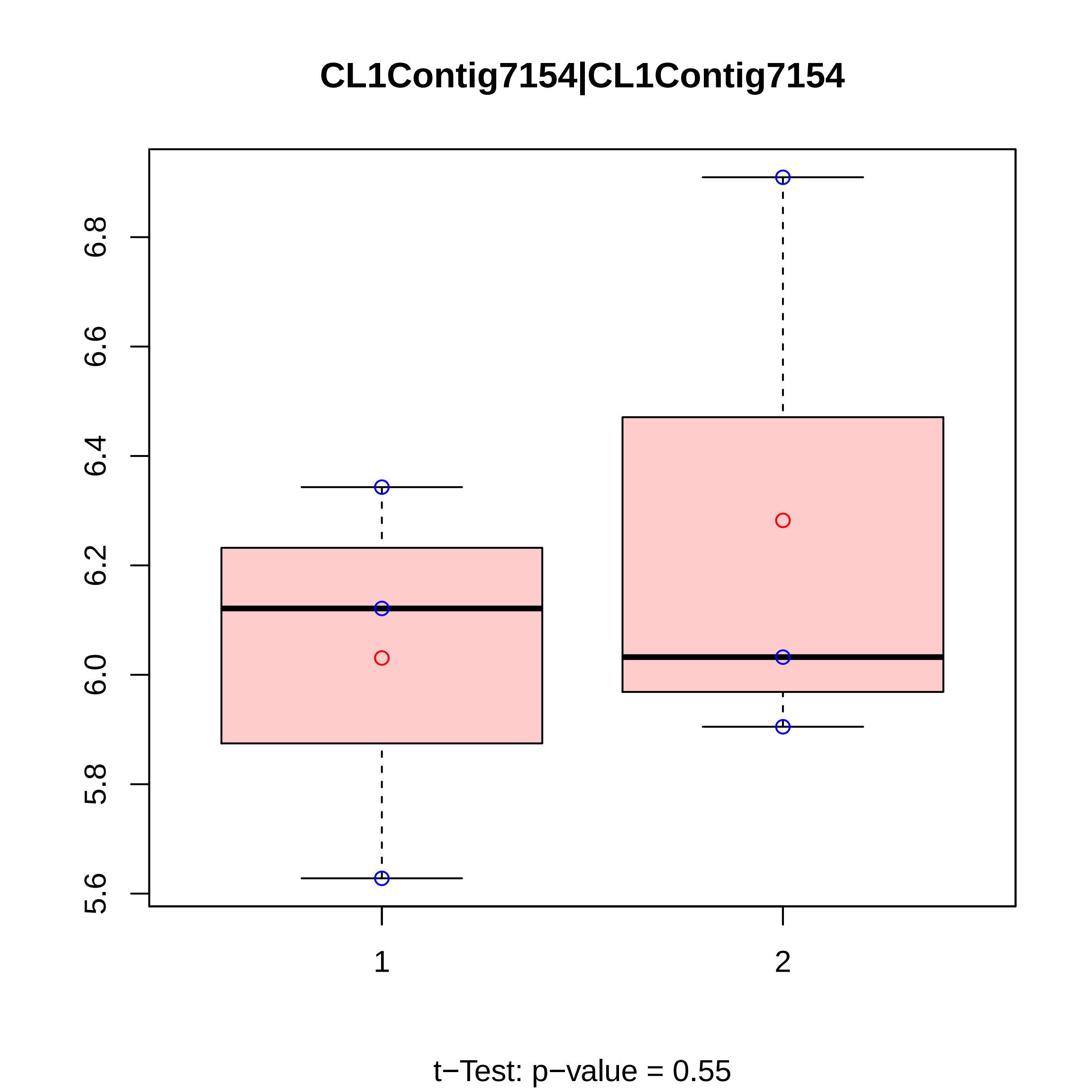

CL1Contig7154|CL1Contig7154
6.8
6.6
6.4
6.2
6.0
5.8
5.6
1
2
t−Test: p−value = 0.55

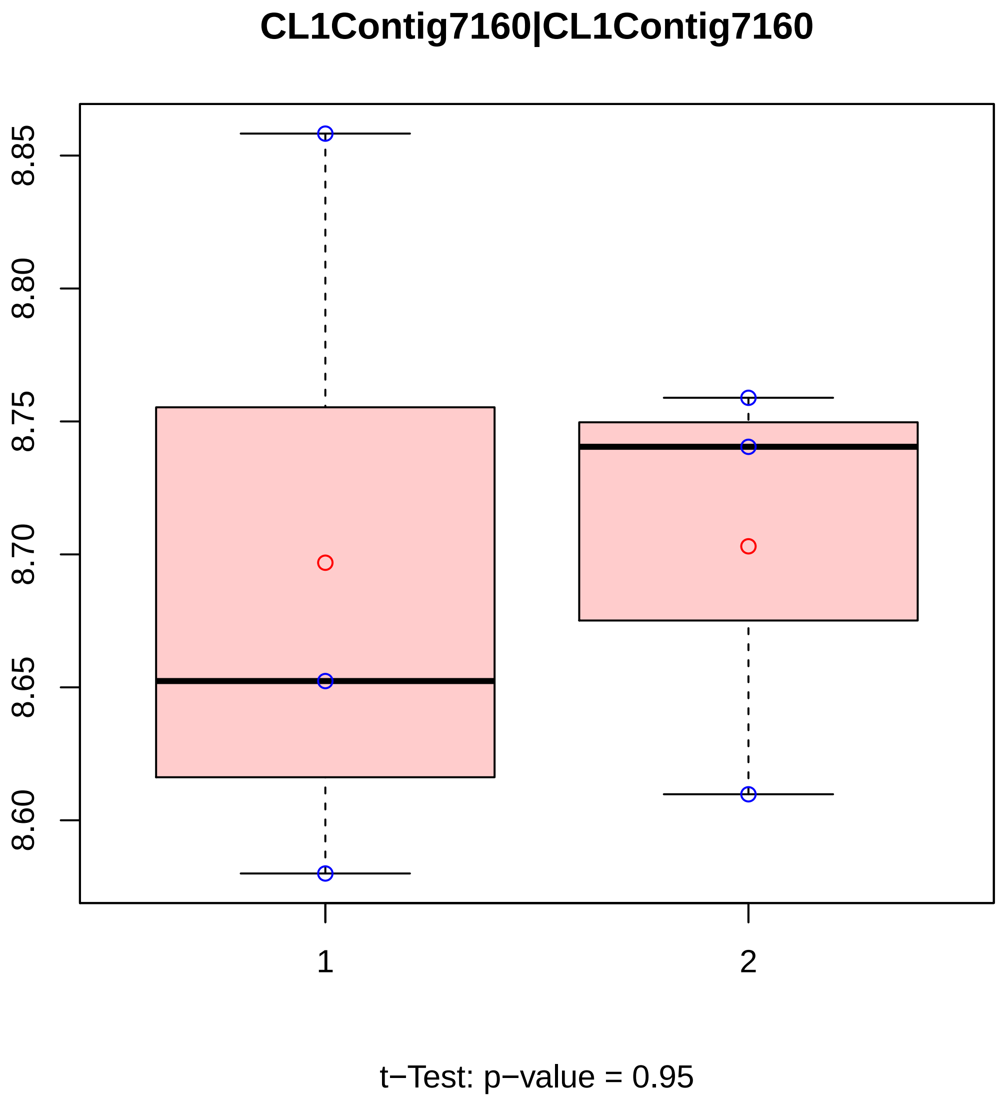
CL1Contig7160|CL1Contig7160
8.60
8.65
8.70
8.75
8.80
8.85
1
2
t−Test: p−value = 0.95

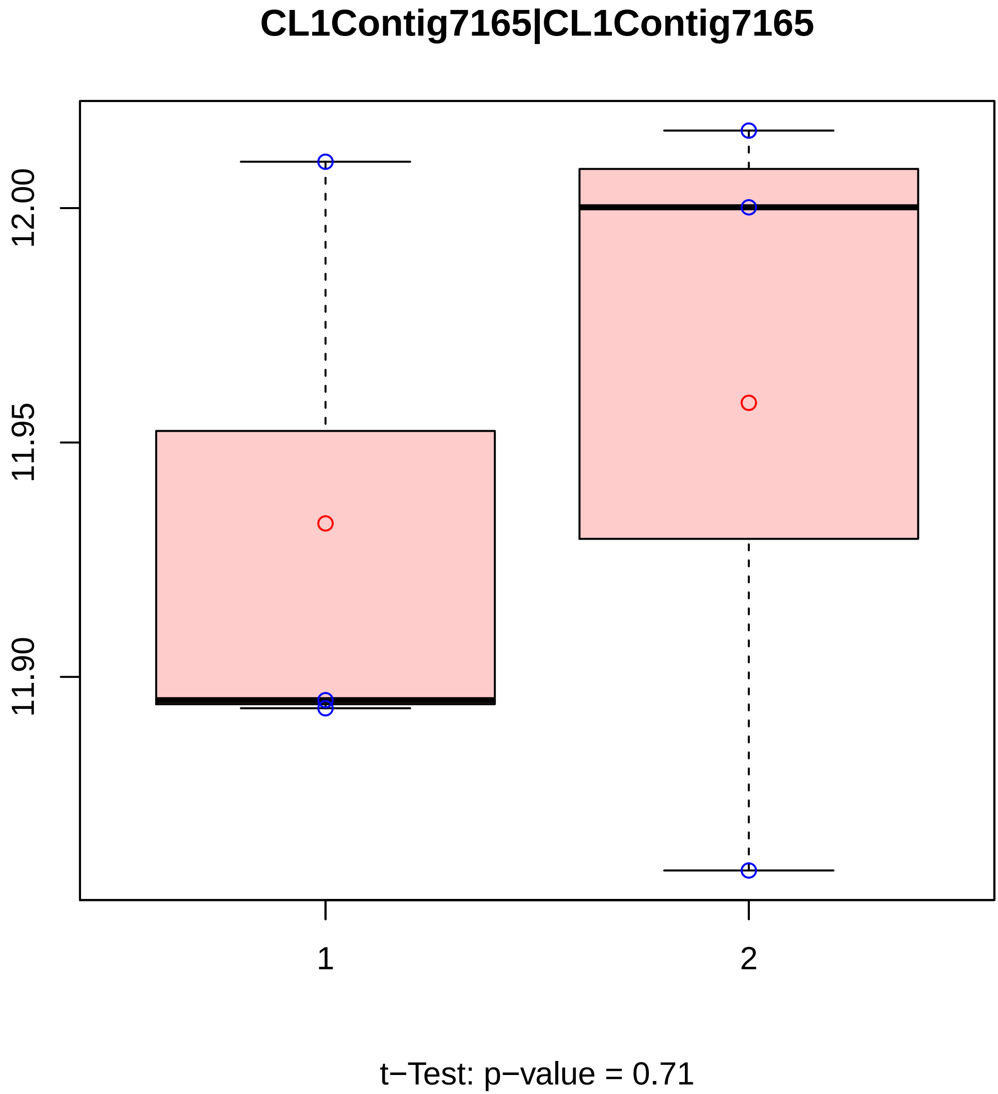
CL1Contig7165|CL1Contig7165
12.00
11.95
11.90
1
2
t−Test: p−value = 0.71

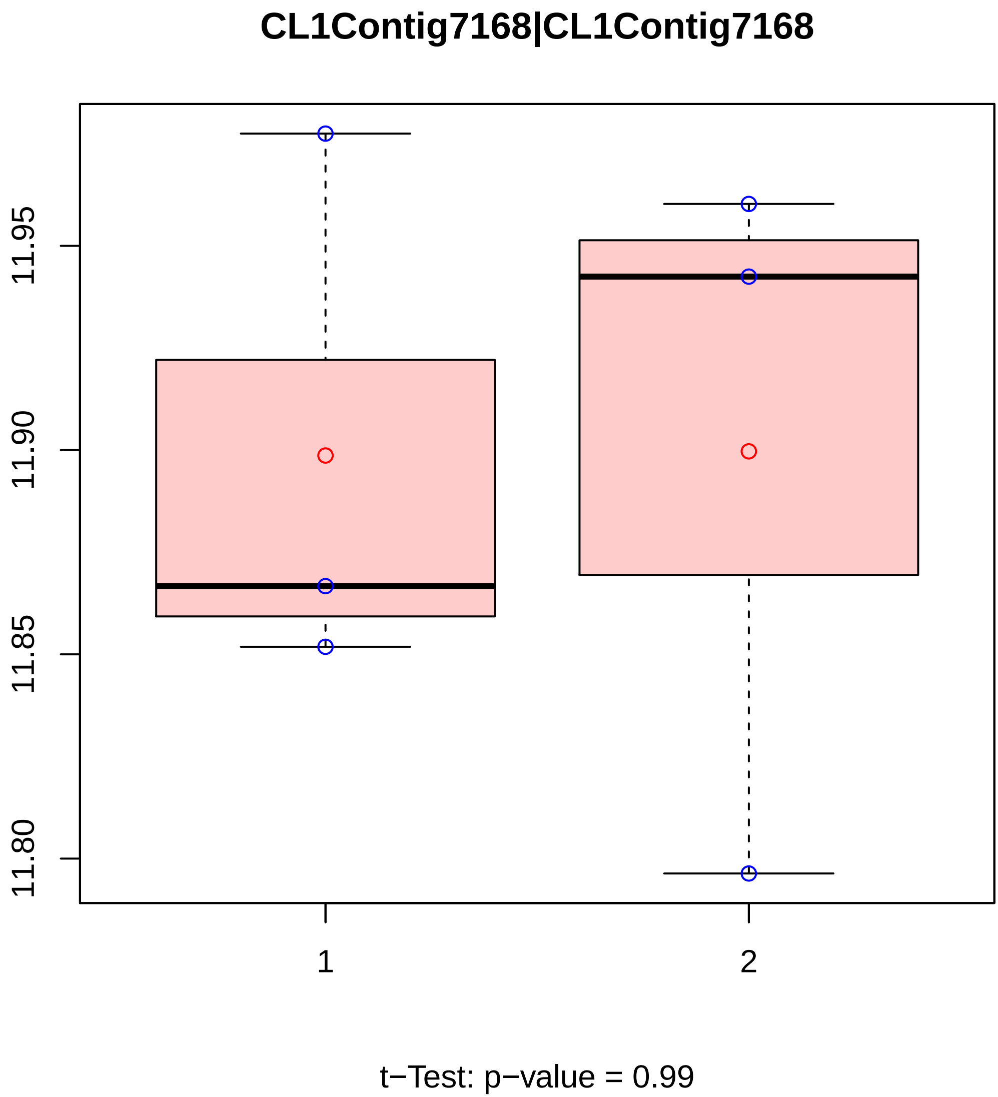

CL1Contig7168|CL1Contig7168
11.95
11.90
11.85
11.80
1
2
t−Test: p−value = 0.99

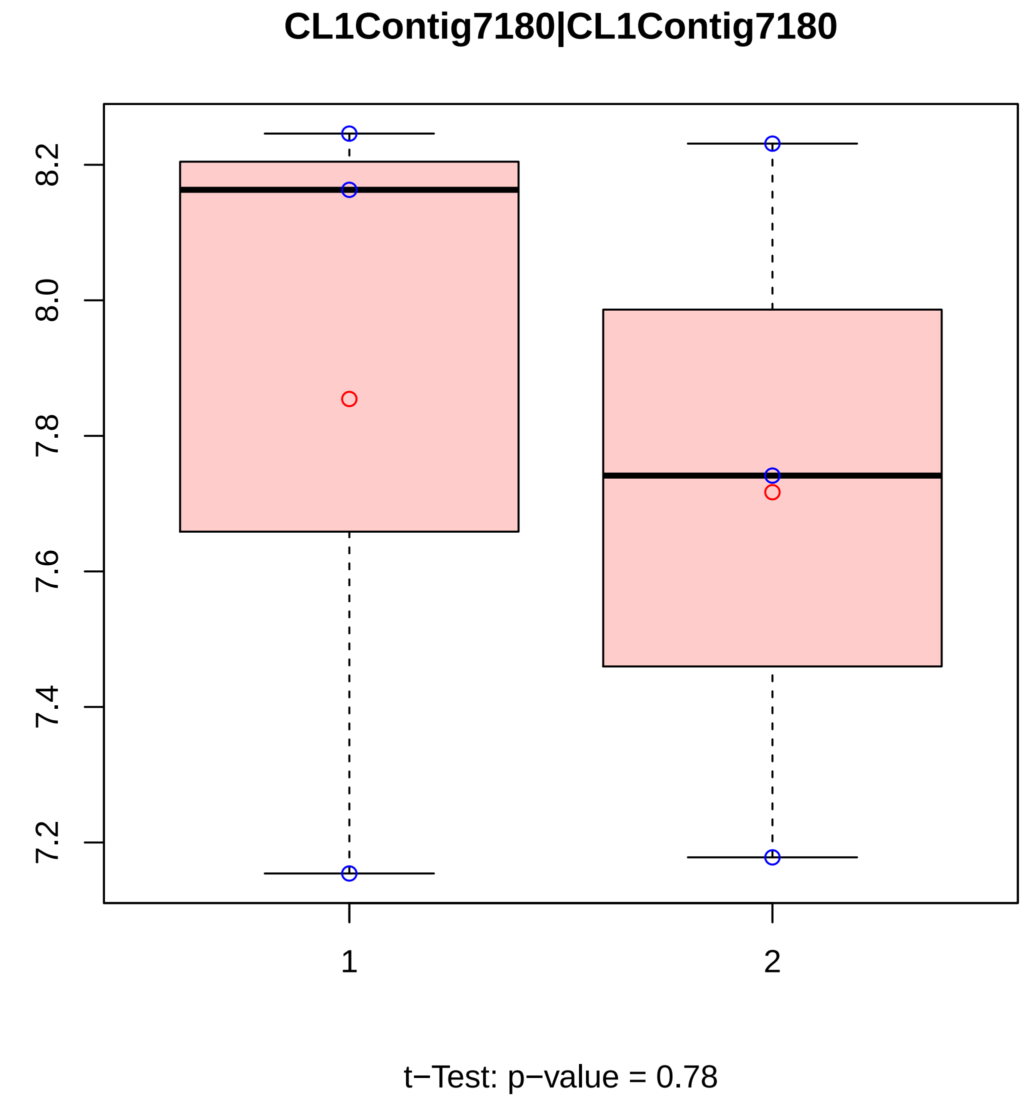
CL1Contig7180|CL1Contig7180
8.2
8.0
7.8
7.6
7.4
7.2
1
2
t−Test: p−value = 0.78

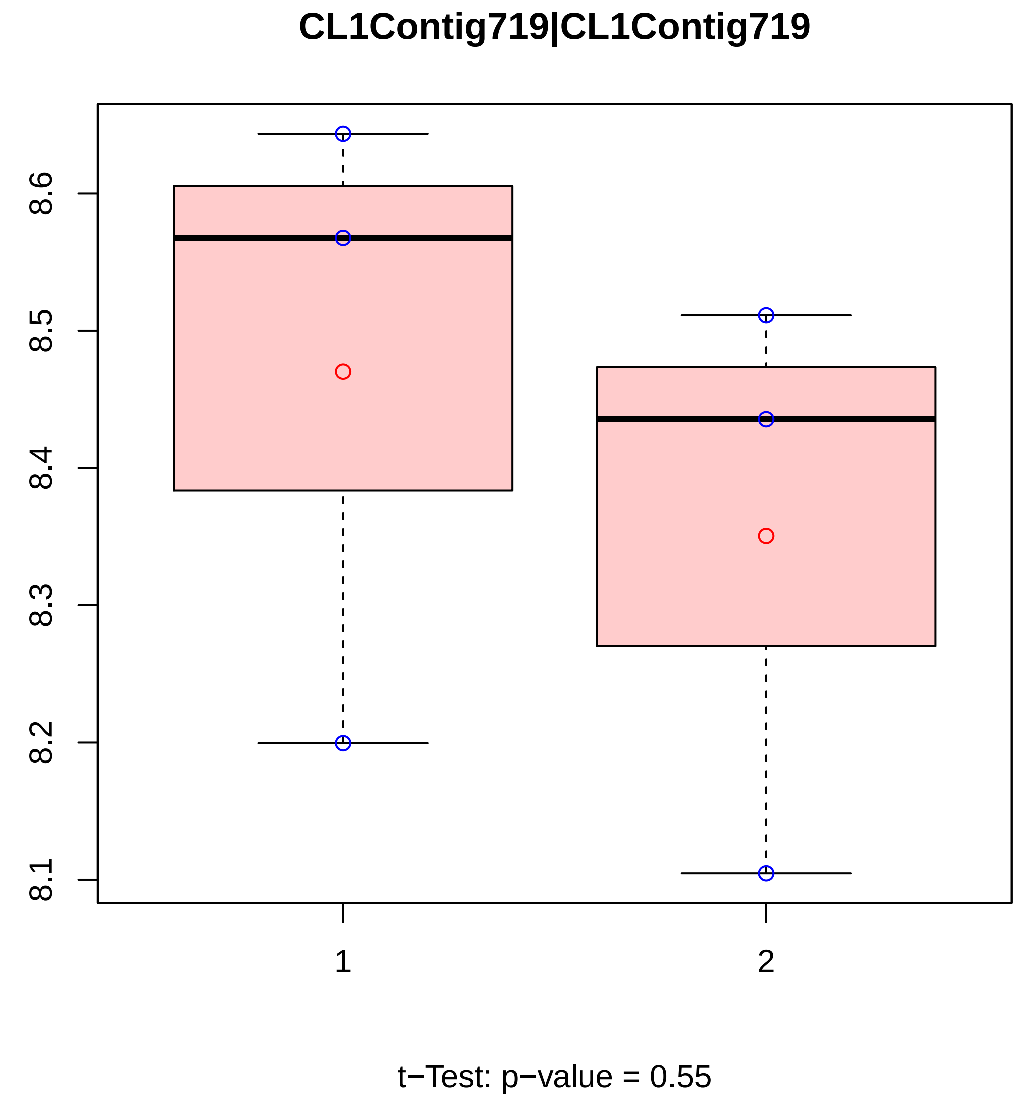
CL1Contig719|CL1Contig719
8.1
8.2
8.3
8.4
8.5
8.6
1
2
t−Test: p−value = 0.55

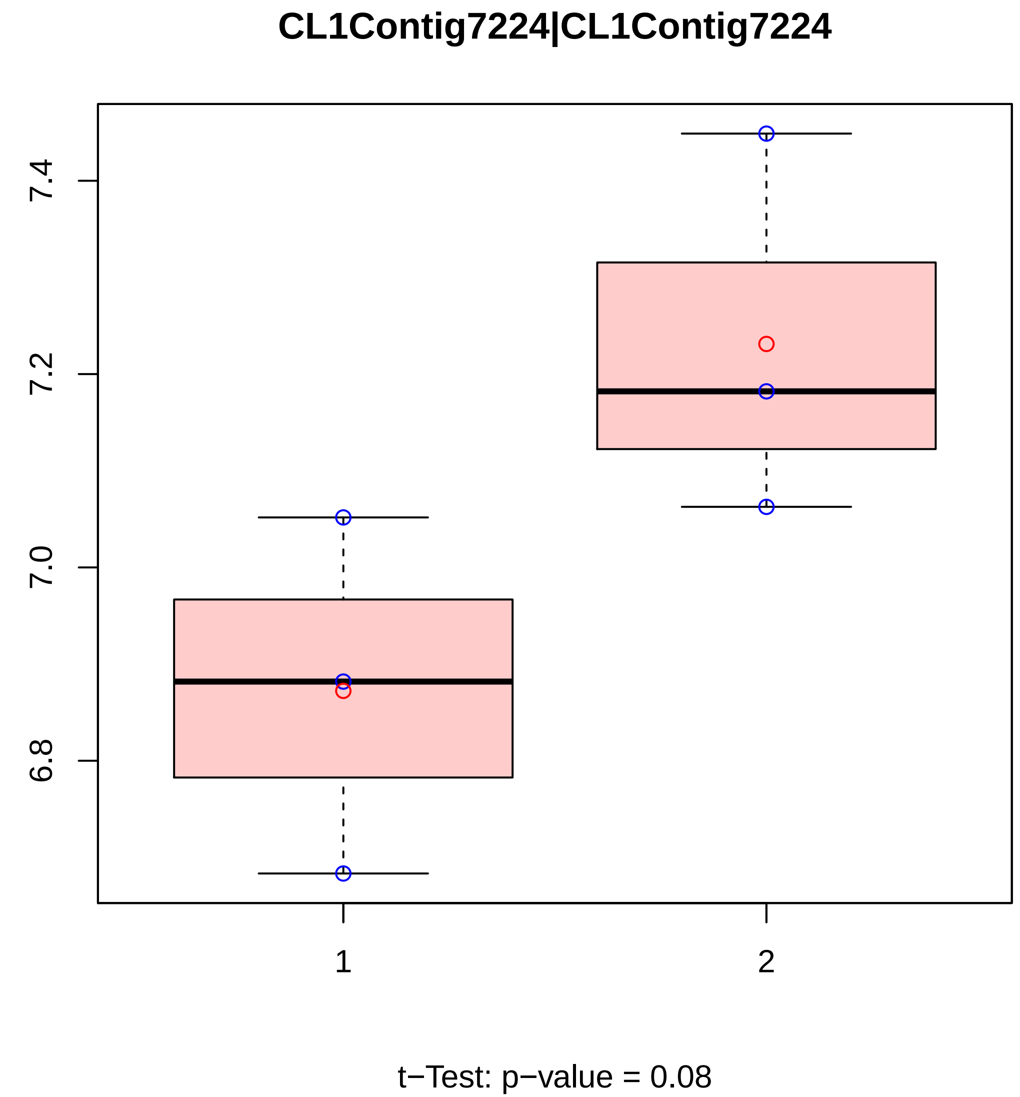
CL1Contig7224|CL1Contig7224
7.4
7.2
7.0
6.8
1
2
t−Test: p−value = 0.08

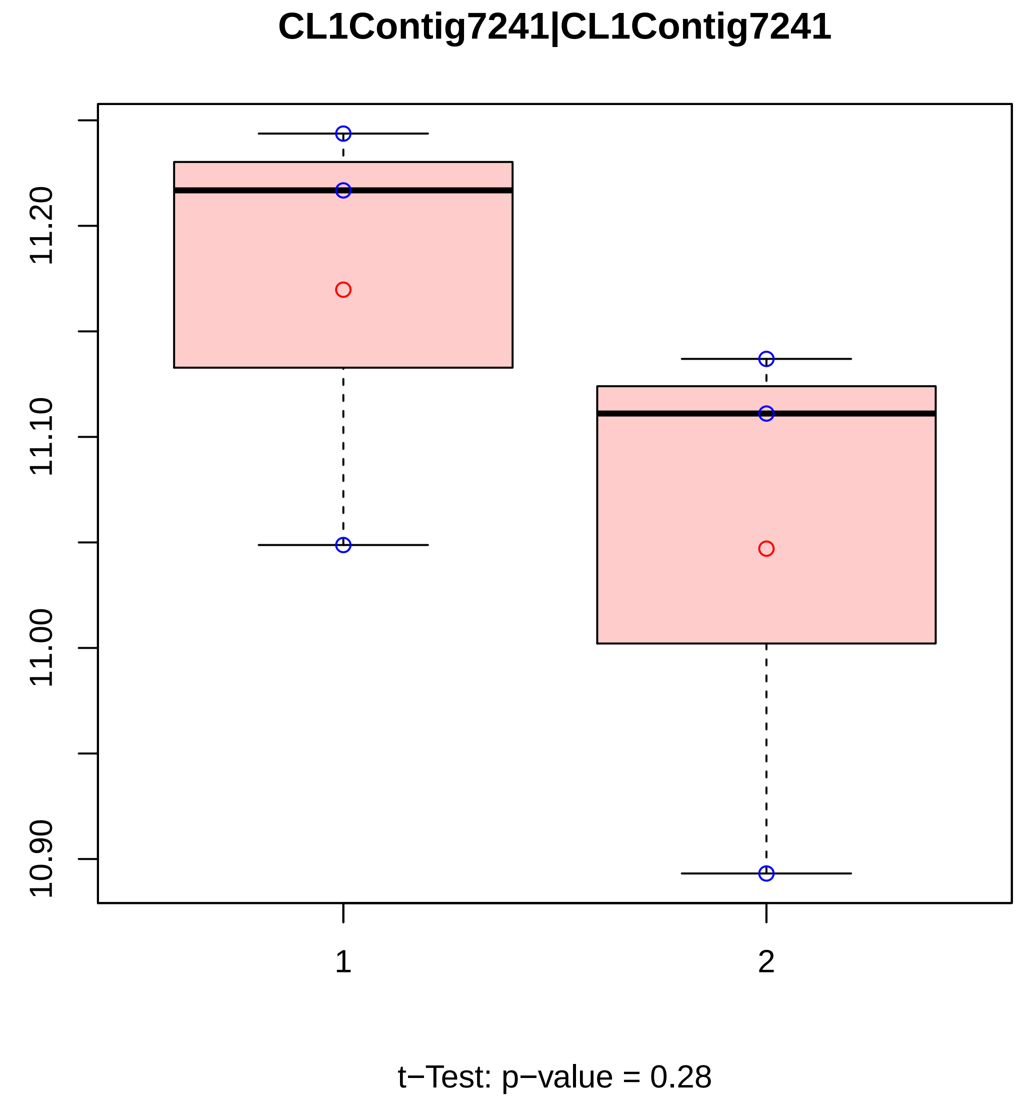
CL1Contig7241|CL1Contig7241
11.20
11.10
11.00
10.90
1
2
t−Test: p−value = 0.28

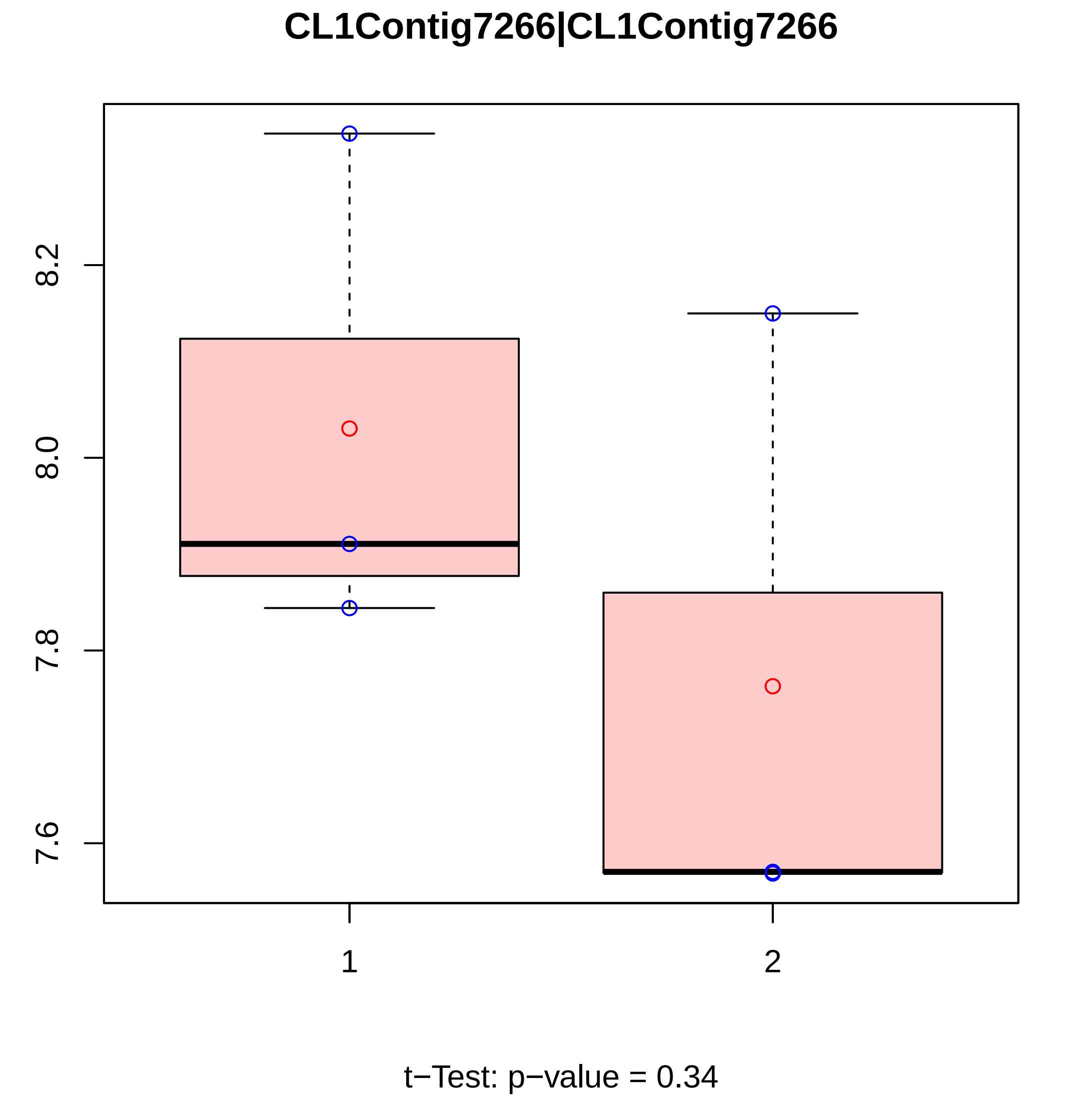

CL1Contig7266|CL1Contig7266
8.2
8.0
7.8
7.6
1
2
t−Test: p−value = 0.34

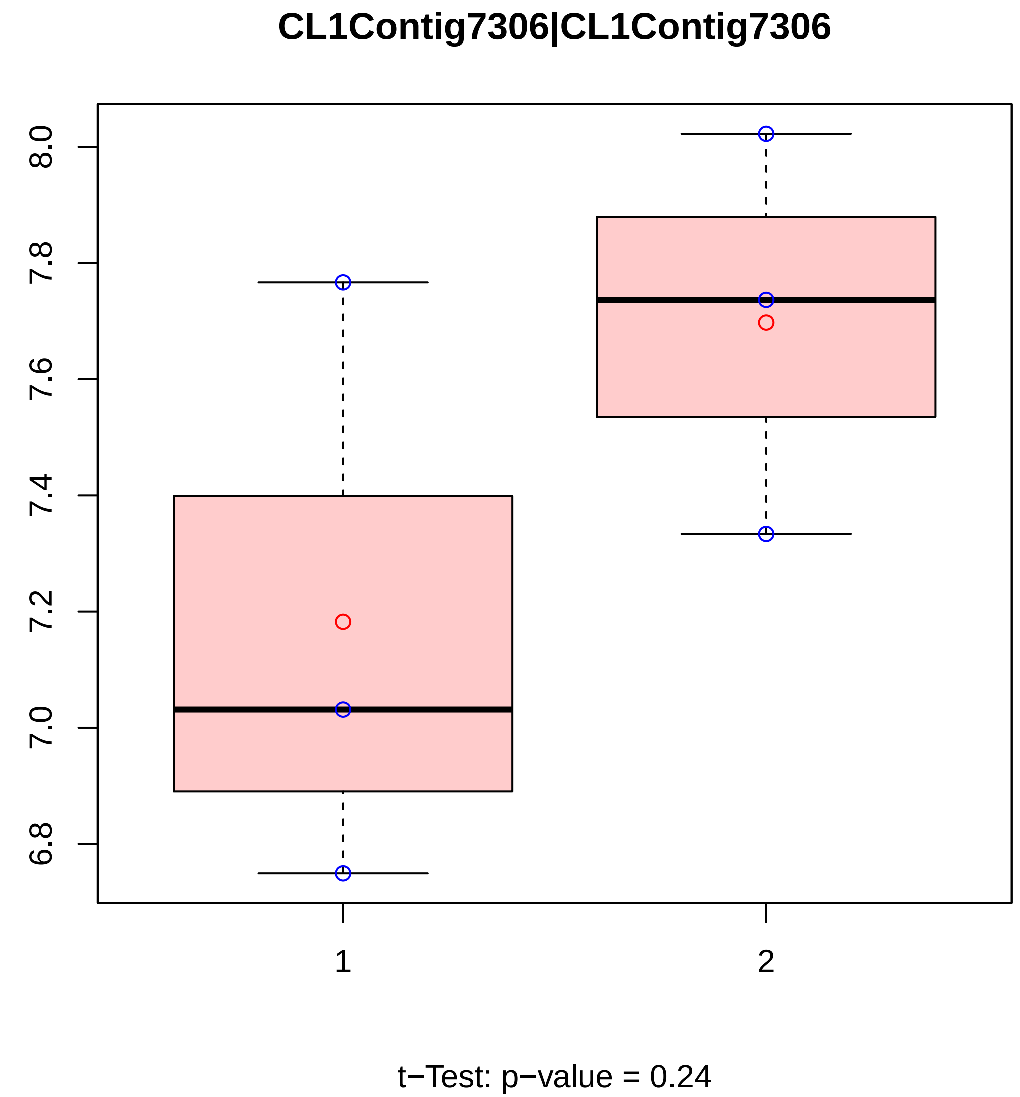

CL1Contig7306|CL1Contig7306
8.0
7.8
7.6
7.4
7.2
7.0
6.8
1
2
t−Test: p−value = 0.24

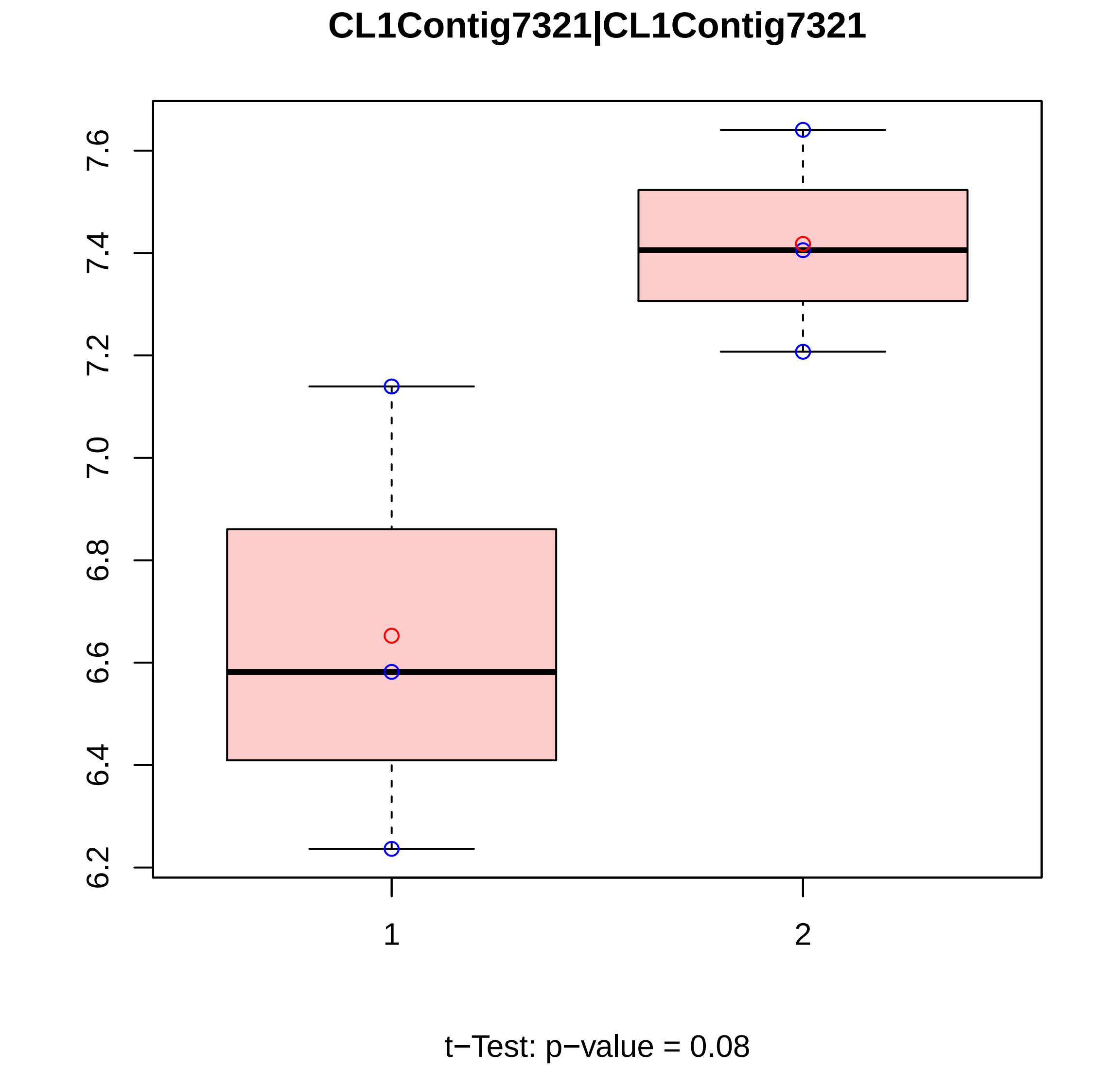

CL1Contig7321|CL1Contig7321
7.6
7.4
7.2
7.0
6.8
6.6
6.4
6.2
1
2
t−Test: p−value = 0.08

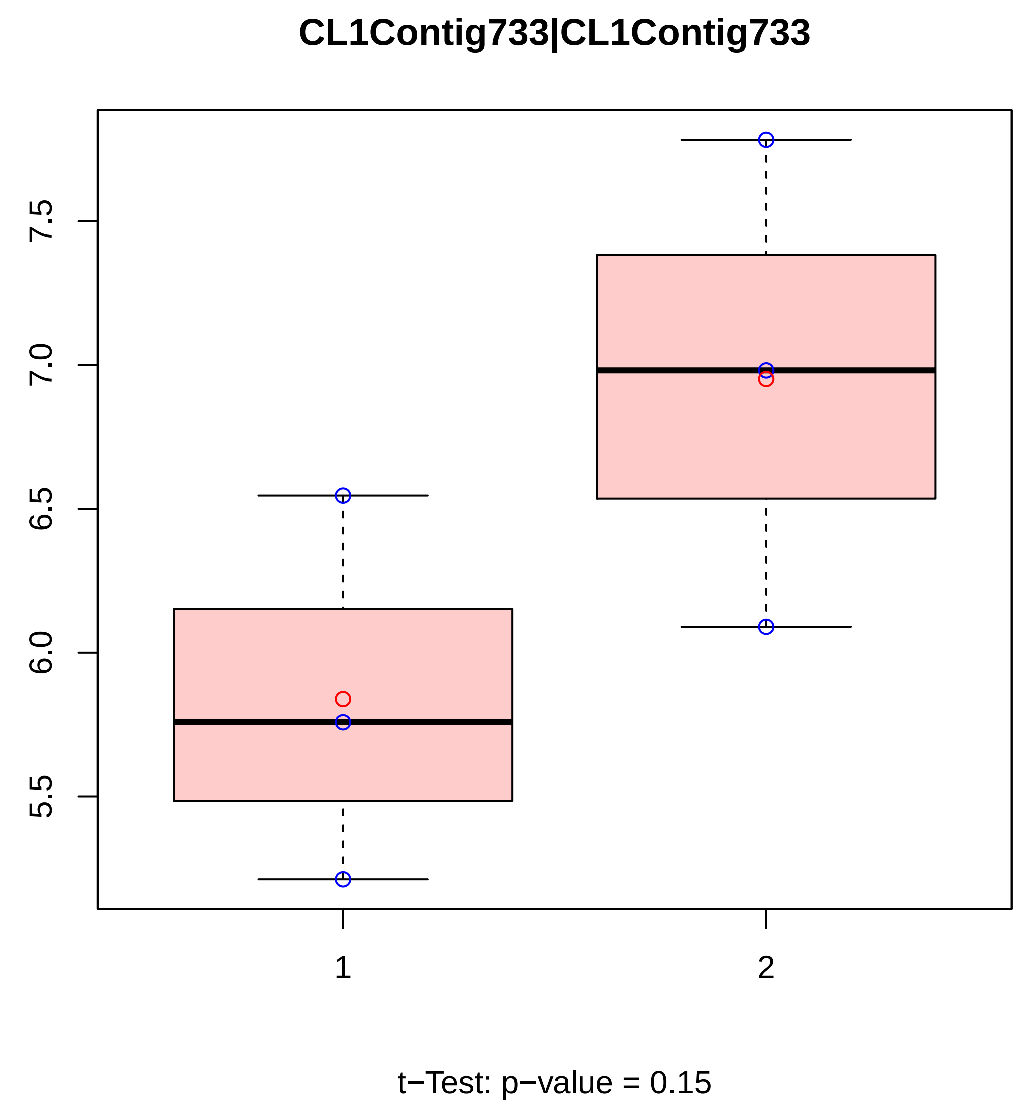
CL1Contig733|CL1Contig733
7.5
7.0
6.5
6.0
5.5
1
2
t−Test: p−value = 0.15

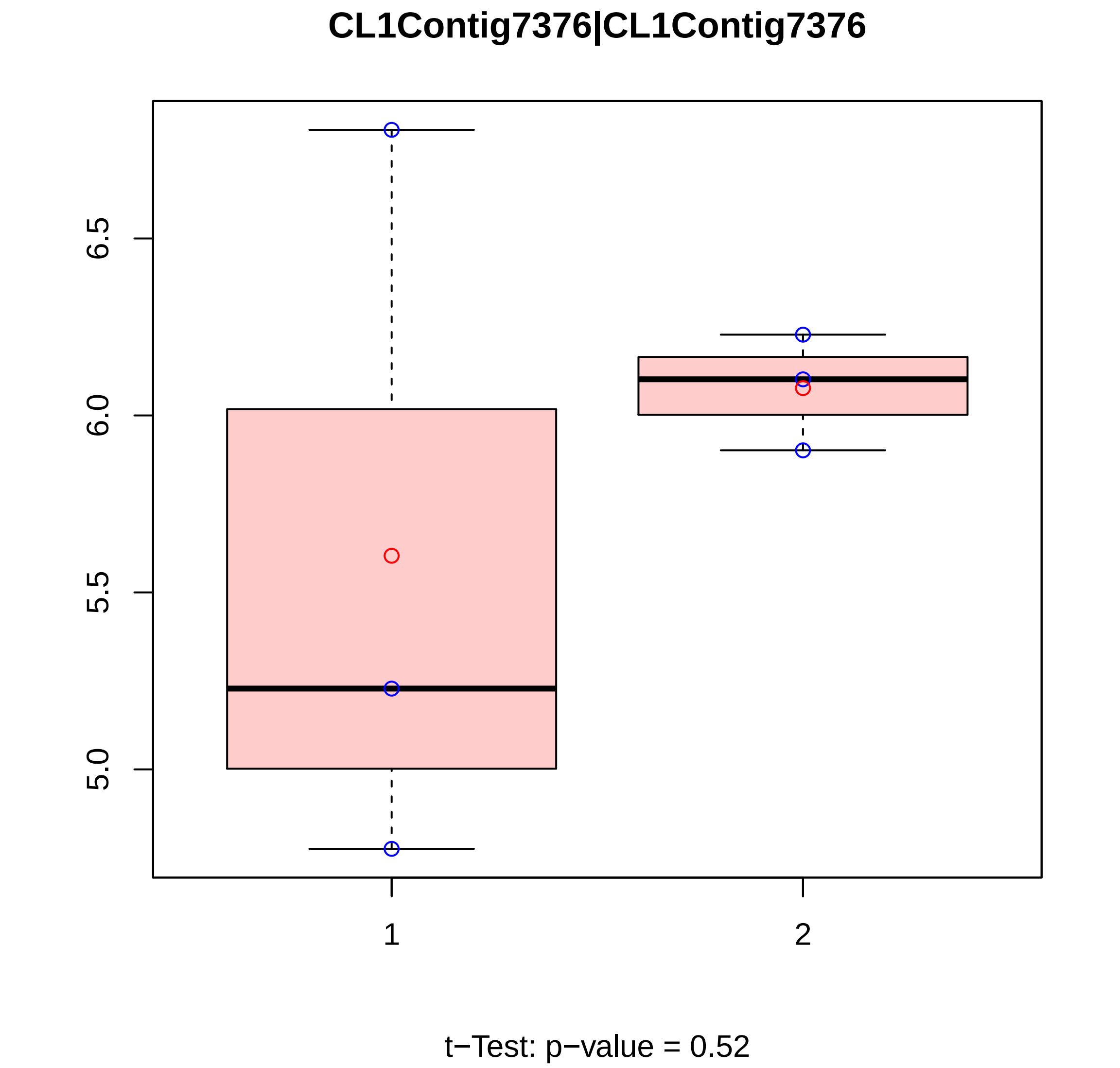
CL1Contig7376|CL1Contig7376
6.5
6.0
5.5
5.0
1
2
t−Test: p−value = 0.52

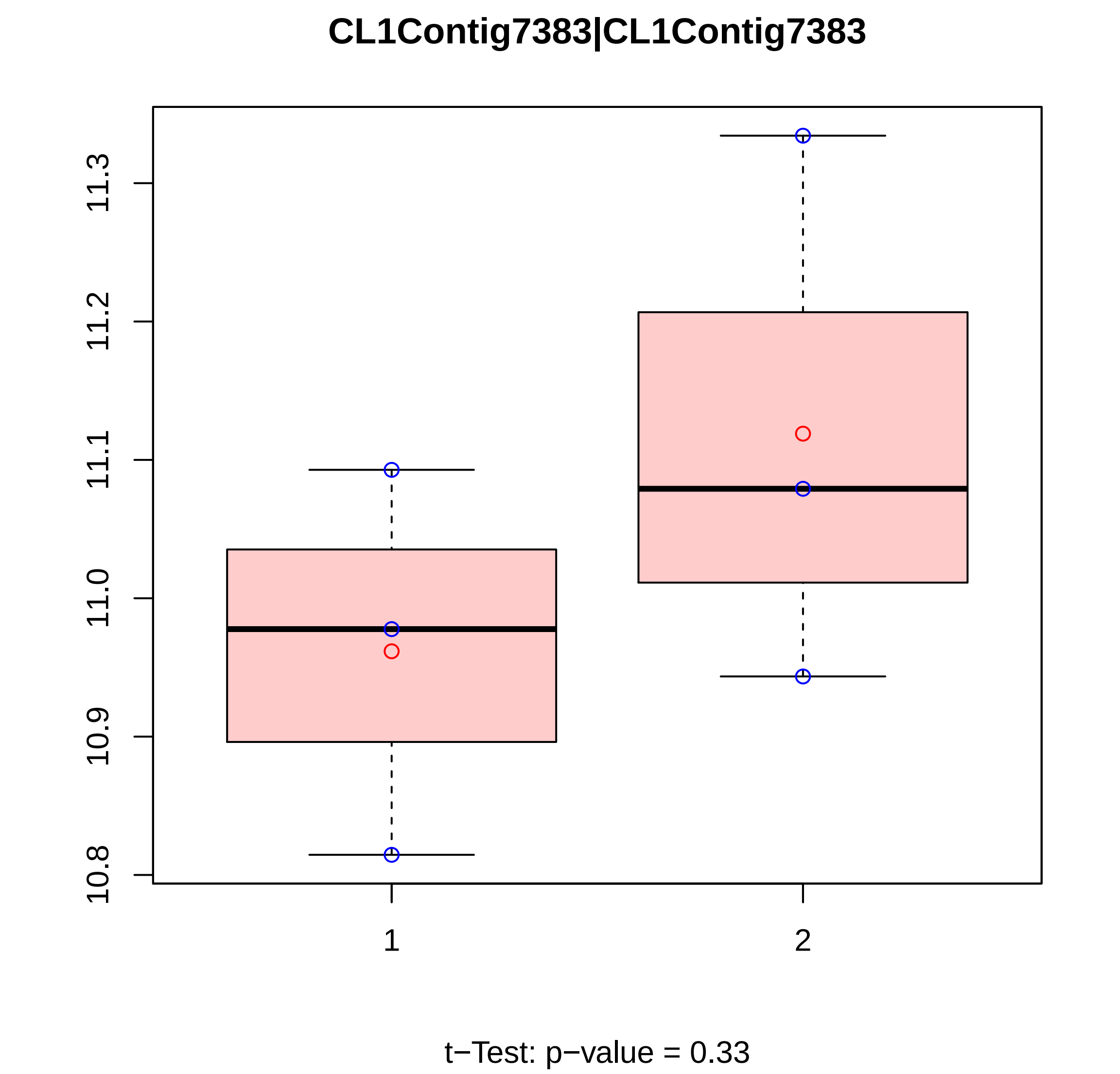
CL1Contig7383|CL1Contig7383
11.3
11.2
11.1
11.0
10.9
10.8
1
2
t−Test: p−value = 0.33

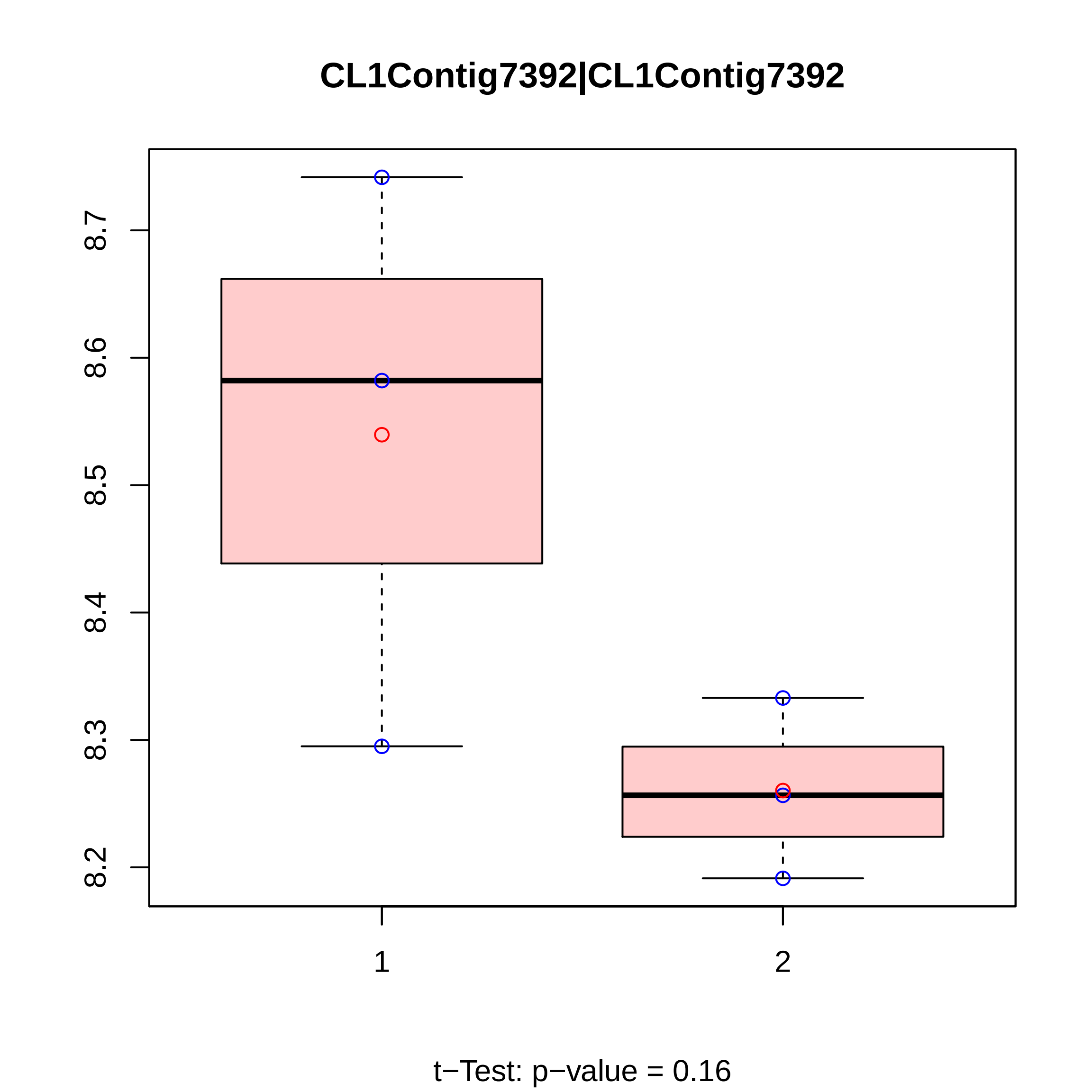

CL1Contig7392|CL1Contig7392
8.2
8.3
8.4
8.5
8.6
8.7
1
2
t−Test: p−value = 0.16

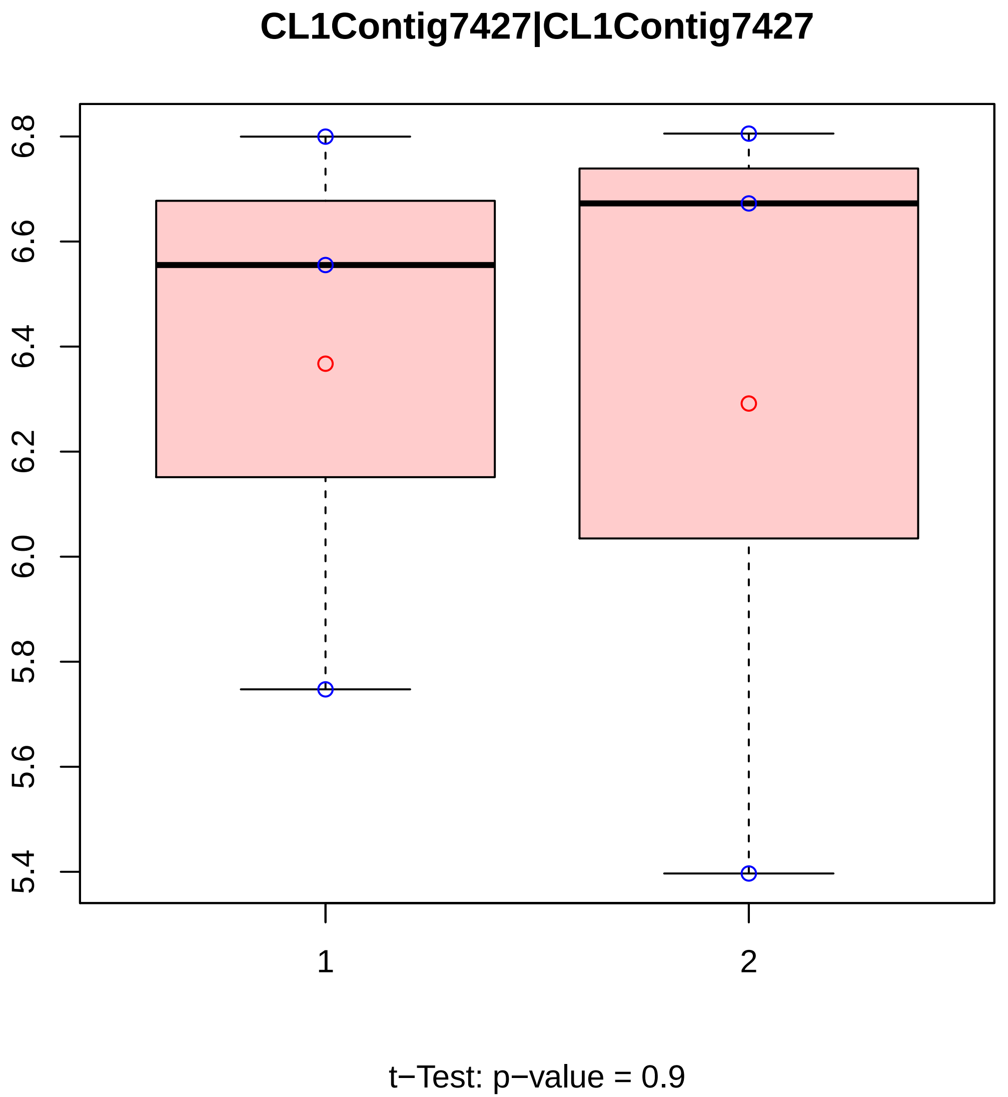
CL1Contig7427|CL1Contig7427
6.8
6.6
6.4
6.2
6.0
5.8
5.6
5.4
1
2
t−Test: p−value = 0.9

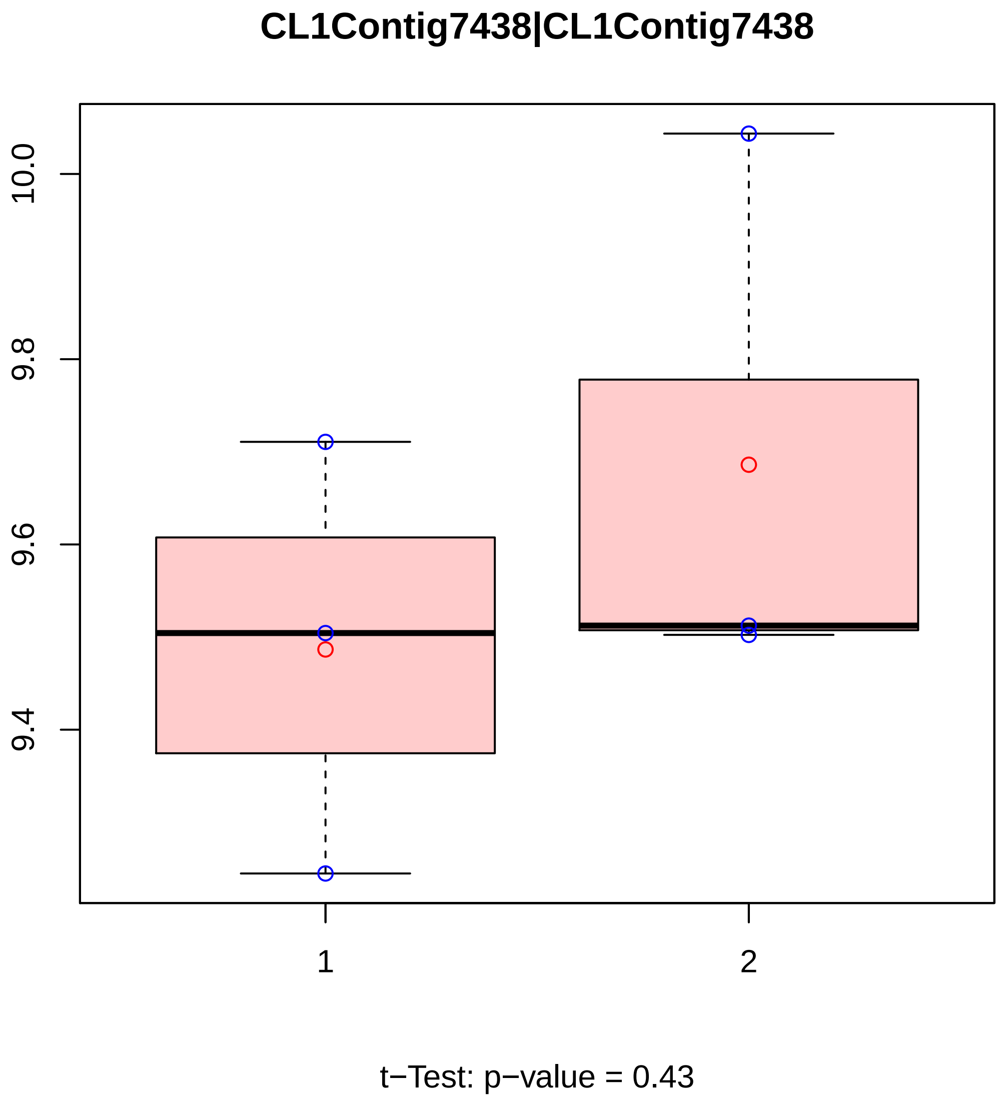

CL1Contig7438|CL1Contig7438
10.0
9.8
9.6
9.4
1
2
t−Test: p−value = 0.43

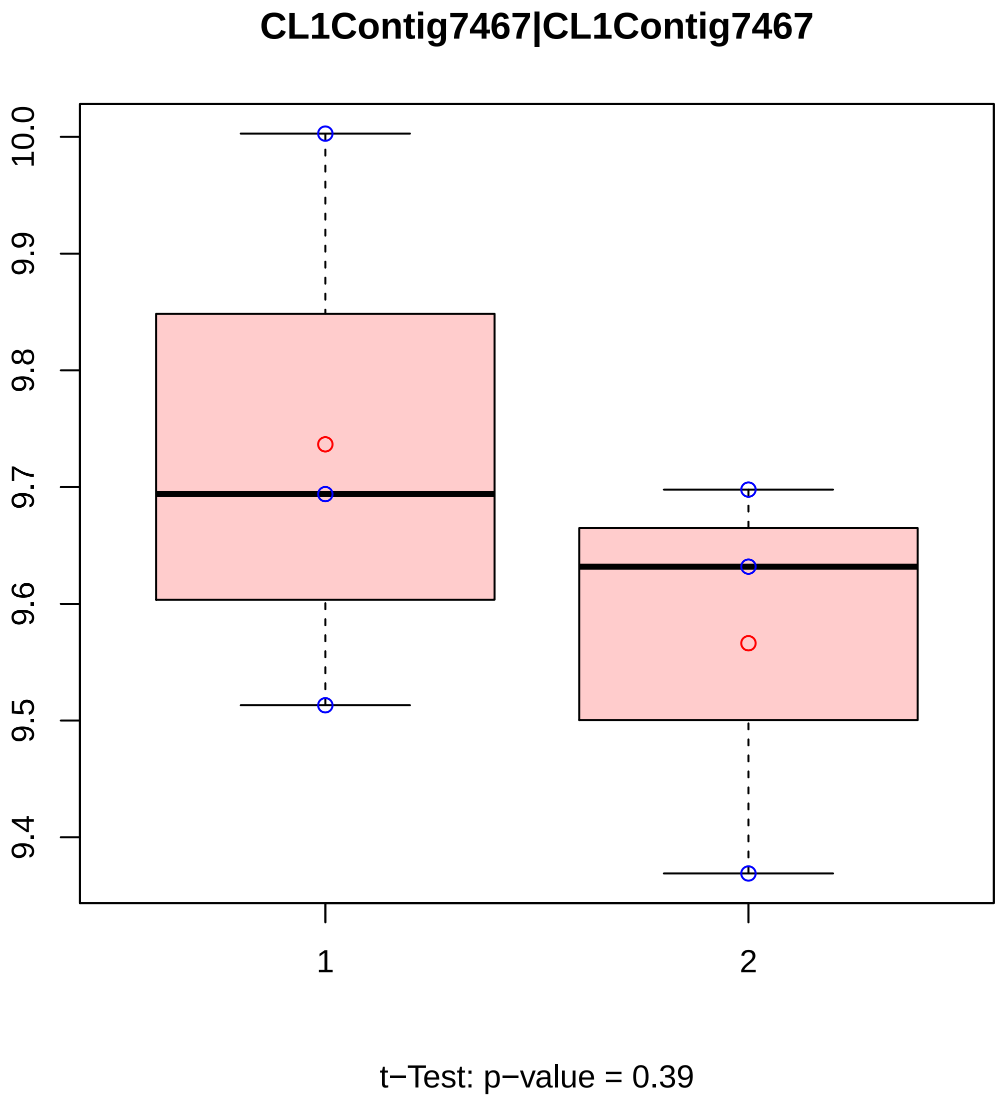

CL1Contig7467|CL1Contig7467
10.0
9.9
9.8
9.7
9.6
9.5
9.4
1
2
t−Test: p−value = 0.39

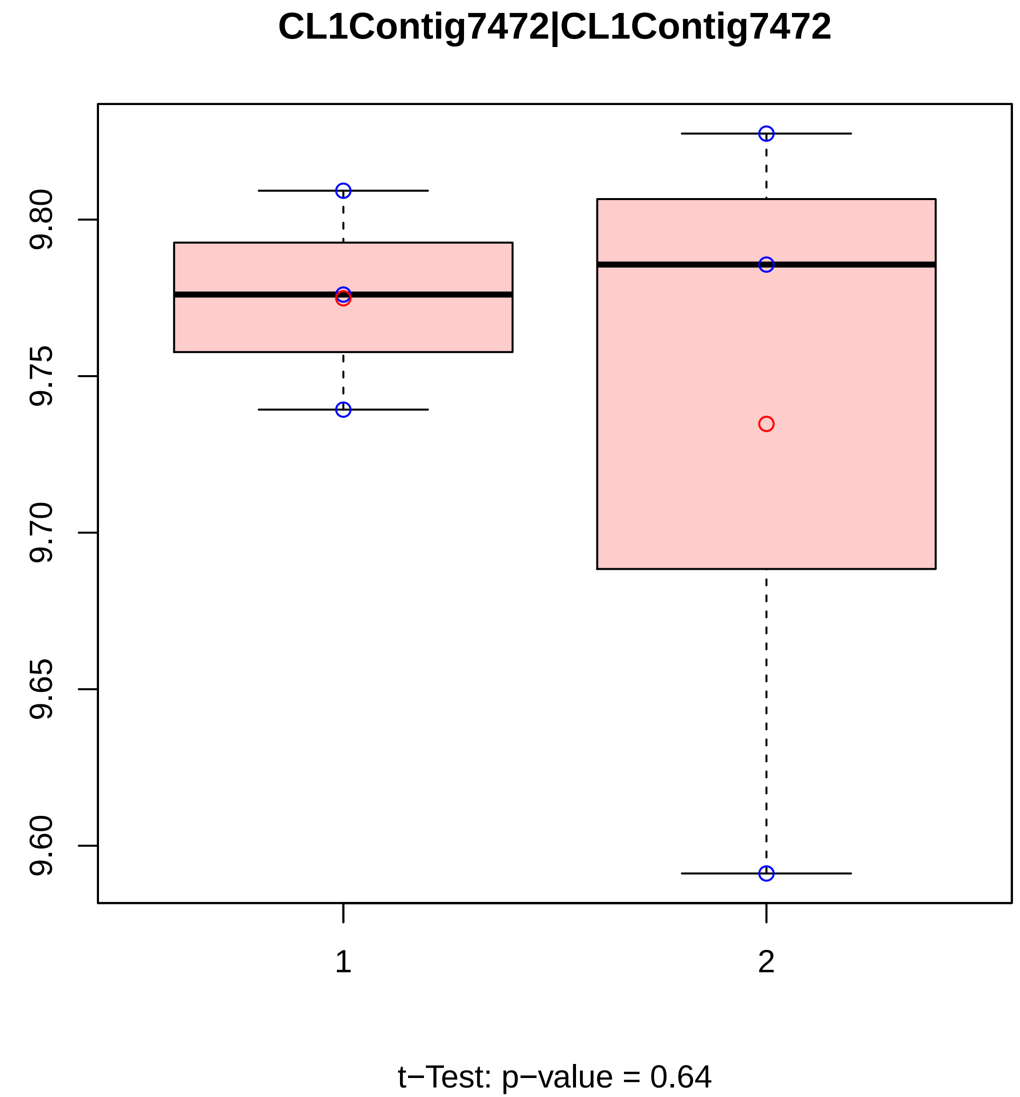
CL1Contig7472|CL1Contig7472
9.60
9.65
9.70
9.75
9.80
1
2
t−Test: p−value = 0.64

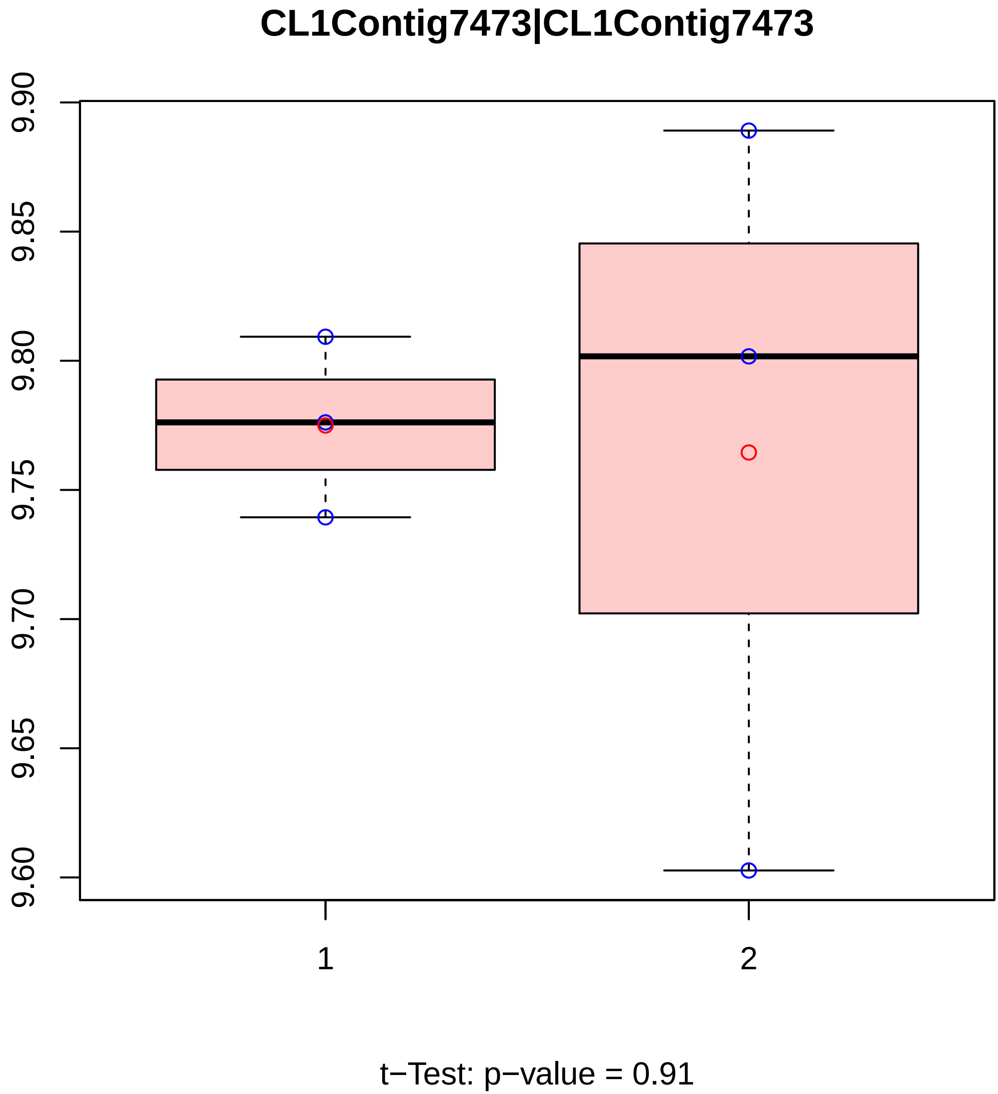

CL1Contig7473|CL1Contig7473
9.90
9.85
9.80
9.75
9.70
9.65
9.60
1
2
t−Test: p−value = 0.91

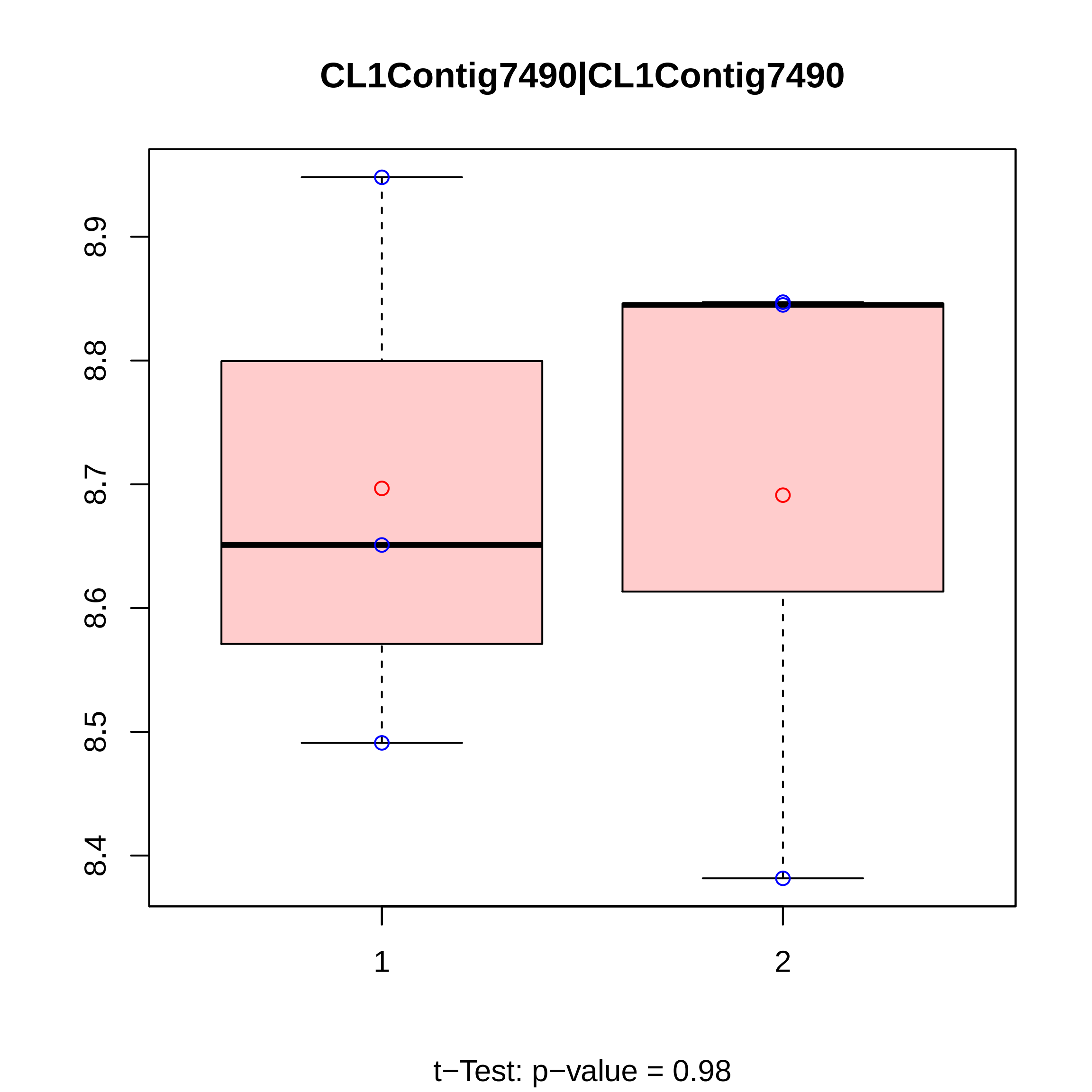

CL1Contig7490|CL1Contig7490
8.4
8.5
8.6
8.7
8.8
8.9
1
2
t−Test: p−value = 0.98

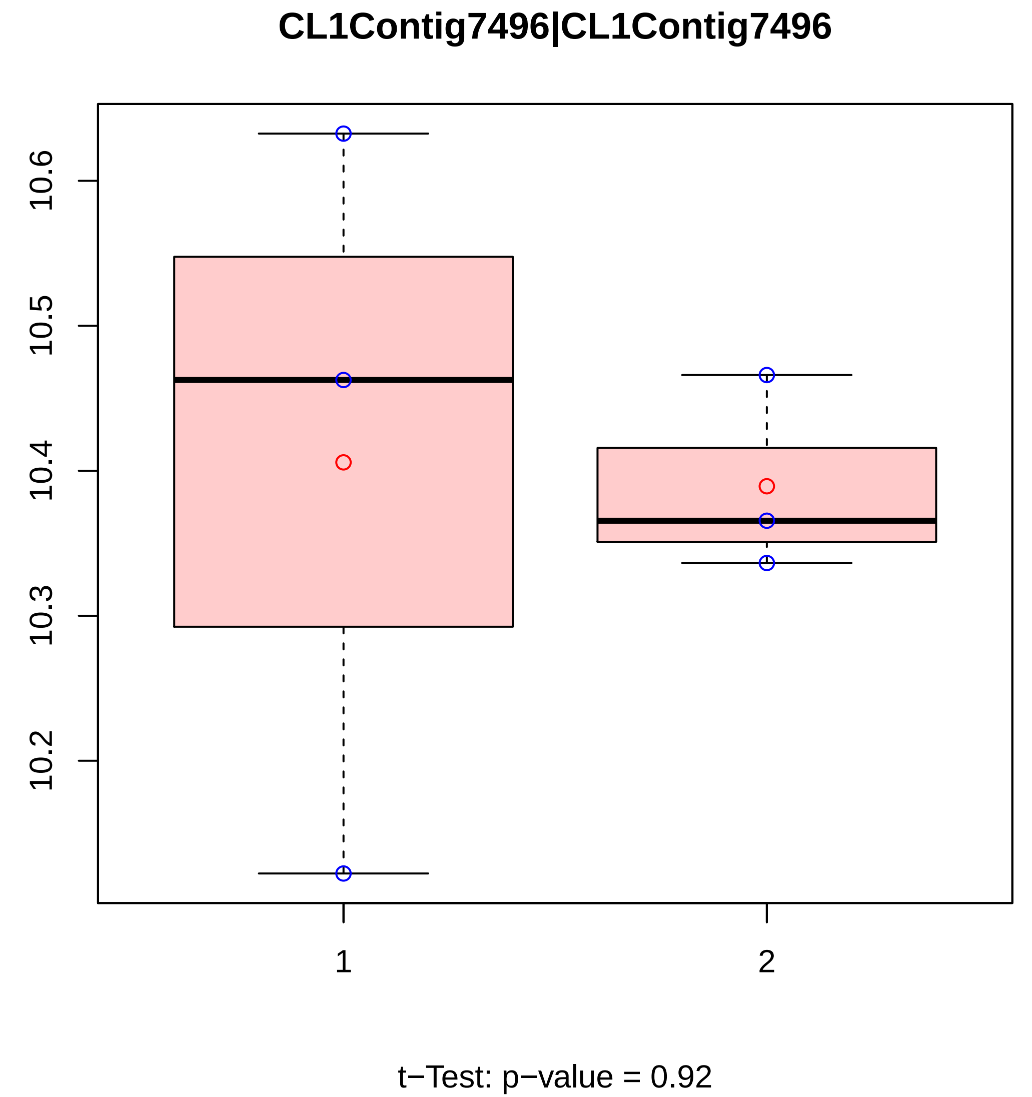
CL1Contig7496|CL1Contig7496
10.2
10.3
10.4
10.5
10.6
1
2
t−Test: p−value = 0.92

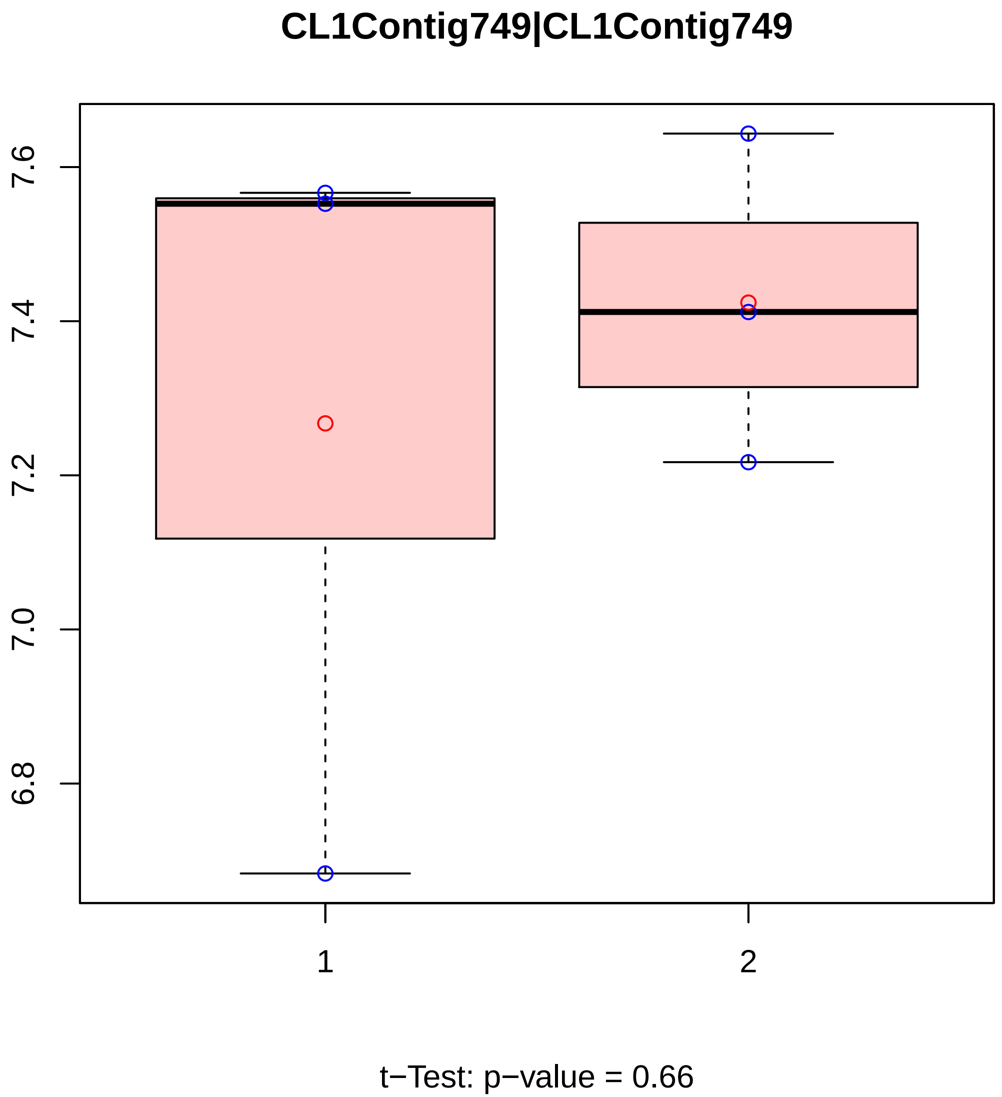

CL1Contig749|CL1Contig749
7.6
7.4
7.2
7.0
6.8
1
2
t−Test: p−value = 0.66

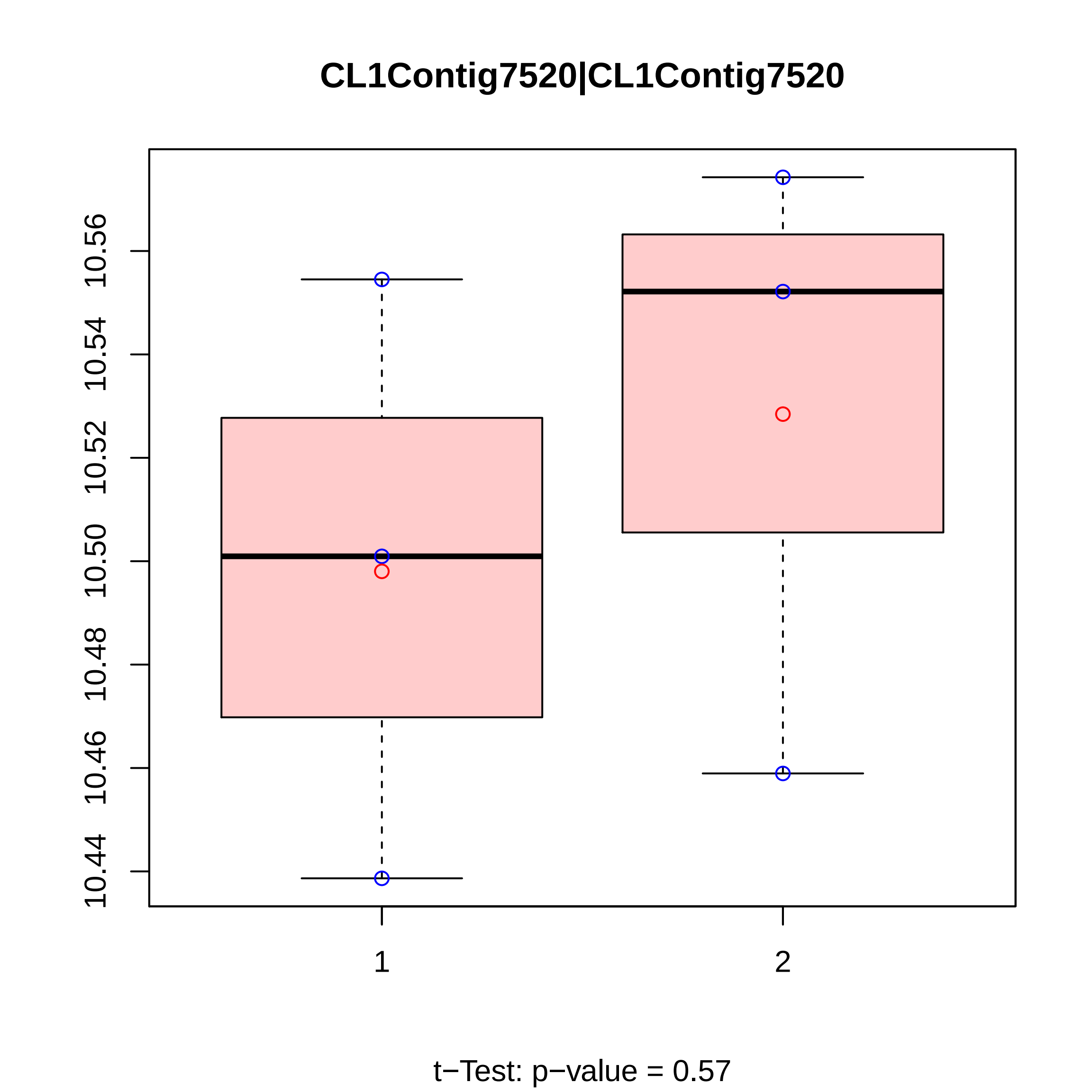
CL1Contig7520|CL1Contig7520
10.44
10.46
10.48
10.50
10.52
10.54
10.56
1
2
t−Test: p−value = 0.57

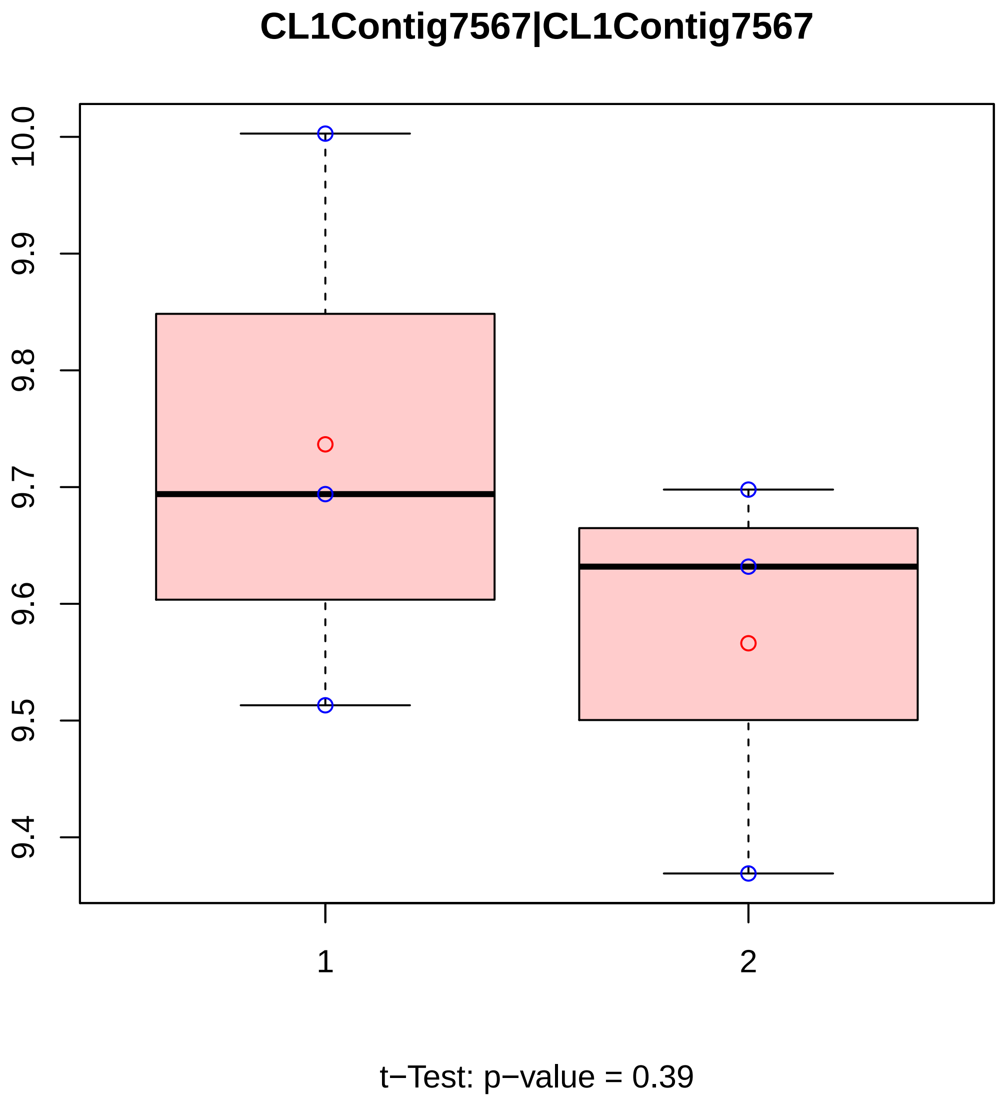
CL1Contig7567|CL1Contig7567
10.0
9.9
9.8
9.7
9.6
9.5
9.4
1
2
t−Test: p−value = 0.39

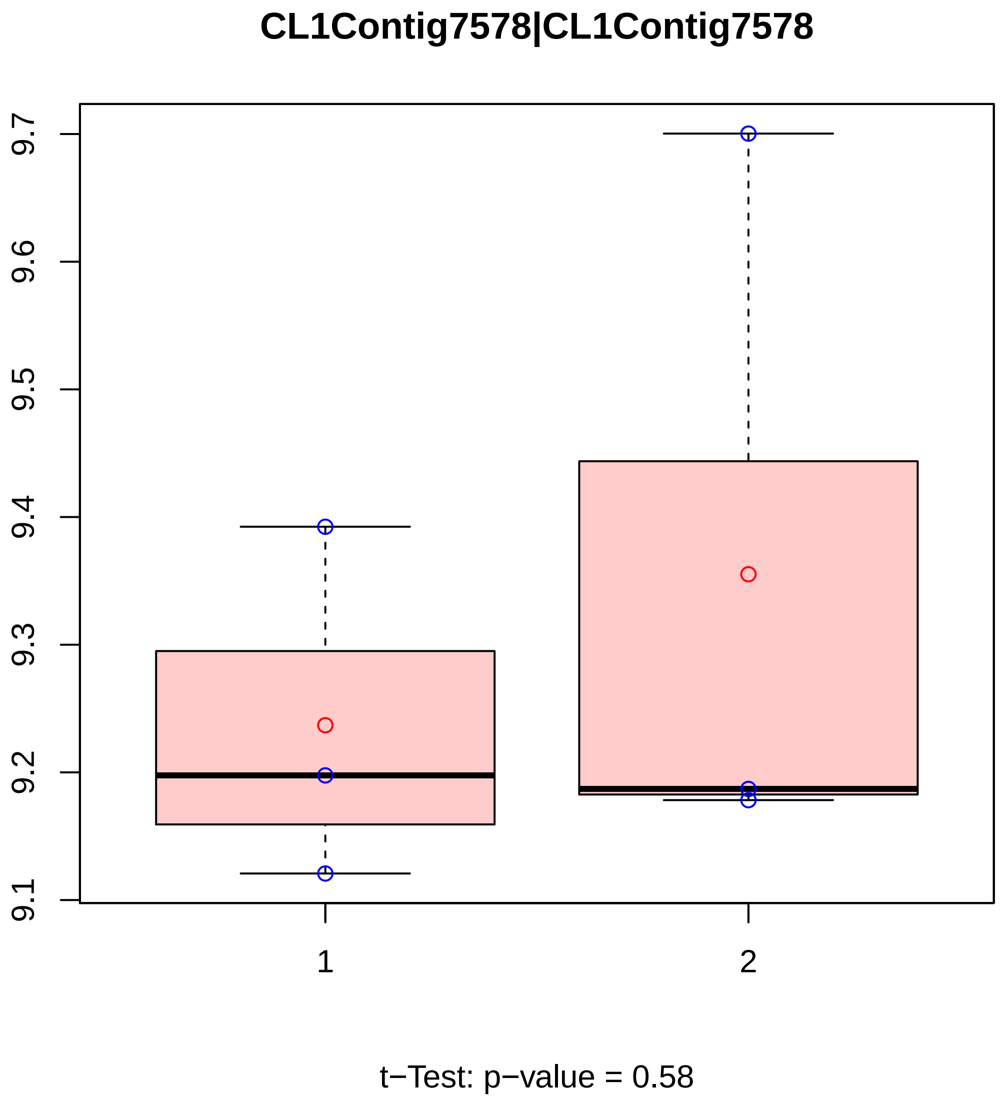

CL1Contig7578|CL1Contig7578
9.1
9.2
9.3
9.4
9.5
9.6
9.7
1
2
t−Test: p−value = 0.58

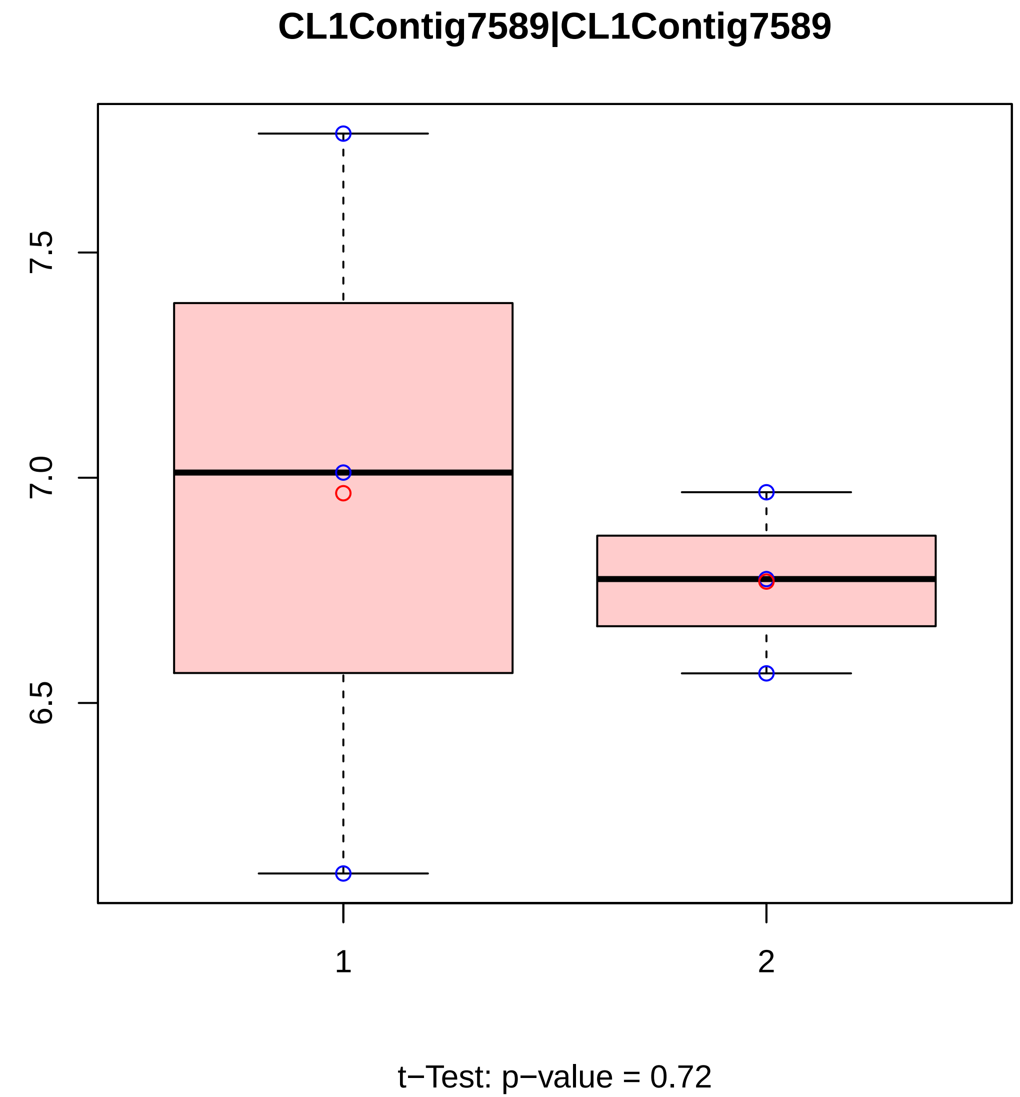

CL1Contig7589|CL1Contig7589
7.5
7.0
6.5
1
2
t−Test: p−value = 0.72

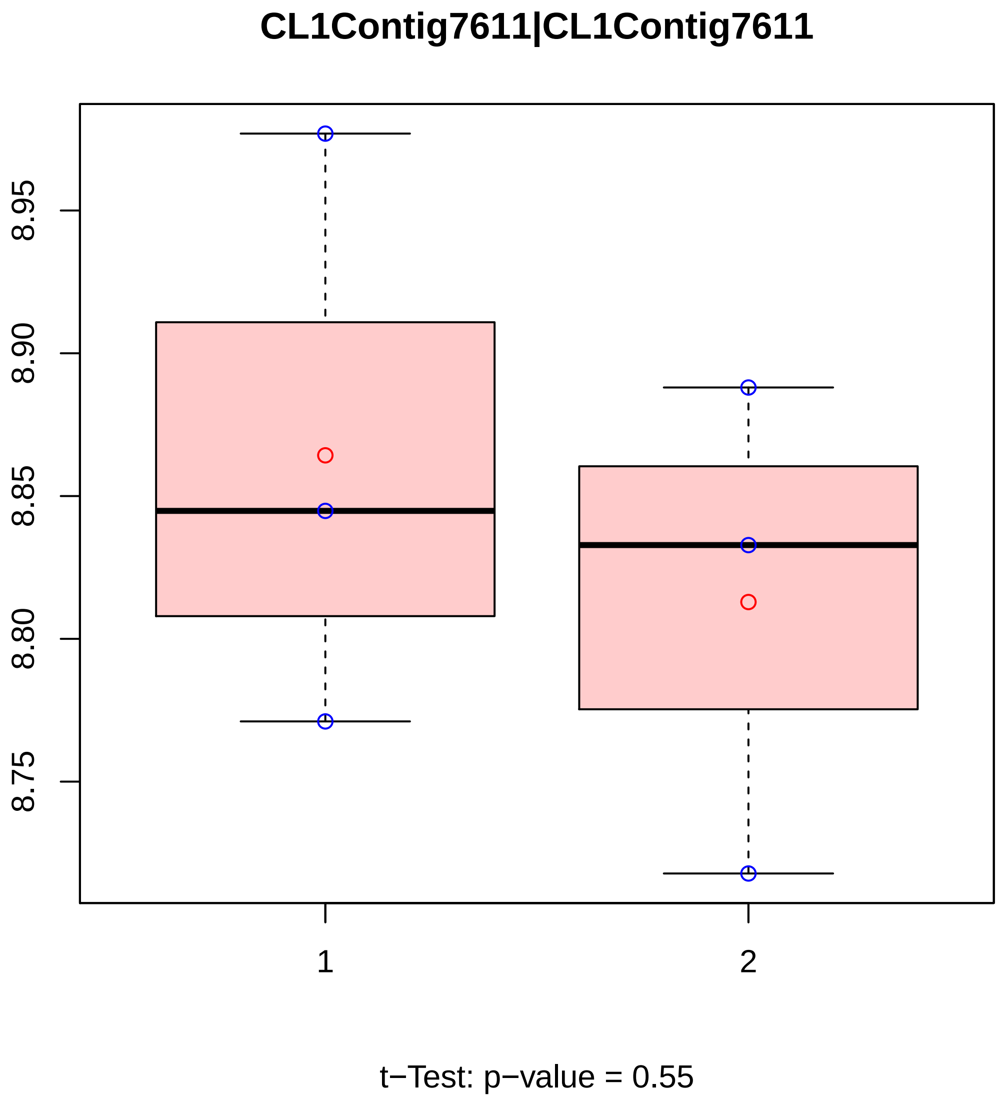

CL1Contig7611|CL1Contig7611
8.95
8.90
8.85
8.80
8.75
1
2
t−Test: p−value = 0.55

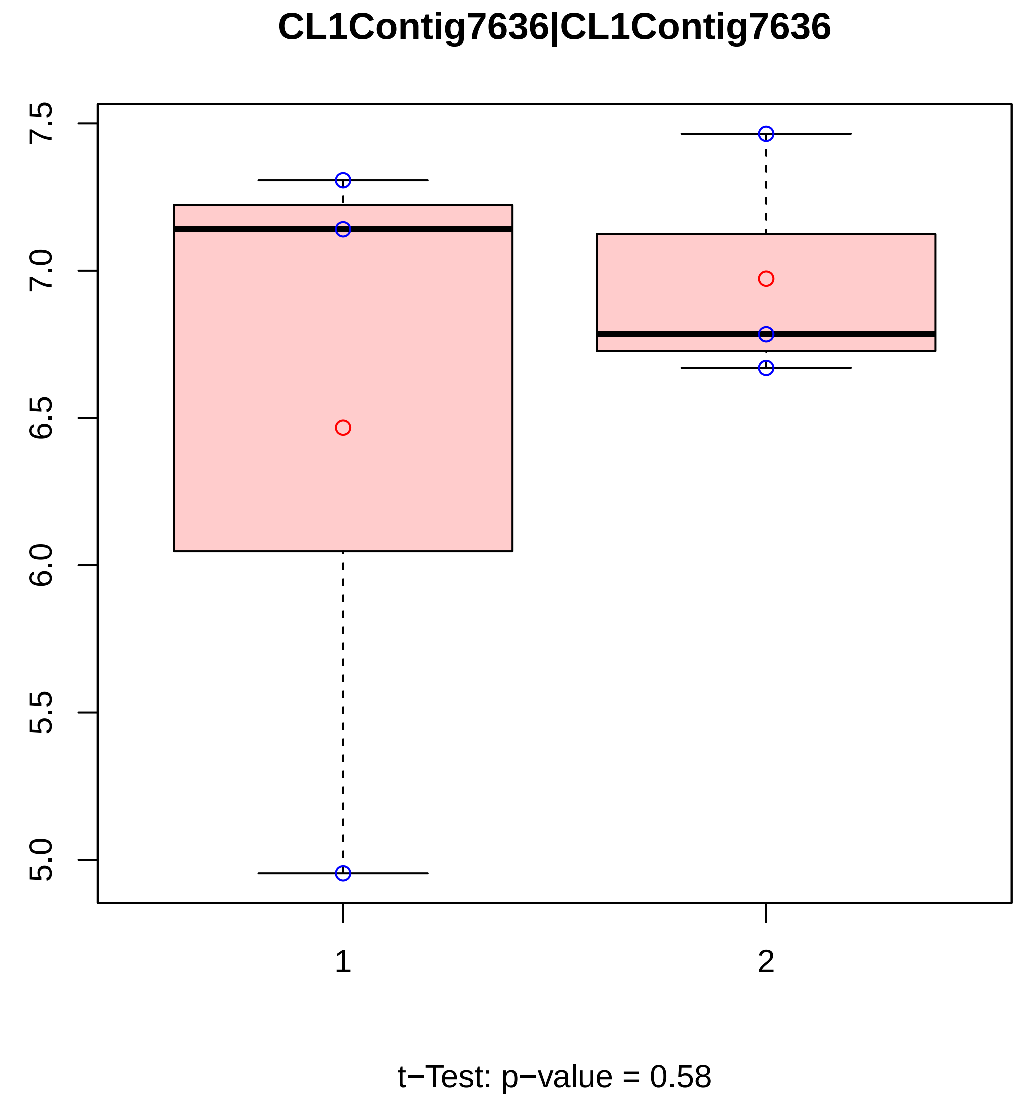
CL1Contig7636|CL1Contig7636
7.5
7.0
6.5
6.0
5.5
5.0
1
2
t−Test: p−value = 0.58

## CL1Contig7657|CL1Contig7657

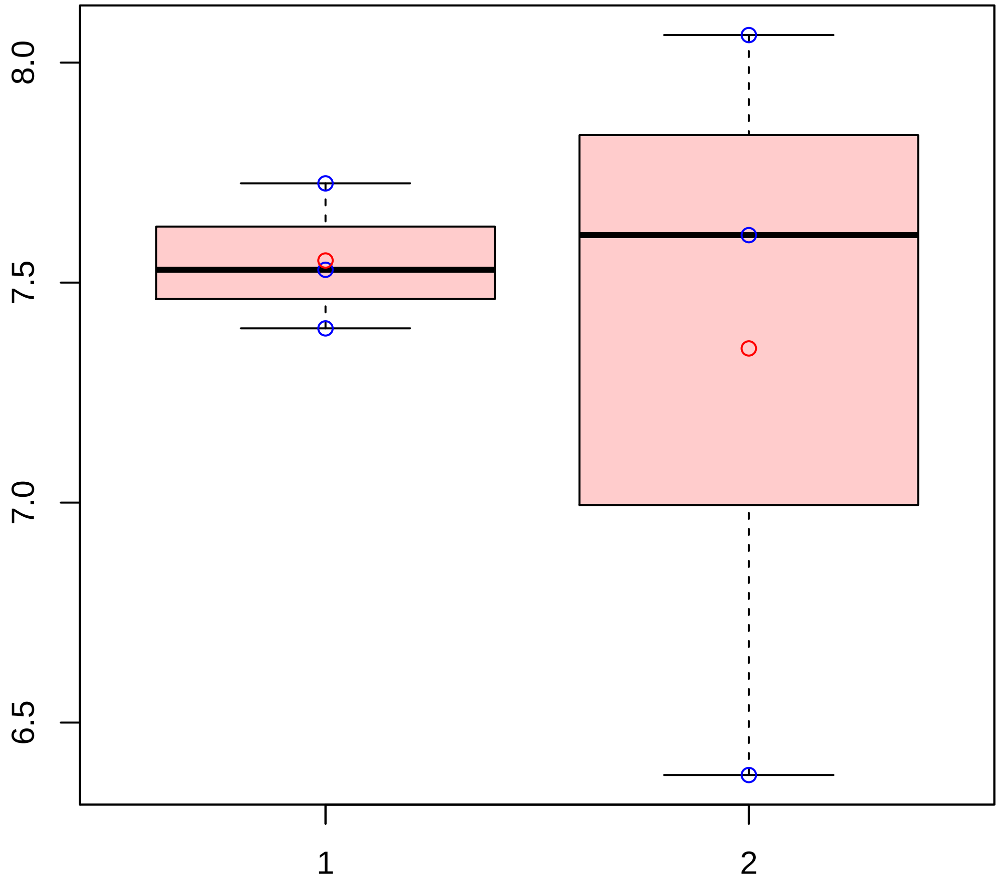


t−Test: p−value = 0.73

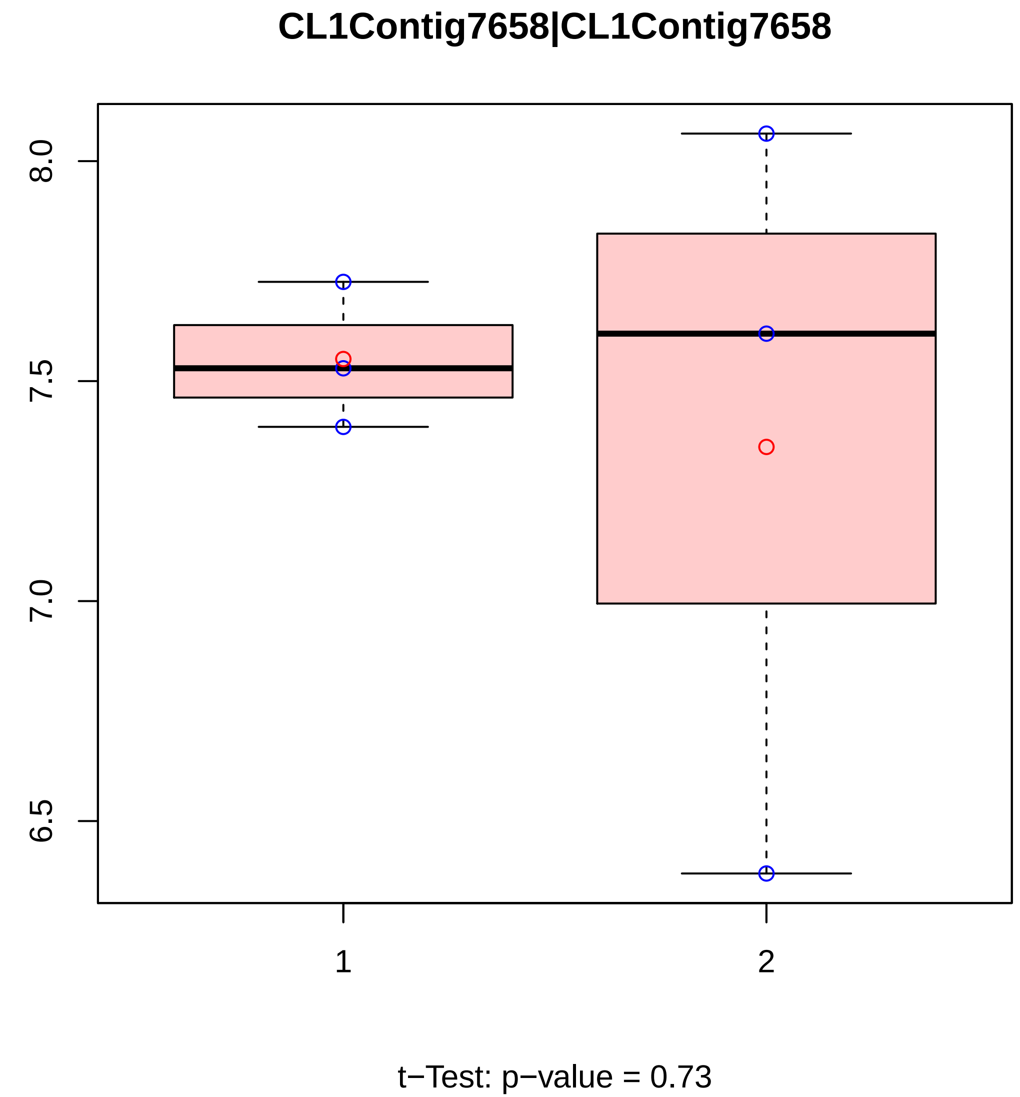
CL1Contig7658|CL1Contig7658
8.0
7.5
7.0
6.5
1
2
t−Test: p−value = 0.73

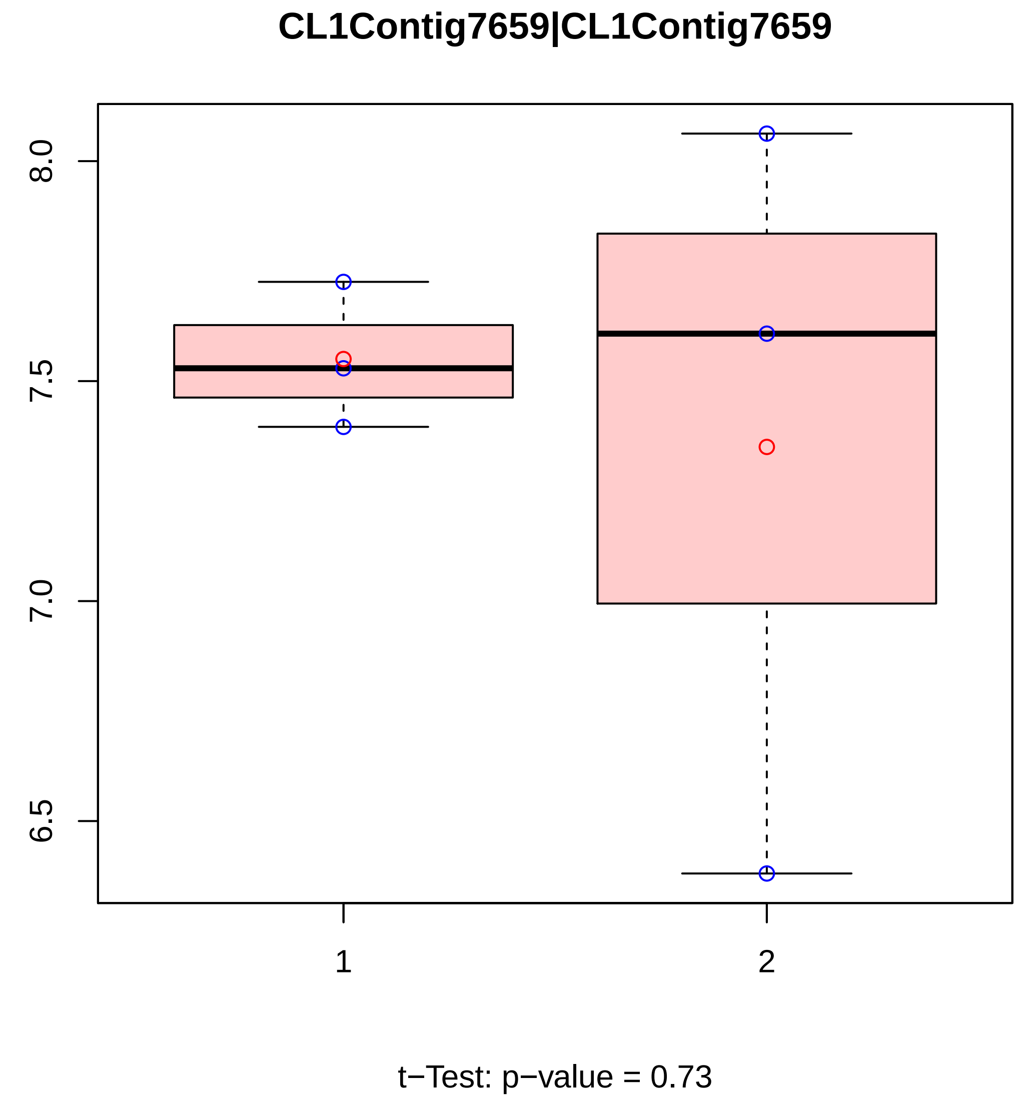
CL1Contig7659|CL1Contig7659
8.0
7.5
7.0
6.5
1
2
t−Test: p−value = 0.73

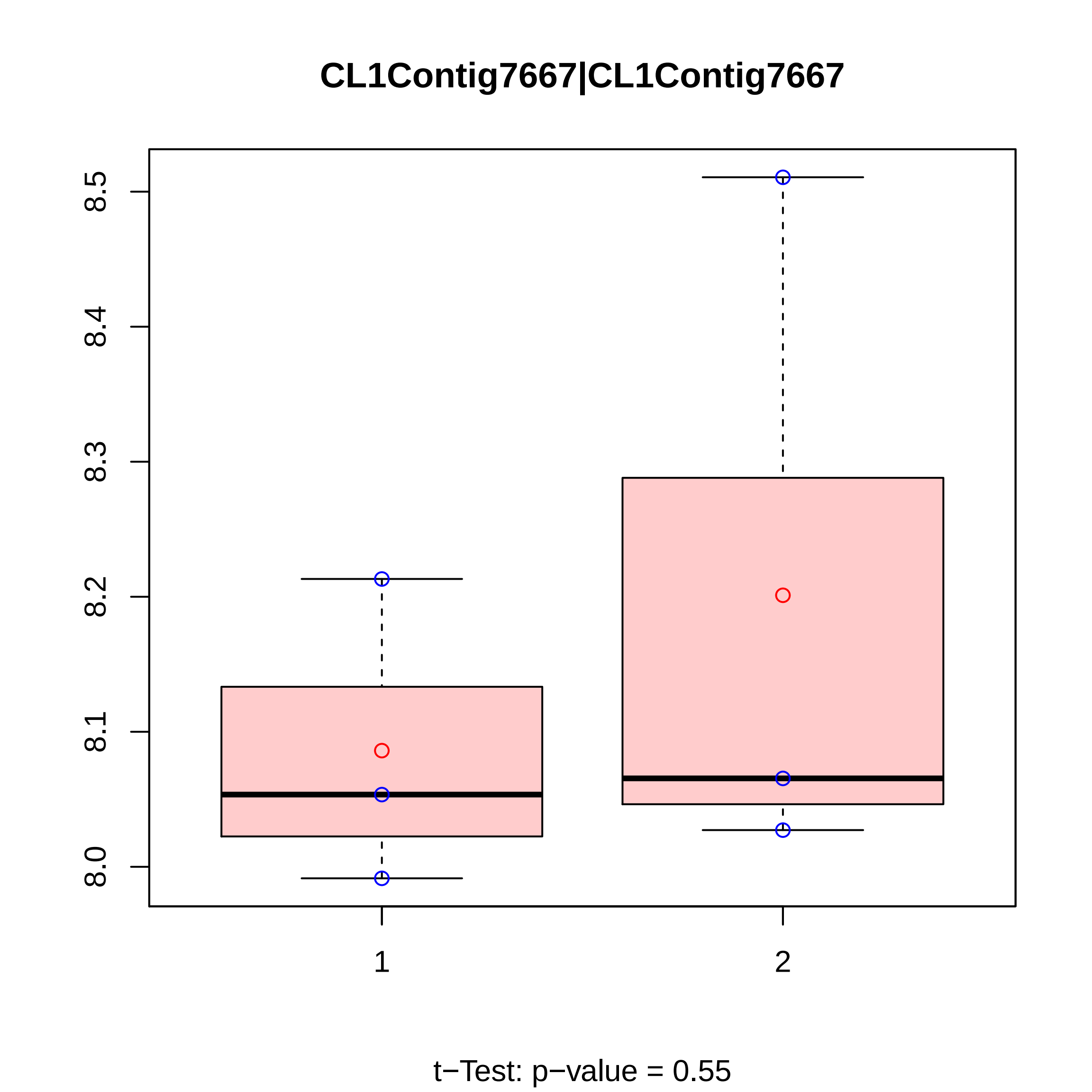

CL1Contig7667|CL1Contig7667
8.0
8.1
8.2
8.3
8.4
8.5
1
2
t−Test: p−value = 0.55

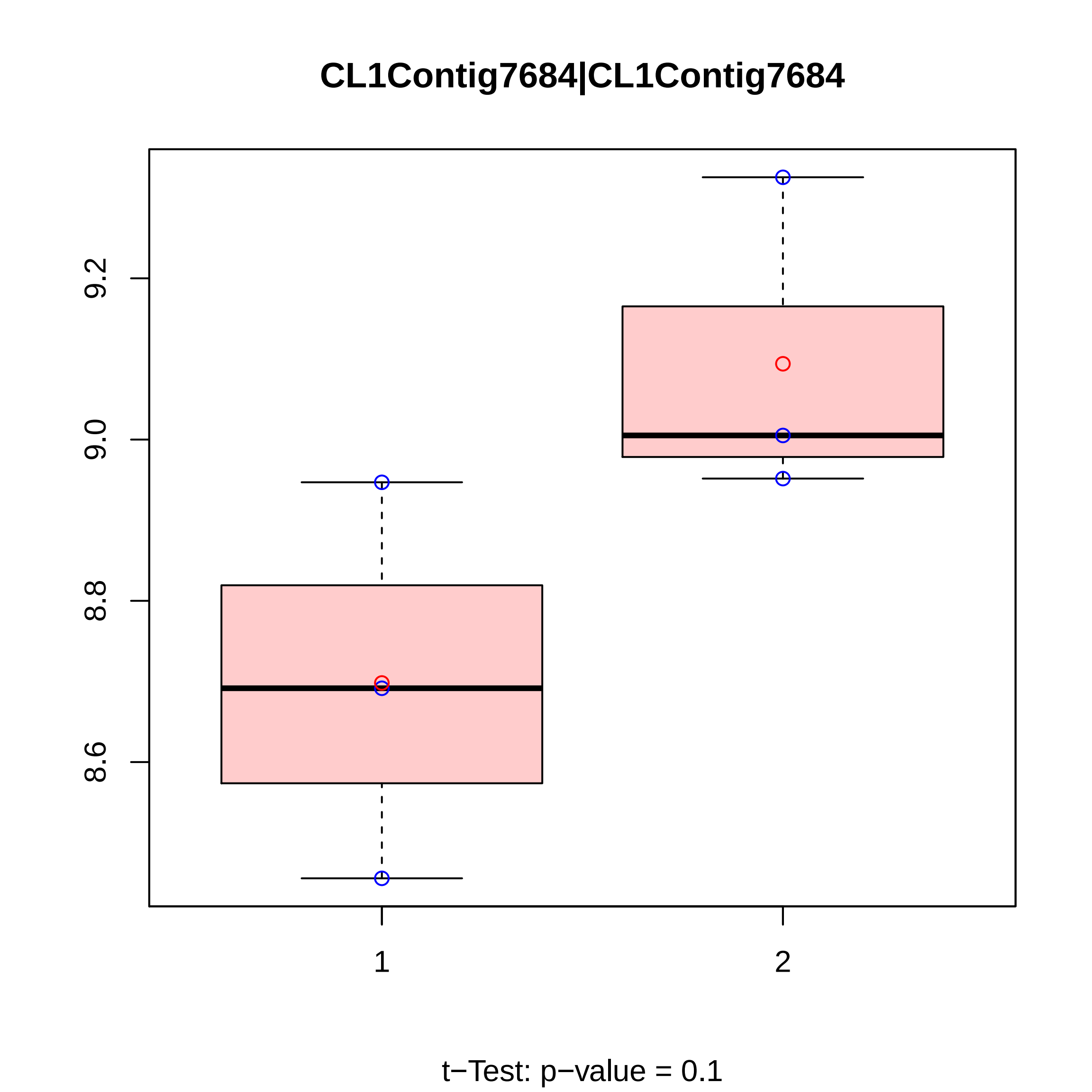
CL1Contig7684|CL1Contig7684
9.2
9.0
8.8
8.6
1
2
t−Test: p−value = 0.1

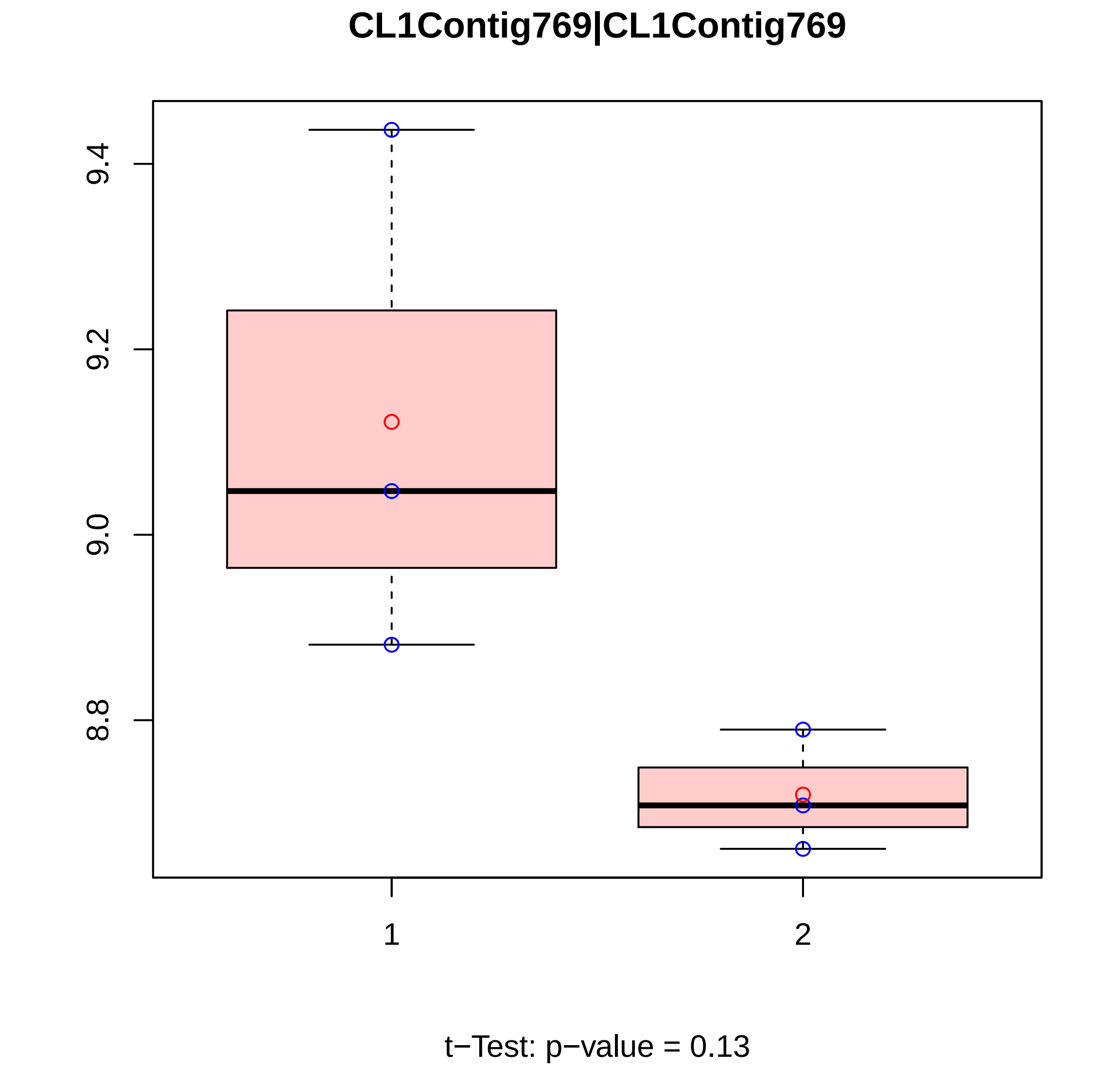

CL1Contig769|CL1Contig769
9.4
9.2
9.0
8.8
1
2
t−Test: p−value = 0.13

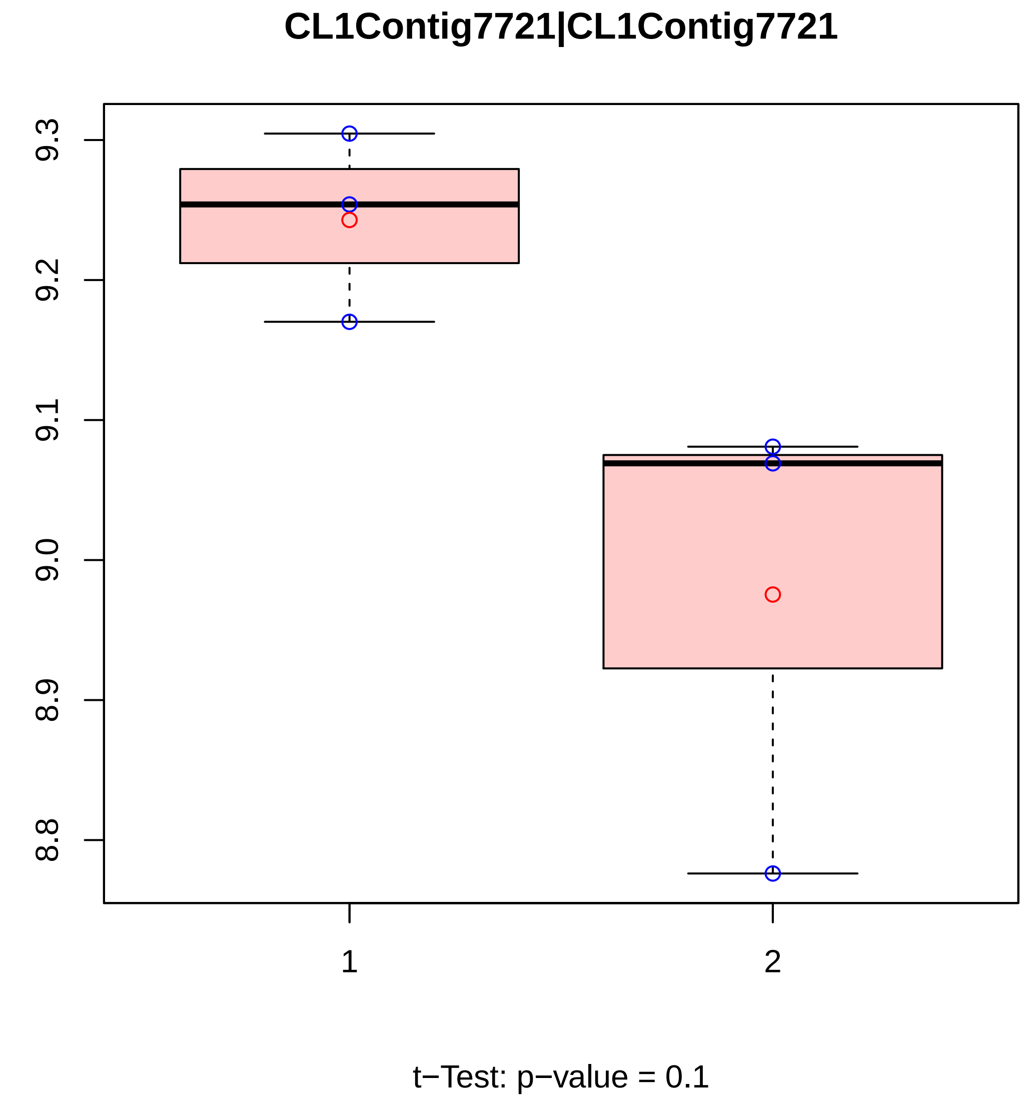
CL1Contig7721|CL1Contig7721
9.3
9.2
9.1
9.0
8.9
8.8
1
2
t−Test: p−value = 0.1

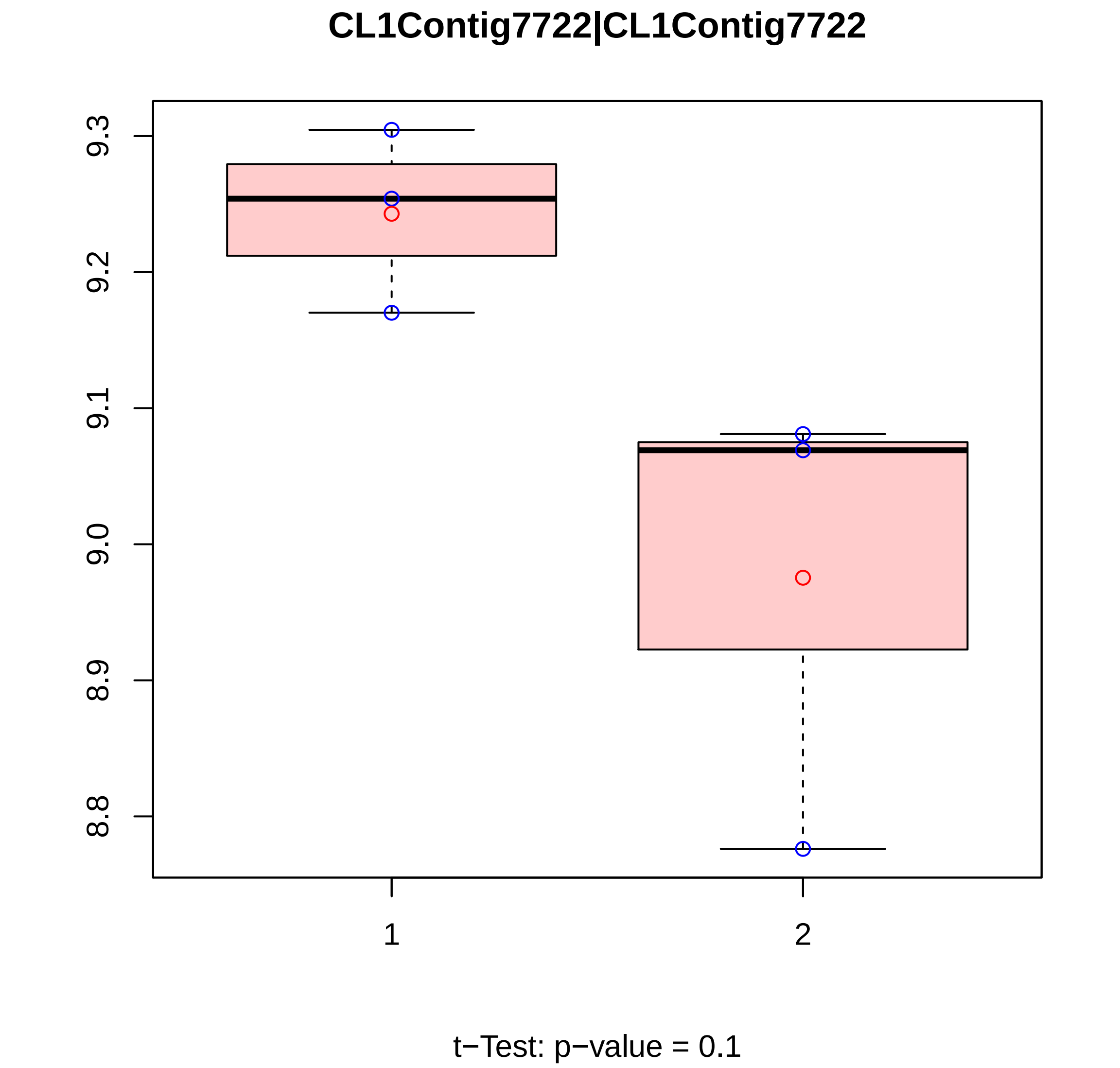
CL1Contig7722|CL1Contig7722
9.3
9.2
9.1
9.0
8.9
8.8
1
2
t−Test: p−value = 0.1

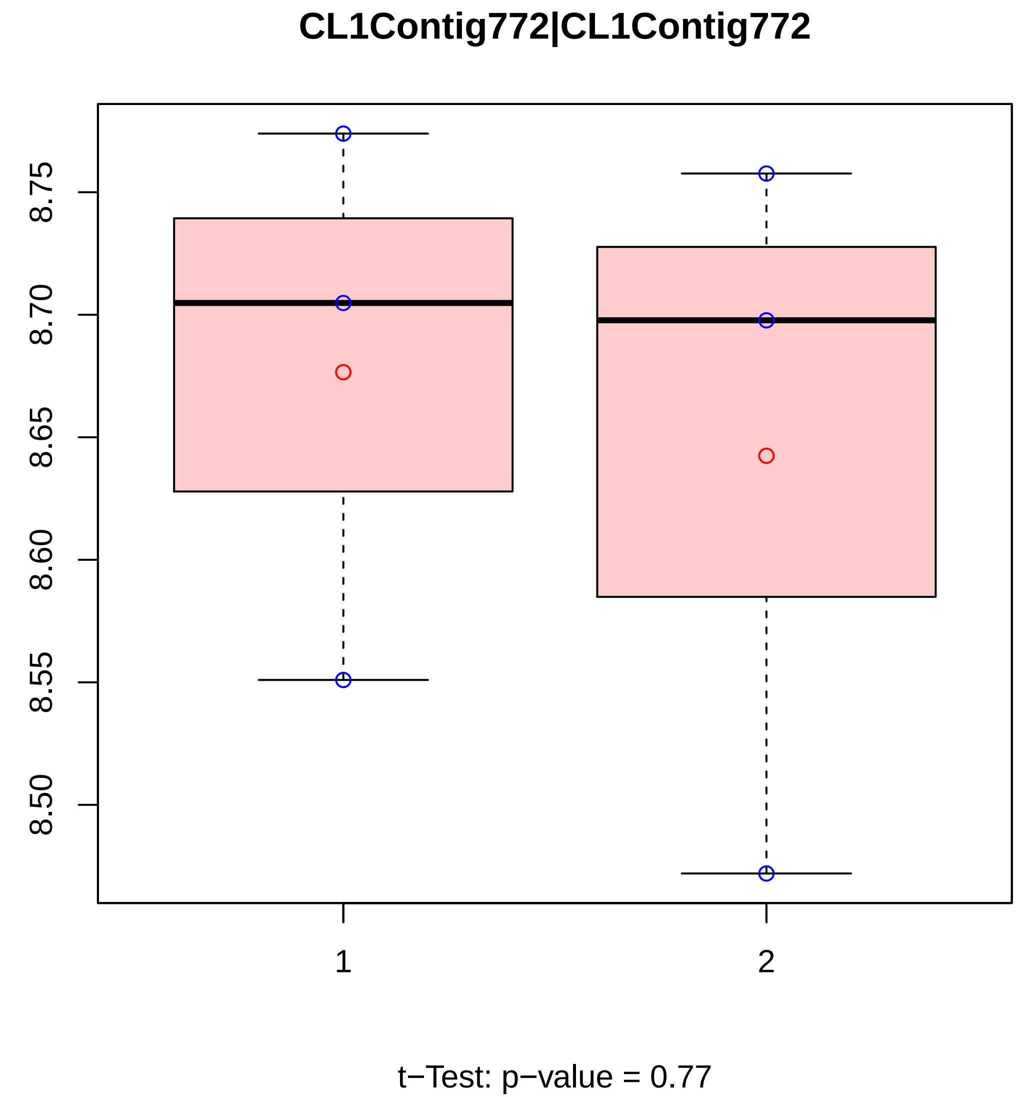

CL1Contig772|CL1Contig772
8.50
8.55
8.60
8.65
8.70
8.75
1
2
t−Test: p−value = 0.77

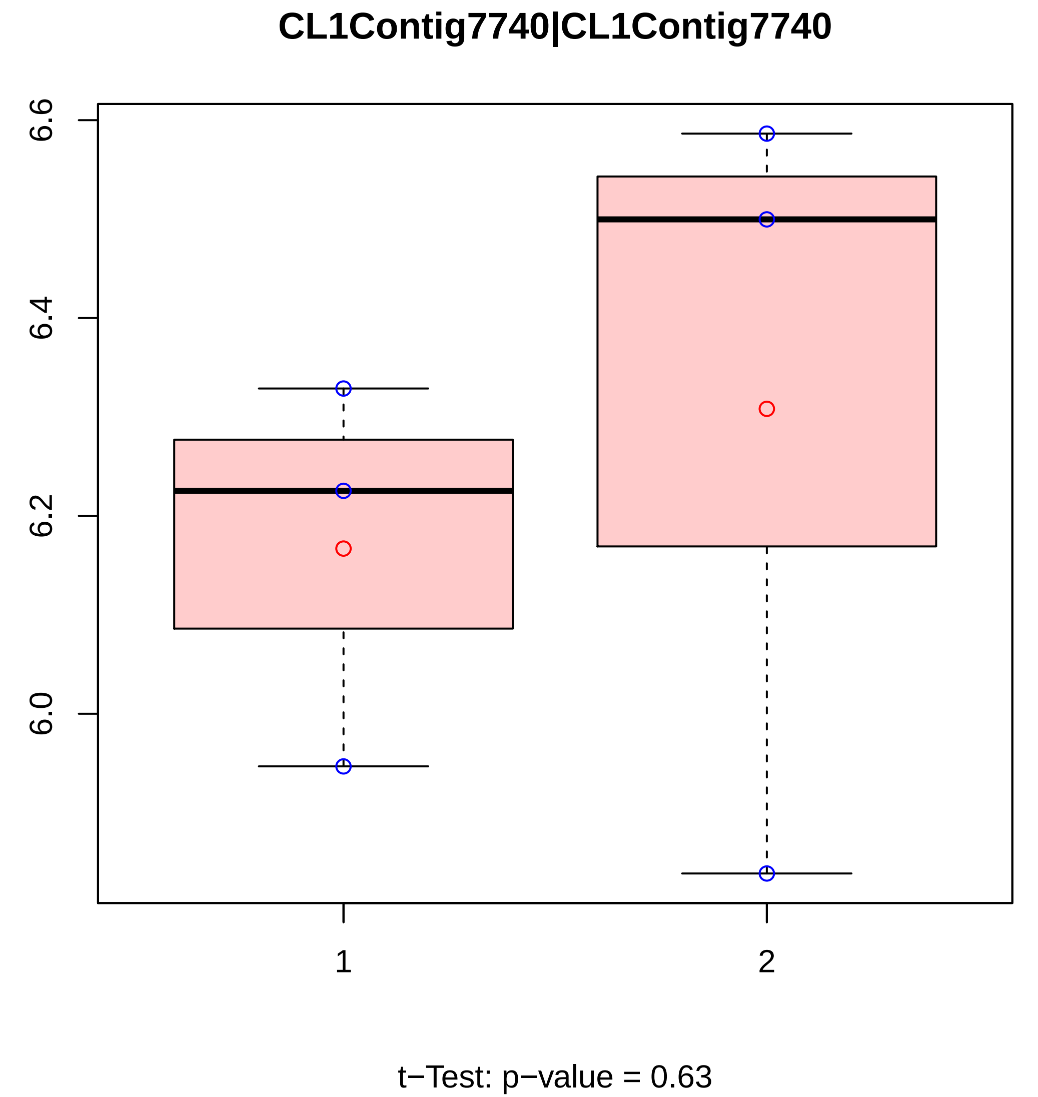
CL1Contig7740|CL1Contig7740
6.0
6.2
6.4
6.6
1
2
t−Test: p−value = 0.63

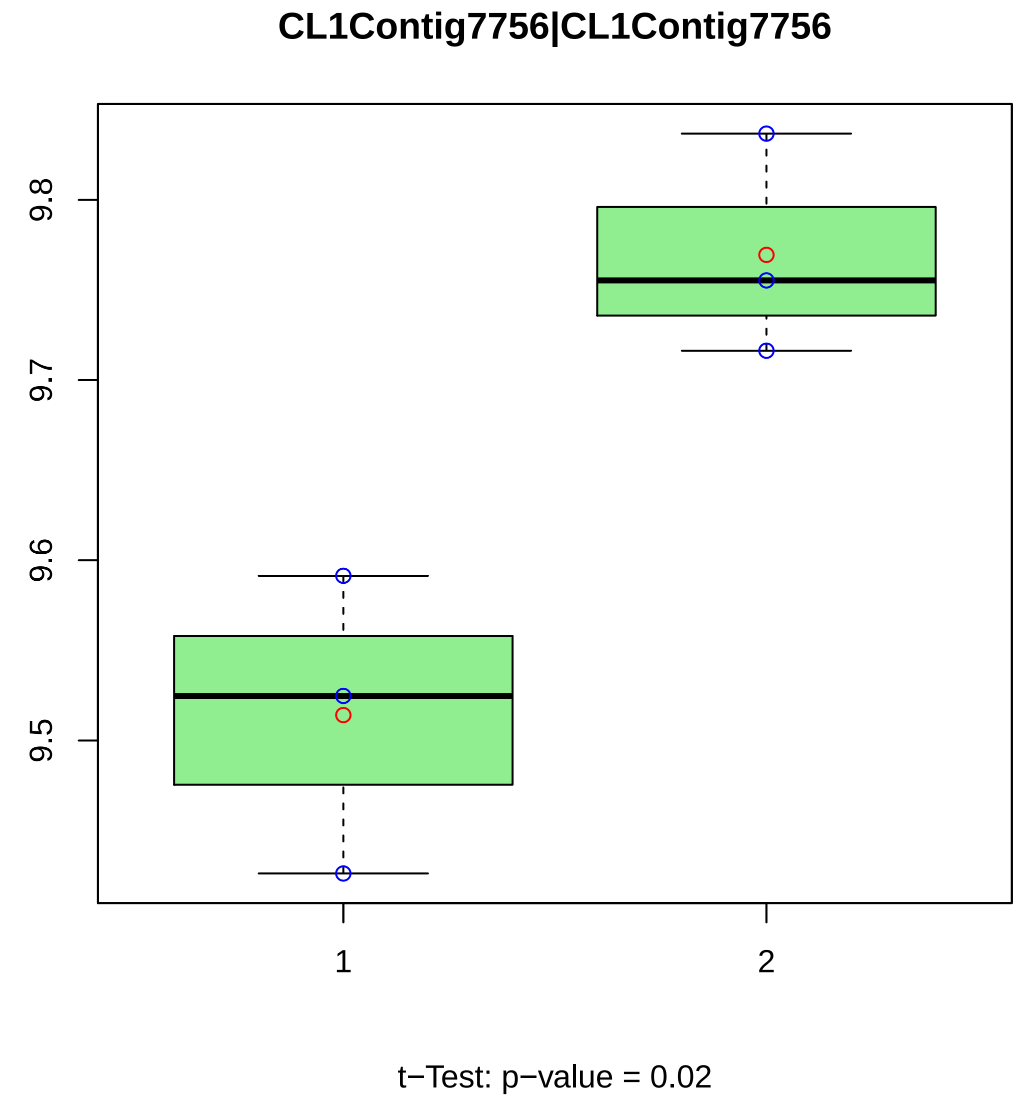
CL1Contig7756|CL1Contig7756
9.5
9.6
9.7
9.8
1
2
t−Test: p−value = 0.02

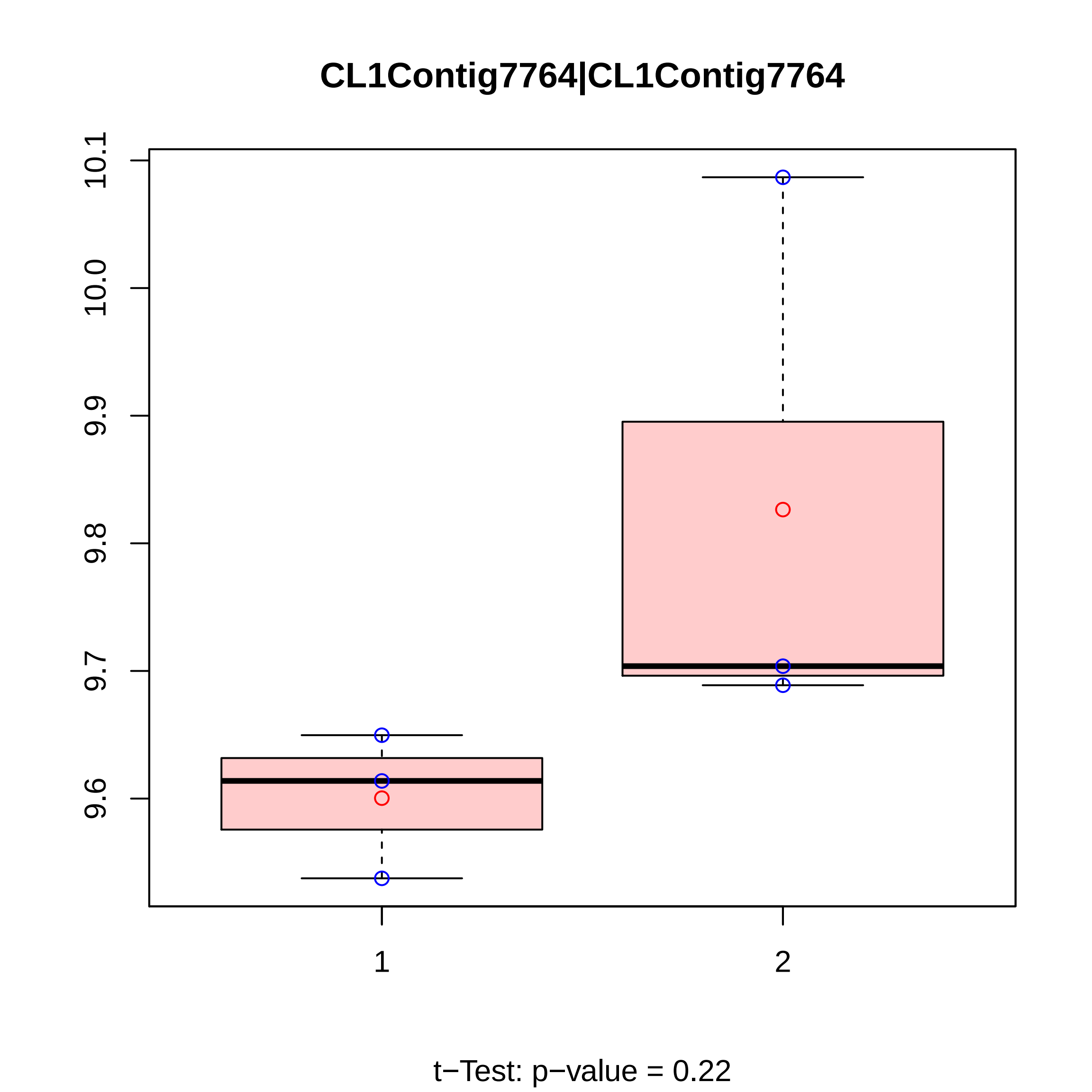

CL1Contig7764|CL1Contig7764
10.1
10.0
9.9
9.8
9.7
9.6
1
2
t−Test: p−value = 0.22

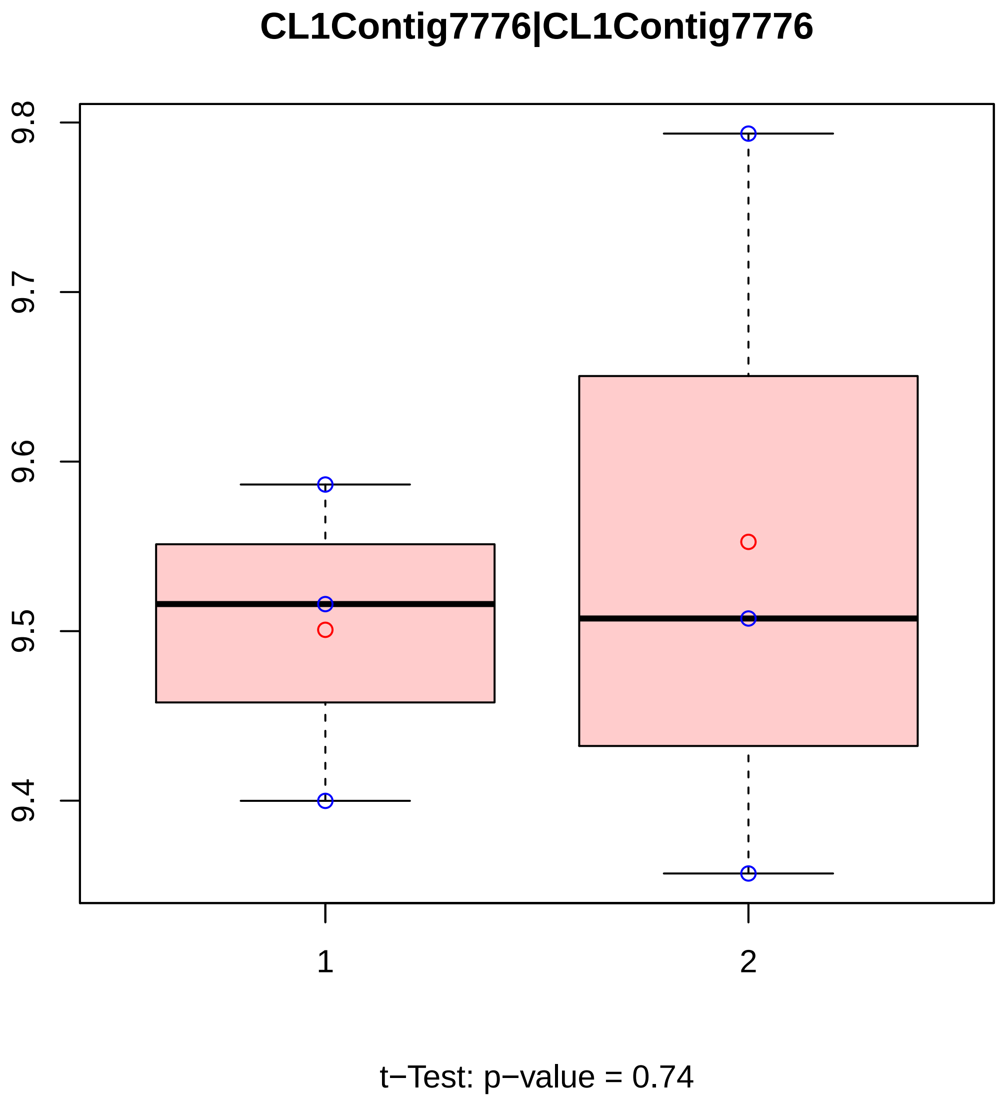
CL1Contig7776|CL1Contig7776
9.8
9.7
9.6
9.5
9.4
1
2
t−Test: p−value = 0.74

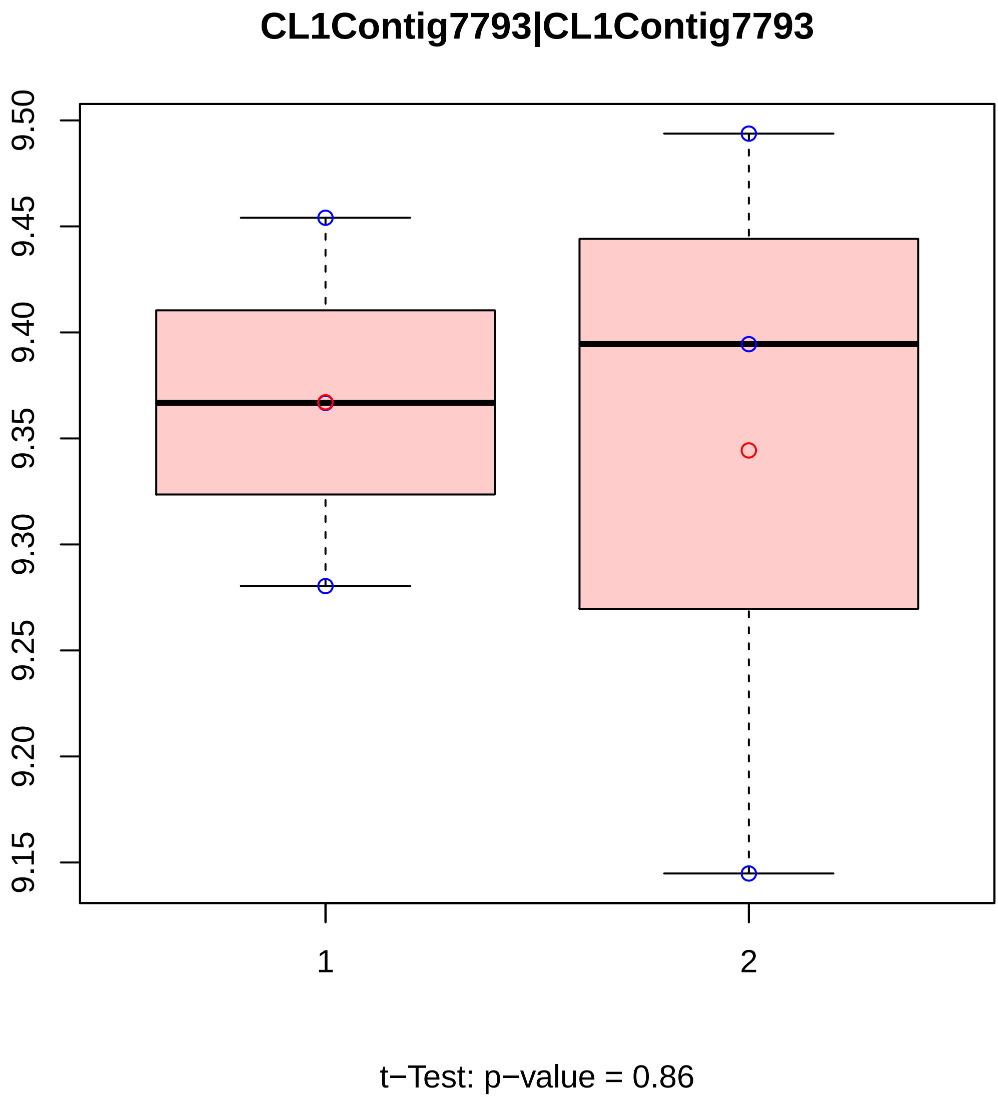
CL1Contig7793|CL1Contig7793
9.50
9.45
9.40
9.35
9.30
9.25
9.20
9.15
1
2
t−Test: p−value = 0.86

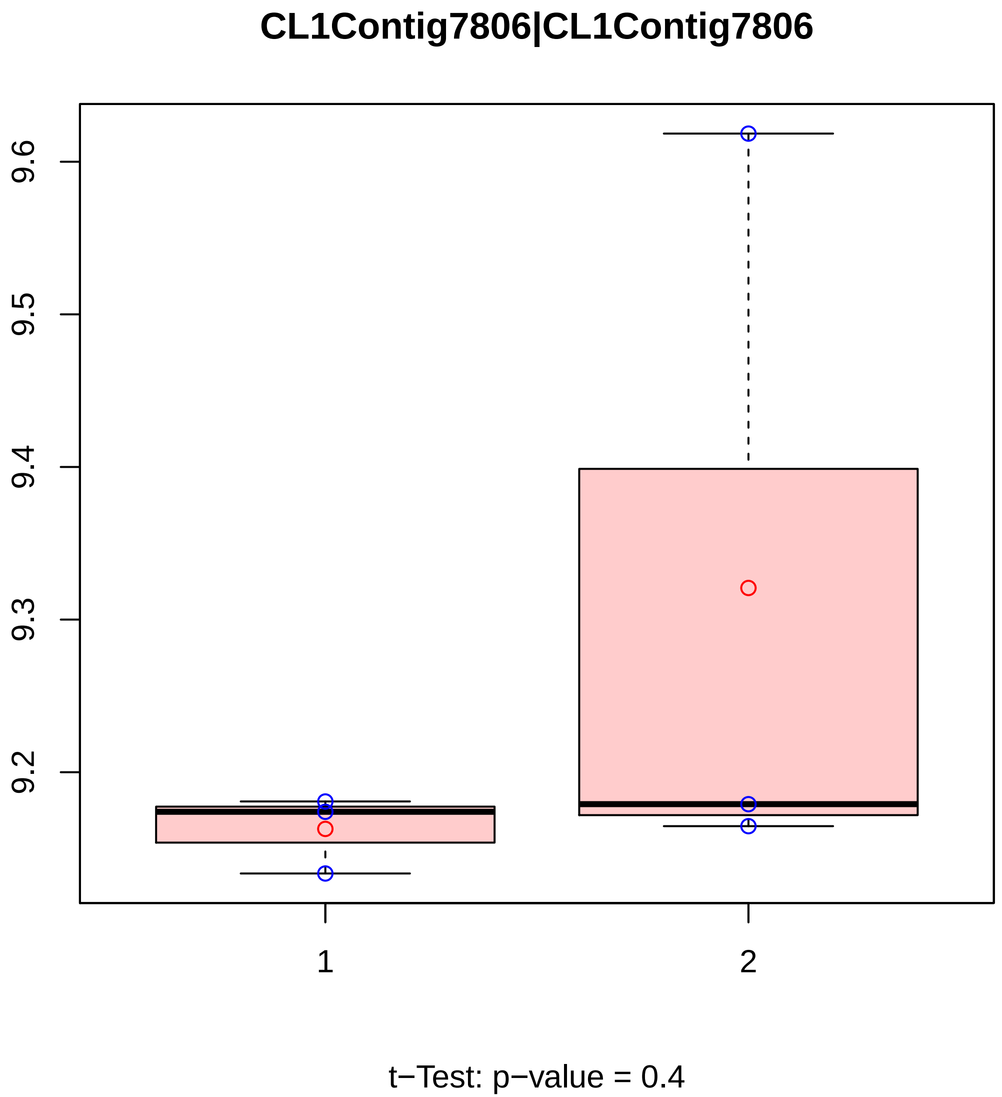
CL1Contig7806|CL1Contig7806
9.2
9.3
9.4
9.5
9.6
1
2
t−Test: p−value = 0.4

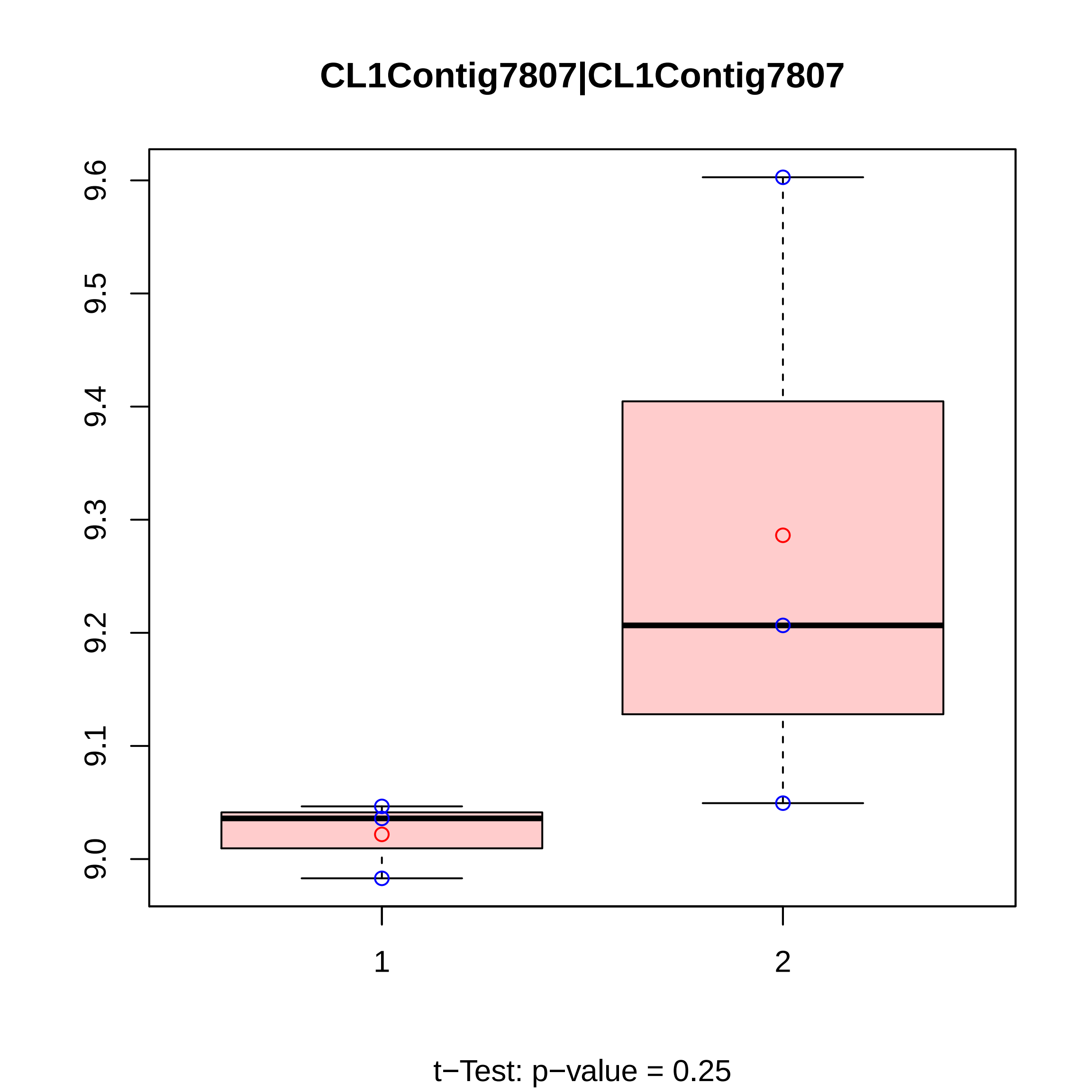

CL1Contig7807|CL1Contig7807
9.0
9.1
9.2
9.3
9.4
9.5
9.6
1
2
t−Test: p−value = 0.25

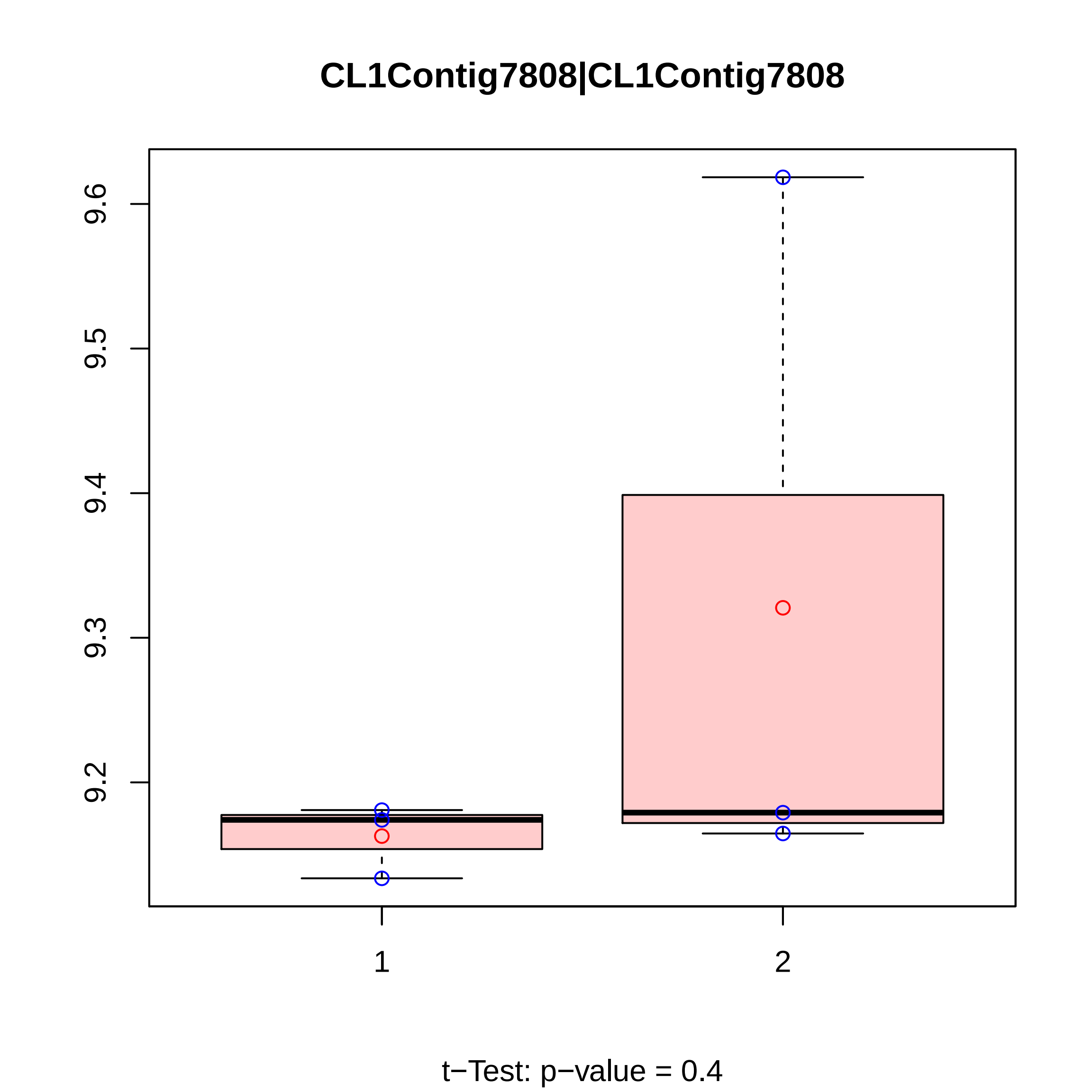
CL1Contig7808|CL1Contig7808
9.6
9.5
9.4
9.3
9.2
1
2
t−Test: p−value = 0.4

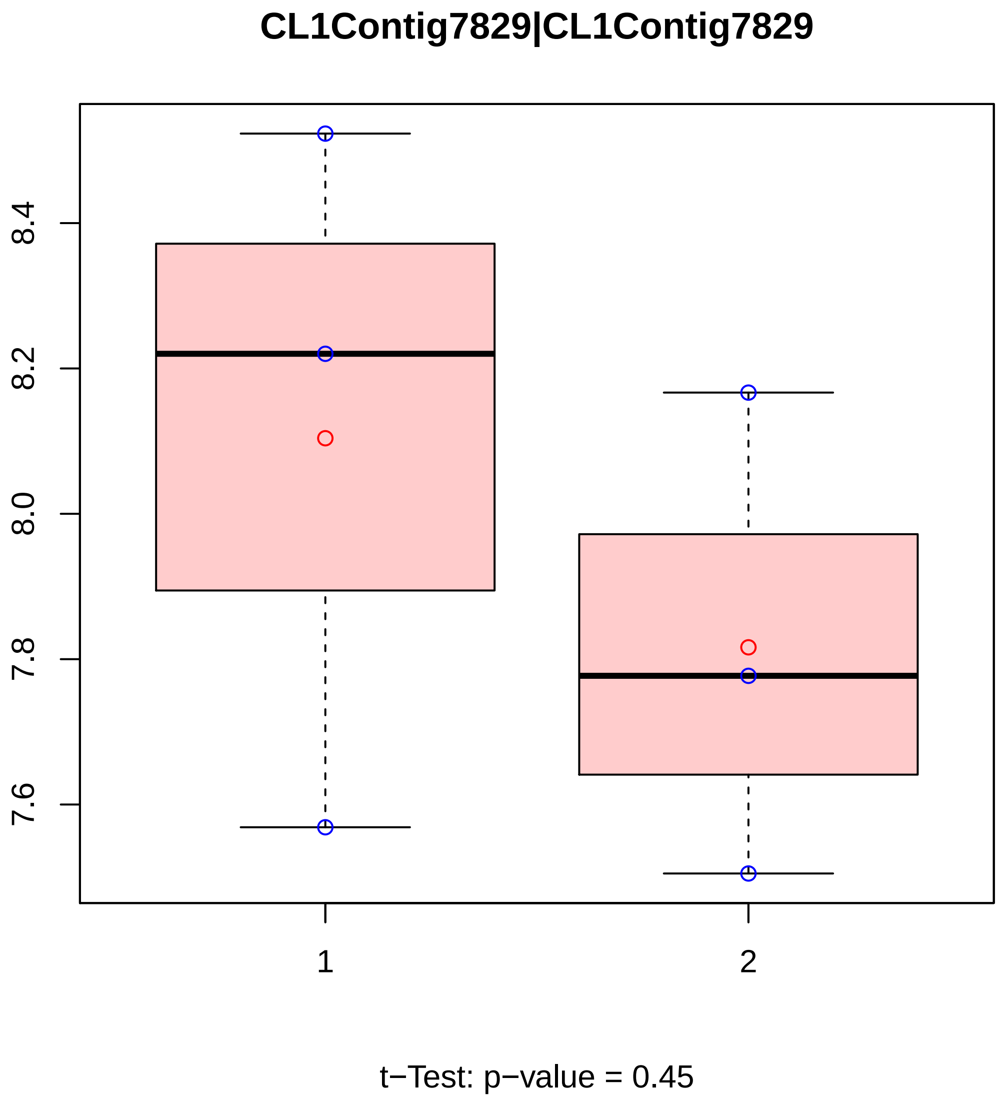
CL1Contig7829|CL1Contig7829
8.4
8.2
8.0
7.8
7.6
1
2
t−Test: p−value = 0.45

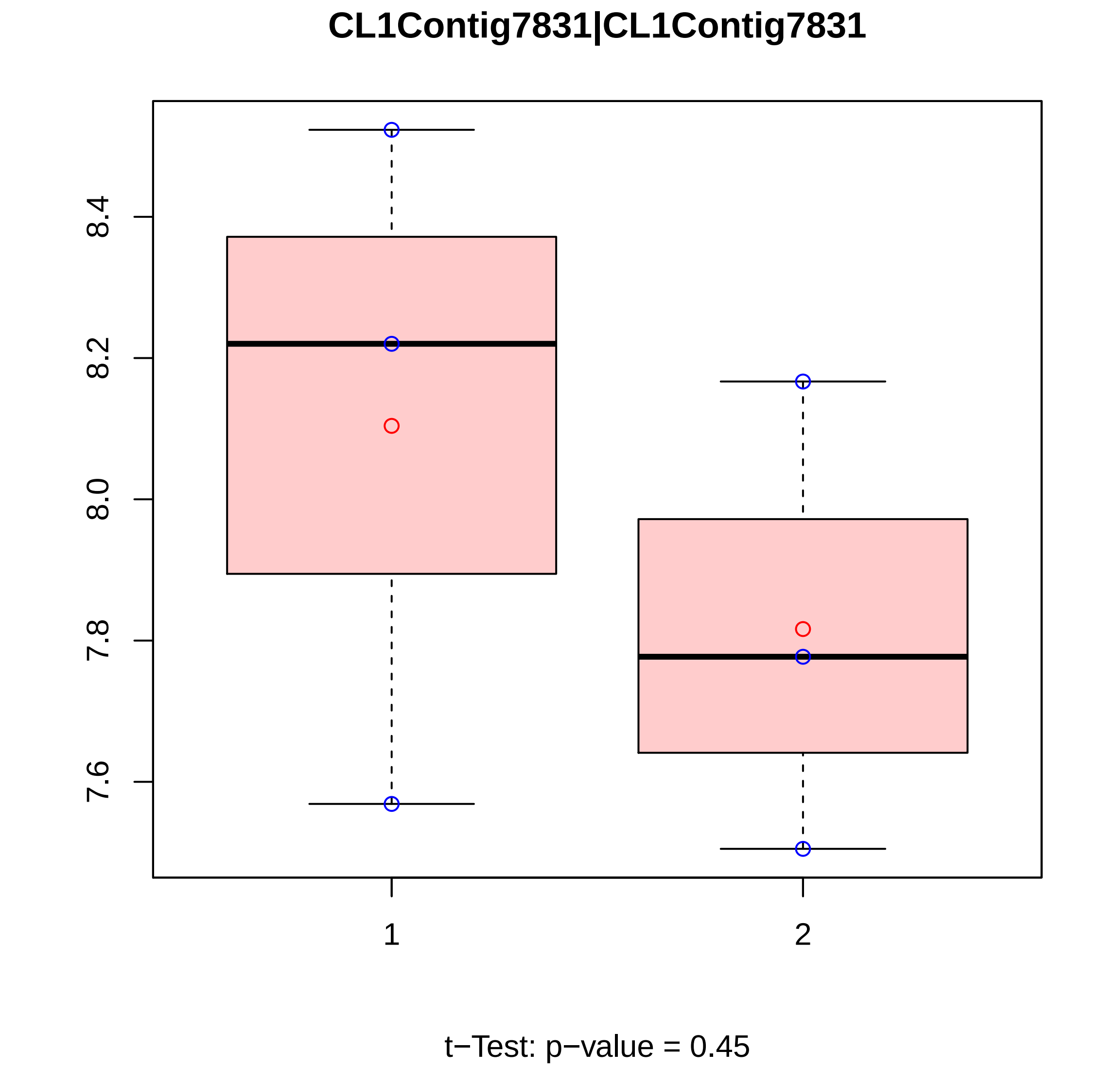

CL1Contig7831|CL1Contig7831
8.4
8.2
8.0
7.8
7.6
1
2
t−Test: p−value = 0.45

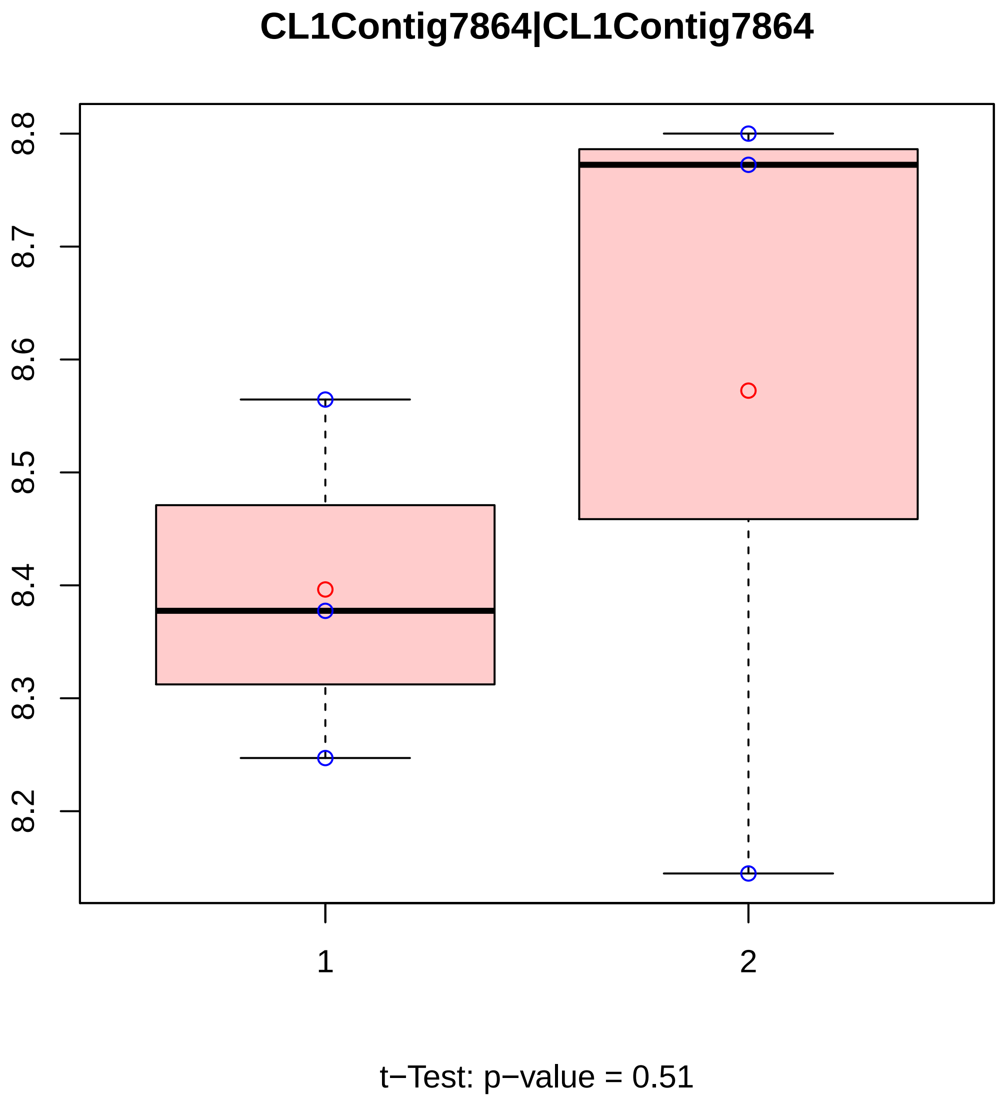
CL1Contig7864|CL1Contig7864
8.2
8.3
8.4
8.5
8.6
8.7
8.8
1
2
t−Test: p−value = 0.51

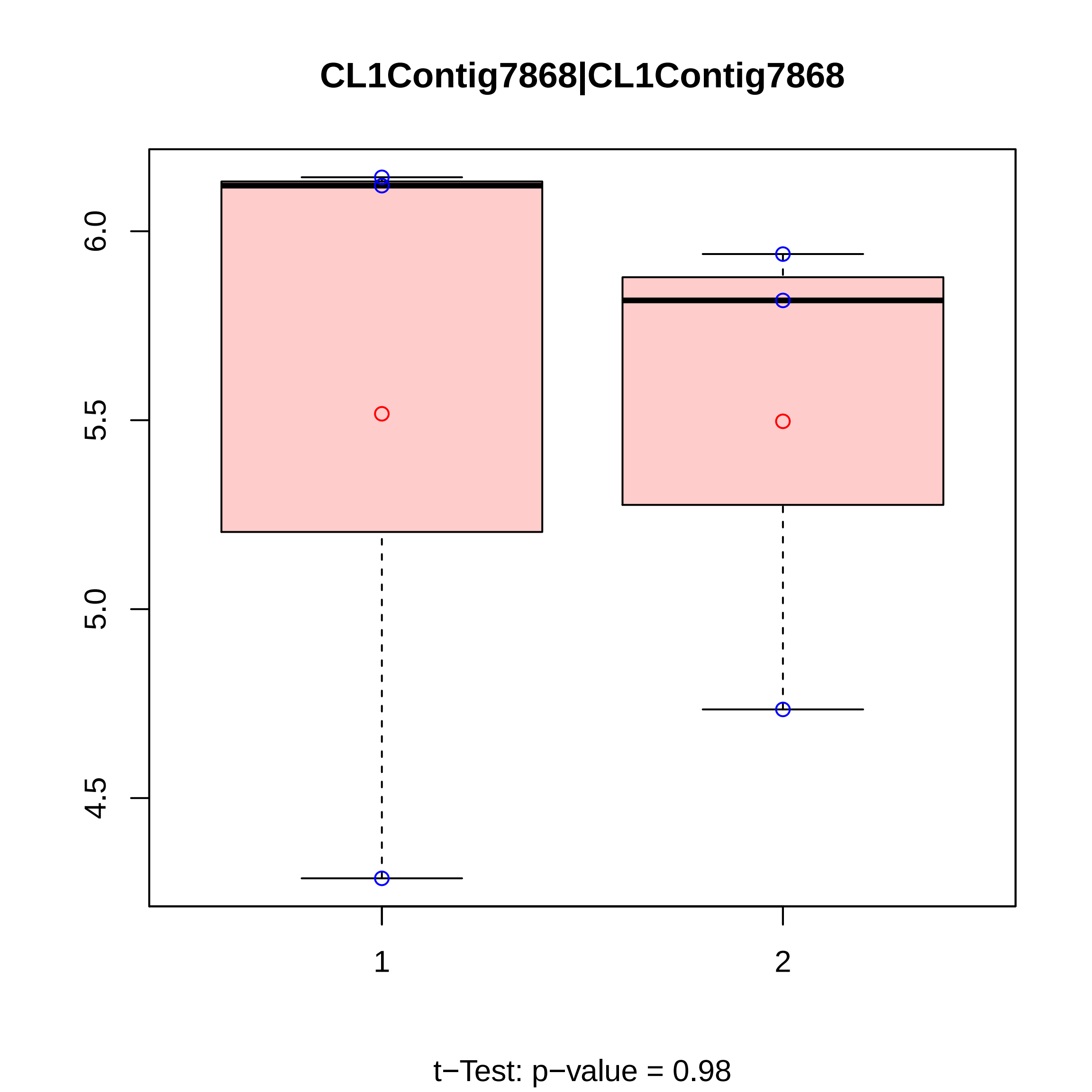

CL1Contig7868|CL1Contig7868
6.0
5.5
5.0
4.5
1
2
t−Test: p−value = 0.98

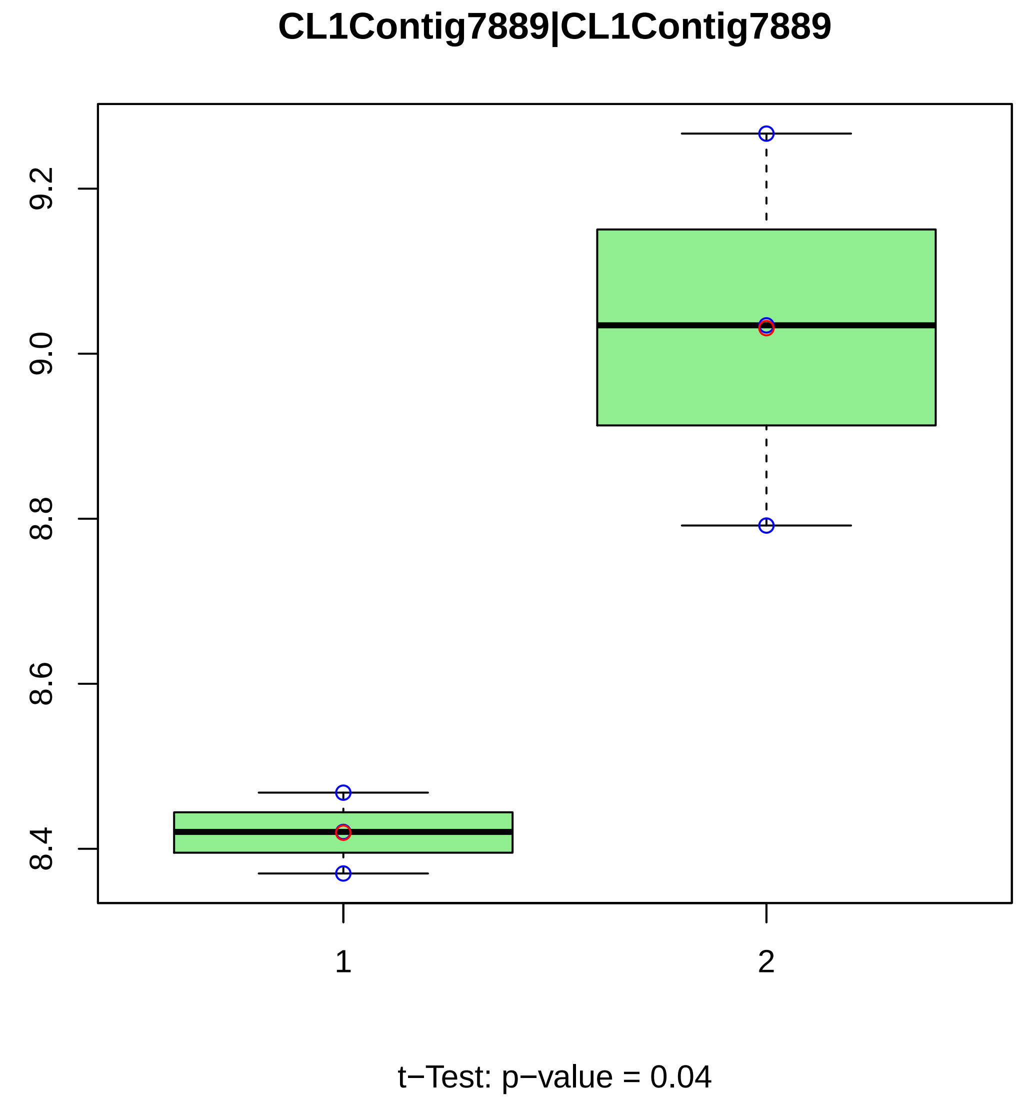
CL1Contig7889|CL1Contig7889
9.2
9.0
8.8
8.6
8.4
1
2
t−Test: p−value = 0.04

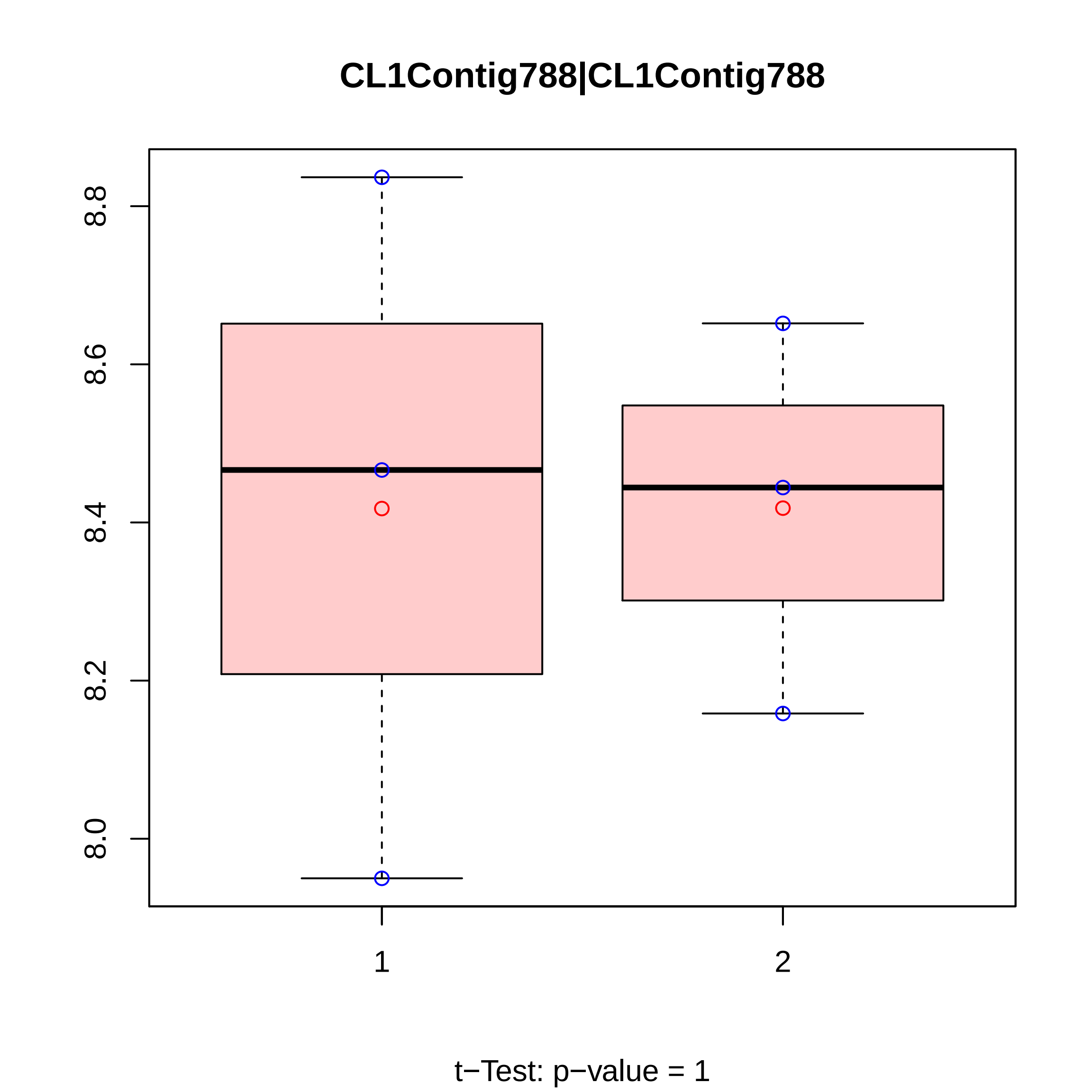

CL1Contig788|CL1Contig788
8.0
8.2
8.4
8.6
8.8
1
2
t−Test: p−value = 1

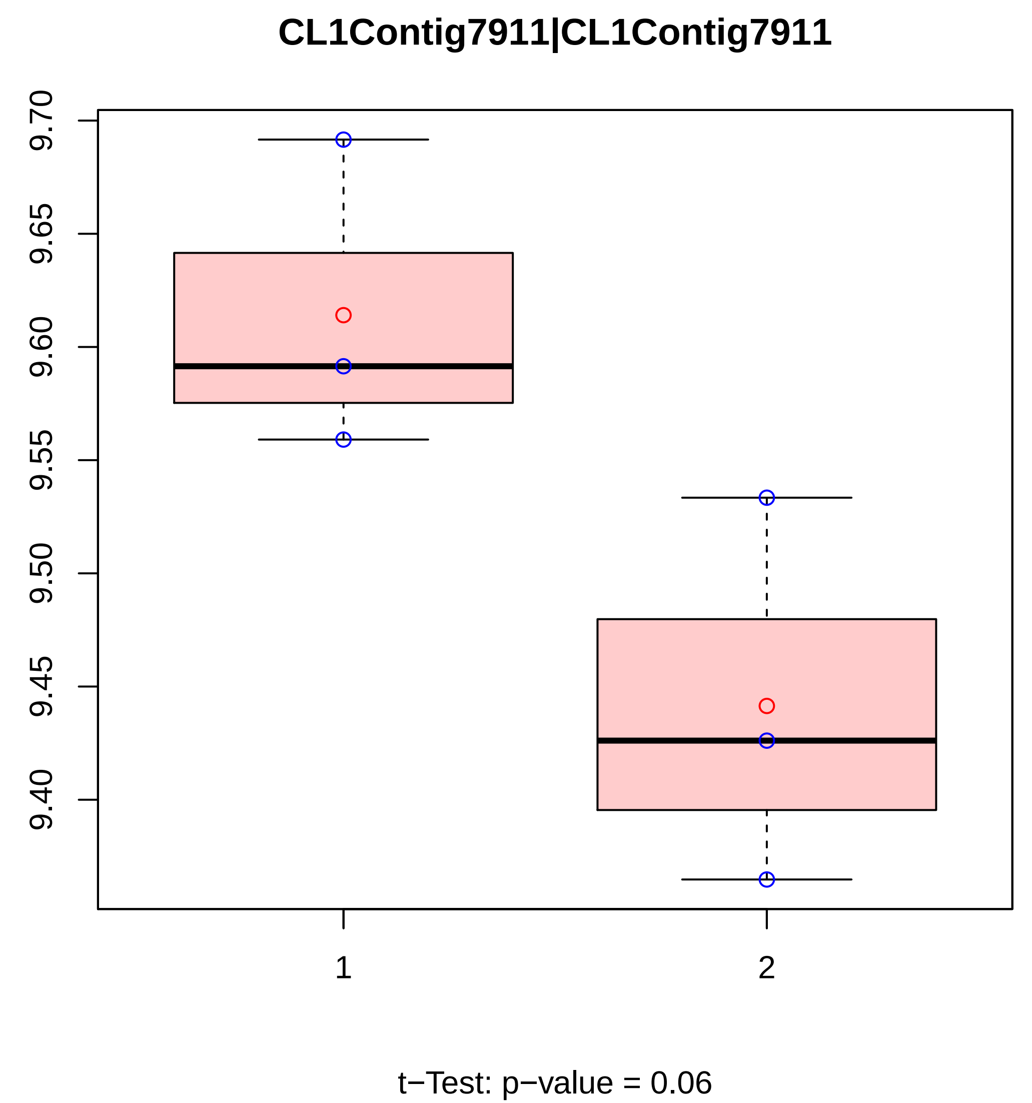
CL1Contig7911|CL1Contig7911
9.70
9.65
9.60
9.55
9.50
9.45
9.40
1
2
t−Test: p−value = 0.06

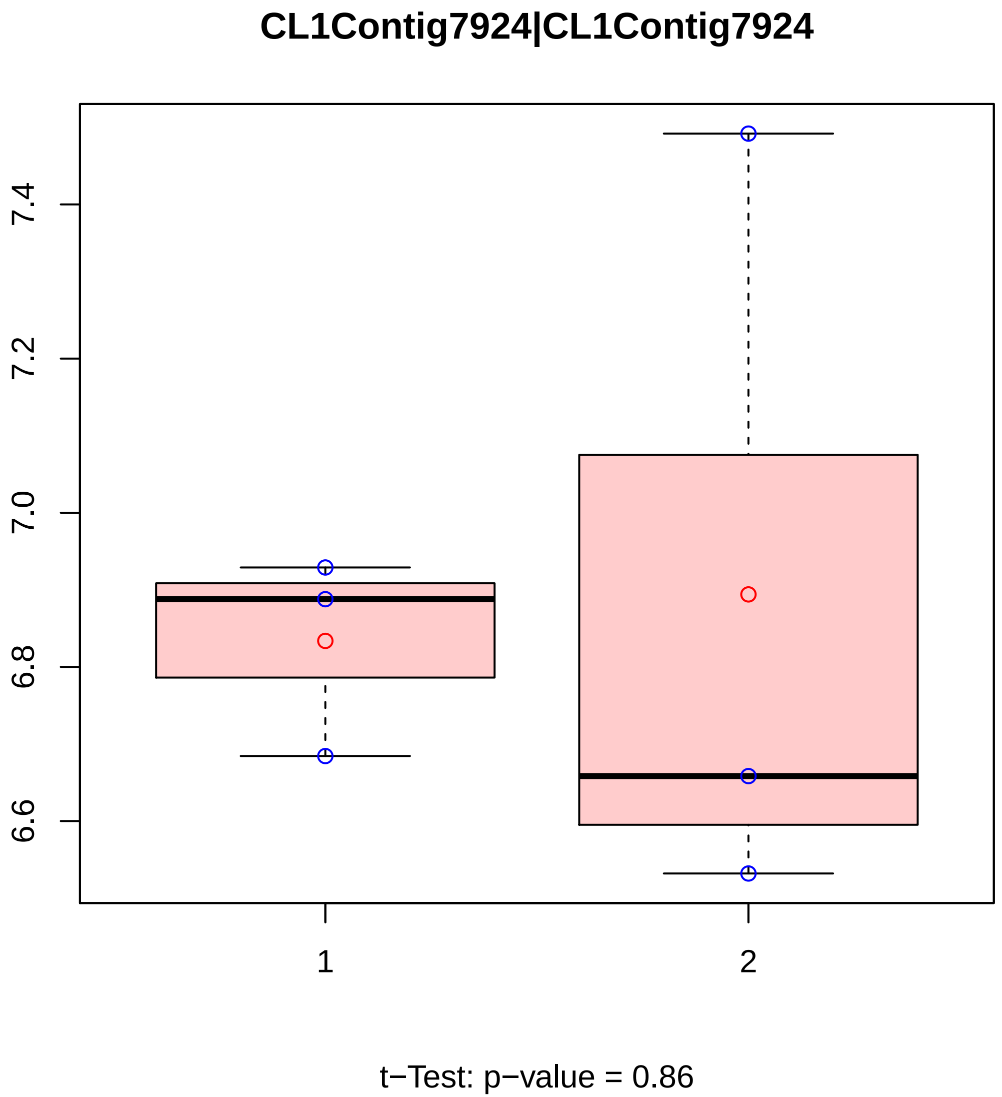
CL1Contig7924|CL1Contig7924
7.4
7.2
7.0
6.8
6.6
1
2
t−Test: p−value = 0.86

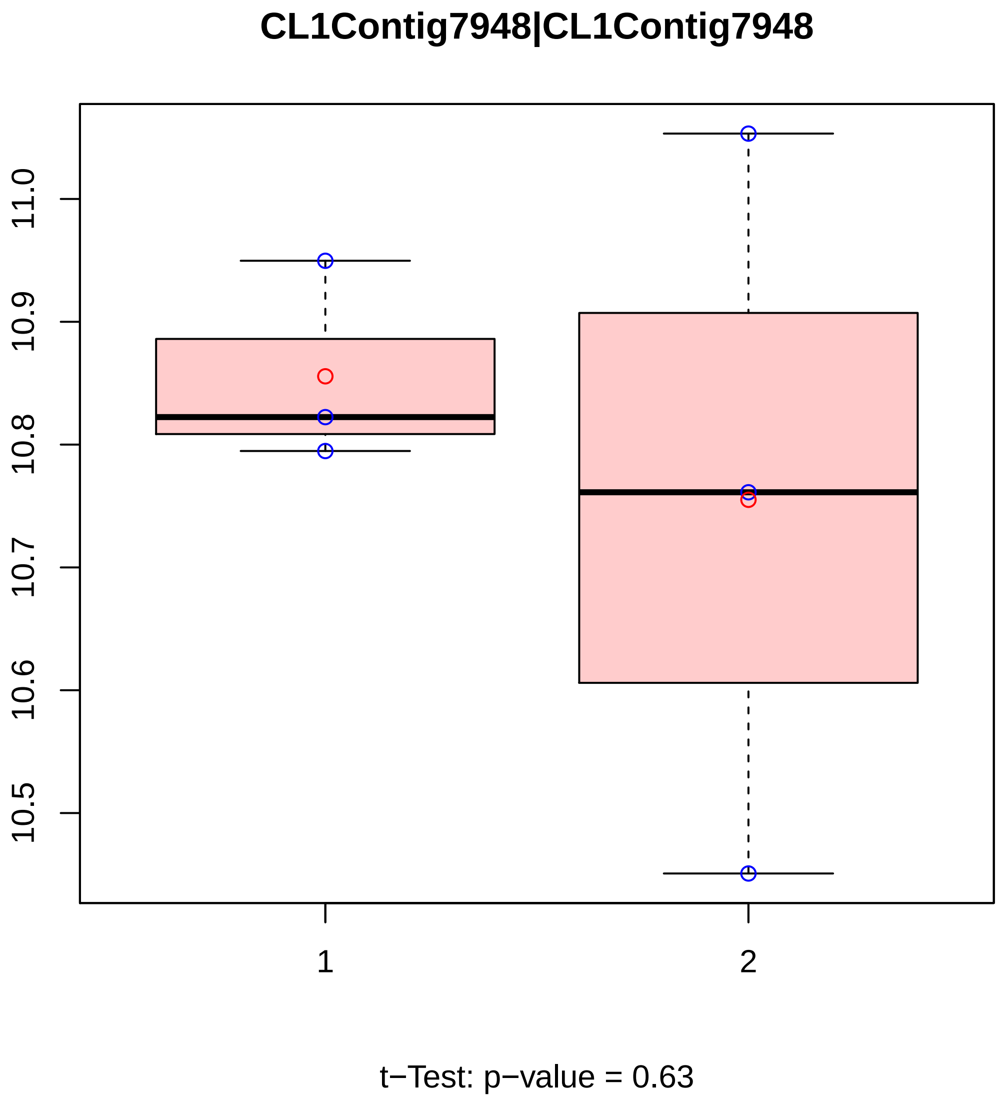
CL1Contig7948|CL1Contig7948
10.5
10.6
10.7
10.8
10.9
11.0
1
2
t−Test: p−value = 0.63

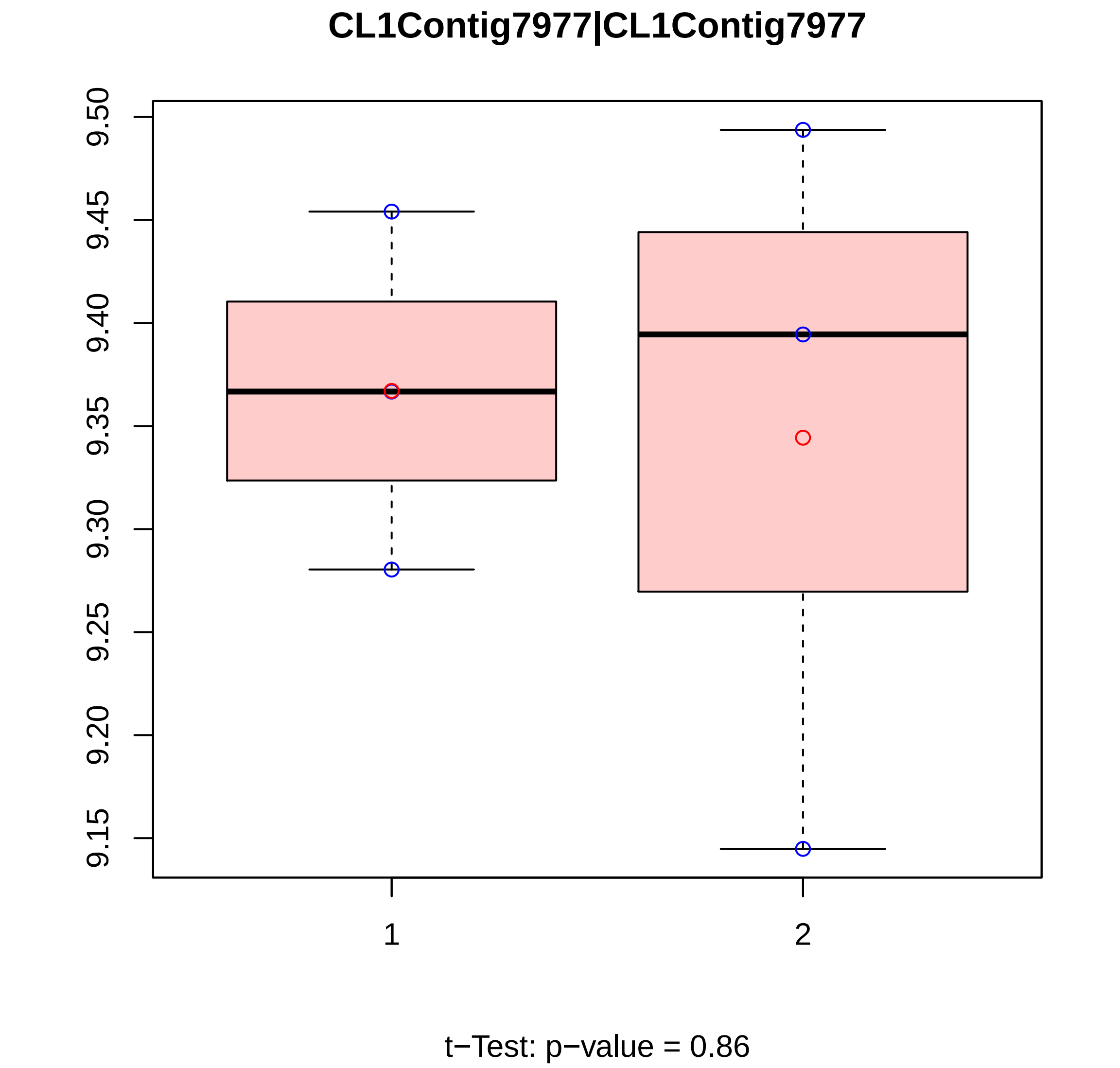
CL1Contig7977|CL1Contig7977
9.50
9.45
9.40
9.35
9.30
9.25
9.20
9.15
1
2
t−Test: p−value = 0.86

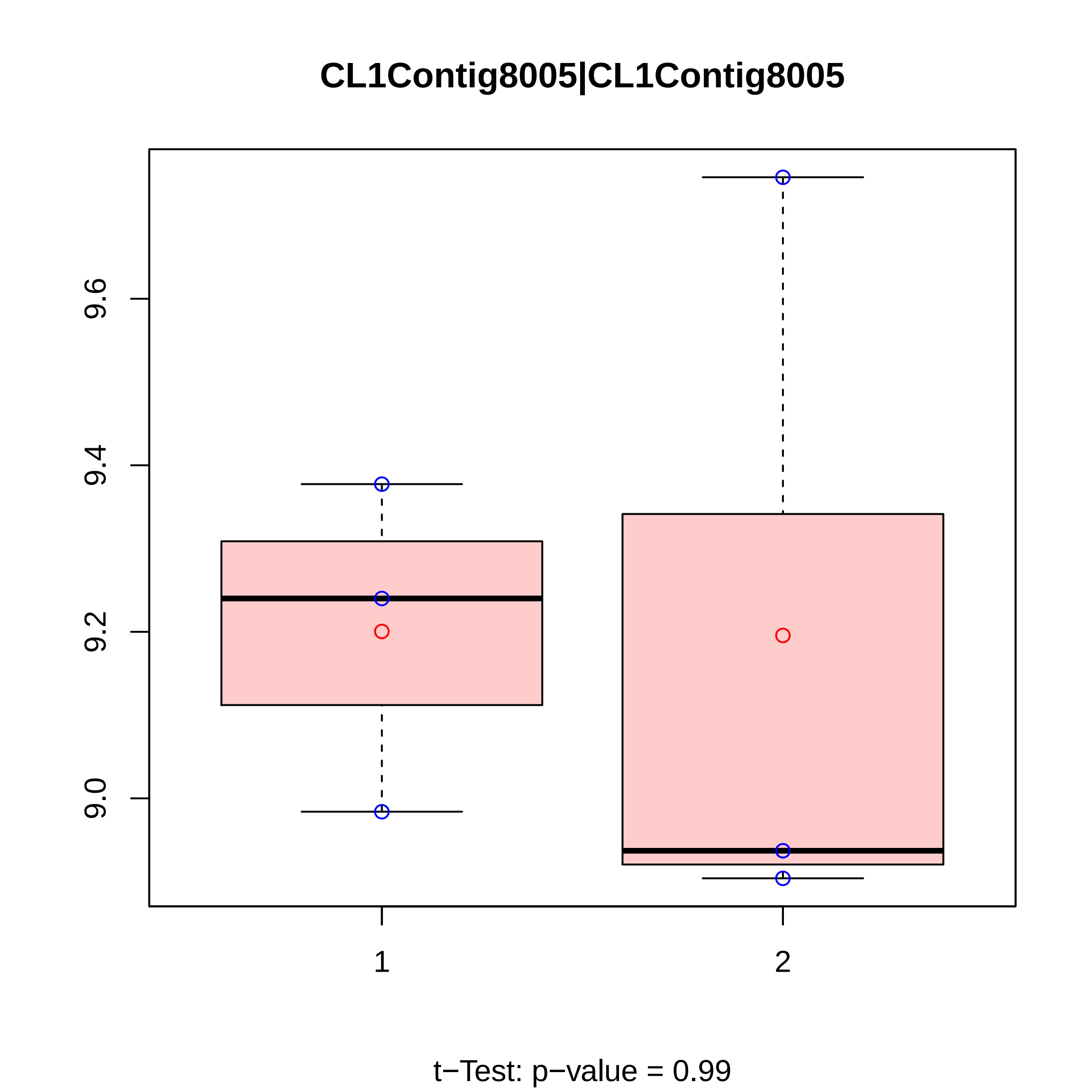

CL1Contig8005|CL1Contig8005
9.0
9.2
9.4
9.6
1
2
t−Test: p−value = 0.99

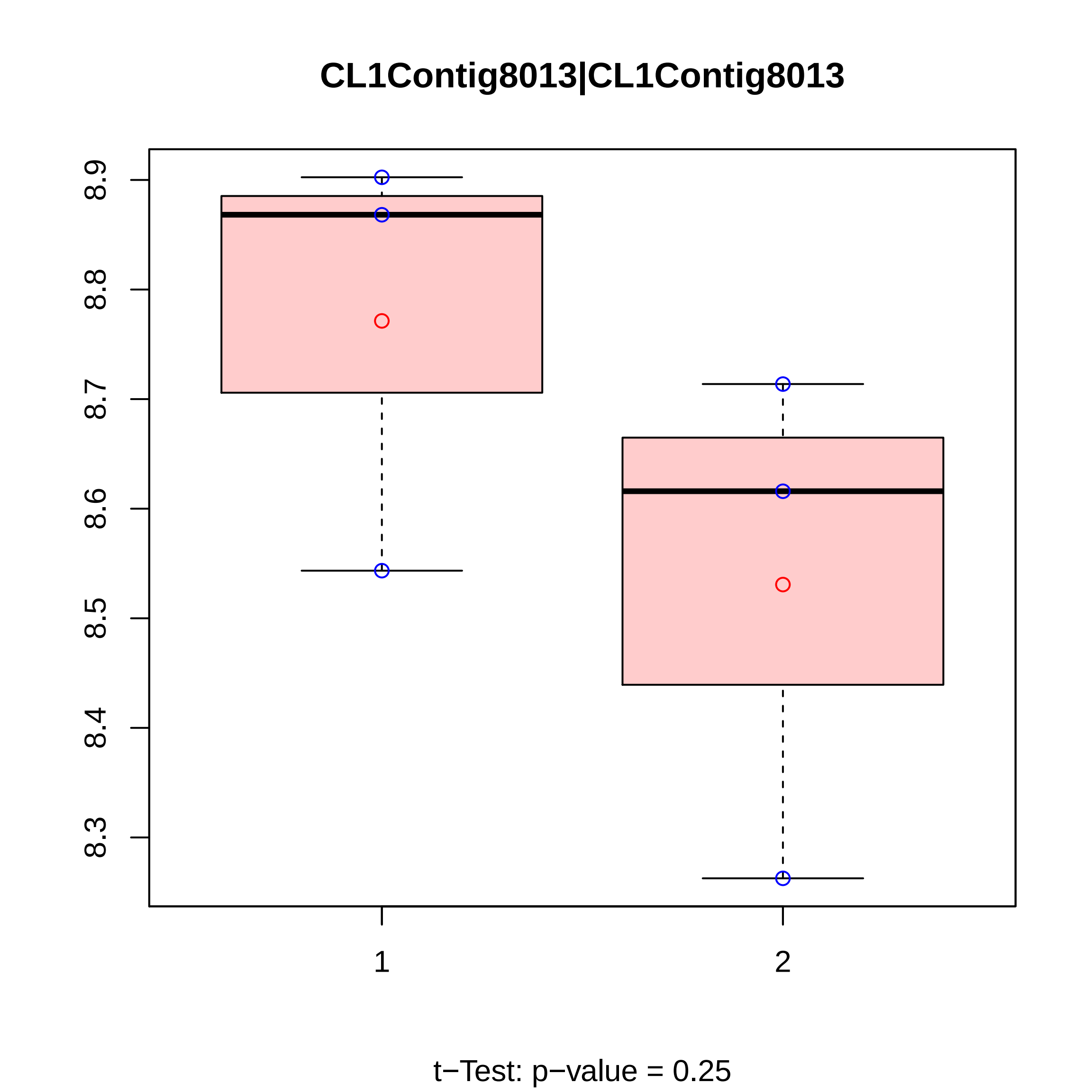
CL1Contig8013|CL1Contig8013
8.9
8.8
8.7
8.6
8.5
8.4
8.3
1
2
t−Test: p−value = 0.25

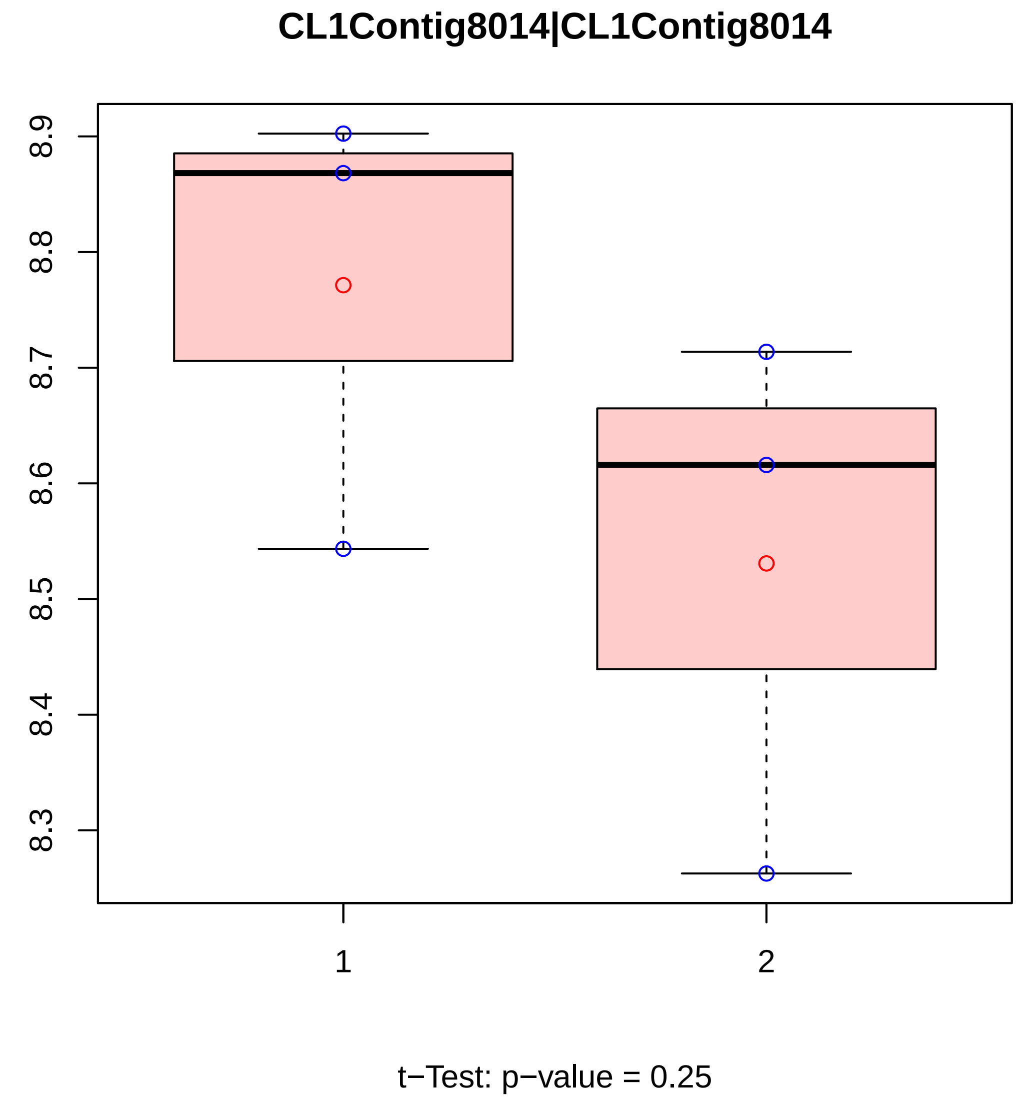

CL1Contig8014|CL1Contig8014
8.3
8.4
8.5
8.6
8.7
8.8
8.9
1
2
t−Test: p−value = 0.25

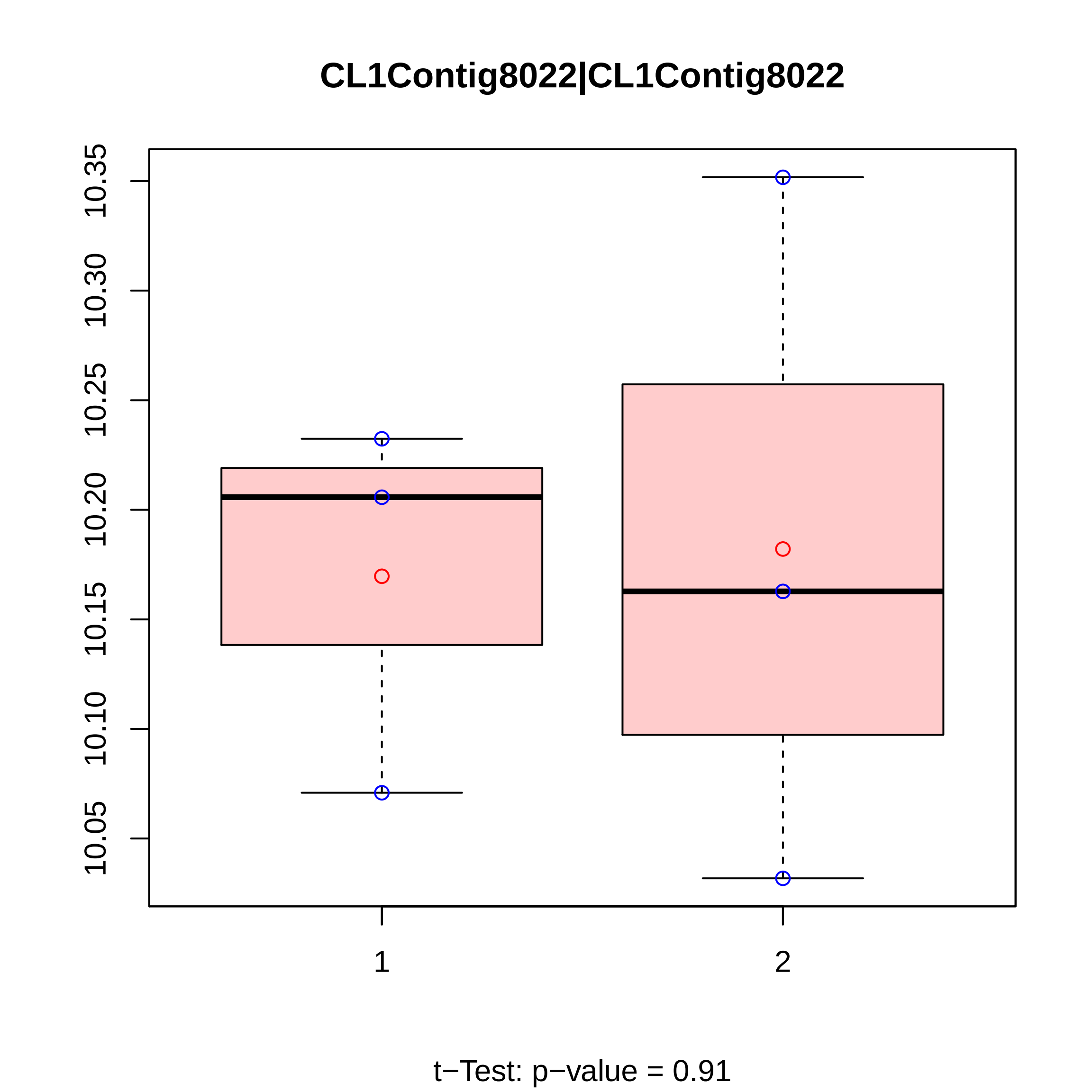

CL1Contig8022|CL1Contig8022
10.05
10.10
10.15
10.20
10.25
10.30
10.35
1
2
t−Test: p−value = 0.91

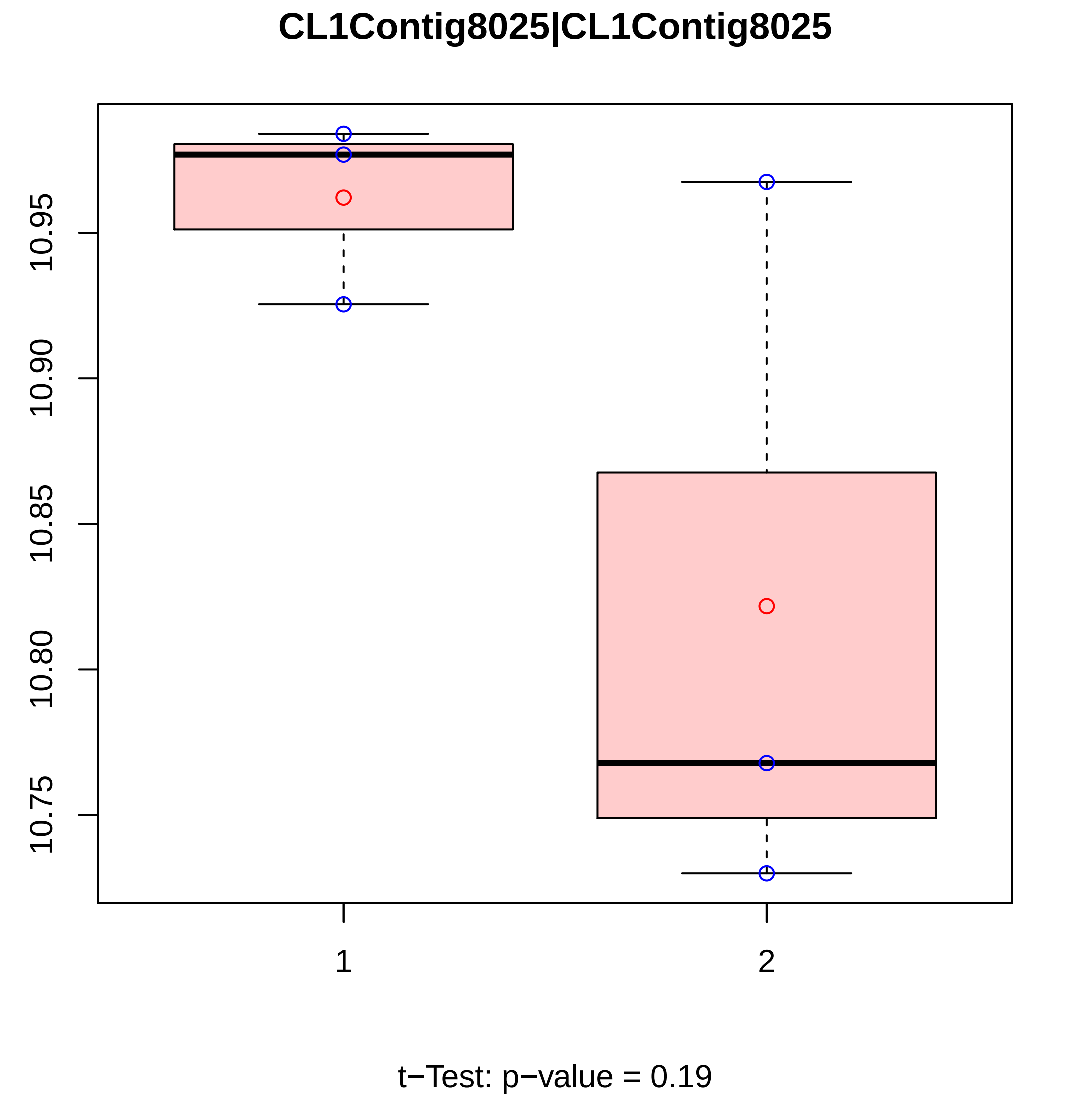
CL1Contig8025|CL1Contig8025
10.75
10.80
10.85
10.90
10.95
1
2
t−Test: p−value = 0.19

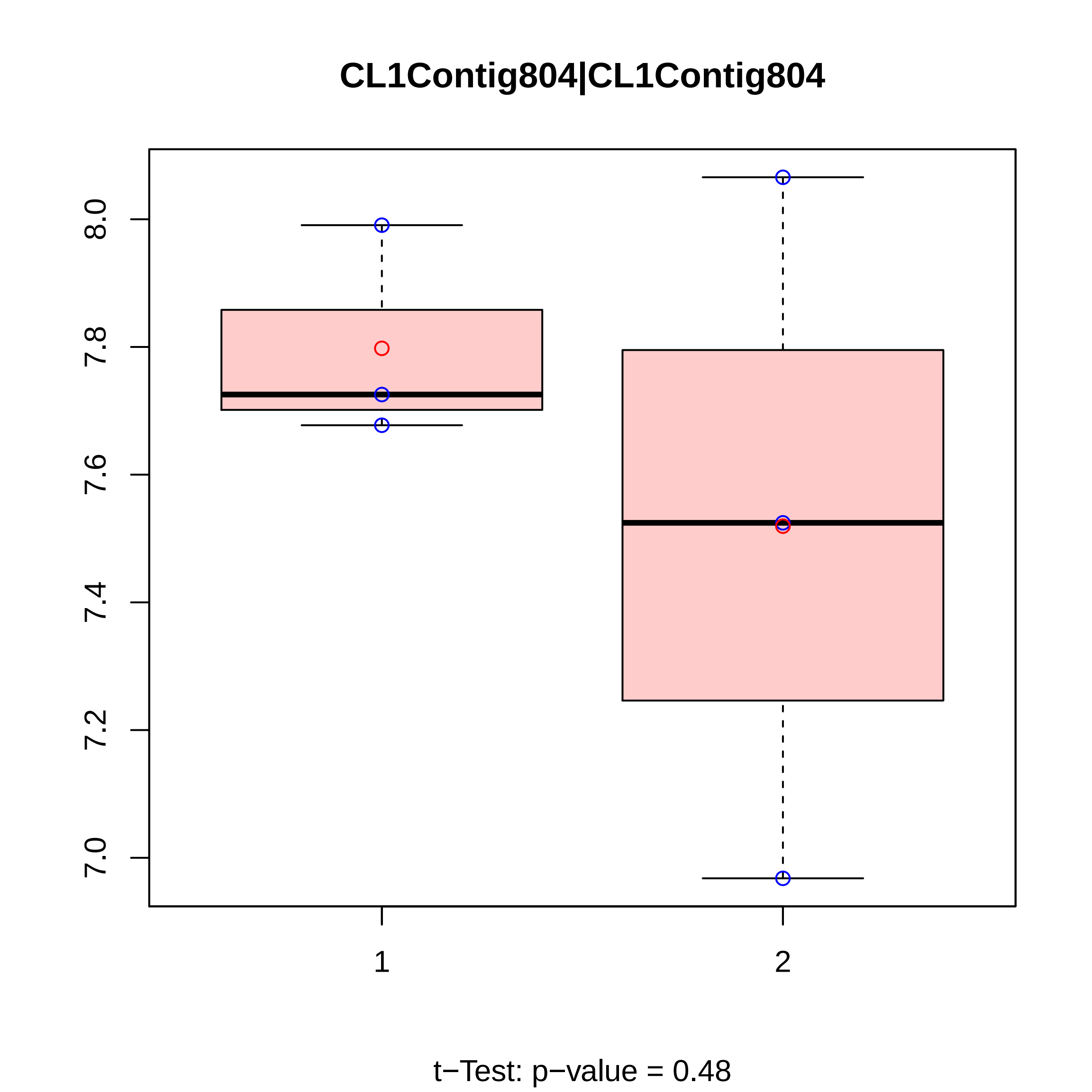

CL1Contig804|CL1Contig804
8.0
7.8
7.6
7.4
7.2
7.0
1
2
t−Test: p−value = 0.48

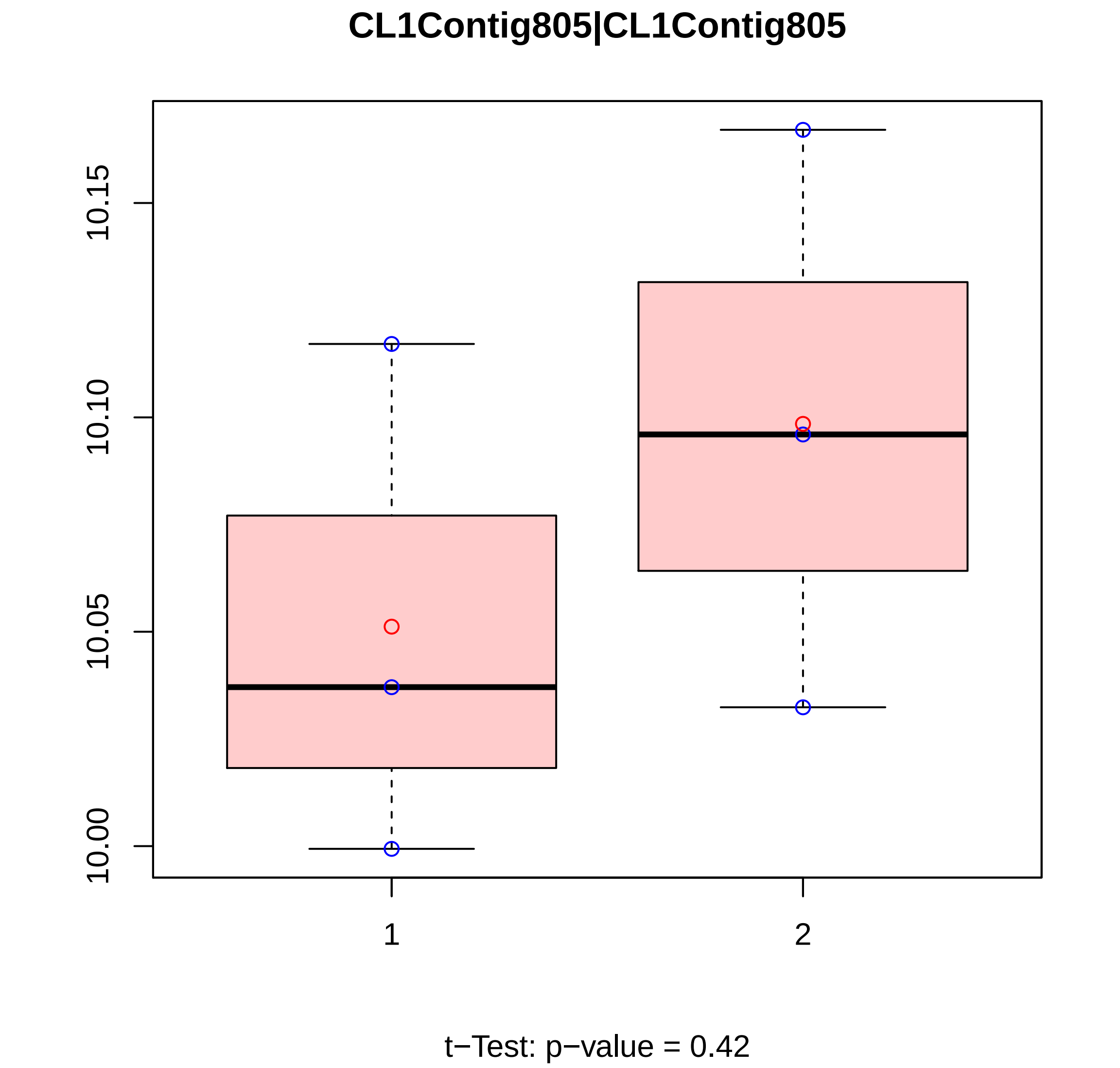
CL1Contig805|CL1Contig805
10.00
10.05
10.10
10.15
1
2
t−Test: p−value = 0.42

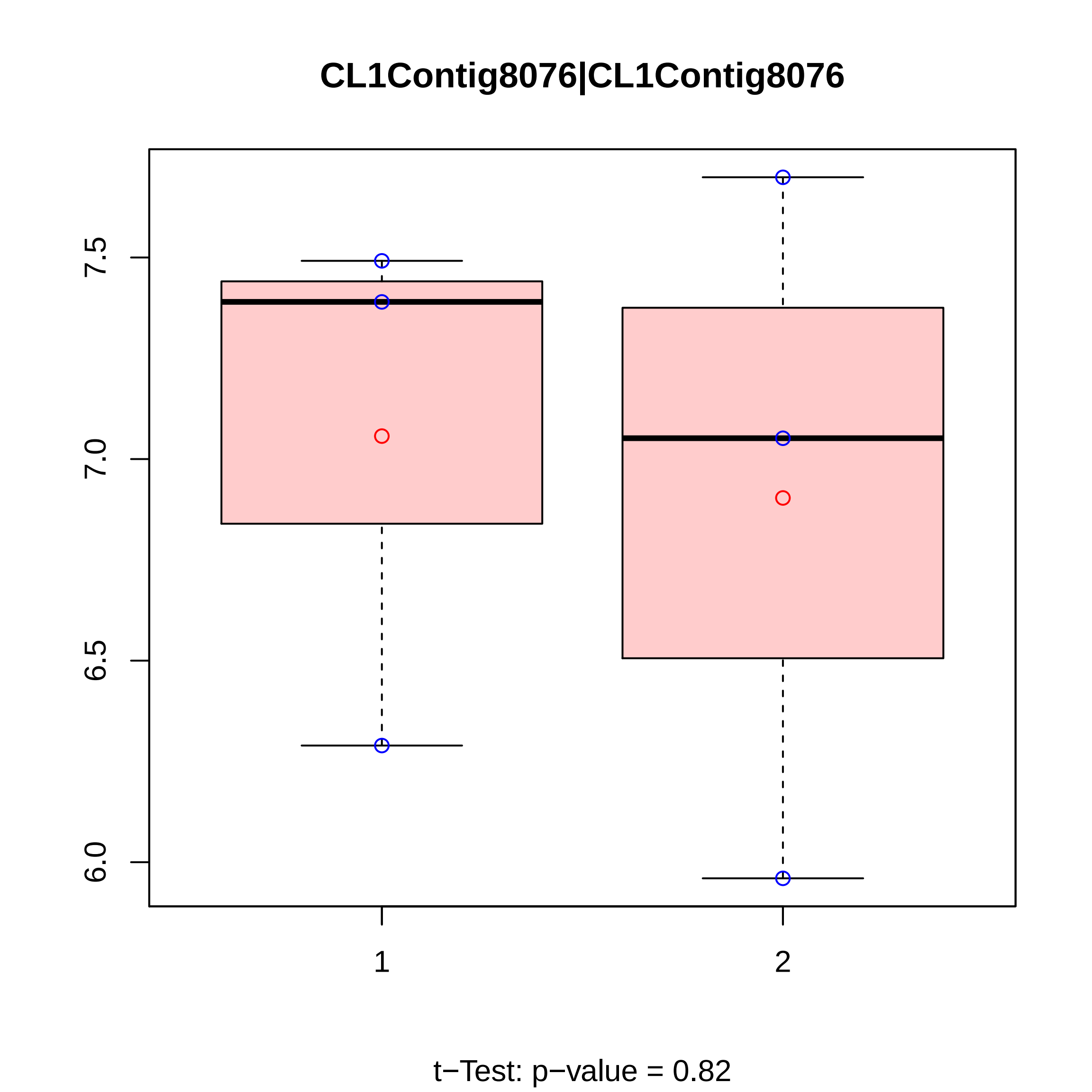

CL1Contig8076|CL1Contig8076
7.5
7.0
6.5
6.0
1
2
t−Test: p−value = 0.82

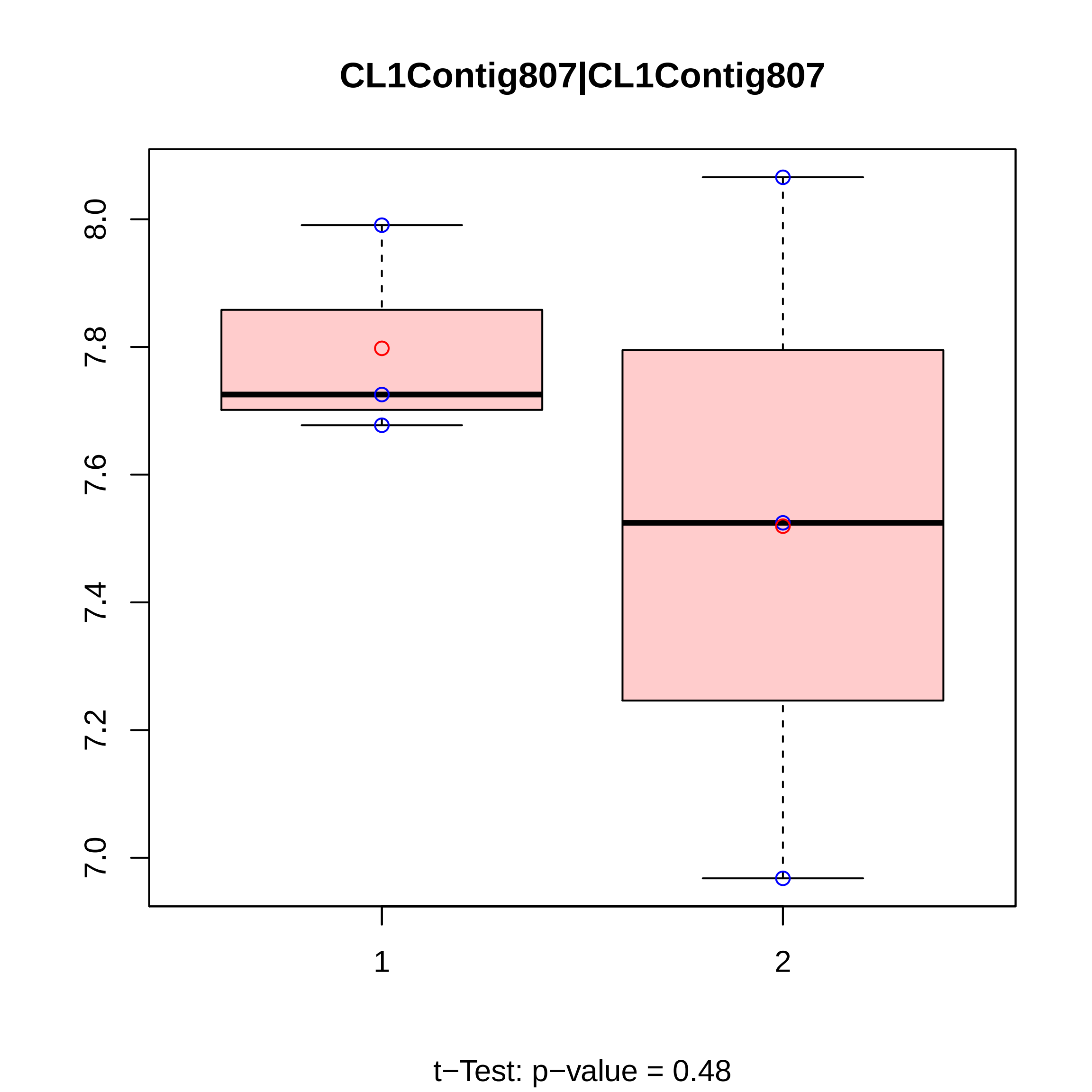
CL1Contig807|CL1Contig807
8.0
7.8
7.6
7.4
7.2
7.0
1
2
t−Test: p−value = 0.48

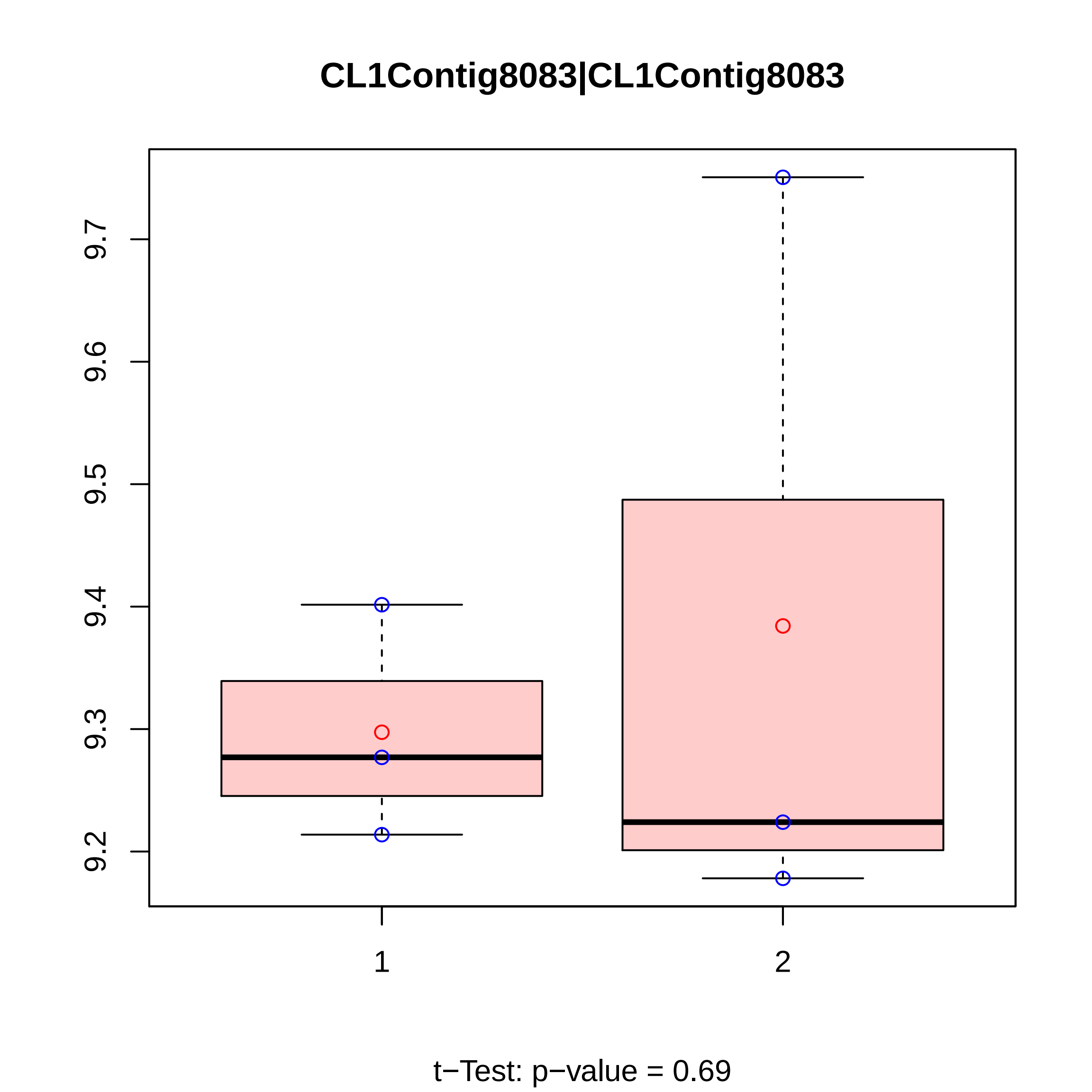

CL1Contig8083|CL1Contig8083
9.2
9.3
9.4
9.5
9.6
9.7
1
2
t−Test: p−value = 0.69

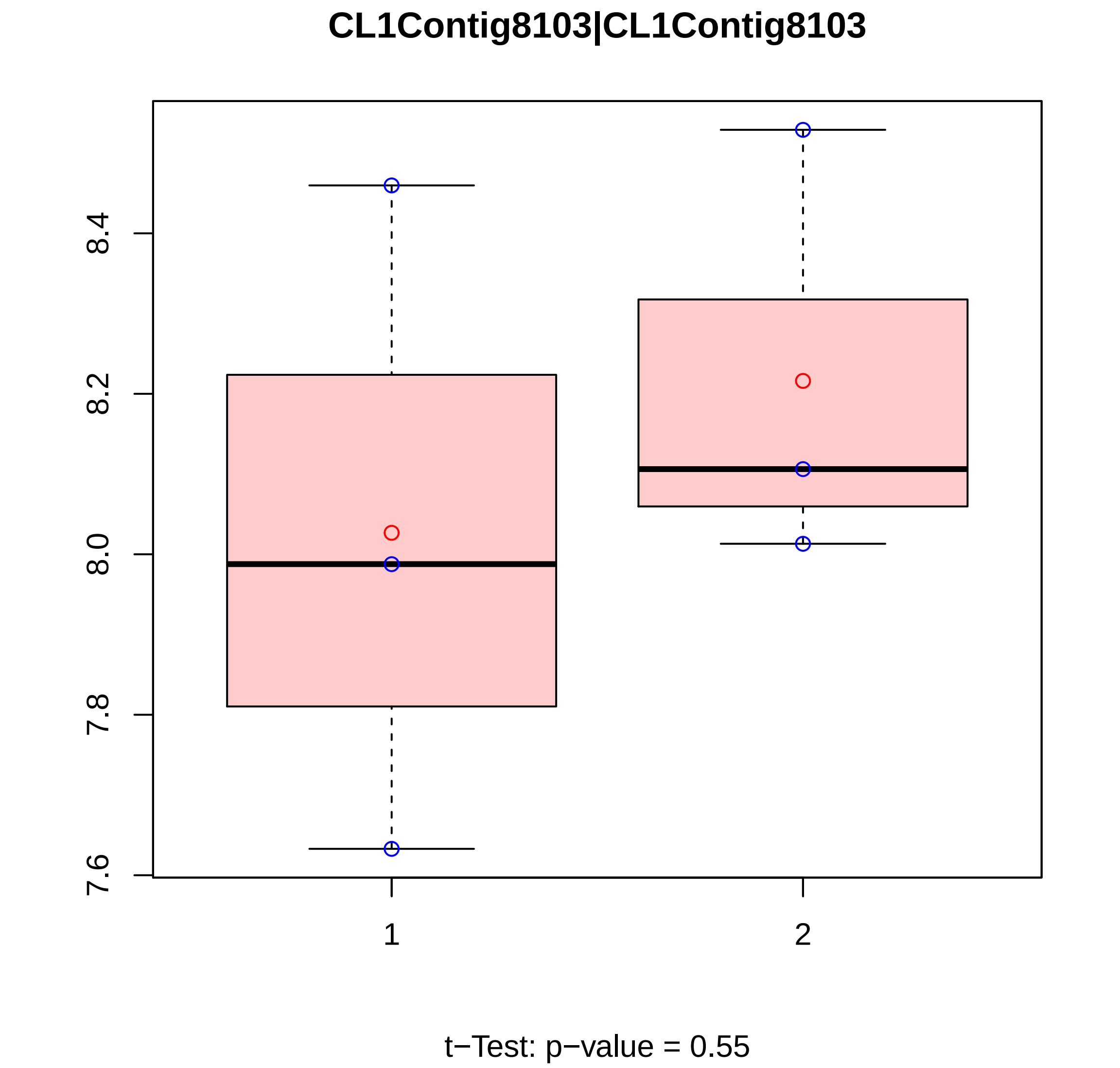
CL1Contig8103|CL1Contig8103
8.4
8.2
8.0
7.8
7.6
1
2
t−Test: p−value = 0.55

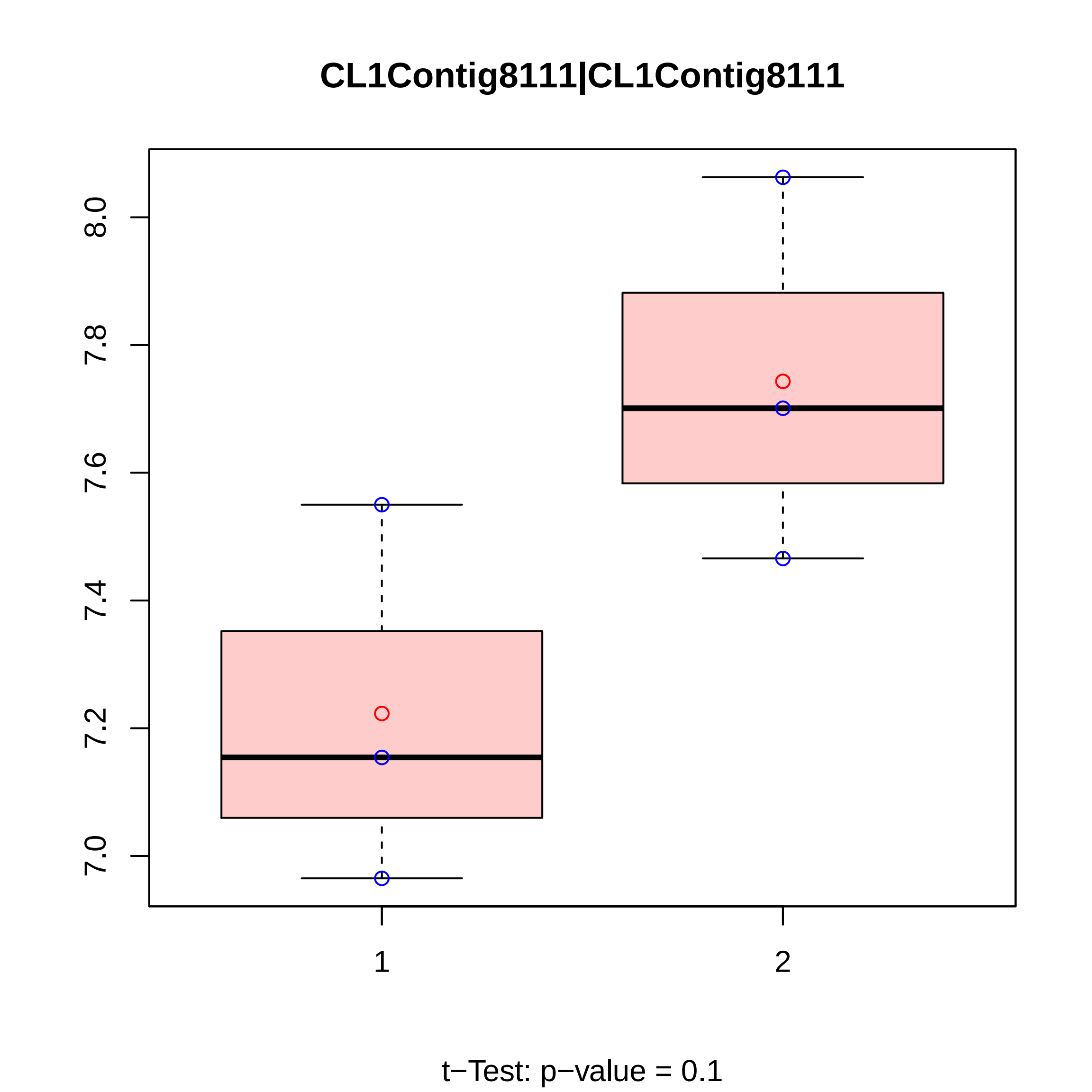

CL1Contig8111|CL1Contig8111
8.0
7.8
7.6
7.4
7.2
7.0
1
2
t−Test: p−value = 0.1

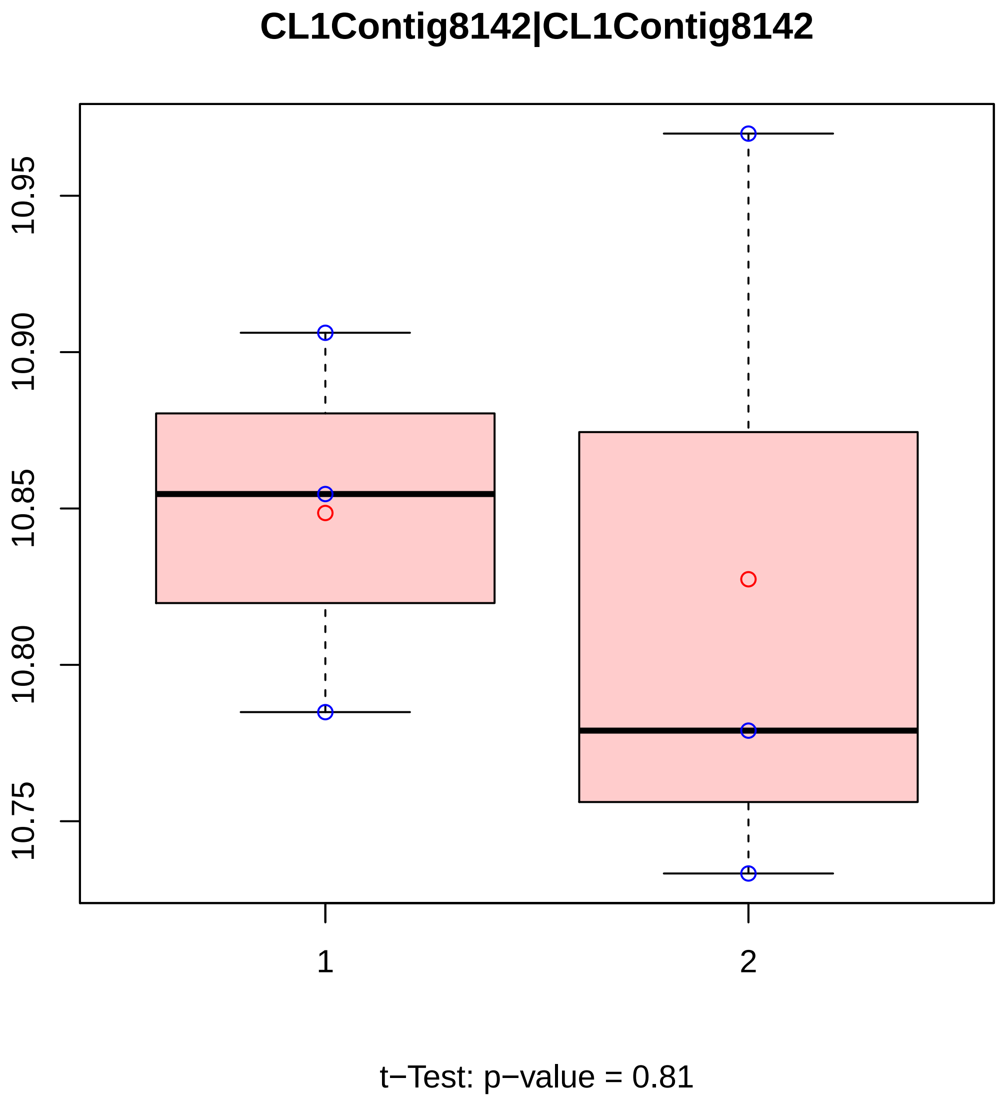

CL1Contig8142|CL1Contig8142
10.75
10.80
10.85
10.90
10.95
1
2
t−Test: p−value = 0.81

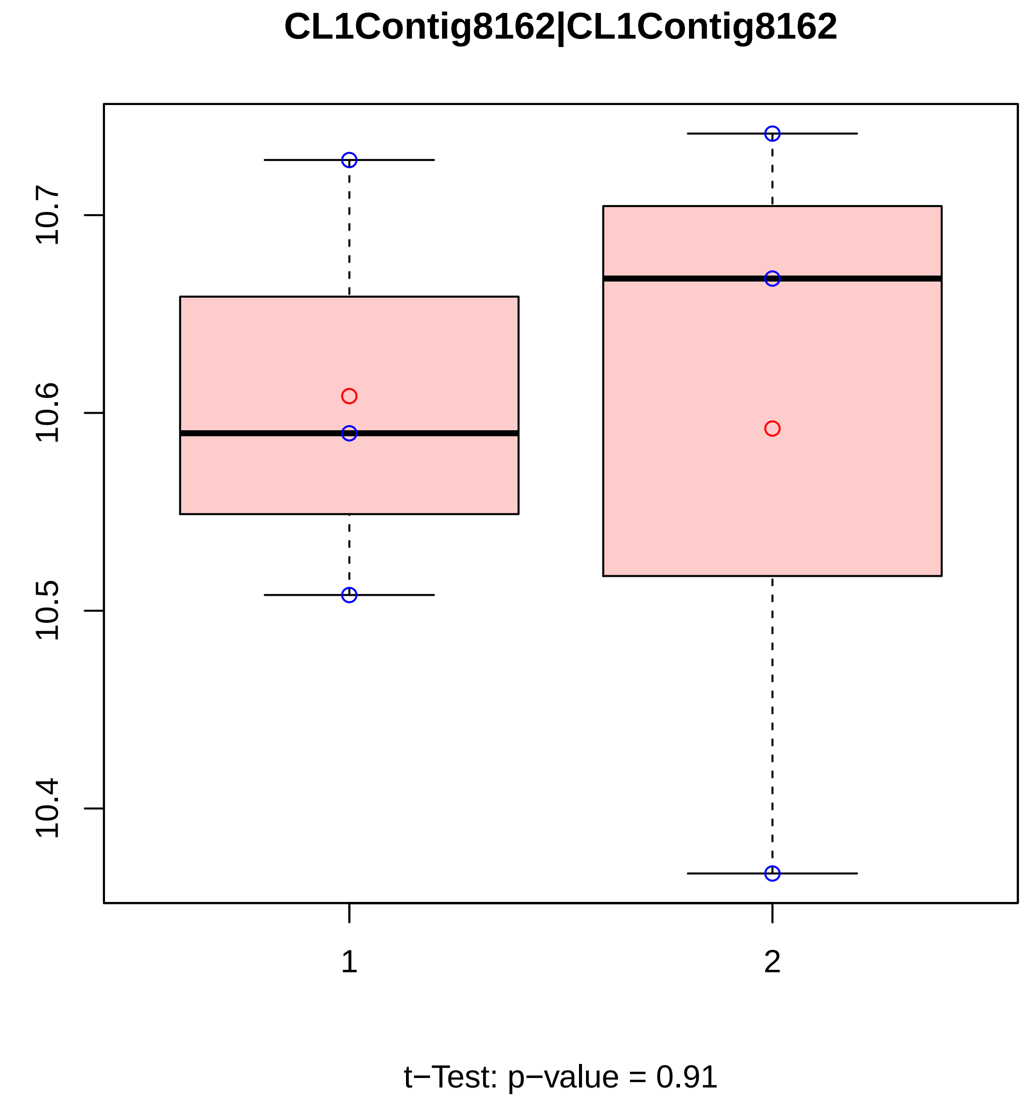
CL1Contig8162|CL1Contig8162
10.4
10.5
10.6
10.7
1
2
t−Test: p−value = 0.91

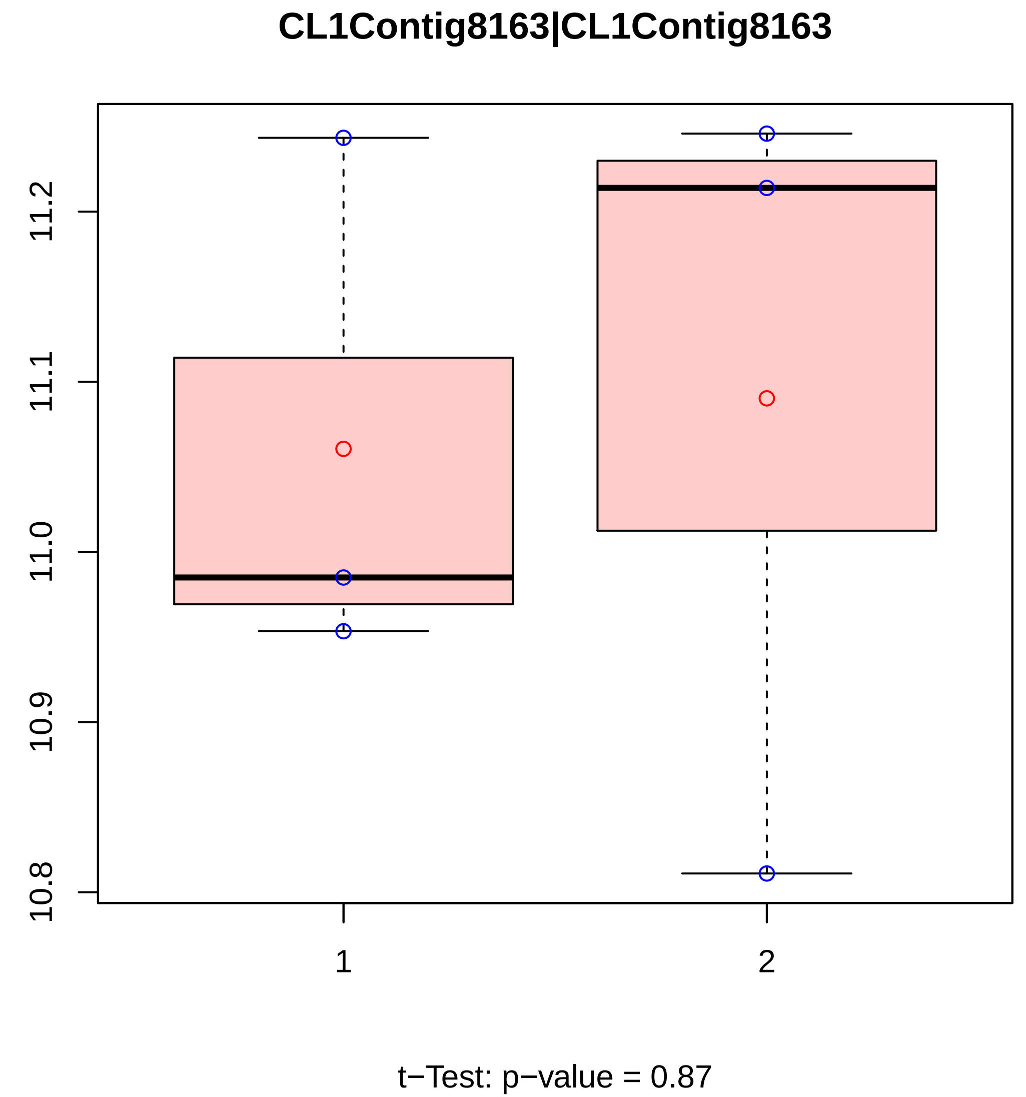
CL1Contig8163|CL1Contig8163
11.2
11.1
11.0
10.9
10.8
1
2
t−Test: p−value = 0.87

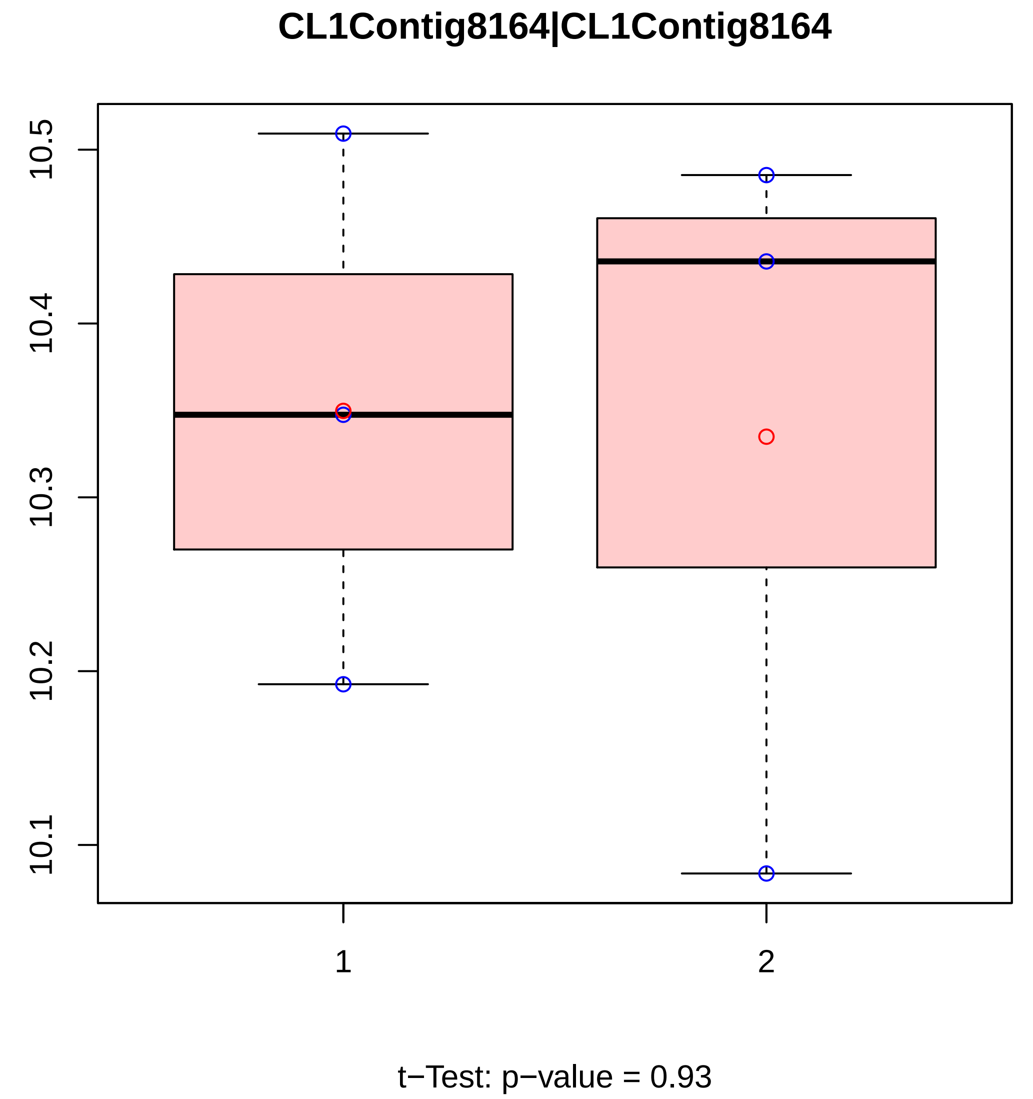
CL1Contig8164|CL1Contig8164
10.5
10.4
10.3
10.2
10.1
1
2
t−Test: p−value = 0.93

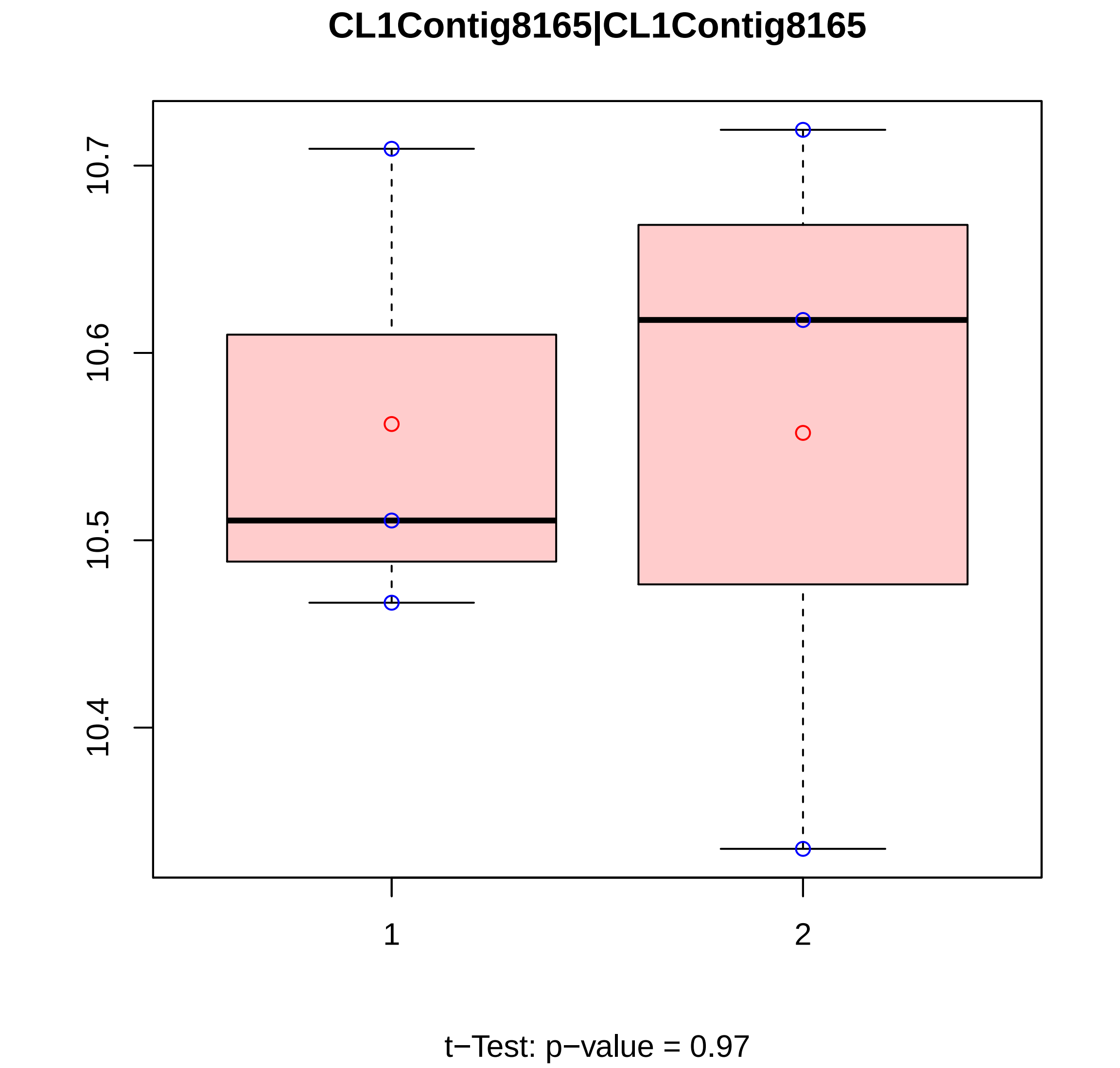

CL1Contig8165|CL1Contig8165
10.4
10.5
10.6
10.7
1
2
t−Test: p−value = 0.97

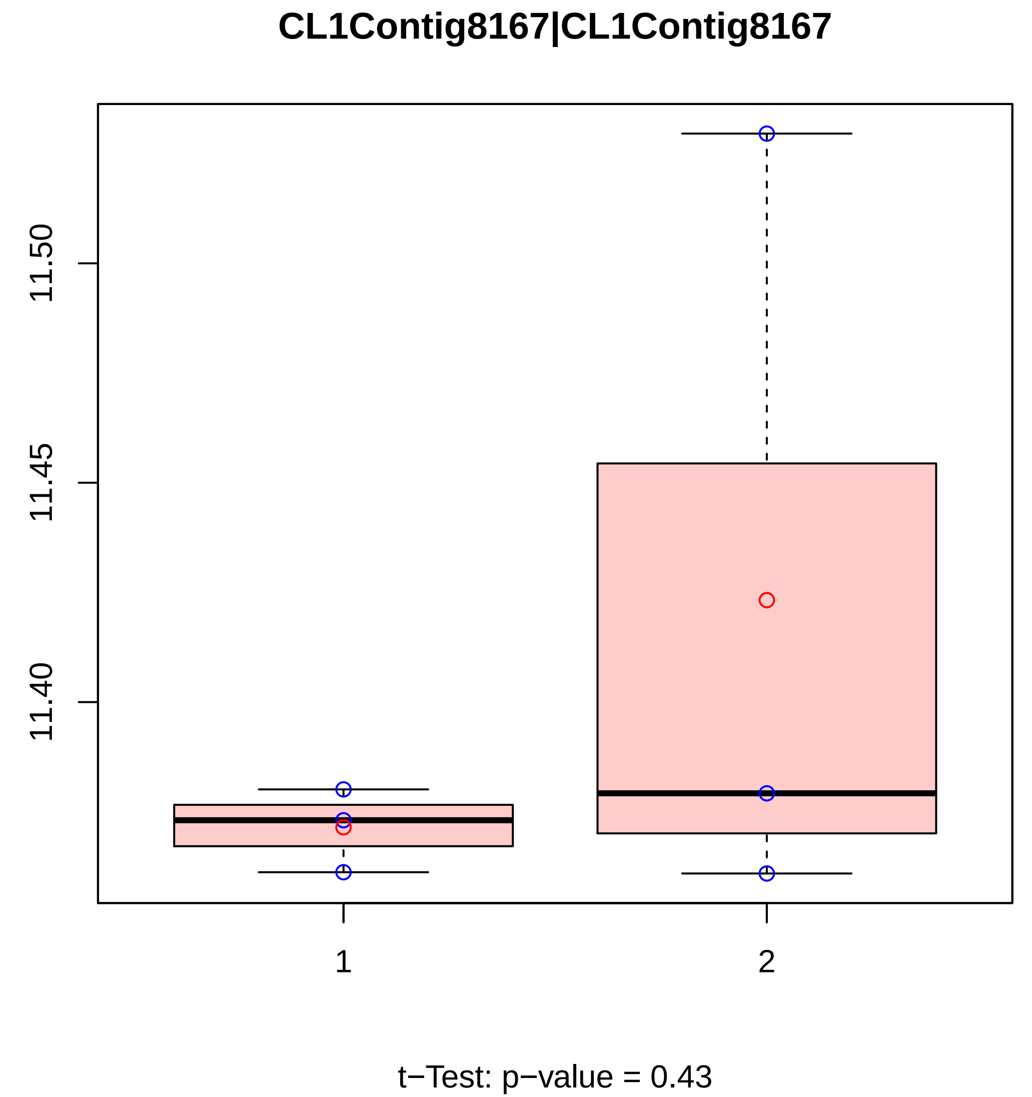
CL1Contig8167|CL1Contig8167
11.40
11.45
11.50
1
2
t−Test: p−value = 0.43

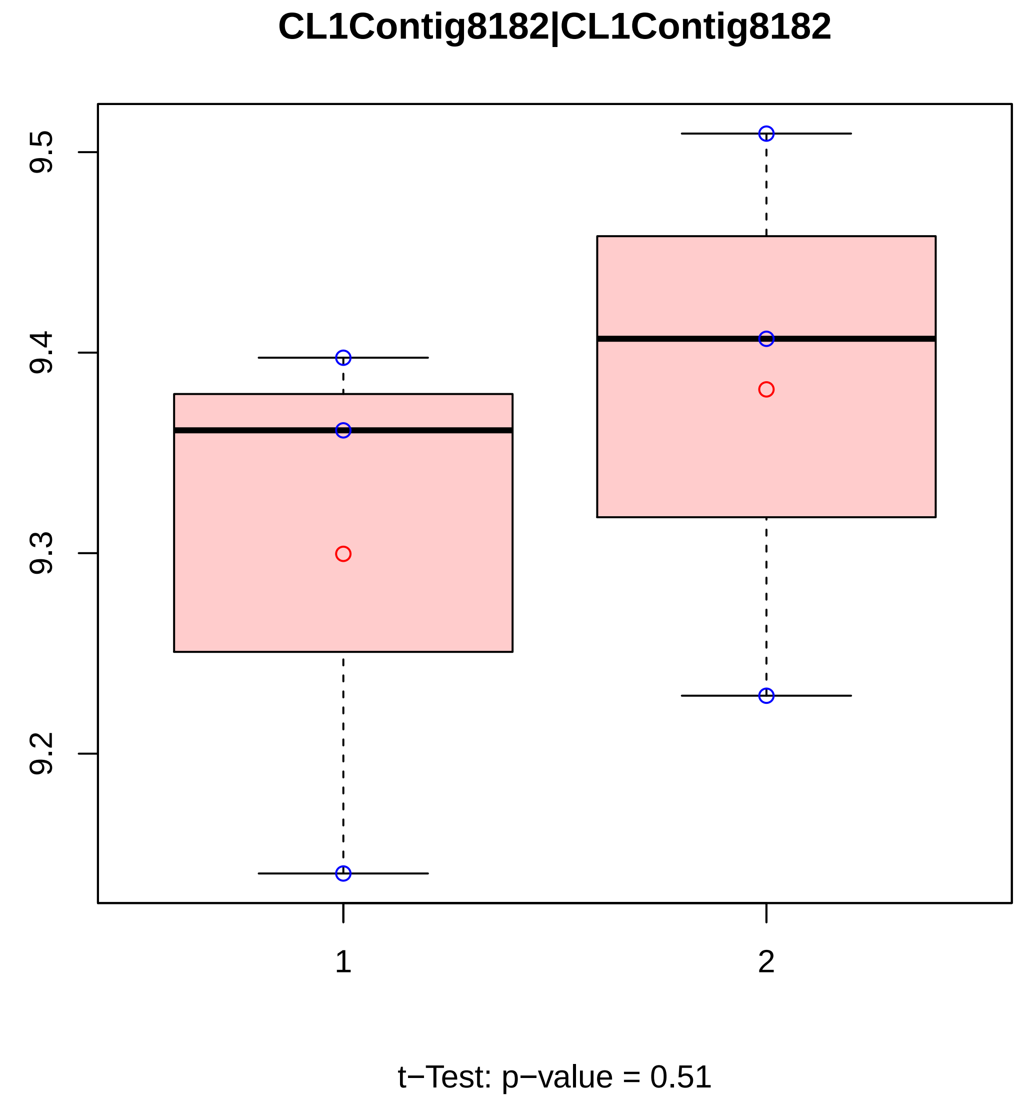
CL1Contig8182|CL1Contig8182
9.5
9.4
9.3
9.2
1
2
t−Test: p−value = 0.51

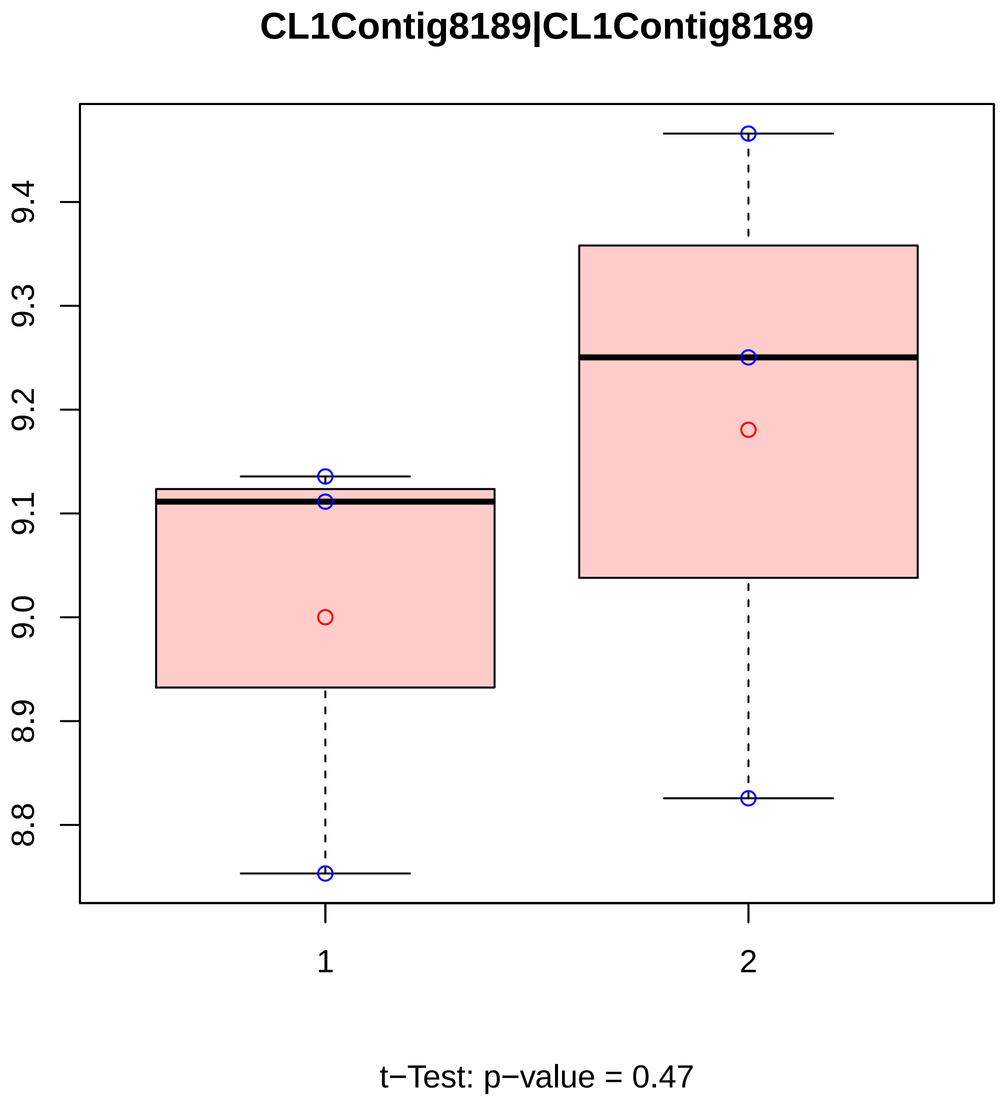
CL1Contig8189|CL1Contig8189
9.4
9.3
9.2
9.1
9.0
8.9
8.8
1
2
t−Test: p−value = 0.47

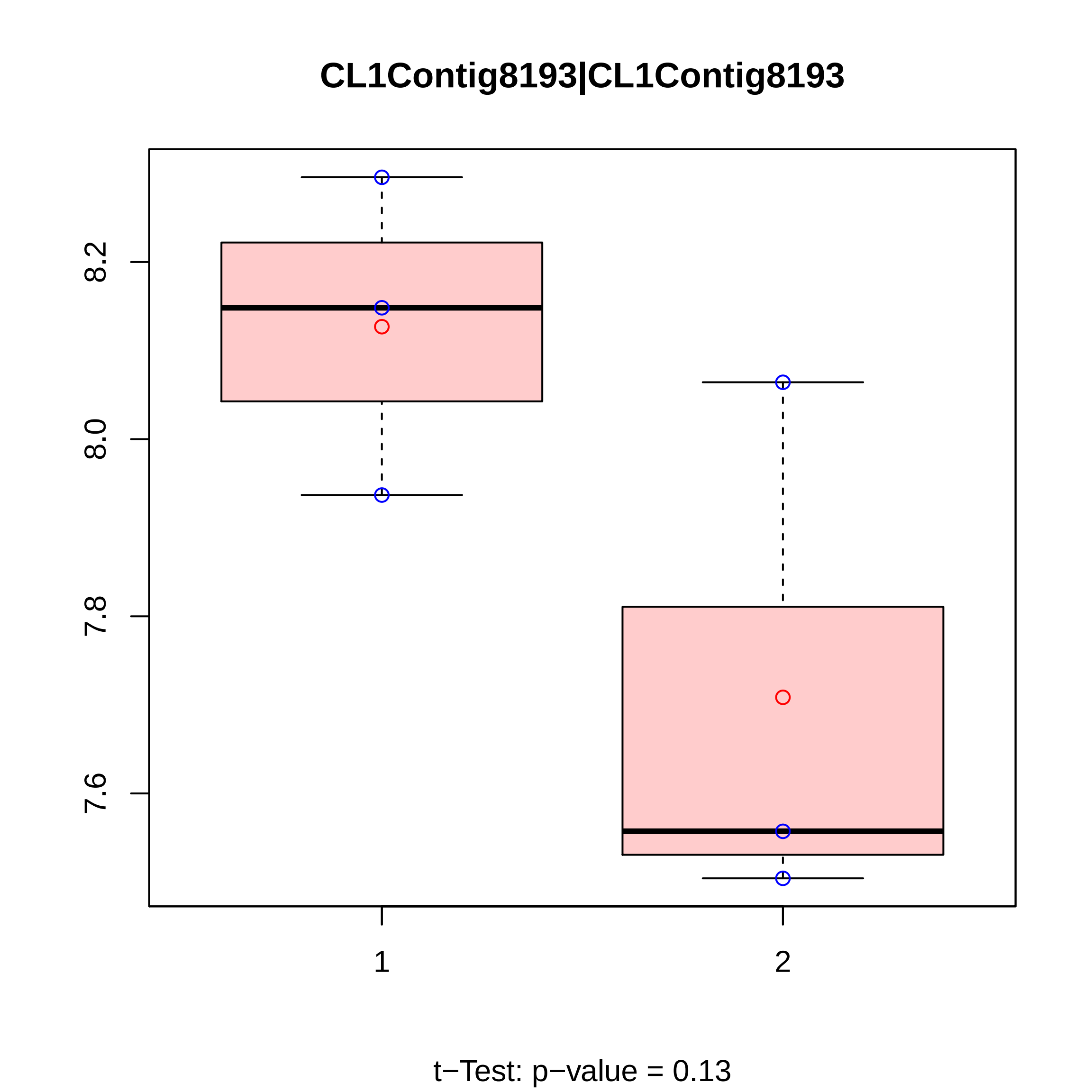
CL1Contig8193|CL1Contig8193
8.2
8.0
7.8
7.6
1
2
t−Test: p−value = 0.13

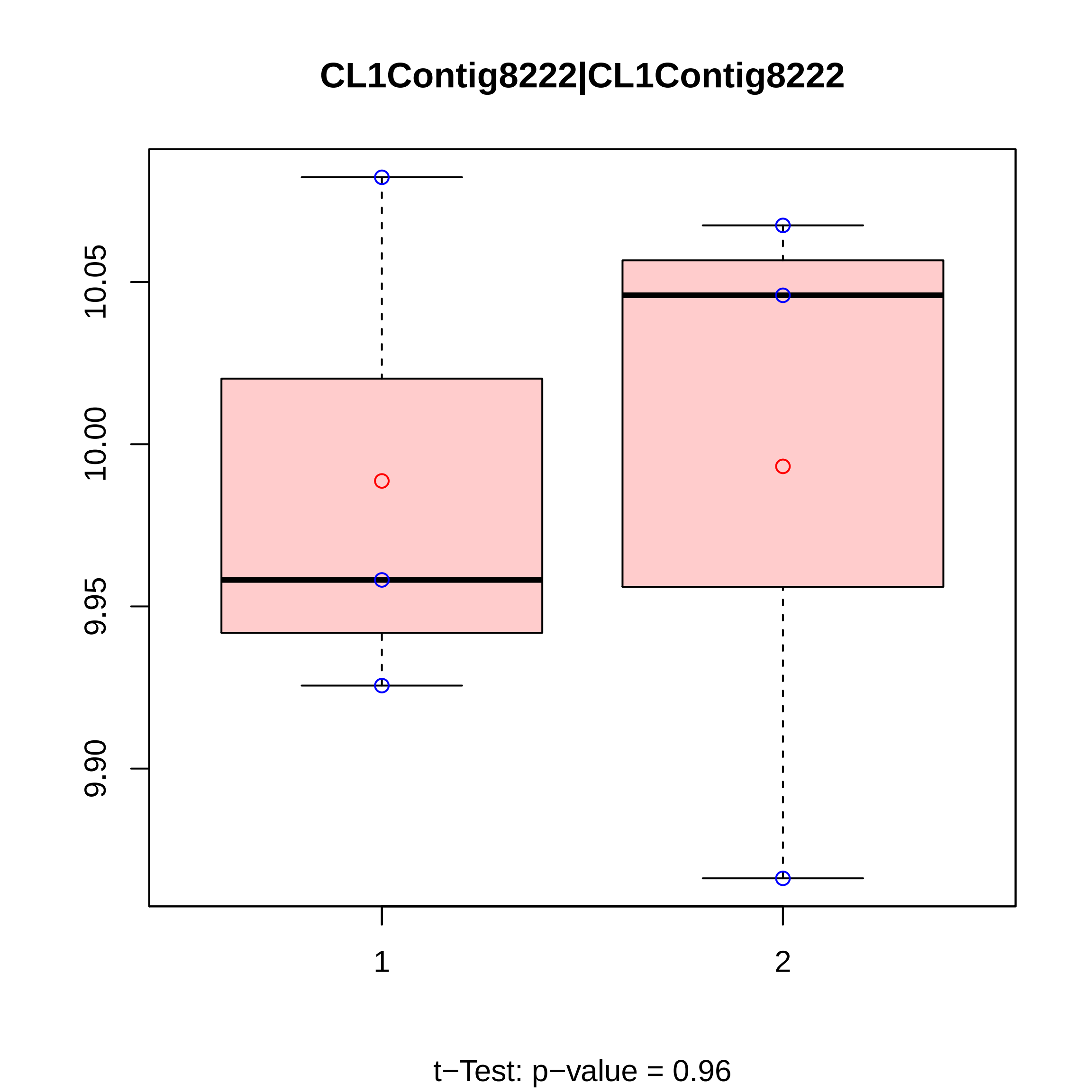
CL1Contig8222|CL1Contig8222
10.05
10.00
9.95
9.90
1
2
t−Test: p−value = 0.96

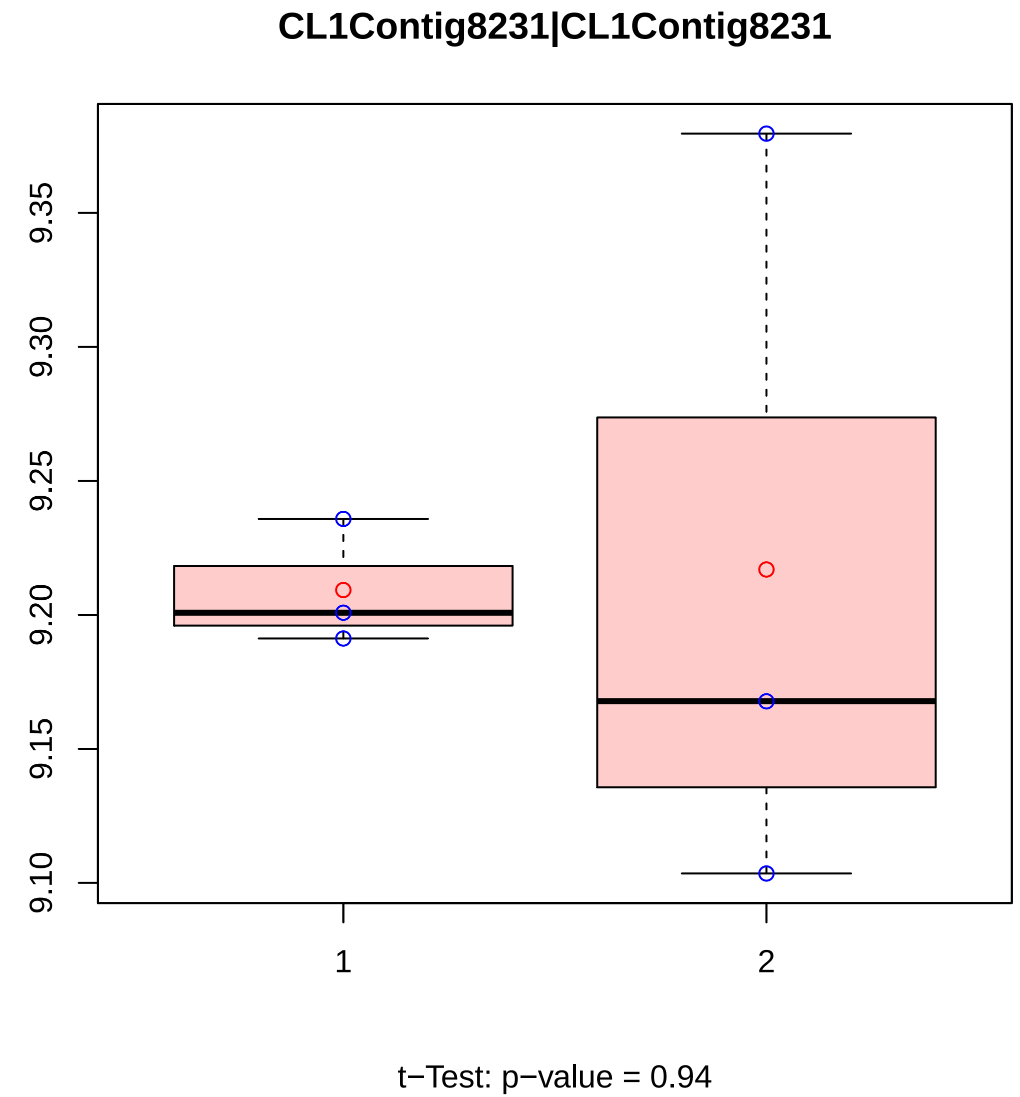

CL1Contig8231|CL1Contig8231
9.10
9.15
9.20
9.25
9.30
9.35
1
2
t−Test: p−value = 0.94

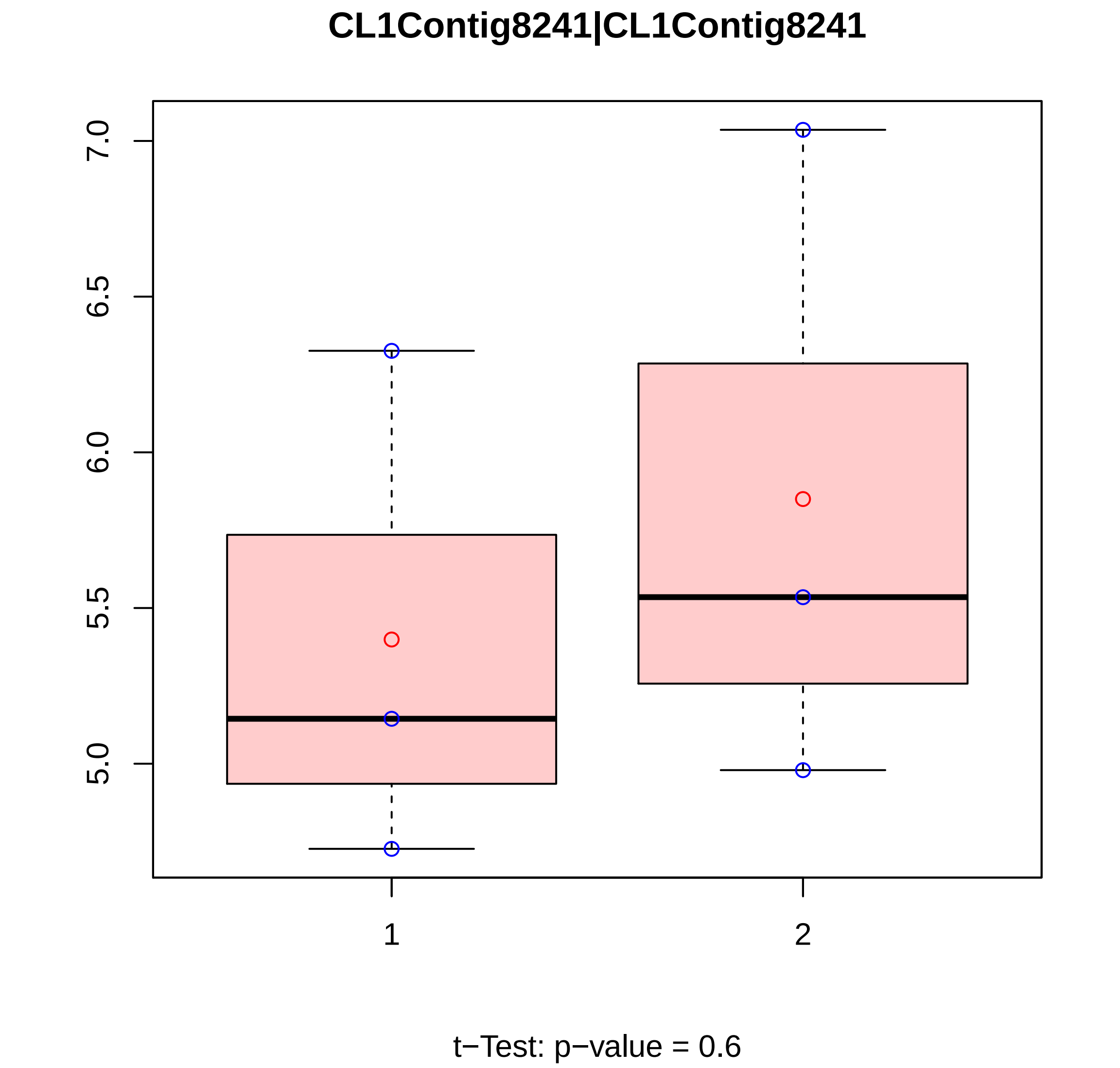
CL1Contig8241|CL1Contig8241
7.0
6.5
6.0
5.5
5.0
1
2
t−Test: p−value = 0.6

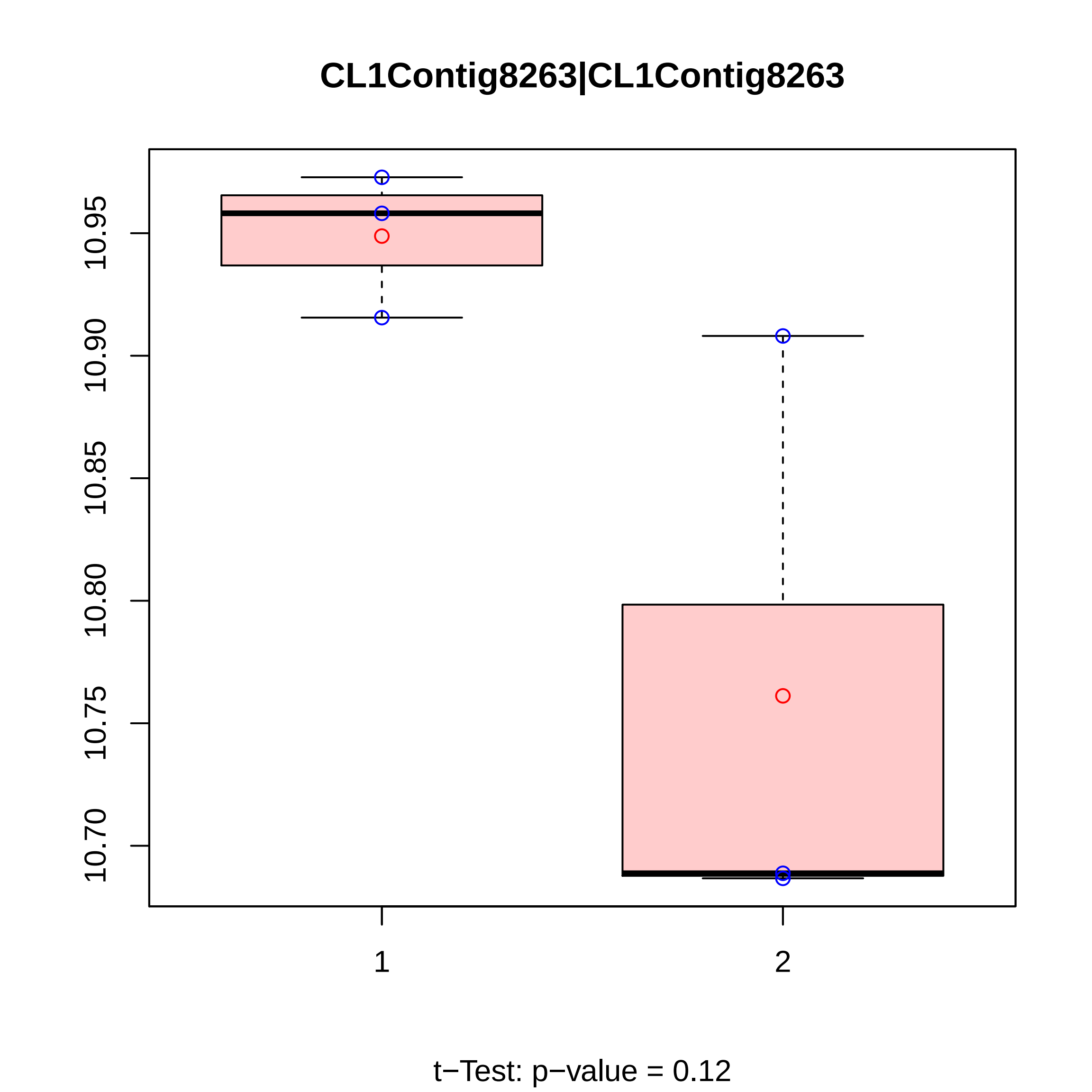

CL1Contig8263|CL1Contig8263
10.70
10.75
10.80
10.85
10.90
10.95
1
2
t−Test: p−value = 0.12

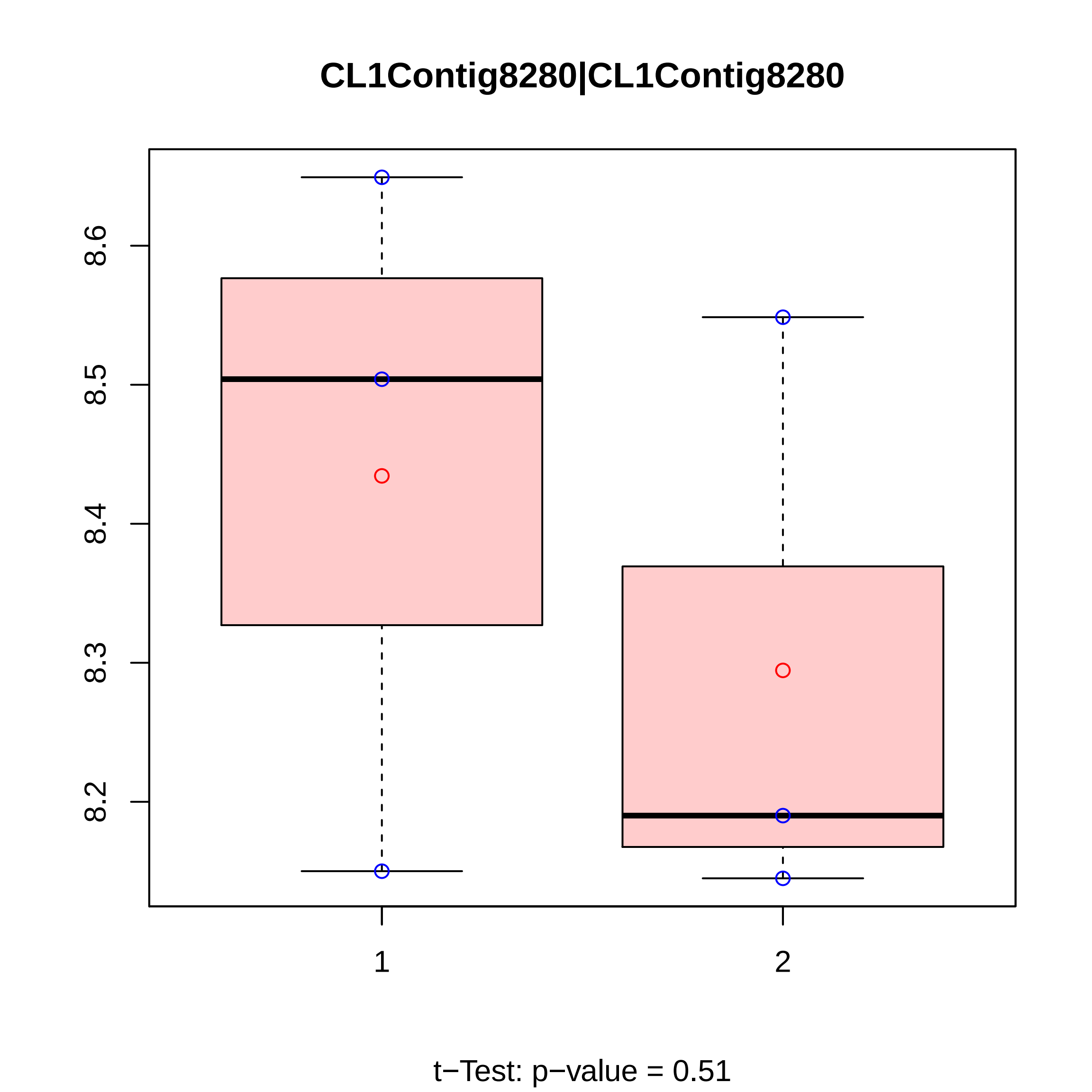

CL1Contig8280|CL1Contig8280
8.6
8.5
8.4
8.3
8.2
1
2
t−Test: p−value = 0.51

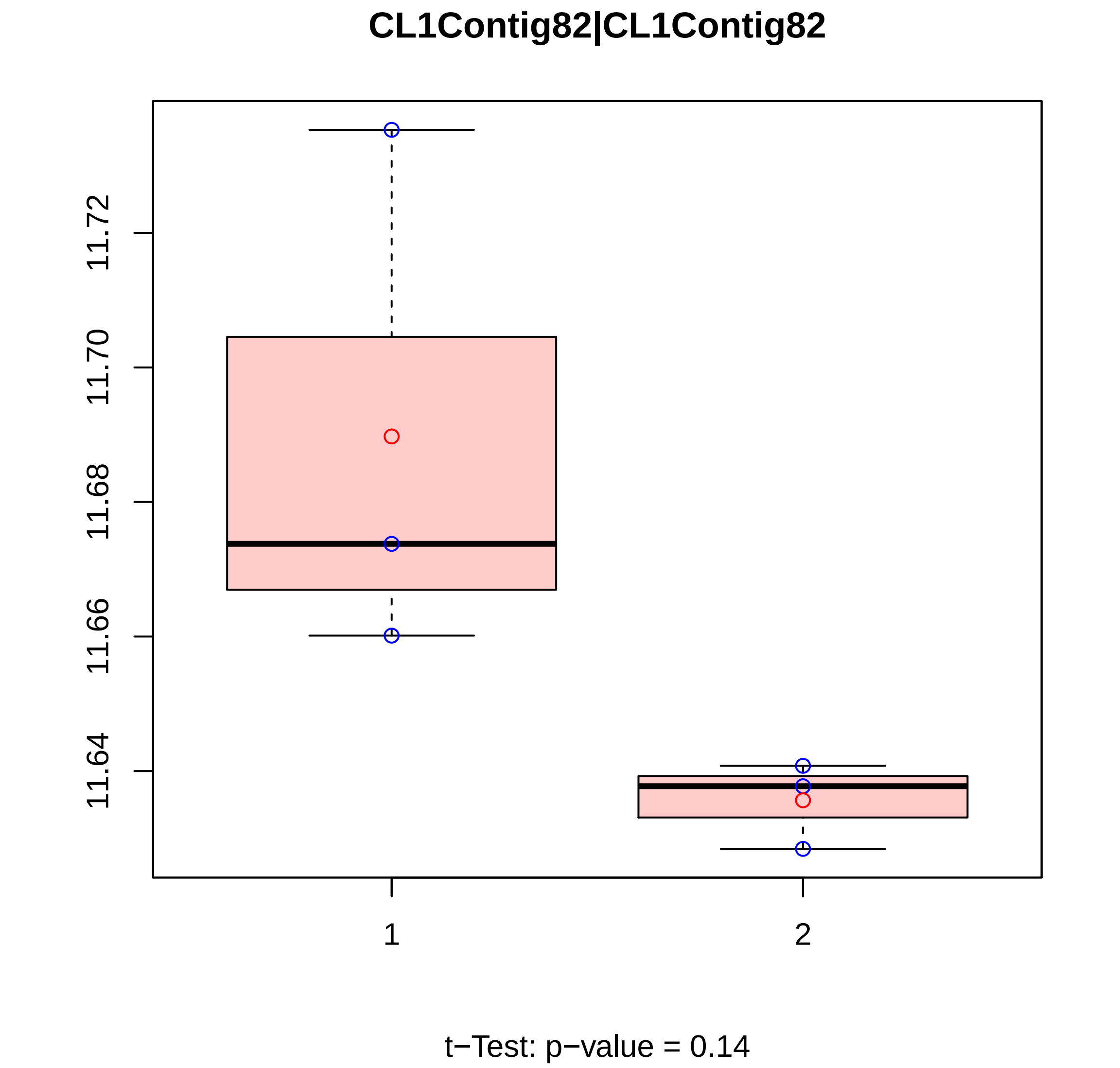
CL1Contig82|CL1Contig82
11.64
11.66
11.68
11.70
11.72
1
2
t−Test: p−value = 0.14

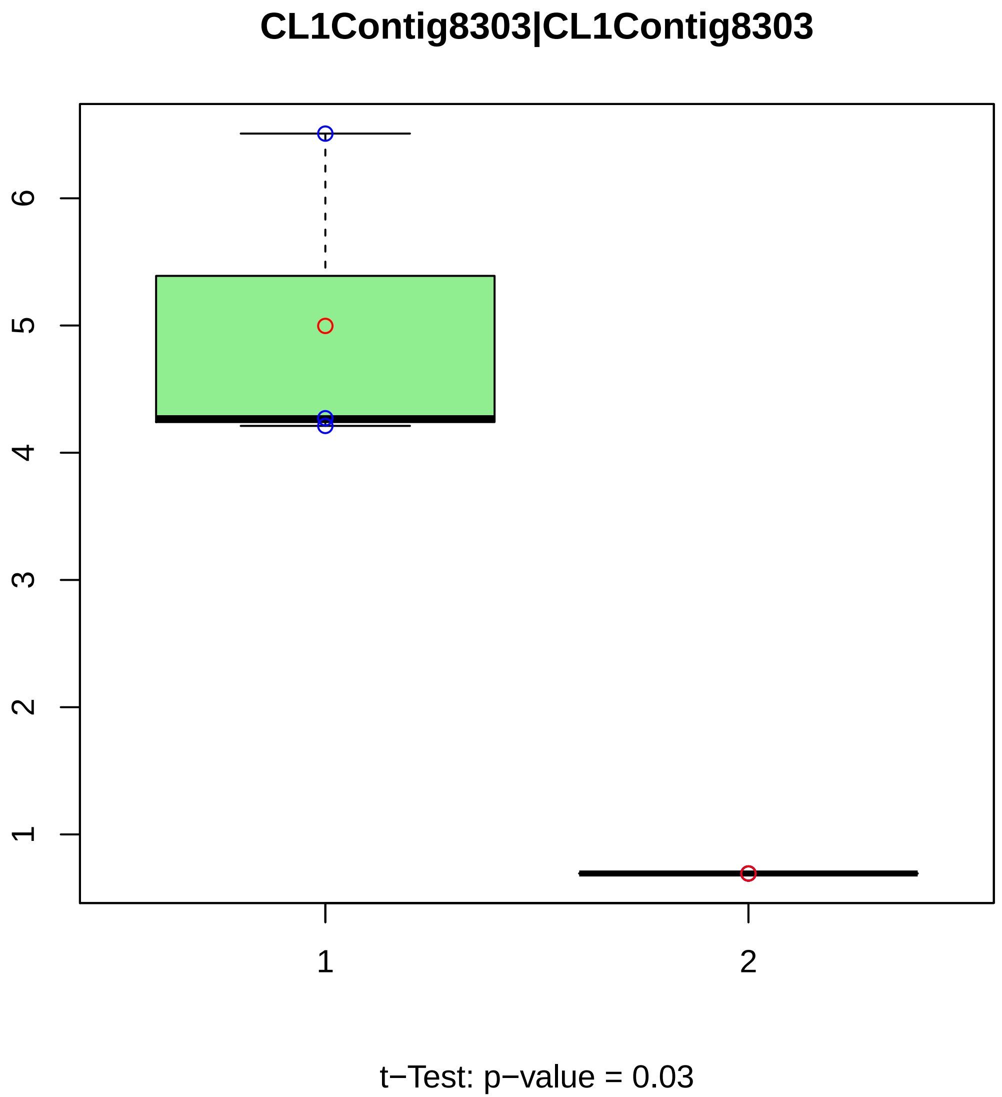
CL1Contig8303|CL1Contig8303
1
2
3
4
5
6
1
2
t−Test: p−value = 0.03

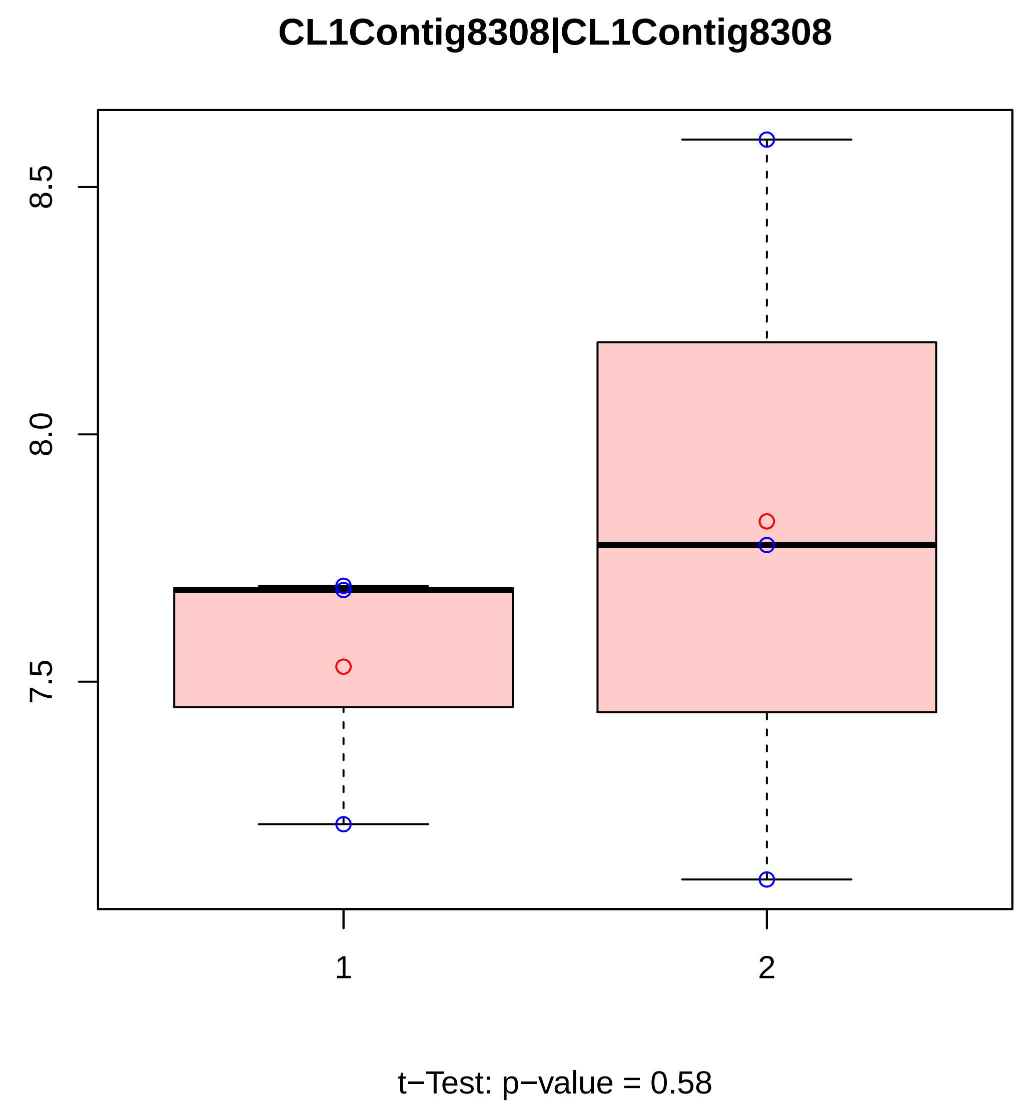
CL1Contig8308|CL1Contig8308
8.5
8.0
7.5
1
2
t−Test: p−value = 0.58

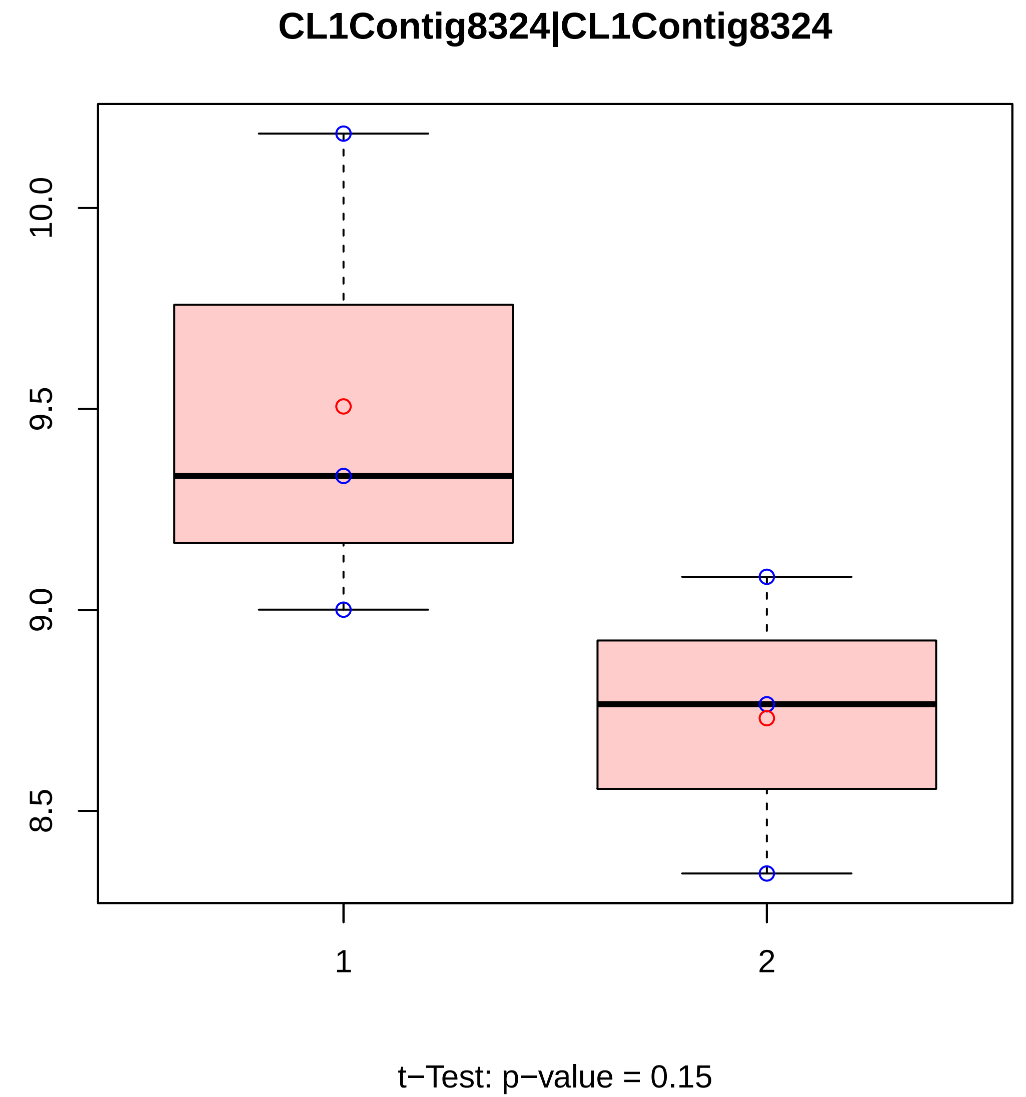
CL1Contig8324|CL1Contig8324
10.0
9.5
9.0
8.5
1
2
t−Test: p−value = 0.15

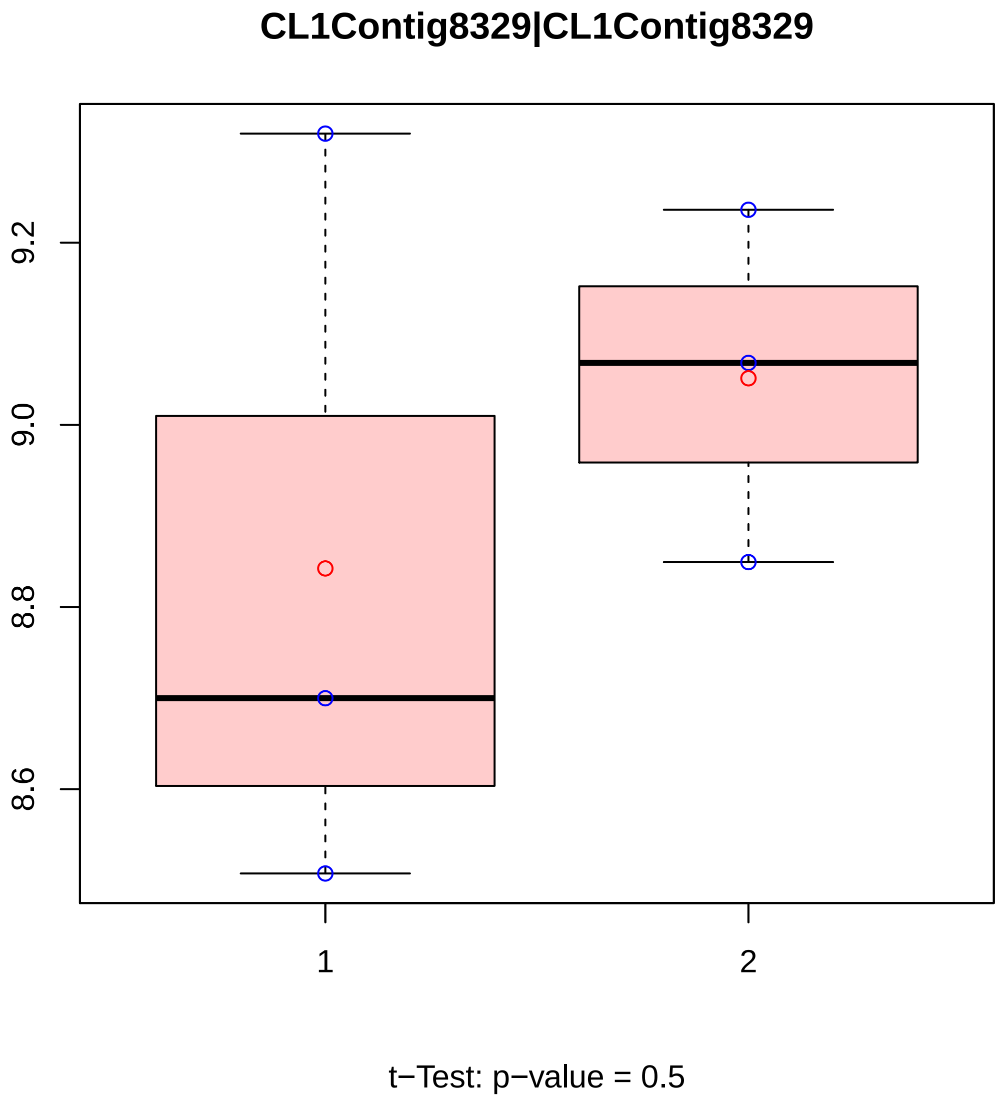
CL1Contig8329|CL1Contig8329
9.2
9.0
8.8
8.6
1
2
t−Test: p−value = 0.5

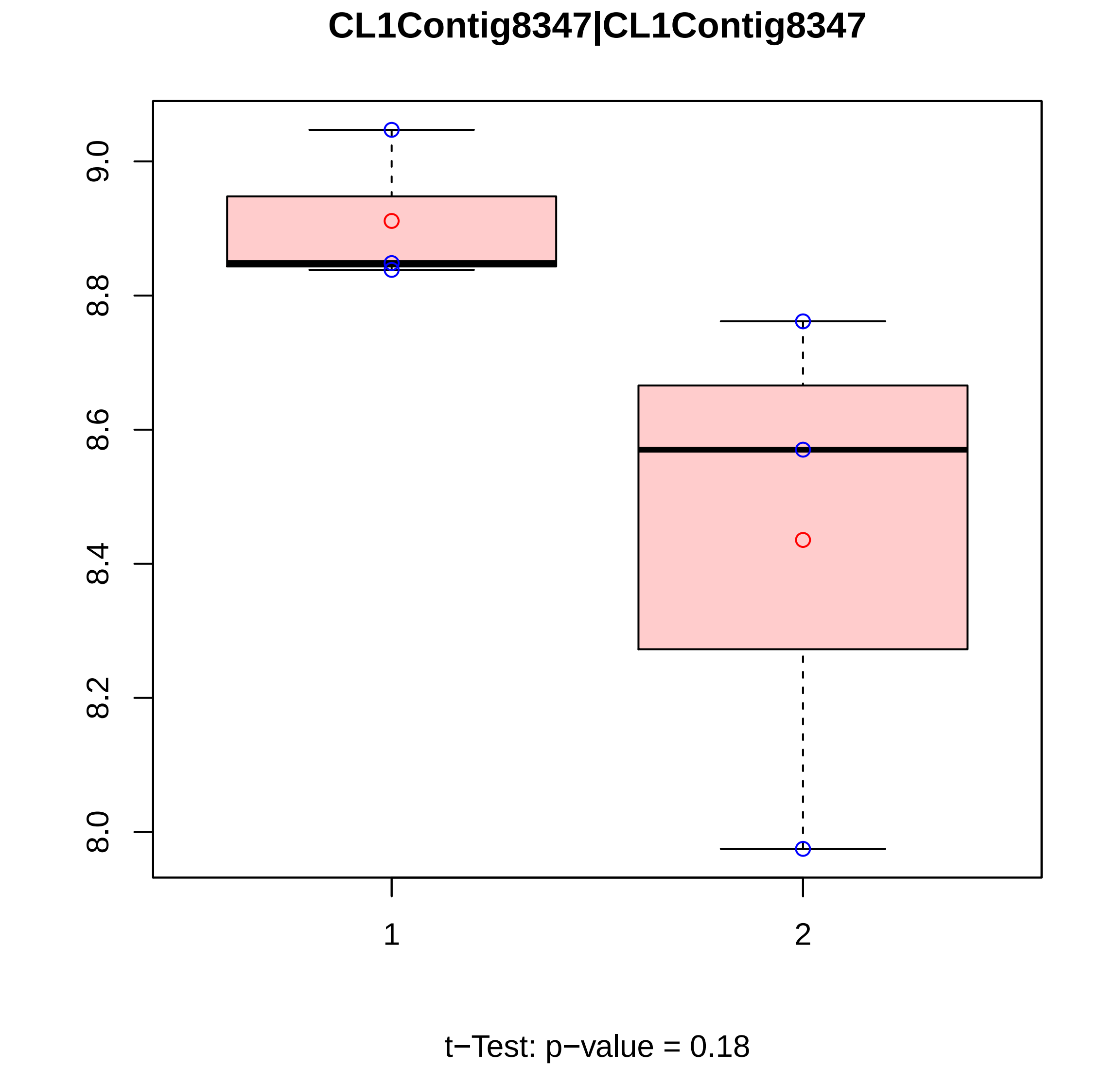
CL1Contig8347|CL1Contig8347
9.0
8.8
8.6
8.4
8.2
8.0
1
2
t−Test: p−value = 0.18

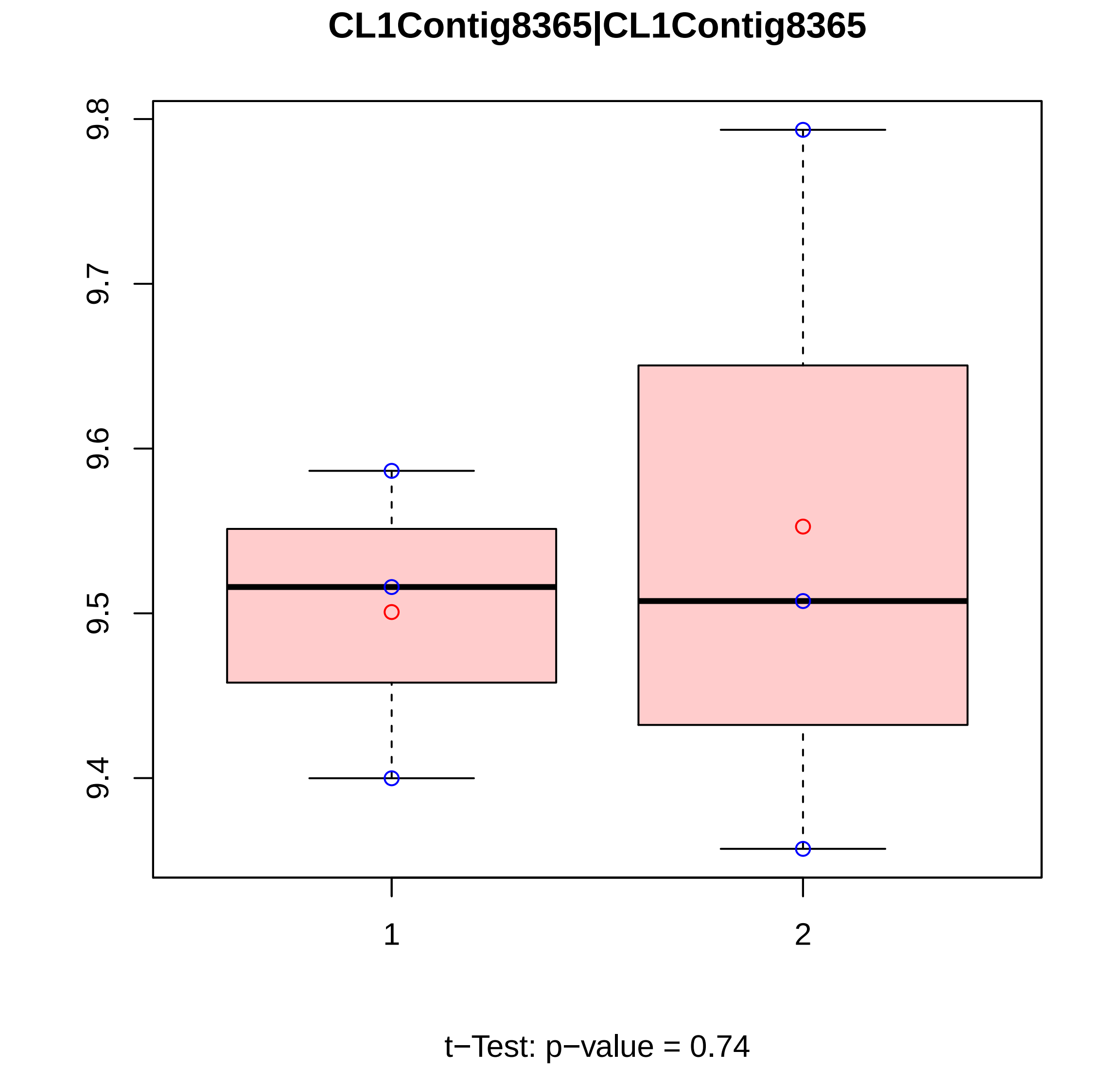

CL1Contig8365|CL1Contig8365
9.4
9.5
9.6
9.7
9.8
1
2
t−Test: p−value = 0.74

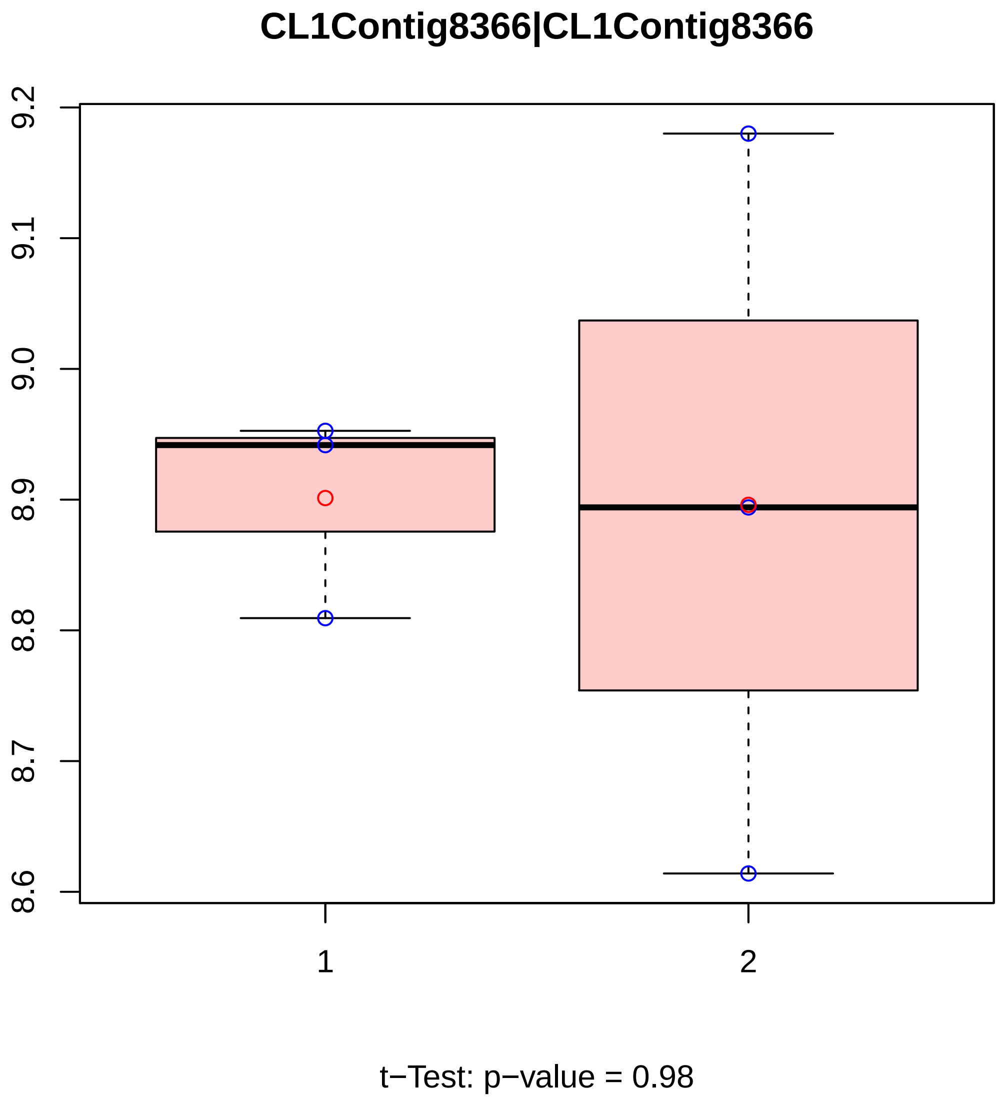
CL1Contig8366|CL1Contig8366
9.2
9.1
9.0
8.9
8.8
8.7
8.6
1
2
t−Test: p−value = 0.98

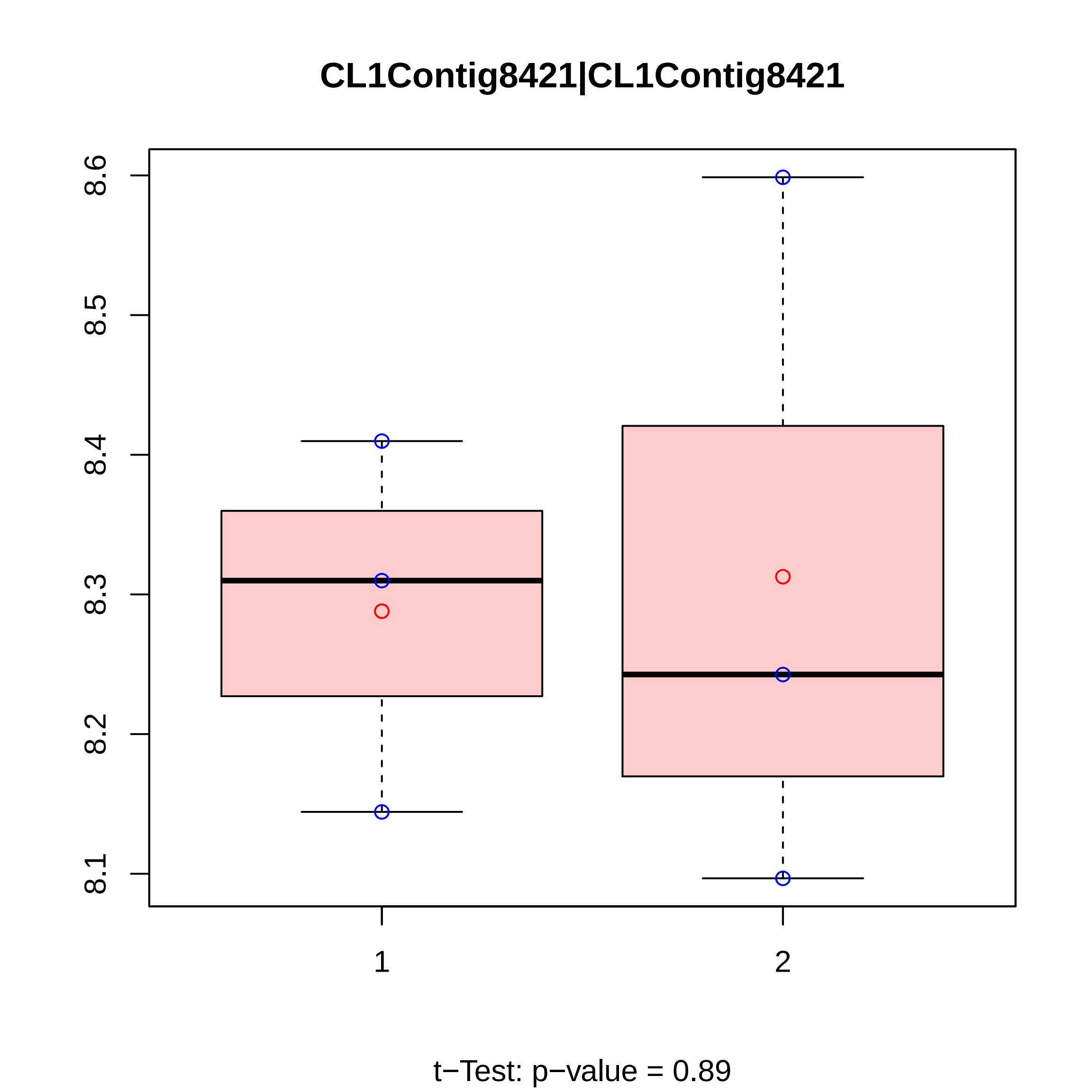

CL1Contig8421|CL1Contig8421
8.1
8.2
8.3
8.4
8.5
8.6
1
2
t−Test: p−value = 0.89

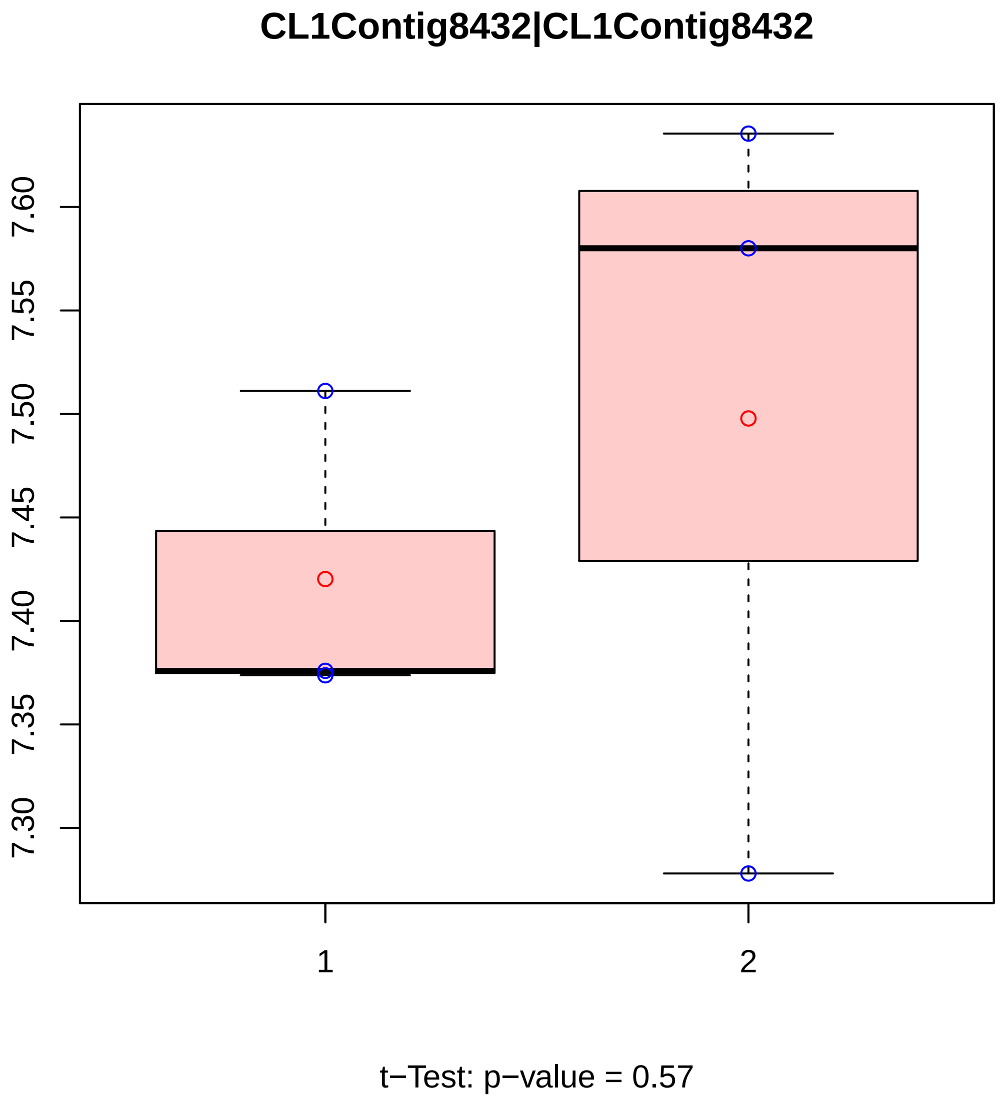

CL1Contig8432|CL1Contig8432
7.30
7.35
7.40
7.45
7.50
7.55
7.60
1
2
t−Test: p−value = 0.57

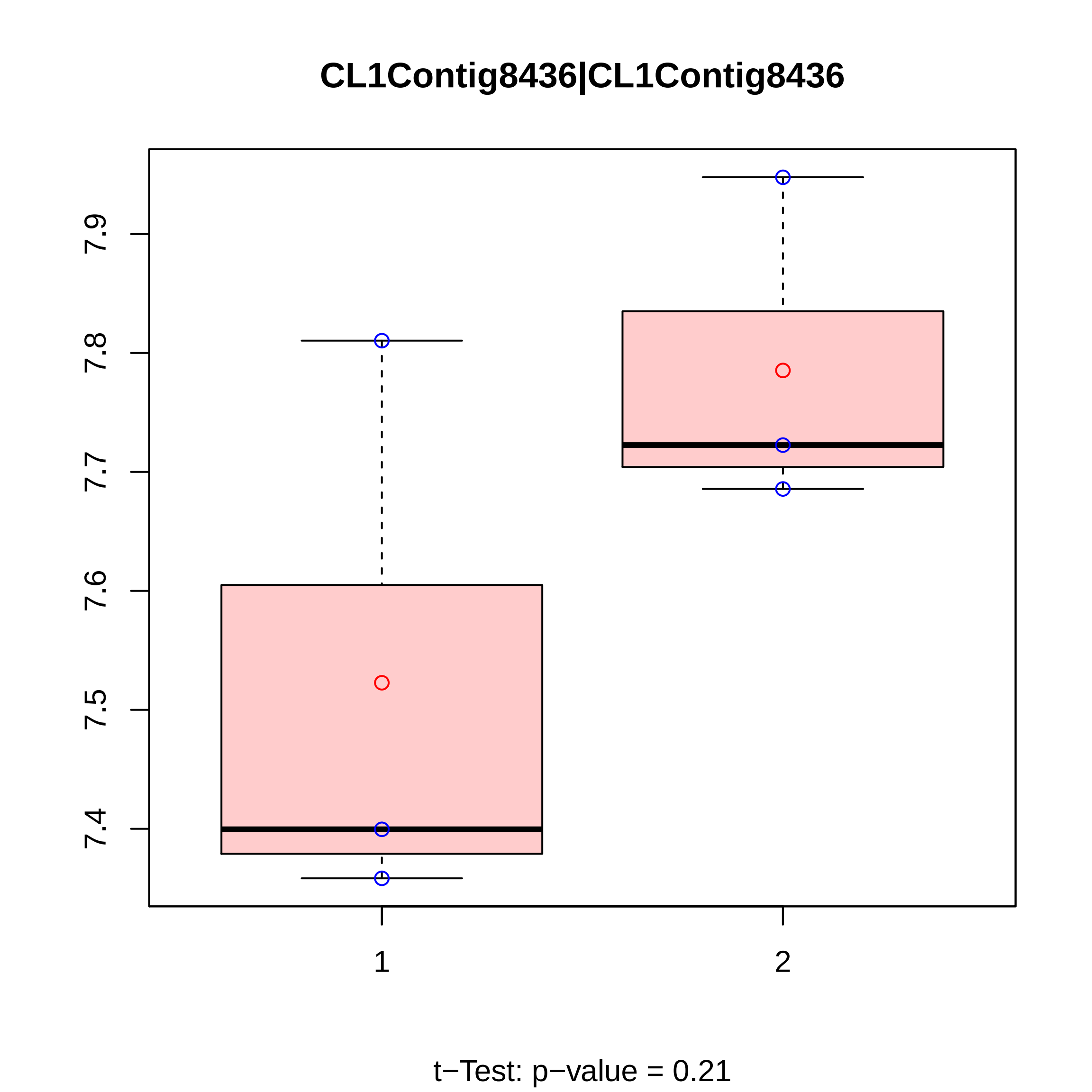
CL1Contig8436|CL1Contig8436
7.4
7.5
7.6
7.7
7.8
7.9
1
2
t−Test: p−value = 0.21

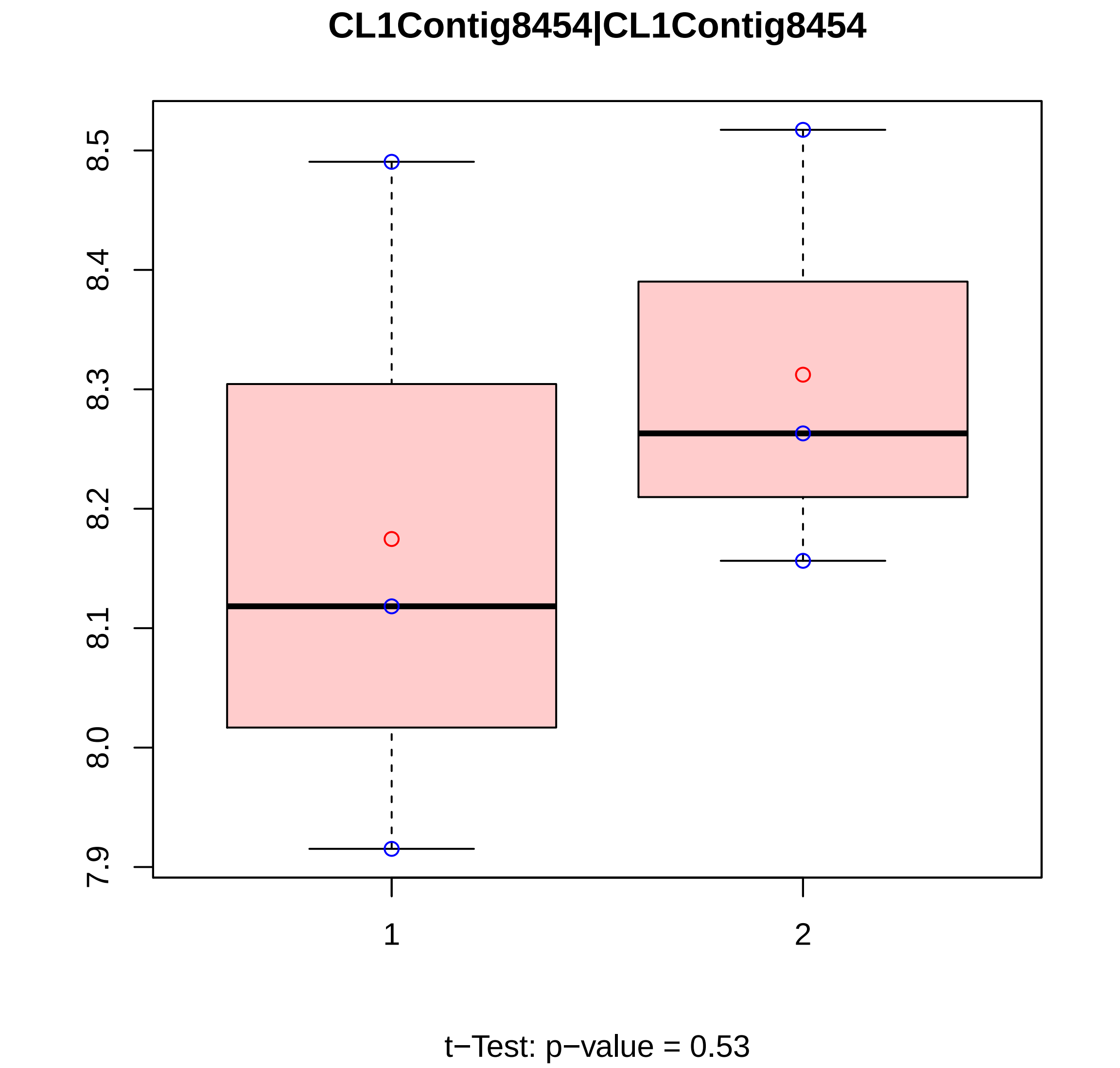
CL1Contig8454|CL1Contig8454
8.5
8.4
8.3
8.2
8.1
8.0
7.9
1
2
t−Test: p−value = 0.53

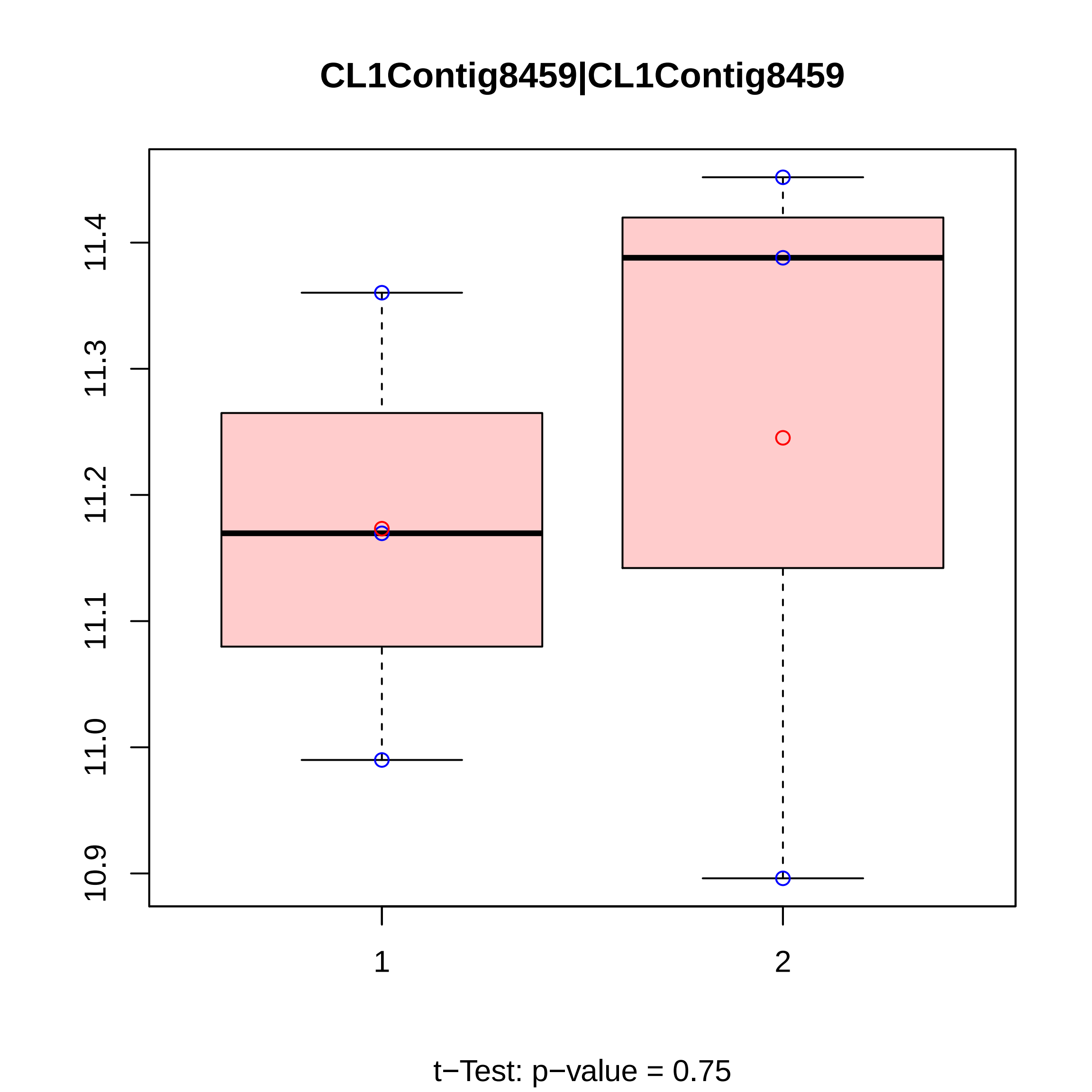
CL1Contig8459|CL1Contig8459
11.4
11.3
11.2
11.1
11.0
10.9
1
2
t−Test: p−value = 0.75

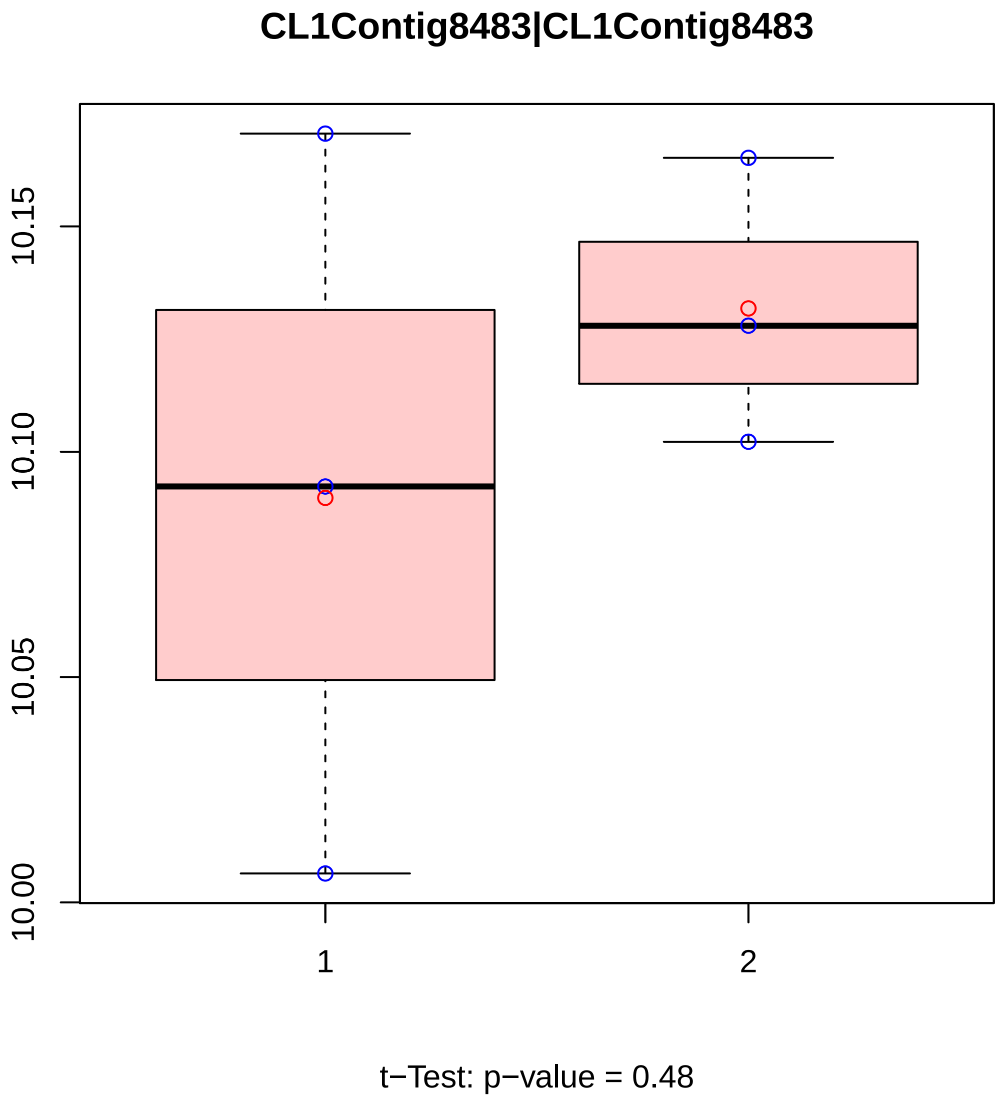
CL1Contig8483|CL1Contig8483
10.00
10.05
10.10
10.15
1
2
t−Test: p−value = 0.48

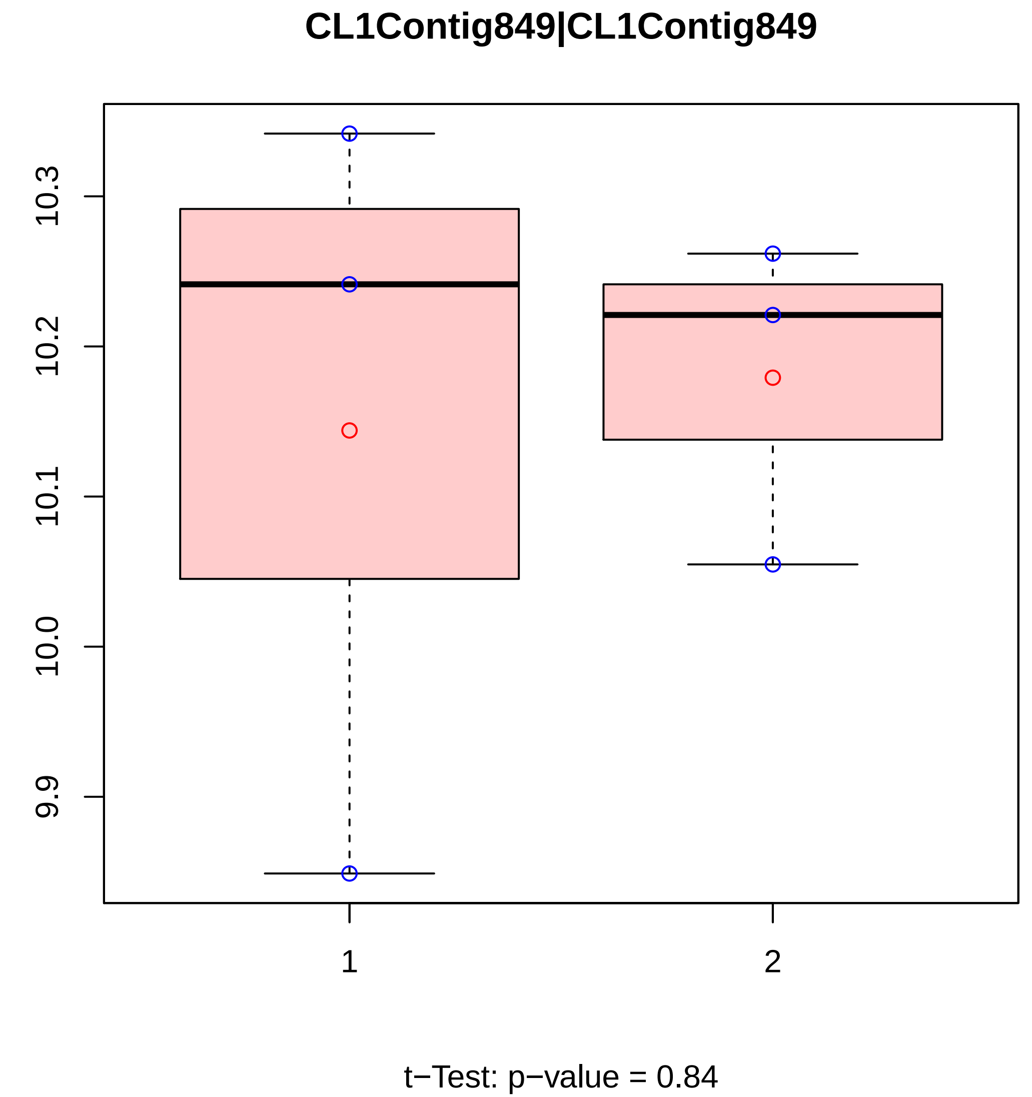

CL1Contig849|CL1Contig849
10.3
10.2
10.1
10.0
9.9
1
2
t−Test: p−value = 0.84

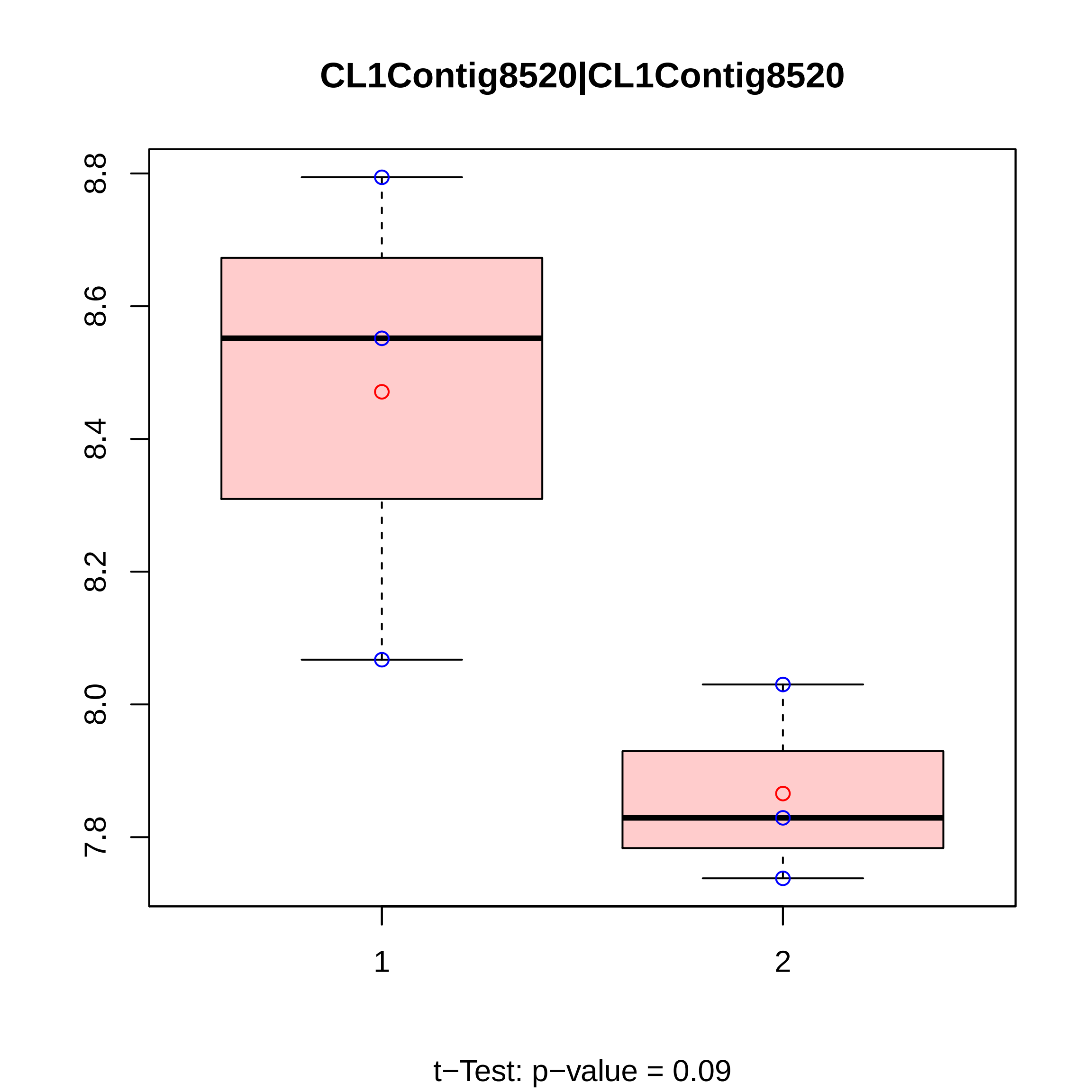

CL1Contig8520|CL1Contig8520
8.8
8.6
8.4
8.2
8.0
7.8
1
2
t−Test: p−value = 0.09

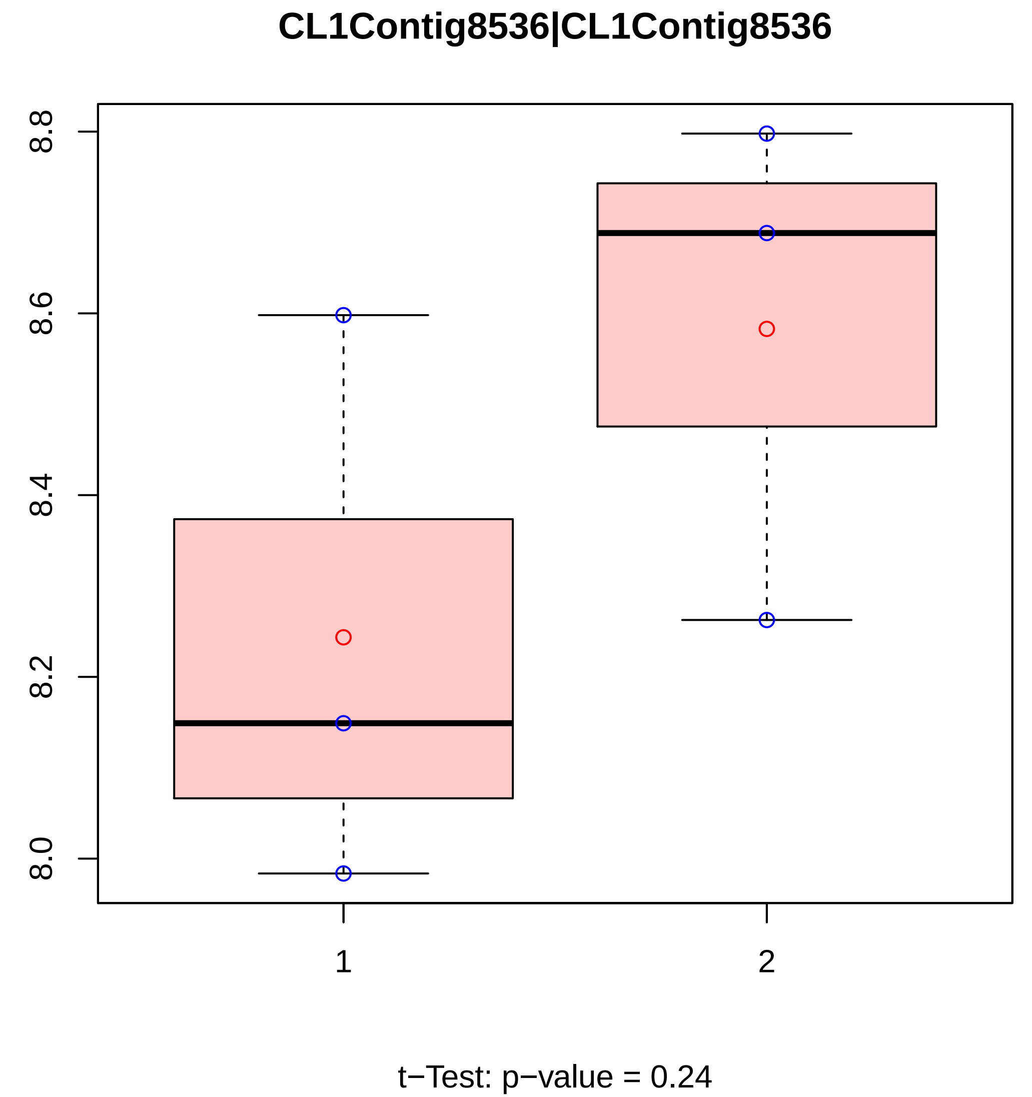
CL1Contig8536|CL1Contig8536
8.0
8.2
8.4
8.6
8.8
1
2
t−Test: p−value = 0.24

# CL1Contig8539|CL1Contig8539

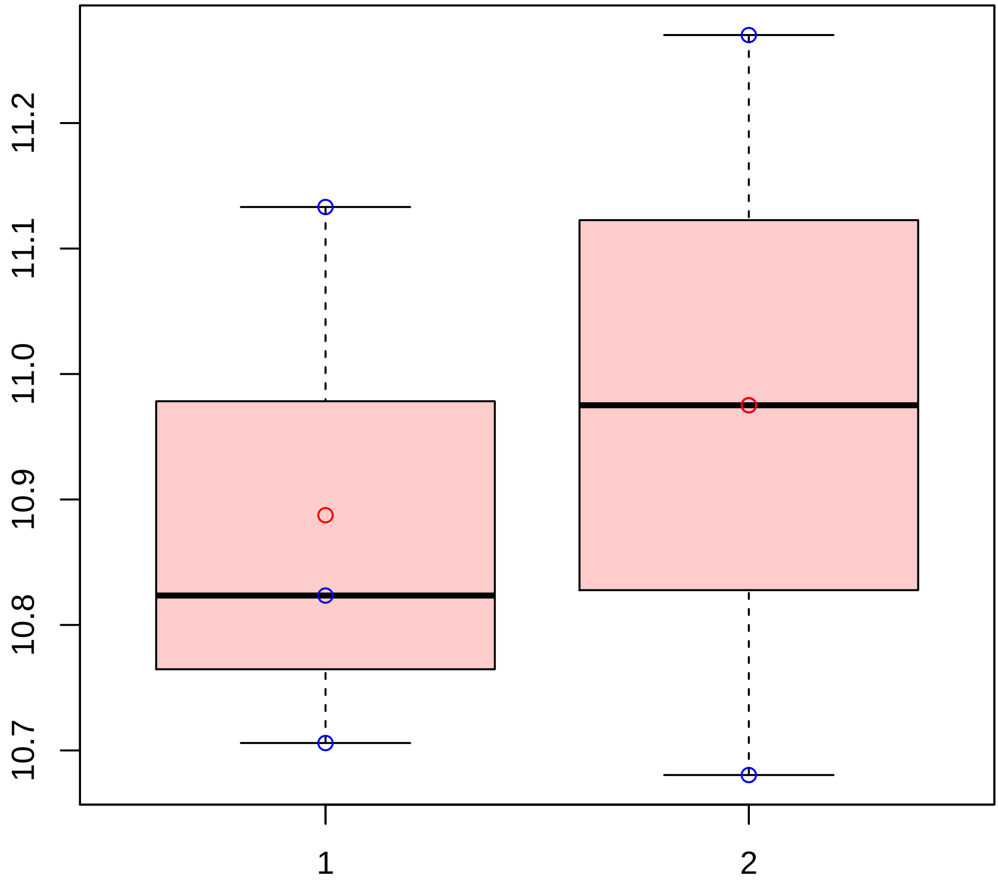


t−Test: p−value = 0.7

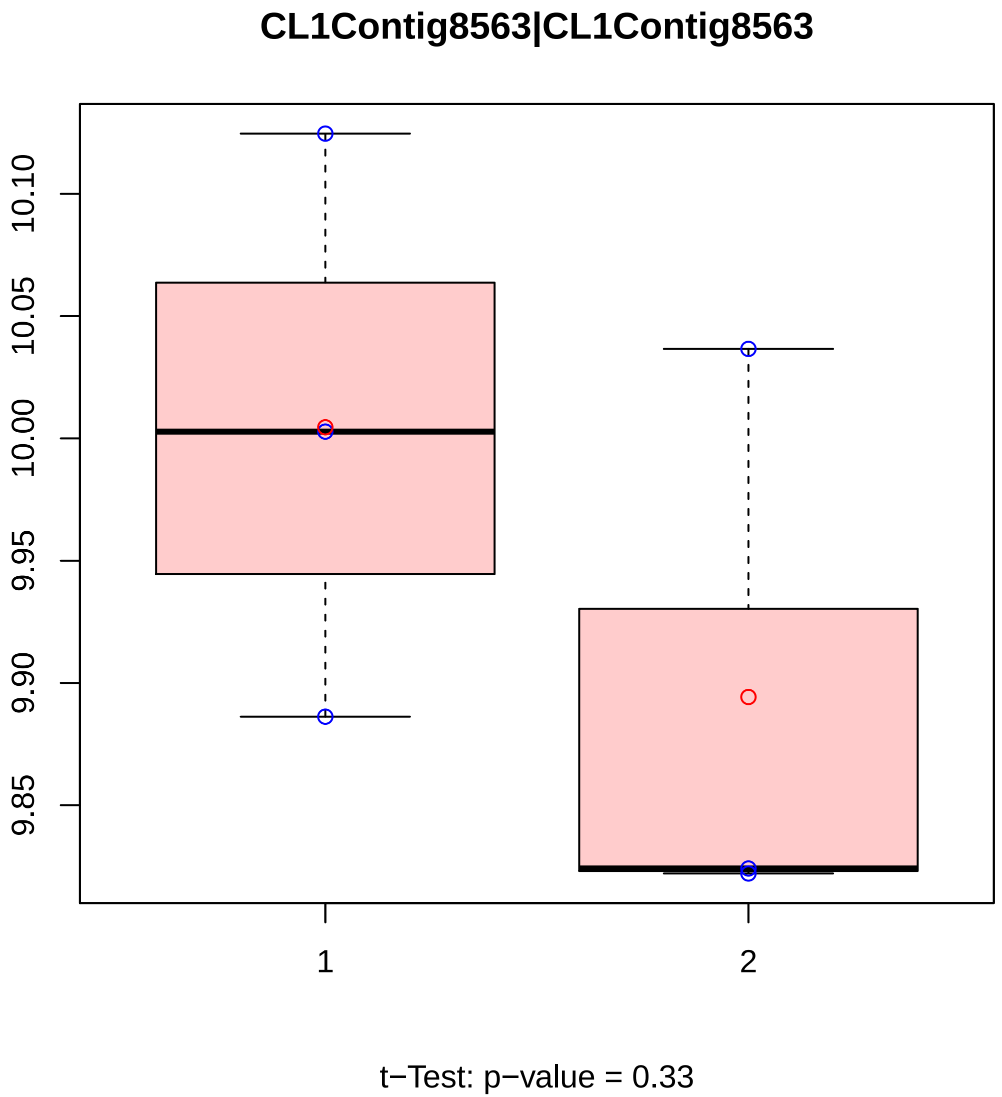
CL1Contig8563|CL1Contig8563
10.10
10.05
10.00
9.95
9.90
9.85
1
2
t−Test: p−value = 0.33

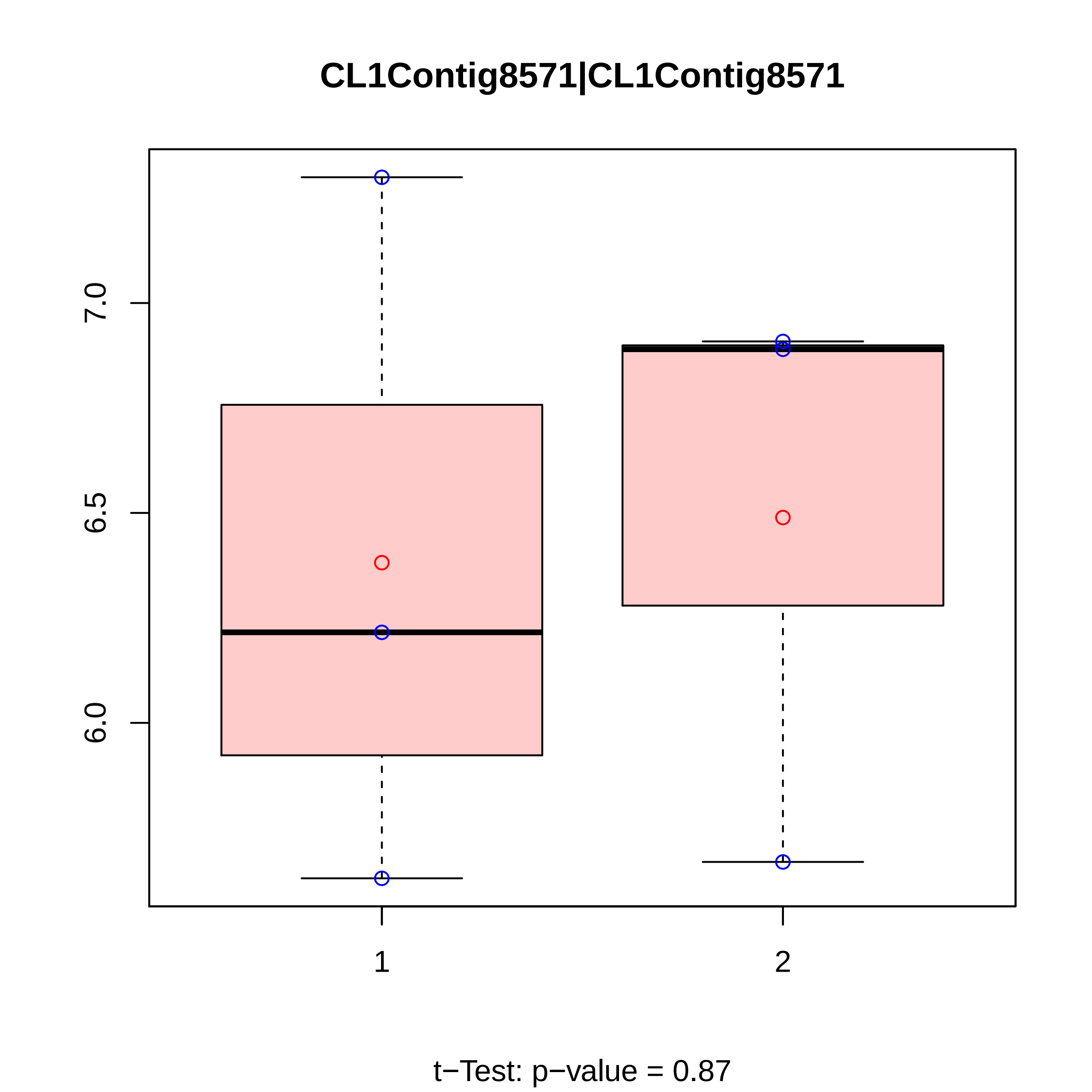
CL1Contig8571|CL1Contig8571
7.0
6.5
6.0
1
2
t−Test: p−value = 0.87

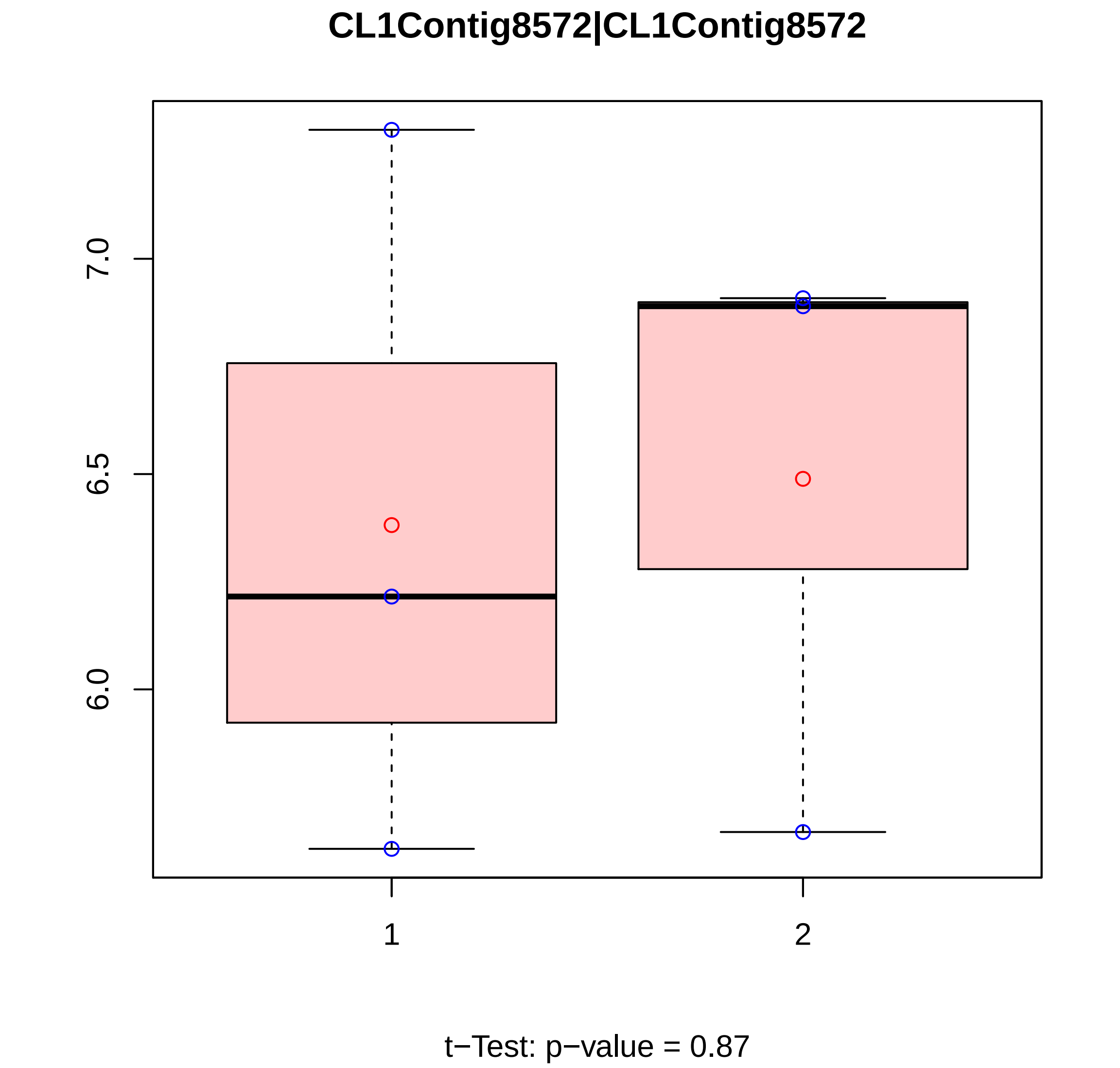
CL1Contig8572|CL1Contig8572
7.0
6.5
6.0
1
2
t−Test: p−value = 0.87

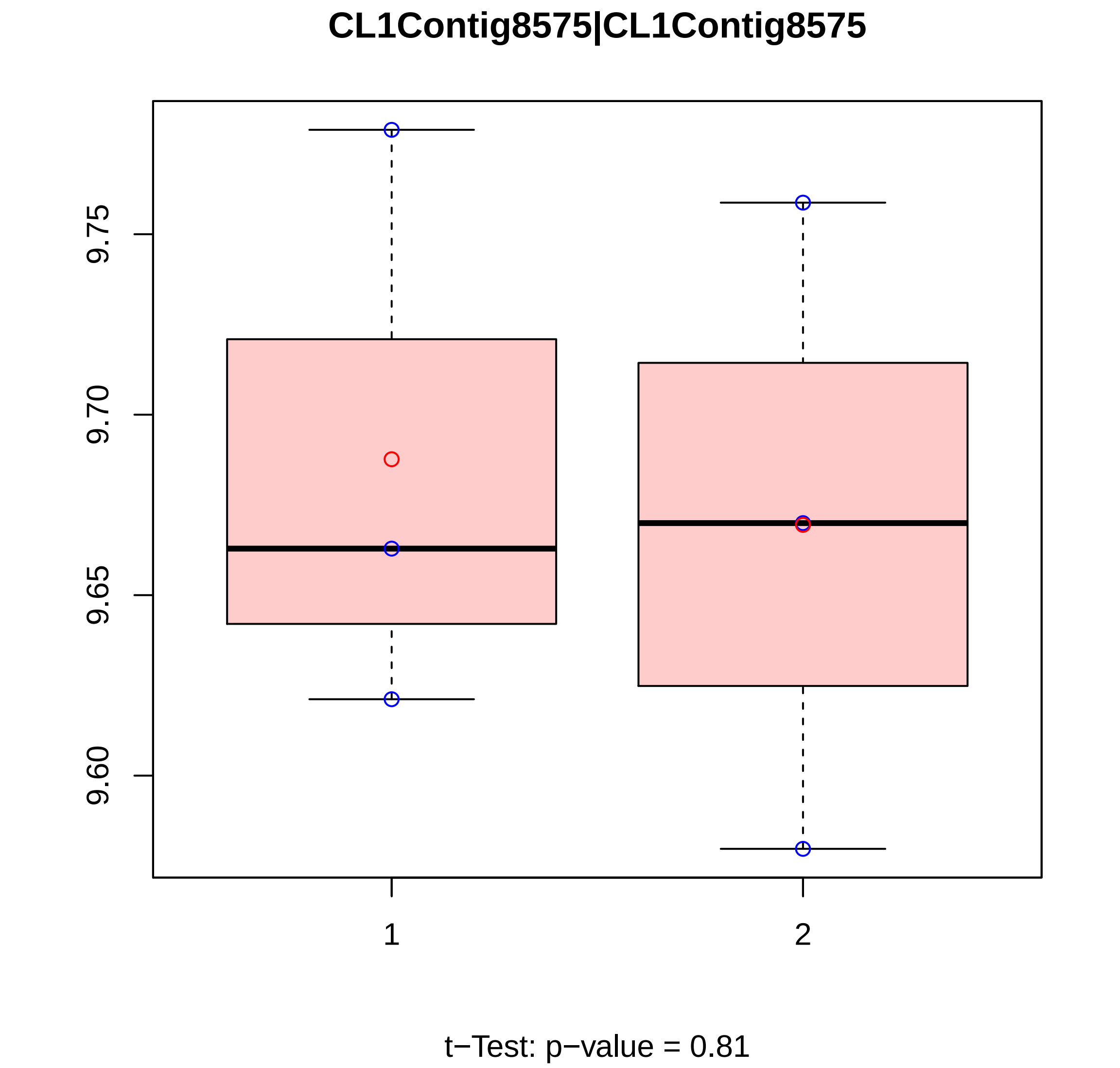

CL1Contig8575|CL1Contig8575
9.60
9.65
9.70
9.75
1
2
t−Test: p−value = 0.81

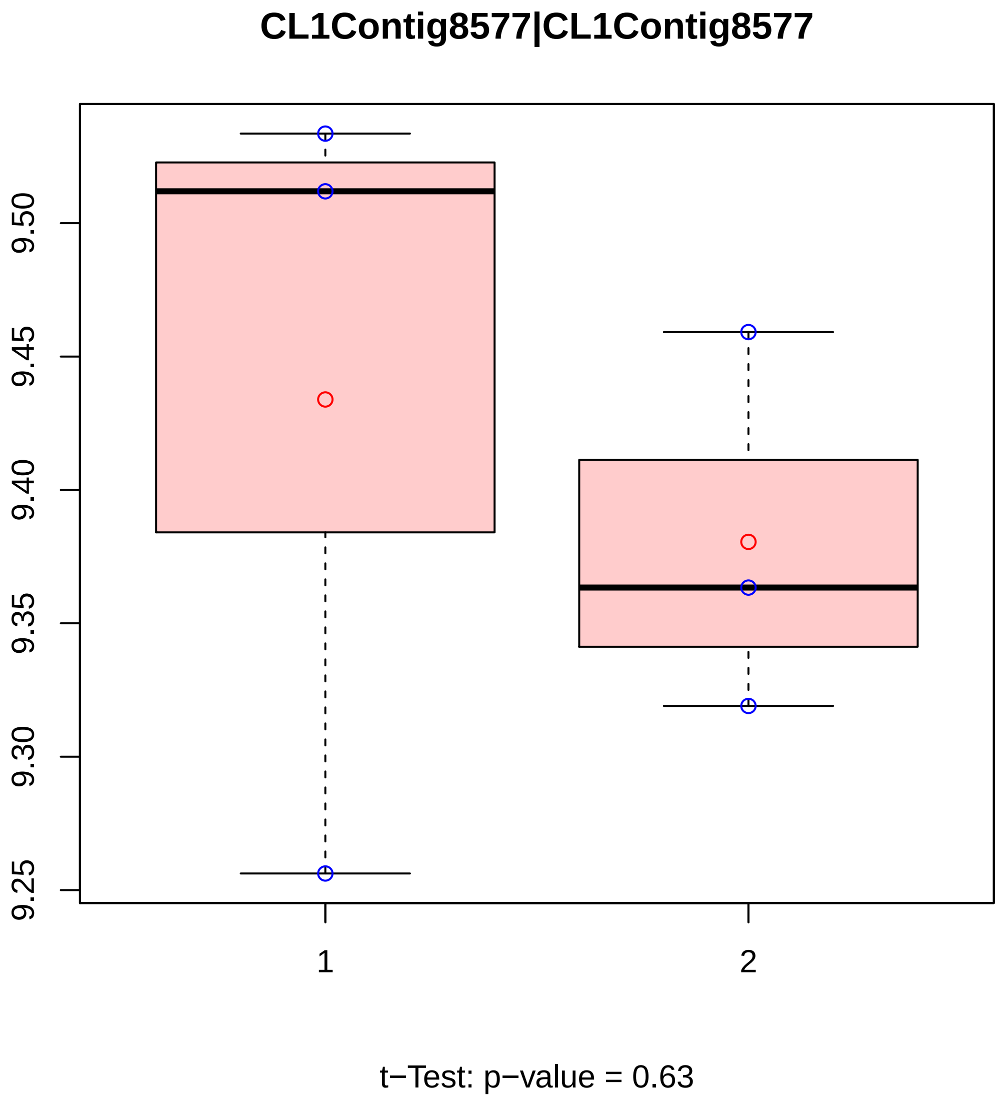
CL1Contig8577|CL1Contig8577
9.25
9.30
9.35
9.40
9.45
9.50
1
2
t−Test: p−value = 0.63

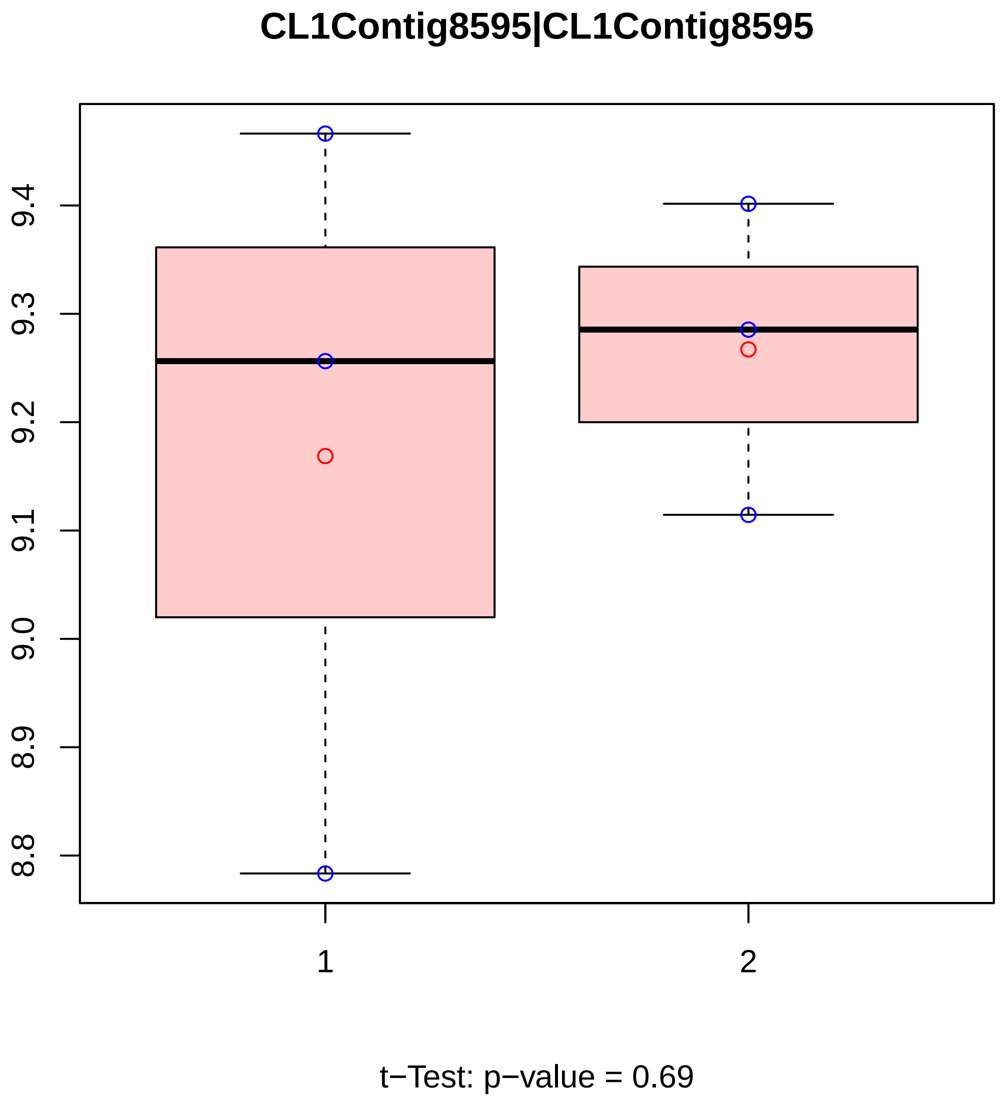
CL1Contig8595|CL1Contig8595
9.4
9.3
9.2
9.1
9.0
8.9
8.8
1
2
t−Test: p−value = 0.69

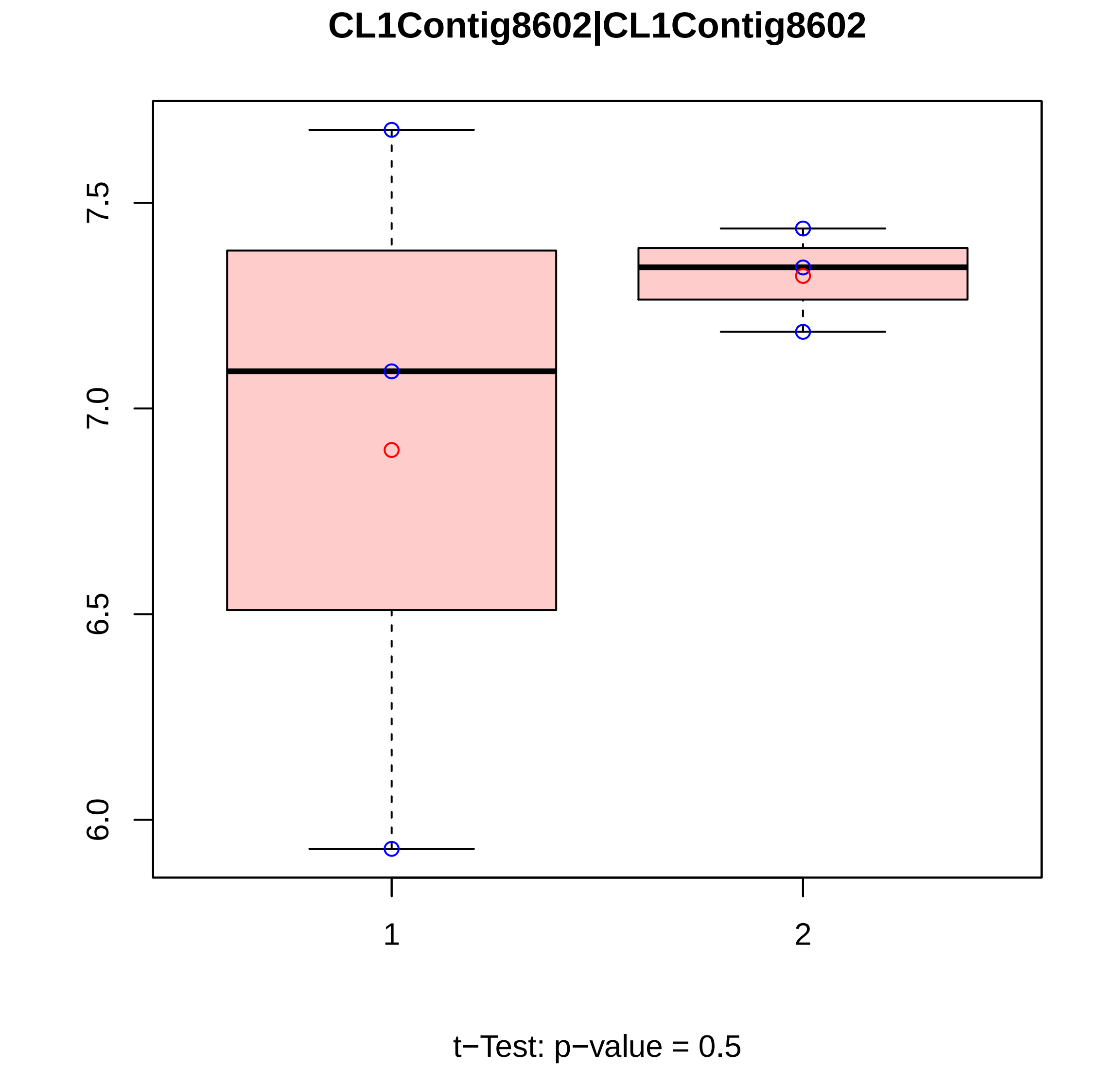
CL1Contig8602|CL1Contig8602
7.5
7.0
6.5
6.0
1
2
t−Test: p−value = 0.5

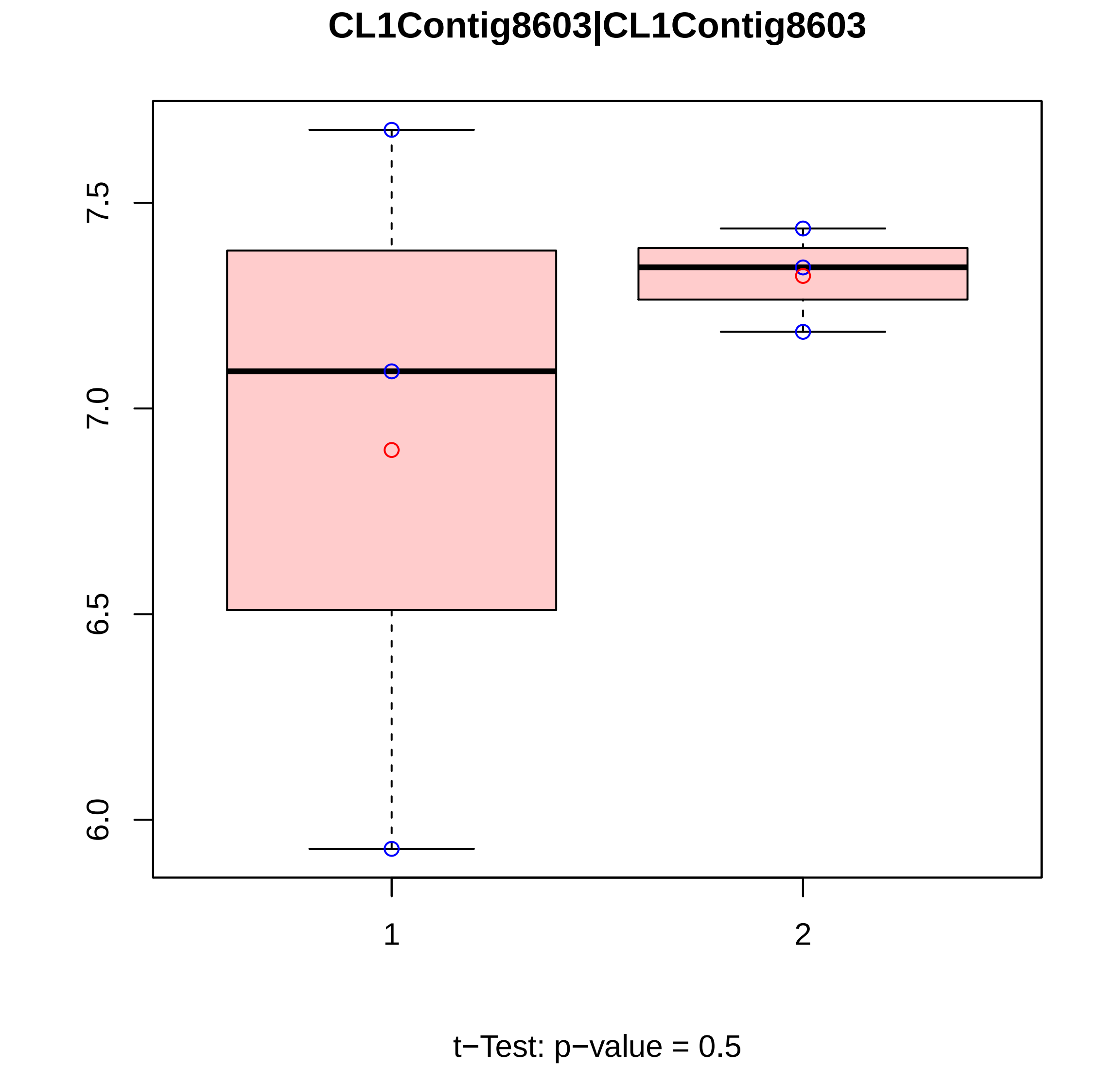
CL1Contig8603|CL1Contig8603
7.5
7.0
6.5
6.0
1
2
t−Test: p−value = 0.5

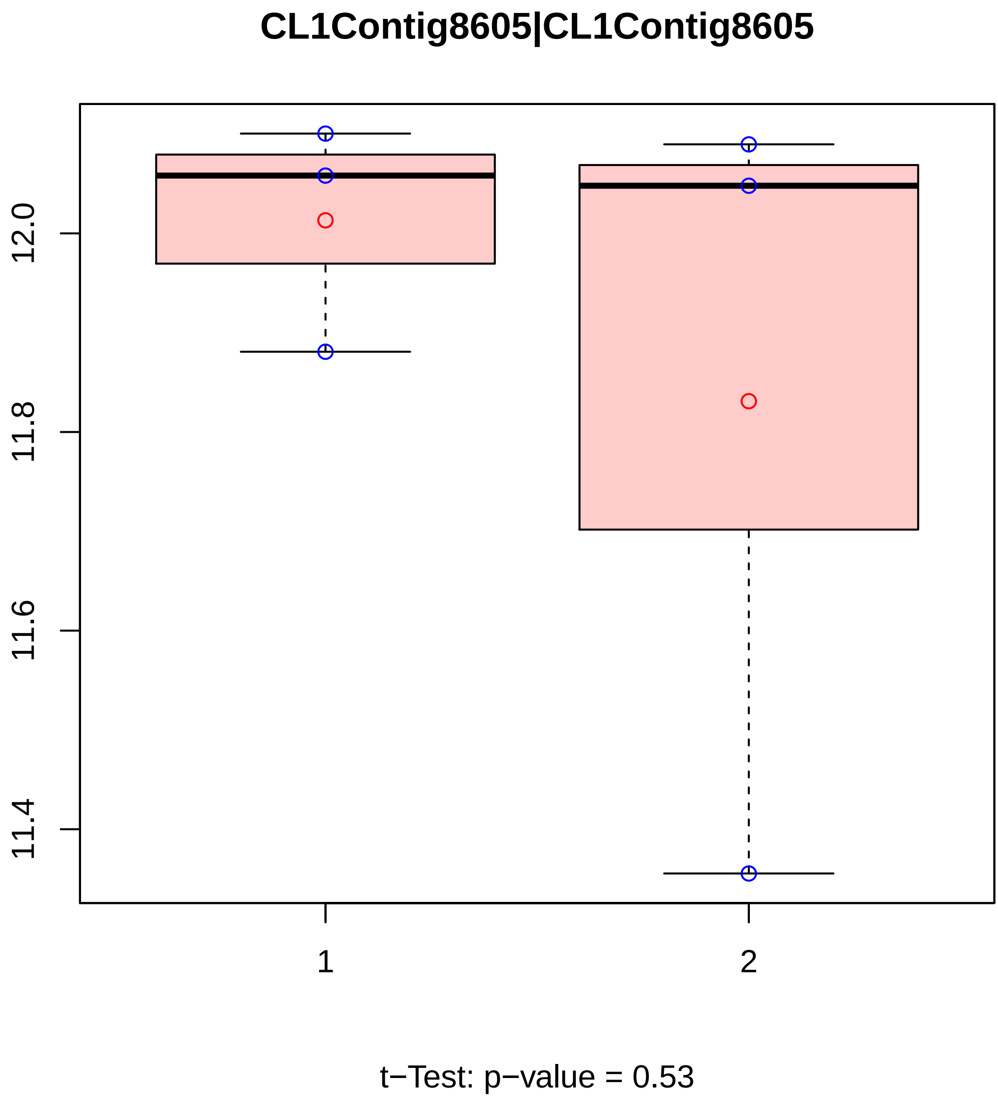
CL1Contig8605|CL1Contig8605
12.0
11.8
11.6
11.4
1
2
t−Test: p−value = 0.53

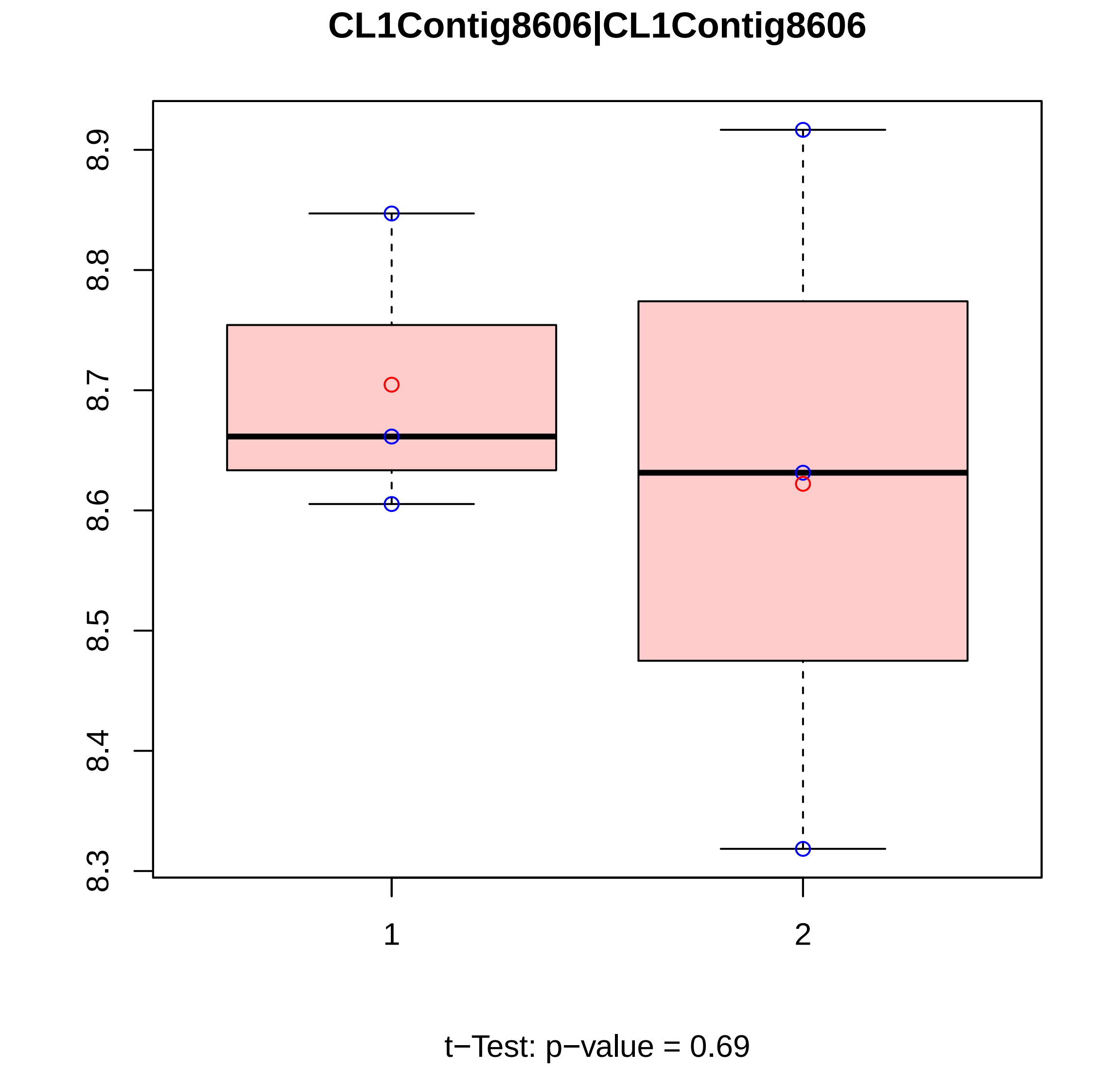
CL1Contig8606|CL1Contig8606
8.3
8.4
8.5
8.6
8.7
8.8
8.9
1
2
t−Test: p−value = 0.69

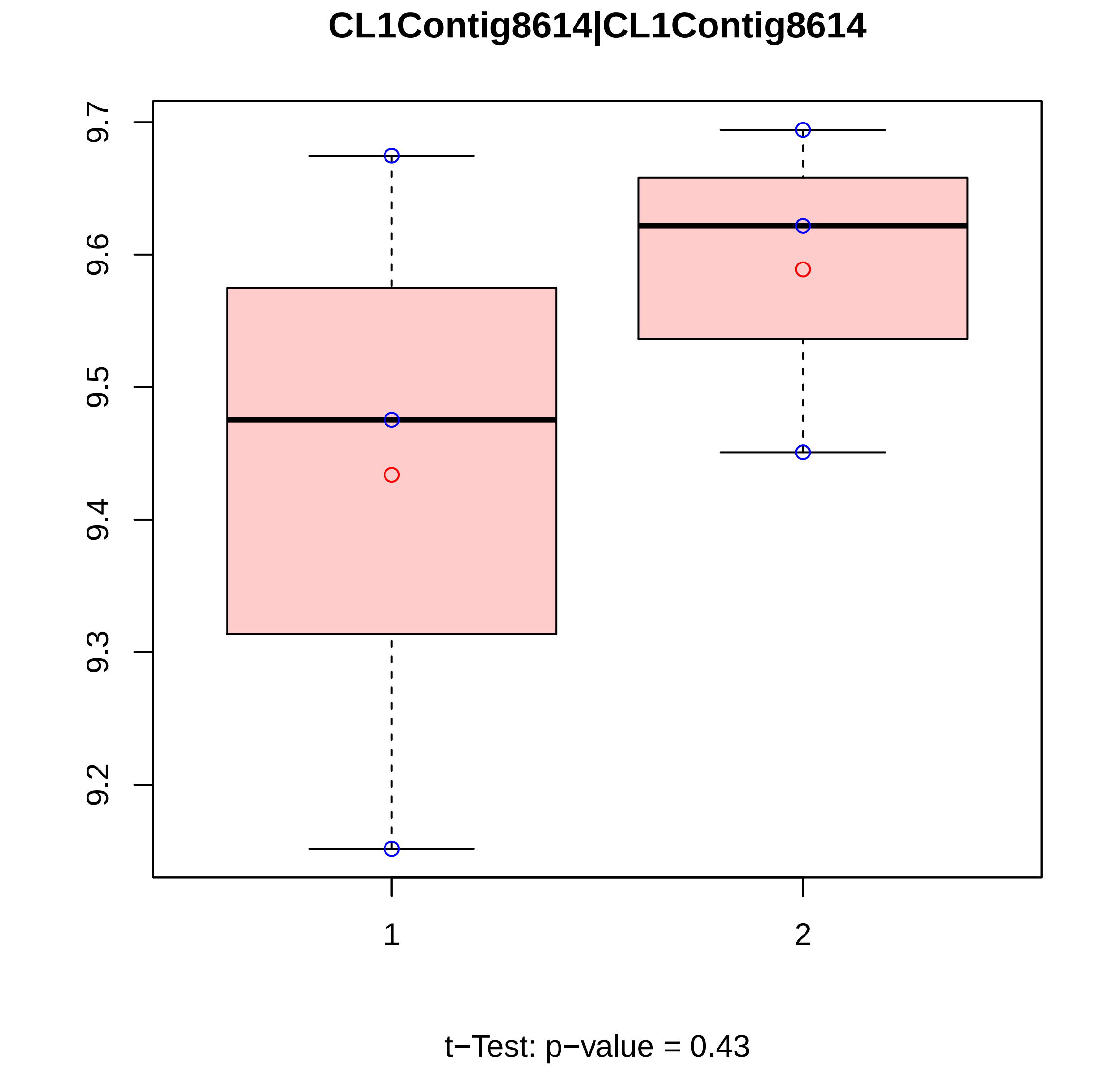
CL1Contig8614|CL1Contig8614
9.7
9.6
9.5
9.4
9.3
9.2
1
2
t−Test: p−value = 0.43

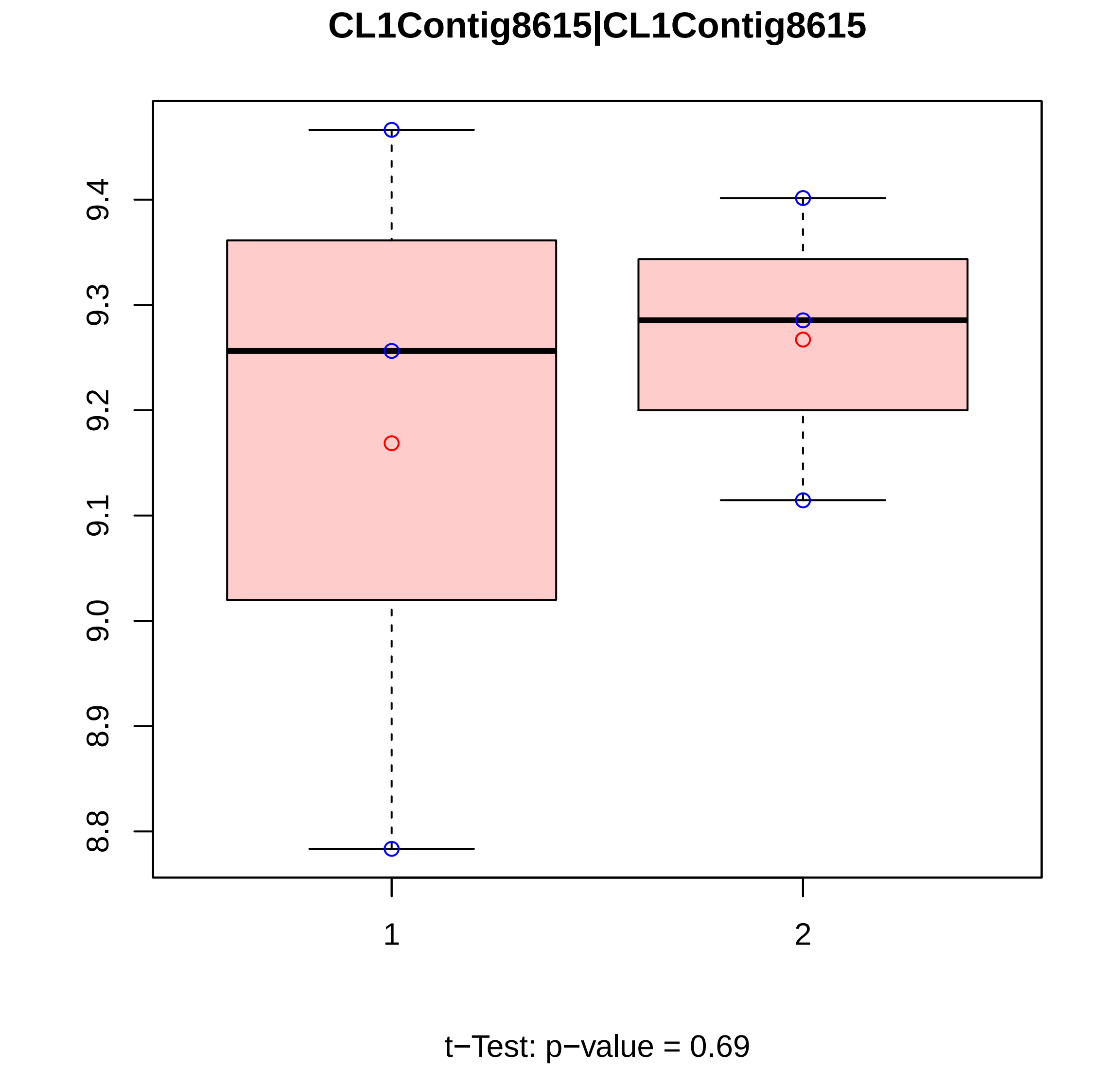

CL1Contig8615|CL1Contig8615
9.4
9.3
9.2
9.1
9.0
8.9
8.8
1
2
t−Test: p−value = 0.69

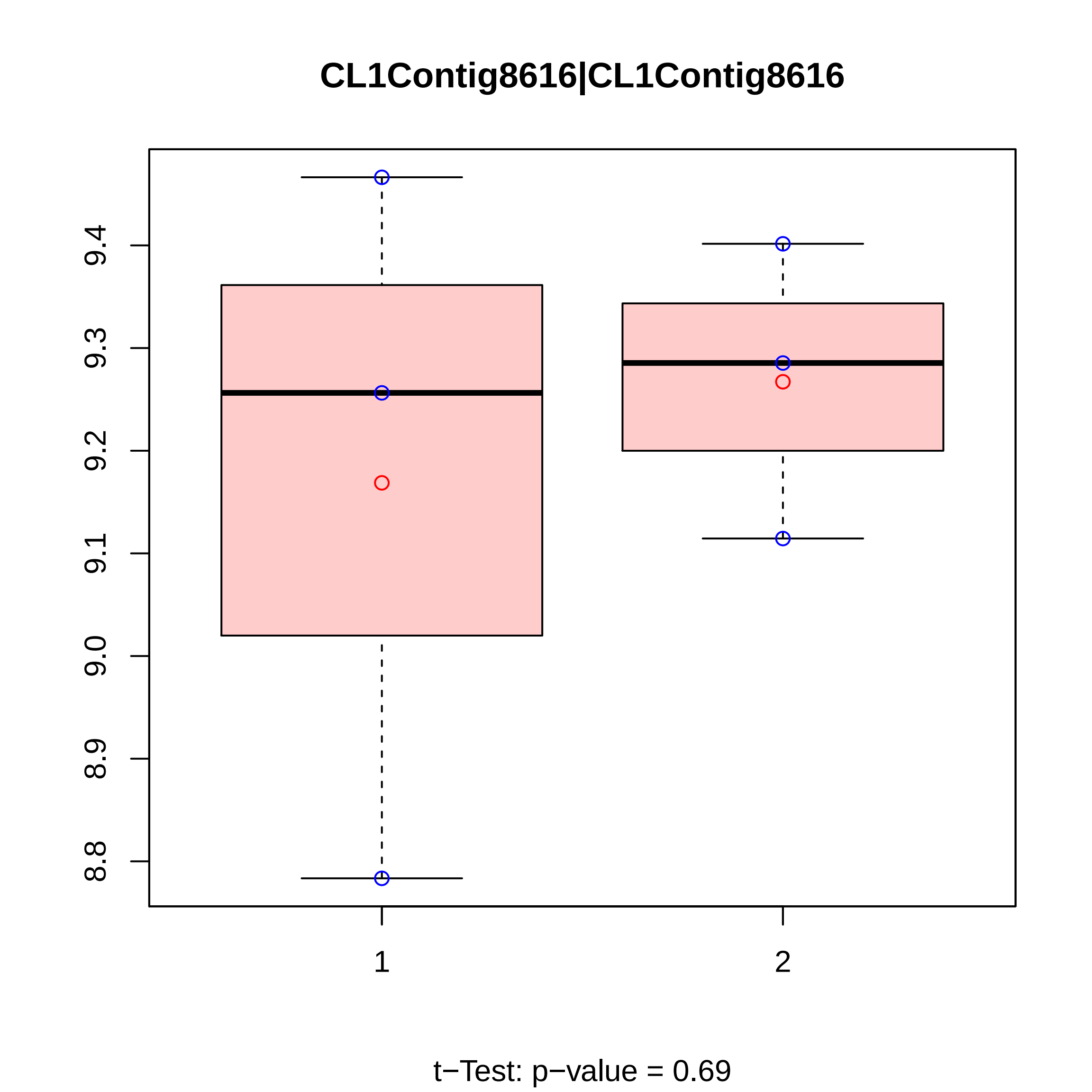
CL1Contig8616|CL1Contig8616
9.4
9.3
9.2
9.1
9.0
8.9
8.8
1
2
t−Test: p−value = 0.69

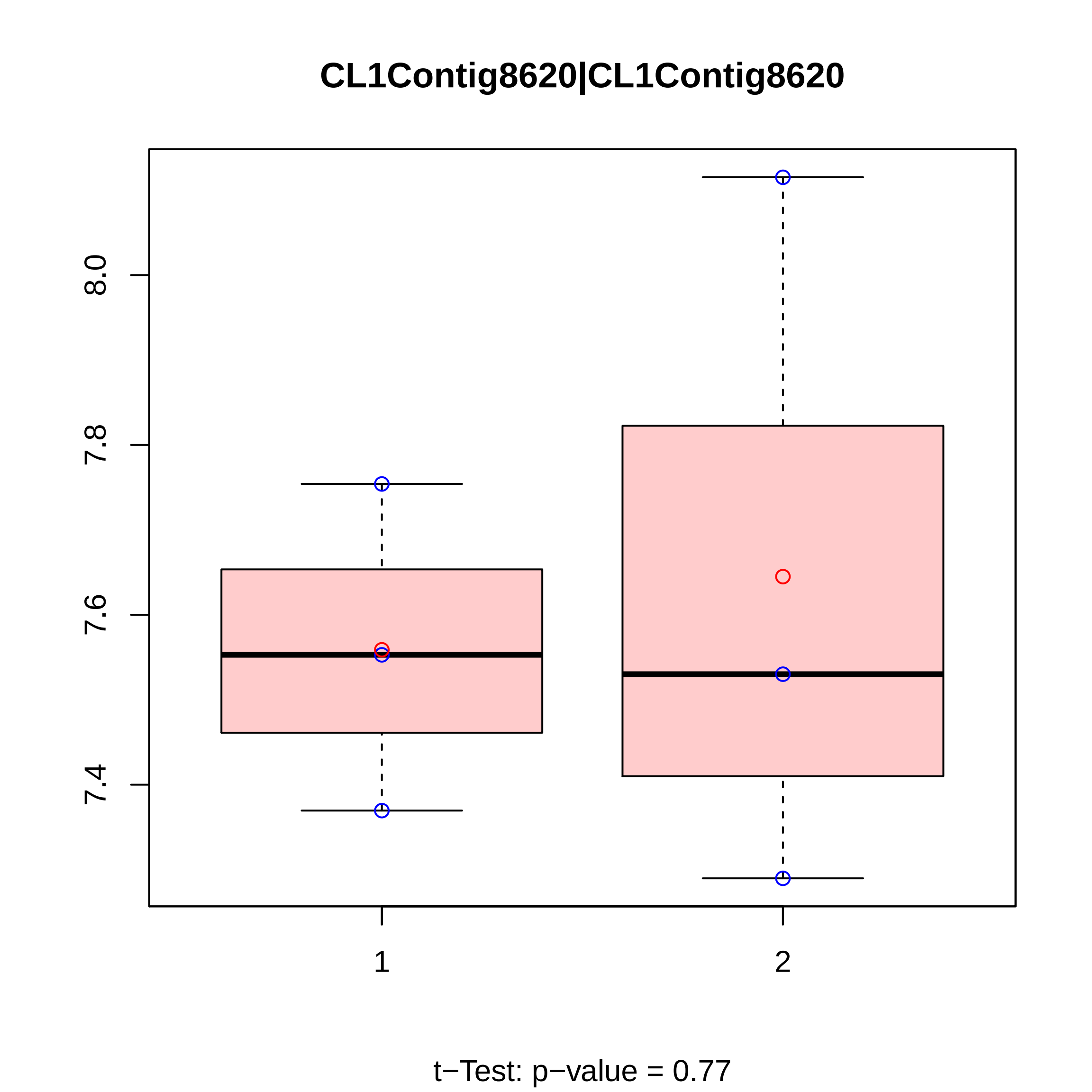
CL1Contig8620|CL1Contig8620
8.0
7.8
7.6
7.4
1
2
t−Test: p−value = 0.77

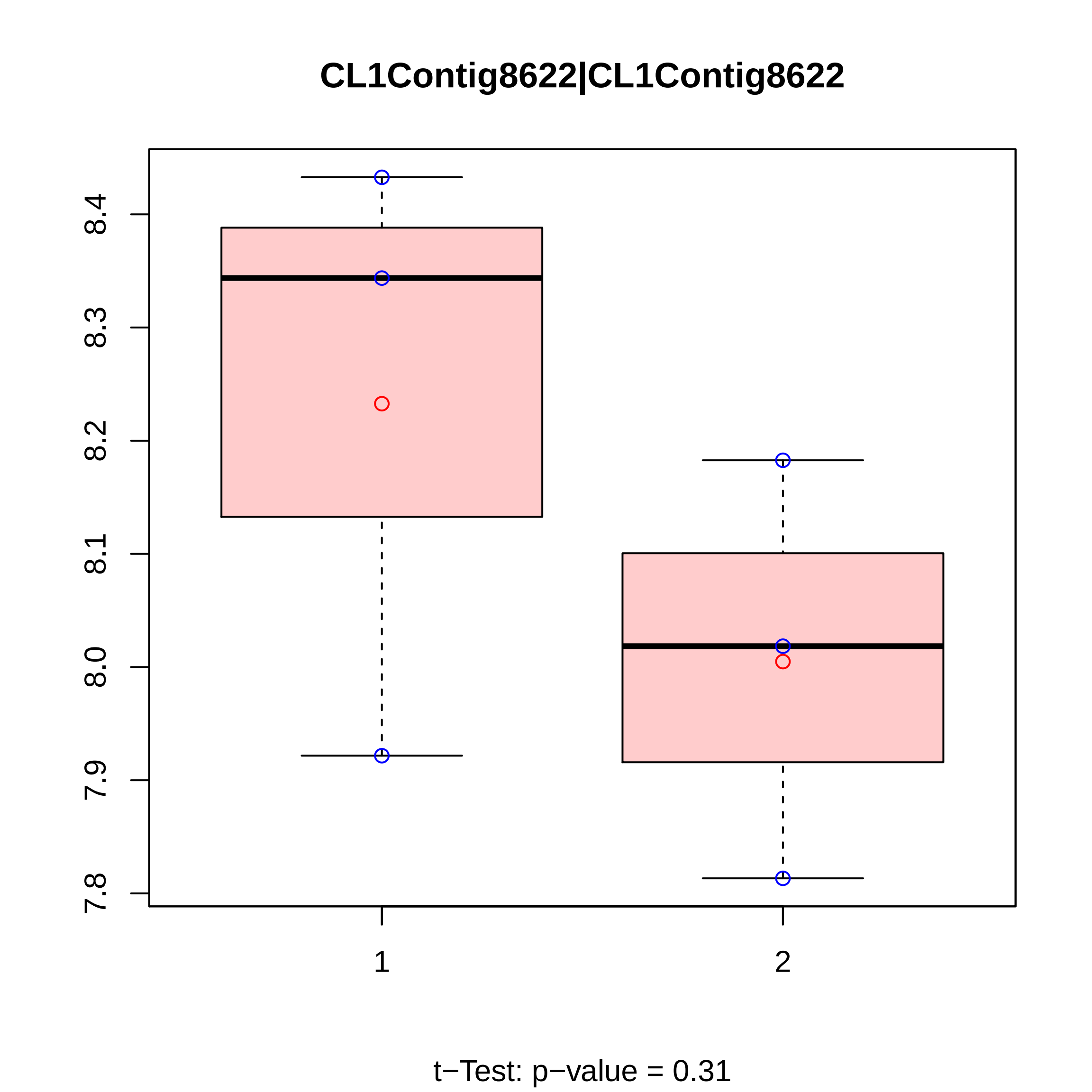

CL1Contig8622|CL1Contig8622
8.4
8.3
8.2
8.1
8.0
7.9
7.8
1
2
t−Test: p−value = 0.31

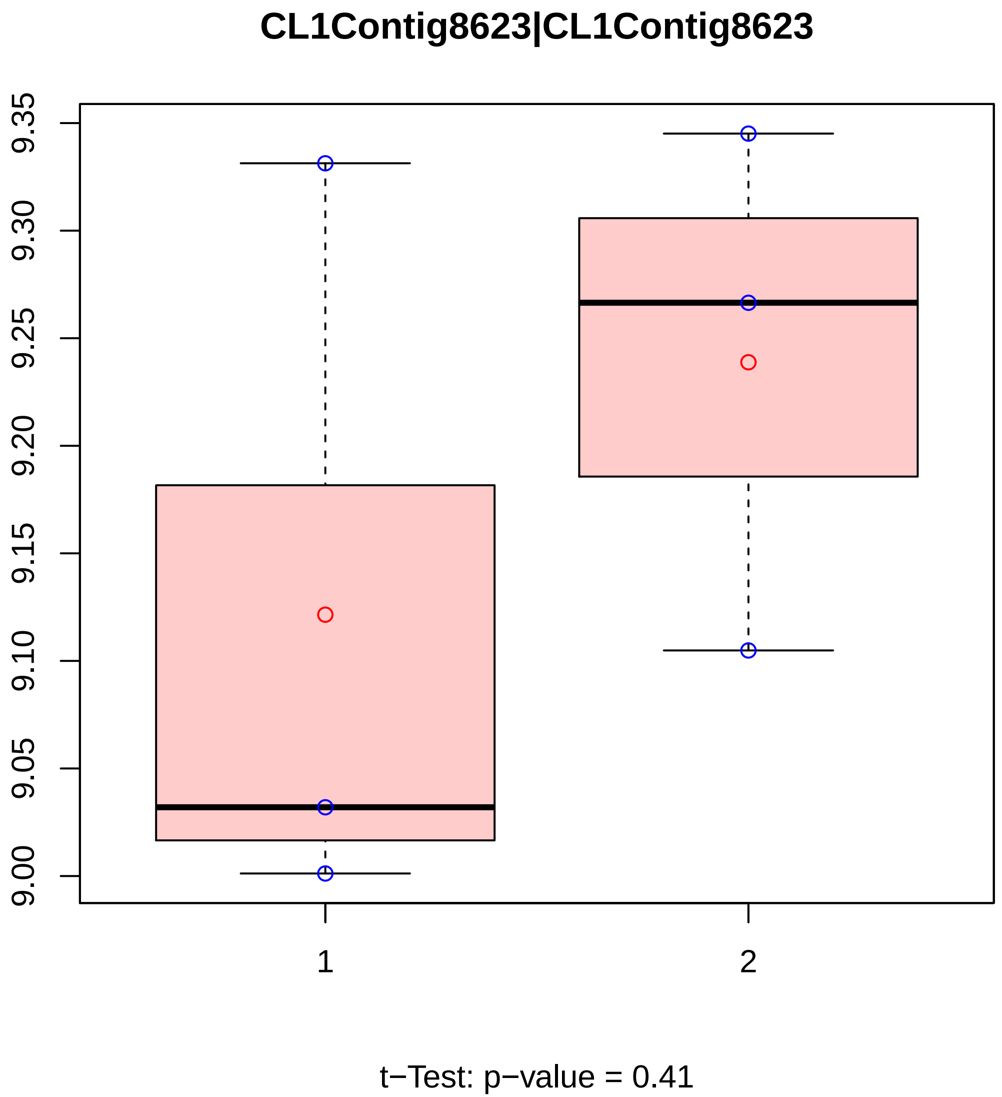
CL1Contig8623|CL1Contig8623
9.00
9.05
9.10
9.15
9.20
9.25
9.30
9.35
1
2
t−Test: p−value = 0.41

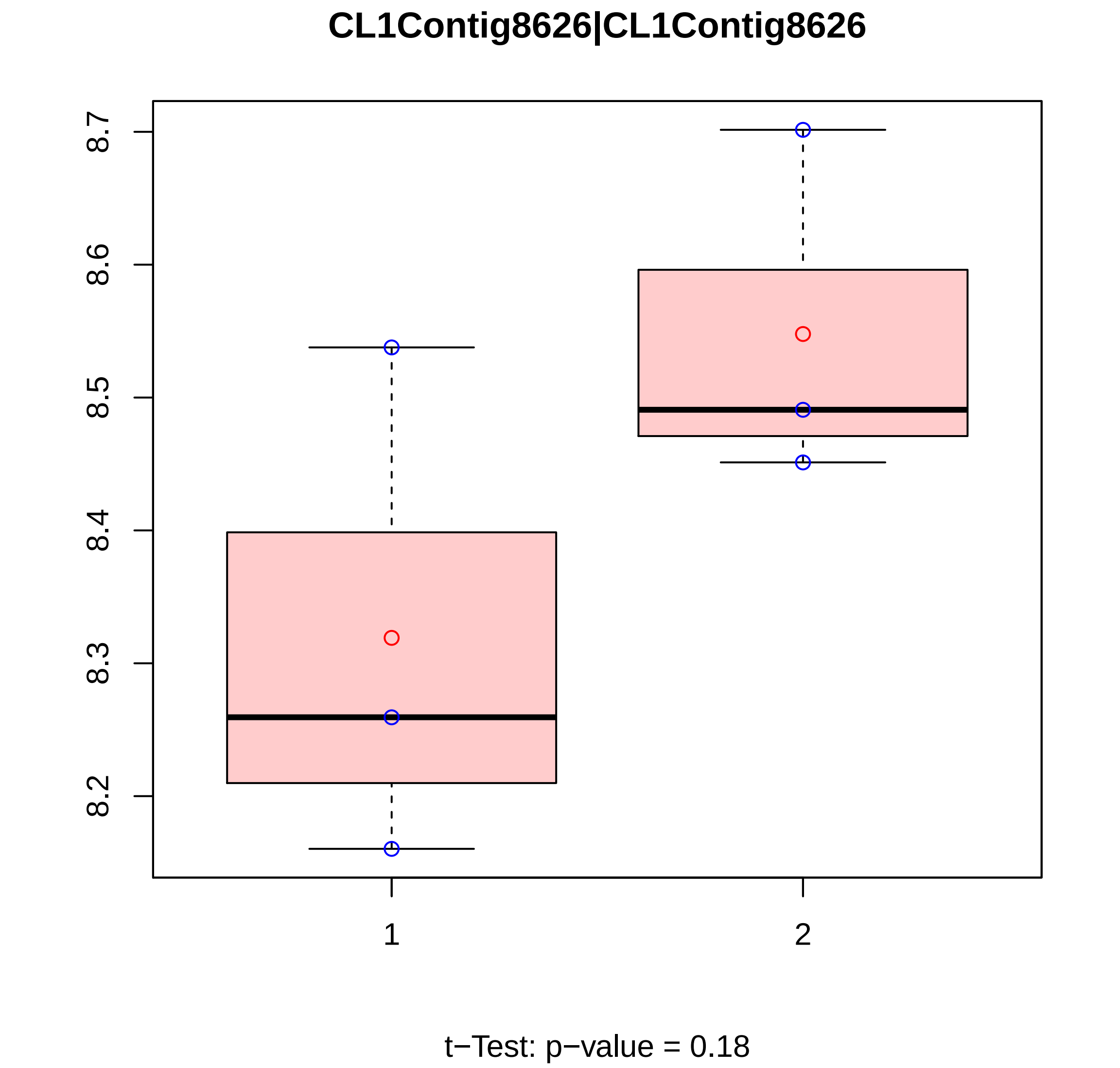
CL1Contig8626|CL1Contig8626
8.2
8.3
8.4
8.5
8.6
8.7
1
2
t−Test: p−value = 0.18

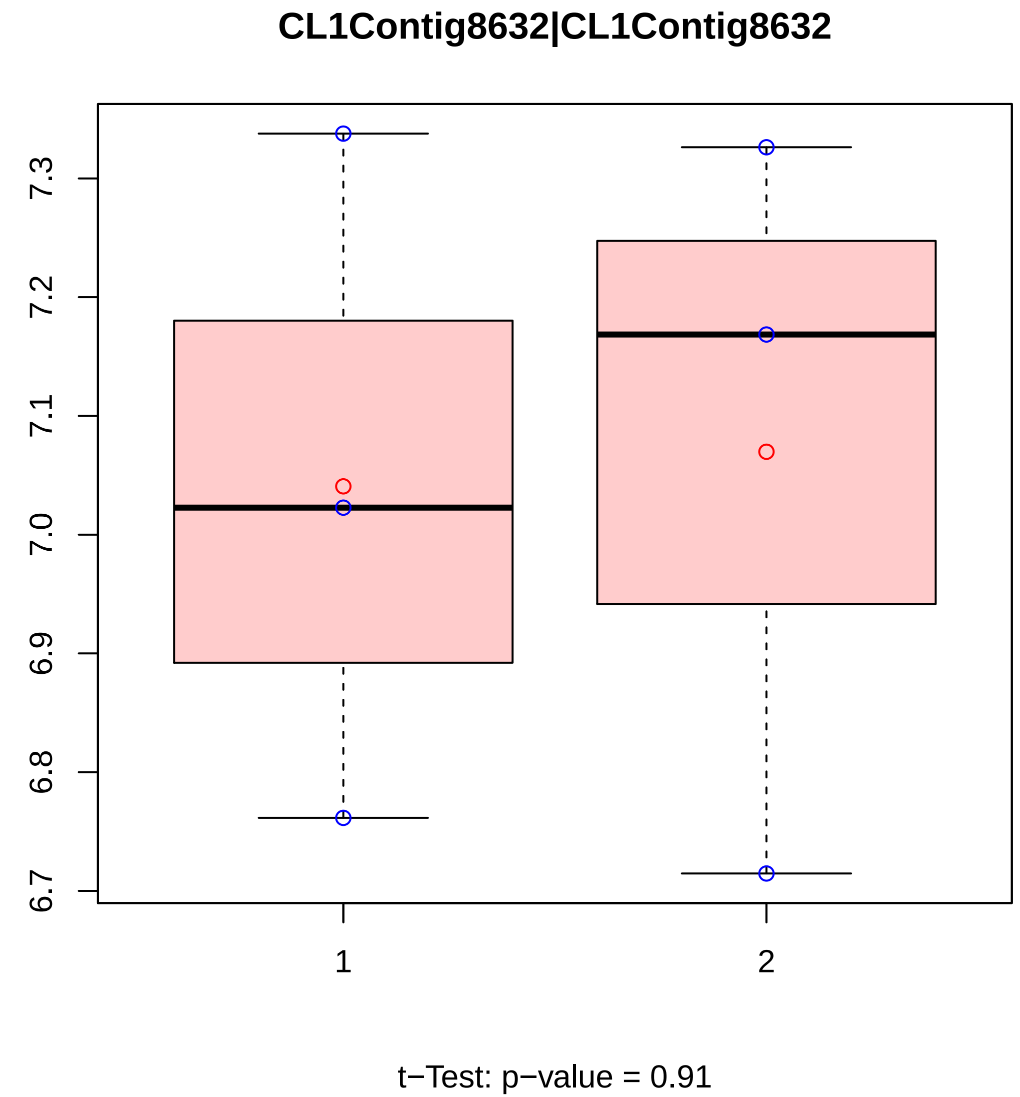

CL1Contig8632|CL1Contig8632
7.3
7.2
7.1
7.0
6.9
6.8
6.7
1
2
t−Test: p−value = 0.91

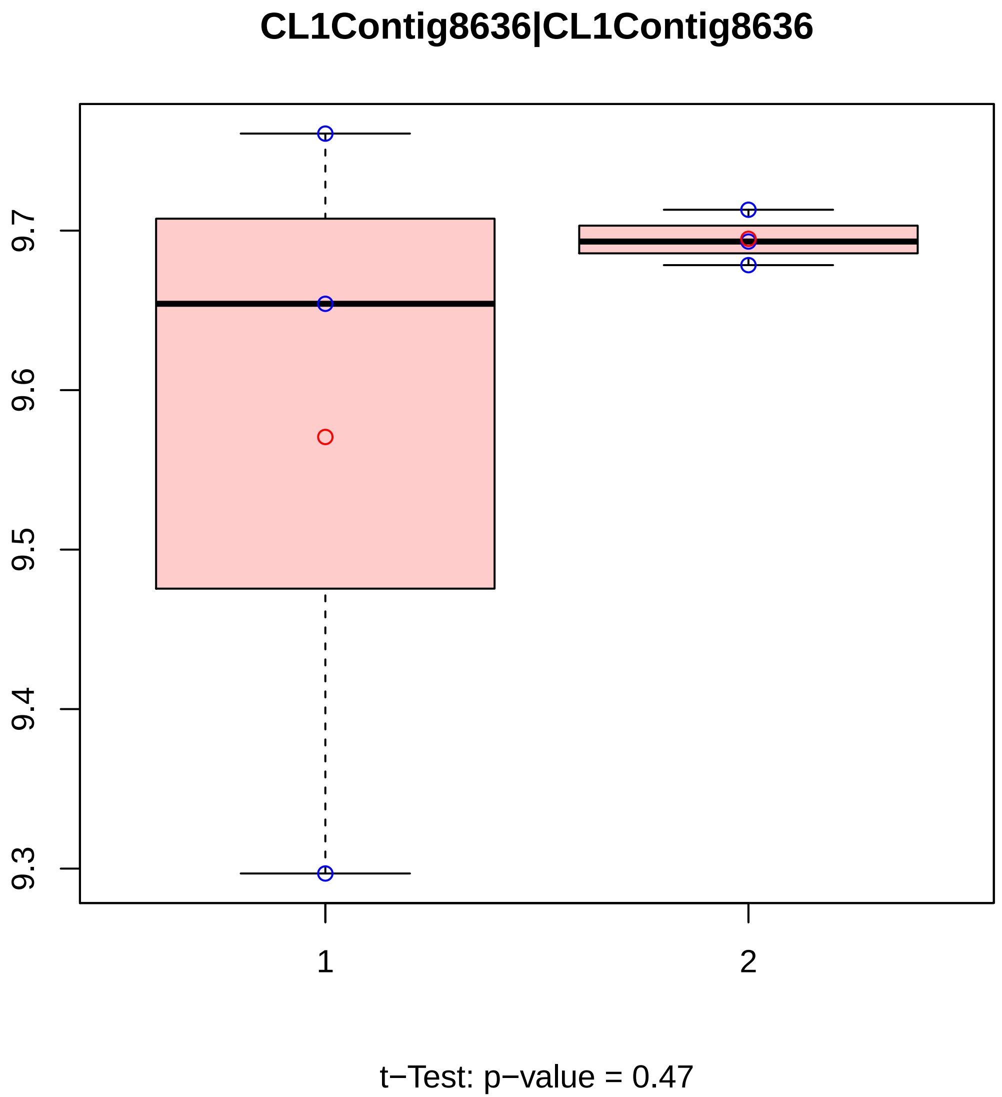
CL1Contig8636|CL1Contig8636
9.3
9.4
9.5
9.6
9.7
1
2
t−Test: p−value = 0.47

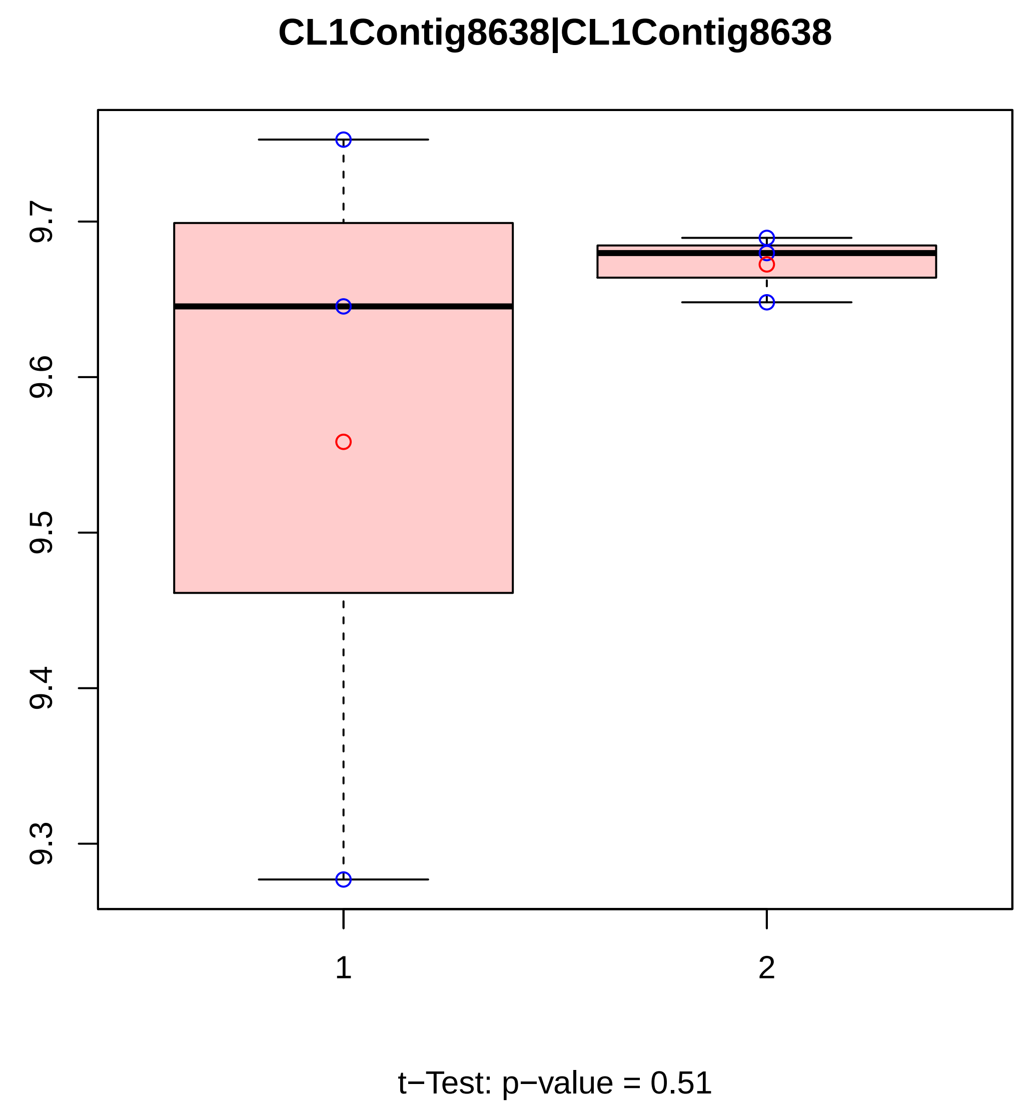
CL1Contig8638|CL1Contig8638
9.3
9.4
9.5
9.6
9.7
1
2
t−Test: p−value = 0.51

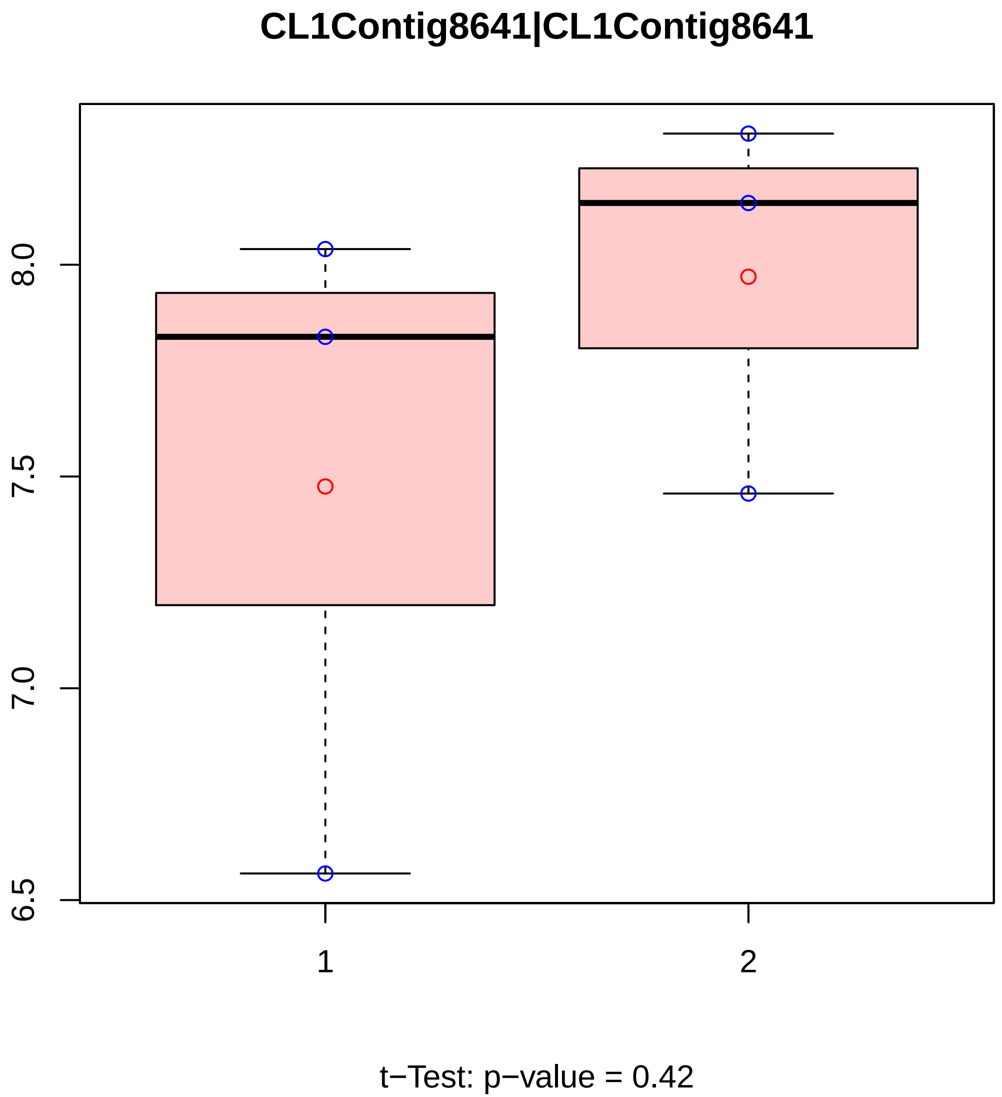
CL1Contig8641|CL1Contig8641
8.0
7.5
7.0
6.5
1
2
t−Test: p−value = 0.42

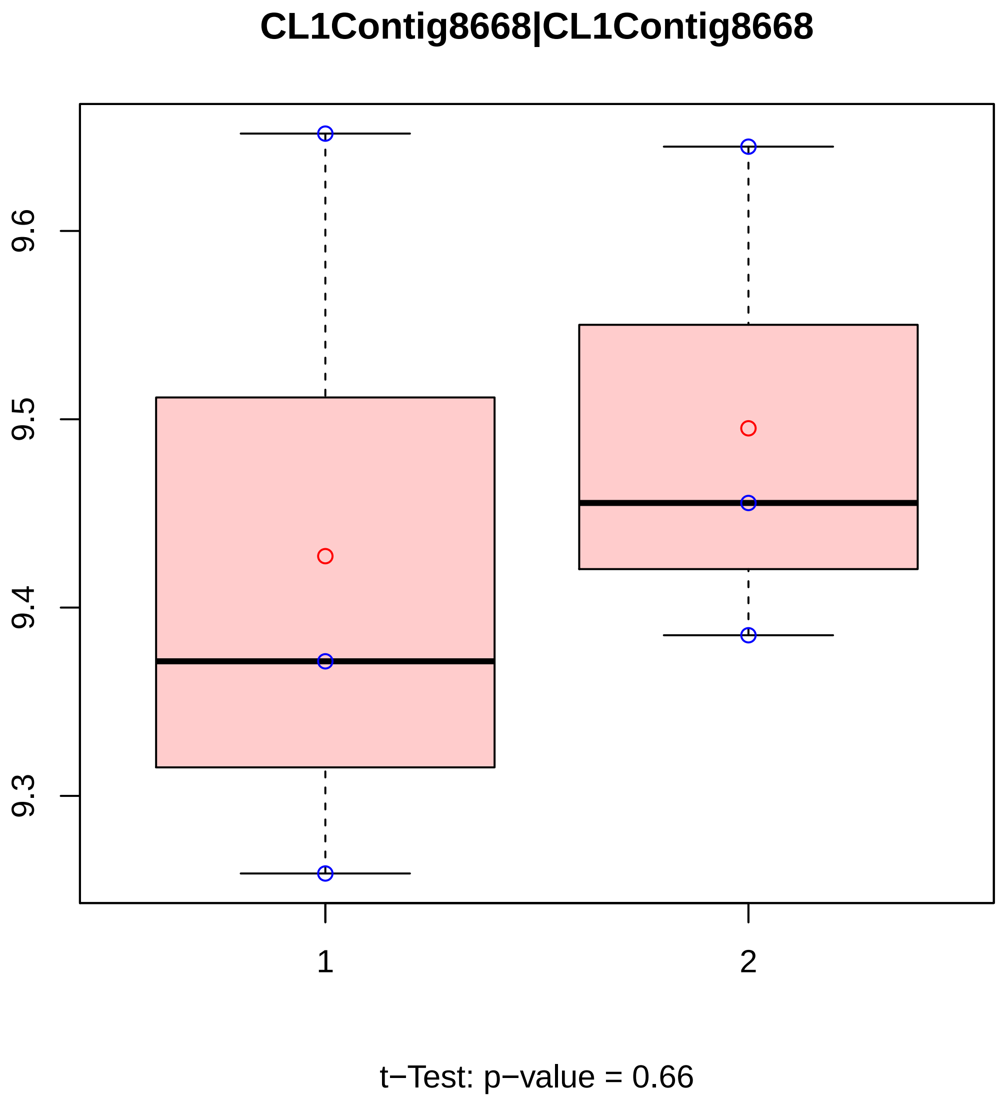

CL1Contig8668|CL1Contig8668
9.3
9.4
9.5
9.6
1
2
t−Test: p−value = 0.66

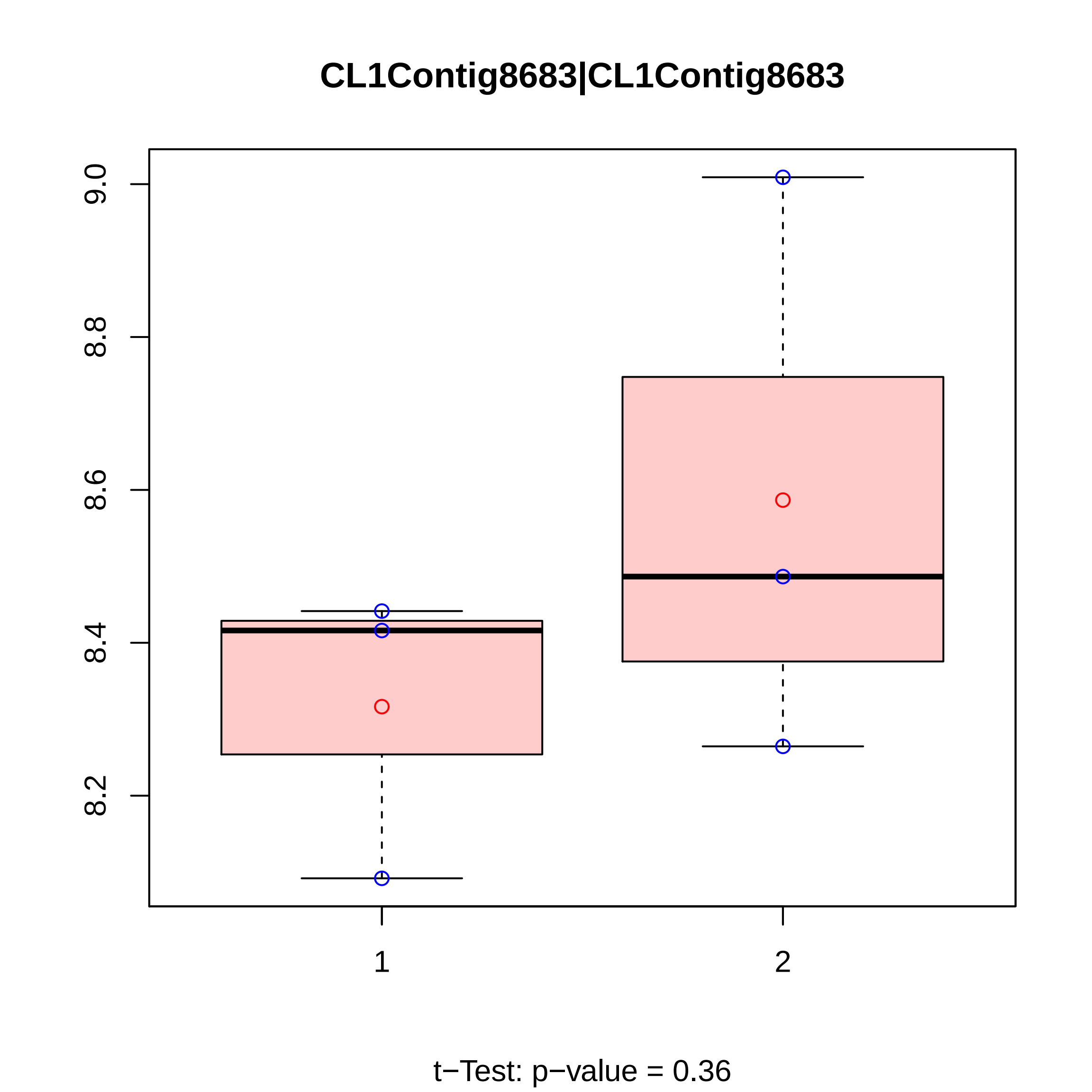
CL1Contig8683|CL1Contig8683
9.0
8.8
8.6
8.4
8.2
1
2
t−Test: p−value = 0.36

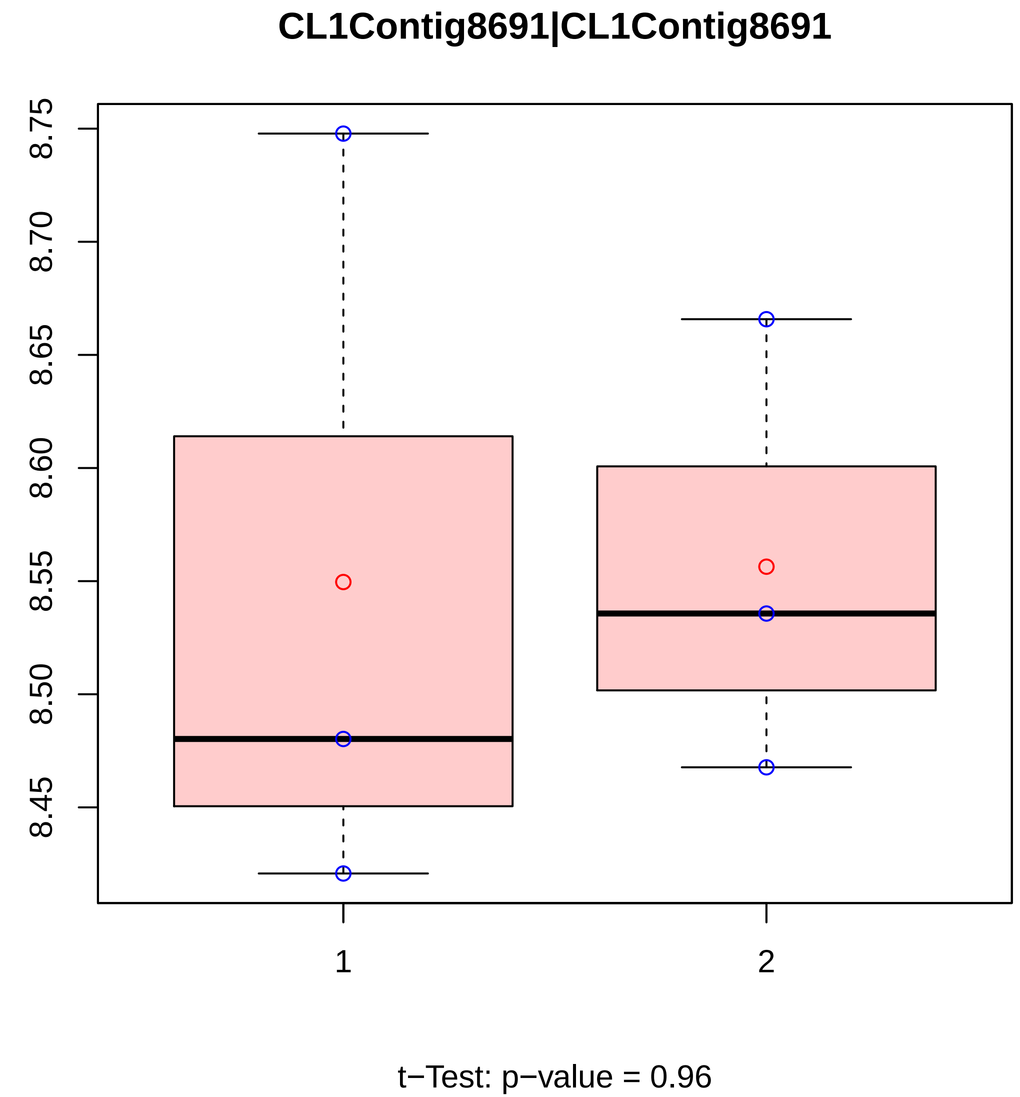
CL1Contig8691|CL1Contig8691
8.45
8.50
8.55
8.60
8.65
8.70
8.75
1
2
t−Test: p−value = 0.96

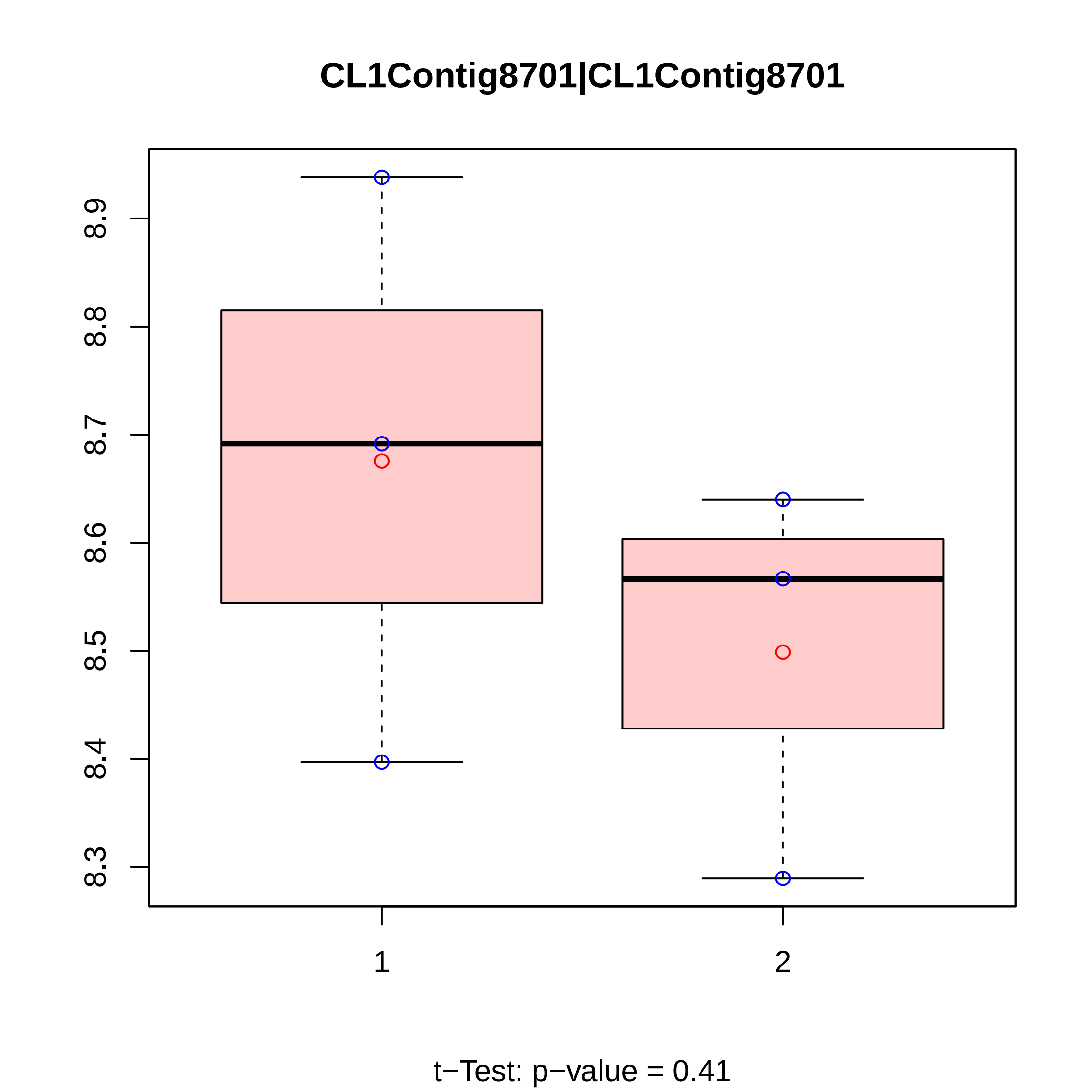

CL1Contig8701|CL1Contig8701
8.9
8.8
8.7
8.6
8.5
8.4
8.3
1
2
t−Test: p−value = 0.41

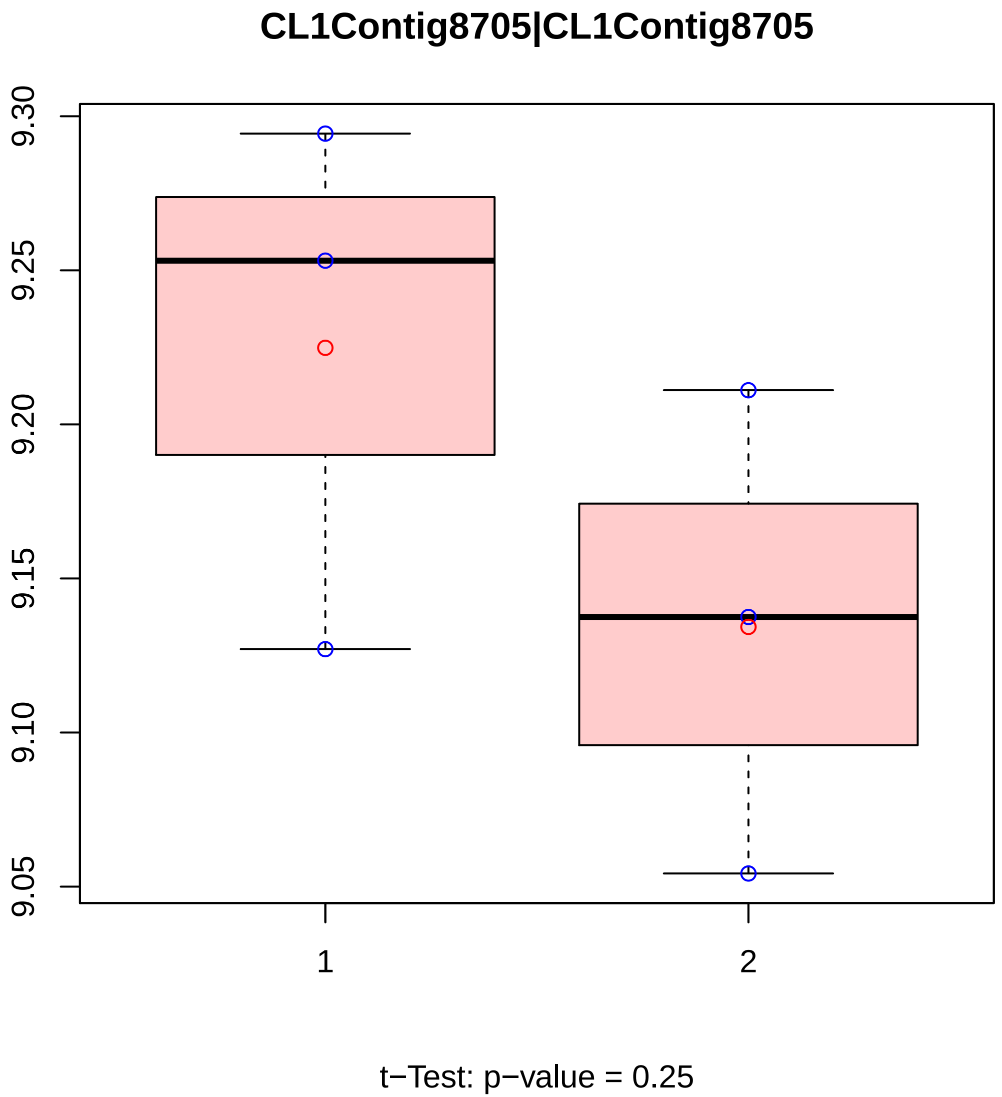

CL1Contig8705|CL1Contig8705
9.30
9.25
9.20
9.15
9.10
9.05
1
2
t−Test: p−value = 0.25

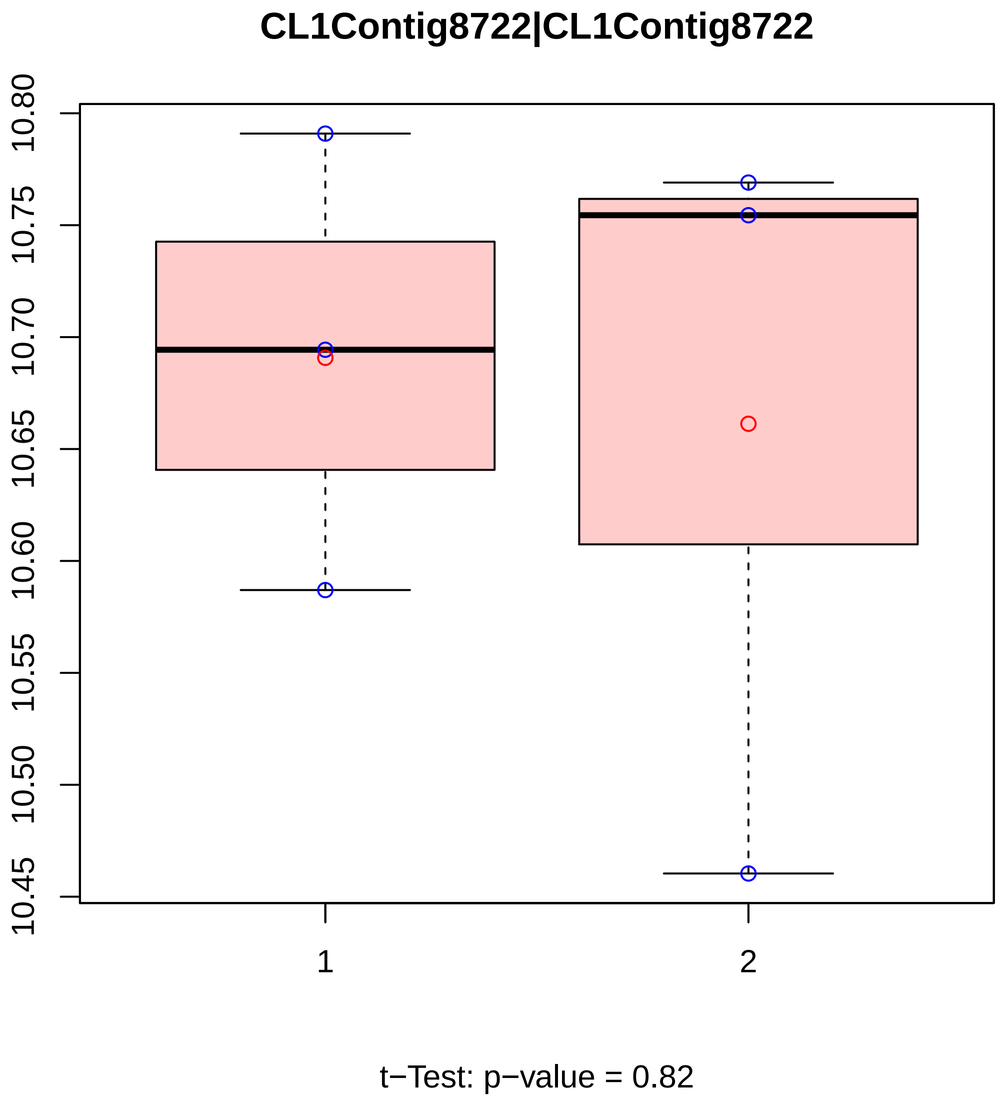

CL1Contig8722|CL1Contig8722
10.45
10.50
10.55
10.60
10.65
10.70
10.75
10.80
1
2
t−Test: p−value = 0.82

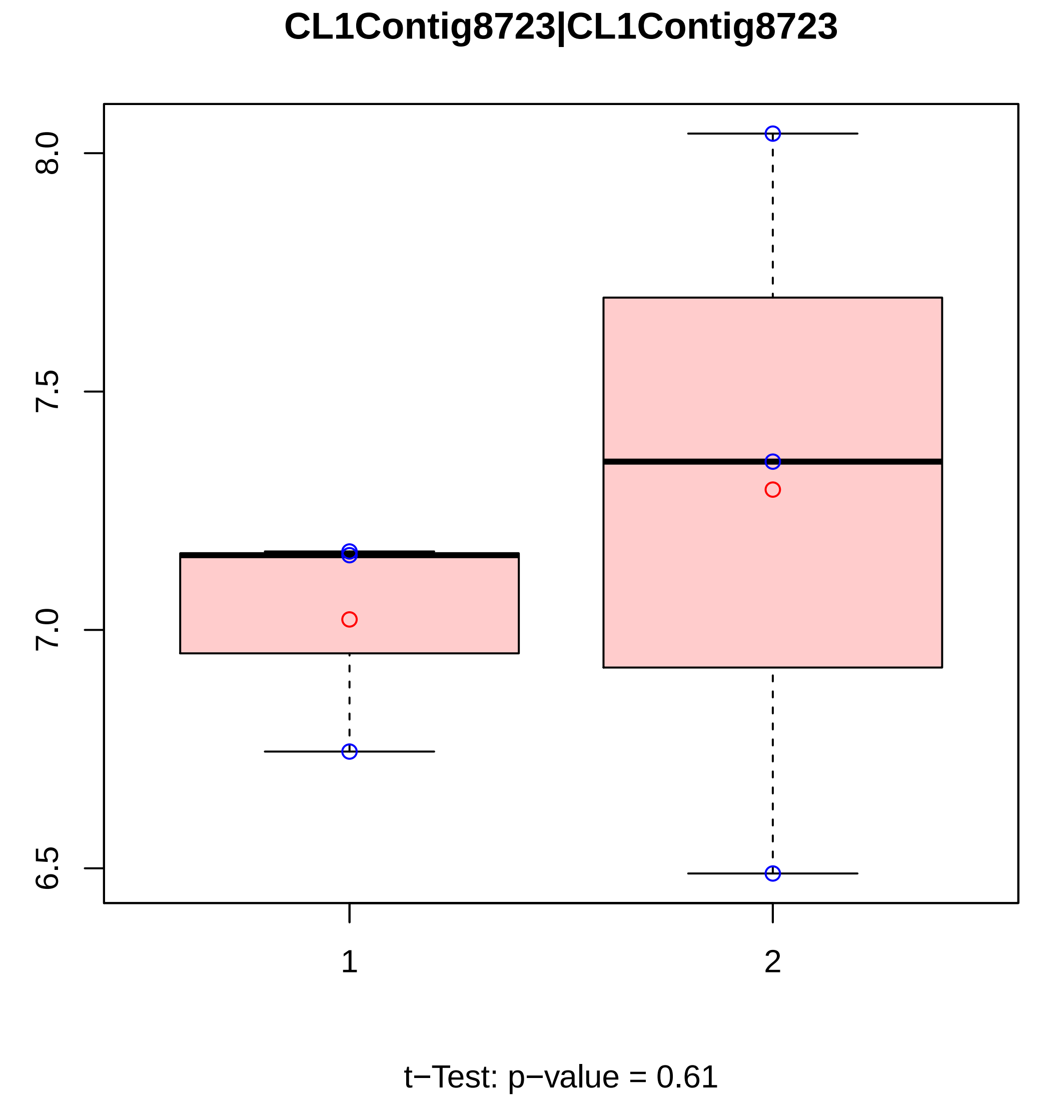

CL1Contig8723|CL1Contig8723
8.0
7.5
7.0
6.5
1
2
t−Test: p−value = 0.61

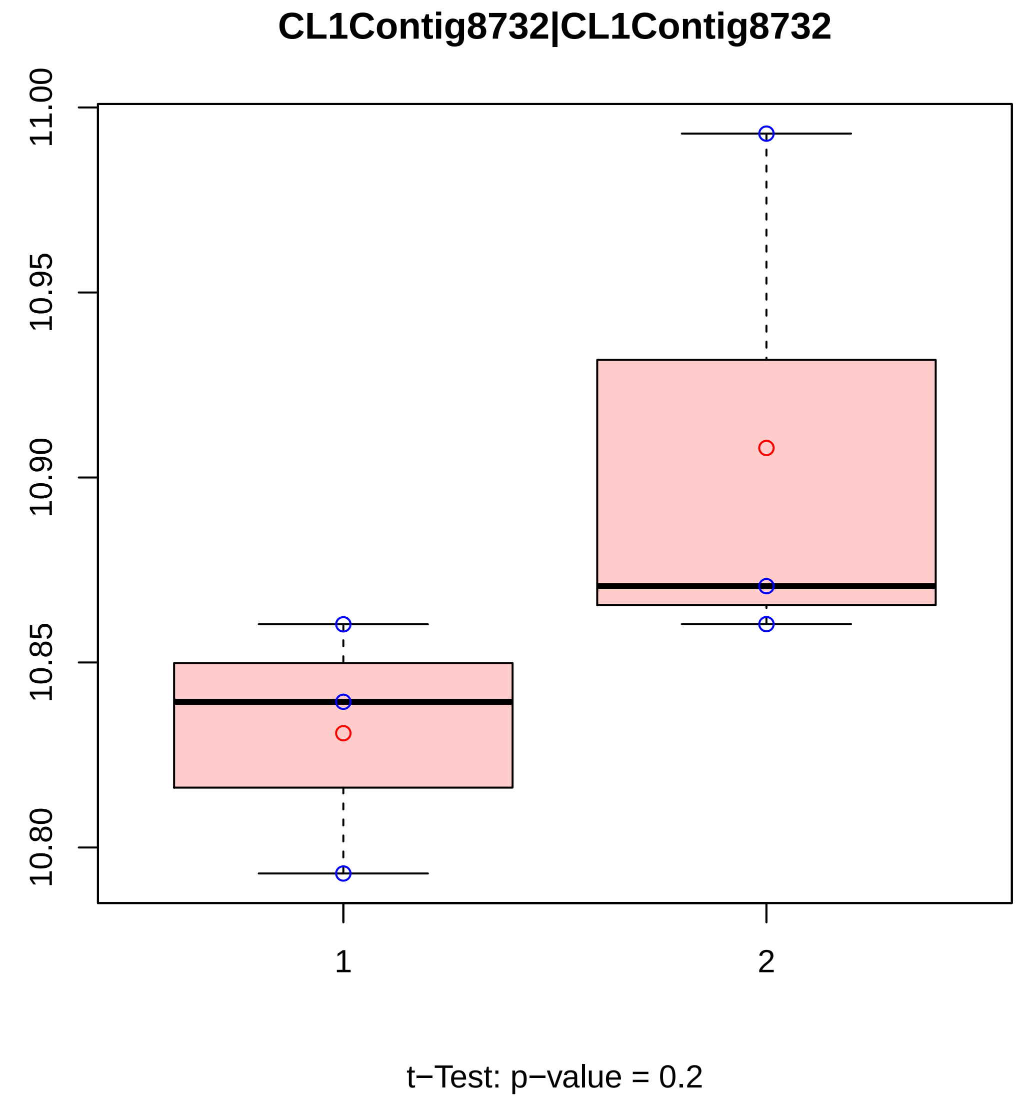
CL1Contig8732|CL1Contig8732
11.00
10.95
10.90
10.85
10.80
1
2
t−Test: p−value = 0.2

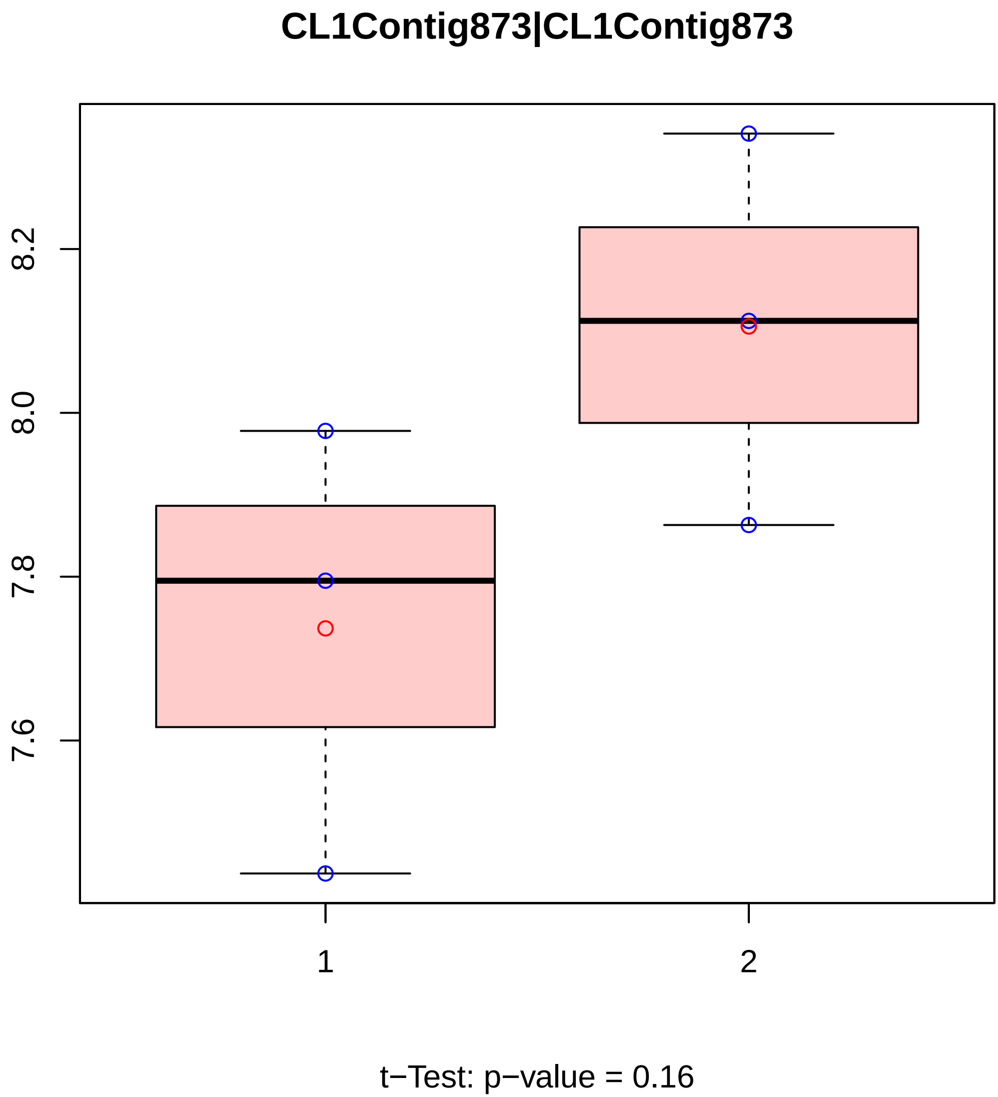

CL1Contig873|CL1Contig873
7.6
7.8
8.0
8.2
1
2
t−Test: p−value = 0.16

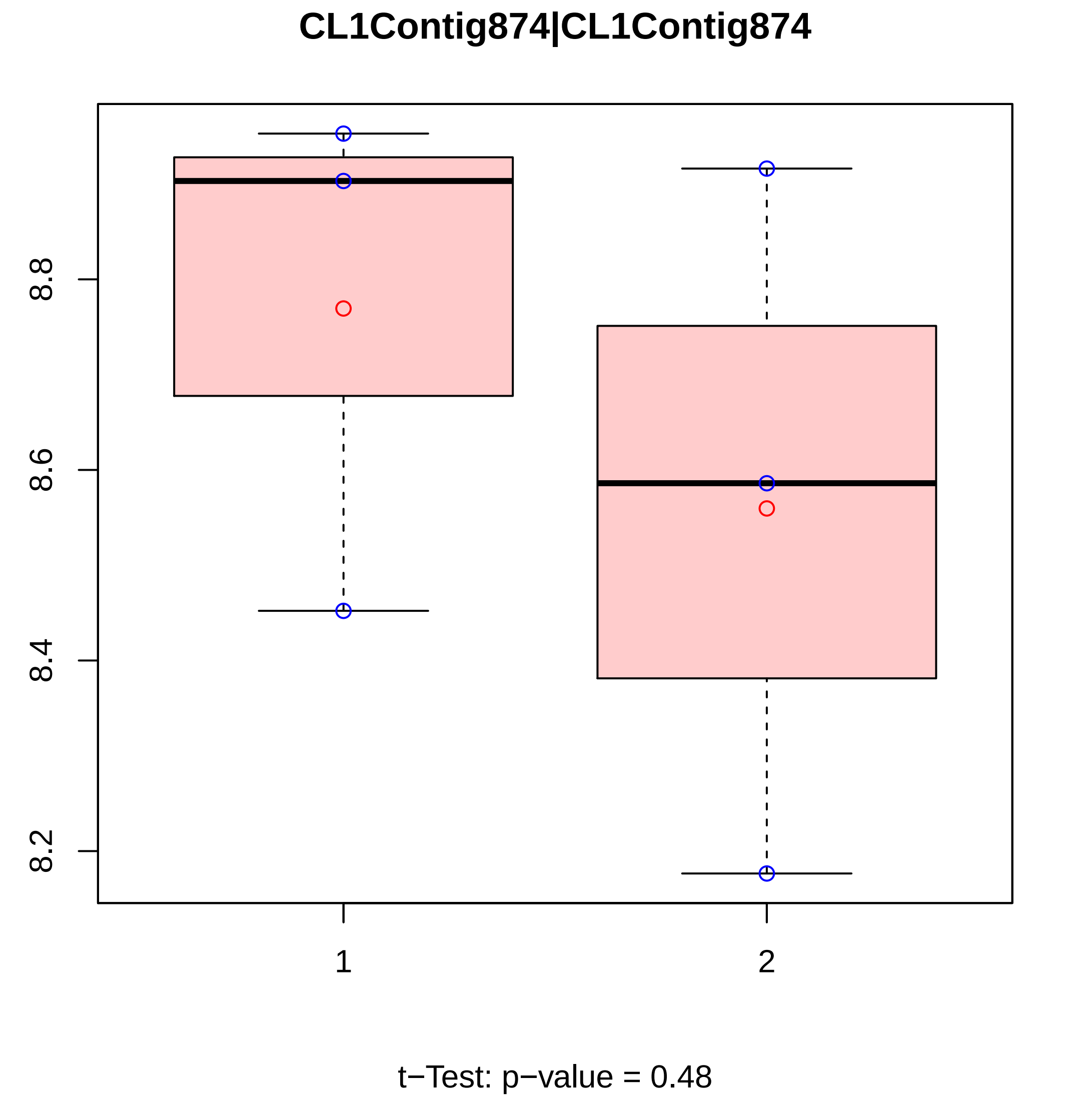

CL1Contig874|CL1Contig874
8.2
8.4
8.6
8.8
1
2
t−Test: p−value = 0.48

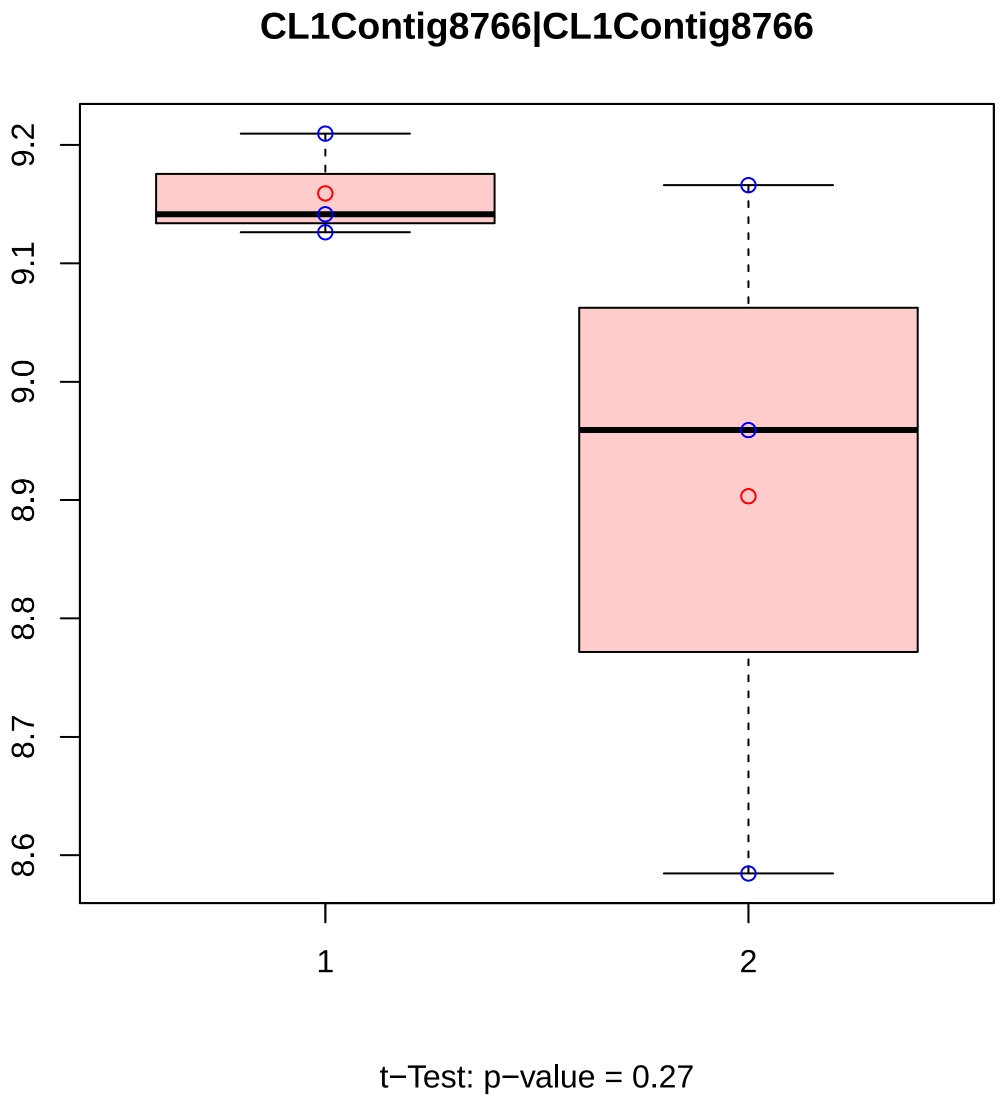

CL1Contig8766|CL1Contig8766
9.2
9.1
9.0
8.9
8.8
8.7
8.6
1
2
t−Test: p−value = 0.27

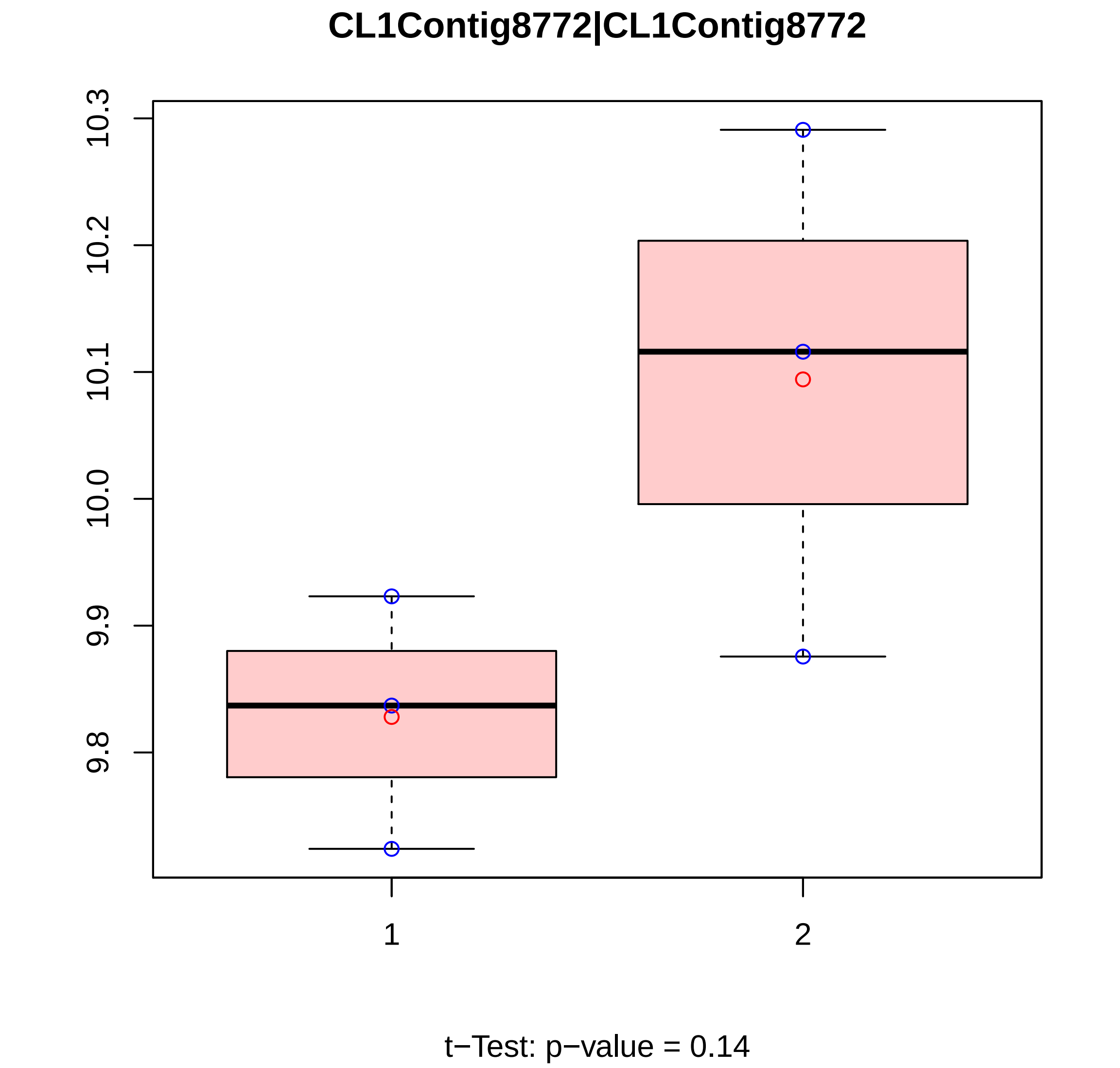
CL1Contig8772|CL1Contig8772
10.3
10.2
10.1
10.0
9.9
9.8
1
2
t−Test: p−value = 0.14

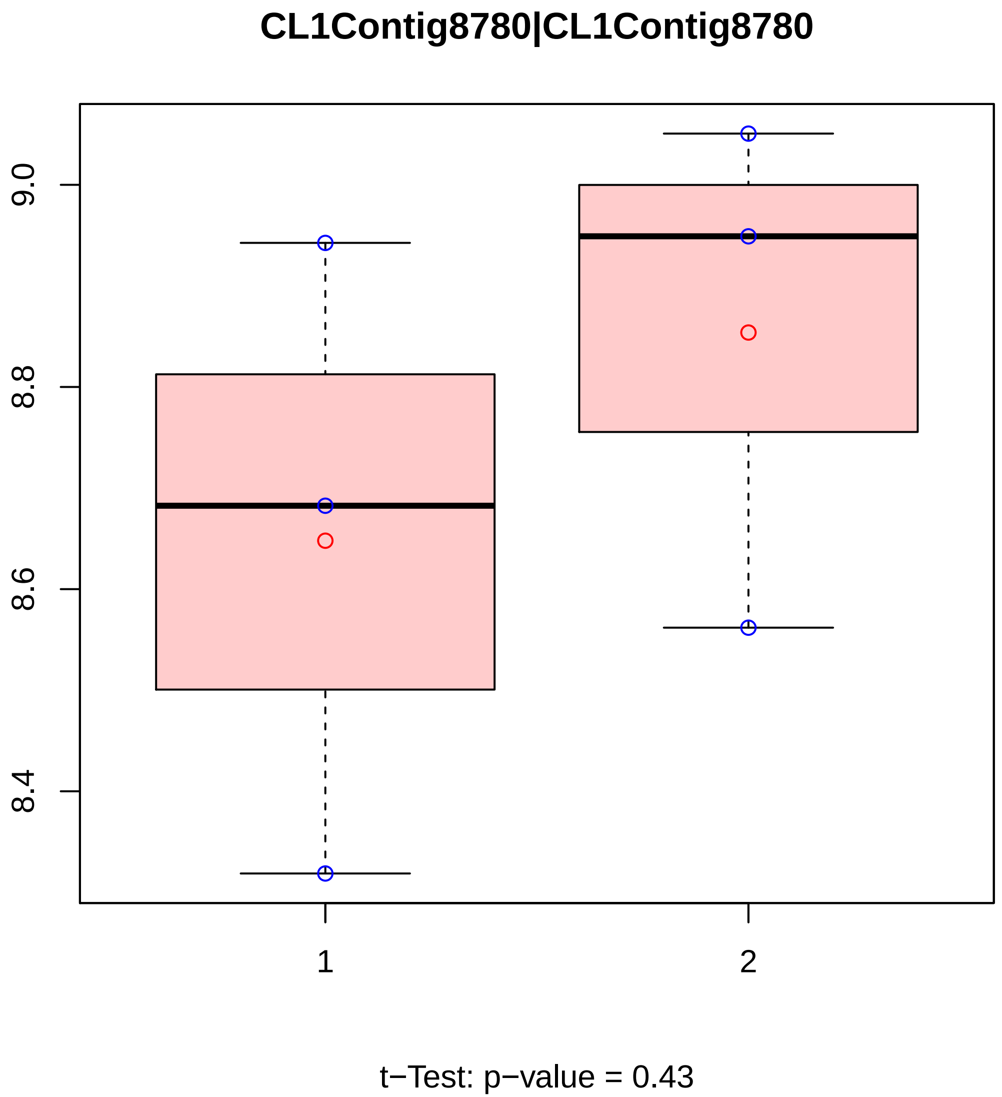

CL1Contig8780|CL1Contig8780
9.0
8.8
8.6
8.4
1
2
t−Test: p−value = 0.43

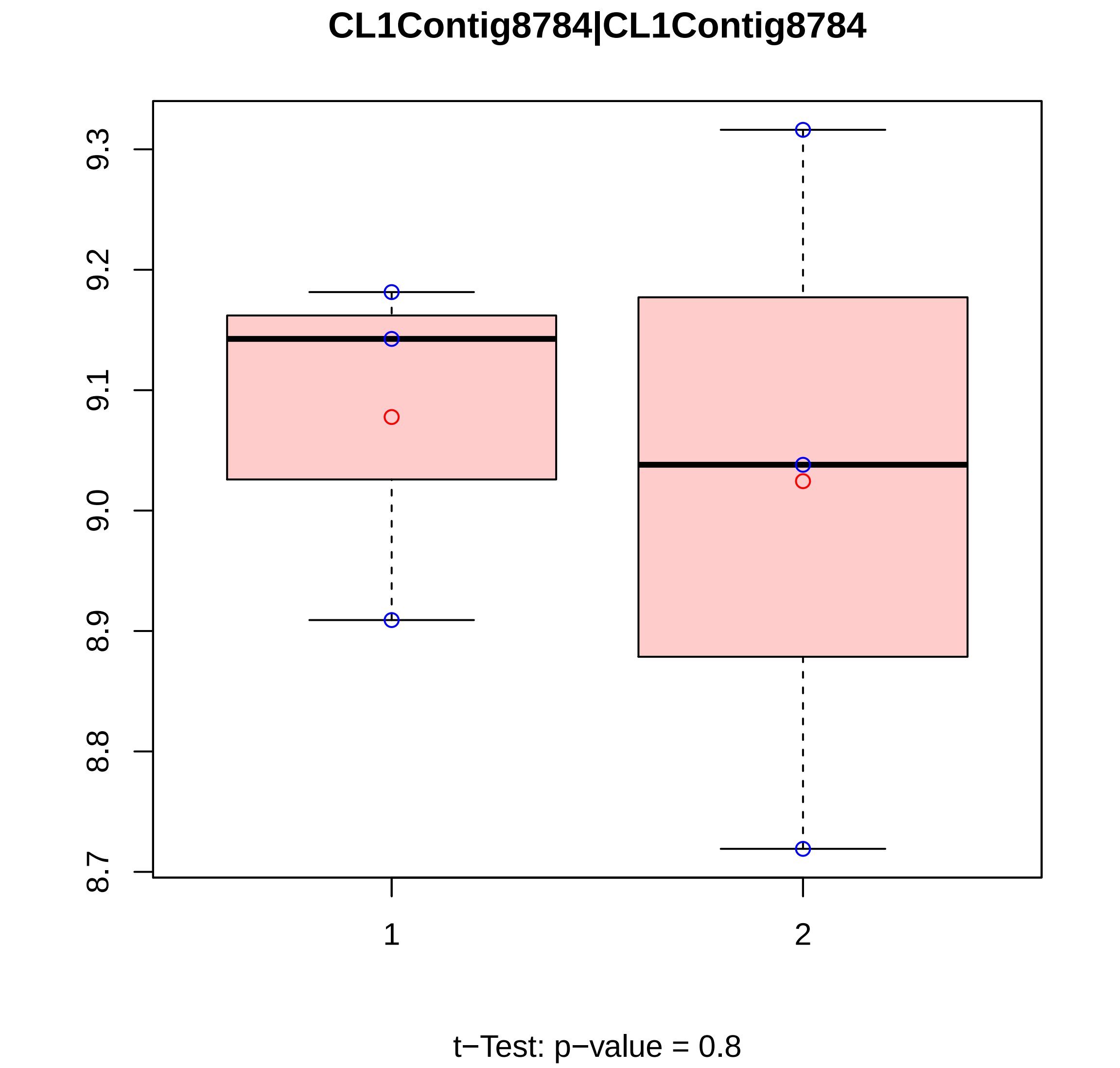

CL1Contig8784|CL1Contig8784
9.3
9.2
9.1
9.0
8.9
8.8
8.7
1
2
t−Test: p−value = 0.8

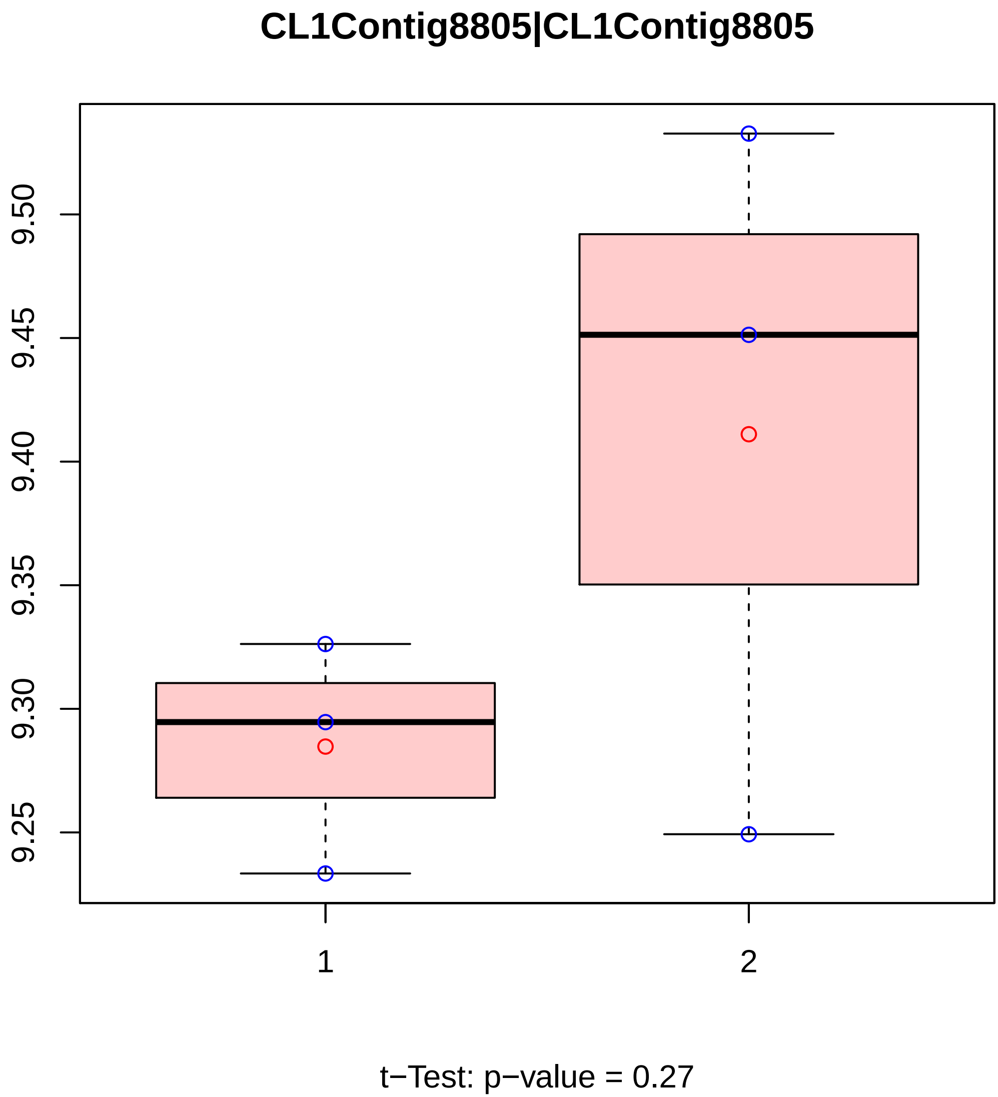
CL1Contig8805|CL1Contig8805
9.25
9.30
9.35
9.40
9.45
9.50
1
2
t−Test: p−value = 0.27

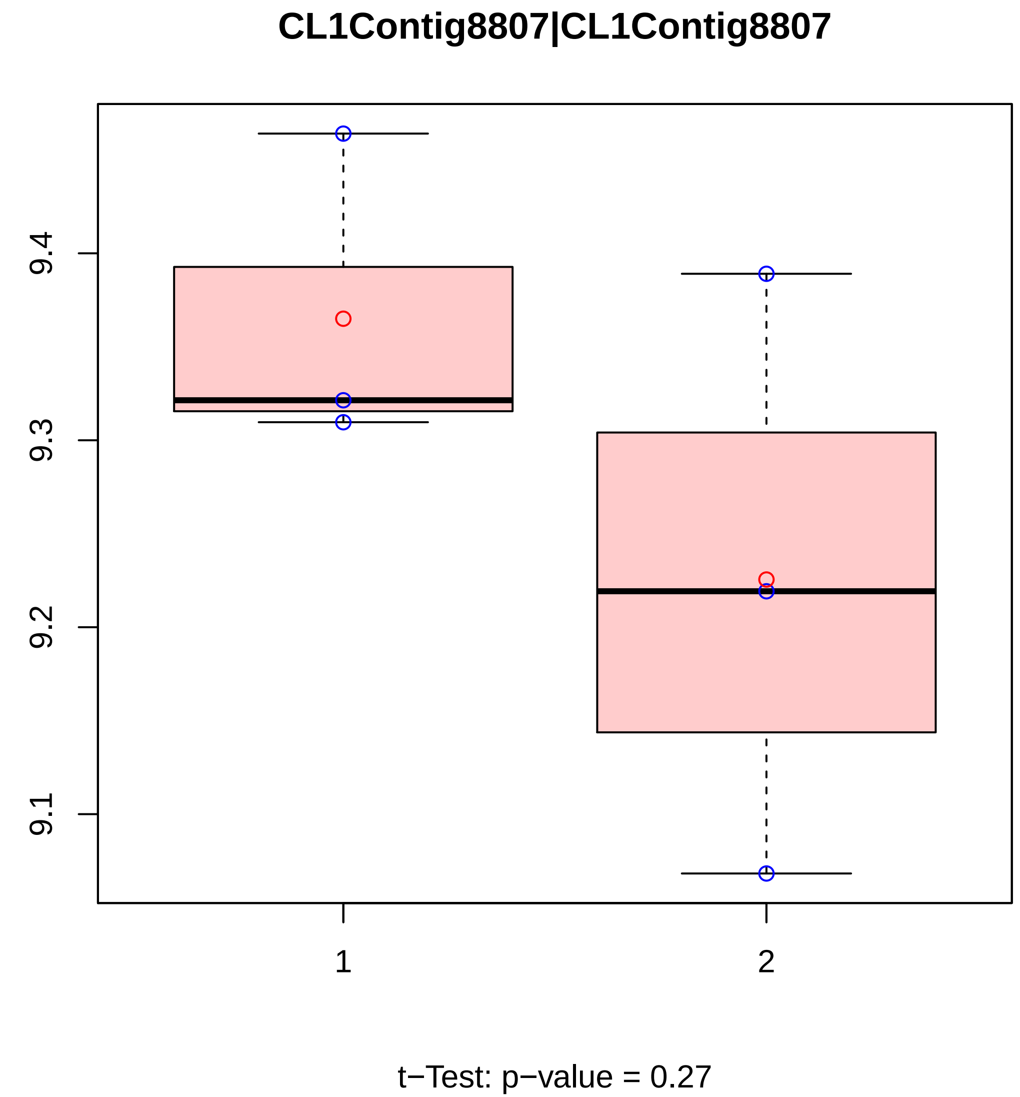

CL1Contig8807|CL1Contig8807
9.4
9.3
9.2
9.1
1
2
t−Test: p−value = 0.27

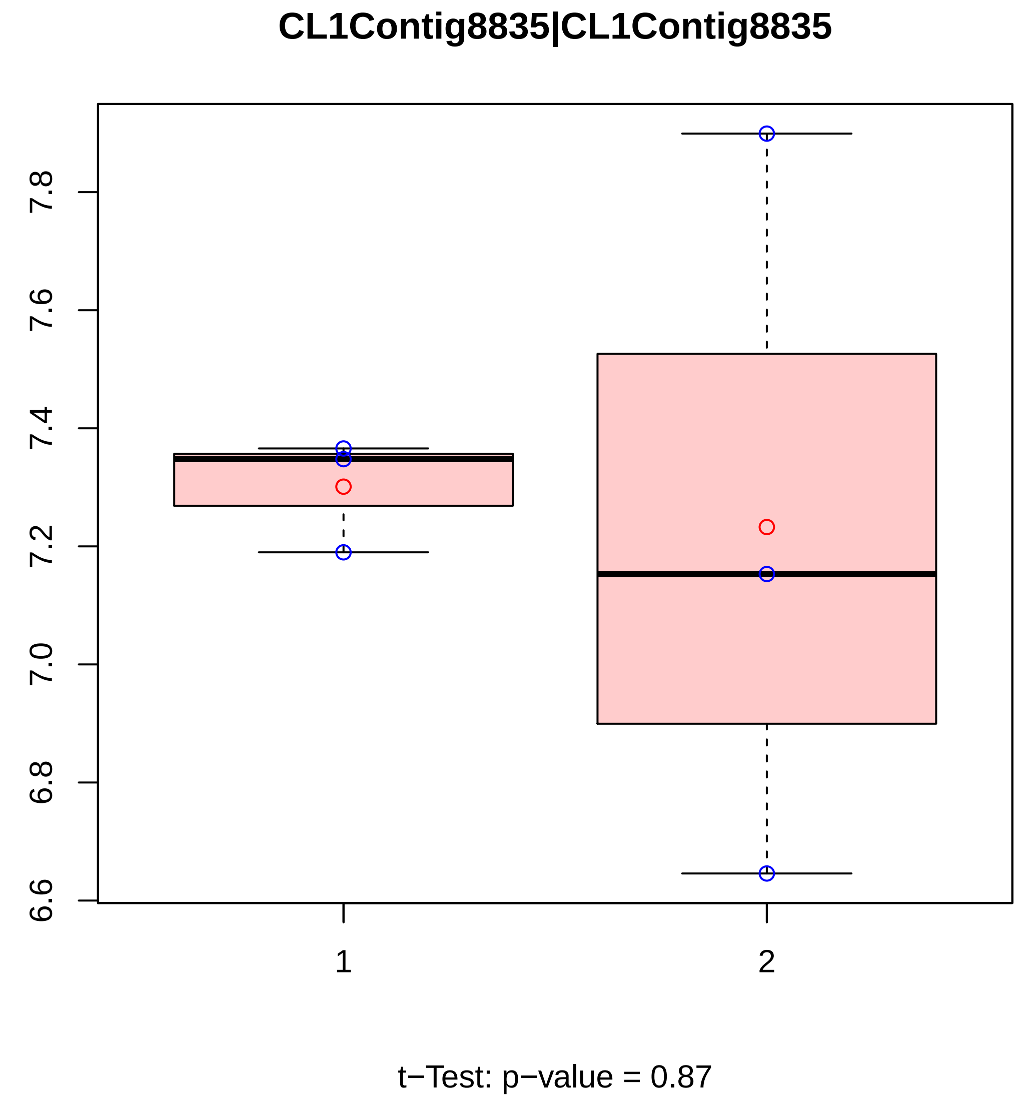
CL1Contig8835|CL1Contig8835
7.8
7.6
7.4
7.2
7.0
6.8
6.6
1
2
t−Test: p−value = 0.87

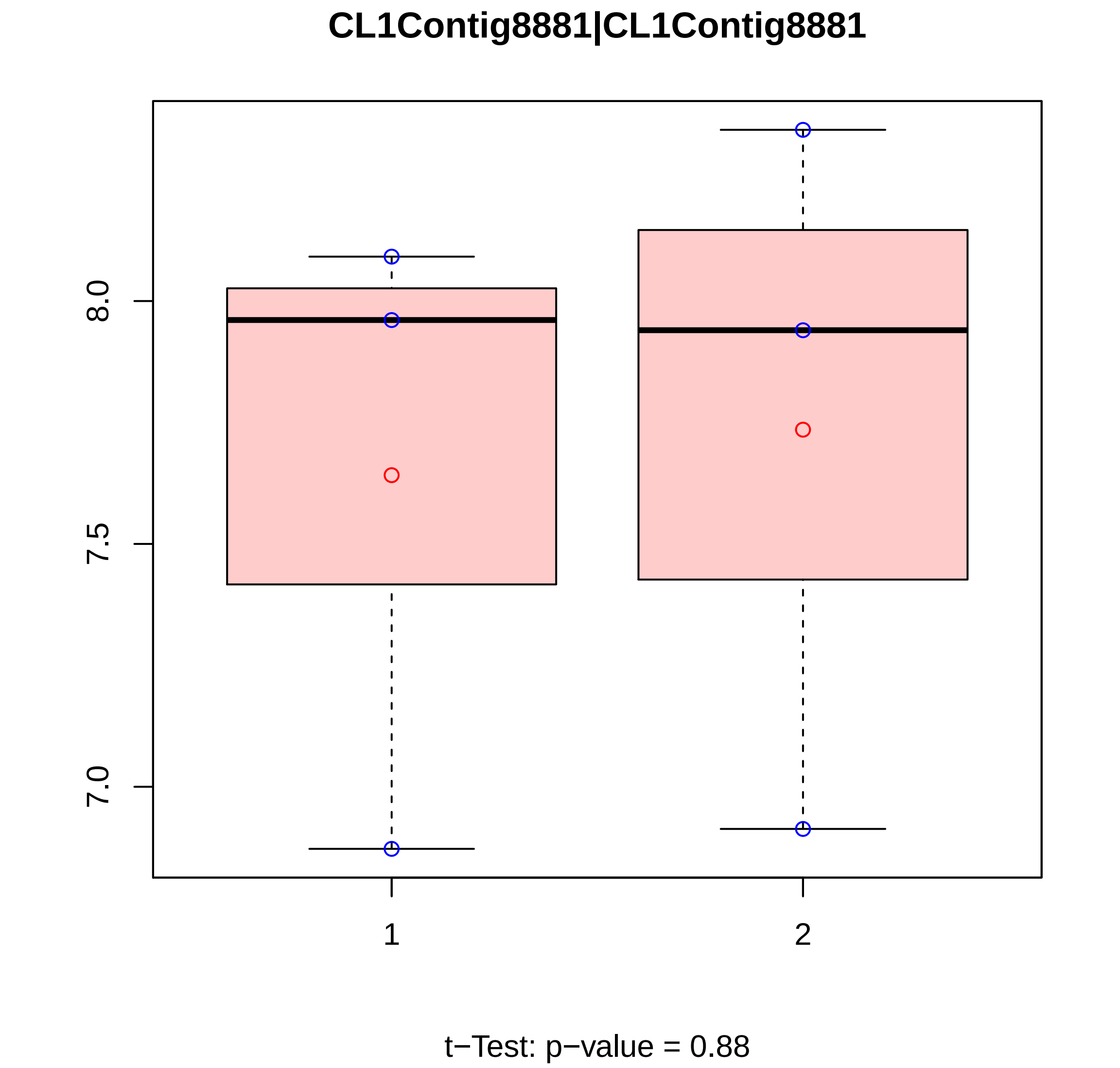
CL1Contig8881|CL1Contig8881
8.0
7.5
7.0
1
2
t−Test: p−value = 0.88

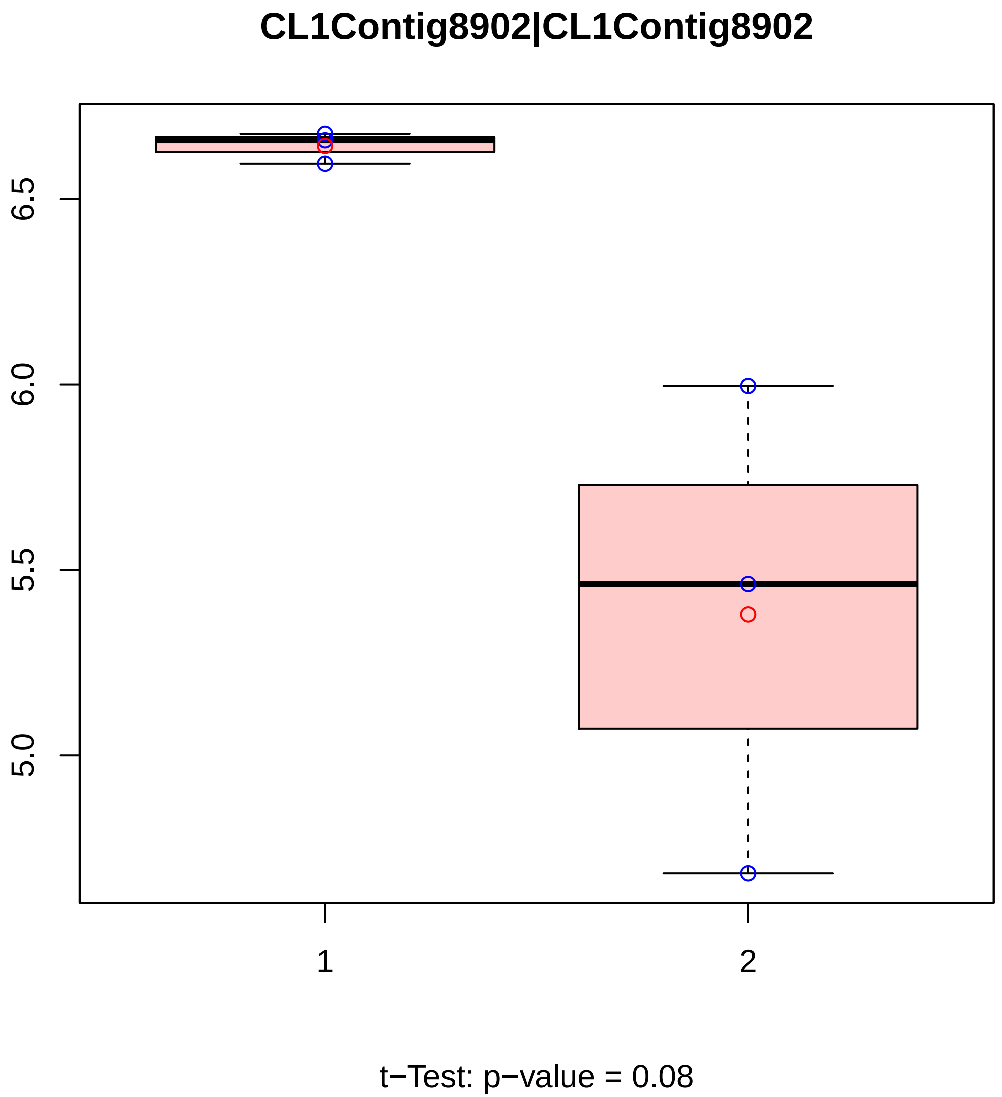
CL1Contig8902|CL1Contig8902
6.5
6.0
5.5
5.0
1
2
t−Test: p−value = 0.08

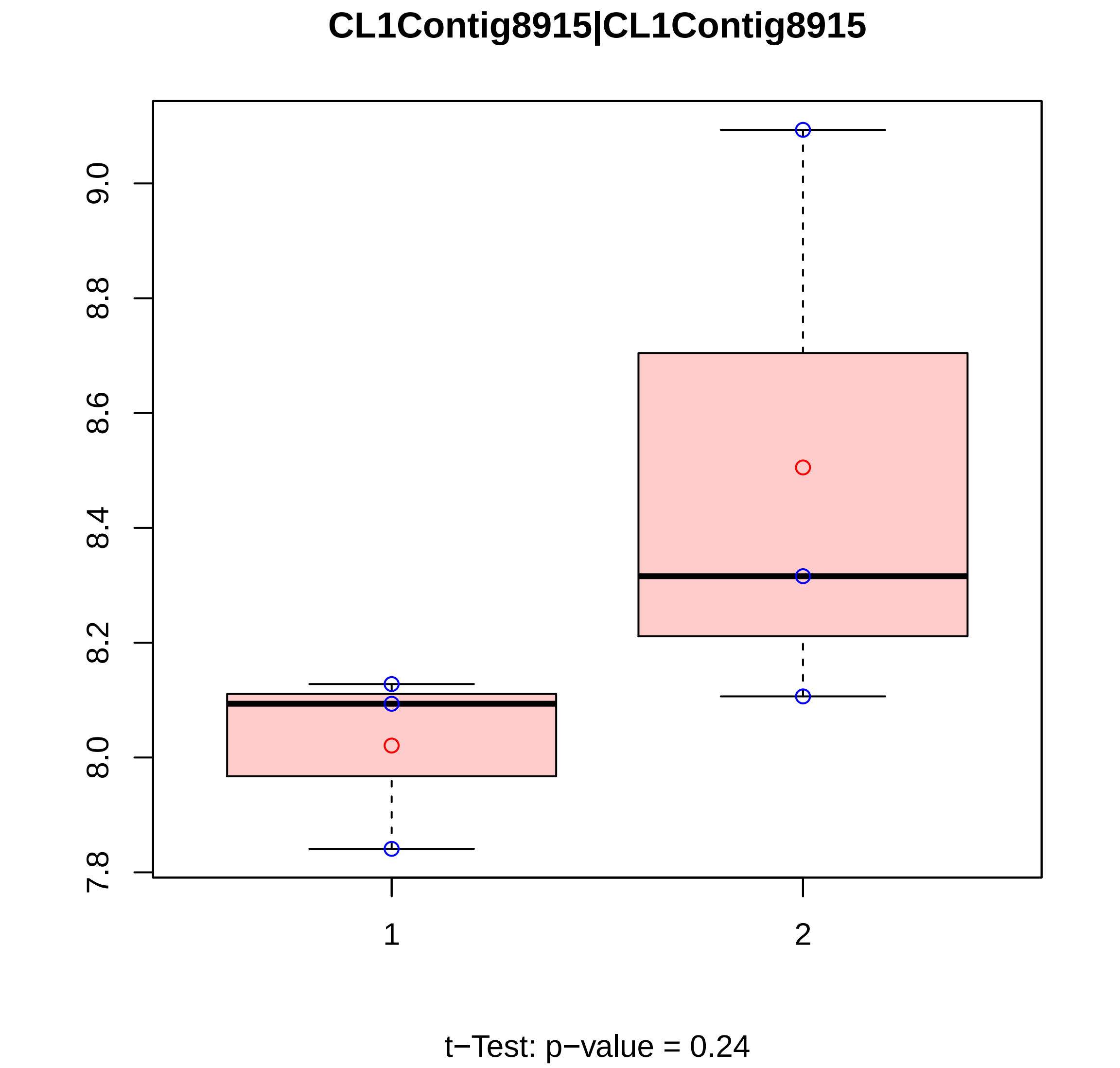
CL1Contig8915|CL1Contig8915
9.0
8.8
8.6
8.4
8.2
8.0
7.8
1
2
t−Test: p−value = 0.24

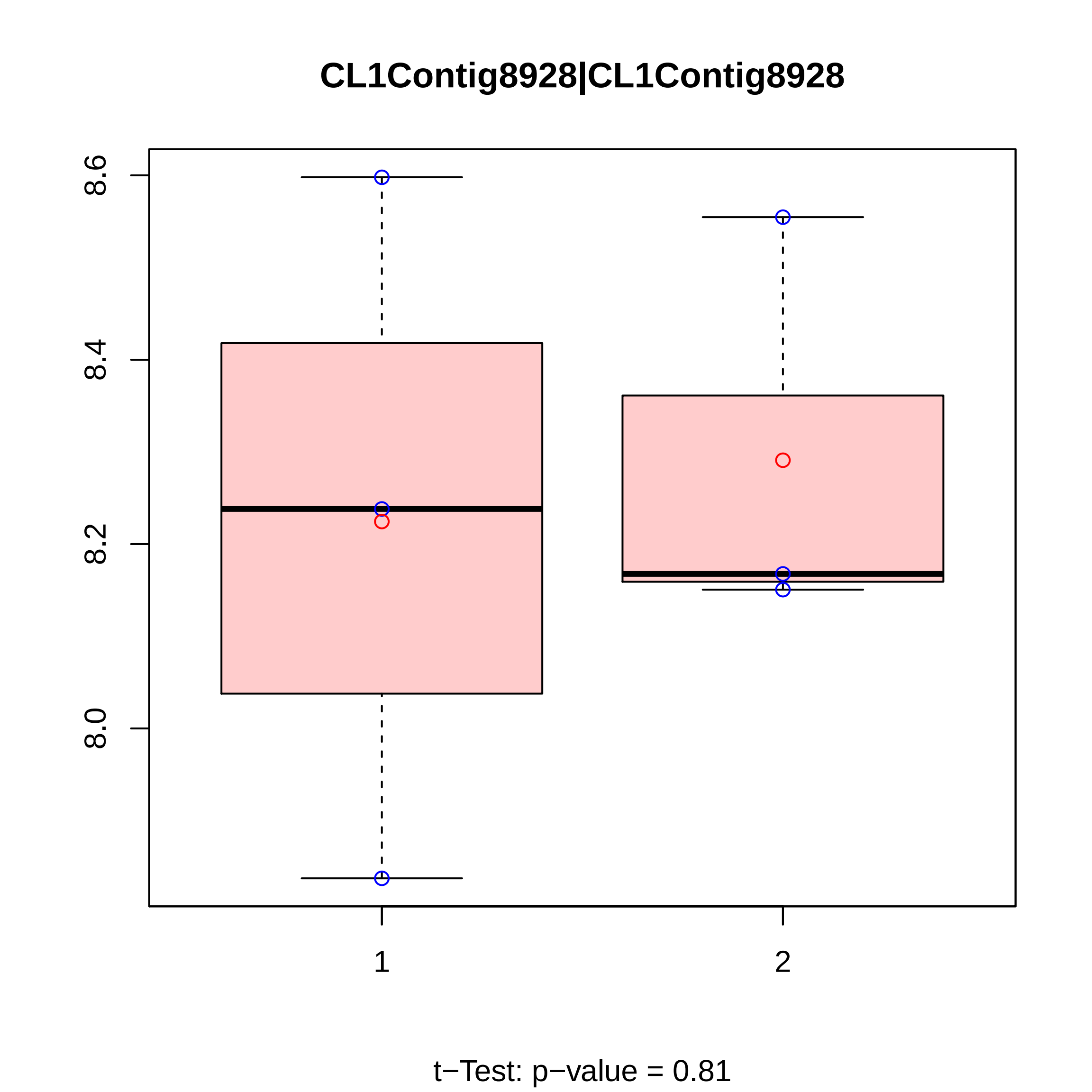
CL1Contig8928|CL1Contig8928
8.6
8.4
8.2
8.0
1
2
t−Test: p−value = 0.81

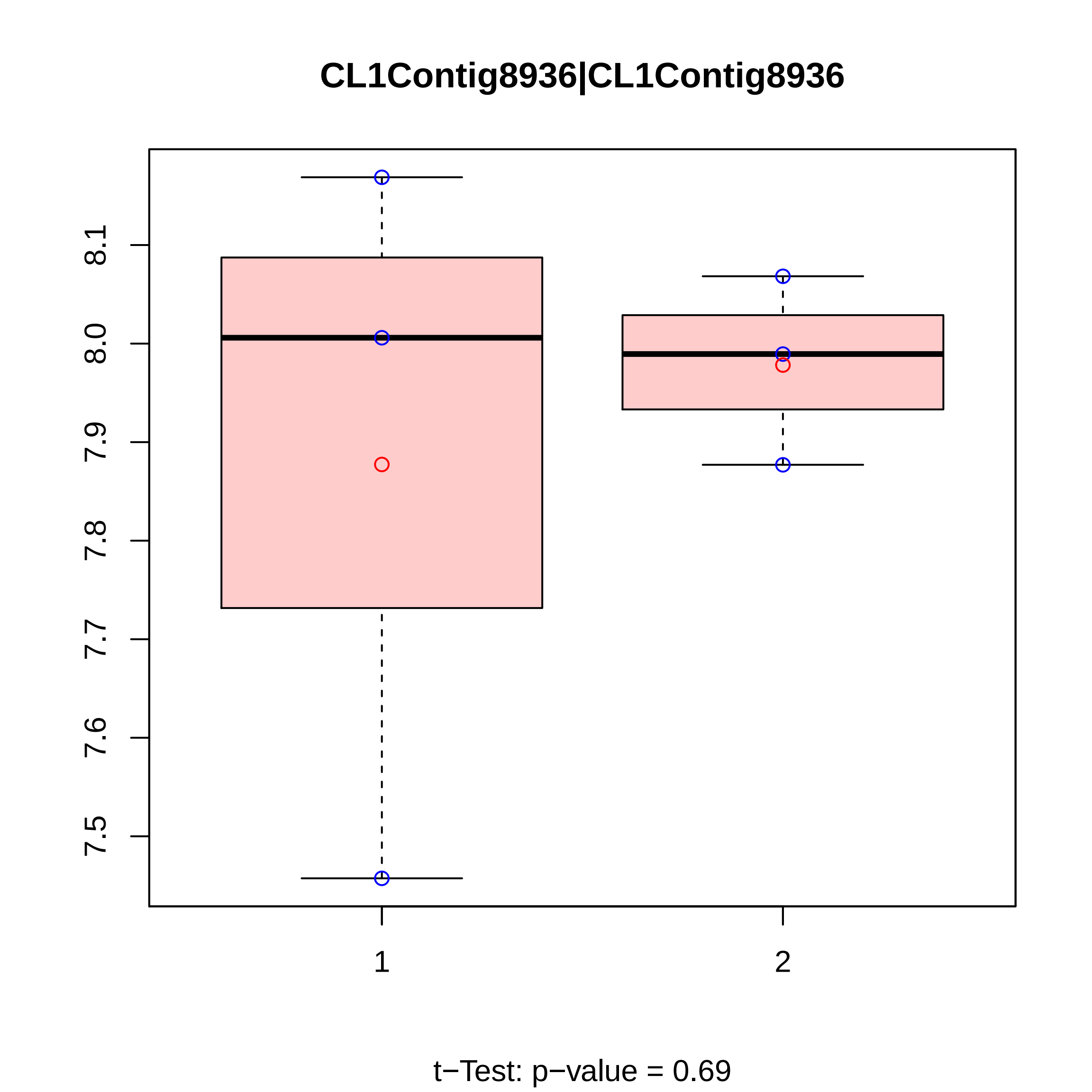
CL1Contig8936|CL1Contig8936
8.1
8.0
7.9
7.8
7.7
7.6
7.5
1
2
t−Test: p−value = 0.69

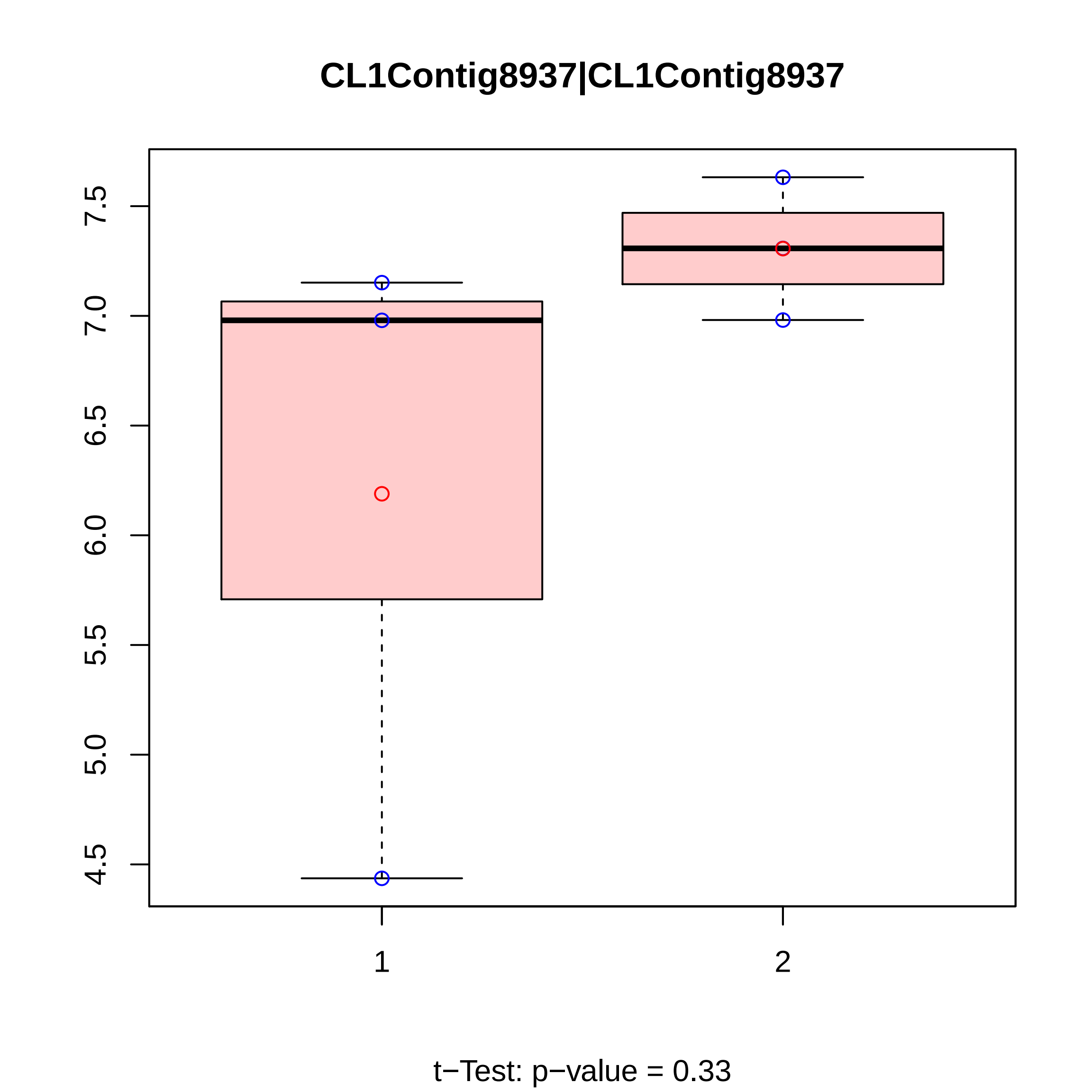

CL1Contig8937|CL1Contig8937
7.5
7.0
6.5
6.0
5.5
5.0
4.5
1
2
t−Test: p−value = 0.33

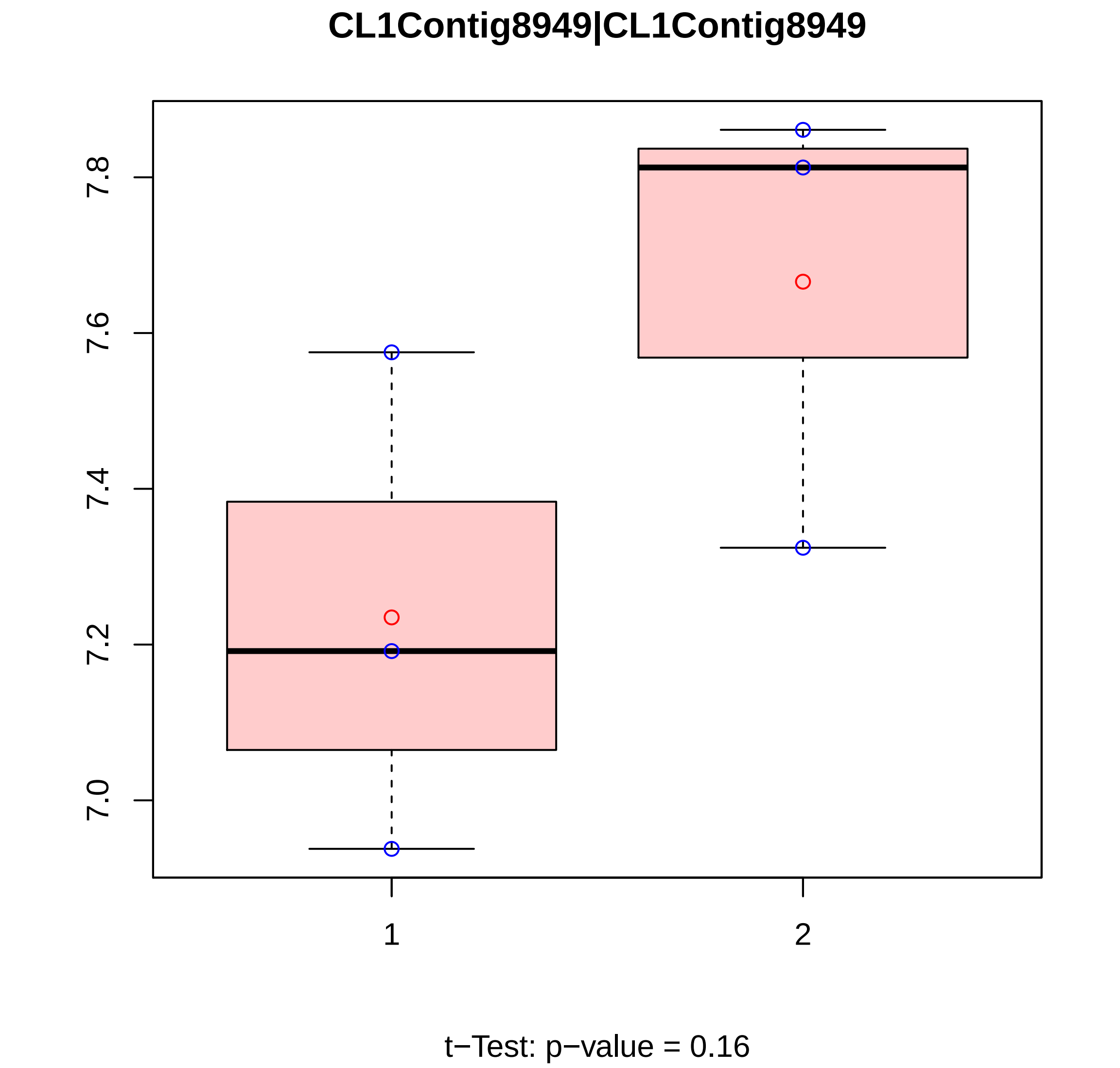

CL1Contig8949|CL1Contig8949
7.0
7.2
7.4
7.6
7.8
1
2
t−Test: p−value = 0.16

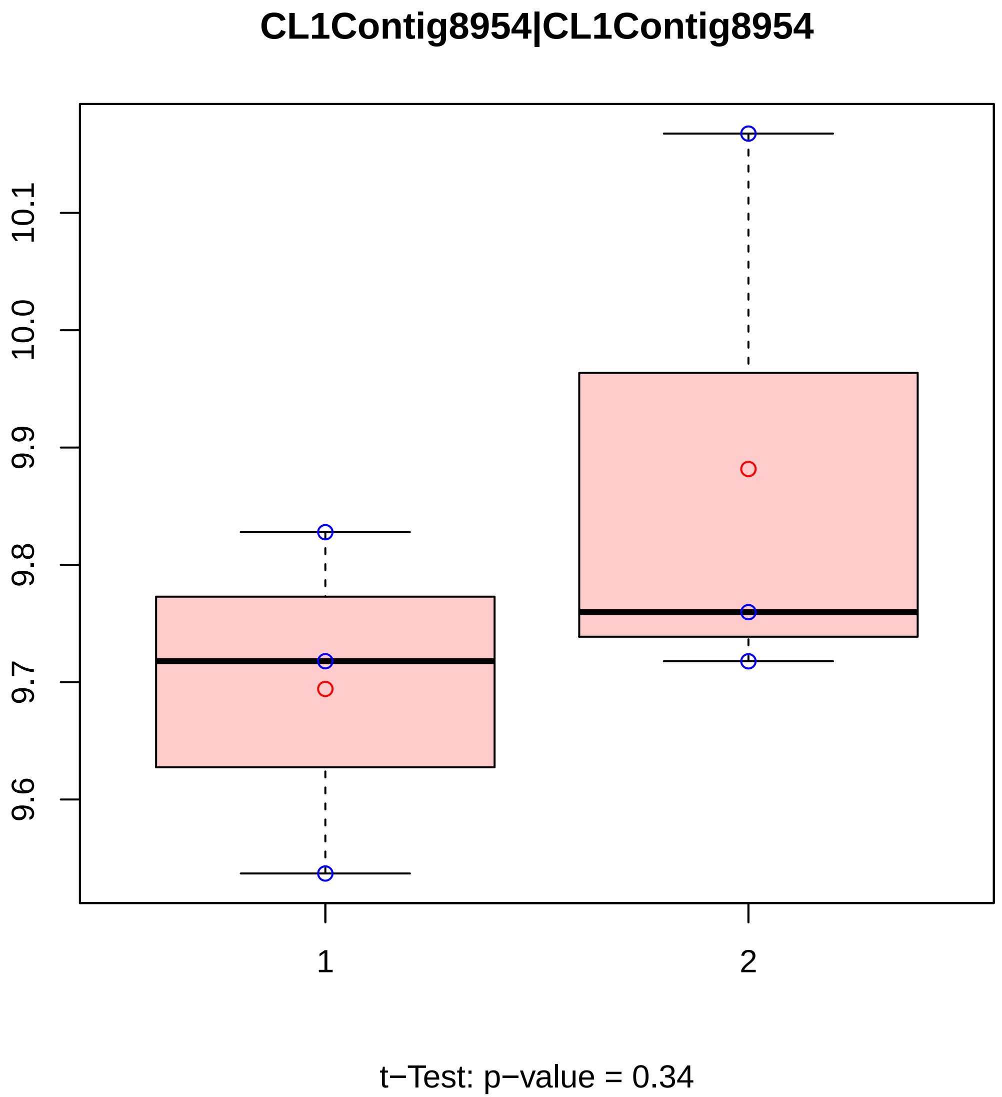
CL1Contig8954|CL1Contig8954
9.6
9.7
9.8
9.9
10.0
10.1
1
2
t−Test: p−value = 0.34

# CL1Contig8960|CL1Contig8960

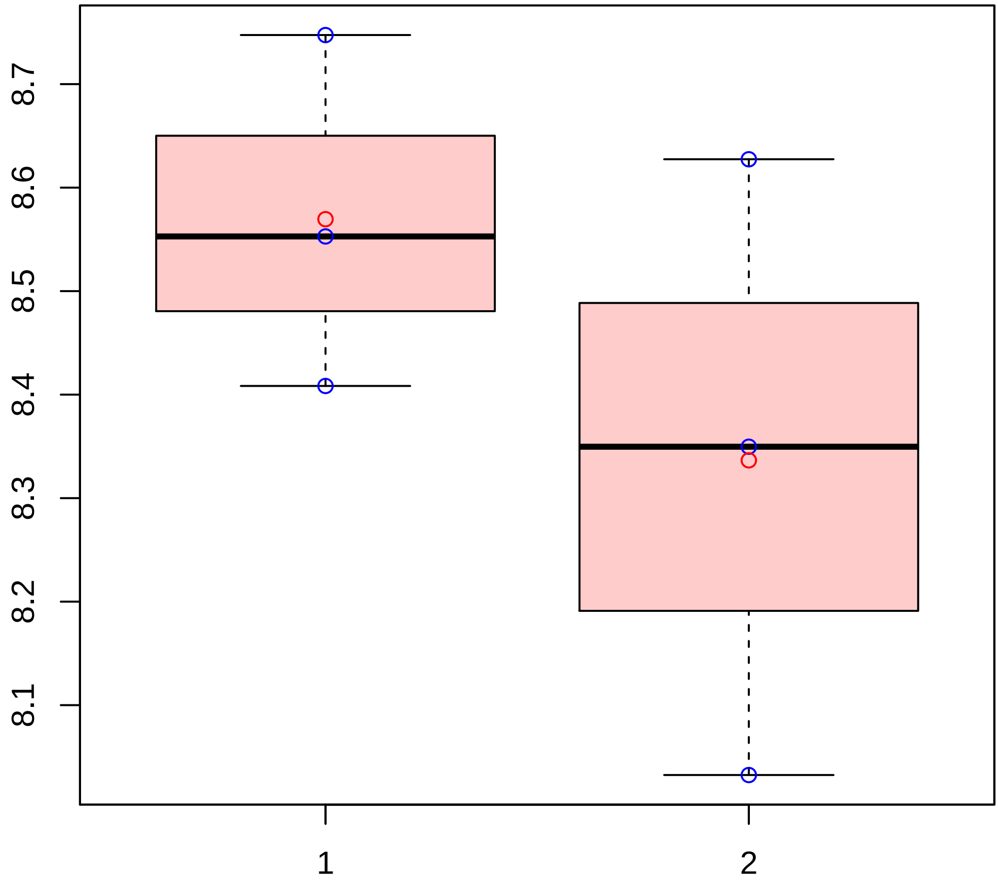


t−Test: p−value = 0.32

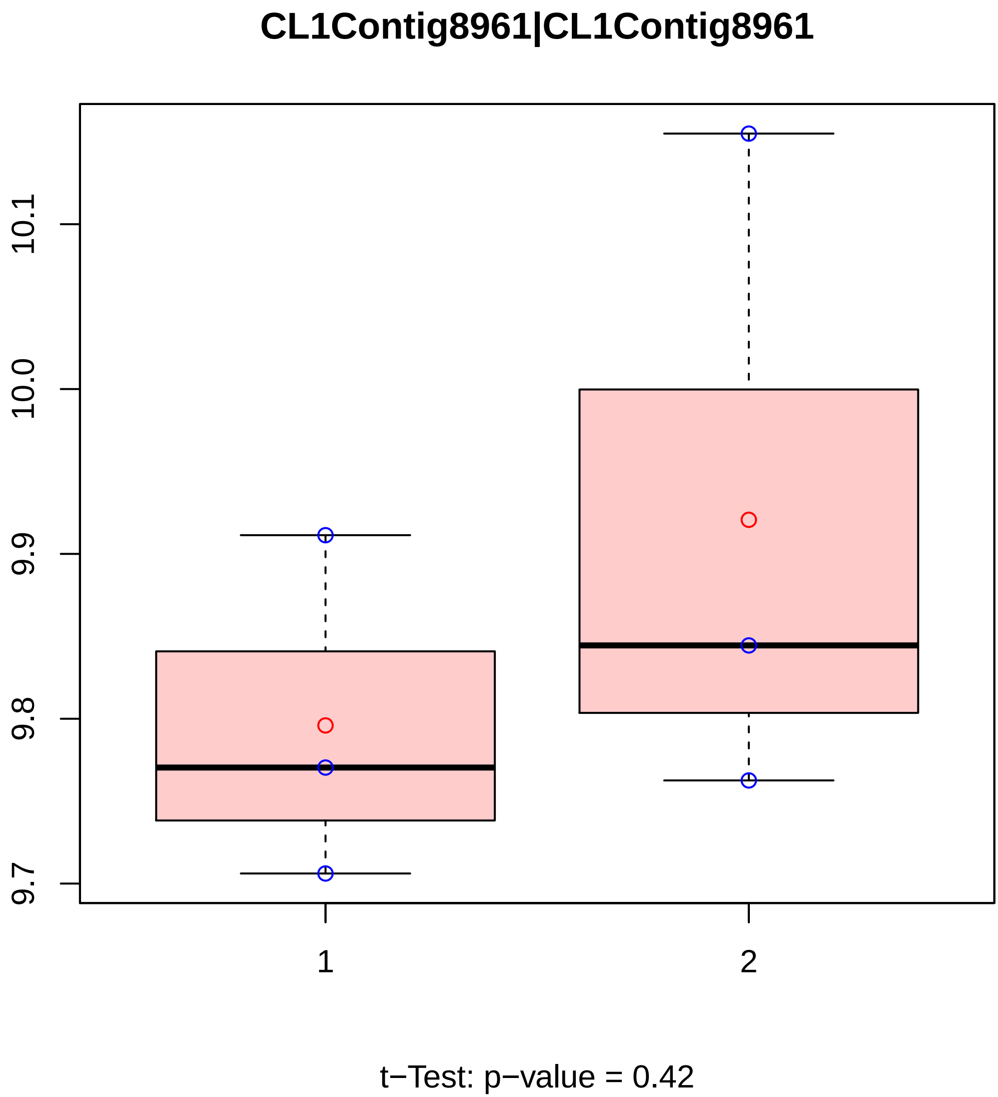
CL1Contig8961|CL1Contig8961
10.1
10.0
9.9
9.8
9.7
1
2
t−Test: p−value = 0.42

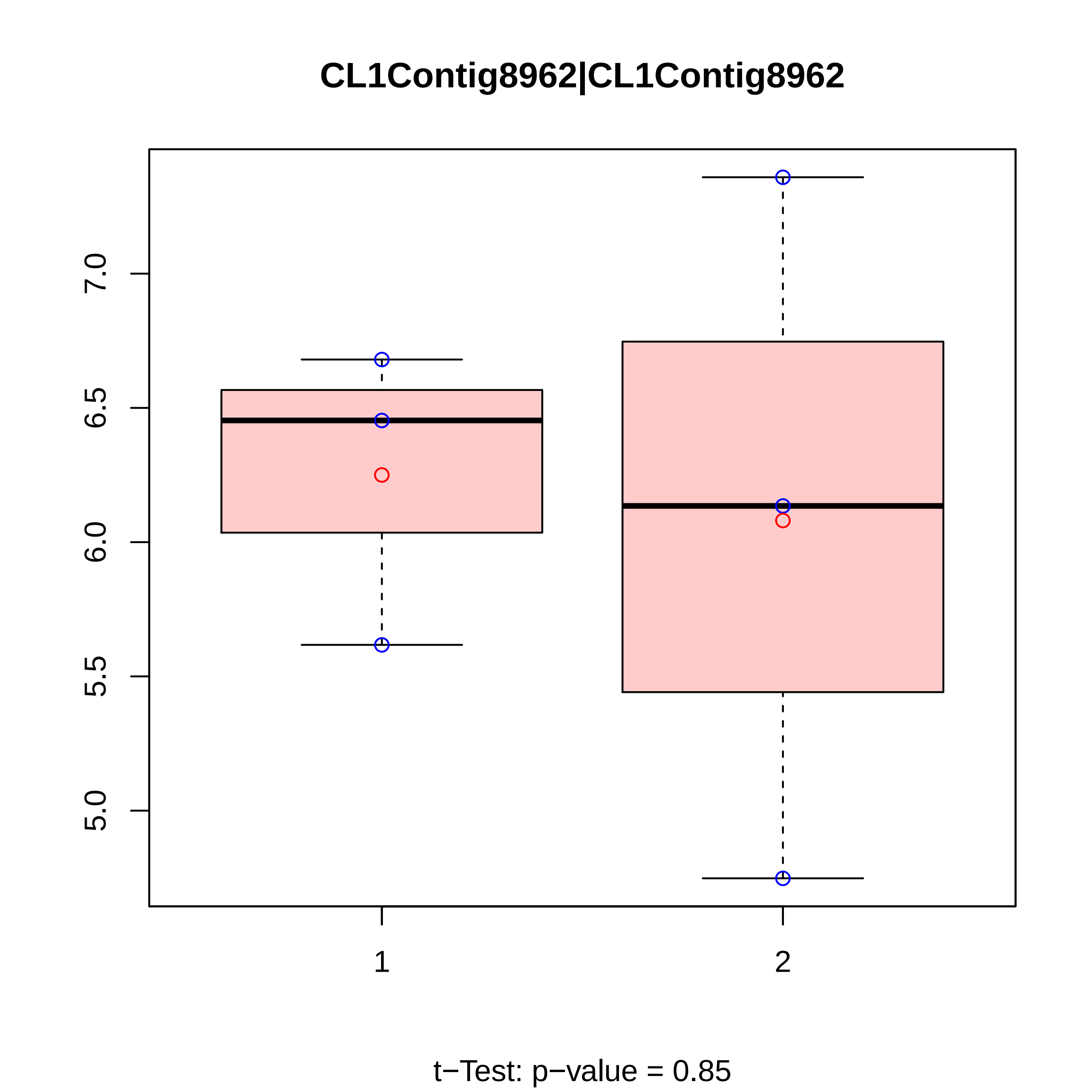

CL1Contig8962|CL1Contig8962
7.0
6.5
6.0
5.5
5.0
1
2
t−Test: p−value = 0.85

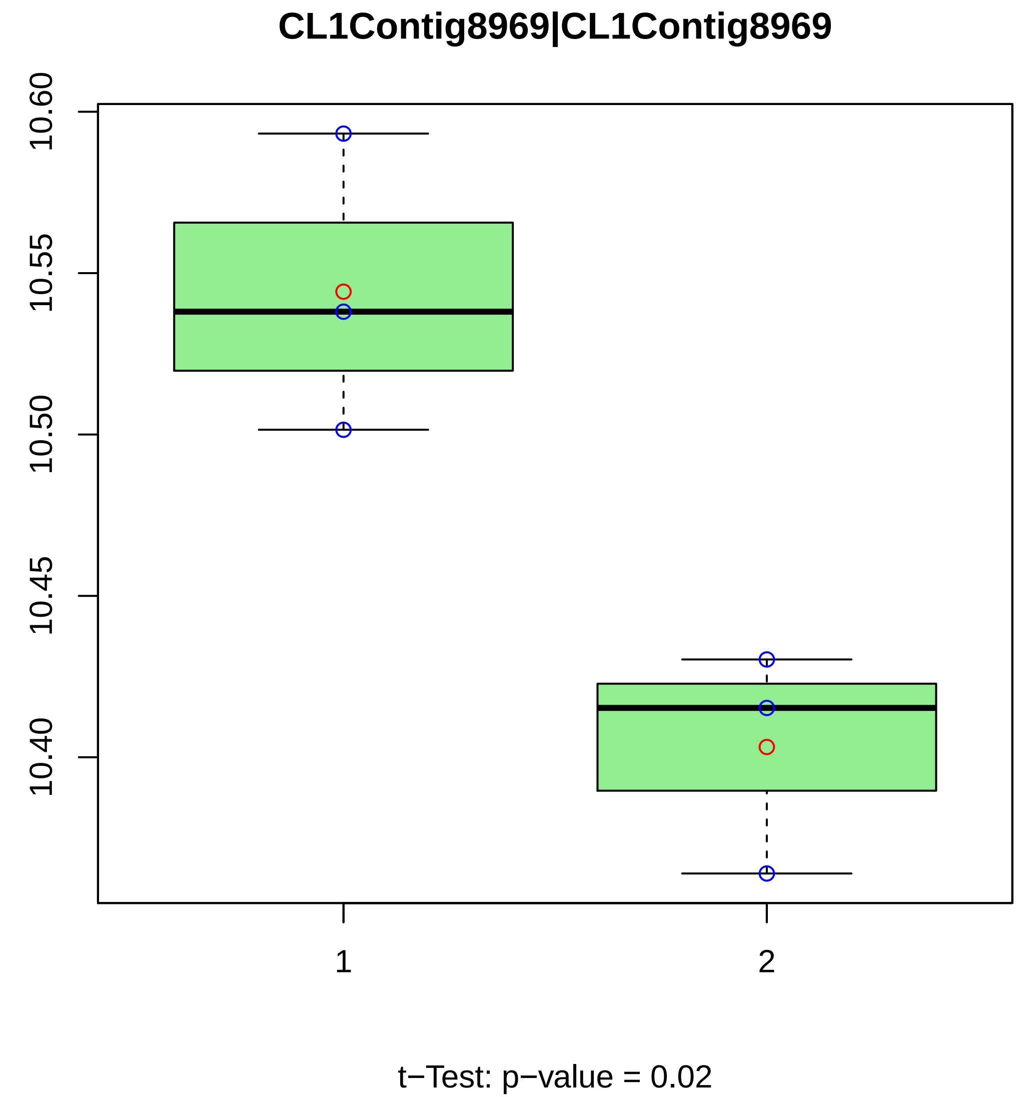
CL1Contig8969|CL1Contig8969
10.60
10.55
10.50
10.45
10.40
1
2
t−Test: p−value = 0.02

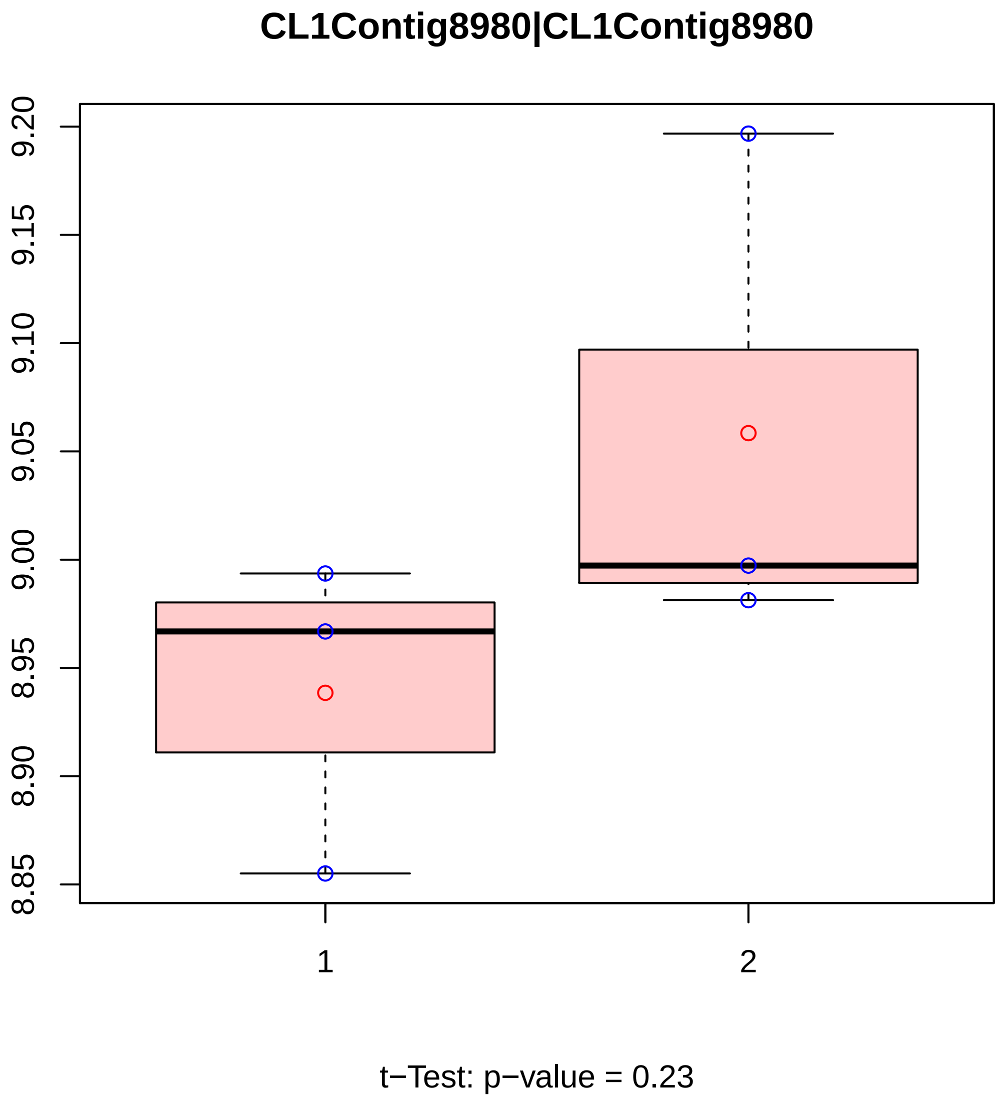
CL1Contig8980|CL1Contig8980
9.20
9.15
9.10
9.05
9.00
8.95
8.90
8.85
1
2
t−Test: p−value = 0.23

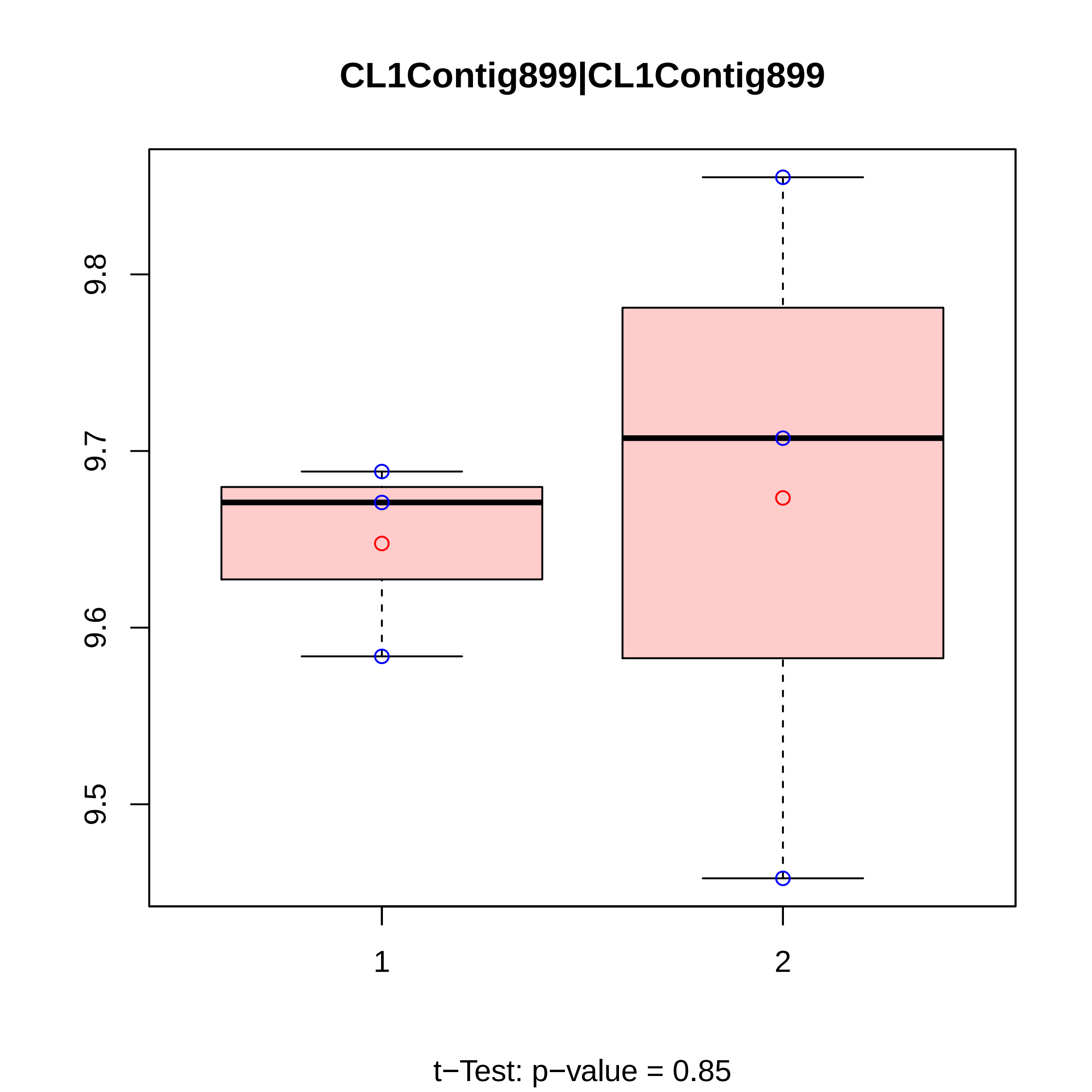
CL1Contig899|CL1Contig899
9.5
9.6
9.7
9.8
1
2
t−Test: p−value = 0.85

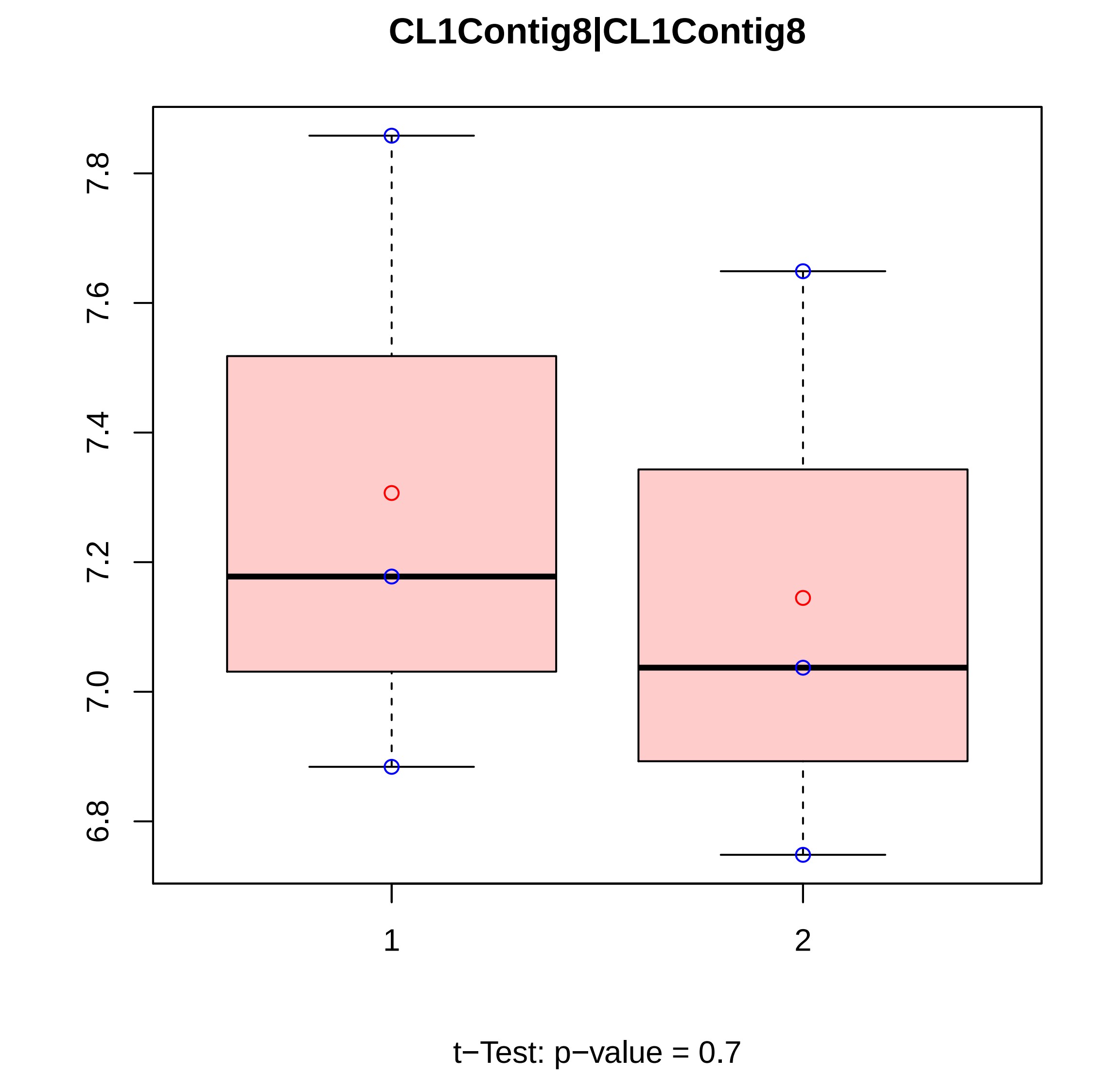
CL1Contig8|CL1Contig8
7.8
7.6
7.4
7.2
7.0
6.8
1
2
t−Test: p−value = 0.7

# CL1Contig9003|CL1Contig9003

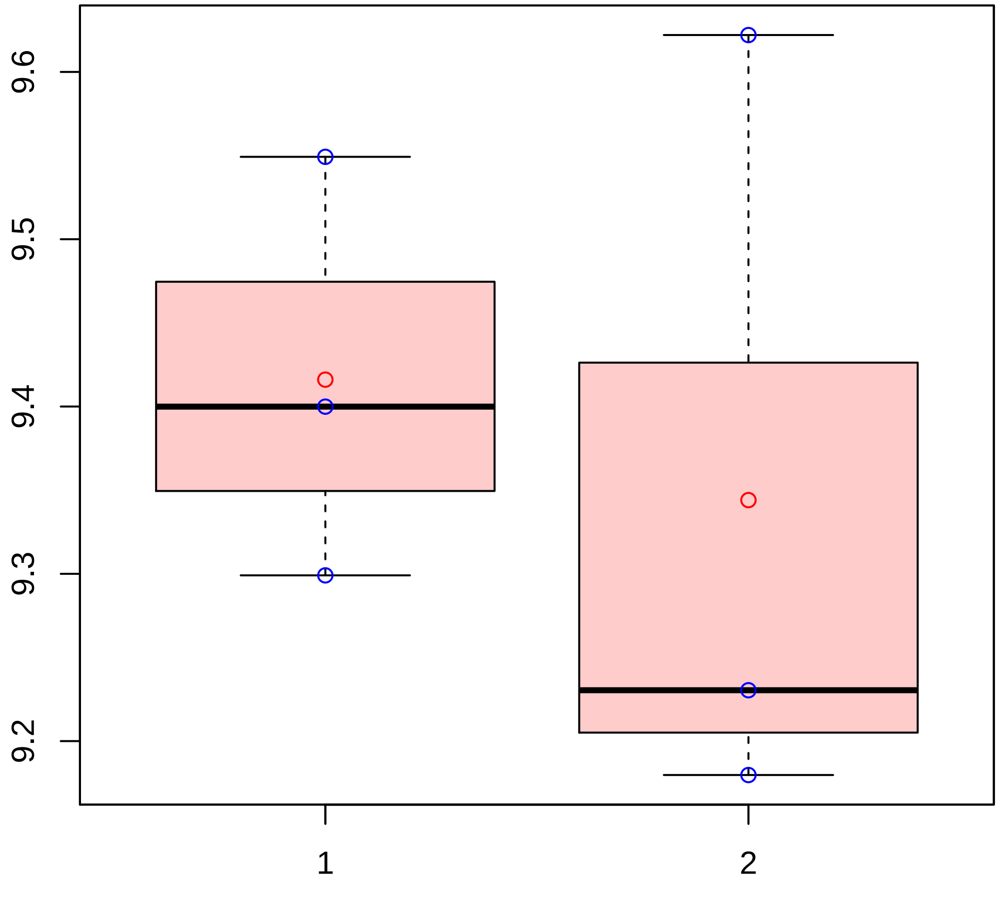


t−Test: p−value = 0.68

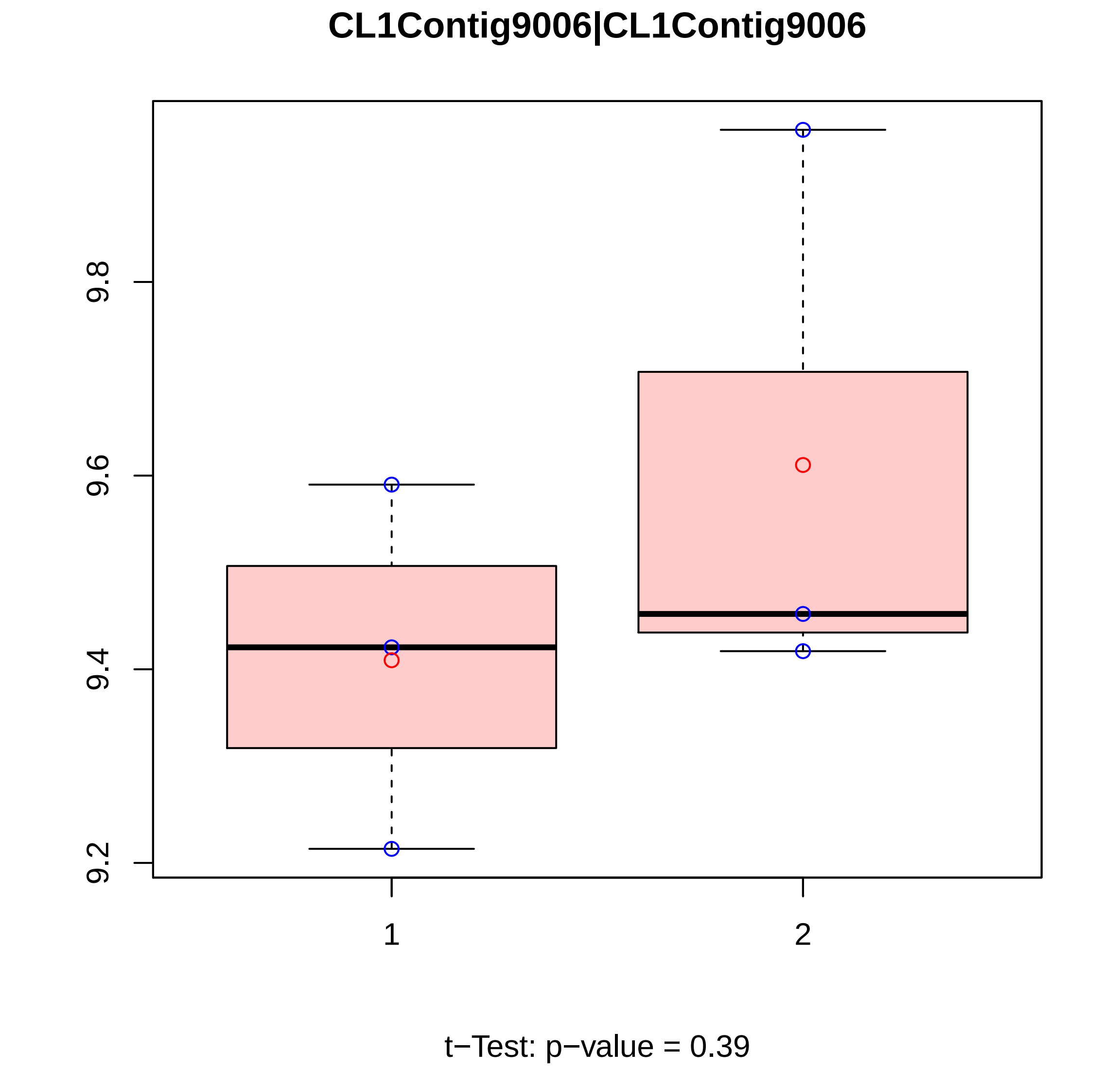
CL1Contig9006|CL1Contig9006
9.2
9.4
9.6
9.8
1
2
t−Test: p−value = 0.39

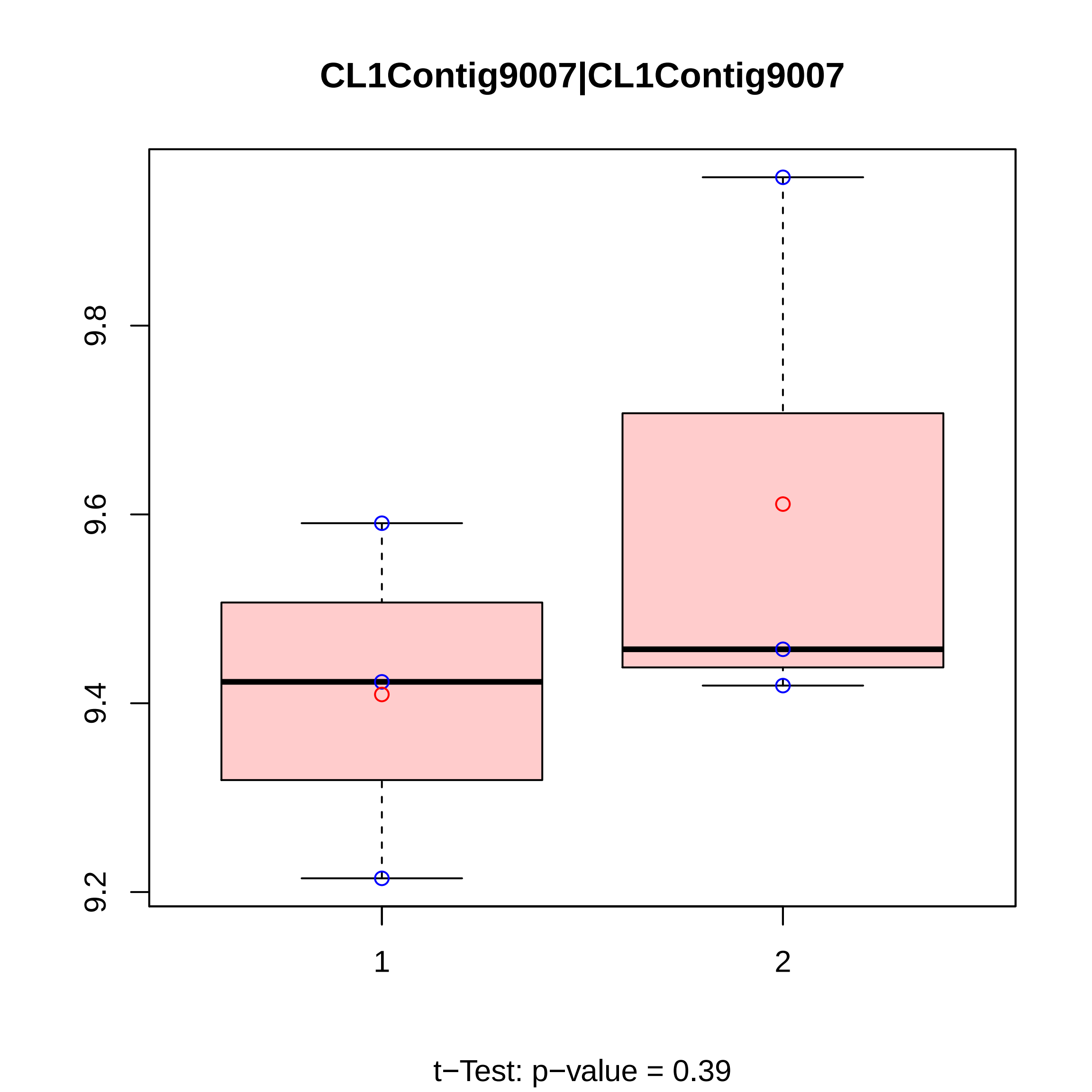
CL1Contig9007|CL1Contig9007
9.2
9.4
9.6
9.8
1
2
t−Test: p−value = 0.39

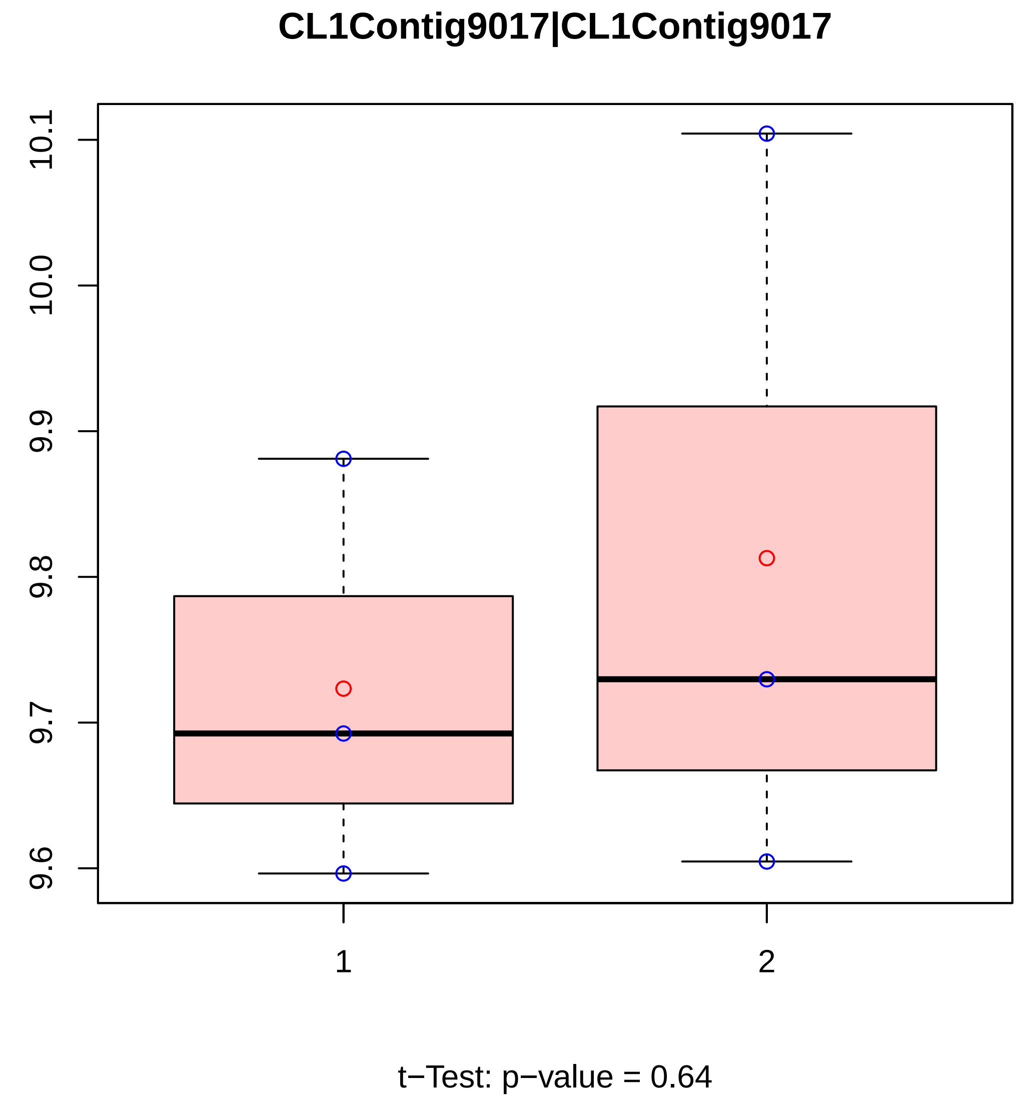

CL1Contig9017|CL1Contig9017
10.1
10.0
9.9
9.8
9.7
9.6
1
2
t−Test: p−value = 0.64

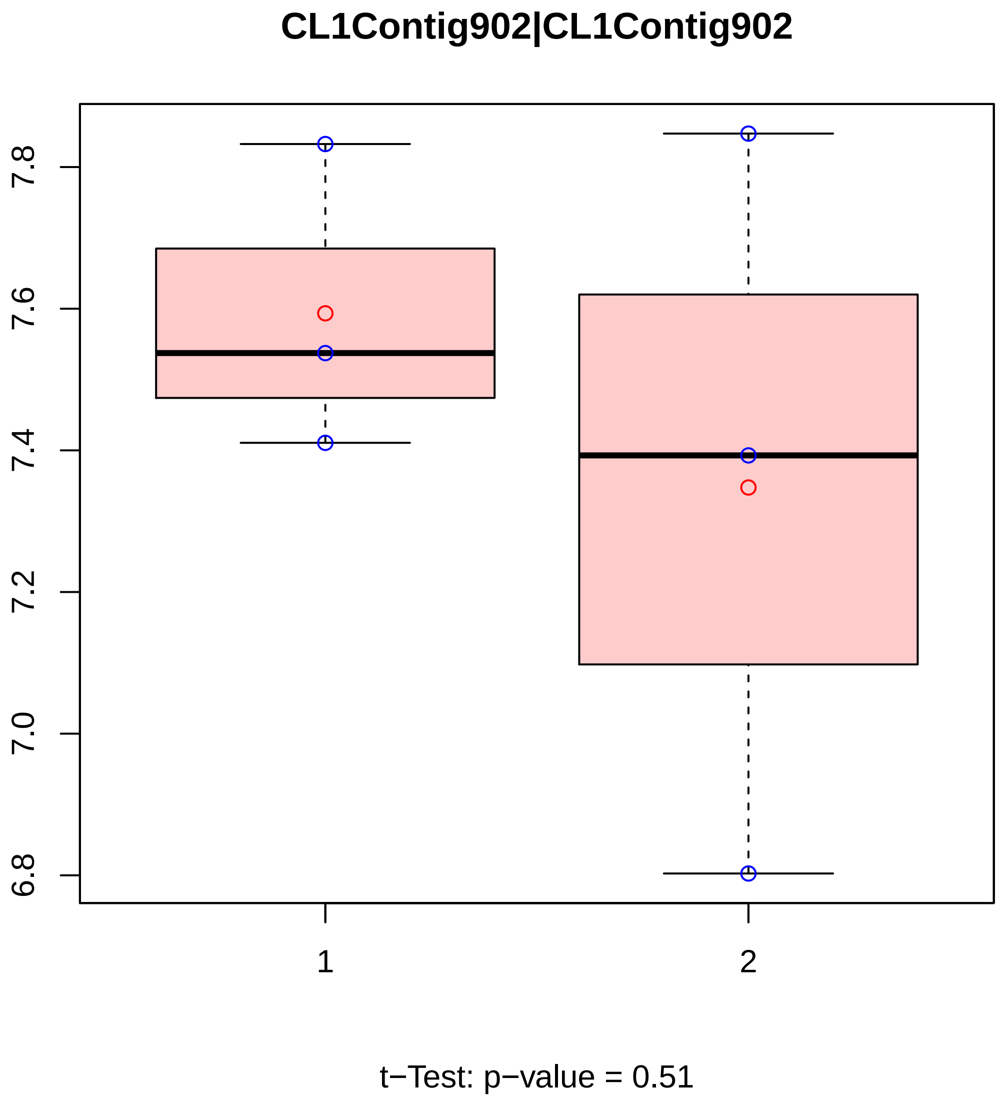
CL1Contig902|CL1Contig902
7.8
7.6
7.4
7.2
7.0
6.8
1
2
t−Test: p−value = 0.51

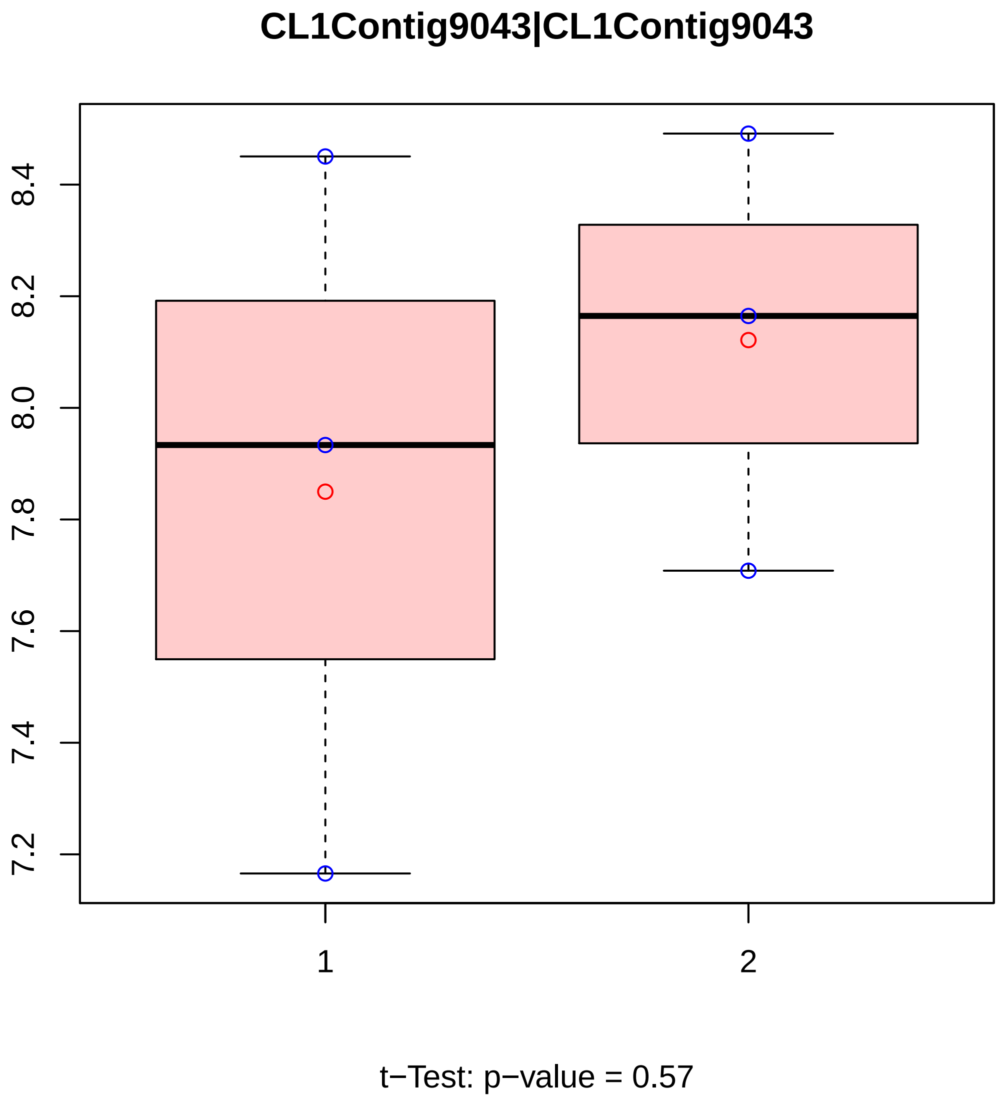

CL1Contig9043|CL1Contig9043
7.2
7.4
7.6
7.8
8.0
8.2
8.4
1
2
t−Test: p−value = 0.57

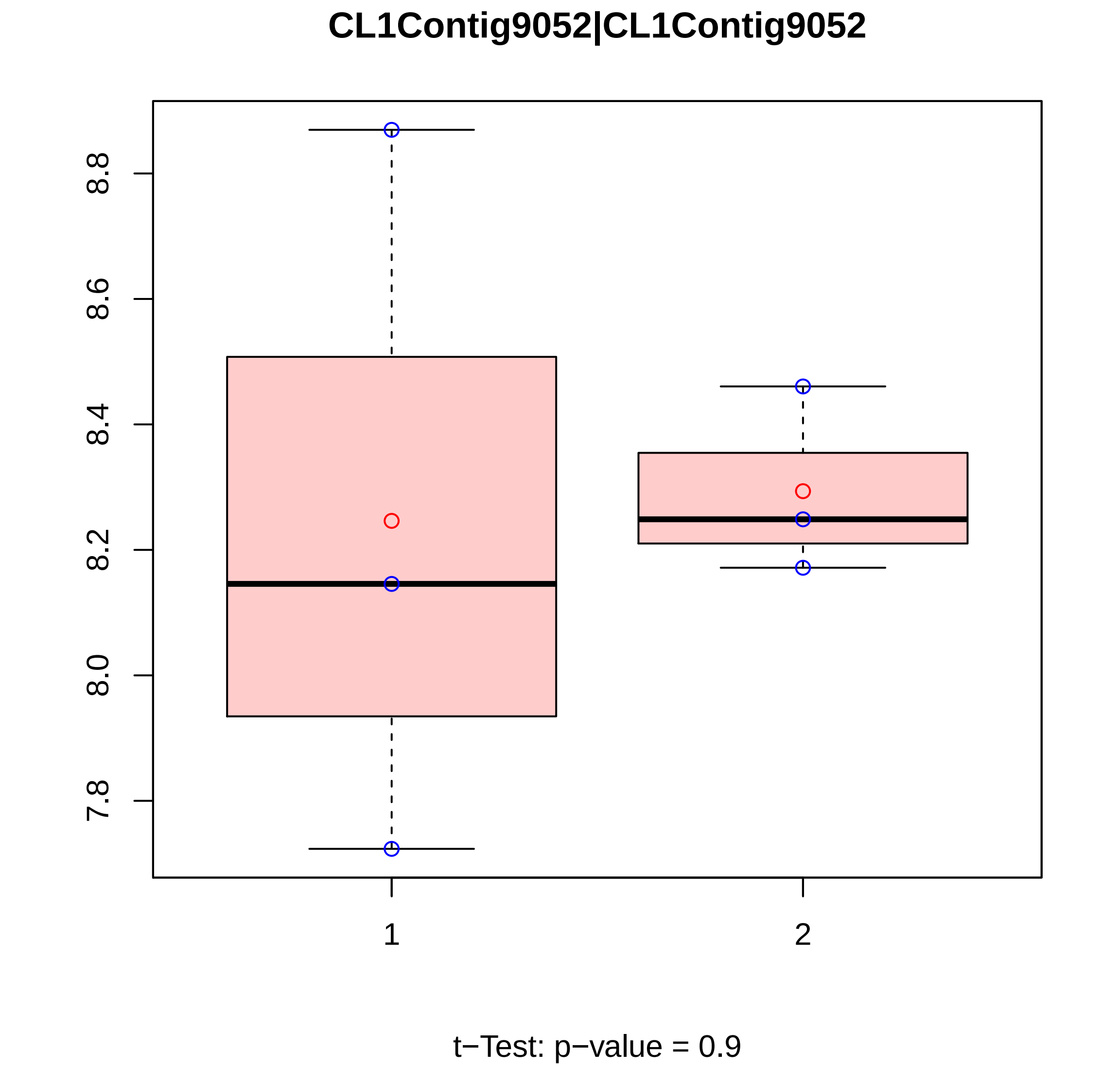
CL1Contig9052|CL1Contig9052
8.8
8.6
8.4
8.2
8.0
7.8
1
2
t−Test: p−value = 0.9

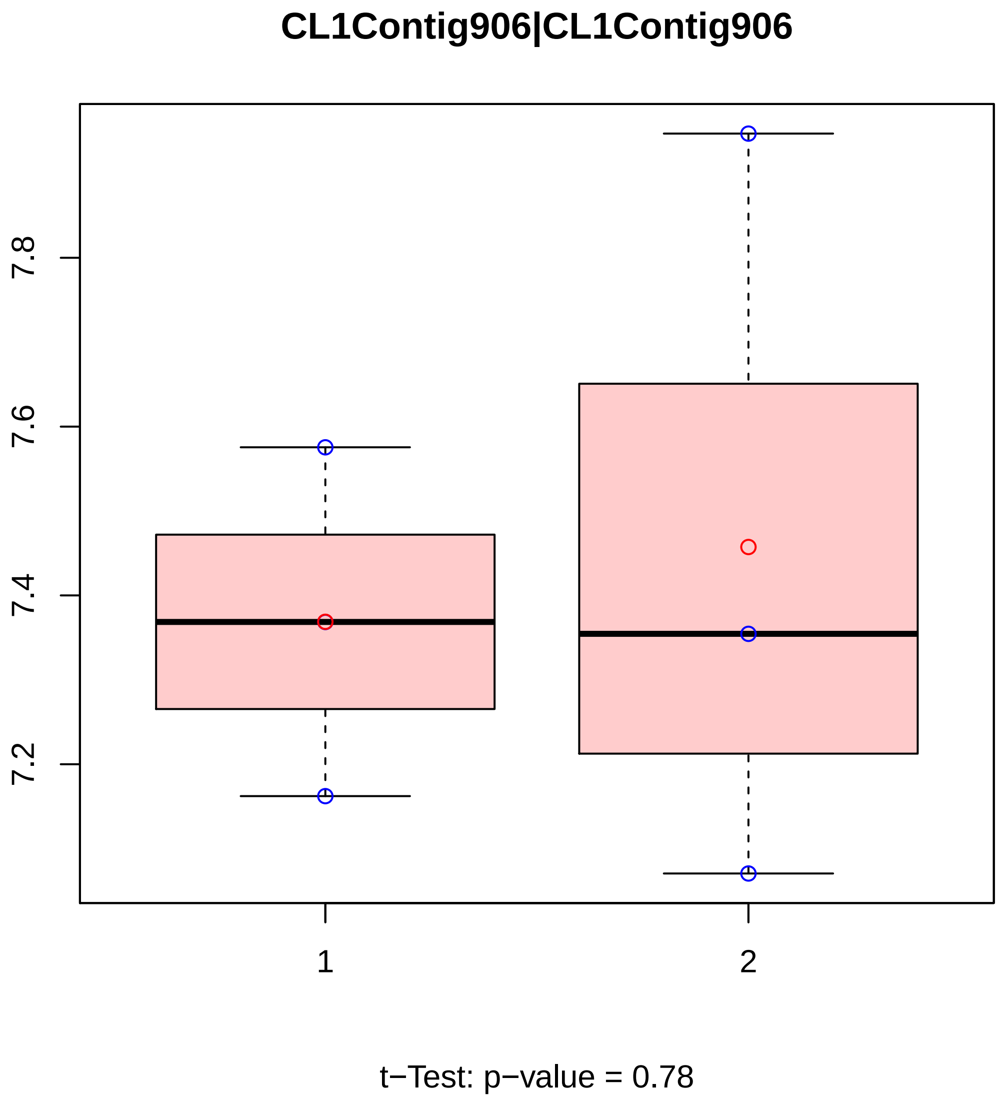

CL1Contig906|CL1Contig906
7.2
7.4
7.6
7.8
1
2
t−Test: p−value = 0.78

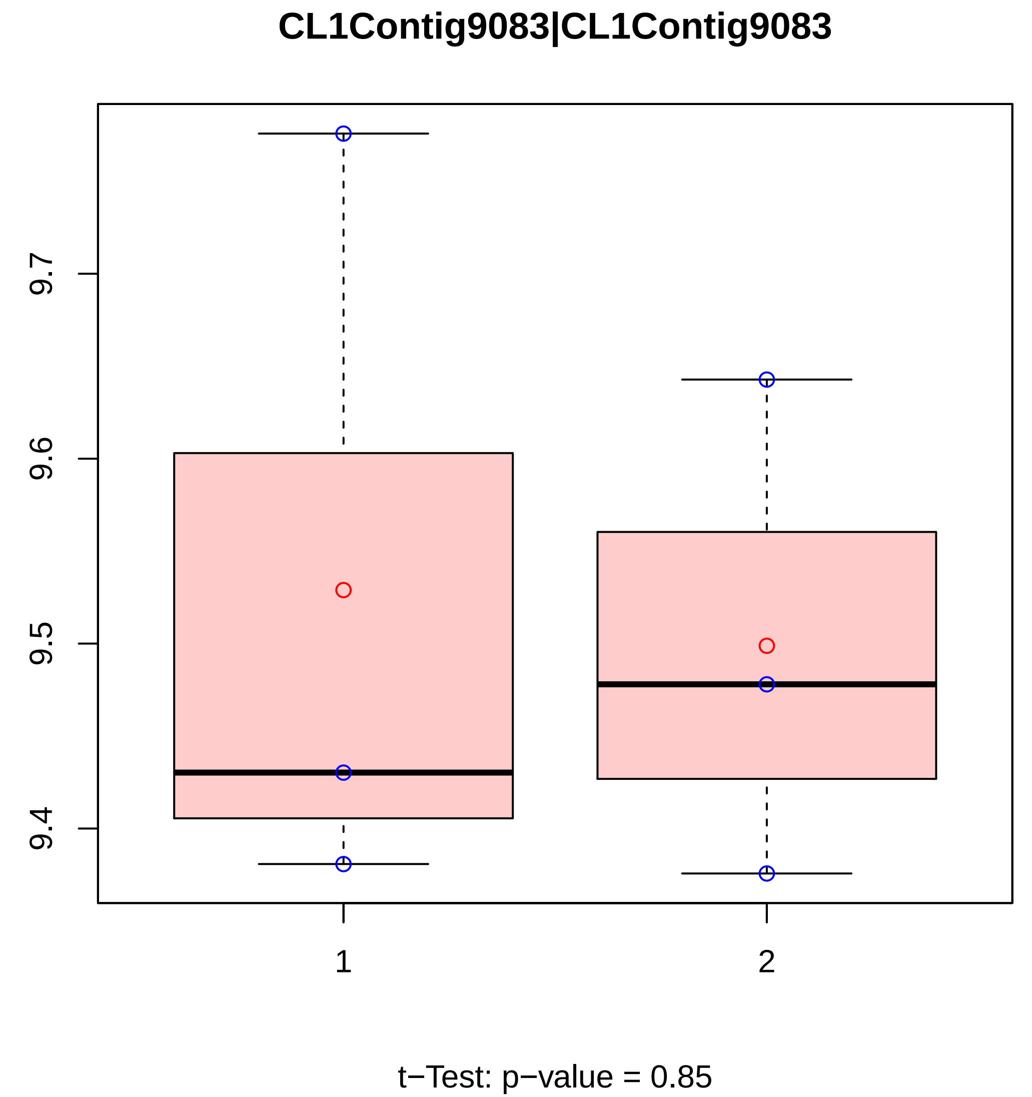
CL1Contig9083|CL1Contig9083
9.4
9.5
9.6
9.7
1
2
t−Test: p−value = 0.85

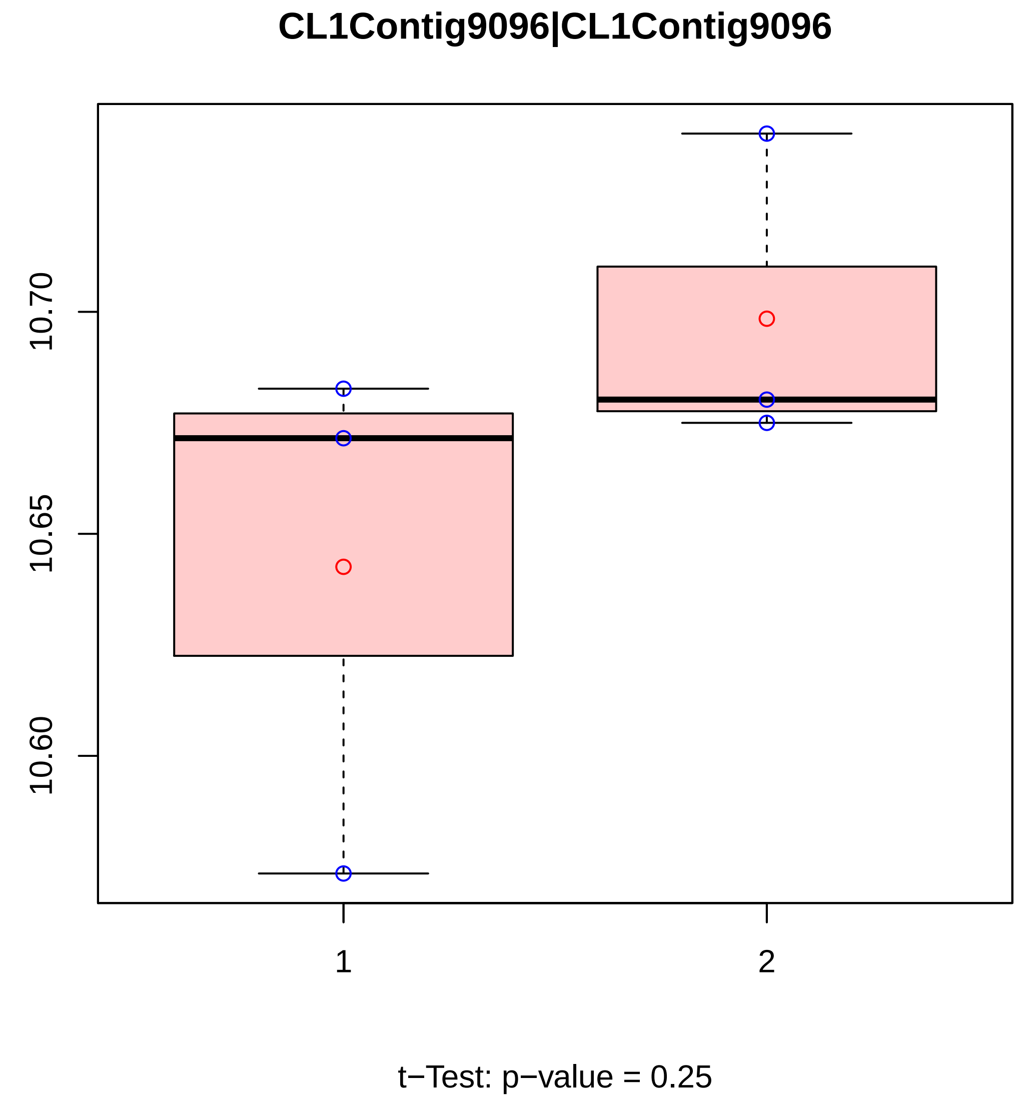
CL1Contig9096|CL1Contig9096
10.60
10.65
10.70
1
2
t−Test: p−value = 0.25

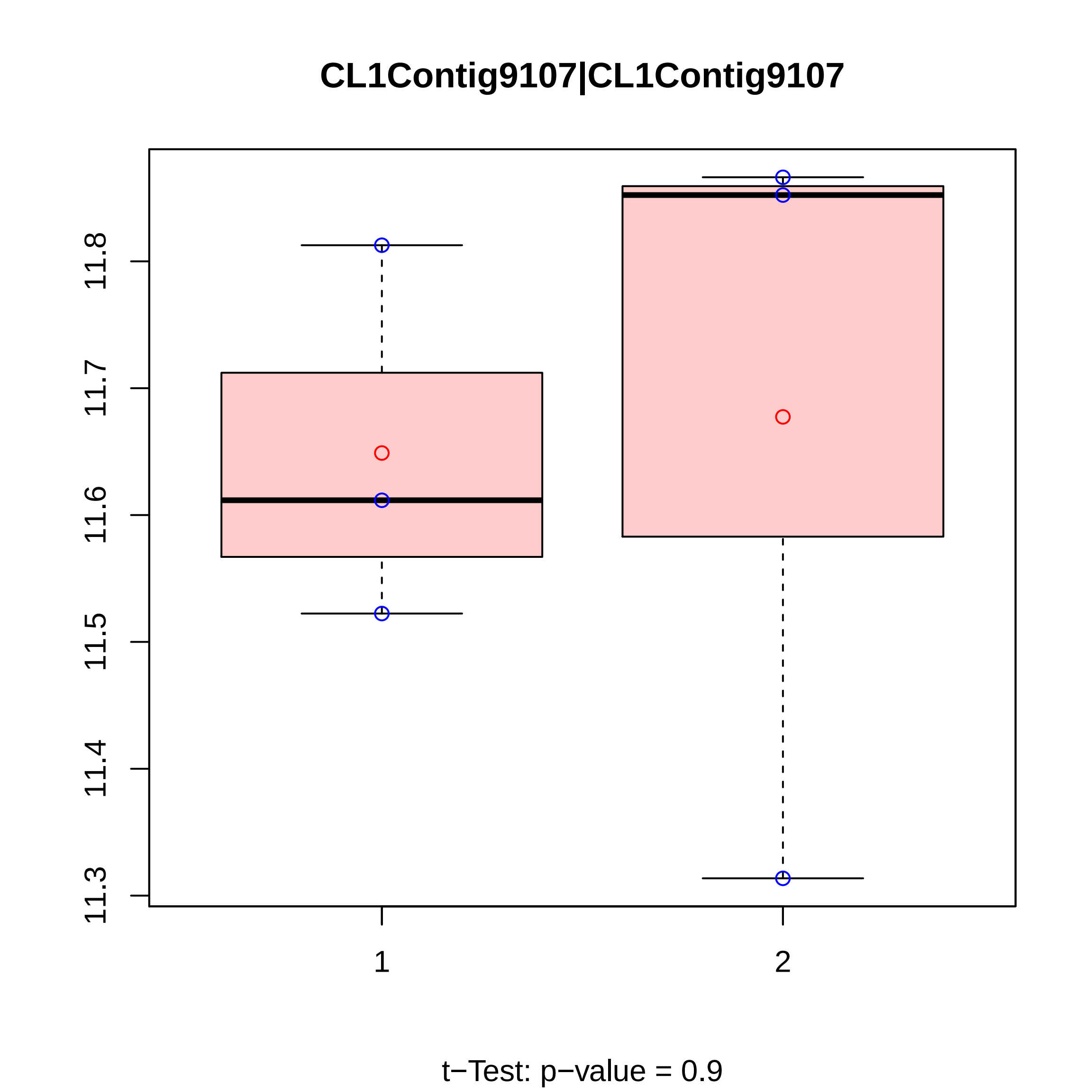
CL1Contig9107|CL1Contig9107
11.3
11.4
11.5
11.6
11.7
11.8
1
2
t−Test: p−value = 0.9

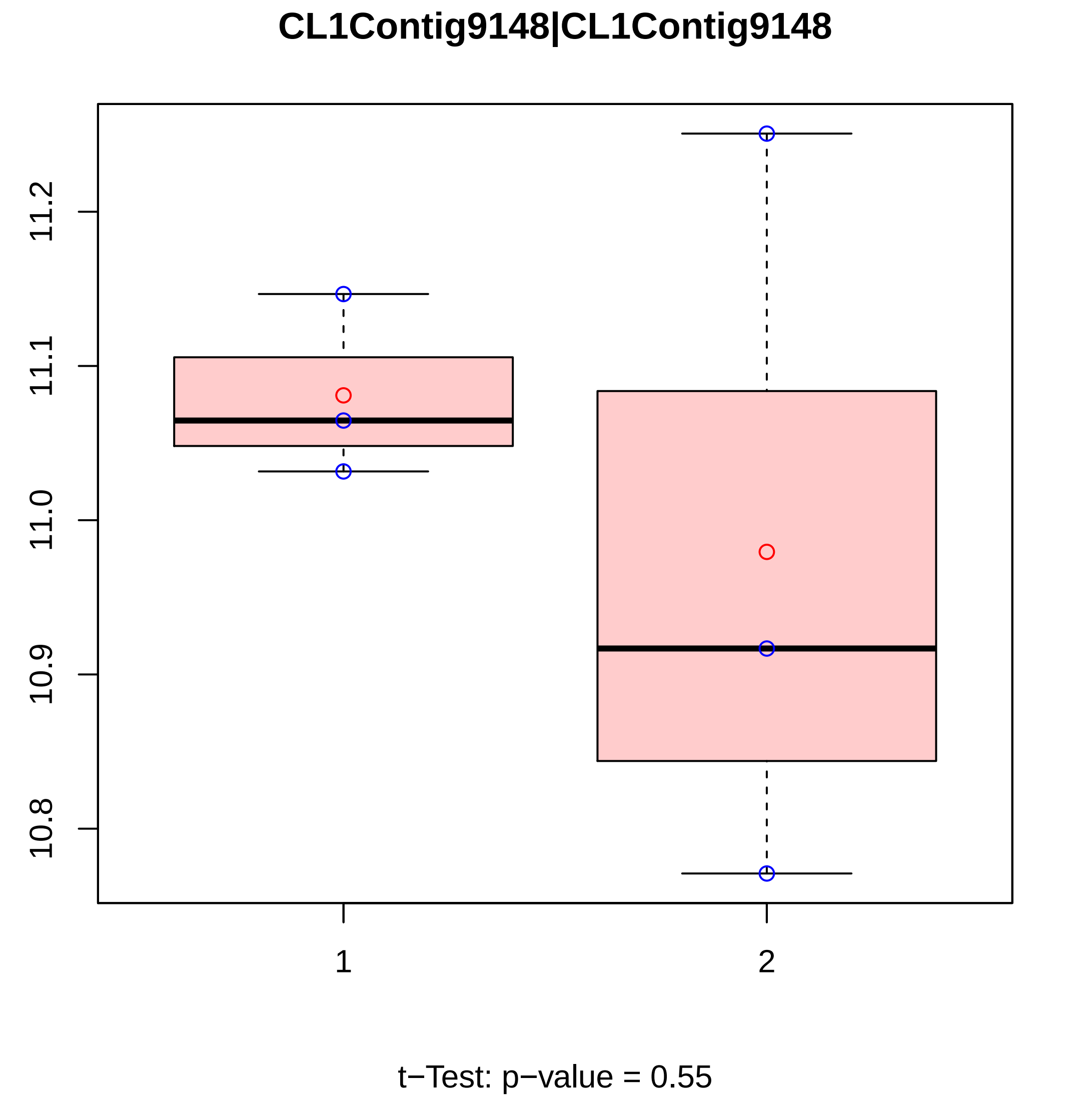

CL1Contig9148|CL1Contig9148
11.2
11.1
11.0
10.9
10.8
1
2
t−Test: p−value = 0.55

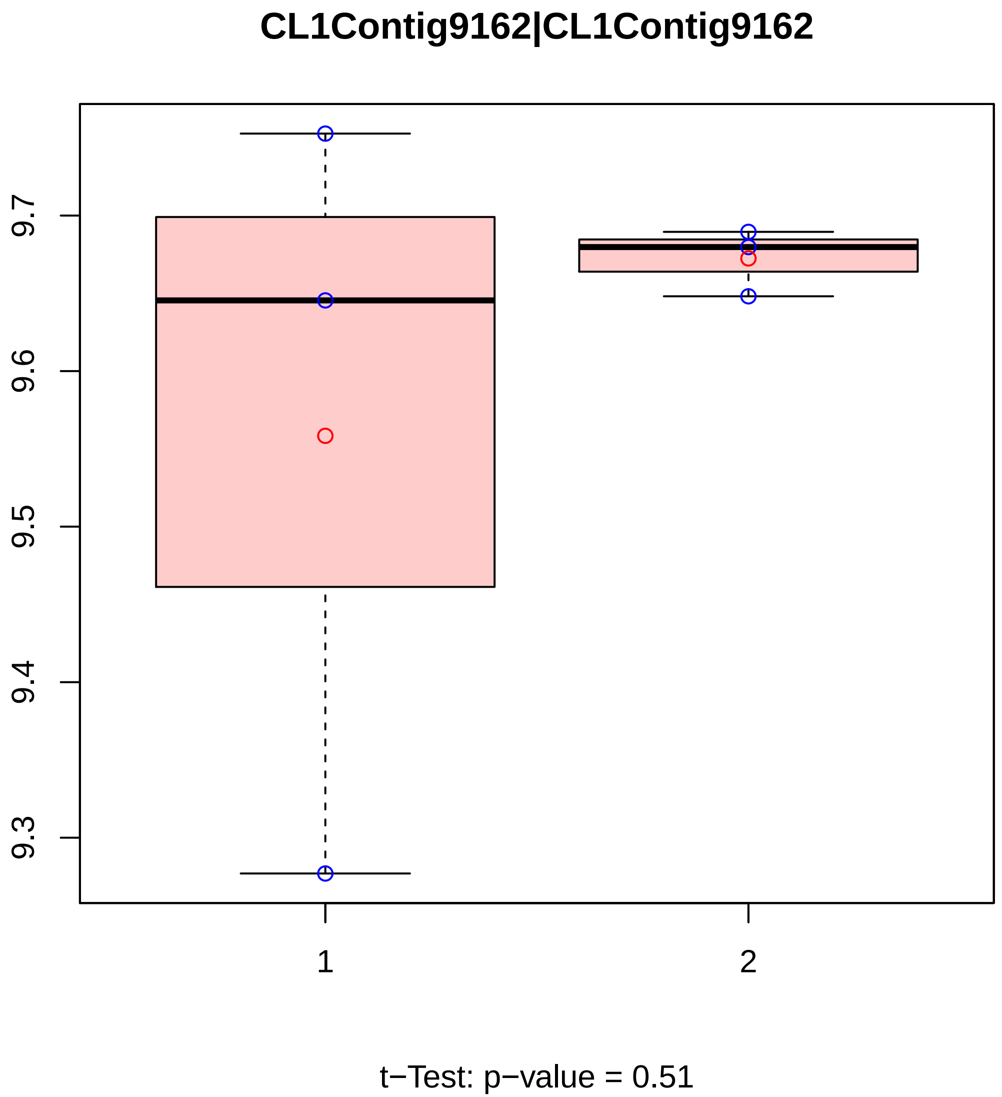
CL1Contig9162|CL1Contig9162
9.3
9.4
9.5
9.6
9.7
1
2
t−Test: p−value = 0.51

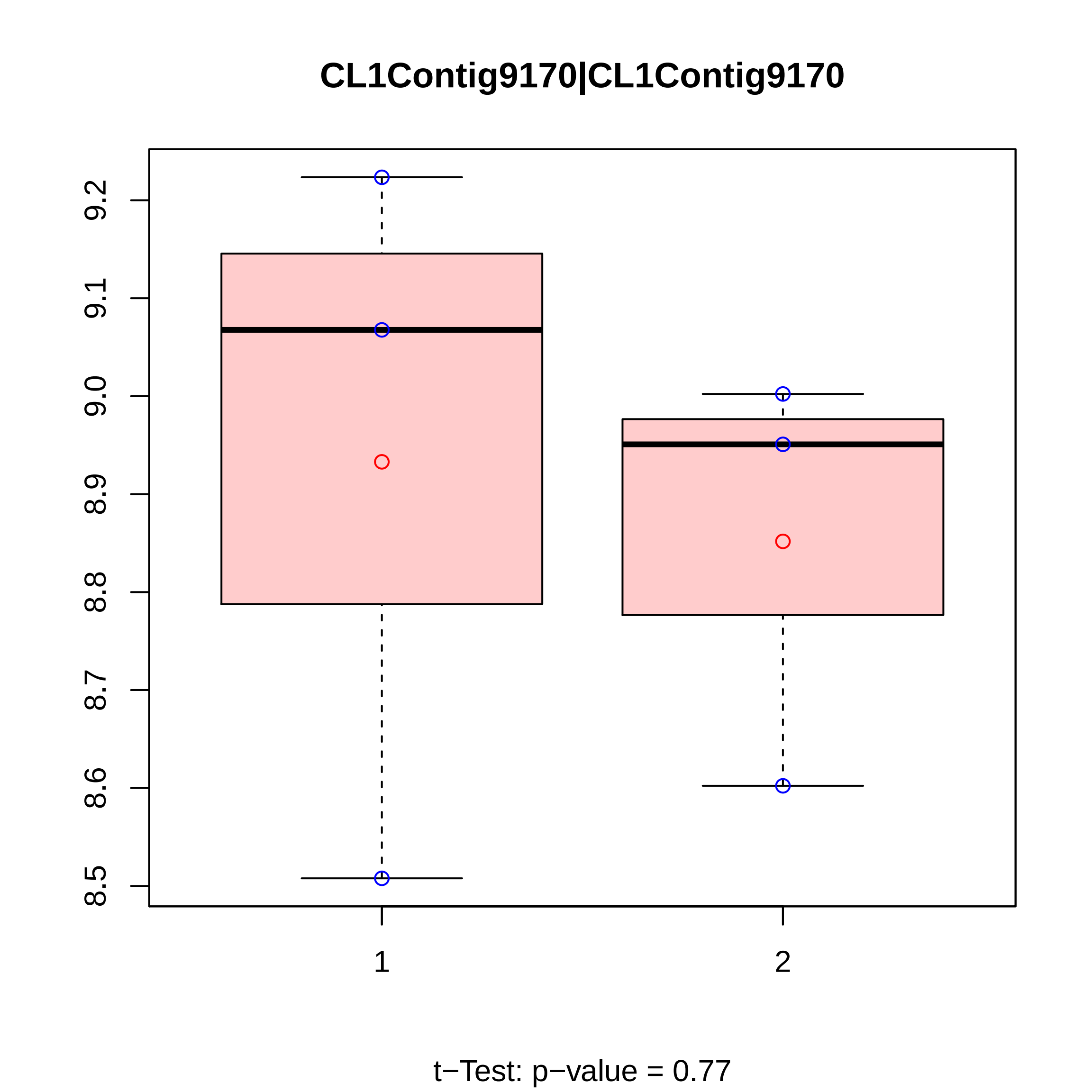
CL1Contig9170|CL1Contig9170
9.2
9.1
9.0
8.9
8.8
8.7
8.6
8.5
1
2
t−Test: p−value = 0.77

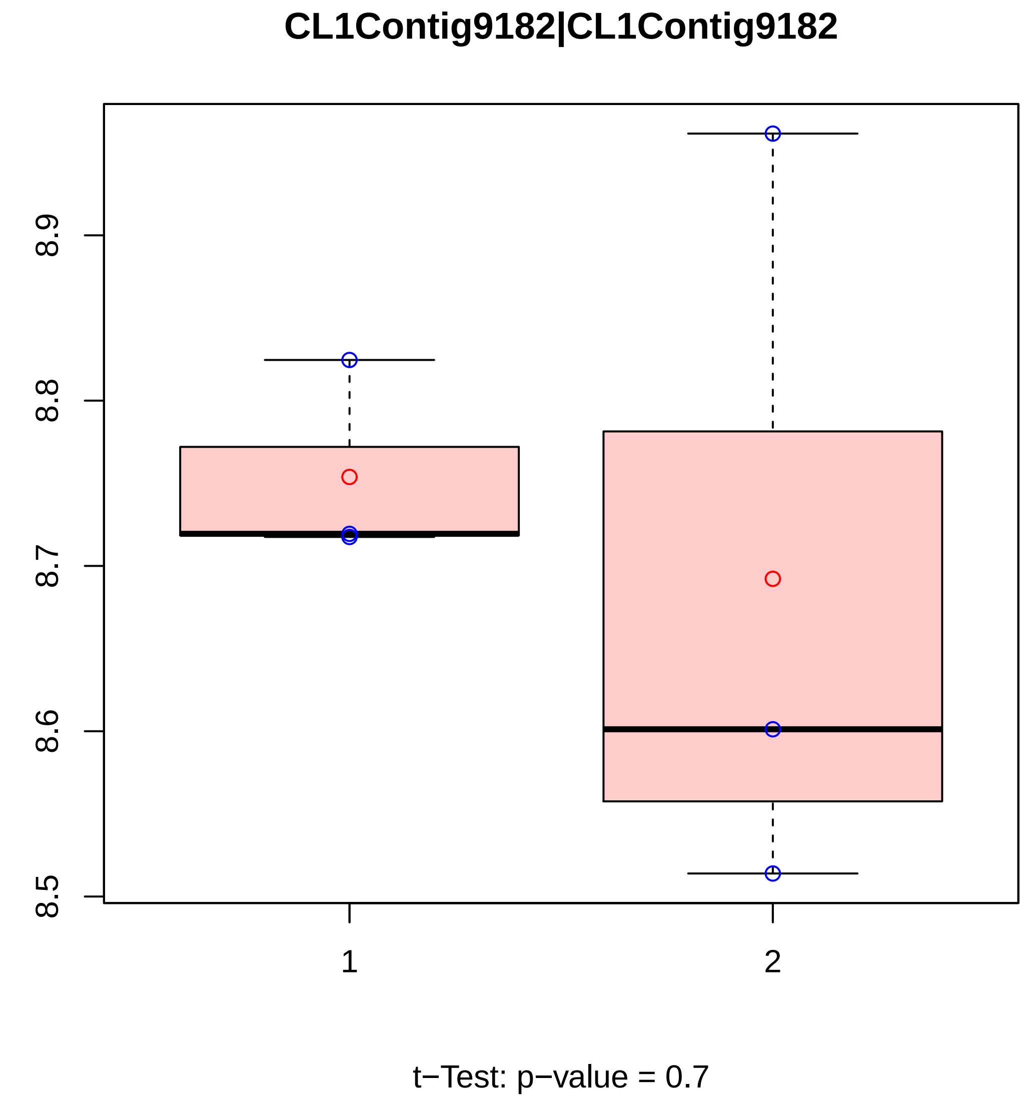
CL1Contig9182|CL1Contig9182
8.5
8.6
8.7
8.8
8.9
1
2
t−Test: p−value = 0.7

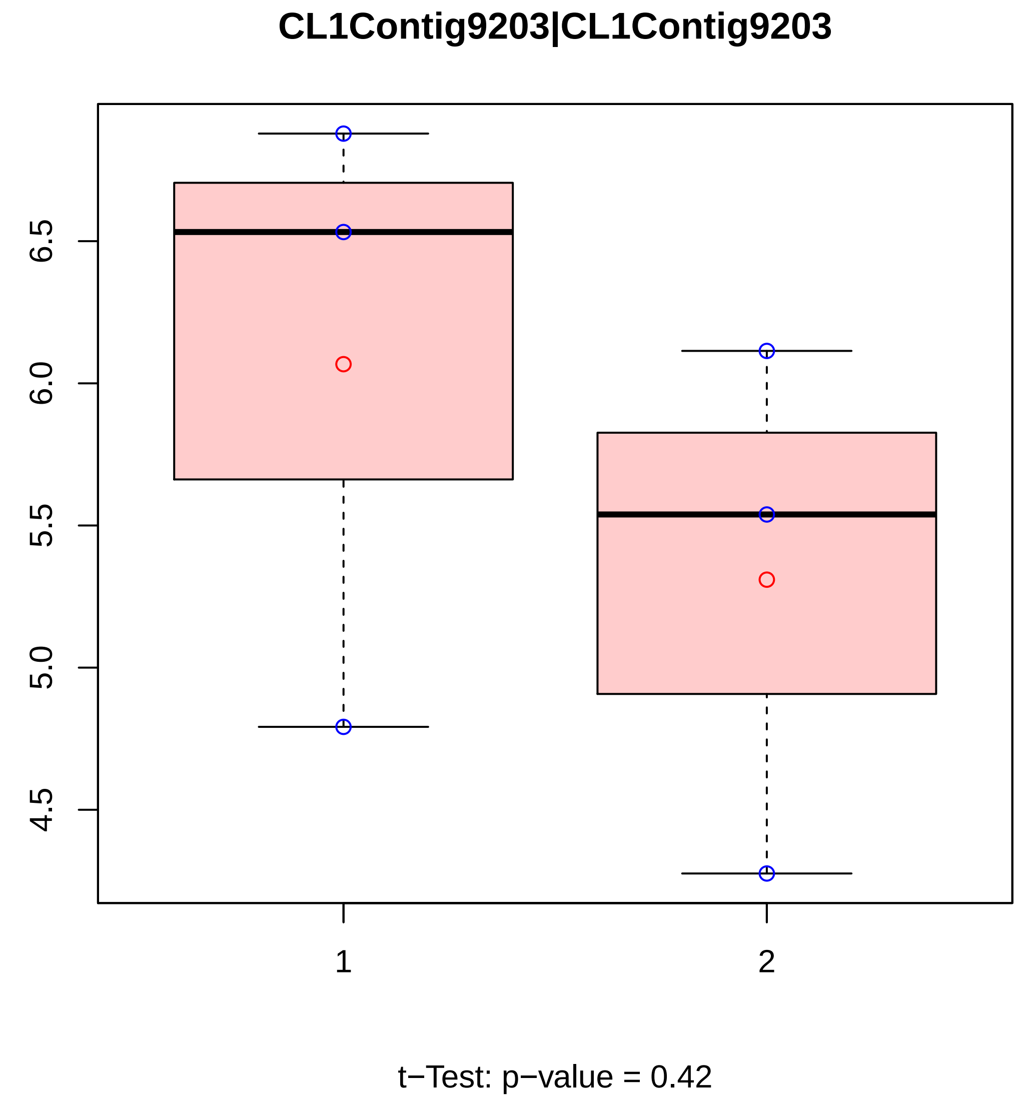
CL1Contig9203|CL1Contig9203
6.5
6.0
5.5
5.0
4.5
1
2
t−Test: p−value = 0.42

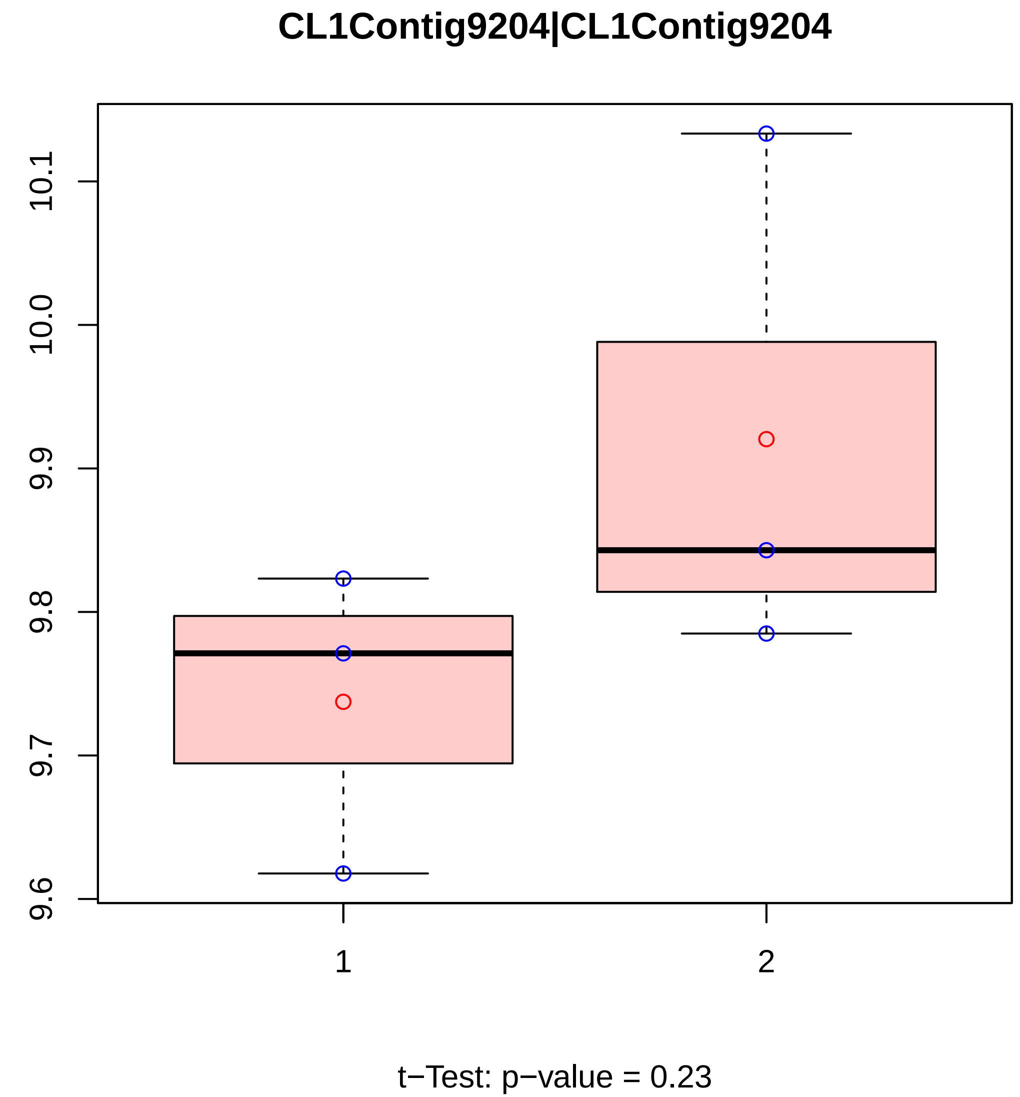

CL1Contig9204|CL1Contig9204
10.1
10.0
9.9
9.8
9.7
9.6
1
2
t−Test: p−value = 0.23

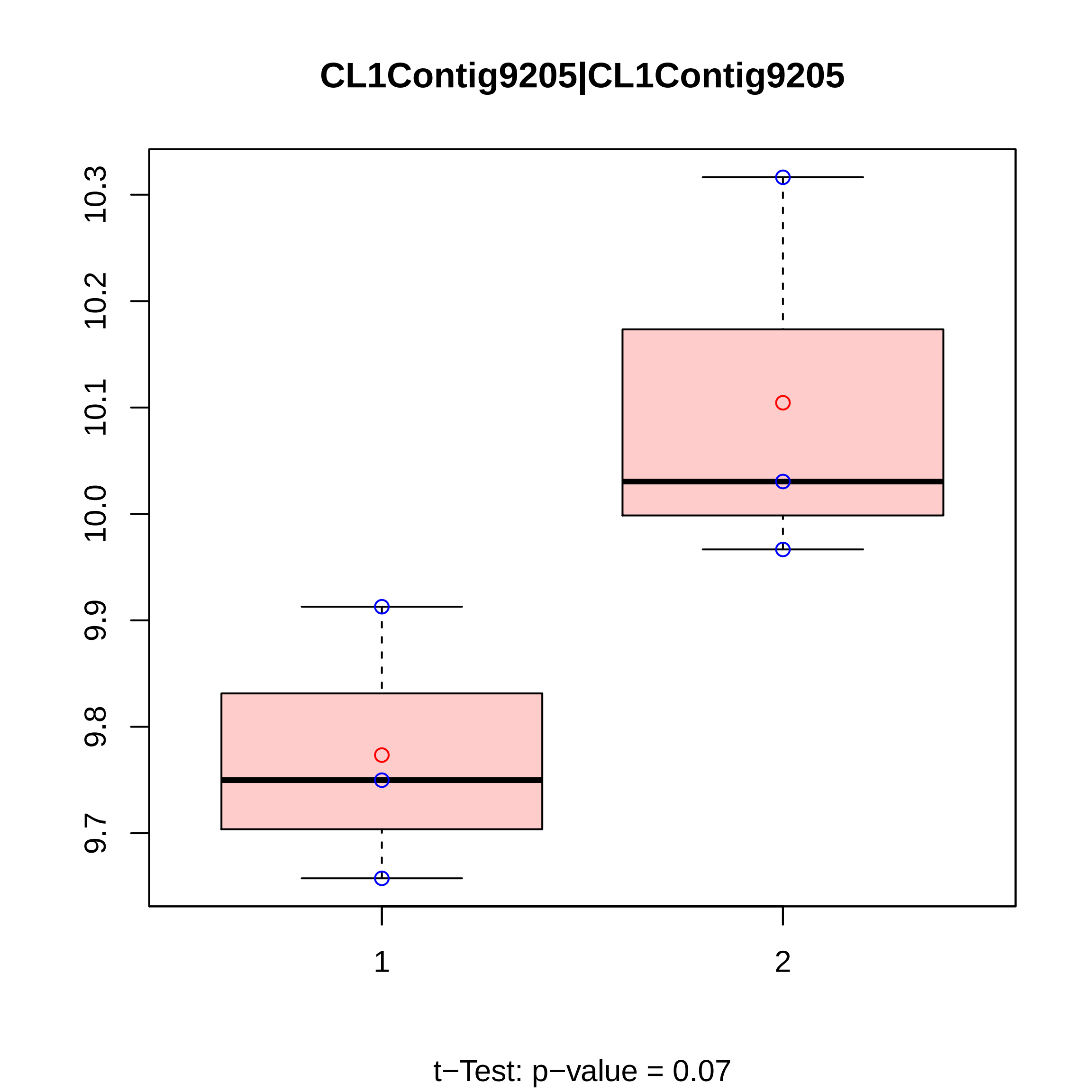
CL1Contig9205|CL1Contig9205
10.3
10.2
10.1
10.0
9.9
9.8
9.7
1
2
t−Test: p−value = 0.07

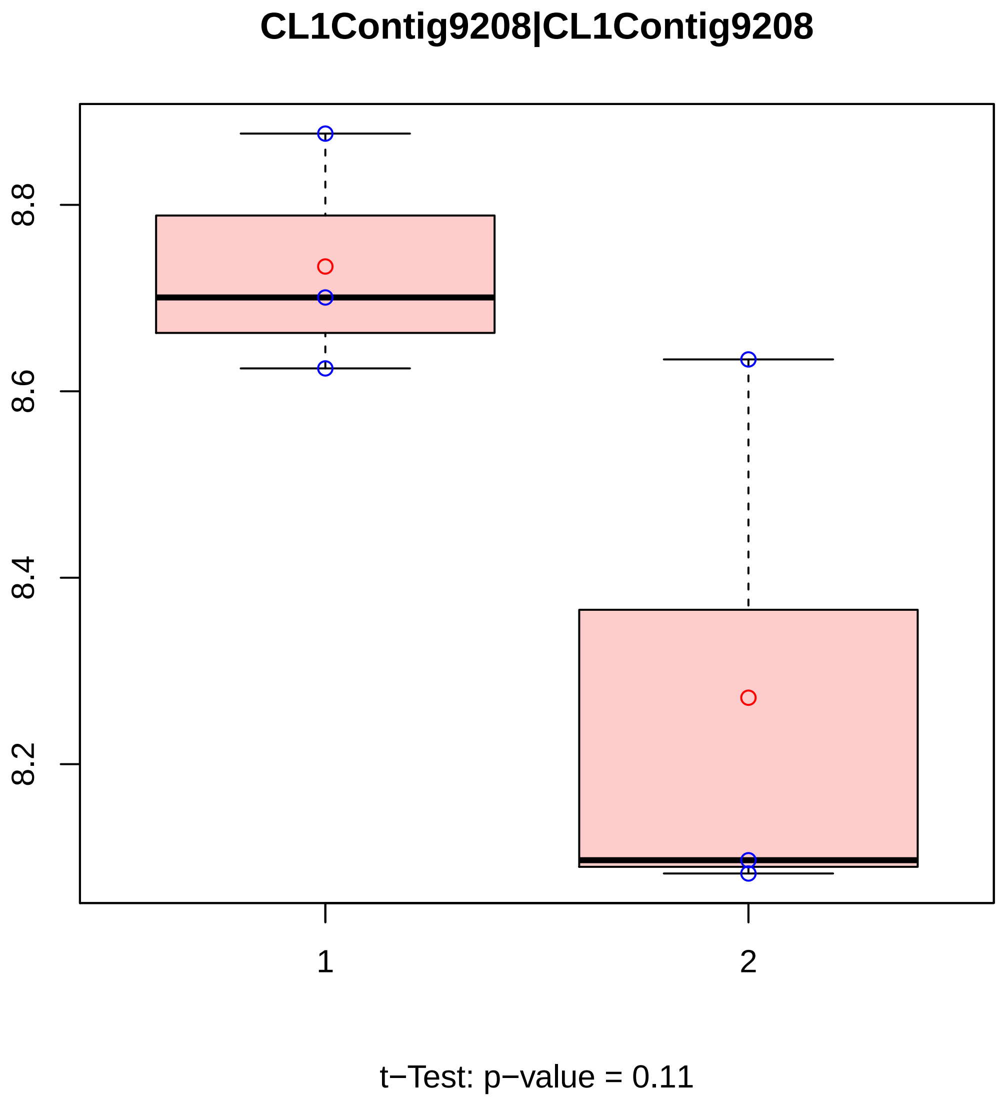

CL1Contig9208|CL1Contig9208
8.2
8.4
8.6
8.8
1
2
t−Test: p−value = 0.11

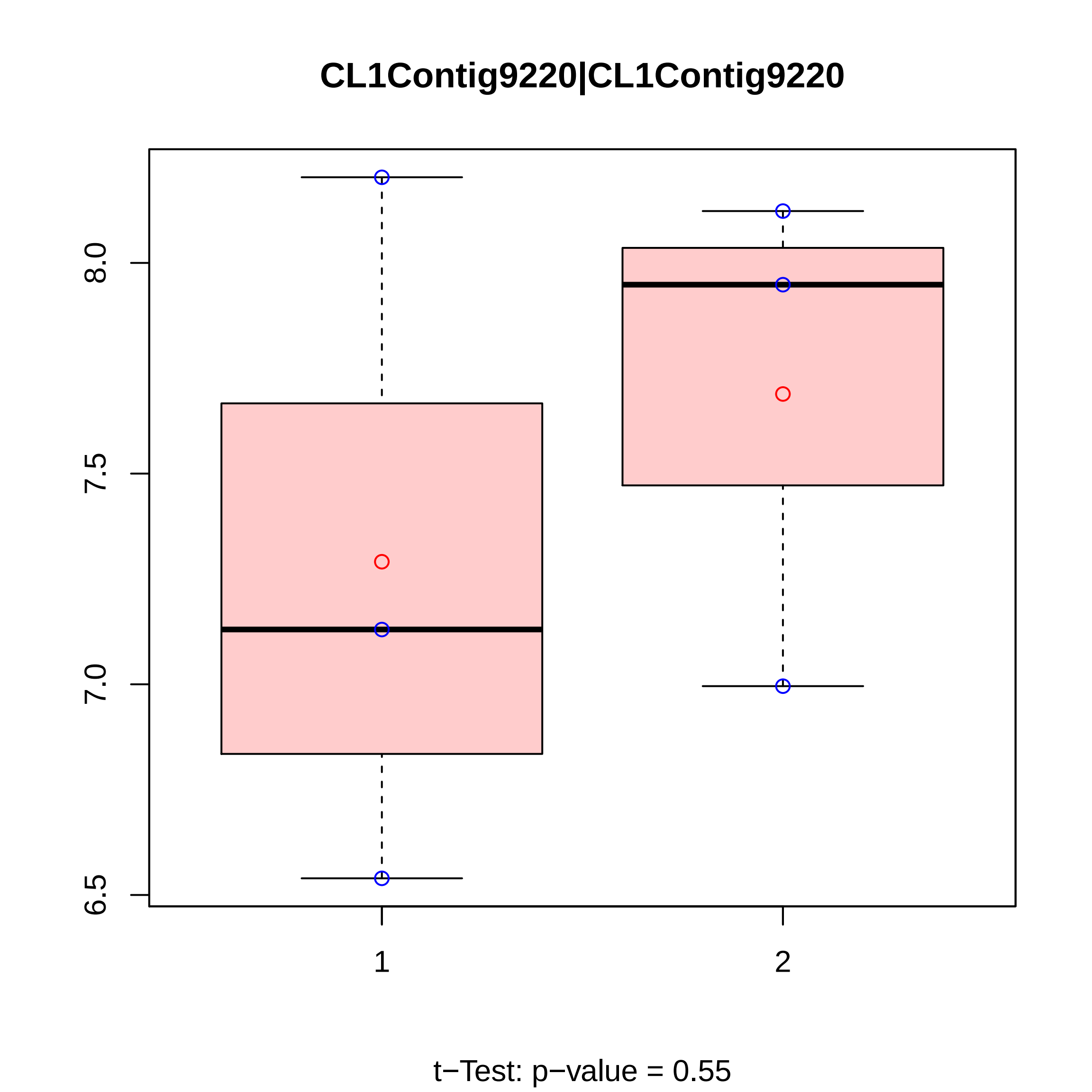
CL1Contig9220|CL1Contig9220
6.5
7.0
7.5
8.0
1
2
t−Test: p−value = 0.55

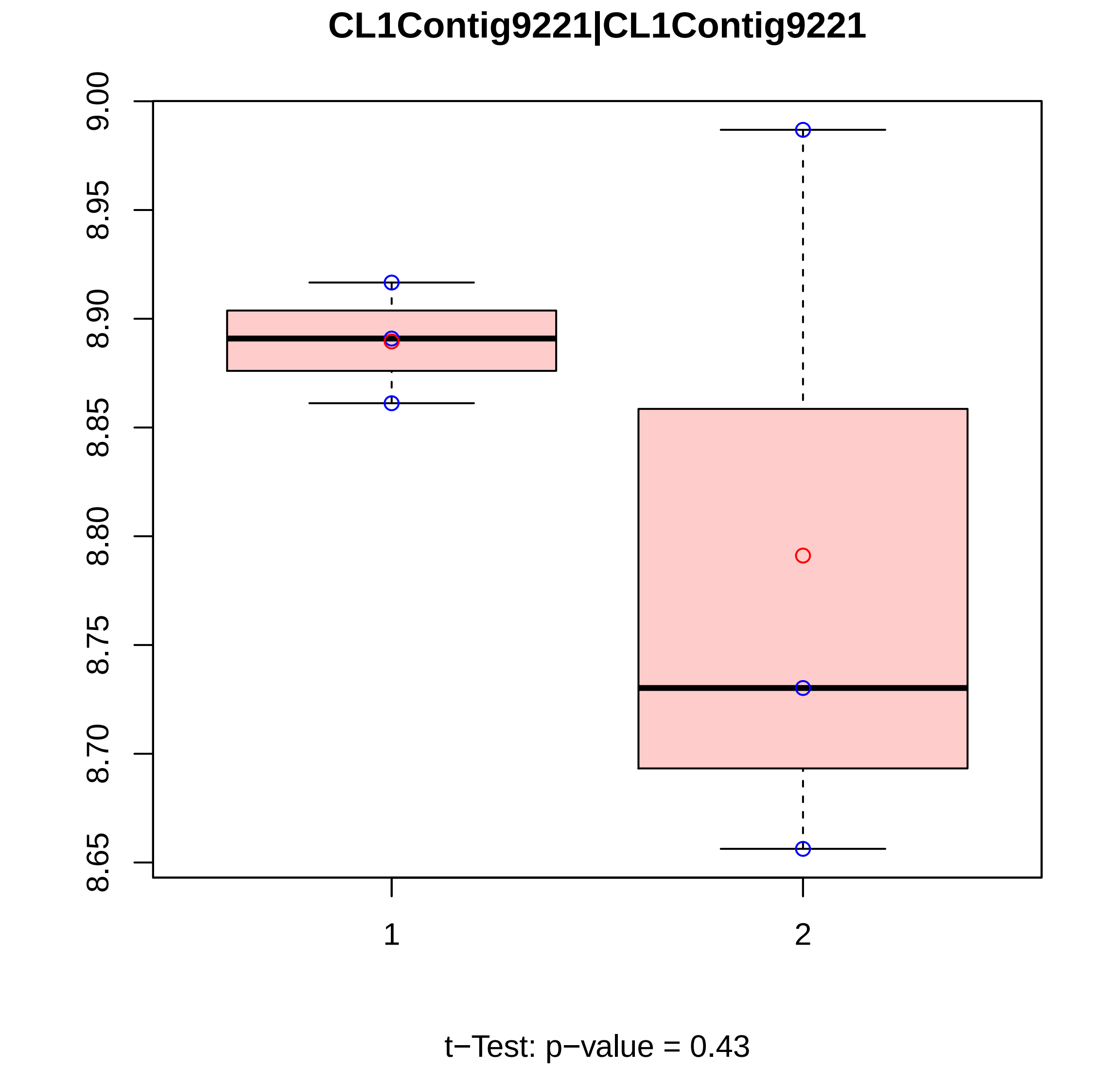

CL1Contig9221|CL1Contig9221
9.00
8.95
8.90
8.85
8.80
8.75
8.70
8.65
1
2
t−Test: p−value = 0.43

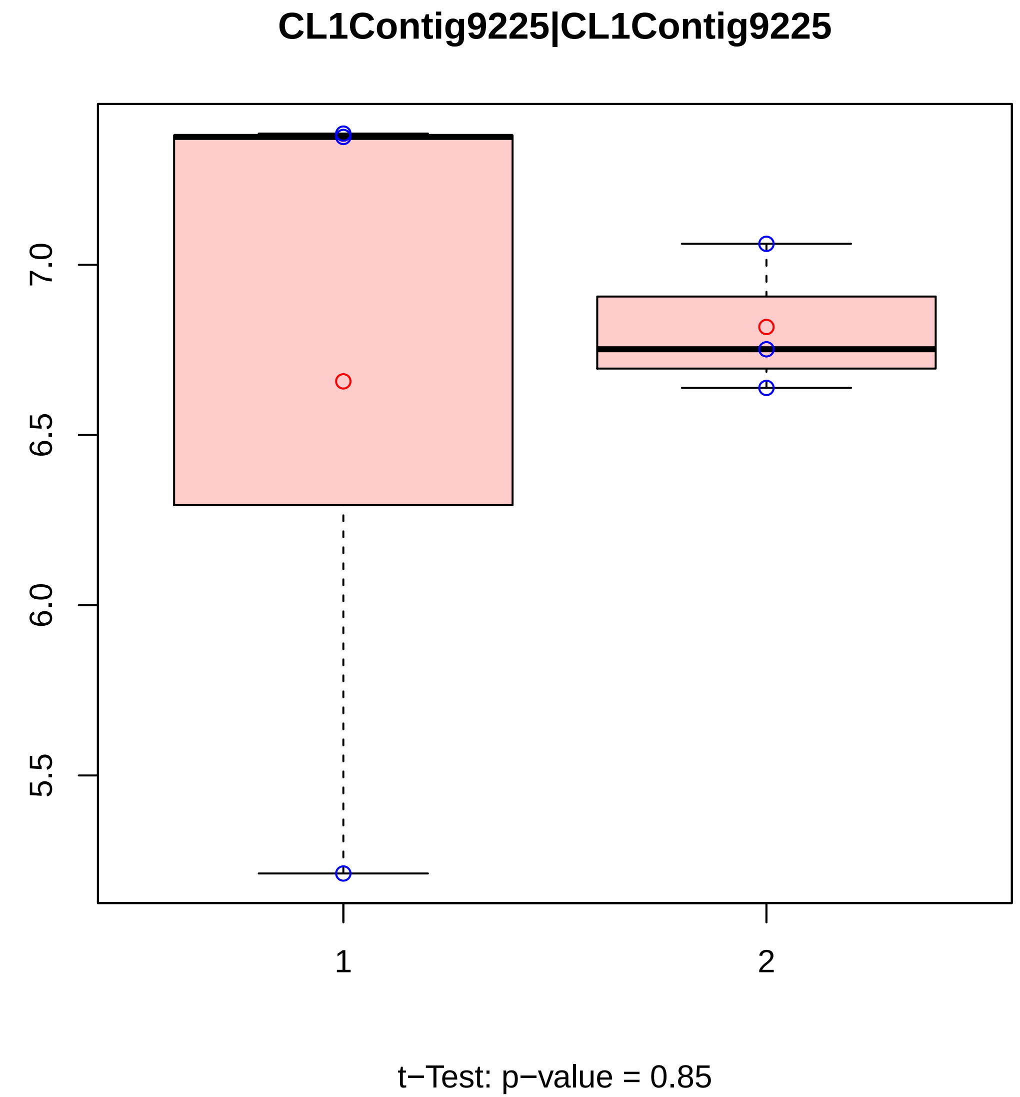
CL1Contig9225|CL1Contig9225
7.0
6.5
6.0
5.5
1
2
t−Test: p−value = 0.85

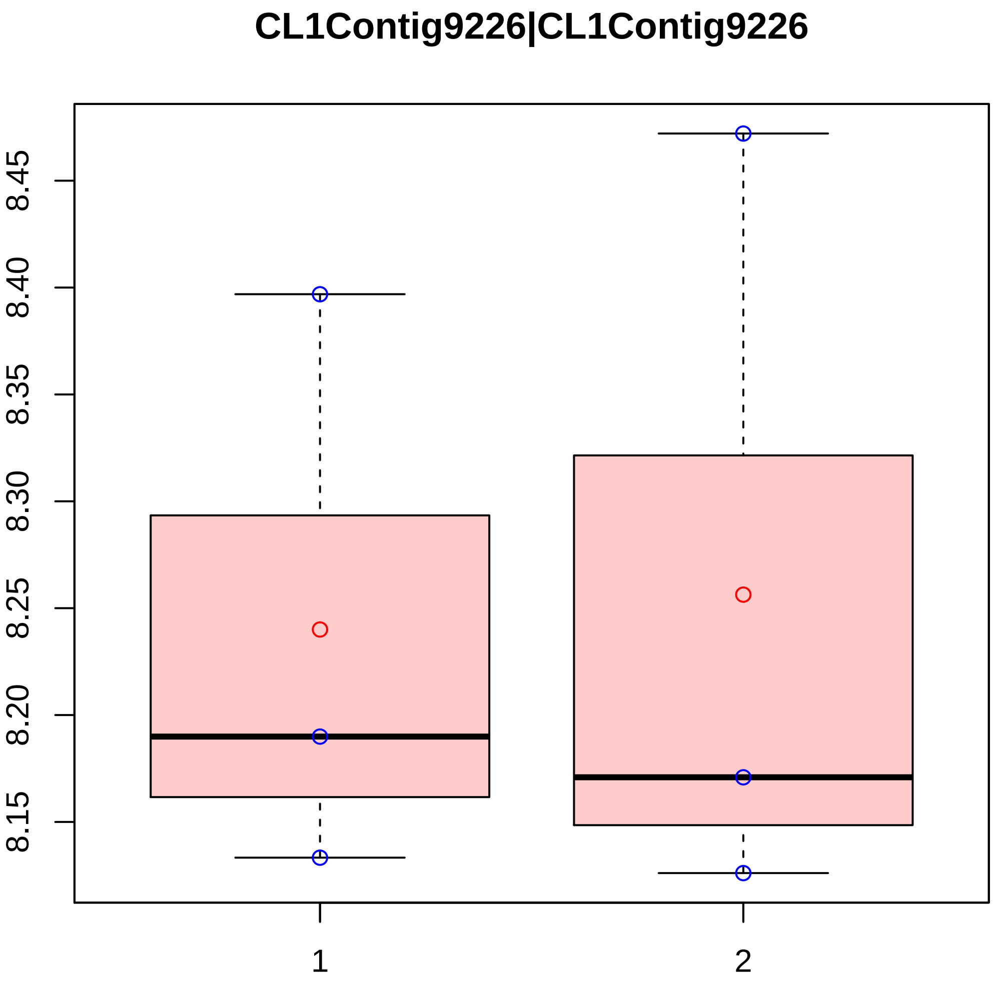


t−Test: p−value = 0.91

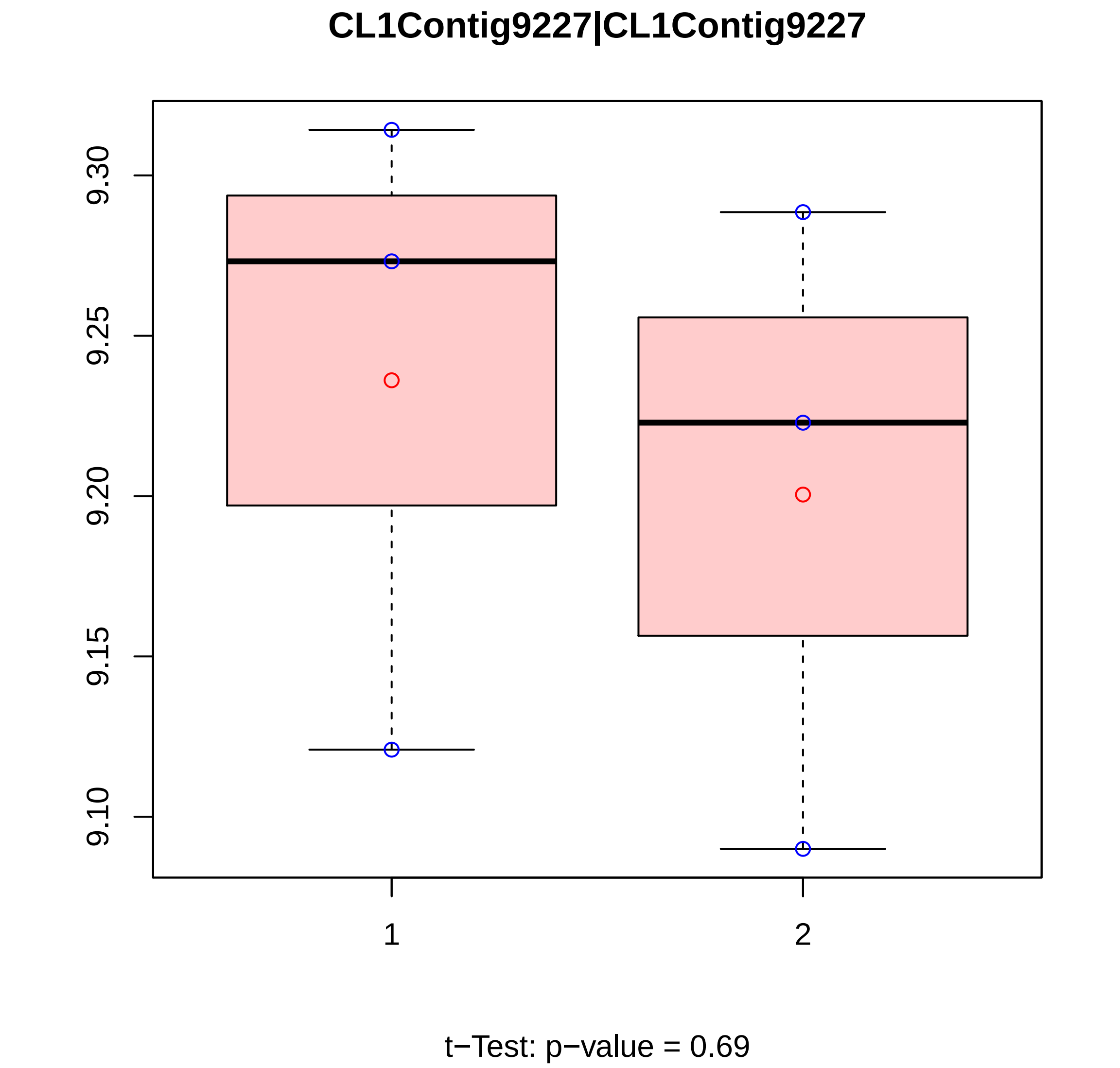

CL1Contig9227|CL1Contig9227
9.30
9.25
9.20
9.15
9.10
1
2
t−Test: p−value = 0.69

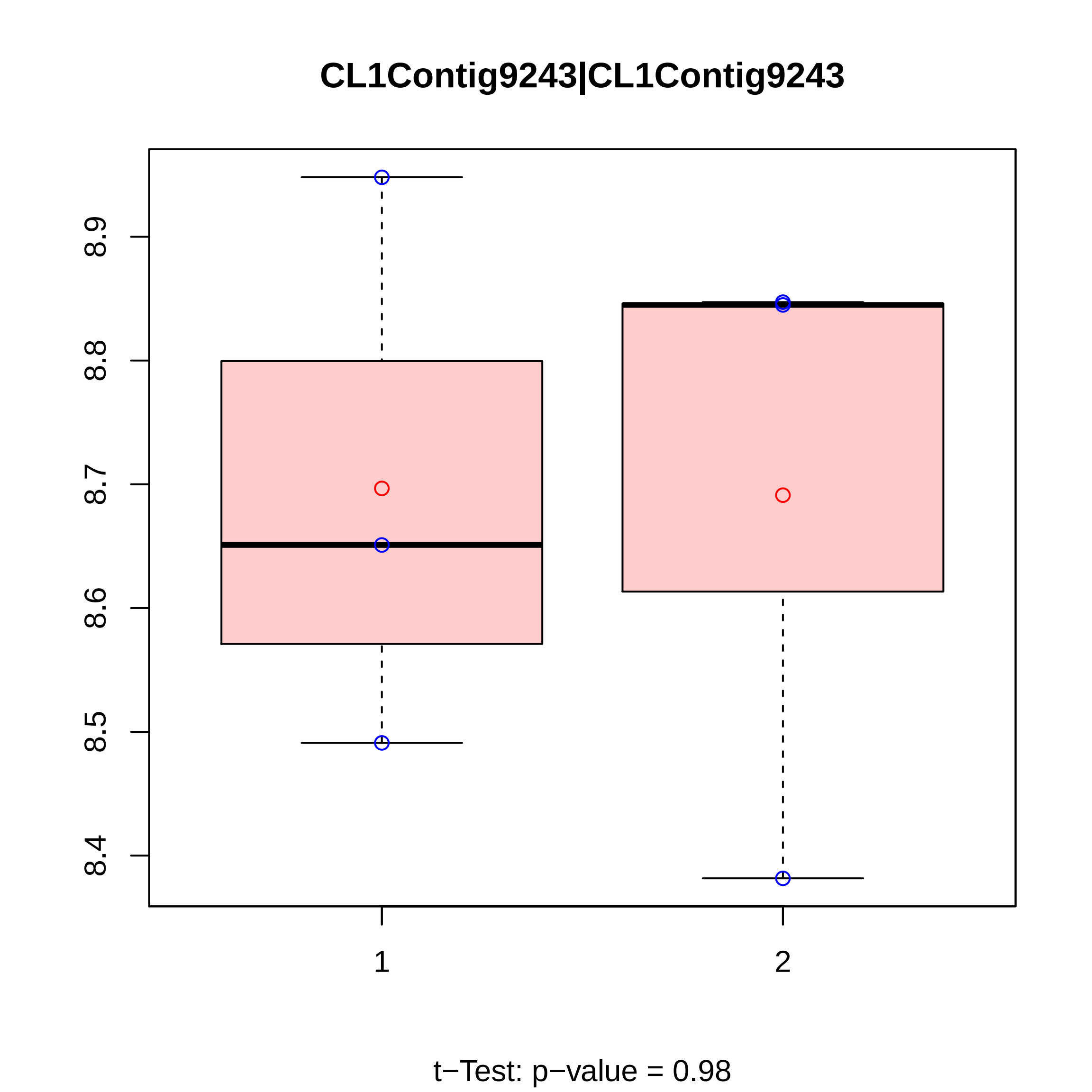
CL1Contig9243|CL1Contig9243
8.4
8.5
8.6
8.7
8.8
8.9
1
2
t−Test: p−value = 0.98

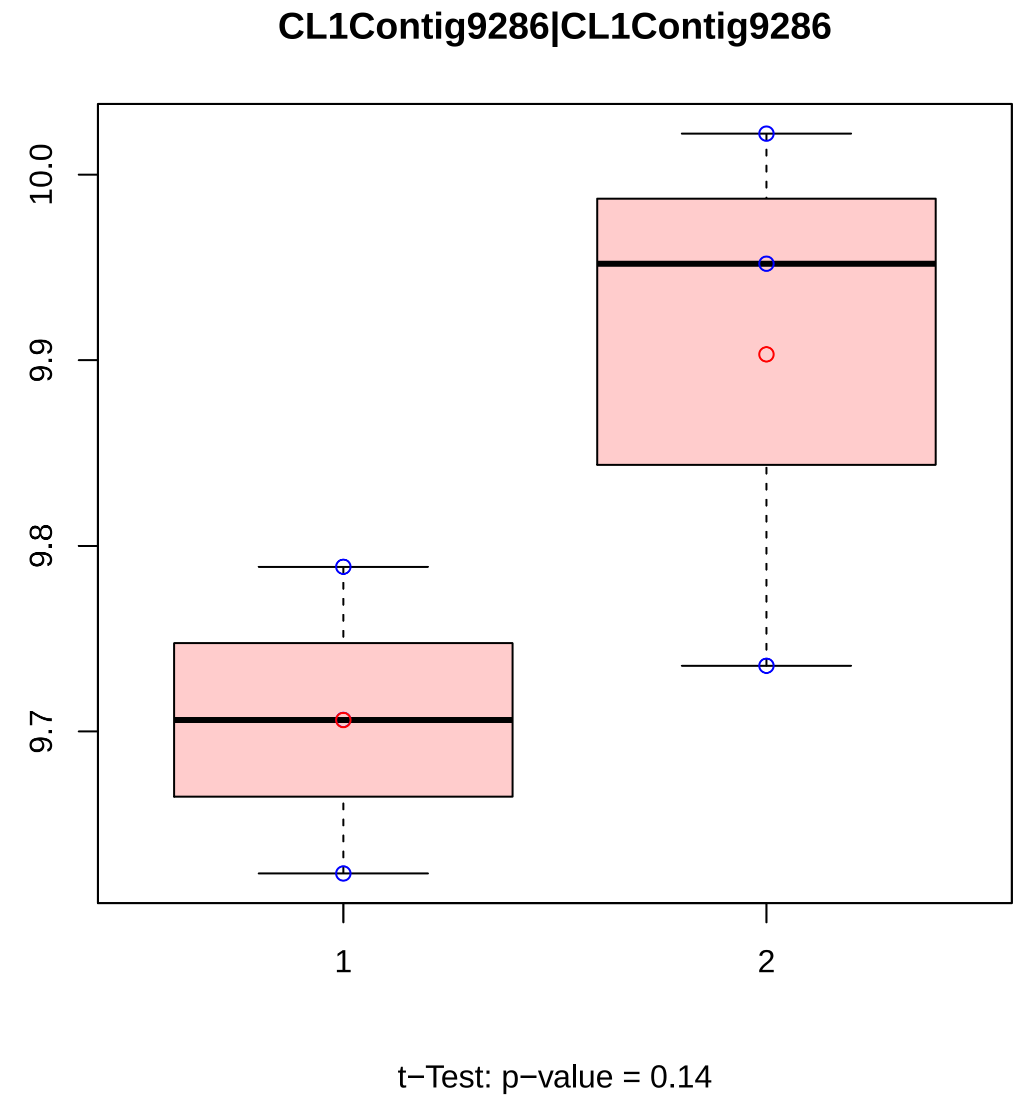
CL1Contig9286|CL1Contig9286
10.0
9.9
9.8
9.7
1
2
t−Test: p−value = 0.14

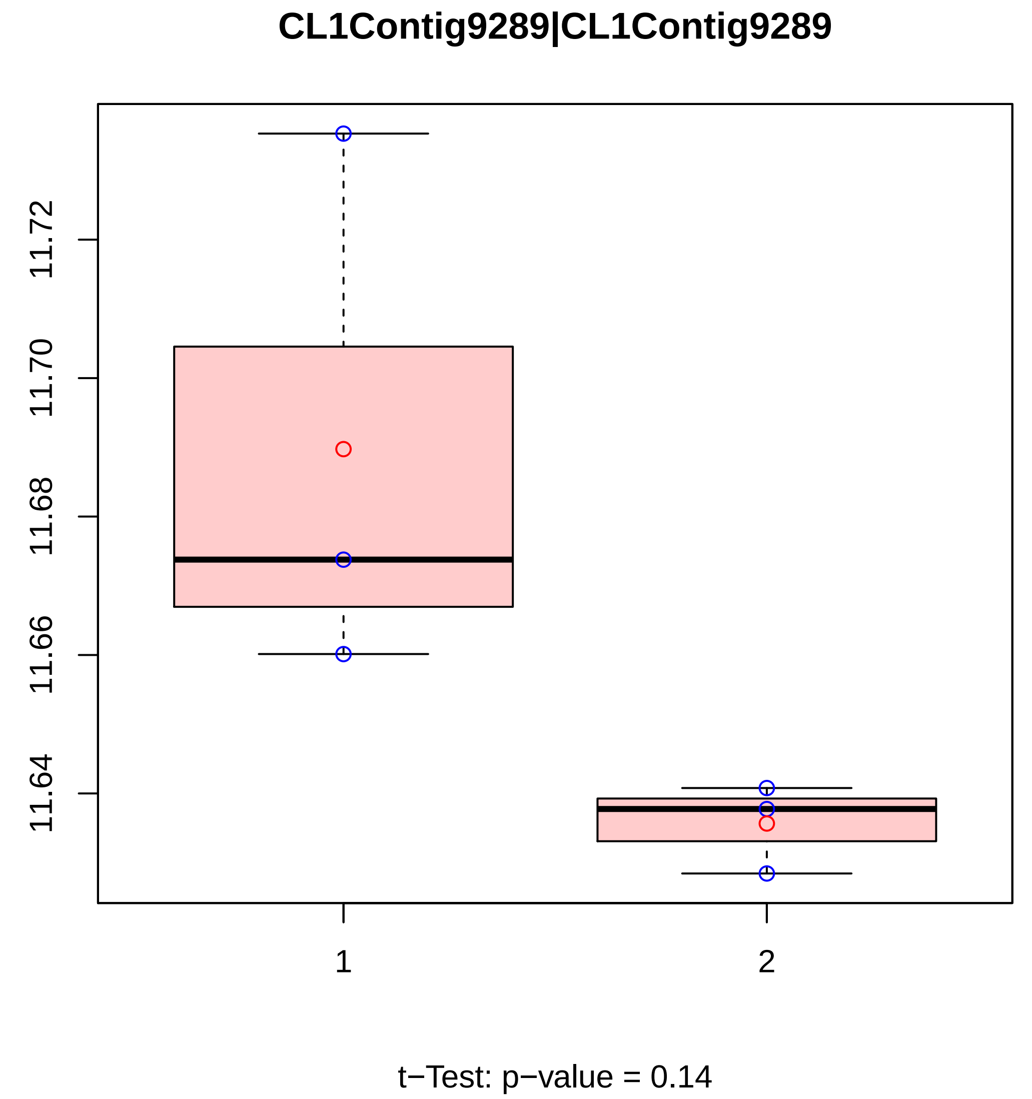
CL1Contig9289|CL1Contig9289
11.64
11.66
11.68
11.70
11.72
1
2
t−Test: p−value = 0.14

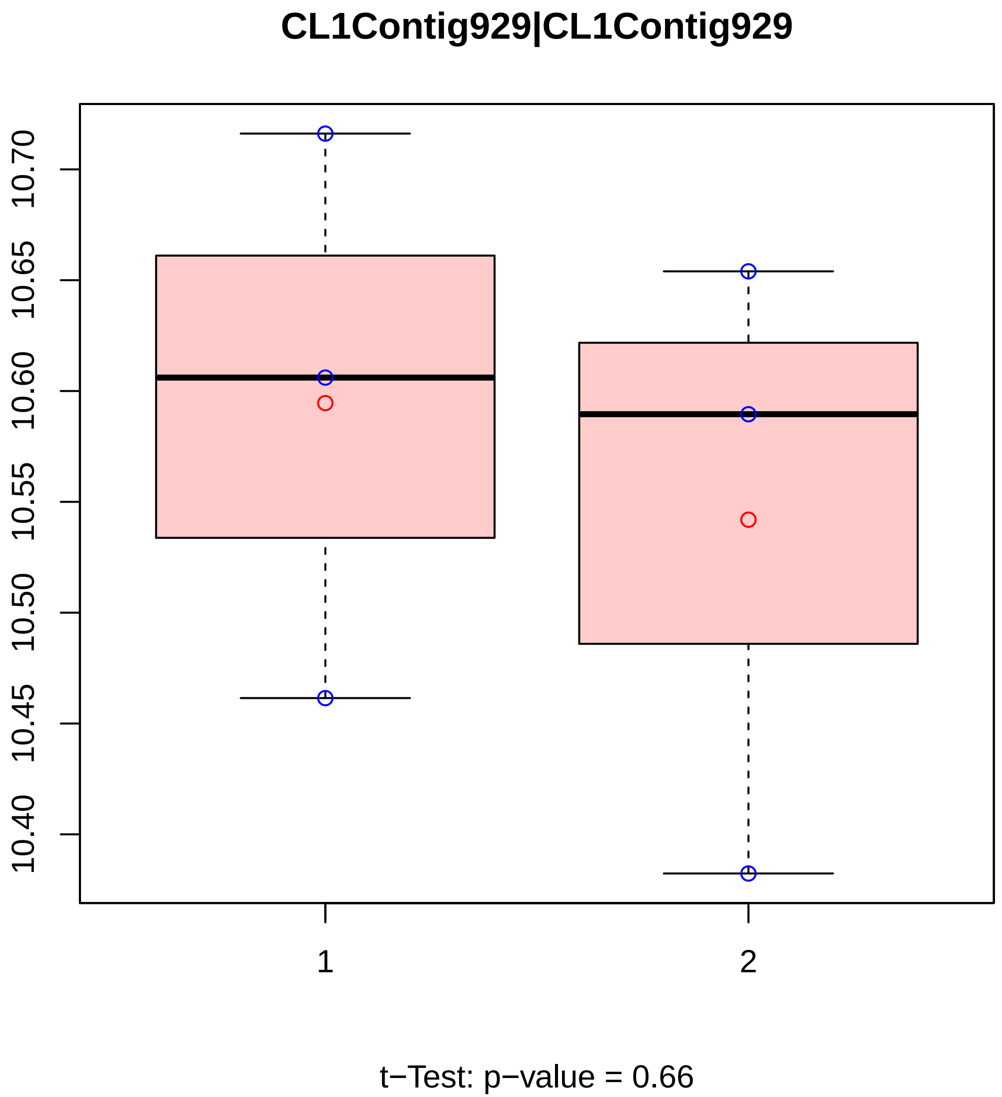
CL1Contig929|CL1Contig929
10.40
10.45
10.50
10.55
10.60
10.65
10.70
1
2
t−Test: p−value = 0.66

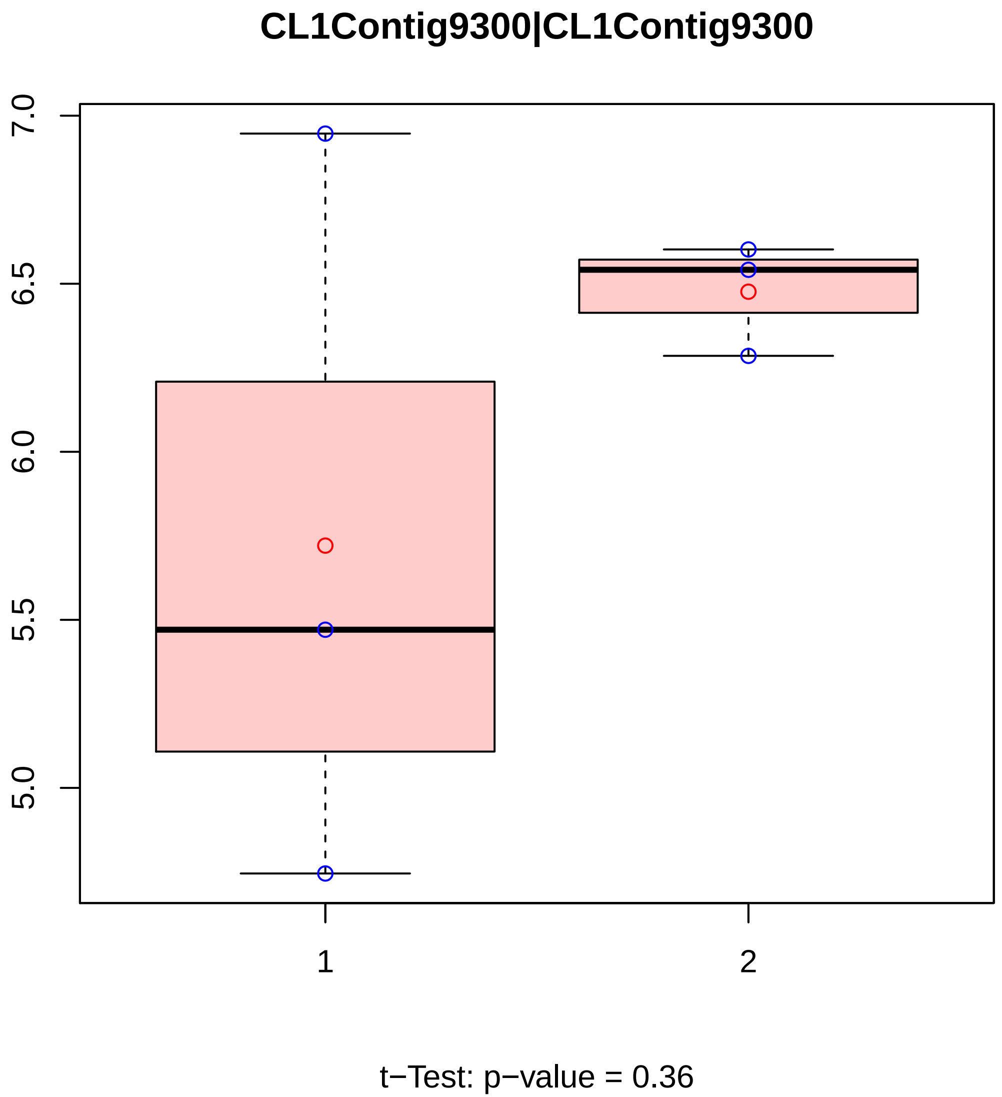
CL1Contig9300|CL1Contig9300
7.0
6.5
6.0
5.5
5.0
1
2
t−Test: p−value = 0.36

# CL1Contig930|CL1Contig930

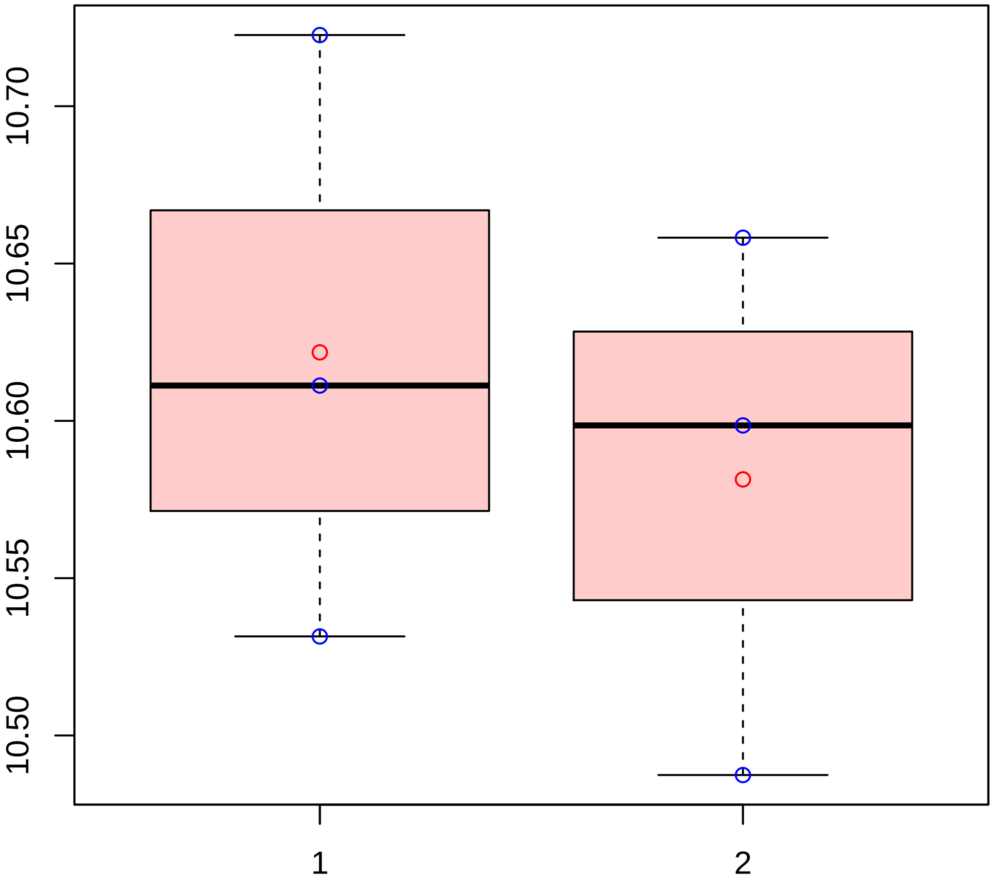


t−Test: p−value = 0.62

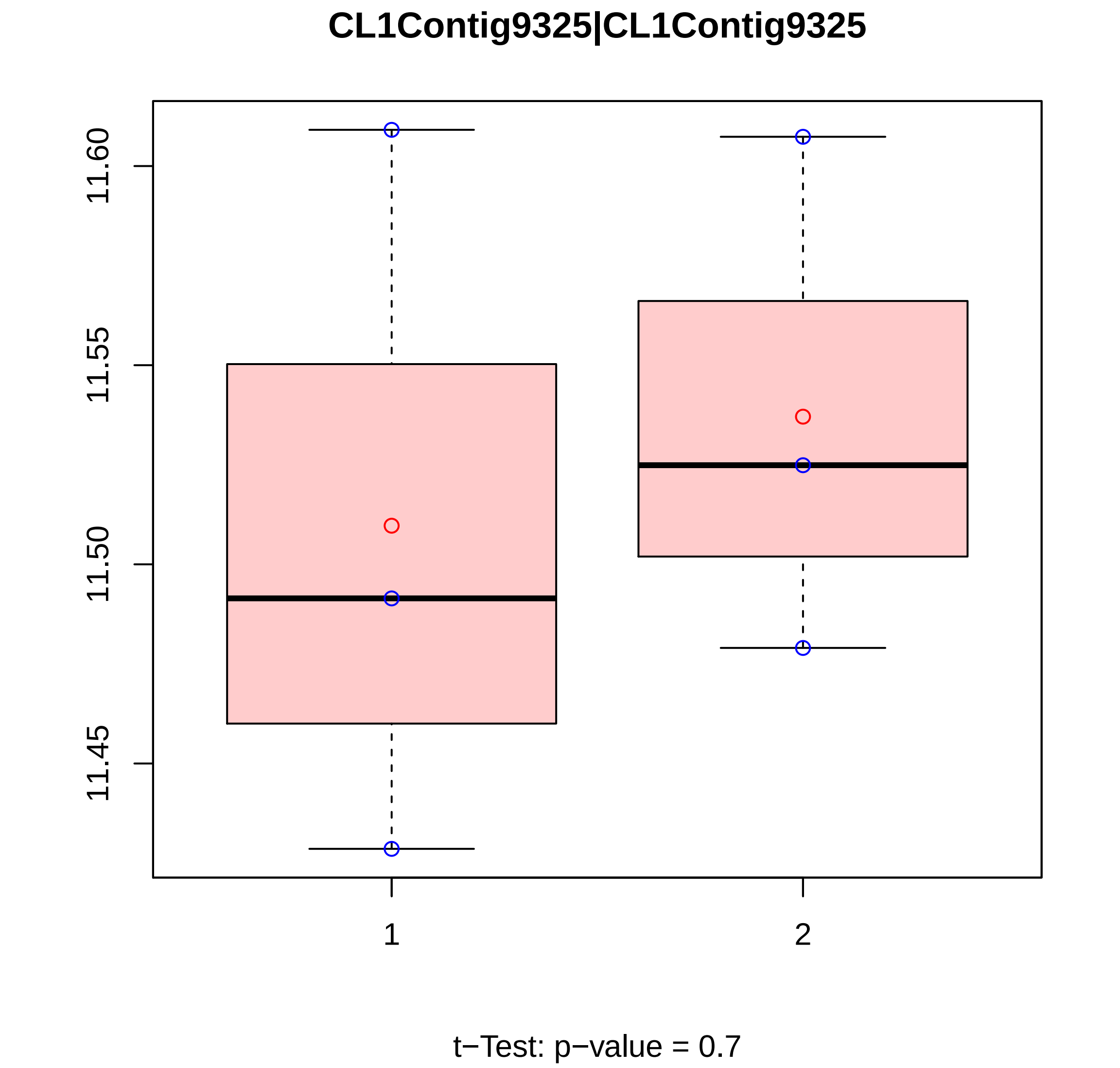
CL1Contig9325|CL1Contig9325
11.60
11.55
11.50
11.45
1
2
t−Test: p−value = 0.7

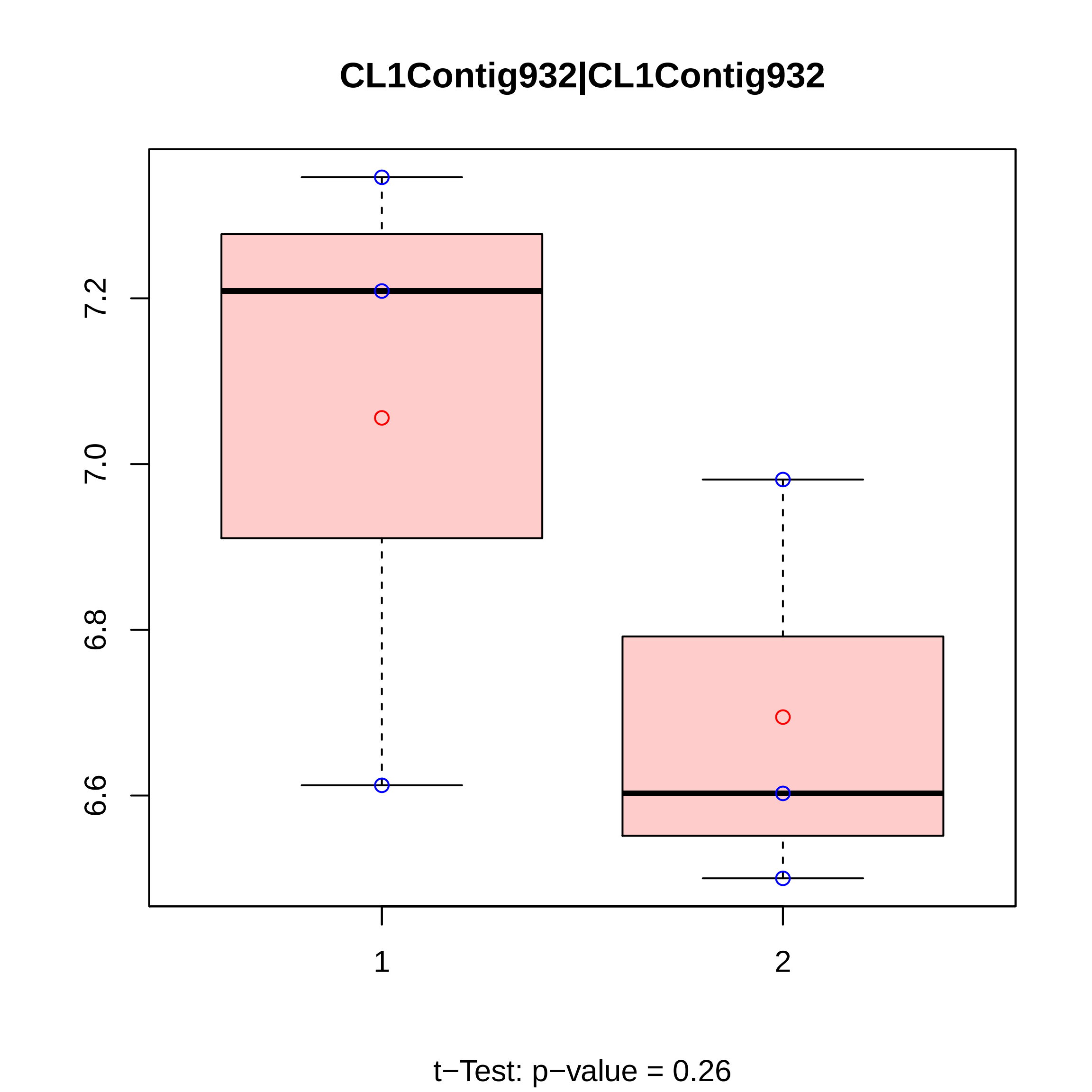

CL1Contig932|CL1Contig932
7.2
7.0
6.8
6.6
1
2
t−Test: p−value = 0.26

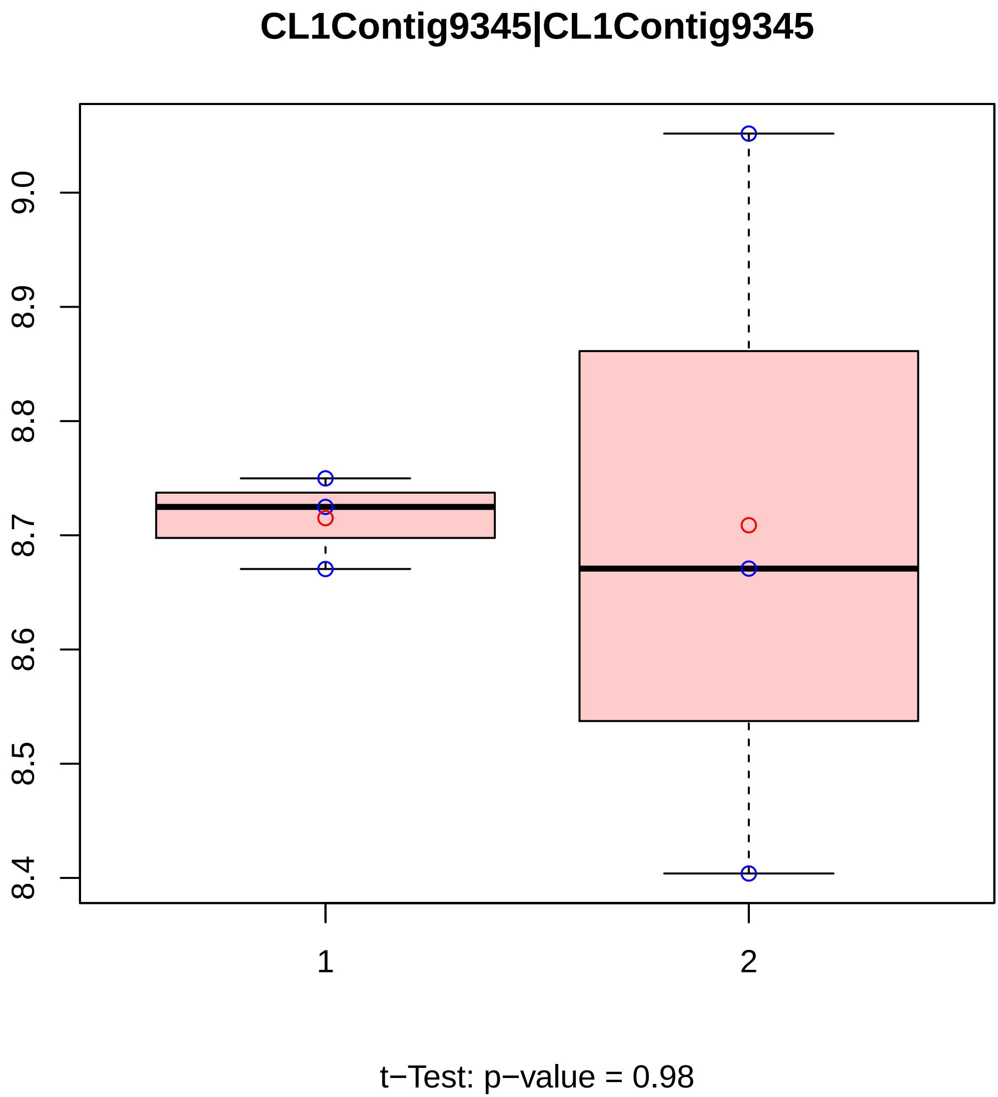
CL1Contig9345|CL1Contig9345
9.0
8.9
8.8
8.7
8.6
8.5
8.4
1
2
t−Test: p−value = 0.98

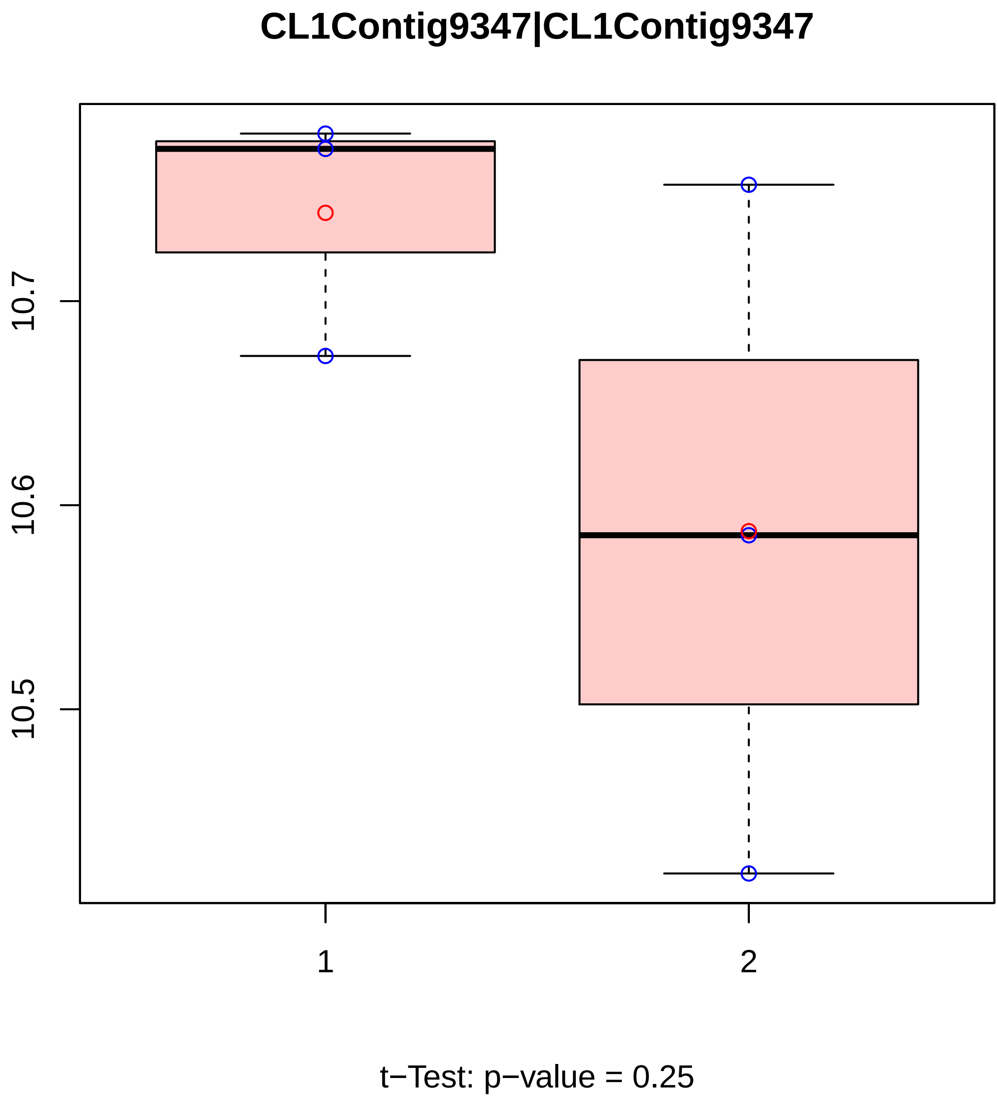
CL1Contig9347|CL1Contig9347
10.5
10.6
10.7
1
2
t−Test: p−value = 0.25

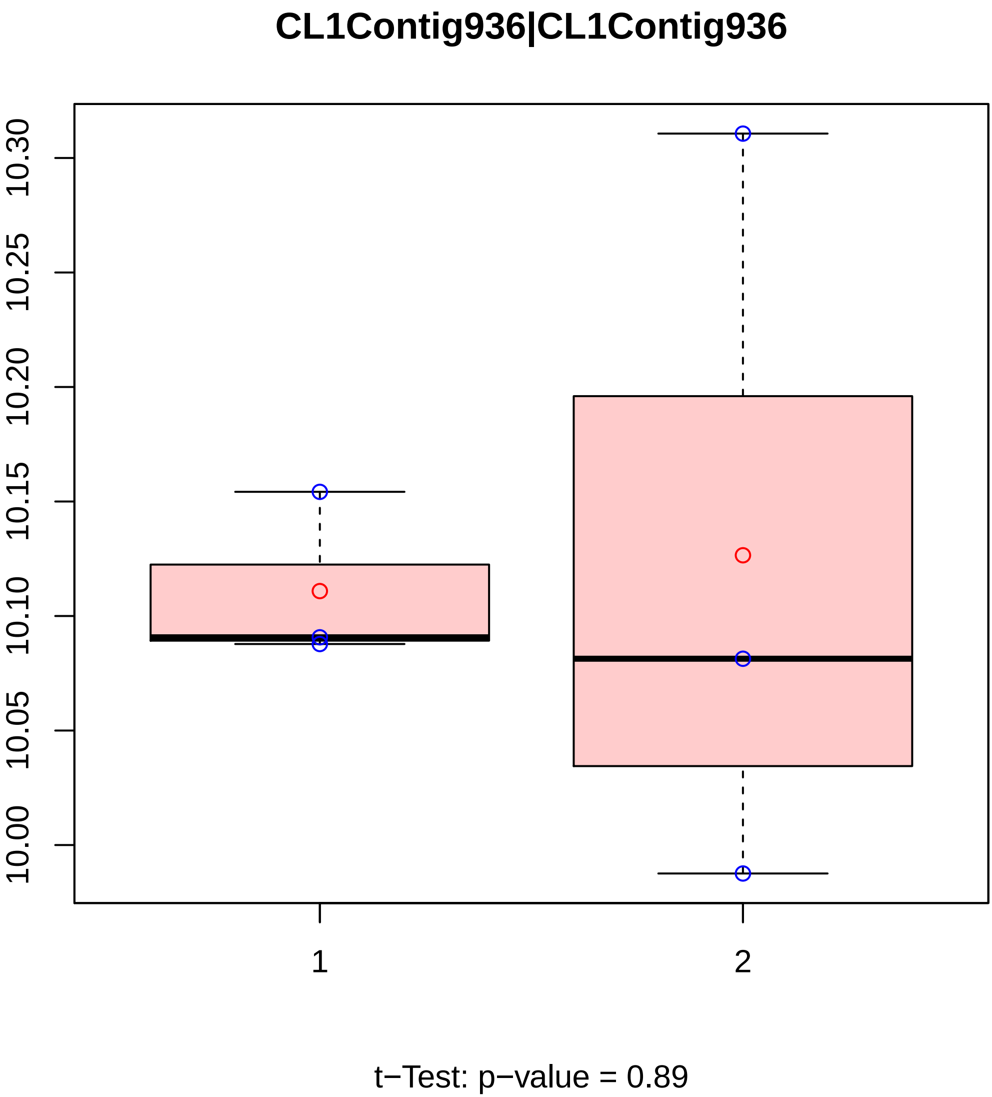
CL1Contig936|CL1Contig936
10.00
10.05
10.10
10.15
10.20
10.25
10.30
1
2
t−Test: p−value = 0.89

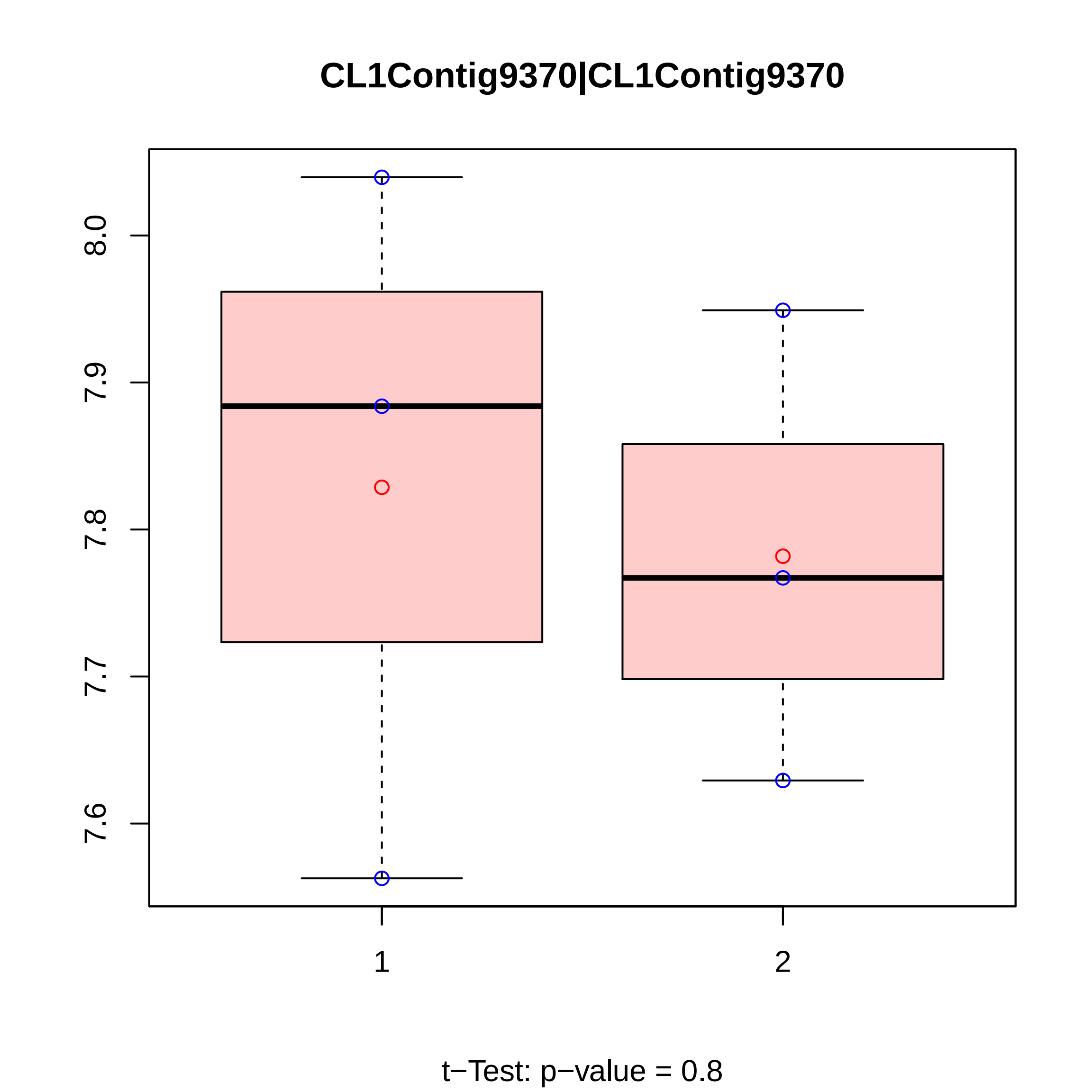

CL1Contig9370|CL1Contig9370
8.0
7.9
7.8
7.7
7.6
1
2
t−Test: p−value = 0.8

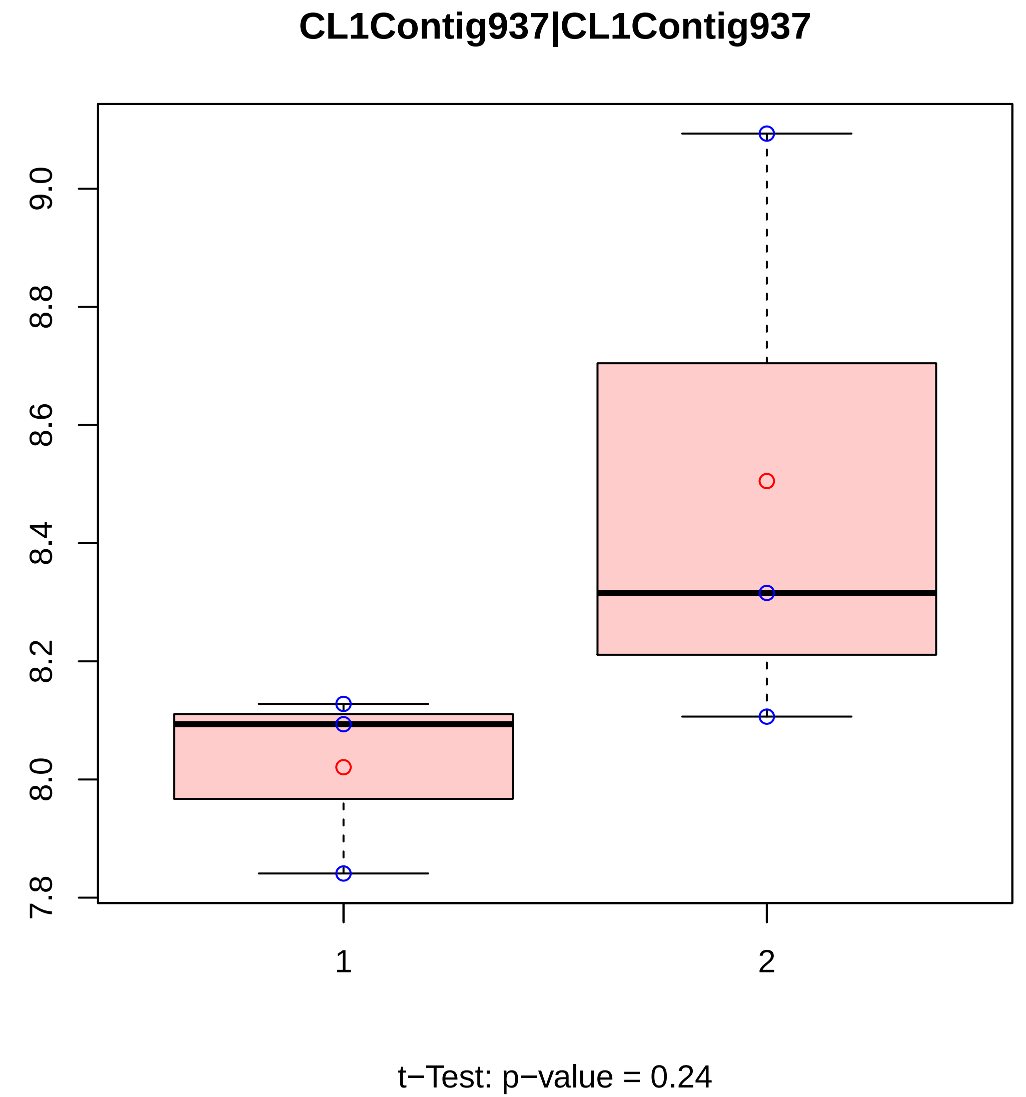

CL1Contig937|CL1Contig937
7.8
8.0
8.2
8.4
8.6
8.8
9.0
1
2
t−Test: p−value = 0.24

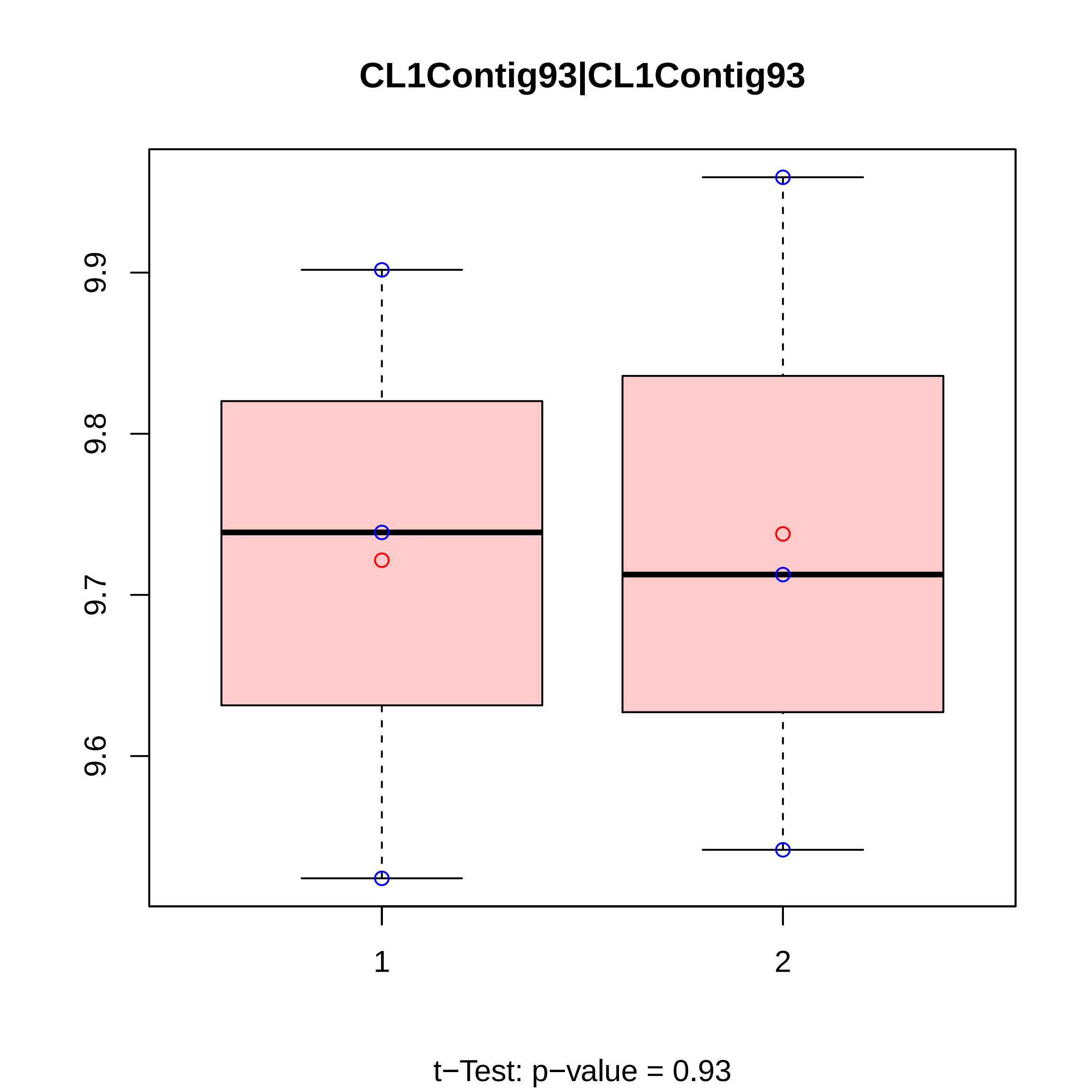
CL1Contig93|CL1Contig93
9.6
9.7
9.8
9.9
1
2
t−Test: p−value = 0.93

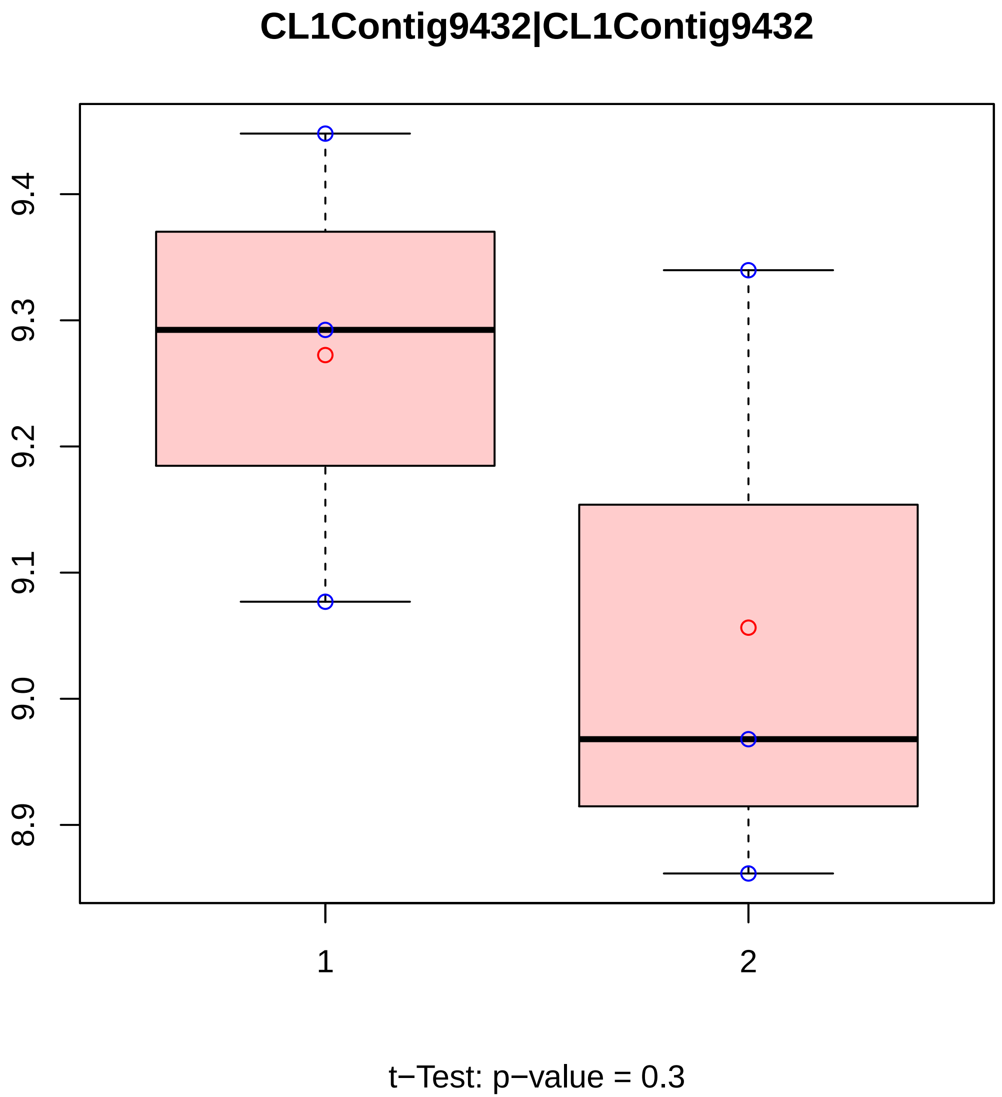

CL1Contig9432|CL1Contig9432
9.4
9.3
9.2
9.1
9.0
8.9
1
2
t−Test: p−value = 0.3

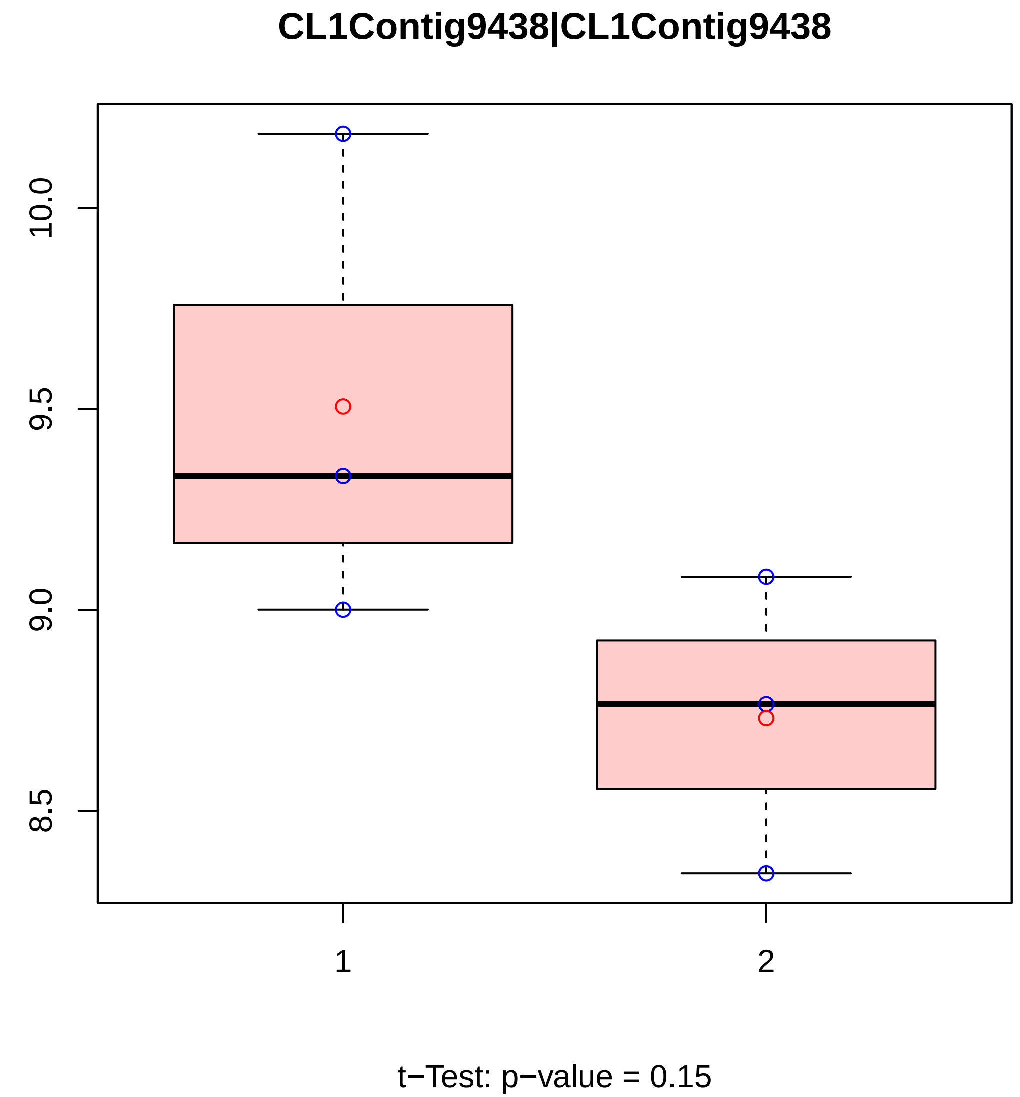
CL1Contig9438|CL1Contig9438
10.0
9.5
9.0
8.5
1
2
t−Test: p−value = 0.15

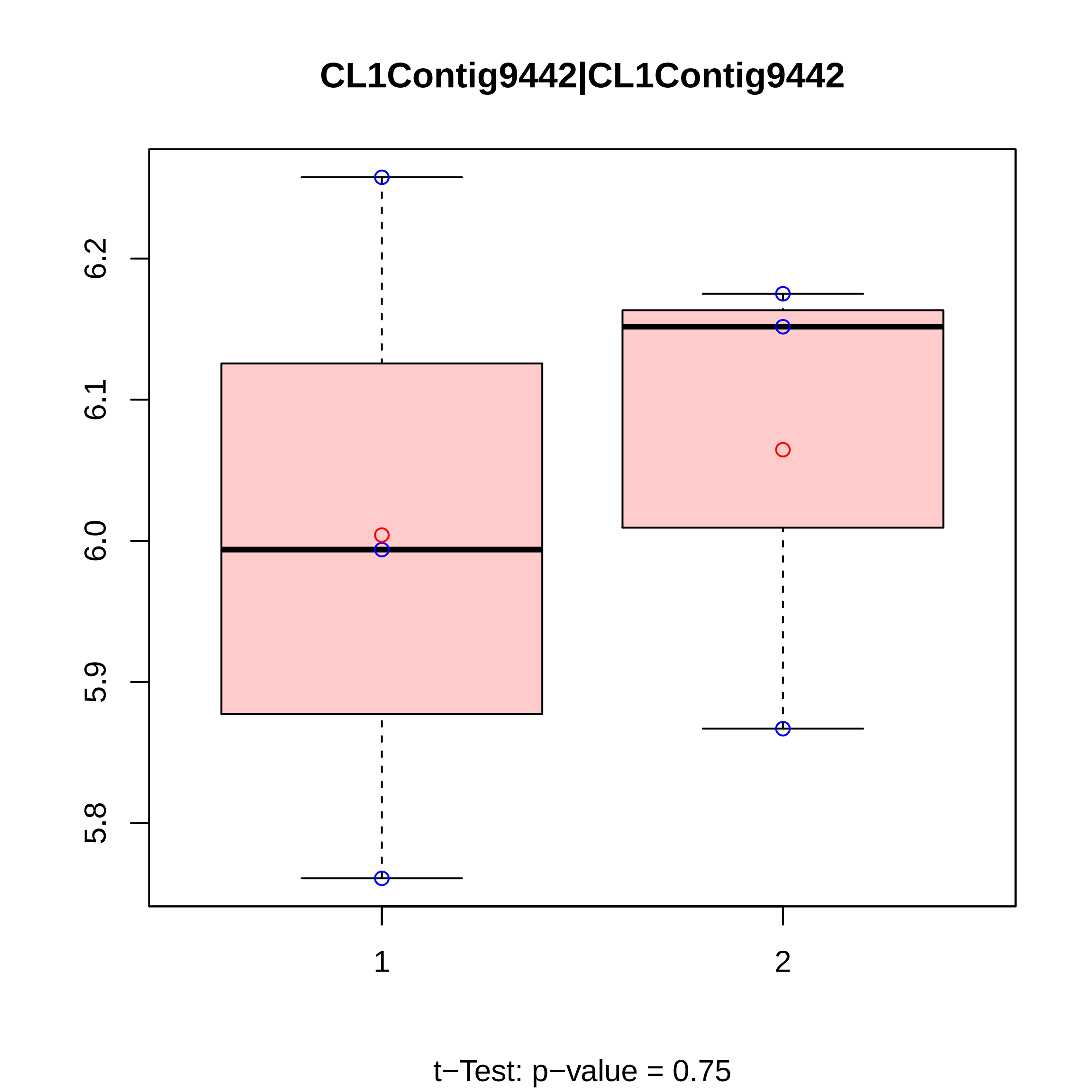
CL1Contig9442|CL1Contig9442
6.2
6.1
6.0
5.9
5.8
1
2
t−Test: p−value = 0.75

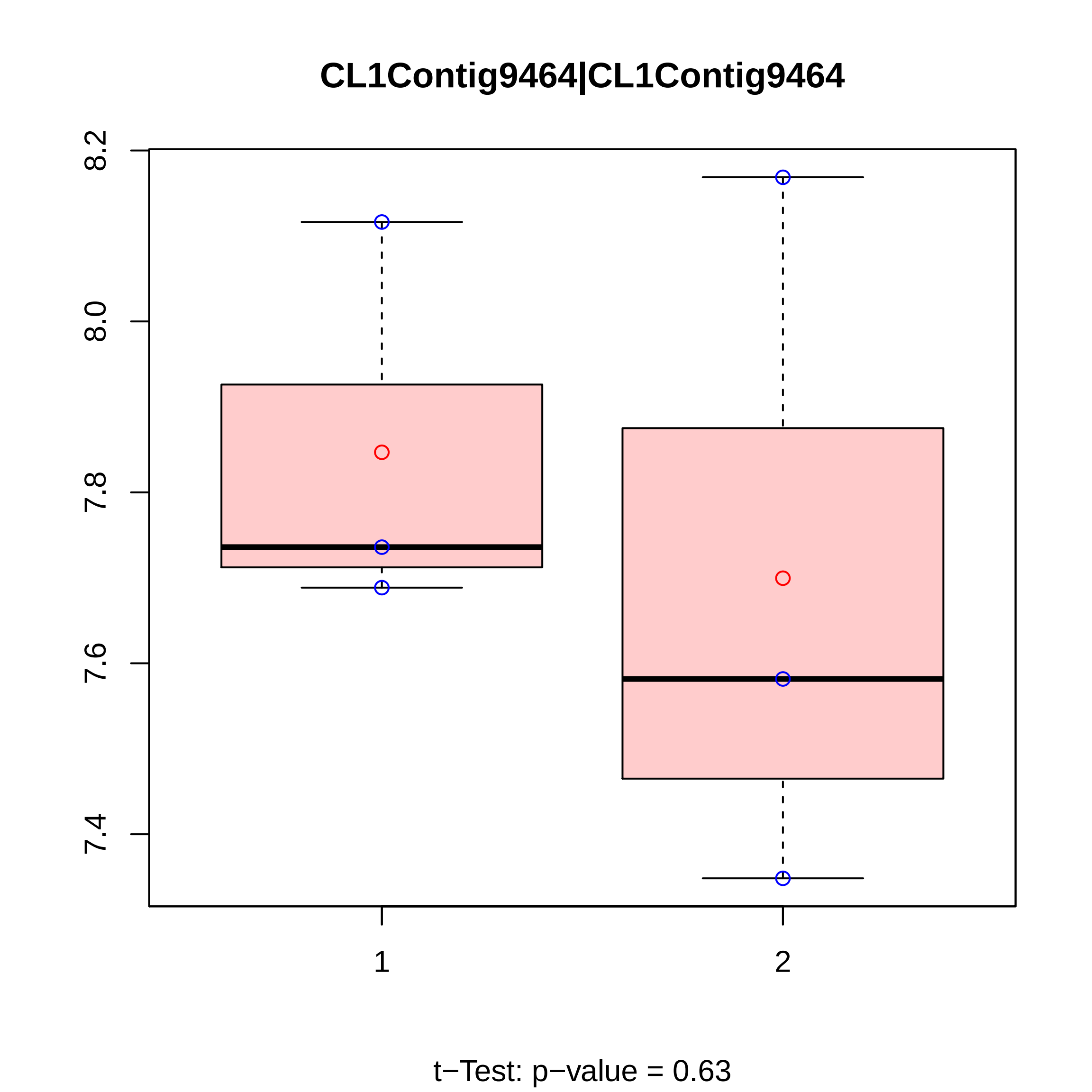
CL1Contig9464|CL1Contig9464
8.2
8.0
7.8
7.6
7.4
1
2
t−Test: p−value = 0.63

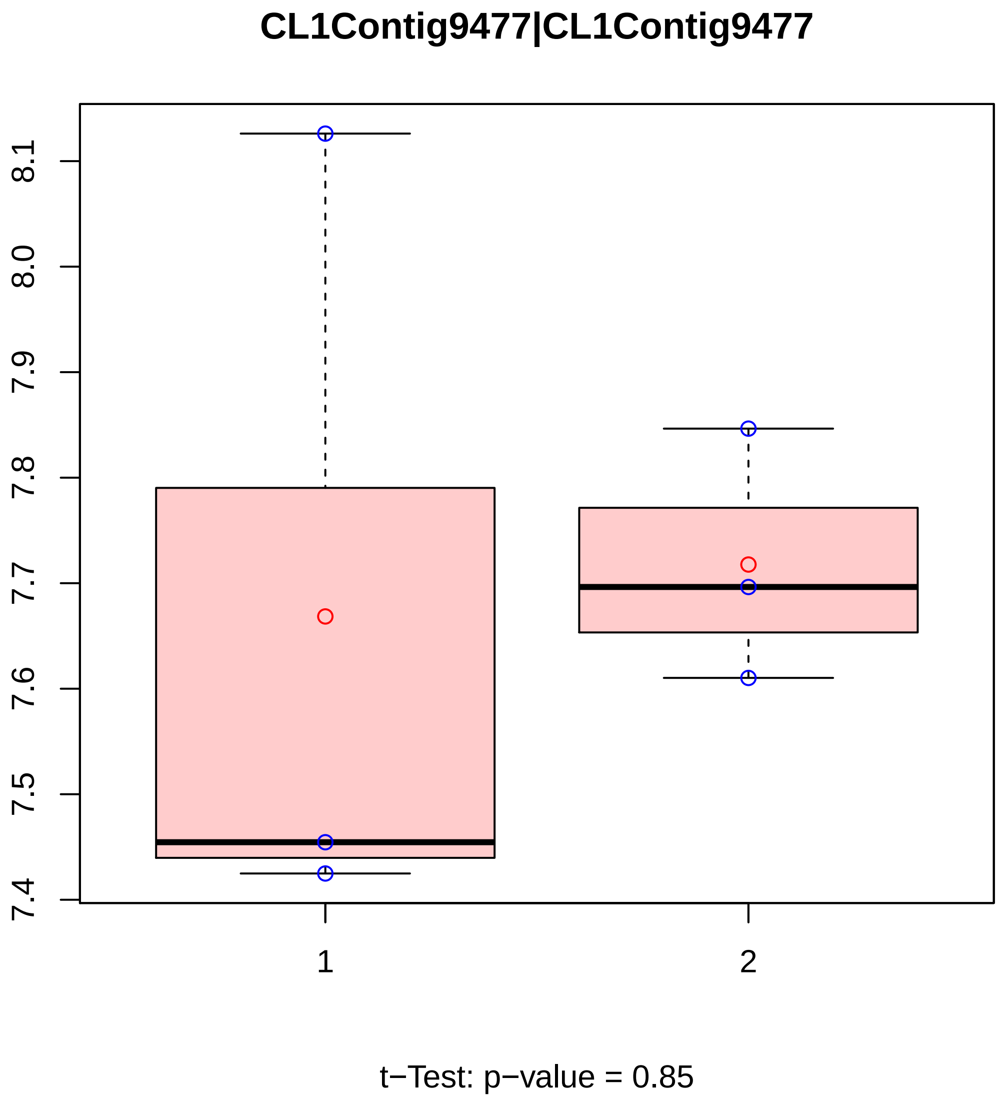
CL1Contig9477|CL1Contig9477
8.1
8.0
7.9
7.8
7.7
7.6
7.5
7.4
1
2
t−Test: p−value = 0.85

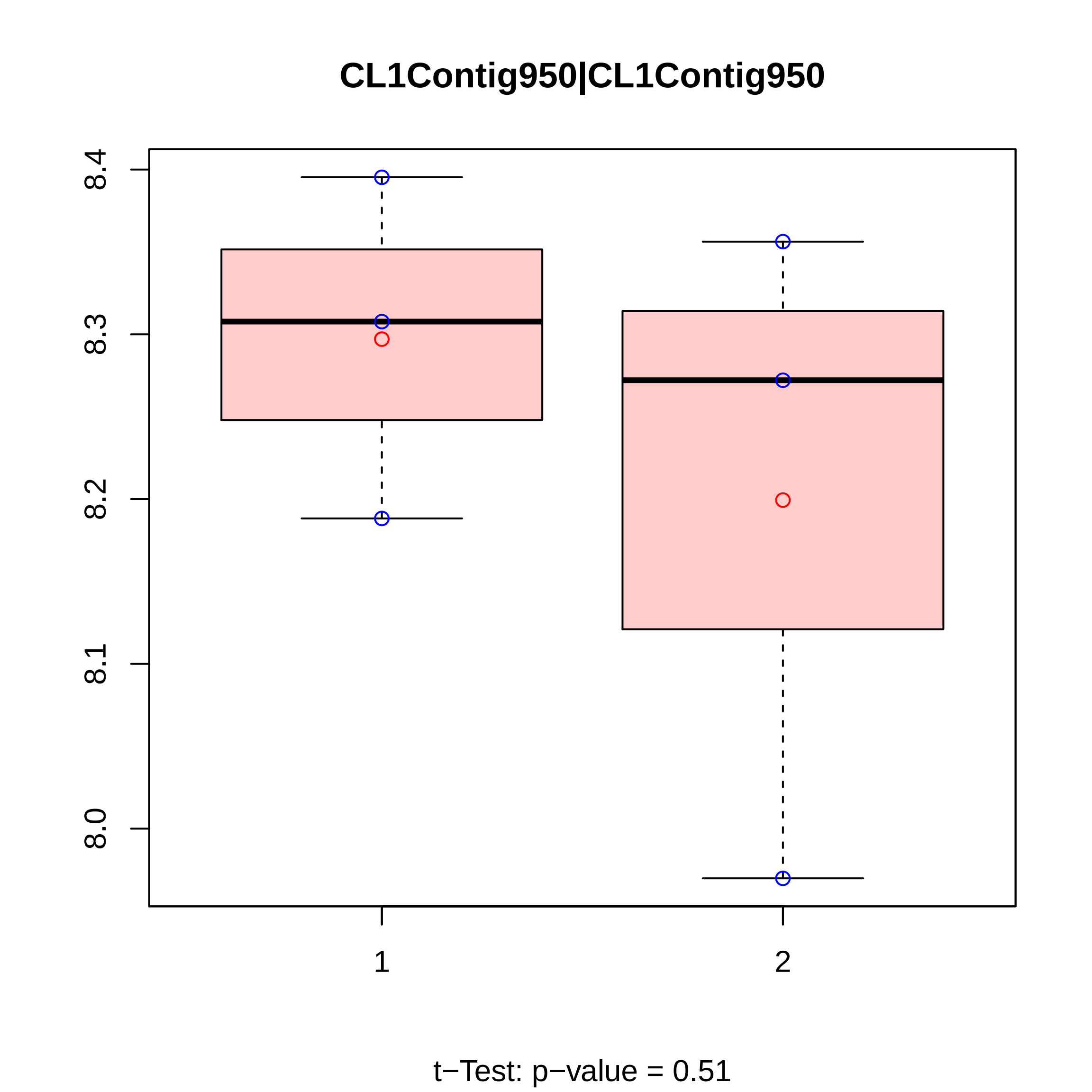

CL1Contig950|CL1Contig950
8.4
8.3
8.2
8.1
8.0
1
2
t−Test: p−value = 0.51

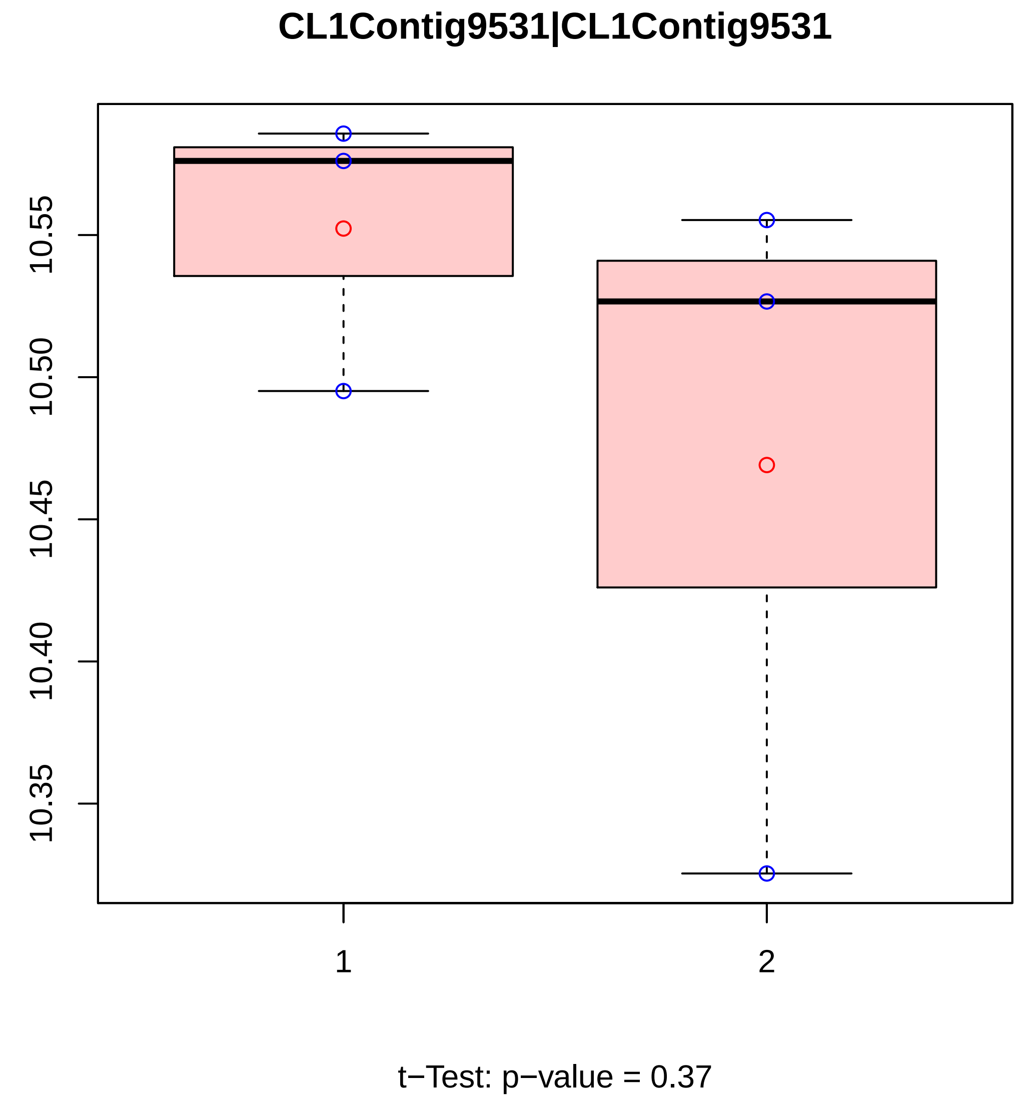

CL1Contig9531|CL1Contig9531
10.35
10.40
10.45
10.50
10.55
1
2
t−Test: p−value = 0.37

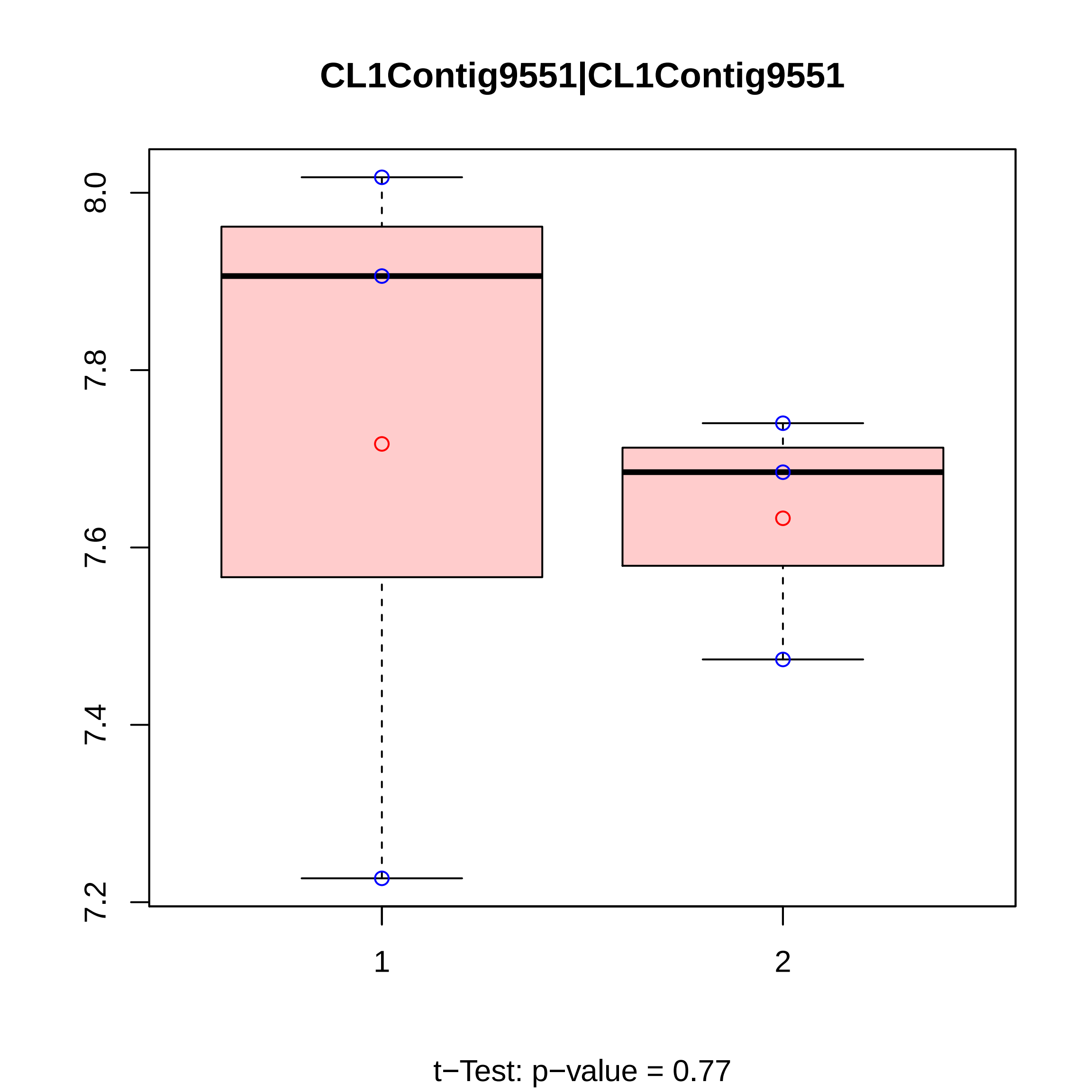
CL1Contig9551|CL1Contig9551
8.0
7.8
7.6
7.4
7.2
1
2
t−Test: p−value = 0.77

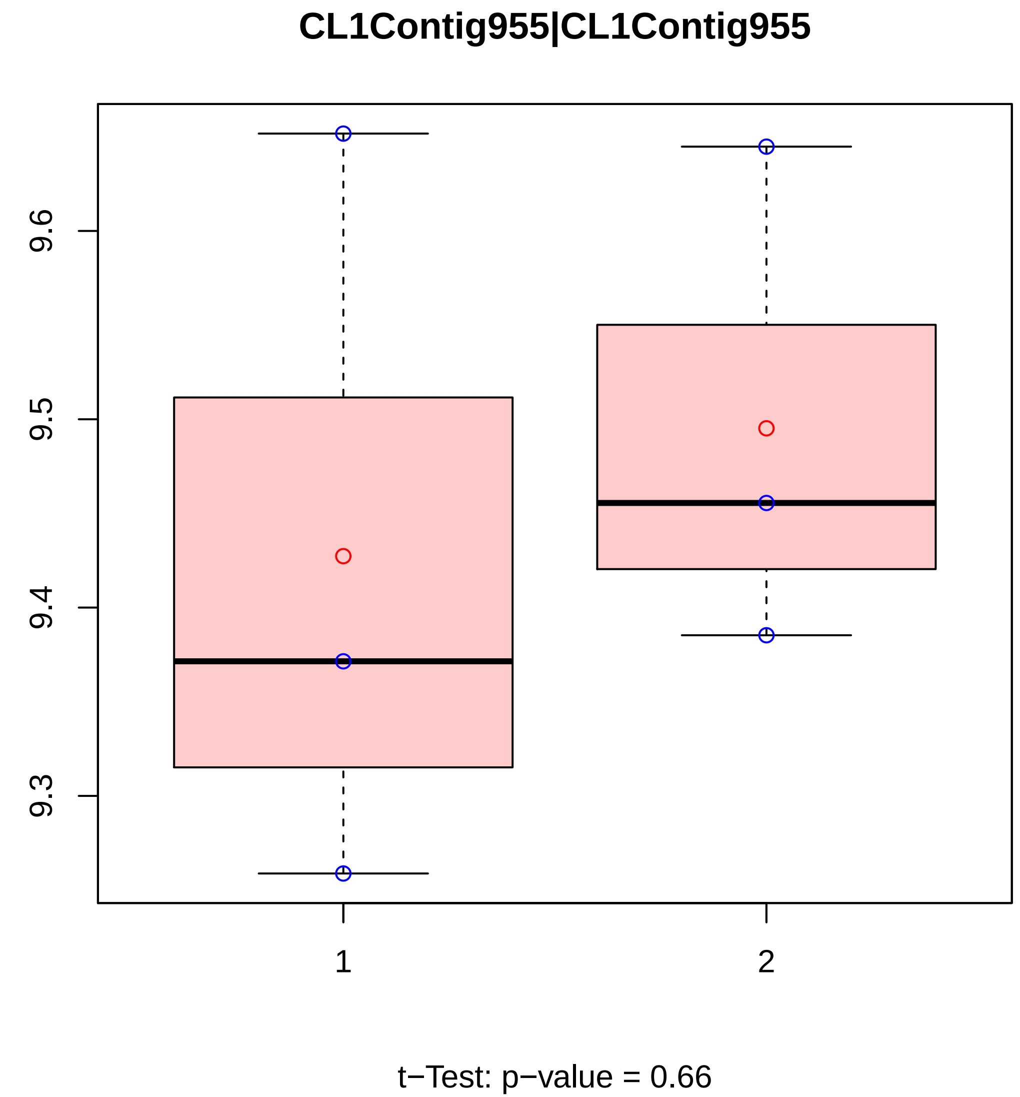

CL1Contig955|CL1Contig955
9.3
9.4
9.5
9.6
1
2
t−Test: p−value = 0.66

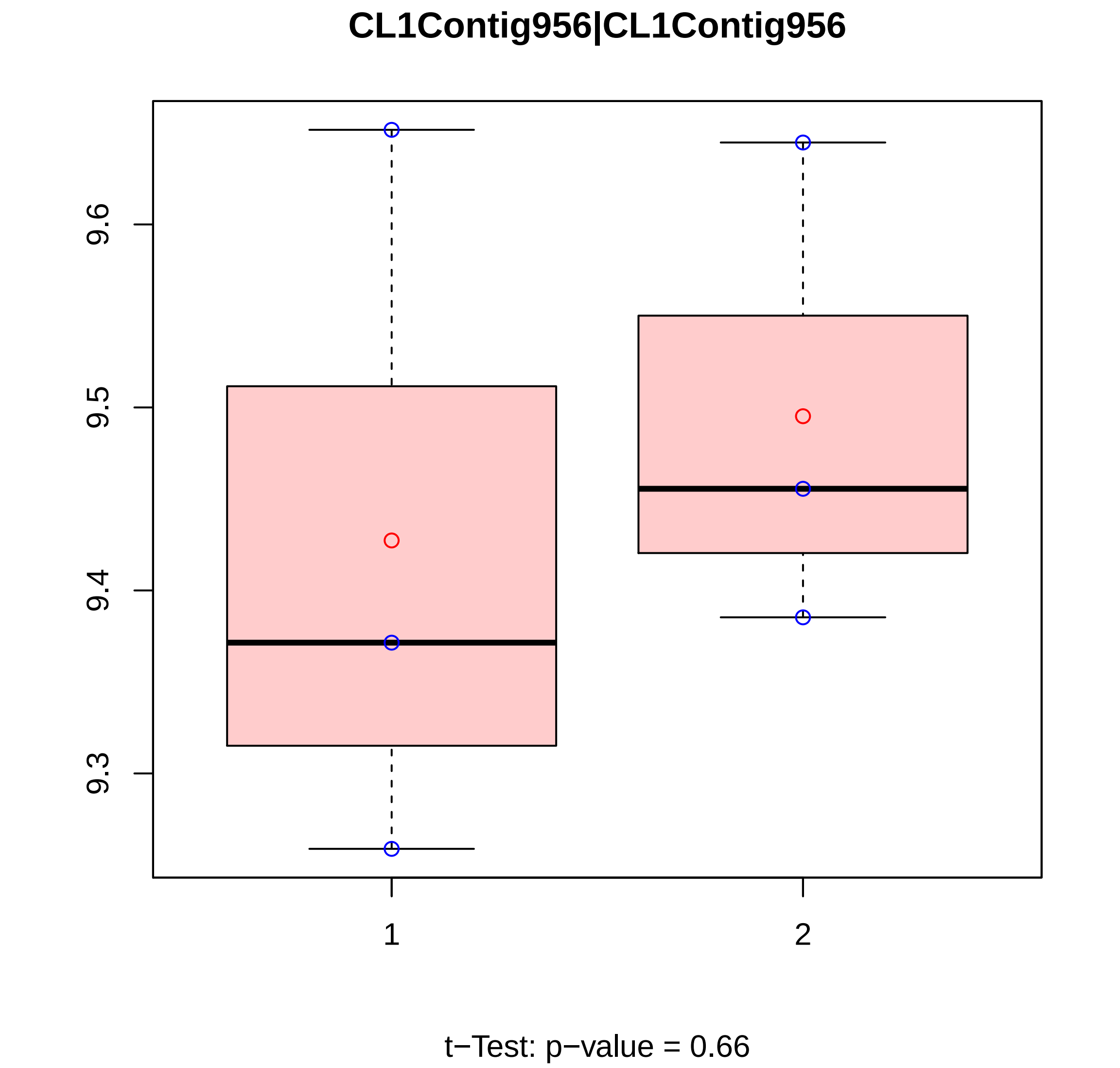
CL1Contig956|CL1Contig956
9.3
9.4
9.5
9.6
1
2
t−Test: p−value = 0.66

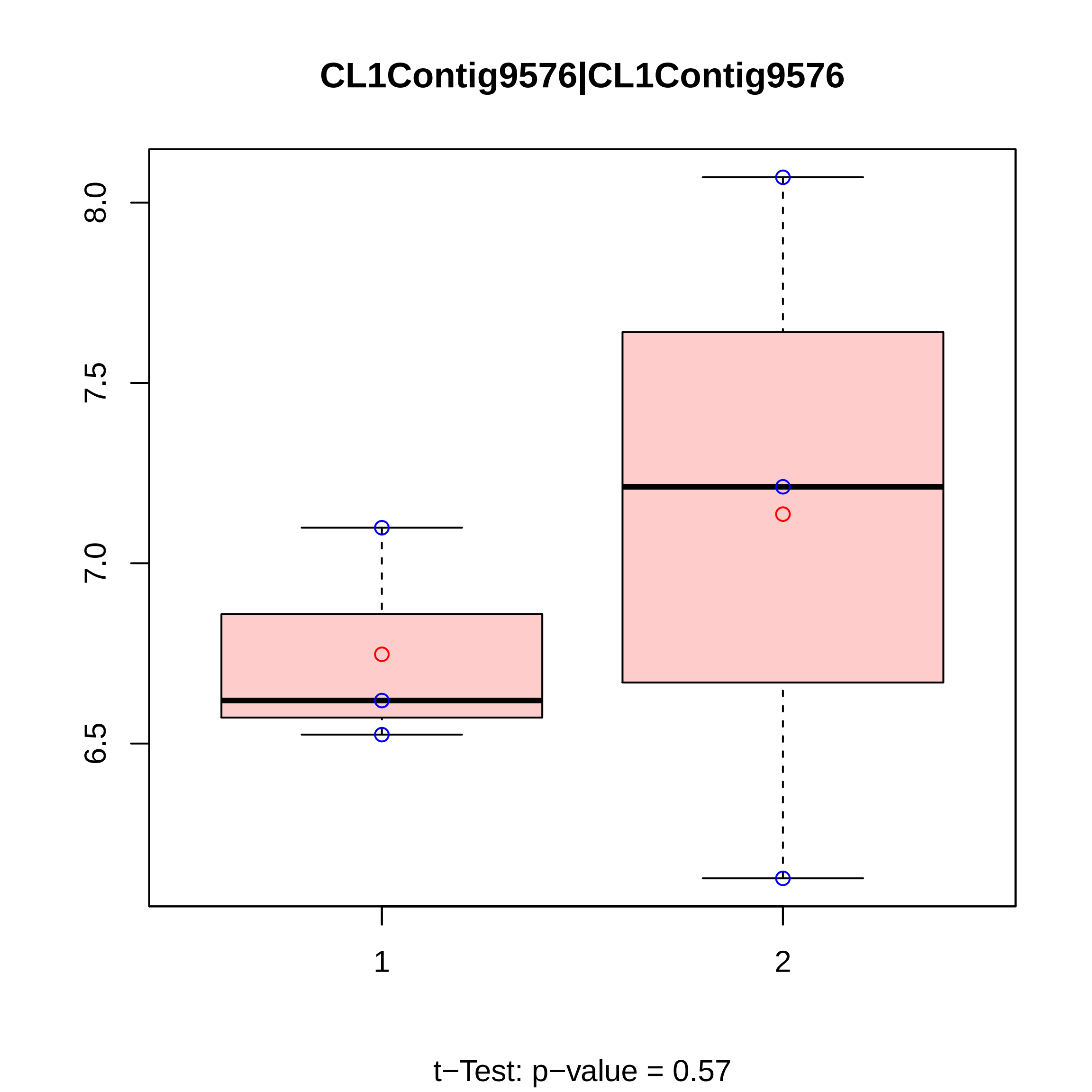

CL1Contig9576|CL1Contig9576
8.0
7.5
7.0
6.5
1
2
t−Test: p−value = 0.57

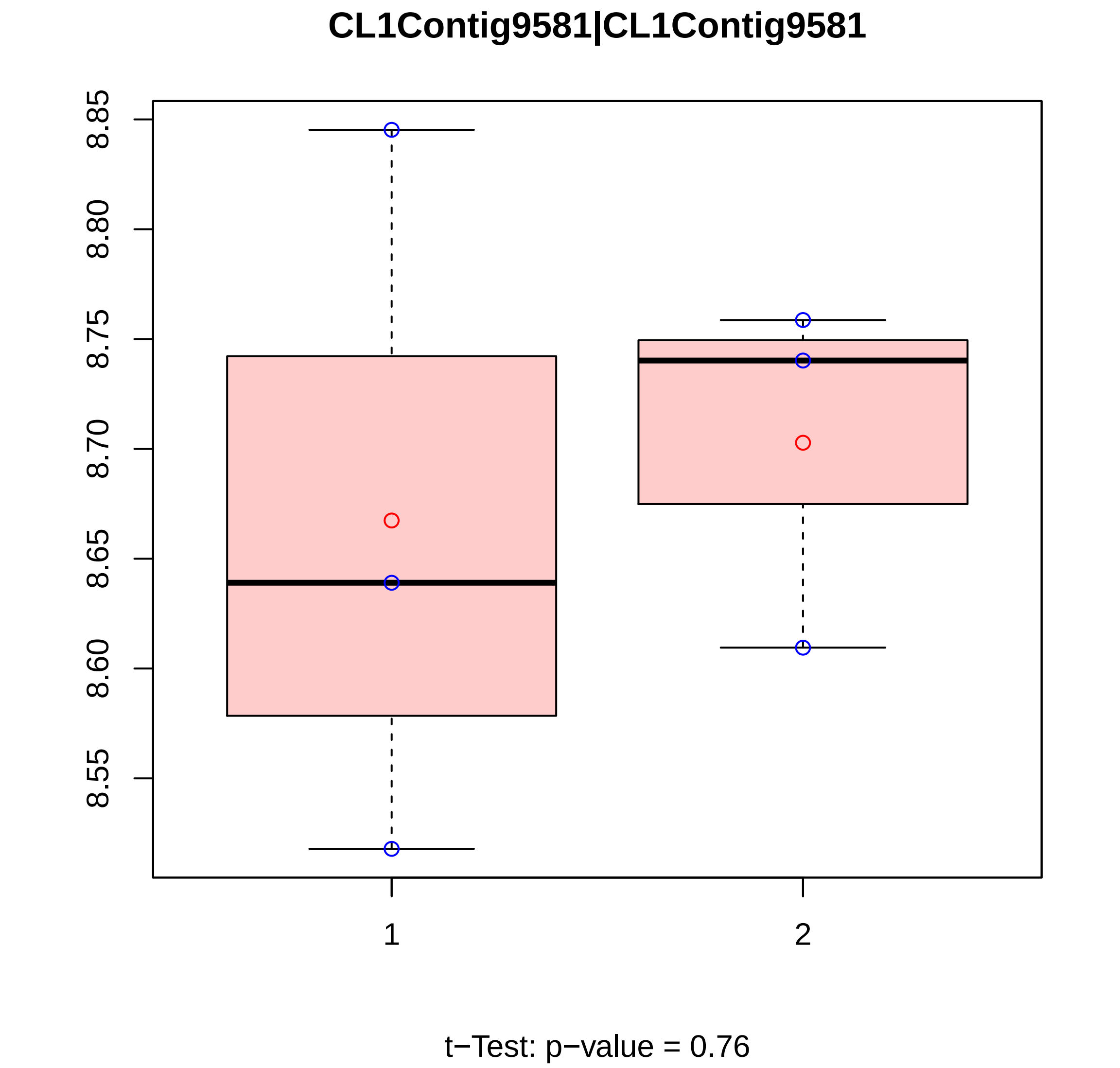
CL1Contig9581|CL1Contig9581
8.55
8.60
8.65
8.70
8.75
8.80
8.85
1
2
t−Test: p−value = 0.76

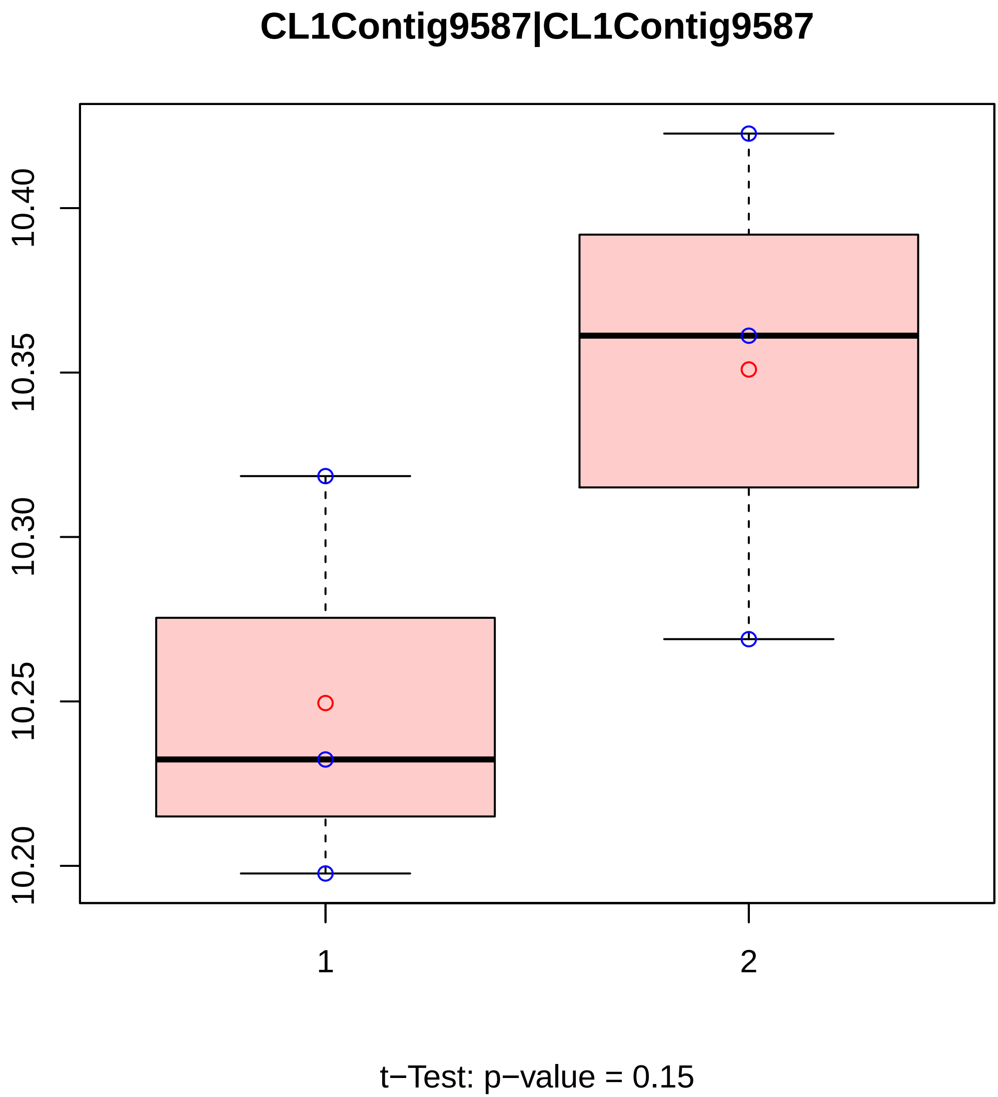

CL1Contig9587|CL1Contig9587
10.20
10.25
10.30
10.35
10.40
1
2
t−Test: p−value = 0.15

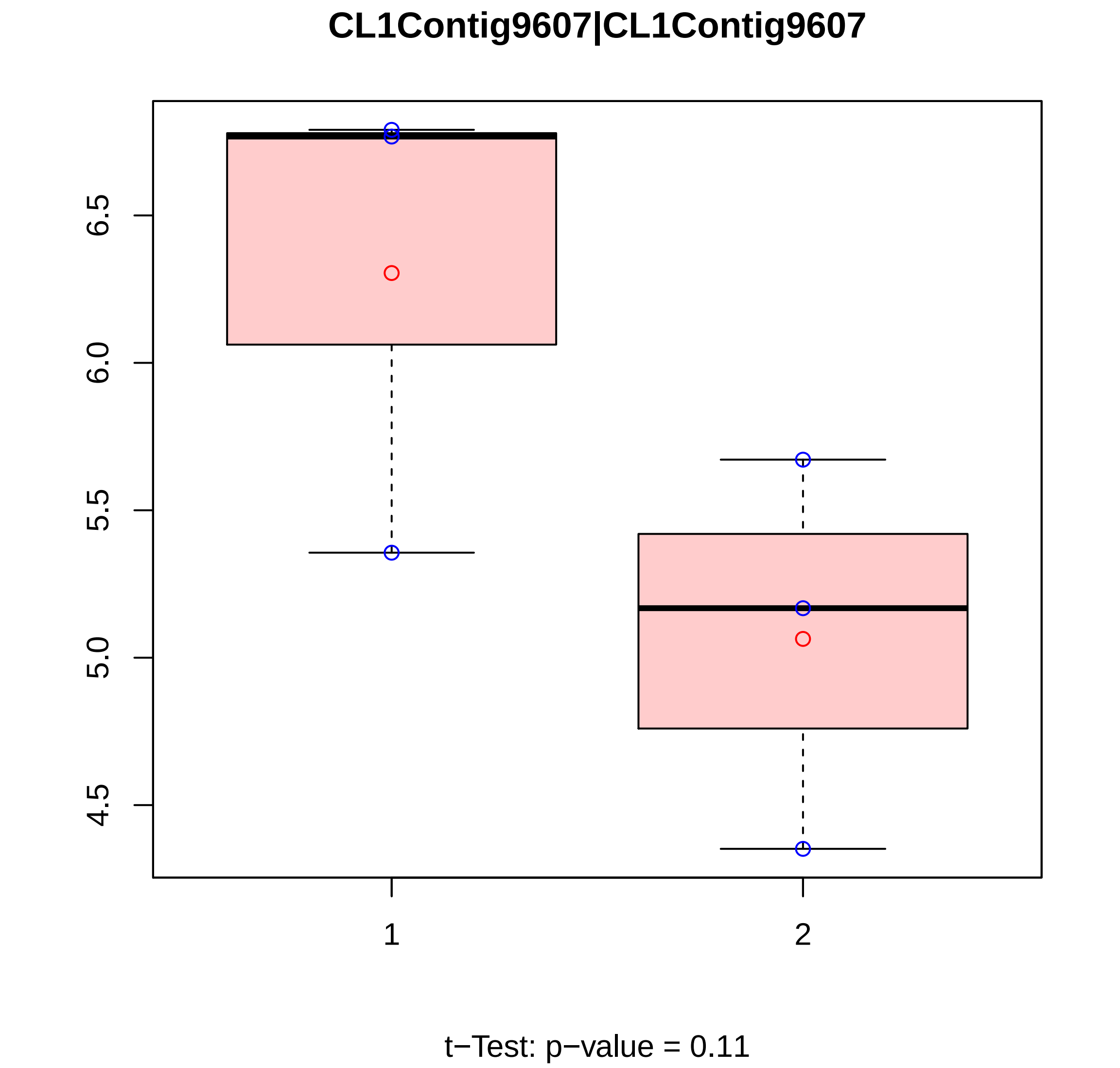
CL1Contig9607|CL1Contig9607
6.5
6.0
5.5
5.0
4.5
1
2
t−Test: p−value = 0.11

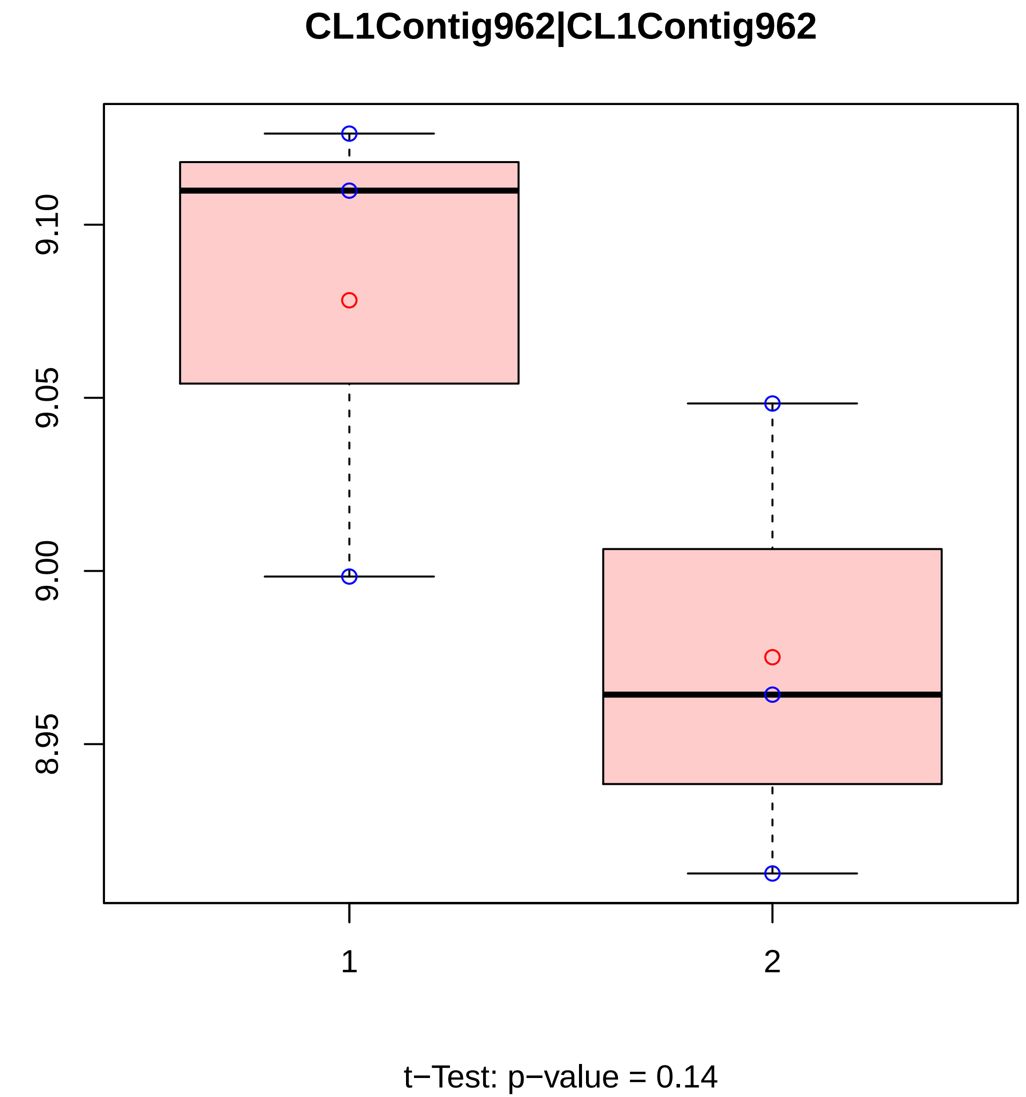
CL1Contig962|CL1Contig962
9.10
9.05
9.00
8.95
1
2
t−Test: p−value = 0.14

# CL1Contig9639|CL1Contig9639

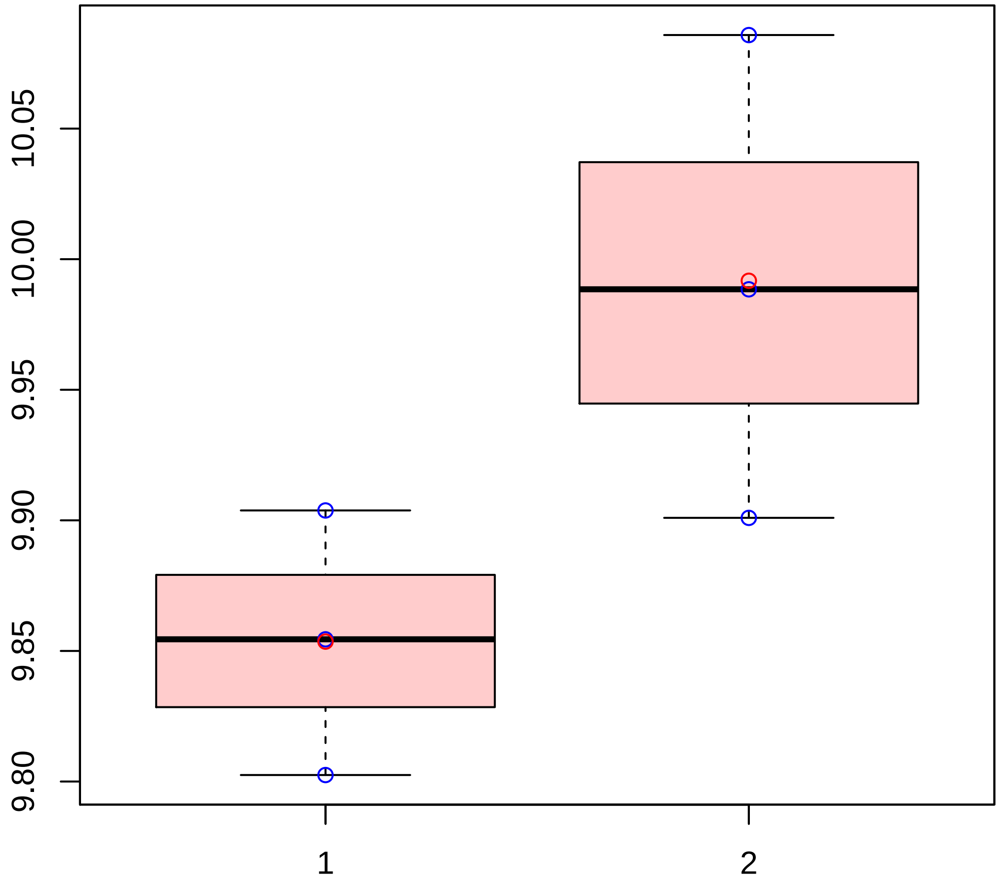


t−Test: p−value = 0.11

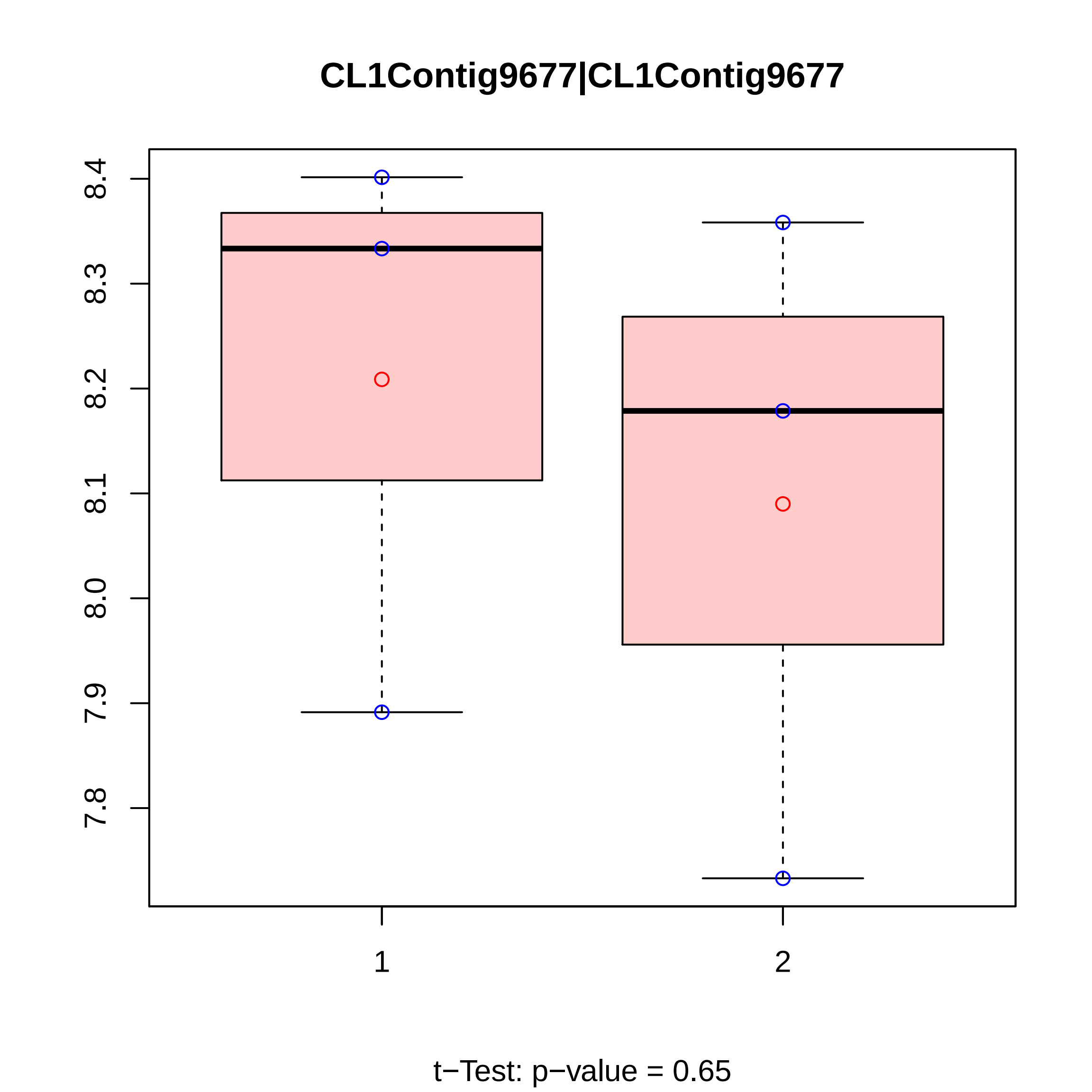

CL1Contig9677|CL1Contig9677
8.4
8.3
8.2
8.1
8.0
7.9
7.8
1
2
t−Test: p−value = 0.65

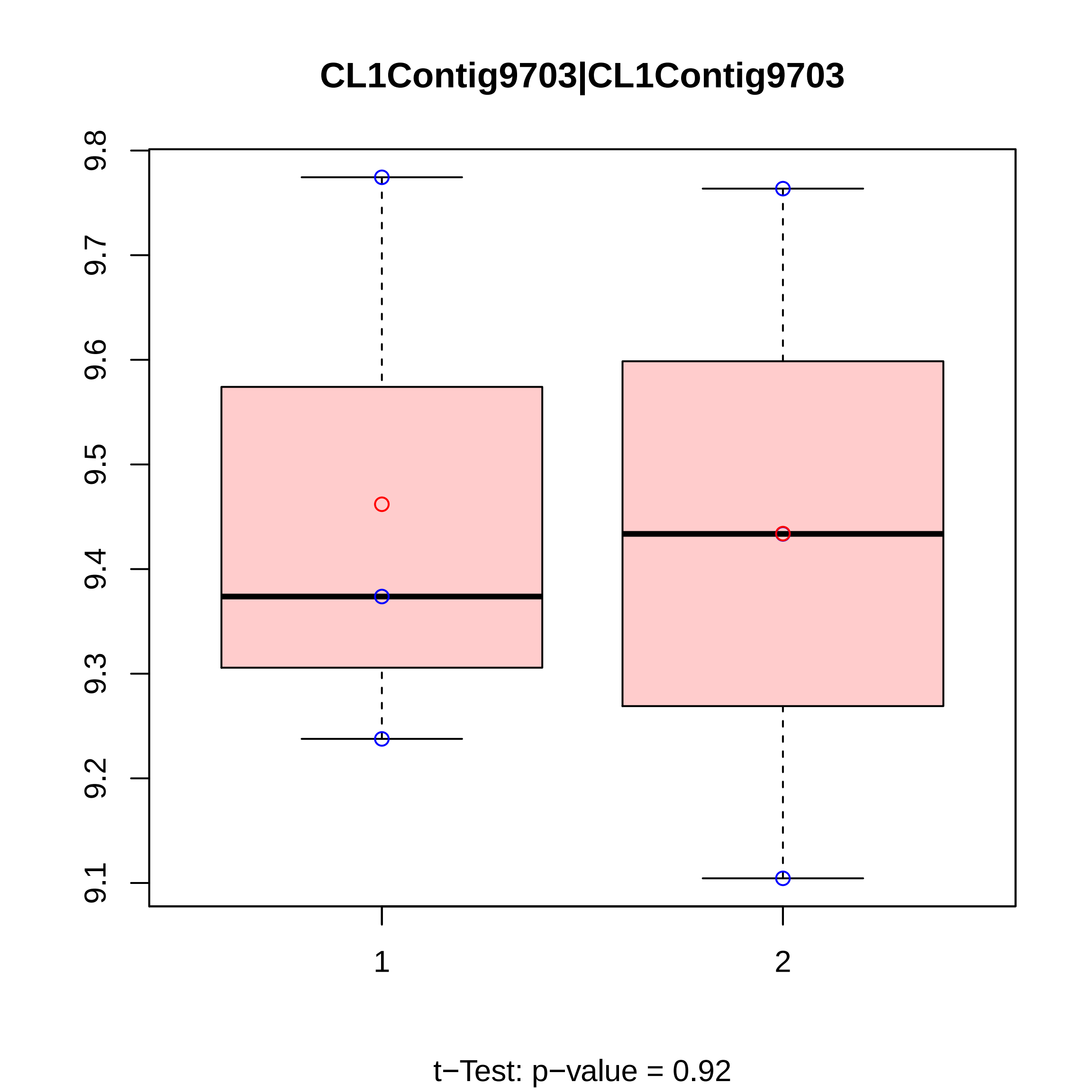
CL1Contig9703|CL1Contig9703
9.8
9.7
9.6
9.5
9.4
9.3
9.2
9.1
1
2
t−Test: p−value = 0.92

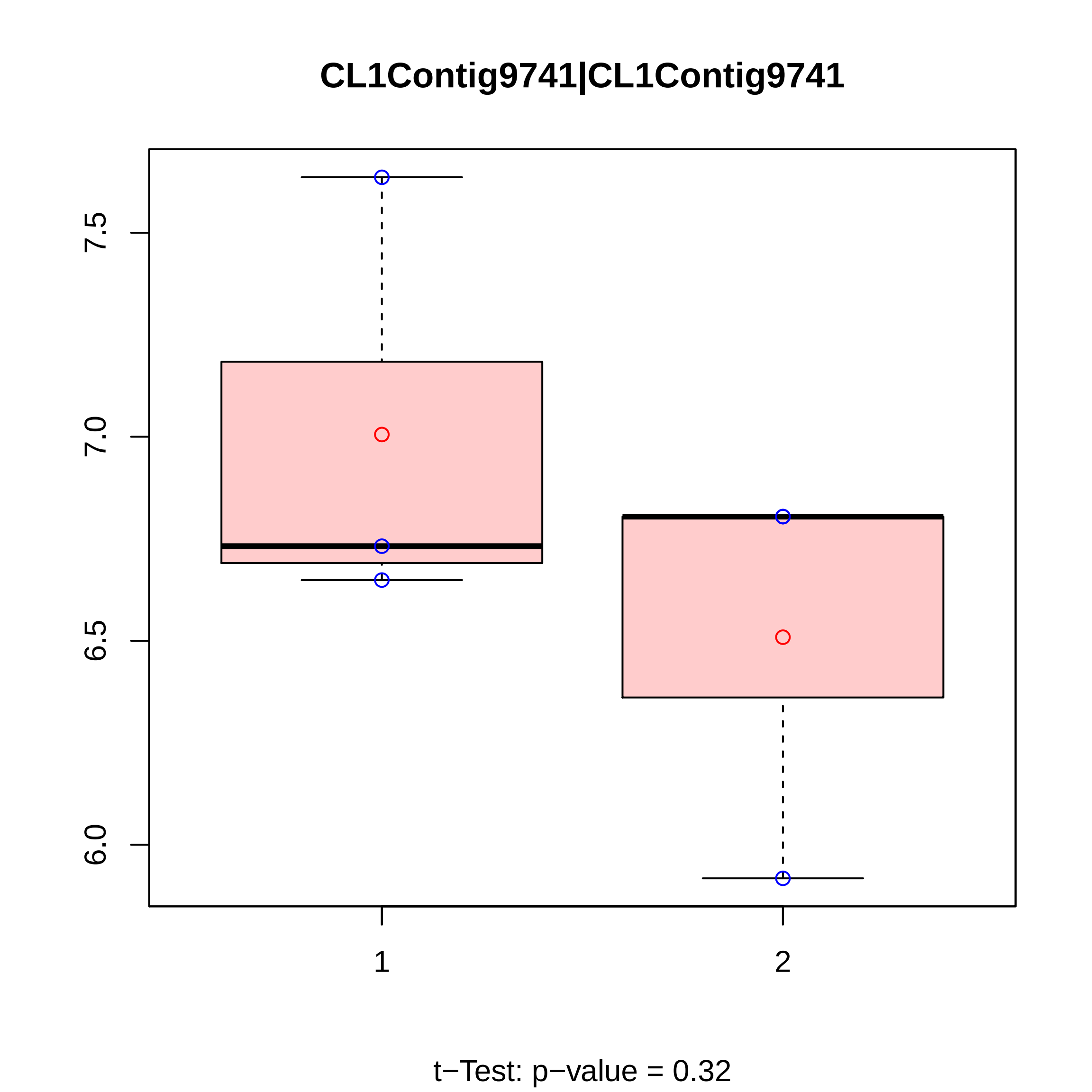

CL1Contig9741|CL1Contig9741
7.5
7.0
6.5
6.0
1
2
t−Test: p−value = 0.32

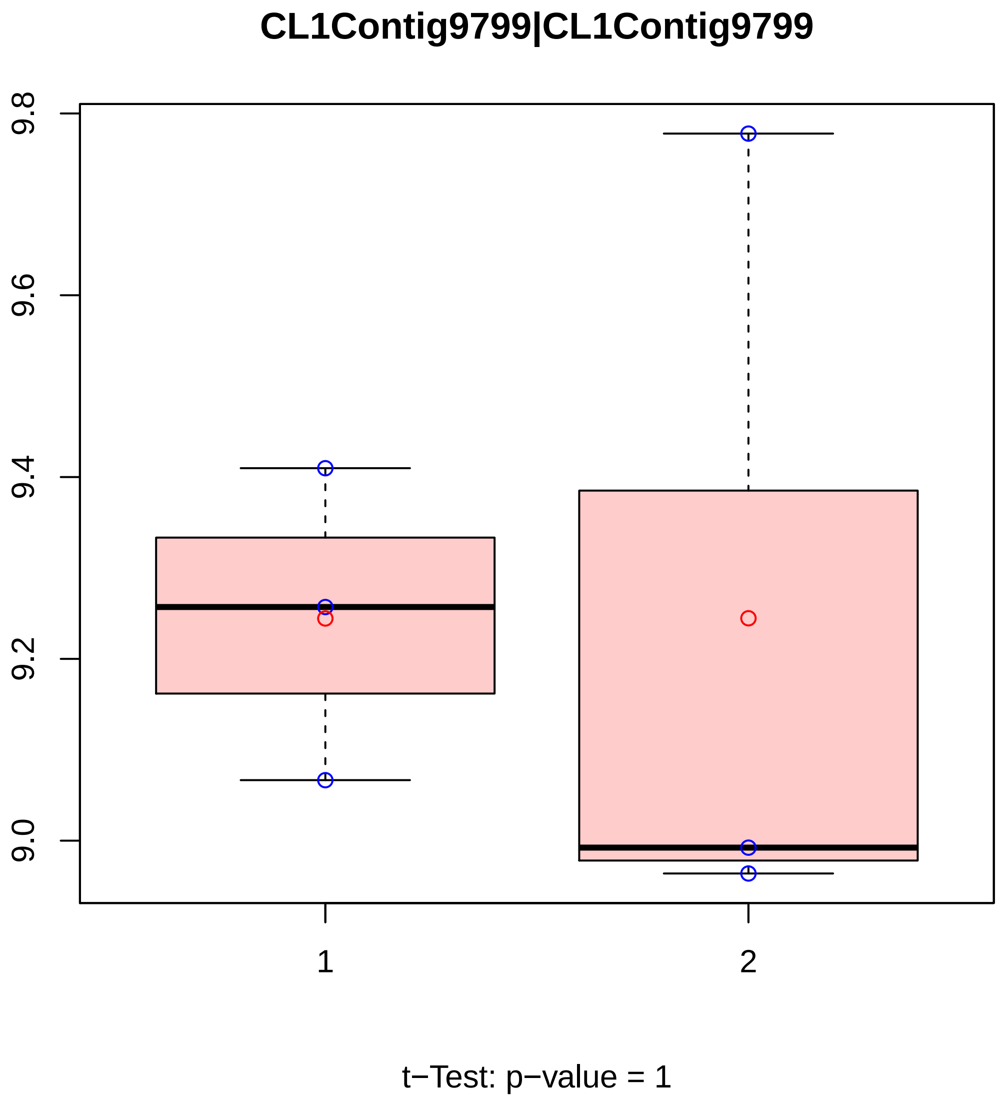
CL1Contig9799|CL1Contig9799
9.0
9.2
9.4
9.6
9.8
1
2
t−Test: p−value = 1

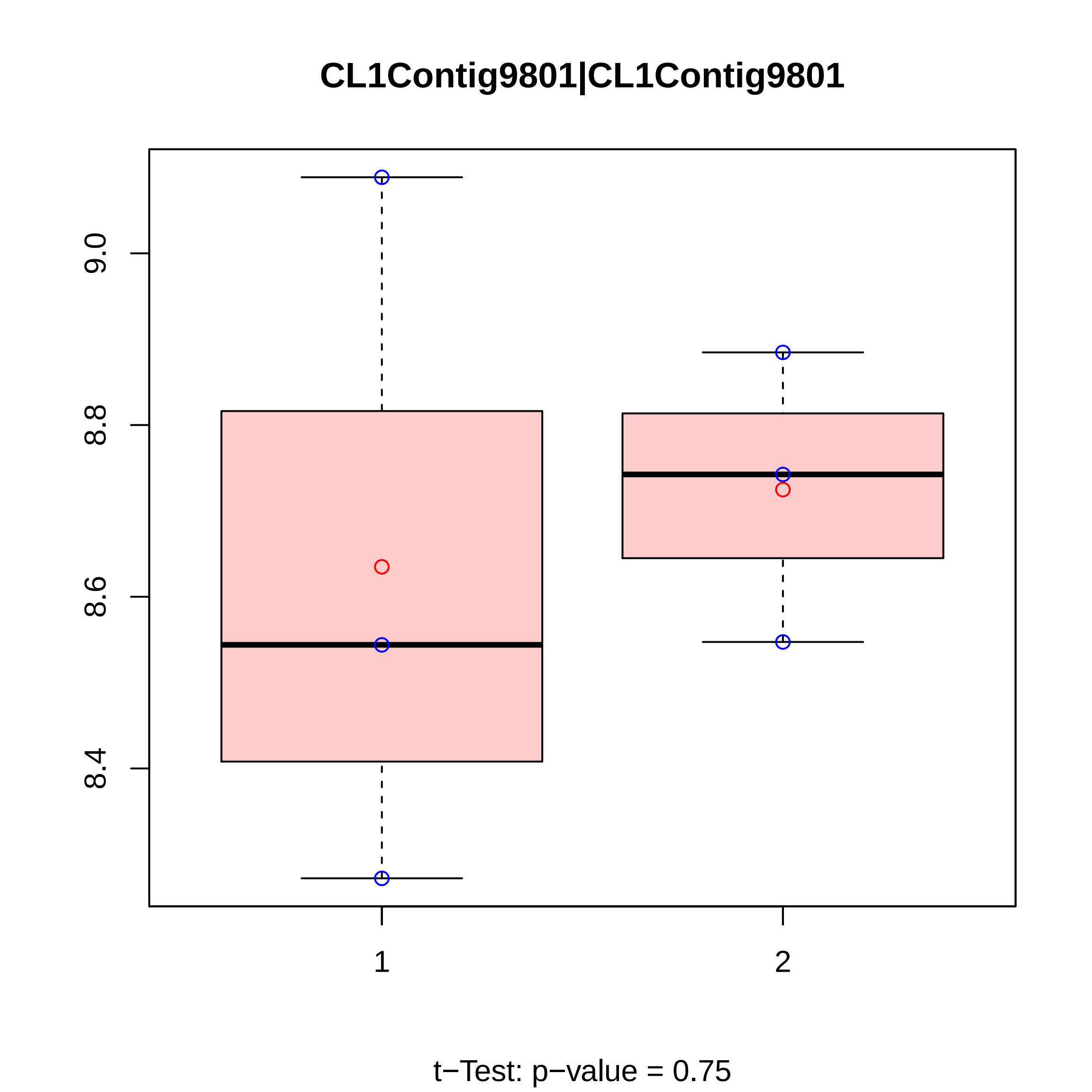

CL1Contig9801|CL1Contig9801
9.0
8.8
8.6
8.4
1
2
t−Test: p−value = 0.75

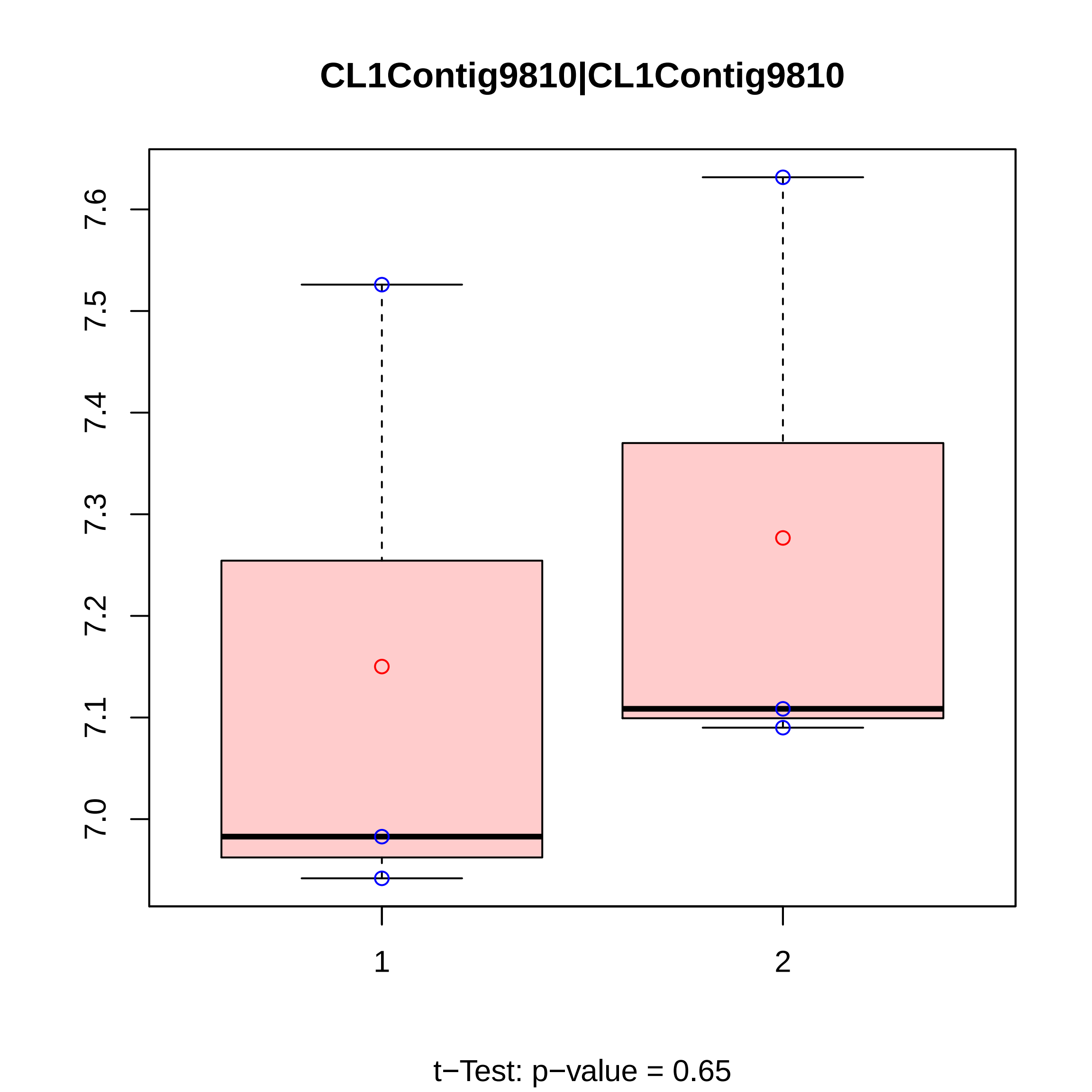
CL1Contig9810|CL1Contig9810
7.0
7.1
7.2
7.3
7.4
7.5
7.6
1
2
t−Test: p−value = 0.65

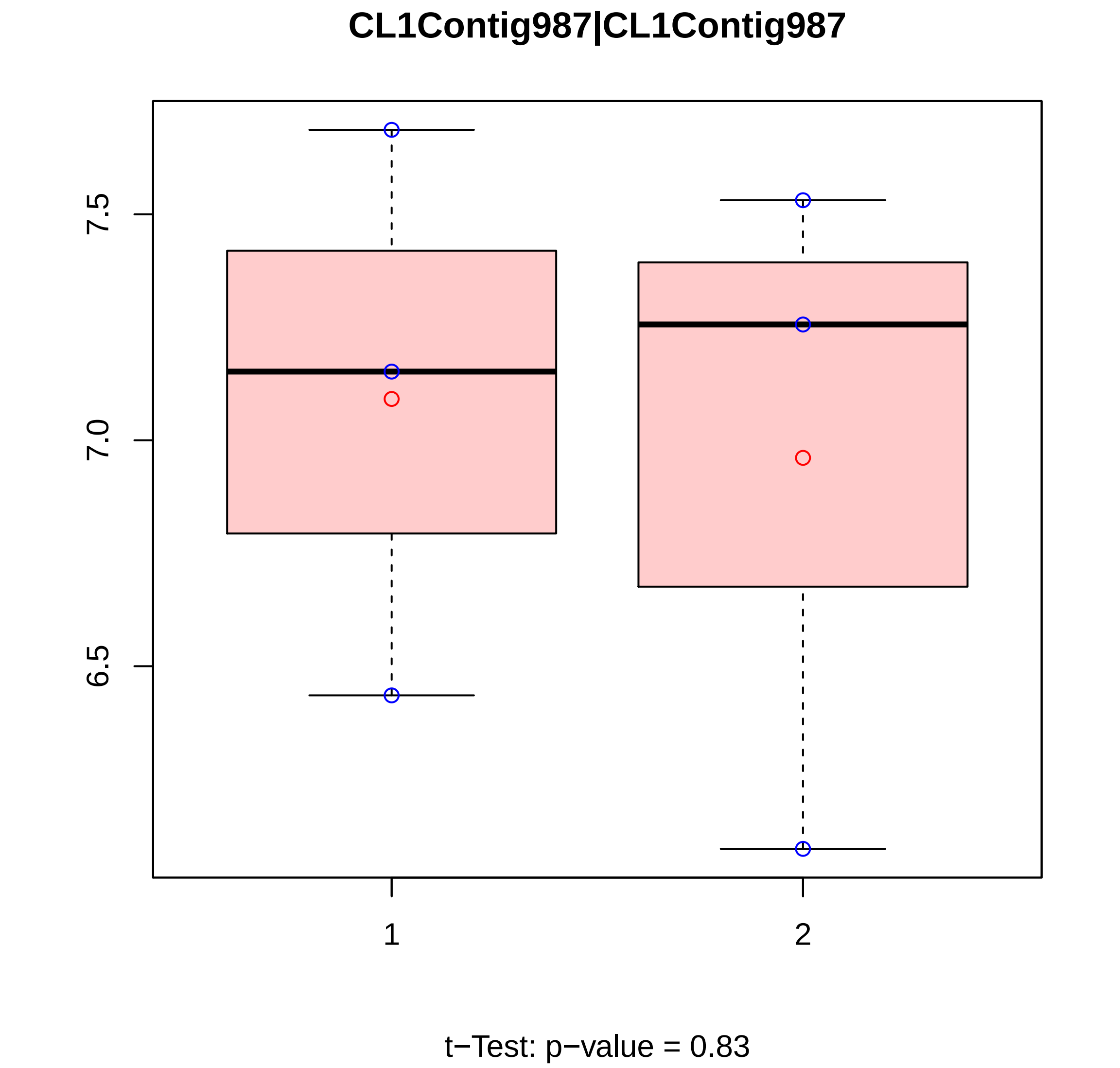
CL1Contig987|CL1Contig987
7.5
7.0
6.5
1
2
t−Test: p−value = 0.83

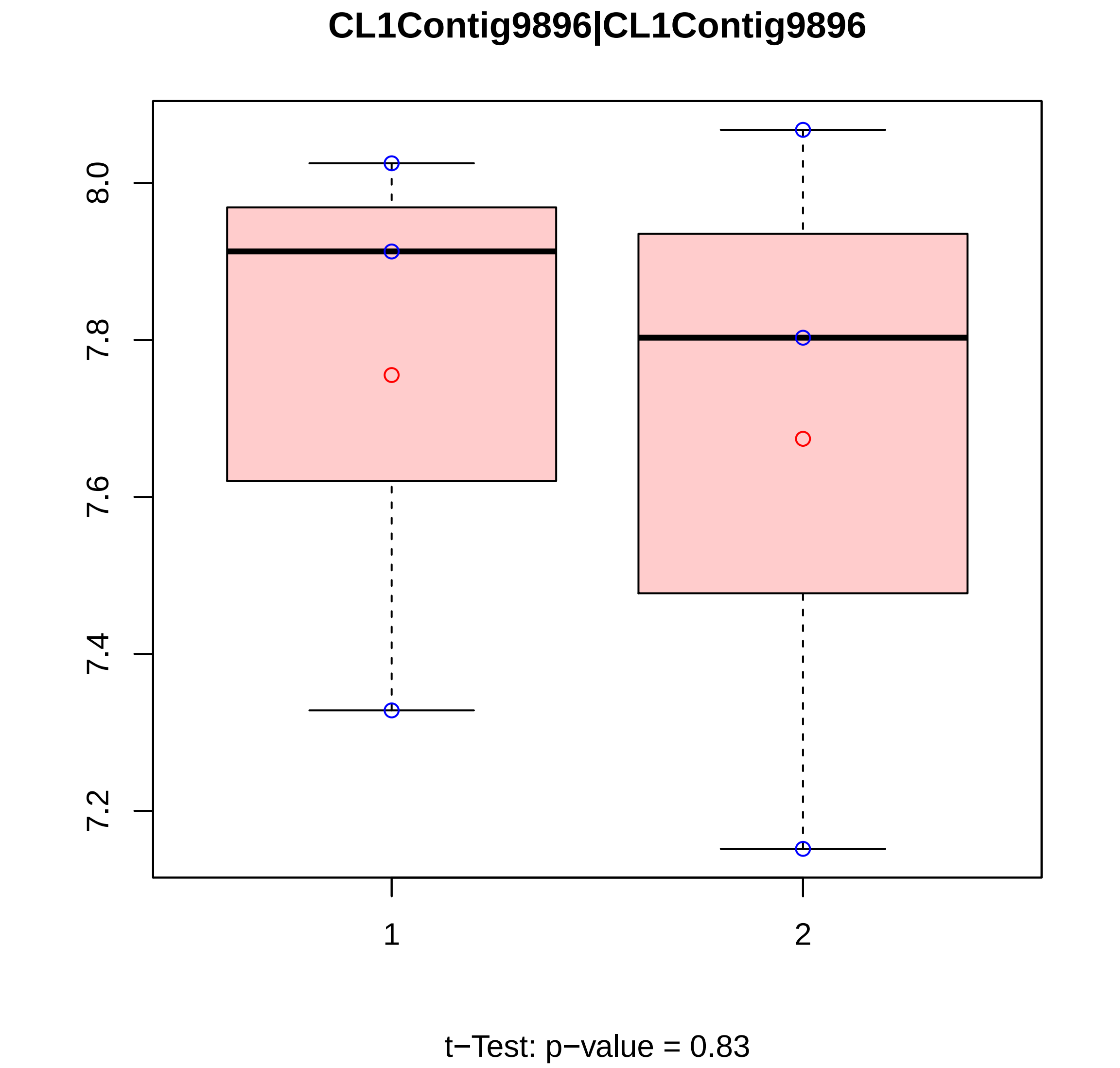
CL1Contig9896|CL1Contig9896
8.0
7.8
7.6
7.4
7.2
1
2
t−Test: p−value = 0.83

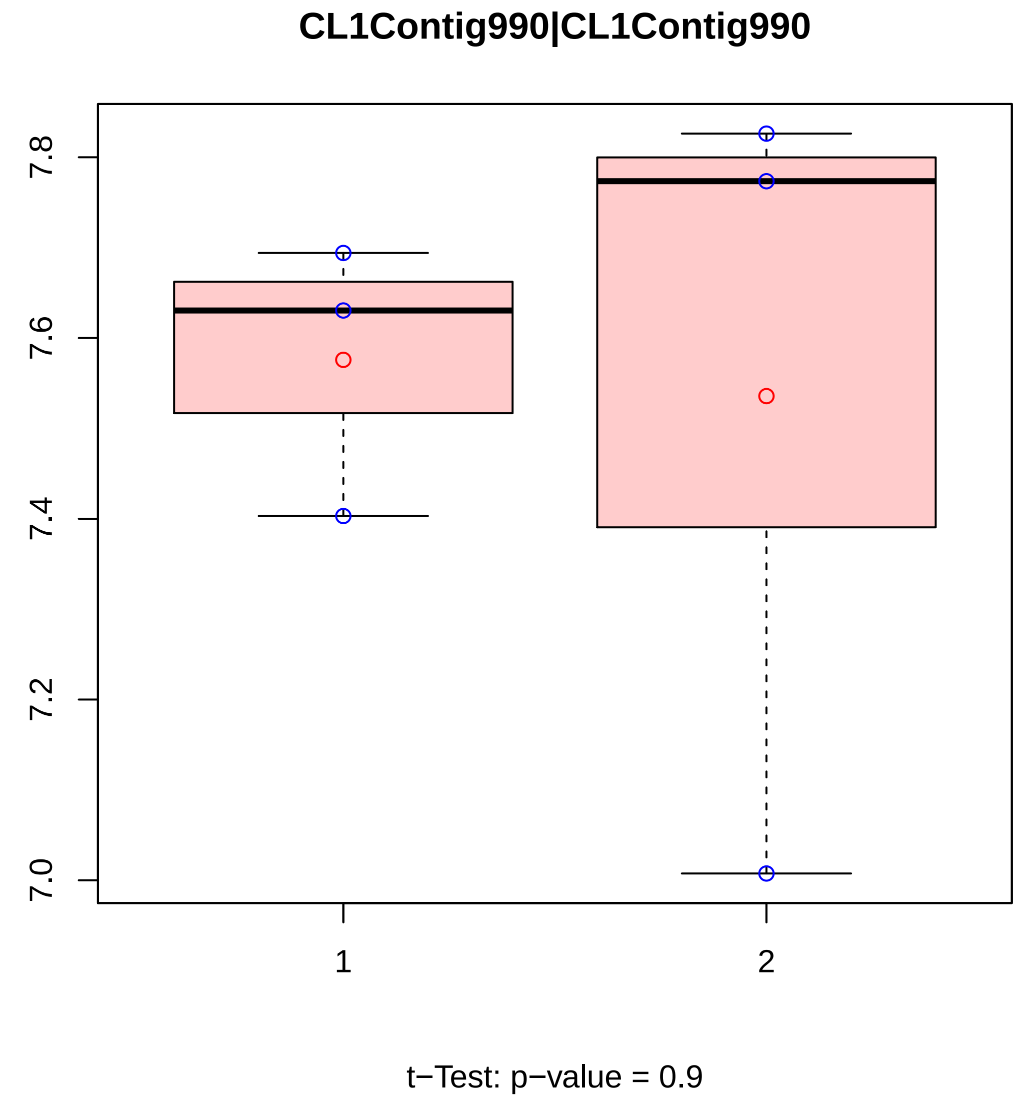
CL1Contig990|CL1Contig990
7.0
7.2
7.4
7.6
7.8
1
2
t−Test: p−value = 0.9

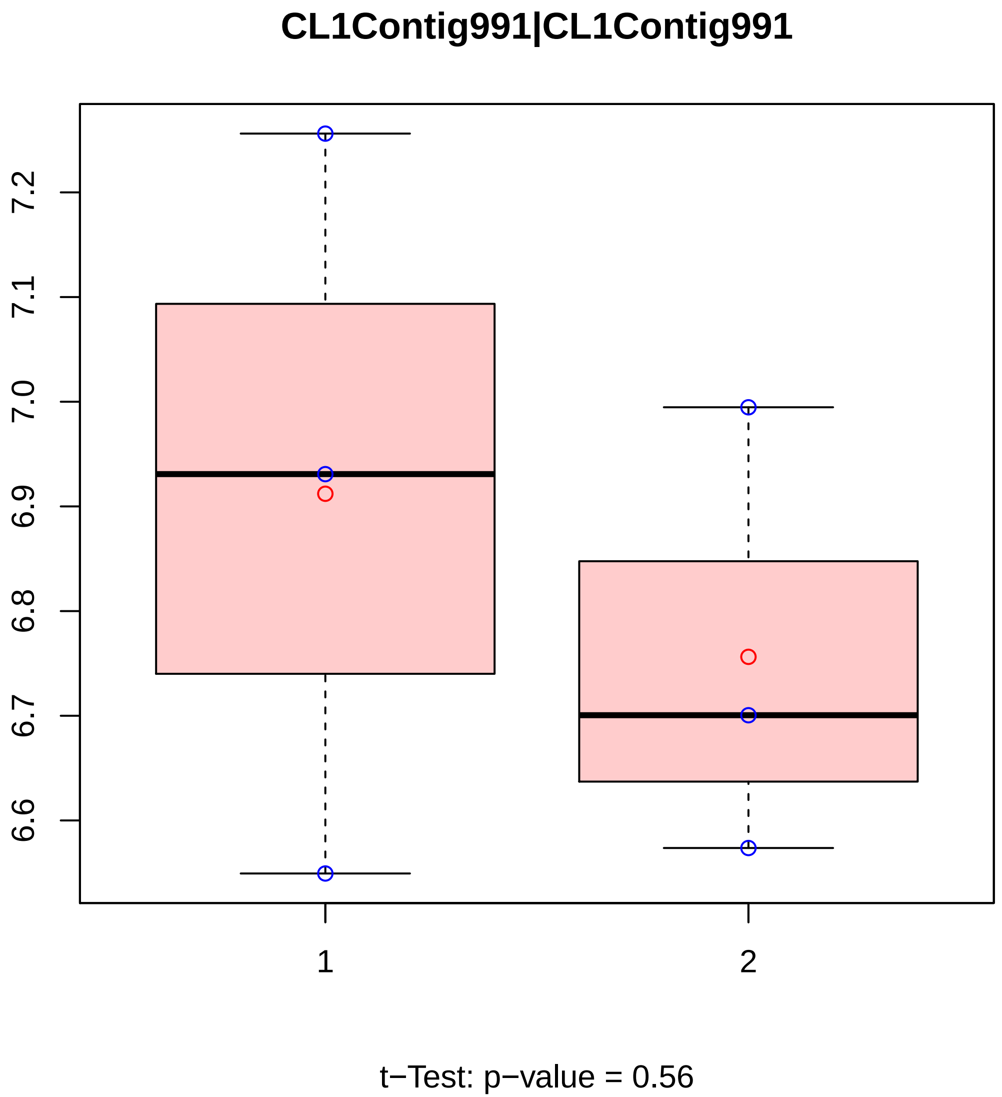
CL1Contig991|CL1Contig991
7.2
7.1
7.0
6.9
6.8
6.7
6.6
1
2
t−Test: p−value = 0.56

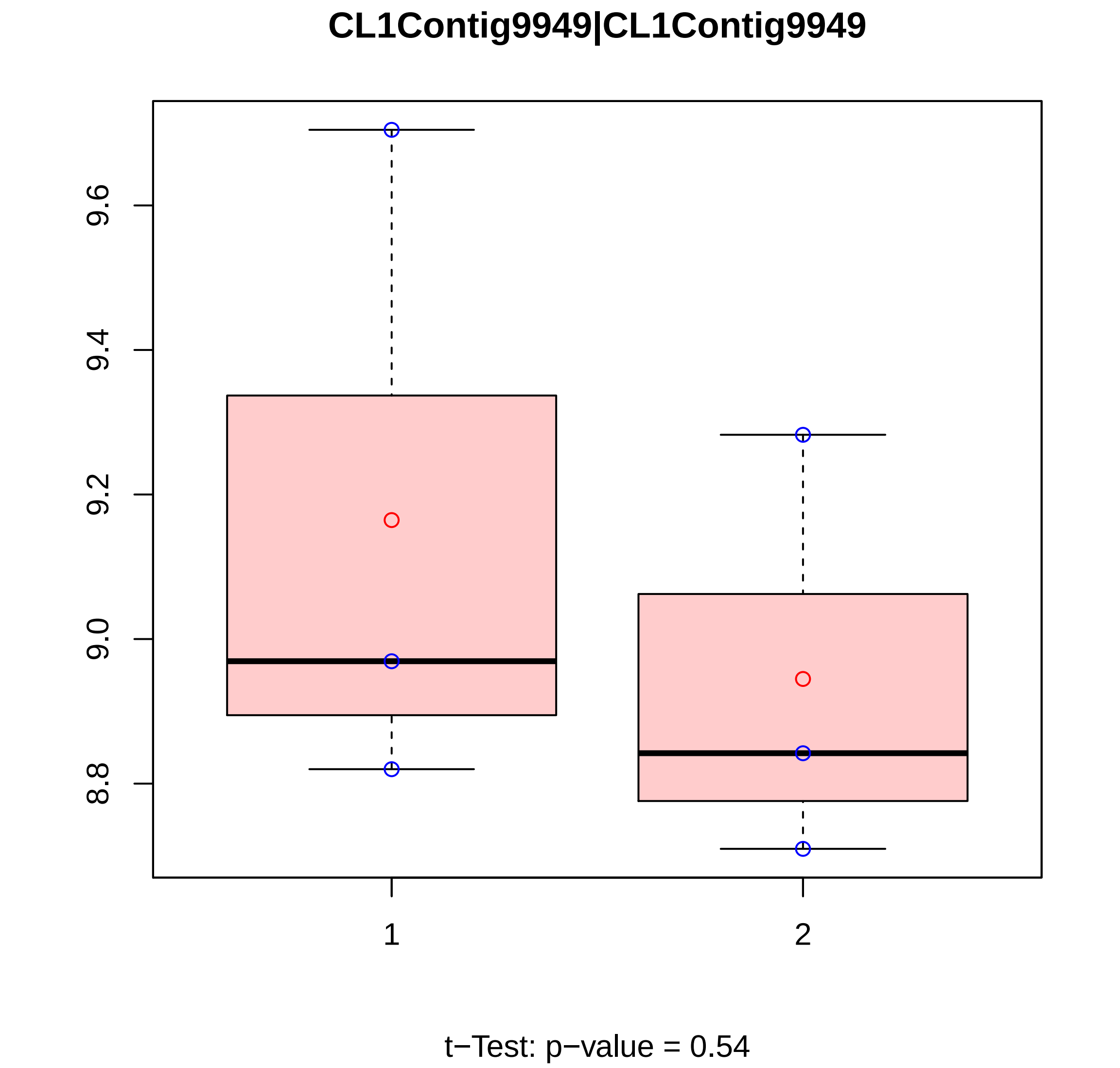
CL1Contig9949|CL1Contig9949
9.6
9.4
9.2
9.0
8.8
1
2
t−Test: p−value = 0.54

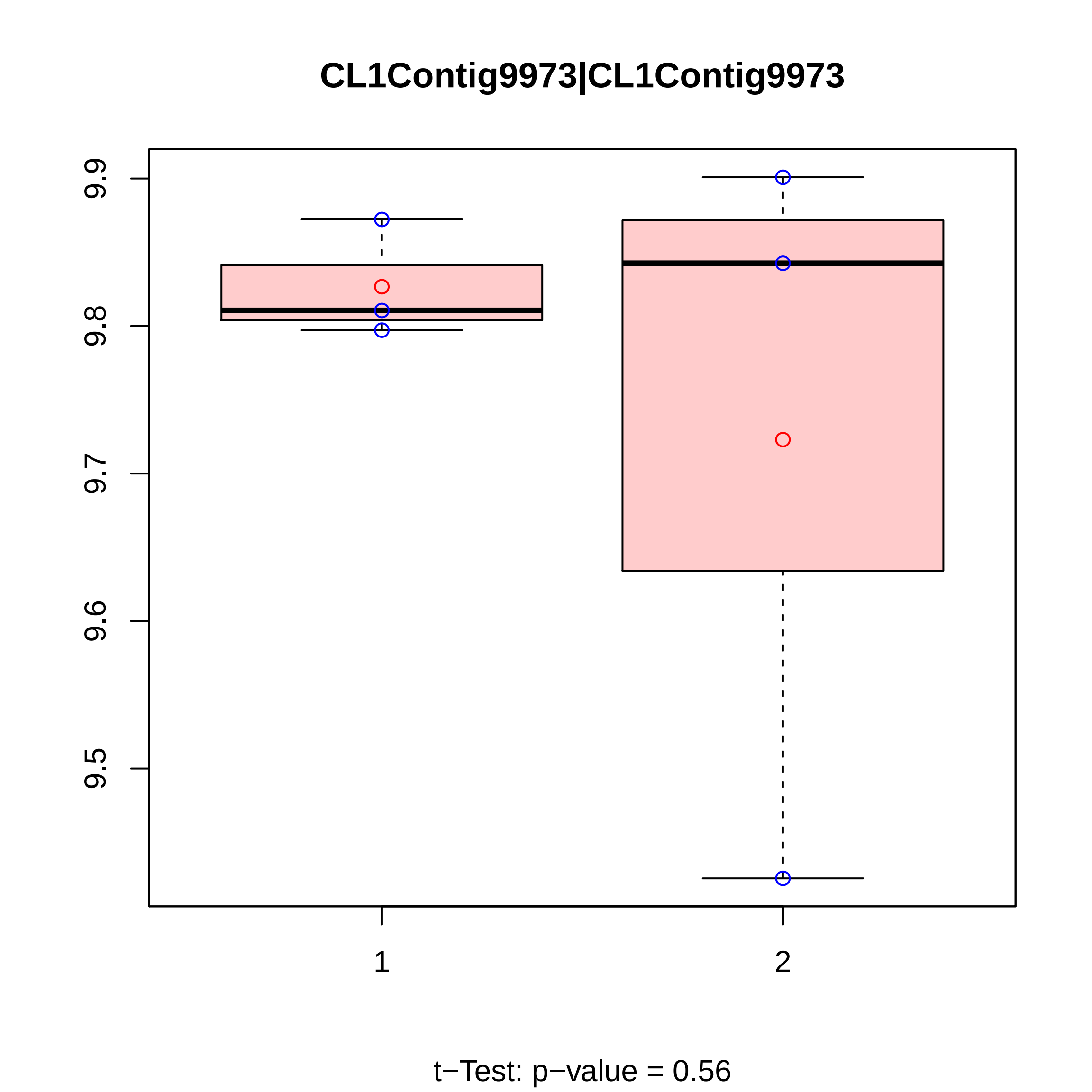
CL1Contig9973|CL1Contig9973
9.5
9.6
9.7
9.8
9.9
1
2
t−Test: p−value = 0.56

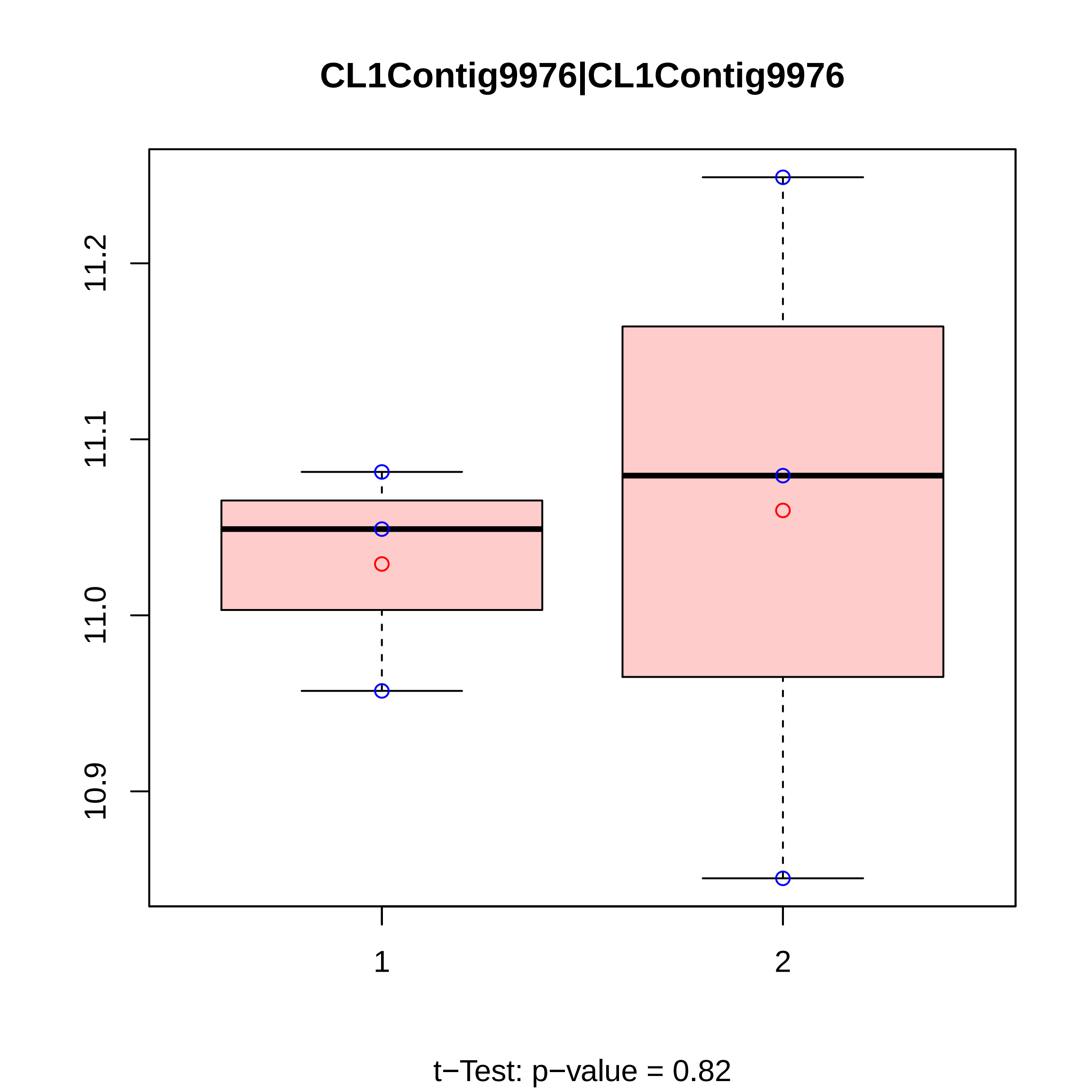
CL1Contig9976|CL1Contig9976
11.2
11.1
11.0
10.9
1
2
t−Test: p−value = 0.82

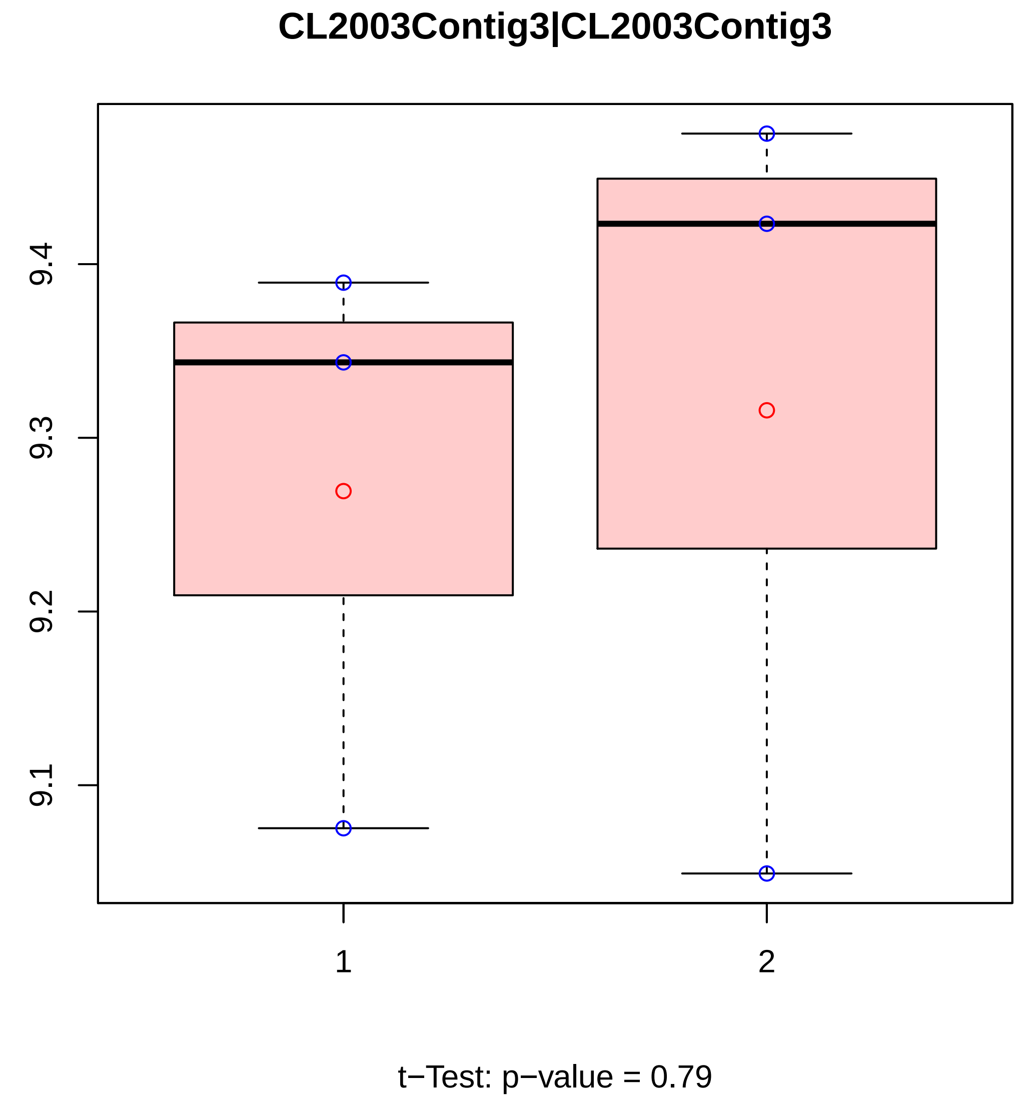
CL2003Contig3|CL2003Contig3
9.1
9.2
9.3
9.4
1
2
t−Test: p−value = 0.79

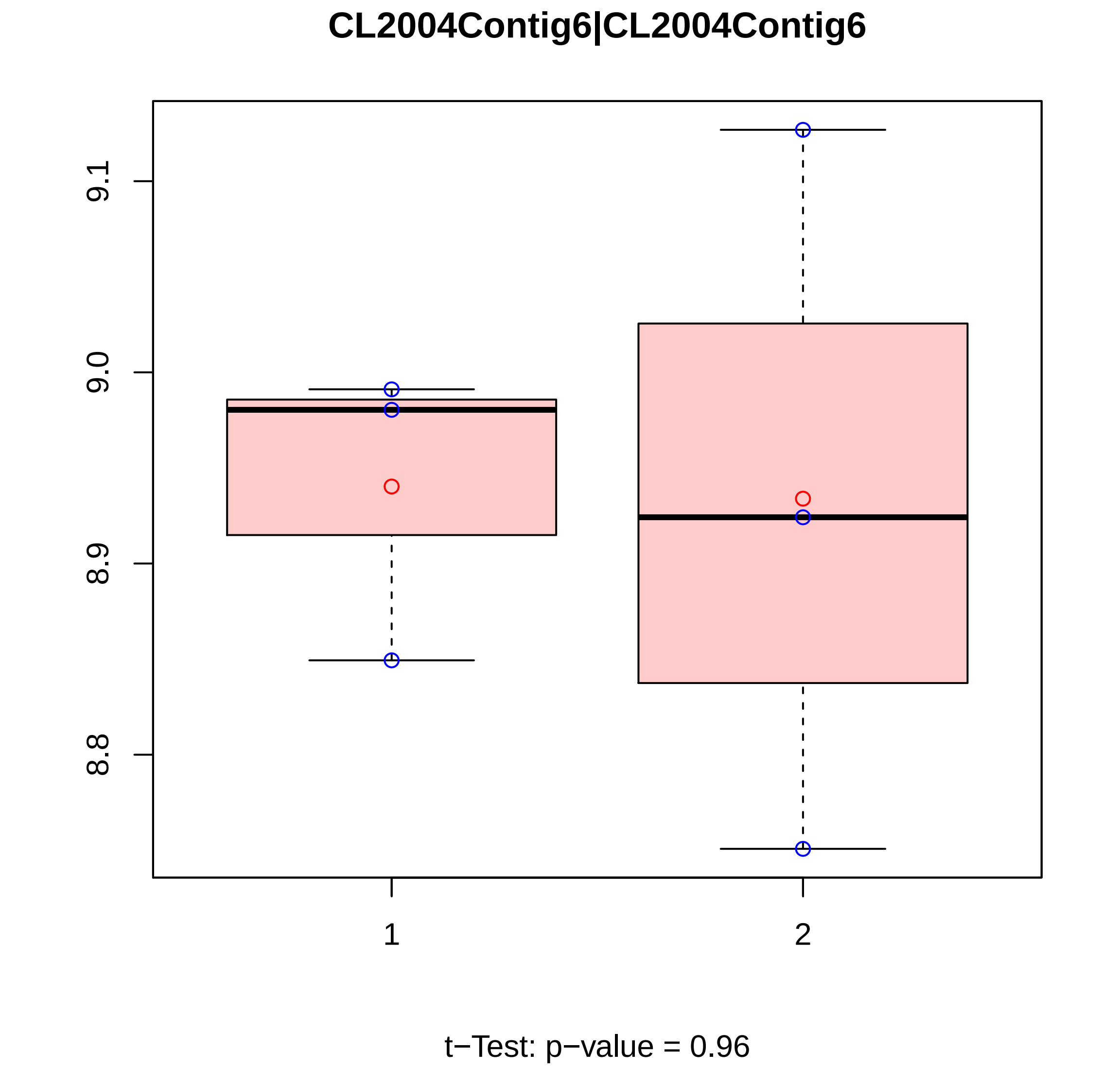
CL2004Contig6|CL2004Contig6
9.1
9.0
8.9
8.8
1
2
t−Test: p−value = 0.96

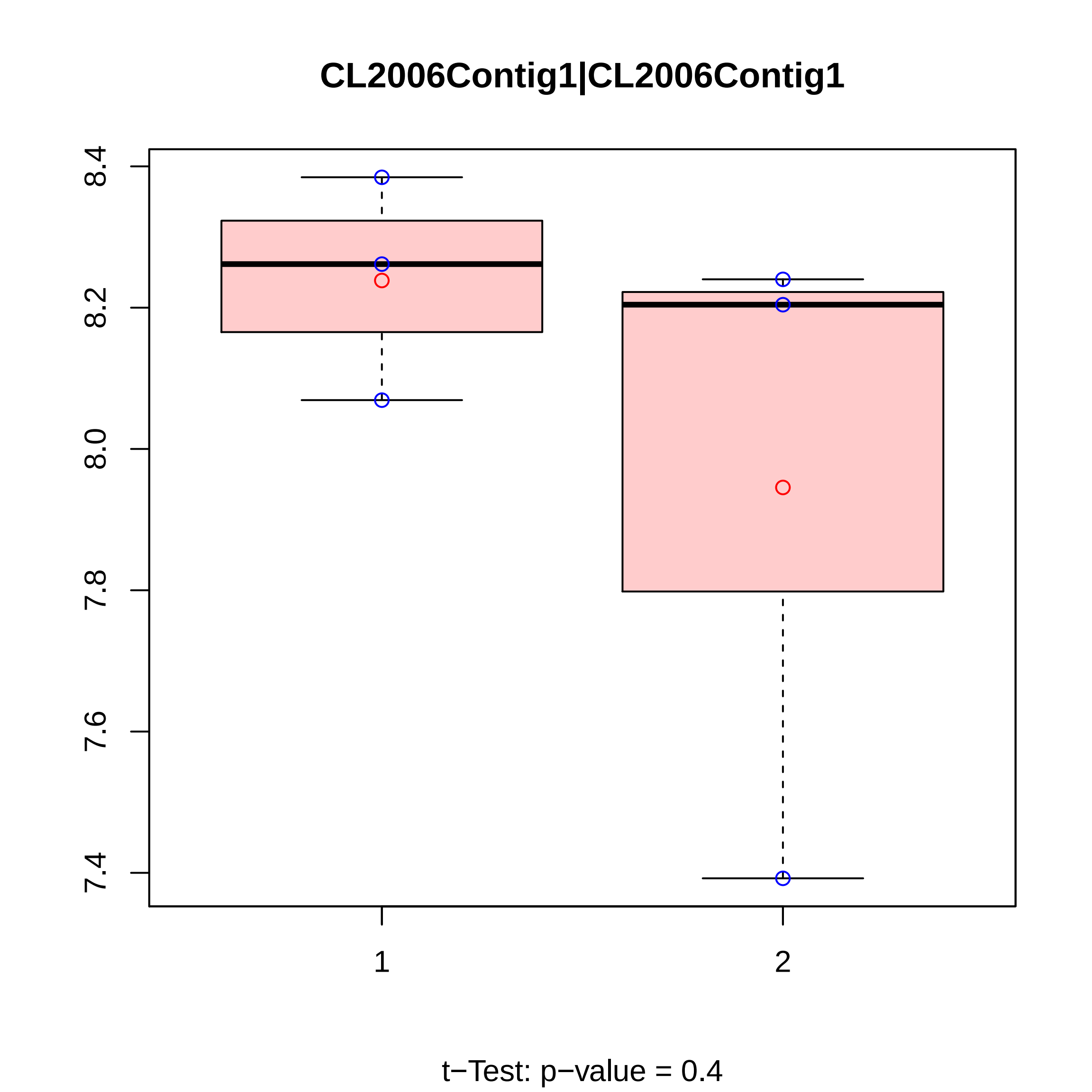

CL2006Contig1|CL2006Contig1
8.4
8.2
8.0
7.8
7.6
7.4
1
2
t−Test: p−value = 0.4

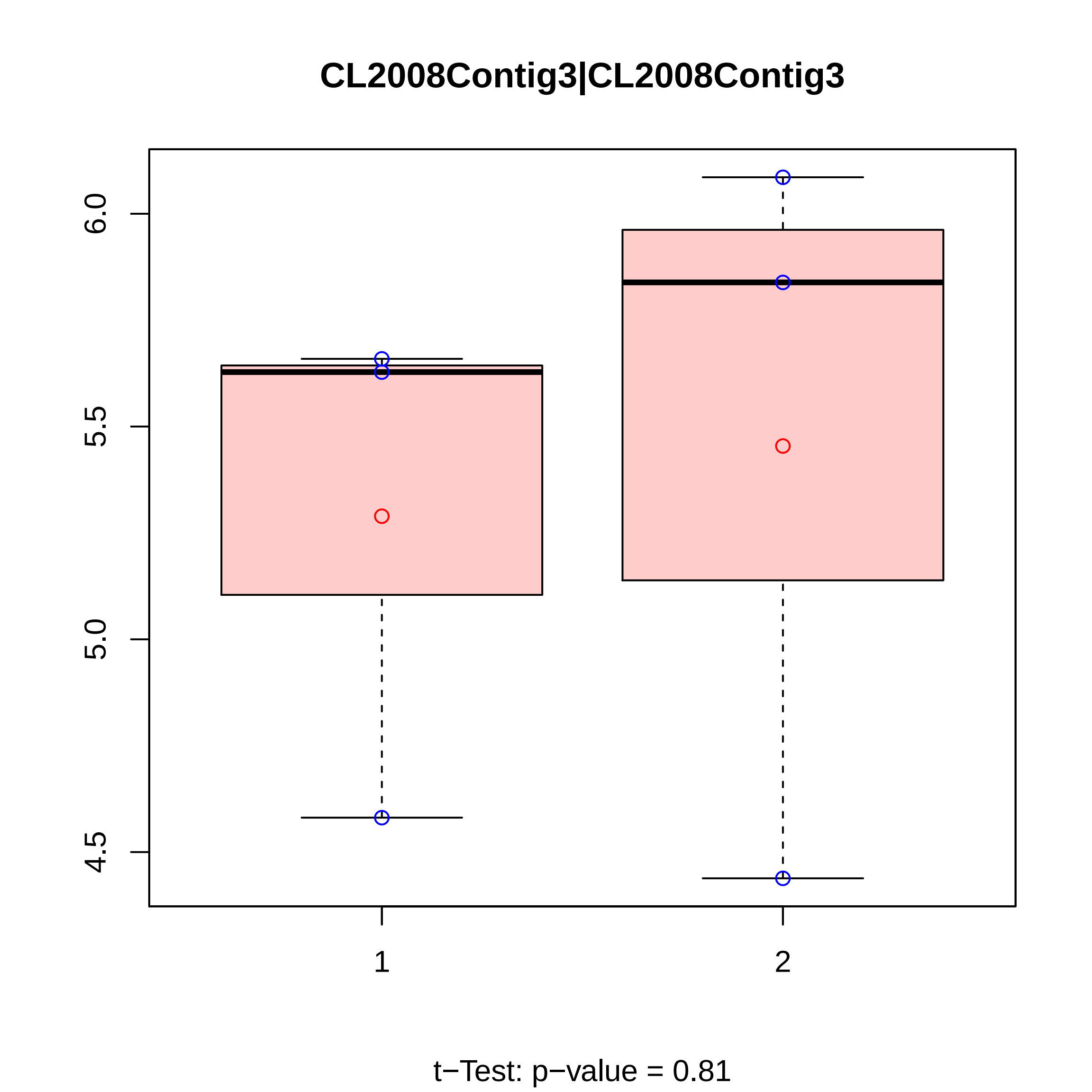

CL2008Contig3|CL2008Contig3
6.0
5.5
5.0
4.5
1
2
t−Test: p−value = 0.81

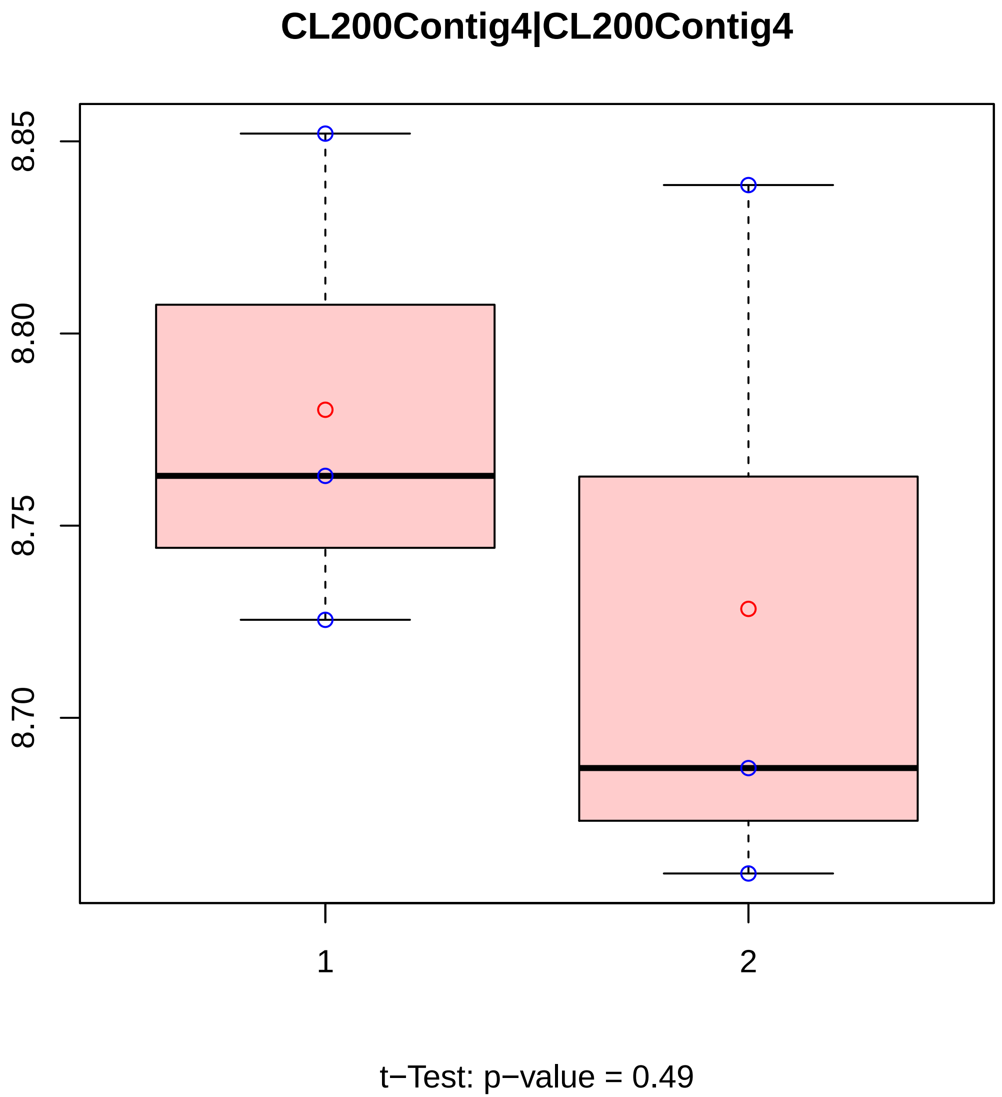
CL200Contig4|CL200Contig4
8.85
8.80
8.75
8.70
1
2
t−Test: p−value = 0.49

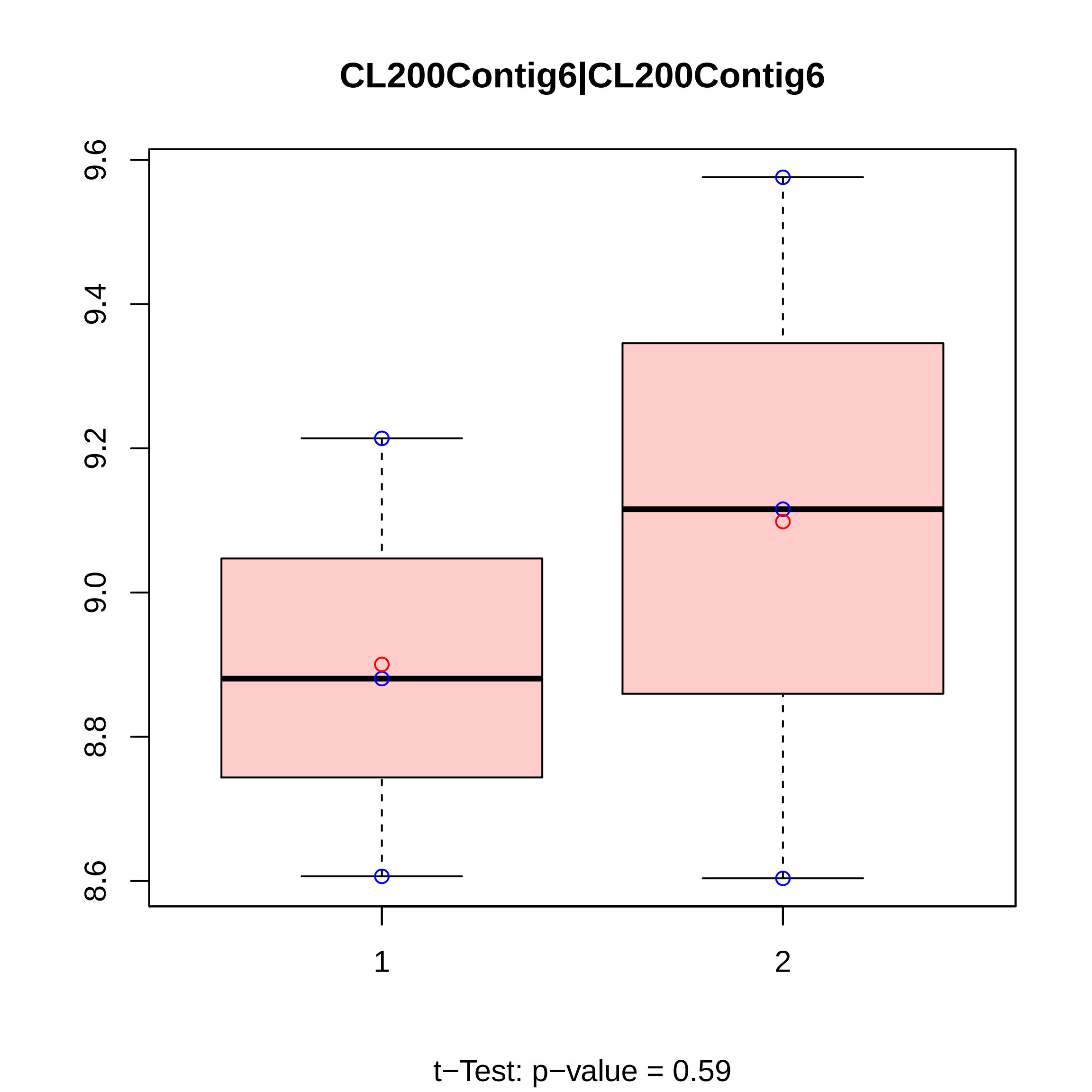
CL200Contig6|CL200Contig6
9.6
9.4
9.2
9.0
8.8
8.6
1
2
t−Test: p−value = 0.59

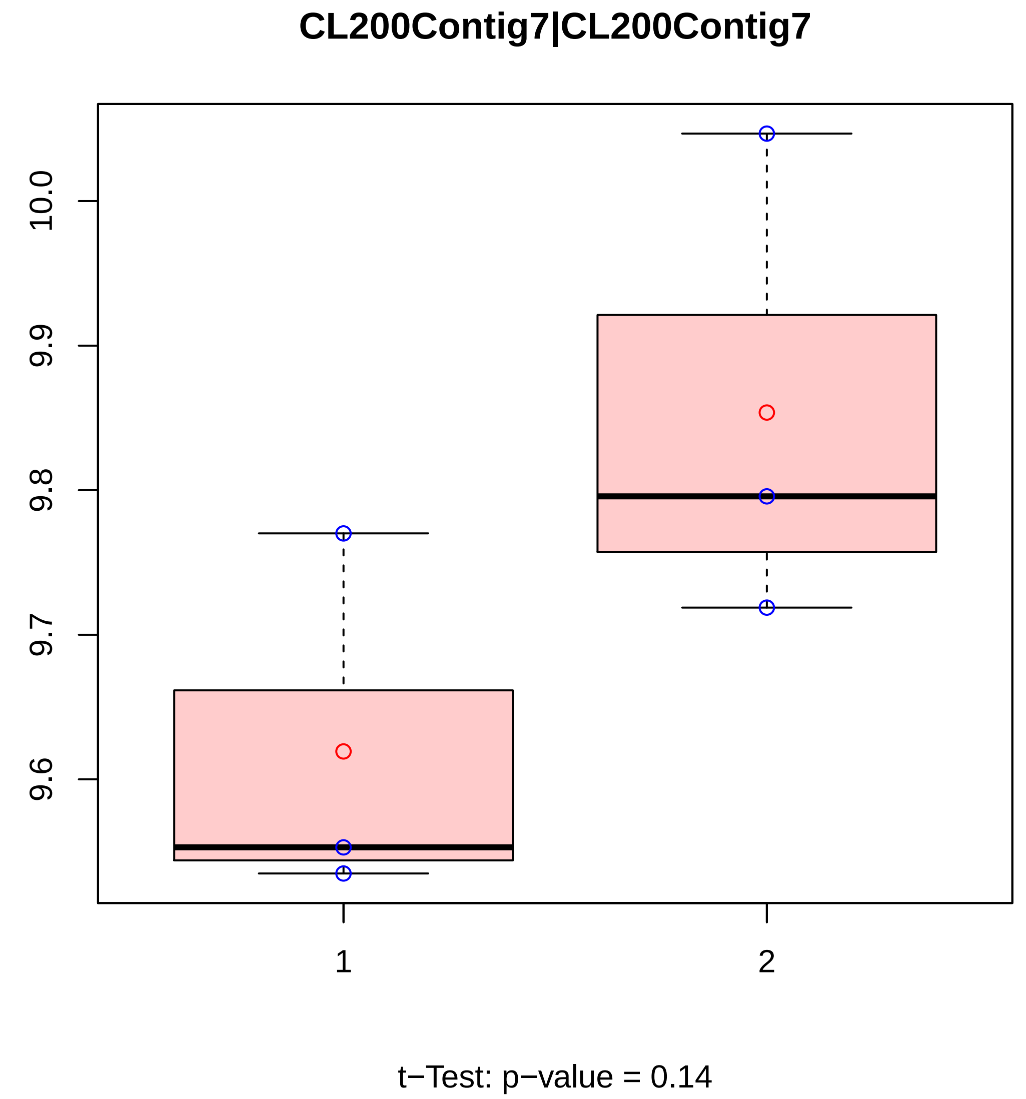
CL200Contig7|CL200Contig7
10.0
9.9
9.8
9.7
9.6
1
2
t−Test: p−value = 0.14

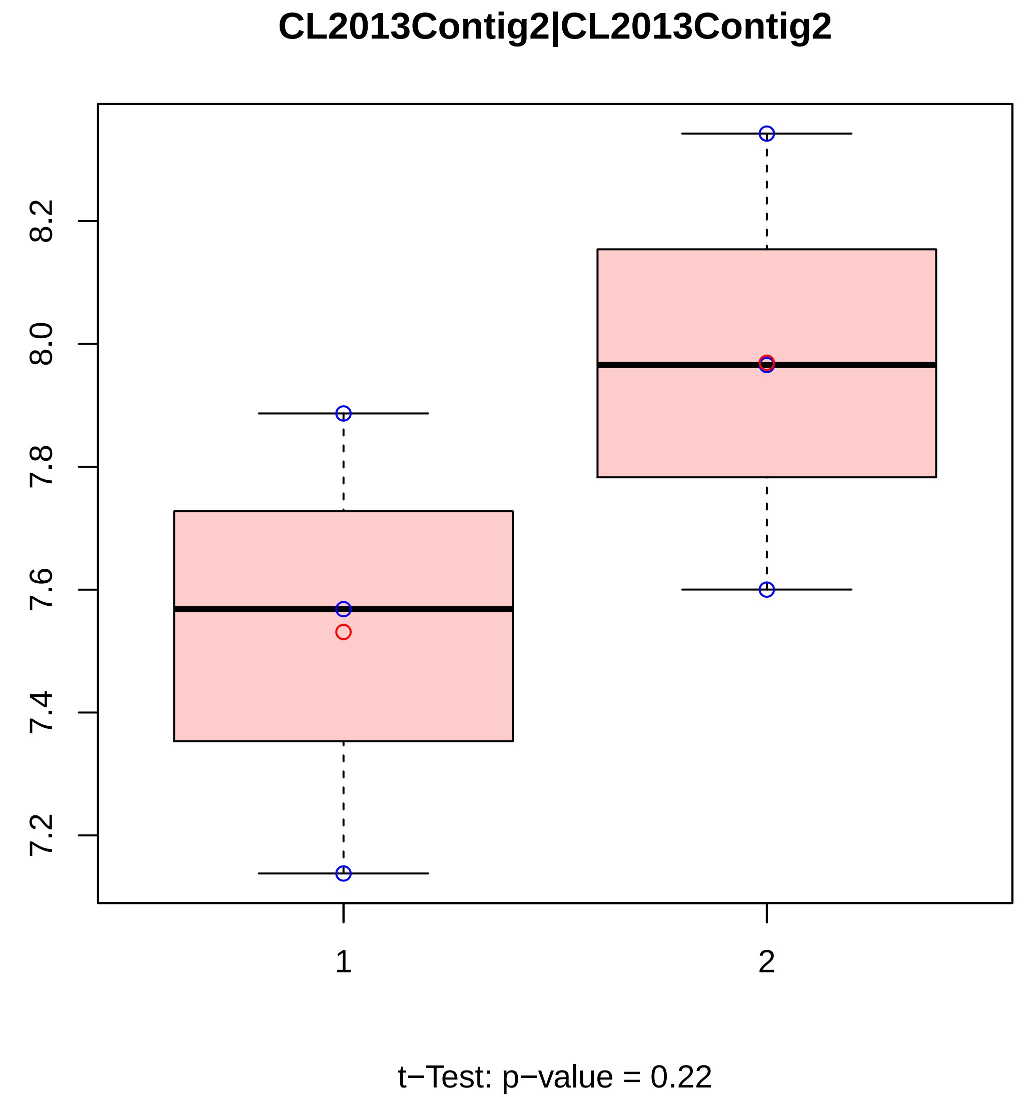
CL2013Contig2|CL2013Contig2
8.2
8.0
7.8
7.6
7.4
7.2
1
2
t−Test: p−value = 0.22

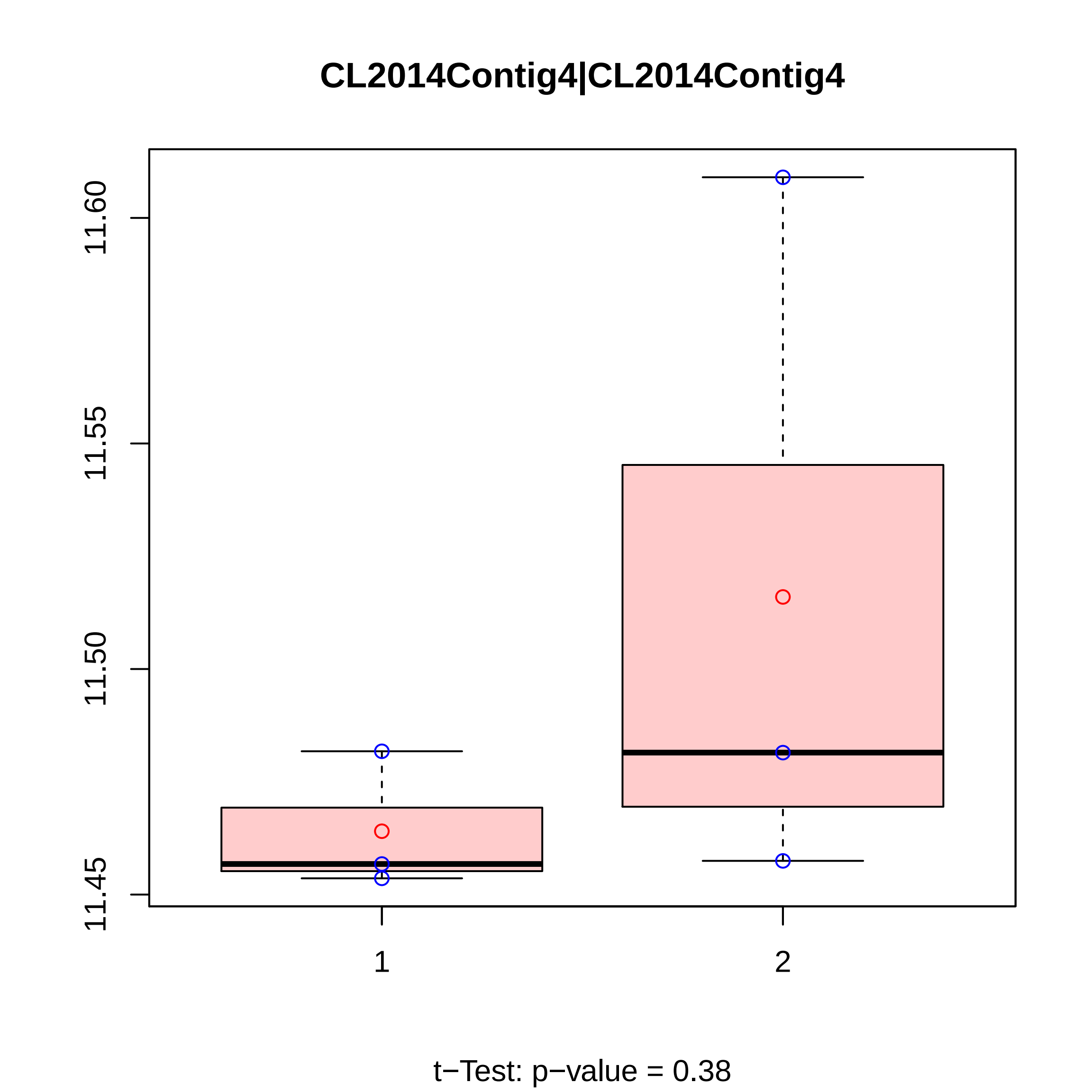

CL2014Contig4|CL2014Contig4
11.45
11.50
11.55
11.60
1
2
t−Test: p−value = 0.38

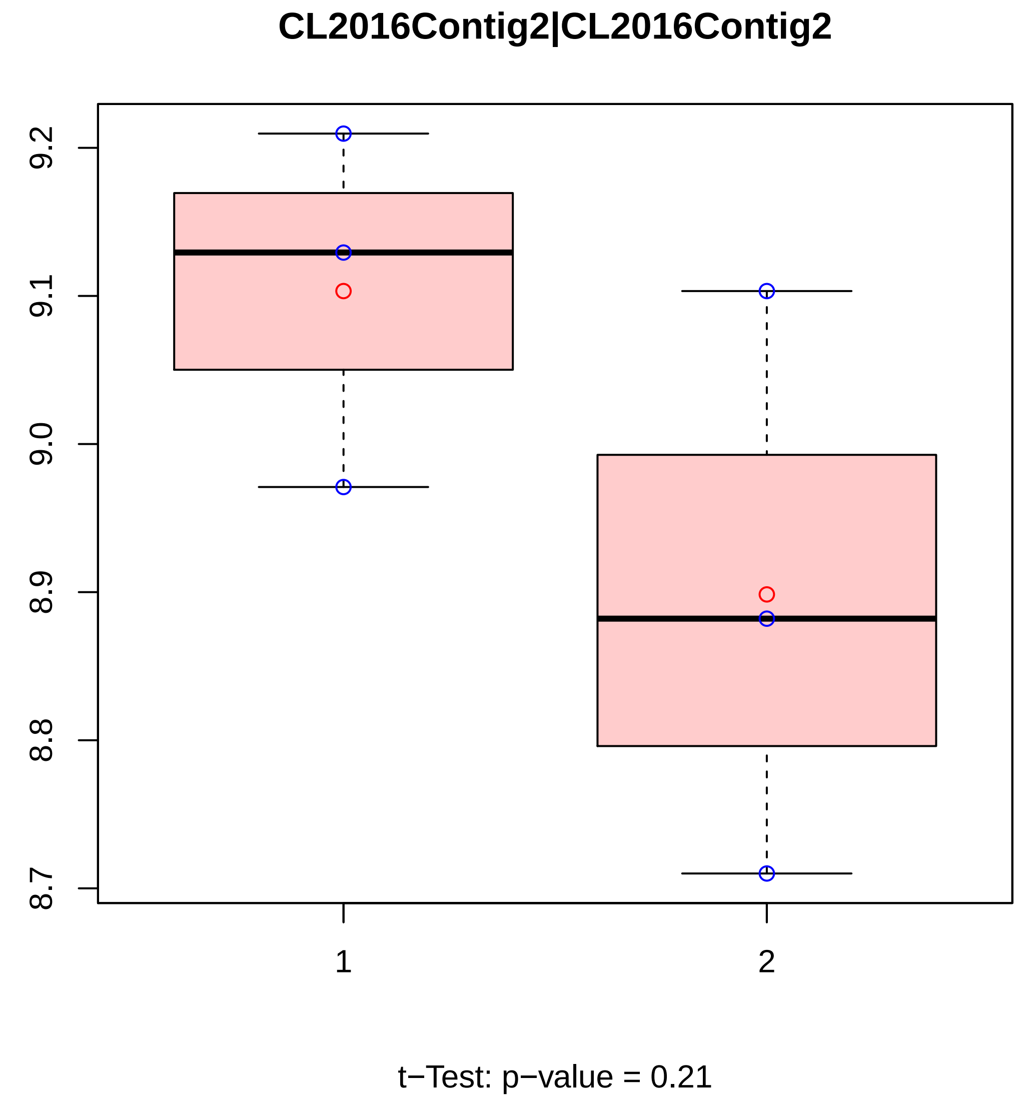
CL2016Contig2|CL2016Contig2
9.2
9.1
9.0
8.9
8.8
8.7
1
2
t−Test: p−value = 0.21

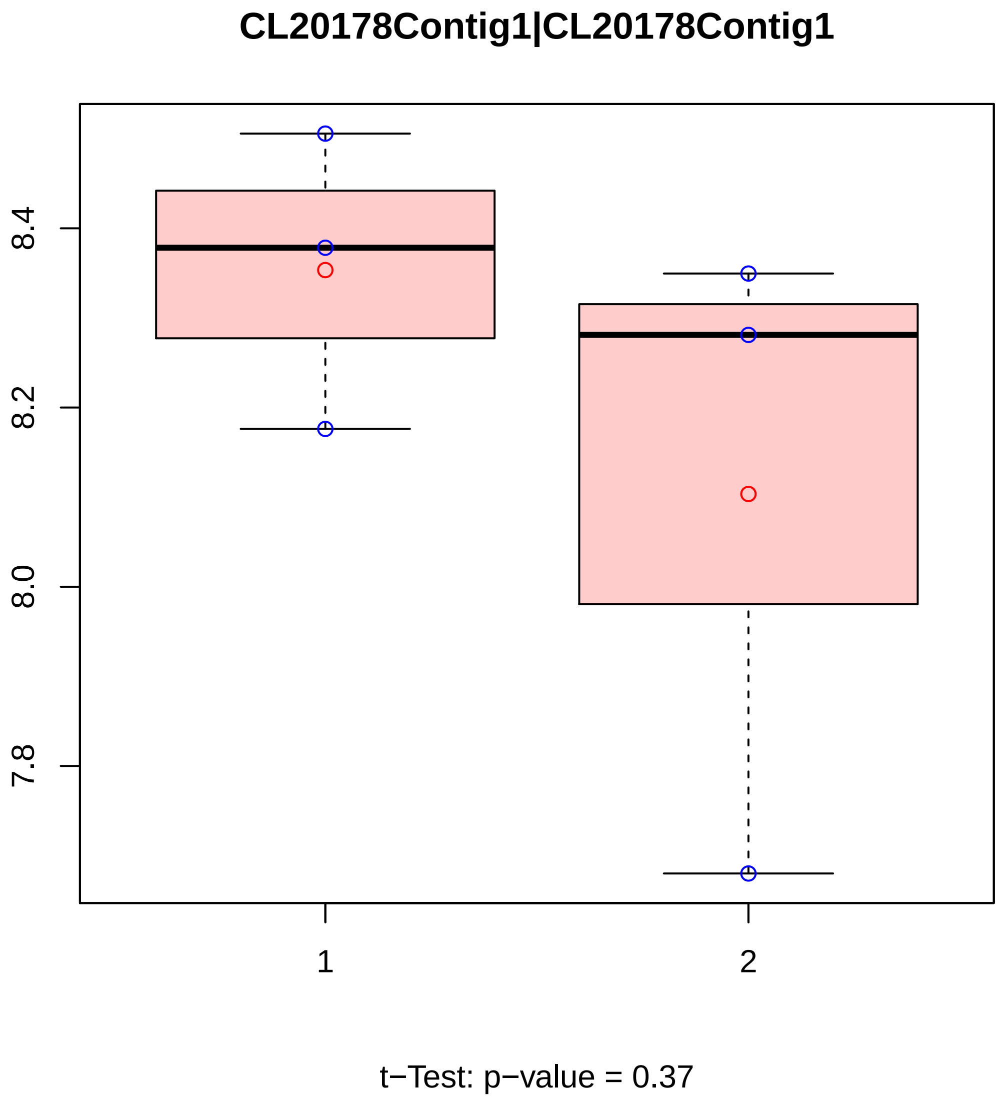
CL20178Contig1|CL20178Contig1
8.4
8.2
8.0
7.8
1
2
t−Test: p−value = 0.37

# CL2017Contig1|CL2017Contig1

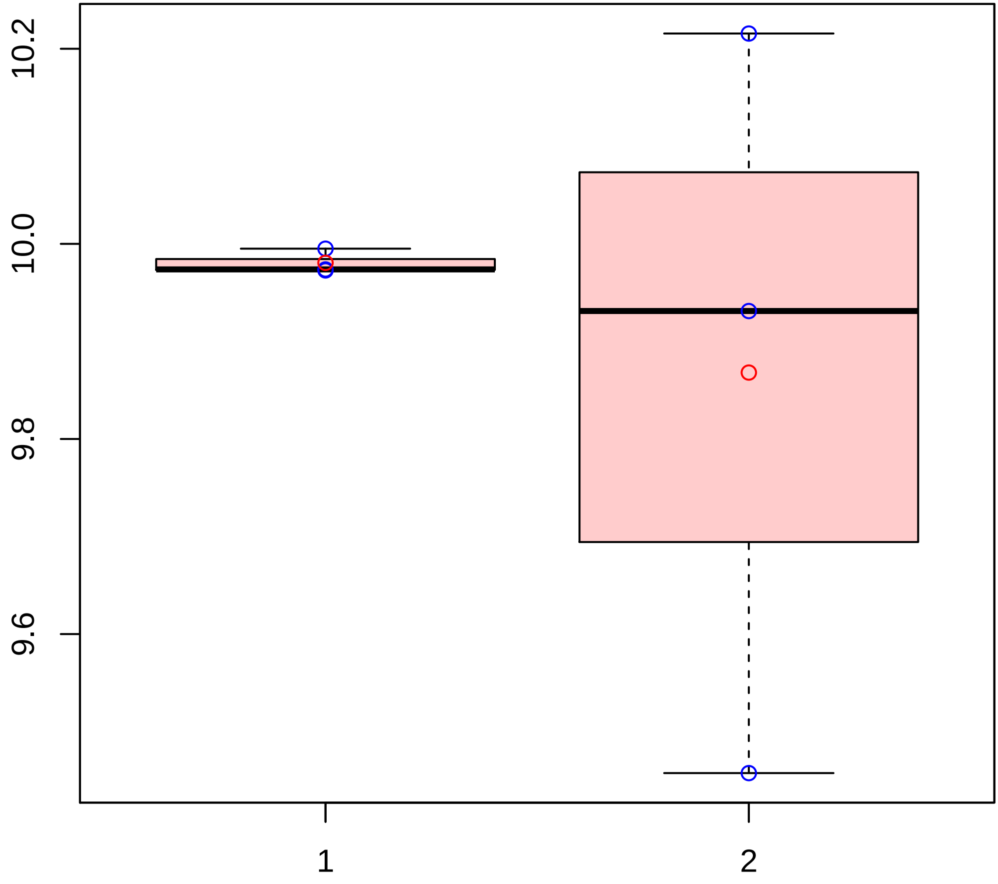


t−Test: p−value = 0.66

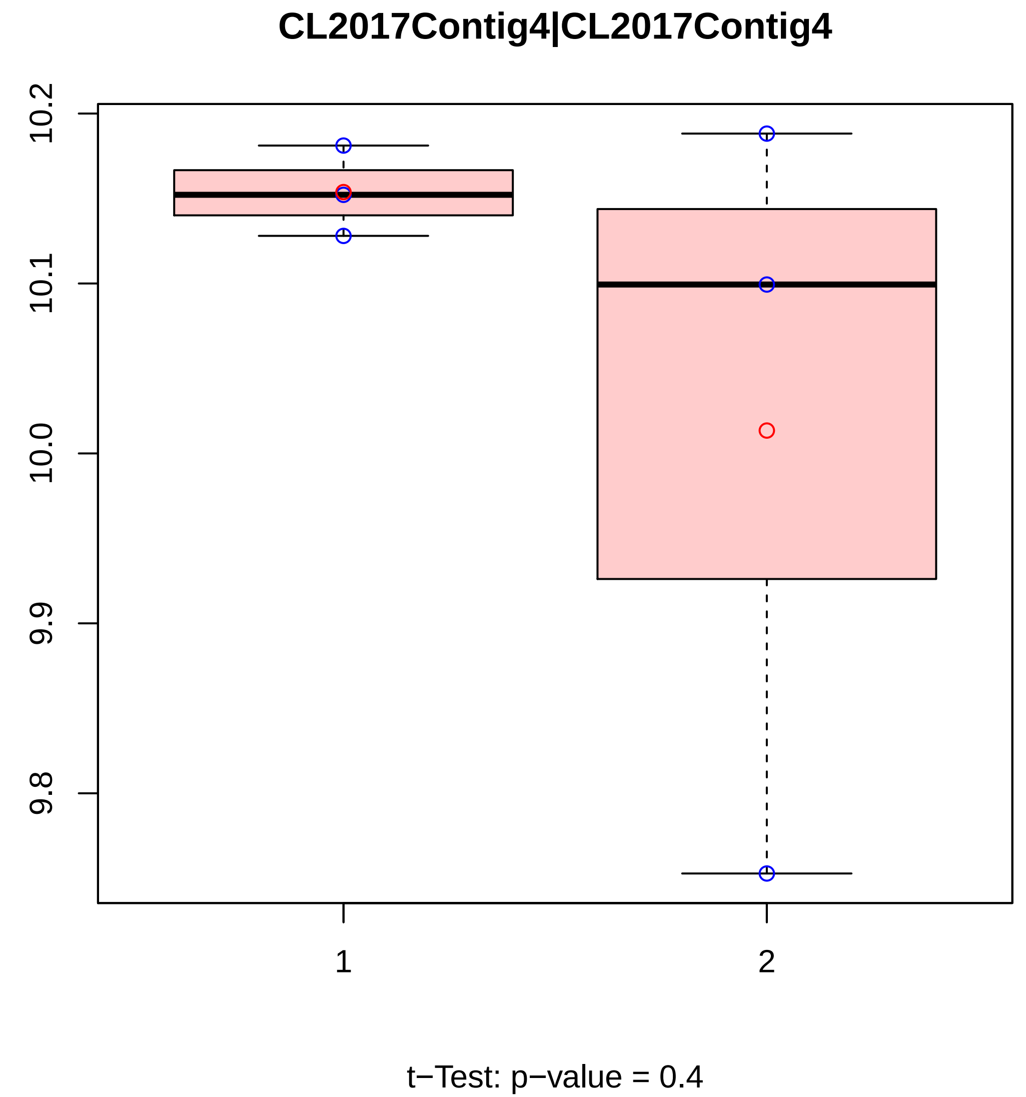

CL2017Contig4|CL2017Contig4
10.2
10.1
10.0
9.9
9.8
1
2
t−Test: p−value = 0.4

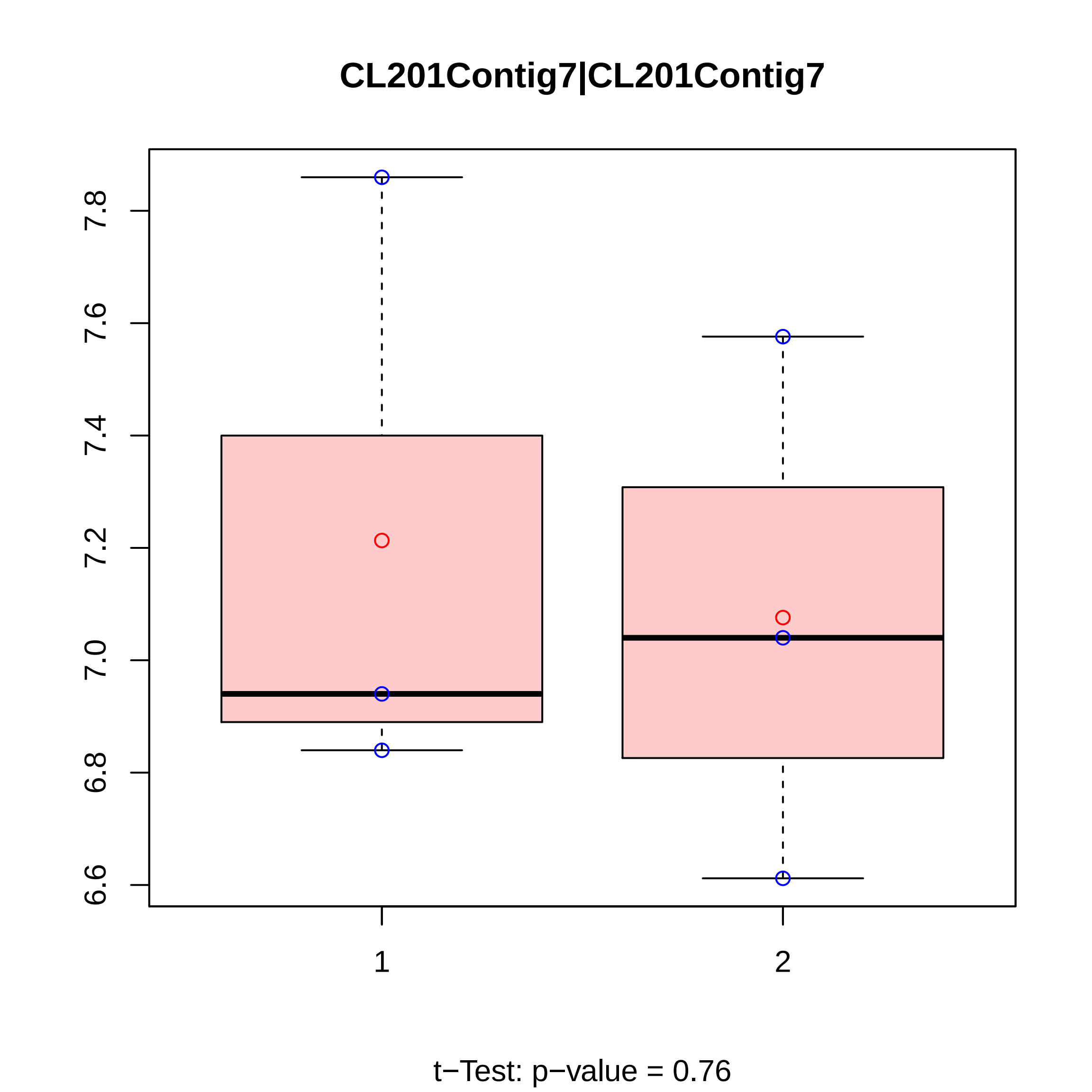

CL201Contig7|CL201Contig7
7.8
7.6
7.4
7.2
7.0
6.8
6.6
1
2
t−Test: p−value = 0.76

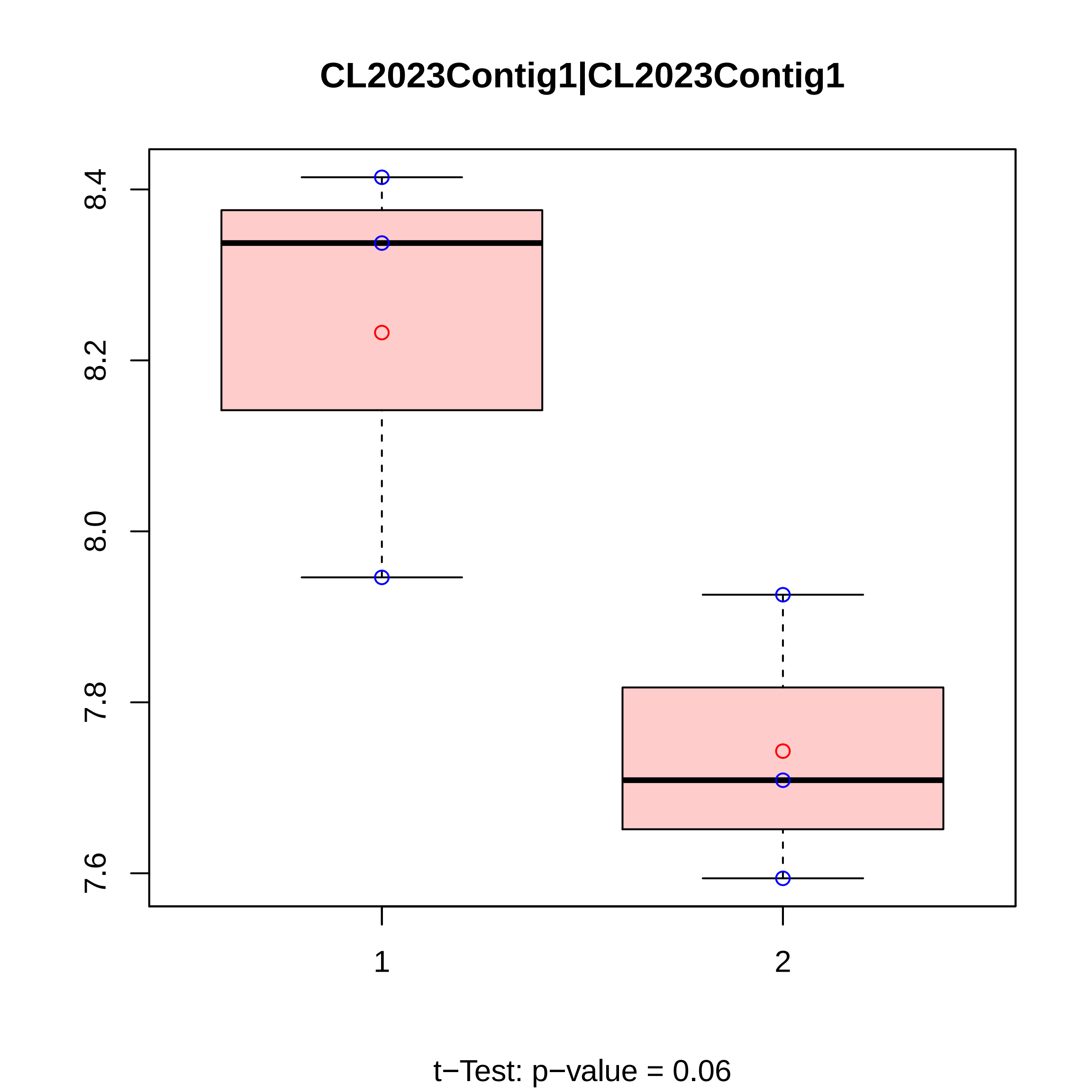
CL2023Contig1|CL2023Contig1
8.4
8.2
8.0
7.8
7.6
1
2
t−Test: p−value = 0.06

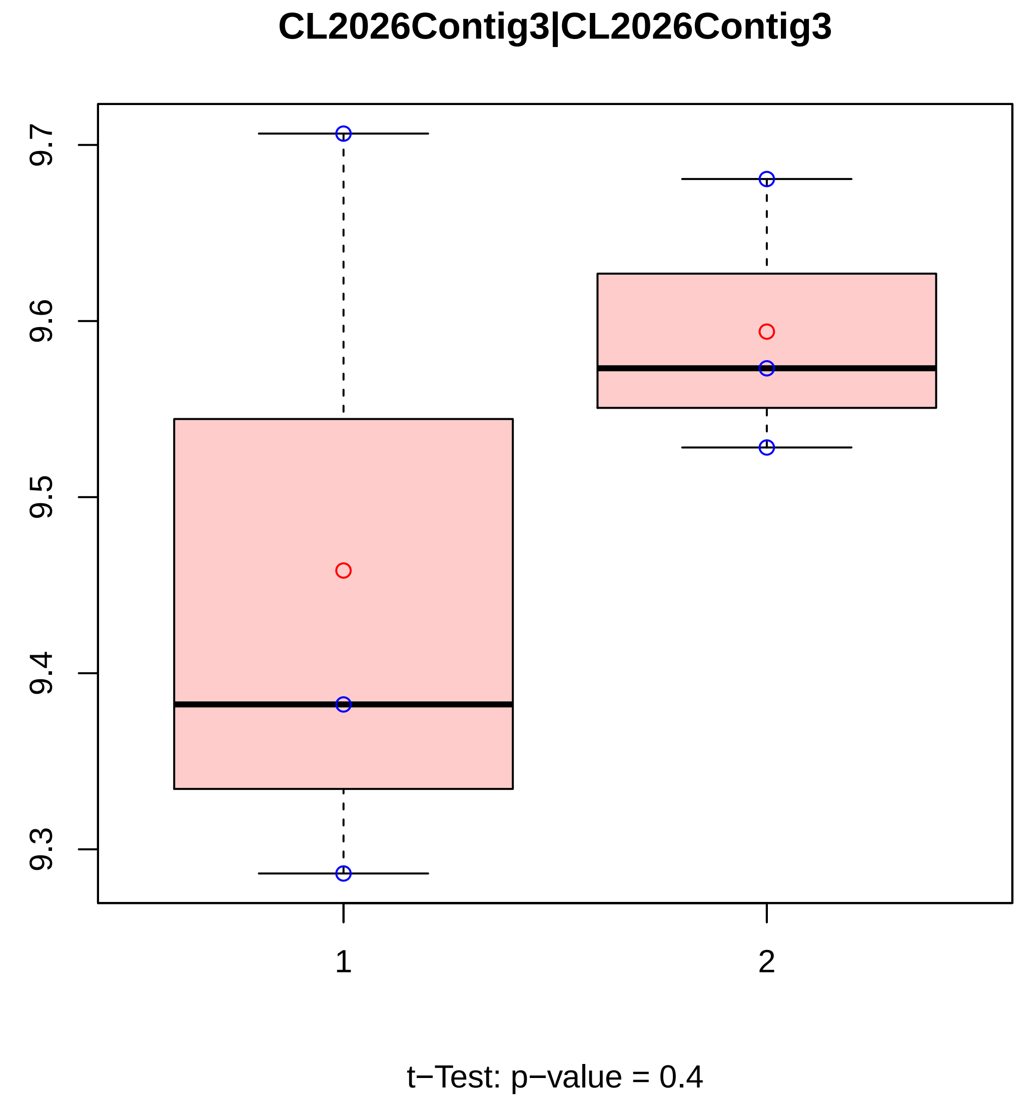
CL2026Contig3|CL2026Contig3
9.7
9.6
9.5
9.4
9.3
1
2
t−Test: p−value = 0.4

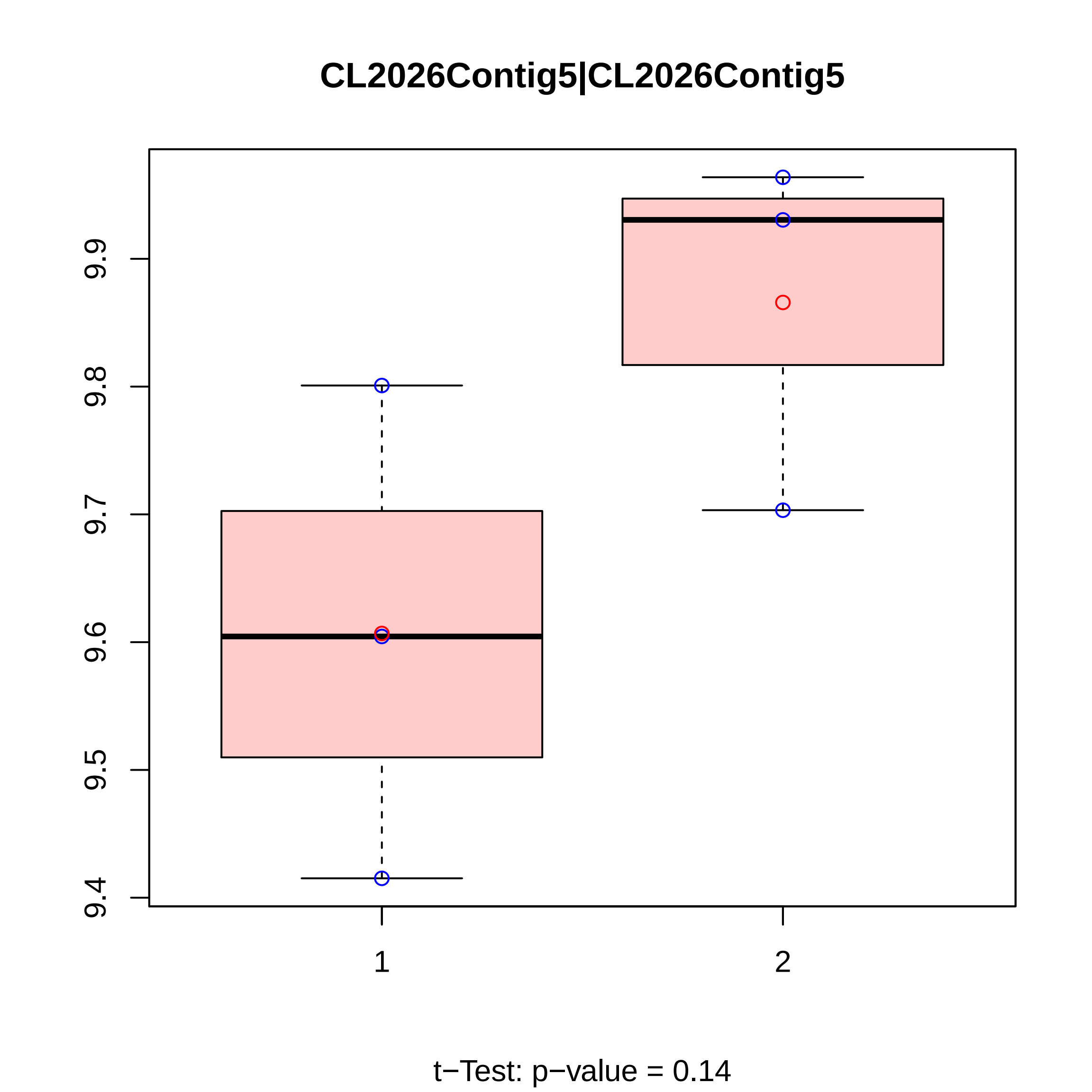
CL2026Contig5|CL2026Contig5
9.4
9.5
9.6
9.7
9.8
9.9
1
2
t−Test: p−value = 0.14

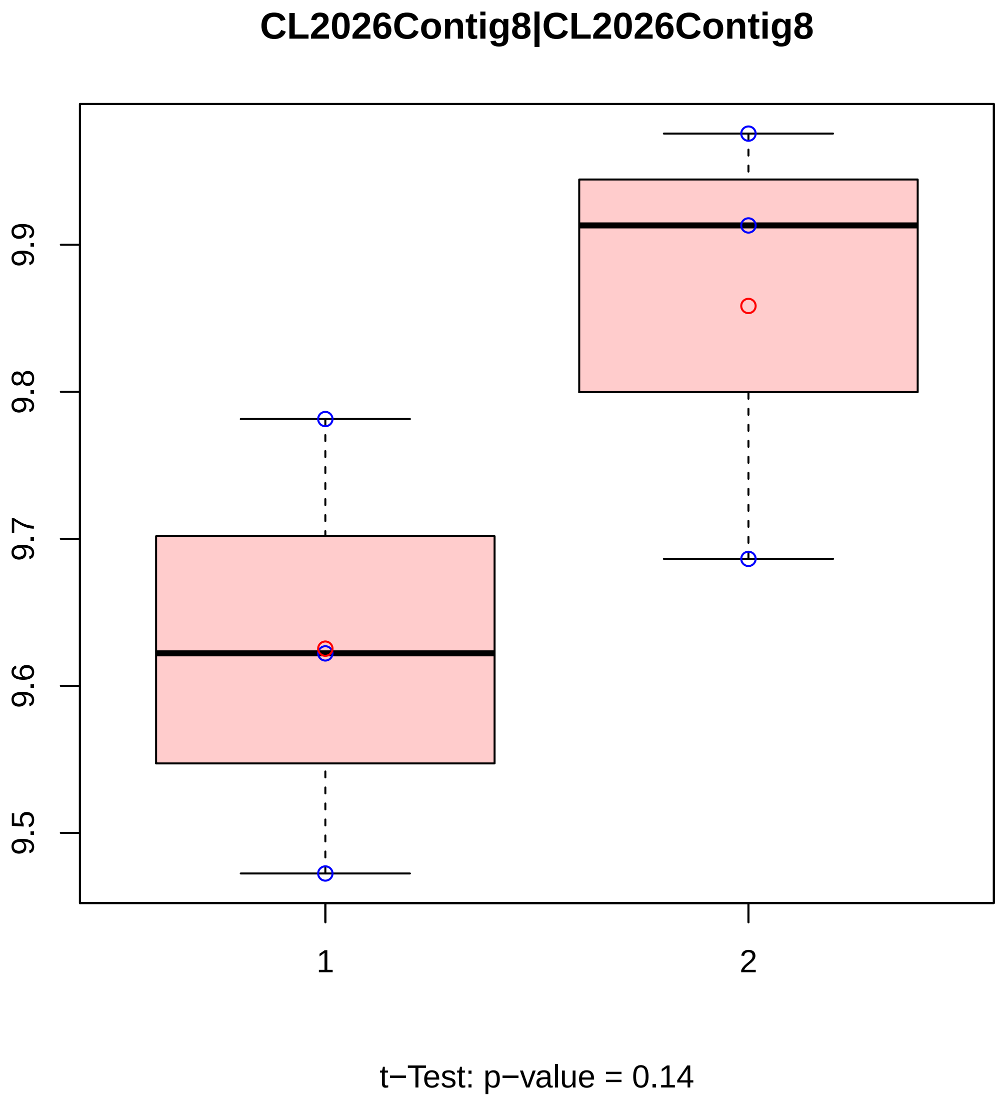

CL2026Contig8|CL2026Contig8
9.5
9.6
9.7
9.8
9.9
1
2
t−Test: p−value = 0.14

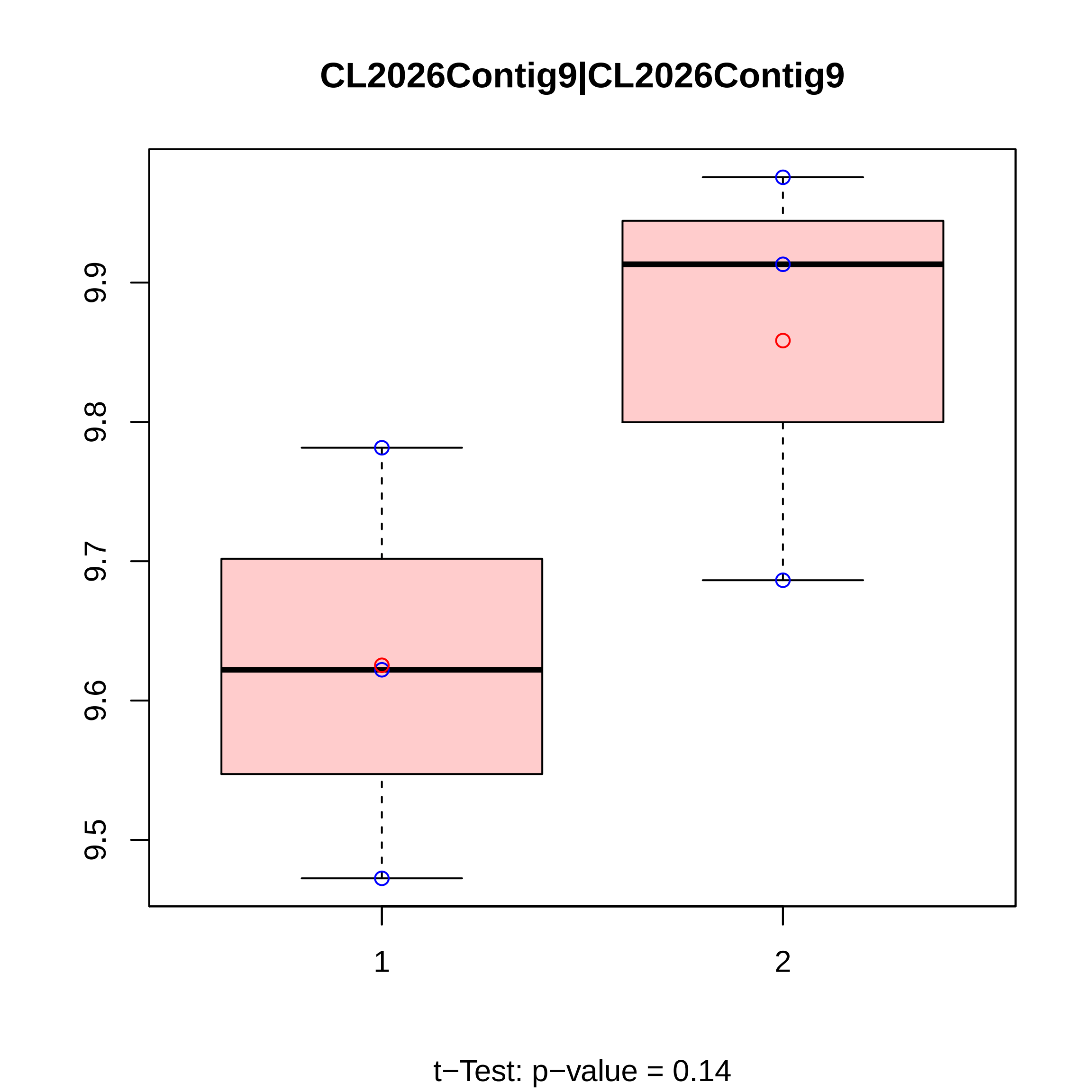
CL2026Contig9|CL2026Contig9
9.5
9.6
9.7
9.8
9.9
1
2
t−Test: p−value = 0.14

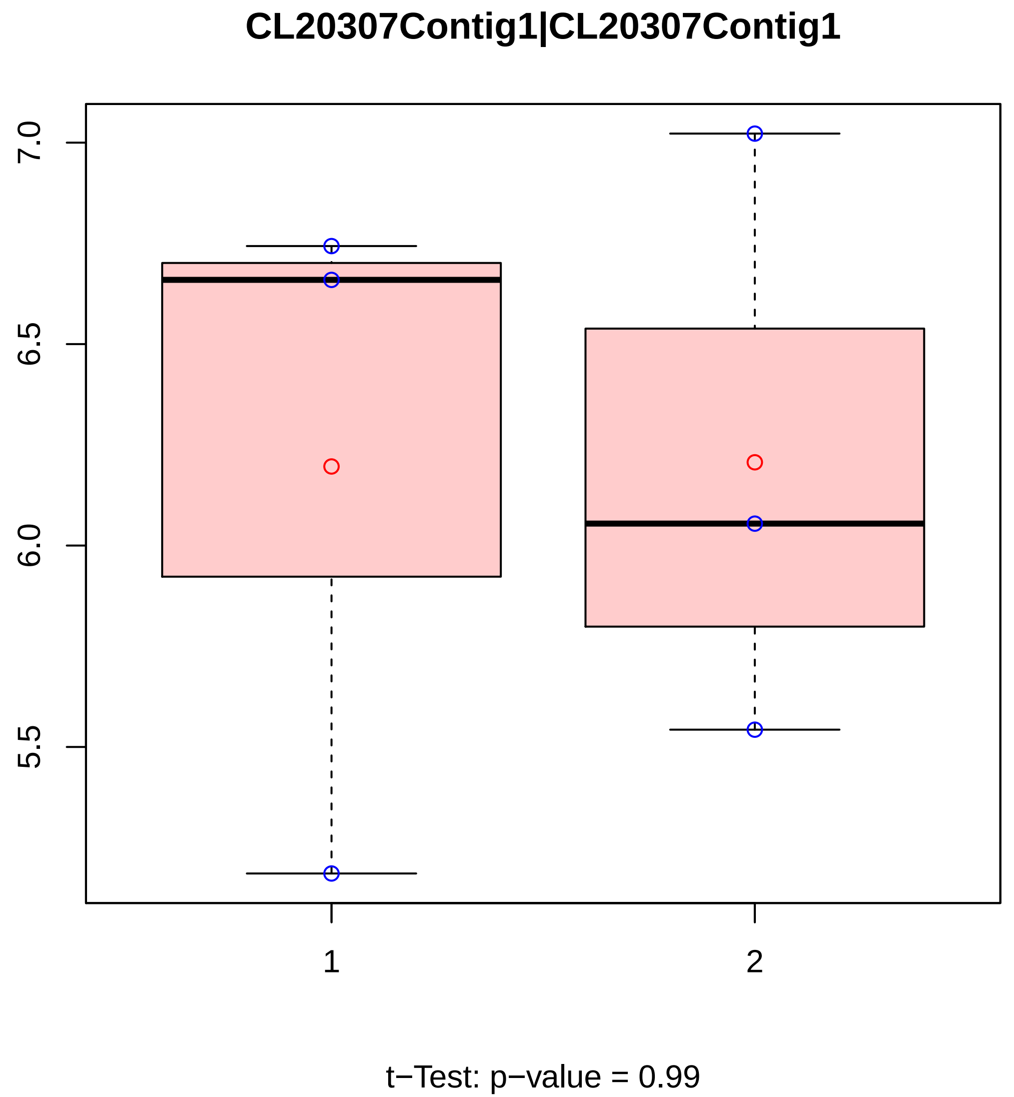

CL20307Contig1|CL20307Contig1
7.0
6.5
6.0
5.5
1
2
t−Test: p−value = 0.99

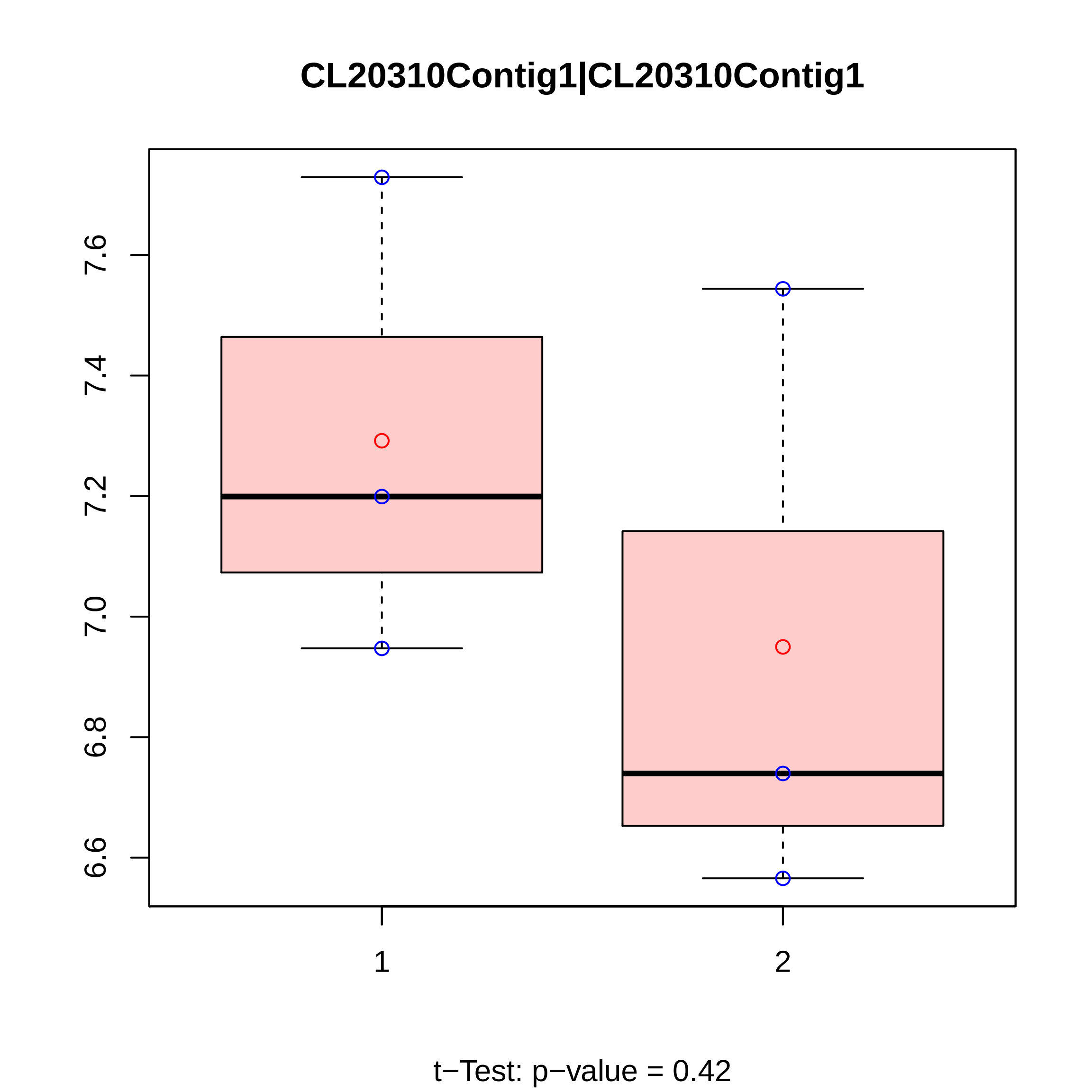
CL20310Contig1|CL20310Contig1
7.6
7.4
7.2
7.0
6.8
6.6
1
2
t−Test: p−value = 0.42

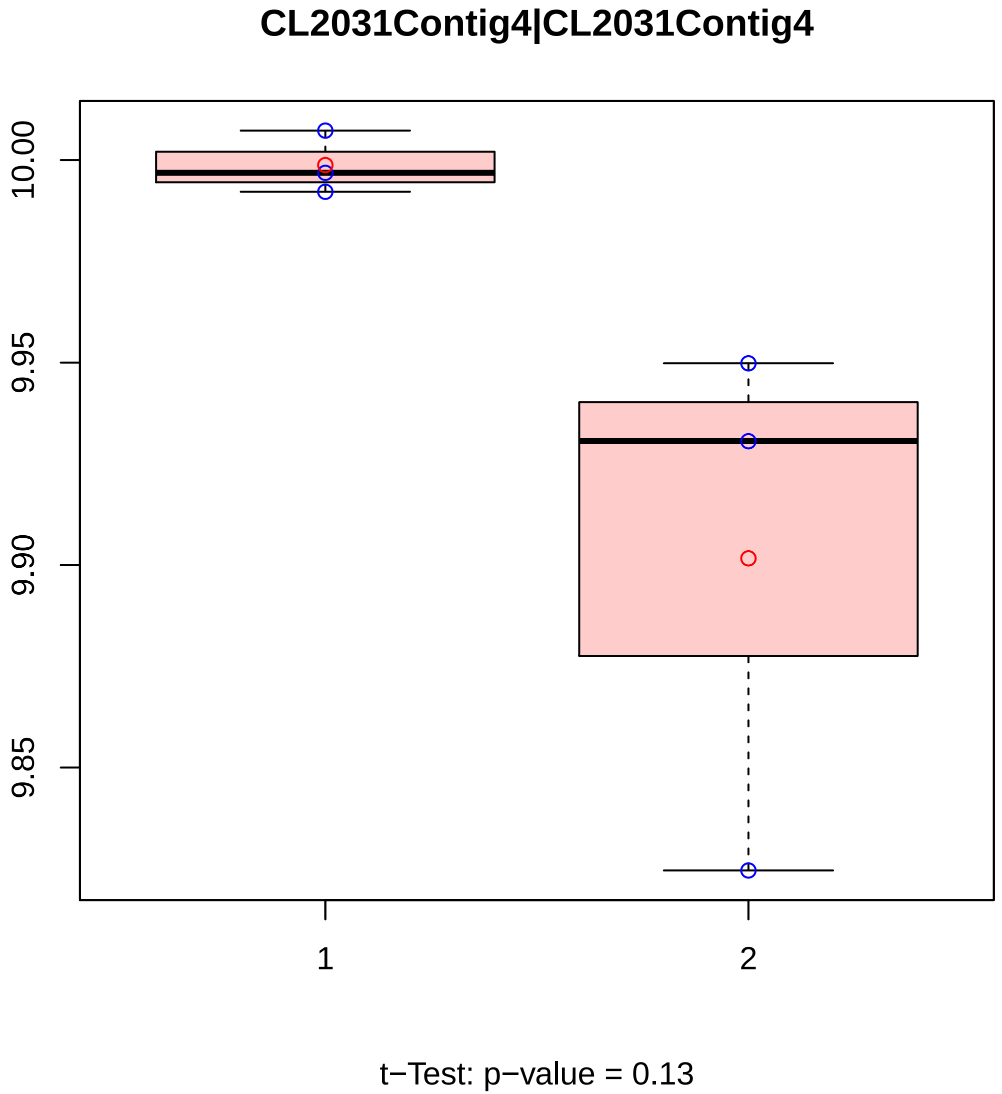

CL2031Contig4|CL2031Contig4
10.00
9.95
9.90
9.85
1
2
t−Test: p−value = 0.13

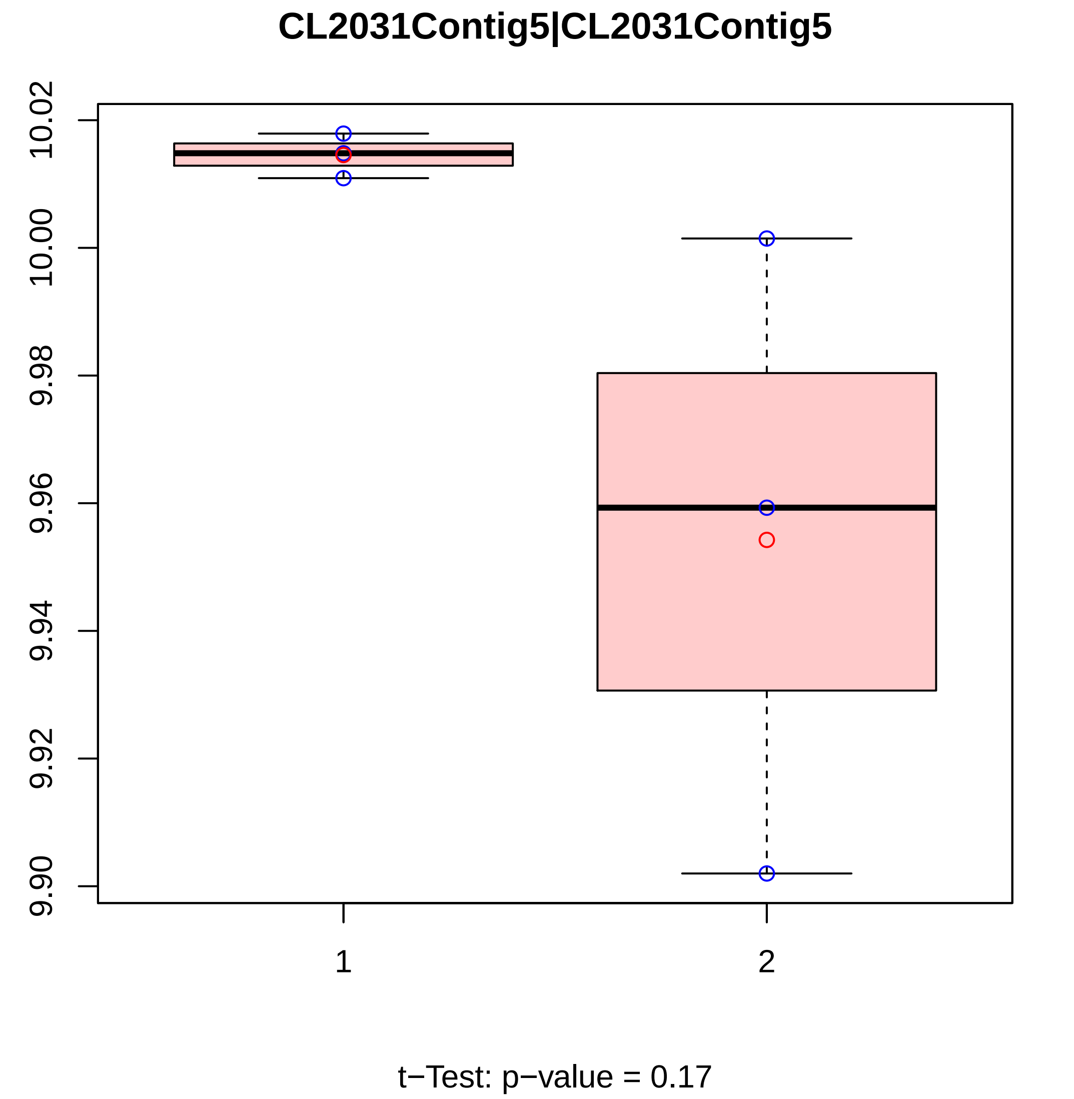
CL2031Contig5|CL2031Contig5
10.02
10.00
9.98
9.96
9.94
9.92
9.90
1
2
t−Test: p−value = 0.17

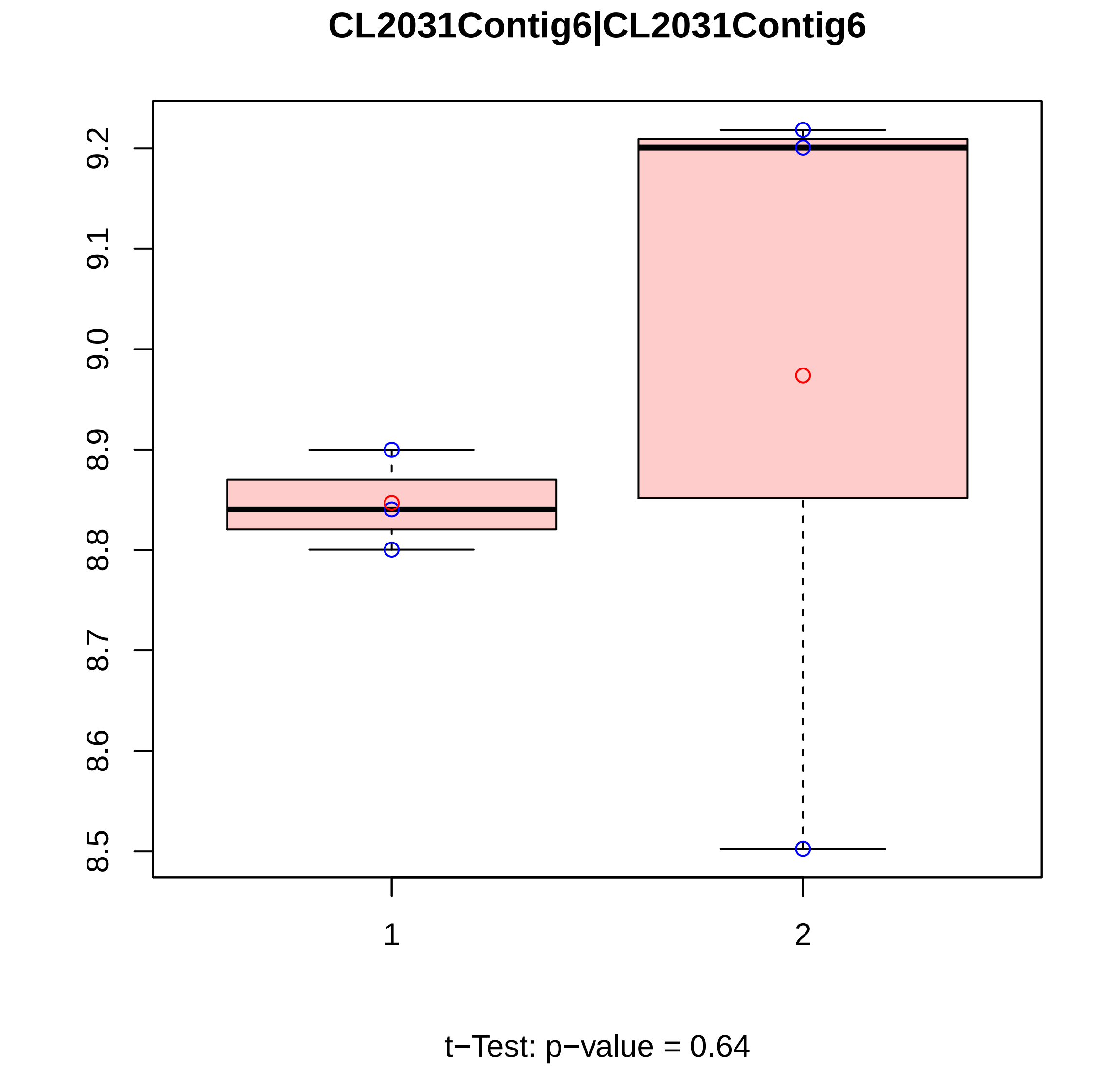
CL2031Contig6|CL2031Contig6
9.2
9.1
9.0
8.9
8.8
8.7
8.6
8.5
1
2
t−Test: p−value = 0.64

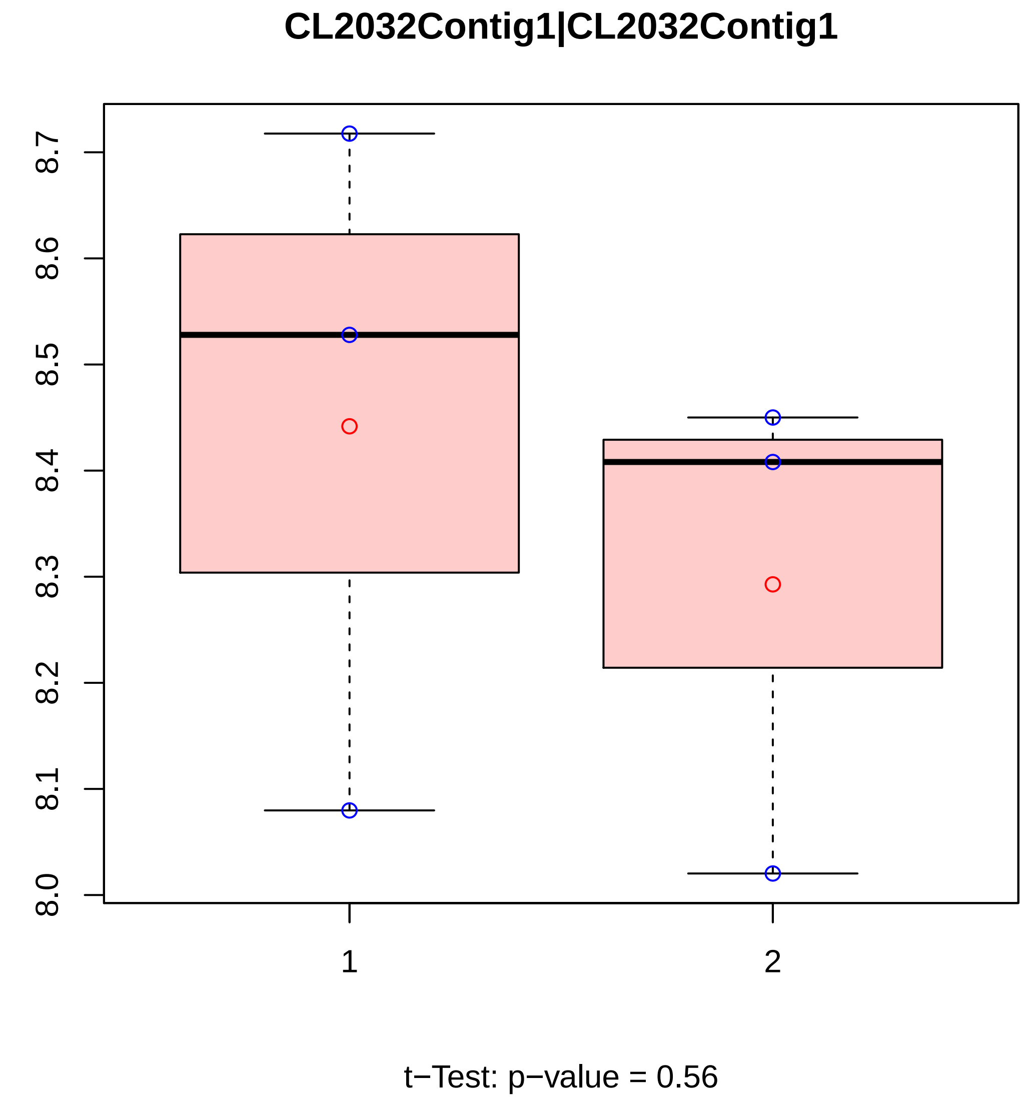

CL2032Contig1|CL2032Contig1
8.0
8.1
8.2
8.3
8.4
8.5
8.6
8.7
1
2
t−Test: p−value = 0.56

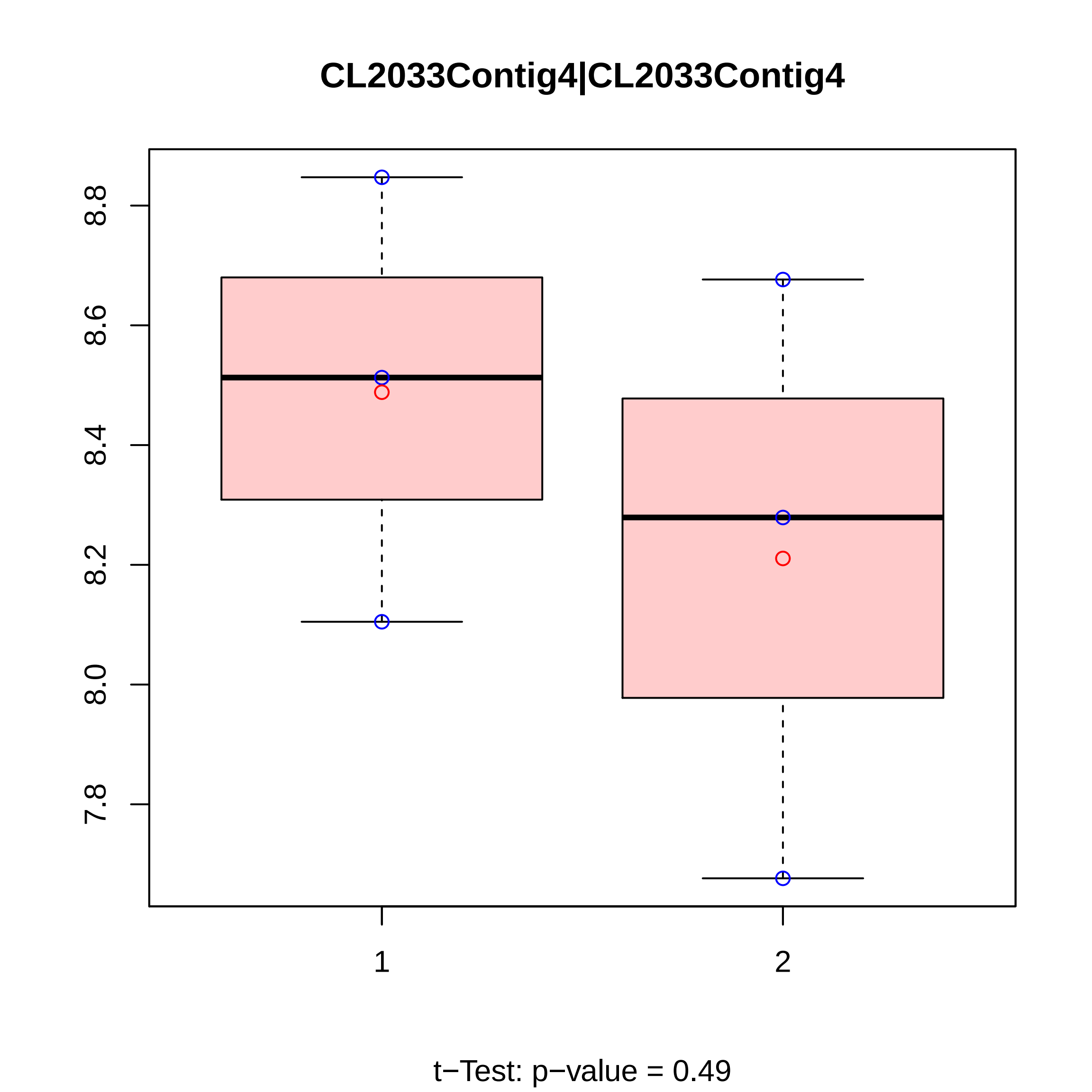

CL2033Contig4|CL2033Contig4
8.8
8.6
8.4
8.2
8.0
7.8
1
2
t−Test: p−value = 0.49

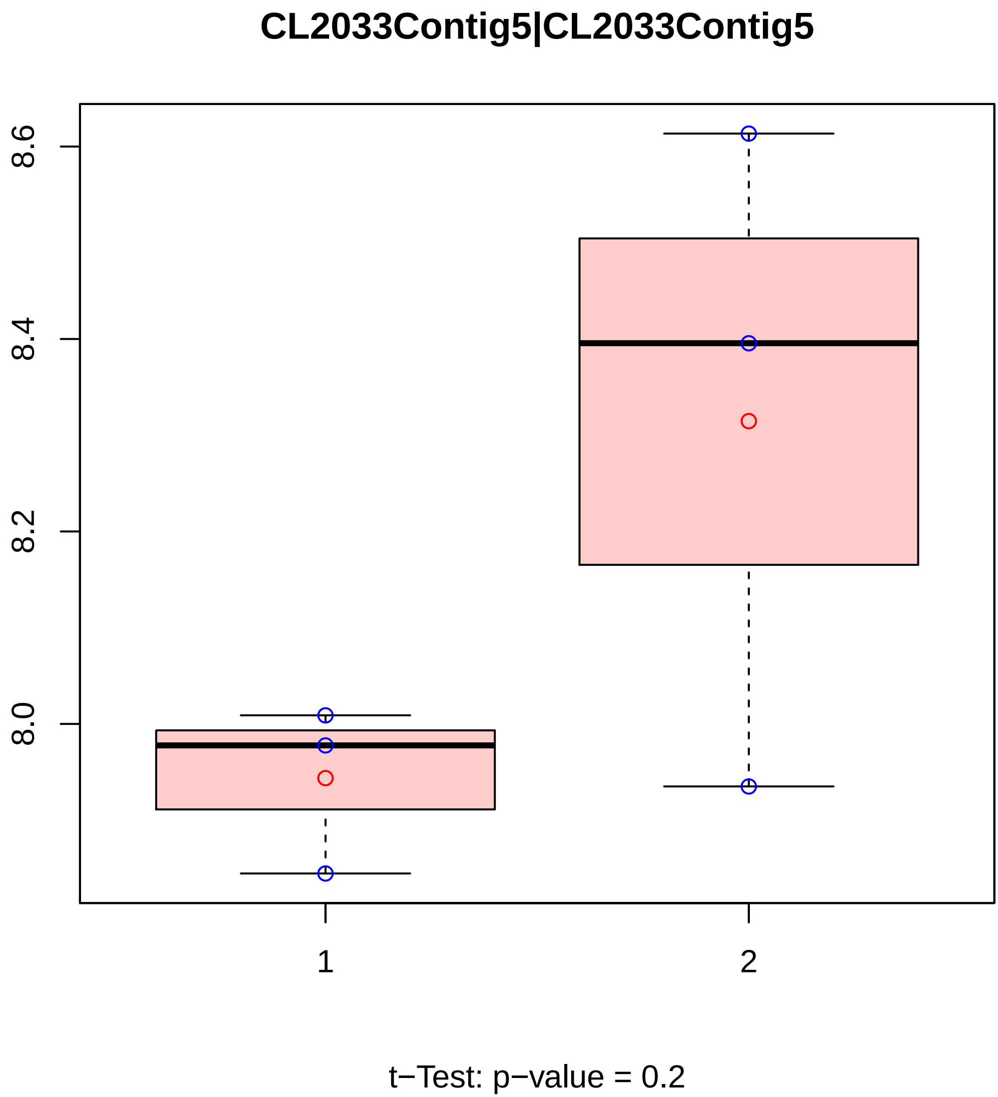

CL2033Contig5|CL2033Contig5
8.6
8.4
8.2
8.0
1
2
t−Test: p−value = 0.2

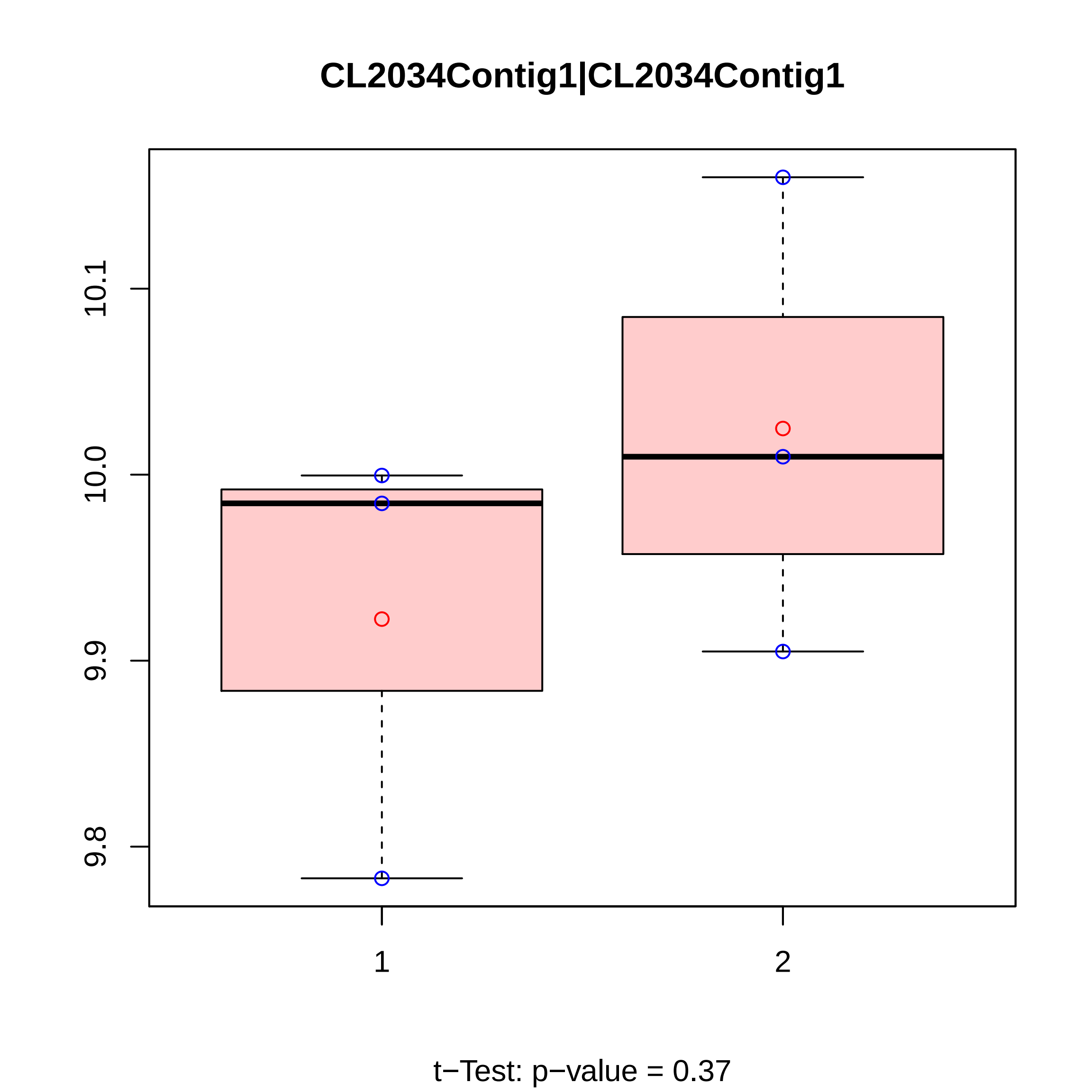
CL2034Contig1|CL2034Contig1
10.1
10.0
9.9
9.8
1
2
t−Test: p−value = 0.37

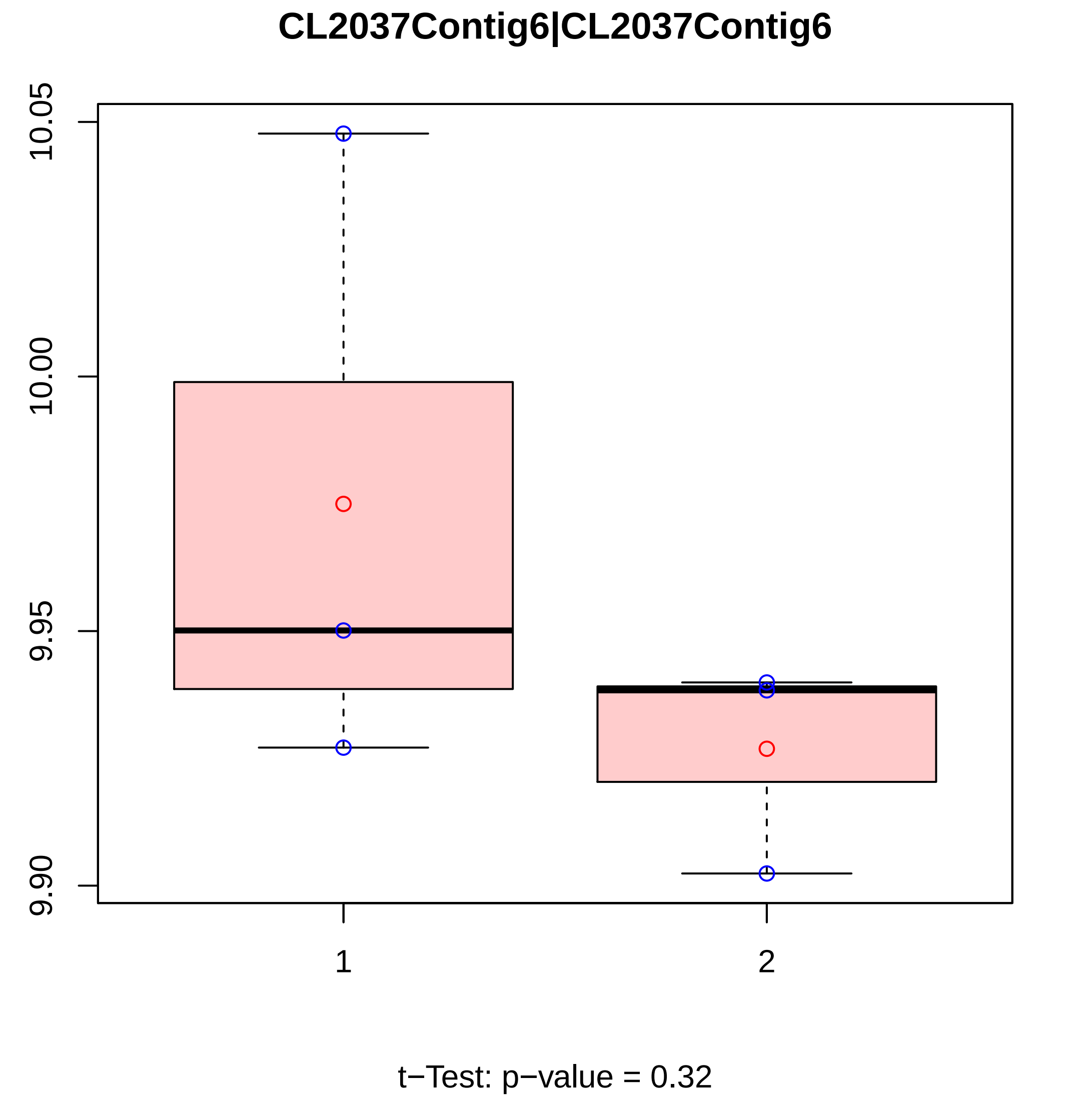
CL2037Contig6|CL2037Contig6
10.05
10.00
9.95
9.90
1
2
t−Test: p−value = 0.32

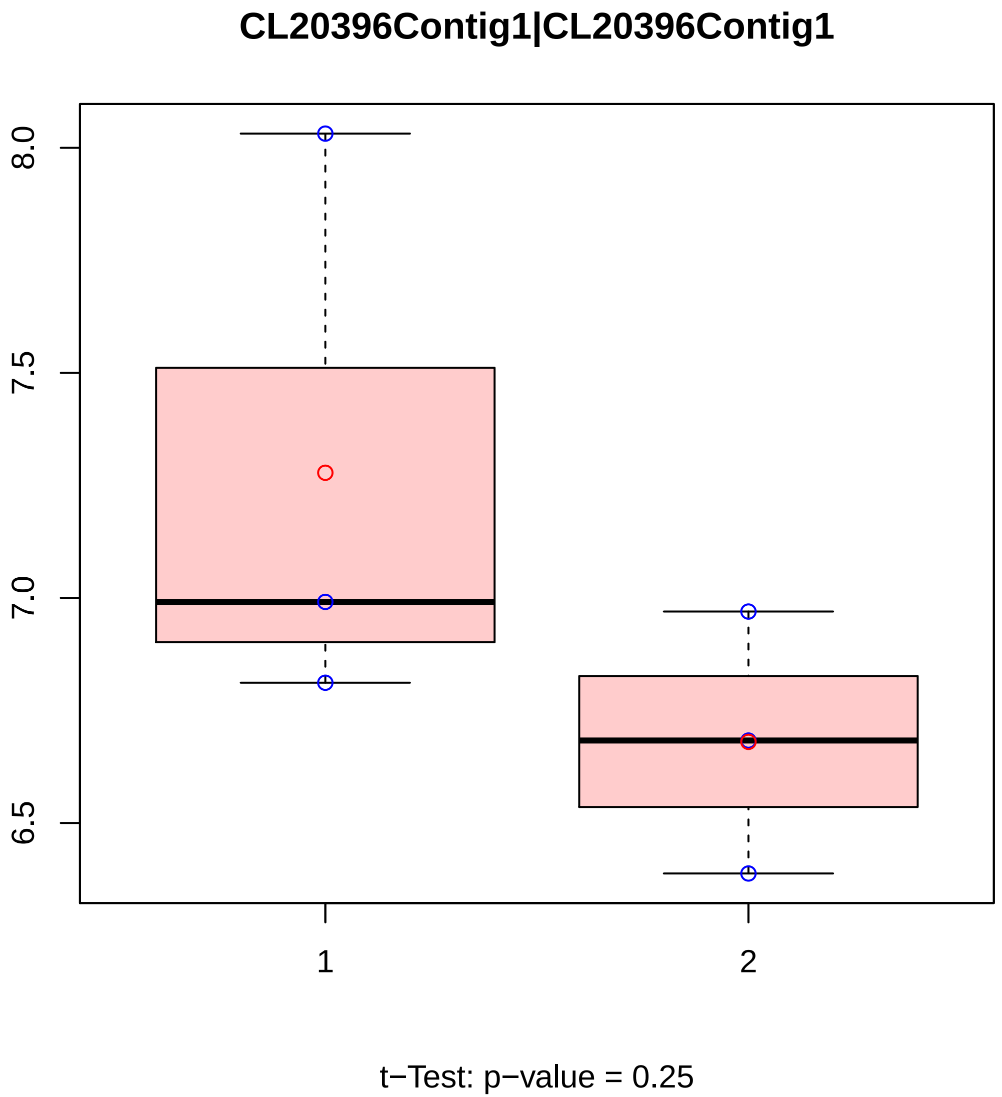
CL20396Contig1|CL20396Contig1
8.0
7.5
7.0
6.5
1
2
t−Test: p−value = 0.25

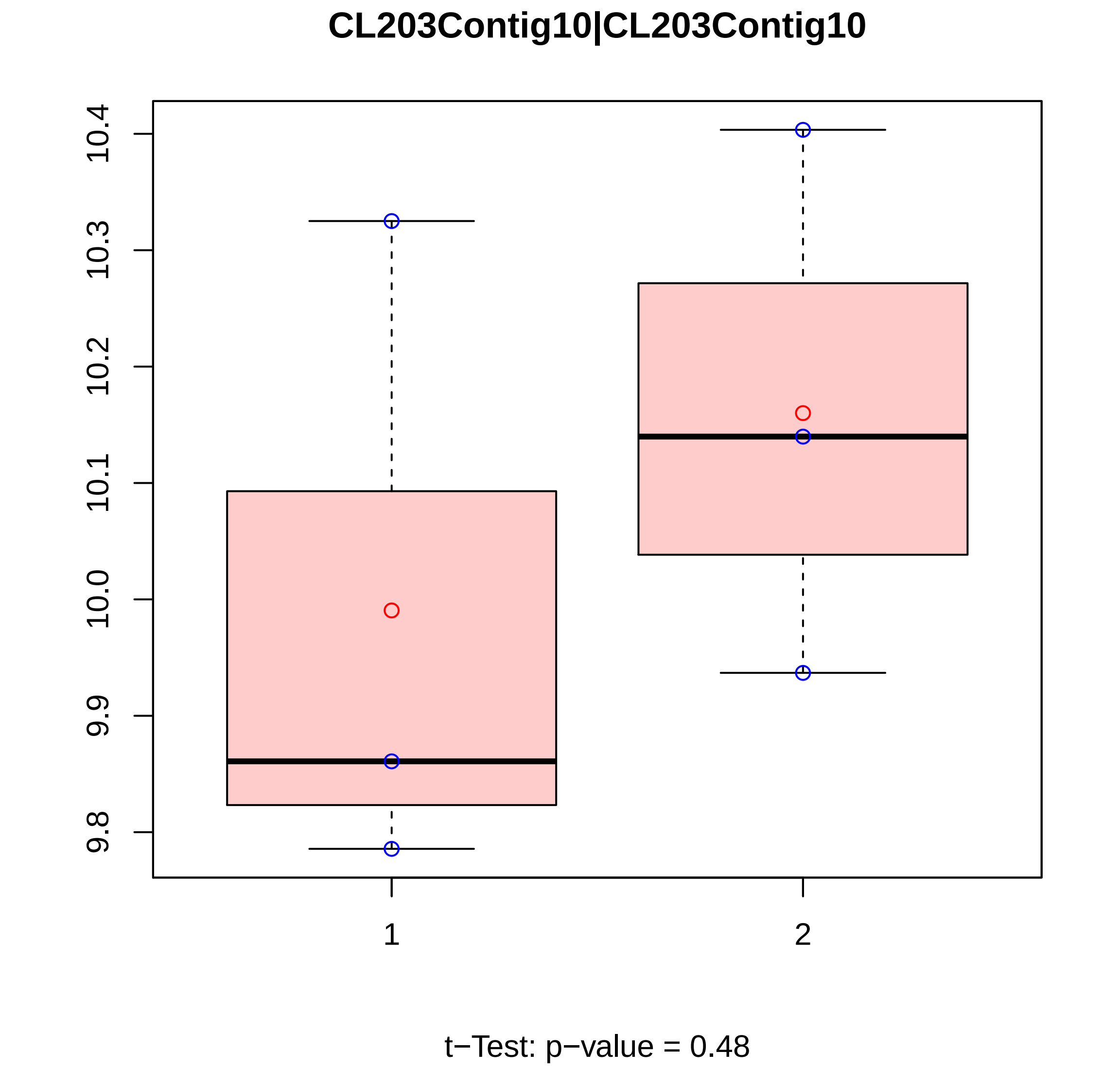

CL203Contig10|CL203Contig10
10.4
10.3
10.2
10.1
10.0
9.9
9.8
1
2
t−Test: p−value = 0.48

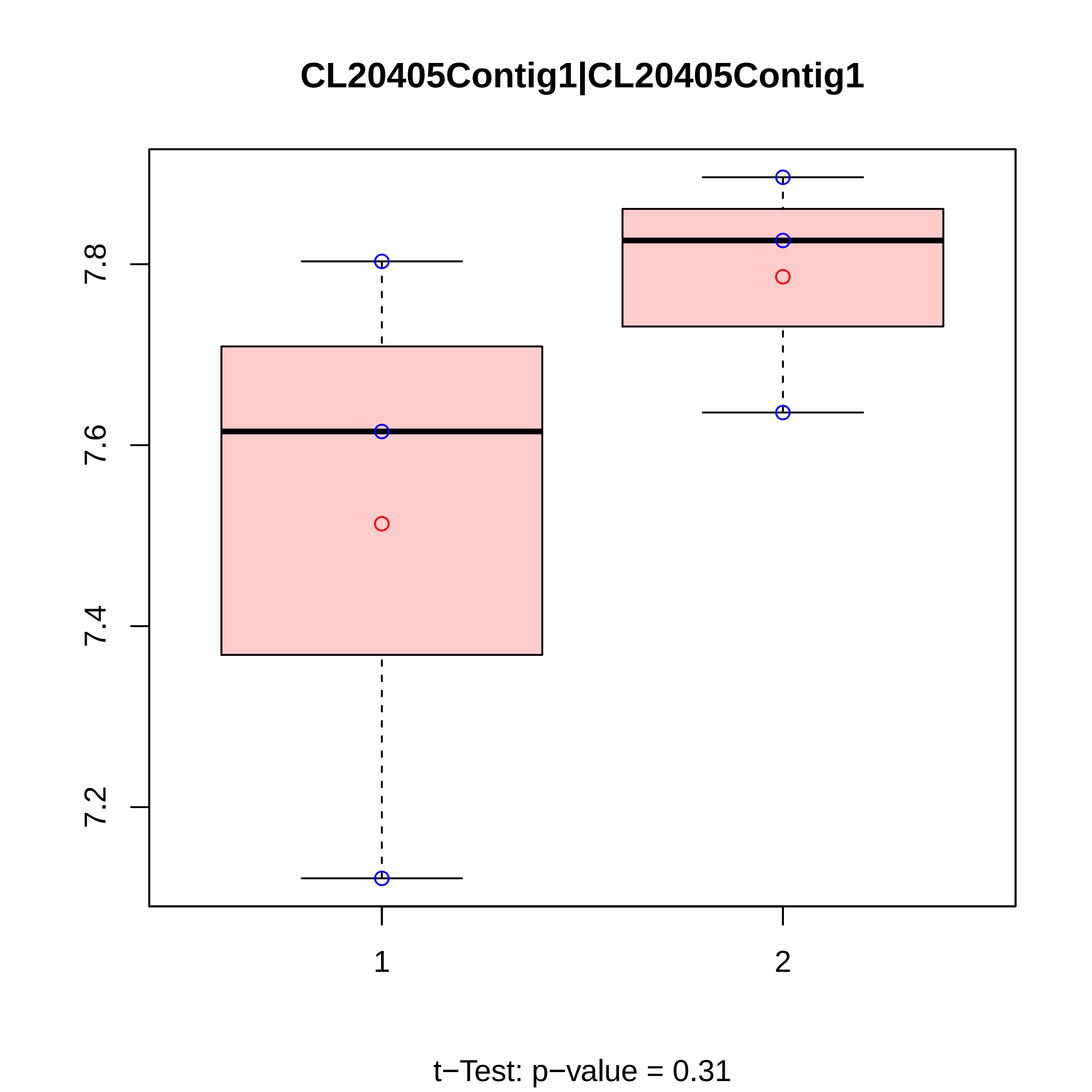

CL20405Contig1|CL20405Contig1
7.2
7.4
7.6
7.8
1
2
t−Test: p−value = 0.31

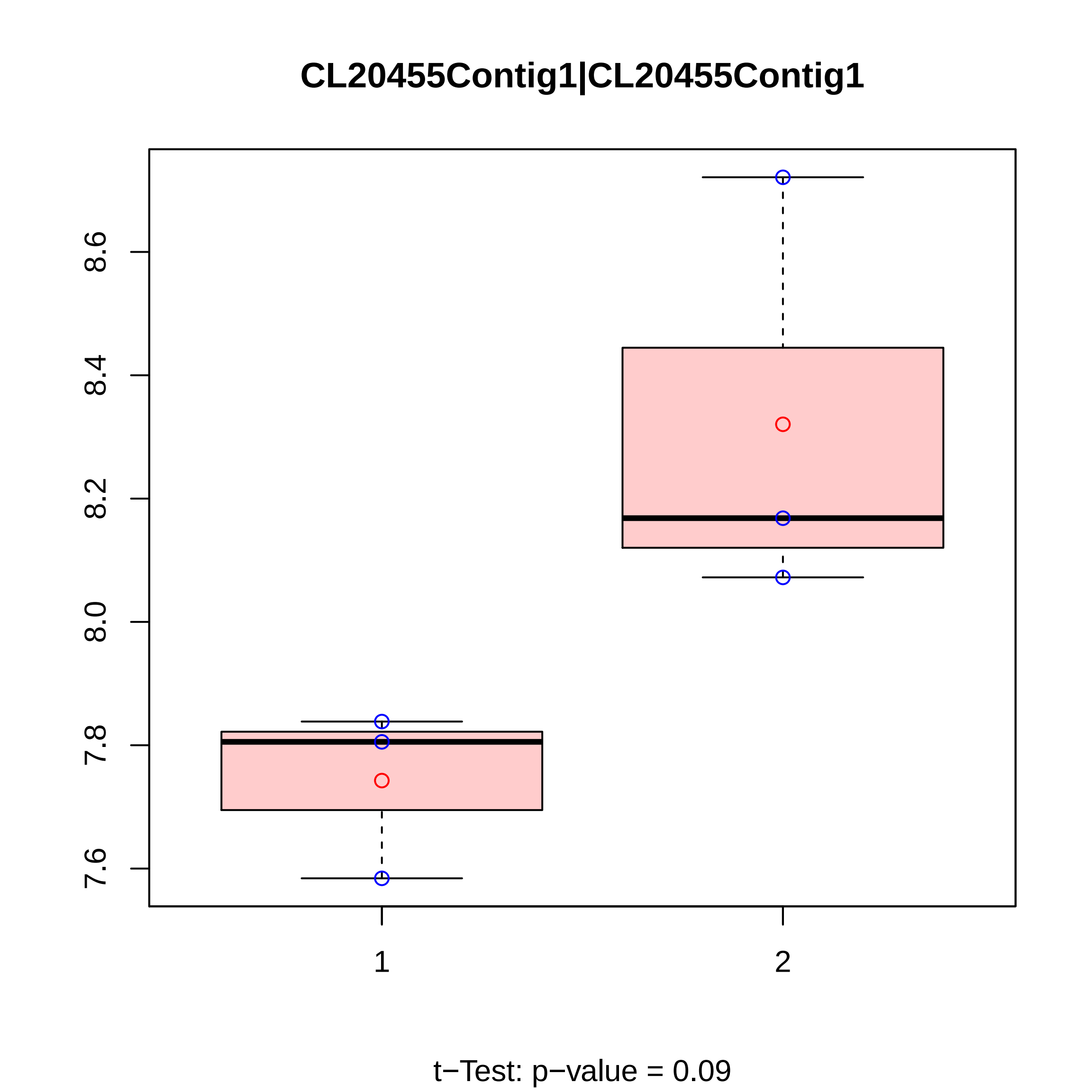

CL20455Contig1|CL20455Contig1
8.6
8.4
8.2
8.0
7.8
7.6
1
2
t−Test: p−value = 0.09

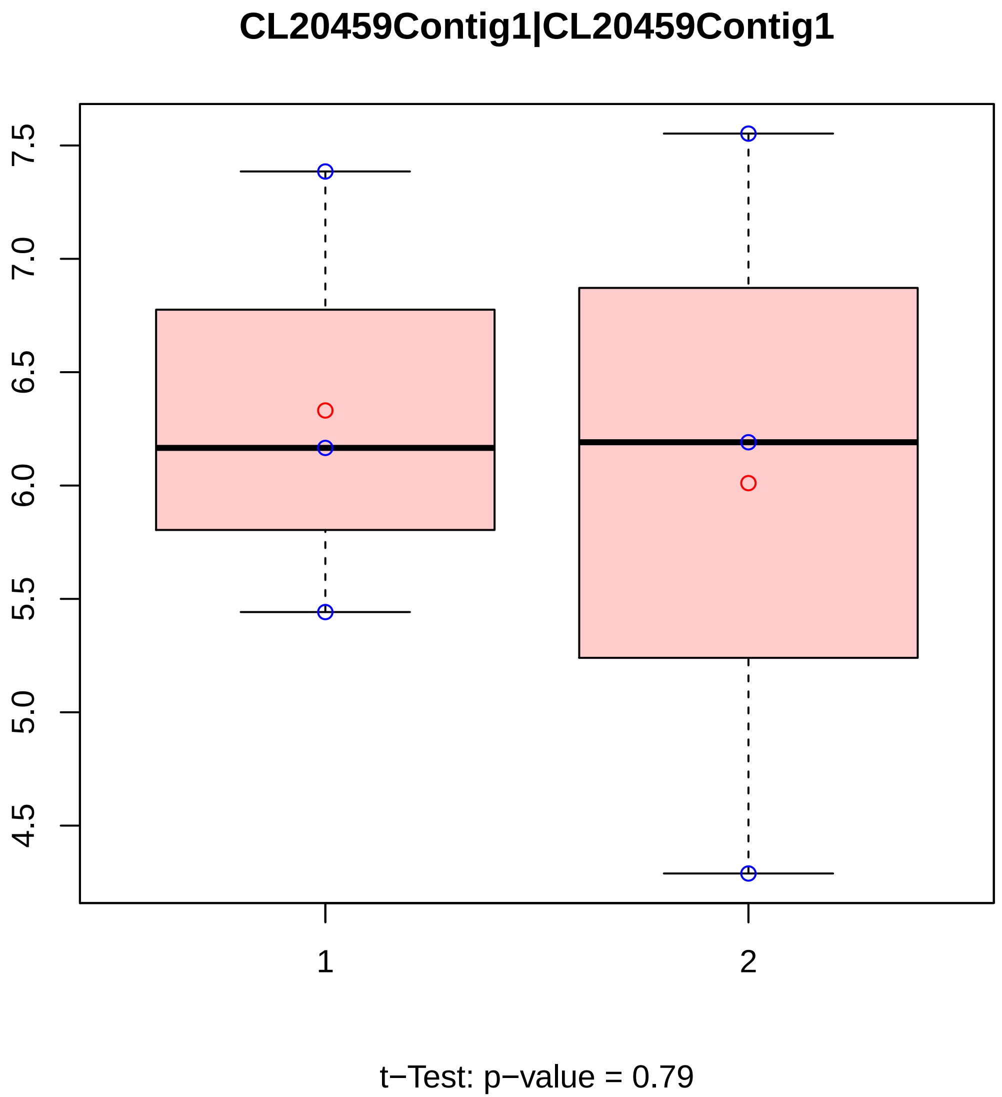
CL20459Contig1|CL20459Contig1
7.5
7.0
6.5
6.0
5.5
5.0
4.5
1
2
t−Test: p−value = 0.79

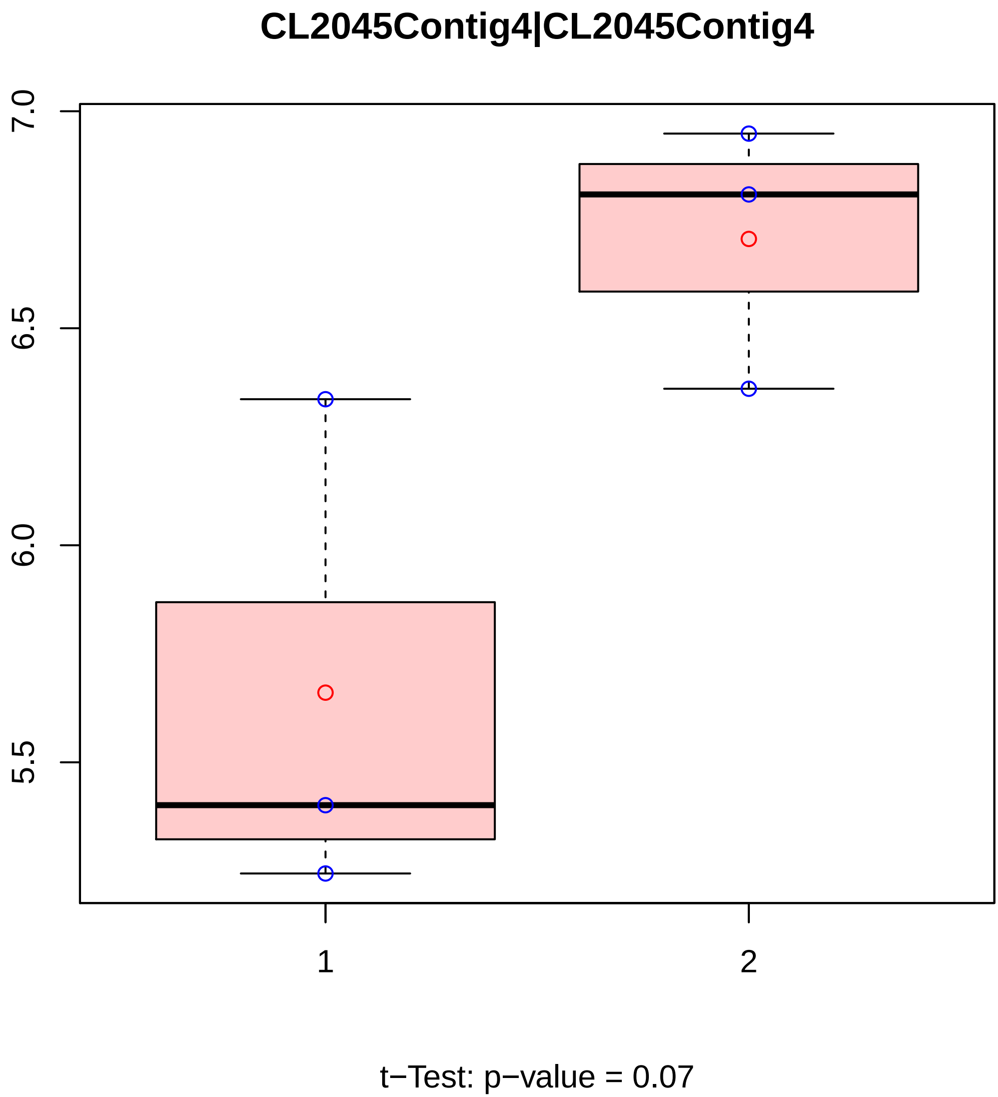
CL2045Contig4|CL2045Contig4
7.0
6.5
6.0
5.5
1
2
t−Test: p−value = 0.07

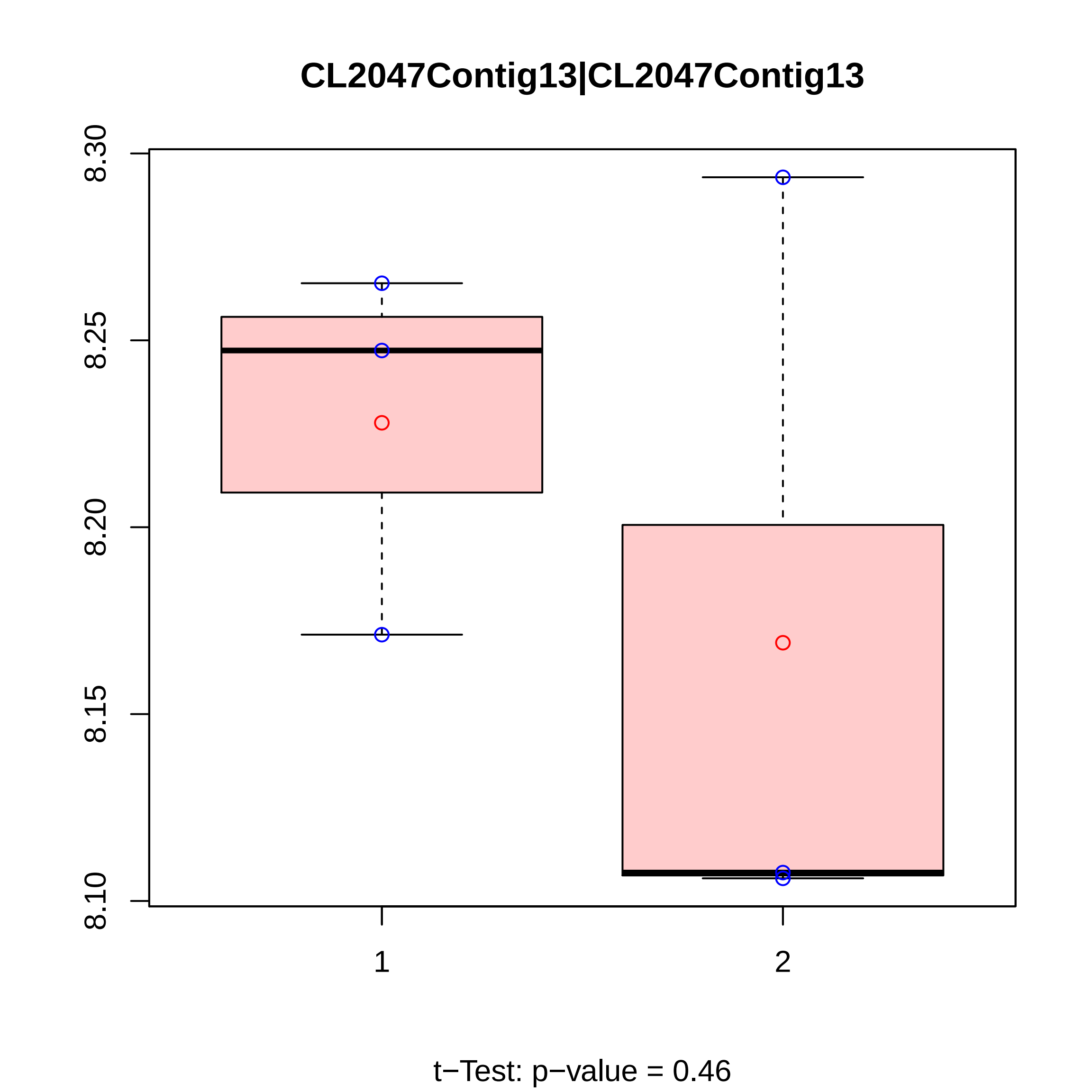

CL2047Contig13|CL2047Contig13
8.30
8.25
8.20
8.15
8.10
1
2
t−Test: p−value = 0.46

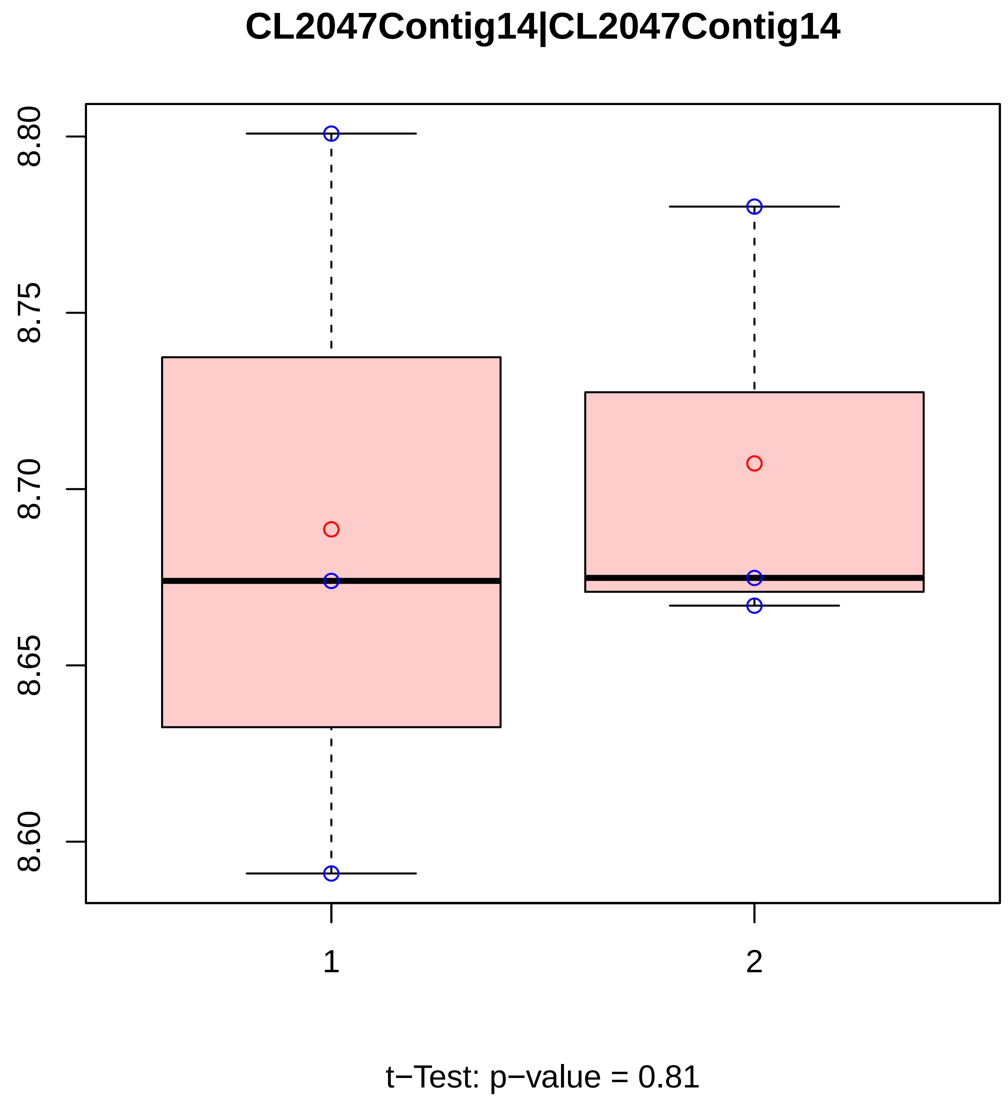

CL2047Contig14|CL2047Contig14
8.80
8.75
8.70
8.65
8.60
1
2
t−Test: p−value = 0.81

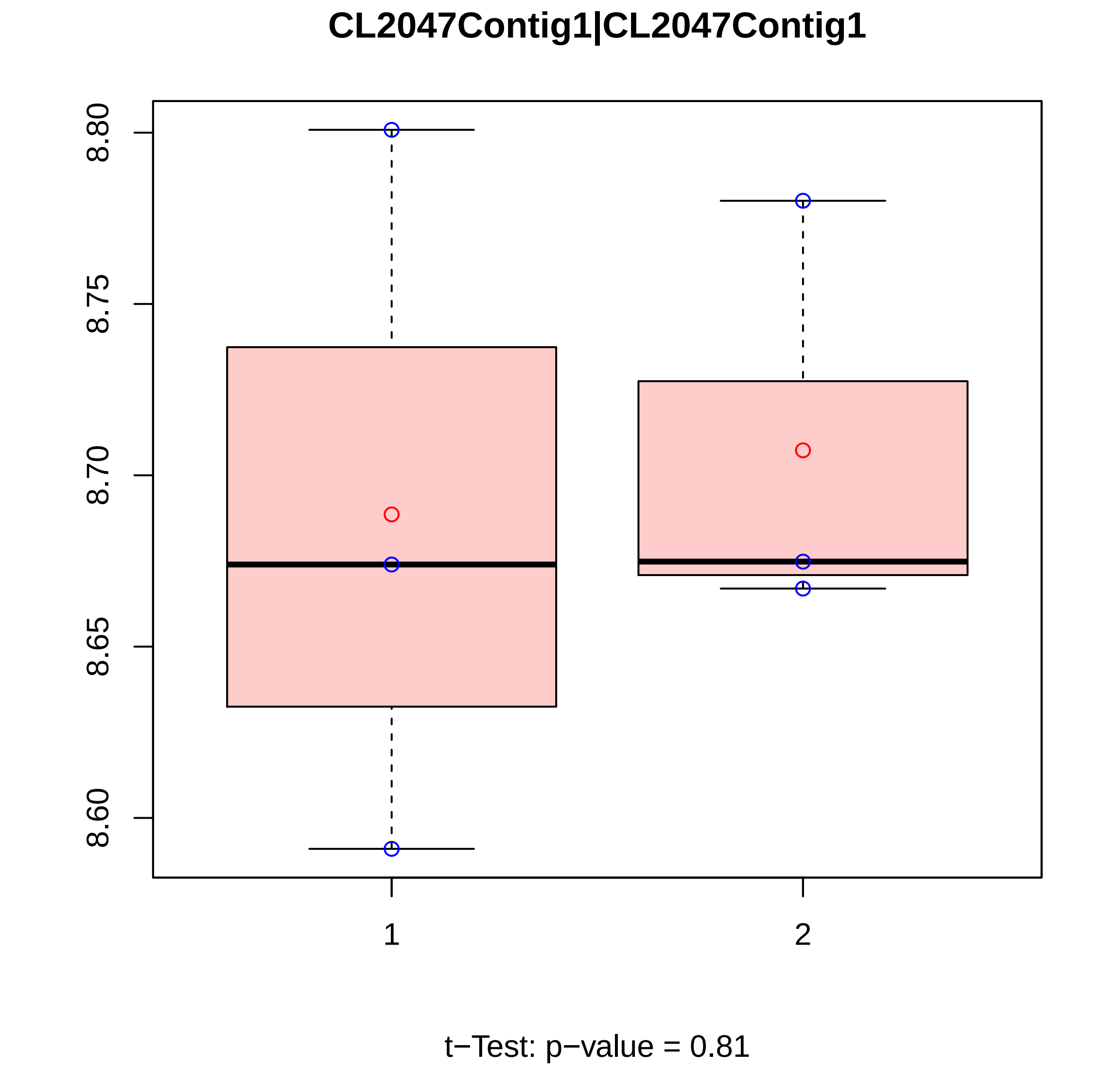

CL2047Contig1|CL2047Contig1
8.80
8.75
8.70
8.65
8.60
1
2
t−Test: p−value = 0.81

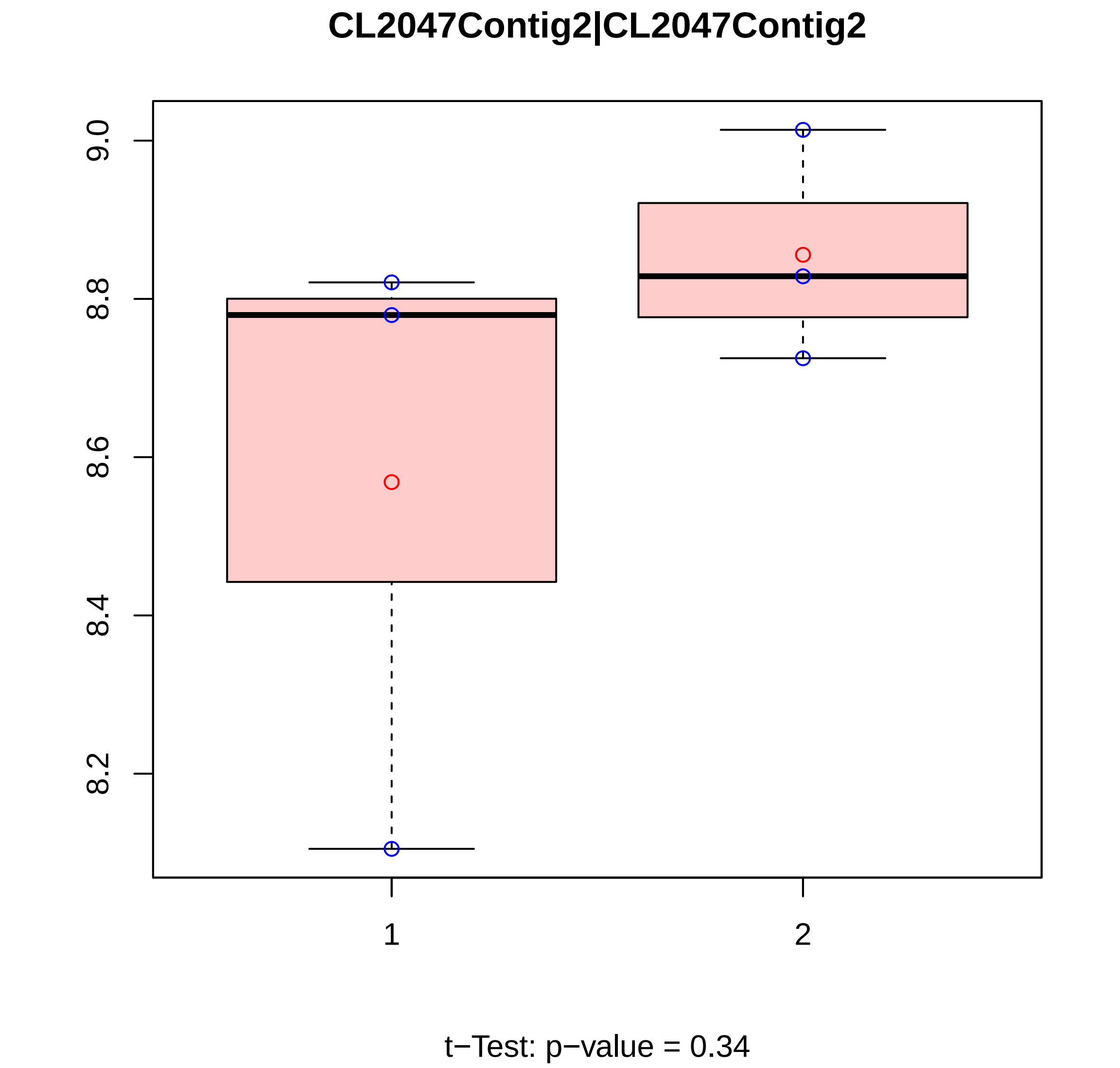
CL2047Contig2|CL2047Contig2
9.0
8.8
8.6
8.4
8.2
1
2
t−Test: p−value = 0.34

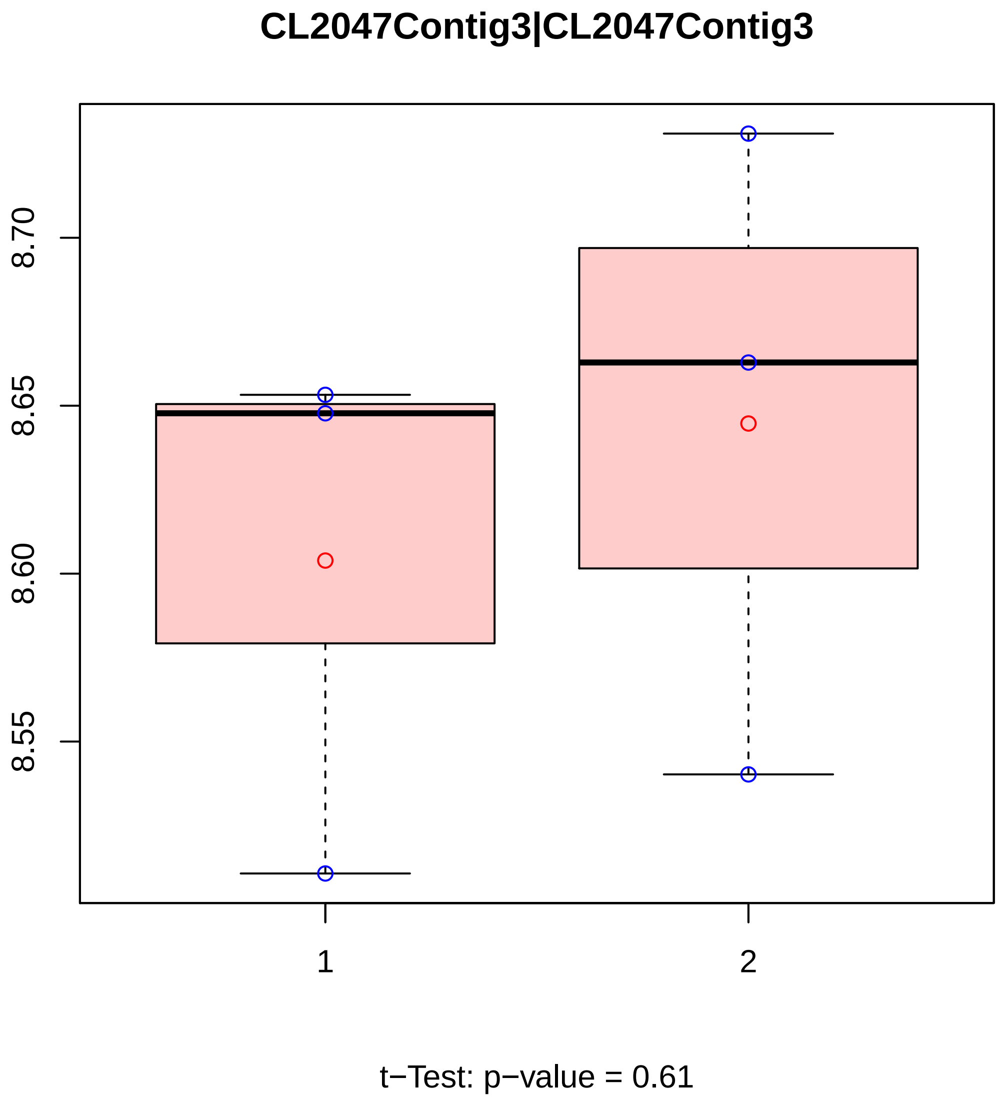

CL2047Contig3|CL2047Contig3
8.55
8.60
8.65
8.70
1
2
t−Test: p−value = 0.61

# CL2047Contig7|CL2047Contig7

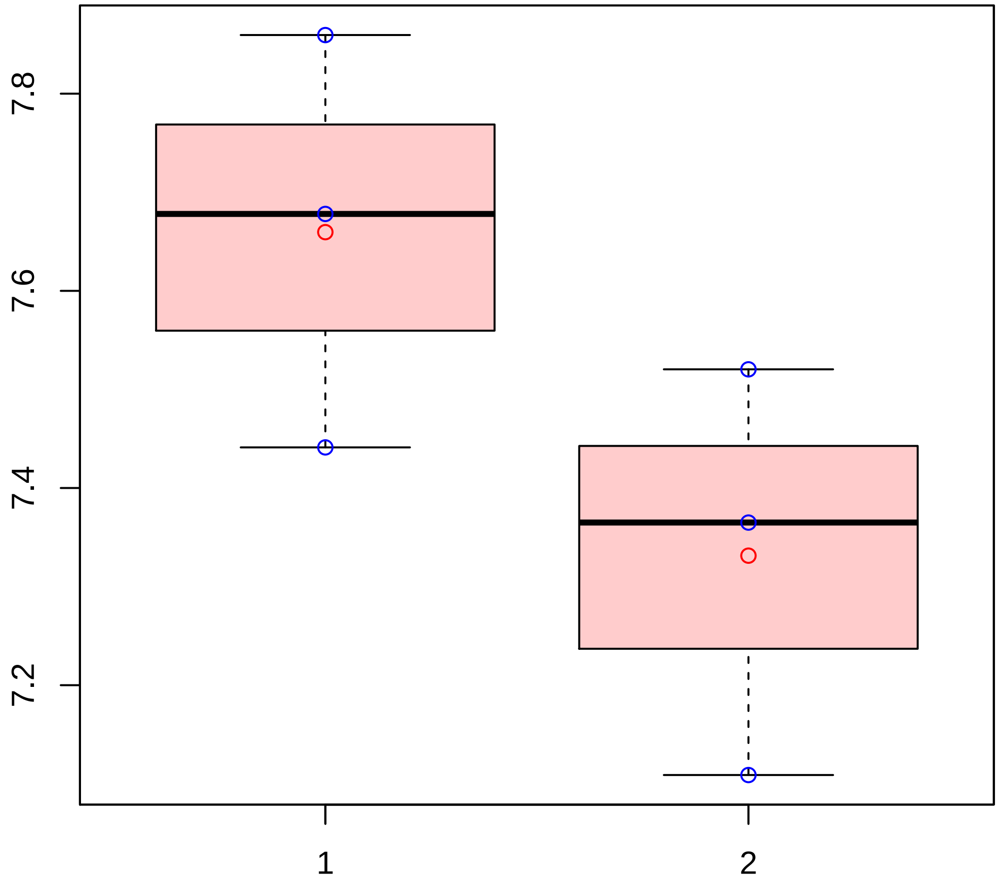


t−Test: p−value = 0.13

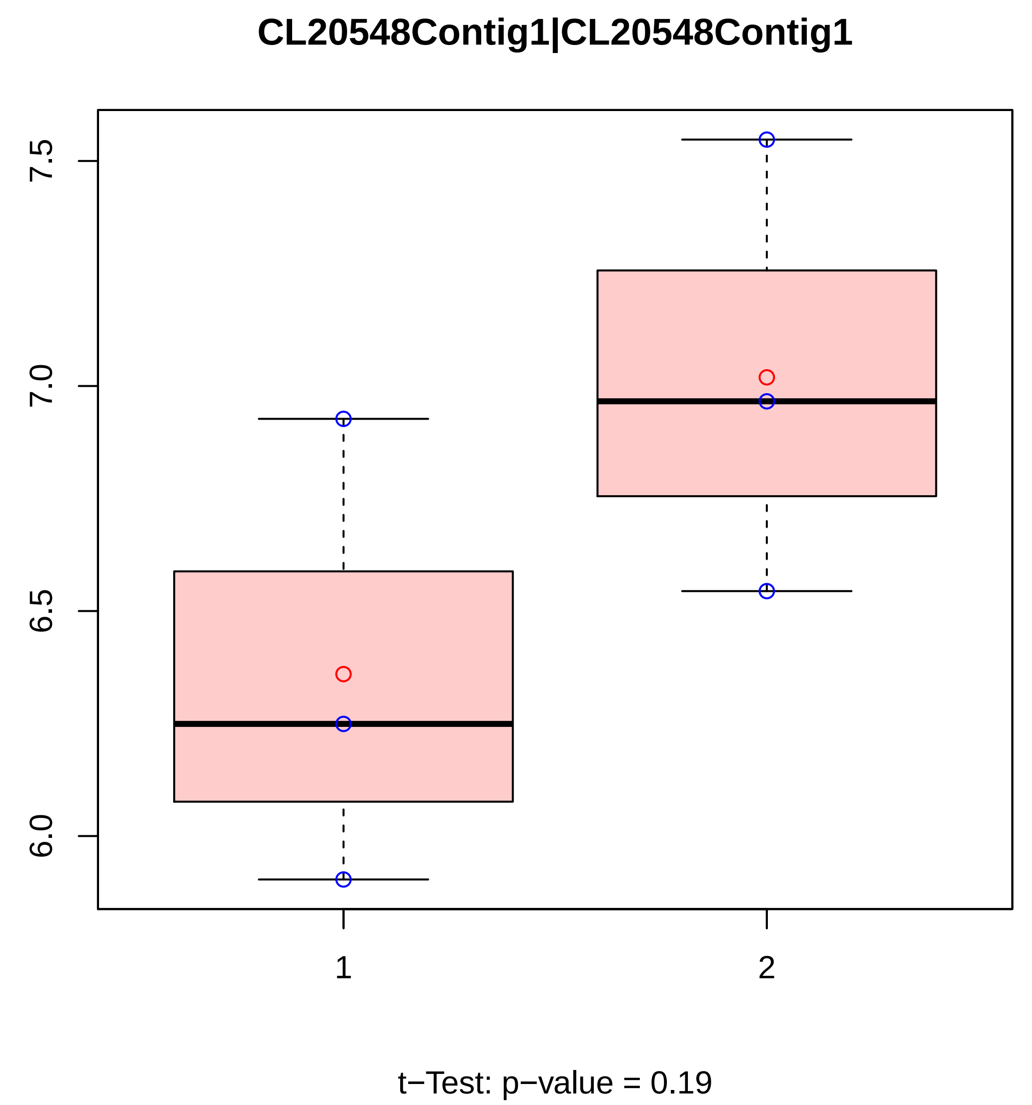
CL20548Contig1|CL20548Contig1
7.5
7.0
6.5
6.0
1
2
t−Test: p−value = 0.19

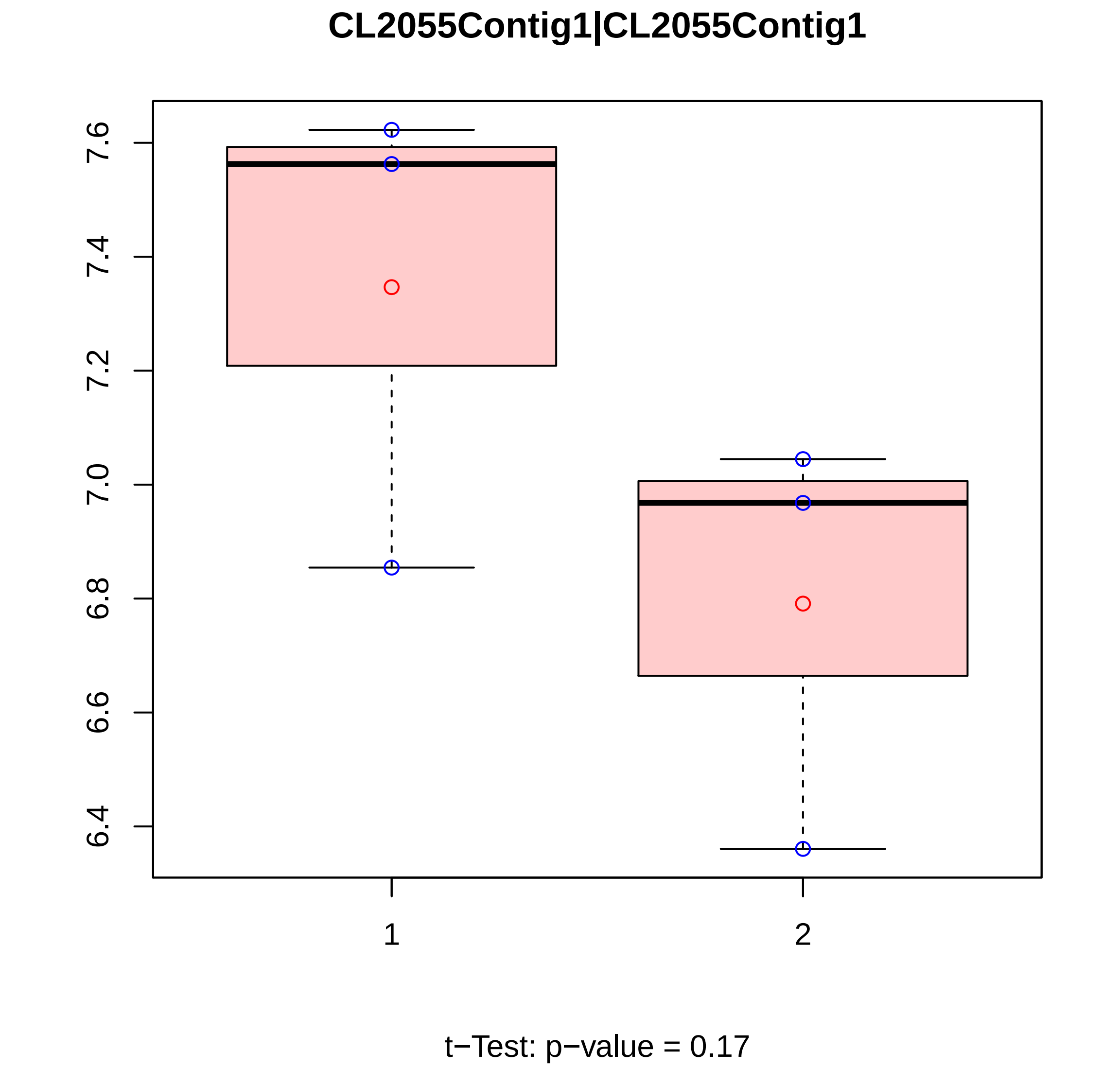
CL2055Contig1|CL2055Contig1
7.6
7.4
7.2
7.0
6.8
6.6
6.4
1
2
t−Test: p−value = 0.17

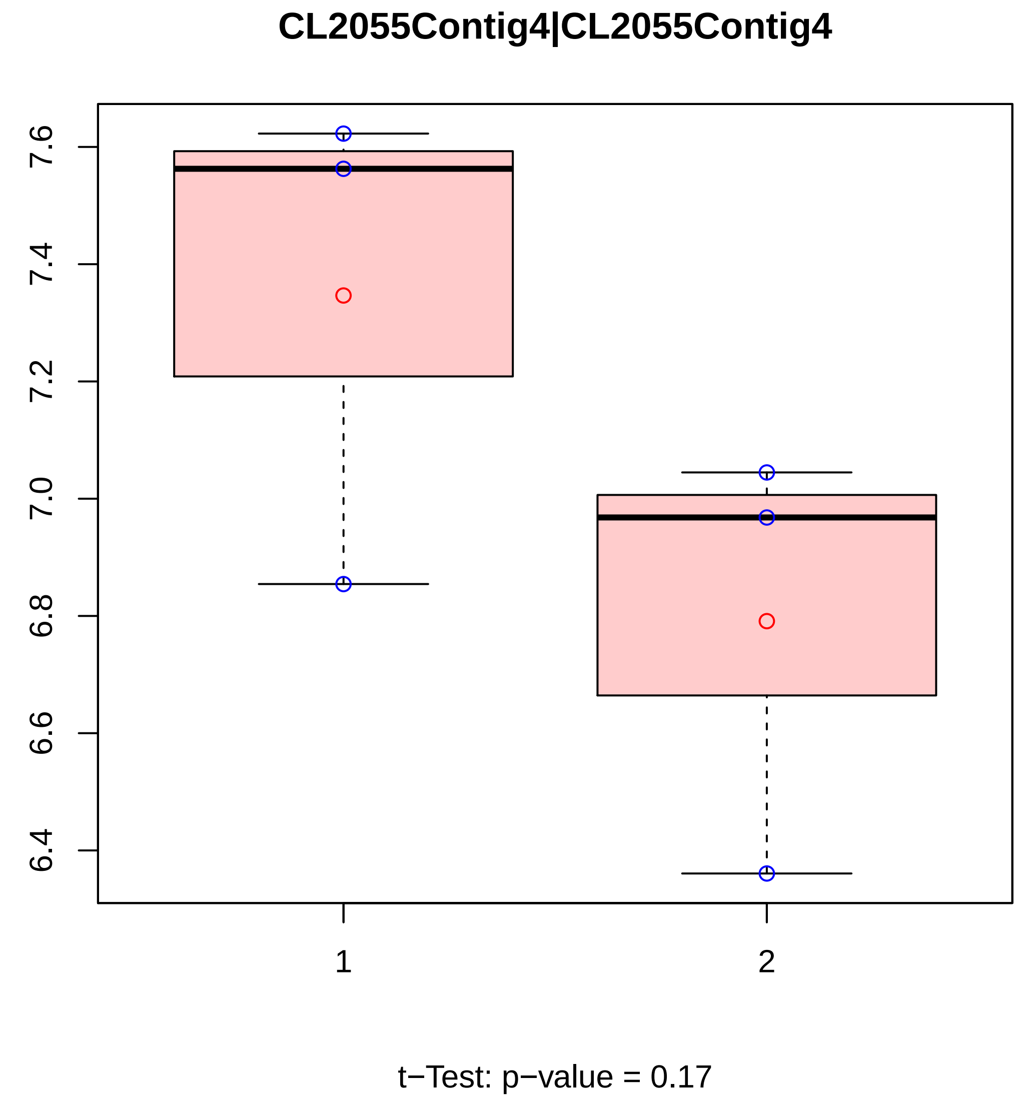
CL2055Contig4|CL2055Contig4
7.6
7.4
7.2
7.0
6.8
6.6
6.4
1
2
t−Test: p−value = 0.17

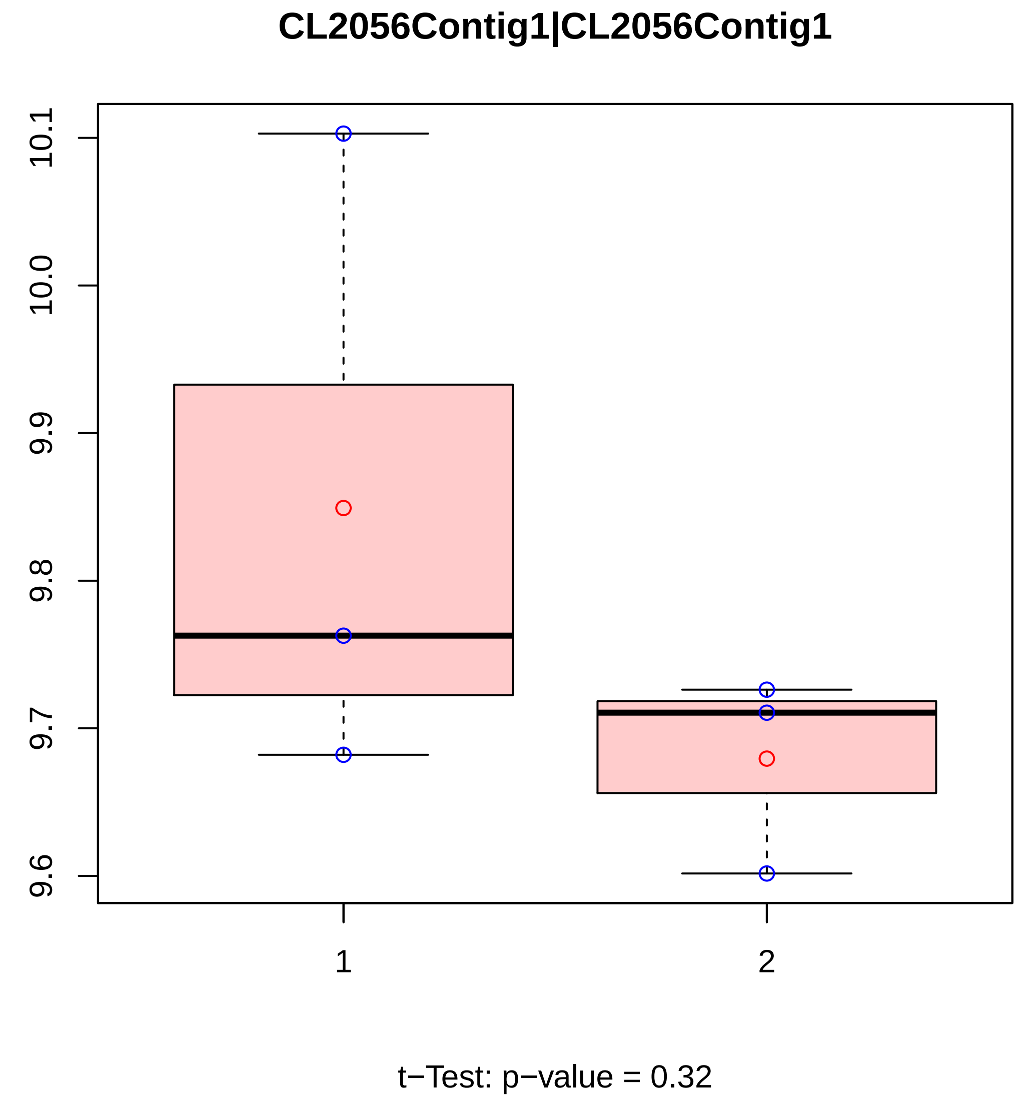

CL2056Contig1|CL2056Contig1
10.1
10.0
9.9
9.8
9.7
9.6
1
2
t−Test: p−value = 0.32

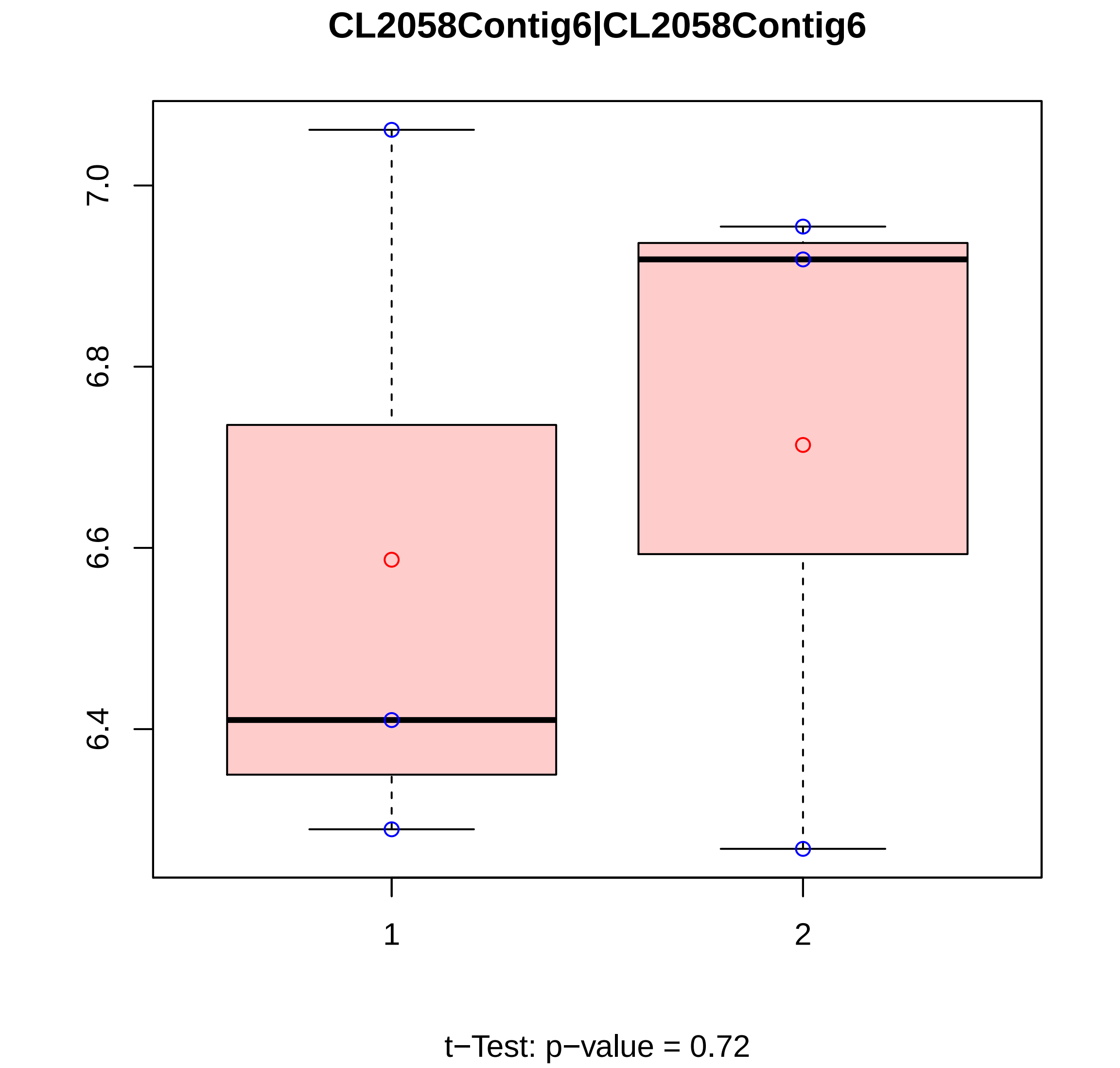

CL2058Contig6|CL2058Contig6
7.0
6.8
6.6
6.4
1
2
t−Test: p−value = 0.72

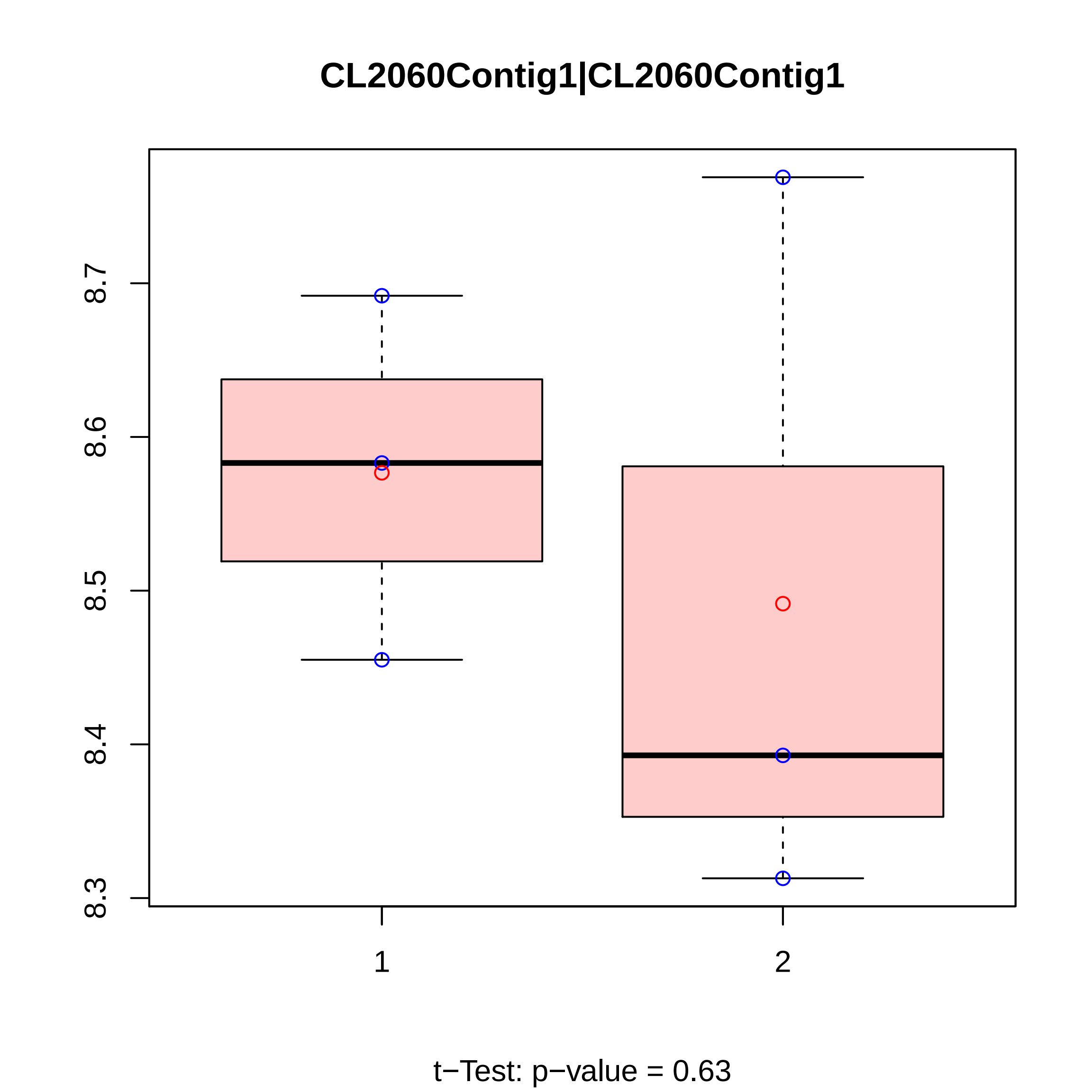
CL2060Contig1|CL2060Contig1
8.3
8.4
8.5
8.6
8.7
1
2
t−Test: p−value = 0.63

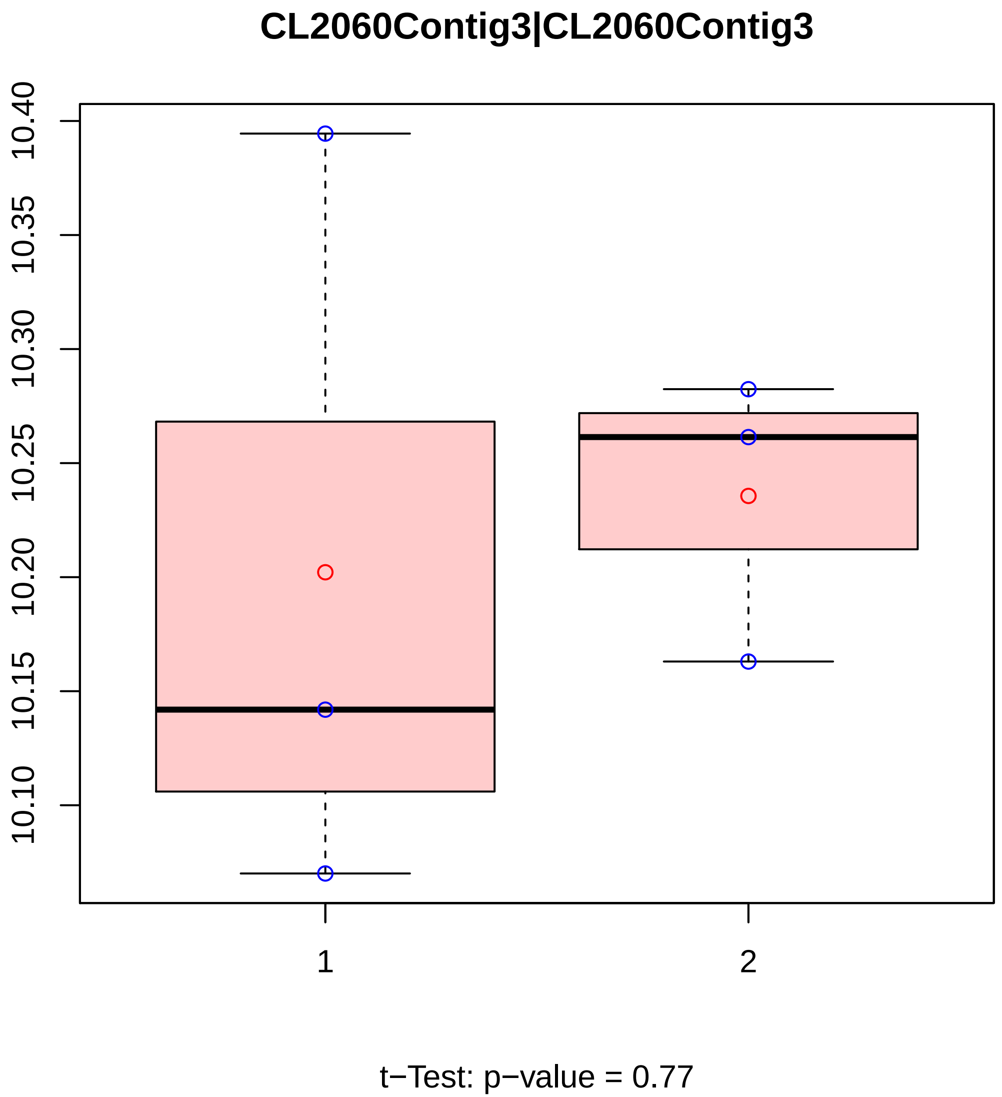
CL2060Contig3|CL2060Contig3
10.40
10.35
10.30
10.25
10.20
10.15
10.10
1
2
t−Test: p−value = 0.77

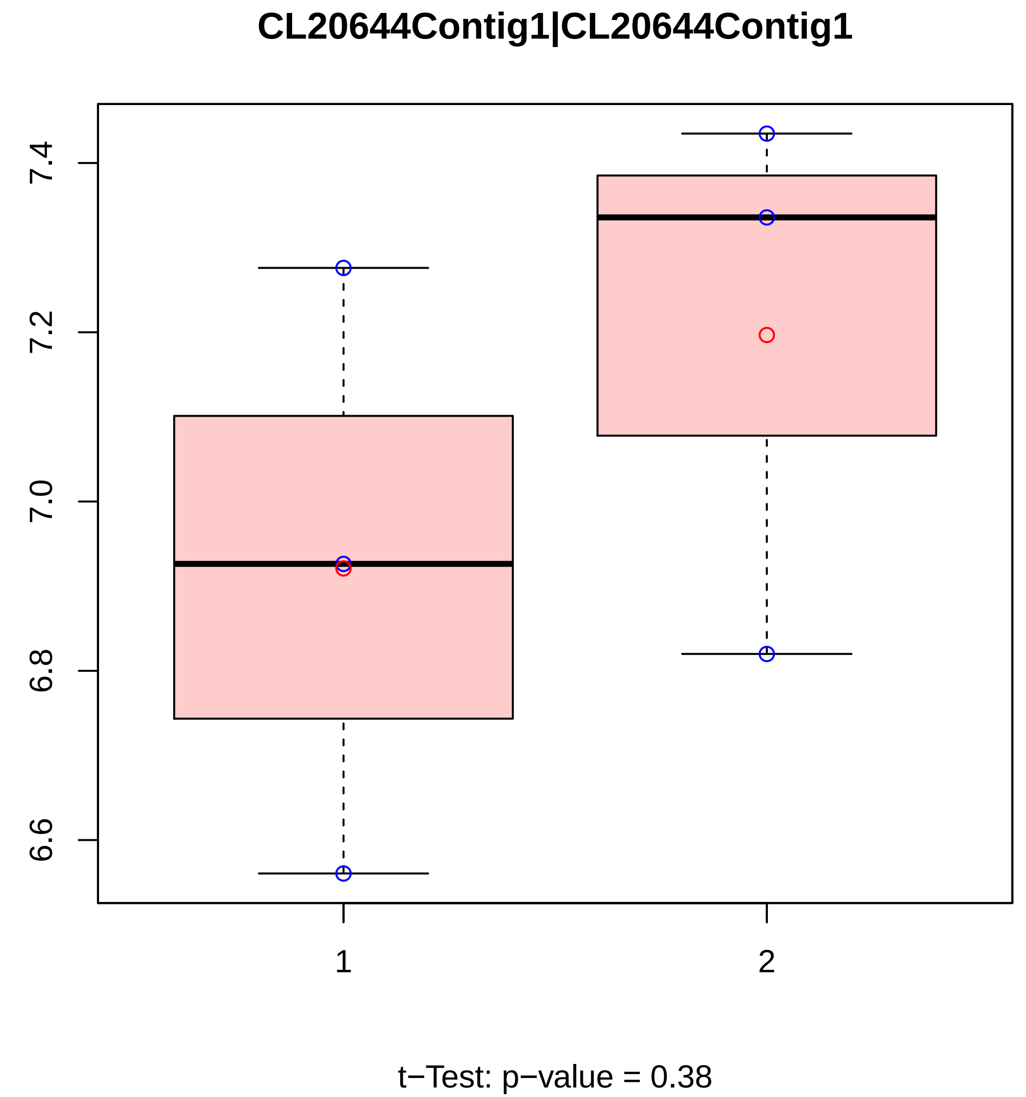
CL20644Contig1|CL20644Contig1
7.4
7.2
7.0
6.8
6.6
1
2
t−Test: p−value = 0.38

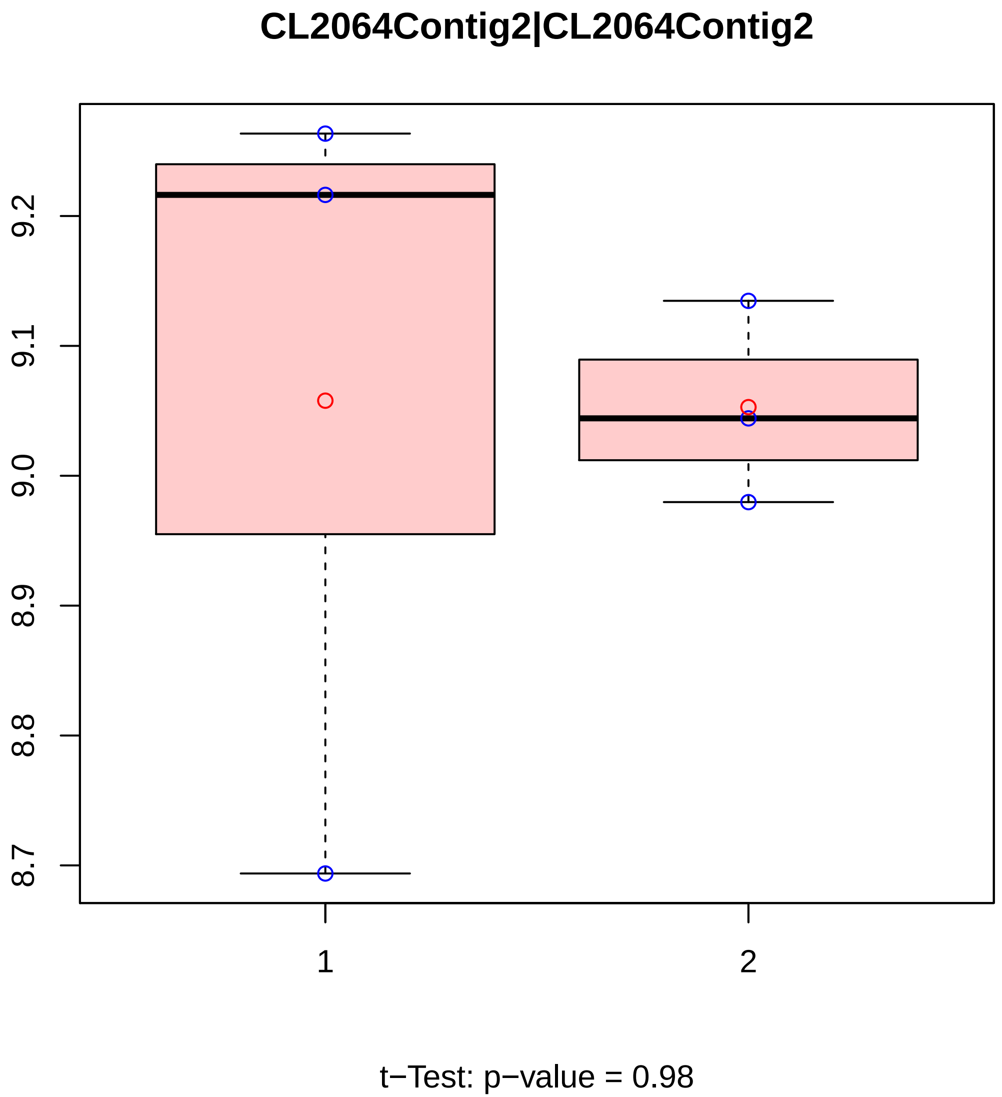

CL2064Contig2|CL2064Contig2
9.2
9.1
9.0
8.9
8.8
8.7
1
2
t−Test: p−value = 0.98

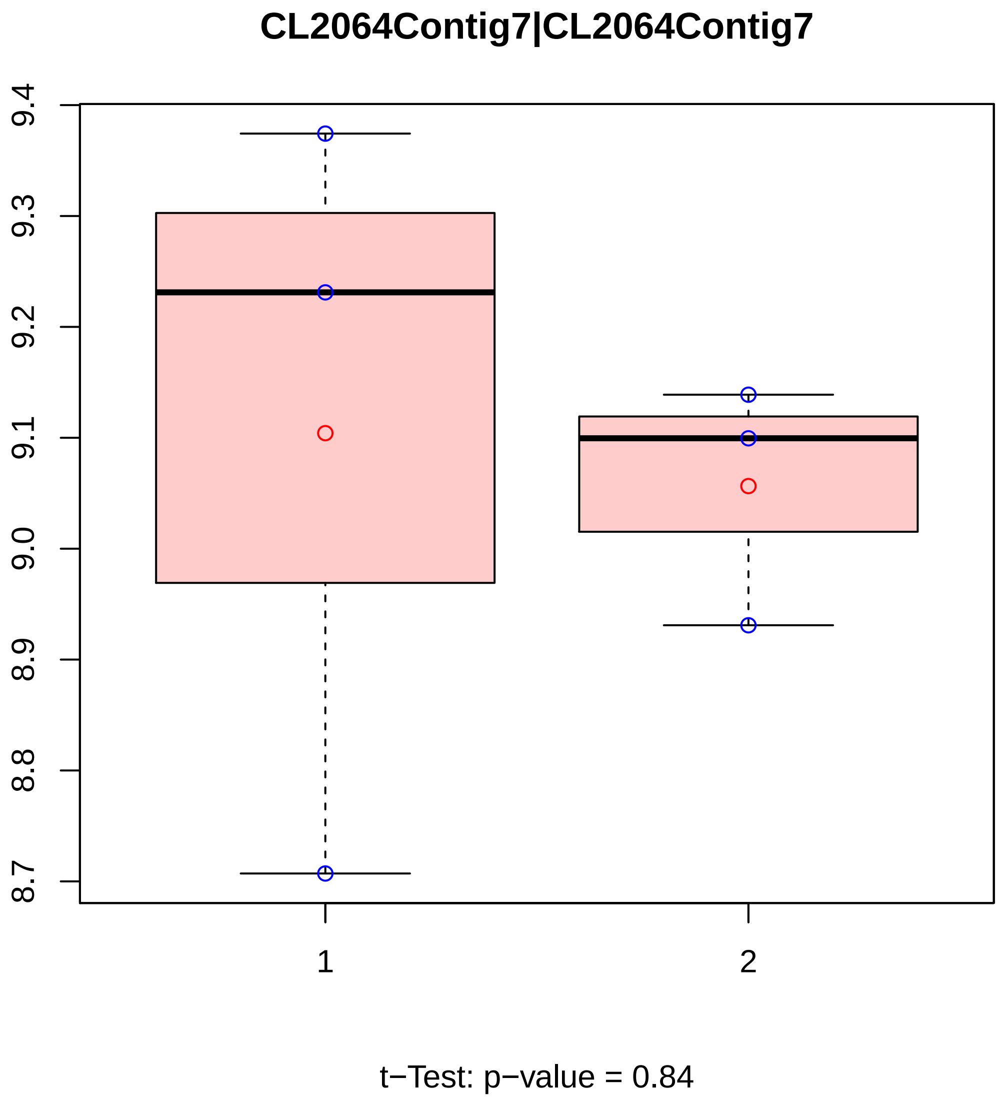
CL2064Contig7|CL2064Contig7
9.4
9.3
9.2
9.1
9.0
8.9
8.8
8.7
1
2
t−Test: p−value = 0.84

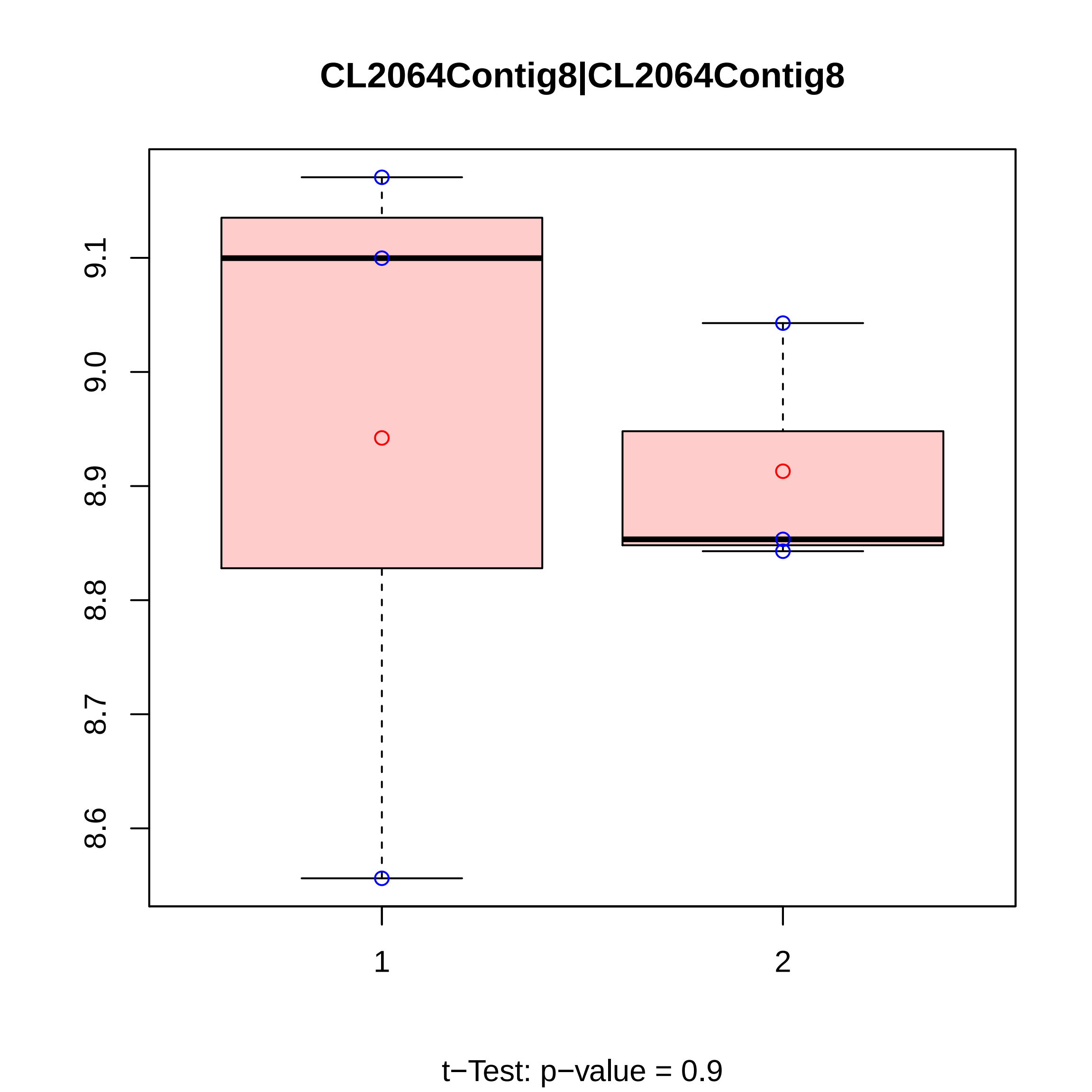

CL2064Contig8|CL2064Contig8
9.1
9.0
8.9
8.8
8.7
8.6
1
2
t−Test: p−value = 0.9

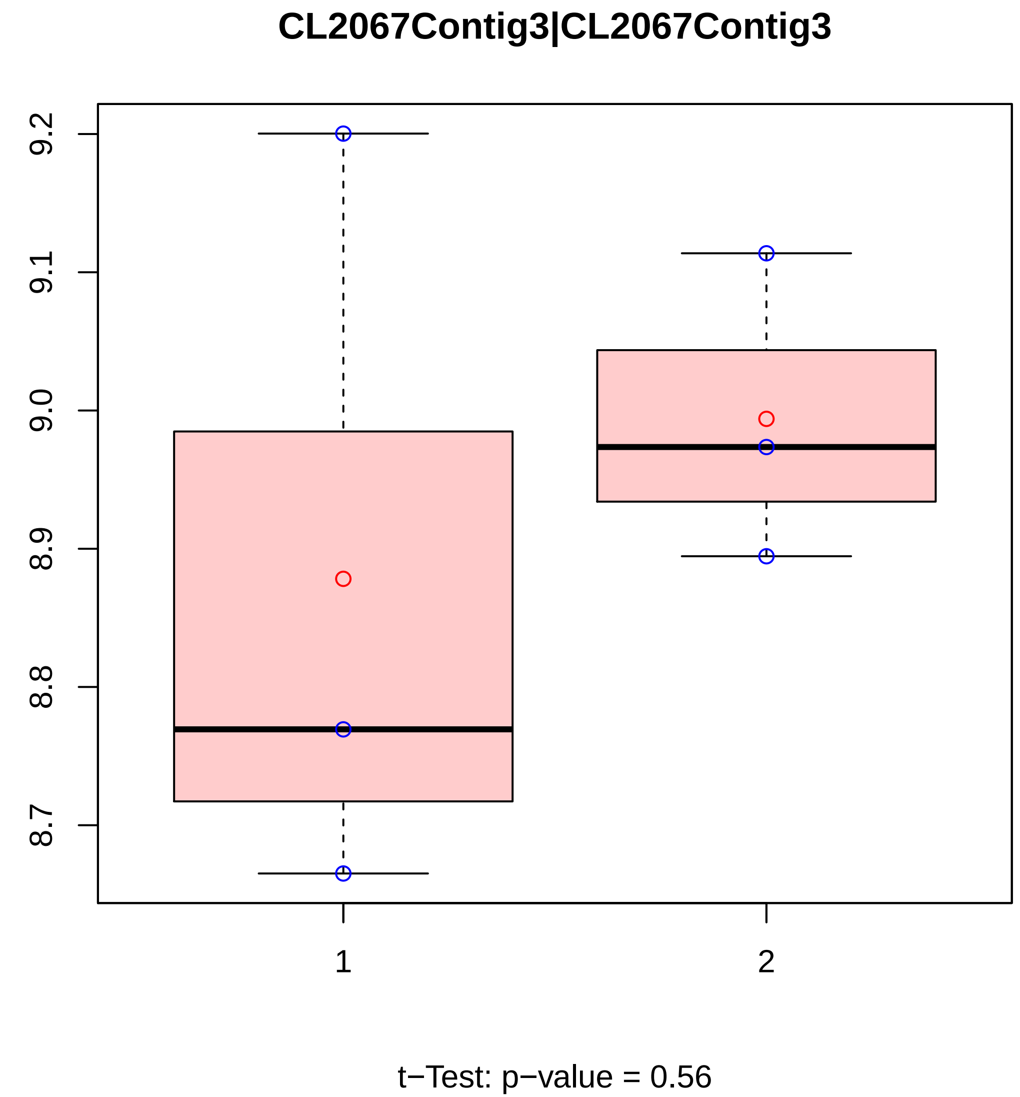
CL2067Contig3|CL2067Contig3
9.2
9.1
9.0
8.9
8.8
8.7
1
2
t−Test: p−value = 0.56

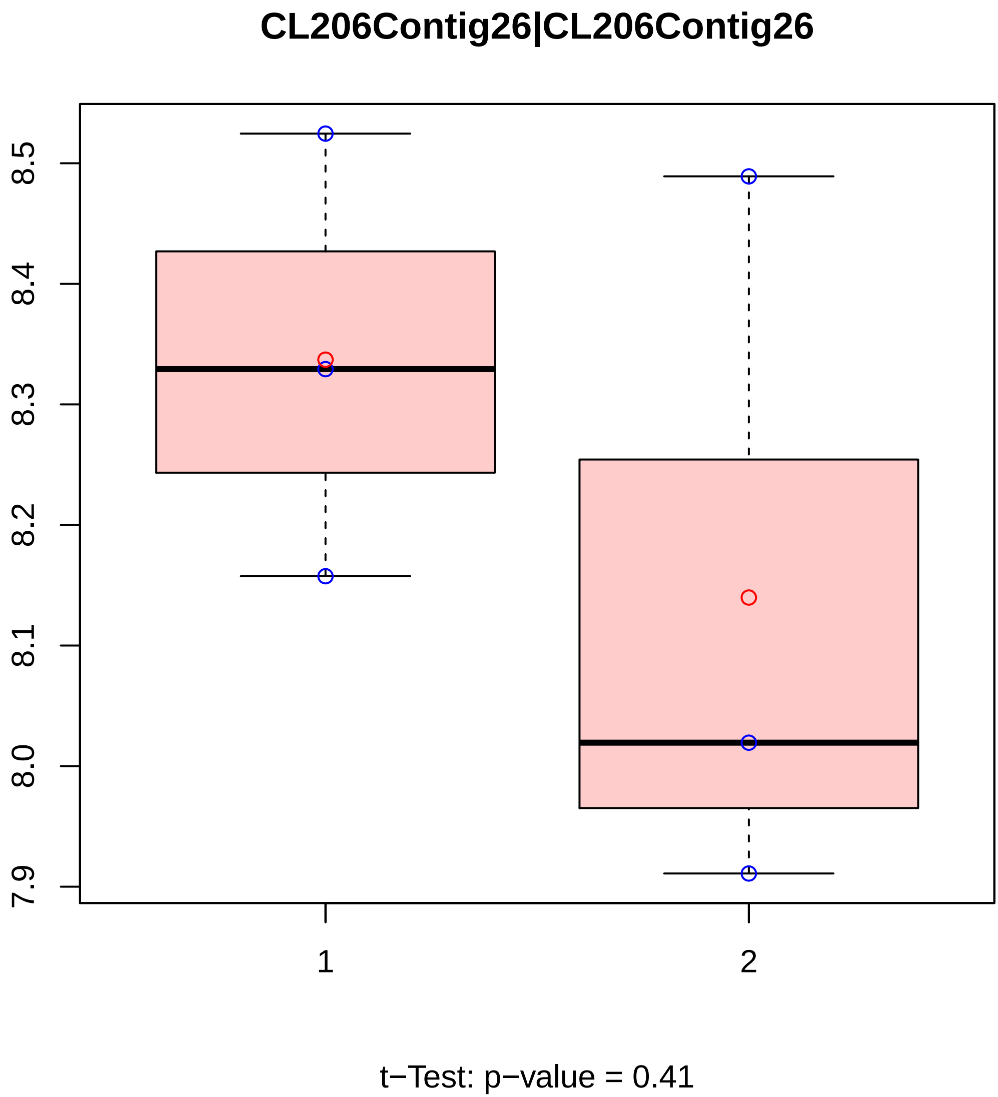
CL206Contig26|CL206Contig26
8.5
8.4
8.3
8.2
8.1
8.0
7.9
1
2
t−Test: p−value = 0.41

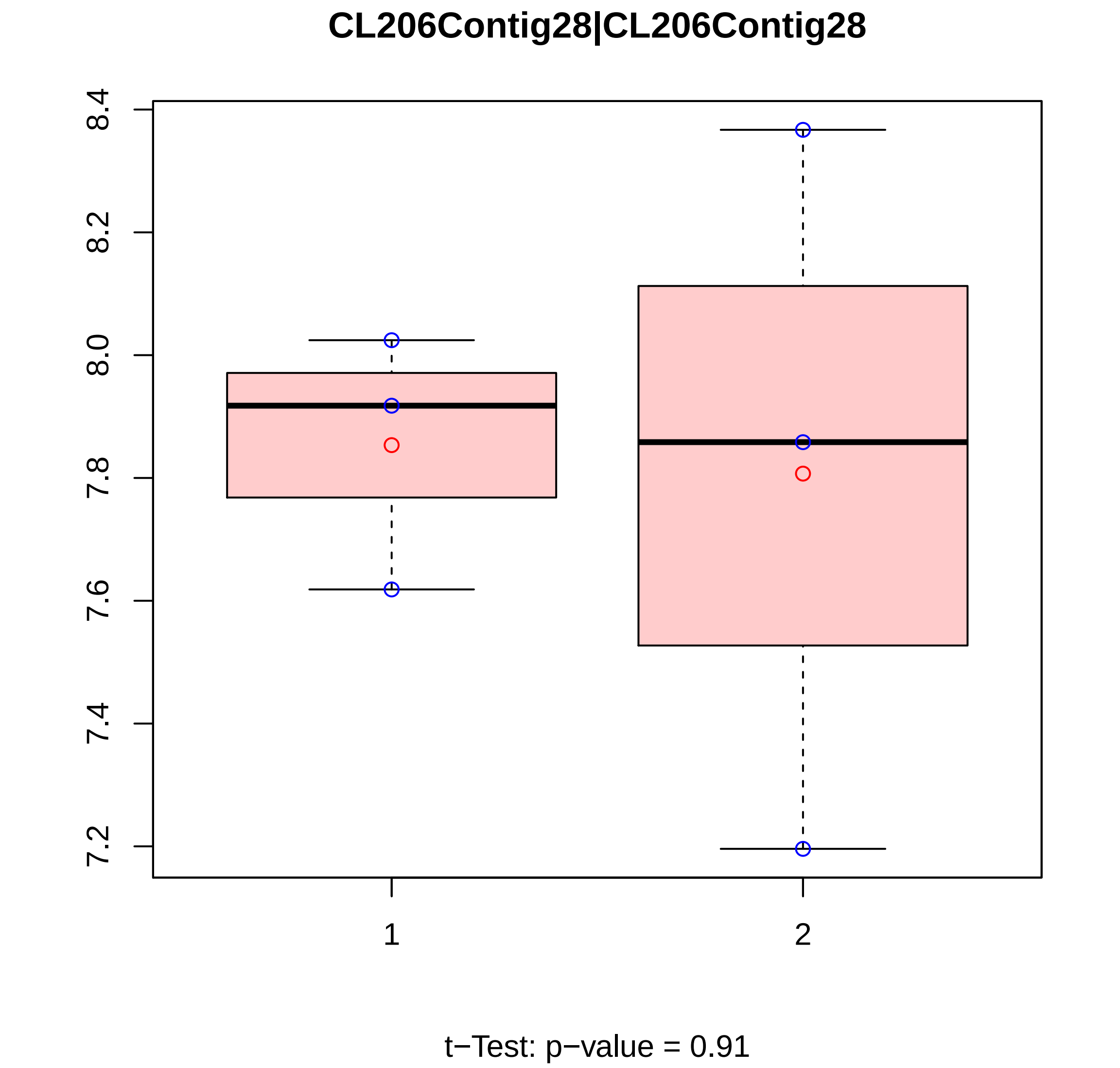
CL206Contig28|CL206Contig28
8.4
8.2
8.0
7.8
7.6
7.4
7.2
1
2
t−Test: p−value = 0.91

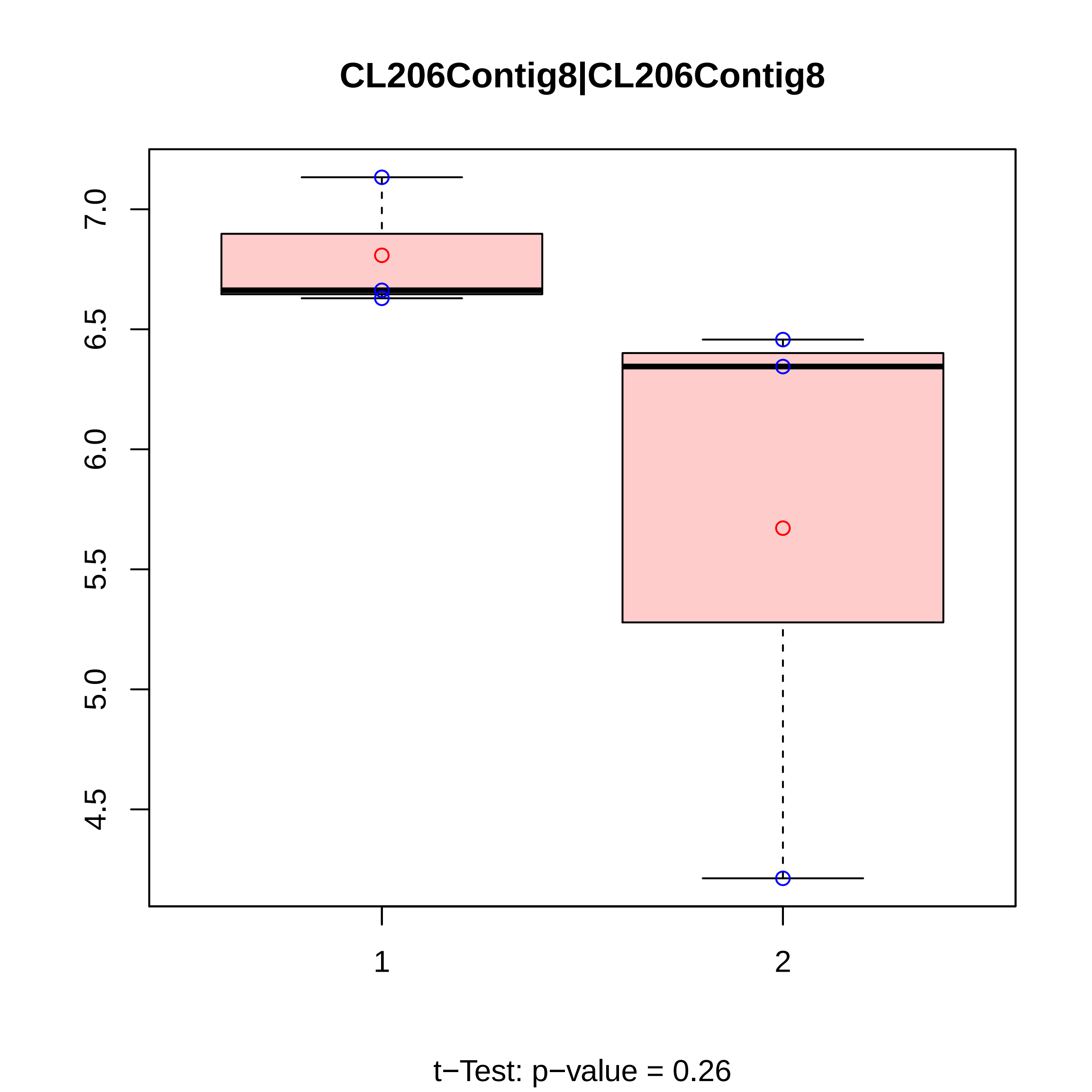
CL206Contig8|CL206Contig8
7.0
6.5
6.0
5.5
5.0
4.5
1
2
t−Test: p−value = 0.26

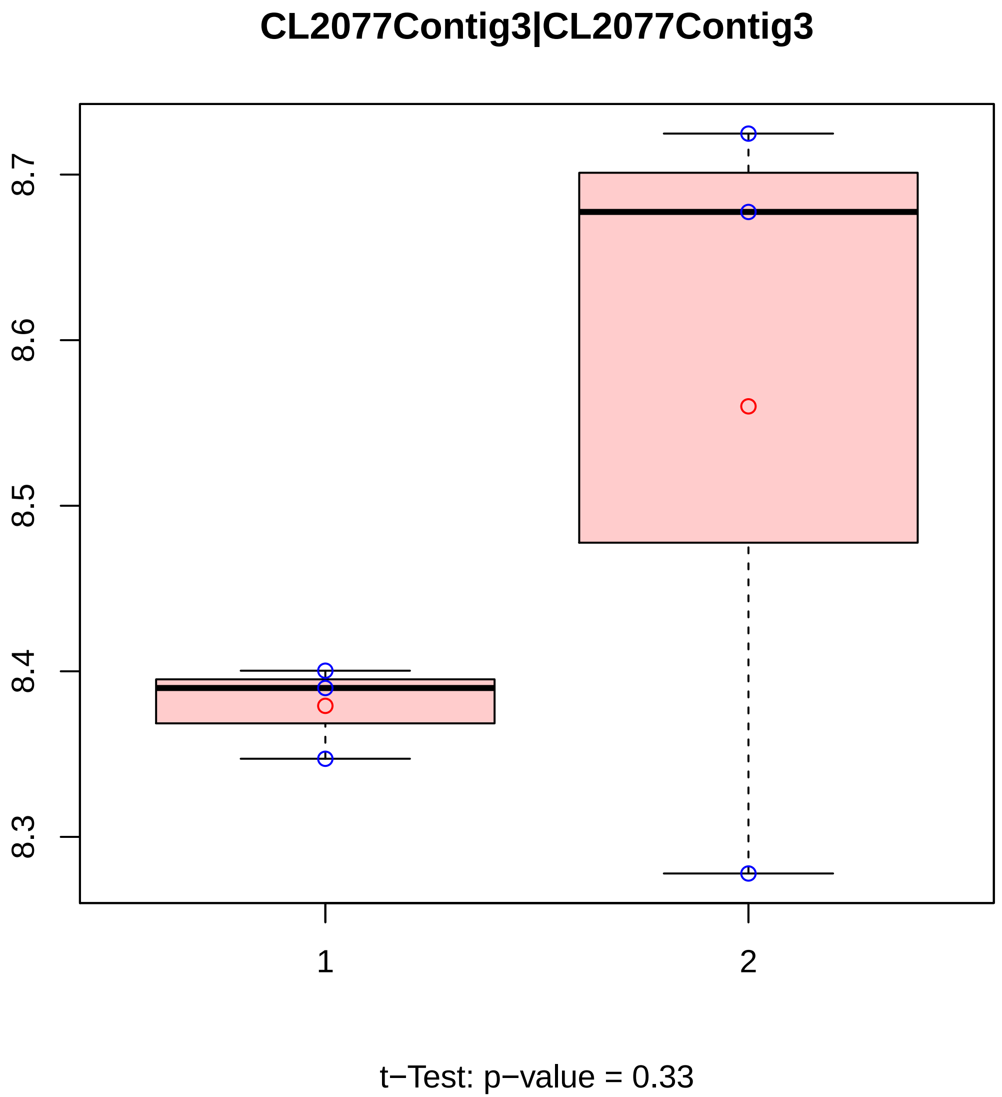
CL2077Contig3|CL2077Contig3
8.7
8.6
8.5
8.4
8.3
1
2
t−Test: p−value = 0.33

## CL2080Contig1|CL2080Contig1

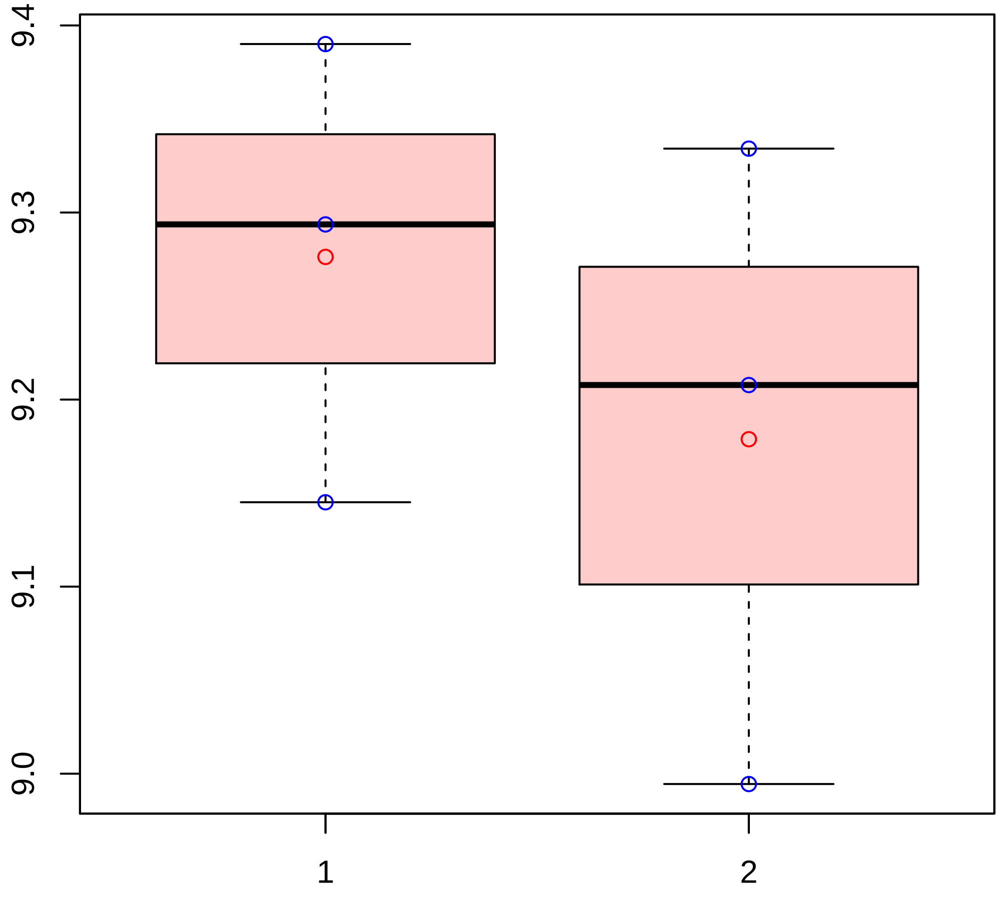


t−Test: p−value = 0.47

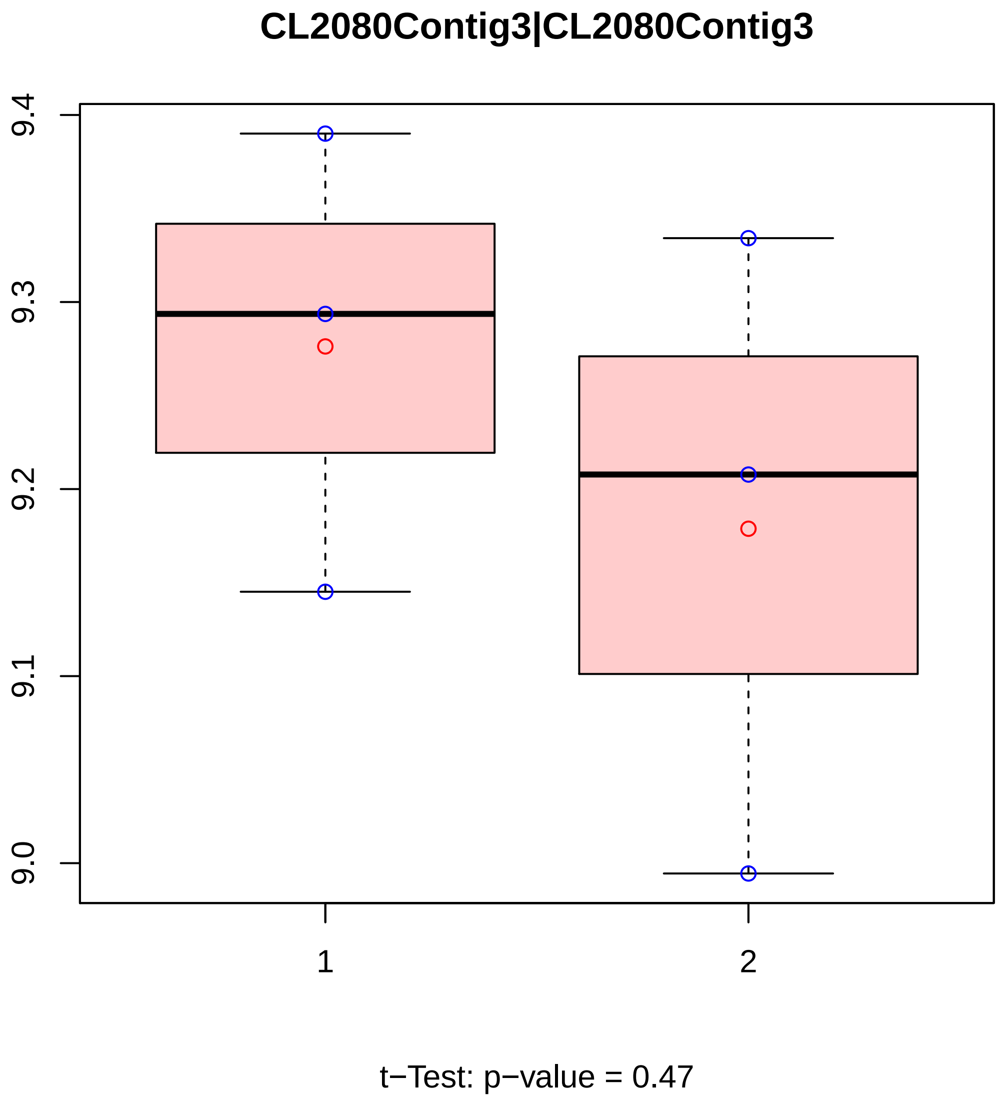
CL2080Contig3|CL2080Contig3
9.4
9.3
9.2
9.1
9.0
1
2
t−Test: p−value = 0.47

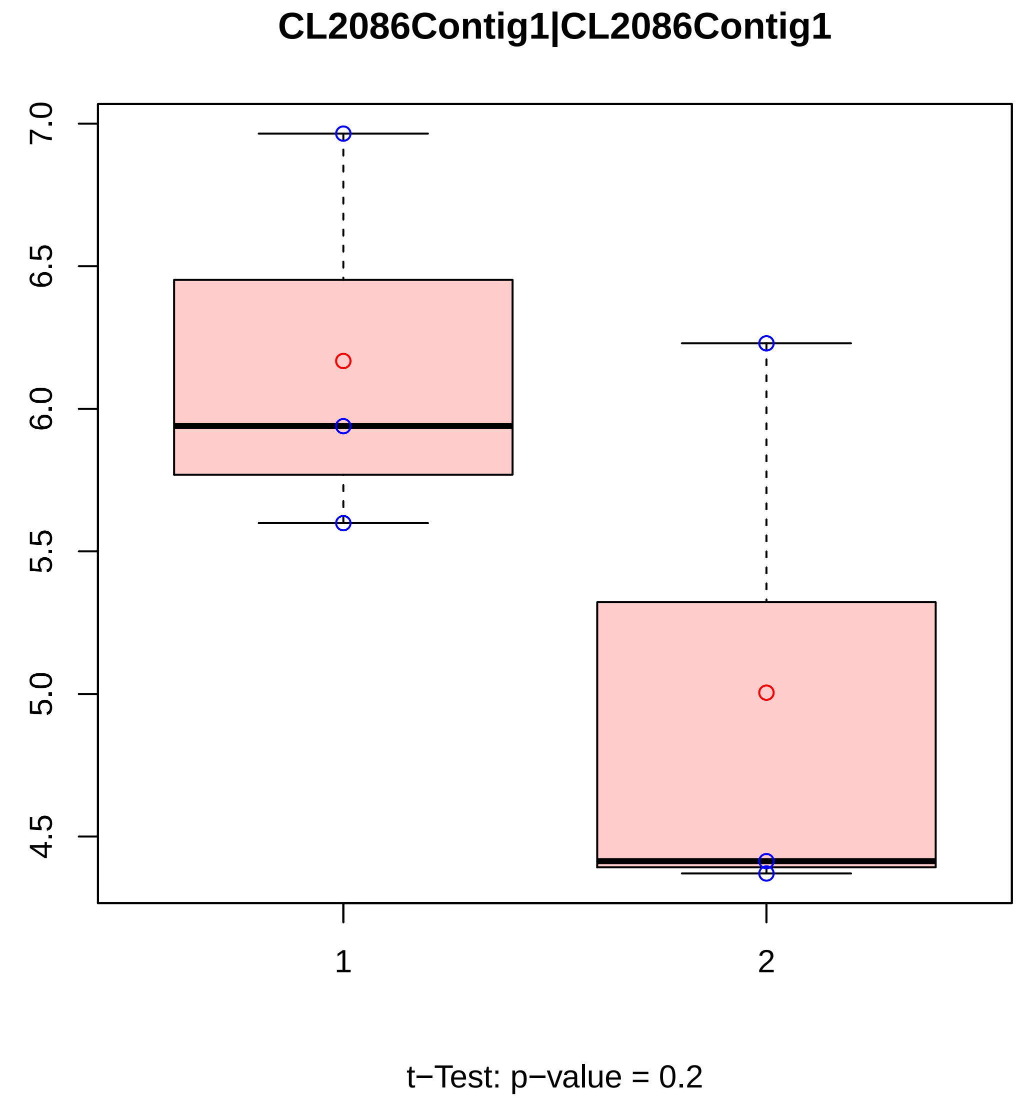

CL2086Contig1|CL2086Contig1
7.0
6.5
6.0
5.5
5.0
4.5
1
2
t−Test: p−value = 0.2

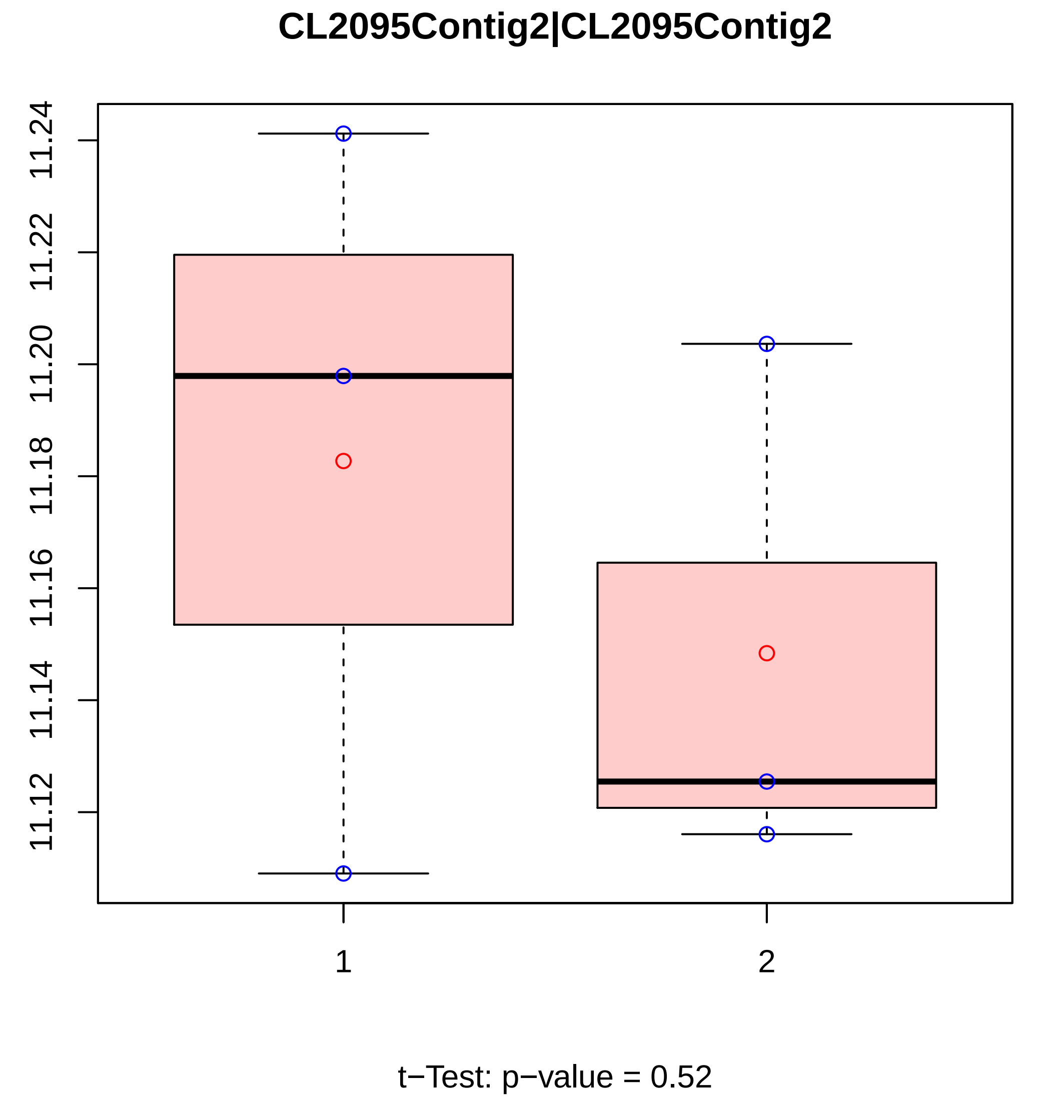
CL2095Contig2|CL2095Contig2
11.24
11.22
11.20
11.18
11.16
11.14
11.12
1
2
t−Test: p−value = 0.52

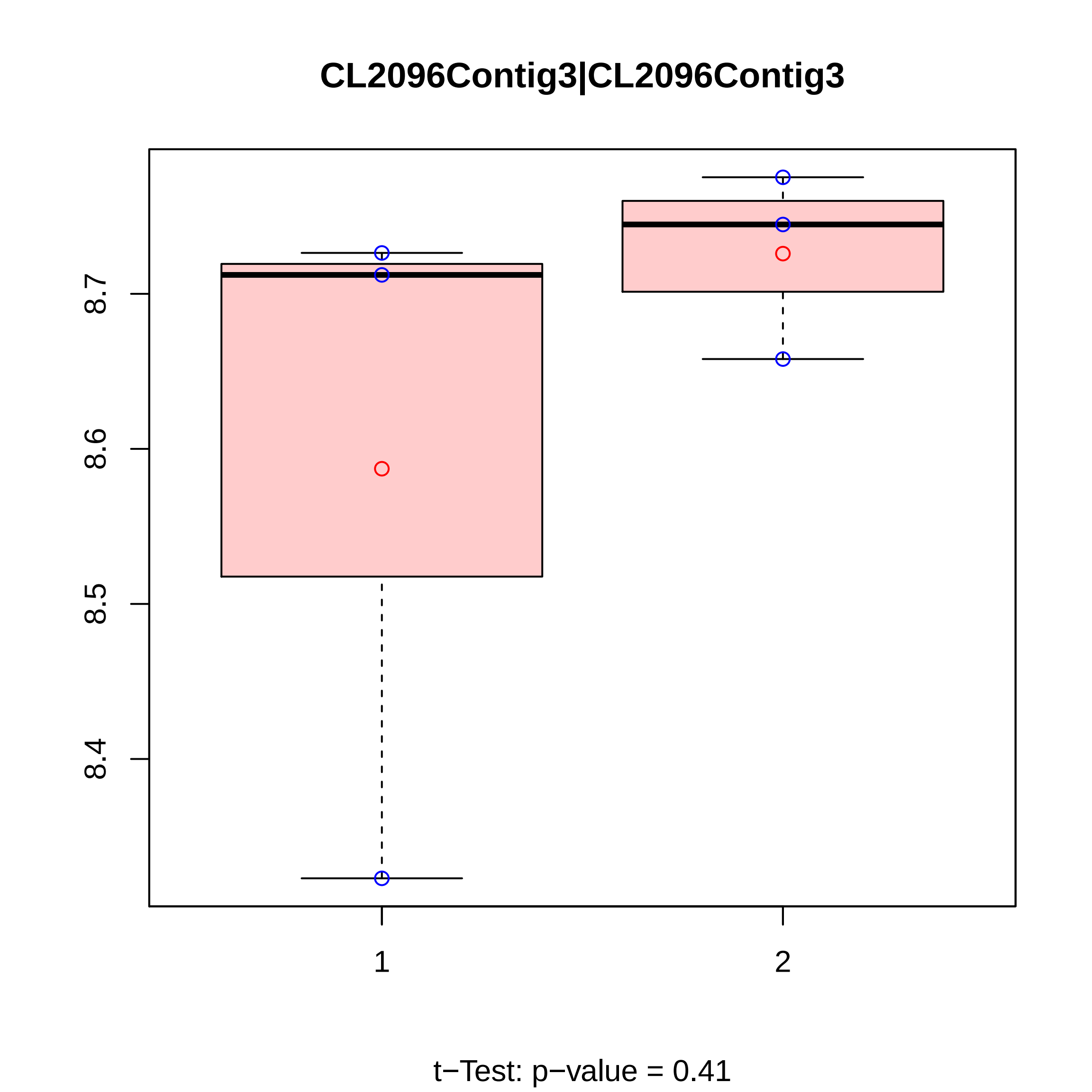

CL2096Contig3|CL2096Contig3
8.4
8.5
8.6
8.7
1
2
t−Test: p−value = 0.41

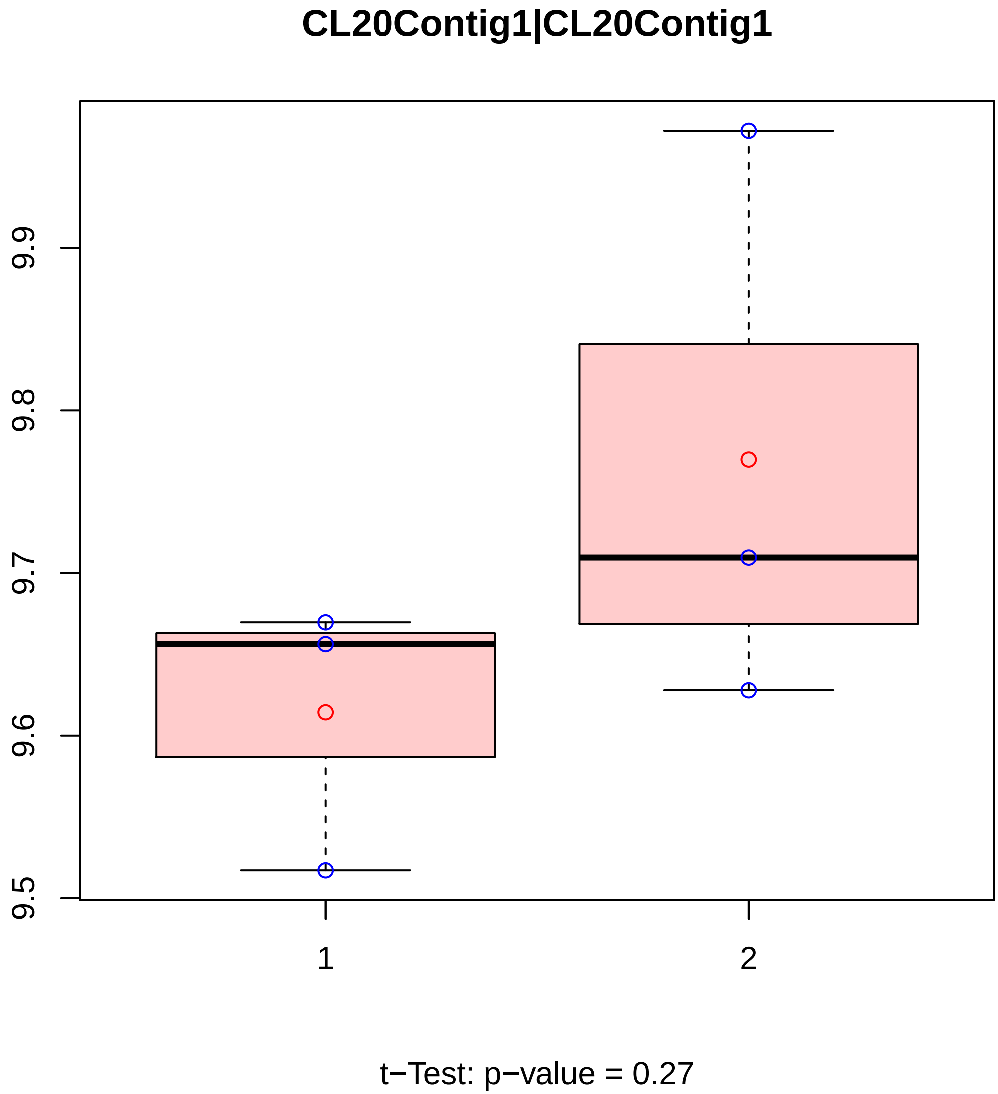
CL20Contig1|CL20Contig1
9.5
9.6
9.7
9.8
9.9
1
2
t−Test: p−value = 0.27

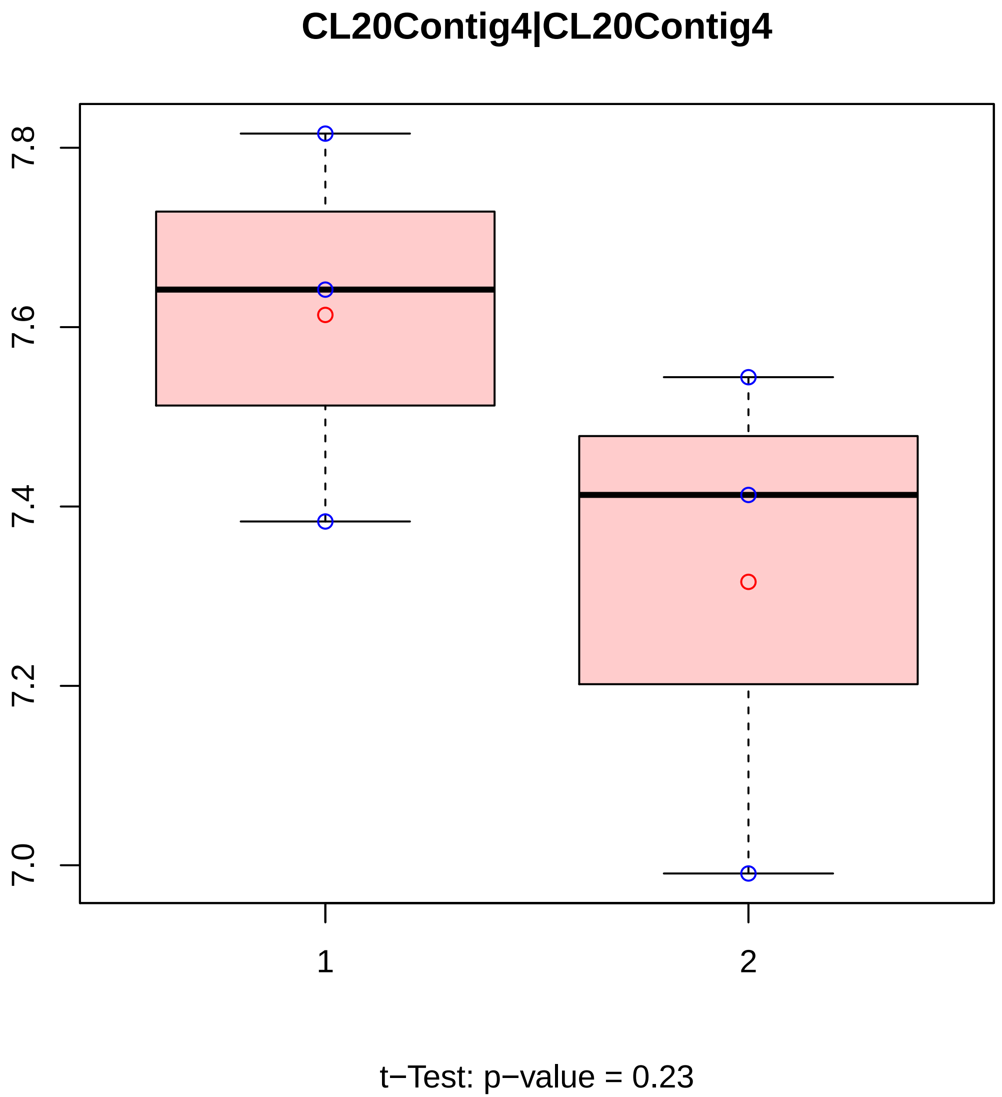
CL20Contig4|CL20Contig4
7.0
7.2
7.4
7.6
7.8
1
2
t−Test: p−value = 0.23

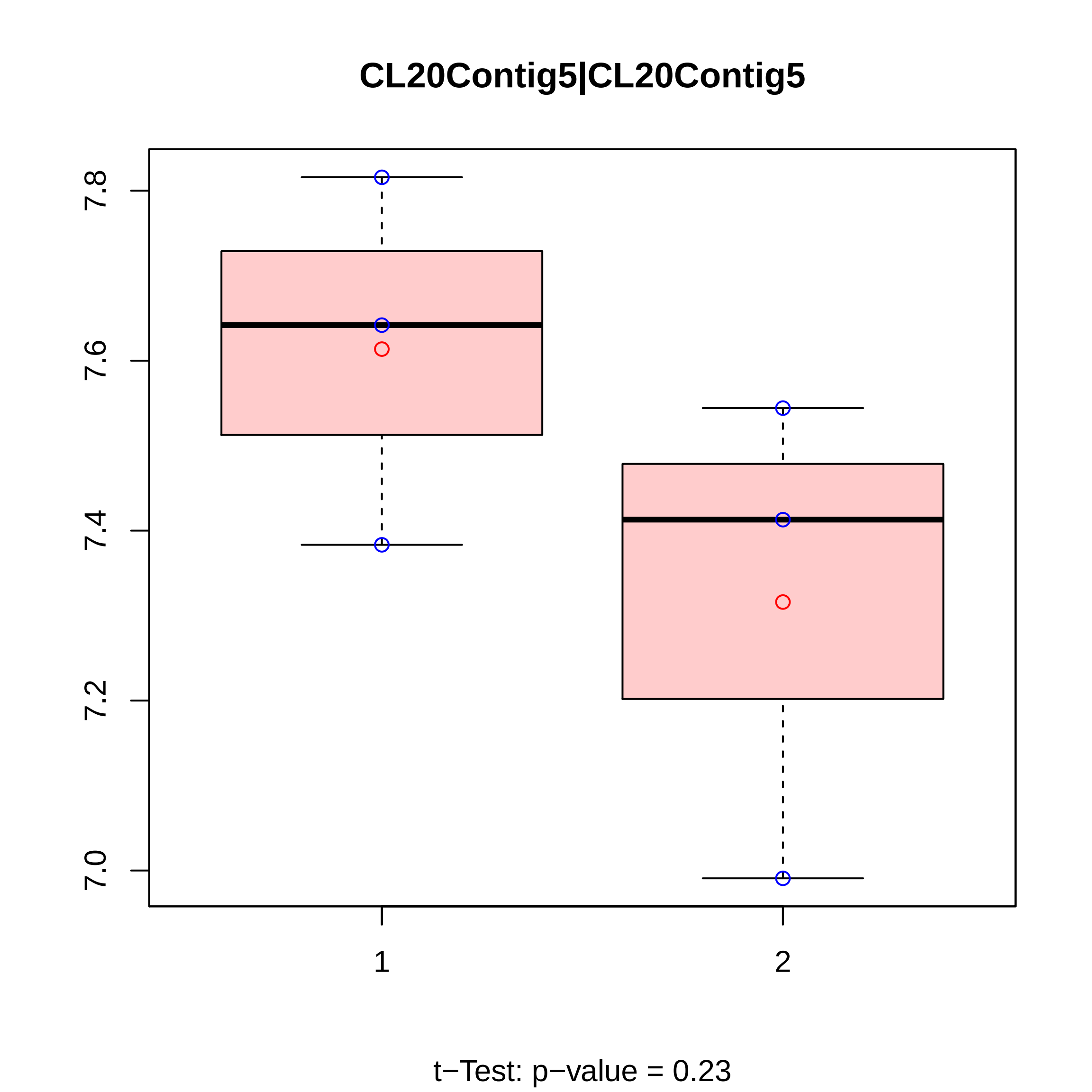

CL20Contig5|CL20Contig5
7.0
7.2
7.4
7.6
7.8
1
2
t−Test: p−value = 0.23

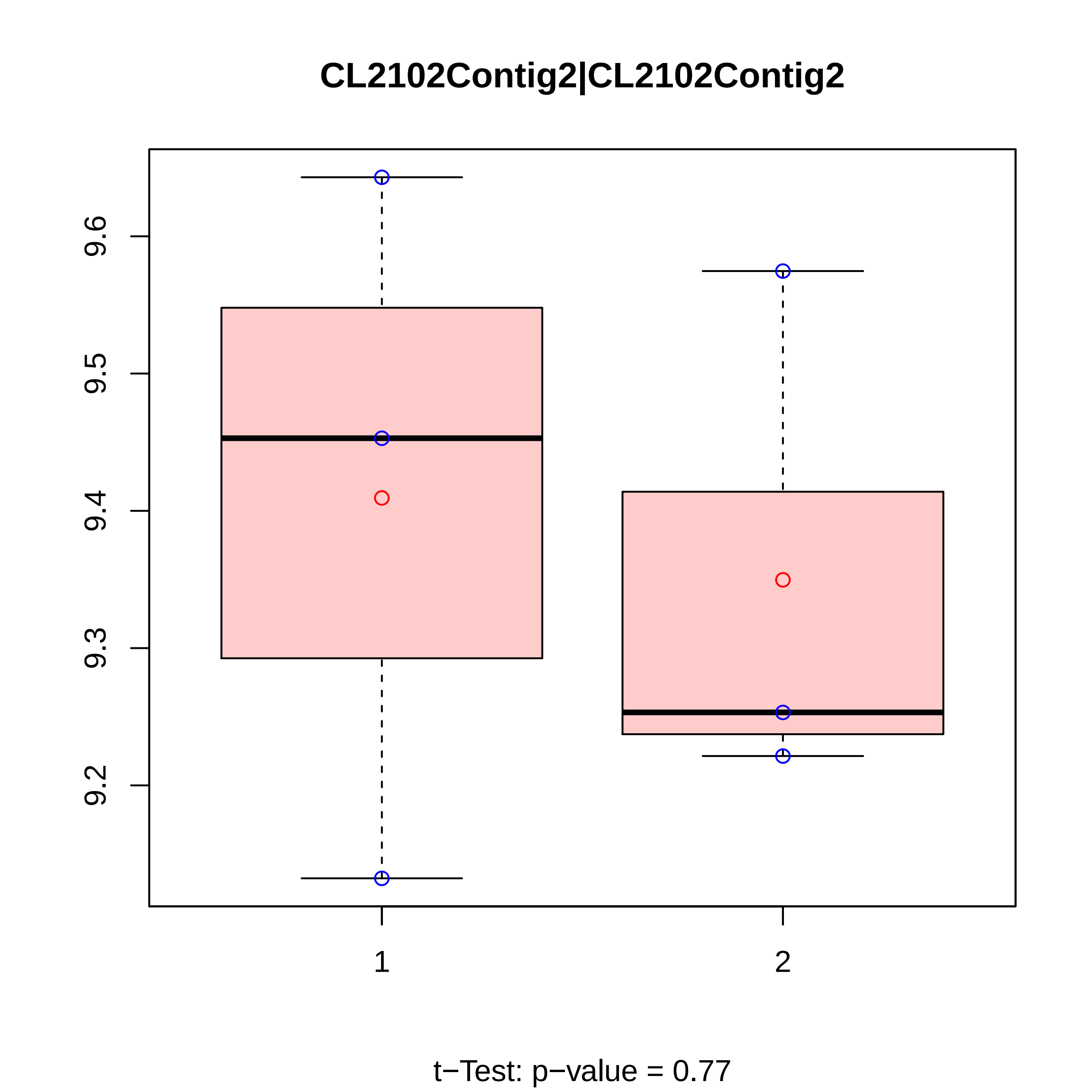
CL2102Contig2|CL2102Contig2
9.2
9.3
9.4
9.5
9.6
1
2
t−Test: p−value = 0.77

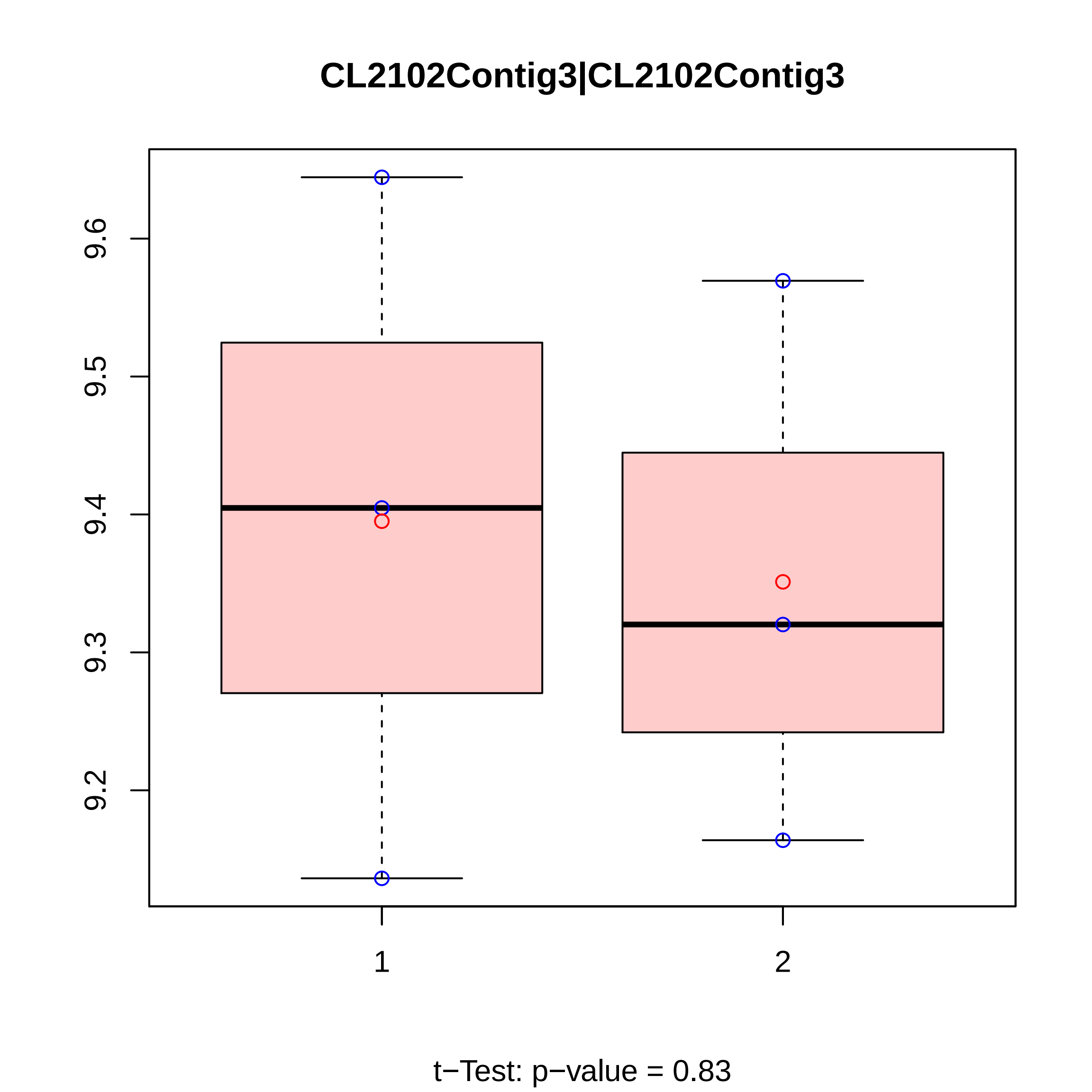

CL2102Contig3|CL2102Contig3
9.2
9.3
9.4
9.5
9.6
1
2
t−Test: p−value = 0.83

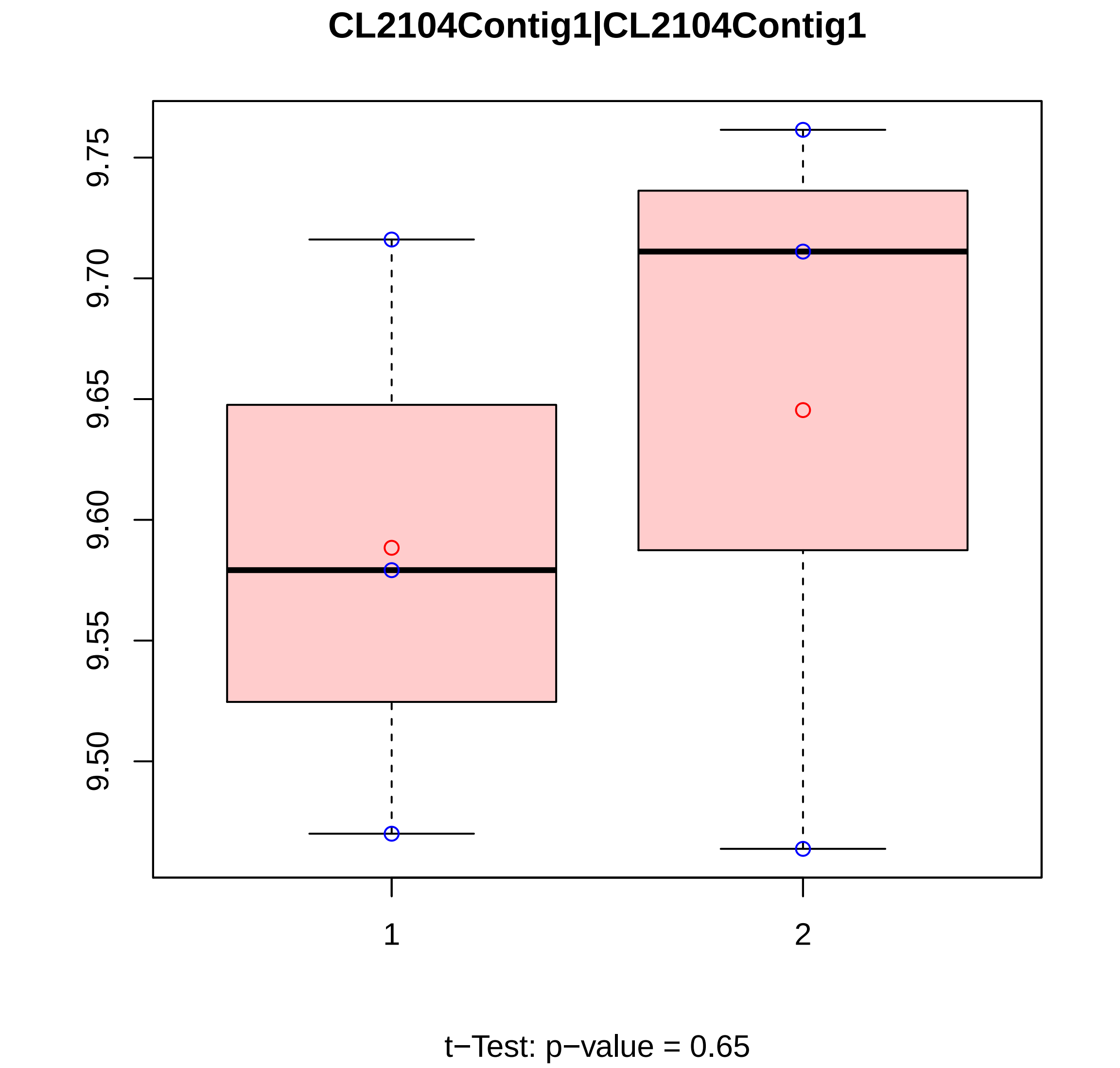
CL2104Contig1|CL2104Contig1
9.50
9.55
9.60
9.65
9.70
9.75
1
2
t−Test: p−value = 0.65

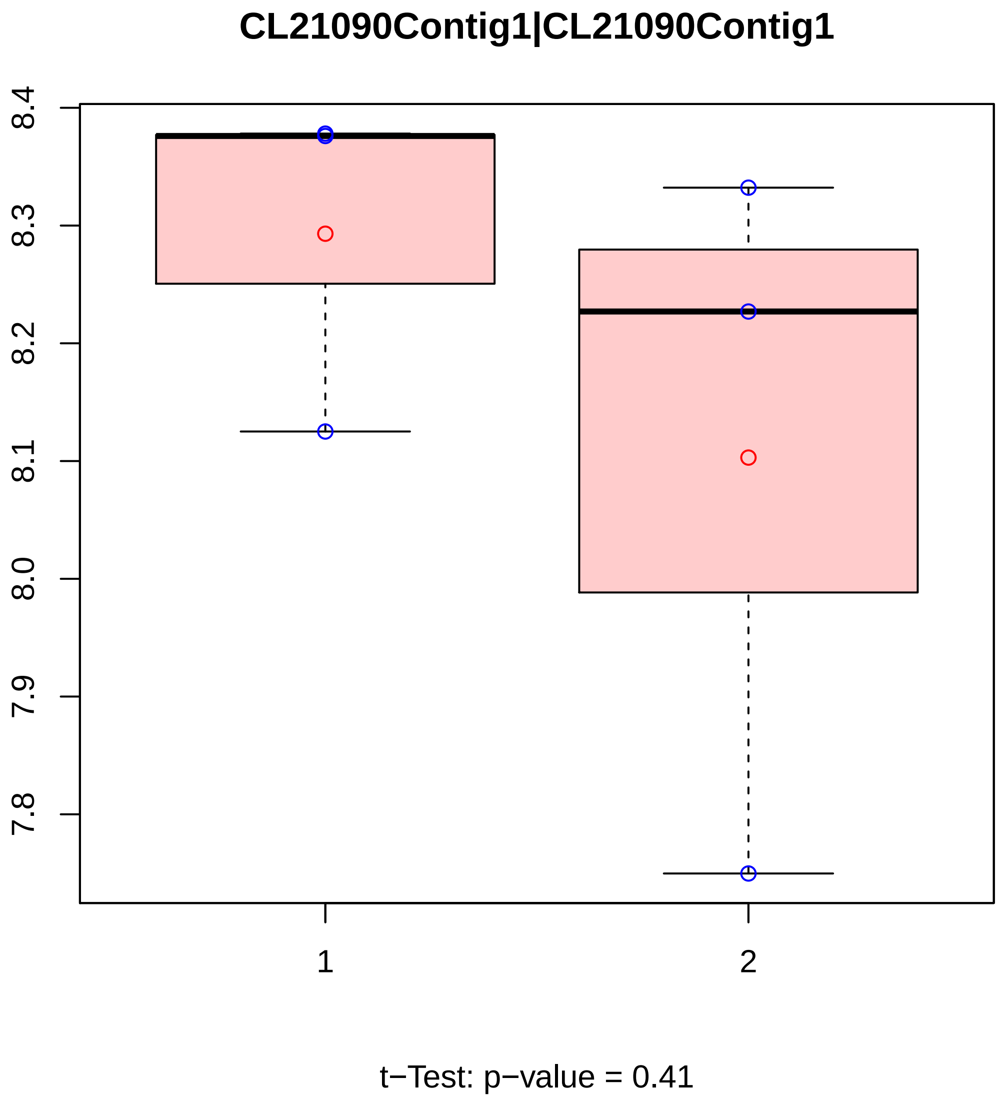

CL21090Contig1|CL21090Contig1
8.4
8.3
8.2
8.1
8.0
7.9
7.8
1
2
t−Test: p−value = 0.41

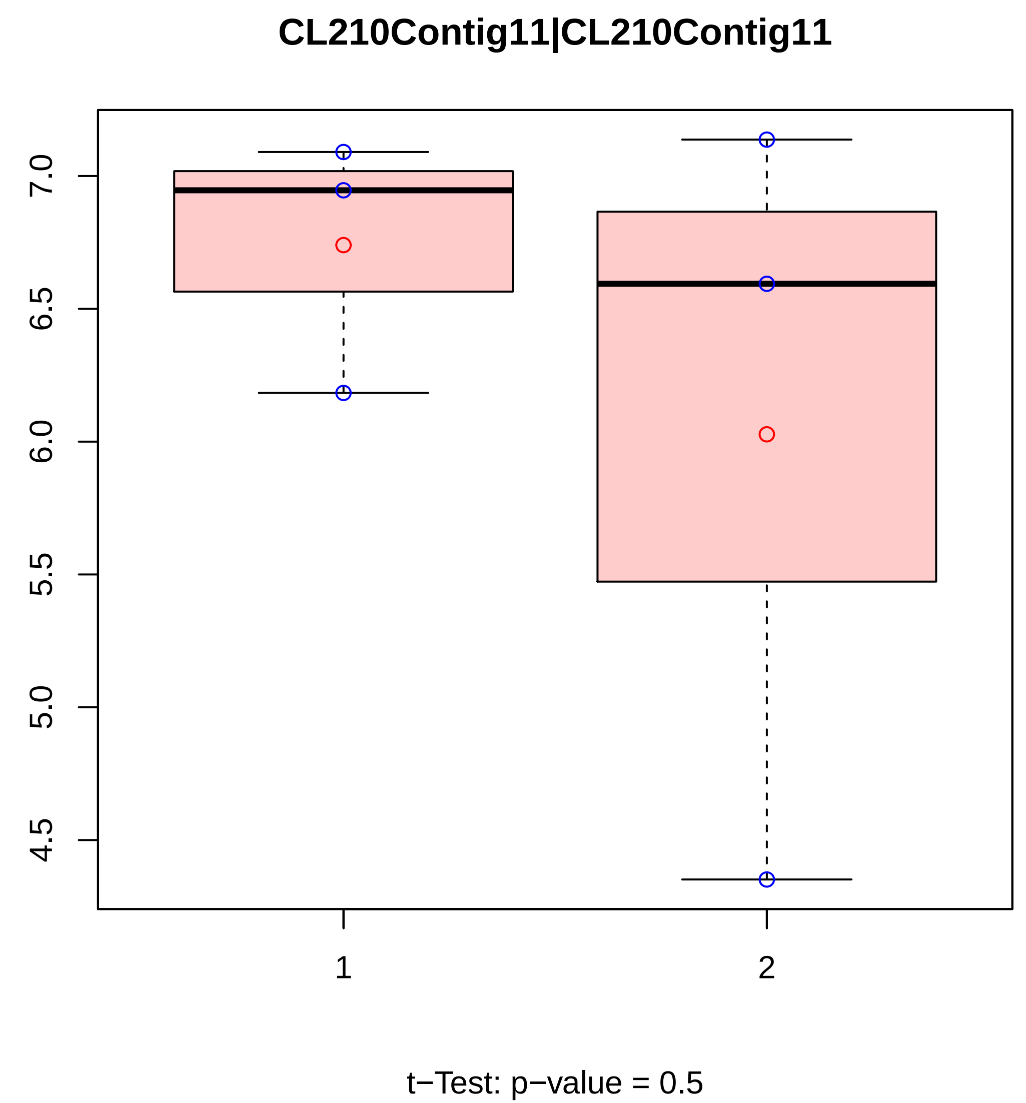
CL210Contig11|CL210Contig11
7.0
6.5
6.0
5.5
5.0
4.5
1
2
t−Test: p−value = 0.5

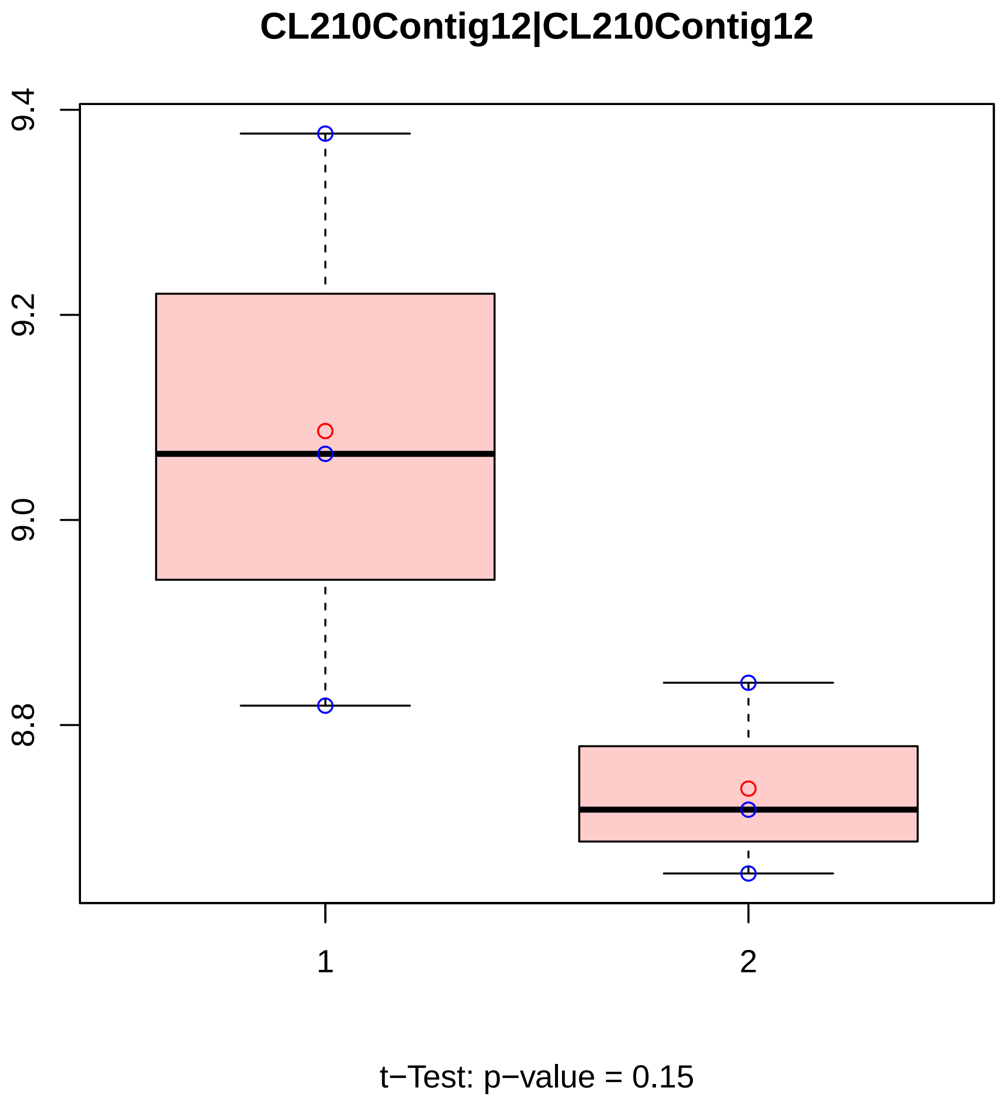

CL210Contig12|CL210Contig12
9.4
9.2
9.0
8.8
1
2
t−Test: p−value = 0.15

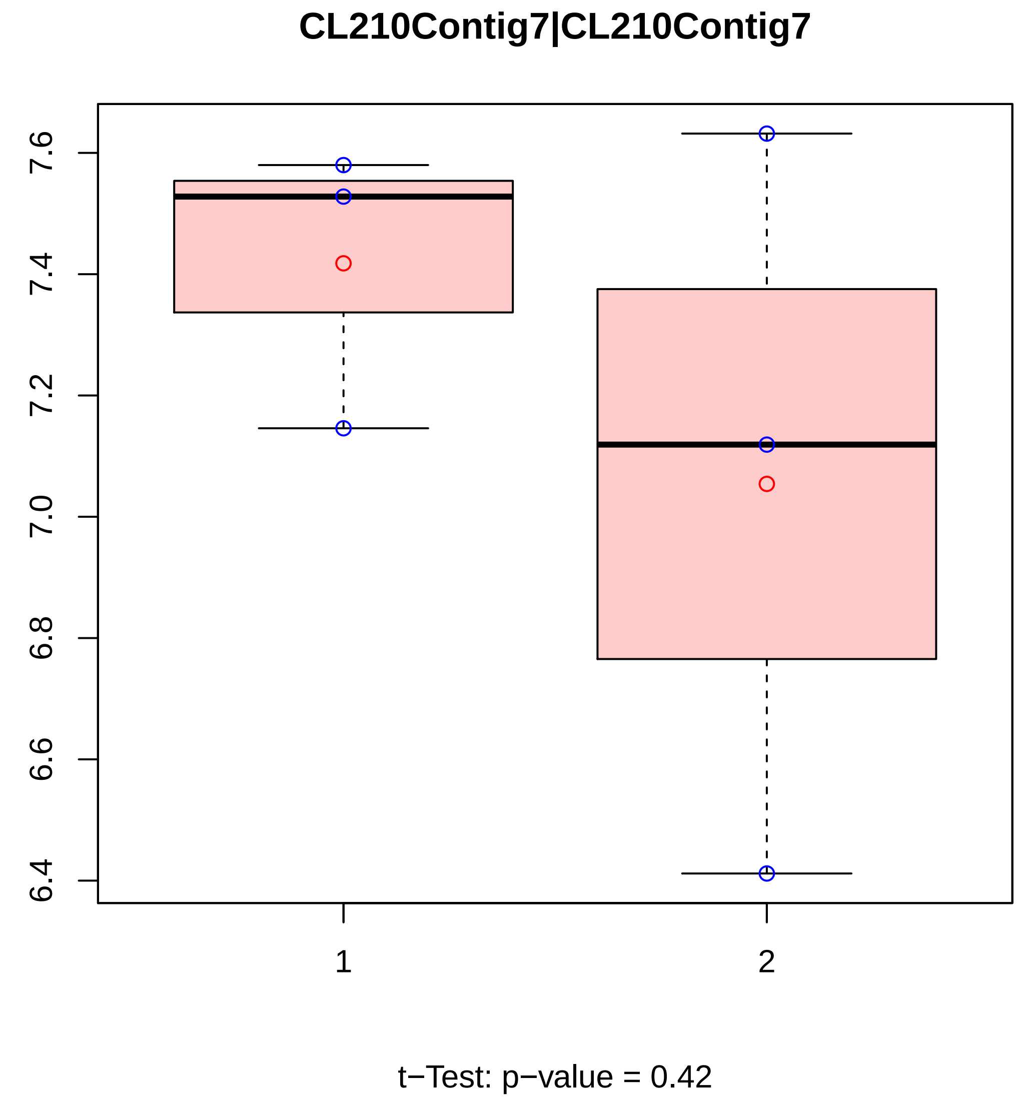
CL210Contig7|CL210Contig7
7.6
7.4
7.2
7.0
6.8
6.6
6.4
1
2
t−Test: p−value = 0.42

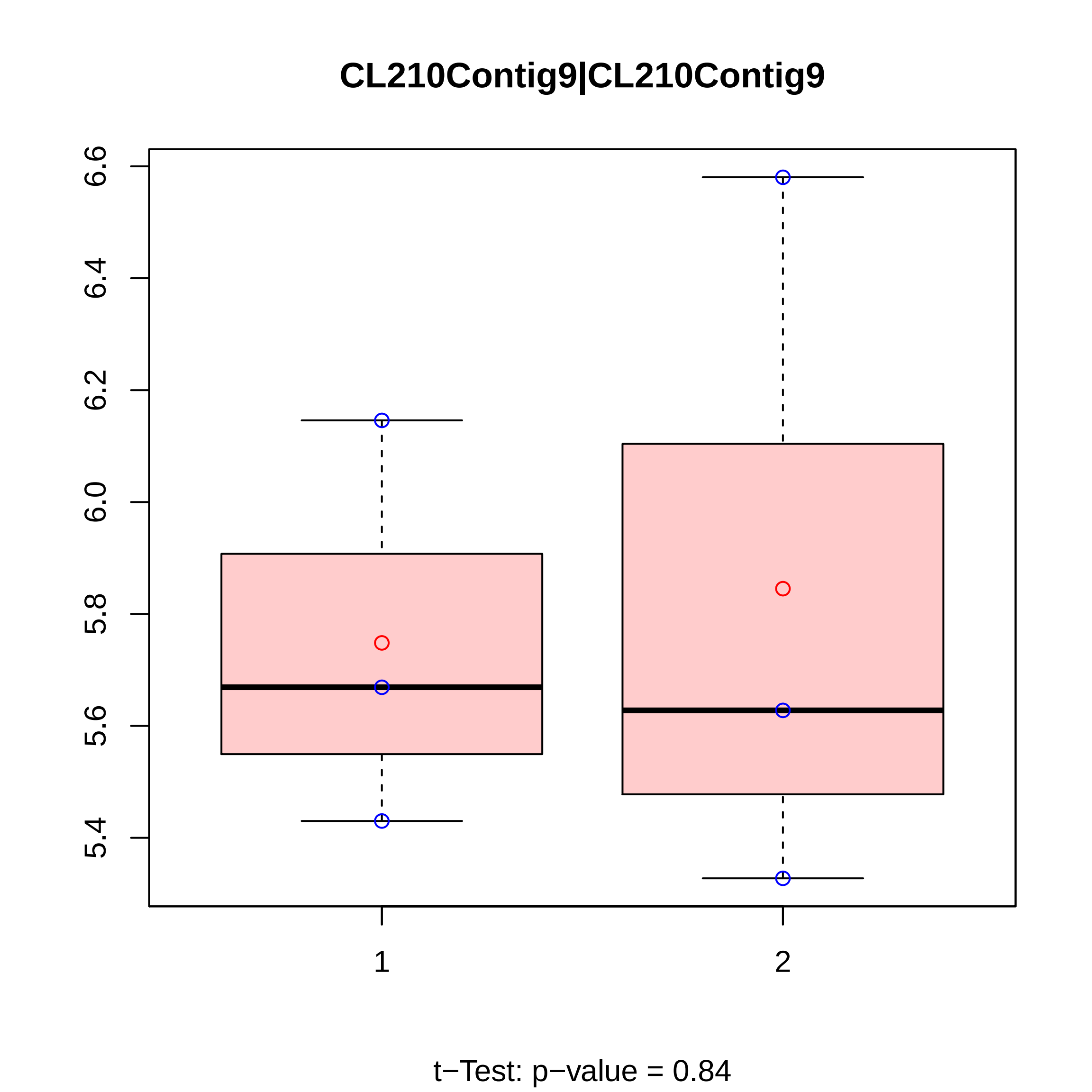
CL210Contig9|CL210Contig9
6.6
6.4
6.2
6.0
5.8
5.6
5.4
1
2
t−Test: p−value = 0.84

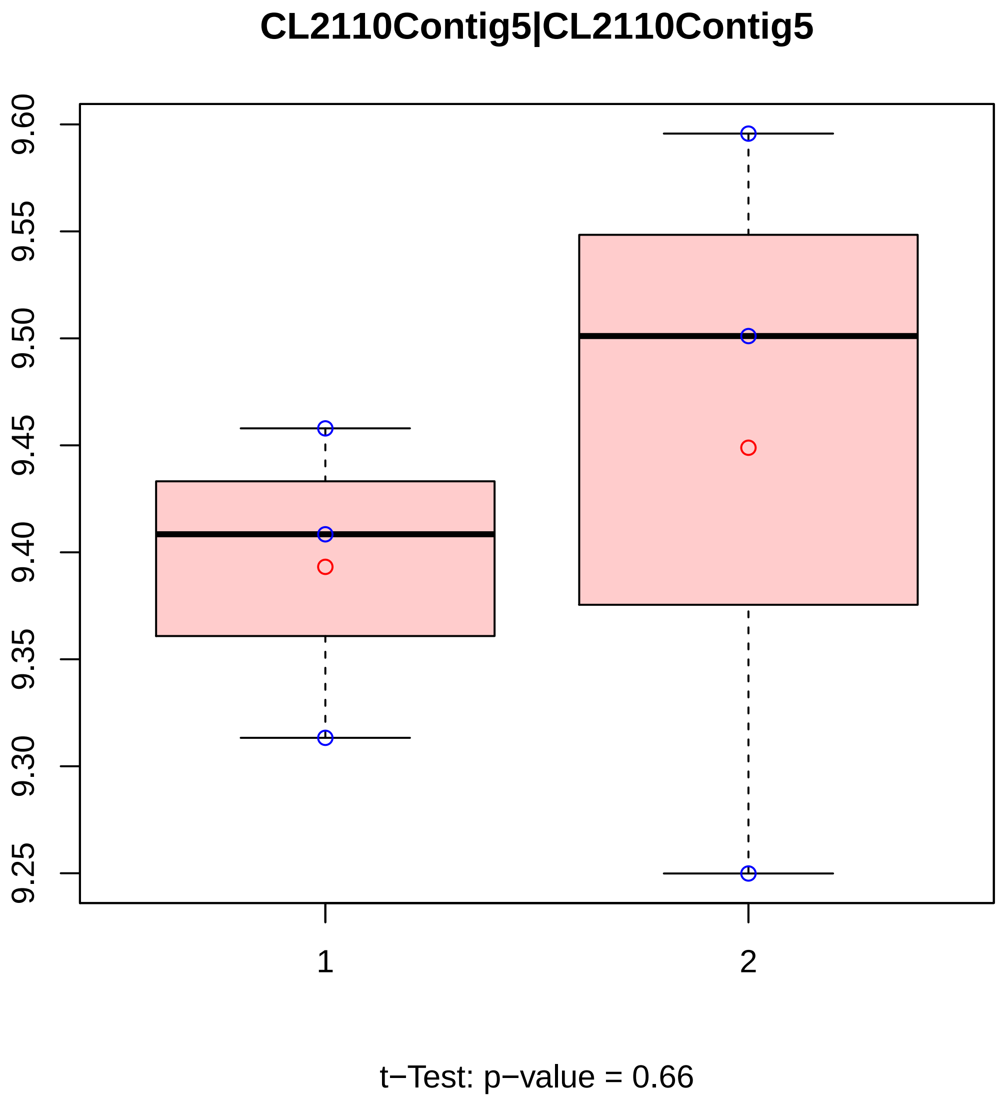
CL2110Contig5|CL2110Contig5
9.60
9.55
9.50
9.45
9.40
9.35
9.30
9.25
1
2
t−Test: p−value = 0.66

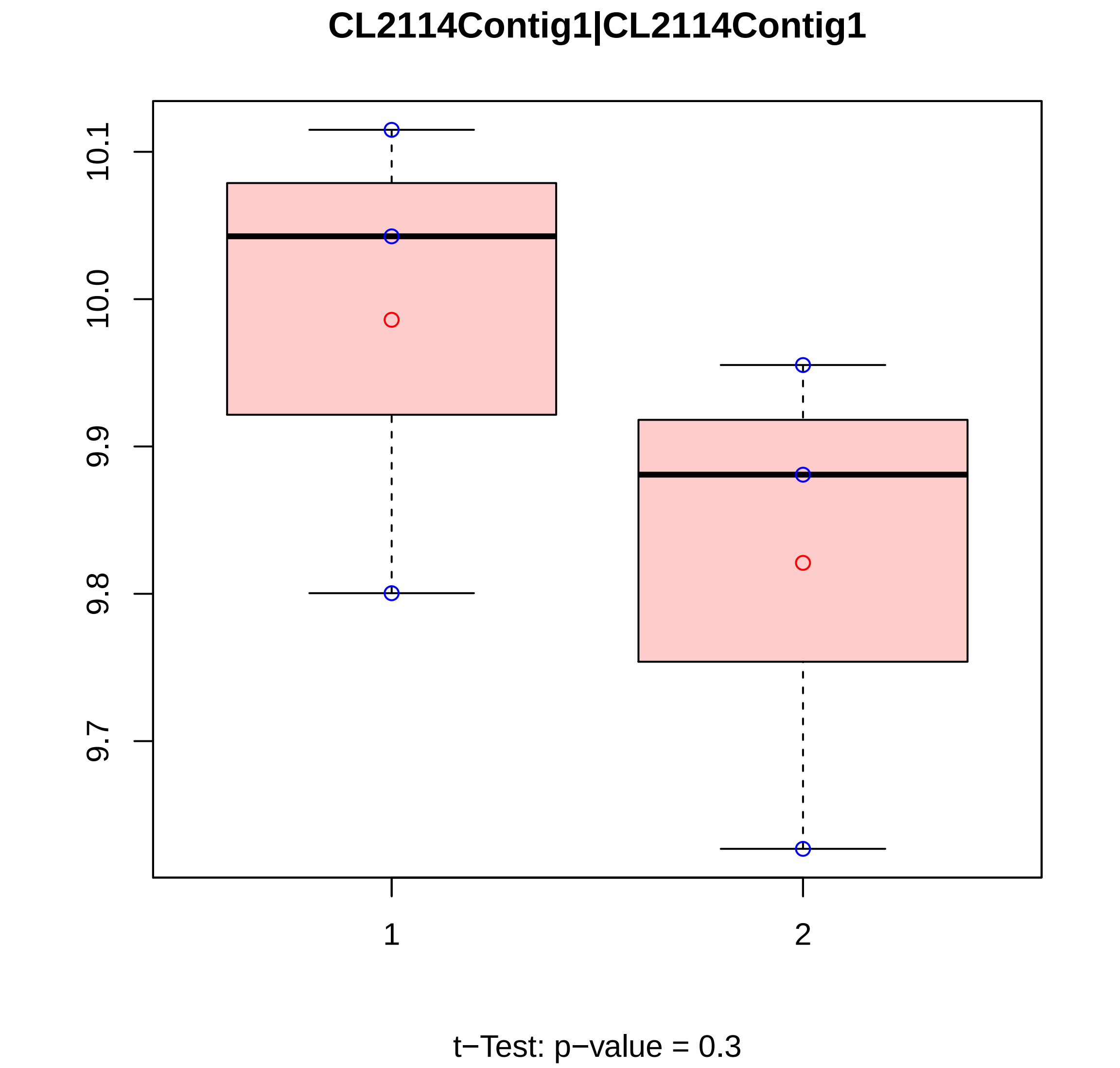

CL2114Contig1|CL2114Contig1
10.1
10.0
9.9
9.8
9.7
1
2
t−Test: p−value = 0.3

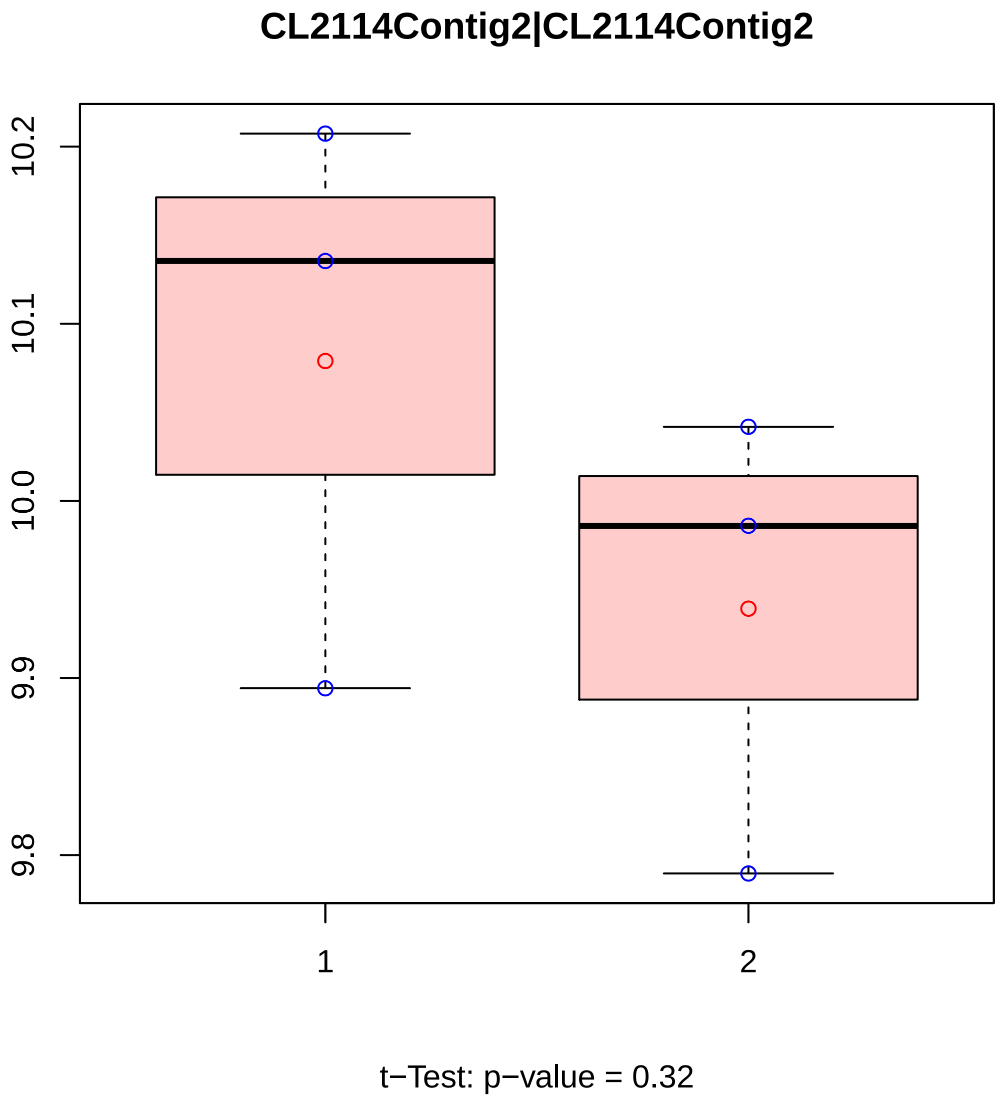
CL2114Contig2|CL2114Contig2
10.2
10.1
10.0
9.9
9.8
1
2
t−Test: p−value = 0.32

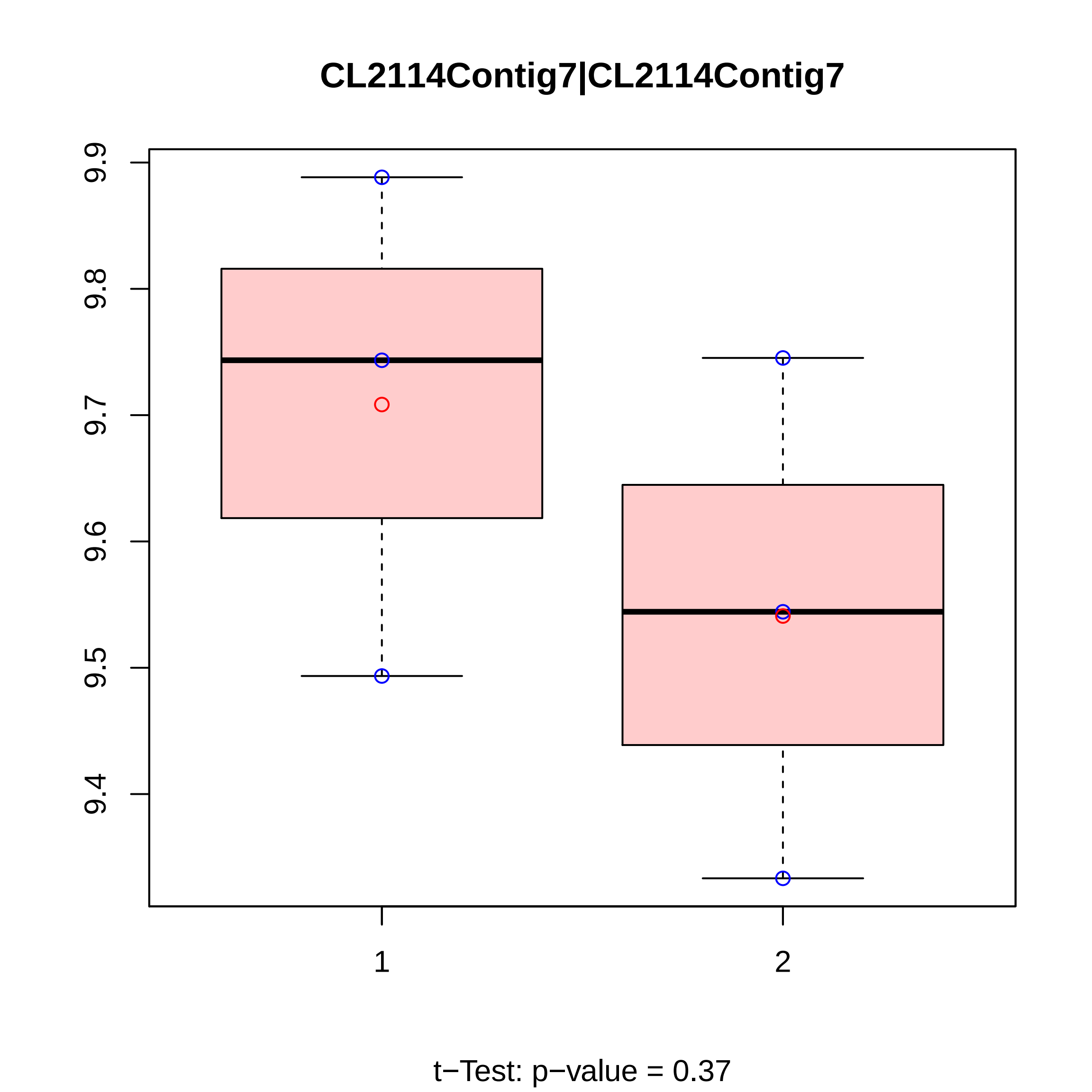

CL2114Contig7|CL2114Contig7
9.9
9.8
9.7
9.6
9.5
9.4
1
2
t−Test: p−value = 0.37

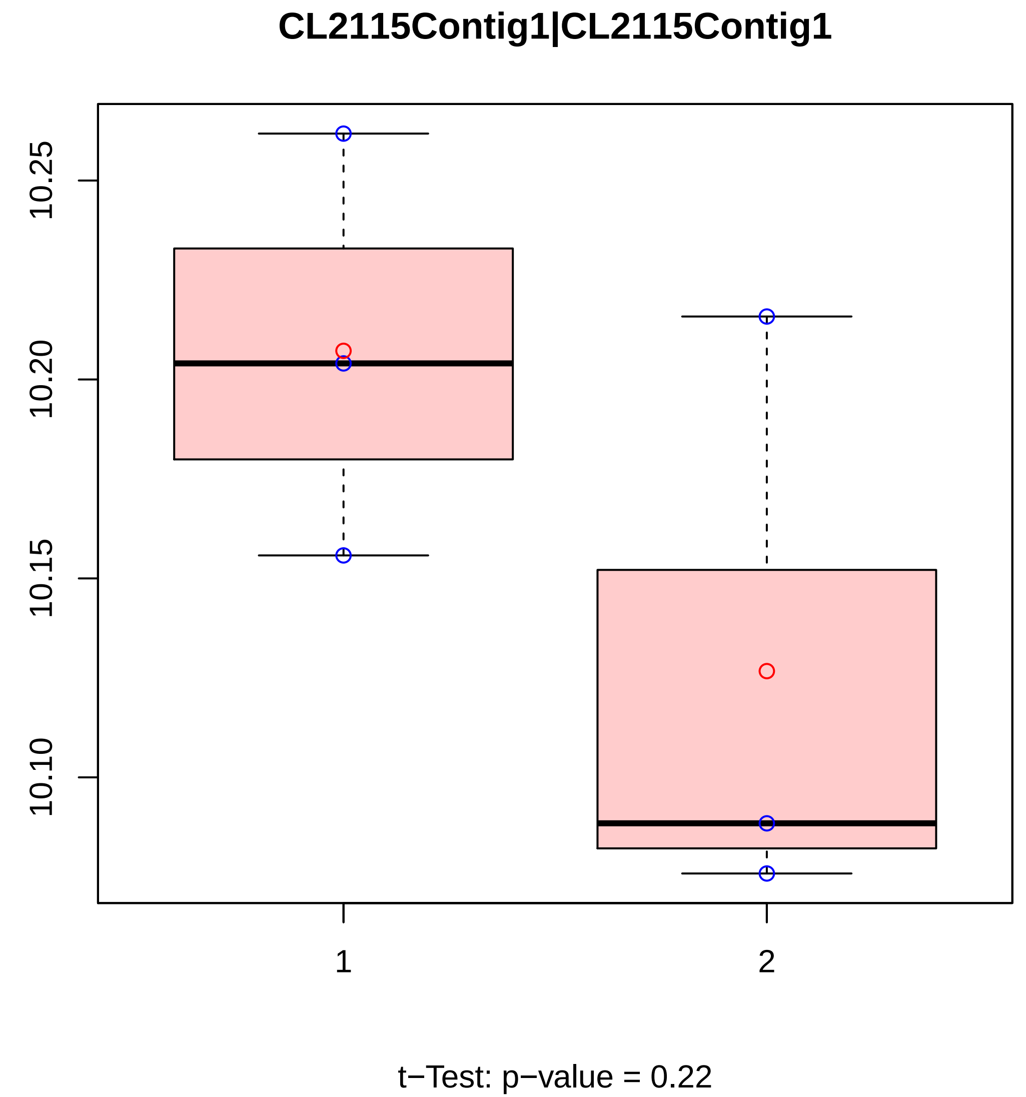
CL2115Contig1|CL2115Contig1
10.25
10.20
10.15
10.10
1
2
t−Test: p−value = 0.22

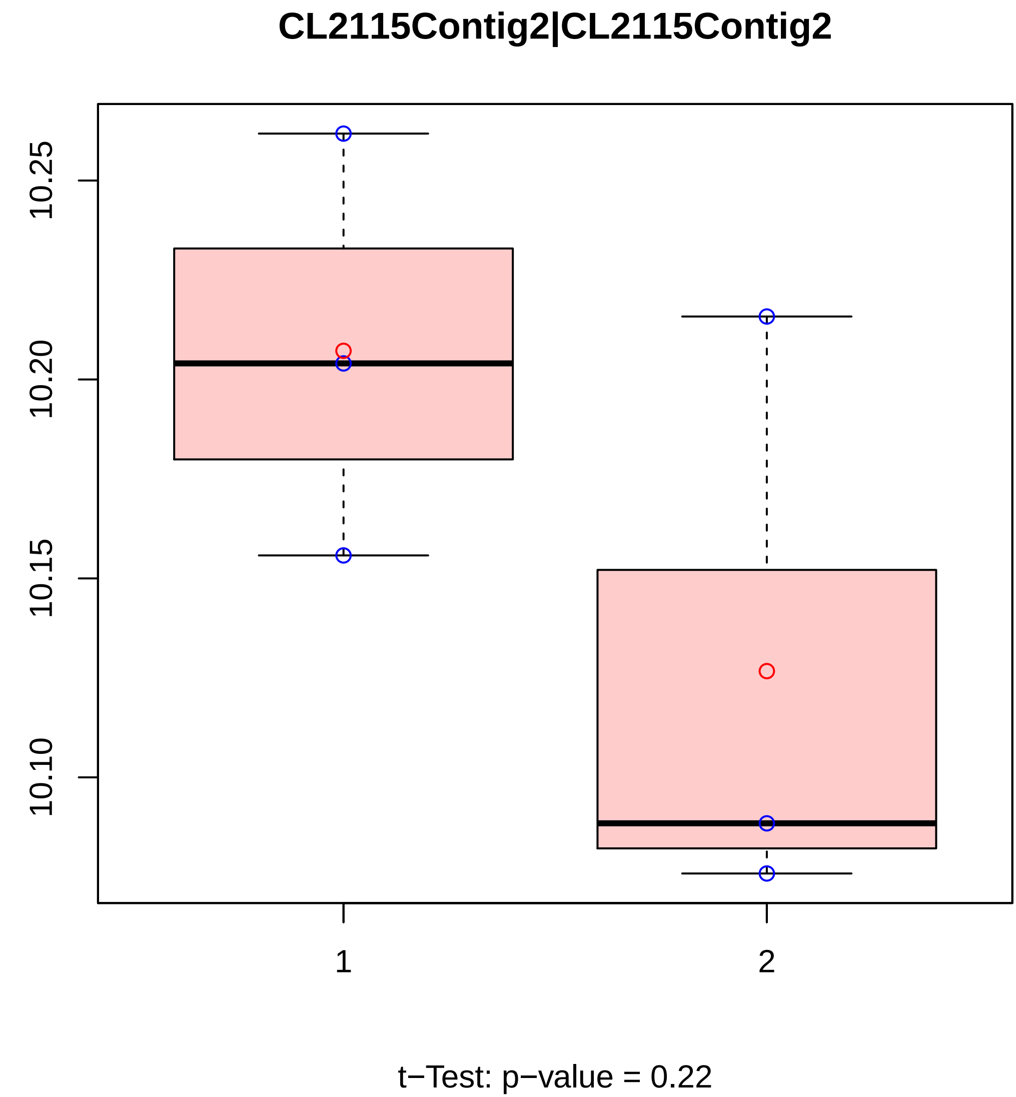

CL2115Contig2|CL2115Contig2
10.10
10.15
10.20
10.25
1
2
t−Test: p−value = 0.22

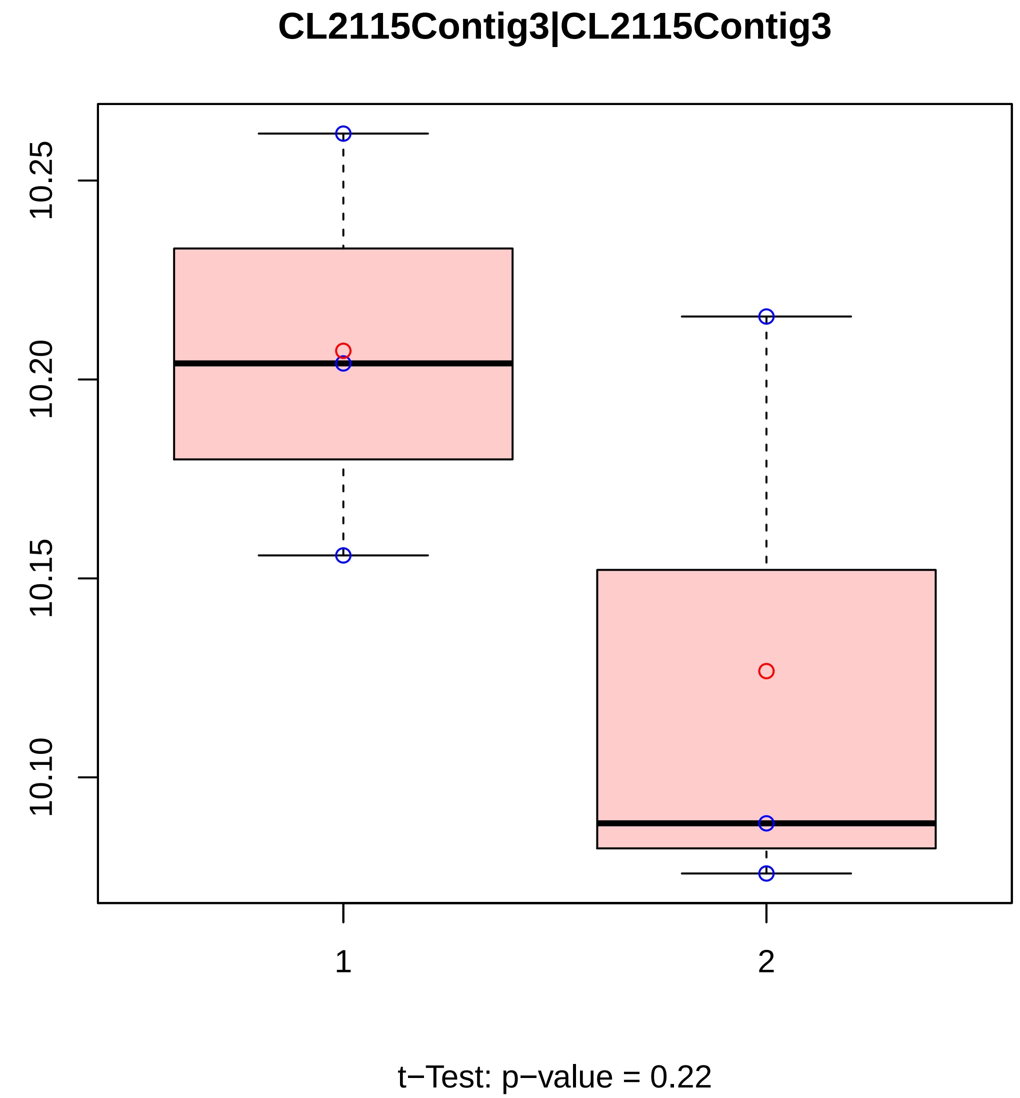
CL2115Contig3|CL2115Contig3
10.25
10.20
10.15
10.10
1
2
t−Test: p−value = 0.22

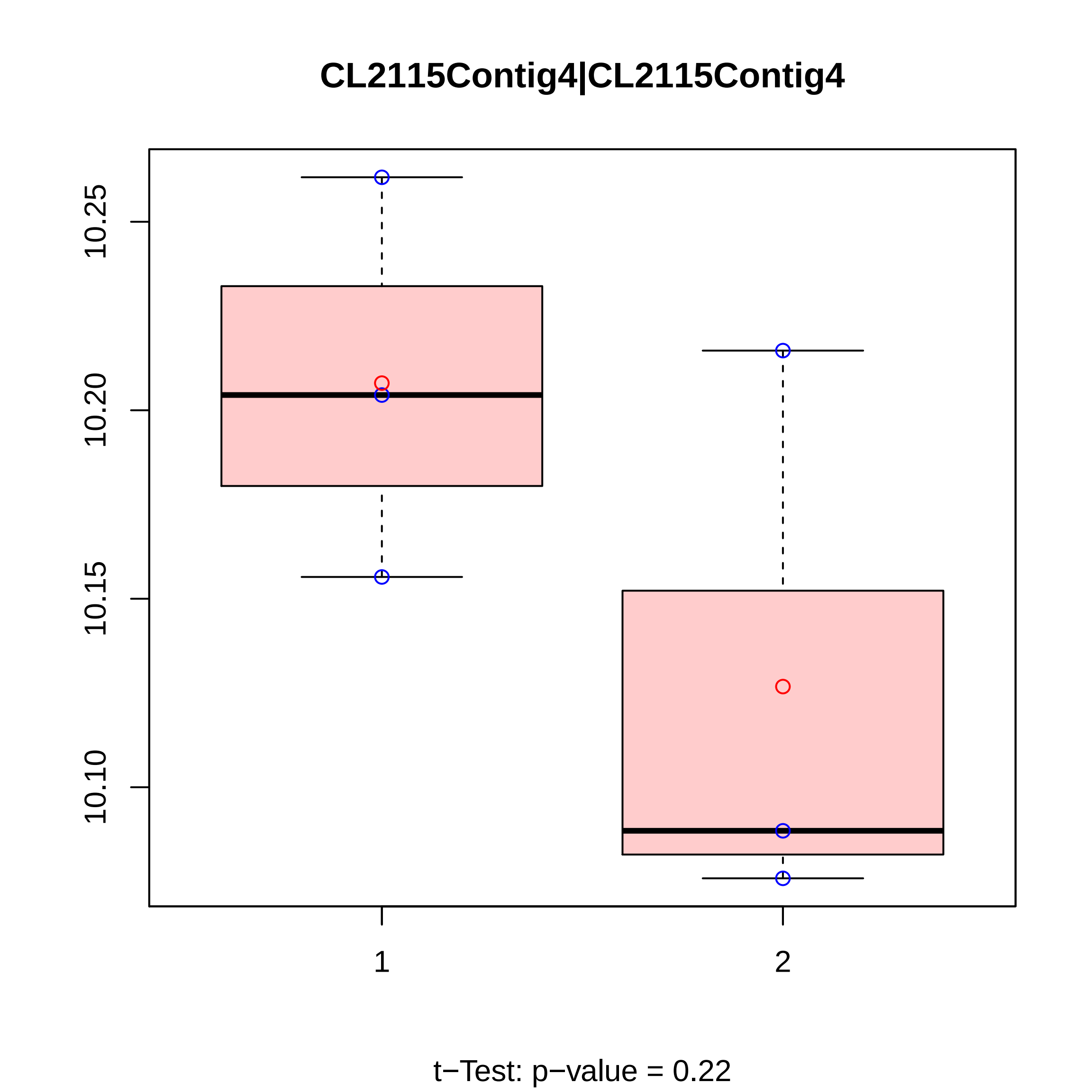

CL2115Contig4|CL2115Contig4
10.10
10.15
10.20
10.25
1
2
t−Test: p−value = 0.22

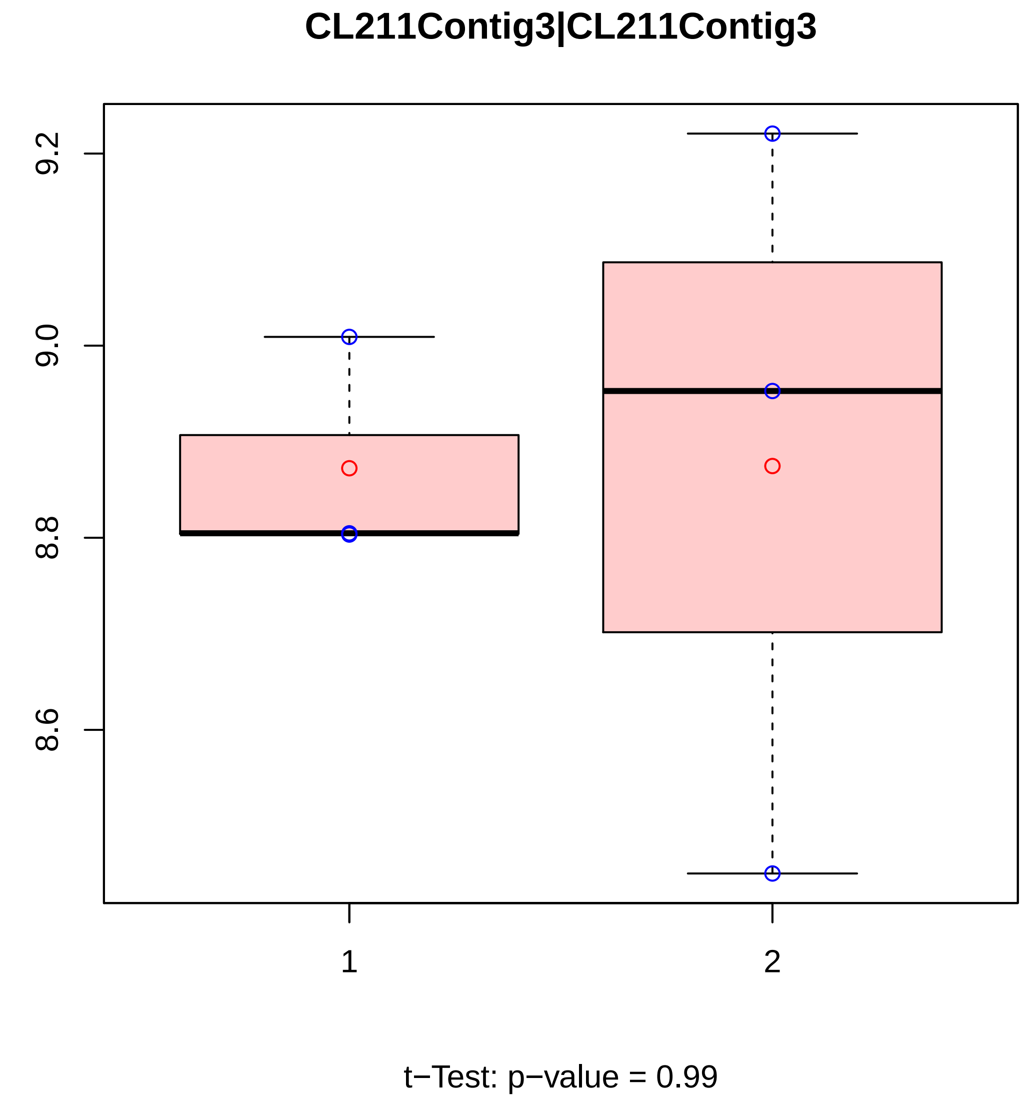
CL211Contig3|CL211Contig3
9.2
9.0
8.8
8.6
1
2
t−Test: p−value = 0.99

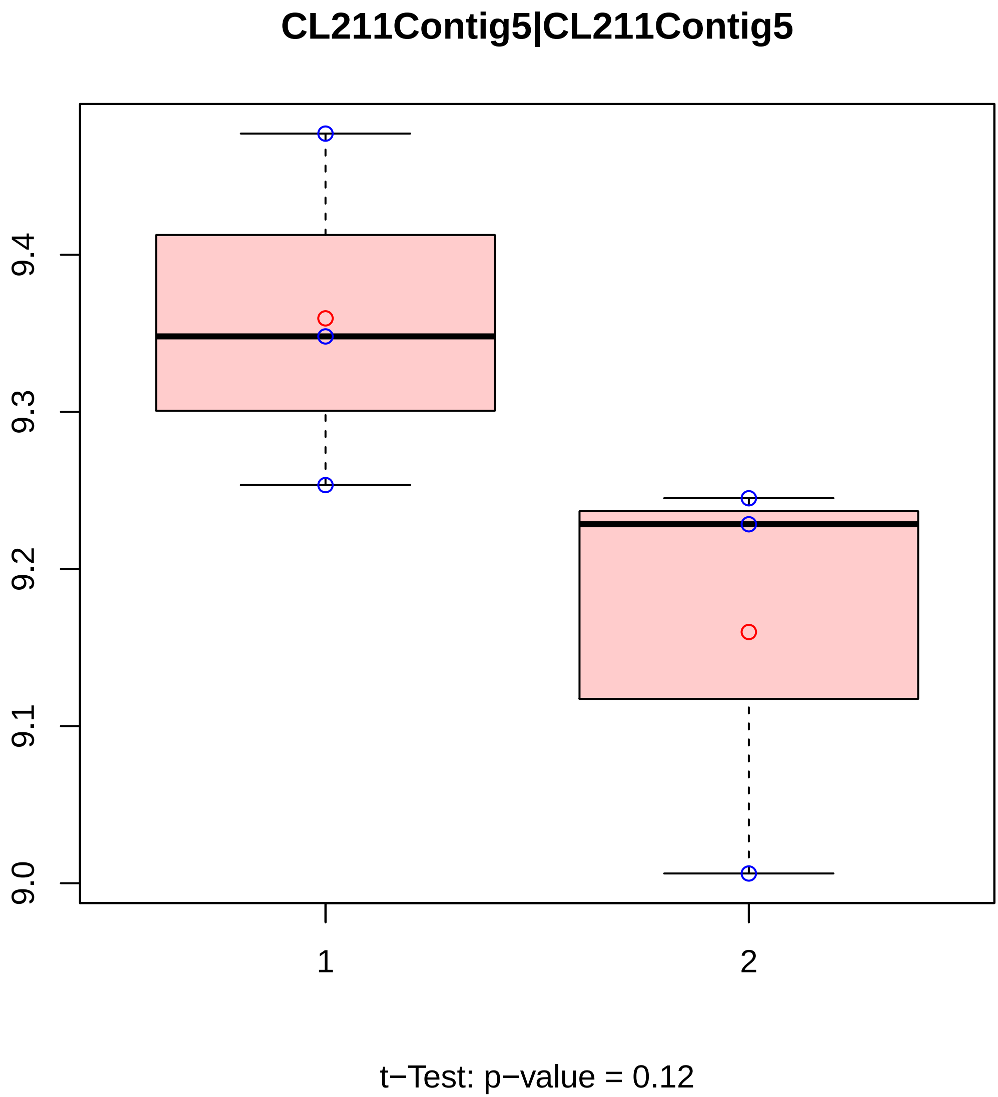
CL211Contig5|CL211Contig5
9.0
9.1
9.2
9.3
9.4
1
2
t−Test: p−value = 0.12

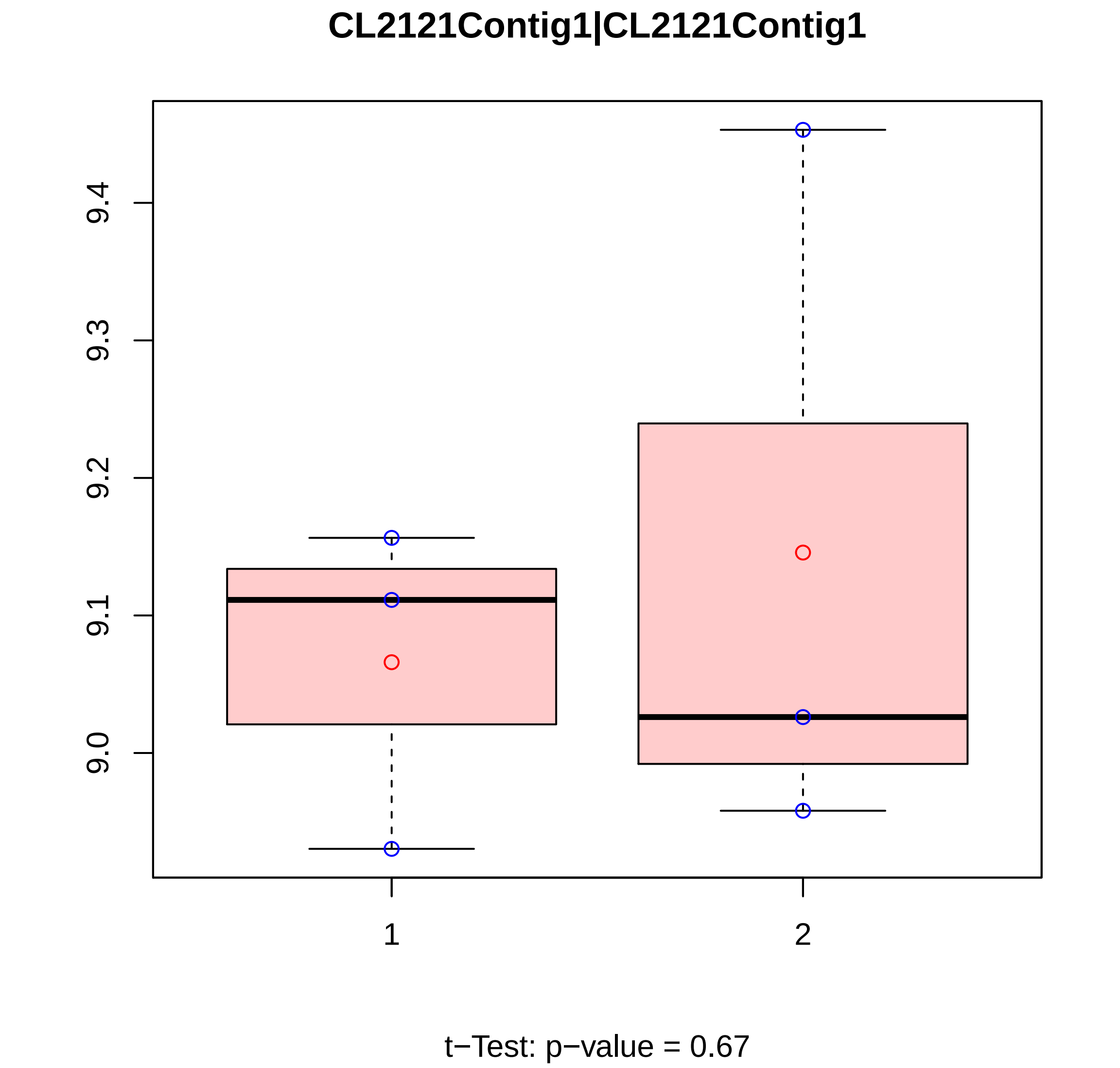

CL2121Contig1|CL2121Contig1
9.0
9.1
9.2
9.3
9.4
1
2
t−Test: p−value = 0.67

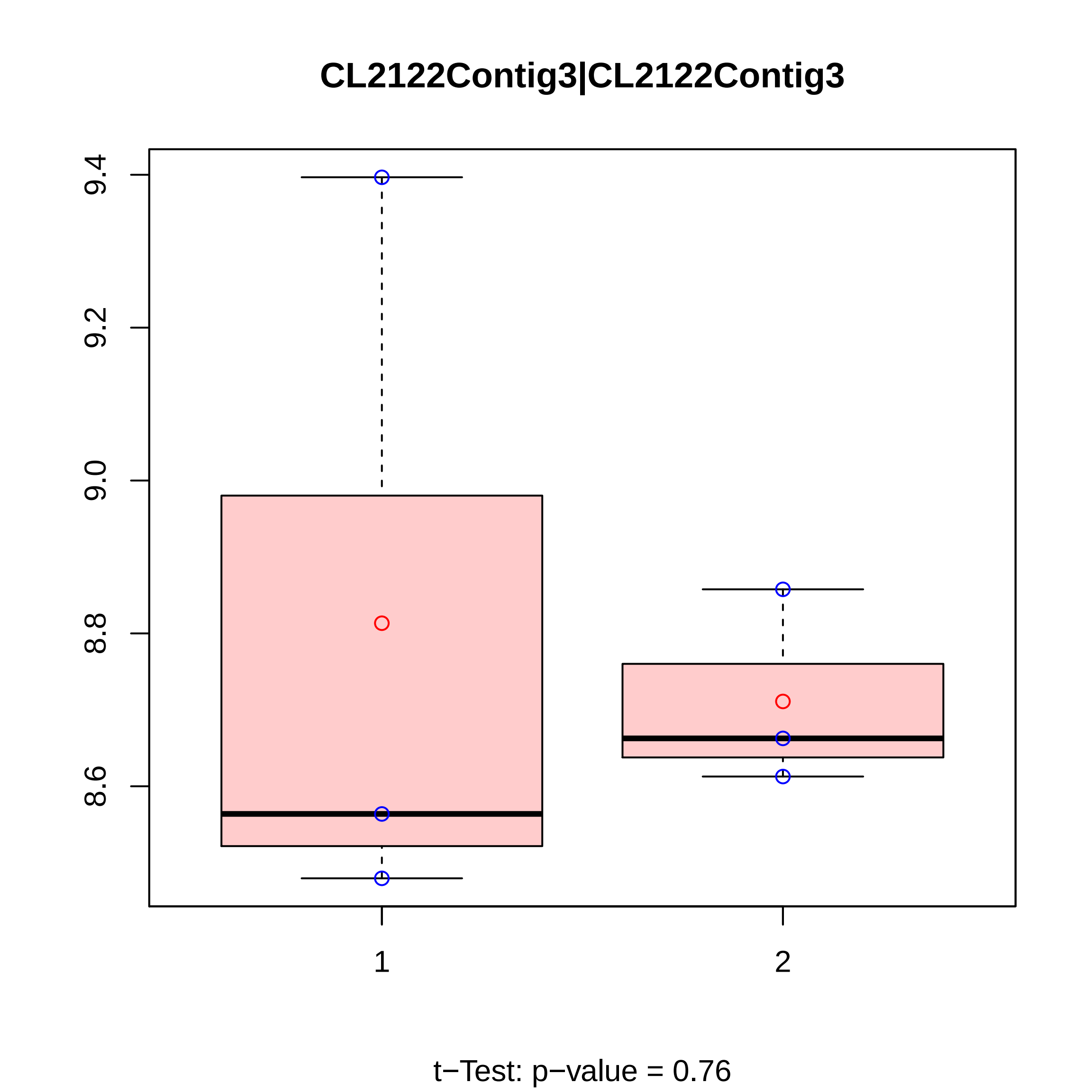

CL2122Contig3|CL2122Contig3
9.4
9.2
9.0
8.8
8.6
1
2
t−Test: p−value = 0.76

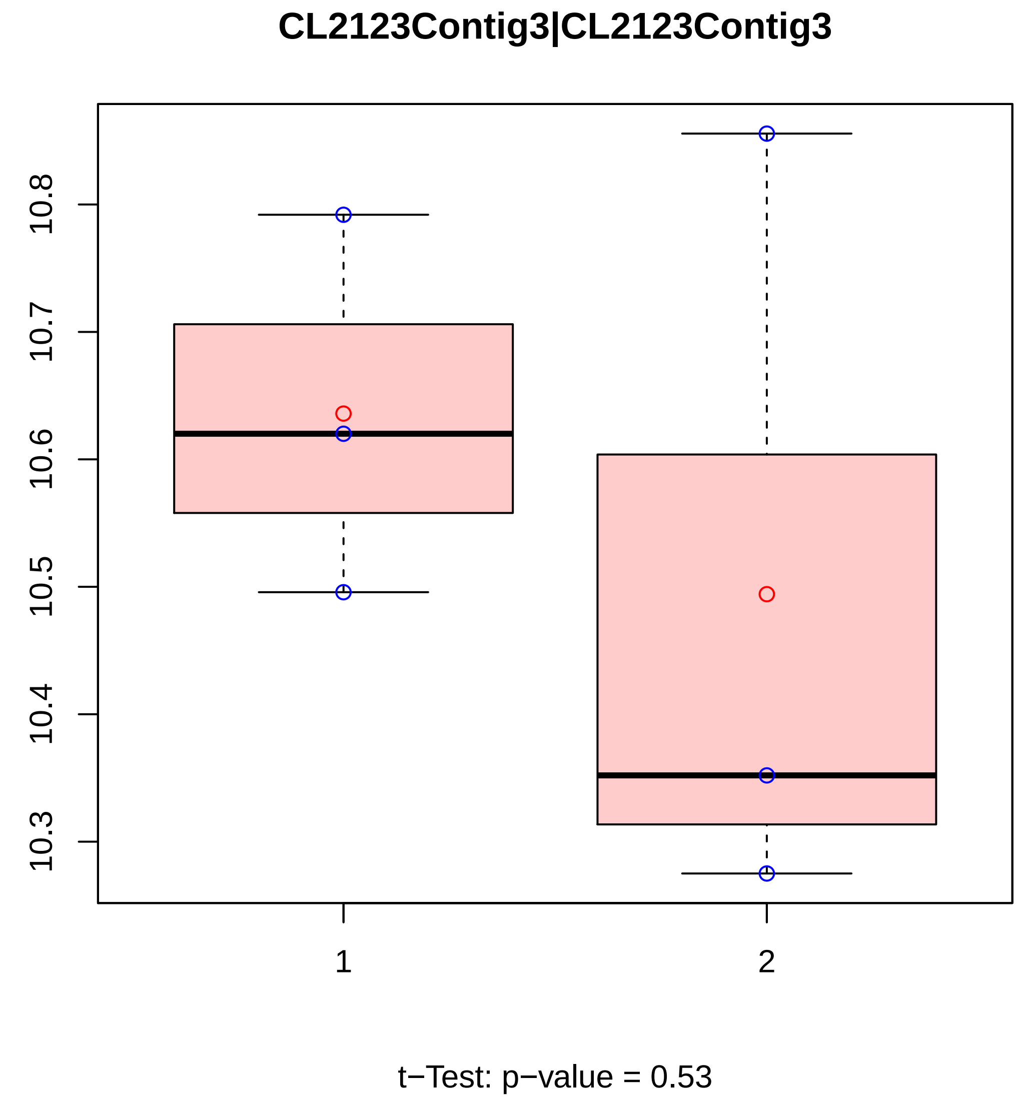
CL2123Contig3|CL2123Contig3
10.3
10.4
10.5
10.6
10.7
10.8
1
2
t−Test: p−value = 0.53

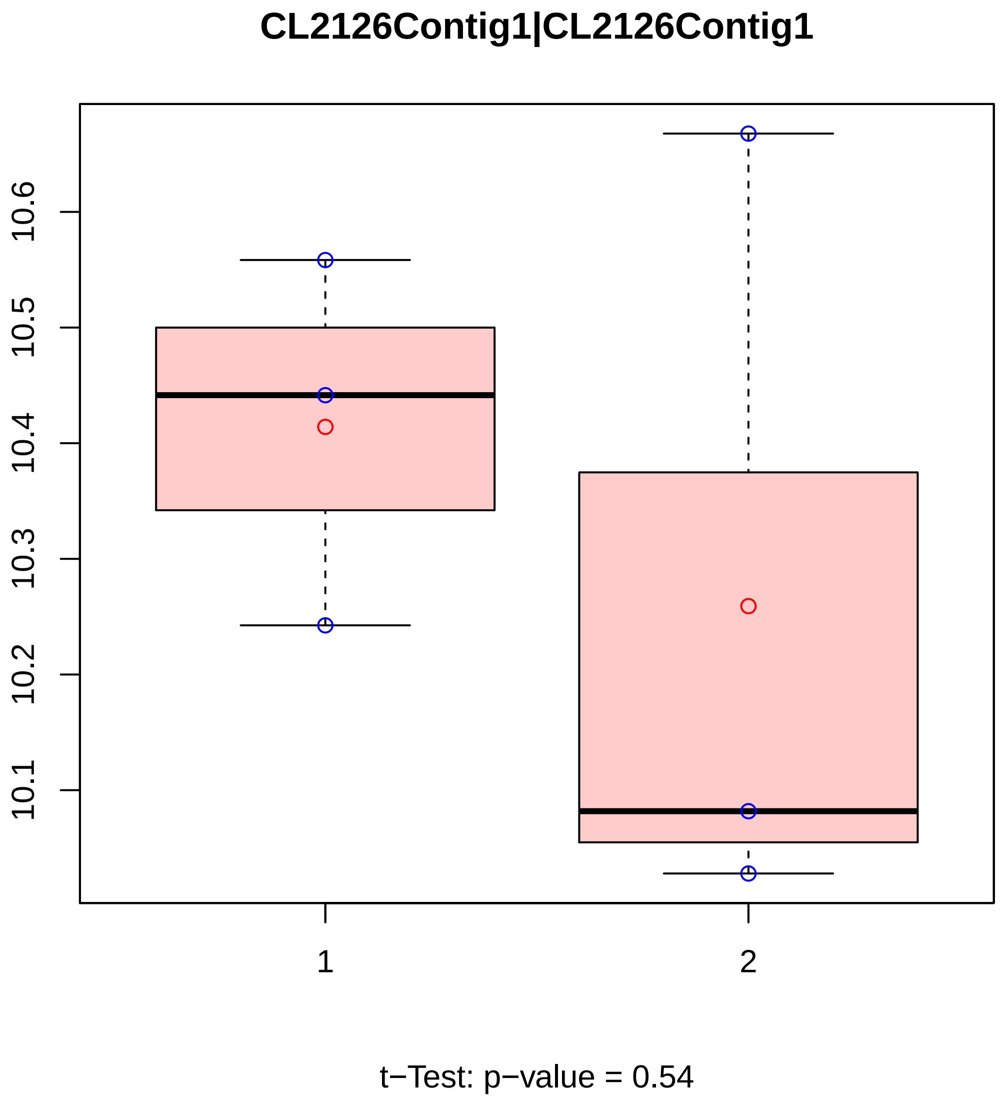
CL2126Contig1|CL2126Contig1
10.1
10.2
10.3
10.4
10.5
10.6
1
2
t−Test: p−value = 0.54

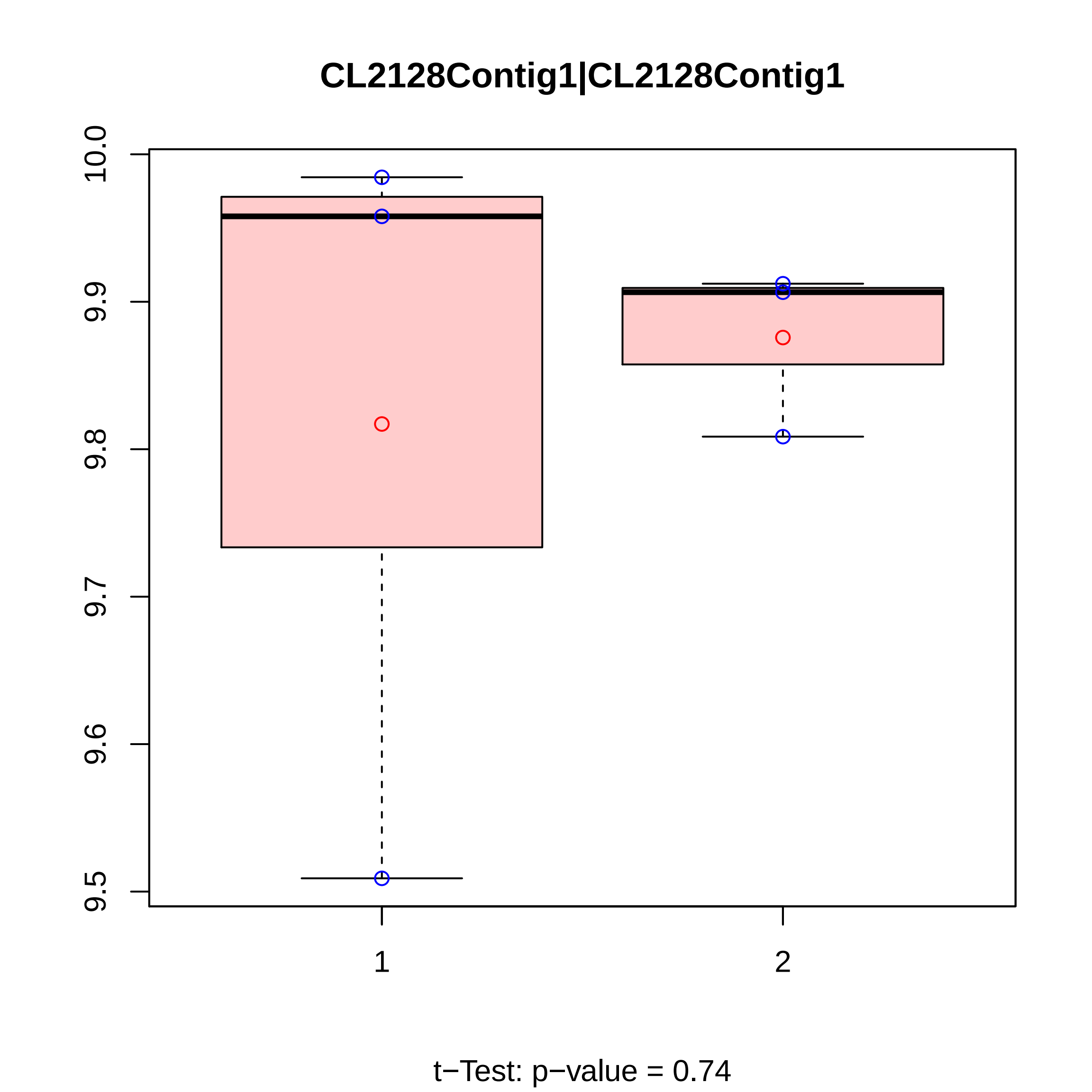

CL2128Contig1|CL2128Contig1
10.0
9.9
9.8
9.7
9.6
9.5
1
2
t−Test: p−value = 0.74

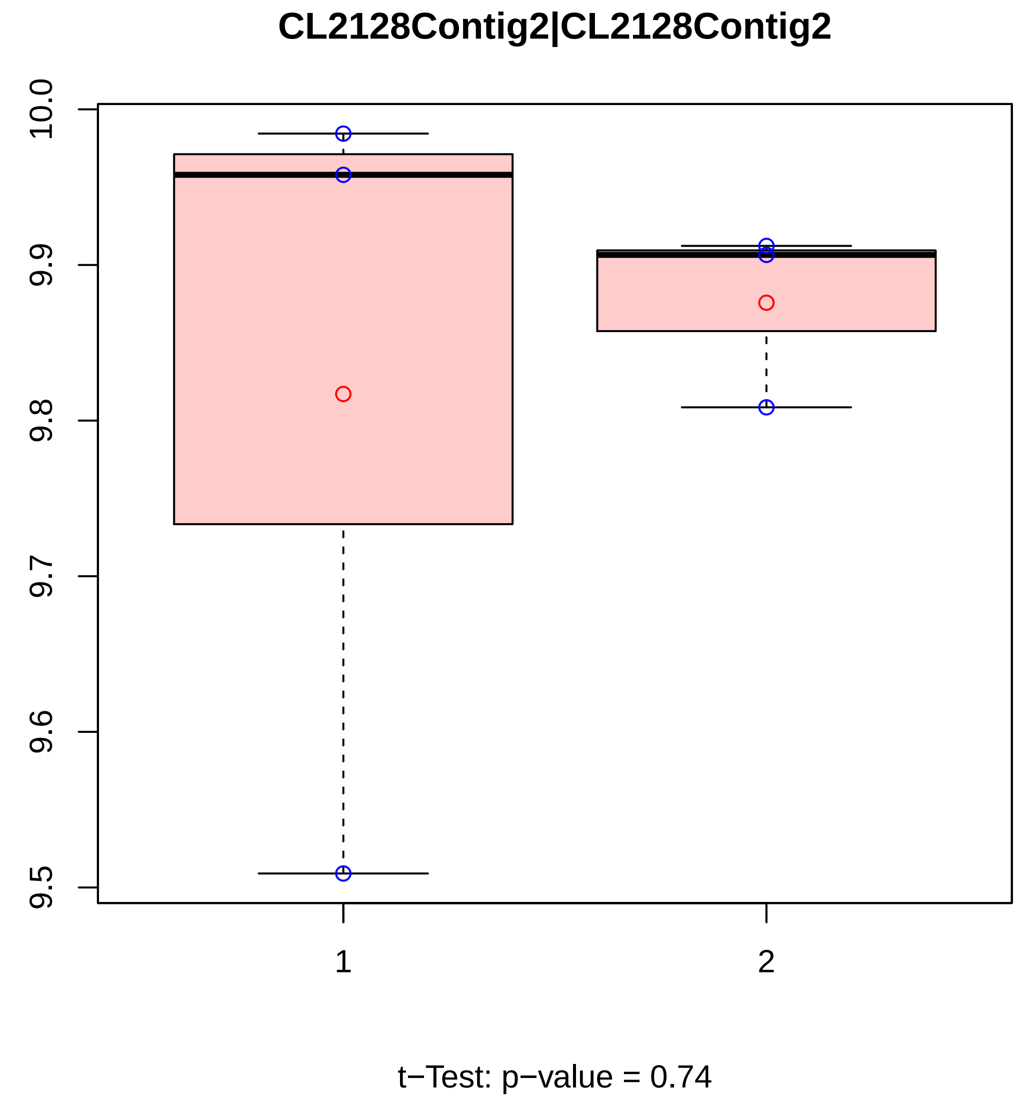
CL2128Contig2|CL2128Contig2
10.0
9.9
9.8
9.7
9.6
9.5
1
2
t−Test: p−value = 0.74

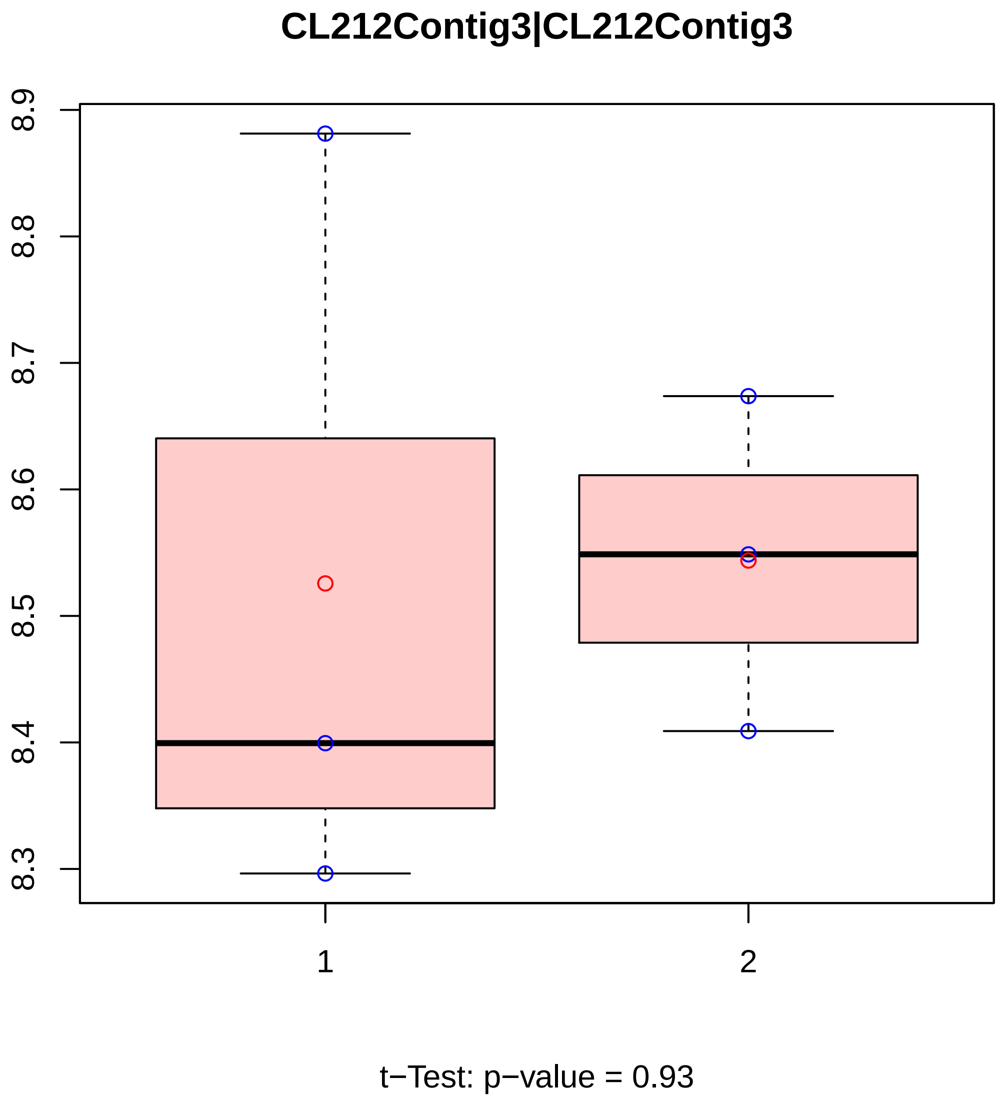
CL212Contig3|CL212Contig3
8.3
8.4
8.5
8.6
8.7
8.8
8.9
1
2
t−Test: p−value = 0.93

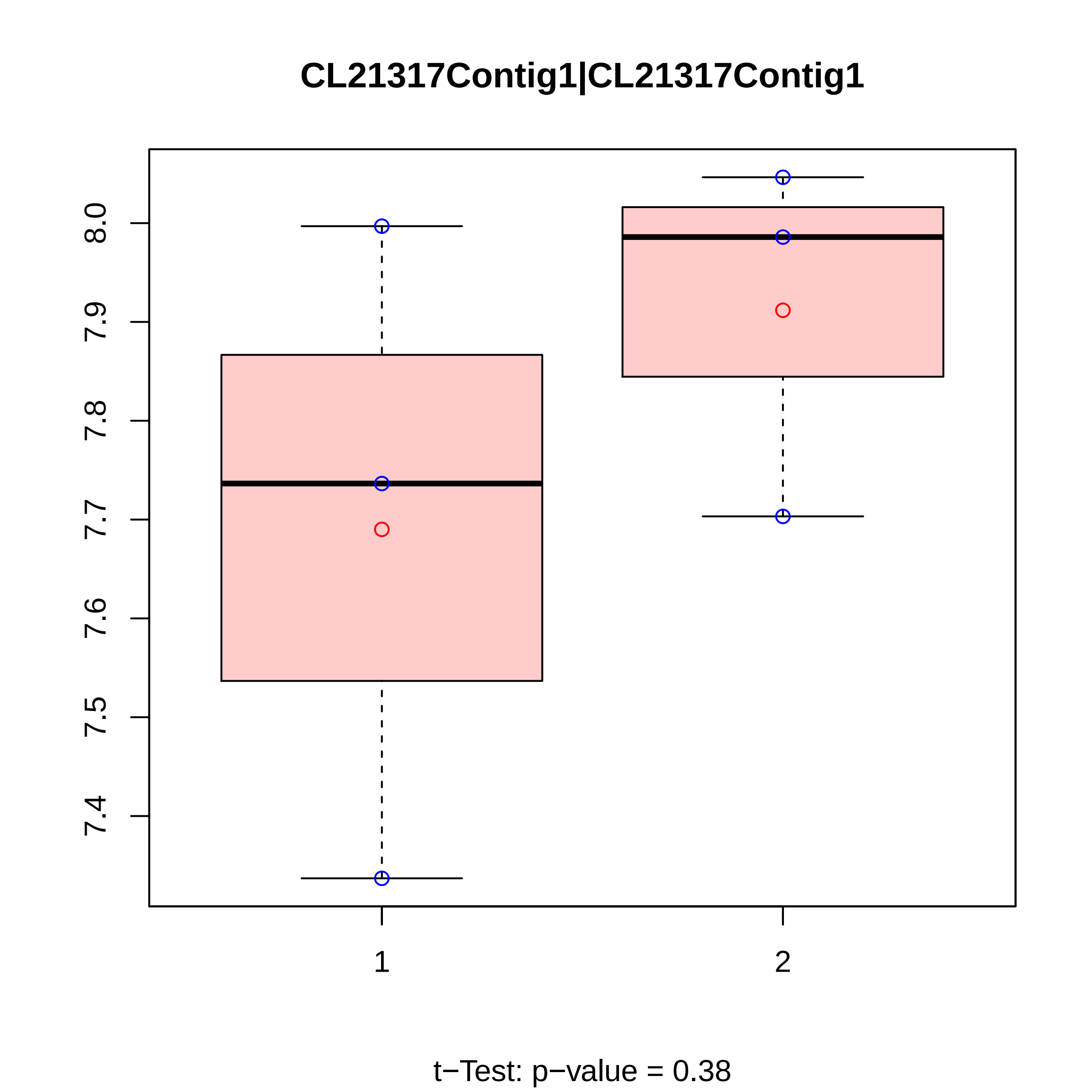
CL21317Contig1|CL21317Contig1
8.0
7.9
7.8
7.7
7.6
7.5
7.4
1
2
t−Test: p−value = 0.38

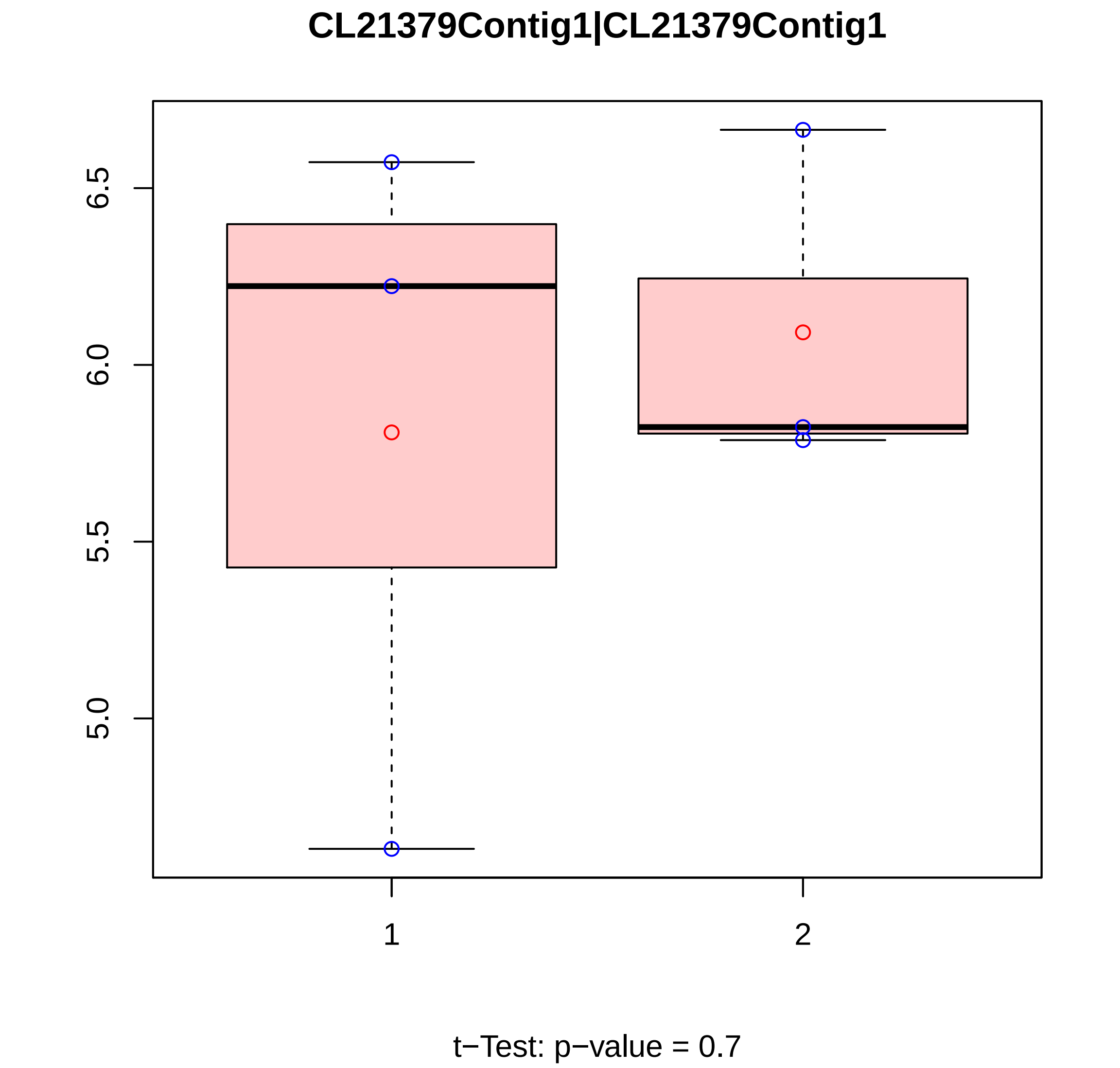
CL21379Contig1|CL21379Contig1
6.5
6.0
5.5
5.0
1
2
t−Test: p−value = 0.7

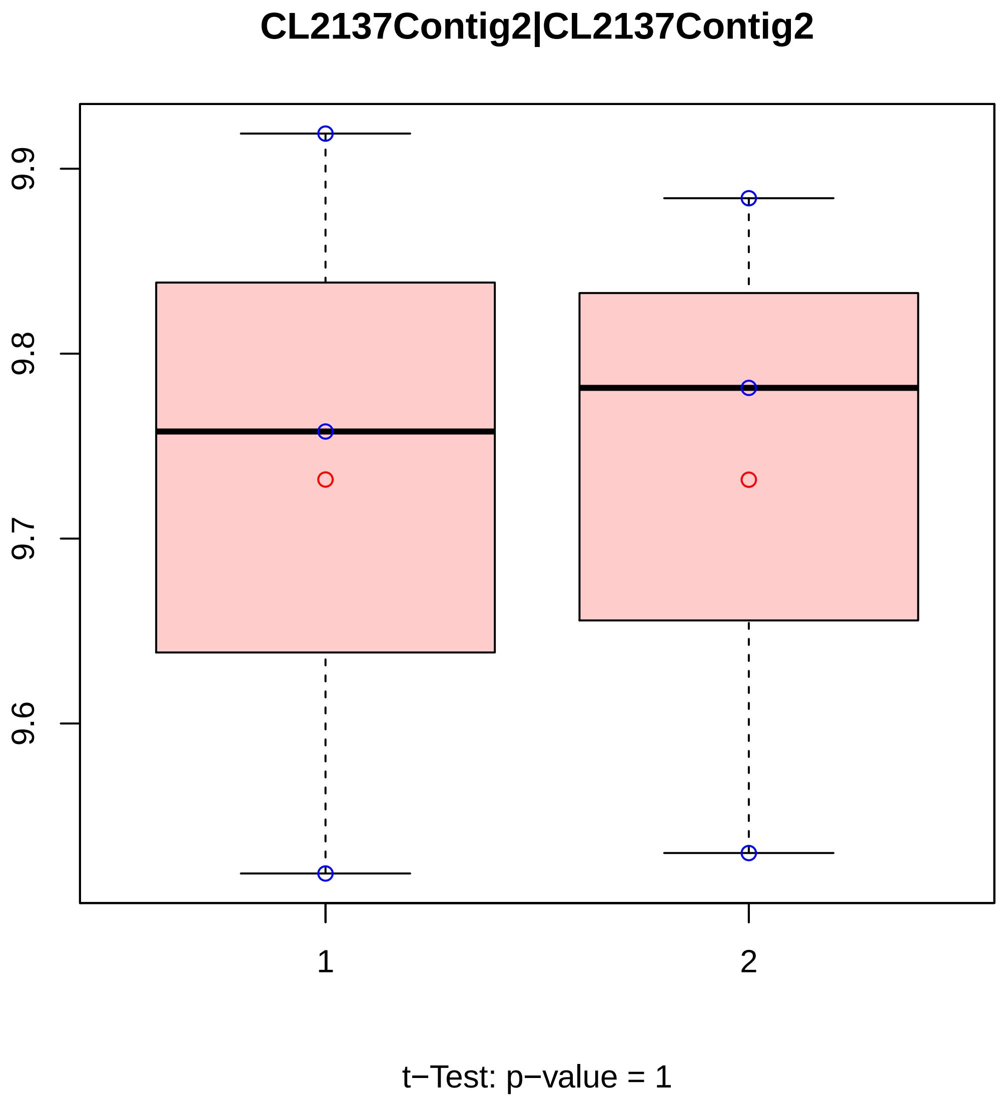

CL2137Contig2|CL2137Contig2
9.9
9.8
9.7
9.6
1
2
t−Test: p−value = 1

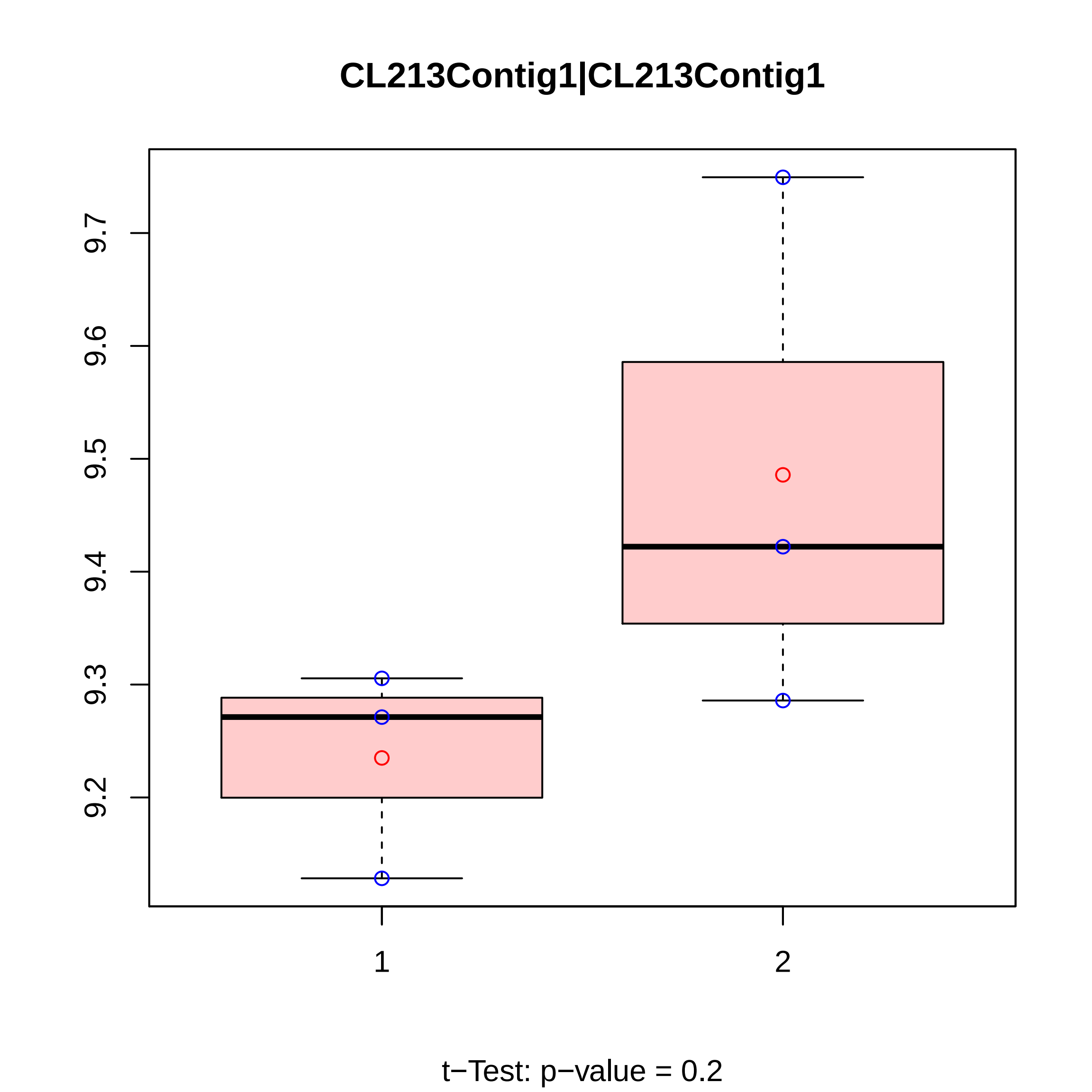
CL213Contig1|CL213Contig1
9.2
9.3
9.4
9.5
9.6
9.7
1
2
t−Test: p−value = 0.2

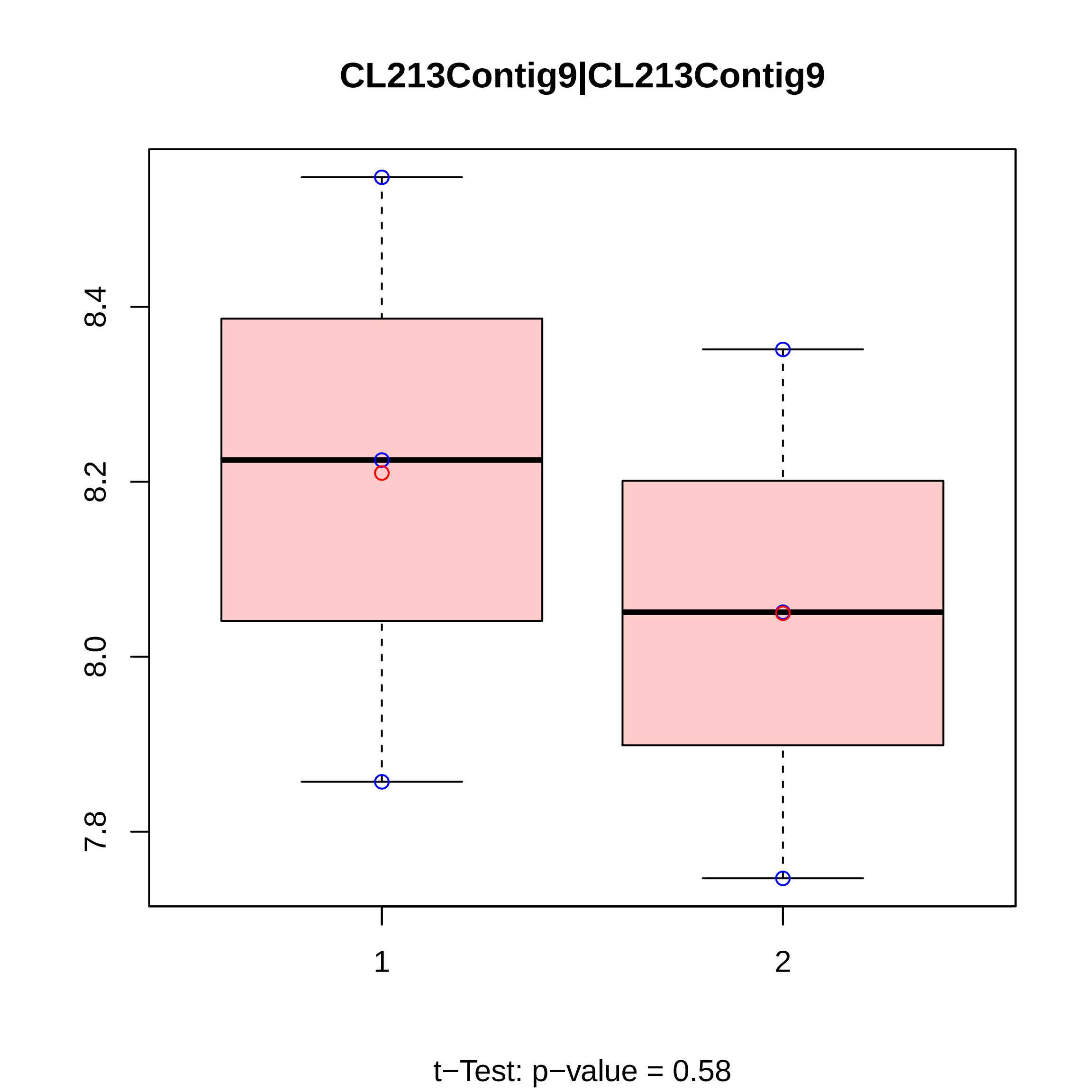
CL213Contig9|CL213Contig9
8.4
8.2
8.0
7.8
1
2
t−Test: p−value = 0.58

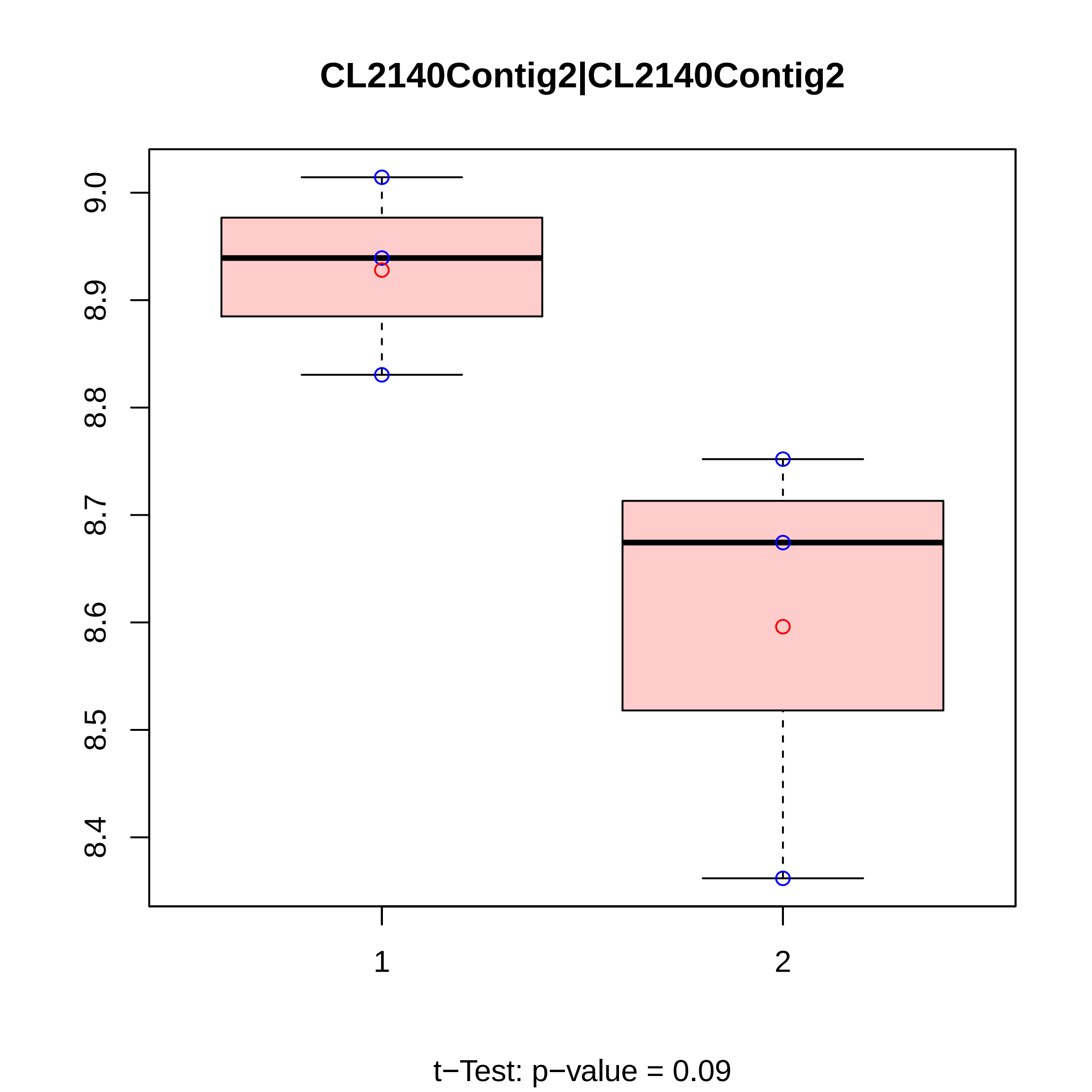

CL2140Contig2|CL2140Contig2
9.0
8.9
8.8
8.7
8.6
8.5
8.4
1
2
t−Test: p−value = 0.09

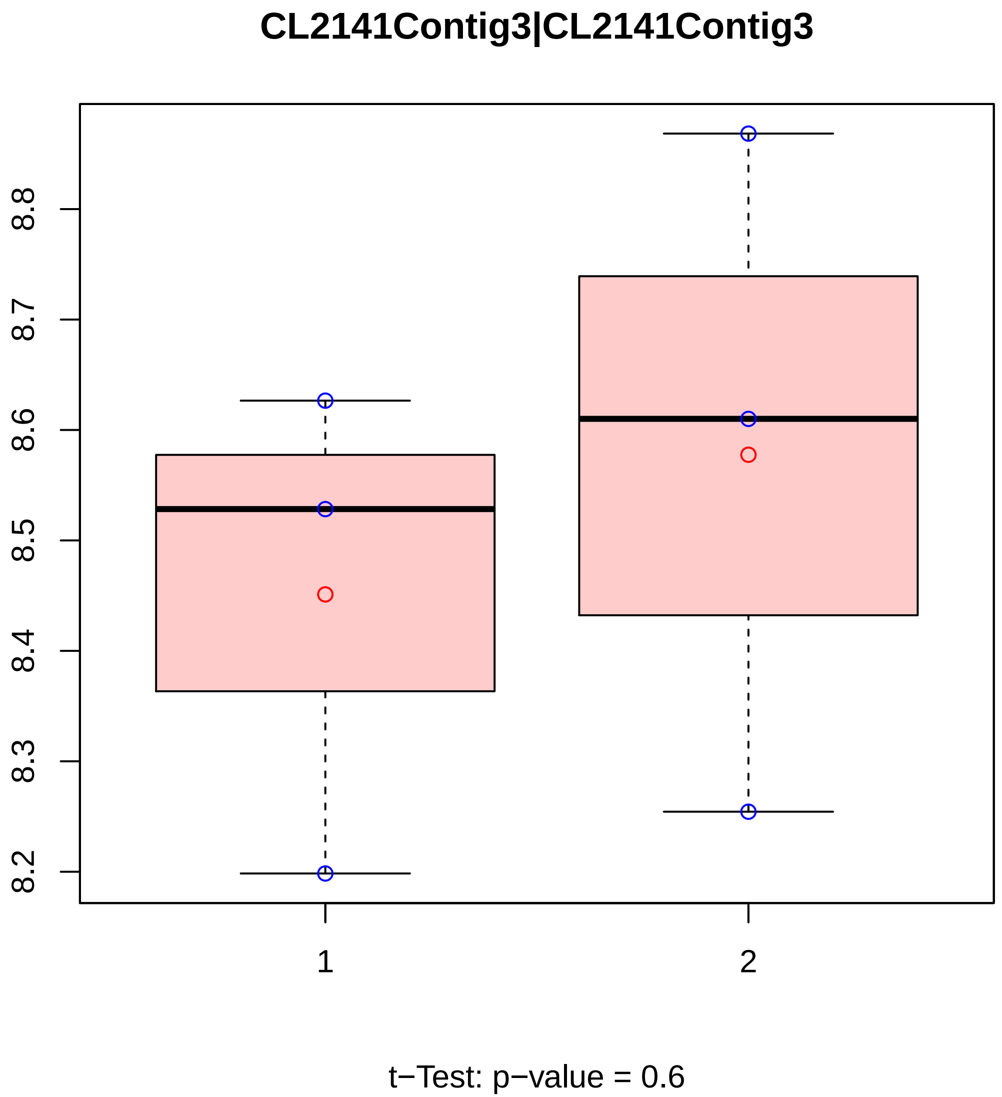
CL2141Contig3|CL2141Contig3
8.2
8.3
8.4
8.5
8.6
8.7
8.8
1
2
t−Test: p−value = 0.6

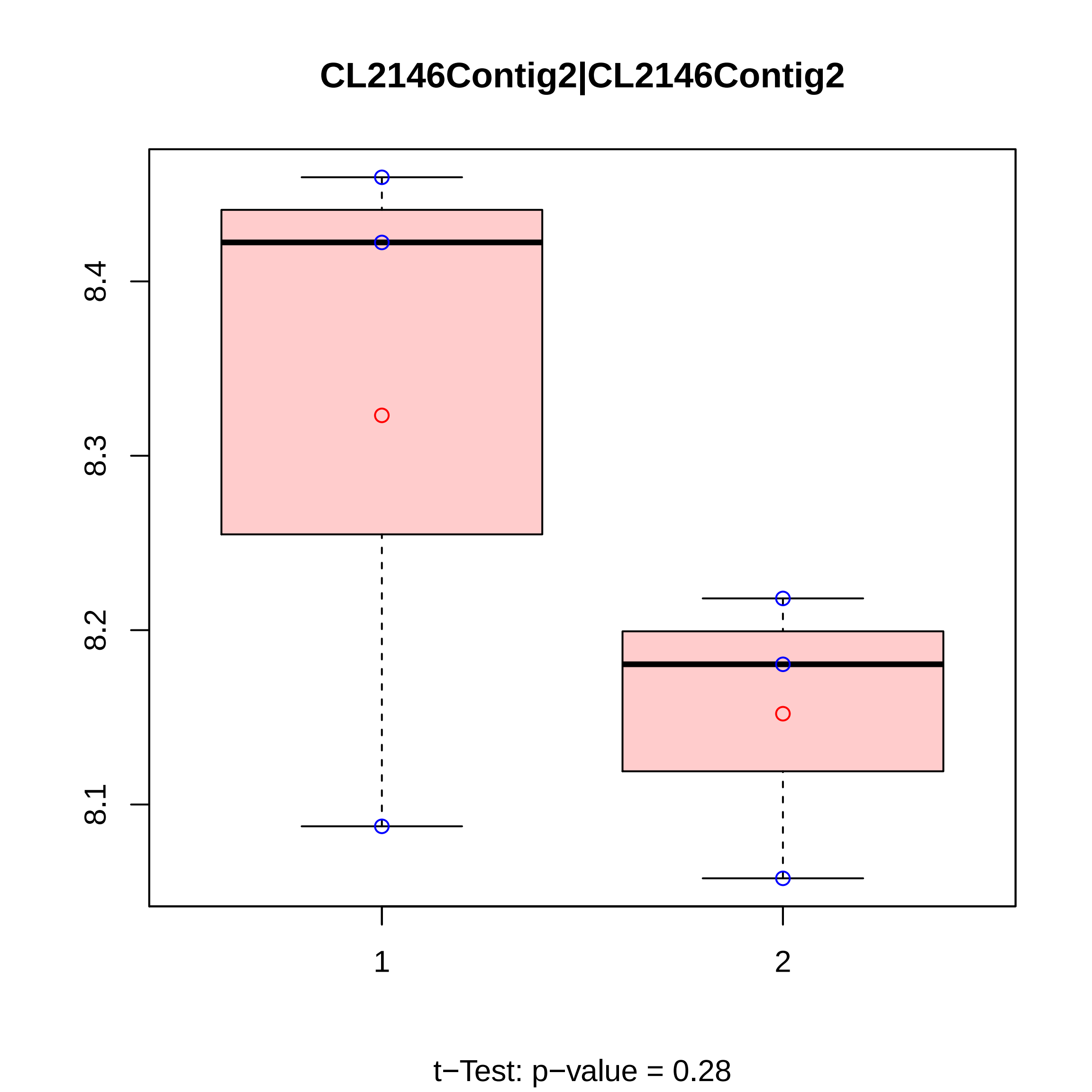

CL2146Contig2|CL2146Contig2
8.1
8.2
8.3
8.4
1
2
t−Test: p−value = 0.28

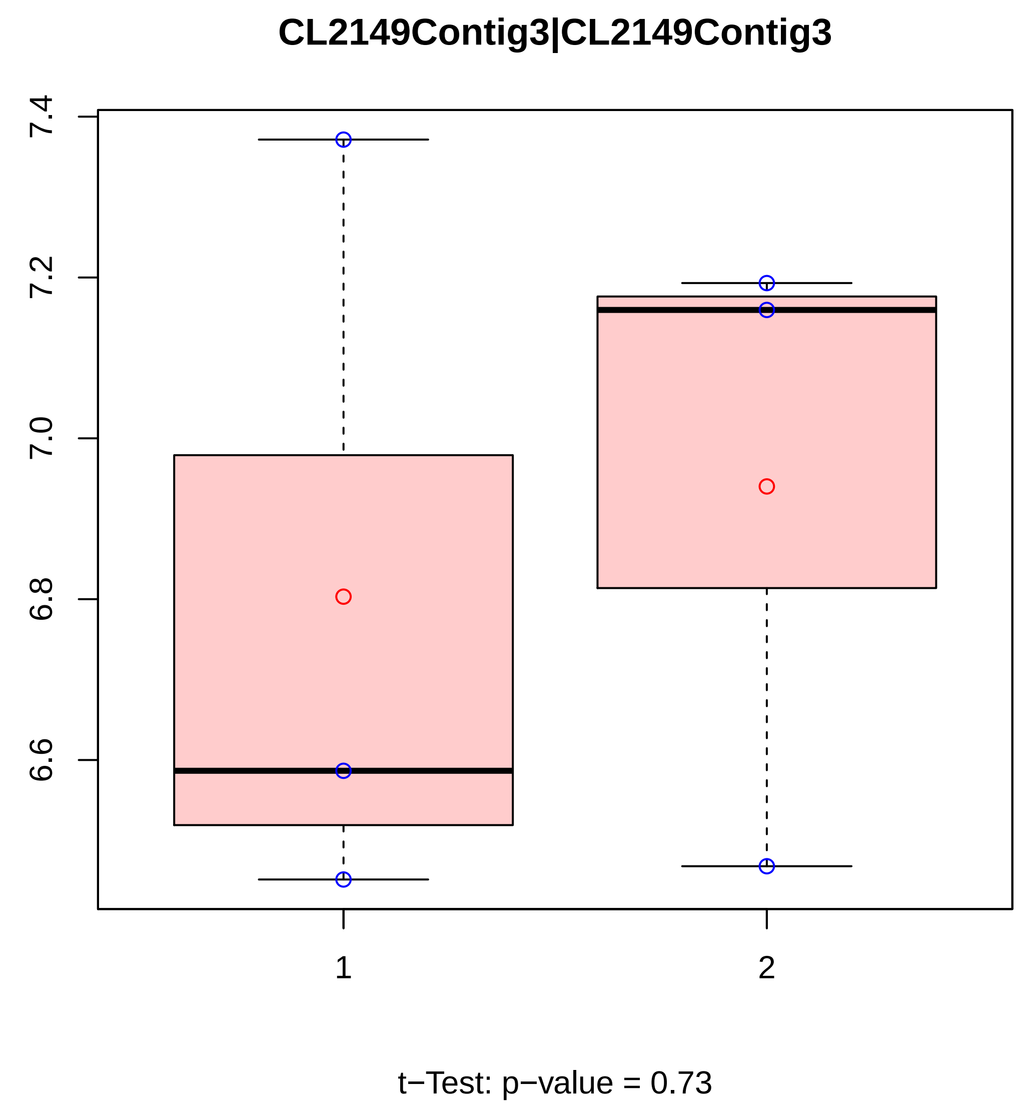
CL2149Contig3|CL2149Contig3
7.4
7.2
7.0
6.8
6.6
1
2
t−Test: p−value = 0.73

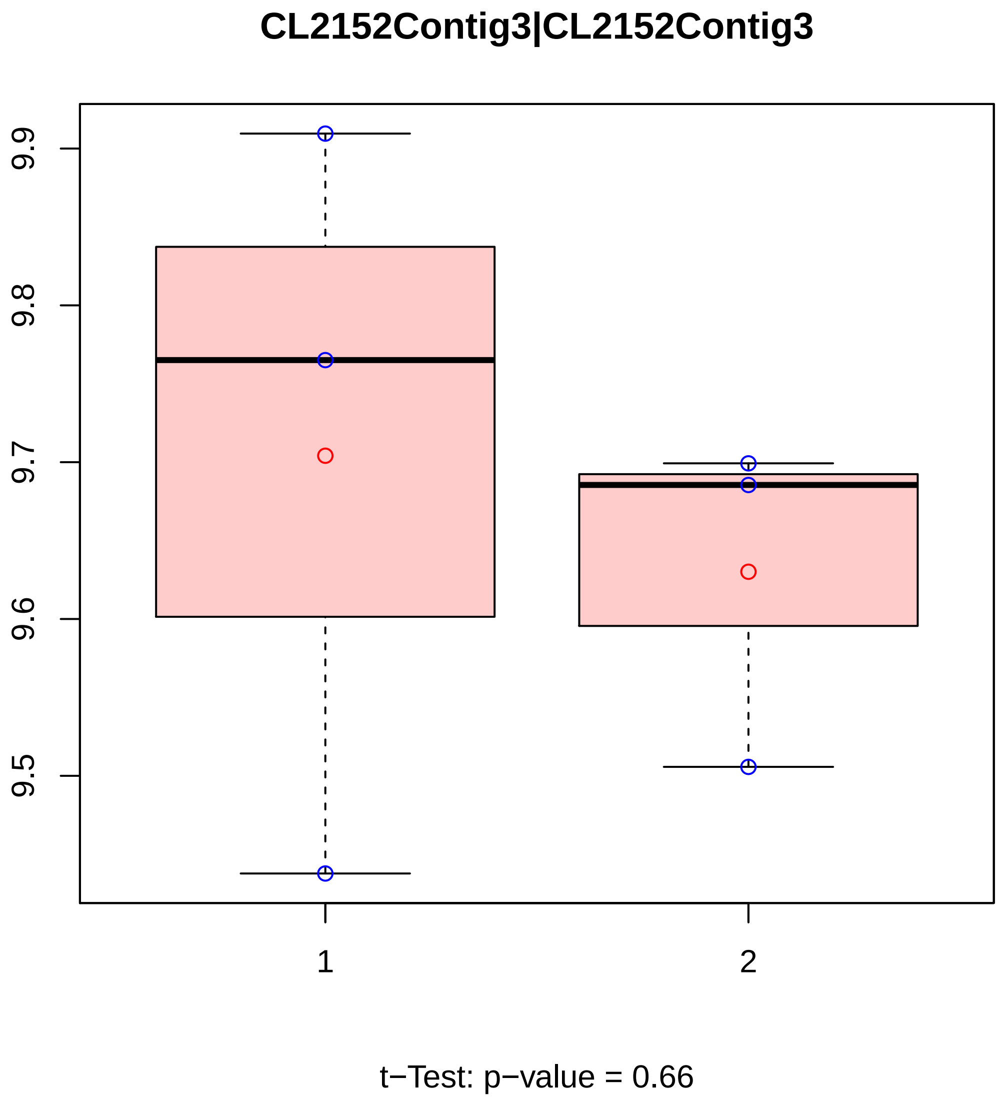
CL2152Contig3|CL2152Contig3
9.9
9.8
9.7
9.6
9.5
1
2
t−Test: p−value = 0.66

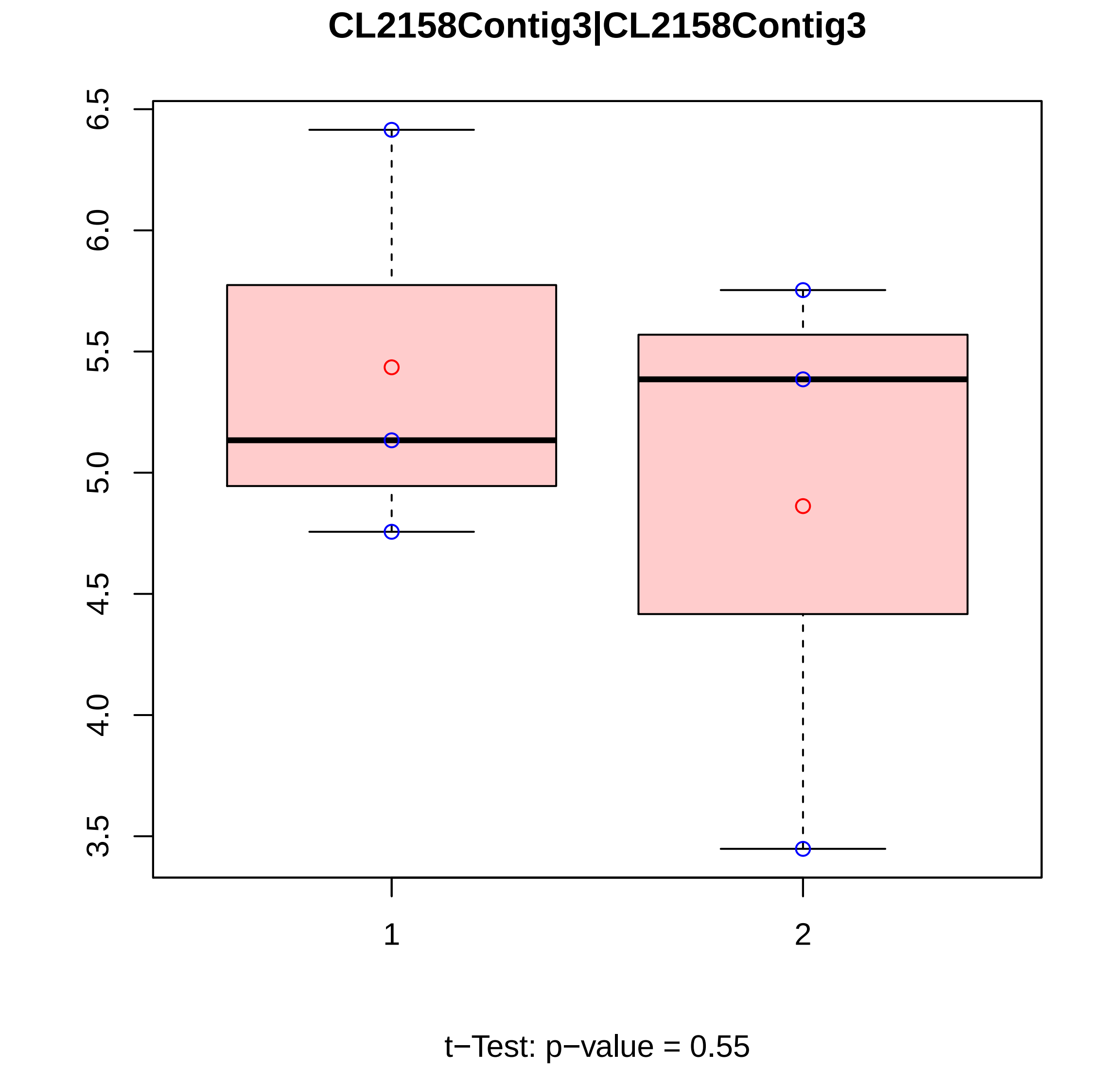
CL2158Contig3|CL2158Contig3
6.5
6.0
5.5
5.0
4.5
4.0
3.5
1
2
t−Test: p−value = 0.55

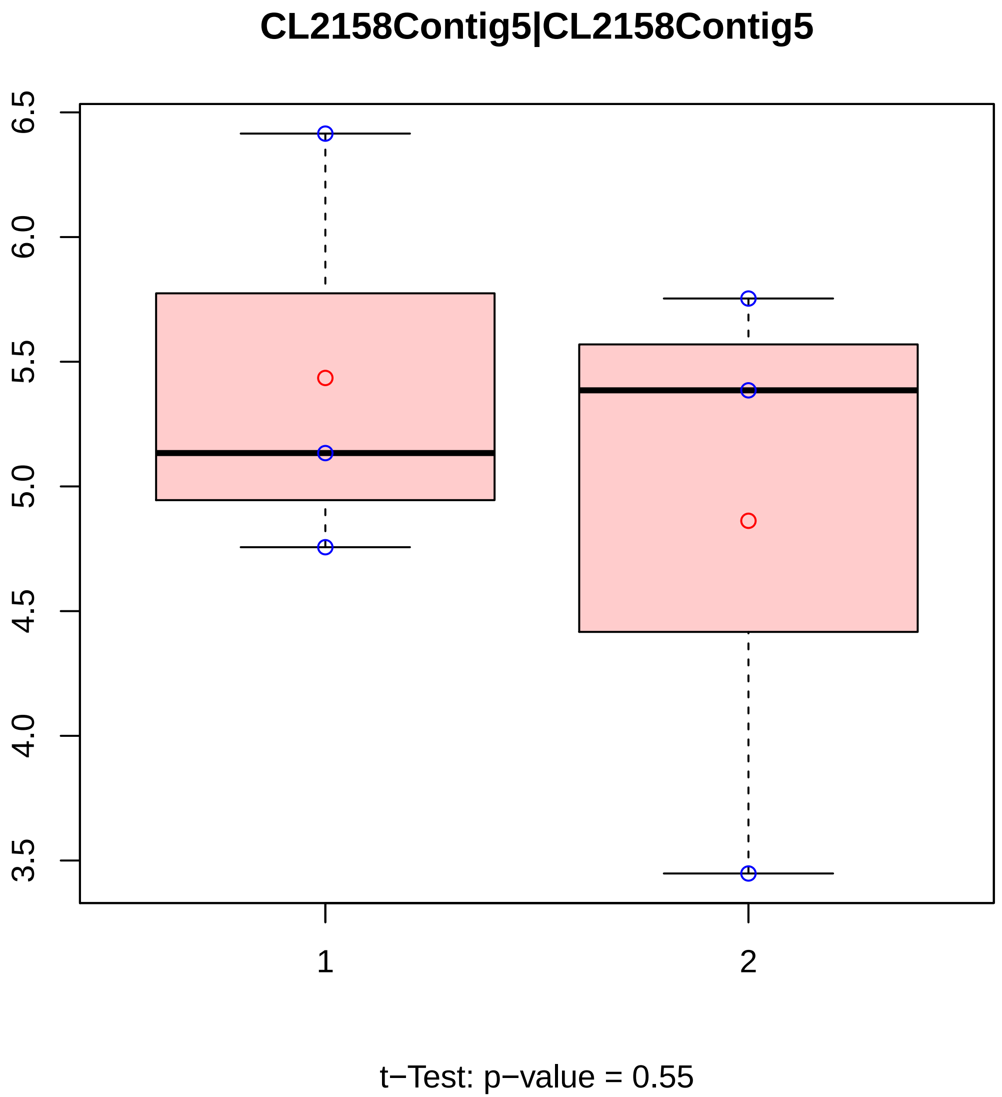
CL2158Contig5|CL2158Contig5
6.5
6.0
5.5
5.0
4.5
4.0
3.5
1
2
t−Test: p−value = 0.55

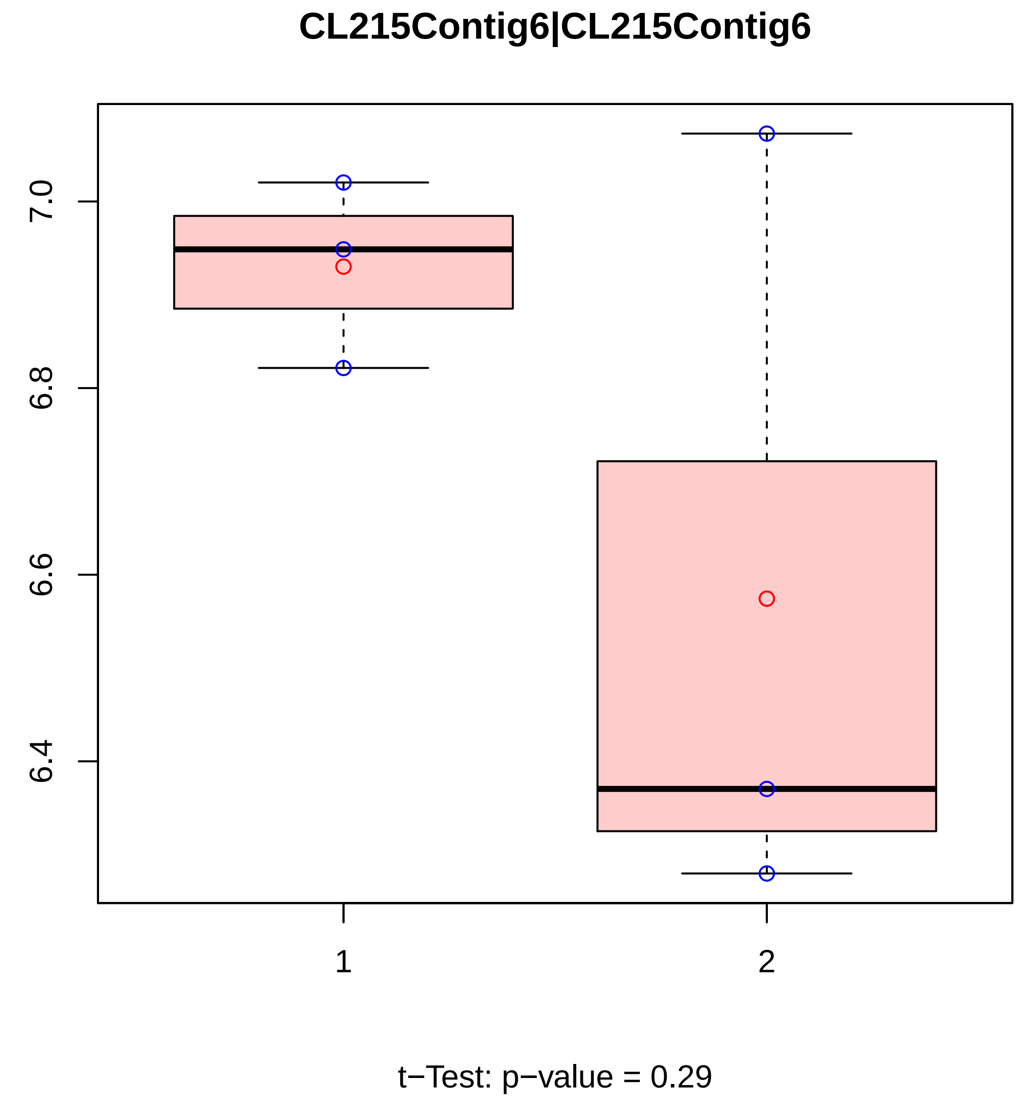
CL215Contig6|CL215Contig6
7.0
6.8
6.6
6.4
1
2
t−Test: p−value = 0.29

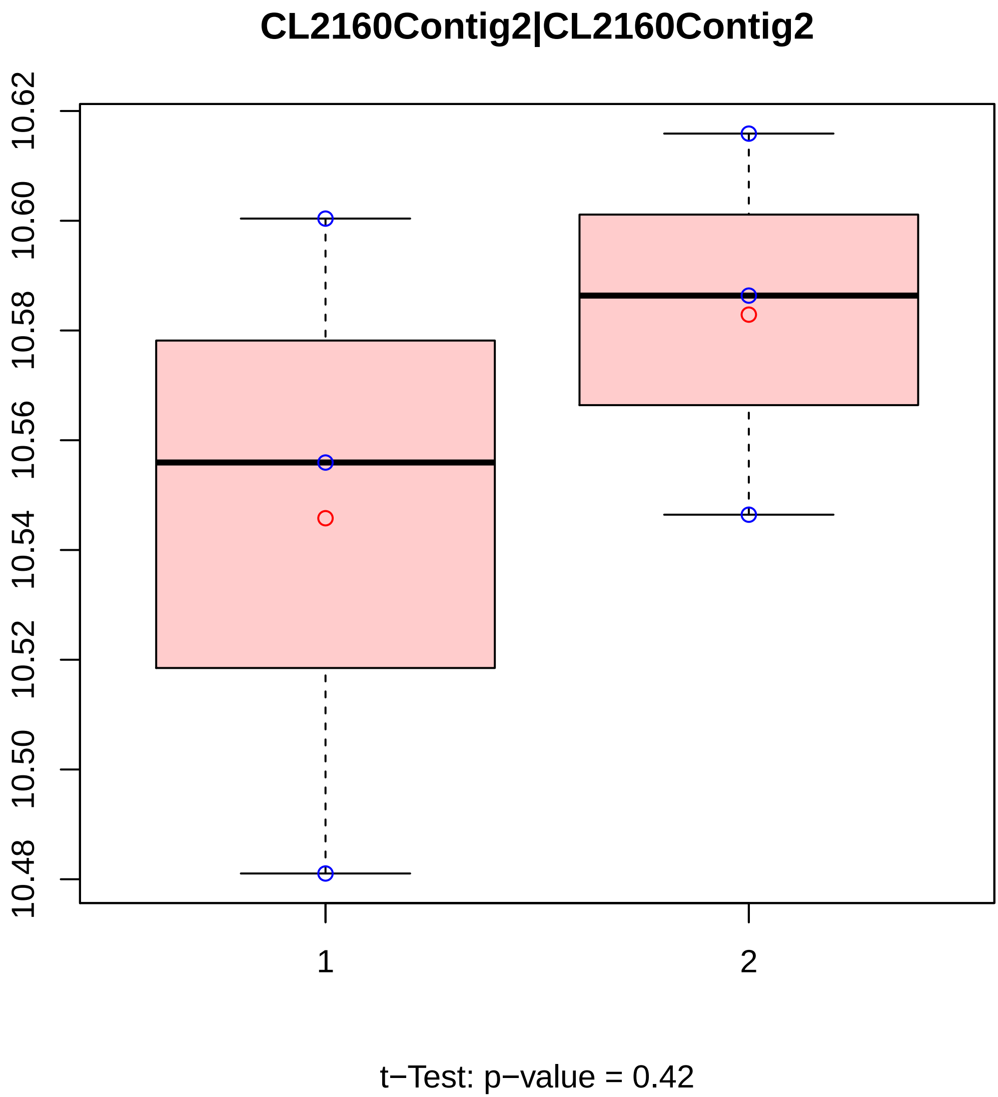
CL2160Contig2|CL2160Contig2
10.48
10.50
10.52
10.54
10.56
10.58
10.60
10.62
1
2
t−Test: p−value = 0.42

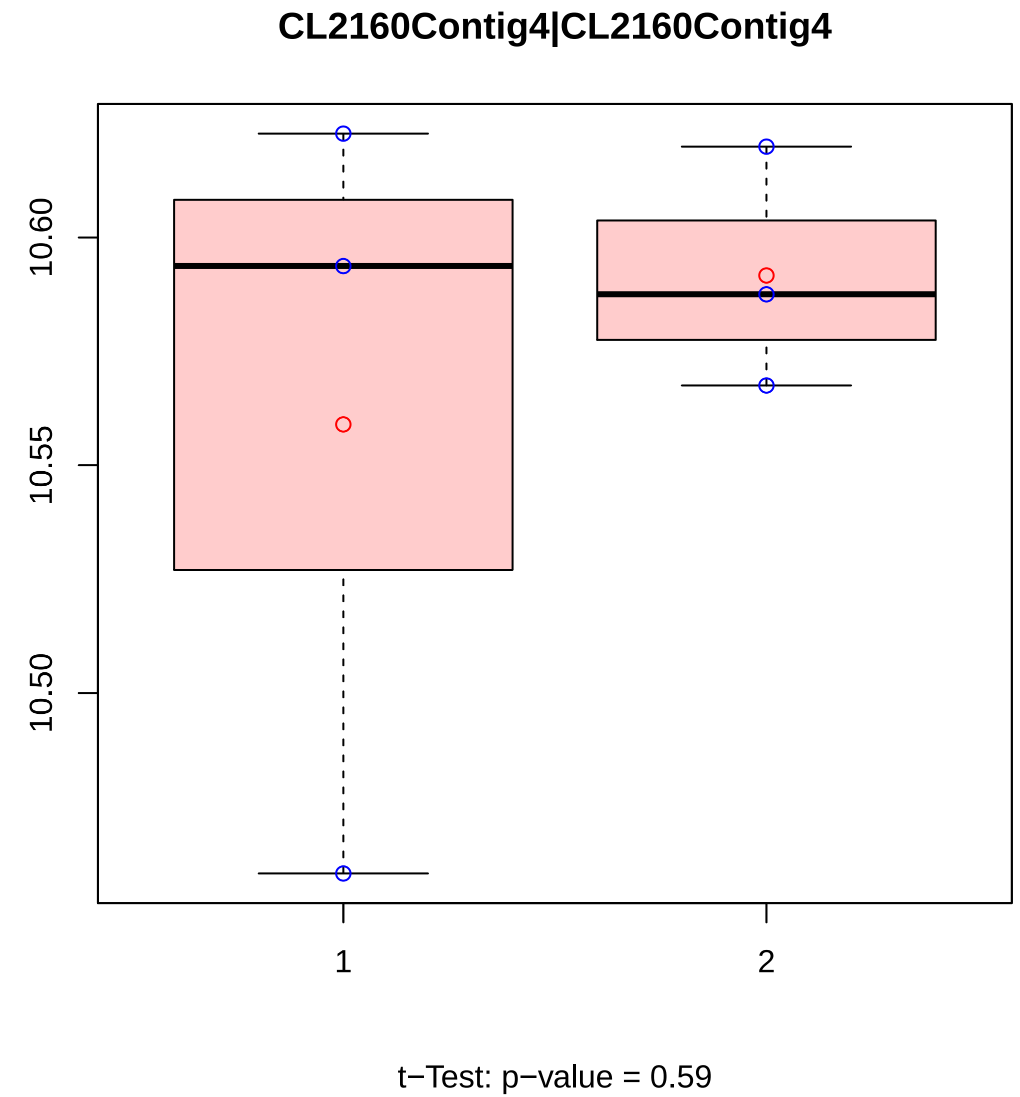
CL2160Contig4|CL2160Contig4
10.60
10.55
10.50
1
2
t−Test: p−value = 0.59

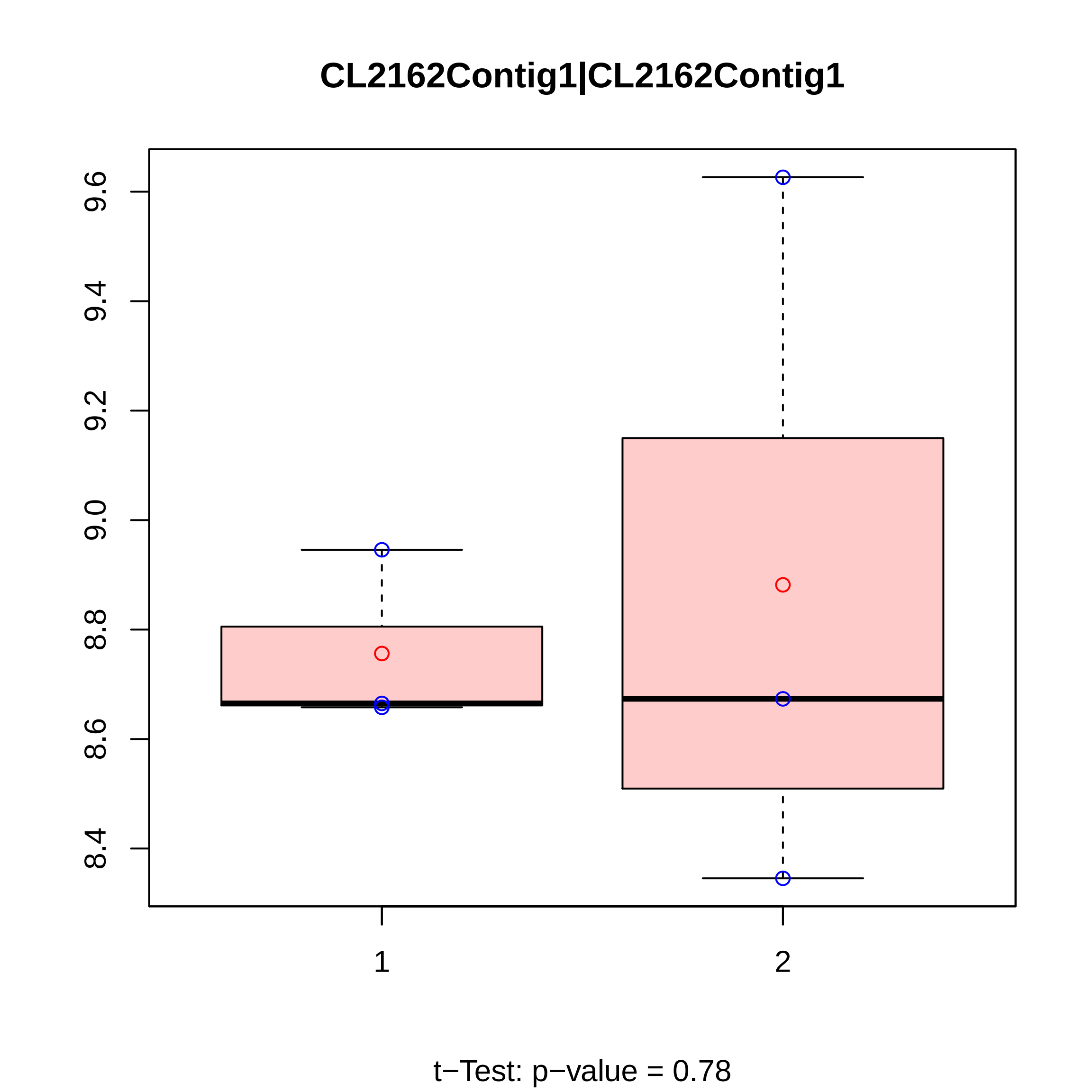

CL2162Contig1|CL2162Contig1
9.6
9.4
9.2
9.0
8.8
8.6
8.4
1
2
t−Test: p−value = 0.78

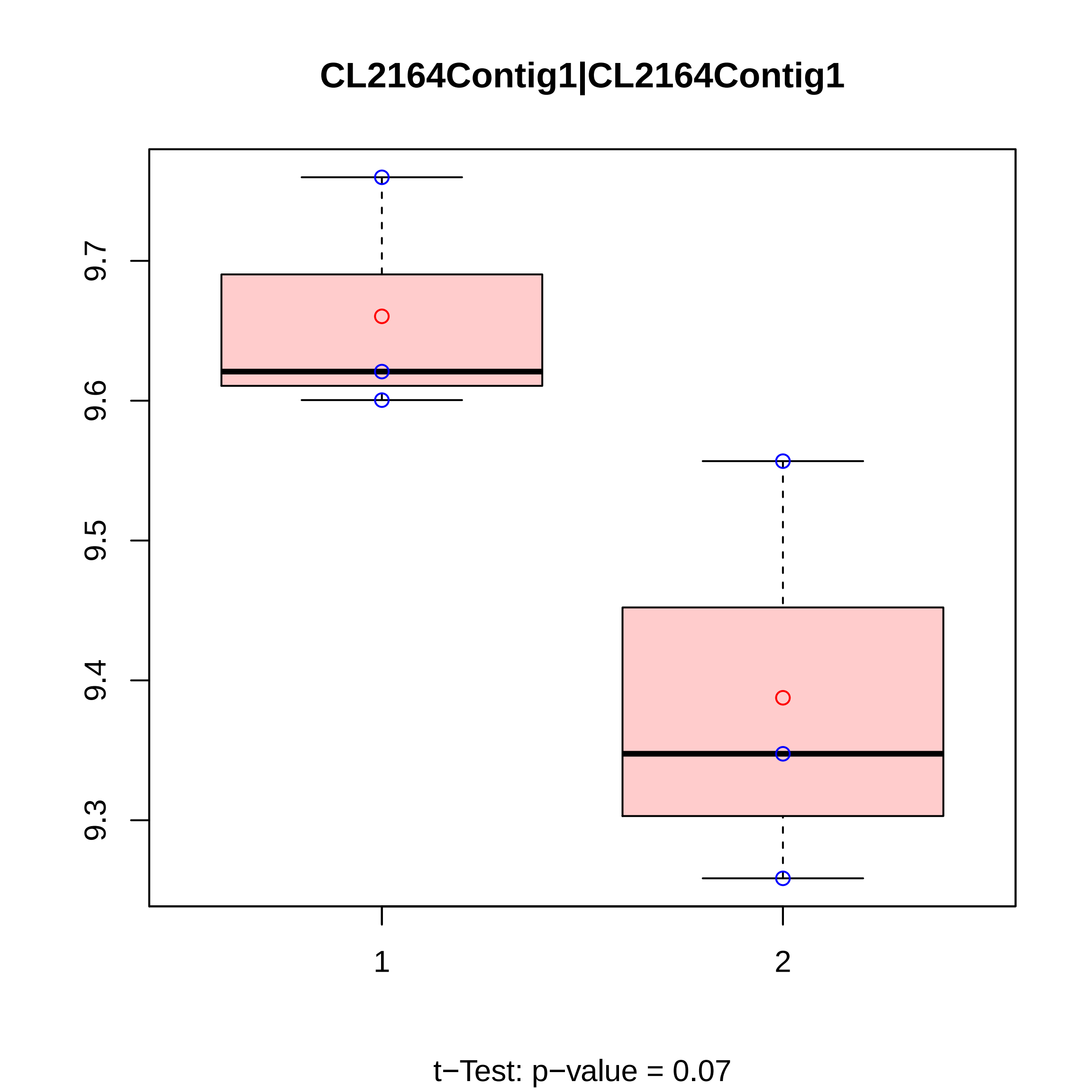
CL2164Contig1|CL2164Contig1
9.3
9.4
9.5
9.6
9.7
1
2
t−Test: p−value = 0.07

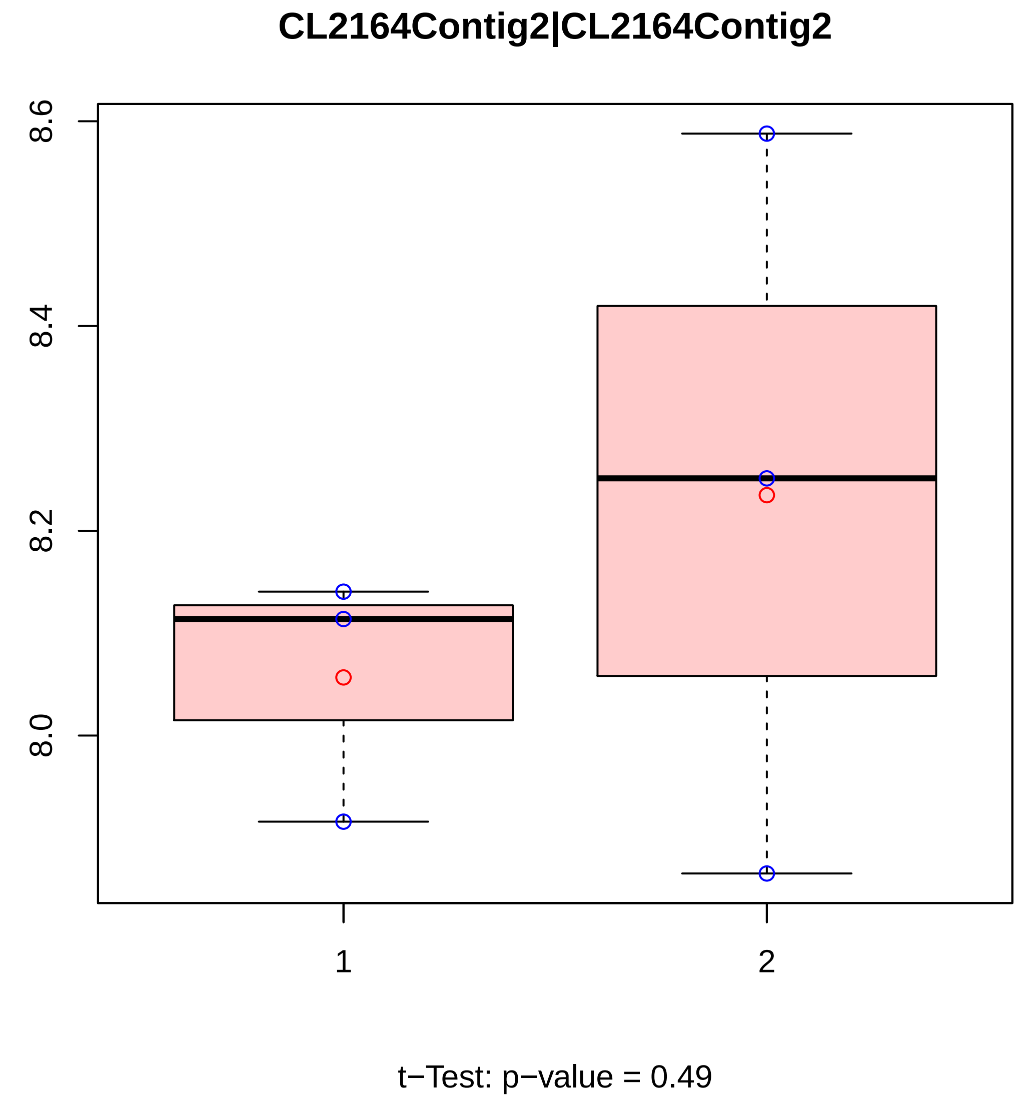
CL2164Contig2|CL2164Contig2
8.6
8.4
8.2
8.0
1
2
t−Test: p−value = 0.49

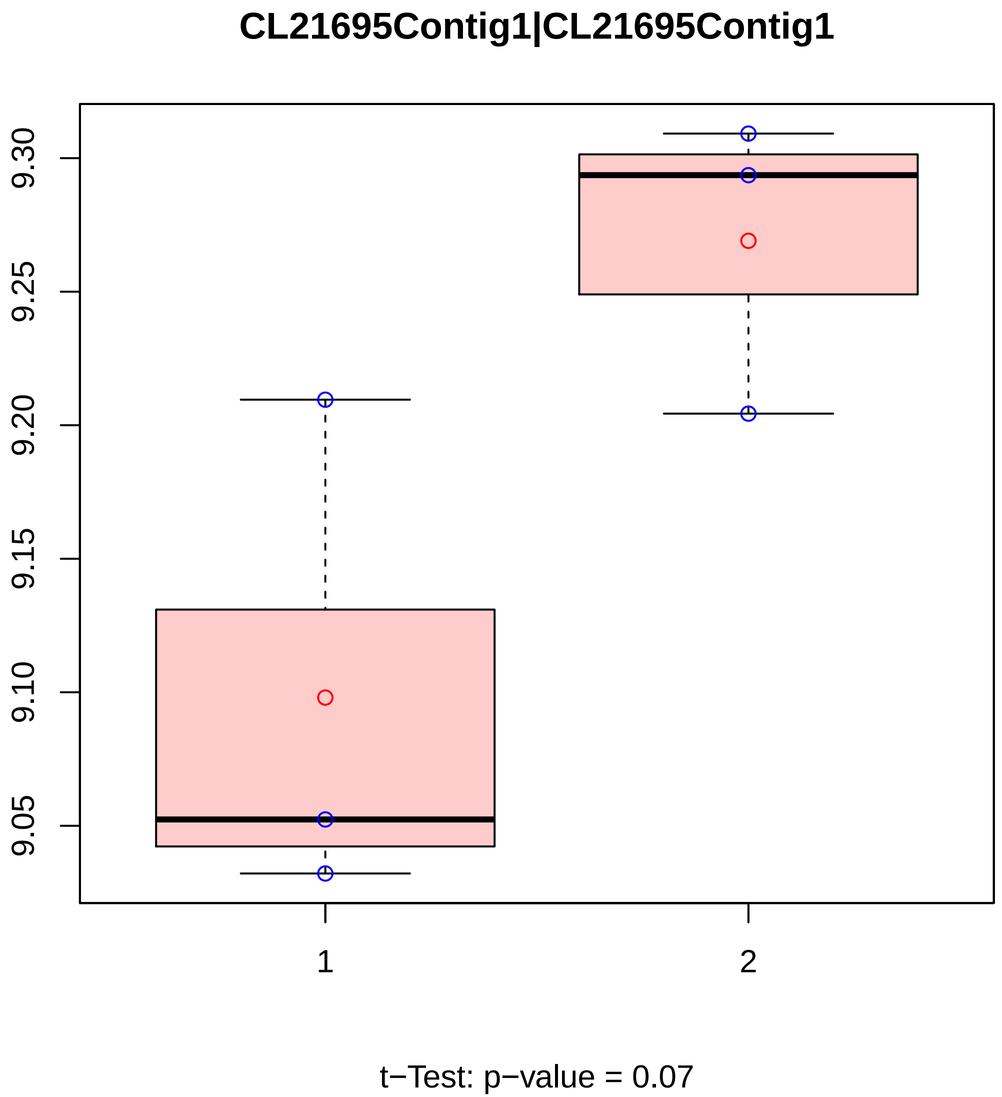

CL21695Contig1|CL21695Contig1
9.05
9.10
9.15
9.20
9.25
9.30
1
2
t−Test: p−value = 0.07

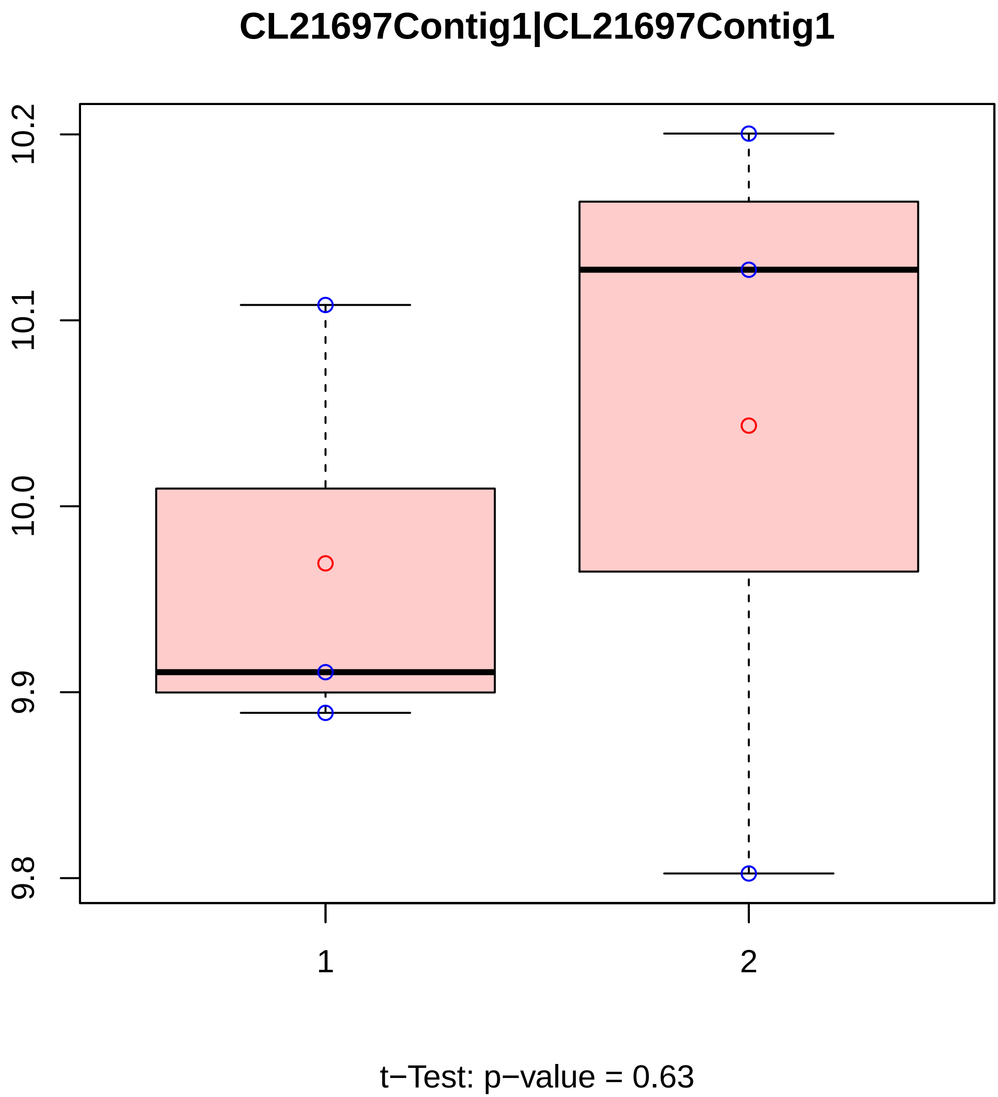

CL21697Contig1|CL21697Contig1
10.2
10.1
10.0
9.9
9.8
1
2
t−Test: p−value = 0.63

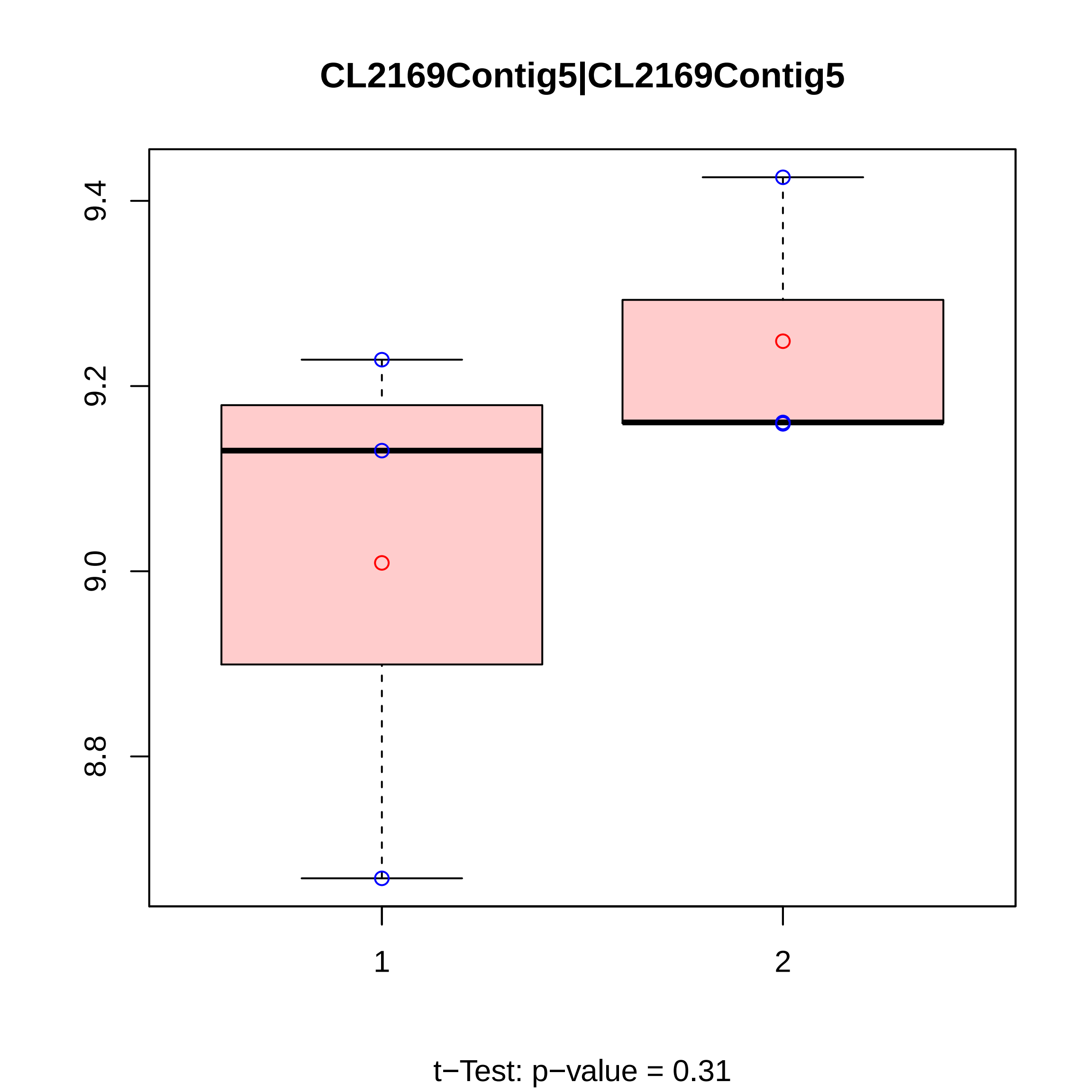

CL2169Contig5|CL2169Contig5
9.4
9.2
9.0
8.8
1
2
t−Test: p−value = 0.31

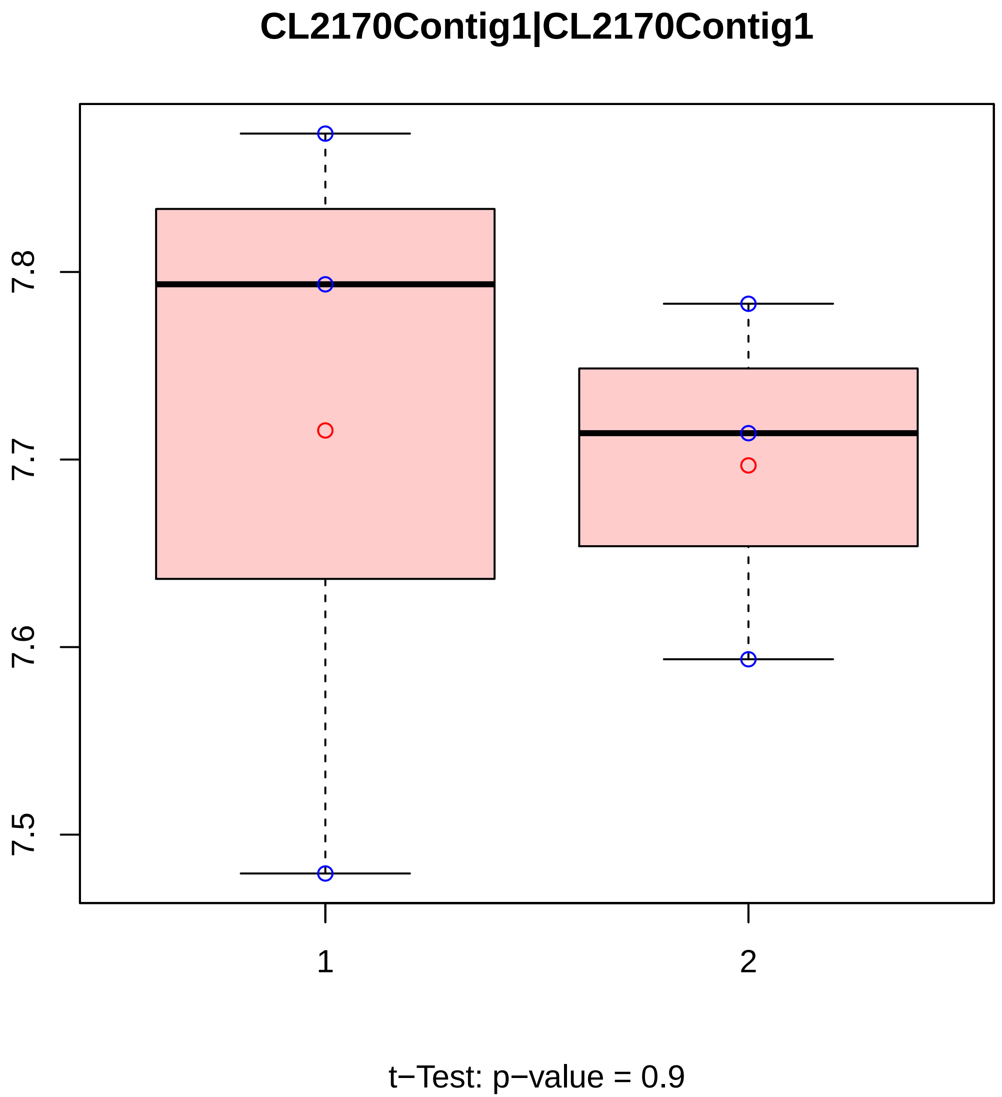
CL2170Contig1|CL2170Contig1
7.5
7.6
7.7
7.8
1
2
t−Test: p−value = 0.9

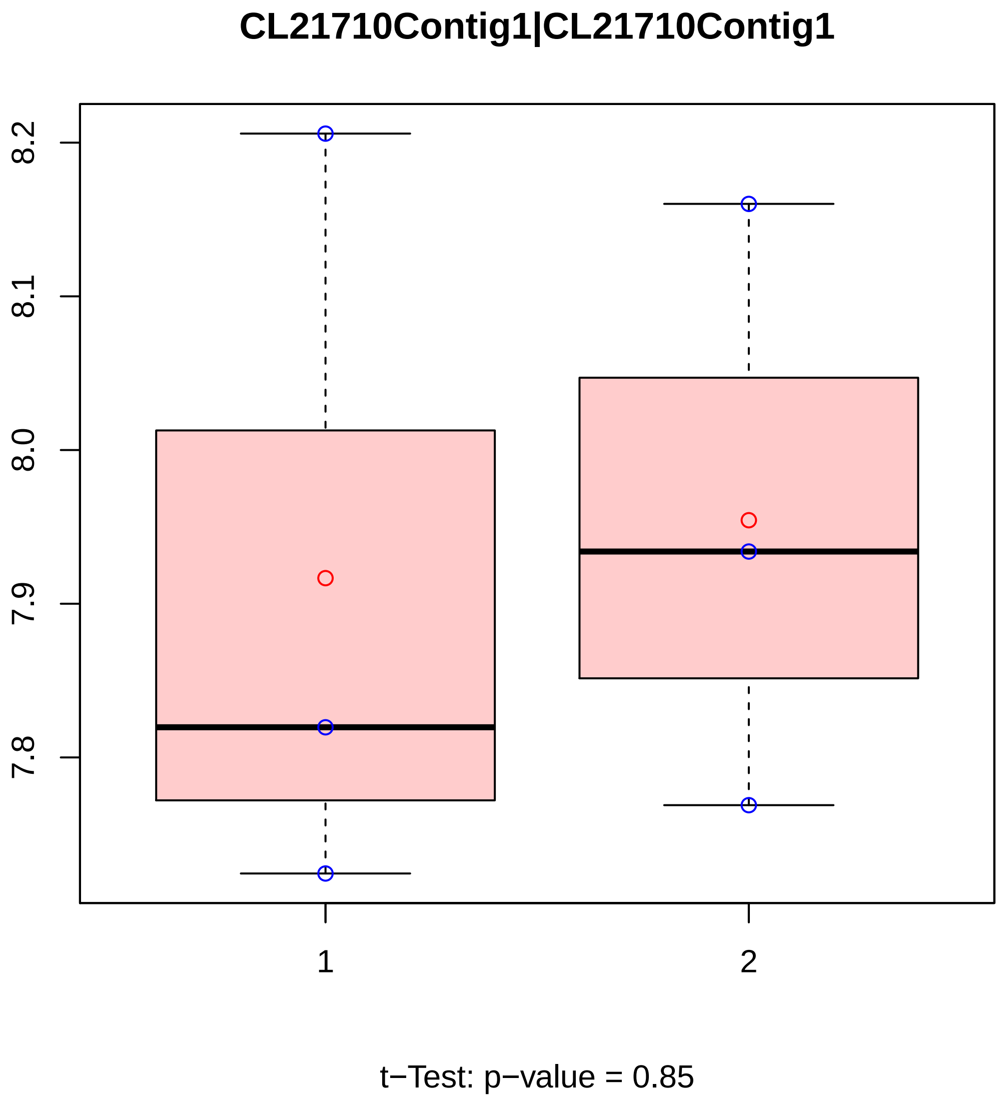

CL21710Contig1|CL21710Contig1
8.2
8.1
8.0
7.9
7.8
1
2
t−Test: p−value = 0.85

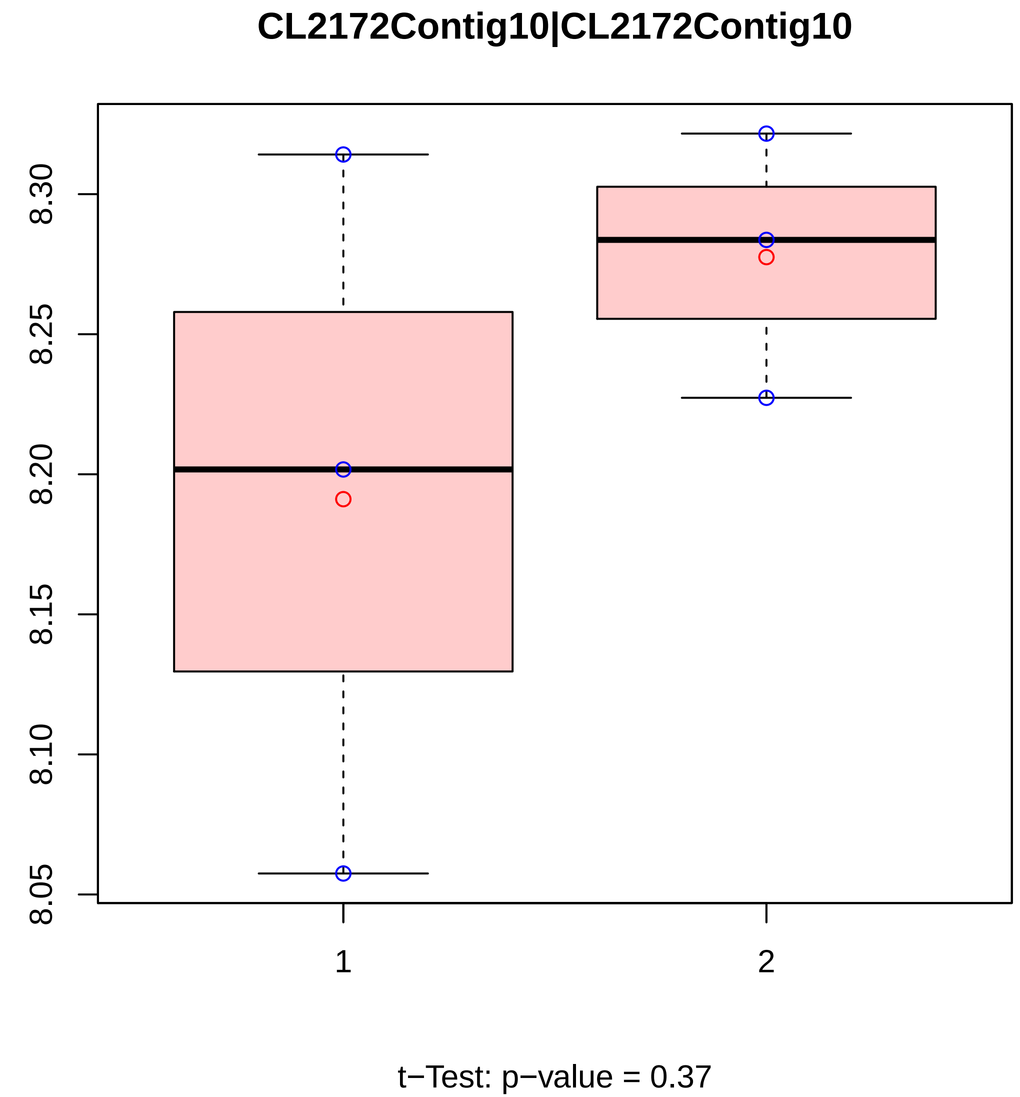
CL2172Contig10|CL2172Contig10
8.05
8.10
8.15
8.20
8.25
8.30
1
2
t−Test: p−value = 0.37

# CL2172Contig1|CL2172Contig1

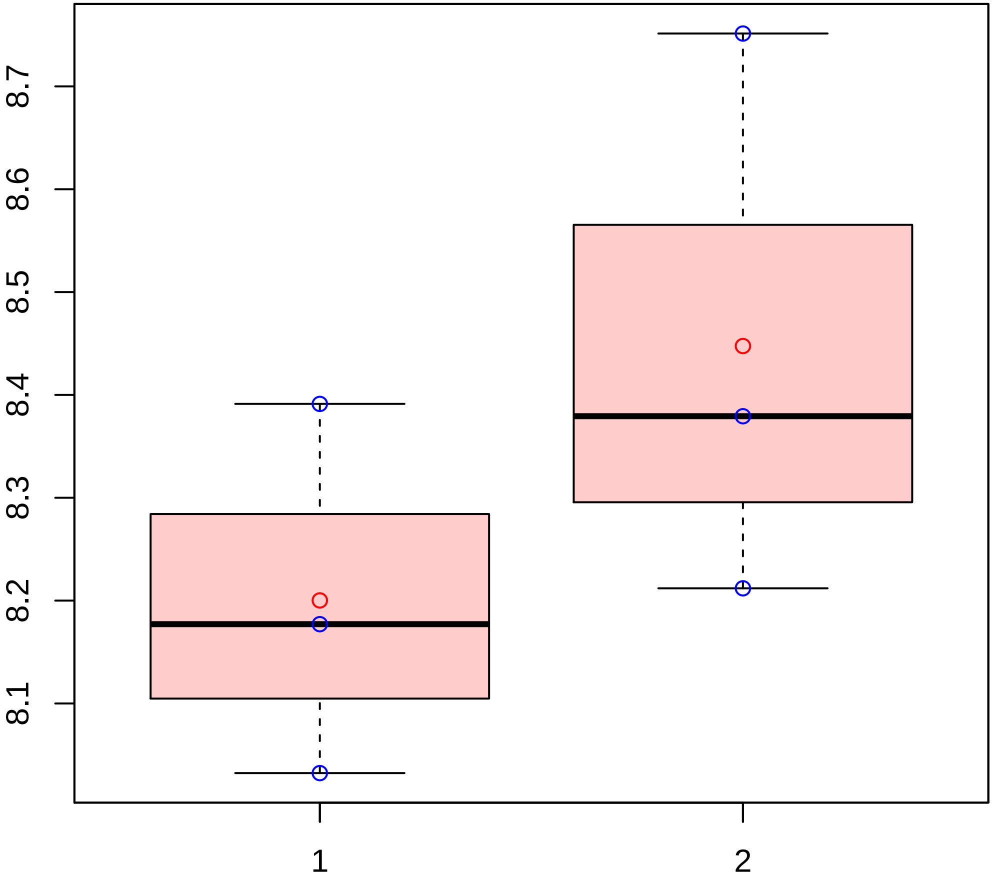


t−Test: p−value = 0.27

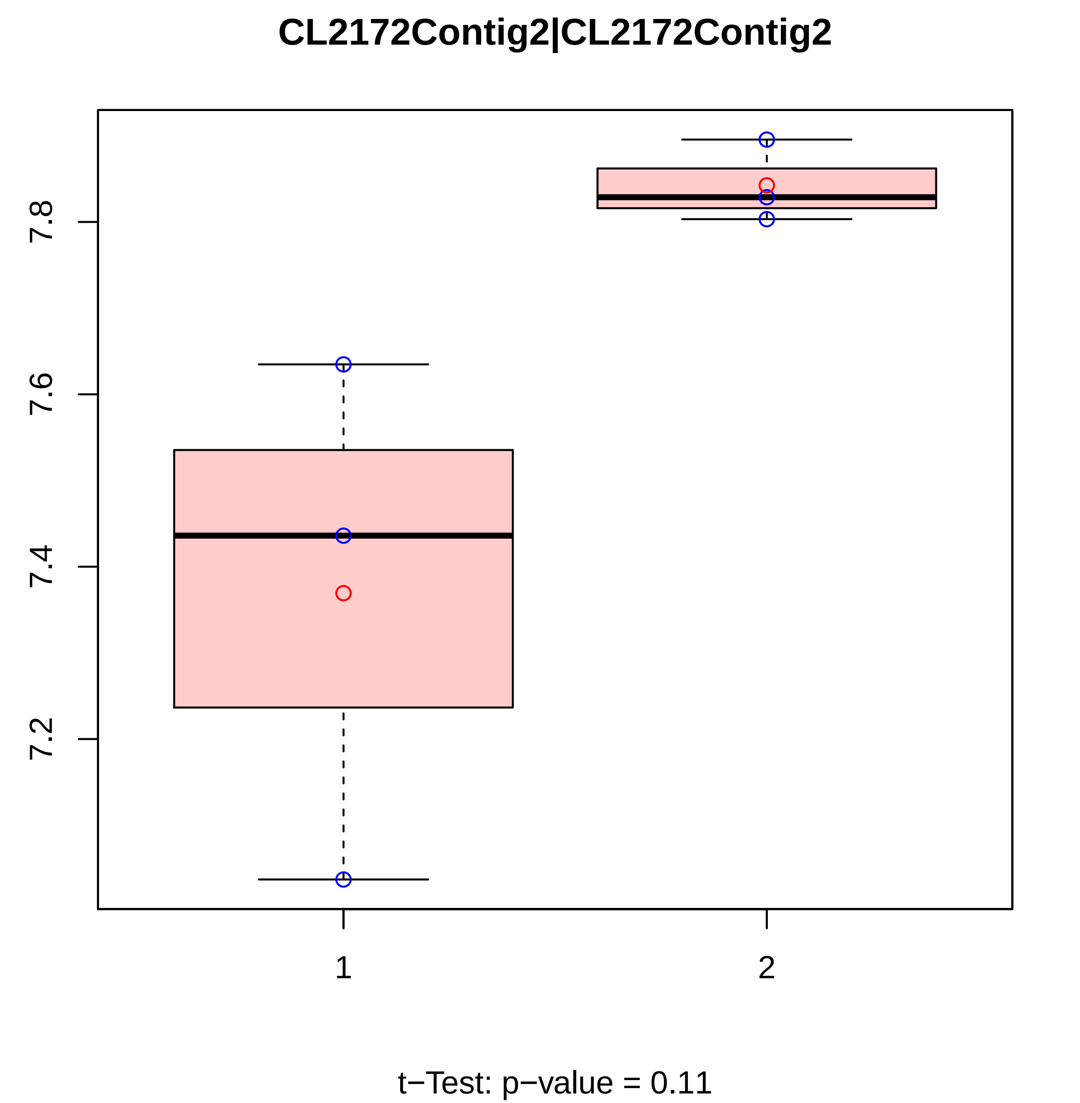
CL2172Contig2|CL2172Contig2
7.2
7.4
7.6
7.8
1
2
t−Test: p−value = 0.11

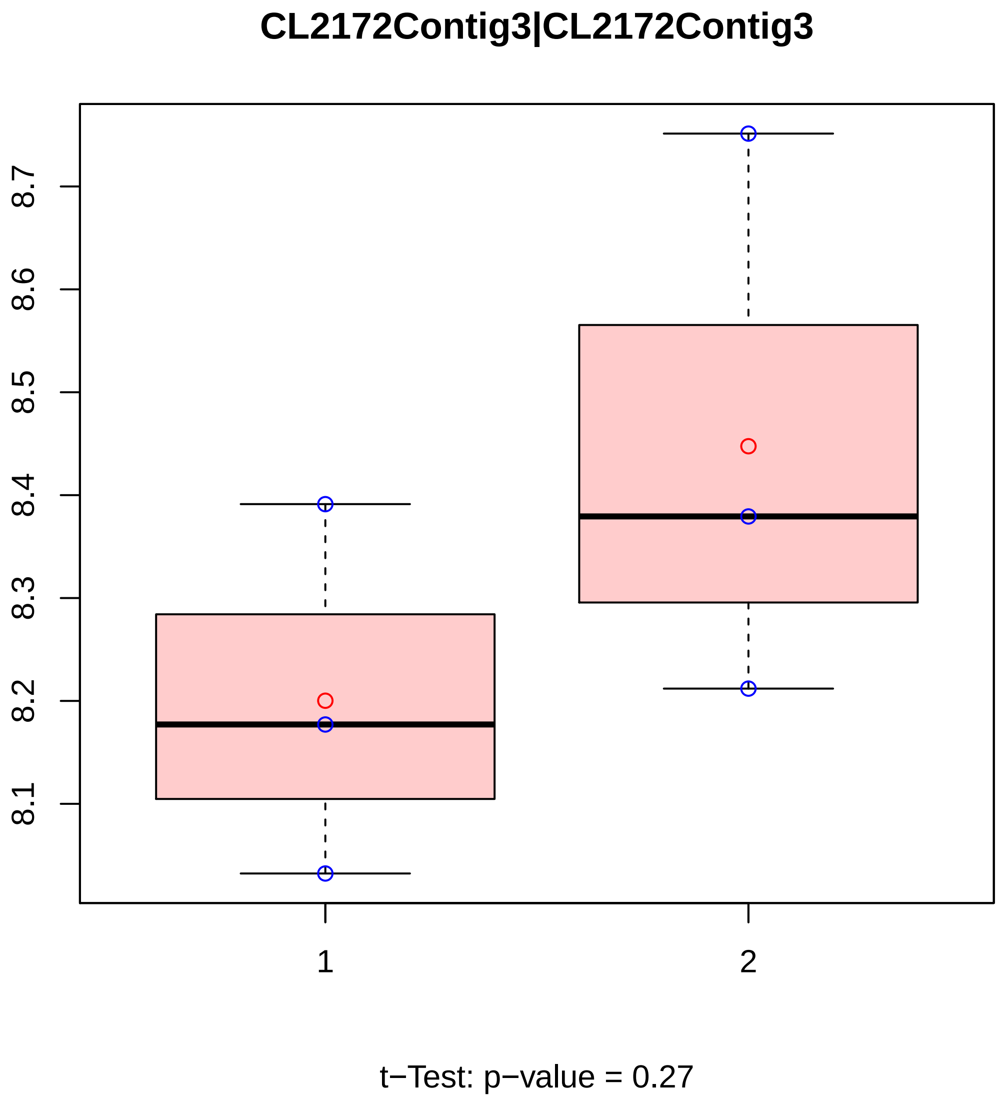
CL2172Contig3|CL2172Contig3
8.1
8.2
8.3
8.4
8.5
8.6
8.7
1
2
t−Test: p−value = 0.27

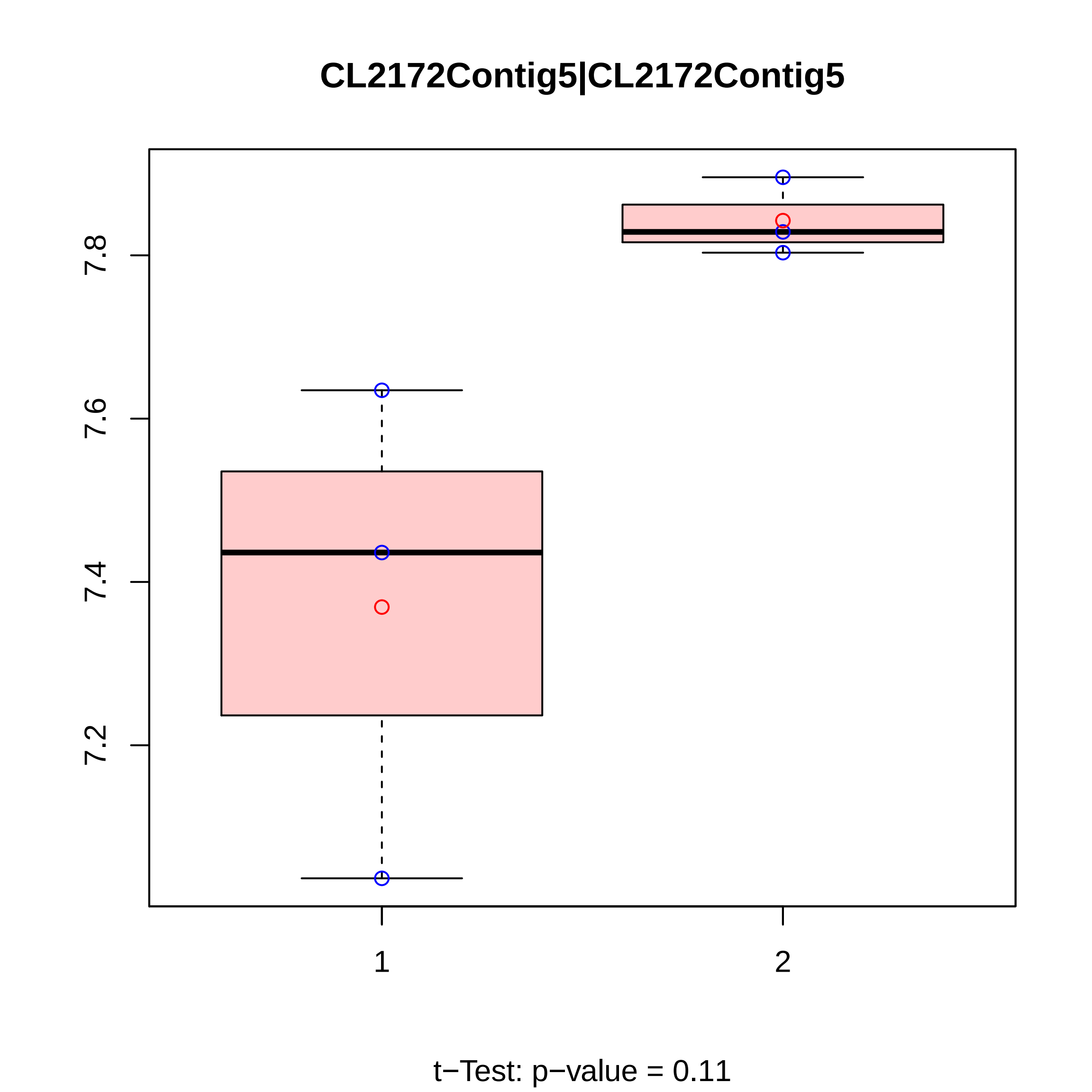

CL2172Contig5|CL2172Contig5
7.8
7.6
7.4
7.2
1
2
t−Test: p−value = 0.11

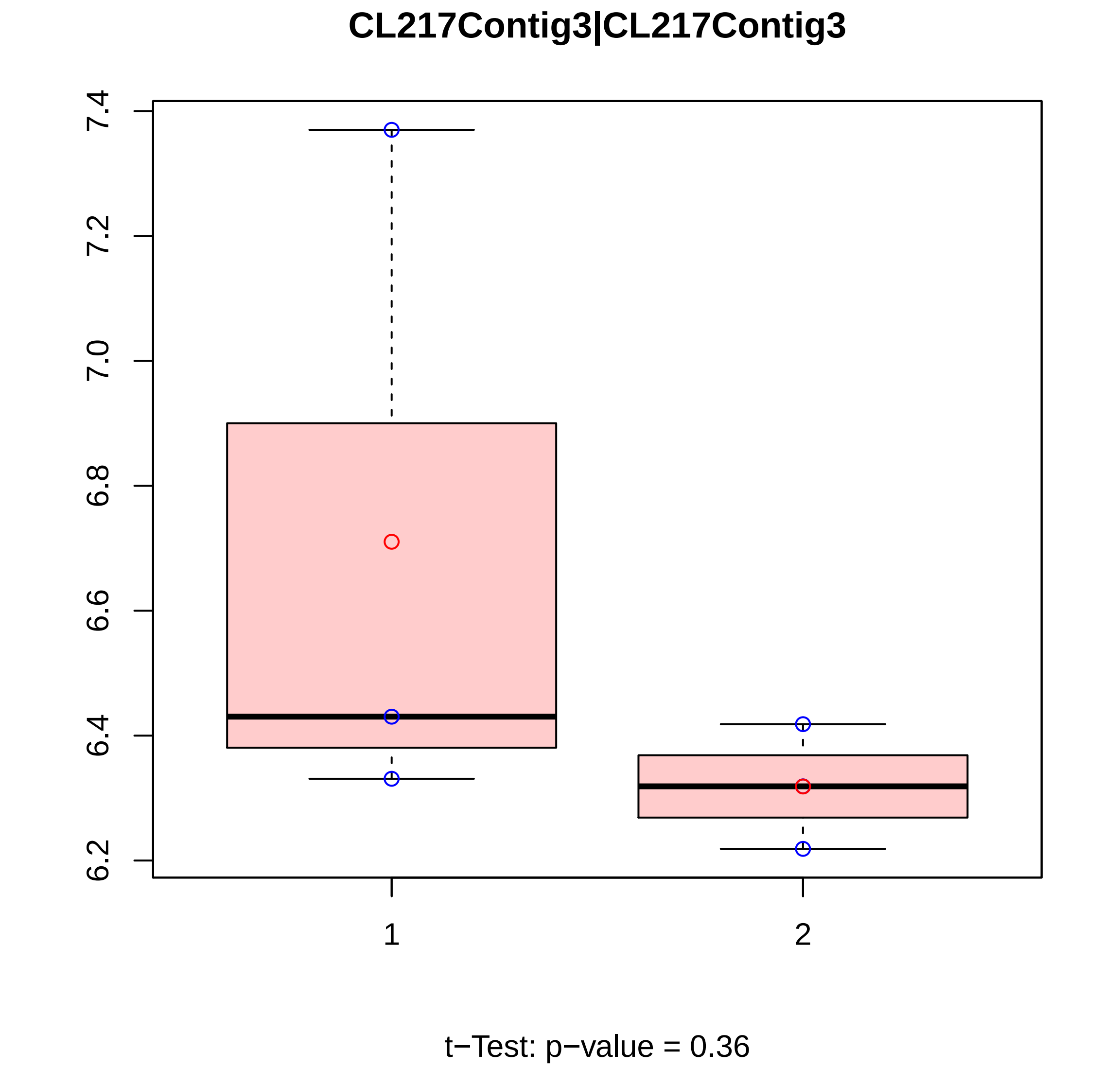

CL217Contig3|CL217Contig3
7.4
7.2
7.0
6.8
6.6
6.4
6.2
1
2
t−Test: p−value = 0.36

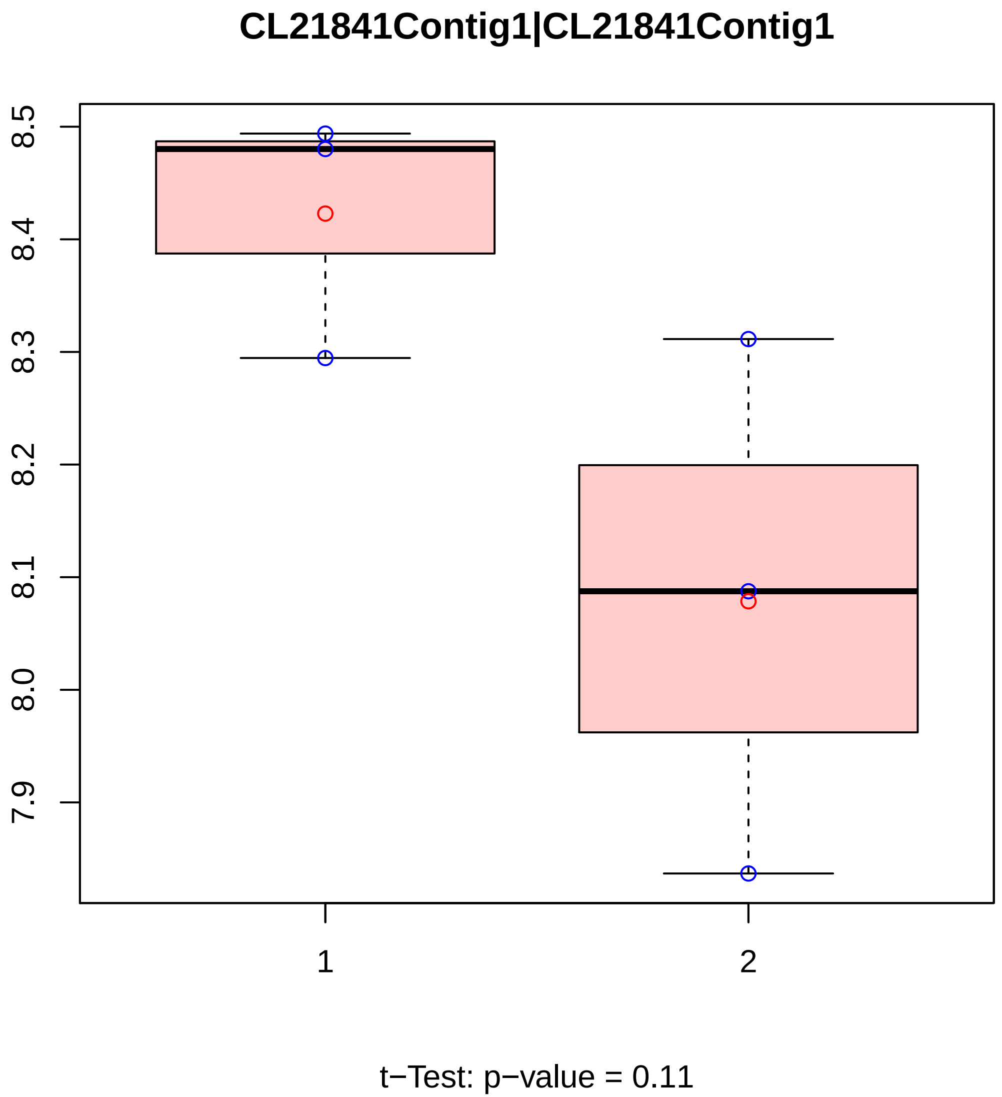
CL21841Contig1|CL21841Contig1
8.5
8.4
8.3
8.2
8.1
8.0
7.9
1
2
t−Test: p−value = 0.11

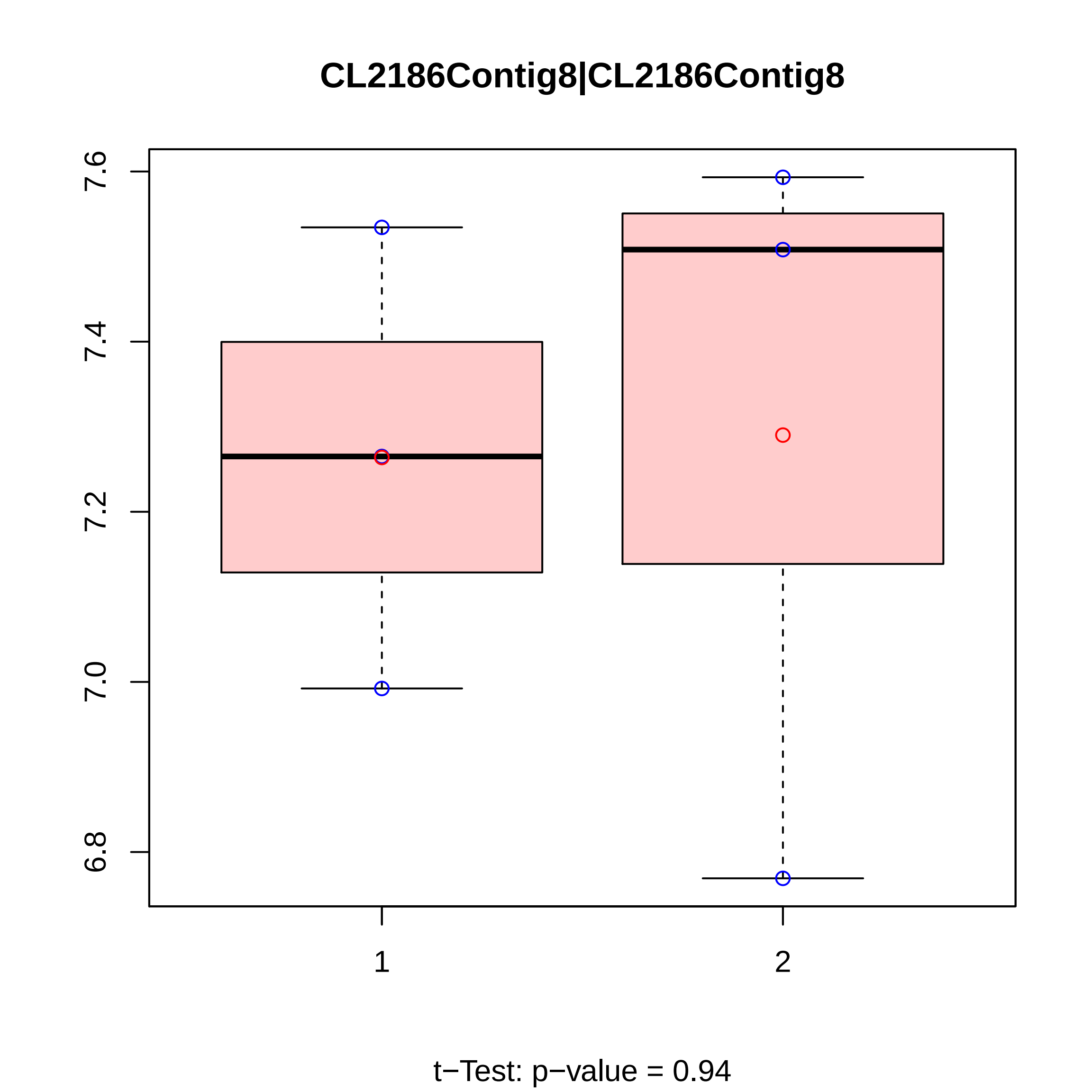

CL2186Contig8|CL2186Contig8
7.6
7.4
7.2
7.0
6.8
1
2
t−Test: p−value = 0.94

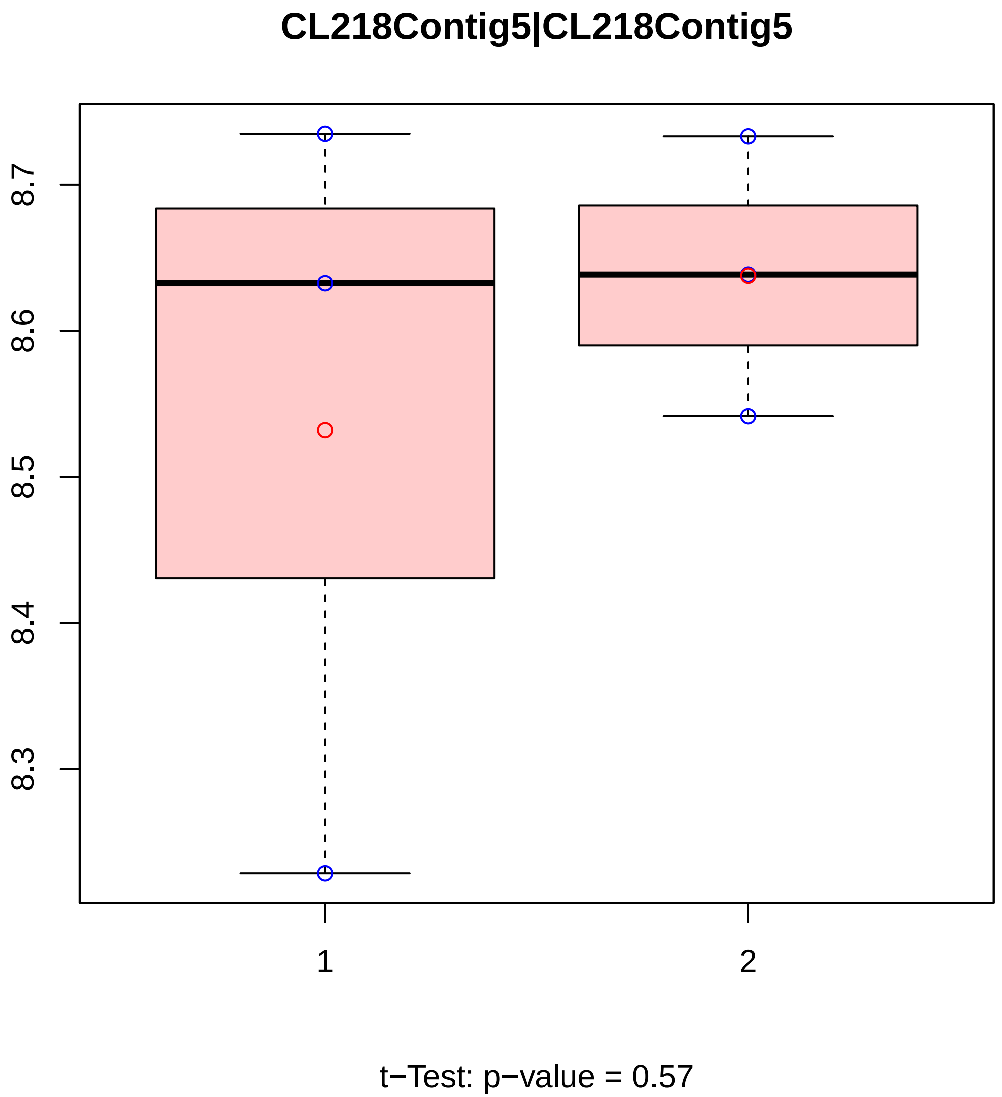
CL218Contig5|CL218Contig5
8.7
8.6
8.5
8.4
8.3
1
2
t−Test: p−value = 0.57

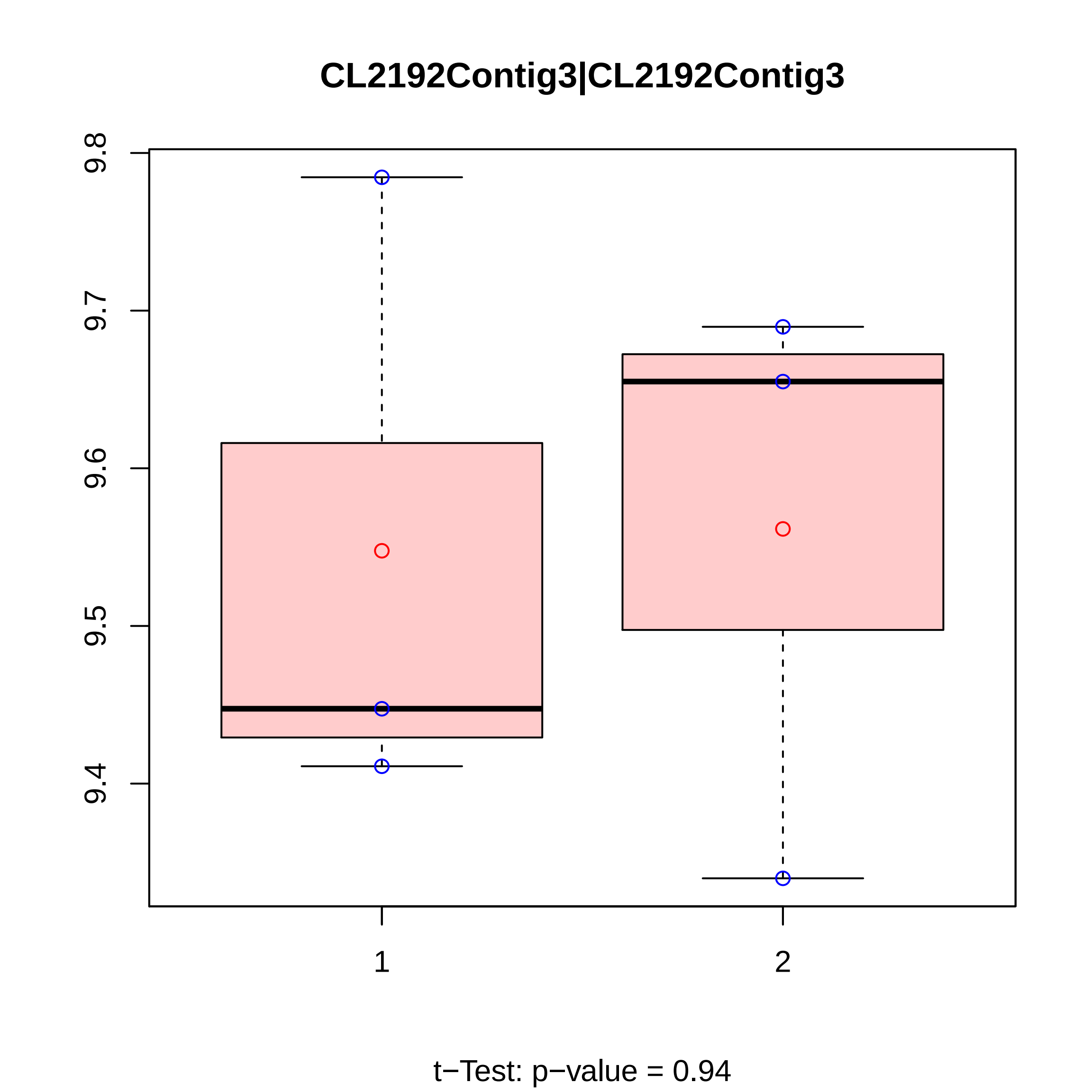
CL2192Contig3|CL2192Contig3
9.4
9.5
9.6
9.7
9.8
1
2
t−Test: p−value = 0.94

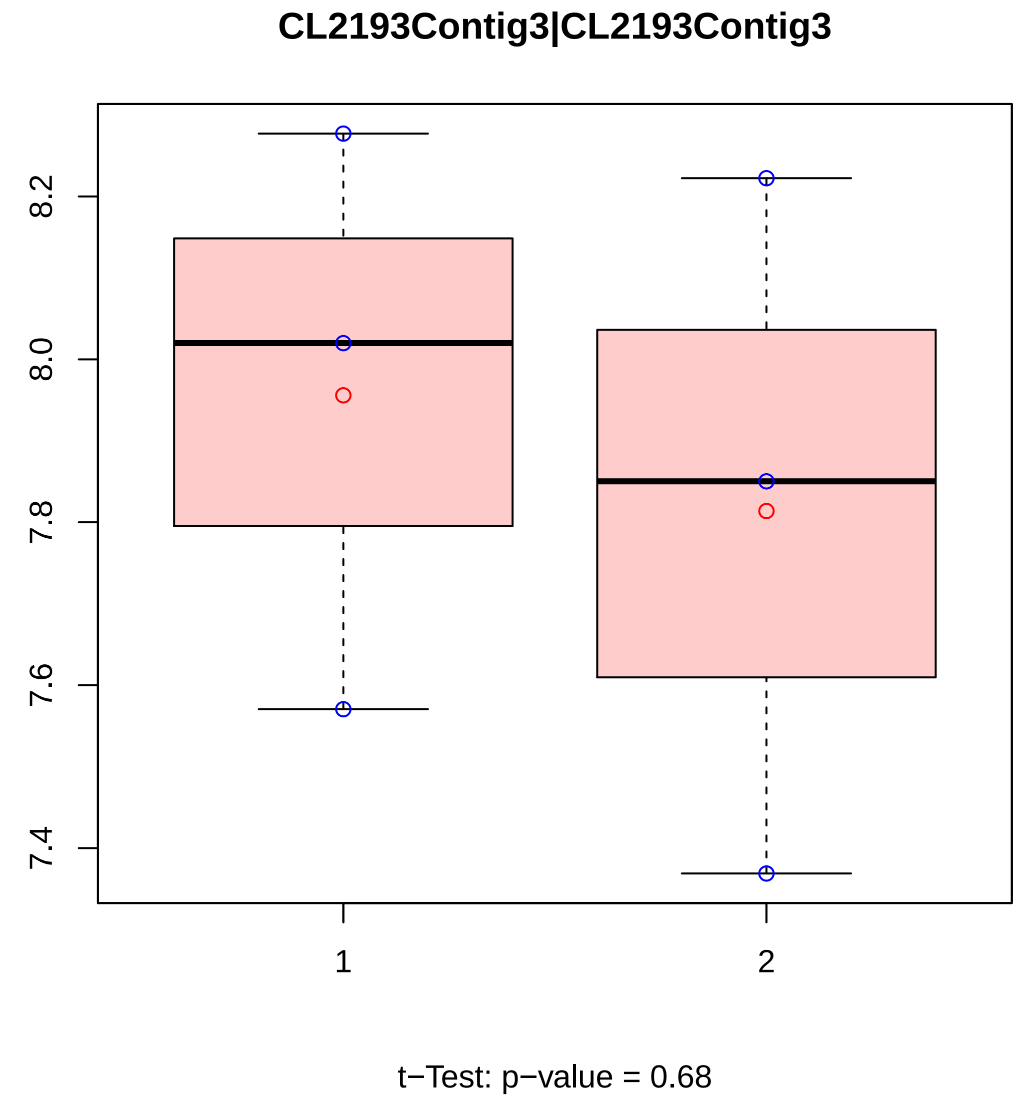

CL2193Contig3|CL2193Contig3
8.2
8.0
7.8
7.6
7.4
1
2
t−Test: p−value = 0.68

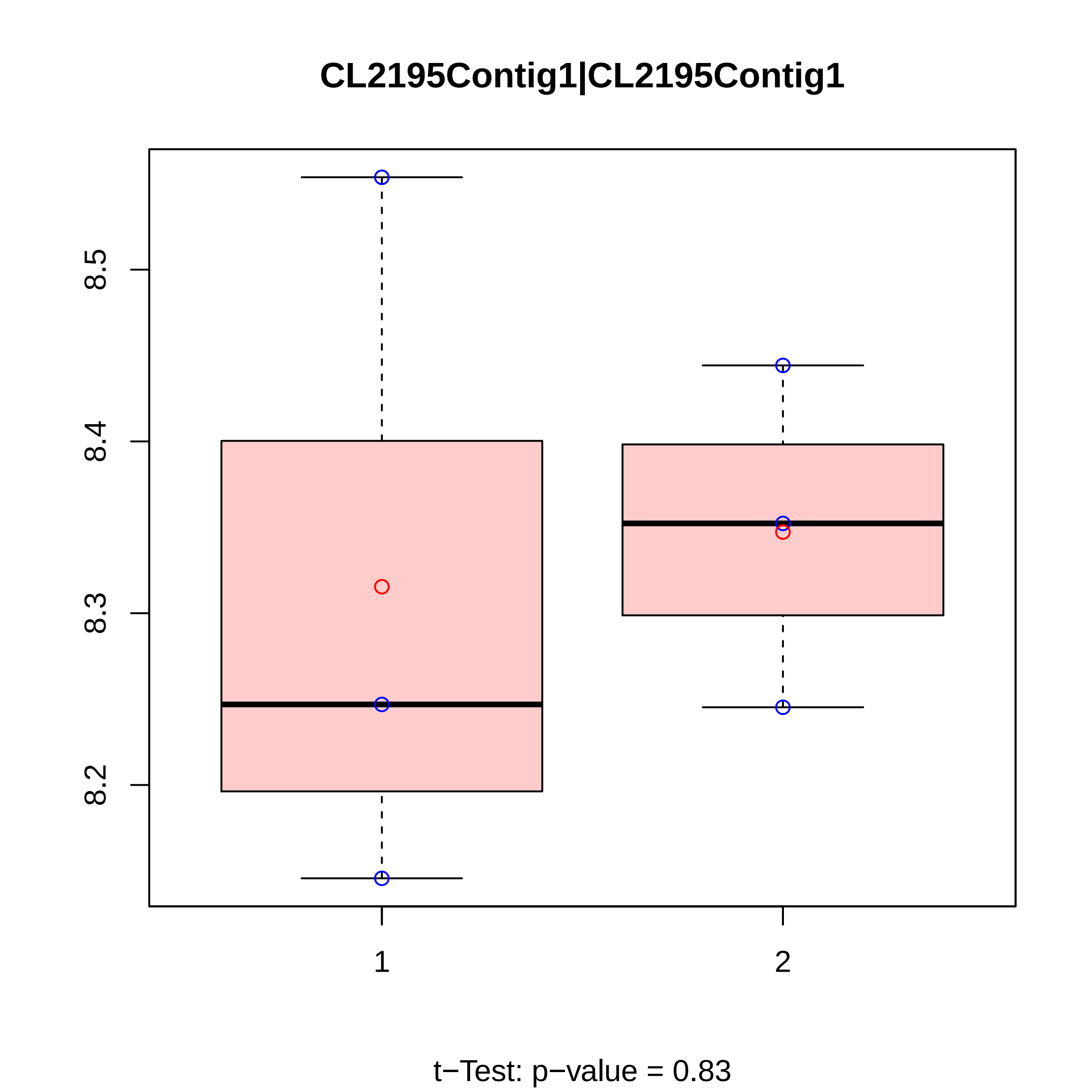
CL2195Contig1|CL2195Contig1
8.2
8.3
8.4
8.5
1
2
t−Test: p−value = 0.83

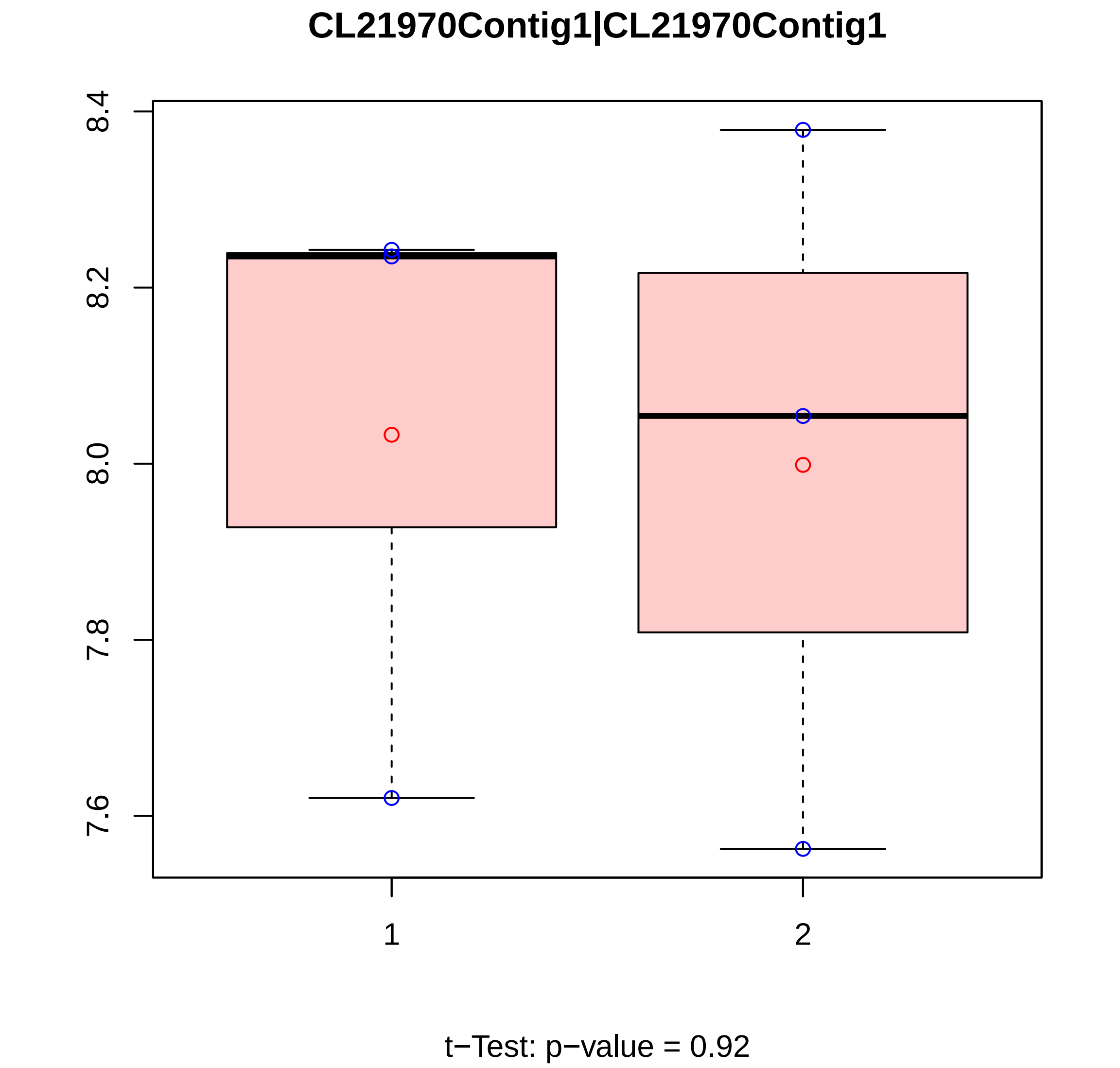
CL21970Contig1|CL21970Contig1
8.4
8.2
8.0
7.8
7.6
1
2
t−Test: p−value = 0.92

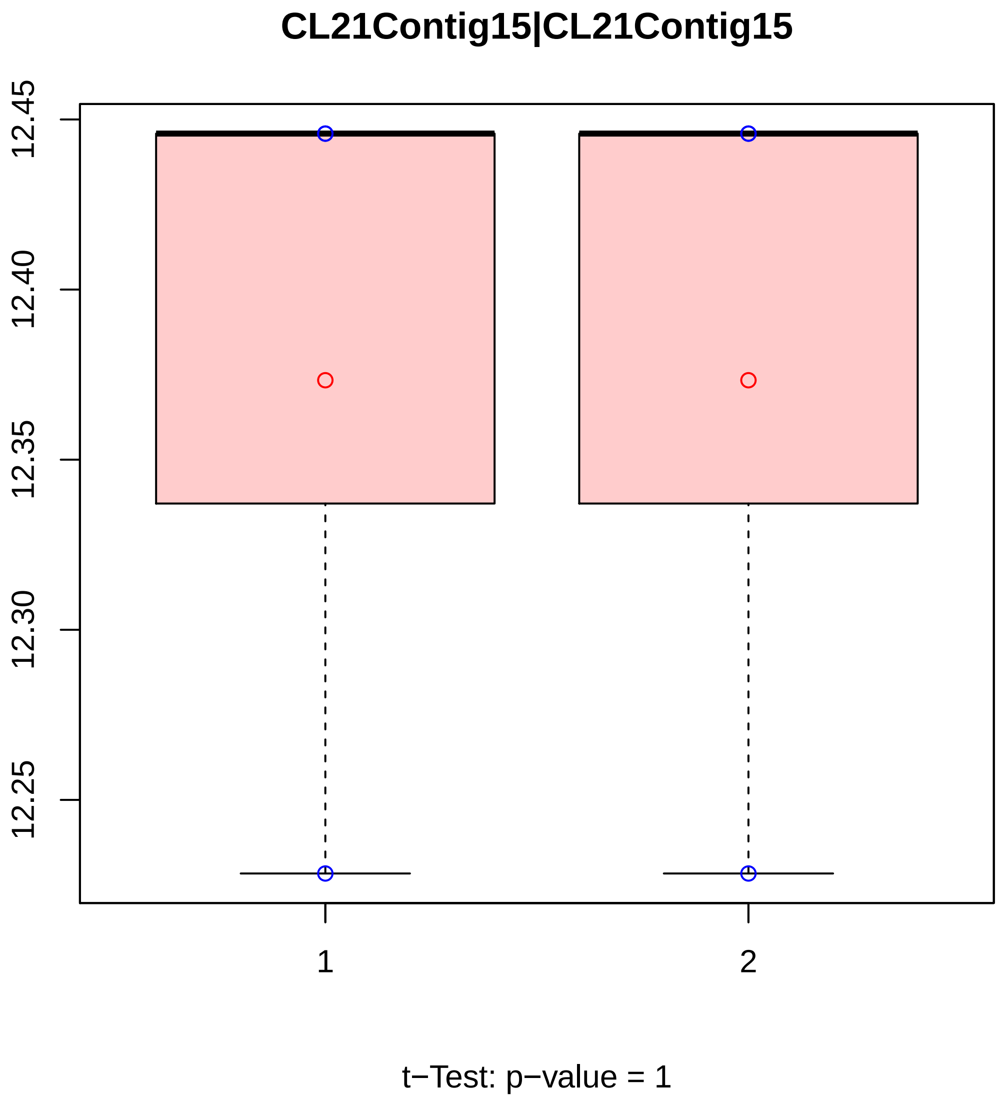
CL21Contig15|CL21Contig15
12.45
12.40
12.35
12.30
12.25
1
2
t−Test: p−value = 1

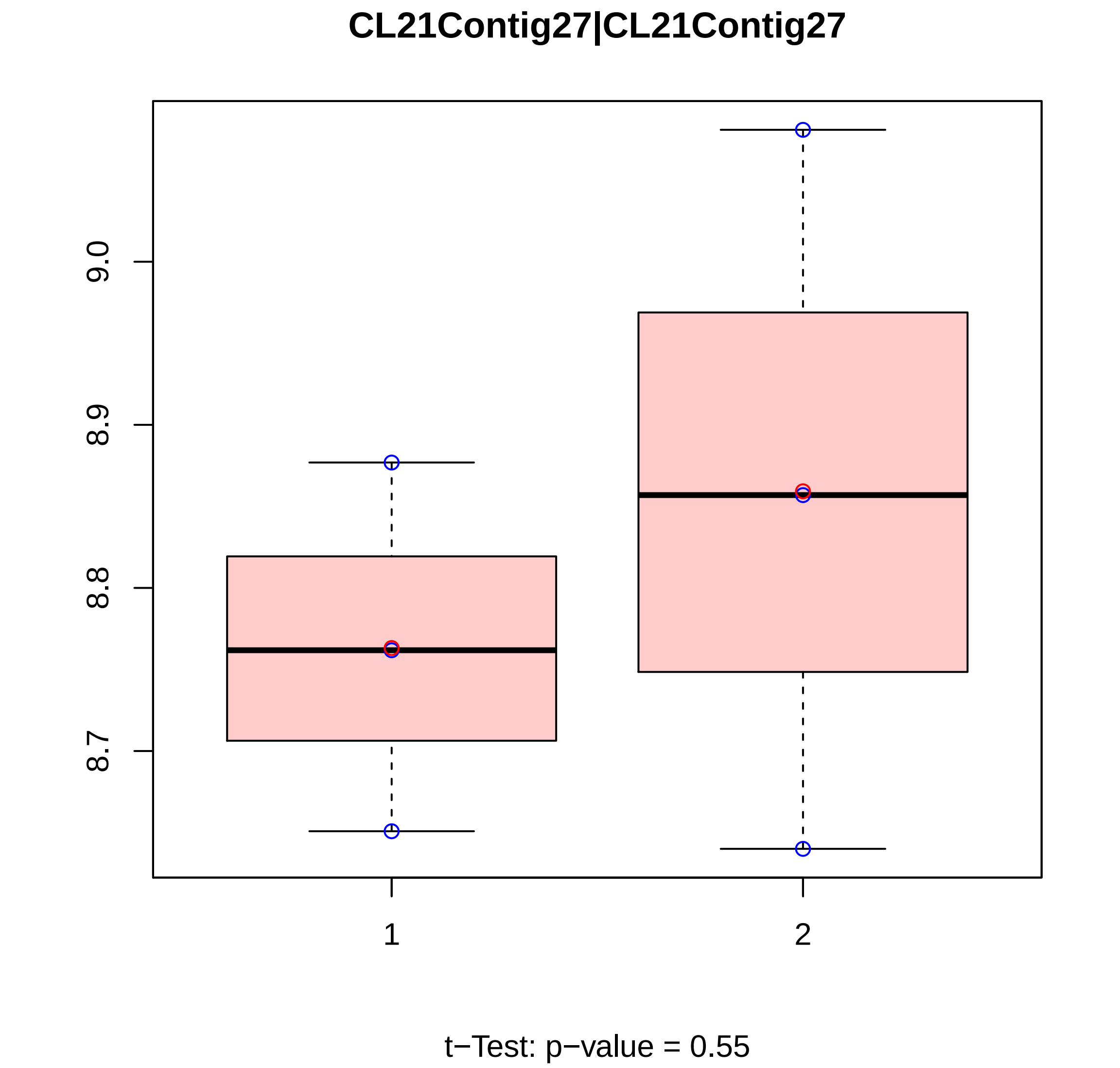
CL21Contig27|CL21Contig27
9.0
8.9
8.8
8.7
1
2
t−Test: p−value = 0.55

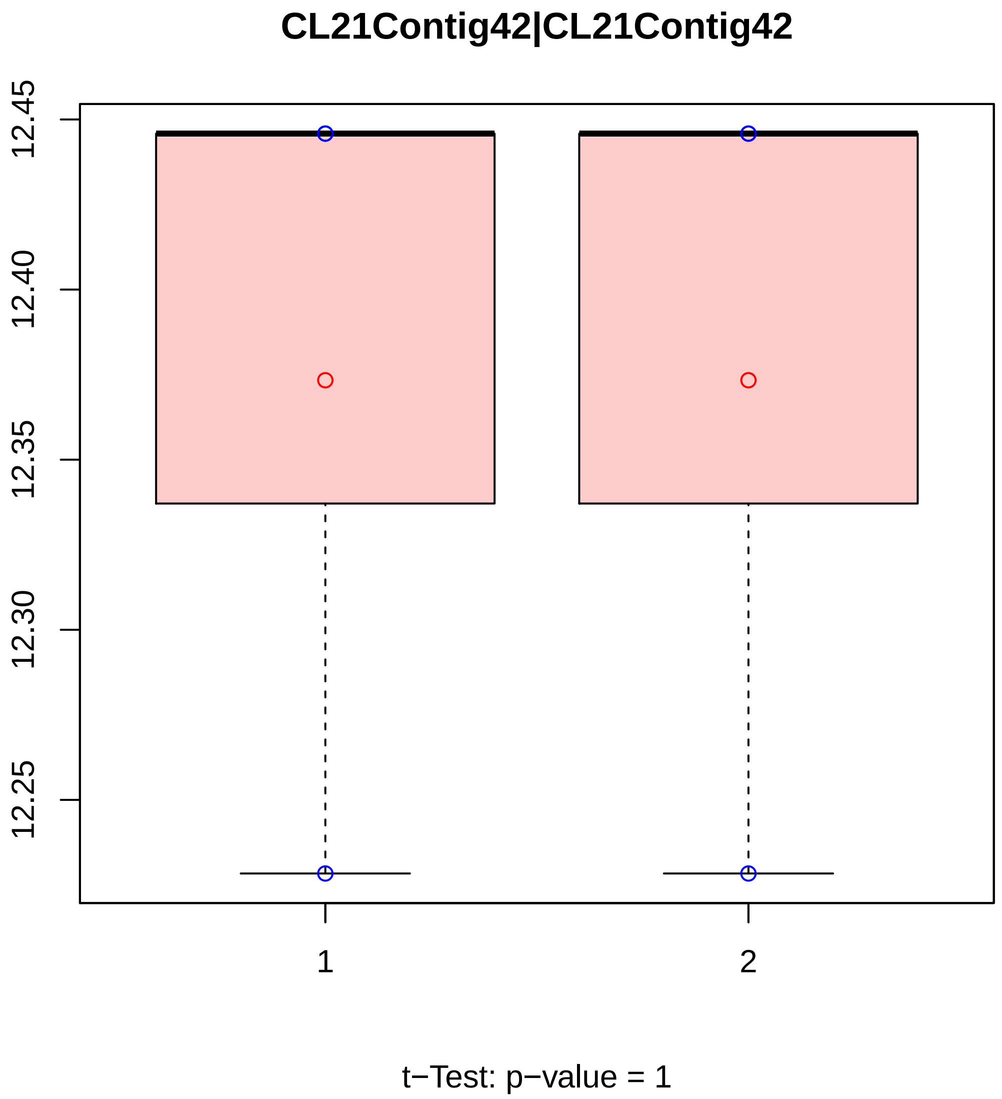
CL21Contig42|CL21Contig42
12.45
12.40
12.35
12.30
12.25
1
2
t−Test: p−value = 1

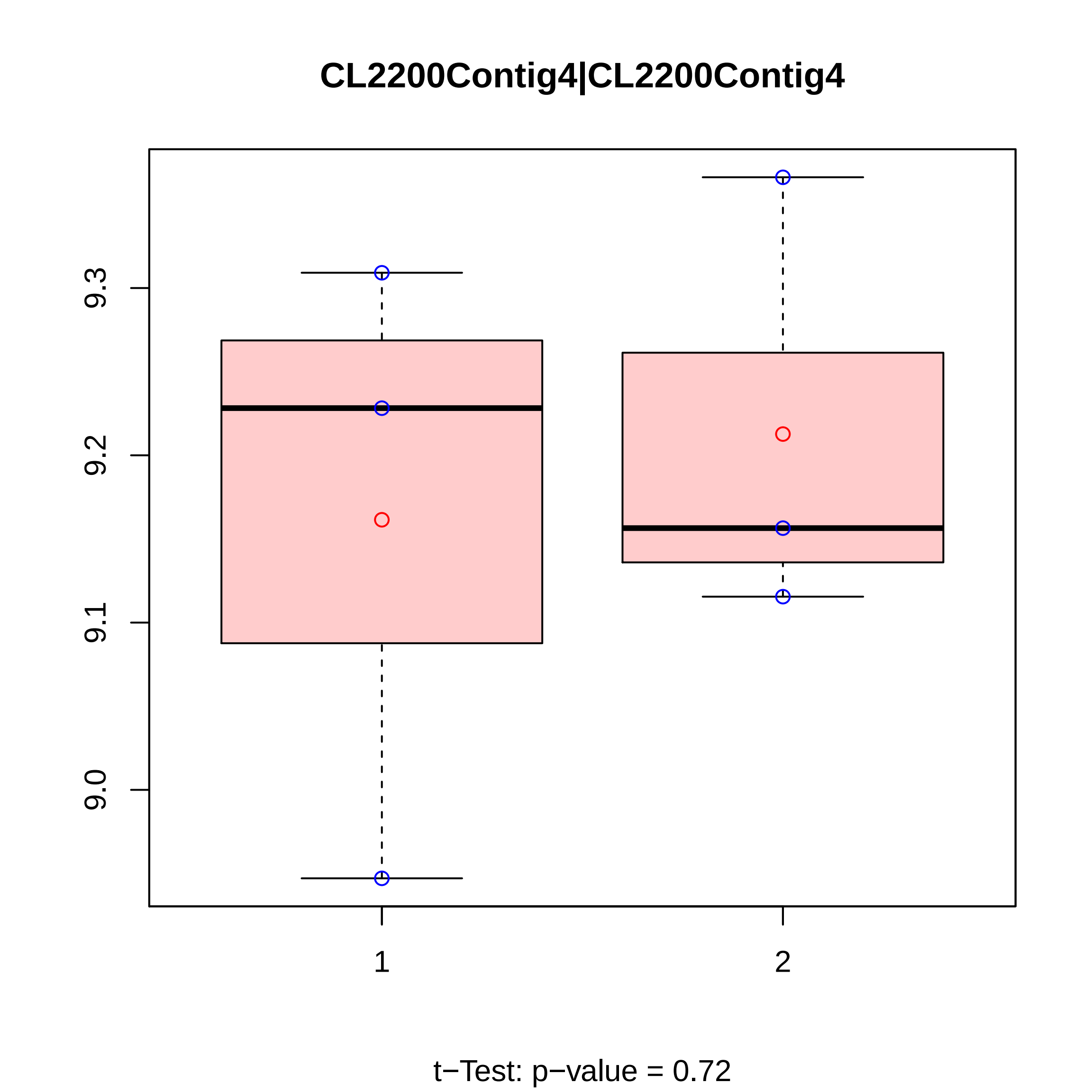

CL2200Contig4|CL2200Contig4
9.0
9.1
9.2
9.3
1
2
t−Test: p−value = 0.72

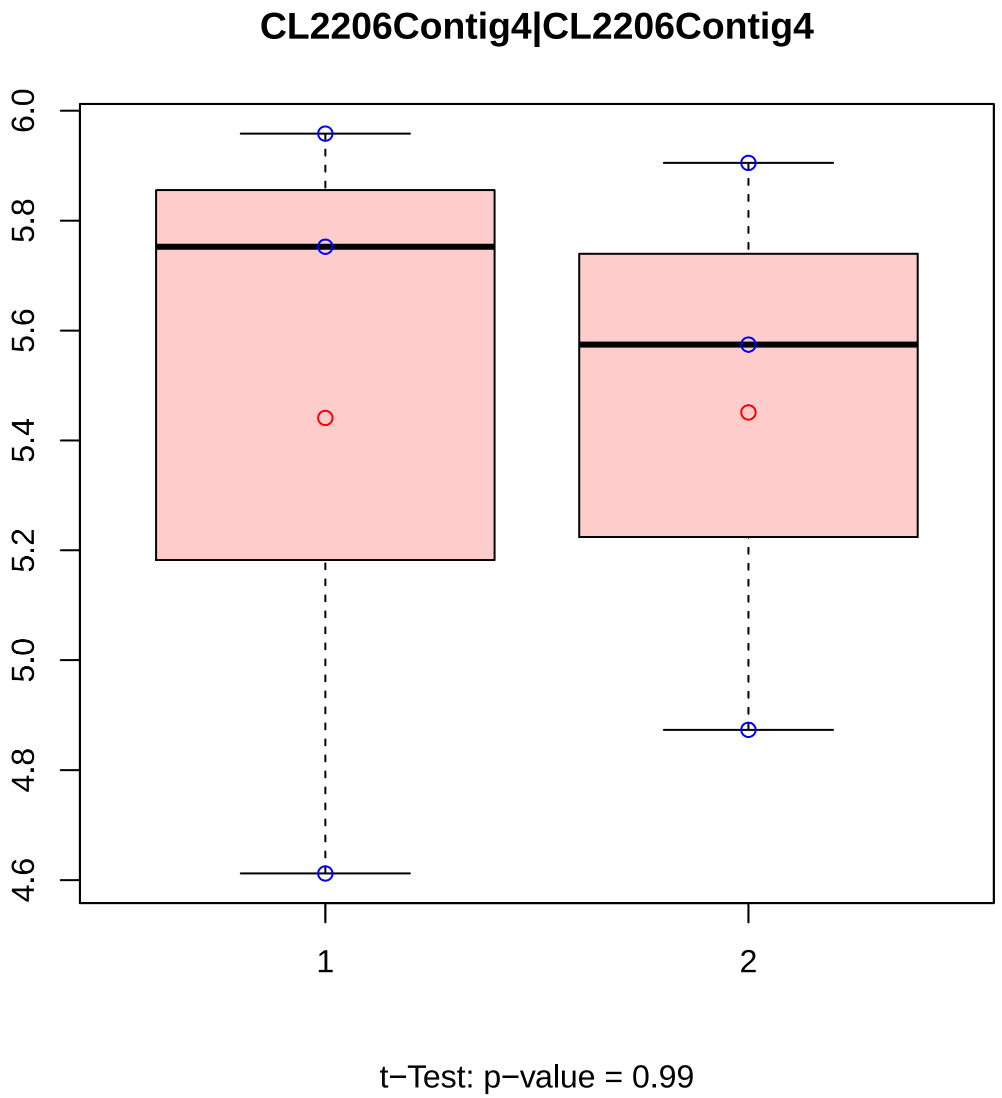

CL2206Contig4|CL2206Contig4
6.0
5.8
5.6
5.4
5.2
5.0
4.8
4.6
1
2
t−Test: p−value = 0.99

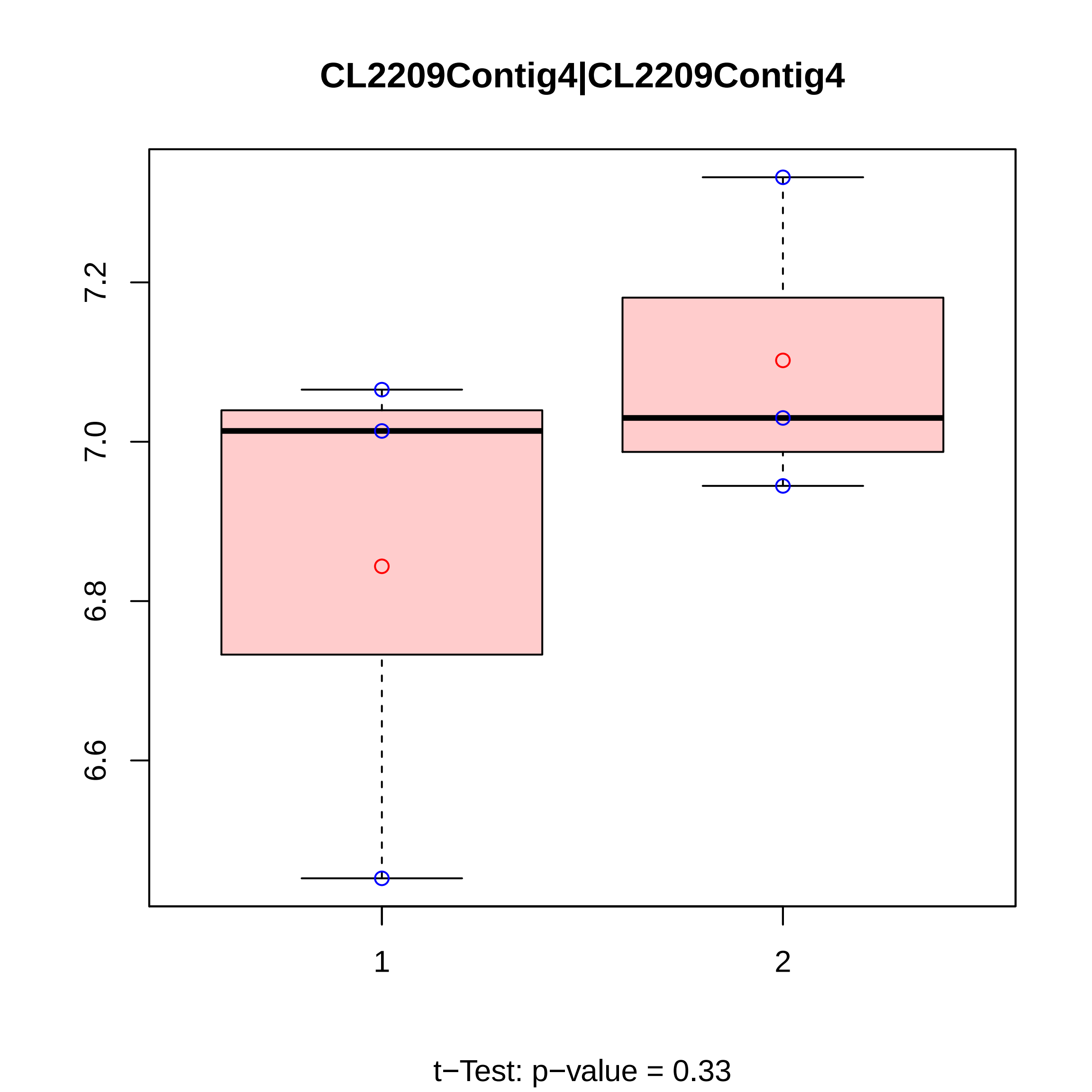
CL2209Contig4|CL2209Contig4
7.2
7.0
6.8
6.6
1
2
t−Test: p−value = 0.33

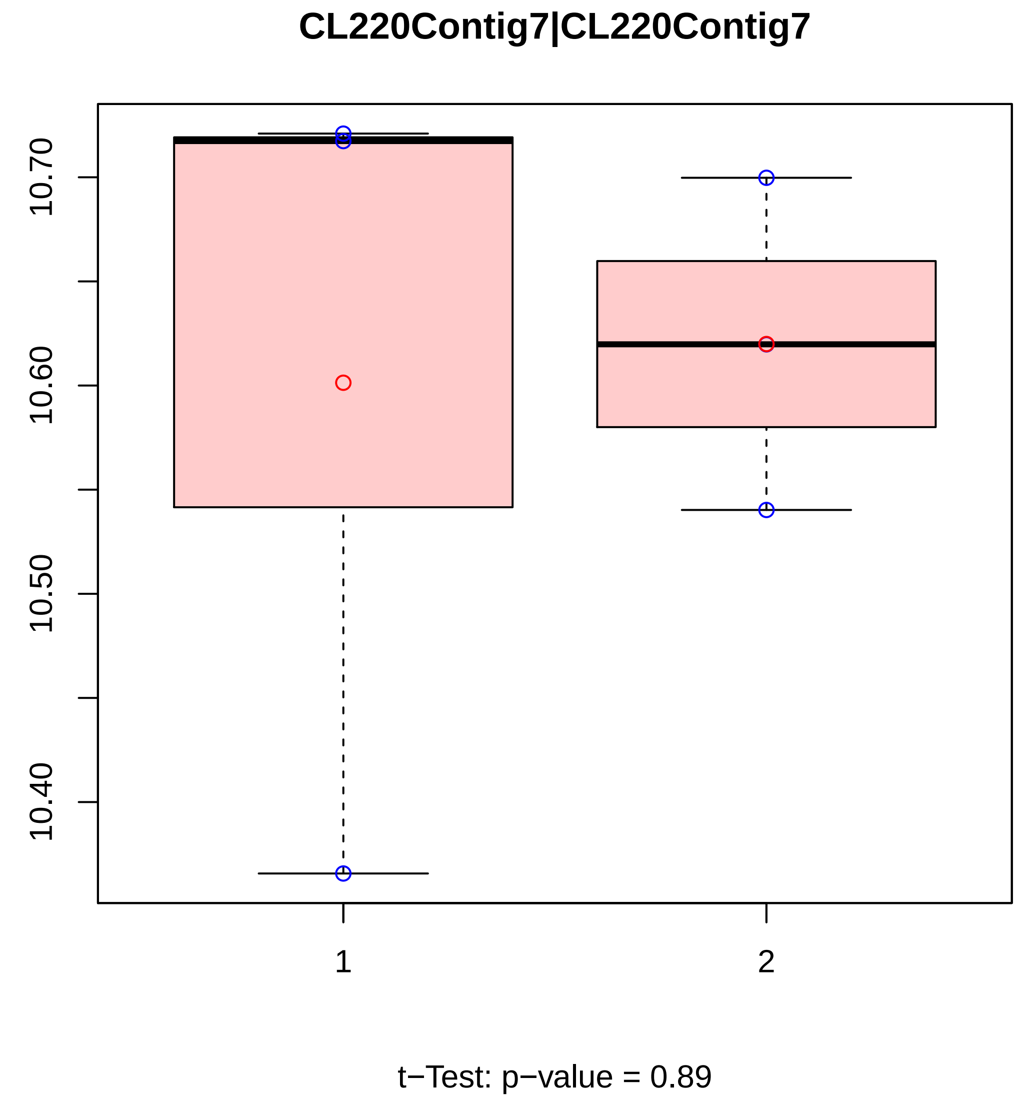
CL220Contig7|CL220Contig7
10.70
10.60
10.50
10.40
1
2
t−Test: p−value = 0.89

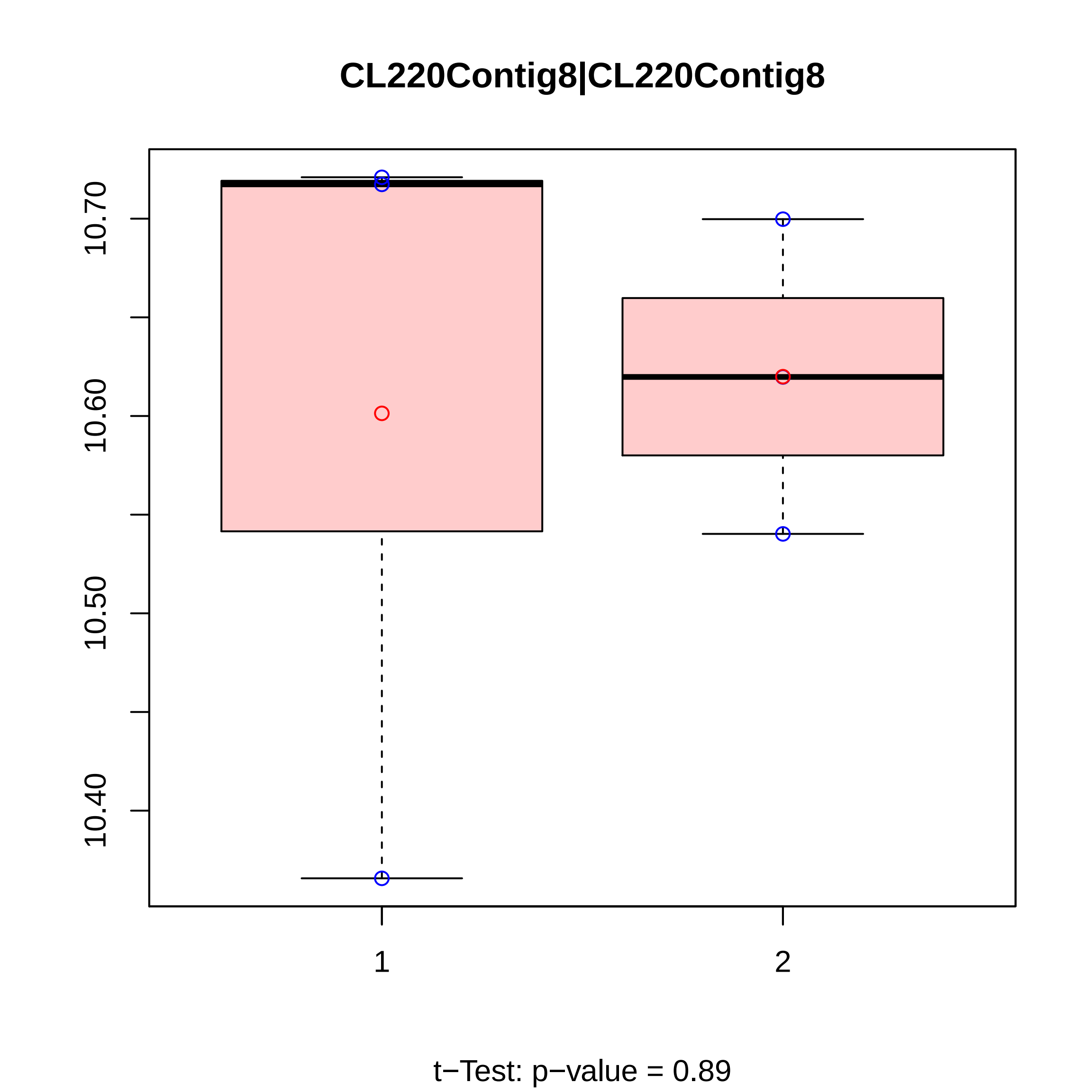
CL220Contig8|CL220Contig8
10.40
10.50
10.60
10.70
1
2
t−Test: p−value = 0.89

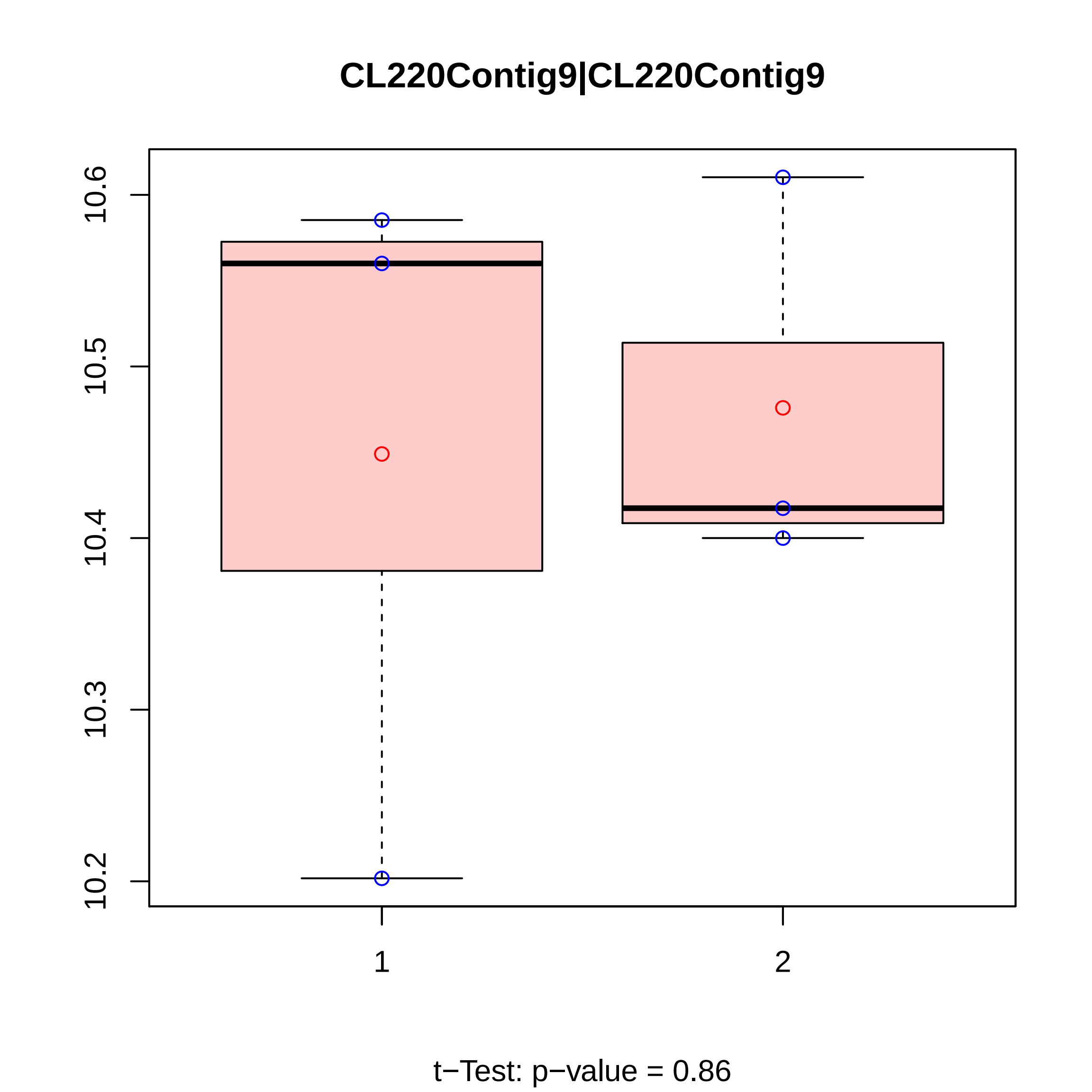
CL220Contig9|CL220Contig9
10.6
10.5
10.4
10.3
10.2
1
2
t−Test: p−value = 0.86

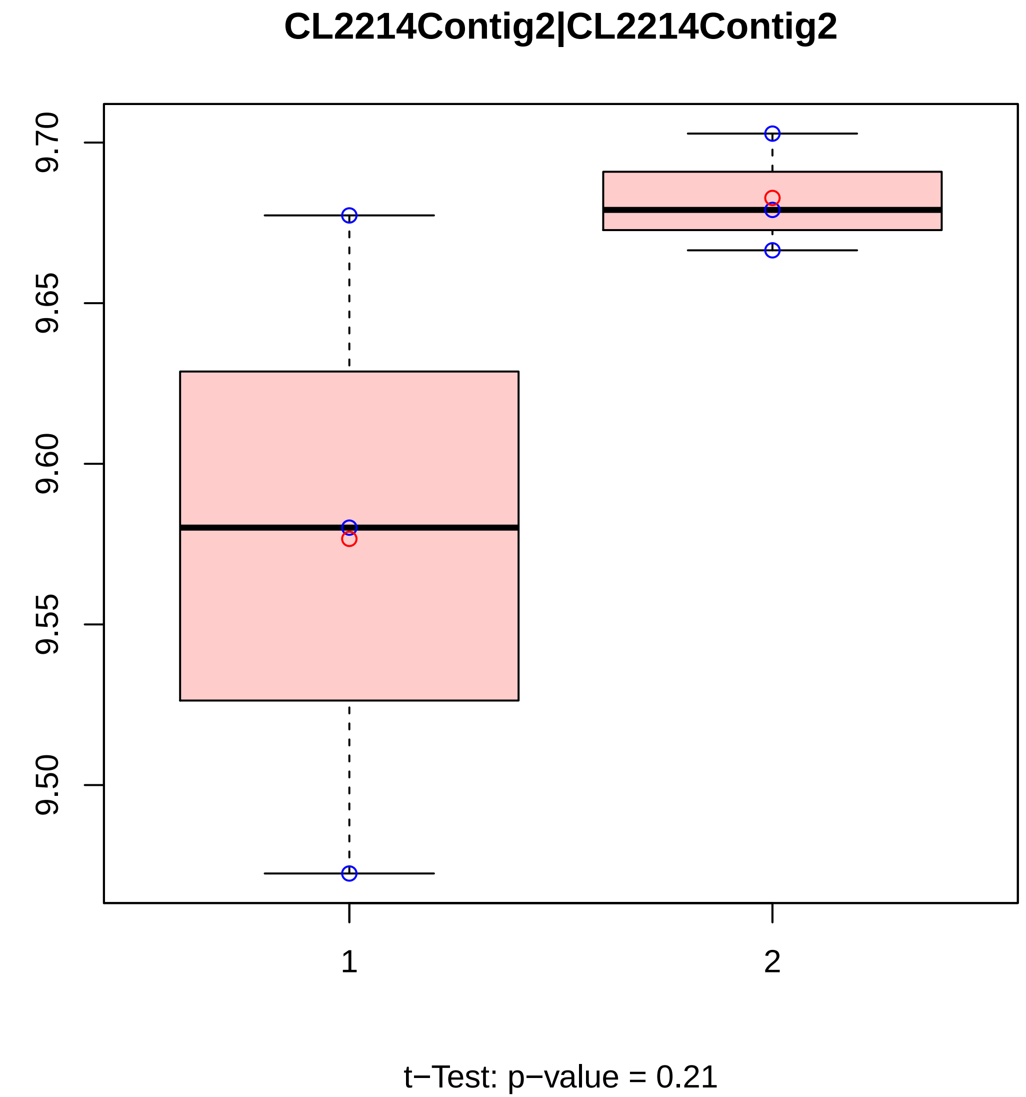
CL2214Contig2|CL2214Contig2
9.70
9.65
9.60
9.55
9.50
1
2
t−Test: p−value = 0.21

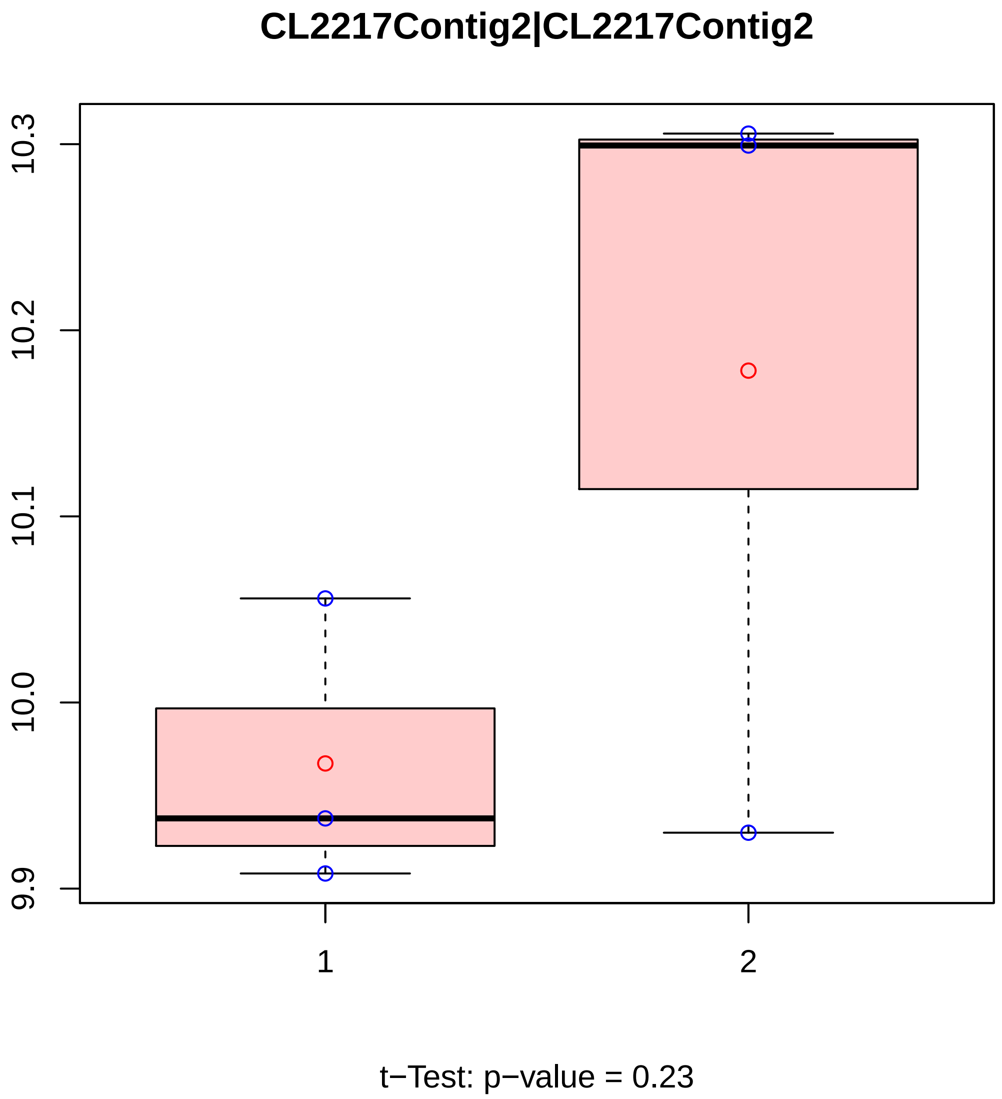
CL2217Contig2|CL2217Contig2
10.3
10.2
10.1
10.0
9.9
1
2
t−Test: p−value = 0.23

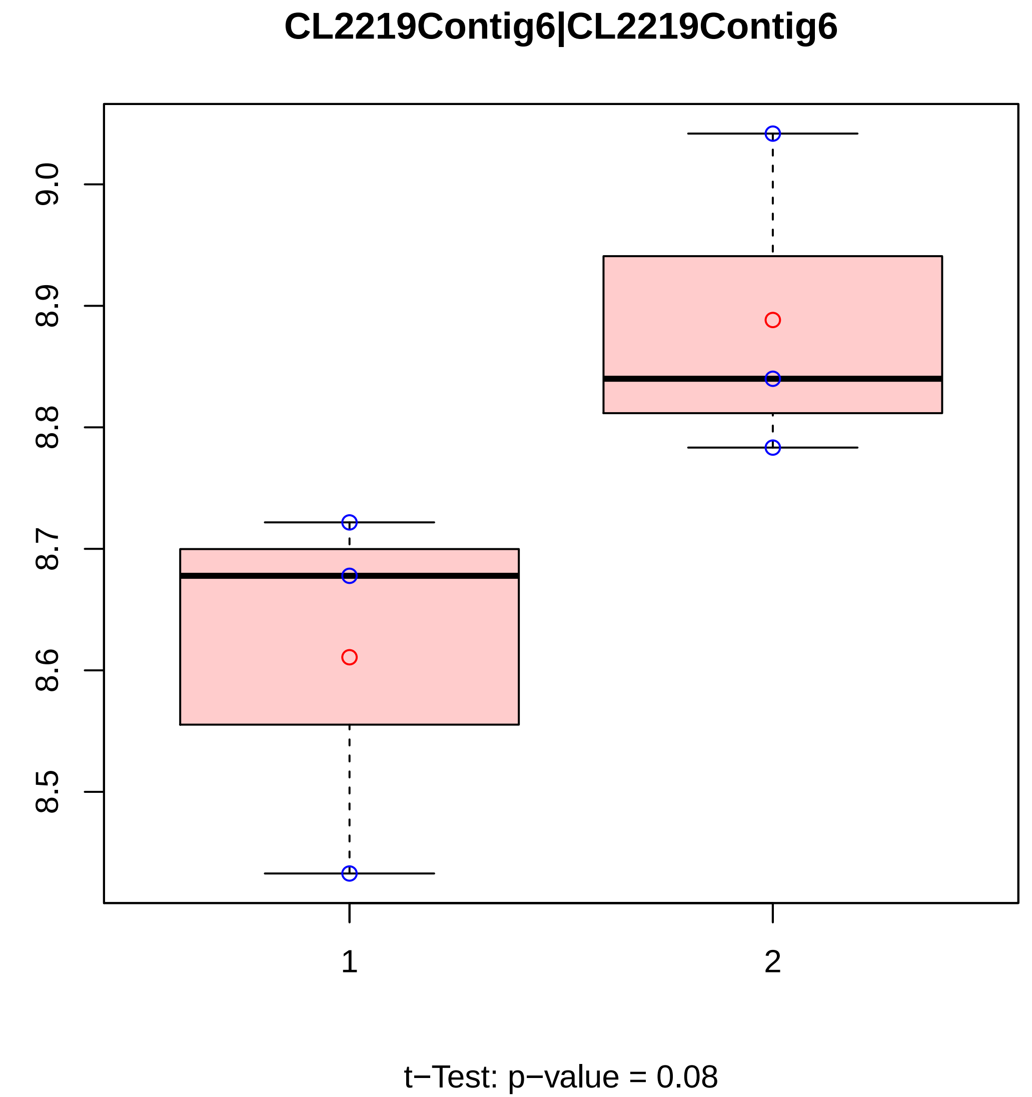
CL2219Contig6|CL2219Contig6
9.0
8.9
8.8
8.7
8.6
8.5
1
2
t−Test: p−value = 0.08

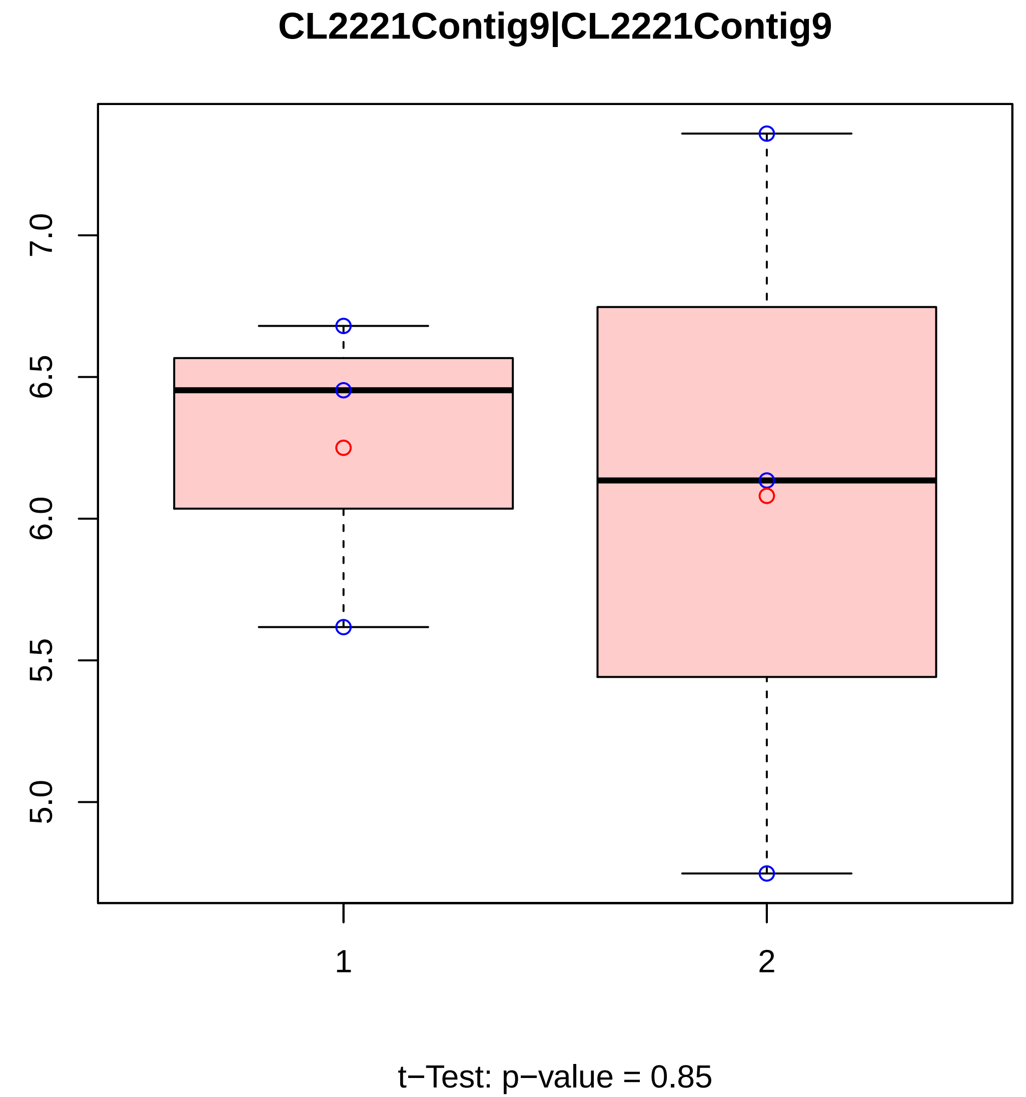
CL2221Contig9|CL2221Contig9
7.0
6.5
6.0
5.5
5.0
1
2
t−Test: p−value = 0.85

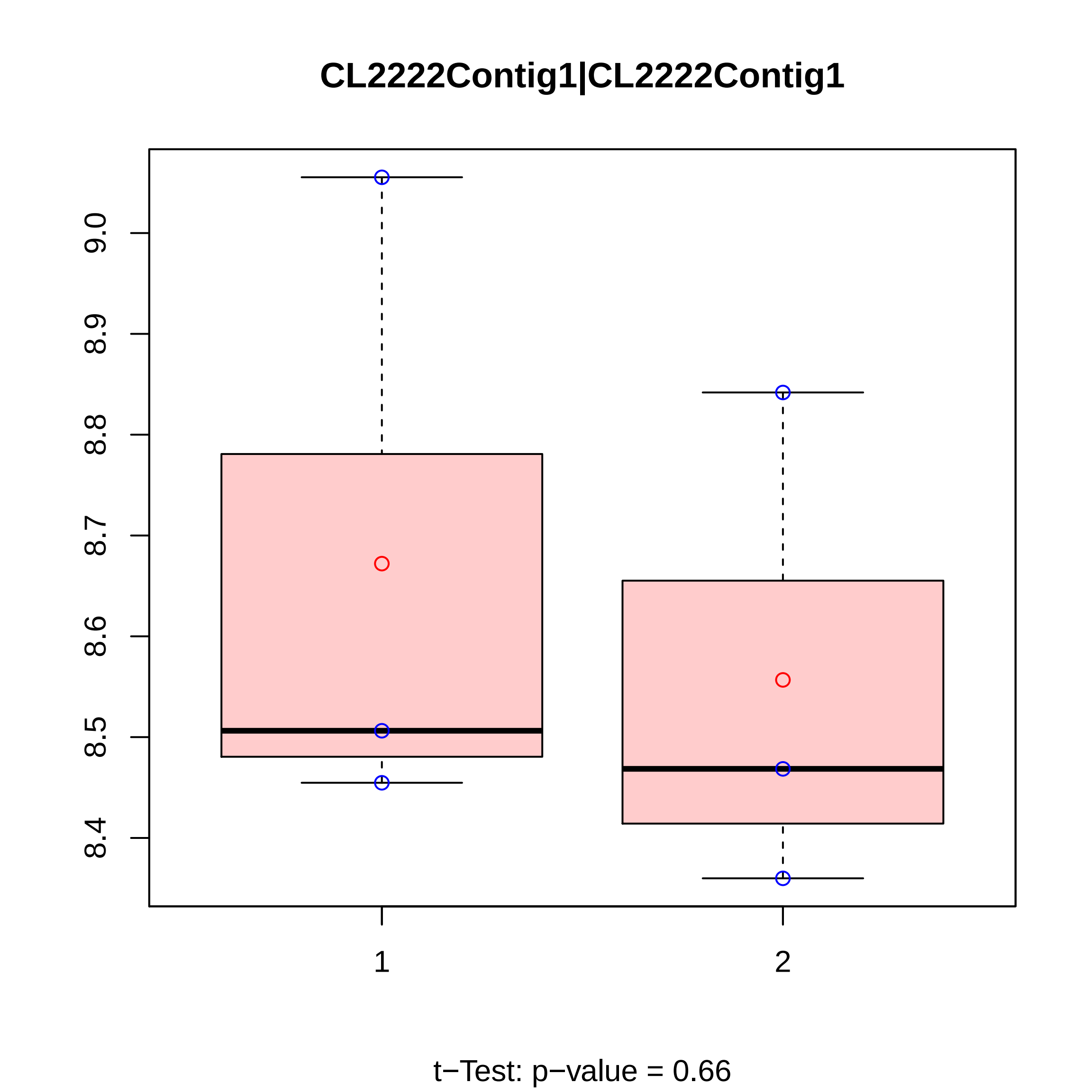
CL2222Contig1|CL2222Contig1
9.0
8.9
8.8
8.7
8.6
8.5
8.4
1
2
t−Test: p−value = 0.66

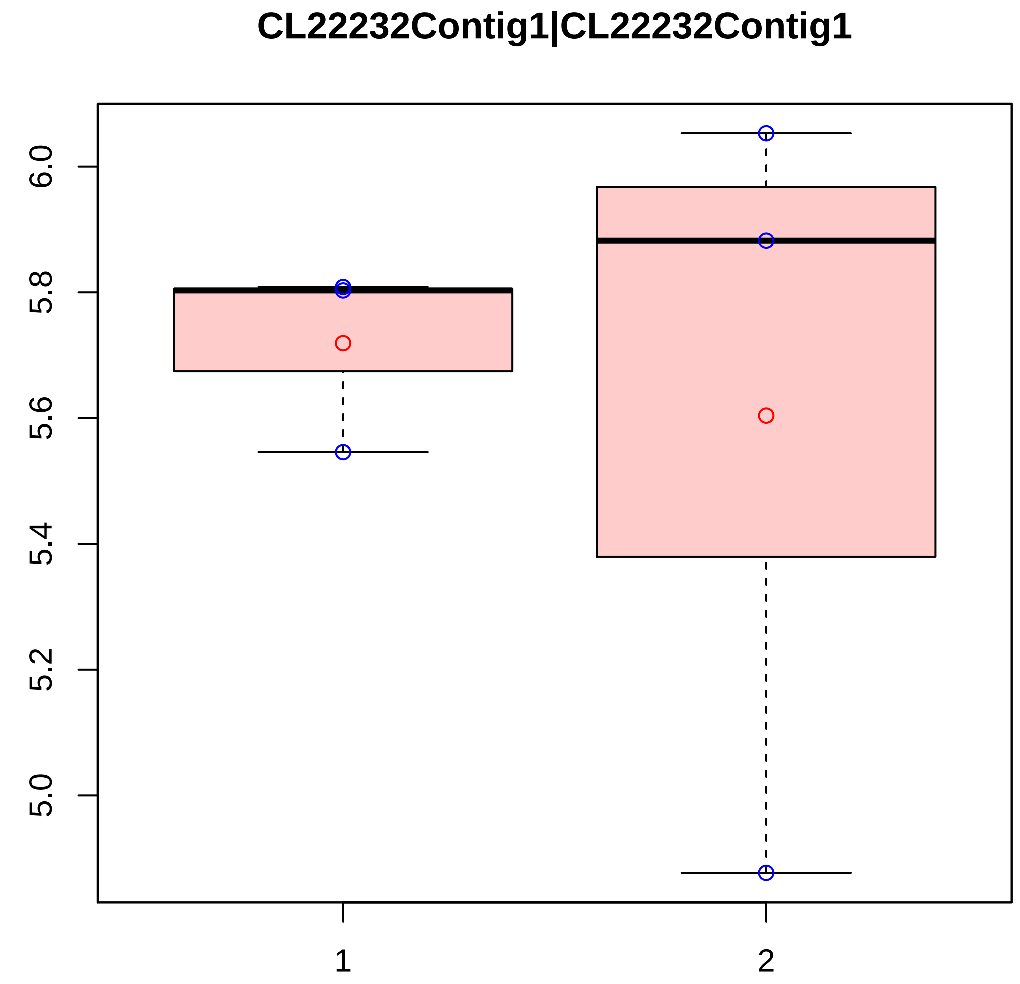


t−Test: p−value = 0.79

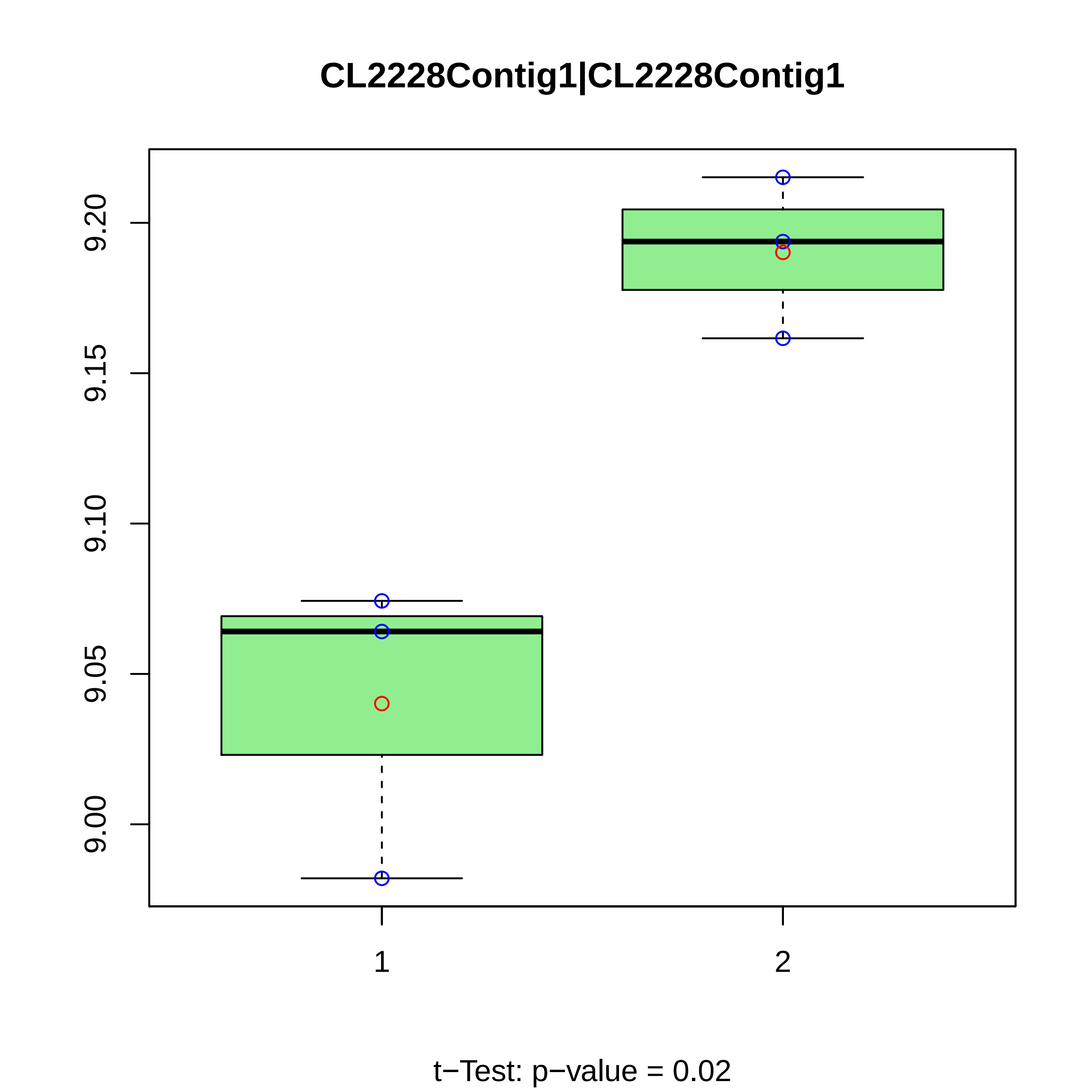
CL2228Contig1|CL2228Contig1
9.00
9.05
9.10
9.15
9.20
1
2
t−Test: p−value = 0.02

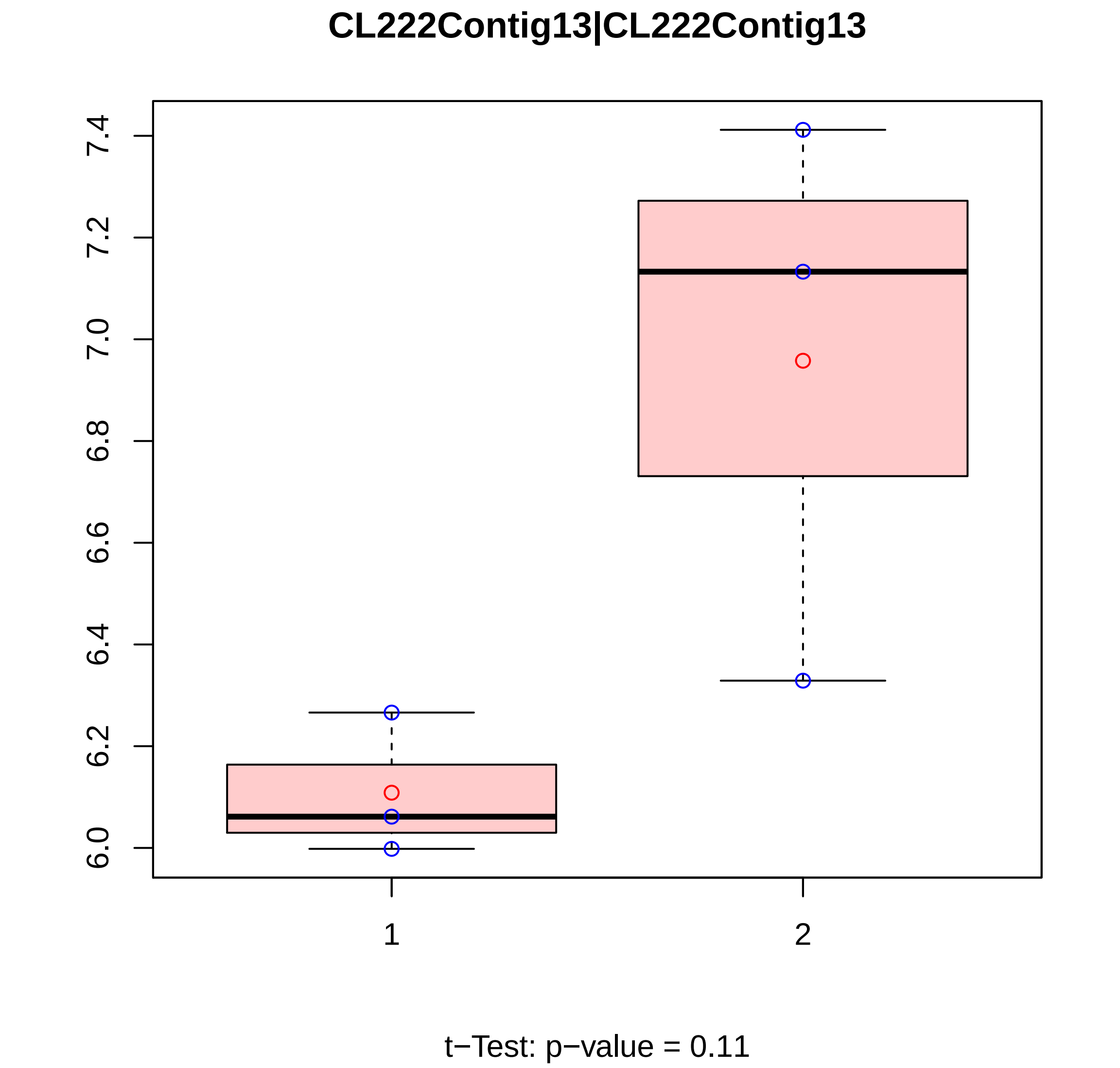
CL222Contig13|CL222Contig13
7.4
7.2
7.0
6.8
6.6
6.4
6.2
6.0
1
2
t−Test: p−value = 0.11

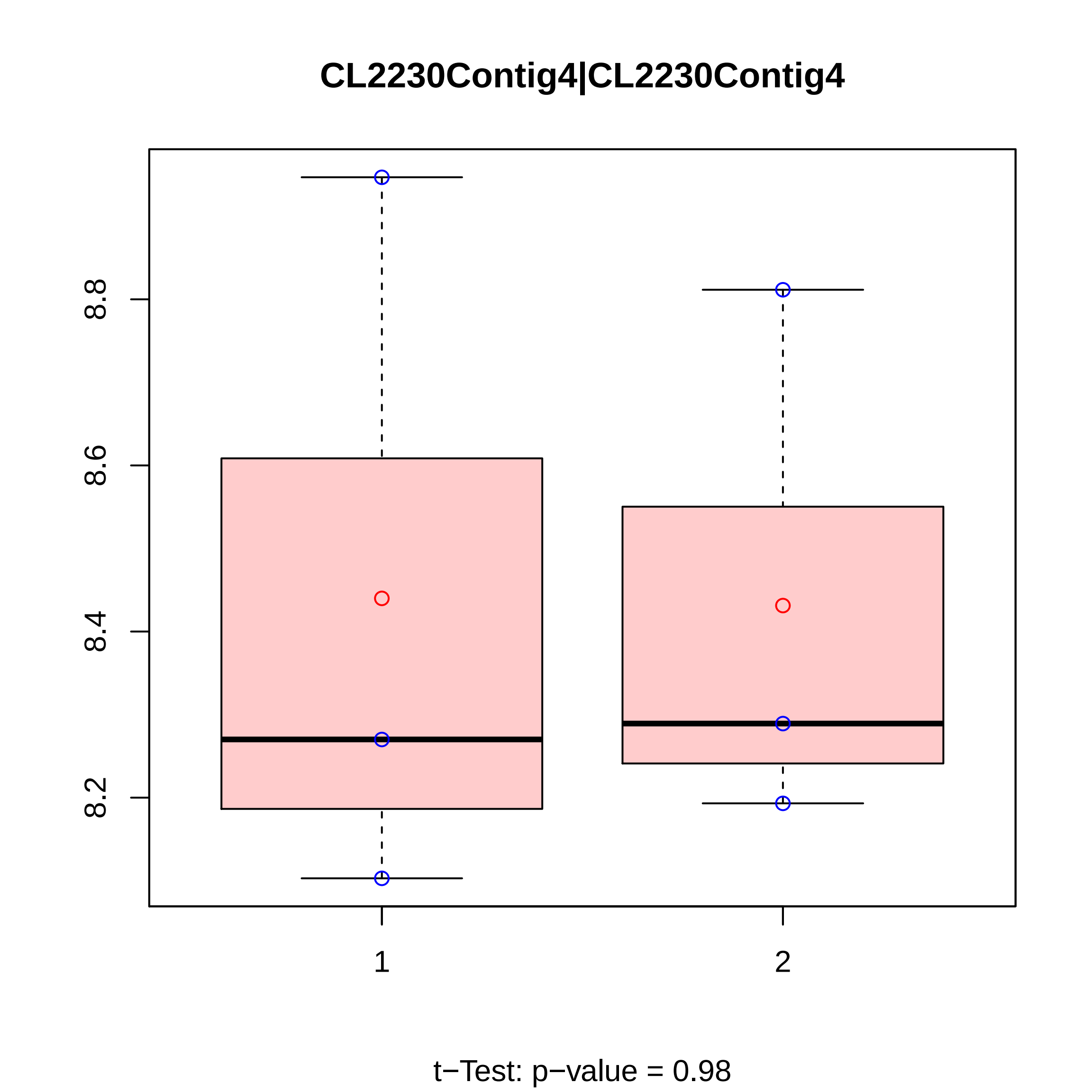
CL2230Contig4|CL2230Contig4
8.2
8.4
8.6
8.8
1
2
t−Test: p−value = 0.98

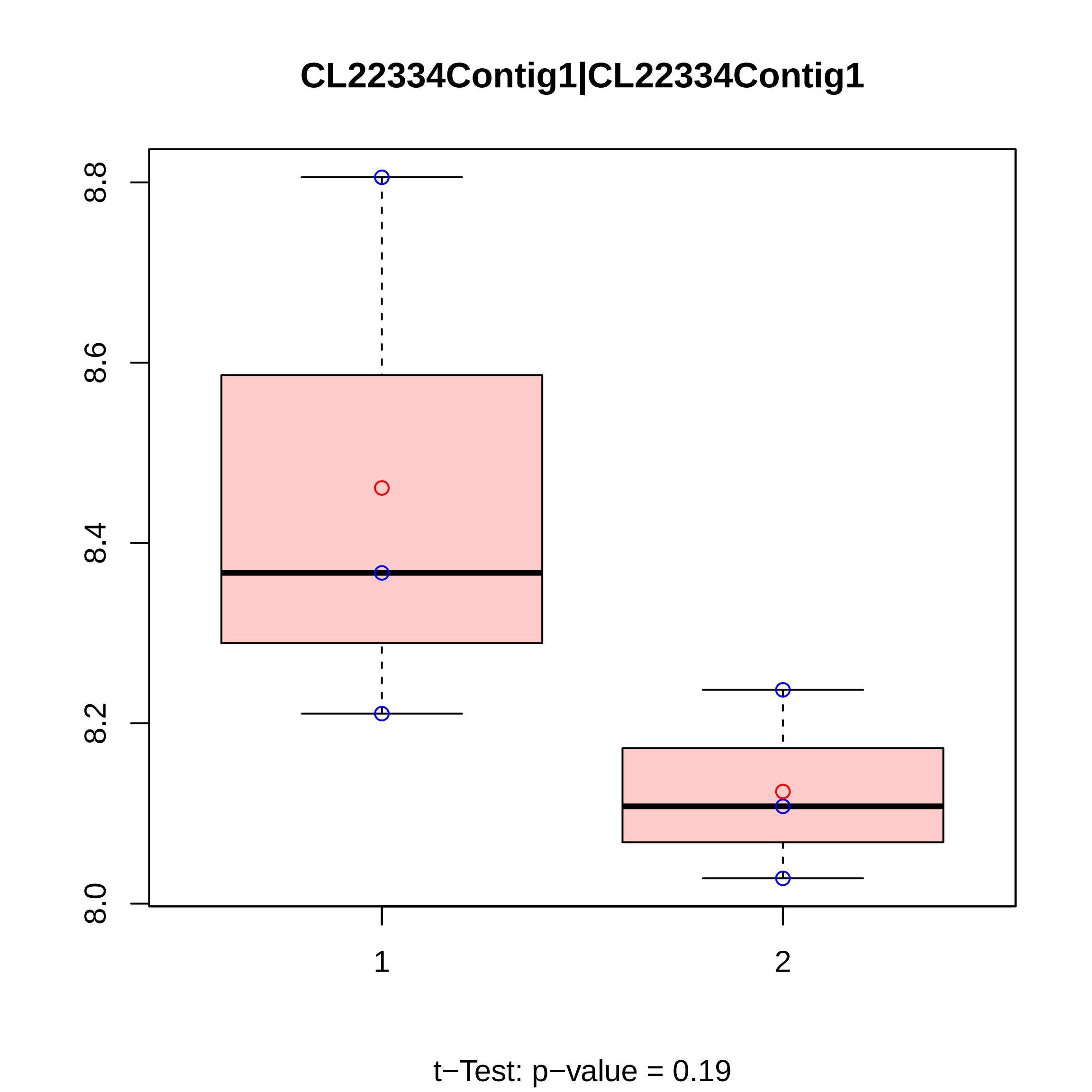
CL22334Contig1|CL22334Contig1
8.0
8.2
8.4
8.6
8.8
1
2
t−Test: p−value = 0.19

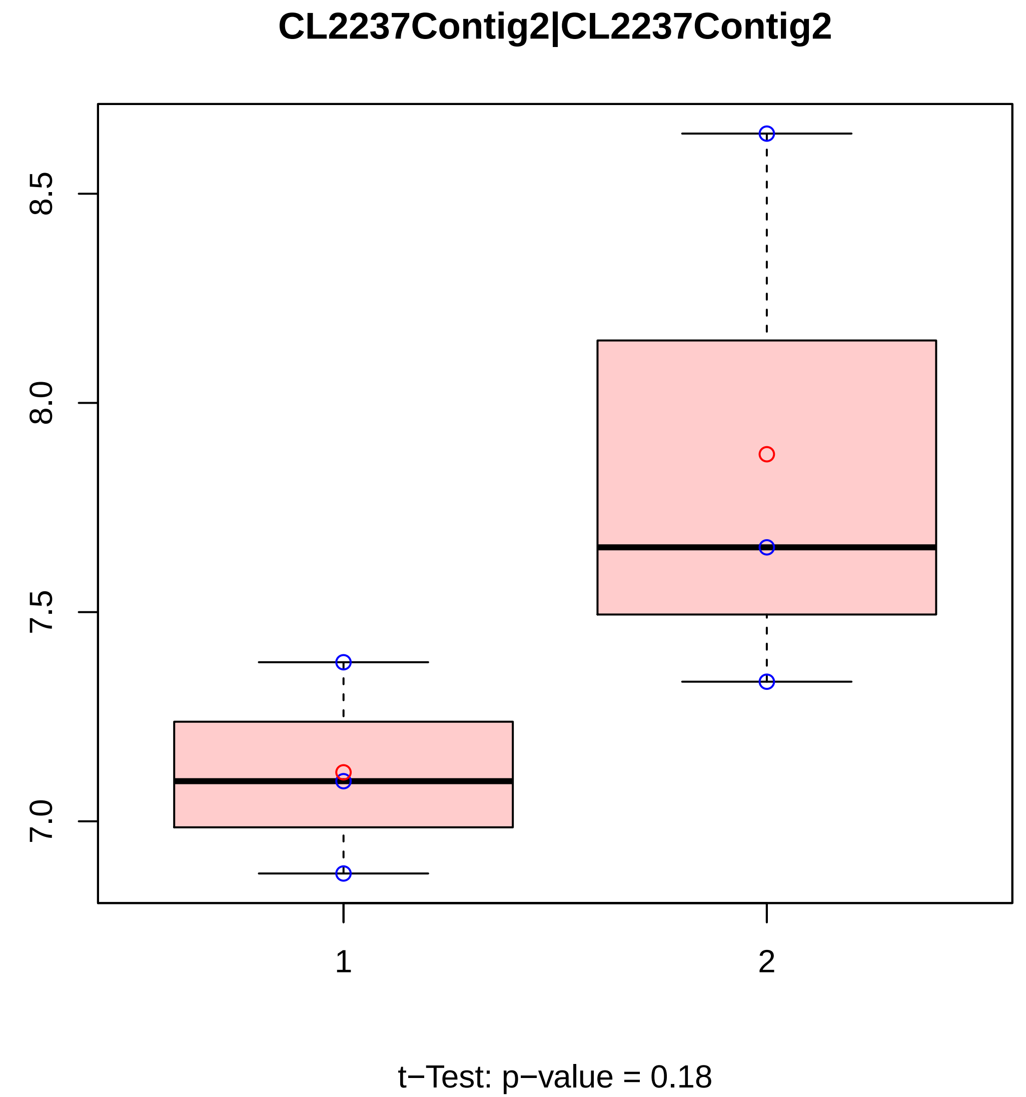

CL2237Contig2|CL2237Contig2
8.5
8.0
7.5
7.0
1
2
t−Test: p−value = 0.18

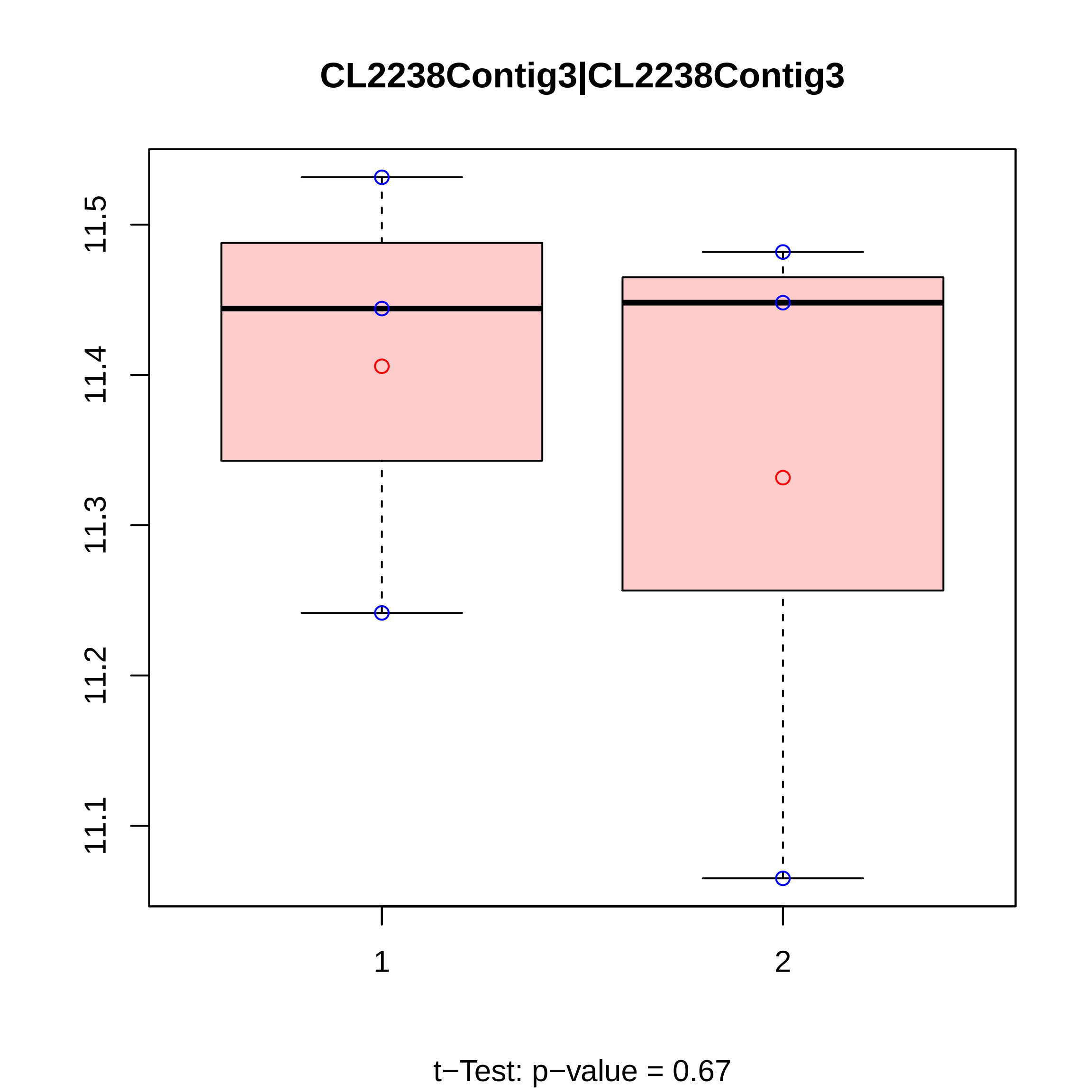

CL2238Contig3|CL2238Contig3
11.1
11.2
11.3
11.4
11.5
1
2
t−Test: p−value = 0.67

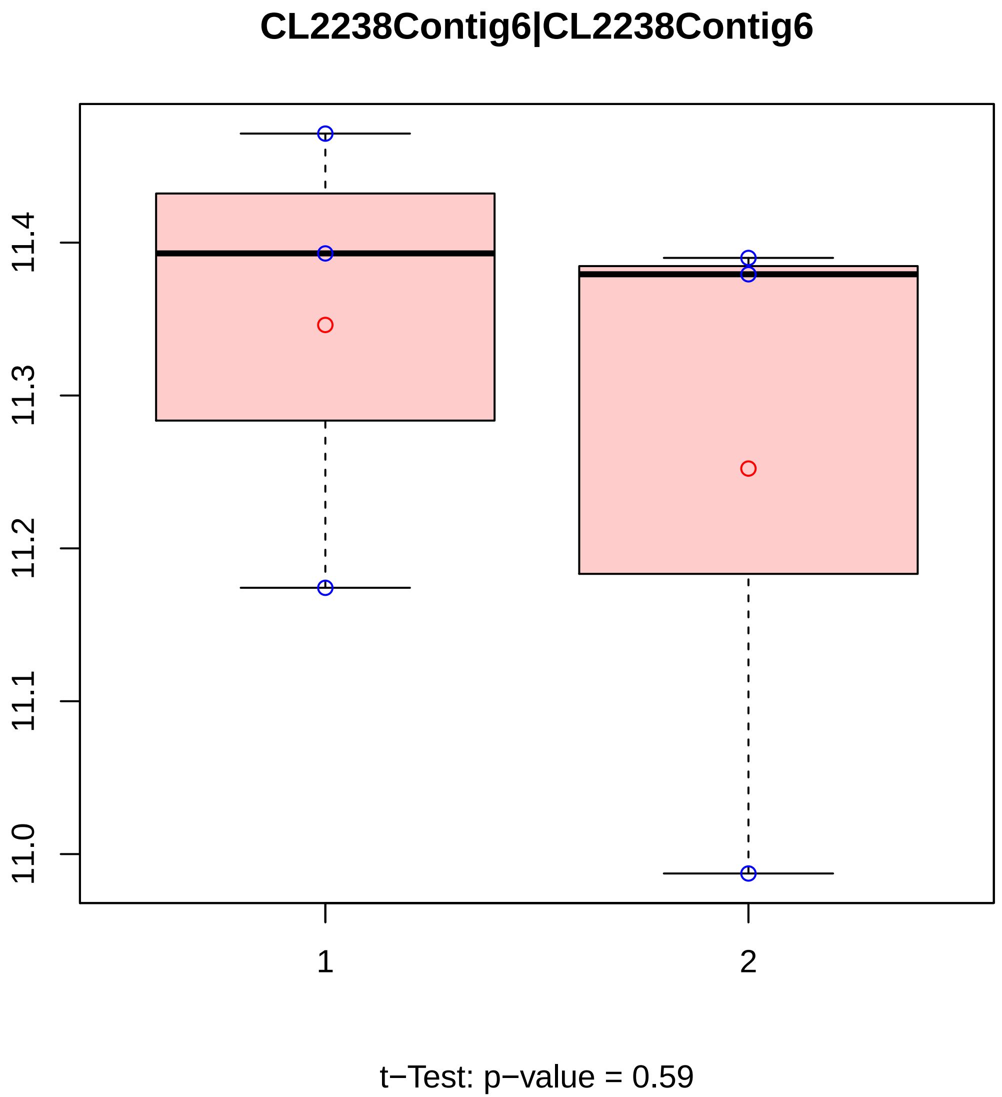

CL2238Contig6|CL2238Contig6
11.0
11.1
11.2
11.3
11.4
1
2
t−Test: p−value = 0.59

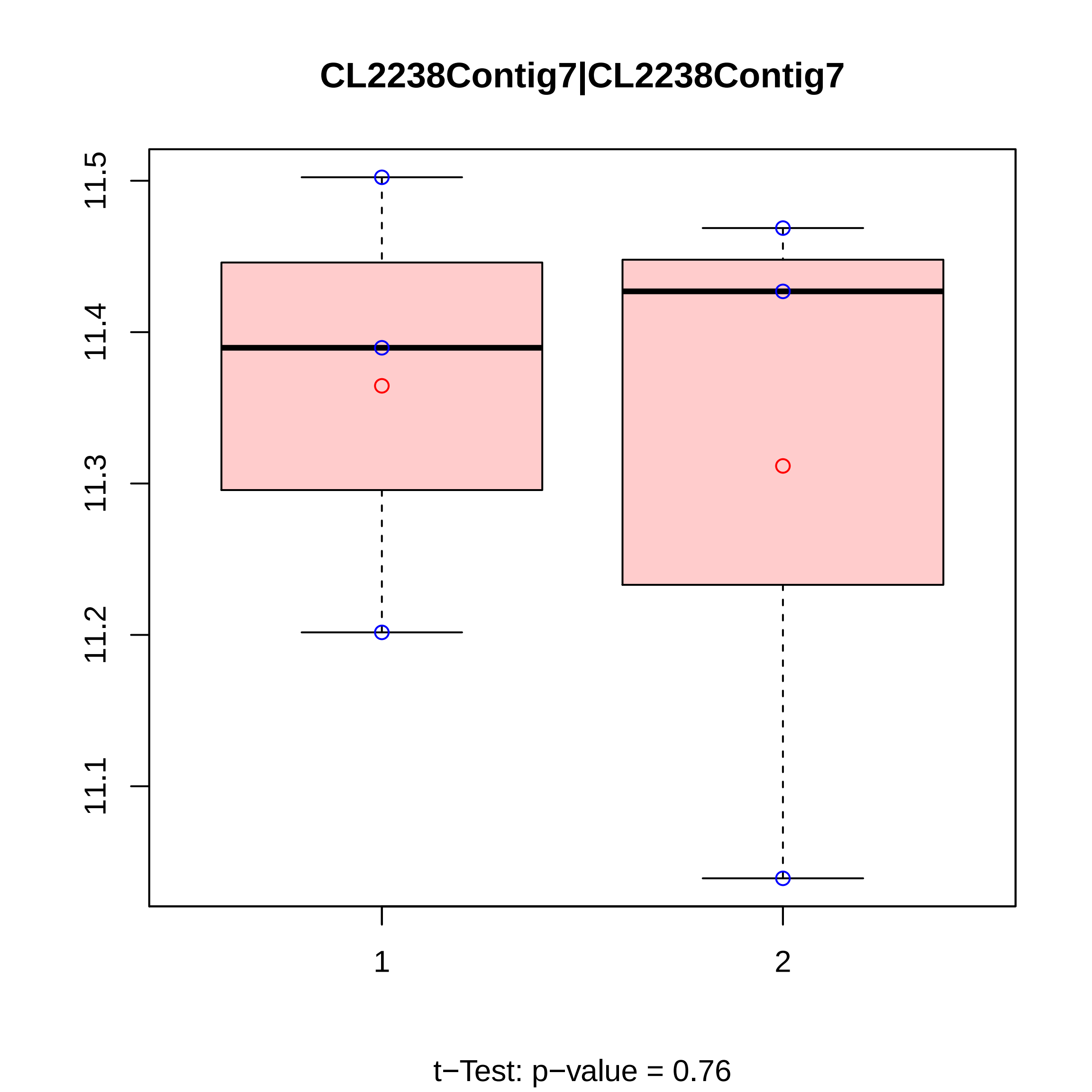

CL2238Contig7|CL2238Contig7
11.1
11.2
11.3
11.4
11.5
1
2
t−Test: p−value = 0.76

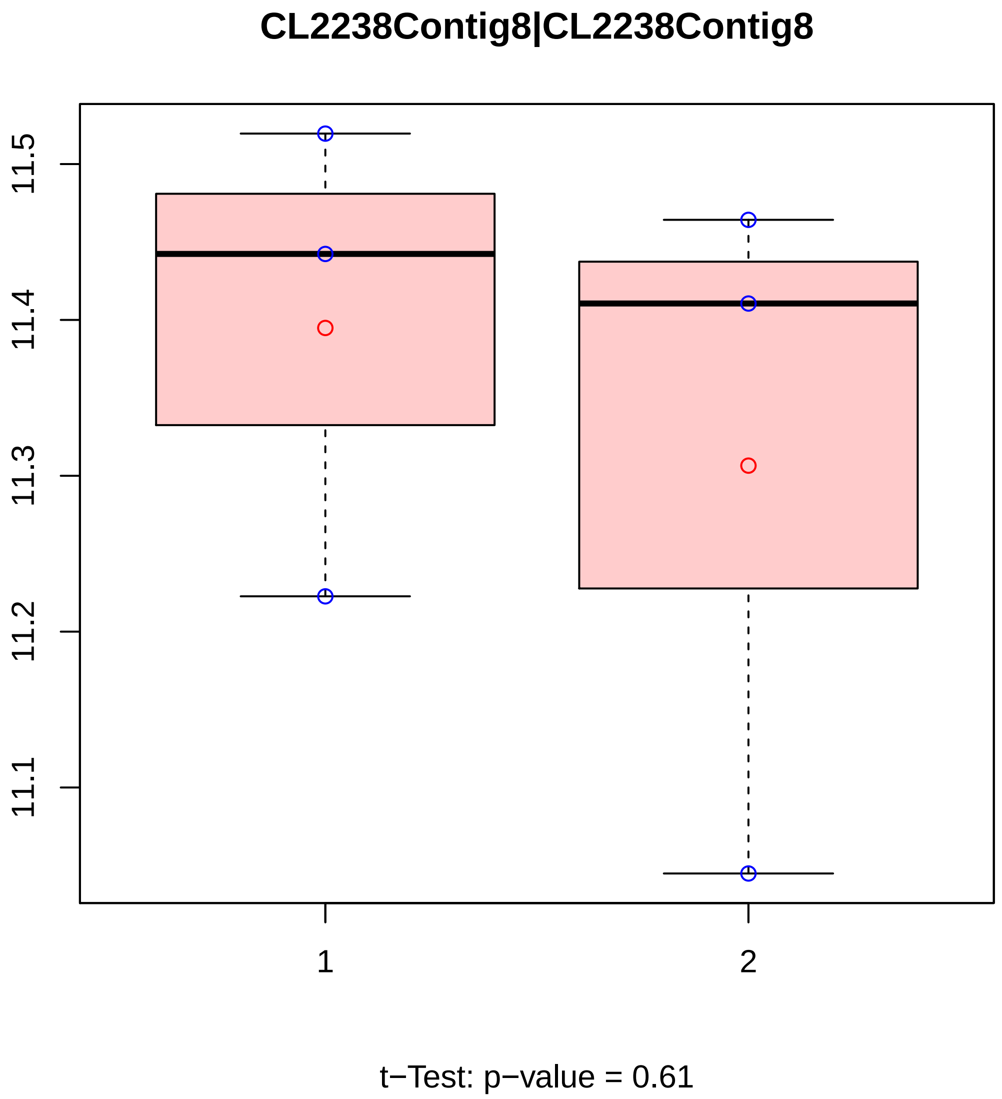
CL2238Contig8|CL2238Contig8
11.1
11.2
11.3
11.4
11.5
1
2
t−Test: p−value = 0.61

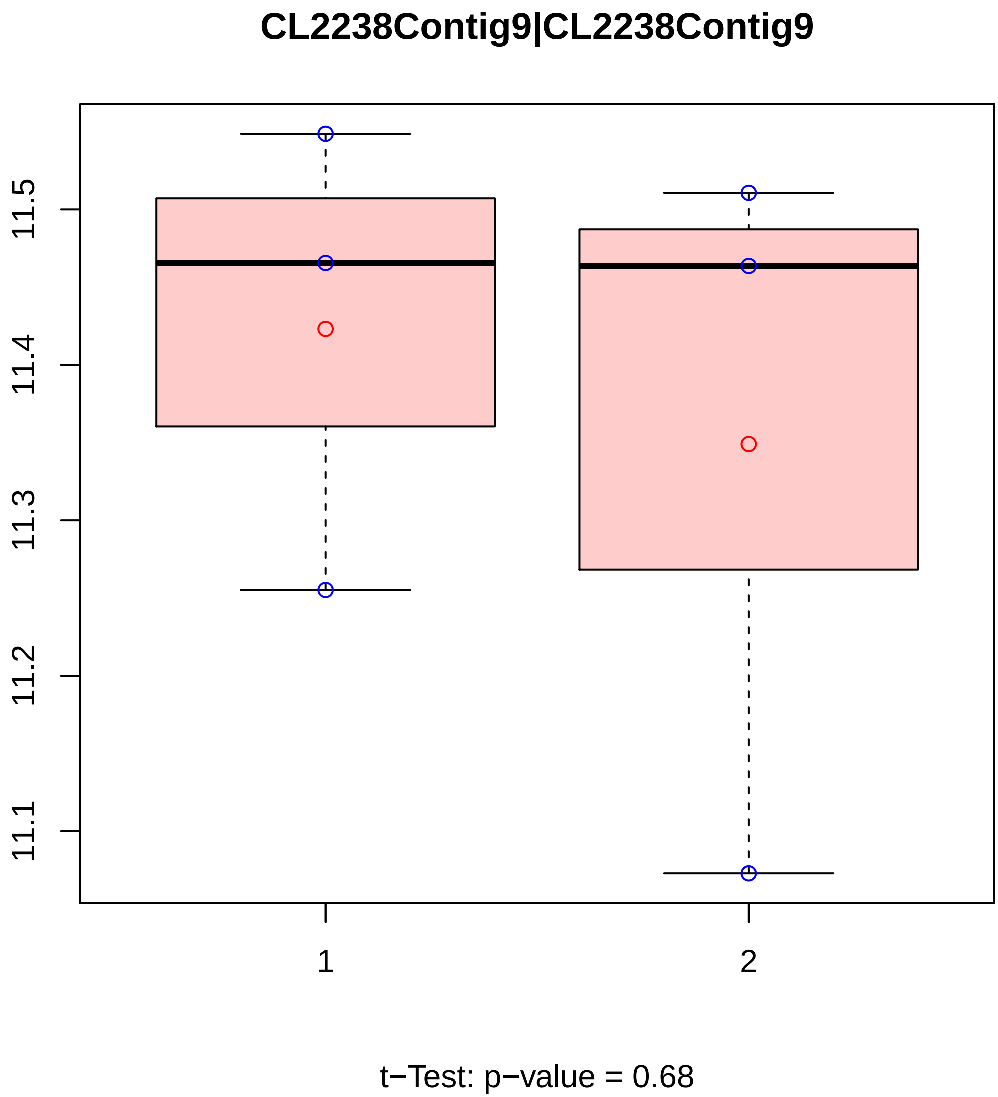

CL2238Contig9|CL2238Contig9
11.1
11.2
11.3
11.4
11.5
1
2
t−Test: p−value = 0.68

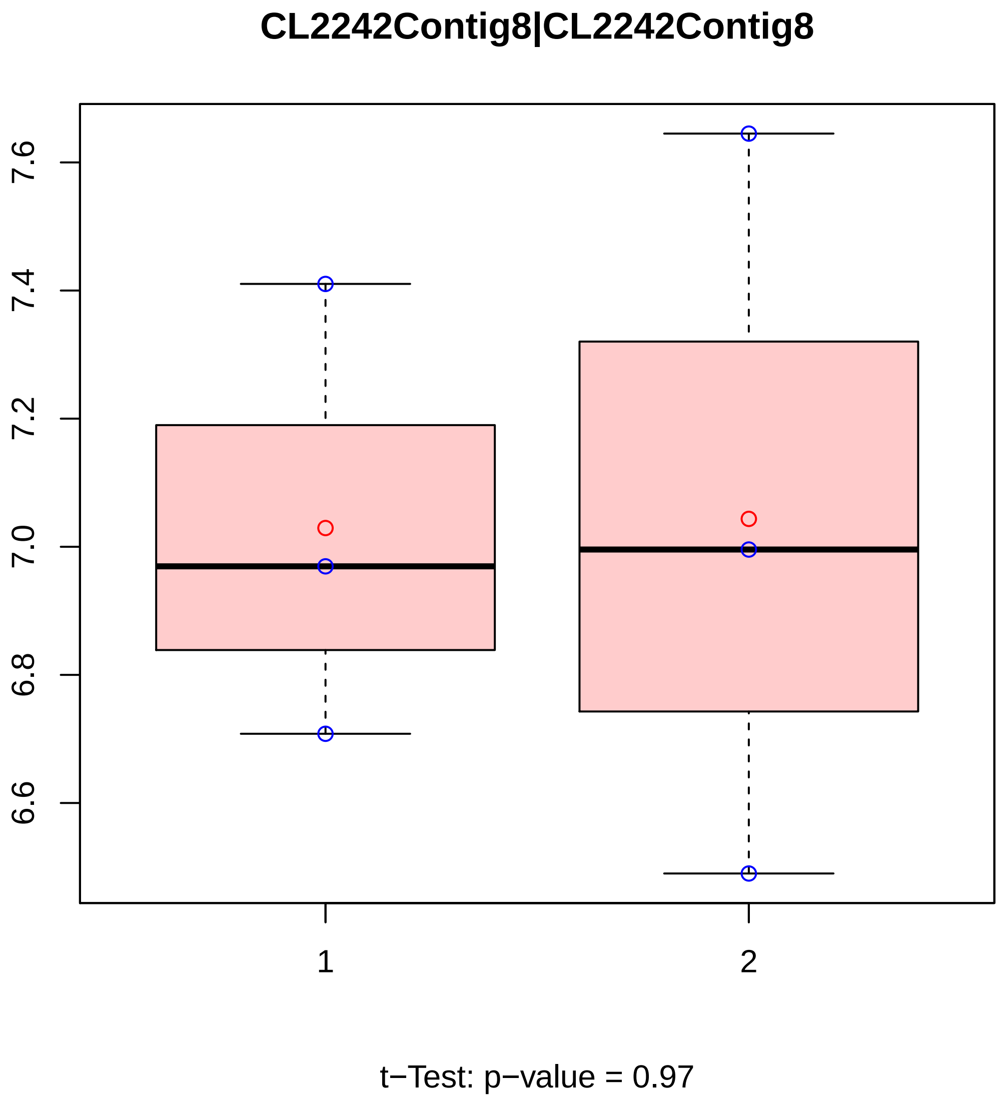
CL2242Contig8|CL2242Contig8
7.6
7.4
7.2
7.0
6.8
6.6
1
2
t−Test: p−value = 0.97

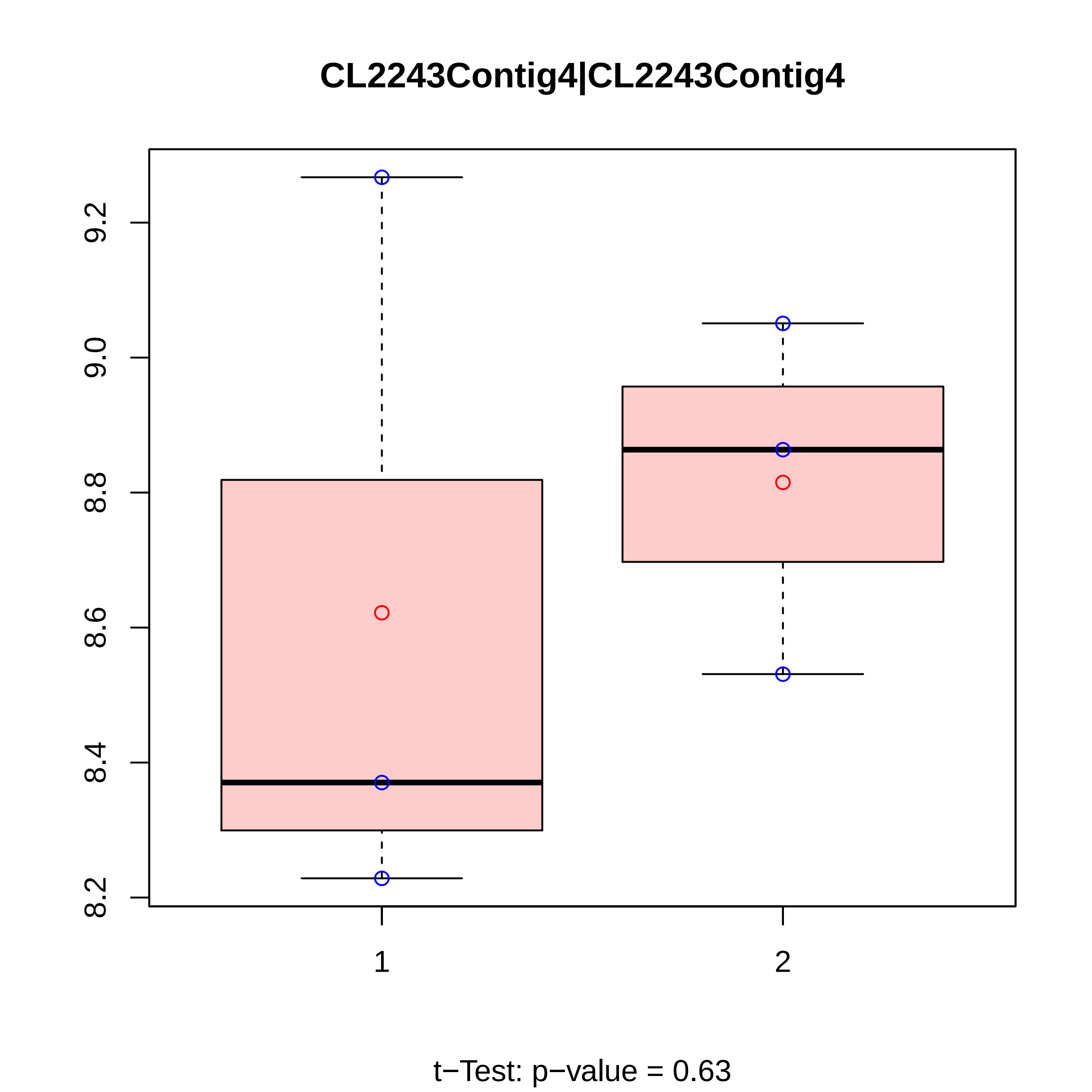

CL2243Contig4|CL2243Contig4
9.2
9.0
8.8
8.6
8.4
8.2
1
2
t−Test: p−value = 0.63

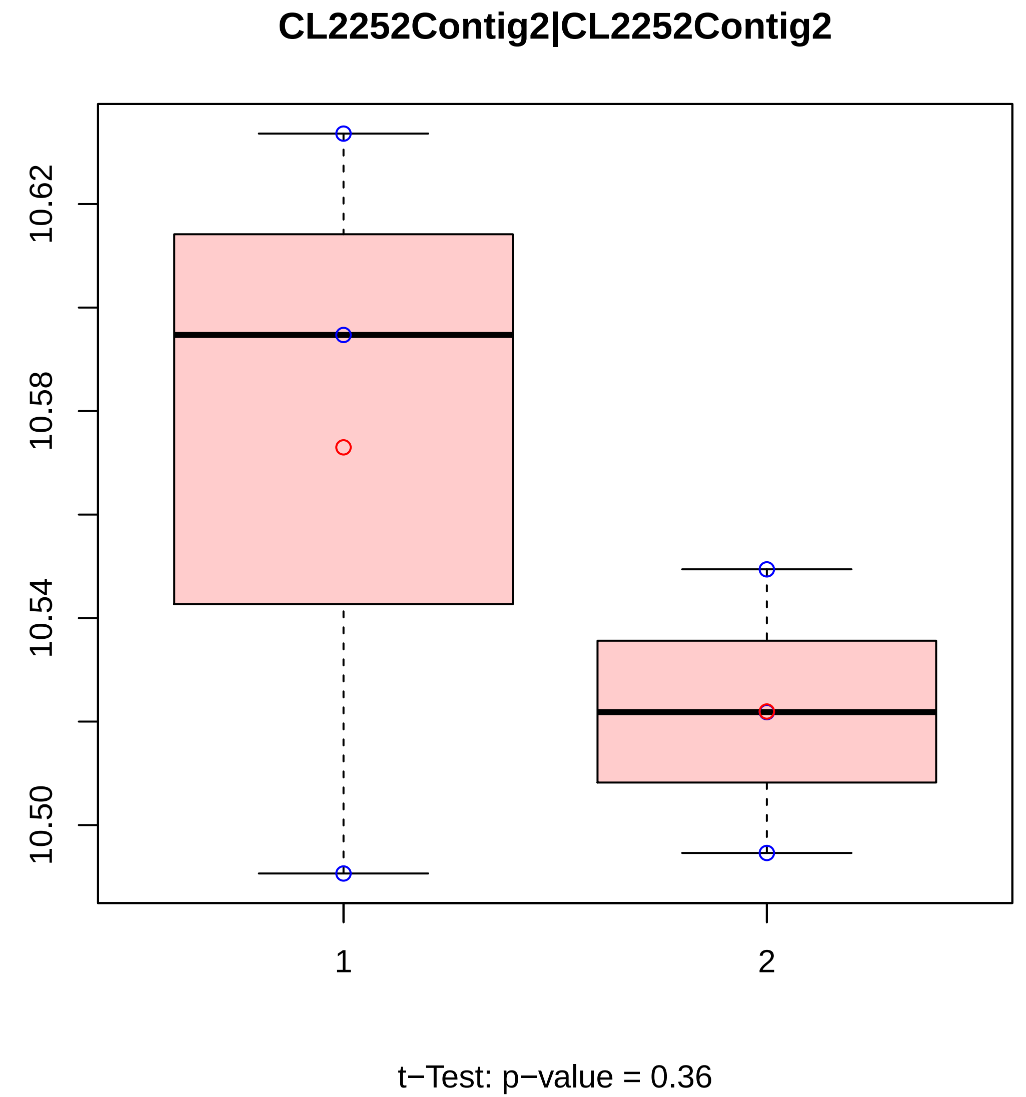

CL2252Contig2|CL2252Contig2
10.62
10.58
10.54
10.50
1
2
t−Test: p−value = 0.36

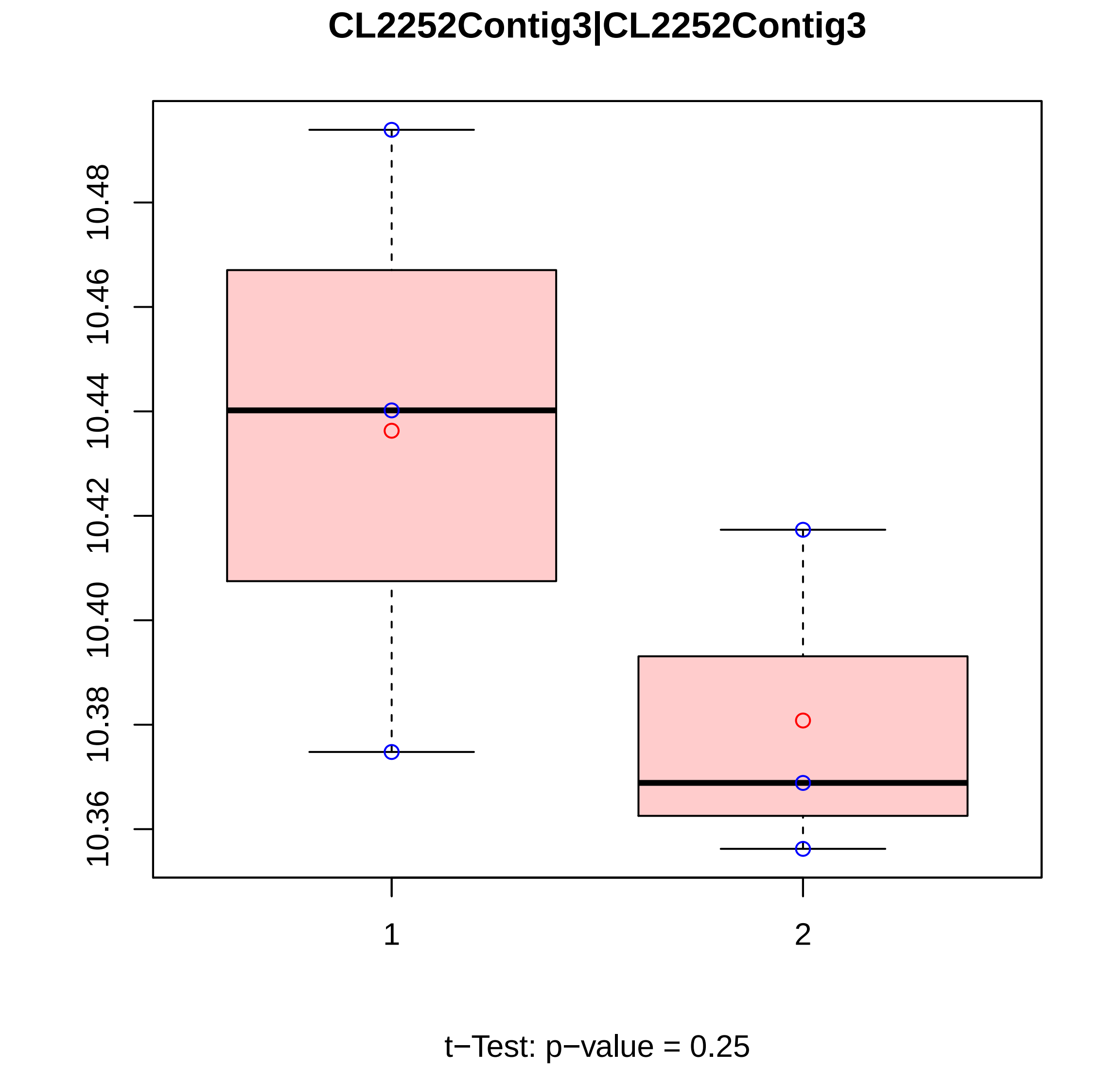

CL2252Contig3|CL2252Contig3
10.36
10.38
10.40
10.42
10.44
10.46
10.48
1
2
t−Test: p−value = 0.25

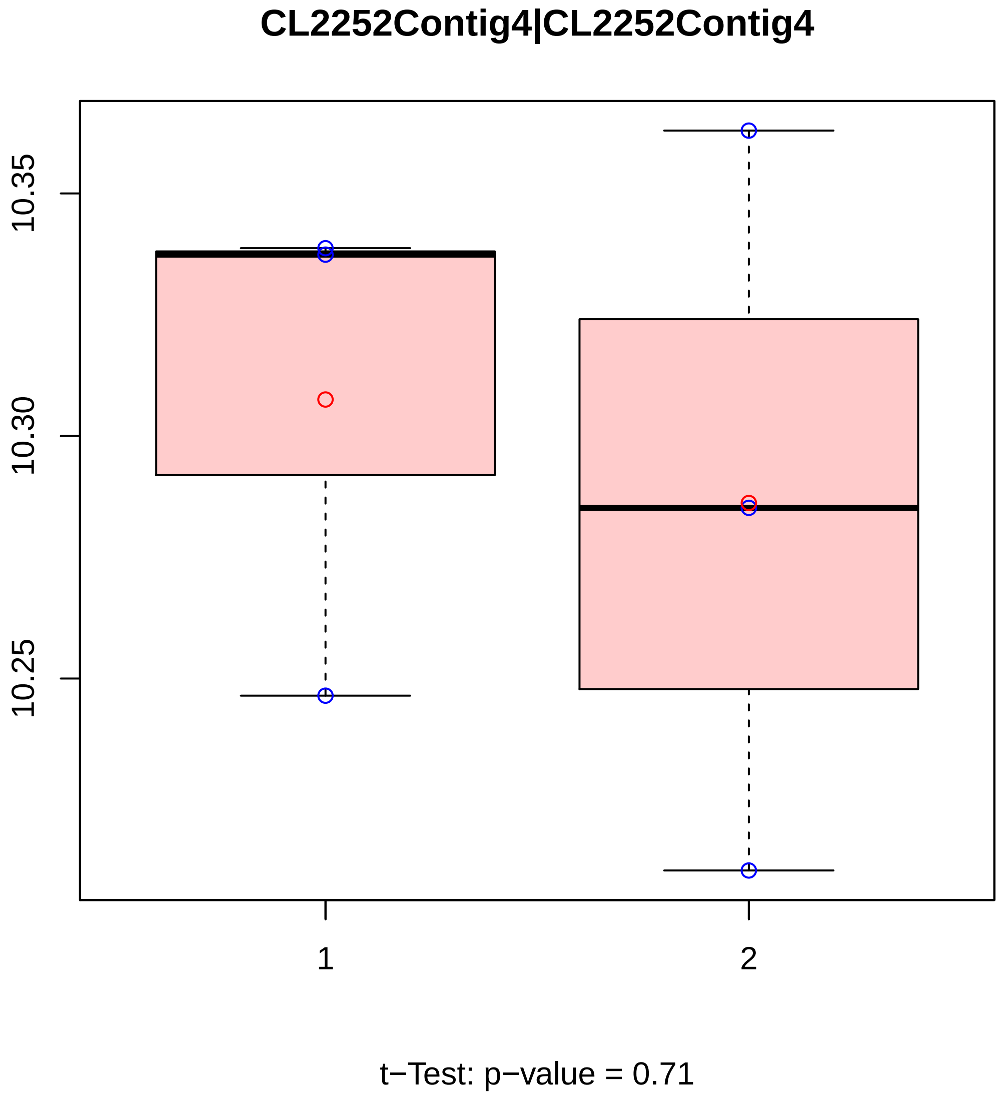
CL2252Contig4|CL2252Contig4
10.35
10.30
10.25
1
2
t−Test: p−value = 0.71

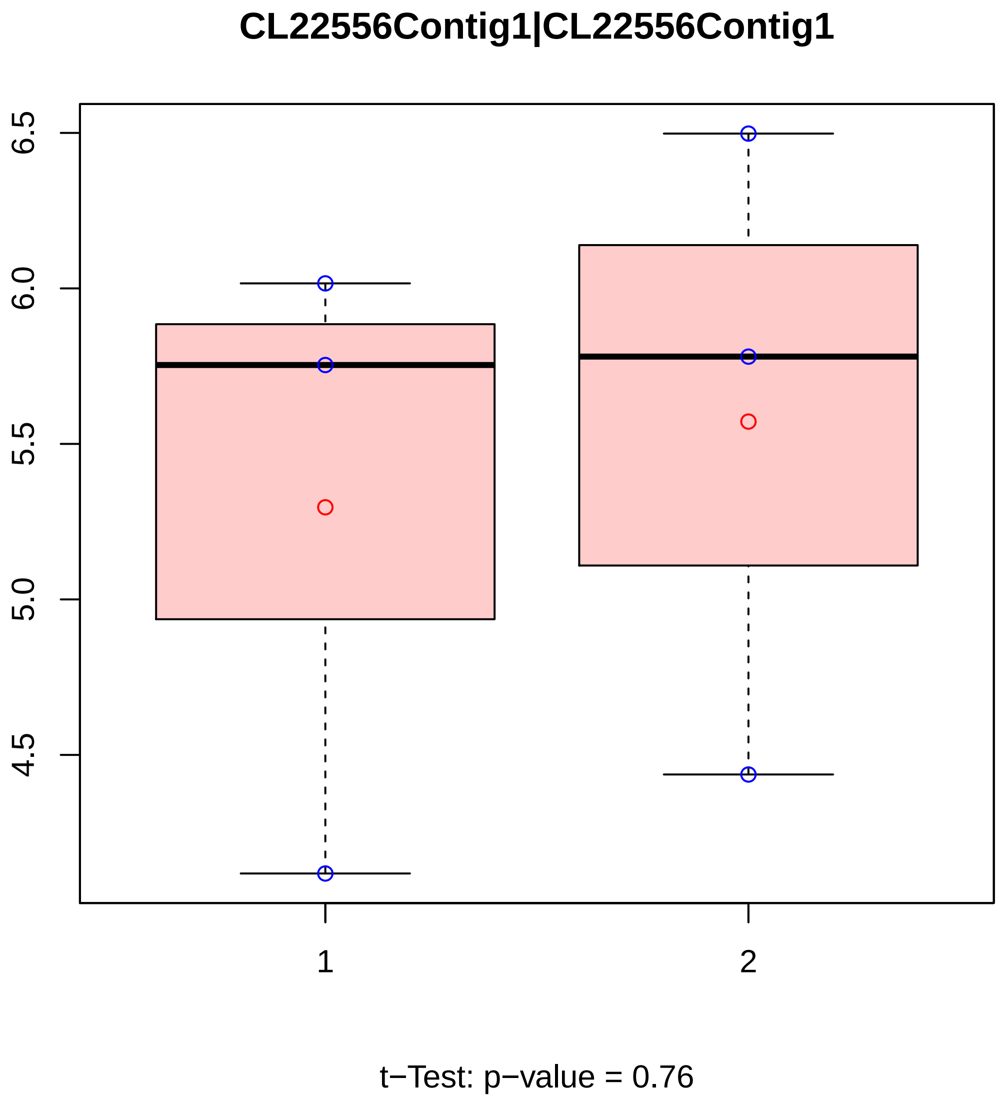
CL22556Contig1|CL22556Contig1
6.5
6.0
5.5
5.0
4.5
1
2
t−Test: p−value = 0.76

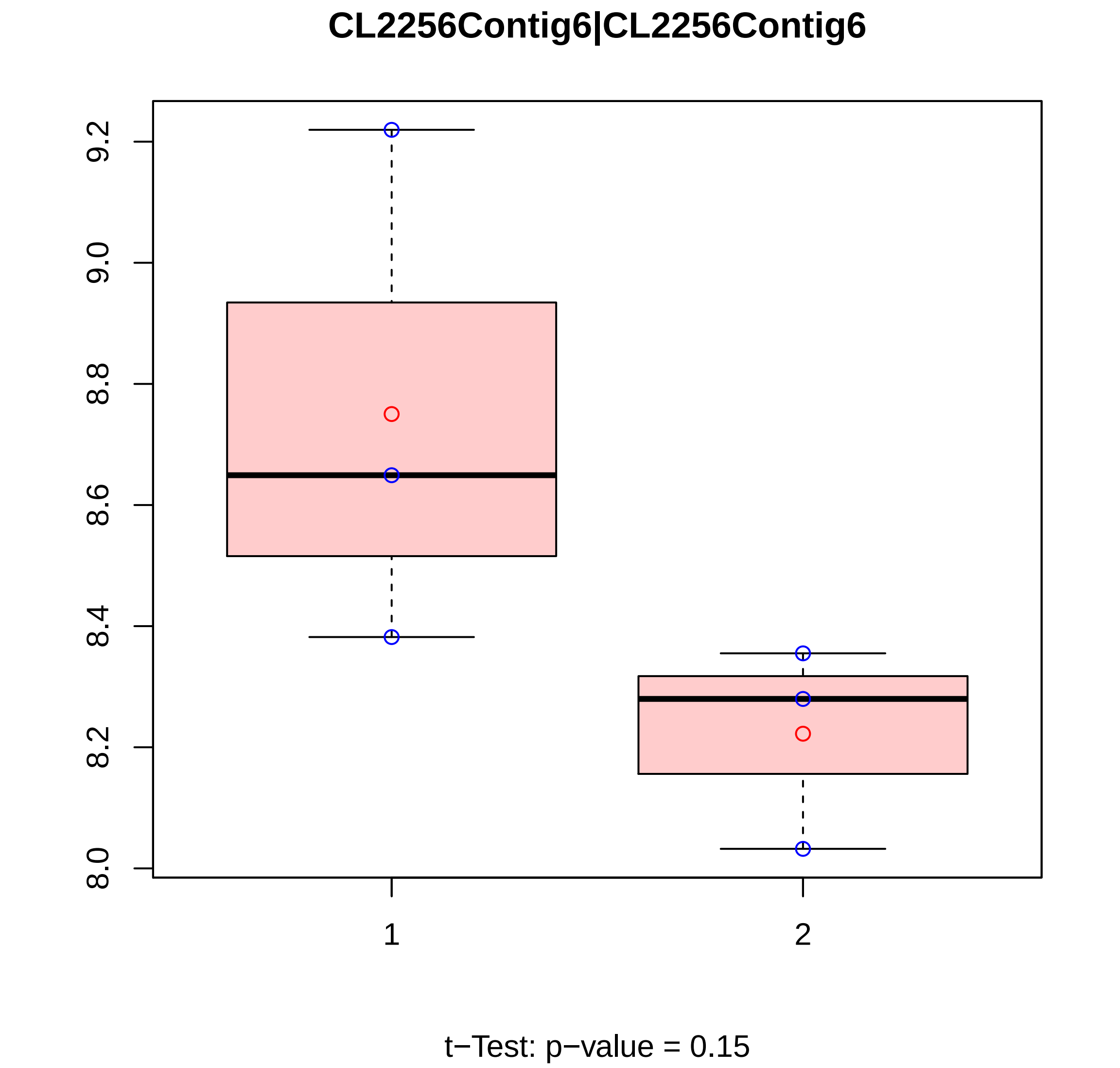
CL2256Contig6|CL2256Contig6
9.2
9.0
8.8
8.6
8.4
8.2
8.0
1
2
t−Test: p−value = 0.15

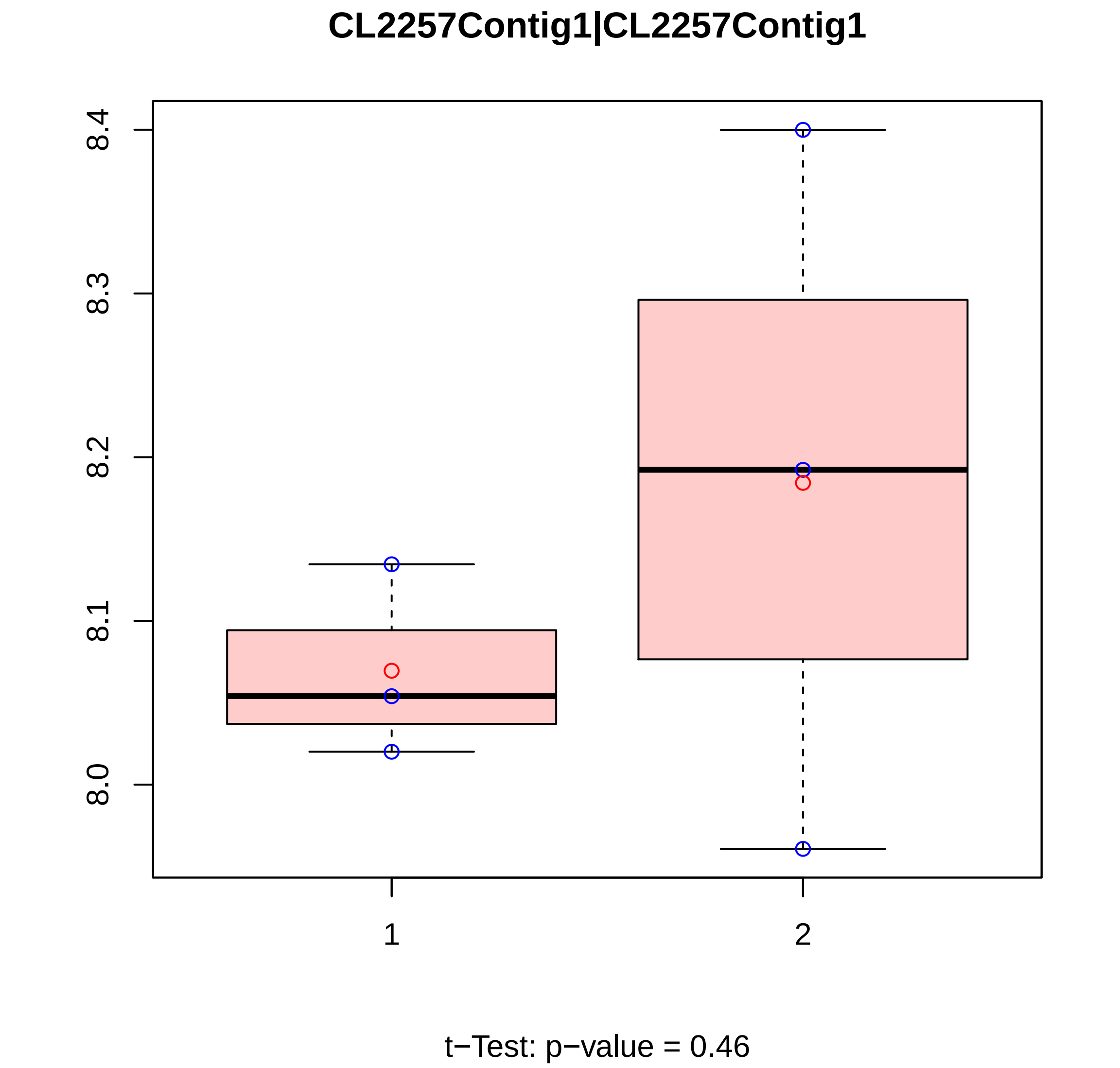
CL2257Contig1|CL2257Contig1
8.0
8.1
8.2
8.3
8.4
1
2
t−Test: p−value = 0.46

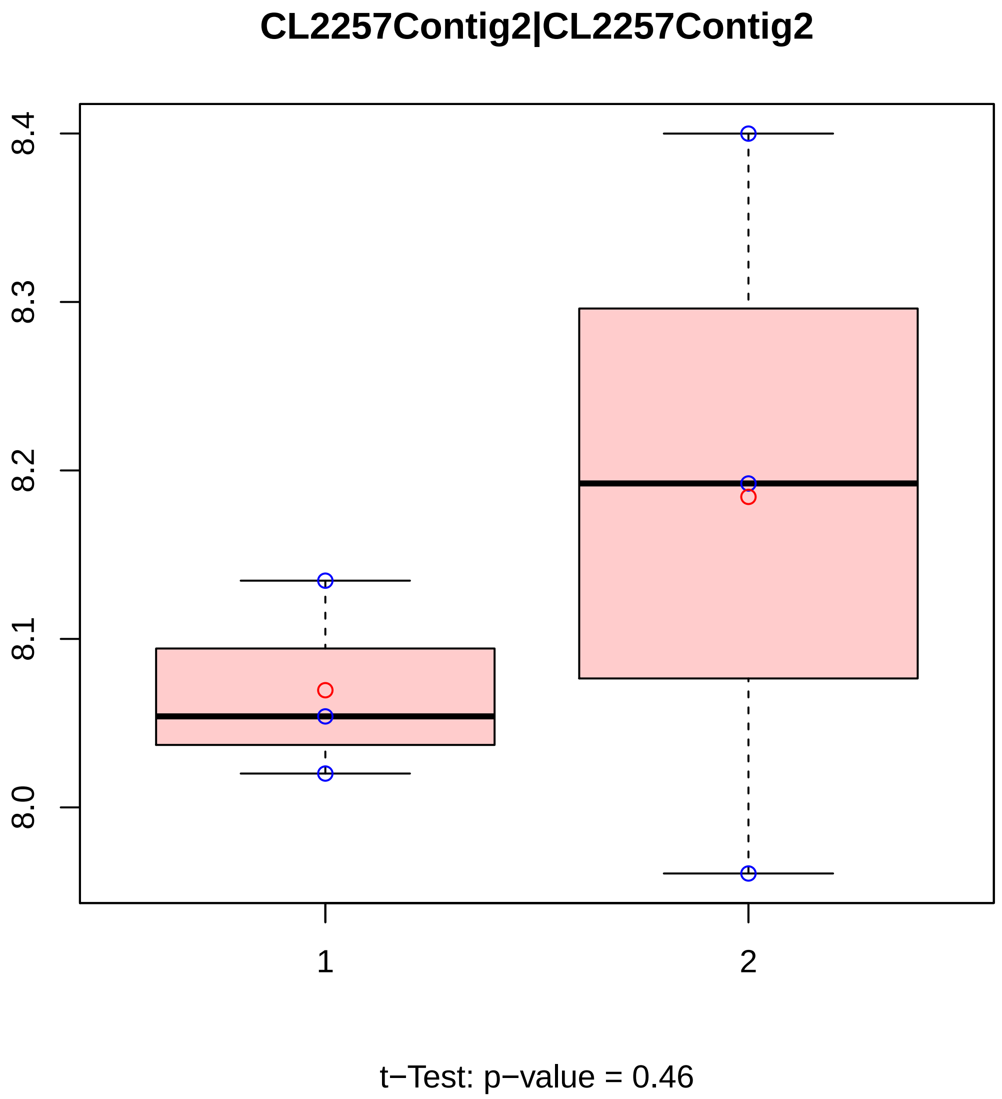
CL2257Contig2|CL2257Contig2
8.0
8.1
8.2
8.3
8.4
1
2
t−Test: p−value = 0.46

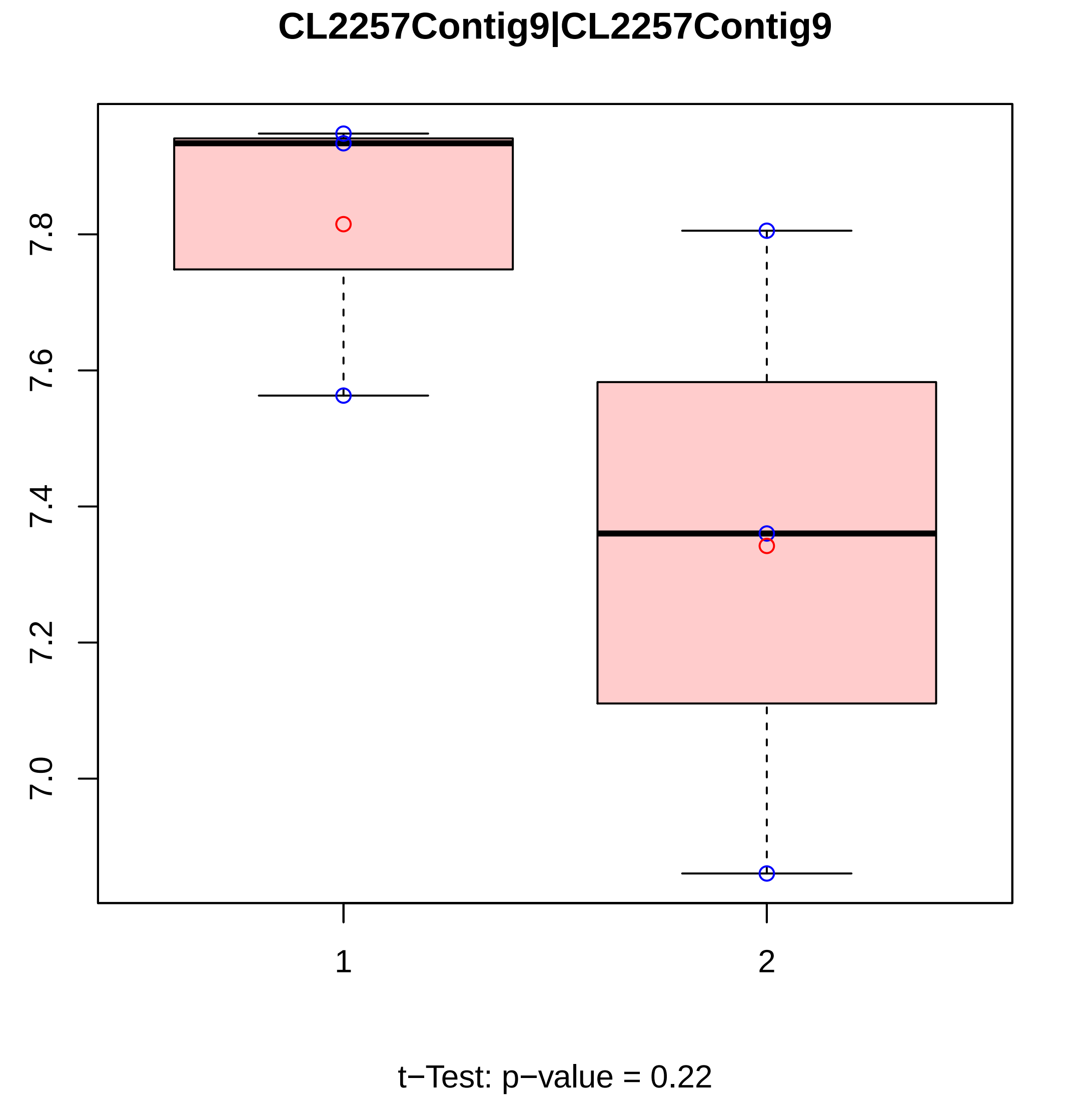

CL2257Contig9|CL2257Contig9
7.0
7.2
7.4
7.6
7.8
1
2
t−Test: p−value = 0.22

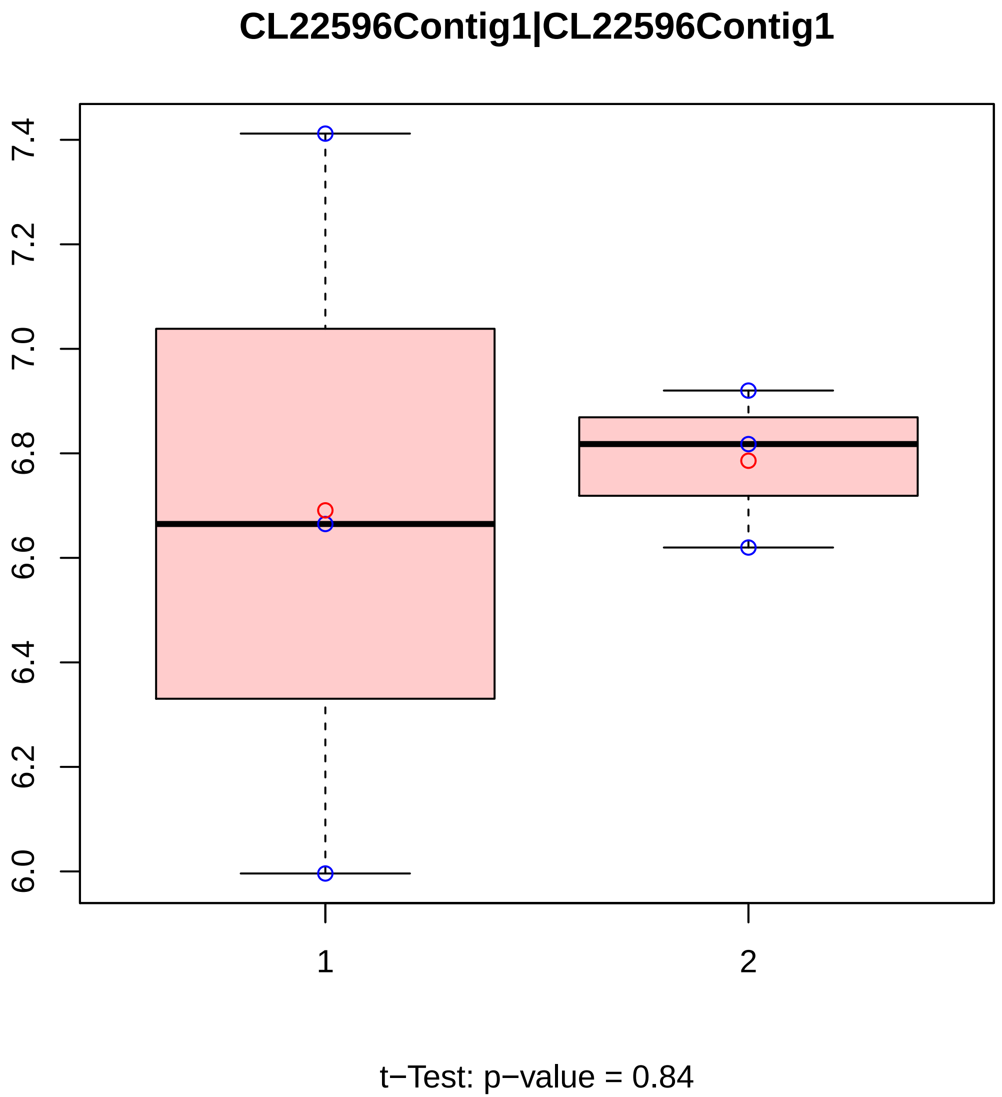

CL22596Contig1|CL22596Contig1
7.4
7.2
7.0
6.8
6.6
6.4
6.2
6.0
1
2
t−Test: p−value = 0.84

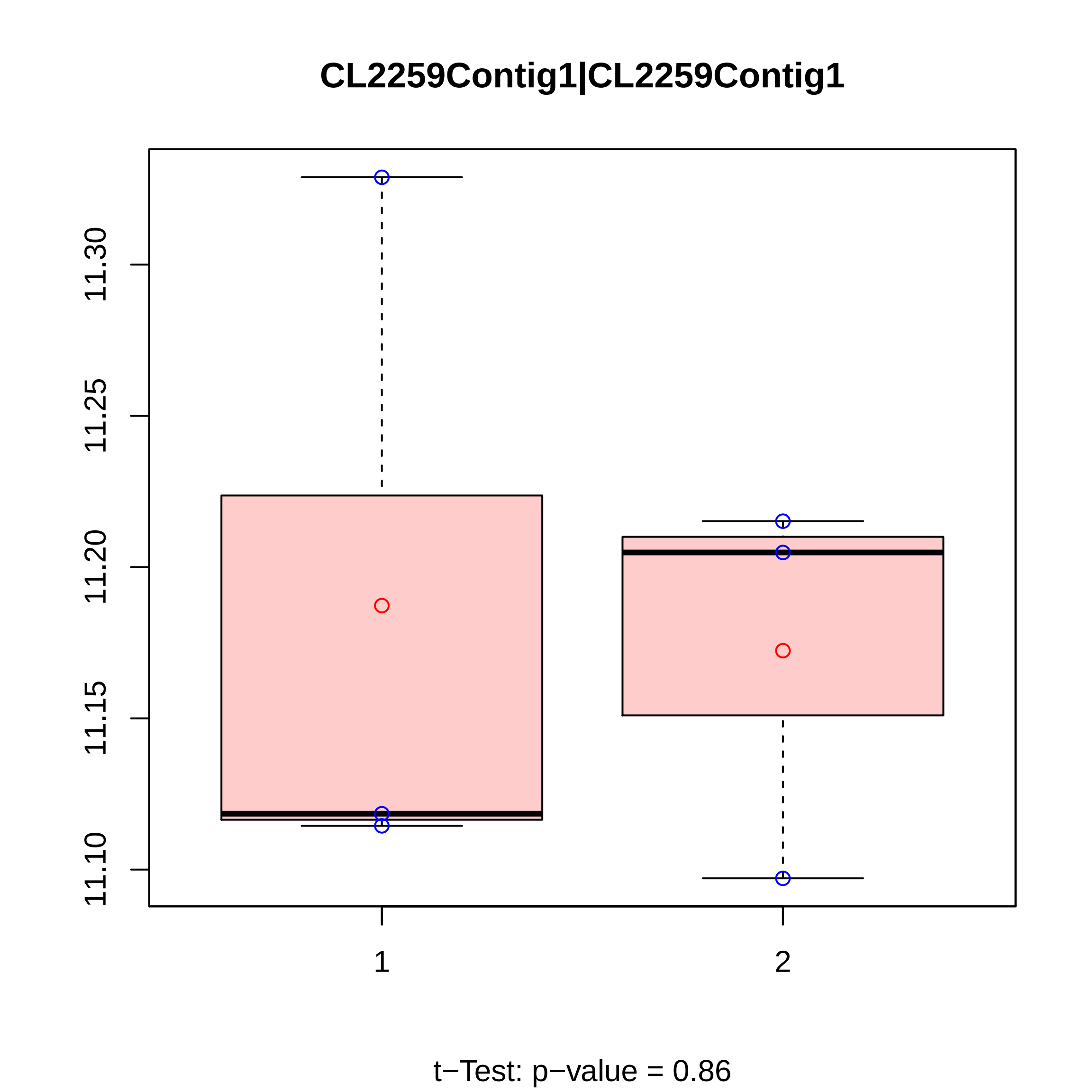

CL2259Contig1|CL2259Contig1
11.30
11.25
11.20
11.15
11.10
1
2
t−Test: p−value = 0.86

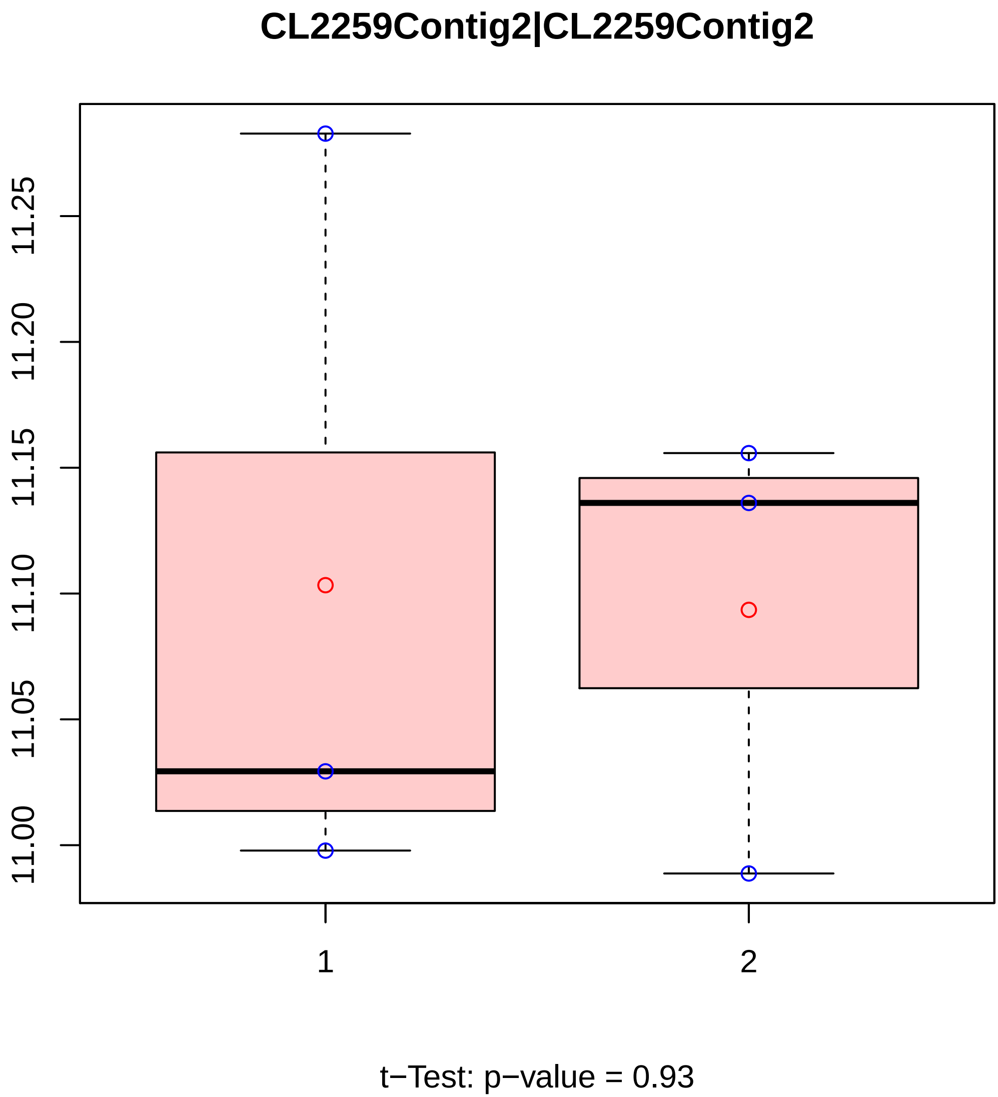
CL2259Contig2|CL2259Contig2
11.00
11.05
11.10
11.15
11.20
11.25
1
2
t−Test: p−value = 0.93

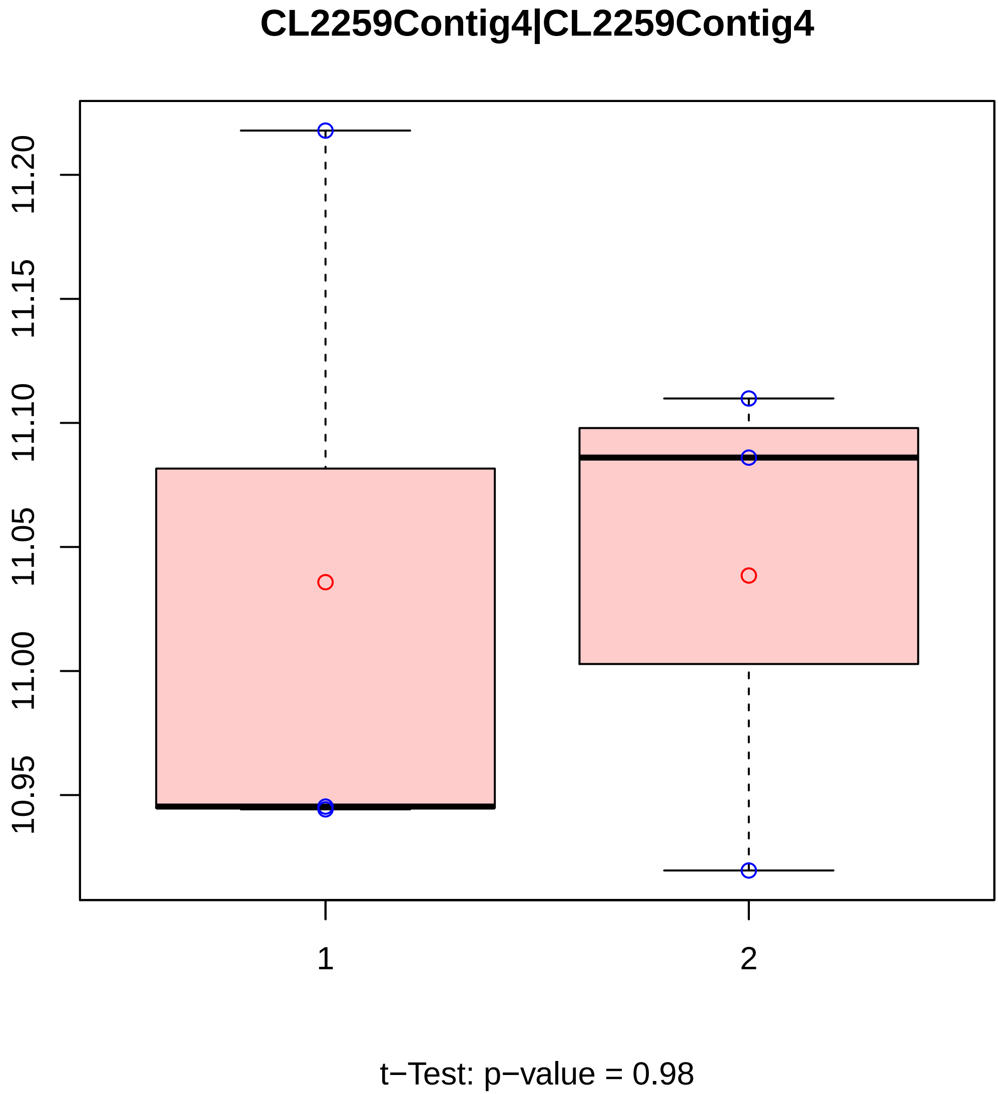
CL2259Contig4|CL2259Contig4
11.20
11.15
11.10
11.05
11.00
10.95
1
2
t−Test: p−value = 0.98

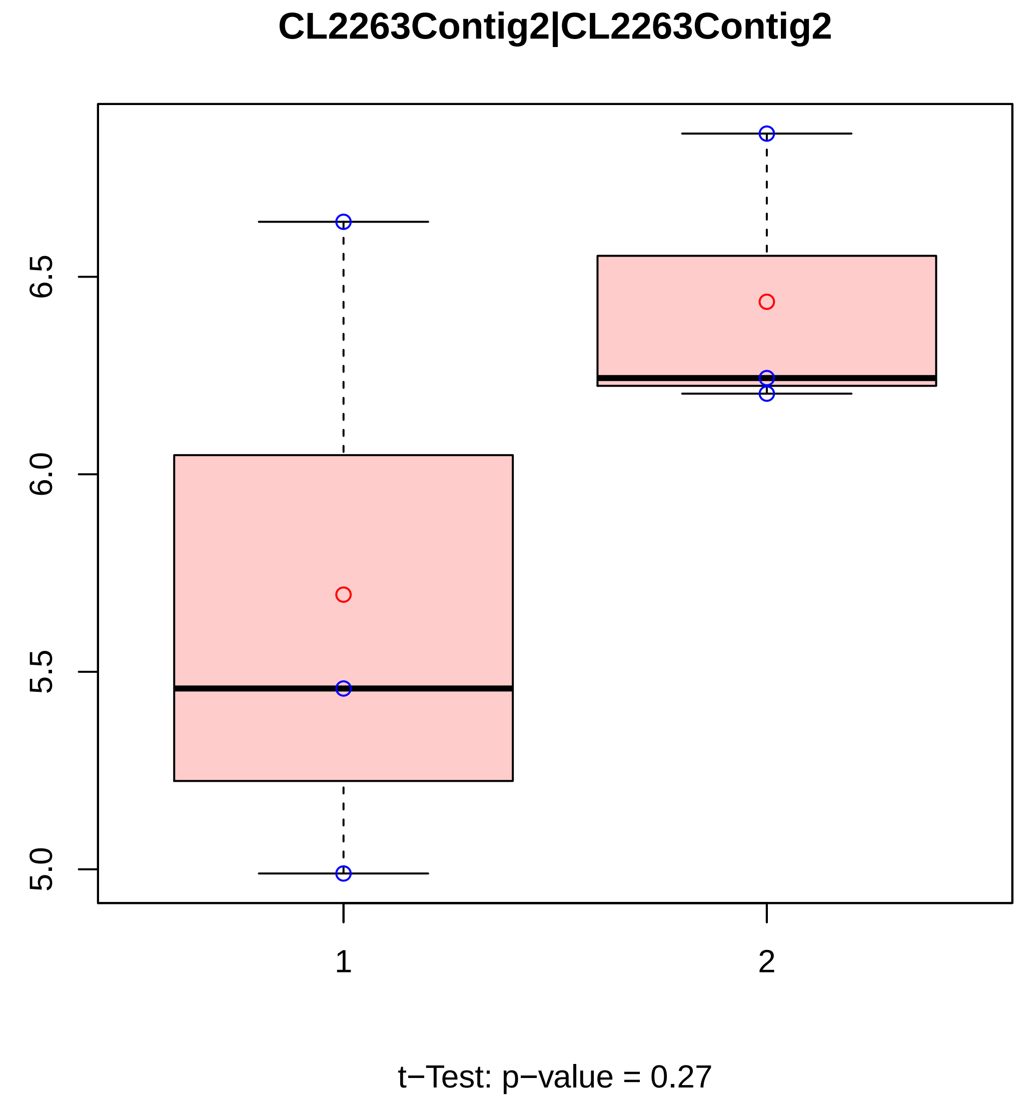
CL2263Contig2|CL2263Contig2
6.5
6.0
5.5
5.0
1
2
t−Test: p−value = 0.27

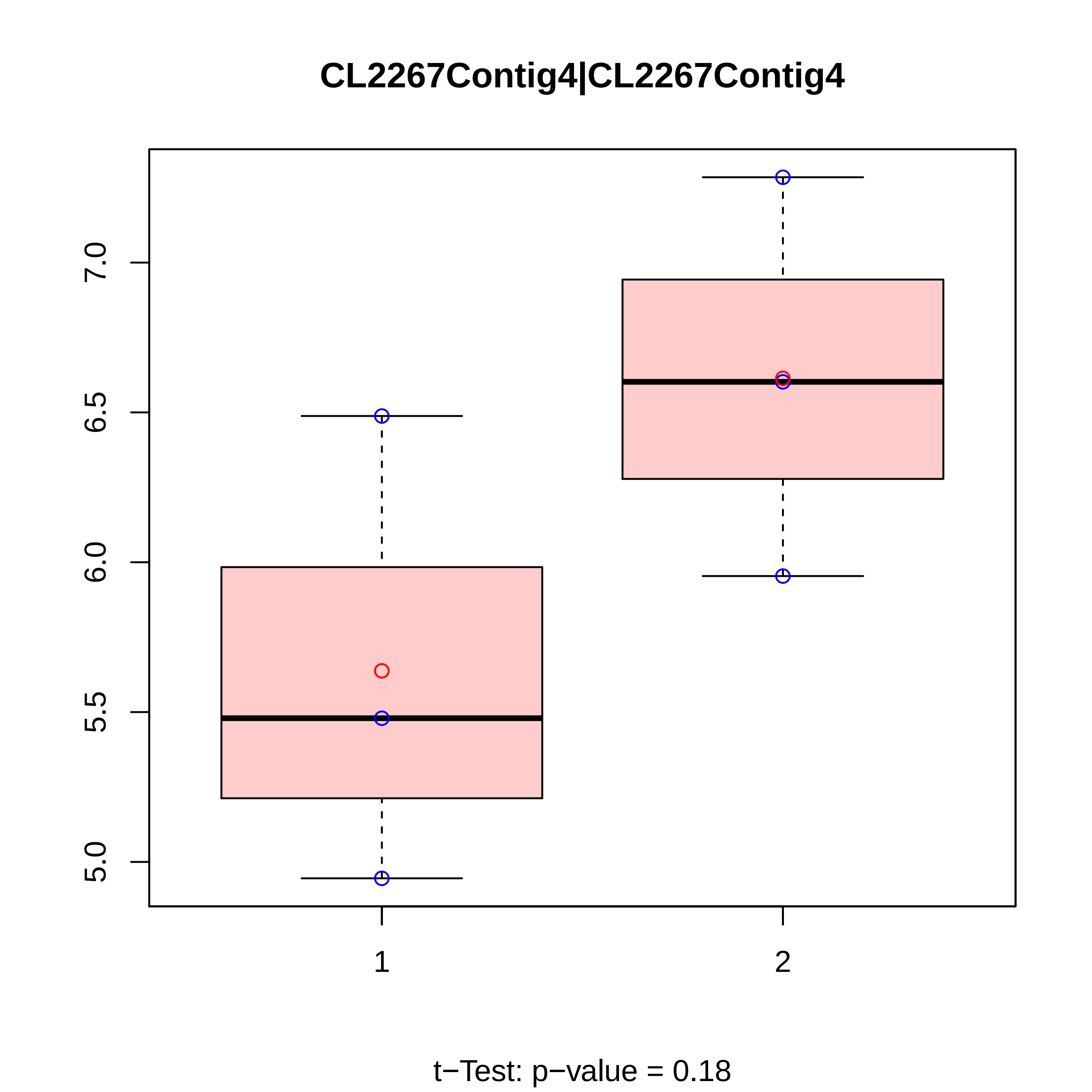
CL2267Contig4|CL2267Contig4
7.0
6.5
6.0
5.5
5.0
1
2
t−Test: p−value = 0.18

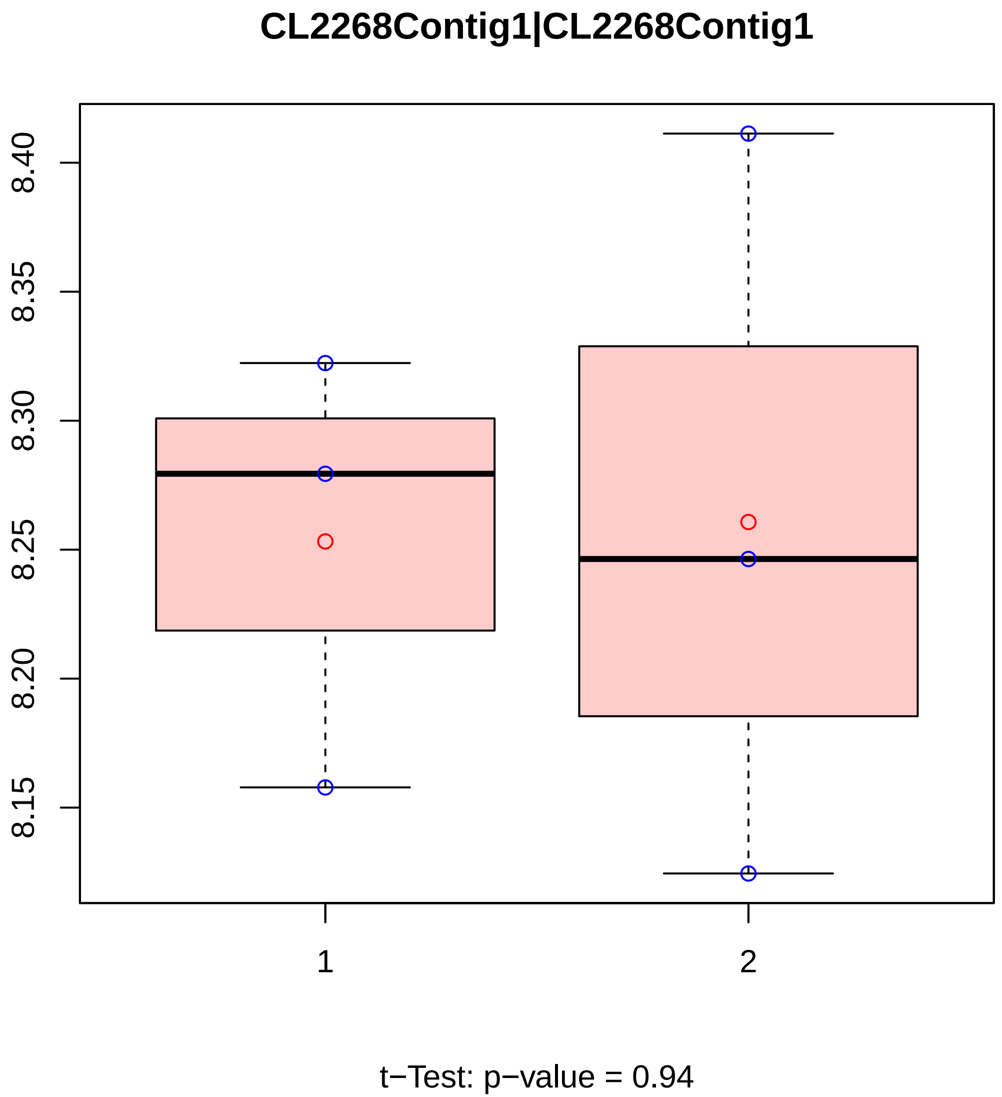
CL2268Contig1|CL2268Contig1
8.15
8.20
8.25
8.30
8.35
8.40
1
2
t−Test: p−value = 0.94

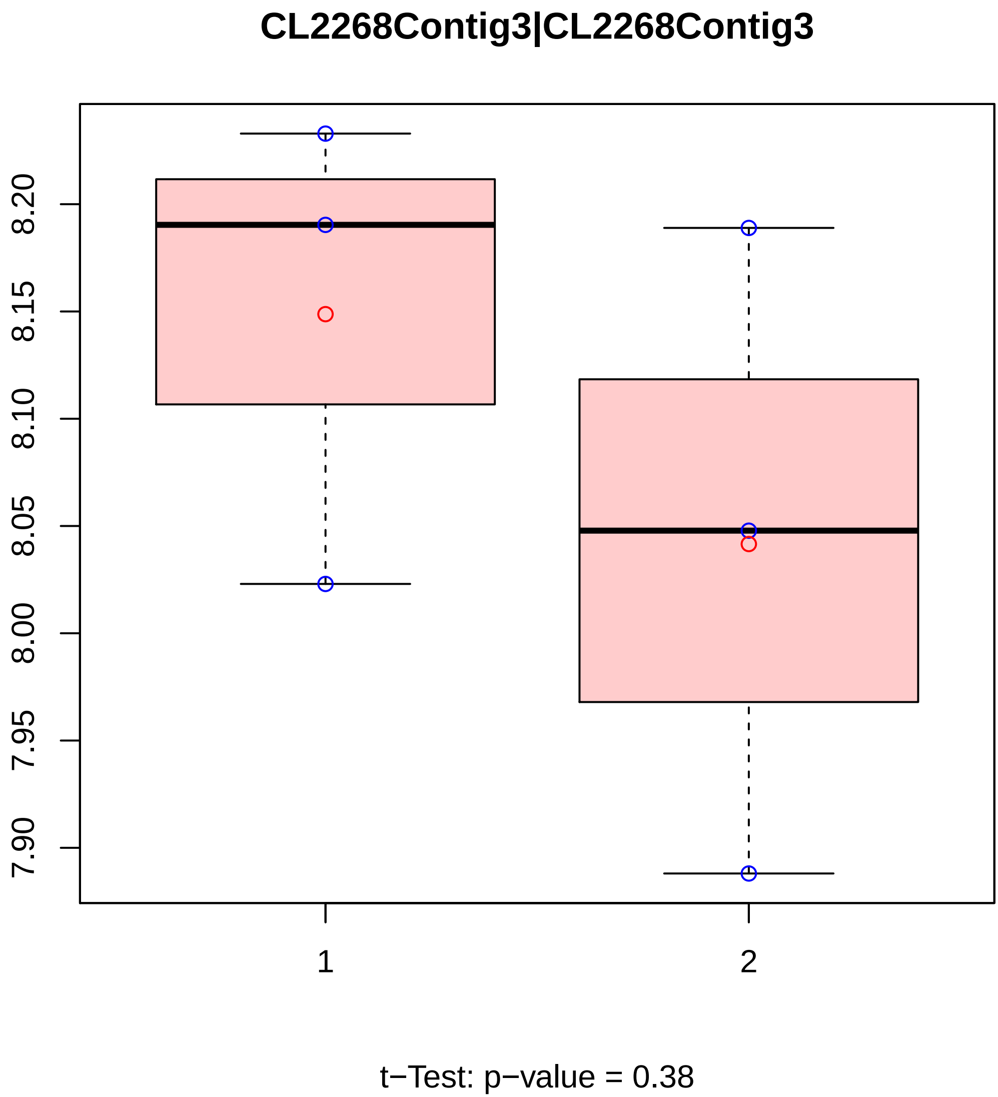
CL2268Contig3|CL2268Contig3
8.20
8.15
8.10
8.05
8.00
7.95
7.90
1
2
t−Test: p−value = 0.38

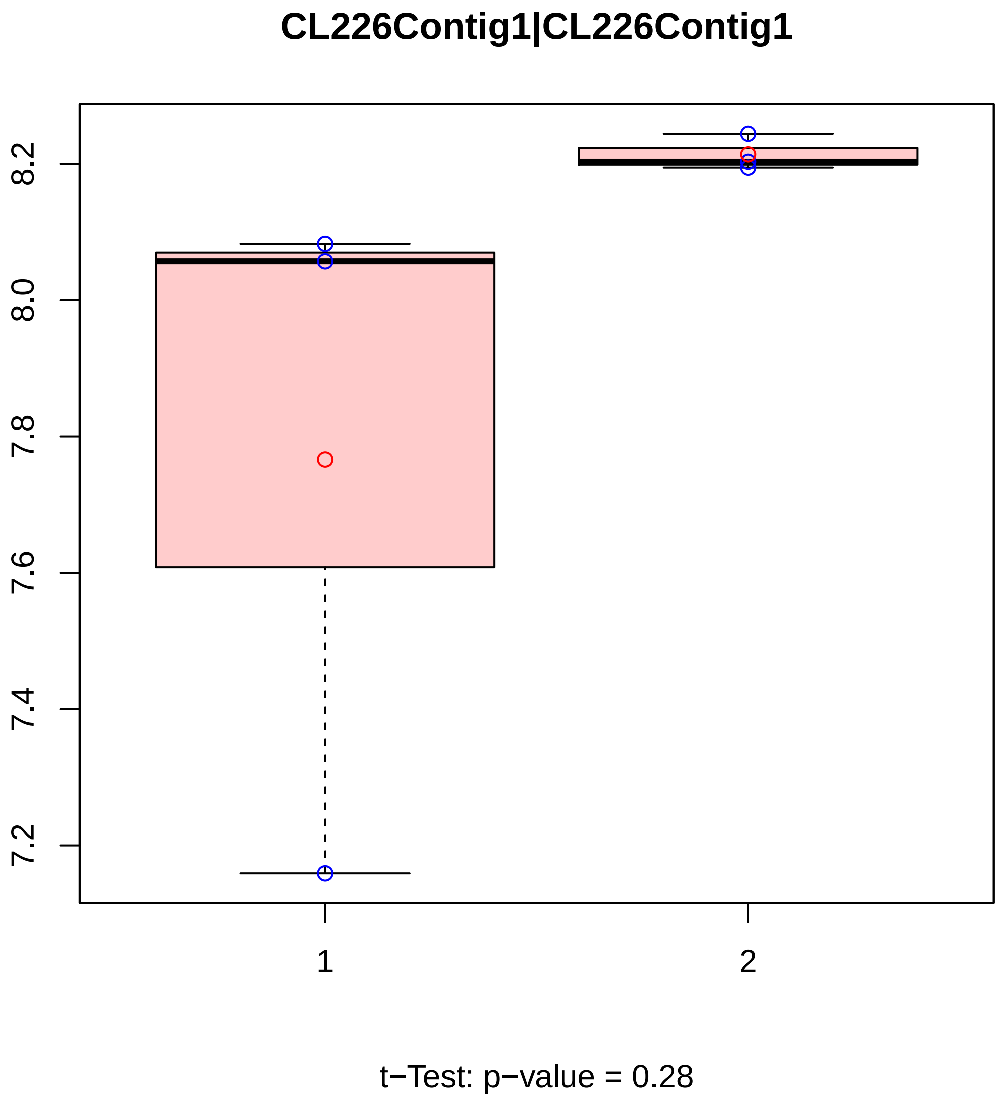

CL226Contig1|CL226Contig1
8.2
8.0
7.8
7.6
7.4
7.2
1
2
t−Test: p−value = 0.28

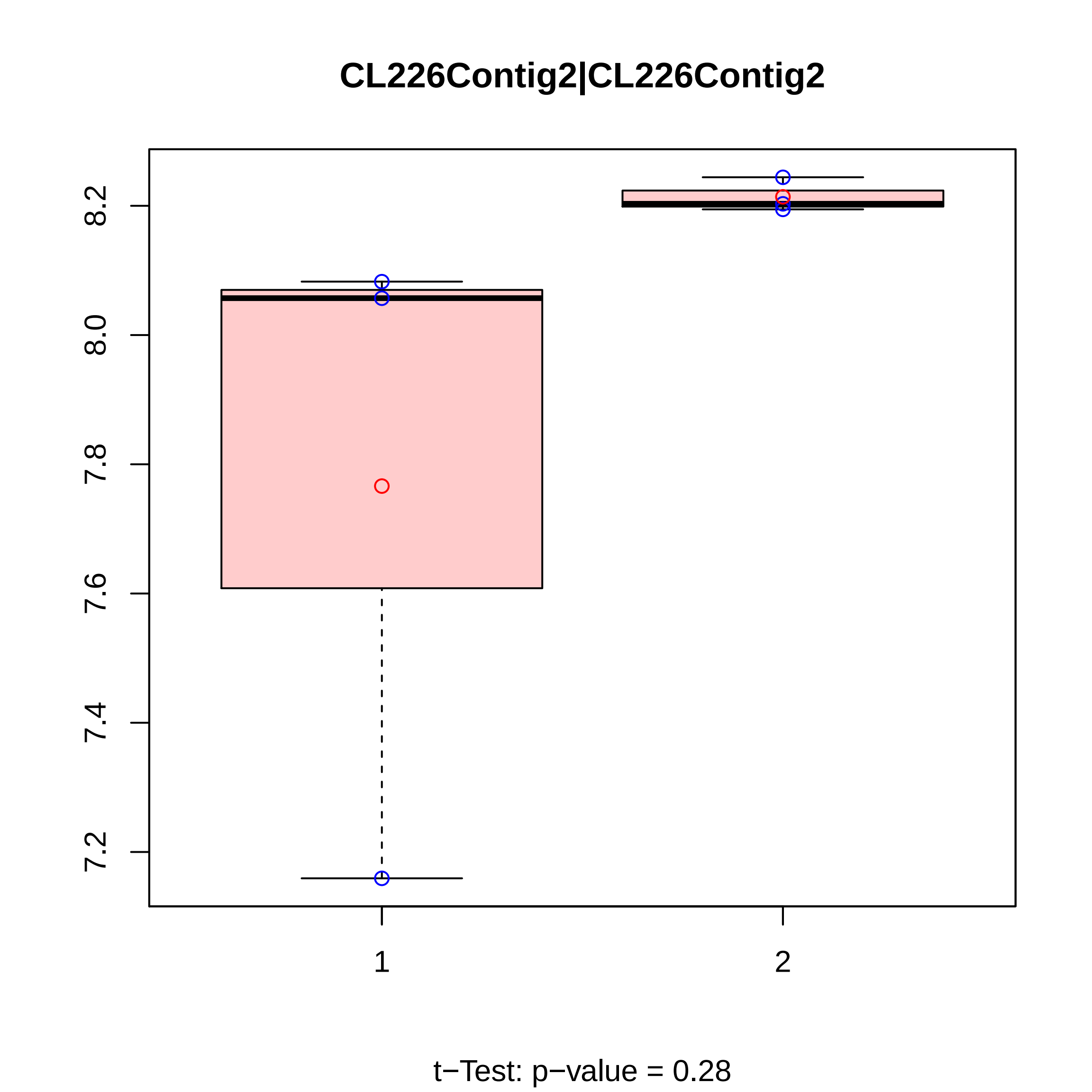

CL226Contig2|CL226Contig2
8.2
8.0
7.8
7.6
7.4
7.2
1
2
t−Test: p−value = 0.28

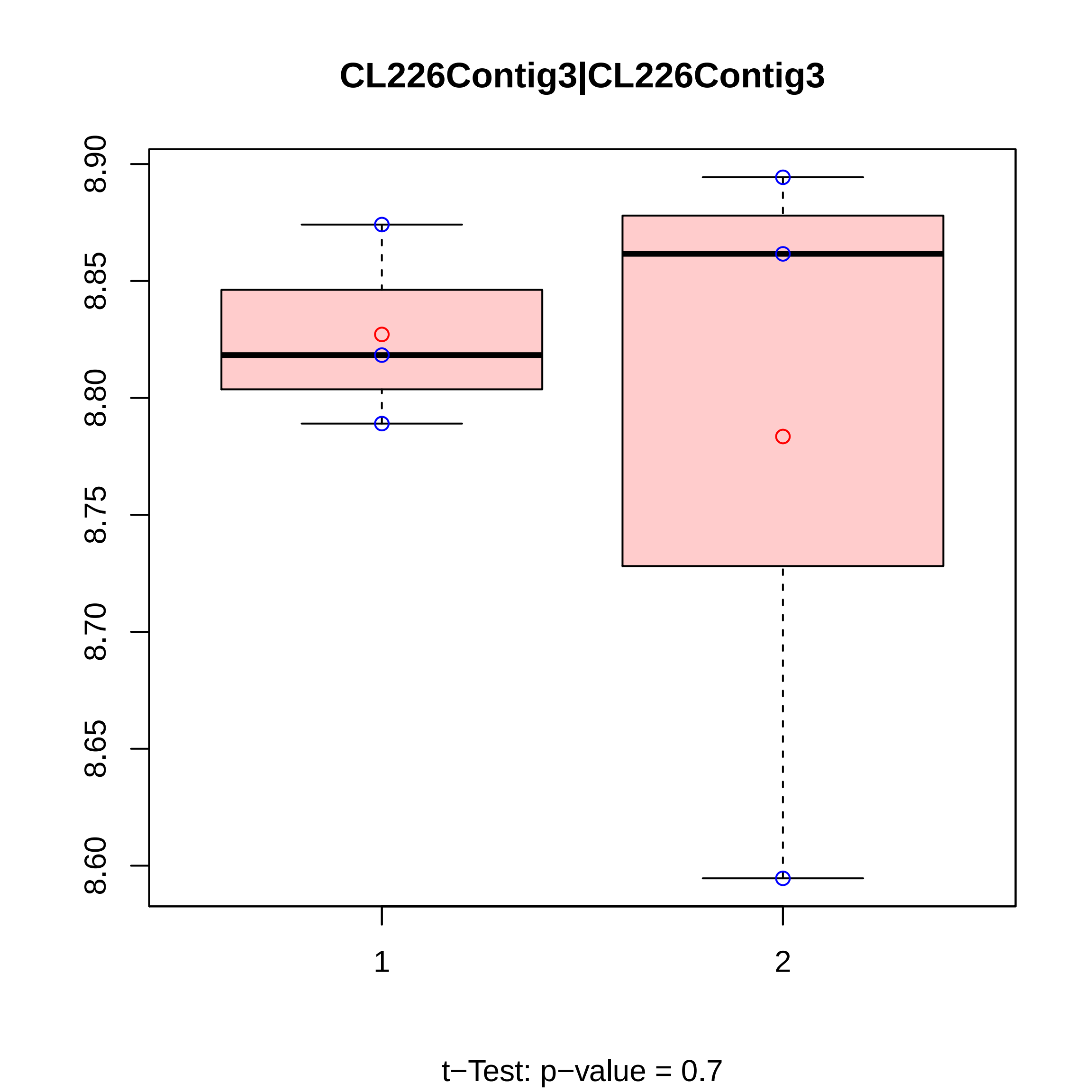
CL226Contig3|CL226Contig3
8.60
8.65
8.70
8.75
8.80
8.85
8.90
1
2
t−Test: p−value = 0.7

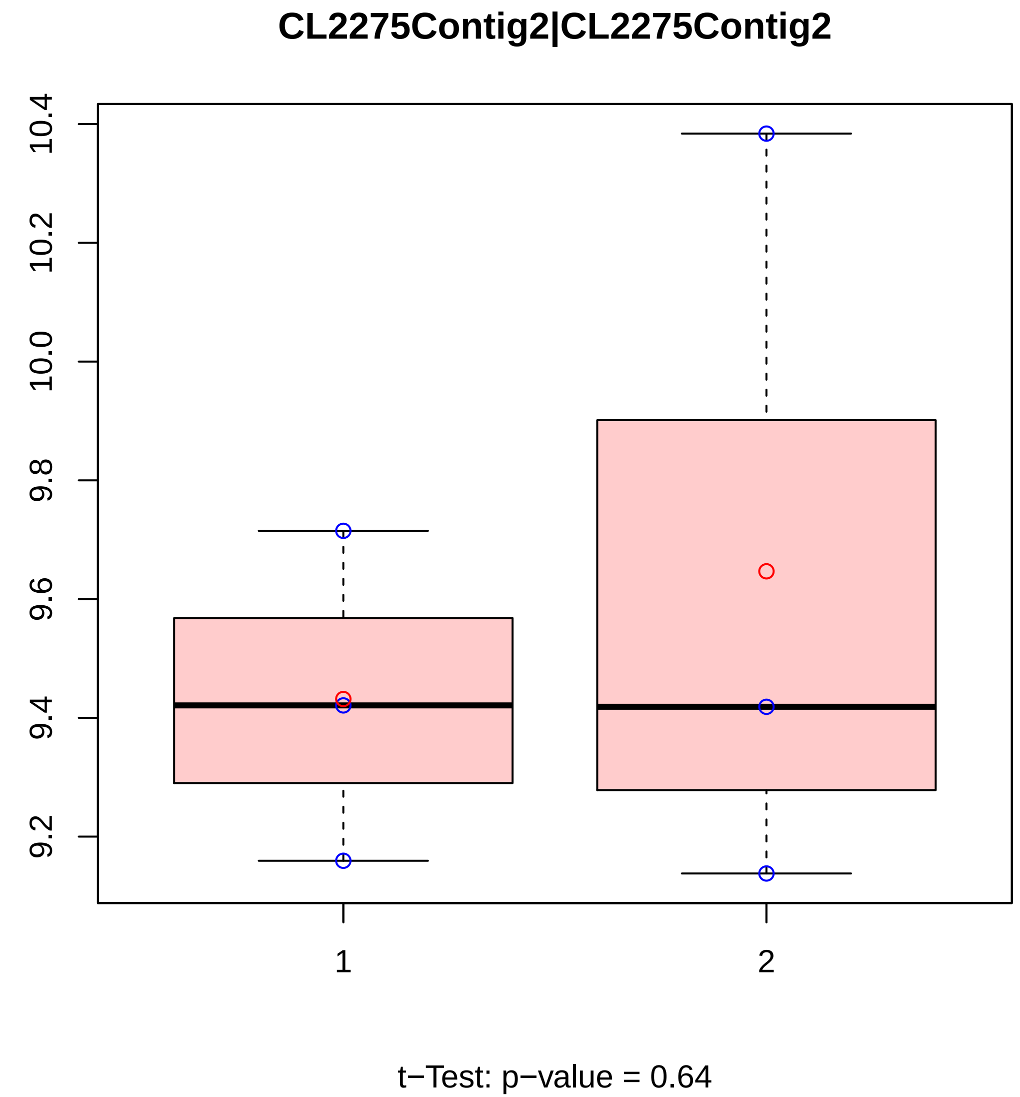
CL2275Contig2|CL2275Contig2
10.4
10.2
10.0
9.8
9.6
9.4
9.2
1
2
t−Test: p−value = 0.64

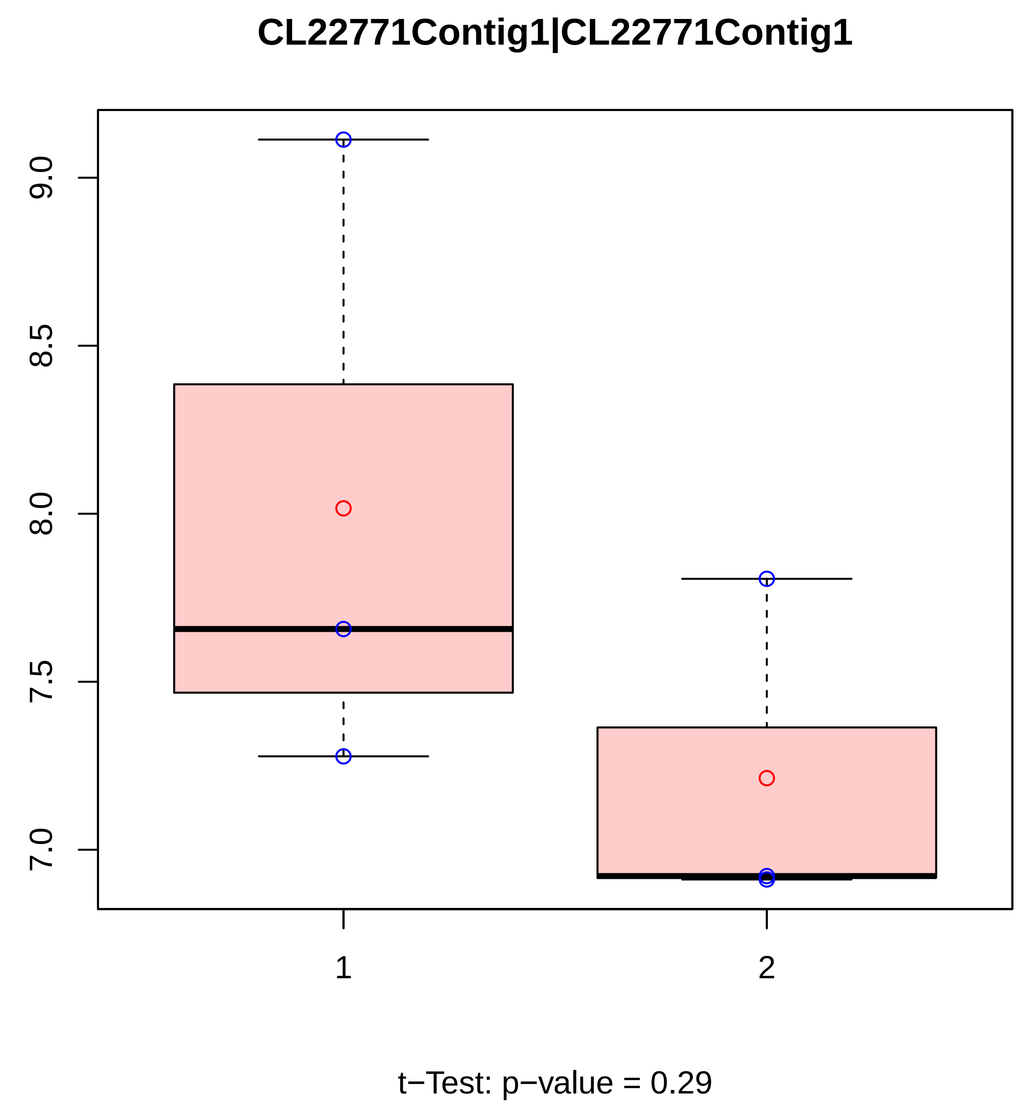
CL22771Contig1|CL22771Contig1
9.0
8.5
8.0
7.5
7.0
1
2
t−Test: p−value = 0.29

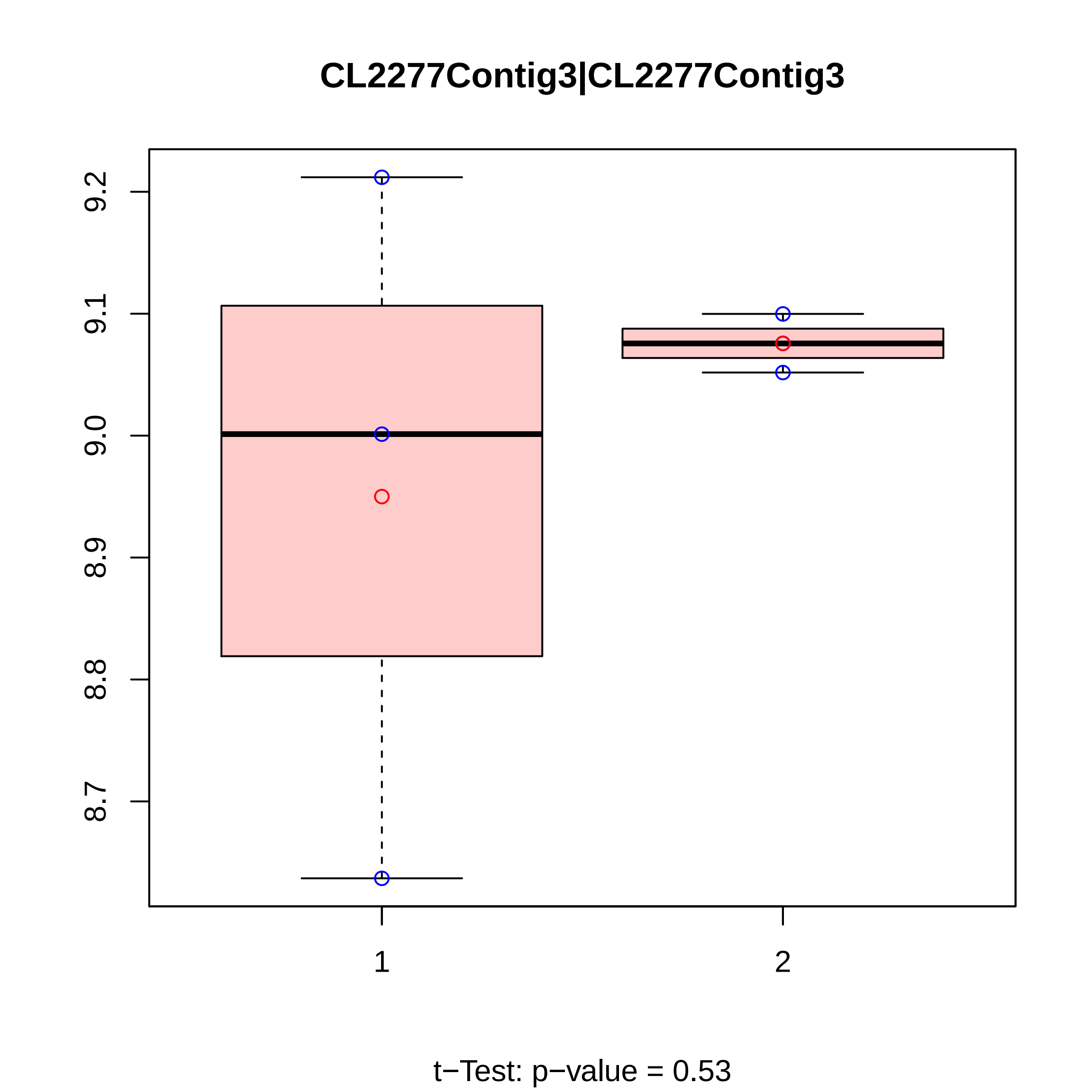
CL2277Contig3|CL2277Contig3
9.2
9.1
9.0
8.9
8.8
8.7
1
2
t−Test: p−value = 0.53

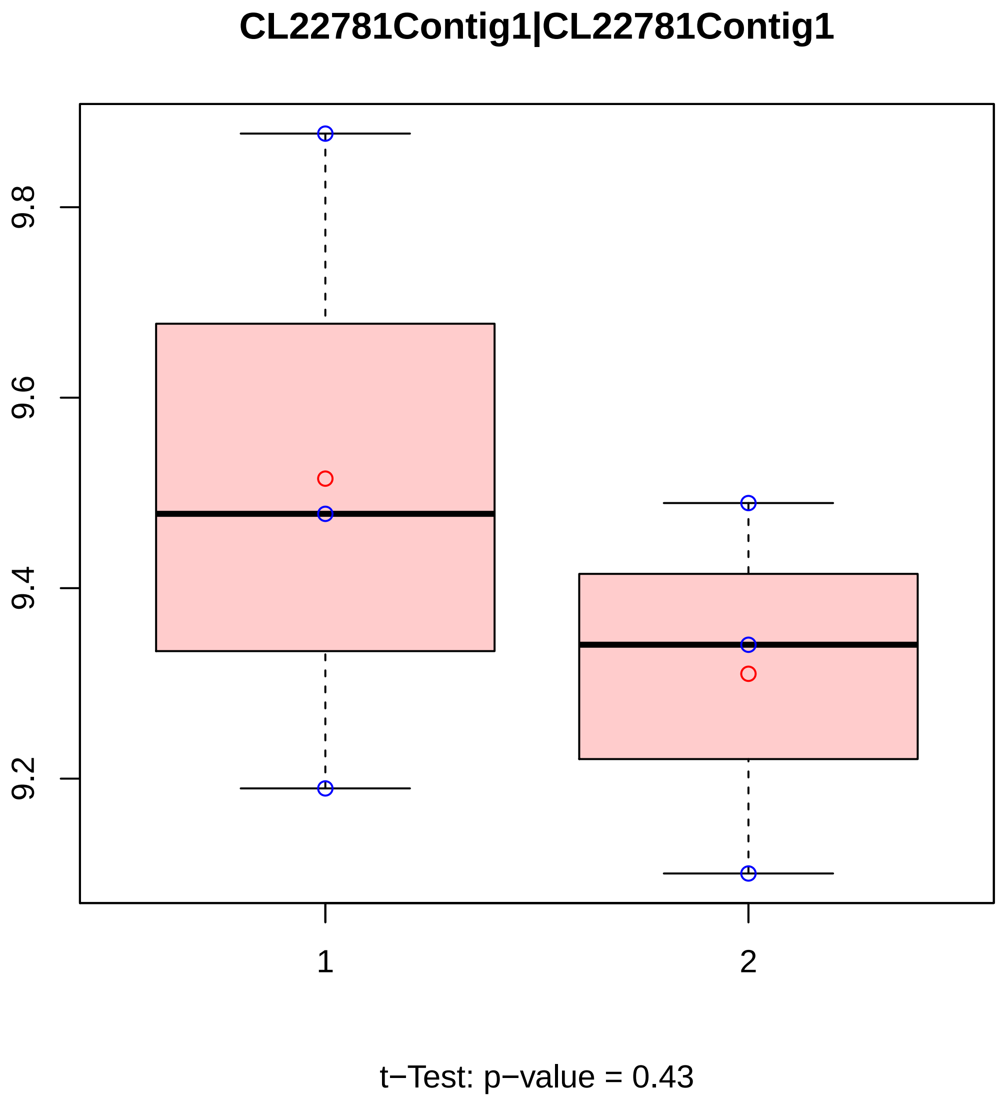
CL22781Contig1|CL22781Contig1
9.2
9.4
9.6
9.8
1
2
t−Test: p−value = 0.43

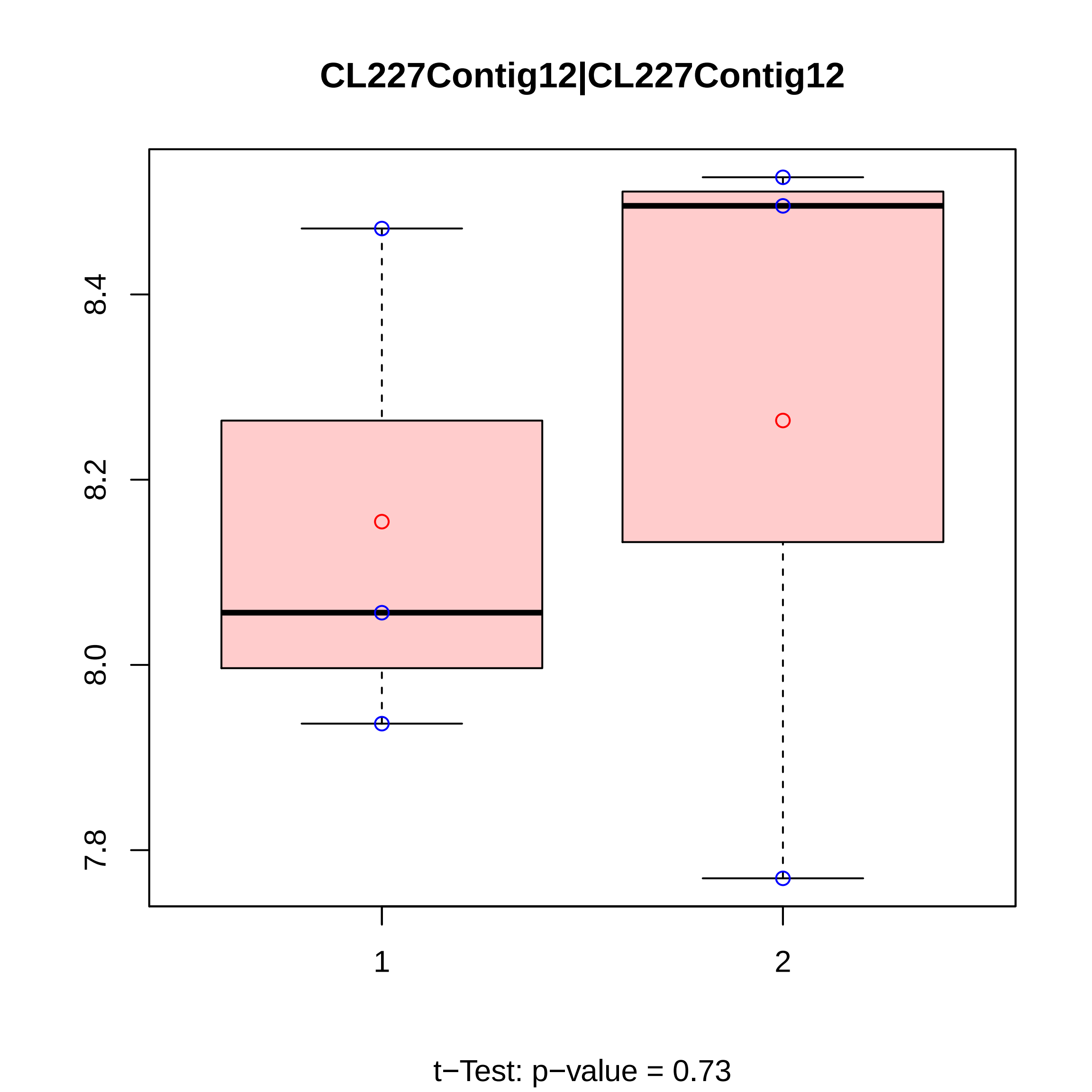
CL227Contig12|CL227Contig12
8.4
8.2
8.0
7.8
1
2
t−Test: p−value = 0.73

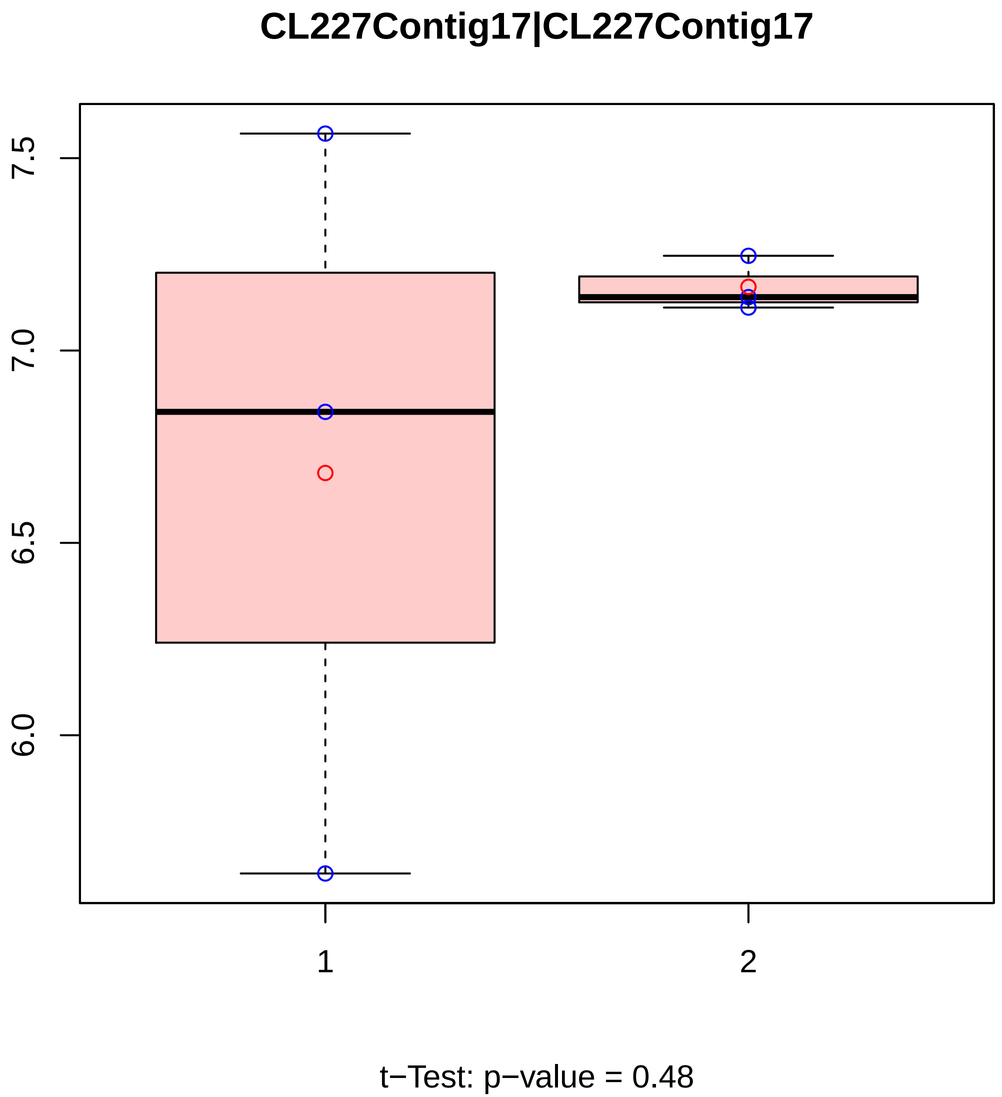
CL227Contig17|CL227Contig17
7.5
7.0
6.5
6.0
1
2
t−Test: p−value = 0.48

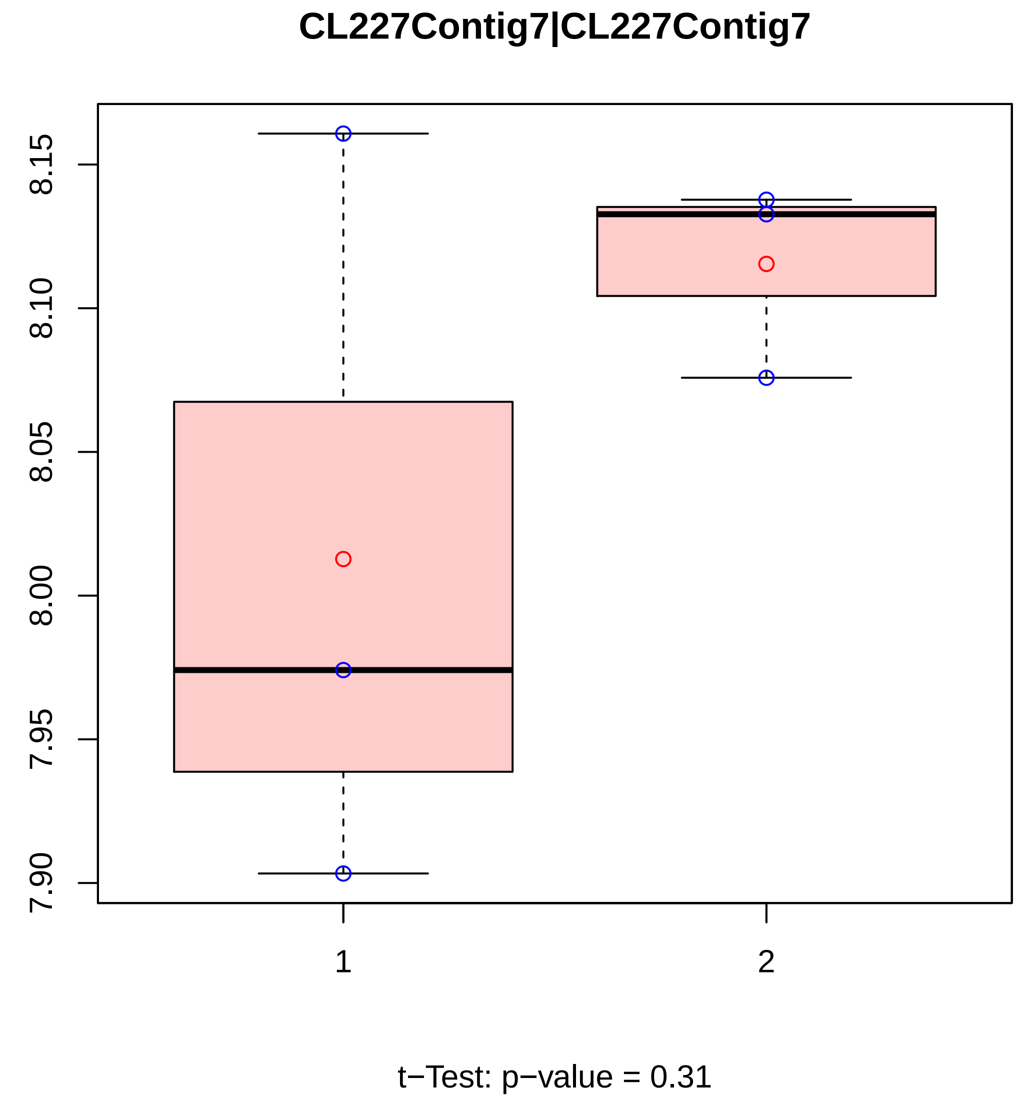
CL227Contig7|CL227Contig7
8.15
8.10
8.05
8.00
7.95
7.90
1
2
t−Test: p−value = 0.31

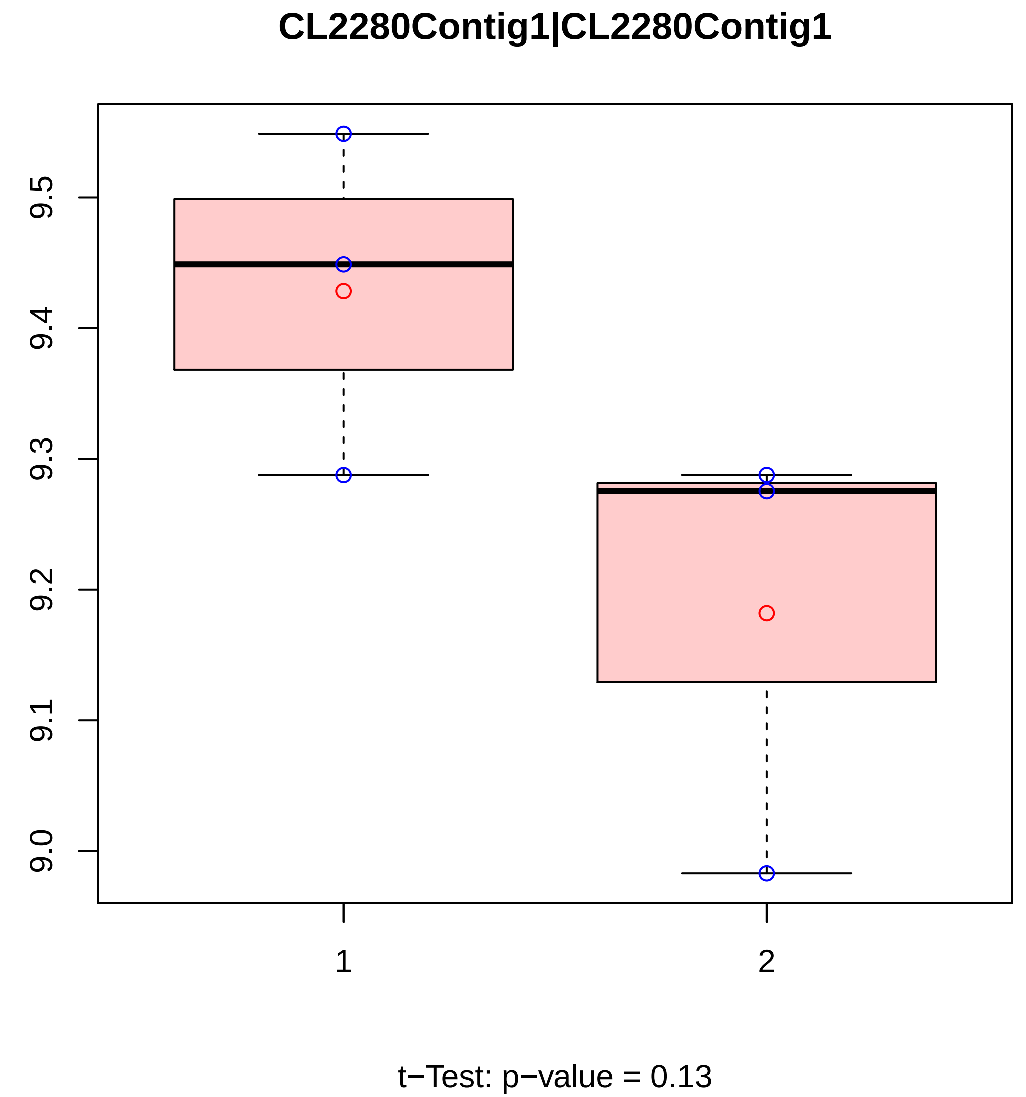
CL2280Contig1|CL2280Contig1
9.0
9.1
9.2
9.3
9.4
9.5
1
2
t−Test: p−value = 0.13

## CL2280Contig3|CL2280Contig3

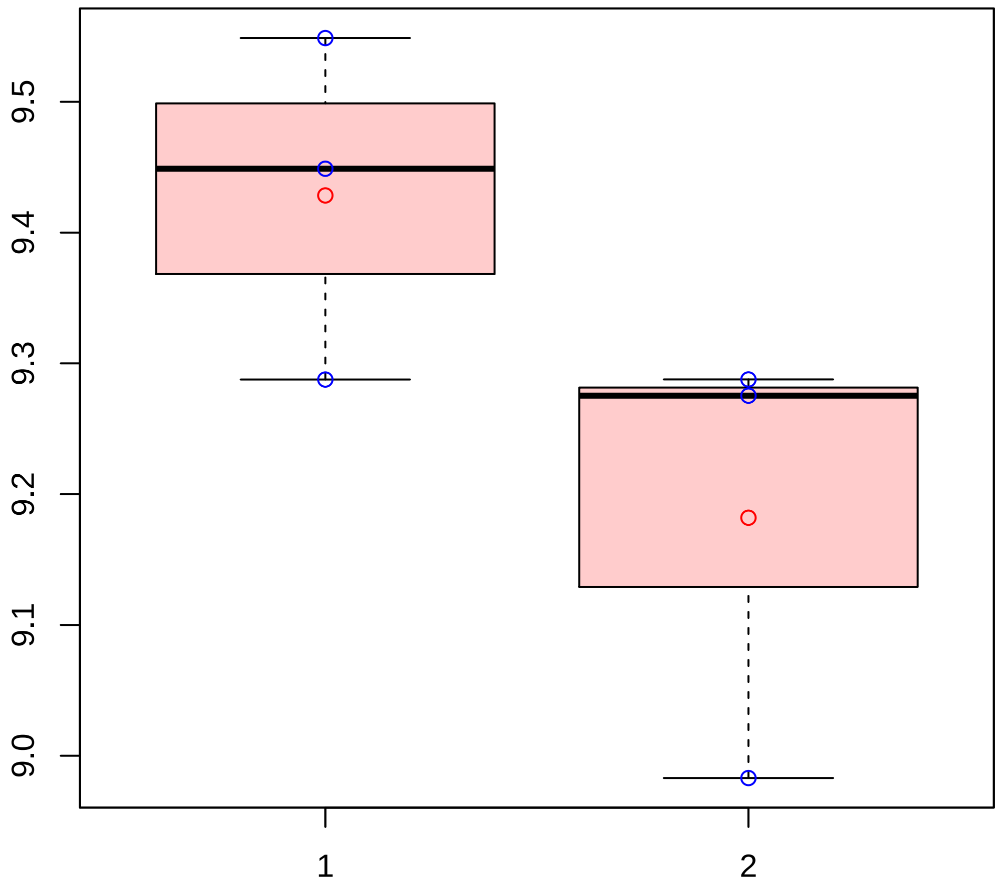


t−Test: p−value = 0.13

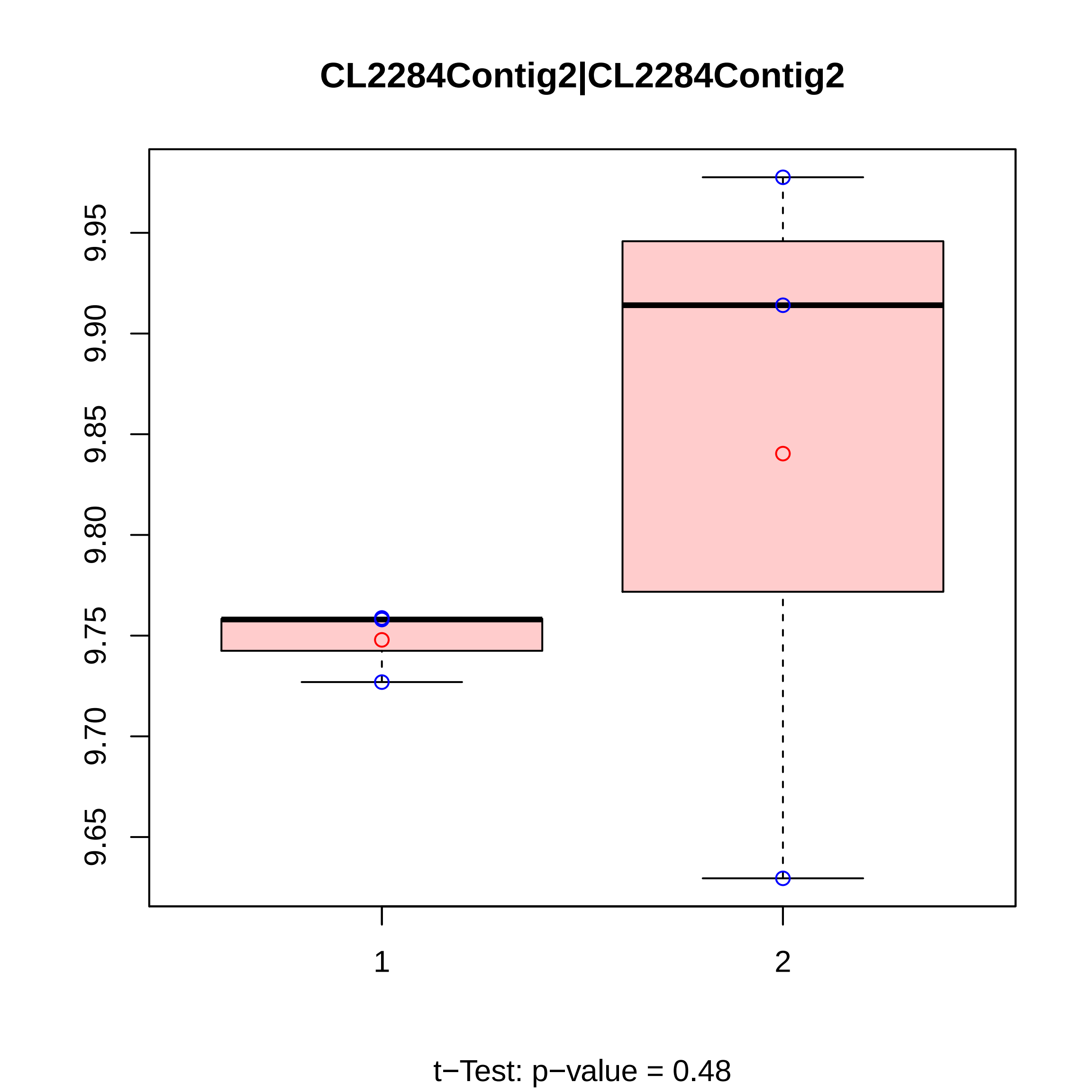
CL2284Contig2|CL2284Contig2
9.65
9.70
9.75
9.80
9.85
9.90
9.95
1
2
t−Test: p−value = 0.48

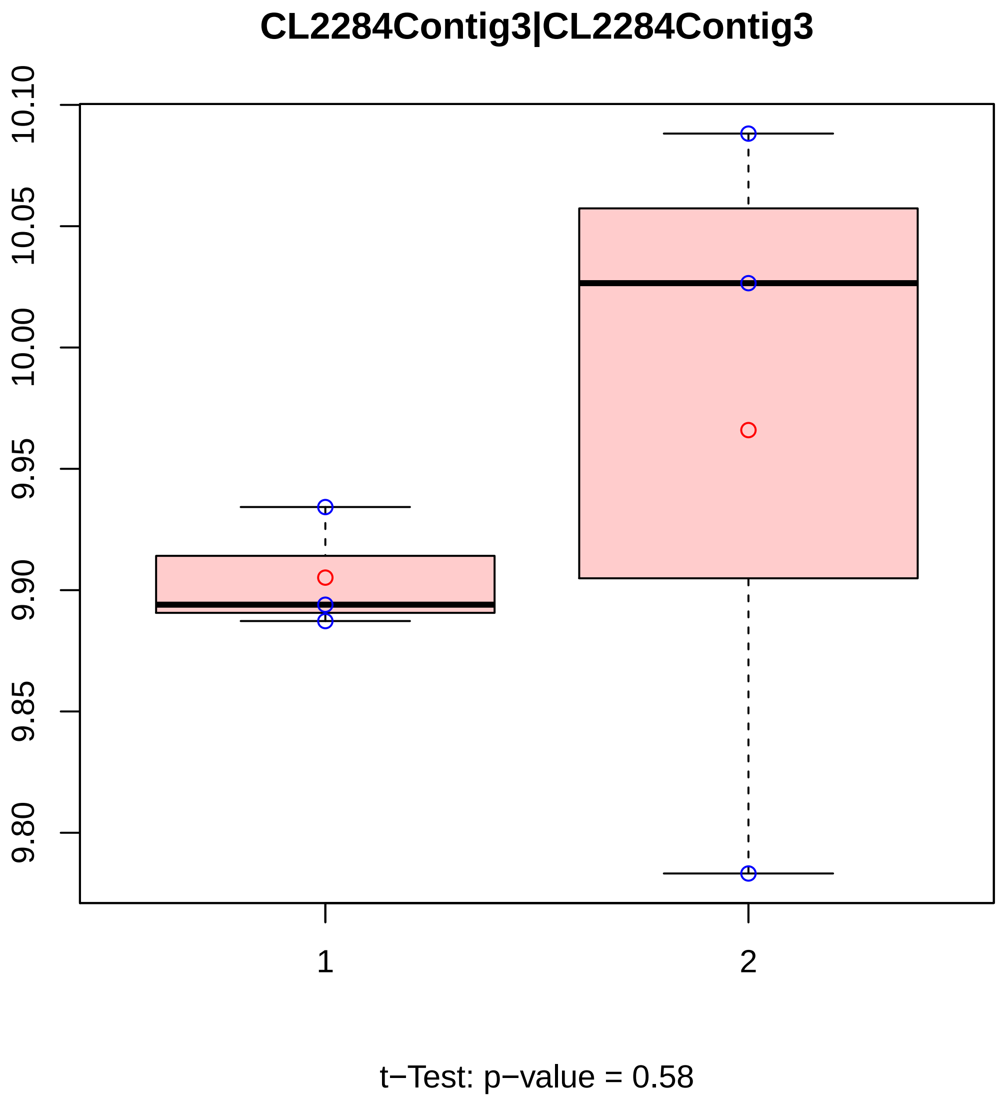
CL2284Contig3|CL2284Contig3
10.10
10.05
10.00
9.95
9.90
9.85
9.80
1
2
t−Test: p−value = 0.58

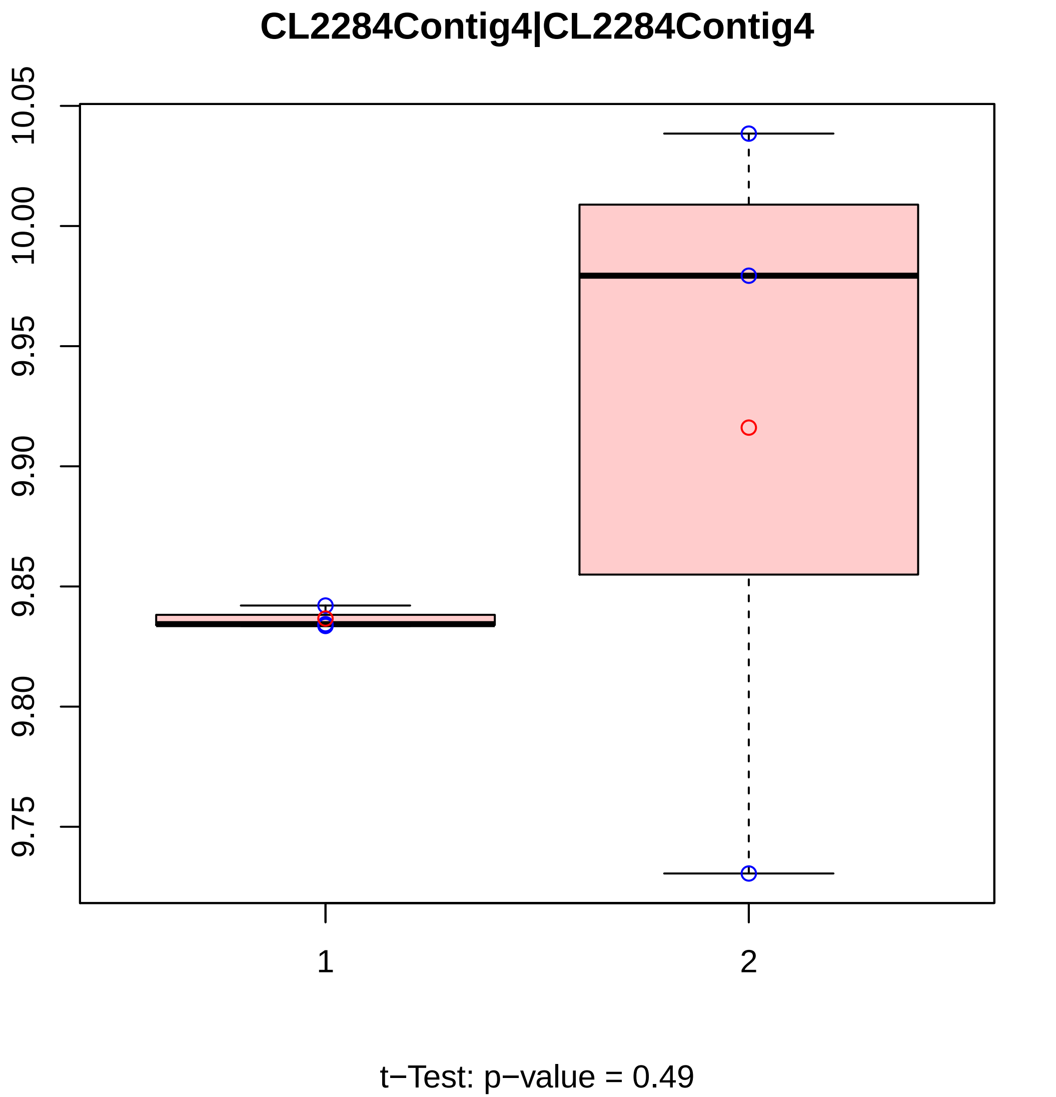
CL2284Contig4|CL2284Contig4
10.05
10.00
9.95
9.90
9.85
9.80
9.75
1
2
t−Test: p−value = 0.49

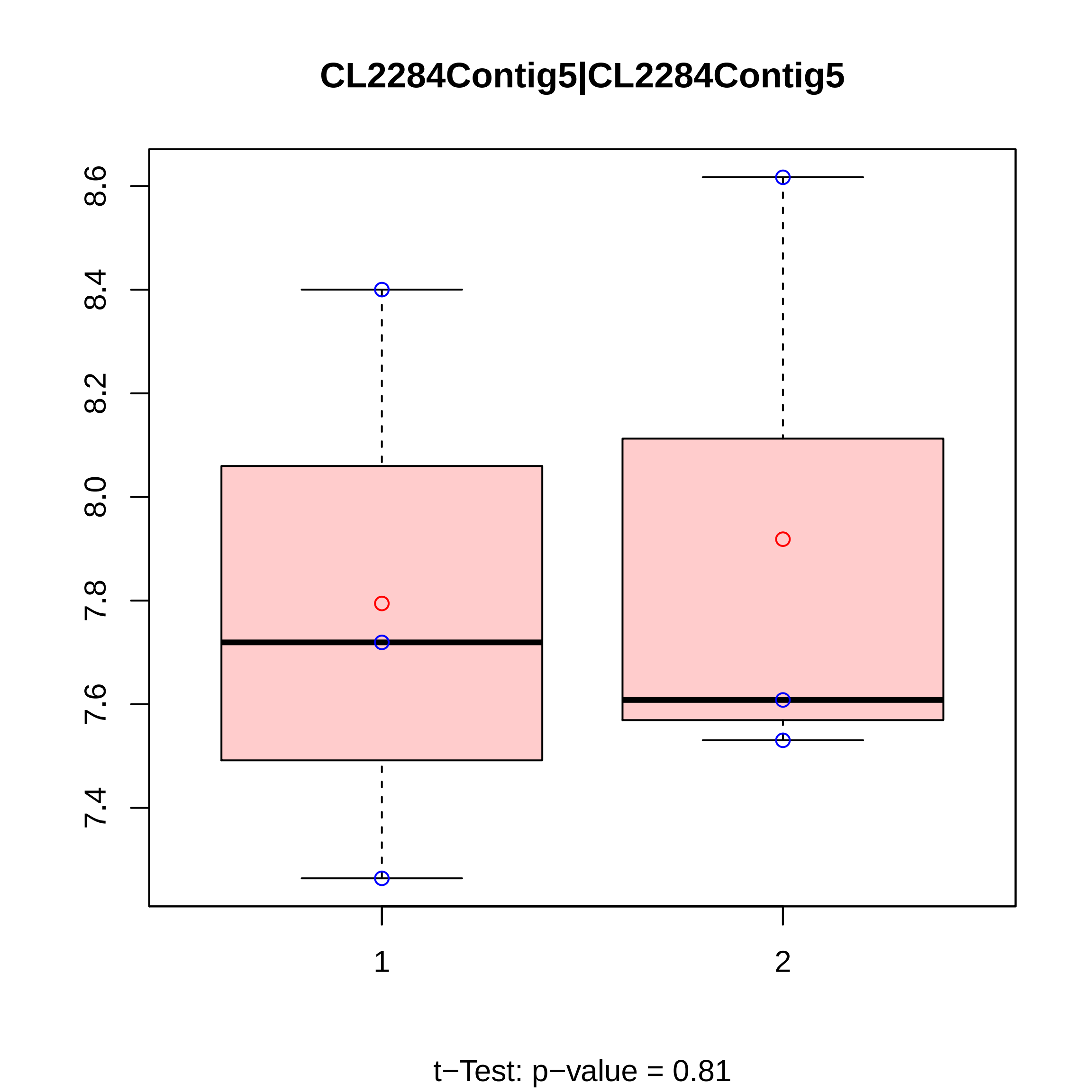
CL2284Contig5|CL2284Contig5
8.6
8.4
8.2
8.0
7.8
7.6
7.4
1
2
t−Test: p−value = 0.81

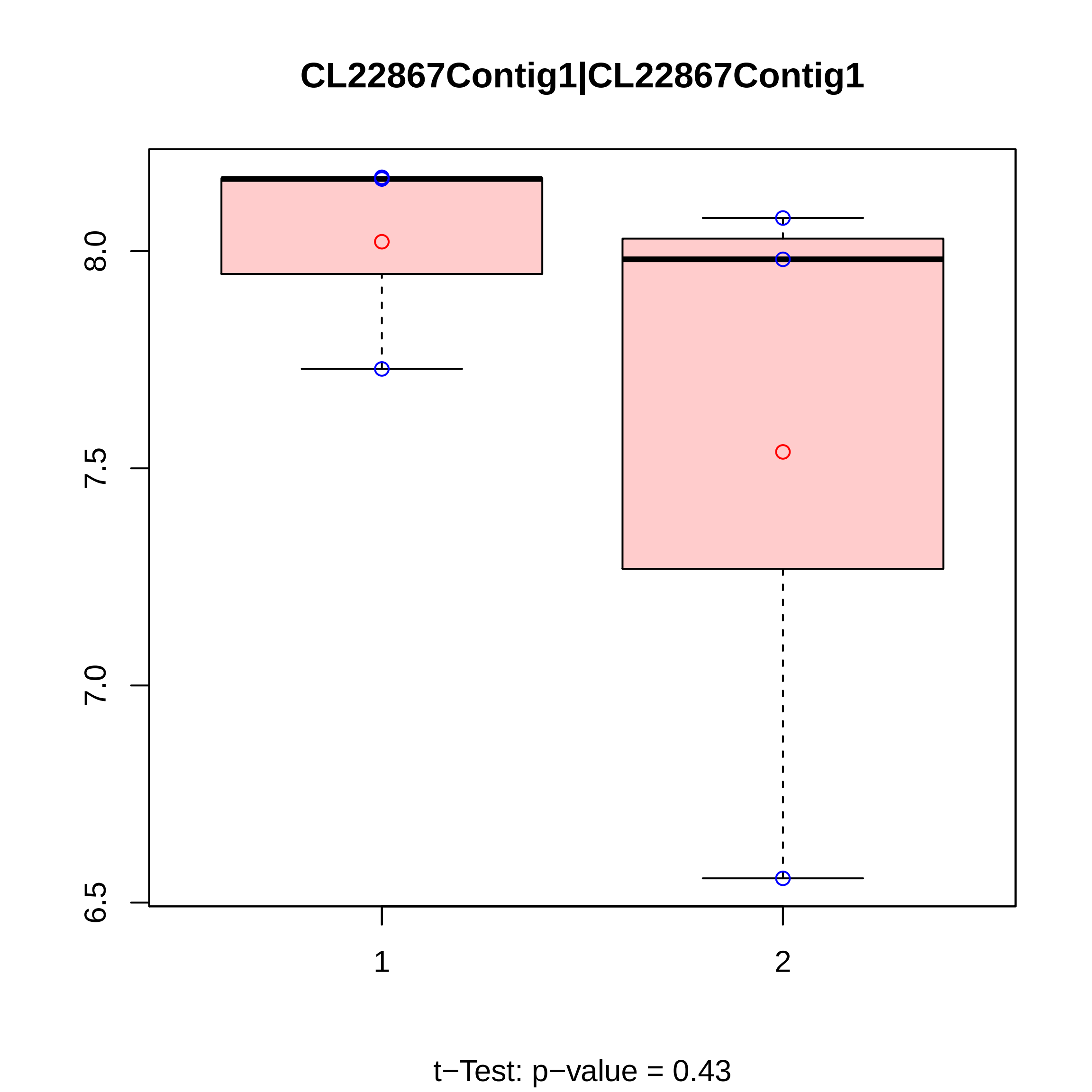
CL22867Contig1|CL22867Contig1
8.0
7.5
7.0
6.5
1
2
t−Test: p−value = 0.43

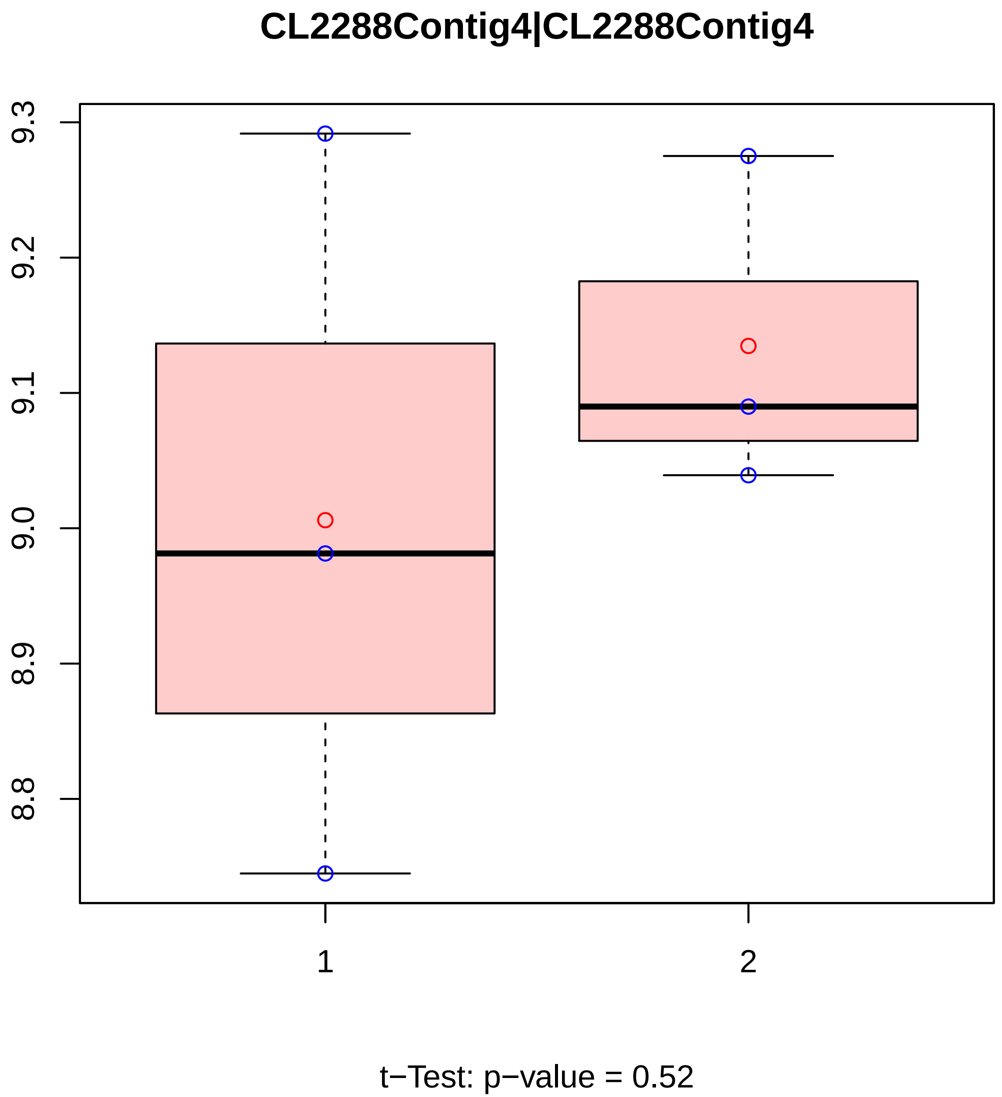
CL2288Contig4|CL2288Contig4
9.3
9.2
9.1
9.0
8.9
8.8
1
2
t−Test: p−value = 0.52

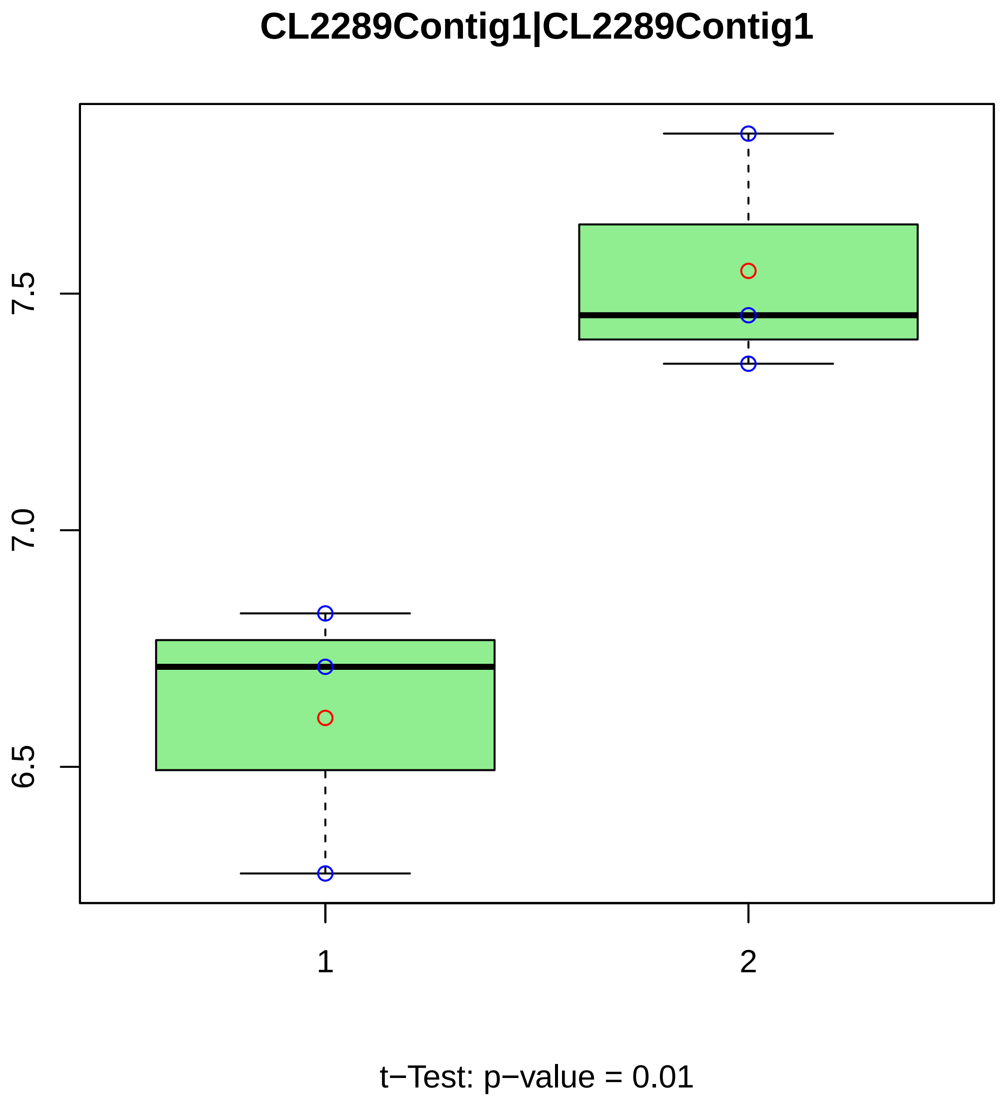
CL2289Contig1|CL2289Contig1
7.5
7.0
6.5
1
2
t−Test: p−value = 0.01

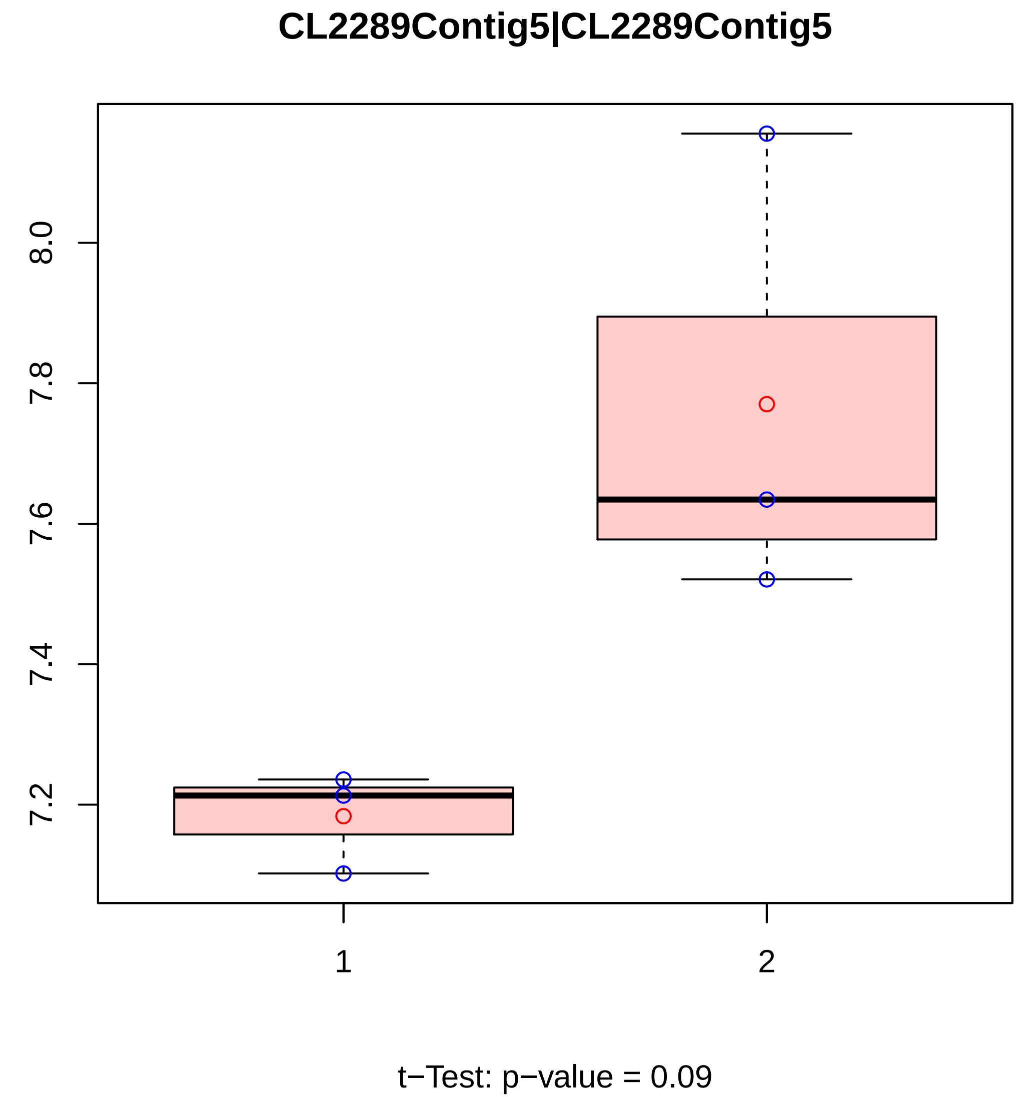
CL2289Contig5|CL2289Contig5
8.0
7.8
7.6
7.4
7.2
1
2
t−Test: p−value = 0.09

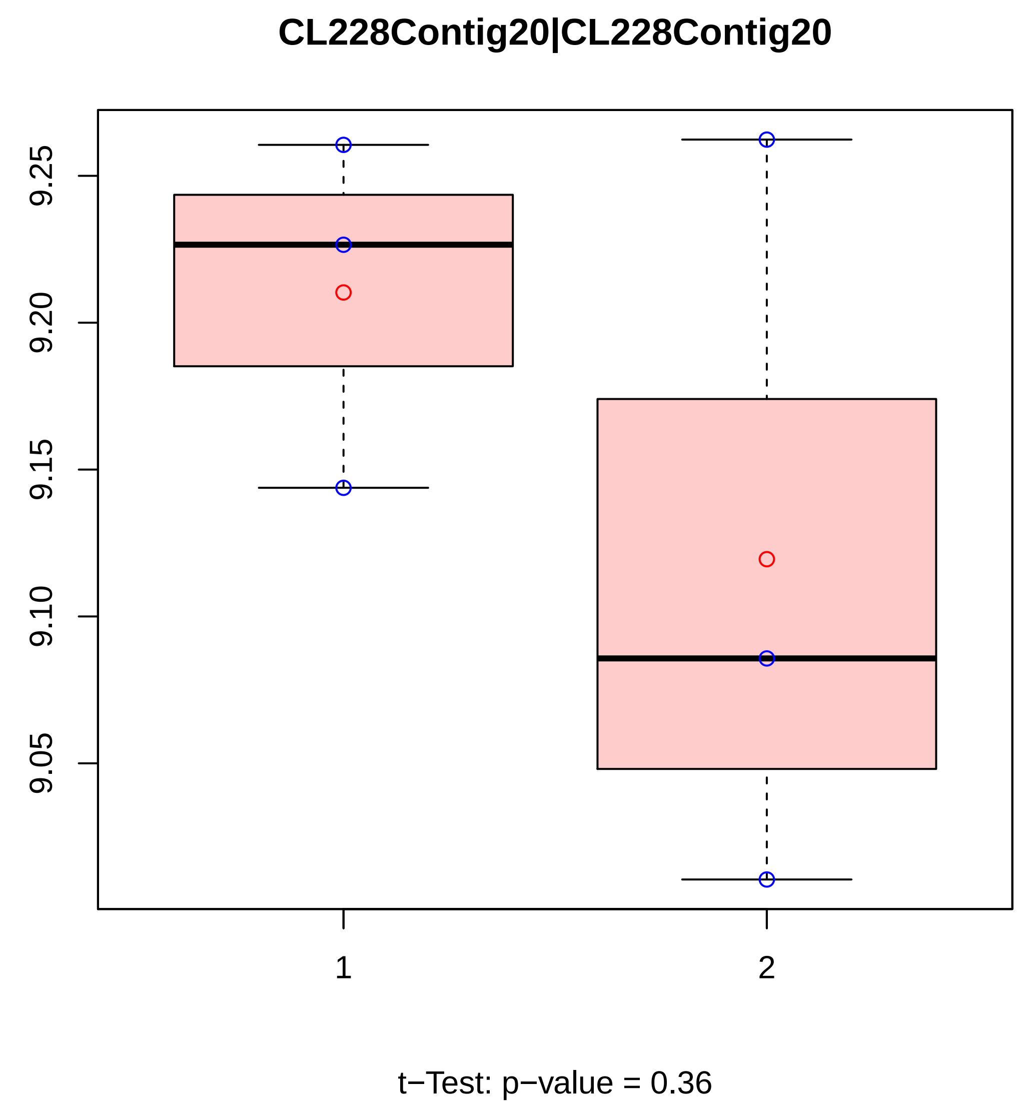

CL228Contig20|CL228Contig20
9.05
9.10
9.15
9.20
9.25
1
2
t−Test: p−value = 0.36

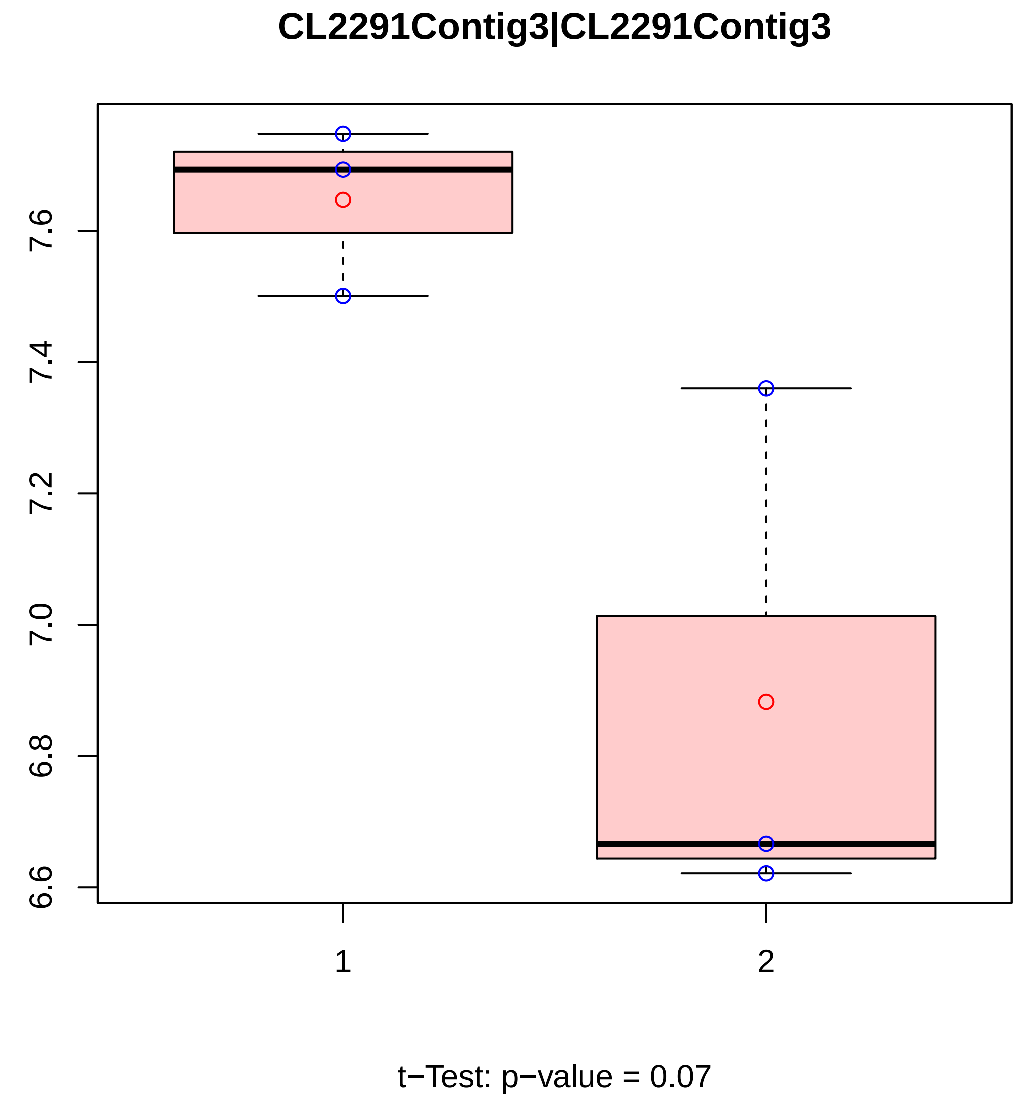
CL2291Contig3|CL2291Contig3
7.6
7.4
7.2
7.0
6.8
6.6
1
2
t−Test: p−value = 0.07

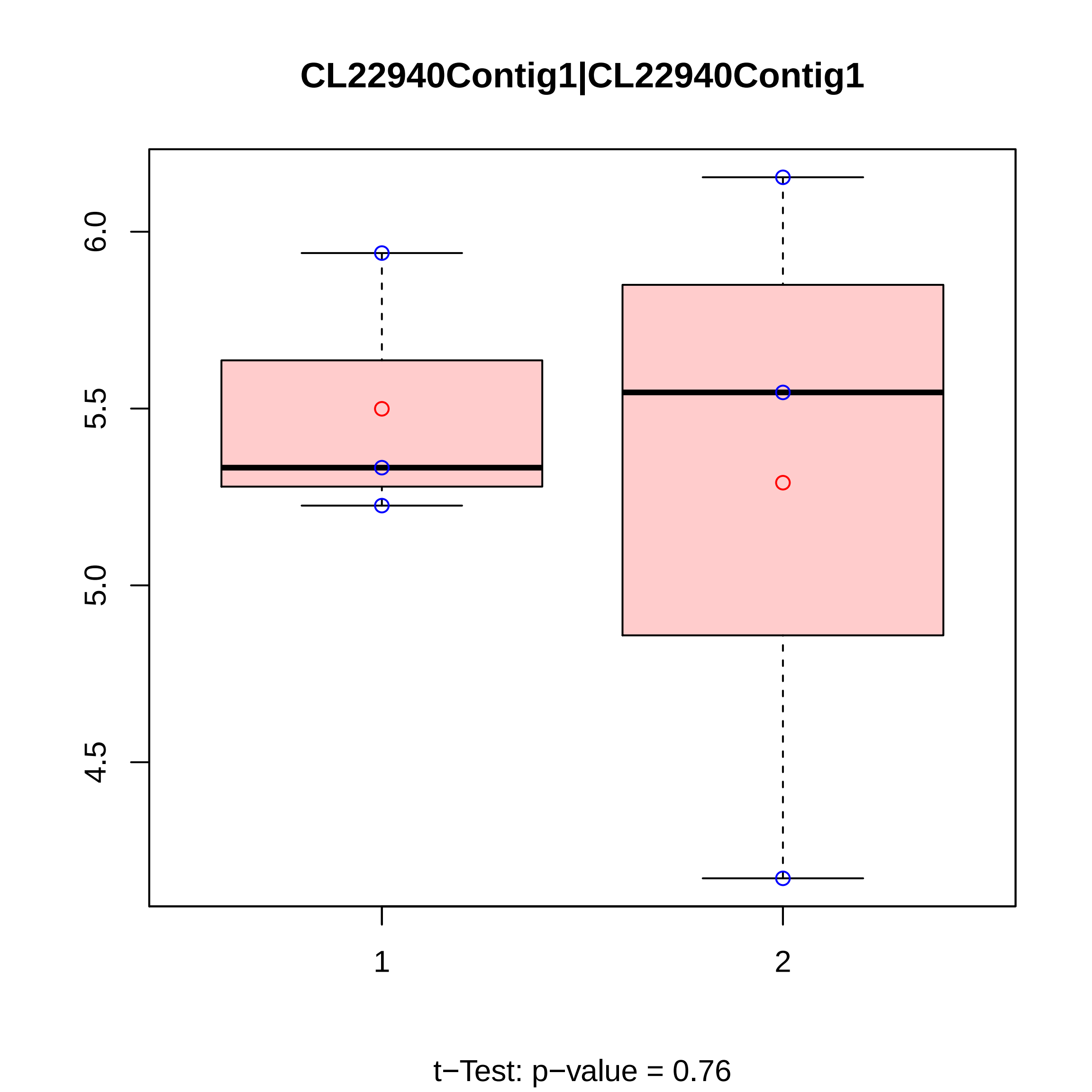
CL22940Contig1|CL22940Contig1
6.0
5.5
5.0
4.5
1
2
t−Test: p−value = 0.76

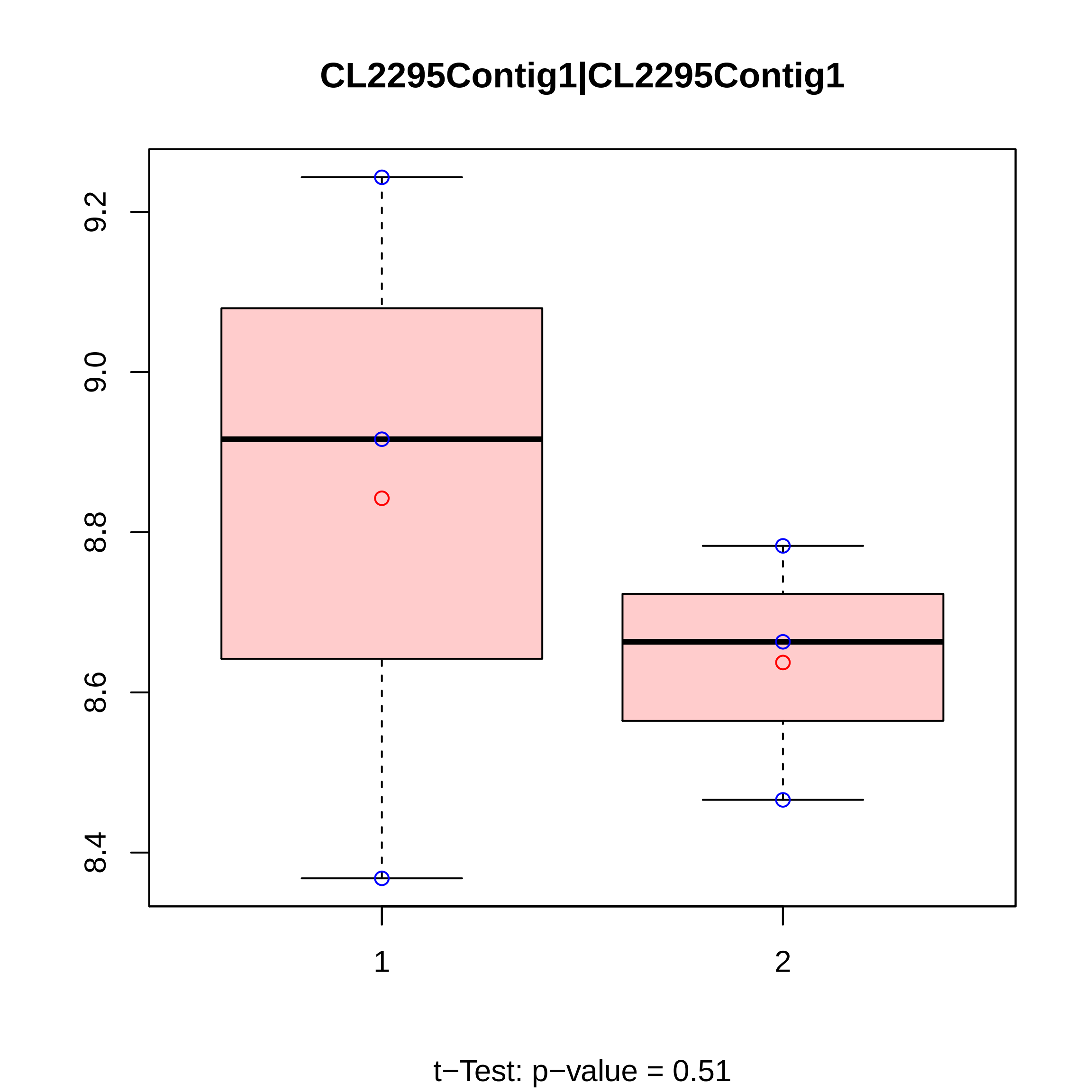

CL2295Contig1|CL2295Contig1
9.2
9.0
8.8
8.6
8.4
1
2
t−Test: p−value = 0.51

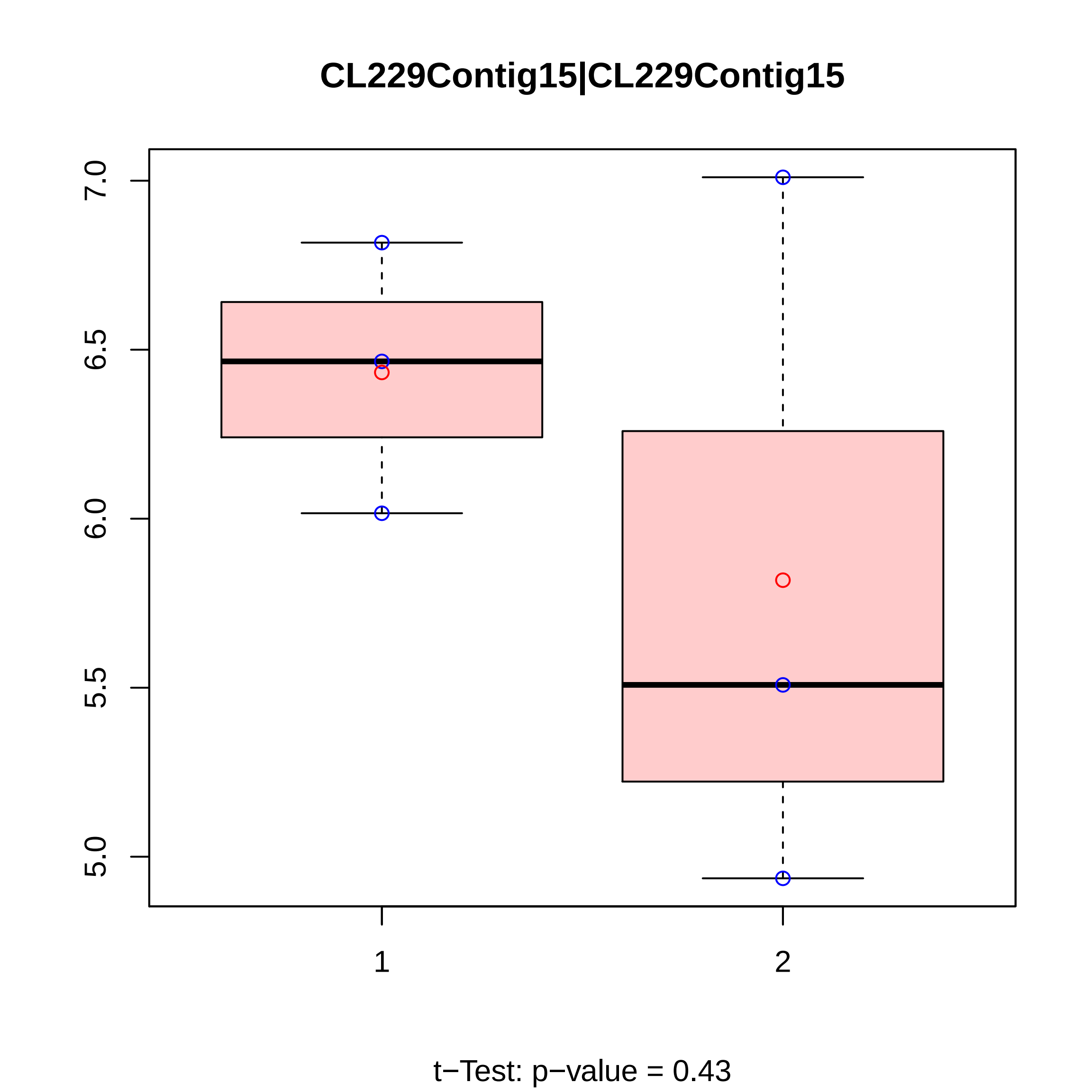

CL229Contig15|CL229Contig15
7.0
6.5
6.0
5.5
5.0
1
2
t−Test: p−value = 0.43

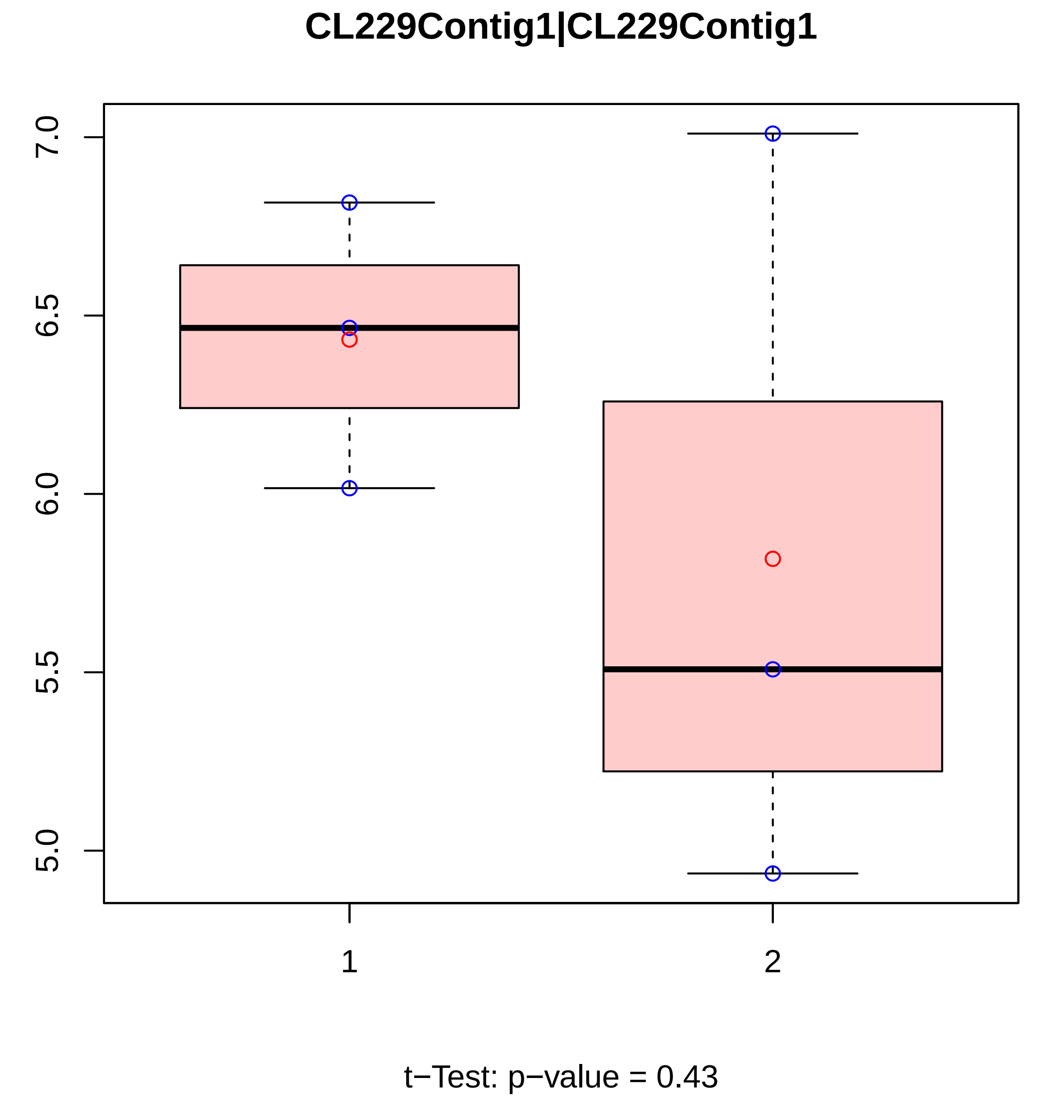
CL229Contig1|CL229Contig1
7.0
6.5
6.0
5.5
5.0
1
2
t−Test: p−value = 0.43

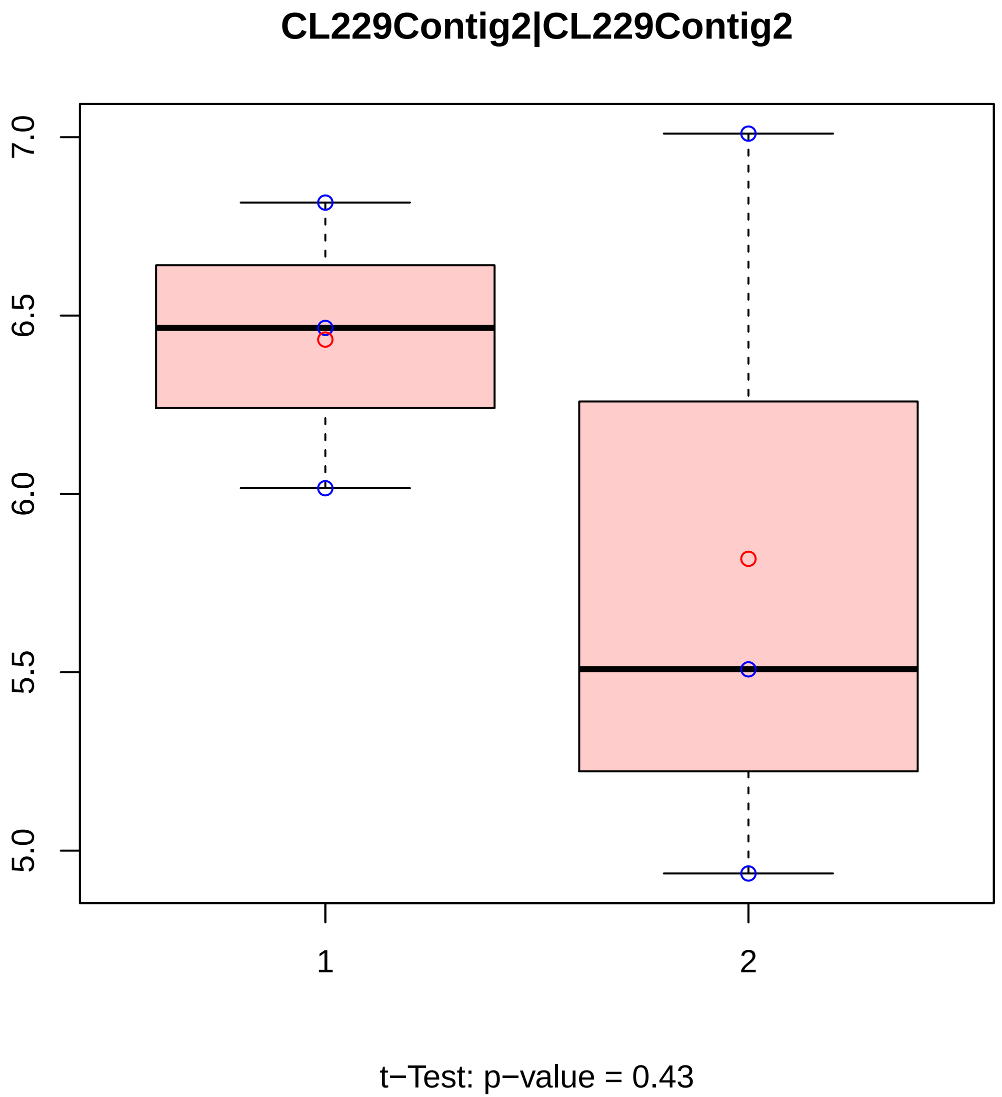

CL229Contig2|CL229Contig2
7.0
6.5
6.0
5.5
5.0
1
2
t−Test: p−value = 0.43

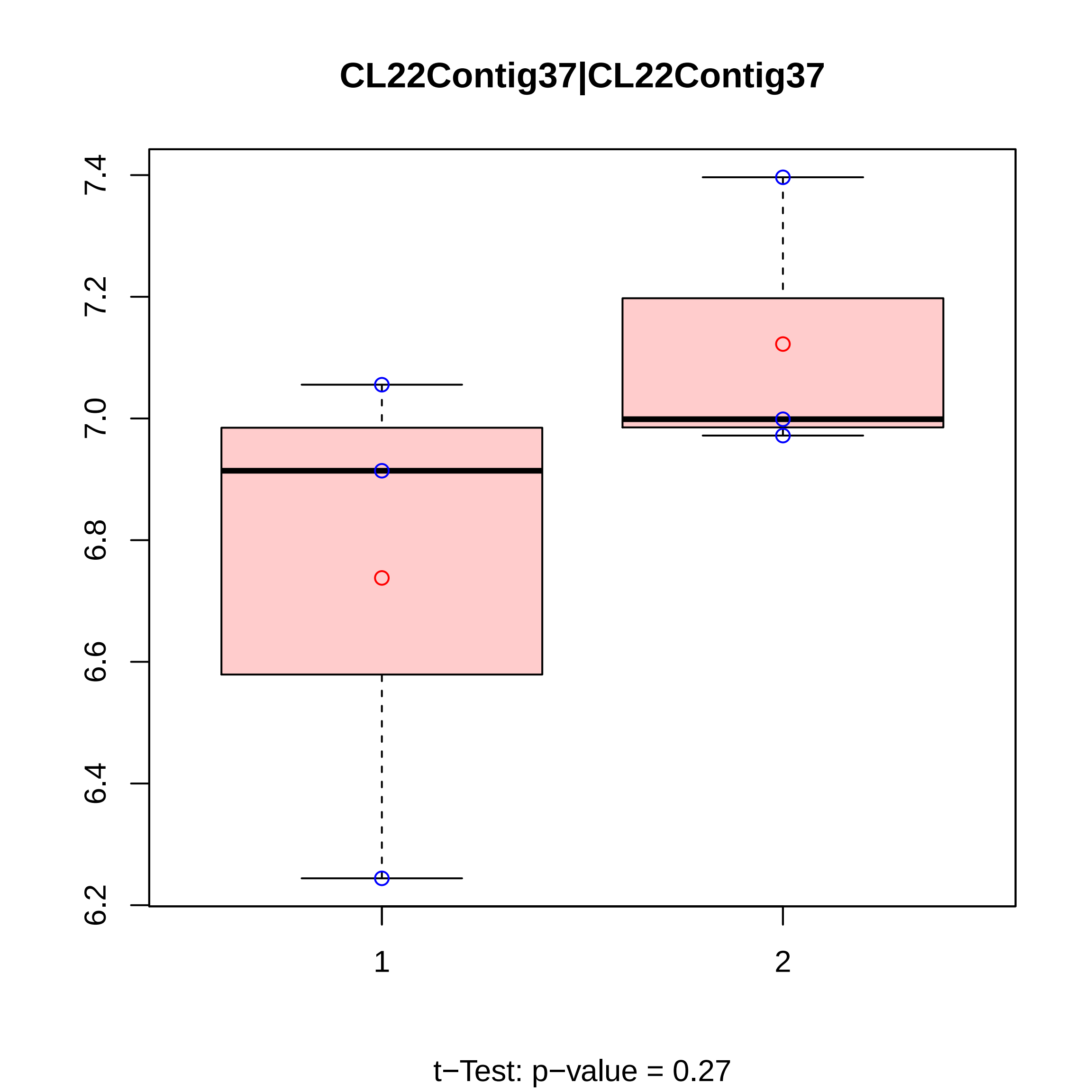

CL22Contig37|CL22Contig37
7.4
7.2
7.0
6.8
6.6
6.4
6.2
1
2
t−Test: p−value = 0.27

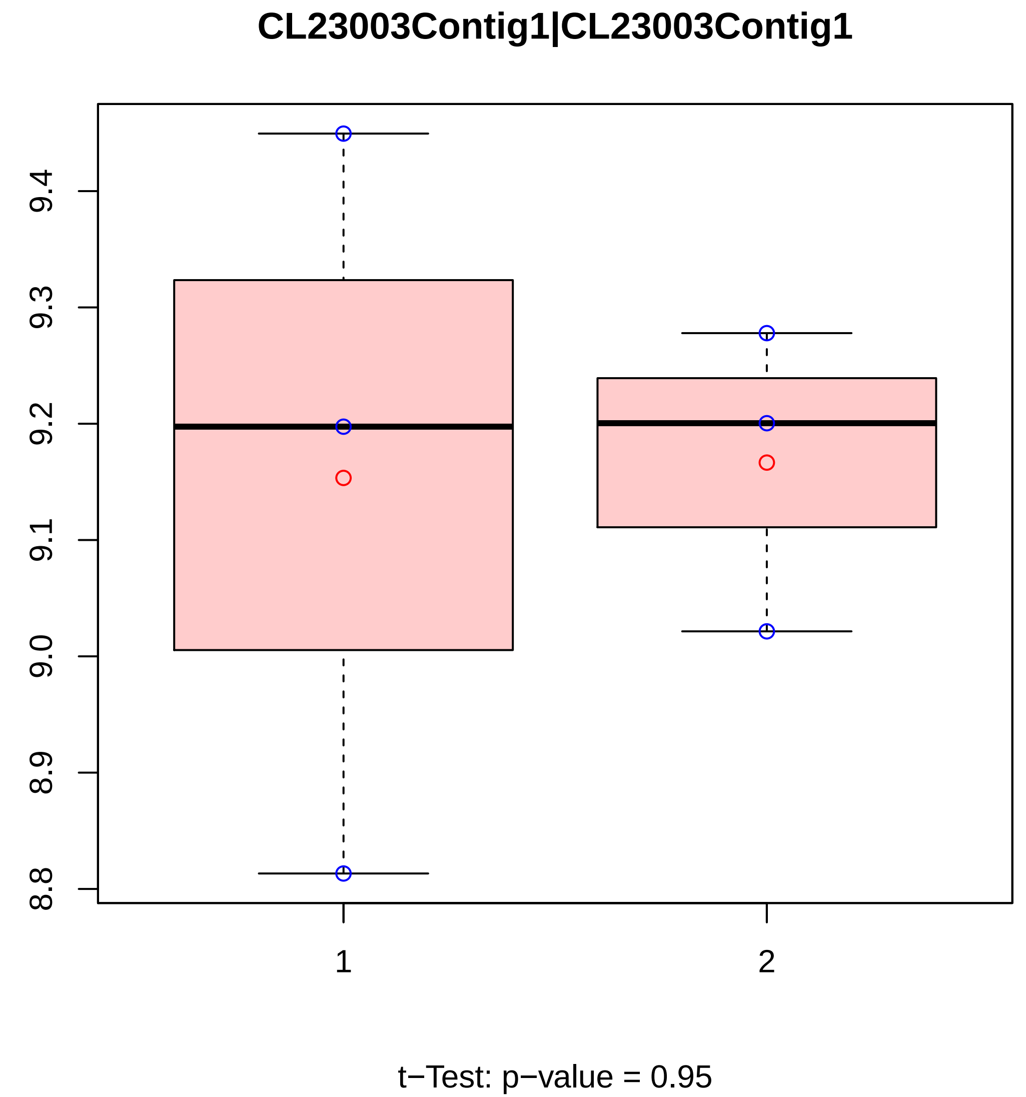
CL23003Contig1|CL23003Contig1
9.4
9.3
9.2
9.1
9.0
8.9
8.8
1
2
t−Test: p−value = 0.95

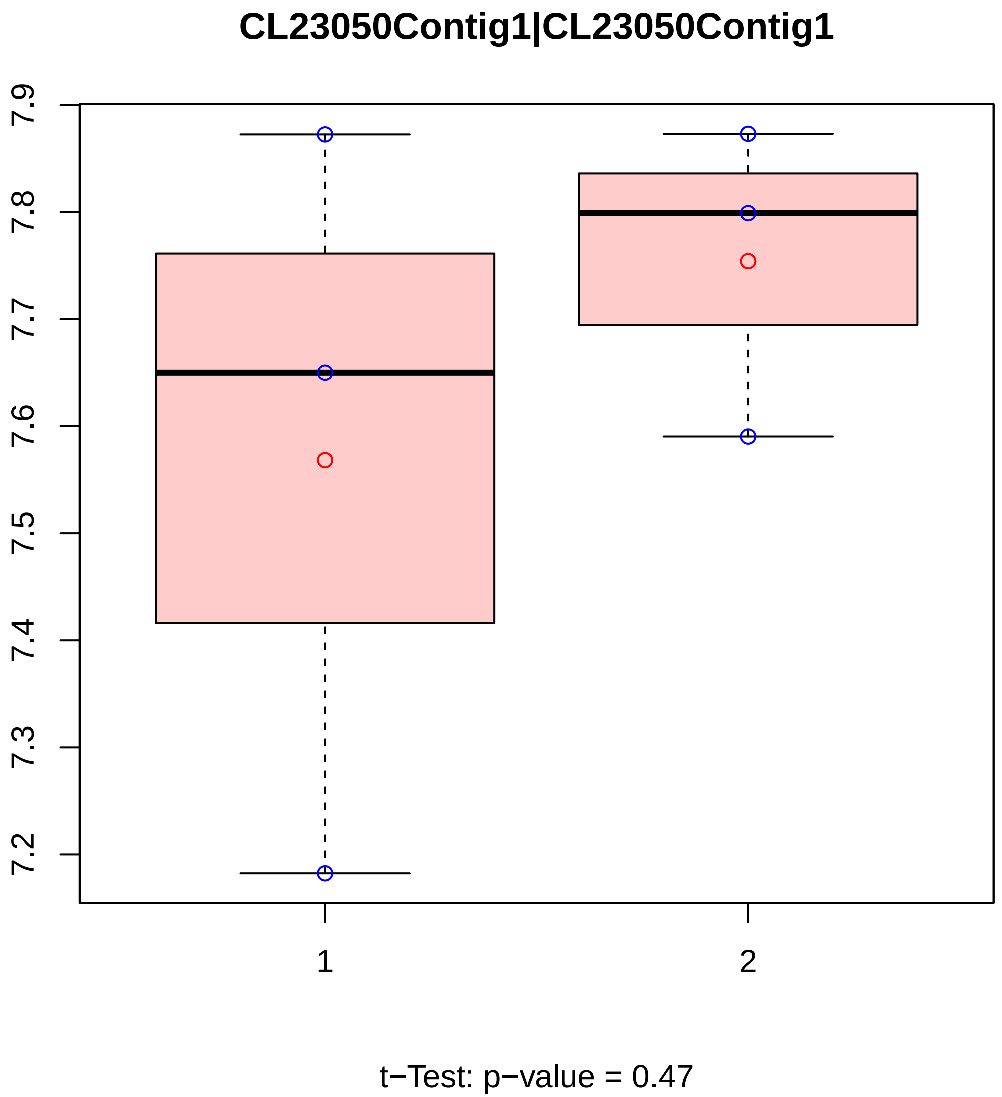

CL23050Contig1|CL23050Contig1
7.9
7.8
7.7
7.6
7.5
7.4
7.3
7.2
1
2
t−Test: p−value = 0.47

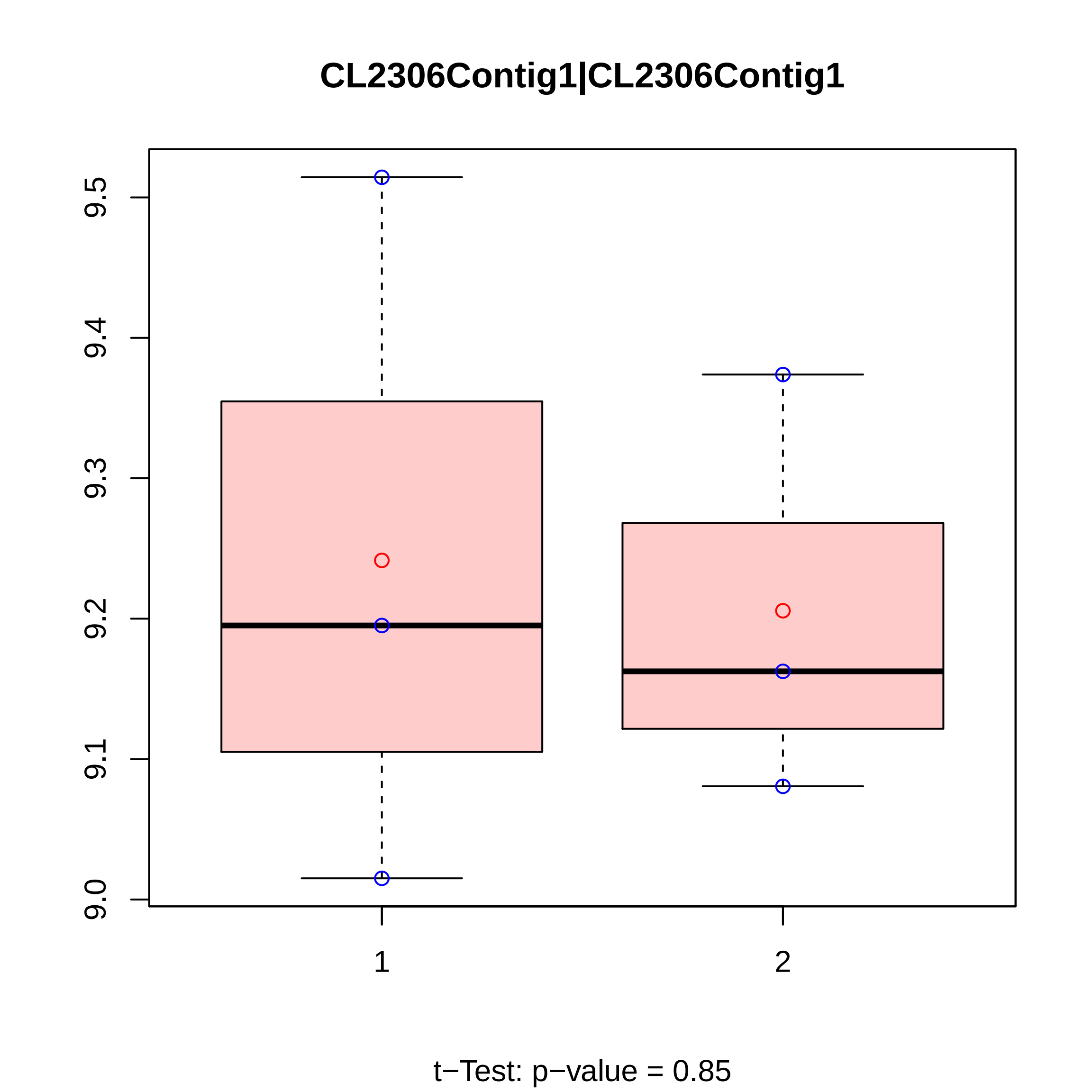

CL2306Contig1|CL2306Contig1
9.0
9.1
9.2
9.3
9.4
9.5
1
2
t−Test: p−value = 0.85

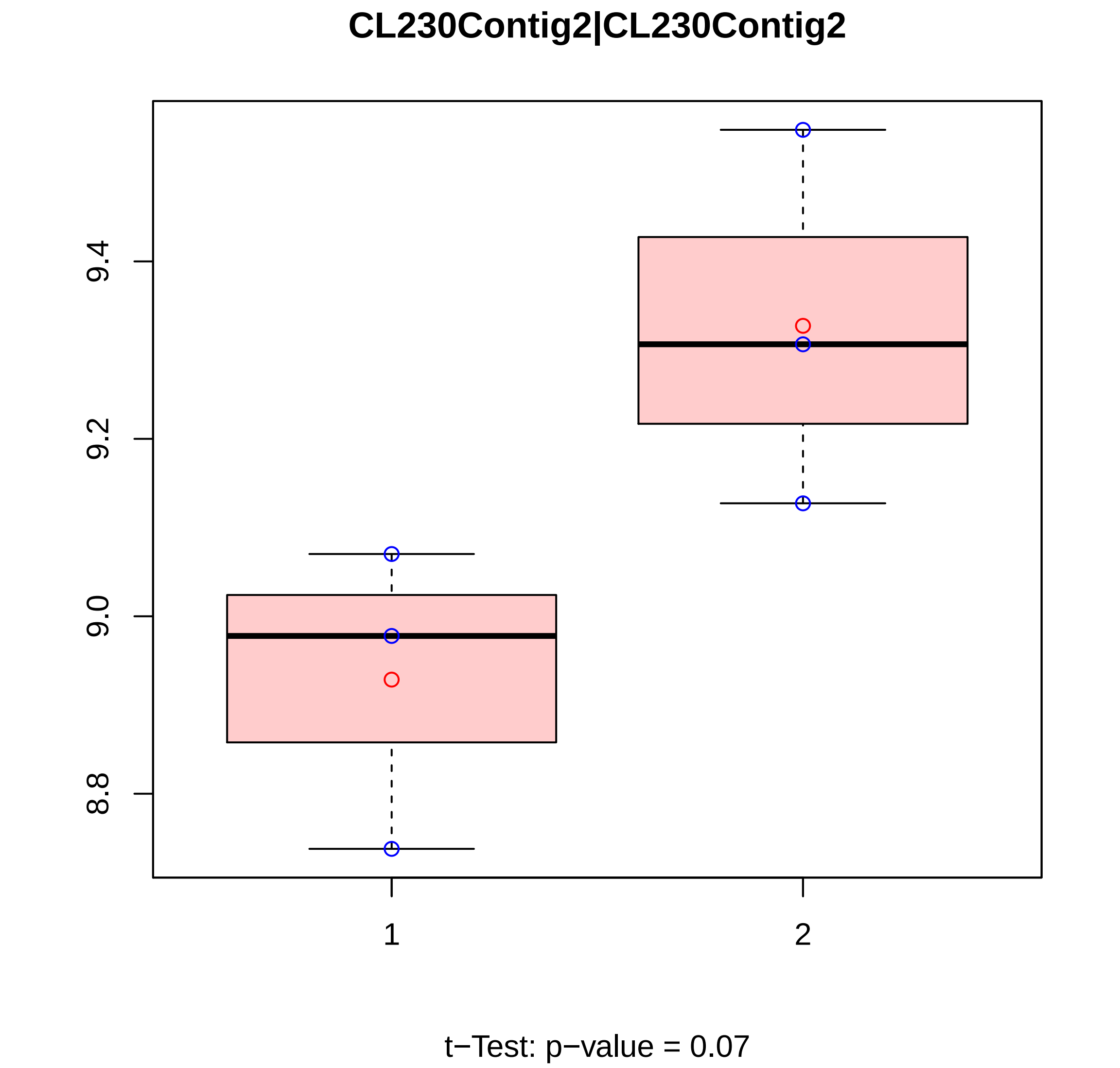
CL230Contig2|CL230Contig2
9.4
9.2
9.0
8.8
1
2
t−Test: p−value = 0.07

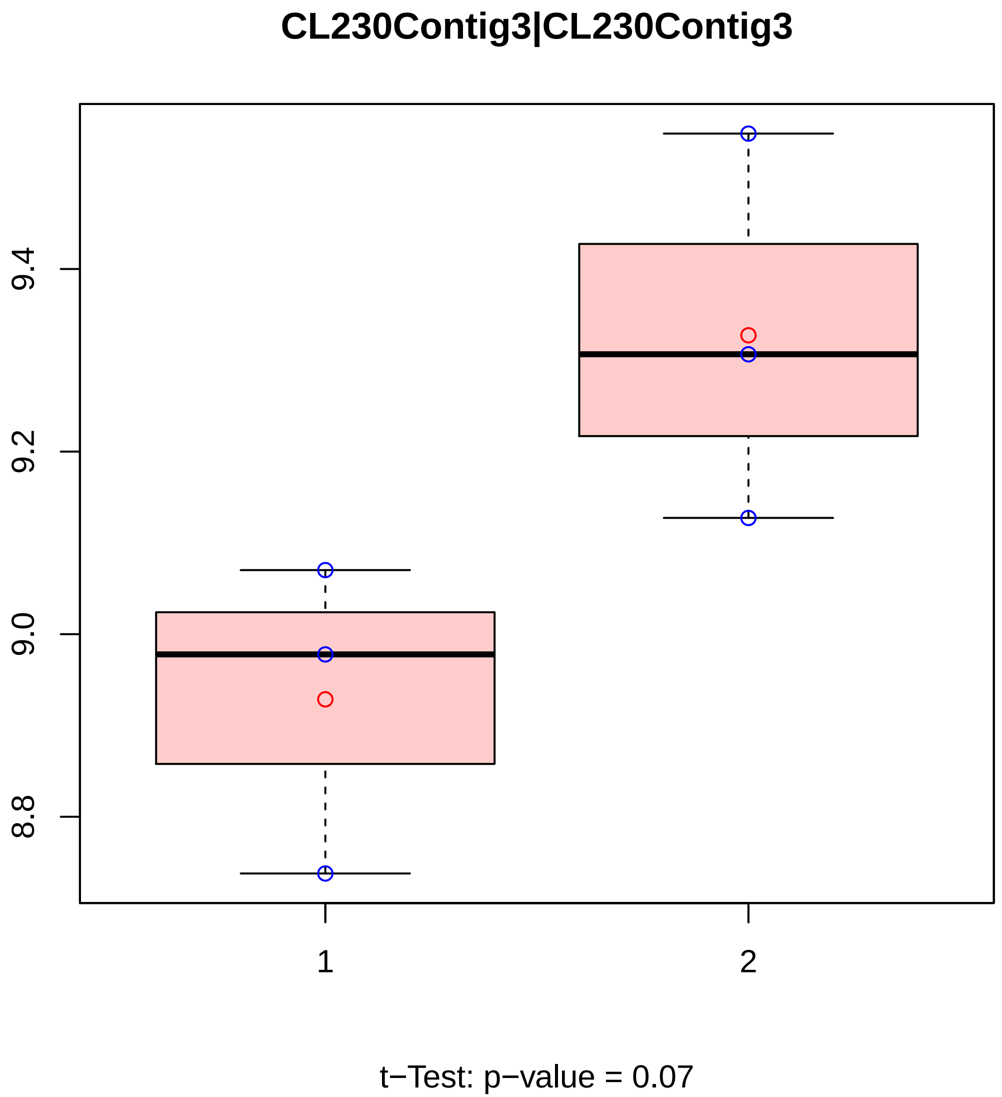
CL230Contig3|CL230Contig3
9.4
9.2
9.0
8.8
1
2
t−Test: p−value = 0.07

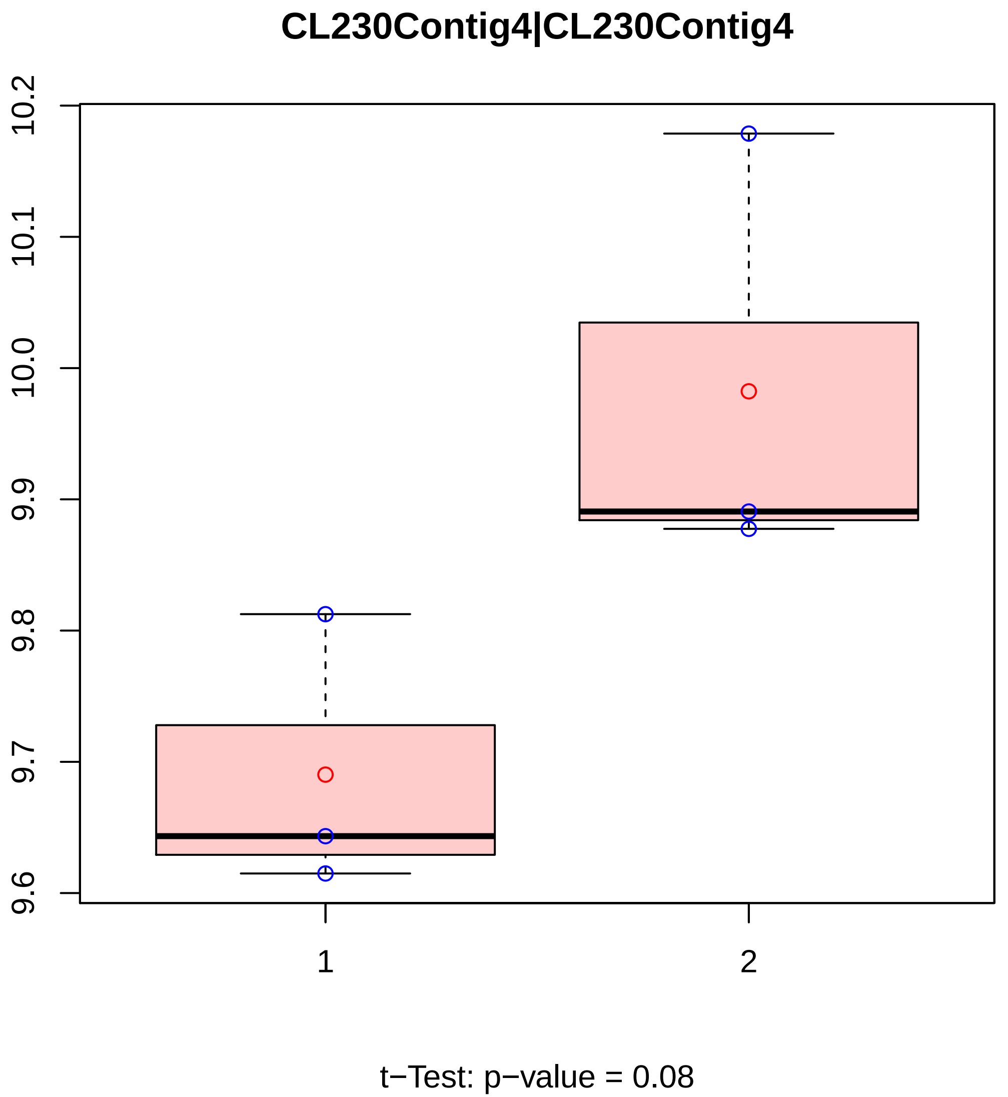

CL230Contig4|CL230Contig4
10.2
10.1
10.0
9.9
9.8
9.7
9.6
1
2
t−Test: p−value = 0.08

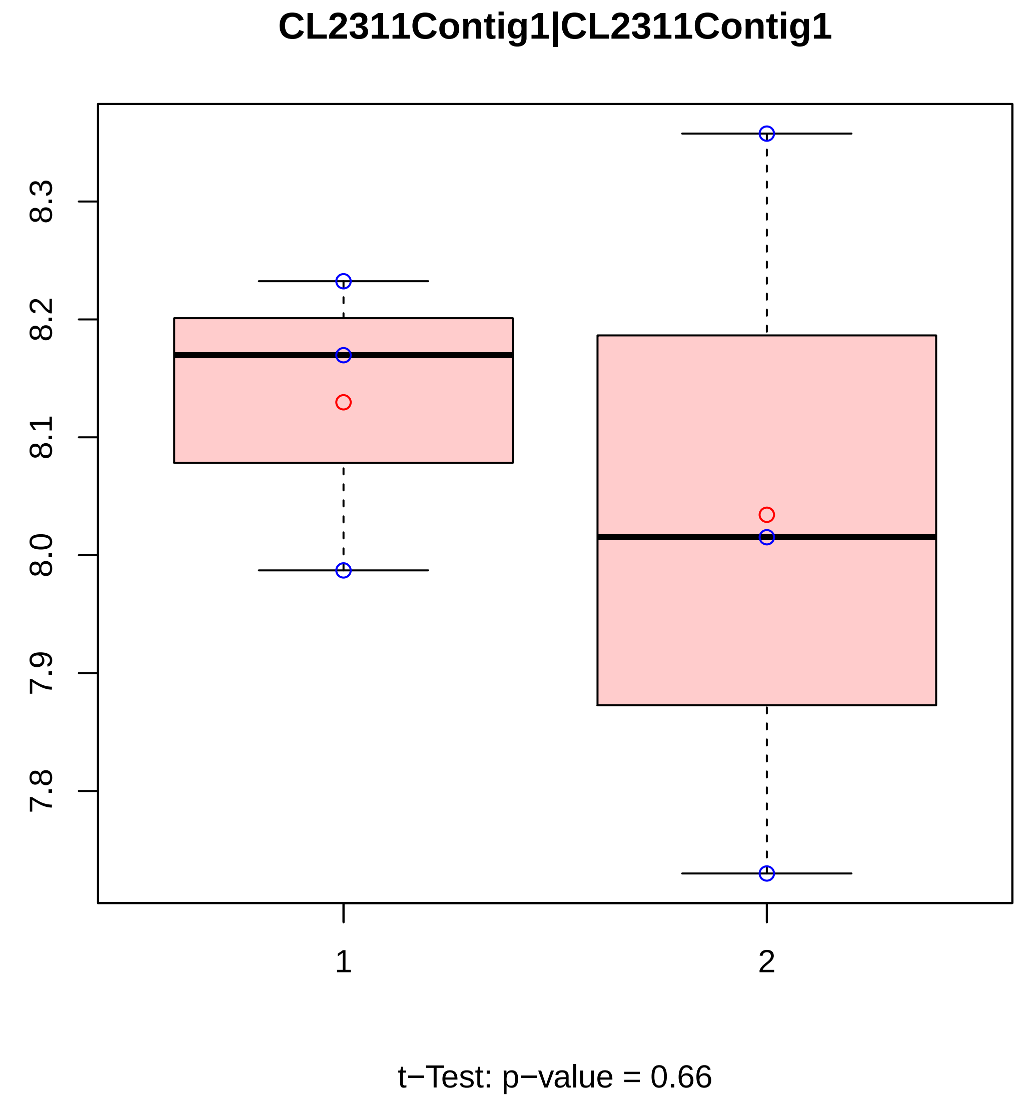
CL2311Contig1|CL2311Contig1
8.3
8.2
8.1
8.0
7.9
7.8
1
2
t−Test: p−value = 0.66

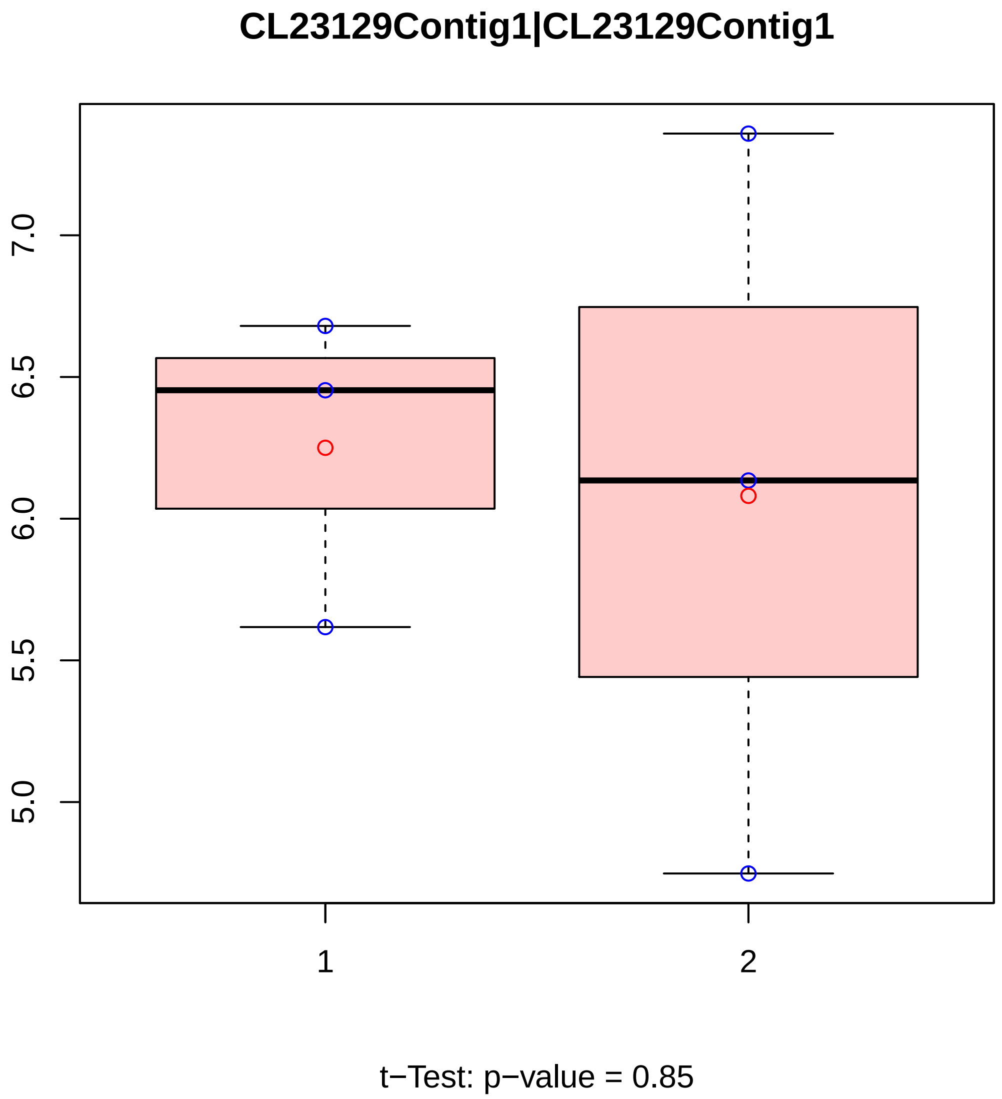

CL23129Contig1|CL23129Contig1
7.0
6.5
6.0
5.5
5.0
1
2
t−Test: p−value = 0.85

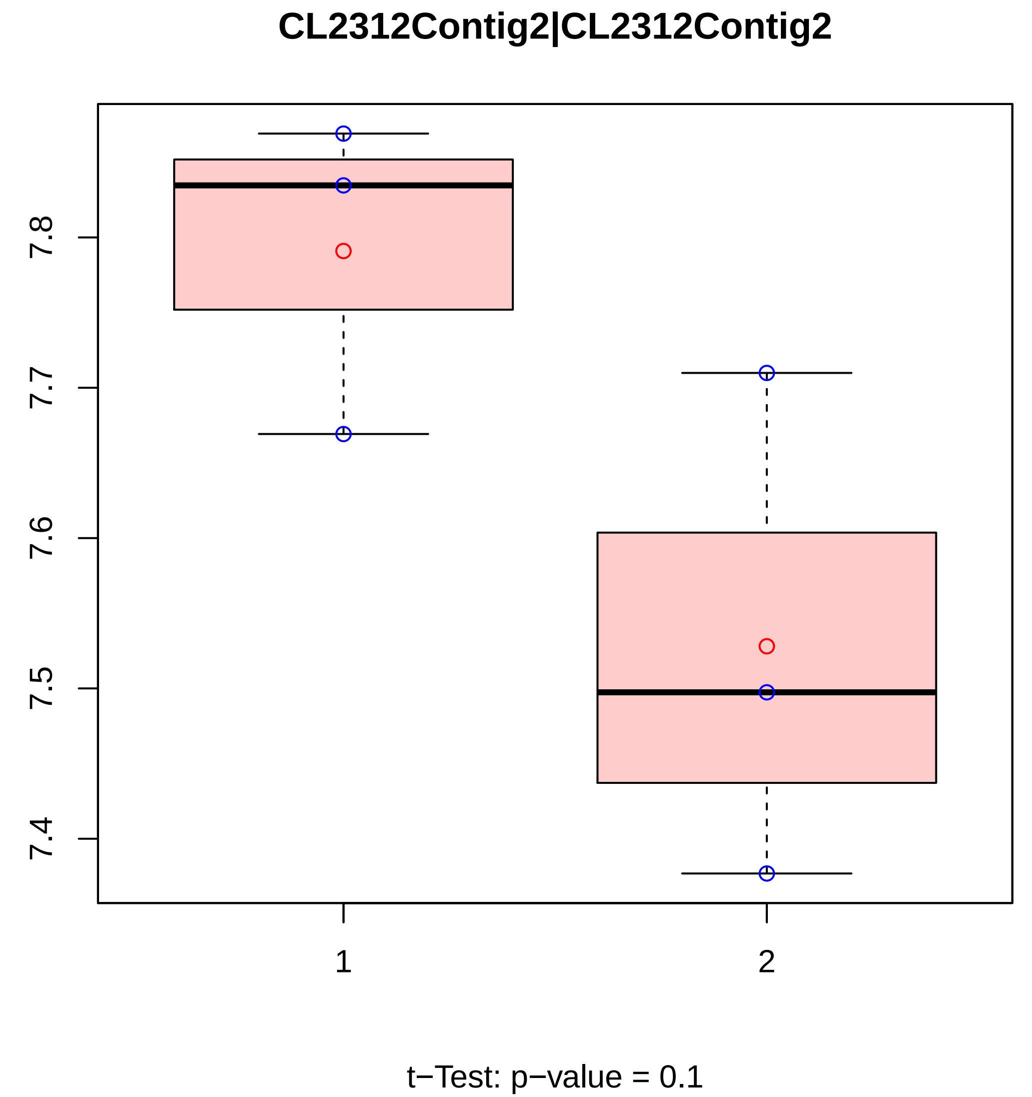

CL2312Contig2|CL2312Contig2
7.4
7.5
7.6
7.7
7.8
1
2
t−Test: p−value = 0.1

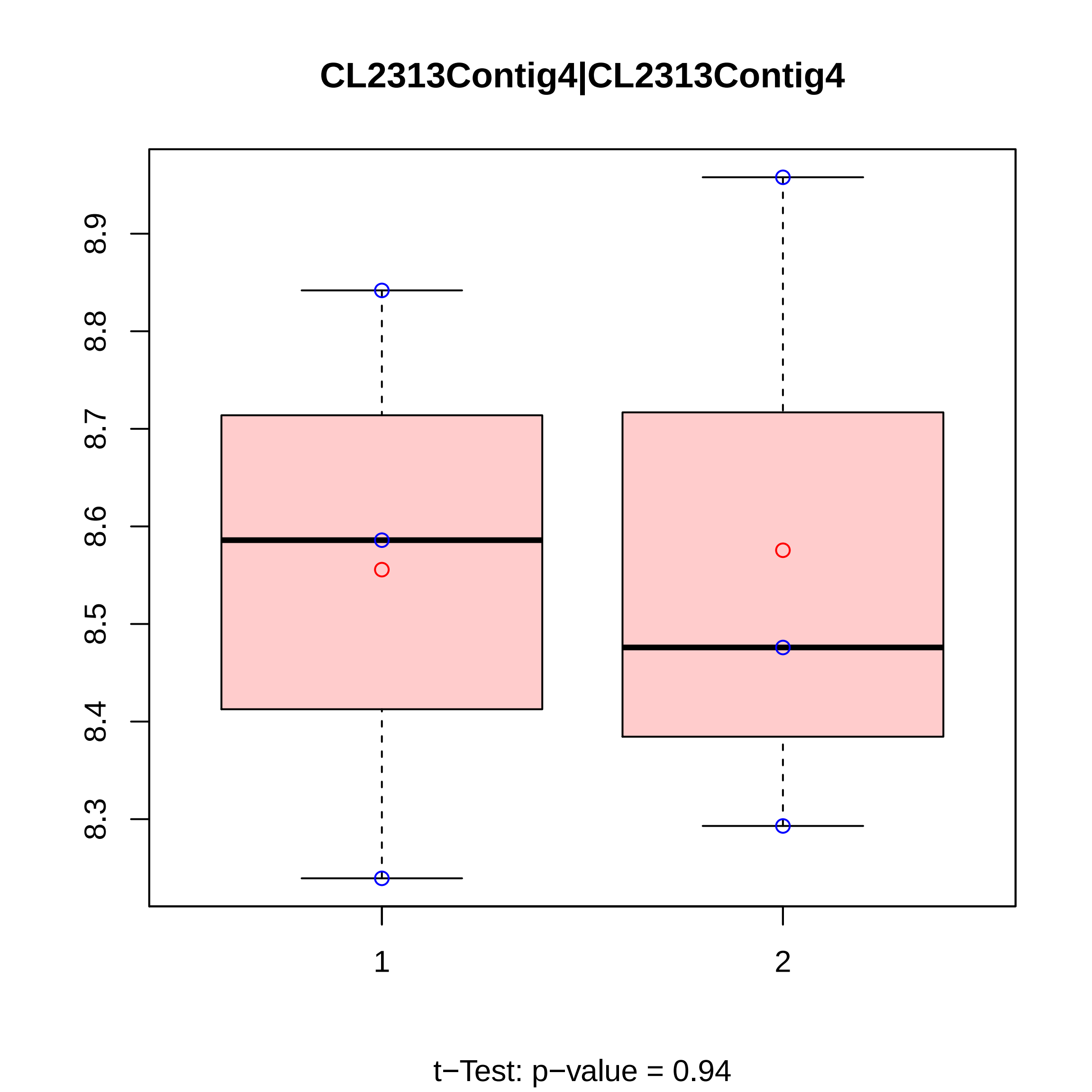

CL2313Contig4|CL2313Contig4
8.3
8.4
8.5
8.6
8.7
8.8
8.9
1
2
t−Test: p−value = 0.94

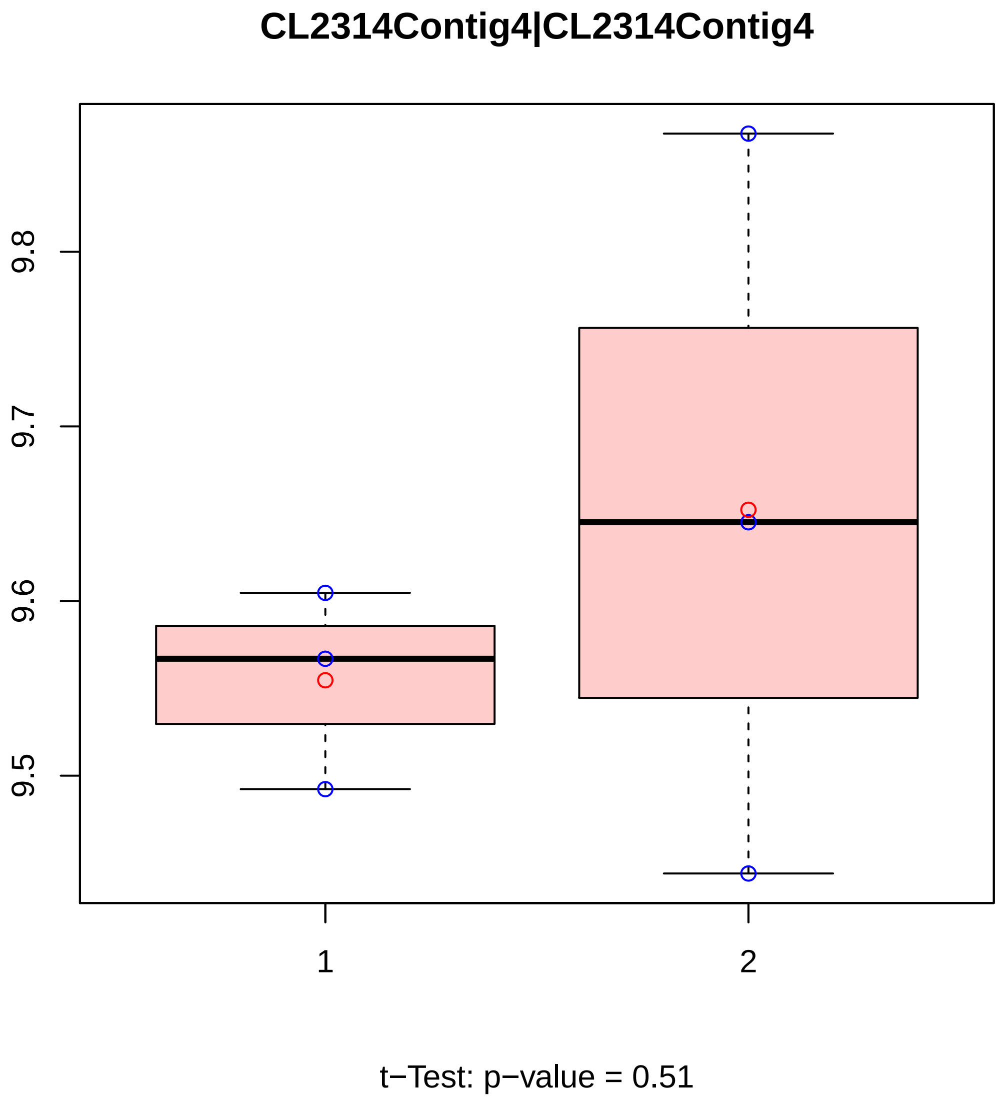

CL2314Contig4|CL2314Contig4
9.5
9.6
9.7
9.8
1
2
t−Test: p−value = 0.51

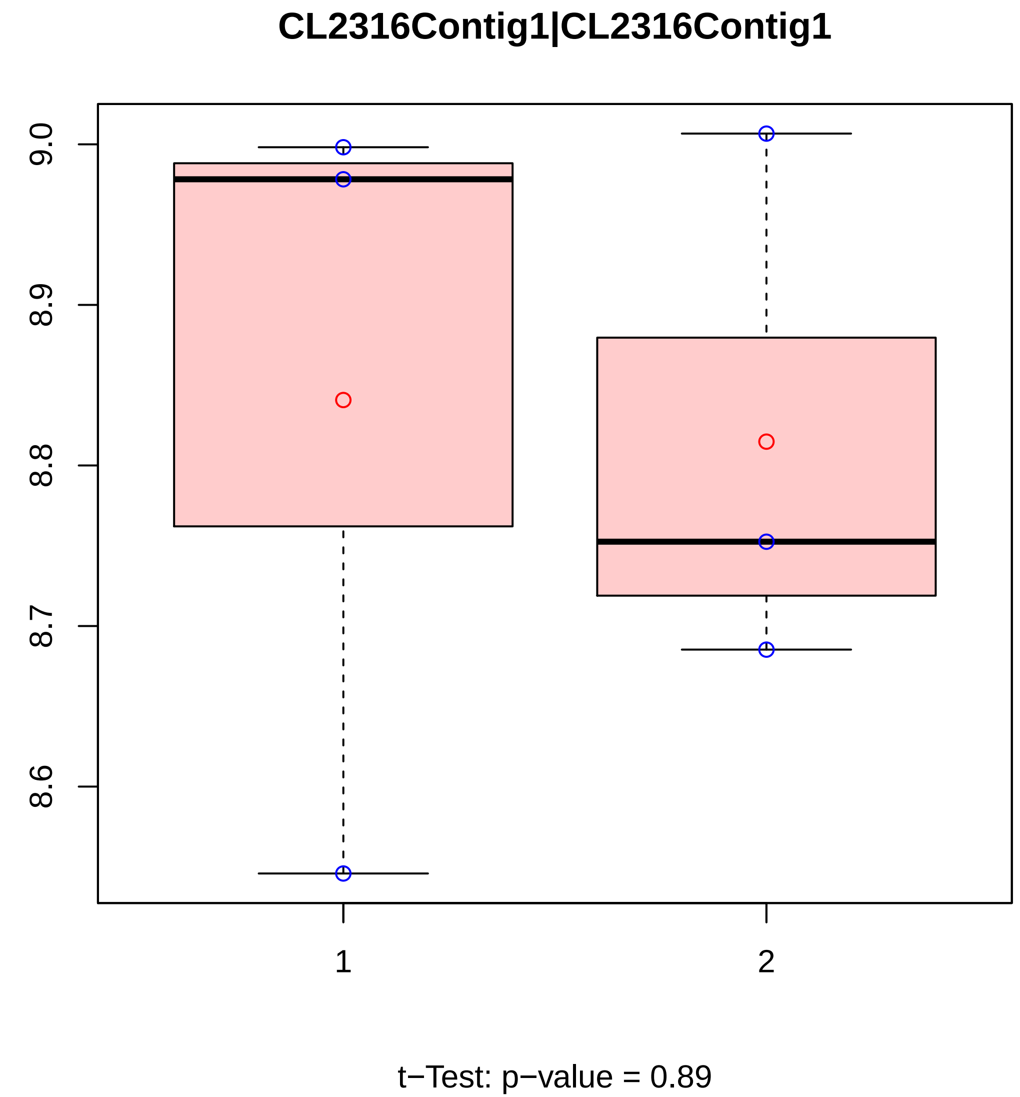

CL2316Contig1|CL2316Contig1
9.0
8.9
8.8
8.7
8.6
1
2
t−Test: p−value = 0.89

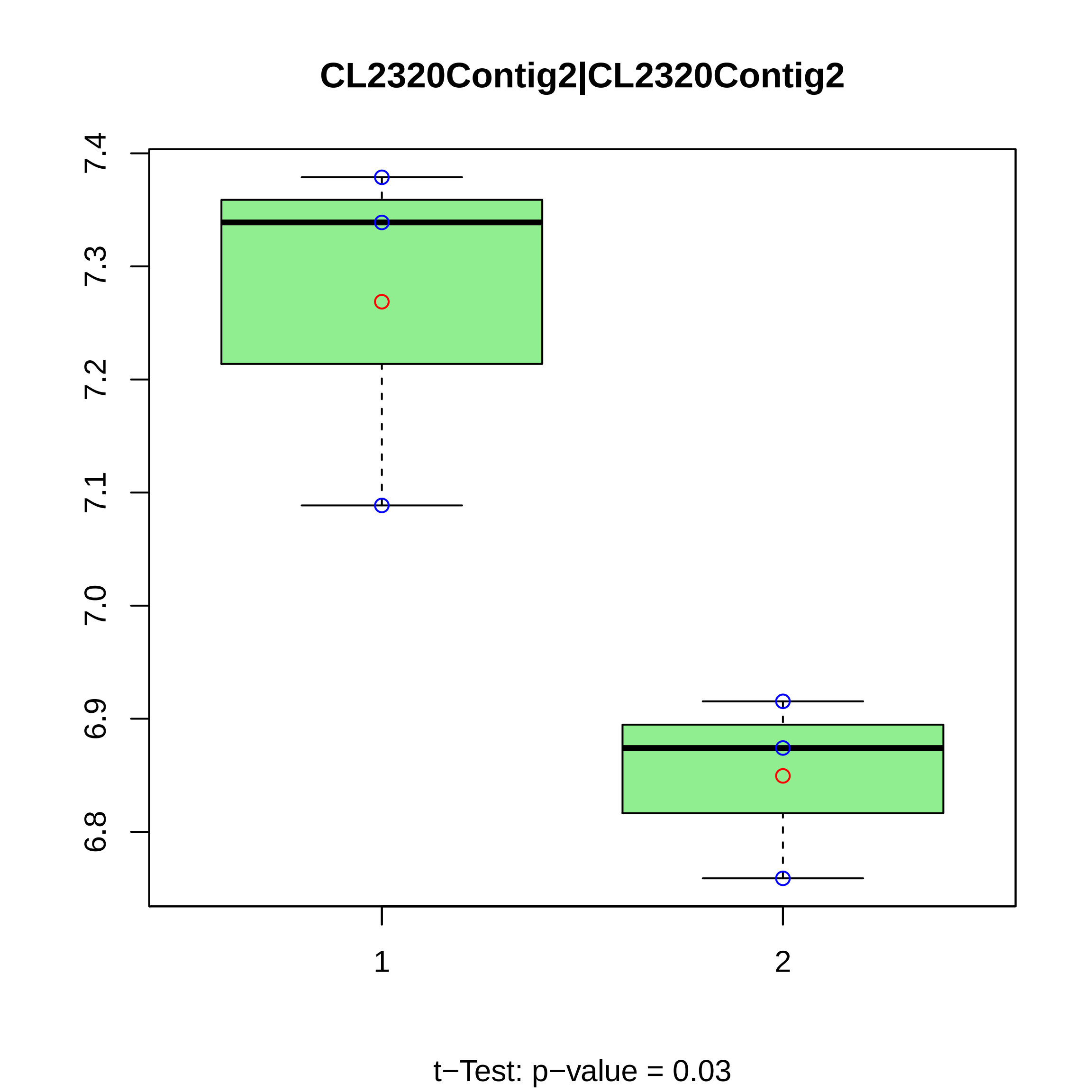
CL2320Contig2|CL2320Contig2
7.4
7.3
7.2
7.1
7.0
6.9
6.8
1
2
t−Test: p−value = 0.03

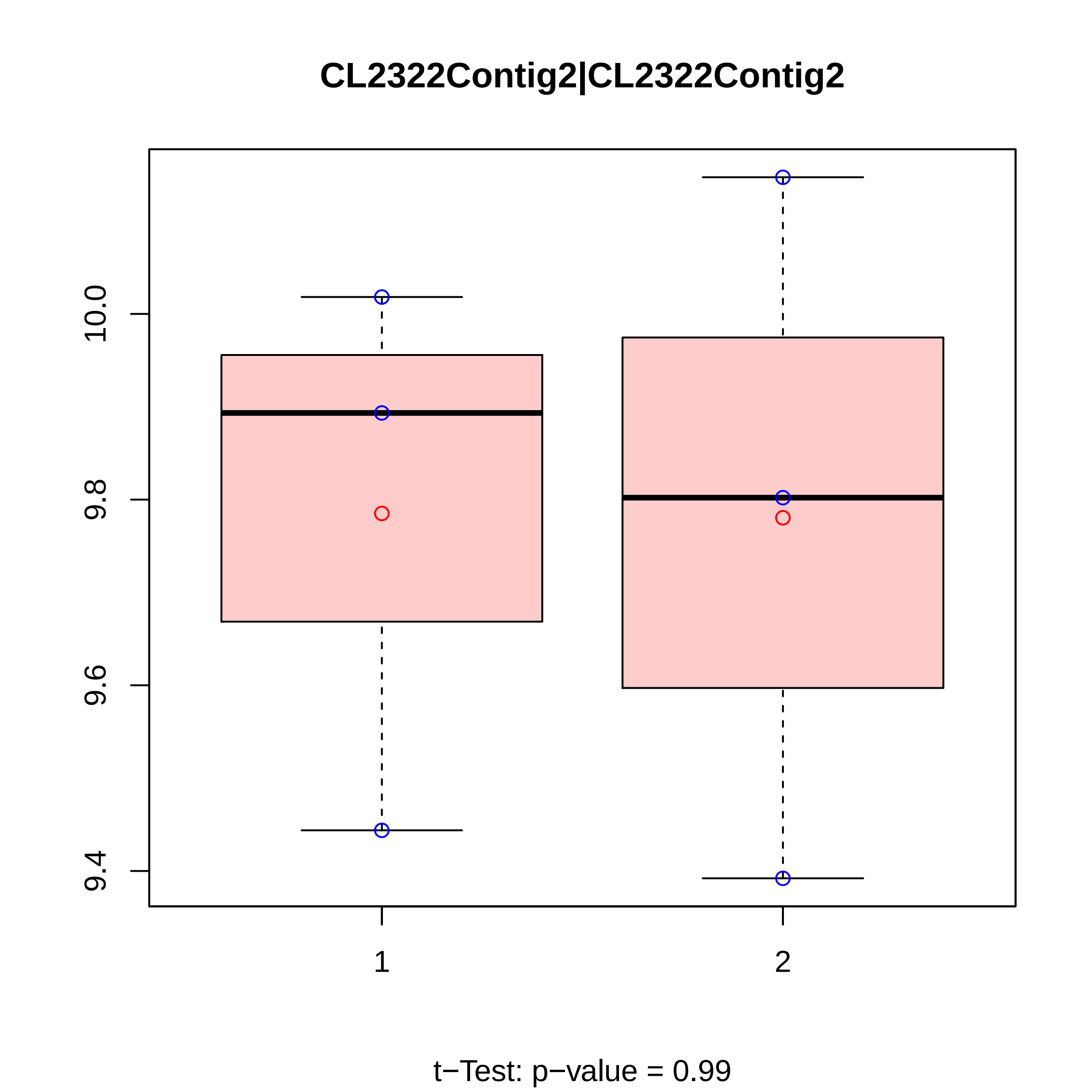

CL2322Contig2|CL2322Contig2
10.0
9.8
9.6
9.4
1
2
t−Test: p−value = 0.99

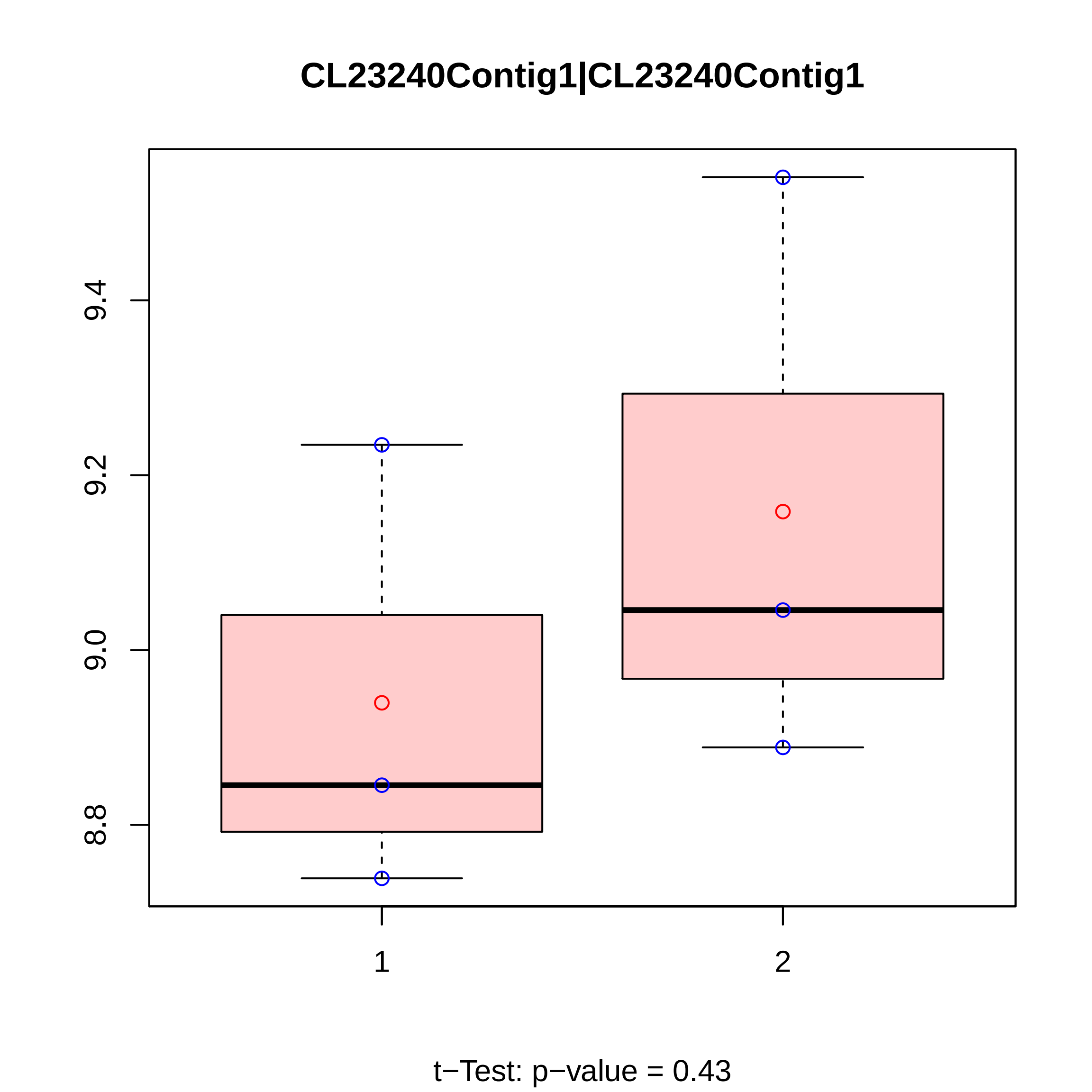
CL23240Contig1|CL23240Contig1
9.4
9.2
9.0
8.8
1
2
t−Test: p−value = 0.43

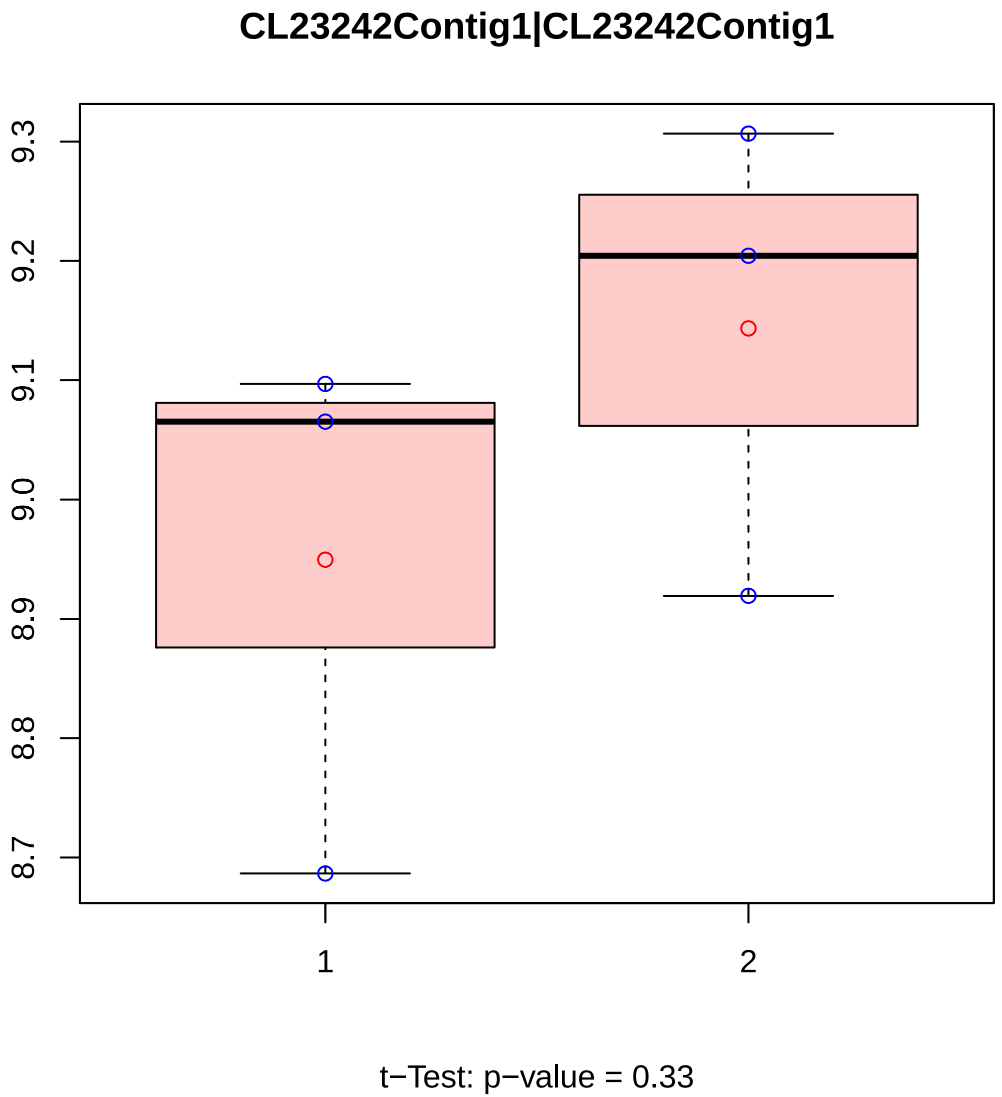

CL23242Contig1|CL23242Contig1
9.3
9.2
9.1
9.0
8.9
8.8
8.7
1
2
t−Test: p−value = 0.33

# CL23265Contig1|CL23265Contig1

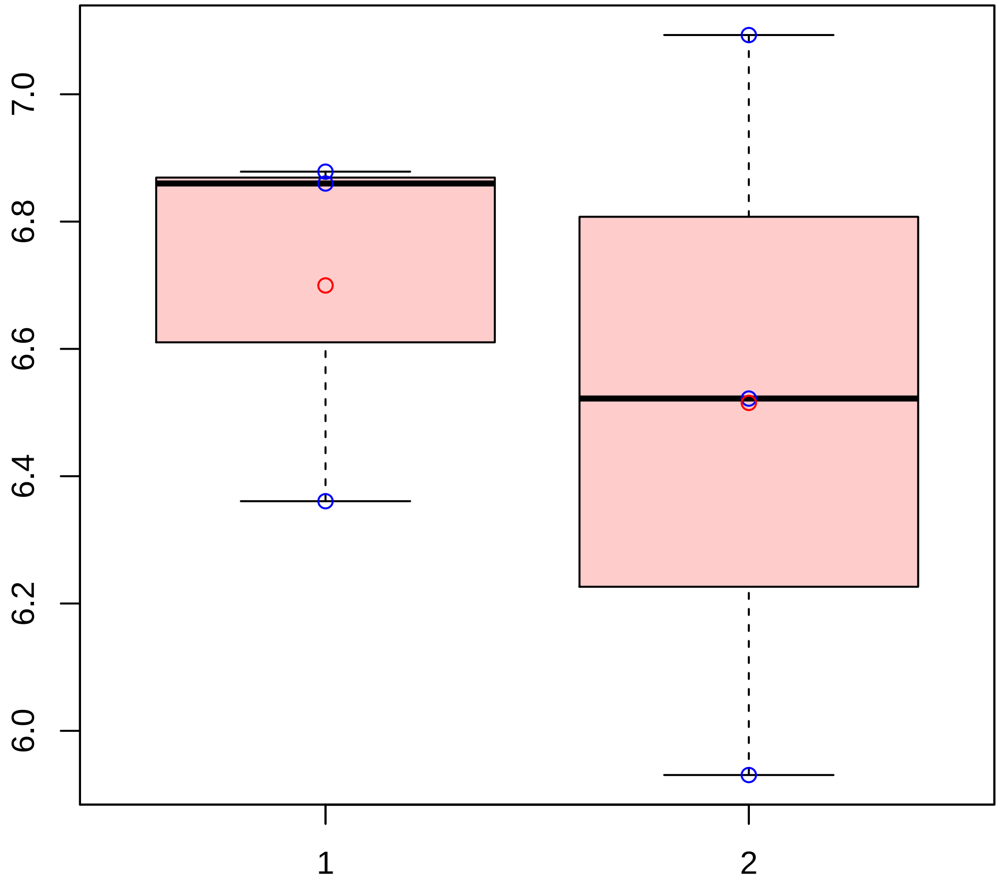


t−Test: p−value = 0.66

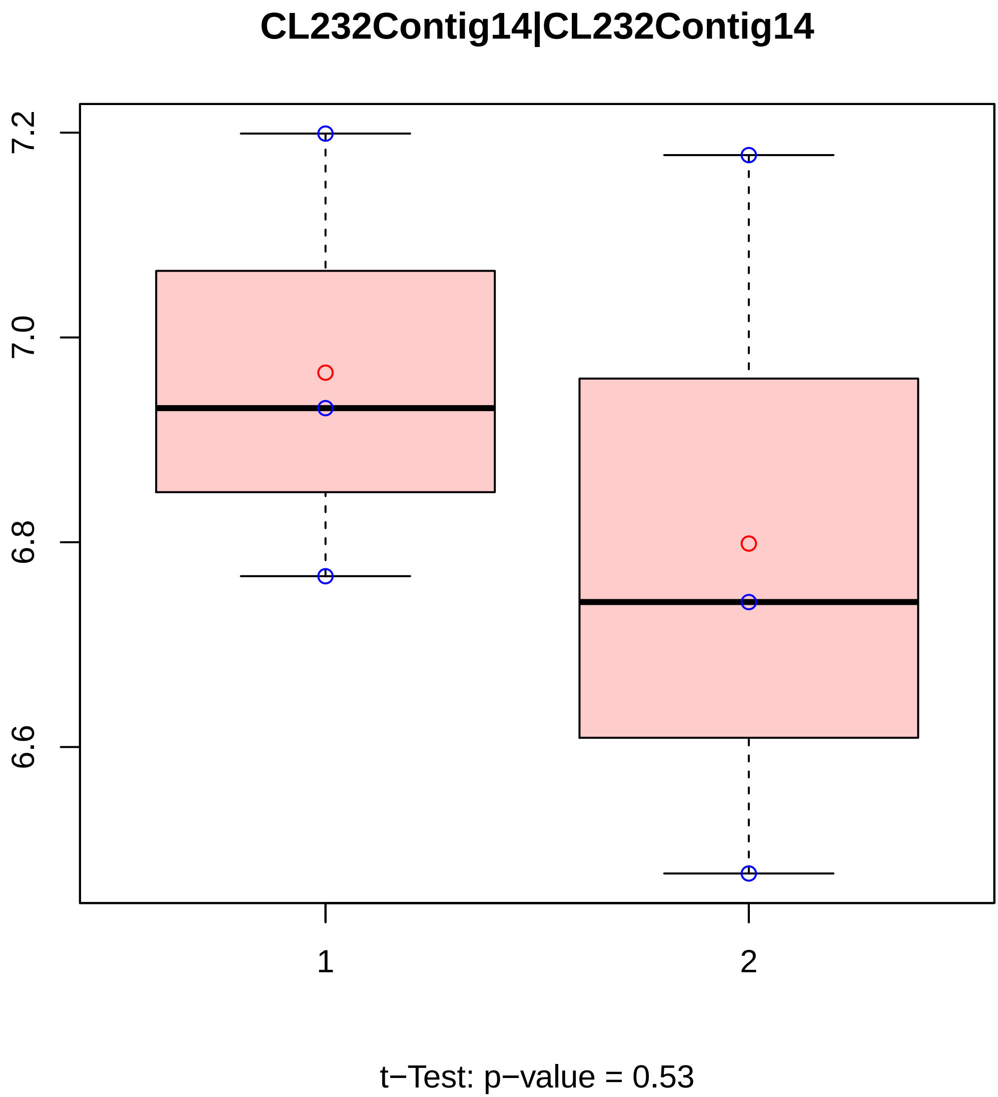

CL232Contig14|CL232Contig14
7.2
7.0
6.8
6.6
1
2
t−Test: p−value = 0.53

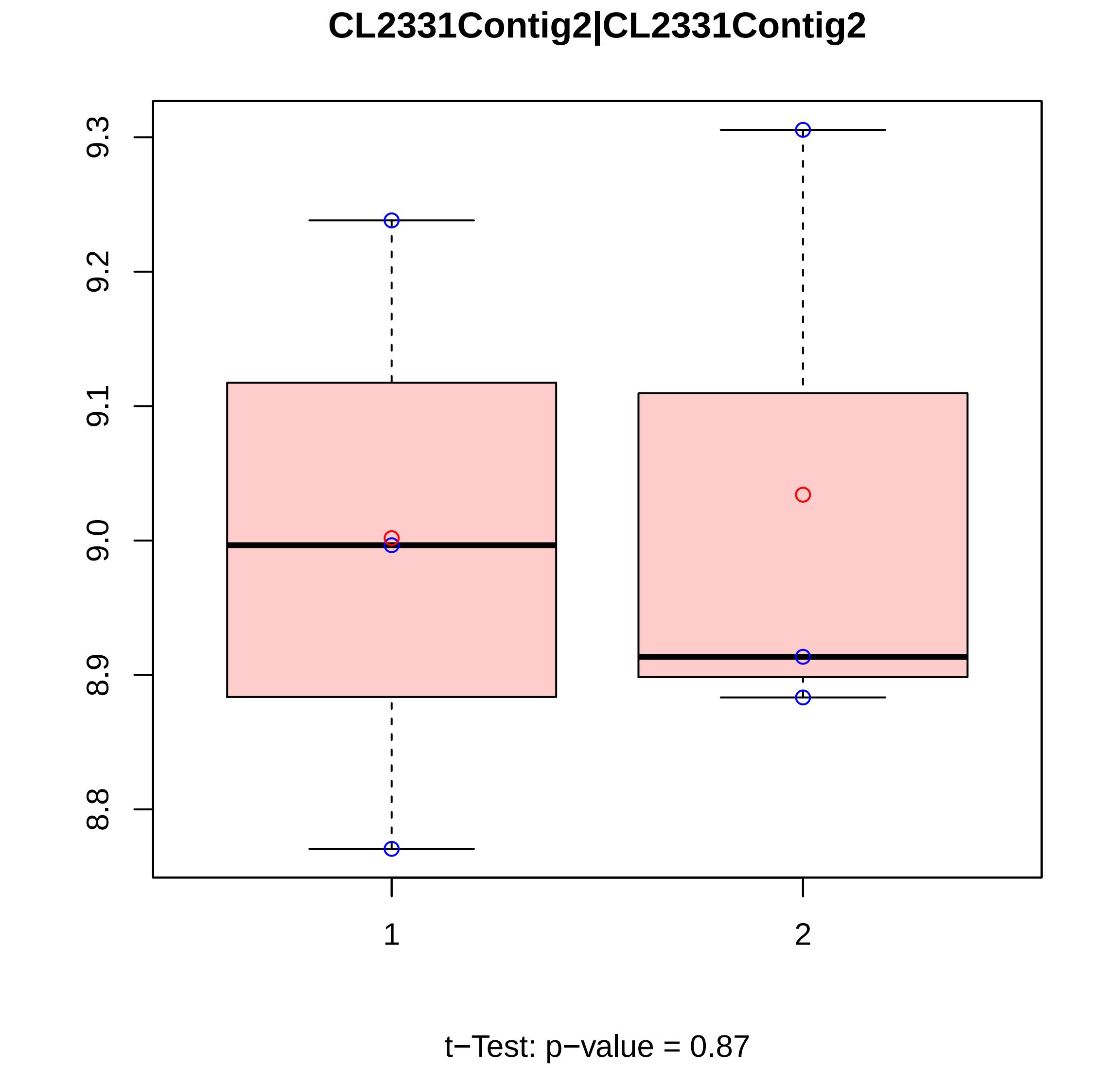

CL2331Contig2|CL2331Contig2
9.3
9.2
9.1
9.0
8.9
8.8
1
2
t−Test: p−value = 0.87

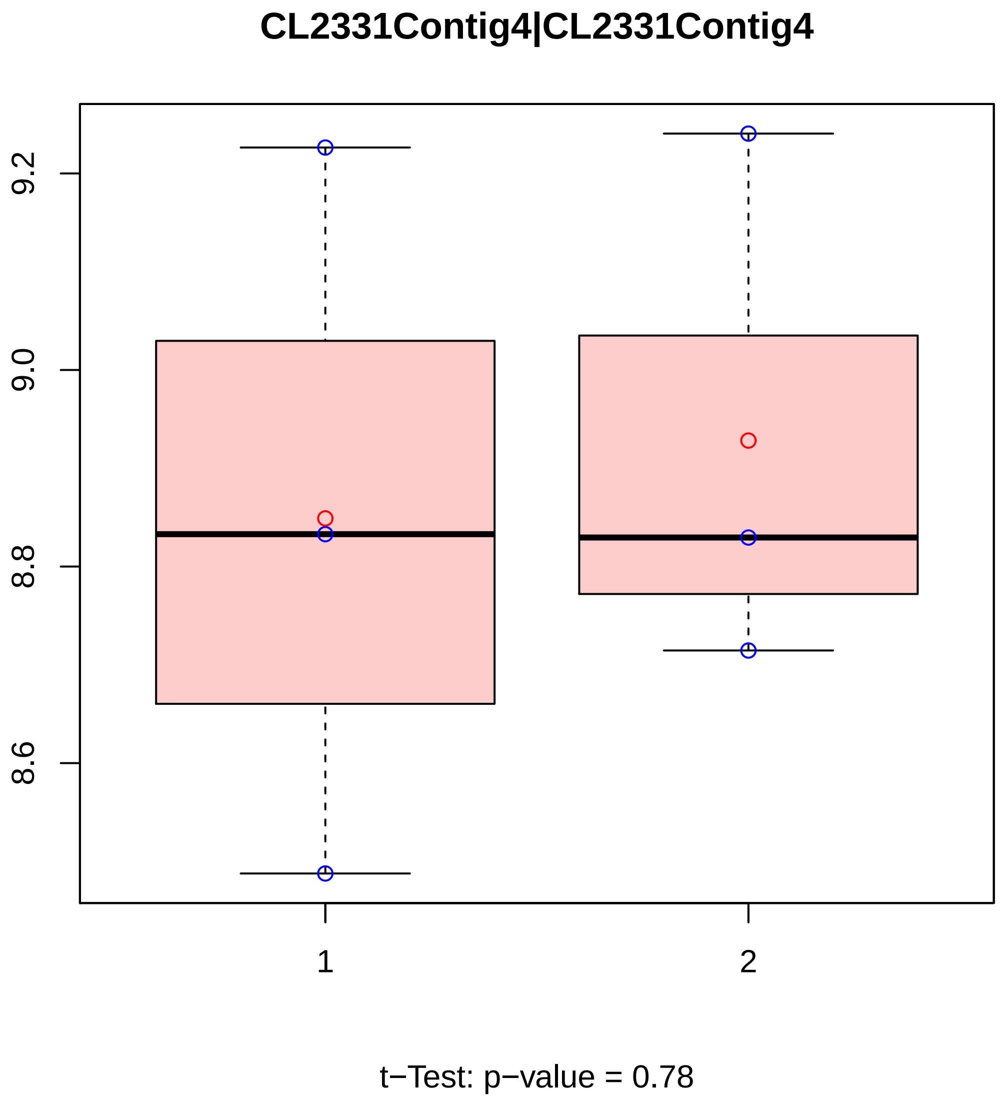

CL2331Contig4|CL2331Contig4
9.2
9.0
8.8
8.6
1
2
t−Test: p−value = 0.78

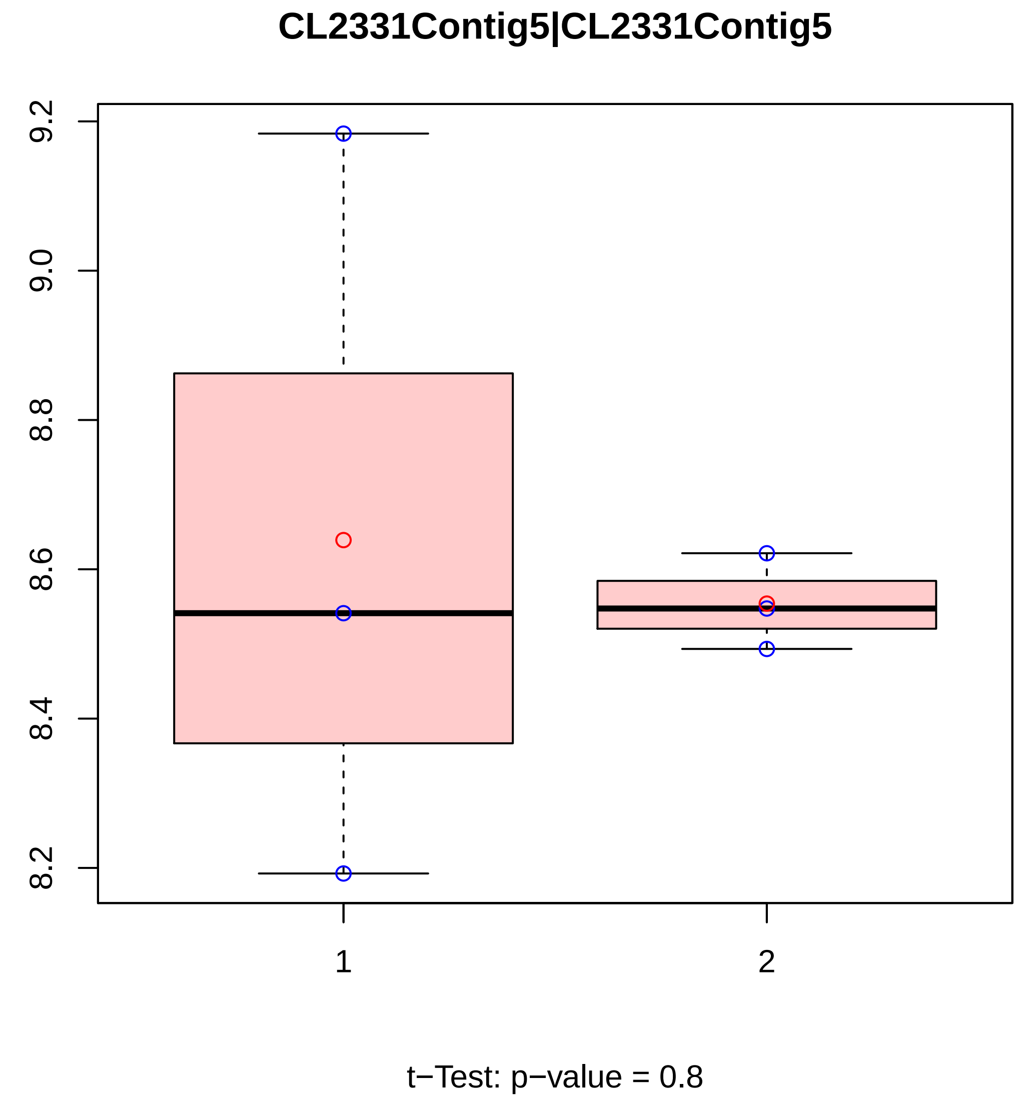
CL2331Contig5|CL2331Contig5
9.2
9.0
8.8
8.6
8.4
8.2
1
2
t−Test: p−value = 0.8

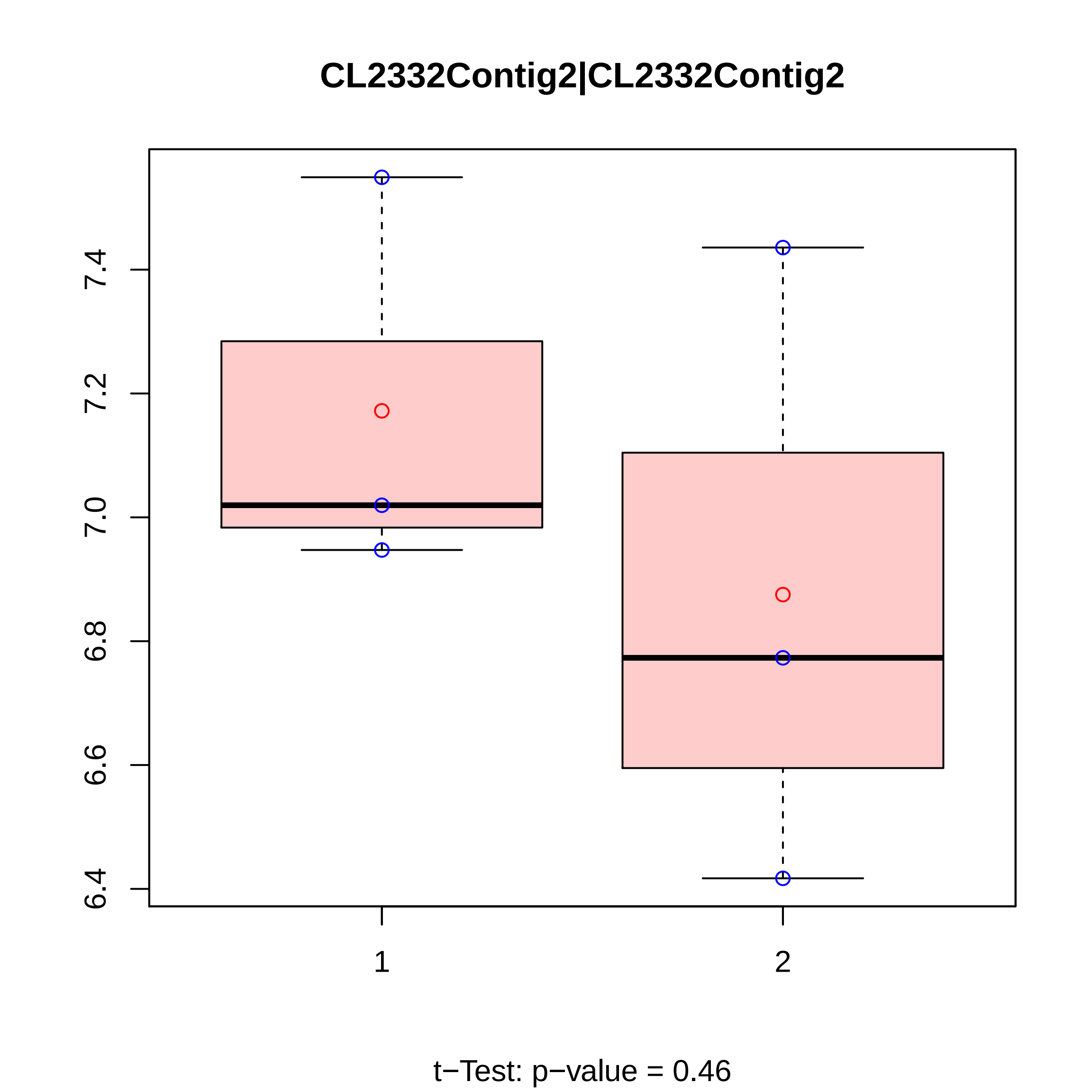

CL2332Contig2|CL2332Contig2
7.4
7.2
7.0
6.8
6.6
6.4
1
2
t−Test: p−value = 0.46

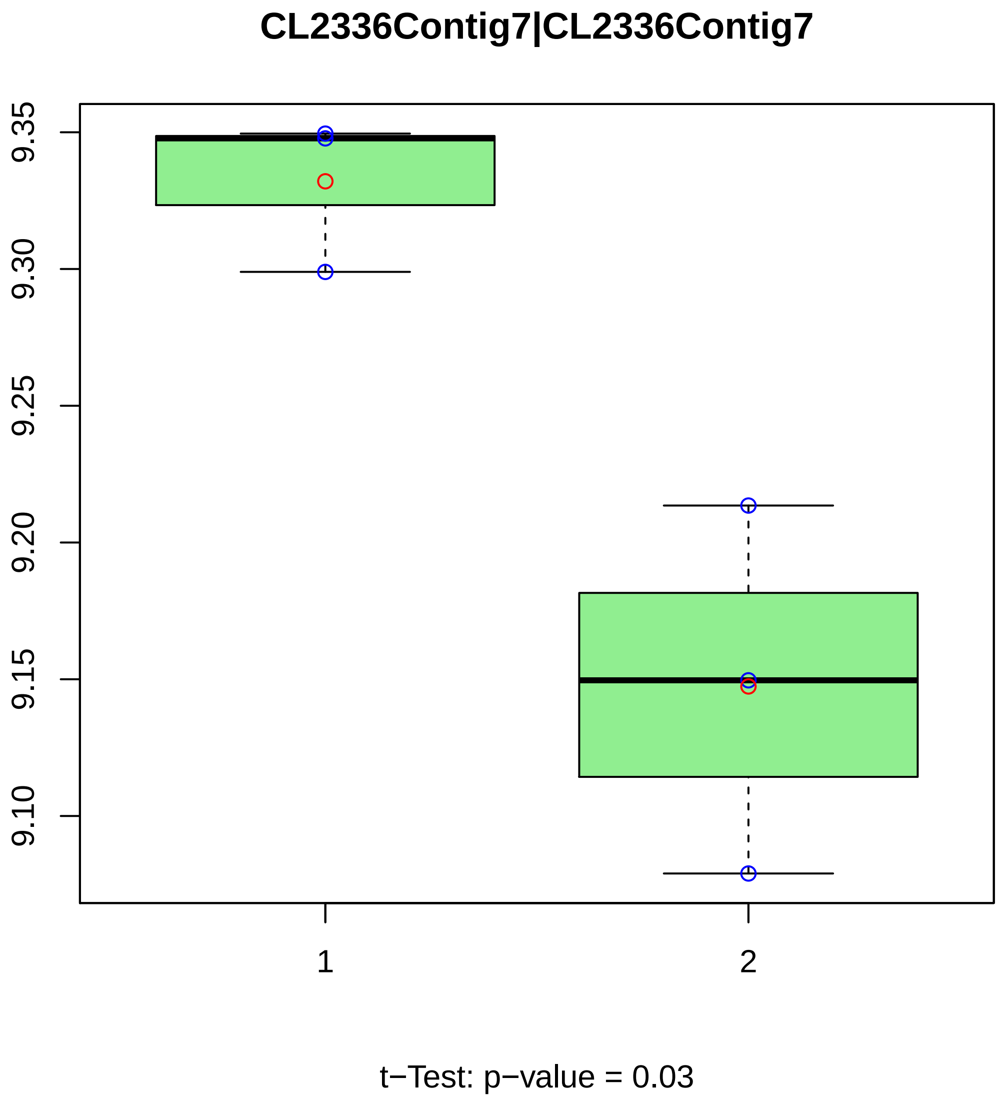

CL2336Contig7|CL2336Contig7
9.35
9.30
9.25
9.20
9.15
9.10
1
2
t−Test: p−value = 0.03

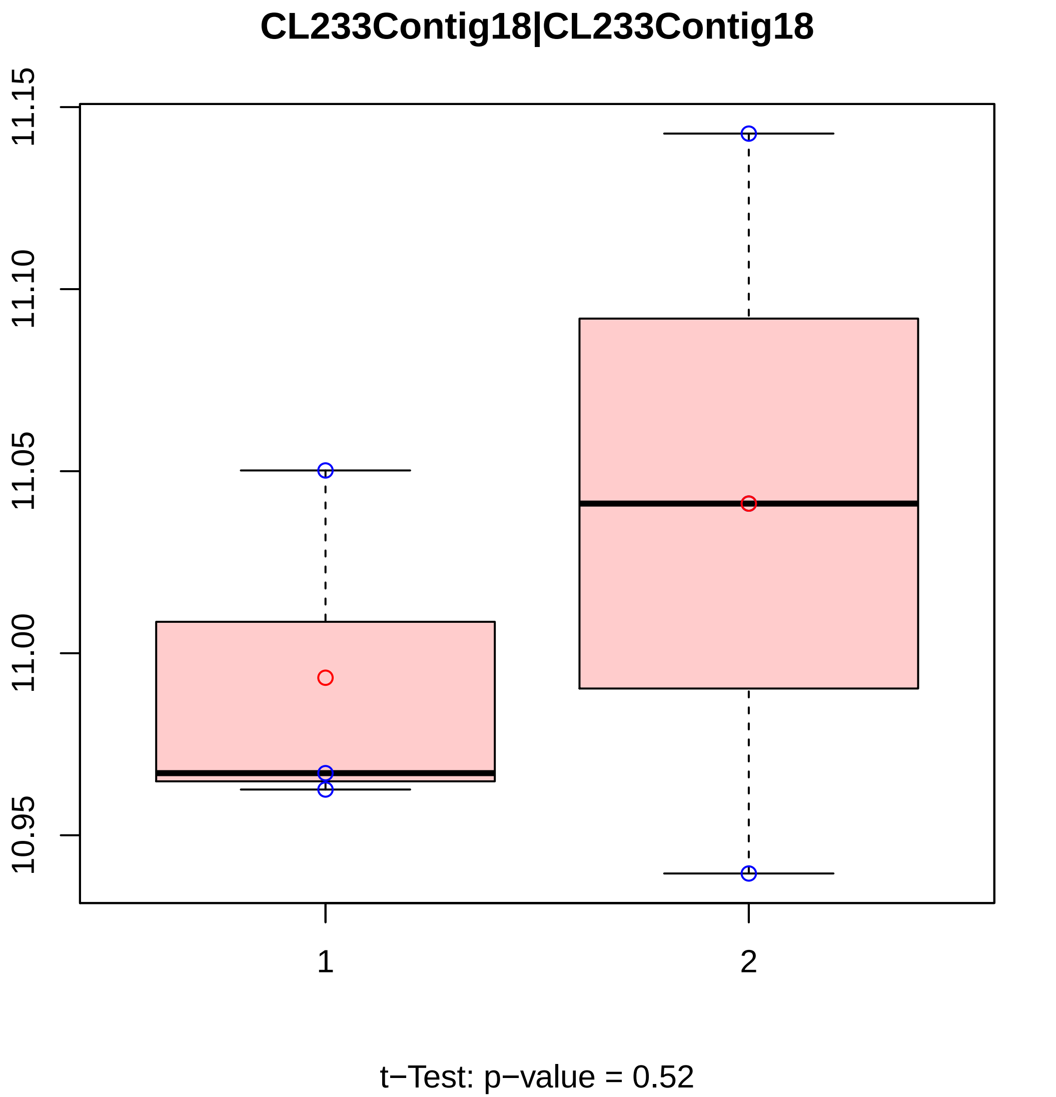

CL233Contig18|CL233Contig18
11.15
11.10
11.05
11.00
10.95
1
2
t−Test: p−value = 0.52

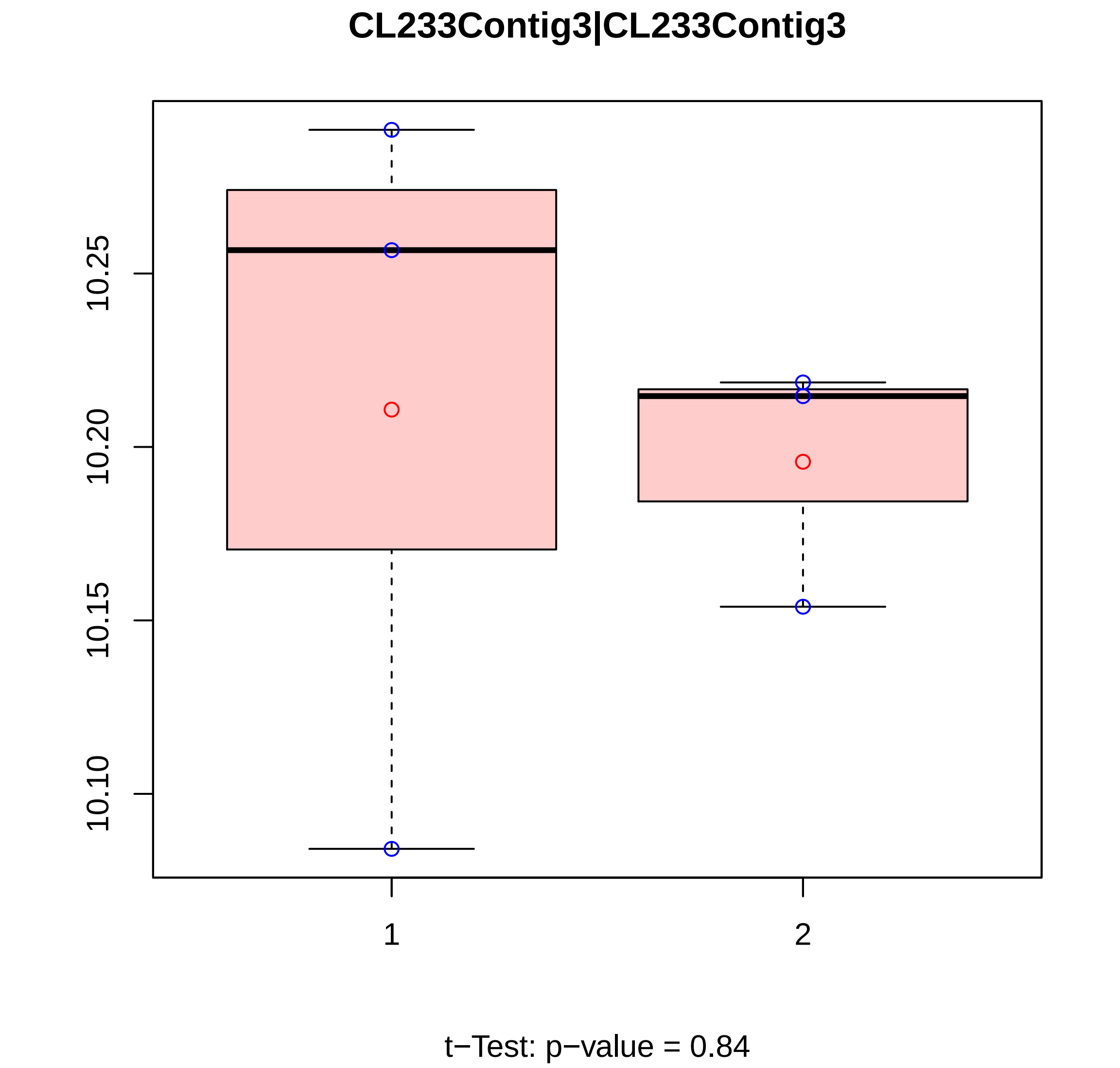
CL233Contig3|CL233Contig3
10.10
10.15
10.20
10.25
1
2
t−Test: p−value = 0.84

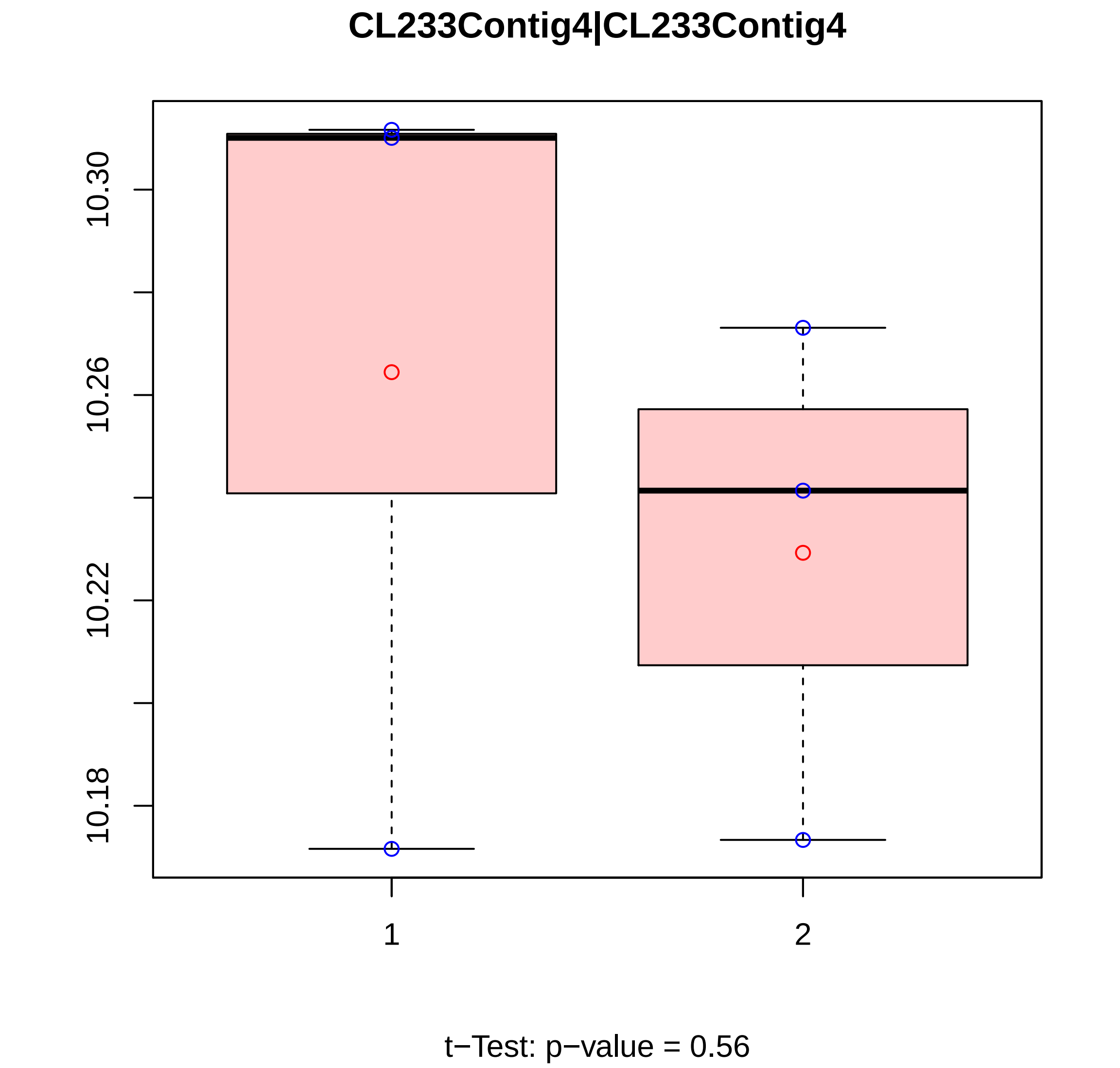

CL233Contig4|CL233Contig4
10.30
10.26
10.22
10.18
1
2
t−Test: p−value = 0.56

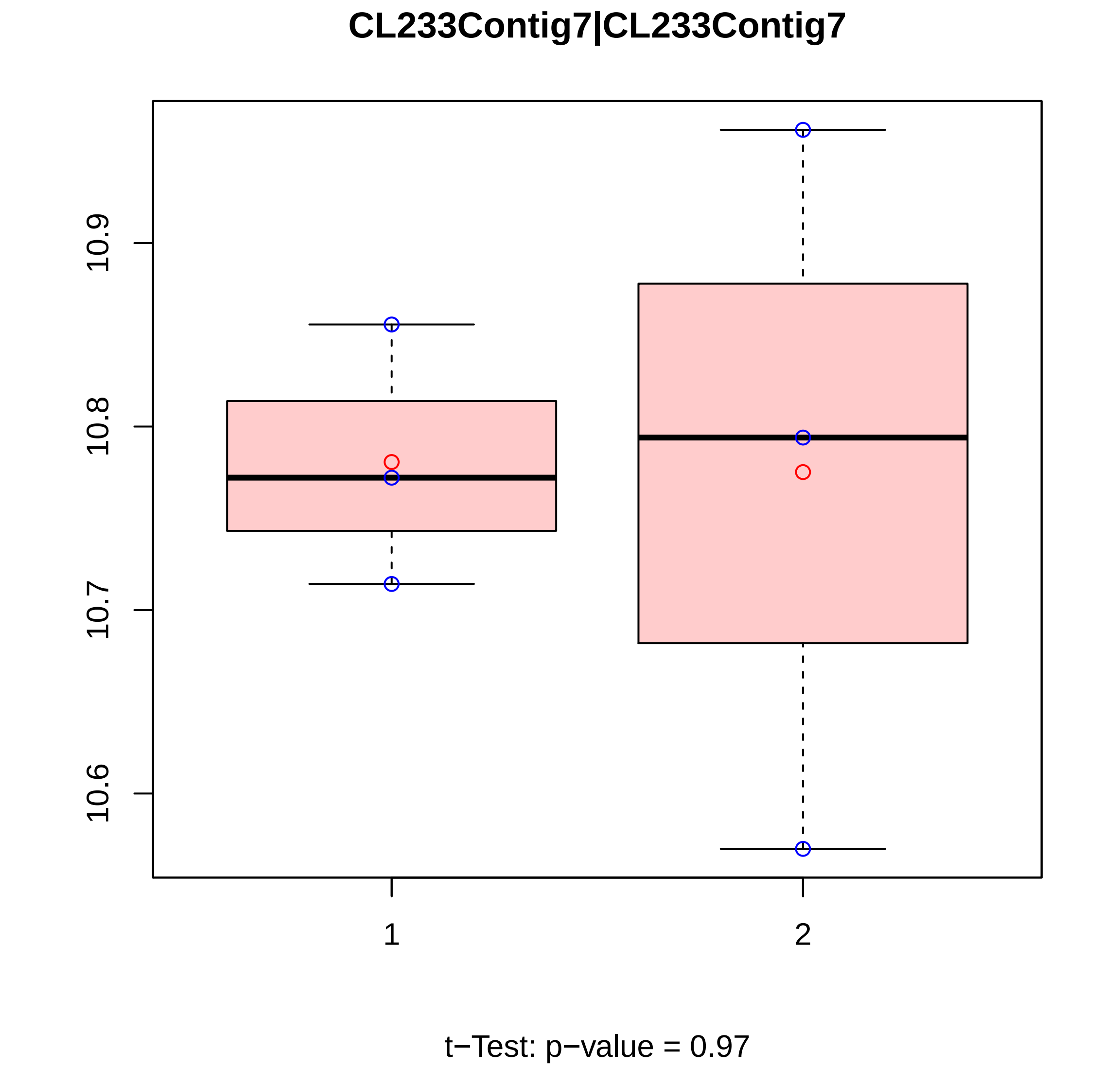
CL233Contig7|CL233Contig7
10.6
10.7
10.8
10.9
1
2
t−Test: p−value = 0.97

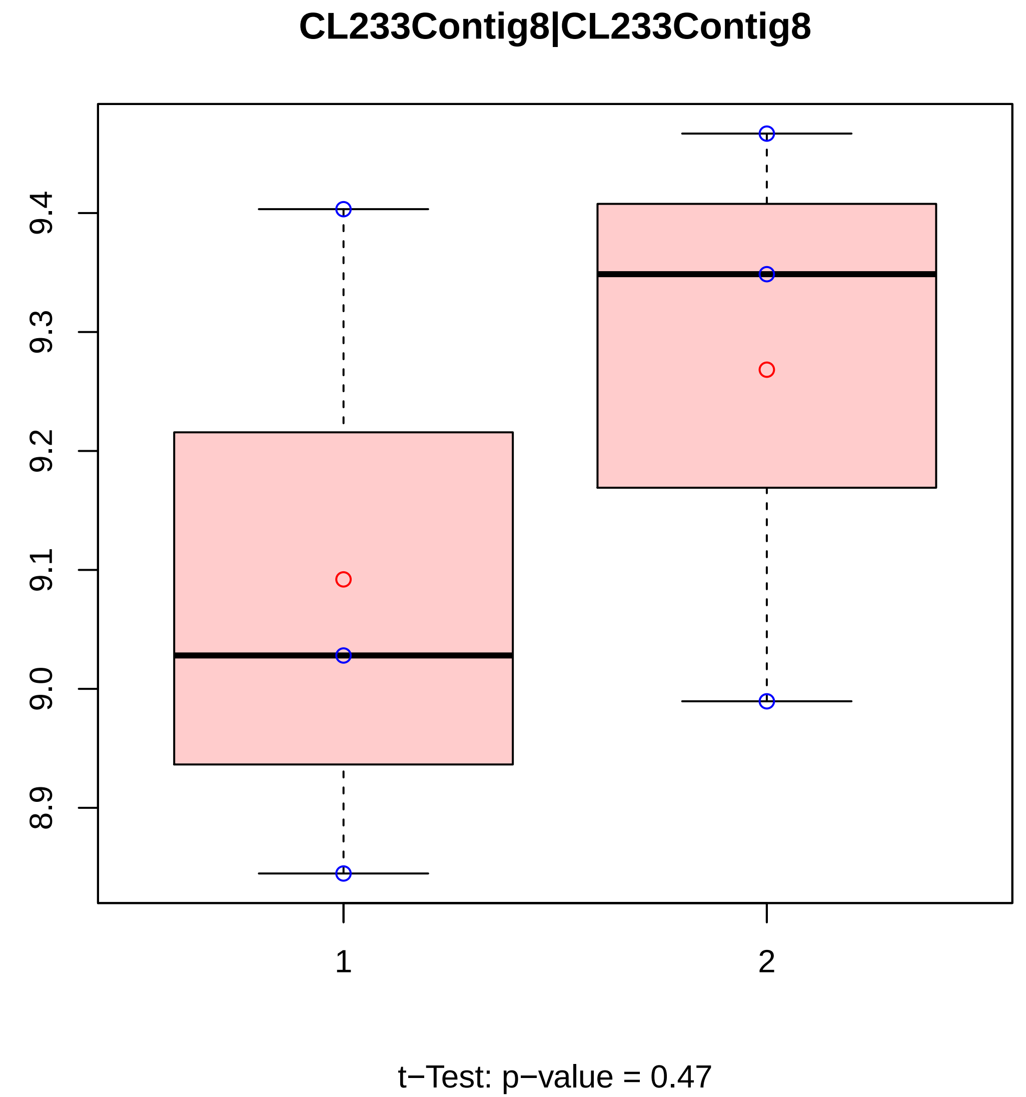

CL233Contig8|CL233Contig8
9.4
9.3
9.2
9.1
9.0
8.9
1
2
t−Test: p−value = 0.47

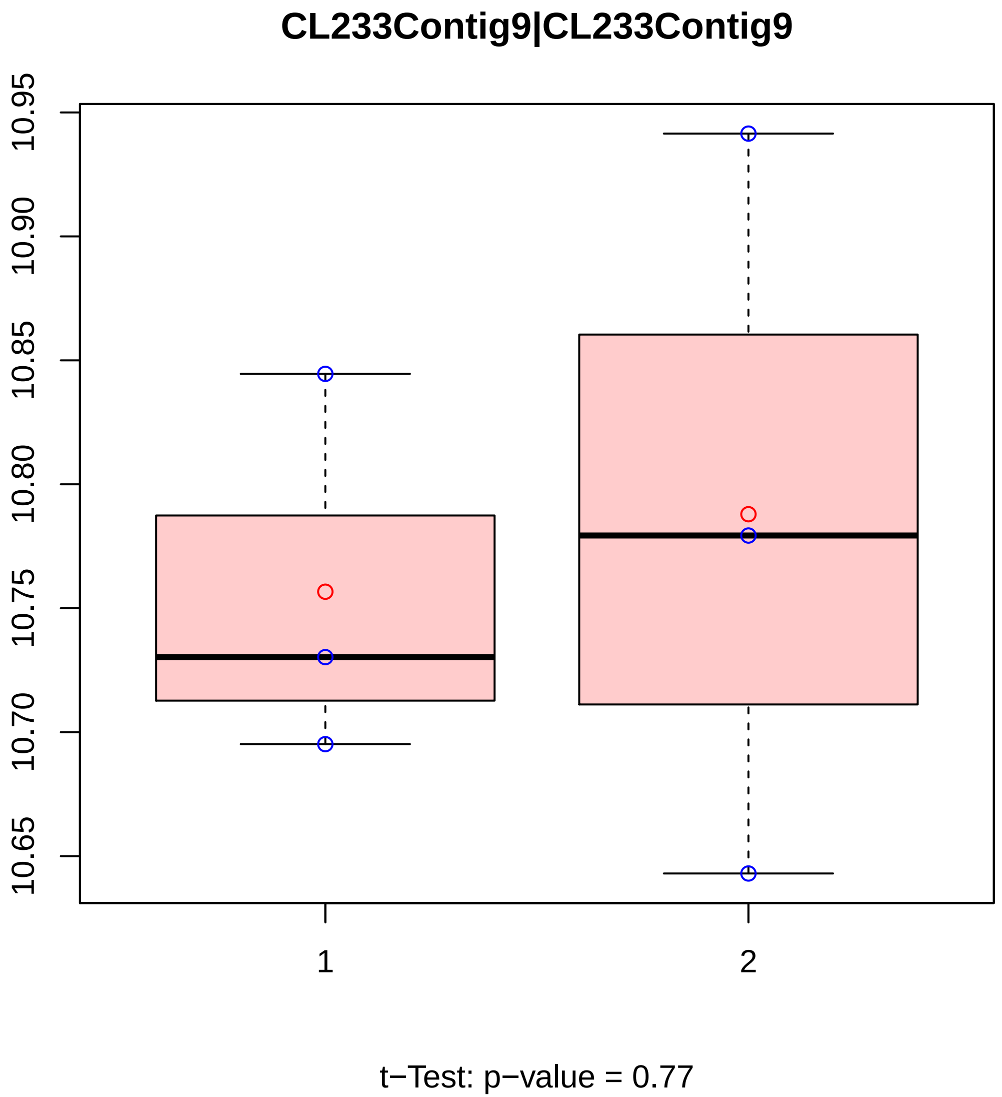

CL233Contig9|CL233Contig9
10.95
10.90
10.85
10.80
10.75
10.70
10.65
1
2
t−Test: p−value = 0.77

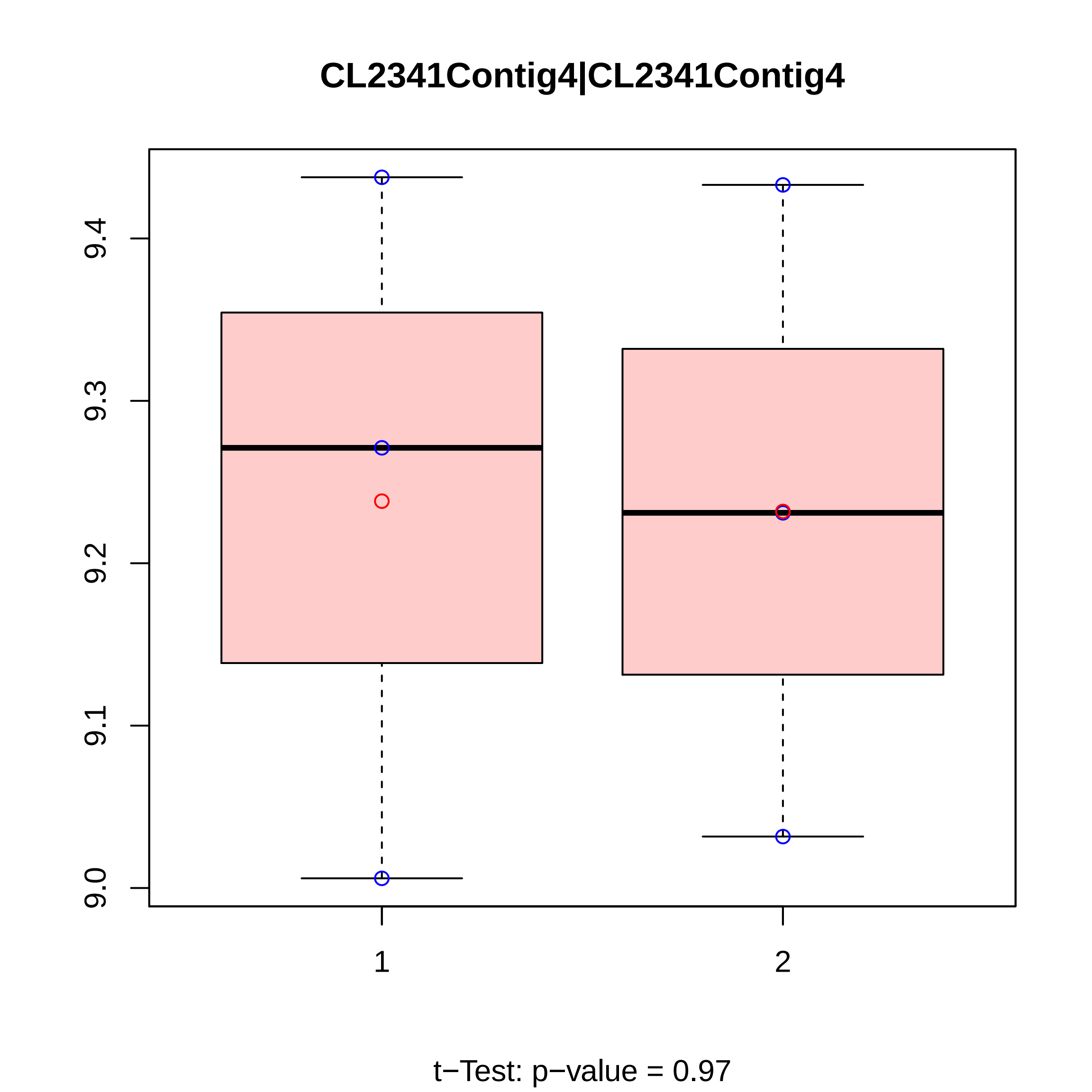

CL2341Contig4|CL2341Contig4
9.0
9.1
9.2
9.3
9.4
1
2
t−Test: p−value = 0.97

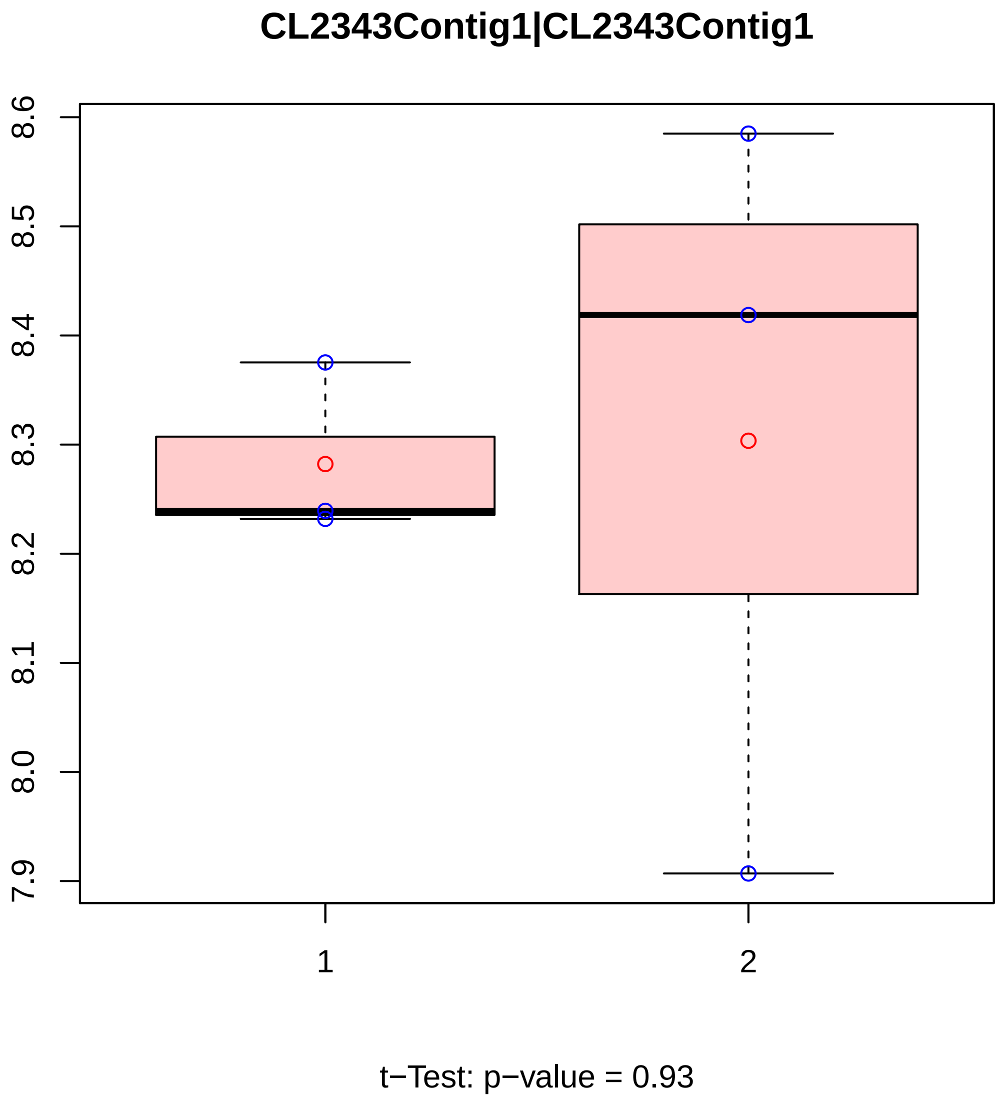

CL2343Contig1|CL2343Contig1
8.6
8.5
8.4
8.3
8.2
8.1
8.0
7.9
1
2
t−Test: p−value = 0.93

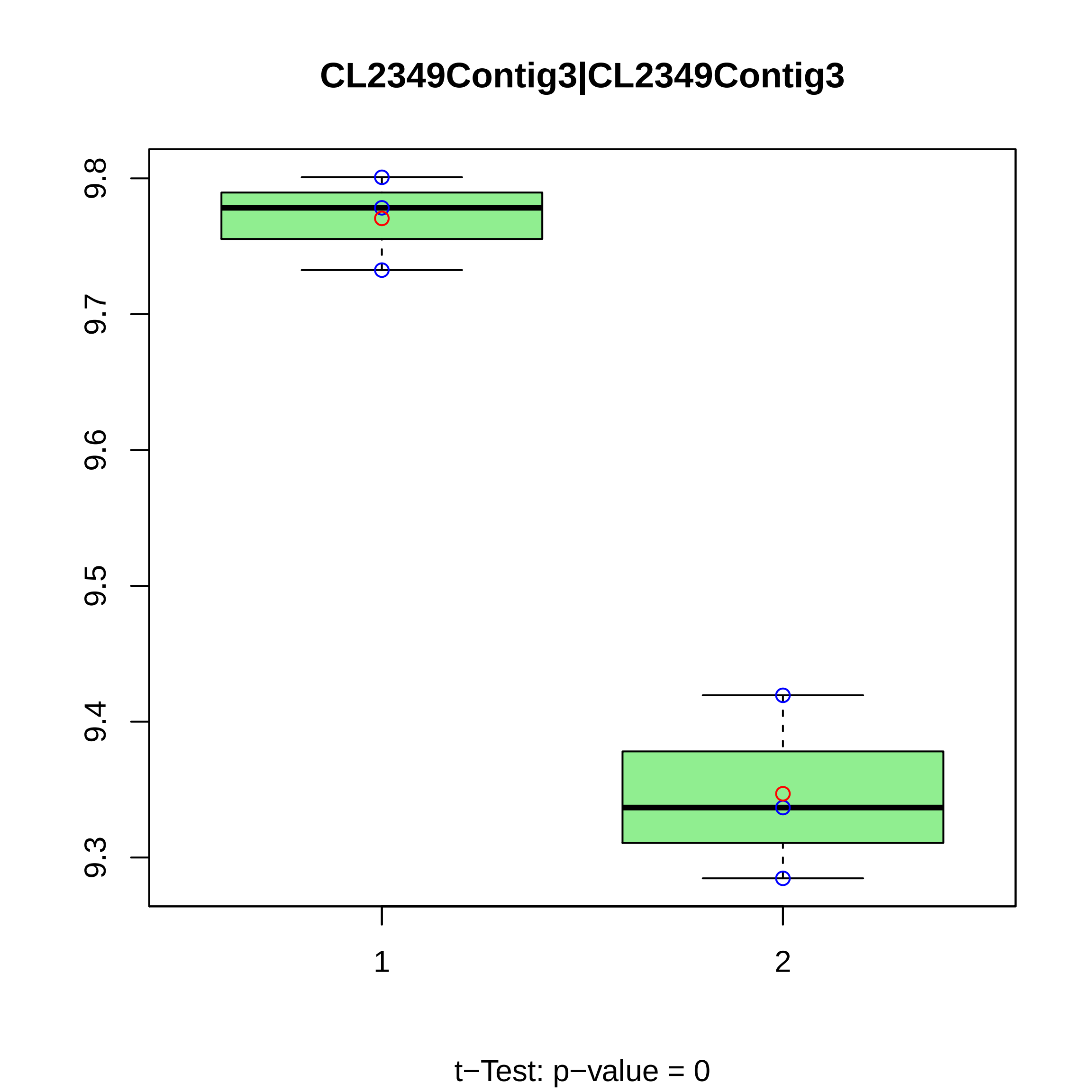

CL2349Contig3|CL2349Contig3
9.8
9.7
9.6
9.5
9.4
9.3
1
2
t−Test: p−value = 0

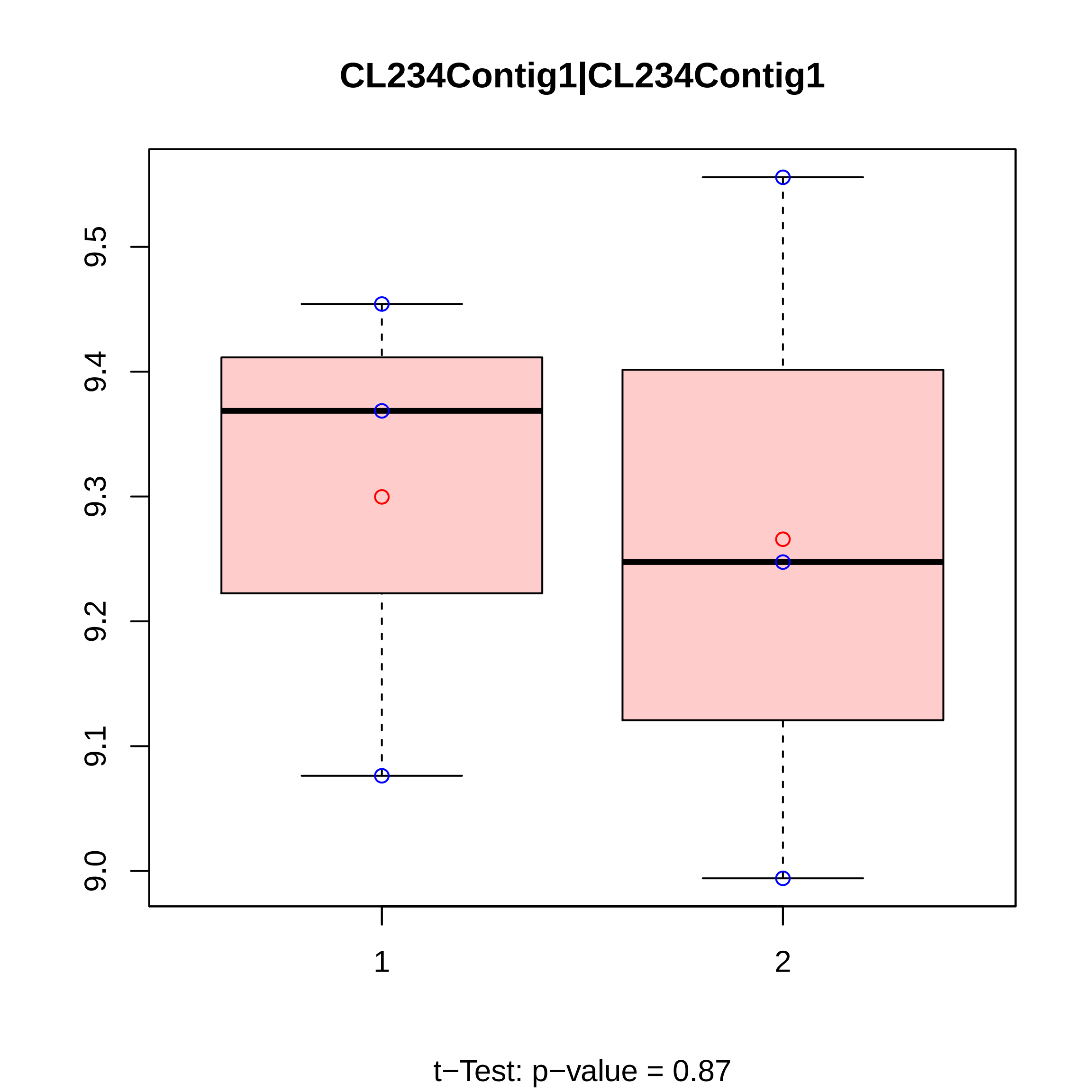

CL234Contig1|CL234Contig1
9.0
9.1
9.2
9.3
9.4
9.5
1
2
t−Test: p−value = 0.87

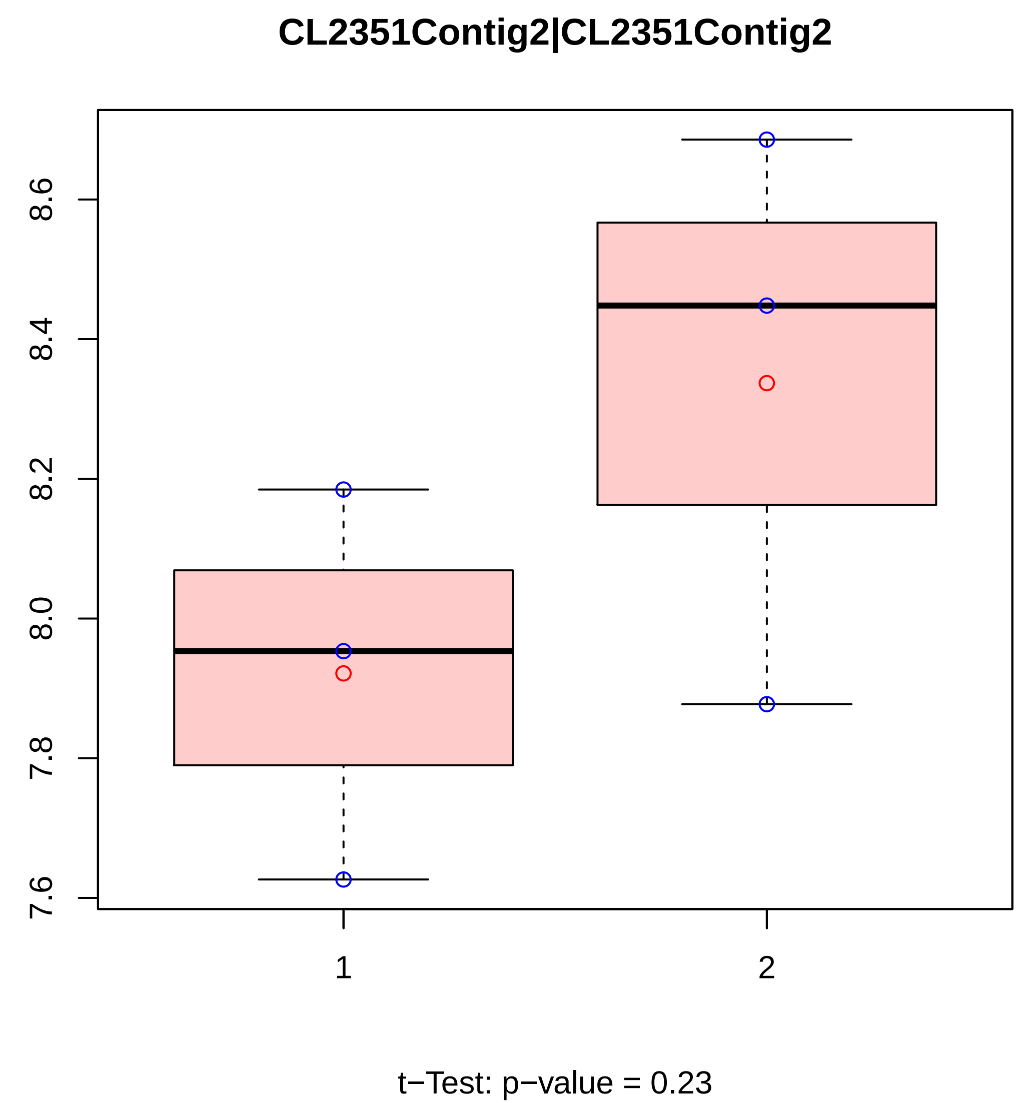

CL2351Contig2|CL2351Contig2
7.6
7.8
8.0
8.2
8.4
8.6
1
2
t−Test: p−value = 0.23

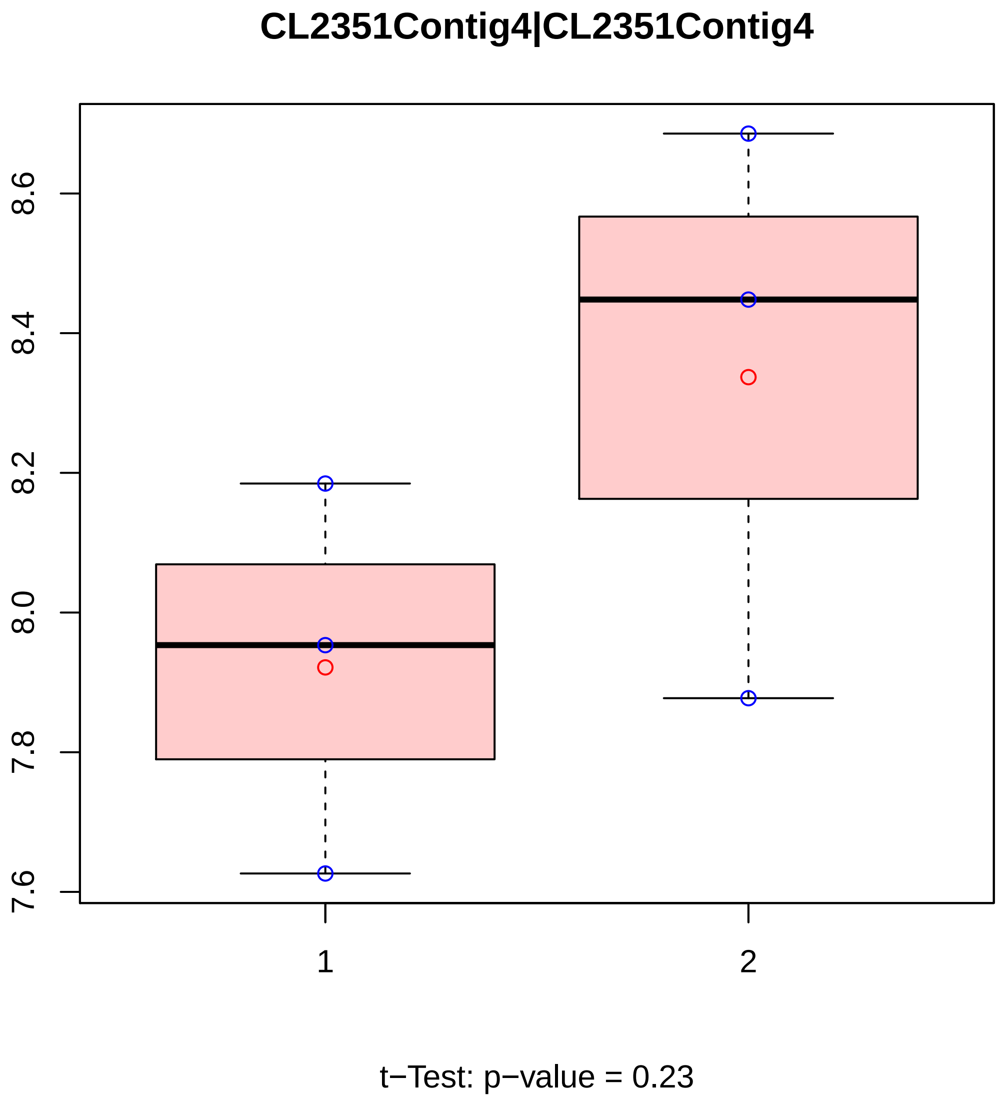
CL2351Contig4|CL2351Contig4
8.6
8.4
8.2
8.0
7.8
7.6
1
2
t−Test: p−value = 0.23

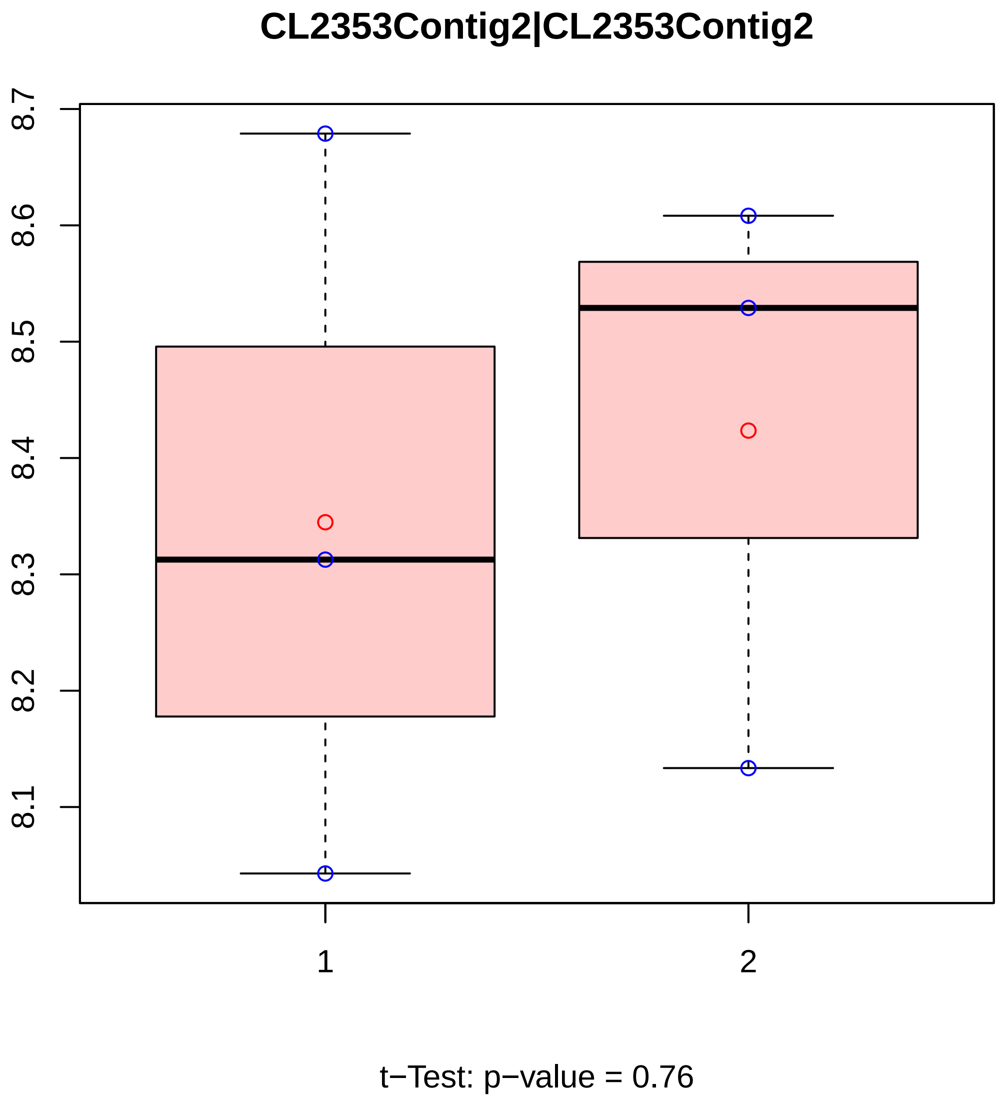
CL2353Contig2|CL2353Contig2
8.1
8.2
8.3
8.4
8.5
8.6
8.7
1
2
t−Test: p−value = 0.76

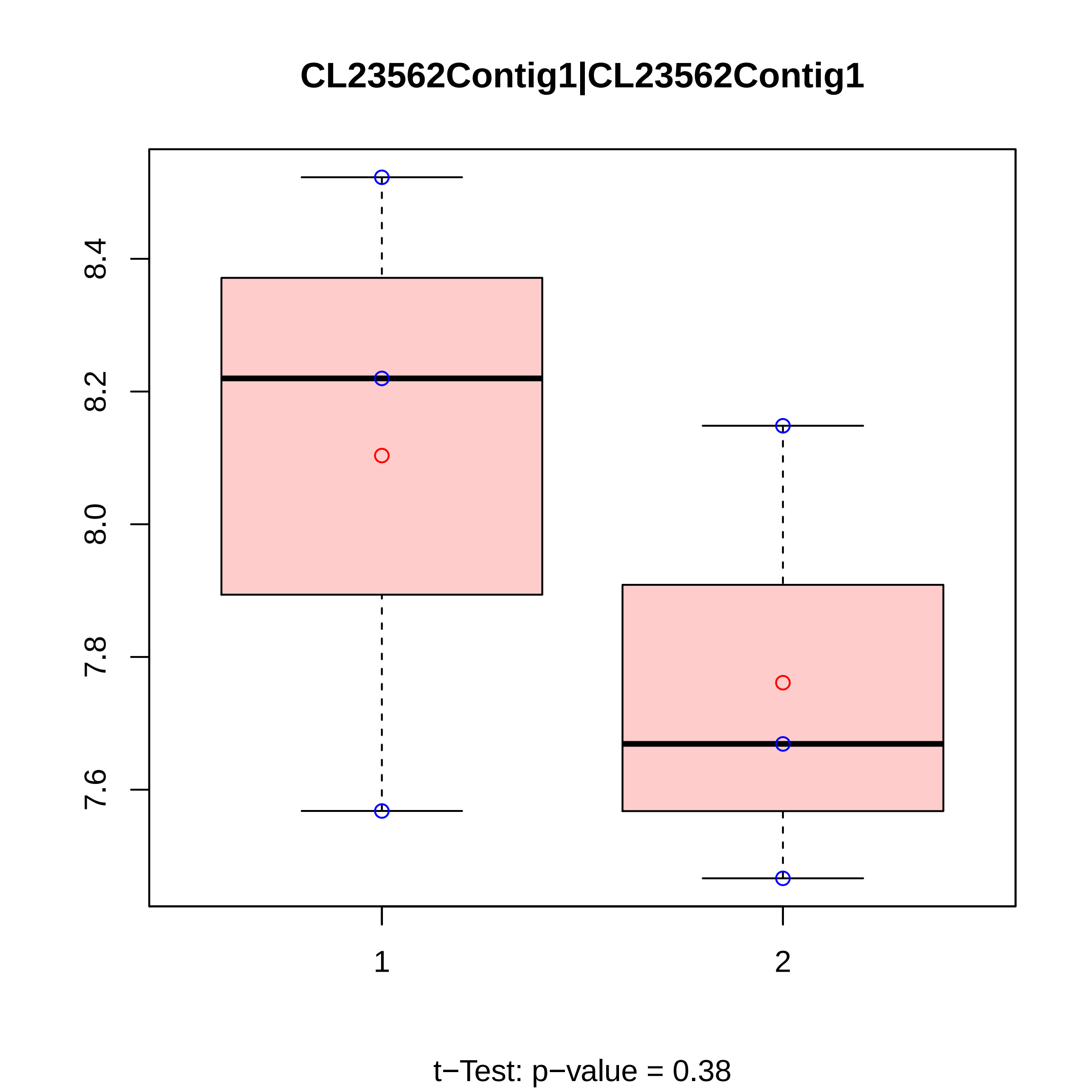
CL23562Contig1|CL23562Contig1
8.4
8.2
8.0
7.8
7.6
1
2
t−Test: p−value = 0.38

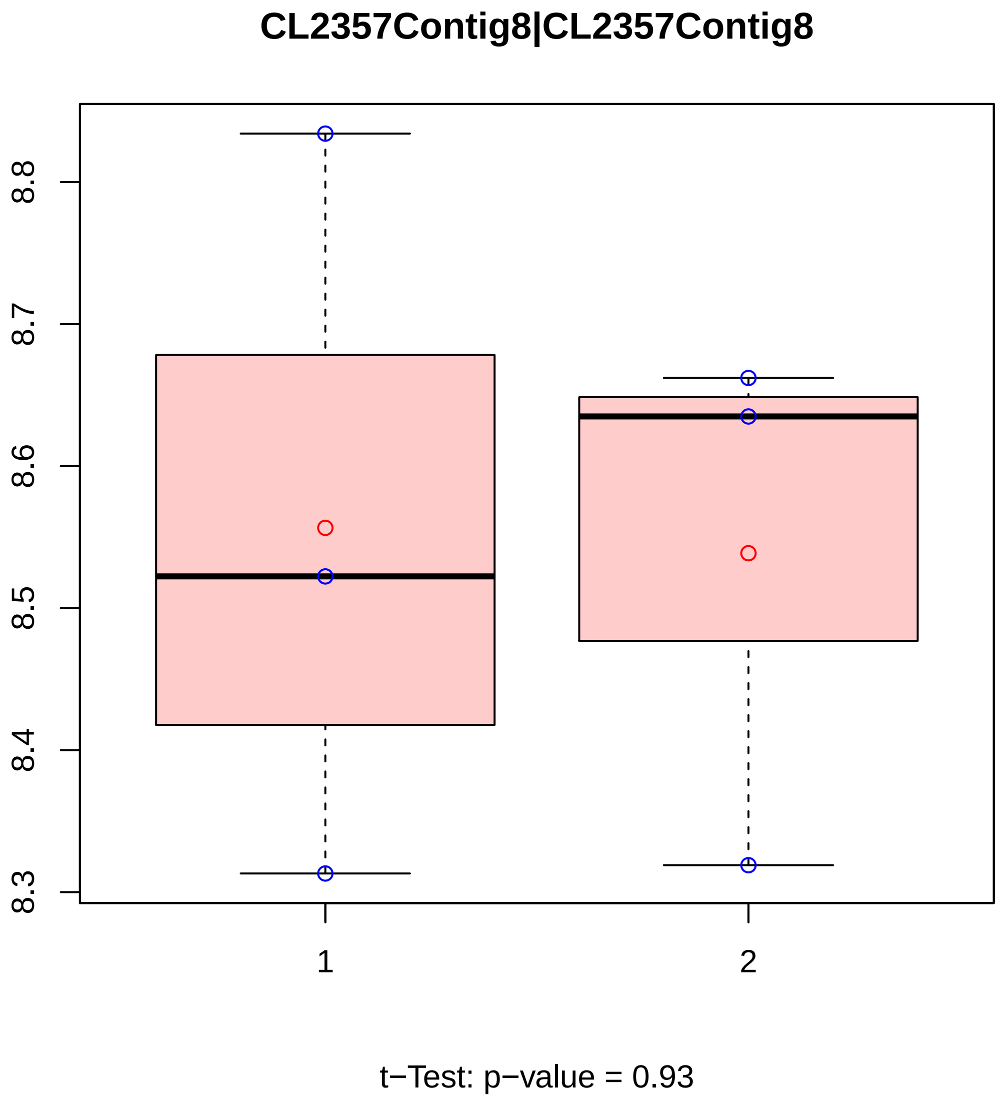
CL2357Contig8|CL2357Contig8
8.3
8.4
8.5
8.6
8.7
8.8
1
2
t−Test: p−value = 0.93

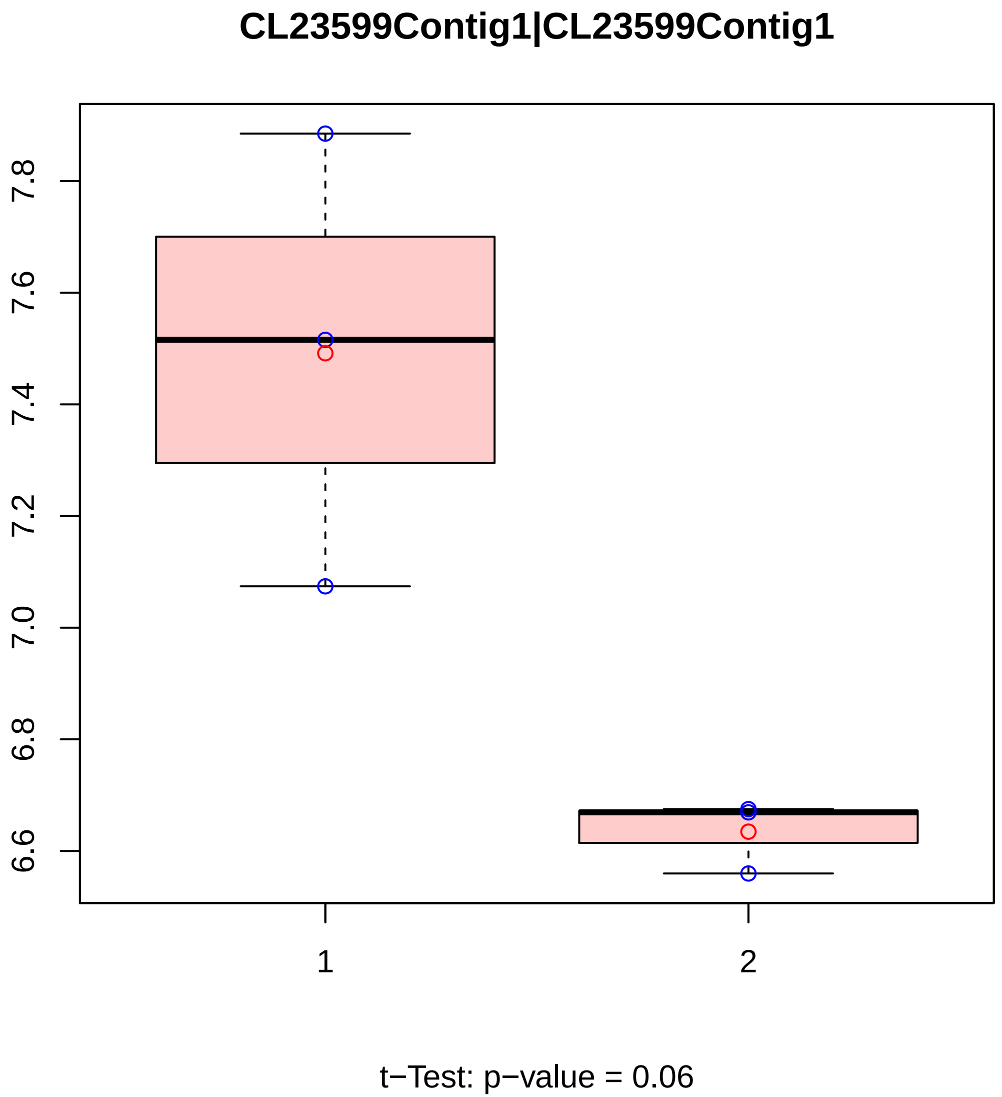
CL23599Contig1|CL23599Contig1
7.8
7.6
7.4
7.2
7.0
6.8
6.6
1
2
t−Test: p−value = 0.06

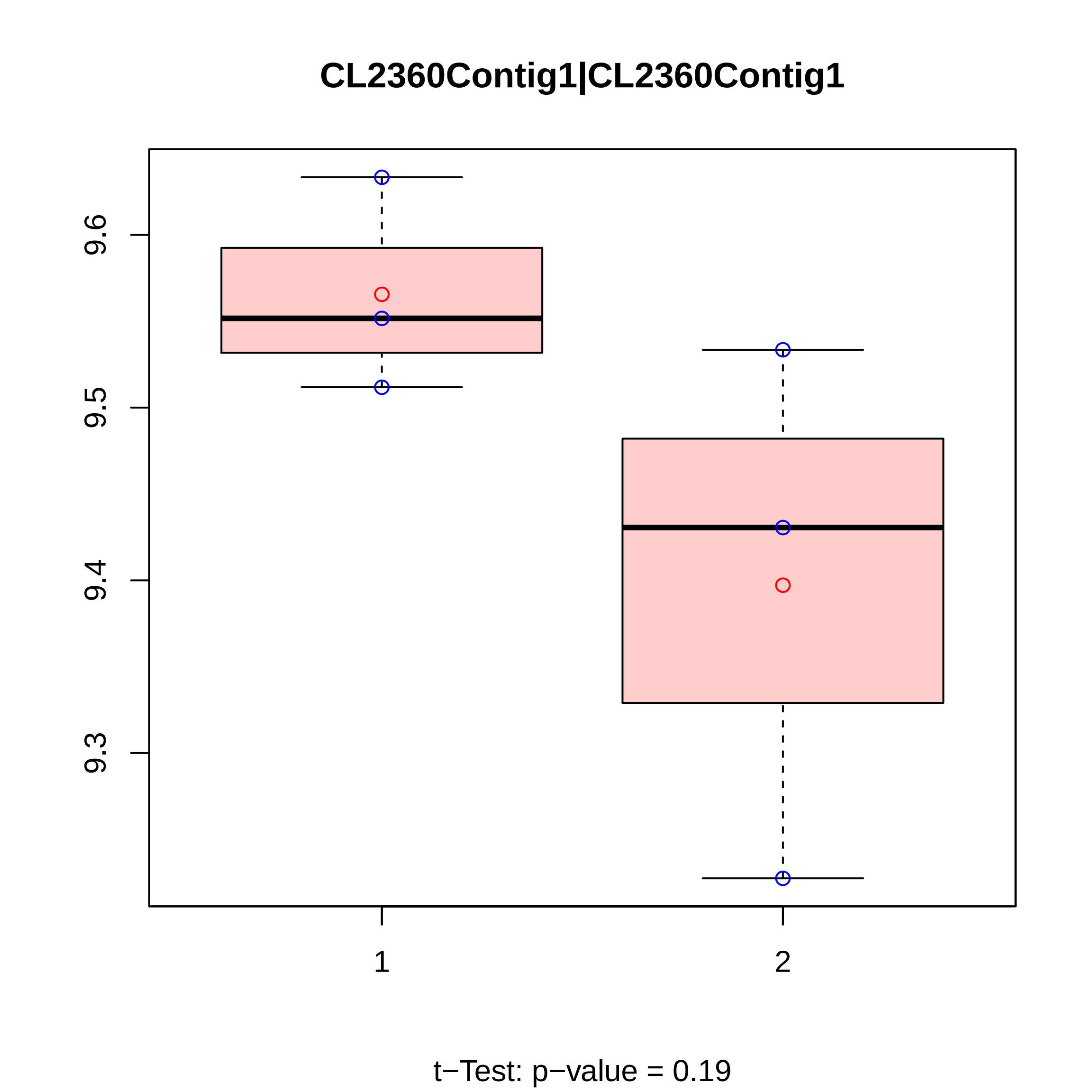
CL2360Contig1|CL2360Contig1
9.6
9.5
9.4
9.3
1
2
t−Test: p−value = 0.19

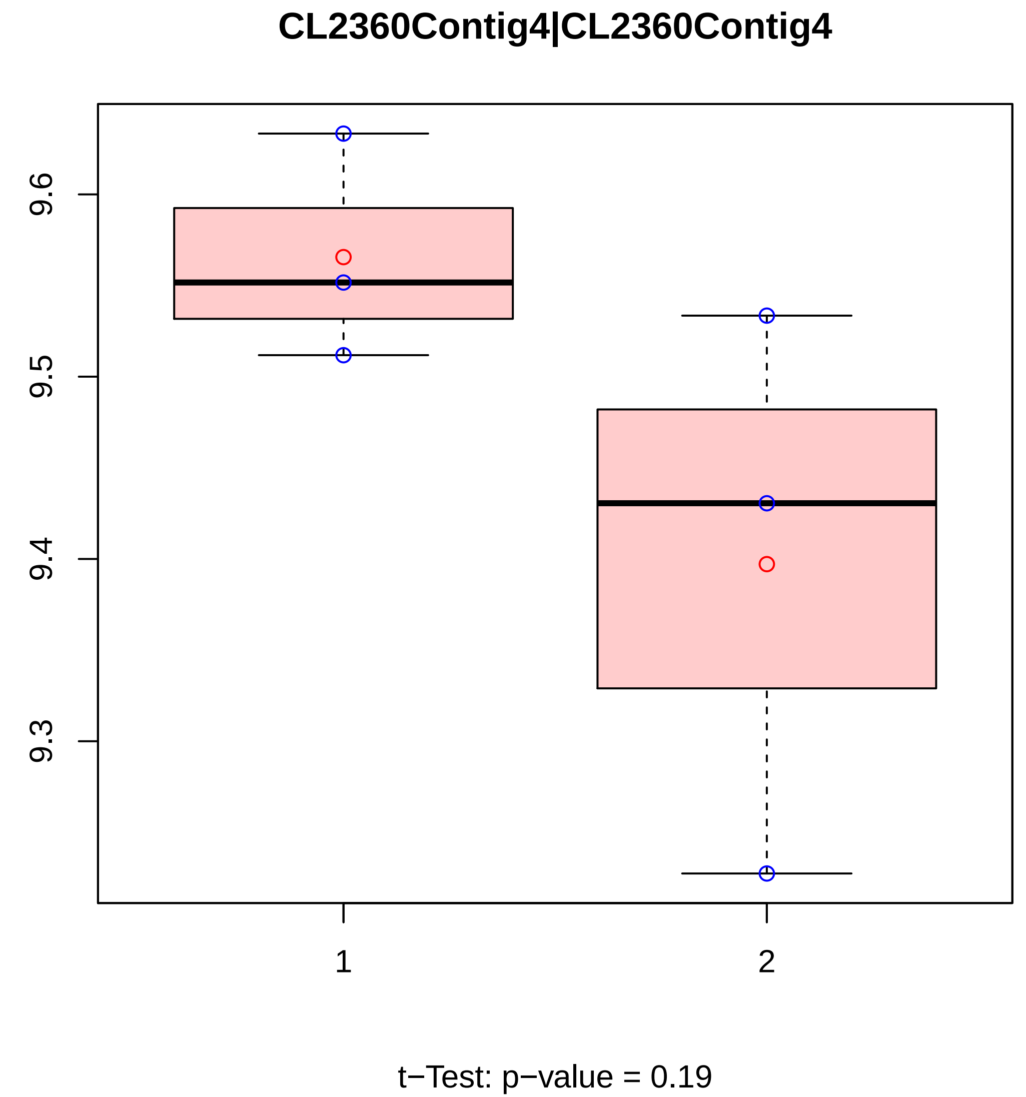
CL2360Contig4|CL2360Contig4
9.6
9.5
9.4
9.3
1
2
t−Test: p−value = 0.19

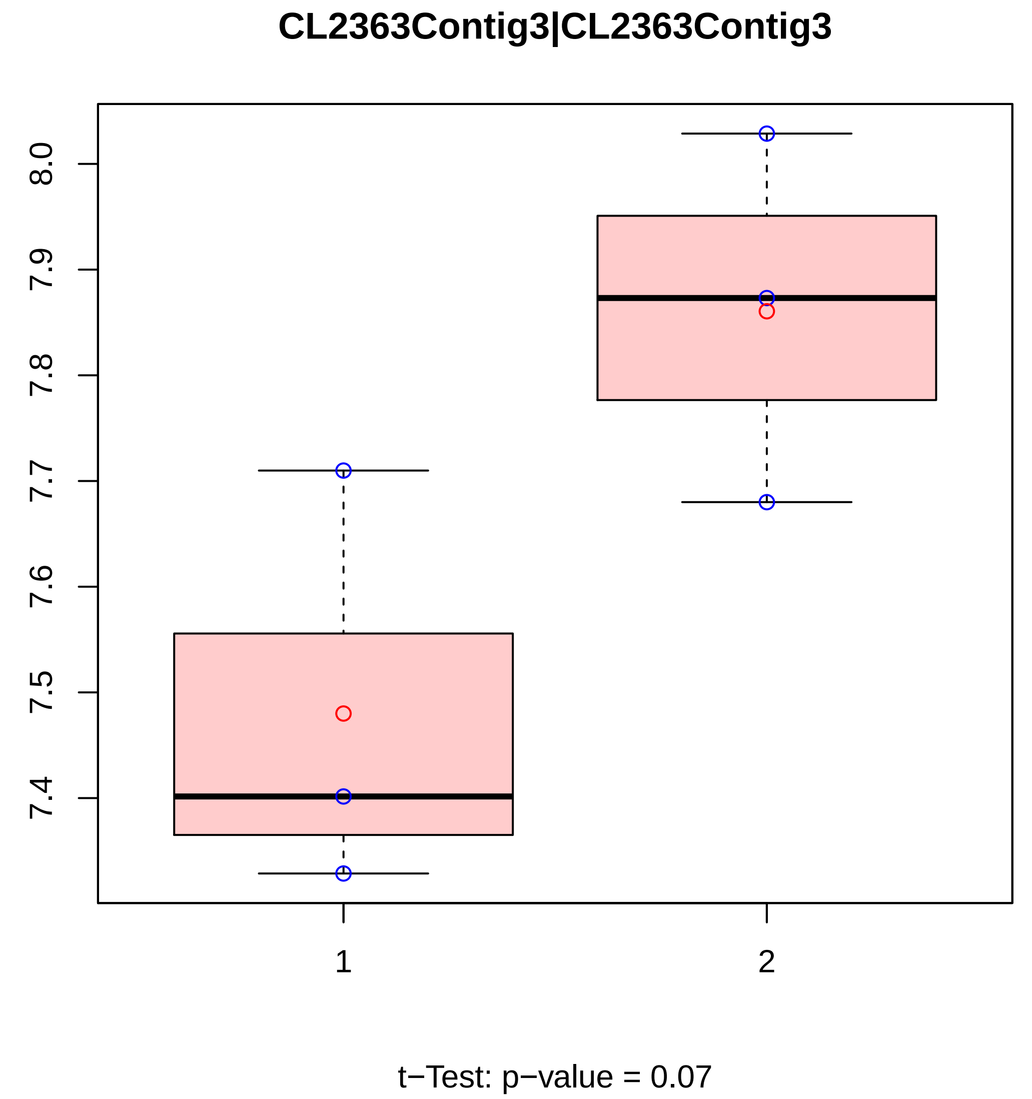

CL2363Contig3|CL2363Contig3
8.0
7.9
7.8
7.7
7.6
7.5
7.4
1
2
t−Test: p−value = 0.07

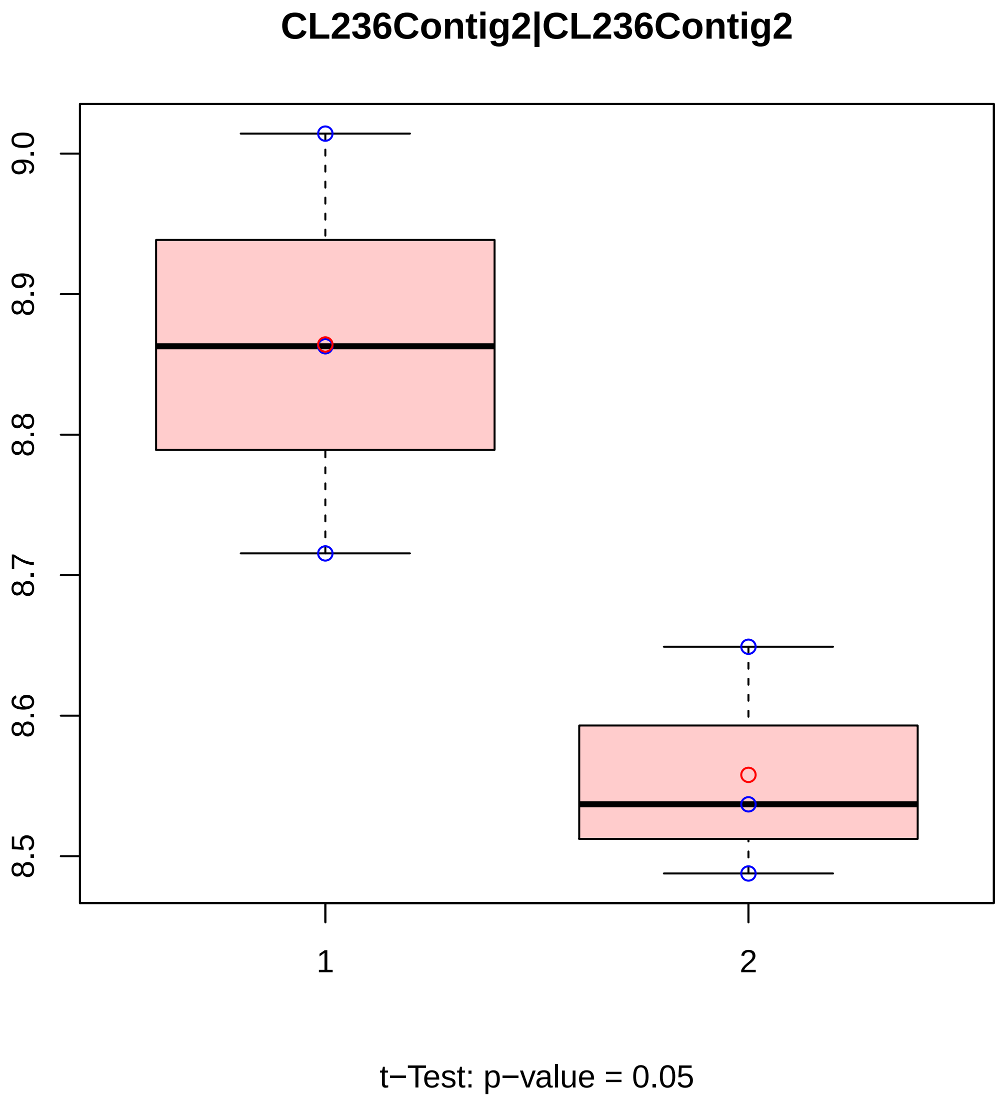

CL236Contig2|CL236Contig2
9.0
8.9
8.8
8.7
8.6
8.5
1
2
t−Test: p−value = 0.05

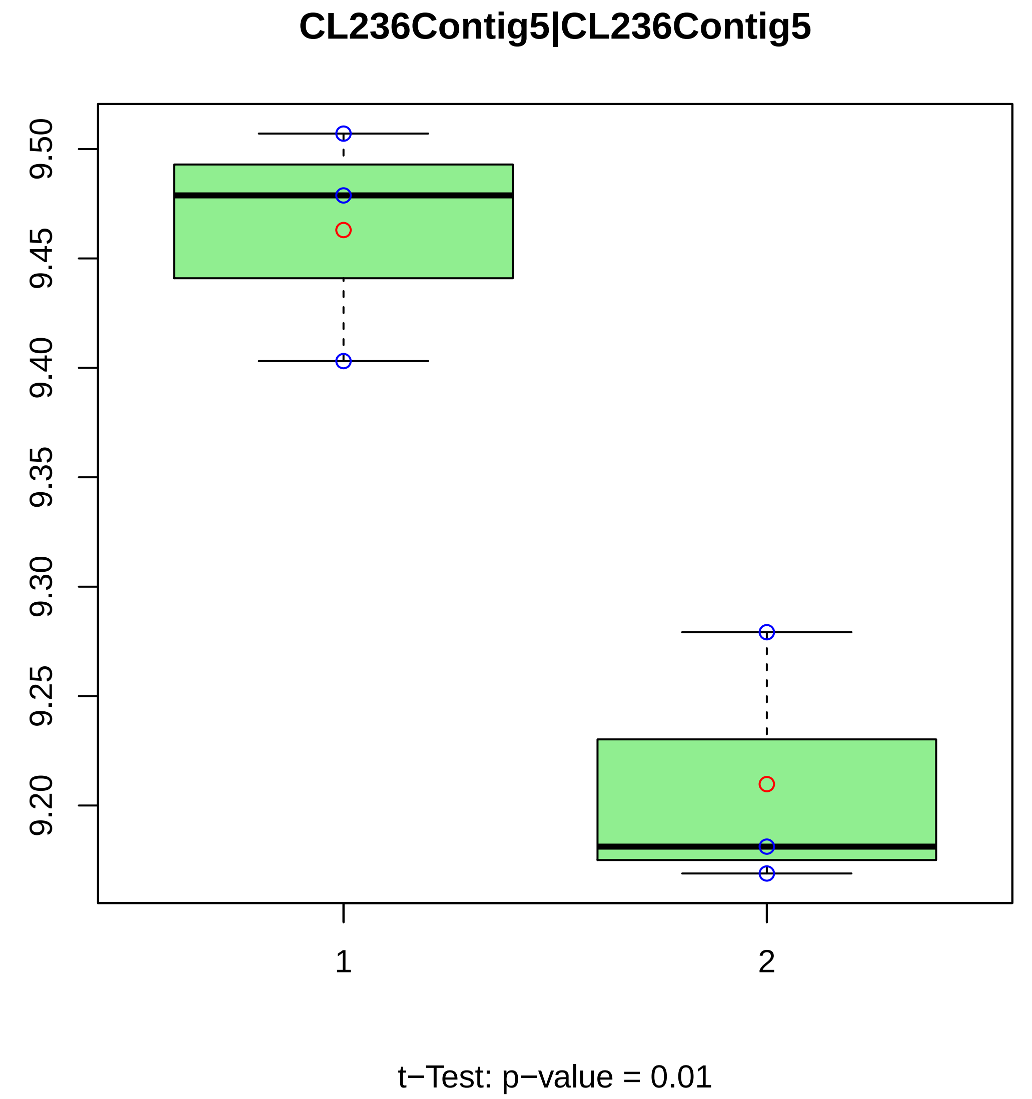
CL236Contig5|CL236Contig5
9.50
9.45
9.40
9.35
9.30
9.25
9.20
1
2
t−Test: p−value = 0.01

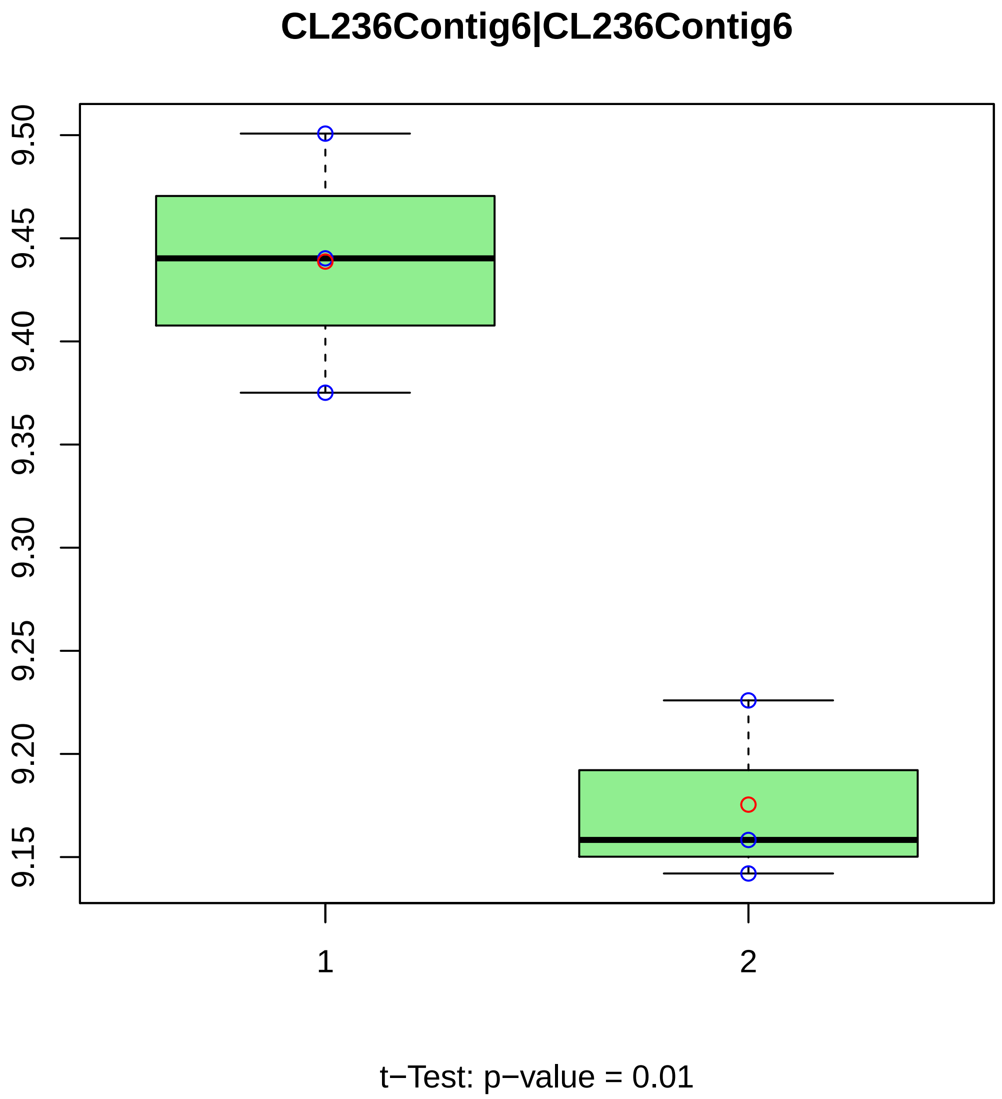

CL236Contig6|CL236Contig6
9.50
9.45
9.40
9.35
9.30
9.25
9.20
9.15
1
2
t−Test: p−value = 0.01

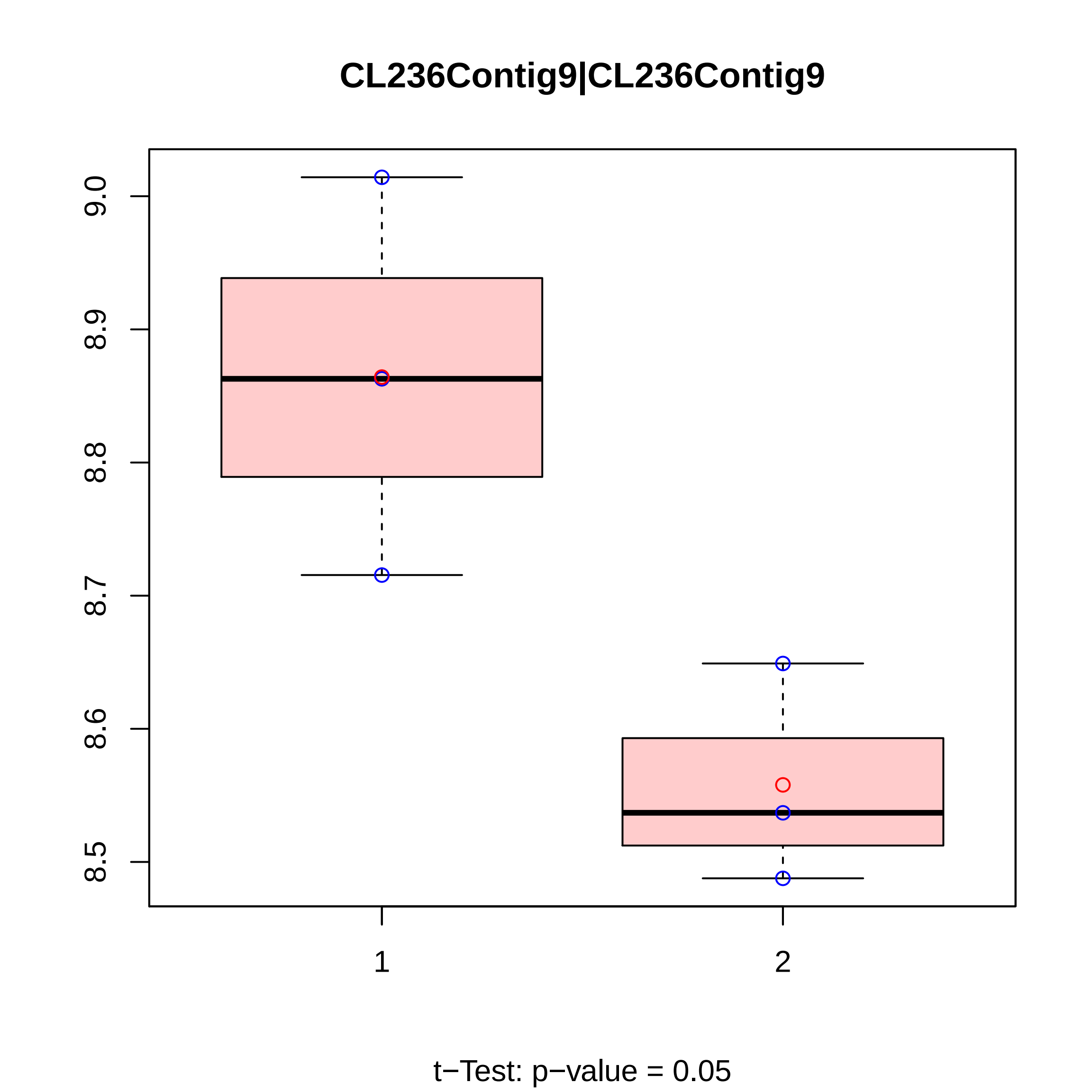
CL236Contig9|CL236Contig9
9.0
8.9
8.8
8.7
8.6
8.5
1
2
t−Test: p−value = 0.05

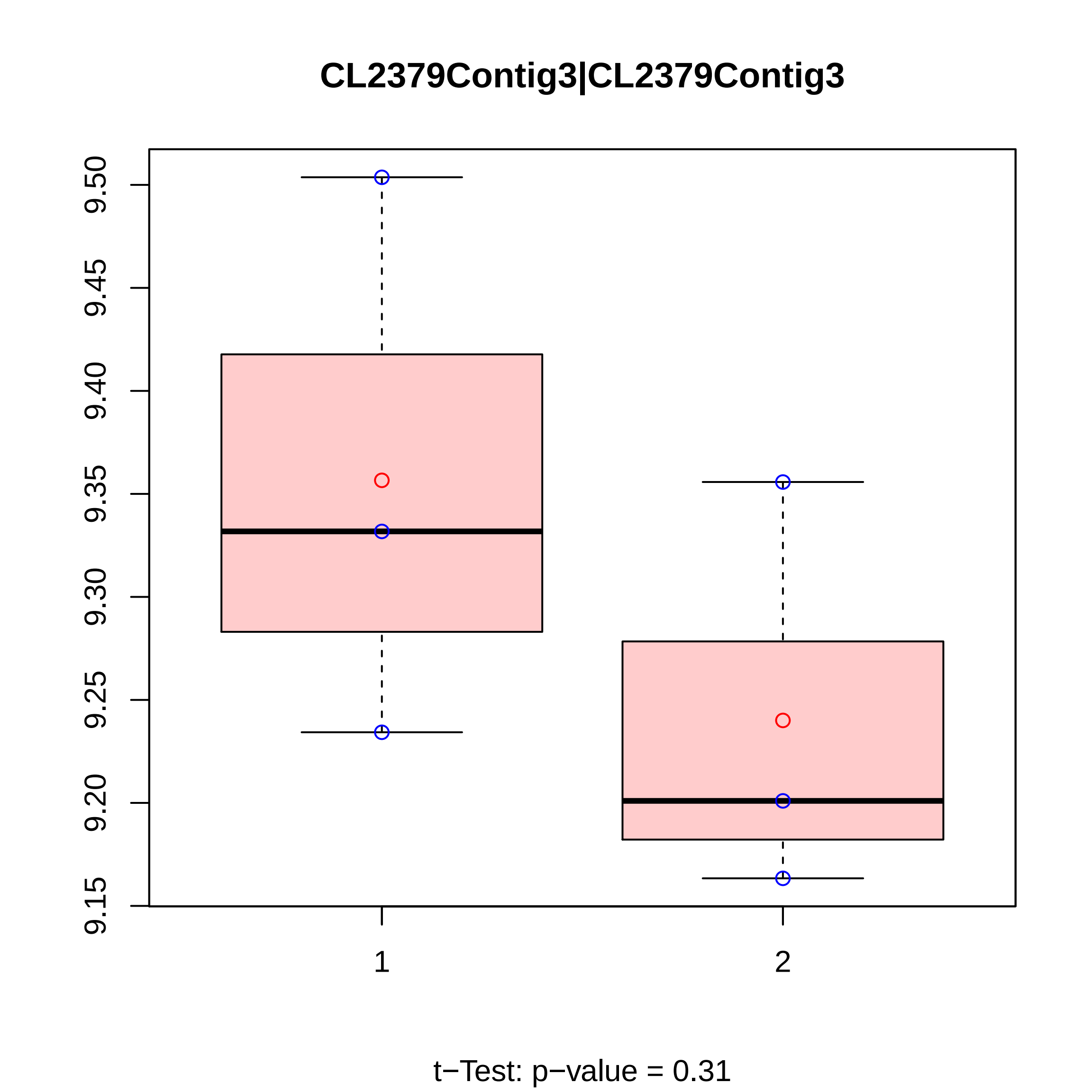

CL2379Contig3|CL2379Contig3
9.50
9.45
9.40
9.35
9.30
9.25
9.20
9.15
1
2
t−Test: p−value = 0.31

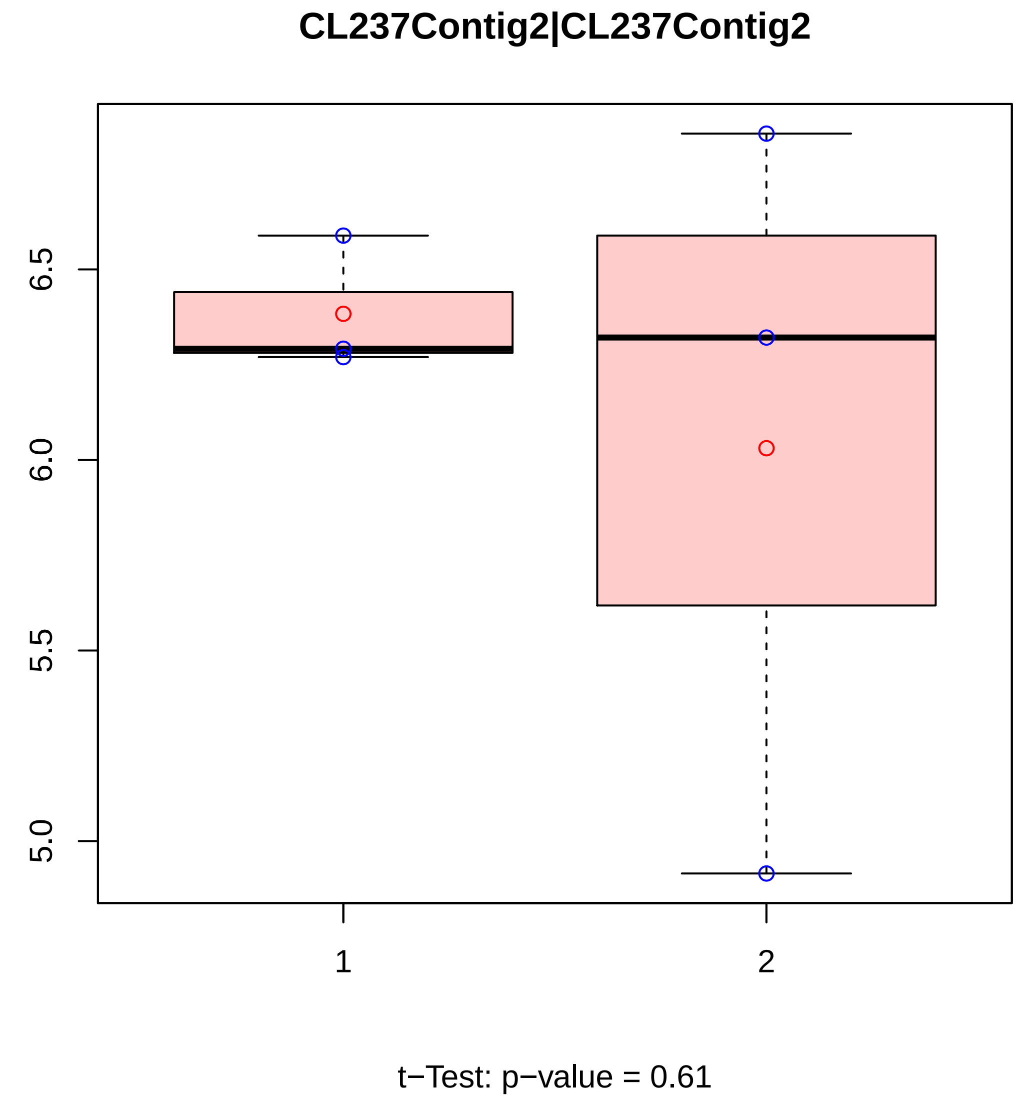
CL237Contig2|CL237Contig2
6.5
6.0
5.5
5.0
1
2
t−Test: p−value = 0.61

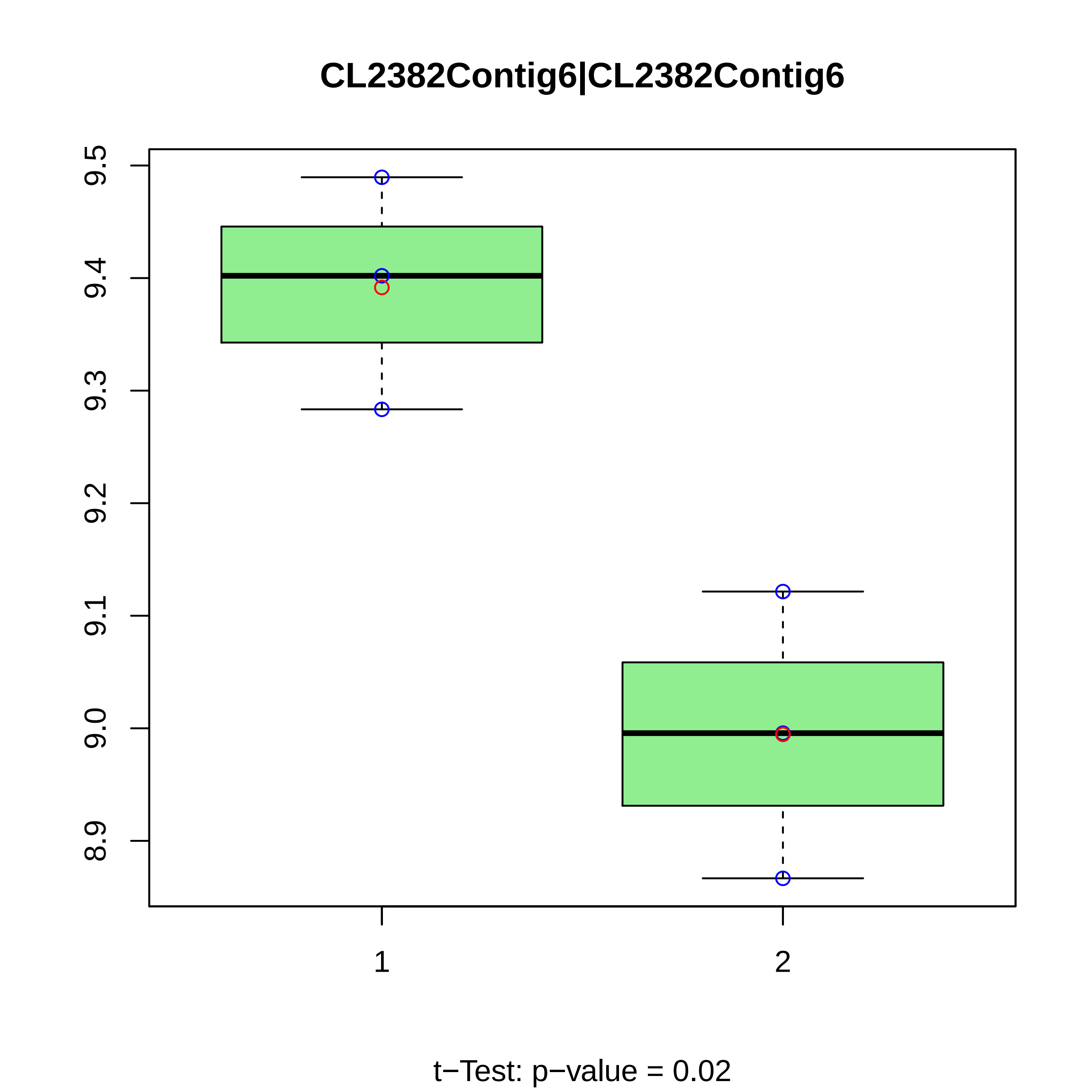
CL2382Contig6|CL2382Contig6
9.5
9.4
9.3
9.2
9.1
9.0
8.9
1
2
t−Test: p−value = 0.02

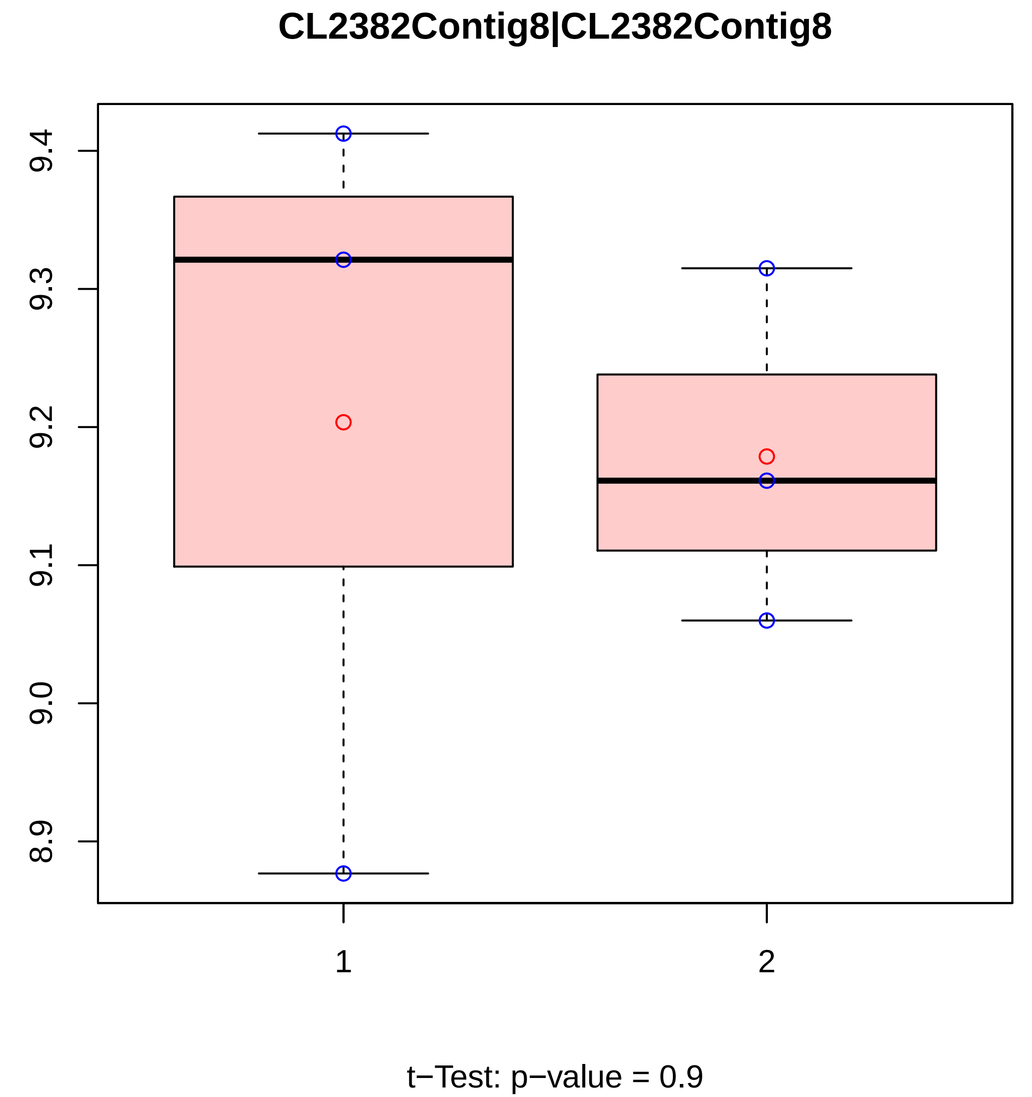
CL2382Contig8|CL2382Contig8
9.4
9.3
9.2
9.1
9.0
8.9
1
2
t−Test: p−value = 0.9

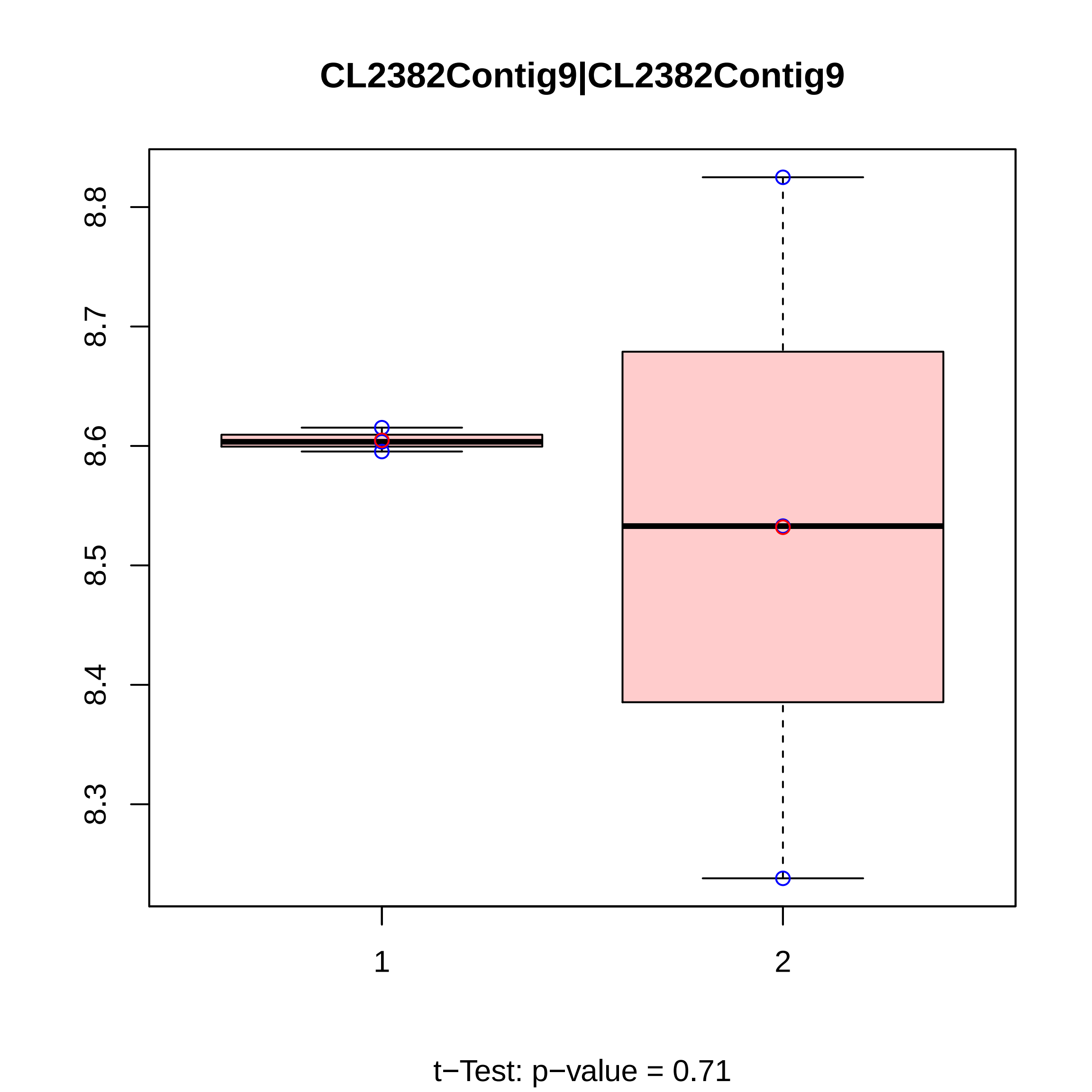
CL2382Contig9|CL2382Contig9
8.3
8.4
8.5
8.6
8.7
8.8
1
2
t−Test: p−value = 0.71

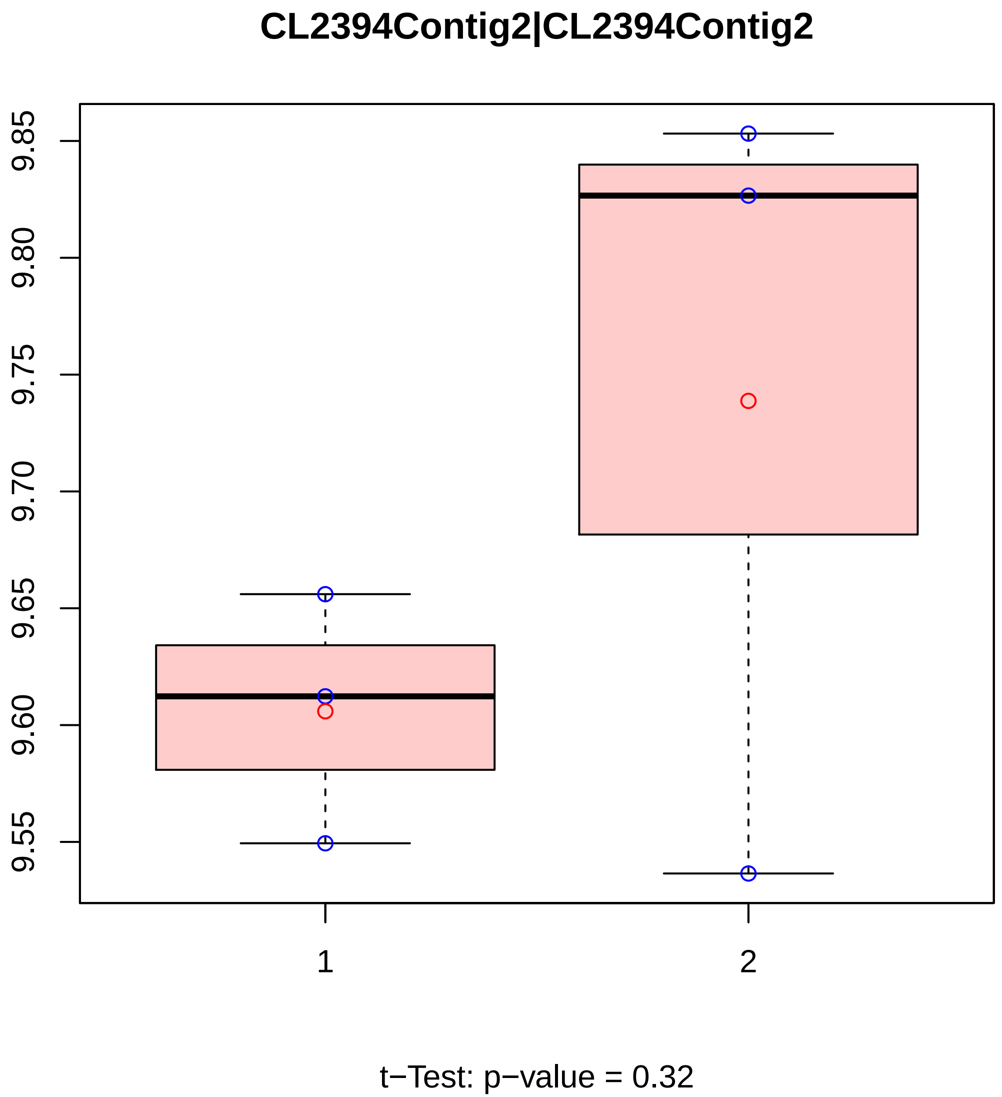
CL2394Contig2|CL2394Contig2
9.55
9.60
9.65
9.70
9.75
9.80
9.85
1
2
t−Test: p−value = 0.32

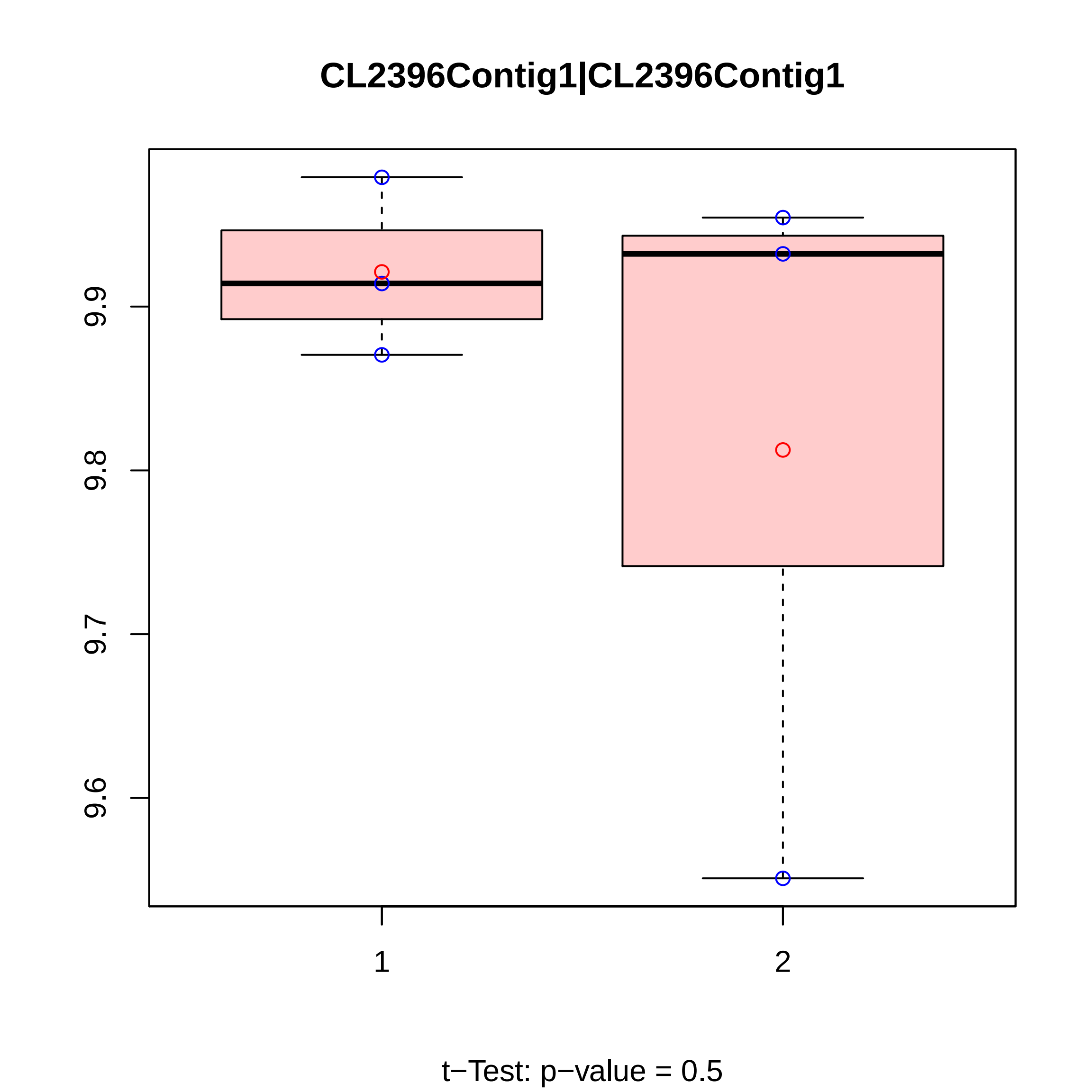

CL2396Contig1|CL2396Contig1
9.9
9.8
9.7
9.6
1
2
t−Test: p−value = 0.5

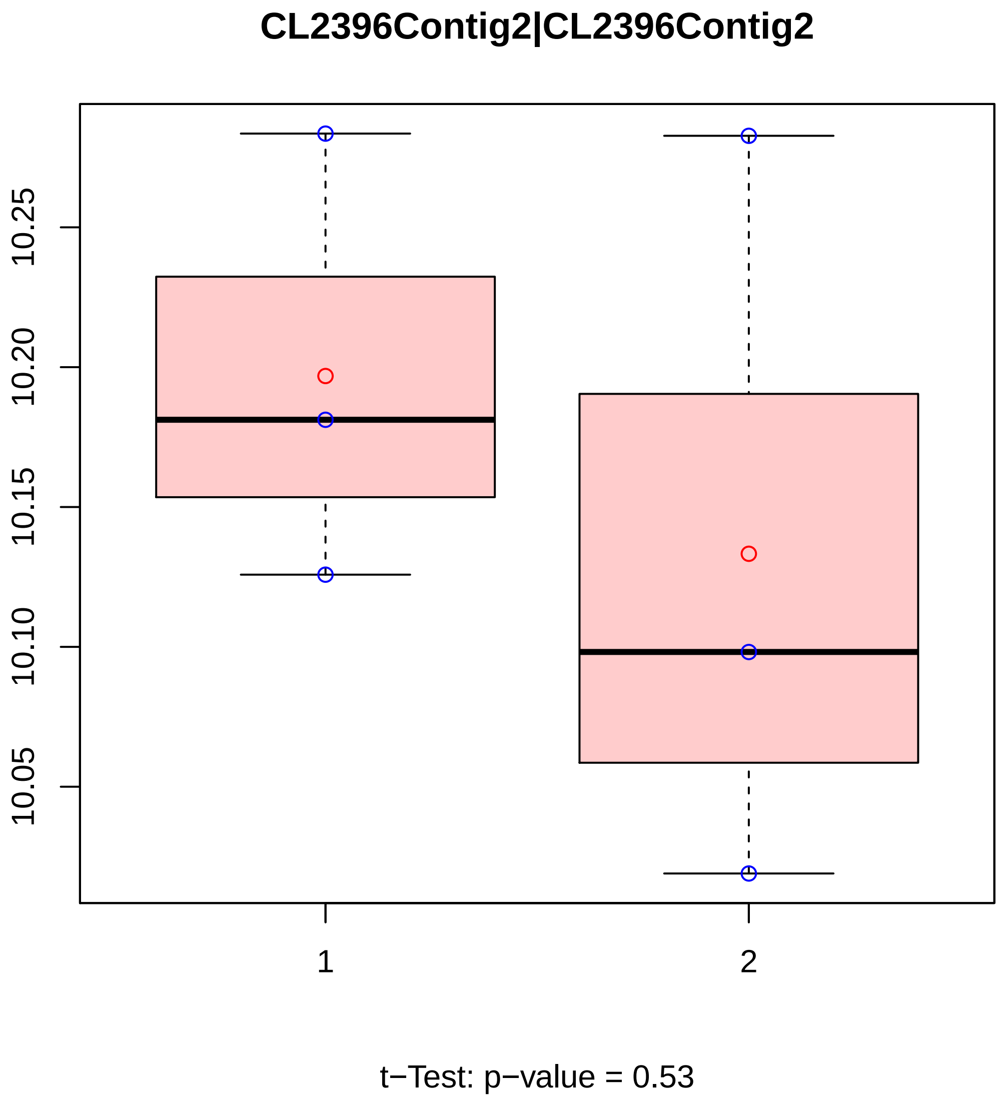

CL2396Contig2|CL2396Contig2
10.05
10.10
10.15
10.20
10.25
1
2
t−Test: p−value = 0.53

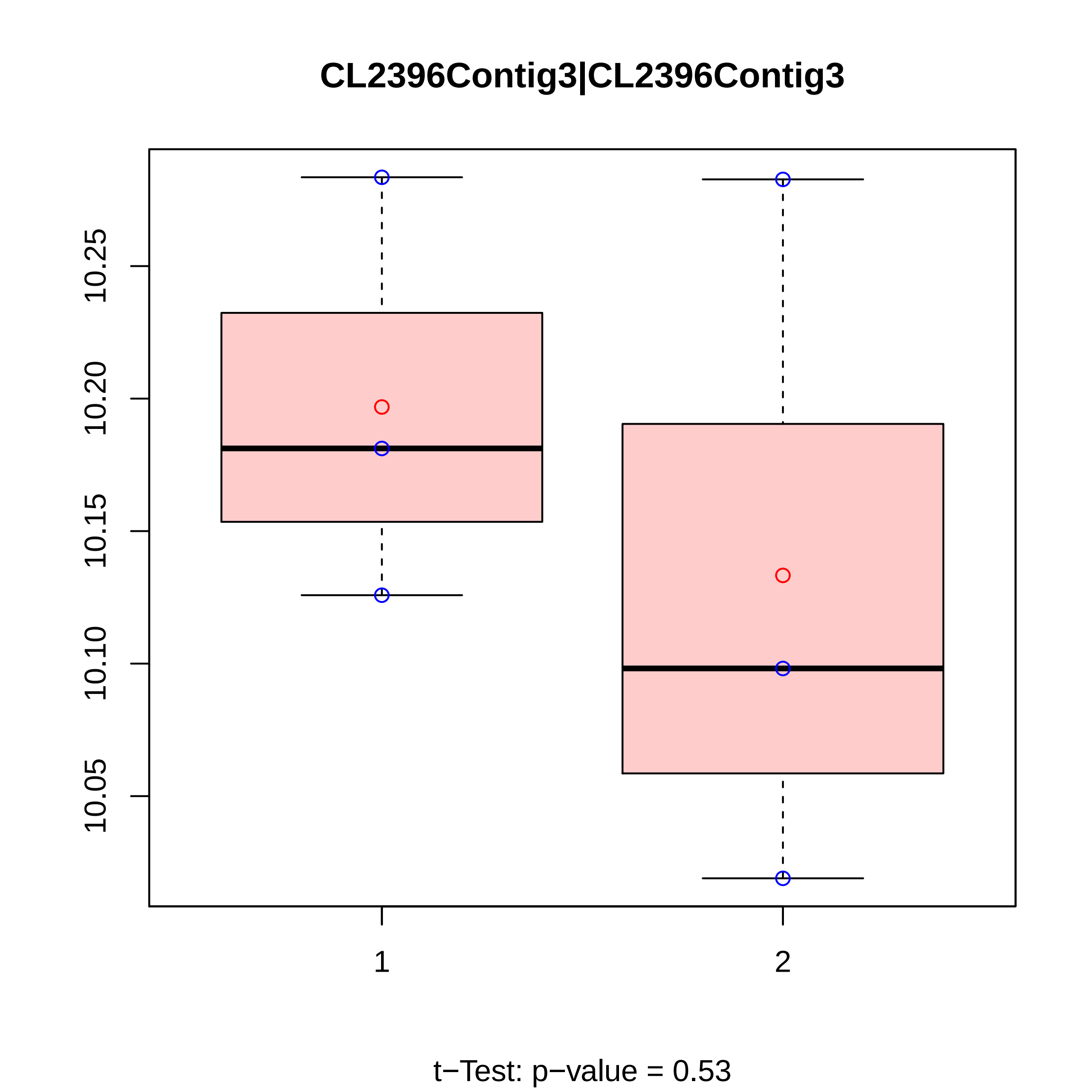
CL2396Contig3|CL2396Contig3
10.05
10.10
10.15
10.20
10.25
1
2
t−Test: p−value = 0.53

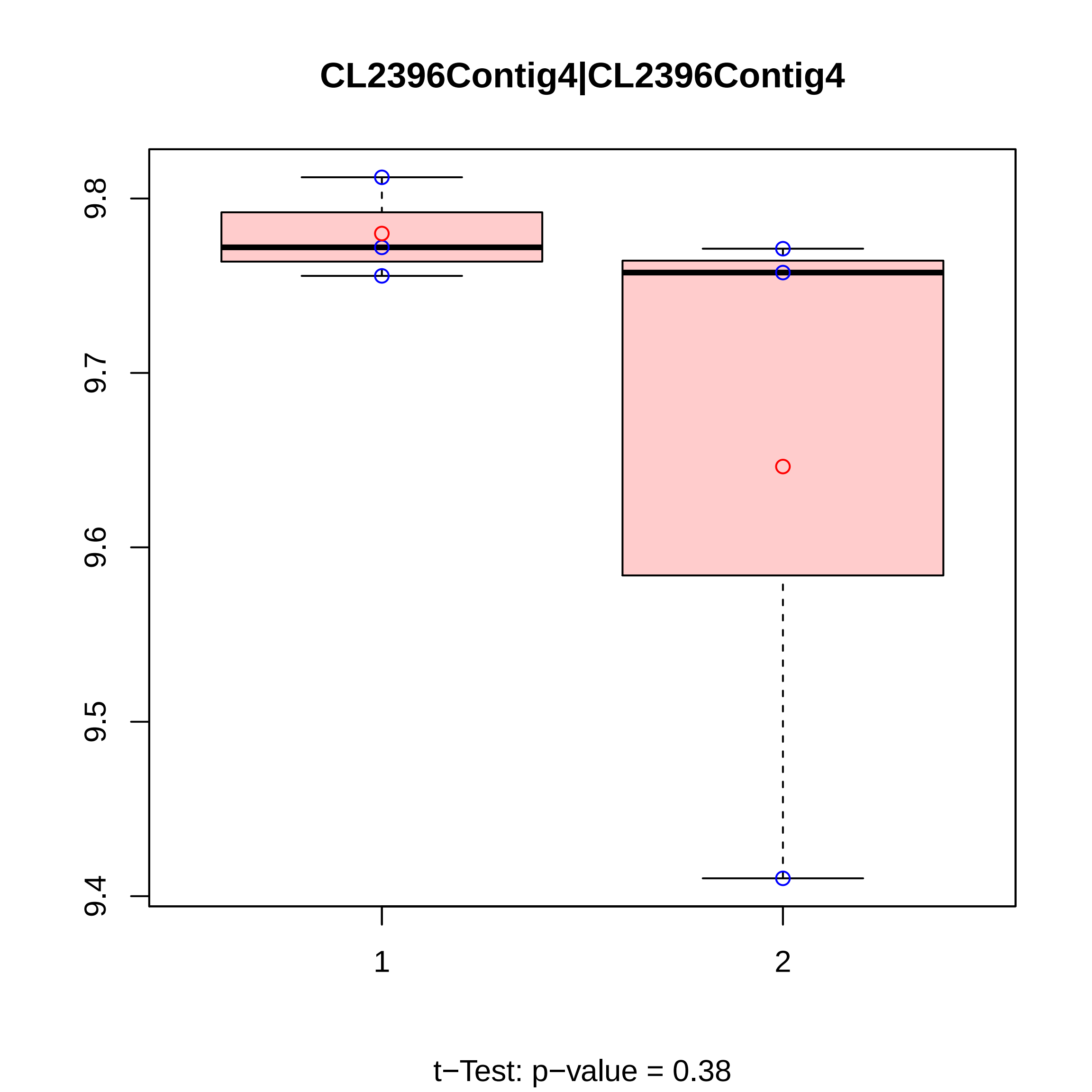

CL2396Contig4|CL2396Contig4
9.8
9.7
9.6
9.5
9.4
1
2
t−Test: p−value = 0.38

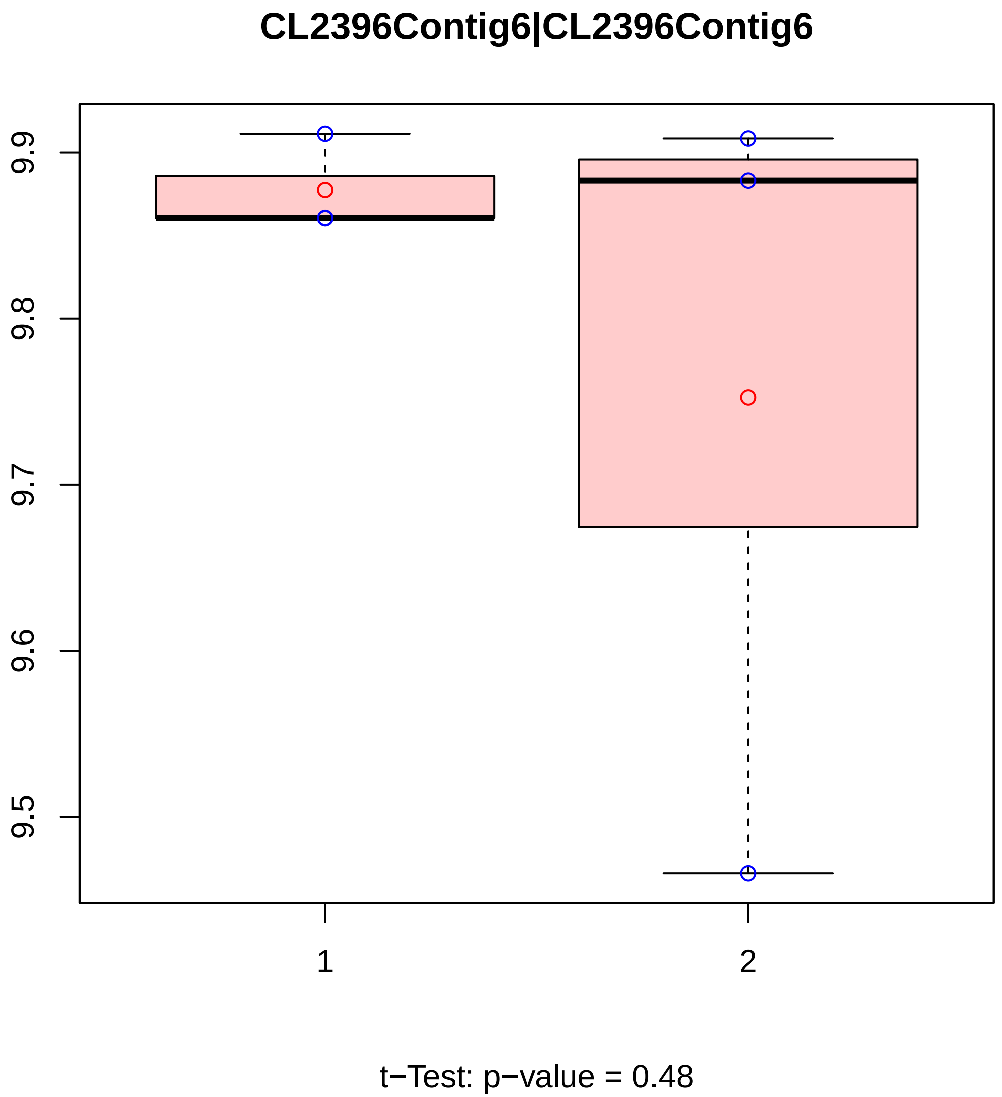

CL2396Contig6|CL2396Contig6
9.5
9.6
9.7
9.8
9.9
1
2
t−Test: p−value = 0.48

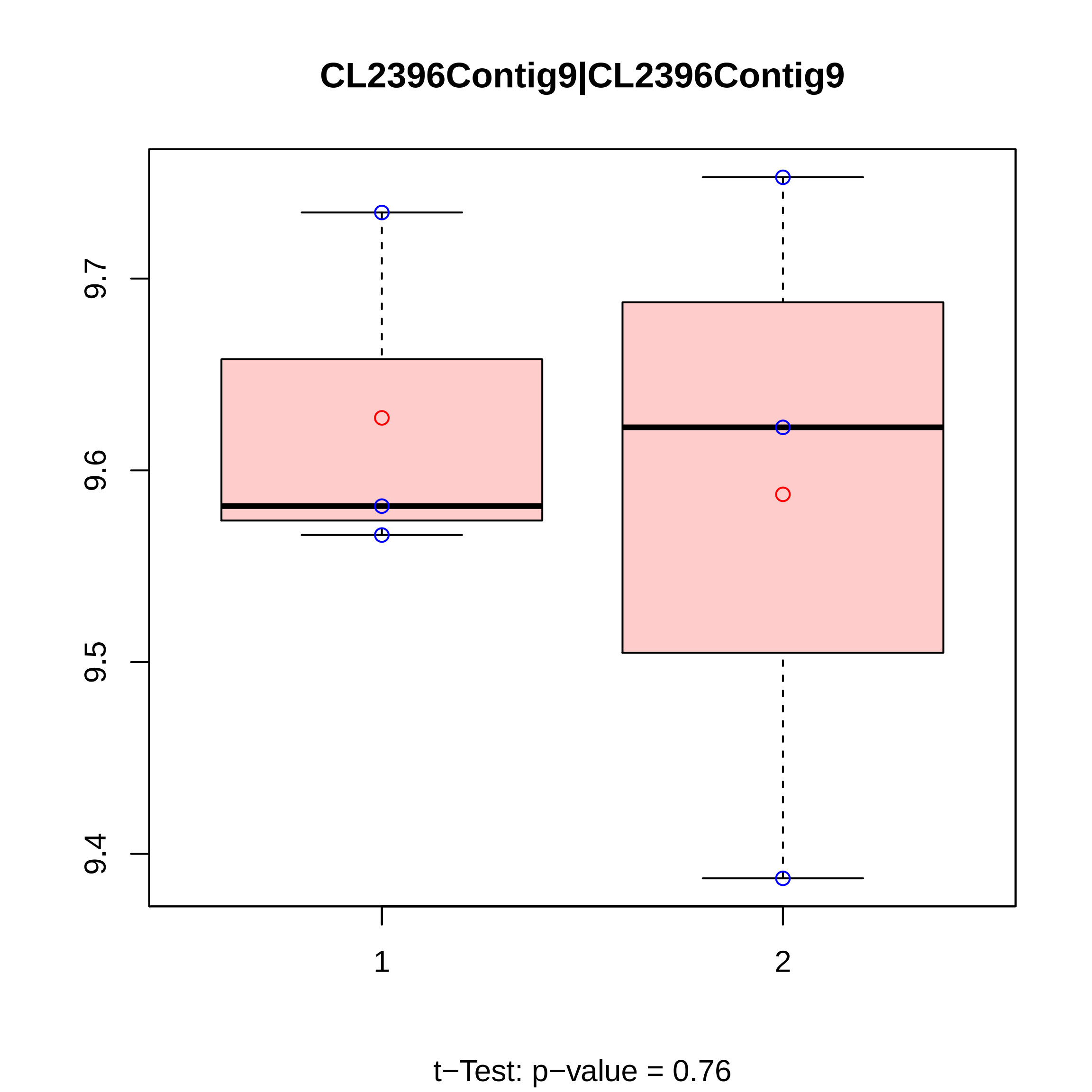

CL2396Contig9|CL2396Contig9
9.4
9.5
9.6
9.7
1
2
t−Test: p−value = 0.76

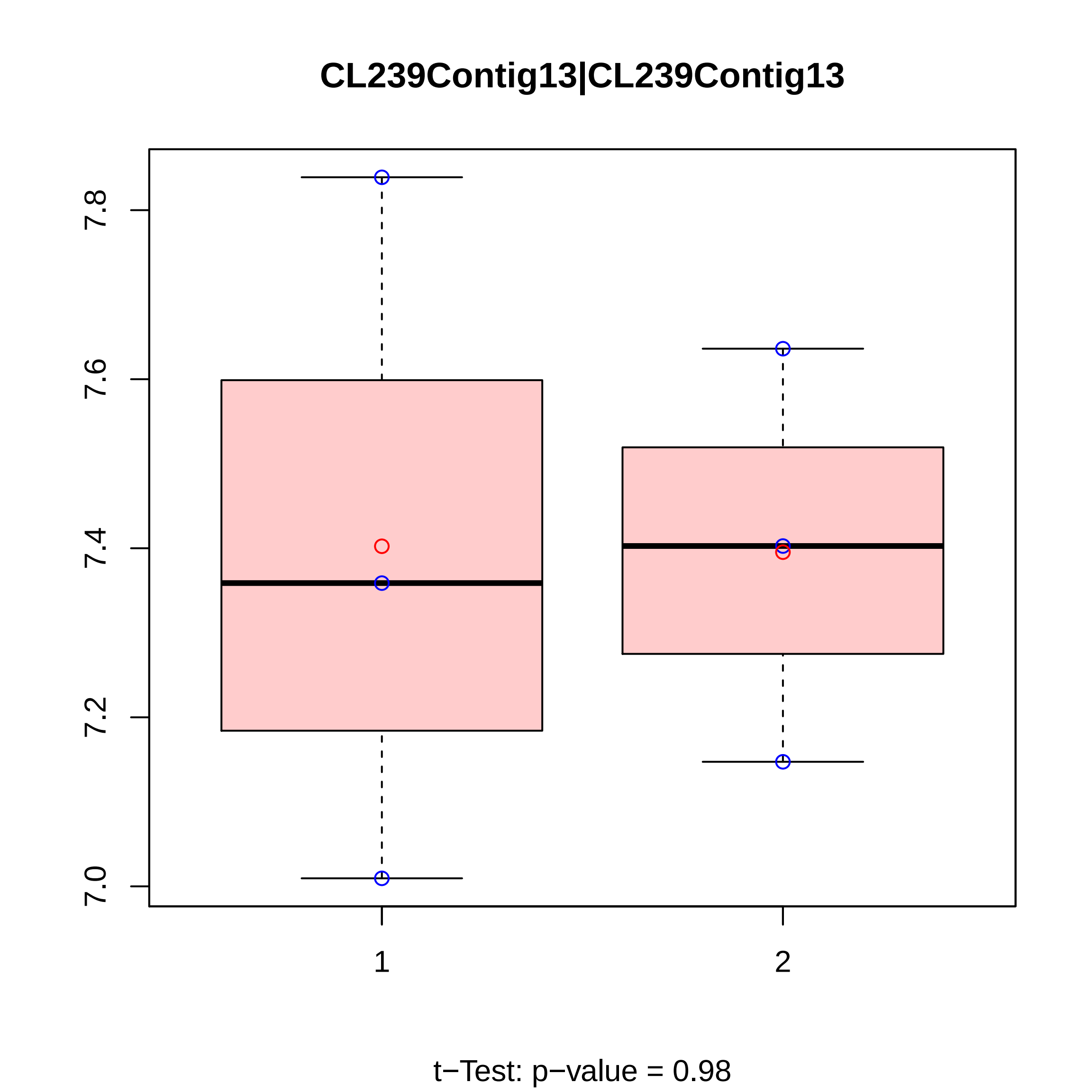
CL239Contig13|CL239Contig13
7.0
7.2
7.4
7.6
7.8
1
2
t−Test: p−value = 0.98

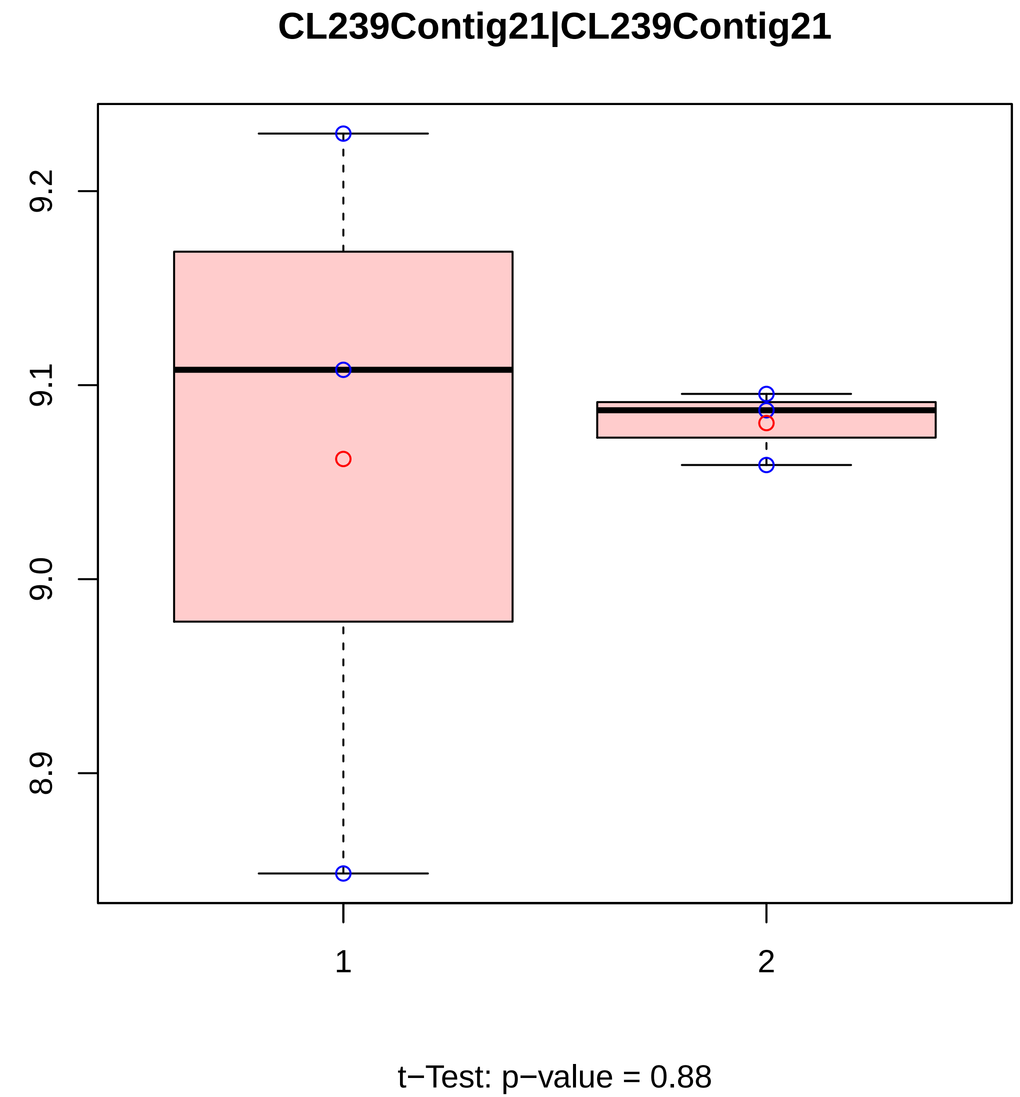
CL239Contig21|CL239Contig21
9.2
9.1
9.0
8.9
1
2
t−Test: p−value = 0.88

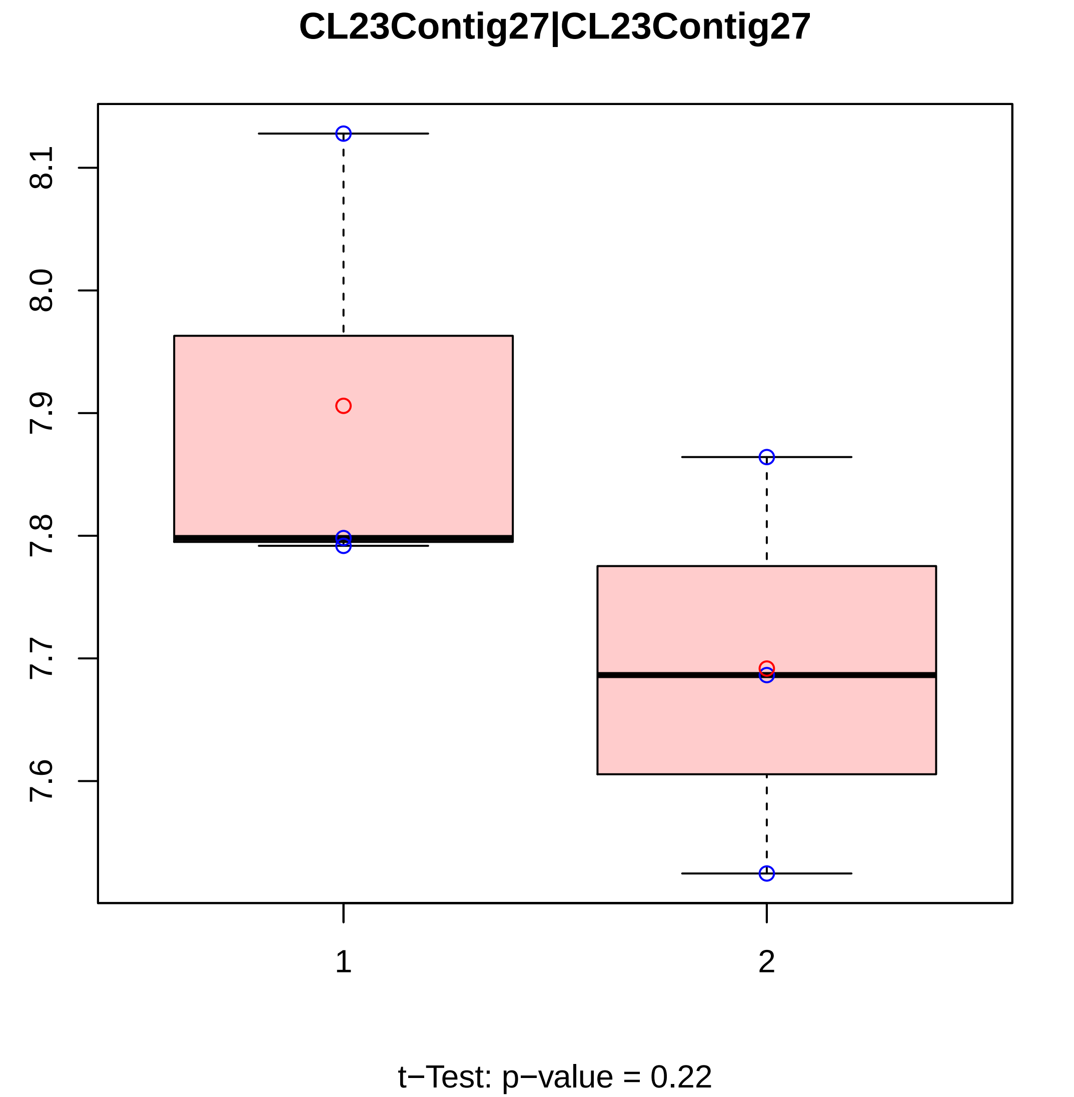
CL23Contig27|CL23Contig27
8.1
8.0
7.9
7.8
7.7
7.6
1
2
t−Test: p−value = 0.22

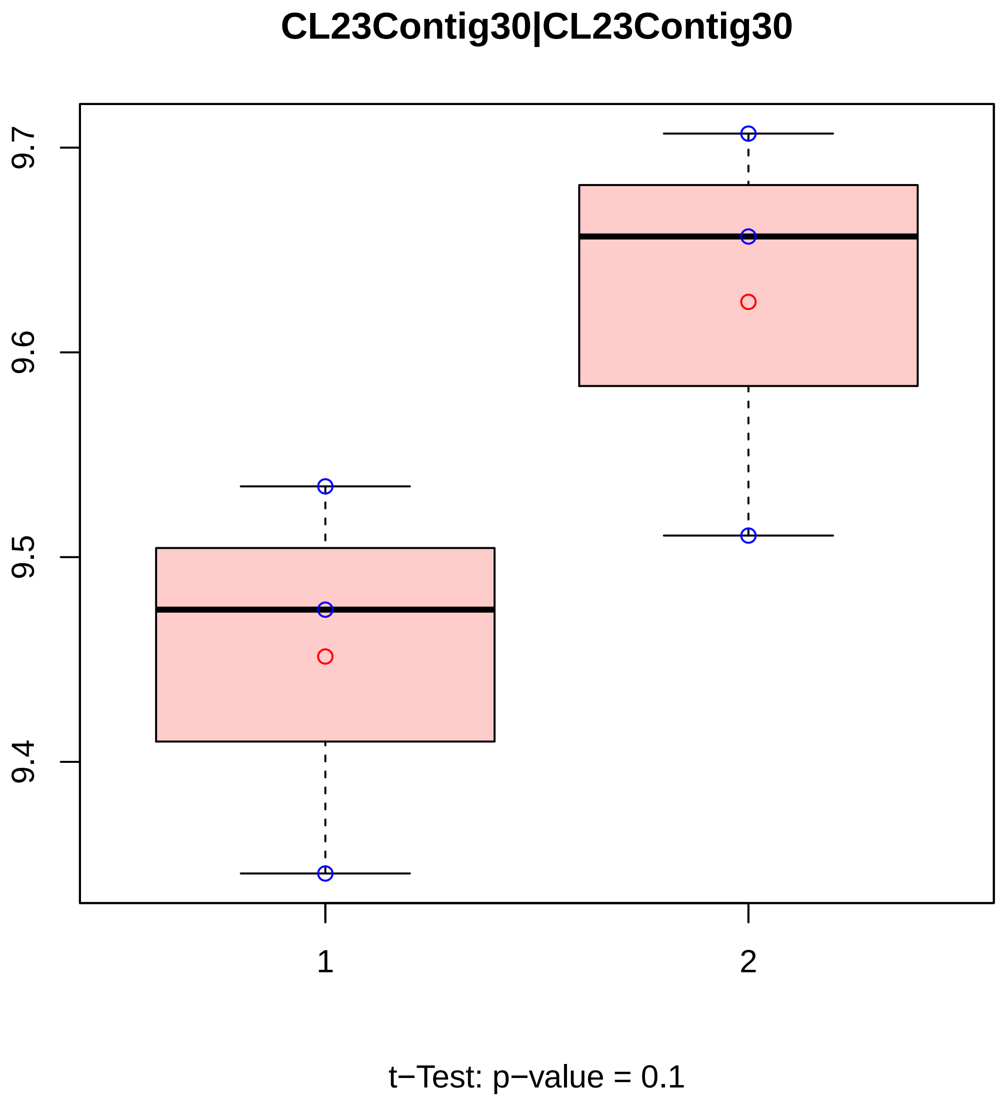

CL23Contig30|CL23Contig30
9.4
9.5
9.6
9.7
1
2
t−Test: p−value = 0.1

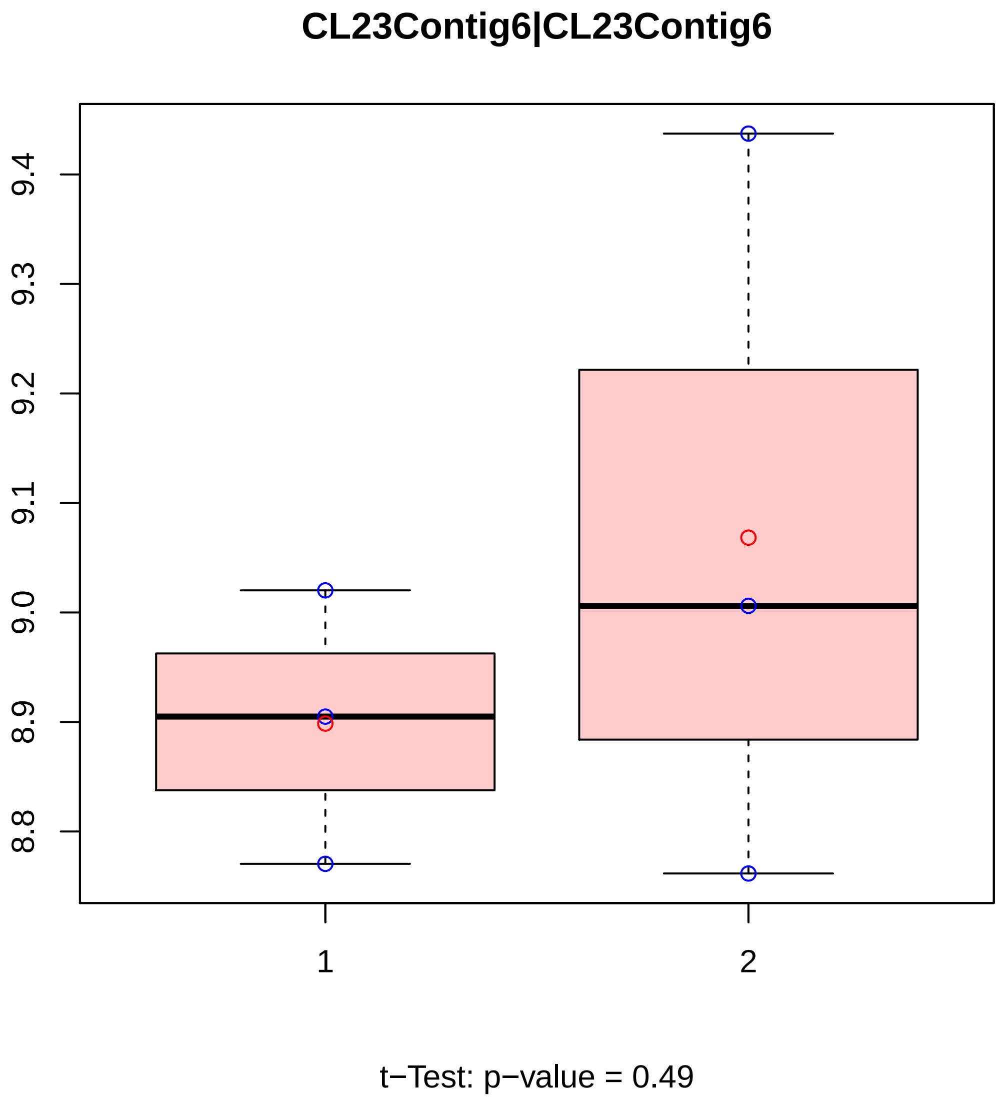
CL23Contig6|CL23Contig6
9.4
9.3
9.2
9.1
9.0
8.9
8.8
1
2
t−Test: p−value = 0.49

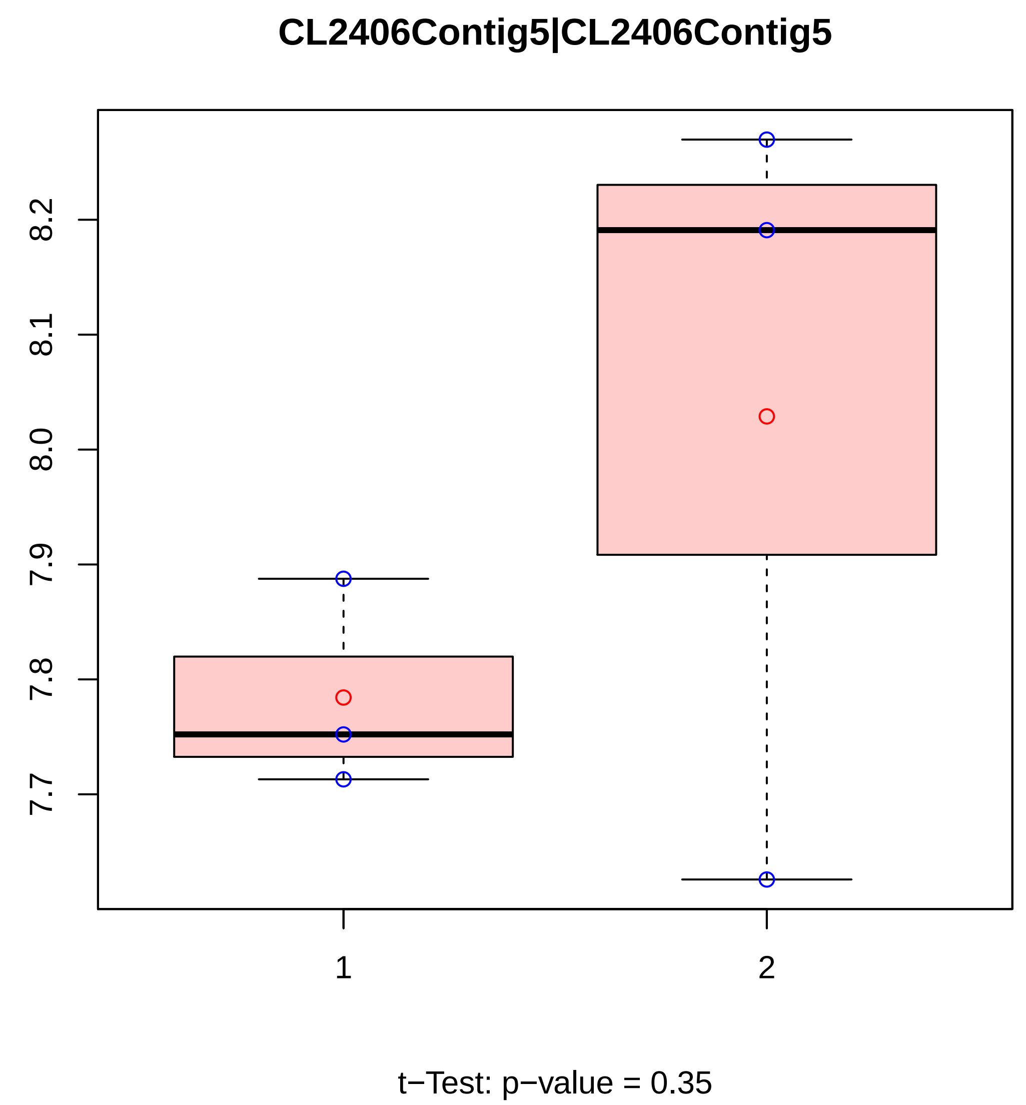
CL2406Contig5|CL2406Contig5
8.2
8.1
8.0
7.9
7.8
7.7
1
2
t−Test: p−value = 0.35

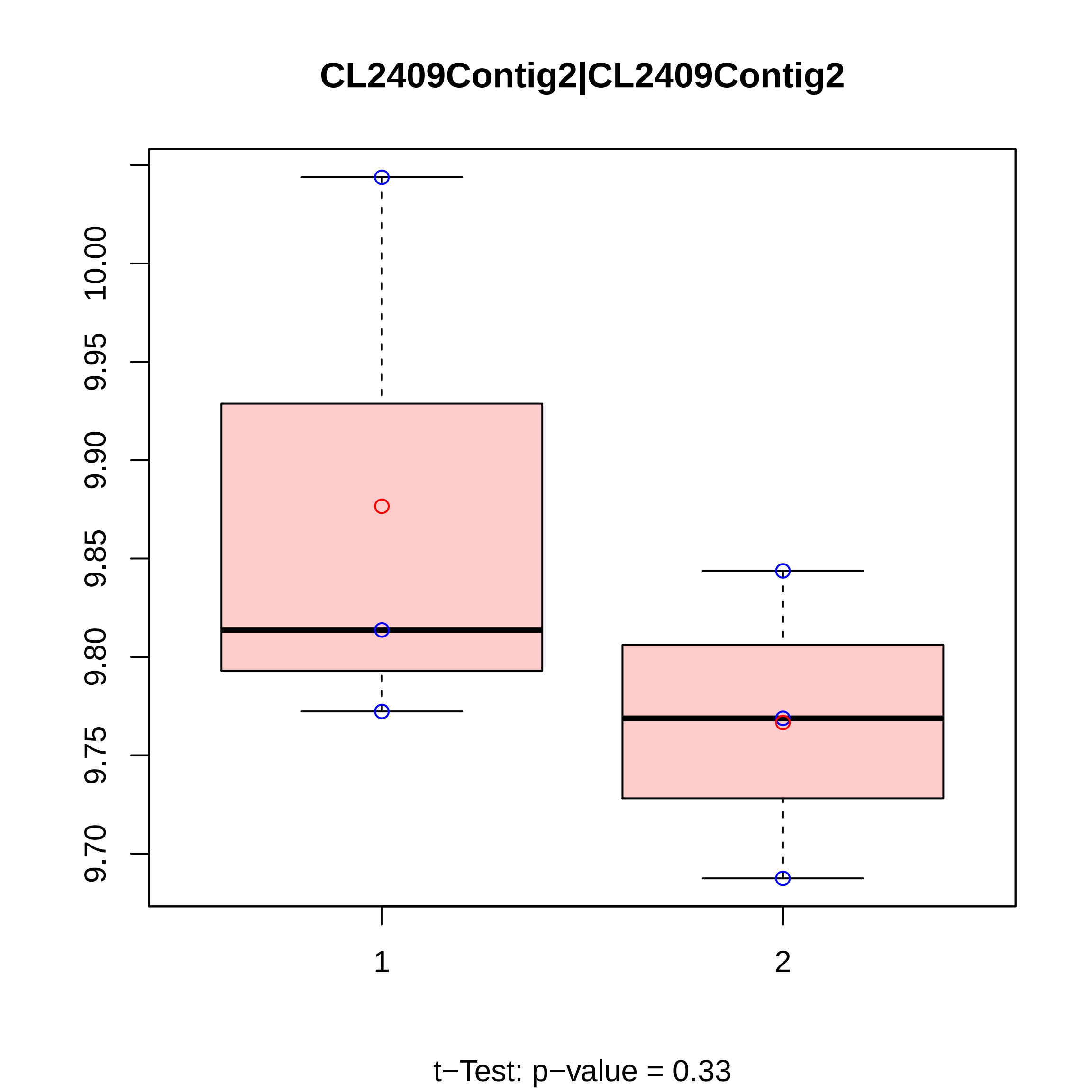

CL2409Contig2|CL2409Contig2
10.00
9.95
9.90
9.85
9.80
9.75
9.70
1
2
t−Test: p−value = 0.33

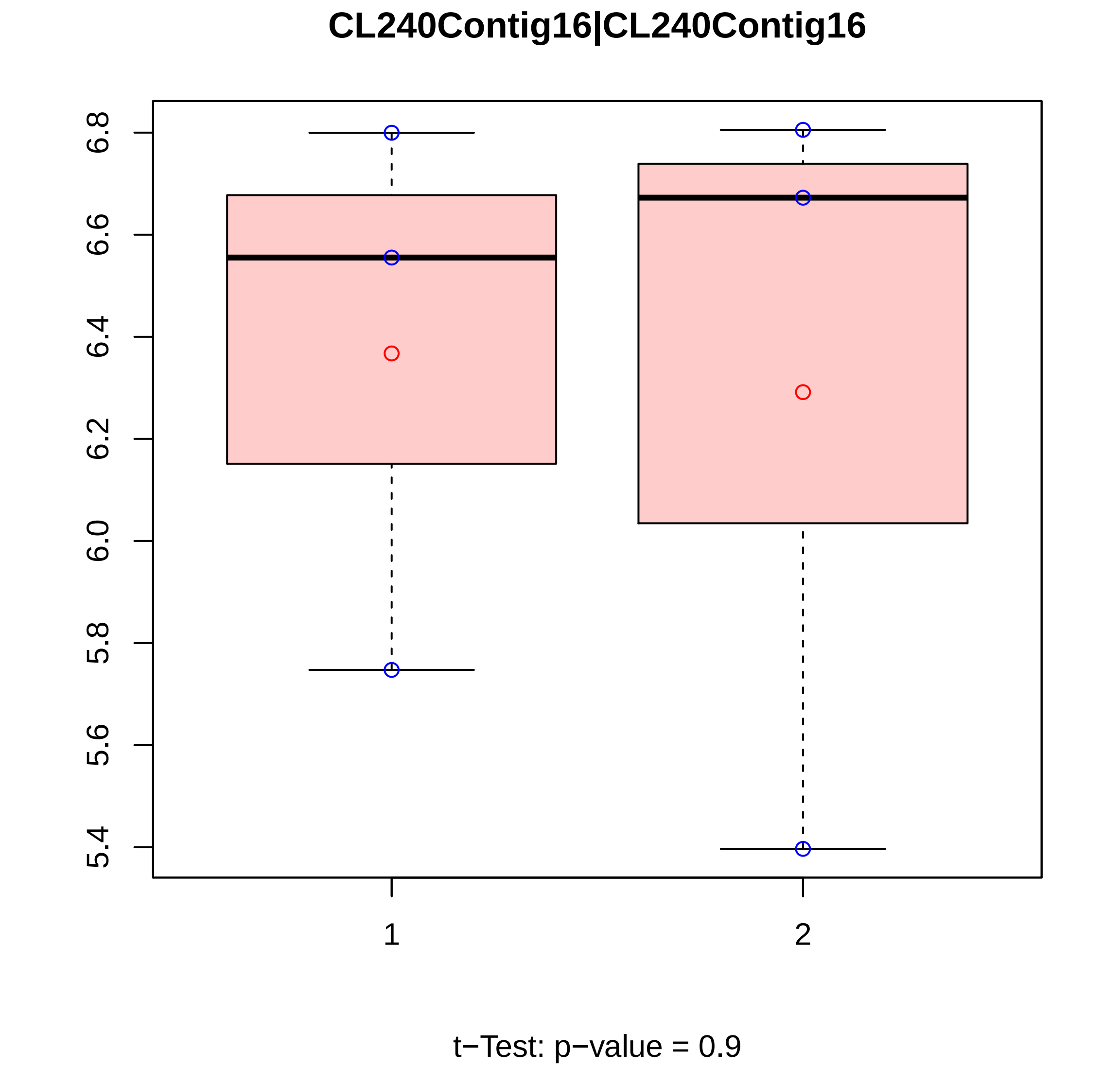
CL240Contig16|CL240Contig16
6.8
6.6
6.4
6.2
6.0
5.8
5.6
5.4
1
2
t−Test: p−value = 0.9

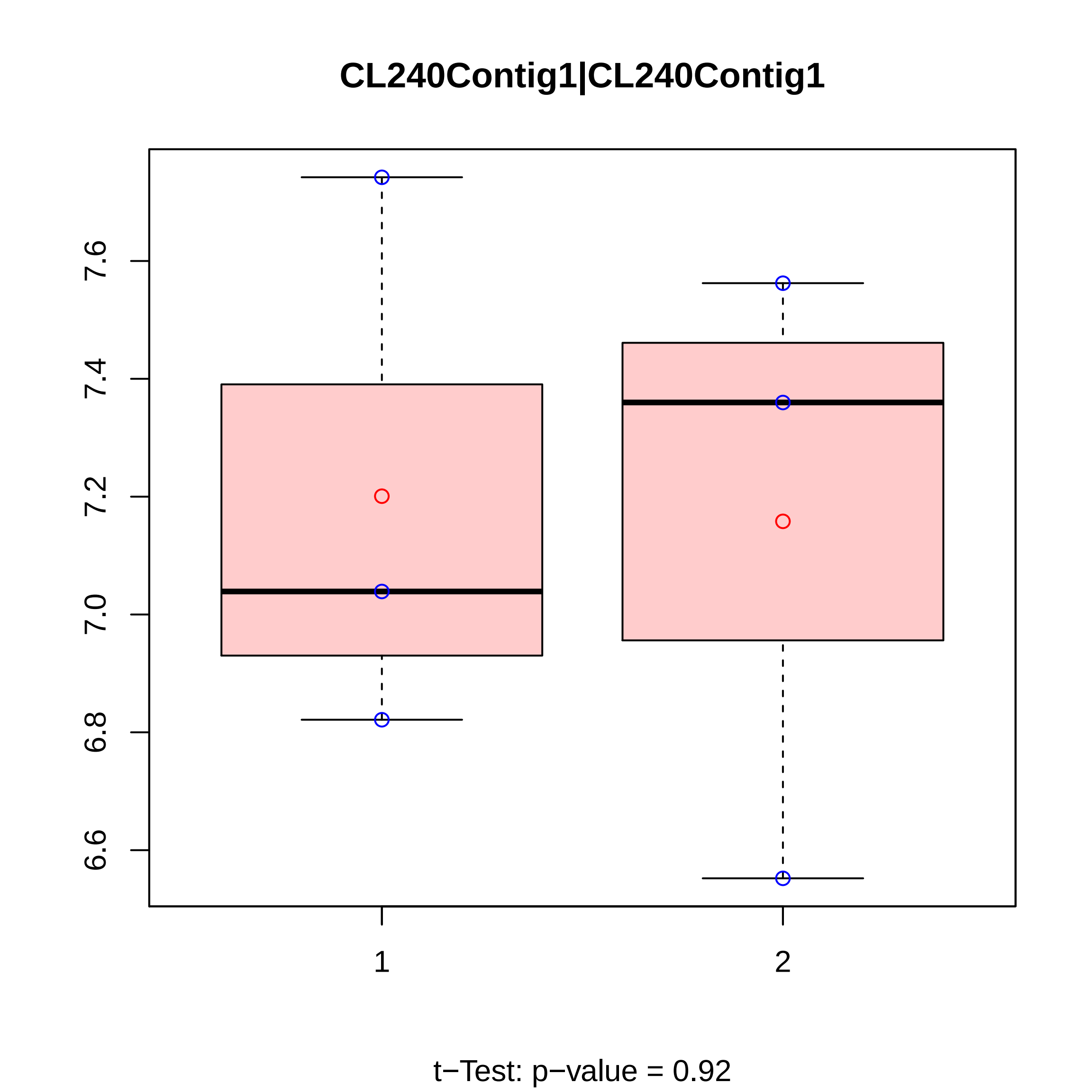

CL240Contig1|CL240Contig1
7.6
7.4
7.2
7.0
6.8
6.6
1
2
t−Test: p−value = 0.92

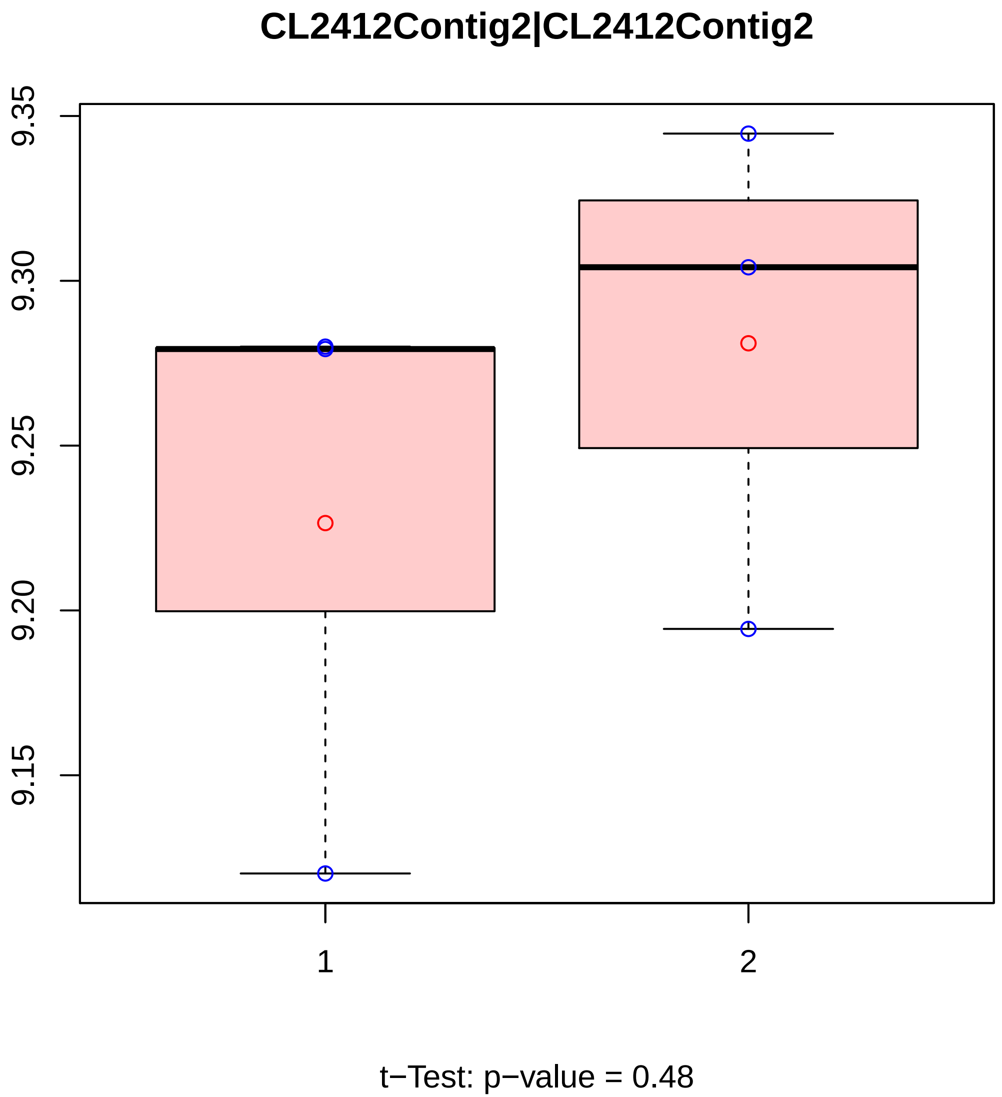
CL2412Contig2|CL2412Contig2
9.35
9.30
9.25
9.20
9.15
1
2
t−Test: p−value = 0.48

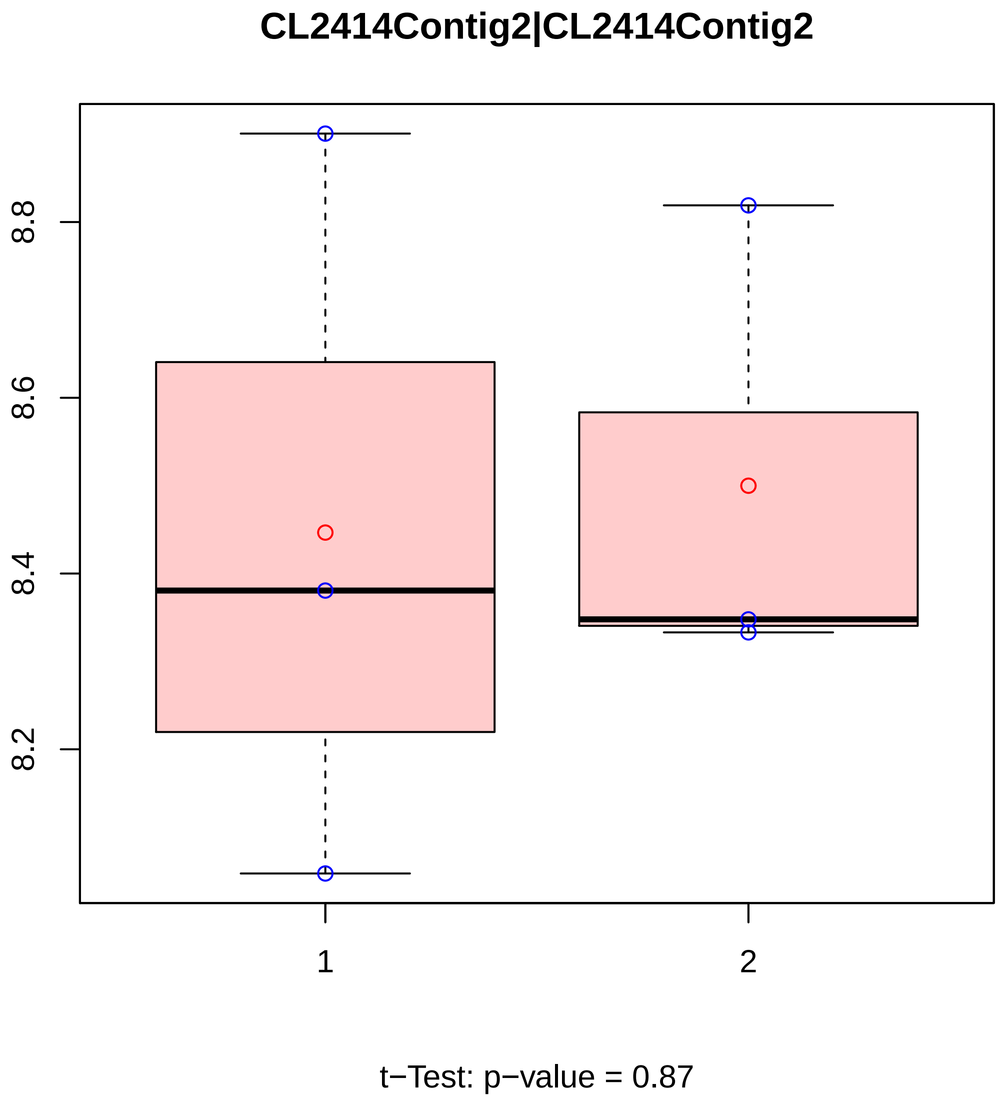

CL2414Contig2|CL2414Contig2
8.8
8.6
8.4
8.2
1
2
t−Test: p−value = 0.87

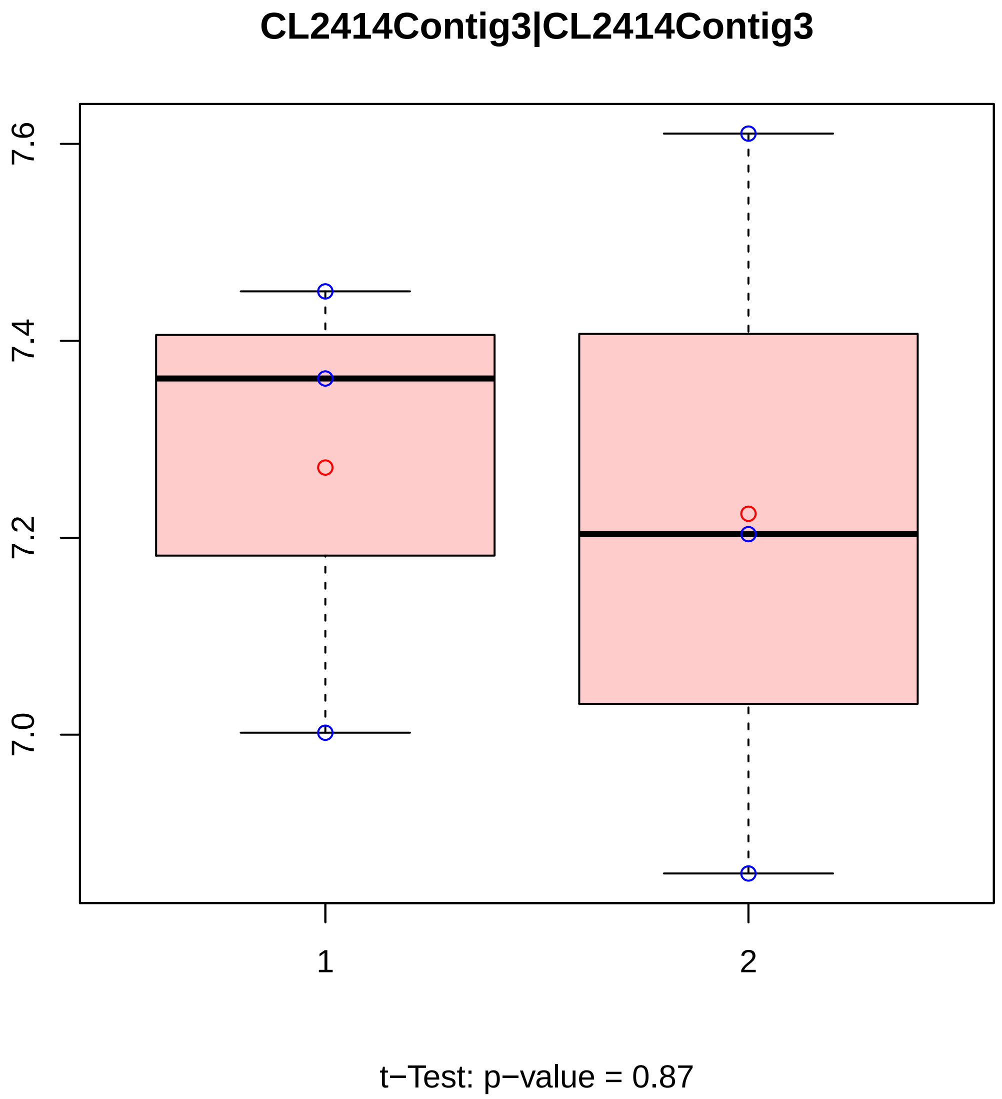
CL2414Contig3|CL2414Contig3
7.6
7.4
7.2
7.0
1
2
t−Test: p−value = 0.87

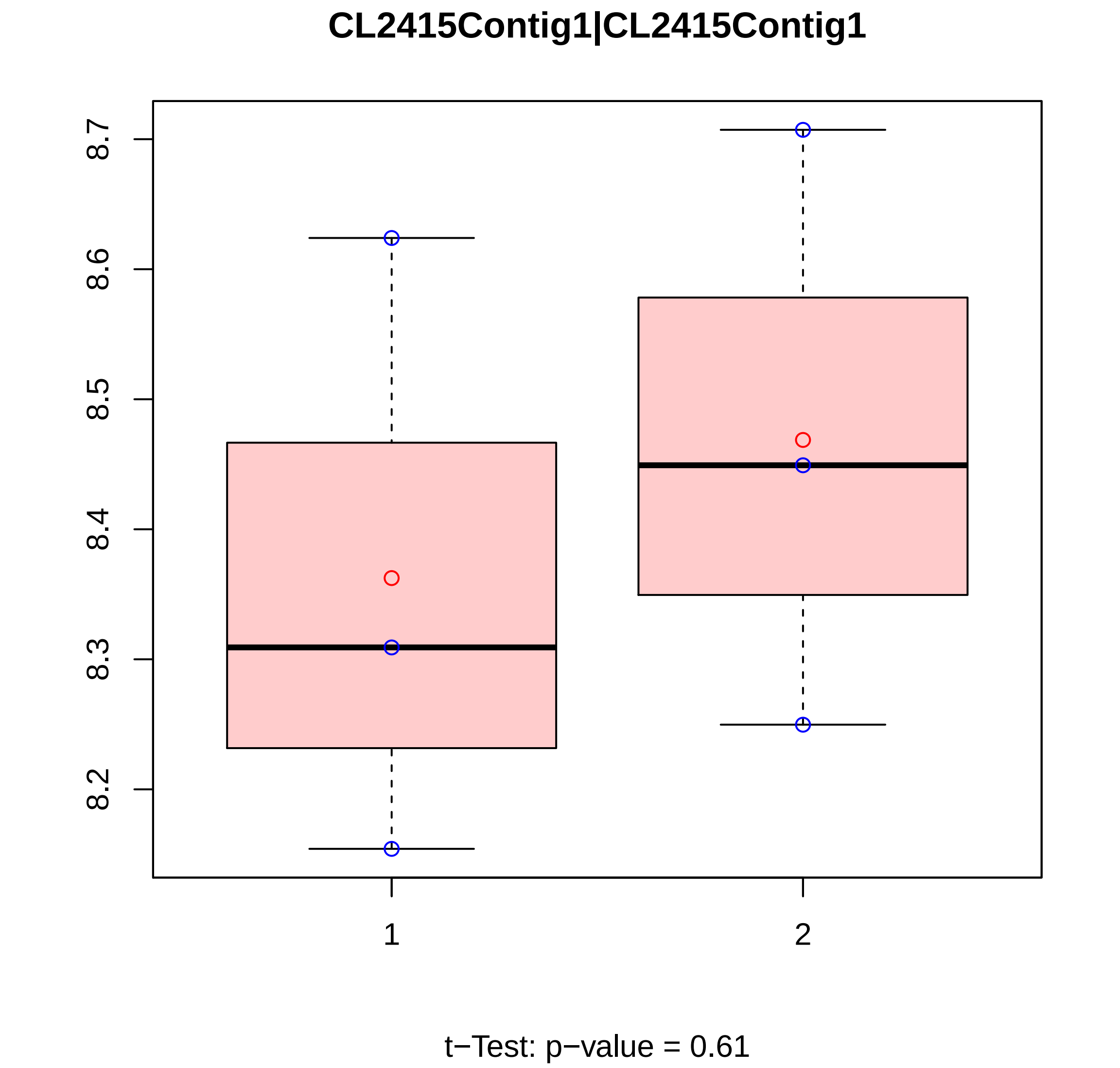
CL2415Contig1|CL2415Contig1
8.7
8.6
8.5
8.4
8.3
8.2
1
2
t−Test: p−value = 0.61

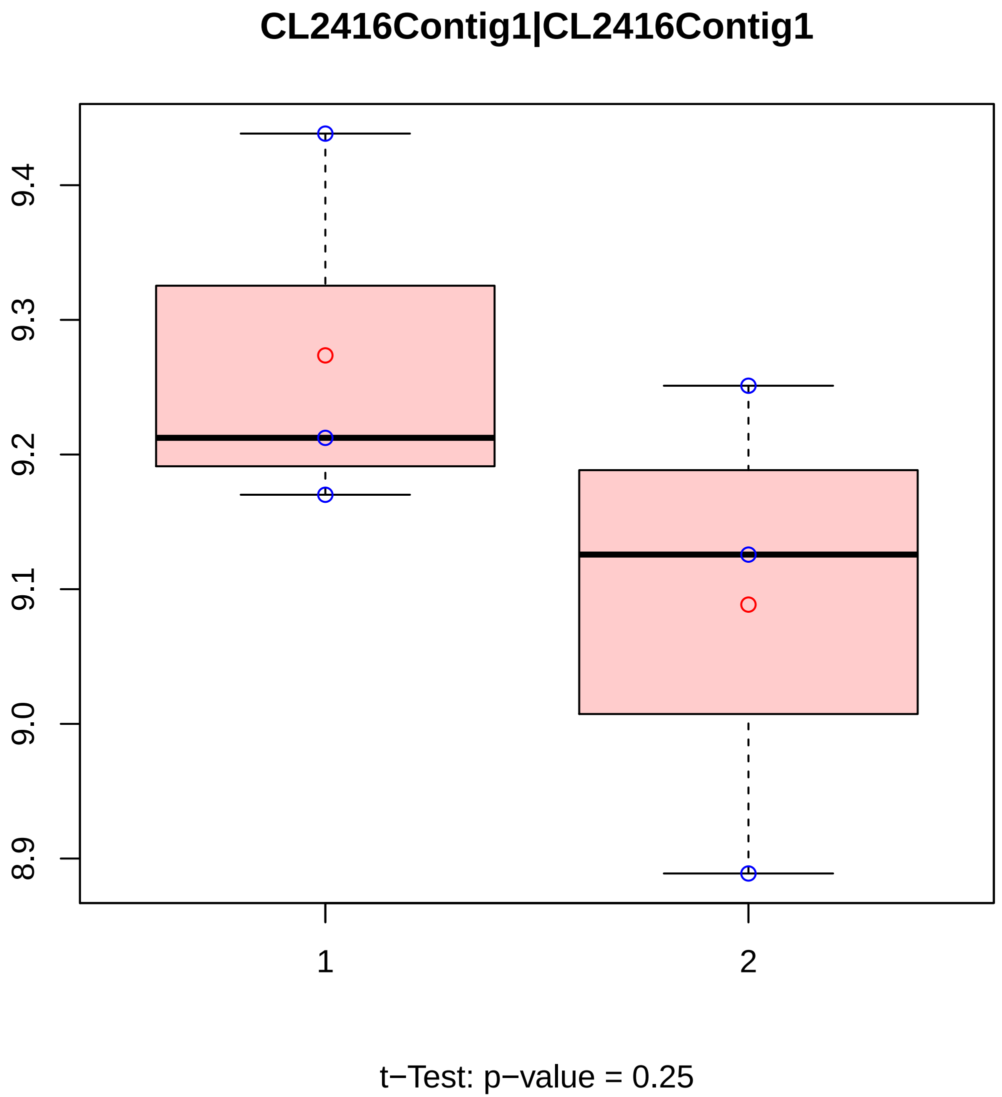

CL2416Contig1|CL2416Contig1
9.4
9.3
9.2
9.1
9.0
8.9
1
2
t−Test: p−value = 0.25

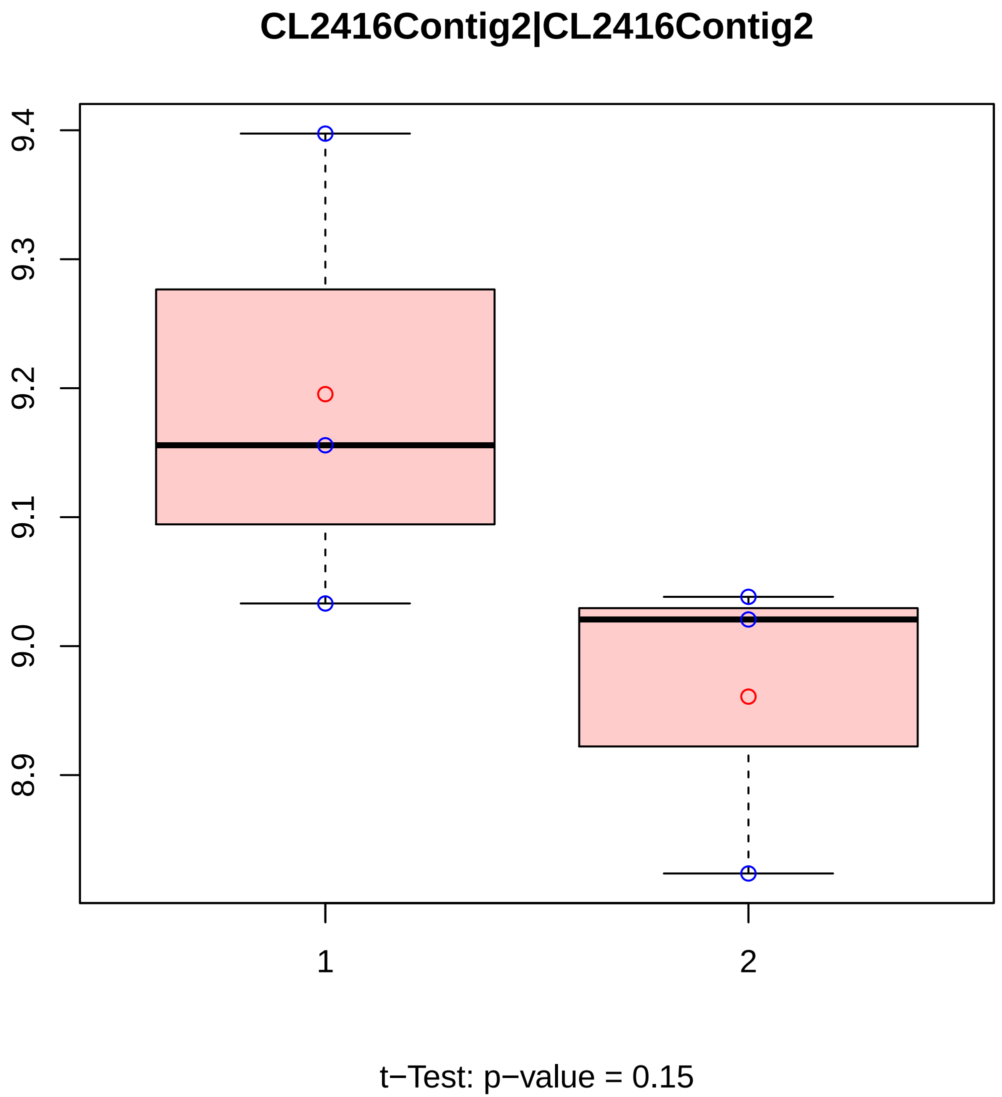
CL2416Contig2|CL2416Contig2
9.4
9.3
9.2
9.1
9.0
8.9
1
2
t−Test: p−value = 0.15

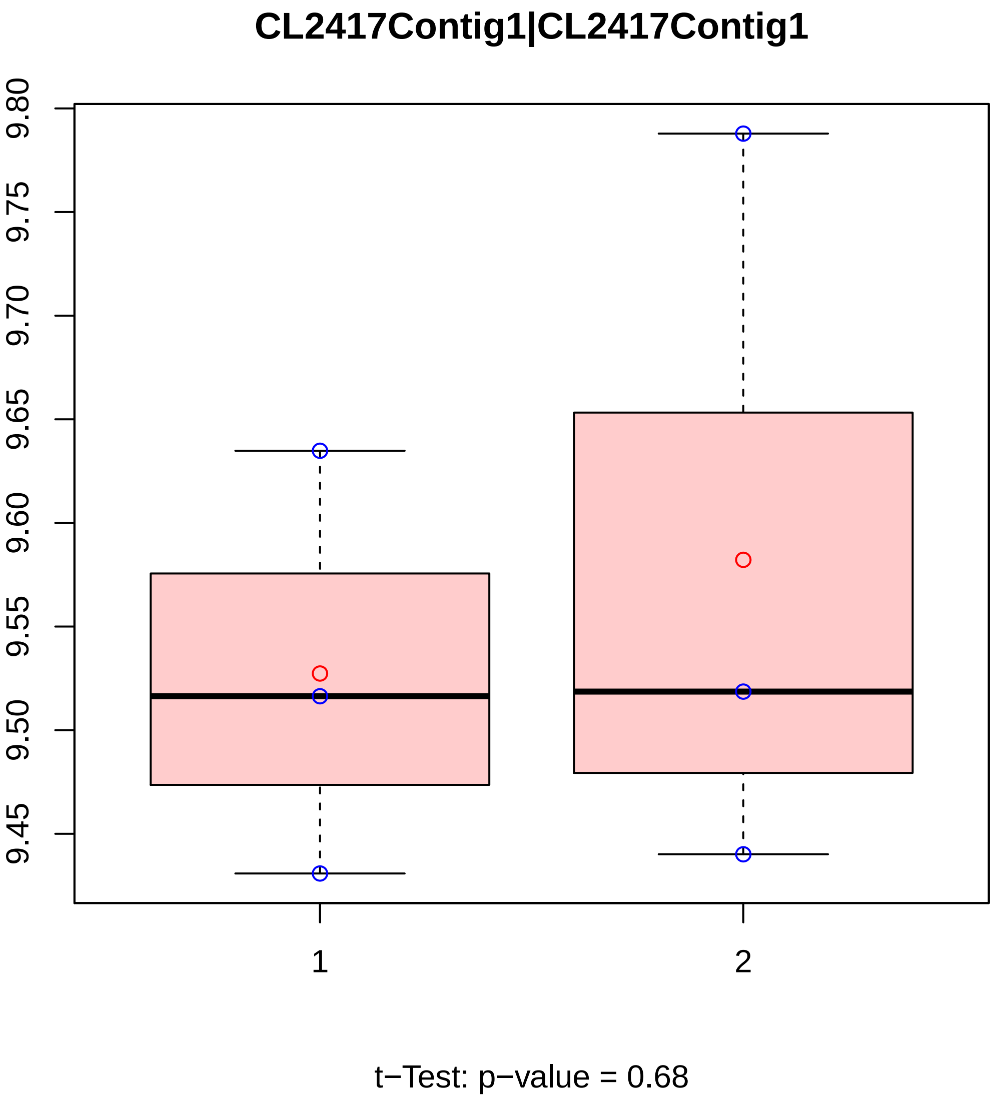
CL2417Contig1|CL2417Contig1
9.80
9.75
9.70
9.65
9.60
9.55
9.50
9.45
1
2
t−Test: p−value = 0.68

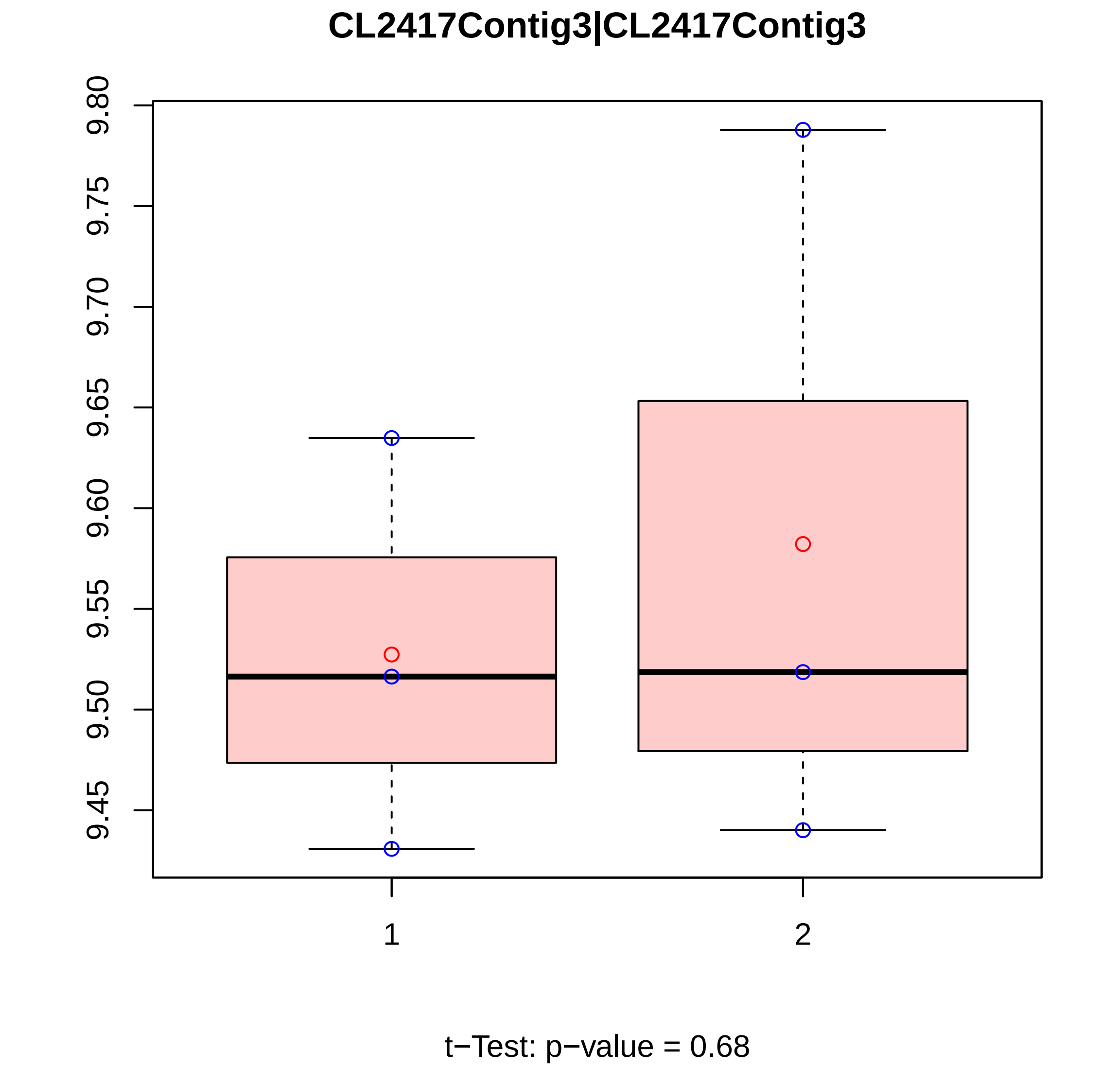
CL2417Contig3|CL2417Contig3
9.80
9.75
9.70
9.65
9.60
9.55
9.50
9.45
1
2
t−Test: p−value = 0.68

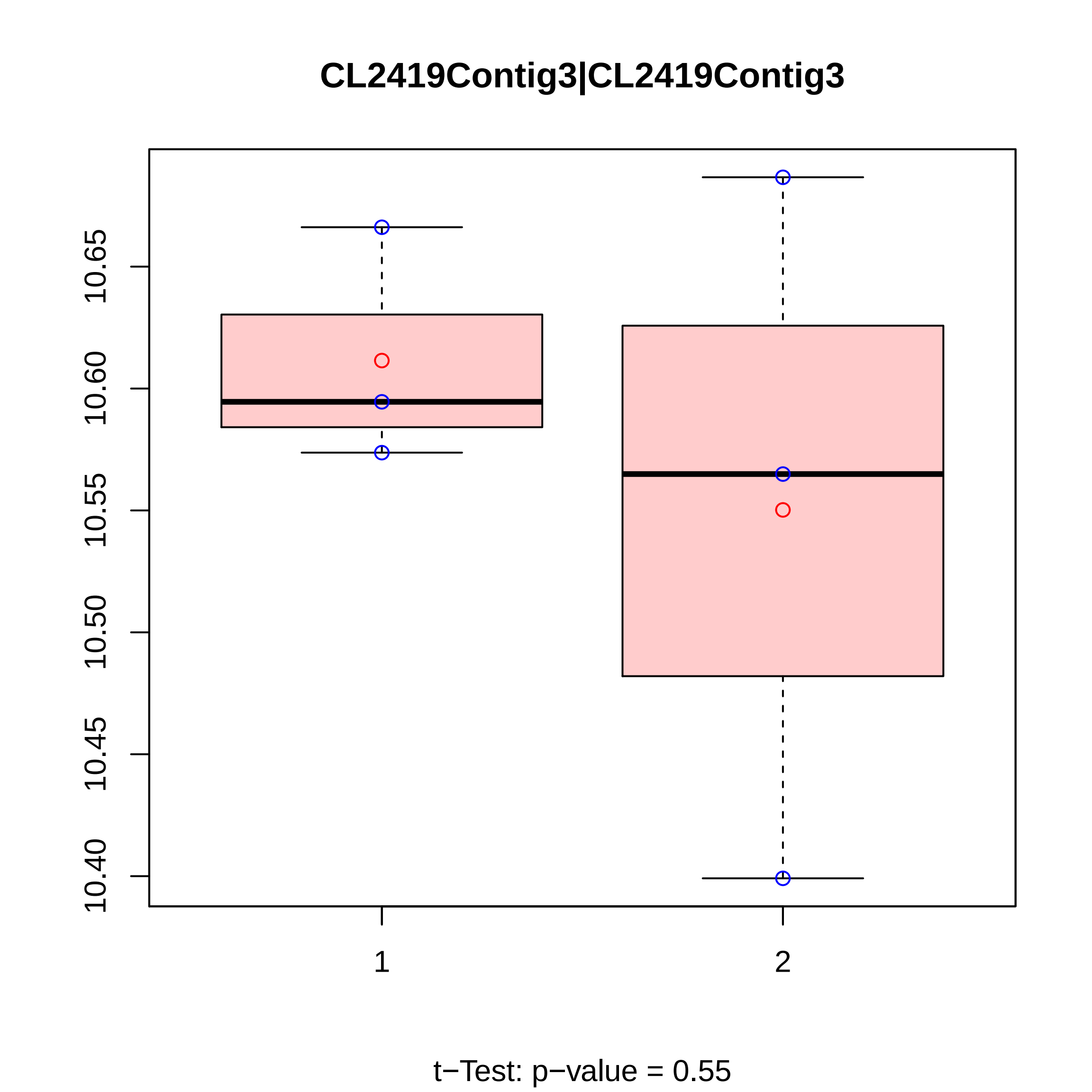

CL2419Contig3|CL2419Contig3
10.40
10.45
10.50
10.55
10.60
10.65
1
2
t−Test: p−value = 0.55

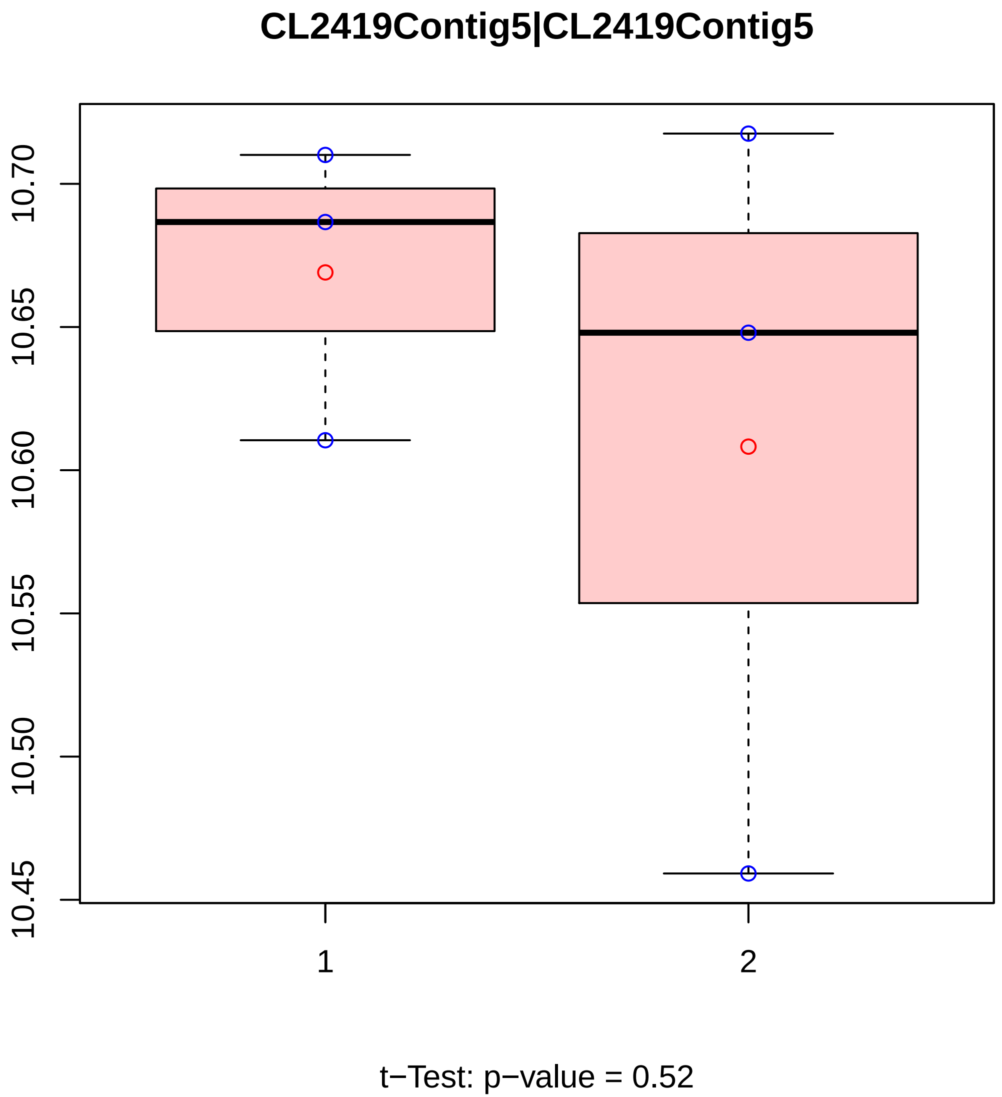

CL2419Contig5|CL2419Contig5
10.45
10.50
10.55
10.60
10.65
10.70
1
2
t−Test: p−value = 0.52

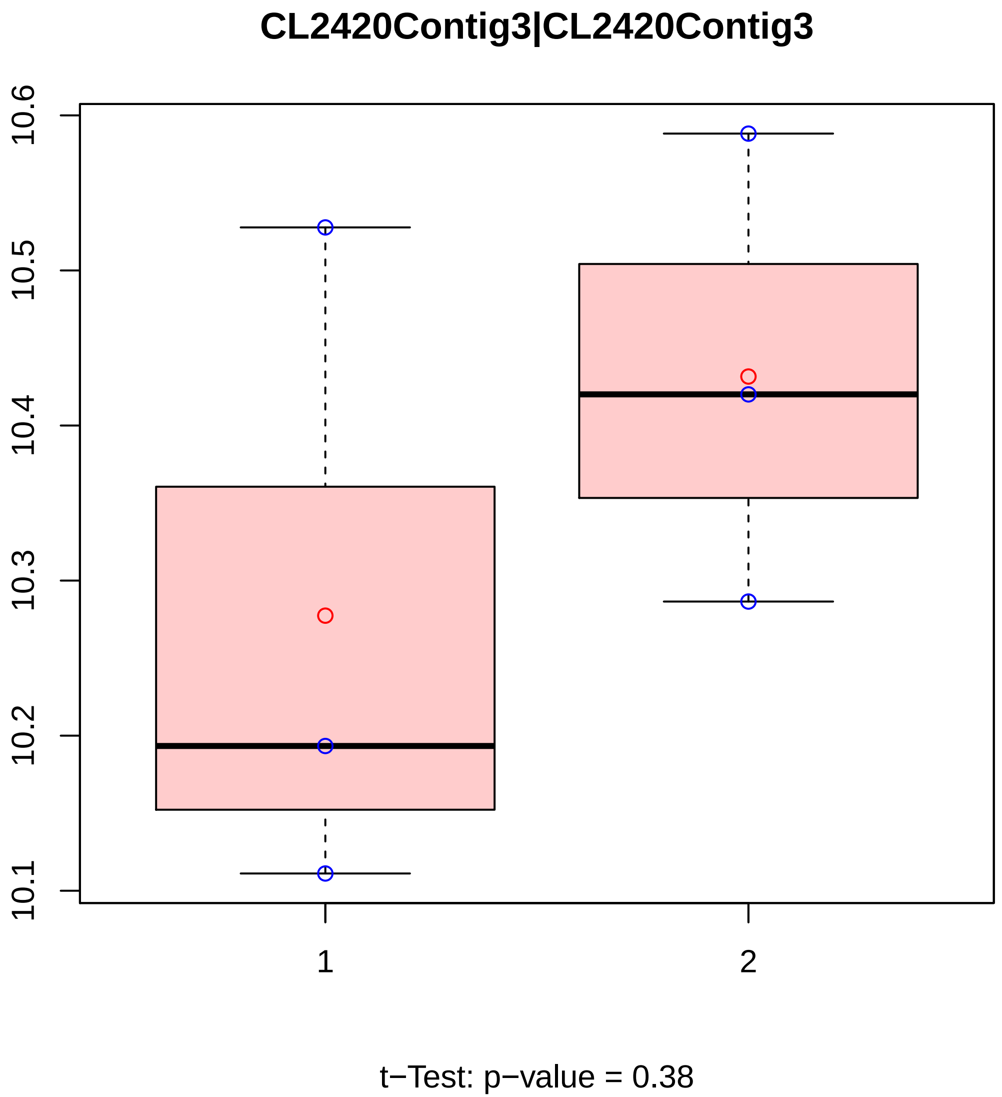
CL2420Contig3|CL2420Contig3
10.6
10.5
10.4
10.3
10.2
10.1
1
2
t−Test: p−value = 0.38

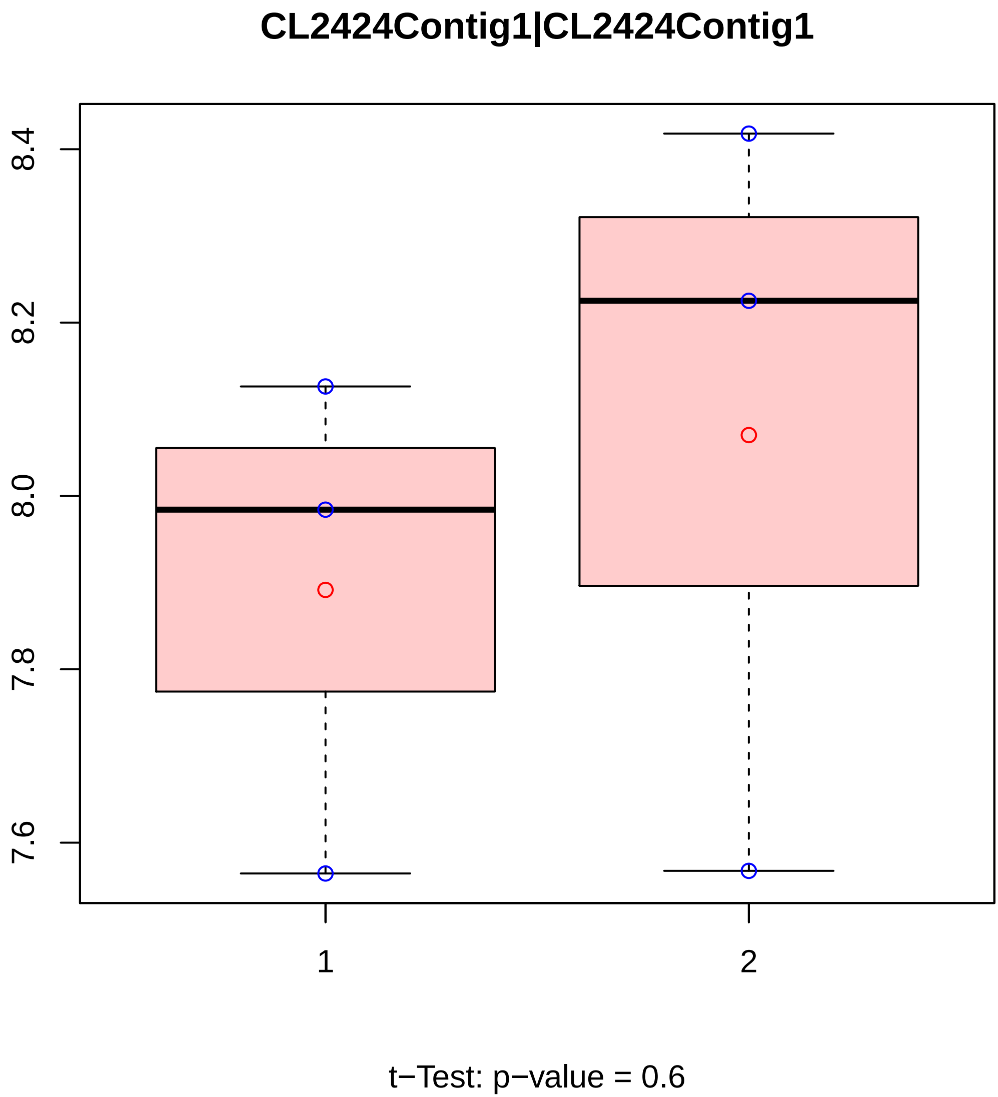
CL2424Contig1|CL2424Contig1
8.4
8.2
8.0
7.8
7.6
1
2
t−Test: p−value = 0.6

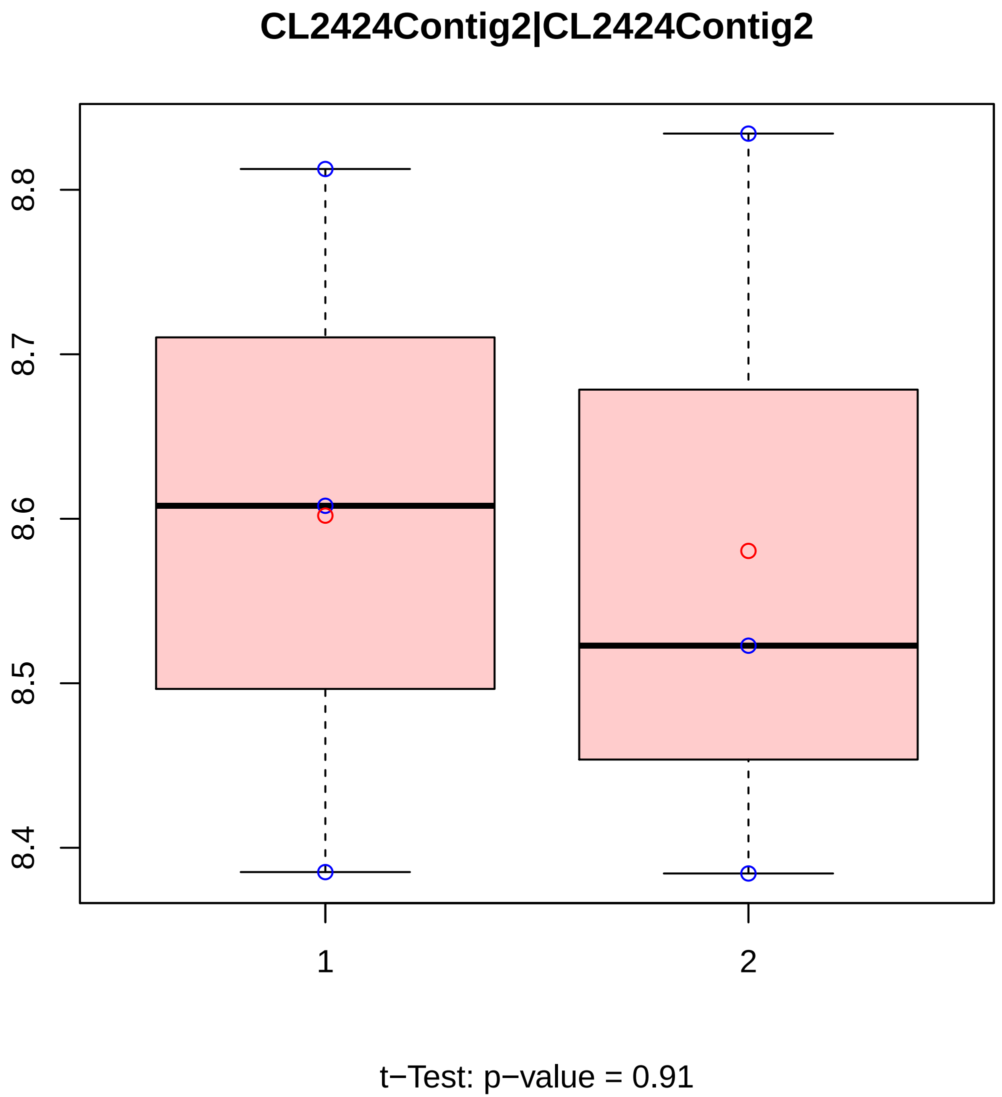
CL2424Contig2|CL2424Contig2
8.4
8.5
8.6
8.7
8.8
1
2
t−Test: p−value = 0.91

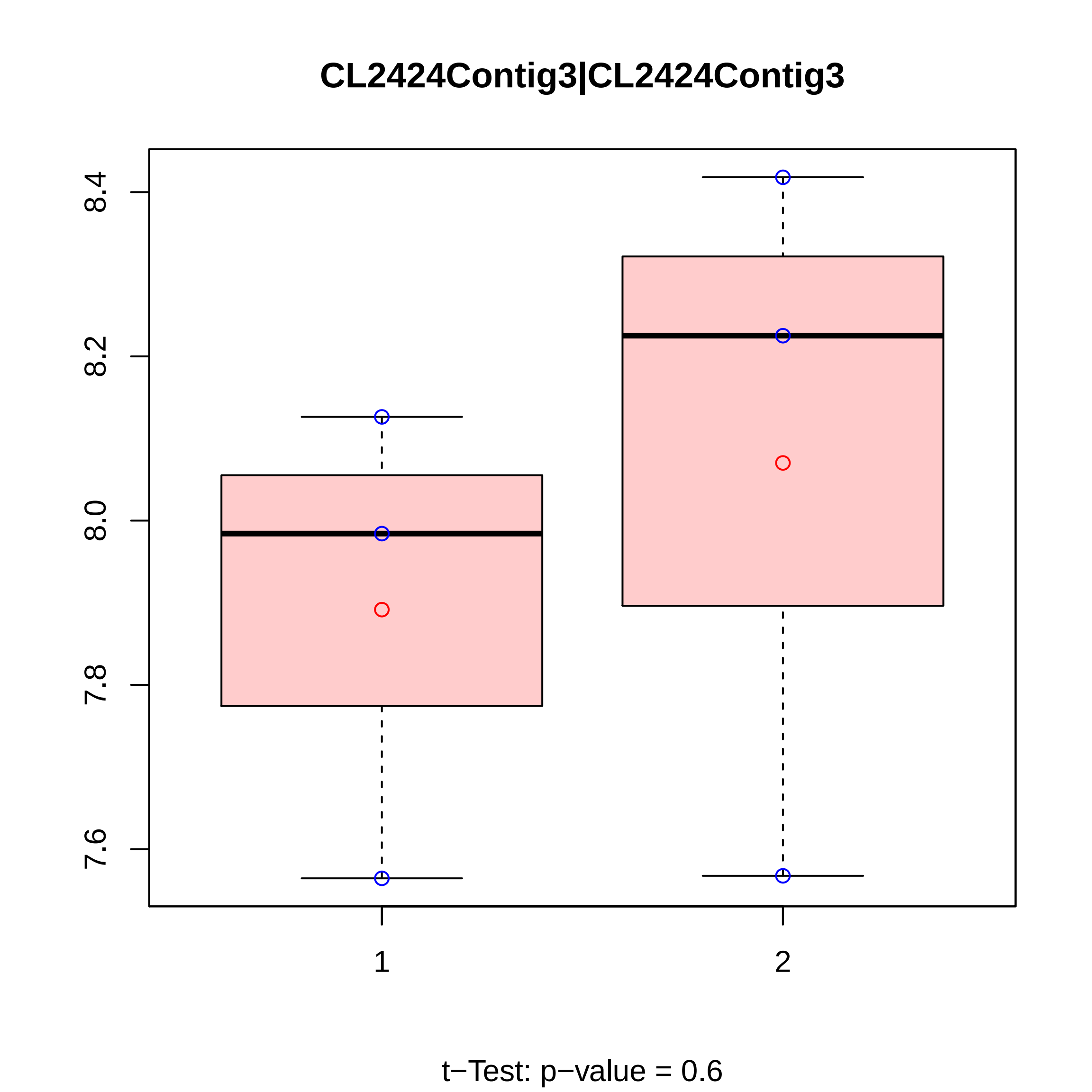
CL2424Contig3|CL2424Contig3
8.4
8.2
8.0
7.8
7.6
1
2
t−Test: p−value = 0.6

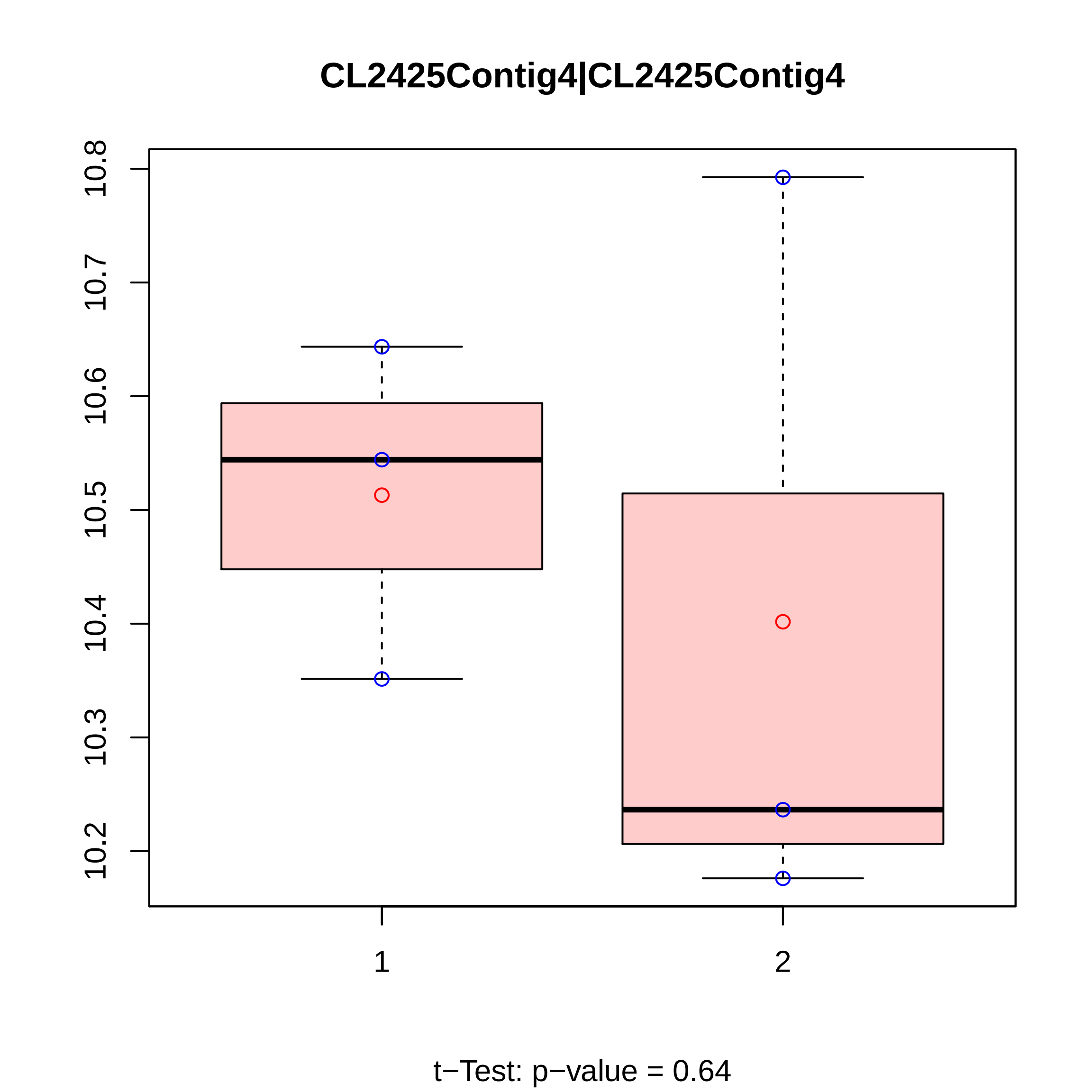

CL2425Contig4|CL2425Contig4
10.2
10.3
10.4
10.5
10.6
10.7
10.8
1
2
t−Test: p−value = 0.64

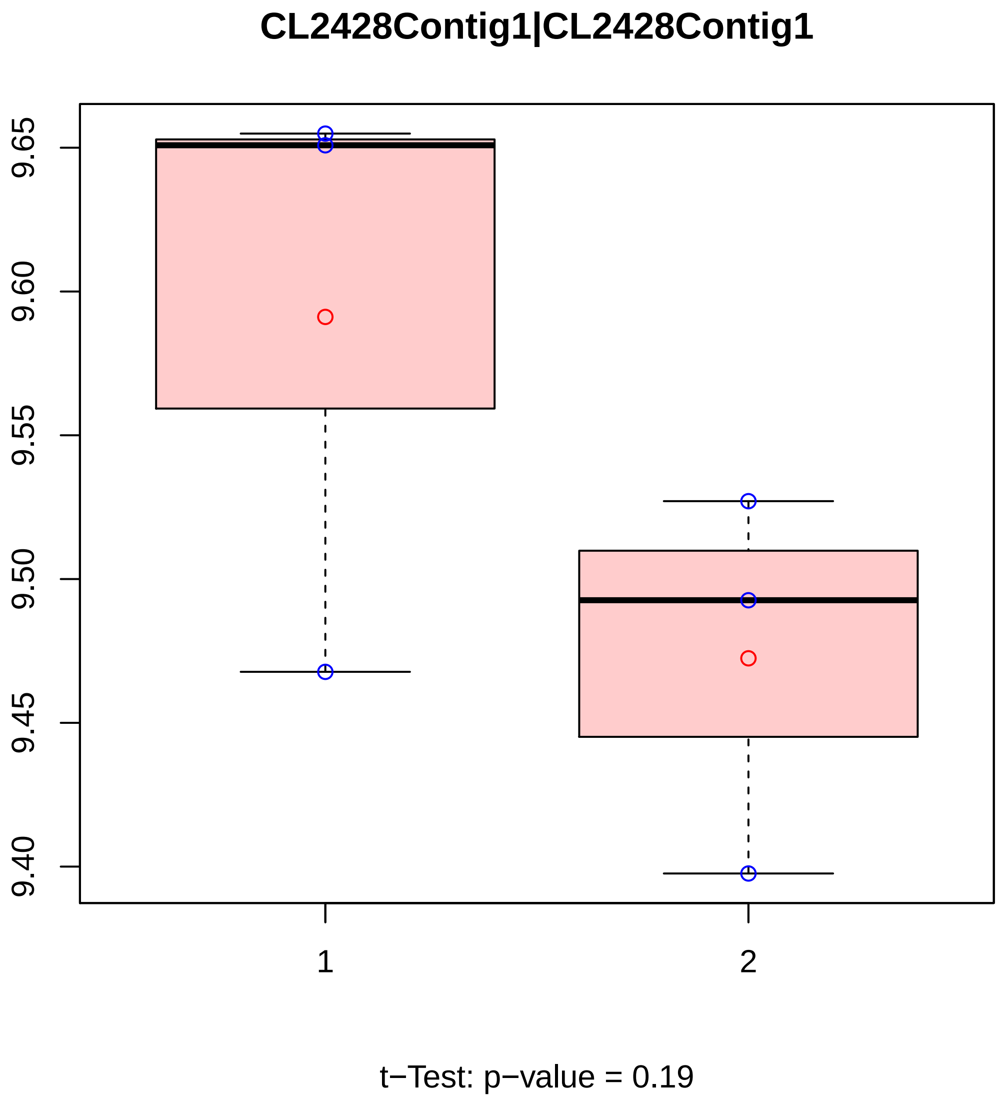
CL2428Contig1|CL2428Contig1
9.65
9.60
9.55
9.50
9.45
9.40
1
2
t−Test: p−value = 0.19

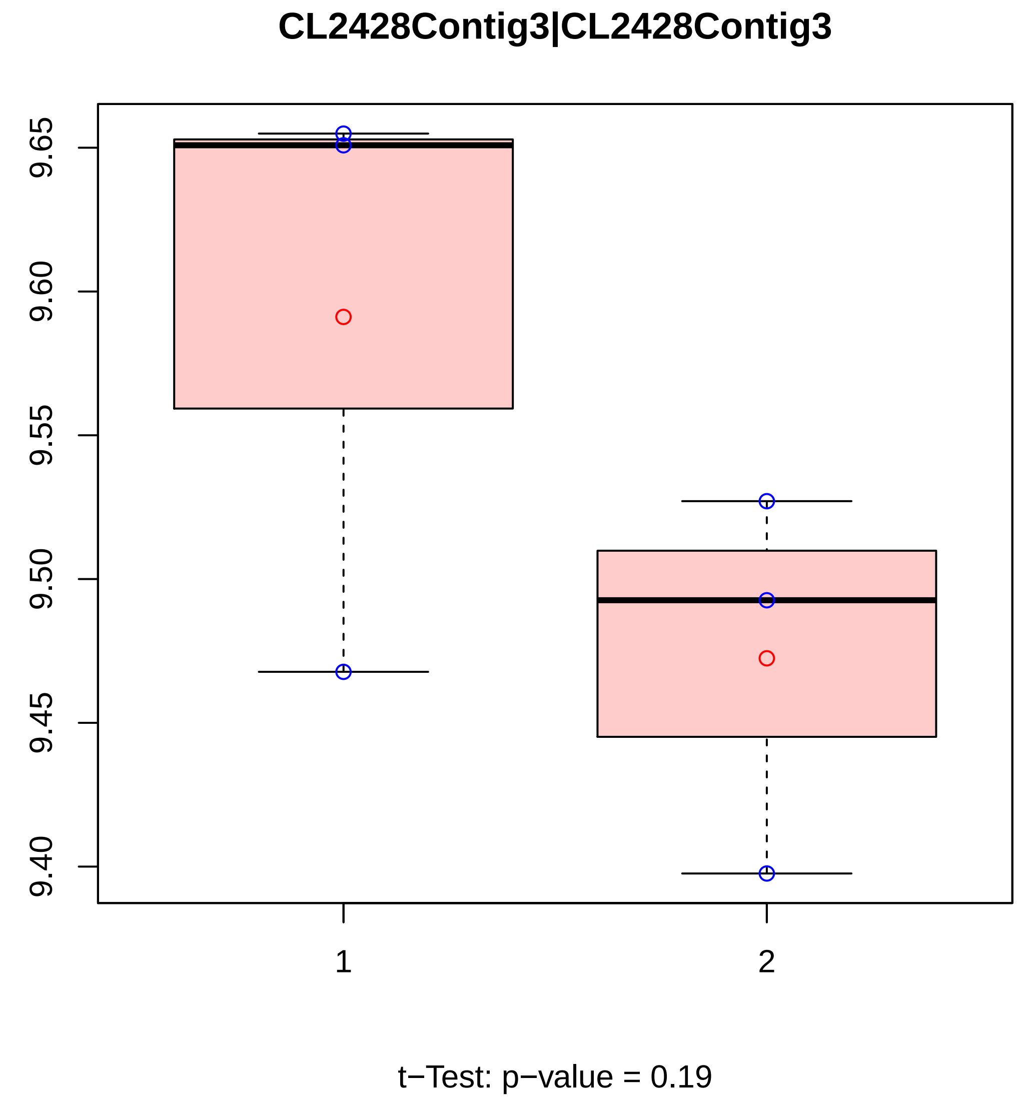
CL2428Contig3|CL2428Contig3
9.65
9.60
9.55
9.50
9.45
9.40
1
2
t−Test: p−value = 0.19

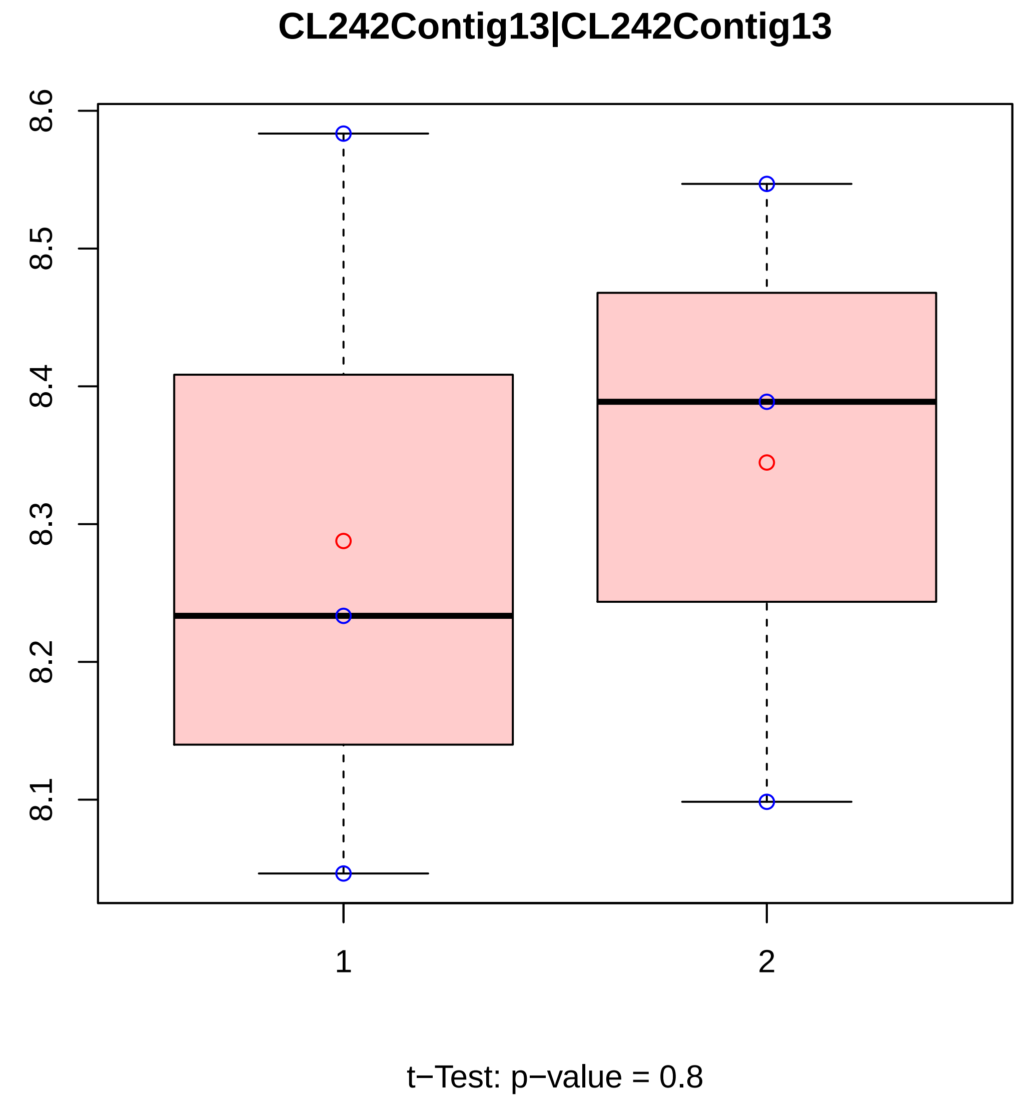
CL242Contig13|CL242Contig13
8.1
8.2
8.3
8.4
8.5
8.6
1
2
t−Test: p−value = 0.8

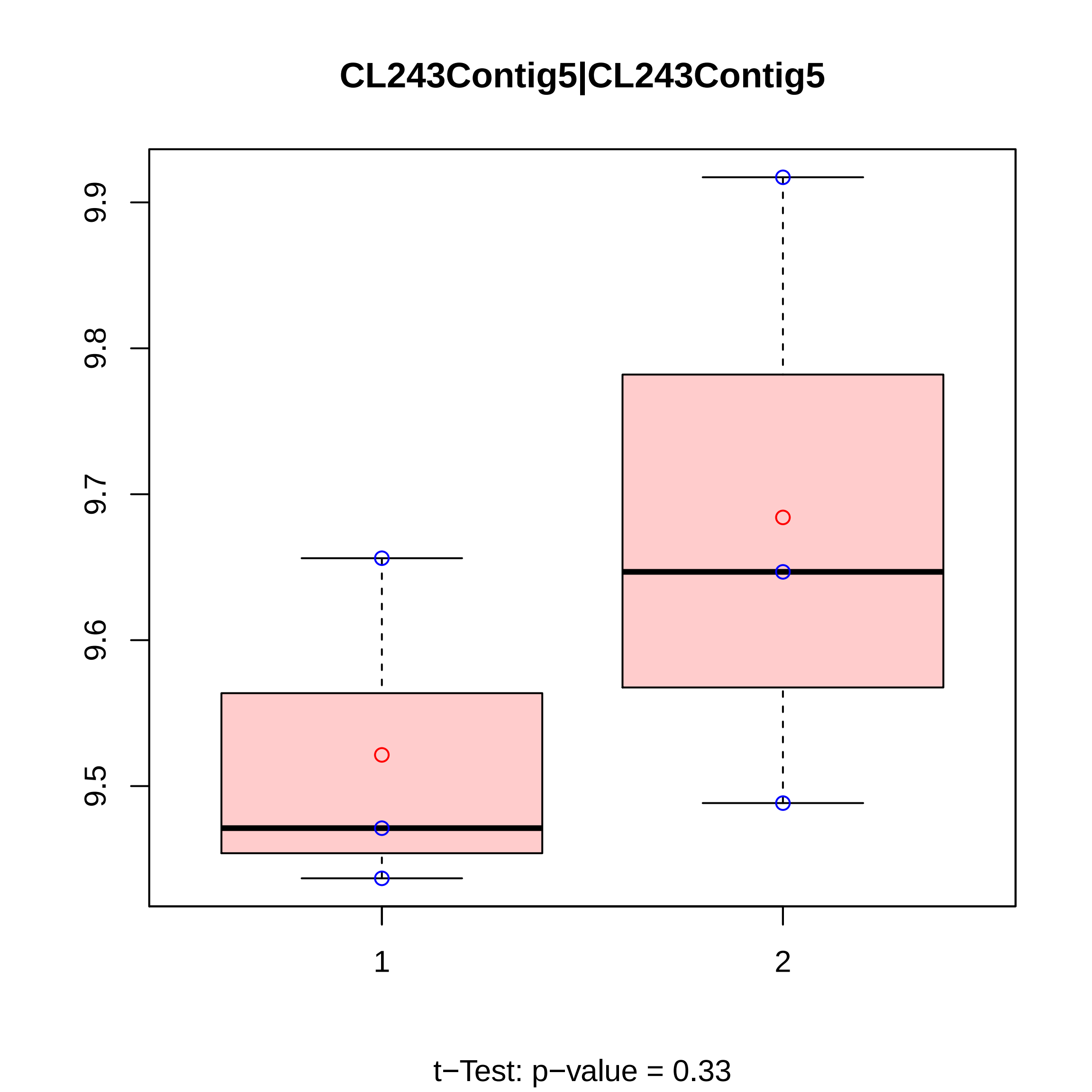
CL243Contig5|CL243Contig5
9.5
9.6
9.7
9.8
9.9
1
2
t−Test: p−value = 0.33

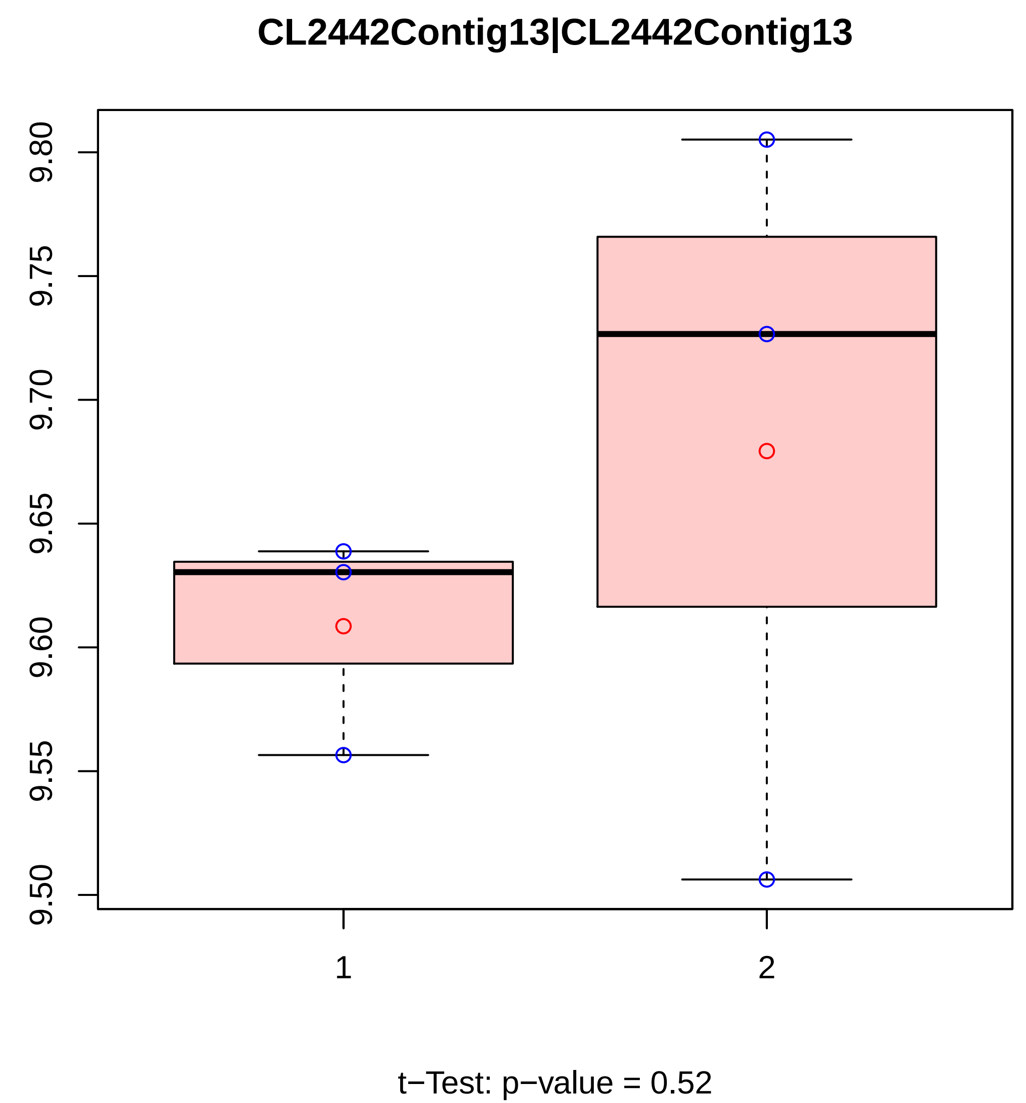
CL2442Contig13|CL2442Contig13
9.50
9.55
9.60
9.65
9.70
9.75
9.80
1
2
t−Test: p−value = 0.52

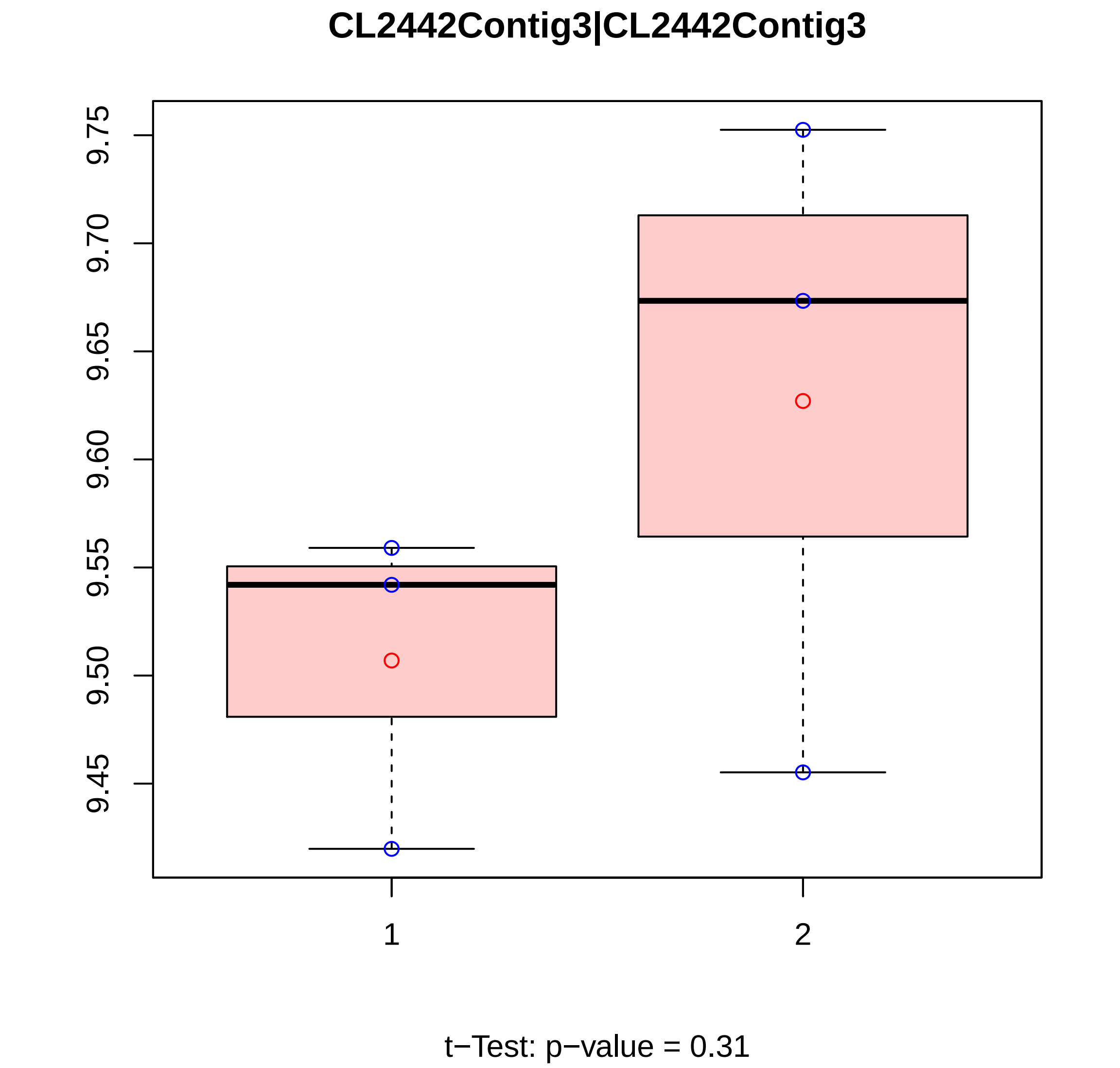

CL2442Contig3|CL2442Contig3
9.45
9.50
9.55
9.60
9.65
9.70
9.75
1
2
t−Test: p−value = 0.31

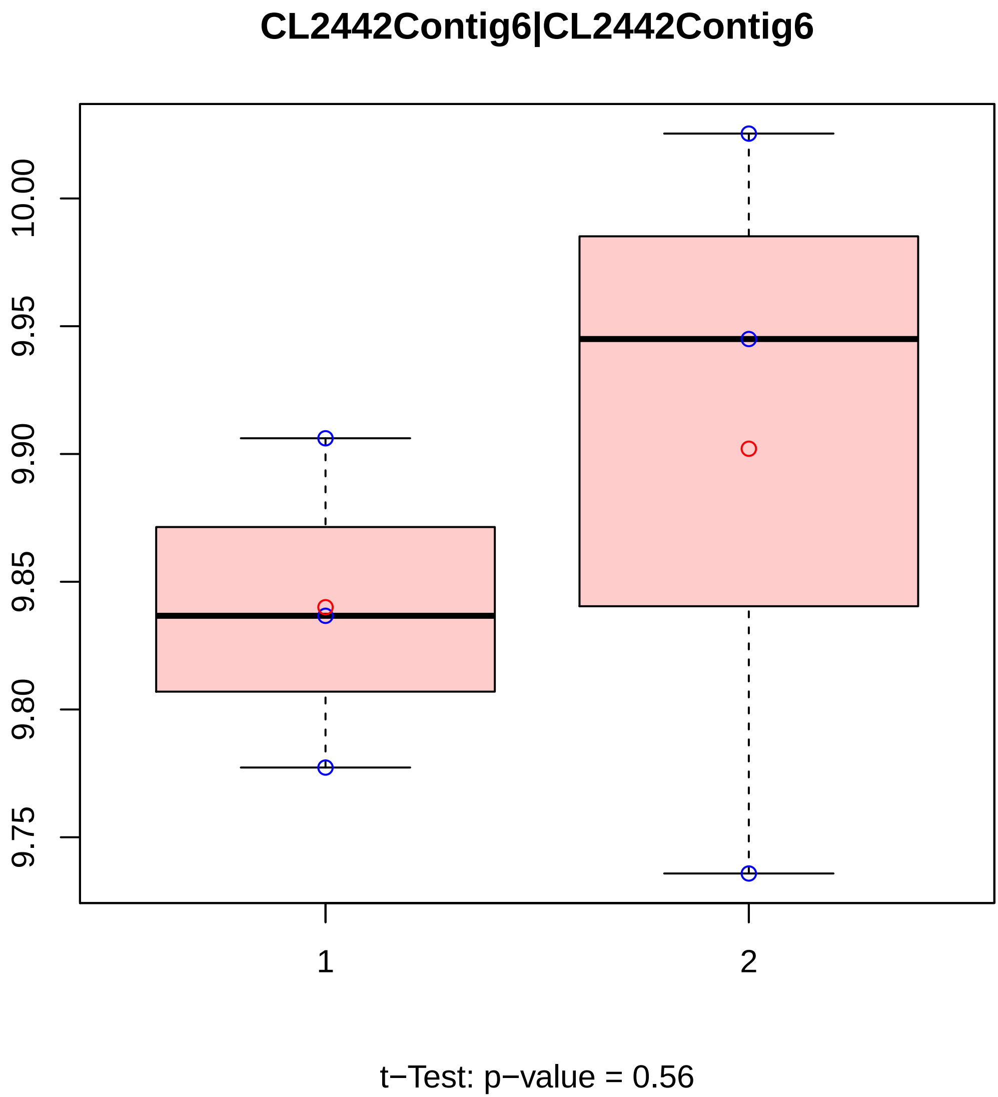
CL2442Contig6|CL2442Contig6
10.00
9.95
9.90
9.85
9.80
9.75
1
2
t−Test: p−value = 0.56

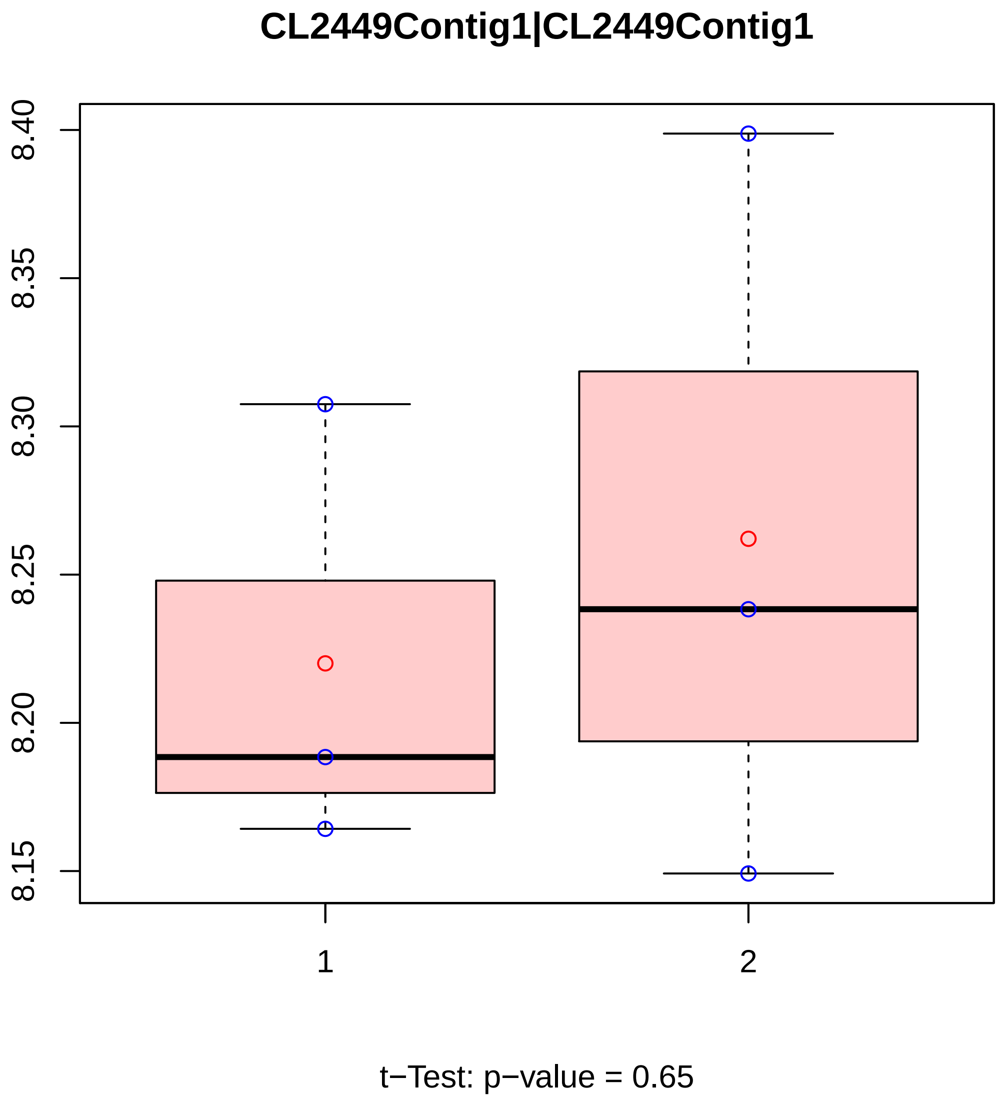

CL2449Contig1|CL2449Contig1
8.15
8.20
8.25
8.30
8.35
8.40
1
2
t−Test: p−value = 0.65

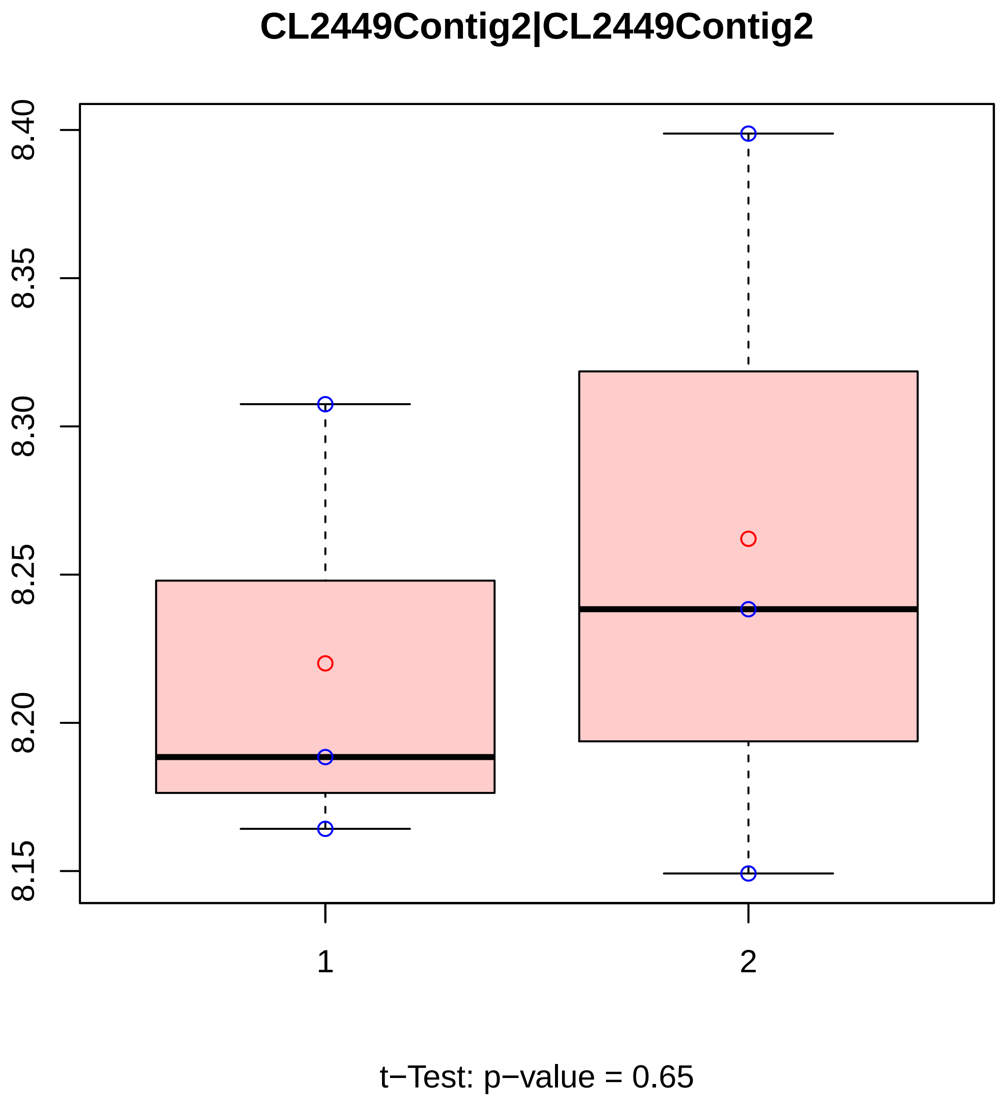

CL2449Contig2|CL2449Contig2
8.40
8.35
8.30
8.25
8.20
8.15
1
2
t−Test: p−value = 0.65

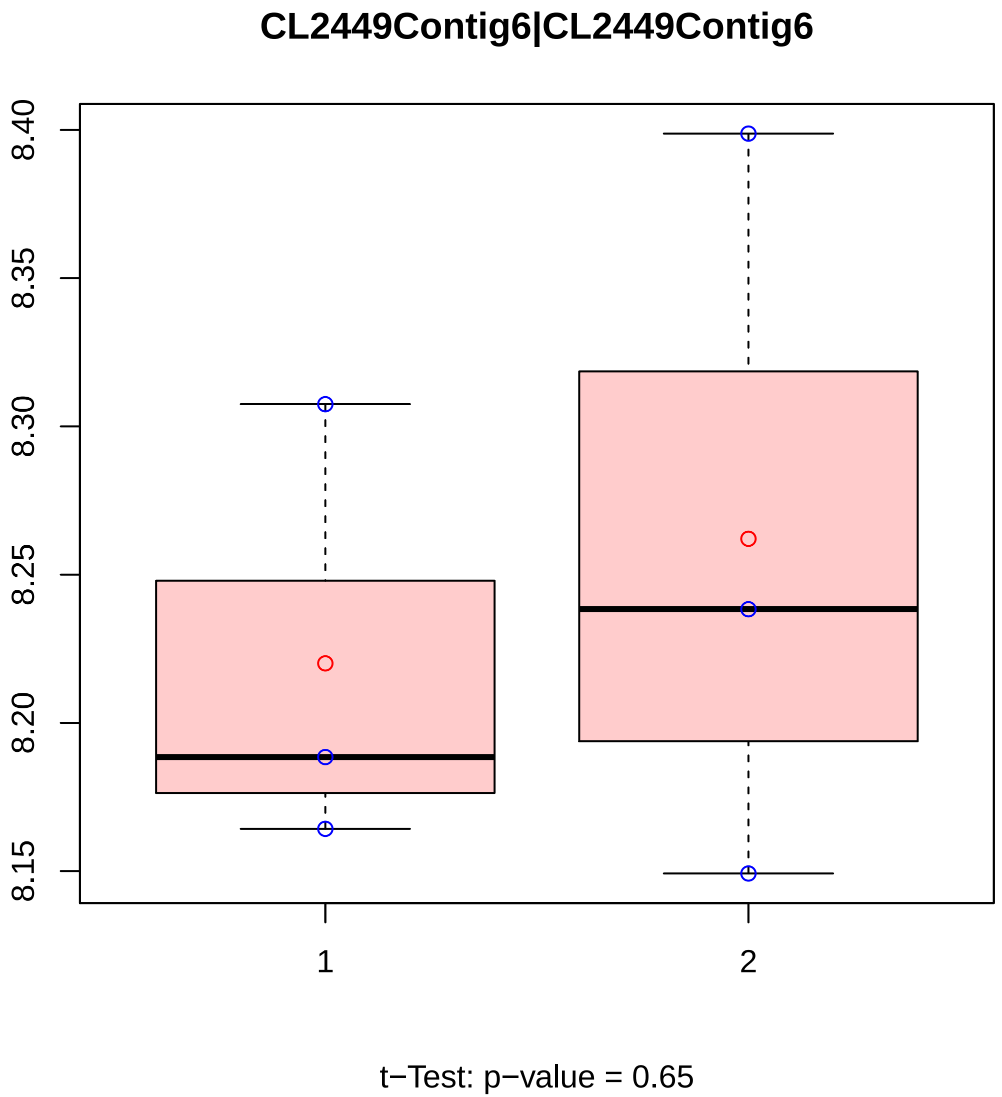
CL2449Contig6|CL2449Contig6
8.40
8.35
8.30
8.25
8.20
8.15
1
2
t−Test: p−value = 0.65

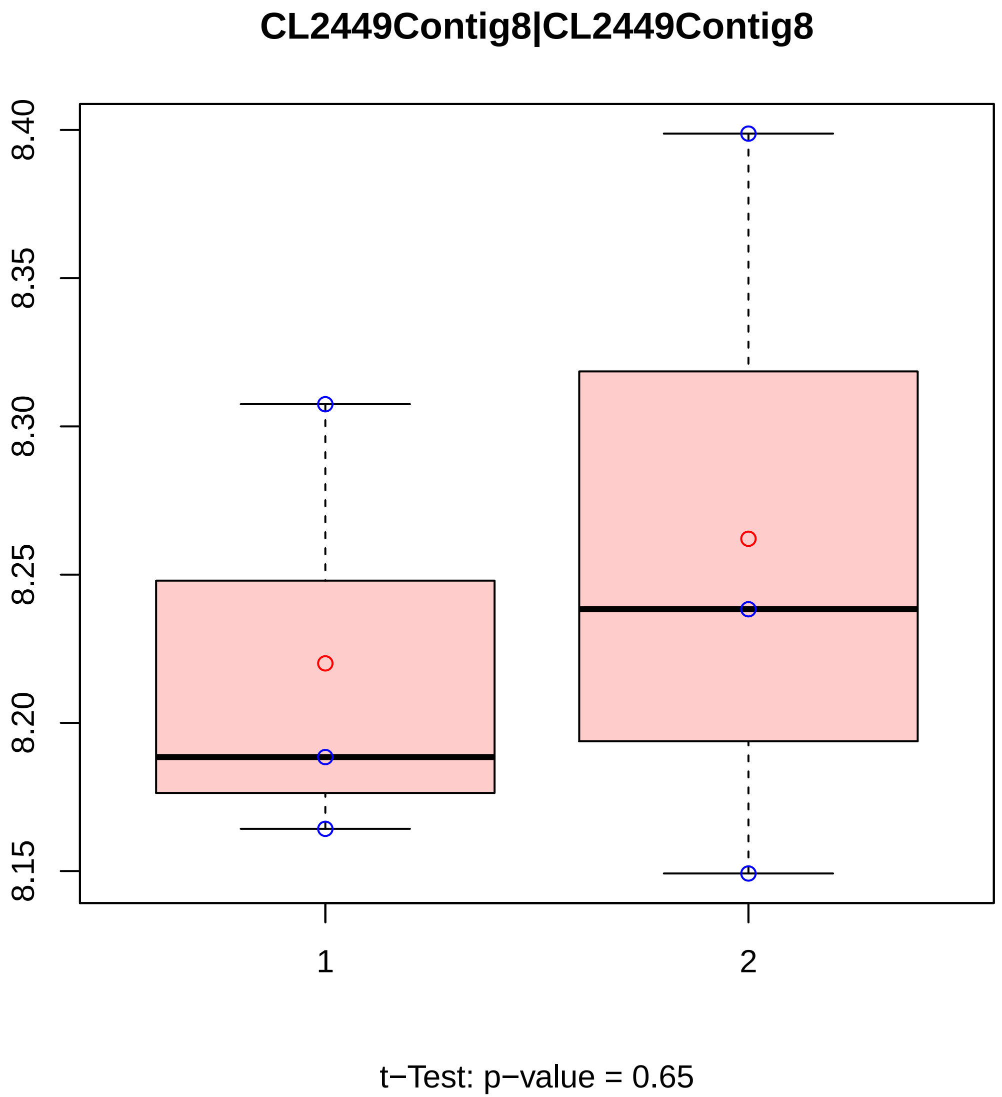

CL2449Contig8|CL2449Contig8
8.40
8.35
8.30
8.25
8.20
8.15
1
2
t−Test: p−value = 0.65

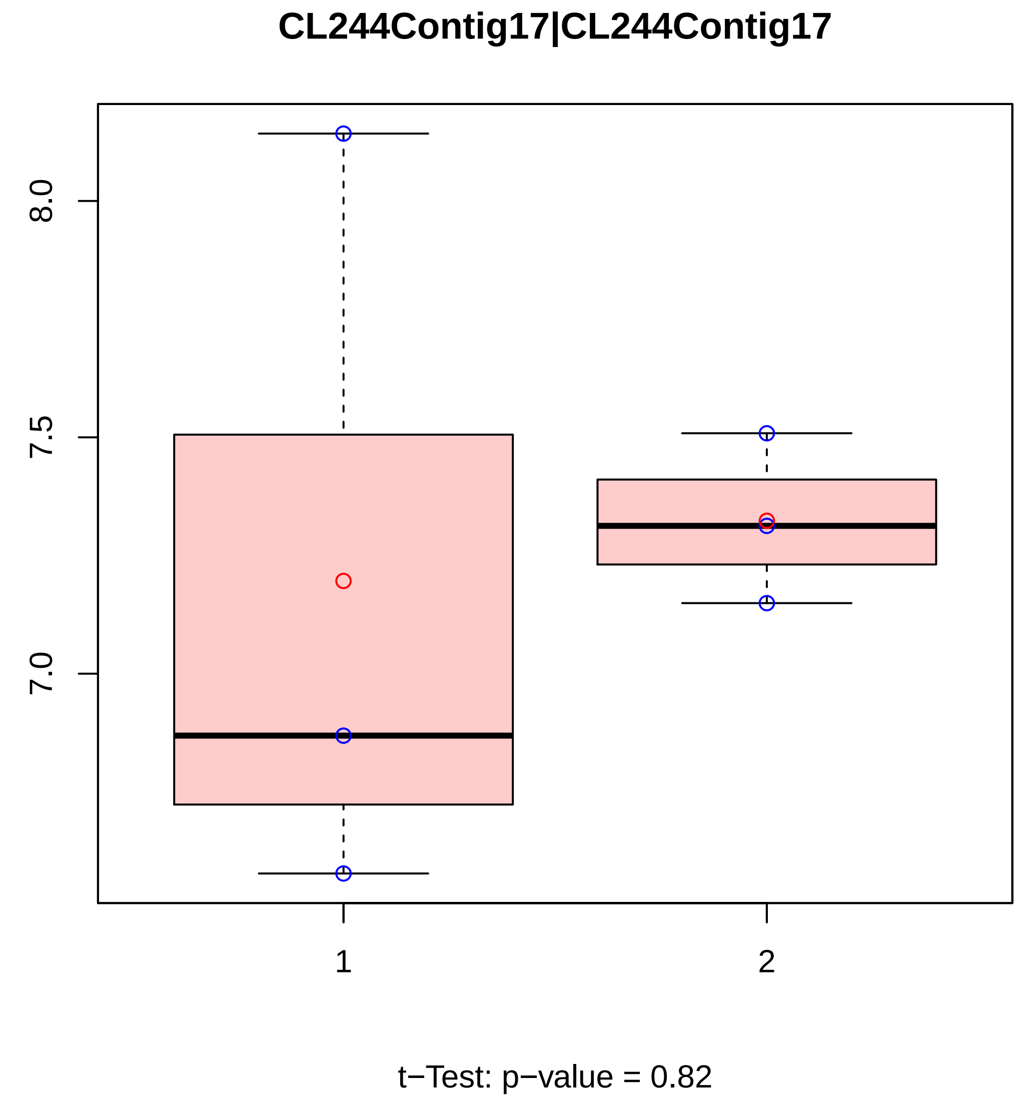
CL244Contig17|CL244Contig17
8.0
7.5
7.0
1
2
t−Test: p−value = 0.82

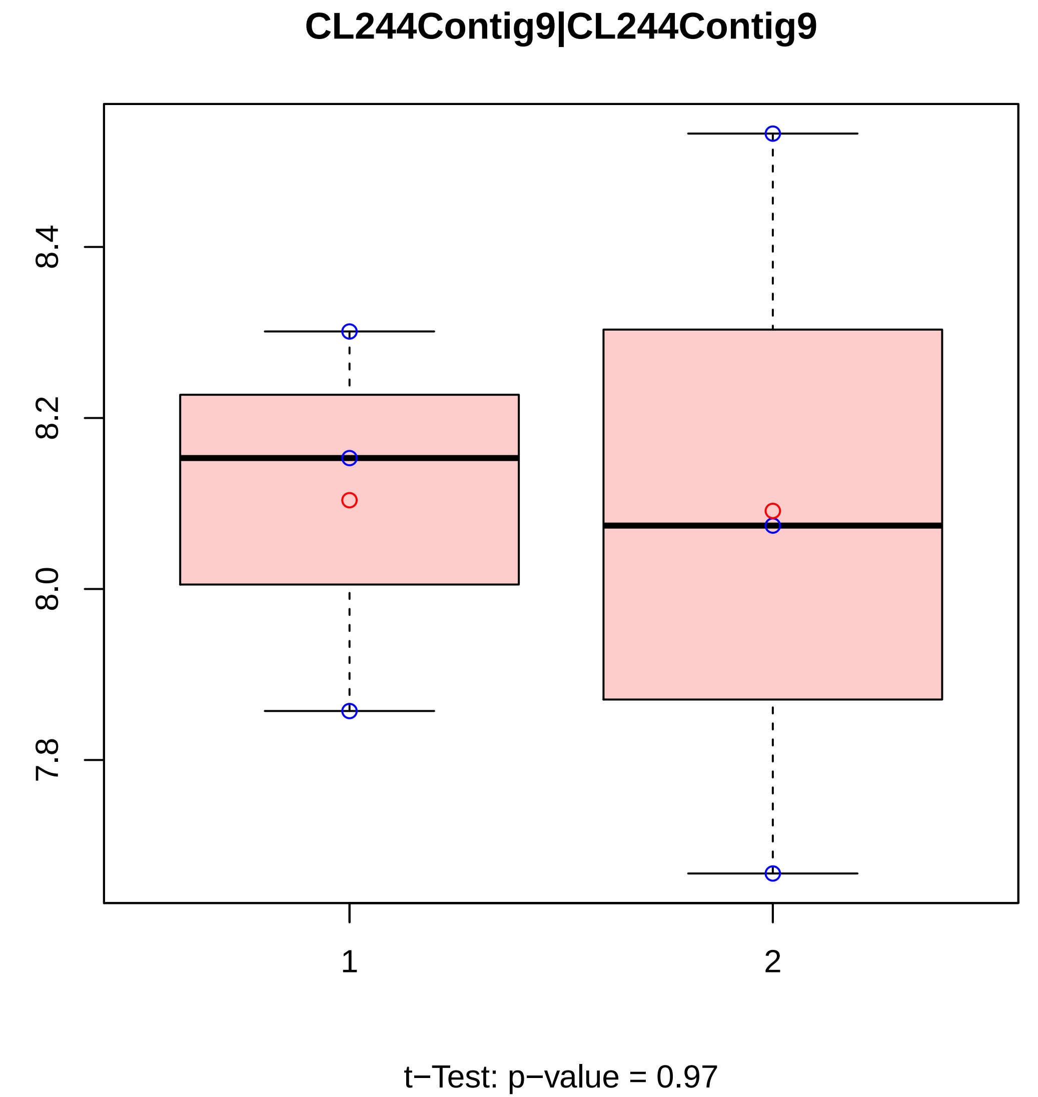
CL244Contig9|CL244Contig9
8.4
8.2
8.0
7.8
1
2
t−Test: p−value = 0.97

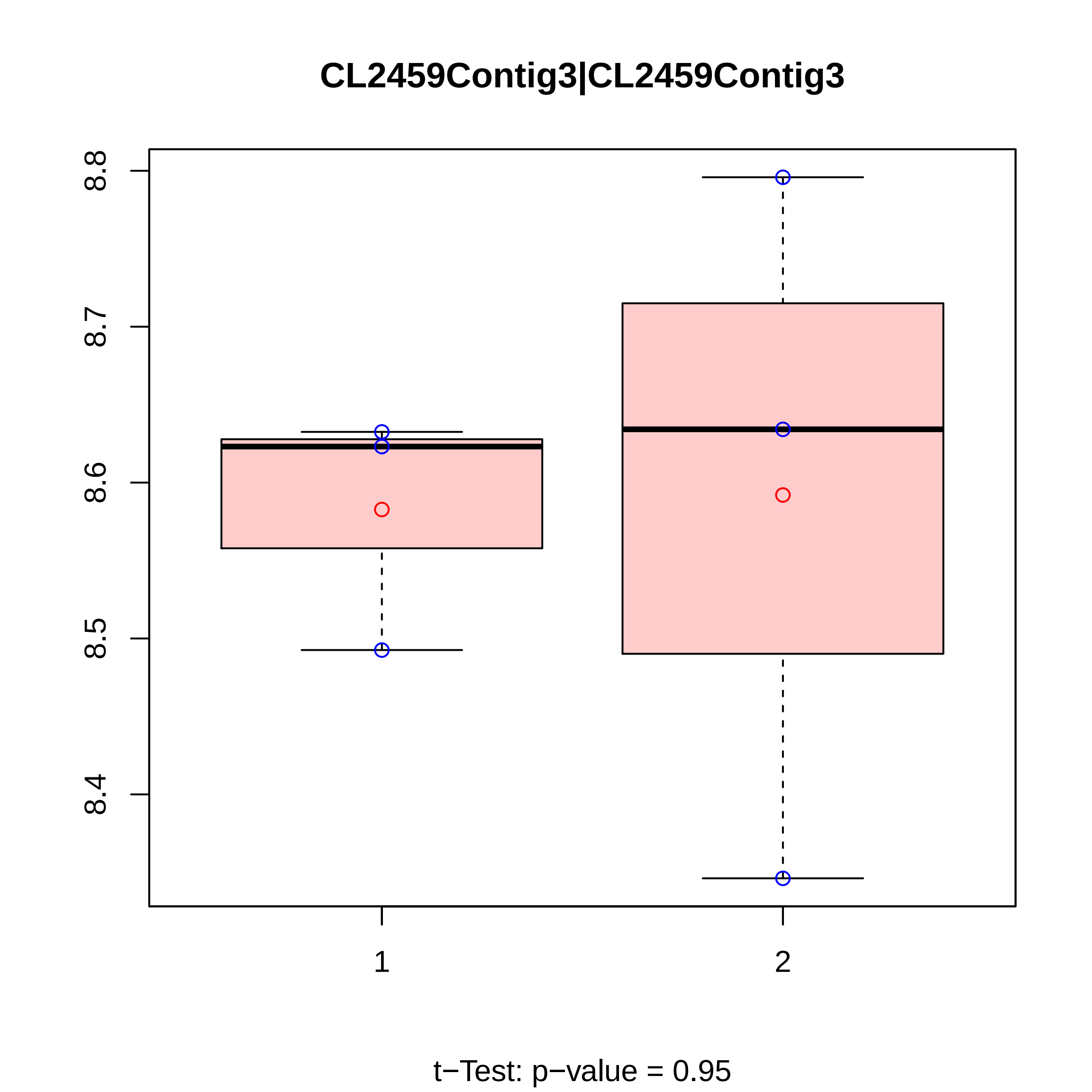

CL2459Contig3|CL2459Contig3
8.8
8.7
8.6
8.5
8.4
1
2
t−Test: p−value = 0.95

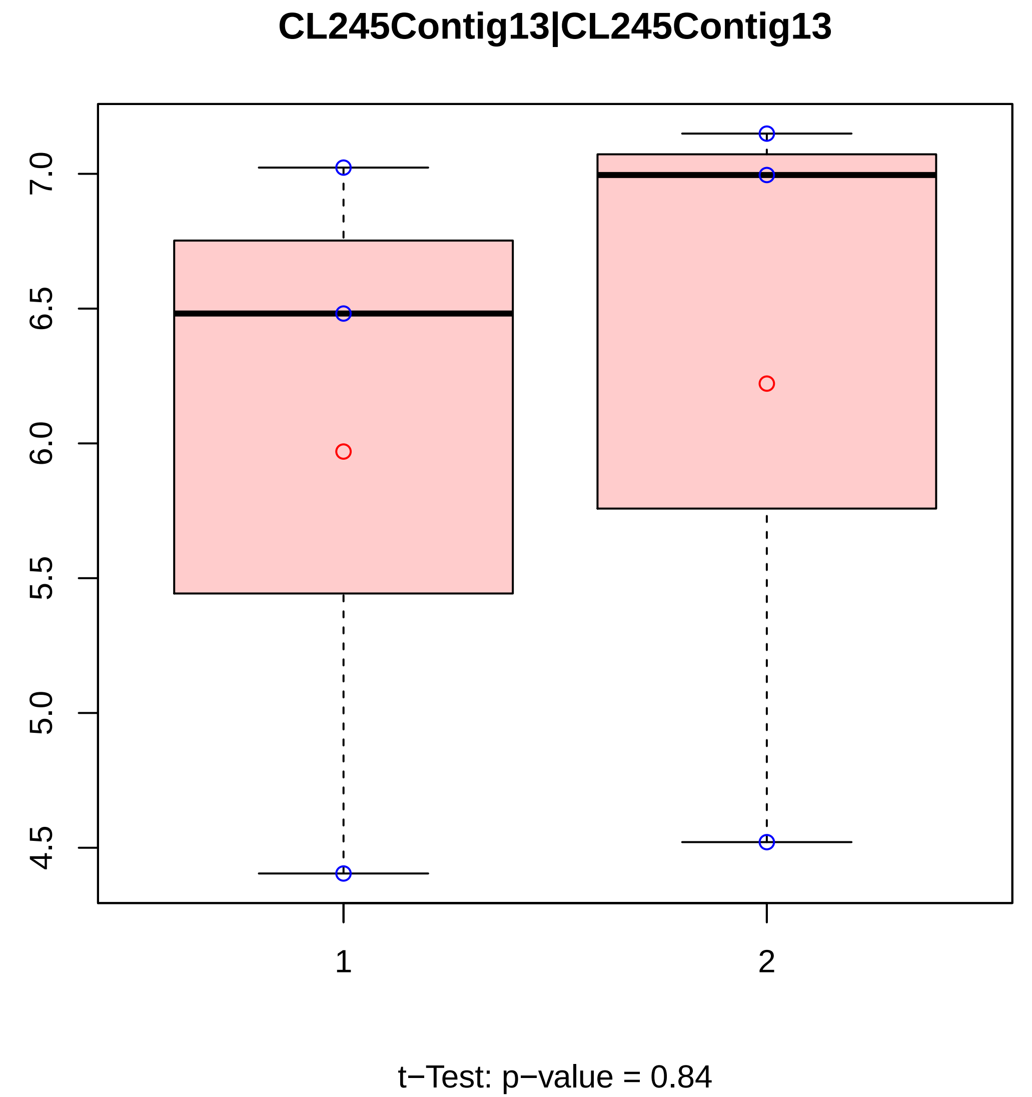
CL245Contig13|CL245Contig13
7.0
6.5
6.0
5.5
5.0
4.5
1
2
t−Test: p−value = 0.84

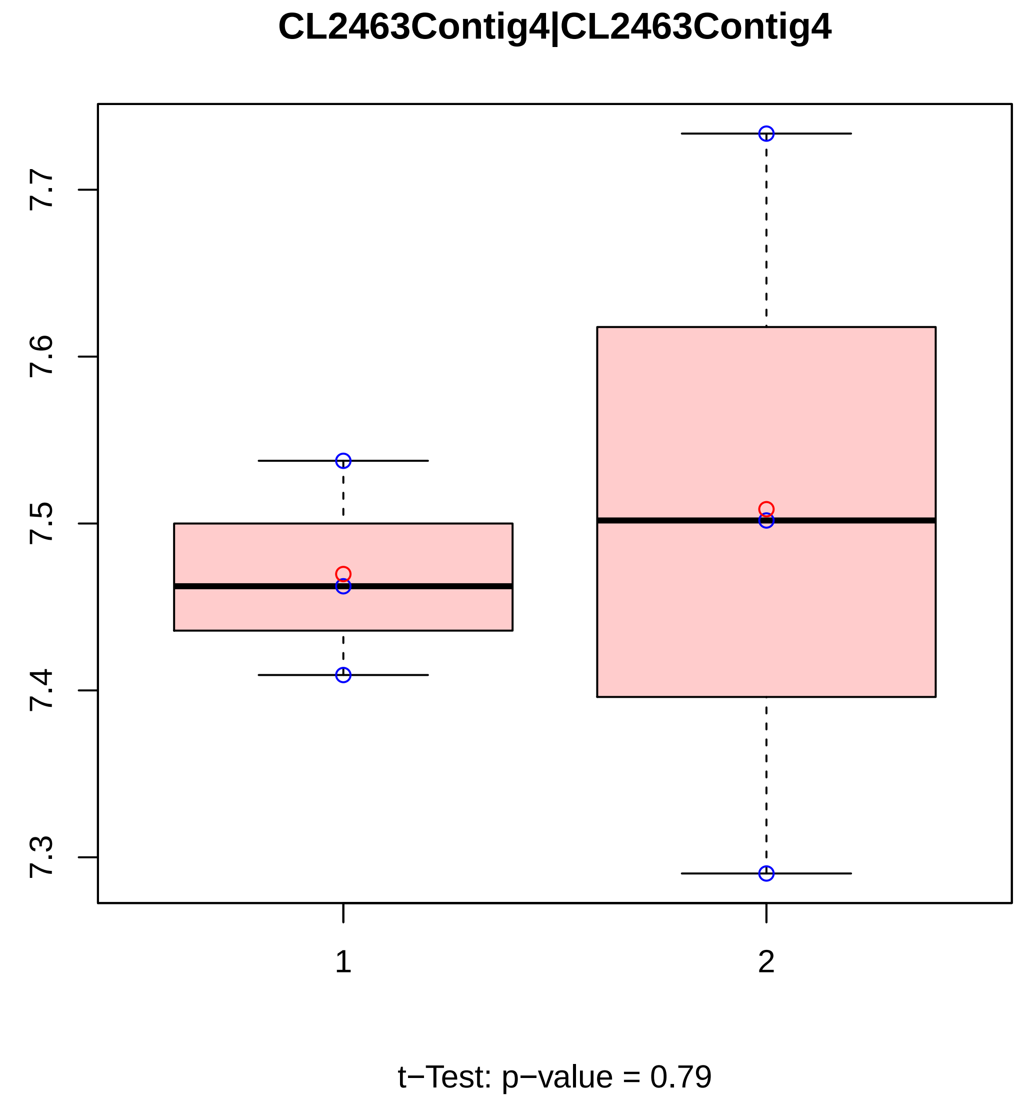
CL2463Contig4|CL2463Contig4
7.3
7.4
7.5
7.6
7.7
1
2
t−Test: p−value = 0.79

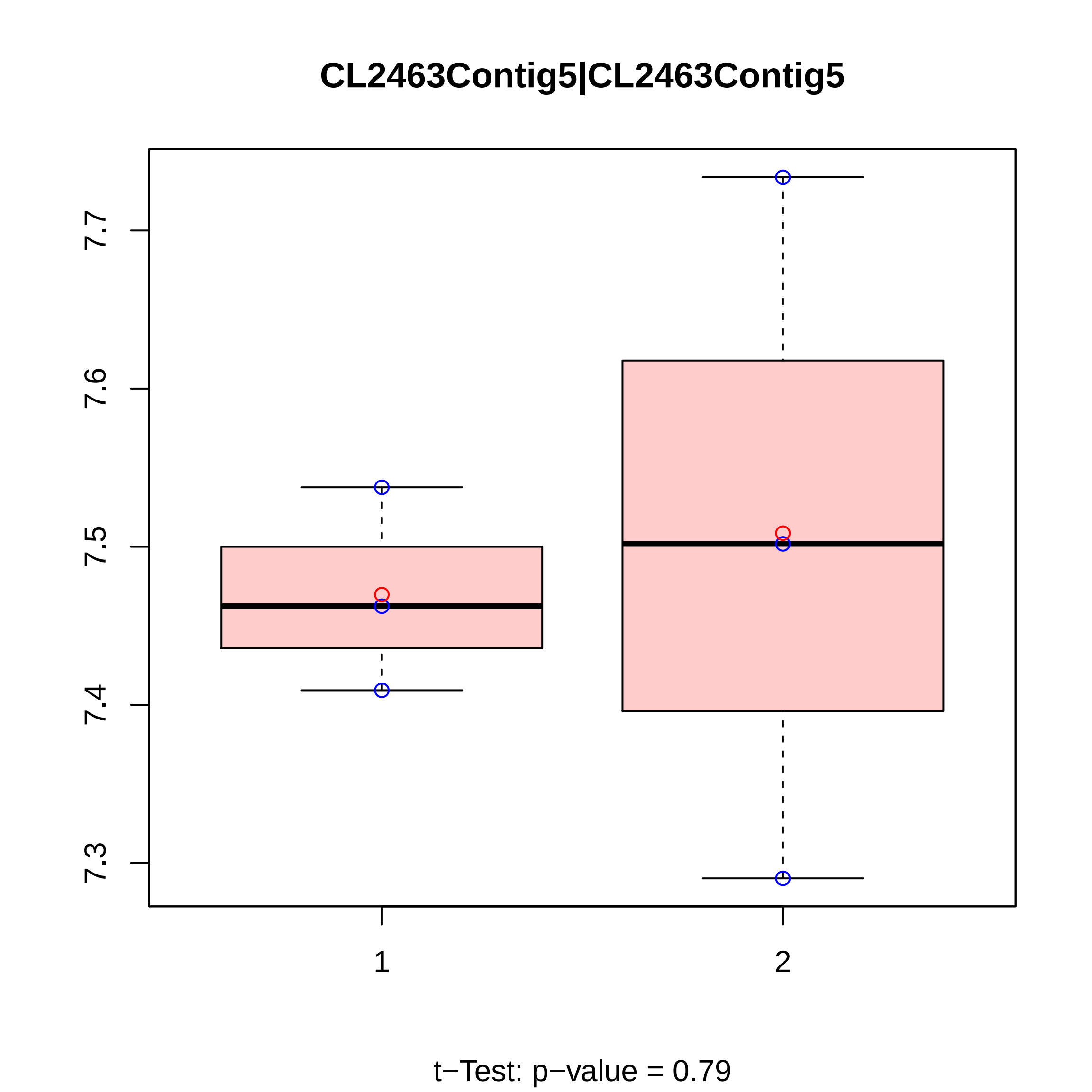

CL2463Contig5|CL2463Contig5
7.3
7.4
7.5
7.6
7.7
1
2
t−Test: p−value = 0.79

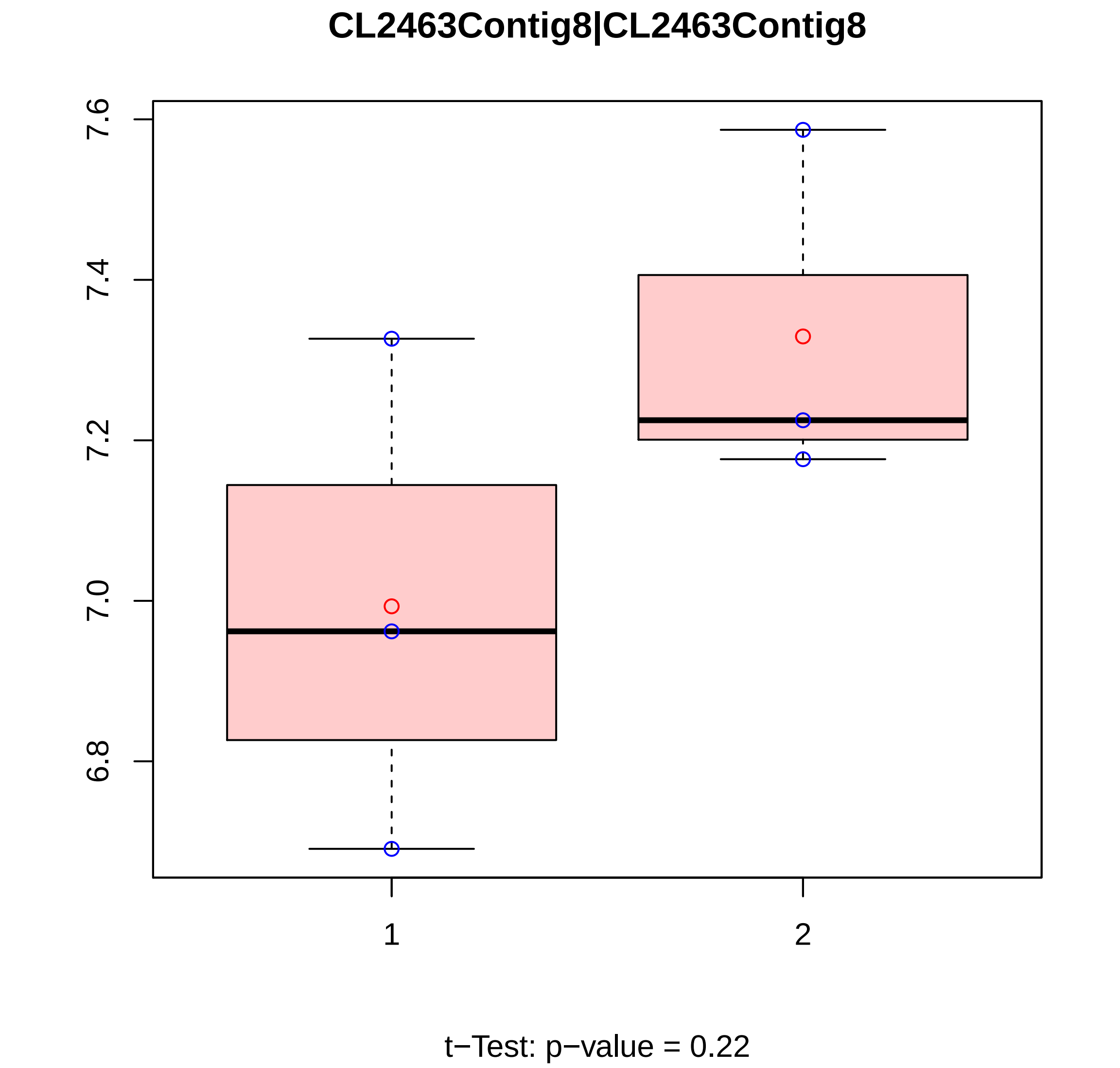
CL2463Contig8|CL2463Contig8
7.6
7.4
7.2
7.0
6.8
1
2
t−Test: p−value = 0.22

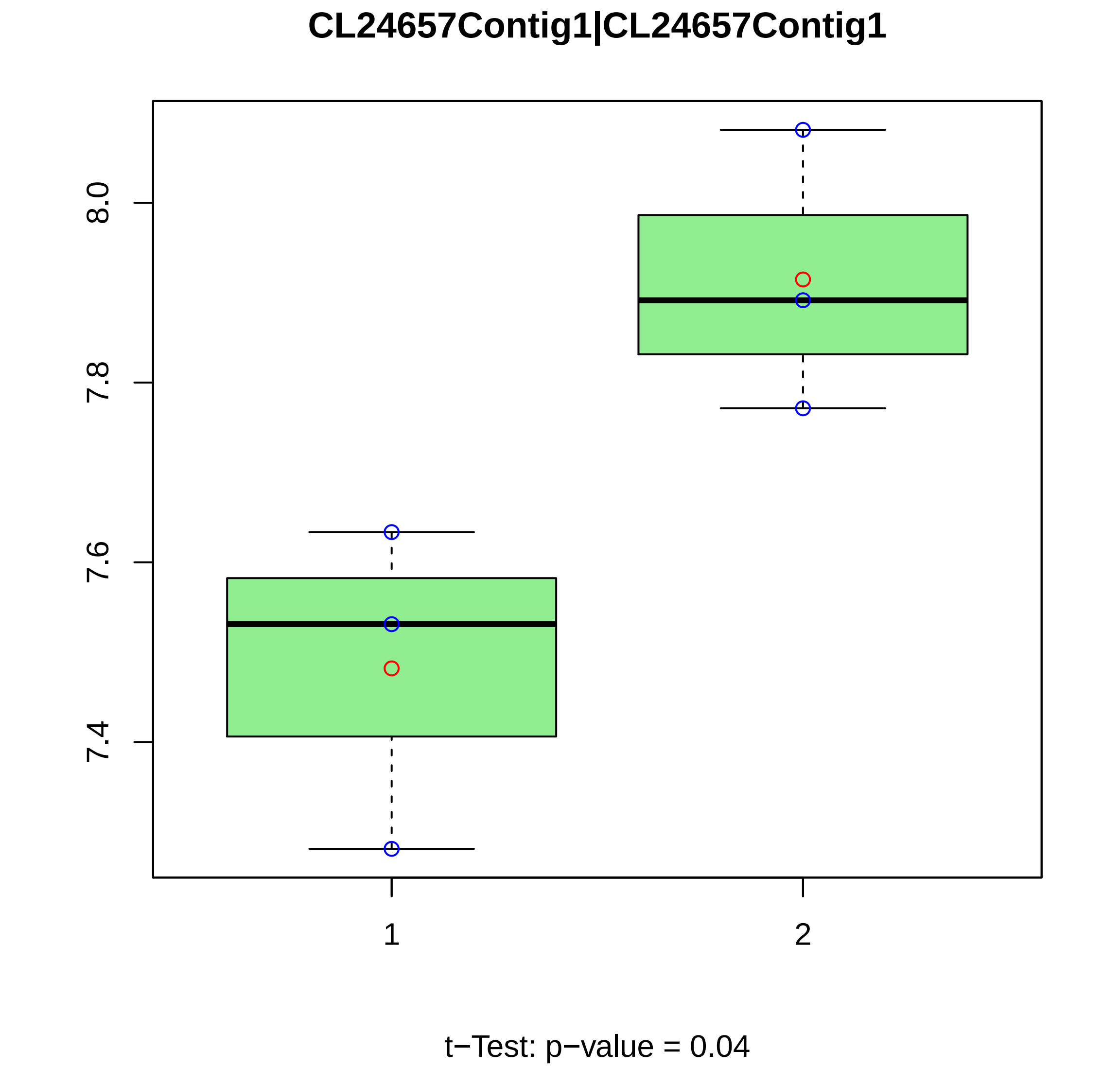

CL24657Contig1|CL24657Contig1
8.0
7.8
7.6
7.4
1
2
t−Test: p−value = 0.04

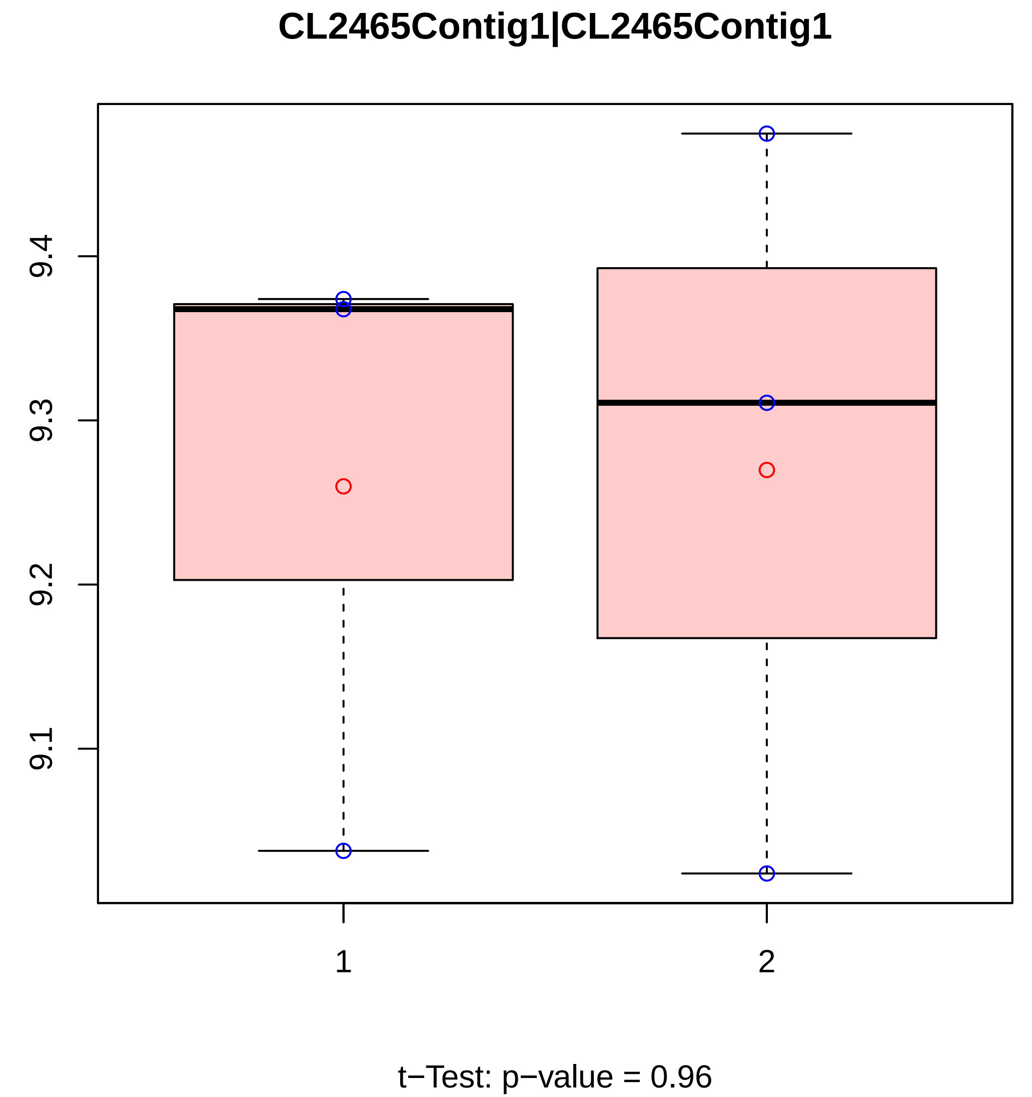
CL2465Contig1|CL2465Contig1
9.1
9.2
9.3
9.4
1
2
t−Test: p−value = 0.96

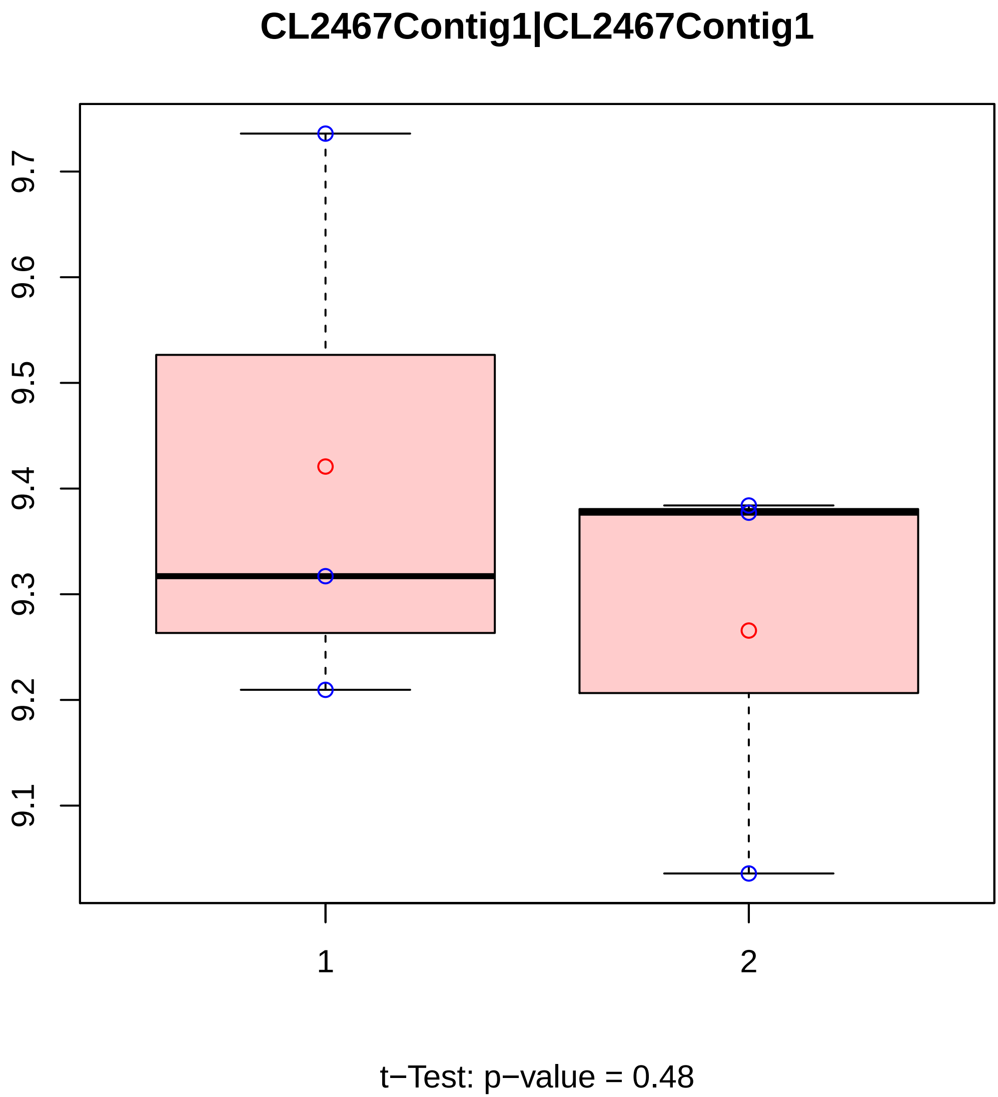

CL2467Contig1|CL2467Contig1
9.1
9.2
9.3
9.4
9.5
9.6
9.7
1
2
t−Test: p−value = 0.48

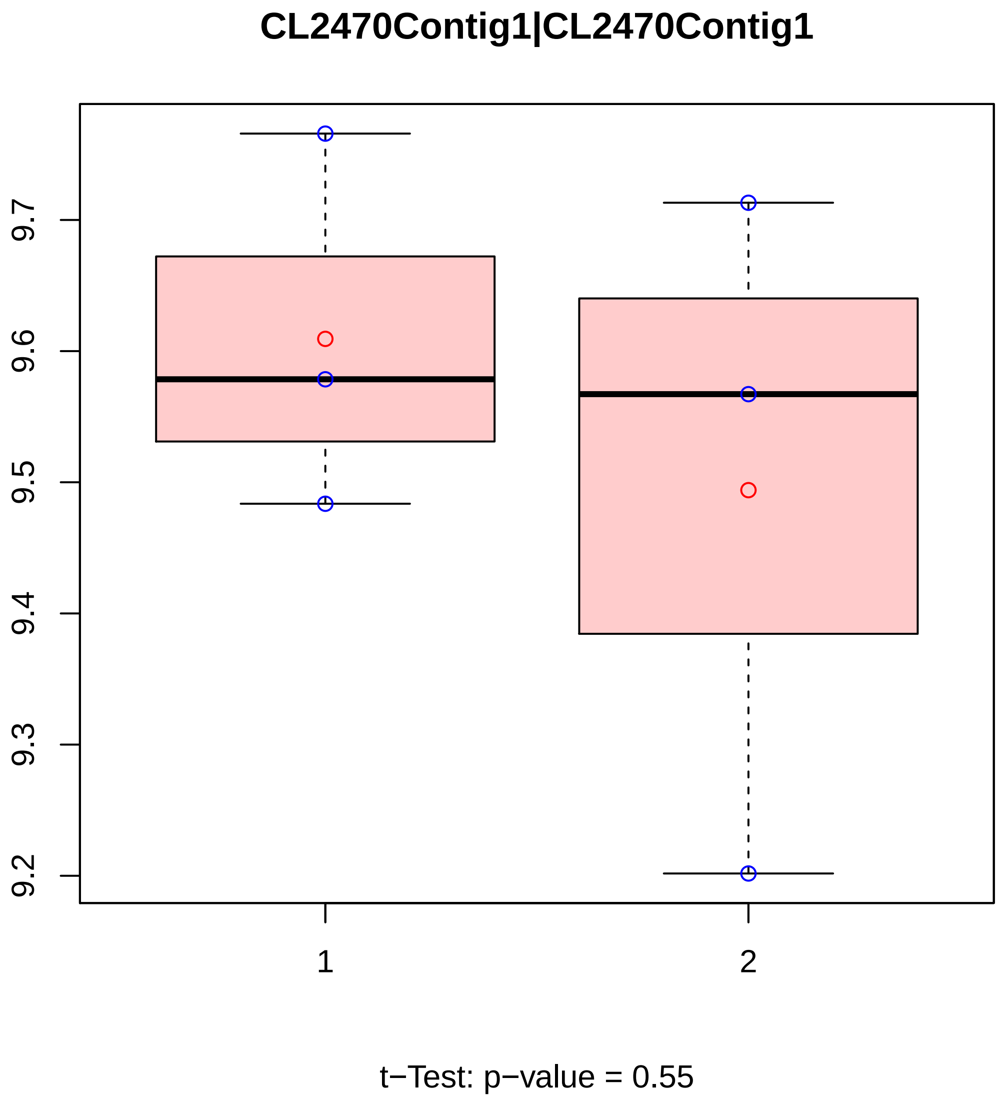

CL2470Contig1|CL2470Contig1
9.2
9.3
9.4
9.5
9.6
9.7
1
2
t−Test: p−value = 0.55

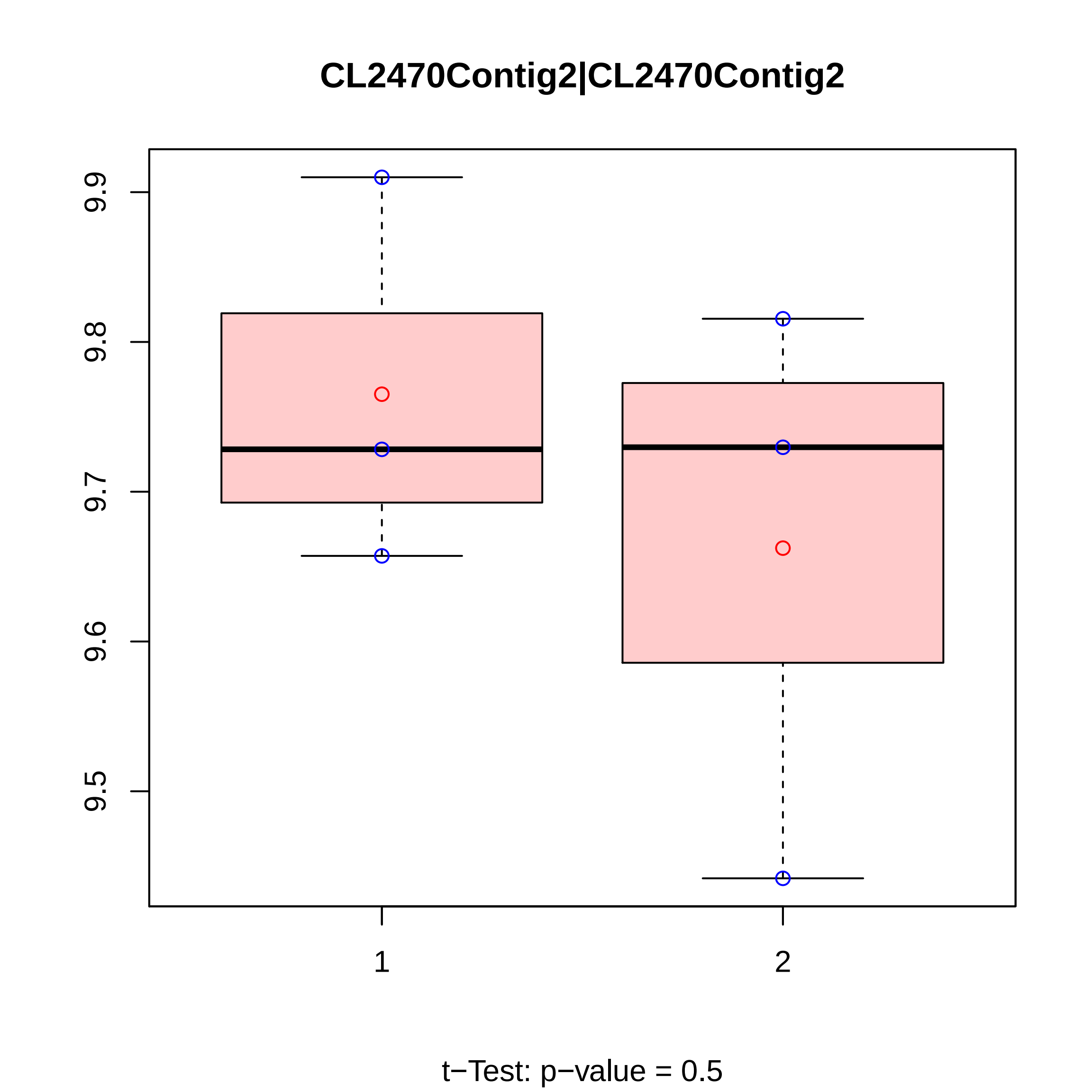

CL2470Contig2|CL2470Contig2
9.5
9.6
9.7
9.8
9.9
1
2
t−Test: p−value = 0.5

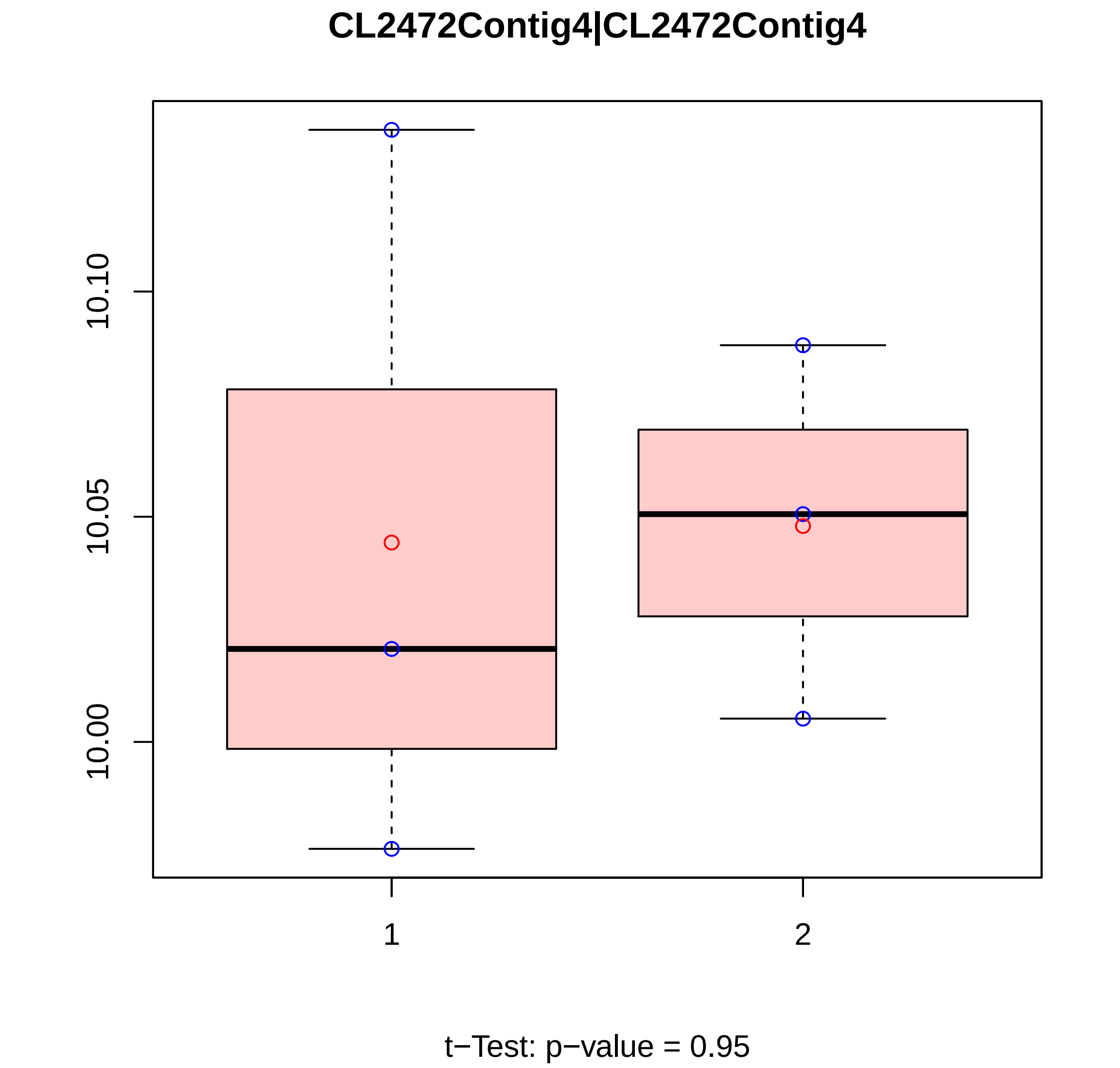
CL2472Contig4|CL2472Contig4
10.10
10.05
10.00
1
2
t−Test: p−value = 0.95

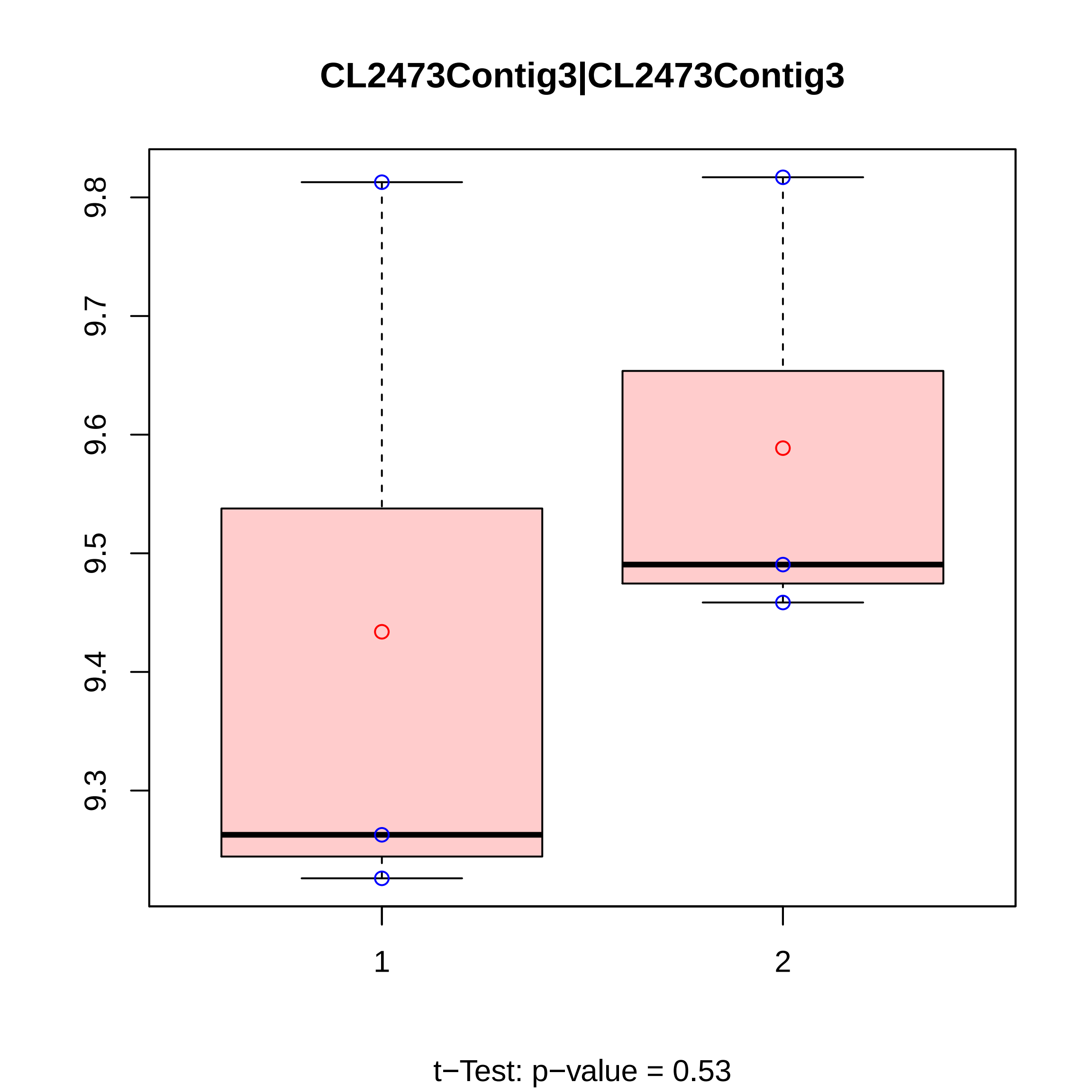
CL2473Contig3|CL2473Contig3
9.3
9.4
9.5
9.6
9.7
9.8
1
2
t−Test: p−value = 0.53

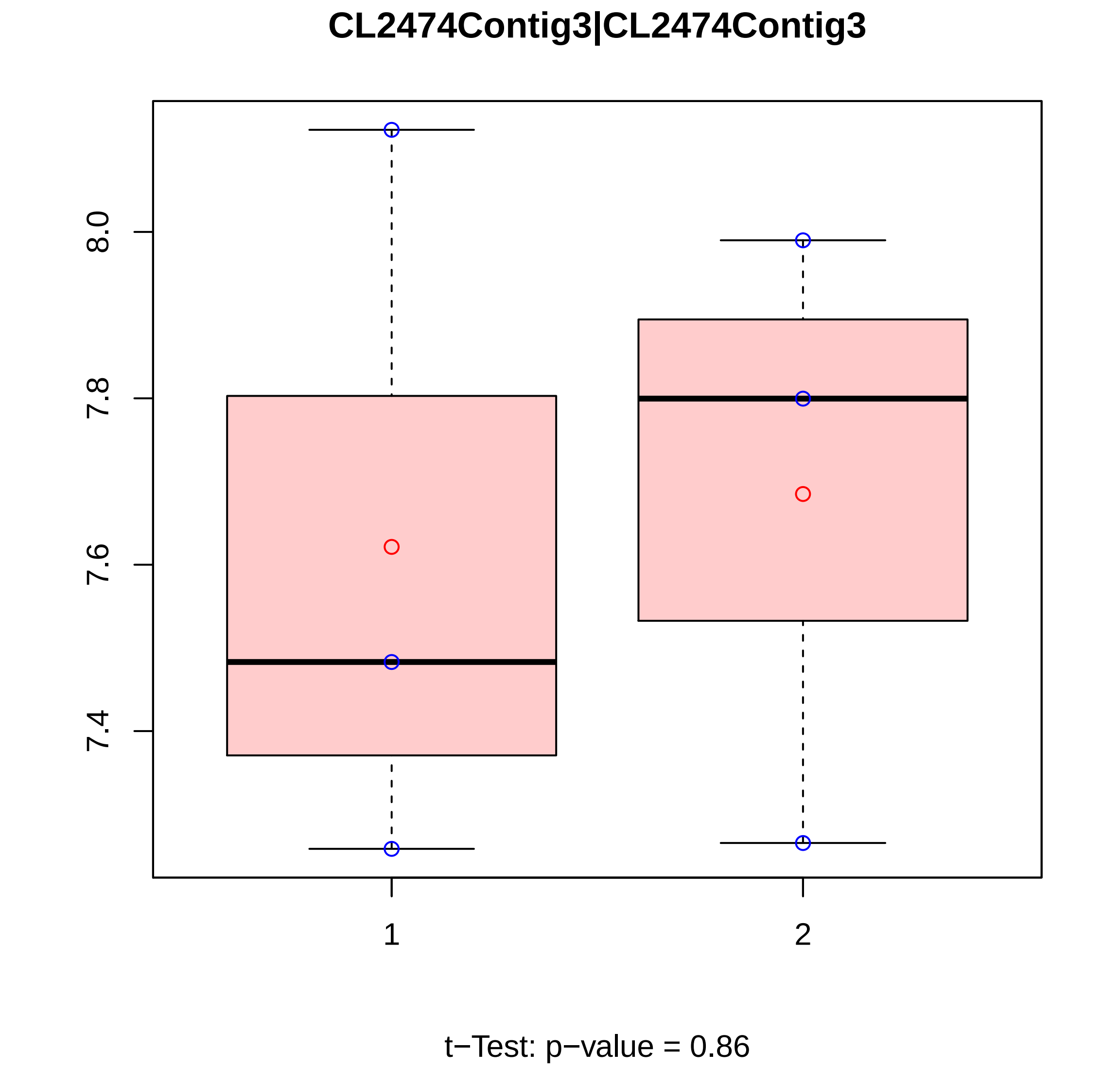
CL2474Contig3|CL2474Contig3
8.0
7.8
7.6
7.4
1
2
t−Test: p−value = 0.86

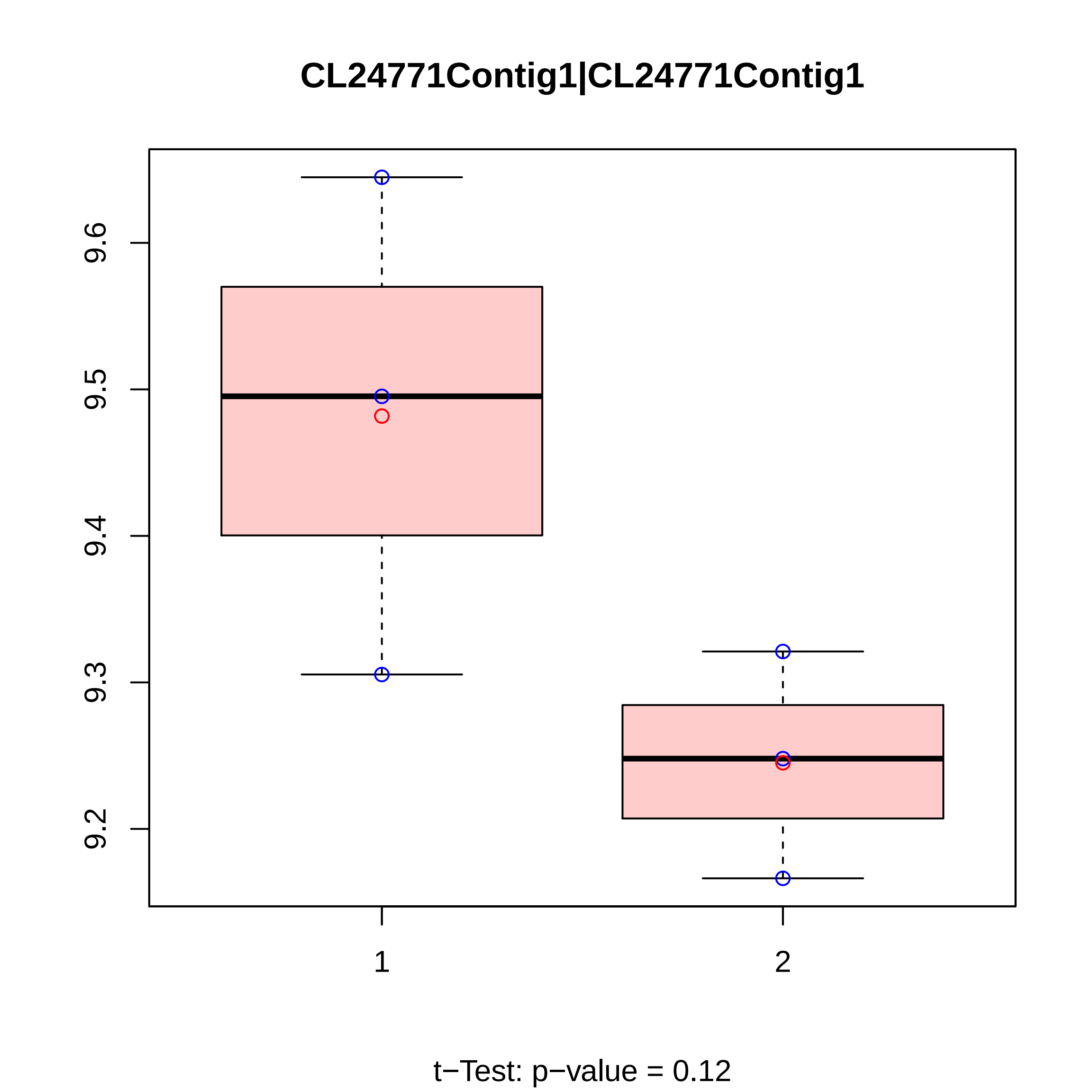

CL24771Contig1|CL24771Contig1
9.2
9.3
9.4
9.5
9.6
1
2
t−Test: p−value = 0.12

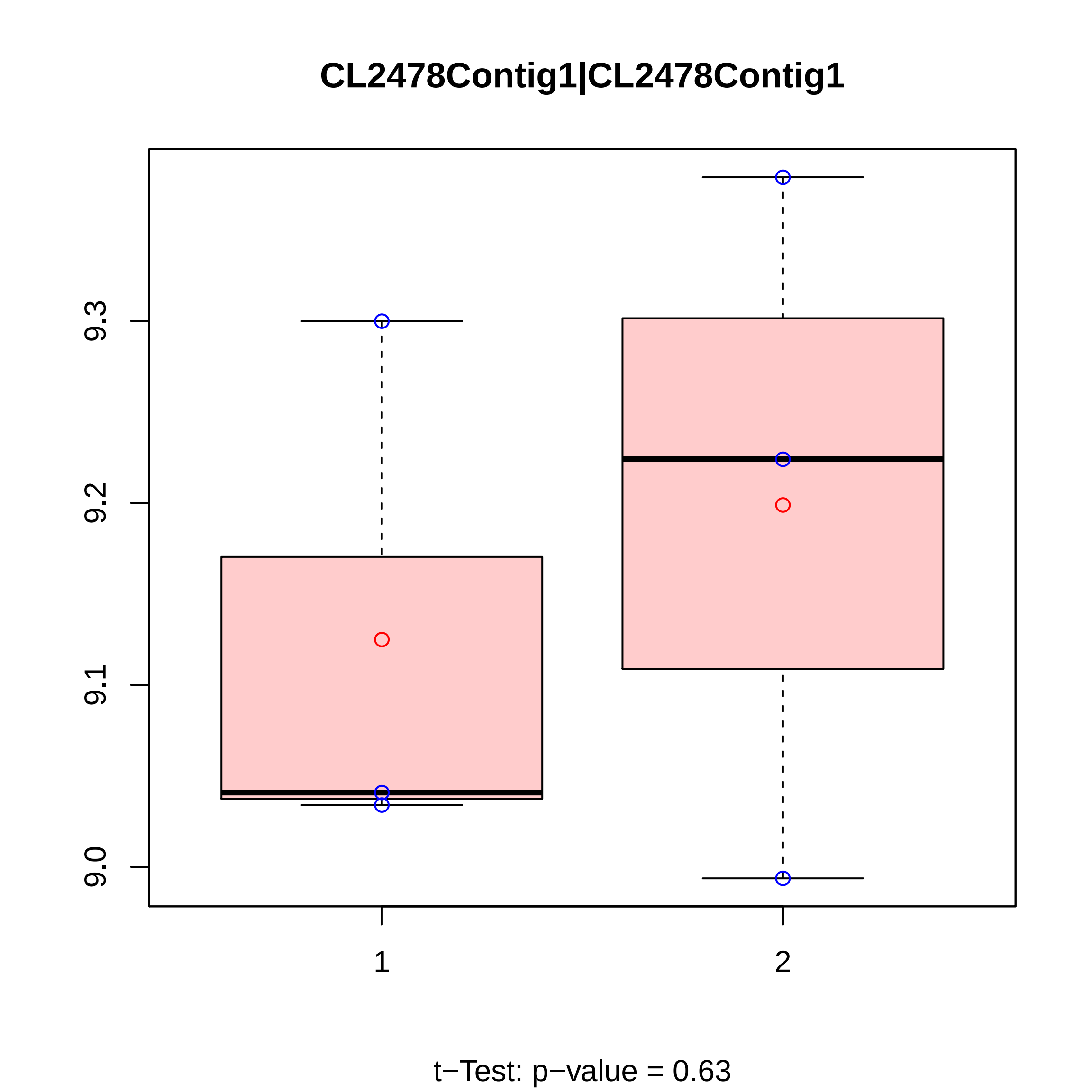
CL2478Contig1|CL2478Contig1
9.0
9.1
9.2
9.3
1
2
t−Test: p−value = 0.63

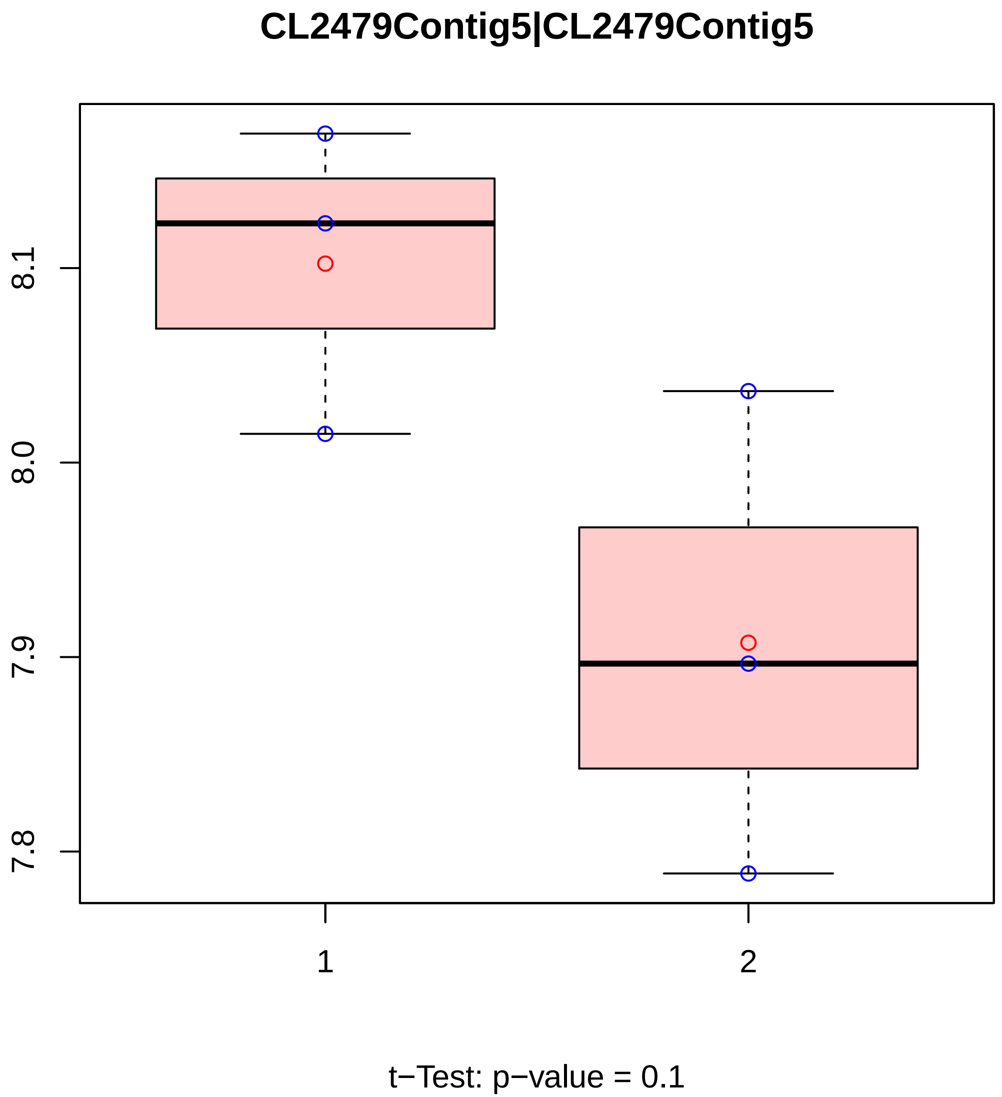
CL2479Contig5|CL2479Contig5
8.1
8.0
7.9
7.8
1
2
t−Test: p−value = 0.1

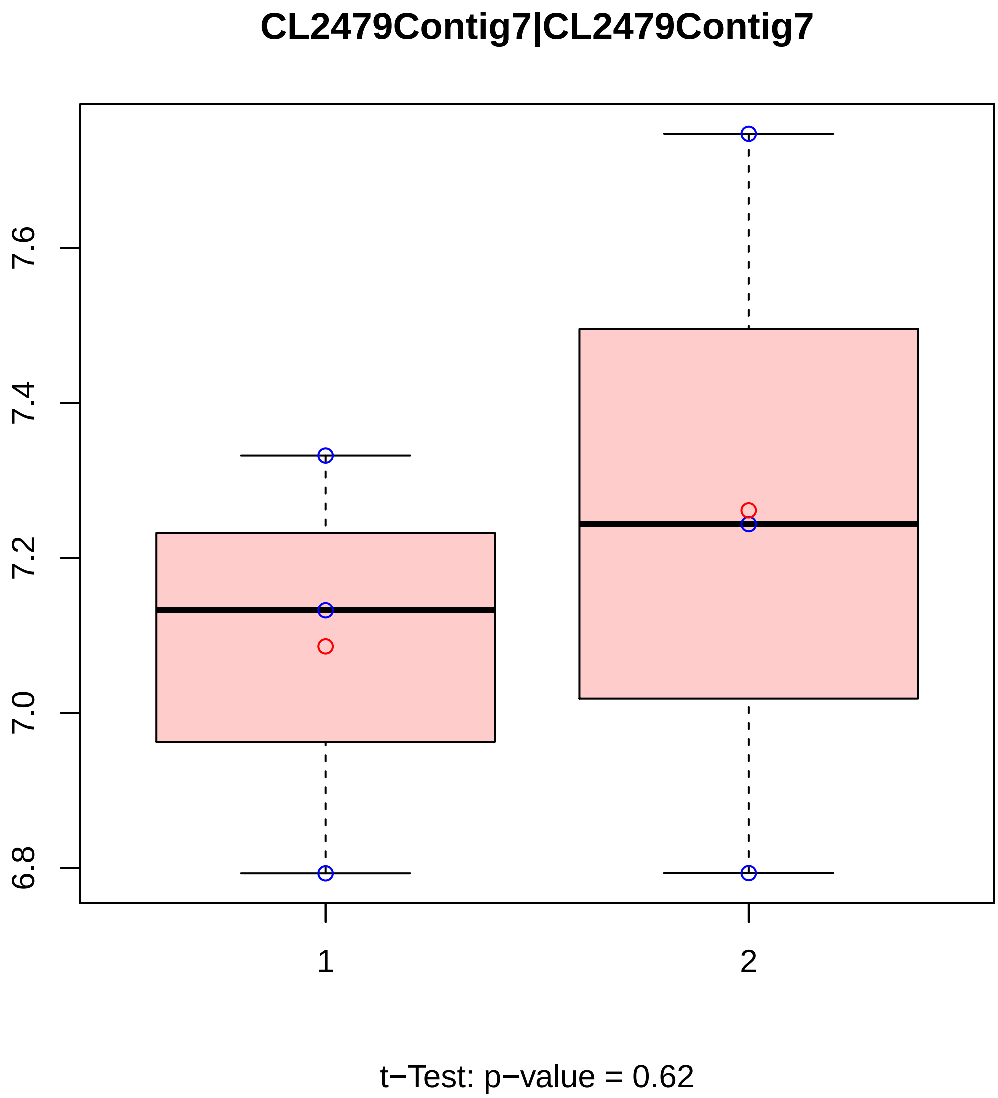
CL2479Contig7|CL2479Contig7
7.6
7.4
7.2
7.0
6.8
1
2
t−Test: p−value = 0.62

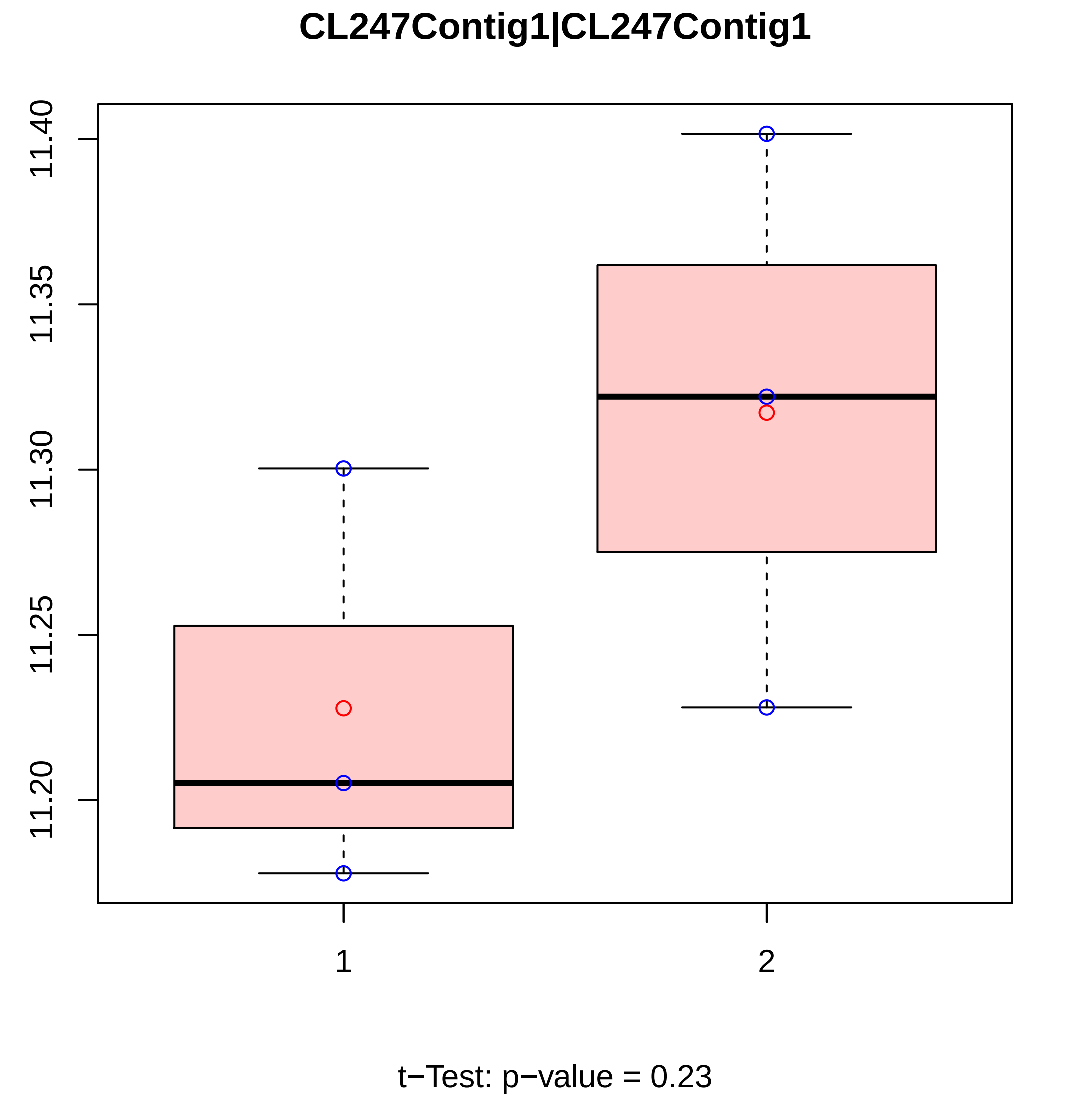
CL247Contig1|CL247Contig1
11.40
11.35
11.30
11.25
11.20
1
2
t−Test: p−value = 0.23

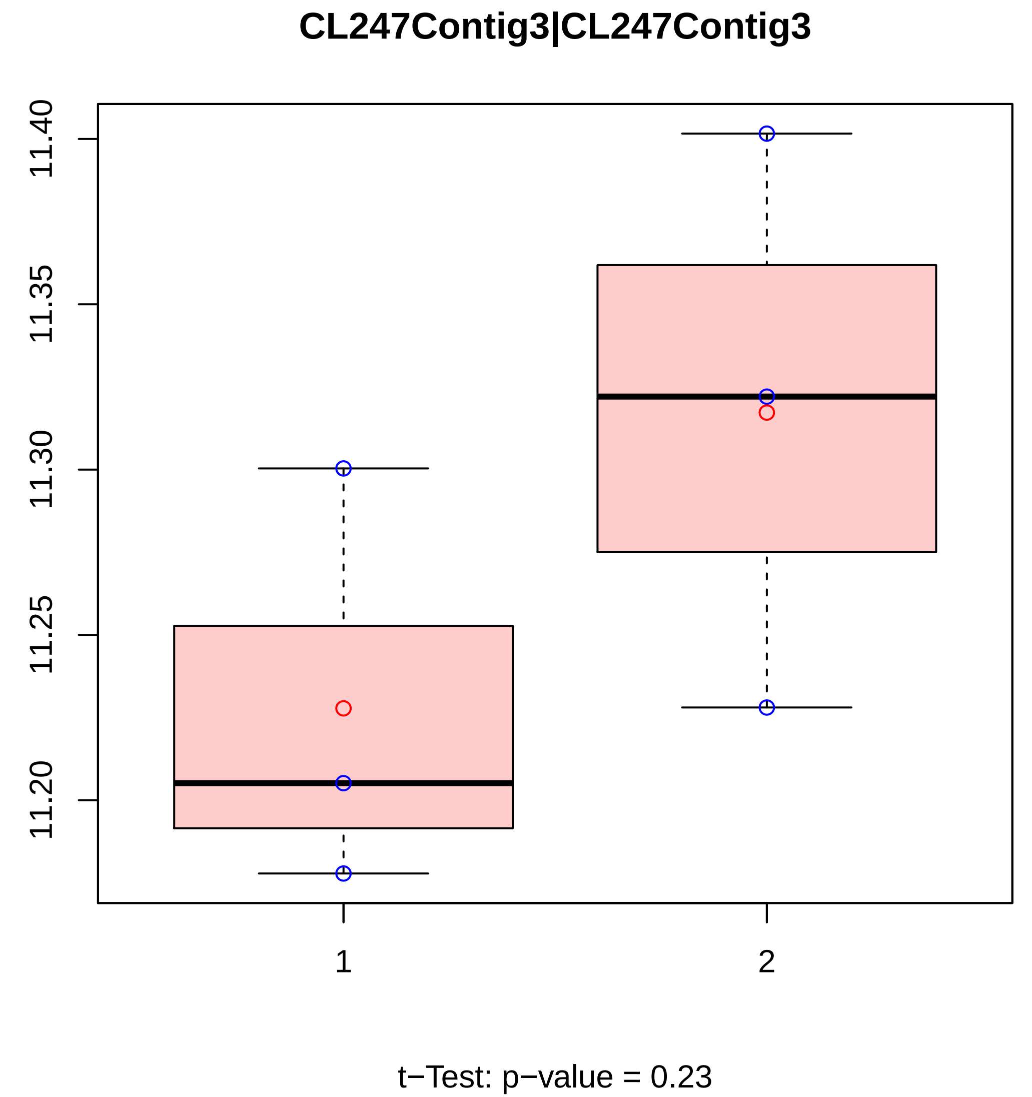
CL247Contig3|CL247Contig3
11.40
11.35
11.30
11.25
11.20
1
2
t−Test: p−value = 0.23

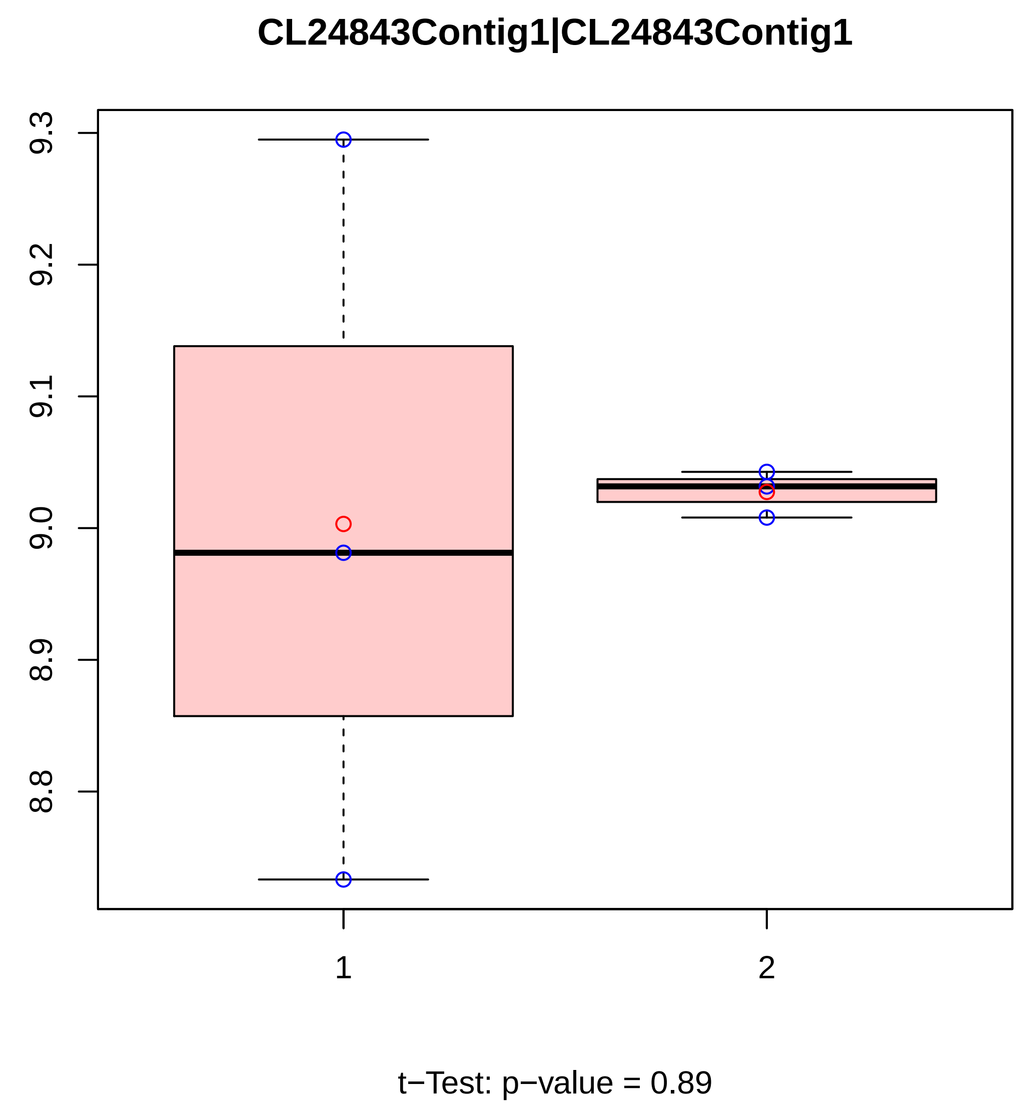
CL24843Contig1|CL24843Contig1
9.3
9.2
9.1
9.0
8.9
8.8
1
2
t−Test: p−value = 0.89

# CL2486Contig1|CL2486Contig1

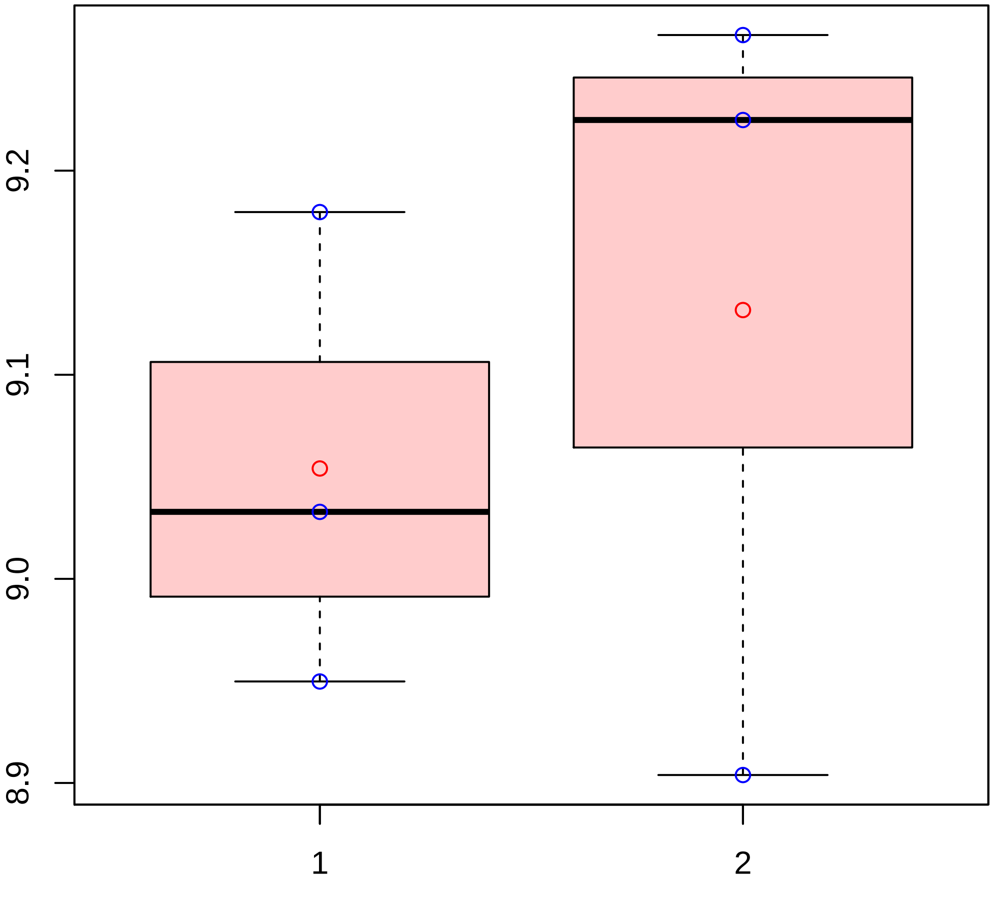


t−Test: p−value = 0.6

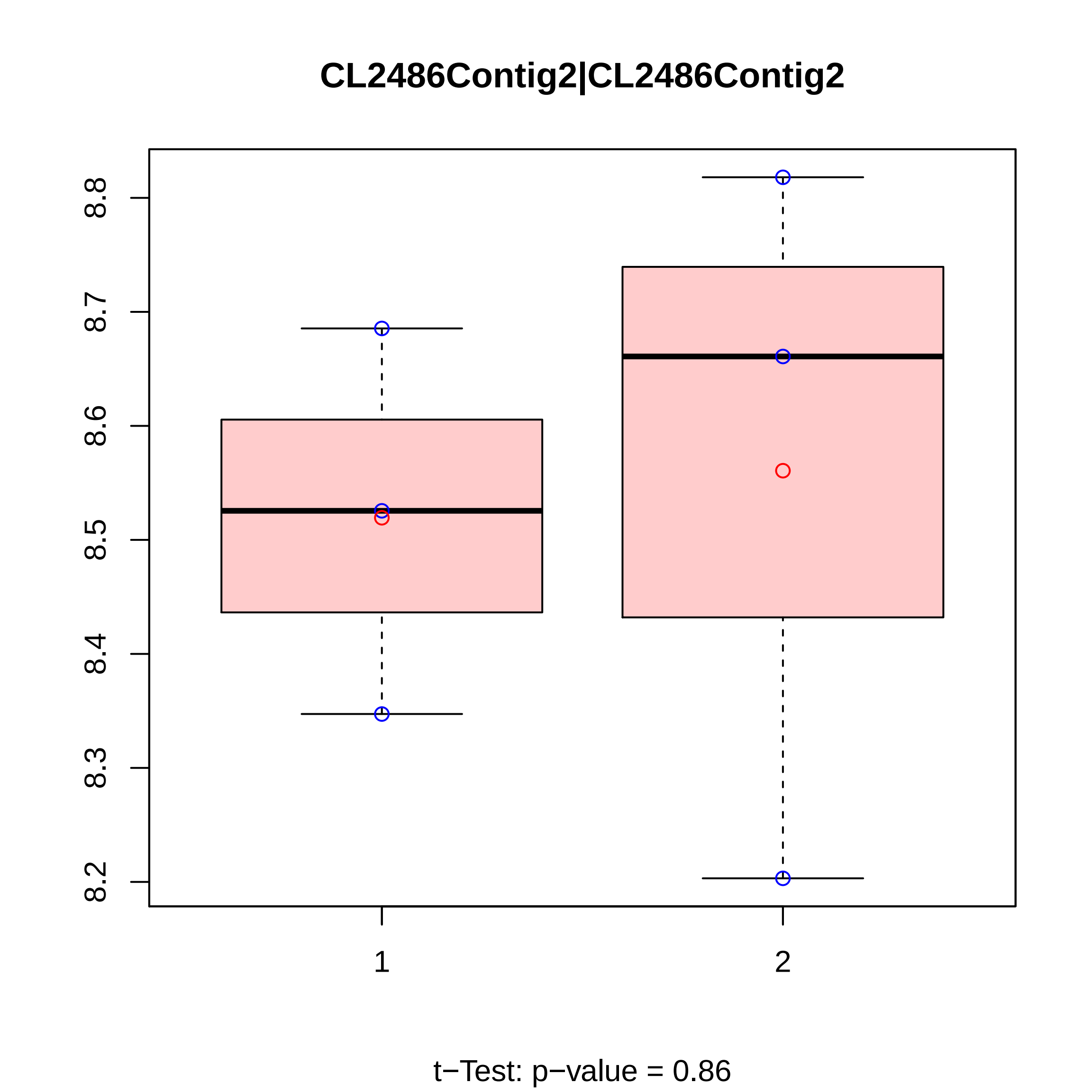

CL2486Contig2|CL2486Contig2
8.2
8.3
8.4
8.5
8.6
8.7
8.8
1
2
t−Test: p−value = 0.86

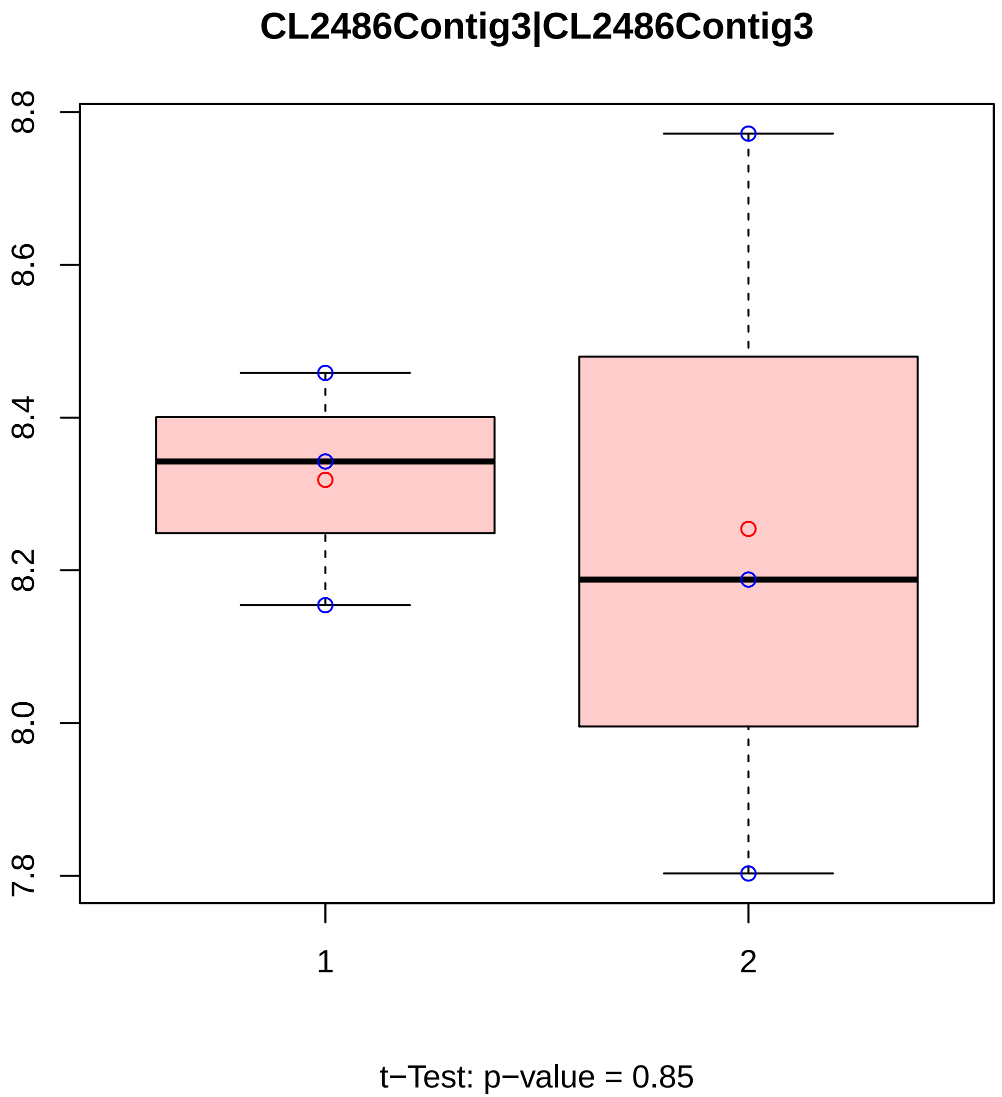

CL2486Contig3|CL2486Contig3
8.8
8.6
8.4
8.2
8.0
7.8
1
2
t−Test: p−value = 0.85

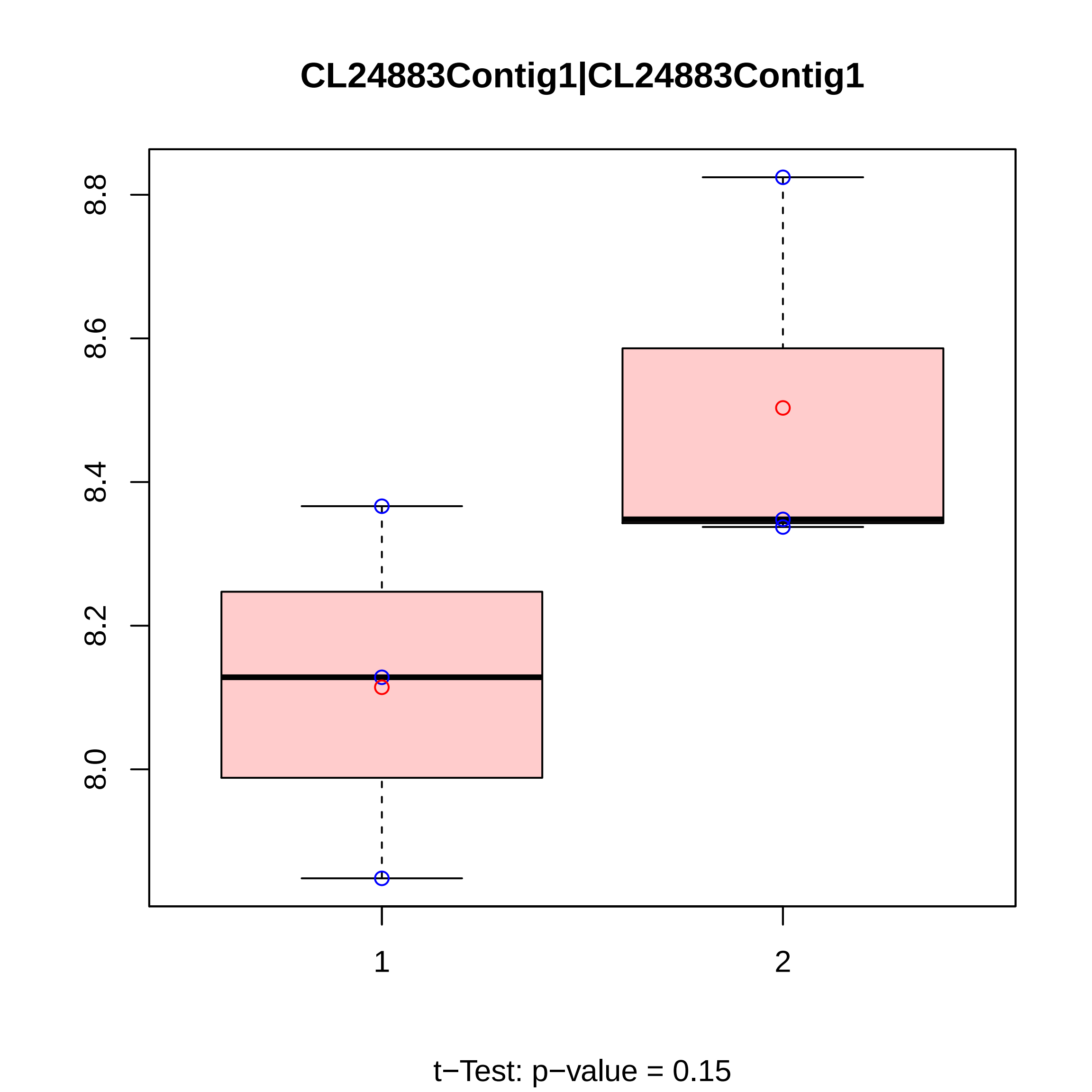

CL24883Contig1|CL24883Contig1
8.0
8.2
8.4
8.6
8.8
1
2
t−Test: p−value = 0.15

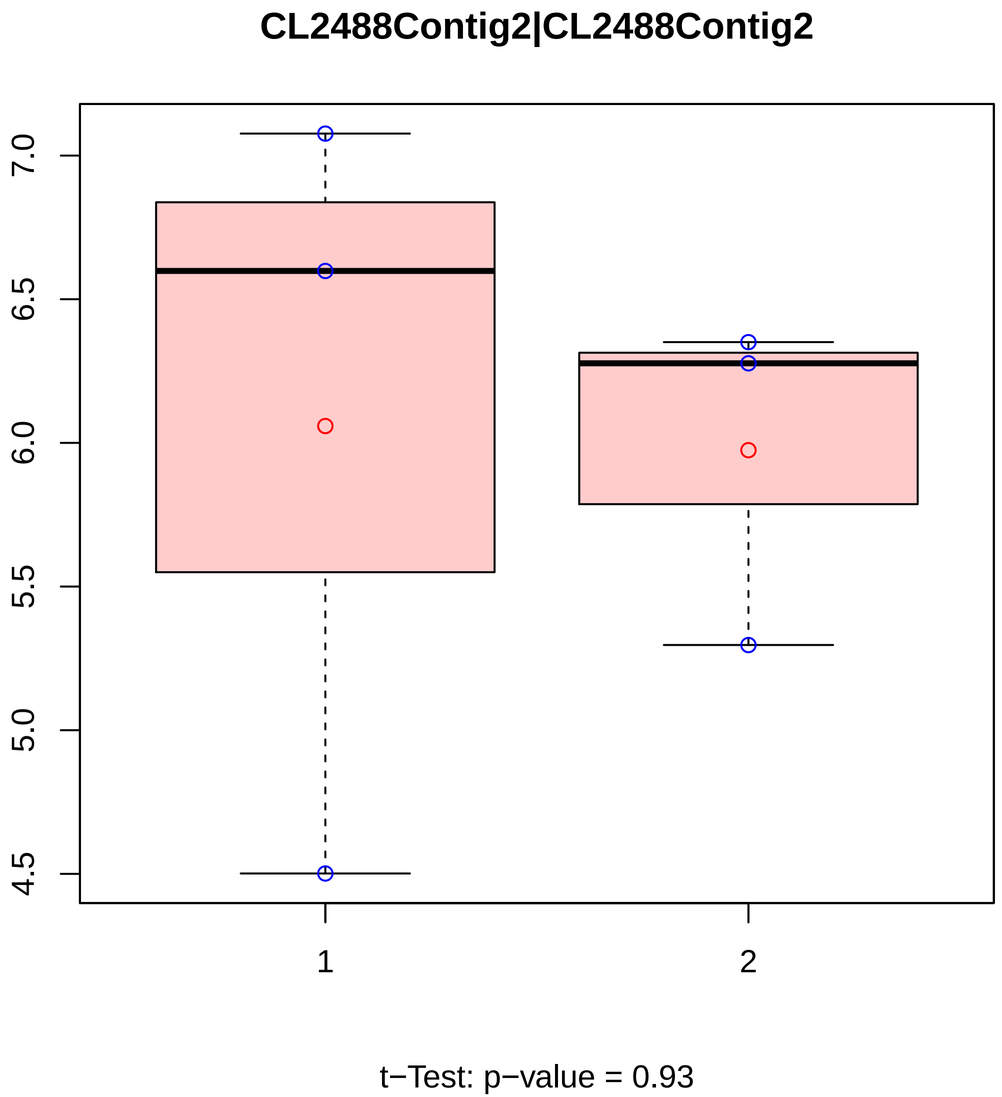
CL2488Contig2|CL2488Contig2
7.0
6.5
6.0
5.5
5.0
4.5
1
2
t−Test: p−value = 0.93

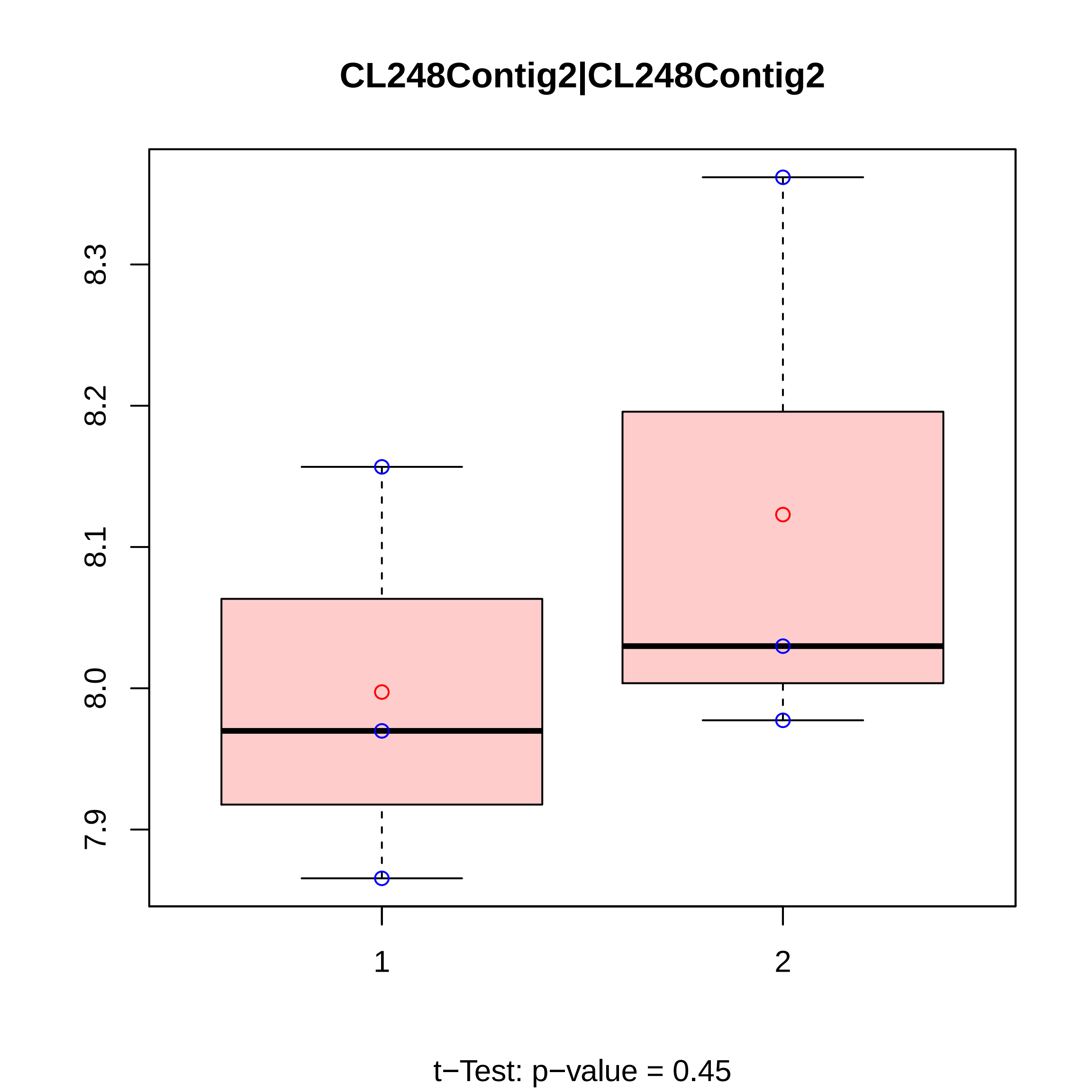

CL248Contig2|CL248Contig2
8.3
8.2
8.1
8.0
7.9
1
2
t−Test: p−value = 0.45

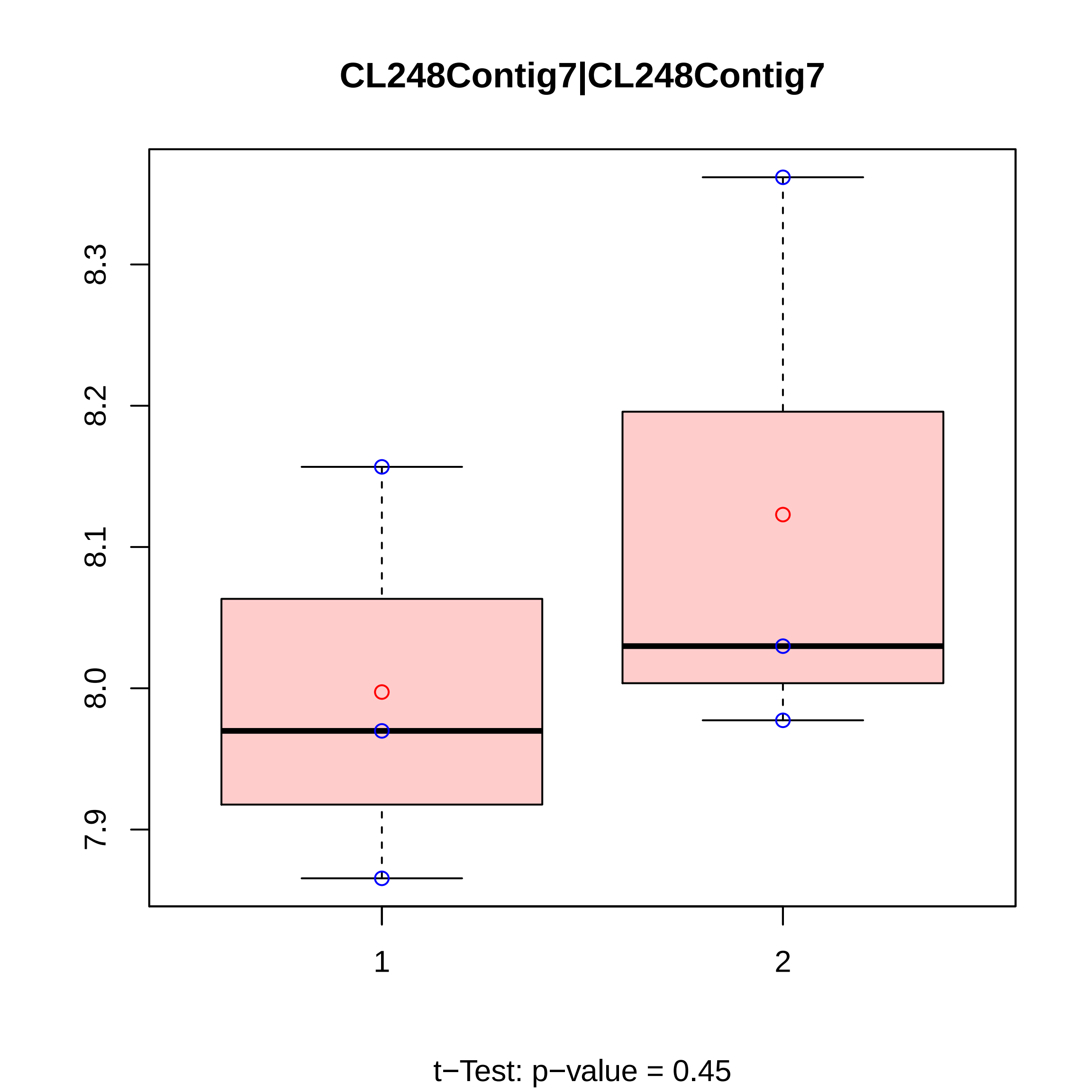
CL248Contig7|CL248Contig7
8.3
8.2
8.1
8.0
7.9
1
2
t−Test: p−value = 0.45

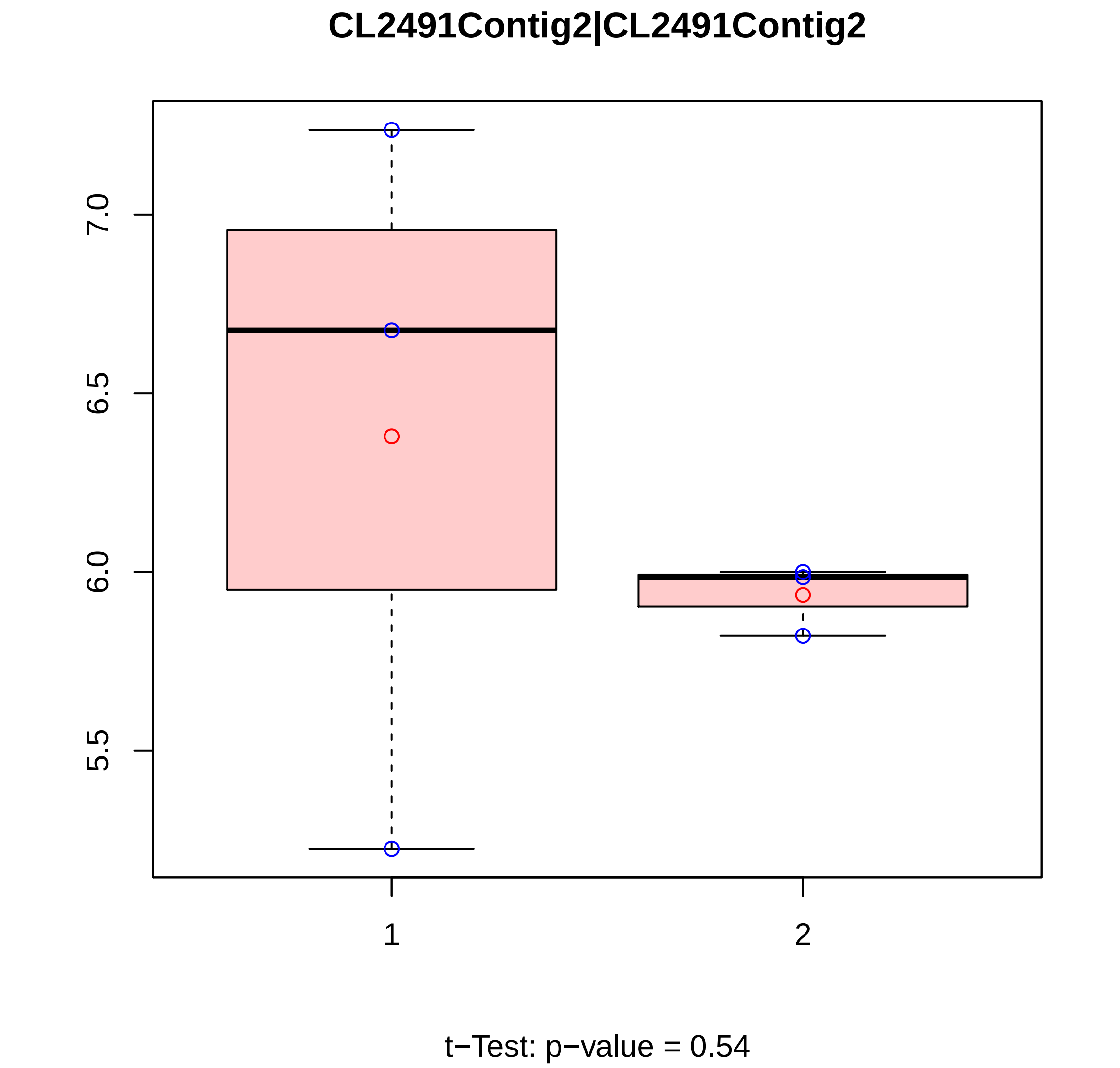

CL2491Contig2|CL2491Contig2
7.0
6.5
6.0
5.5
1
2
t−Test: p−value = 0.54

## CL2494Contig1|CL2494Contig1

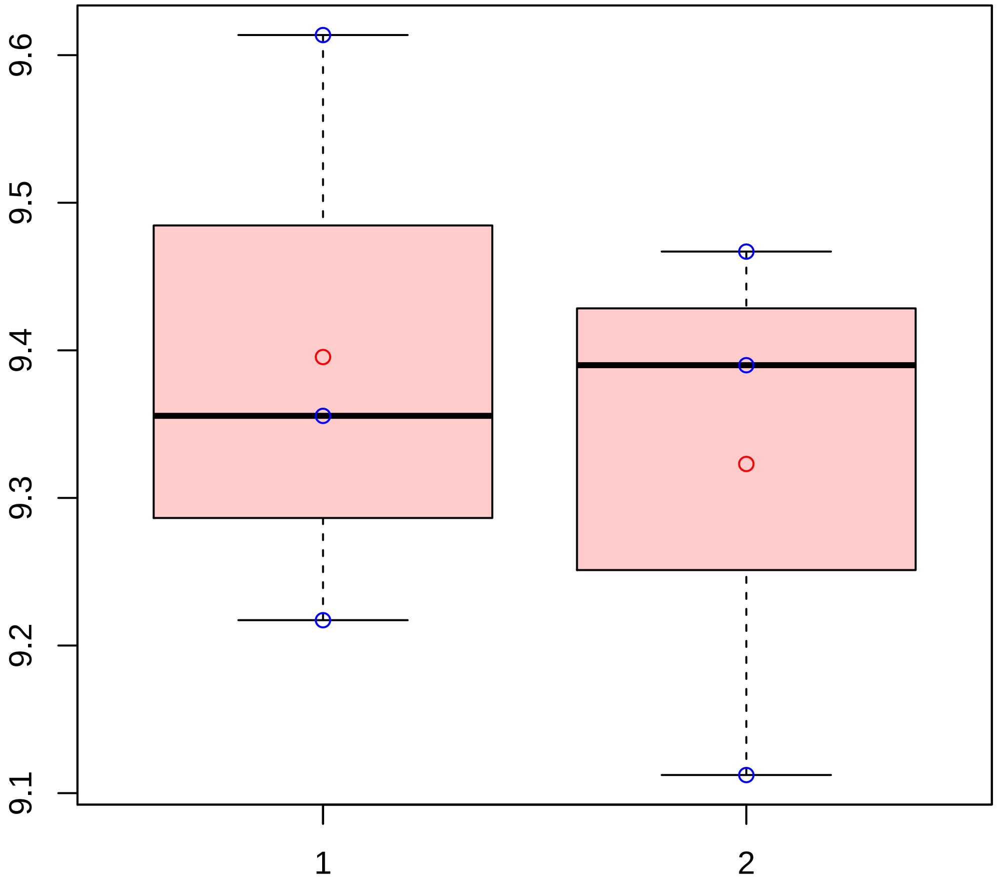


t−Test: p−value = 0.67

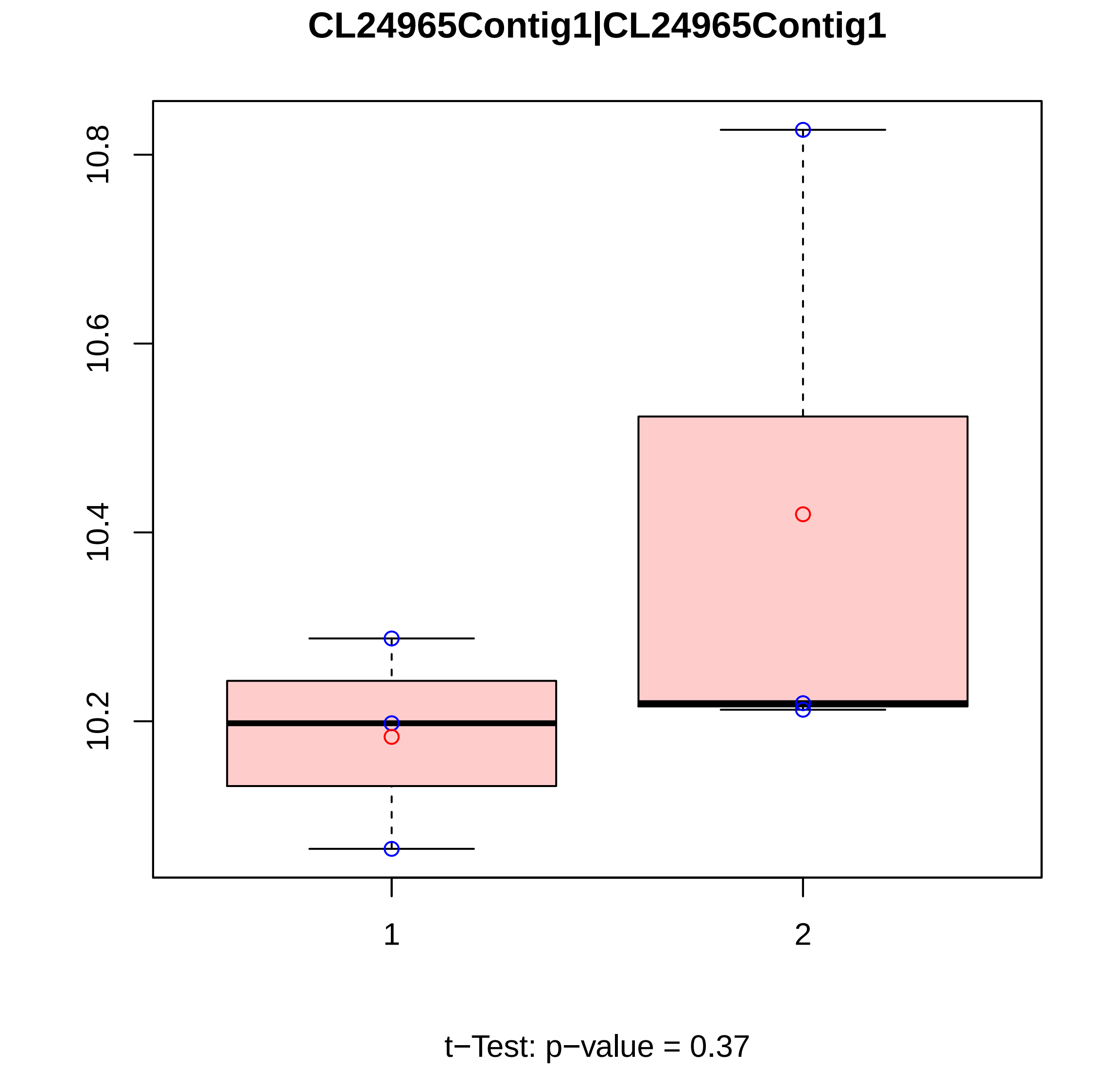
CL24965Contig1|CL24965Contig1
10.8
10.6
10.4
10.2
1
2
t−Test: p−value = 0.37

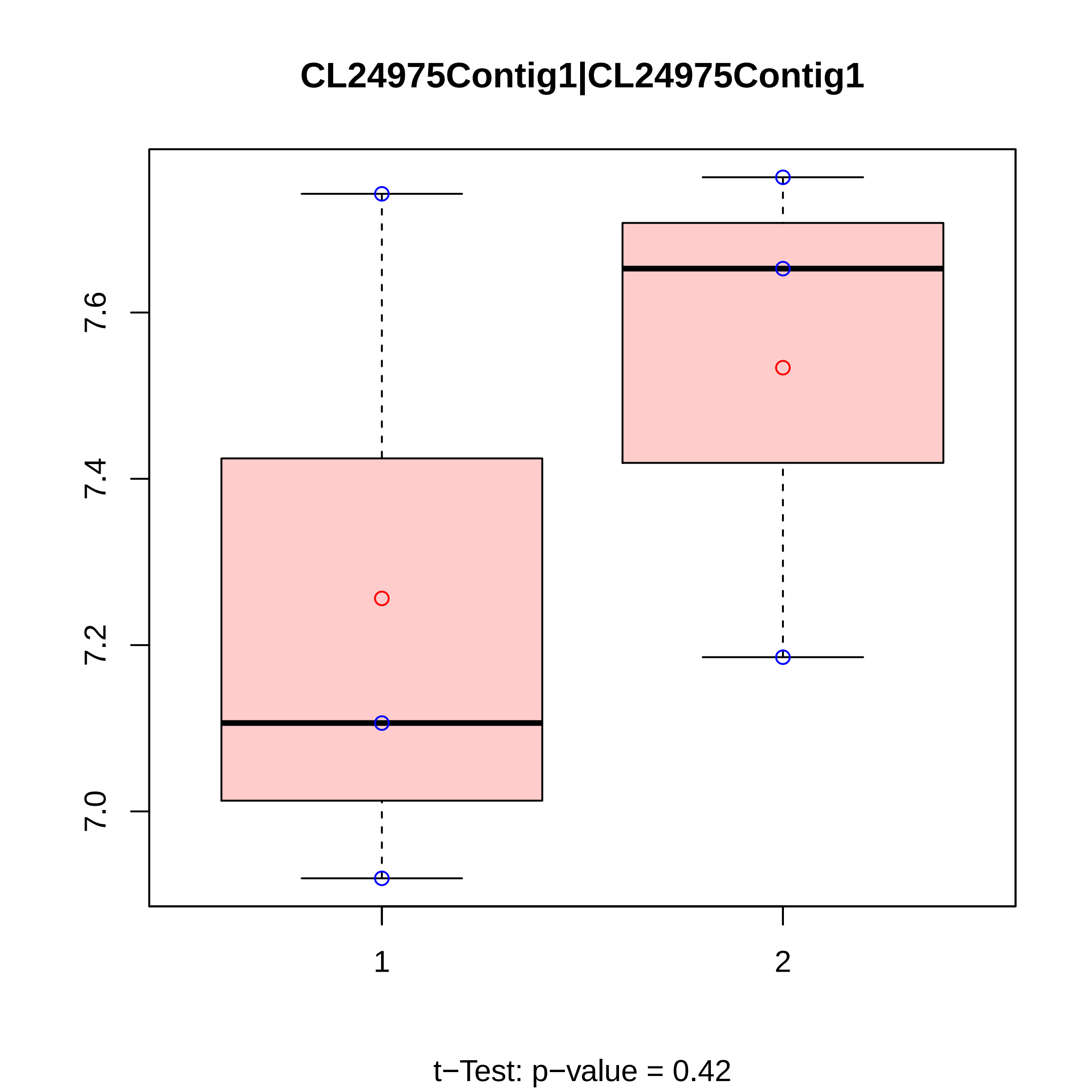
CL24975Contig1|CL24975Contig1
7.0
7.2
7.4
7.6
1
2
t−Test: p−value = 0.42

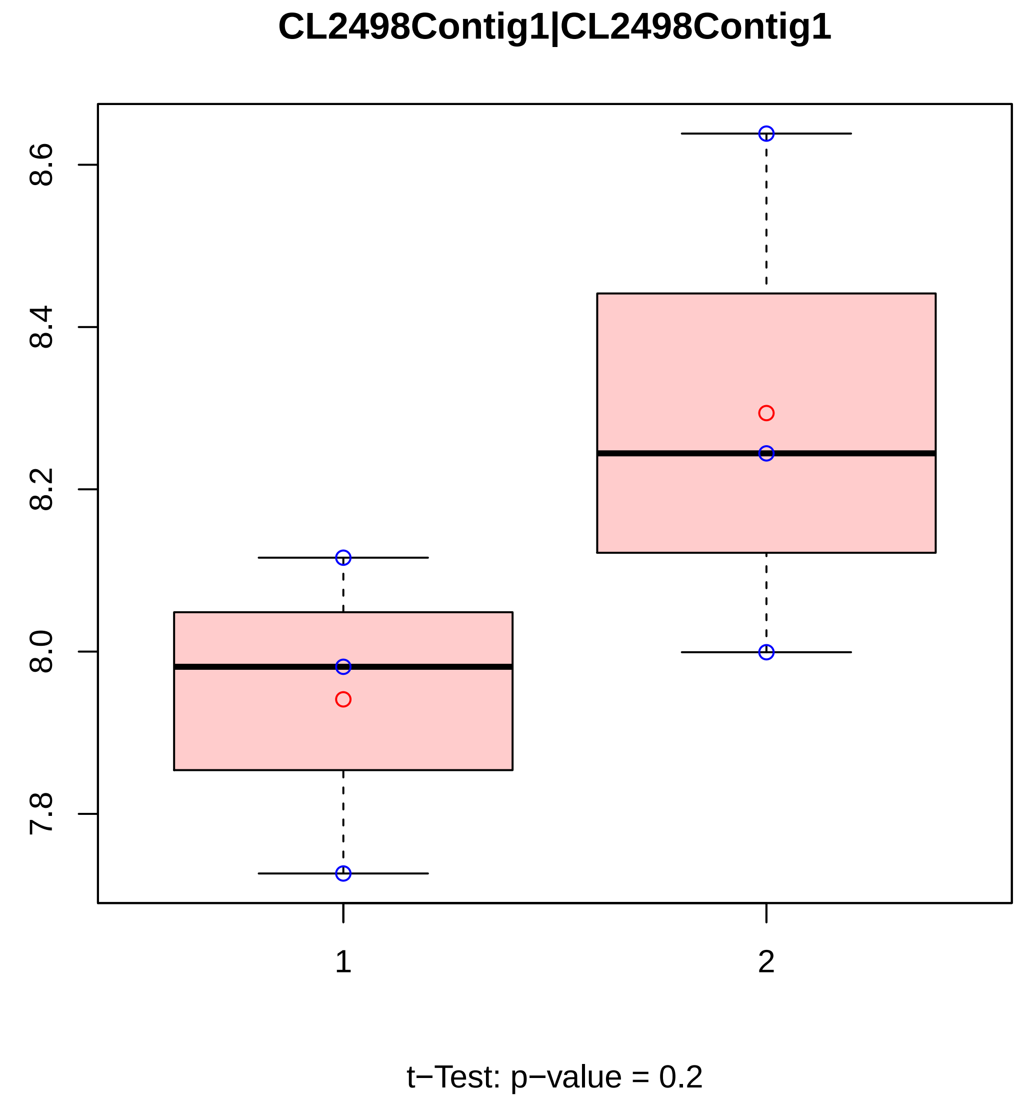
CL2498Contig1|CL2498Contig1
8.6
8.4
8.2
8.0
7.8
1
2
t−Test: p−value = 0.2

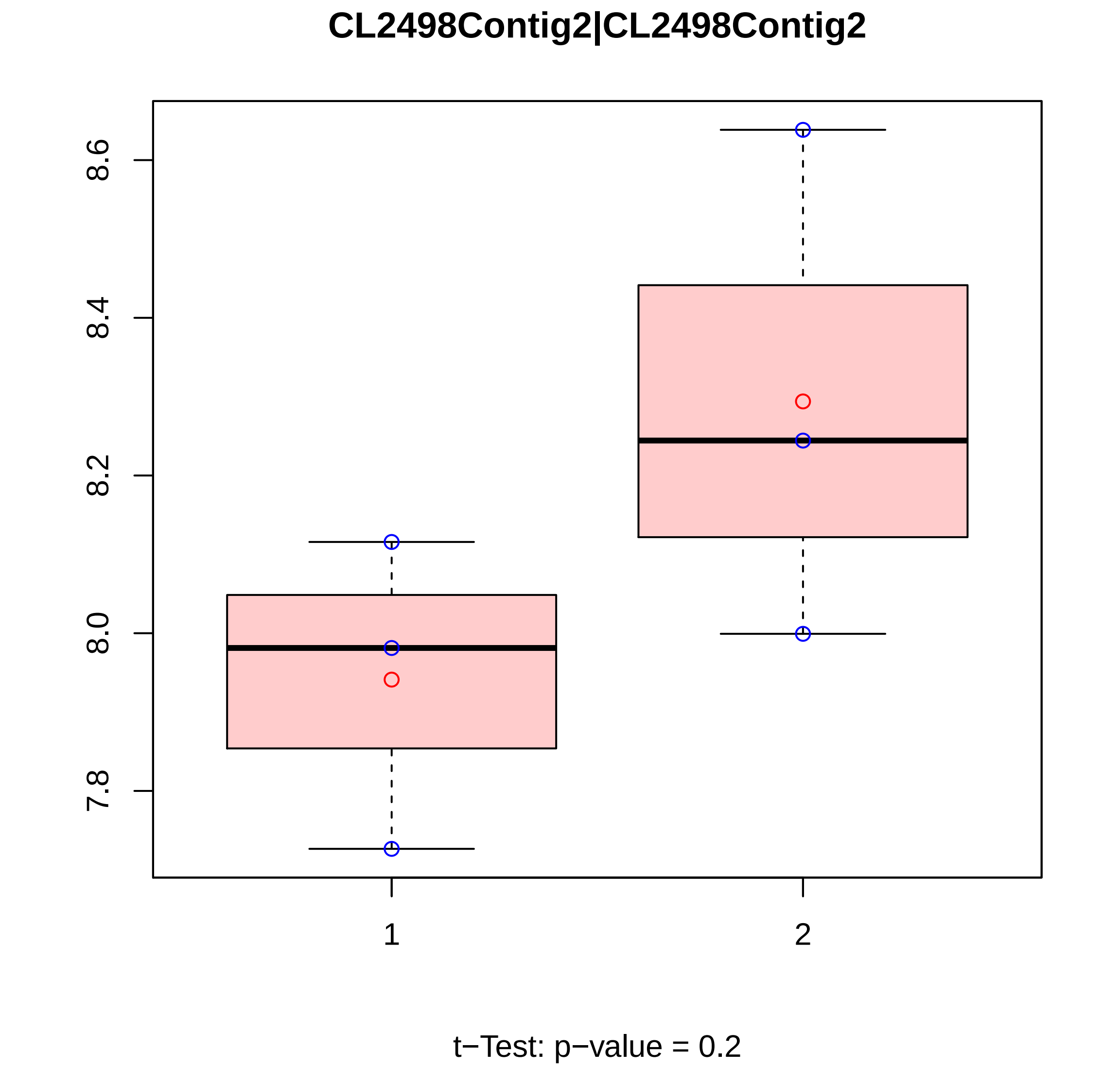

CL2498Contig2|CL2498Contig2
8.6
8.4
8.2
8.0
7.8
1
2
t−Test: p−value = 0.2

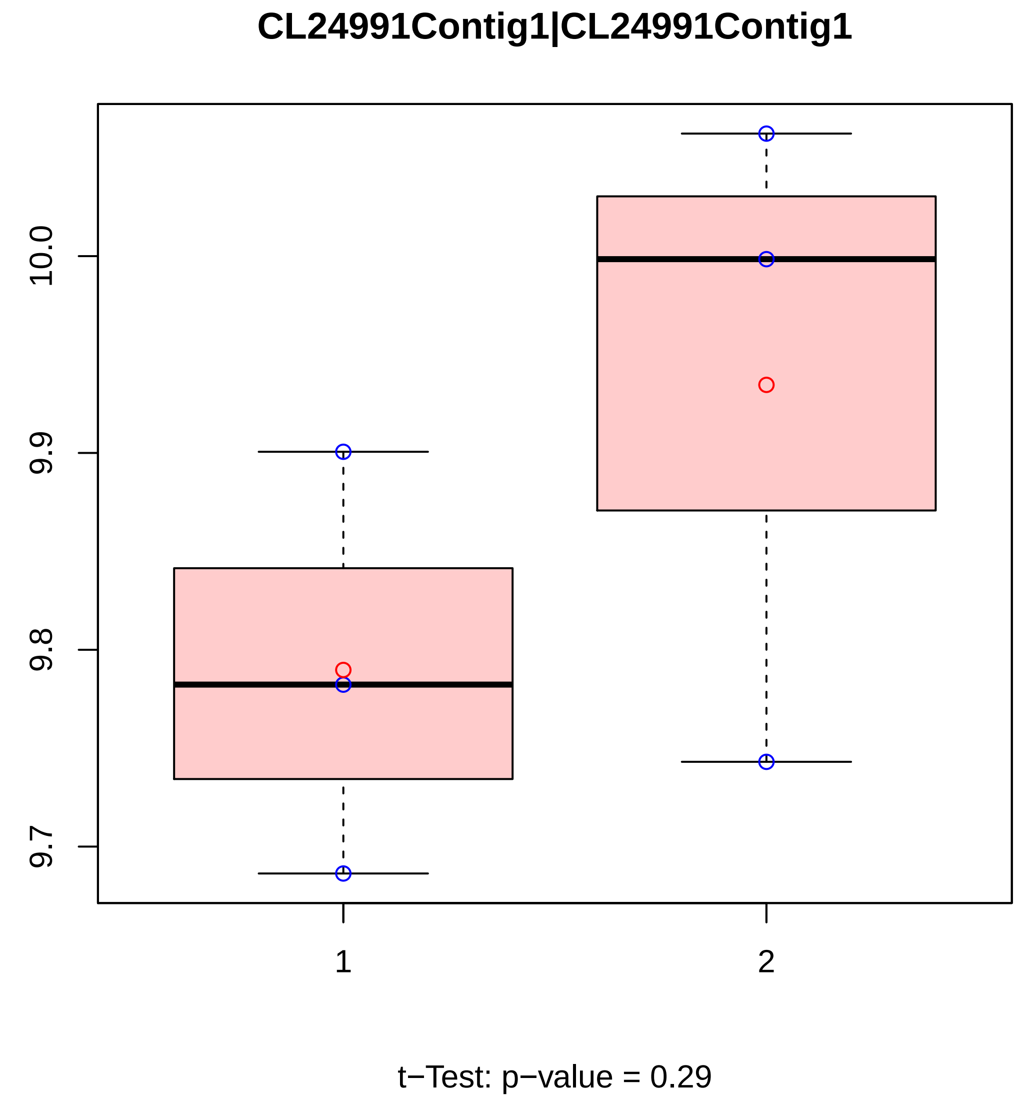
CL24991Contig1|CL24991Contig1
10.0
9.9
9.8
9.7
1
2
t−Test: p−value = 0.29

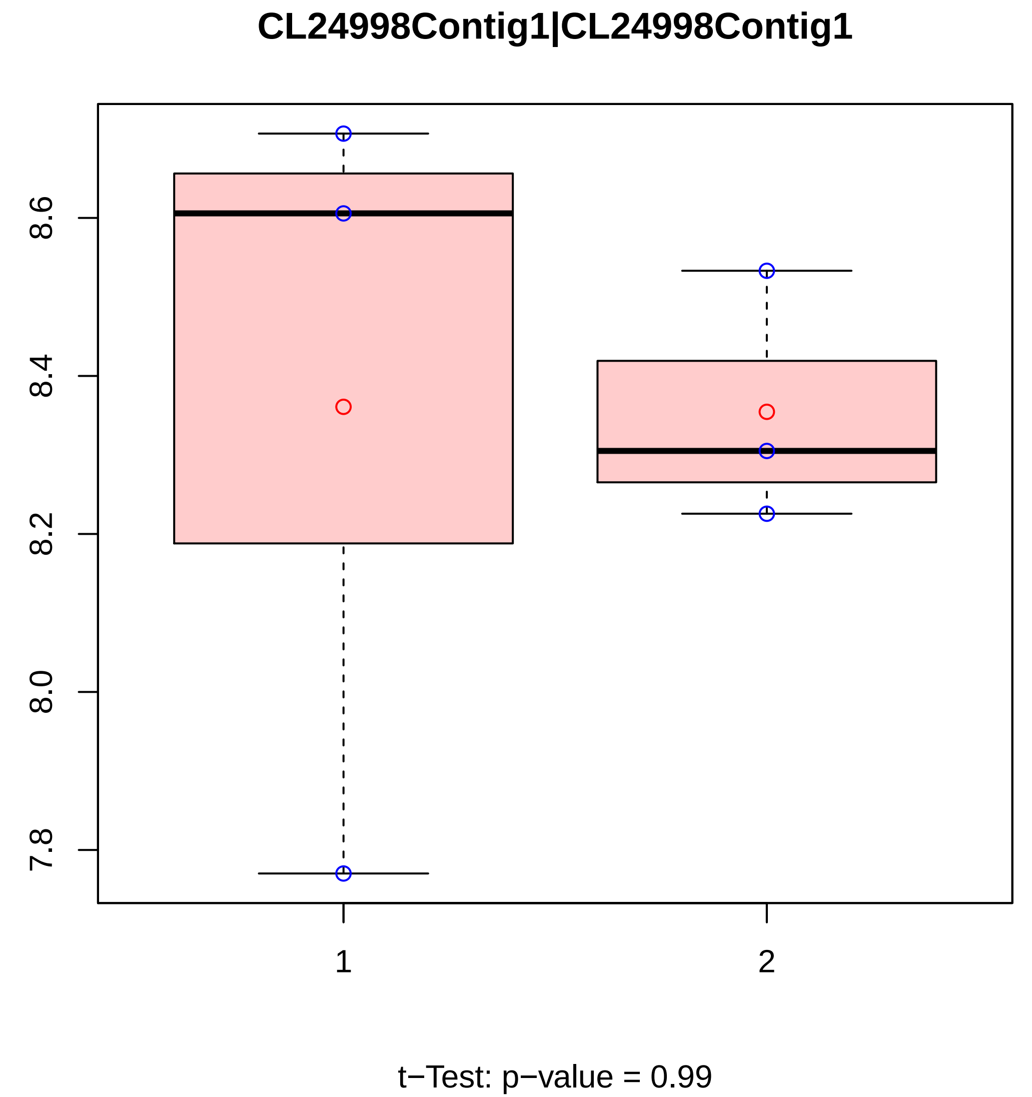
CL24998Contig1|CL24998Contig1
8.6
8.4
8.2
8.0
7.8
1
2
t−Test: p−value = 0.99

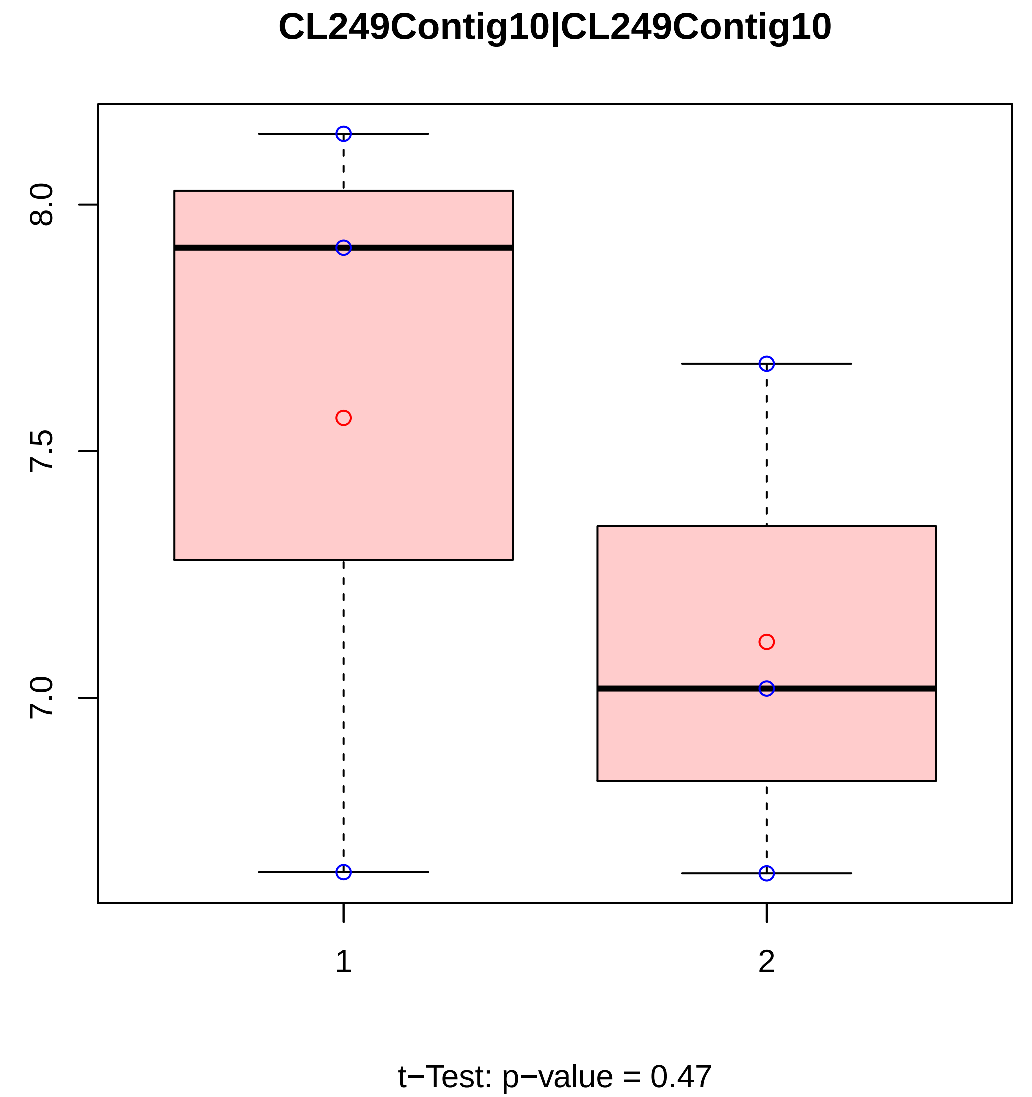
CL249Contig10|CL249Contig10
8.0
7.5
7.0
1
2
t−Test: p−value = 0.47

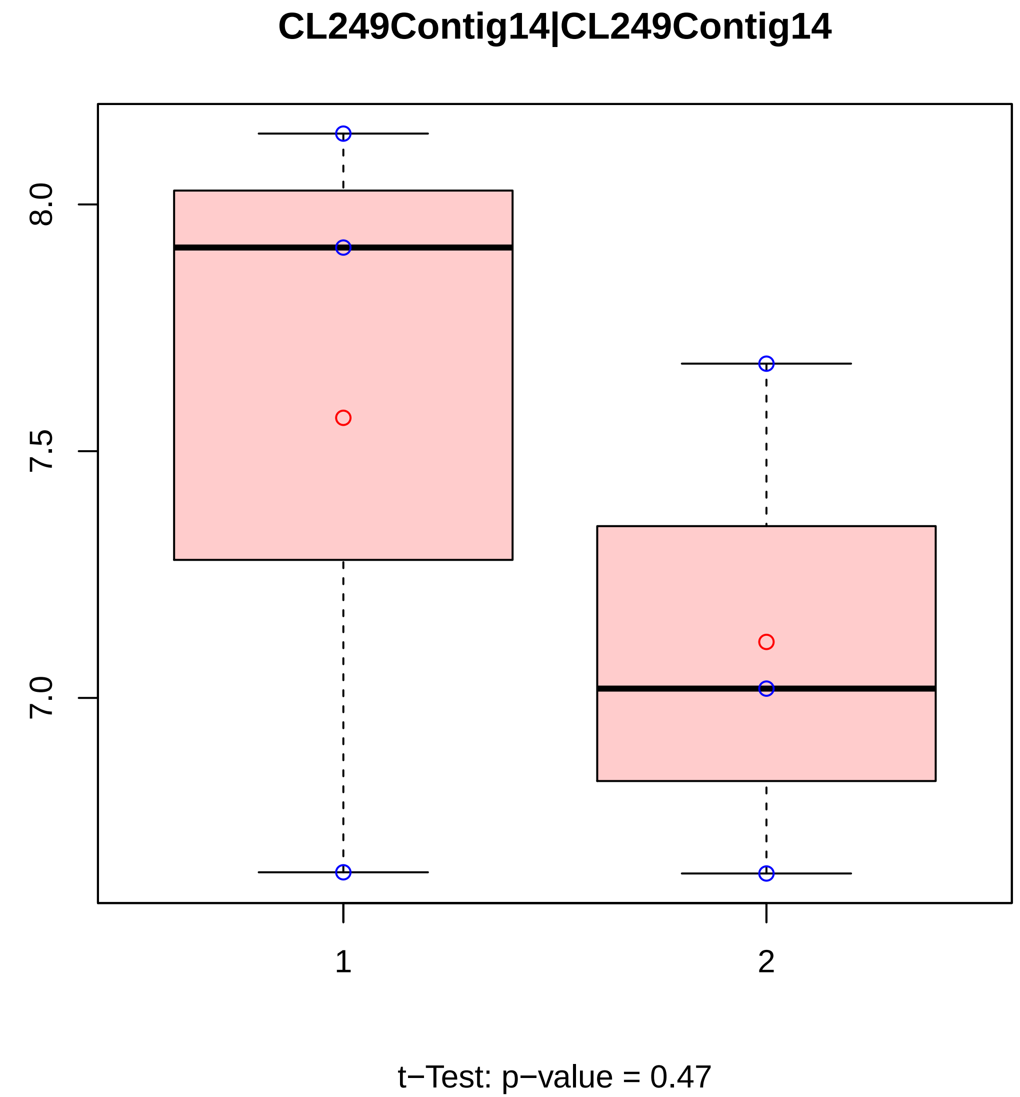
CL249Contig14|CL249Contig14
8.0
7.5
7.0
1
2
t−Test: p−value = 0.47

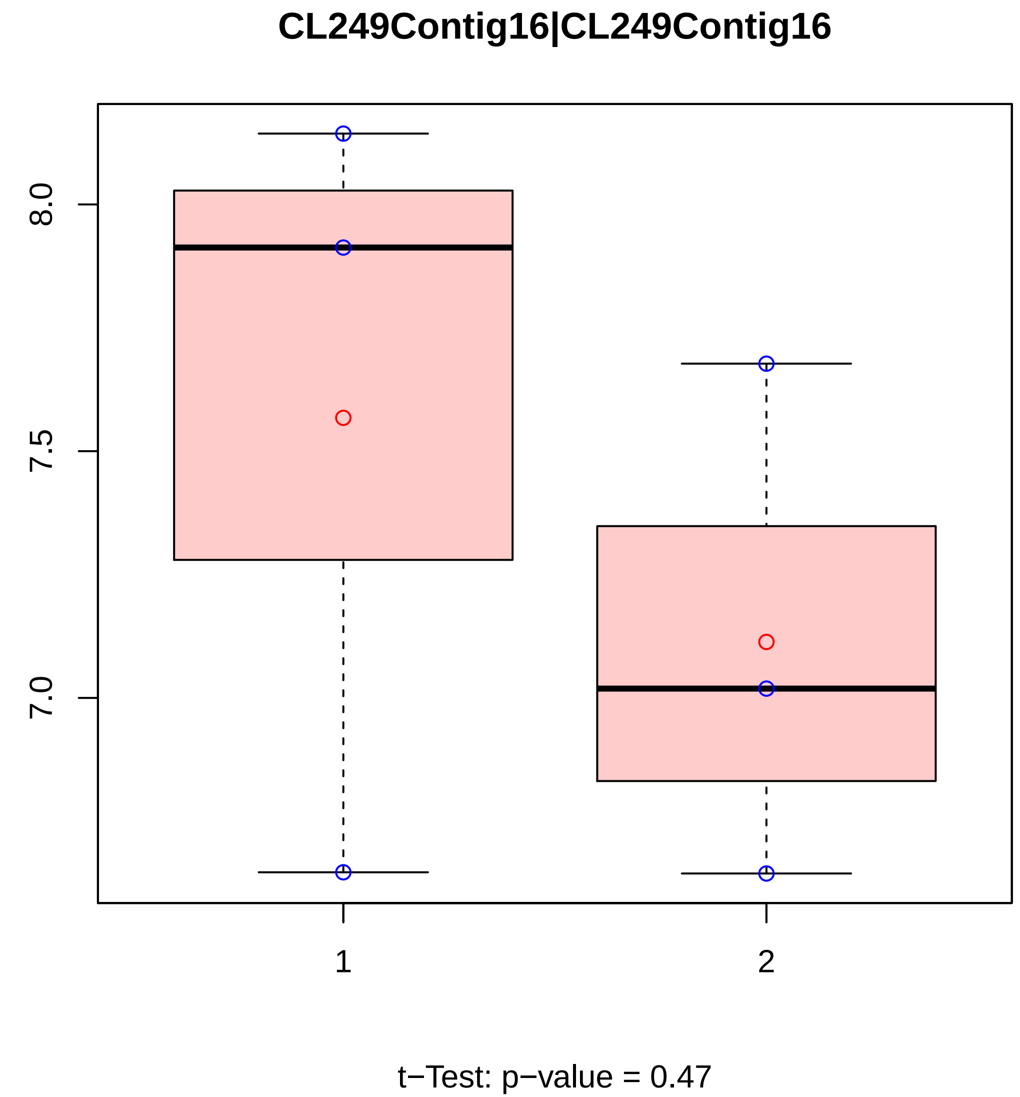

CL249Contig16|CL249Contig16
8.0
7.5
7.0
1
2
t−Test: p−value = 0.47

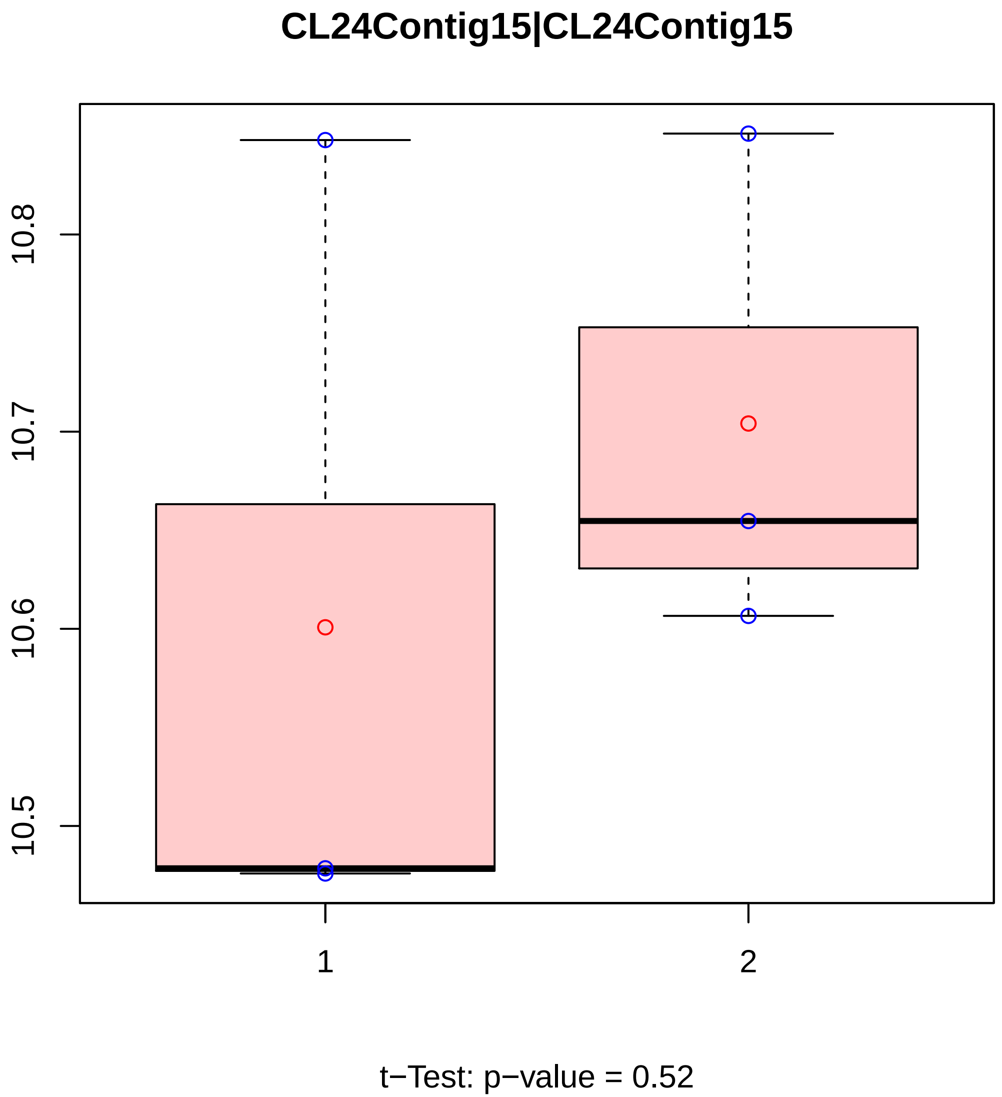

CL24Contig15|CL24Contig15
10.5
10.6
10.7
10.8
1
2
t−Test: p−value = 0.52

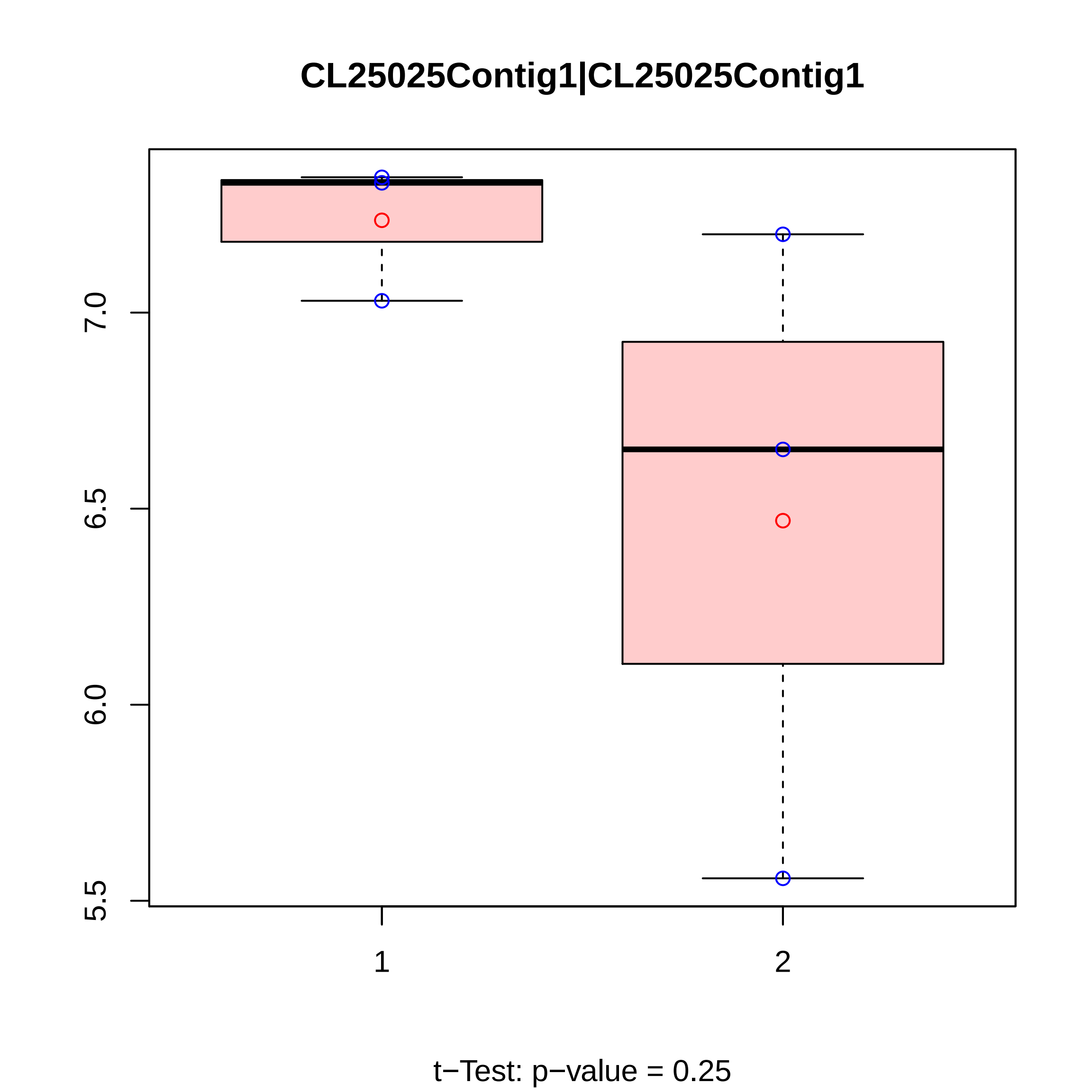
CL25025Contig1|CL25025Contig1
7.0
6.5
6.0
5.5
1
2
t−Test: p−value = 0.25

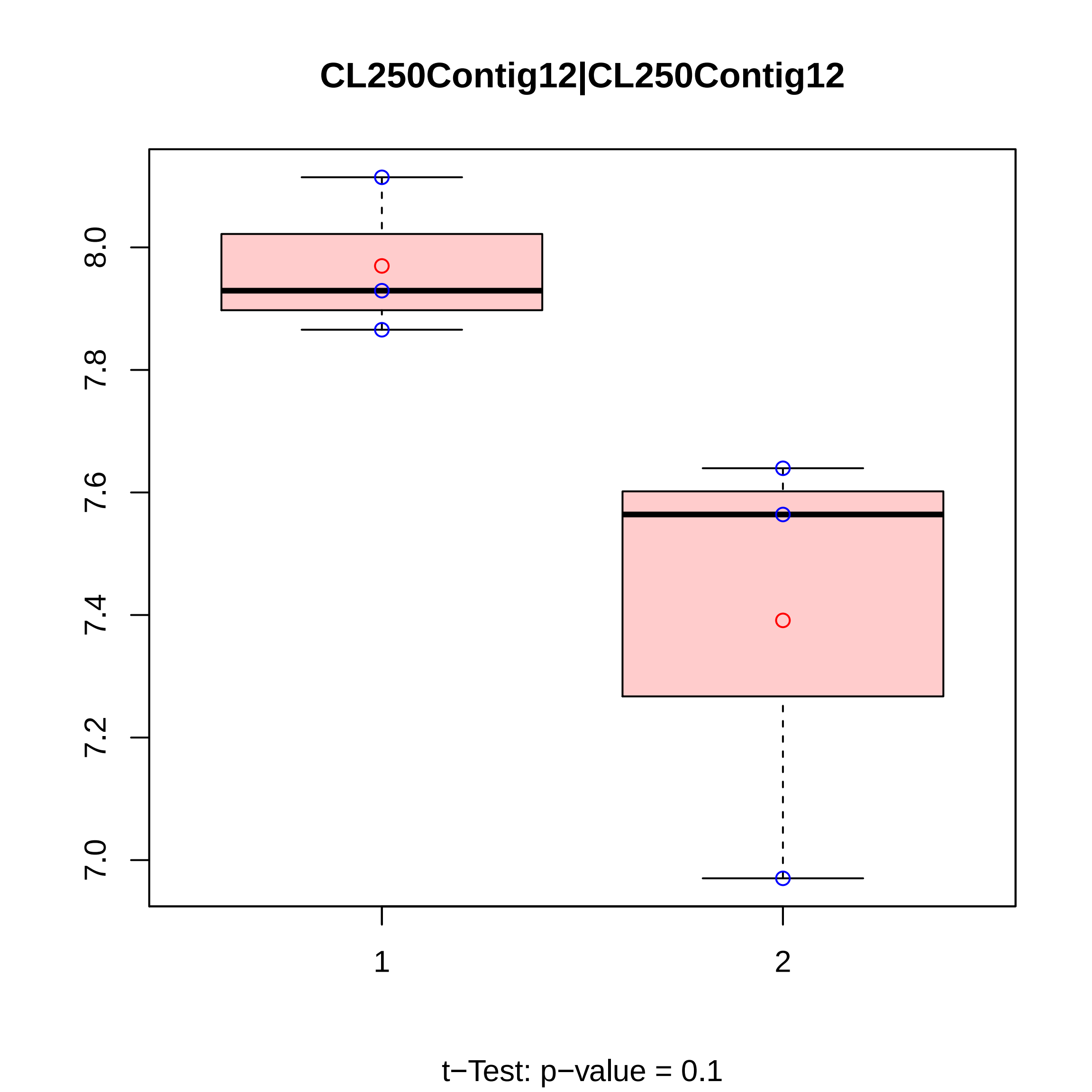

CL250Contig12|CL250Contig12
8.0
7.8
7.6
7.4
7.2
7.0
1
2
t−Test: p−value = 0.1

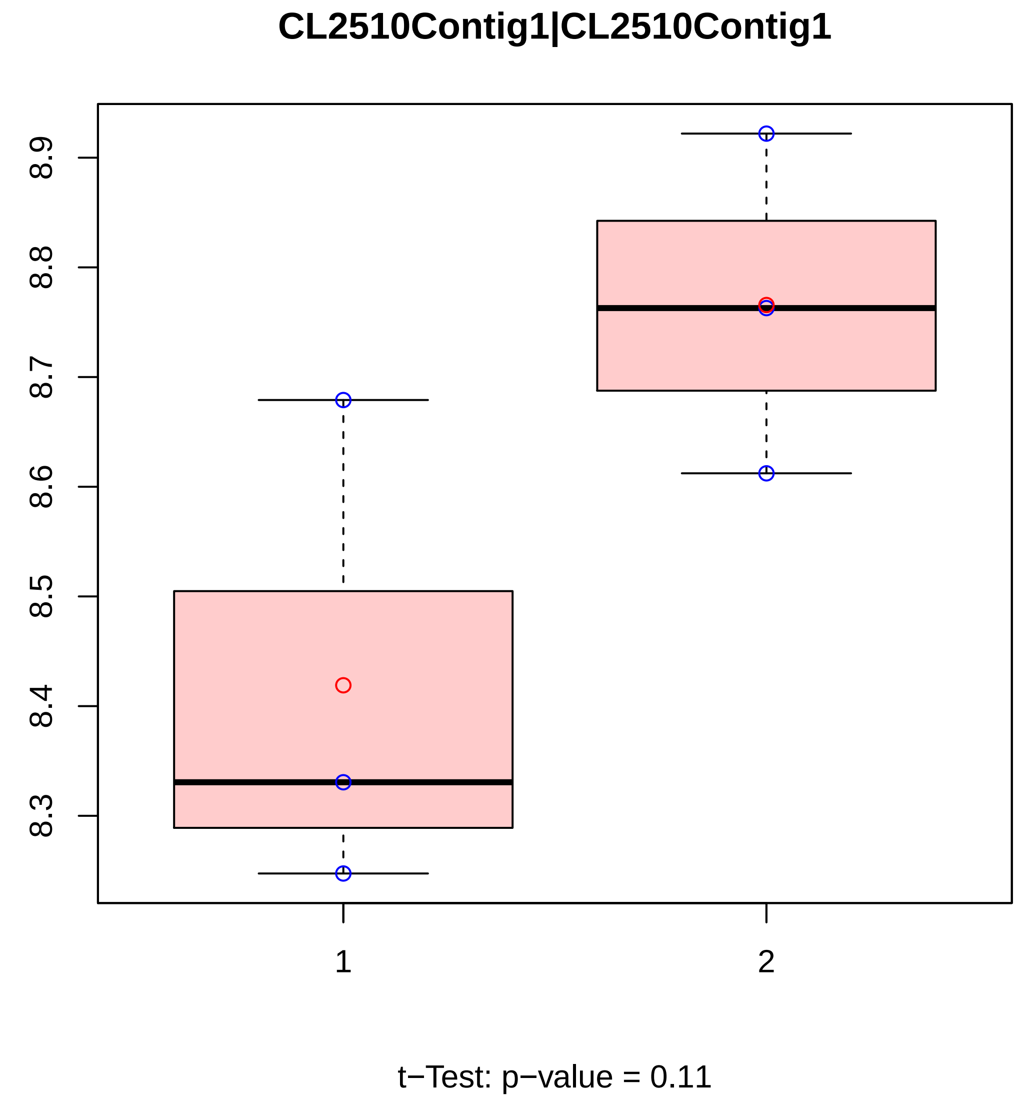
CL2510Contig1|CL2510Contig1
8.3
8.4
8.5
8.6
8.7
8.8
8.9
1
2
t−Test: p−value = 0.11

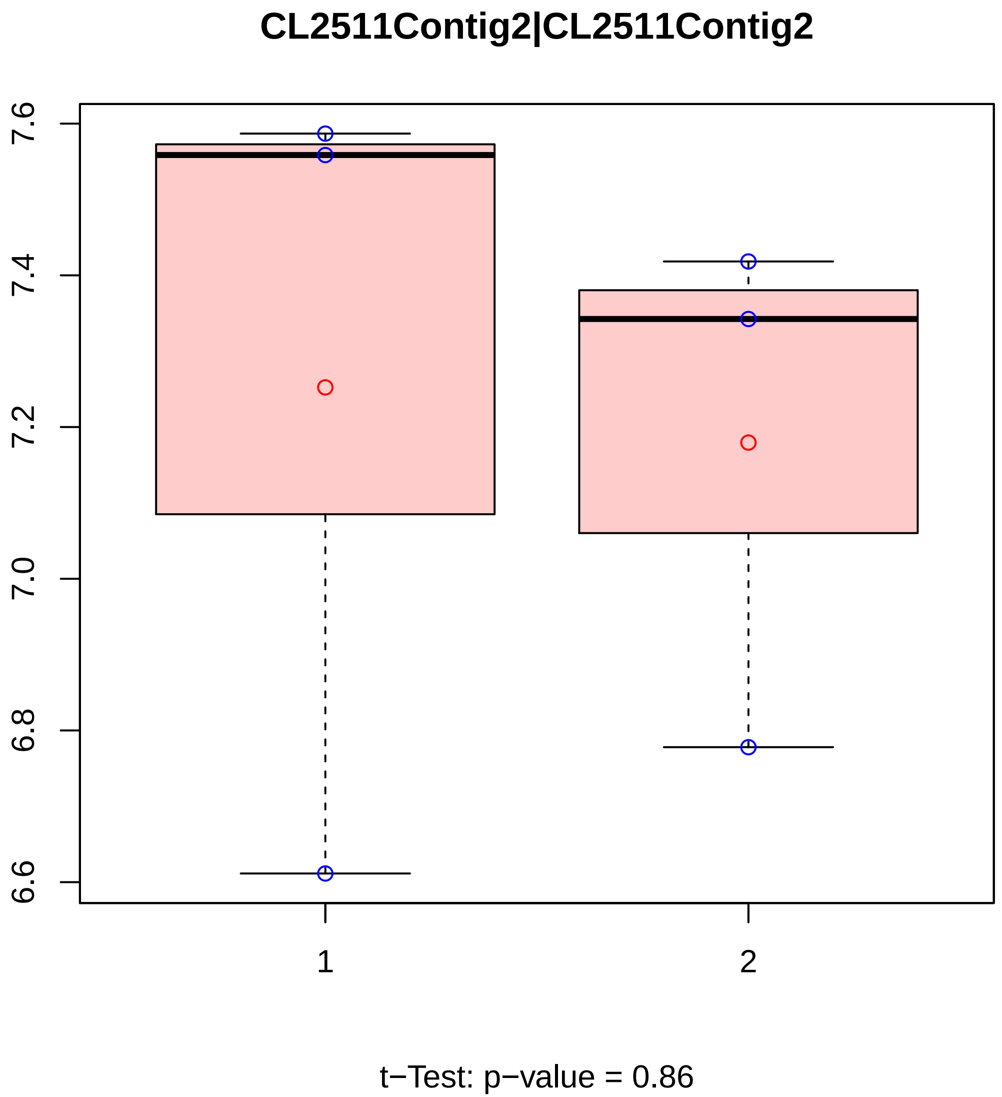
CL2511Contig2|CL2511Contig2
7.6
7.4
7.2
7.0
6.8
6.6
1
2
t−Test: p−value = 0.86

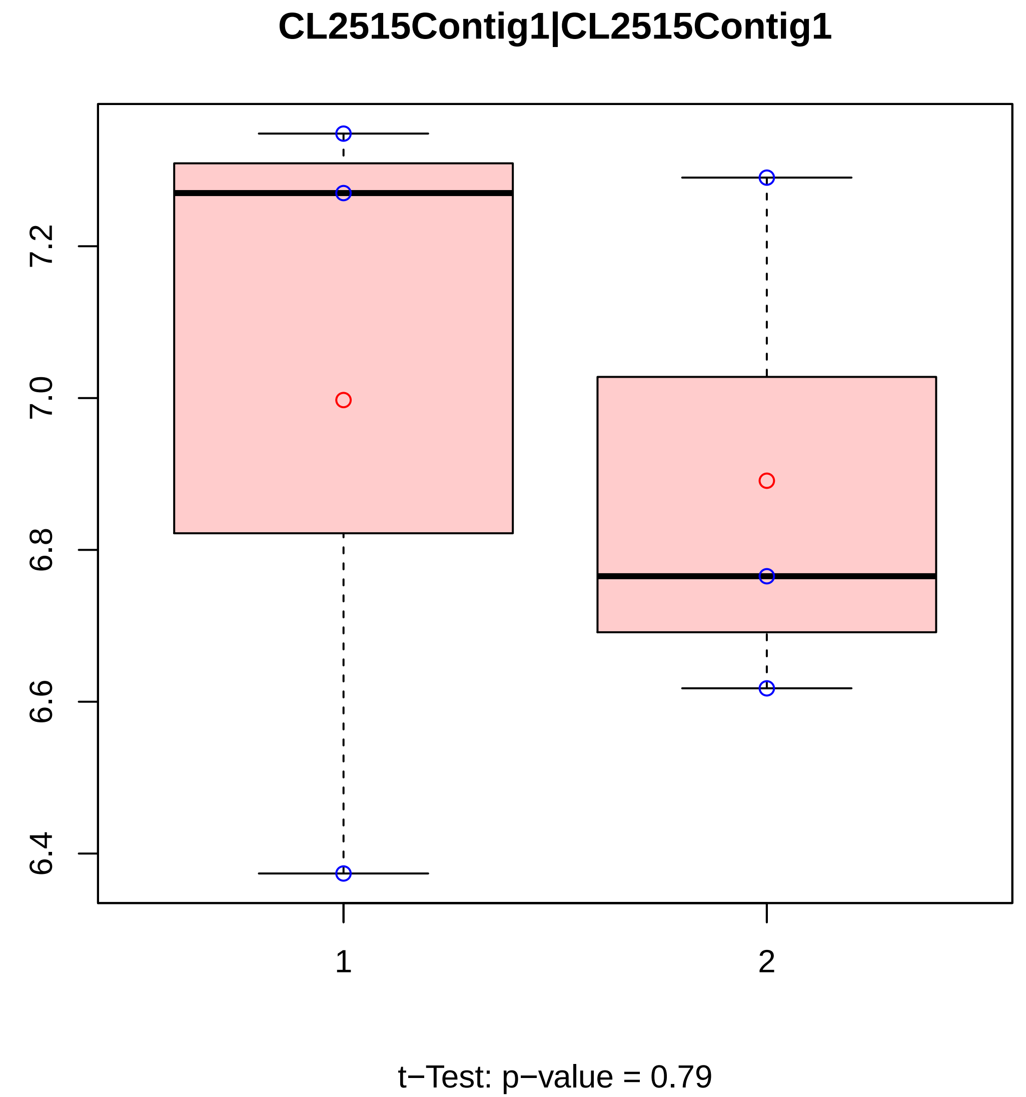
CL2515Contig1|CL2515Contig1
7.2
7.0
6.8
6.6
6.4
1
2
t−Test: p−value = 0.79

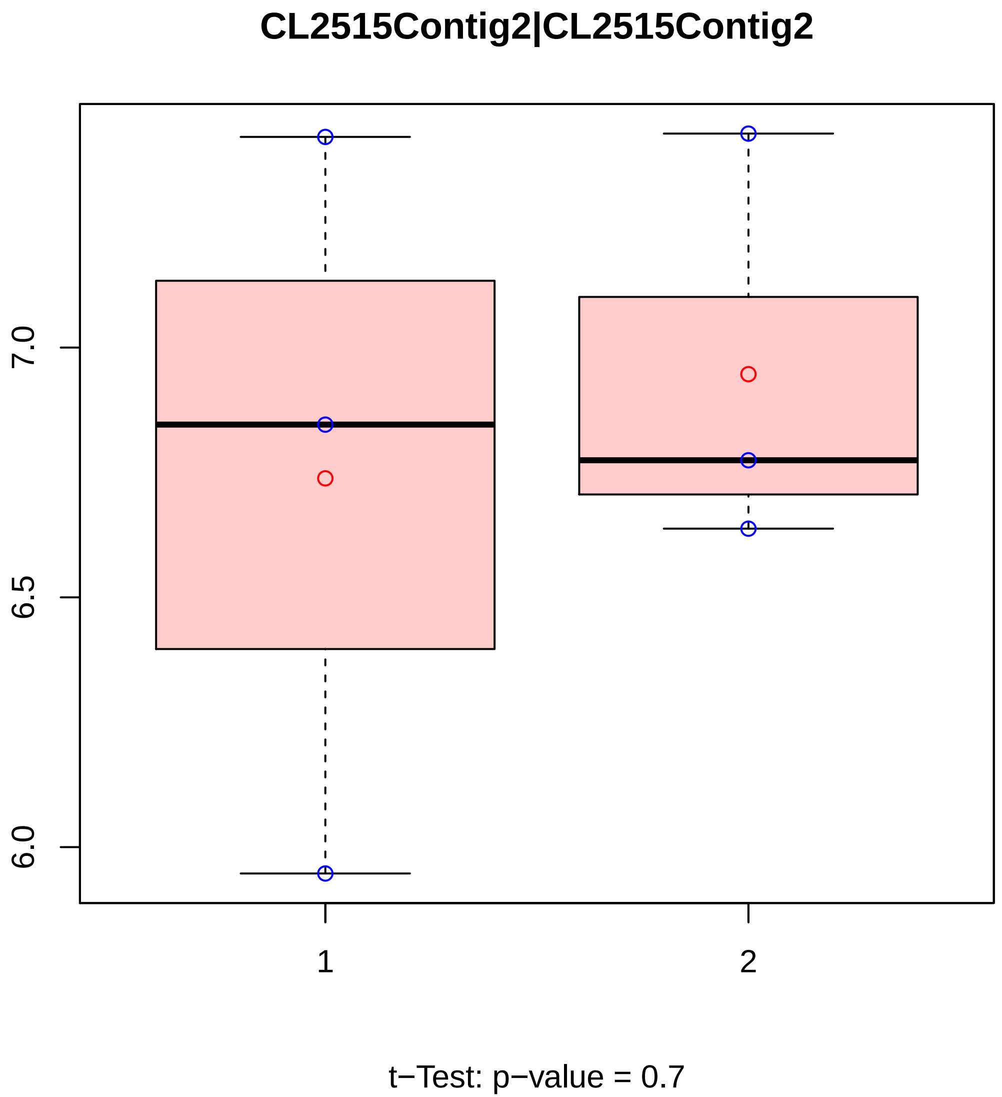

CL2515Contig2|CL2515Contig2
7.0
6.5
6.0
1
2
t−Test: p−value = 0.7

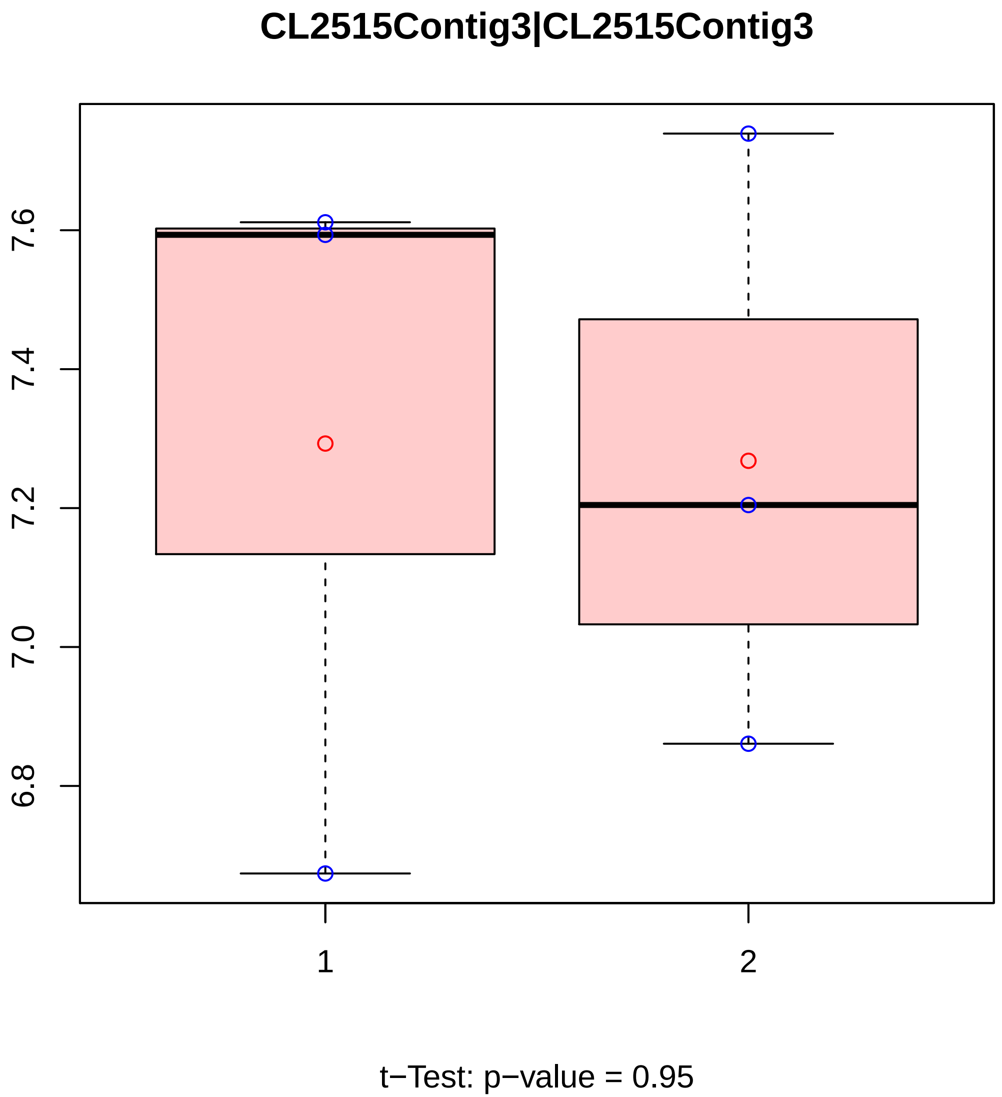
CL2515Contig3|CL2515Contig3
7.6
7.4
7.2
7.0
6.8
1
2
t−Test: p−value = 0.95

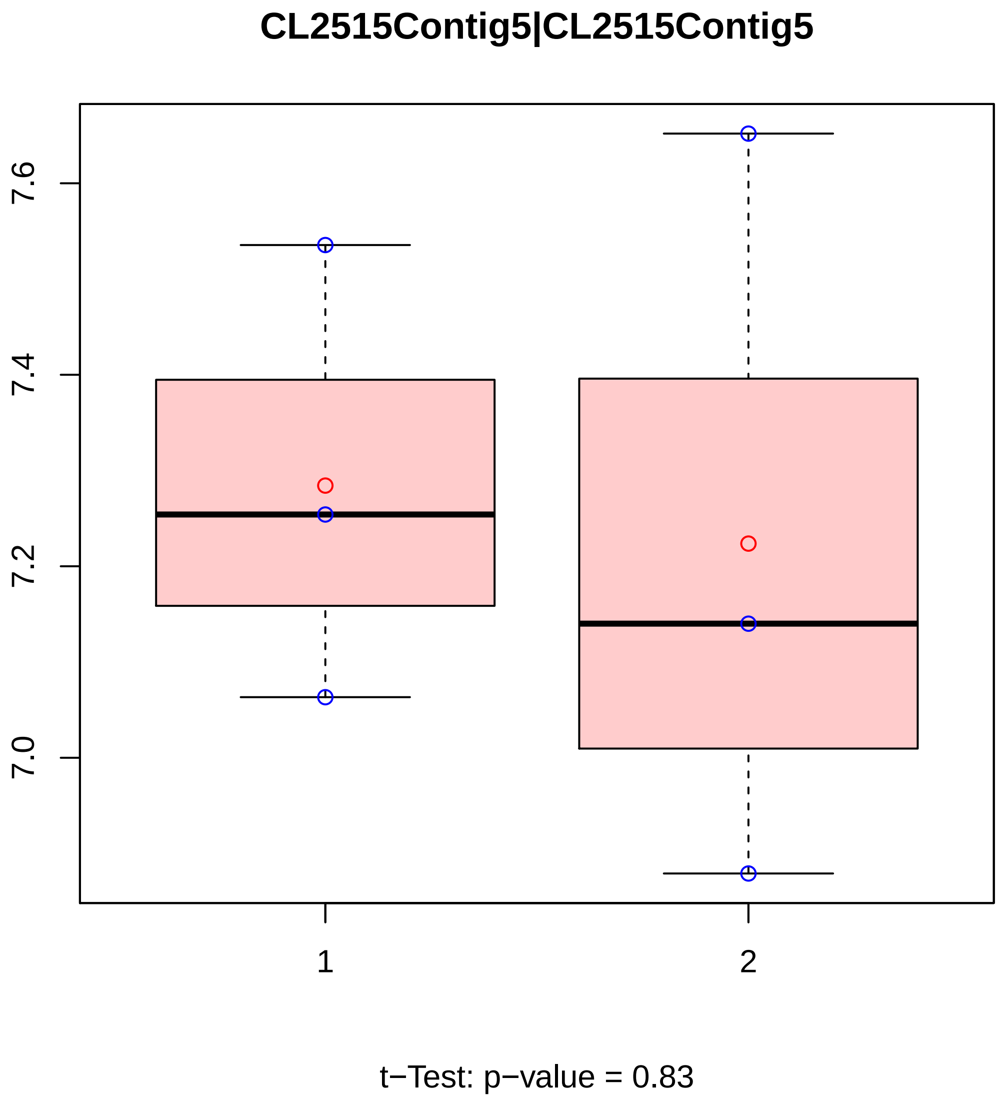
CL2515Contig5|CL2515Contig5
7.0
7.2
7.4
7.6
1
2
t−Test: p−value = 0.83

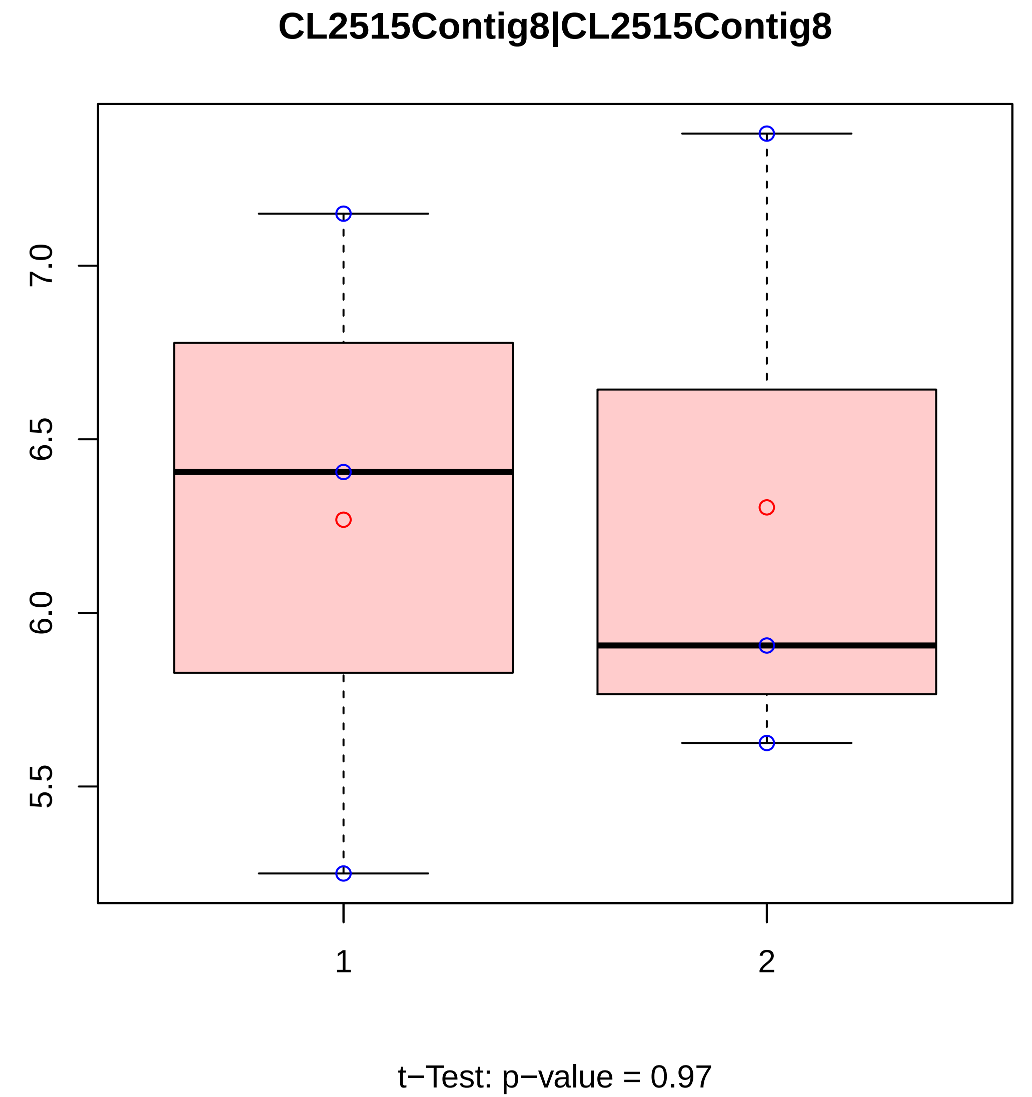

CL2515Contig8|CL2515Contig8
7.0
6.5
6.0
5.5
1
2
t−Test: p−value = 0.97

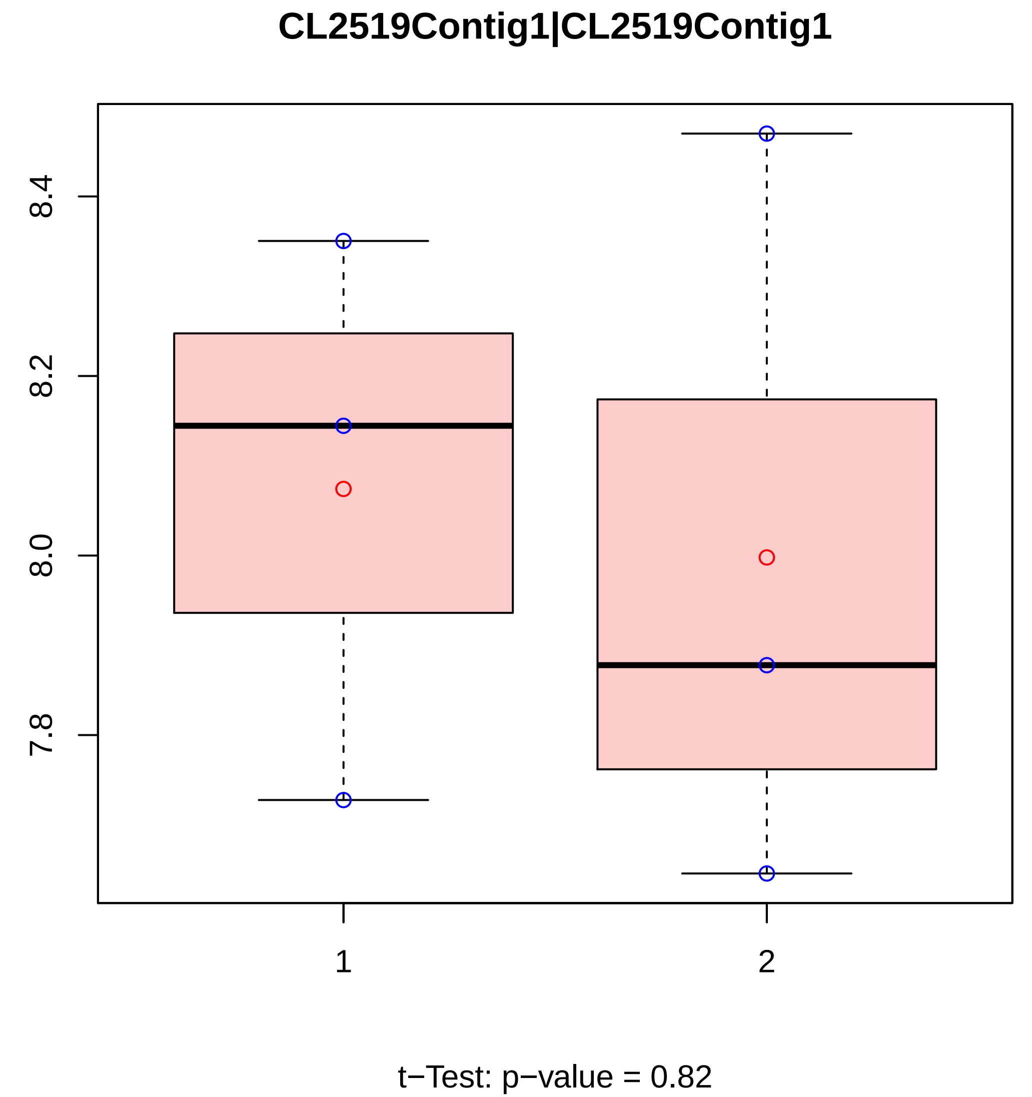

CL2519Contig1|CL2519Contig1
8.4
8.2
8.0
7.8
1
2
t−Test: p−value = 0.82

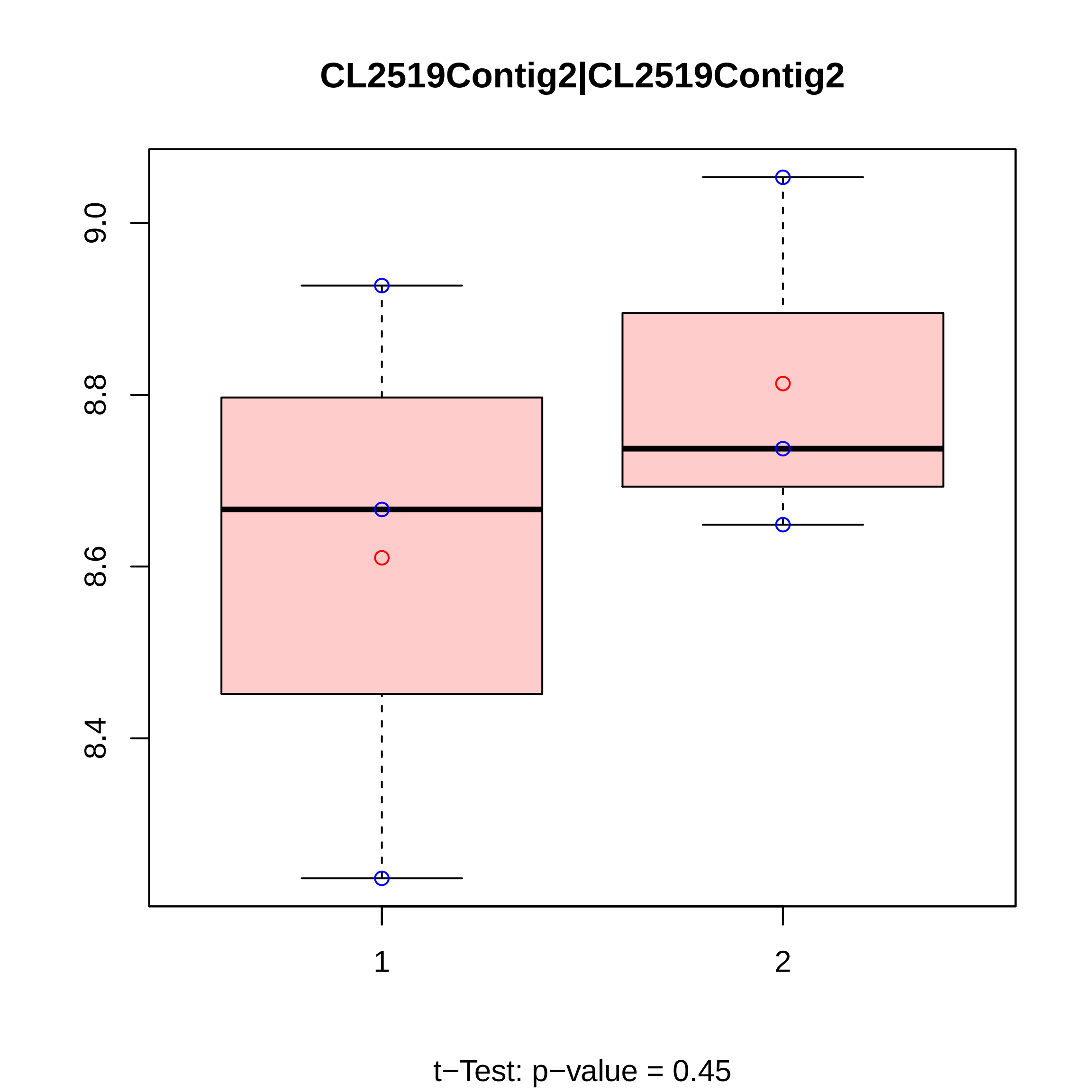

CL2519Contig2|CL2519Contig2
9.0
8.8
8.6
8.4
1
2
t−Test: p−value = 0.45

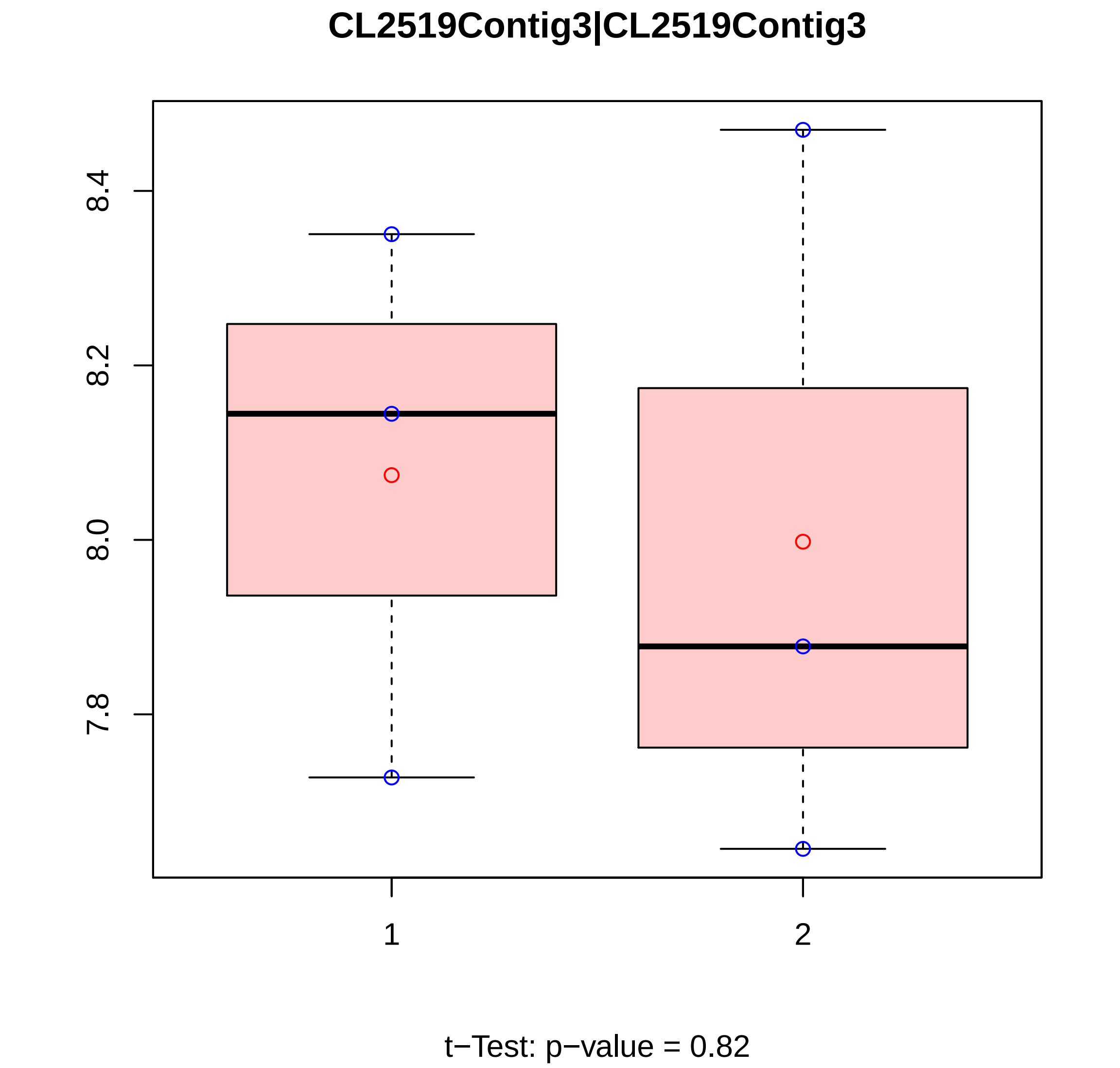
CL2519Contig3|CL2519Contig3
8.4
8.2
8.0
7.8
1
2
t−Test: p−value = 0.82

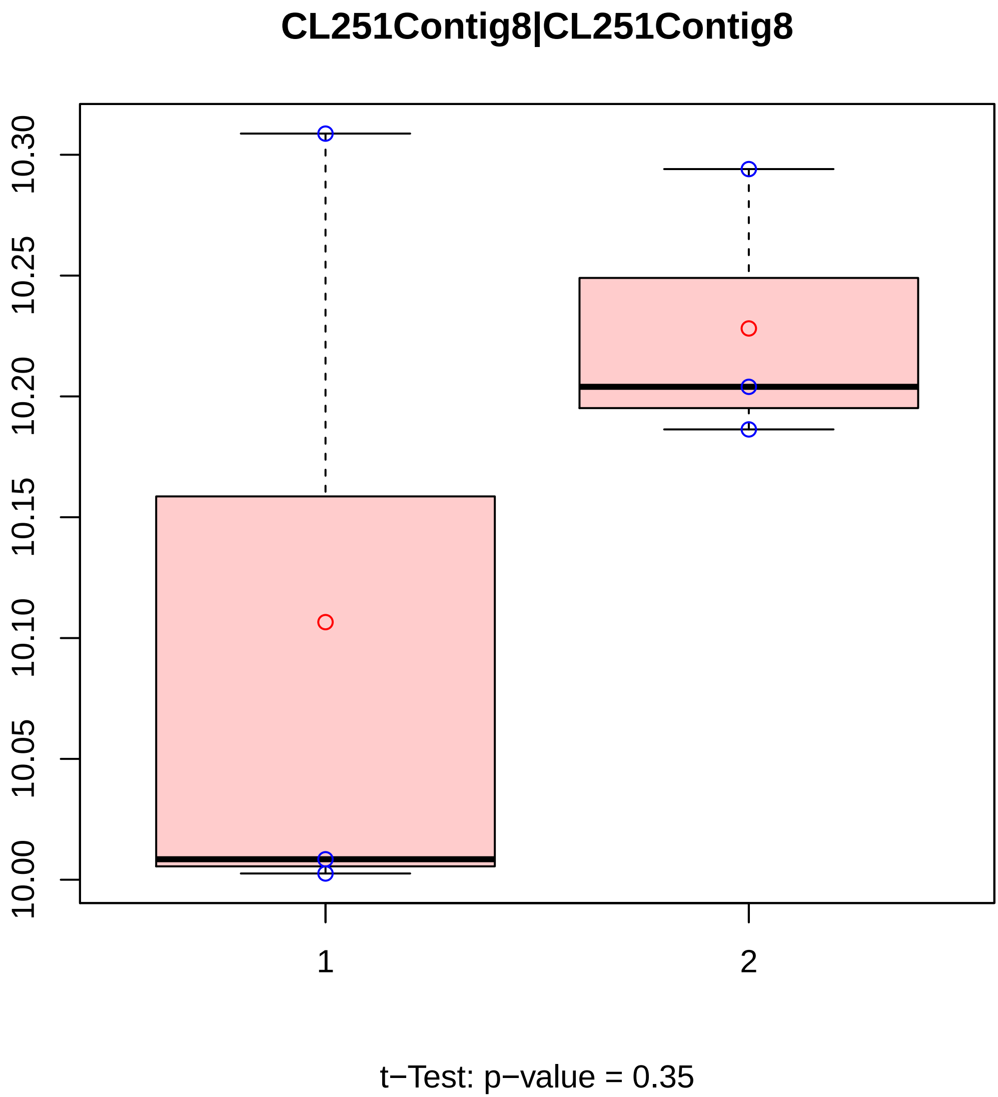

CL251Contig8|CL251Contig8
10.00
10.05
10.10
10.15
10.20
10.25
10.30
1
2
t−Test: p−value = 0.35

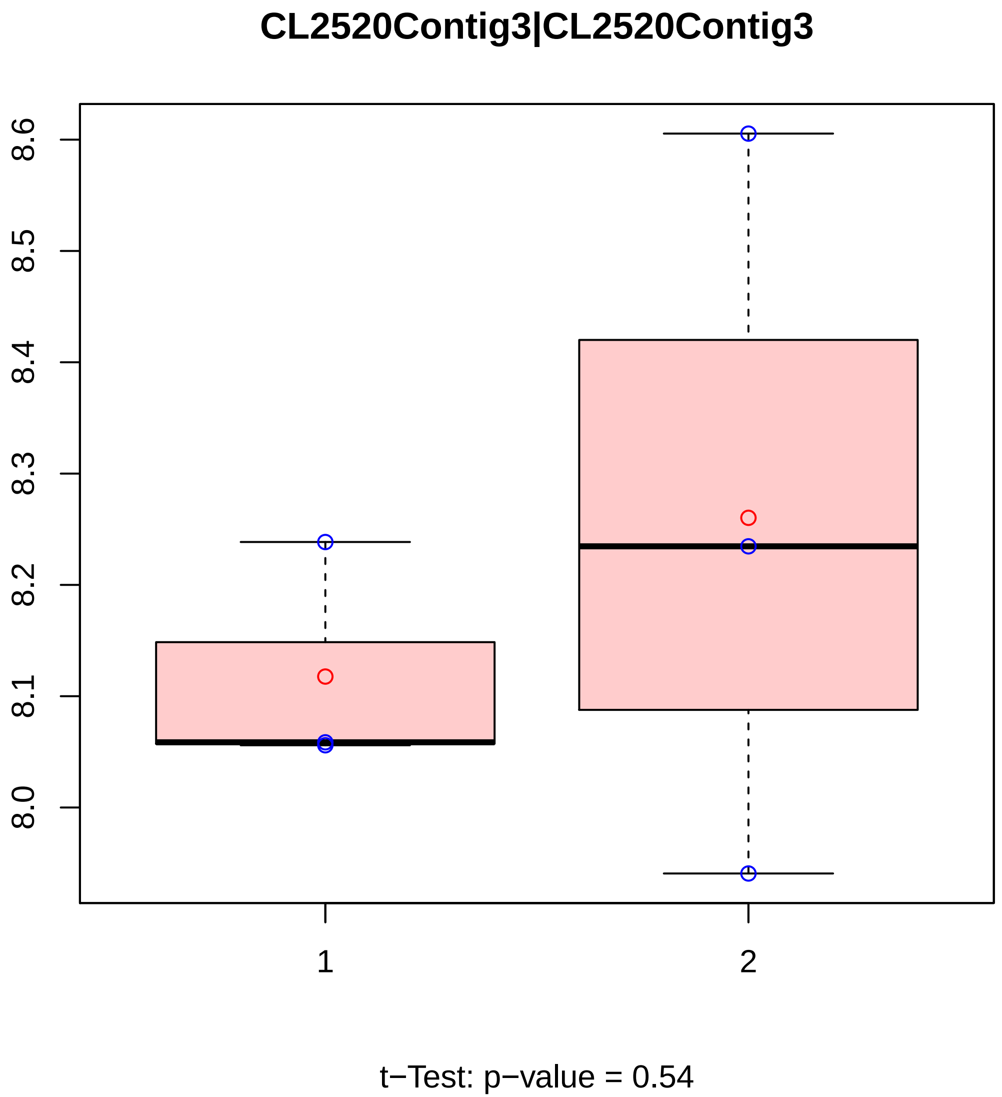
CL2520Contig3|CL2520Contig3
8.0
8.1
8.2
8.3
8.4
8.5
8.6
1
2
t−Test: p−value = 0.54

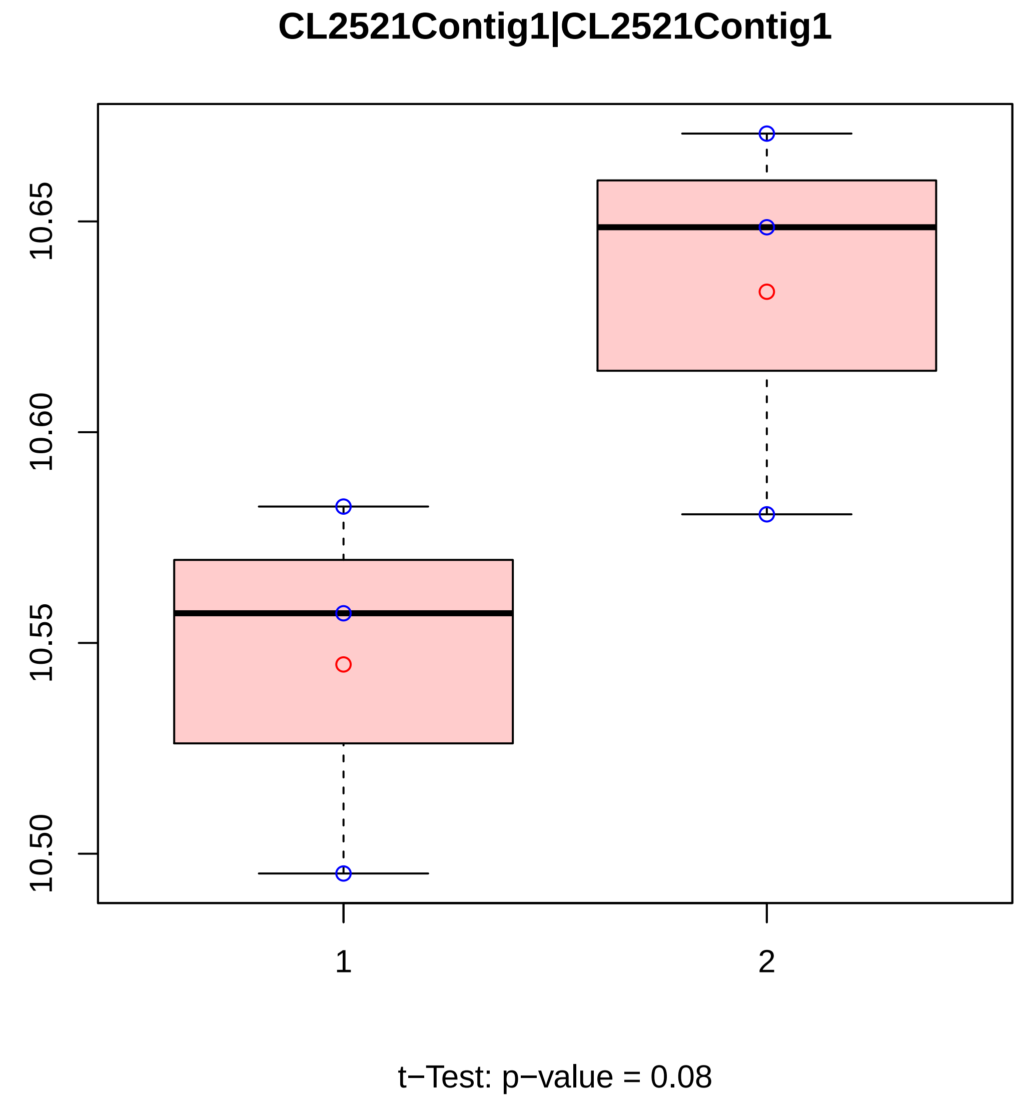

CL2521Contig1|CL2521Contig1
10.50
10.55
10.60
10.65
1
2
t−Test: p−value = 0.08

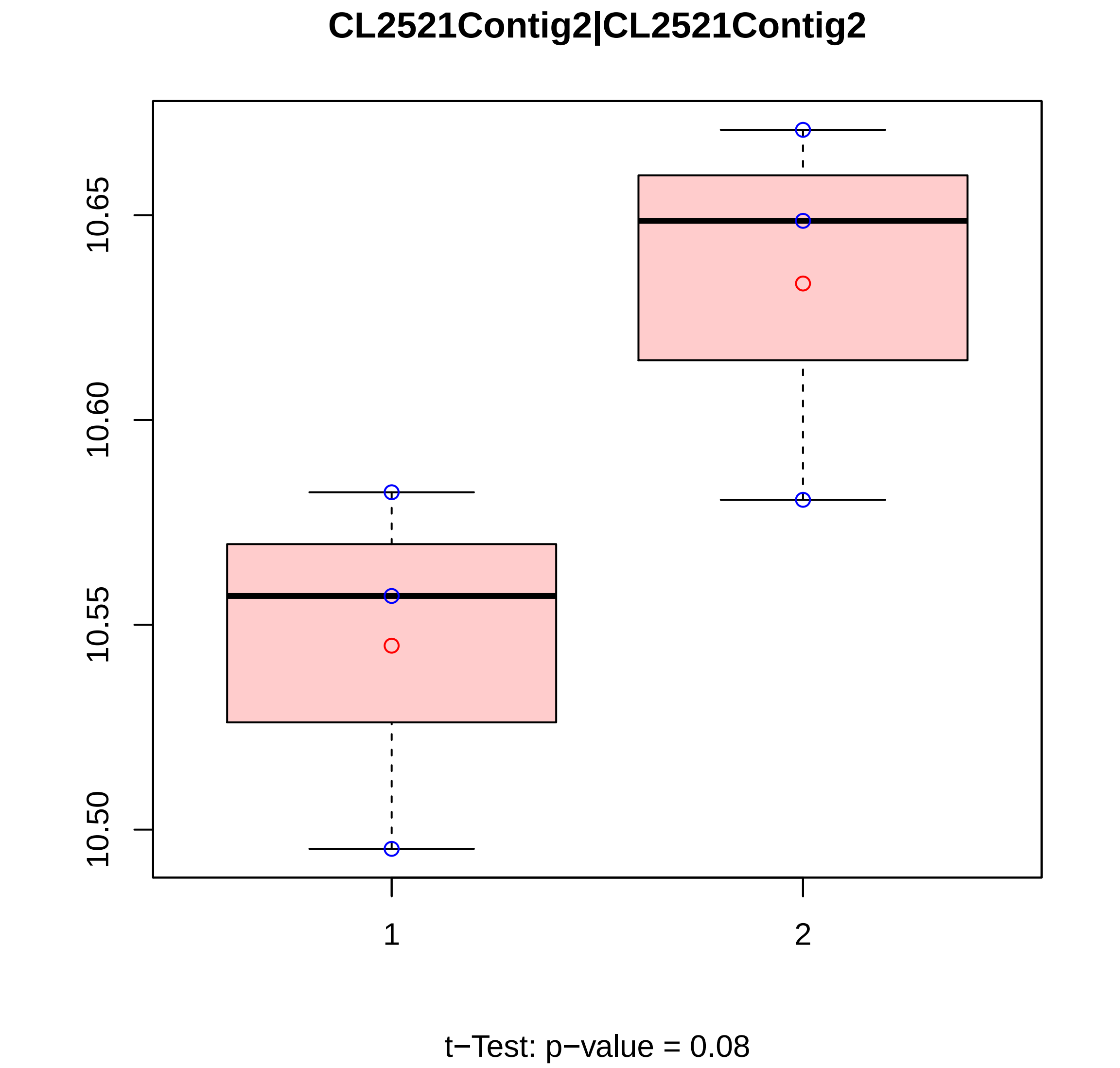

CL2521Contig2|CL2521Contig2
10.50
10.55
10.60
10.65
1
2
t−Test: p−value = 0.08

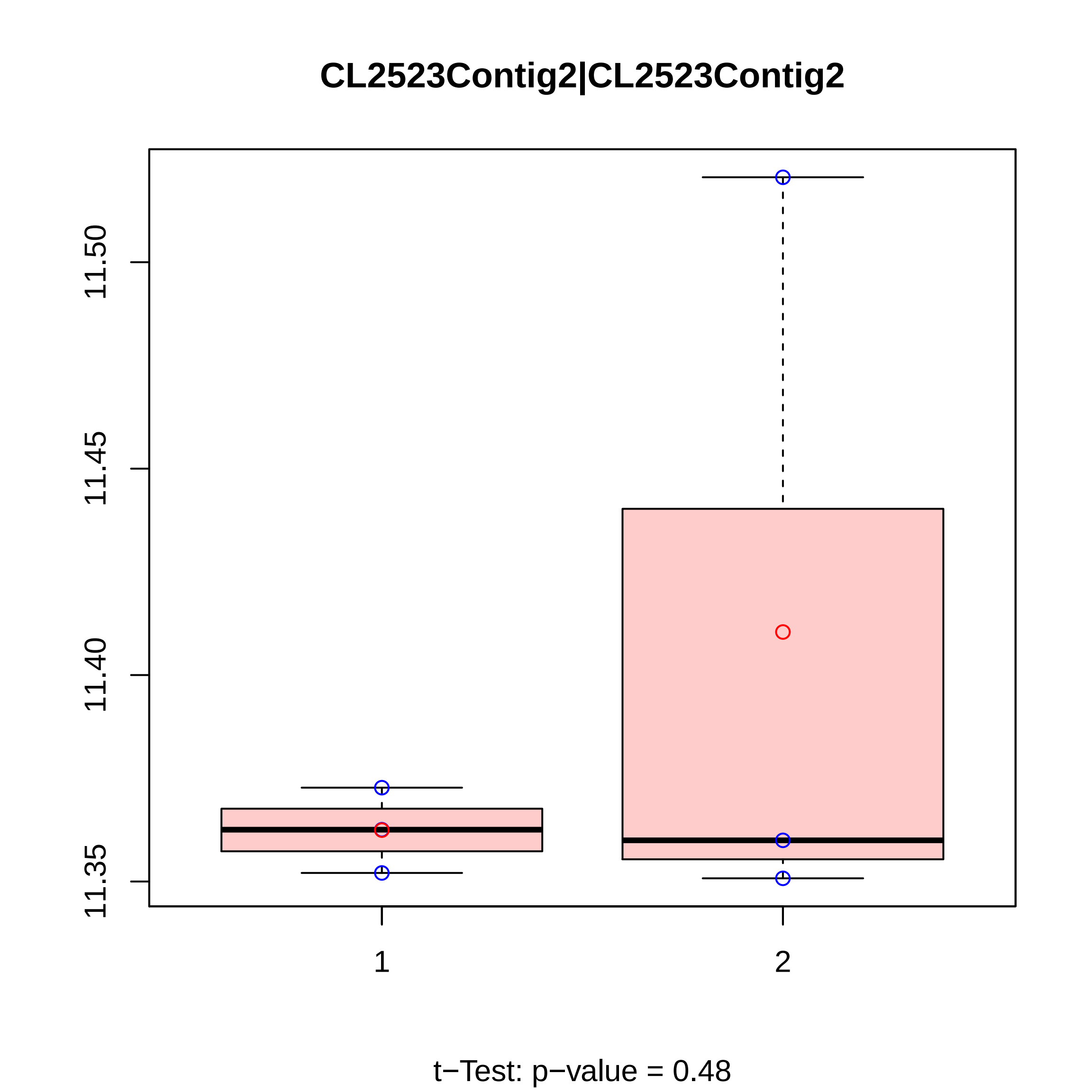

CL2523Contig2|CL2523Contig2
11.35
11.40
11.45
11.50
1
2
t−Test: p−value = 0.48

# CL25246Contig1|CL25246Contig1

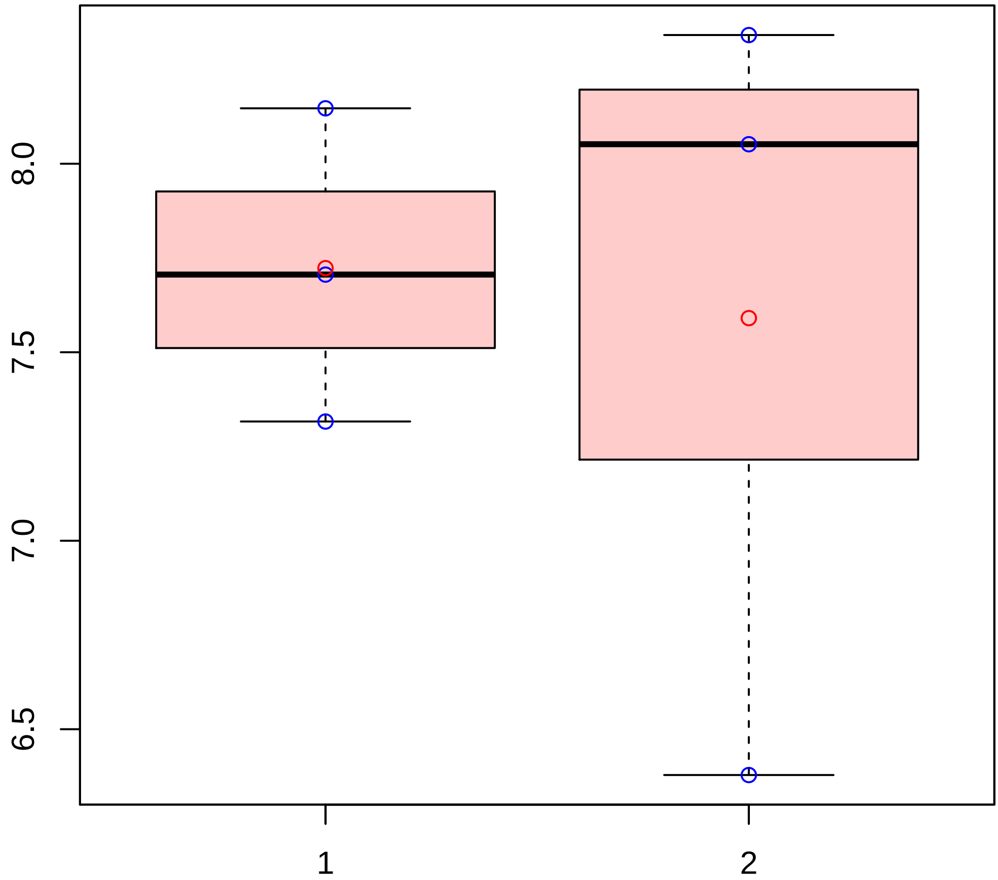


t−Test: p−value = 0.85

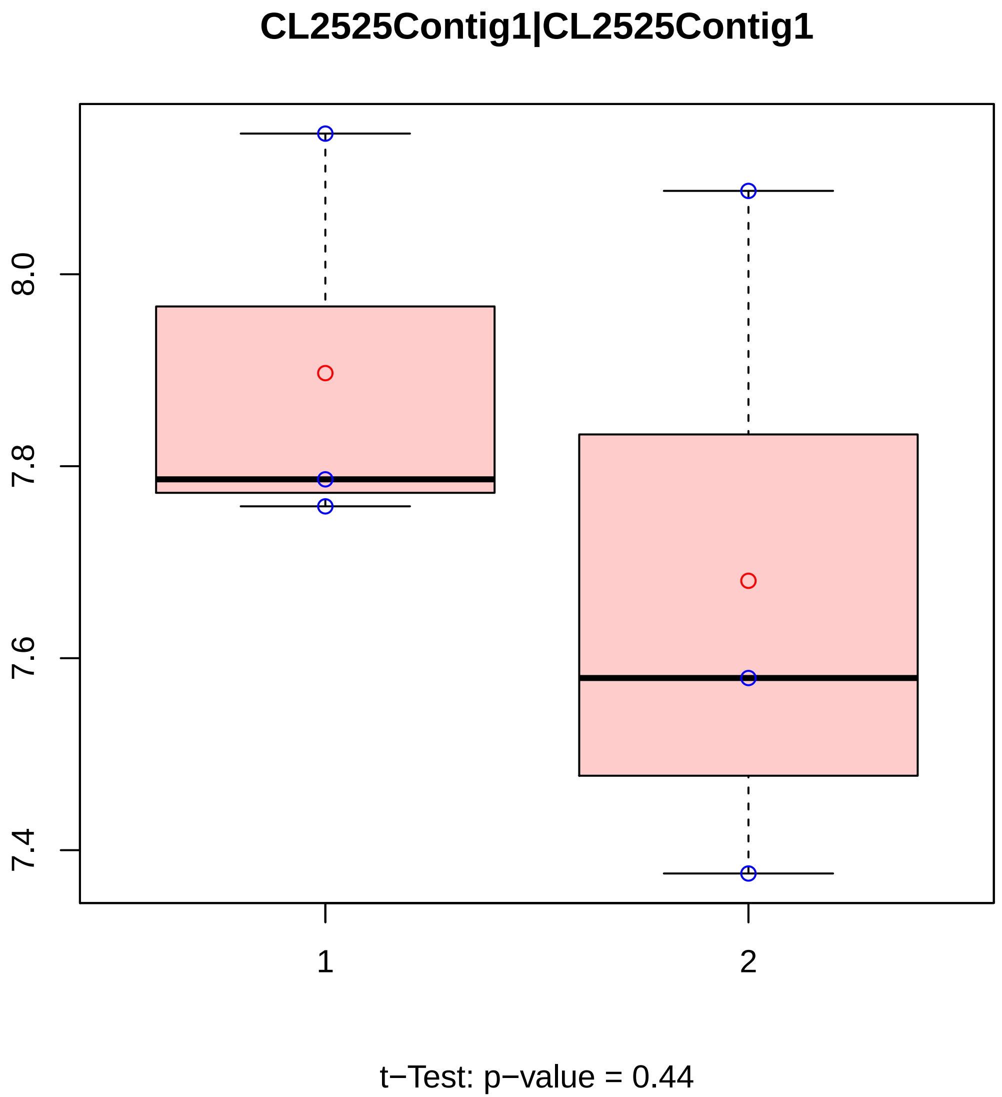
CL2525Contig1|CL2525Contig1
8.0
7.8
7.6
7.4
1
2
t−Test: p−value = 0.44

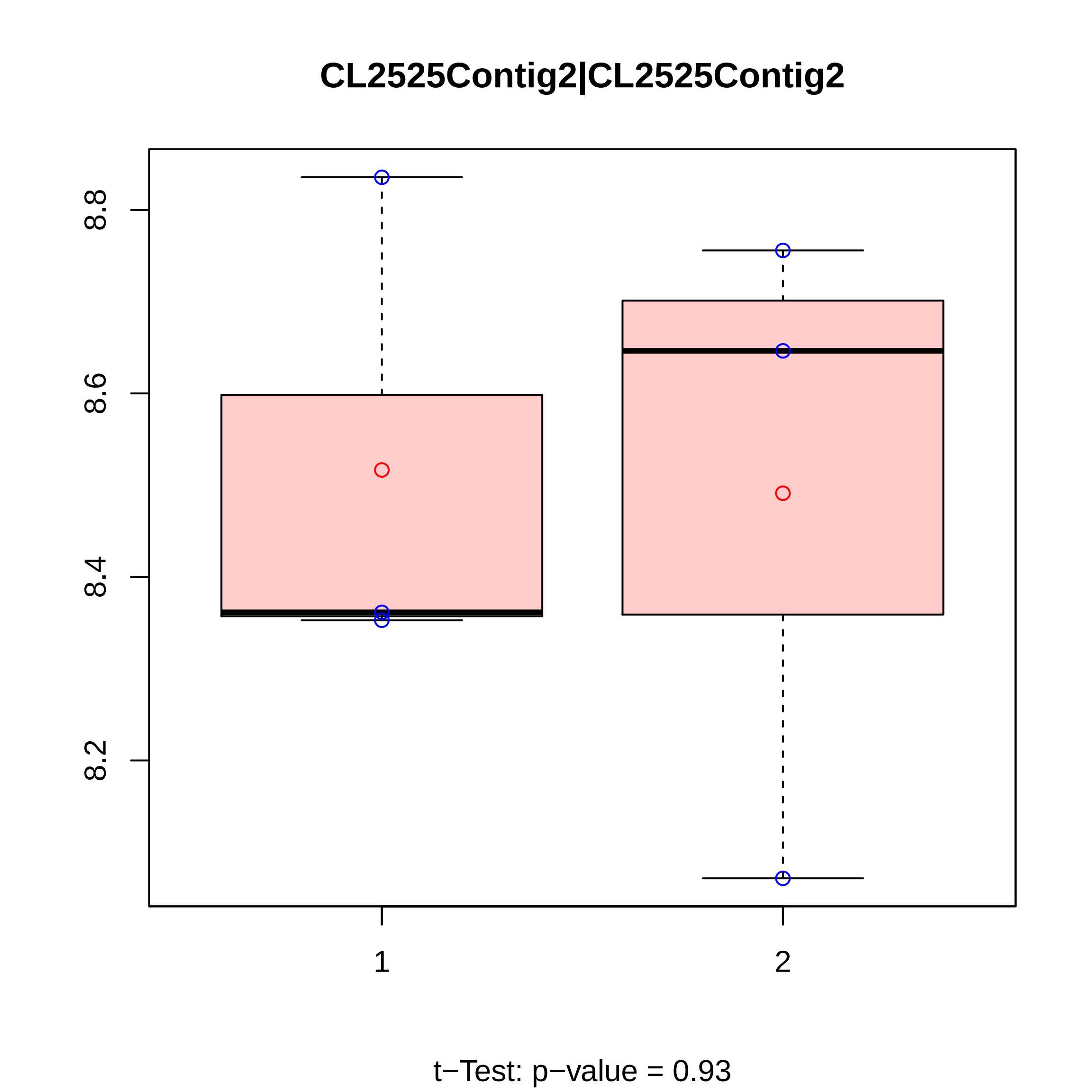
CL2525Contig2|CL2525Contig2
8.8
8.6
8.4
8.2
1
2
t−Test: p−value = 0.93

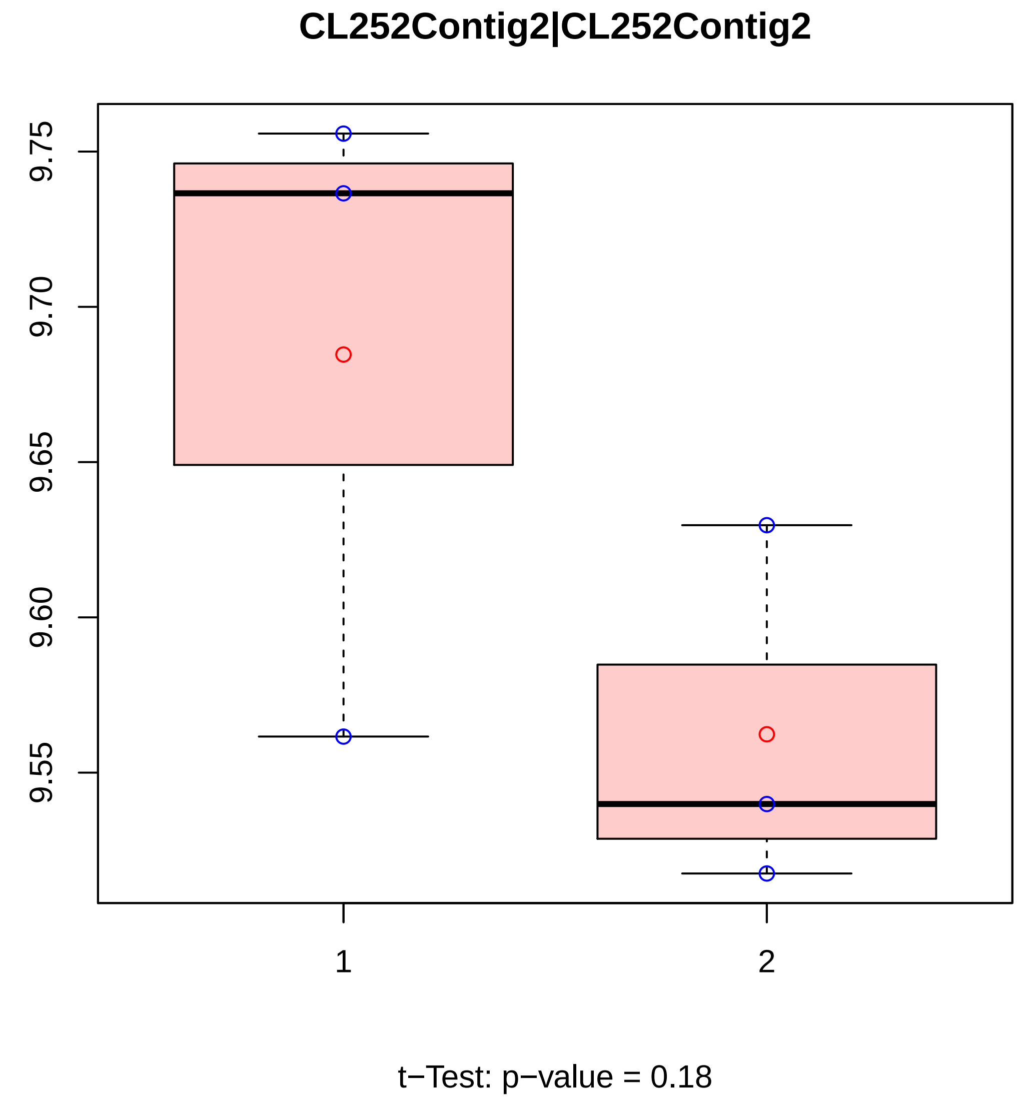
CL252Contig2|CL252Contig2
9.75
9.70
9.65
9.60
9.55
1
2
t−Test: p−value = 0.18

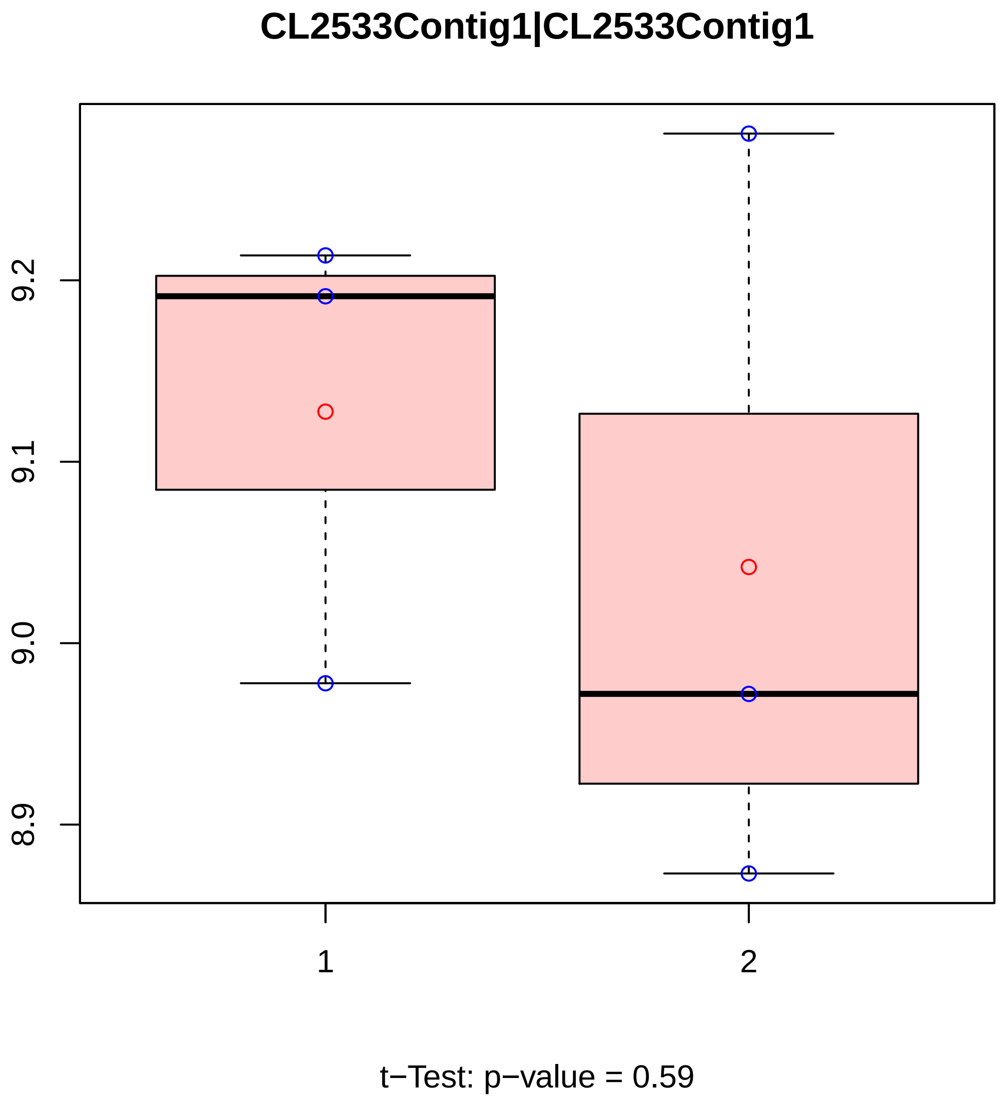
CL2533Contig1|CL2533Contig1
9.2
9.1
9.0
8.9
1
2
t−Test: p−value = 0.59

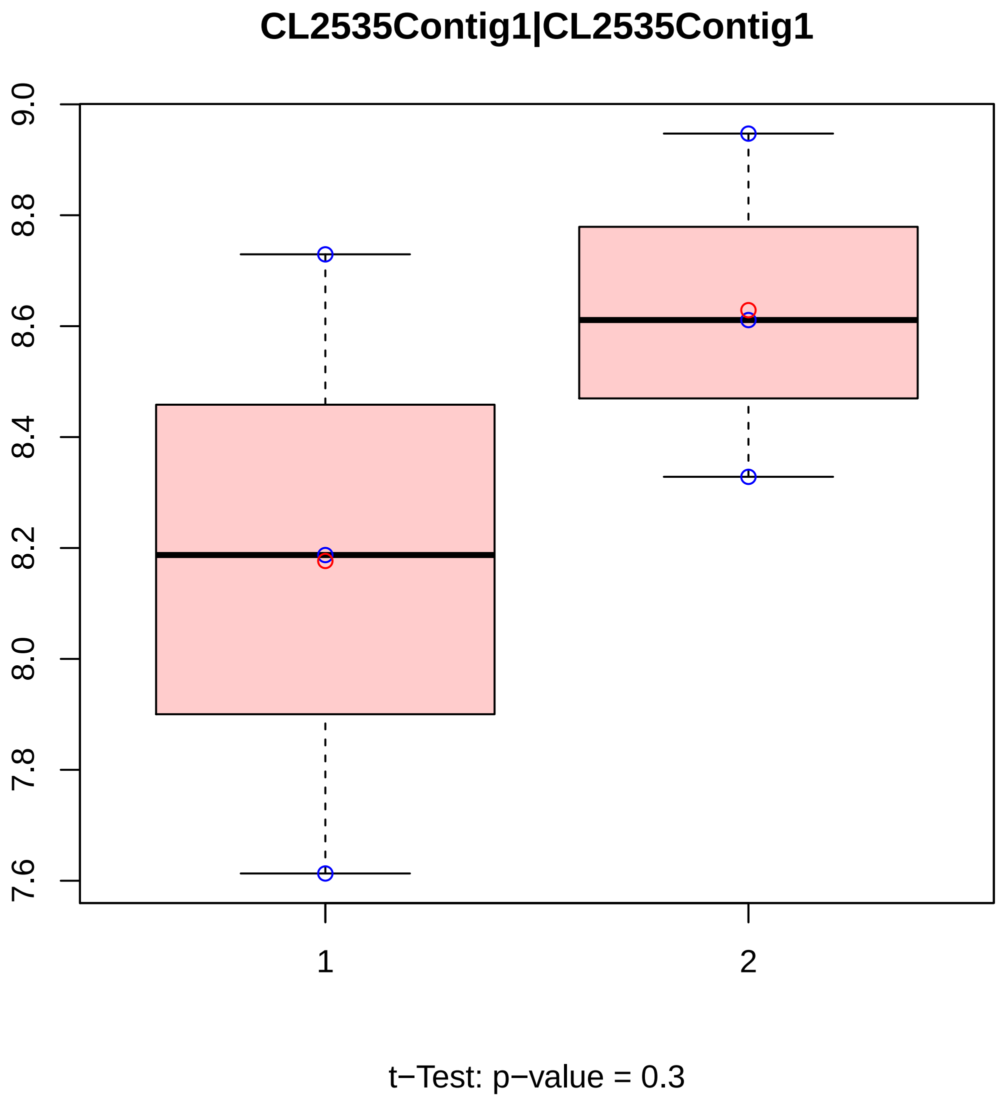
CL2535Contig1|CL2535Contig1
9.0
8.8
8.6
8.4
8.2
8.0
7.8
7.6
1
2
t−Test: p−value = 0.3

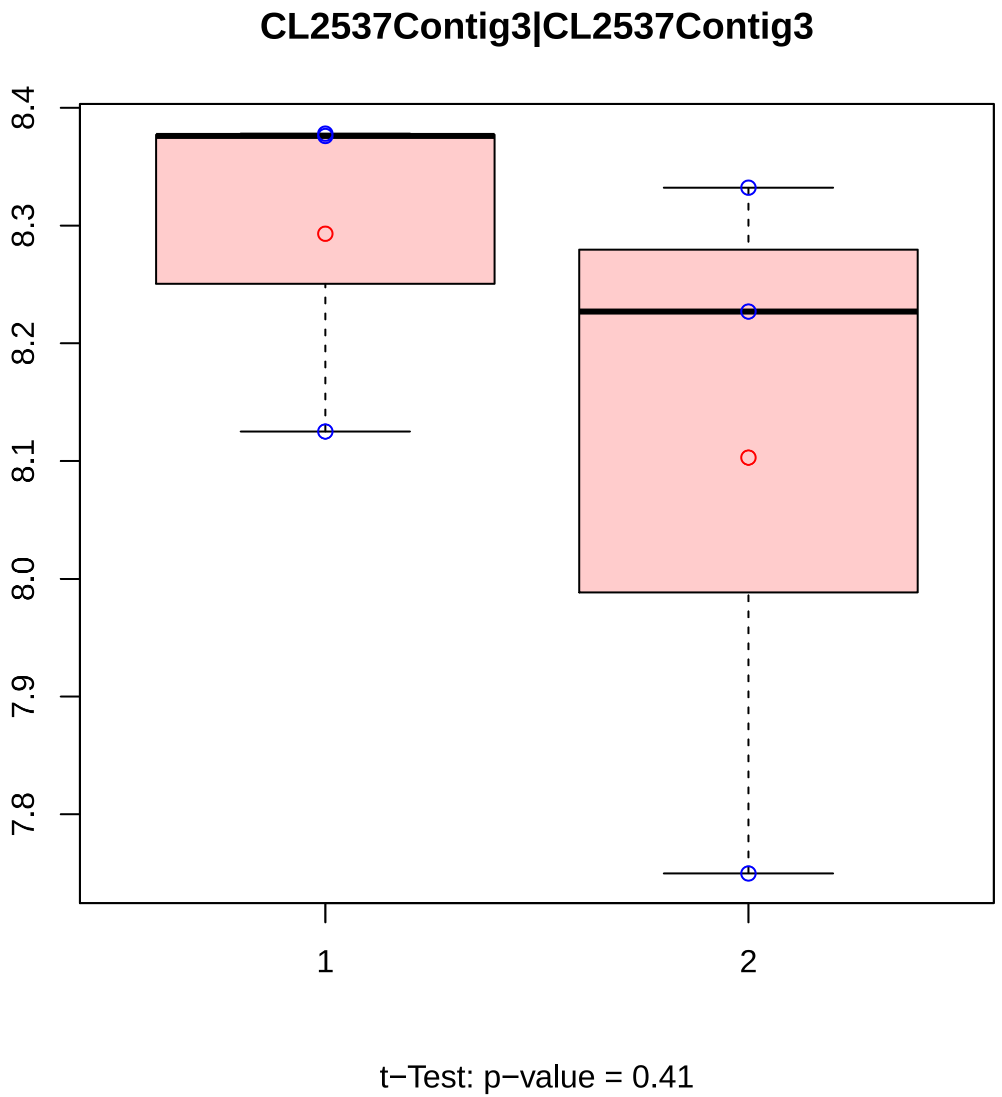
CL2537Contig3|CL2537Contig3
8.4
8.3
8.2
8.1
8.0
7.9
7.8
1
2
t−Test: p−value = 0.41

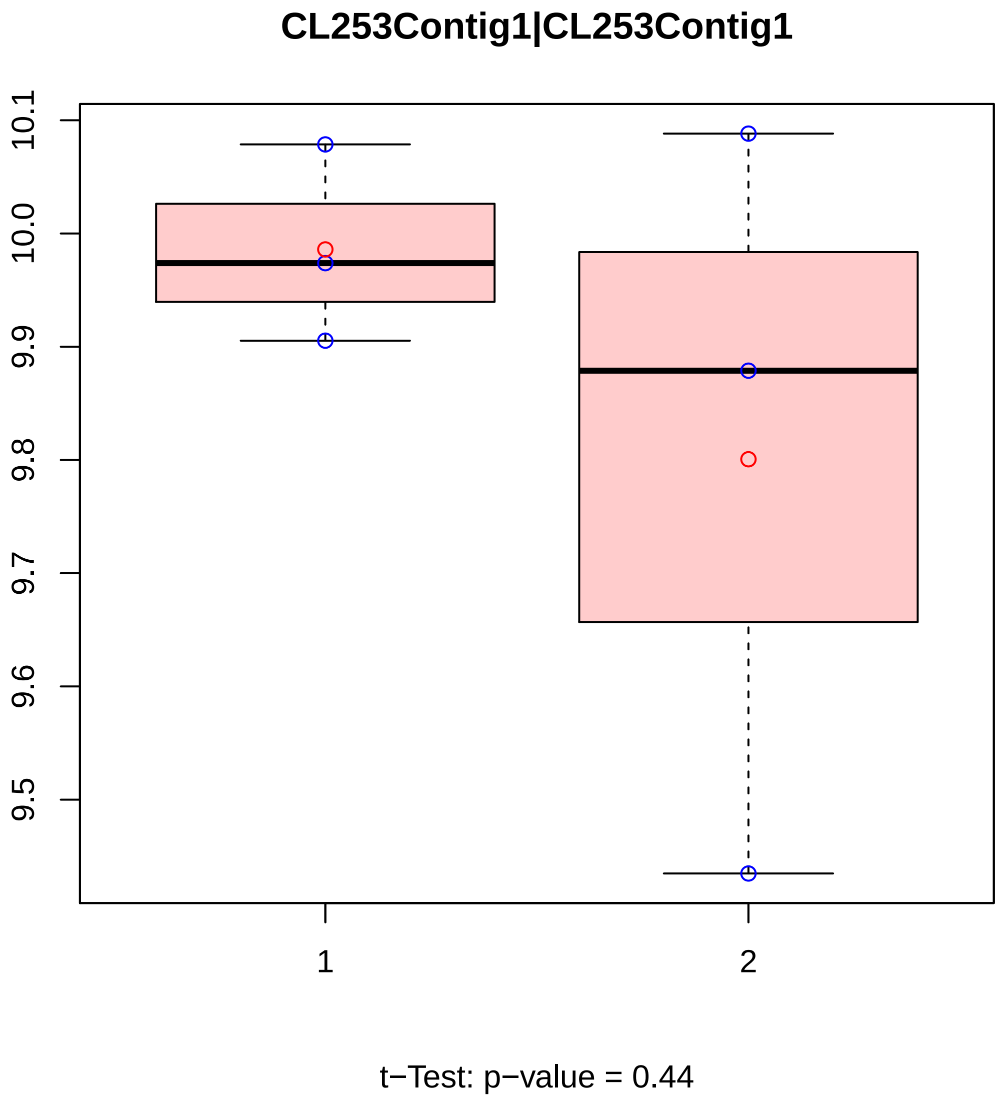
CL253Contig1|CL253Contig1
10.1
10.0
9.9
9.8
9.7
9.6
9.5
1
2
t−Test: p−value = 0.44

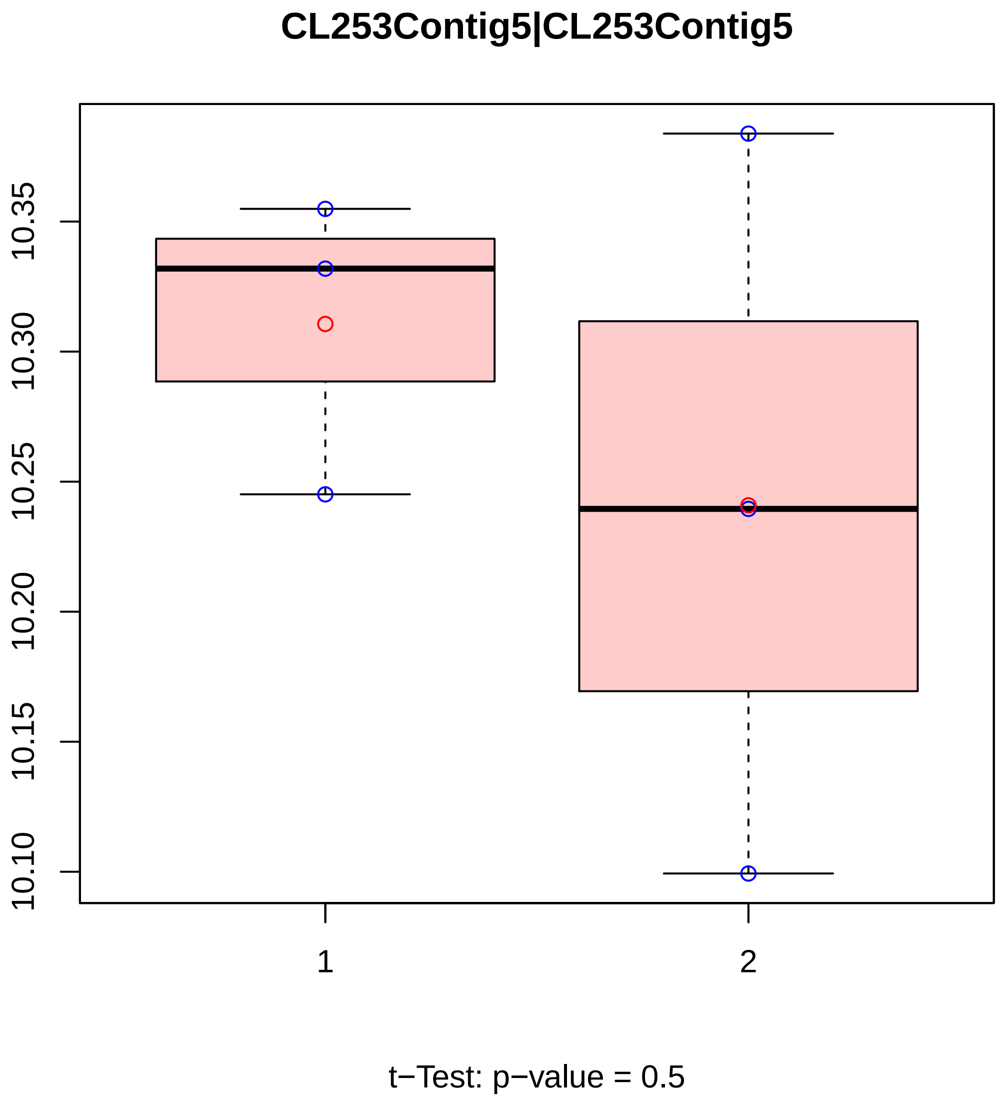
CL253Contig5|CL253Contig5
10.10
10.15
10.20
10.25
10.30
10.35
1
2
t−Test: p−value = 0.5

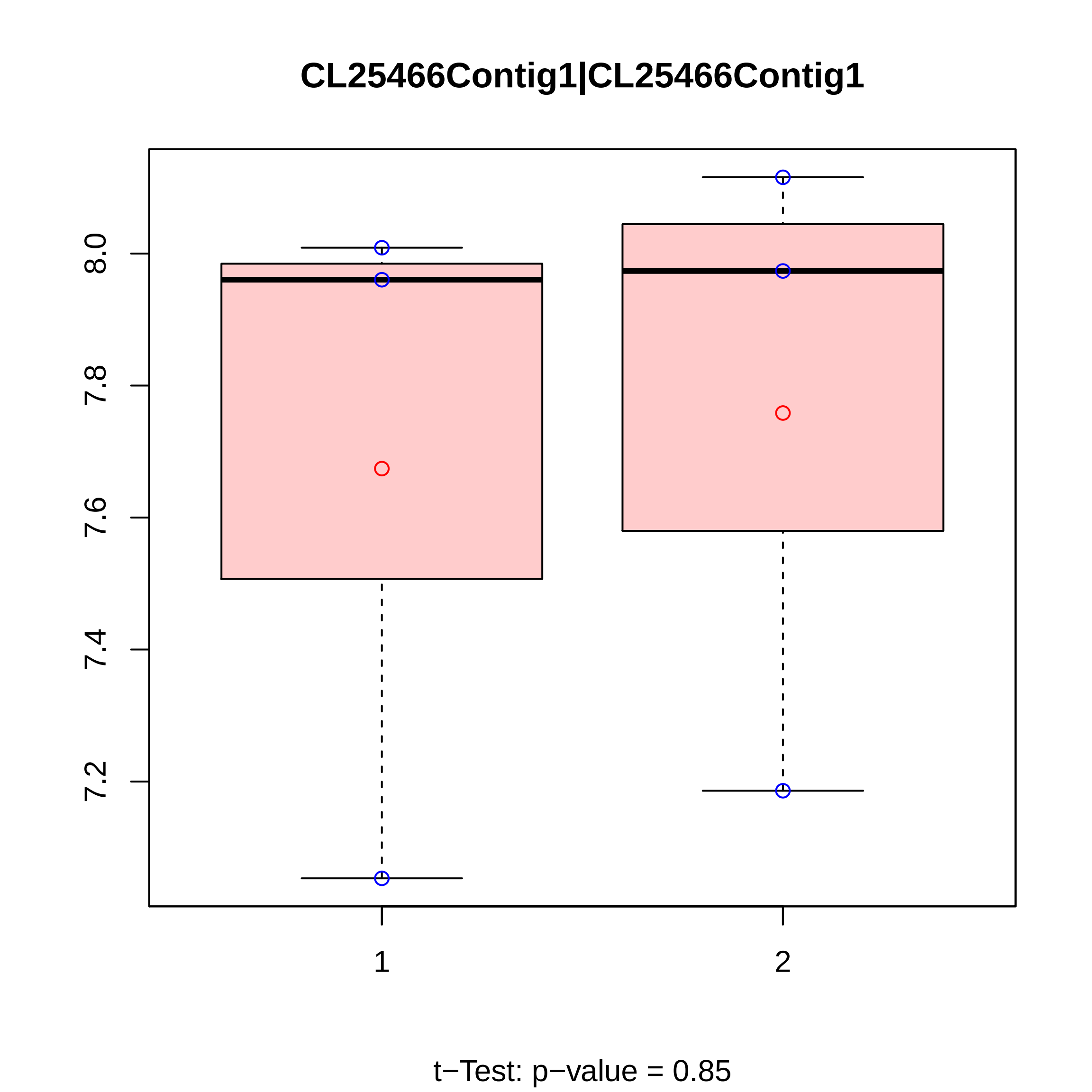

CL25466Contig1|CL25466Contig1
8.0
7.8
7.6
7.4
7.2
1
2
t−Test: p−value = 0.85

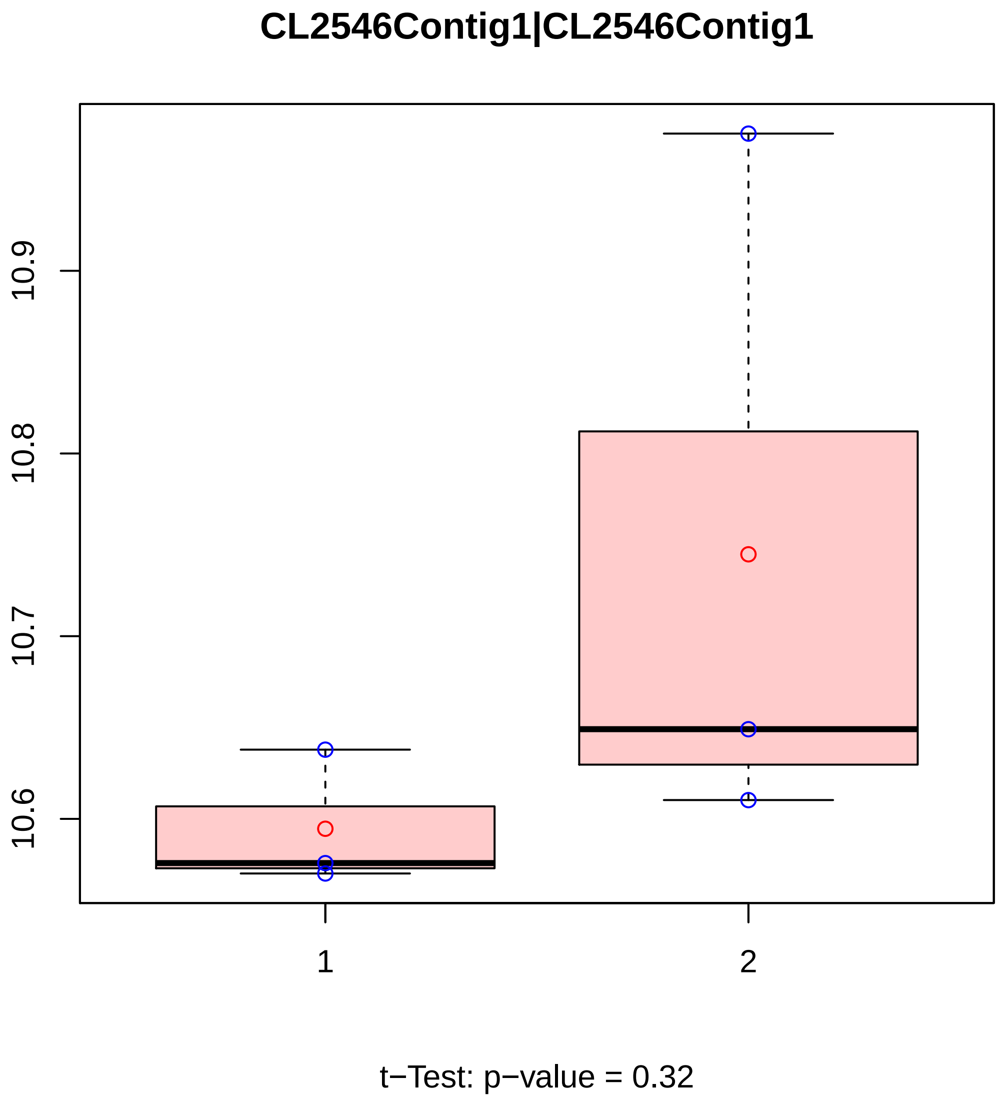

CL2546Contig1|CL2546Contig1
10.6
10.7
10.8
10.9
1
2
t−Test: p−value = 0.32

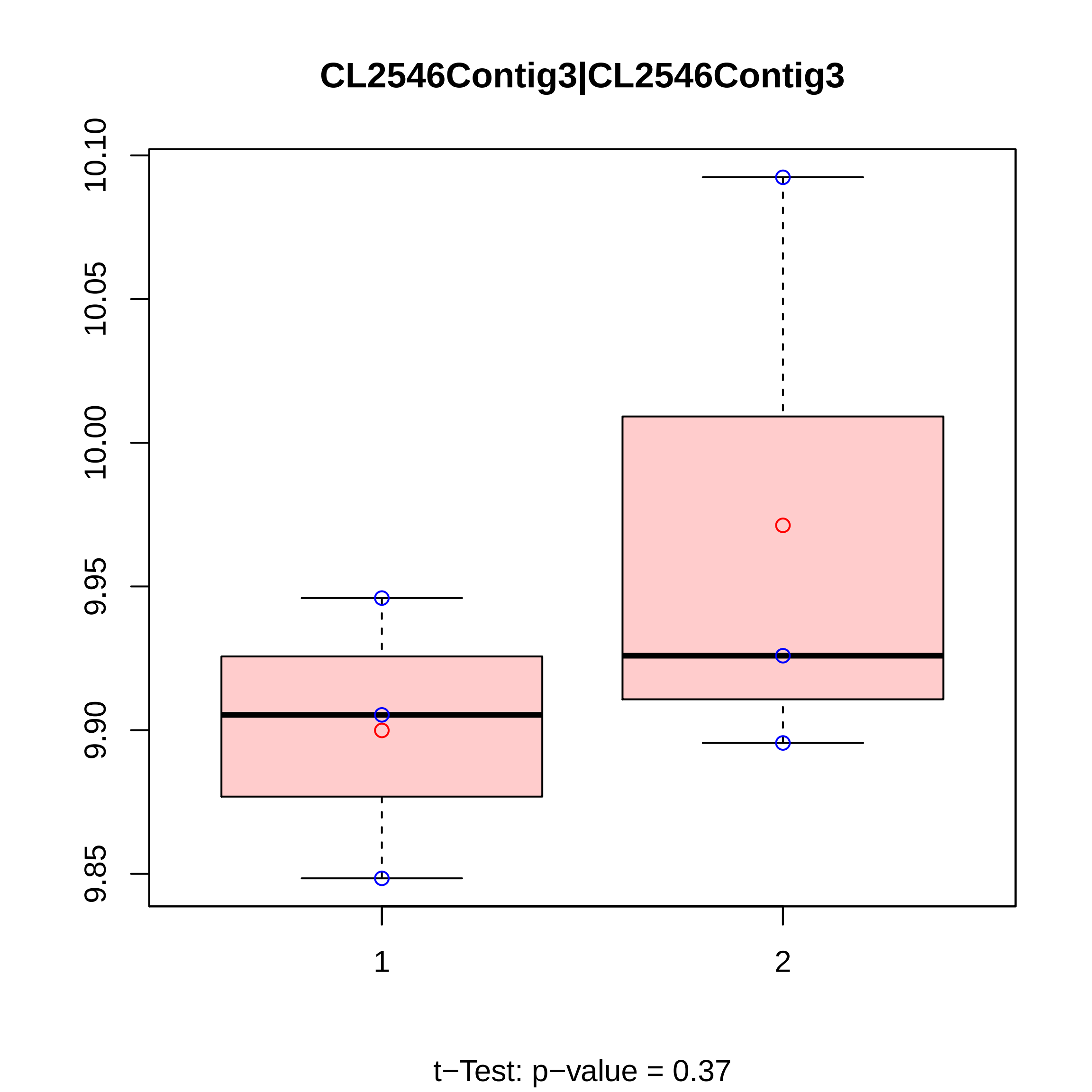

CL2546Contig3|CL2546Contig3
10.10
10.05
10.00
9.95
9.90
9.85
1
2
t−Test: p−value = 0.37

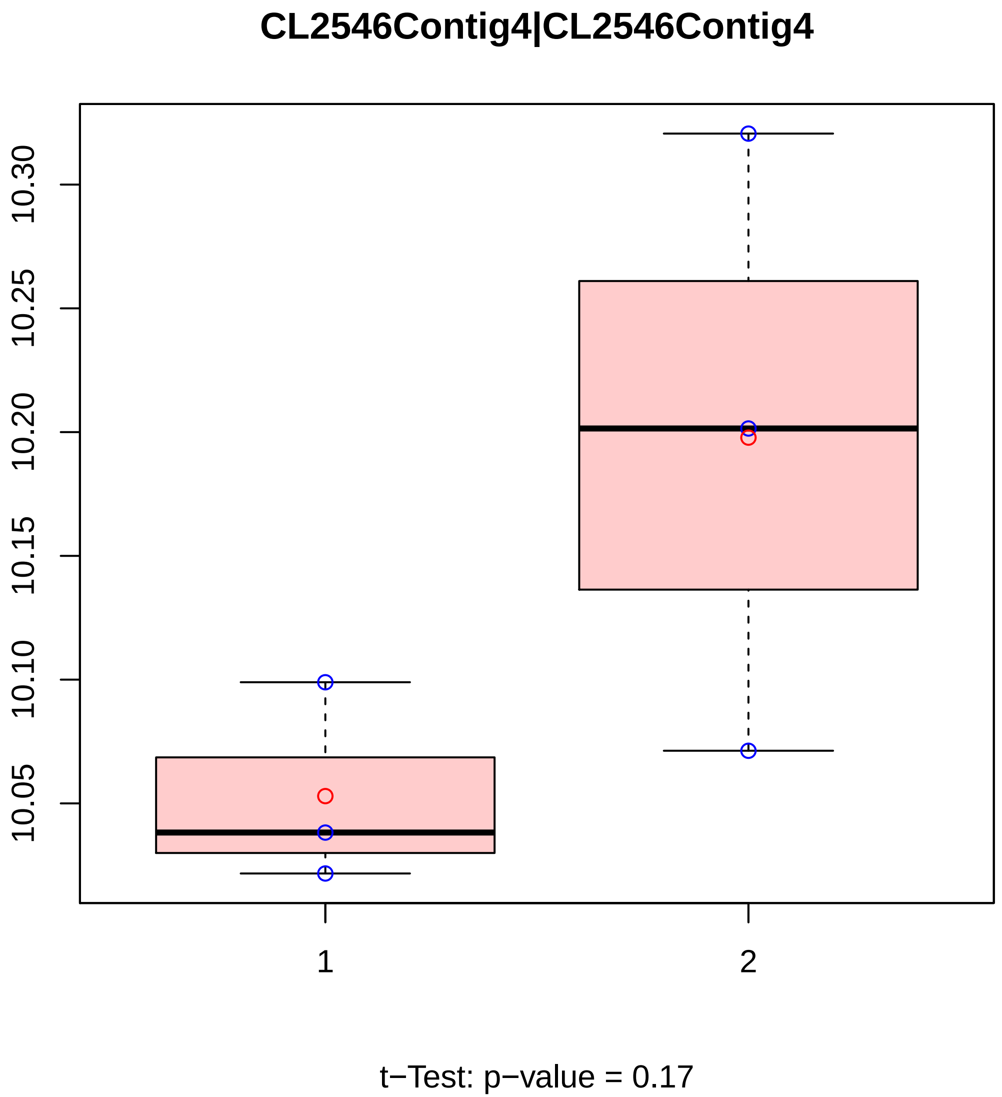

CL2546Contig4|CL2546Contig4
10.05
10.10
10.15
10.20
10.25
10.30
1
2
t−Test: p−value = 0.17

# CL2546Contig5|CL2546Contig5

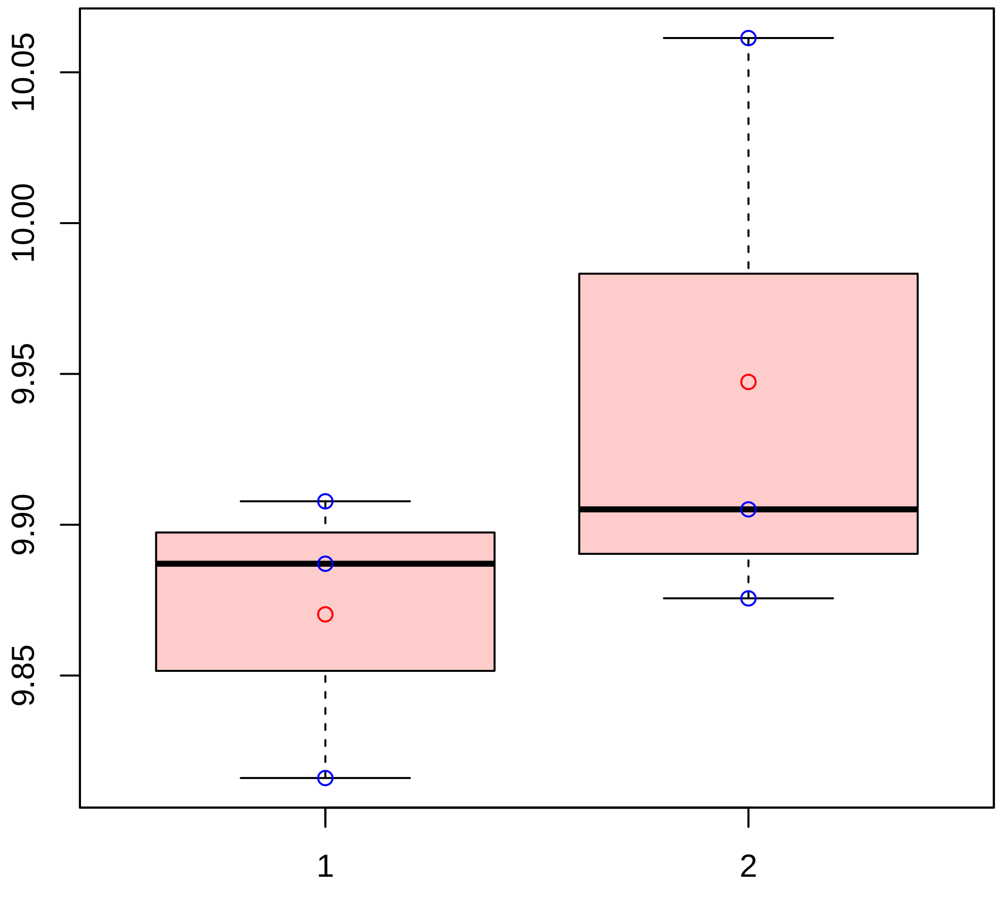


t−Test: p−value = 0.32

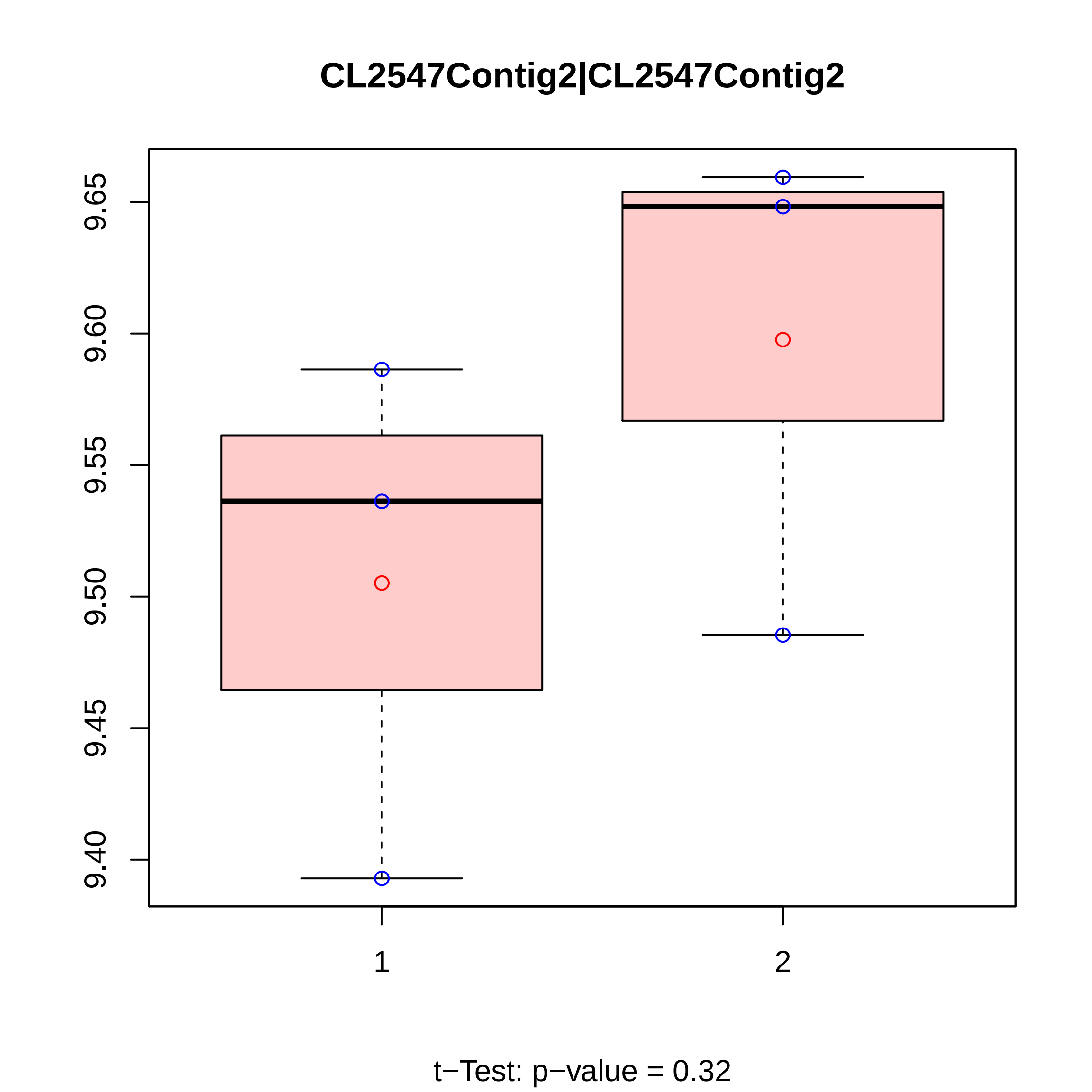

CL2547Contig2|CL2547Contig2
9.40
9.45
9.50
9.55
9.60
9.65
1
2
t−Test: p−value = 0.32

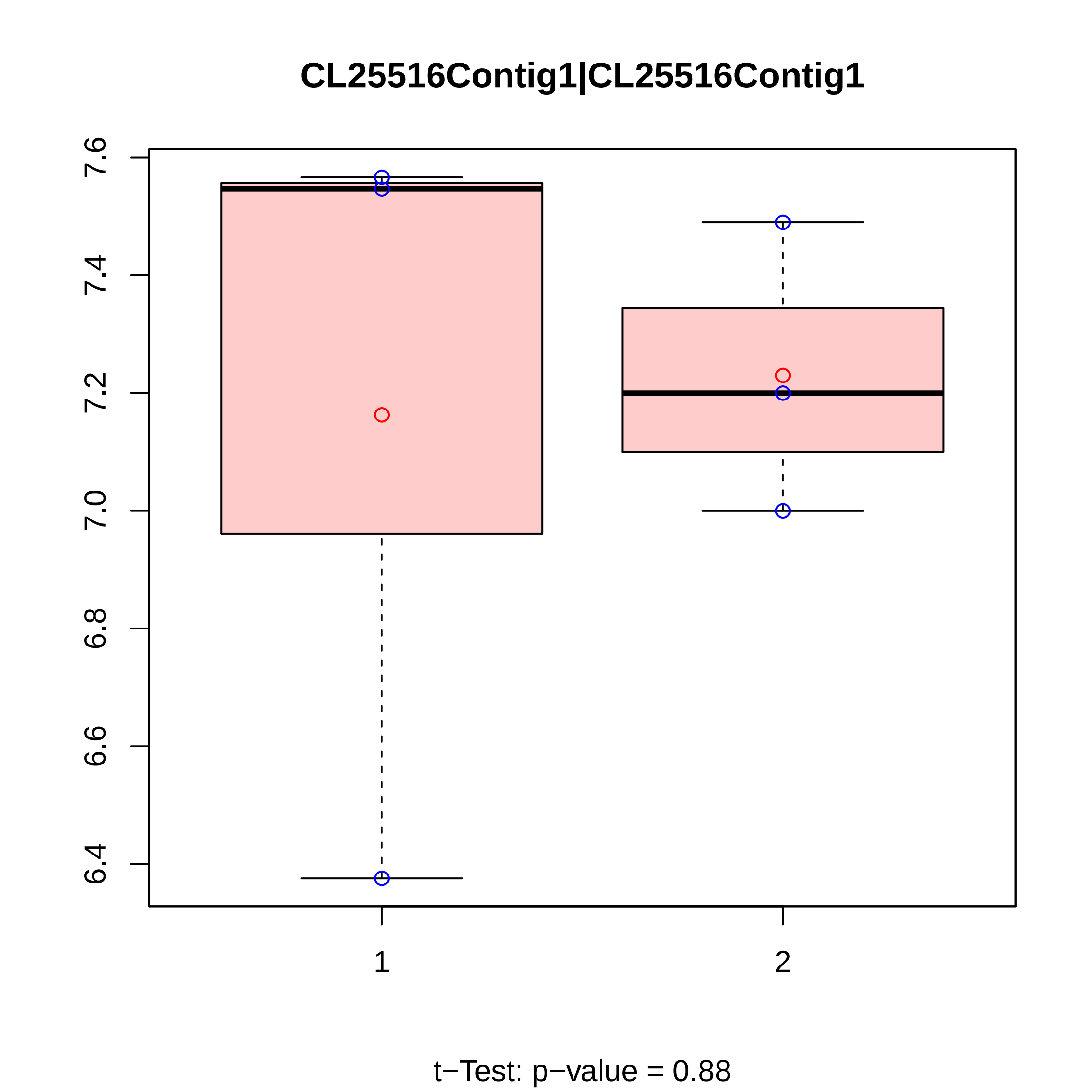

CL25516Contig1|CL25516Contig1
7.6
7.4
7.2
7.0
6.8
6.6
6.4
1
2
t−Test: p−value = 0.88

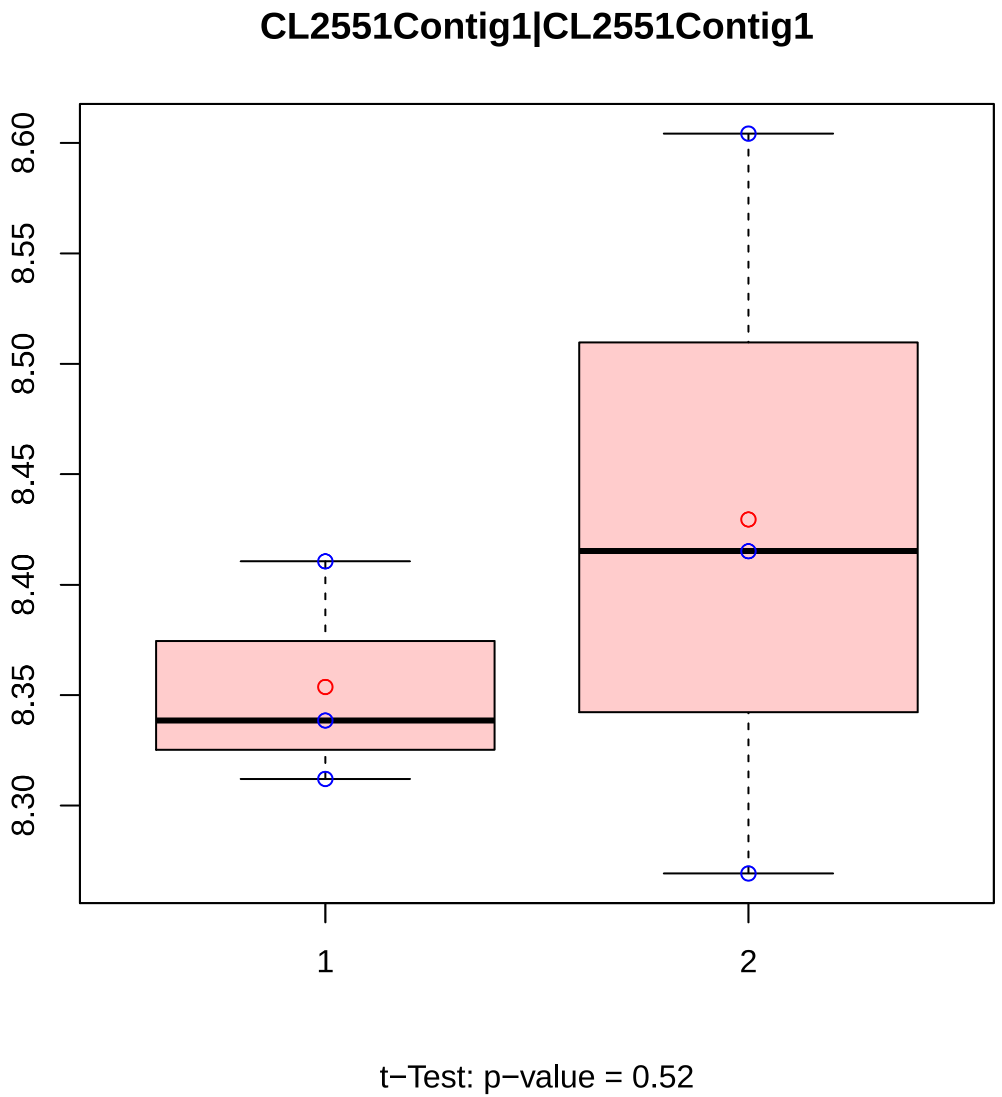
CL2551Contig1|CL2551Contig1
8.60
8.55
8.50
8.45
8.40
8.35
8.30
1
2
t−Test: p−value = 0.52

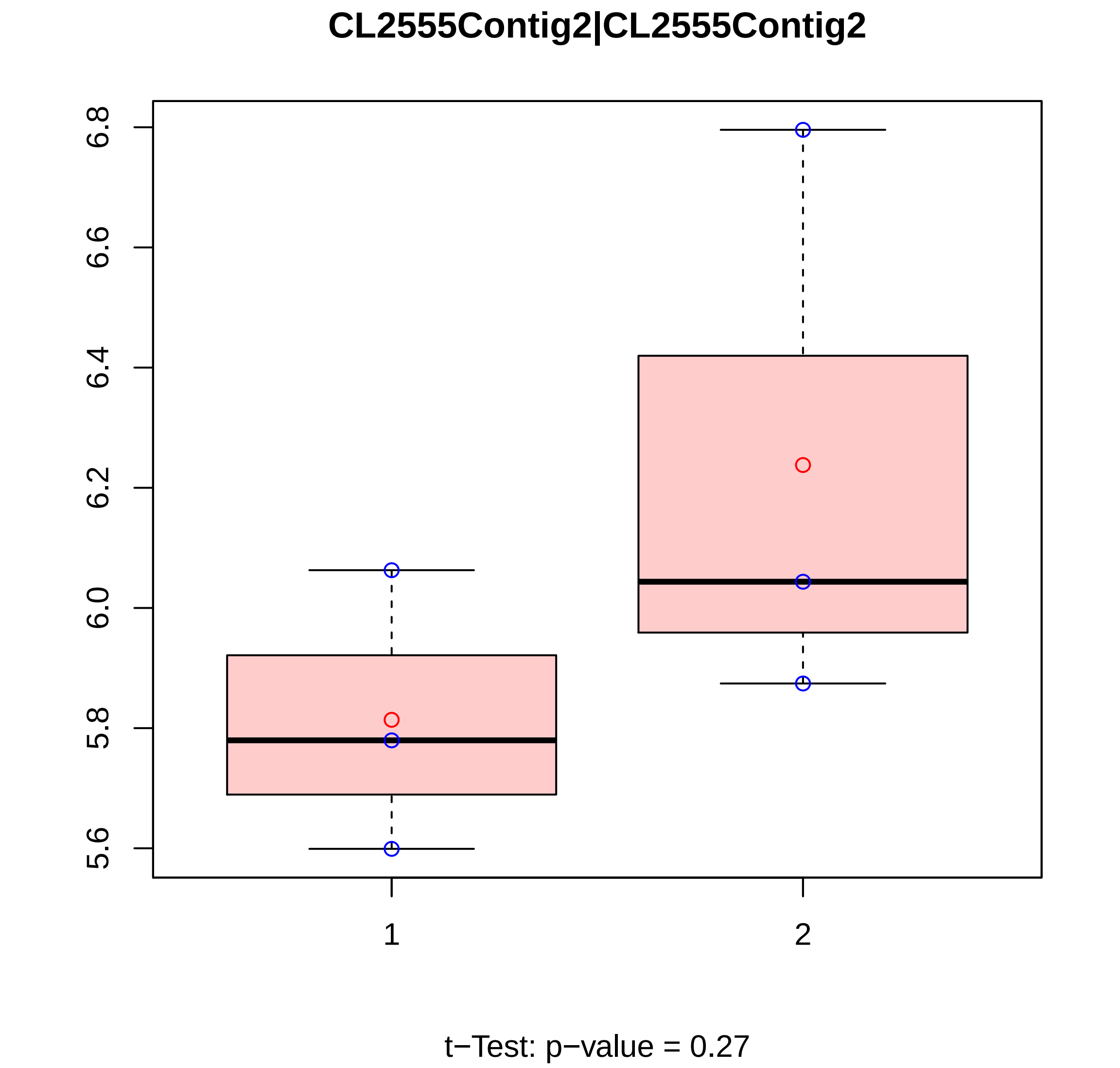
CL2555Contig2|CL2555Contig2
6.8
6.6
6.4
6.2
6.0
5.8
5.6
1
2
t−Test: p−value = 0.27

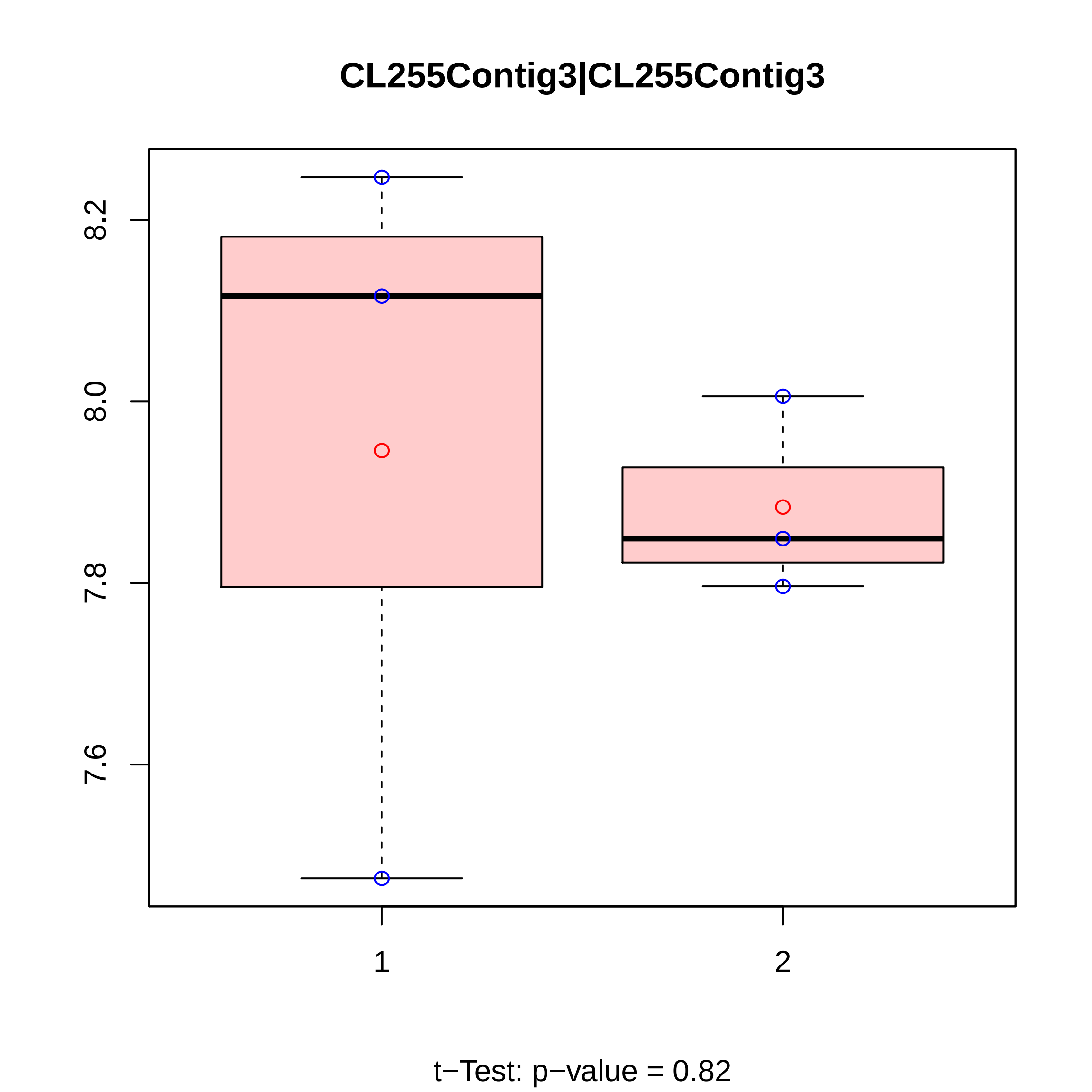

CL255Contig3|CL255Contig3
8.2
8.0
7.8
7.6
1
2
t−Test: p−value = 0.82

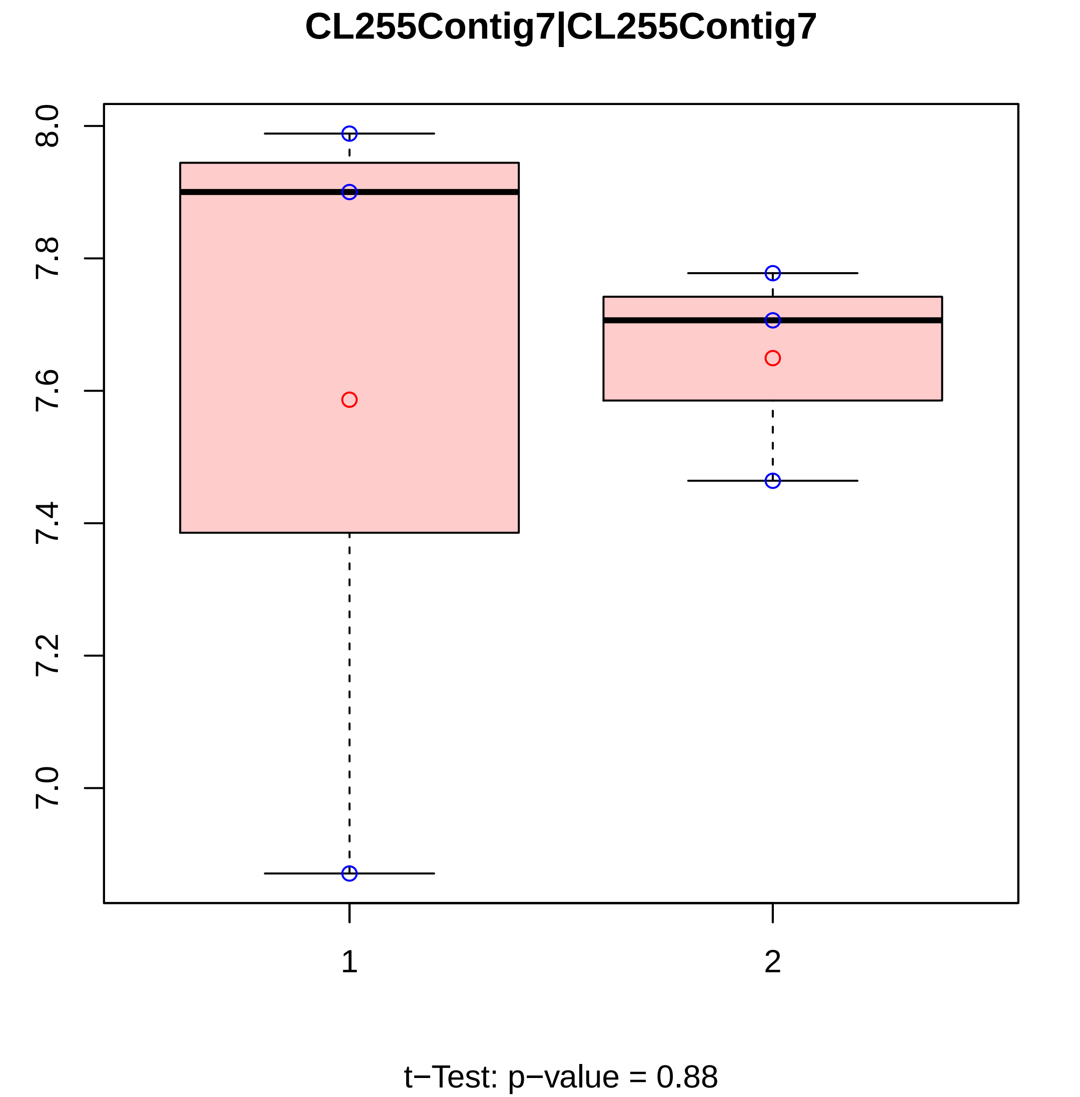
CL255Contig7|CL255Contig7
8.0
7.8
7.6
7.4
7.2
7.0
1
2
t−Test: p−value = 0.88

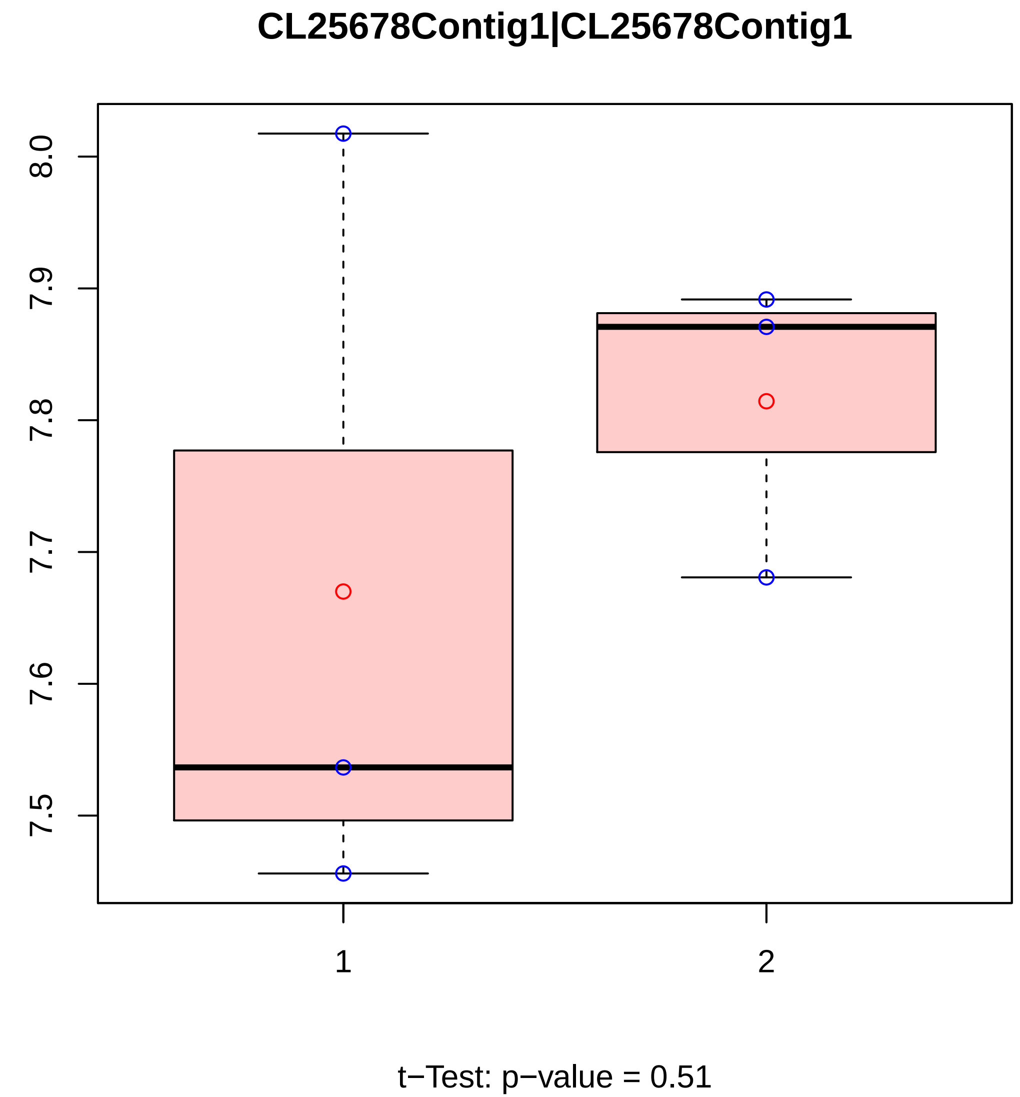
CL25678Contig1|CL25678Contig1
8.0
7.9
7.8
7.7
7.6
7.5
1
2
t−Test: p−value = 0.51

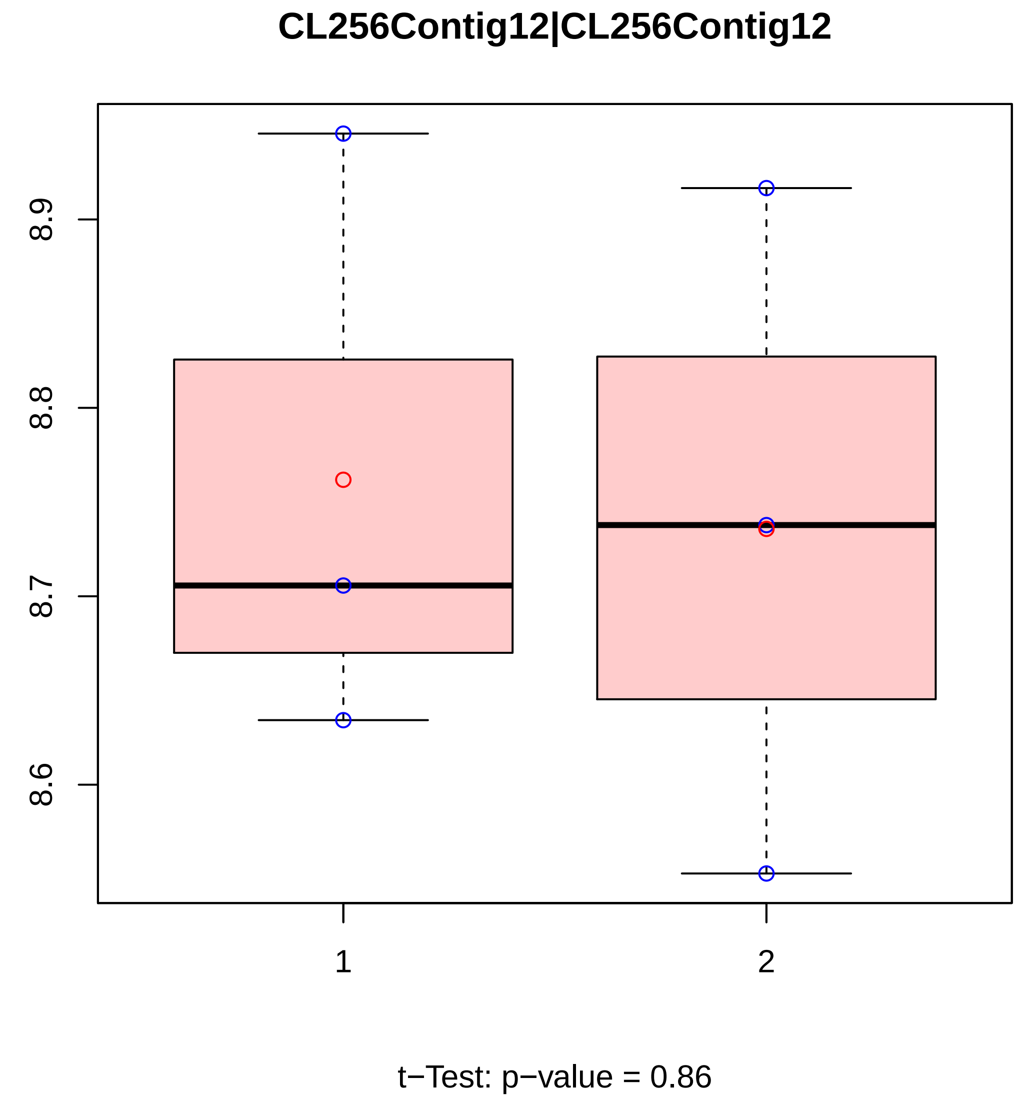
CL256Contig12|CL256Contig12
8.6
8.7
8.8
8.9
1
2
t−Test: p−value = 0.86

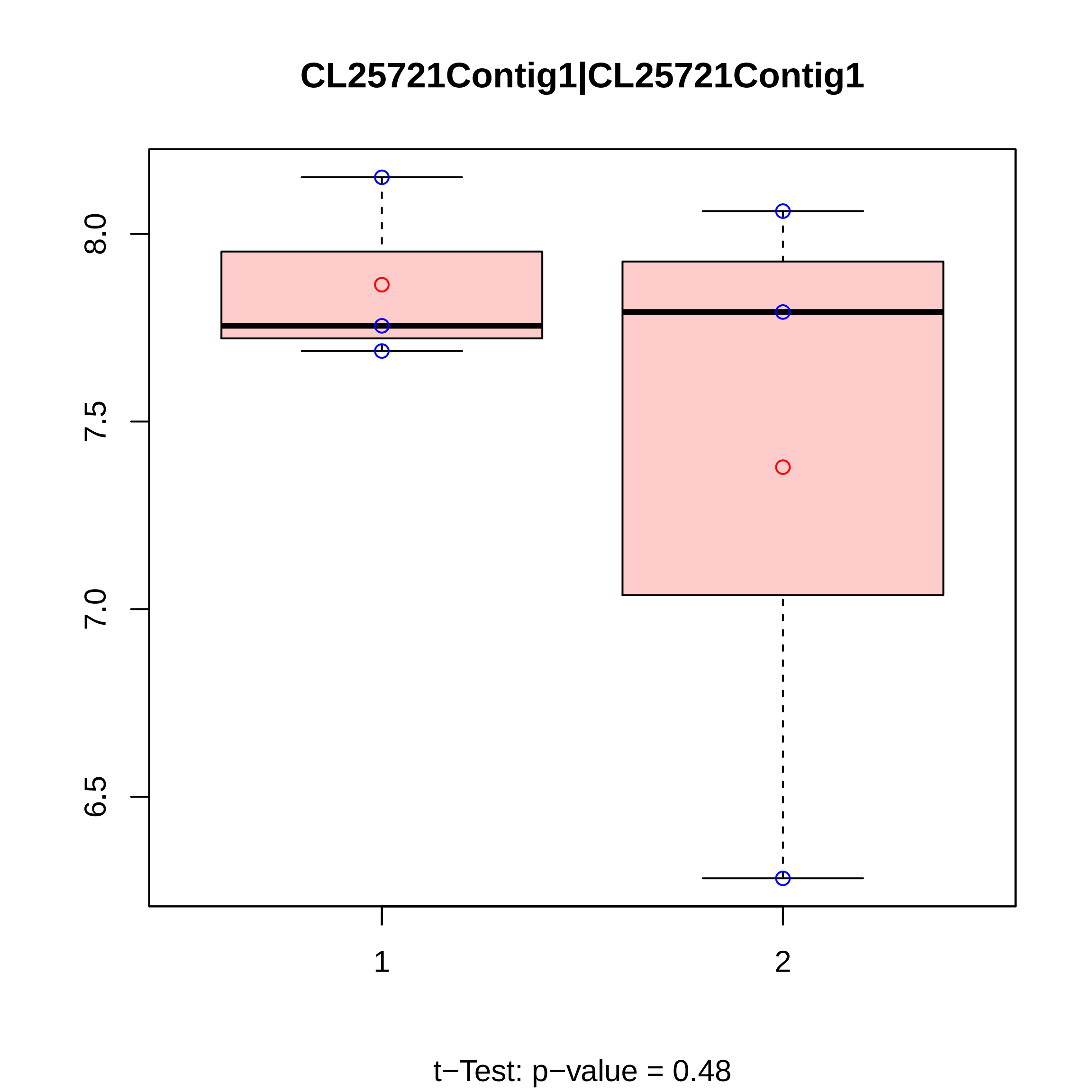
CL25721Contig1|CL25721Contig1
8.0
7.5
7.0
6.5
1
2
t−Test: p−value = 0.48

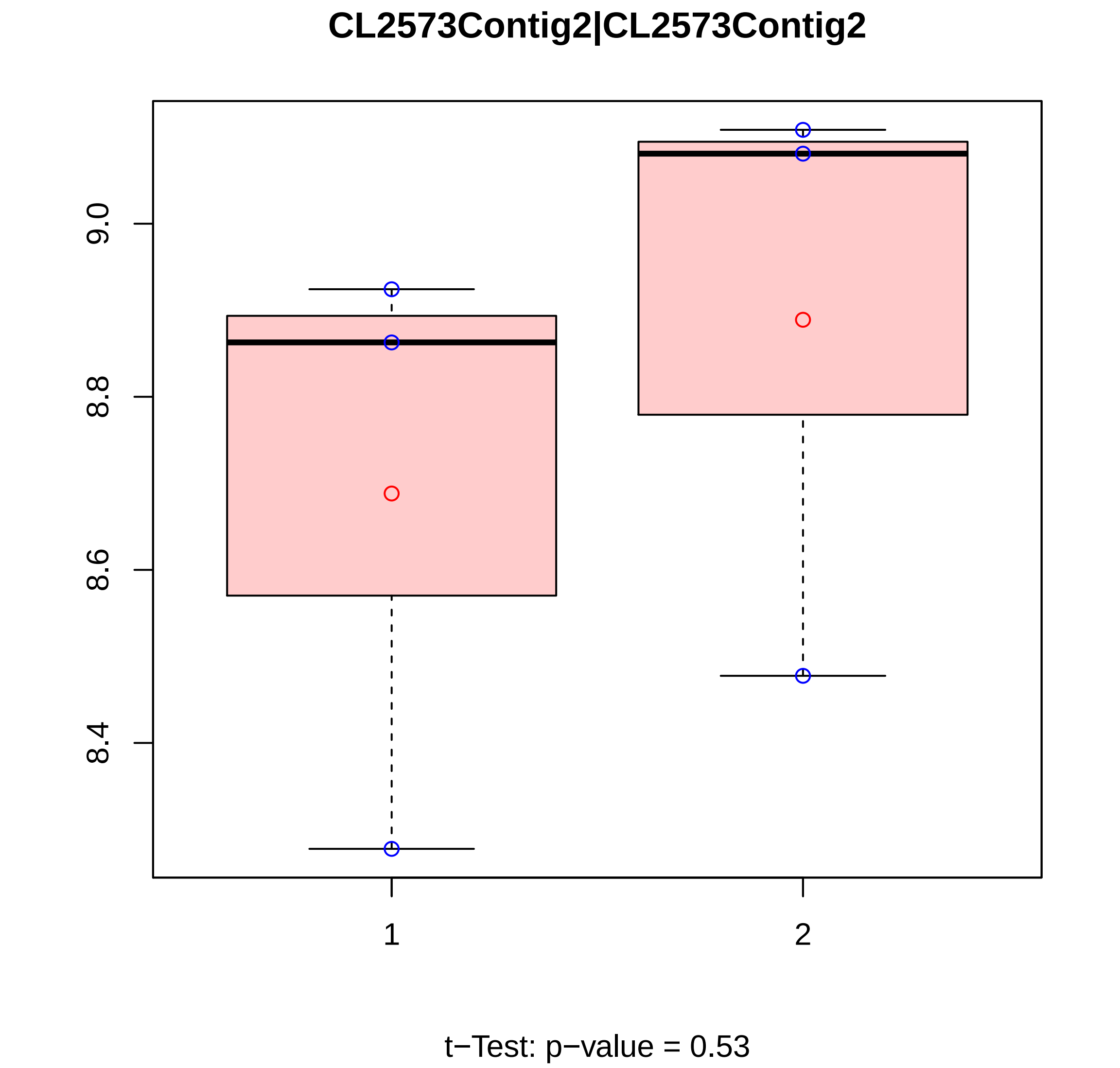
CL2573Contig2|CL2573Contig2
9.0
8.8
8.6
8.4
1
2
t−Test: p−value = 0.53

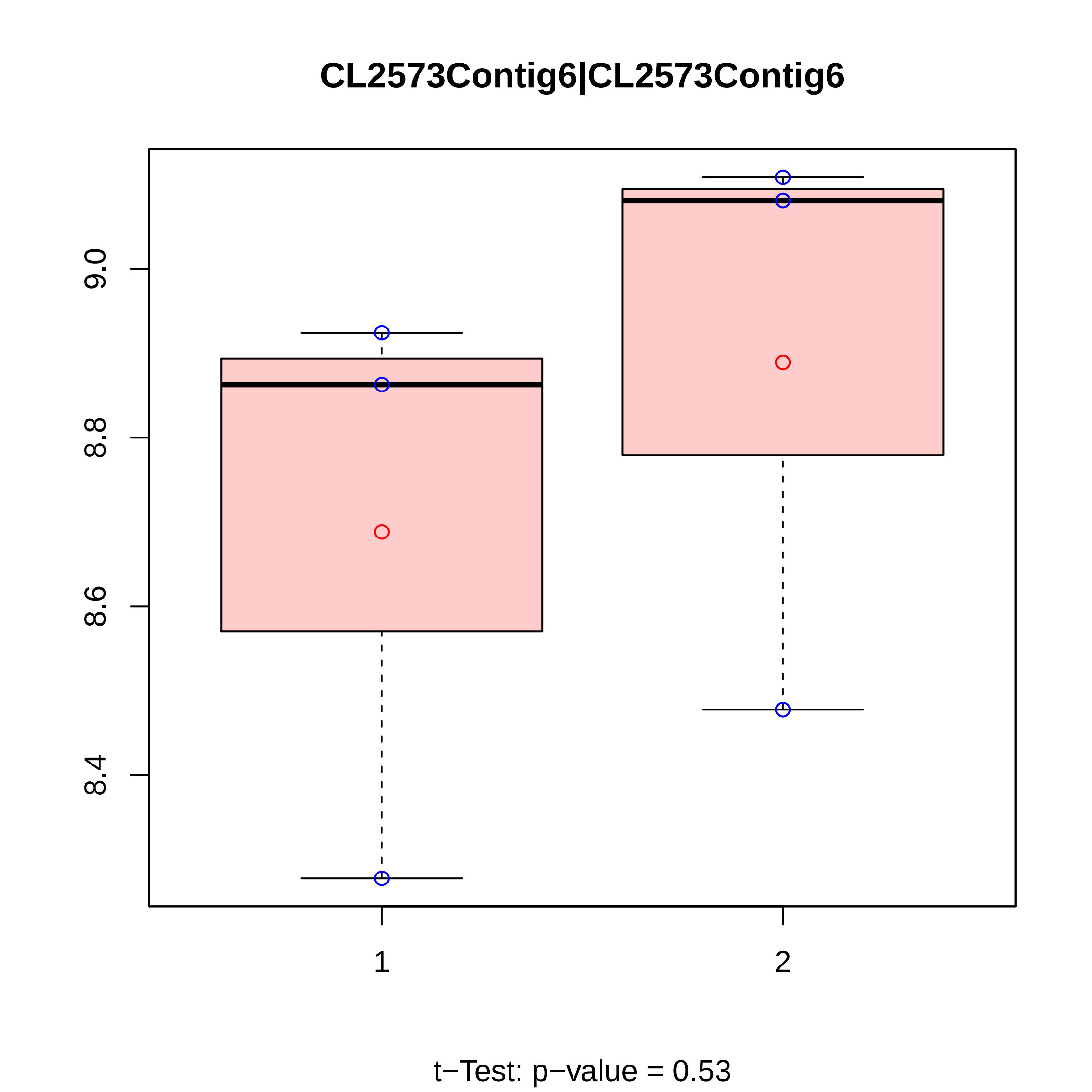
CL2573Contig6|CL2573Contig6
9.0
8.8
8.6
8.4
1
2
t−Test: p−value = 0.53

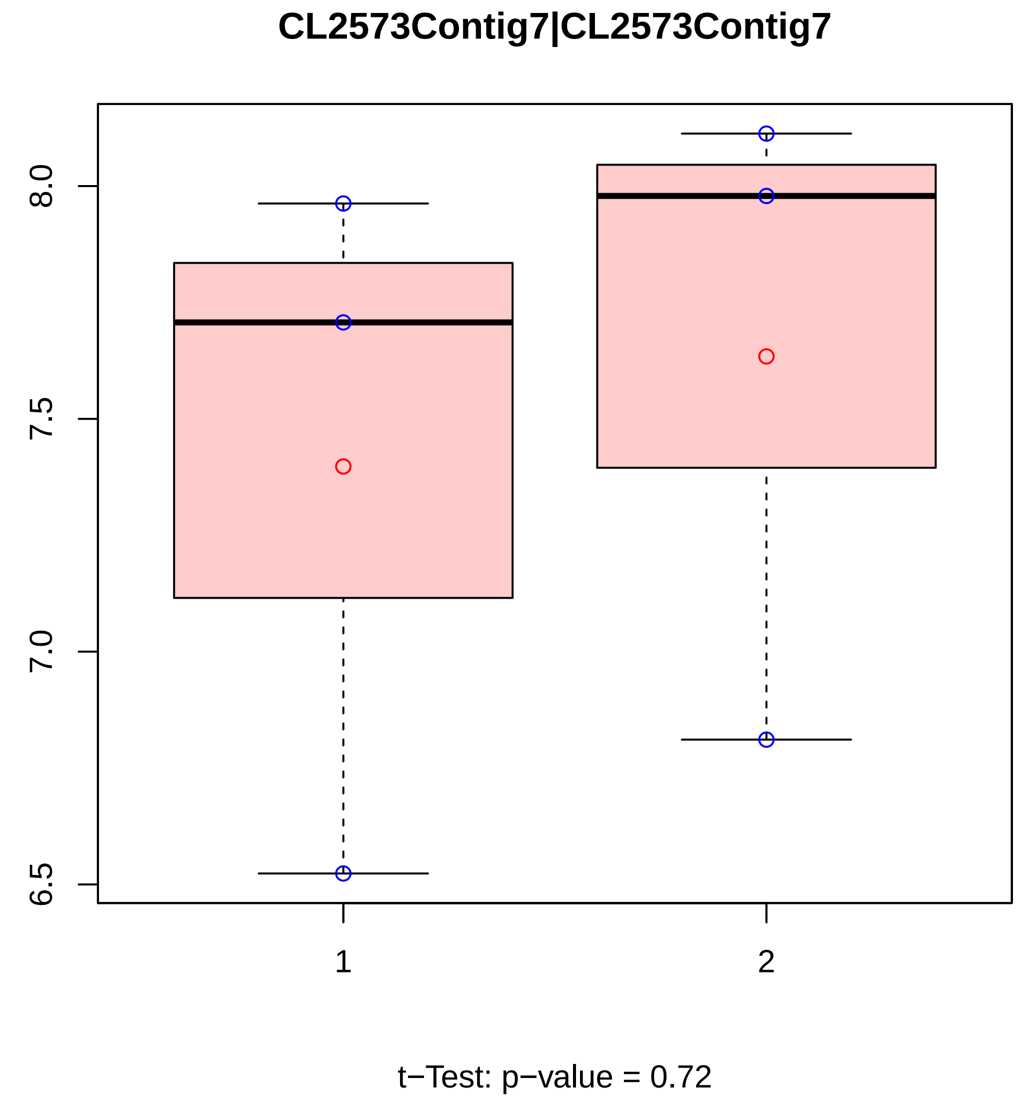
CL2573Contig7|CL2573Contig7
8.0
7.5
7.0
6.5
1
2
t−Test: p−value = 0.72

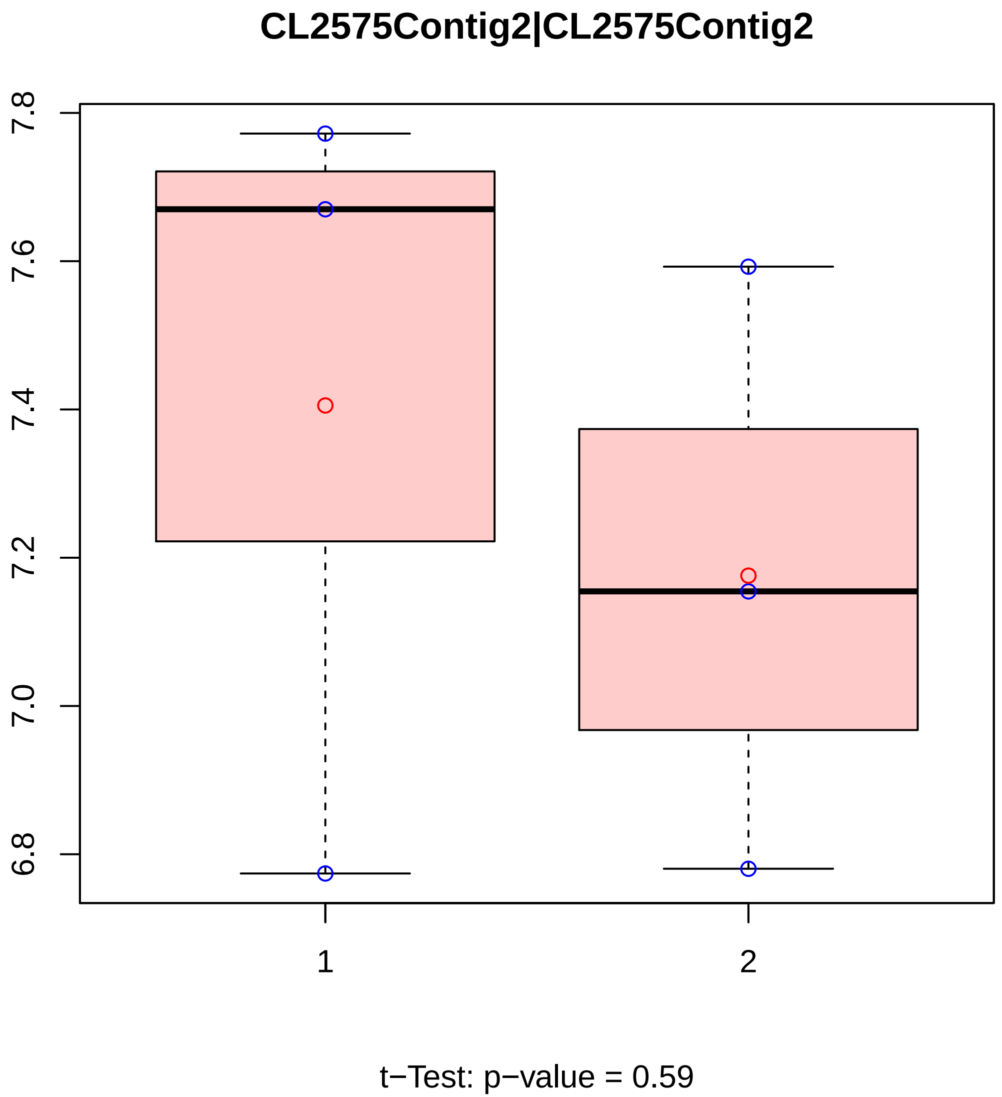

CL2575Contig2|CL2575Contig2
7.8
7.6
7.4
7.2
7.0
6.8
1
2
t−Test: p−value = 0.59

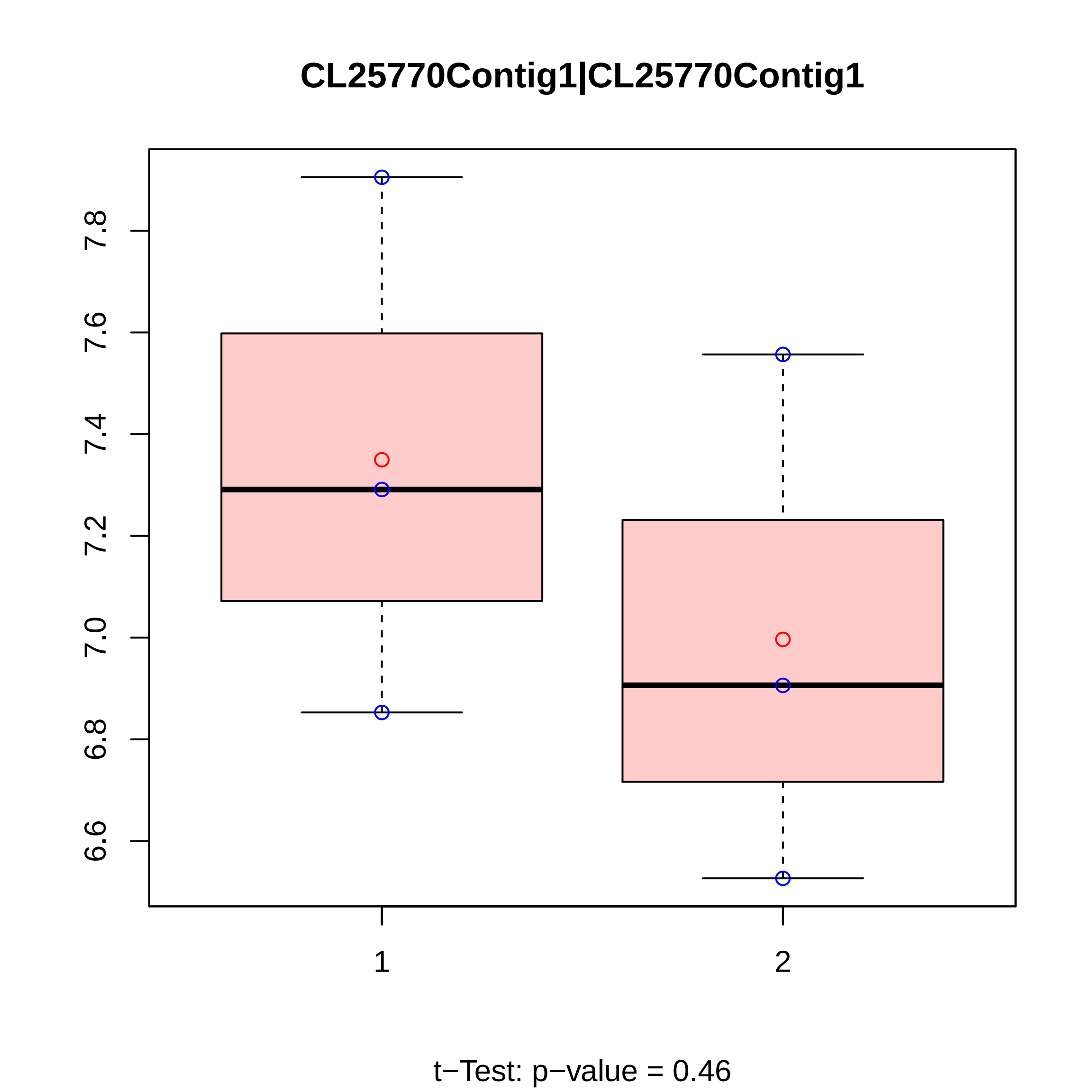

CL25770Contig1|CL25770Contig1
7.8
7.6
7.4
7.2
7.0
6.8
6.6
1
2
t−Test: p−value = 0.46

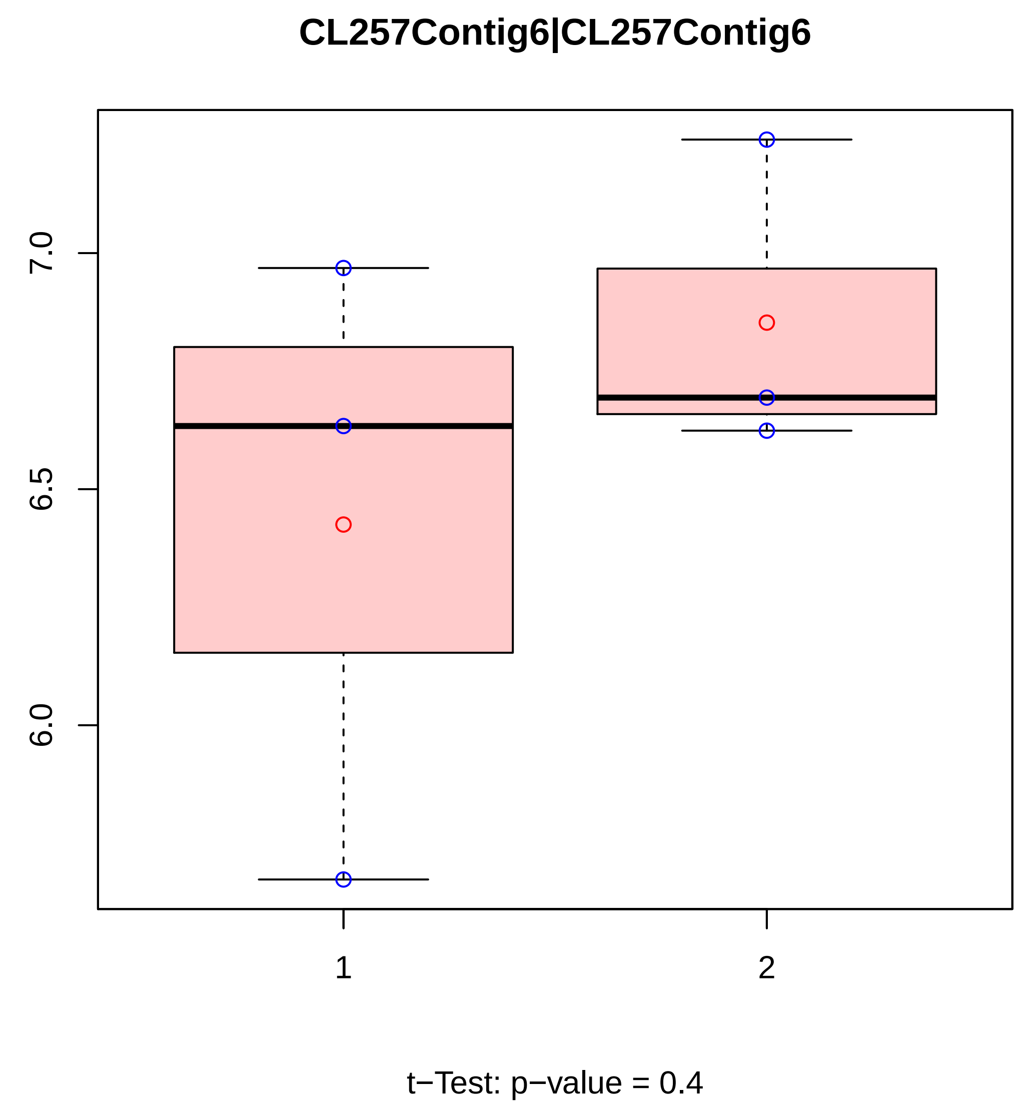
CL257Contig6|CL257Contig6
7.0
6.5
6.0
1
2
t−Test: p−value = 0.4

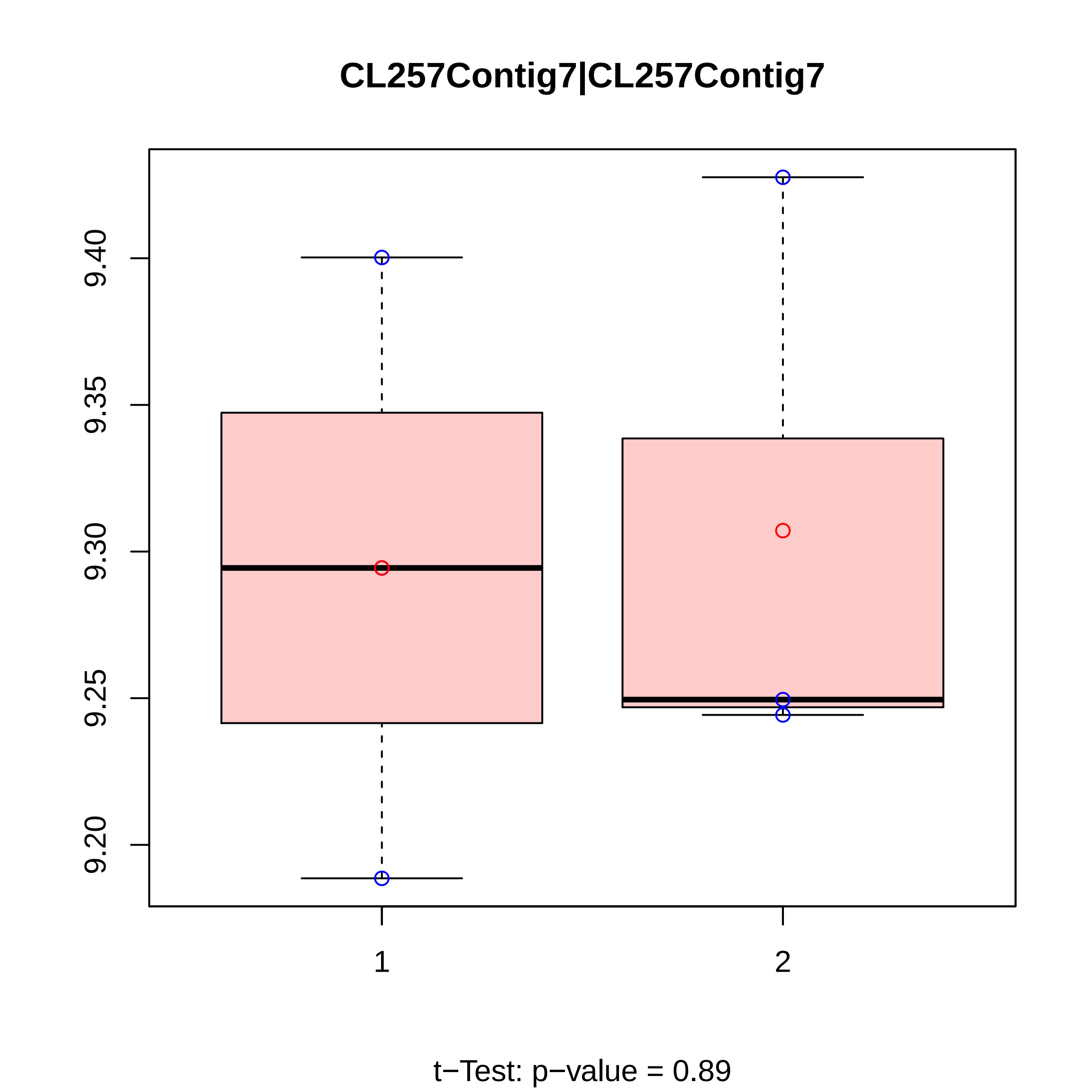
CL257Contig7|CL257Contig7
9.20
9.25
9.30
9.35
9.40
1
2
t−Test: p−value = 0.89

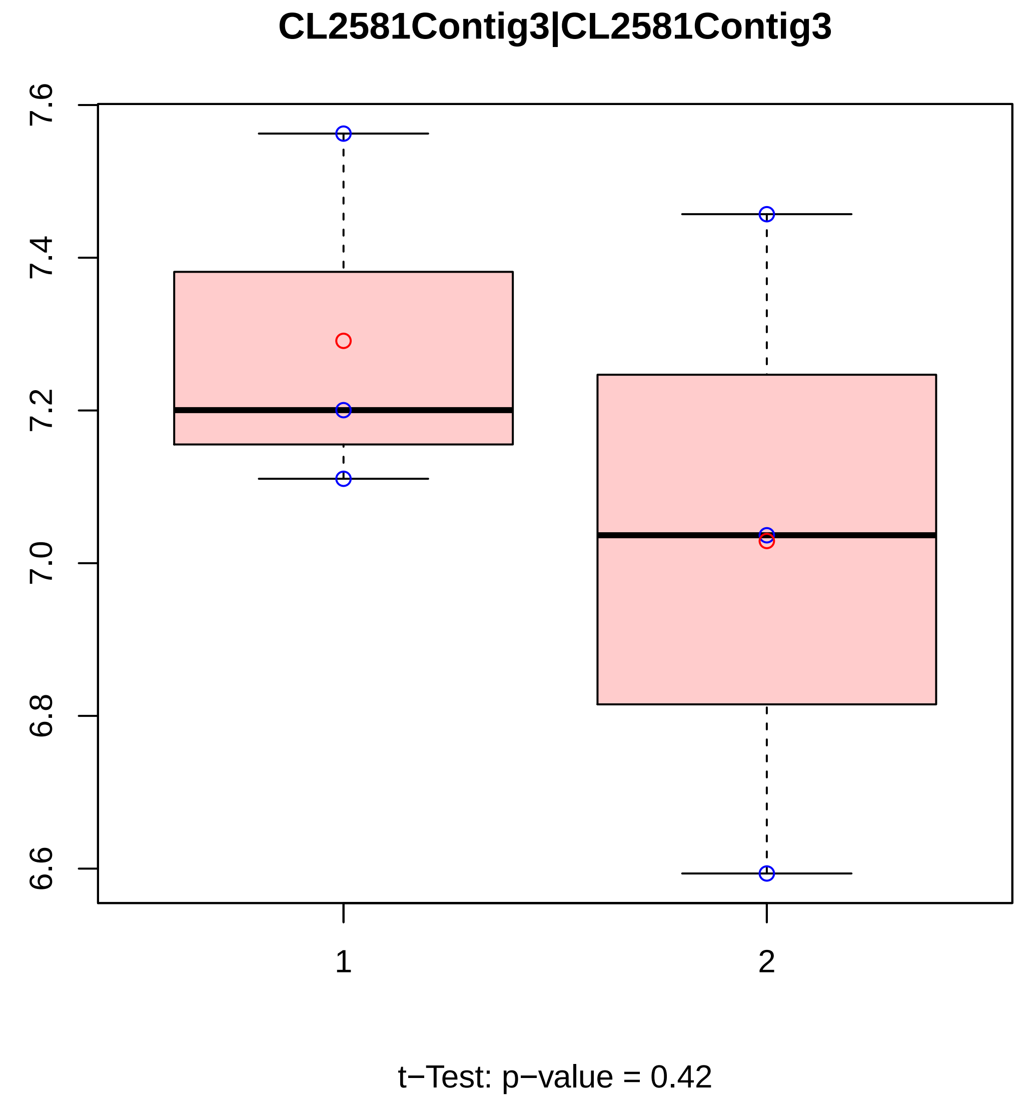
CL2581Contig3|CL2581Contig3
7.6
7.4
7.2
7.0
6.8
6.6
1
2
t−Test: p−value = 0.42

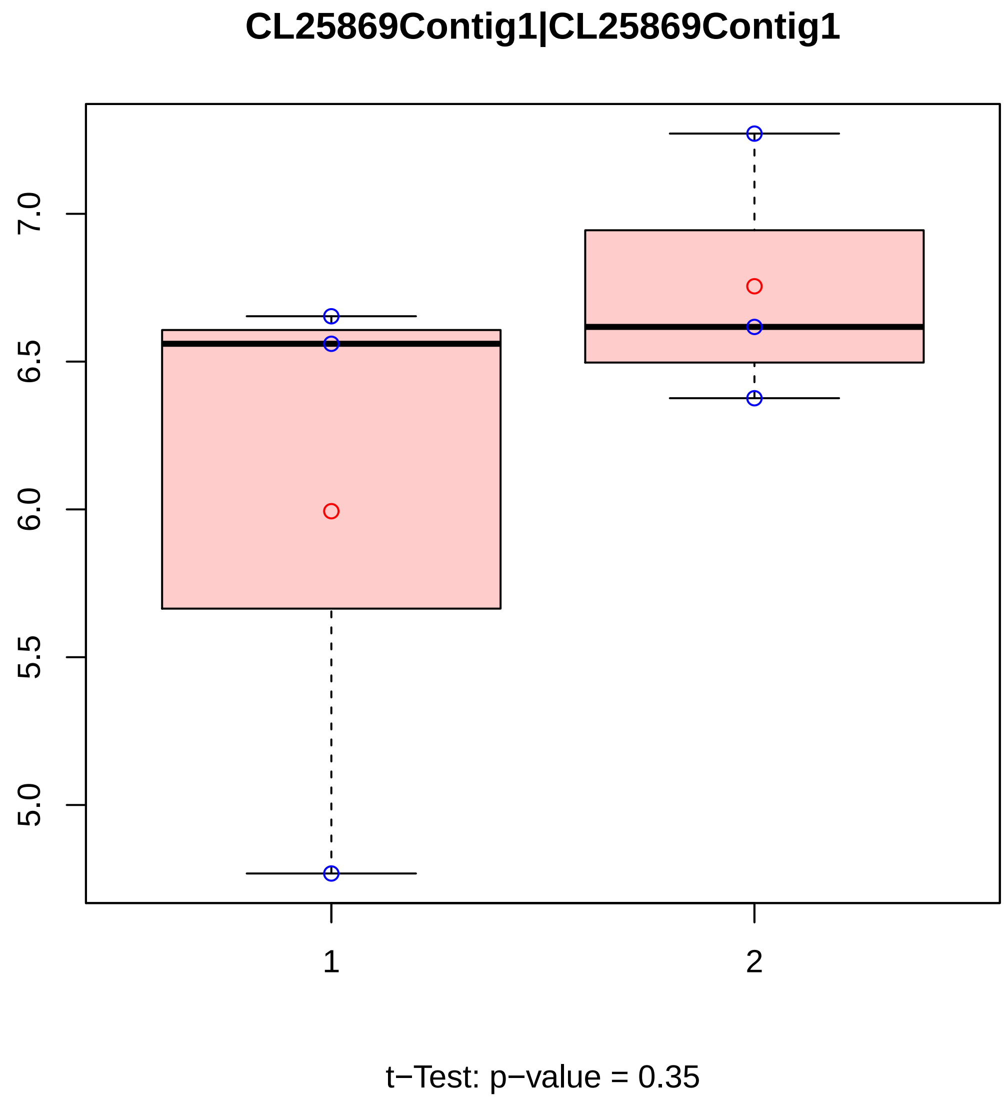

CL25869Contig1|CL25869Contig1
7.0
6.5
6.0
5.5
5.0
1
2
t−Test: p−value = 0.35

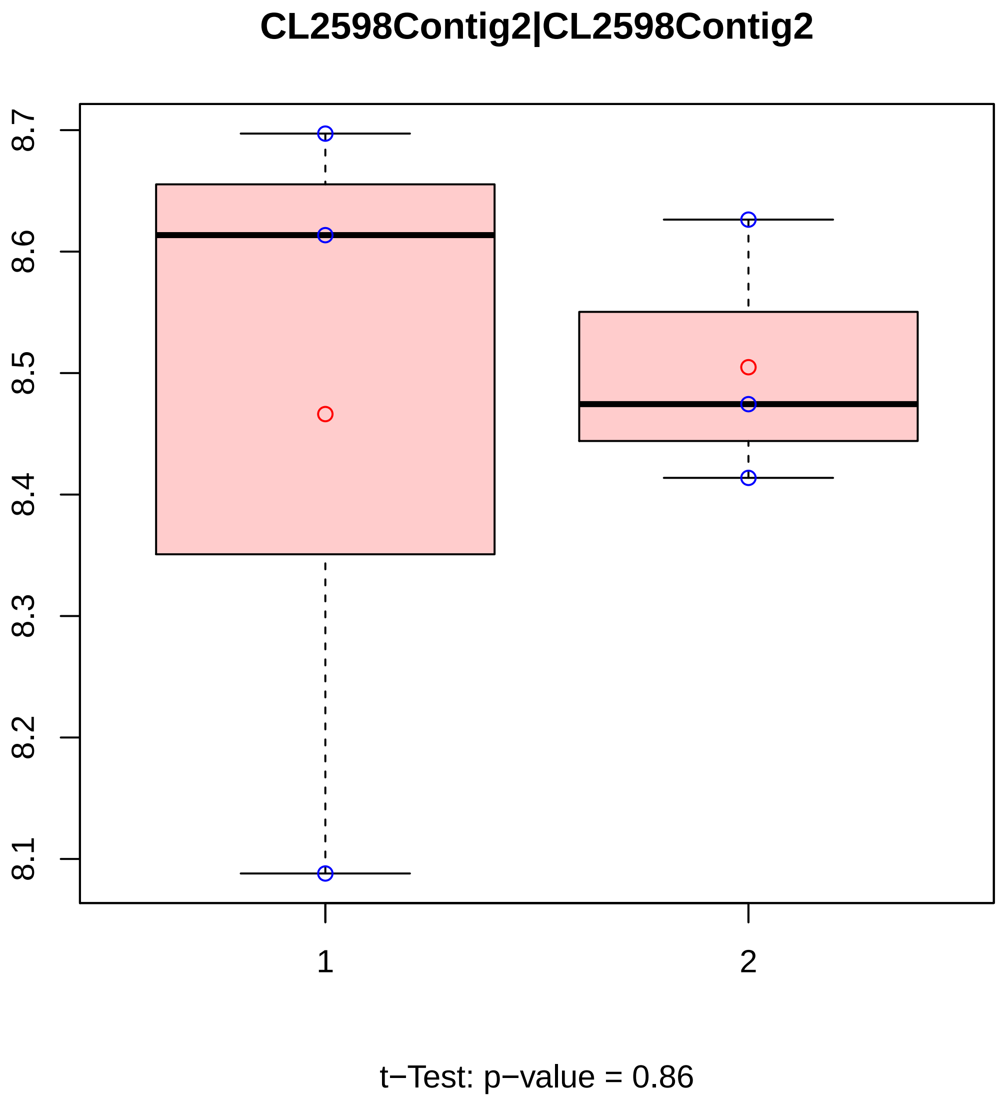

CL2598Contig2|CL2598Contig2
8.1
8.2
8.3
8.4
8.5
8.6
8.7
1
2
t−Test: p−value = 0.86

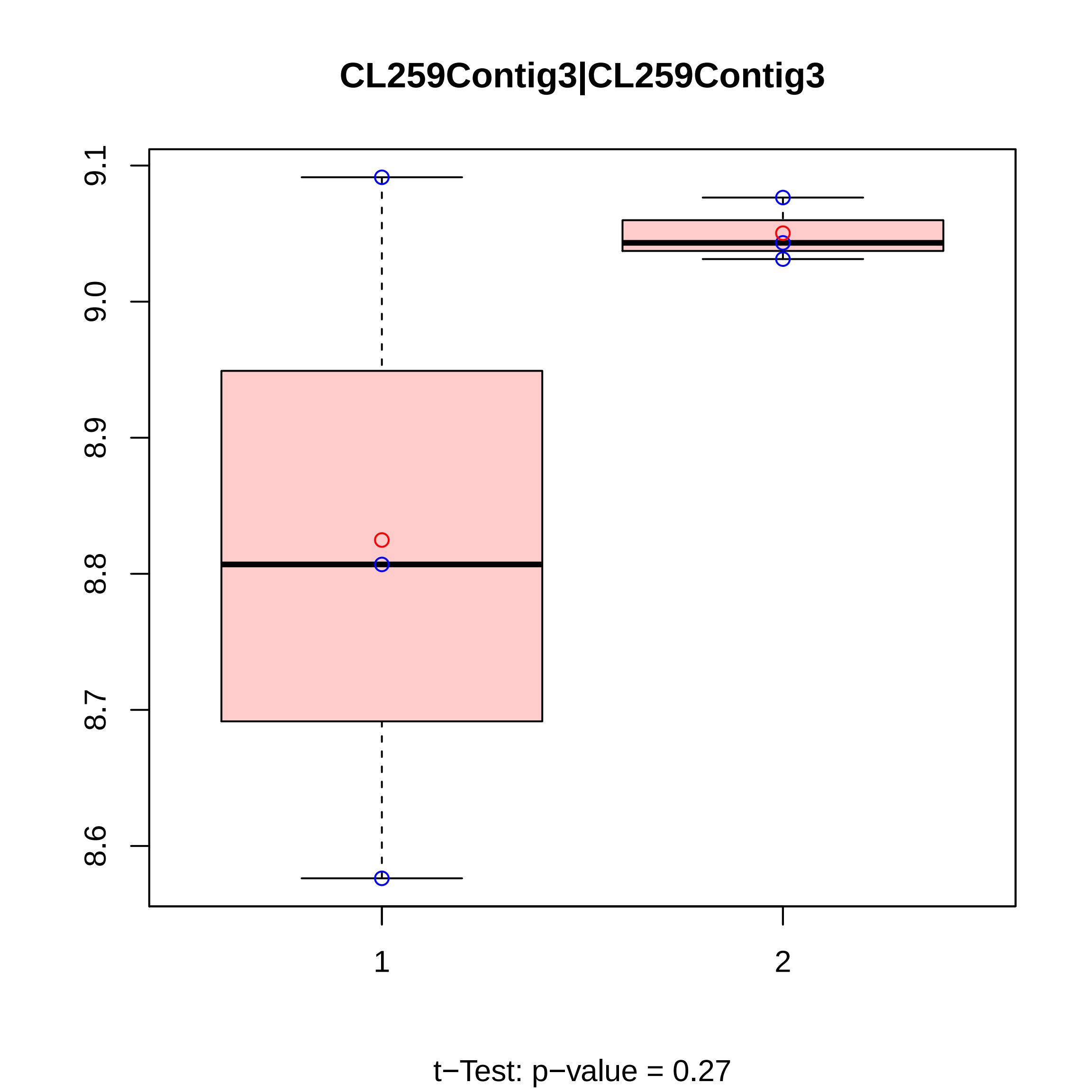
CL259Contig3|CL259Contig3
9.1
9.0
8.9
8.8
8.7
8.6
1
2
t−Test: p−value = 0.27

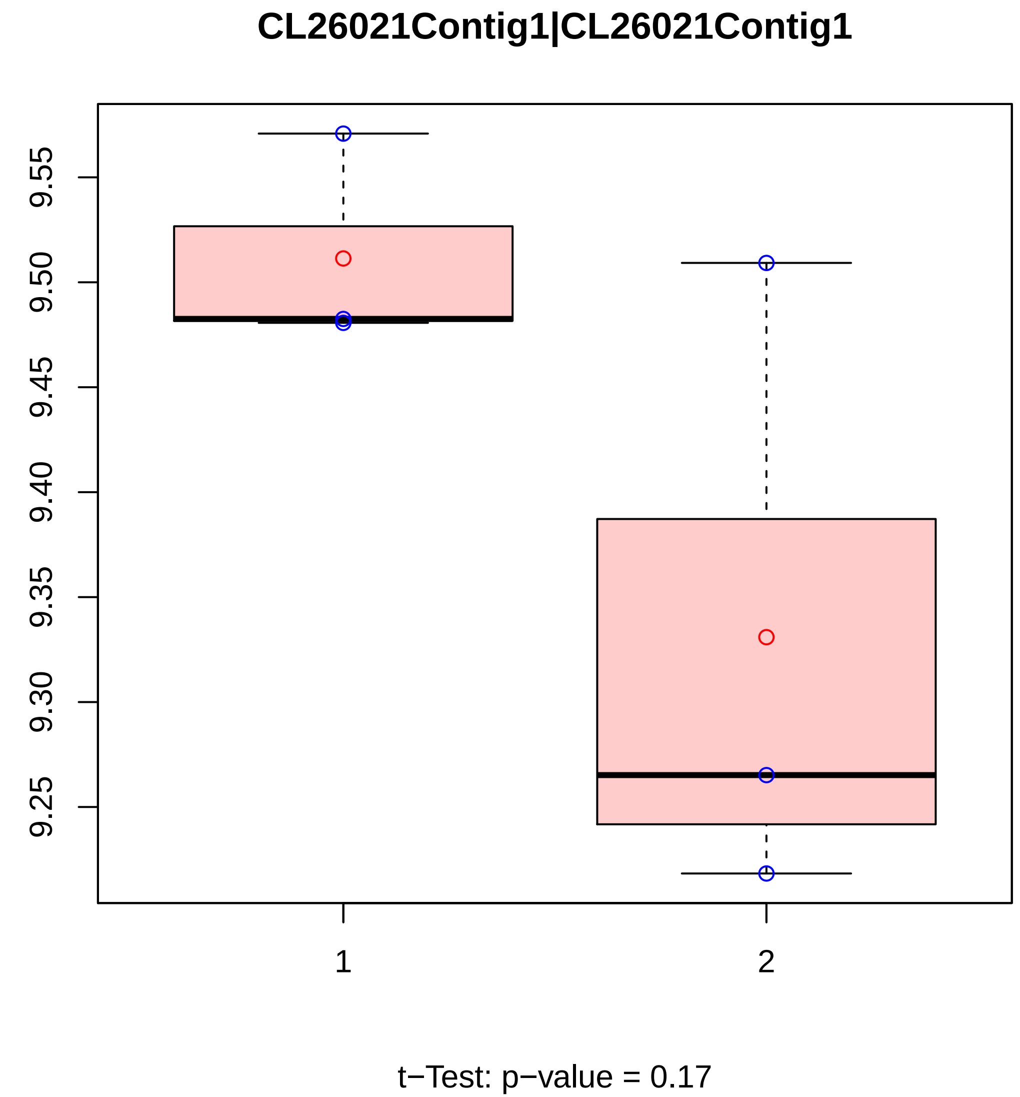

CL26021Contig1|CL26021Contig1
9.25
9.30
9.35
9.40
9.45
9.50
9.55
1
2
t−Test: p−value = 0.17

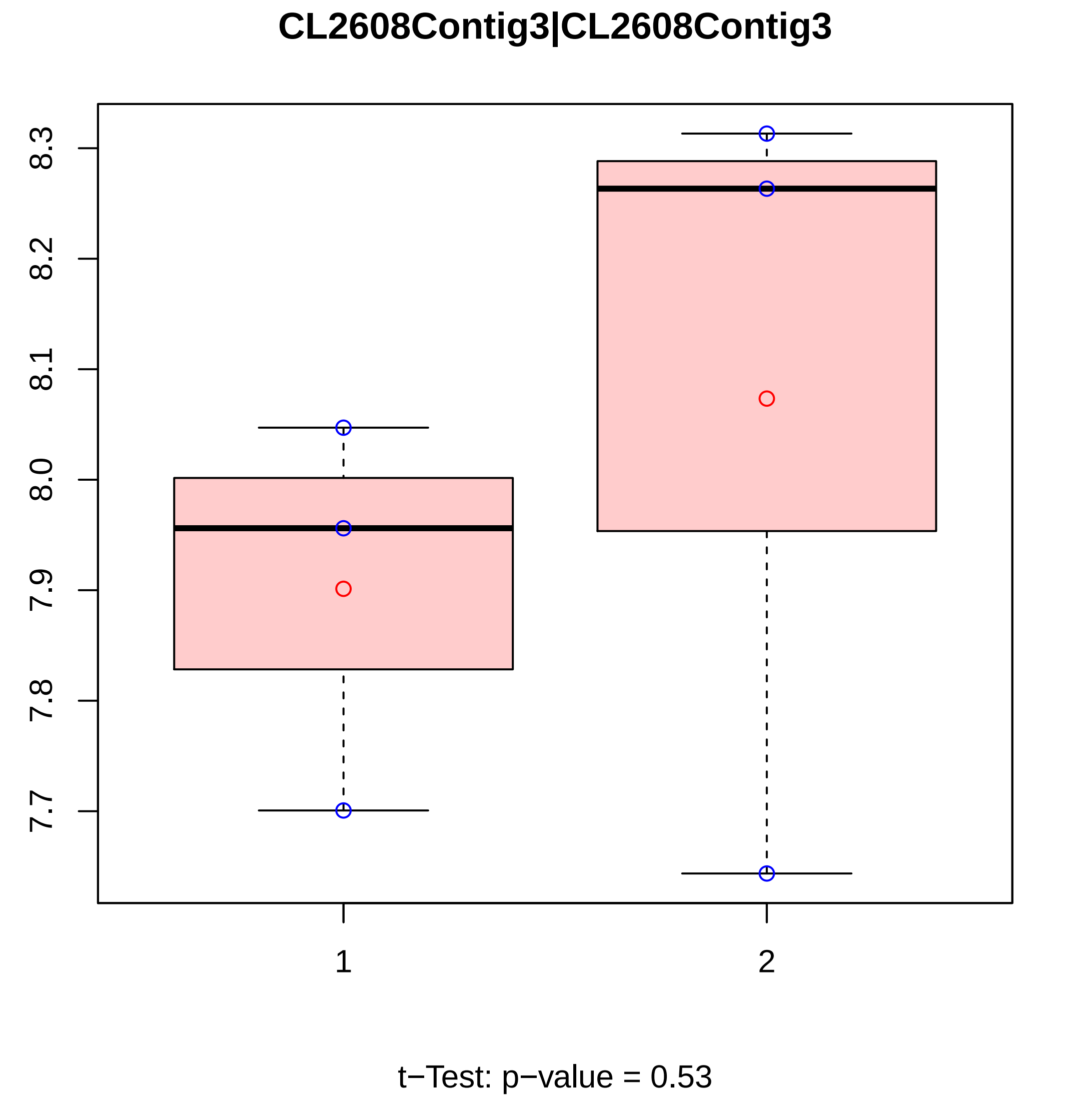
CL2608Contig3|CL2608Contig3
8.3
8.2
8.1
8.0
7.9
7.8
7.7
1
2
t−Test: p−value = 0.53

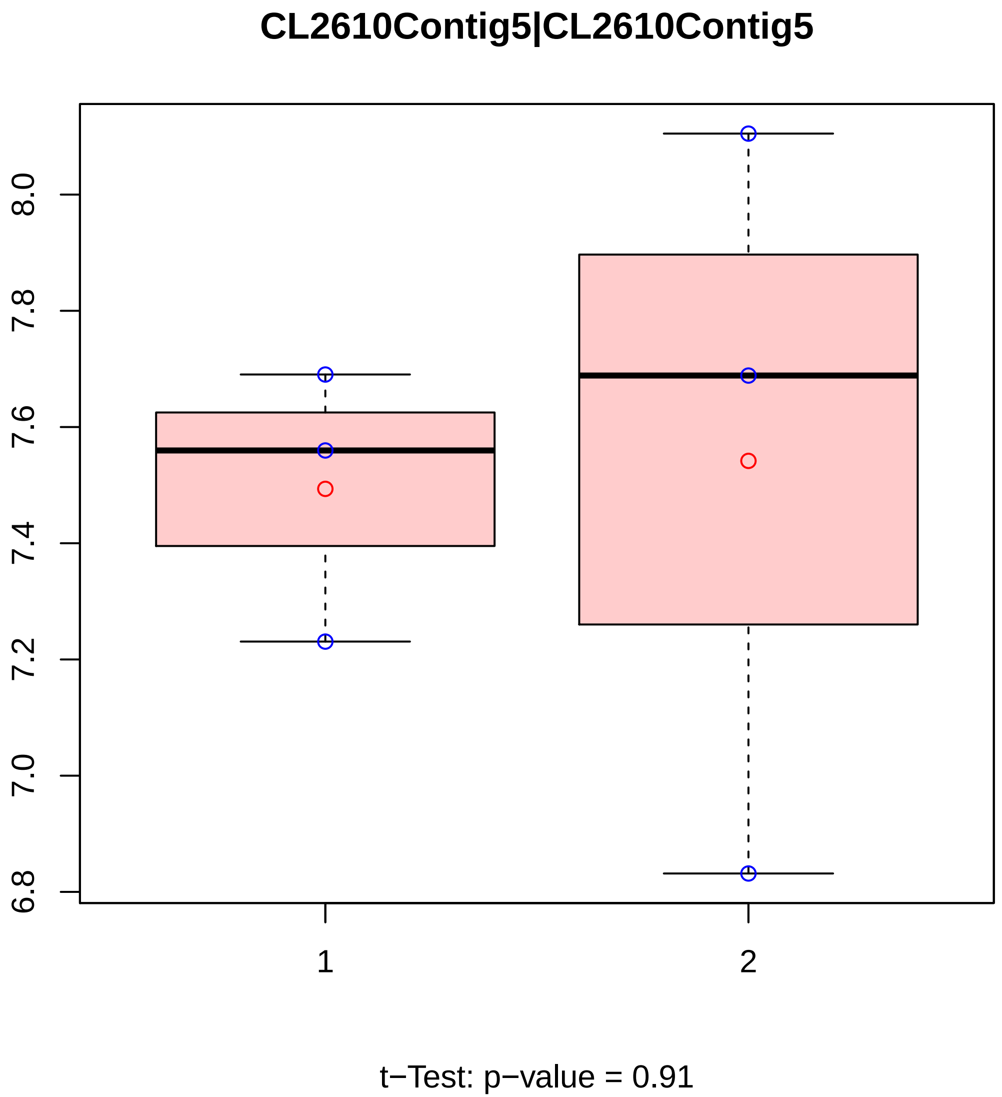

CL2610Contig5|CL2610Contig5
8.0
7.8
7.6
7.4
7.2
7.0
6.8
1
2
t−Test: p−value = 0.91

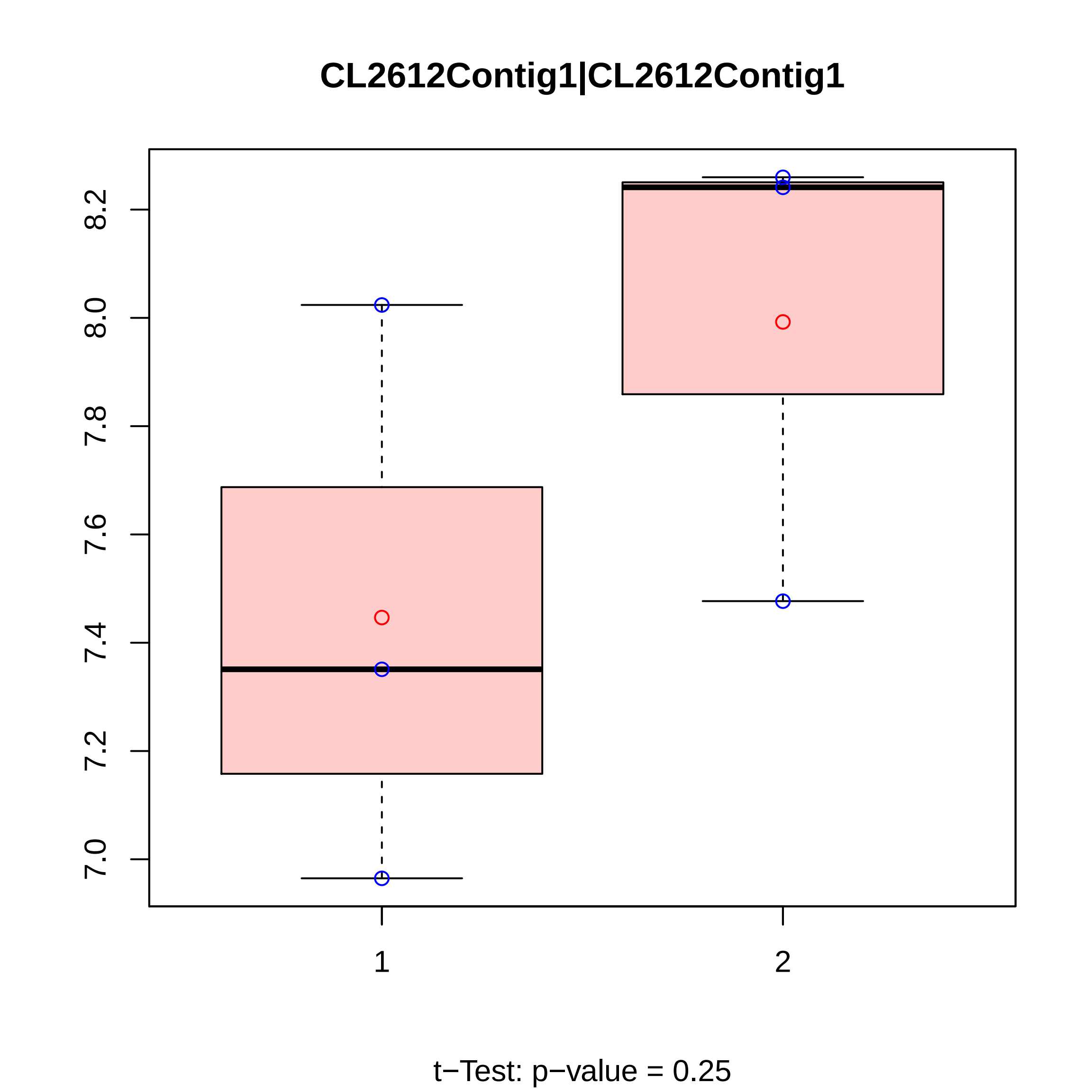
CL2612Contig1|CL2612Contig1
8.2
8.0
7.8
7.6
7.4
7.2
7.0
1
2
t−Test: p−value = 0.25

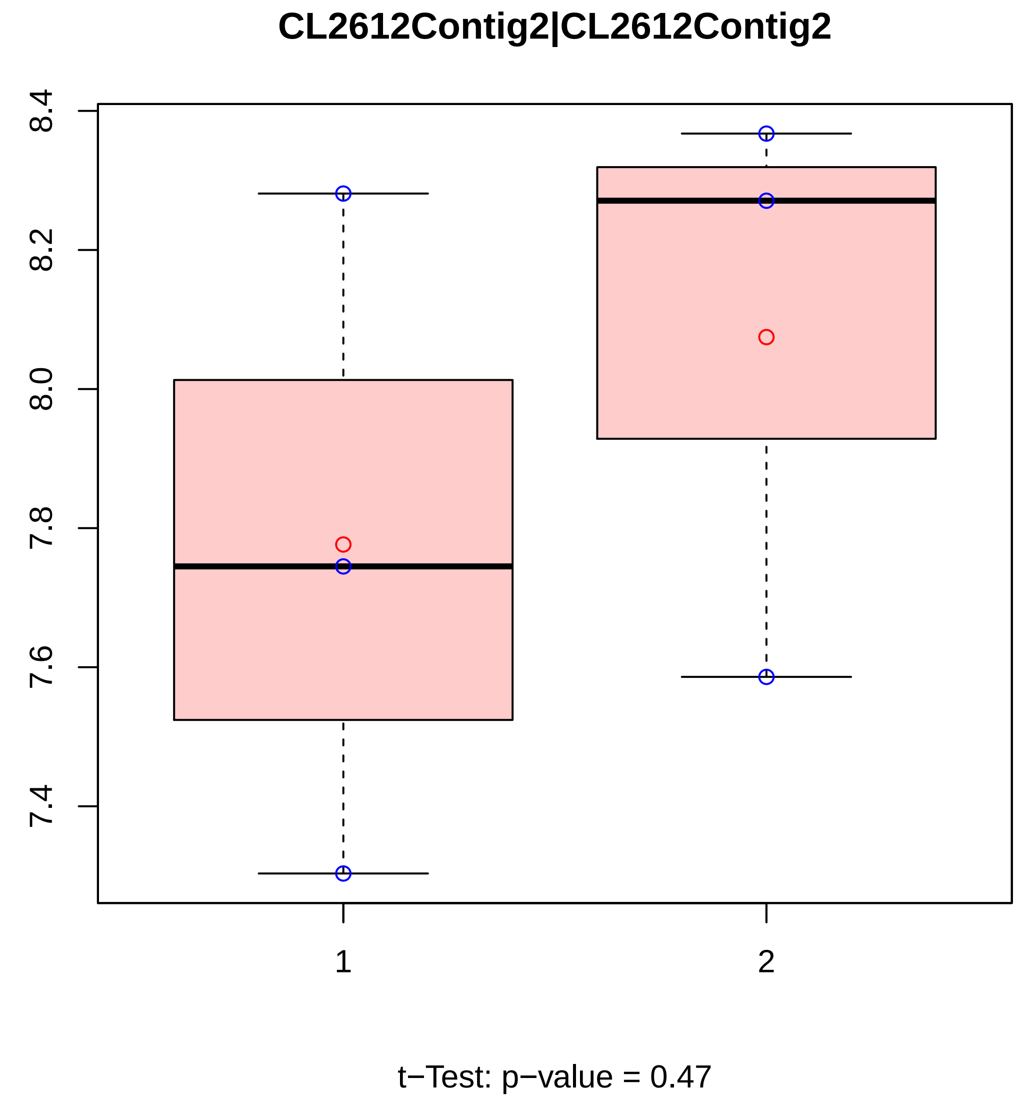
CL2612Contig2|CL2612Contig2
8.4
8.2
8.0
7.8
7.6
7.4
1
2
t−Test: p−value = 0.47

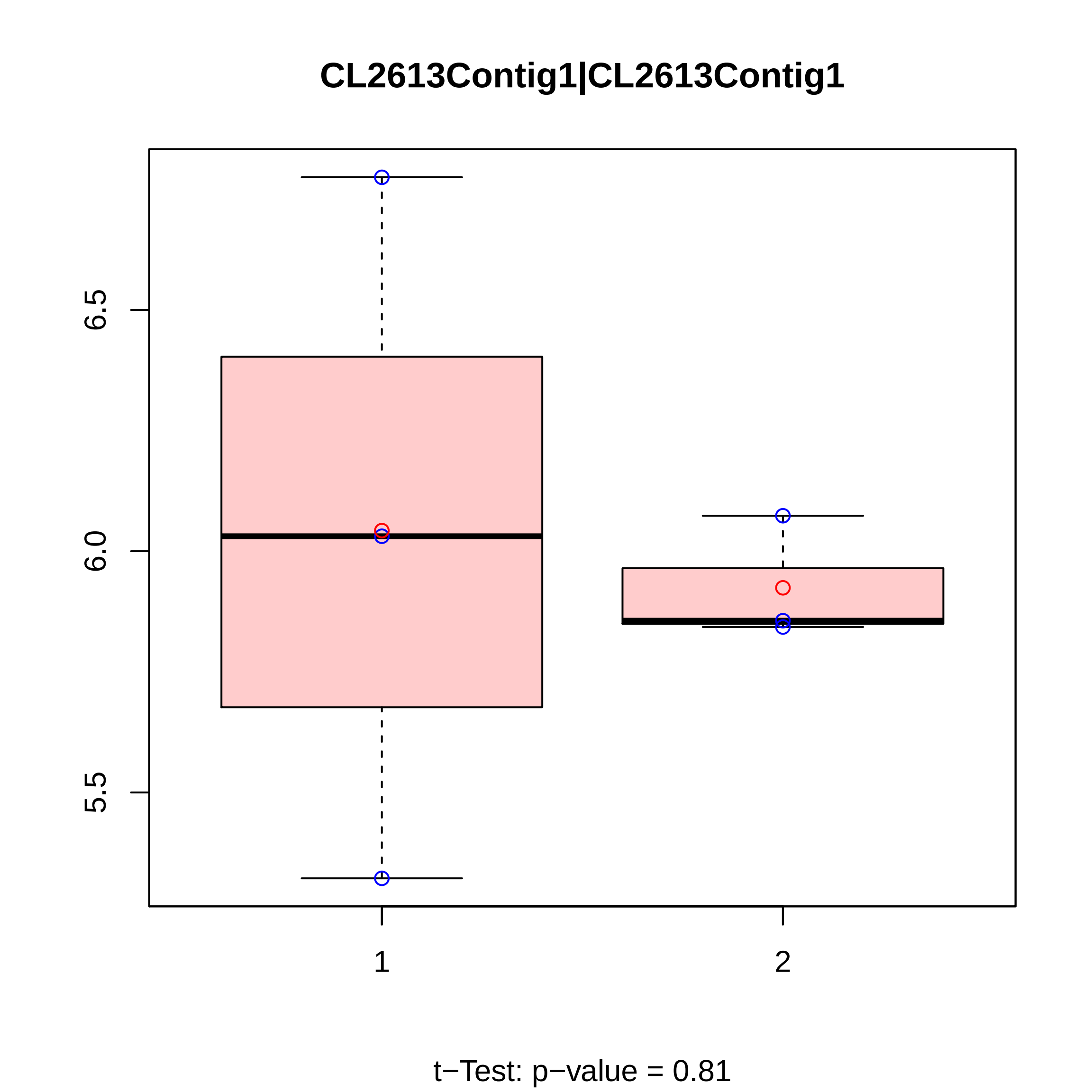
CL2613Contig1|CL2613Contig1
6.5
6.0
5.5
1
2
t−Test: p−value = 0.81

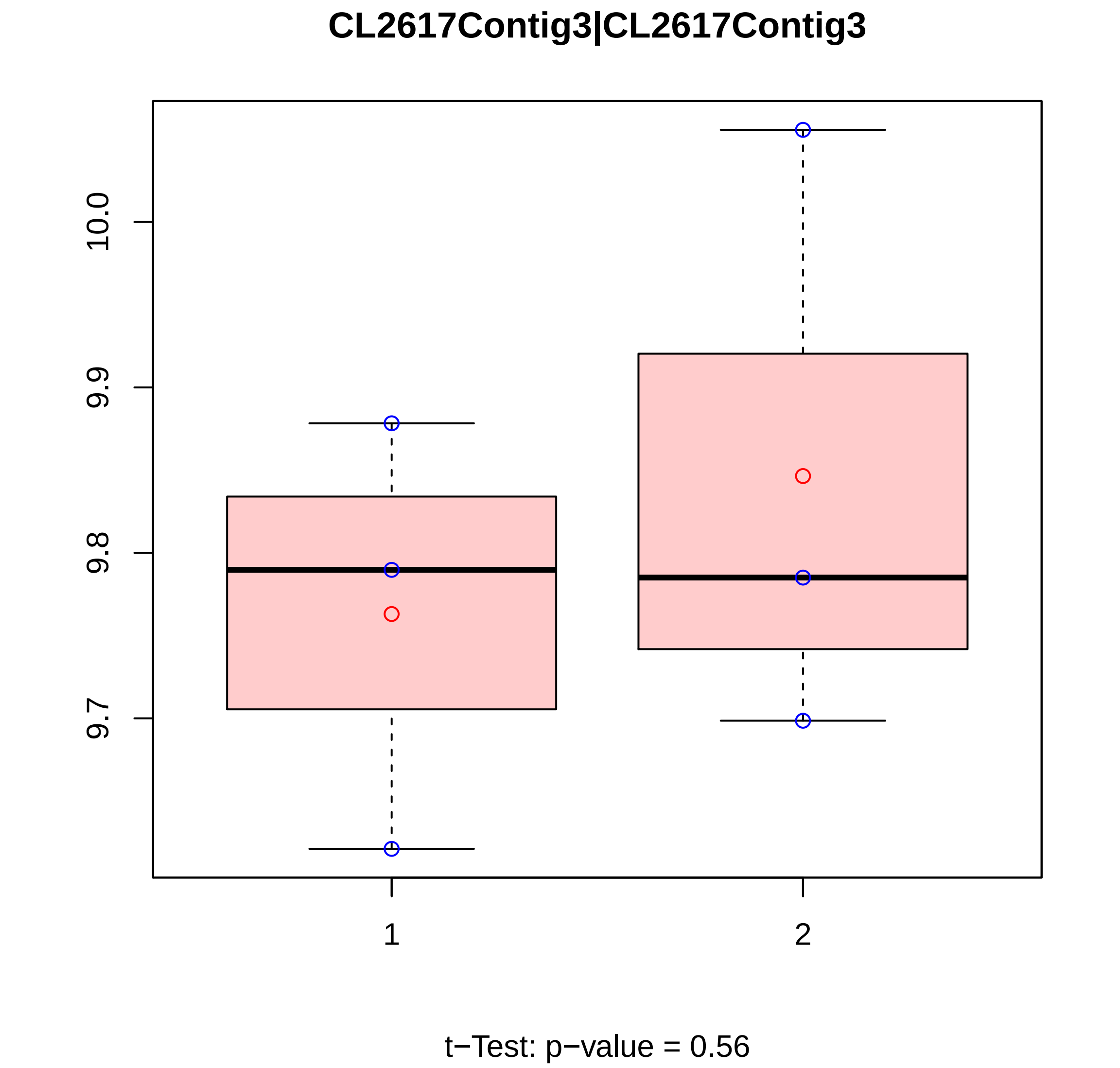
CL2617Contig3|CL2617Contig3
10.0
9.9
9.8
9.7
1
2
t−Test: p−value = 0.56

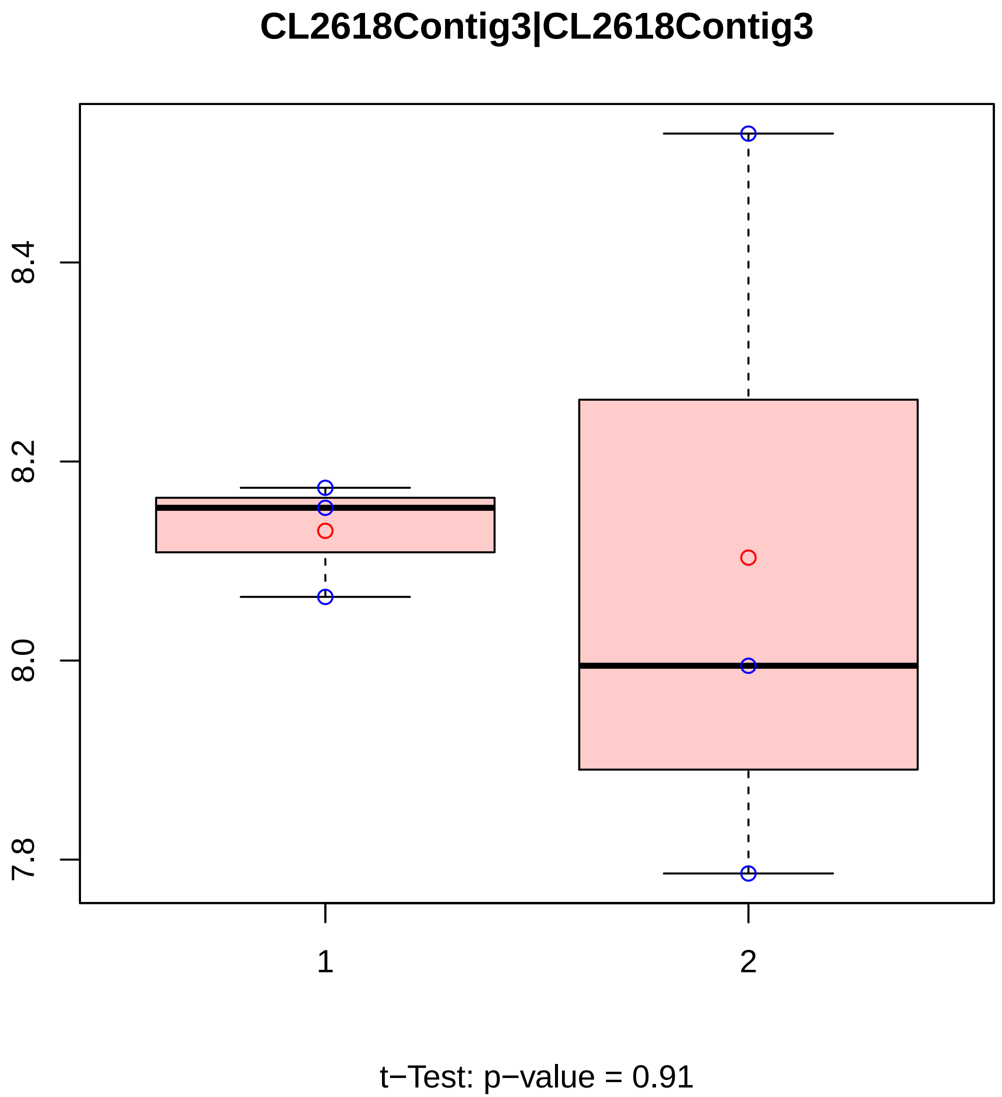
CL2618Contig3|CL2618Contig3
8.4
8.2
8.0
7.8
1
2
t−Test: p−value = 0.91

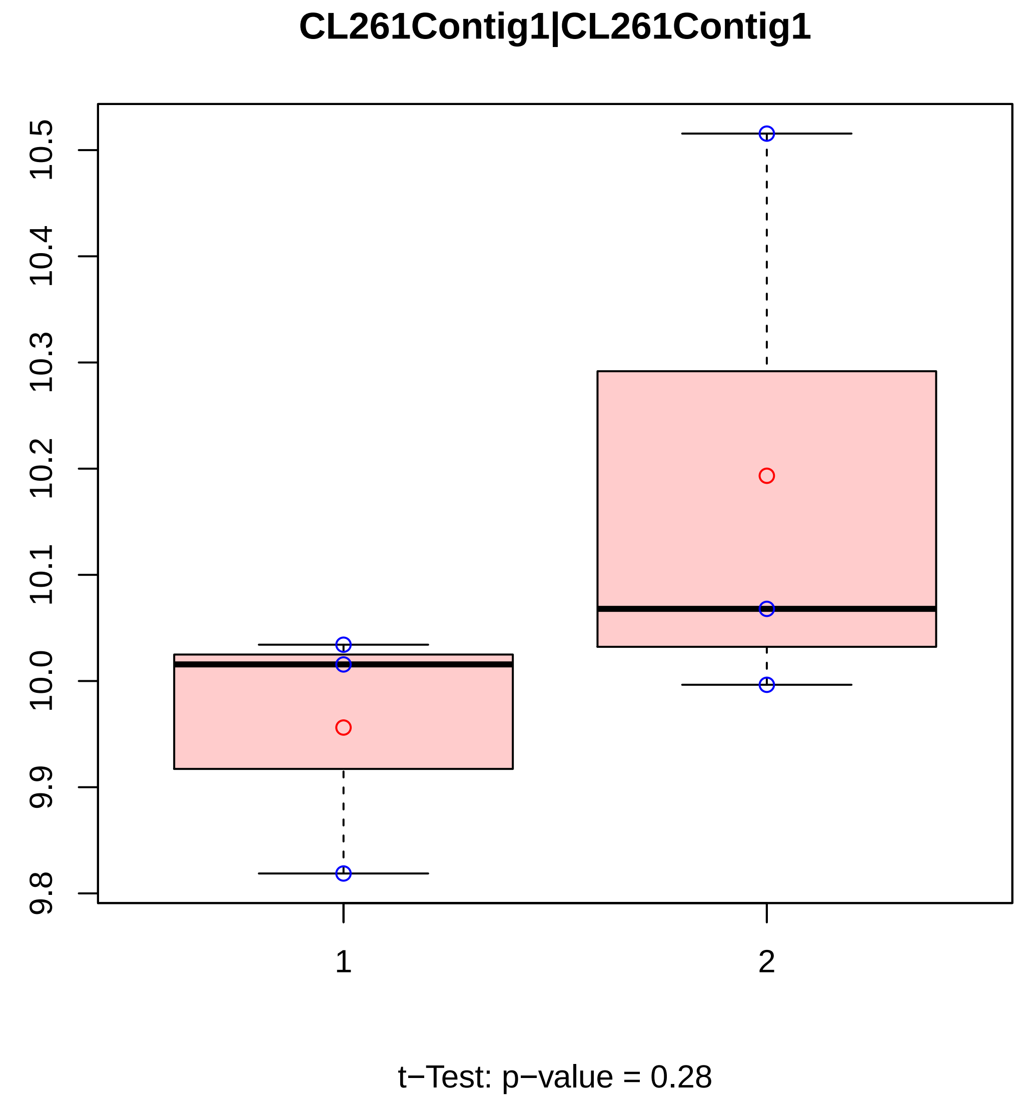

CL261Contig1|CL261Contig1
10.5
10.4
10.3
10.2
10.1
10.0
9.9
9.8
1
2
t−Test: p−value = 0.28

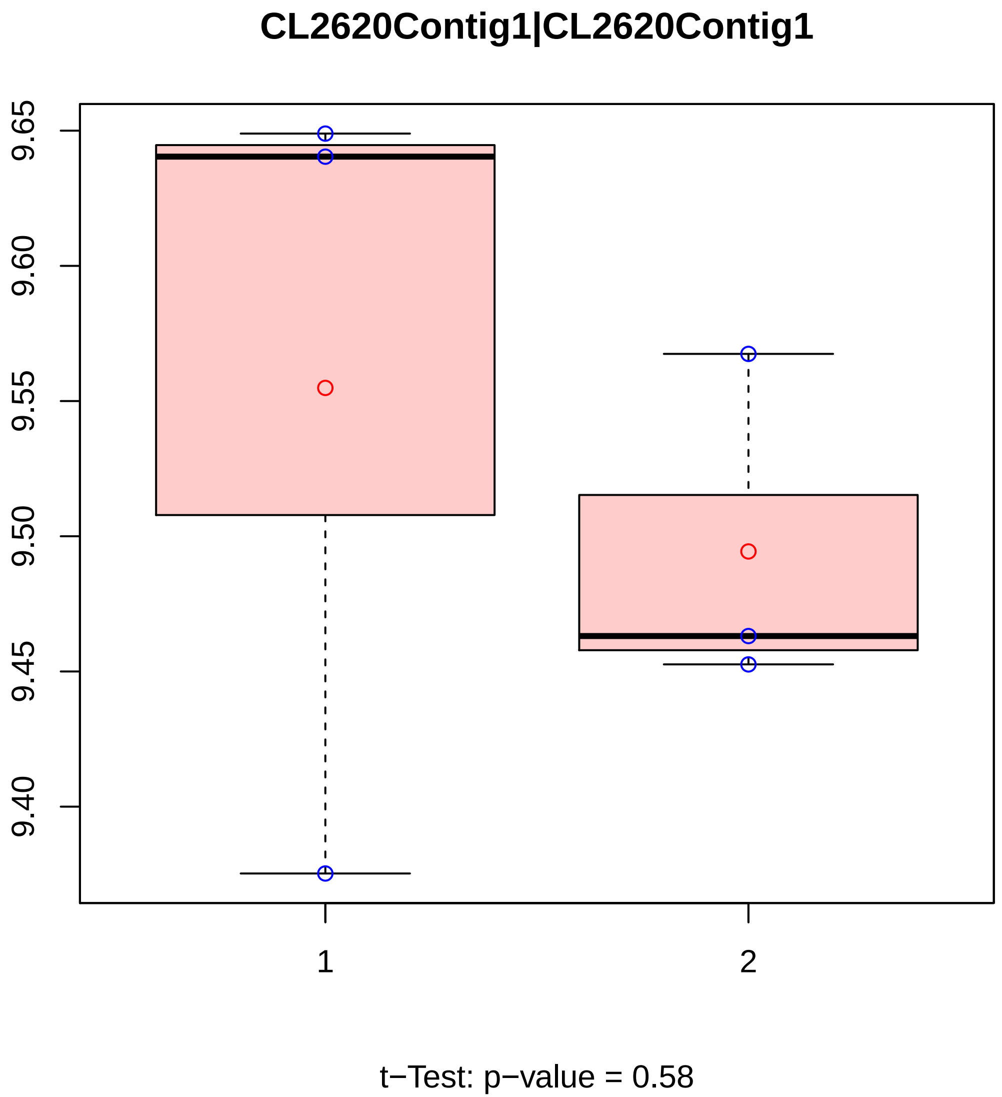
CL2620Contig1|CL2620Contig1
9.40
9.45
9.50
9.55
9.60
9.65
1
2
t−Test: p−value = 0.58

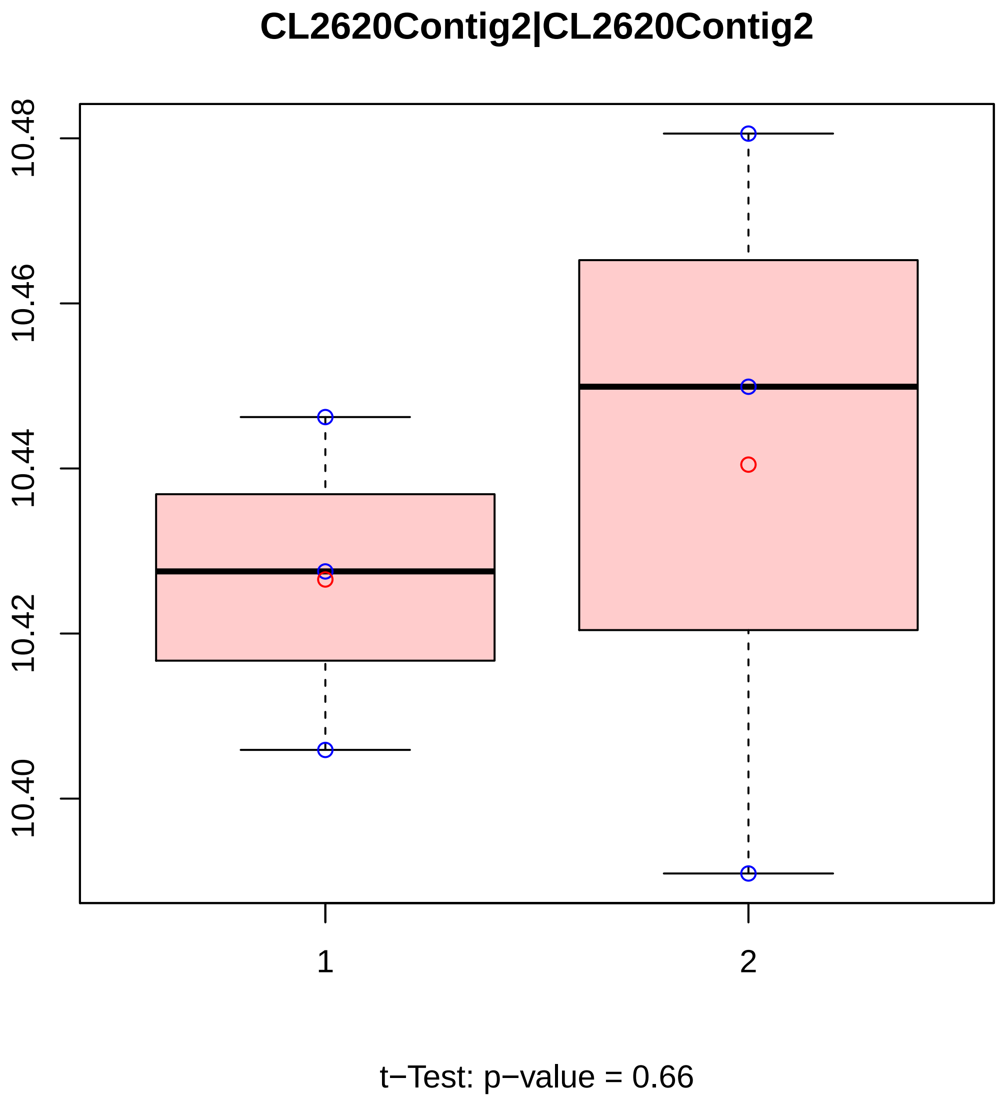
CL2620Contig2|CL2620Contig2
10.40
10.42
10.44
10.46
10.48
1
2
t−Test: p−value = 0.66

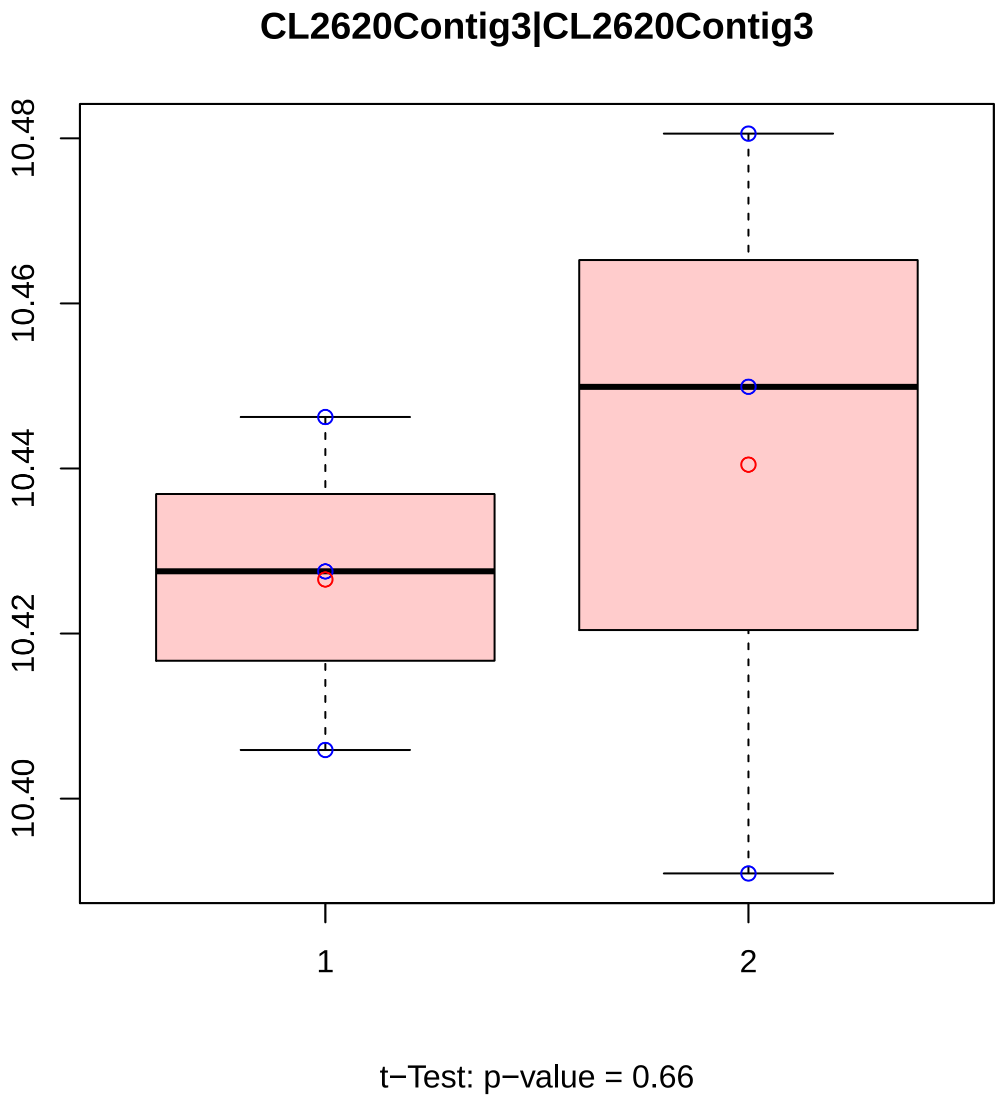
CL2620Contig3|CL2620Contig3
10.48
10.46
10.44
10.42
10.40
1
2
t−Test: p−value = 0.66

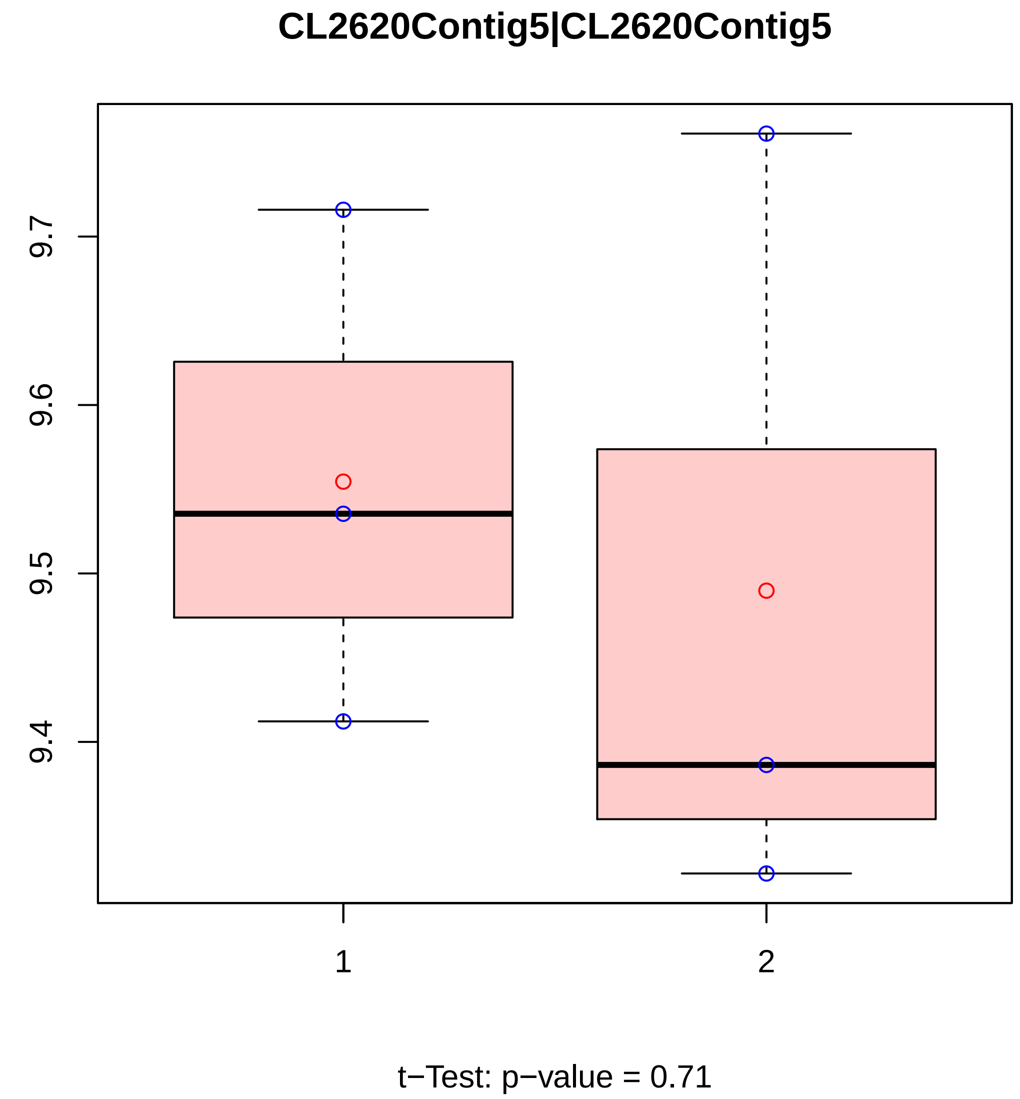

CL2620Contig5|CL2620Contig5
9.7
9.6
9.5
9.4
1
2
t−Test: p−value = 0.71

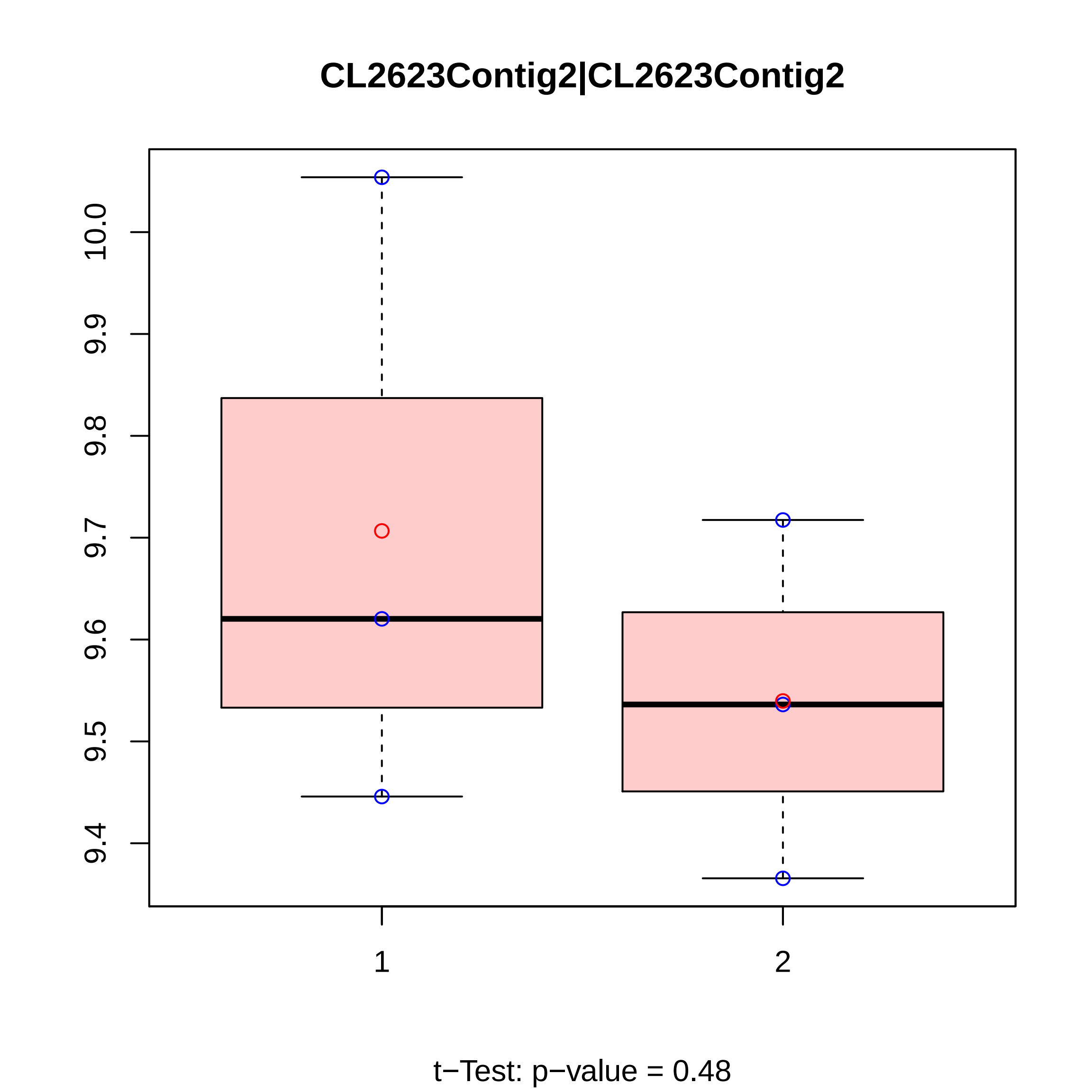
CL2623Contig2|CL2623Contig2
10.0
9.9
9.8
9.7
9.6
9.5
9.4
1
2
t−Test: p−value = 0.48

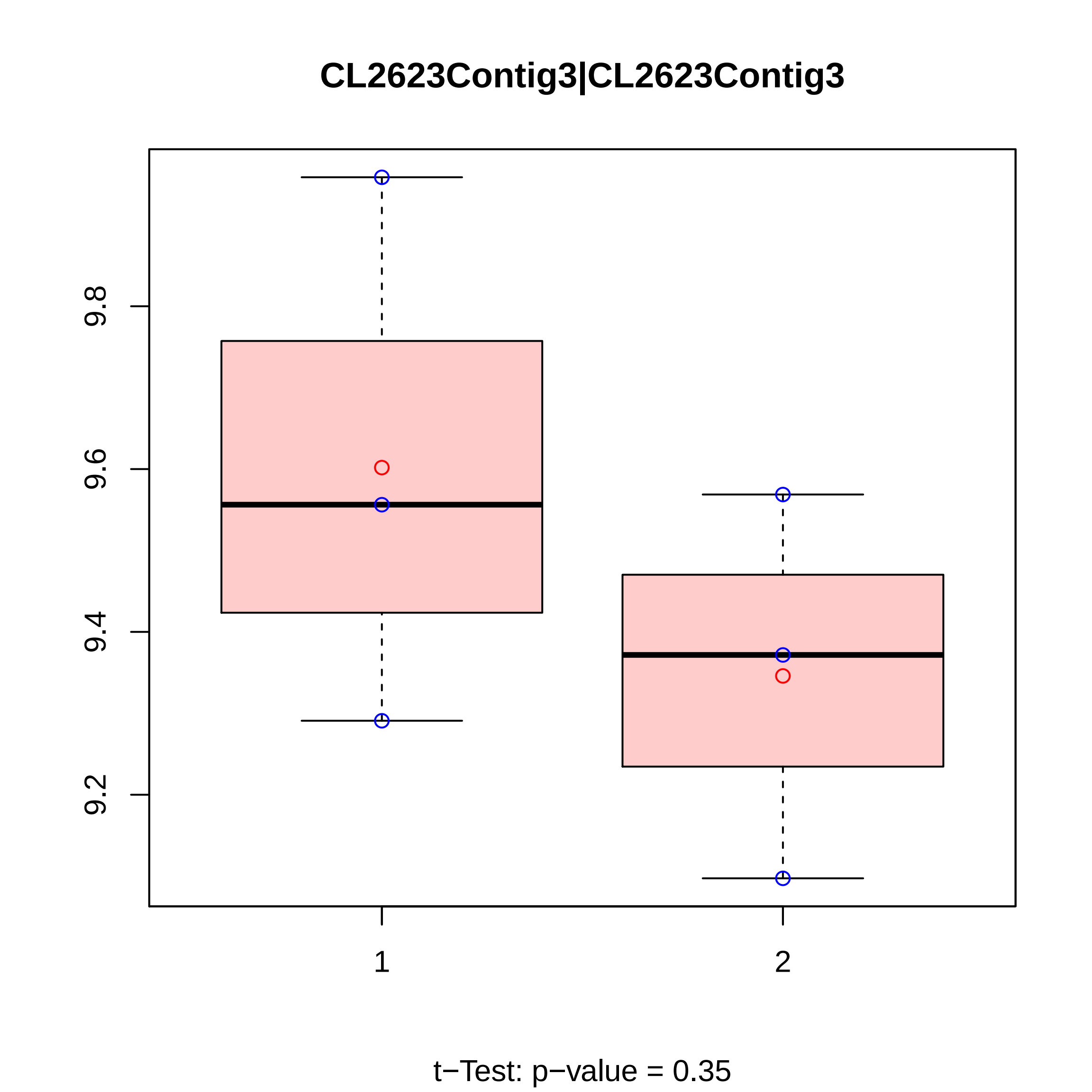

CL2623Contig3|CL2623Contig3
9.2
9.4
9.6
9.8
1
2
t−Test: p−value = 0.35

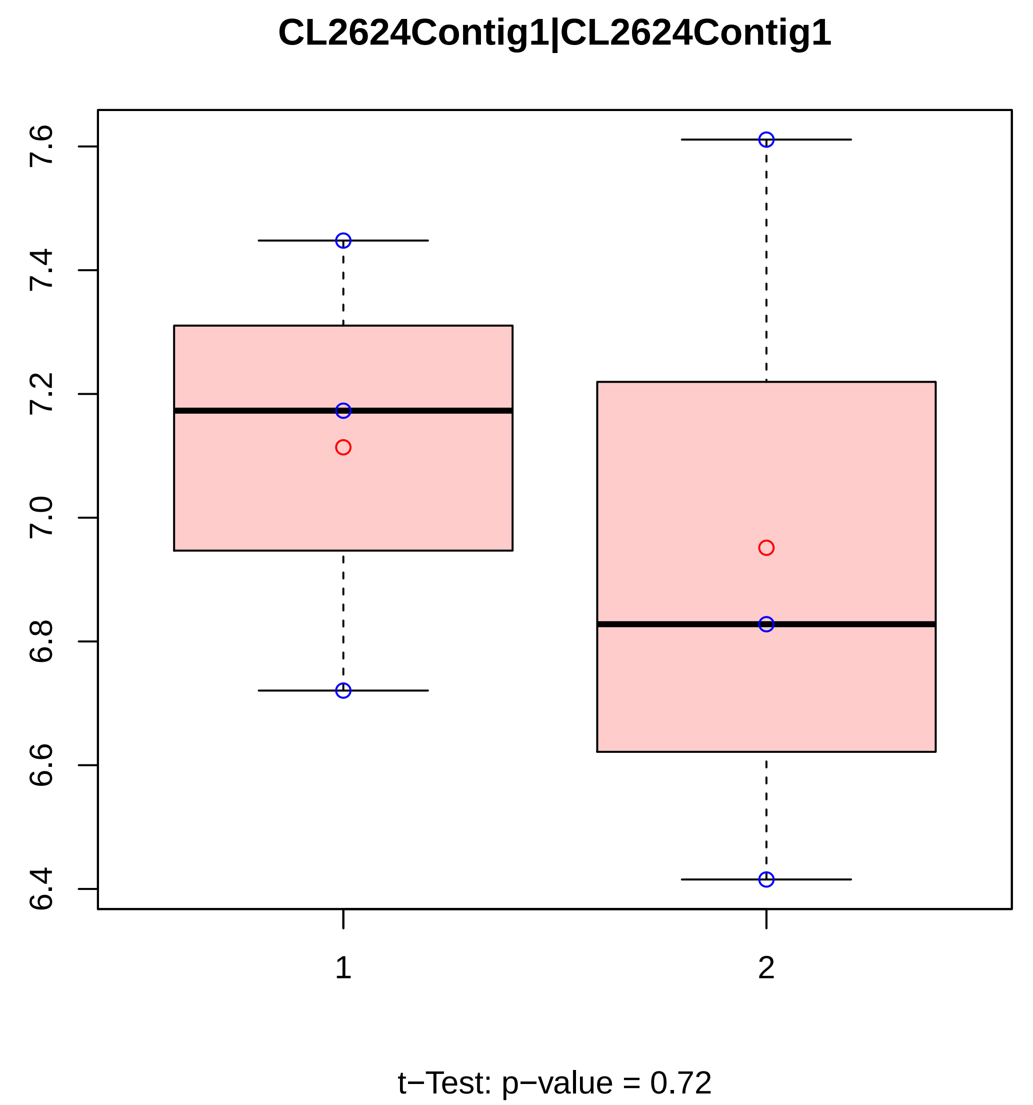
CL2624Contig1|CL2624Contig1
7.6
7.4
7.2
7.0
6.8
6.6
6.4
1
2
t−Test: p−value = 0.72

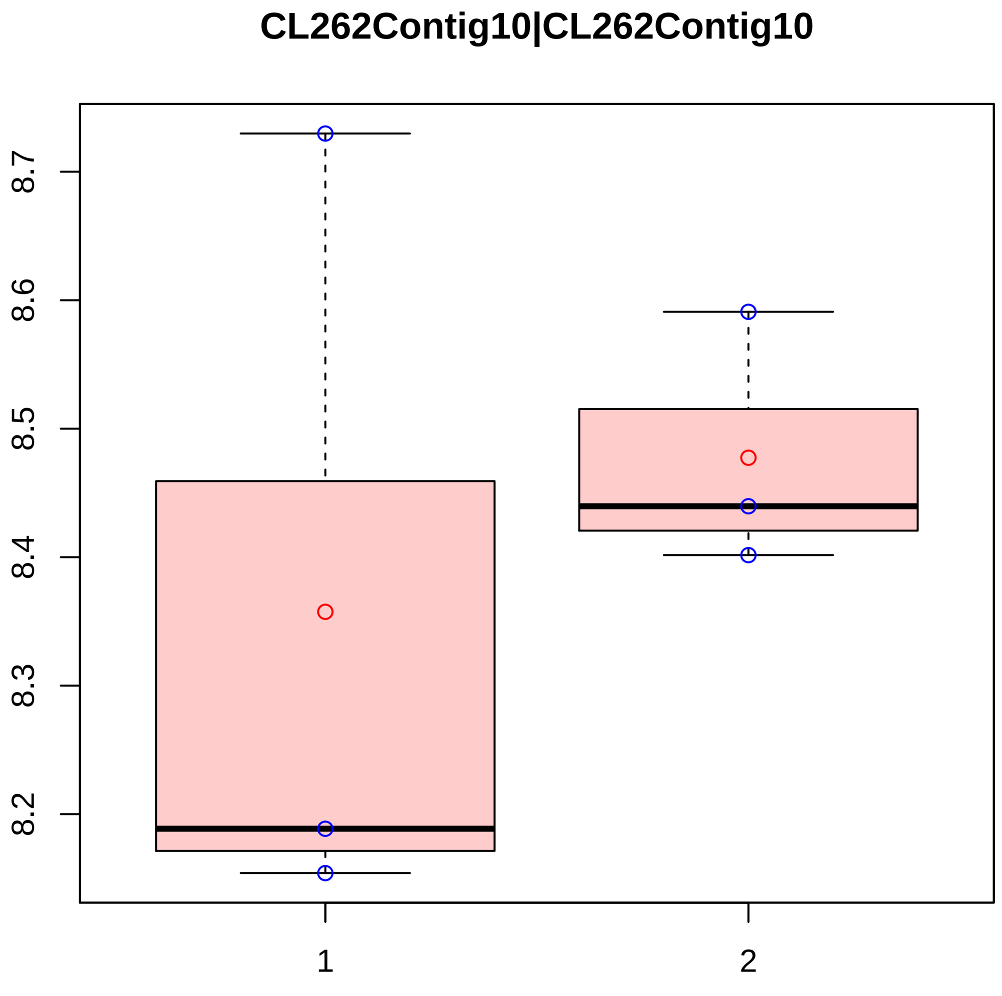


t−Test: p−value = 0.59

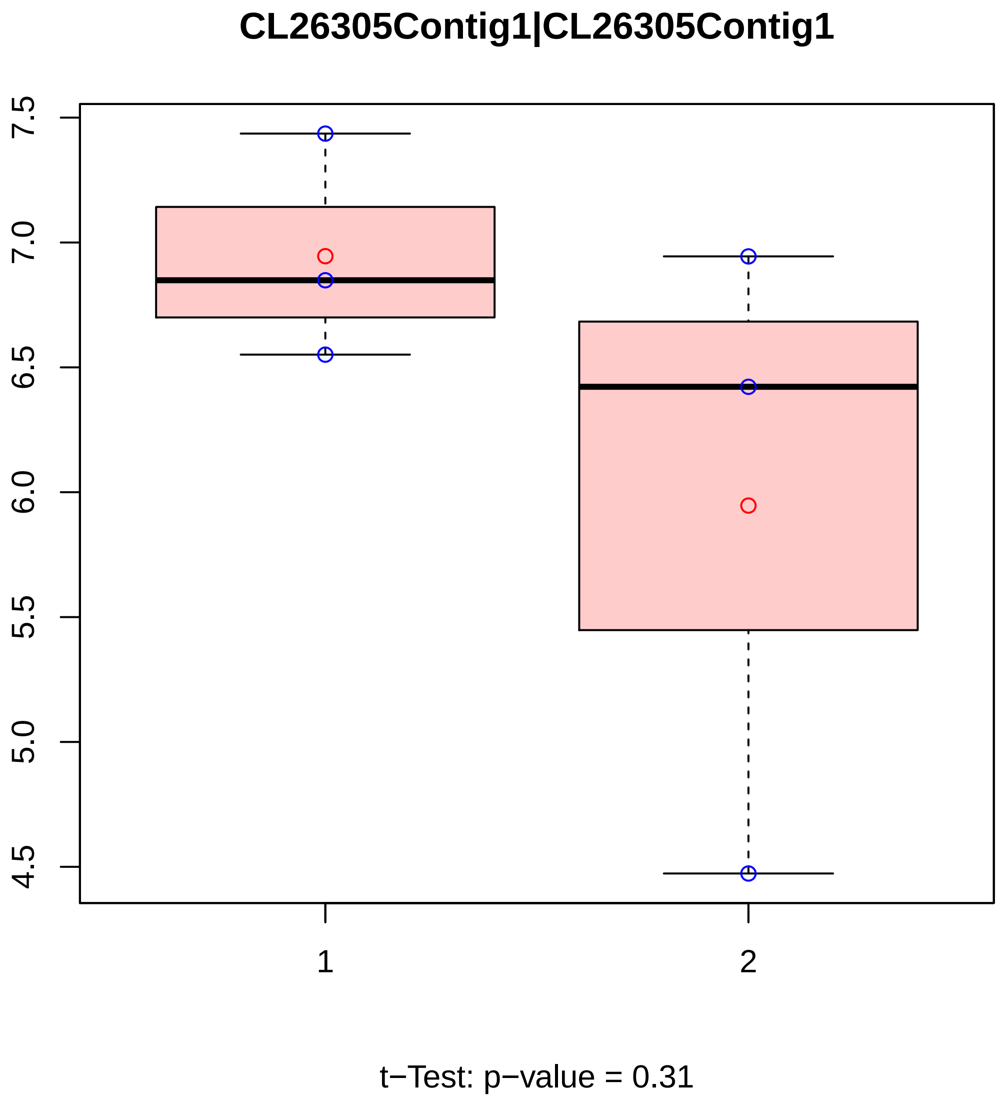
CL26305Contig1|CL26305Contig1
7.5
7.0
6.5
6.0
5.5
5.0
4.5
1
2
t−Test: p−value = 0.31

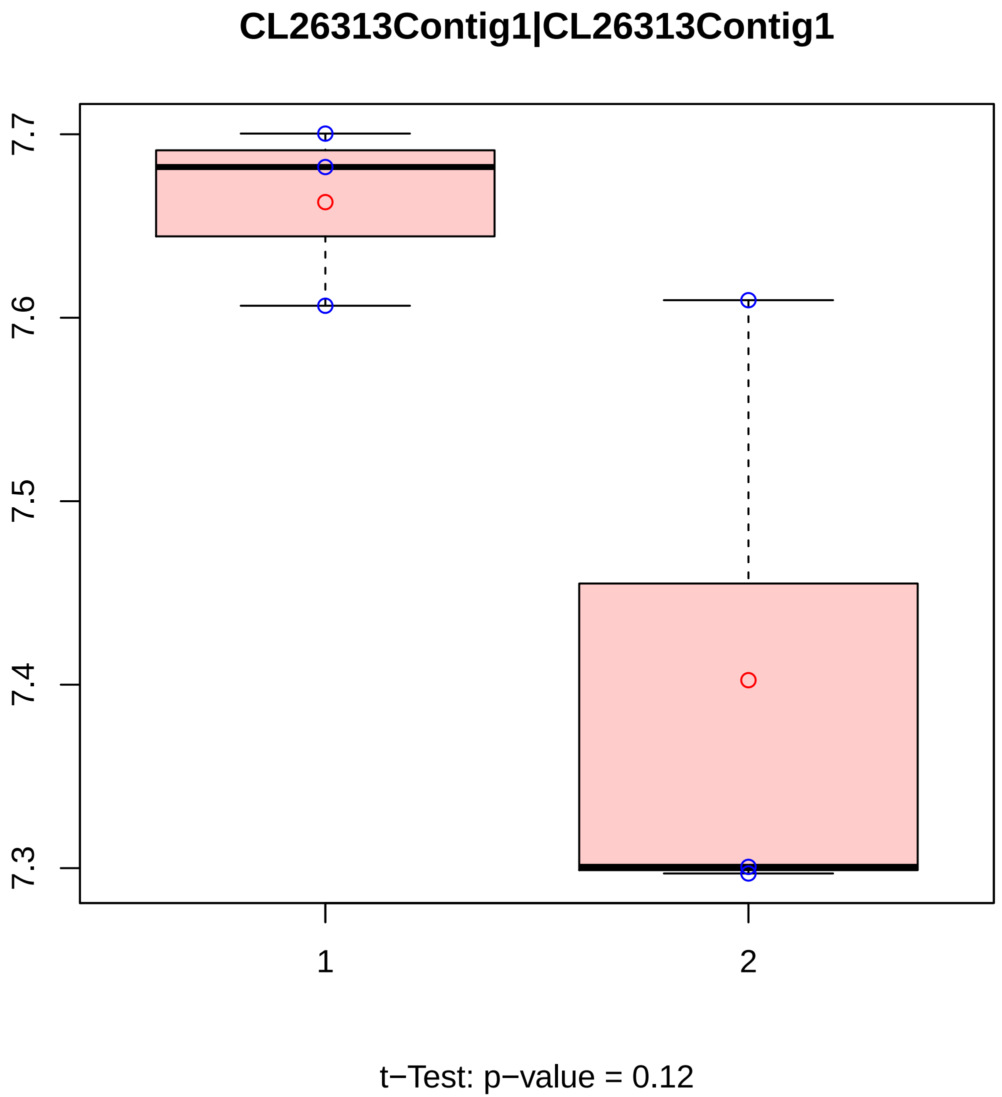
CL26313Contig1|CL26313Contig1
7.7
7.6
7.5
7.4
7.3
1
2
t−Test: p−value = 0.12

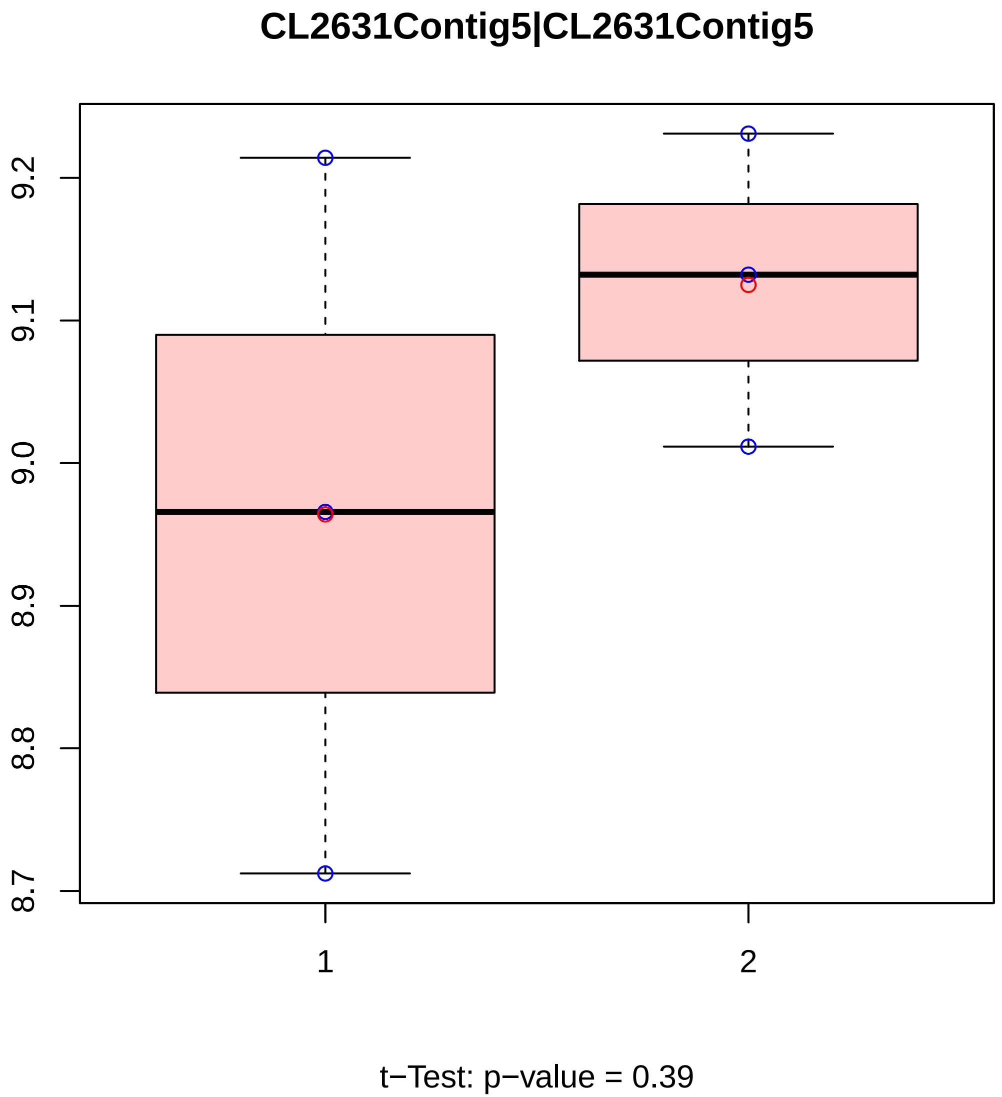
CL2631Contig5|CL2631Contig5
9.2
9.1
9.0
8.9
8.8
8.7
1
2
t−Test: p−value = 0.39

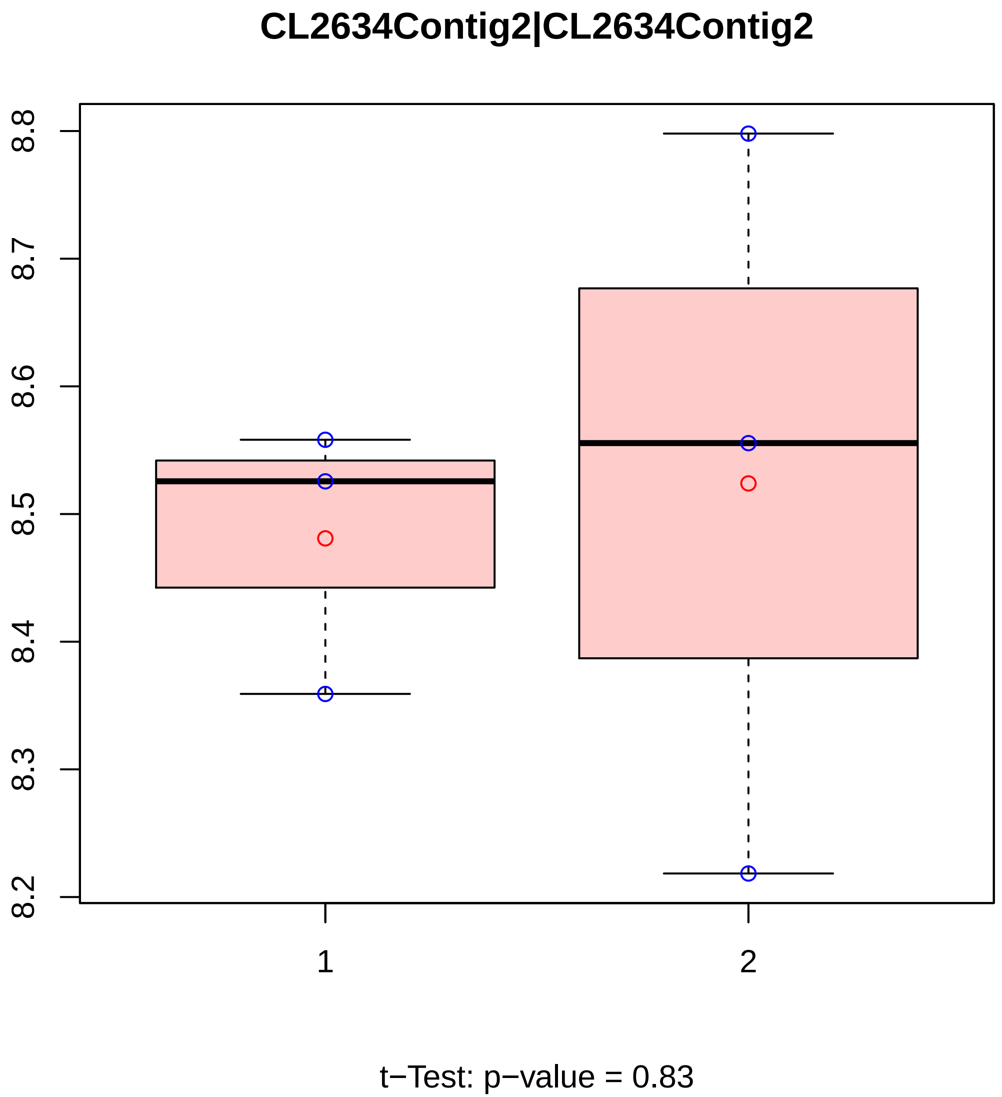
CL2634Contig2|CL2634Contig2
8.2
8.3
8.4
8.5
8.6
8.7
8.8
1
2
t−Test: p−value = 0.83

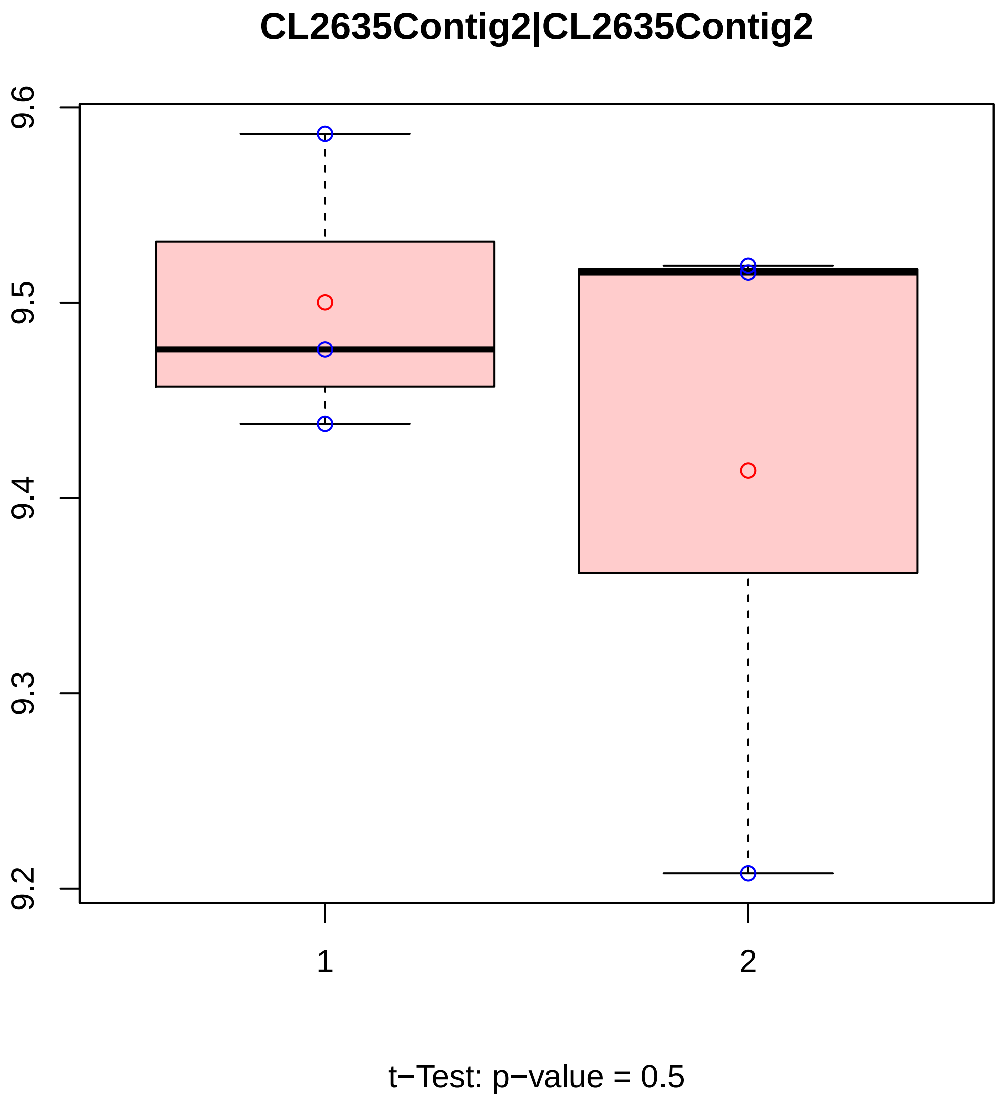
CL2635Contig2|CL2635Contig2
9.6
9.5
9.4
9.3
9.2
1
2
t−Test: p−value = 0.5

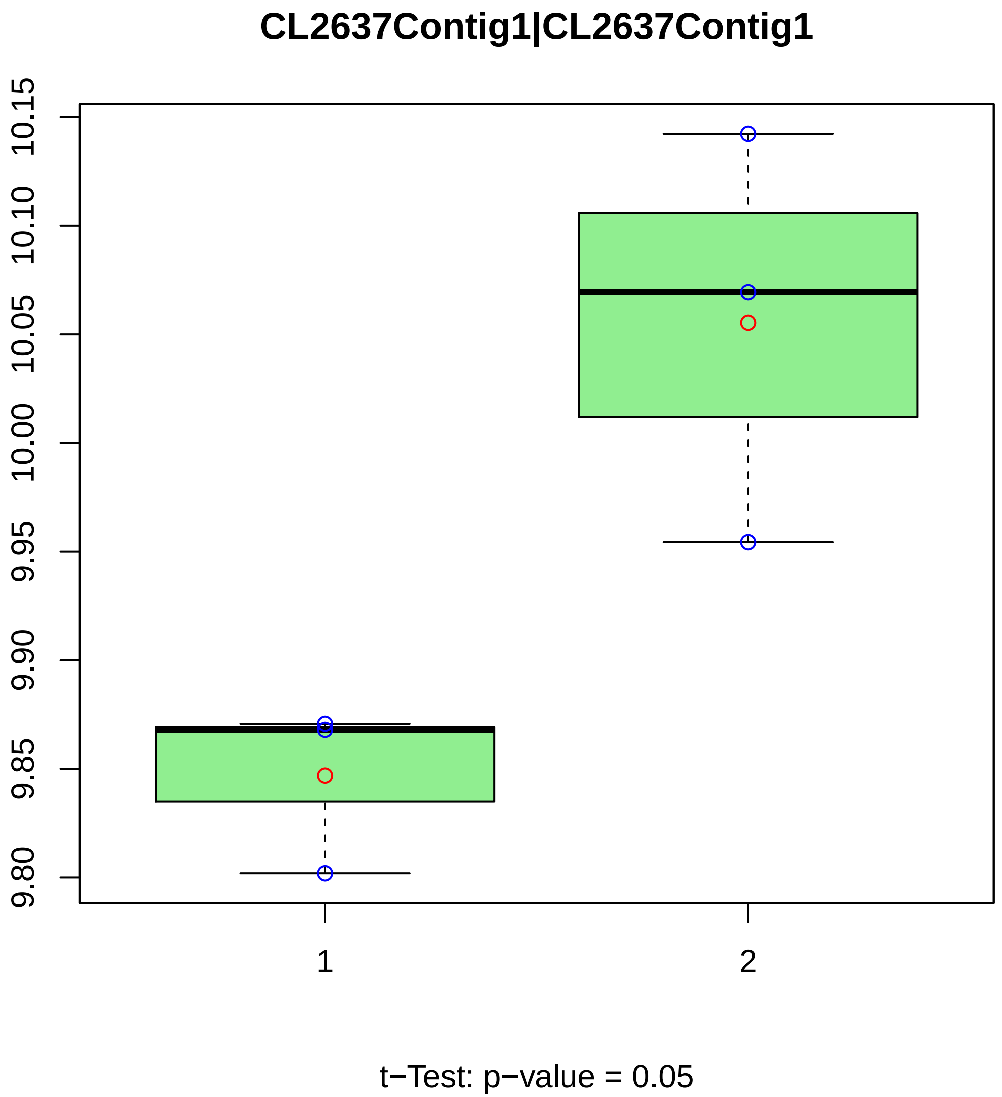
CL2637Contig1|CL2637Contig1
10.15
10.10
10.05
10.00
9.95
9.90
9.85
9.80
1
2
t−Test: p−value = 0.05

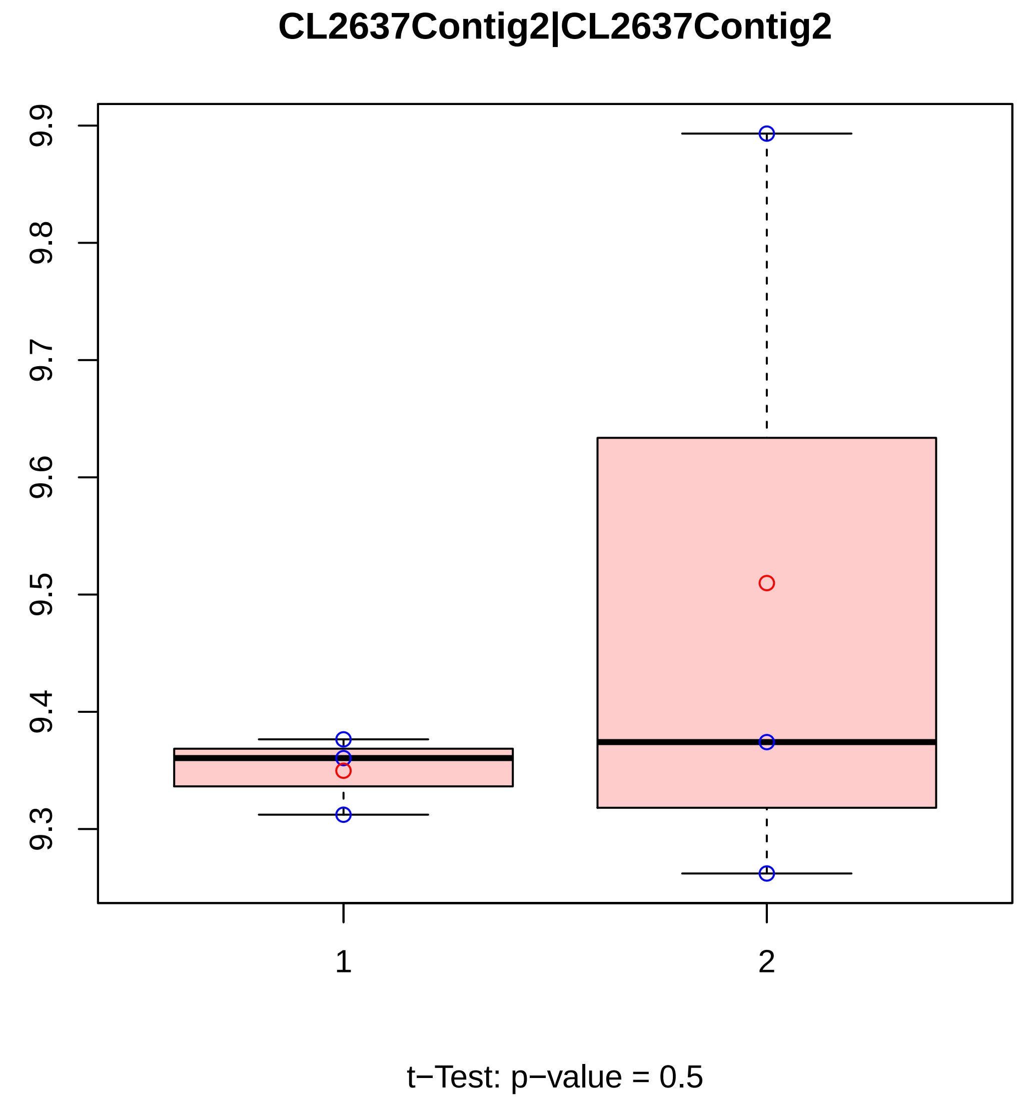
CL2637Contig2|CL2637Contig2
9.3
9.4
9.5
9.6
9.7
9.8
9.9
1
2
t−Test: p−value = 0.5

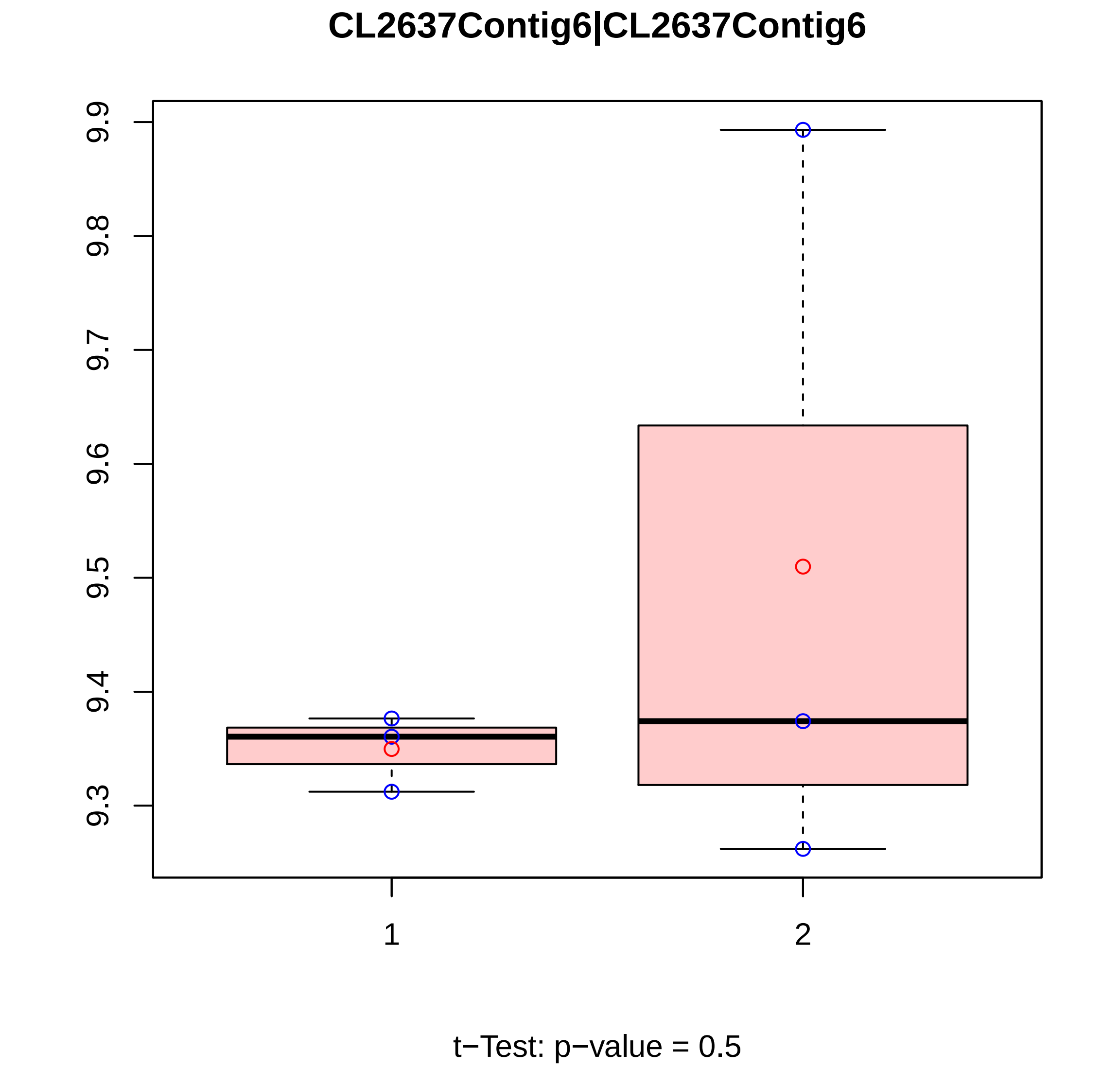
CL2637Contig6|CL2637Contig6
9.3
9.4
9.5
9.6
9.7
9.8
9.9
1
2
t−Test: p−value = 0.5

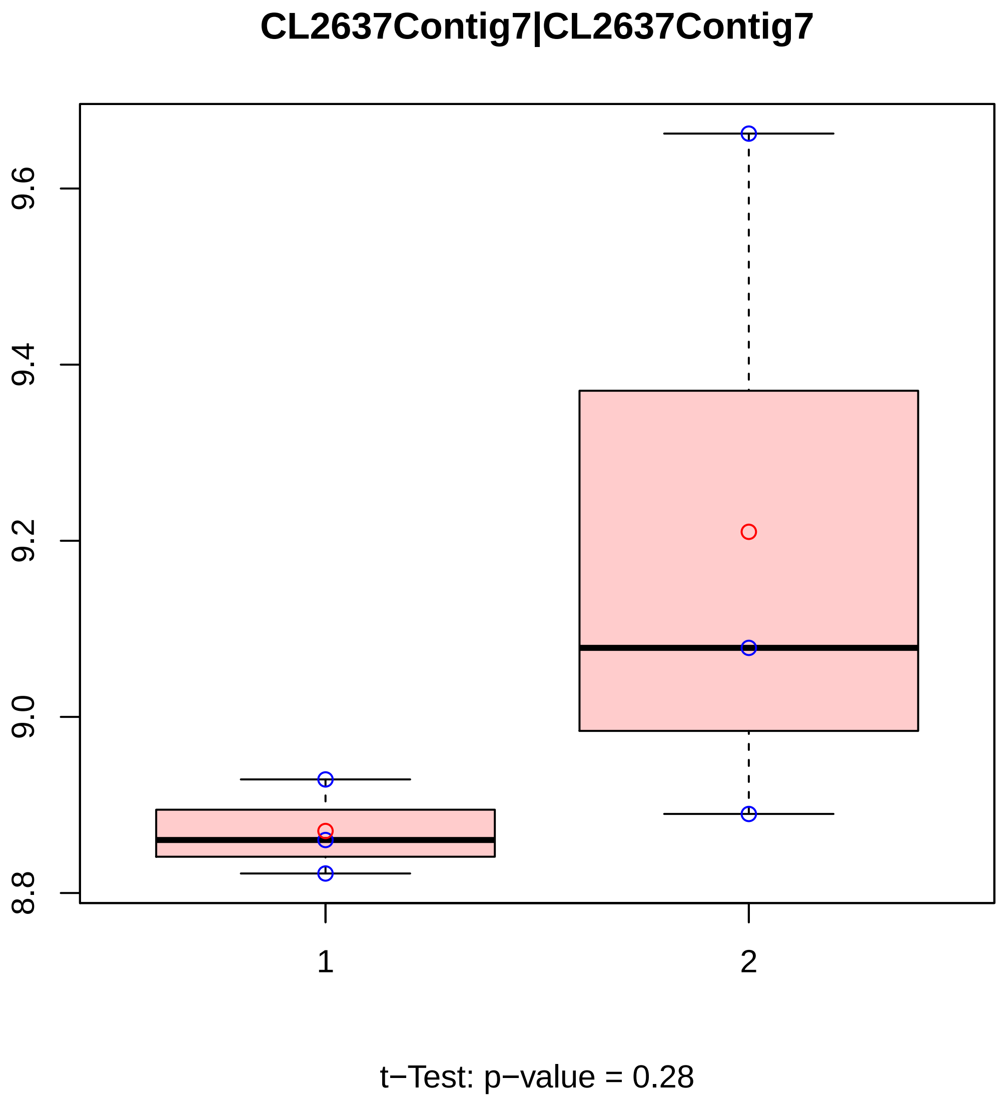
CL2637Contig7|CL2637Contig7
9.6
9.4
9.2
9.0
8.8
1
2
t−Test: p−value = 0.28

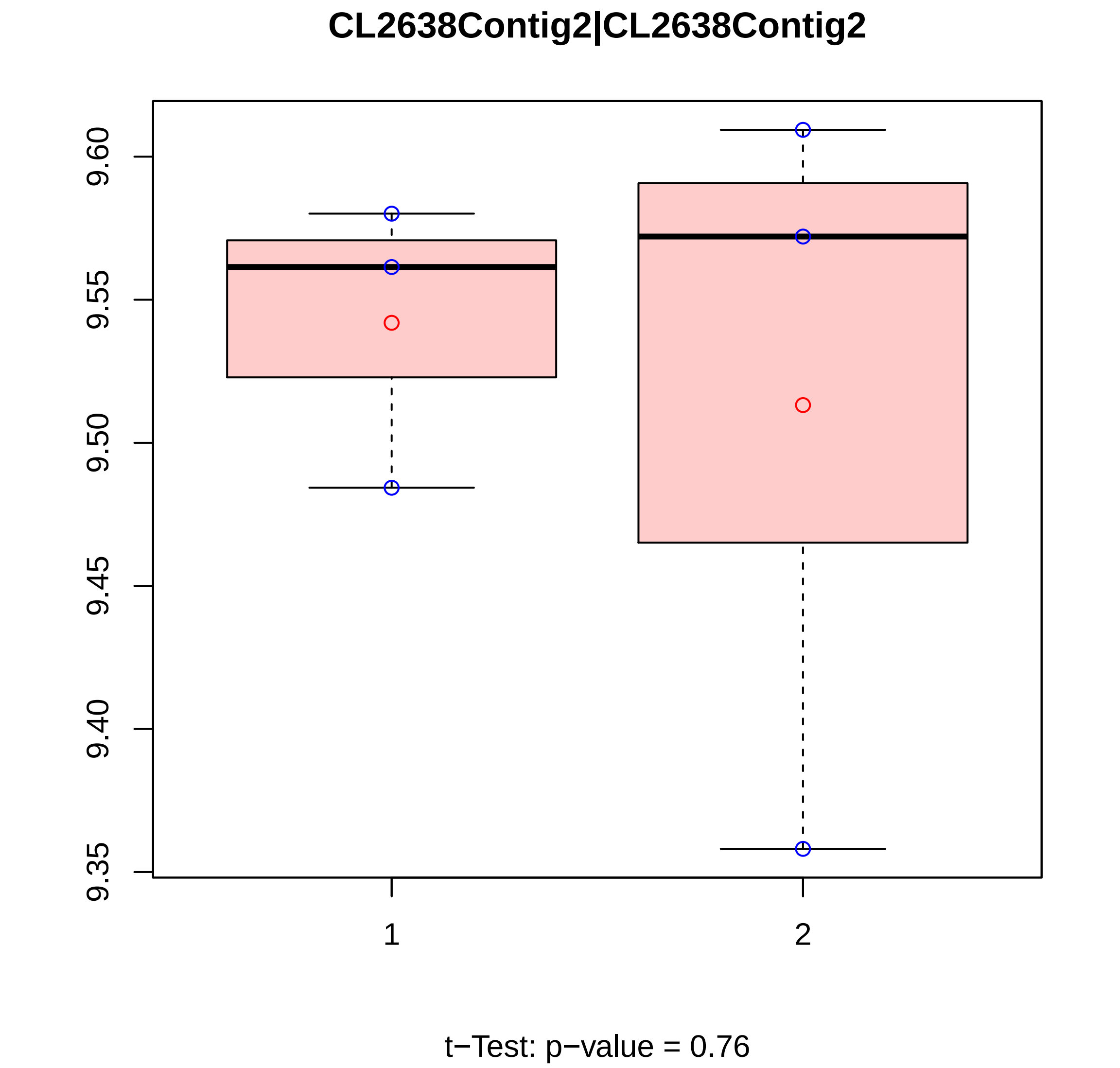
CL2638Contig2|CL2638Contig2
9.60
9.55
9.50
9.45
9.40
9.35
1
2
t−Test: p−value = 0.76

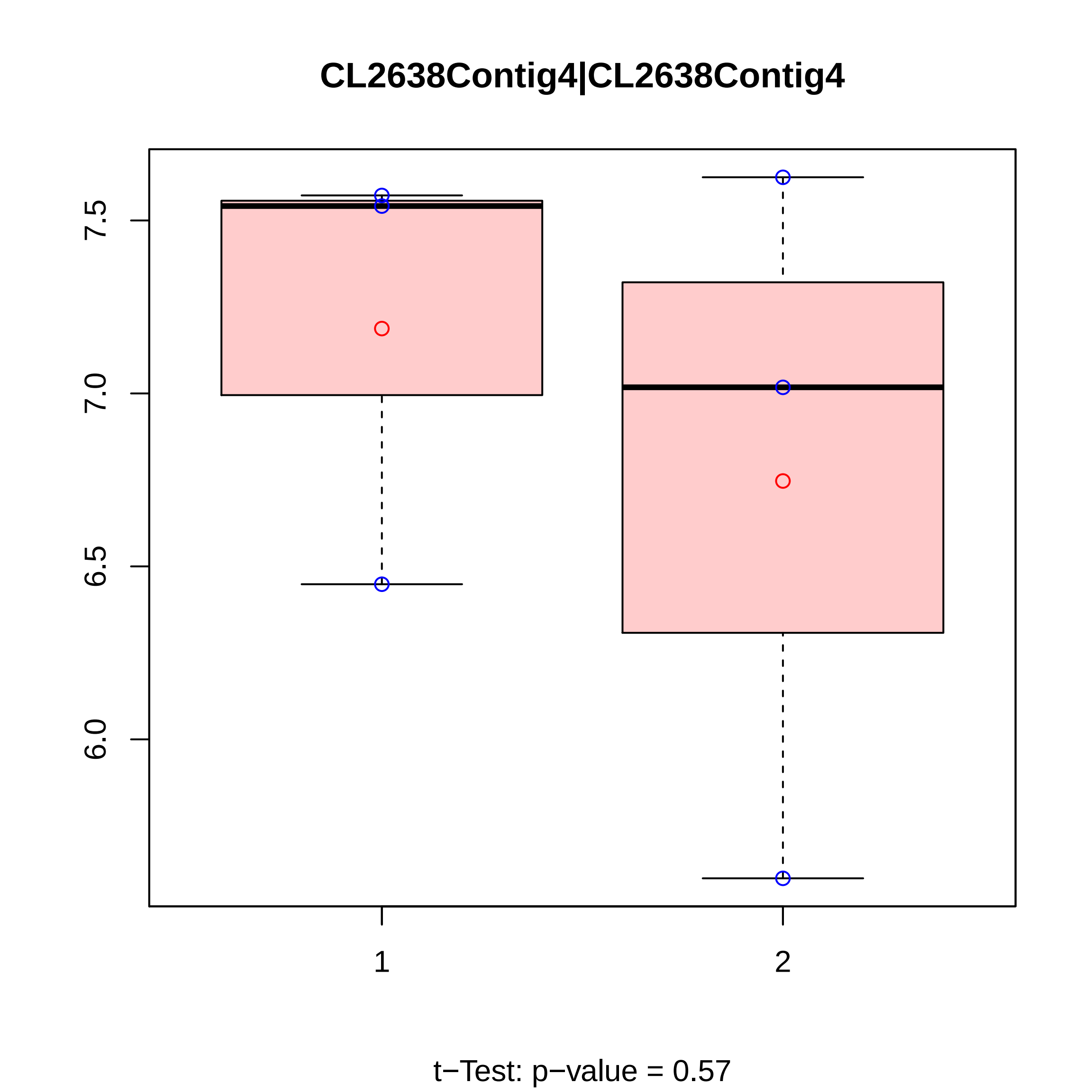
CL2638Contig4|CL2638Contig4
7.5
7.0
6.5
6.0
1
2
t−Test: p−value = 0.57

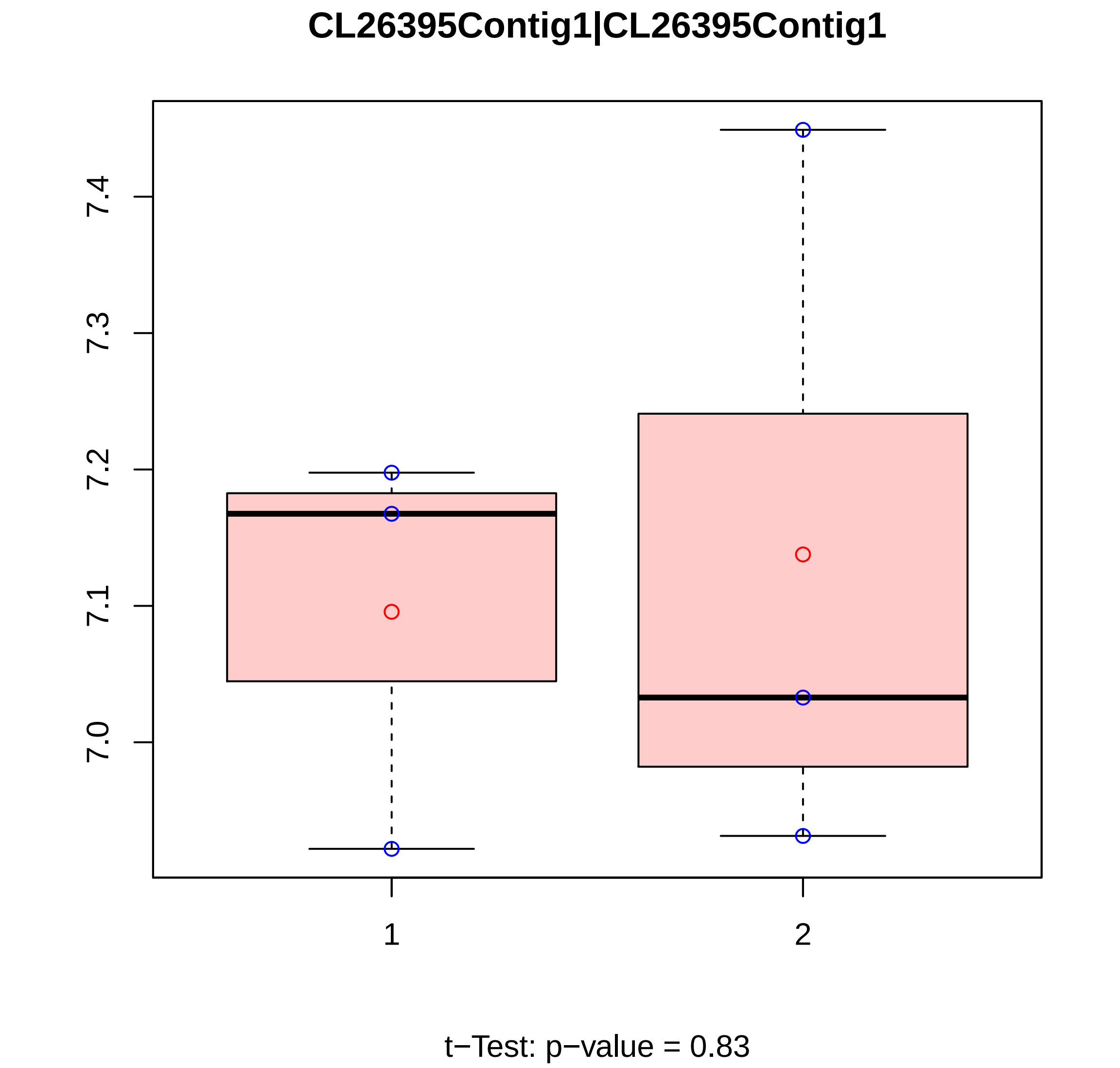
CL26395Contig1|CL26395Contig1
7.0
7.1
7.2
7.3
7.4
1
2
t−Test: p−value = 0.83

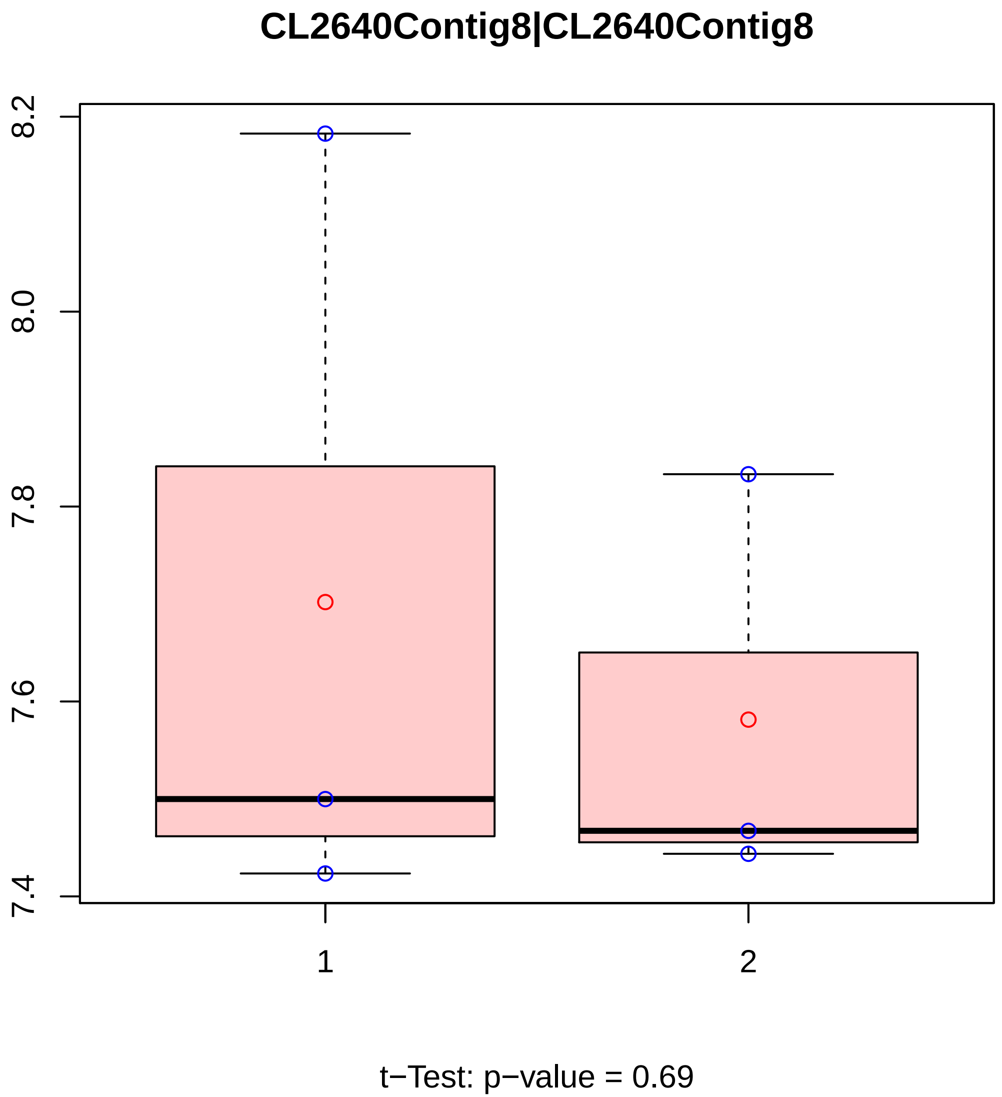
CL2640Contig8|CL2640Contig8
8.2
8.0
7.8
7.6
7.4
1
2
t−Test: p−value = 0.69

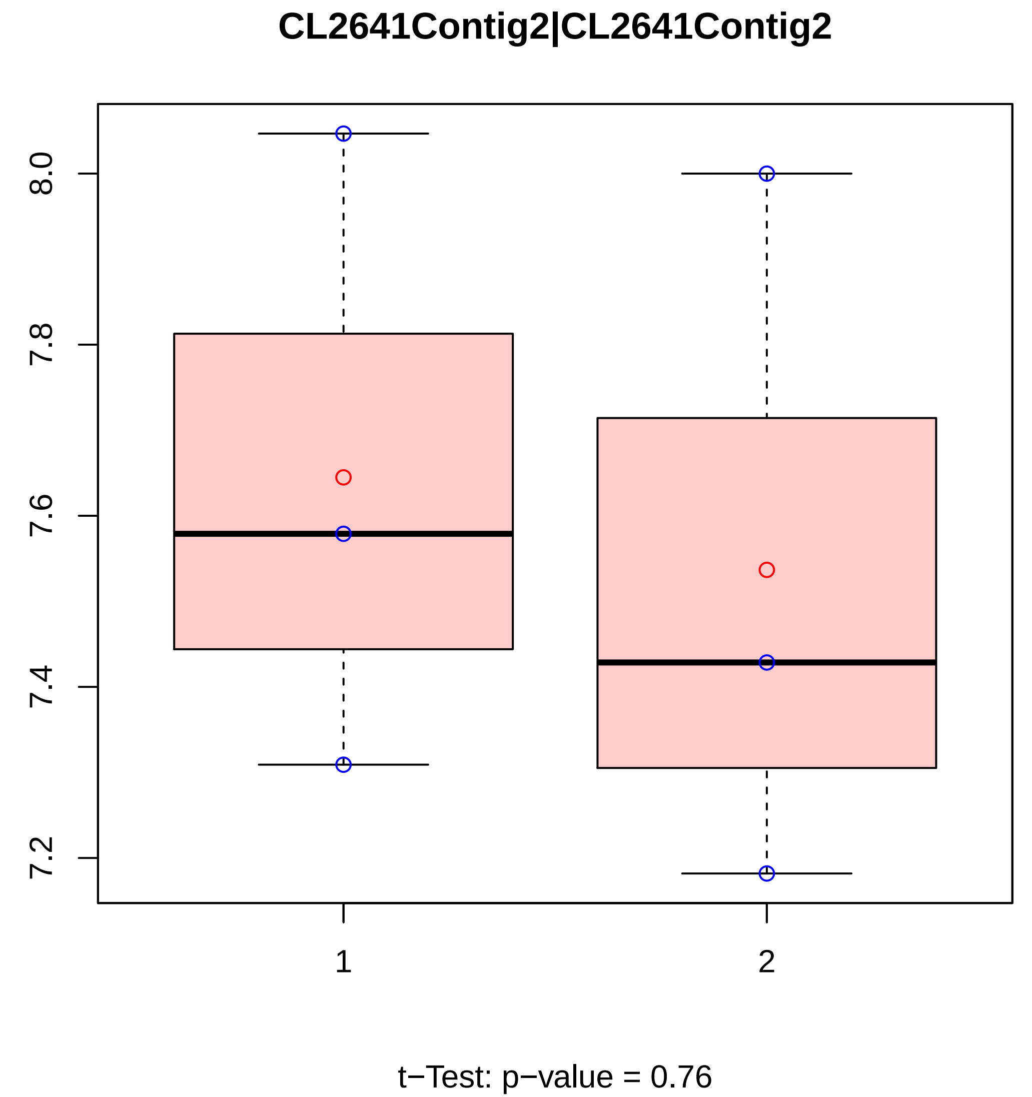
CL2641Contig2|CL2641Contig2
8.0
7.8
7.6
7.4
7.2
1
2
t−Test: p−value = 0.76

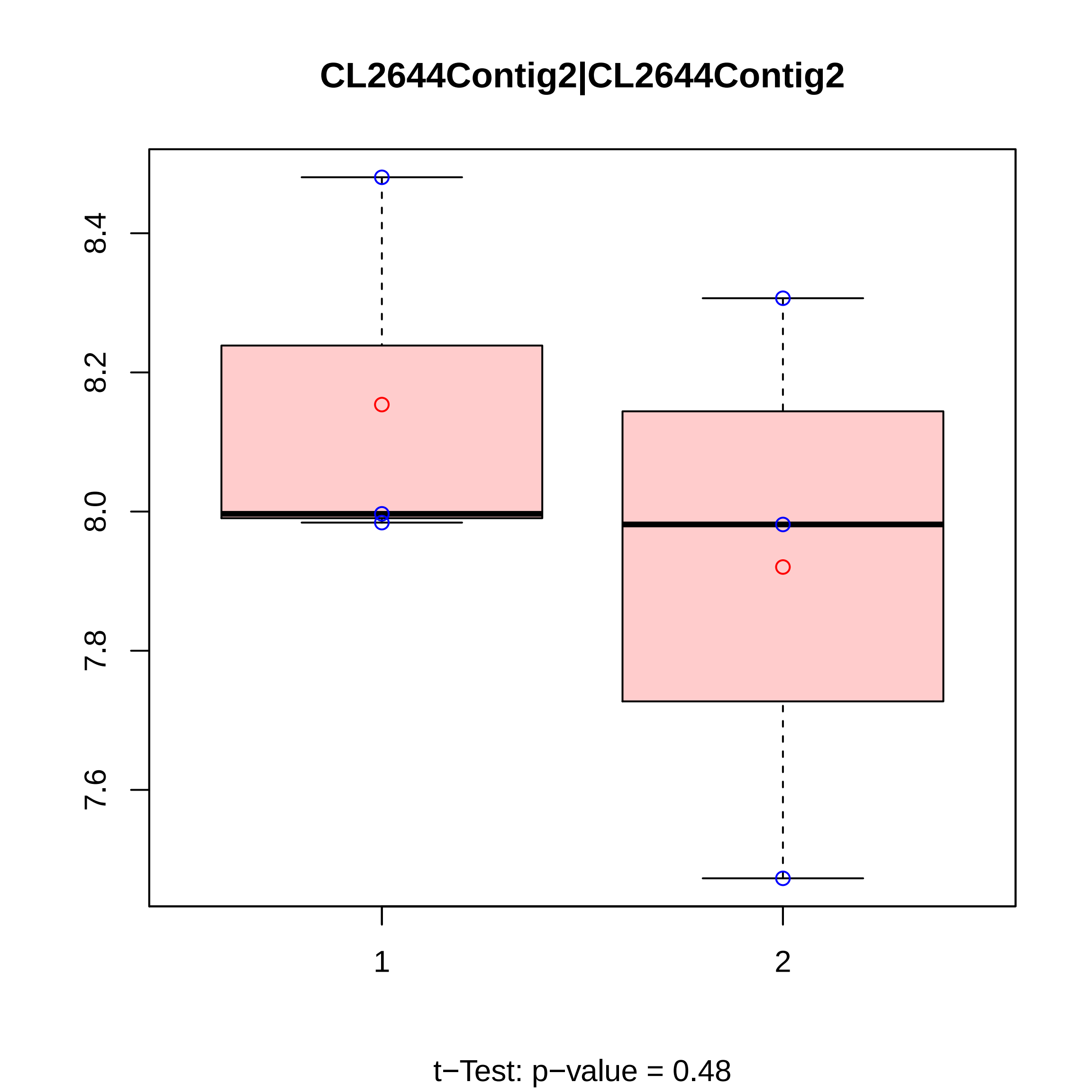
CL2644Contig2|CL2644Contig2
8.4
8.2
8.0
7.8
7.6
1
2
t−Test: p−value = 0.48

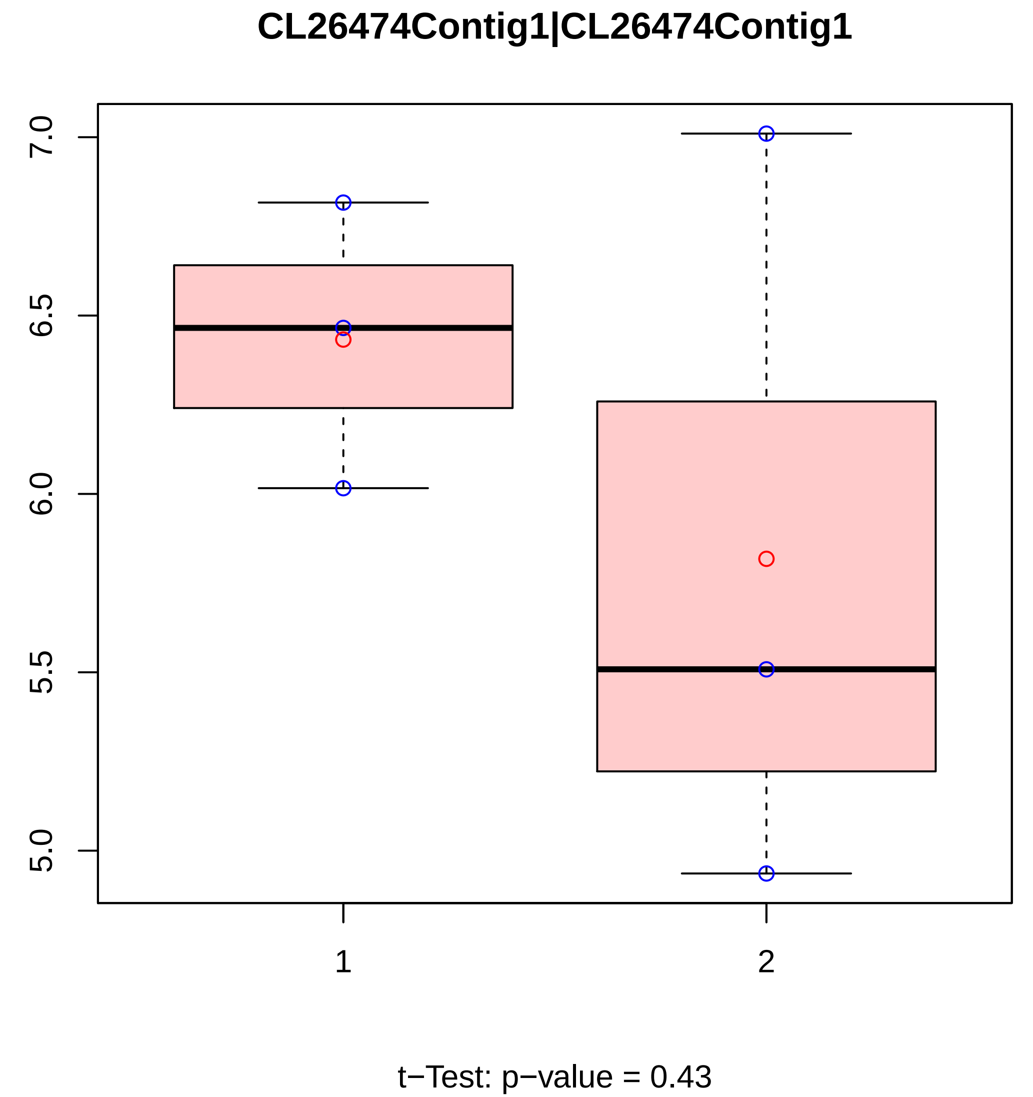
CL26474Contig1|CL26474Contig1
7.0
6.5
6.0
5.5
5.0
1
2
t−Test: p−value = 0.43

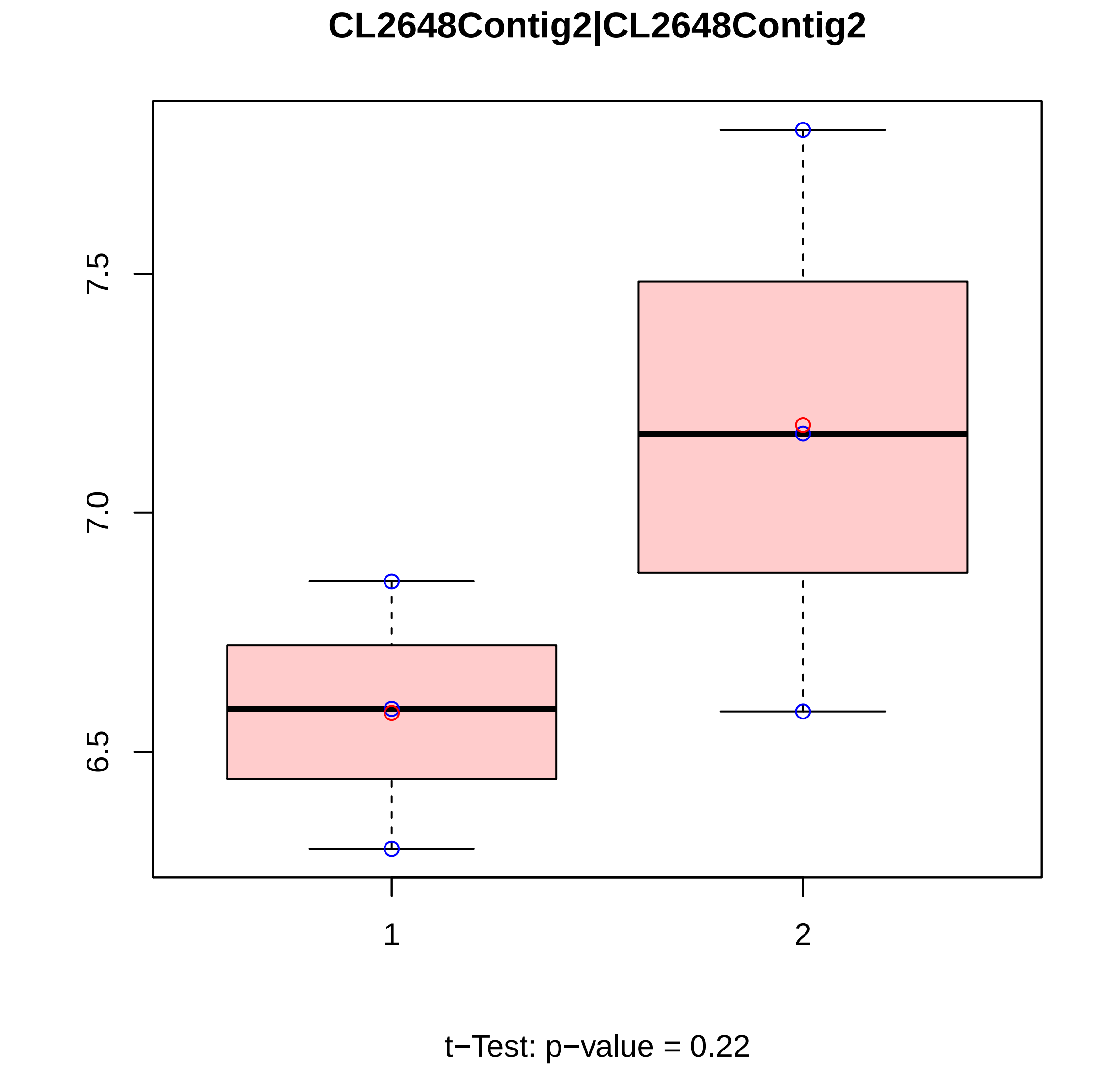

CL2648Contig2|CL2648Contig2
6.5
7.0
7.5
1
2
t−Test: p−value = 0.22

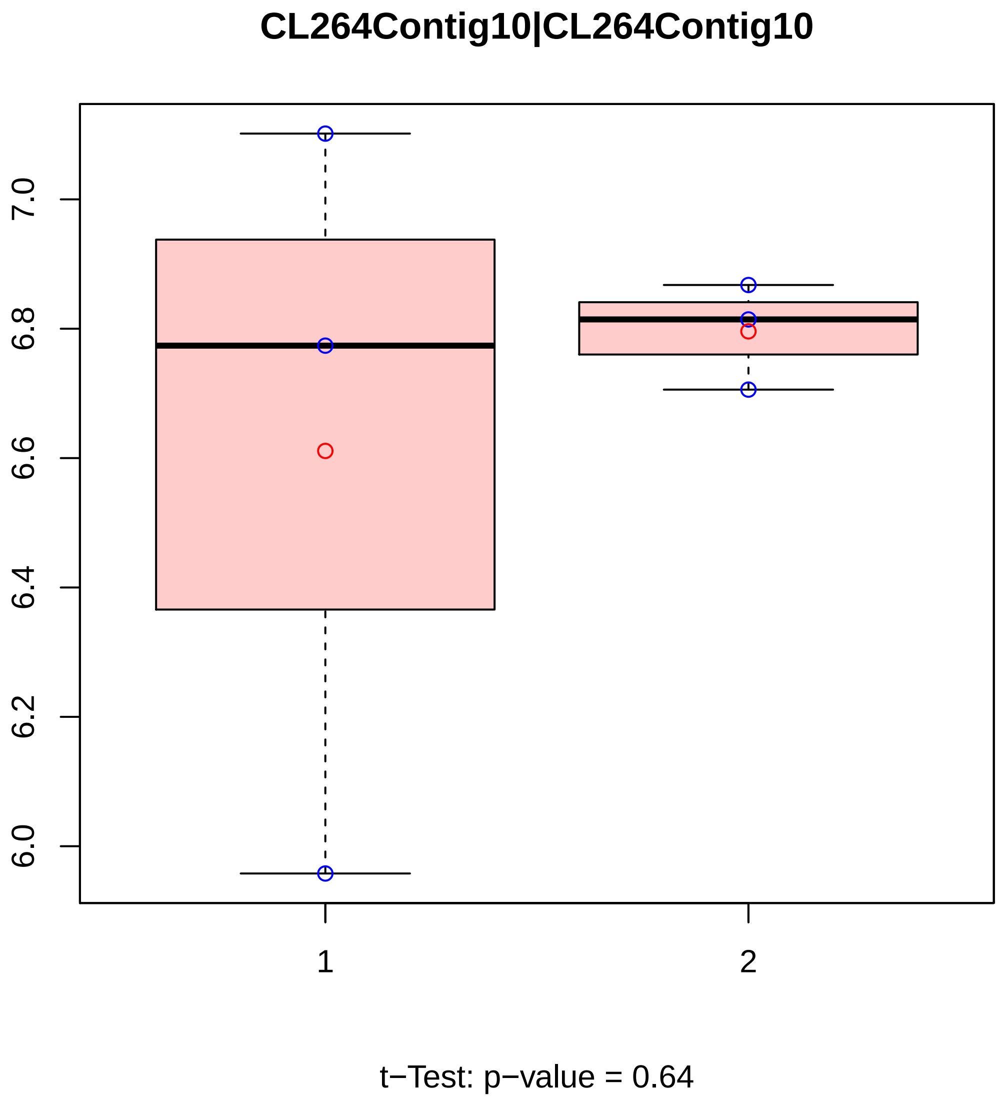
CL264Contig10|CL264Contig10
7.0
6.8
6.6
6.4
6.2
6.0
1
2
t−Test: p−value = 0.64

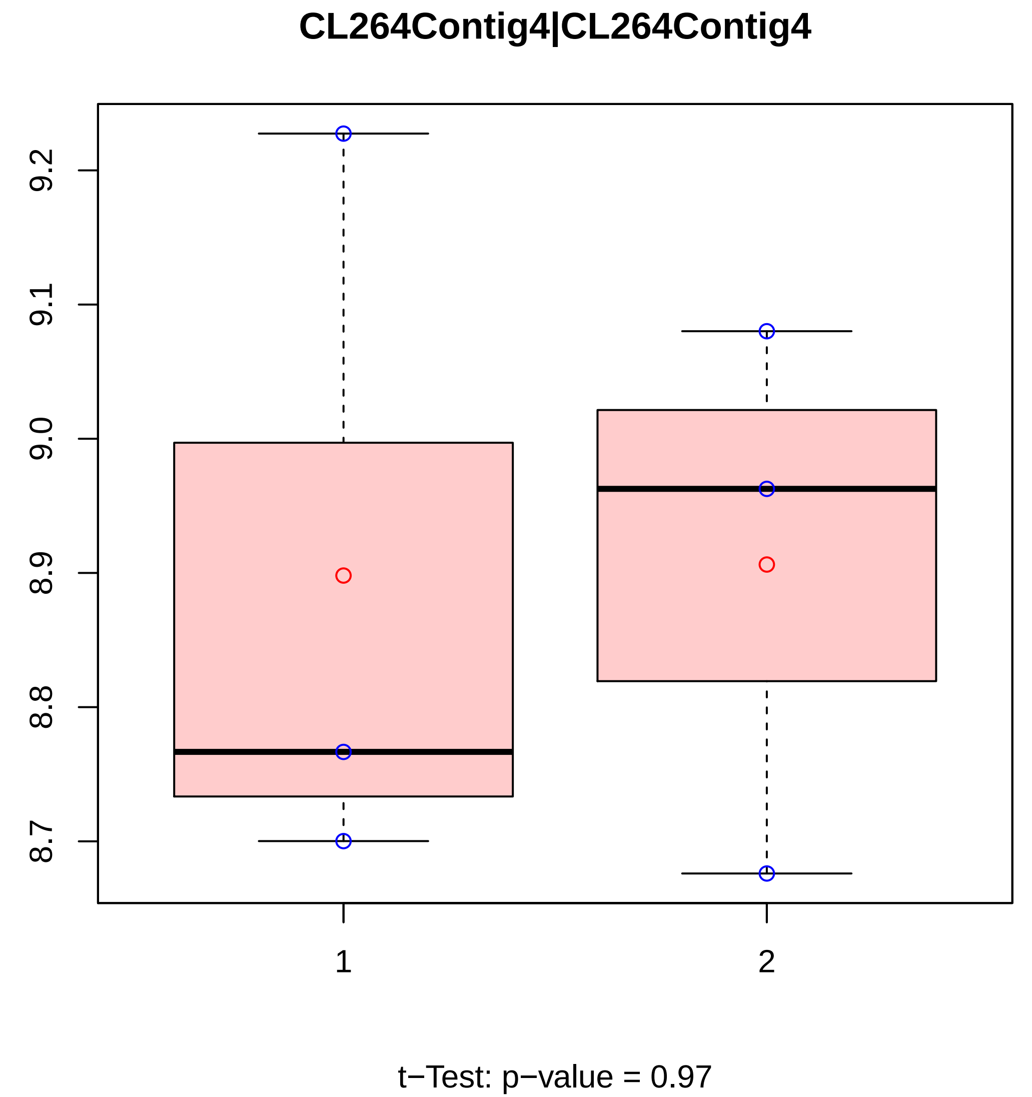

CL264Contig4|CL264Contig4
9.2
9.1
9.0
8.9
8.8
8.7
1
2
t−Test: p−value = 0.97

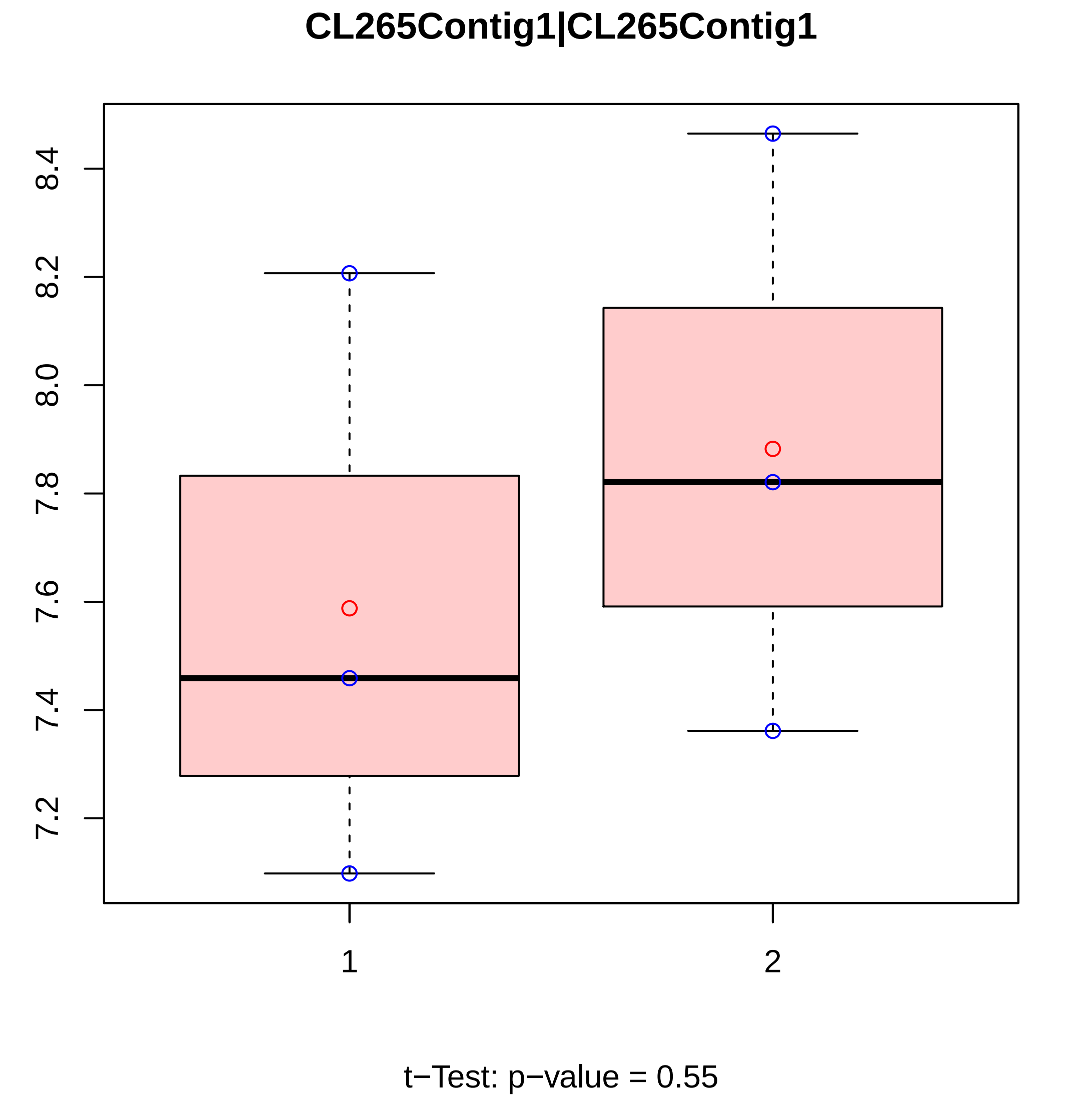
CL265Contig1|CL265Contig1
8.4
8.2
8.0
7.8
7.6
7.4
7.2
1
2
t−Test: p−value = 0.55

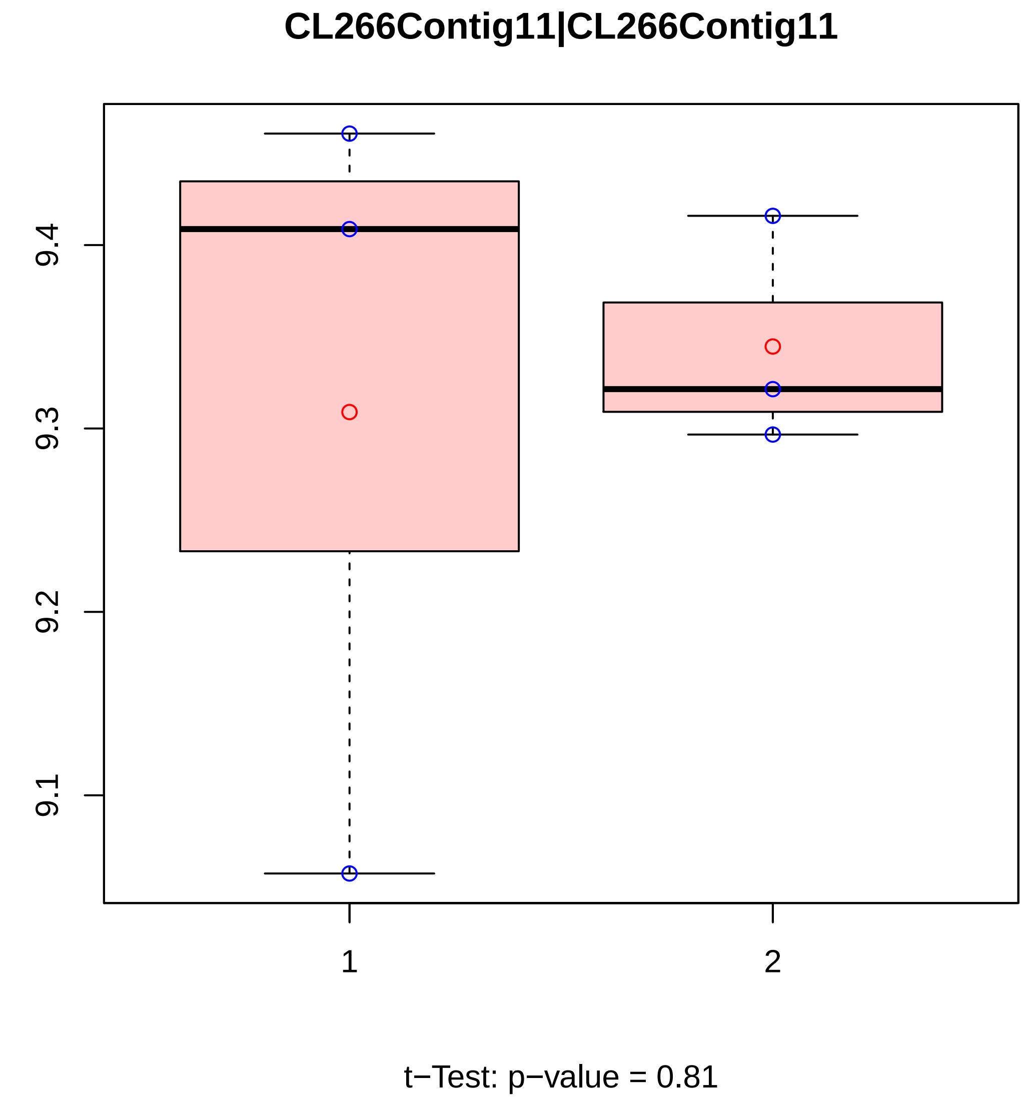
CL266Contig11|CL266Contig11
9.1
9.2
9.3
9.4
1
2
t−Test: p−value = 0.81

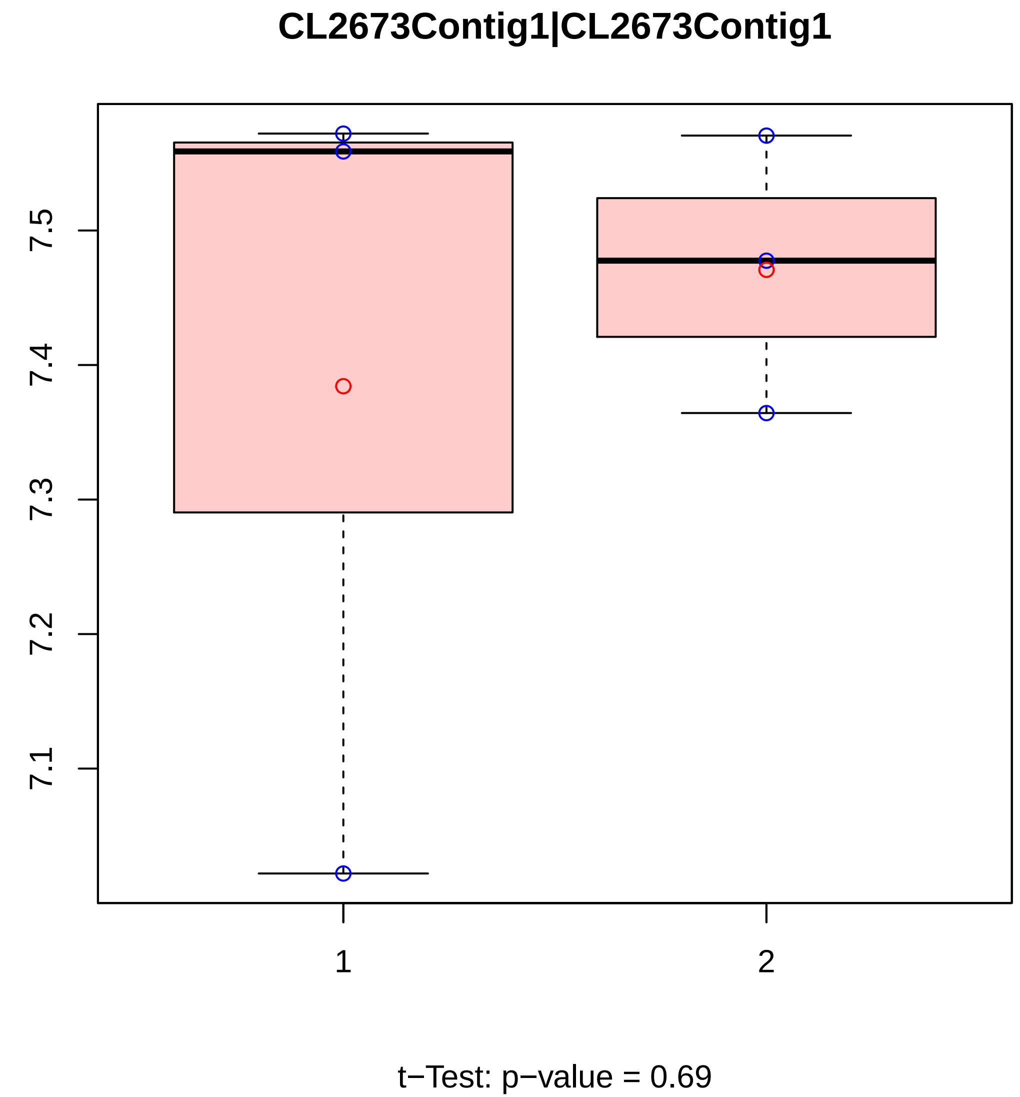
CL2673Contig1|CL2673Contig1
7.1
7.2
7.3
7.4
7.5
1
2
t−Test: p−value = 0.69

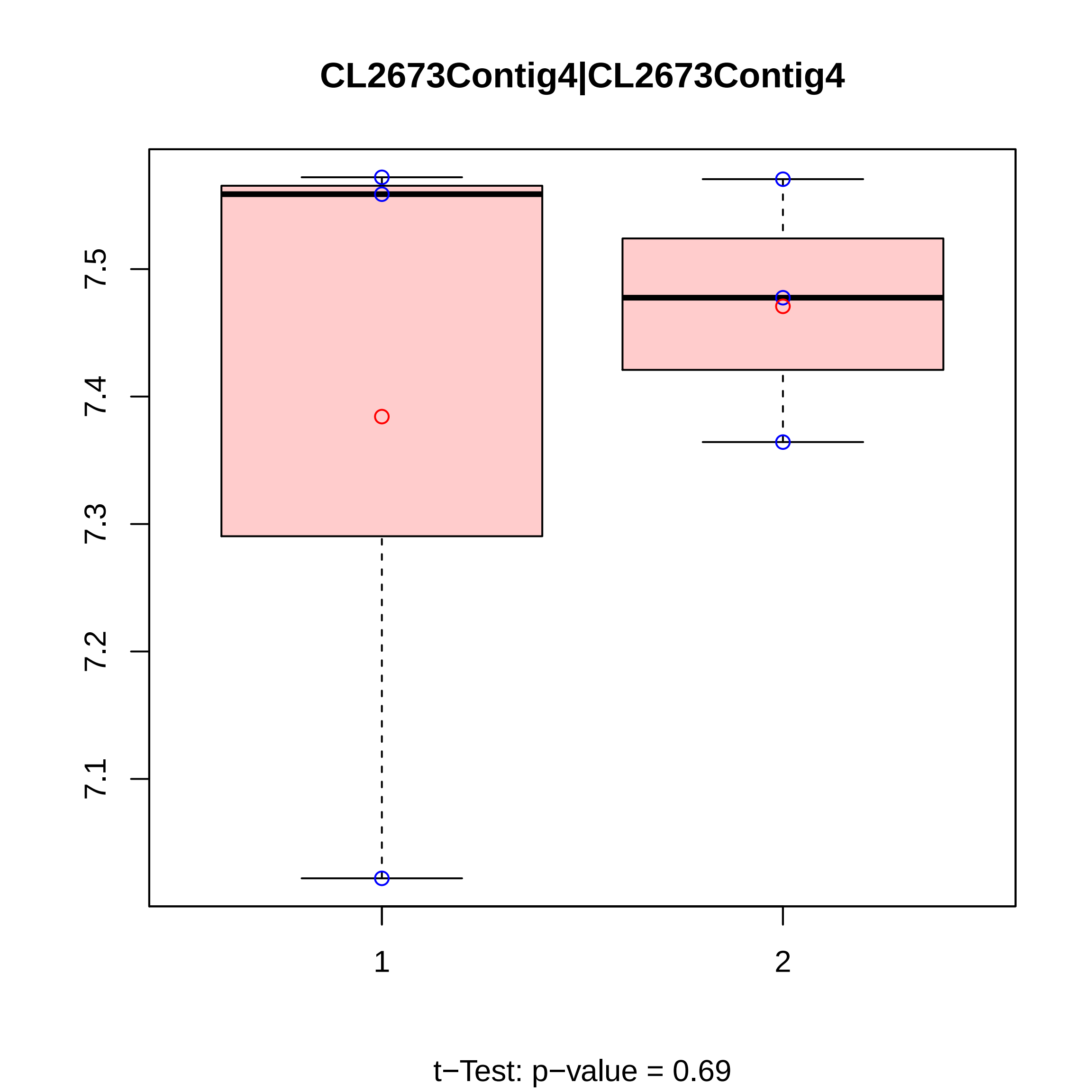
CL2673Contig4|CL2673Contig4
7.1
7.2
7.3
7.4
7.5
1
2
t−Test: p−value = 0.69

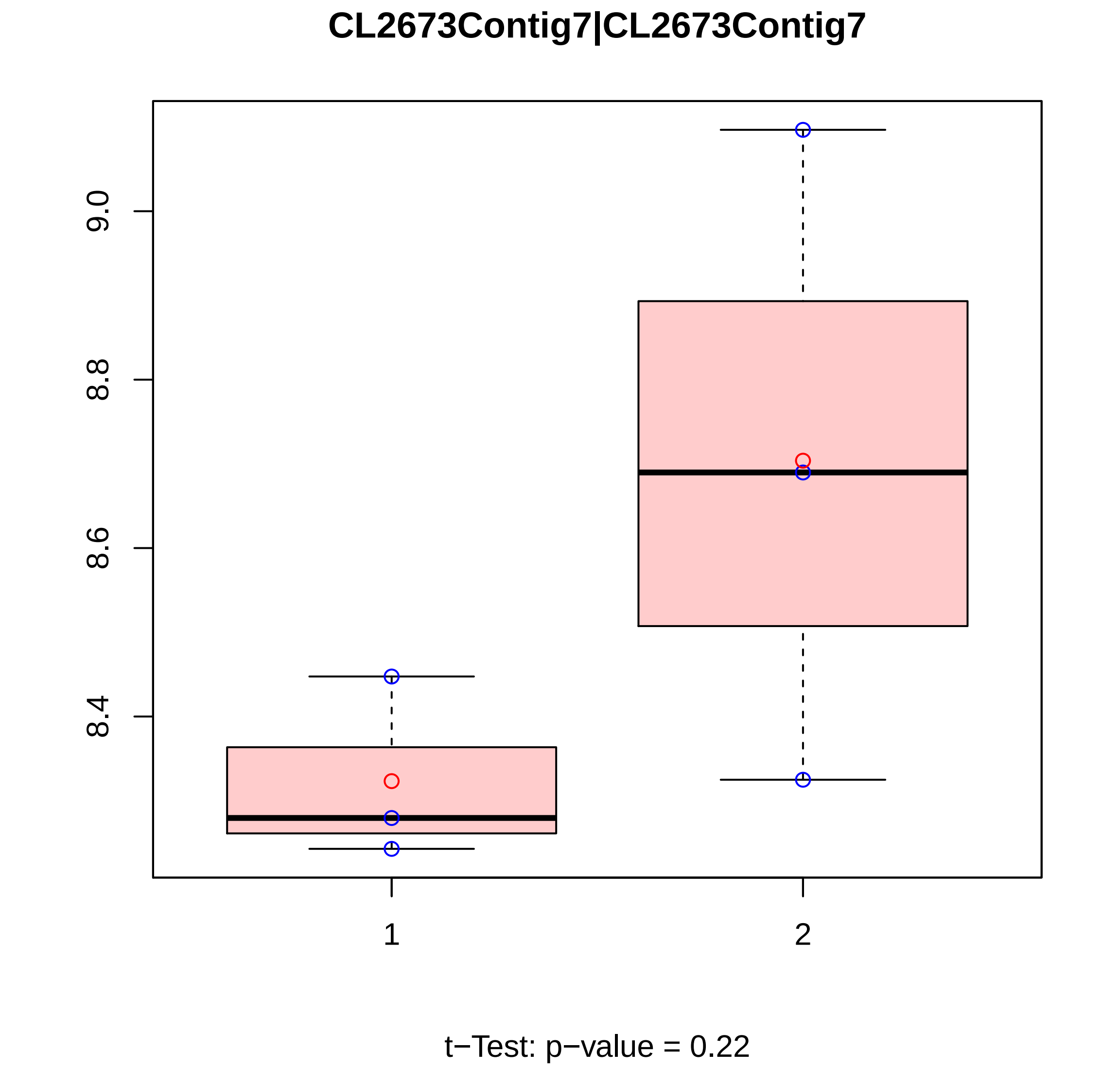
CL2673Contig7|CL2673Contig7
9.0
8.8
8.6
8.4
1
2
t−Test: p−value = 0.22

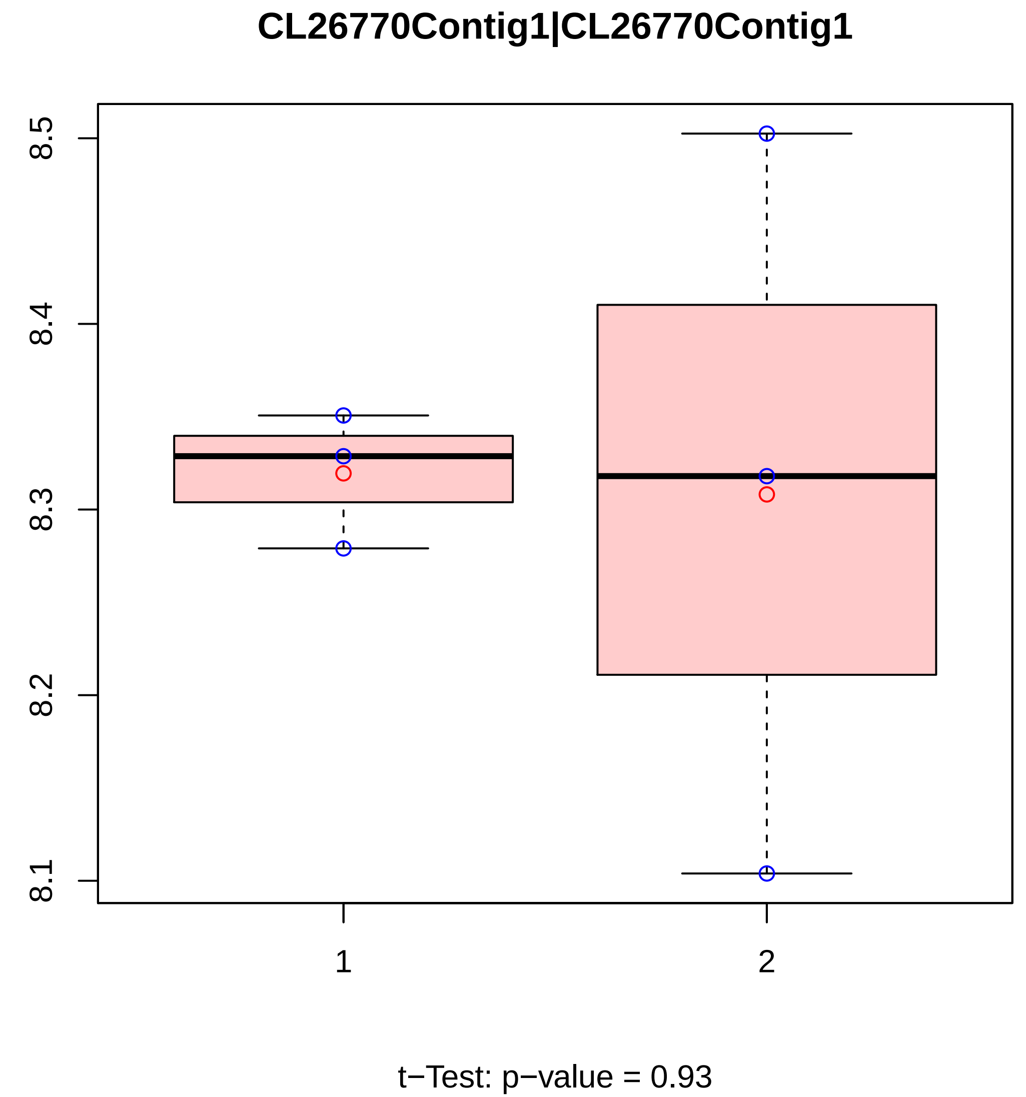
CL26770Contig1|CL26770Contig1
8.5
8.4
8.3
8.2
8.1
1
2
t−Test: p−value = 0.93

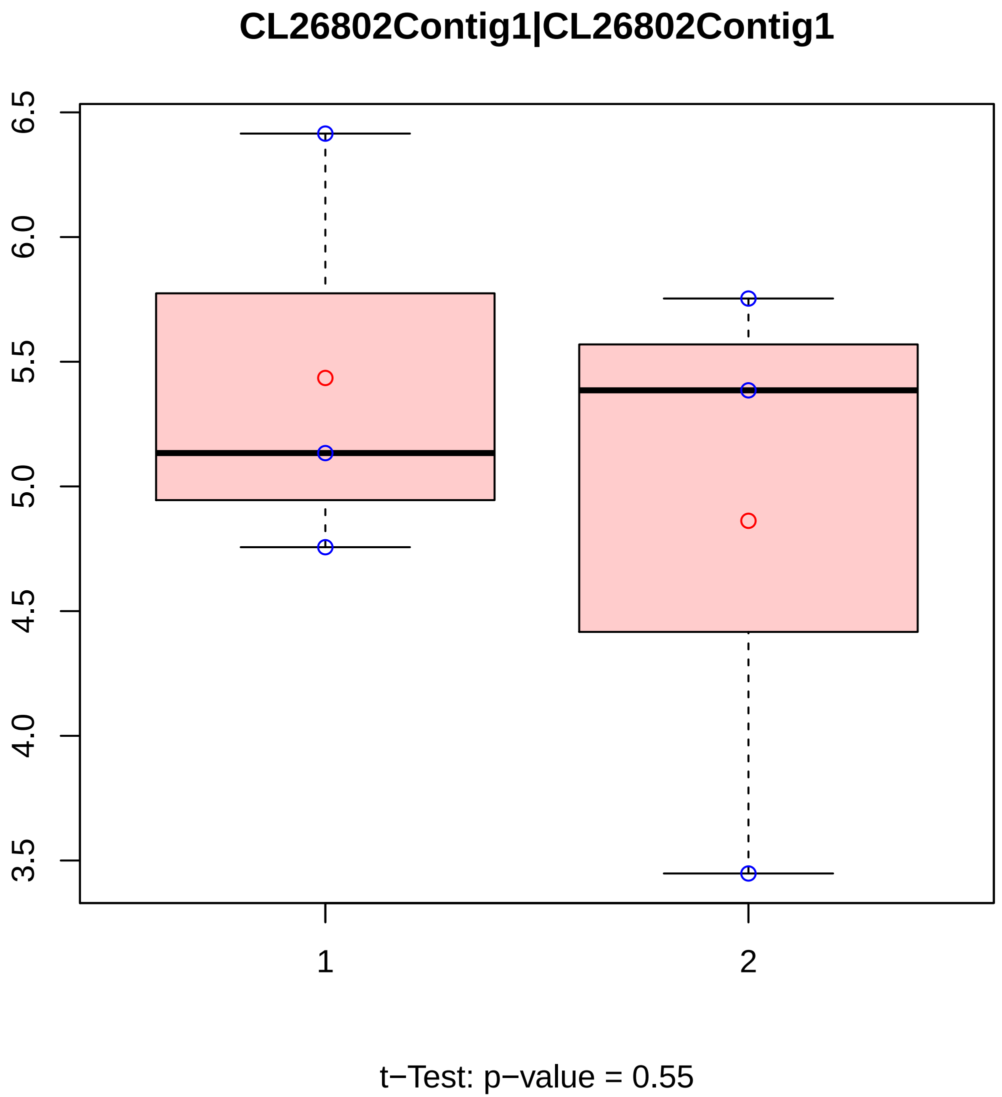

CL26802Contig1|CL26802Contig1
6.5
6.0
5.5
5.0
4.5
4.0
3.5
1
2
t−Test: p−value = 0.55

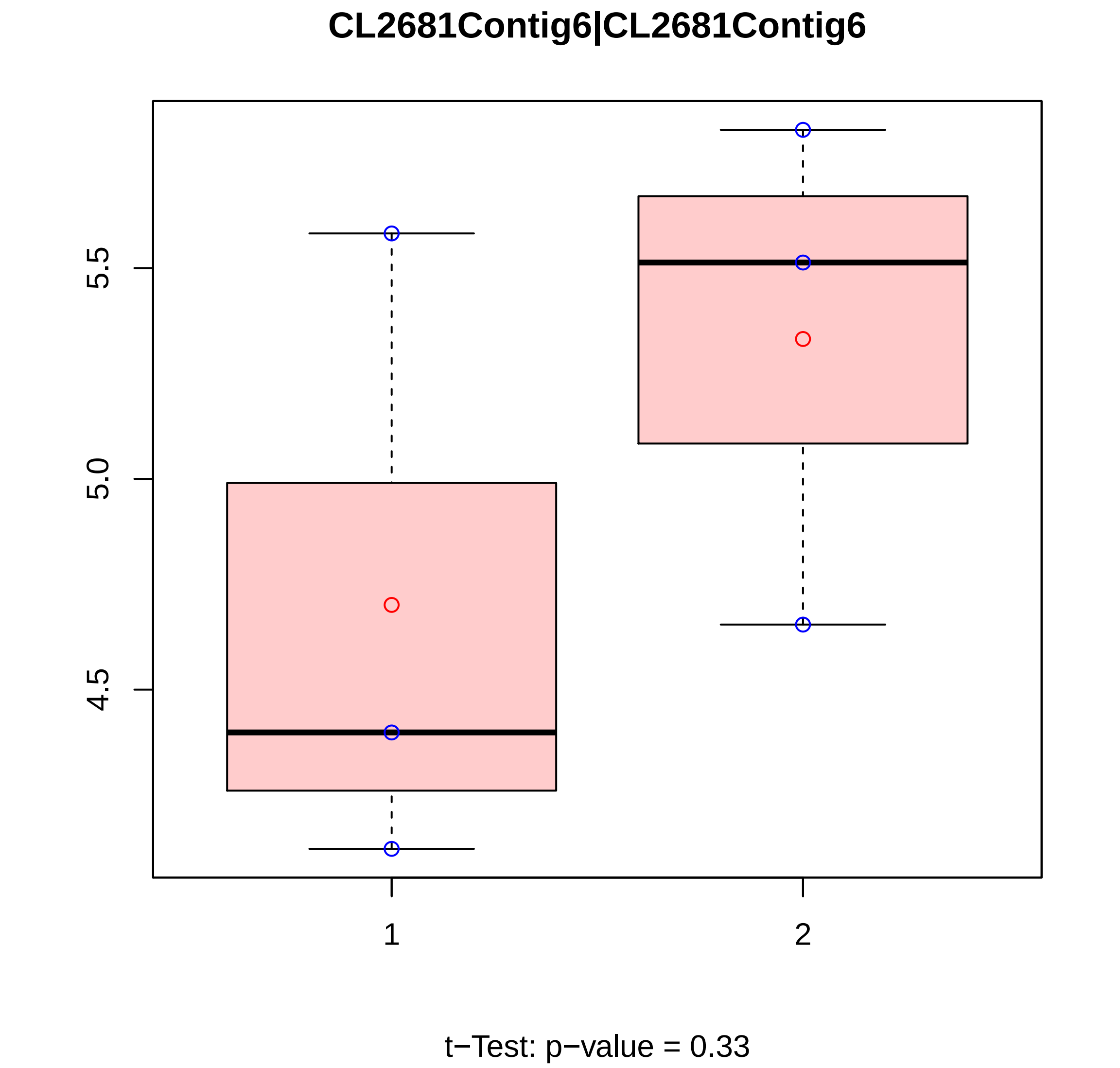
CL2681Contig6|CL2681Contig6
5.5
5.0
4.5
1
2
t−Test: p−value = 0.33

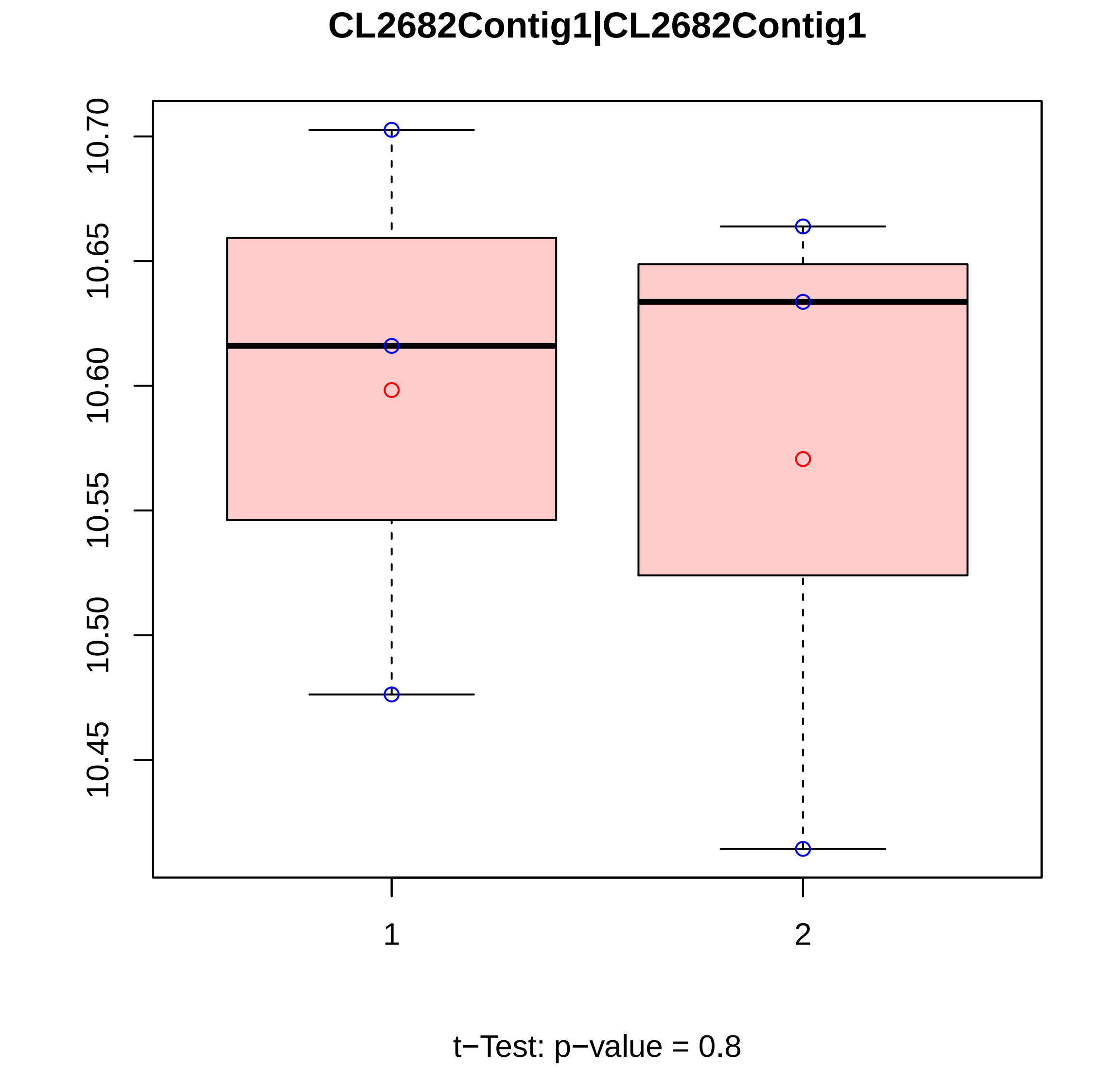
CL2682Contig1|CL2682Contig1
10.70
10.65
10.60
10.55
10.50
10.45
1
2
t−Test: p−value = 0.8

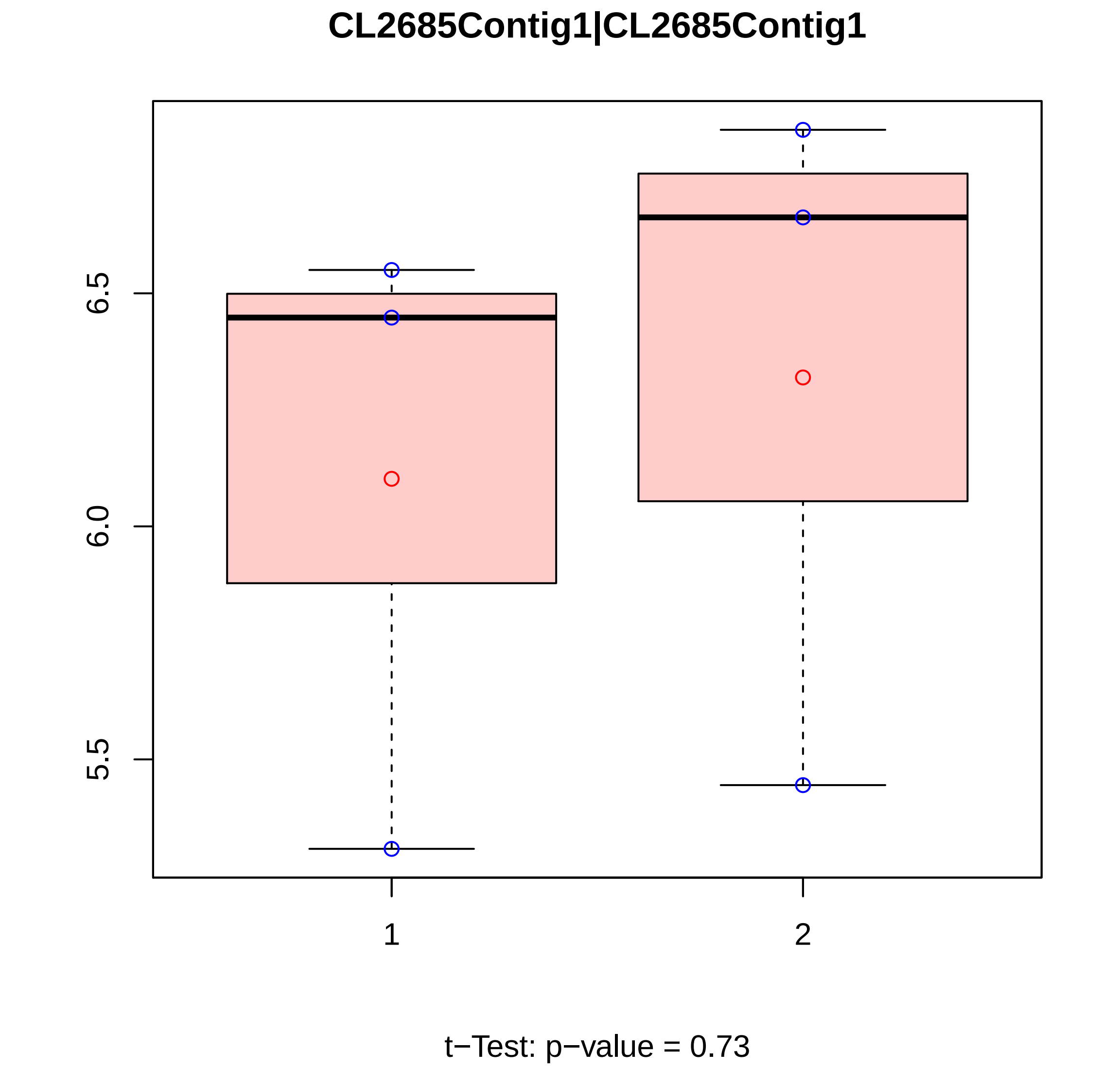

CL2685Contig1|CL2685Contig1
6.5
6.0
5.5
1
2
t−Test: p−value = 0.73

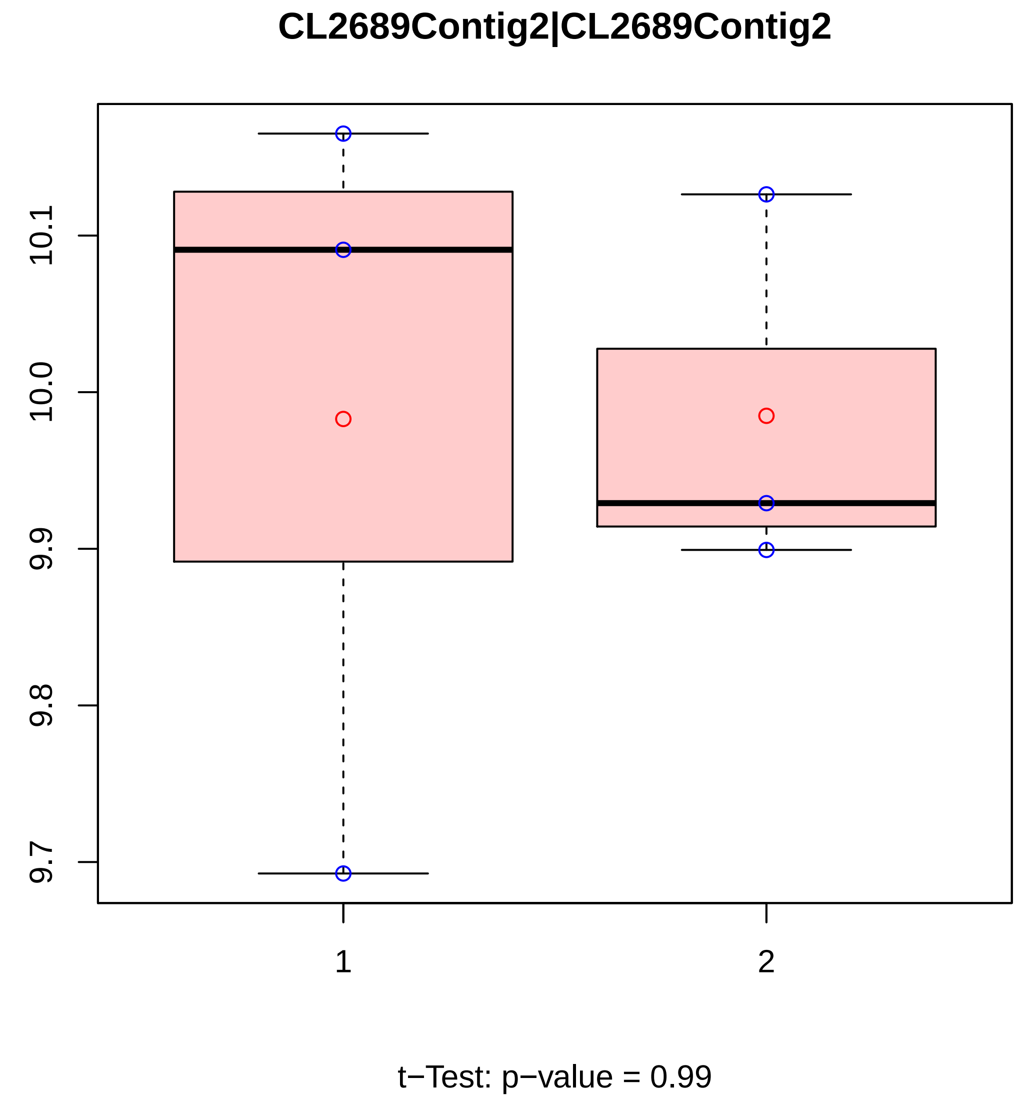

CL2689Contig2|CL2689Contig2
10.1
10.0
9.9
9.8
9.7
1
2
t−Test: p−value = 0.99

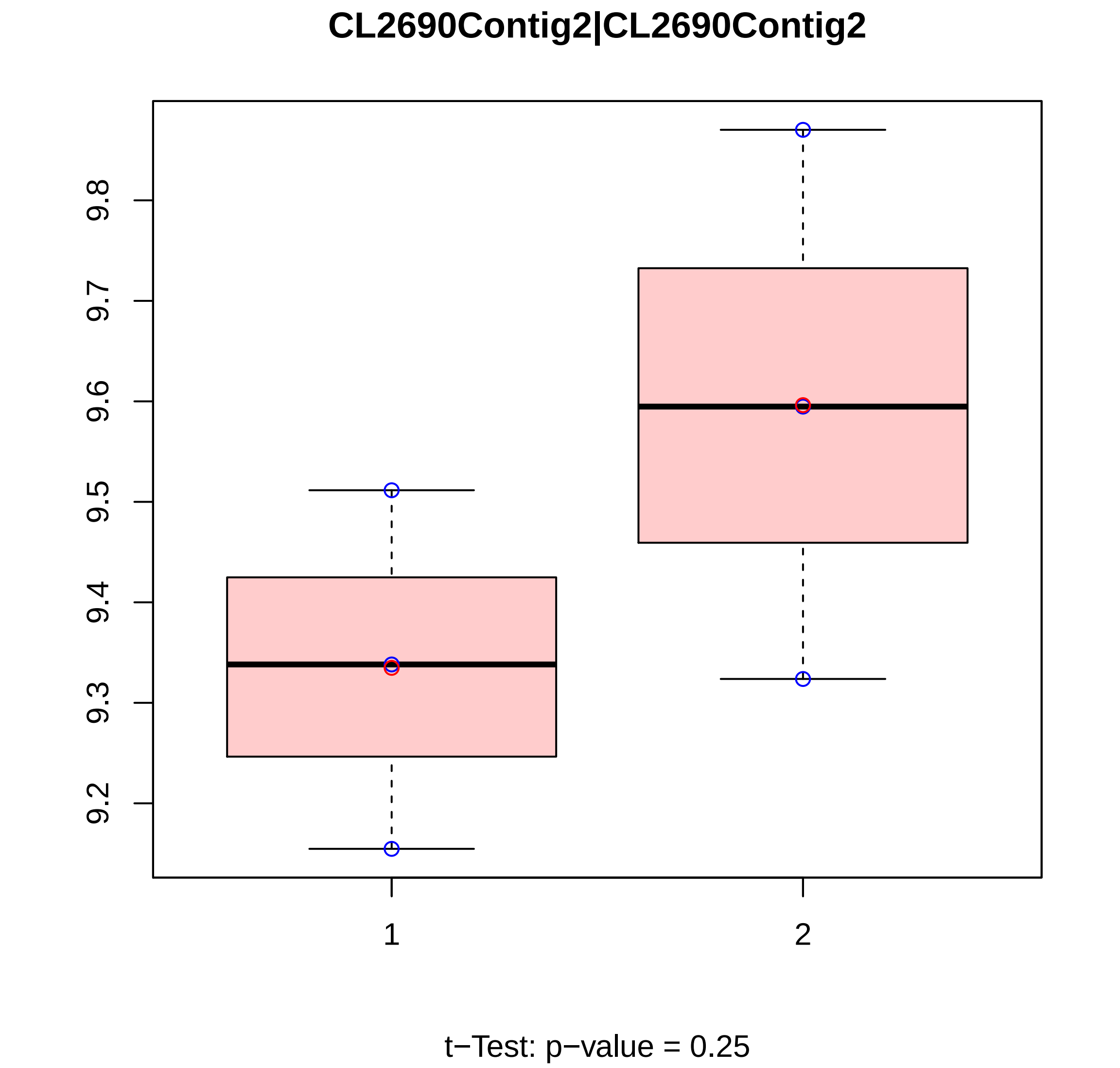
CL2690Contig2|CL2690Contig2
9.2
9.3
9.4
9.5
9.6
9.7
9.8
1
2
t−Test: p−value = 0.25

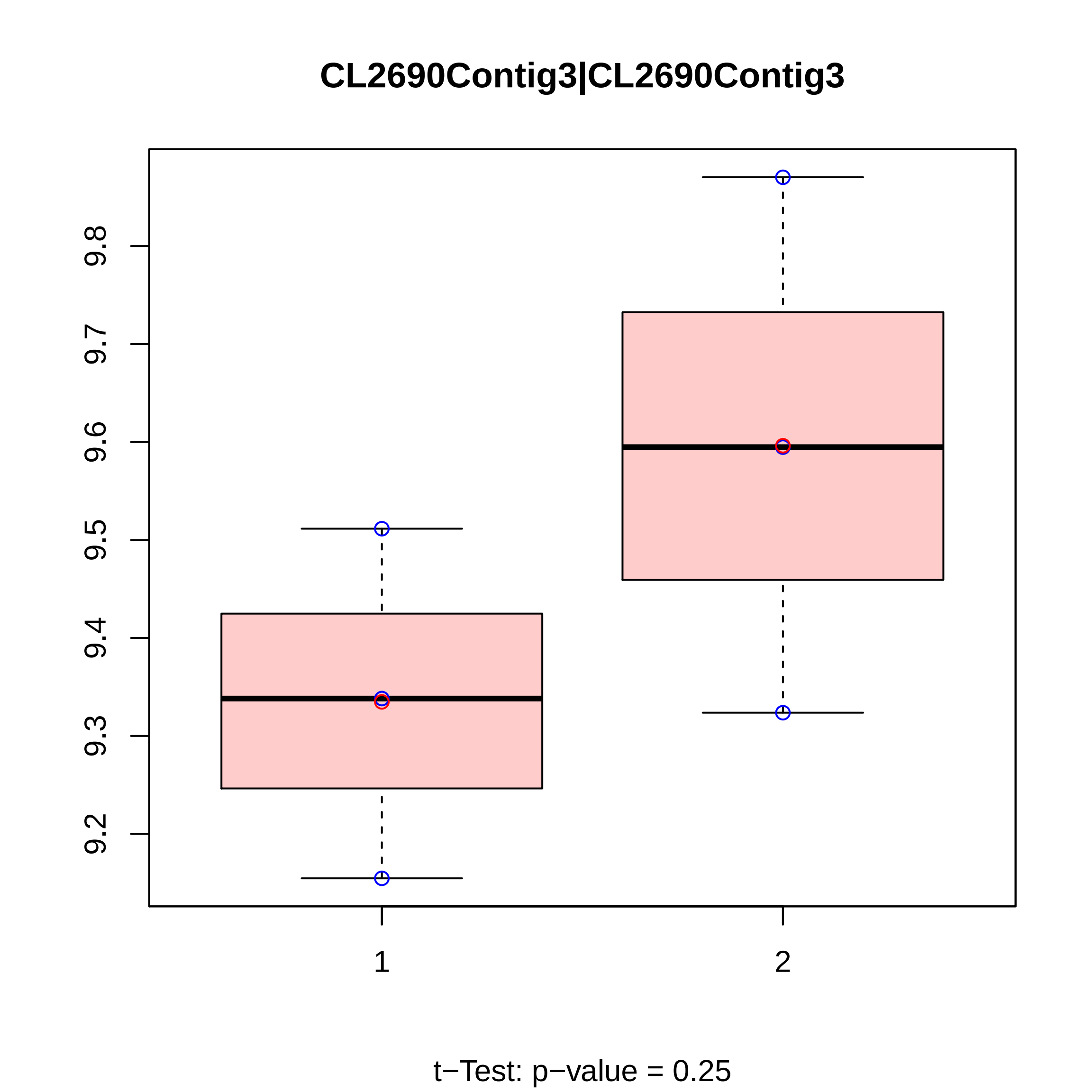

CL2690Contig3|CL2690Contig3
9.2
9.3
9.4
9.5
9.6
9.7
9.8
1
2
t−Test: p−value = 0.25

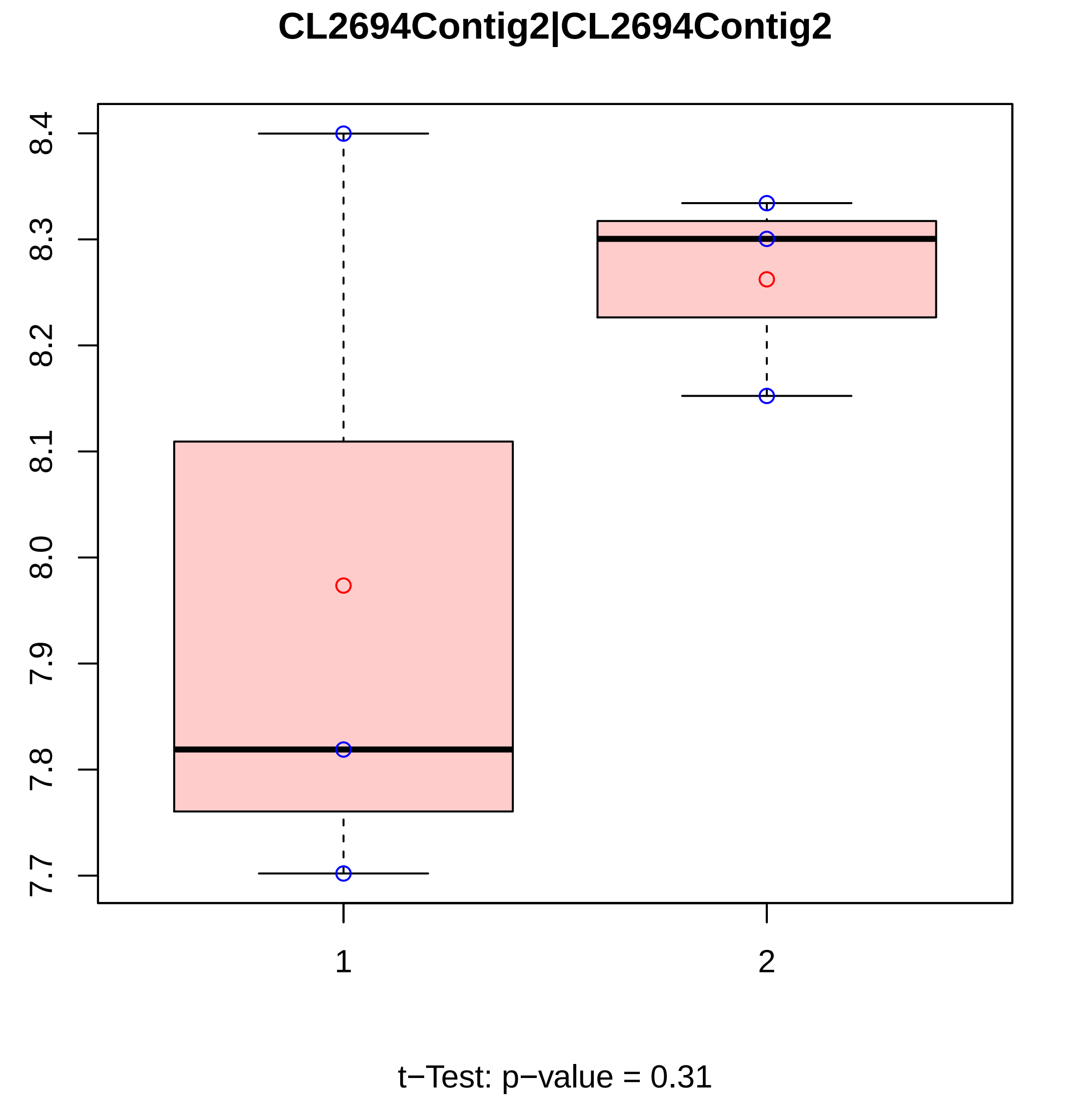
CL2694Contig2|CL2694Contig2
8.4
8.3
8.2
8.1
8.0
7.9
7.8
7.7
1
2
t−Test: p−value = 0.31

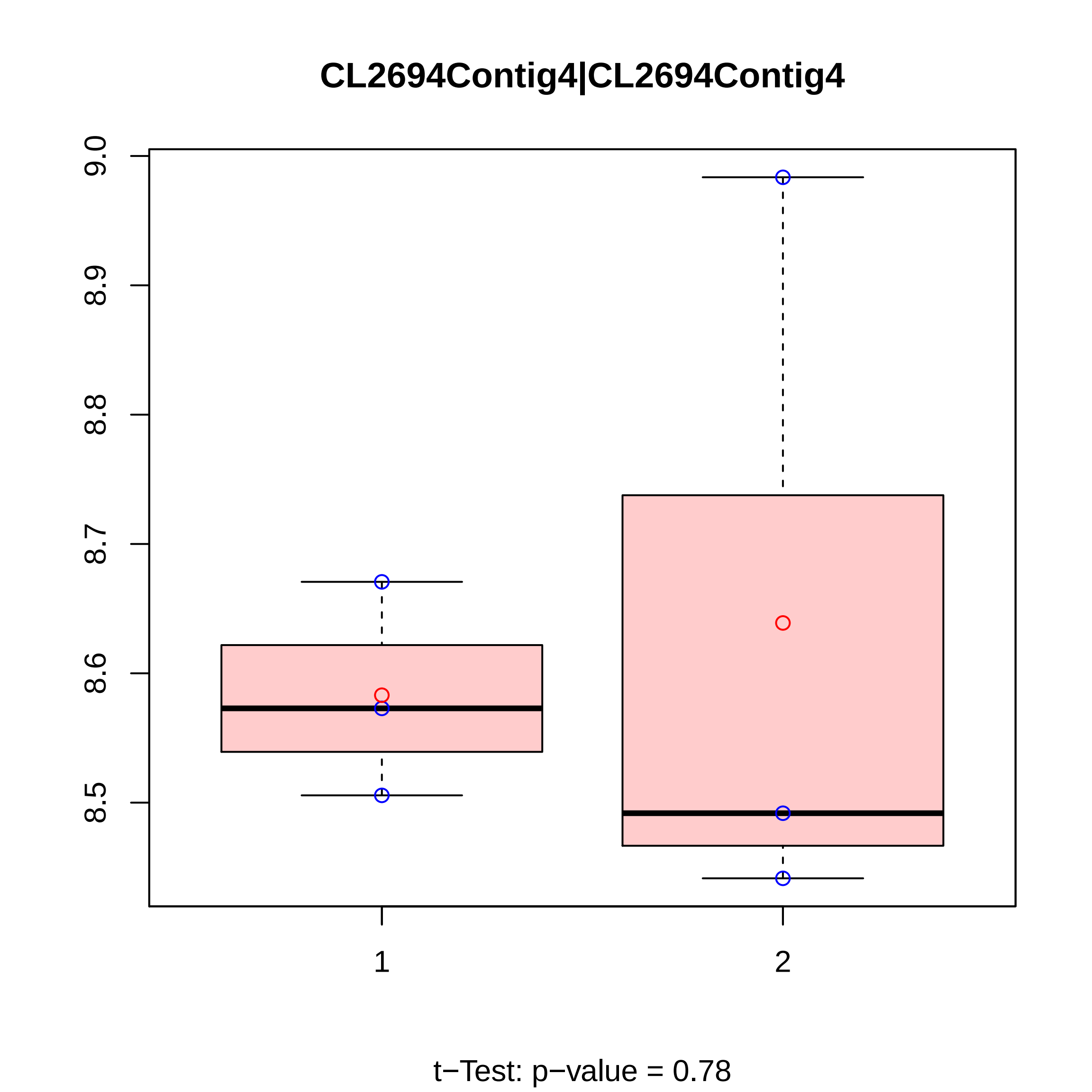
CL2694Contig4|CL2694Contig4
9.0
8.9
8.8
8.7
8.6
8.5
1
2
t−Test: p−value = 0.78

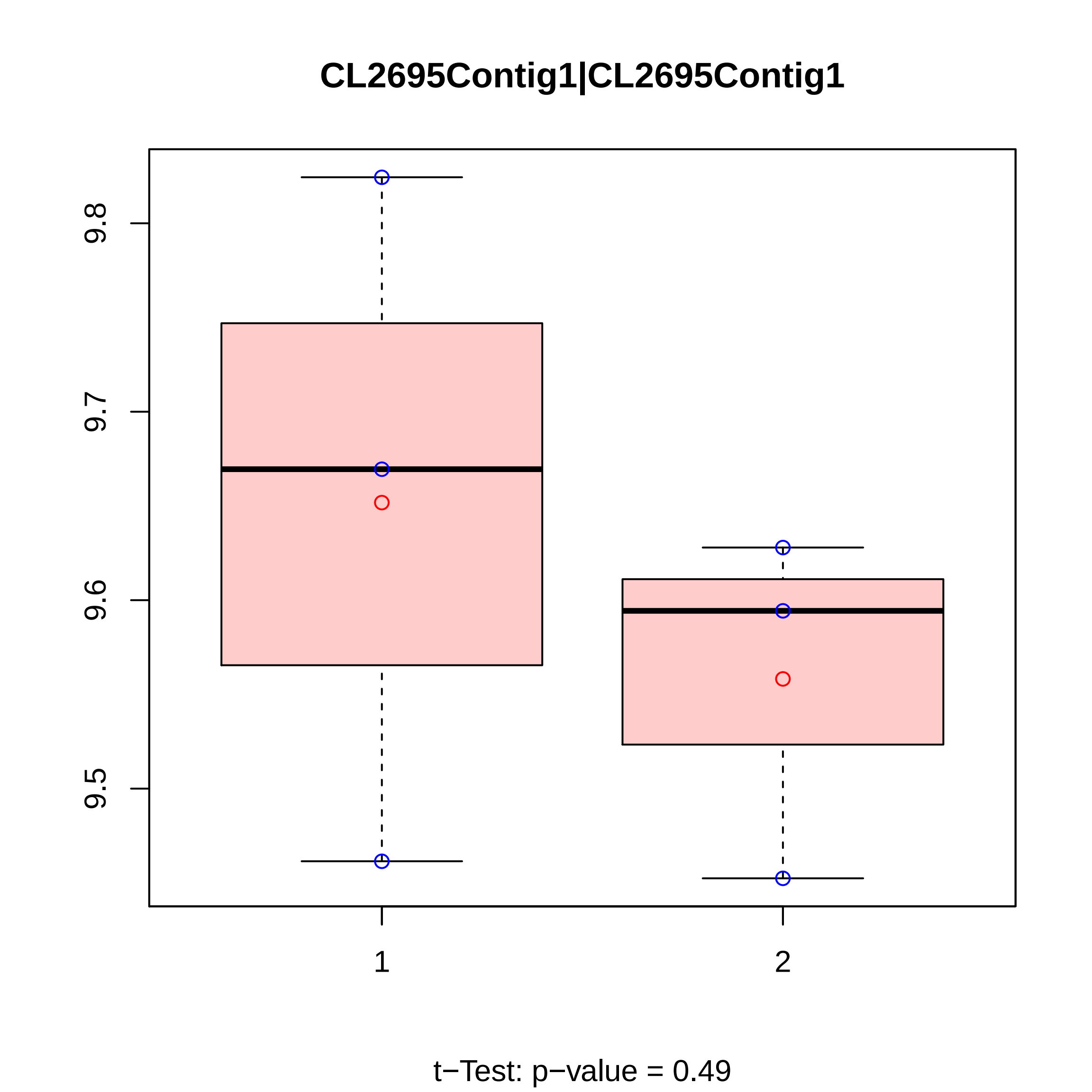

CL2695Contig1|CL2695Contig1
9.8
9.7
9.6
9.5
1
2
t−Test: p−value = 0.49

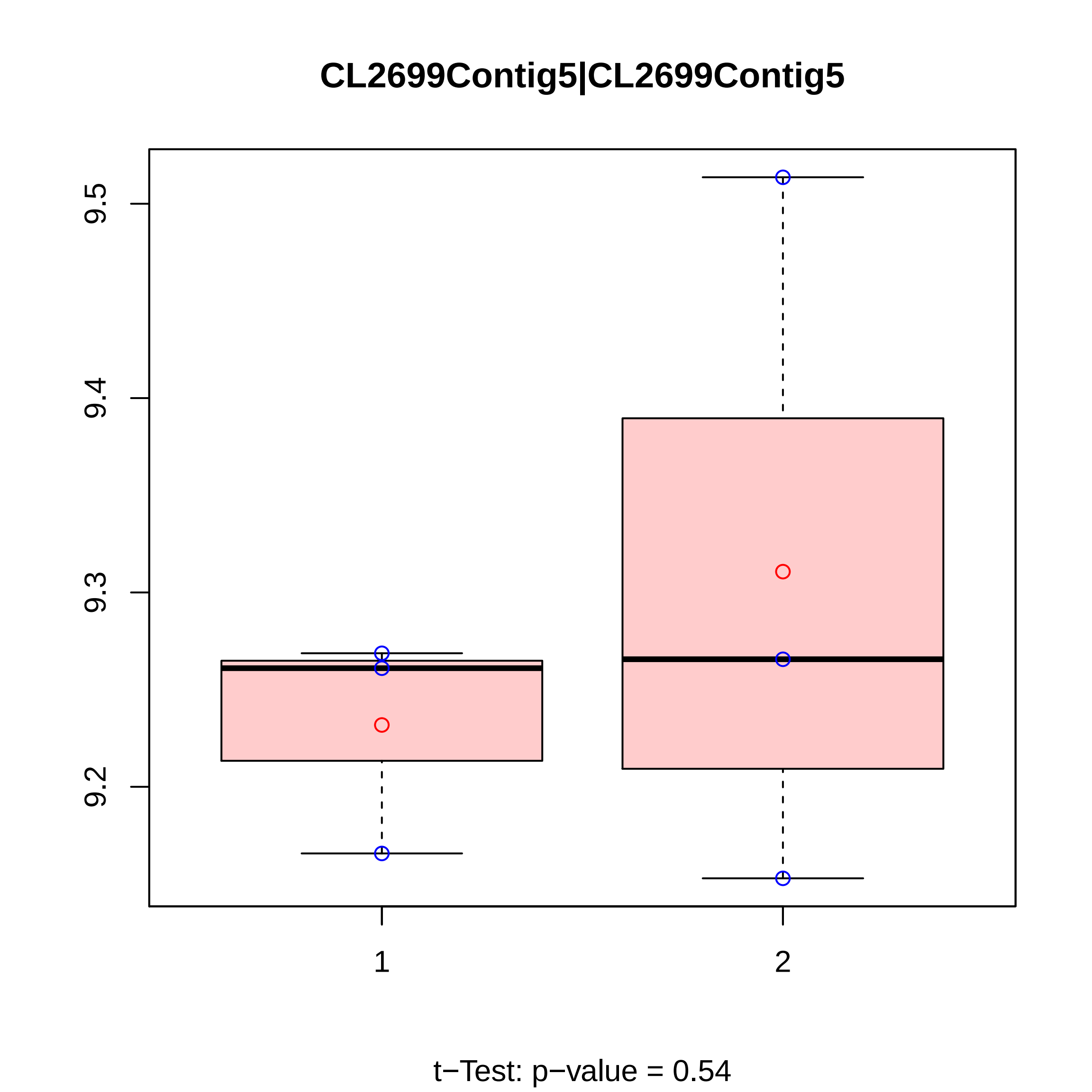
CL2699Contig5|CL2699Contig5
9.2
9.3
9.4
9.5
1
2
t−Test: p−value = 0.54

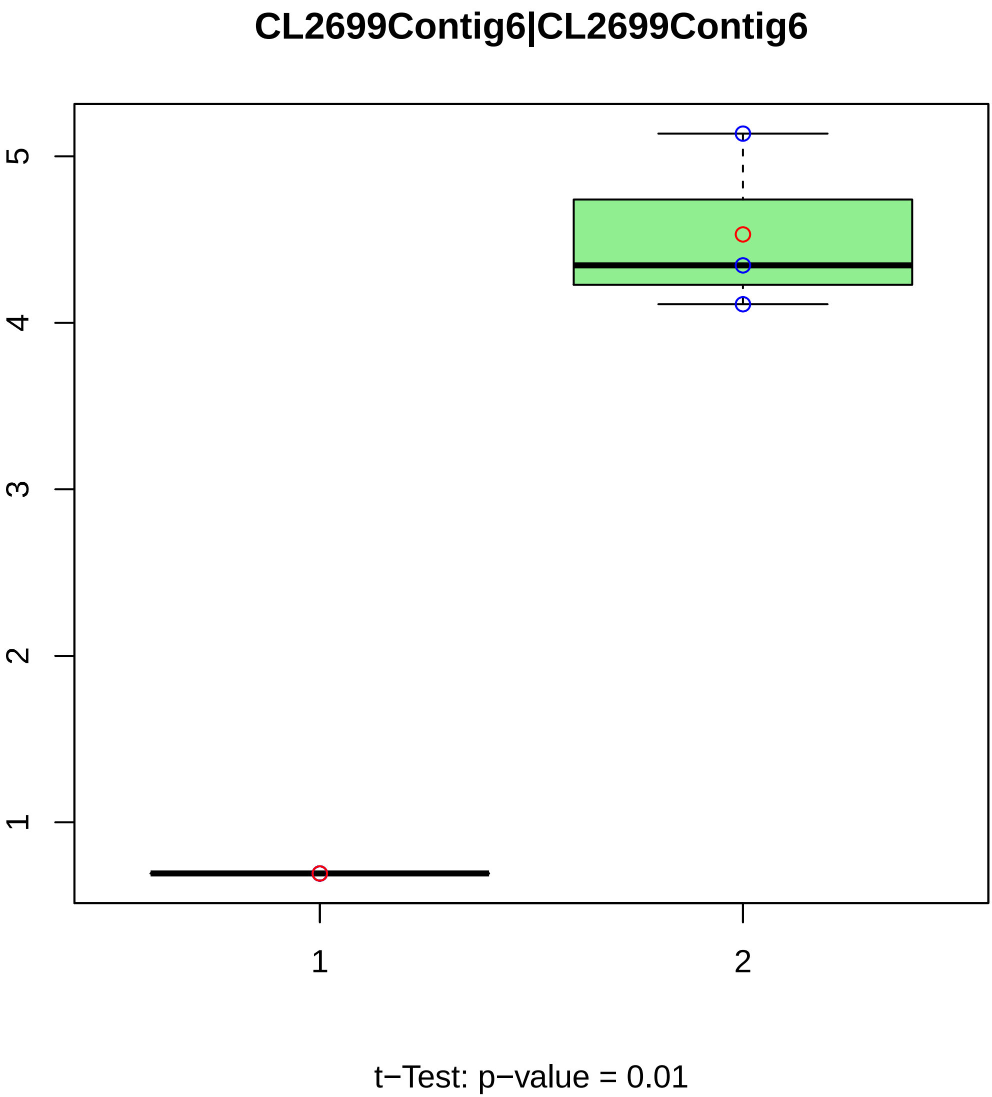
CL2699Contig6|CL2699Contig6
1
2
3
4
5
1
2
t−Test: p−value = 0.01

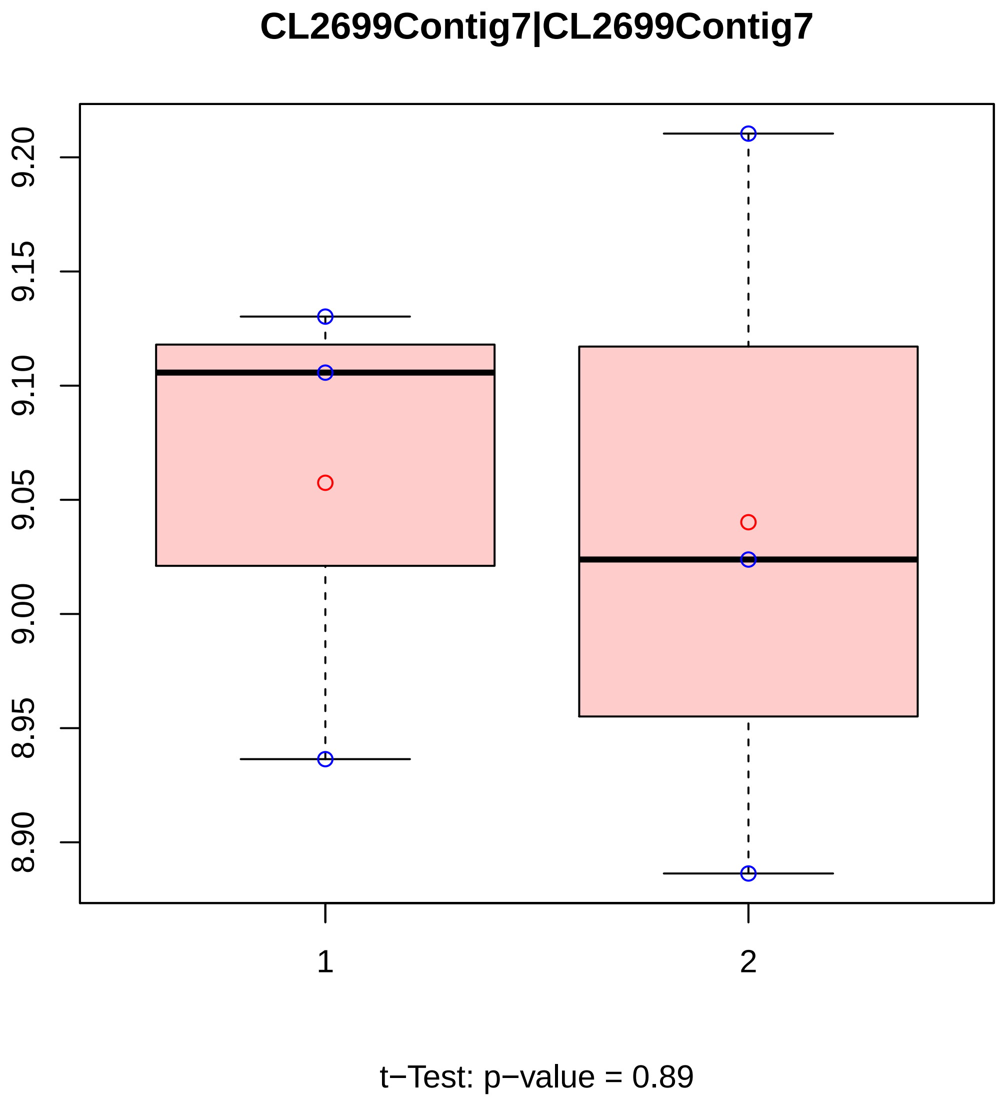
CL2699Contig7|CL2699Contig7
9.20
9.15
9.10
9.05
9.00
8.95
8.90
1
2
t−Test: p−value = 0.89

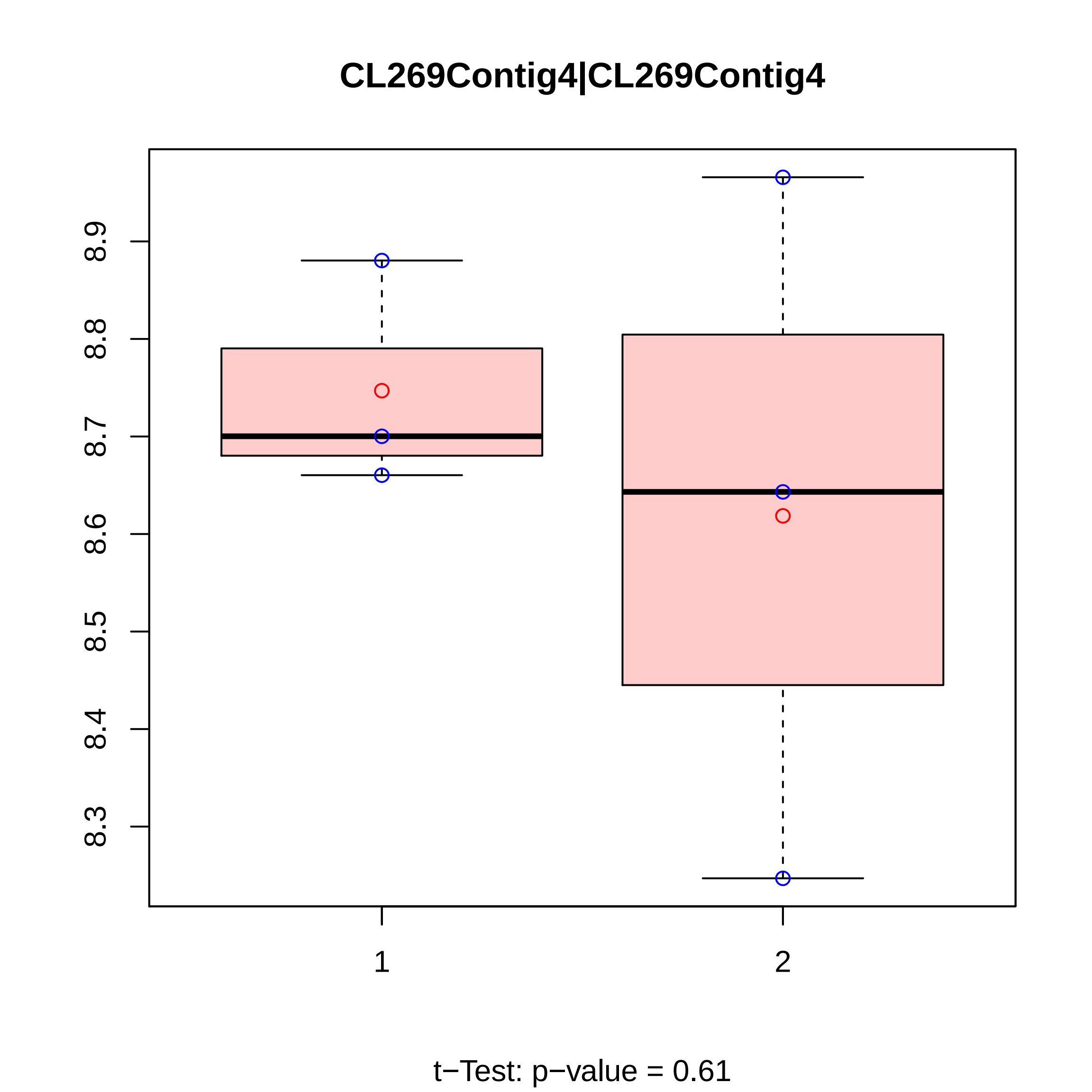
CL269Contig4|CL269Contig4
8.3
8.4
8.5
8.6
8.7
8.8
8.9
1
2
t−Test: p−value = 0.61

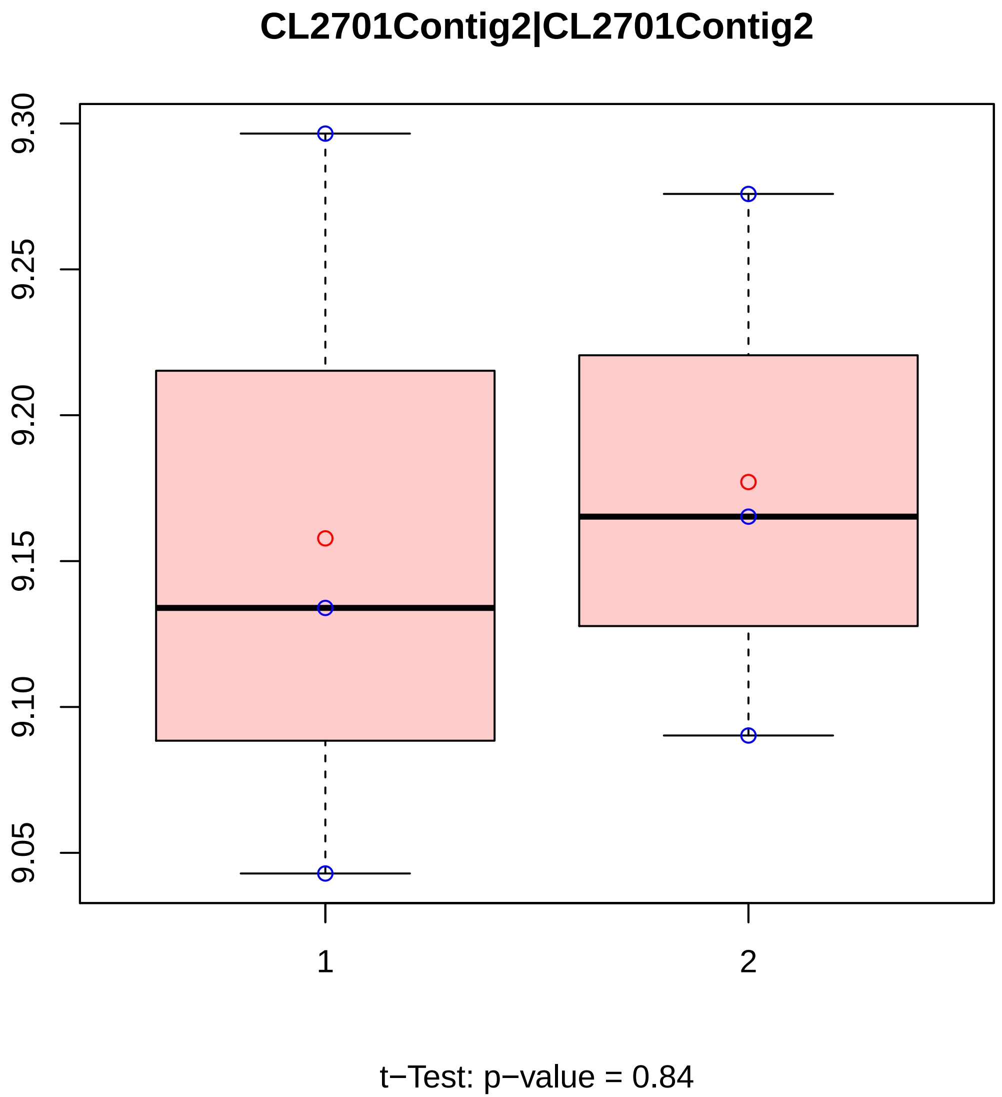
CL2701Contig2|CL2701Contig2
9.30
9.25
9.20
9.15
9.10
9.05
1
2
t−Test: p−value = 0.84

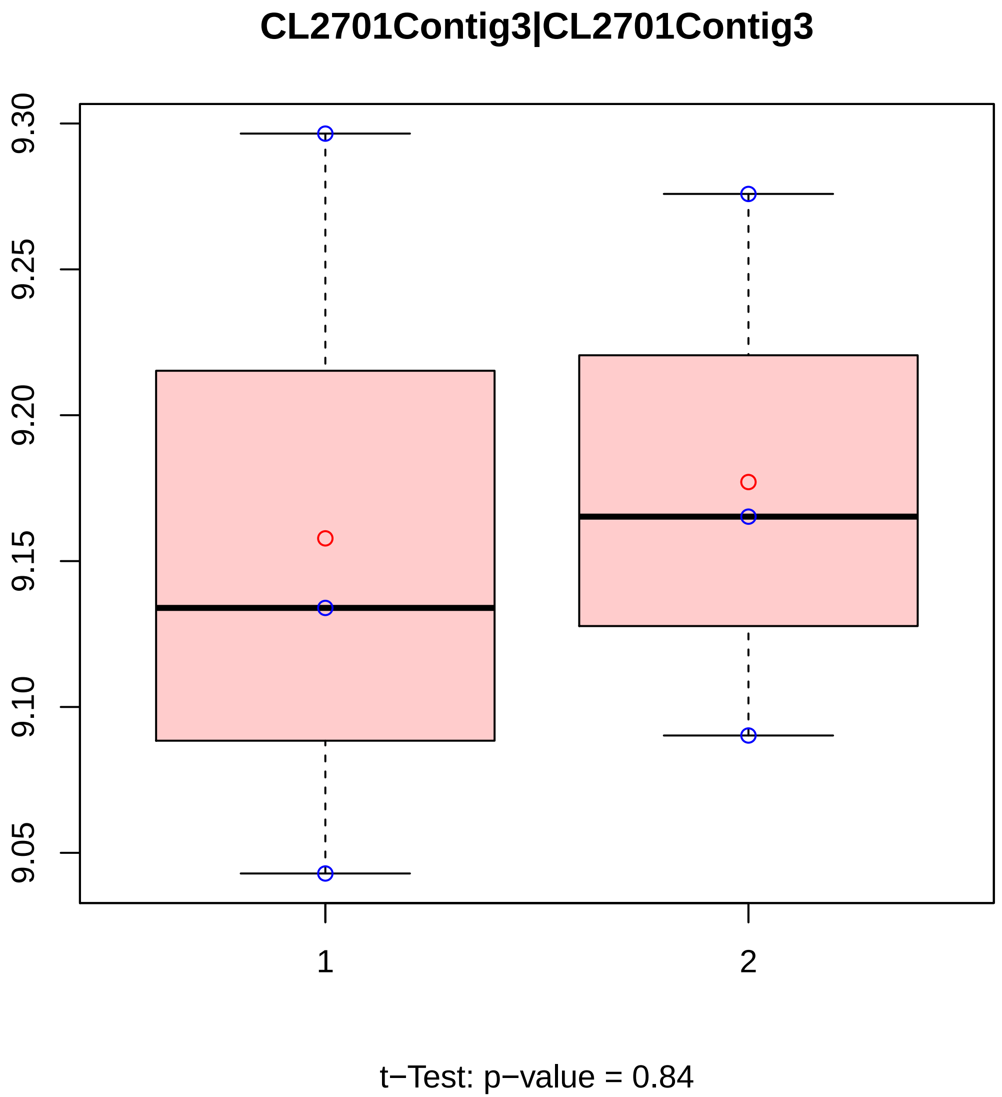
CL2701Contig3|CL2701Contig3
9.30
9.25
9.20
9.15
9.10
9.05
1
2
t−Test: p−value = 0.84

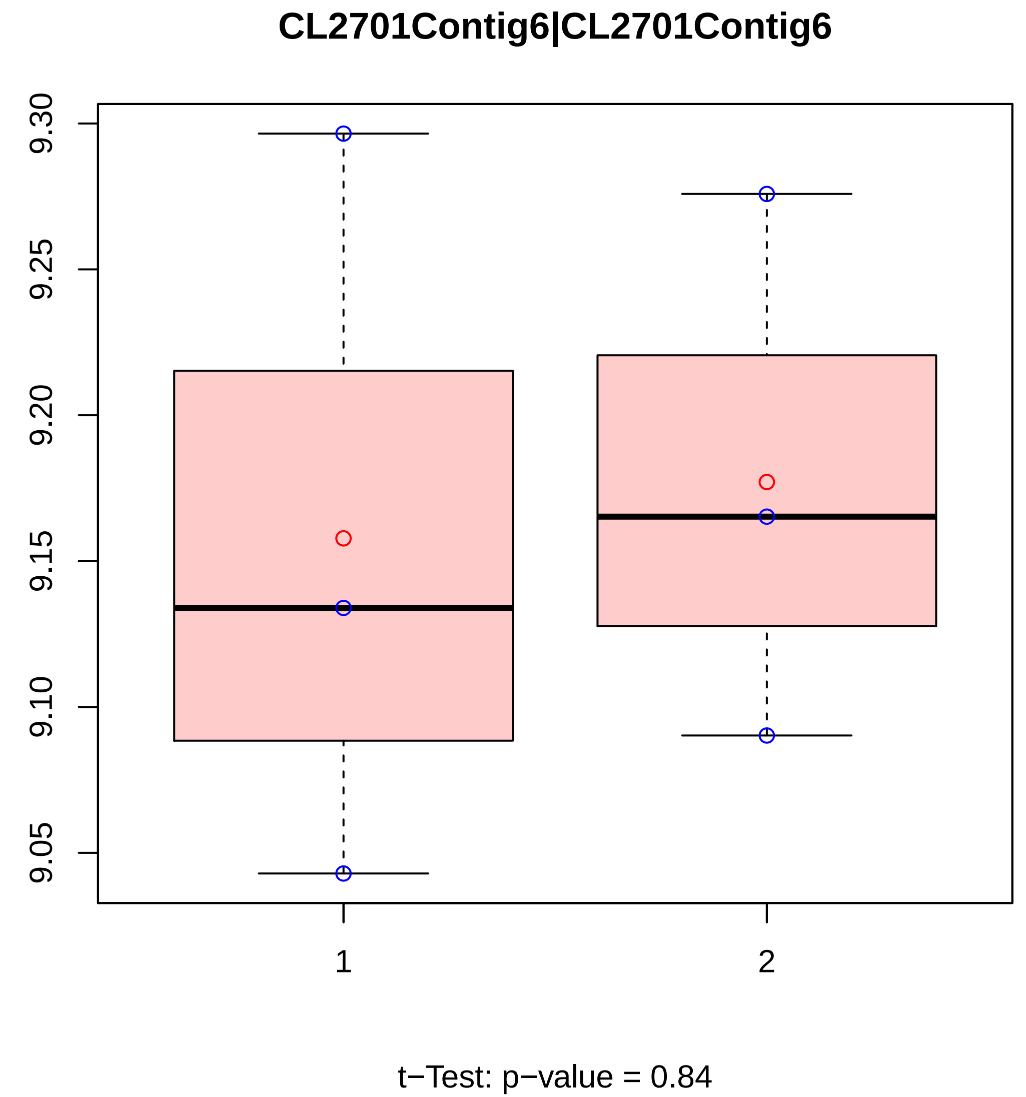
CL2701Contig6|CL2701Contig6
9.30
9.25
9.20
9.15
9.10
9.05
1
2
t−Test: p−value = 0.84

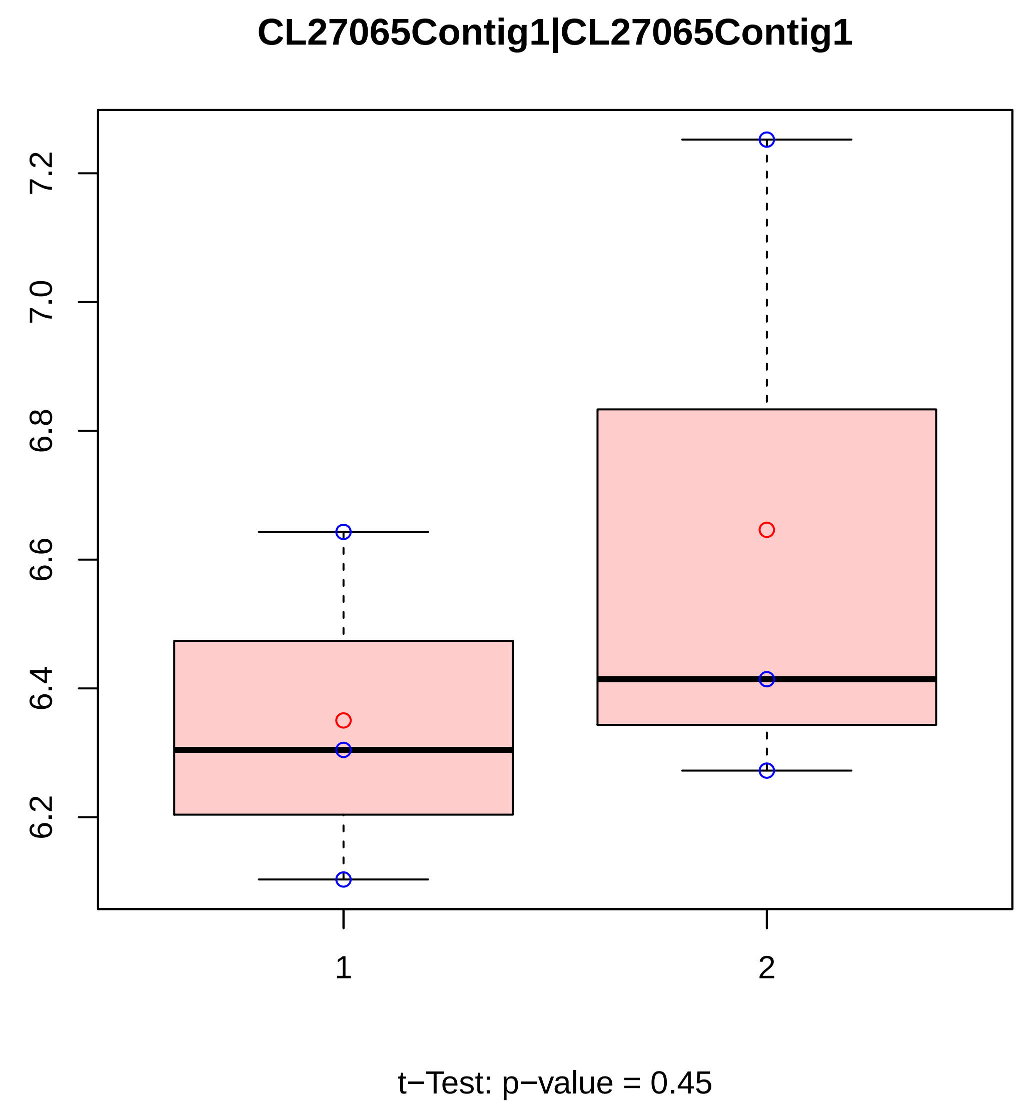
CL27065Contig1|CL27065Contig1
7.2
7.0
6.8
6.6
6.4
6.2
1
2
t−Test: p−value = 0.45

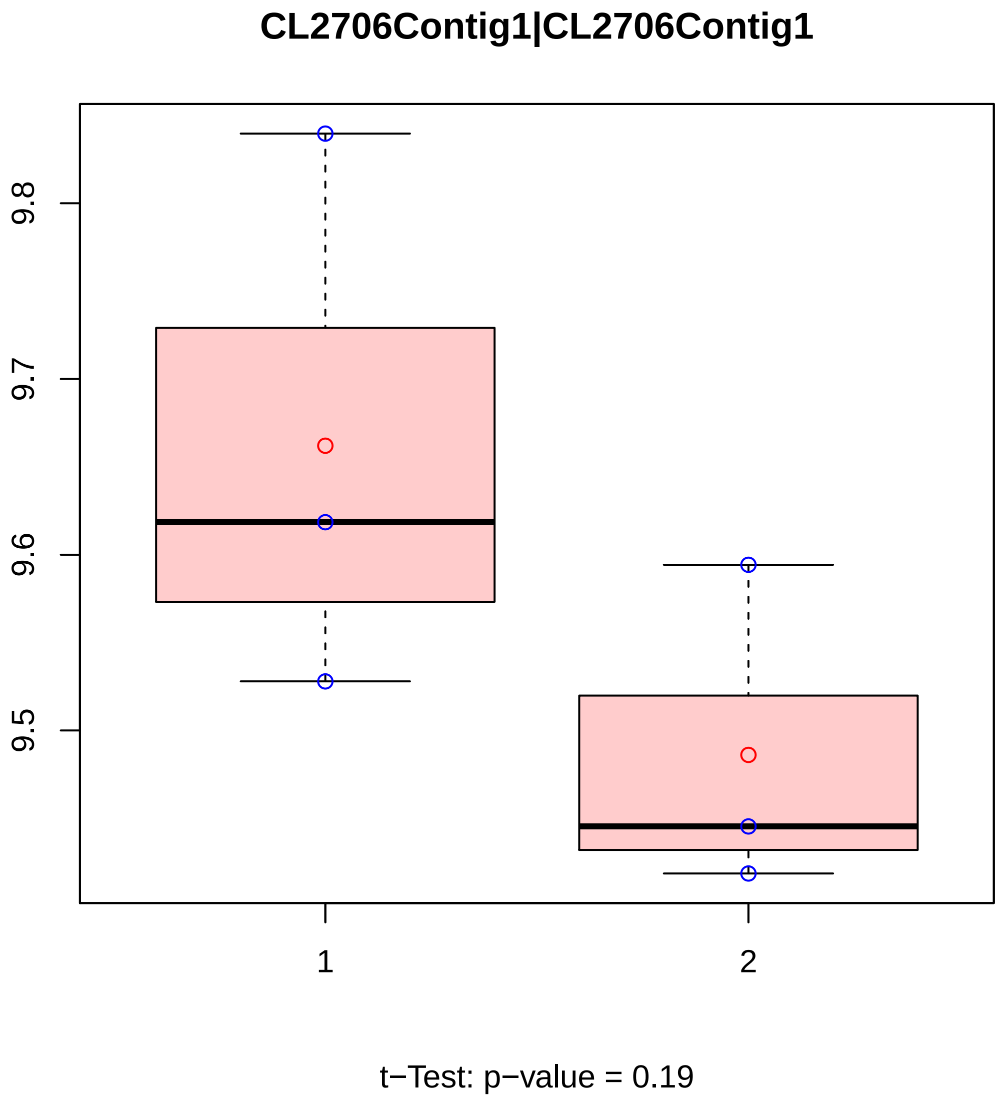

CL2706Contig1|CL2706Contig1
9.5
9.6
9.7
9.8
1
2
t−Test: p−value = 0.19

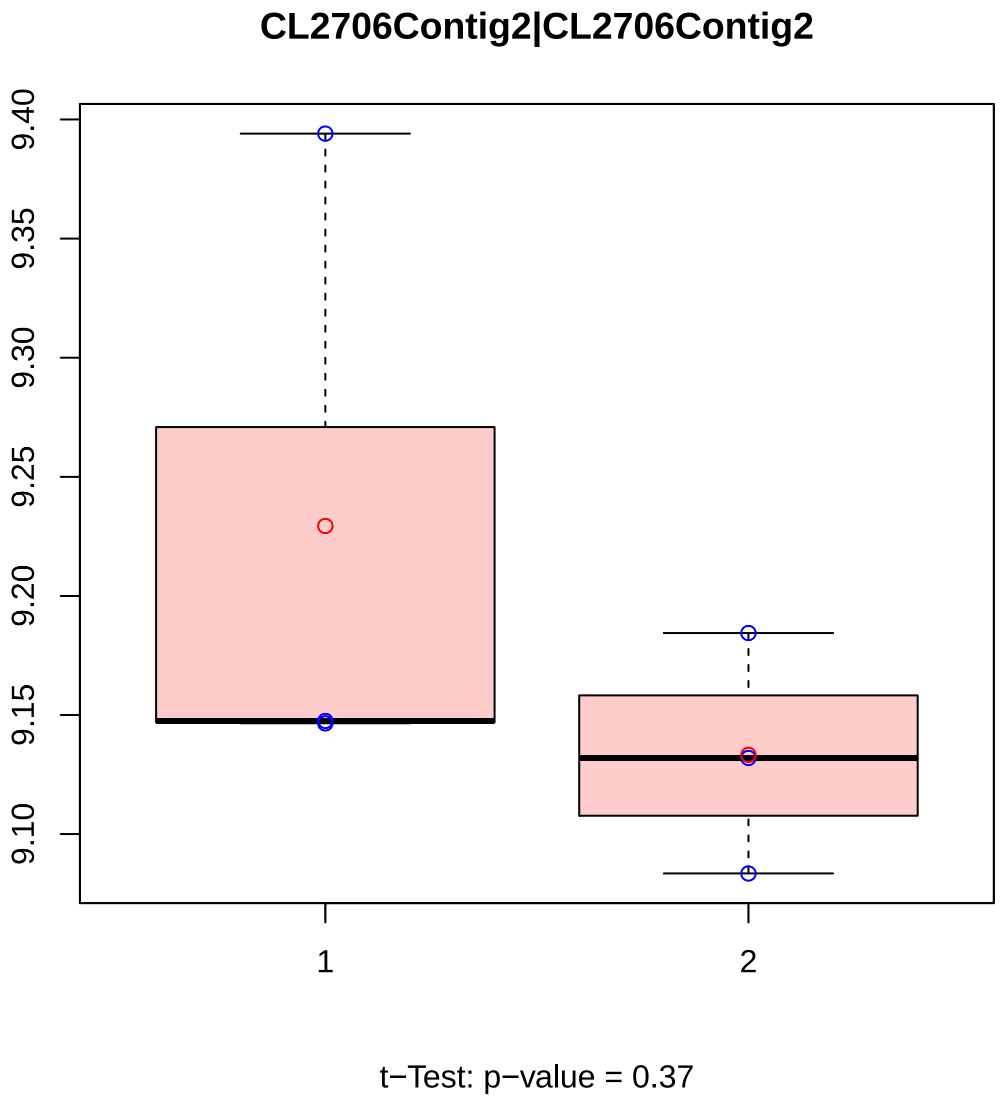

CL2706Contig2|CL2706Contig2
9.40
9.35
9.30
9.25
9.20
9.15
9.10
1
2
t−Test: p−value = 0.37

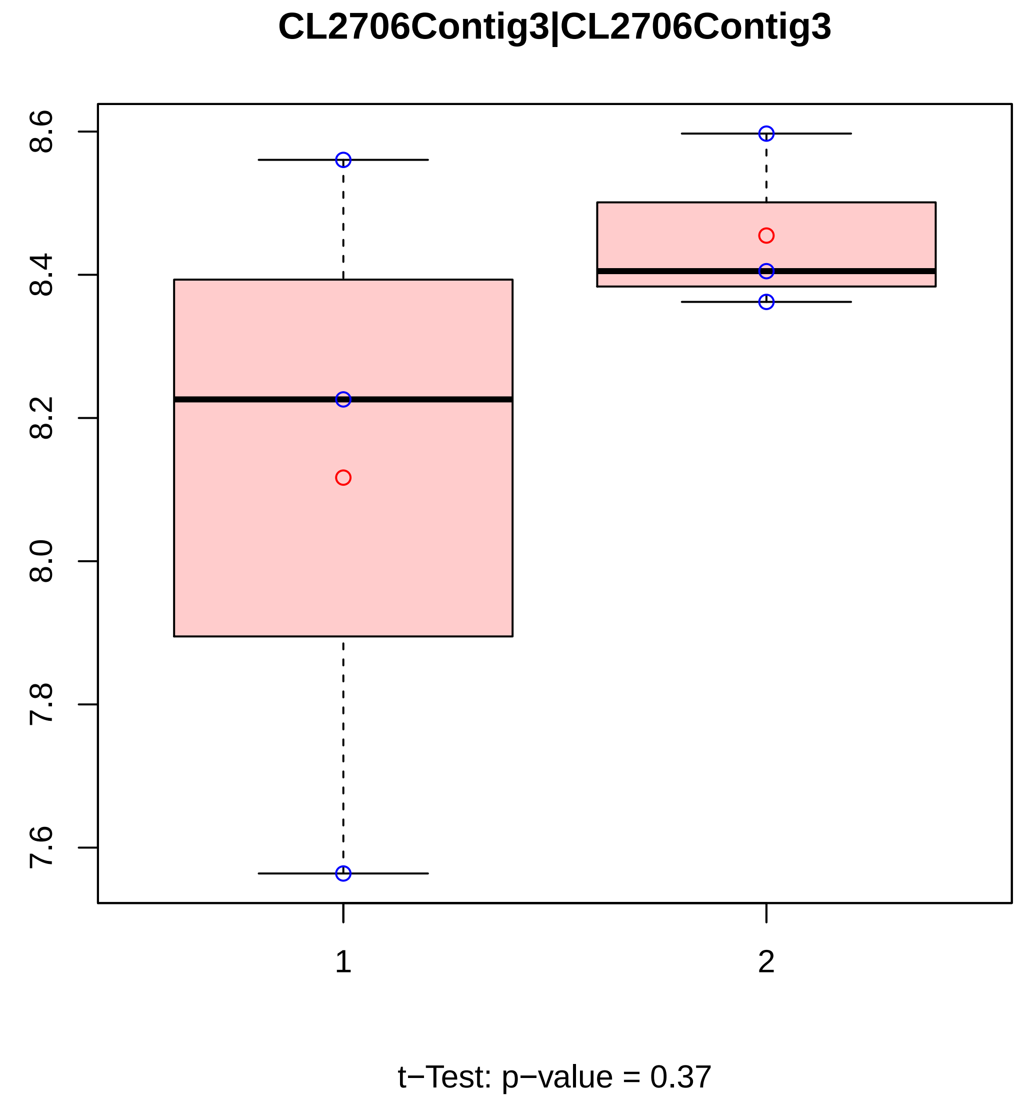
CL2706Contig3|CL2706Contig3
8.6
8.4
8.2
8.0
7.8
7.6
1
2
t−Test: p−value = 0.37

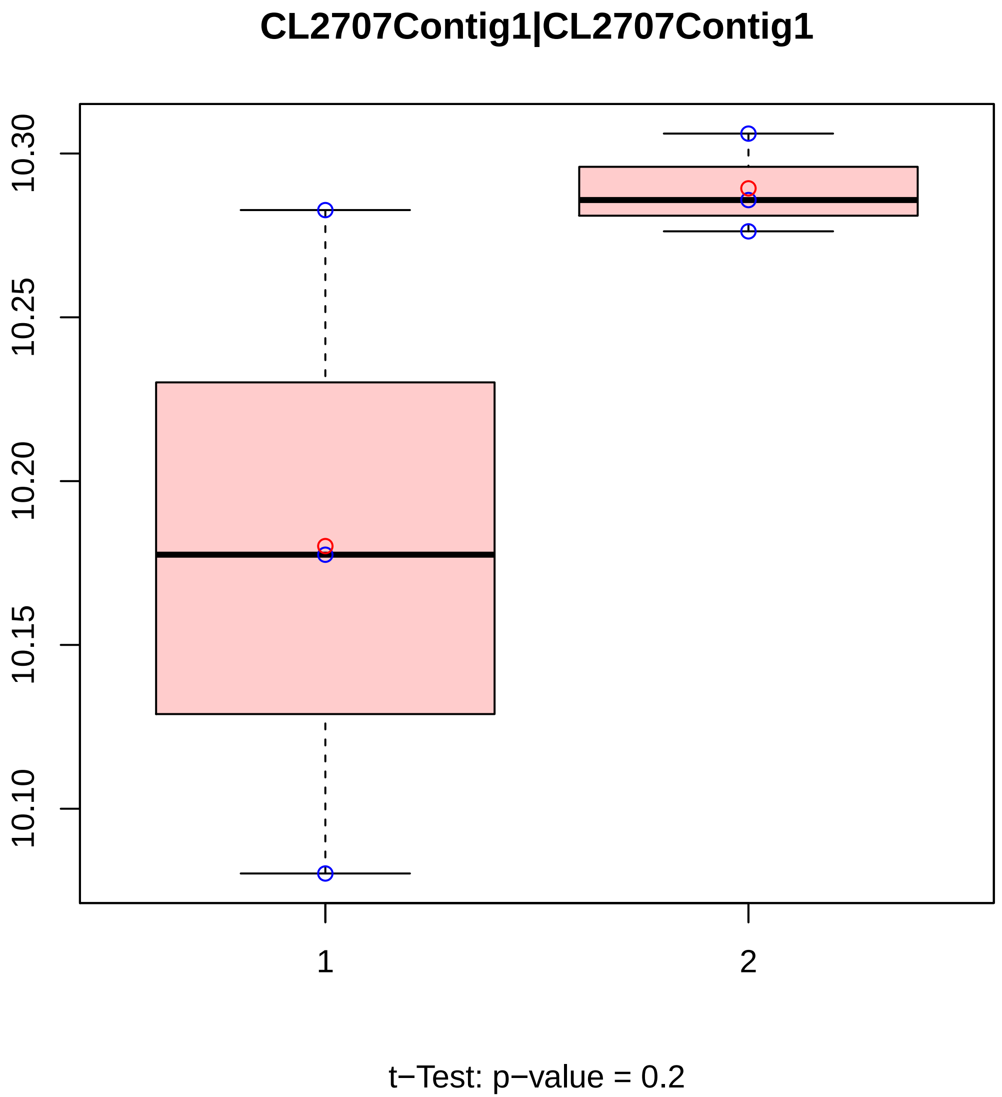

CL2707Contig1|CL2707Contig1
10.10
10.15
10.20
10.25
10.30
1
2
t−Test: p−value = 0.2

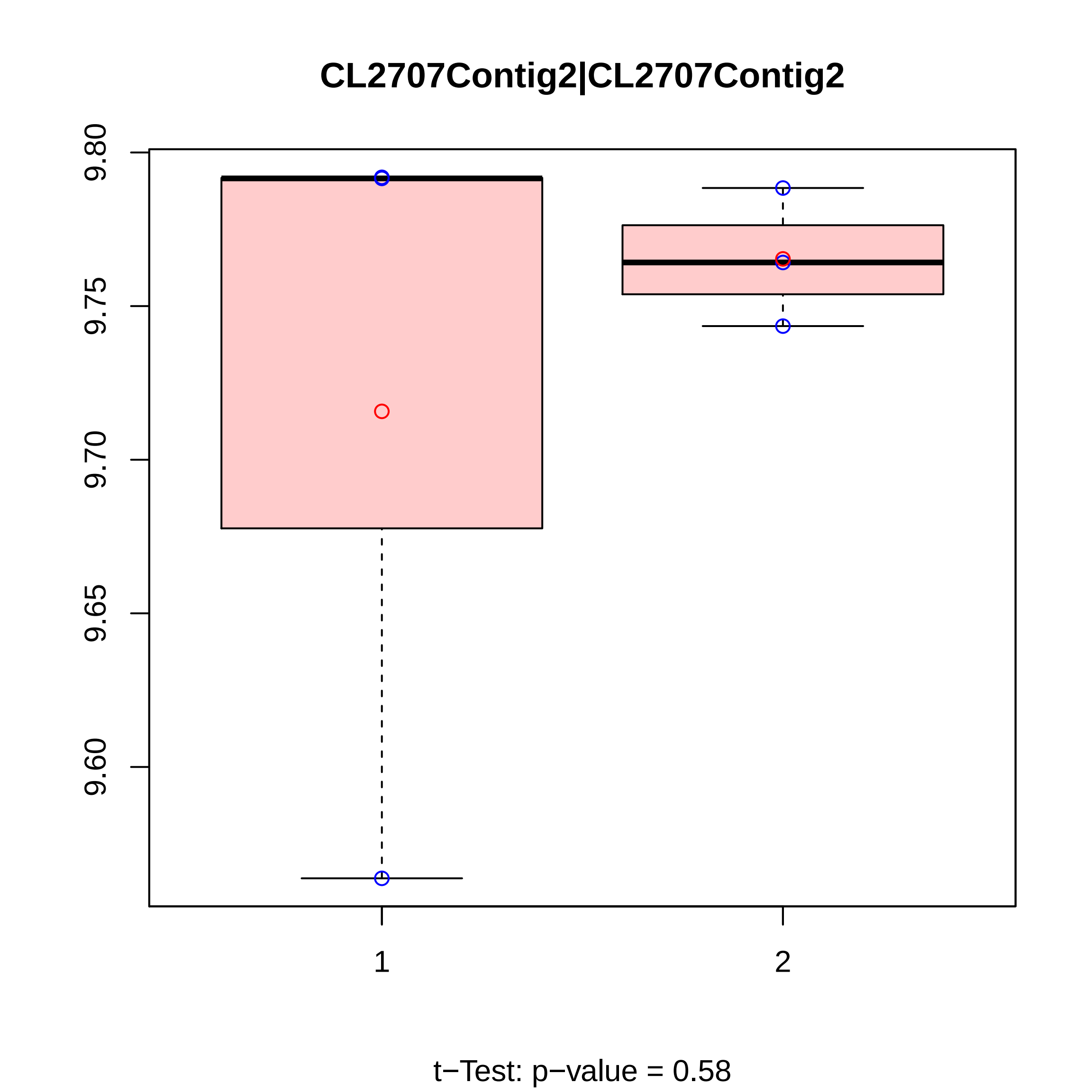

CL2707Contig2|CL2707Contig2
9.80
9.75
9.70
9.65
9.60
1
2
t−Test: p−value = 0.58

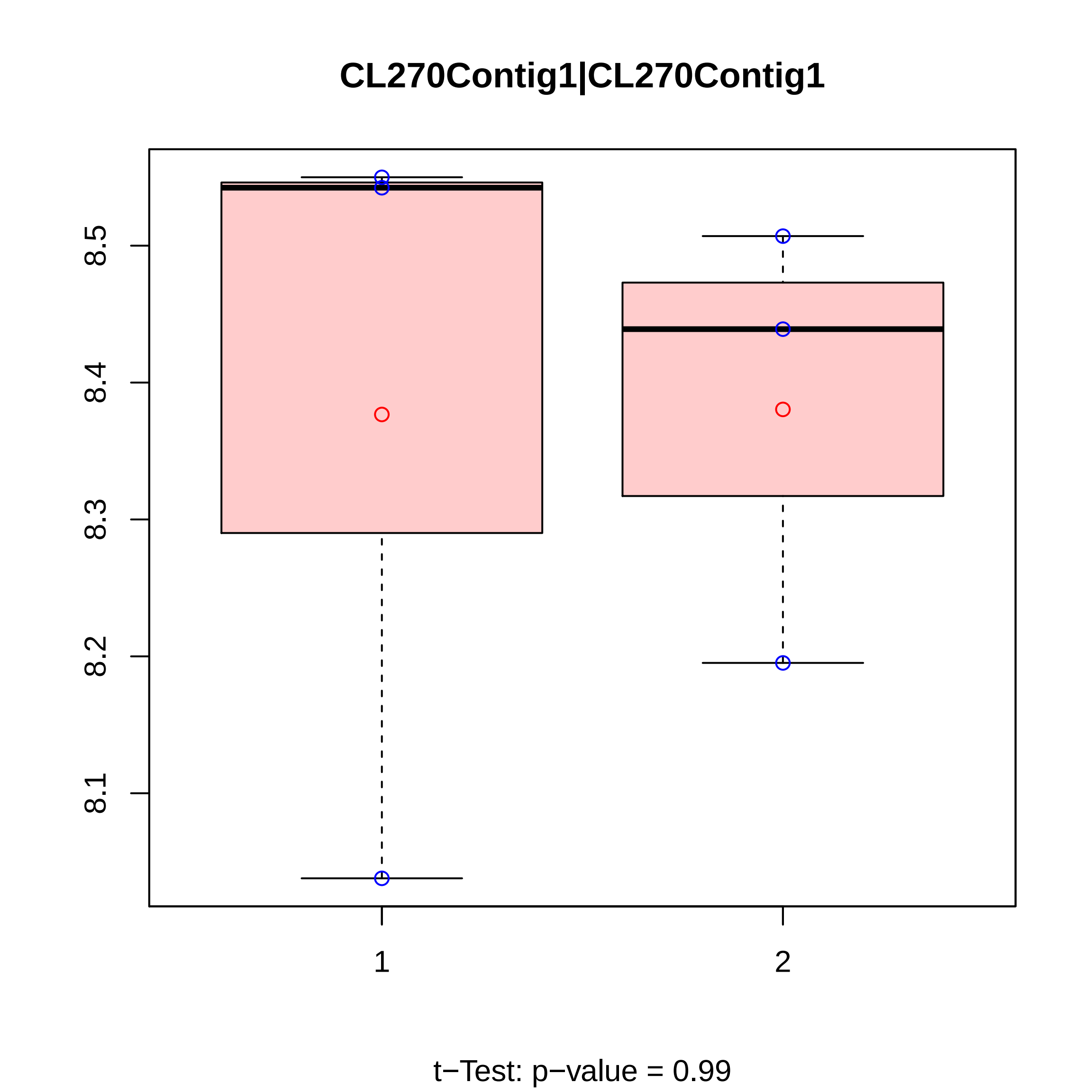
CL270Contig1|CL270Contig1
8.1
8.2
8.3
8.4
8.5
1
2
t−Test: p−value = 0.99

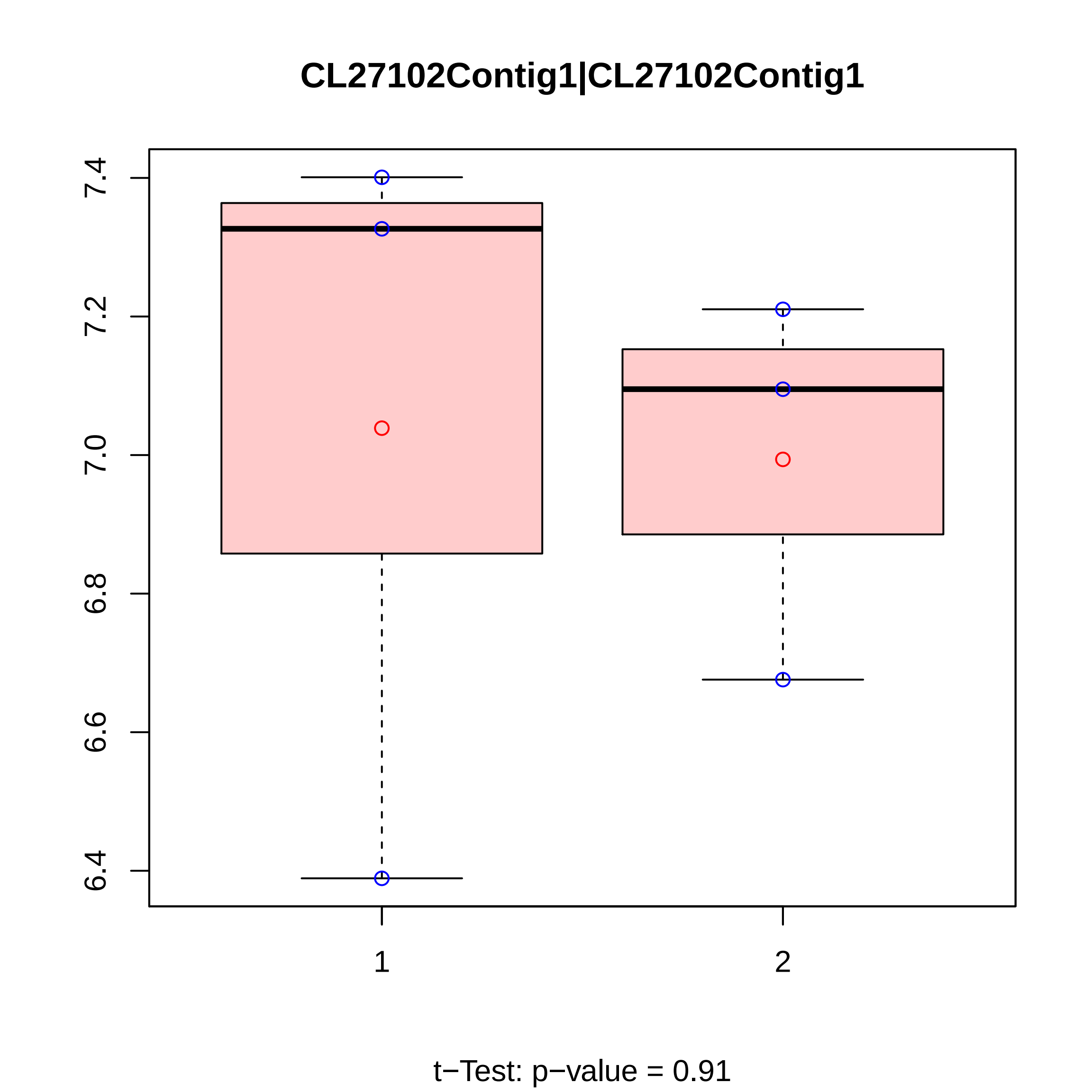
CL27102Contig1|CL27102Contig1
7.4
7.2
7.0
6.8
6.6
6.4
1
2
t−Test: p−value = 0.91

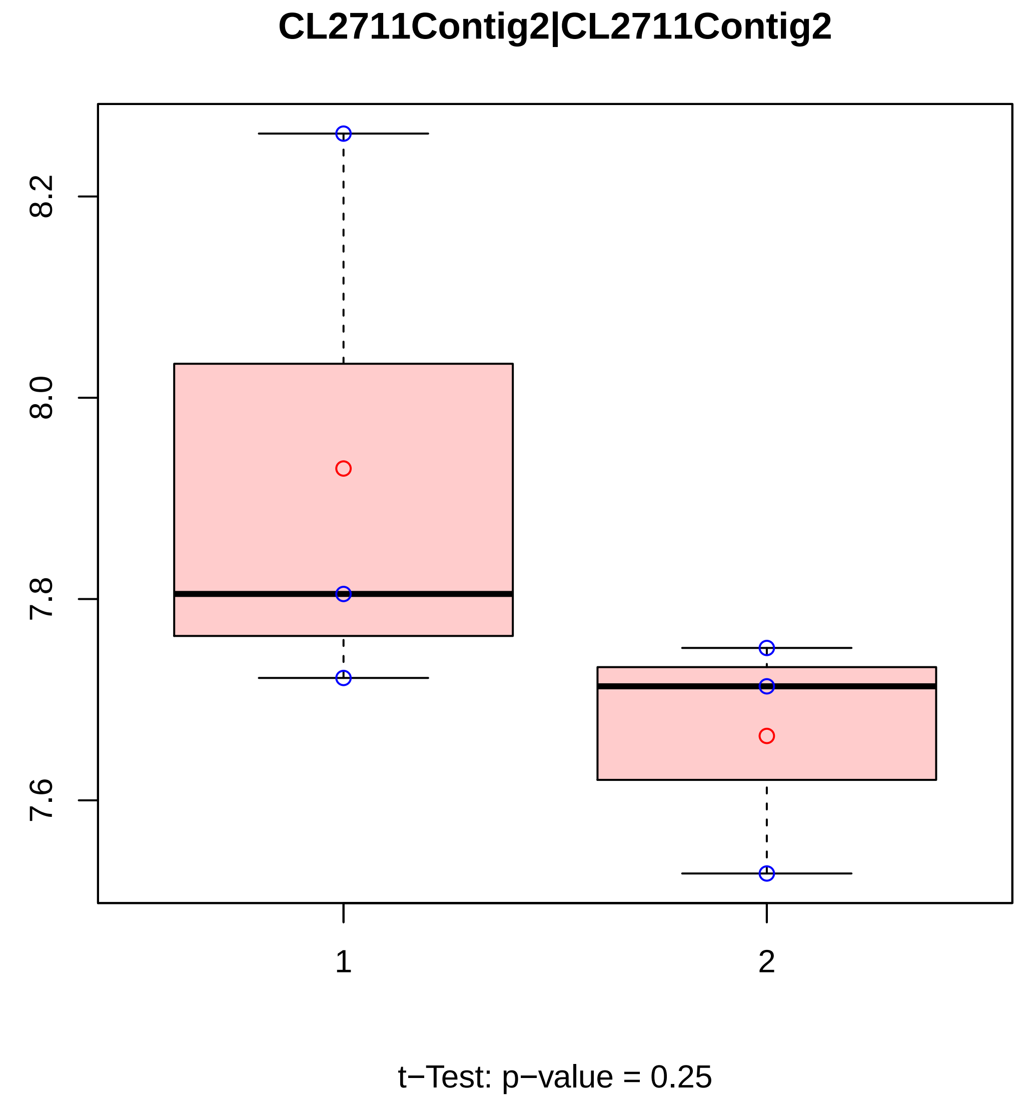
CL2711Contig2|CL2711Contig2
8.2
8.0
7.8
7.6
1
2
t−Test: p−value = 0.25

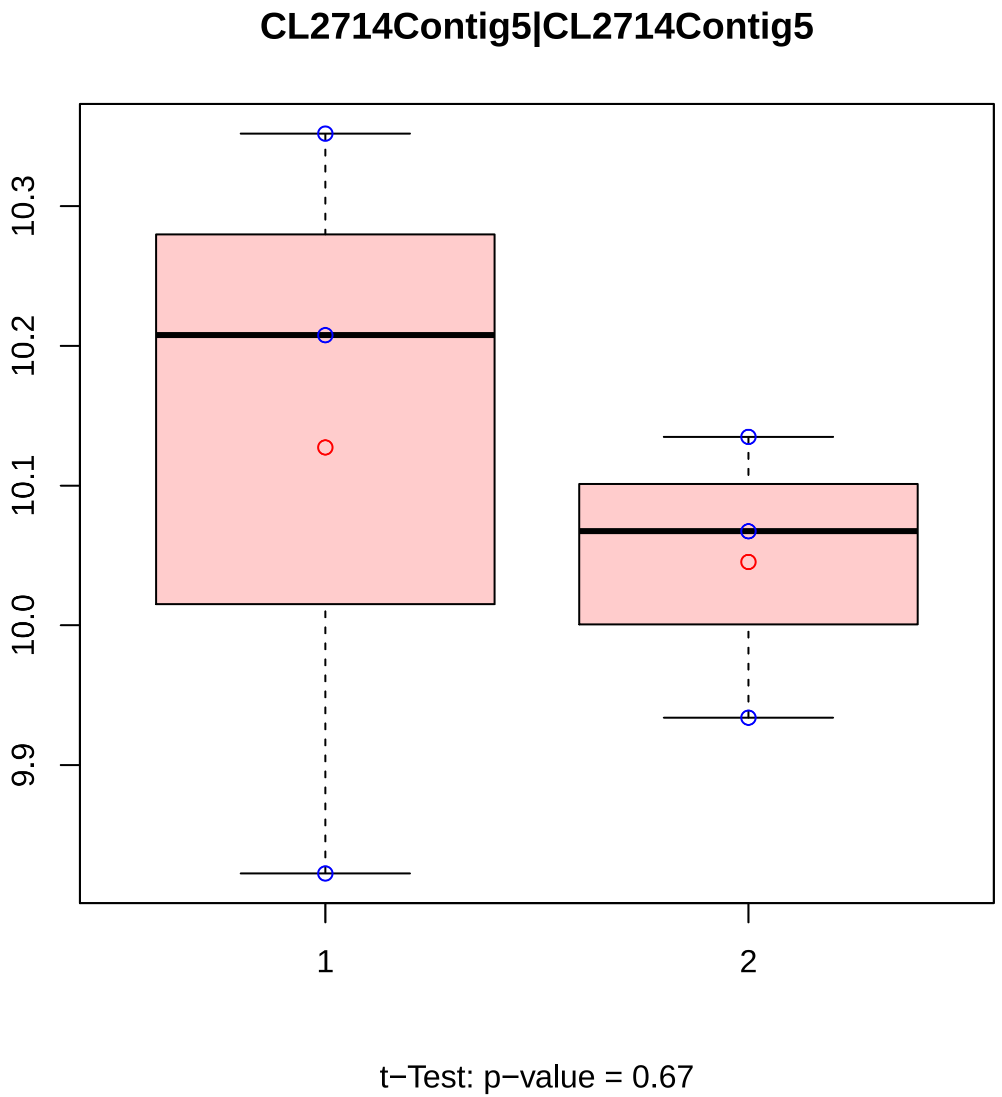
CL2714Contig5|CL2714Contig5
10.3
10.2
10.1
10.0
9.9
1
2
t−Test: p−value = 0.67

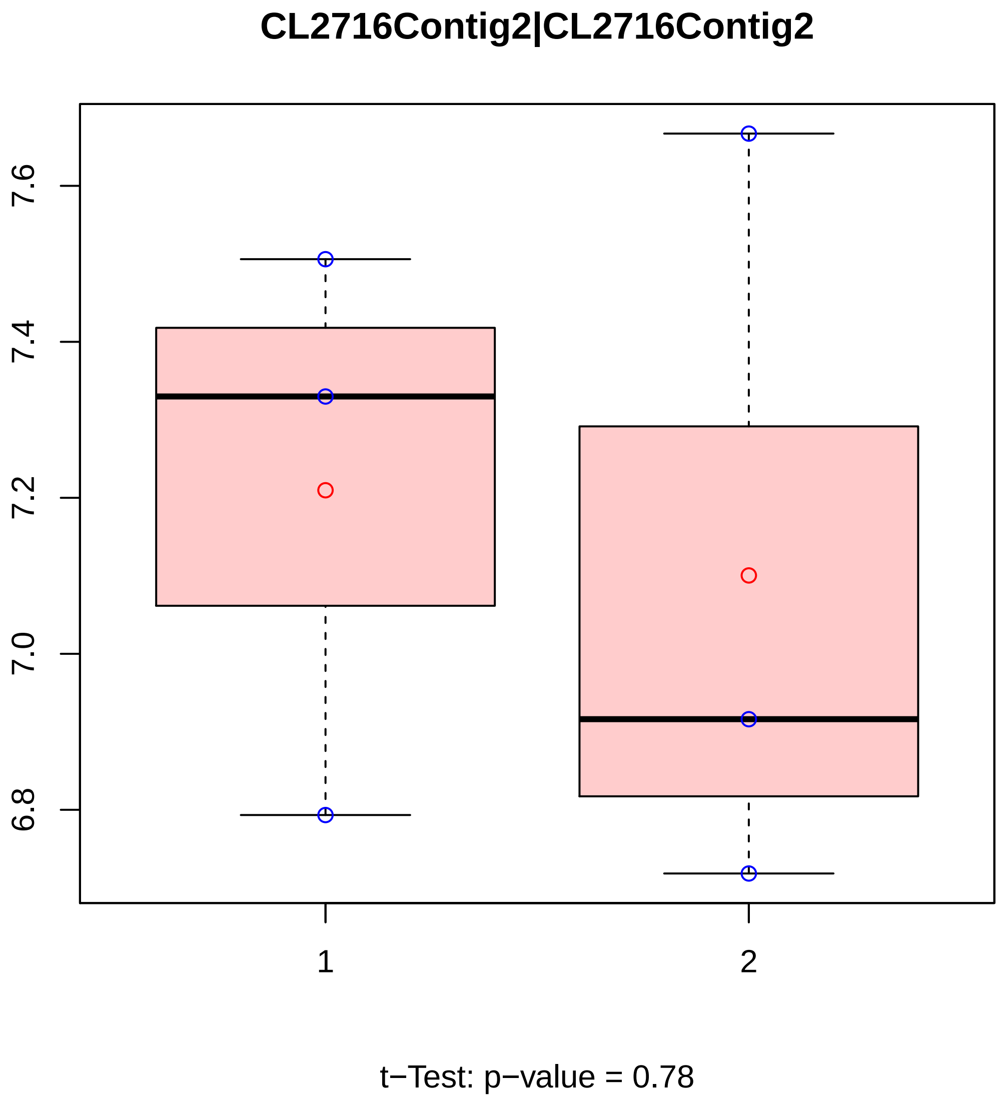

CL2716Contig2|CL2716Contig2
7.6
7.4
7.2
7.0
6.8
1
2
t−Test: p−value = 0.78

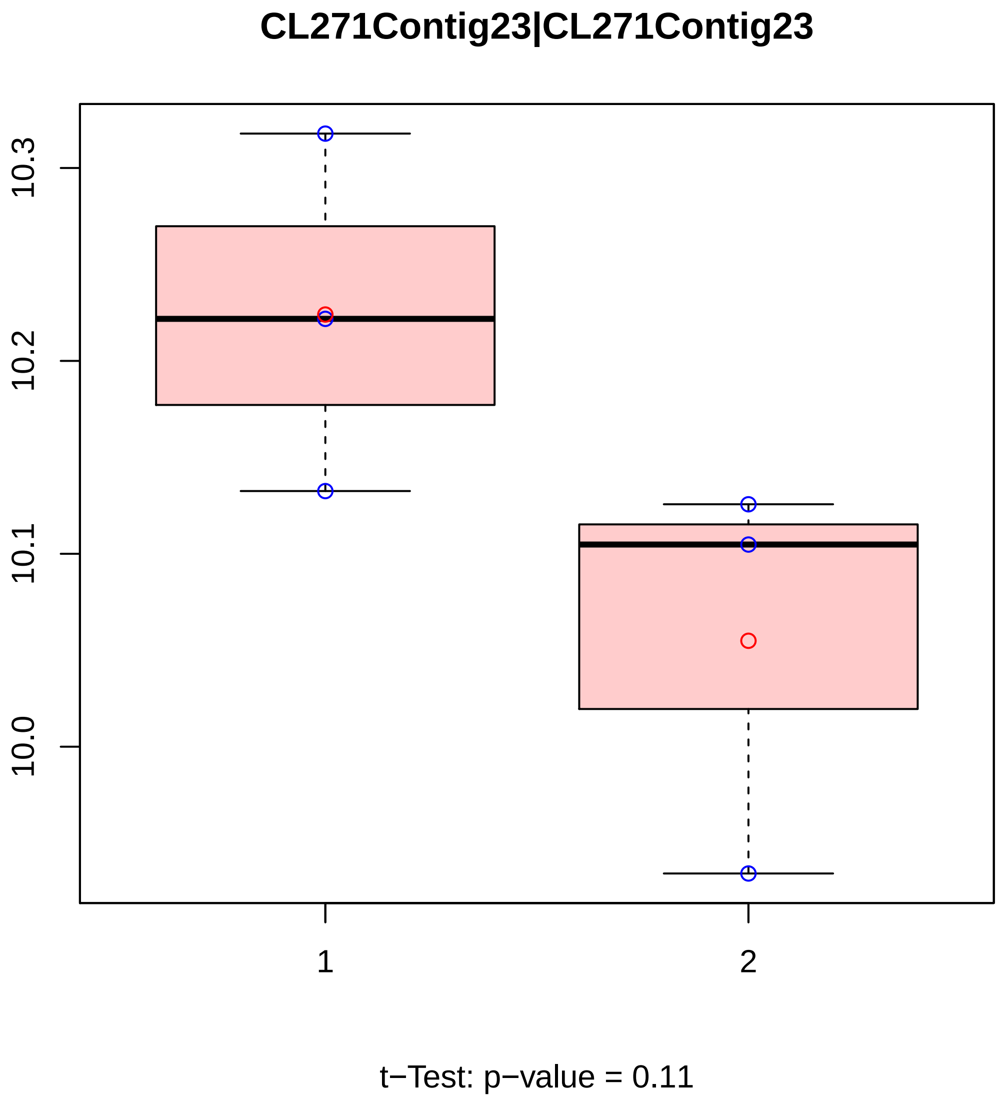

CL271Contig23|CL271Contig23
10.3
10.2
10.1
10.0
1
2
t−Test: p−value = 0.11

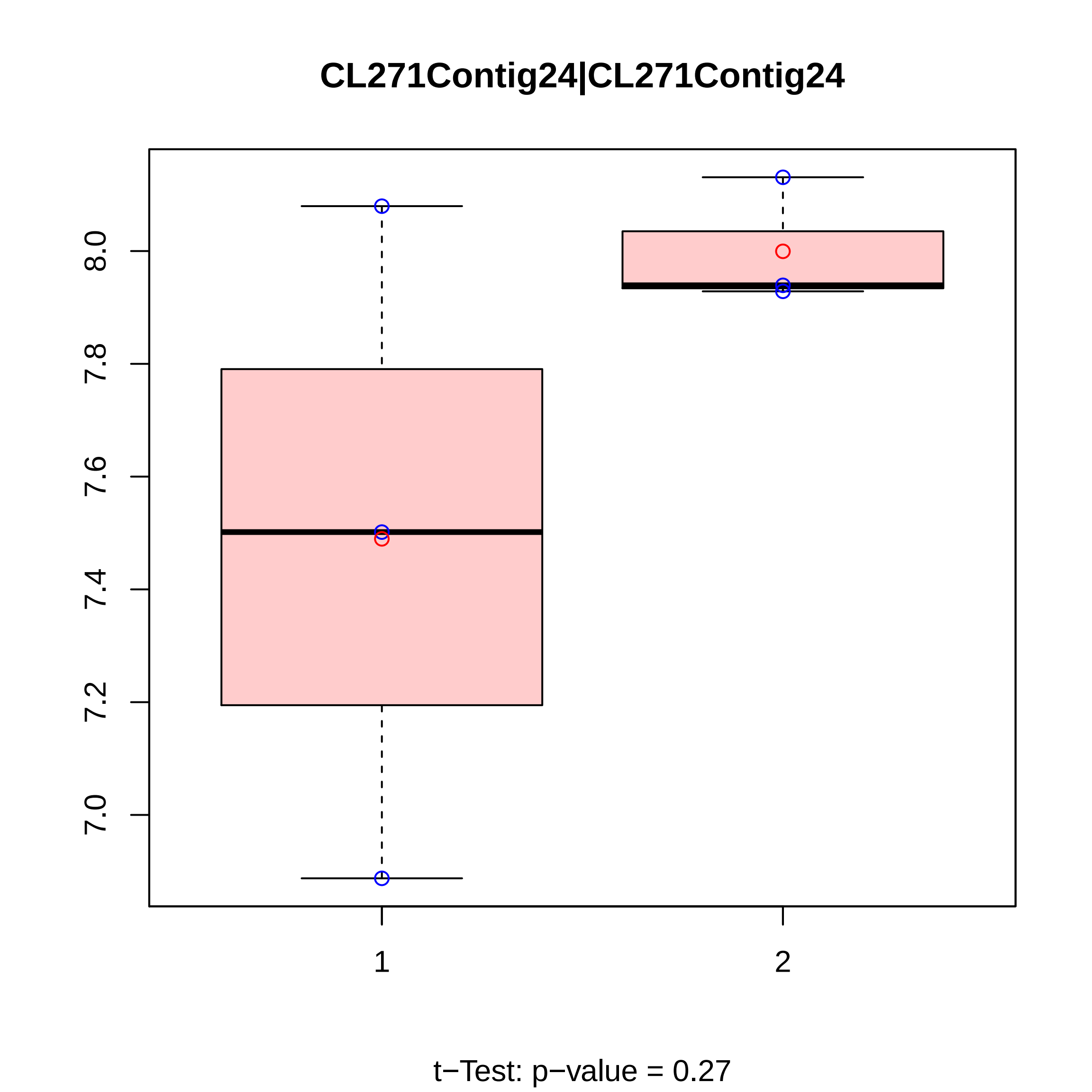

CL271Contig24|CL271Contig24
8.0
7.8
7.6
7.4
7.2
7.0
1
2
t−Test: p−value = 0.27

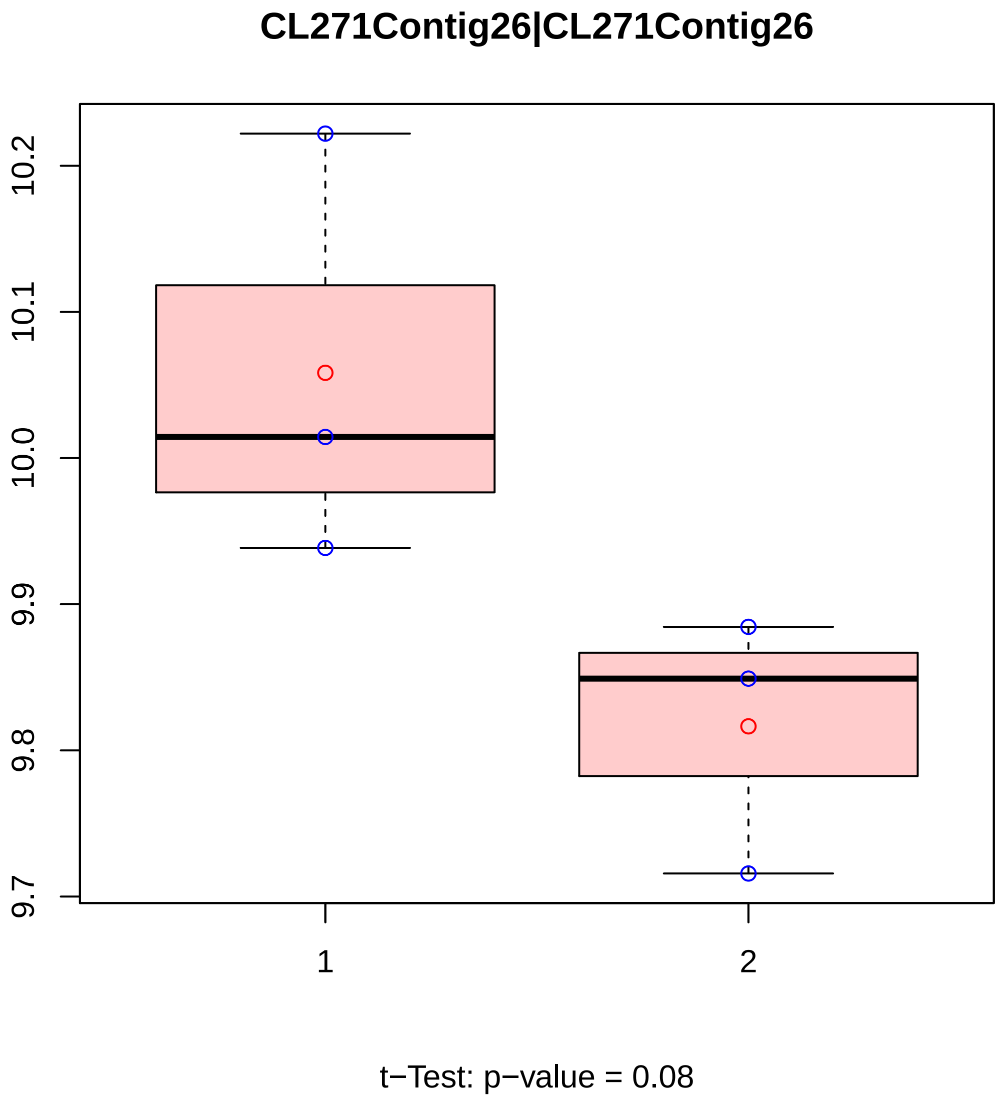

CL271Contig26|CL271Contig26
10.2
10.1
10.0
9.9
9.8
9.7
1
2
t−Test: p−value = 0.08

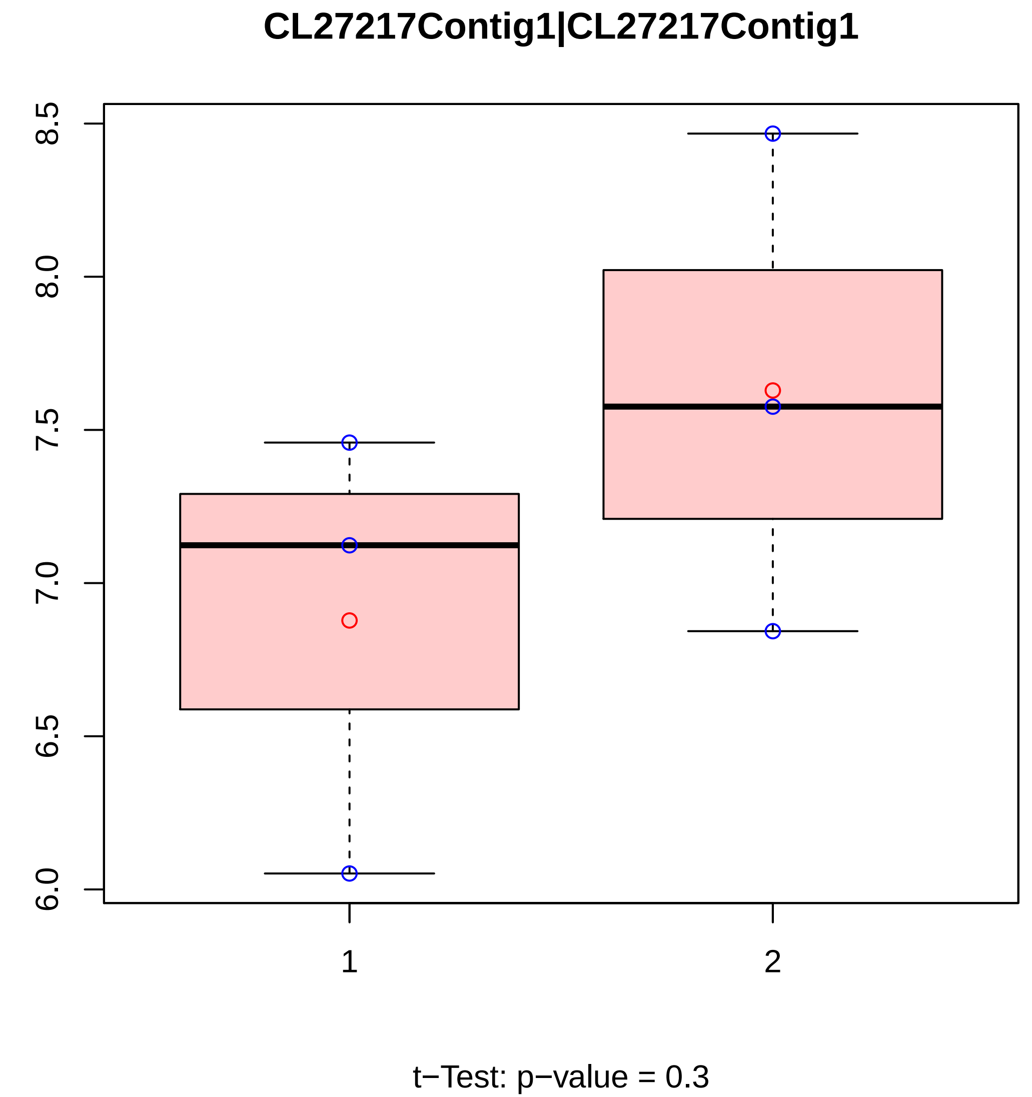
CL27217Contig1|CL27217Contig1
8.5
8.0
7.5
7.0
6.5
6.0
1
2
t−Test: p−value = 0.3

## CL2723Contig3|CL2723Contig3

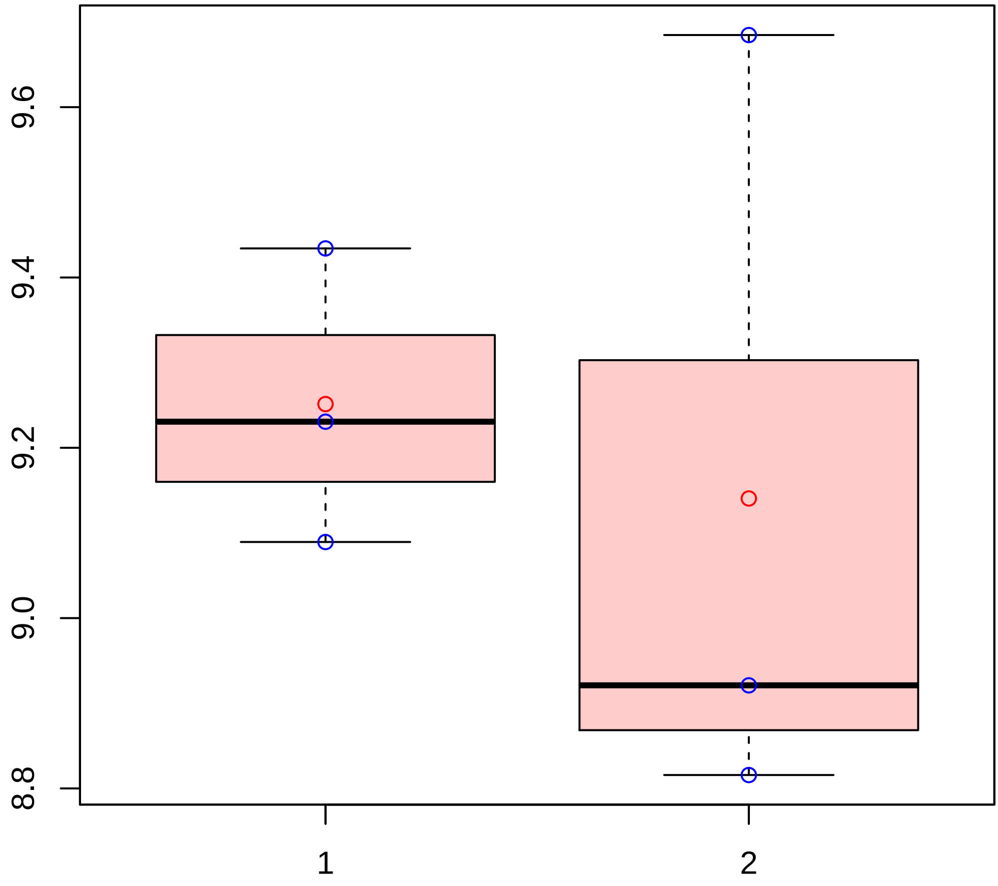


t−Test: p−value = 0.73

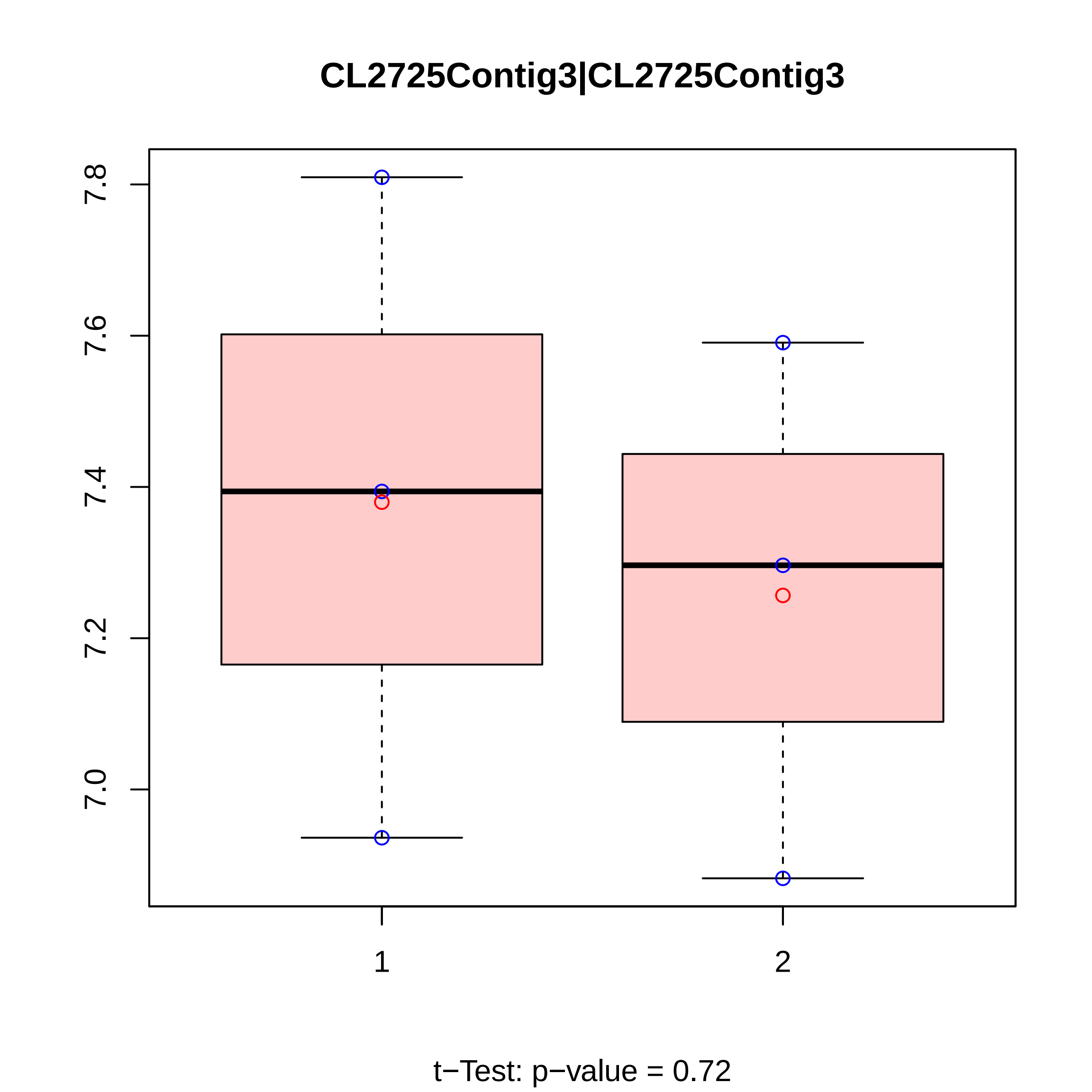
CL2725Contig3|CL2725Contig3
7.8
7.6
7.4
7.2
7.0
1
2
t−Test: p−value = 0.72

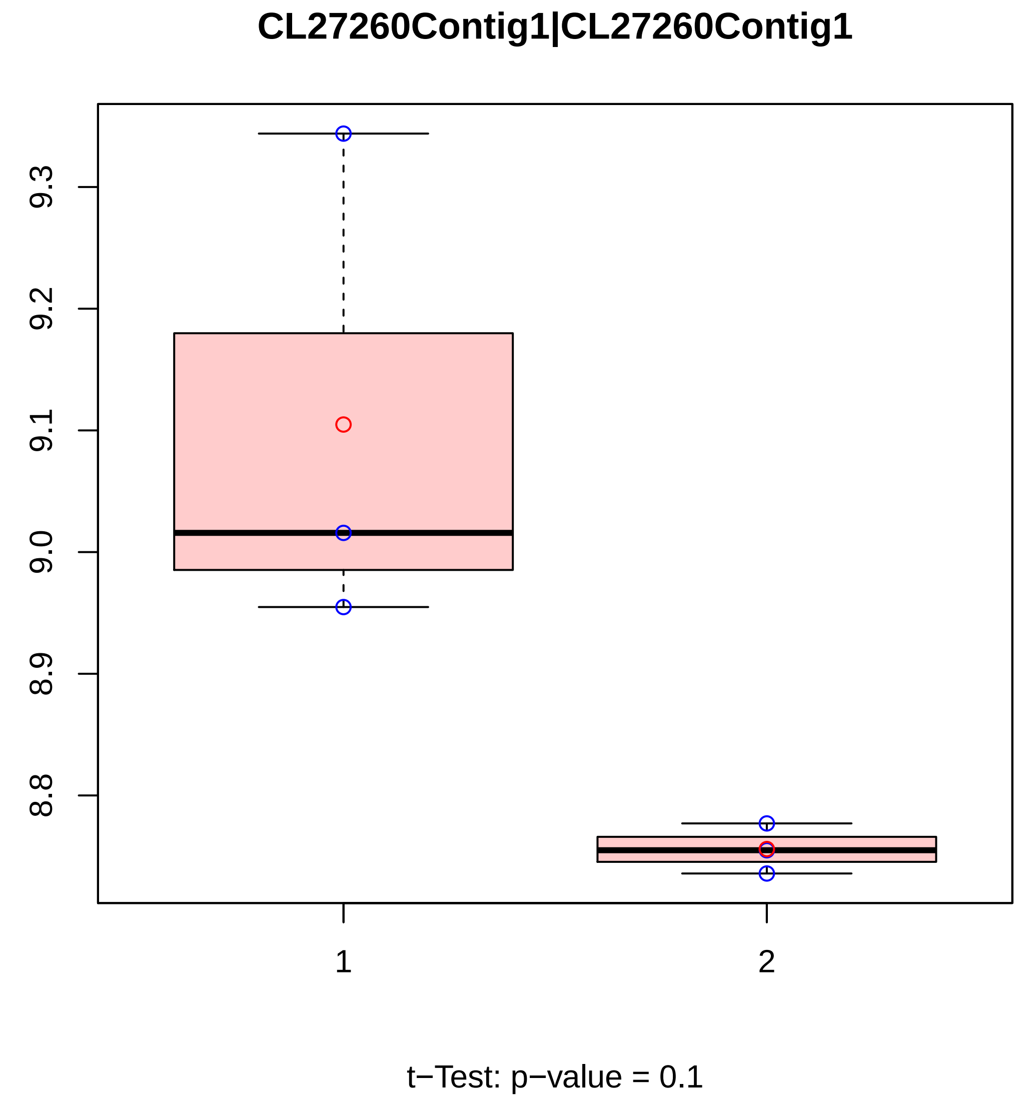

CL27260Contig1|CL27260Contig1
9.3
9.2
9.1
9.0
8.9
8.8
1
2
t−Test: p−value = 0.1

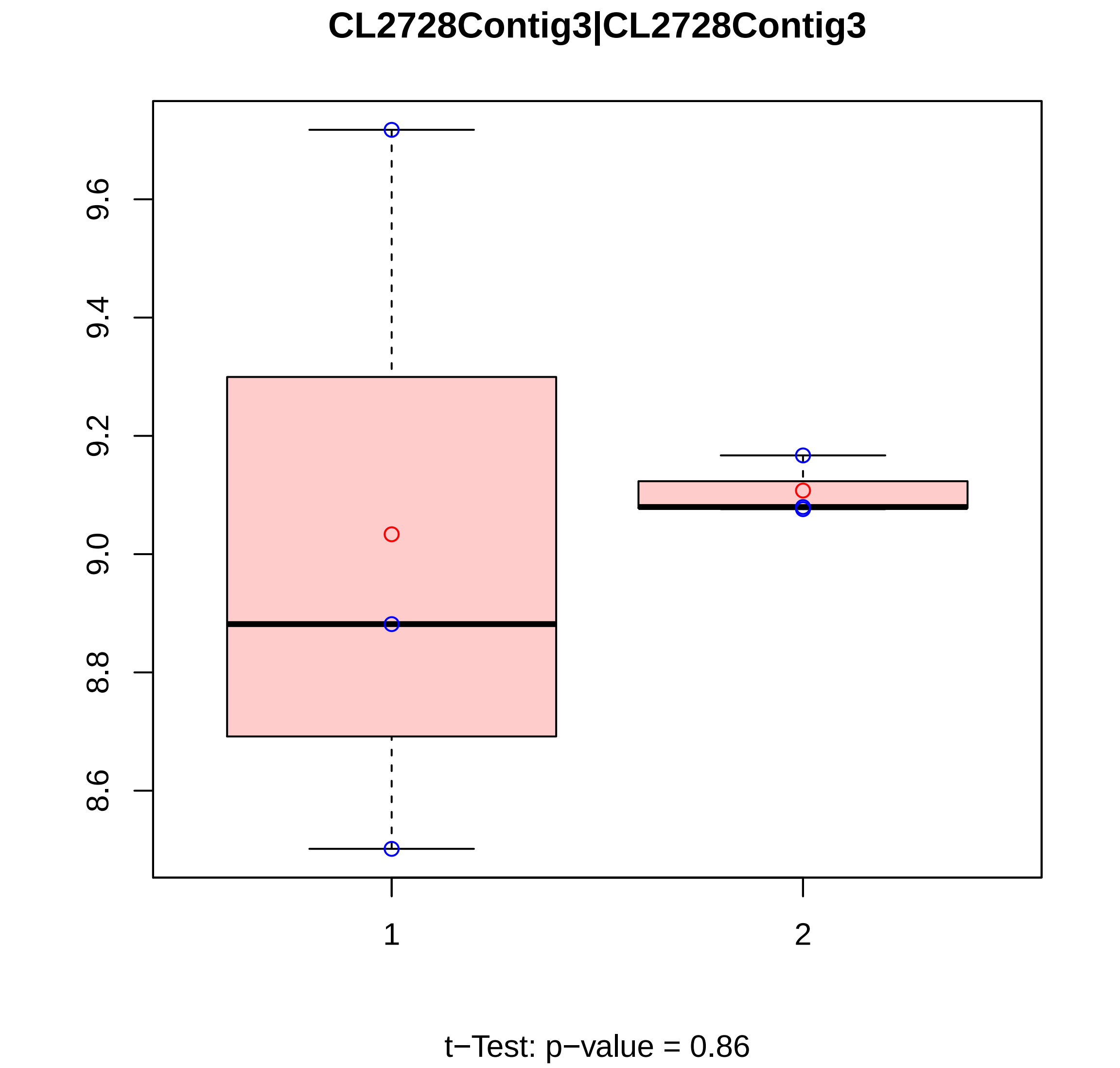
CL2728Contig3|CL2728Contig3
9.6
9.4
9.2
9.0
8.8
8.6
1
2
t−Test: p−value = 0.86

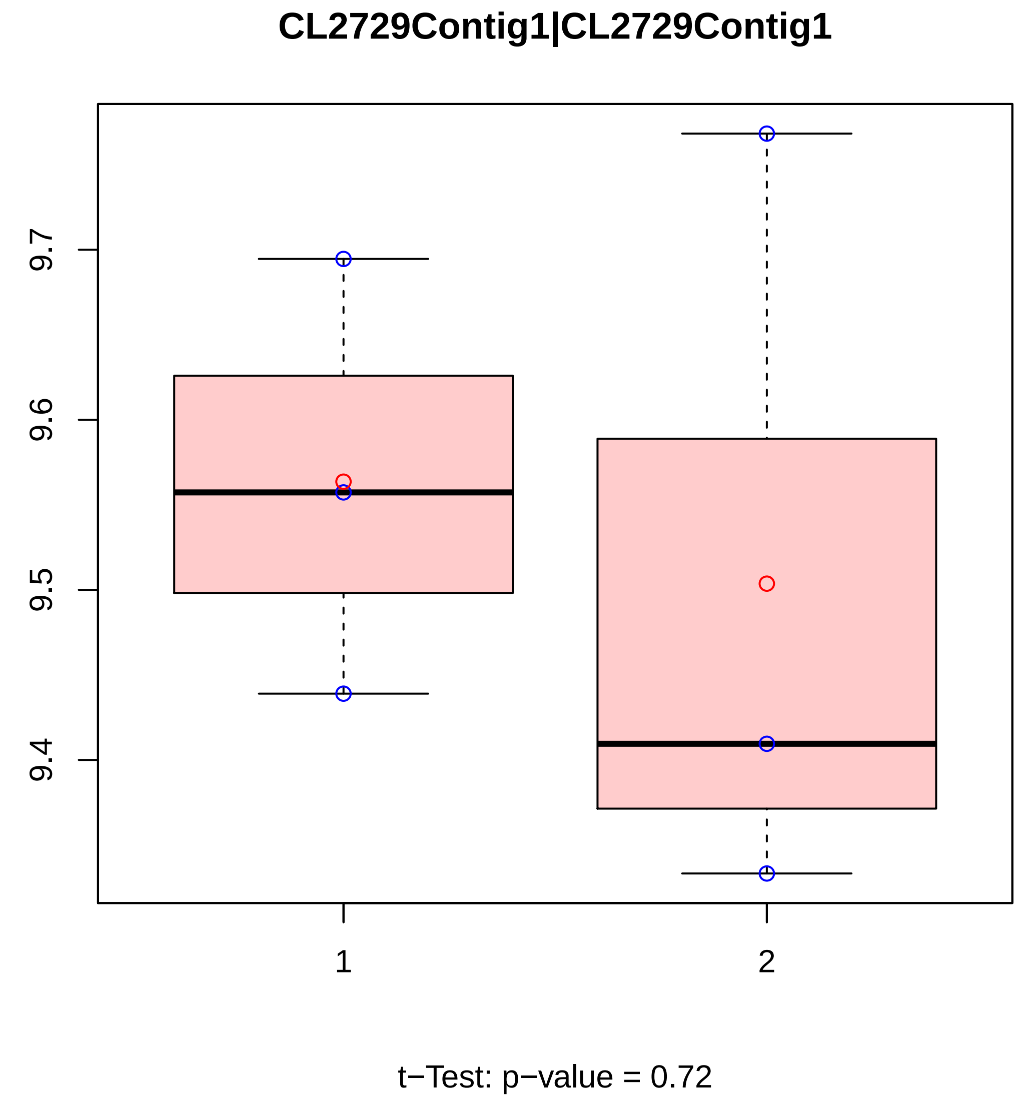

CL2729Contig1|CL2729Contig1
9.4
9.5
9.6
9.7
1
2
t−Test: p−value = 0.72

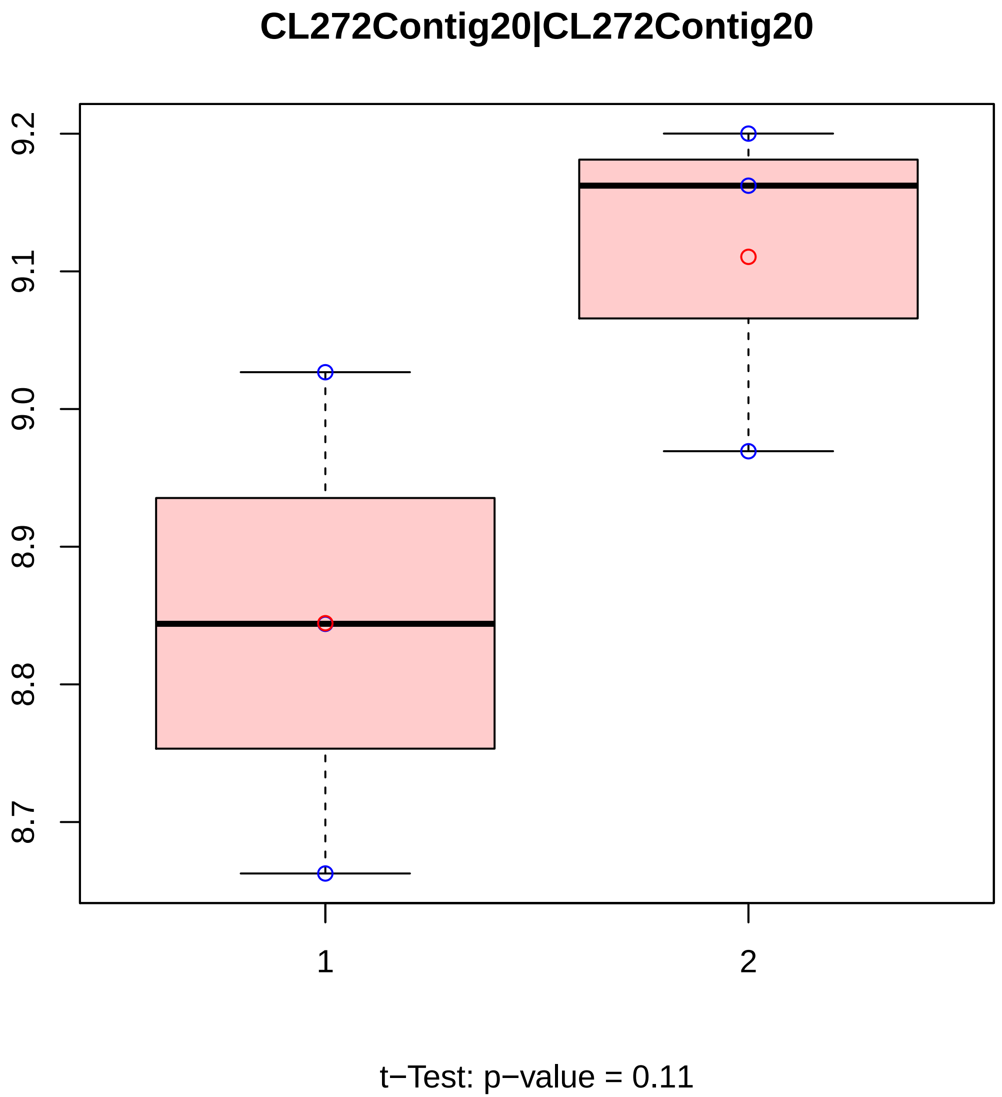
CL272Contig20|CL272Contig20
9.2
9.1
9.0
8.9
8.8
8.7
1
2
t−Test: p−value = 0.11

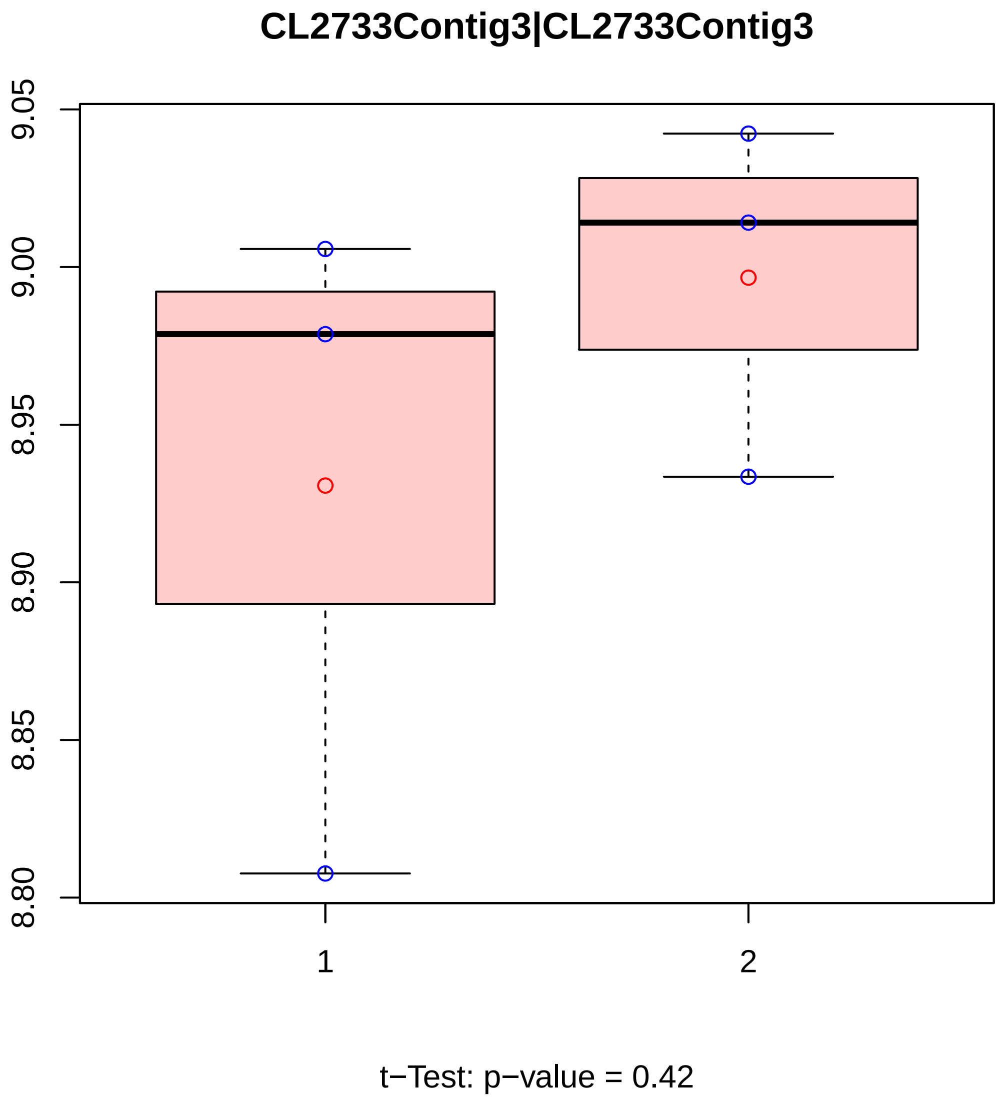
CL2733Contig3|CL2733Contig3
9.05
9.00
8.95
8.90
8.85
8.80
1
2
t−Test: p−value = 0.42

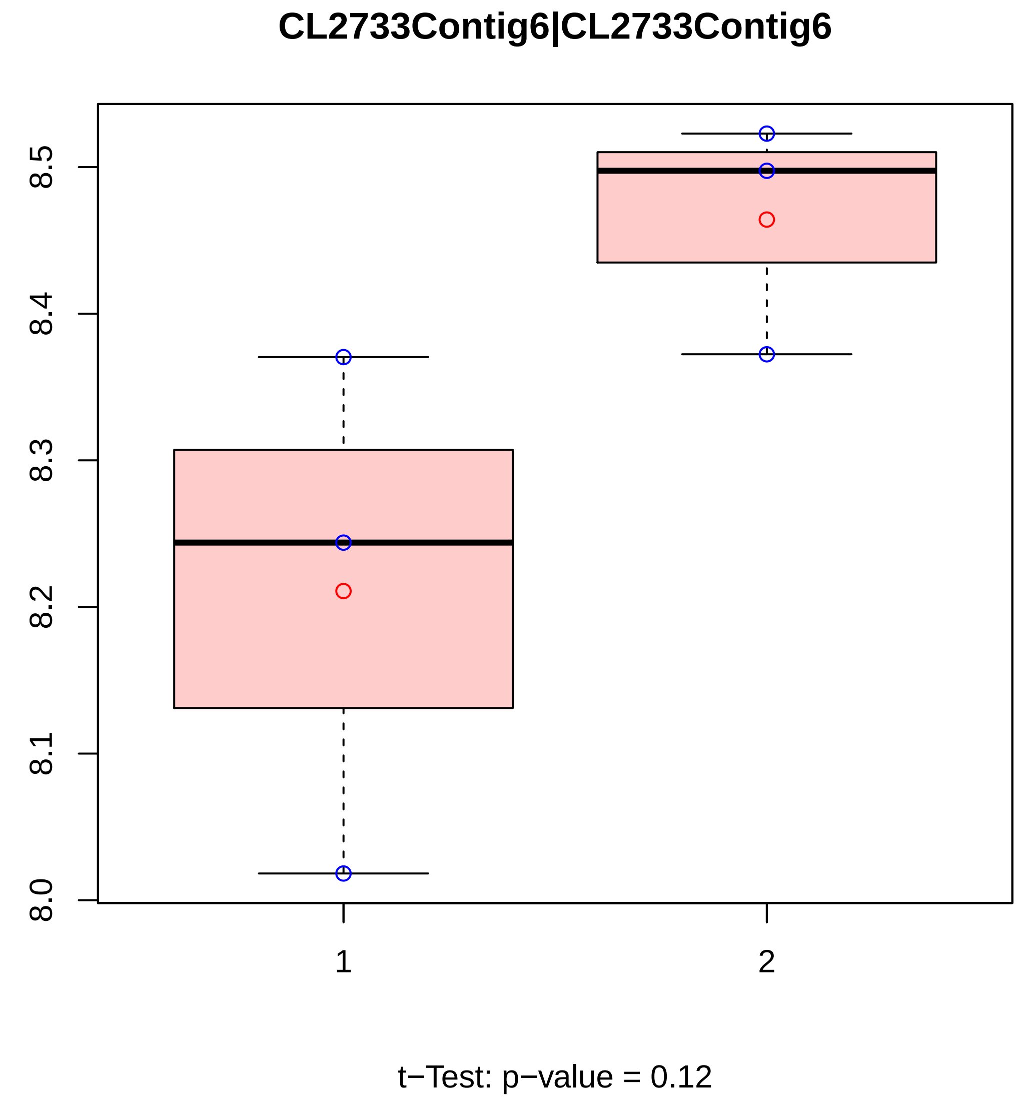
CL2733Contig6|CL2733Contig6
8.0
8.1
8.2
8.3
8.4
8.5
1
2
t−Test: p−value = 0.12

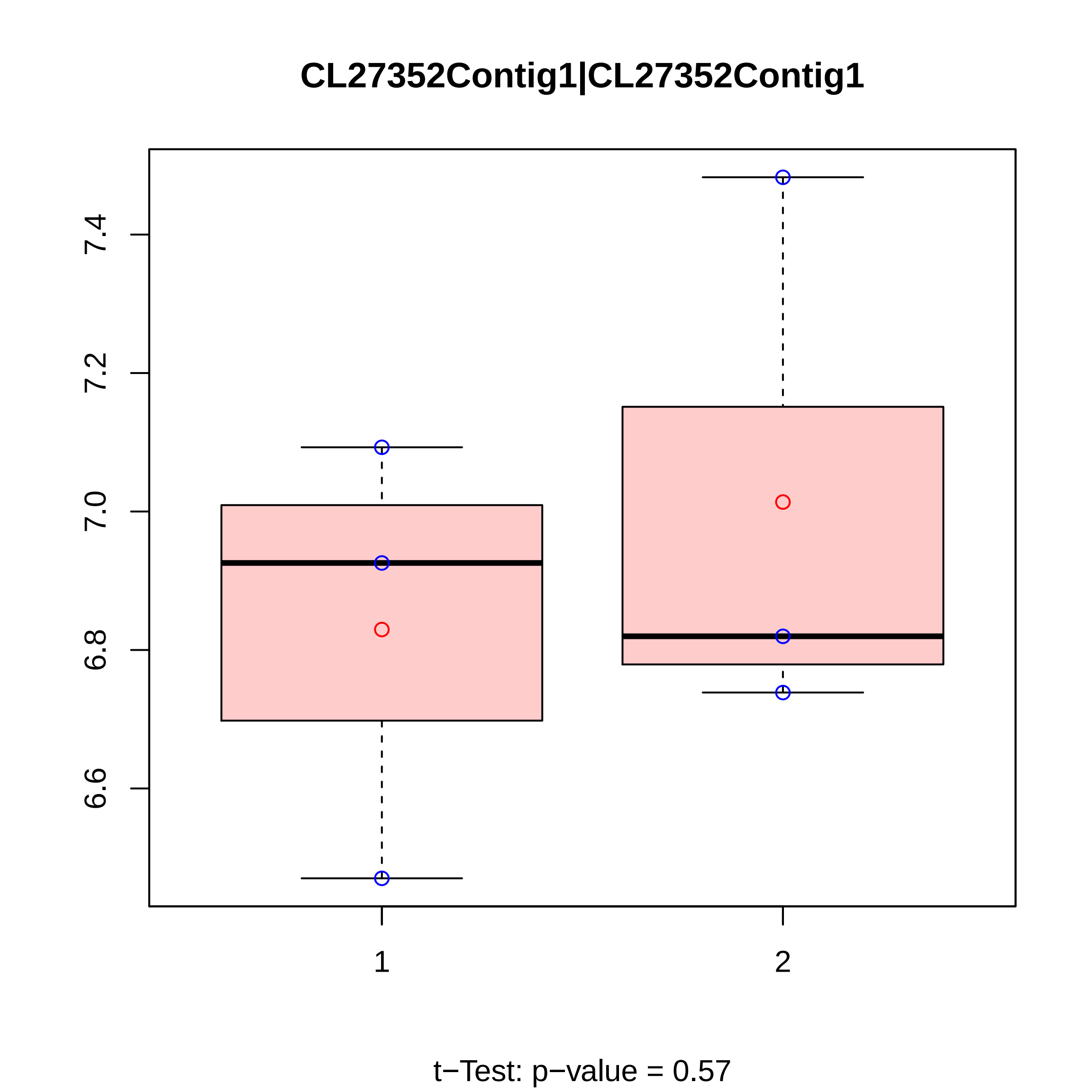

CL27352Contig1|CL27352Contig1
7.4
7.2
7.0
6.8
6.6
1
2
t−Test: p−value = 0.57

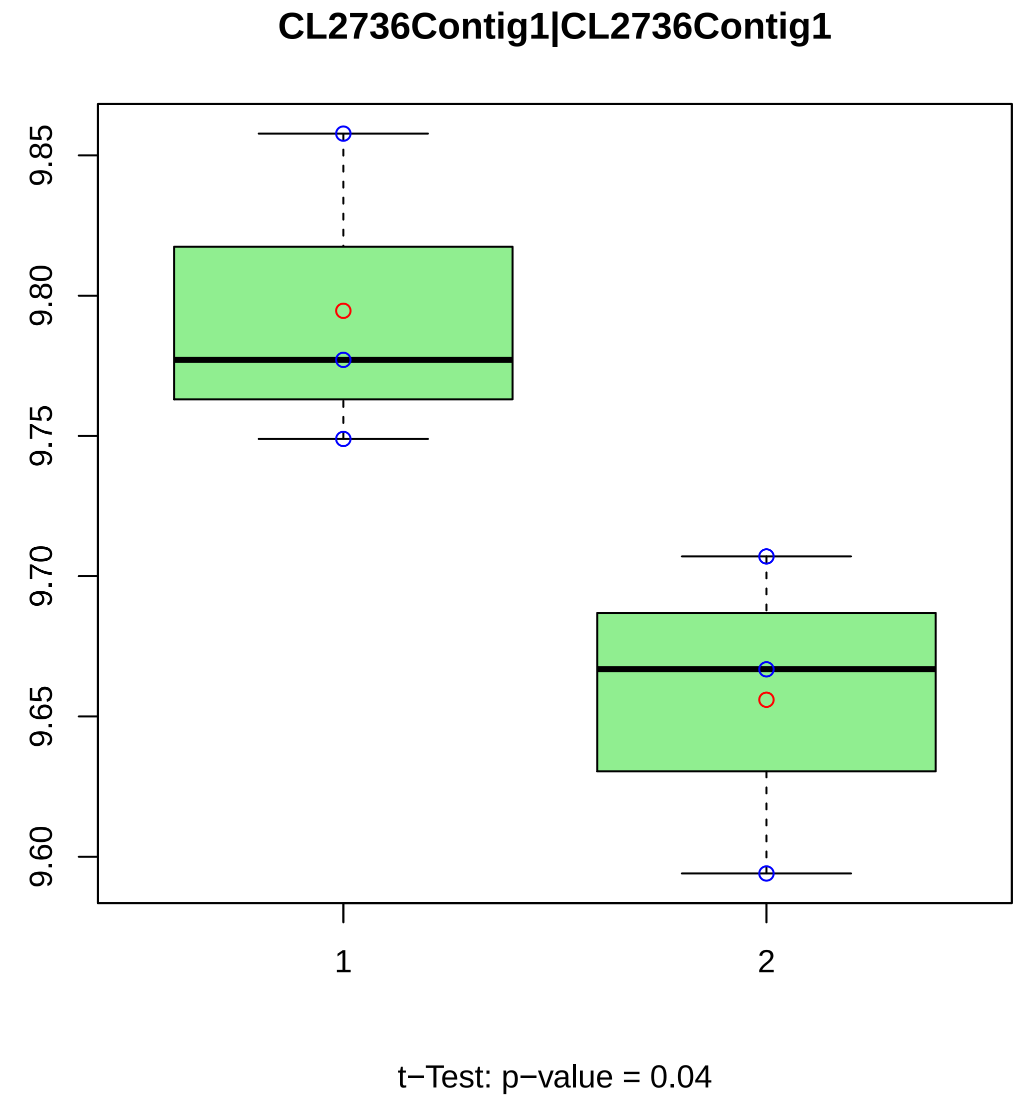
CL2736Contig1|CL2736Contig1
9.85
9.80
9.75
9.70
9.65
9.60
1
2
t−Test: p−value = 0.04

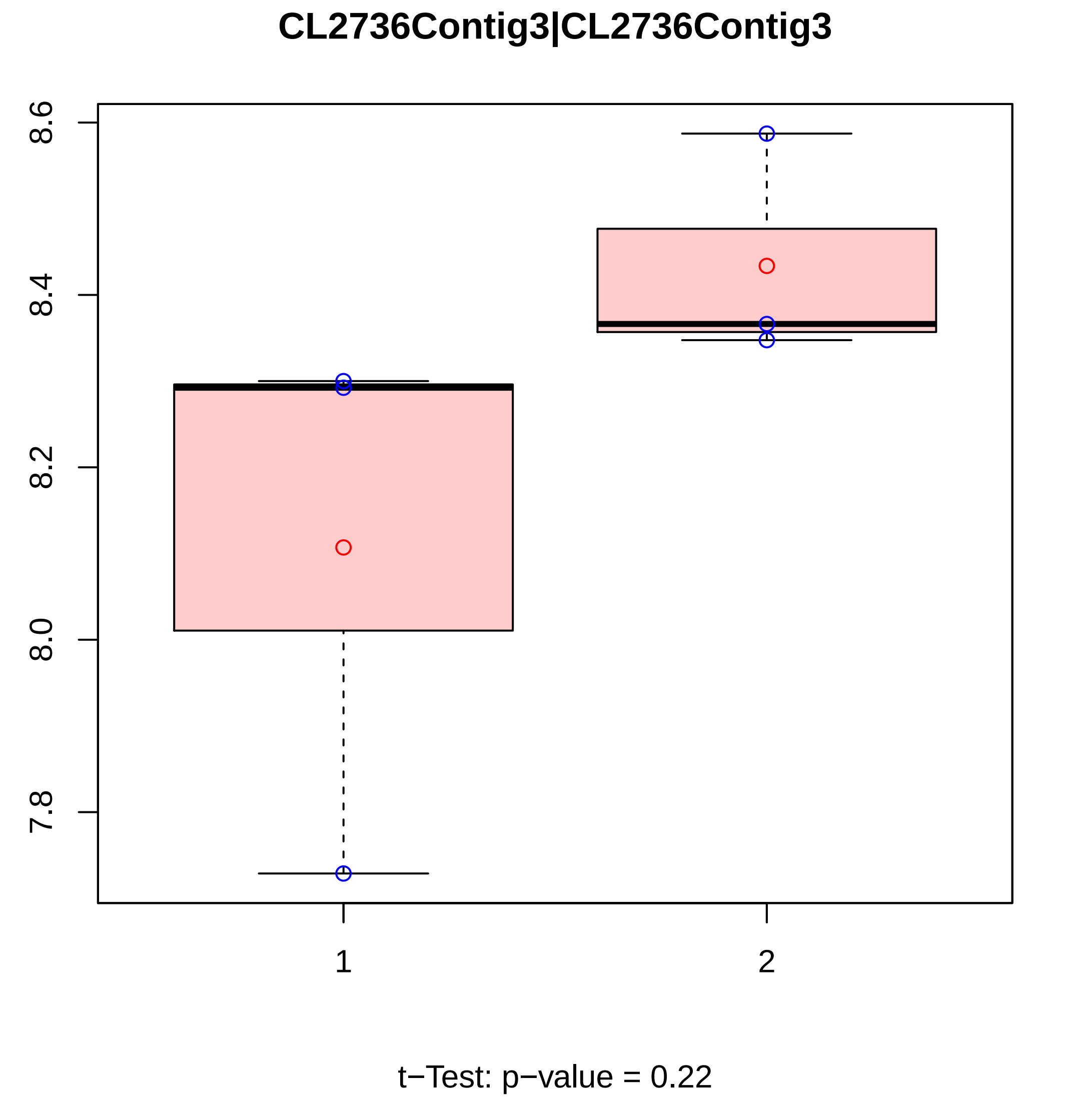
CL2736Contig3|CL2736Contig3
8.6
8.4
8.2
8.0
7.8
1
2
t−Test: p−value = 0.22

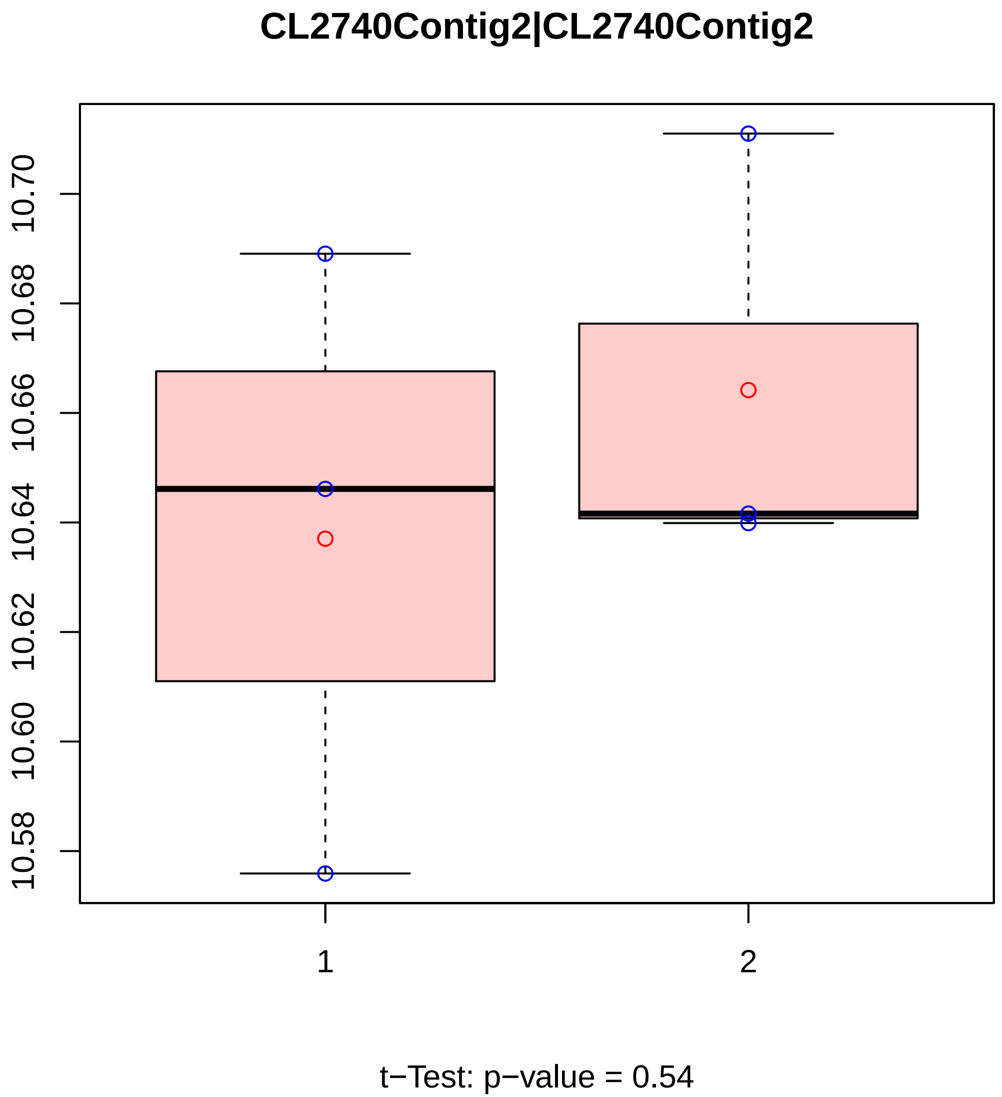
CL2740Contig2|CL2740Contig2
10.58
10.60
10.62
10.64
10.66
10.68
10.70
1
2
t−Test: p−value = 0.54

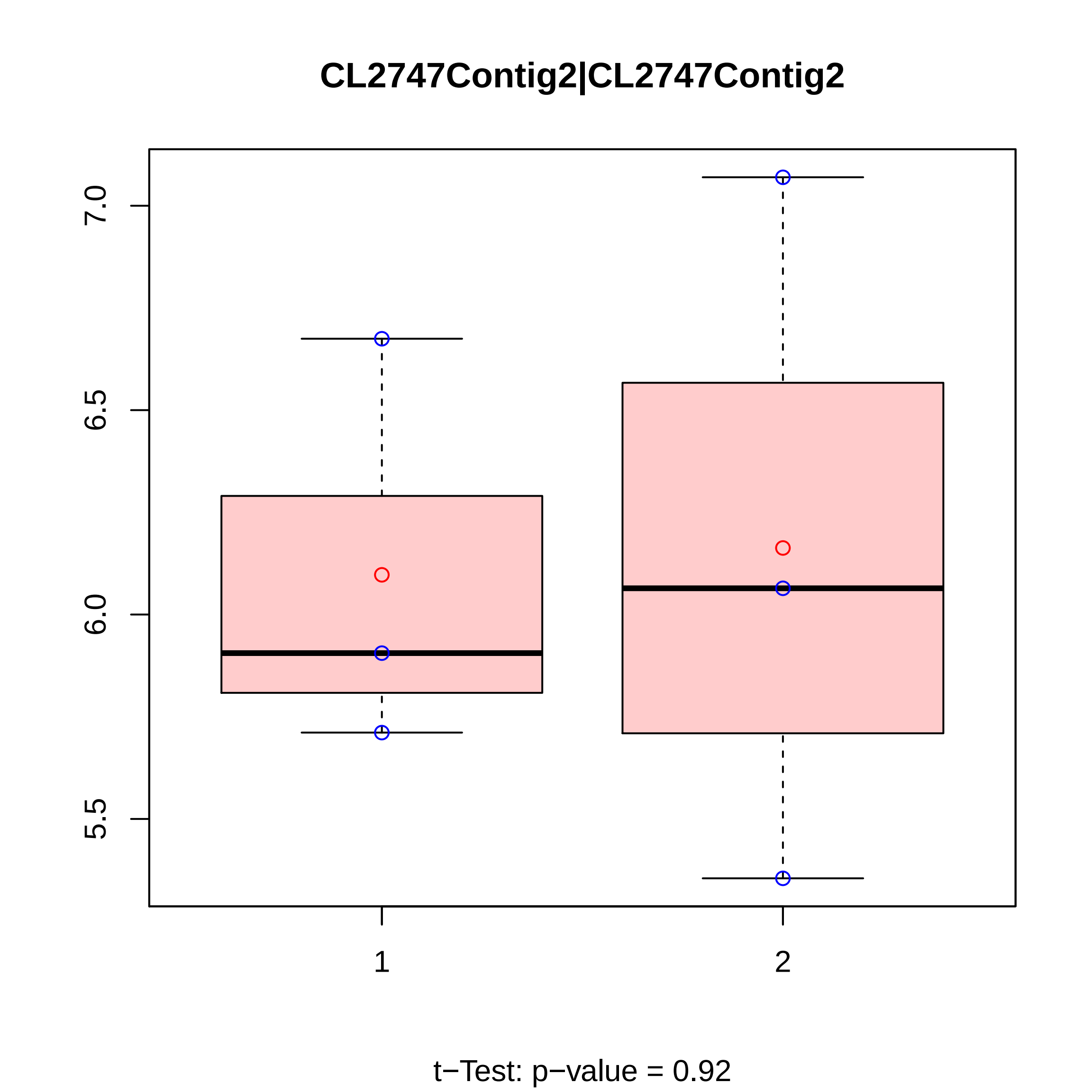
CL2747Contig2|CL2747Contig2
7.0
6.5
6.0
5.5
1
2
t−Test: p−value = 0.92

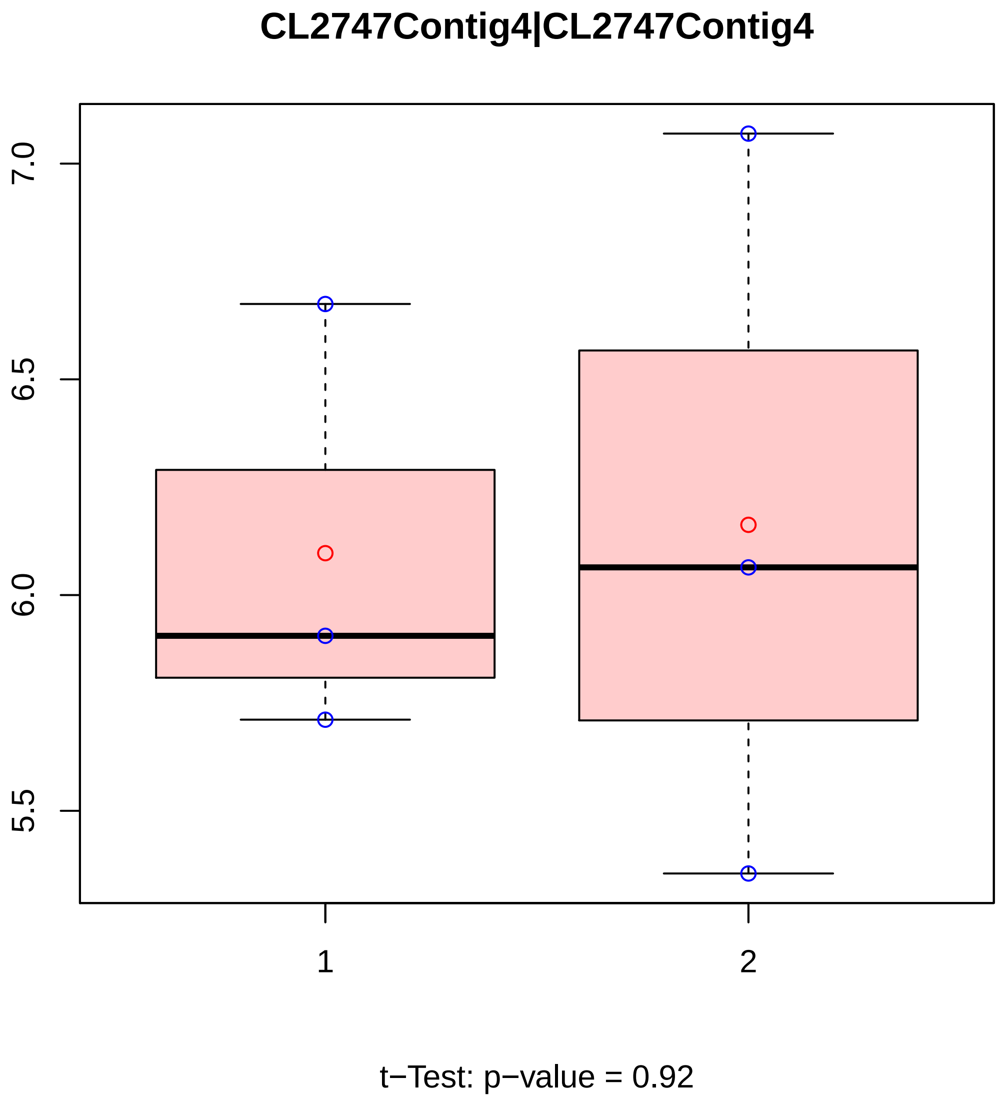
CL2747Contig4|CL2747Contig4
7.0
6.5
6.0
5.5
1
2
t−Test: p−value = 0.92

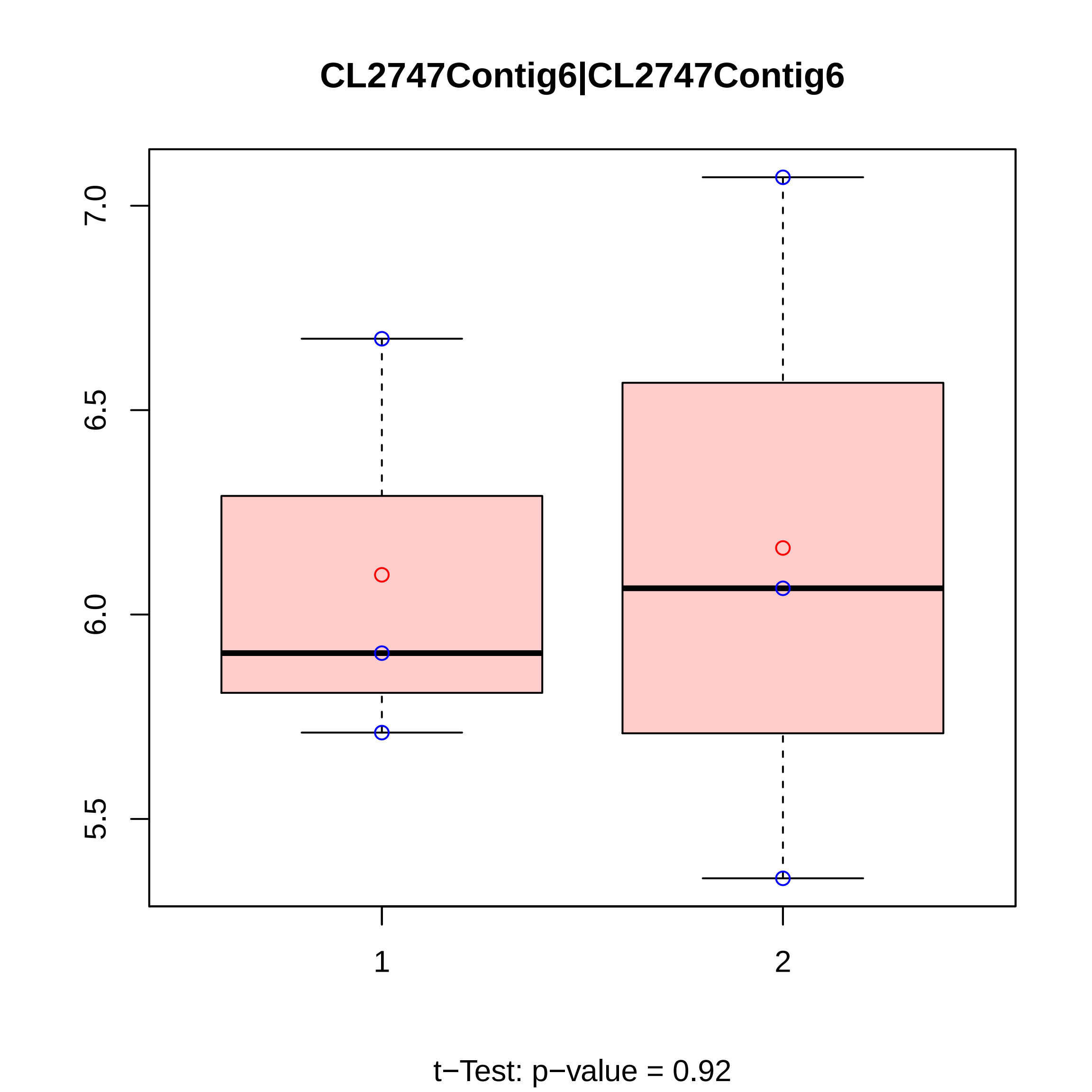

CL2747Contig6|CL2747Contig6
7.0
6.5
6.0
5.5
1
2
t−Test: p−value = 0.92

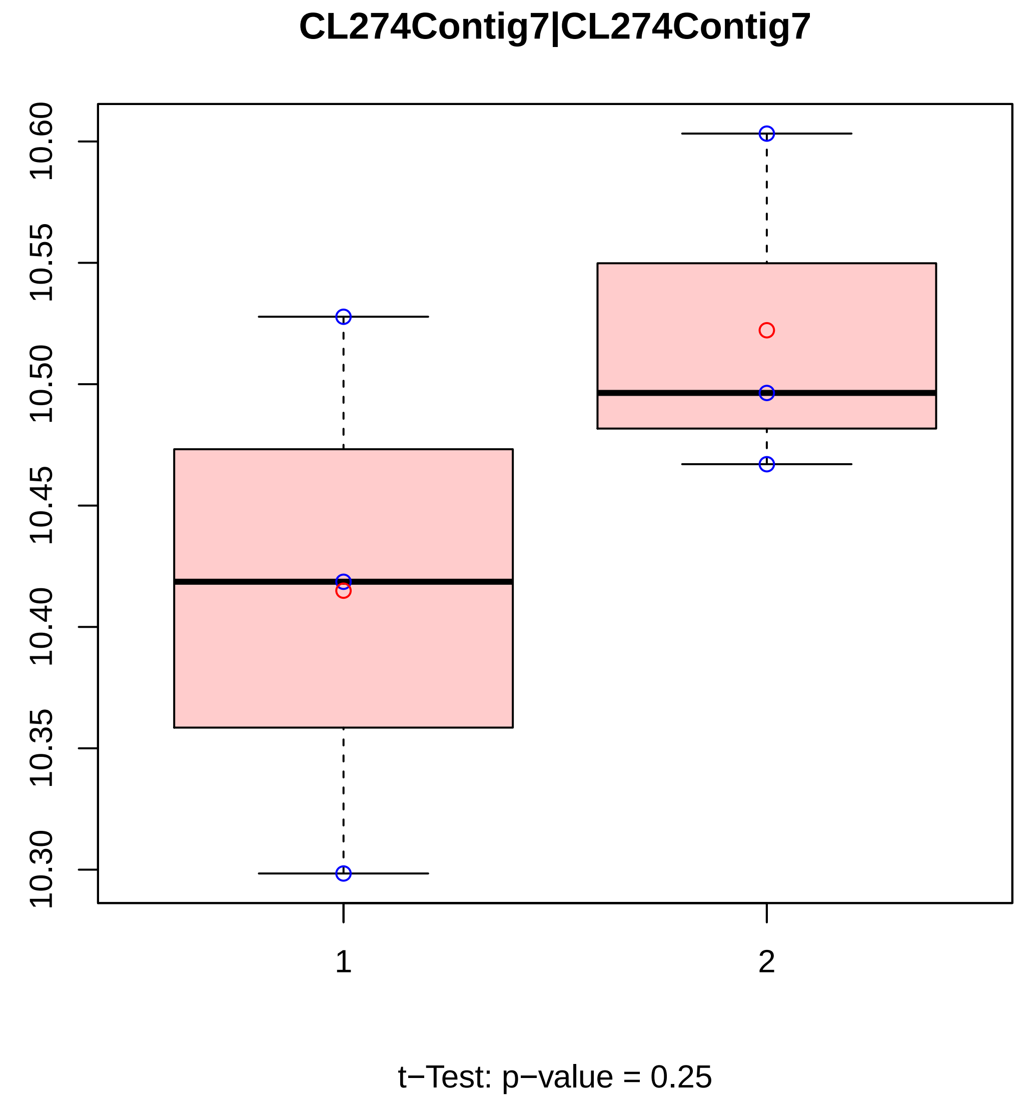
CL274Contig7|CL274Contig7
10.60
10.55
10.50
10.45
10.40
10.35
10.30
1
2
t−Test: p−value = 0.25

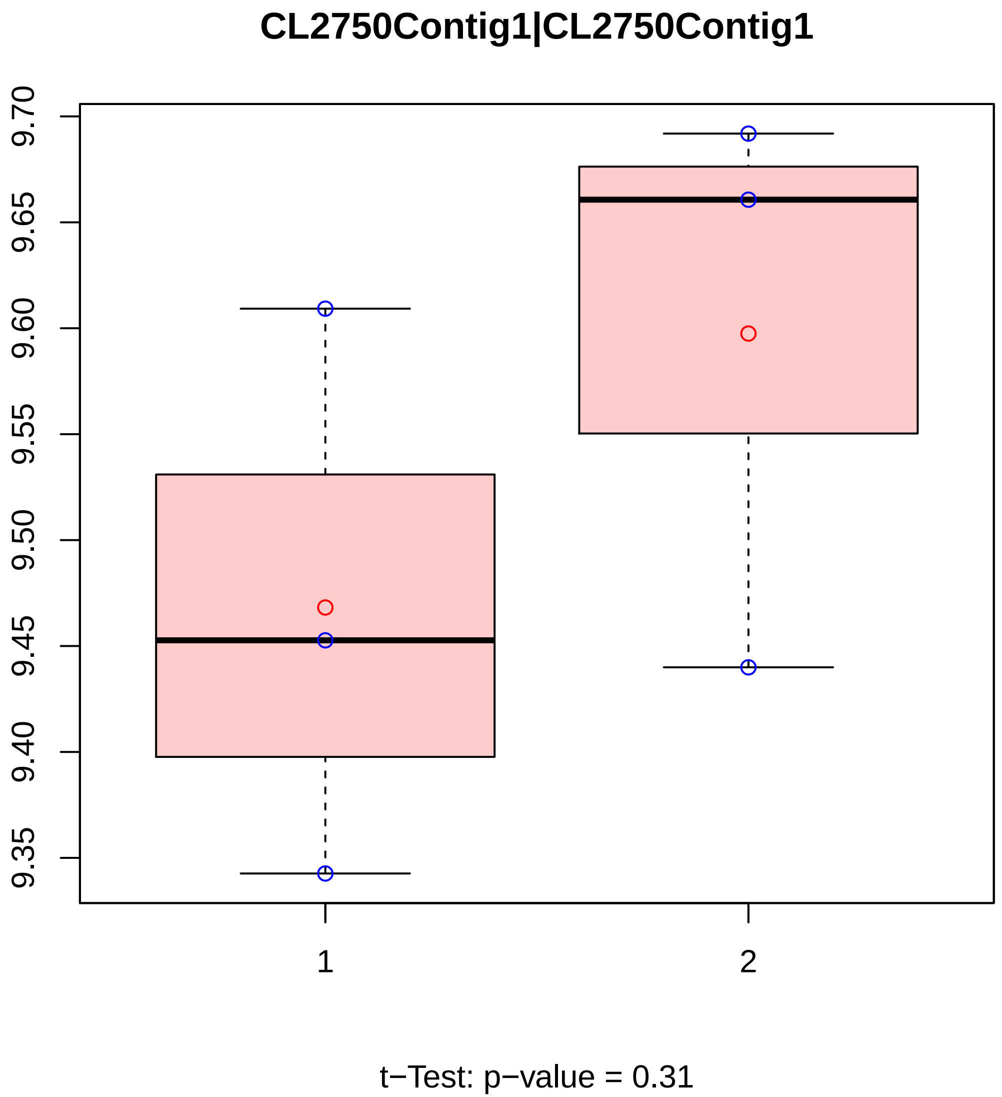
CL2750Contig1|CL2750Contig1
9.70
9.65
9.60
9.55
9.50
9.45
9.40
9.35
1
2
t−Test: p−value = 0.31

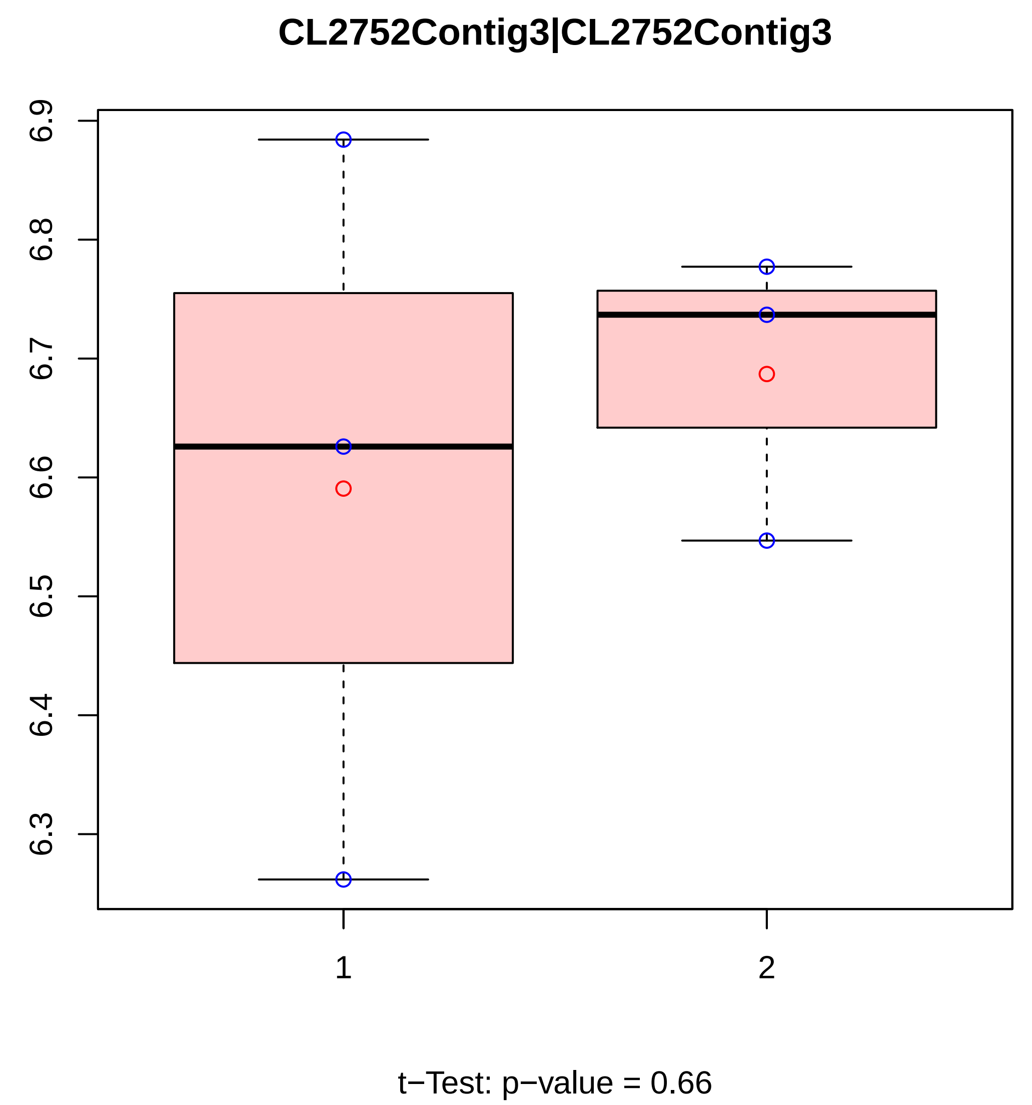
CL2752Contig3|CL2752Contig3
6.3
6.4
6.5
6.6
6.7
6.8
6.9
1
2
t−Test: p−value = 0.66

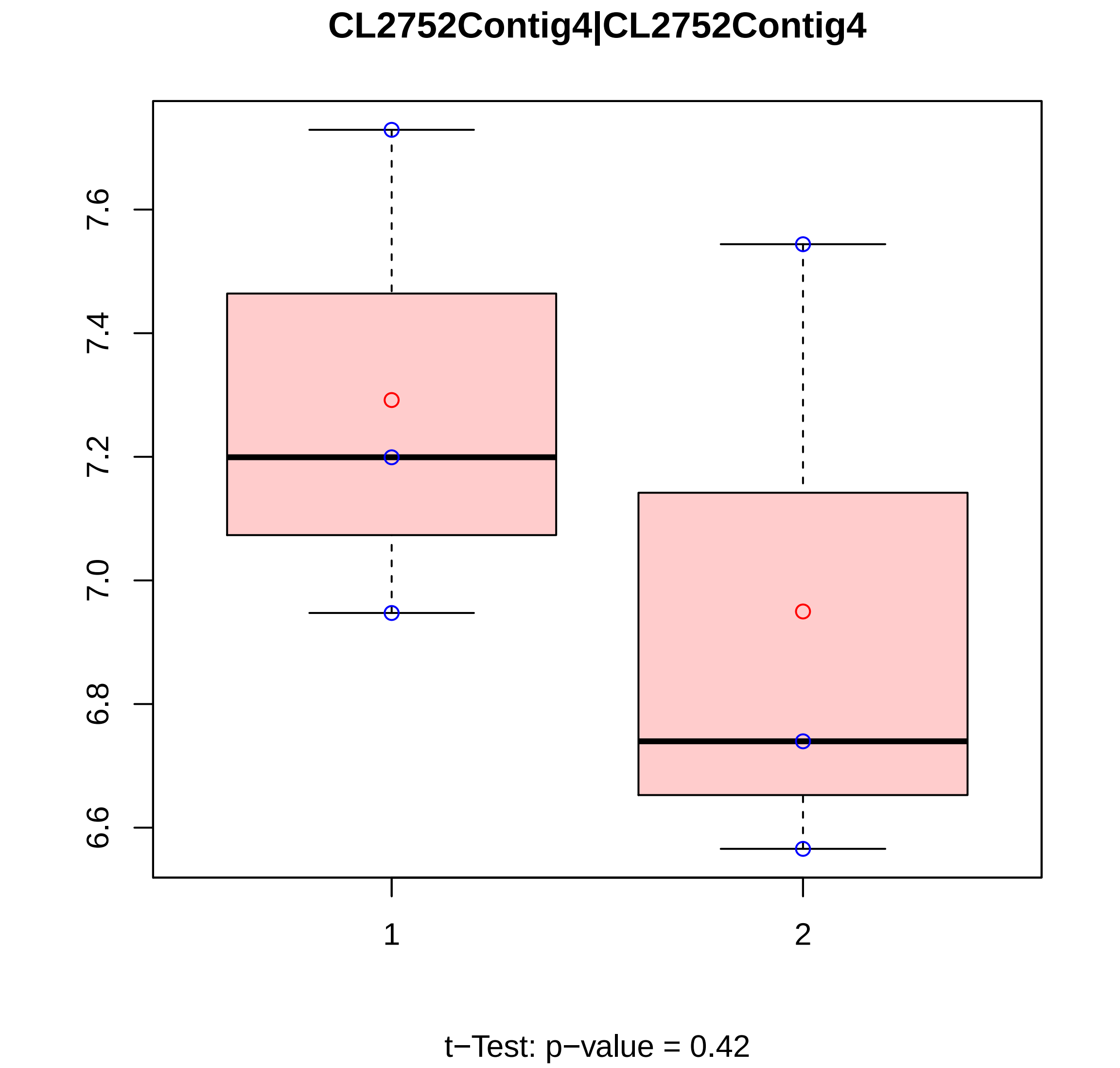
CL2752Contig4|CL2752Contig4
7.6
7.4
7.2
7.0
6.8
6.6
1
2
t−Test: p−value = 0.42

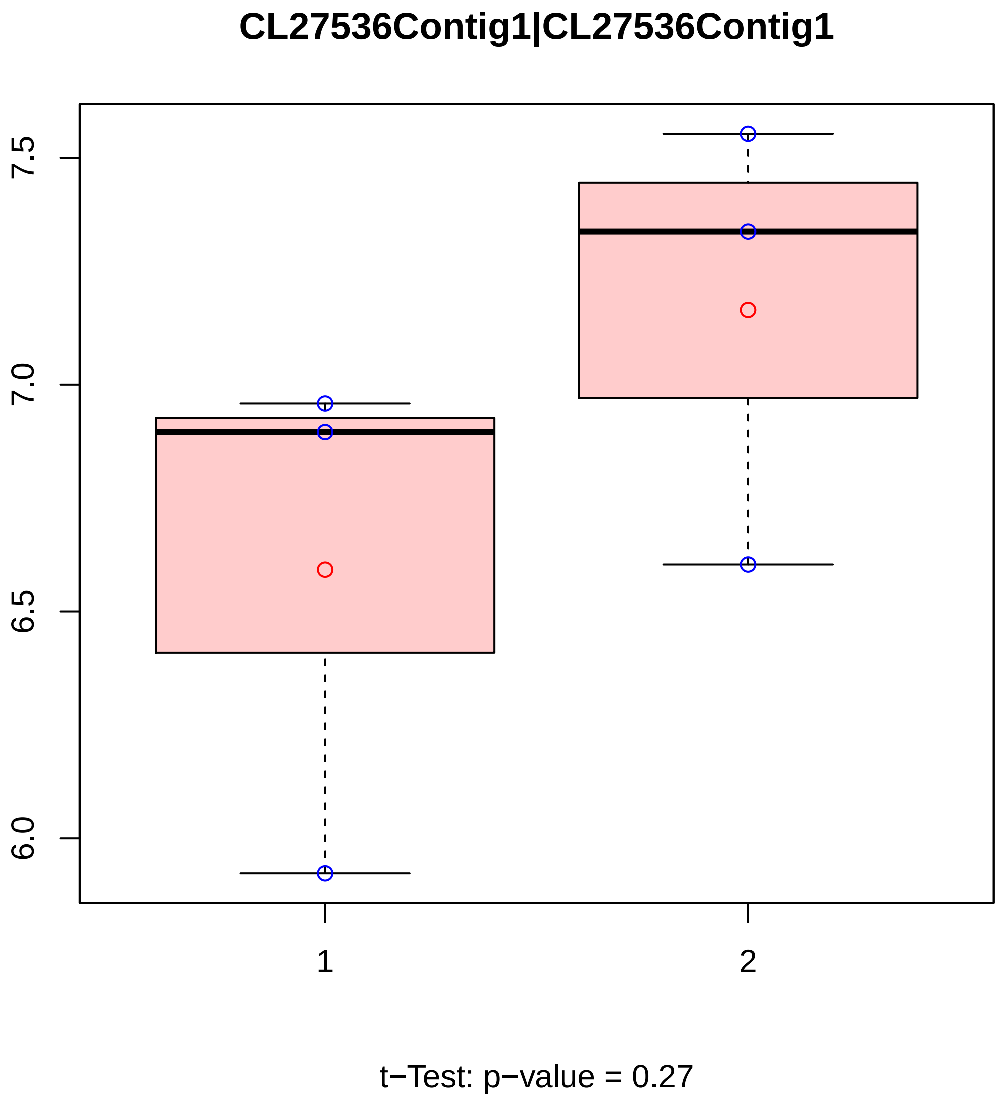
CL27536Contig1|CL27536Contig1
7.5
7.0
6.5
6.0
1
2
t−Test: p−value = 0.27

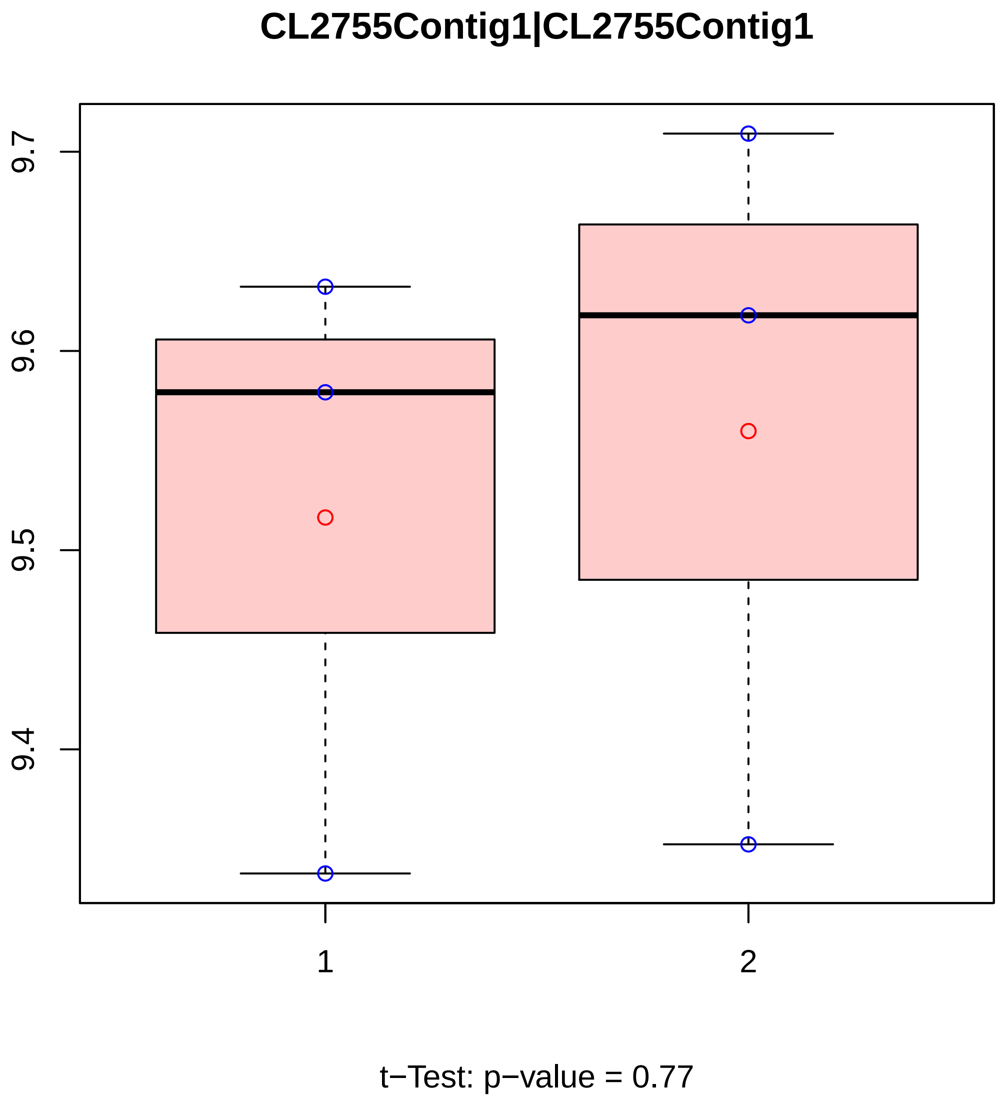
CL2755Contig1|CL2755Contig1
9.4
9.5
9.6
9.7
1
2
t−Test: p−value = 0.77

## CL2756Contig5|CL2756Contig5

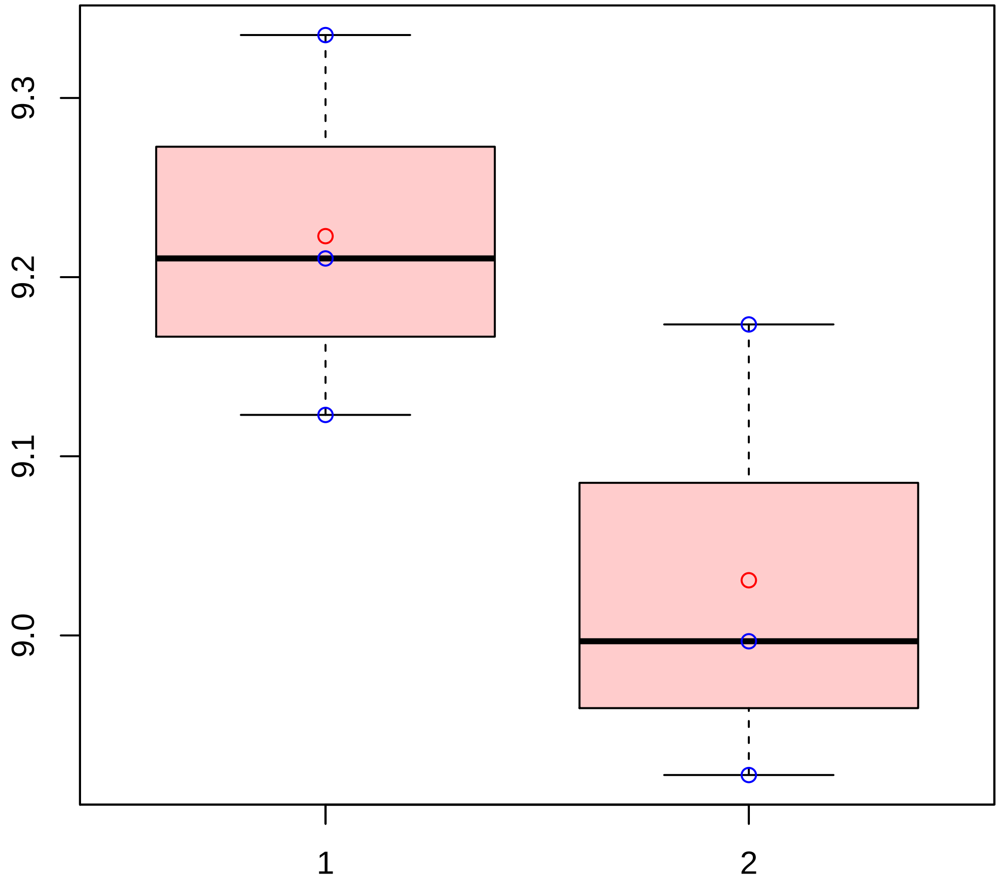


t−Test: p−value = 0.12

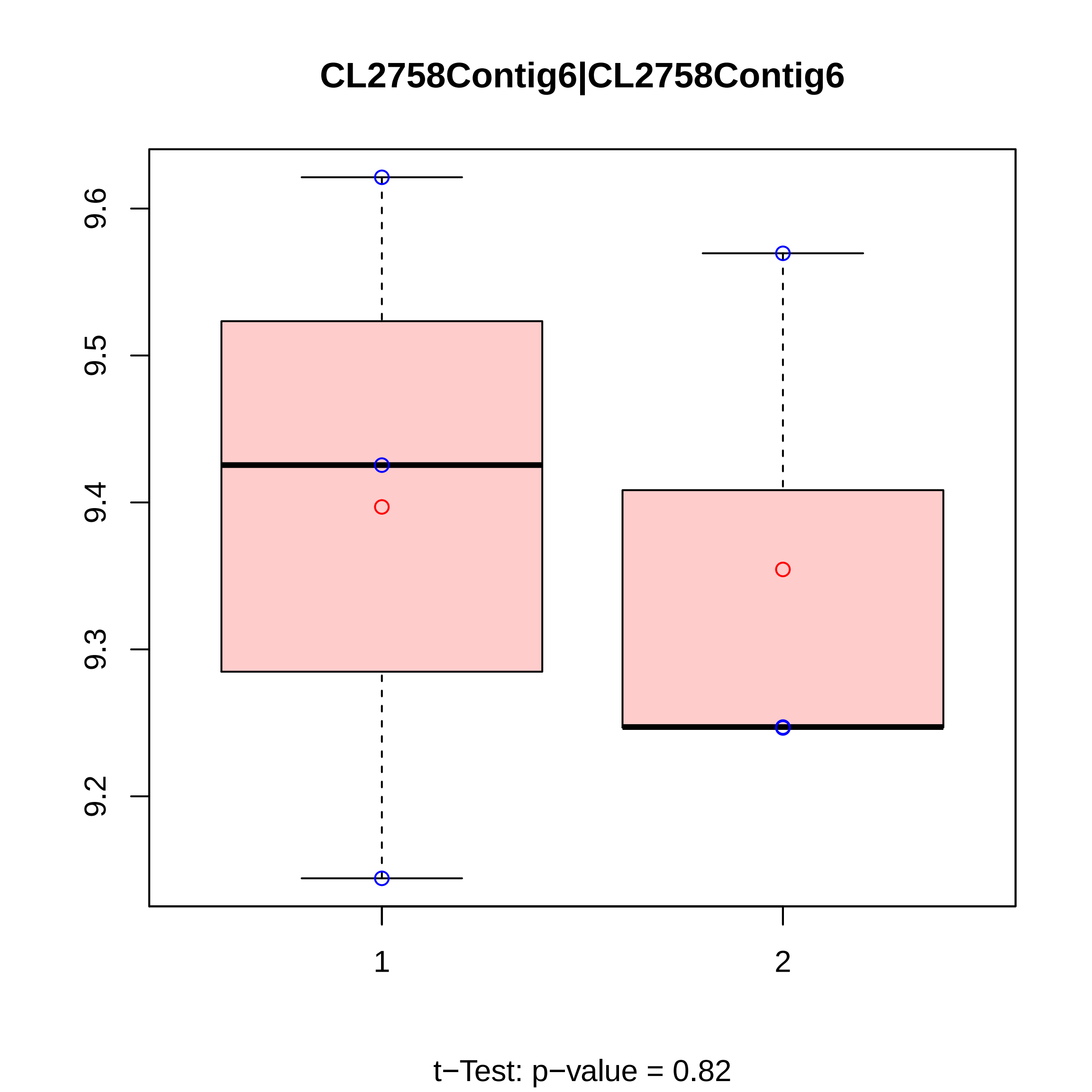

CL2758Contig6|CL2758Contig6
9.2
9.3
9.4
9.5
9.6
1
2
t−Test: p−value = 0.82

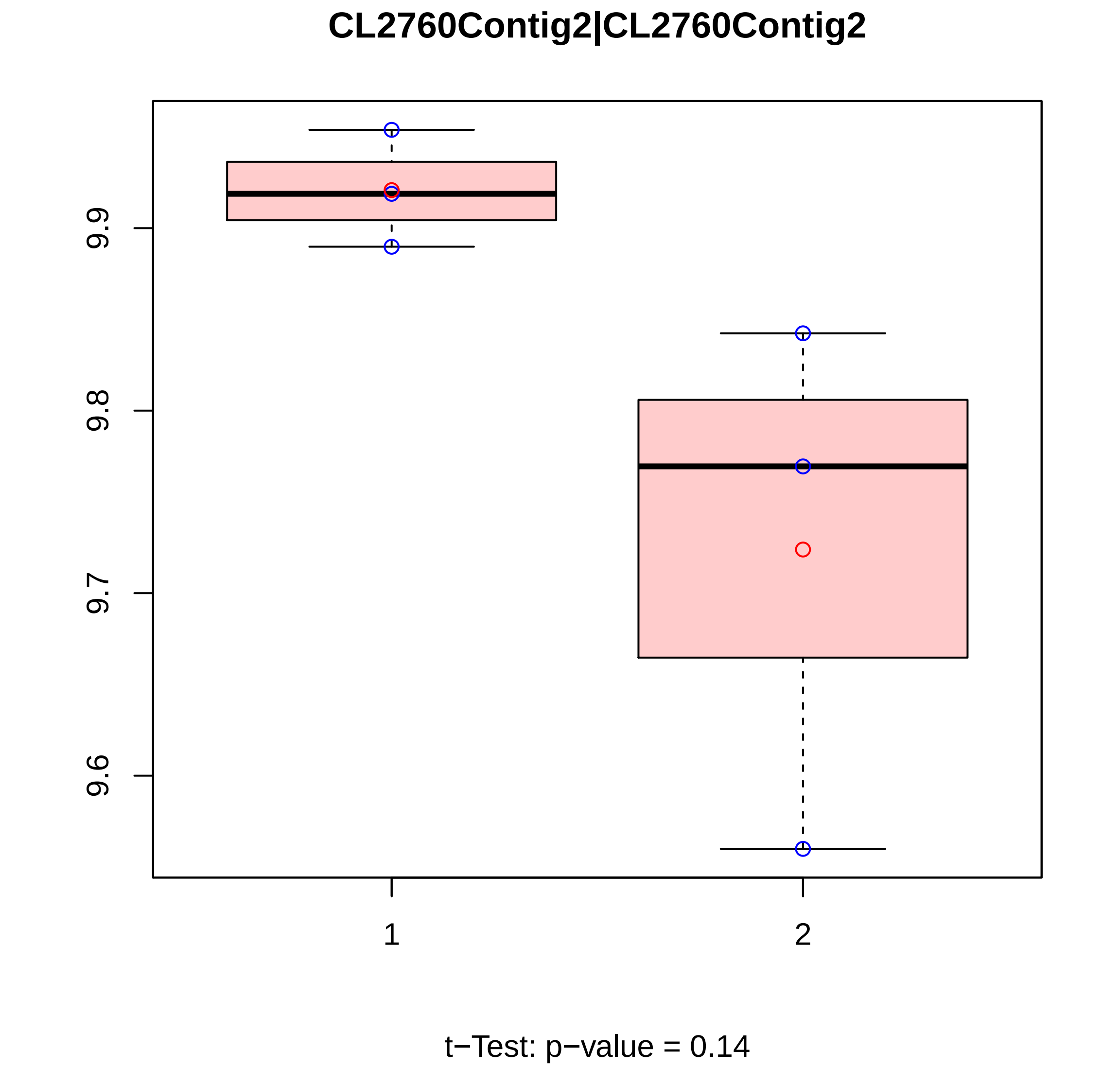
CL2760Contig2|CL2760Contig2
9.6
9.7
9.8
9.9
1
2
t−Test: p−value = 0.14

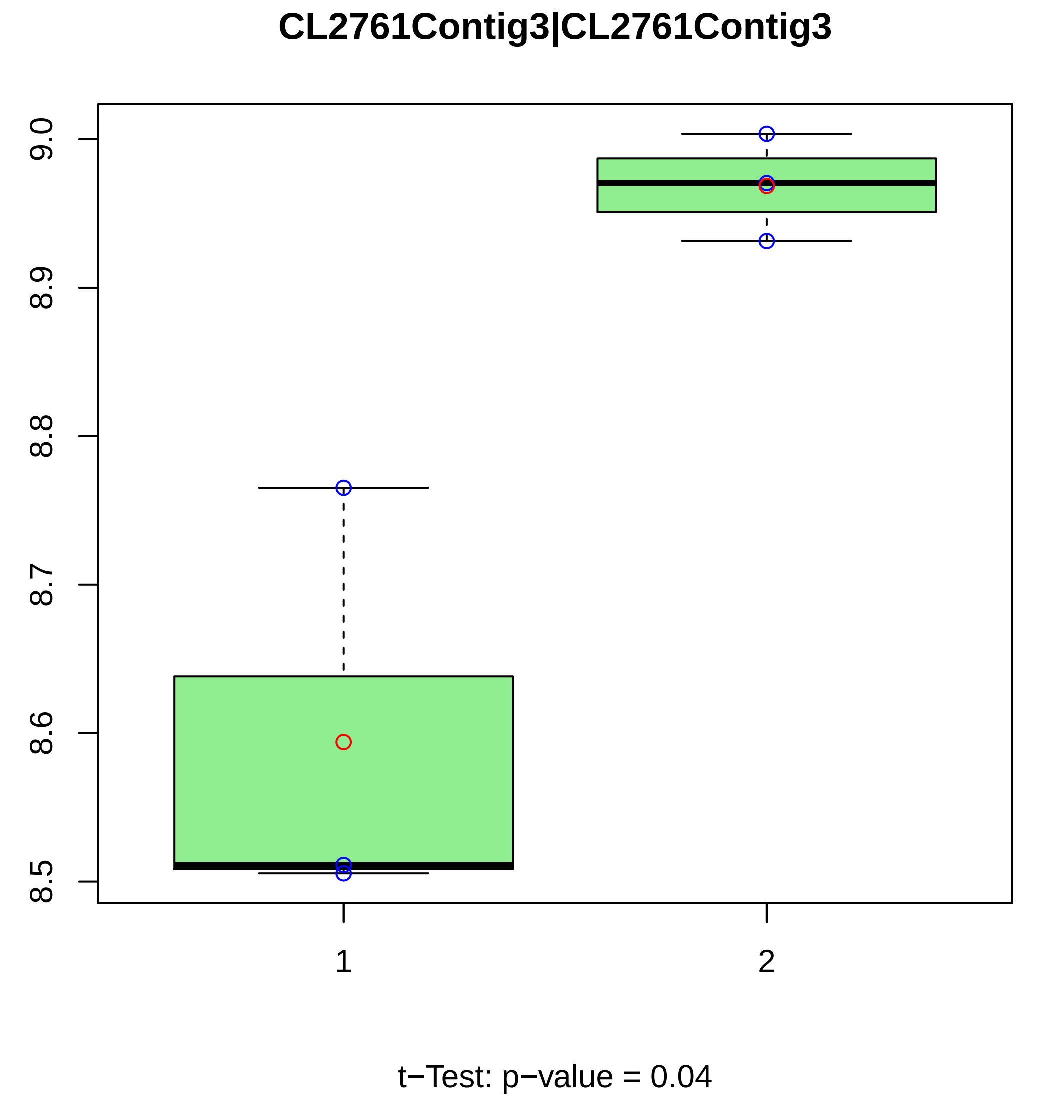
CL2761Contig3|CL2761Contig3
9.0
8.9
8.8
8.7
8.6
8.5
1
2
t−Test: p−value = 0.04

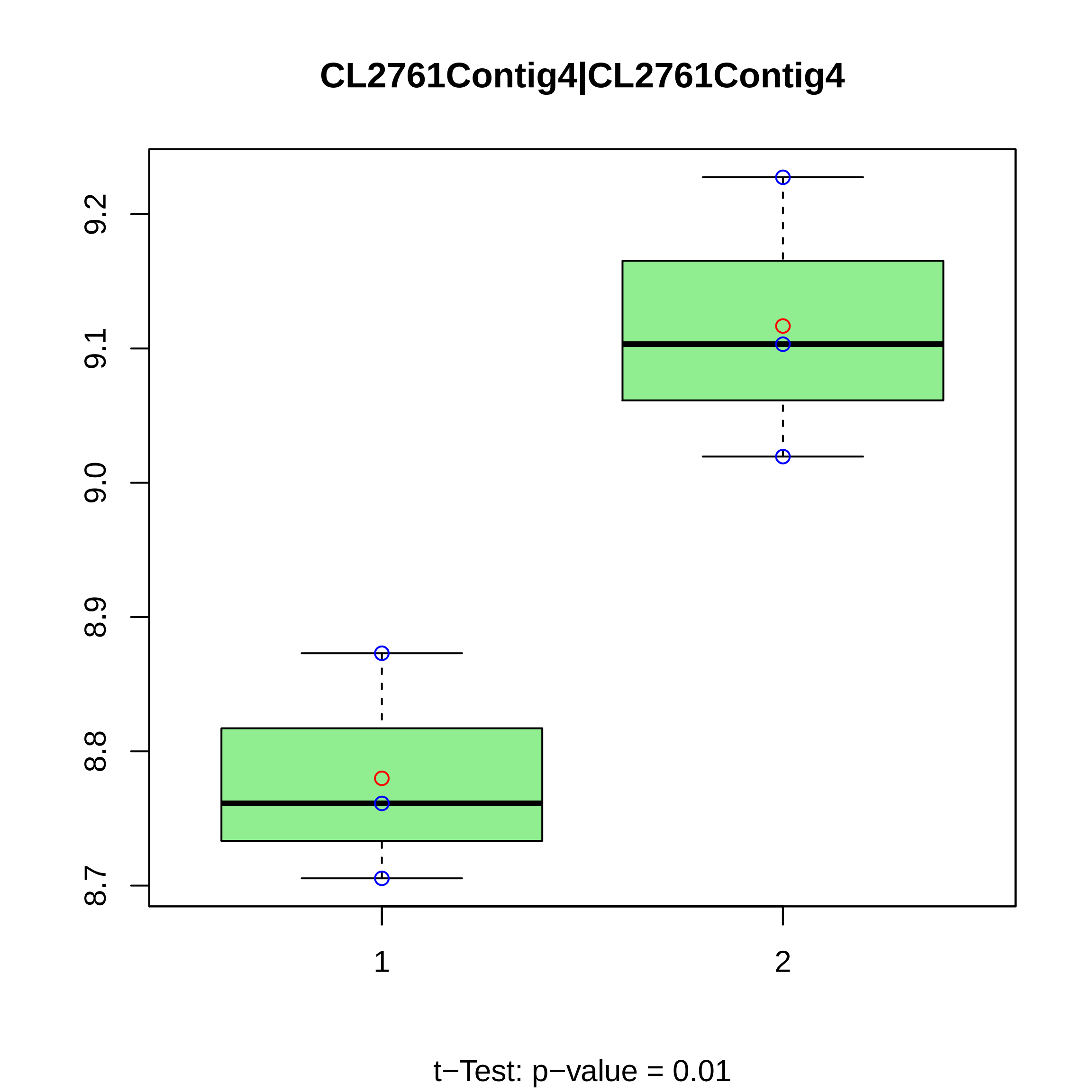
CL2761Contig4|CL2761Contig4
9.2
9.1
9.0
8.9
8.8
8.7
1
2
t−Test: p−value = 0.01

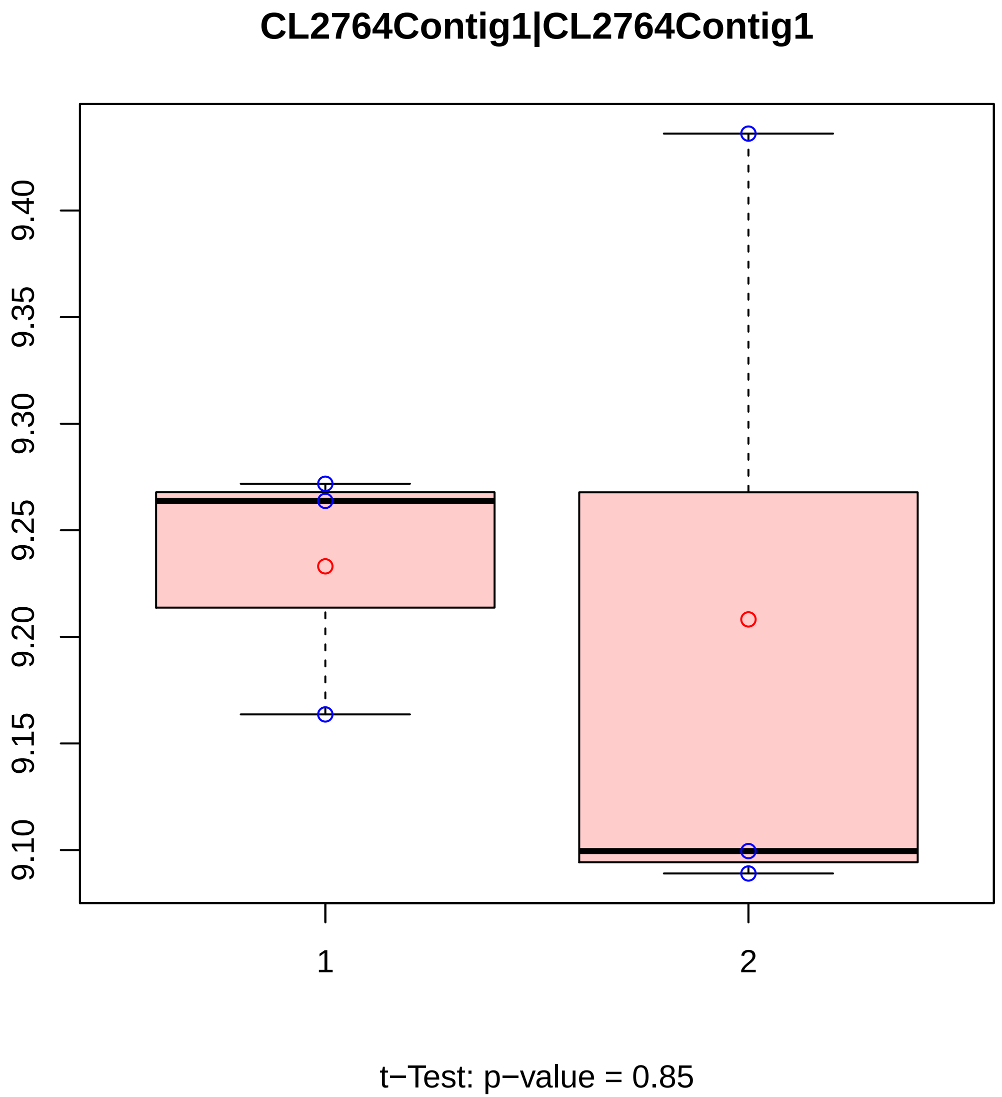

CL2764Contig1|CL2764Contig1
9.10
9.15
9.20
9.25
9.30
9.35
9.40
1
2
t−Test: p−value = 0.85

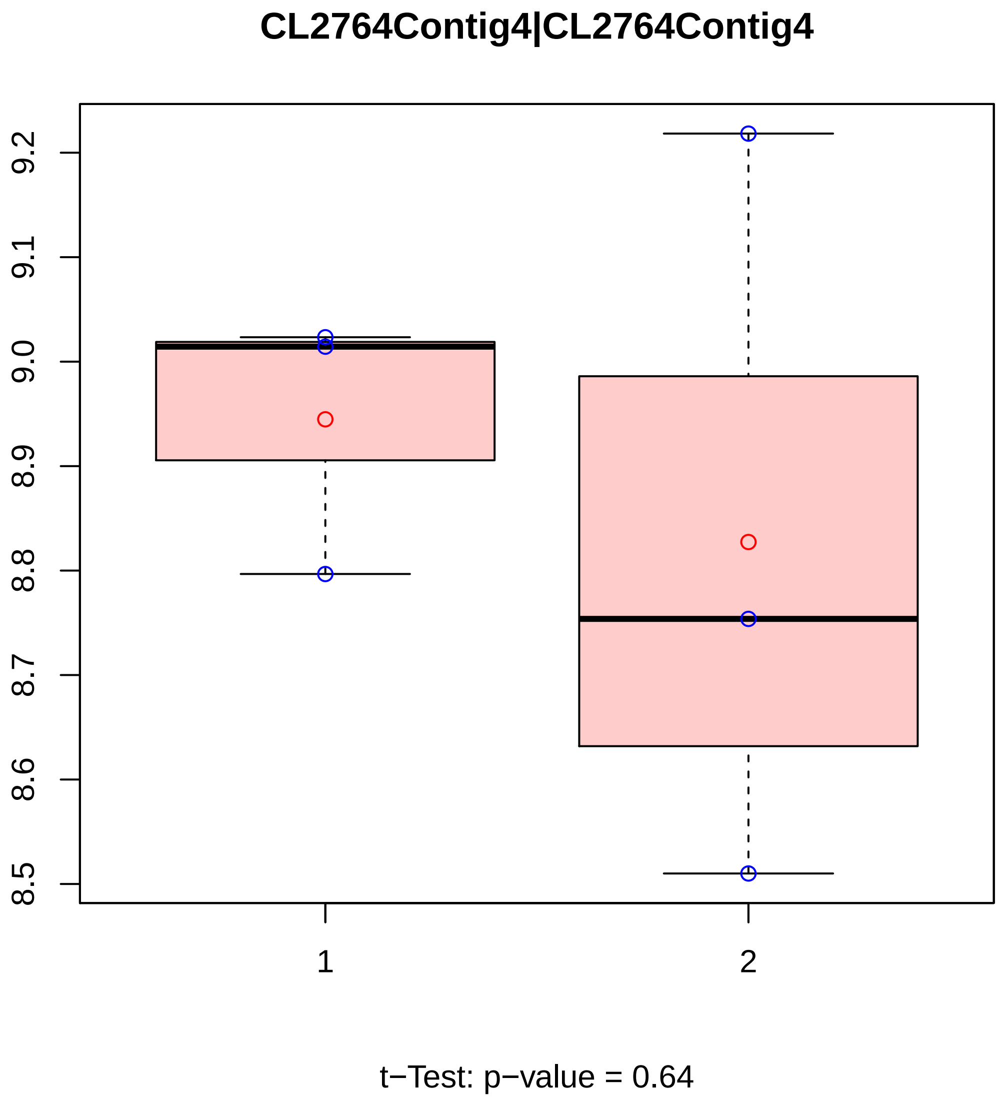

CL2764Contig4|CL2764Contig4
9.2
9.1
9.0
8.9
8.8
8.7
8.6
8.5
1
2
t−Test: p−value = 0.64

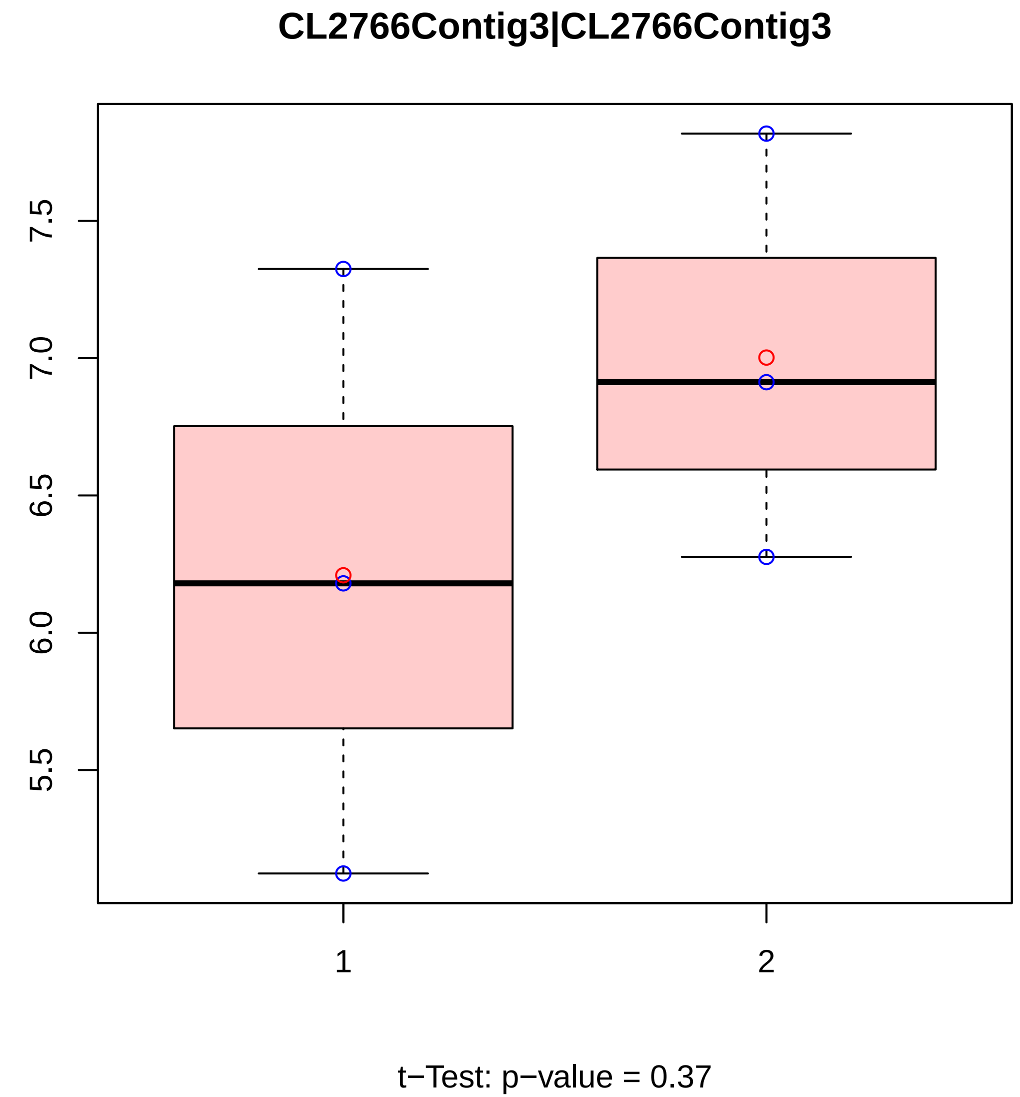

CL2766Contig3|CL2766Contig3
7.5
7.0
6.5
6.0
5.5
1
2
t−Test: p−value = 0.37

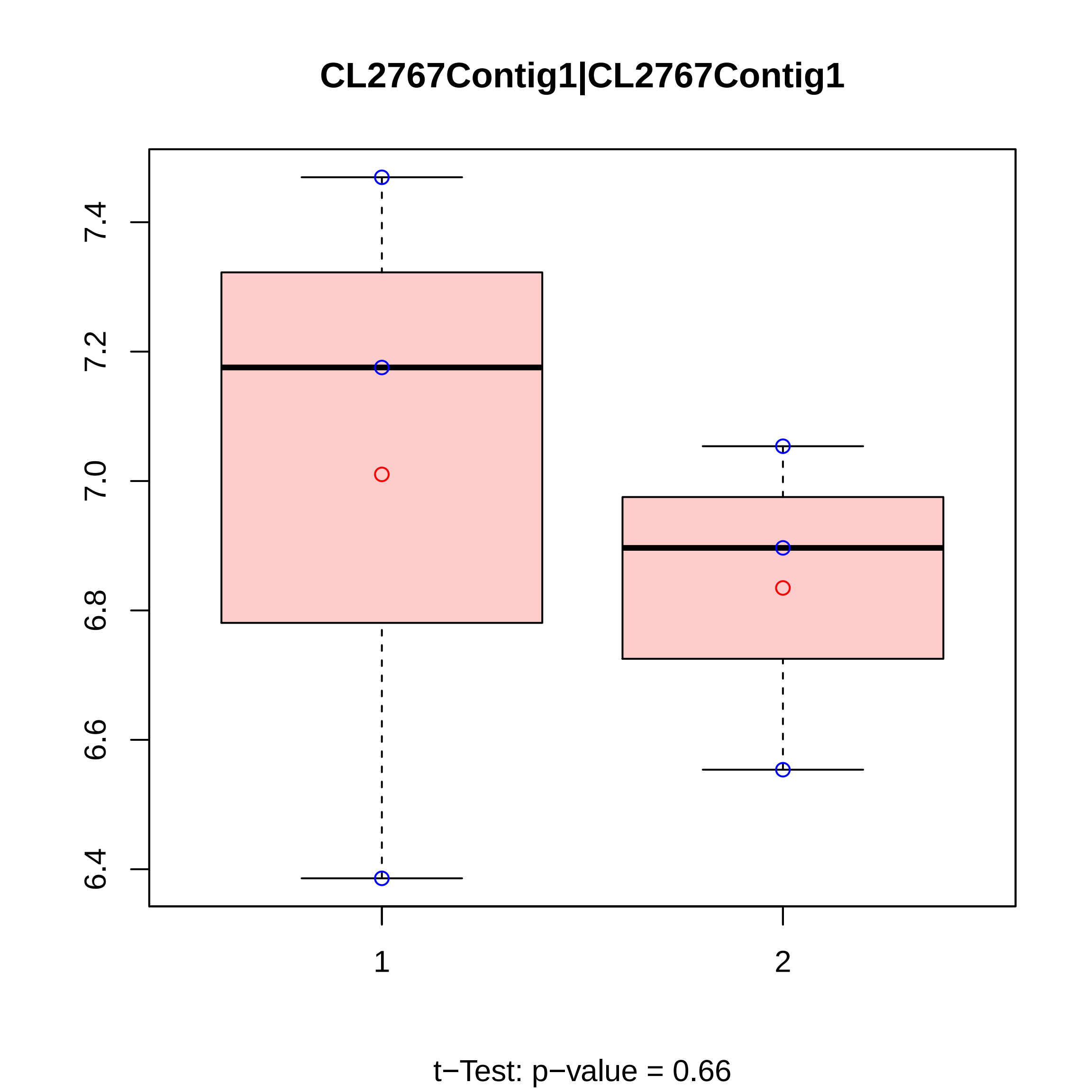
CL2767Contig1|CL2767Contig1
7.4
7.2
7.0
6.8
6.6
6.4
1
2
t−Test: p−value = 0.66

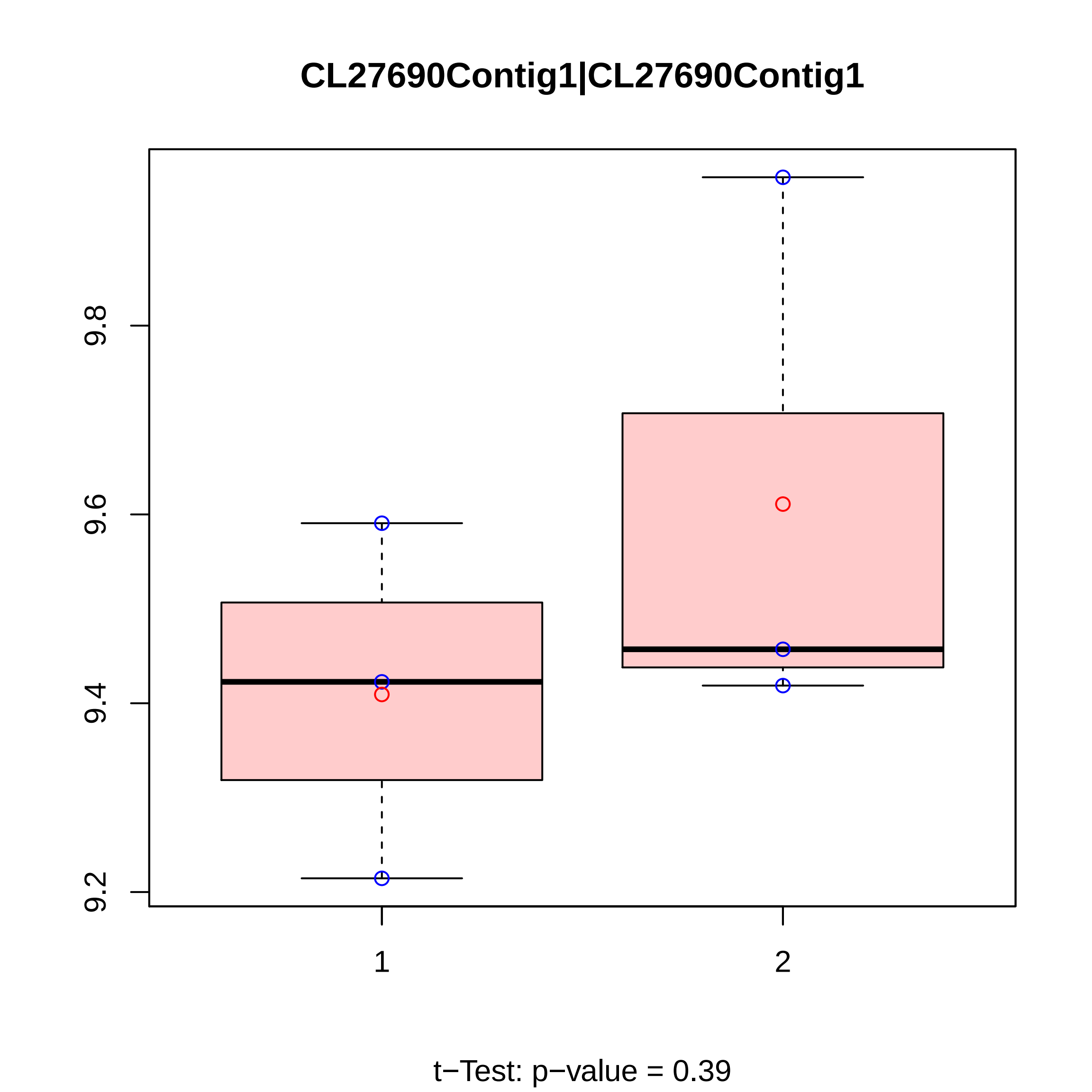

CL27690Contig1|CL27690Contig1
9.2
9.4
9.6
9.8
1
2
t−Test: p−value = 0.39

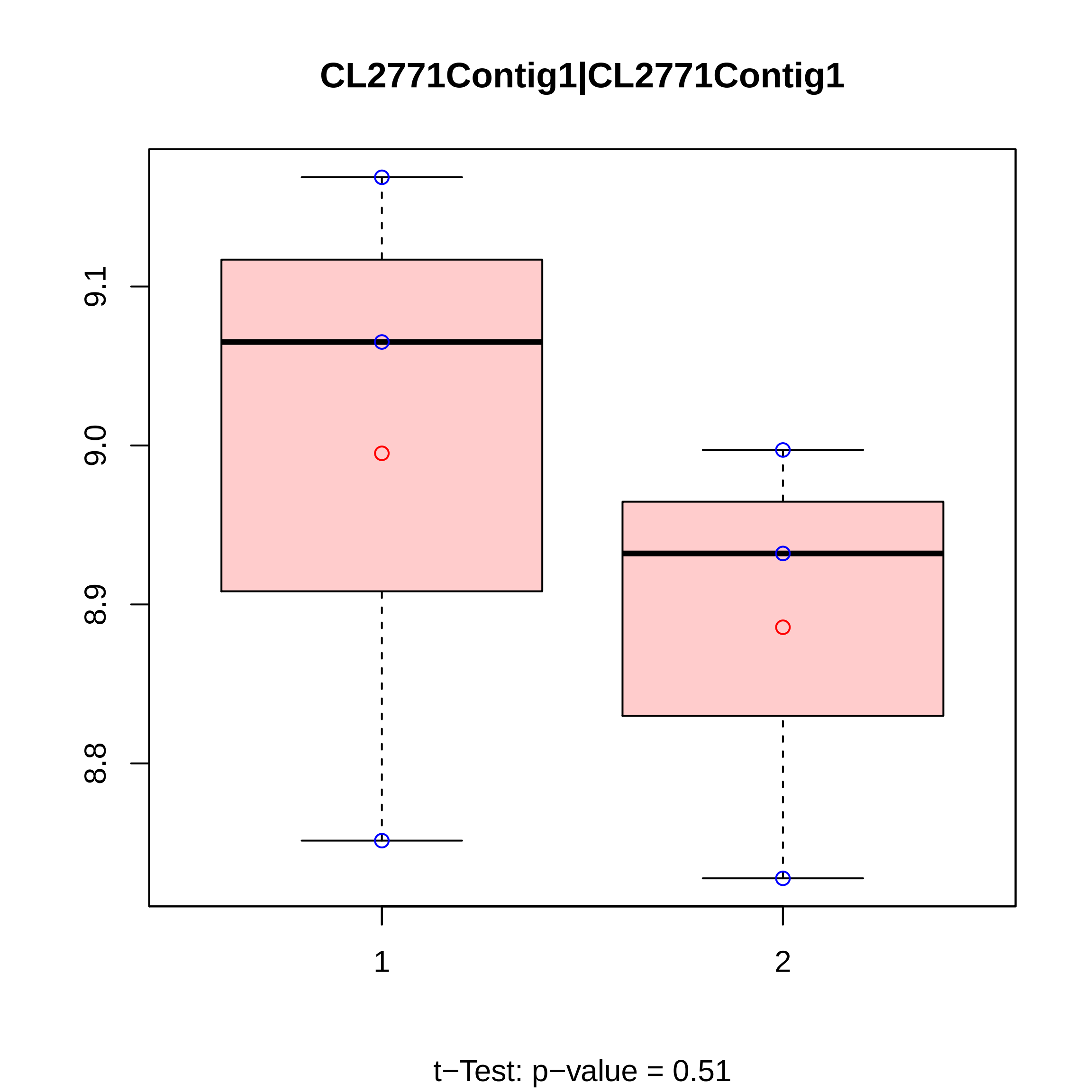
CL2771Contig1|CL2771Contig1
9.1
9.0
8.9
8.8
1
2
t−Test: p−value = 0.51

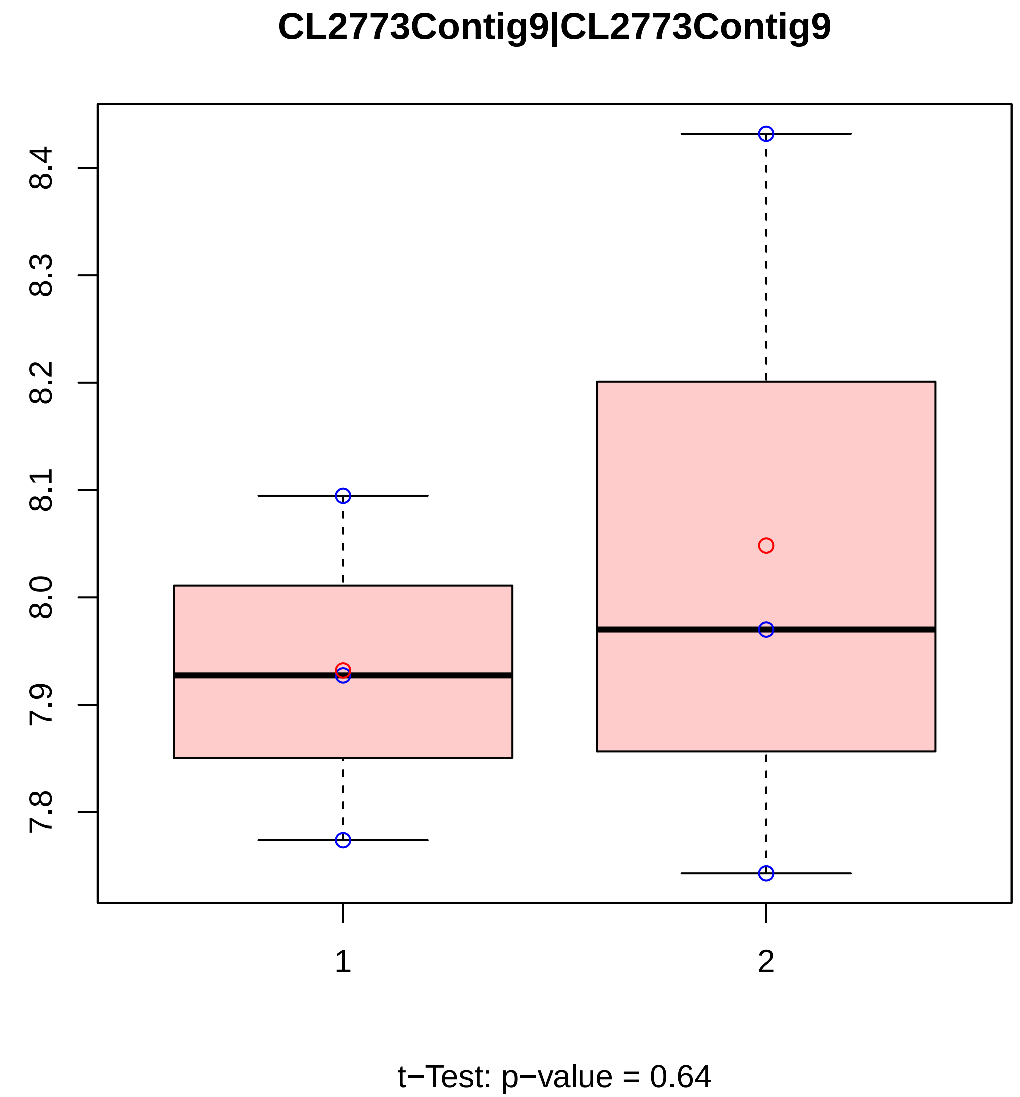
CL2773Contig9|CL2773Contig9
8.4
8.3
8.2
8.1
8.0
7.9
7.8
1
2
t−Test: p−value = 0.64

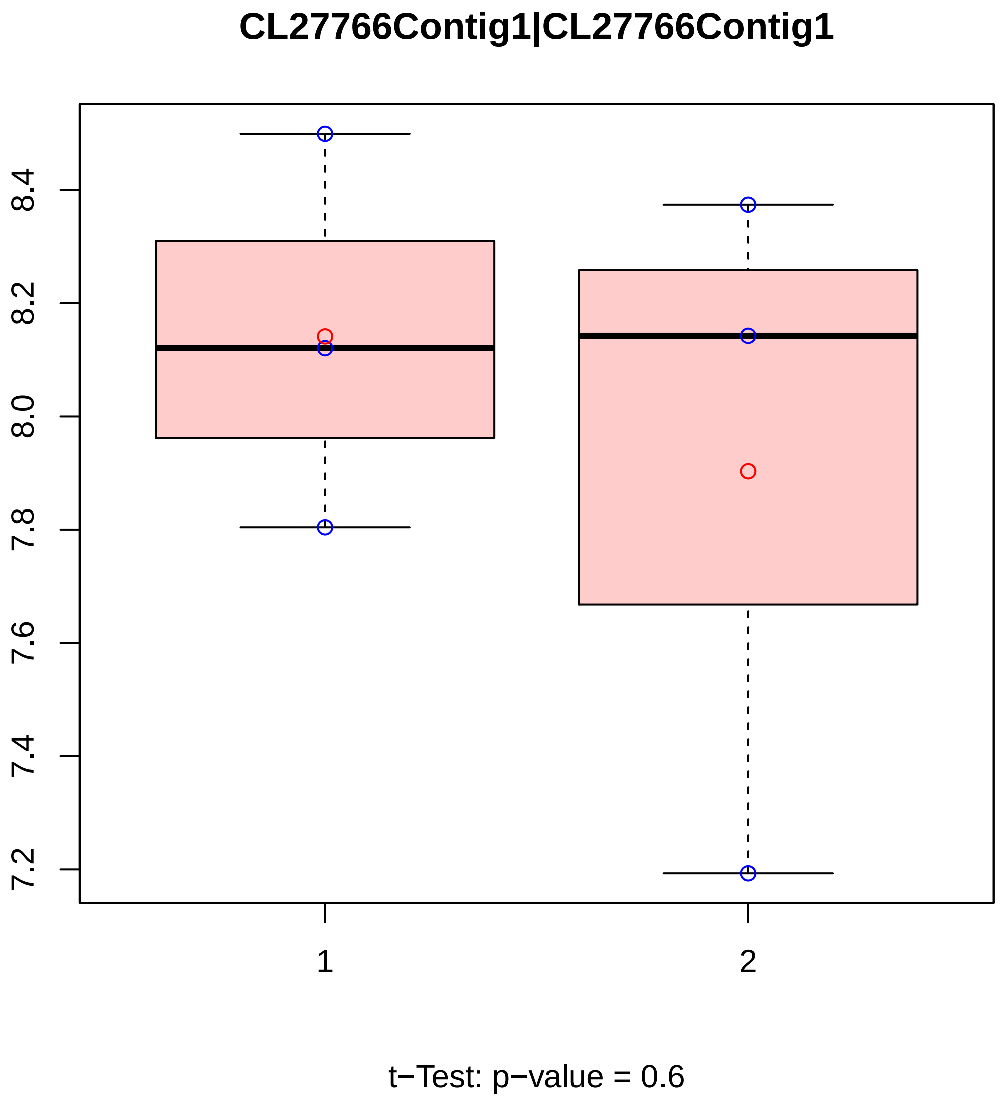
CL27766Contig1|CL27766Contig1
8.4
8.2
8.0
7.8
7.6
7.4
7.2
1
2
t−Test: p−value = 0.6

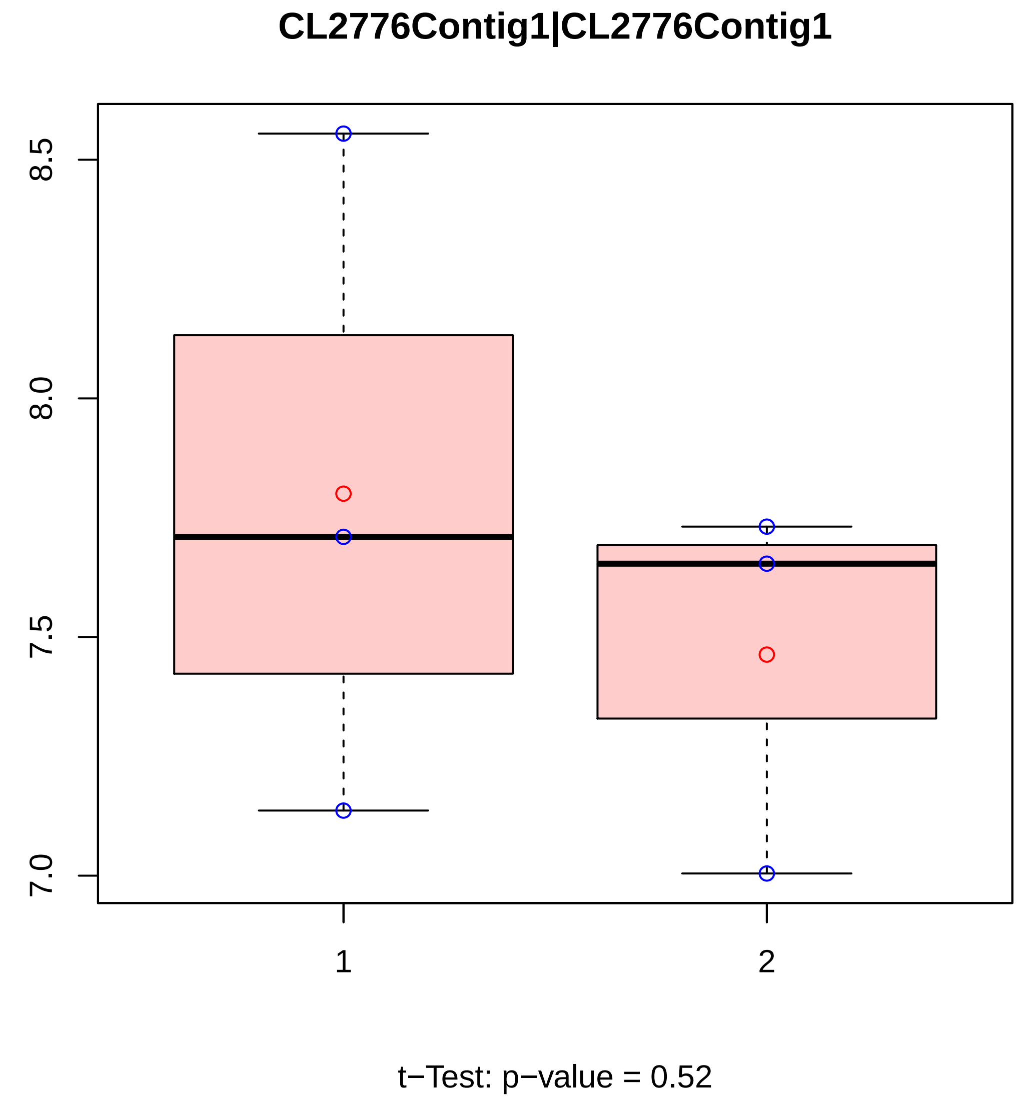

CL2776Contig1|CL2776Contig1
8.5
8.0
7.5
7.0
1
2
t−Test: p−value = 0.52

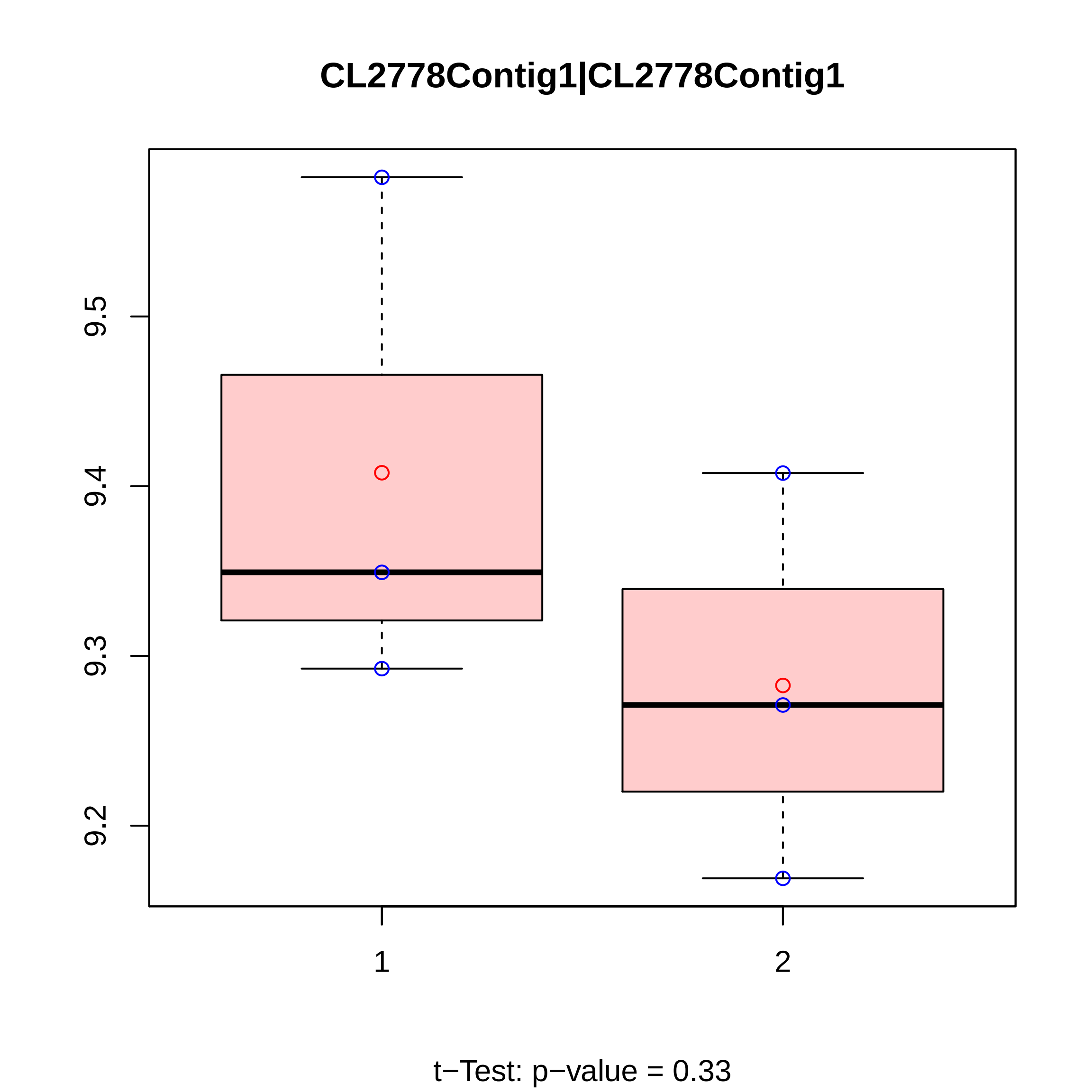

CL2778Contig1|CL2778Contig1
9.2
9.3
9.4
9.5
1
2
t−Test: p−value = 0.33

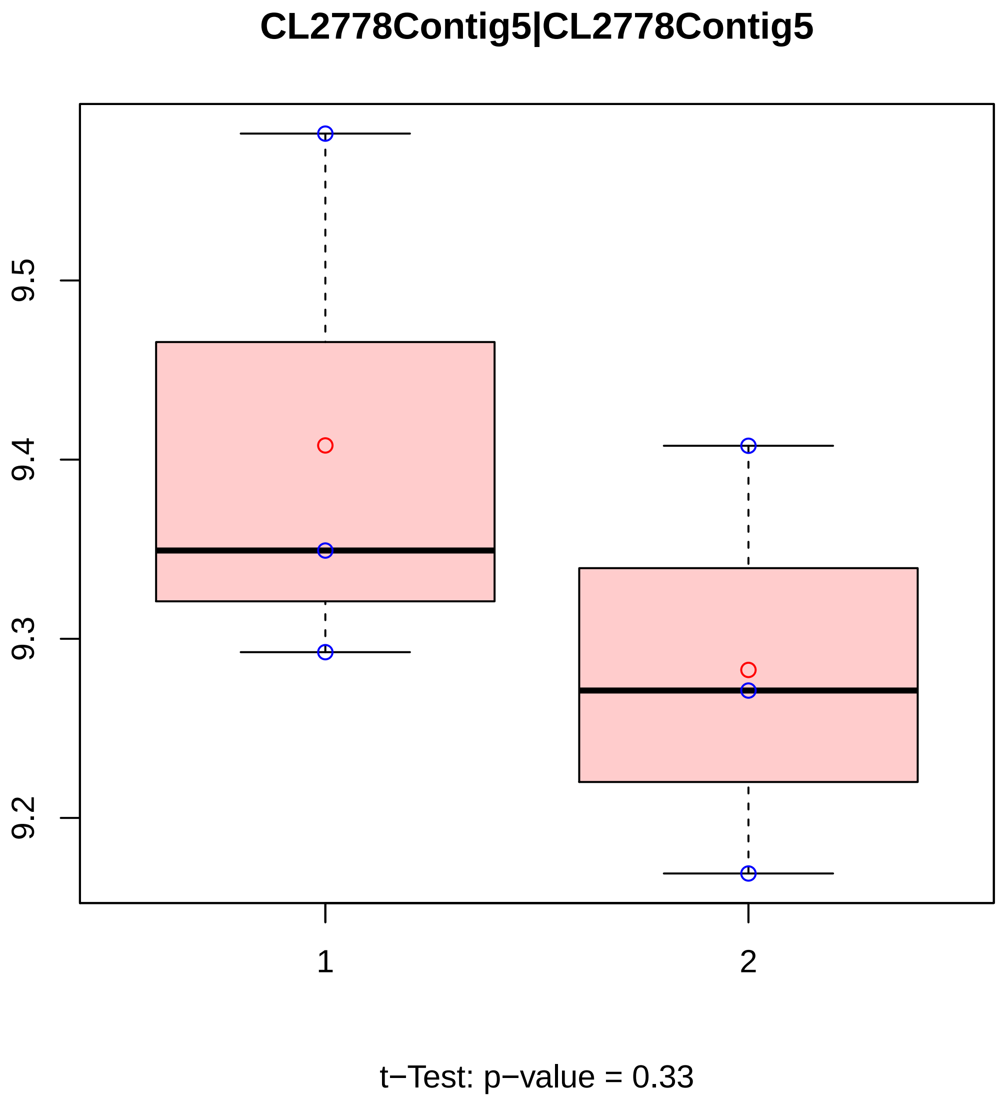

CL2778Contig5|CL2778Contig5
9.2
9.3
9.4
9.5
1
2
t−Test: p−value = 0.33

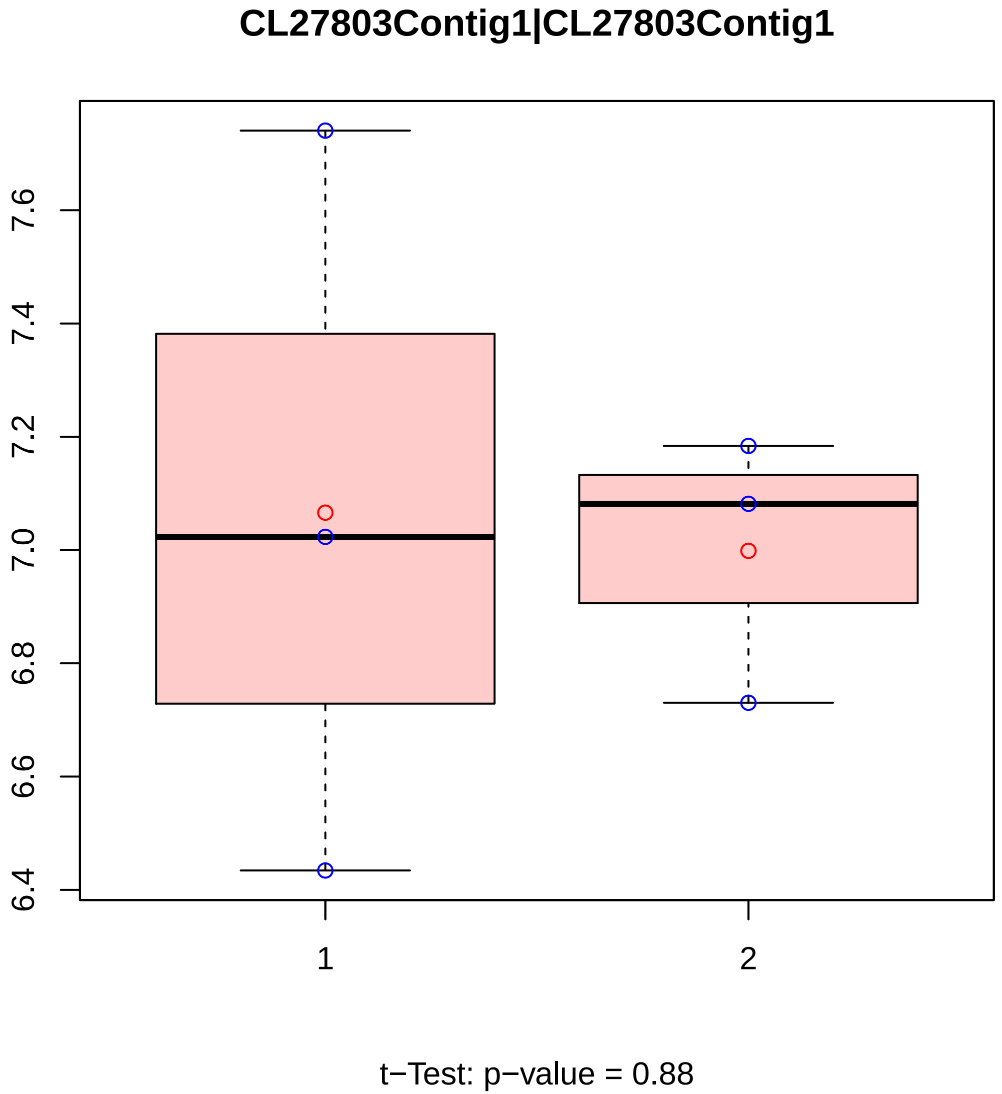

CL27803Contig1|CL27803Contig1
7.6
7.4
7.2
7.0
6.8
6.6
6.4
1
2
t−Test: p−value = 0.88

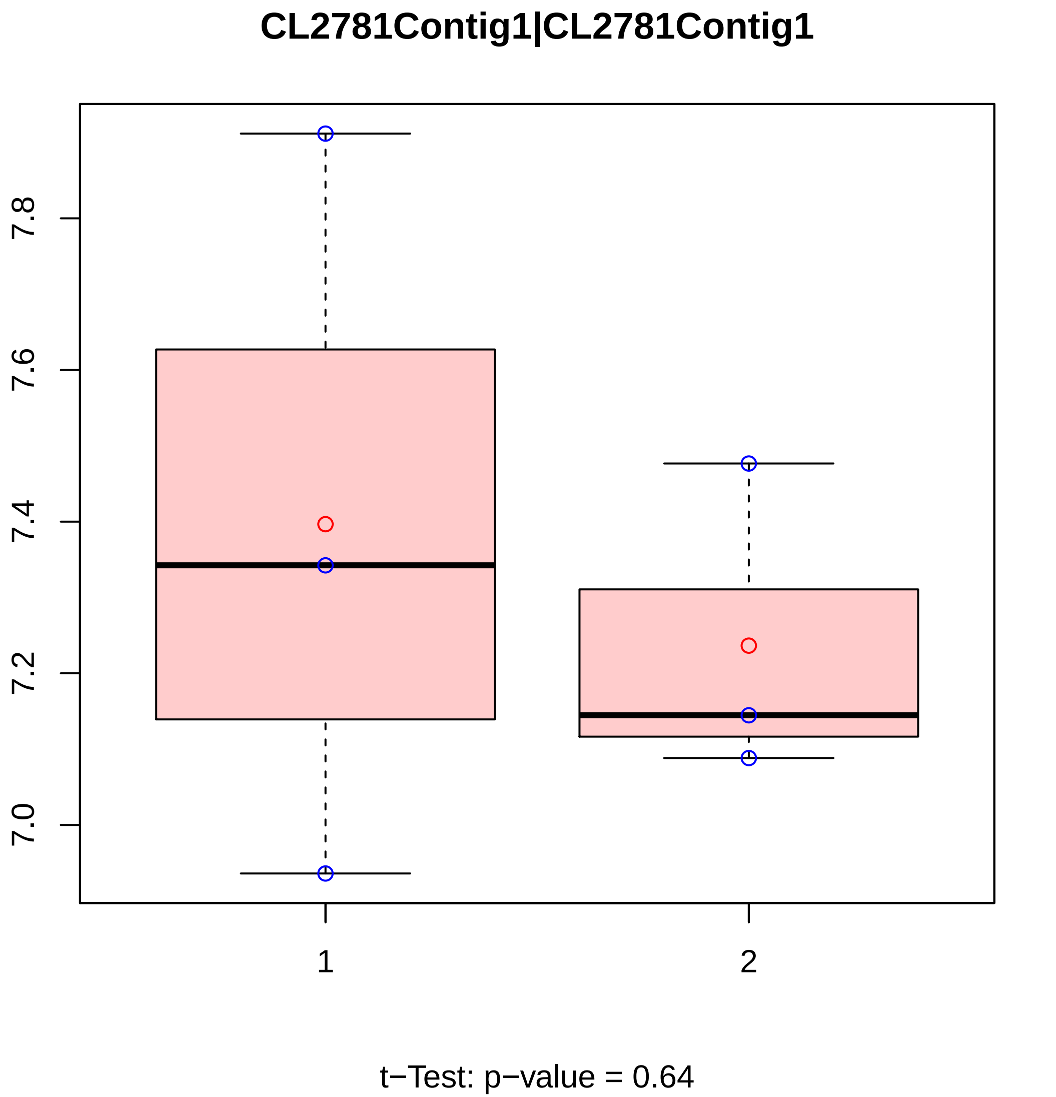

CL2781Contig1|CL2781Contig1
7.0
7.2
7.4
7.6
7.8
1
2
t−Test: p−value = 0.64

# CL2783Contig1|CL2783Contig1

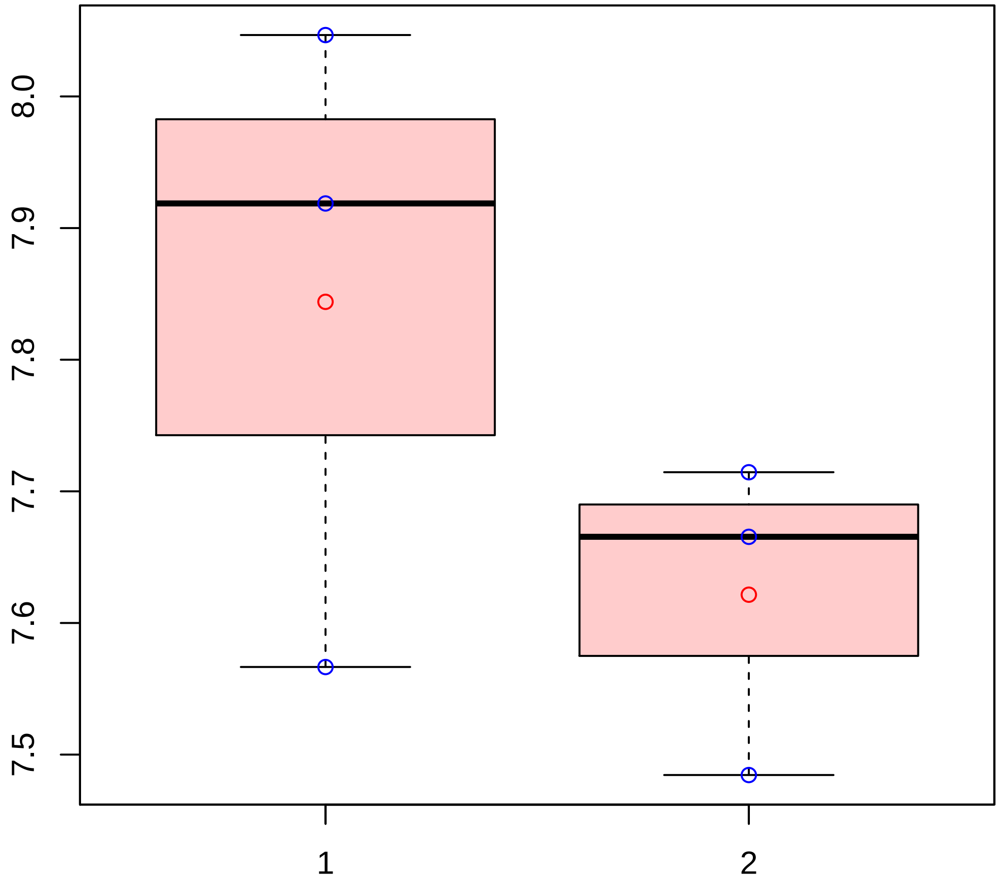


t−Test: p−value = 0.26

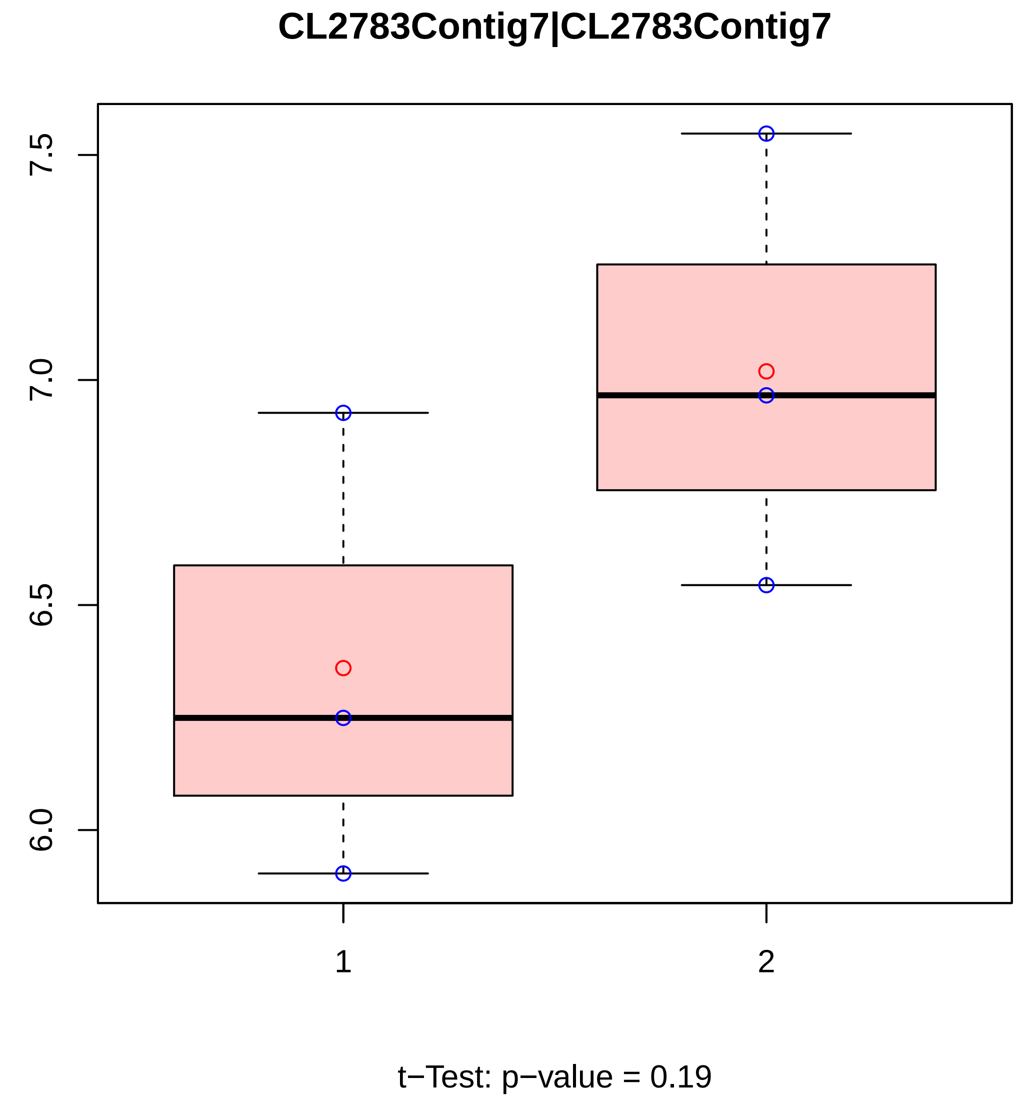
CL2783Contig7|CL2783Contig7
7.5
7.0
6.5
6.0
1
2
t−Test: p−value = 0.19

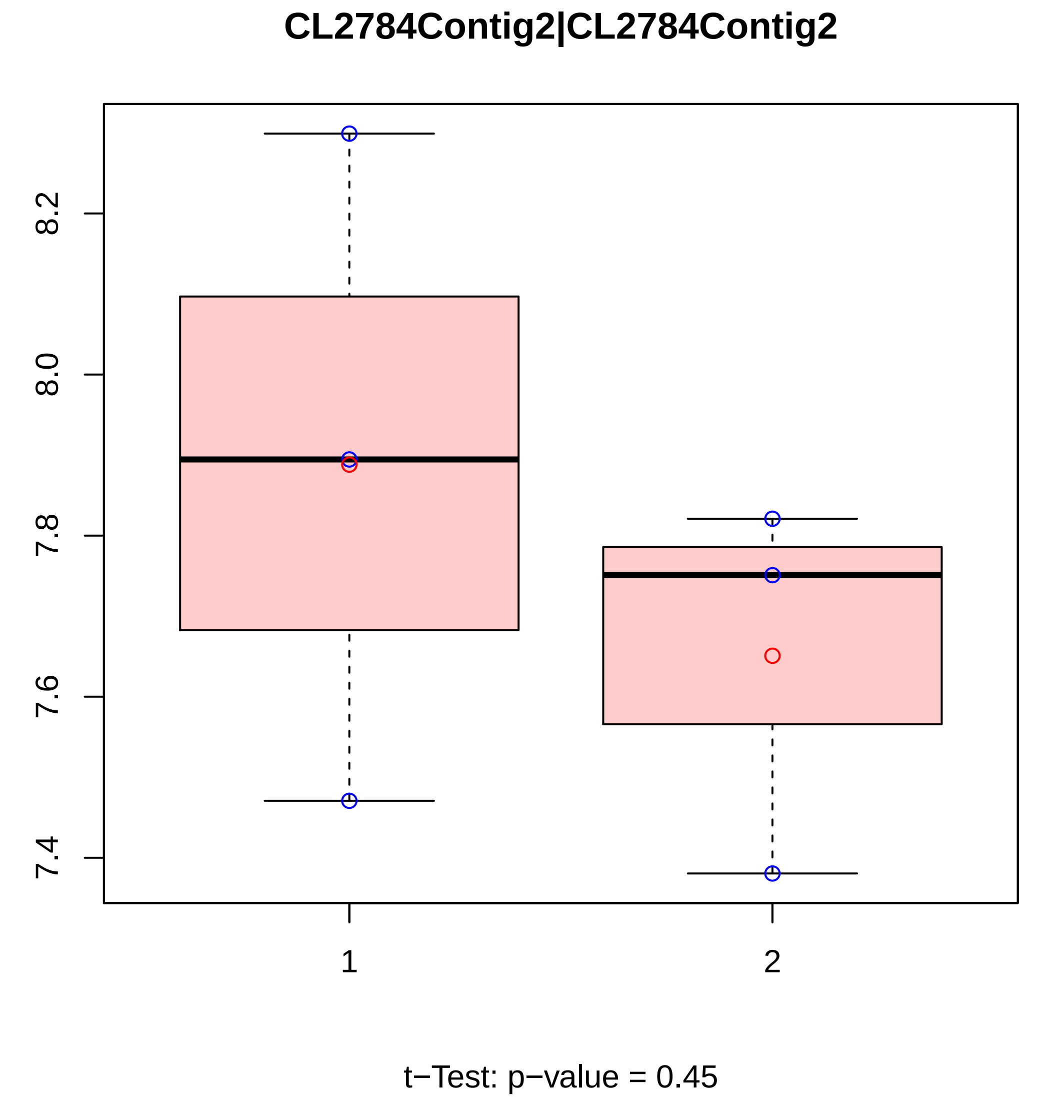
CL2784Contig2|CL2784Contig2
8.2
8.0
7.8
7.6
7.4
1
2
t−Test: p−value = 0.45

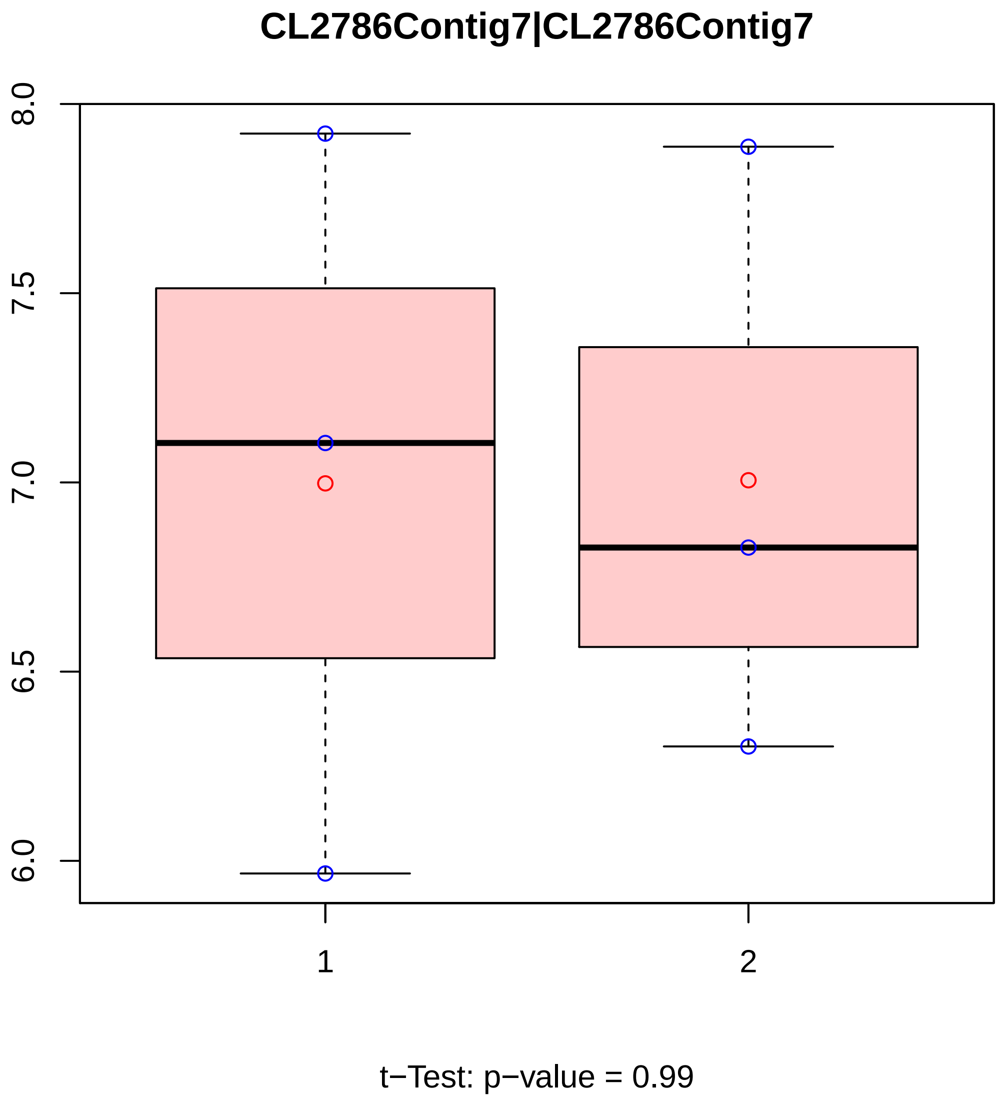

CL2786Contig7|CL2786Contig7
8.0
7.5
7.0
6.5
6.0
1
2
t−Test: p−value = 0.99

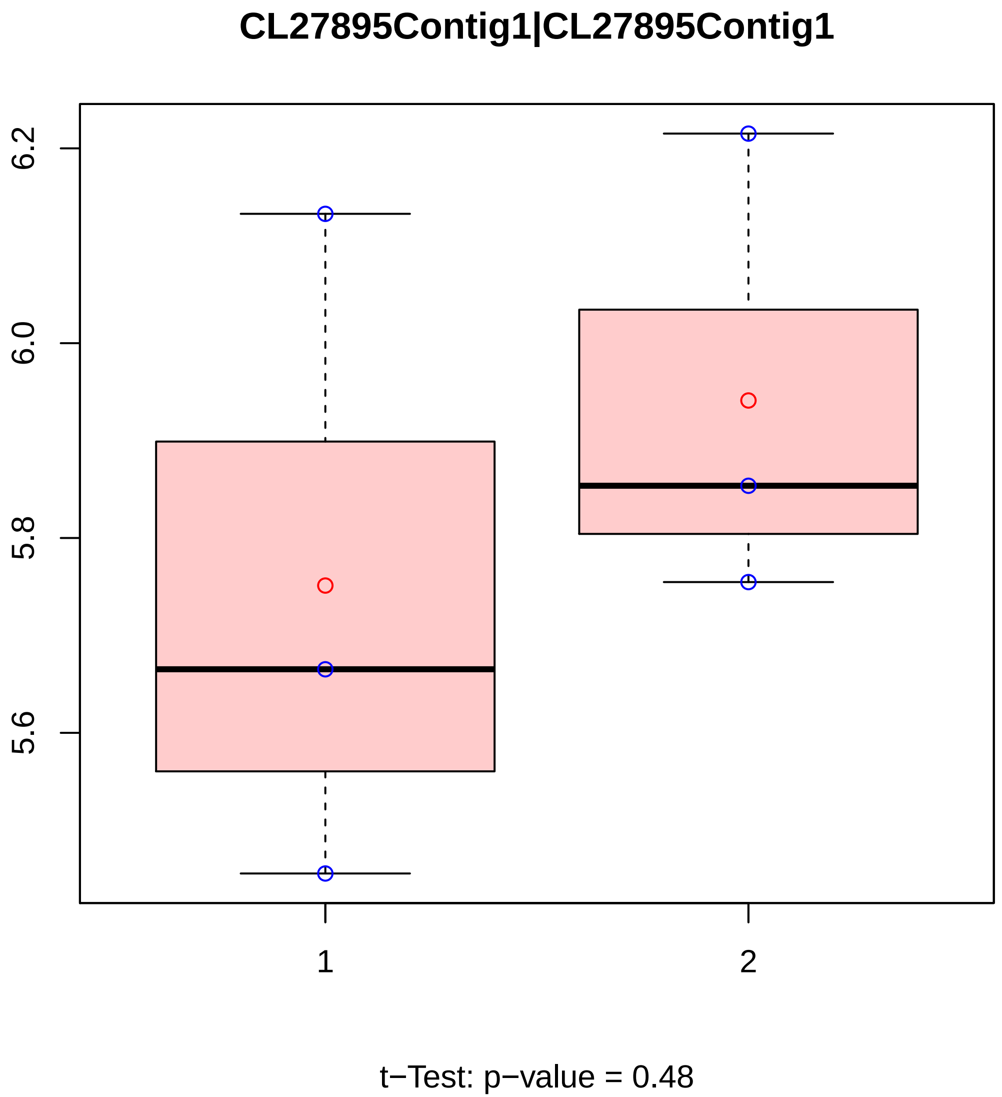
CL27895Contig1|CL27895Contig1
6.2
6.0
5.8
5.6
1
2
t−Test: p−value = 0.48

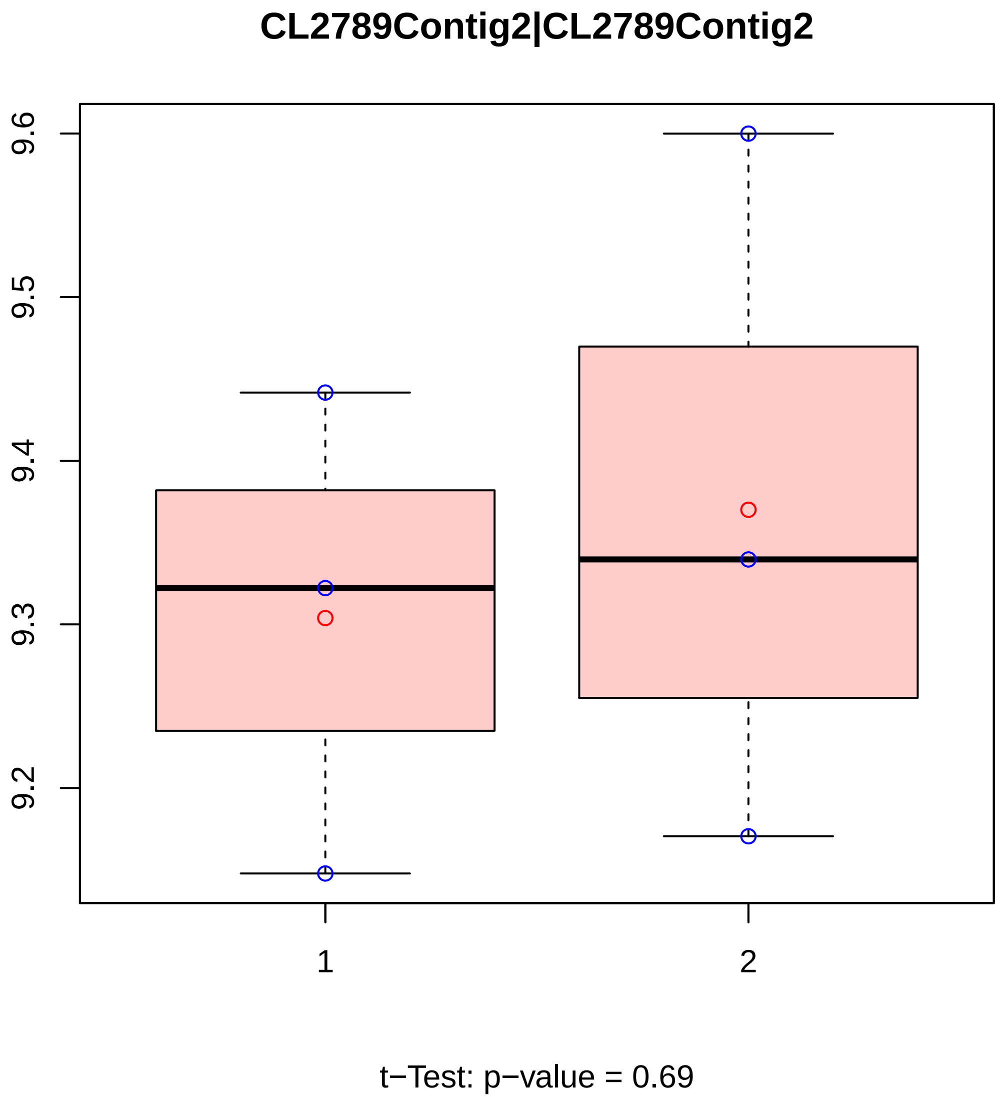

CL2789Contig2|CL2789Contig2
9.6
9.5
9.4
9.3
9.2
1
2
t−Test: p−value = 0.69

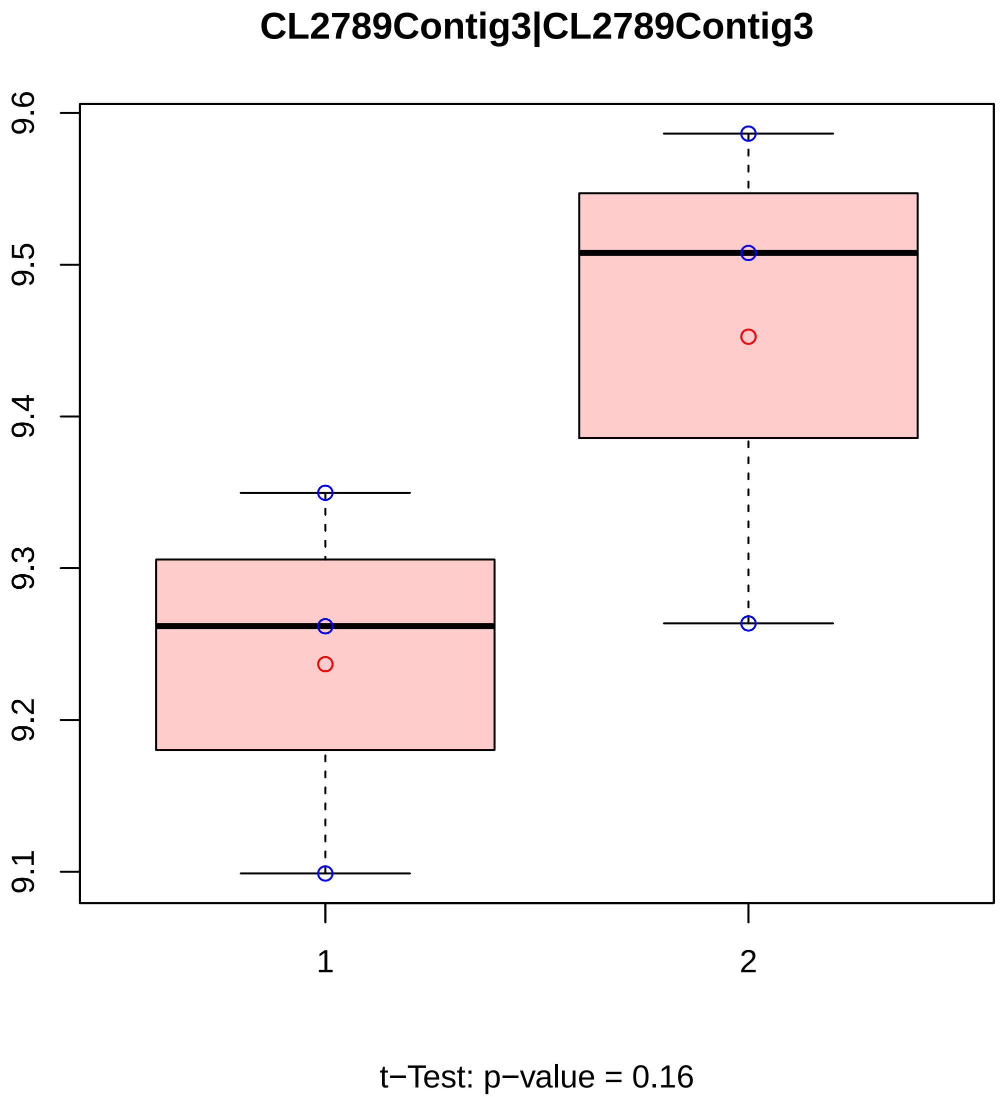
CL2789Contig3|CL2789Contig3
9.1
9.2
9.3
9.4
9.5
9.6
1
2
t−Test: p−value = 0.16

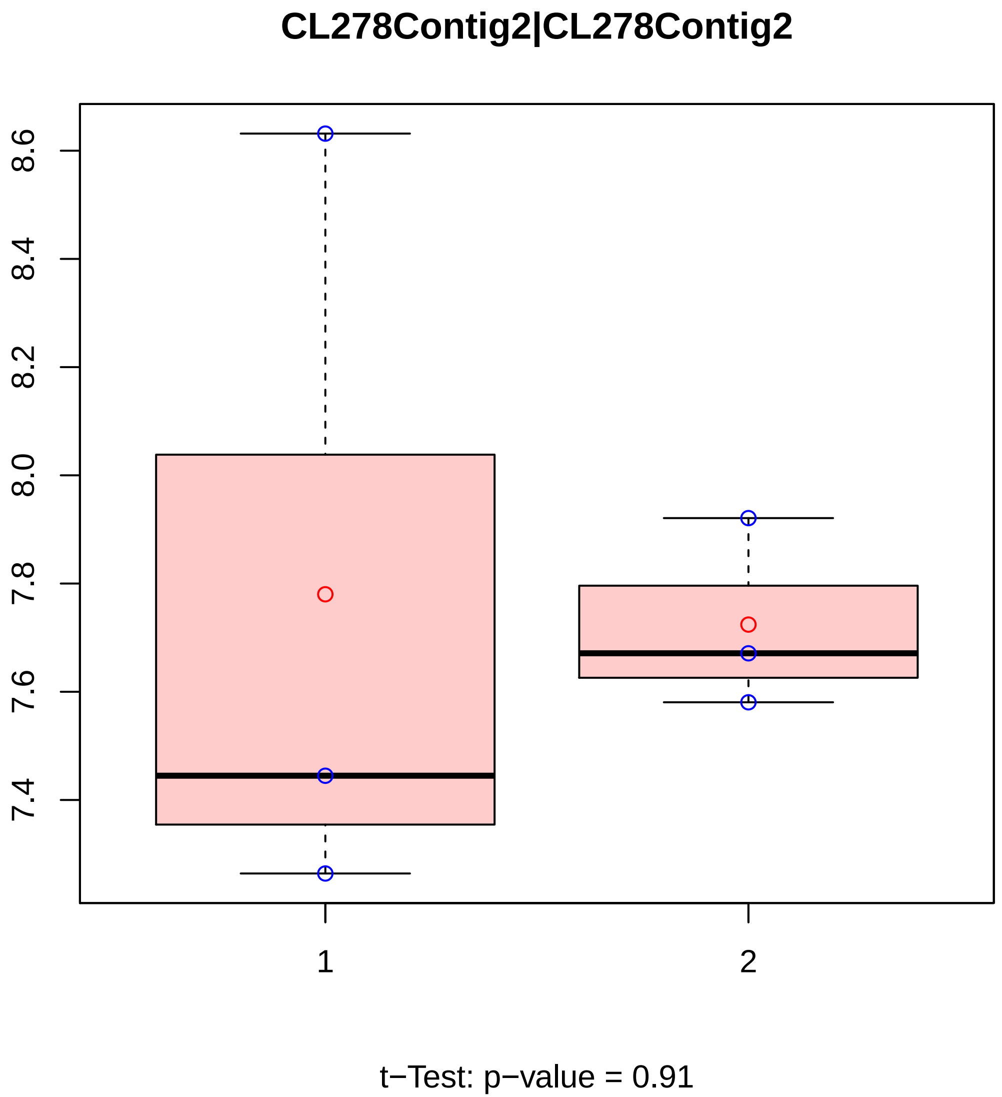
CL278Contig2|CL278Contig2
8.6
8.4
8.2
8.0
7.8
7.6
7.4
1
2
t−Test: p−value = 0.91

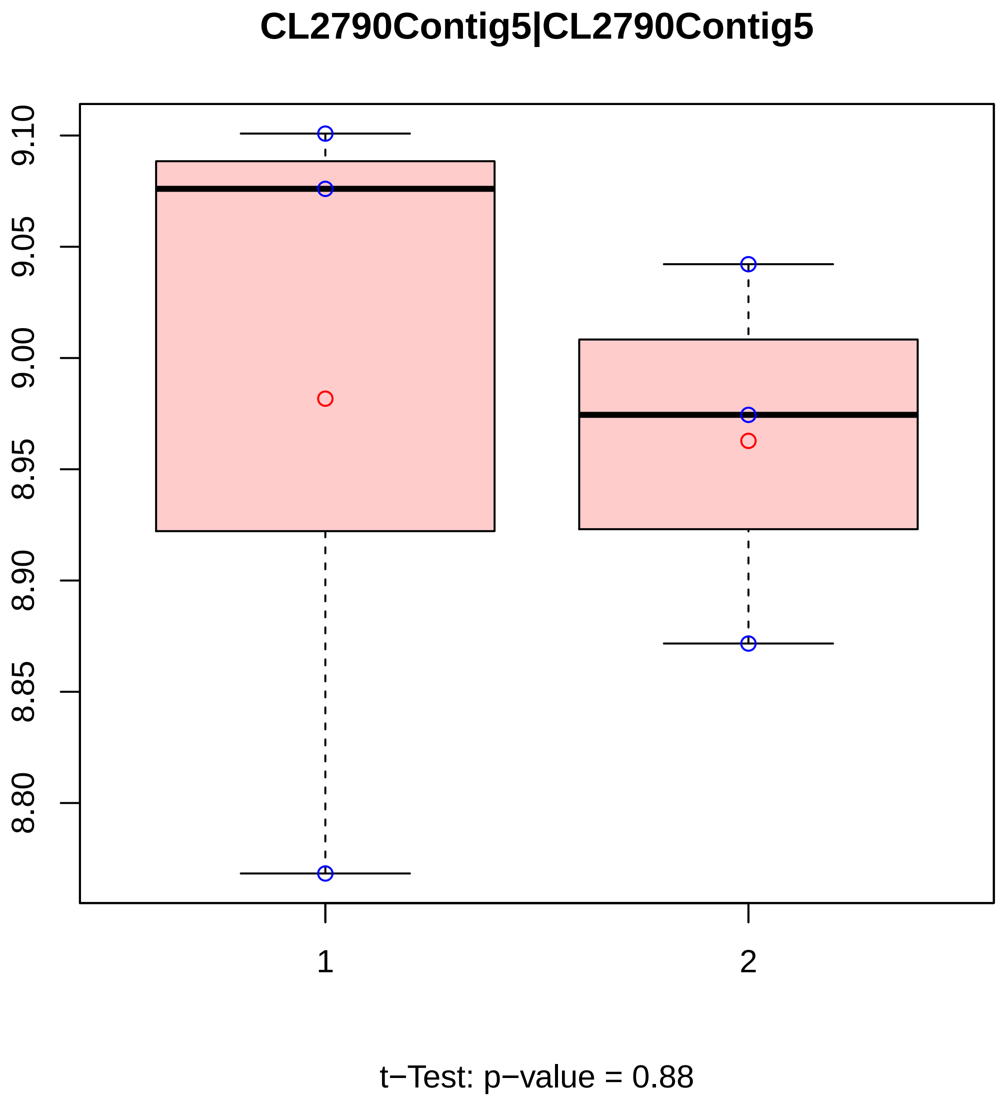

CL2790Contig5|CL2790Contig5
9.10
9.05
9.00
8.95
8.90
8.85
8.80
1
2
t−Test: p−value = 0.88

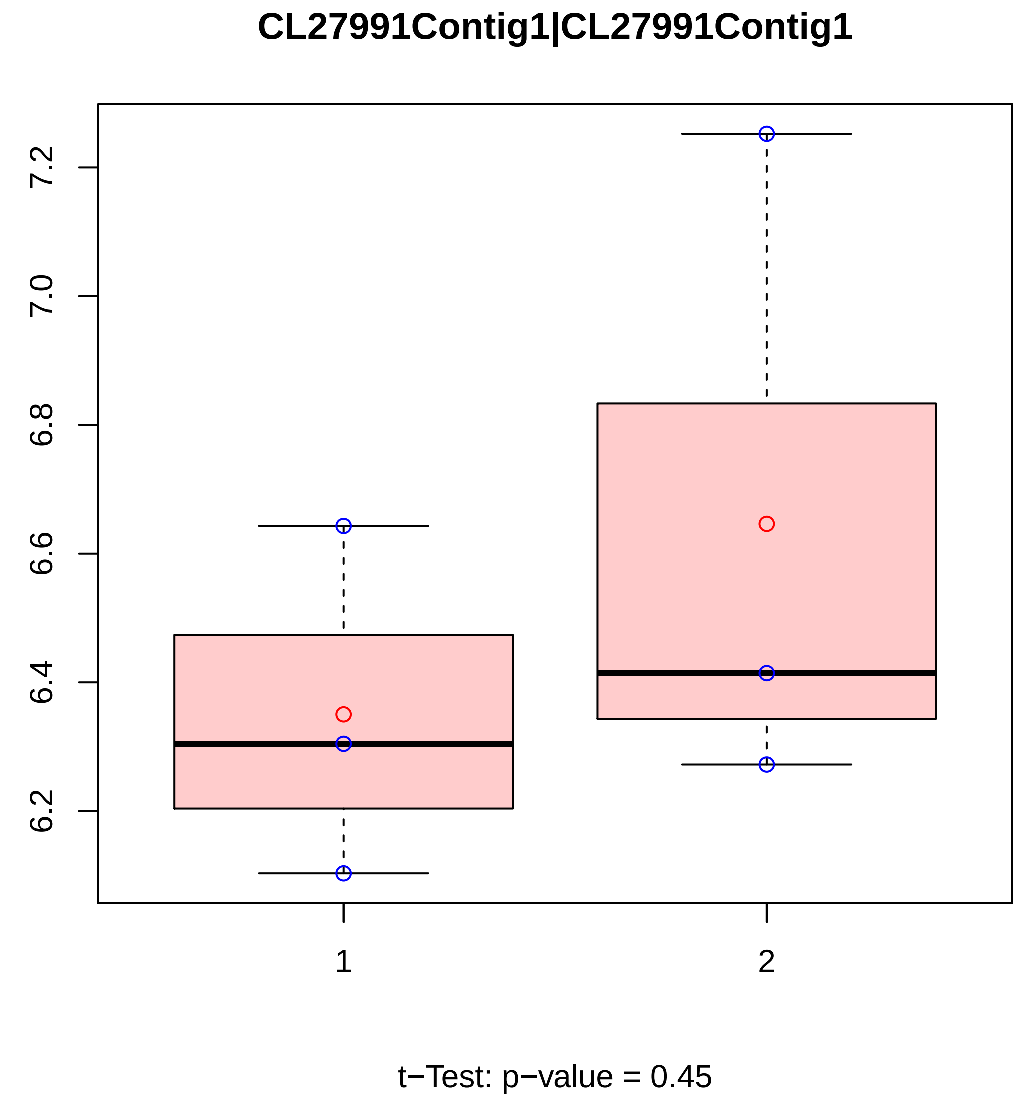

CL27991Contig1|CL27991Contig1
6.2
6.4
6.6
6.8
7.0
7.2
1
2
t−Test: p−value = 0.45

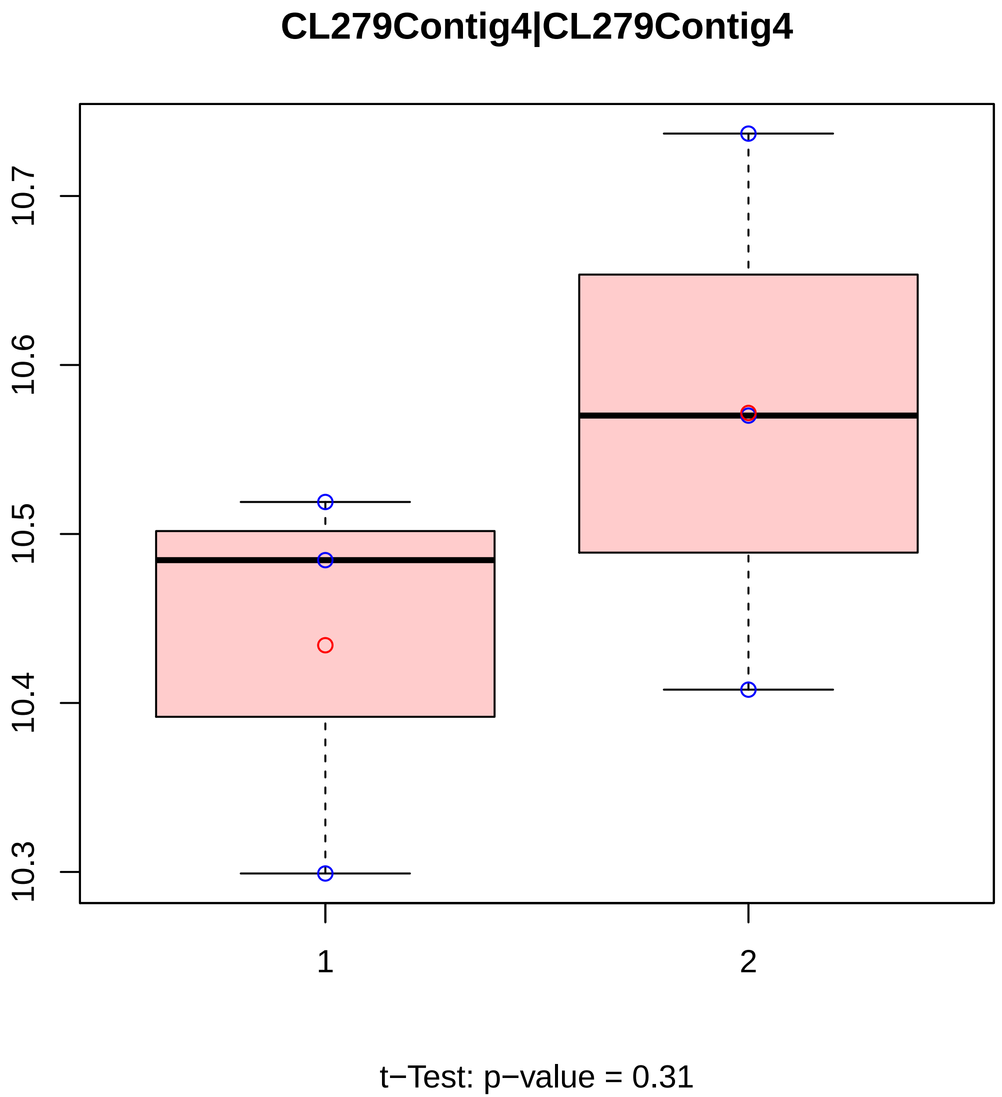

CL279Contig4|CL279Contig4
10.3
10.4
10.5
10.6
10.7
1
2
t−Test: p−value = 0.31

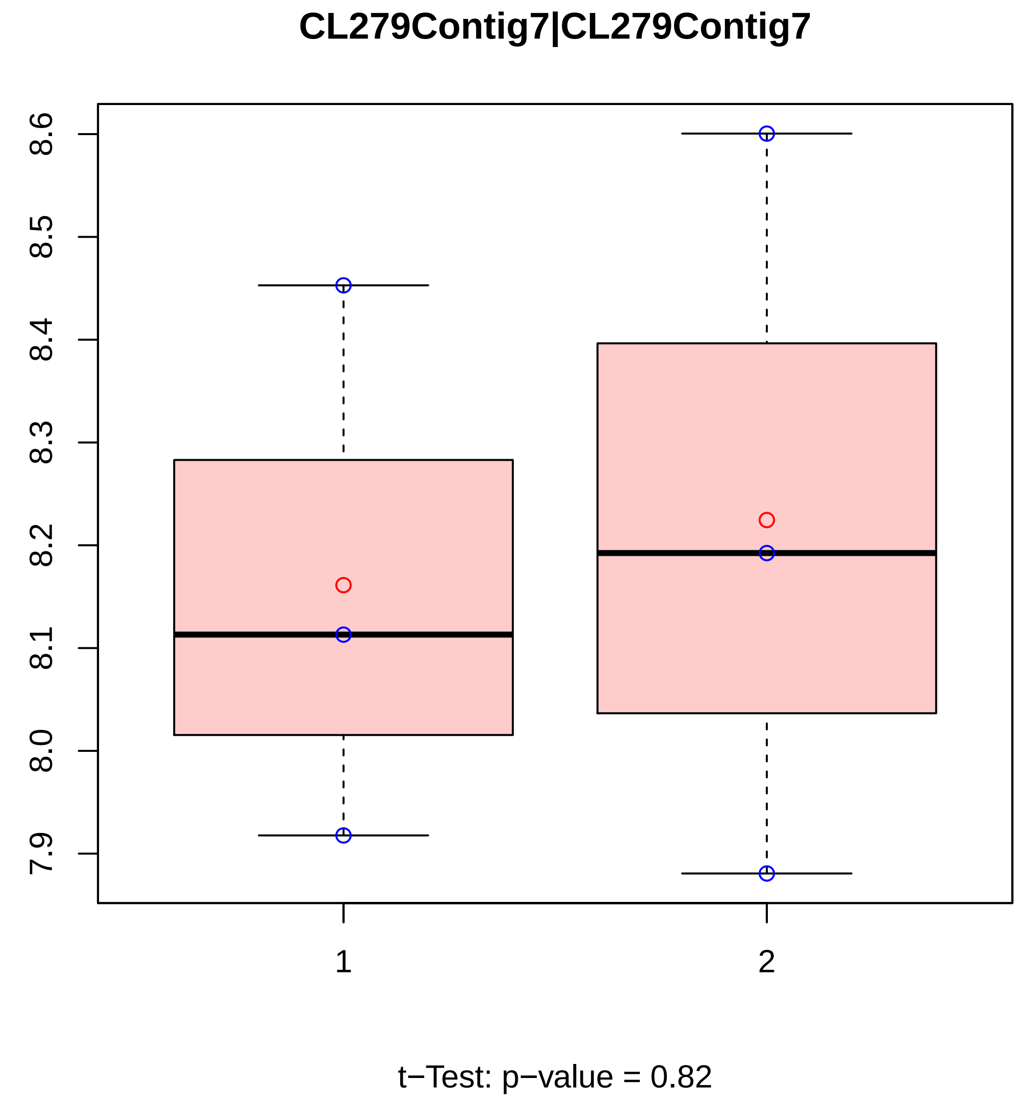
CL279Contig7|CL279Contig7
8.6
8.5
8.4
8.3
8.2
8.1
8.0
7.9
1
2
t−Test: p−value = 0.82

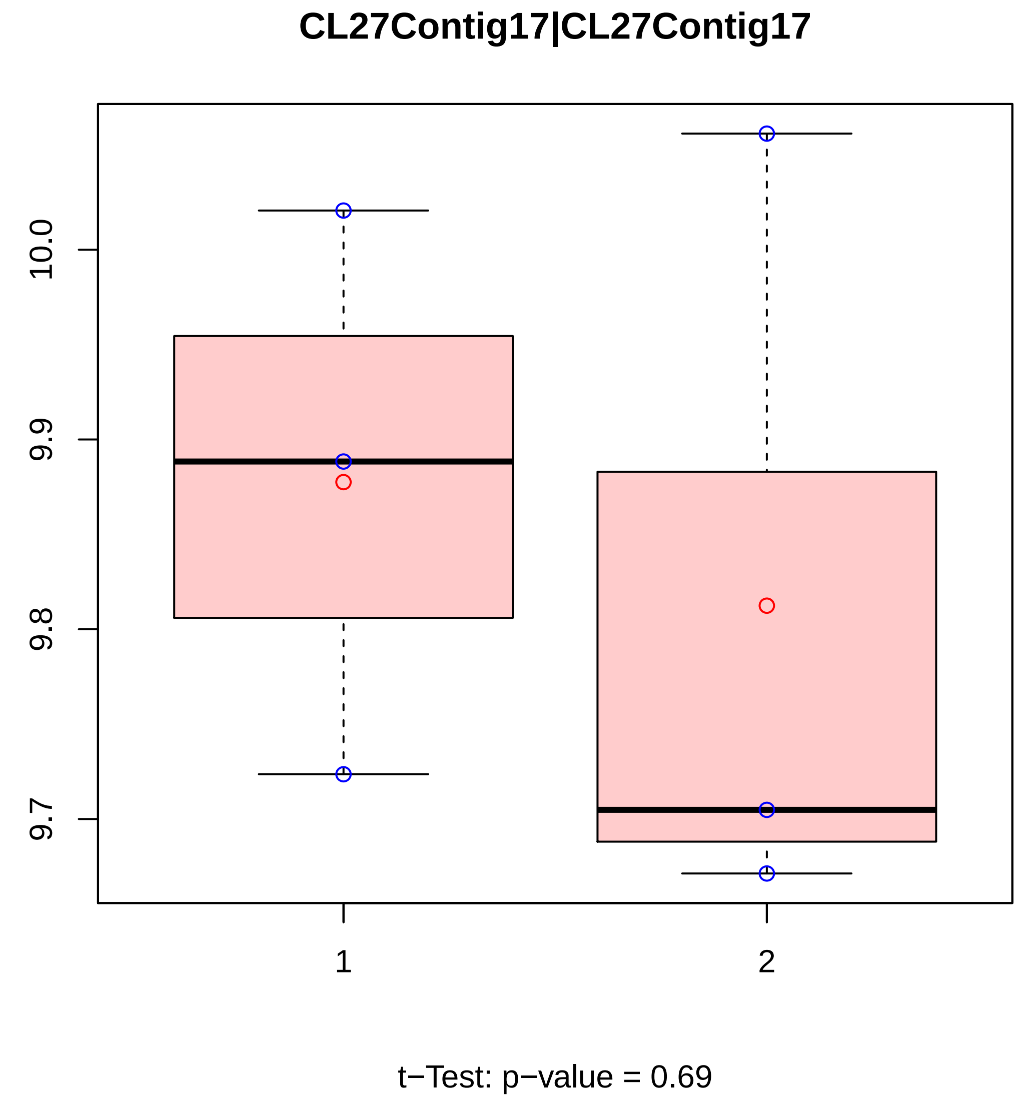

CL27Contig17|CL27Contig17
10.0
9.9
9.8
9.7
1
2
t−Test: p−value = 0.69

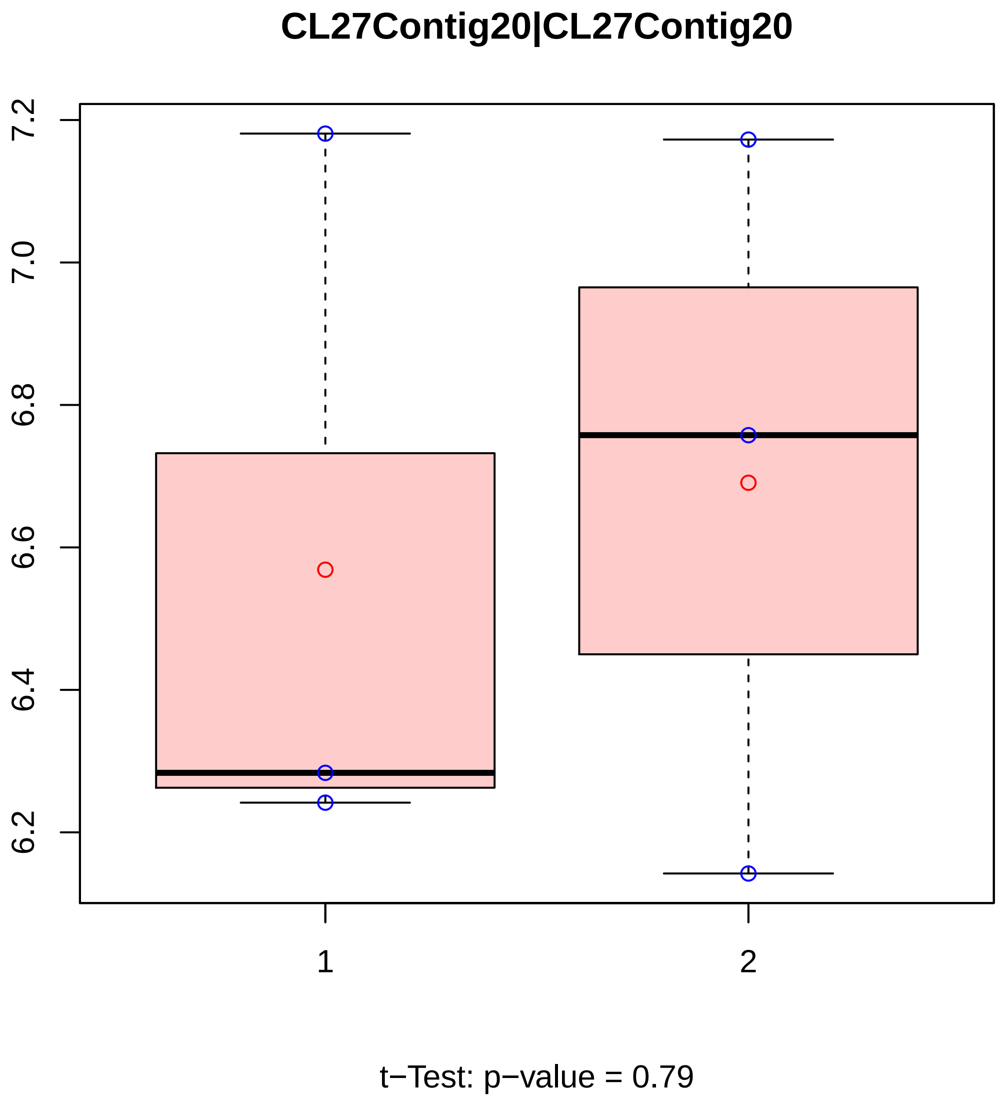
CL27Contig20|CL27Contig20
7.2
7.0
6.8
6.6
6.4
6.2
1
2
t−Test: p−value = 0.79

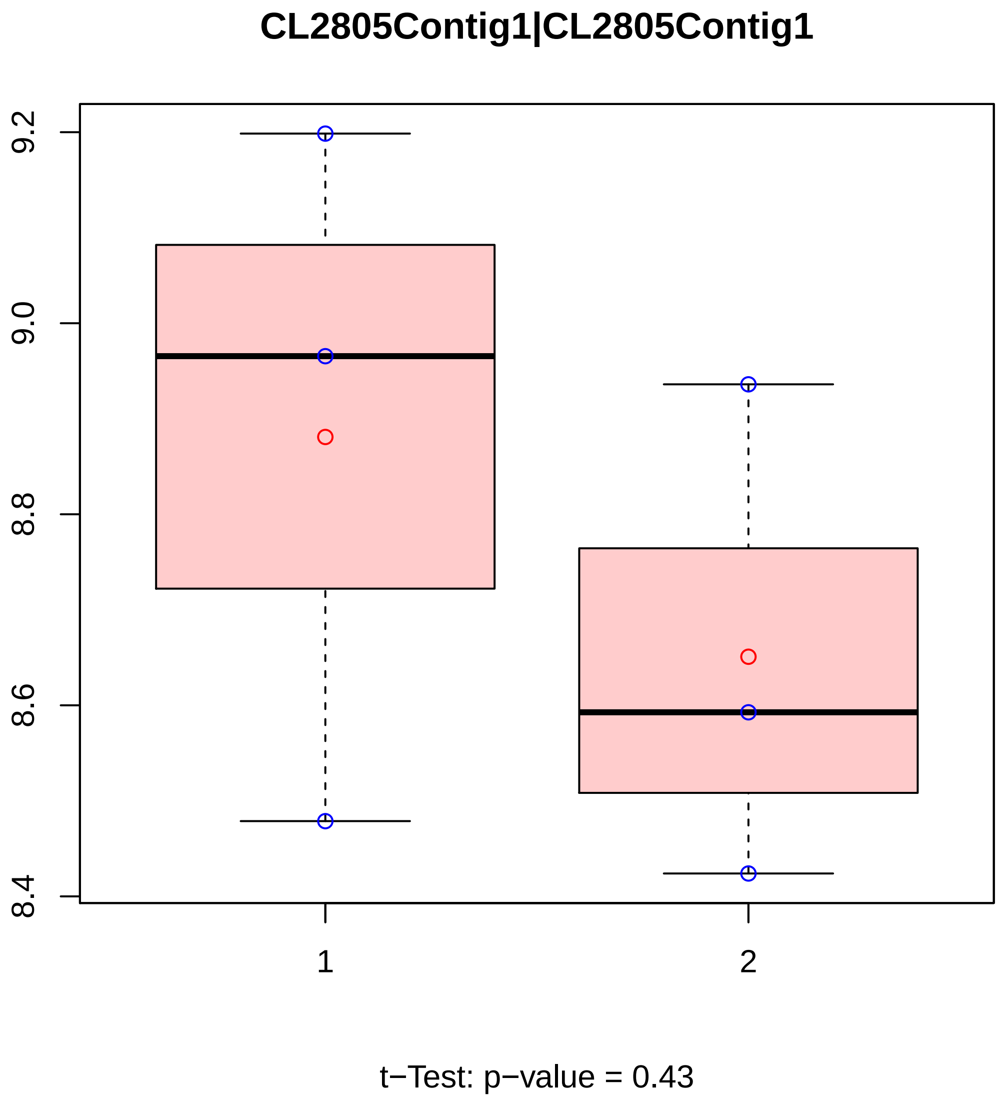

CL2805Contig1|CL2805Contig1
9.2
9.0
8.8
8.6
8.4
1
2
t−Test: p−value = 0.43

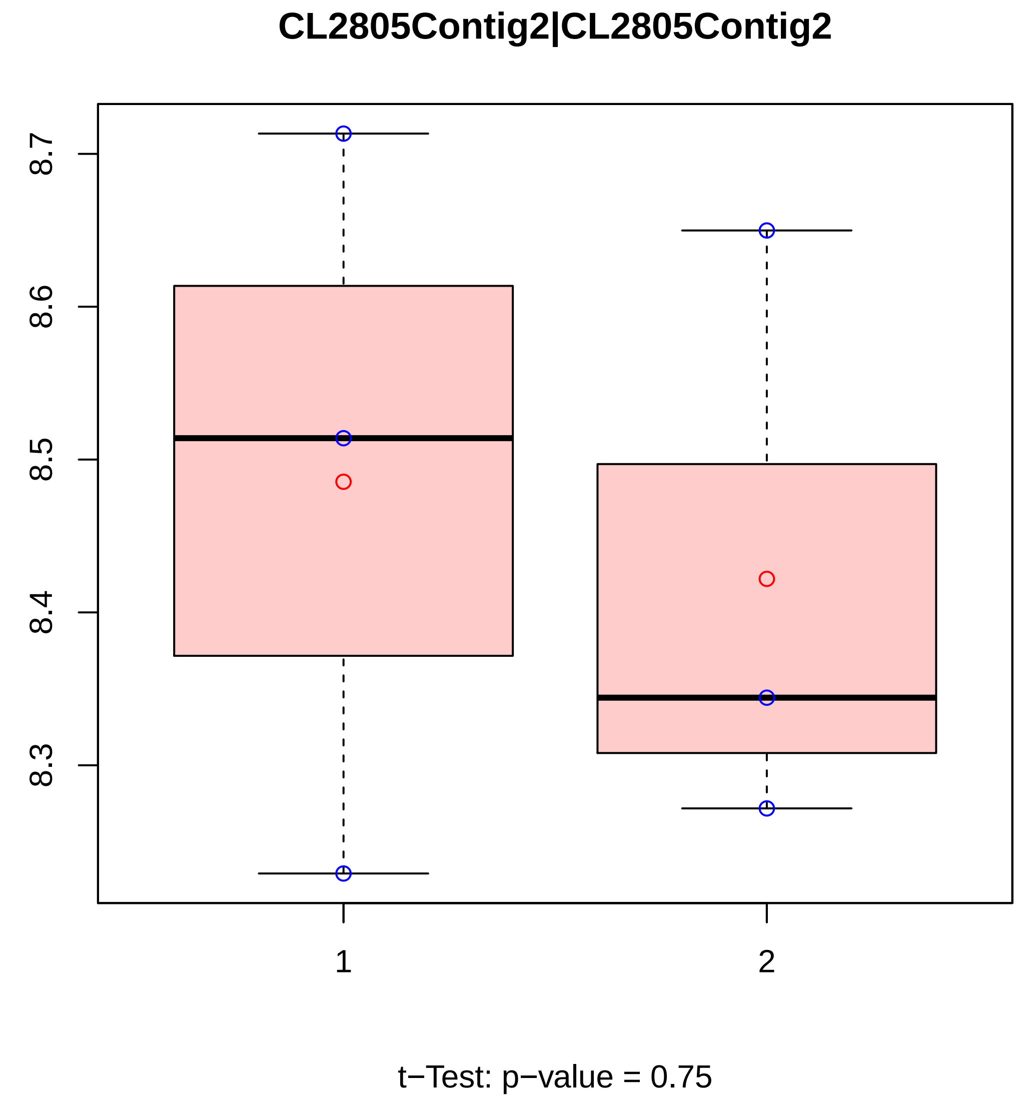
CL2805Contig2|CL2805Contig2
8.3
8.4
8.5
8.6
8.7
1
2
t−Test: p−value = 0.75

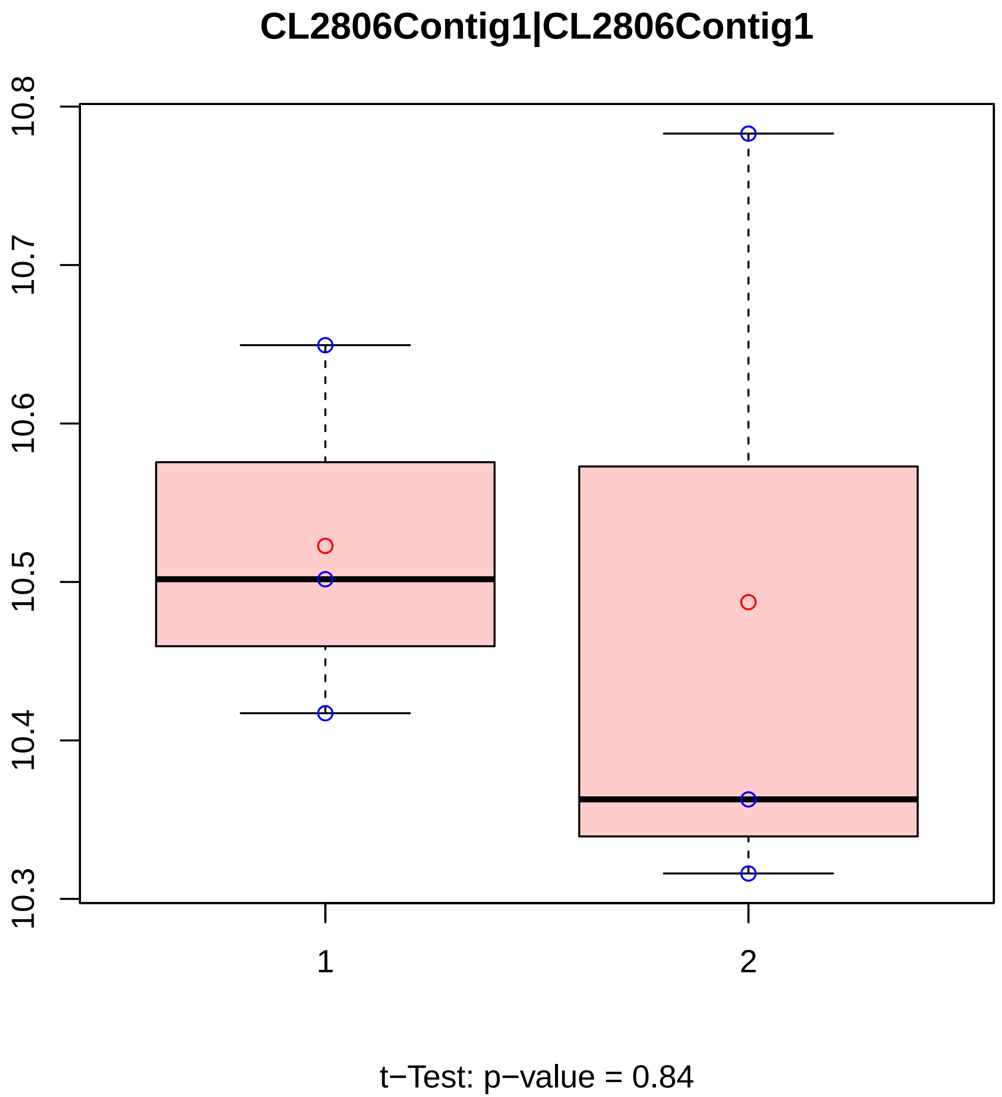
CL2806Contig1|CL2806Contig1
10.8
10.7
10.6
10.5
10.4
10.3
1
2
t−Test: p−value = 0.84

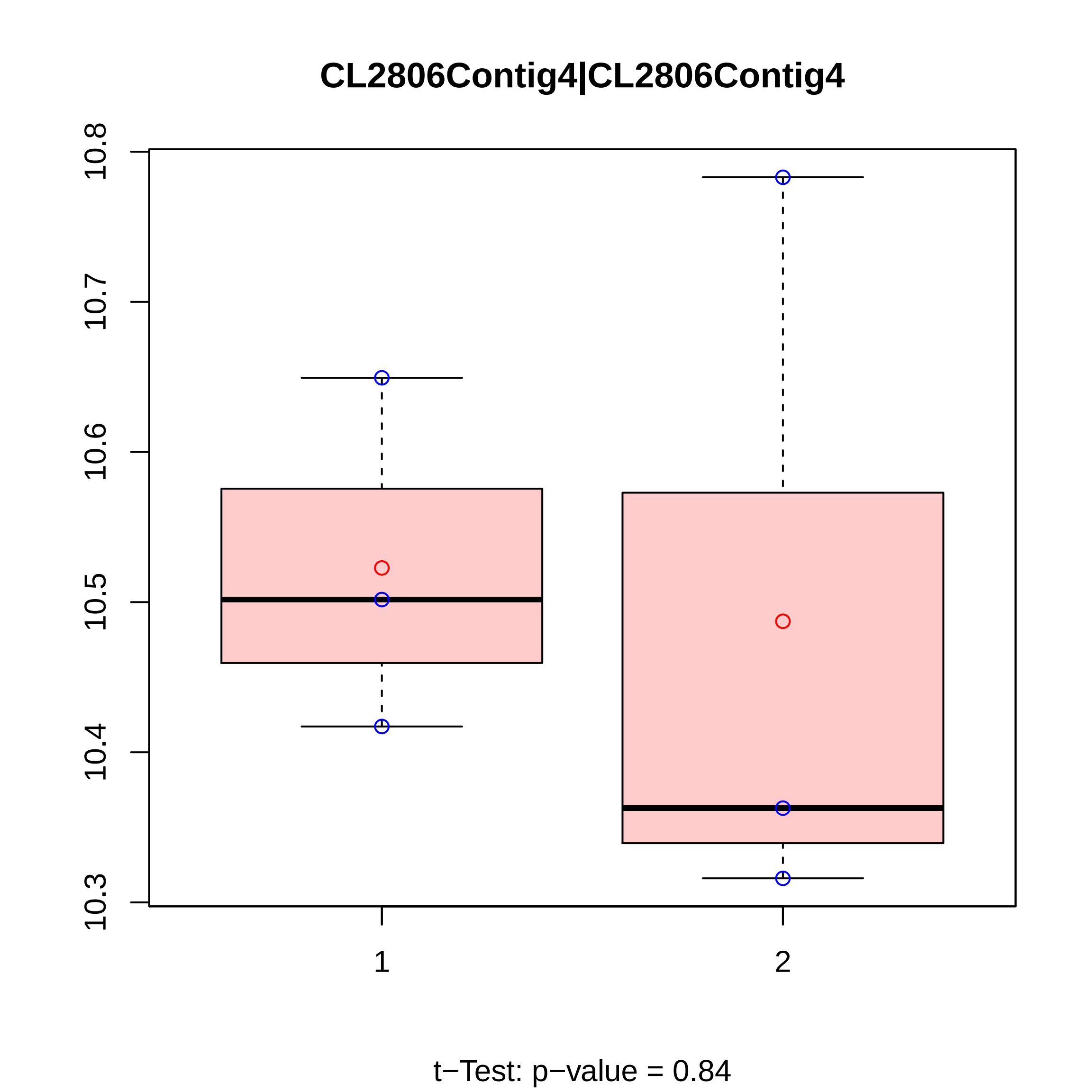

CL2806Contig4|CL2806Contig4
10.8
10.7
10.6
10.5
10.4
10.3
1
2
t−Test: p−value = 0.84

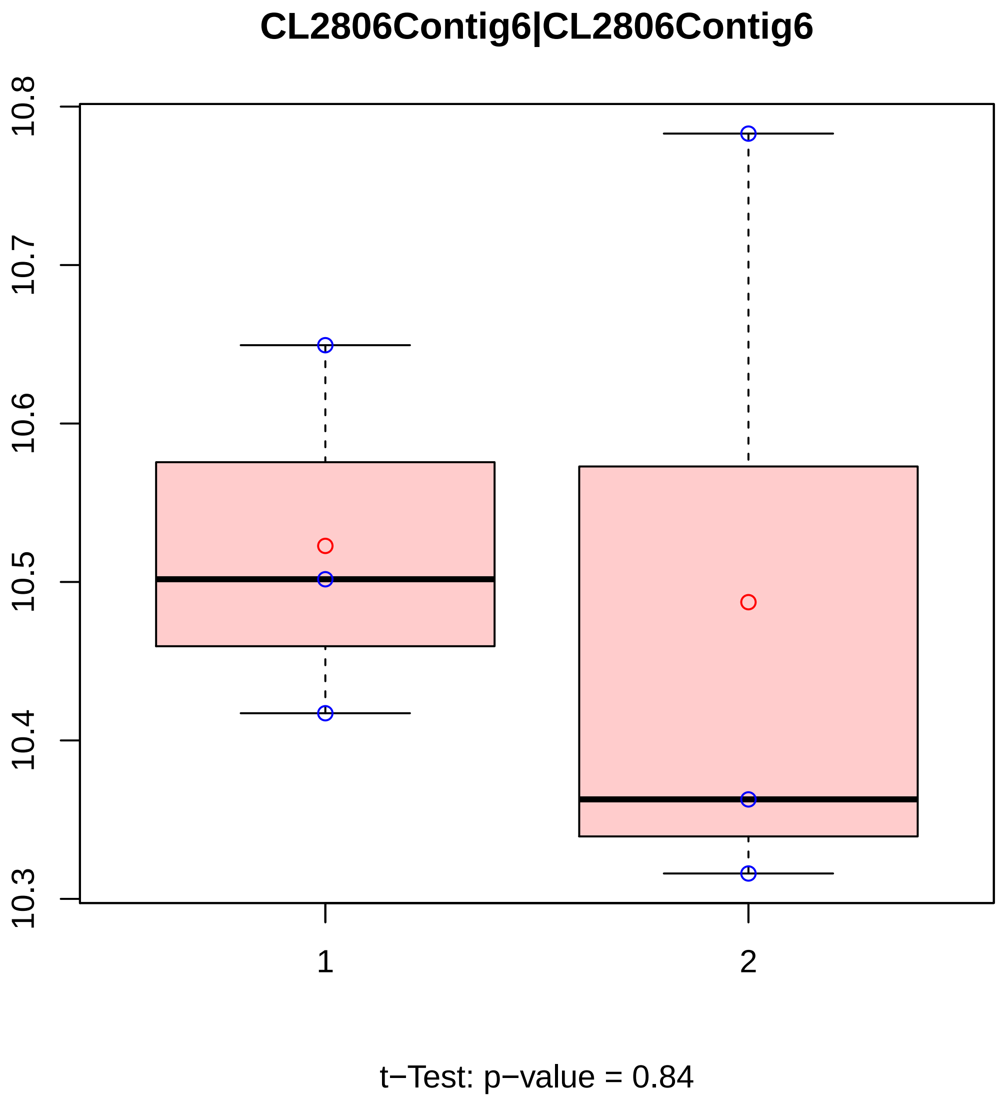
CL2806Contig6|CL2806Contig6
10.3
10.4
10.5
10.6
10.7
10.8
1
2
t−Test: p−value = 0.84

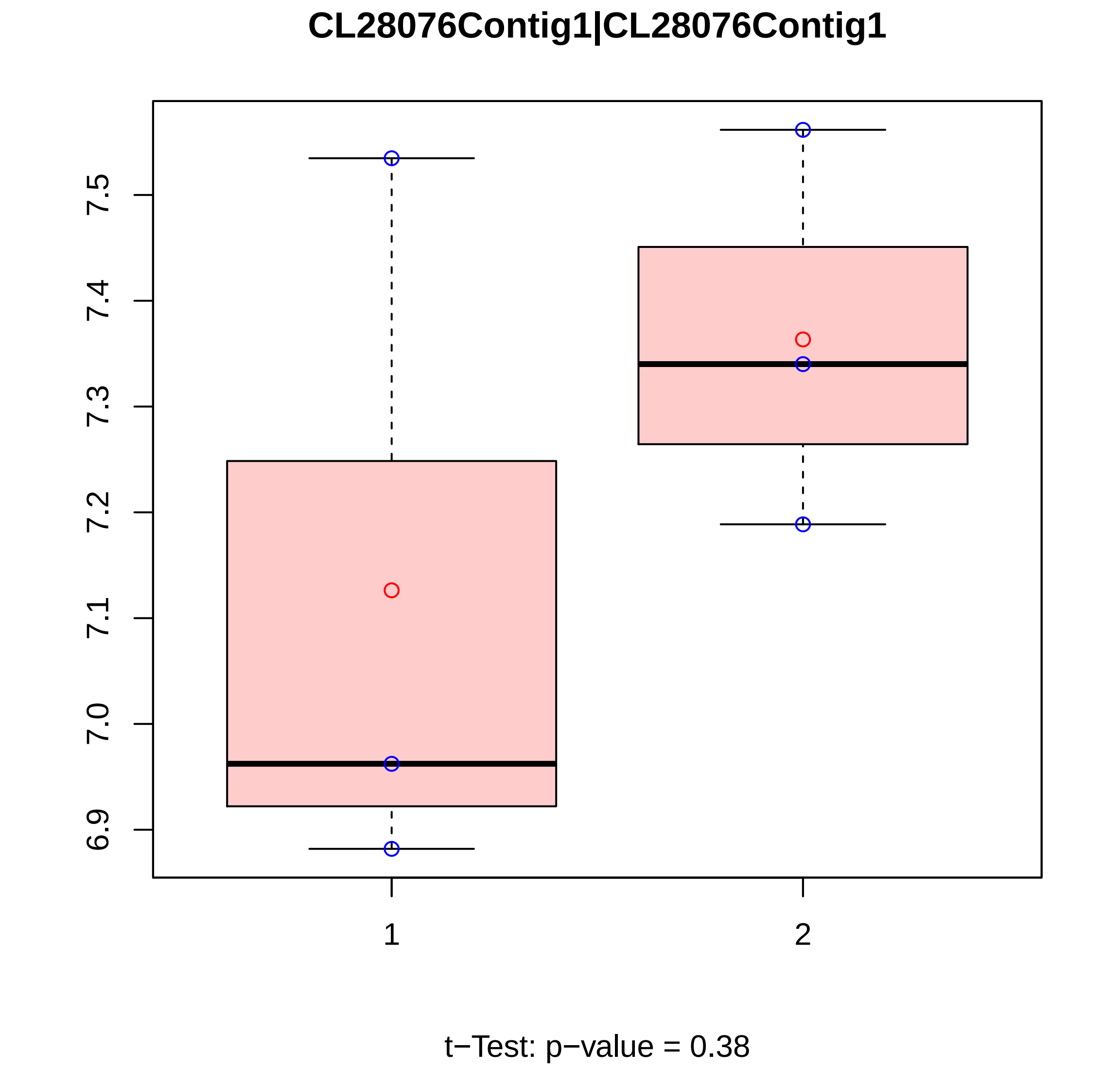

CL28076Contig1|CL28076Contig1
7.5
7.4
7.3
7.2
7.1
7.0
6.9
1
2
t−Test: p−value = 0.38

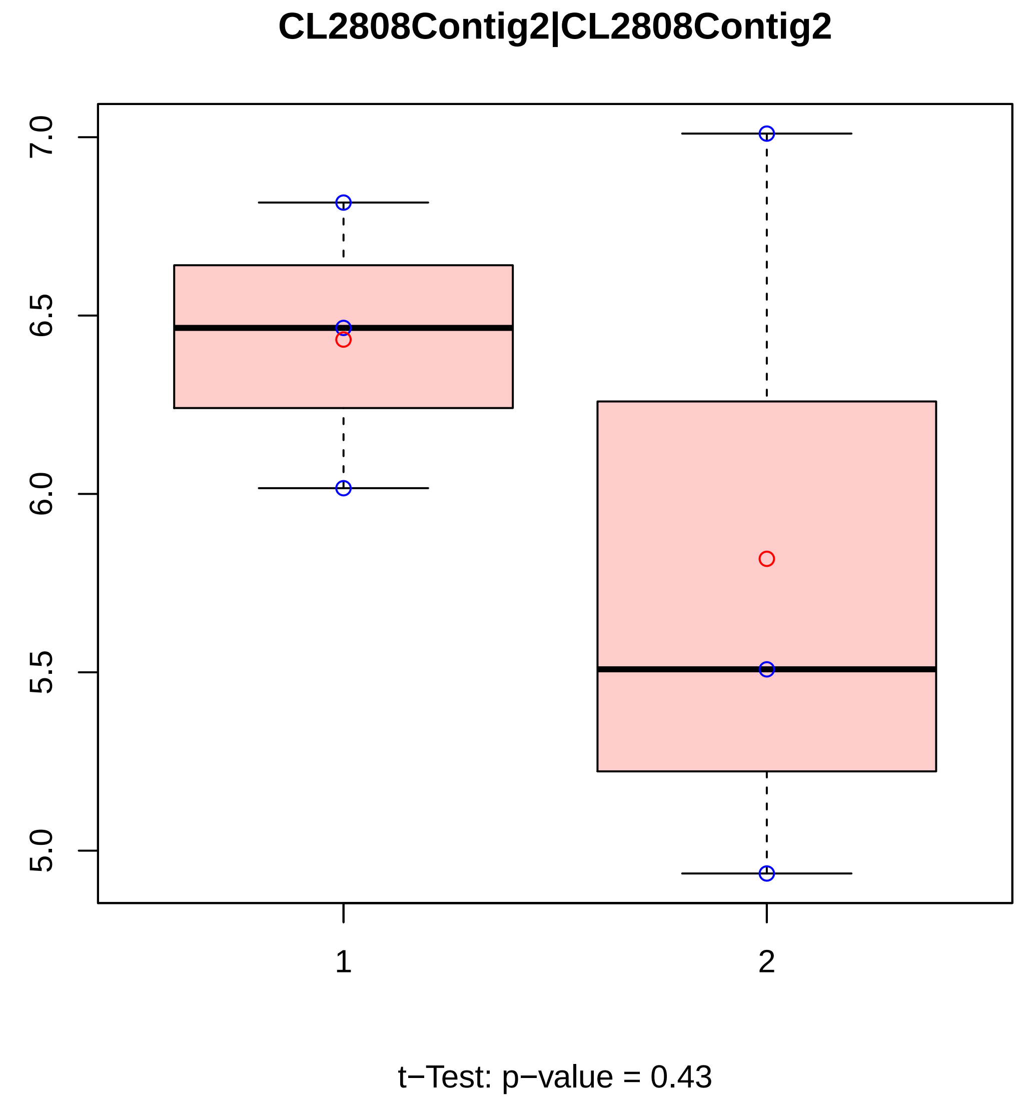
CL2808Contig2|CL2808Contig2
7.0
6.5
6.0
5.5
5.0
1
2
t−Test: p−value = 0.43

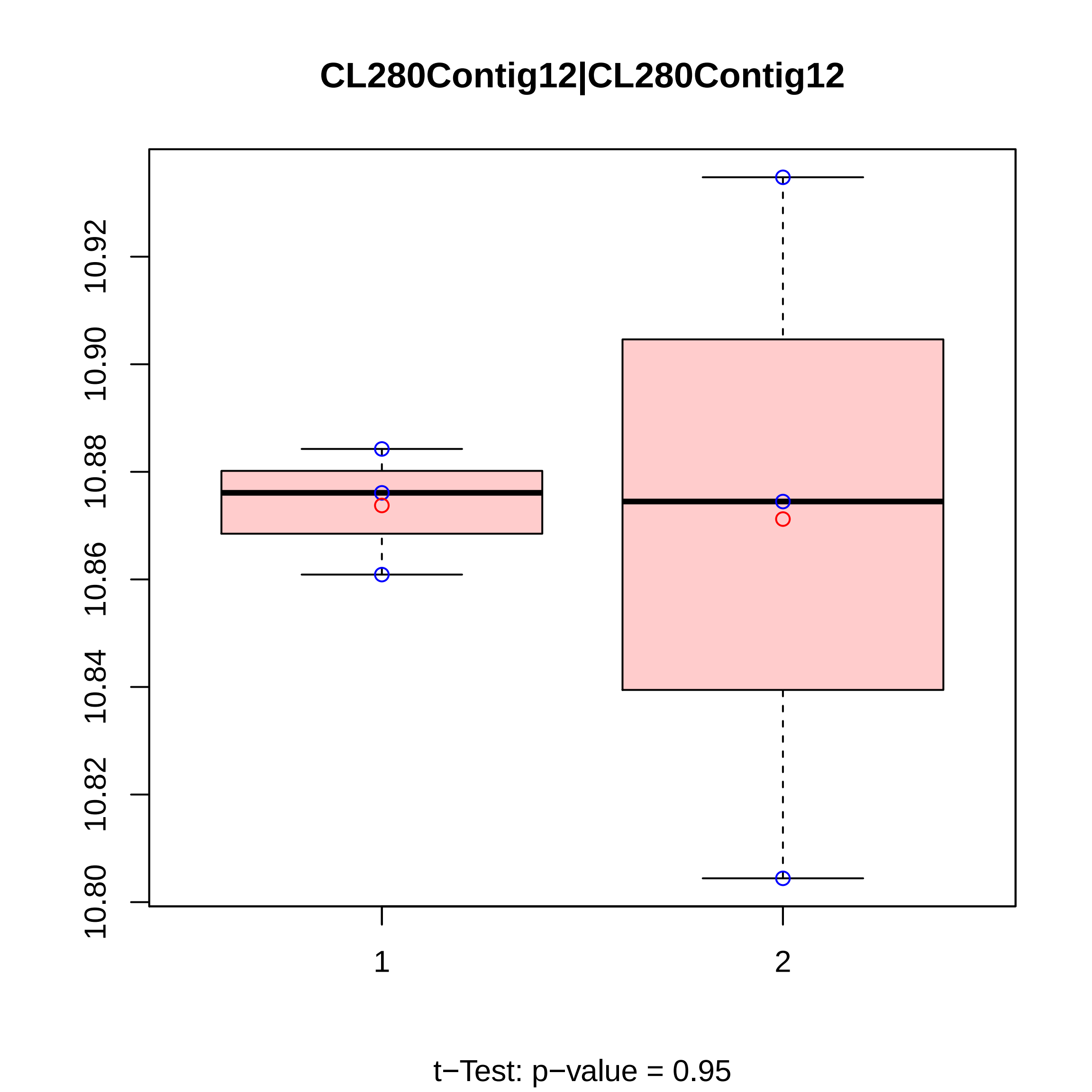

CL280Contig12|CL280Contig12
10.80
10.82
10.84
10.86
10.88
10.90
10.92
1
2
t−Test: p−value = 0.95

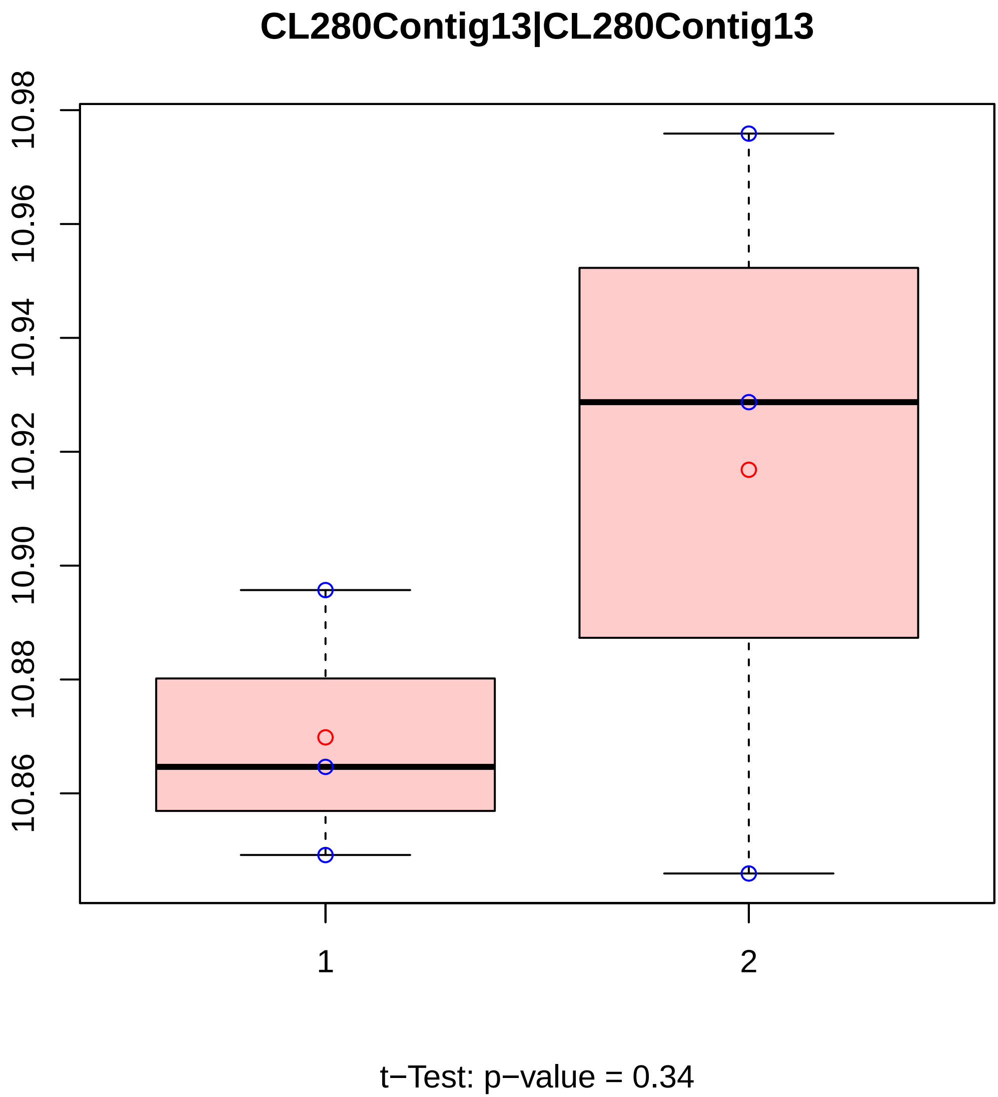

CL280Contig13|CL280Contig13
10.98
10.96
10.94
10.92
10.90
10.88
10.86
1
2
t−Test: p−value = 0.34

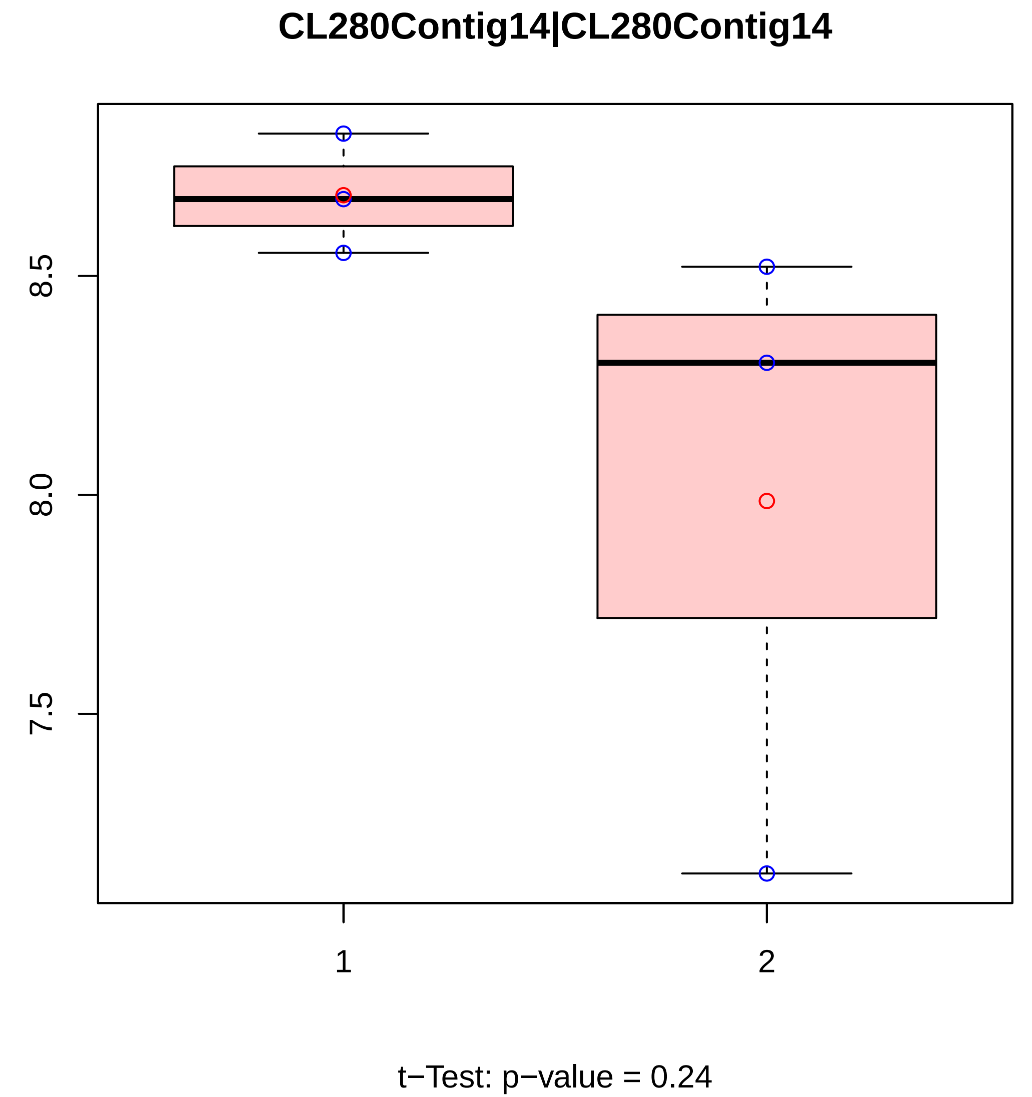
CL280Contig14|CL280Contig14
8.5
8.0
7.5
1
2
t−Test: p−value = 0.24

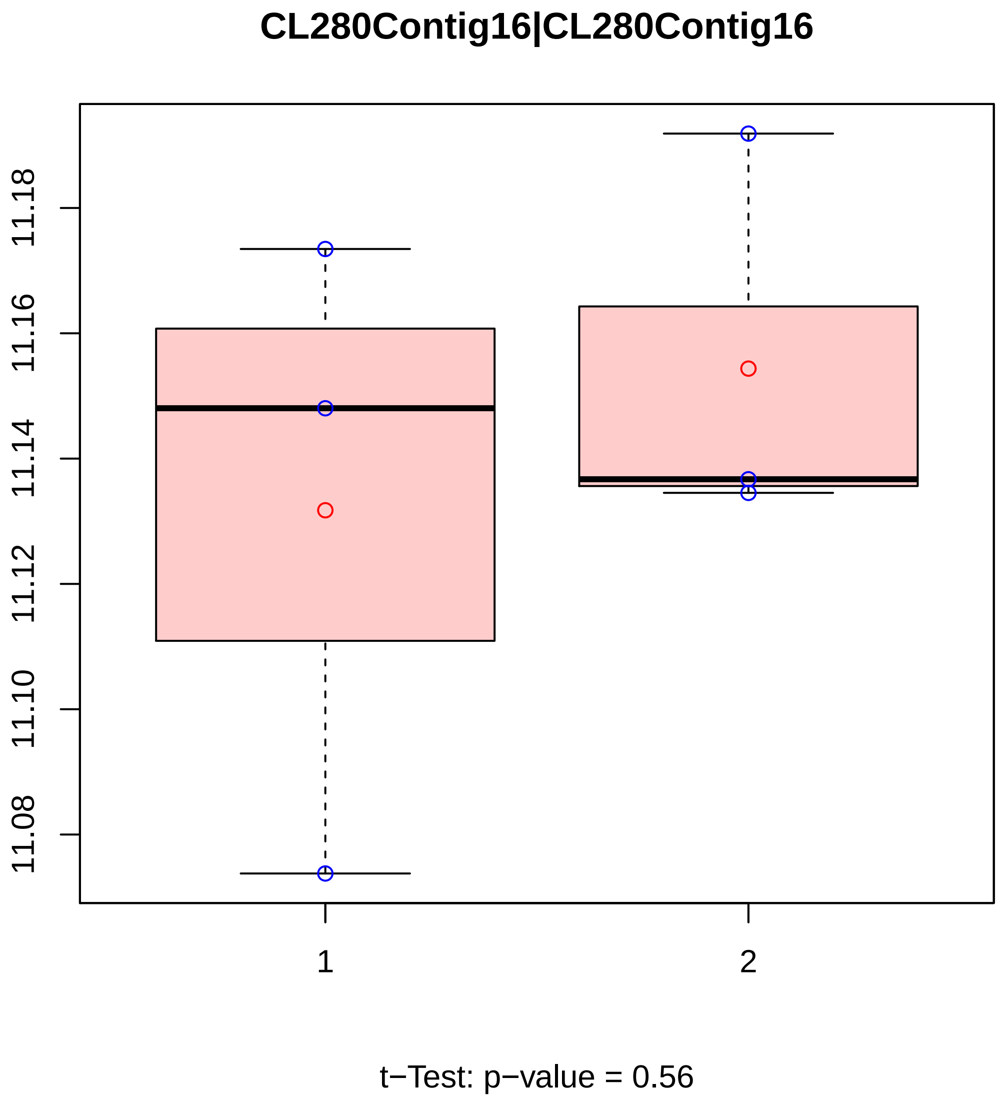
CL280Contig16|CL280Contig16
11.08
11.10
11.12
11.14
11.16
11.18
1
2
t−Test: p−value = 0.56

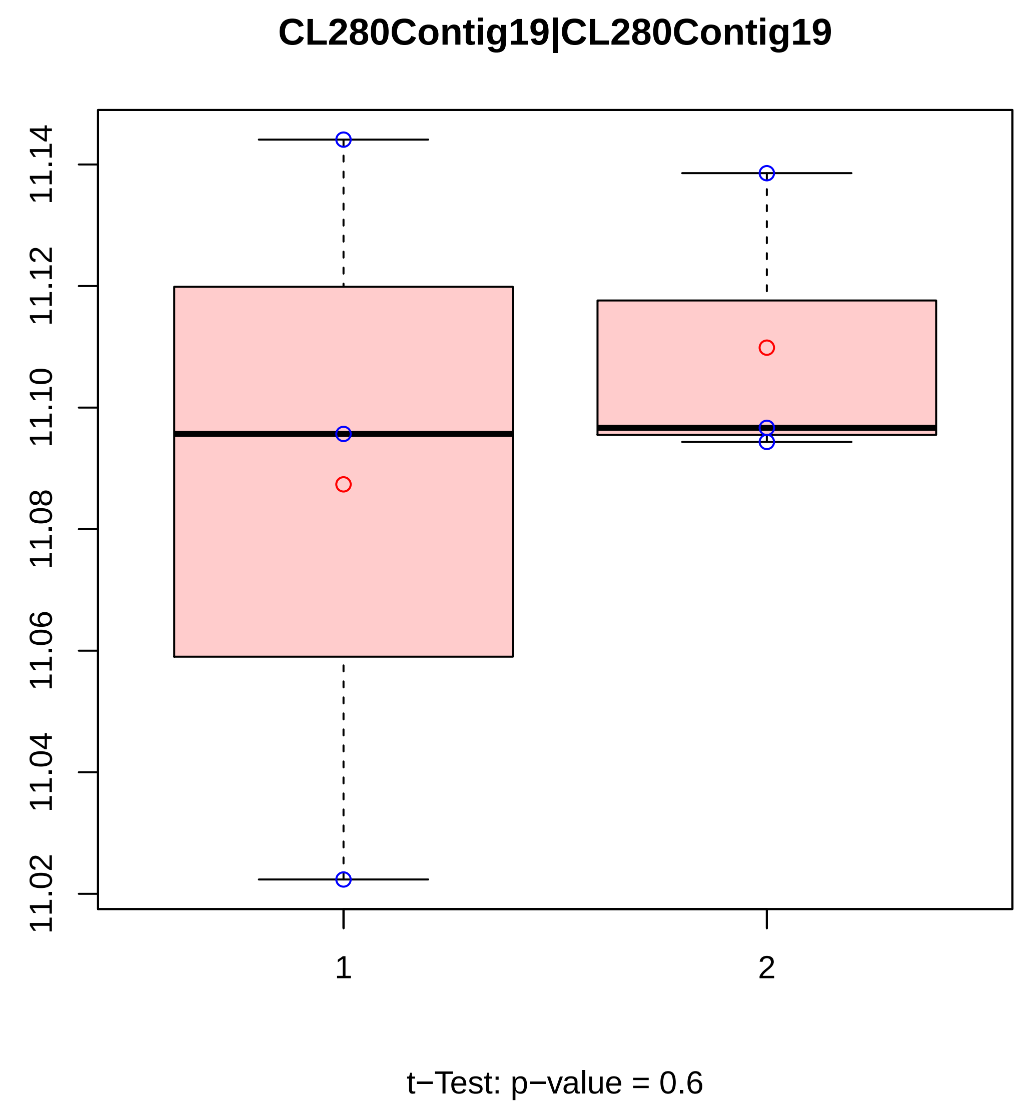

CL280Contig19|CL280Contig19
11.02
11.04
11.06
11.08
11.10
11.12
11.14
1
2
t−Test: p−value = 0.6

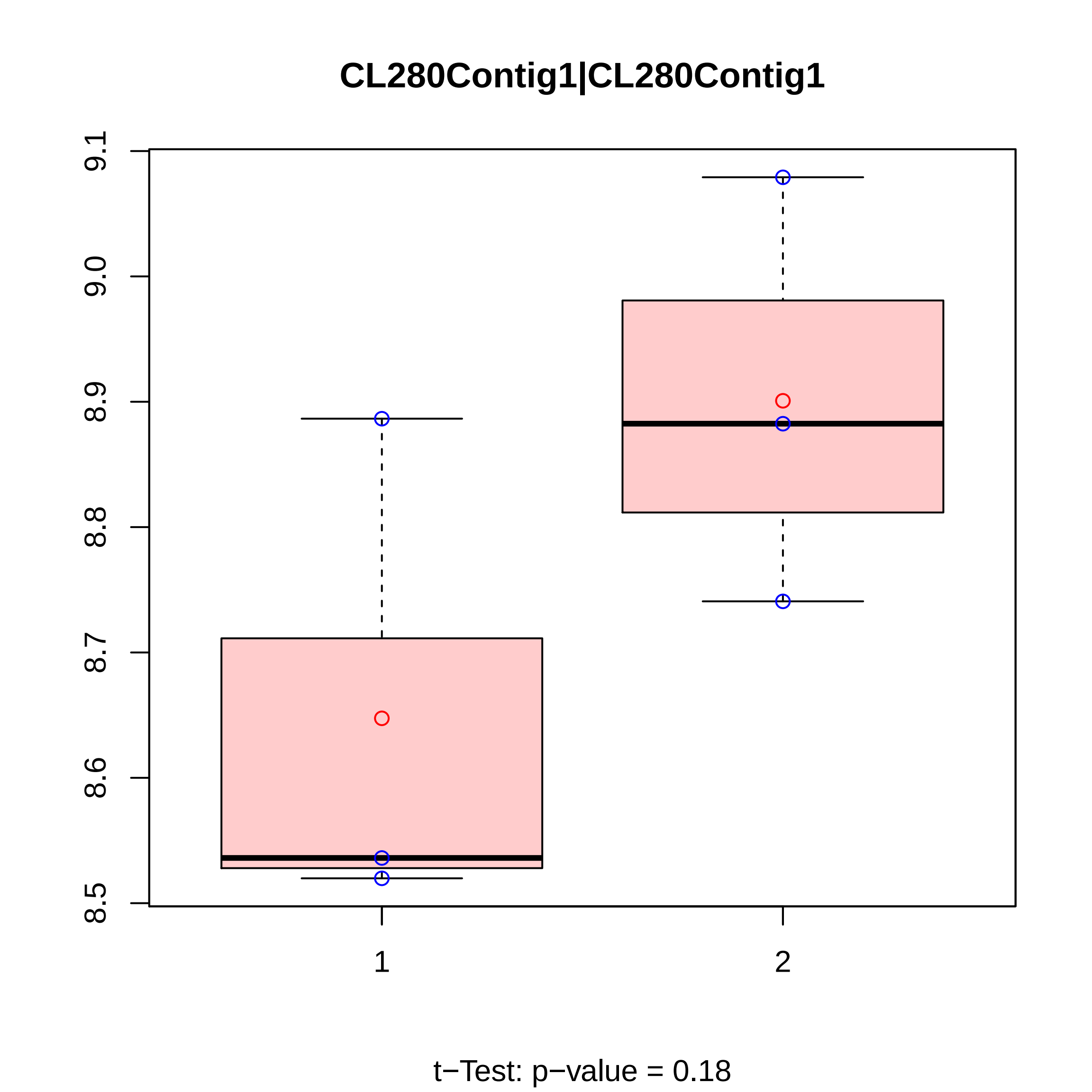

CL280Contig1|CL280Contig1
9.1
9.0
8.9
8.8
8.7
8.6
8.5
1
2
t−Test: p−value = 0.18

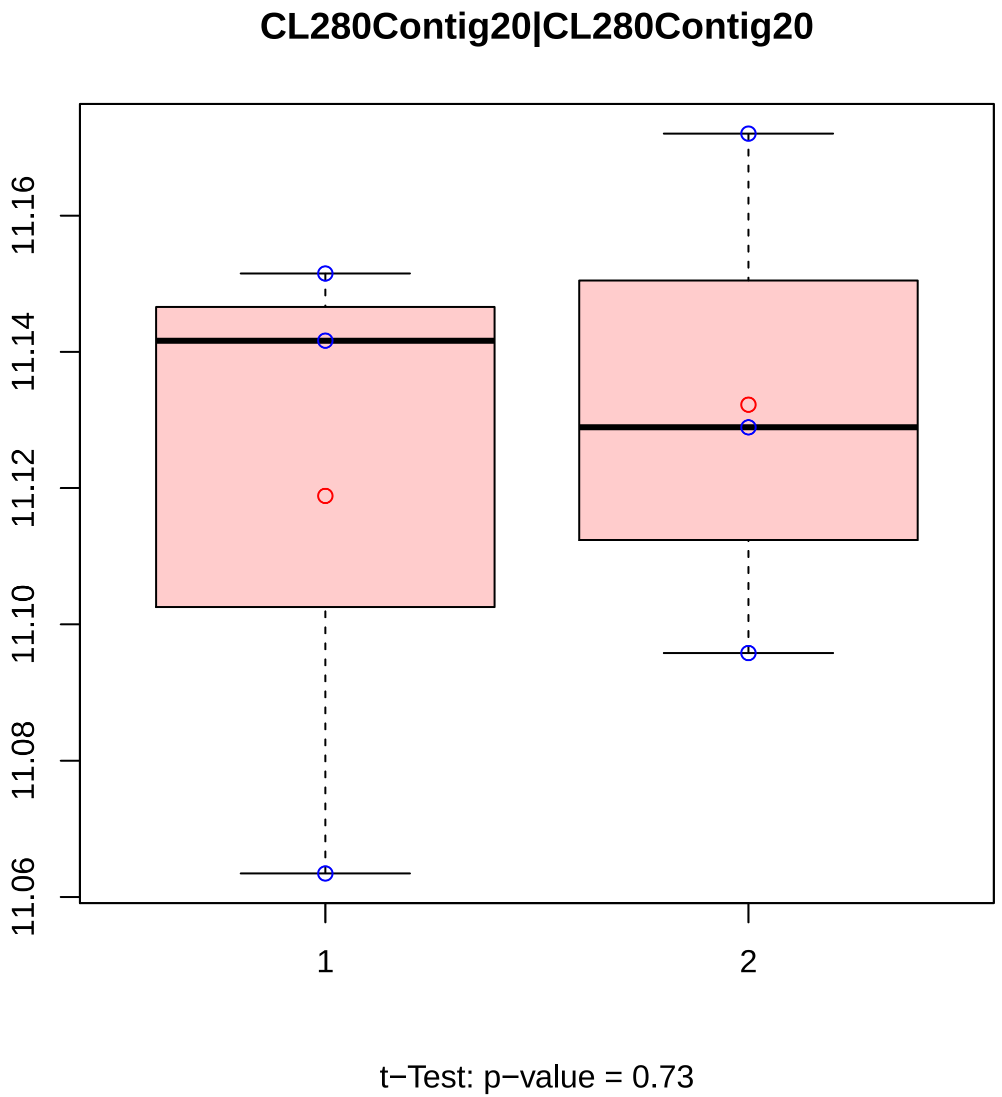

CL280Contig20|CL280Contig20
11.06
11.08
11.10
11.12
11.14
11.16
1
2
t−Test: p−value = 0.73

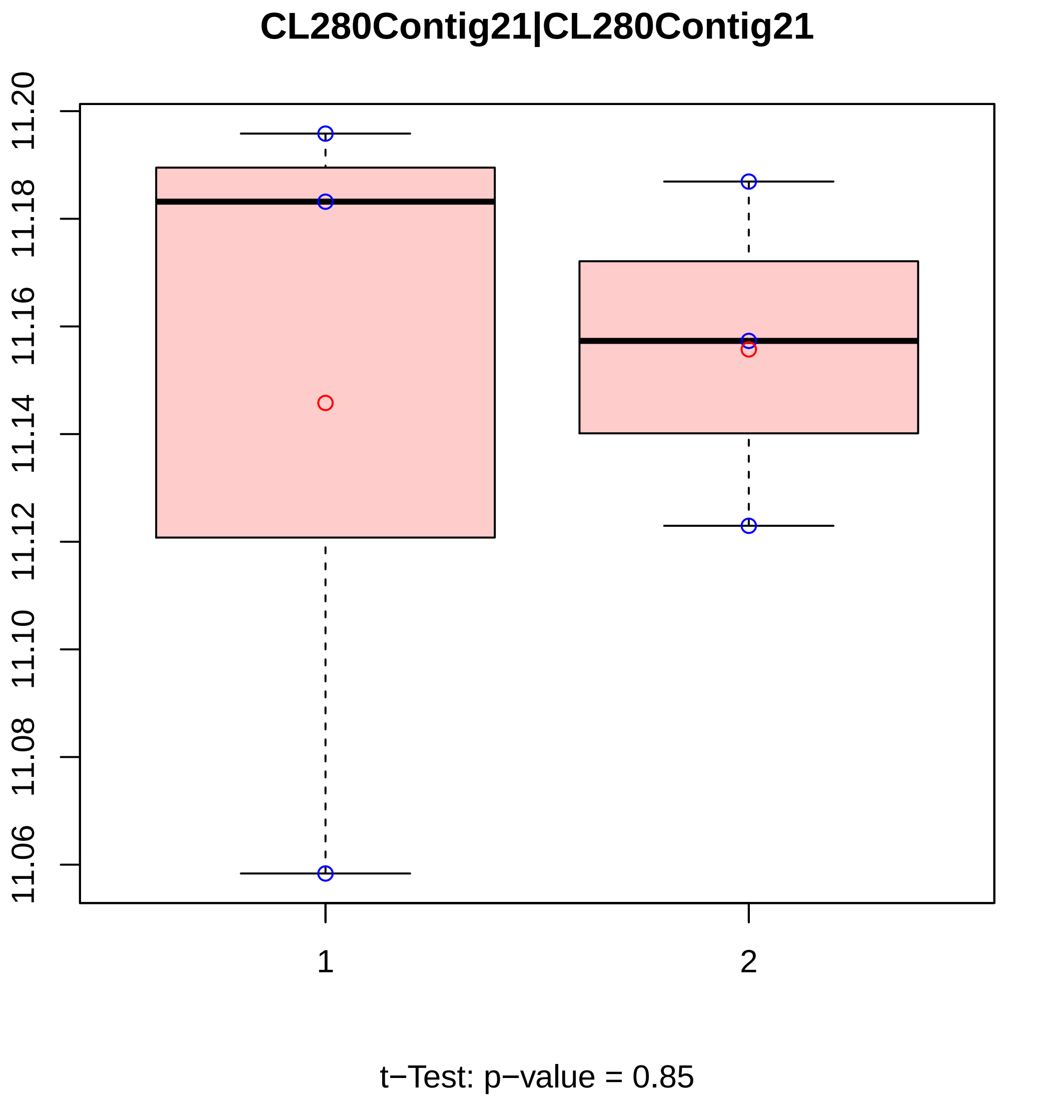

CL280Contig21|CL280Contig21
11.06
11.08
11.10
11.12
11.14
11.16
11.18
11.20
1
2
t−Test: p−value = 0.85

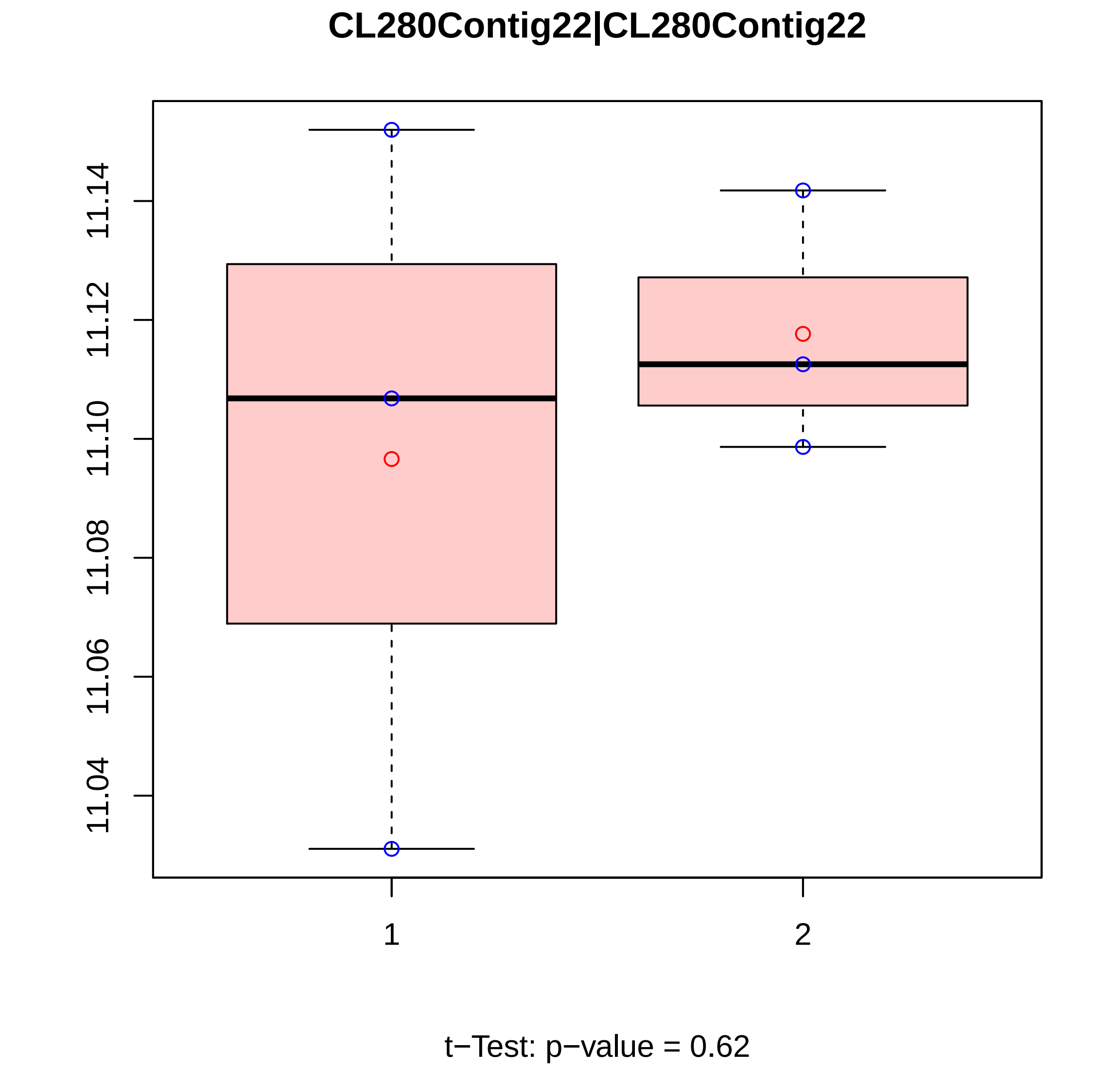
CL280Contig22|CL280Contig22
11.04
11.06
11.08
11.10
11.12
11.14
1
2
t−Test: p−value = 0.62

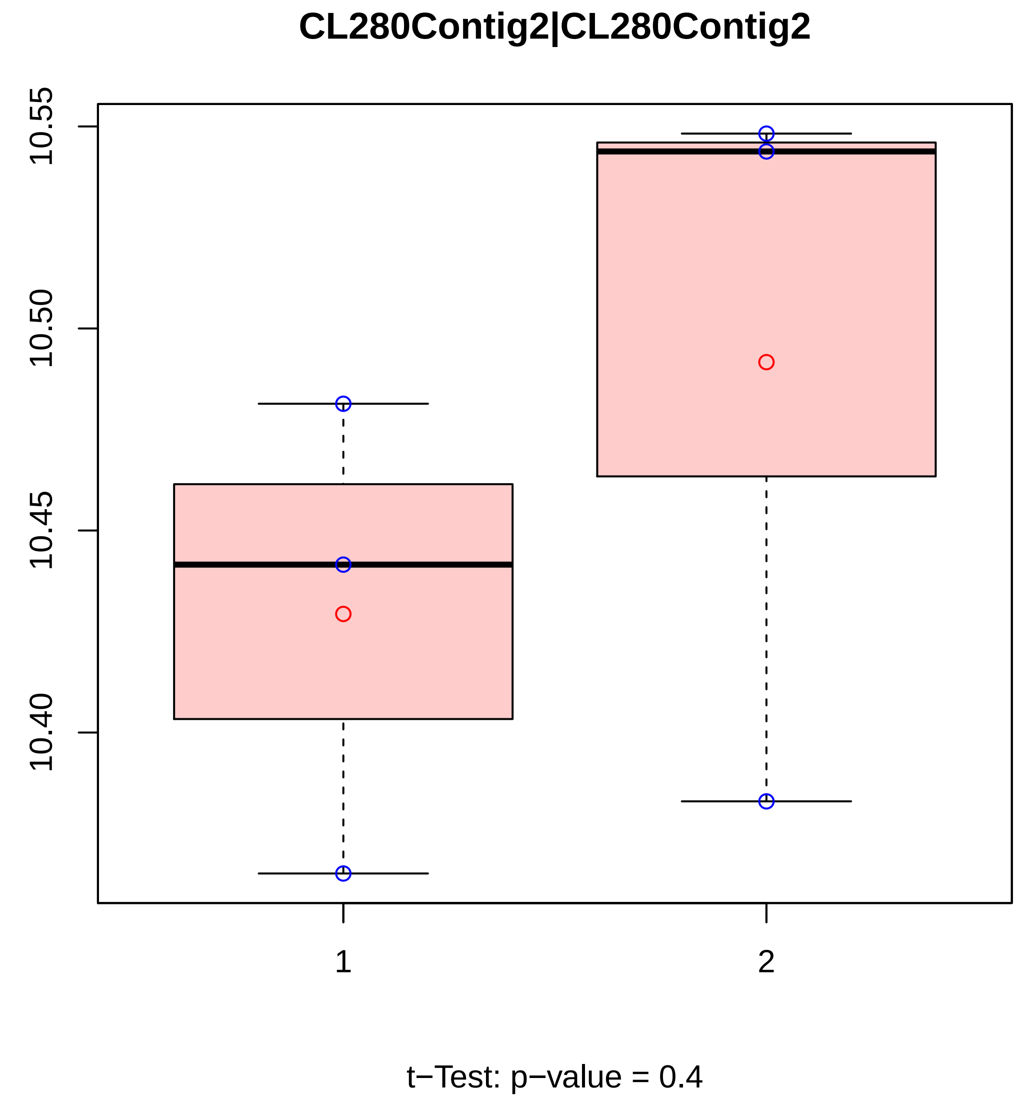
CL280Contig2|CL280Contig2
10.40
10.45
10.50
10.55
1
2
t−Test: p−value = 0.4

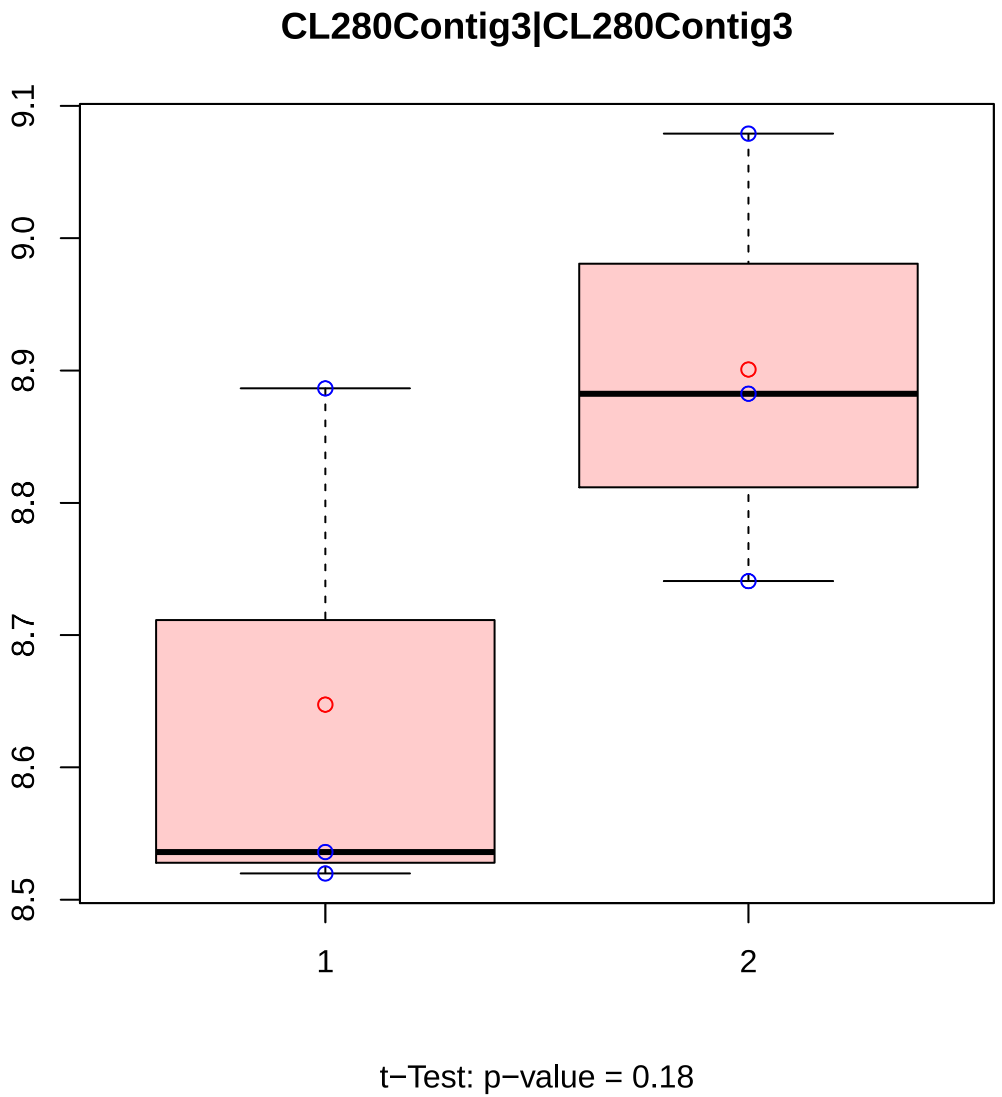
CL280Contig3|CL280Contig3
9.1
9.0
8.9
8.8
8.7
8.6
8.5
1
2
t−Test: p−value = 0.18

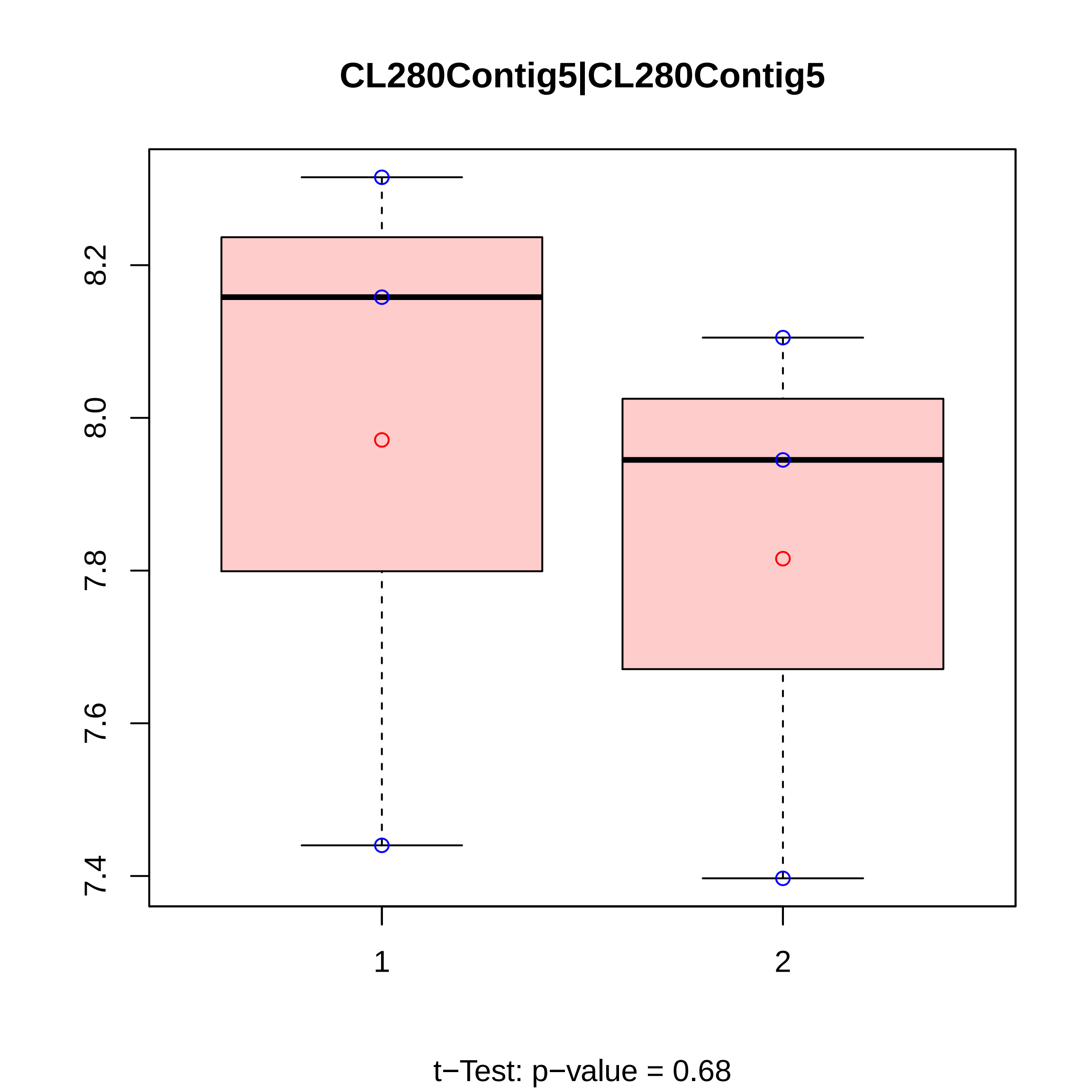

CL280Contig5|CL280Contig5
8.2
8.0
7.8
7.6
7.4
1
2
t−Test: p−value = 0.68

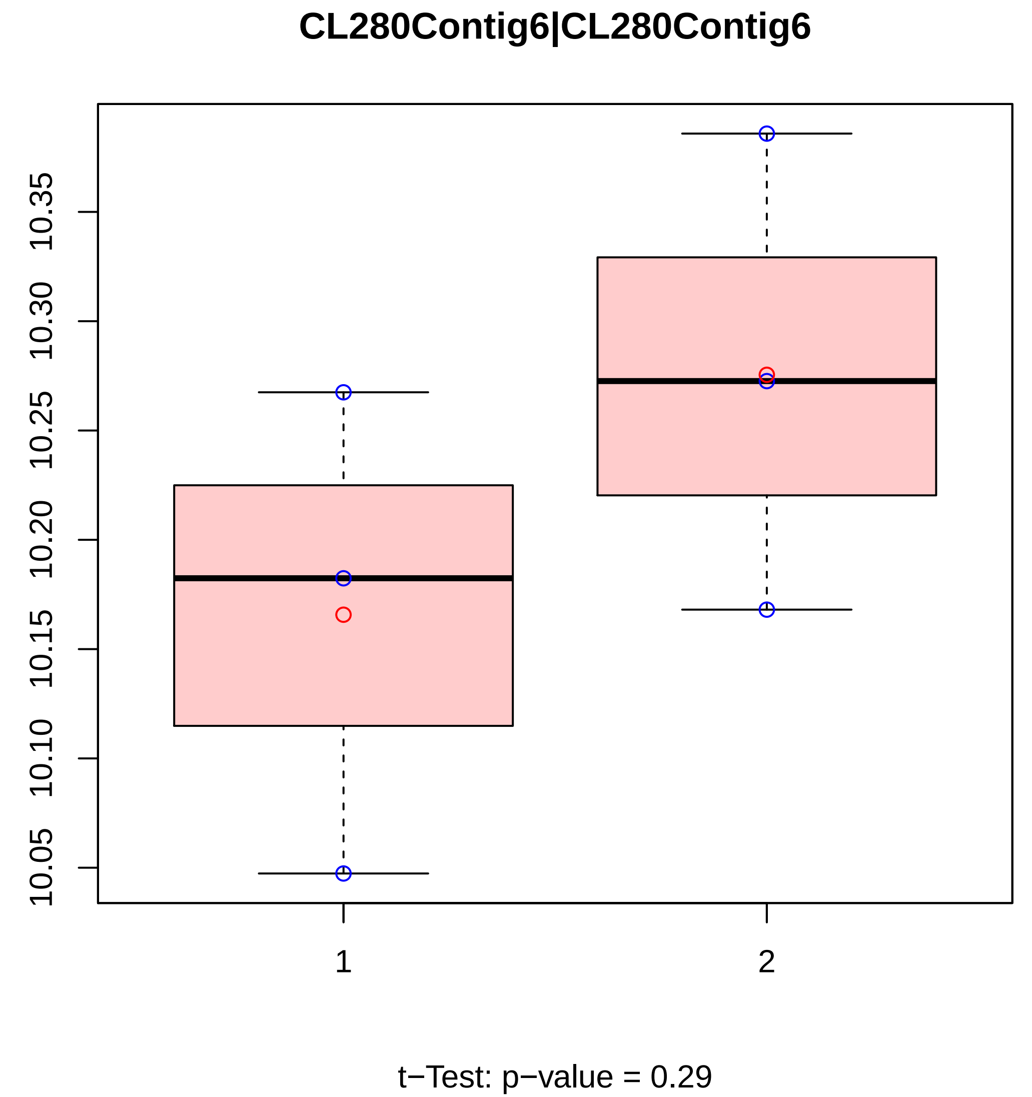

CL280Contig6|CL280Contig6
10.05
10.10
10.15
10.20
10.25
10.30
10.35
1
2
t−Test: p−value = 0.29

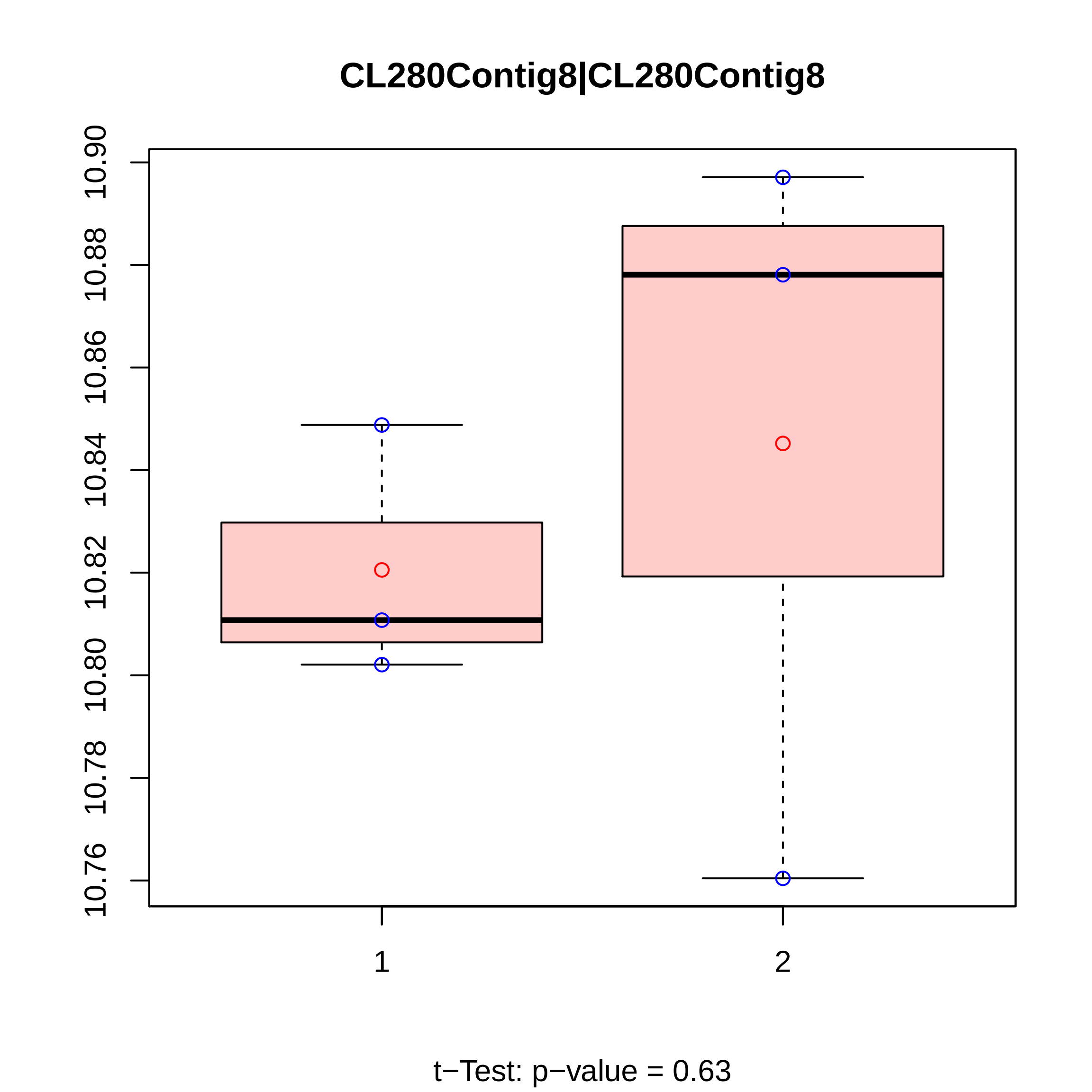
CL280Contig8|CL280Contig8
10.76
10.78
10.80
10.82
10.84
10.86
10.88
10.90
1
2
t−Test: p−value = 0.63

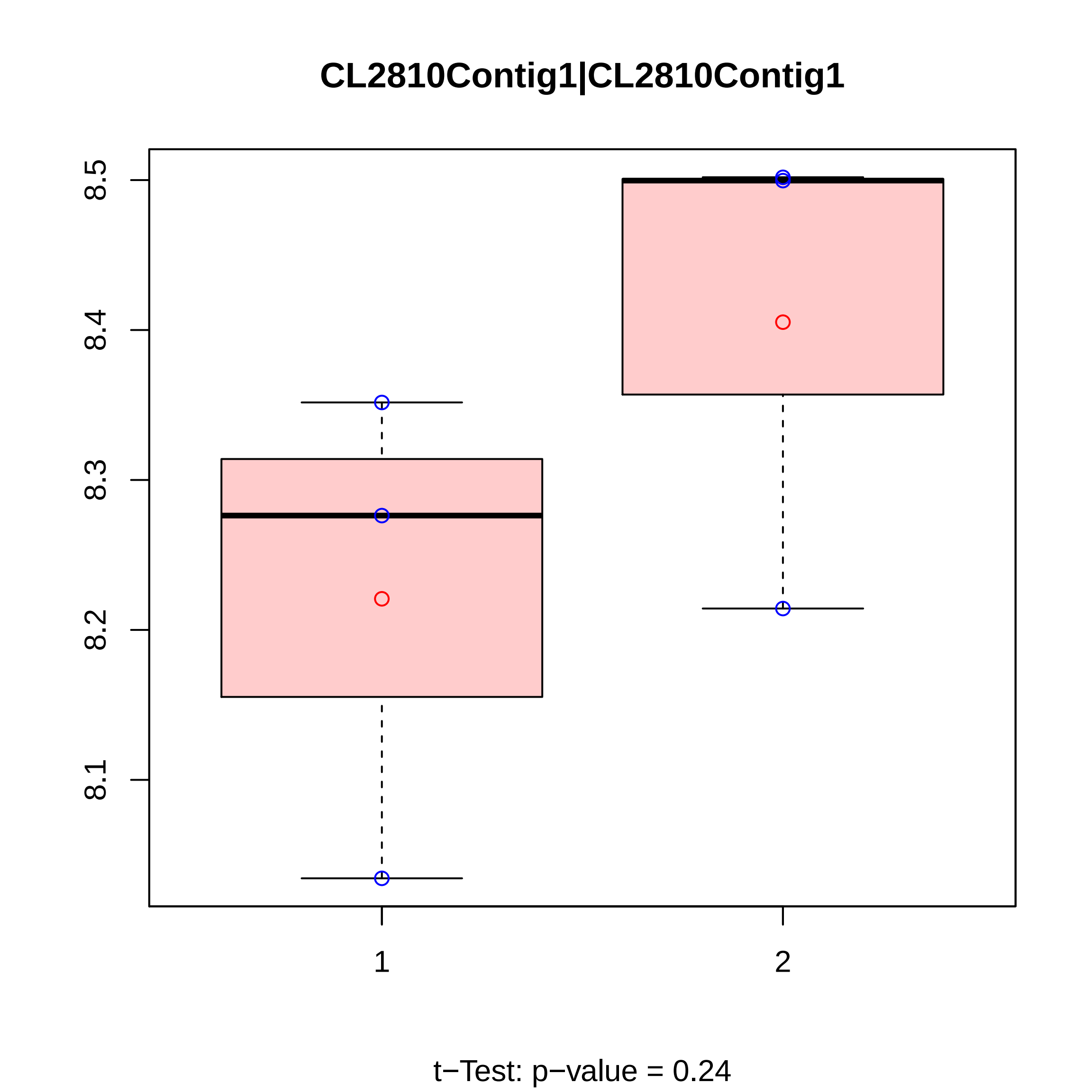

CL2810Contig1|CL2810Contig1
8.1
8.2
8.3
8.4
8.5
1
2
t−Test: p−value = 0.24

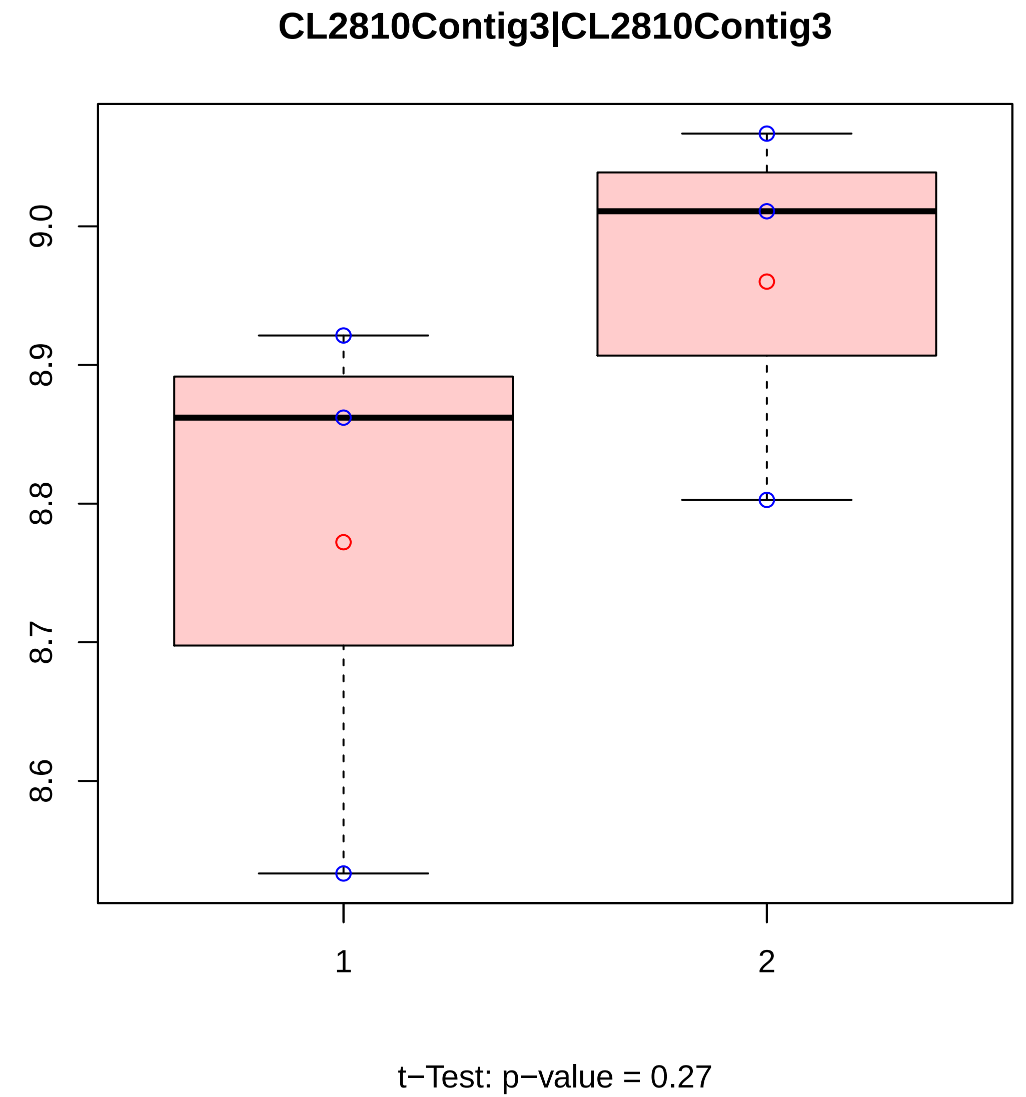

CL2810Contig3|CL2810Contig3
9.0
8.9
8.8
8.7
8.6
1
2
t−Test: p−value = 0.27

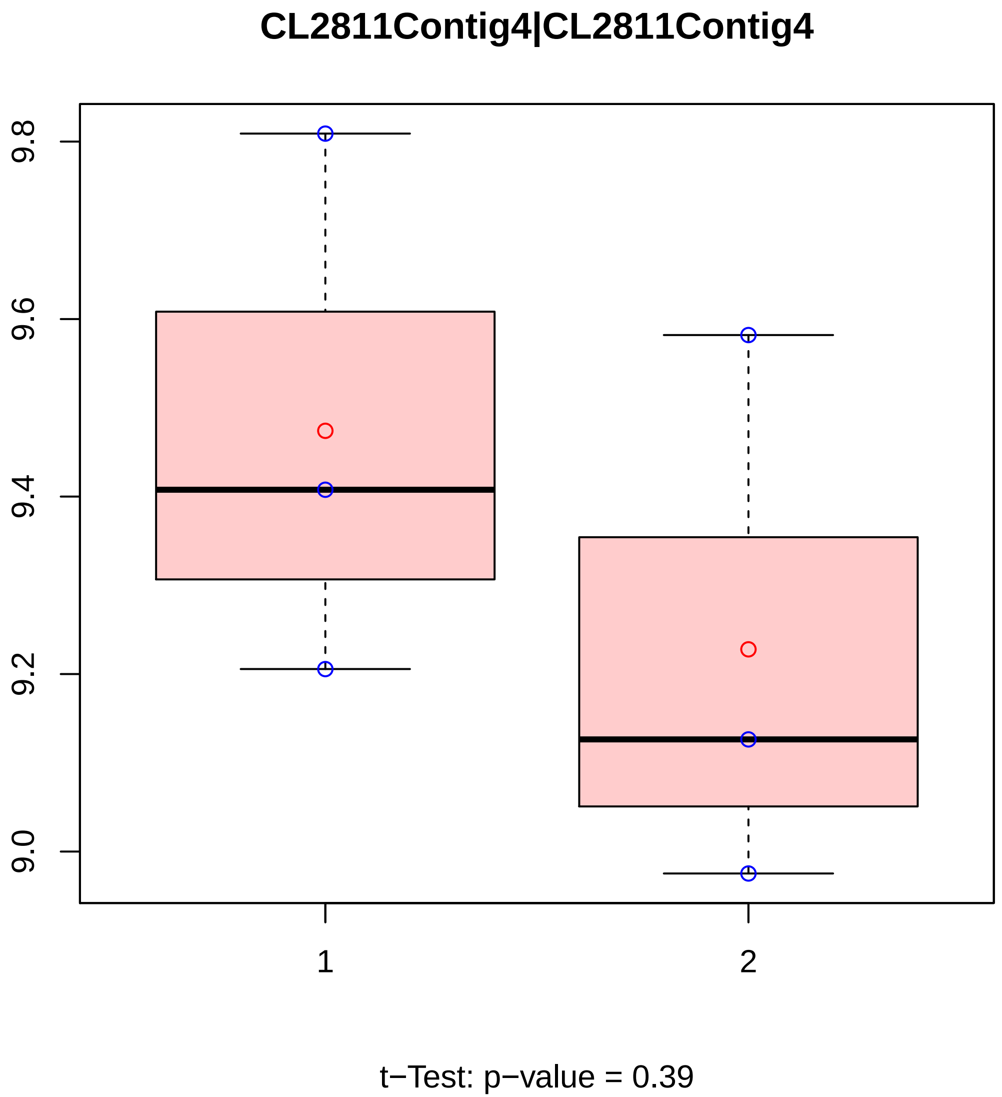
CL2811Contig4|CL2811Contig4
9.0
9.2
9.4
9.6
9.8
1
2
t−Test: p−value = 0.39

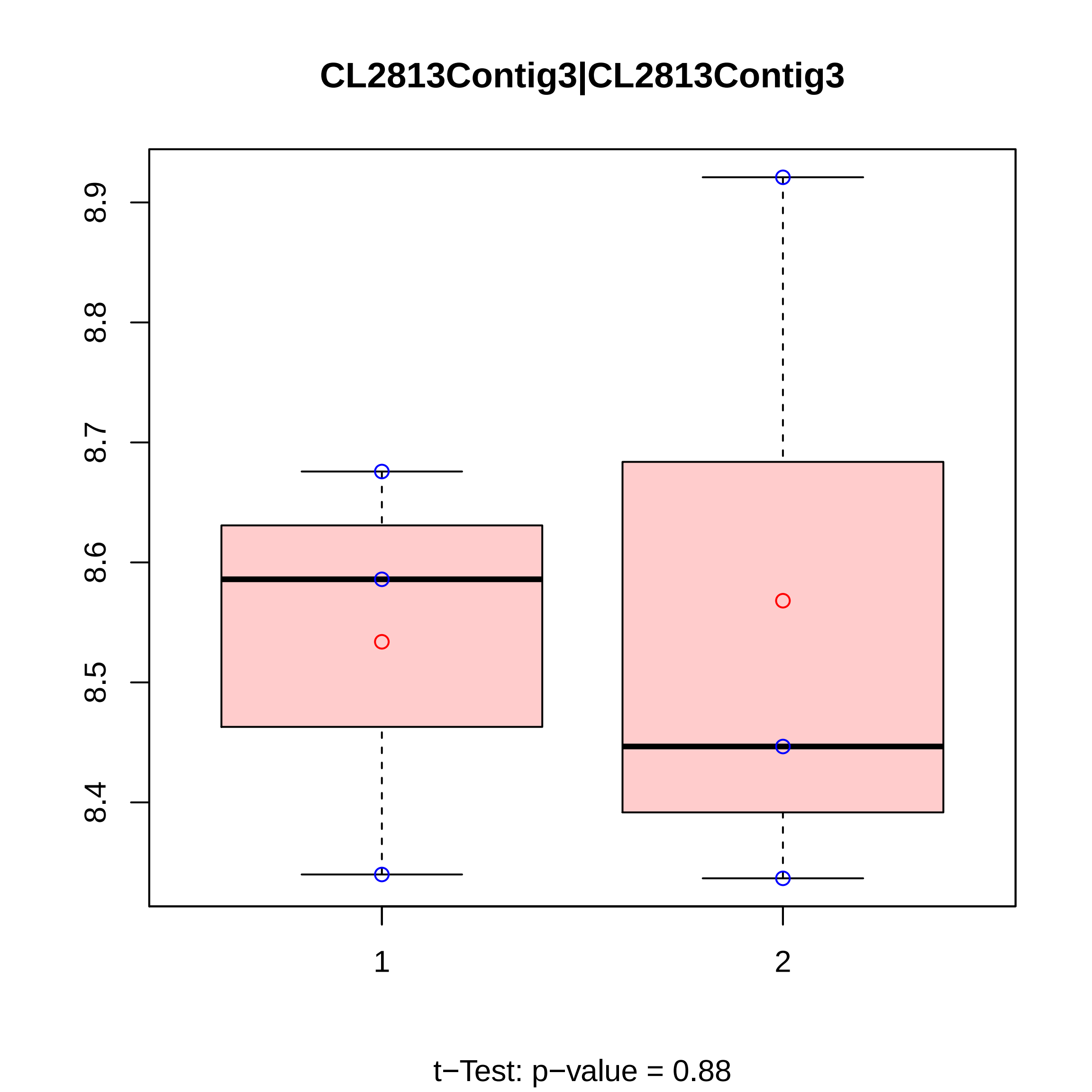
CL2813Contig3|CL2813Contig3
8.4
8.5
8.6
8.7
8.8
8.9
1
2
t−Test: p−value = 0.88

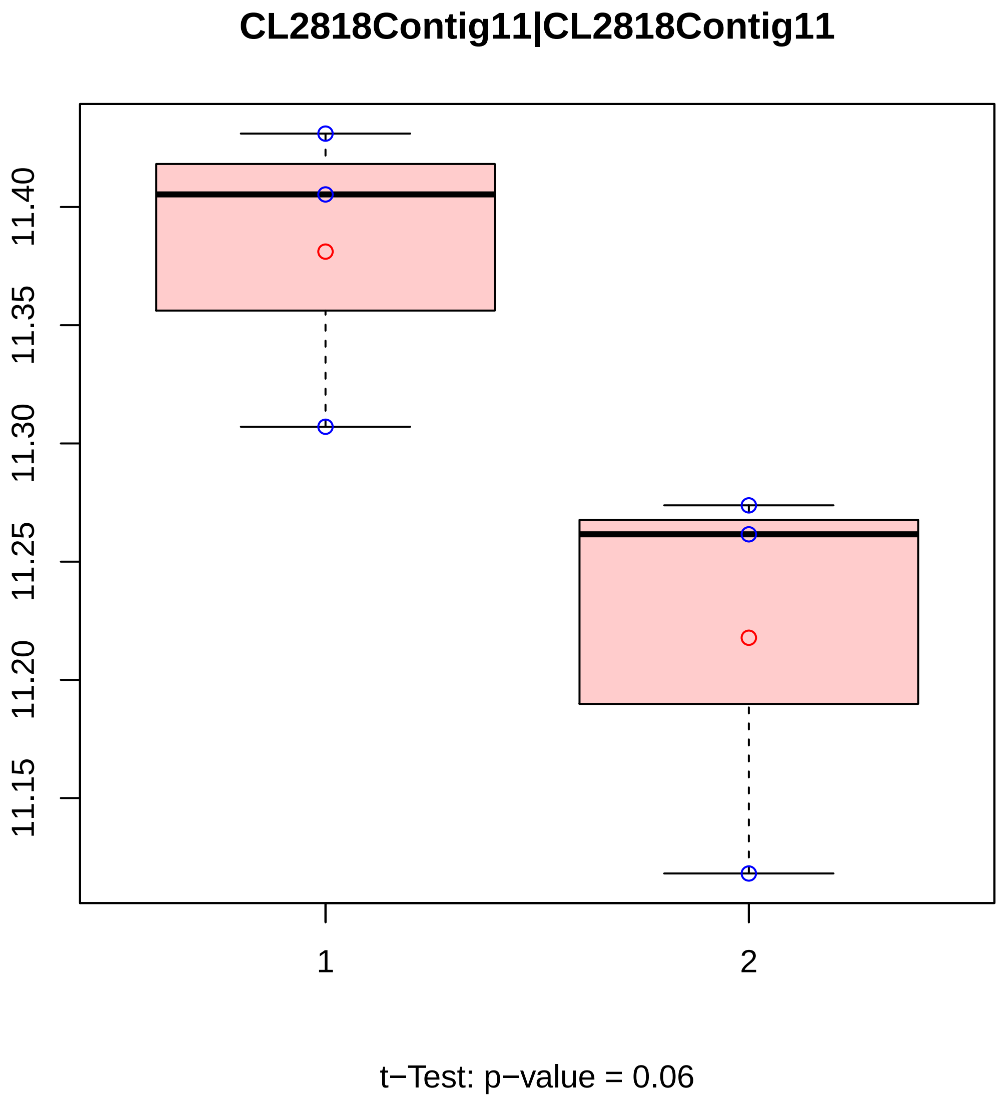
CL2818Contig11|CL2818Contig11
11.15
11.20
11.25
11.30
11.35
11.40
1
2
t−Test: p−value = 0.06

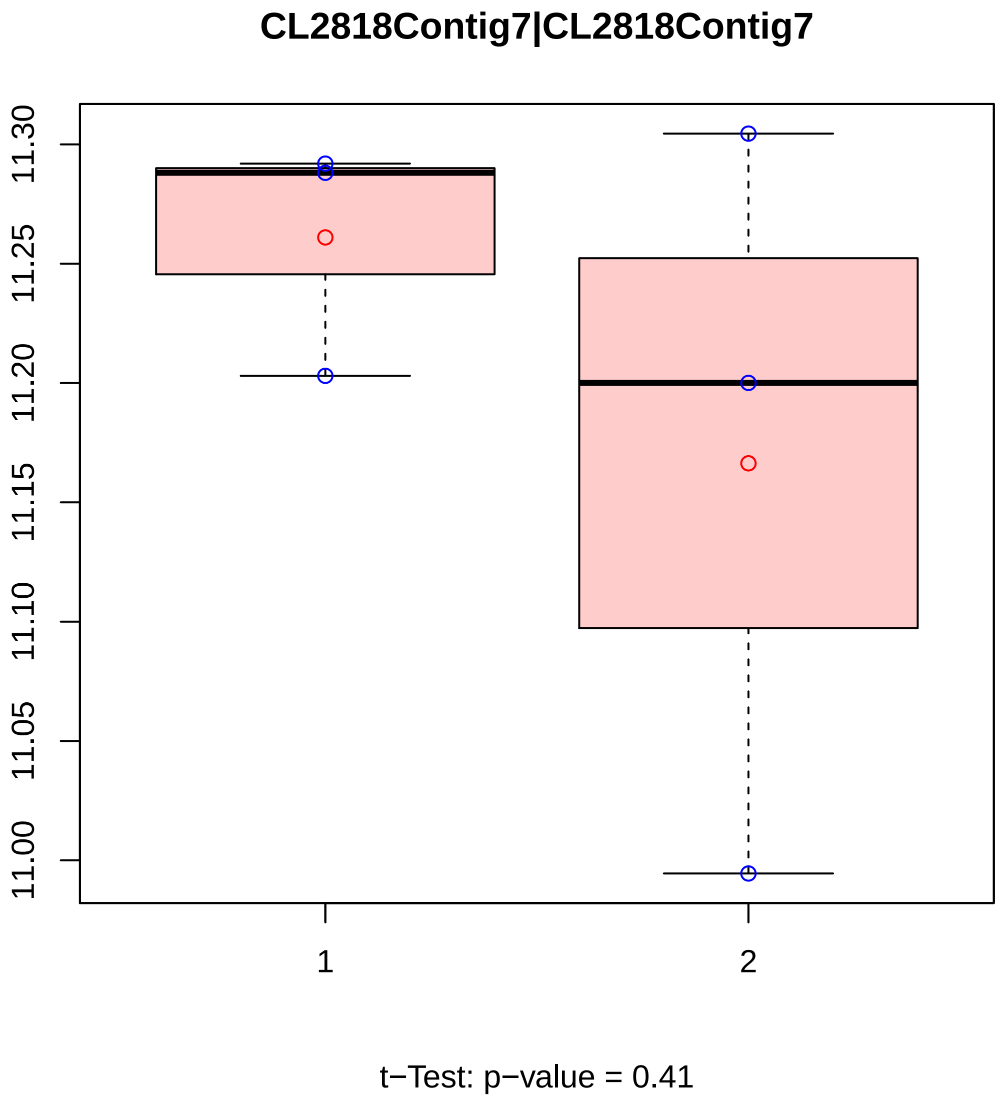
CL2818Contig7|CL2818Contig7
11.00
11.05
11.10
11.15
11.20
11.25
11.30
1
2
t−Test: p−value = 0.41

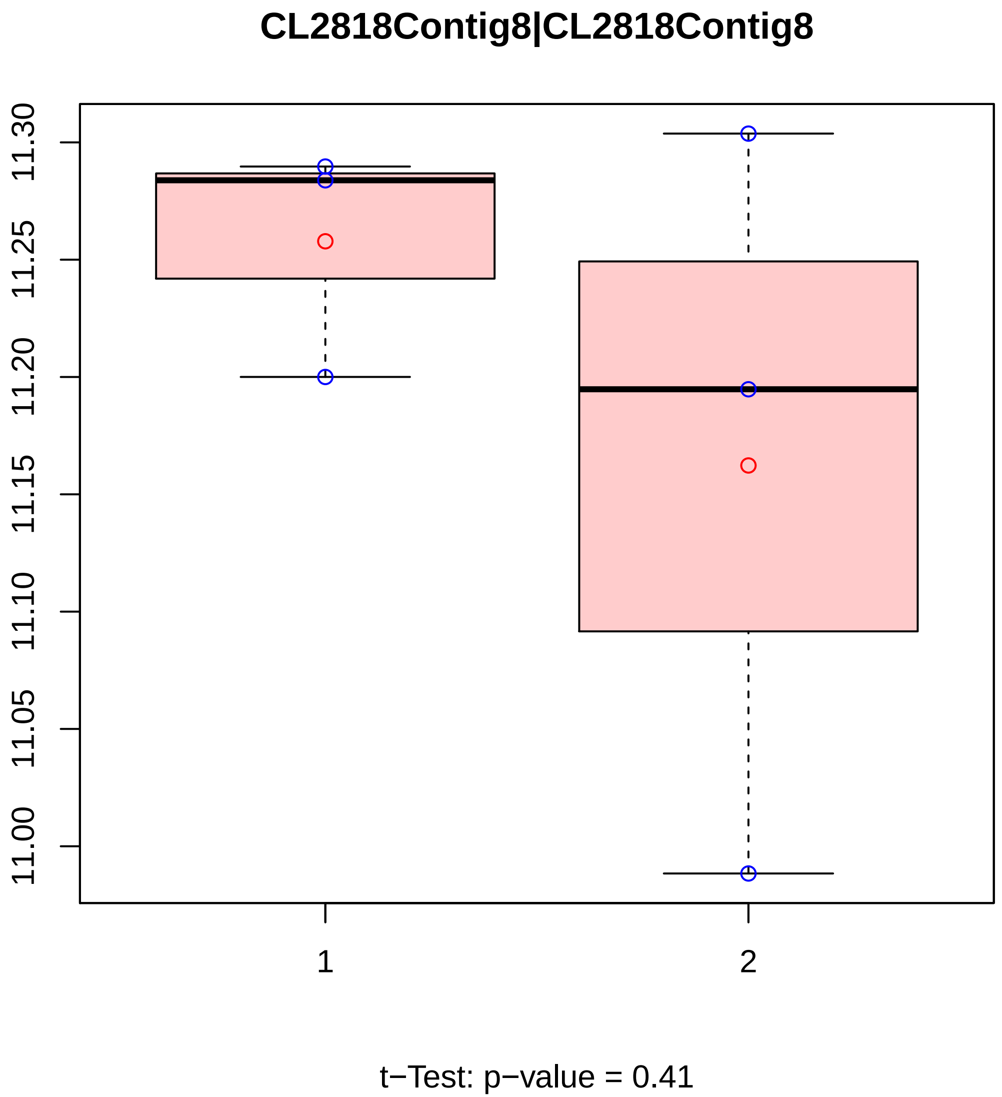

CL2818Contig8|CL2818Contig8
11.30
11.25
11.20
11.15
11.10
11.05
11.00
1
2
t−Test: p−value = 0.41

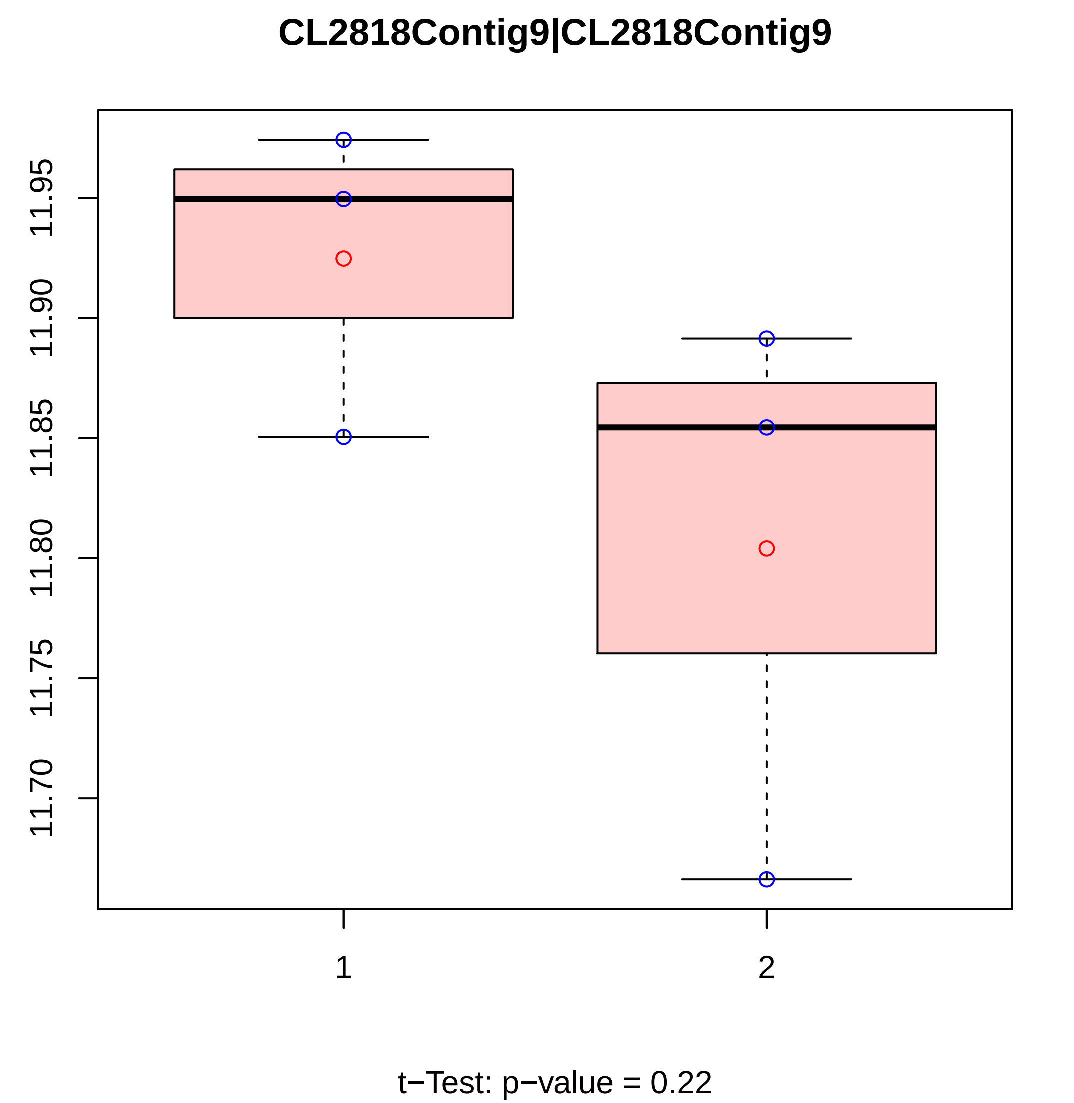

CL2818Contig9|CL2818Contig9
11.70
11.75
11.80
11.85
11.90
11.95
1
2
t−Test: p−value = 0.22

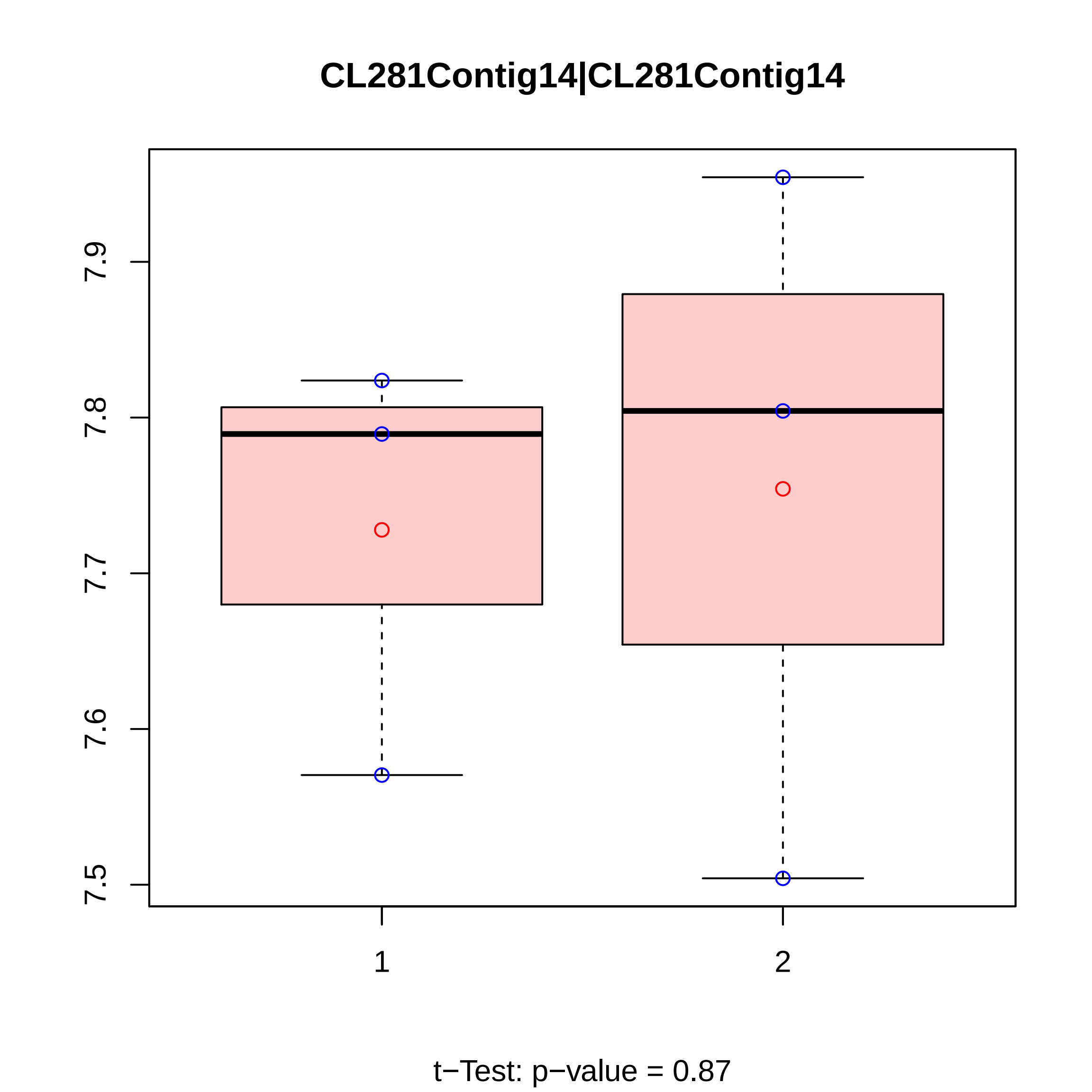

CL281Contig14|CL281Contig14
7.5
7.6
7.7
7.8
7.9
1
2
t−Test: p−value = 0.87

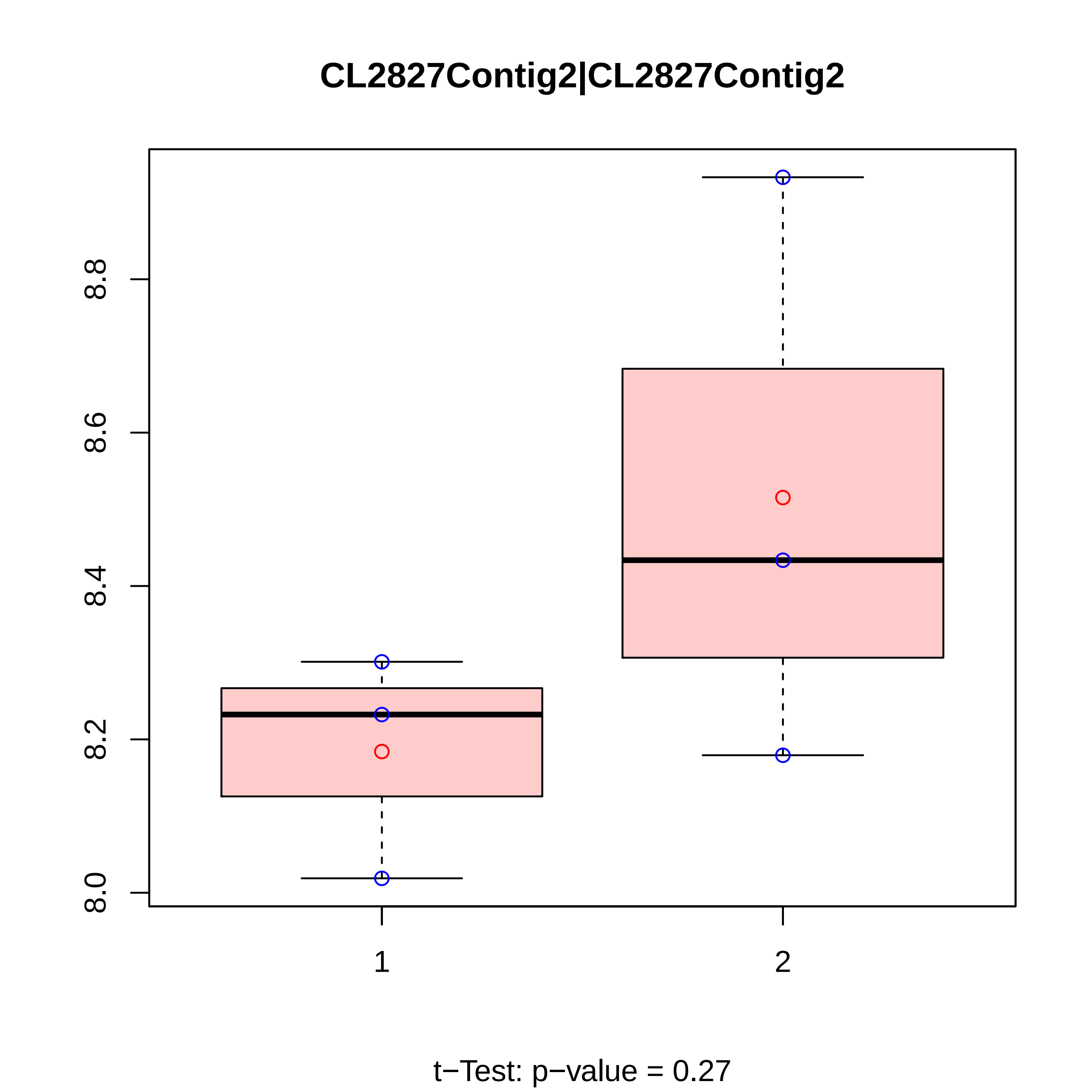
CL2827Contig2|CL2827Contig2
8.0
8.2
8.4
8.6
8.8
1
2
t−Test: p−value = 0.27

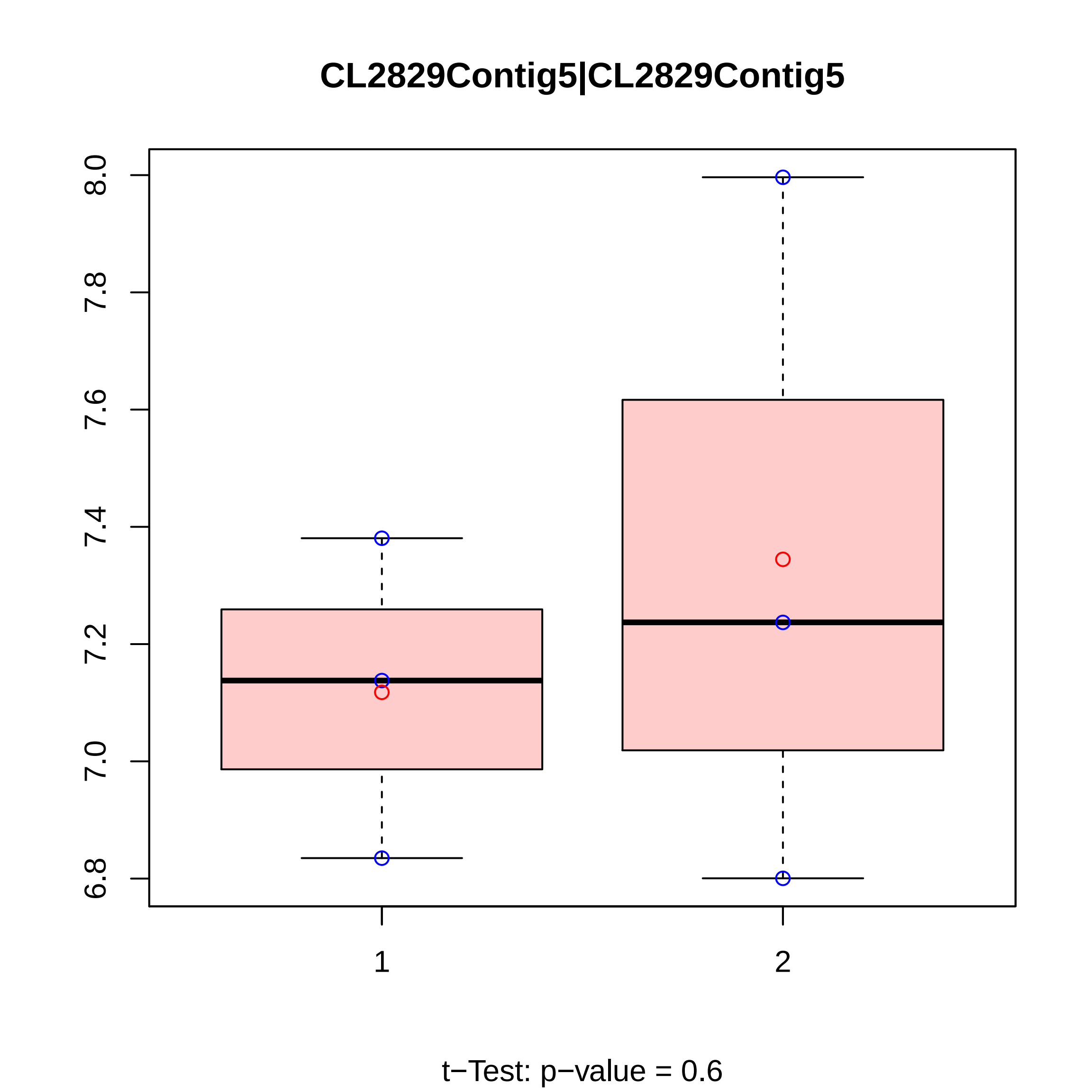

CL2829Contig5|CL2829Contig5
8.0
7.8
7.6
7.4
7.2
7.0
6.8
1
2
t−Test: p−value = 0.6

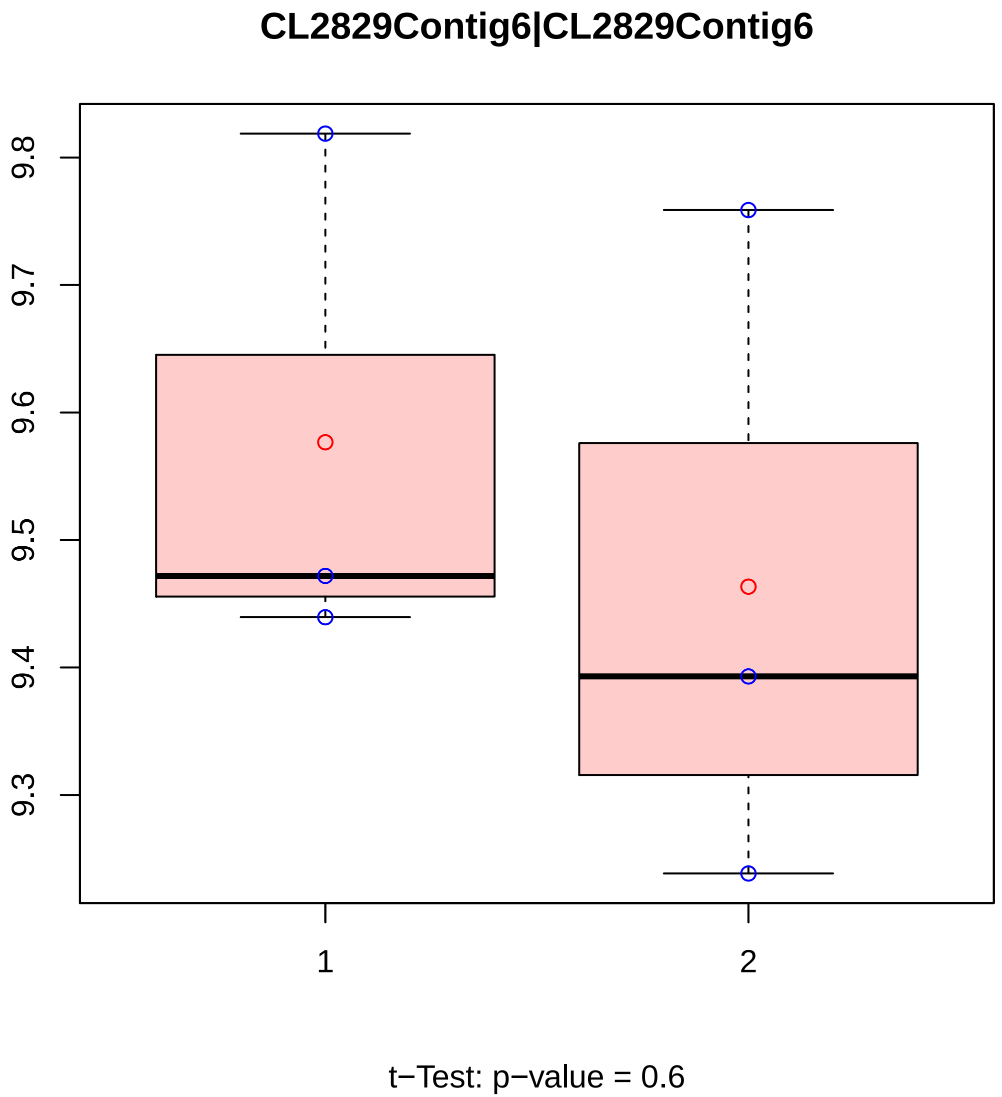
CL2829Contig6|CL2829Contig6
9.3
9.4
9.5
9.6
9.7
9.8
1
2
t−Test: p−value = 0.6

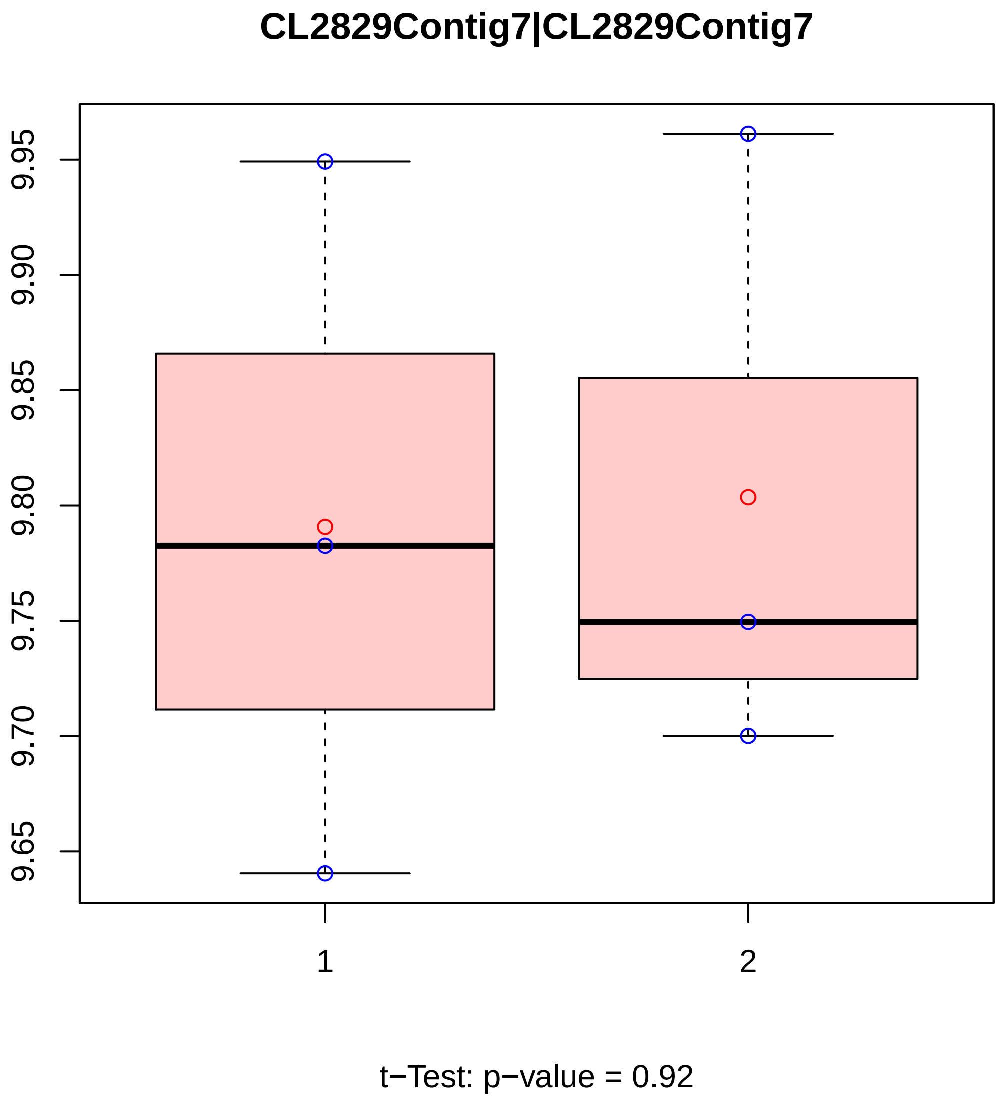

CL2829Contig7|CL2829Contig7
9.65
9.70
9.75
9.80
9.85
9.90
9.95
1
2
t−Test: p−value = 0.92

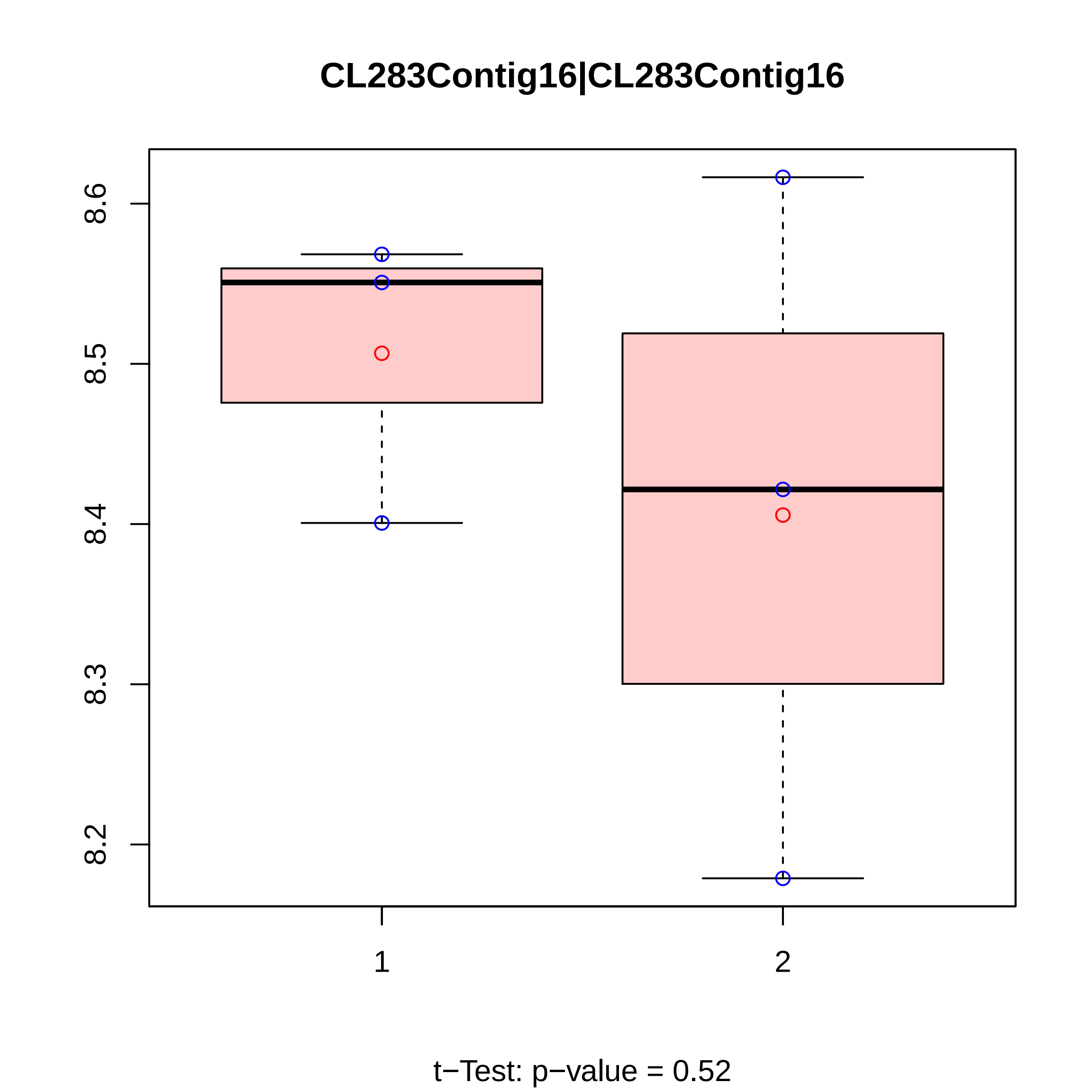
CL283Contig16|CL283Contig16
8.2
8.3
8.4
8.5
8.6
1
2
t−Test: p−value = 0.52

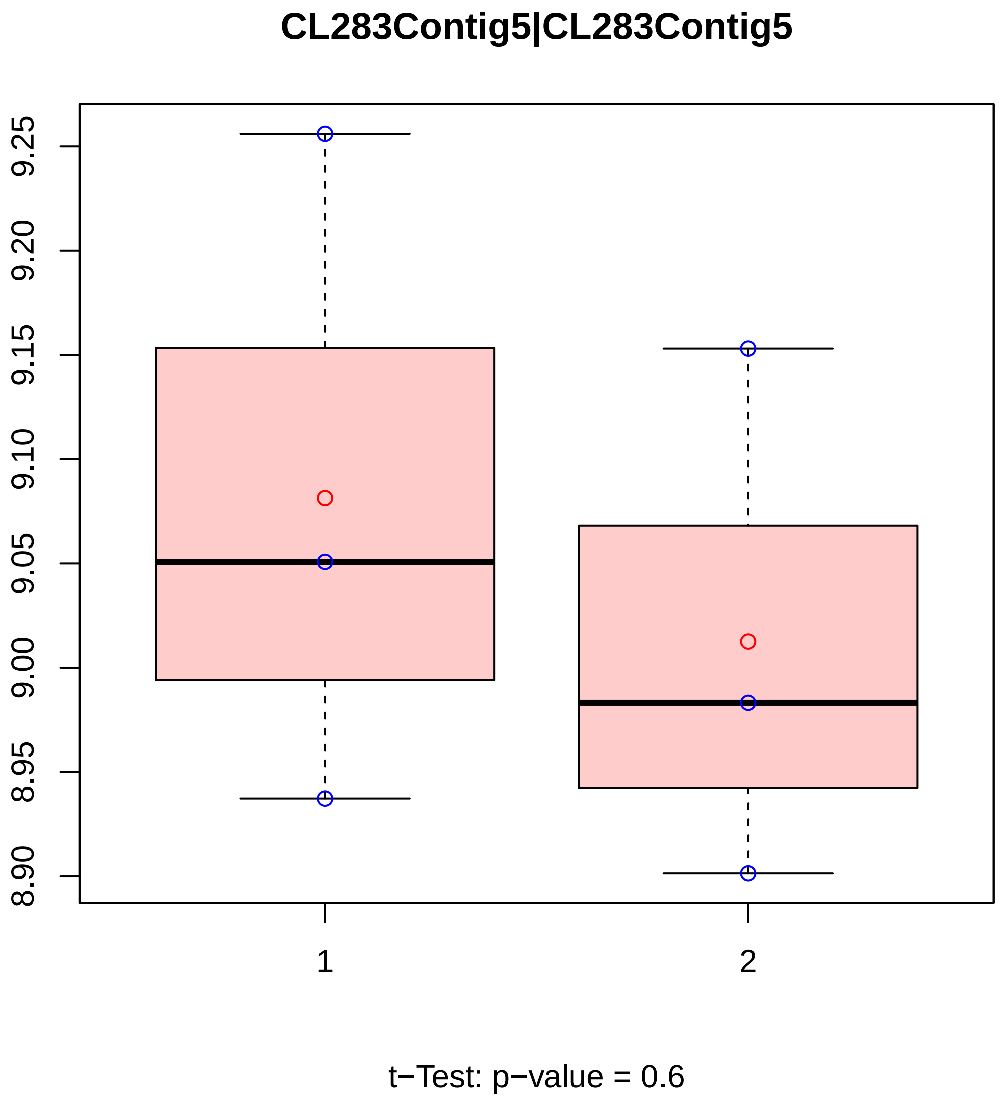

CL283Contig5|CL283Contig5
9.25
9.20
9.15
9.10
9.05
9.00
8.95
8.90
1
2
t−Test: p−value = 0.6

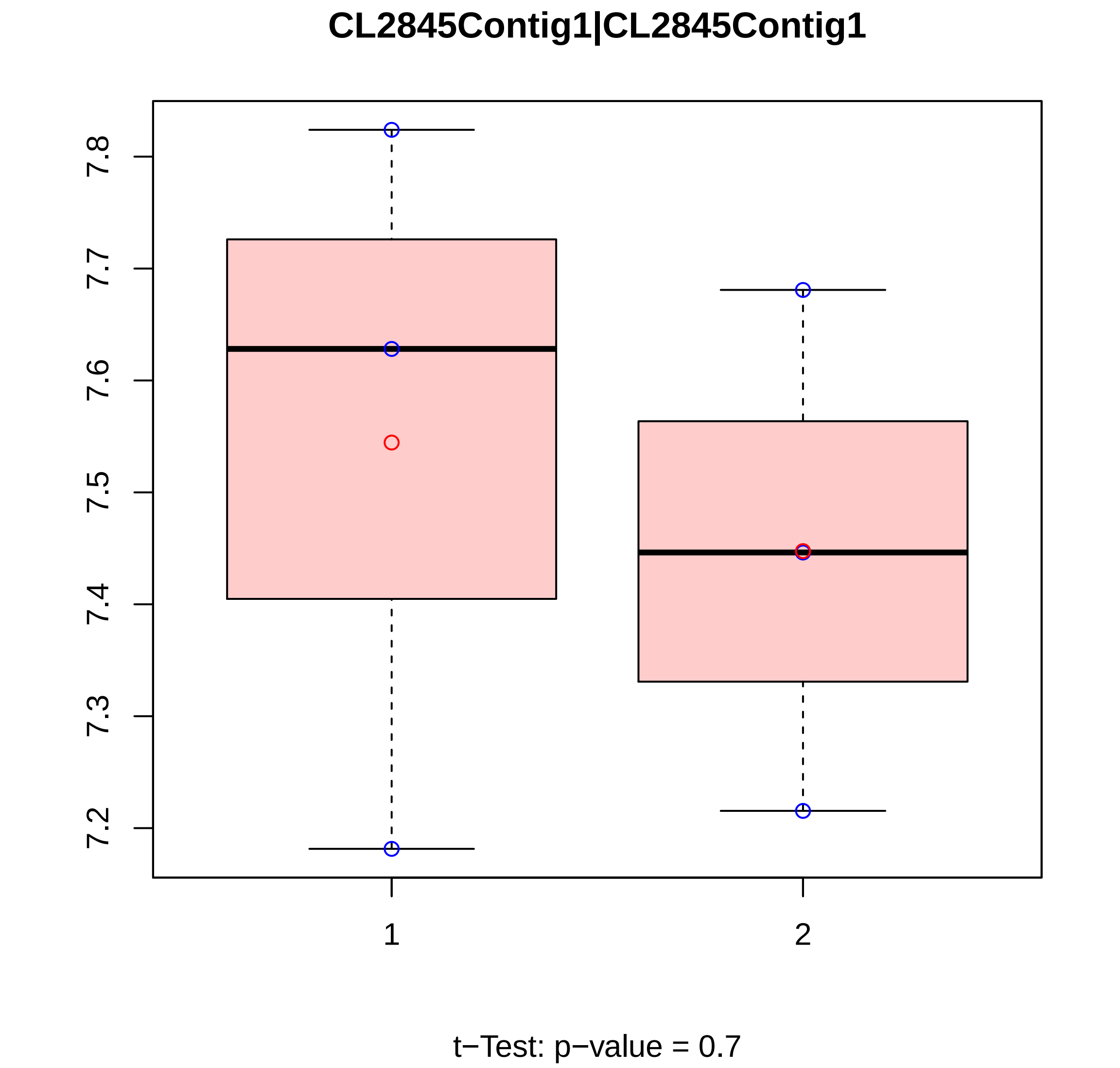
CL2845Contig1|CL2845Contig1
7.2
7.3
7.4
7.5
7.6
7.7
7.8
1
2
t−Test: p−value = 0.7

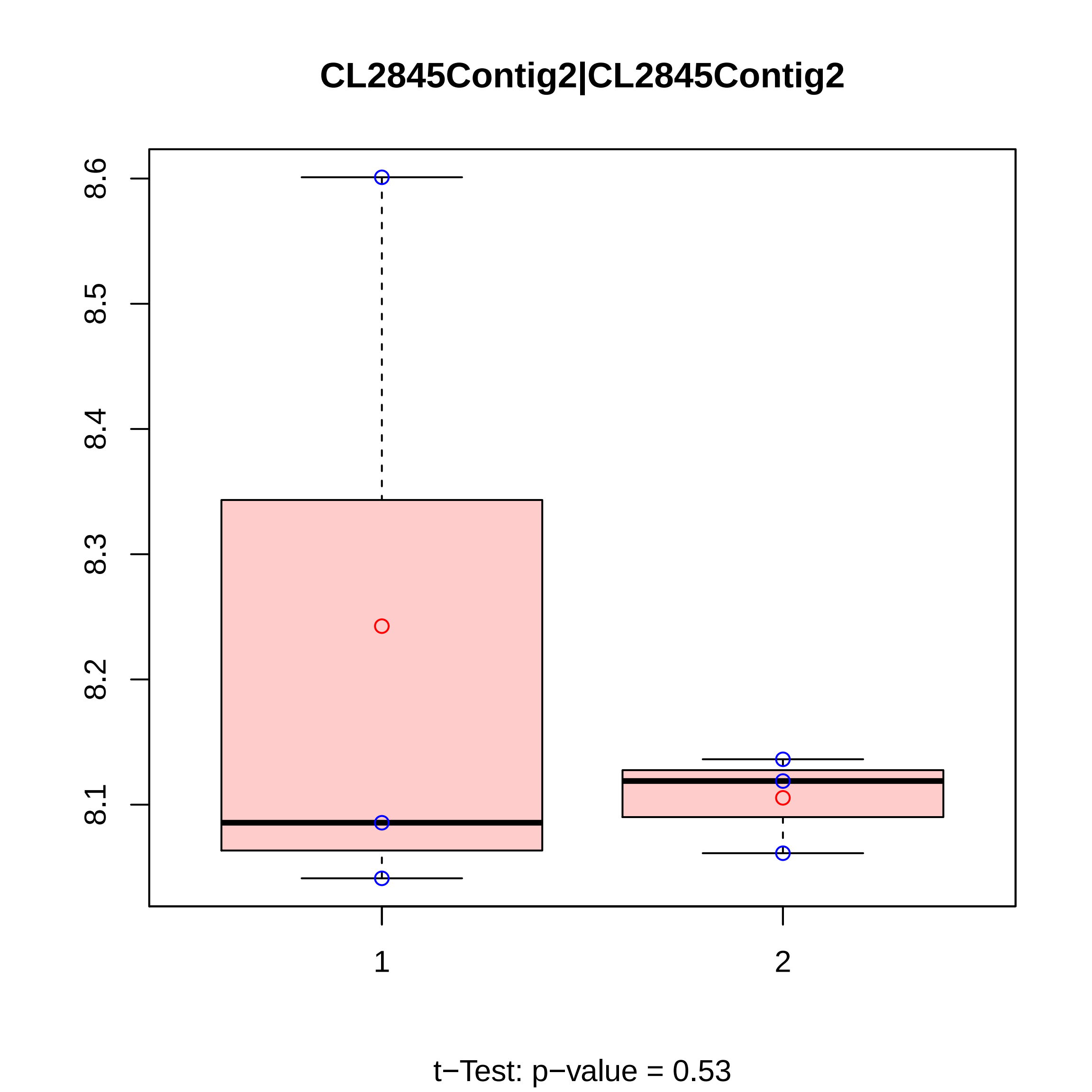

CL2845Contig2|CL2845Contig2
8.6
8.5
8.4
8.3
8.2
8.1
1
2
t−Test: p−value = 0.53

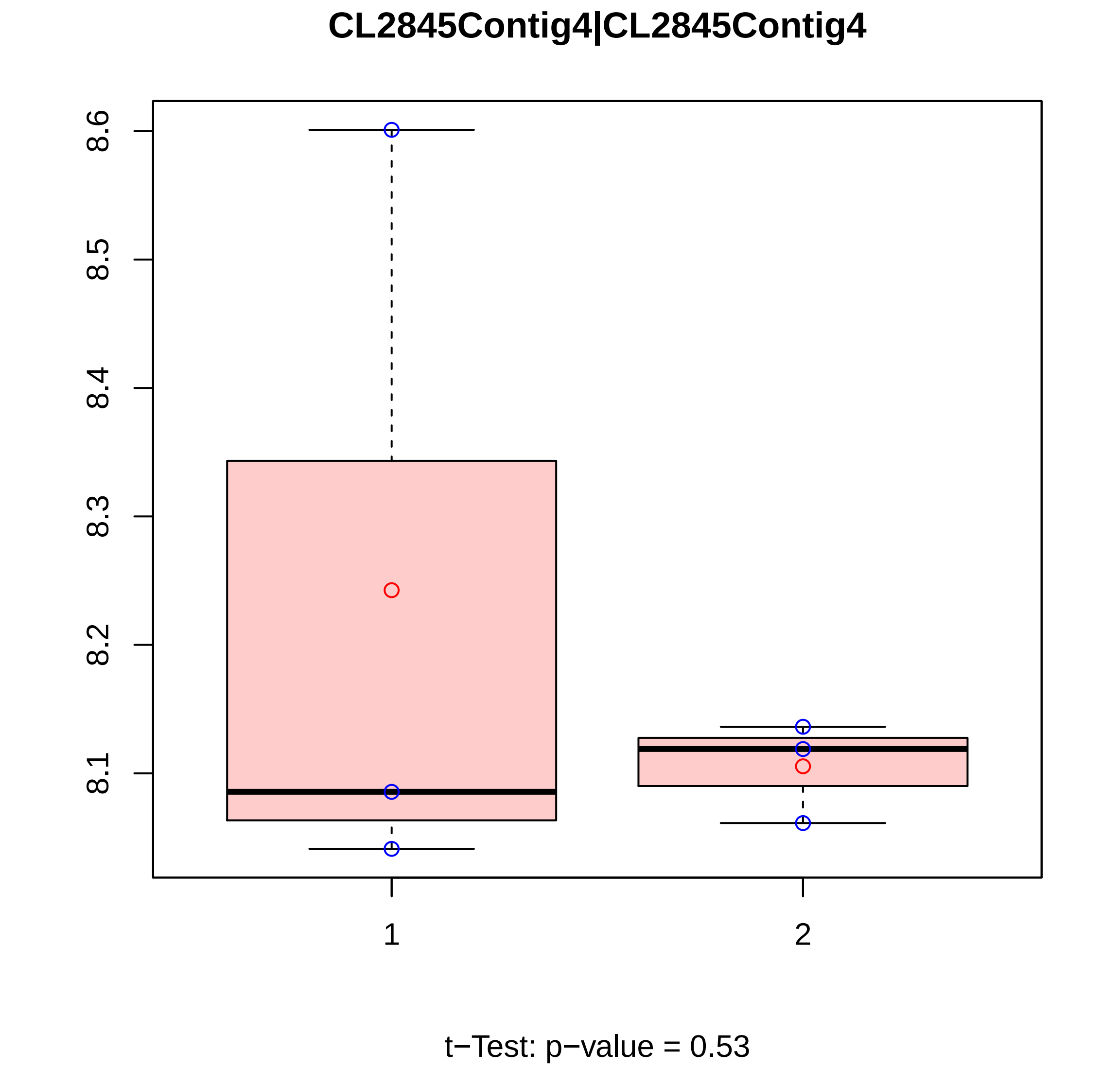
CL2845Contig4|CL2845Contig4
8.6
8.5
8.4
8.3
8.2
8.1
1
2
t−Test: p−value = 0.53

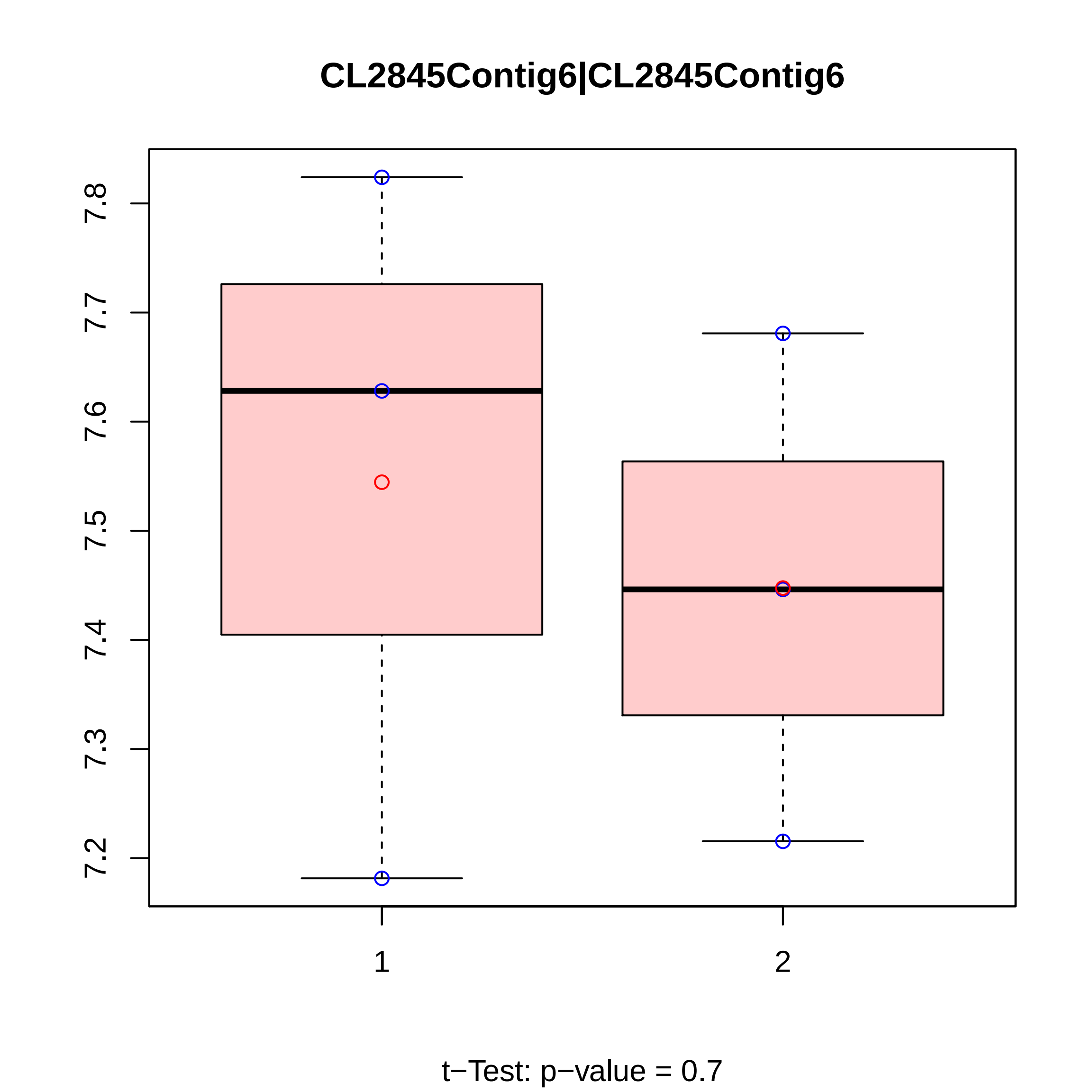

CL2845Contig6|CL2845Contig6
7.2
7.3
7.4
7.5
7.6
7.7
7.8
1
2
t−Test: p−value = 0.7

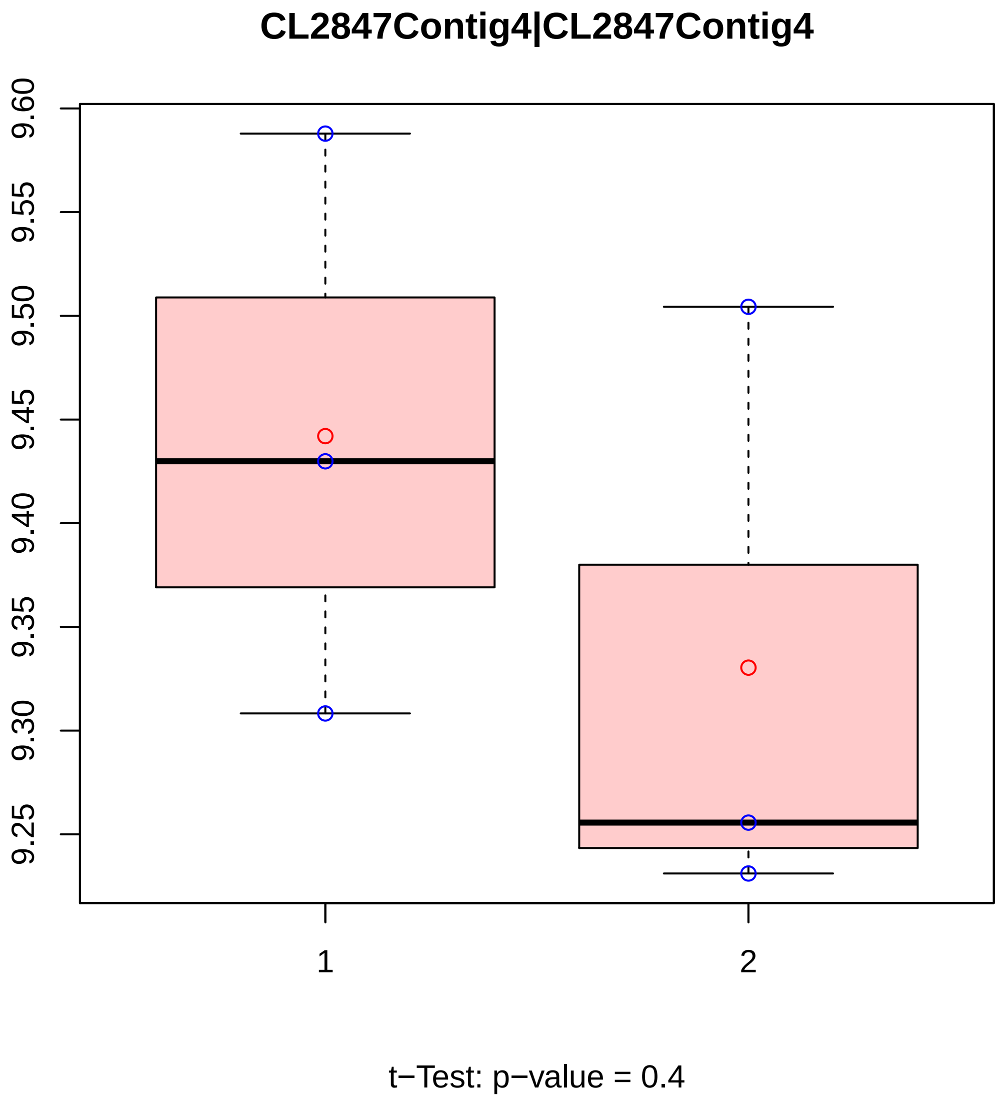

CL2847Contig4|CL2847Contig4
9.60
9.55
9.50
9.45
9.40
9.35
9.30
9.25
1
2
t−Test: p−value = 0.4

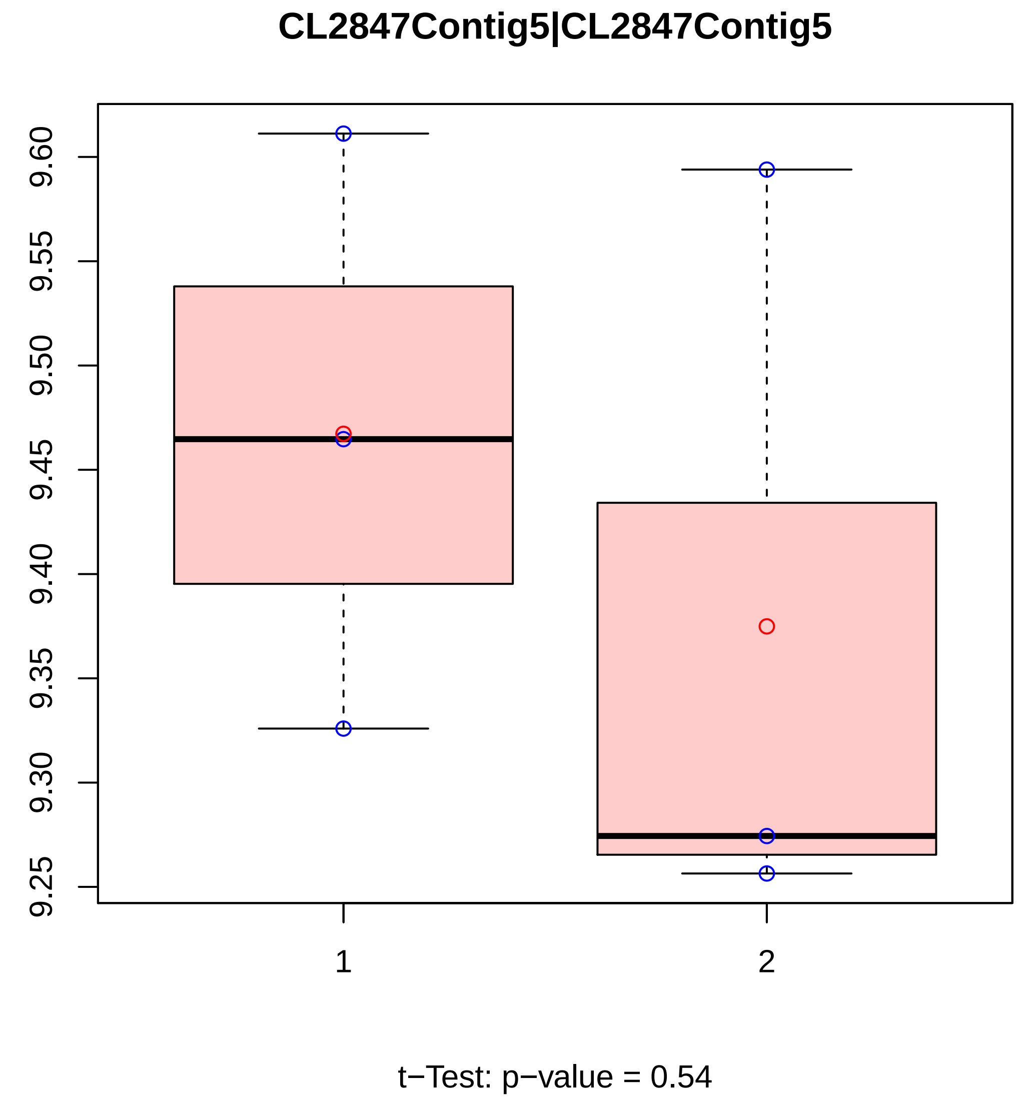
CL2847Contig5|CL2847Contig5
9.60
9.55
9.50
9.45
9.40
9.35
9.30
9.25
1
2
t−Test: p−value = 0.54

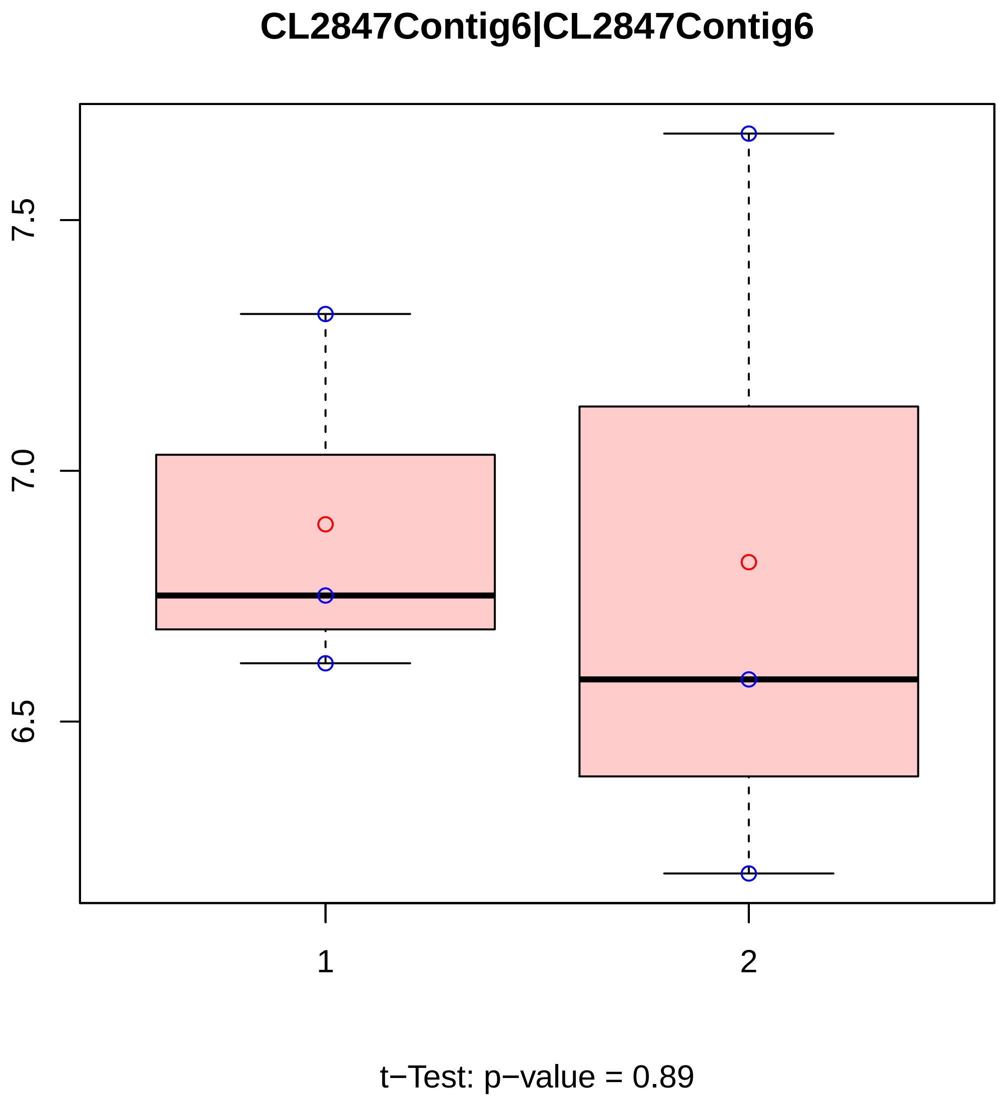
CL2847Contig6|CL2847Contig6
7.5
7.0
6.5
1
2
t−Test: p−value = 0.89

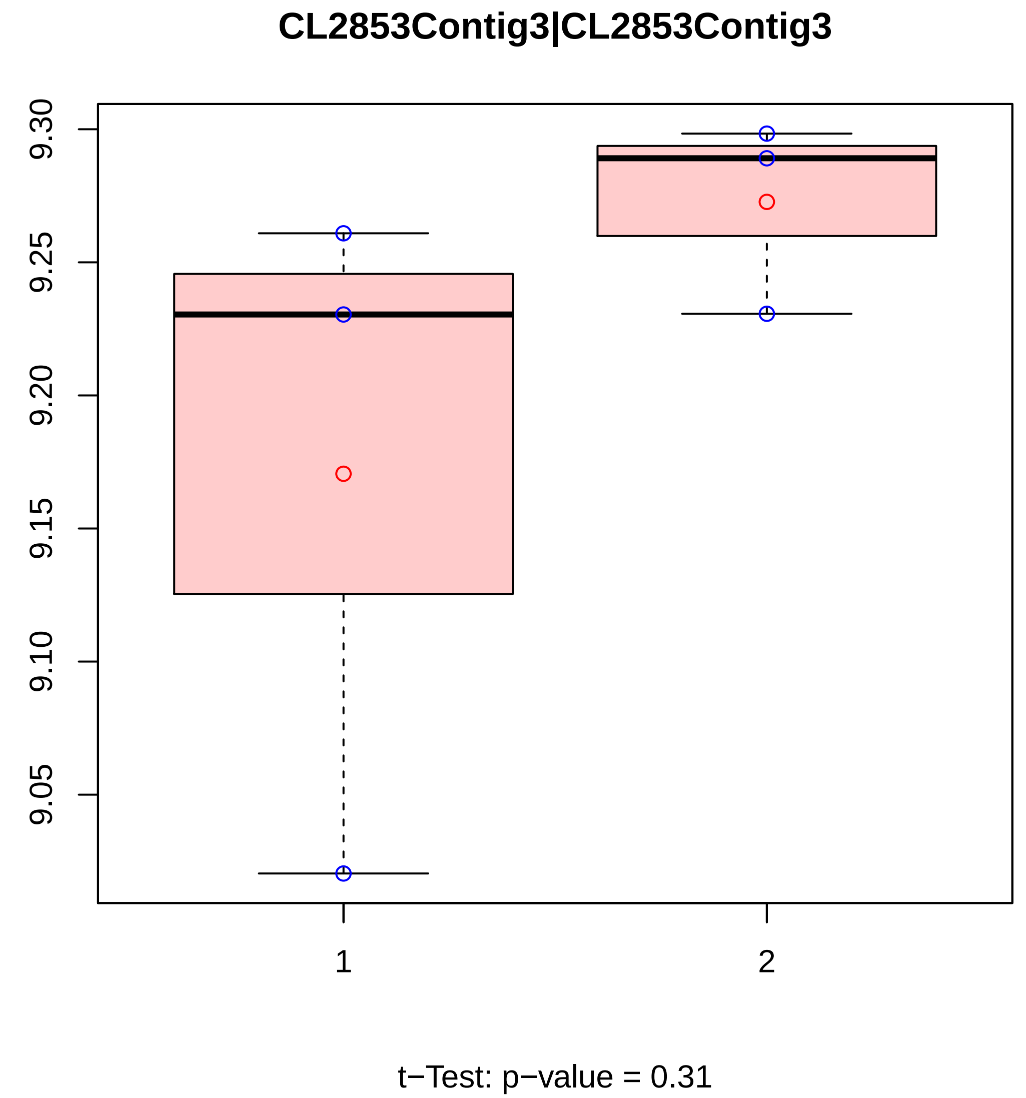
CL2853Contig3|CL2853Contig3
9.30
9.25
9.20
9.15
9.10
9.05
1
2
t−Test: p−value = 0.31

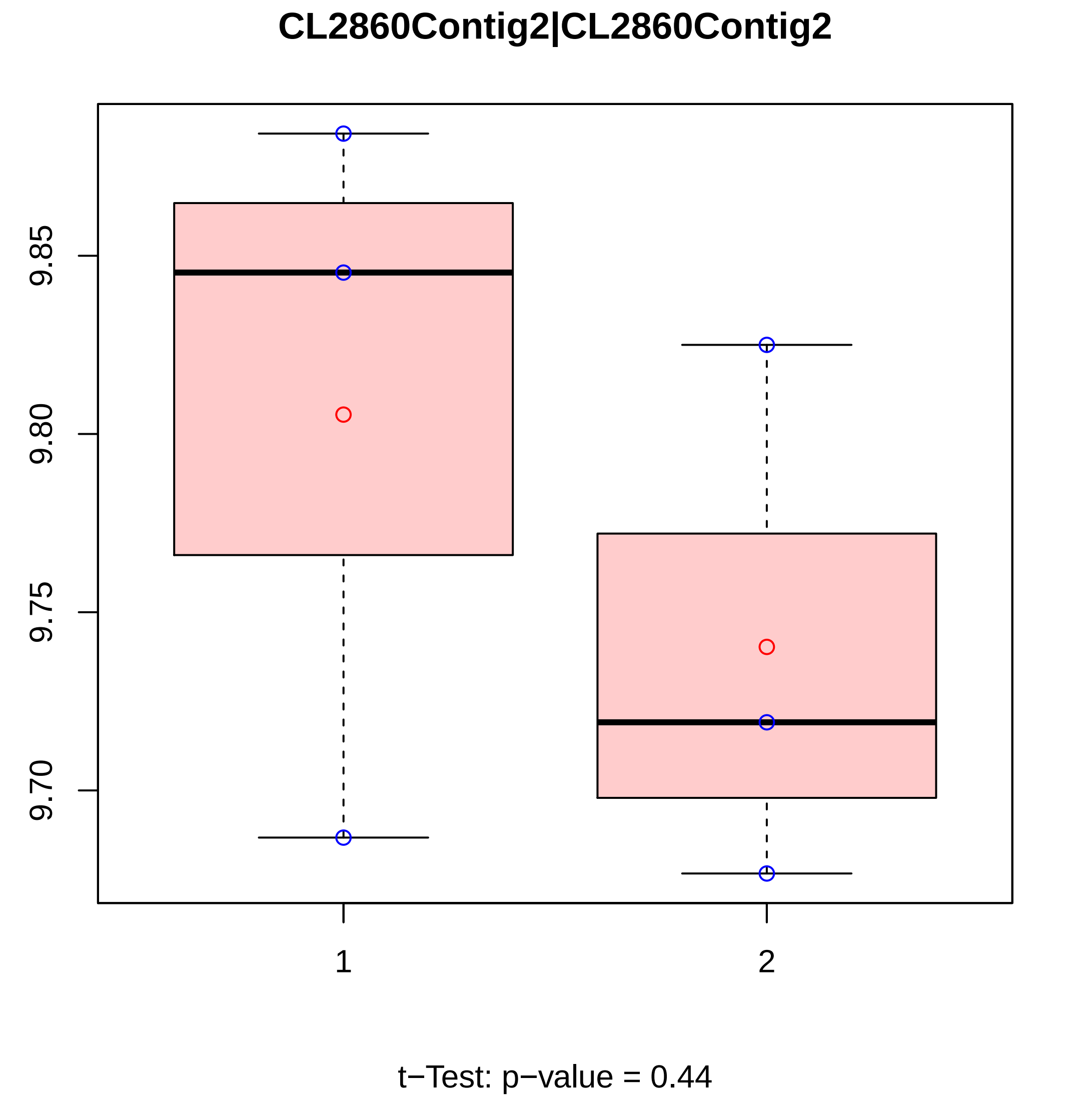
CL2860Contig2|CL2860Contig2
9.85
9.80
9.75
9.70
1
2
t−Test: p−value = 0.44

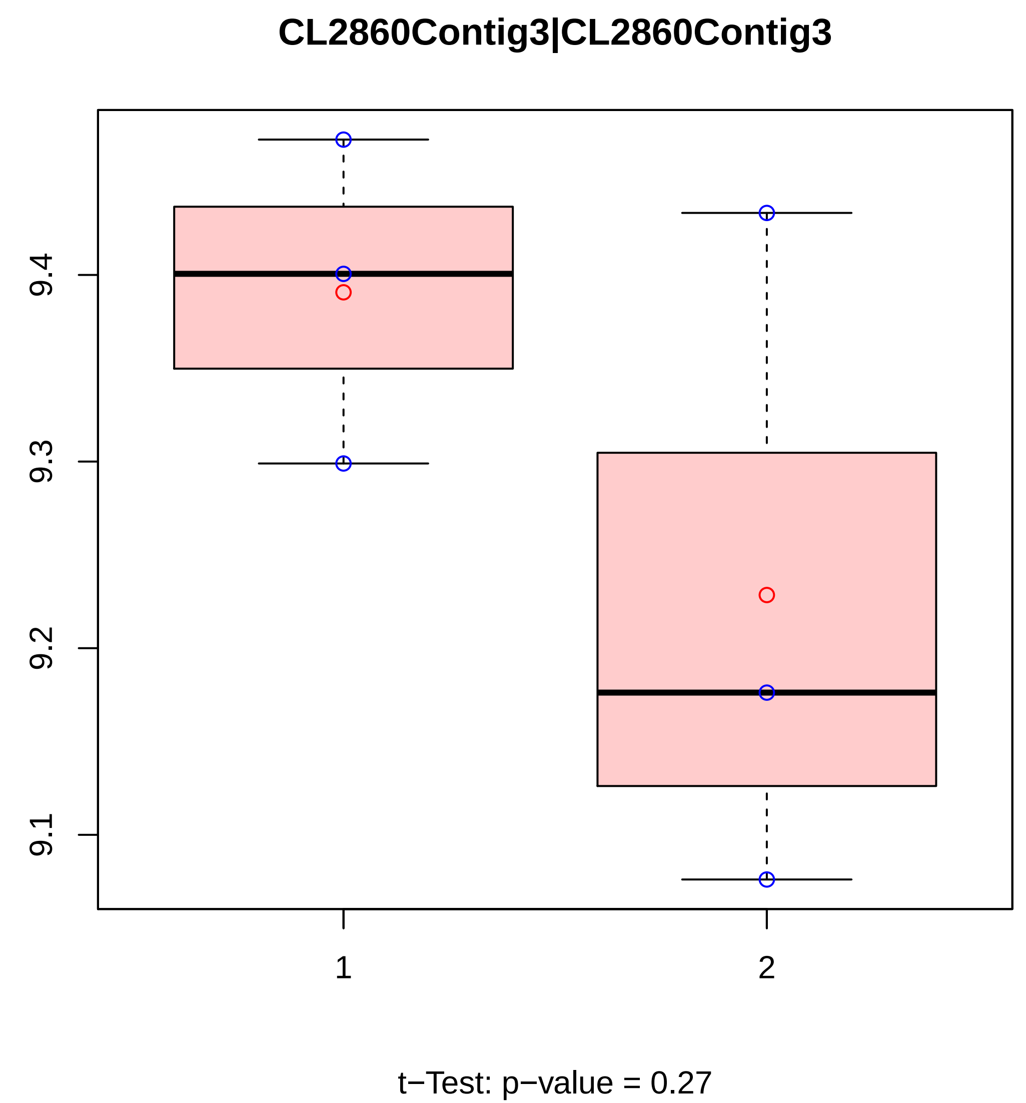

CL2860Contig3|CL2860Contig3
9.1
9.2
9.3
9.4
1
2
t−Test: p−value = 0.27

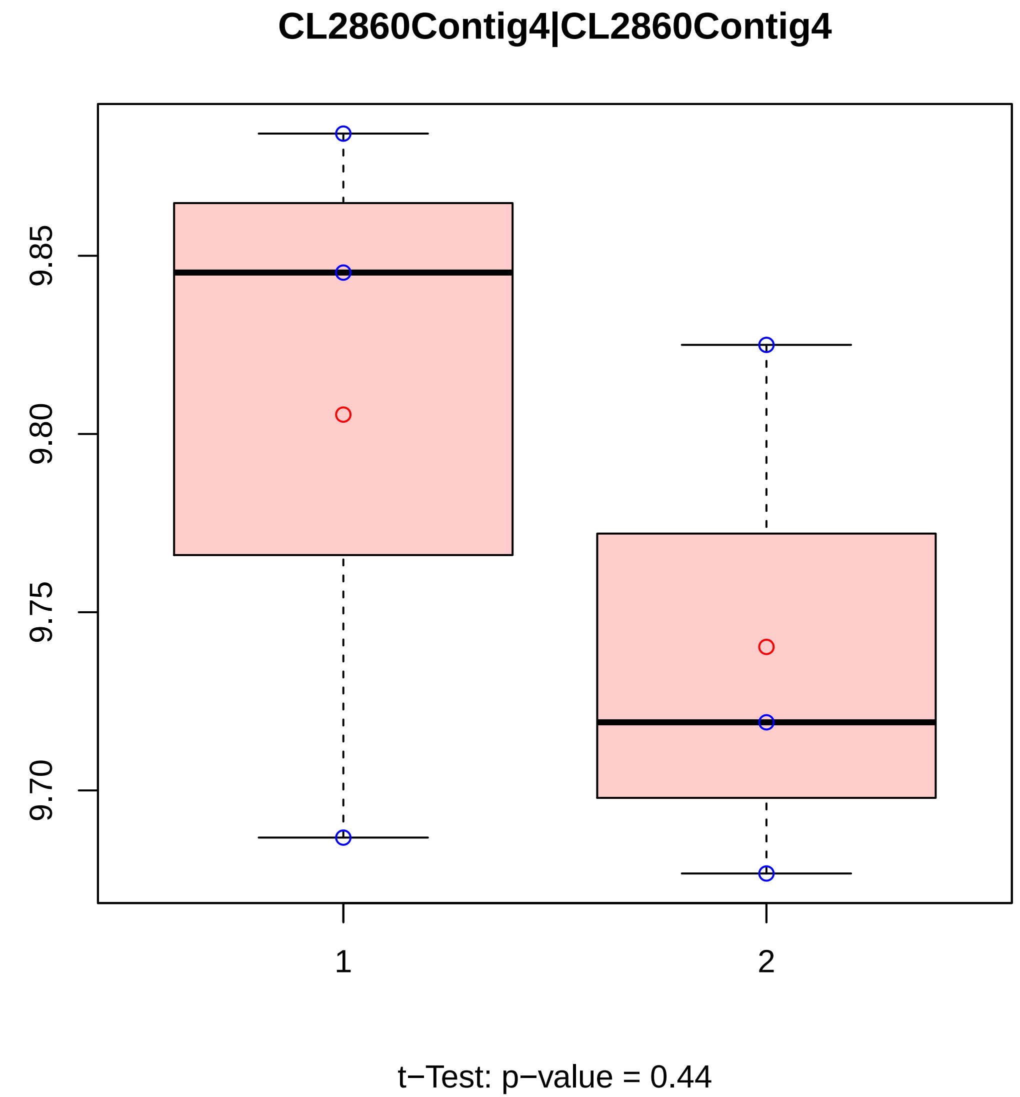
CL2860Contig4|CL2860Contig4
9.70
9.75
9.80
9.85
1
2
t−Test: p−value = 0.44

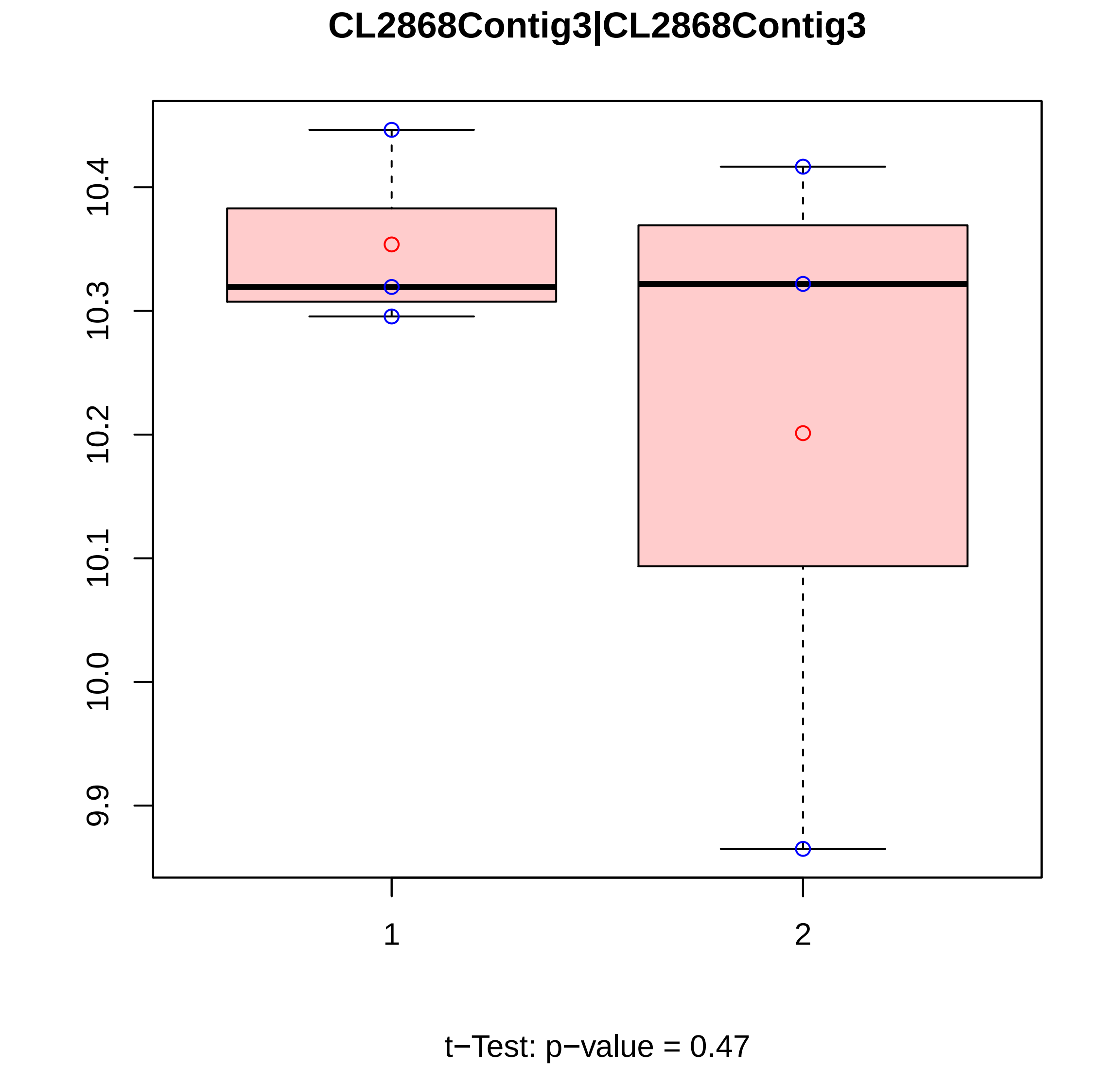
CL2868Contig3|CL2868Contig3
10.4
10.3
10.2
10.1
10.0
9.9
1
2
t−Test: p−value = 0.47

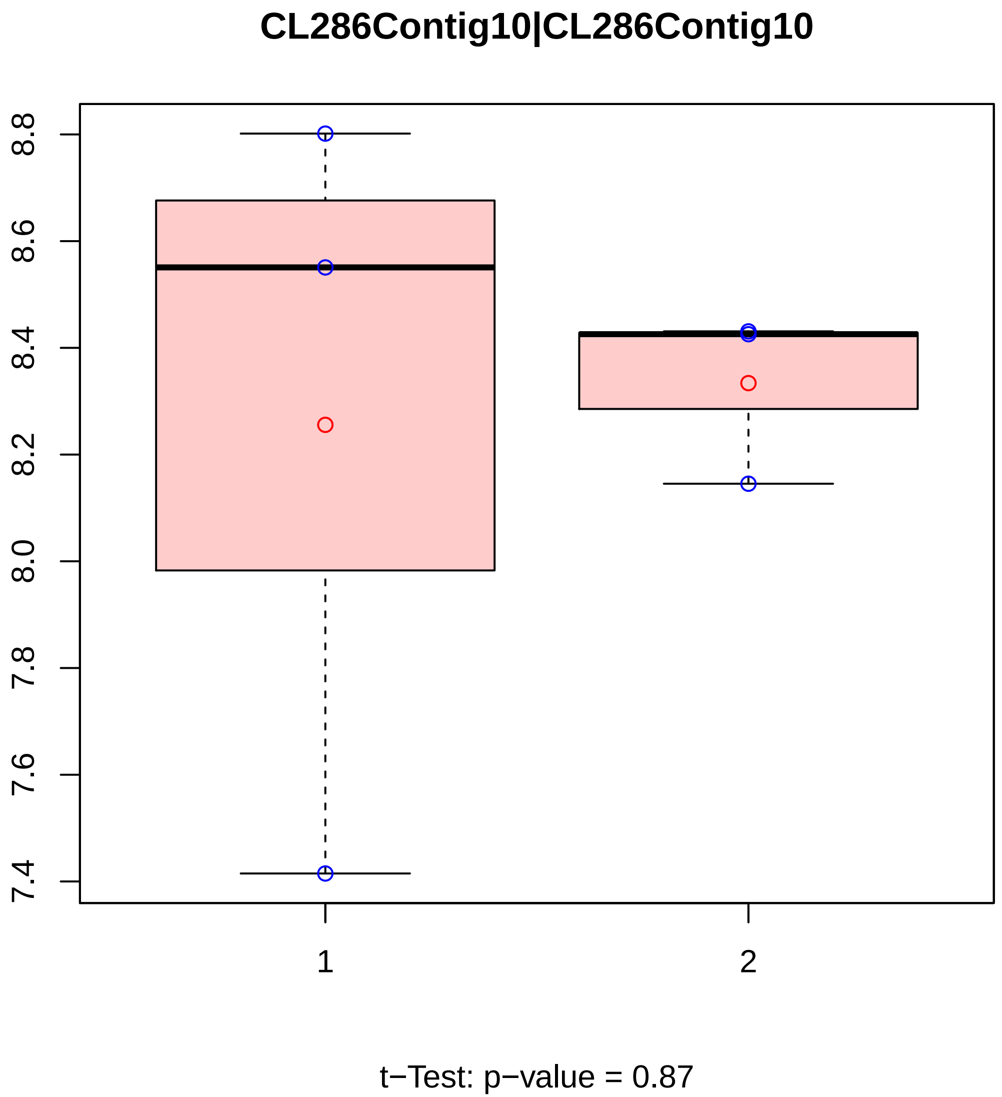

CL286Contig10|CL286Contig10
8.8
8.6
8.4
8.2
8.0
7.8
7.6
7.4
1
2
t−Test: p−value = 0.87

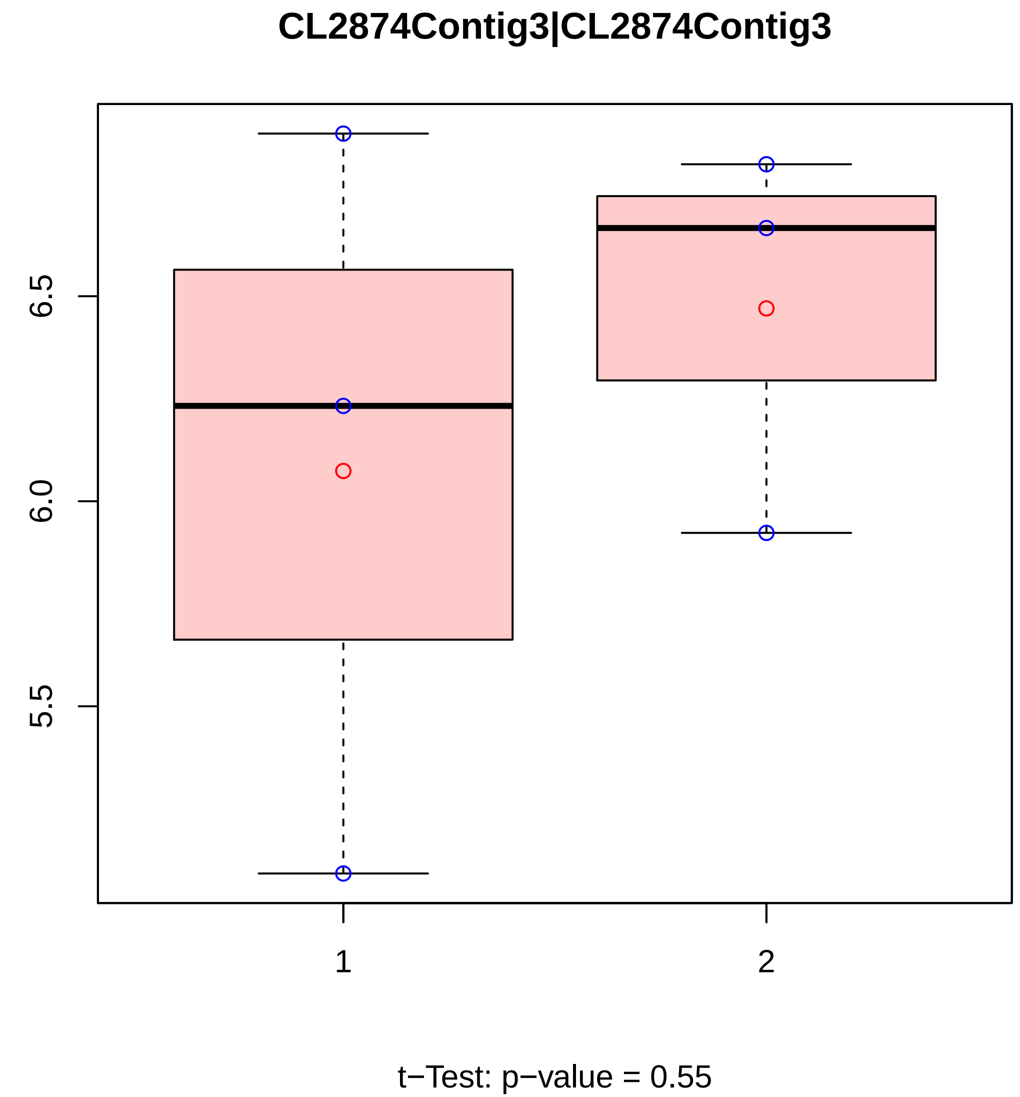

CL2874Contig3|CL2874Contig3
6.5
6.0
5.5
1
2
t−Test: p−value = 0.55

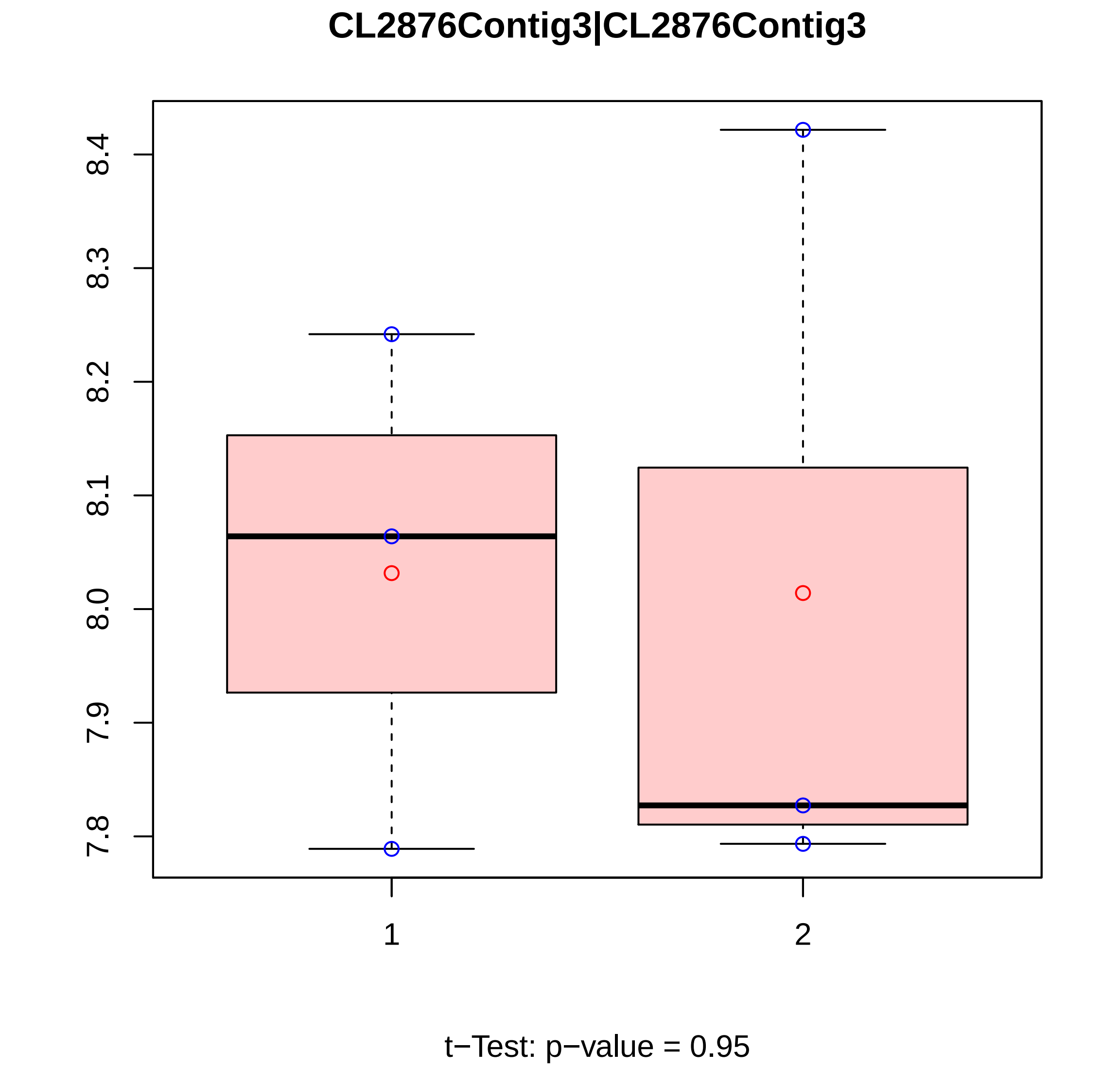
CL2876Contig3|CL2876Contig3
8.4
8.3
8.2
8.1
8.0
7.9
7.8
1
2
t−Test: p−value = 0.95

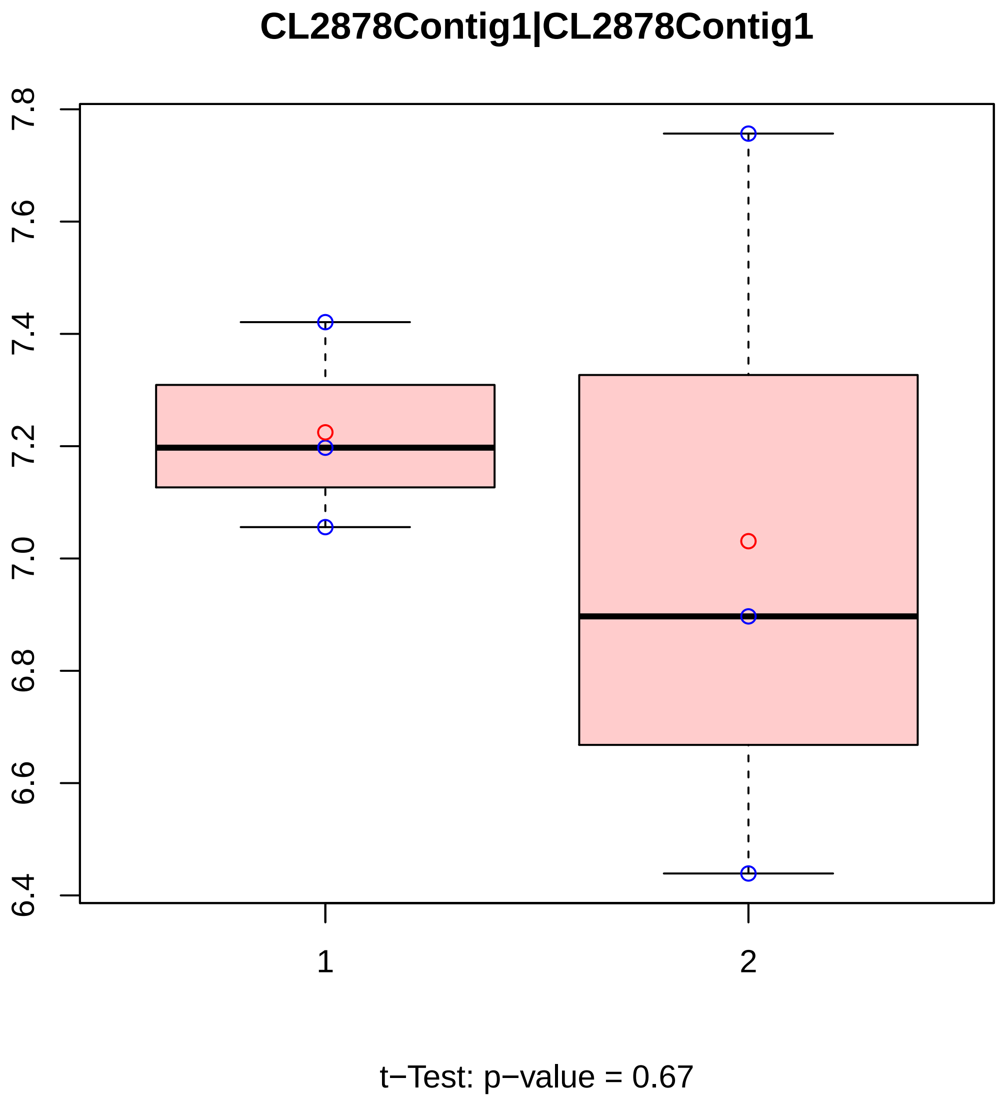

CL2878Contig1|CL2878Contig1
7.8
7.6
7.4
7.2
7.0
6.8
6.6
6.4
1
2
t−Test: p−value = 0.67

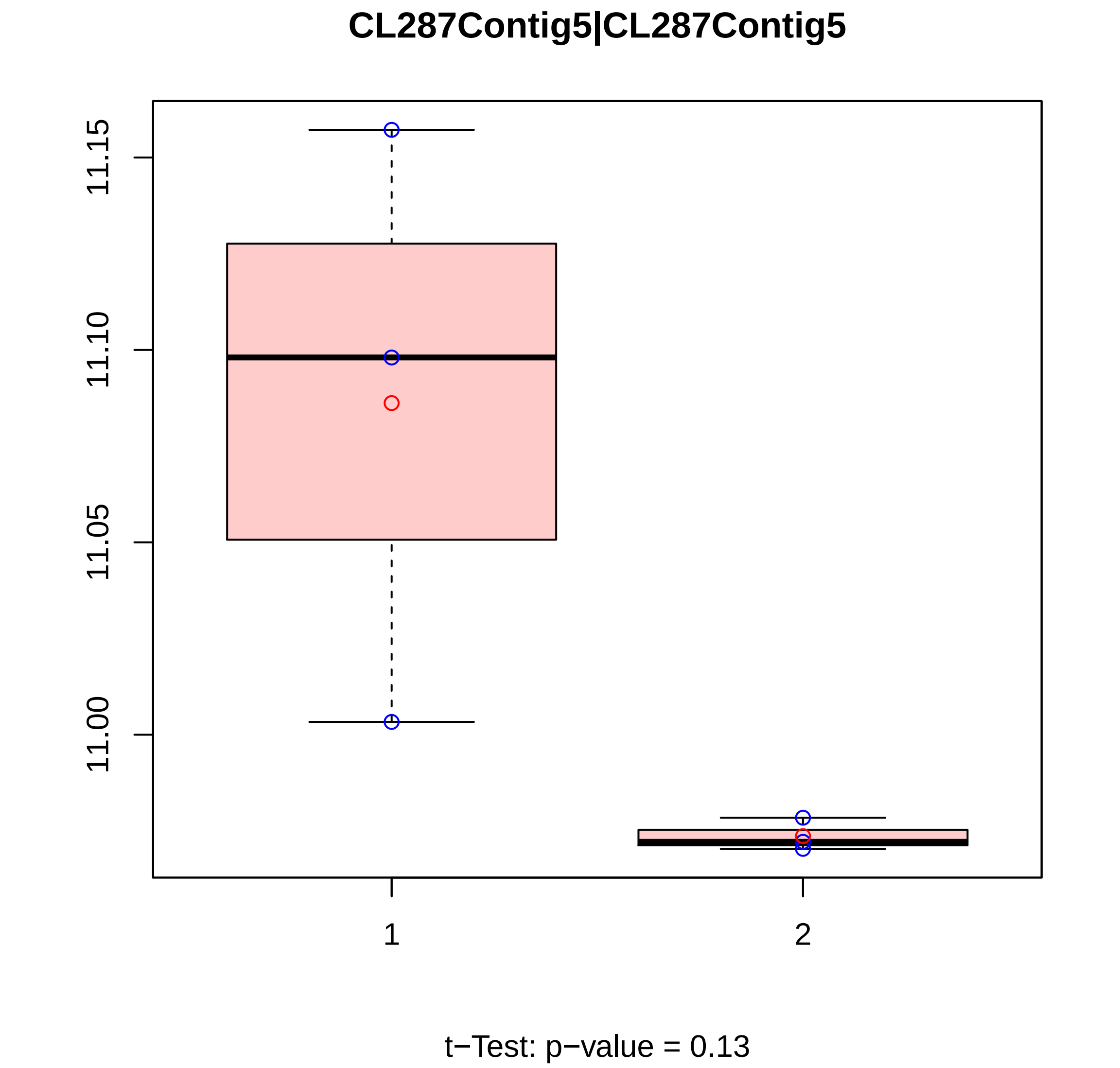

CL287Contig5|CL287Contig5
11.00
11.05
11.10
11.15
1
2
t−Test: p−value = 0.13

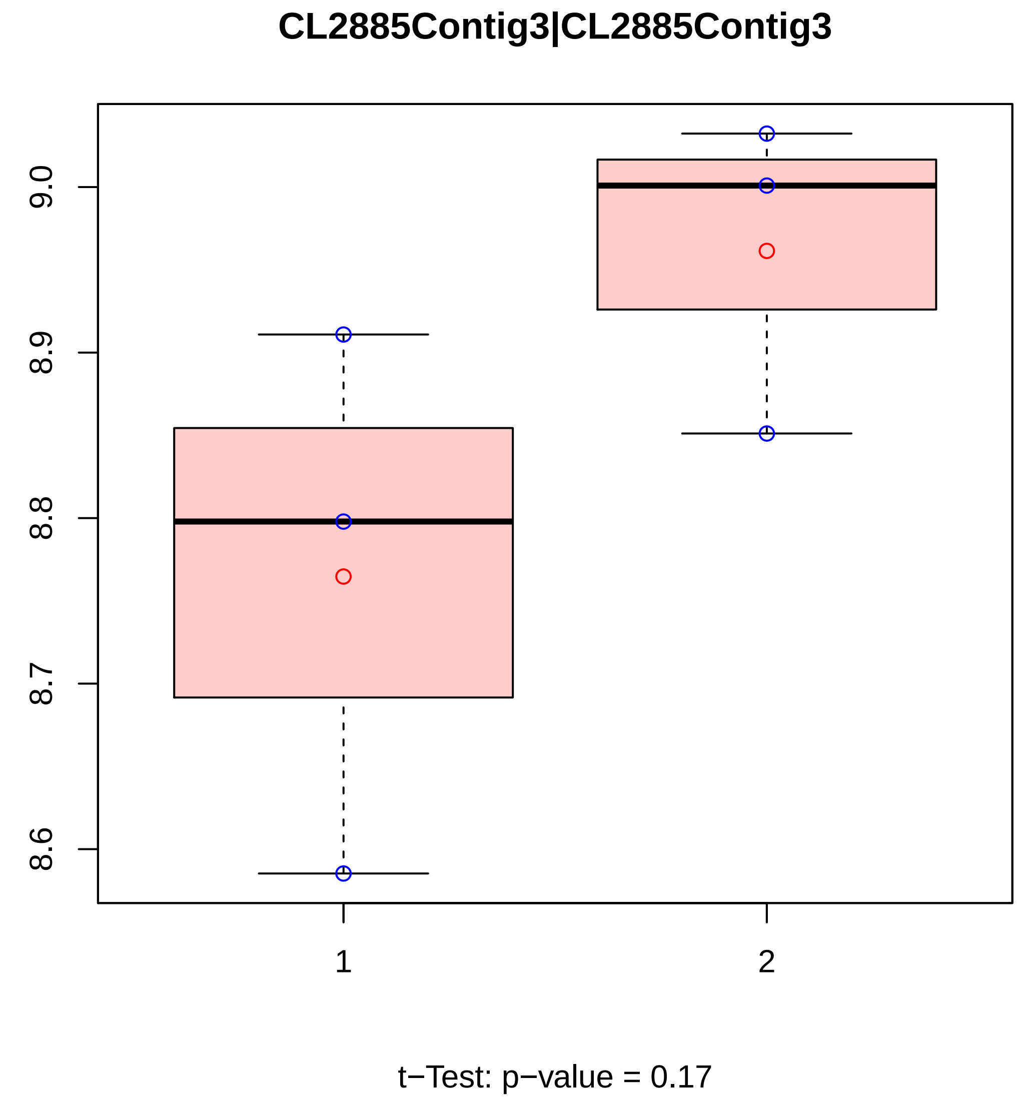
CL2885Contig3|CL2885Contig3
9.0
8.9
8.8
8.7
8.6
1
2
t−Test: p−value = 0.17

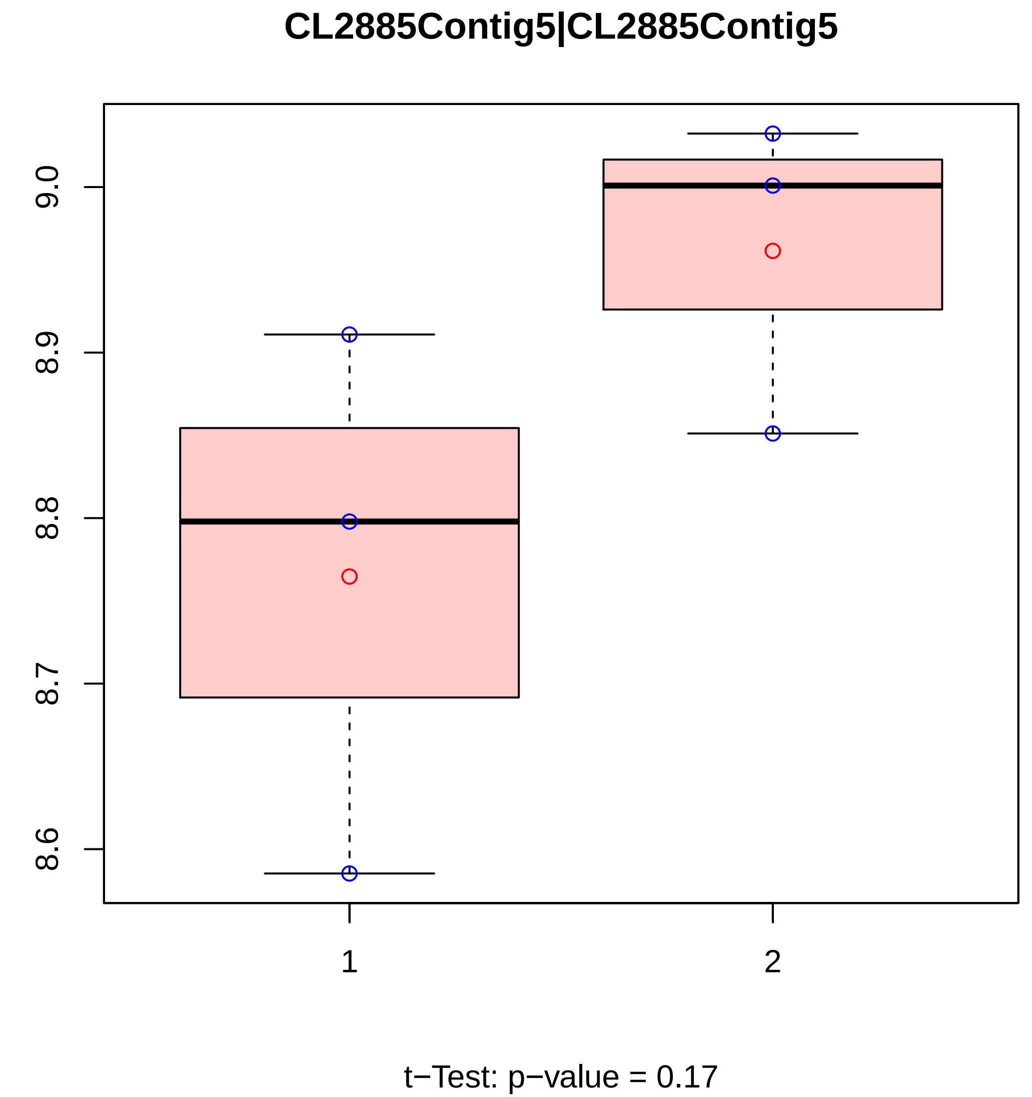
CL2885Contig5|CL2885Contig5
9.0
8.9
8.8
8.7
8.6
1
2
t−Test: p−value = 0.17

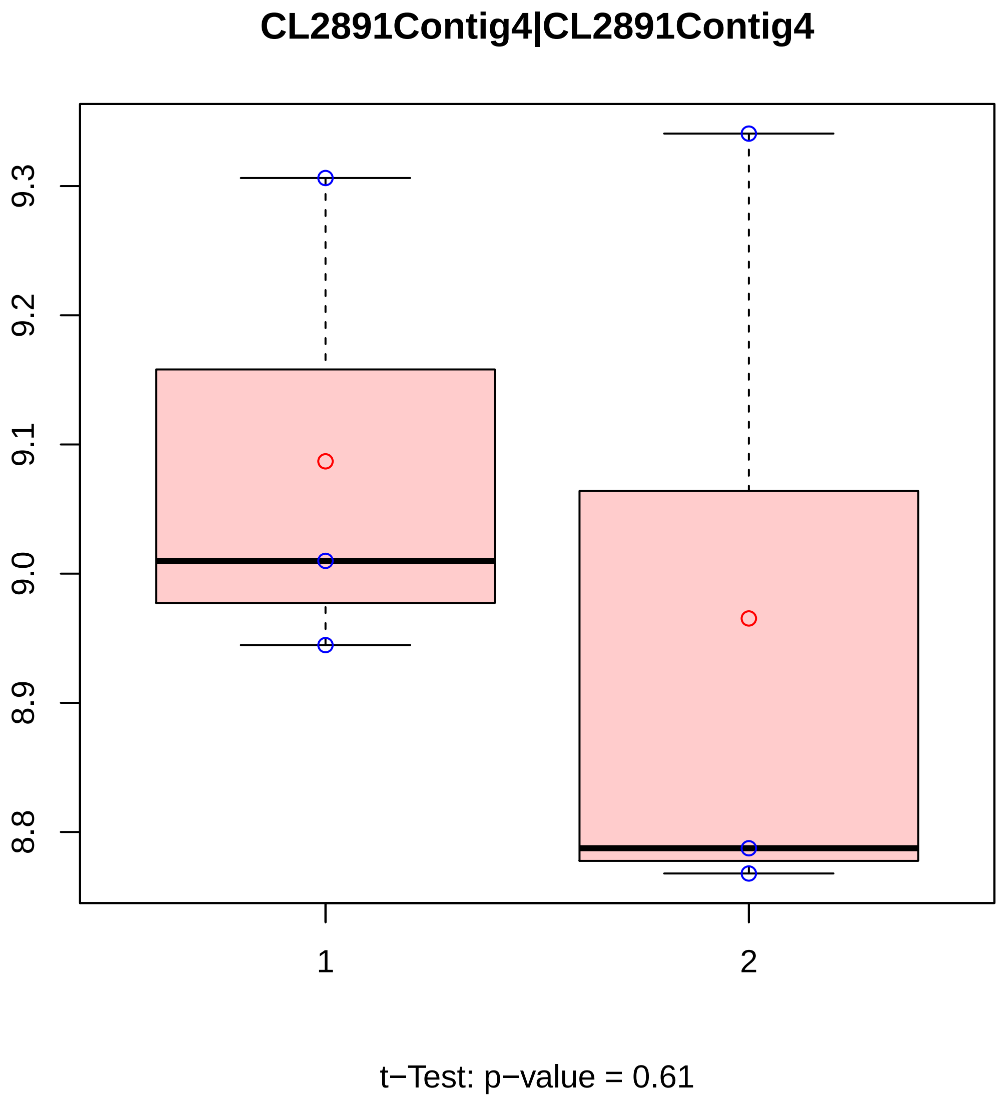

CL2891Contig4|CL2891Contig4
9.3
9.2
9.1
9.0
8.9
8.8
1
2
t−Test: p−value = 0.61

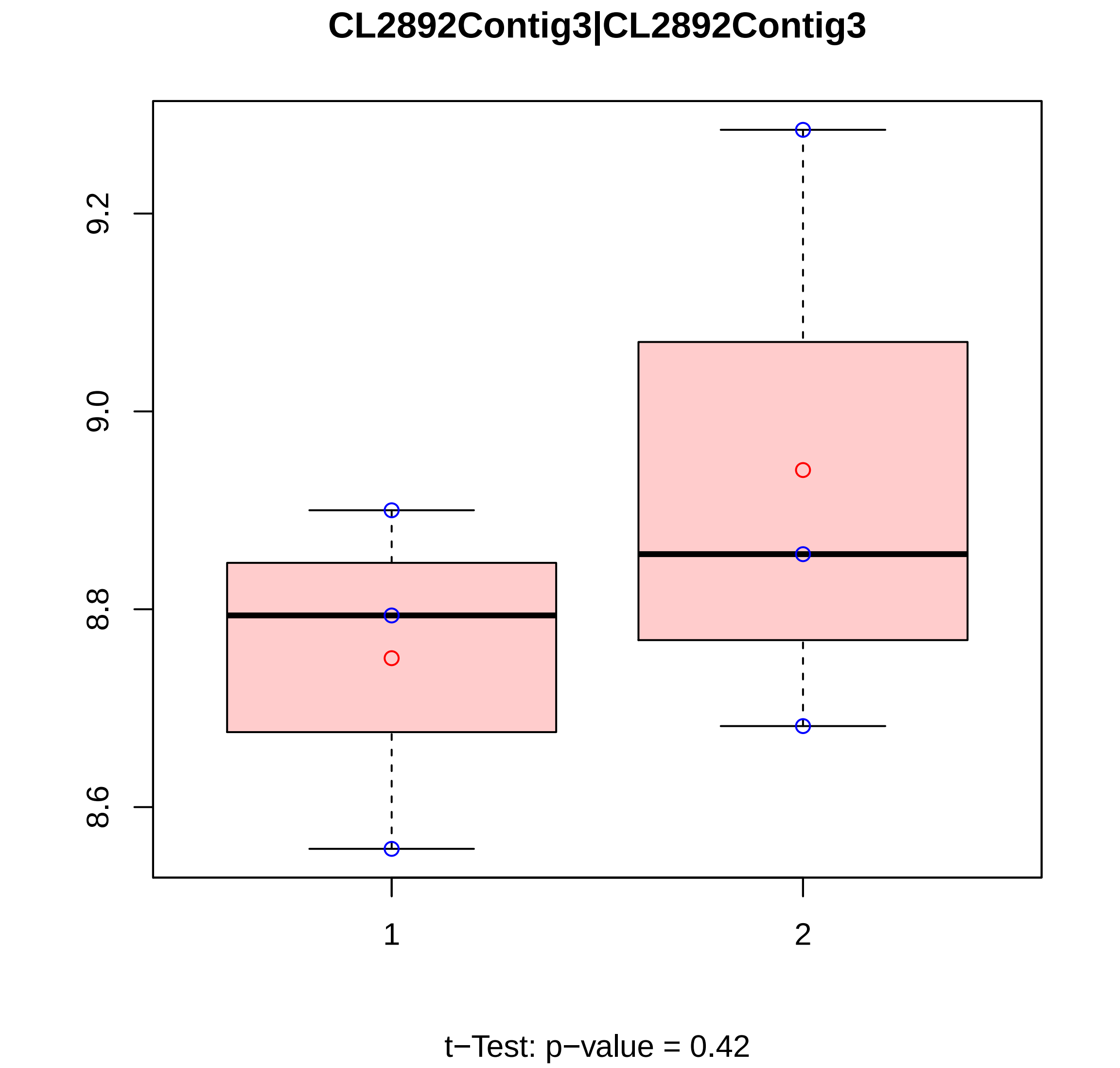

CL2892Contig3|CL2892Contig3
9.2
9.0
8.8
8.6
1
2
t−Test: p−value = 0.42

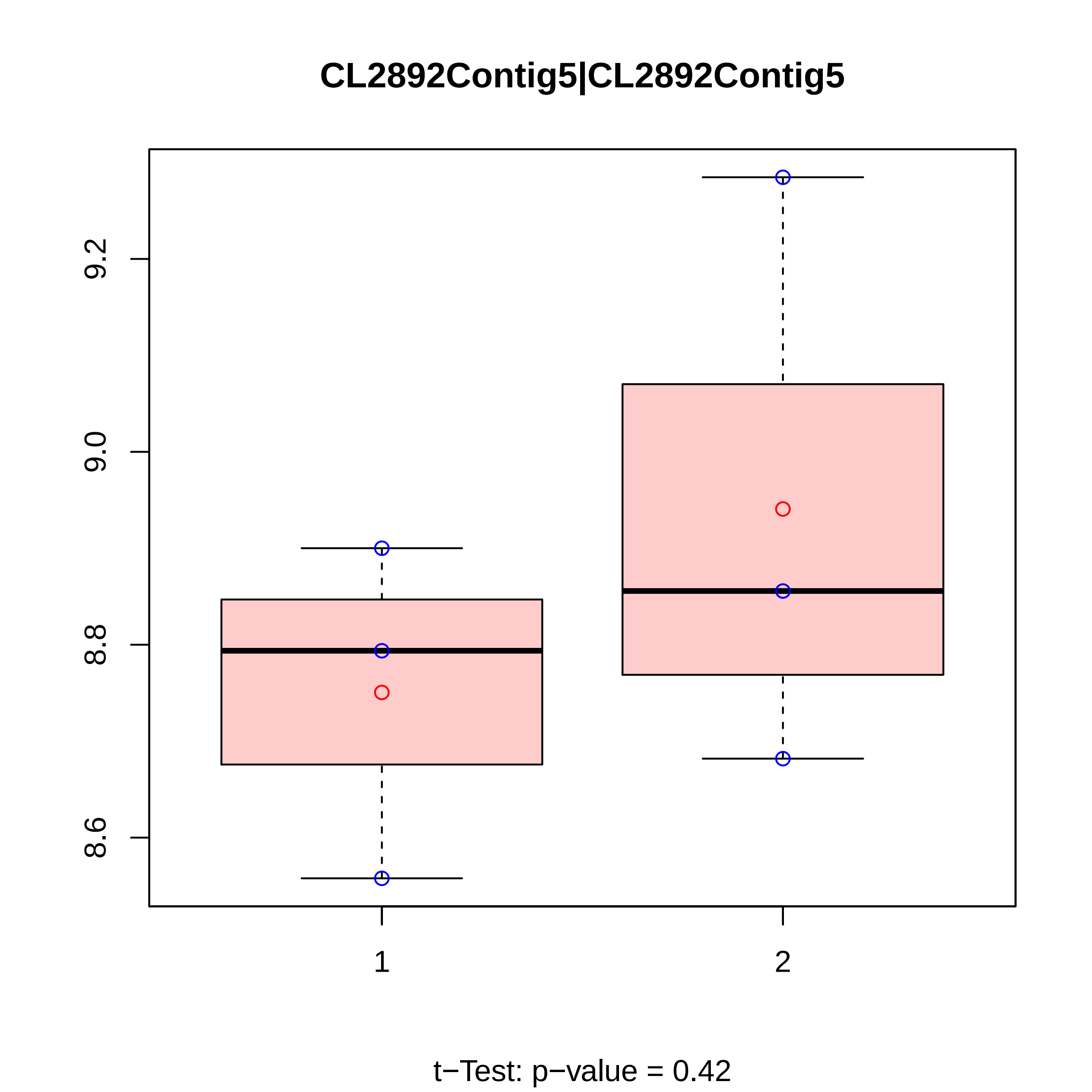

CL2892Contig5|CL2892Contig5
9.2
9.0
8.8
8.6
1
2
t−Test: p−value = 0.42

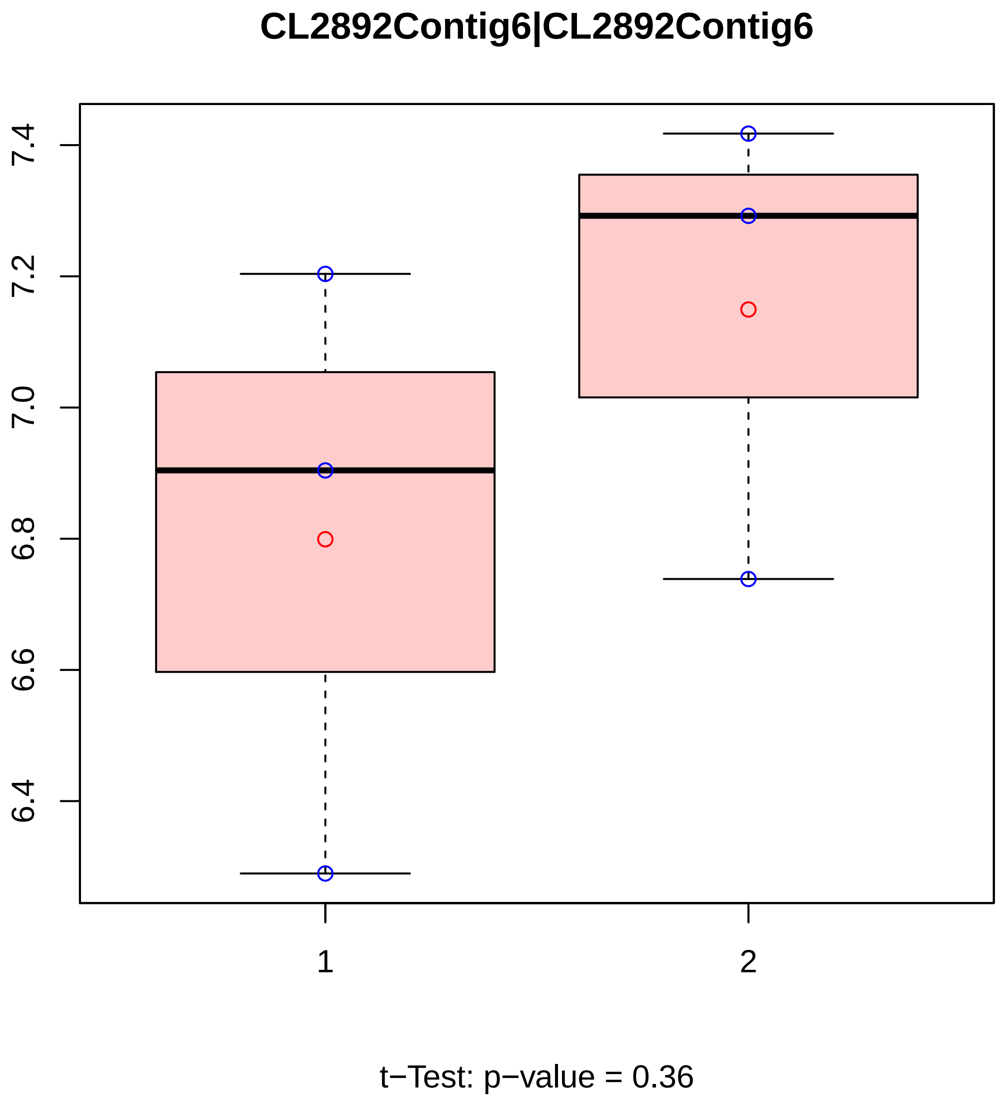
CL2892Contig6|CL2892Contig6
7.4
7.2
7.0
6.8
6.6
6.4
1
2
t−Test: p−value = 0.36

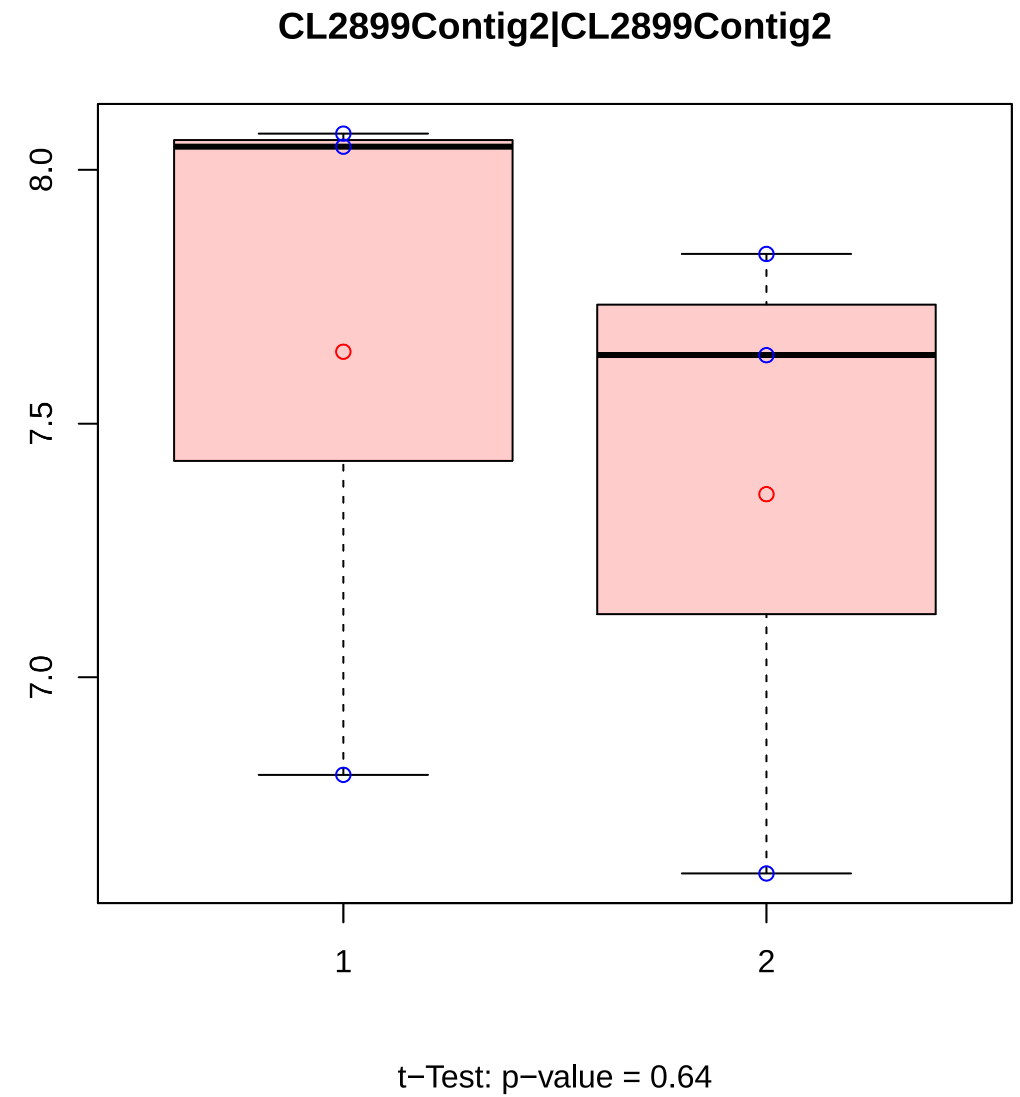
CL2899Contig2|CL2899Contig2
8.0
7.5
7.0
1
2
t−Test: p−value = 0.64

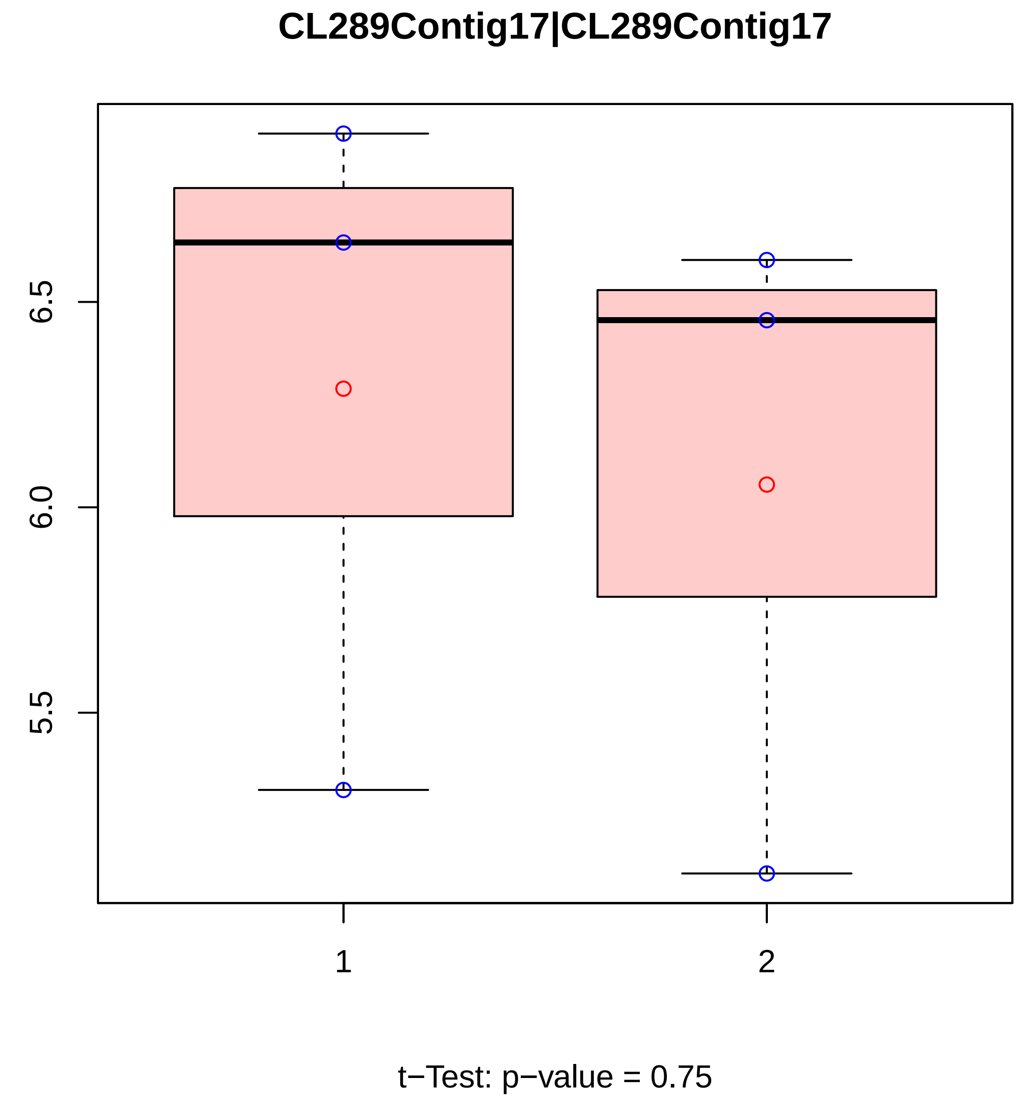
CL289Contig17|CL289Contig17
6.5
6.0
5.5
1
2
t−Test: p−value = 0.75

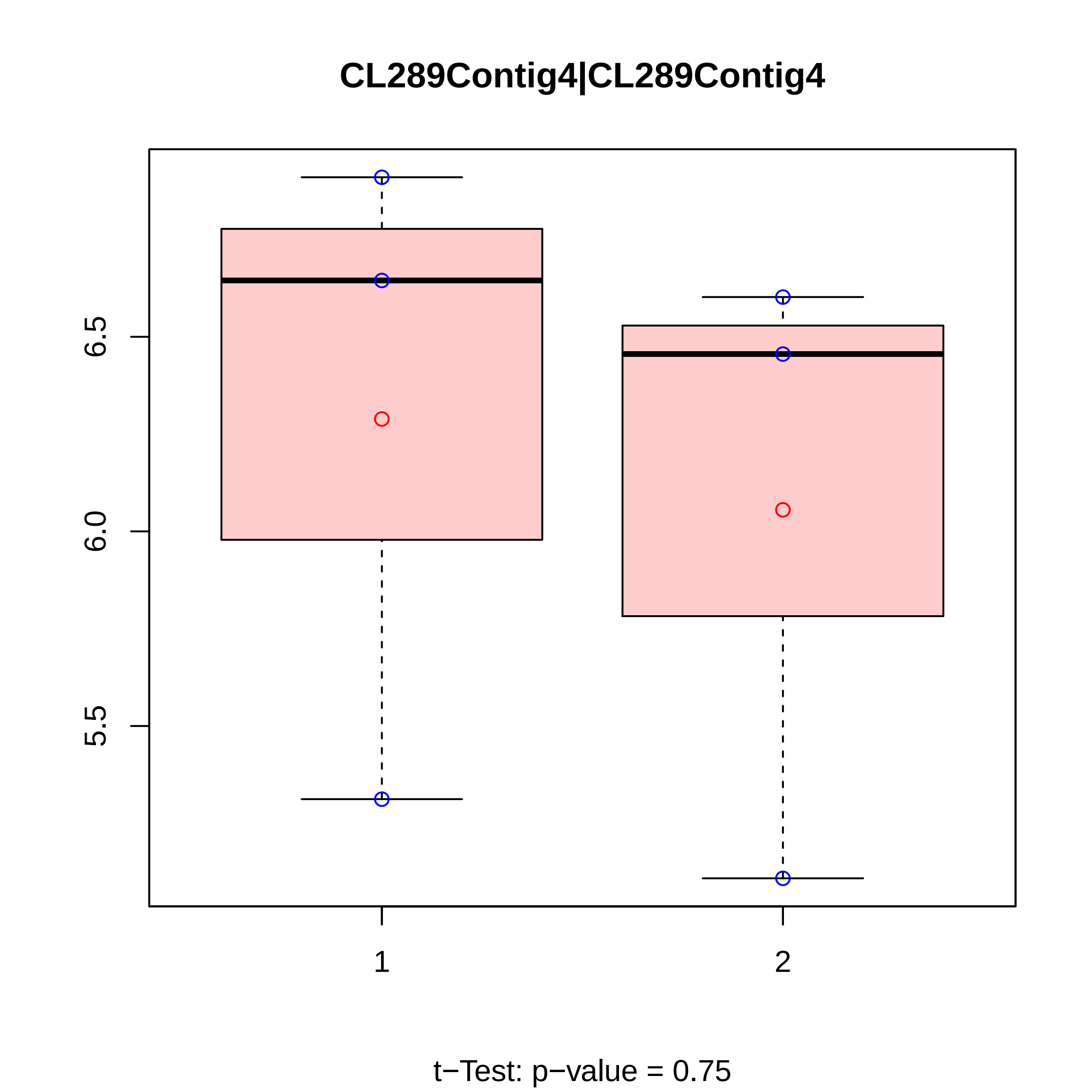

CL289Contig4|CL289Contig4
6.5
6.0
5.5
1
2
t−Test: p−value = 0.75

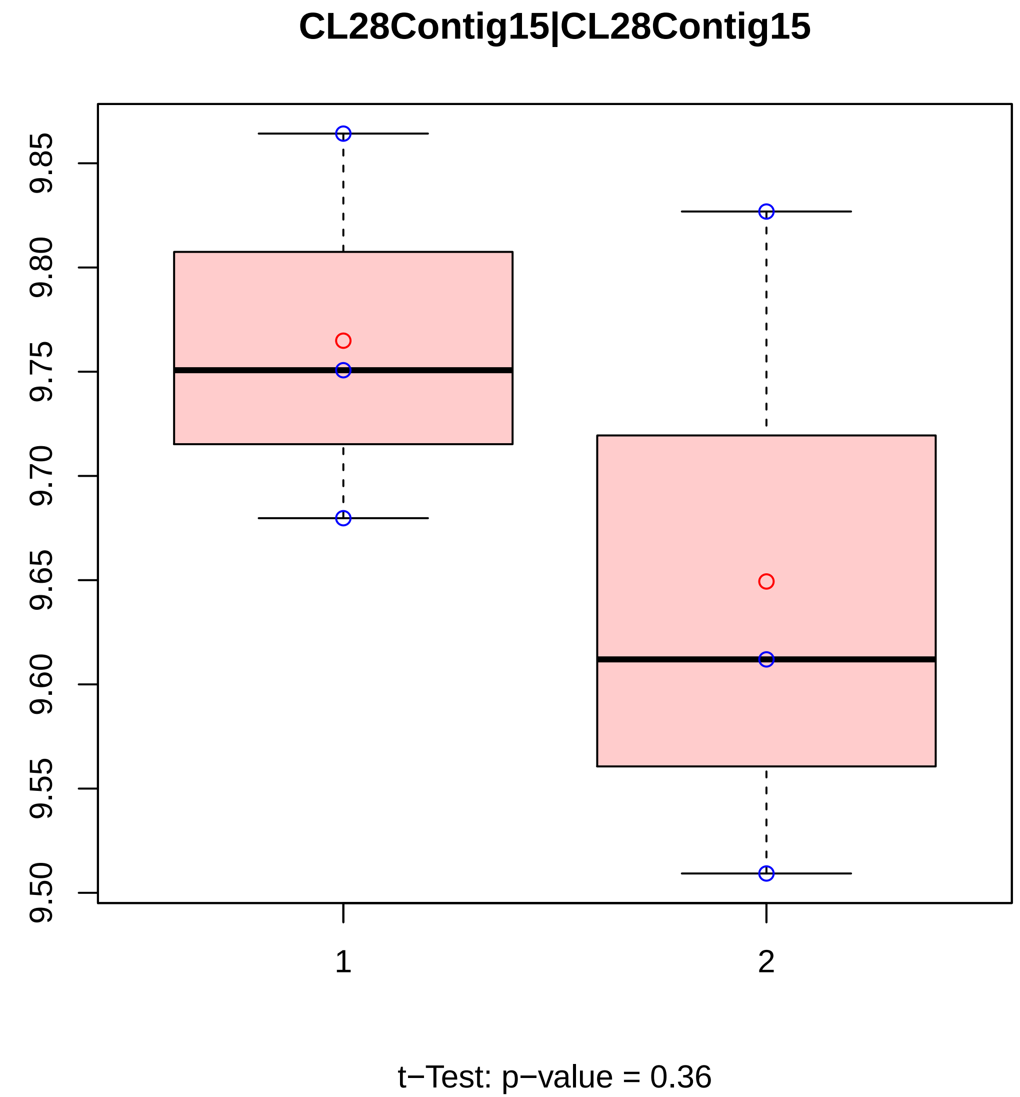
CL28Contig15|CL28Contig15
9.50
9.55
9.60
9.65
9.70
9.75
9.80
9.85
1
2
t−Test: p−value = 0.36

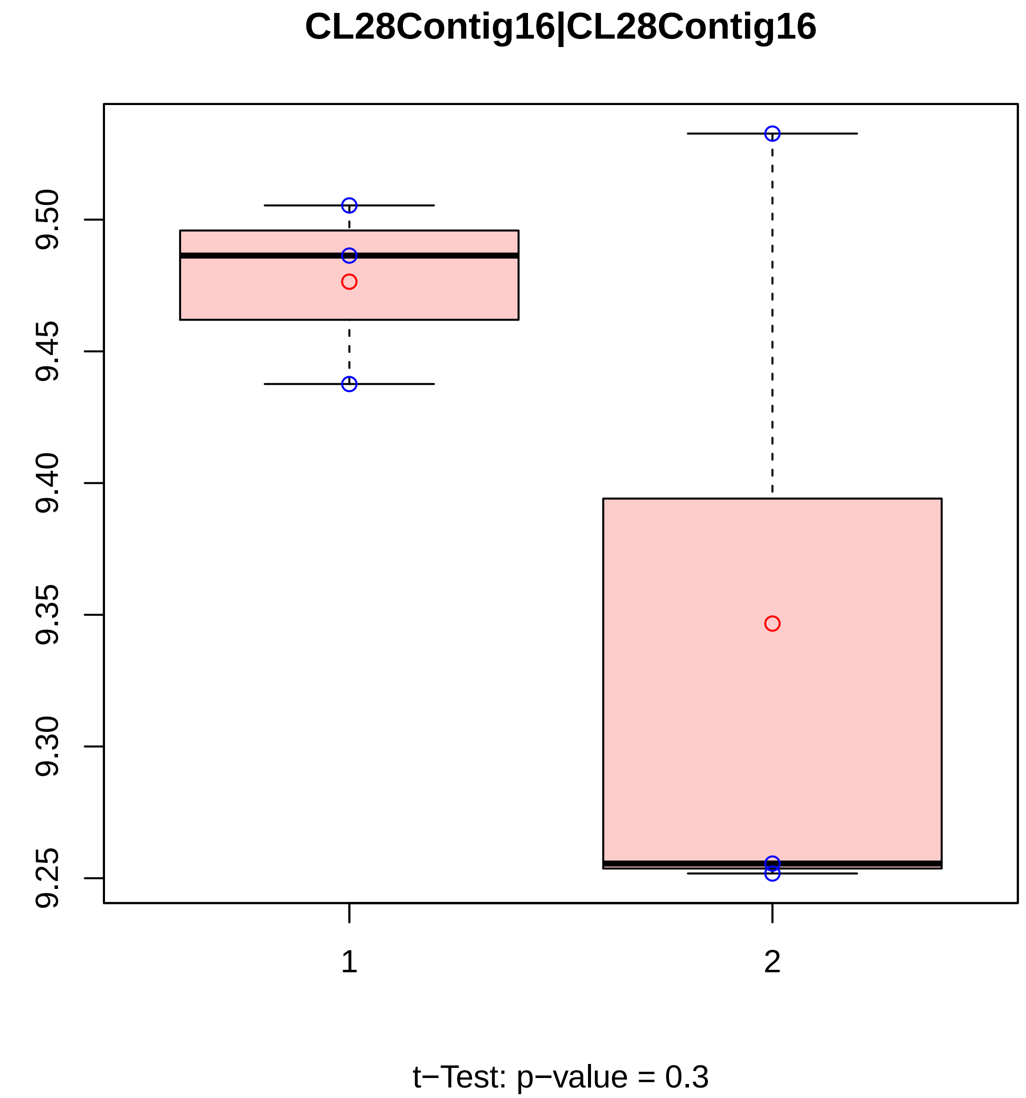
CL28Contig16|CL28Contig16
9.25
9.30
9.35
9.40
9.45
9.50
1
2
t−Test: p−value = 0.3

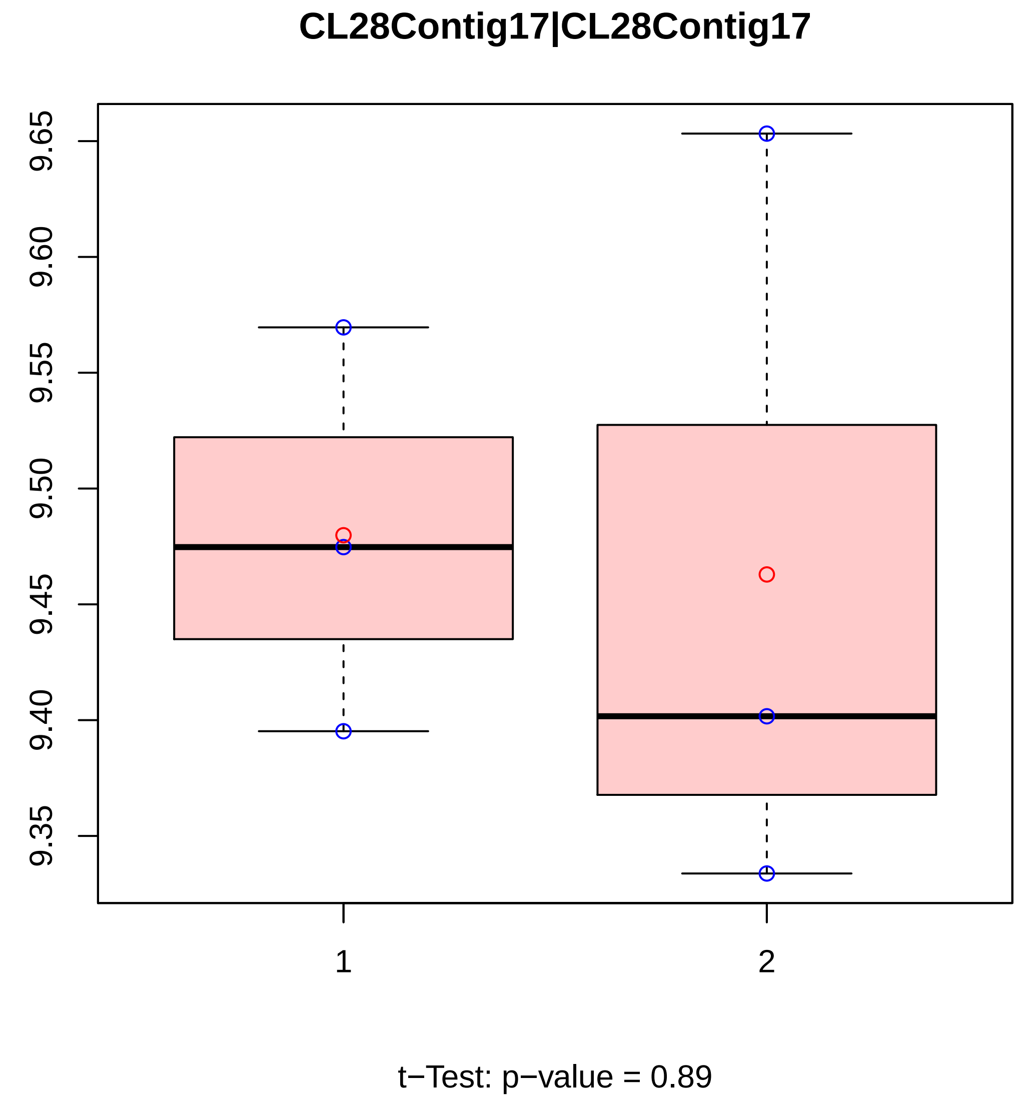
CL28Contig17|CL28Contig17
9.35
9.40
9.45
9.50
9.55
9.60
9.65
1
2
t−Test: p−value = 0.89

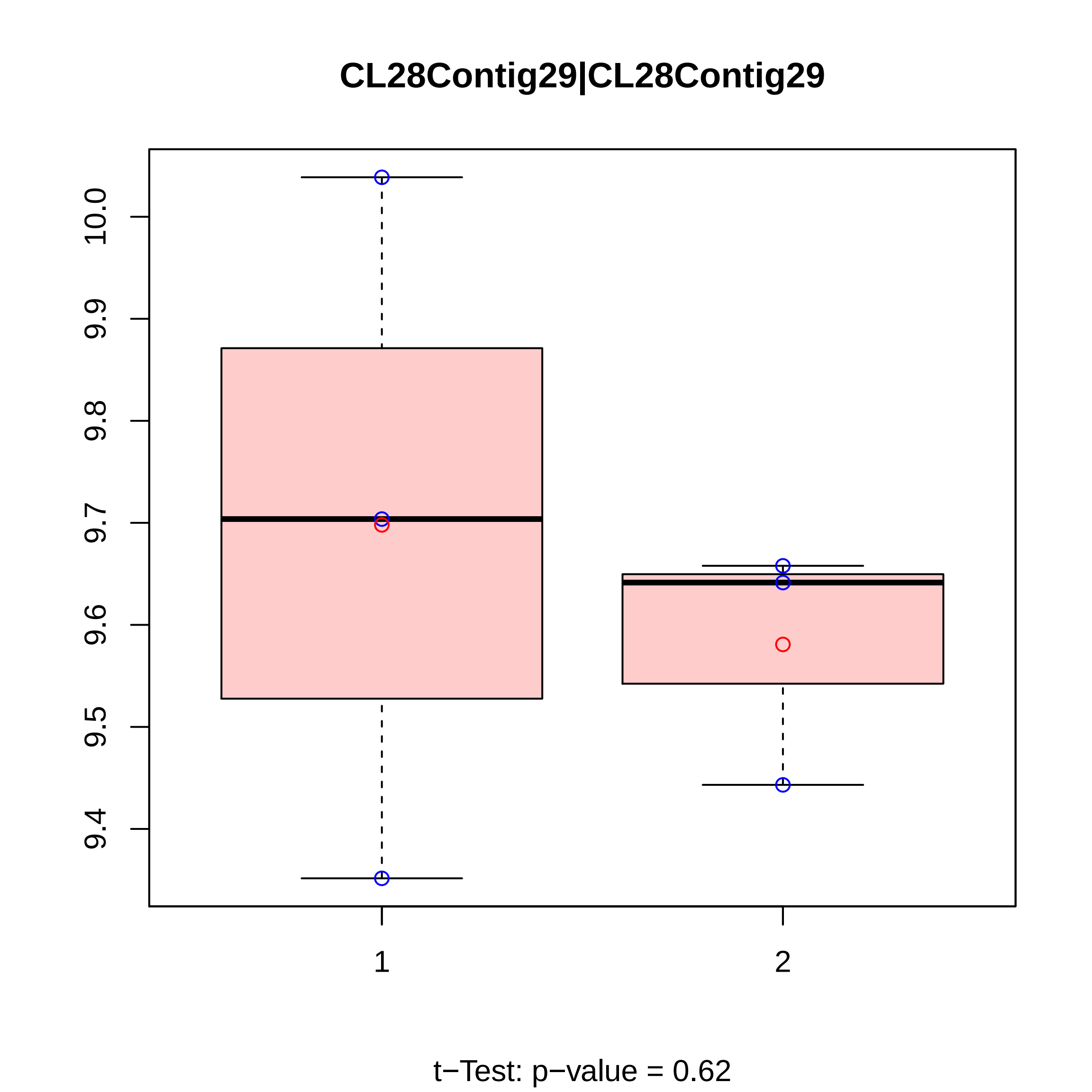

CL28Contig29|CL28Contig29
10.0
9.9
9.8
9.7
9.6
9.5
9.4
1
2
t−Test: p−value = 0.62

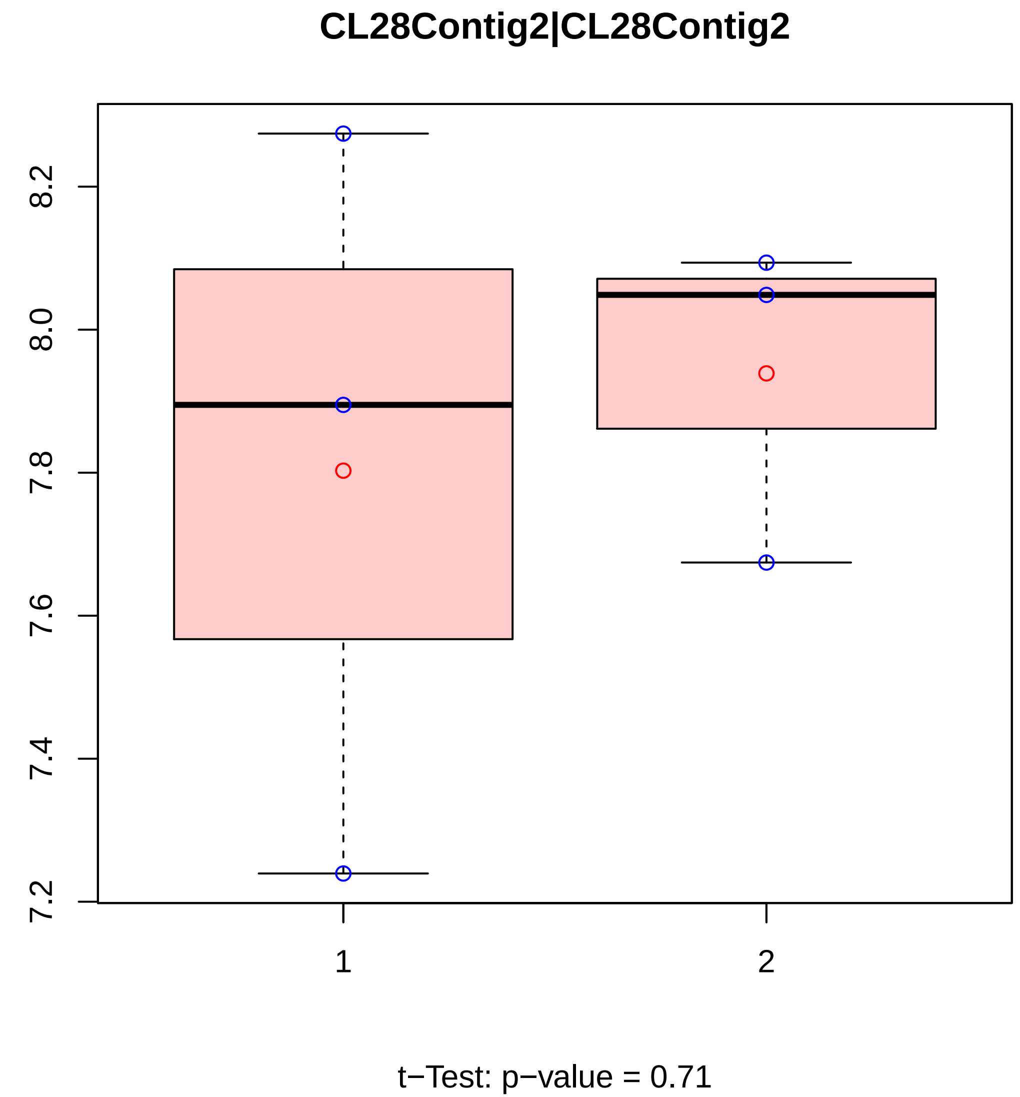

CL28Contig2|CL28Contig2
8.2
8.0
7.8
7.6
7.4
7.2
1
2
t−Test: p−value = 0.71

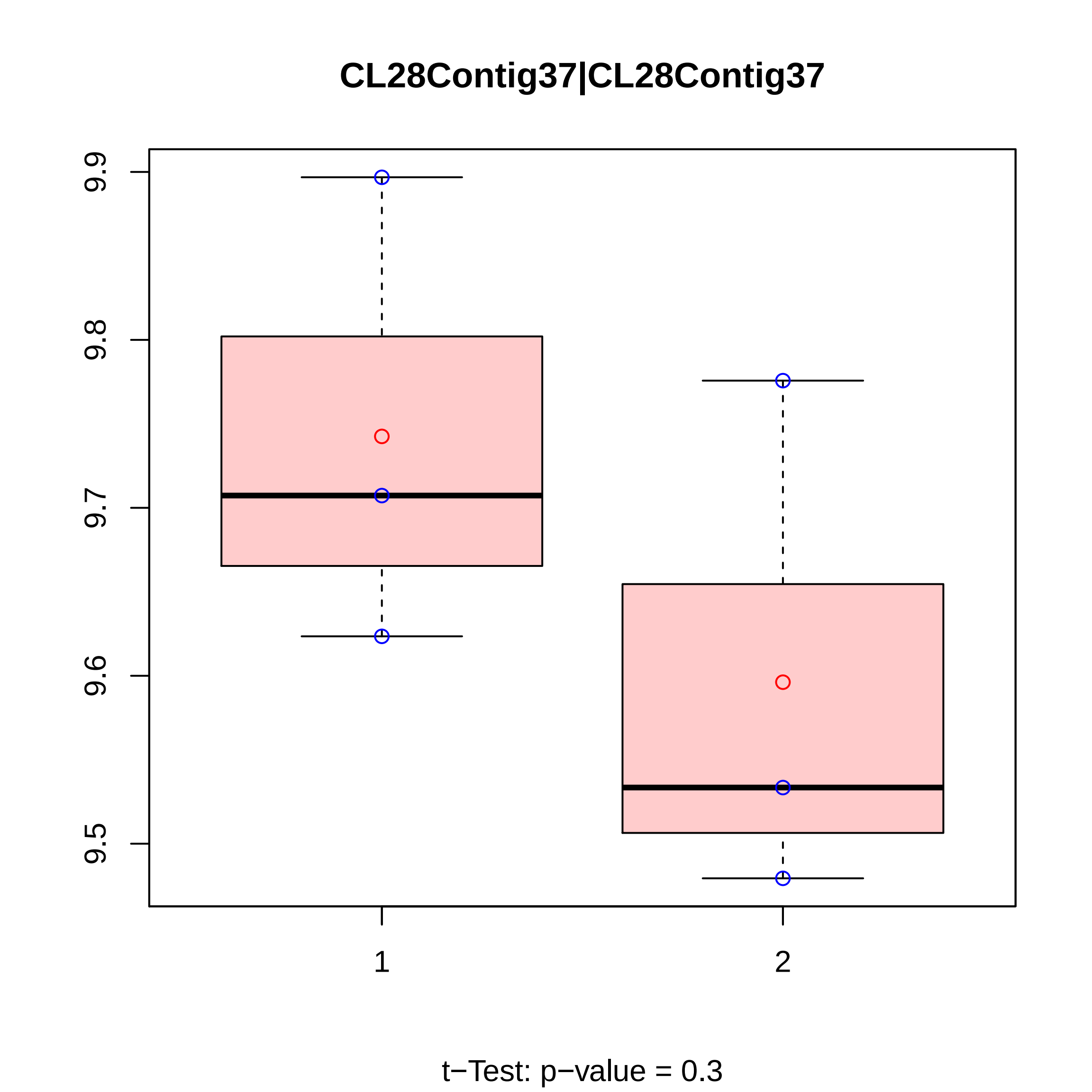

CL28Contig37|CL28Contig37
9.5
9.6
9.7
9.8
9.9
1
2
t−Test: p−value = 0.3

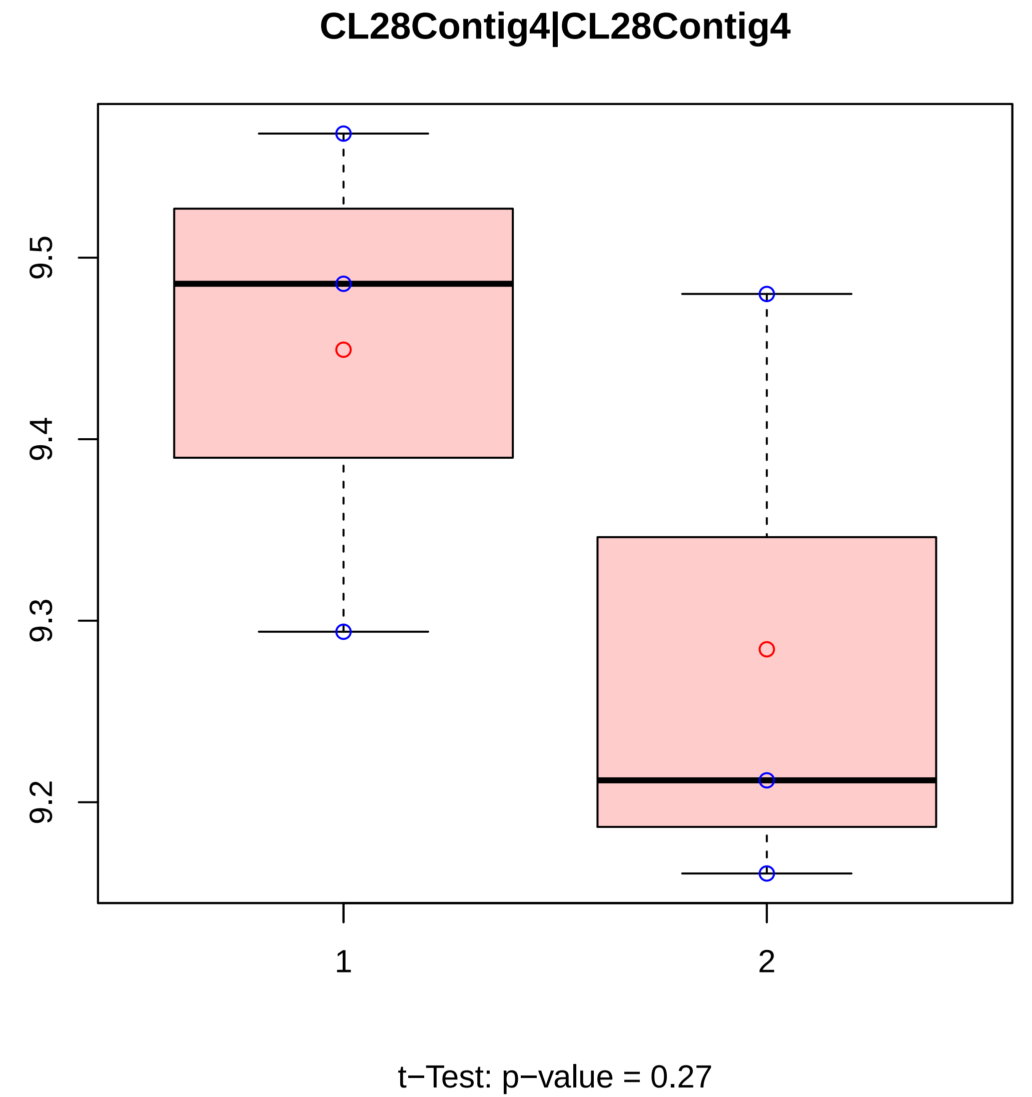
CL28Contig4|CL28Contig4
9.2
9.3
9.4
9.5
1
2
t−Test: p−value = 0.27

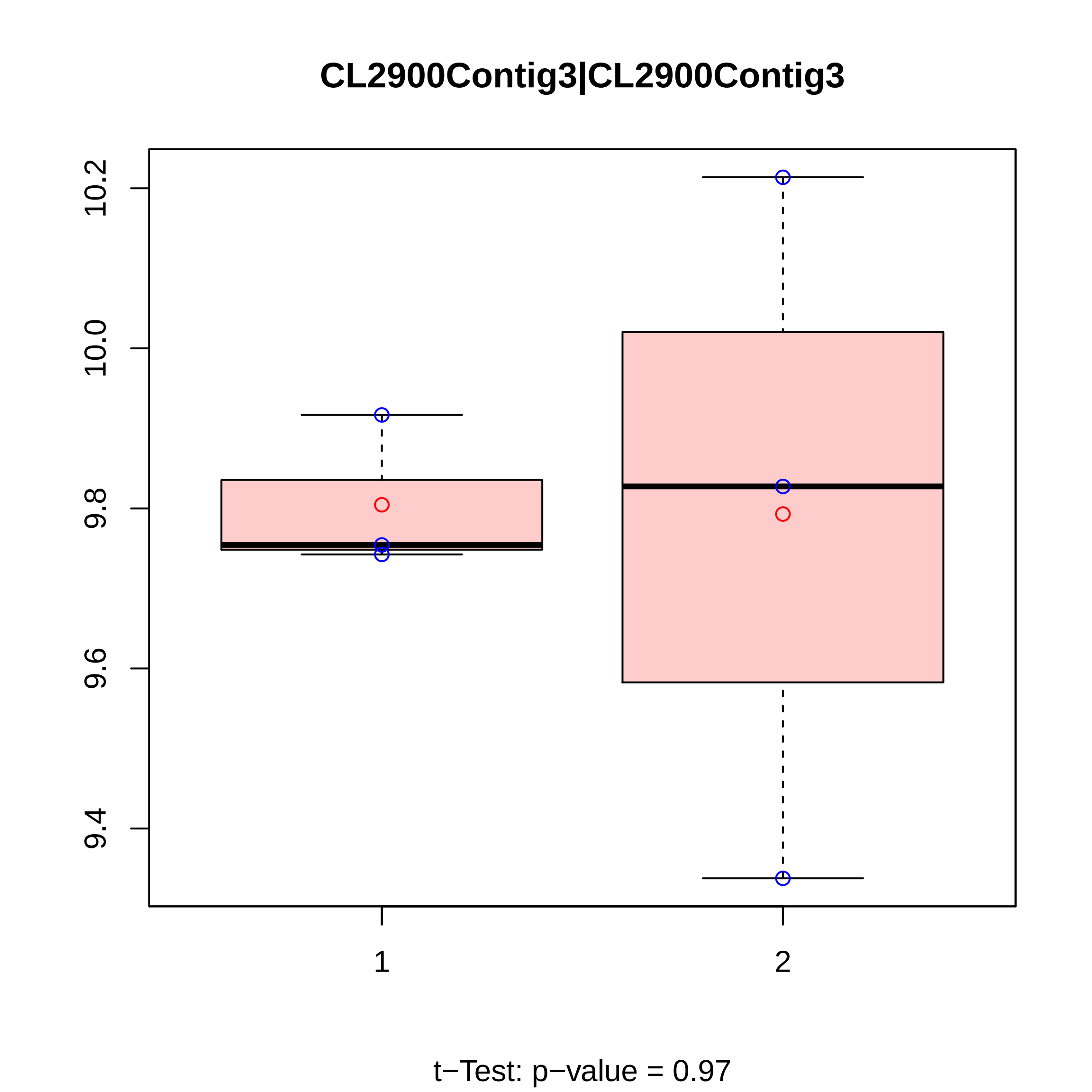

CL2900Contig3|CL2900Contig3
10.2
10.0
9.8
9.6
9.4
1
2
t−Test: p−value = 0.97

# CL2900Contig4|CL2900Contig4

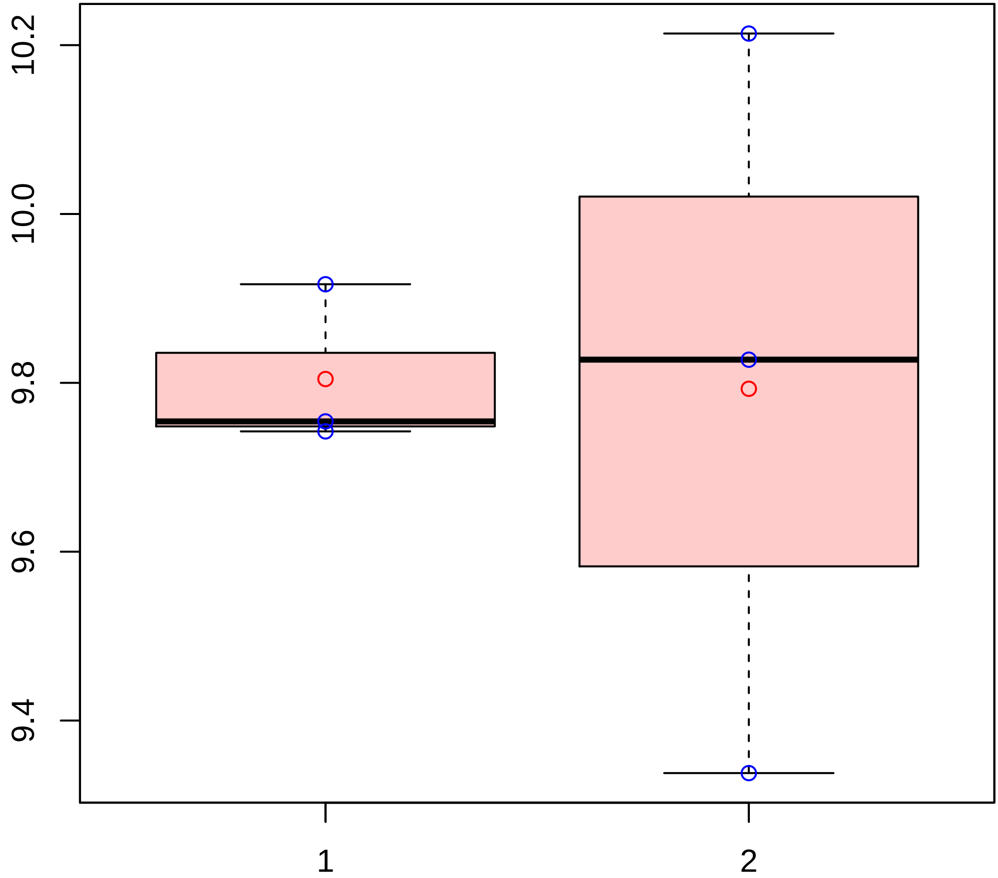


t−Test: p−value = 0.97

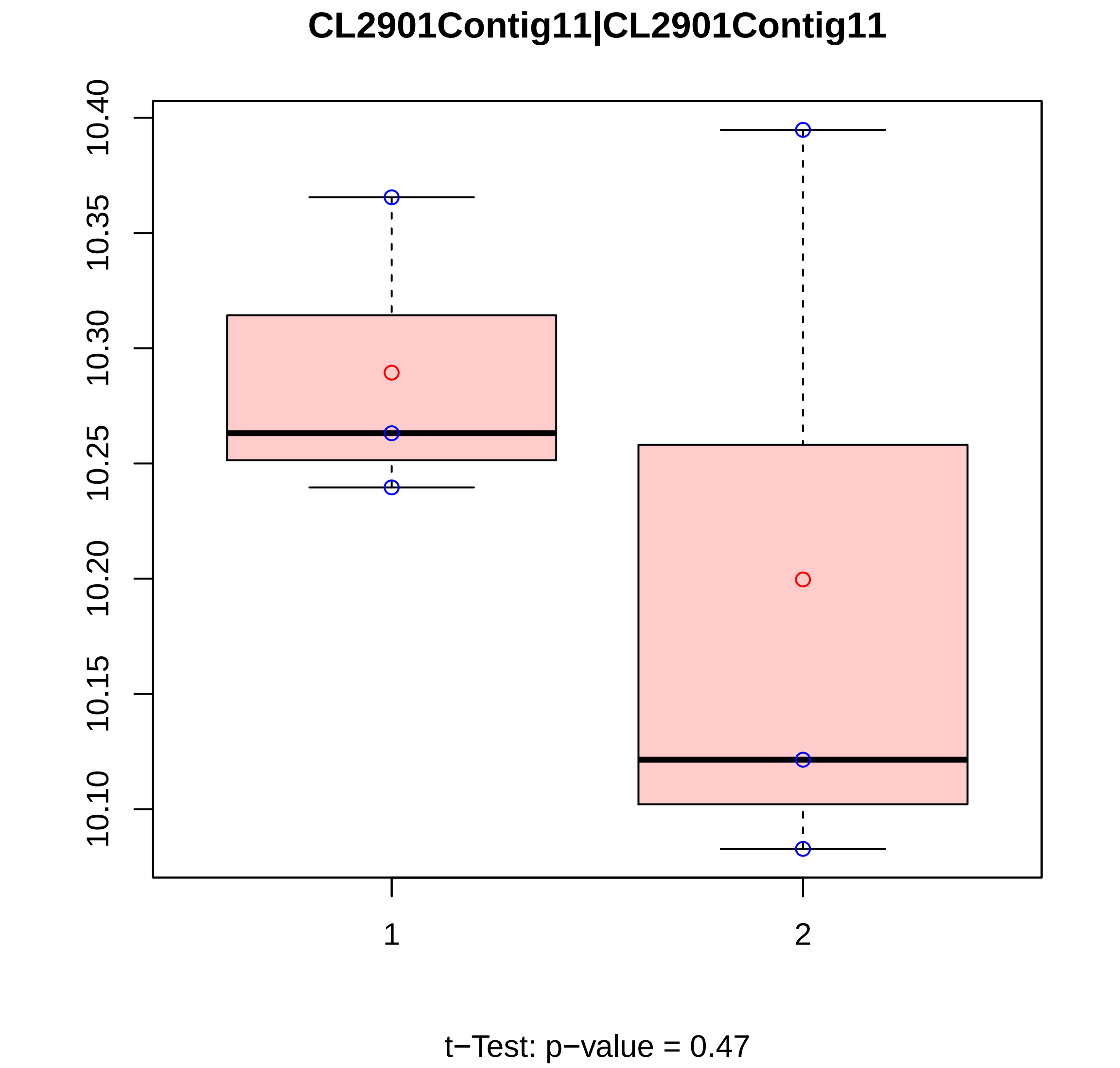
CL2901Contig11|CL2901Contig11
10.40
10.35
10.30
10.25
10.20
10.15
10.10
1
2
t−Test: p−value = 0.47

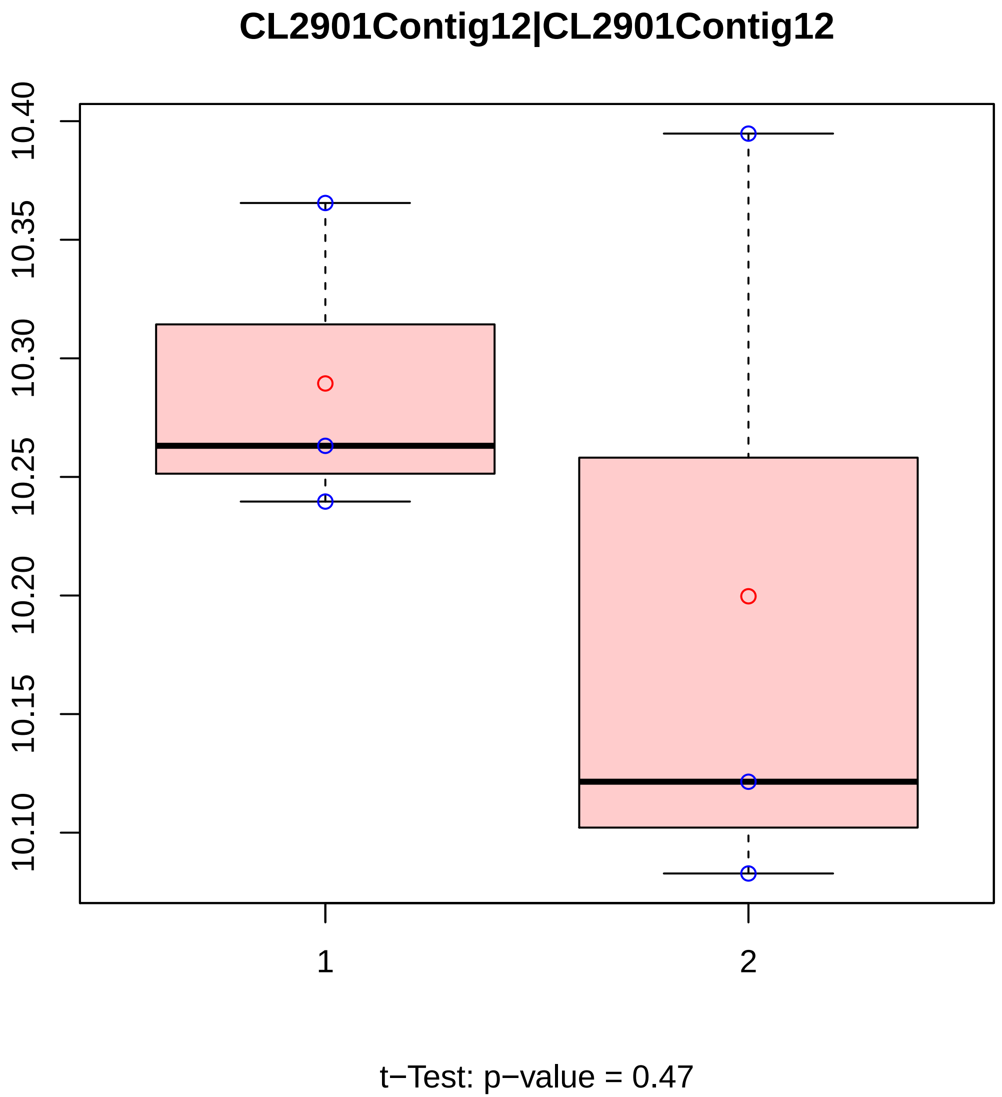

CL2901Contig12|CL2901Contig12
10.10
10.15
10.20
10.25
10.30
10.35
10.40
1
2
t−Test: p−value = 0.47

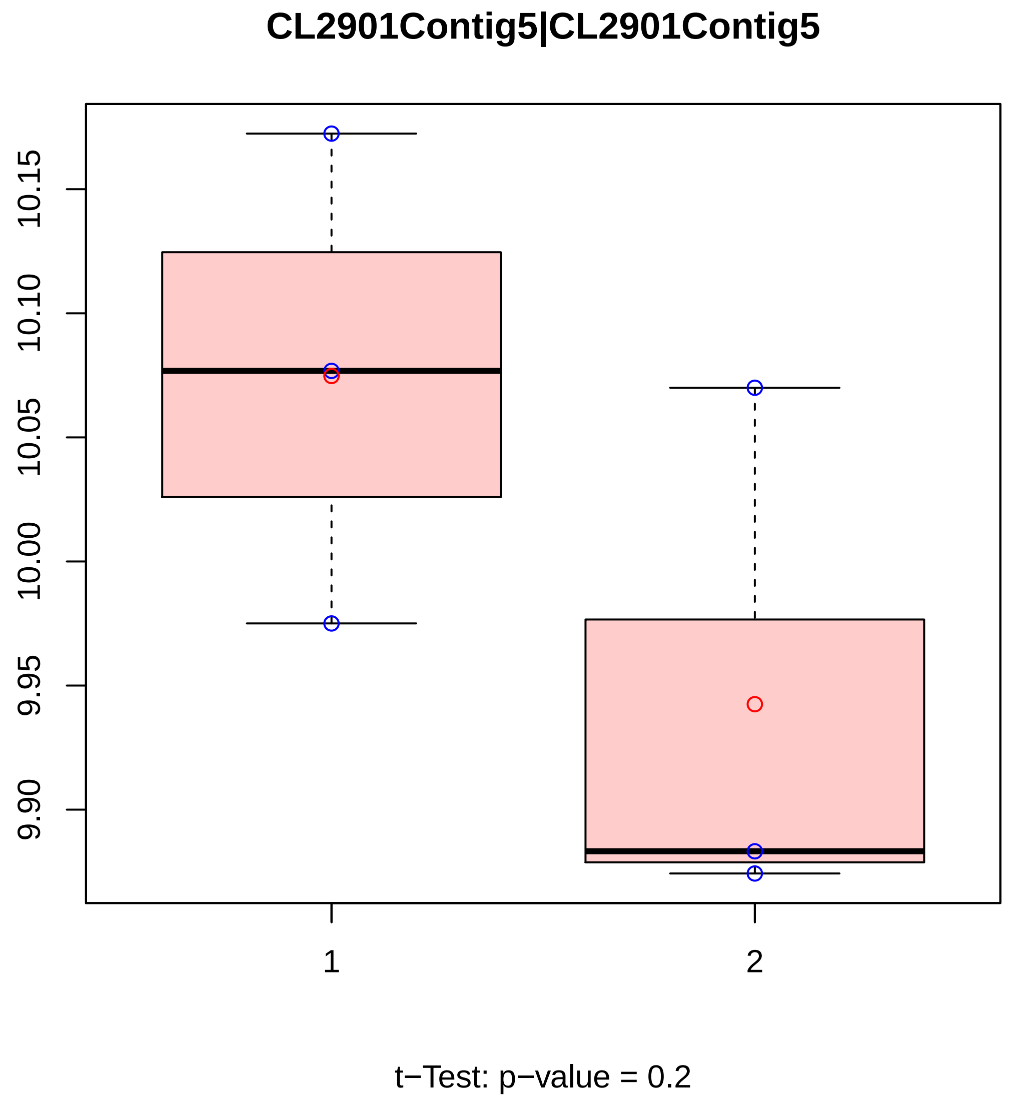

CL2901Contig5|CL2901Contig5
10.15
10.10
10.05
10.00
9.95
9.90
1
2
t−Test: p−value = 0.2

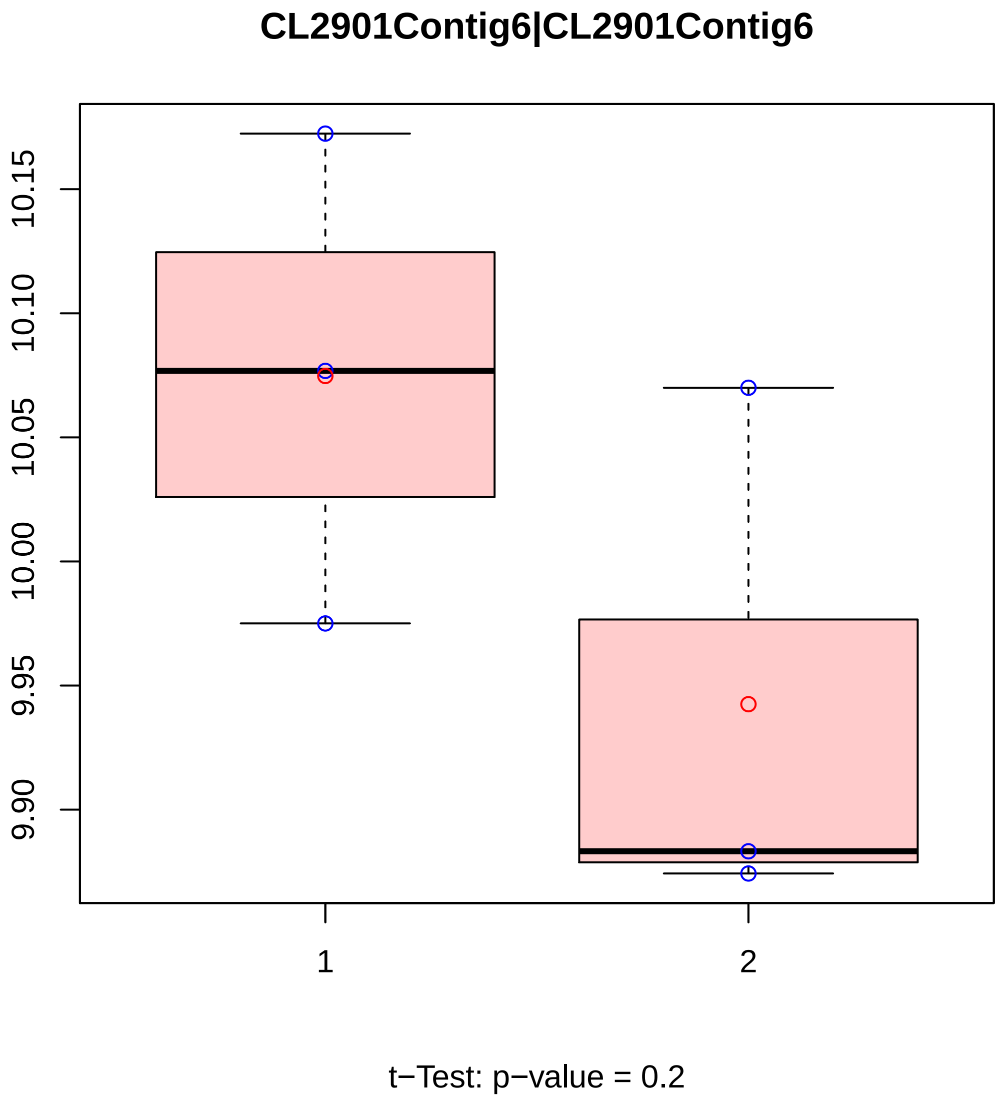

CL2901Contig6|CL2901Contig6
10.15
10.10
10.05
10.00
9.95
9.90
1
2
t−Test: p−value = 0.2

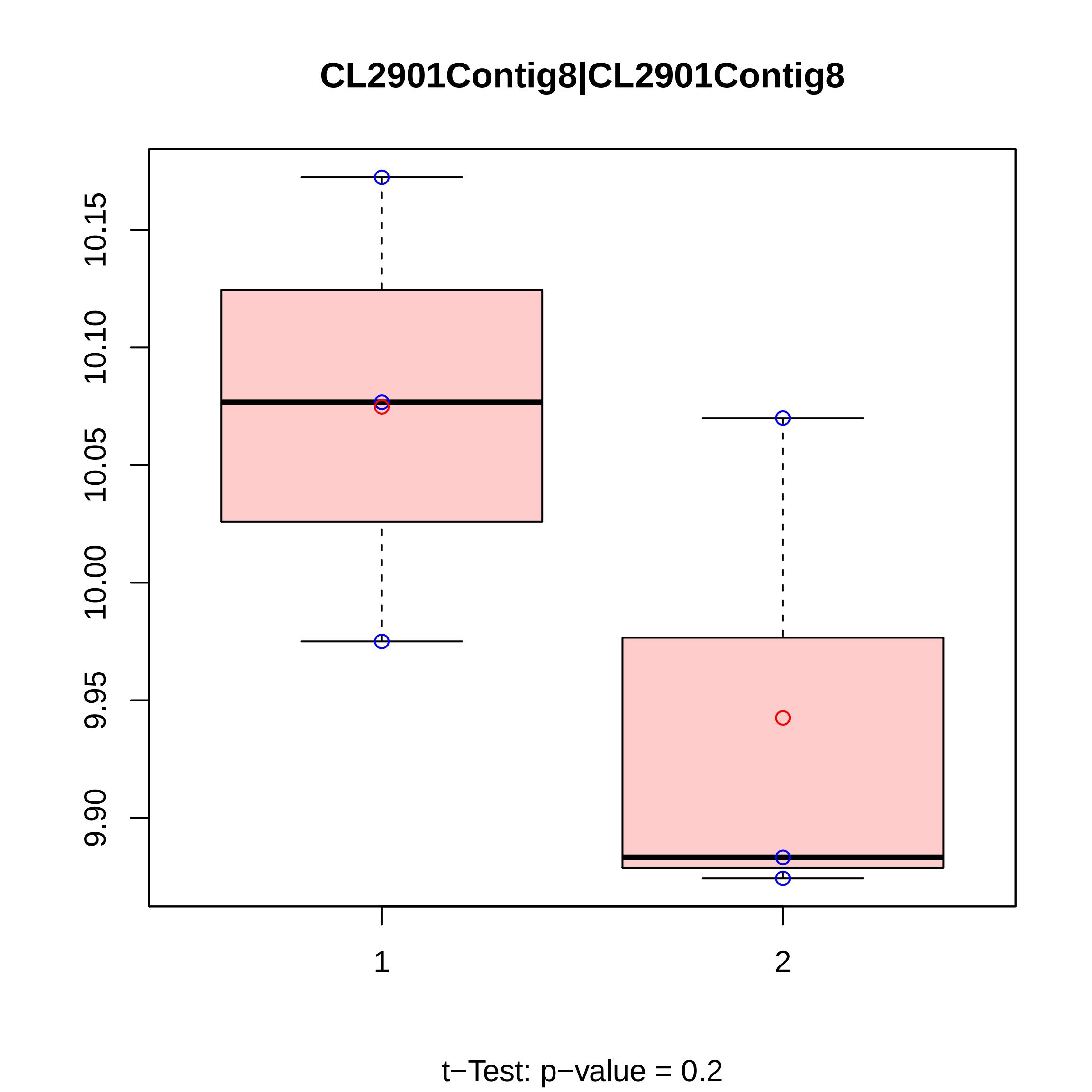

CL2901Contig8|CL2901Contig8
10.15
10.10
10.05
10.00
9.95
9.90
1
2
t−Test: p−value = 0.2

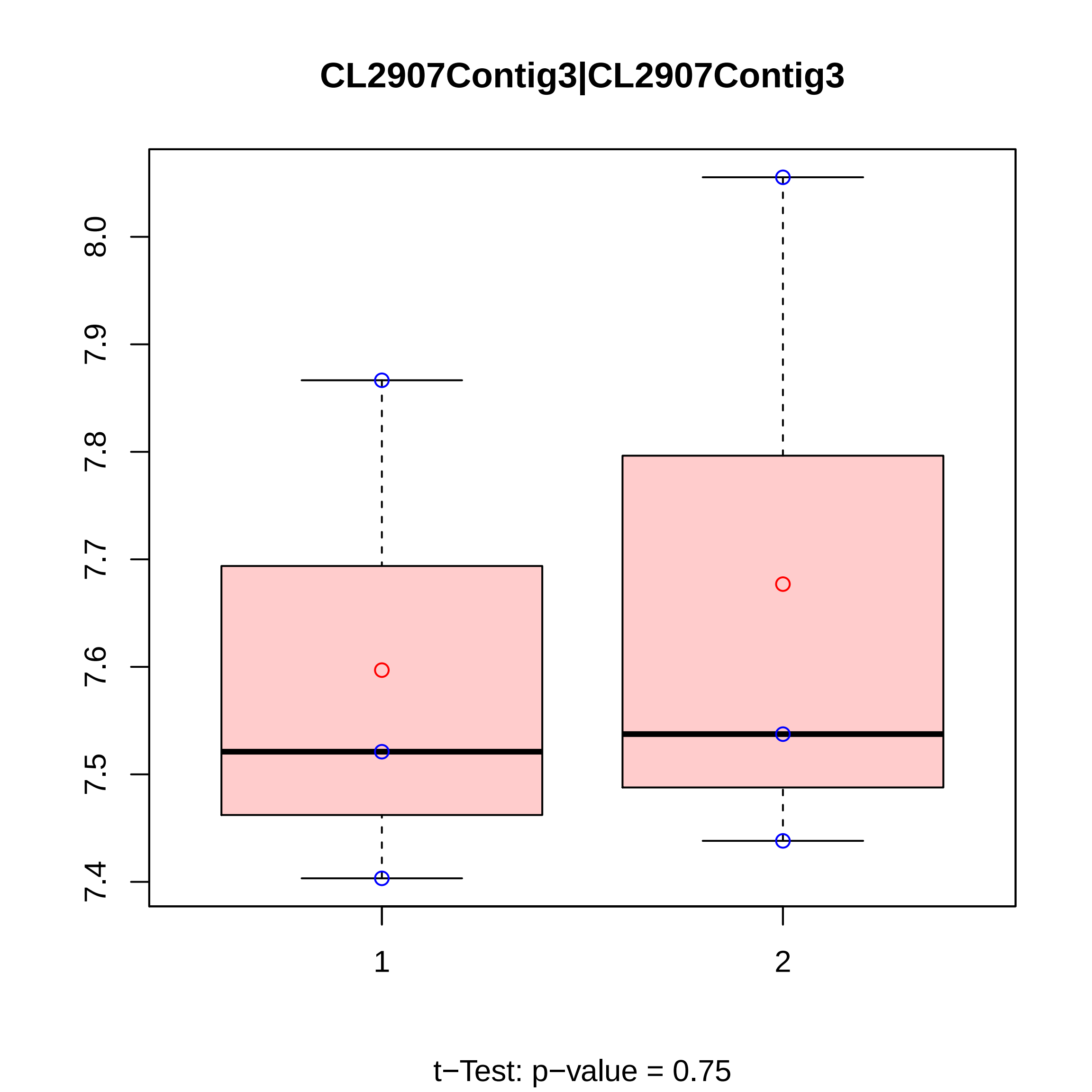
CL2907Contig3|CL2907Contig3
8.0
7.9
7.8
7.7
7.6
7.5
7.4
1
2
t−Test: p−value = 0.75

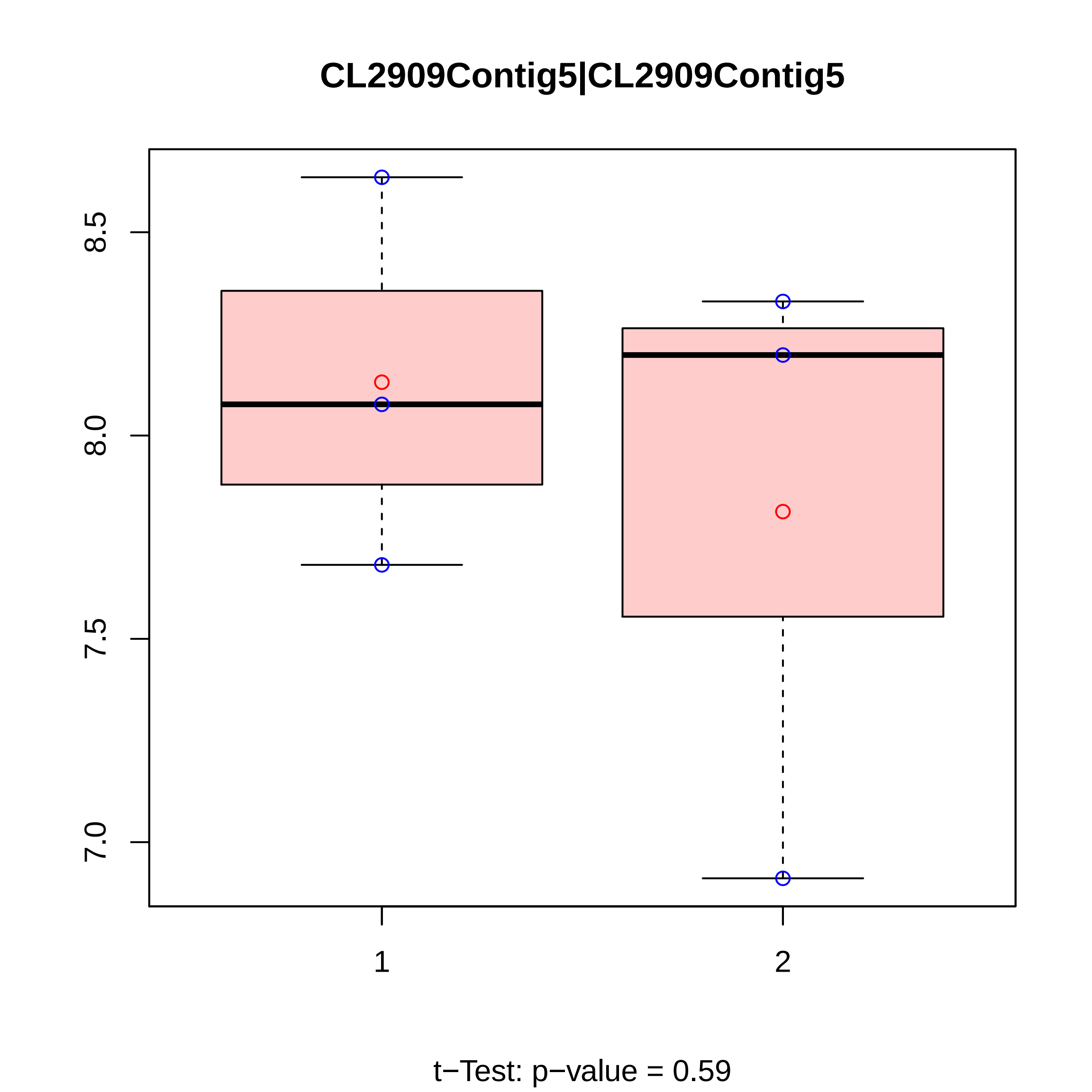
CL2909Contig5|CL2909Contig5
8.5
8.0
7.5
7.0
1
2
t−Test: p−value = 0.59

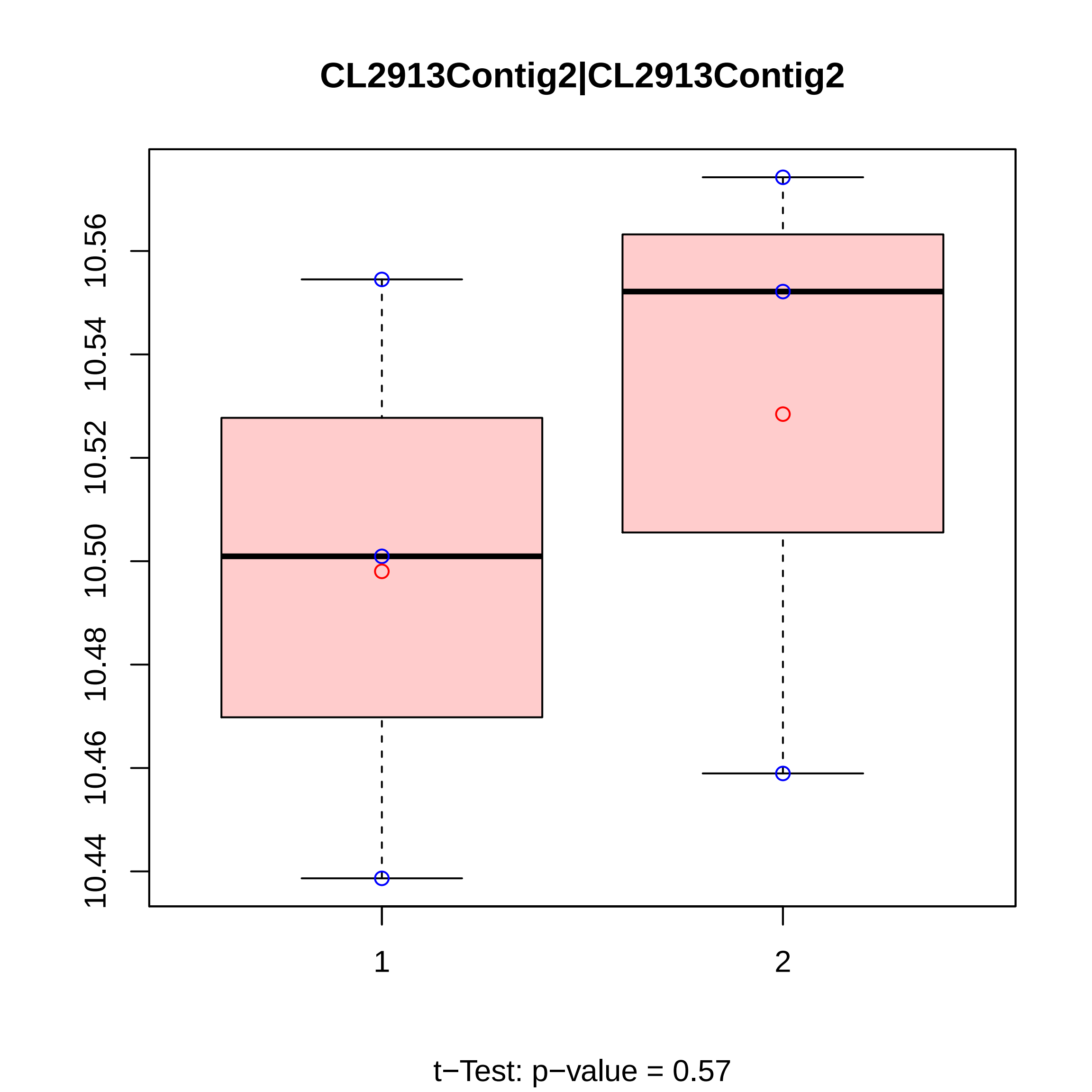
CL2913Contig2|CL2913Contig2
10.44
10.46
10.48
10.50
10.52
10.54
10.56
1
2
t−Test: p−value = 0.57

# CL2916Contig2|CL2916Contig2

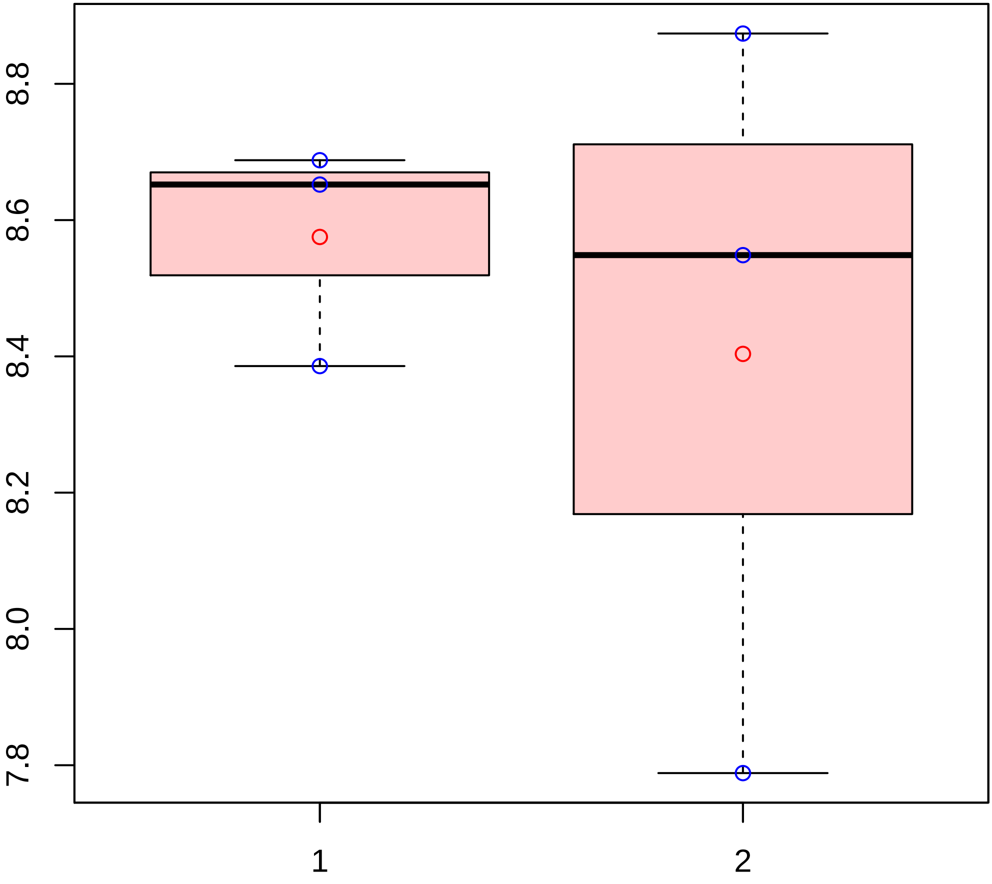


t−Test: p−value = 0.65

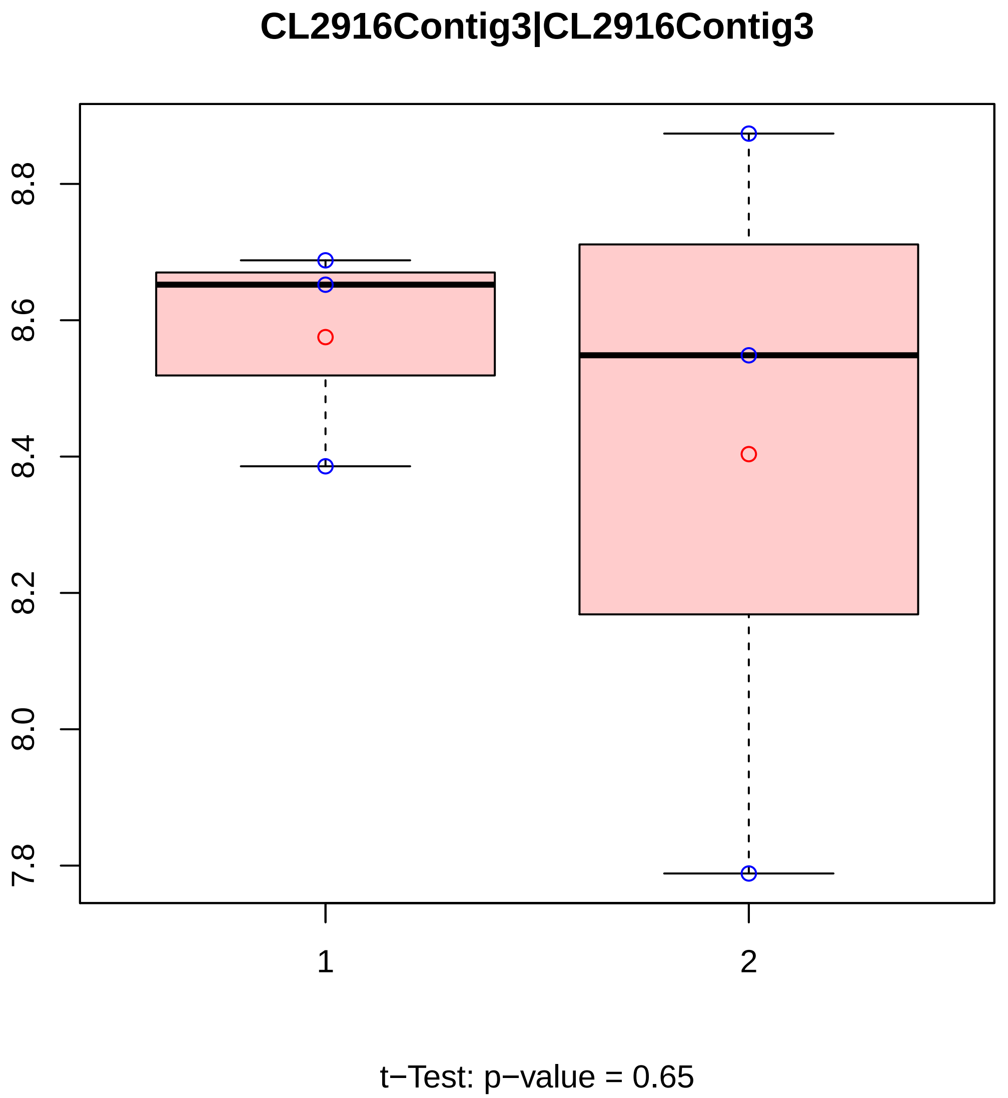
CL2916Contig3|CL2916Contig3
8.8
8.6
8.4
8.2
8.0
7.8
1
2
t−Test: p−value = 0.65

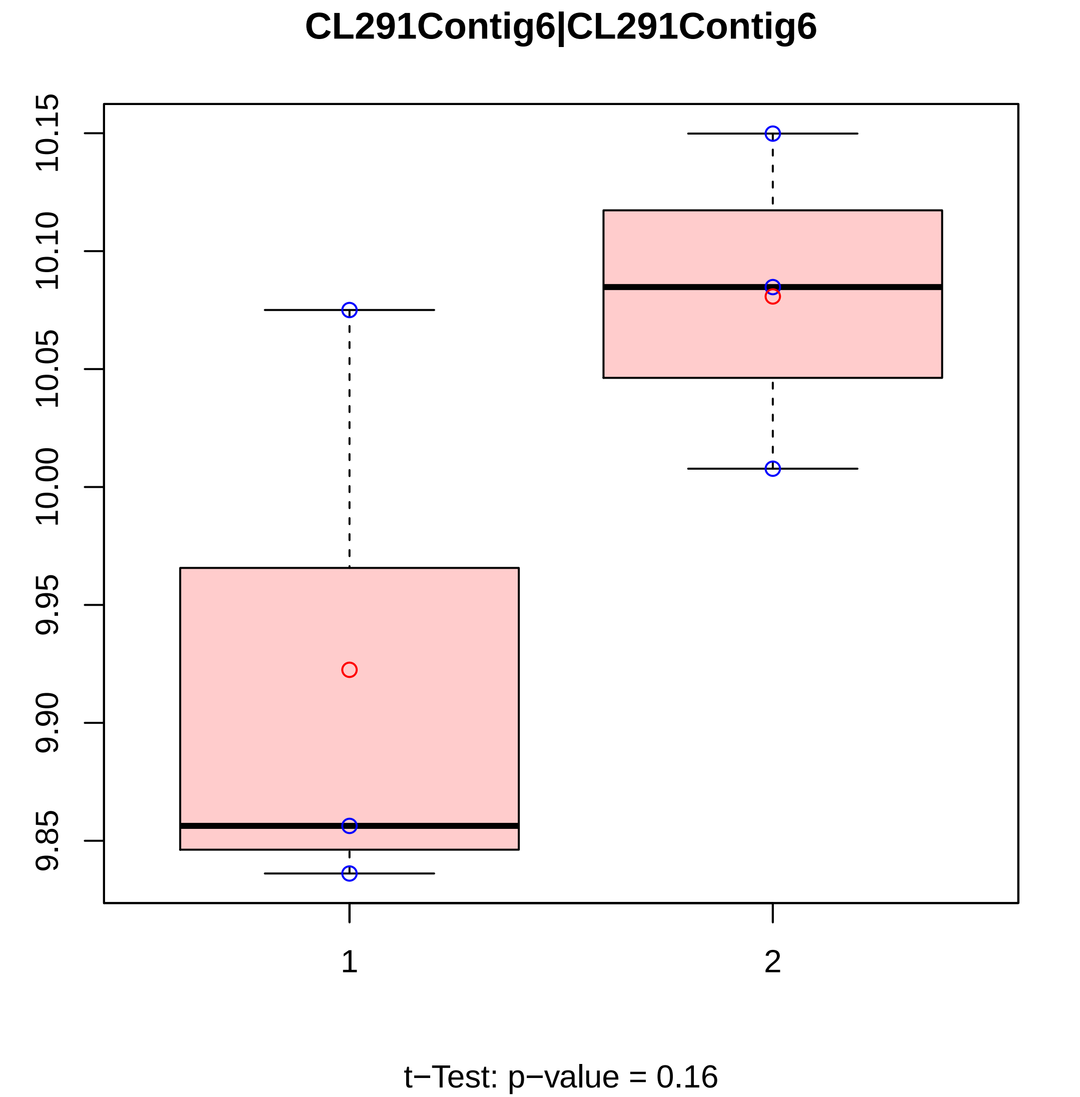

CL291Contig6|CL291Contig6
10.15
10.10
10.05
10.00
9.95
9.90
9.85
1
2
t−Test: p−value = 0.16

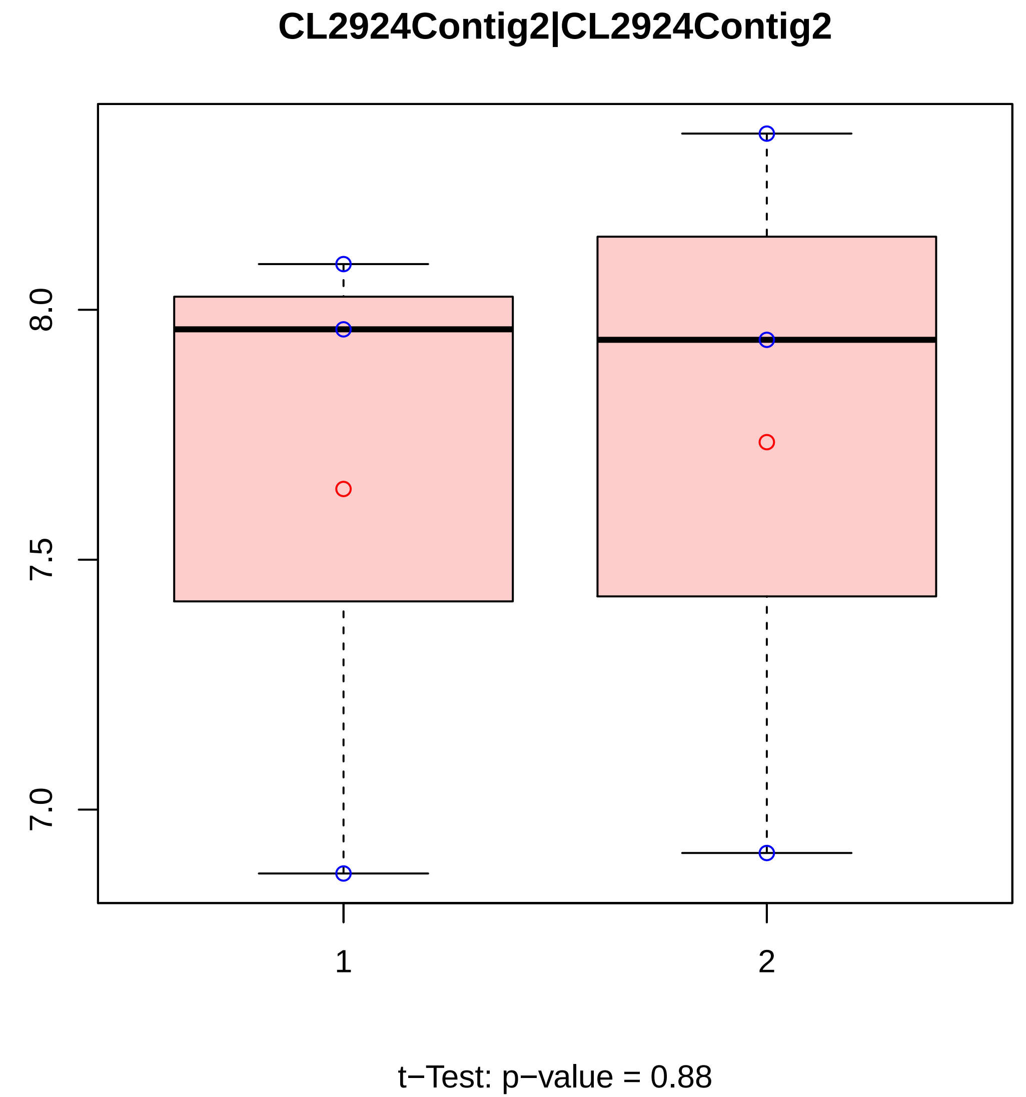

CL2924Contig2|CL2924Contig2
8.0
7.5
7.0
1
2
t−Test: p−value = 0.88

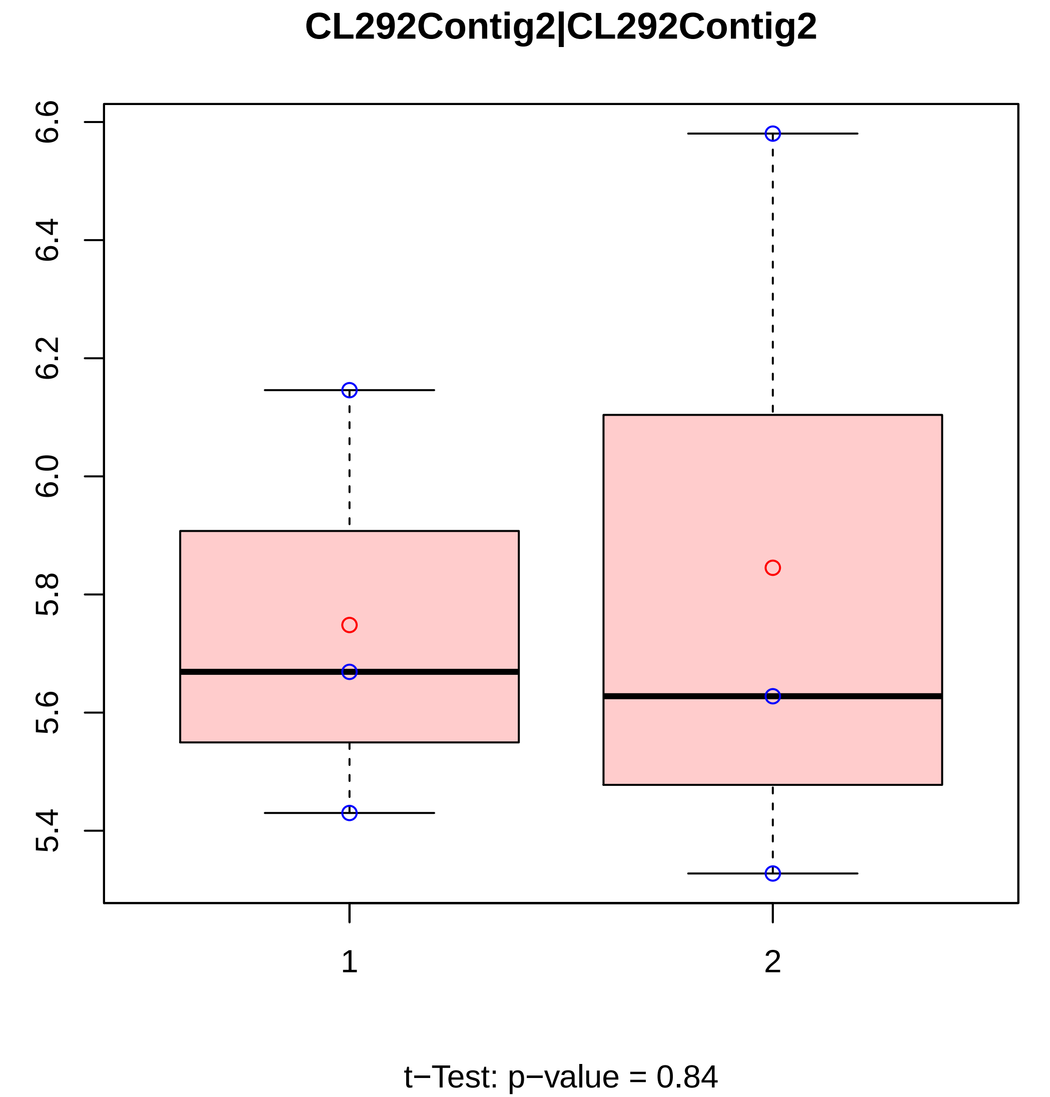
CL292Contig2|CL292Contig2
6.6
6.4
6.2
6.0
5.8
5.6
5.4
1
2
t−Test: p−value = 0.84

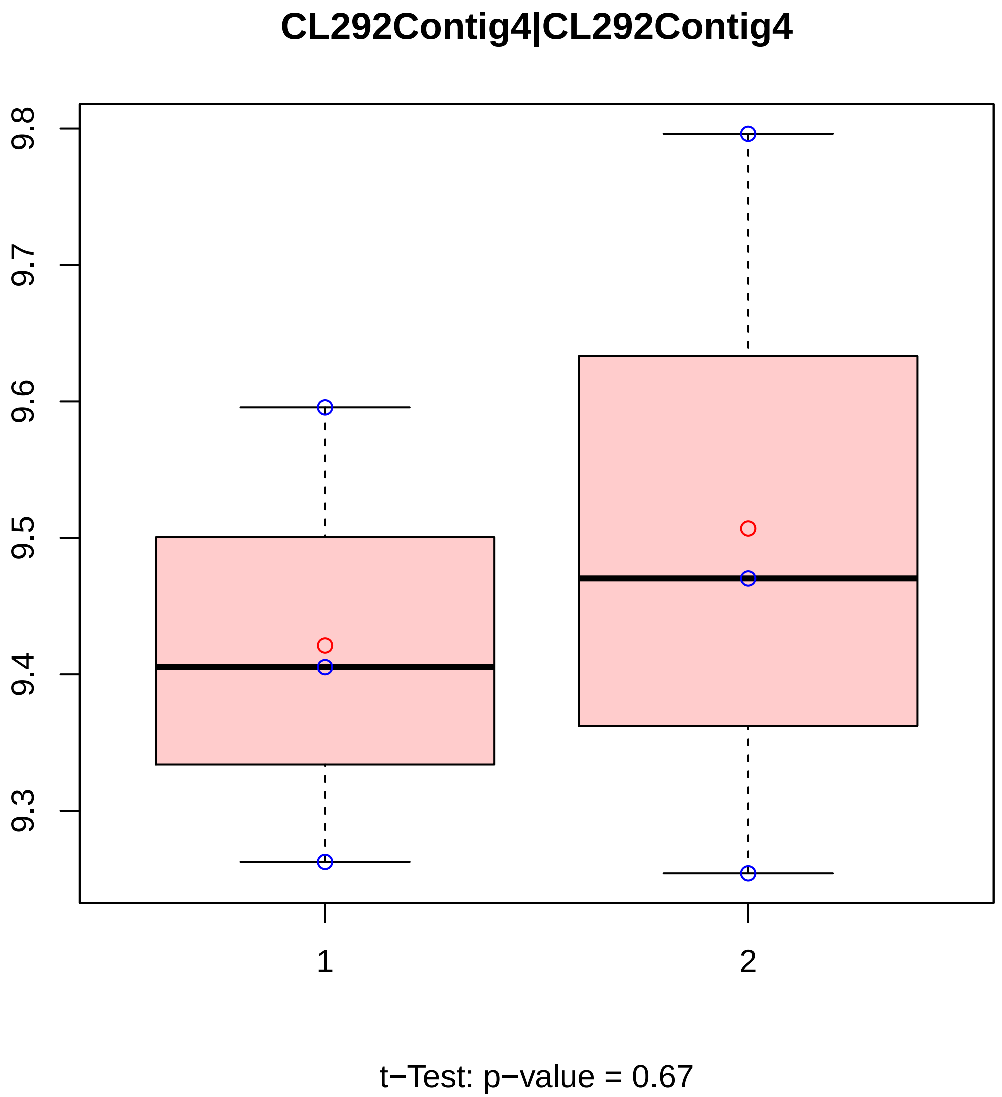

CL292Contig4|CL292Contig4
9.3
9.4
9.5
9.6
9.7
9.8
1
2
t−Test: p−value = 0.67

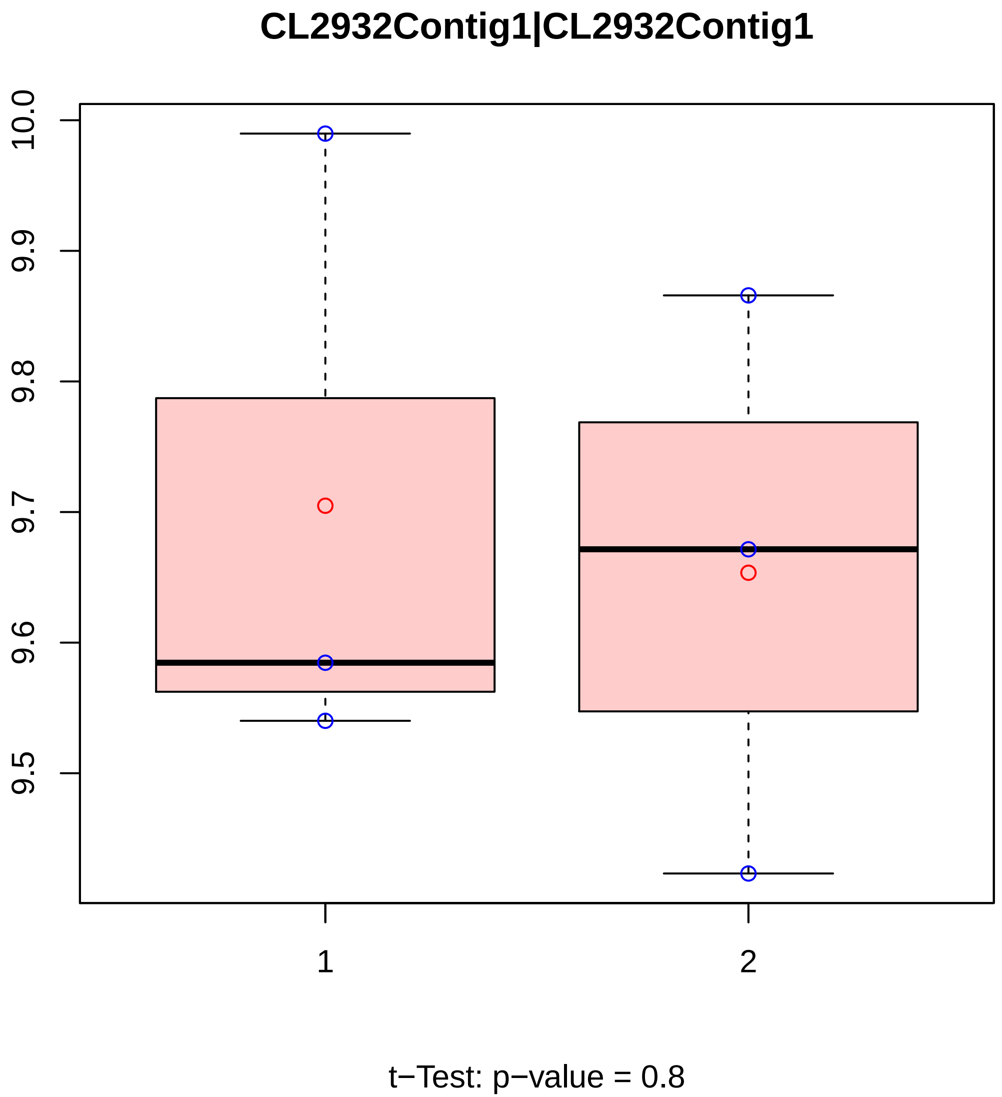

CL2932Contig1|CL2932Contig1
10.0
9.9
9.8
9.7
9.6
9.5
1
2
t−Test: p−value = 0.8

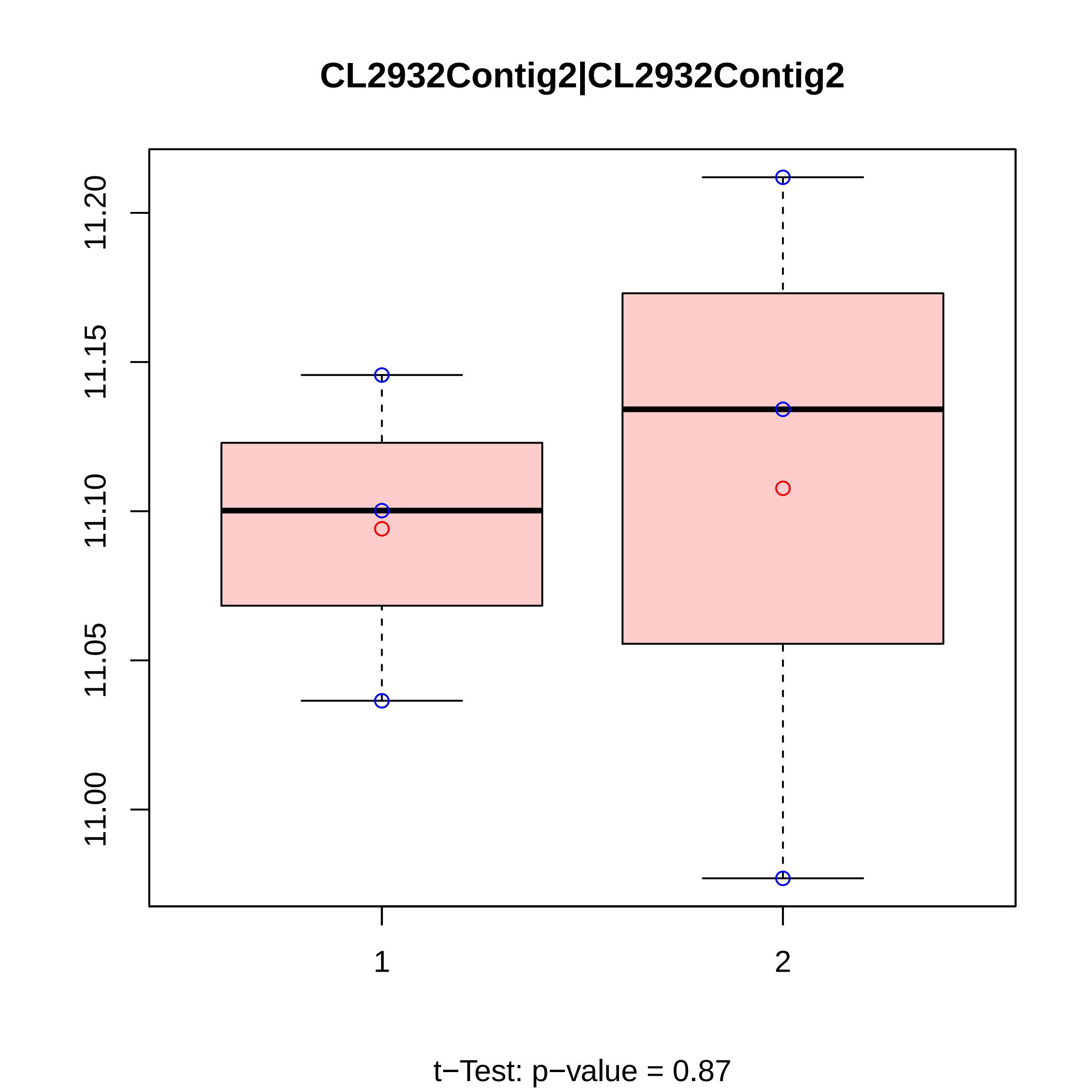
CL2932Contig2|CL2932Contig2
11.00
11.05
11.10
11.15
11.20
1
2
t−Test: p−value = 0.87

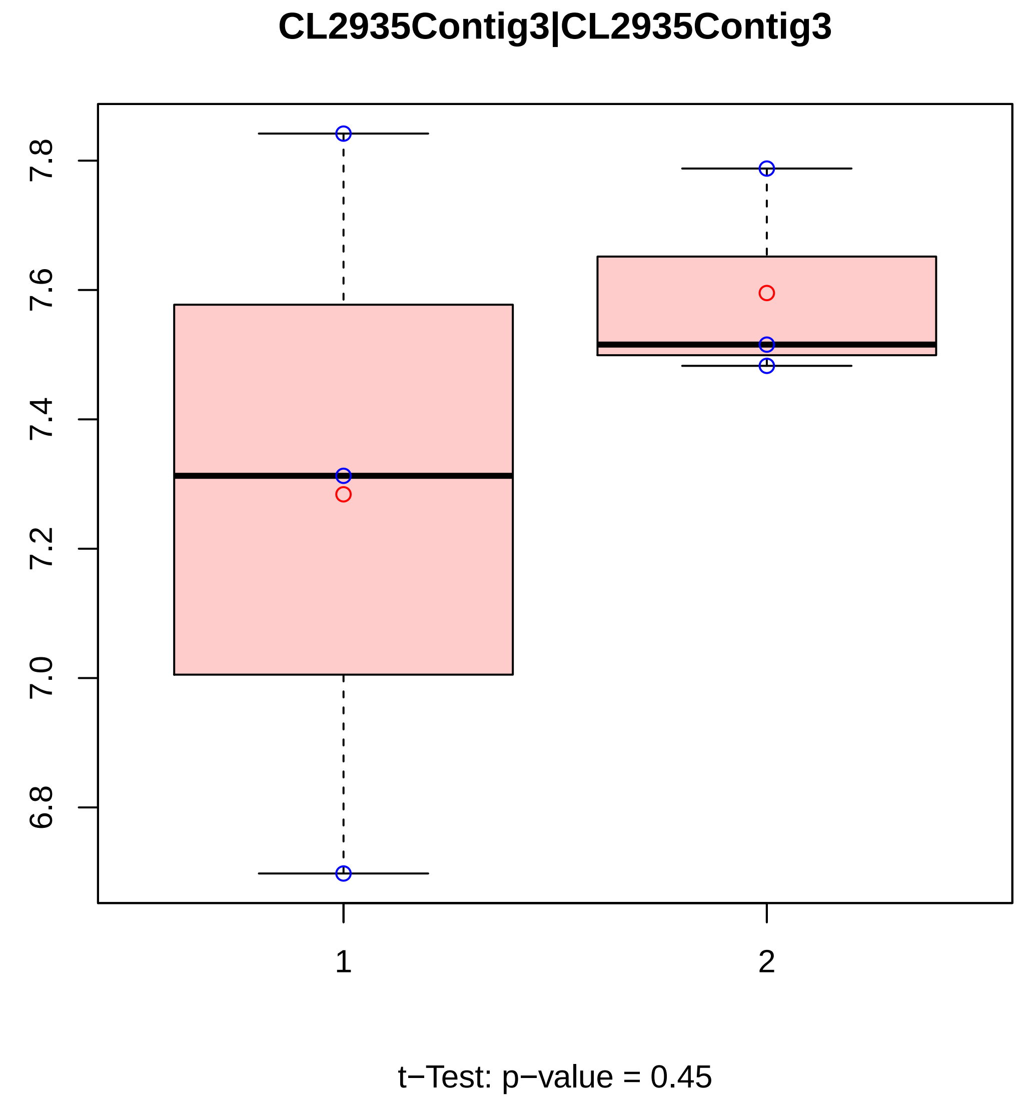
CL2935Contig3|CL2935Contig3
7.8
7.6
7.4
7.2
7.0
6.8
1
2
t−Test: p−value = 0.45

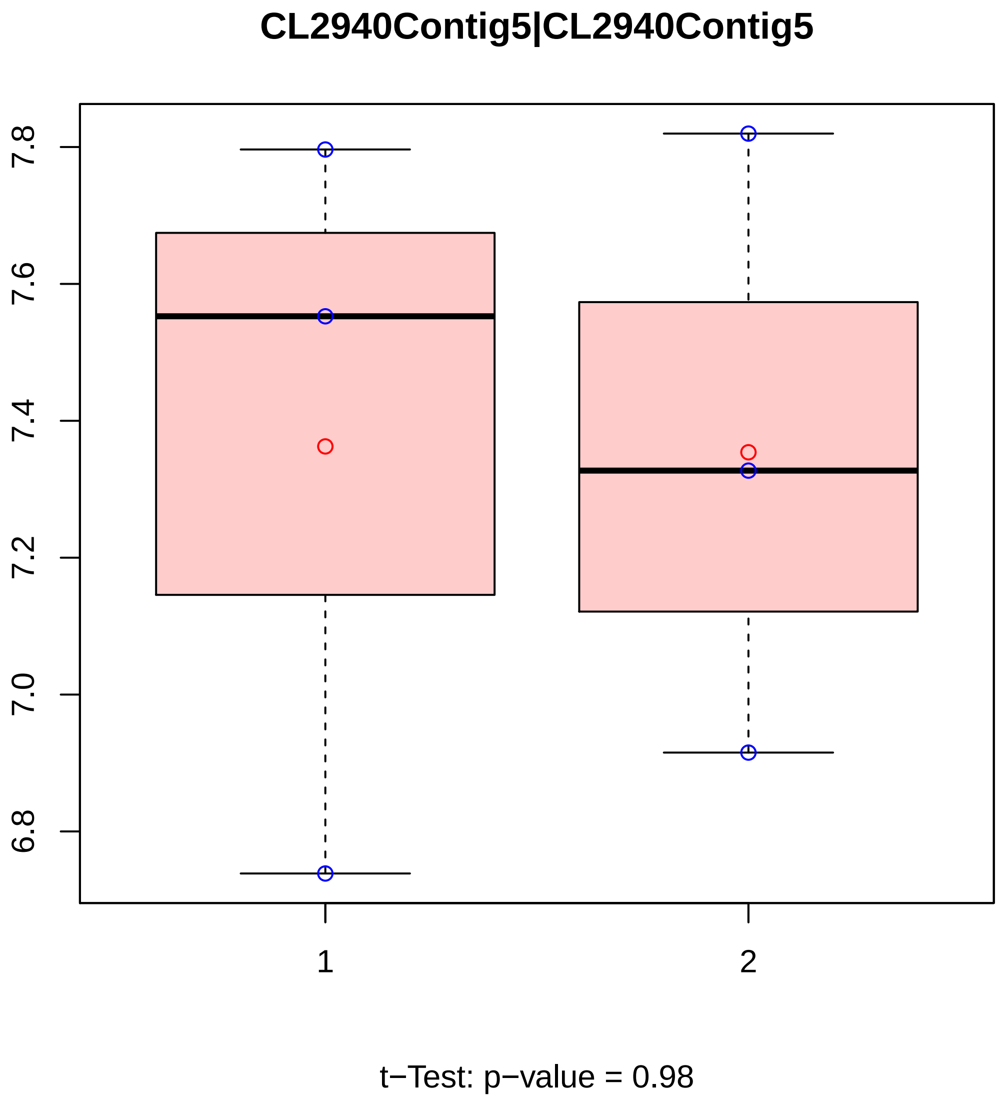
CL2940Contig5|CL2940Contig5
7.8
7.6
7.4
7.2
7.0
6.8
1
2
t−Test: p−value = 0.98

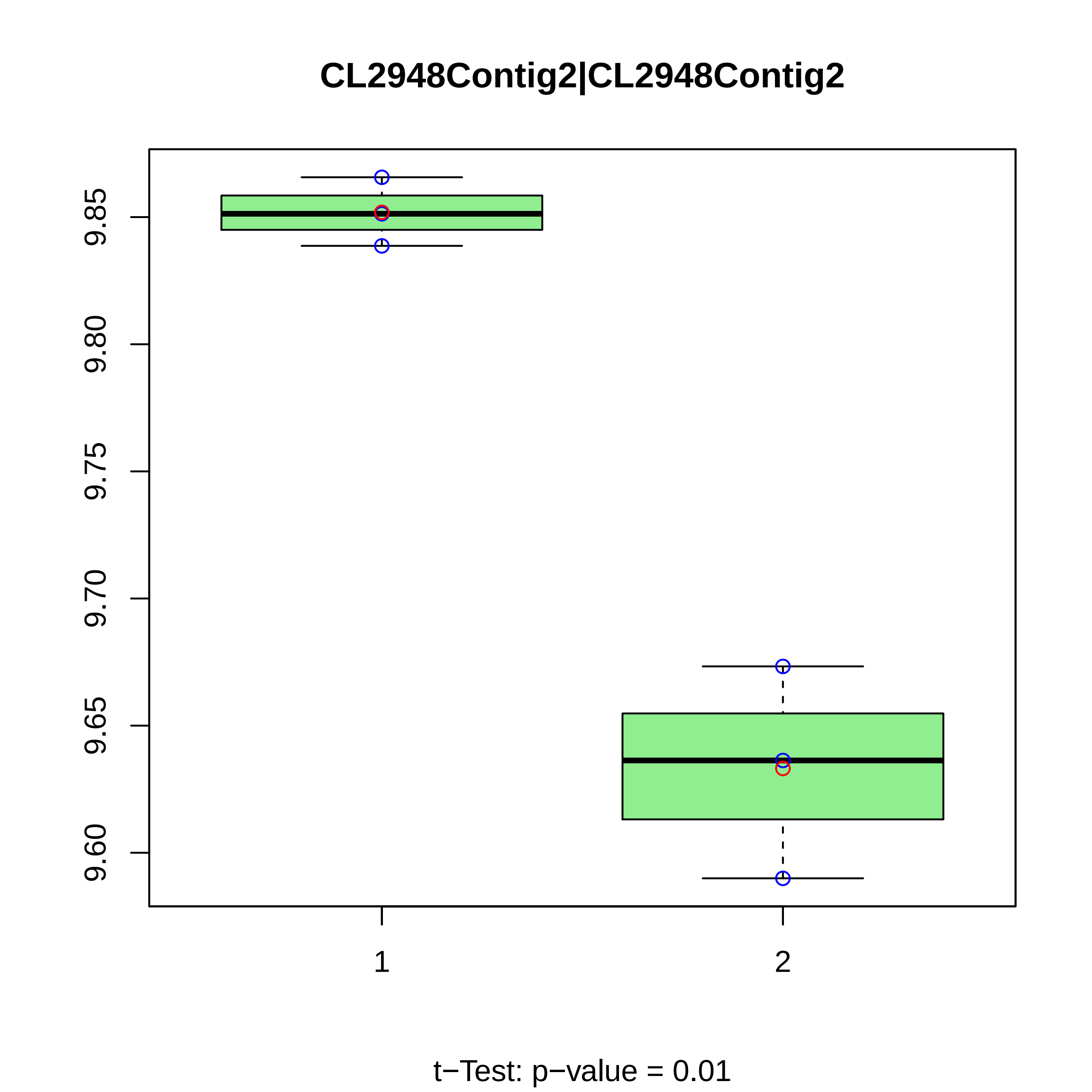

CL2948Contig2|CL2948Contig2
9.60
9.65
9.70
9.75
9.80
9.85
1
2
t−Test: p−value = 0.01

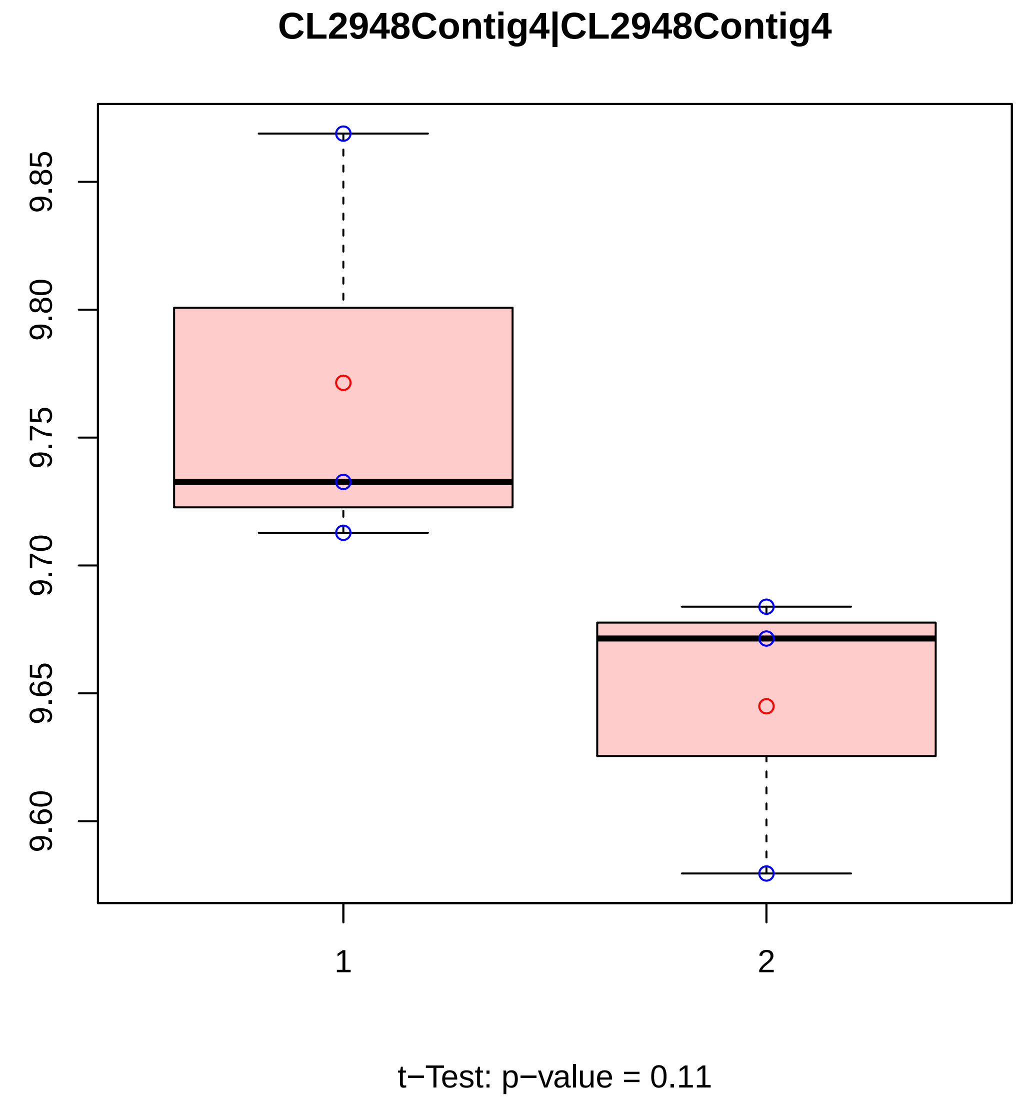
CL2948Contig4|CL2948Contig4
9.60
9.65
9.70
9.75
9.80
9.85
1
2
t−Test: p−value = 0.11

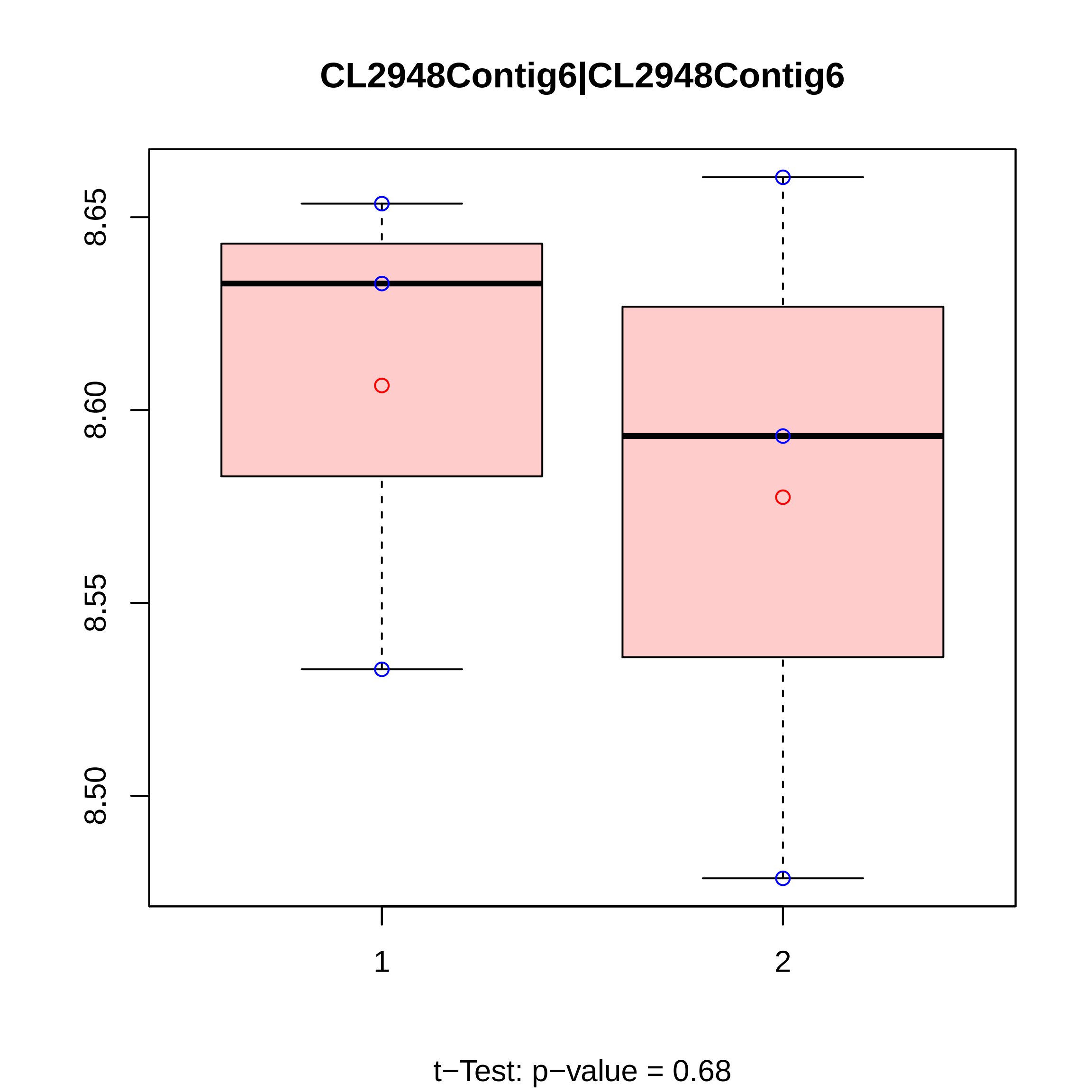

CL2948Contig6|CL2948Contig6
8.50
8.55
8.60
8.65
1
2
t−Test: p−value = 0.68

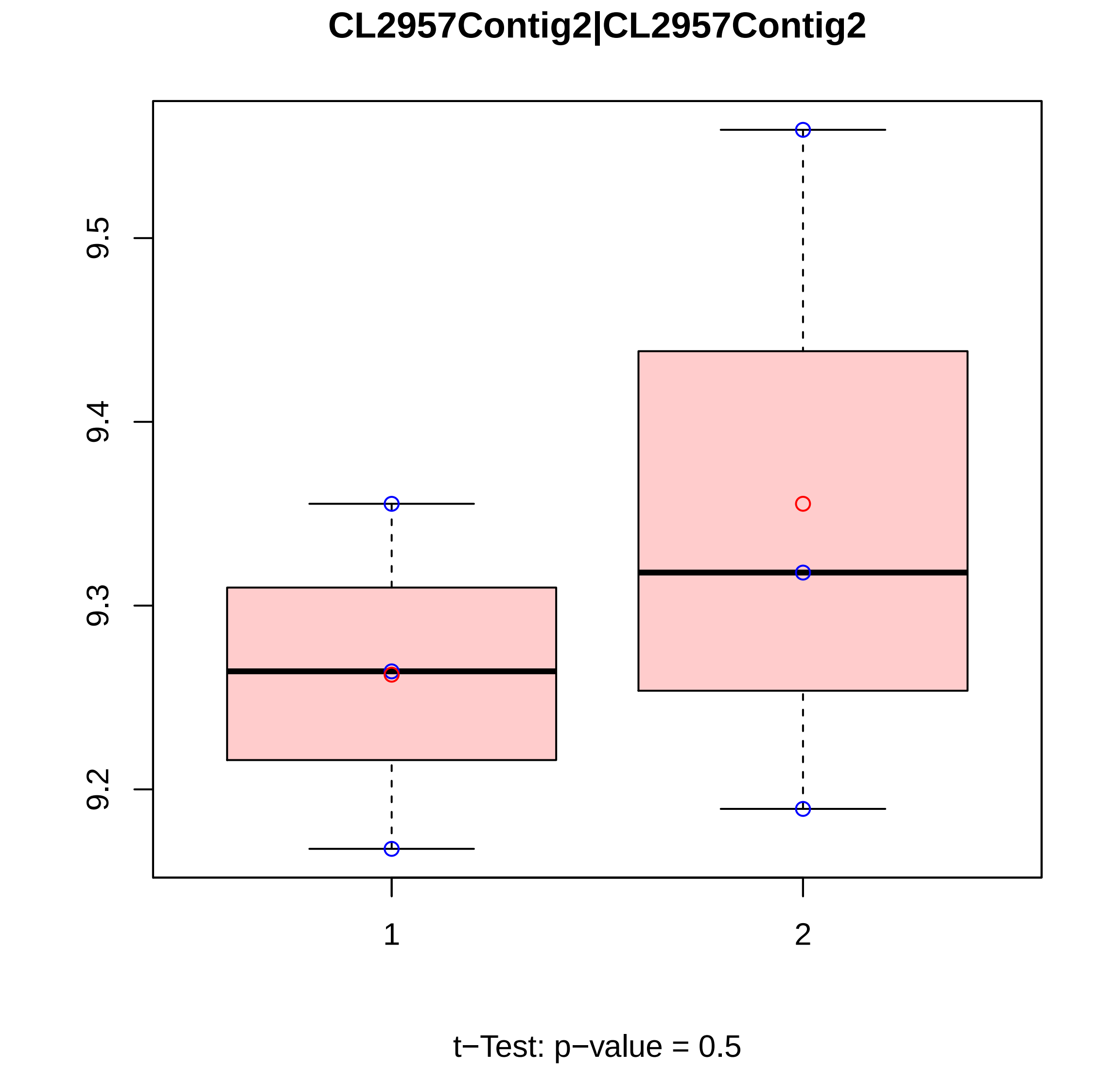

CL2957Contig2|CL2957Contig2
9.2
9.3
9.4
9.5
1
2
t−Test: p−value = 0.5

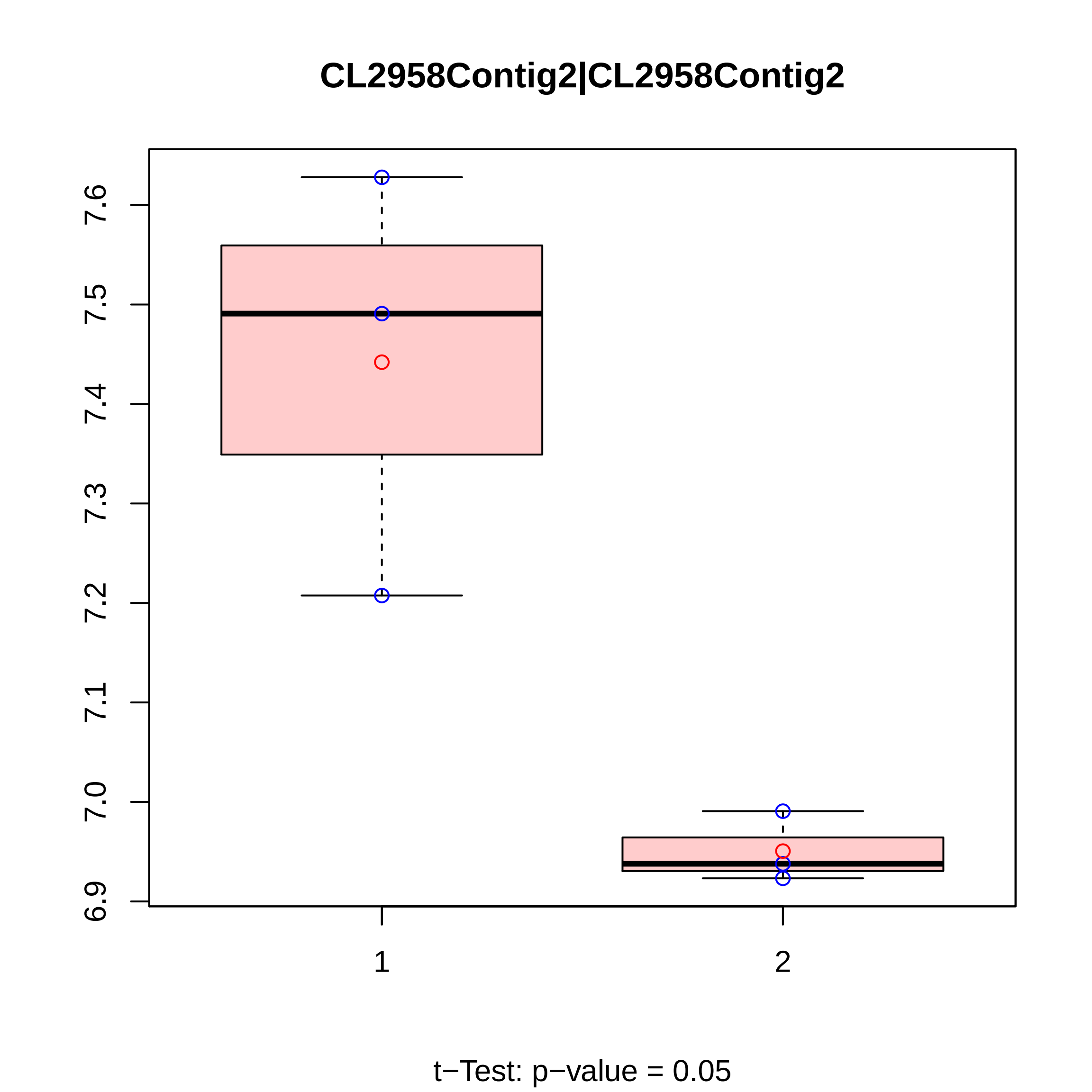
CL2958Contig2|CL2958Contig2
7.6
7.5
7.4
7.3
7.2
7.1
7.0
6.9
1
2
t−Test: p−value = 0.05

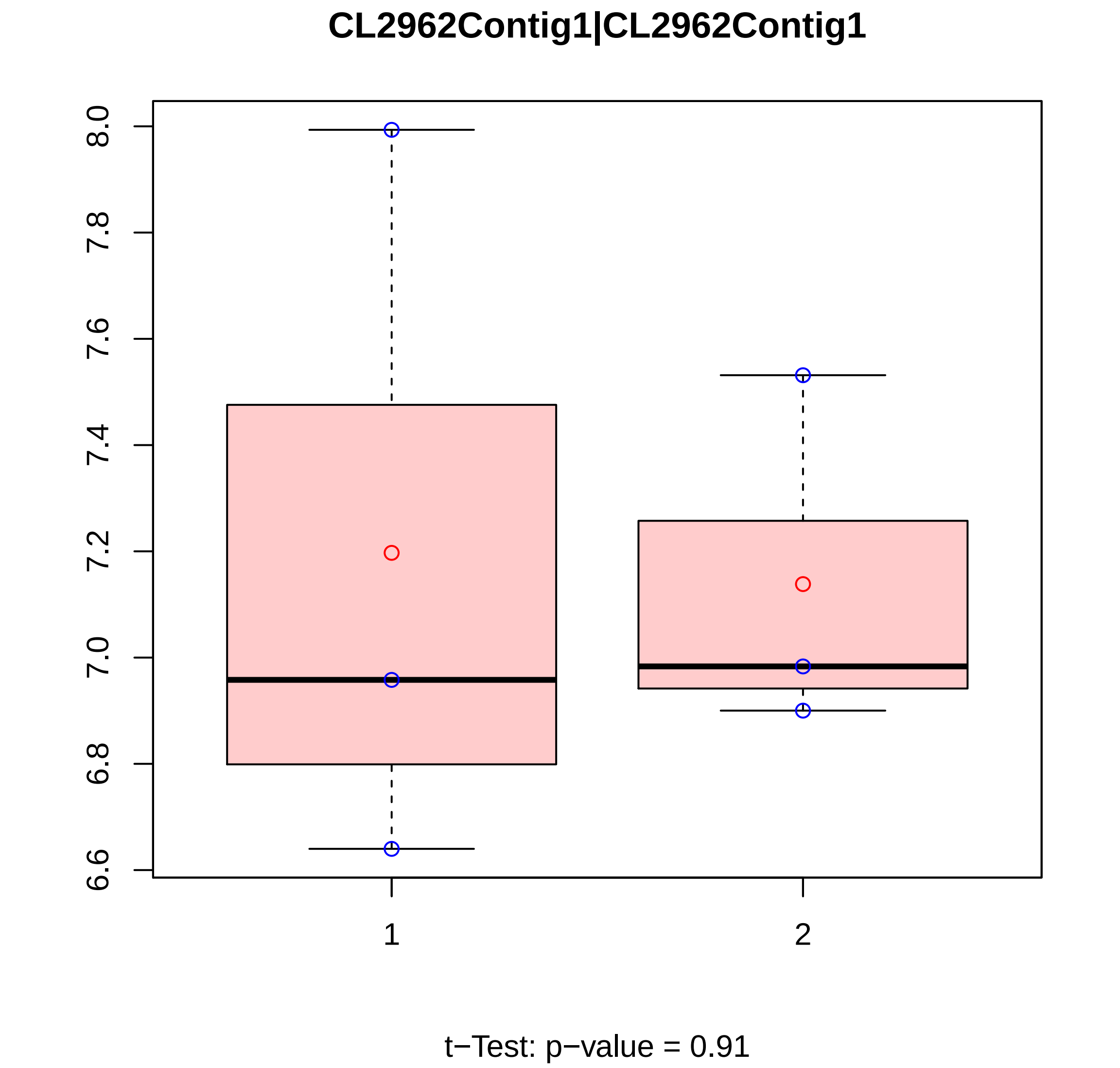
CL2962Contig1|CL2962Contig1
8.0
7.8
7.6
7.4
7.2
7.0
6.8
6.6
1
2
t−Test: p−value = 0.91

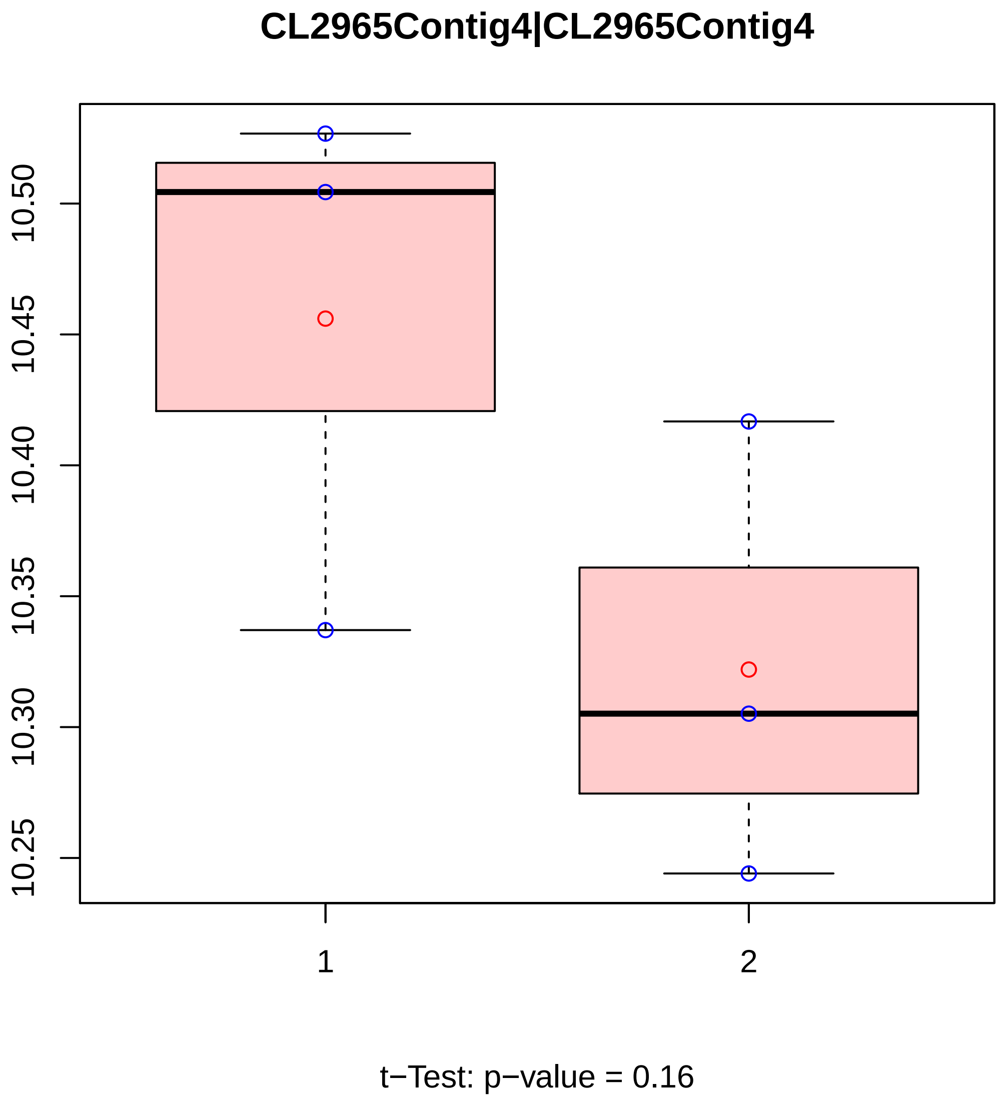
CL2965Contig4|CL2965Contig4
10.25
10.30
10.35
10.40
10.45
10.50
1
2
t−Test: p−value = 0.16

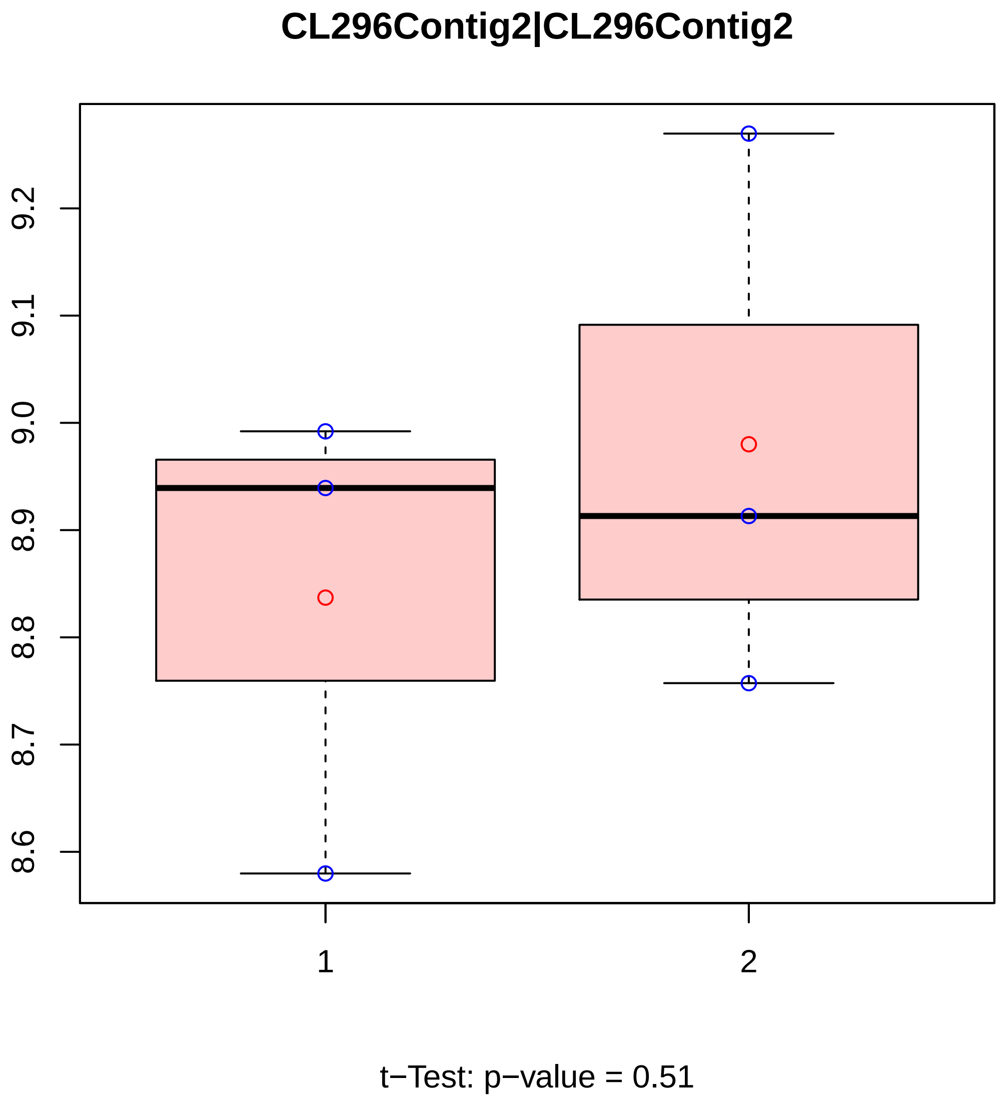

CL296Contig2|CL296Contig2
9.2
9.1
9.0
8.9
8.8
8.7
8.6
1
2
t−Test: p−value = 0.51

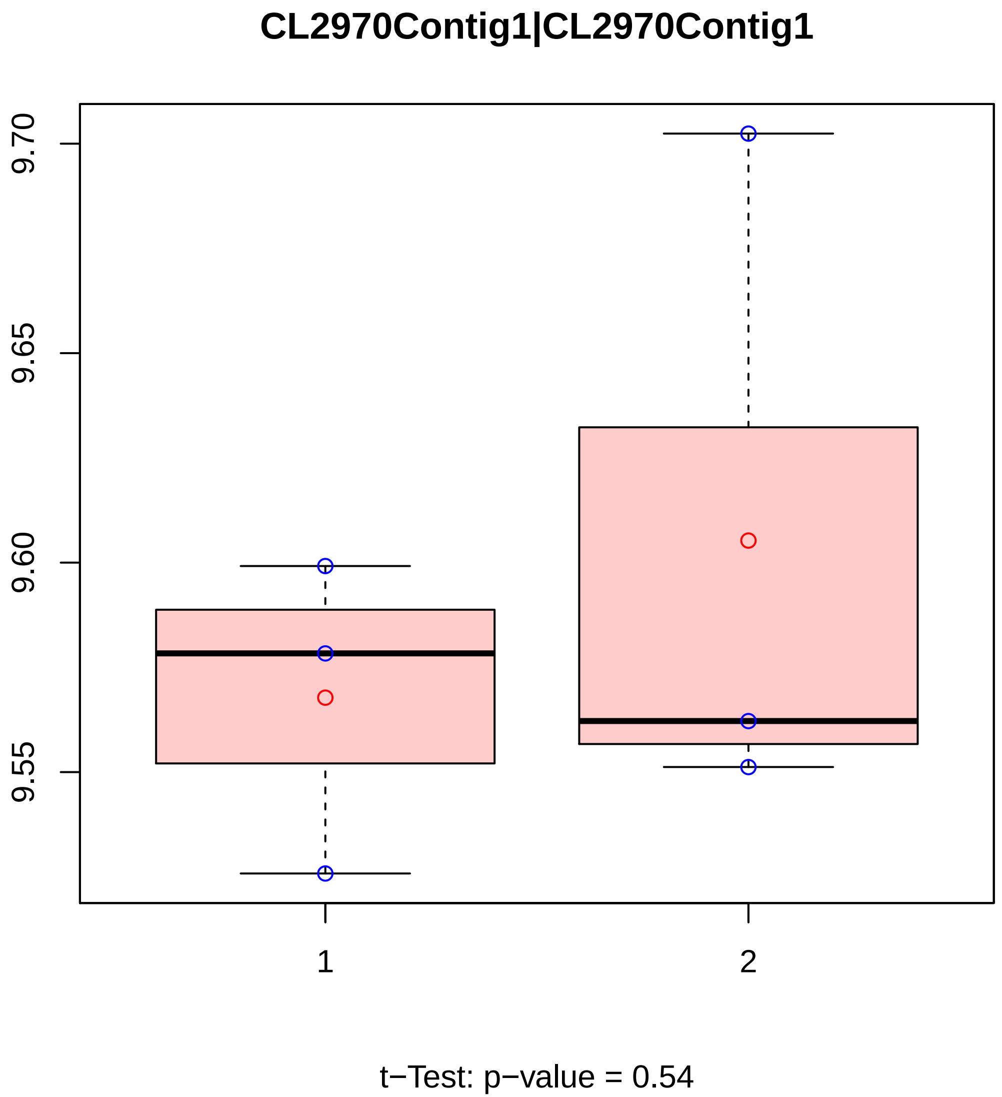
CL2970Contig1|CL2970Contig1
9.70
9.65
9.60
9.55
1
2
t−Test: p−value = 0.54

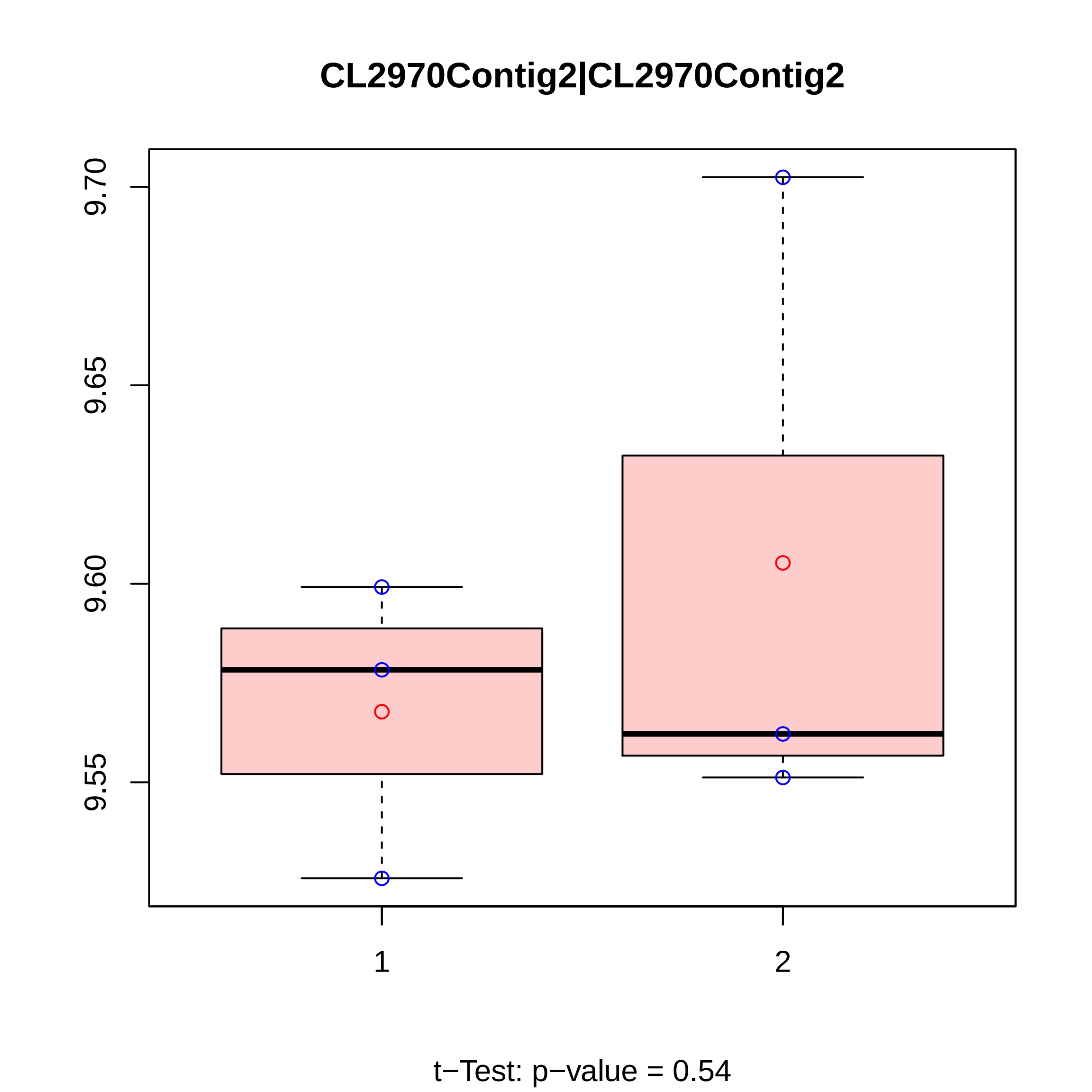

CL2970Contig2|CL2970Contig2
9.70
9.65
9.60
9.55
1
2
t−Test: p−value = 0.54

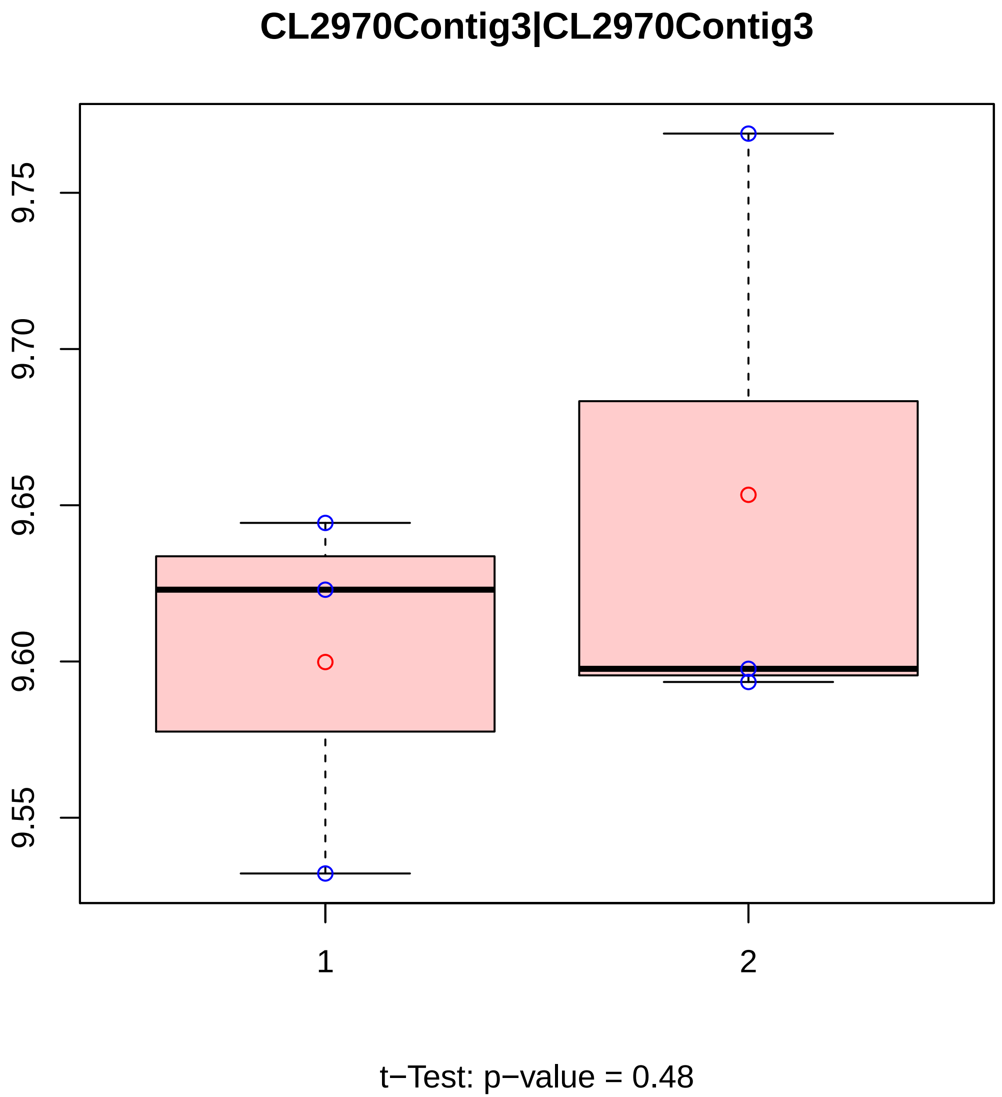

CL2970Contig3|CL2970Contig3
9.55
9.60
9.65
9.70
9.75
1
2
t−Test: p−value = 0.48

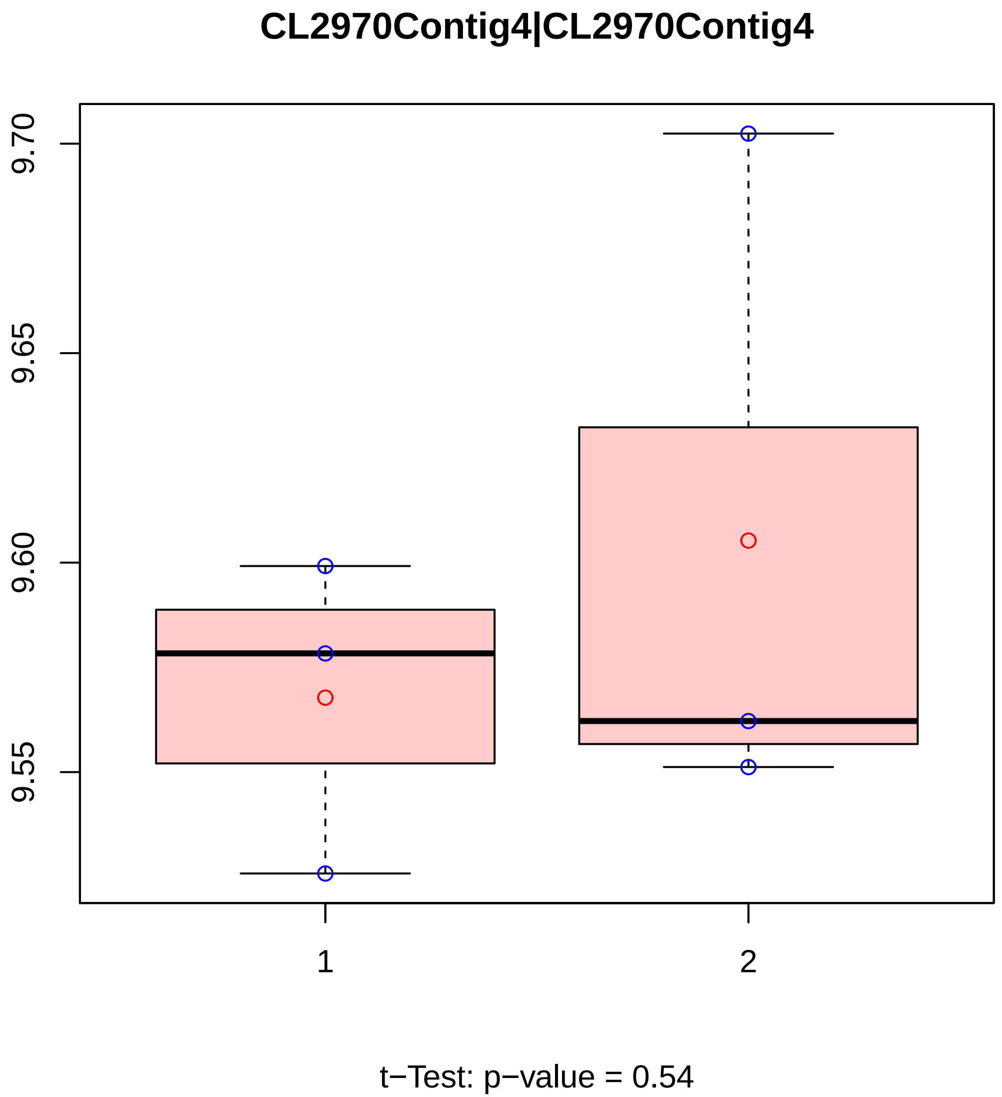

CL2970Contig4|CL2970Contig4
9.55
9.60
9.65
9.70
1
2
t−Test: p−value = 0.54

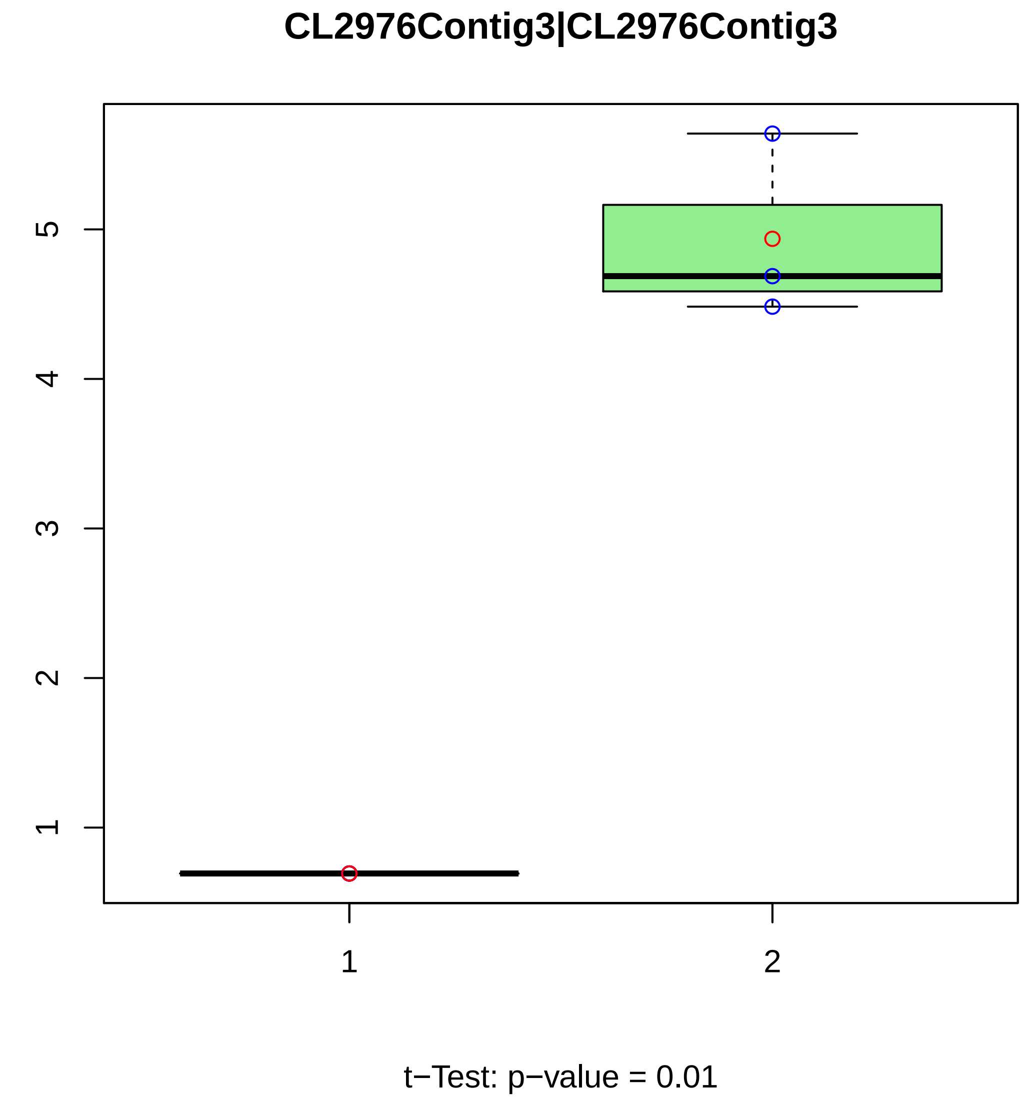

CL2976Contig3|CL2976Contig3
1
2
3
4
5
1
2
t−Test: p−value = 0.01

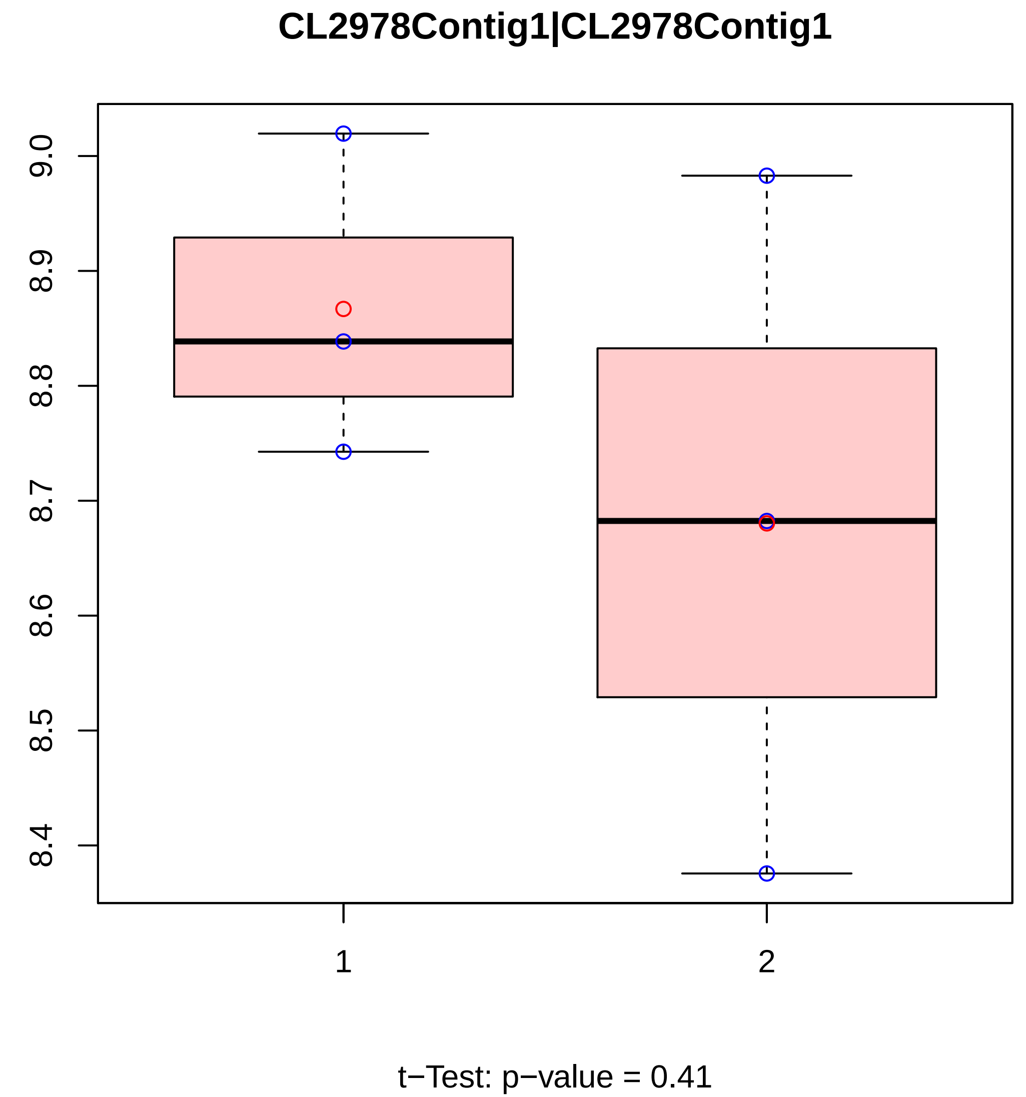
CL2978Contig1|CL2978Contig1
9.0
8.9
8.8
8.7
8.6
8.5
8.4
1
2
t−Test: p−value = 0.41

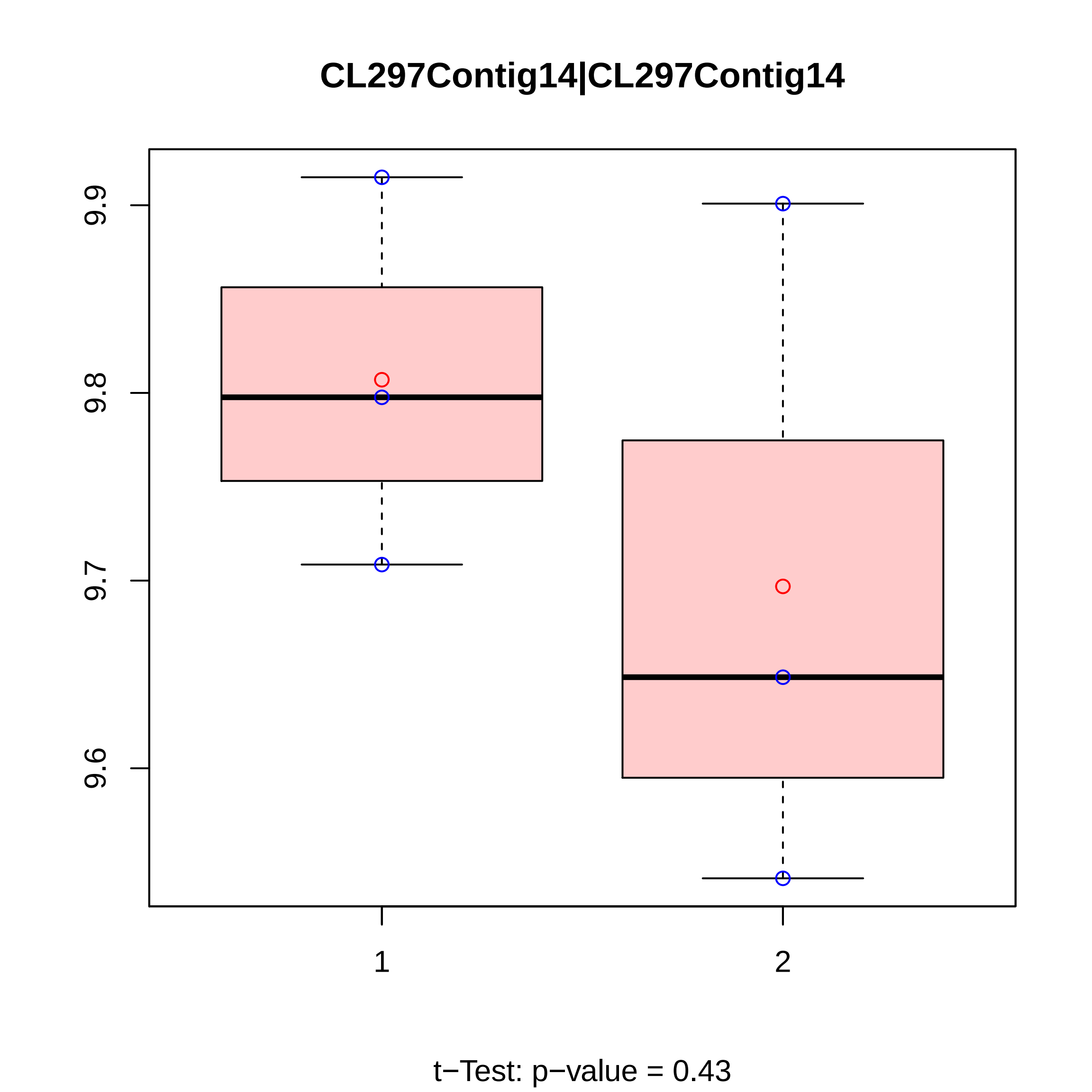

CL297Contig14|CL297Contig14
9.9
9.8
9.7
9.6
1
2
t−Test: p−value = 0.43

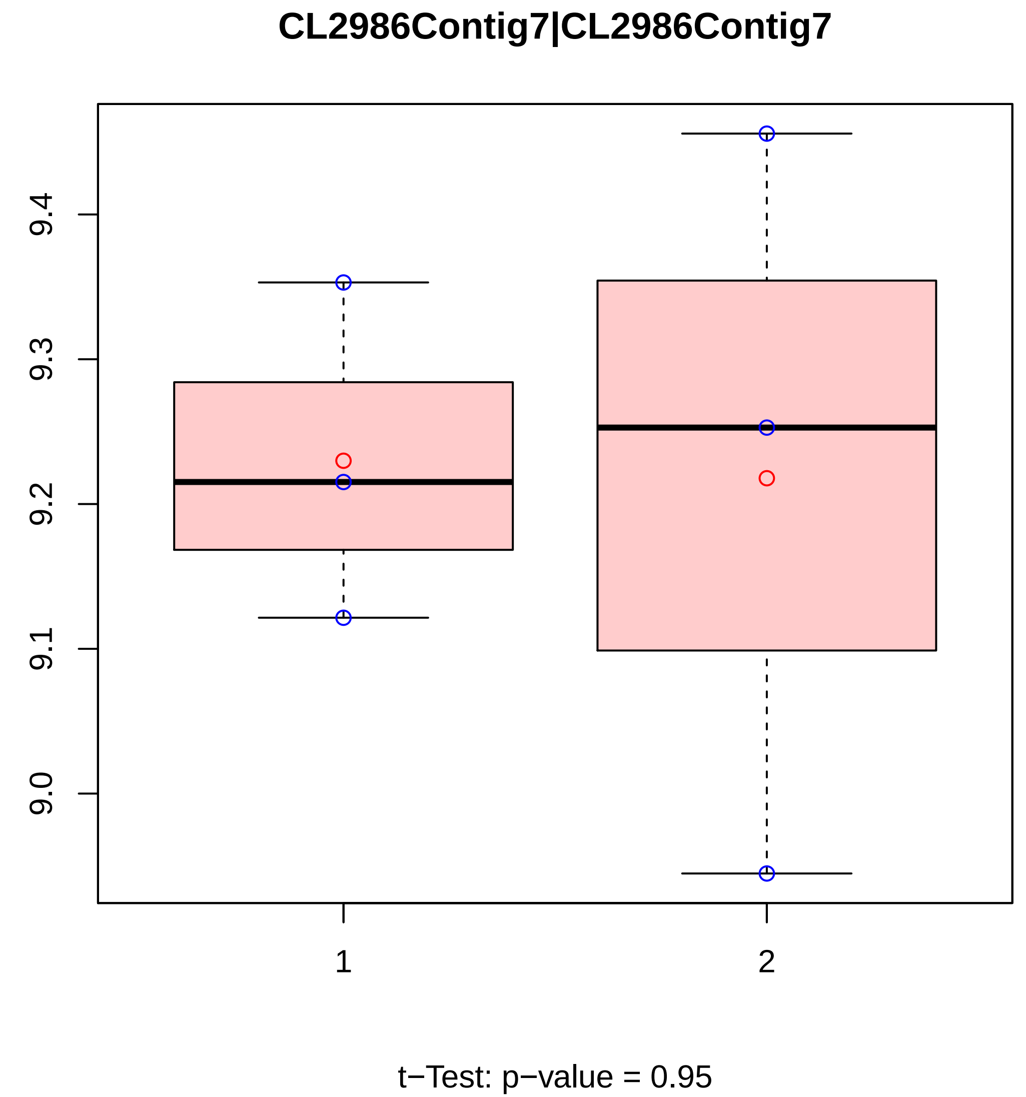

CL2986Contig7|CL2986Contig7
9.0
9.1
9.2
9.3
9.4
1
2
t−Test: p−value = 0.95

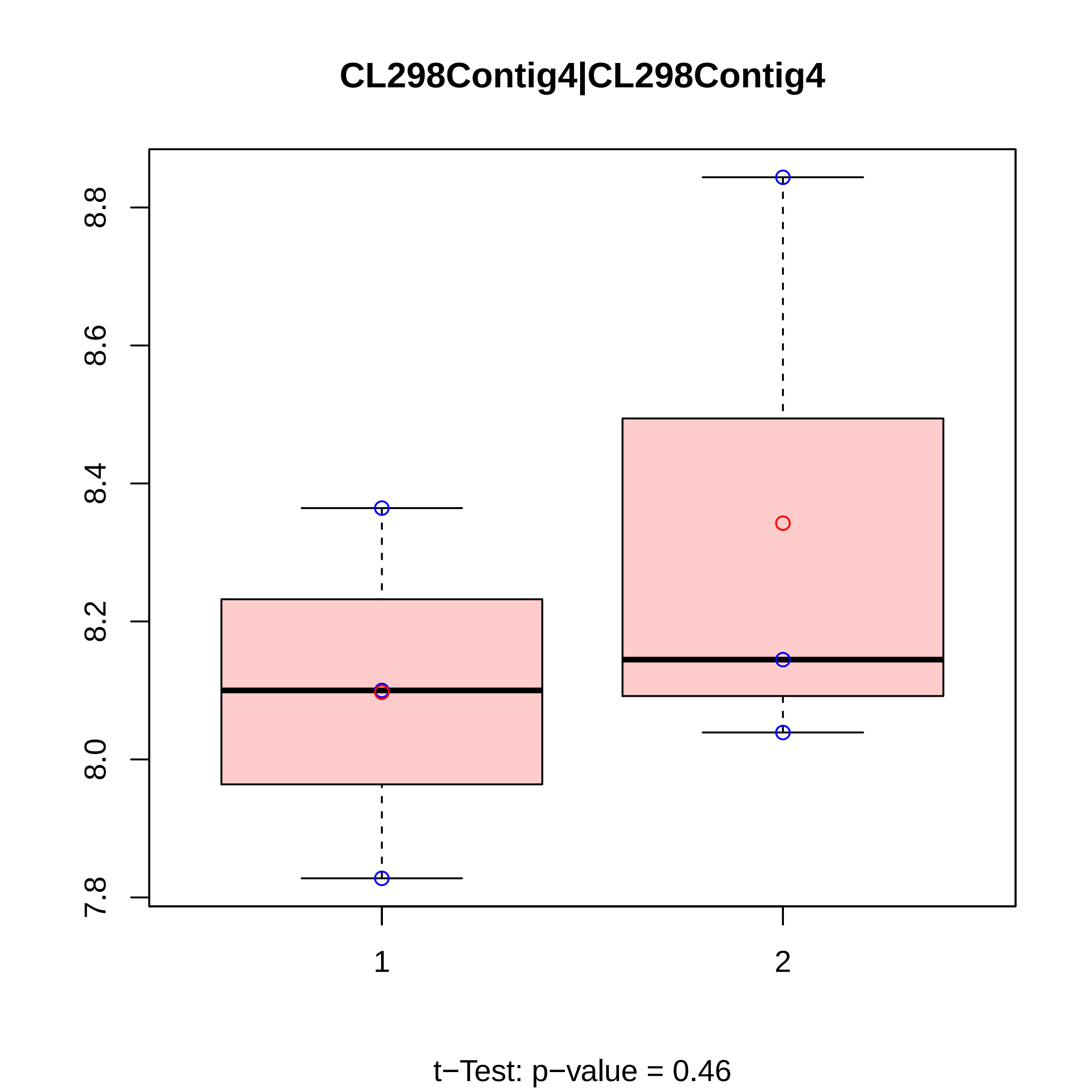
CL298Contig4|CL298Contig4
8.8
8.6
8.4
8.2
8.0
7.8
1
2
t−Test: p−value = 0.46

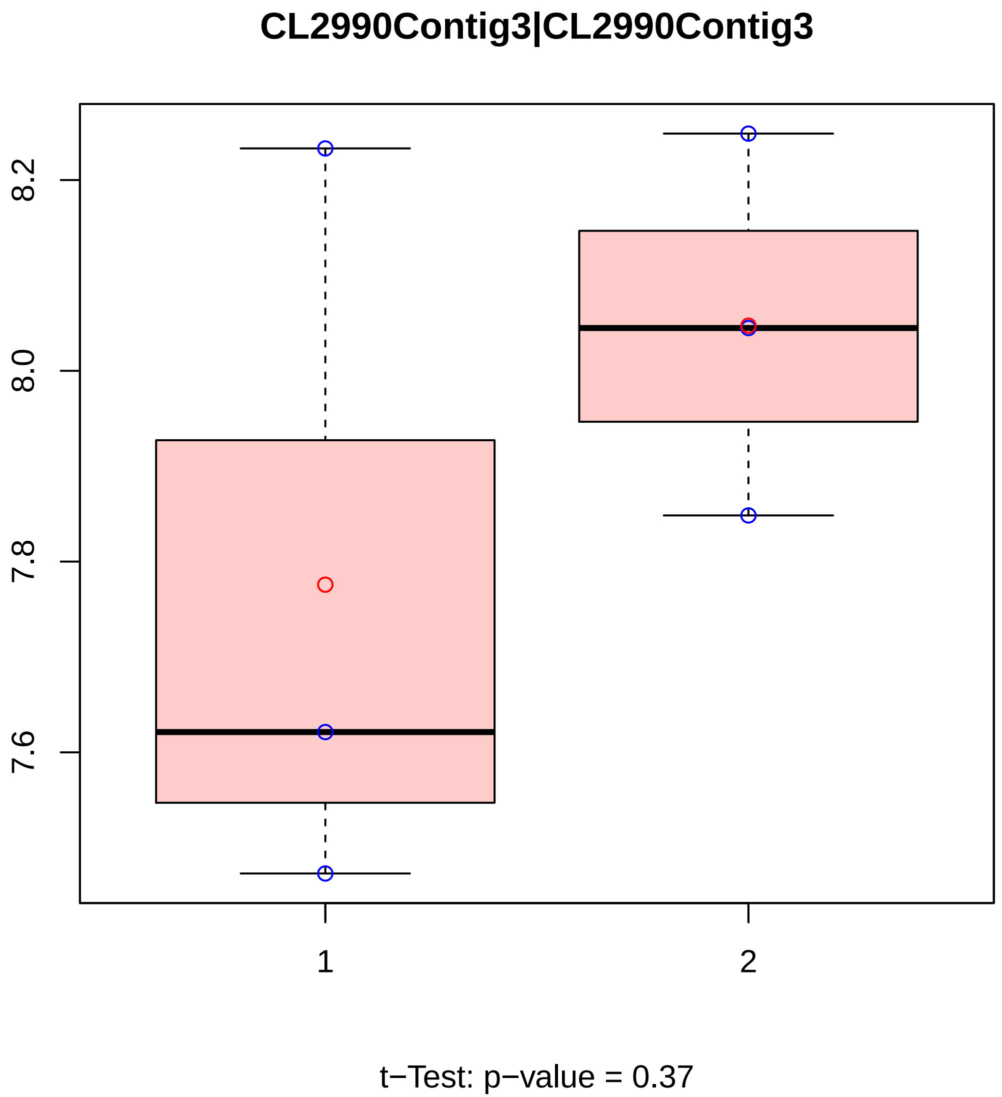
CL2990Contig3|CL2990Contig3
8.2
8.0
7.8
7.6
1
2
t−Test: p−value = 0.37

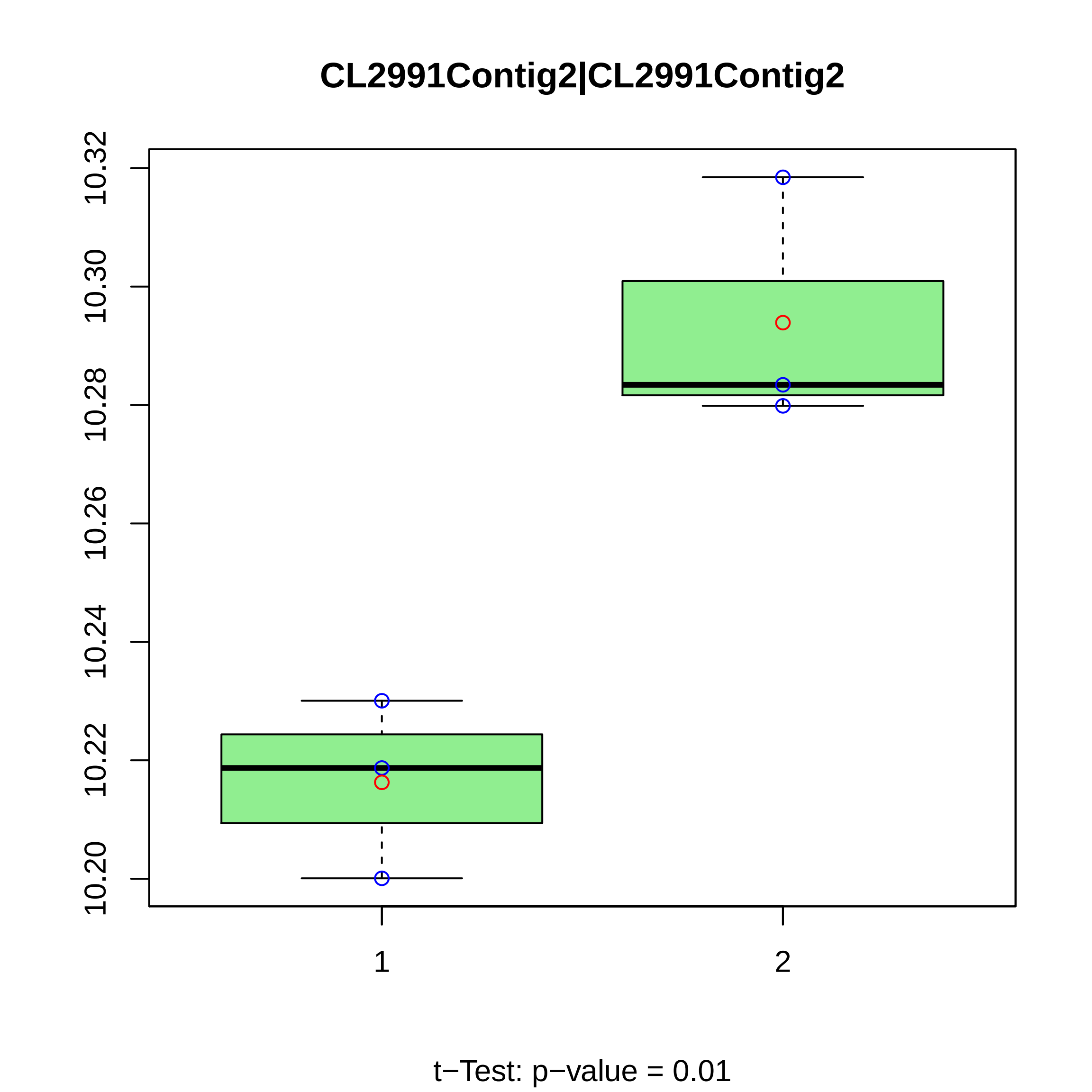

CL2991Contig2|CL2991Contig2
10.20
10.22
10.24
10.26
10.28
10.30
10.32
1
2
t−Test: p−value = 0.01

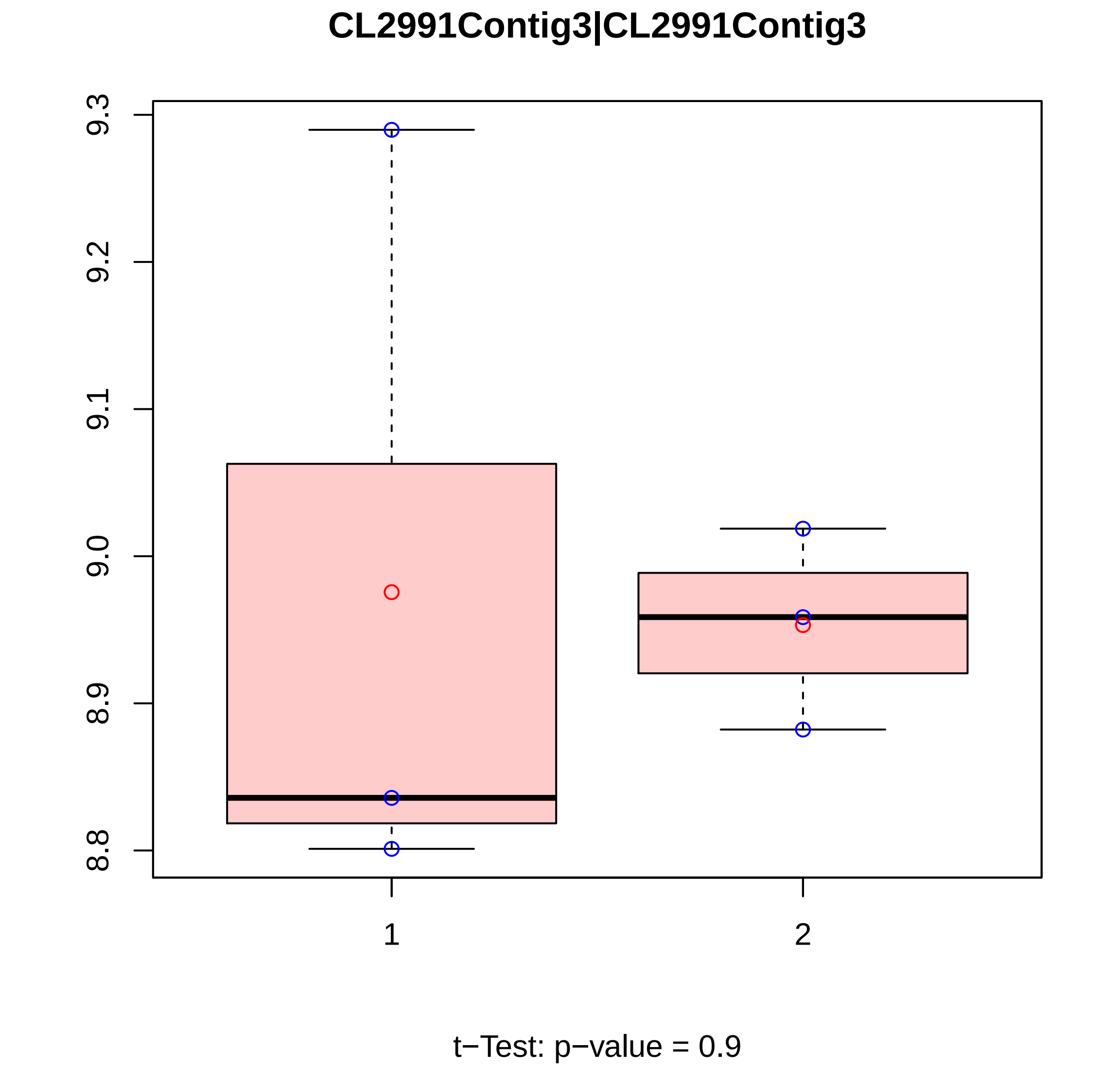
CL2991Contig3|CL2991Contig3
9.3
9.2
9.1
9.0
8.9
8.8
1
2
t−Test: p−value = 0.9

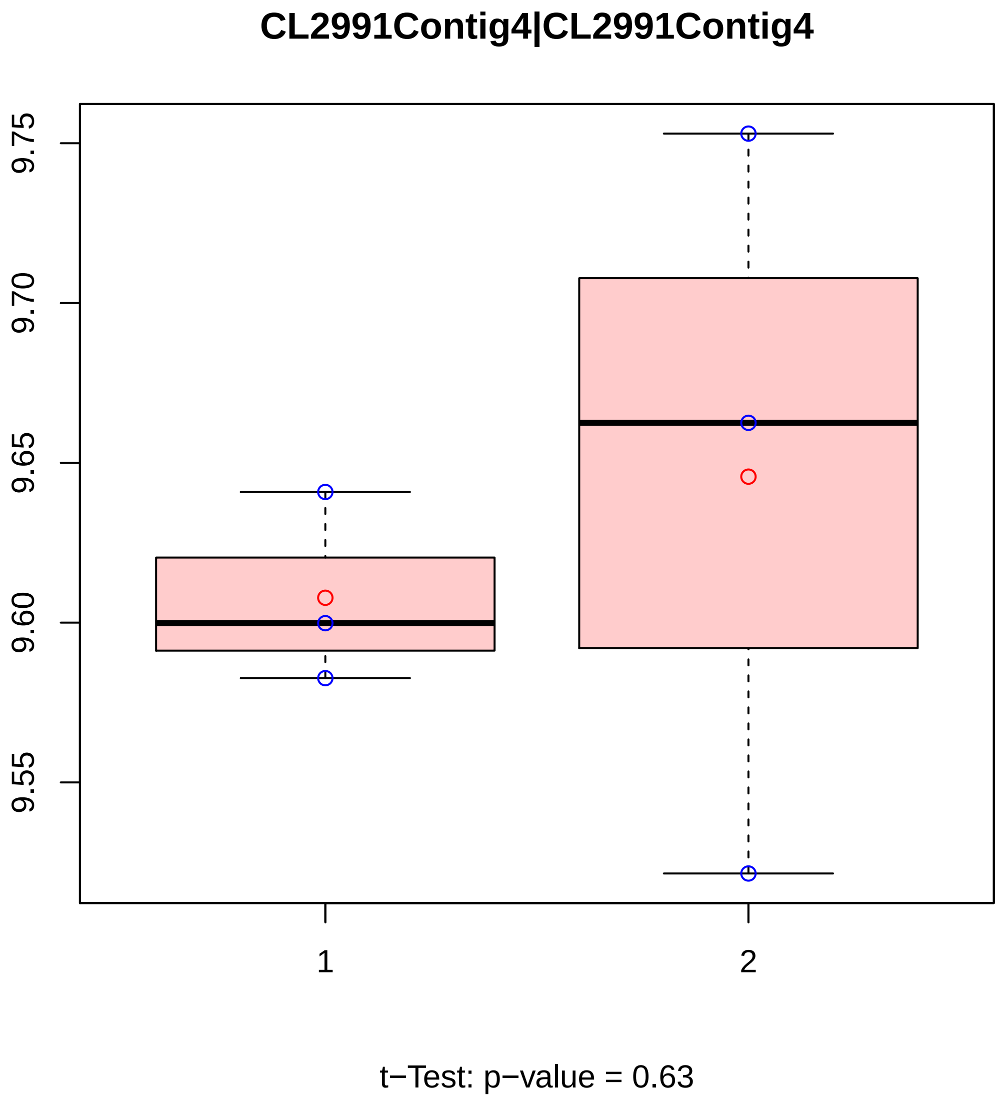
CL2991Contig4|CL2991Contig4
9.55
9.60
9.65
9.70
9.75
1
2
t−Test: p−value = 0.63

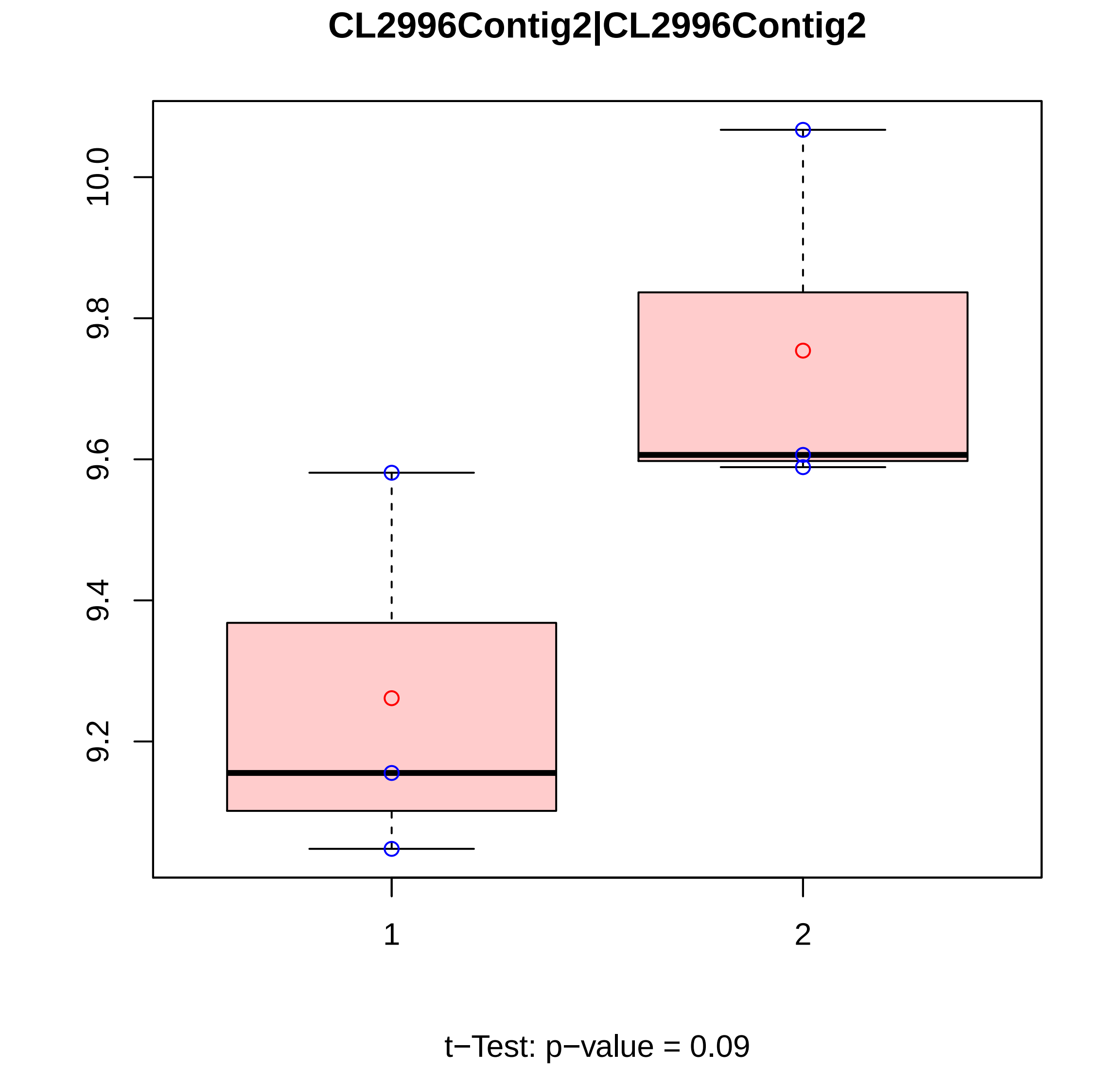
CL2996Contig2|CL2996Contig2
10.0
9.8
9.6
9.4
9.2
1
2
t−Test: p−value = 0.09

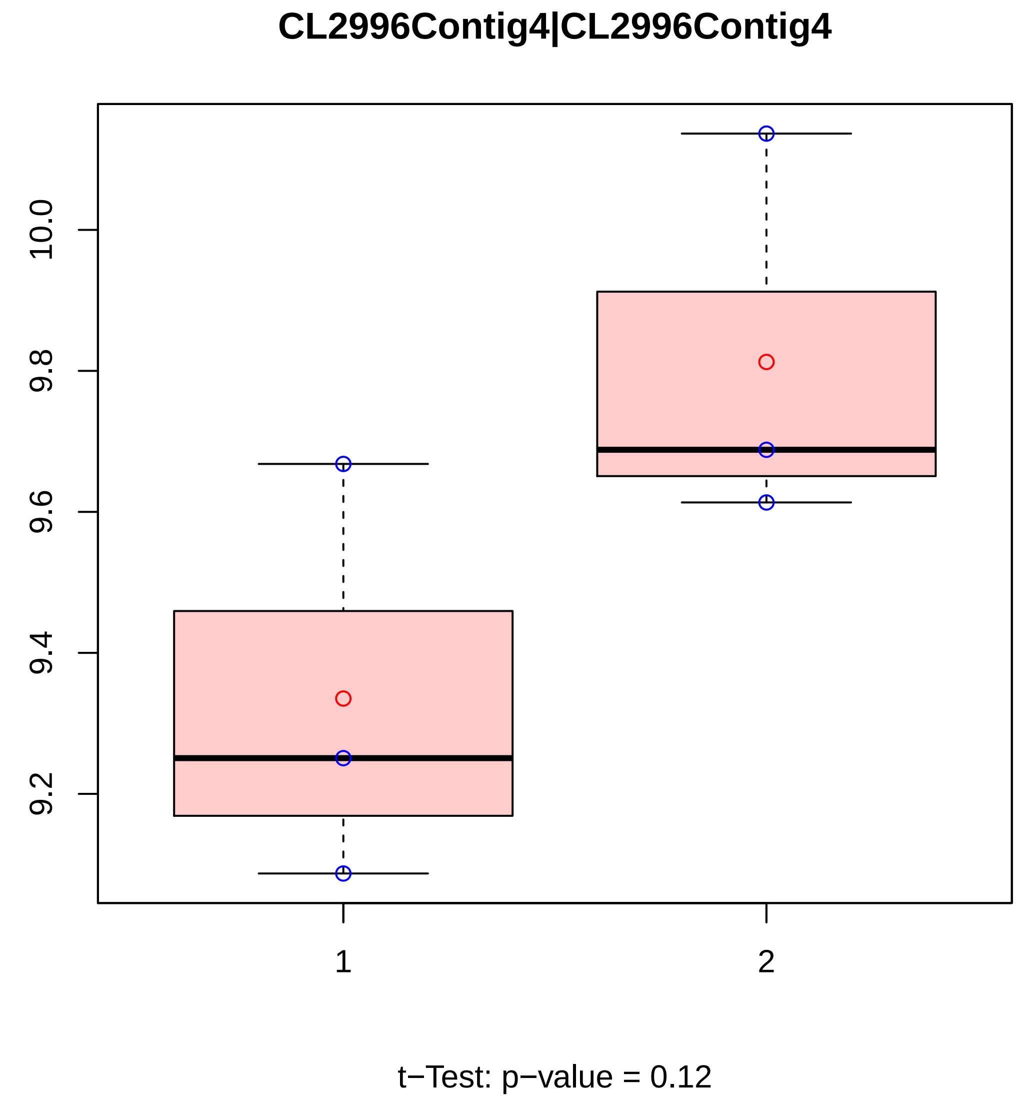

CL2996Contig4|CL2996Contig4
10.0
9.8
9.6
9.4
9.2
1
2
t−Test: p−value = 0.12

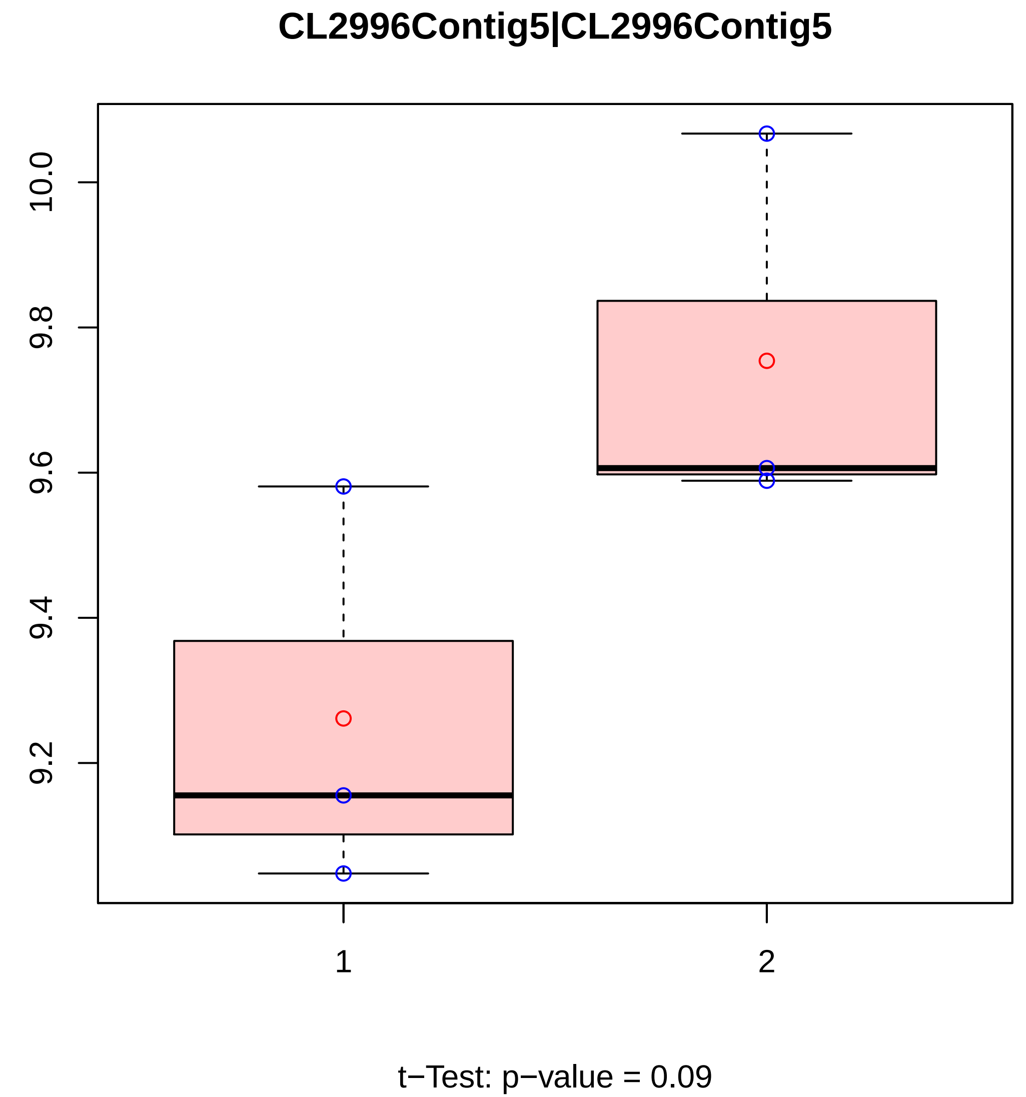
CL2996Contig5|CL2996Contig5
10.0
9.8
9.6
9.4
9.2
1
2
t−Test: p−value = 0.09

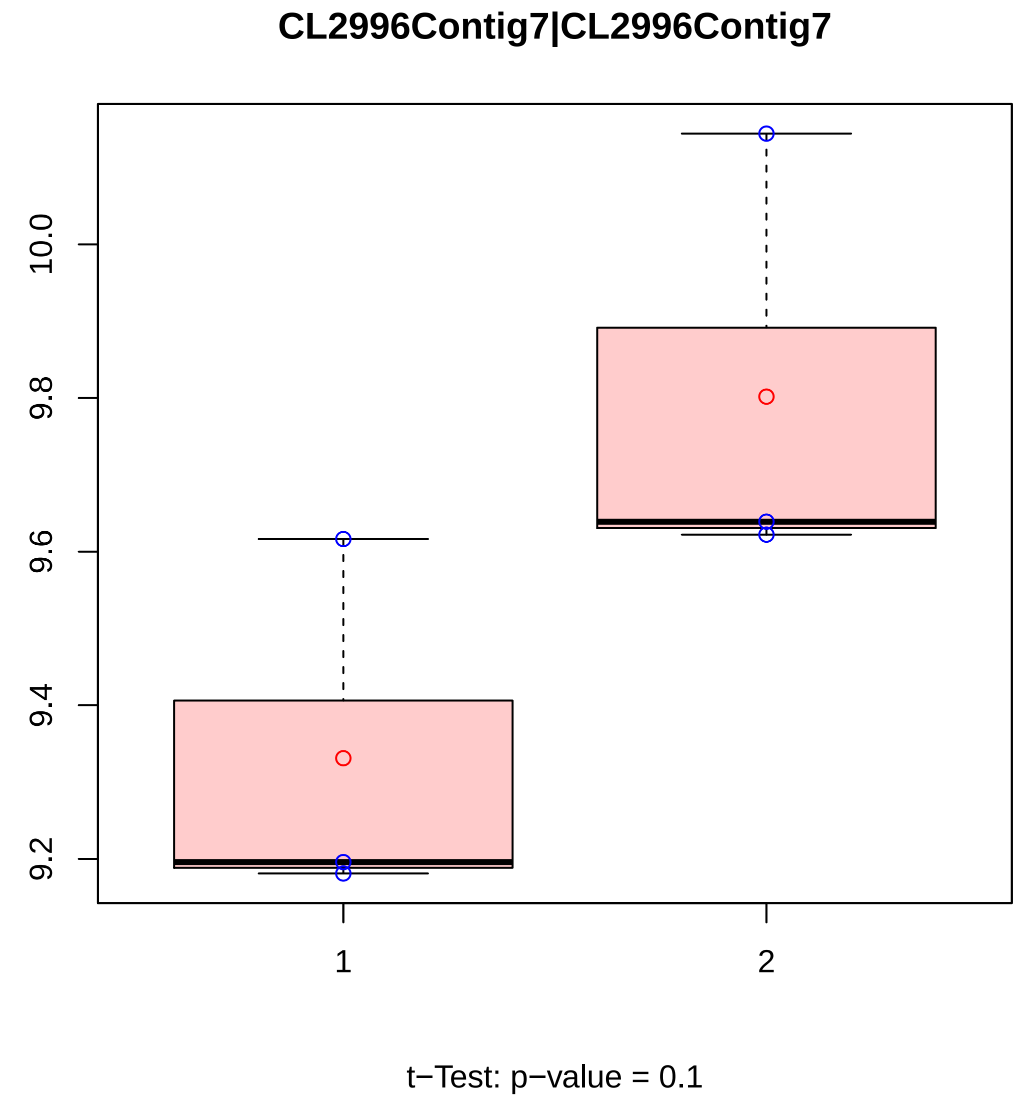
CL2996Contig7|CL2996Contig7
10.0
9.8
9.6
9.4
9.2
1
2
t−Test: p−value = 0.1

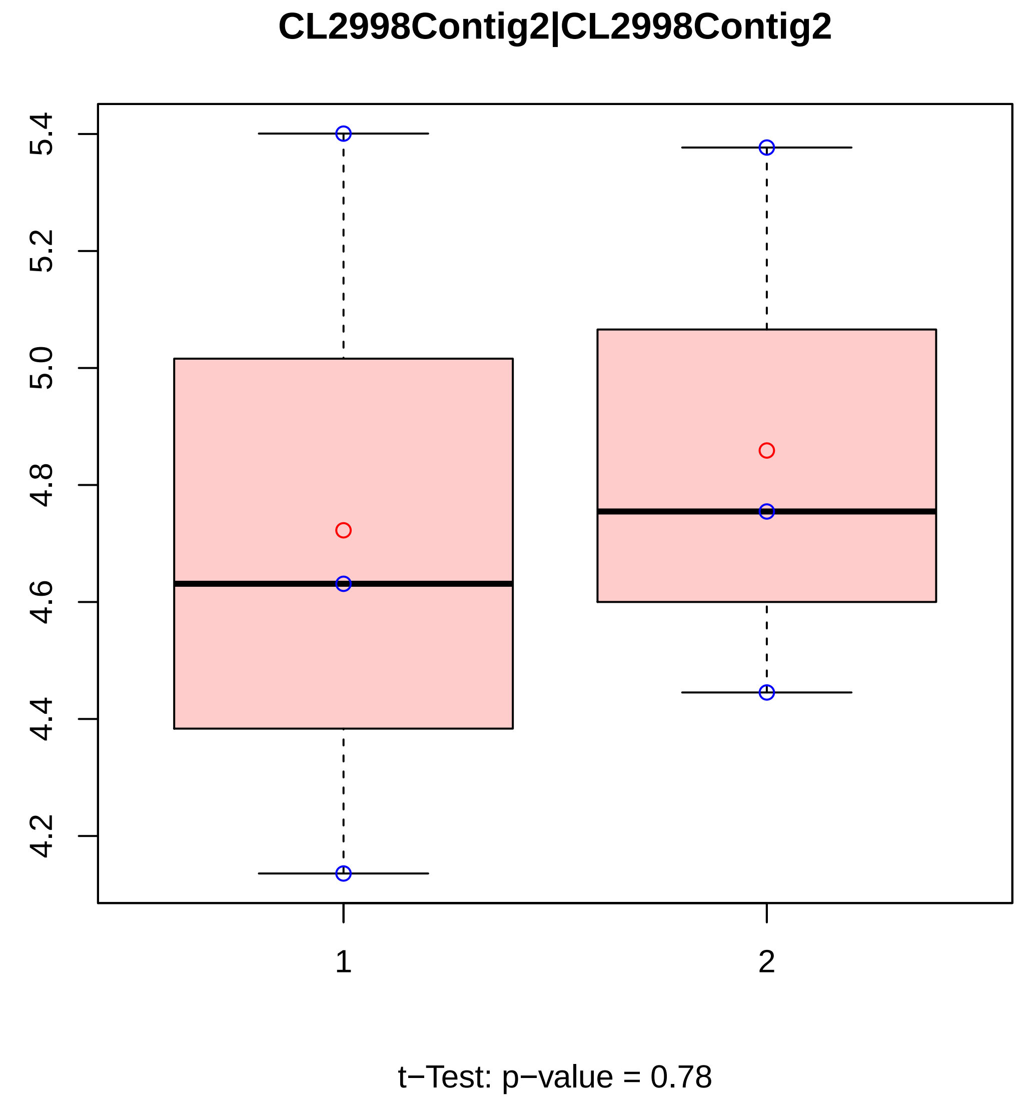
CL2998Contig2|CL2998Contig2
5.4
5.2
5.0
4.8
4.6
4.4
4.2
1
2
t−Test: p−value = 0.78

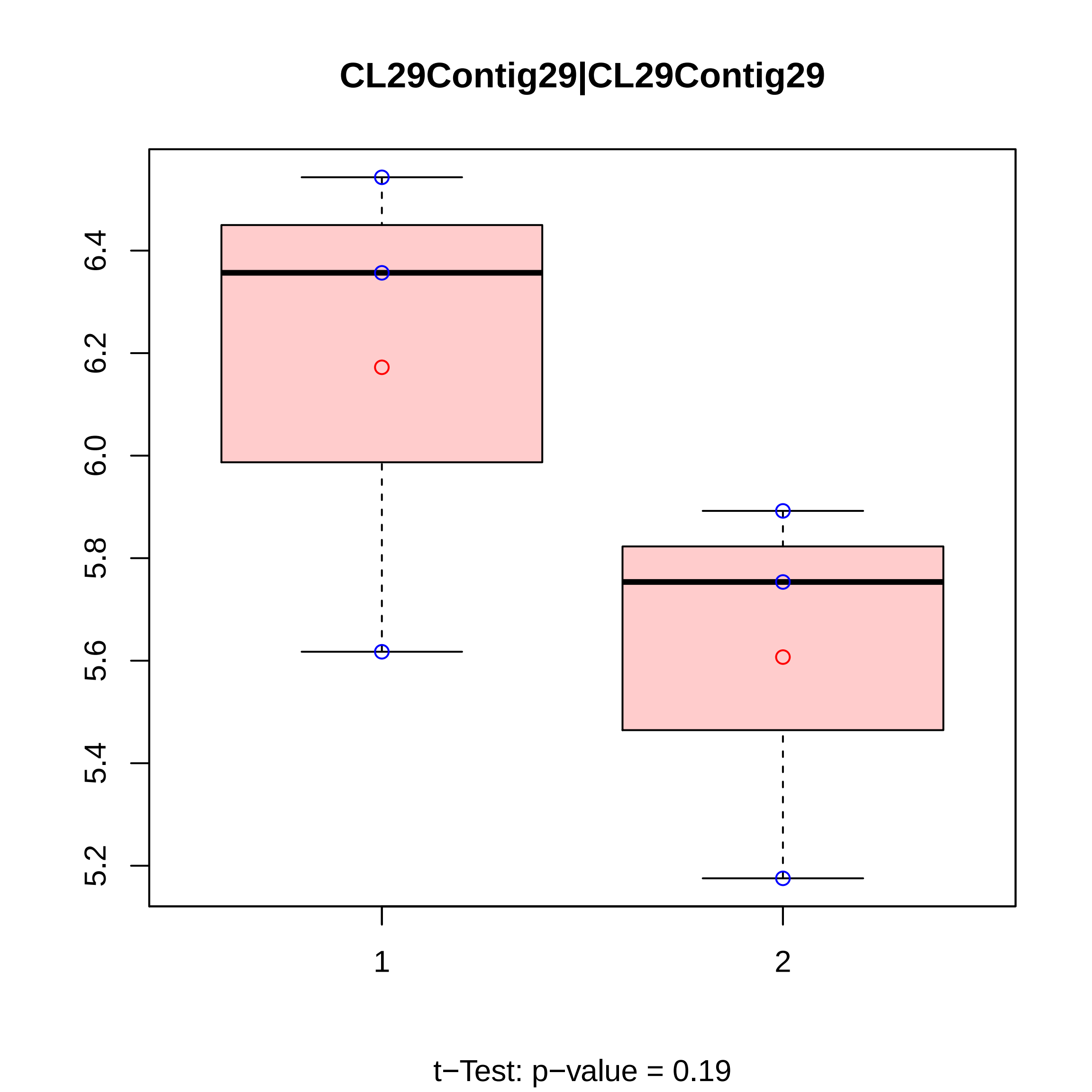
CL29Contig29|CL29Contig29
6.4
6.2
6.0
5.8
5.6
5.4
5.2
1
2
t−Test: p−value = 0.19

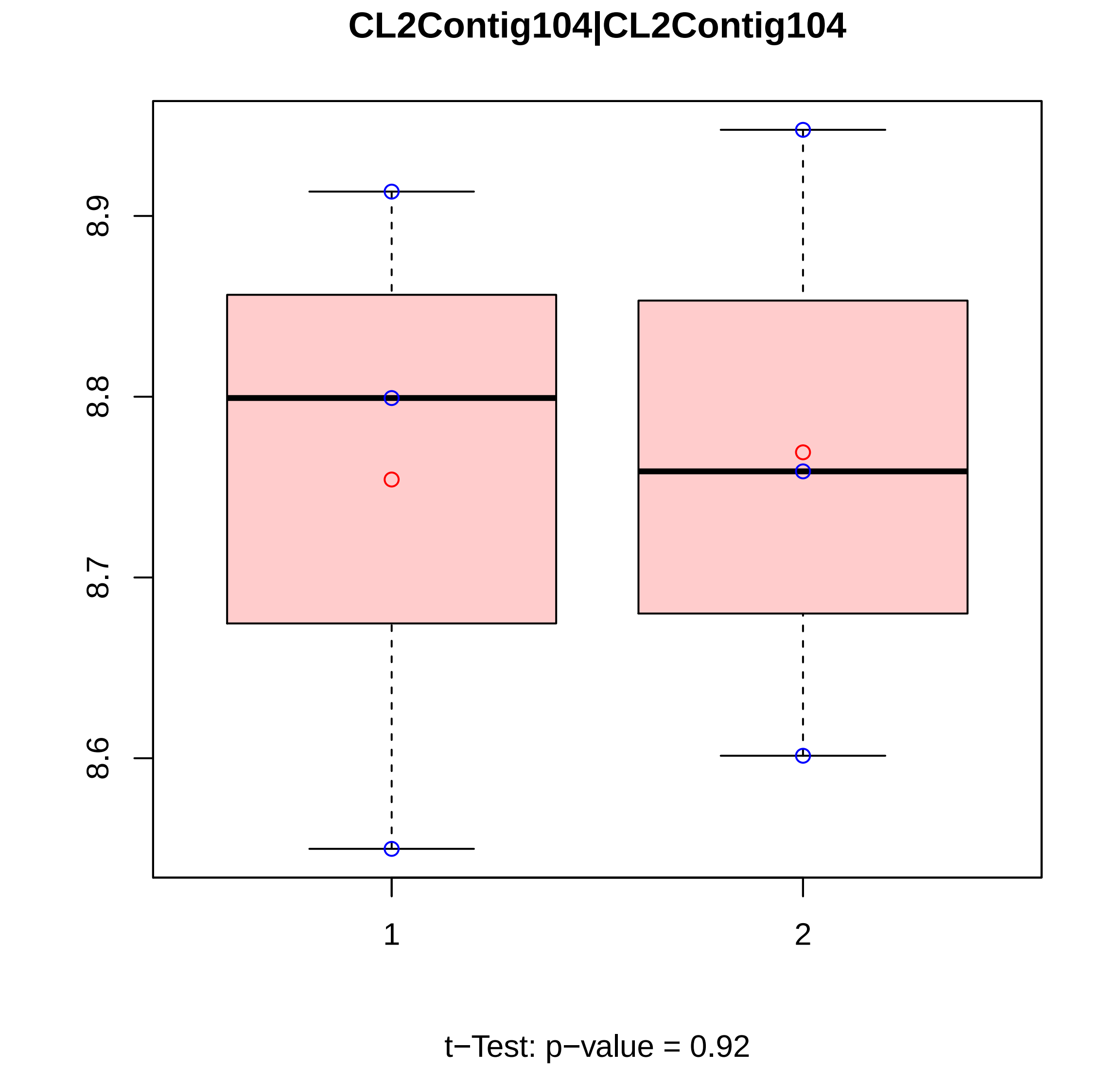

CL2Contig104|CL2Contig104
8.9
8.8
8.7
8.6
1
2
t−Test: p−value = 0.92

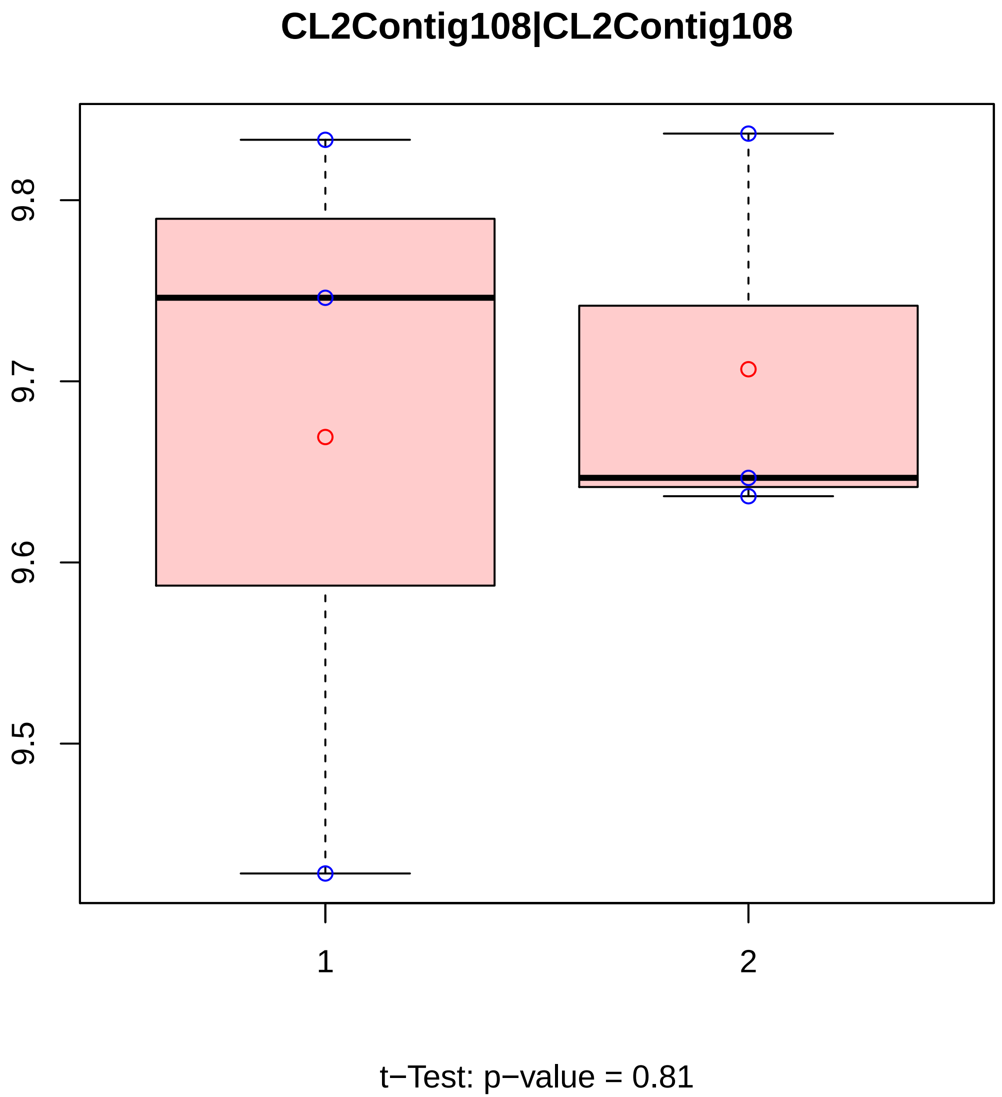
CL2Contig108|CL2Contig108
9.5
9.6
9.7
9.8
1
2
t−Test: p−value = 0.81

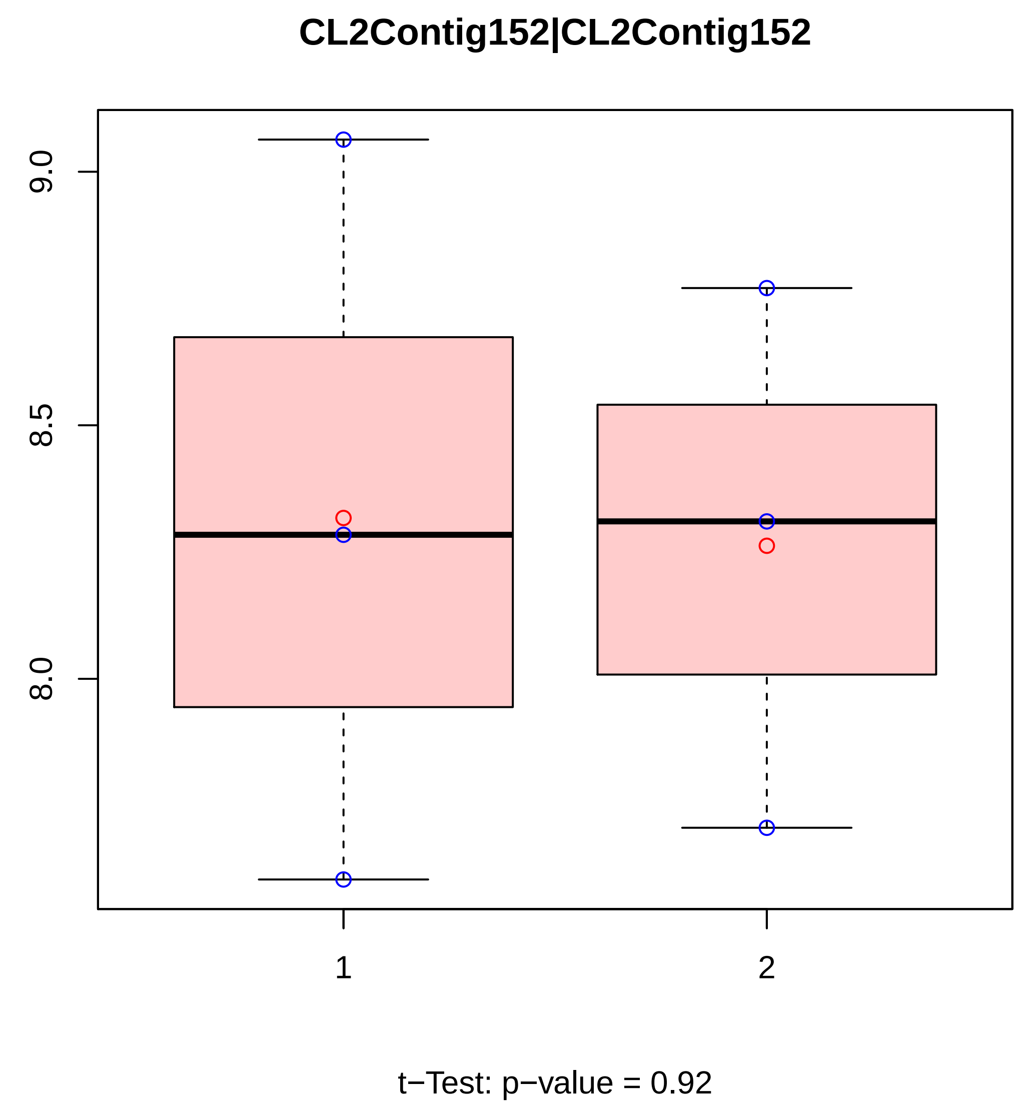
CL2Contig152|CL2Contig152
9.0
8.5
8.0
1
2
t−Test: p−value = 0.92

# CL2Contig153|CL2Contig153

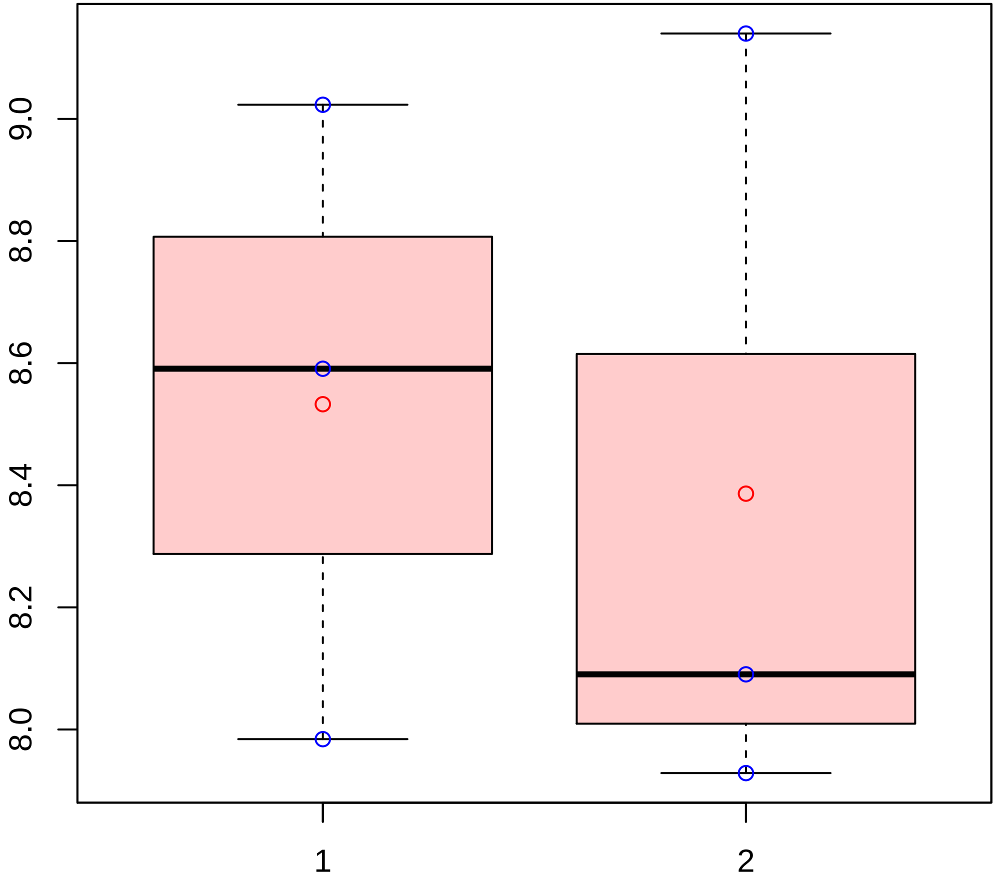


t−Test: p−value = 0.78

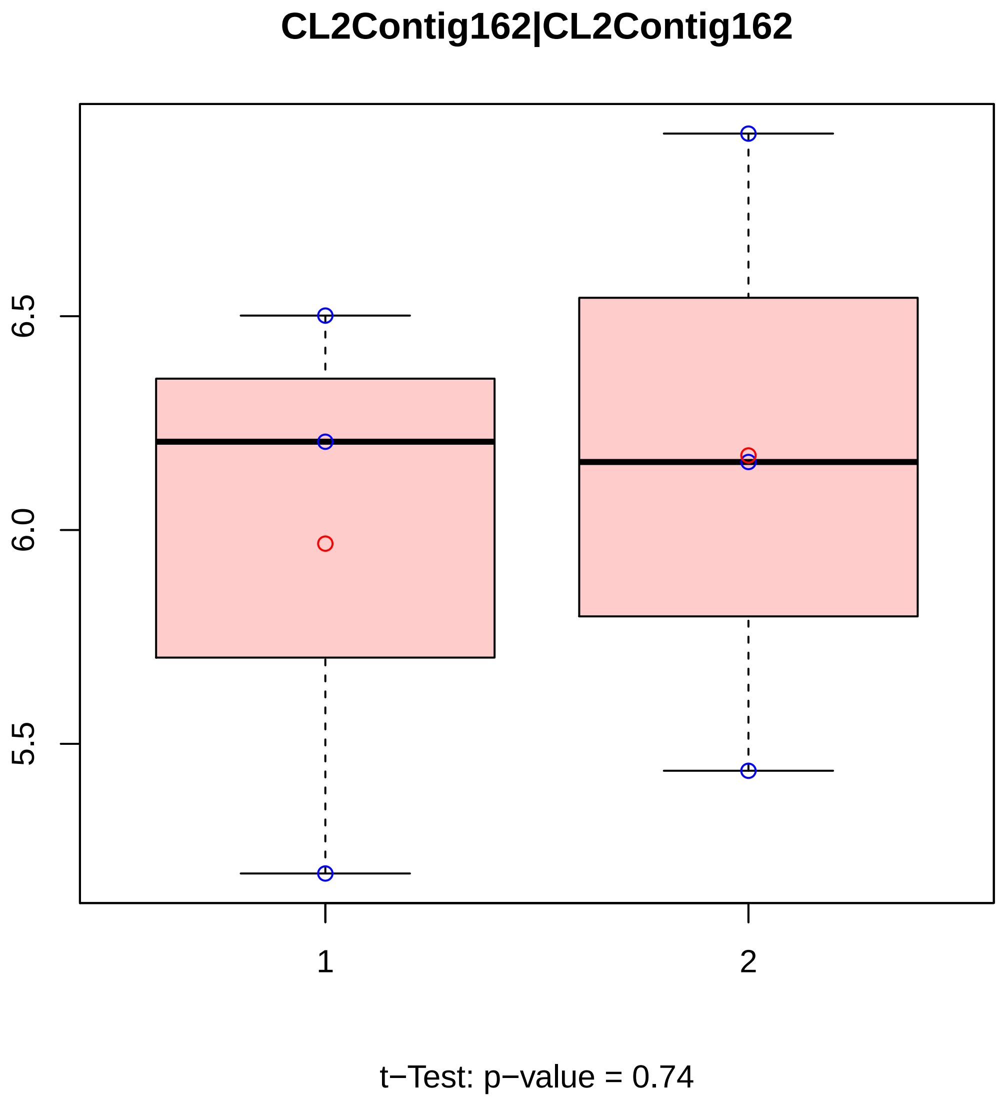
CL2Contig162|CL2Contig162
6.5
6.0
5.5
1
2
t−Test: p−value = 0.74

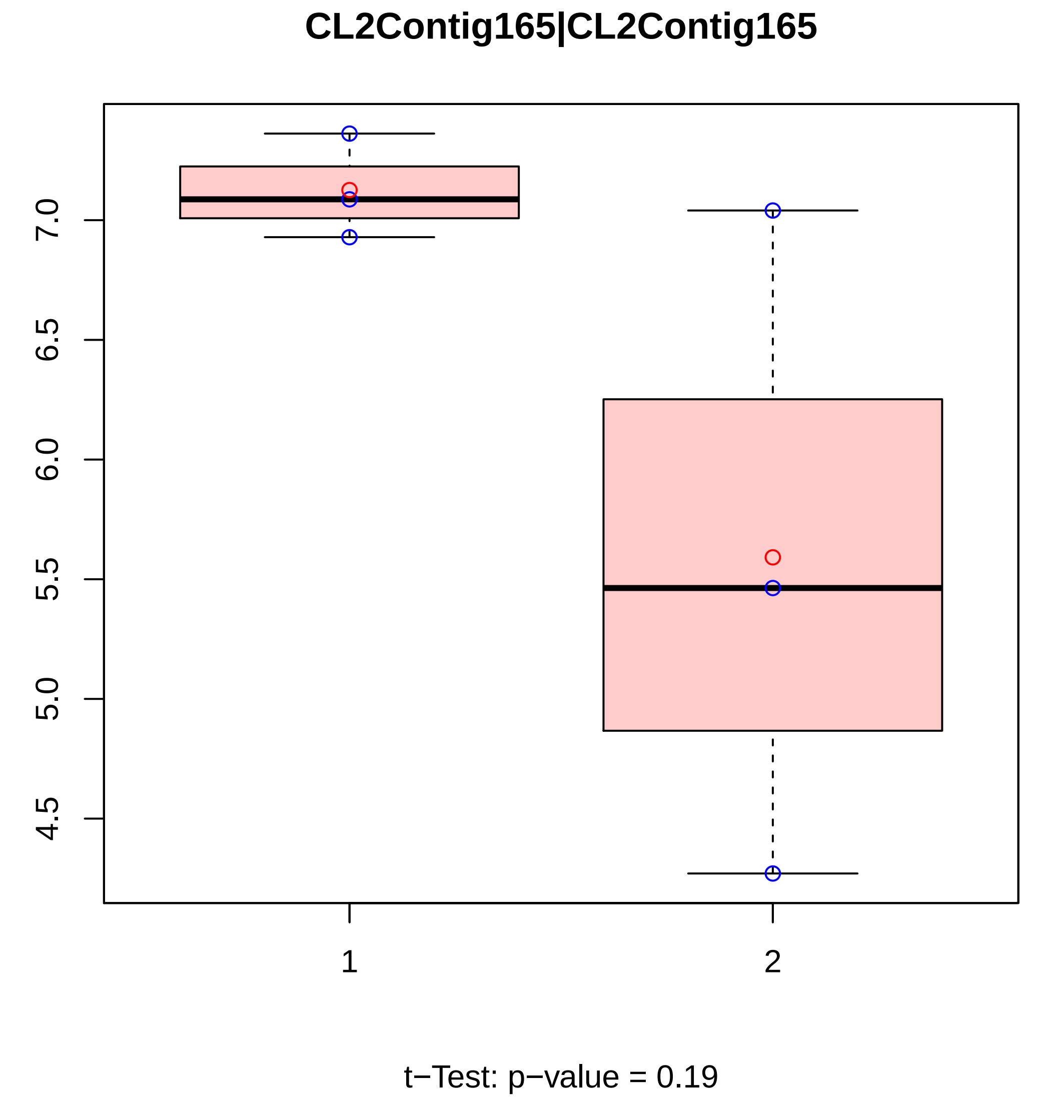
CL2Contig165|CL2Contig165
7.0
6.5
6.0
5.5
5.0
4.5
1
2
t−Test: p−value = 0.19

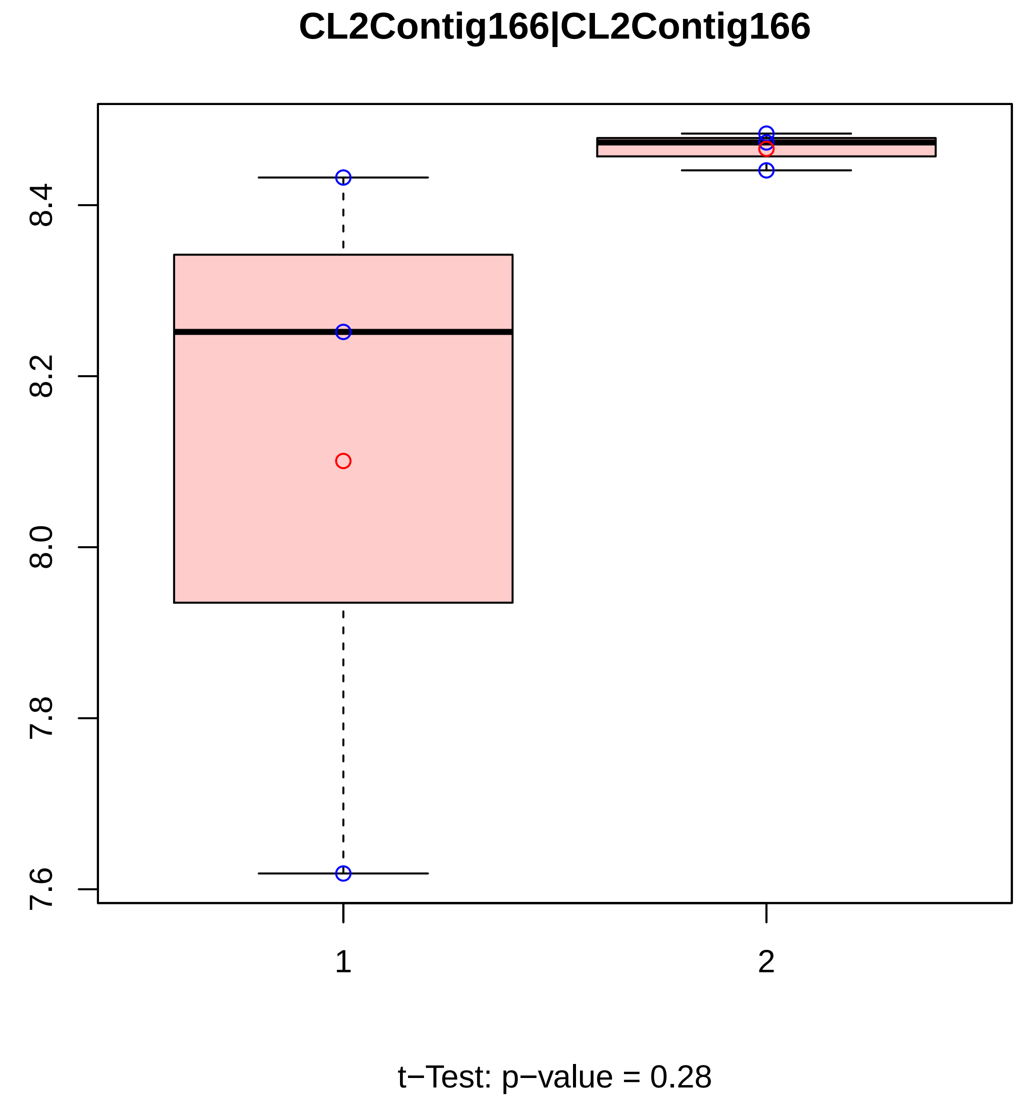
CL2Contig166|CL2Contig166
8.4
8.2
8.0
7.8
7.6
1
2
t−Test: p−value = 0.28

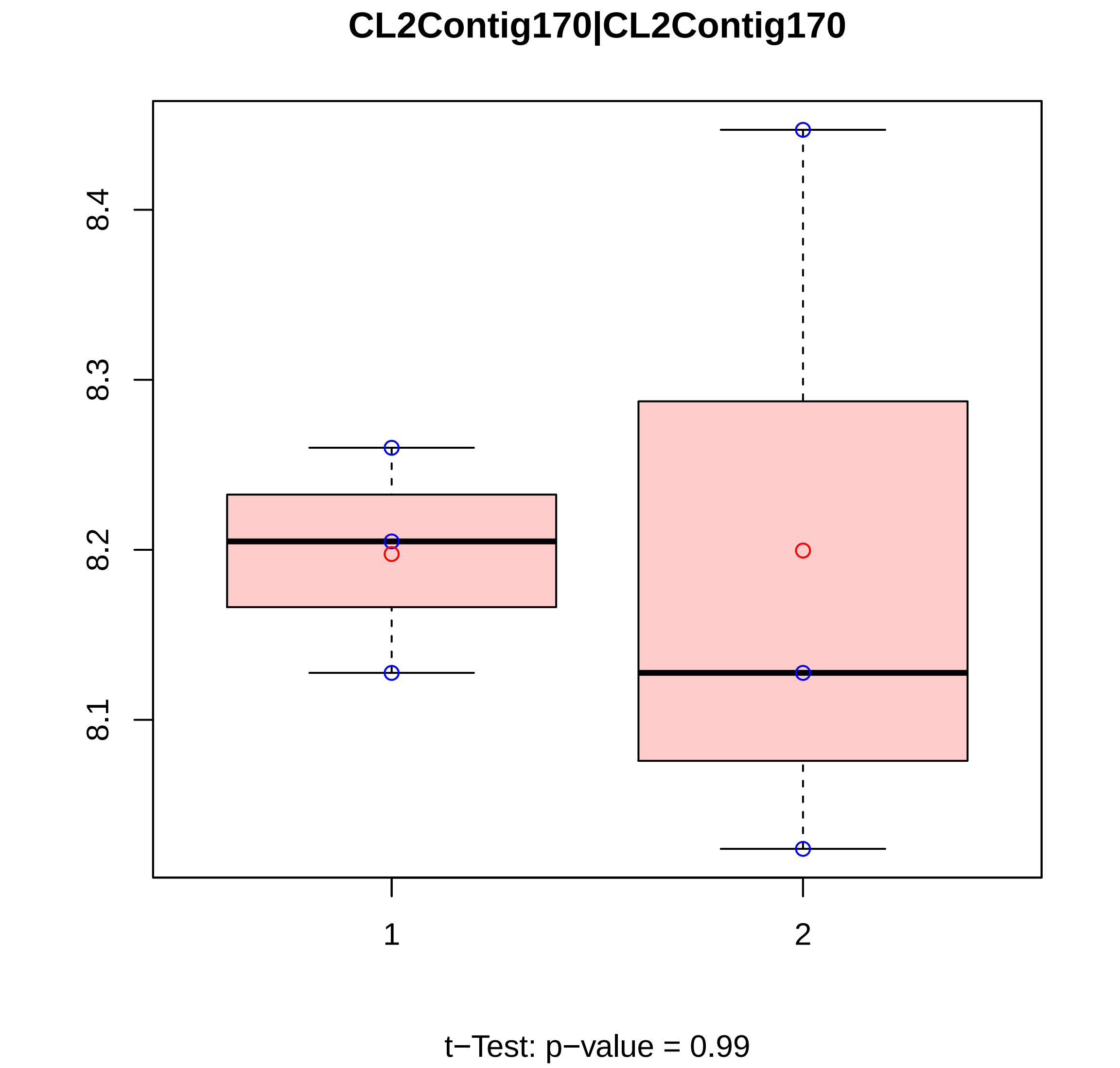
CL2Contig170|CL2Contig170
8.1
8.2
8.3
8.4
1
2
t−Test: p−value = 0.99

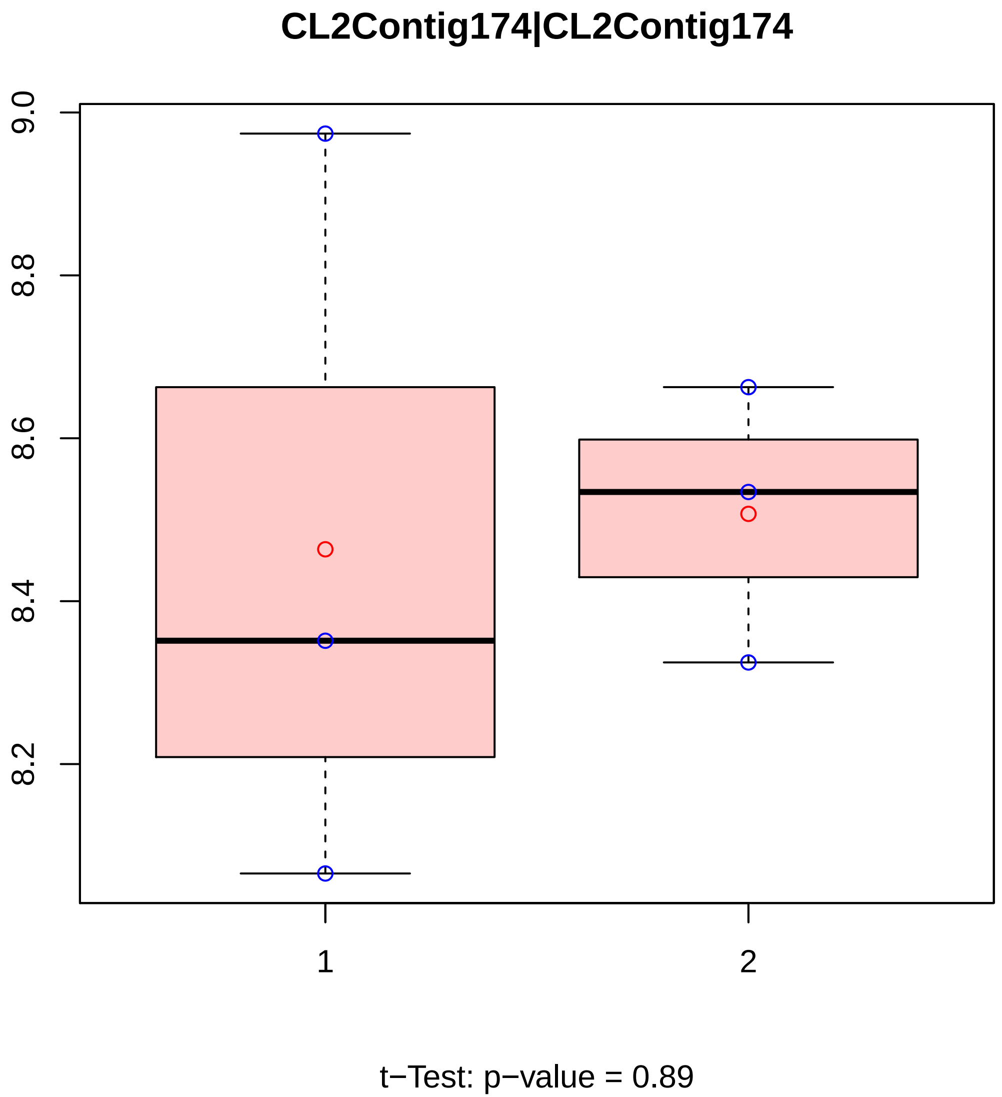

CL2Contig174|CL2Contig174
9.0
8.8
8.6
8.4
8.2
1
2
t−Test: p−value = 0.89

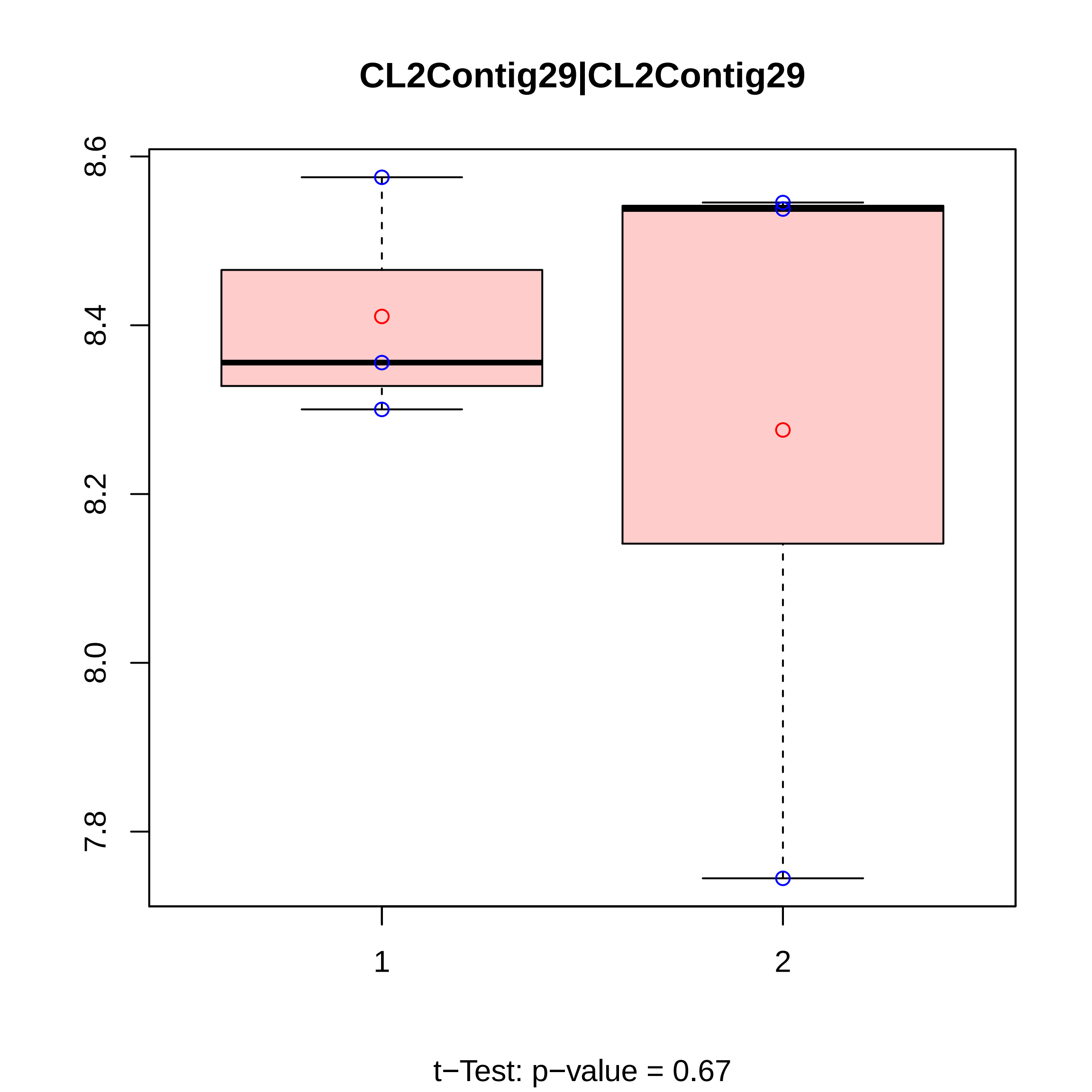

CL2Contig29|CL2Contig29
8.6
8.4
8.2
8.0
7.8
1
2
t−Test: p−value = 0.67

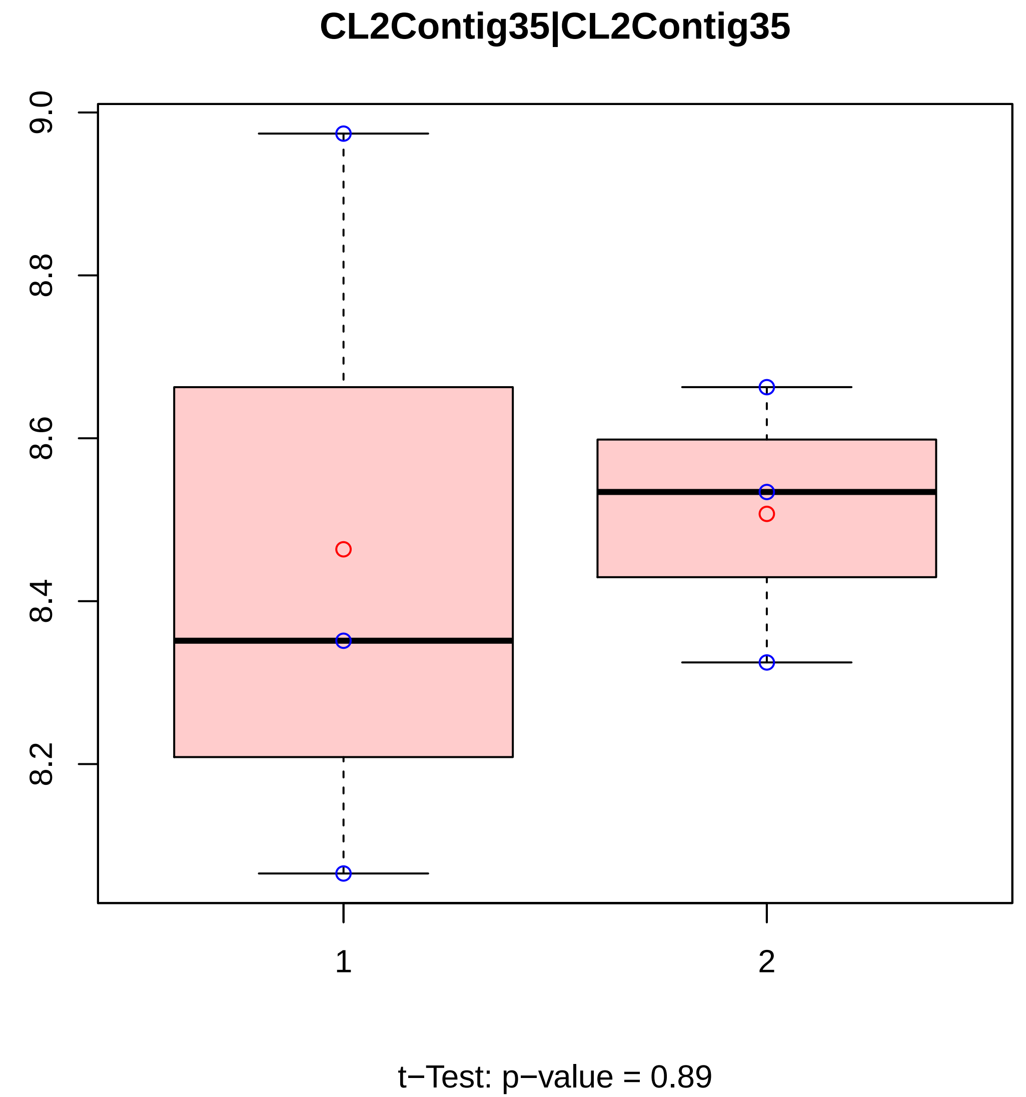
CL2Contig35|CL2Contig35
9.0
8.8
8.6
8.4
8.2
1
2
t−Test: p−value = 0.89

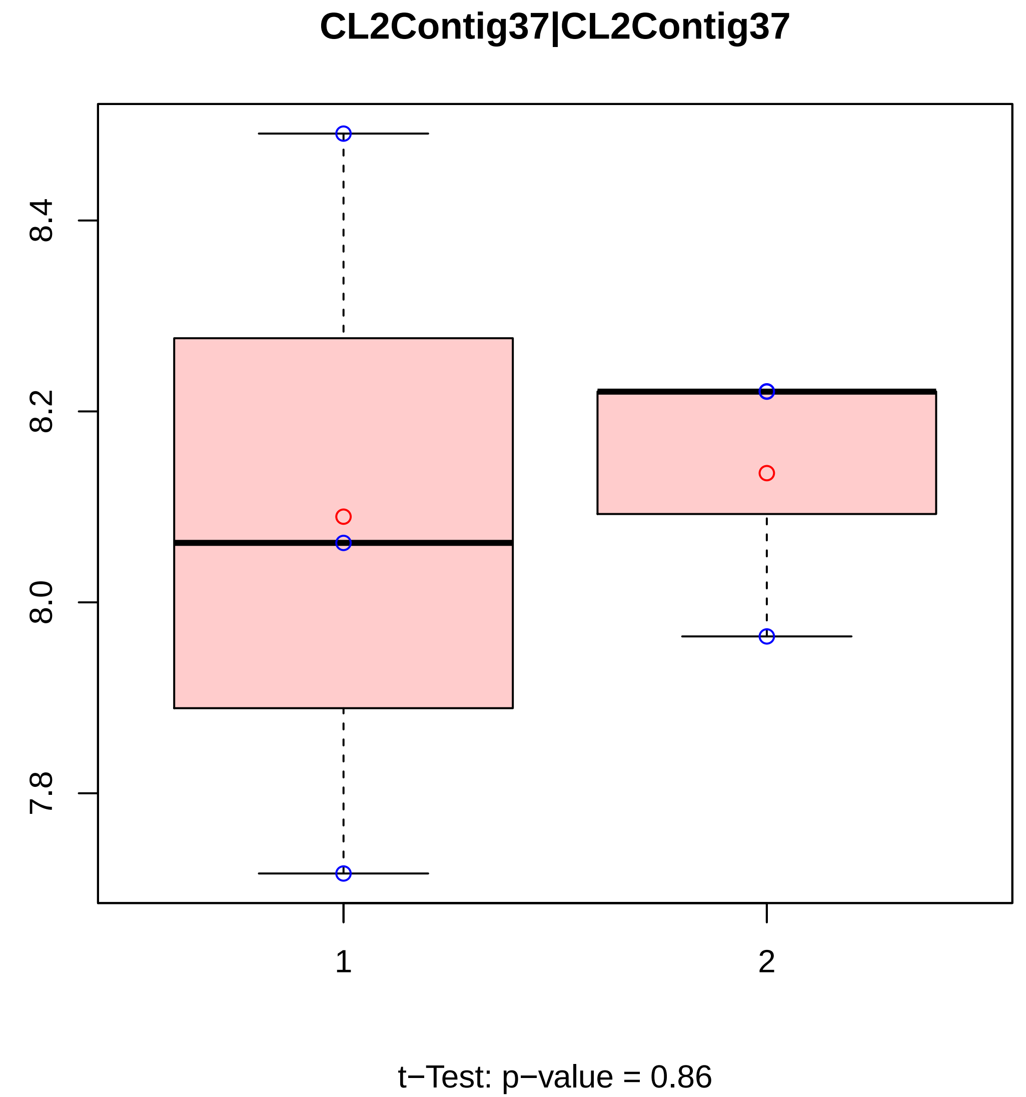
CL2Contig37|CL2Contig37
8.4
8.2
8.0
7.8
1
2
t−Test: p−value = 0.86

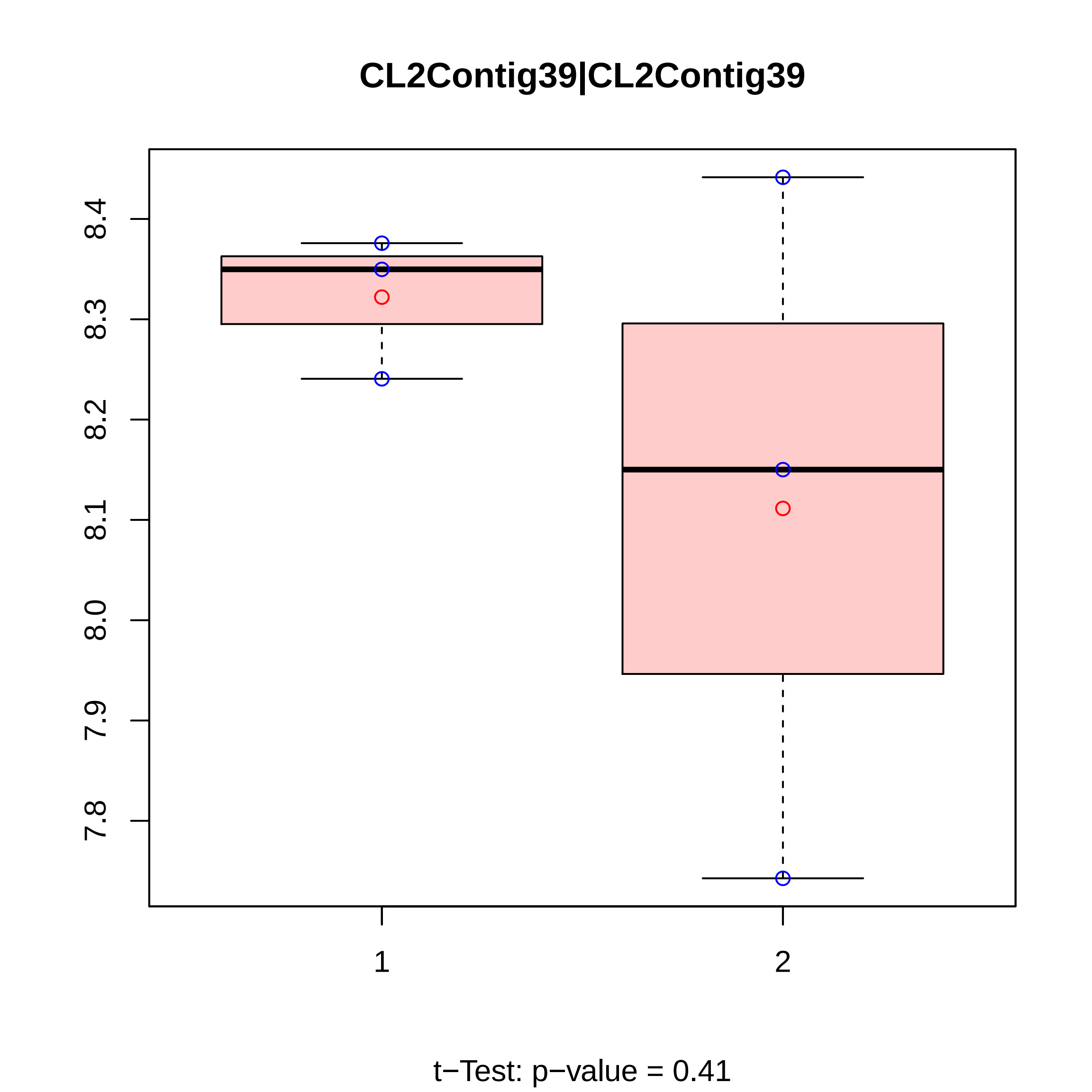
CL2Contig39|CL2Contig39
8.4
8.3
8.2
8.1
8.0
7.9
7.8
1
2
t−Test: p−value = 0.41

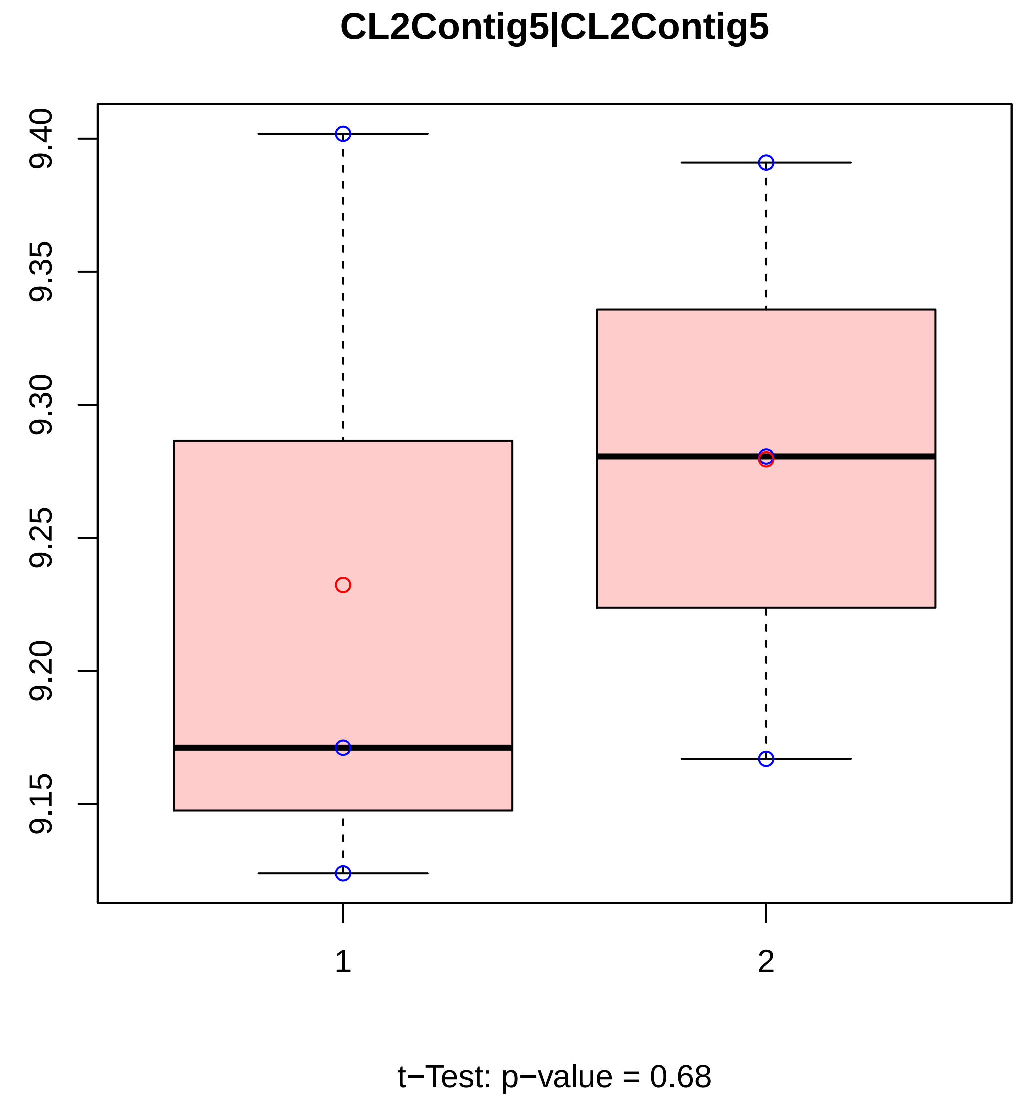
CL2Contig5|CL2Contig5
9.40
9.35
9.30
9.25
9.20
9.15
1
2
t−Test: p−value = 0.68

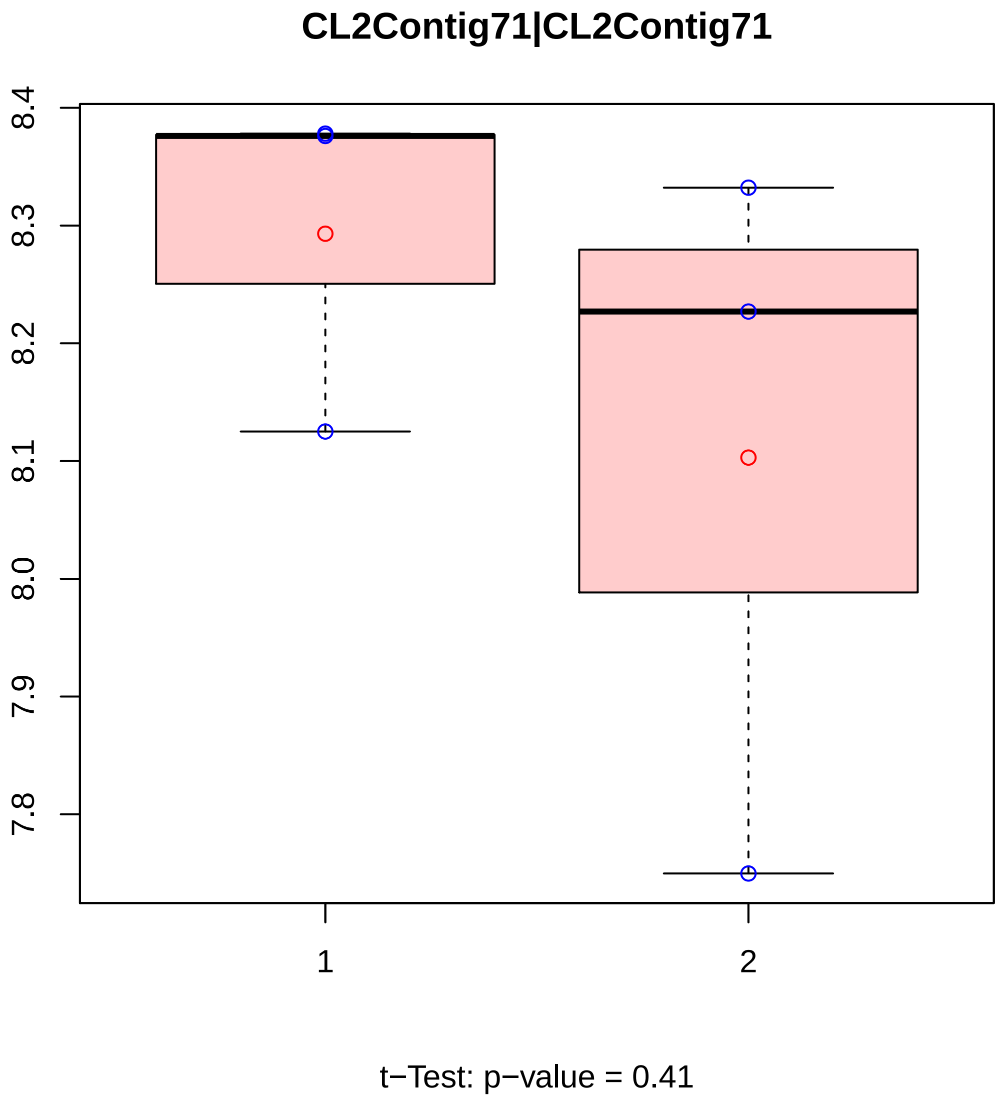
CL2Contig71|CL2Contig71
8.4
8.3
8.2
8.1
8.0
7.9
7.8
1
2
t−Test: p−value = 0.41

# CL2Contig8|CL2Contig8

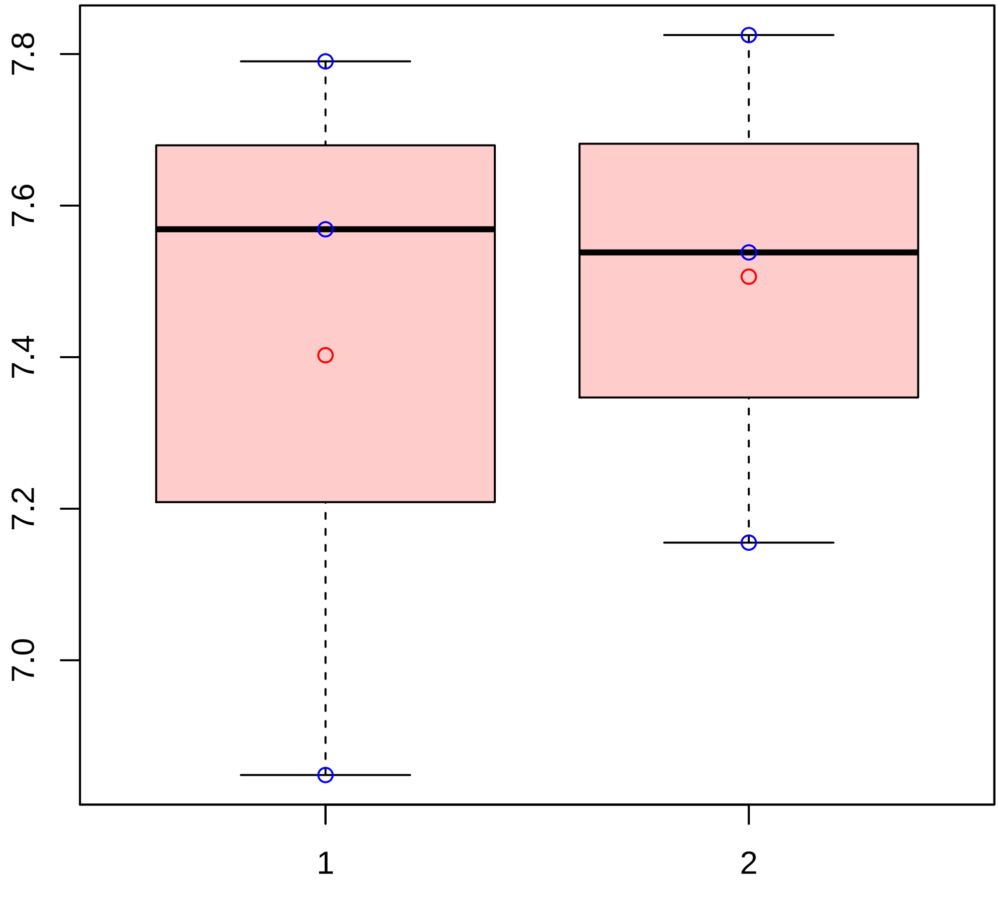


t−Test: p−value = 0.78

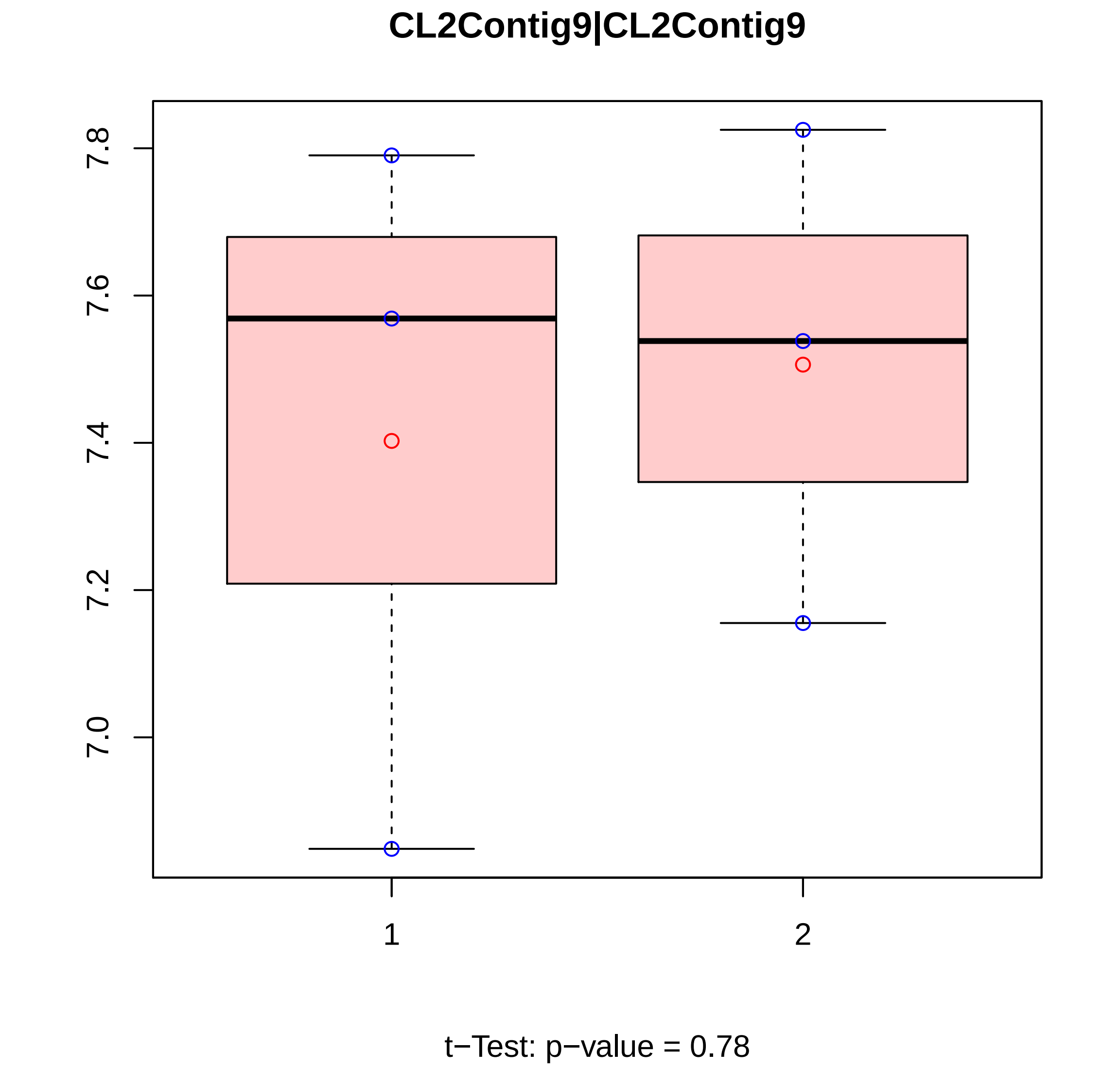
CL2Contig9|CL2Contig9
7.0
7.2
7.4
7.6
7.8
1
2
t−Test: p−value = 0.78

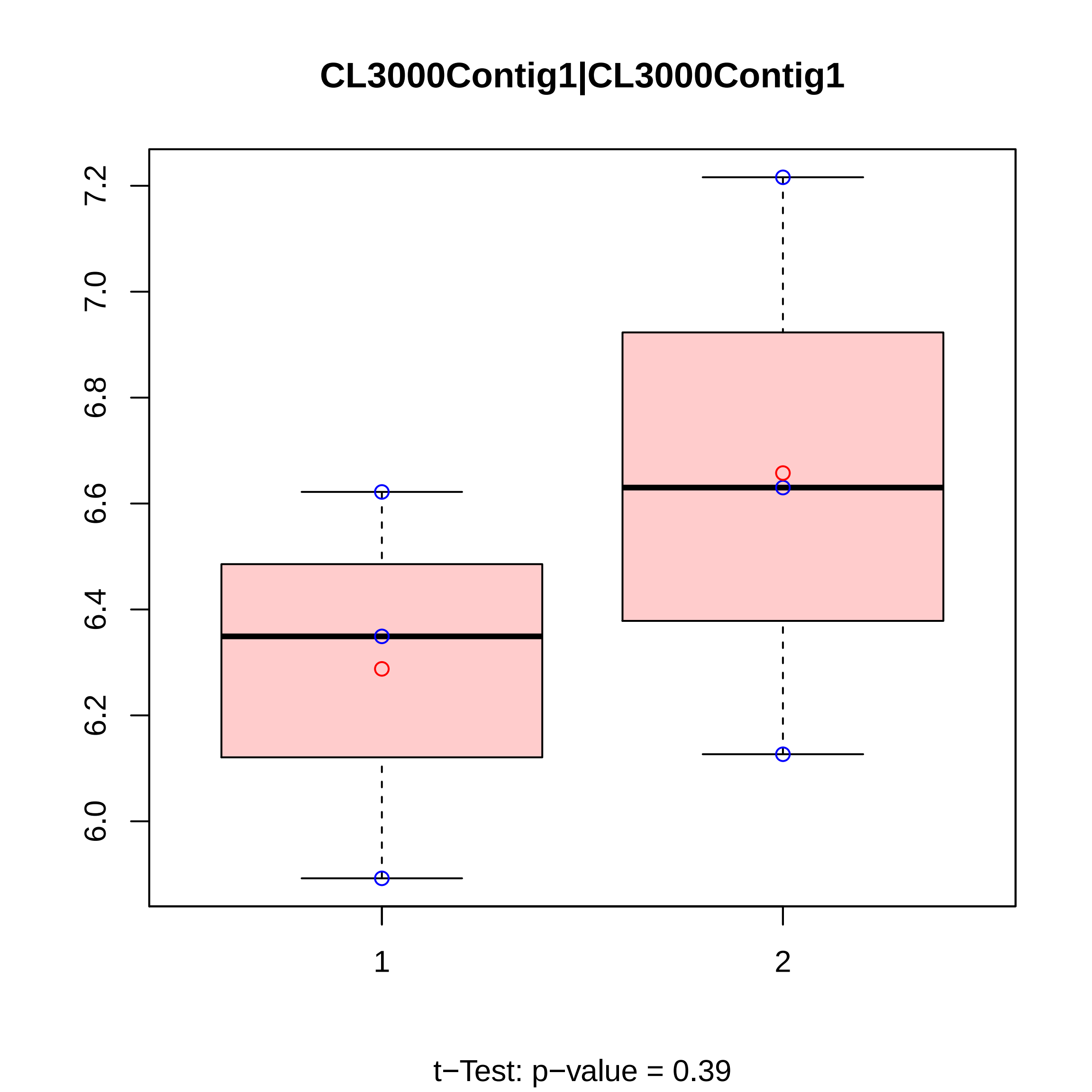

CL3000Contig1|CL3000Contig1
7.2
7.0
6.8
6.6
6.4
6.2
6.0
1
2
t−Test: p−value = 0.39

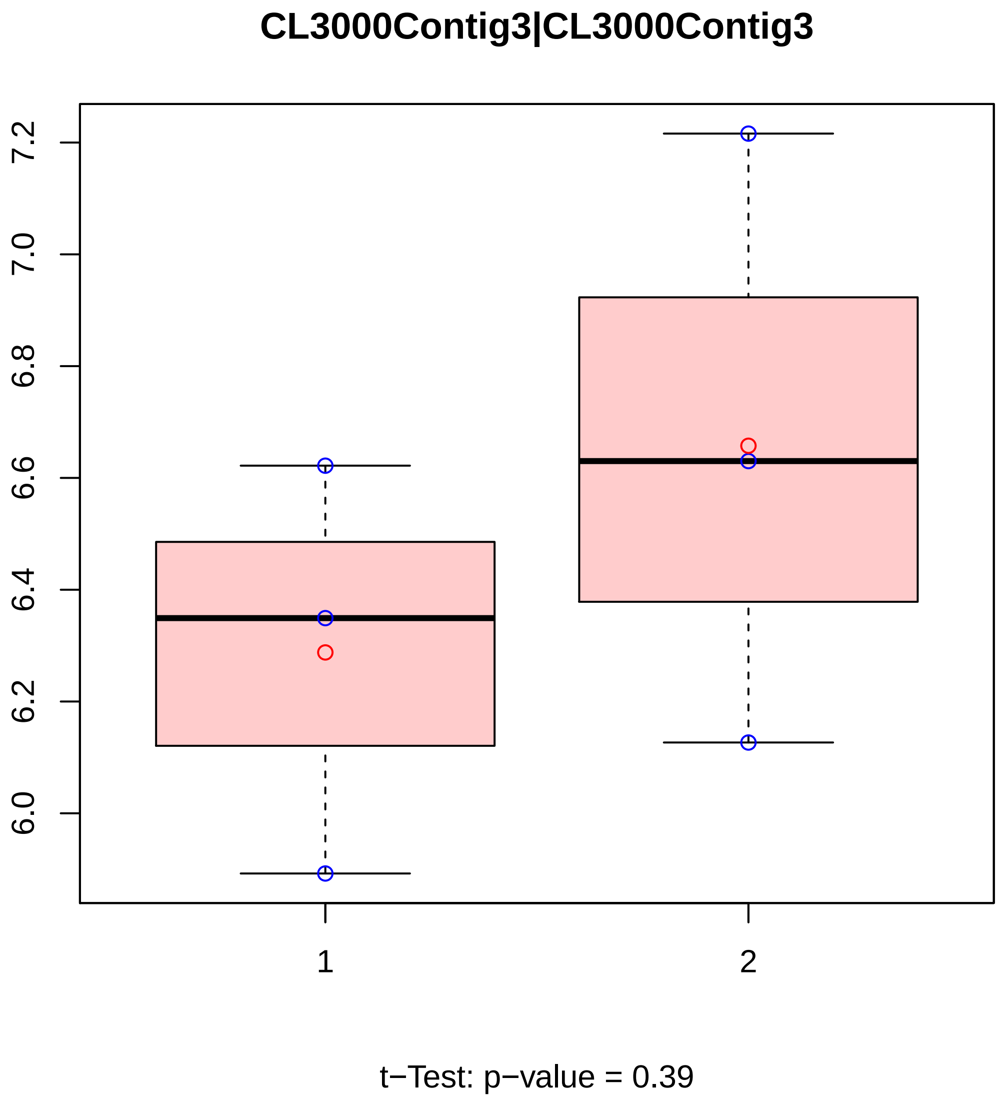

CL3000Contig3|CL3000Contig3
7.2
7.0
6.8
6.6
6.4
6.2
6.0
1
2
t−Test: p−value = 0.39

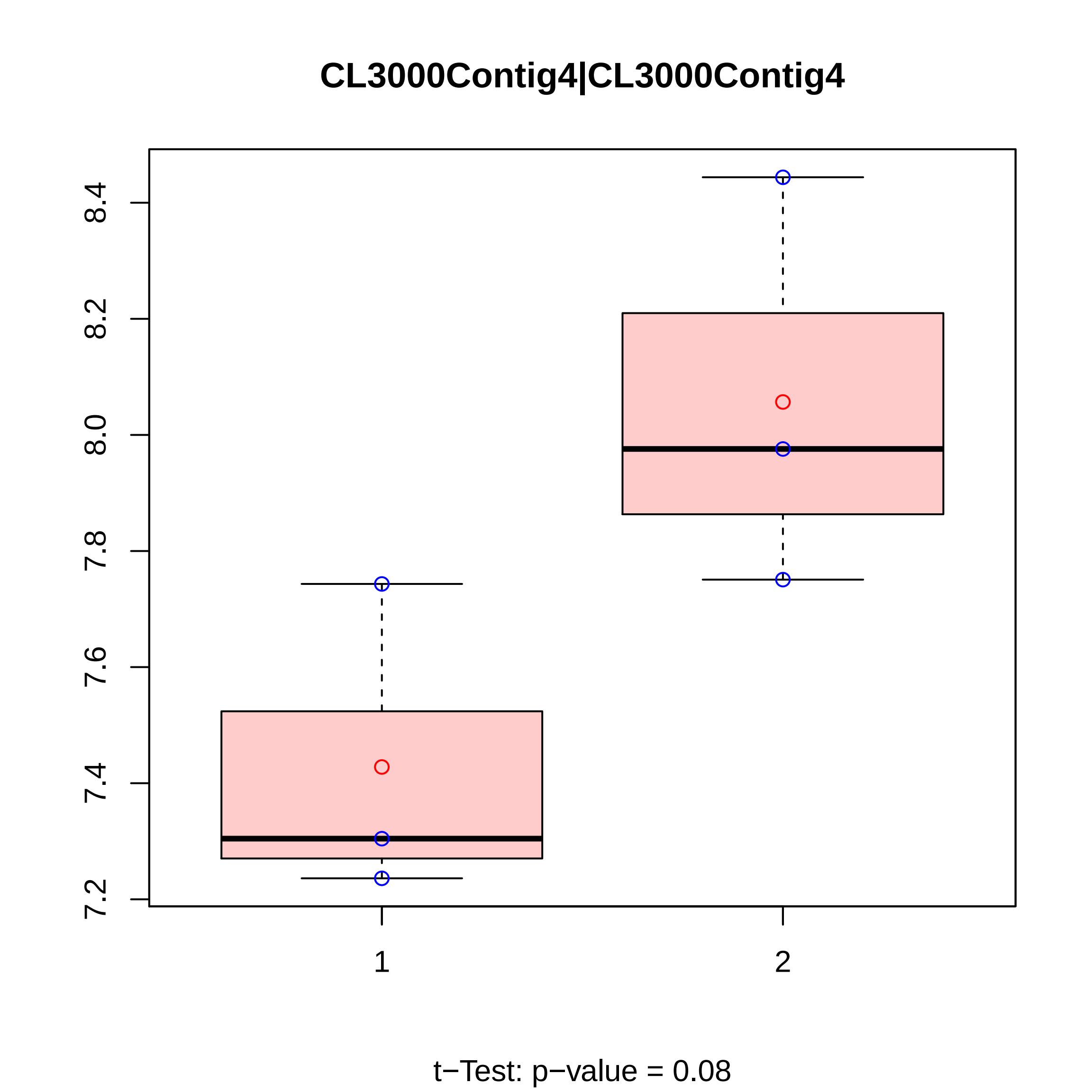

CL3000Contig4|CL3000Contig4
8.4
8.2
8.0
7.8
7.6
7.4
7.2
1
2
t−Test: p−value = 0.08

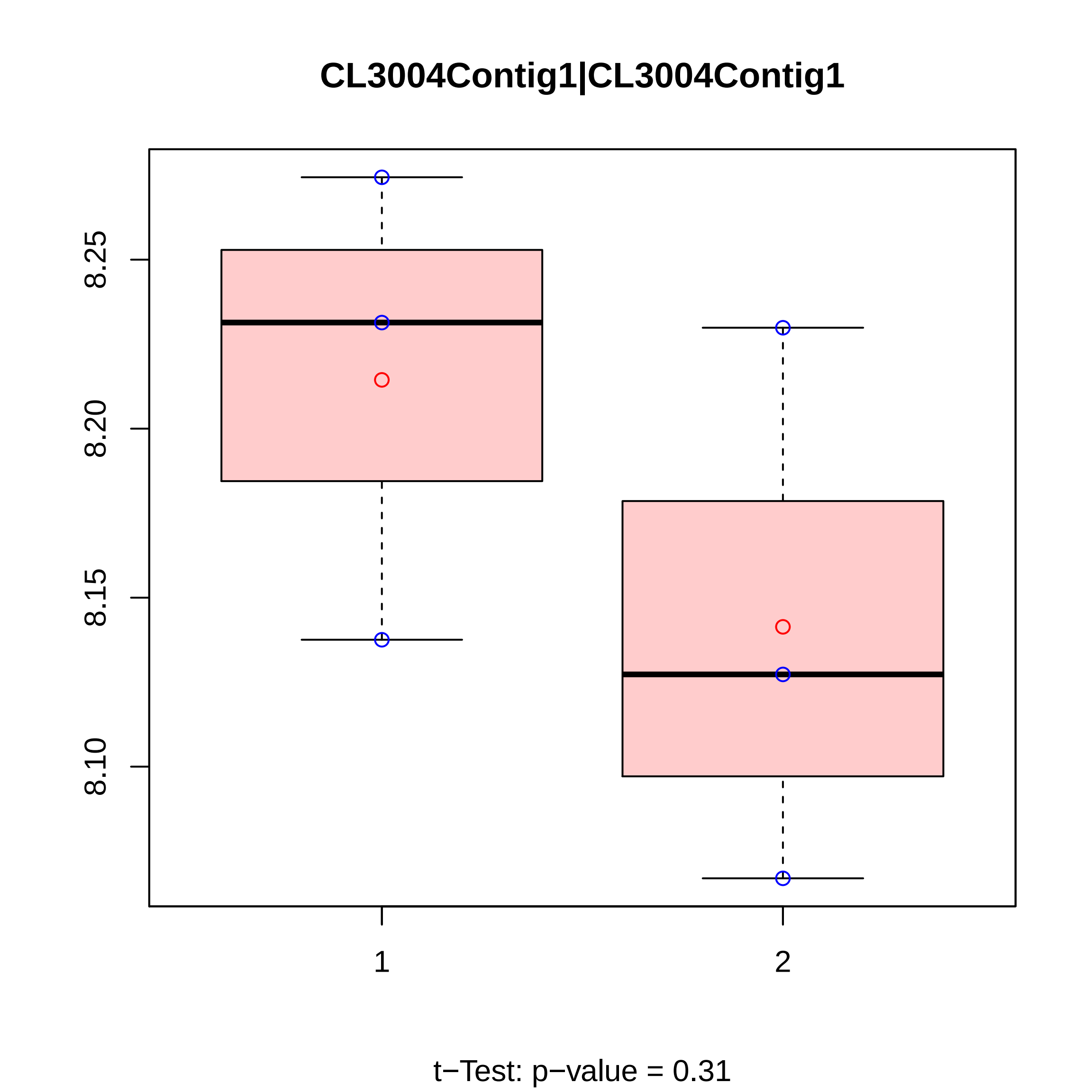
CL3004Contig1|CL3004Contig1
8.25
8.20
8.15
8.10
1
2
t−Test: p−value = 0.31

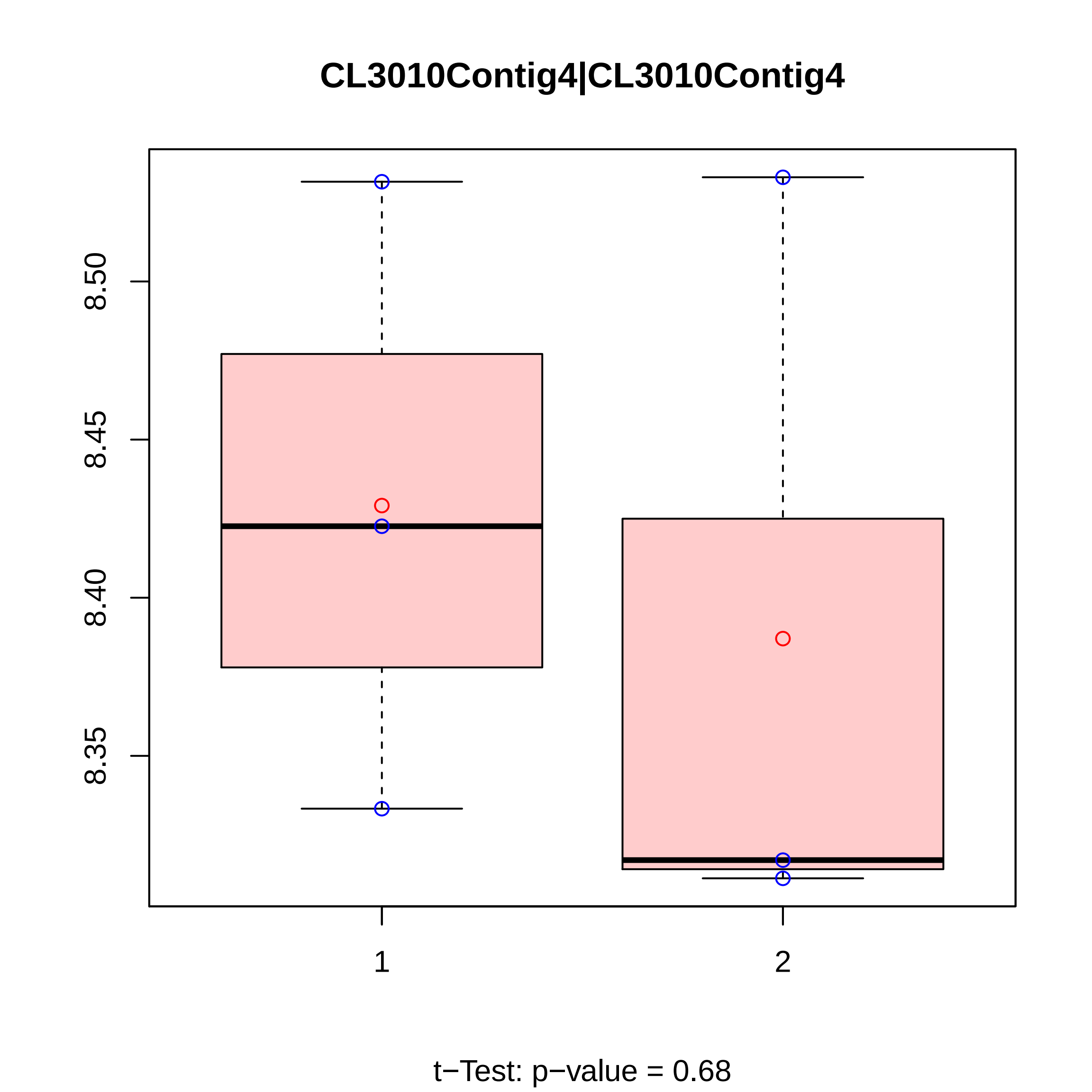

CL3010Contig4|CL3010Contig4
8.35
8.40
8.45
8.50
1
2
t−Test: p−value = 0.68

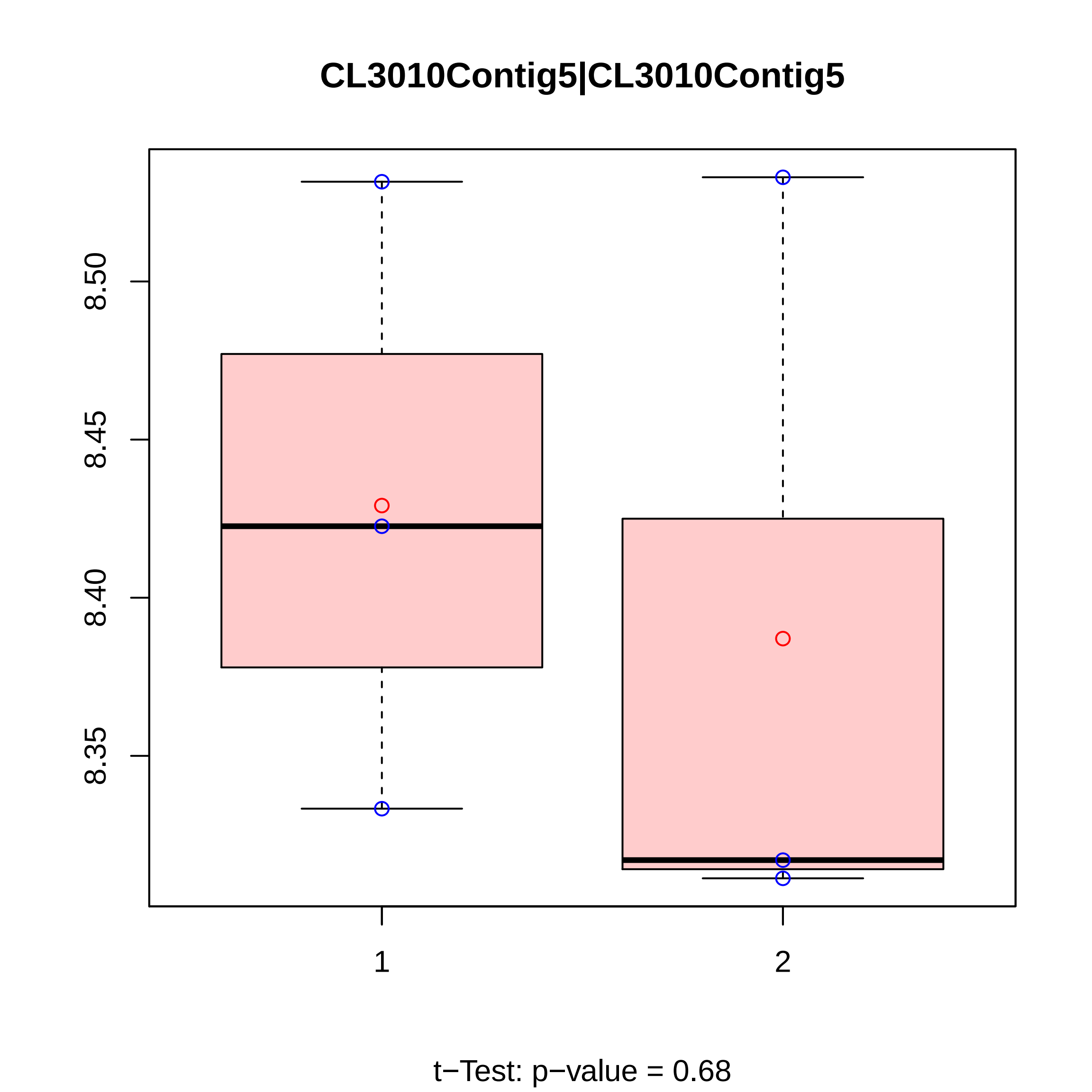

CL3010Contig5|CL3010Contig5
8.35
8.40
8.45
8.50
1
2
t−Test: p−value = 0.68

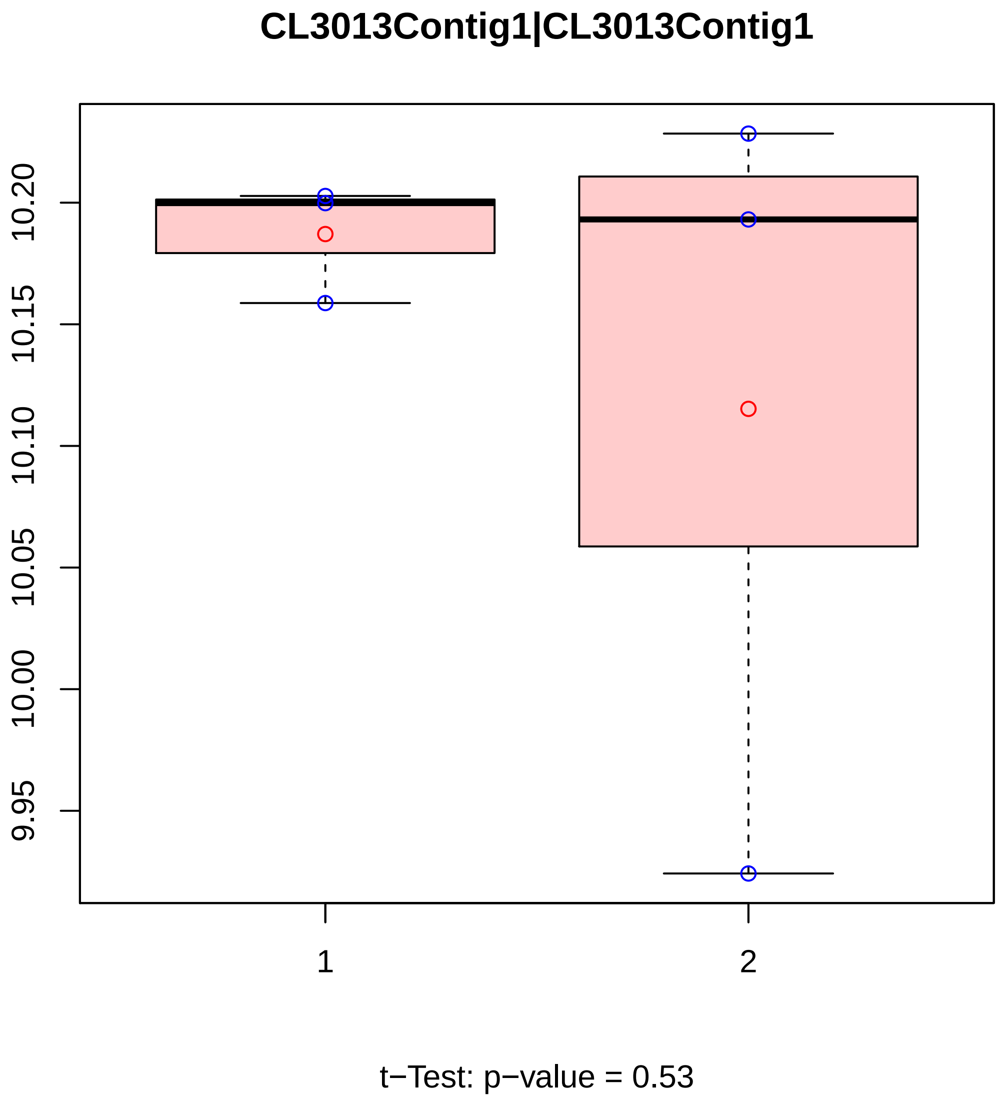
CL3013Contig1|CL3013Contig1
10.20
10.15
10.10
10.05
10.00
9.95
1
2
t−Test: p−value = 0.53

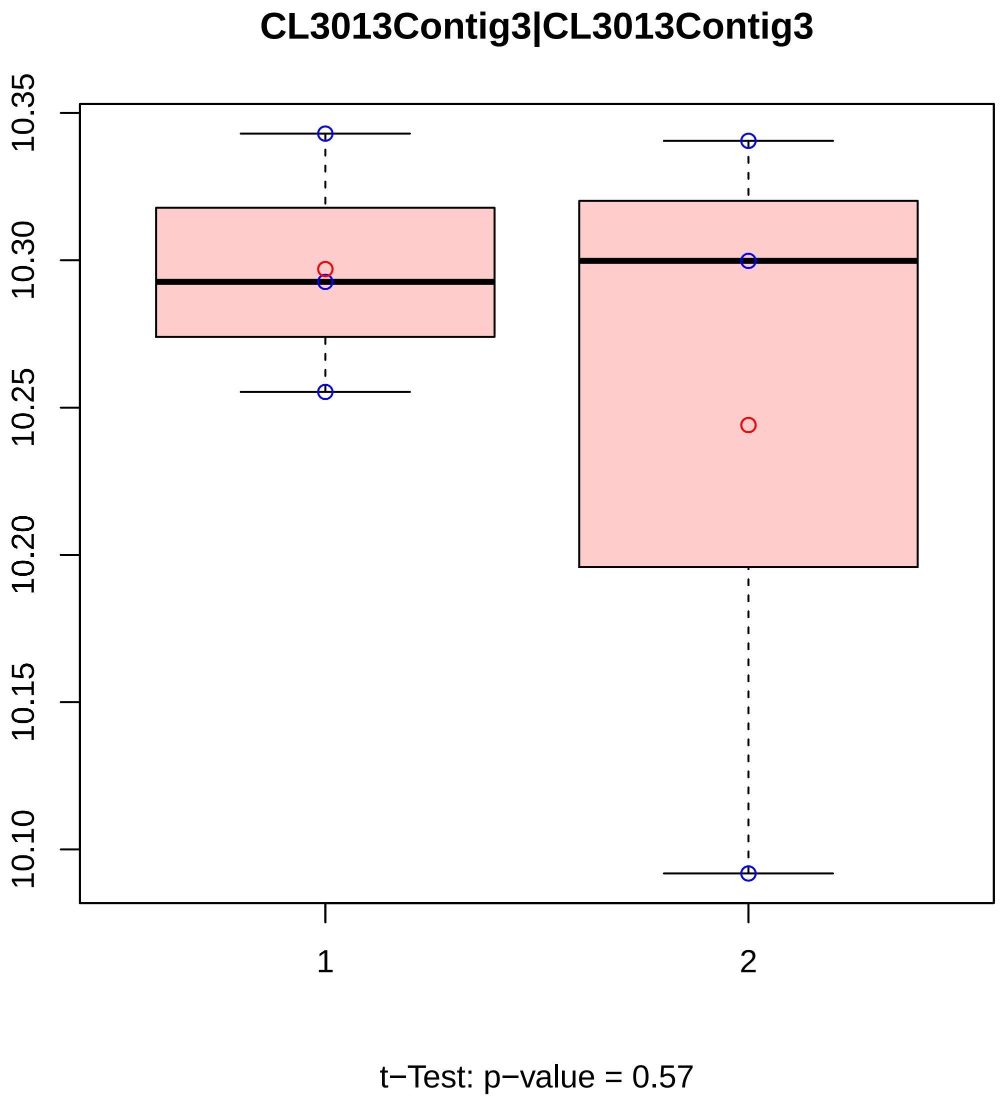
CL3013Contig3|CL3013Contig3
10.35
10.30
10.25
10.20
10.15
10.10
1
2
t−Test: p−value = 0.57

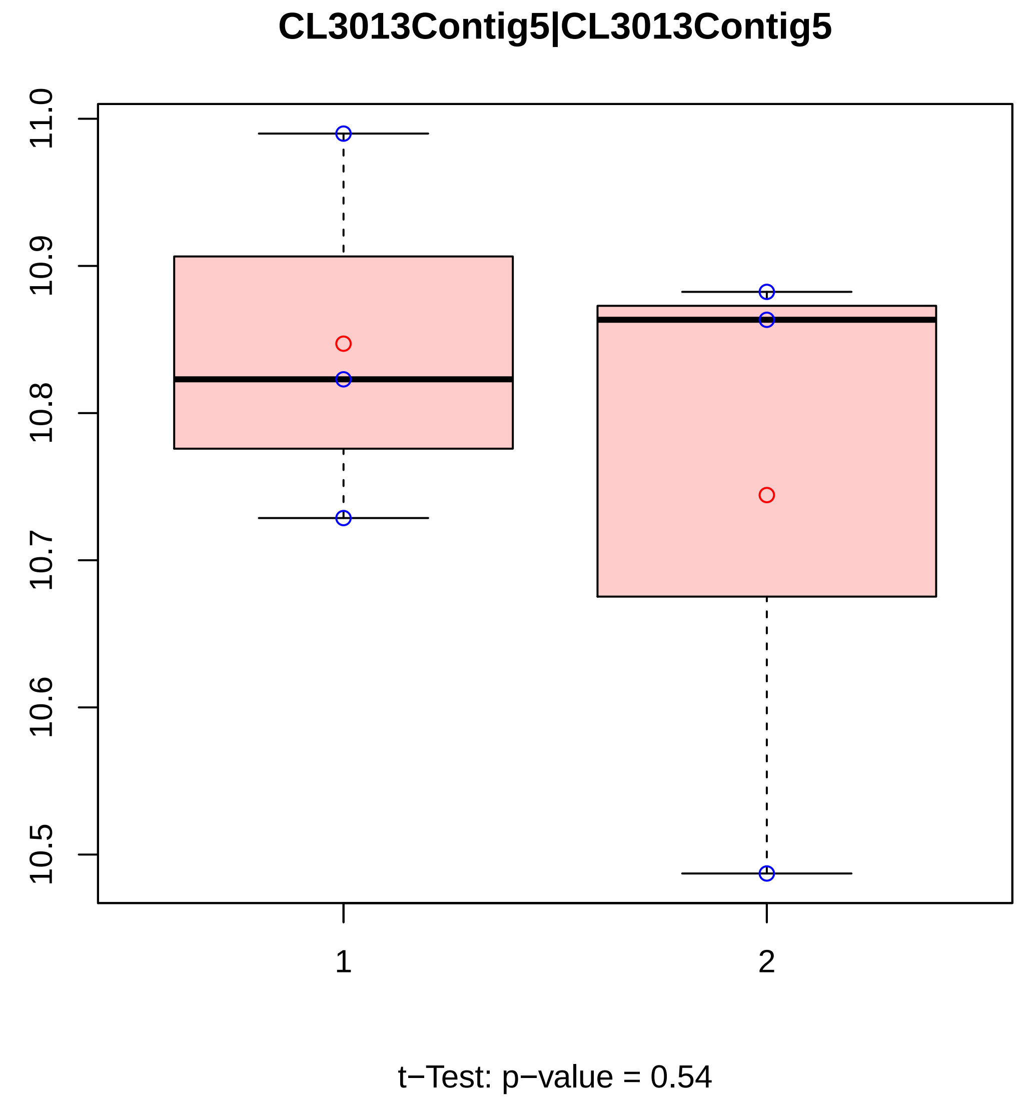
CL3013Contig5|CL3013Contig5
11.0
10.9
10.8
10.7
10.6
10.5
1
2
t−Test: p−value = 0.54

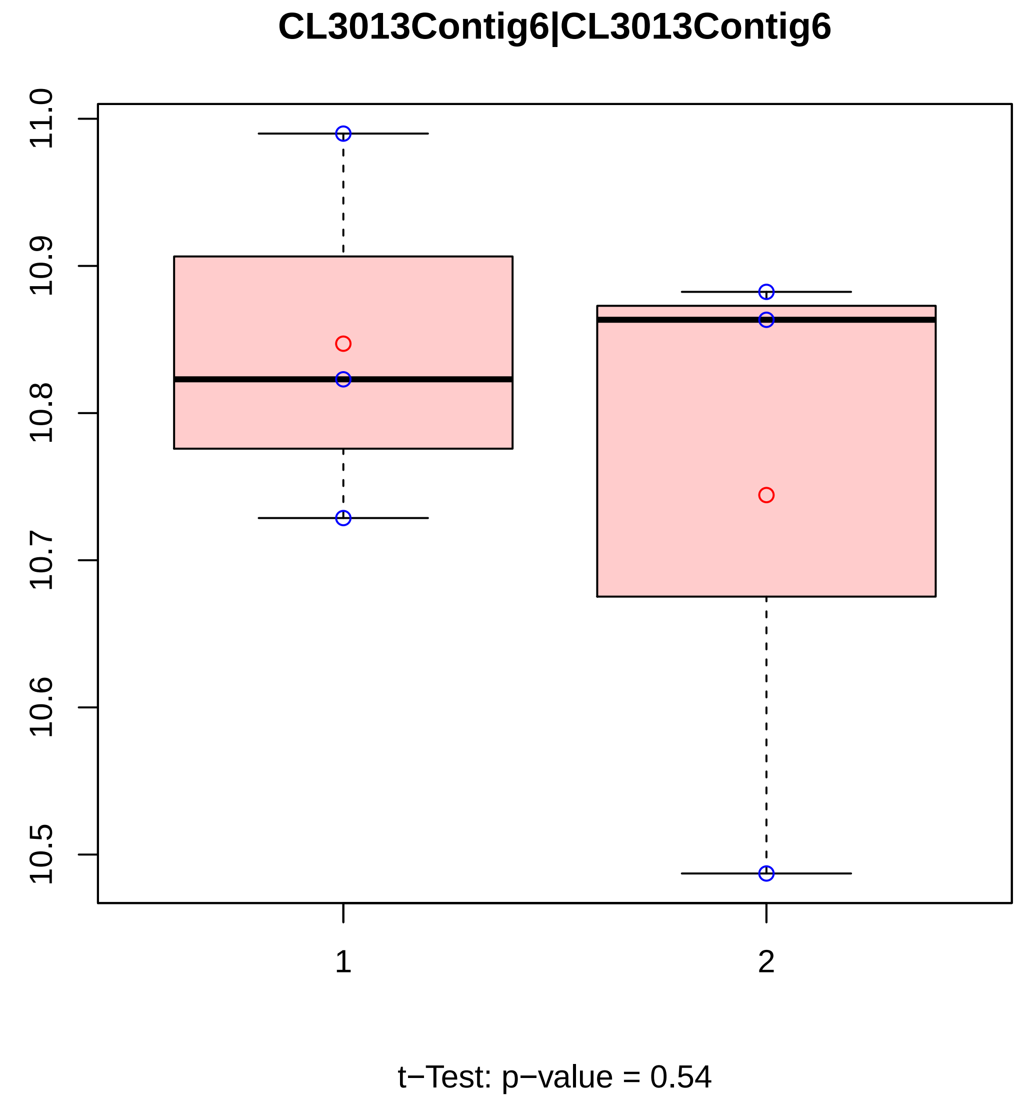
CL3013Contig6|CL3013Contig6
11.0
10.9
10.8
10.7
10.6
10.5
1
2
t−Test: p−value = 0.54

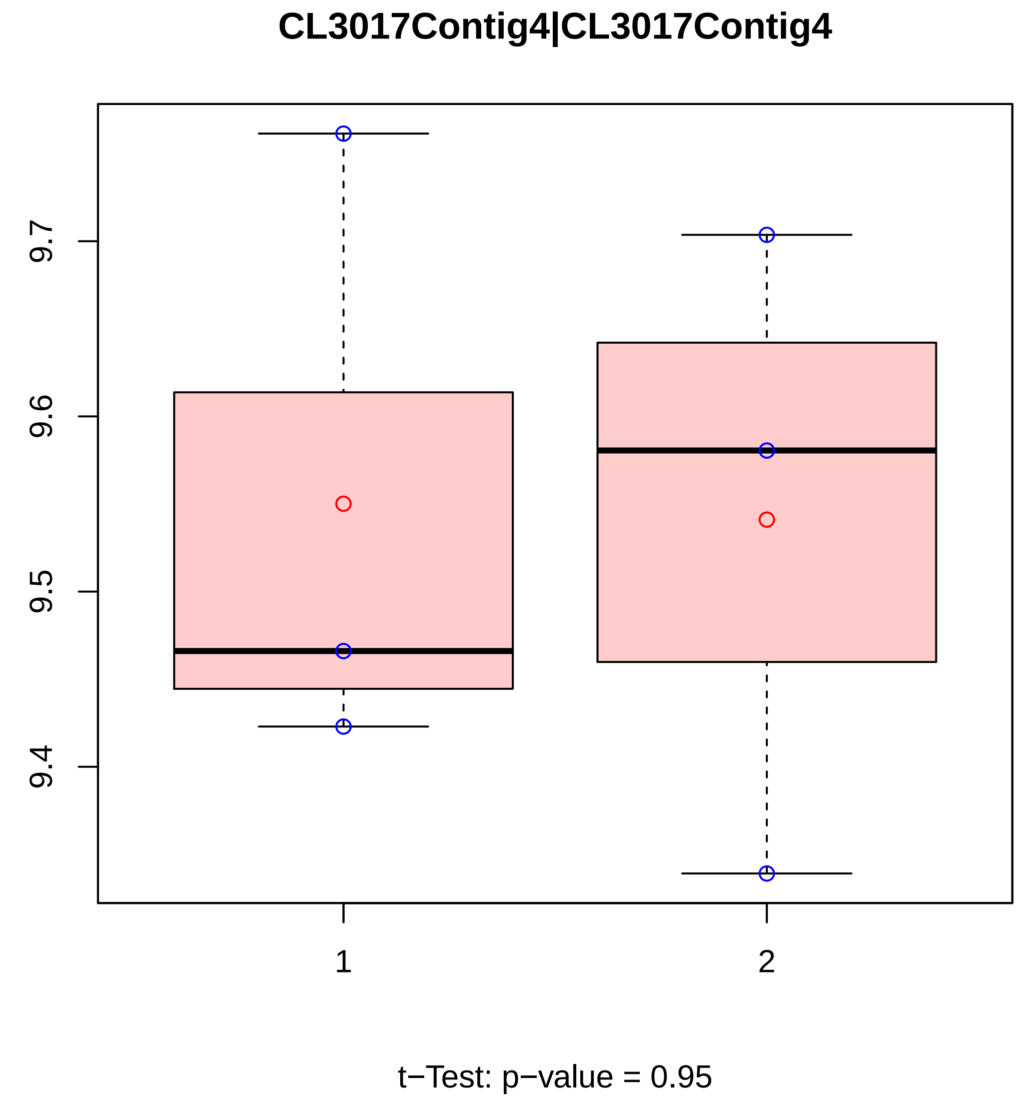
CL3017Contig4|CL3017Contig4
9.4
9.5
9.6
9.7
1
2
t−Test: p−value = 0.95

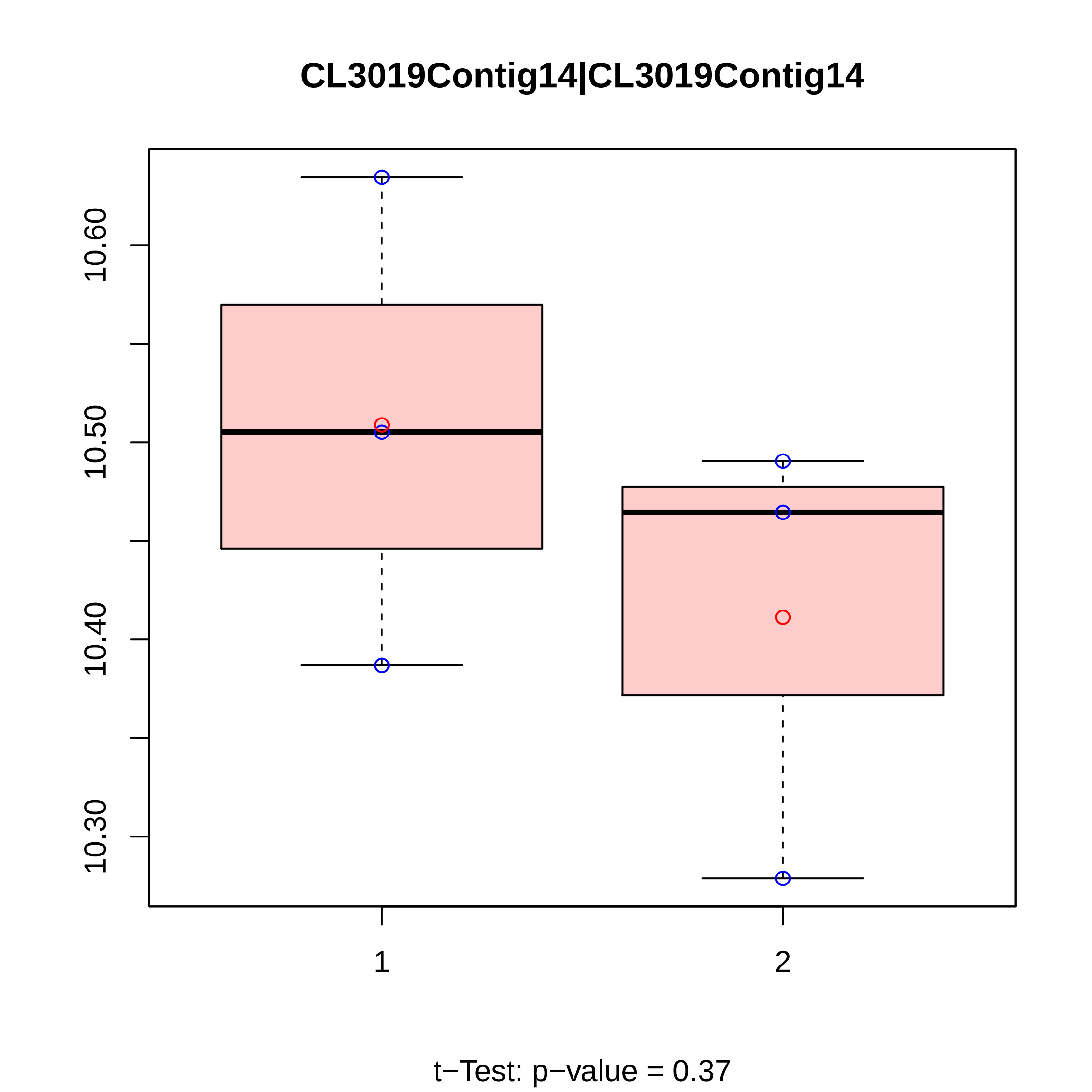

CL3019Contig14|CL3019Contig14
10.60
10.50
10.40
10.30
1
2
t−Test: p−value = 0.37

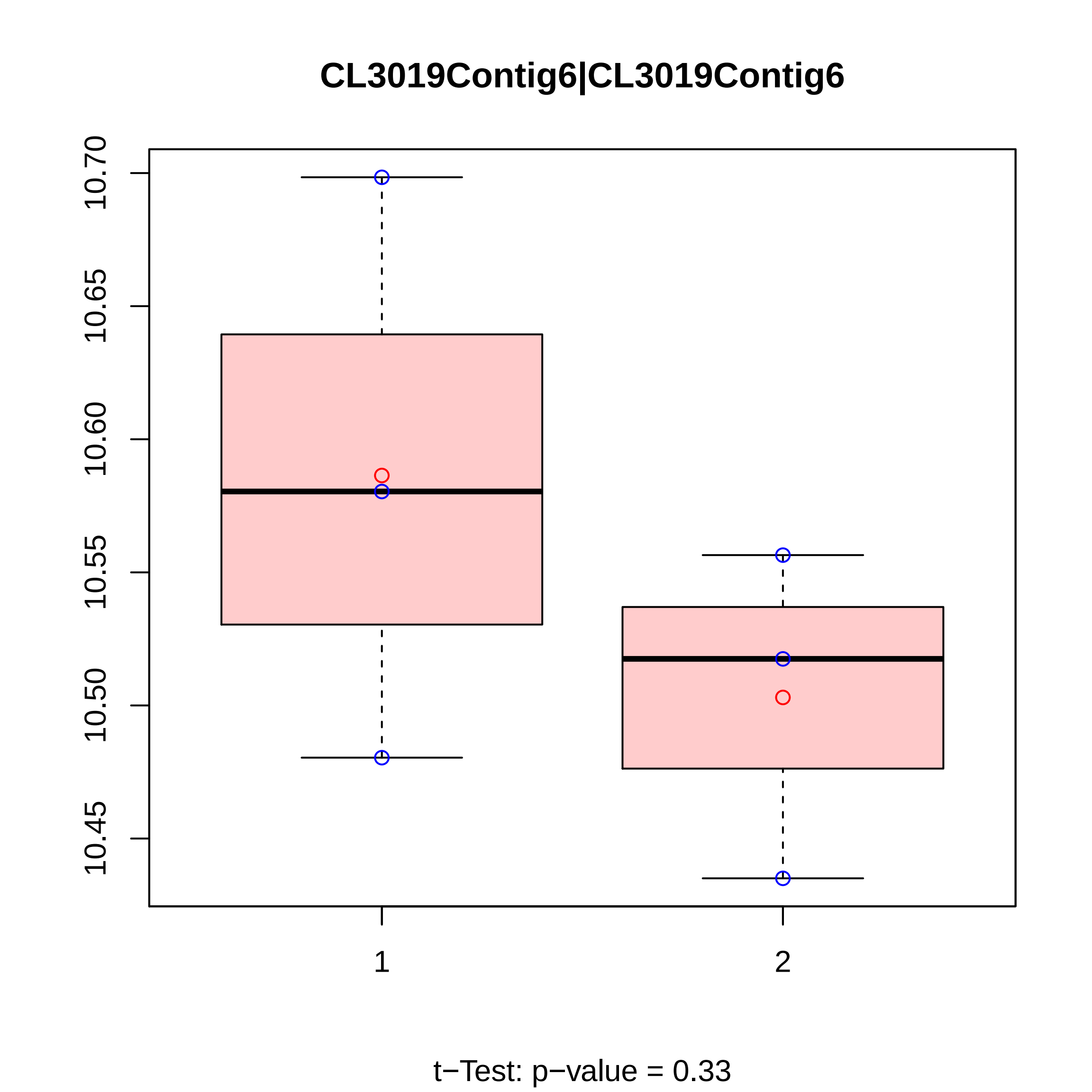

CL3019Contig6|CL3019Contig6
10.70
10.65
10.60
10.55
10.50
10.45
1
2
t−Test: p−value = 0.33

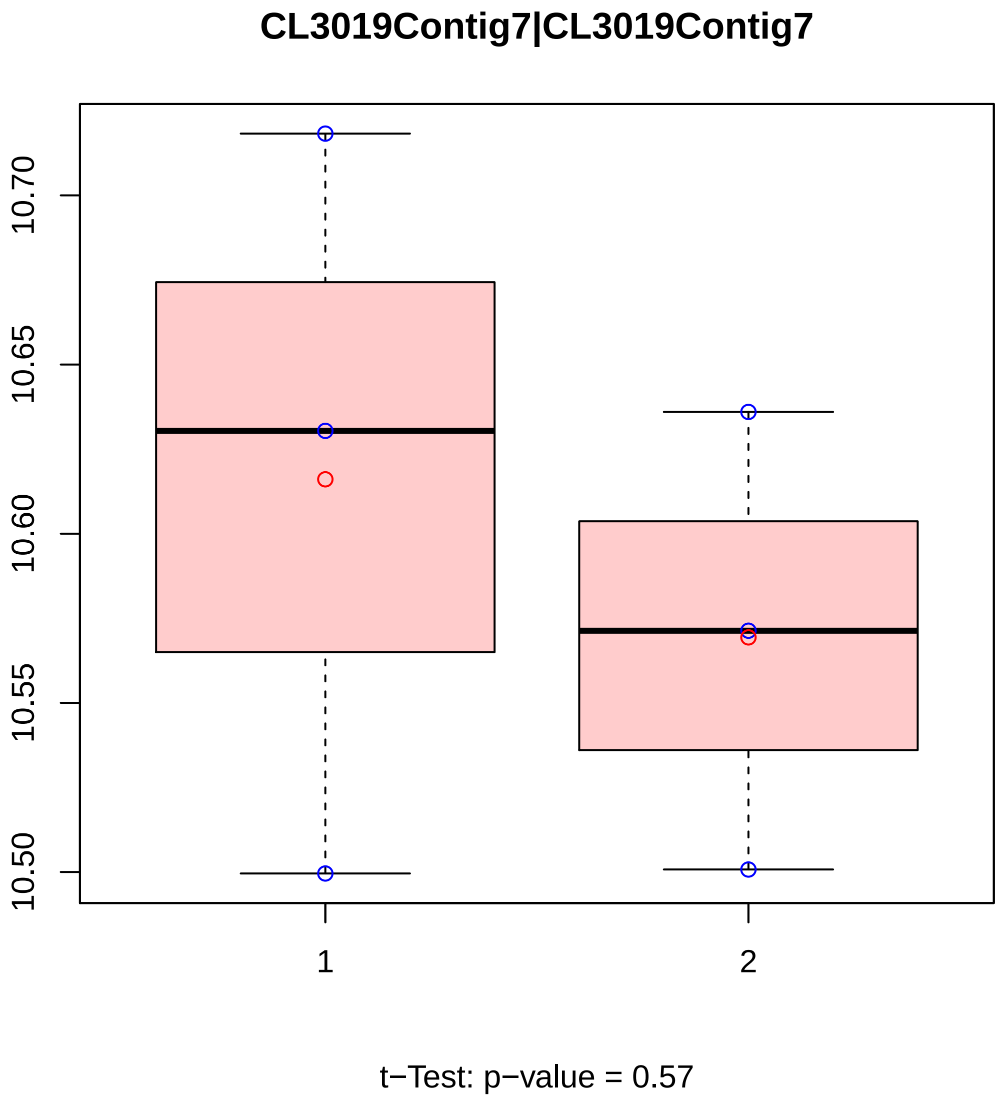
CL3019Contig7|CL3019Contig7
10.70
10.65
10.60
10.55
10.50
1
2
t−Test: p−value = 0.57

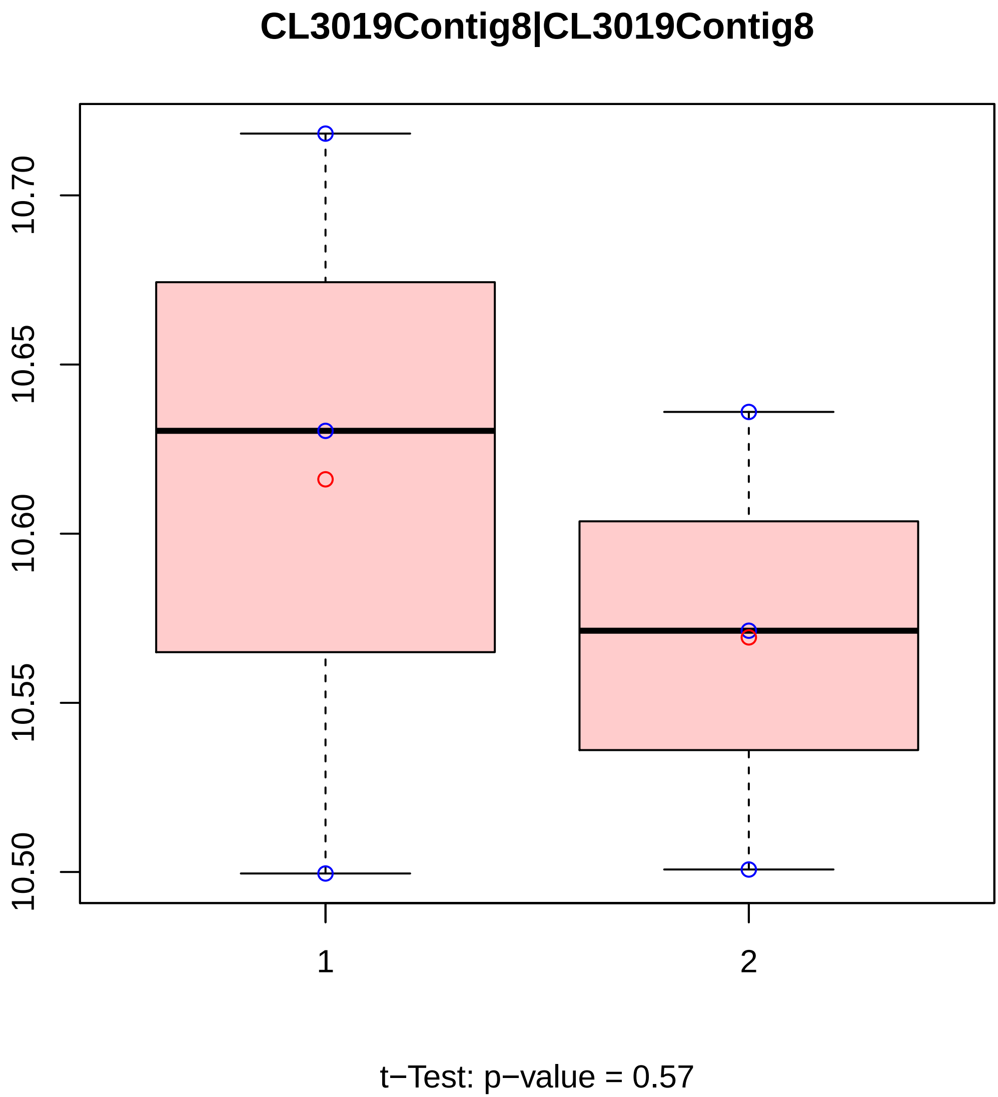

CL3019Contig8|CL3019Contig8
10.50
10.55
10.60
10.65
10.70
1
2
t−Test: p−value = 0.57

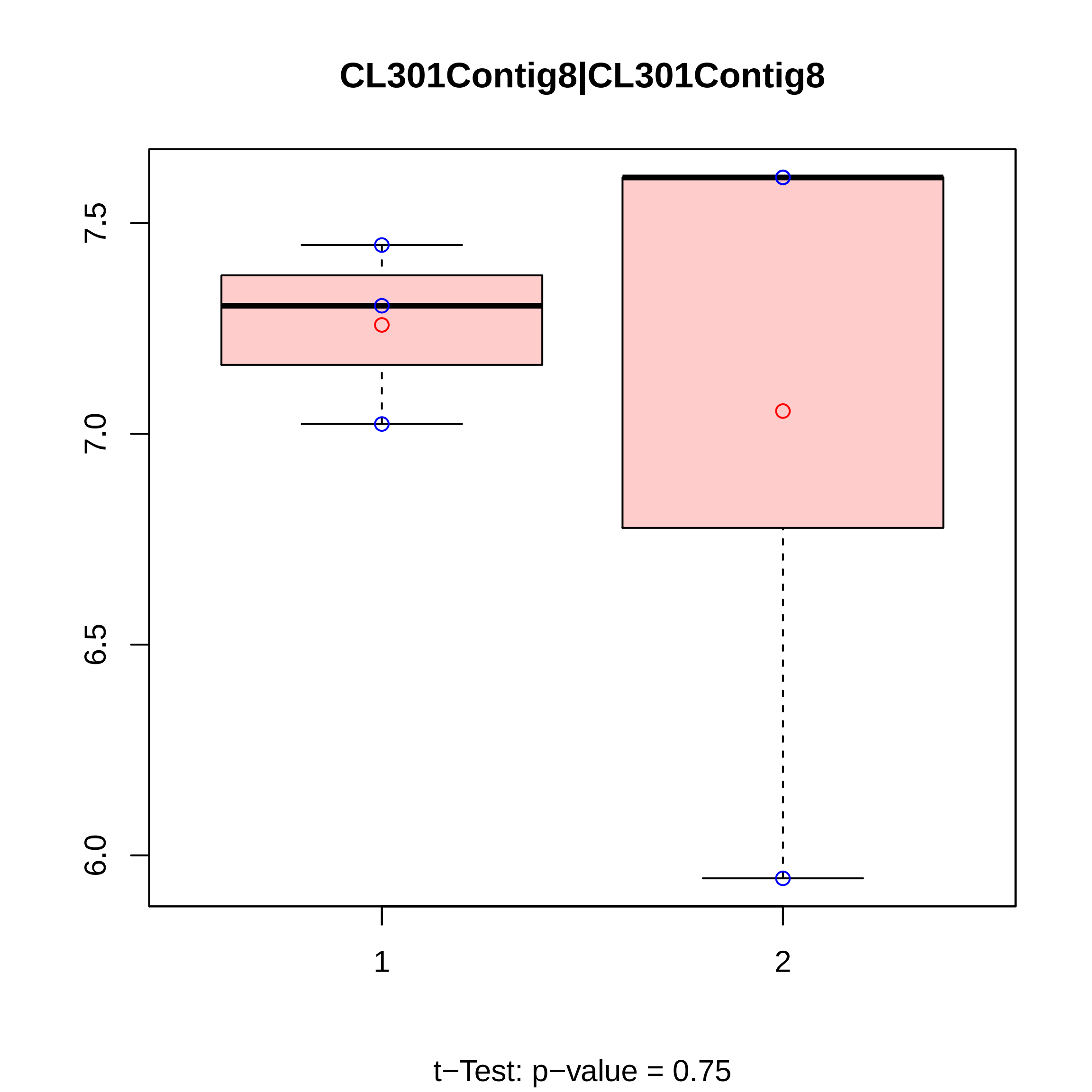

CL301Contig8|CL301Contig8
7.5
7.0
6.5
6.0
1
2
t−Test: p−value = 0.75

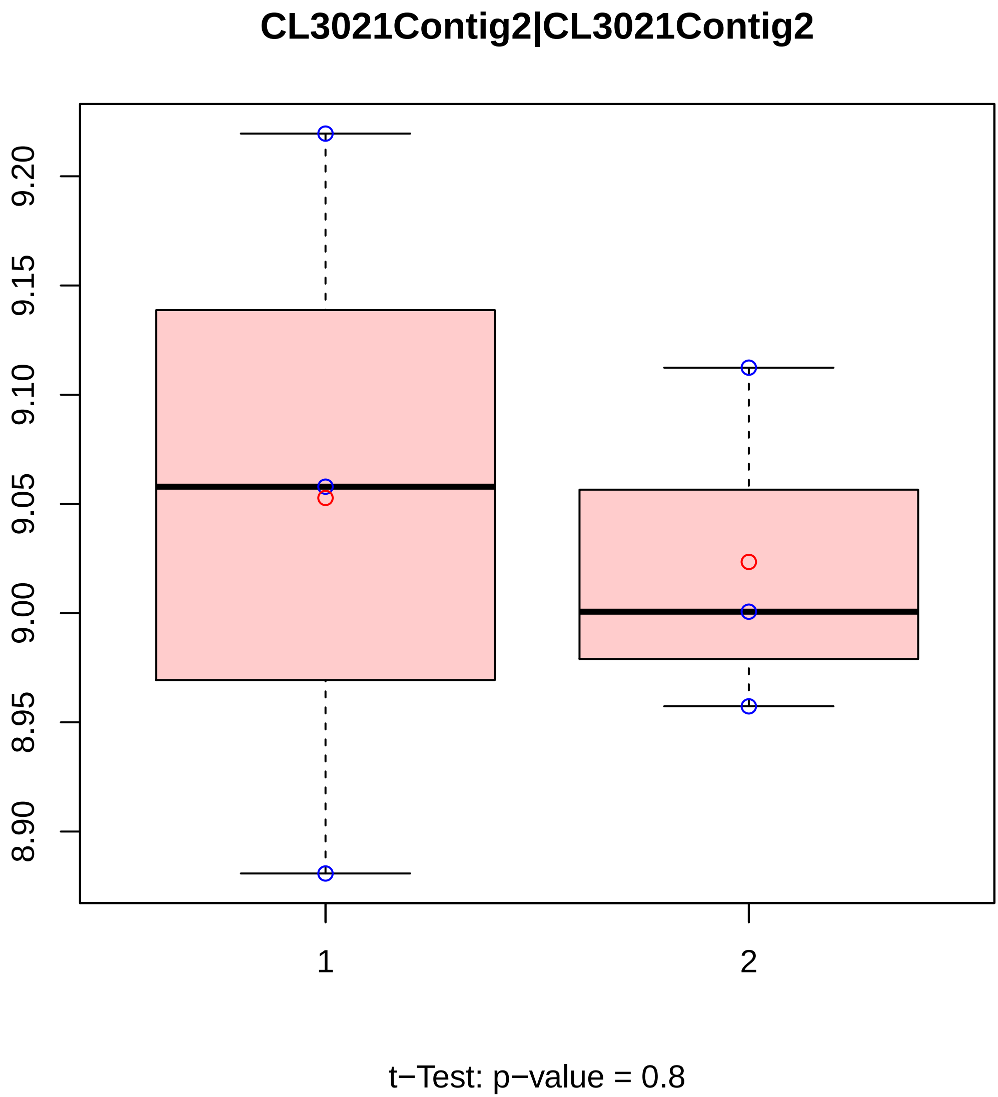

CL3021Contig2|CL3021Contig2
9.20
9.15
9.10
9.05
9.00
8.95
8.90
1
2
t−Test: p−value = 0.8

## CL3023Contig1|CL3023Contig1

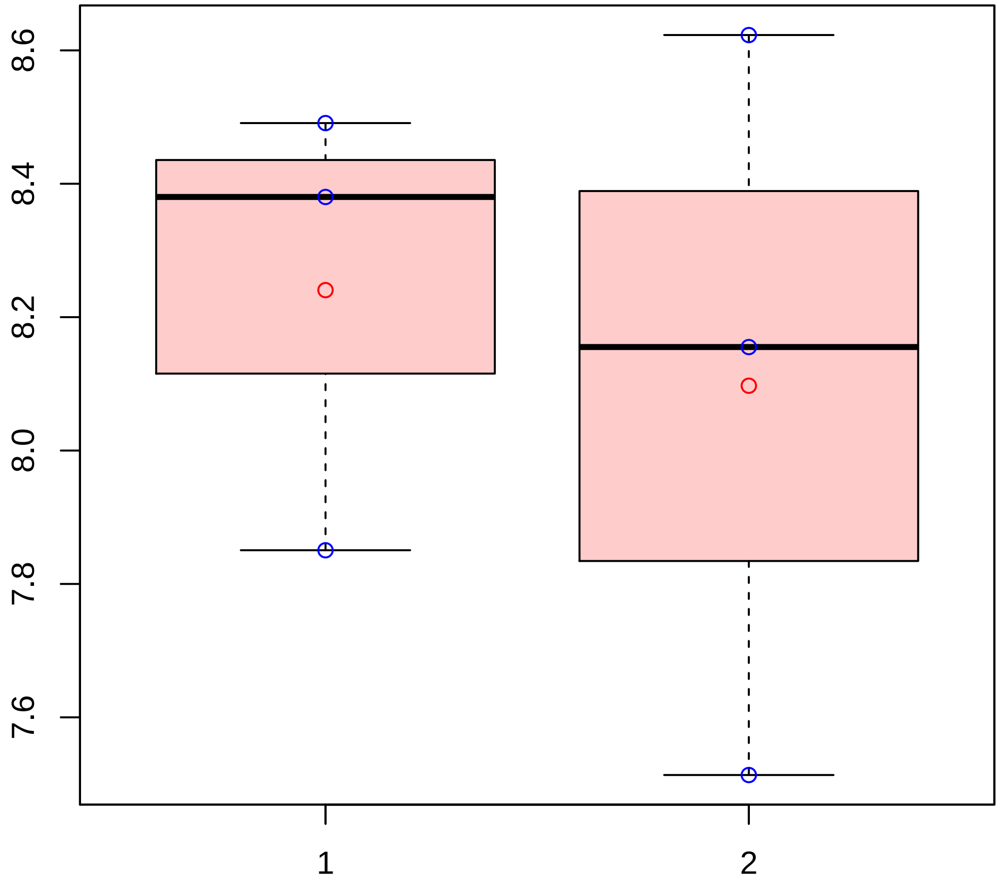


t−Test: p−value = 0.73

## CL3024Contig1|CL3024Contig1

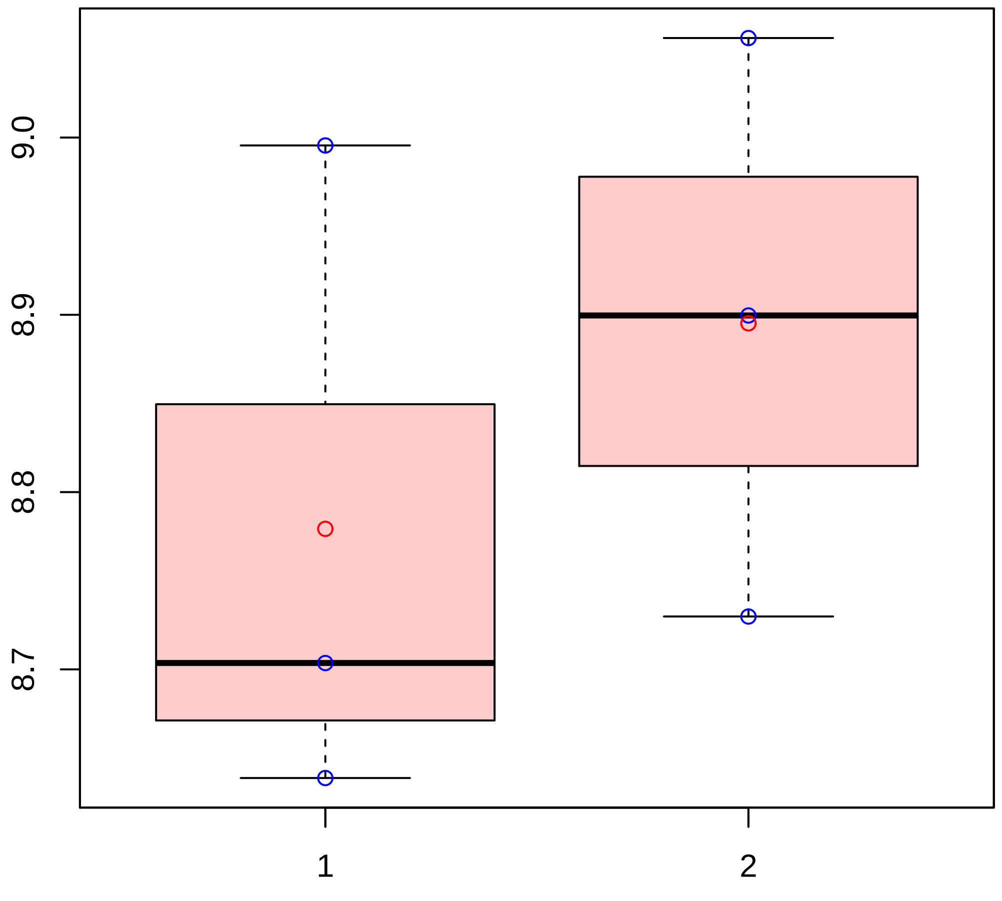


t−Test: p−value = 0.47

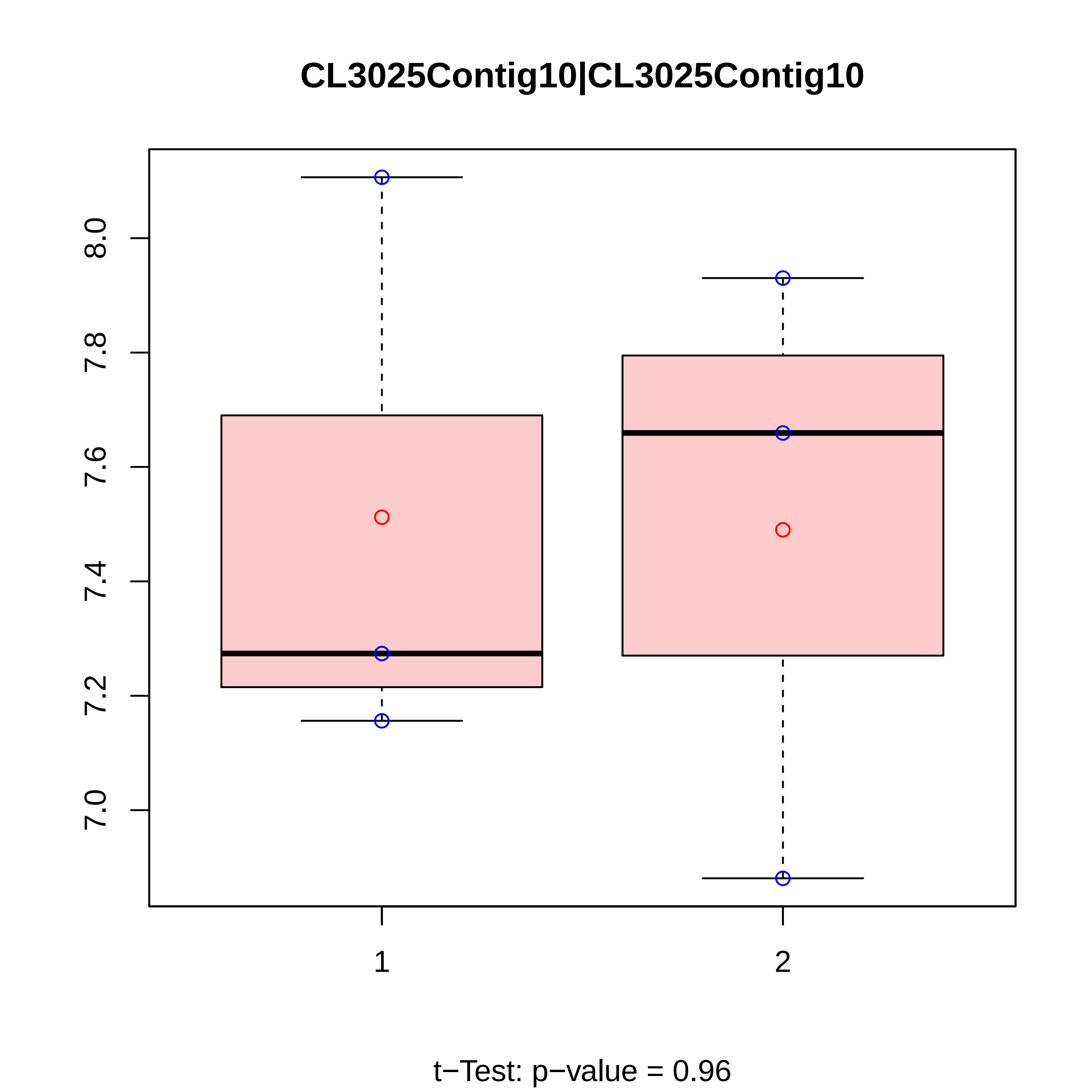

CL3025Contig10|CL3025Contig10
8.0
7.8
7.6
7.4
7.2
7.0
1
2
t−Test: p−value = 0.96

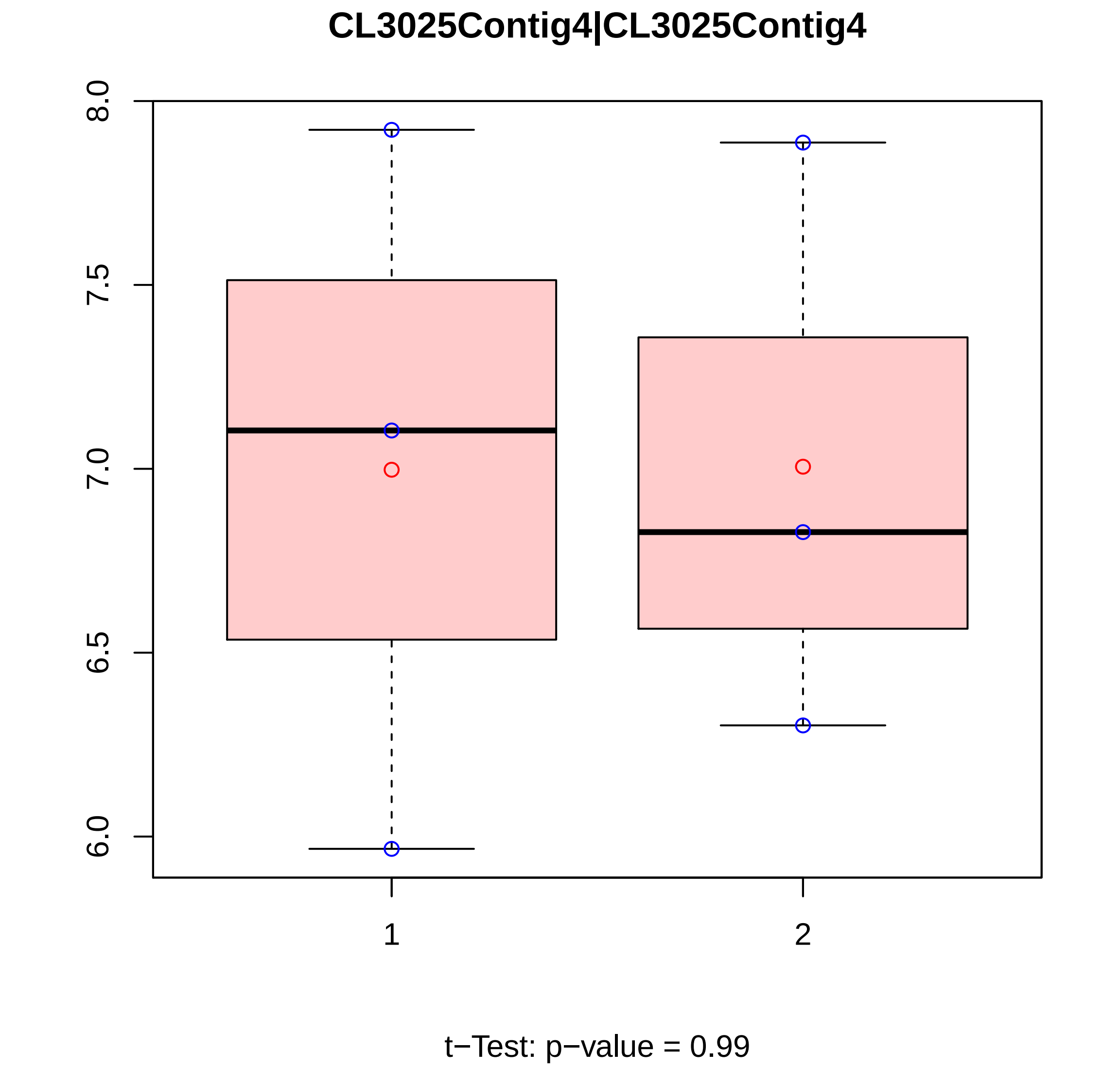

CL3025Contig4|CL3025Contig4
8.0
7.5
7.0
6.5
6.0
1
2
t−Test: p−value = 0.99

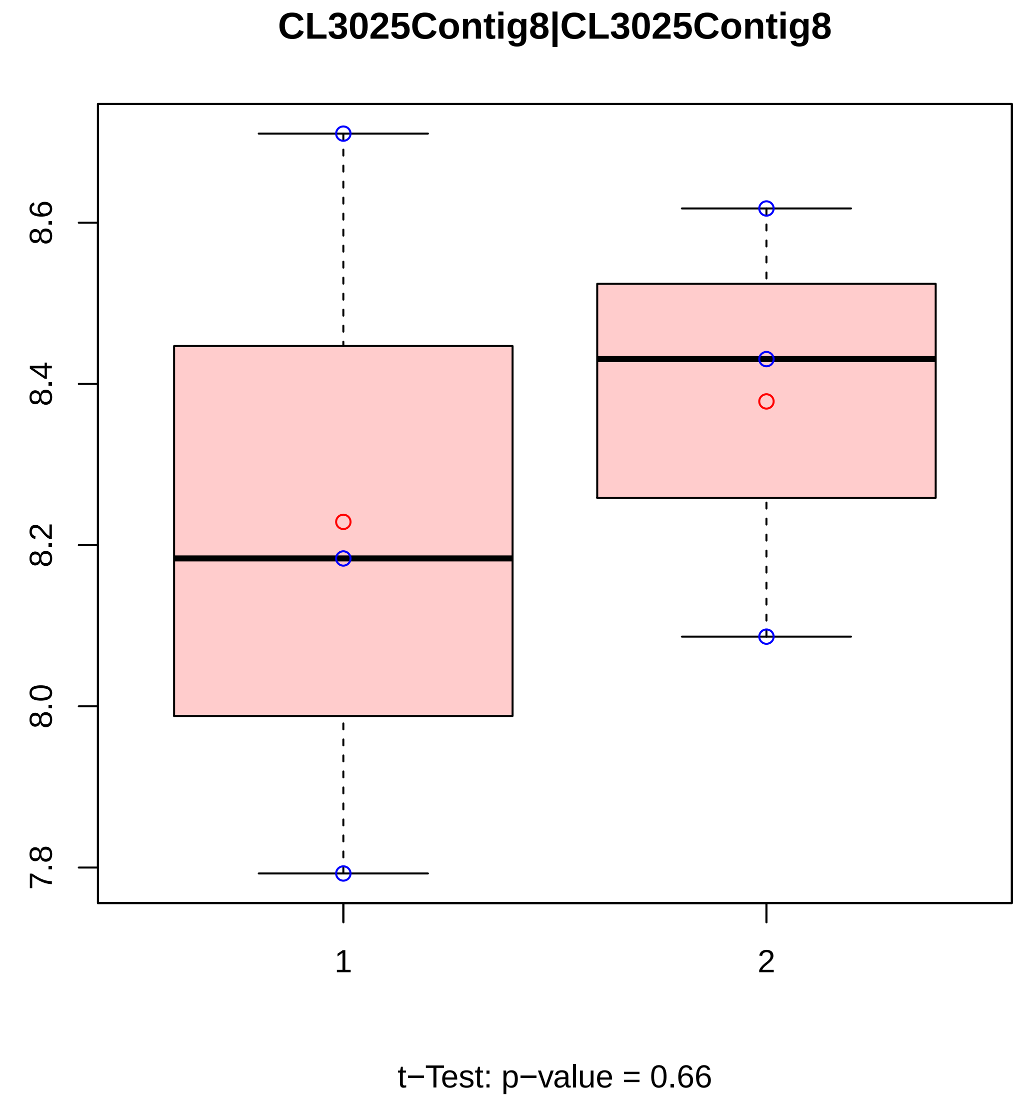
CL3025Contig8|CL3025Contig8
7.8
8.0
8.2
8.4
8.6
1
2
t−Test: p−value = 0.66

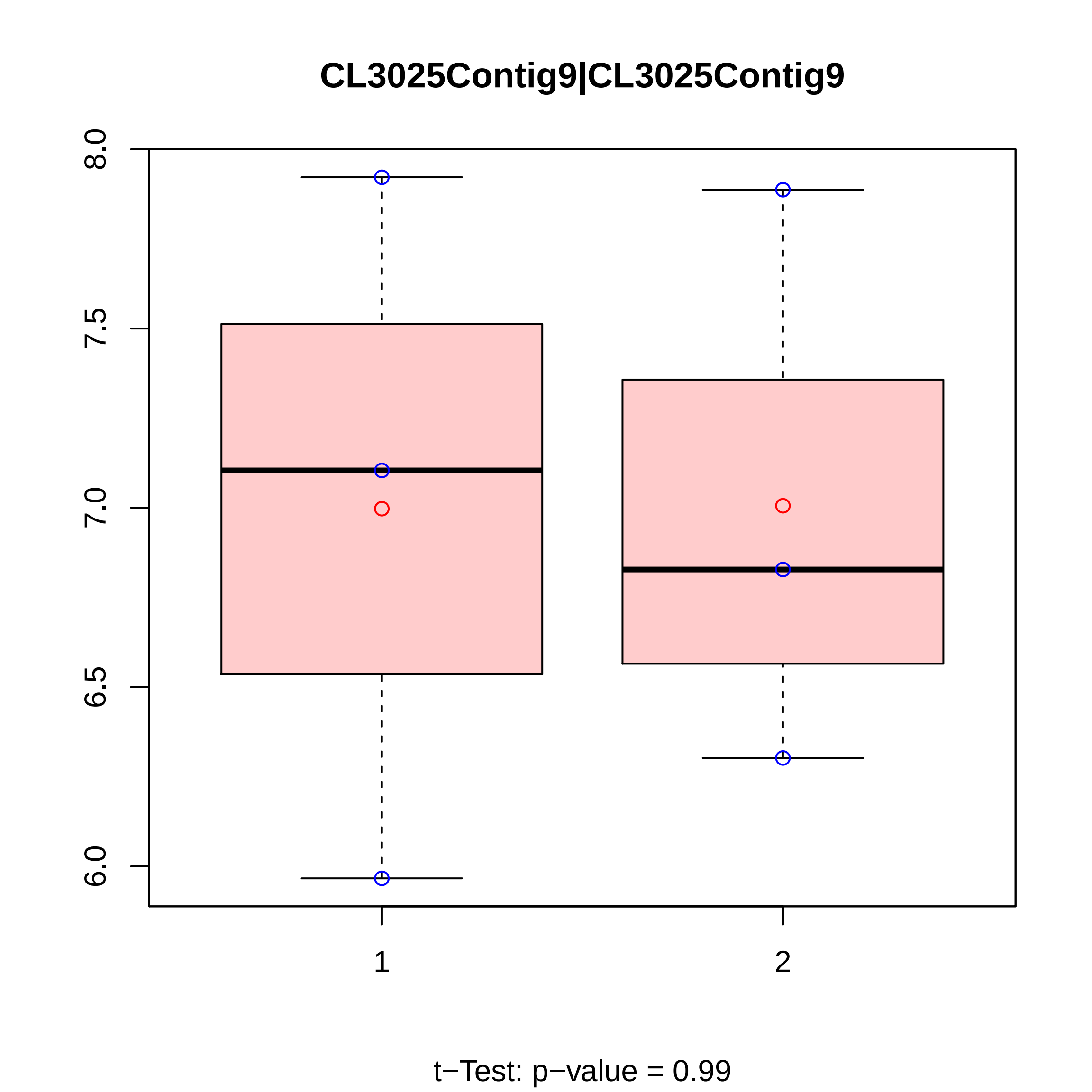

CL3025Contig9|CL3025Contig9
8.0
7.5
7.0
6.5
6.0
1
2
t−Test: p−value = 0.99

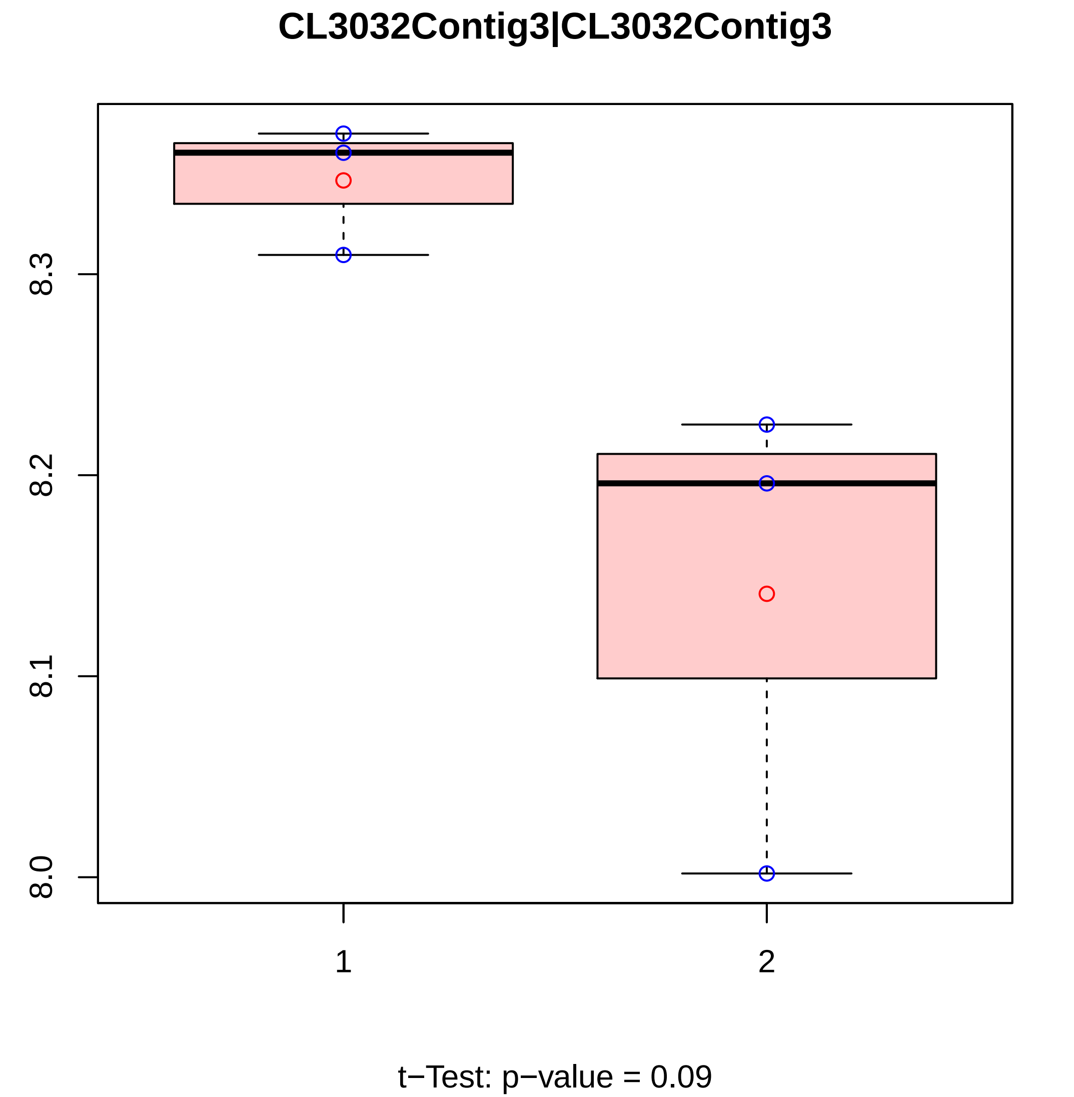
CL3032Contig3|CL3032Contig3
8.0
8.1
8.2
8.3
1
2
t−Test: p−value = 0.09

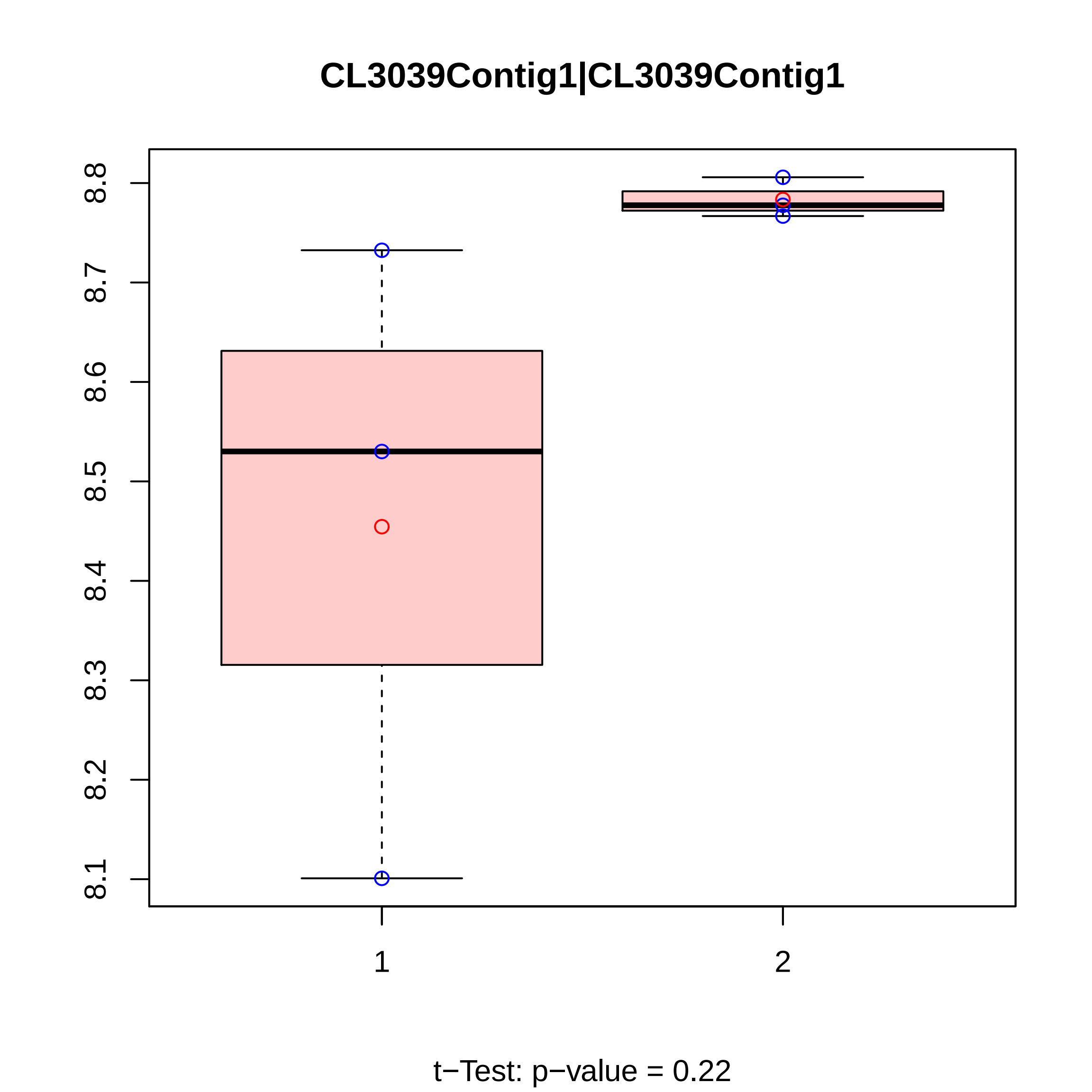

CL3039Contig1|CL3039Contig1
8.1
8.2
8.3
8.4
8.5
8.6
8.7
8.8
1
2
t−Test: p−value = 0.22

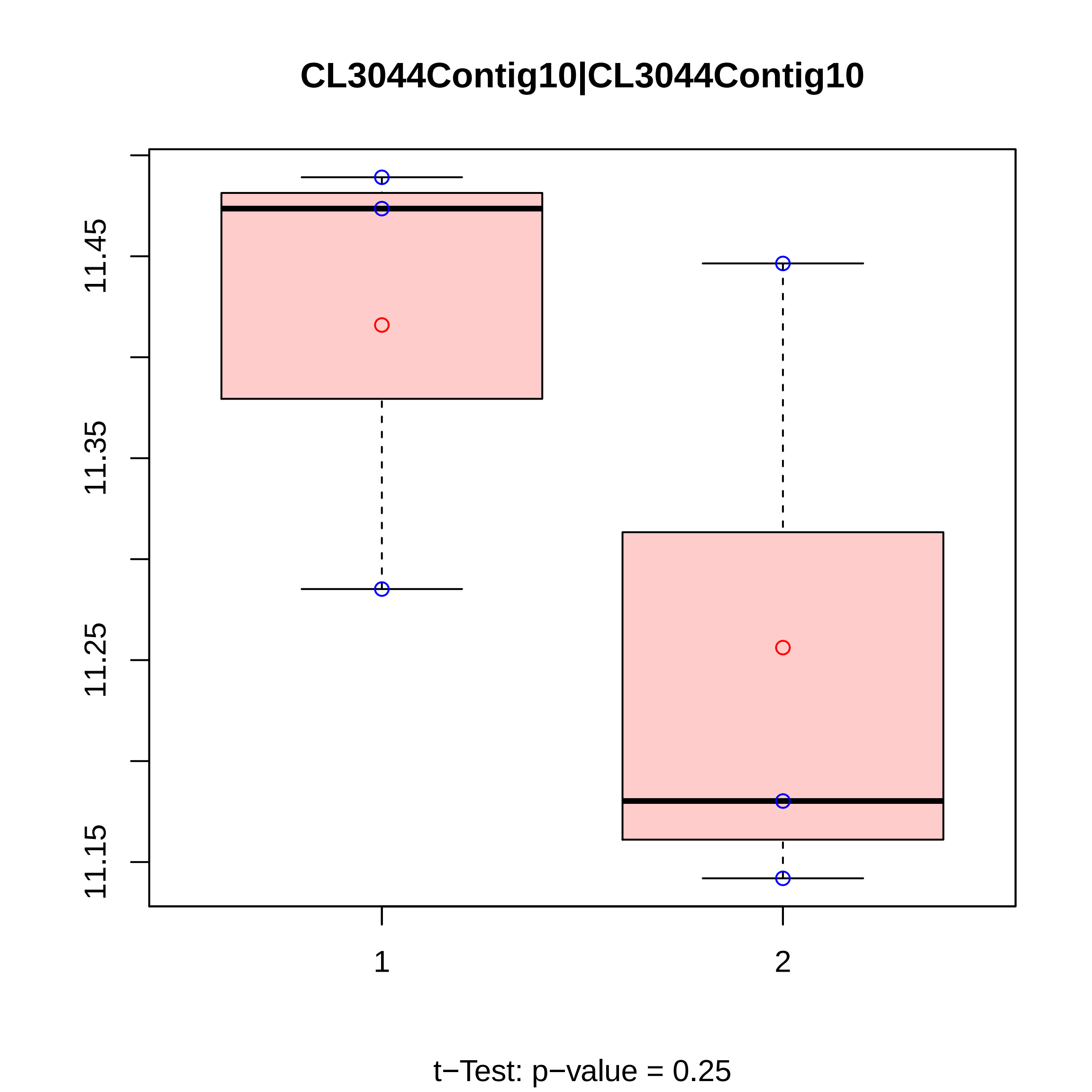
CL3044Contig10|CL3044Contig10
11.45
11.35
11.25
11.15
1
2
t−Test: p−value = 0.25

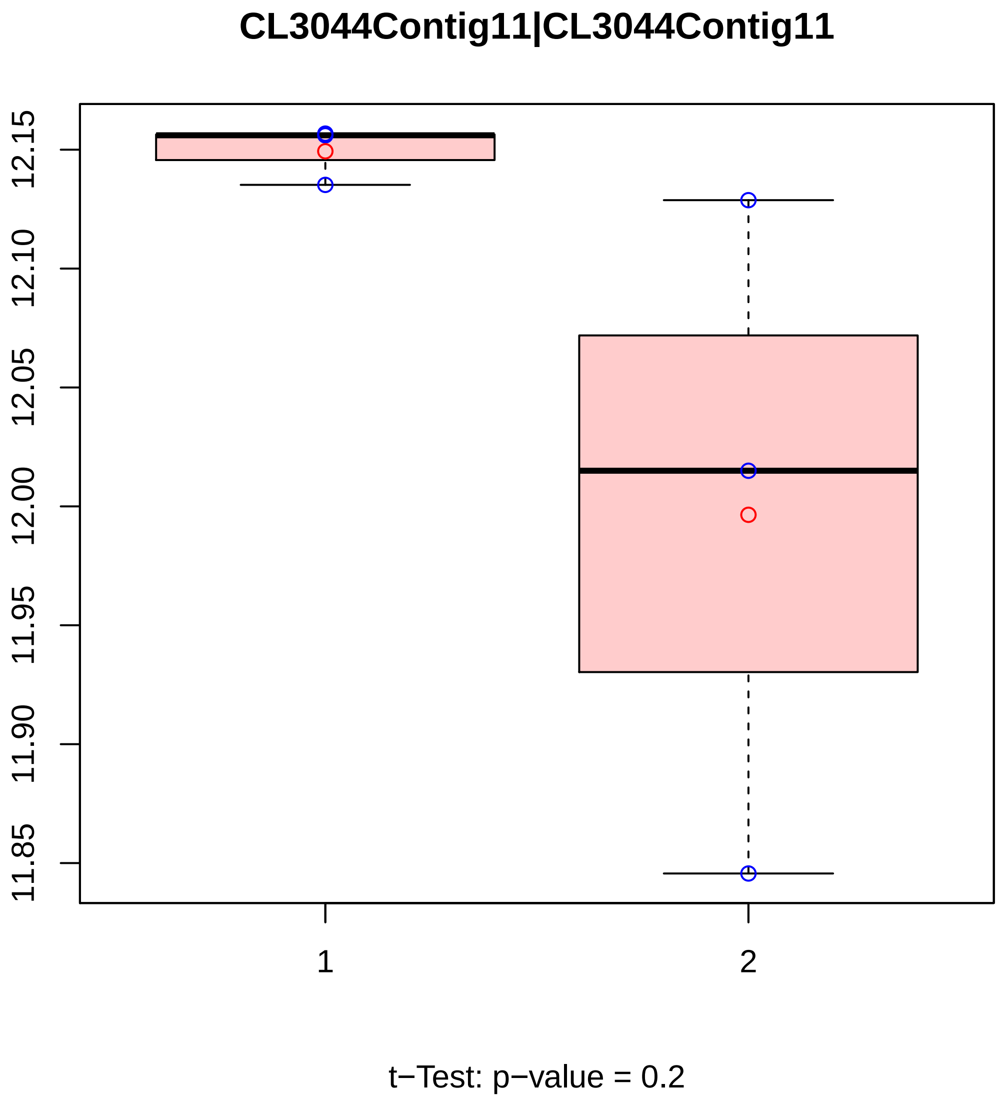

CL3044Contig11|CL3044Contig11
12.15
12.10
12.05
12.00
11.95
11.90
11.85
1
2
t−Test: p−value = 0.2

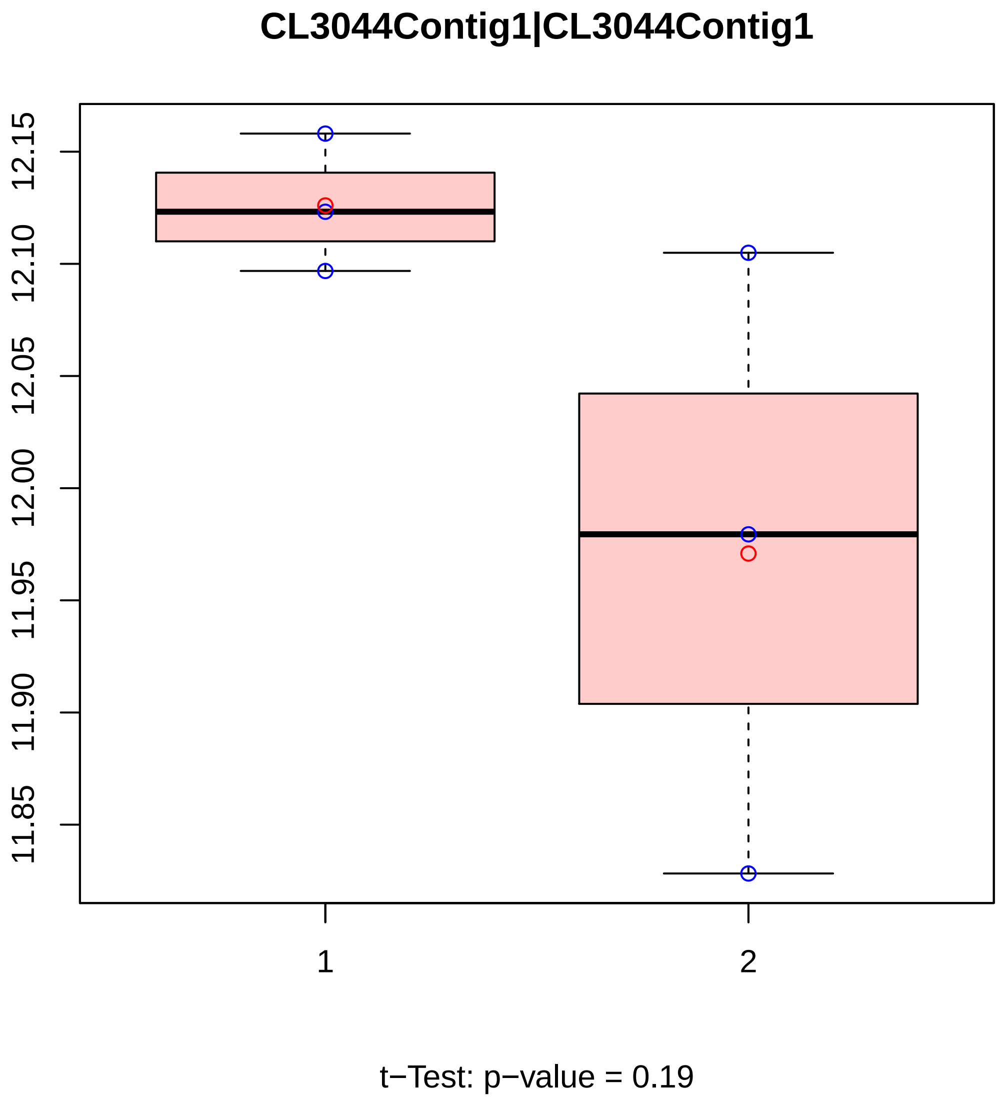

CL3044Contig1|CL3044Contig1
12.15
12.10
12.05
12.00
11.95
11.90
11.85
1
2
t−Test: p−value = 0.19

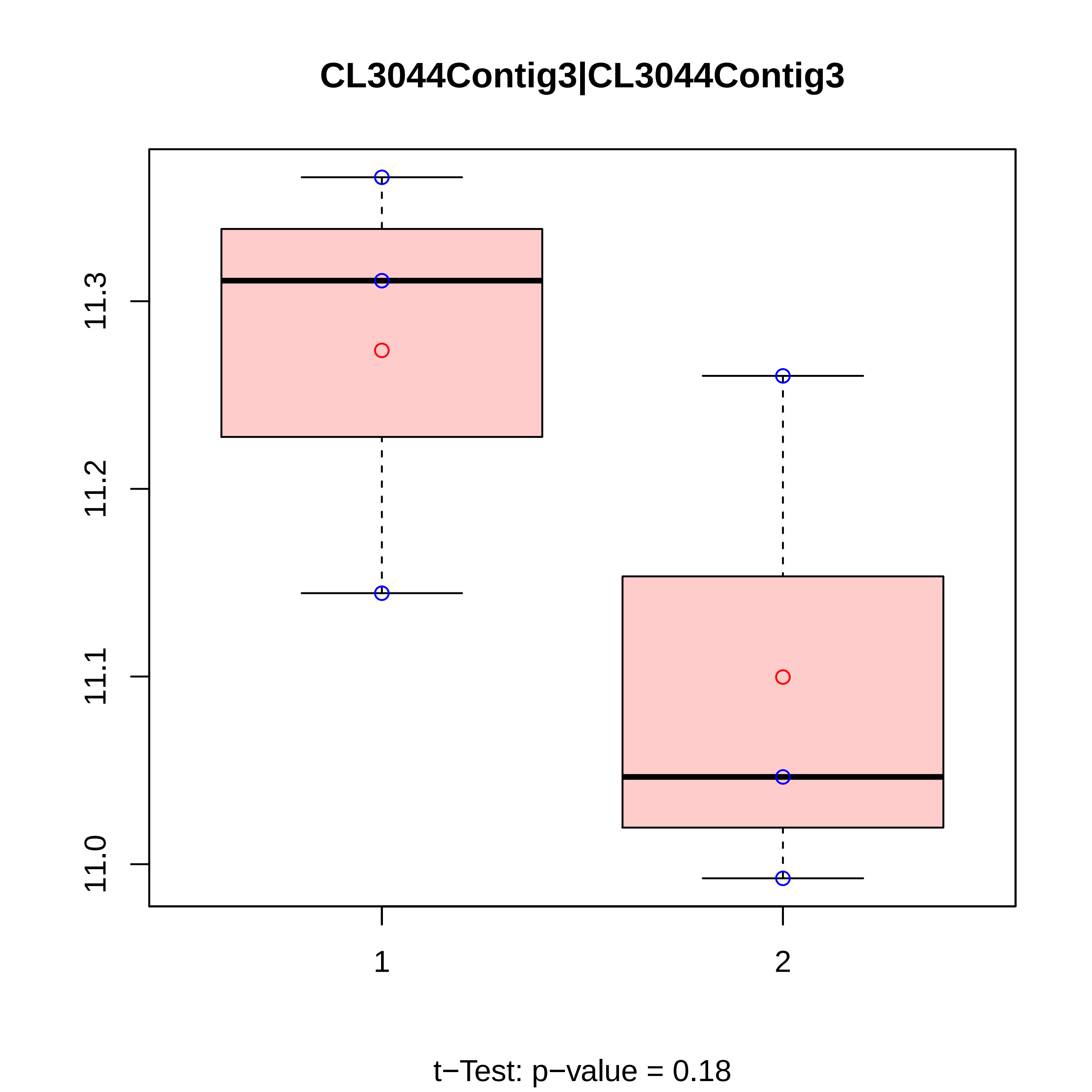
CL3044Contig3|CL3044Contig3
11.0
11.1
11.2
11.3
1
2
t−Test: p−value = 0.18

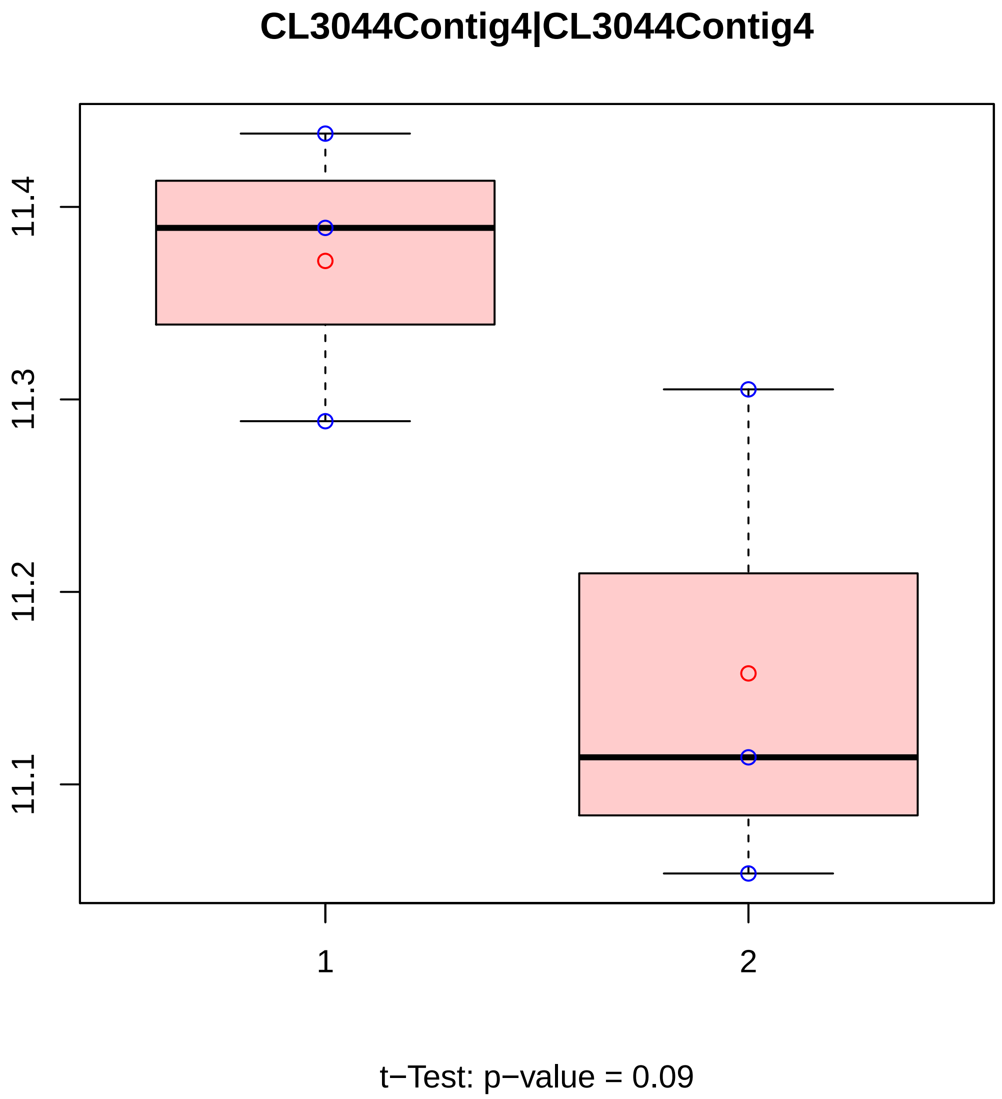
CL3044Contig4|CL3044Contig4
11.4
11.3
11.2
11.1
1
2
t−Test: p−value = 0.09

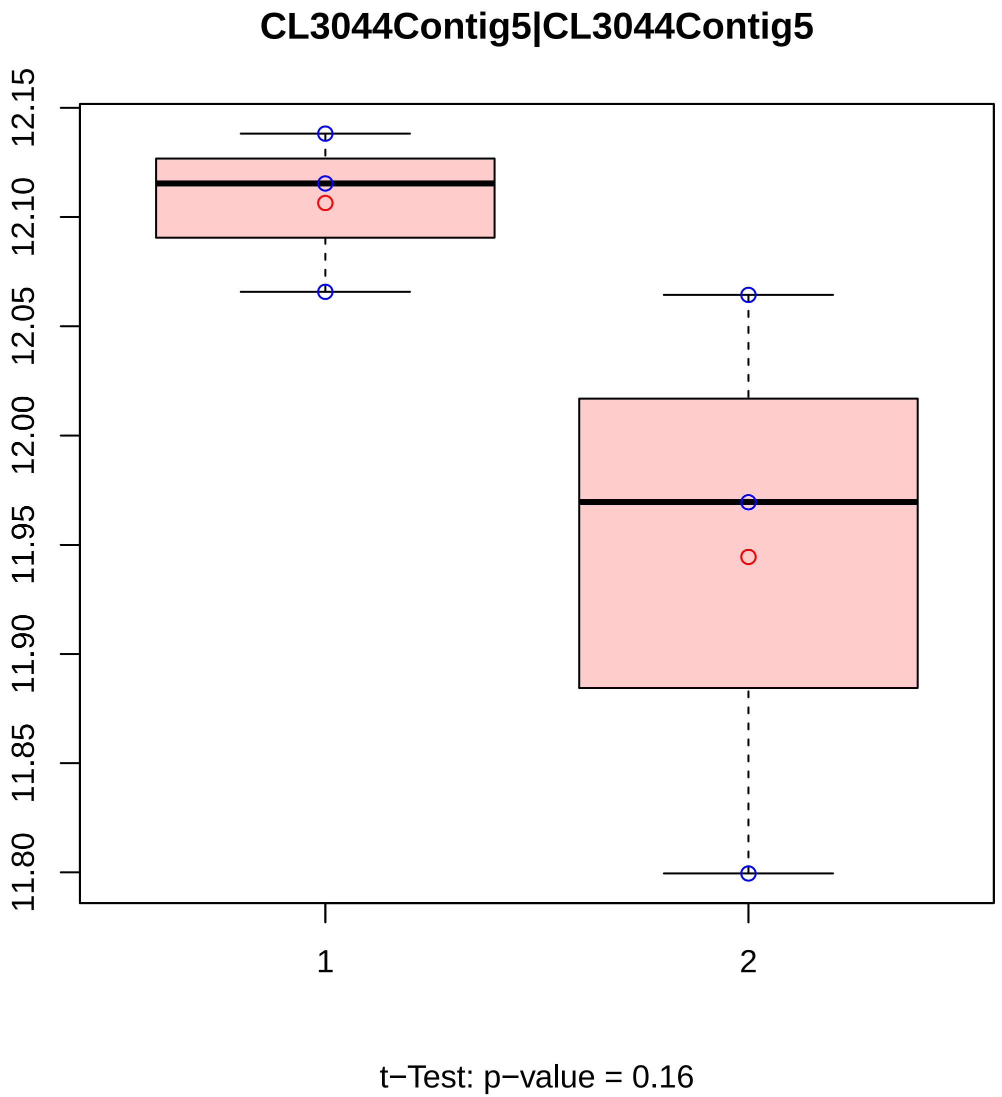

CL3044Contig5|CL3044Contig5
12.15
12.10
12.05
12.00
11.95
11.90
11.85
11.80
1
2
t−Test: p−value = 0.16

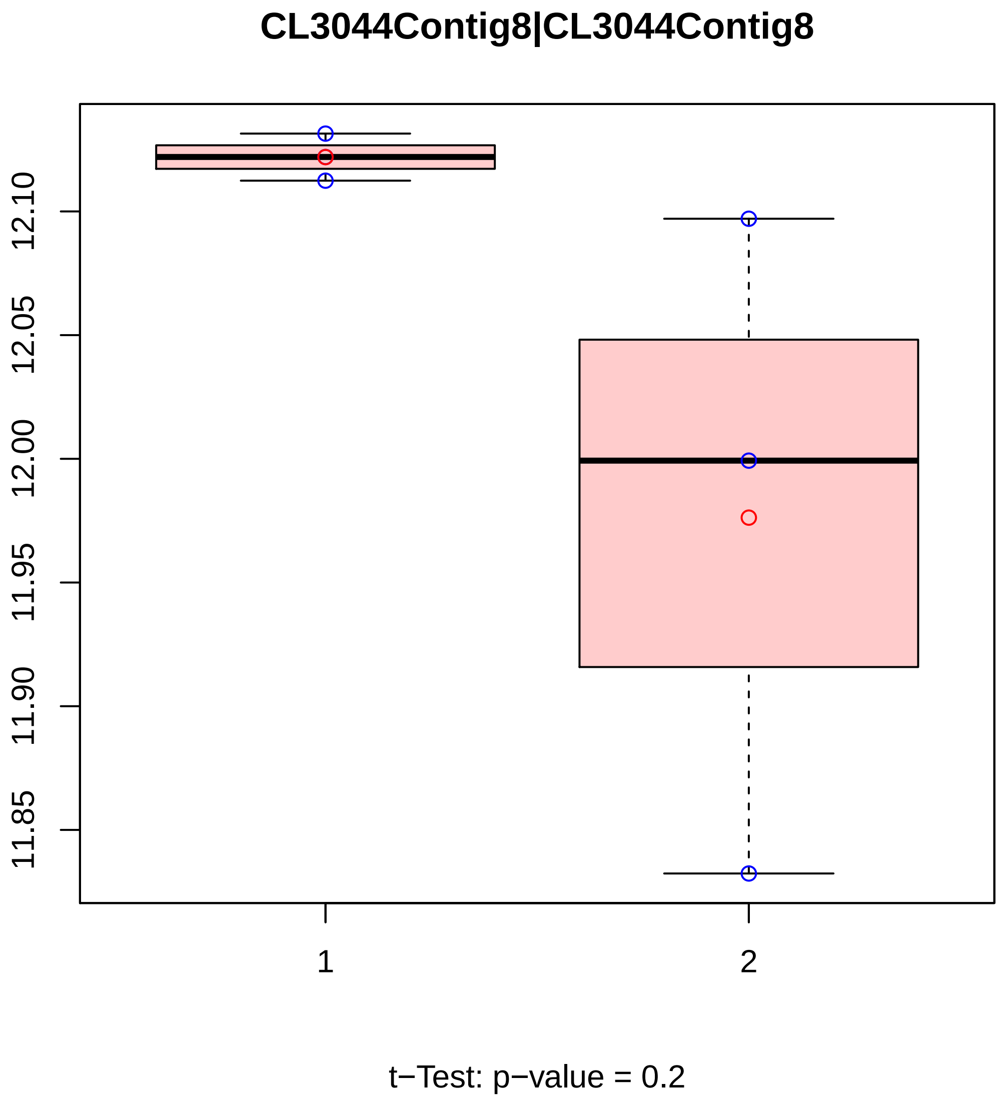

CL3044Contig8|CL3044Contig8
12.10
12.05
12.00
11.95
11.90
11.85
1
2
t−Test: p−value = 0.2

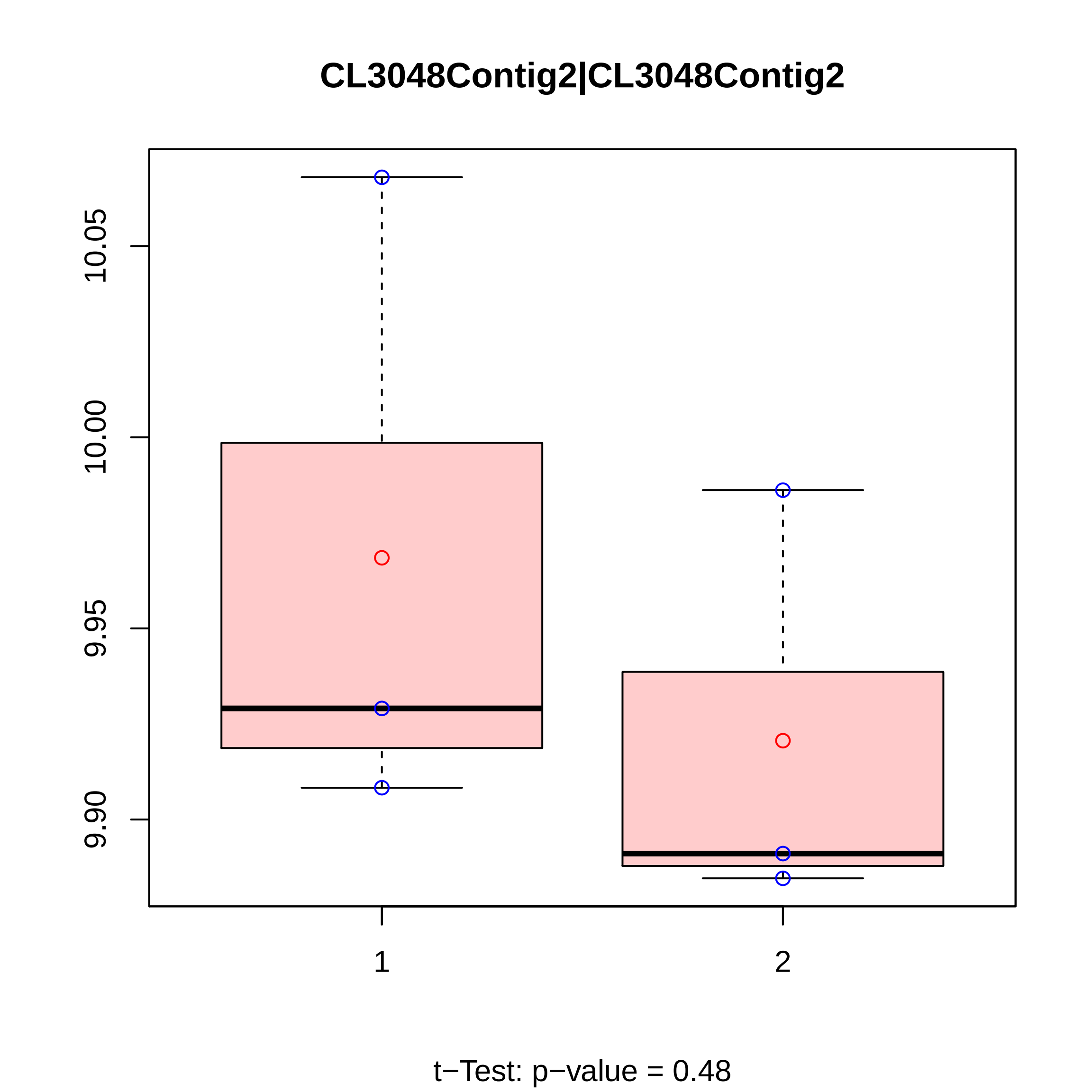

CL3048Contig2|CL3048Contig2
10.05
10.00
9.95
9.90
1
2
t−Test: p−value = 0.48

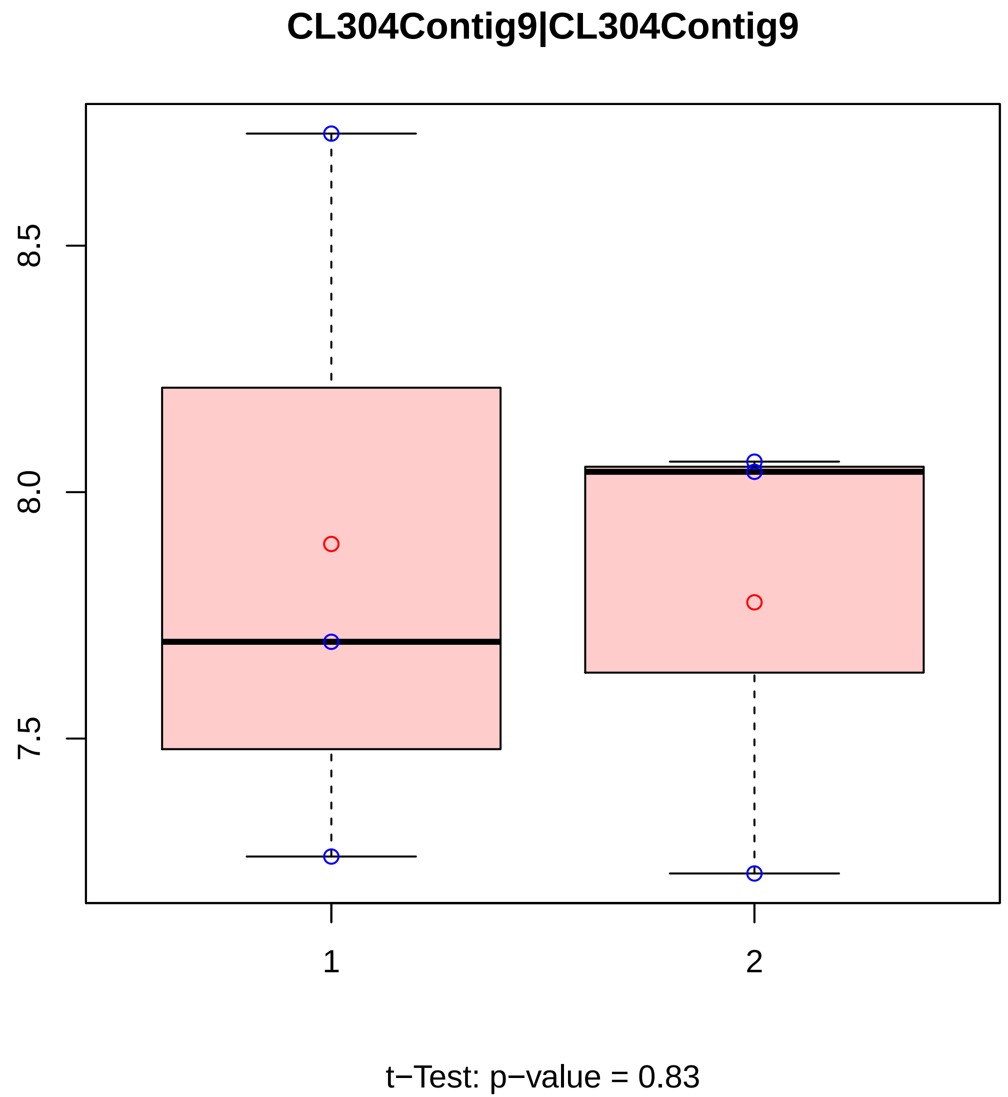
CL304Contig9|CL304Contig9
8.5
8.0
7.5
1
2
t−Test: p−value = 0.83

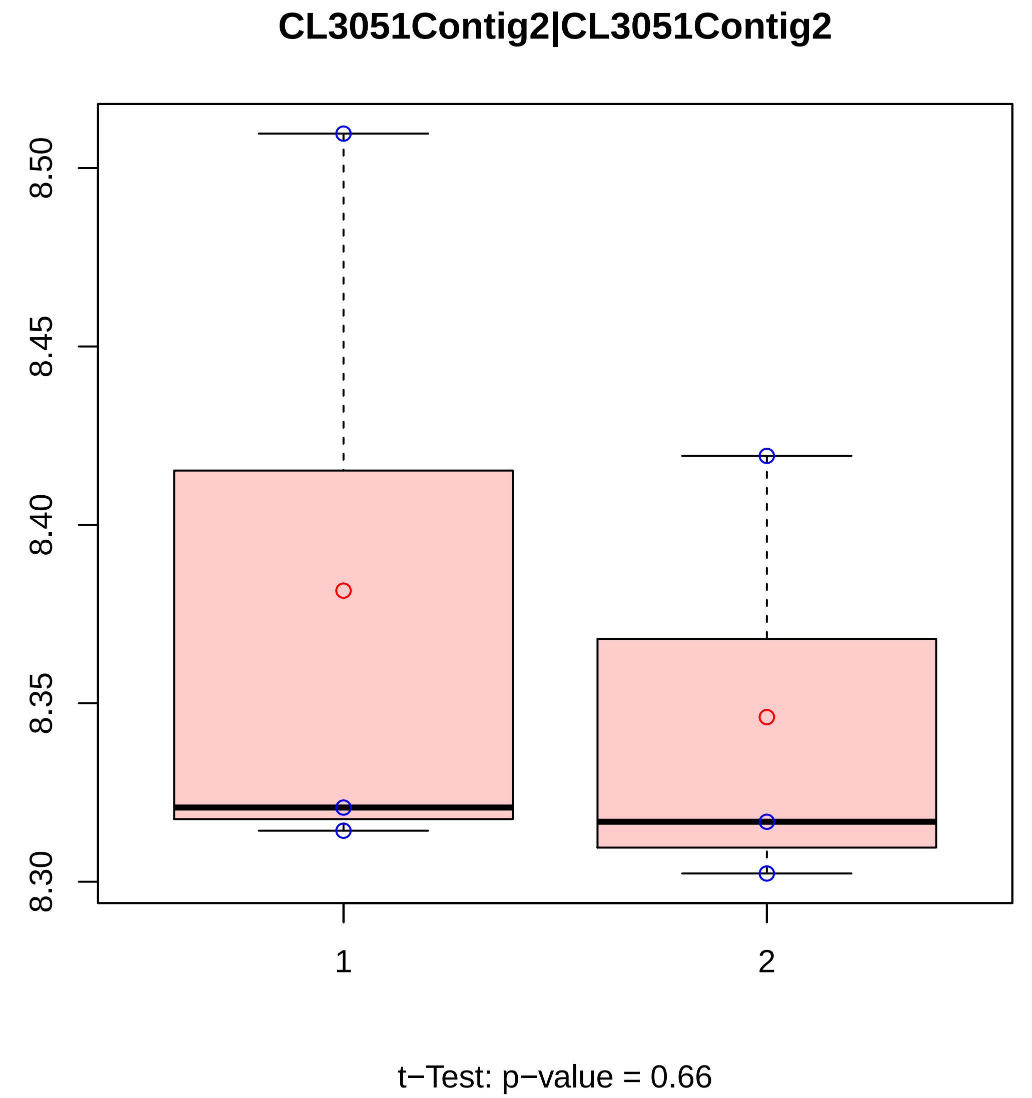
CL3051Contig2|CL3051Contig2
8.50
8.45
8.40
8.35
8.30
1
2
t−Test: p−value = 0.66

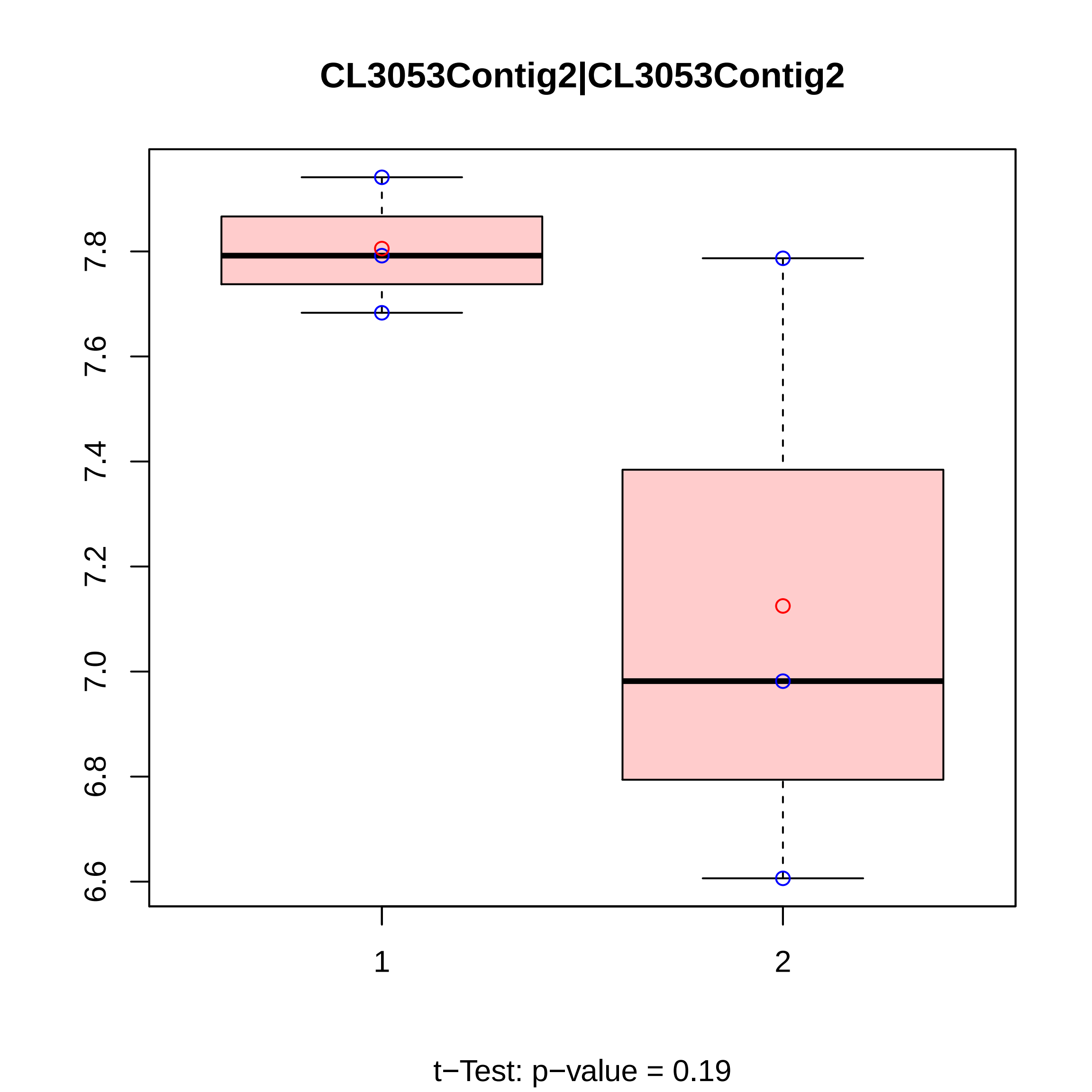

CL3053Contig2|CL3053Contig2
7.8
7.6
7.4
7.2
7.0
6.8
6.6
1
2
t−Test: p−value = 0.19

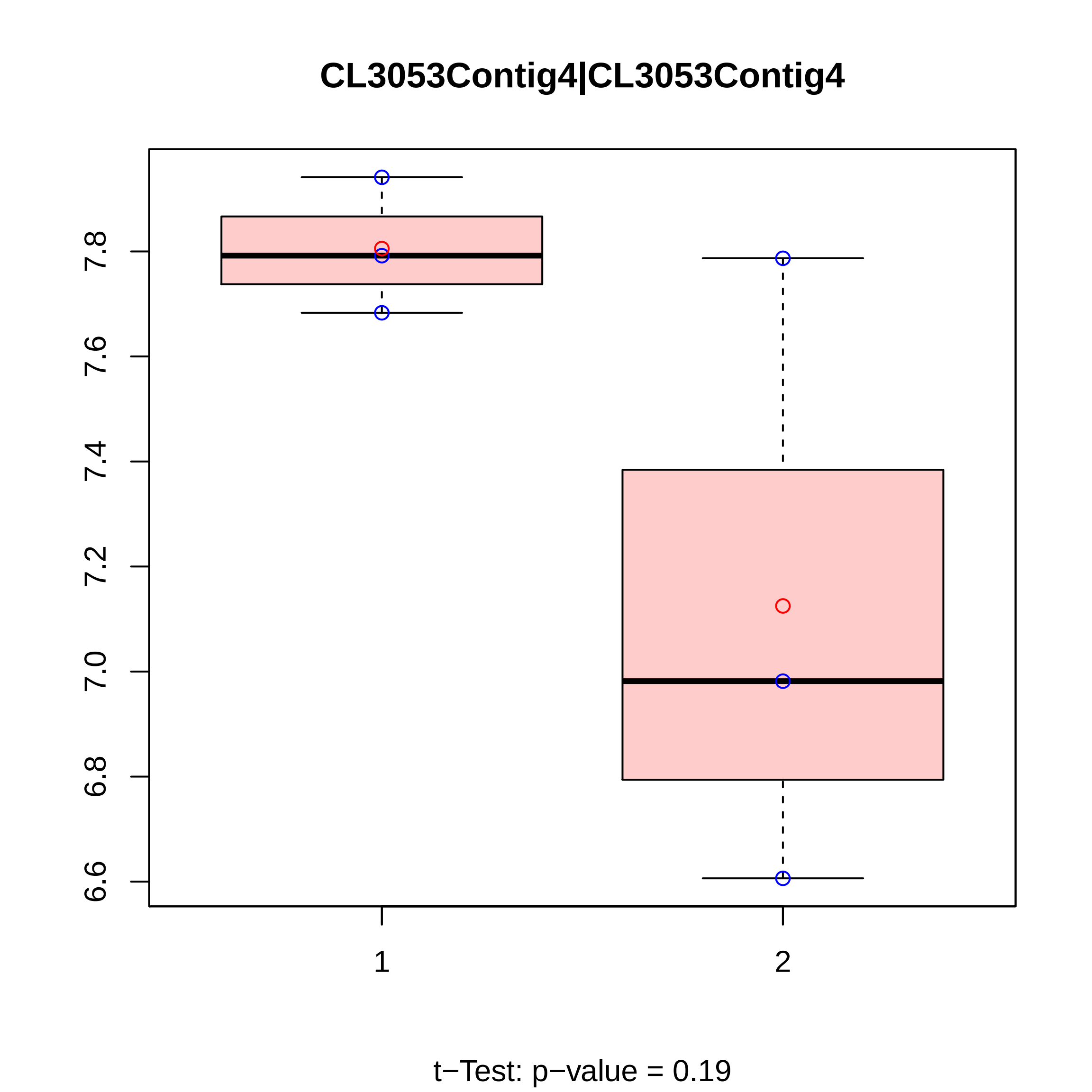
CL3053Contig4|CL3053Contig4
7.8
7.6
7.4
7.2
7.0
6.8
6.6
1
2
t−Test: p−value = 0.19

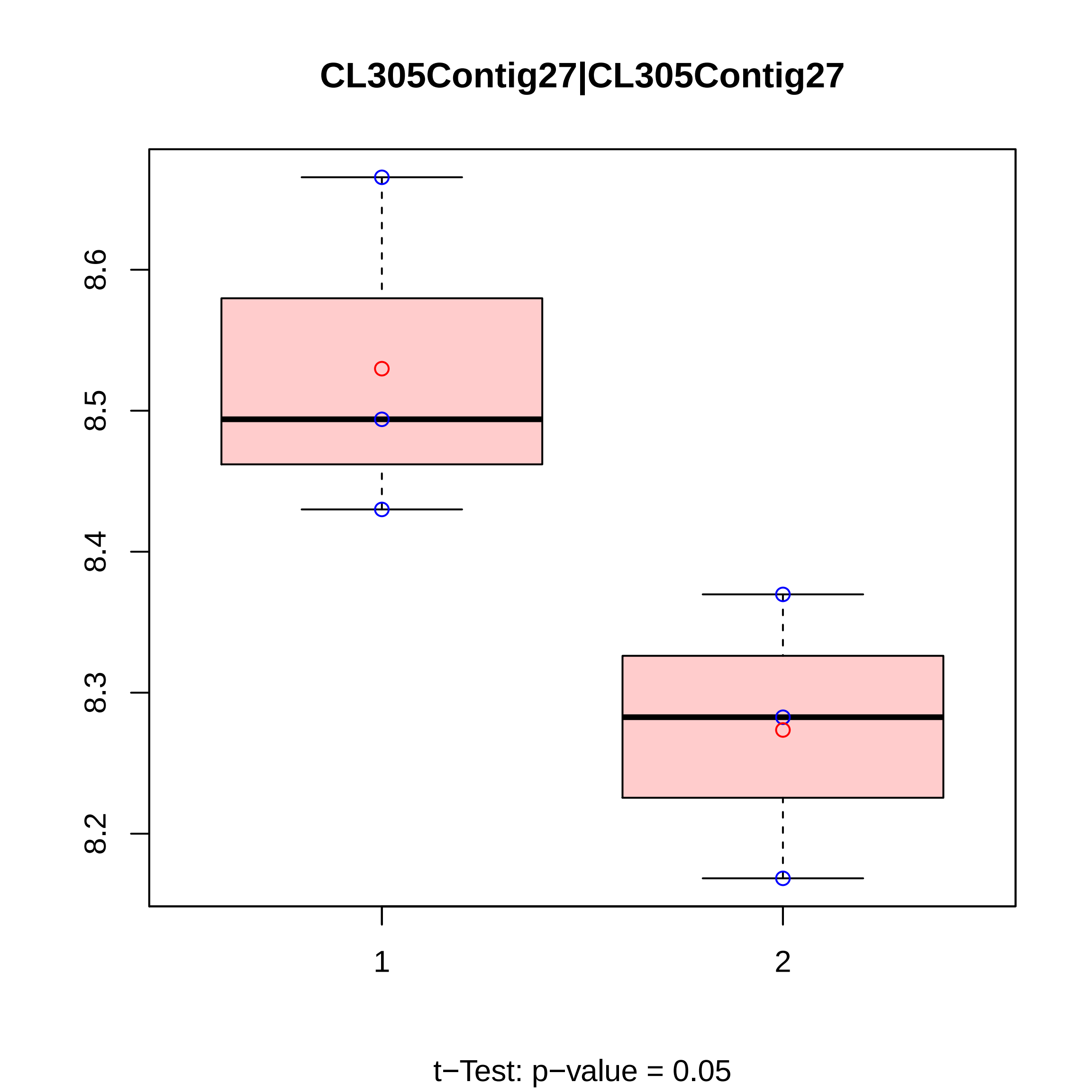
CL305Contig27|CL305Contig27
8.2
8.3
8.4
8.5
8.6
1
2
t−Test: p−value = 0.05

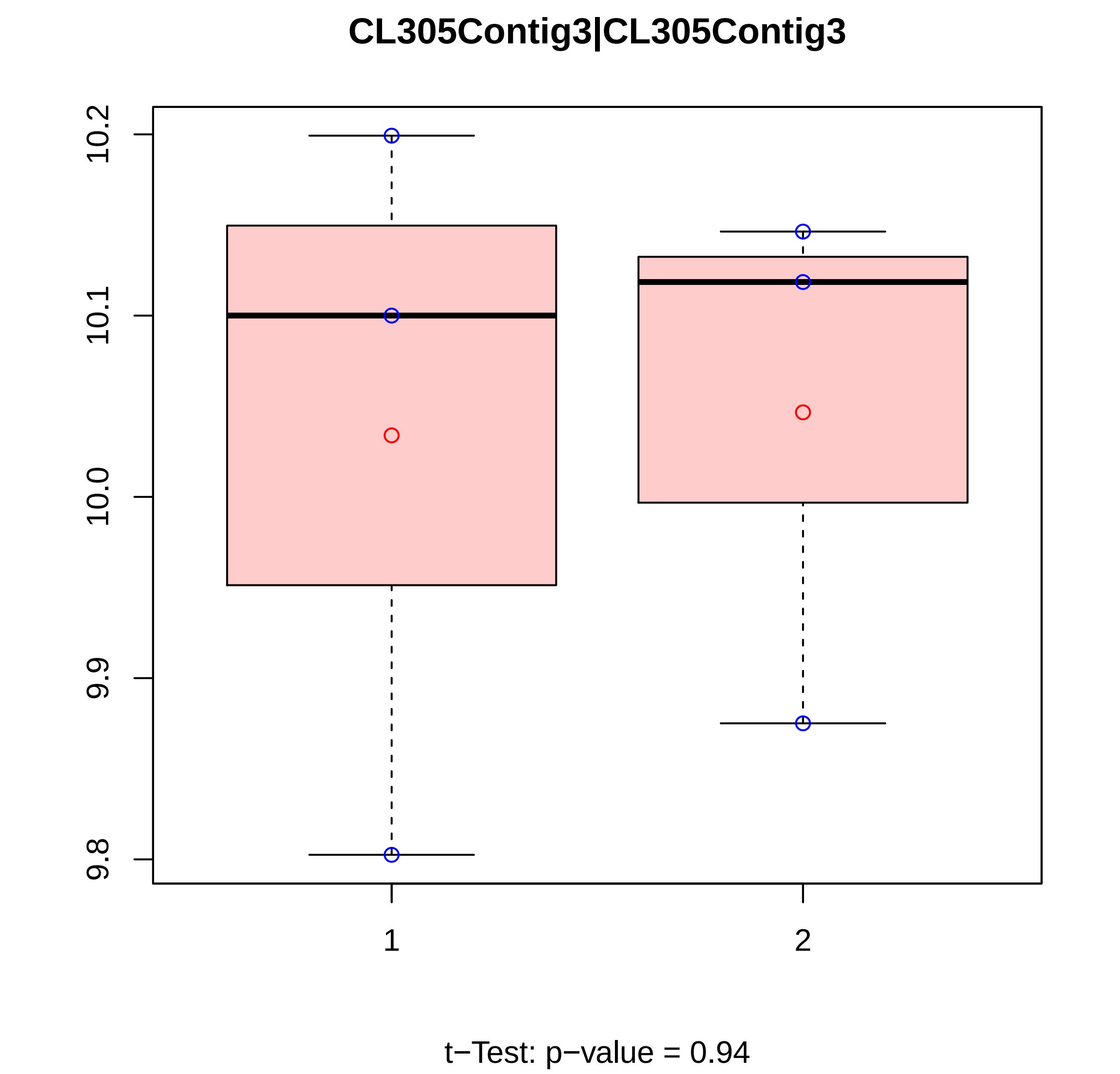

CL305Contig3|CL305Contig3
10.2
10.1
10.0
9.9
9.8
1
2
t−Test: p−value = 0.94

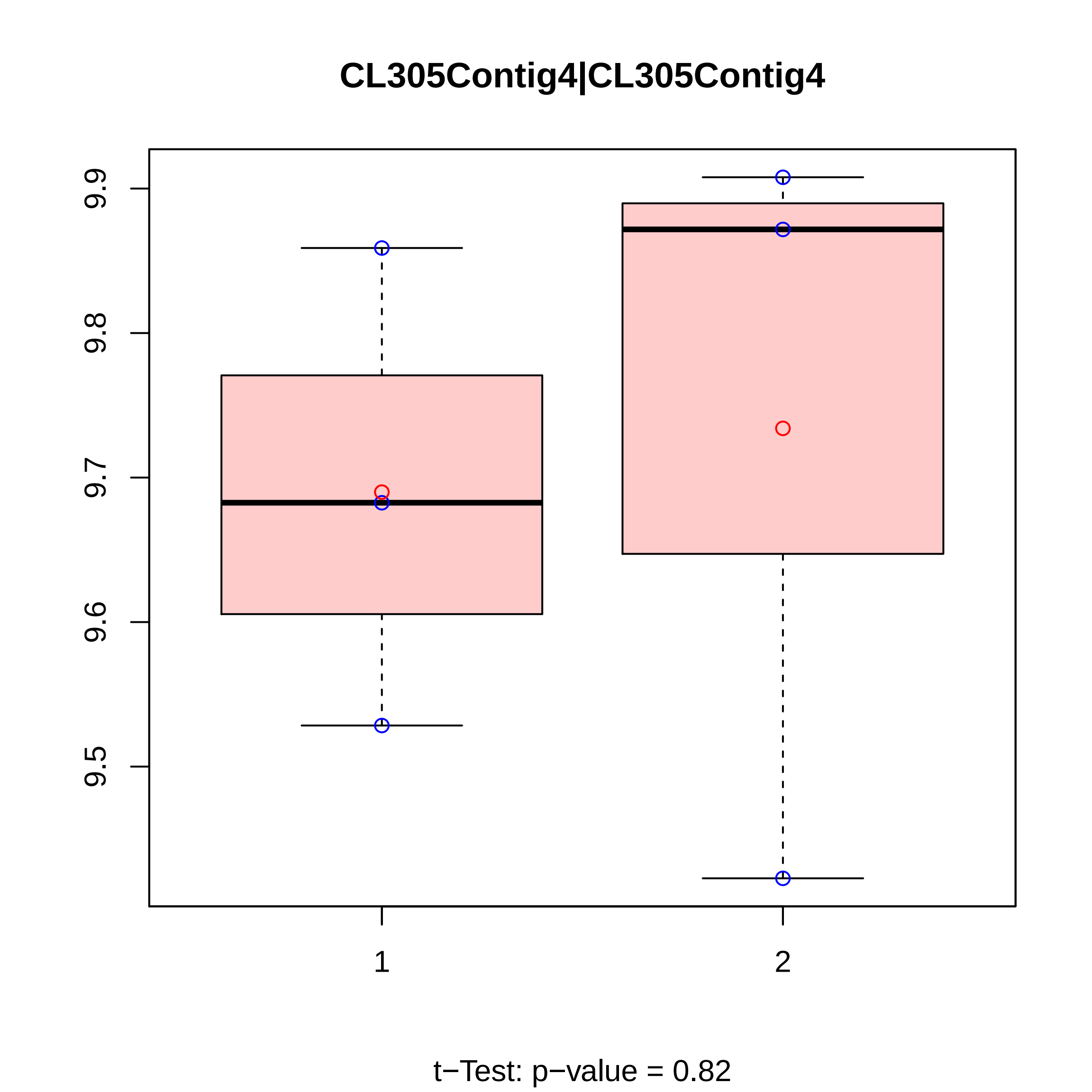

CL305Contig4|CL305Contig4
9.5
9.6
9.7
9.8
9.9
1
2
t−Test: p−value = 0.82

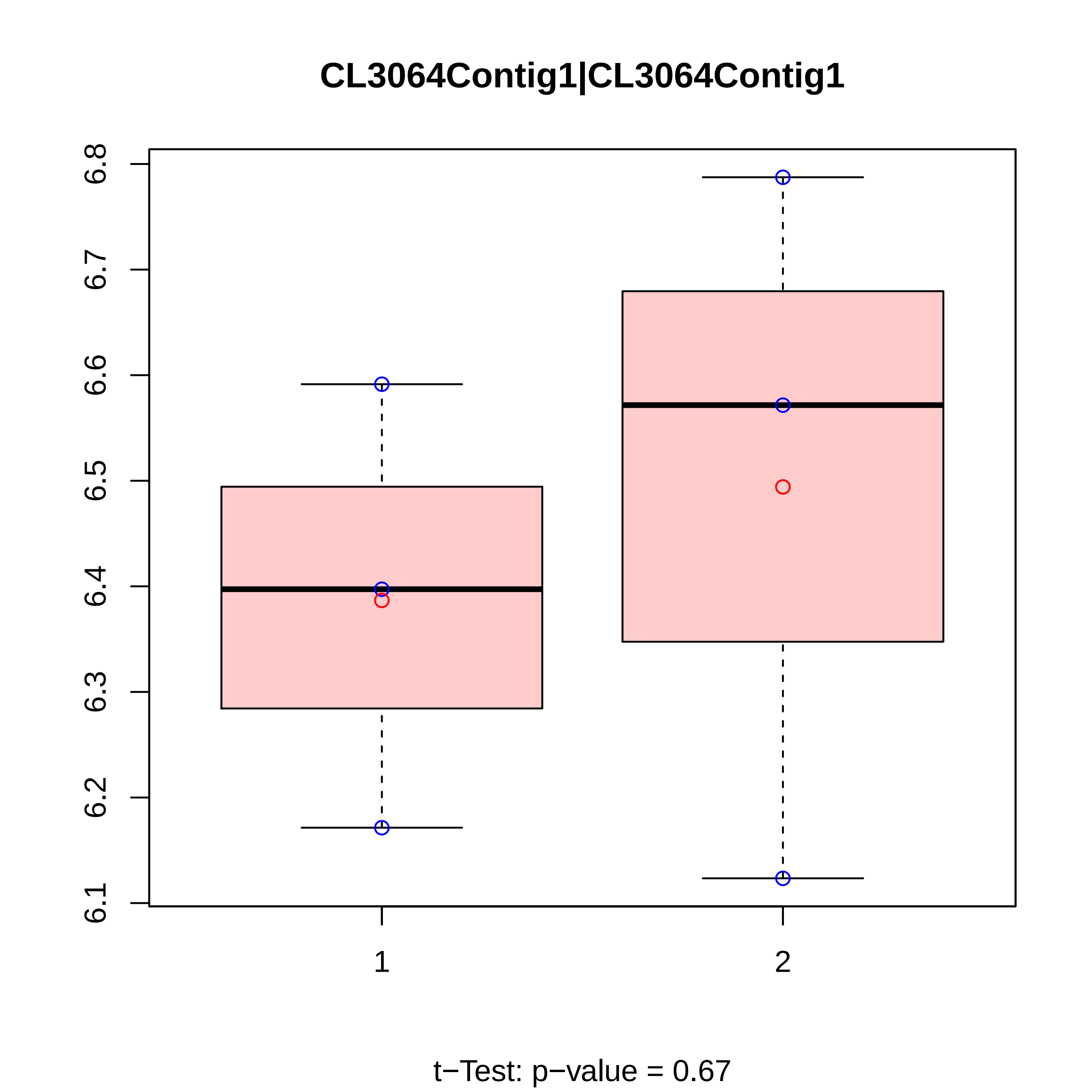
CL3064Contig1|CL3064Contig1
6.1
6.2
6.3
6.4
6.5
6.6
6.7
6.8
1
2
t−Test: p−value = 0.67

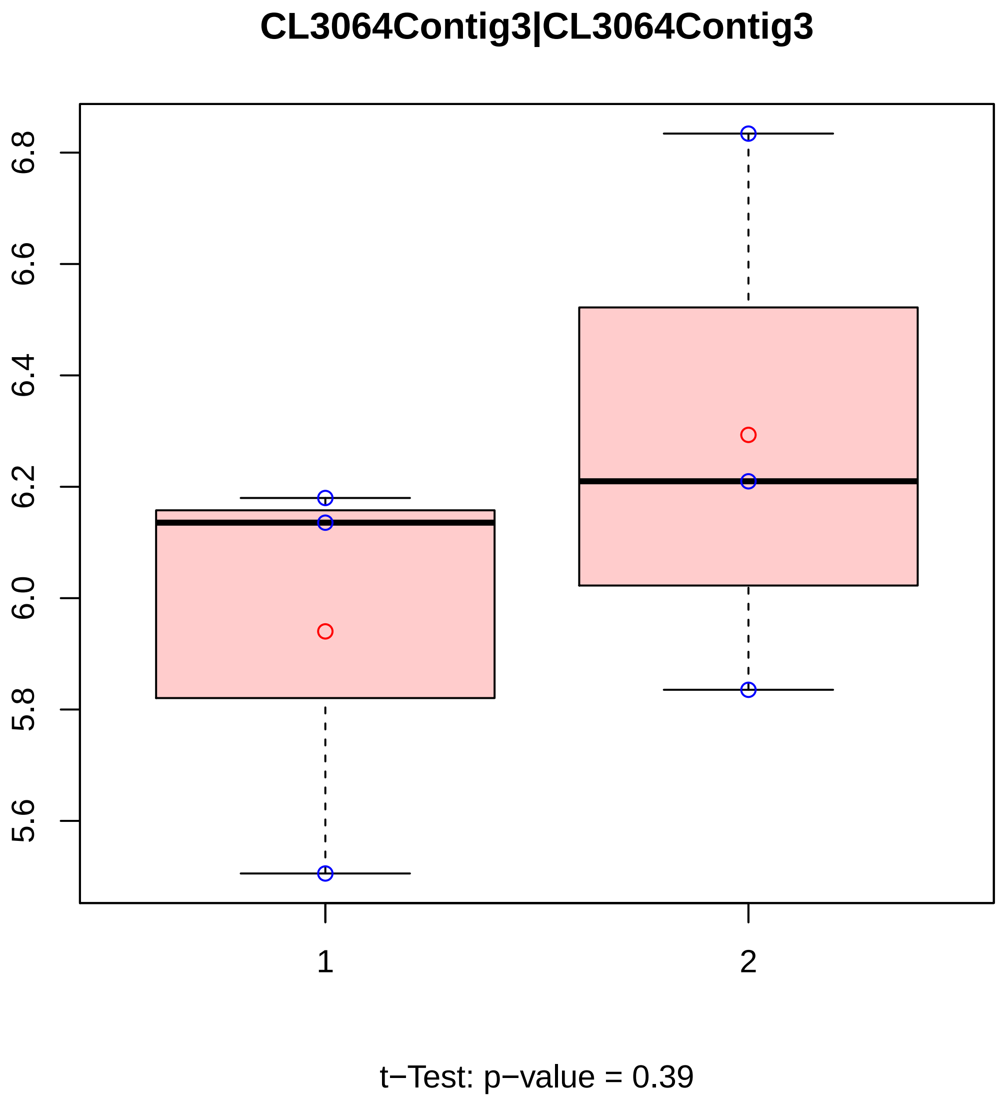

CL3064Contig3|CL3064Contig3
6.8
6.6
6.4
6.2
6.0
5.8
5.6
1
2
t−Test: p−value = 0.39

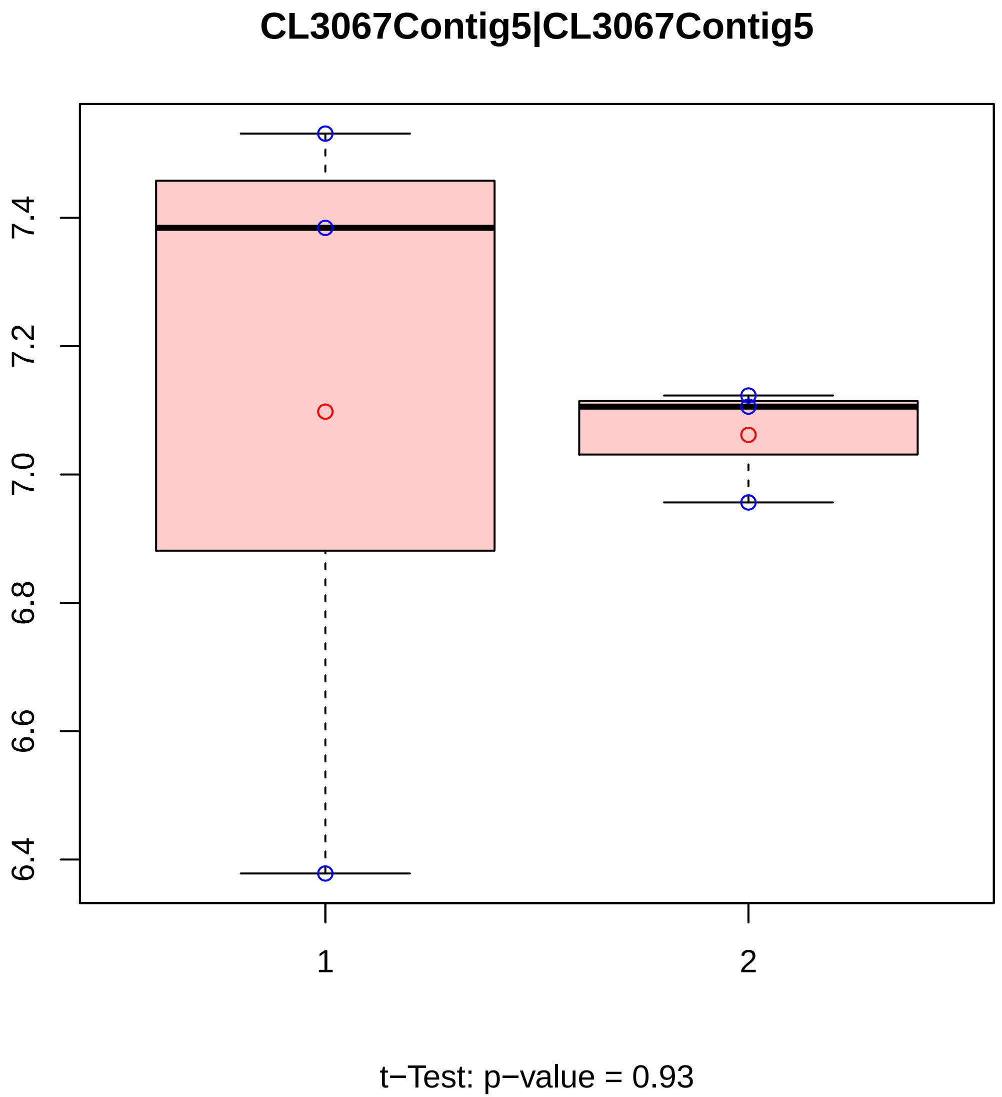
CL3067Contig5|CL3067Contig5
7.4
7.2
7.0
6.8
6.6
6.4
1
2
t−Test: p−value = 0.93

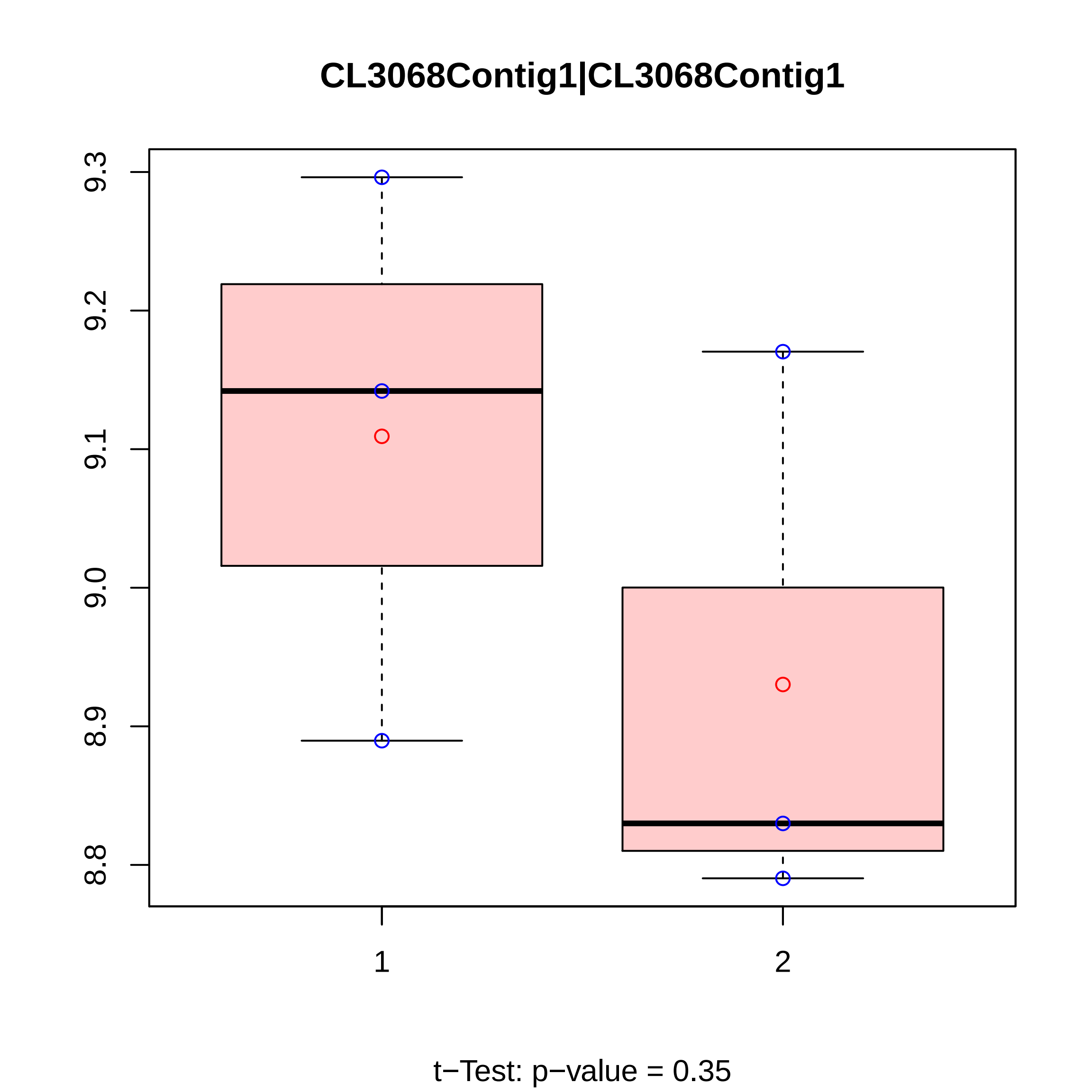

CL3068Contig1|CL3068Contig1
9.3
9.2
9.1
9.0
8.9
8.8
1
2
t−Test: p−value = 0.35

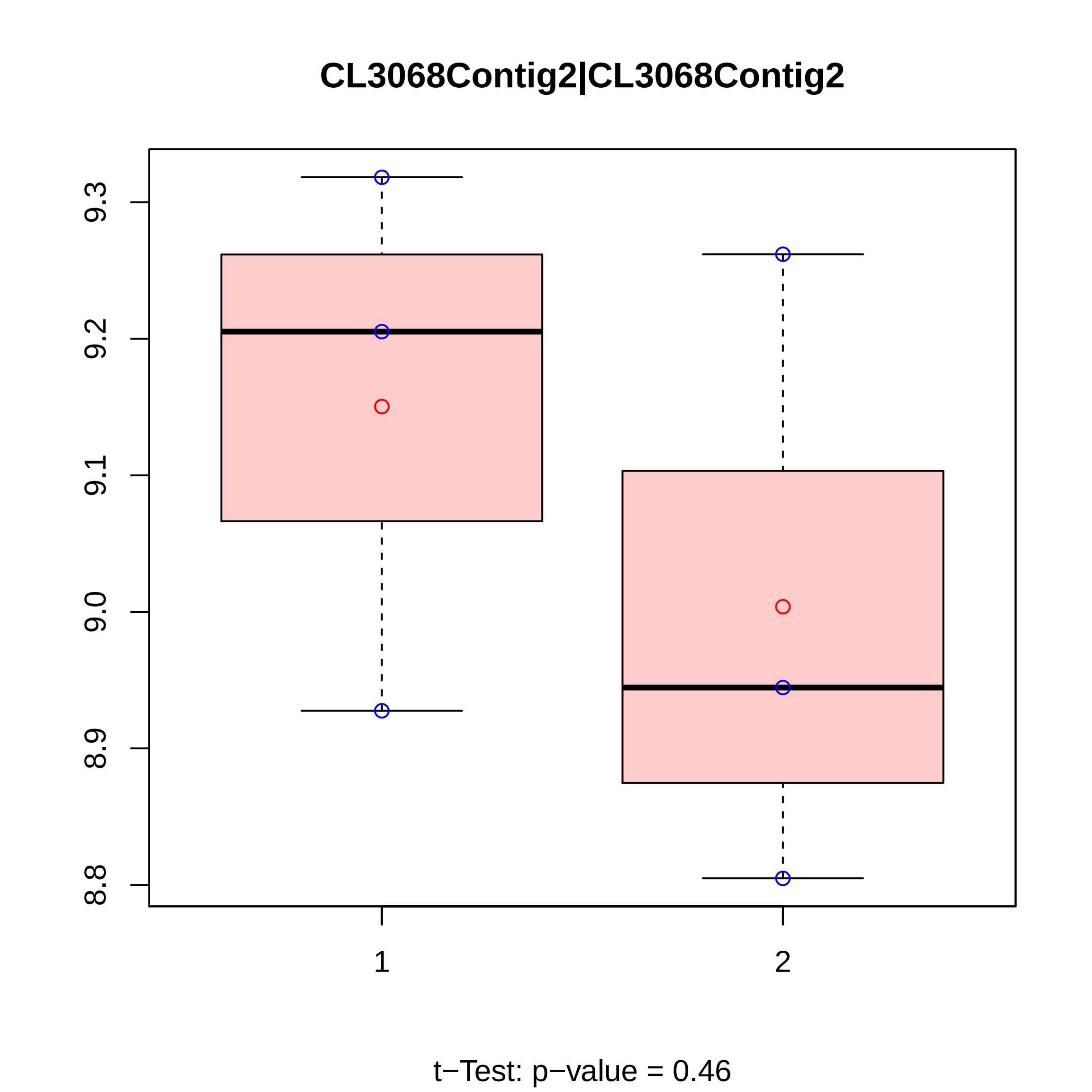

CL3068Contig2|CL3068Contig2
9.3
9.2
9.1
9.0
8.9
8.8
1
2
t−Test: p−value = 0.46

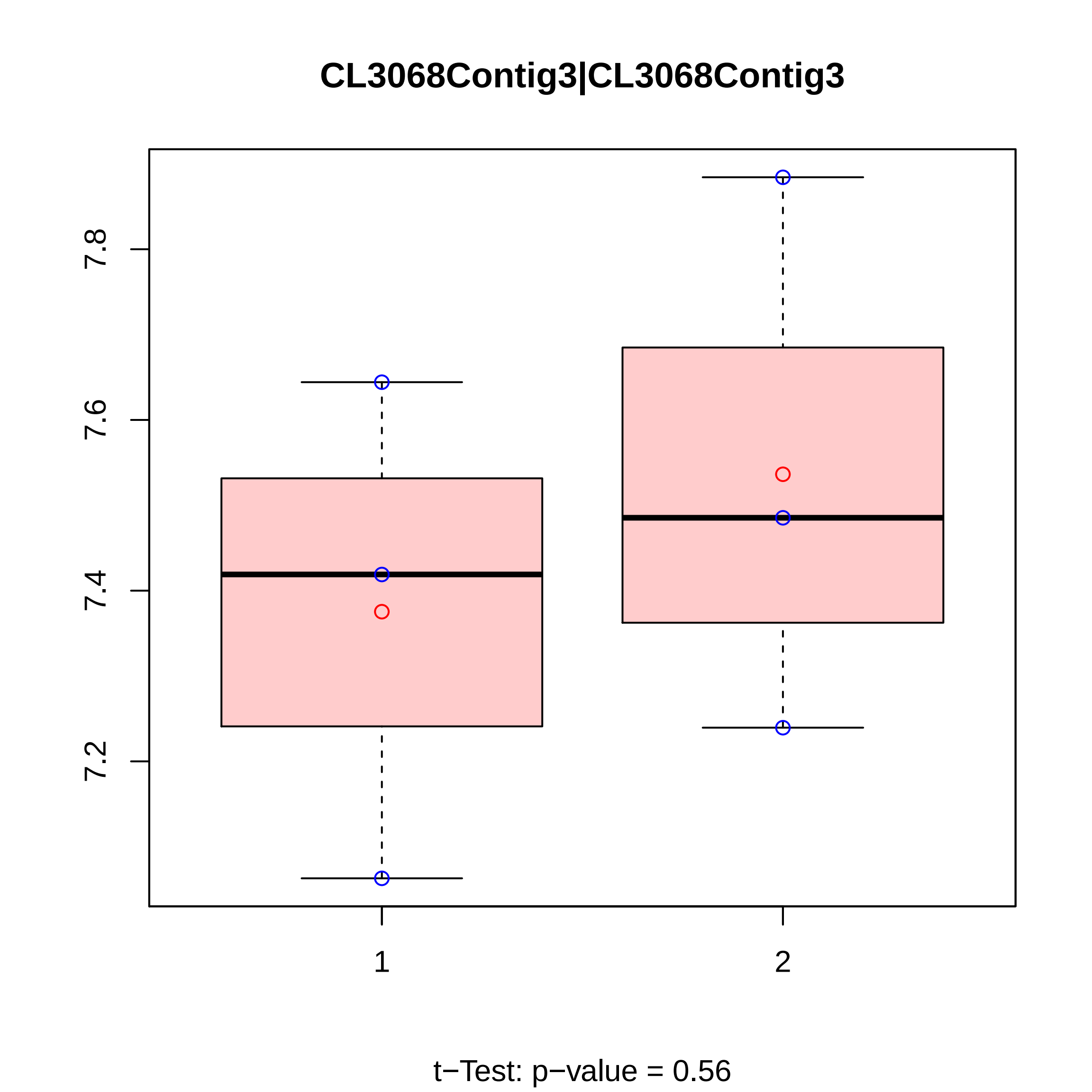
CL3068Contig3|CL3068Contig3
7.2
7.4
7.6
7.8
1
2
t−Test: p−value = 0.56

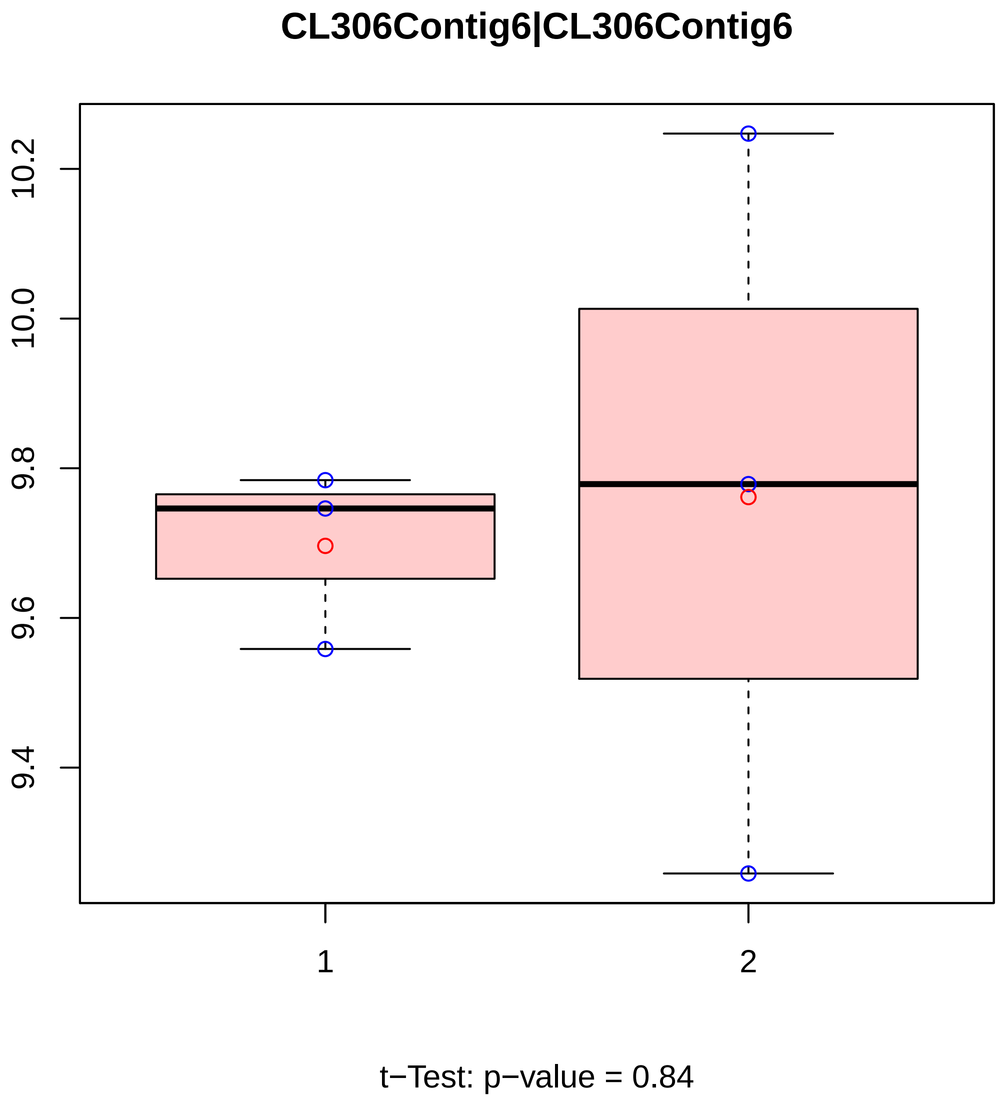

CL306Contig6|CL306Contig6
10.2
10.0
9.8
9.6
9.4
1
2
t−Test: p−value = 0.84

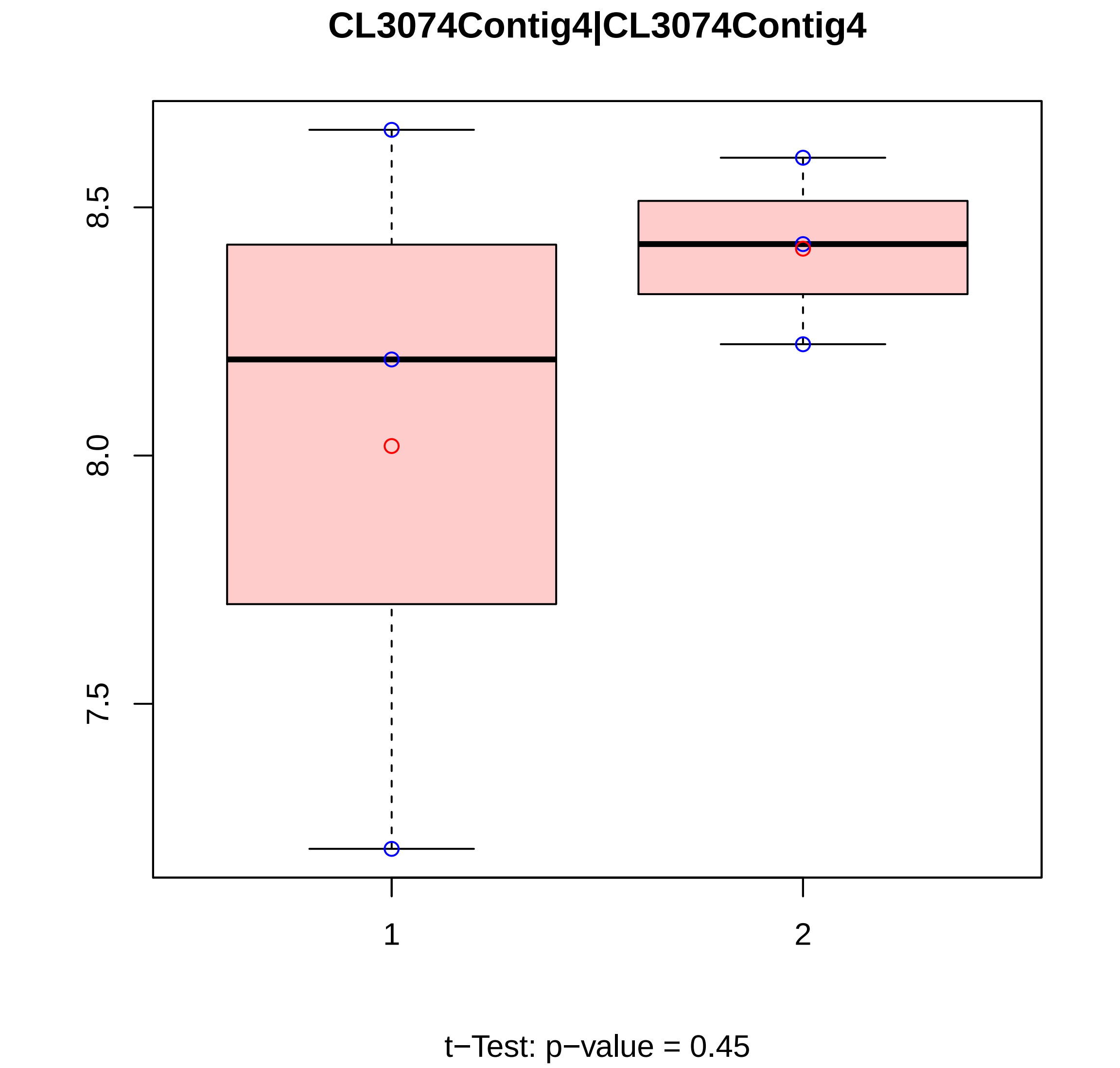
CL3074Contig4|CL3074Contig4
8.5
8.0
7.5
1
2
t−Test: p−value = 0.45

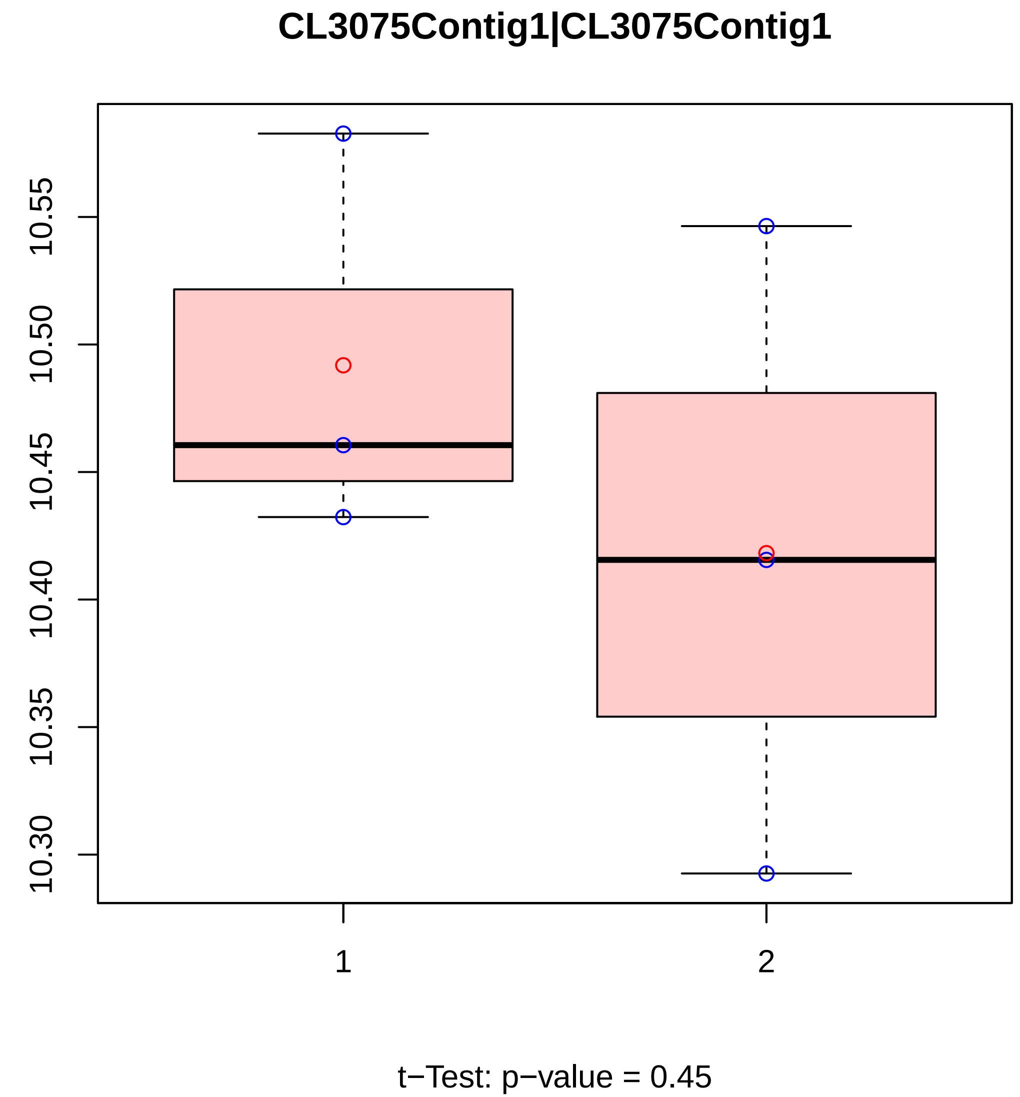
CL3075Contig1|CL3075Contig1
10.30
10.35
10.40
10.45
10.50
10.55
1
2
t−Test: p−value = 0.45

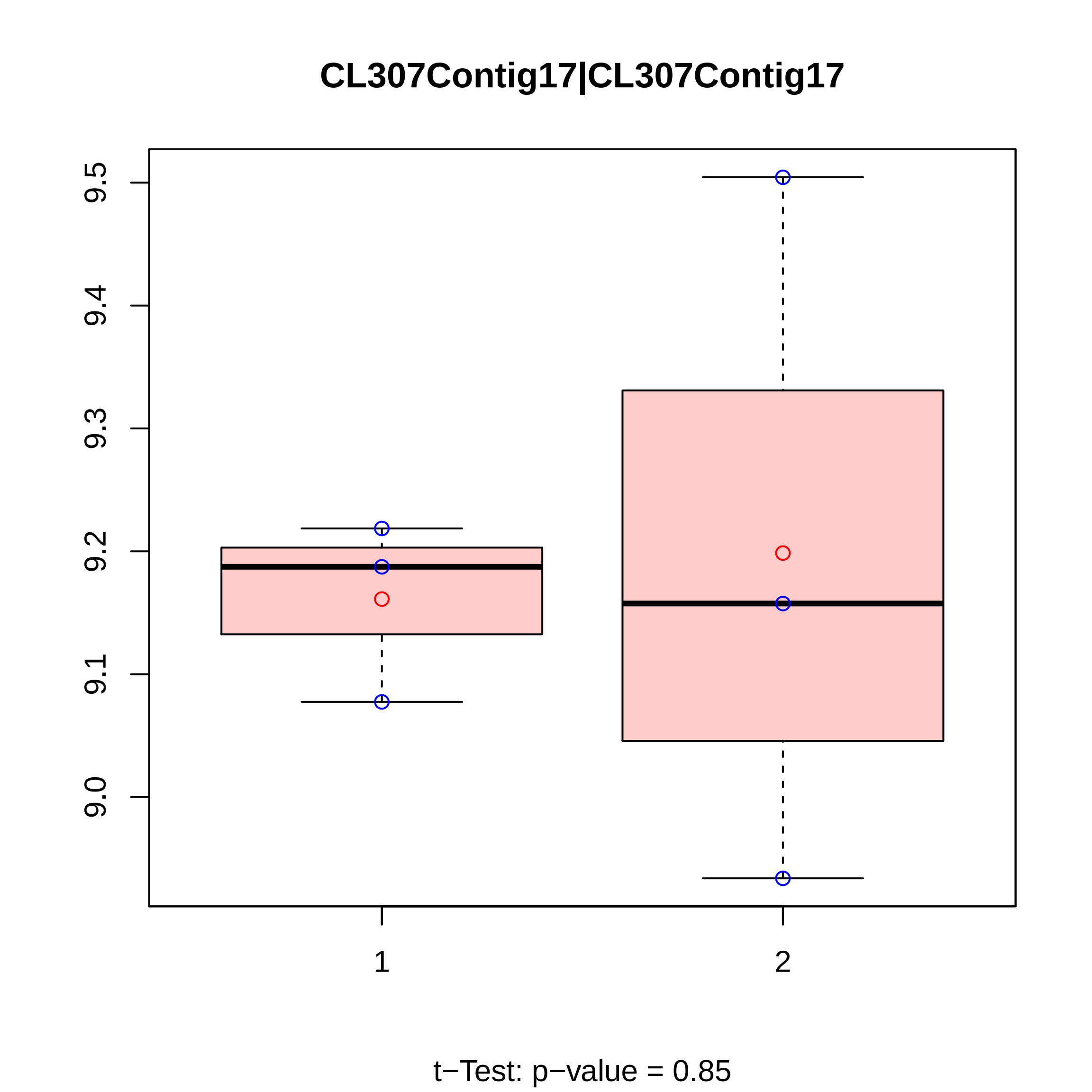

CL307Contig17|CL307Contig17
9.0
9.1
9.2
9.3
9.4
9.5
1
2
t−Test: p−value = 0.85

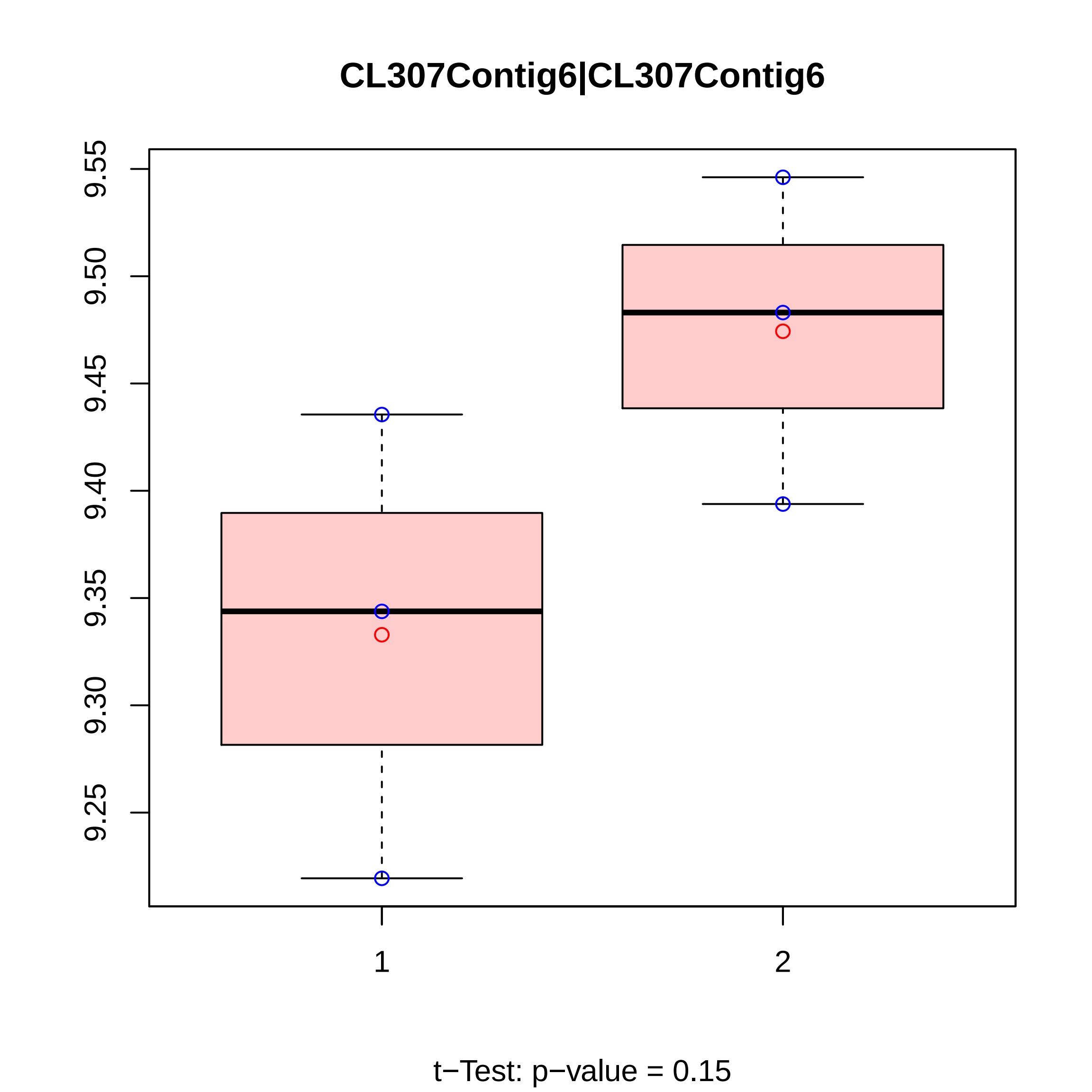
CL307Contig6|CL307Contig6
9.25
9.30
9.35
9.40
9.45
9.50
9.55
1
2
t−Test: p−value = 0.15

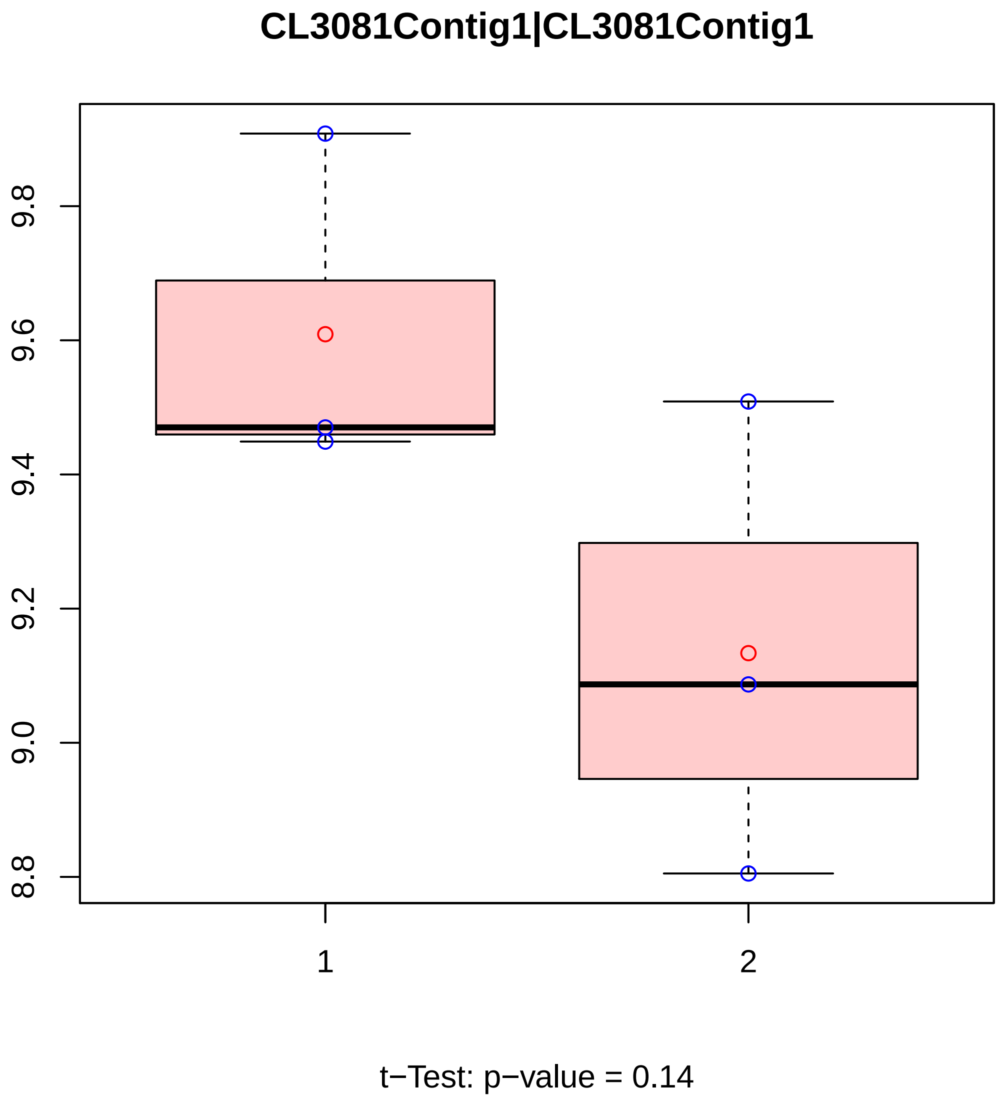

CL3081Contig1|CL3081Contig1
9.8
9.6
9.4
9.2
9.0
8.8
1
2
t−Test: p−value = 0.14

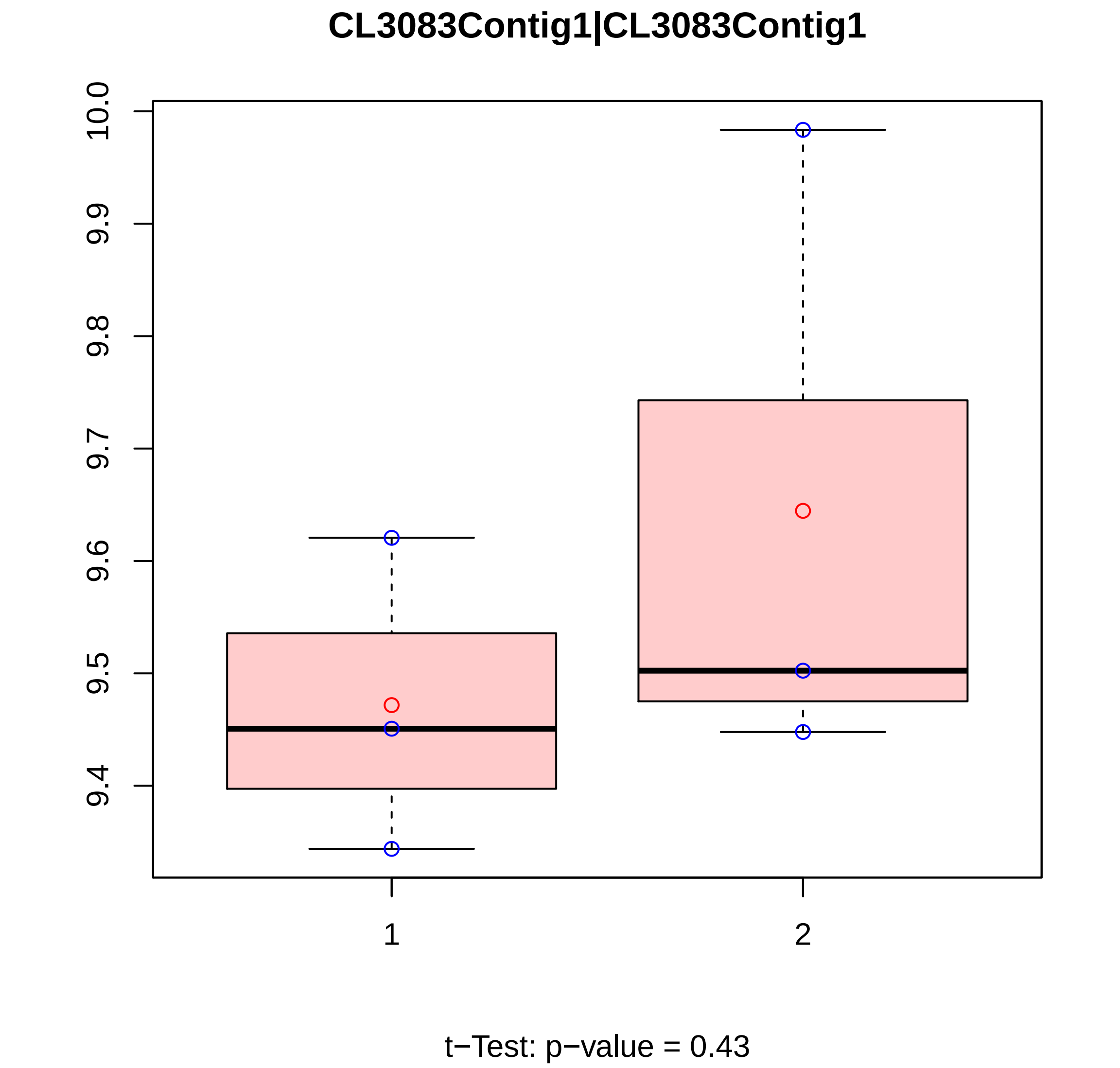
CL3083Contig1|CL3083Contig1
10.0
9.9
9.8
9.7
9.6
9.5
9.4
1
2
t−Test: p−value = 0.43

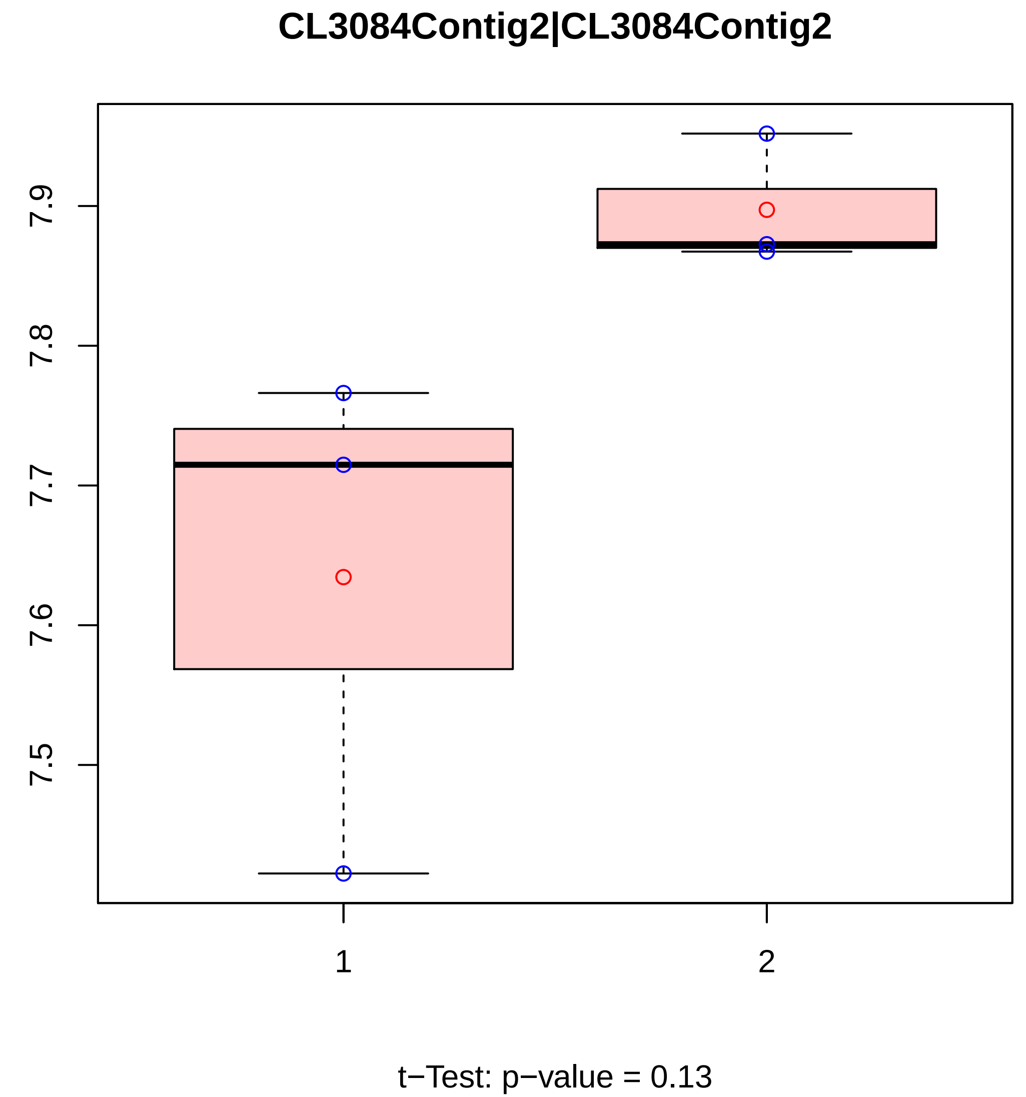
CL3084Contig2|CL3084Contig2
7.5
7.6
7.7
7.8
7.9
1
2
t−Test: p−value = 0.13

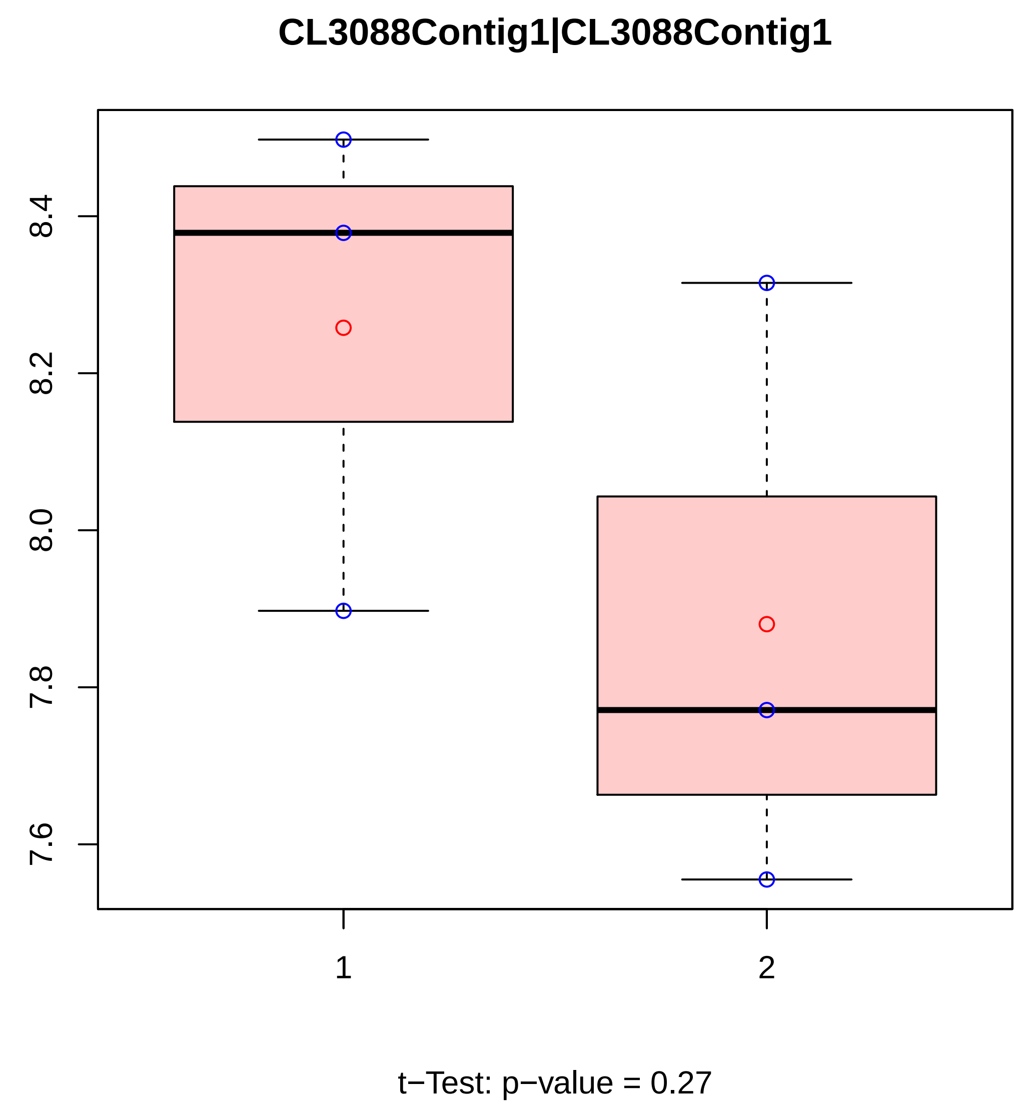
CL3088Contig1|CL3088Contig1
8.4
8.2
8.0
7.8
7.6
1
2
t−Test: p−value = 0.27

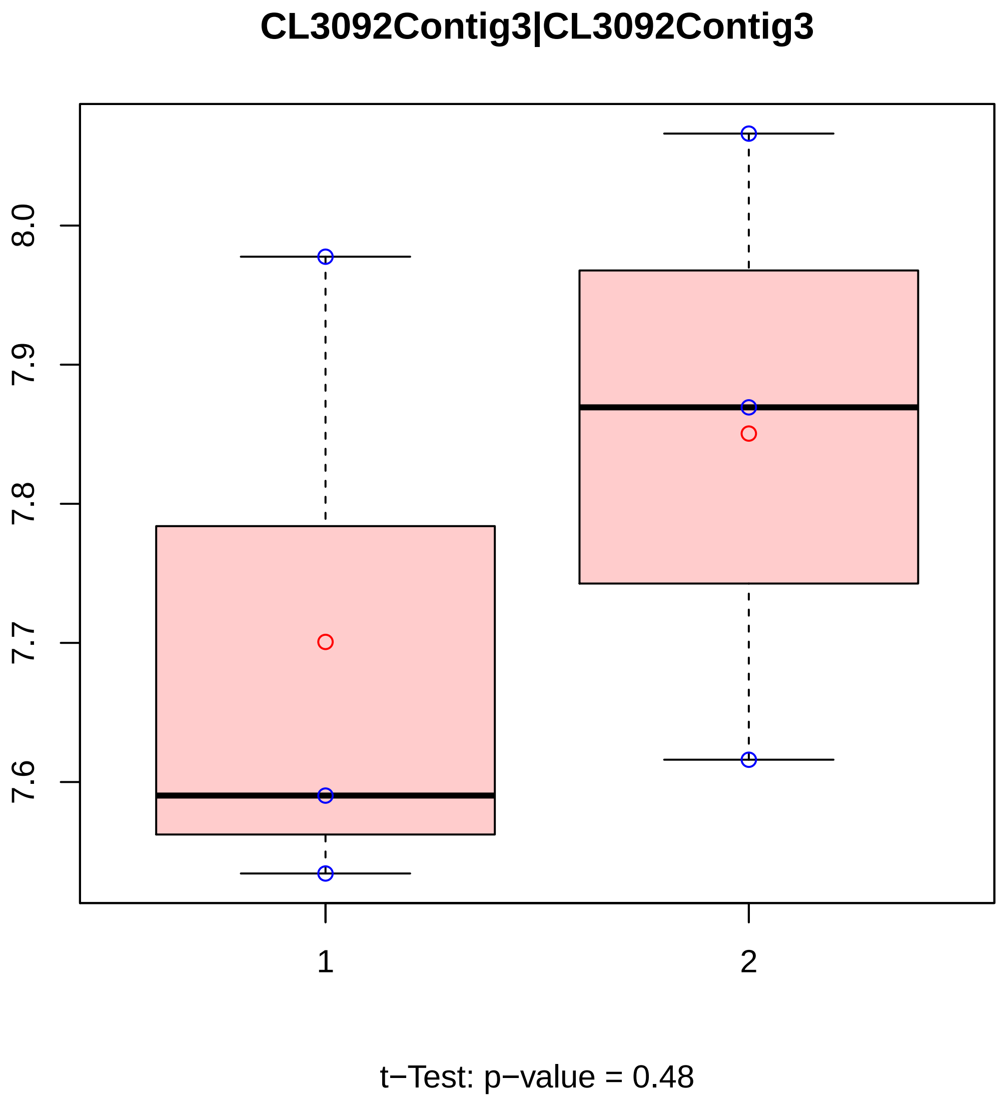
CL3092Contig3|CL3092Contig3
8.0
7.9
7.8
7.7
7.6
1
2
t−Test: p−value = 0.48

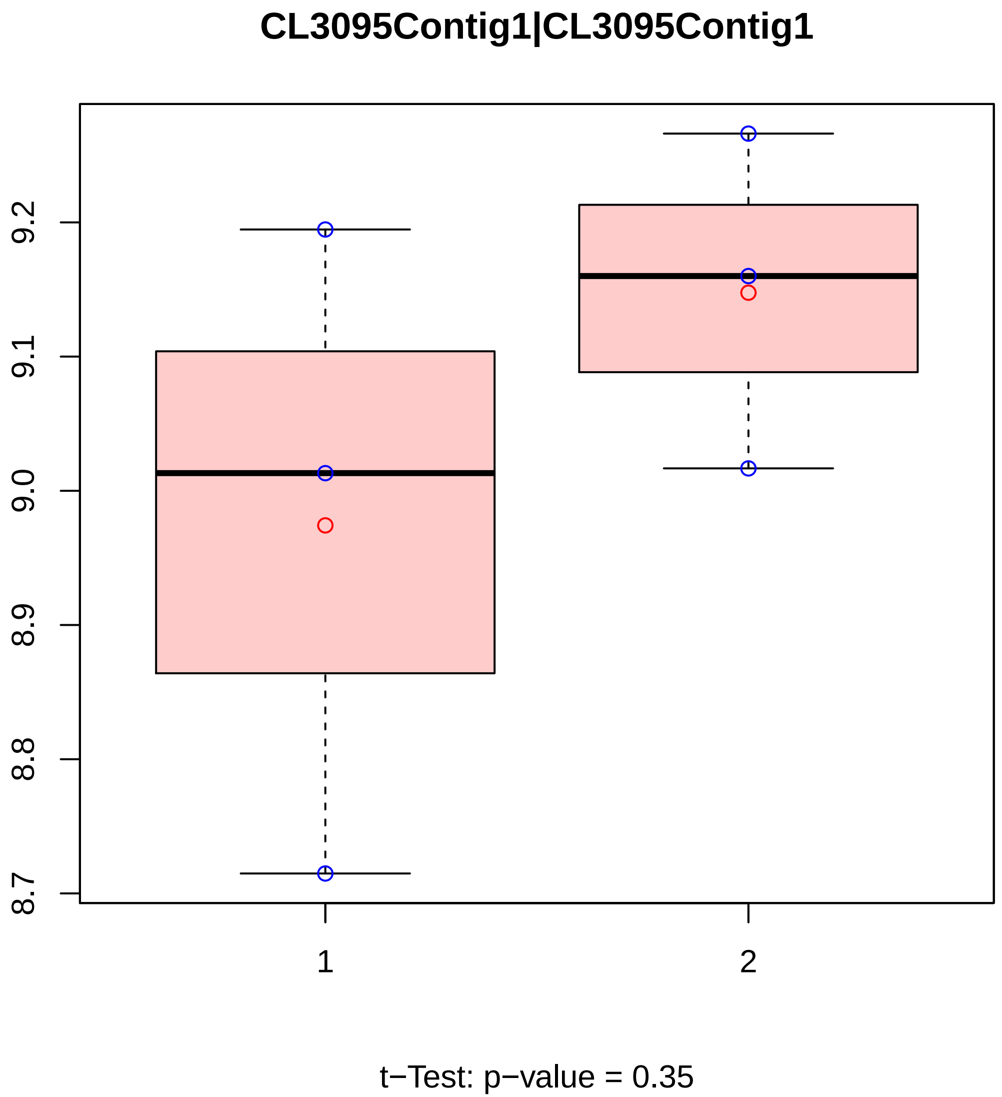

CL3095Contig1|CL3095Contig1
9.2
9.1
9.0
8.9
8.8
8.7
1
2
t−Test: p−value = 0.35

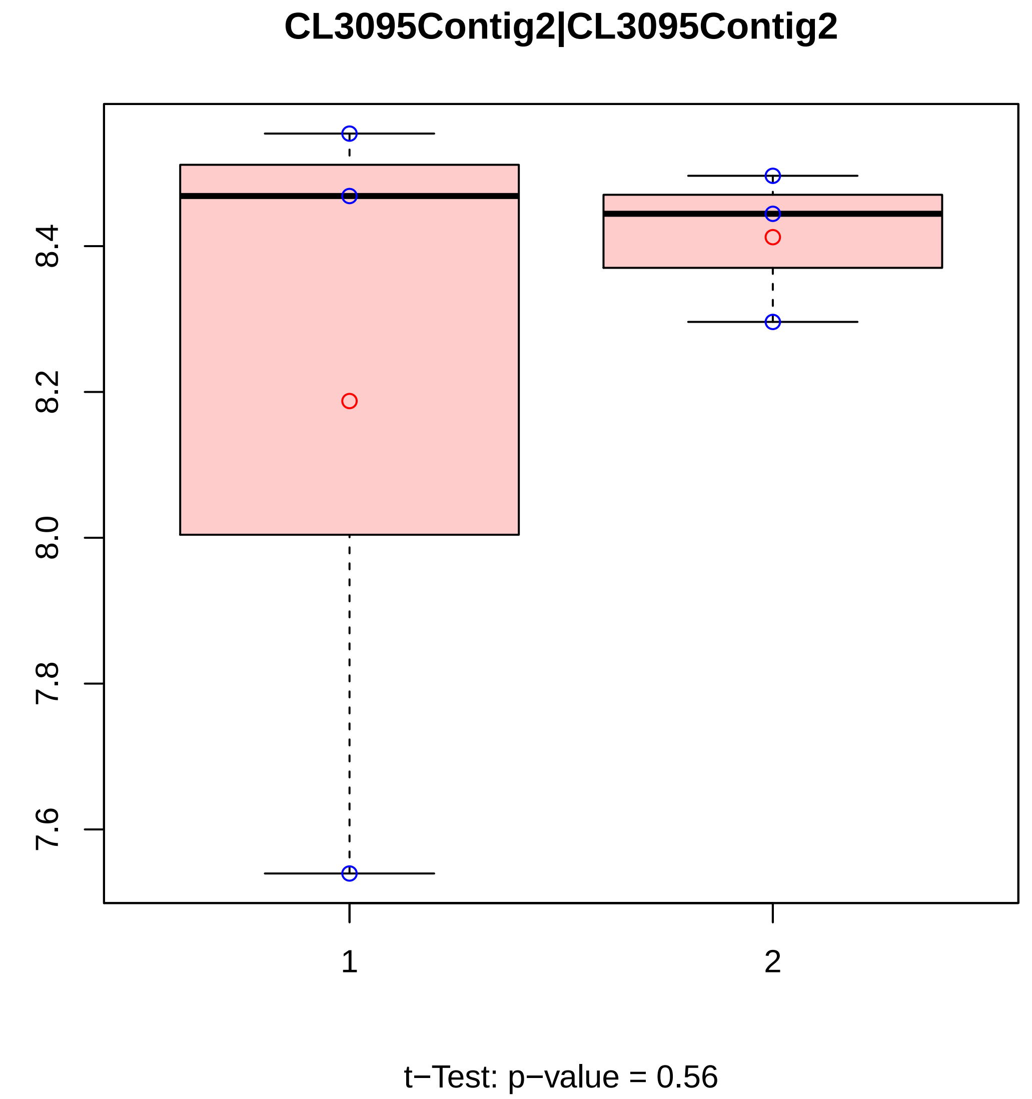
CL3095Contig2|CL3095Contig2
8.4
8.2
8.0
7.8
7.6
1
2
t−Test: p−value = 0.56

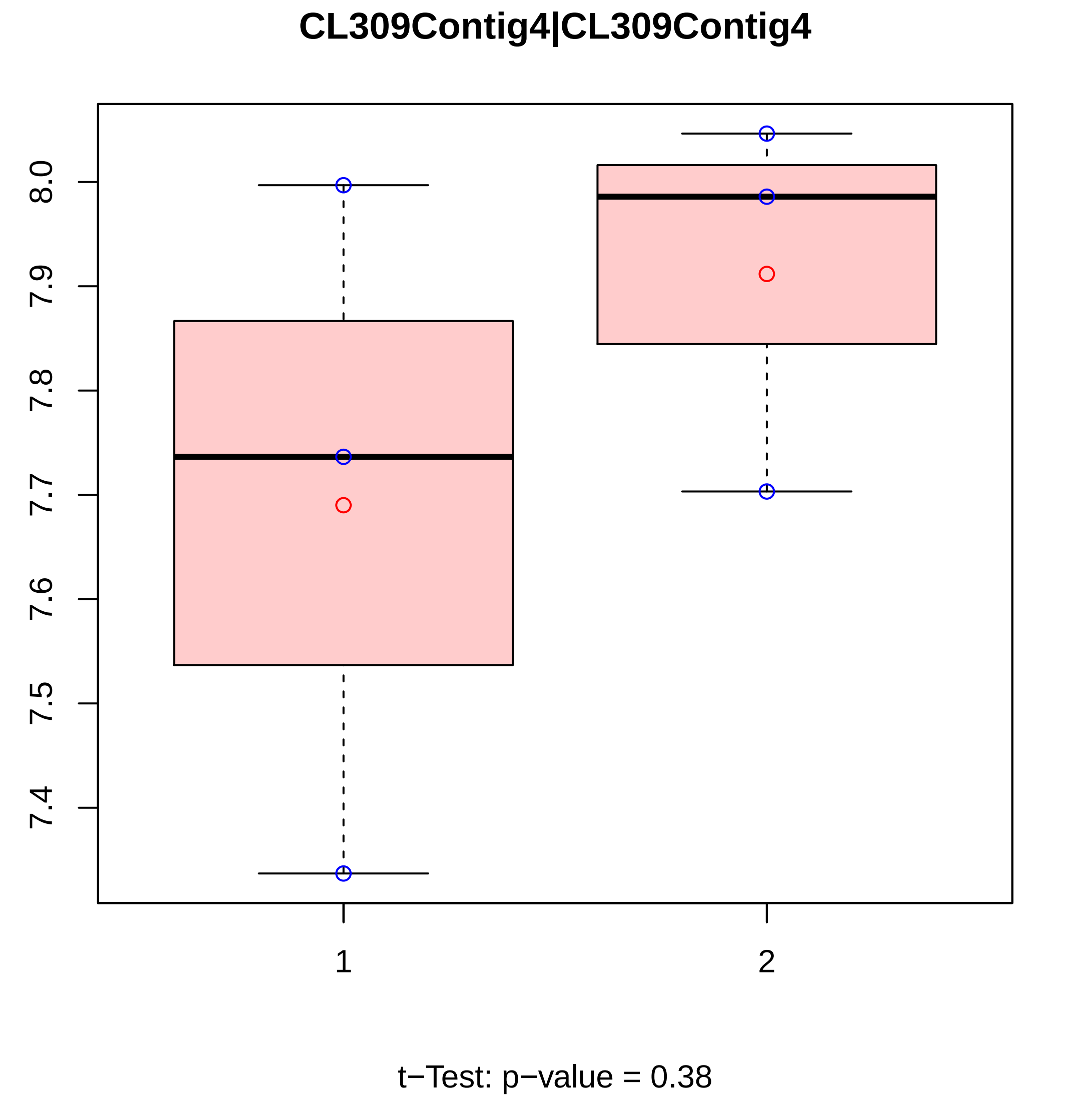

CL309Contig4|CL309Contig4
8.0
7.9
7.8
7.7
7.6
7.5
7.4
1
2
t−Test: p−value = 0.38

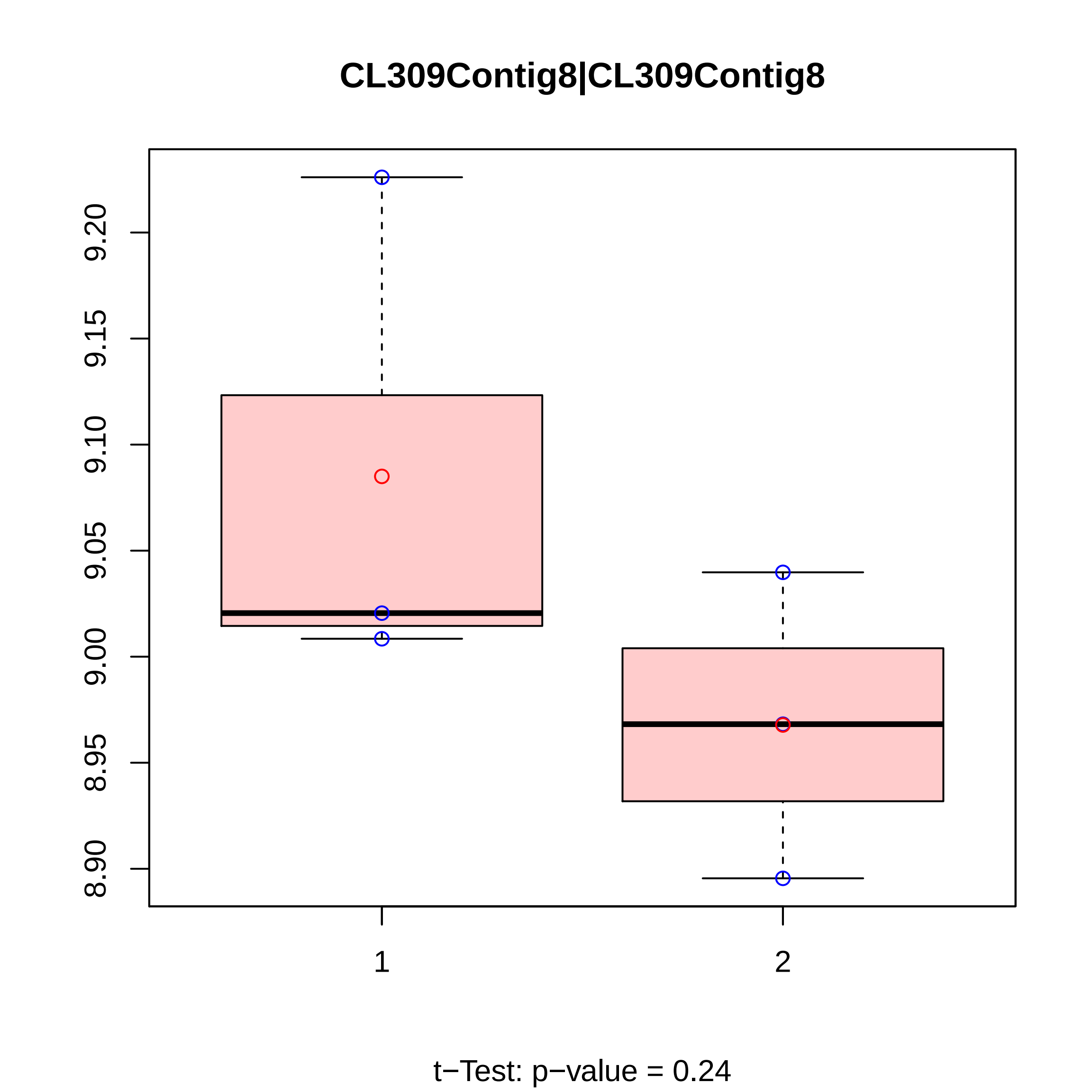

CL309Contig8|CL309Contig8
9.20
9.15
9.10
9.05
9.00
8.95
8.90
1
2
t−Test: p−value = 0.24

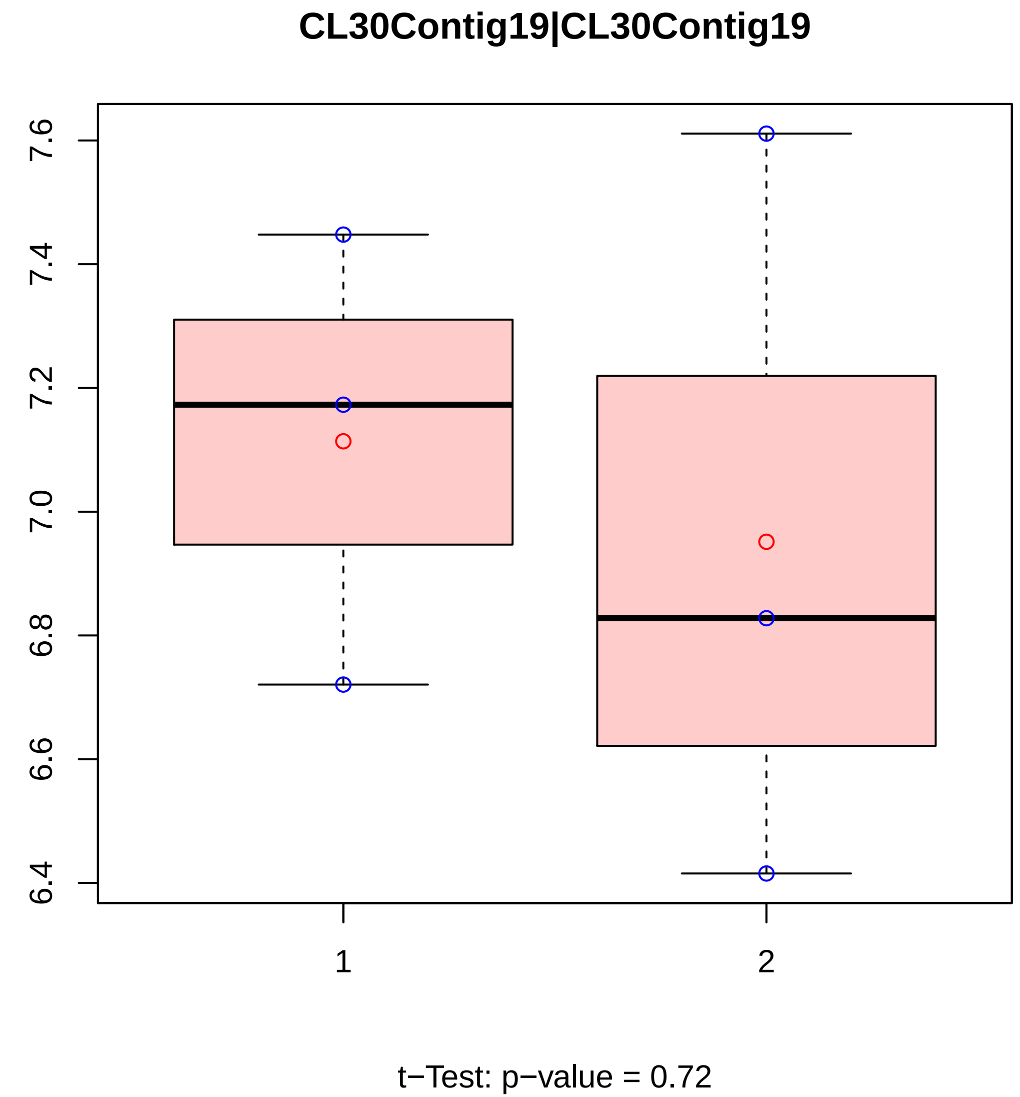
CL30Contig19|CL30Contig19
7.6
7.4
7.2
7.0
6.8
6.6
6.4
1
2
t−Test: p−value = 0.72

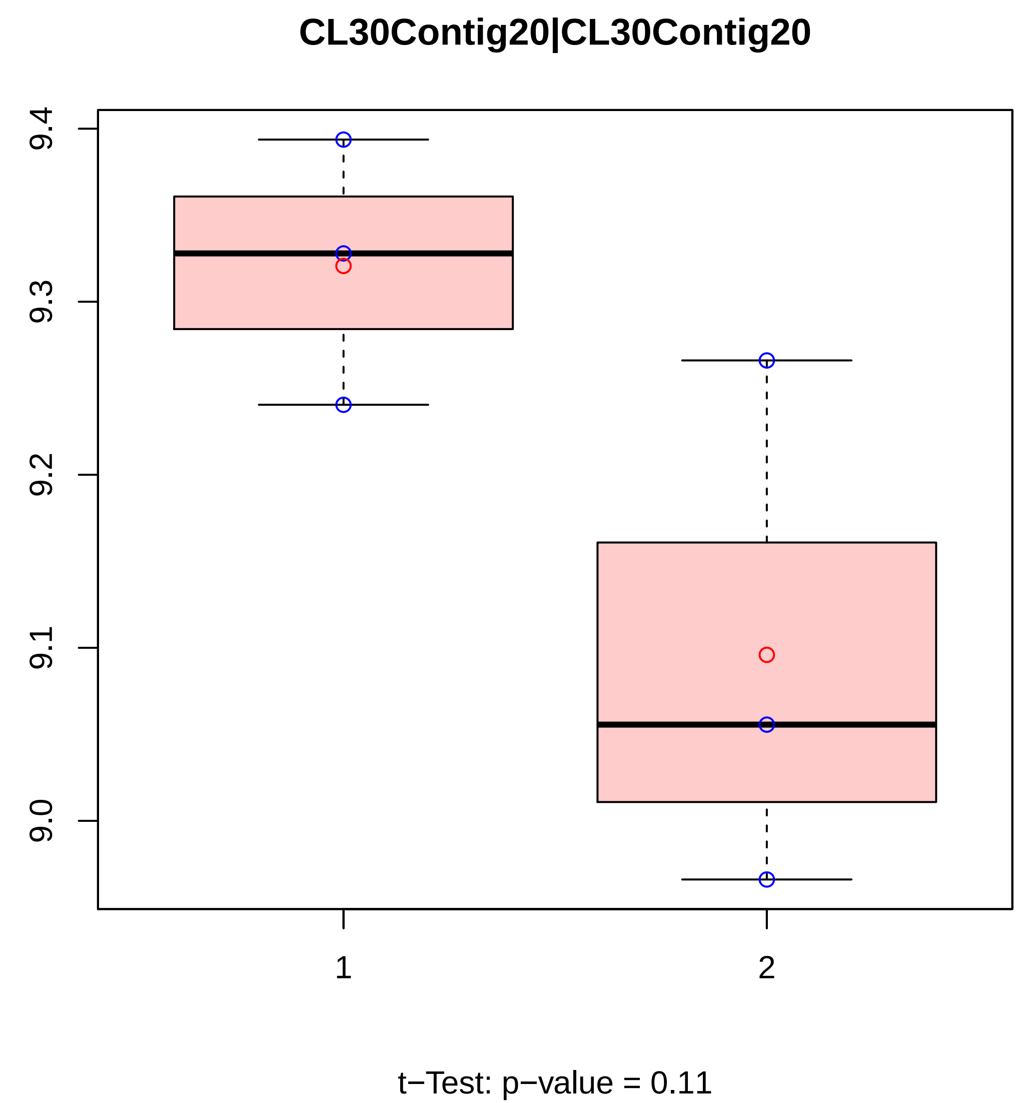

CL30Contig20|CL30Contig20
9.0
9.1
9.2
9.3
9.4
1
2
t−Test: p−value = 0.11

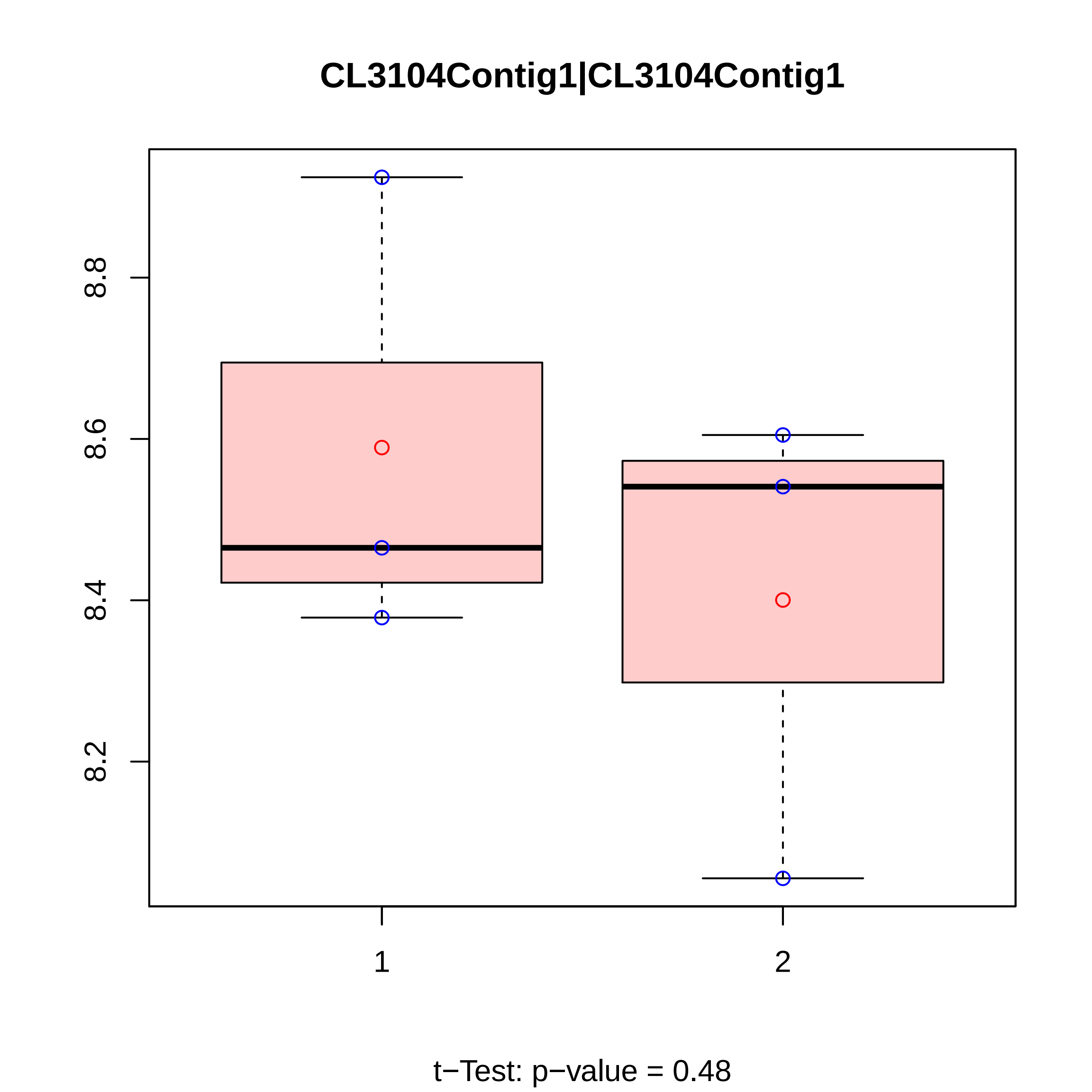

CL3104Contig1|CL3104Contig1
8.2
8.4
8.6
8.8
1
2
t−Test: p−value = 0.48

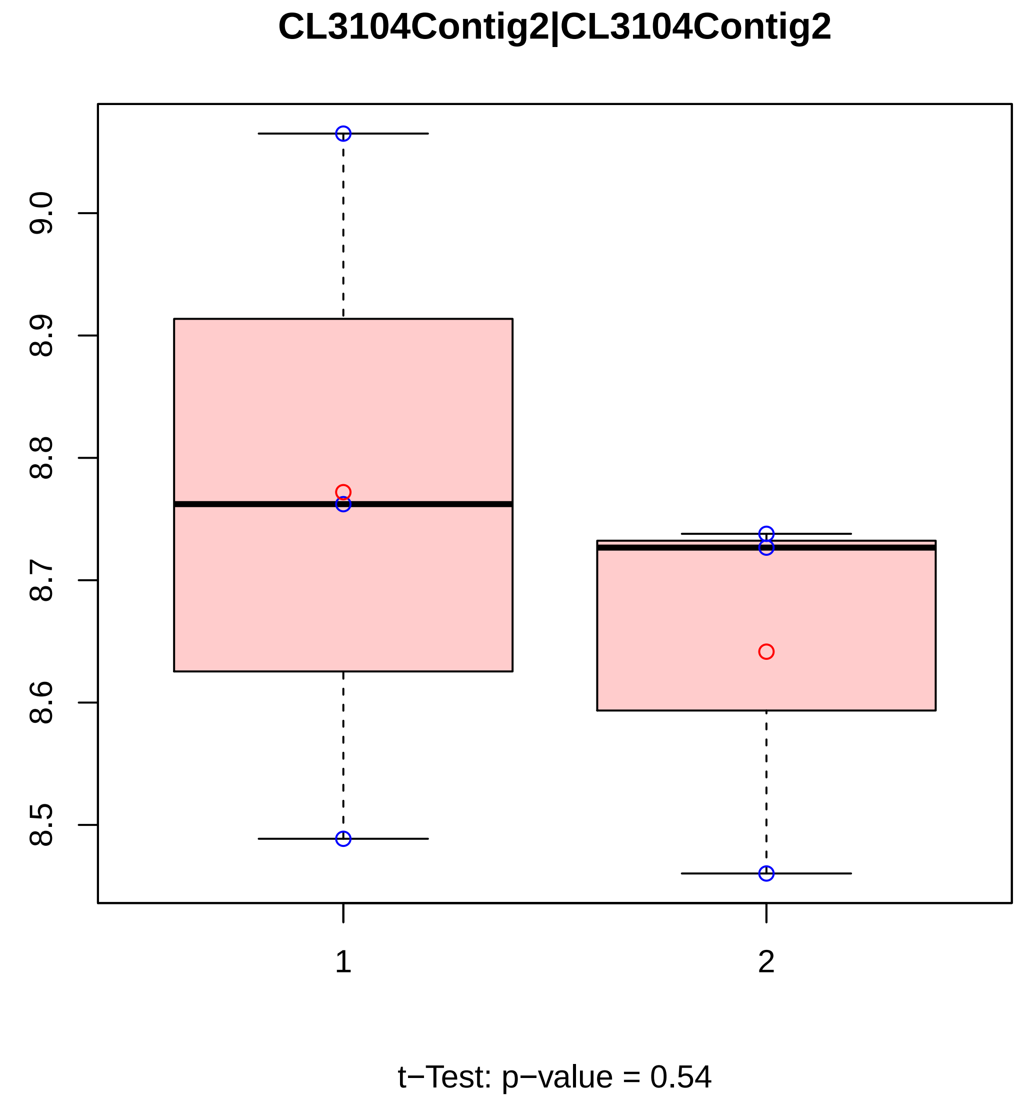
CL3104Contig2|CL3104Contig2
9.0
8.9
8.8
8.7
8.6
8.5
1
2
t−Test: p−value = 0.54

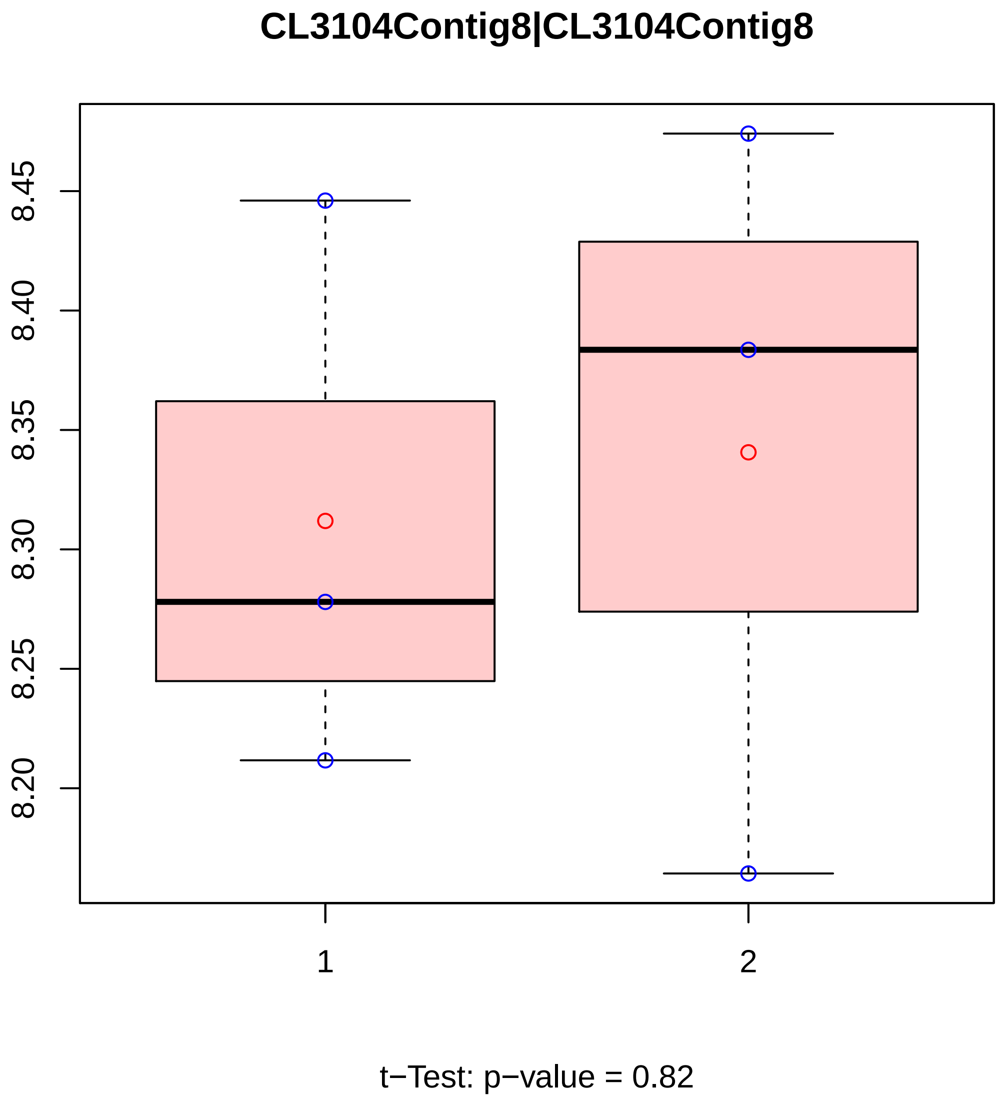

CL3104Contig8|CL3104Contig8
8.20
8.25
8.30
8.35
8.40
8.45
1
2
t−Test: p−value = 0.82

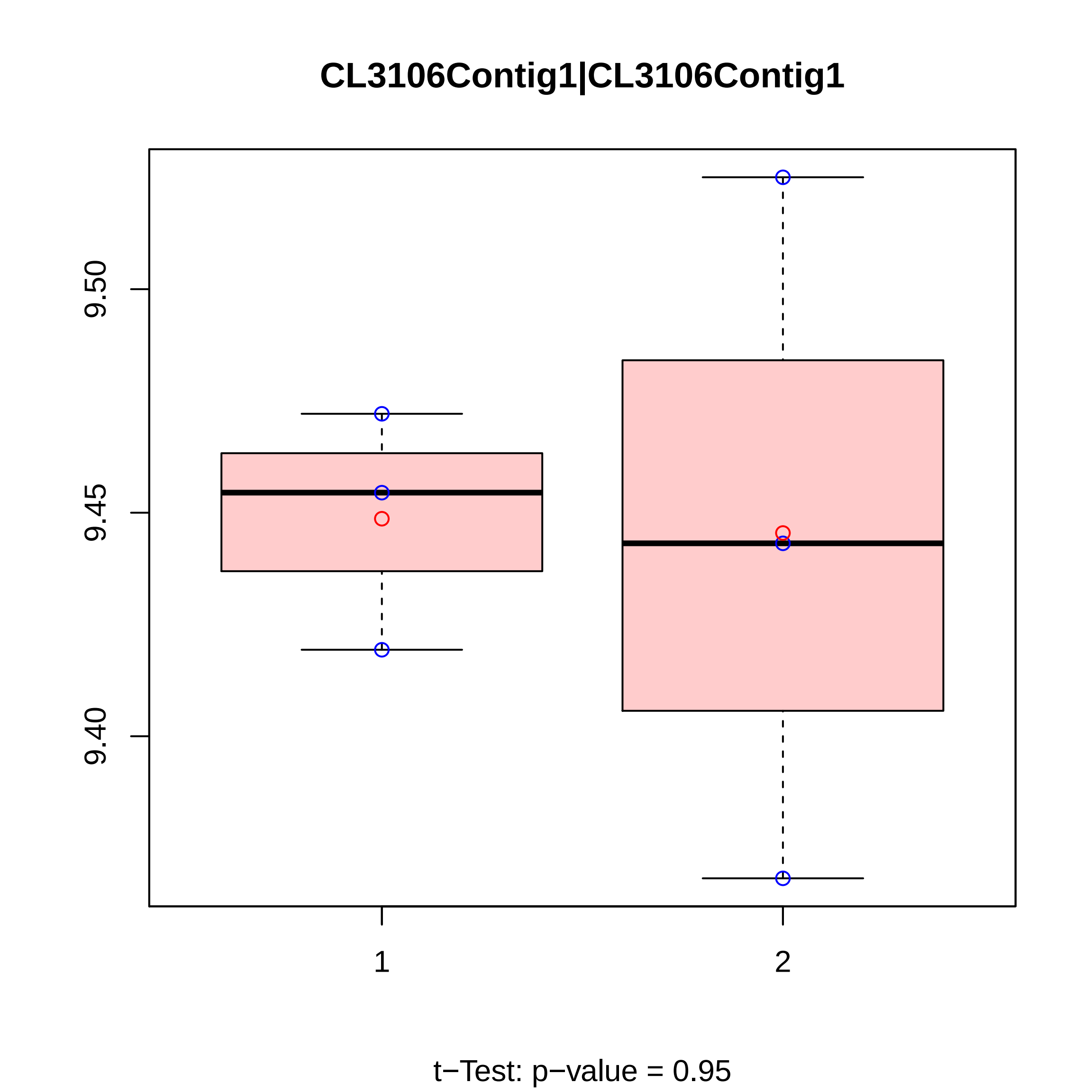
CL3106Contig1|CL3106Contig1
9.50
9.45
9.40
1
2
t−Test: p−value = 0.95

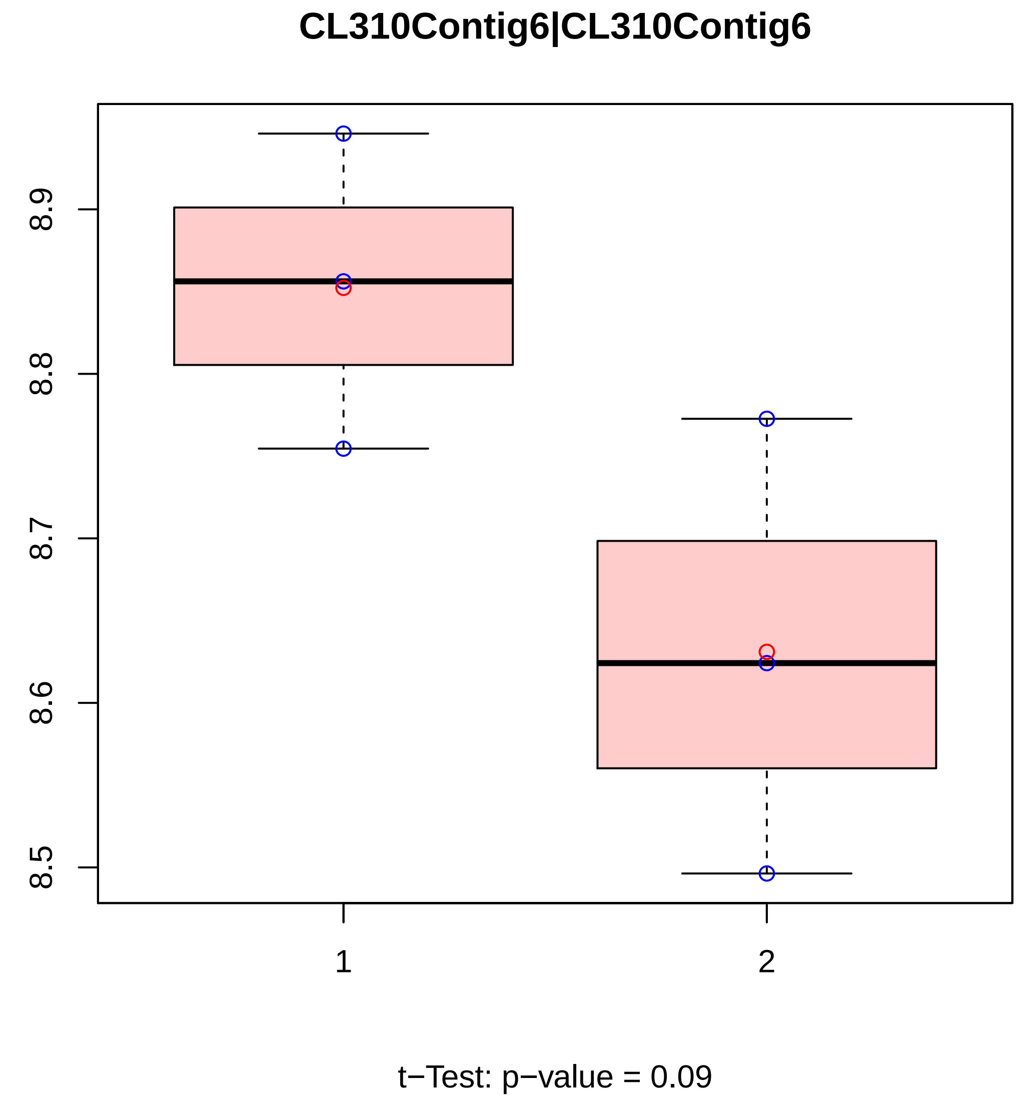

CL310Contig6|CL310Contig6
8.5
8.6
8.7
8.8
8.9
1
2
t−Test: p−value = 0.09

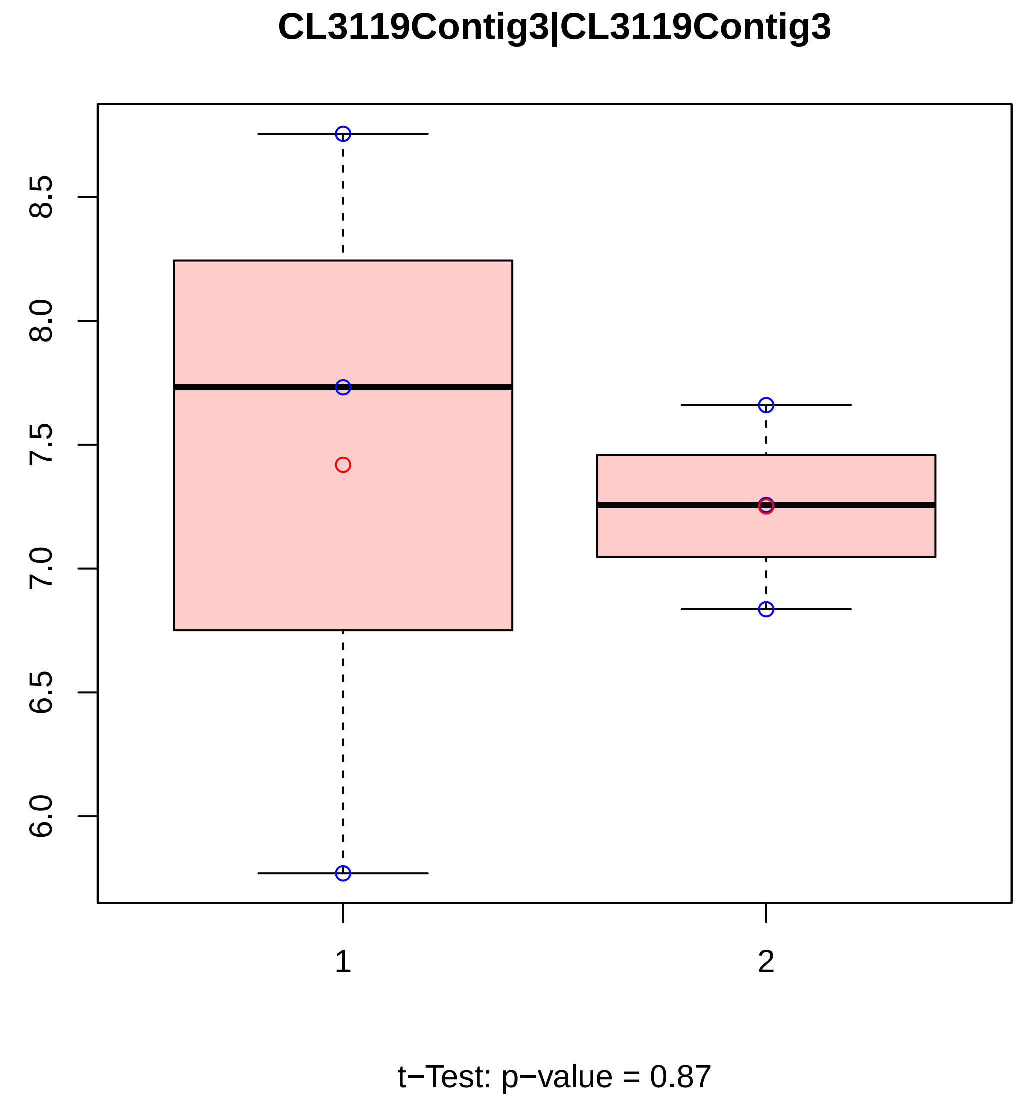
CL3119Contig3|CL3119Contig3
8.5
8.0
7.5
7.0
6.5
6.0
1
2
t−Test: p−value = 0.87

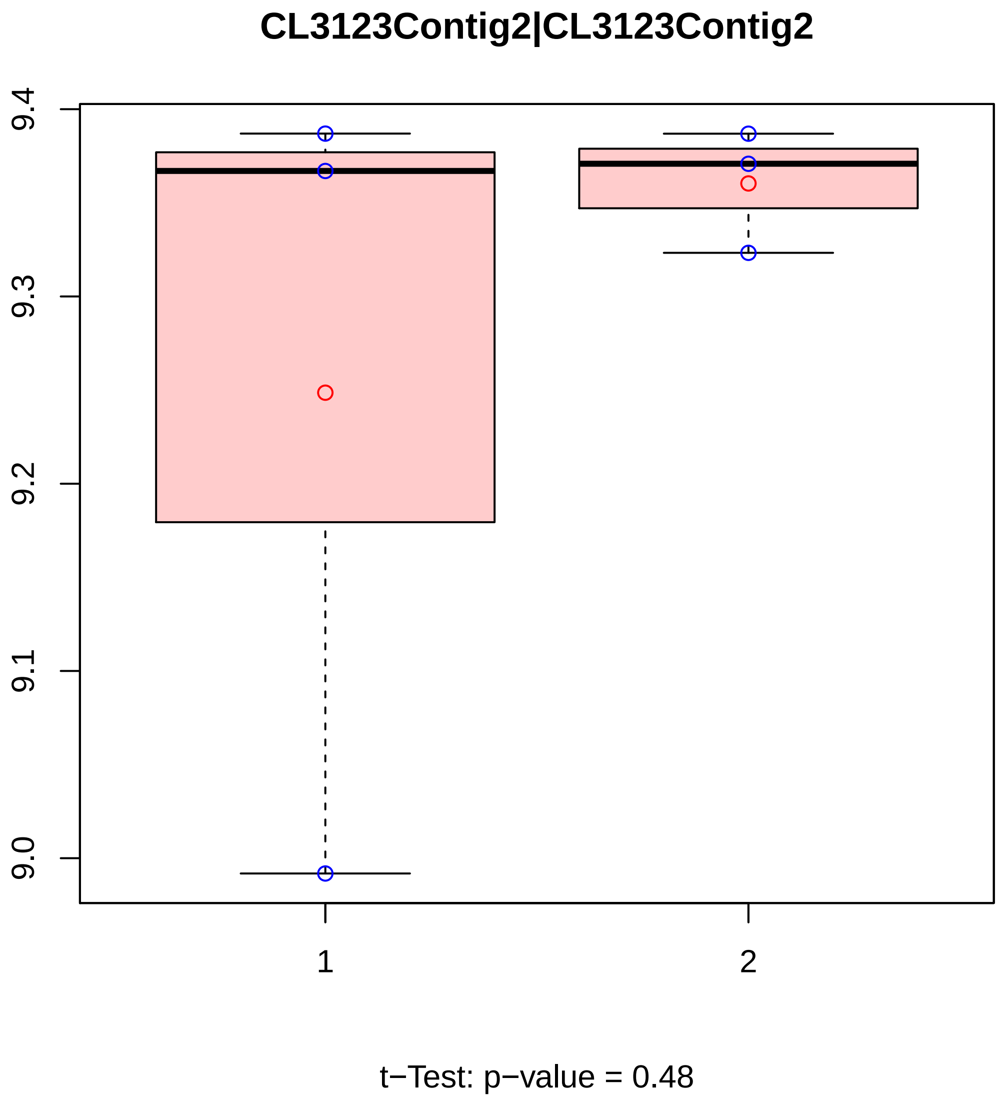
CL3123Contig2|CL3123Contig2
9.0
9.1
9.2
9.3
9.4
1
2
t−Test: p−value = 0.48

**CL3123Contig4|CL3123Contig4**

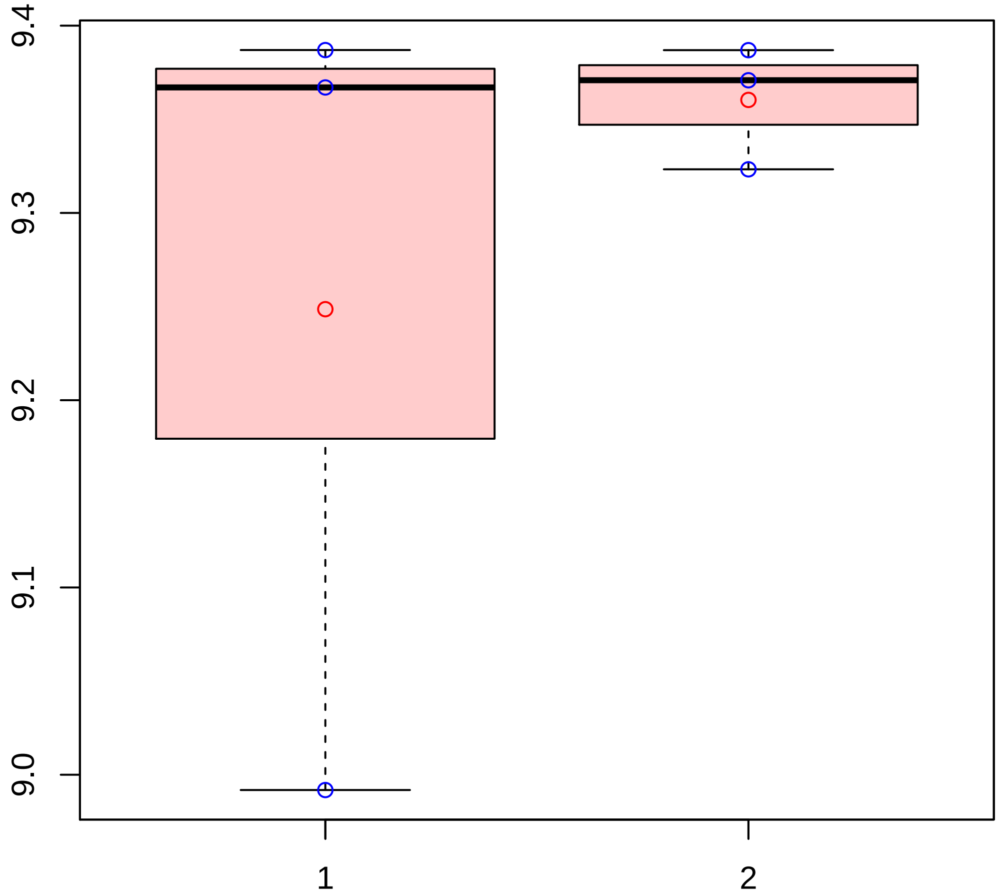


t−Test: p−value = 0.48

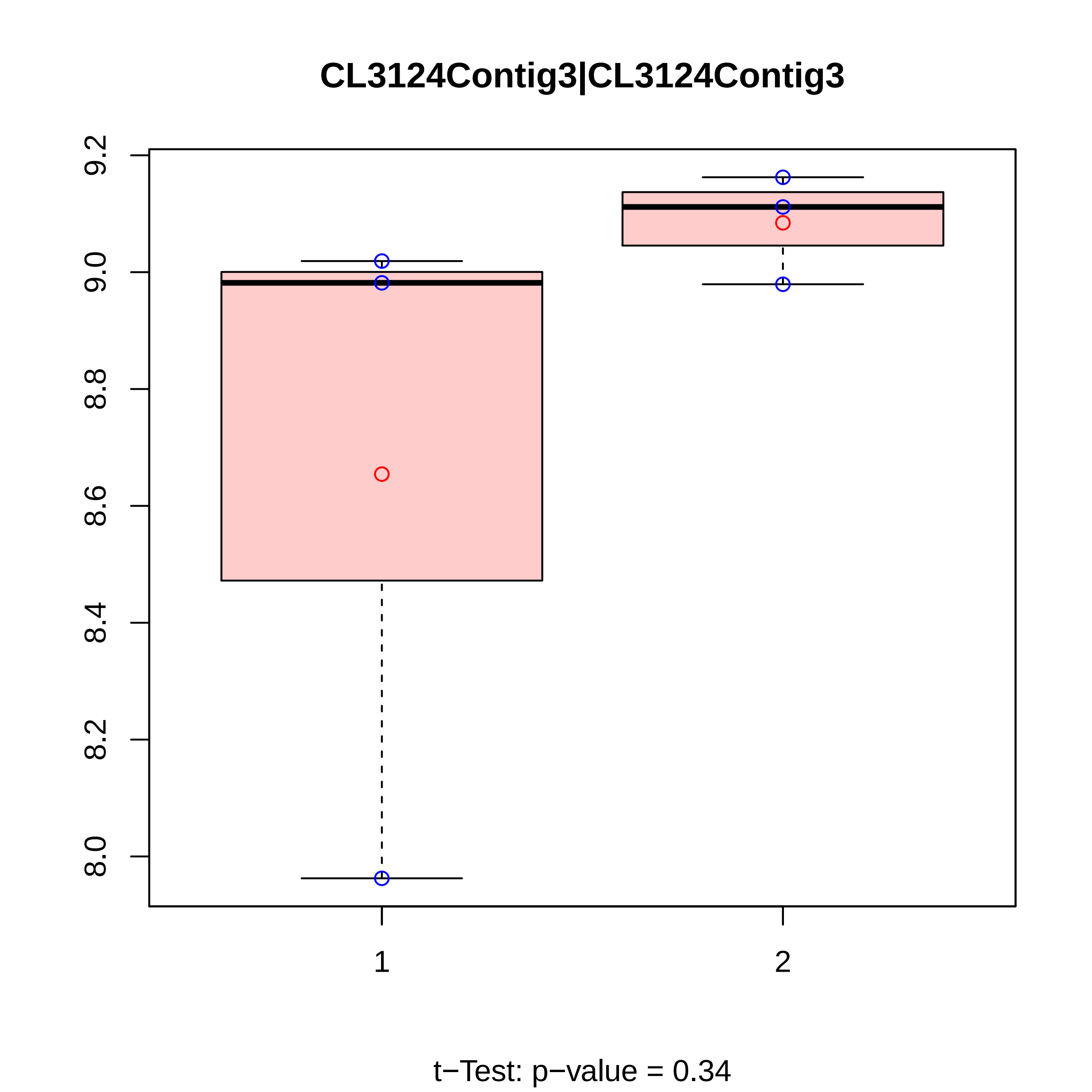

CL3124Contig3|CL3124Contig3
9.2
9.0
8.8
8.6
8.4
8.2
8.0
1
2
t−Test: p−value = 0.34

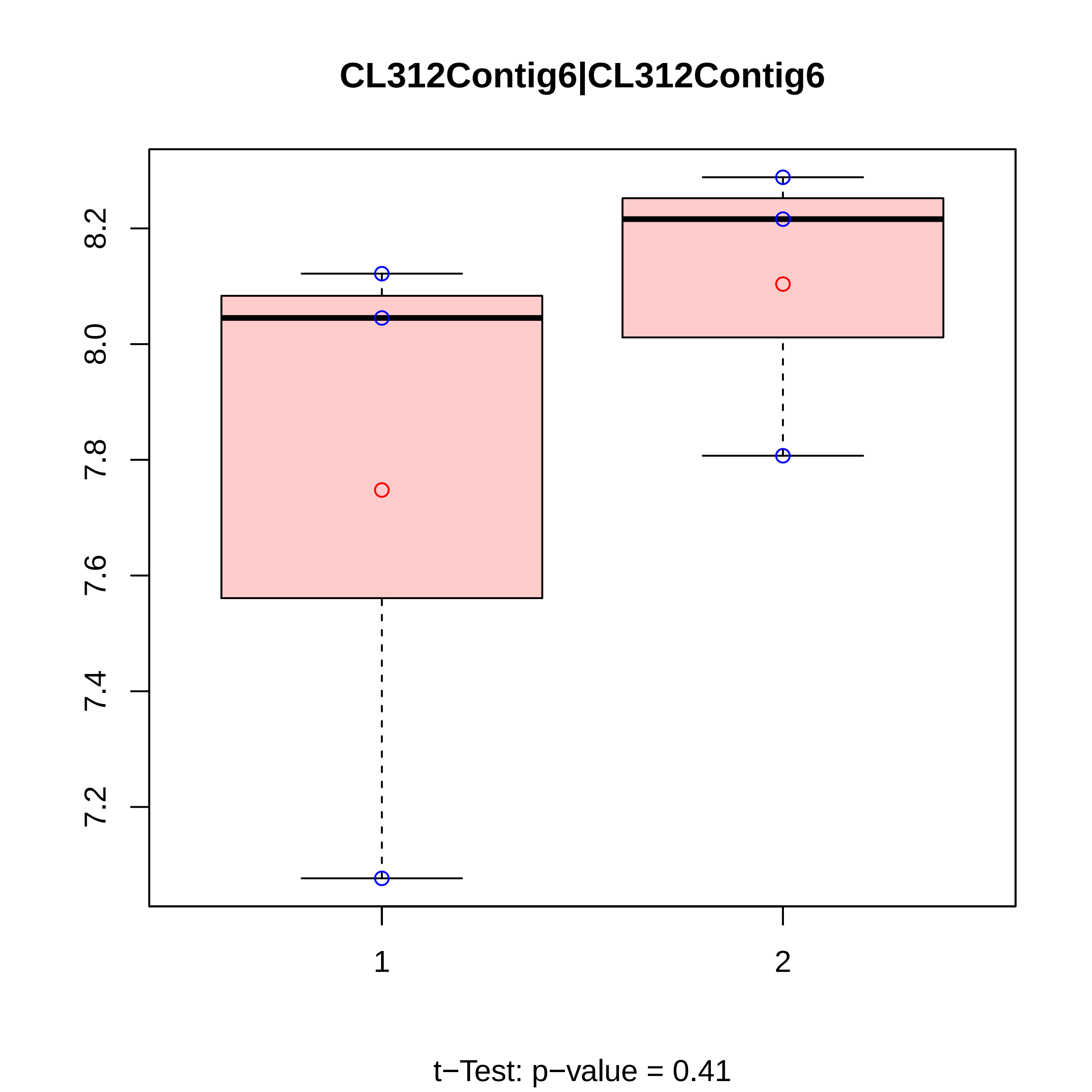
CL312Contig6|CL312Contig6
8.2
8.0
7.8
7.6
7.4
7.2
1
2
t−Test: p−value = 0.41

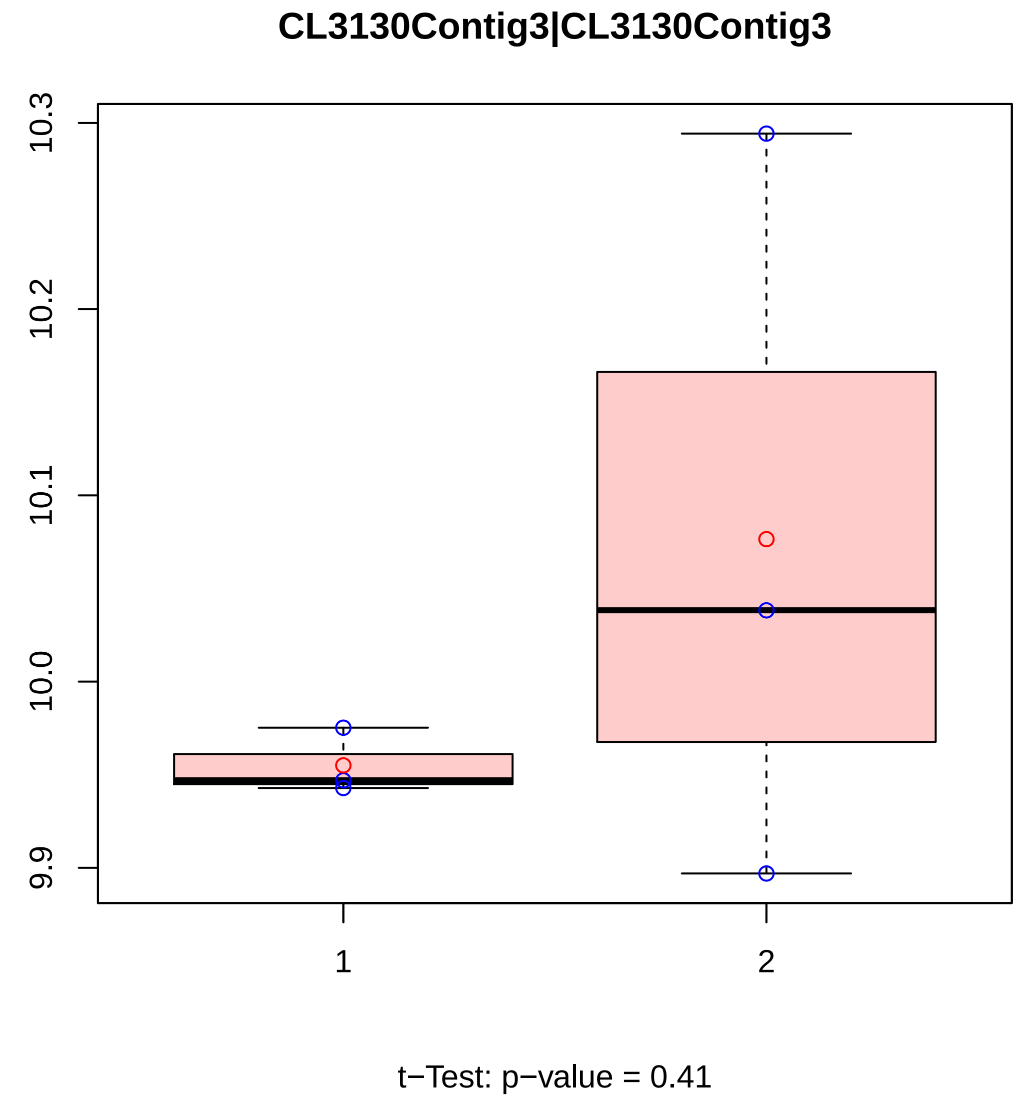
CL3130Contig3|CL3130Contig3
10.3
10.2
10.1
10.0
9.9
1
2
t−Test: p−value = 0.41

# CL3130Contig4|CL3130Contig4

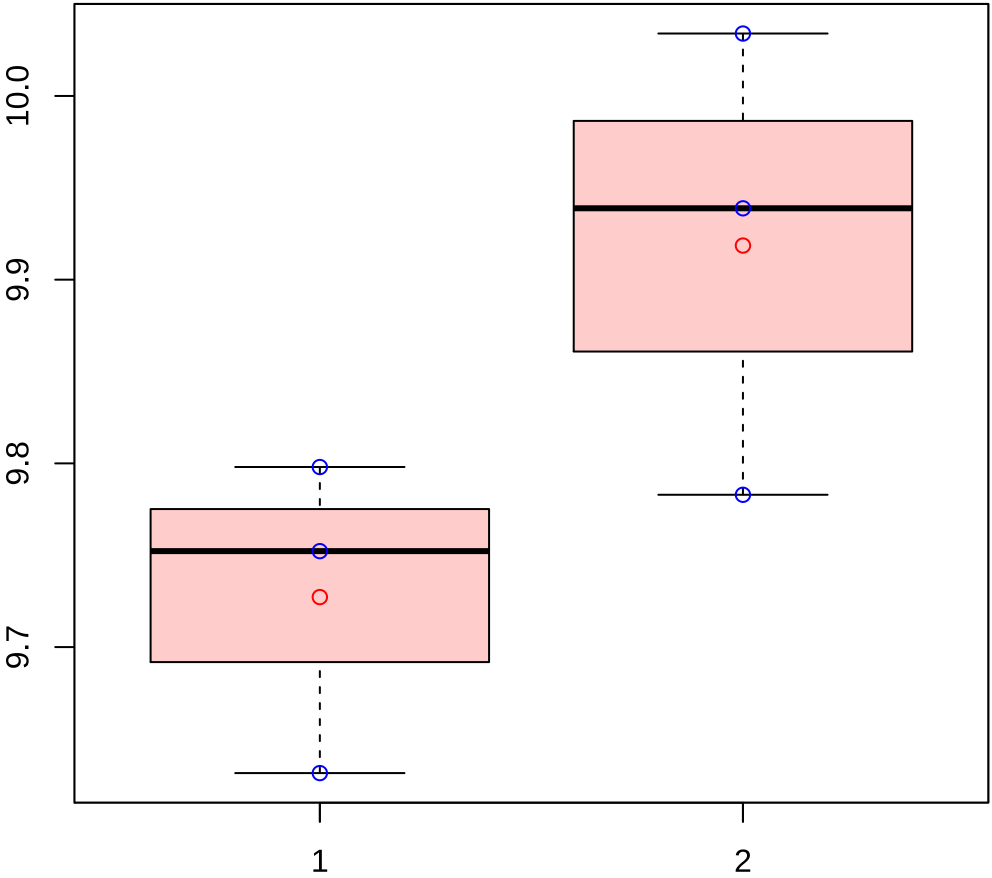


t−Test: p−value = 0.11

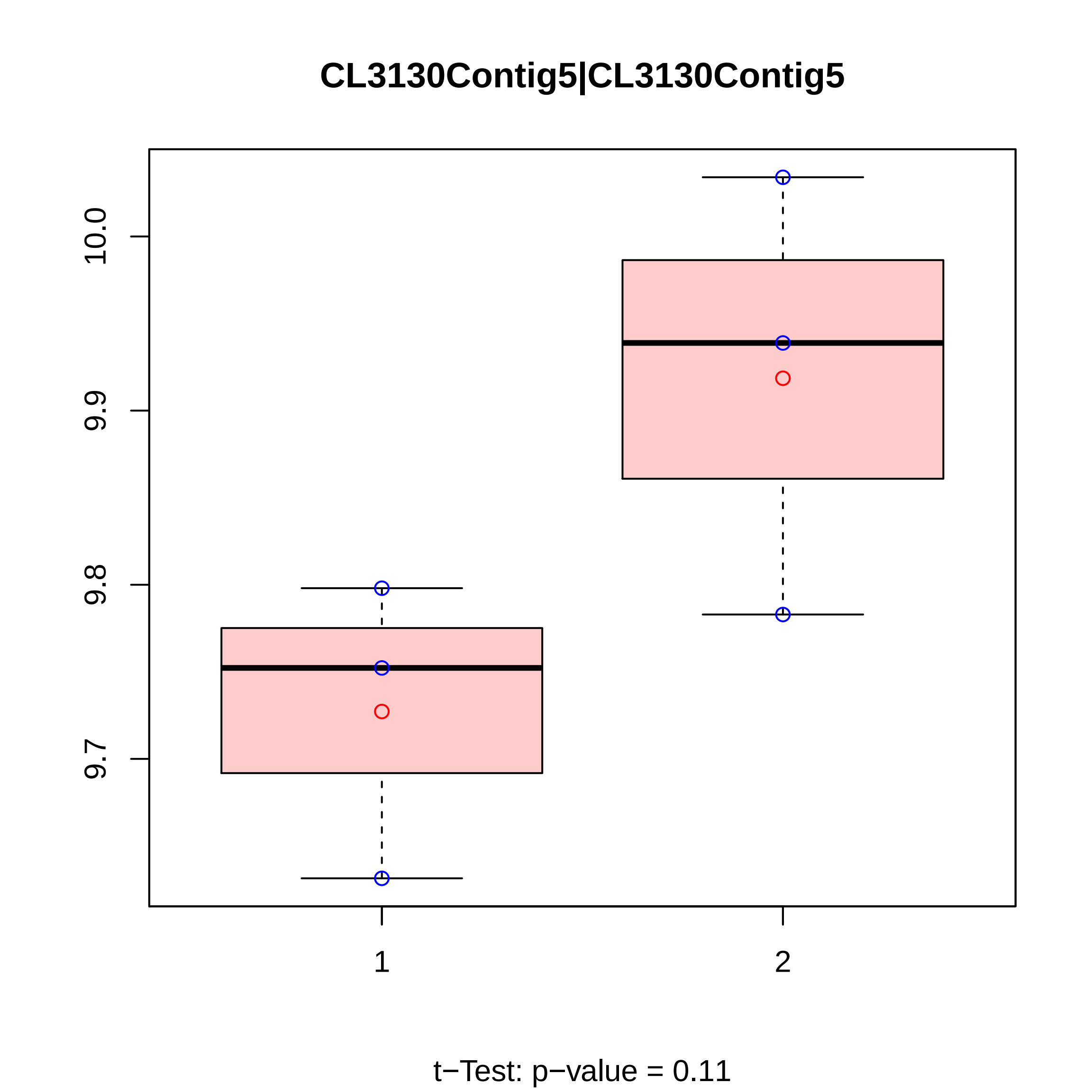

CL3130Contig5|CL3130Contig5
10.0
9.9
9.8
9.7
1
2
t−Test: p−value = 0.11

# CL3133Contig1|CL3133Contig1

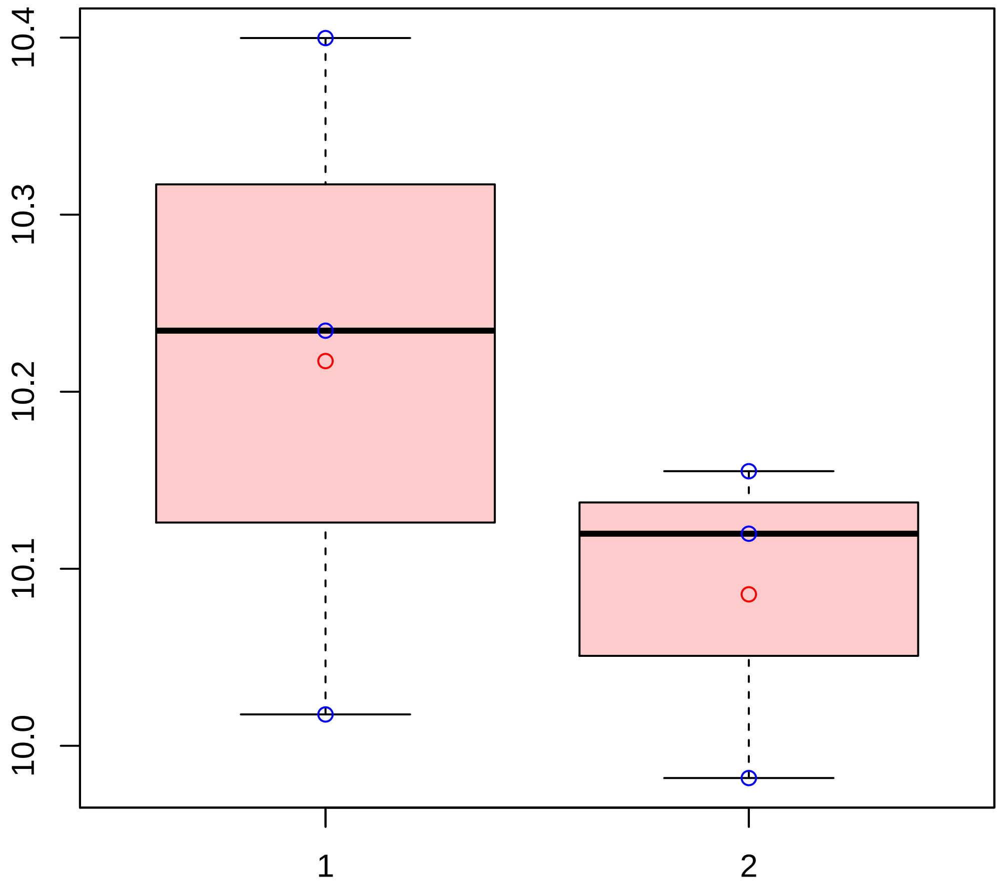


t−Test: p−value = 0.36

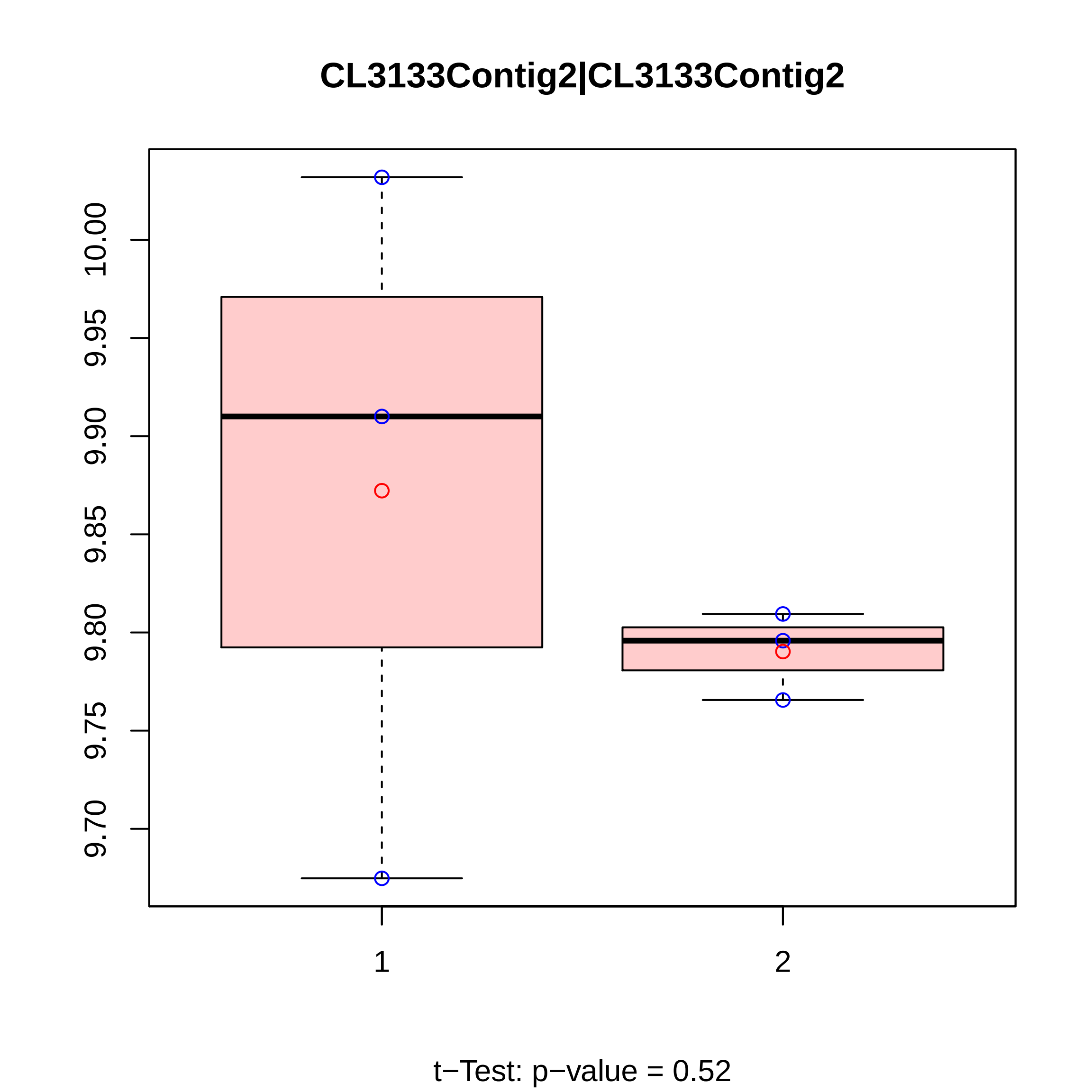
CL3133Contig2|CL3133Contig2
10.00
9.95
9.90
9.85
9.80
9.75
9.70
1
2
t−Test: p−value = 0.52

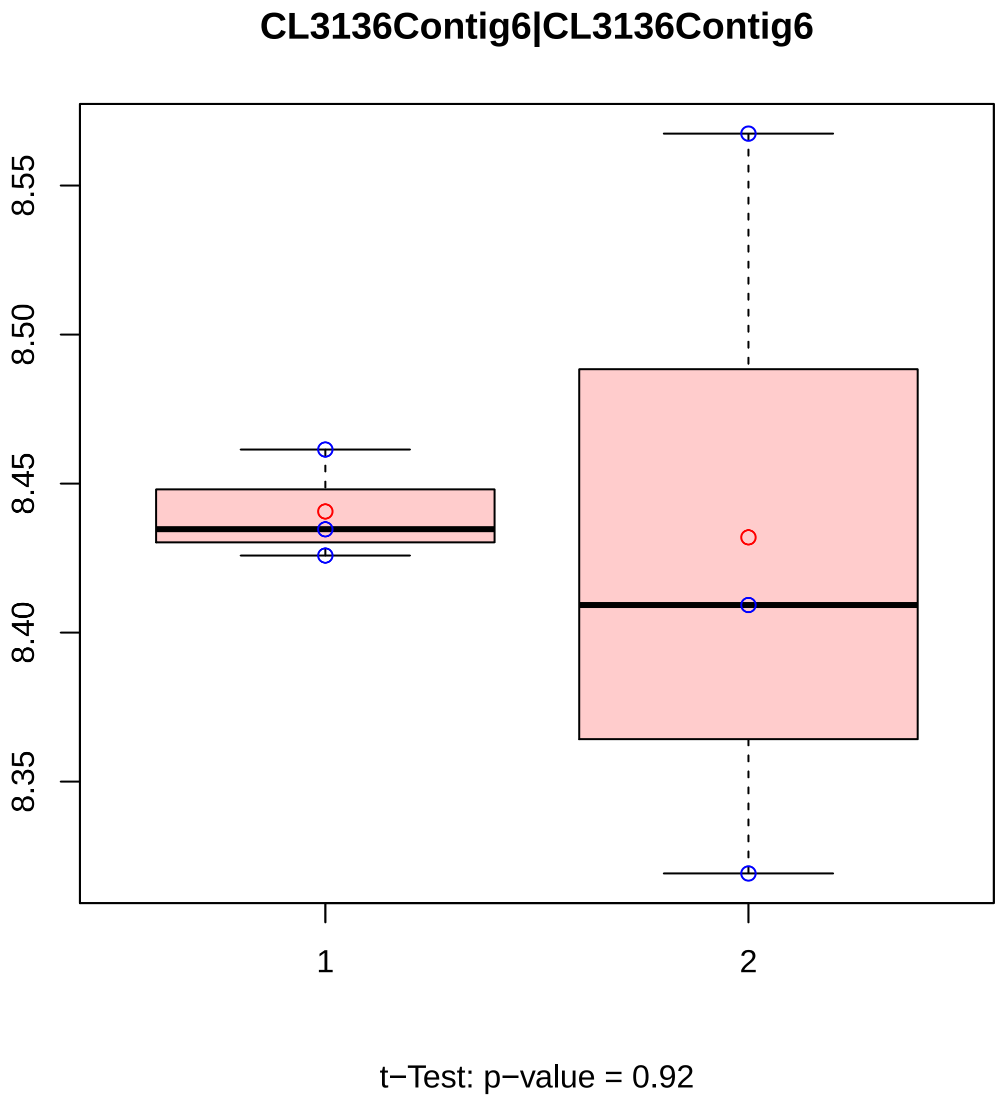
CL3136Contig6|CL3136Contig6
8.35
8.40
8.45
8.50
8.55
1
2
t−Test: p−value = 0.92

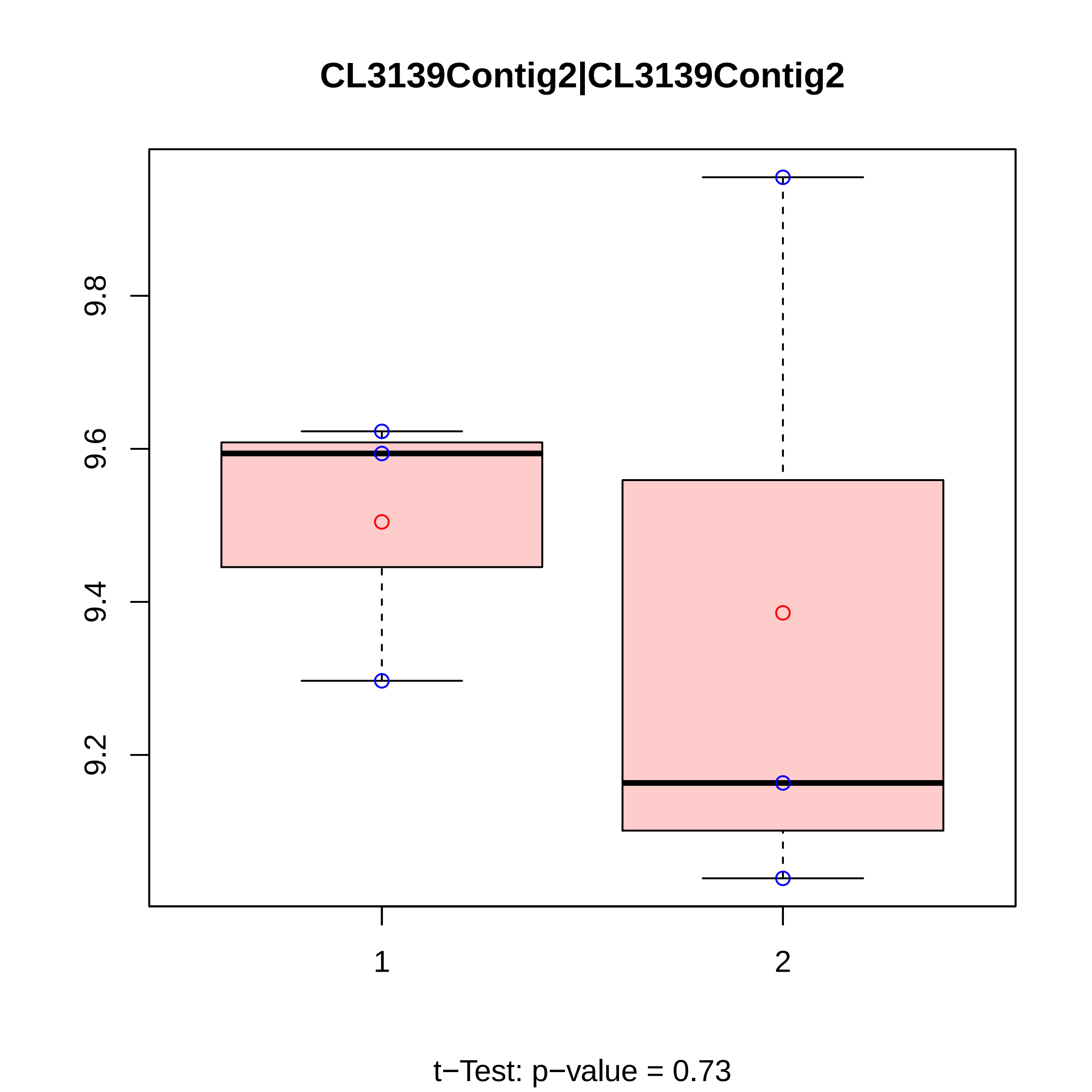

CL3139Contig2|CL3139Contig2
9.2
9.4
9.6
9.8
1
2
t−Test: p−value = 0.73

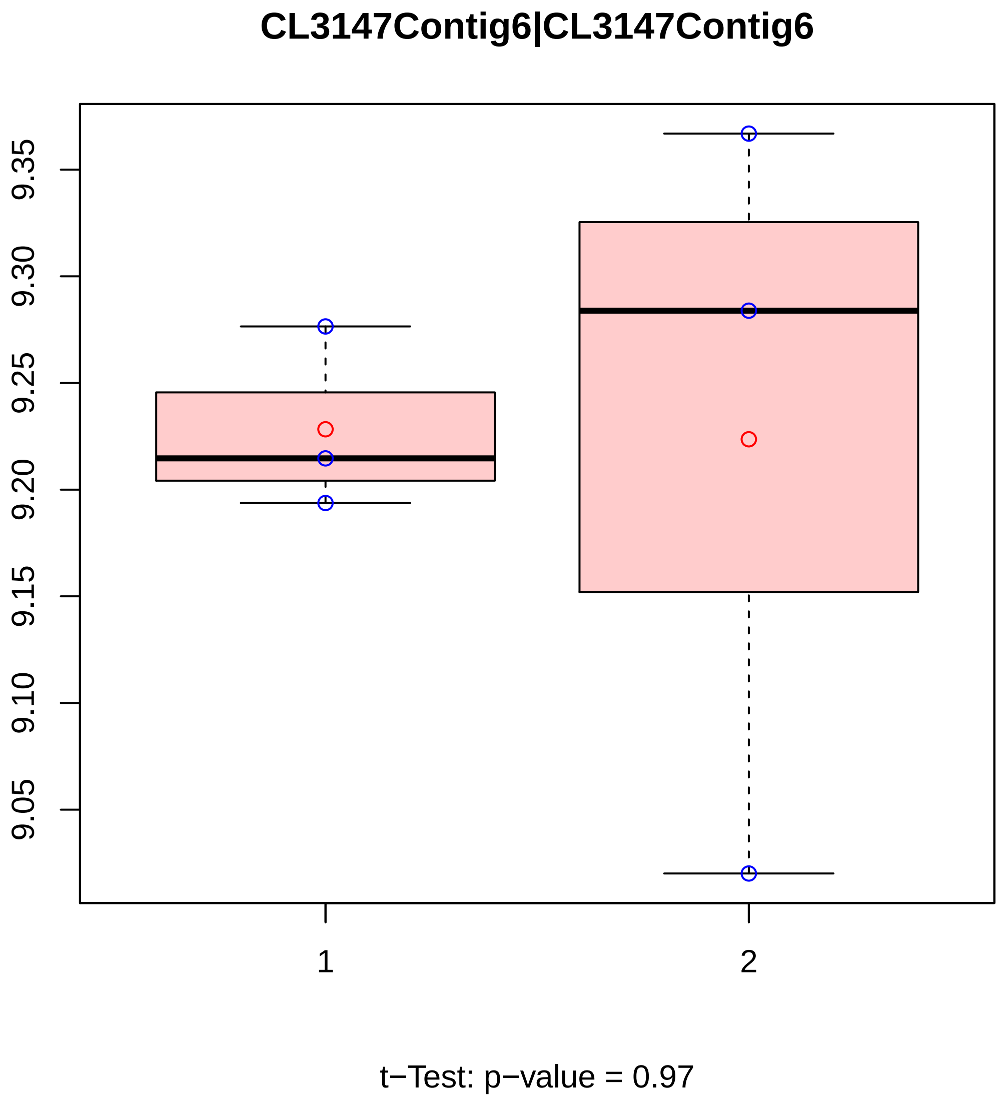

CL3147Contig6|CL3147Contig6
9.05
9.10
9.15
9.20
9.25
9.30
9.35
1
2
t−Test: p−value = 0.97

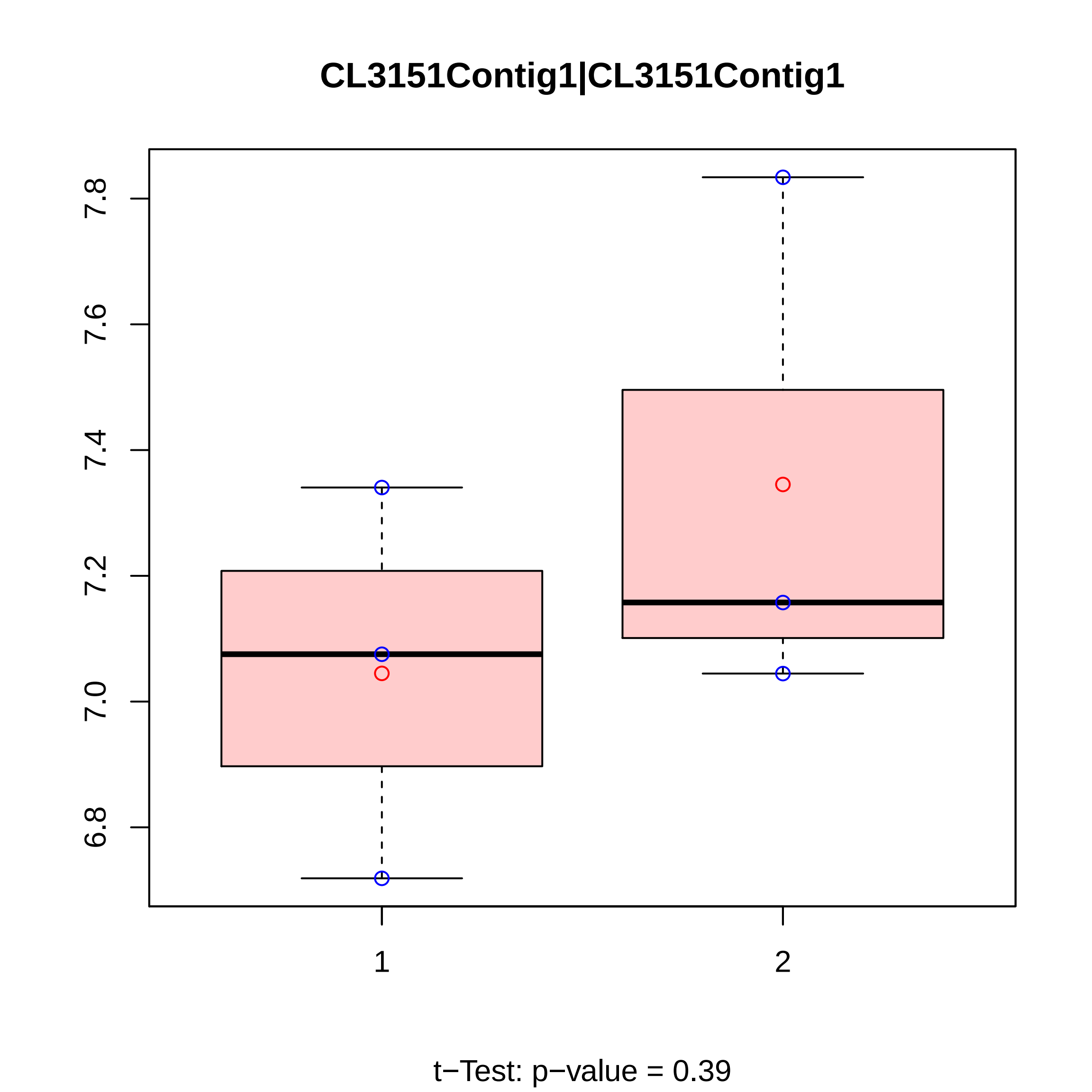

CL3151Contig1|CL3151Contig1
7.8
7.6
7.4
7.2
7.0
6.8
1
2
t−Test: p−value = 0.39

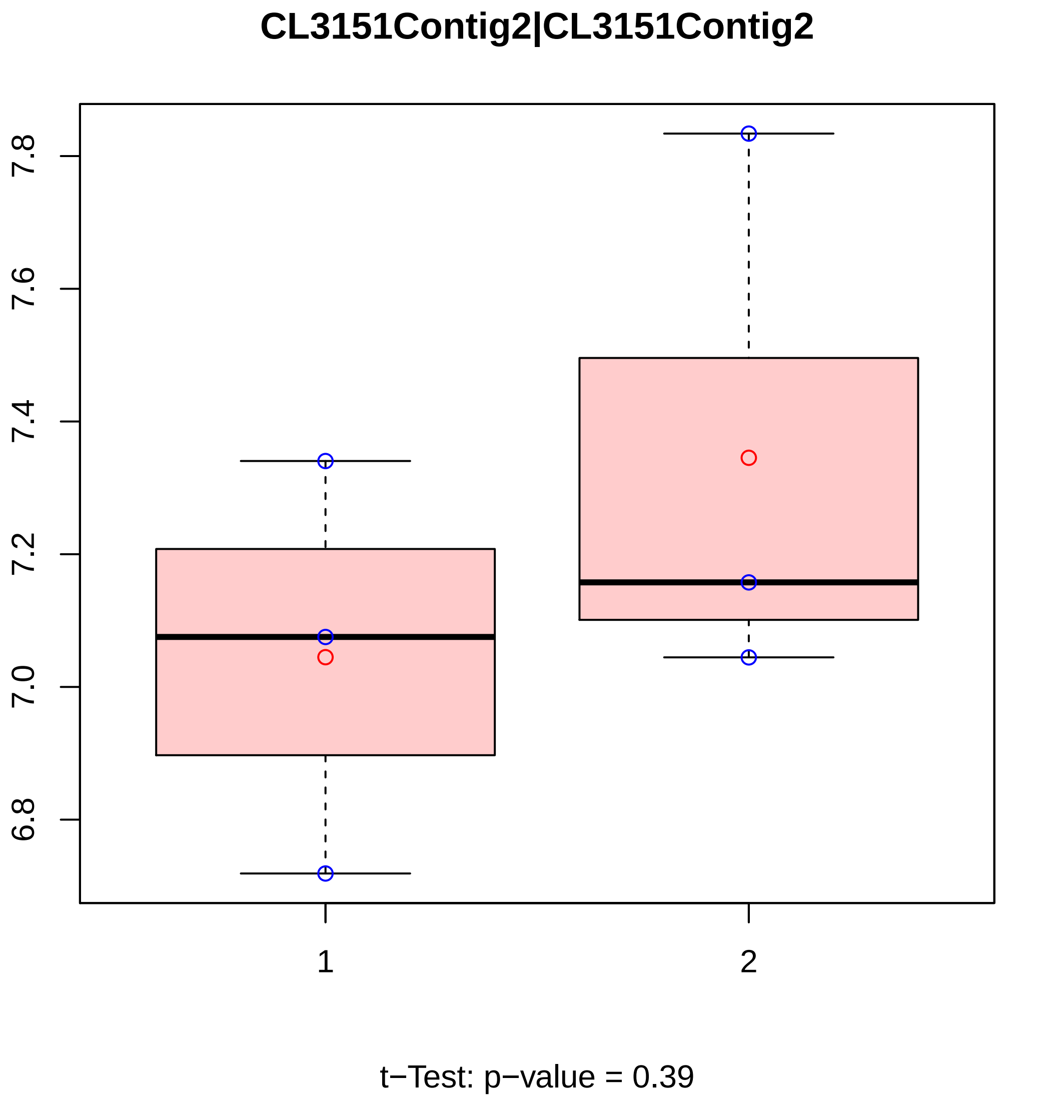
CL3151Contig2|CL3151Contig2
7.8
7.6
7.4
7.2
7.0
6.8
1
2
t−Test: p−value = 0.39

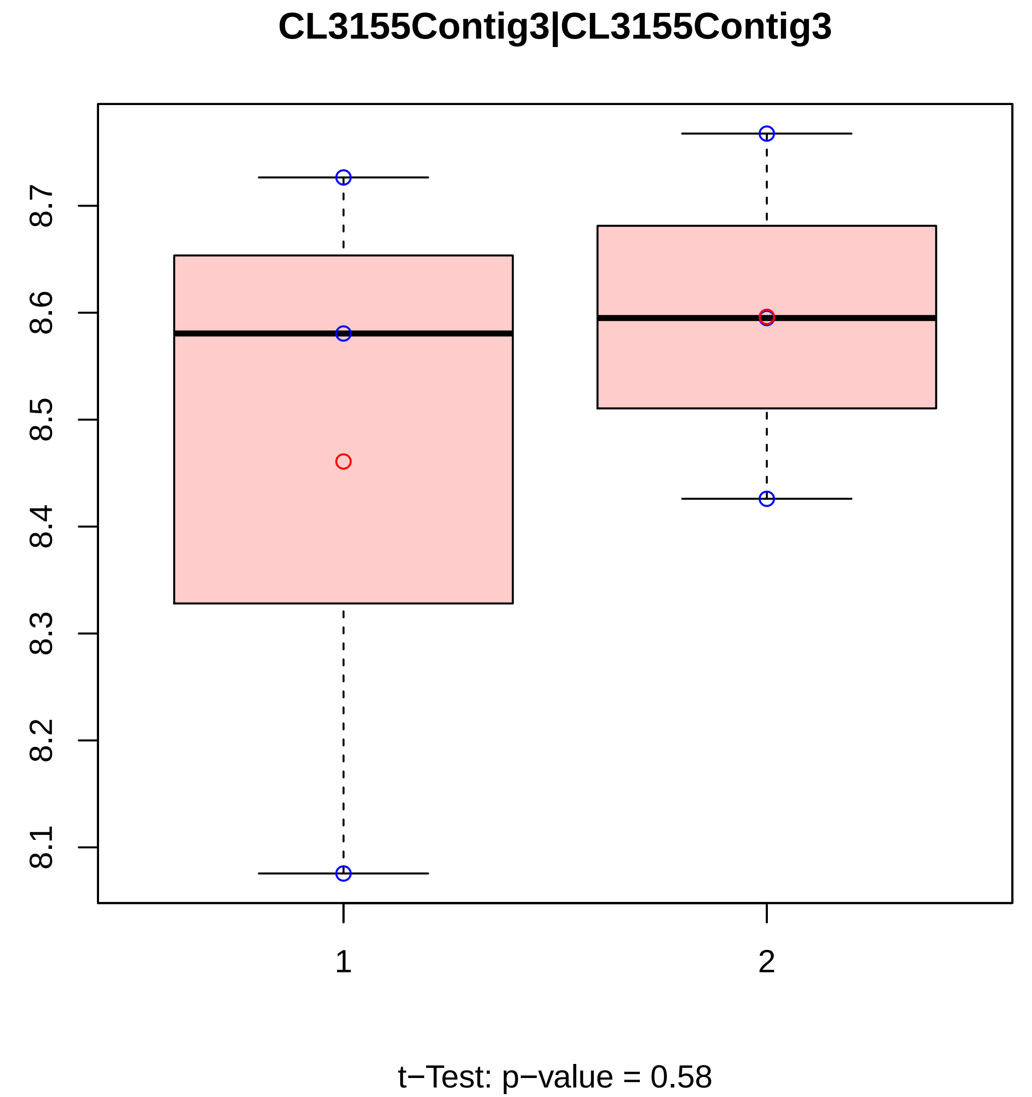

CL3155Contig3|CL3155Contig3
8.1
8.2
8.3
8.4
8.5
8.6
8.7
1
2
t−Test: p−value = 0.58

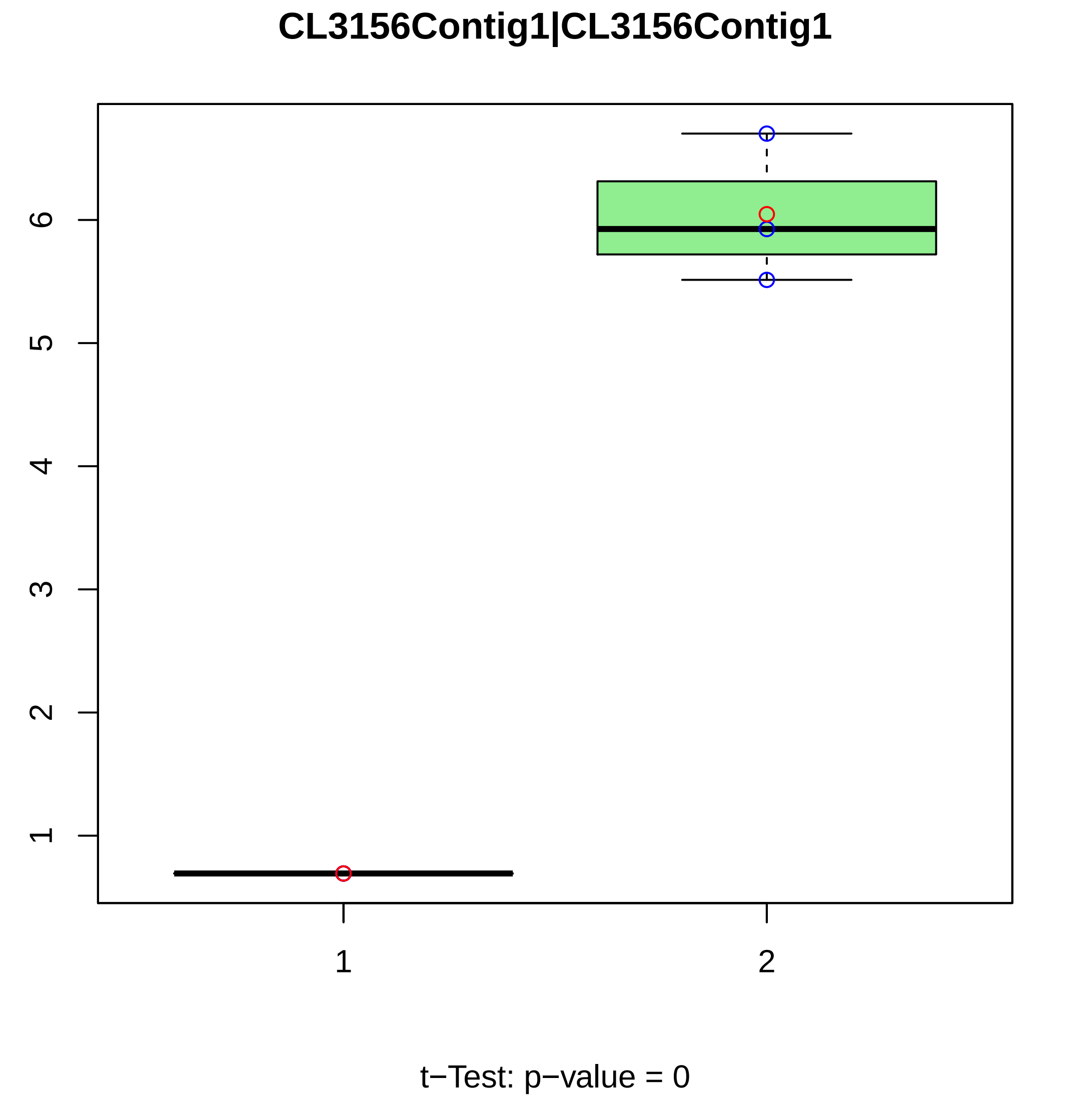

CL3156Contig1|CL3156Contig1
1
2
3
4
5
6
1
2
t−Test: p−value = 0

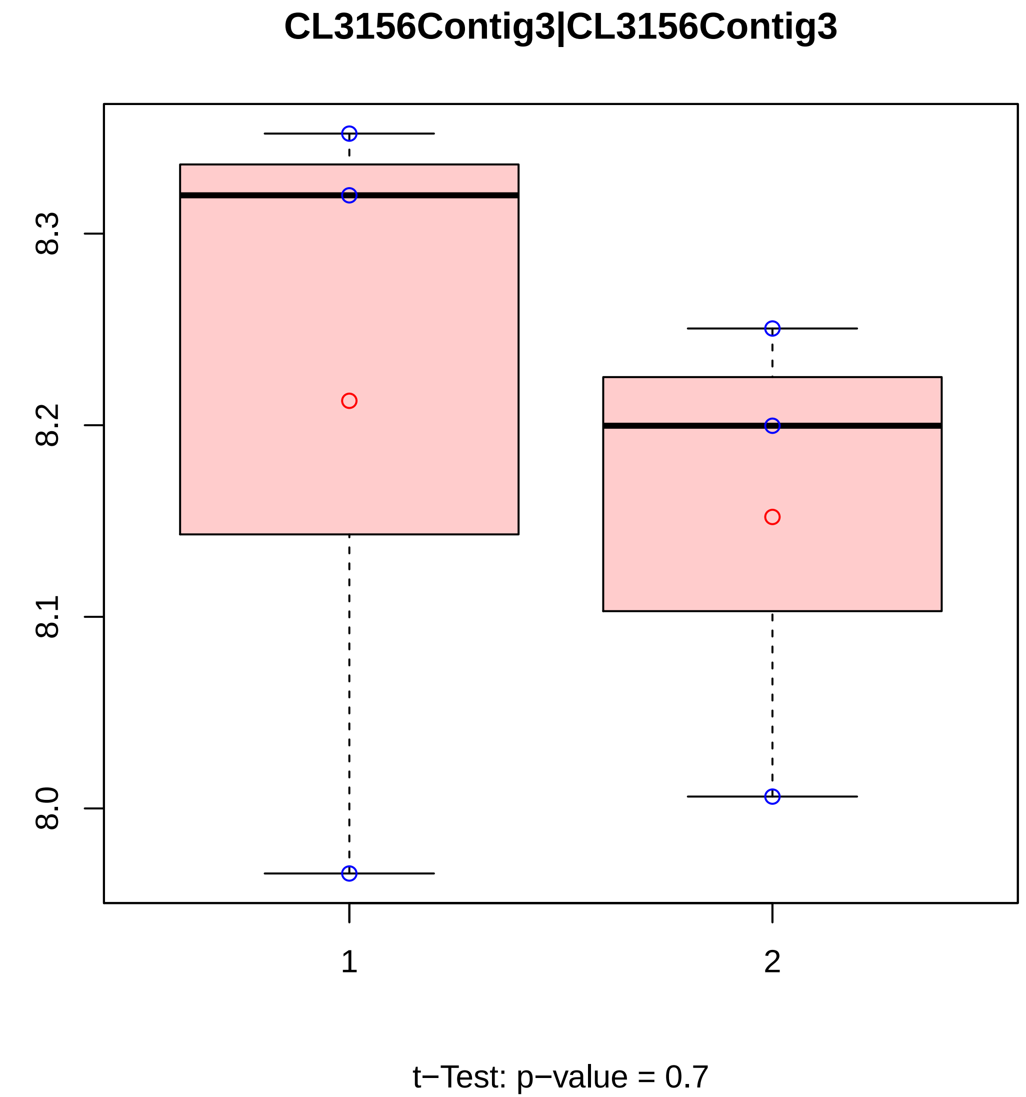

CL3156Contig3|CL3156Contig3
8.0
8.1
8.2
8.3
1
2
t−Test: p−value = 0.7

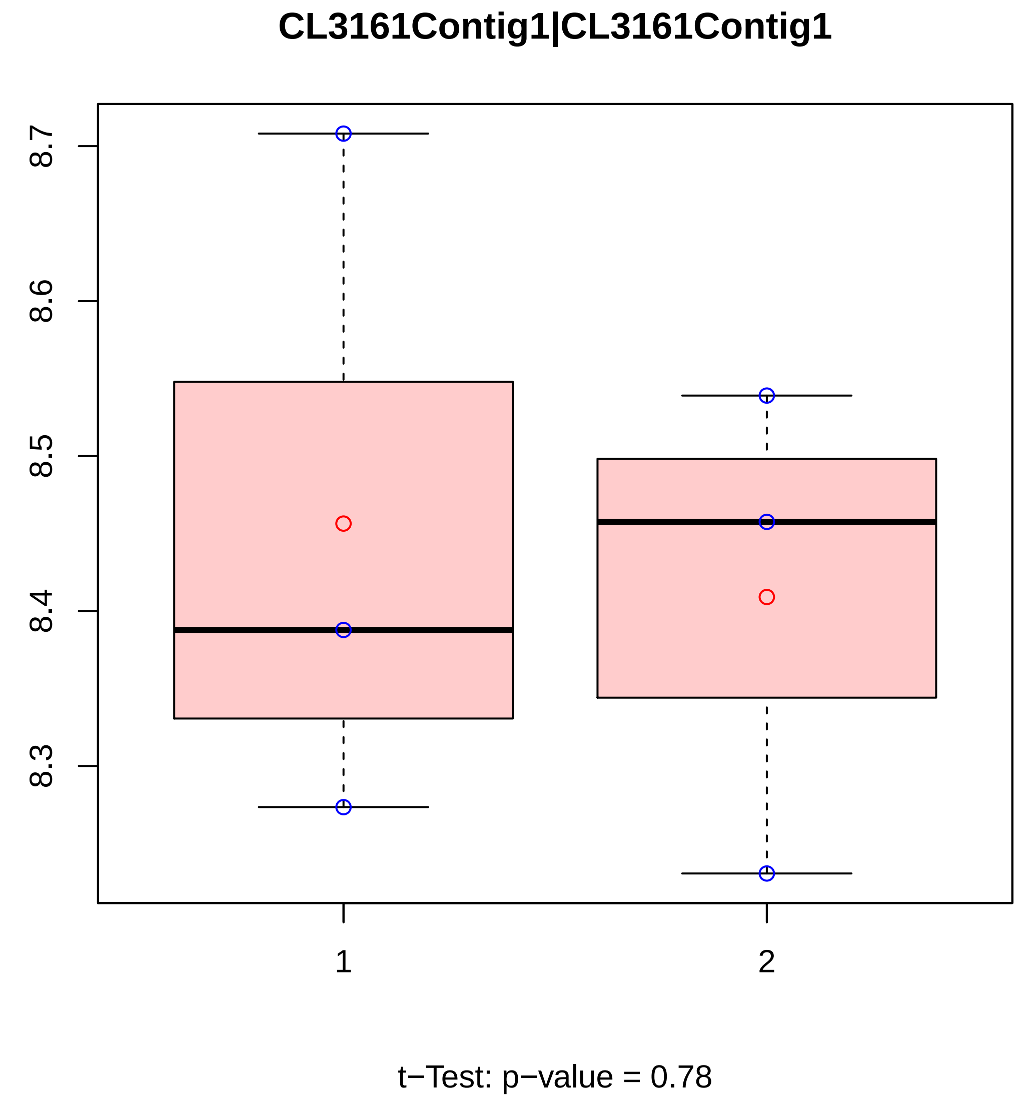

CL3161Contig1|CL3161Contig1
8.7
8.6
8.5
8.4
8.3
1
2
t−Test: p−value = 0.78

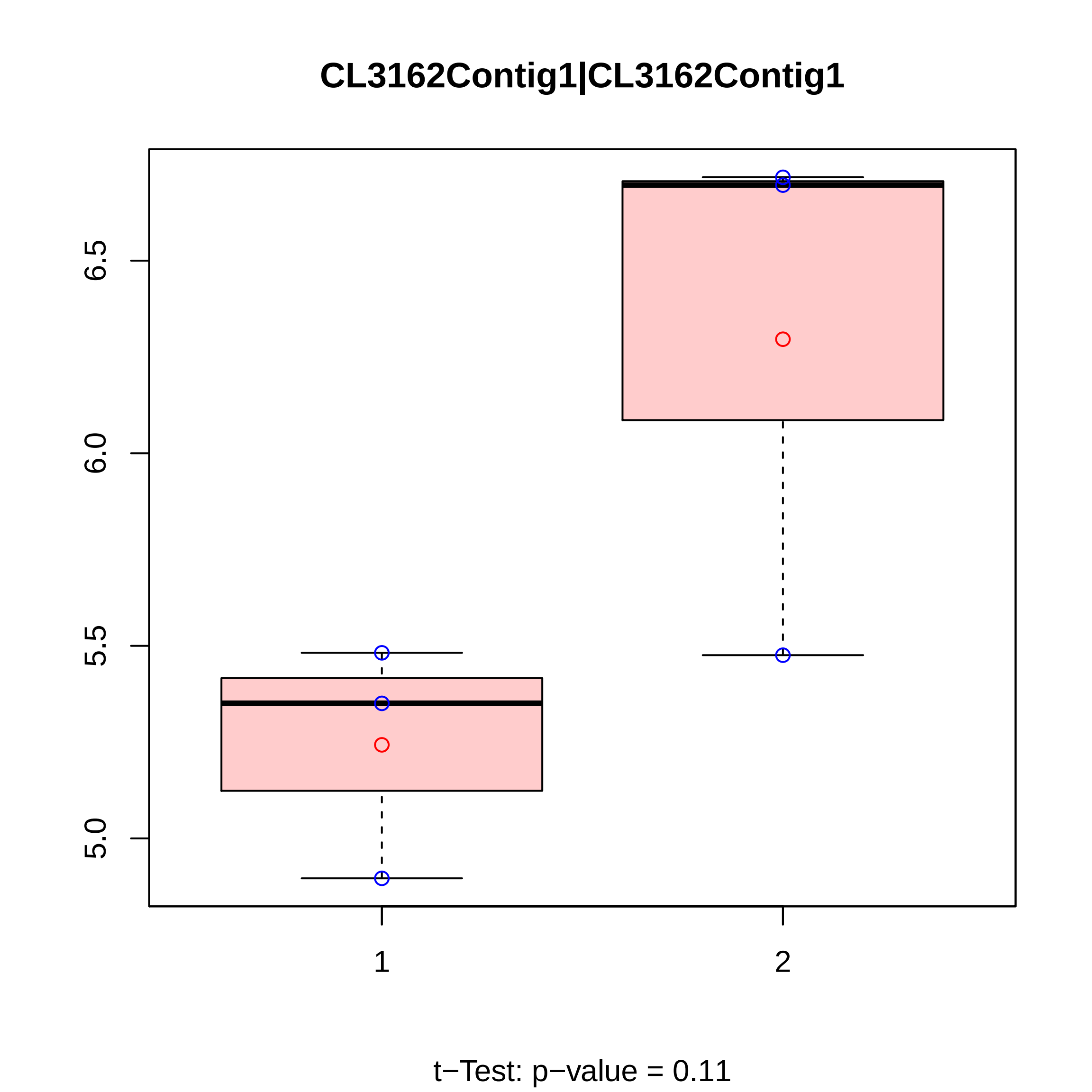

CL3162Contig1|CL3162Contig1
6.5
6.0
5.5
5.0
1
2
t−Test: p−value = 0.11

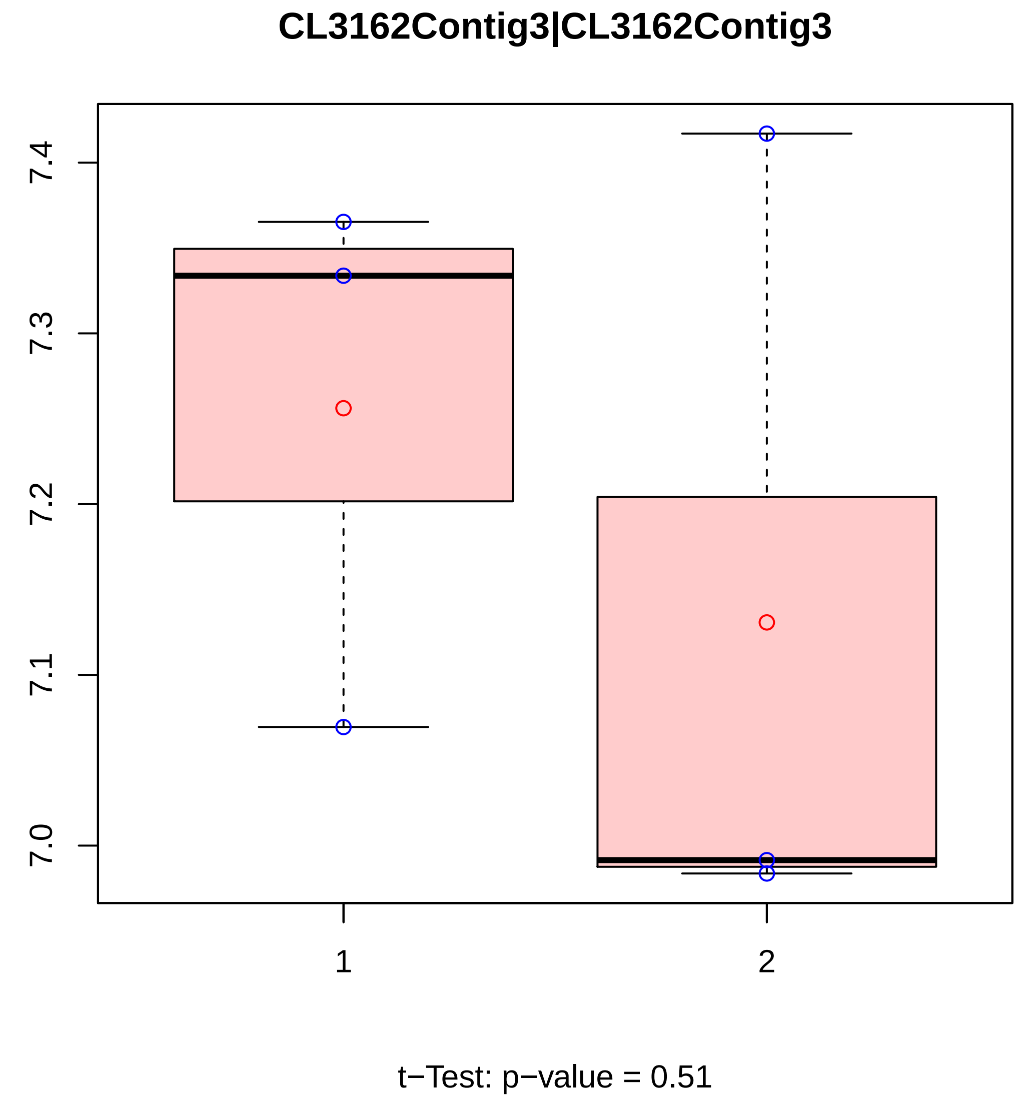

CL3162Contig3|CL3162Contig3
7.0
7.1
7.2
7.3
7.4
1
2
t−Test: p−value = 0.51

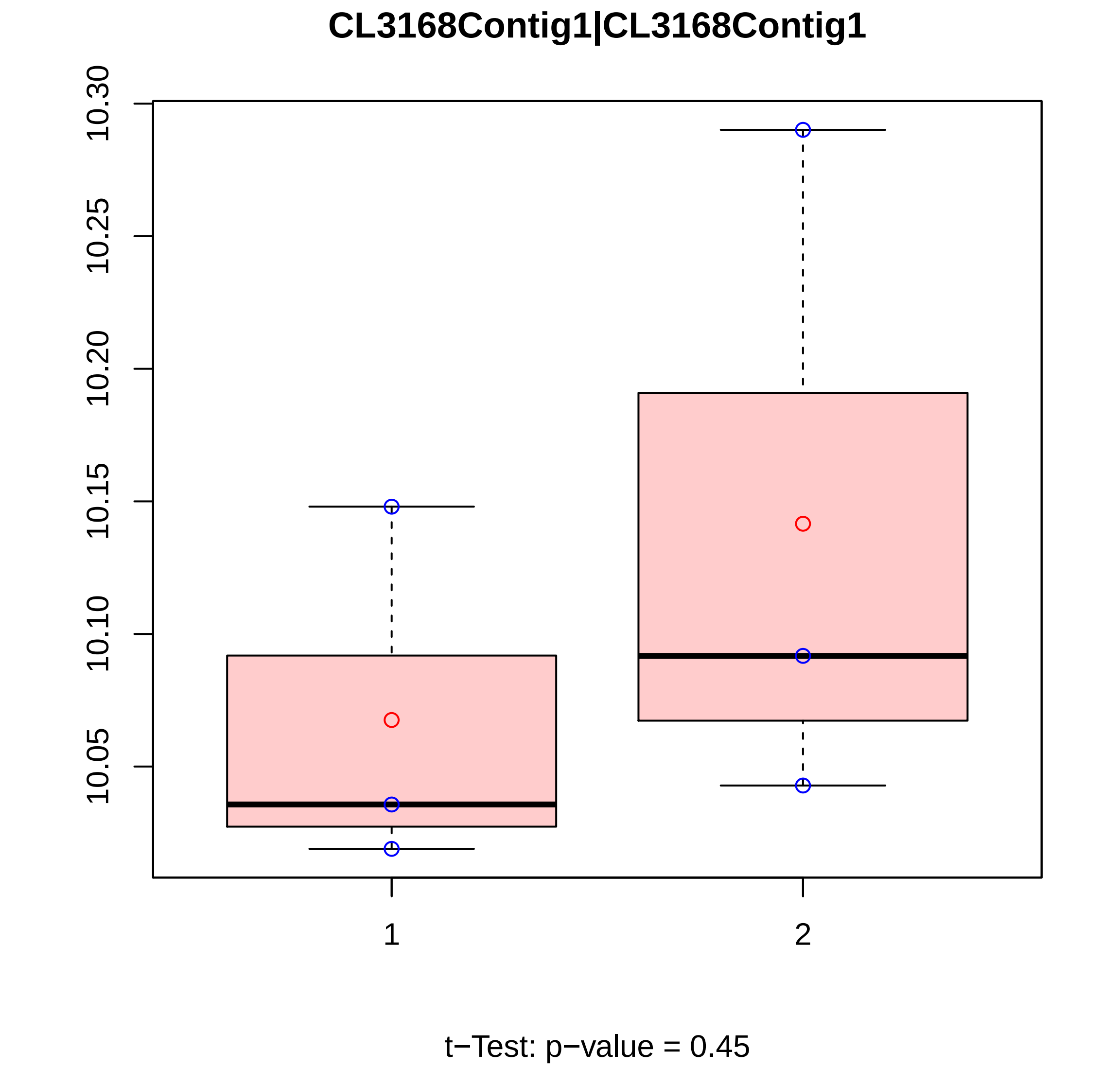
CL3168Contig1|CL3168Contig1
10.30
10.25
10.20
10.15
10.10
10.05
1
2
t−Test: p−value = 0.45

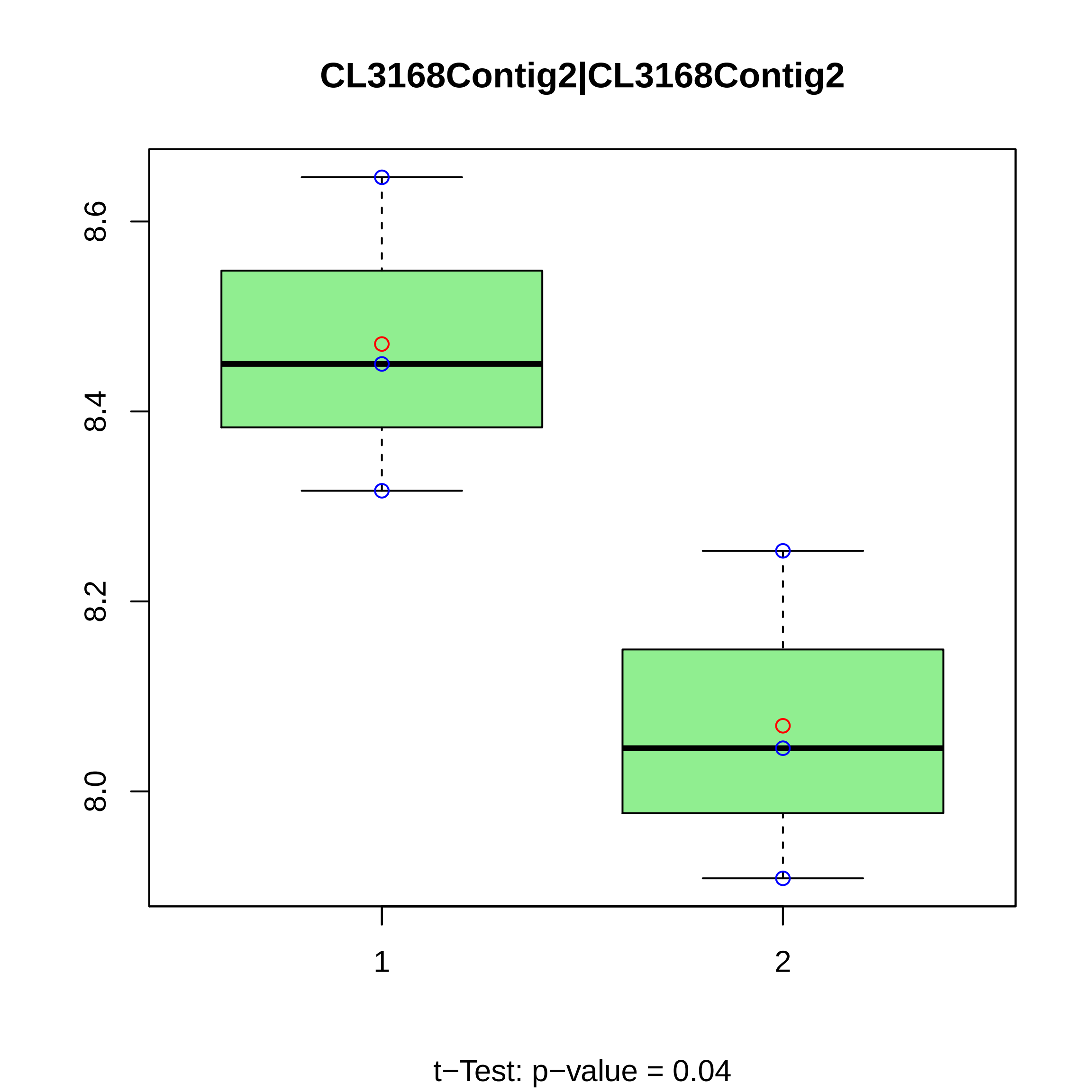
CL3168Contig2|CL3168Contig2
8.0
8.2
8.4
8.6
1
2
t−Test: p−value = 0.04

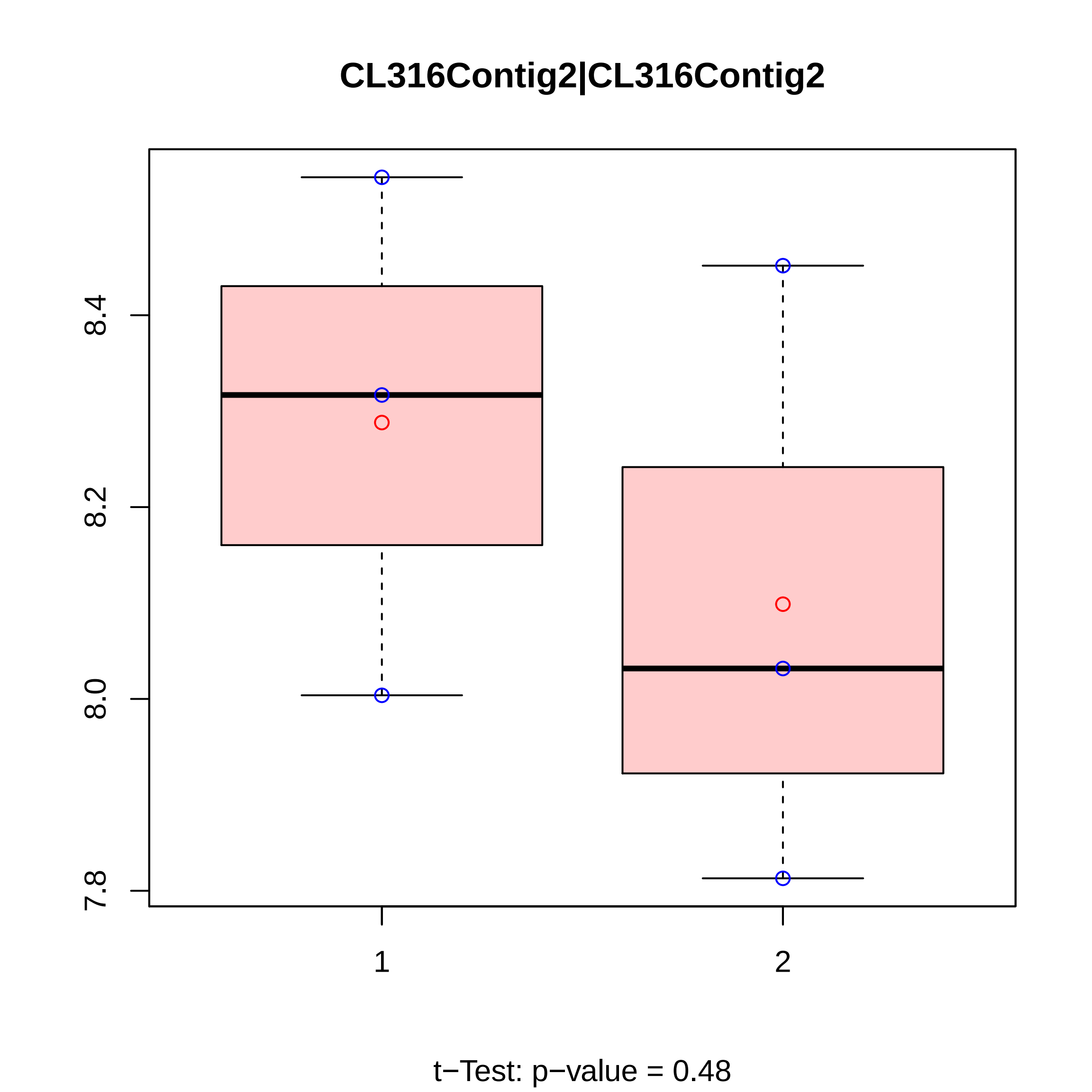
CL316Contig2|CL316Contig2
8.4
8.2
8.0
7.8
1
2
t−Test: p−value = 0.48

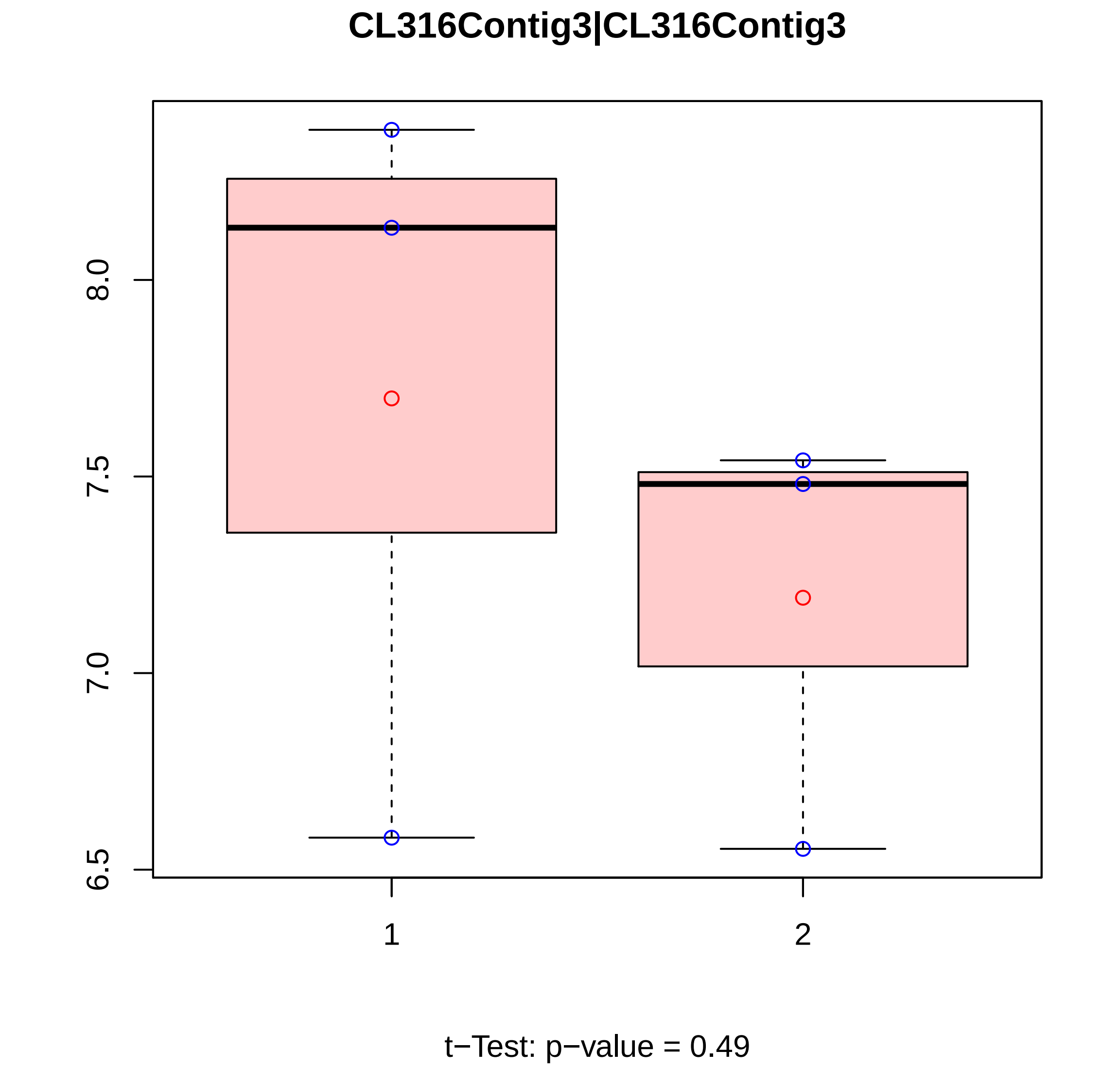
CL316Contig3|CL316Contig3
8.0
7.5
7.0
6.5
1
2
t−Test: p−value = 0.49

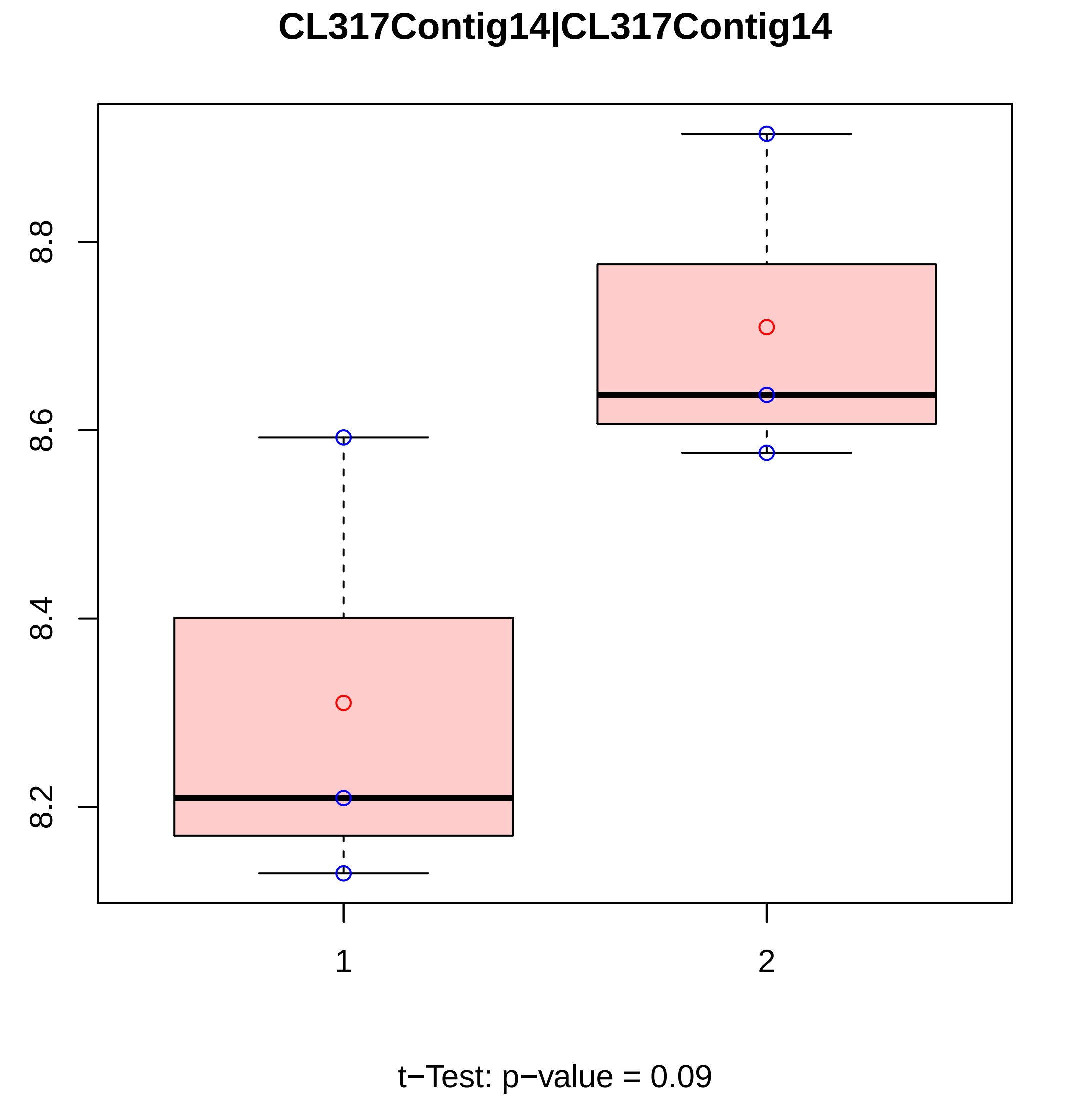
CL317Contig14|CL317Contig14
8.2
8.4
8.6
8.8
1
2
t−Test: p−value = 0.09

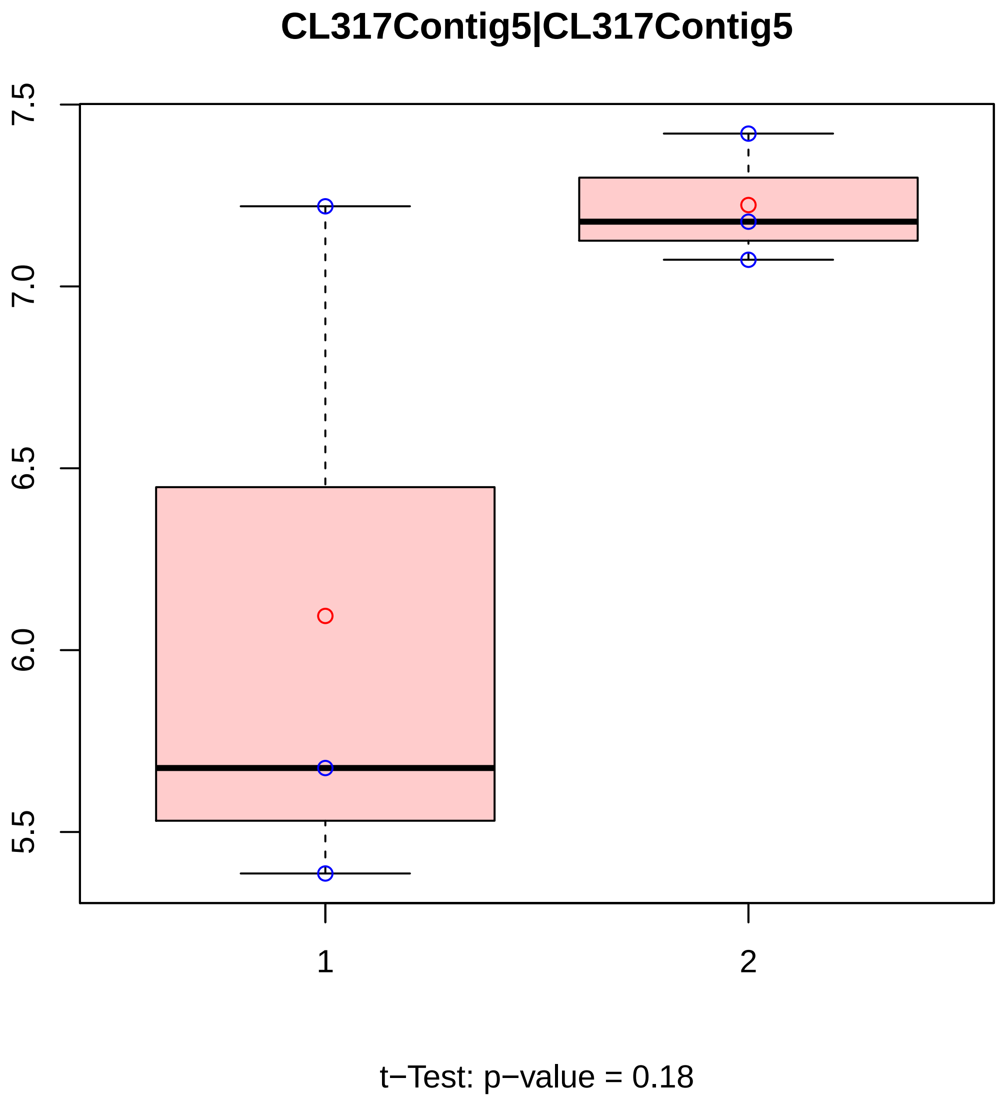
CL317Contig5|CL317Contig5
7.5
7.0
6.5
6.0
5.5
1
2
t−Test: p−value = 0.18

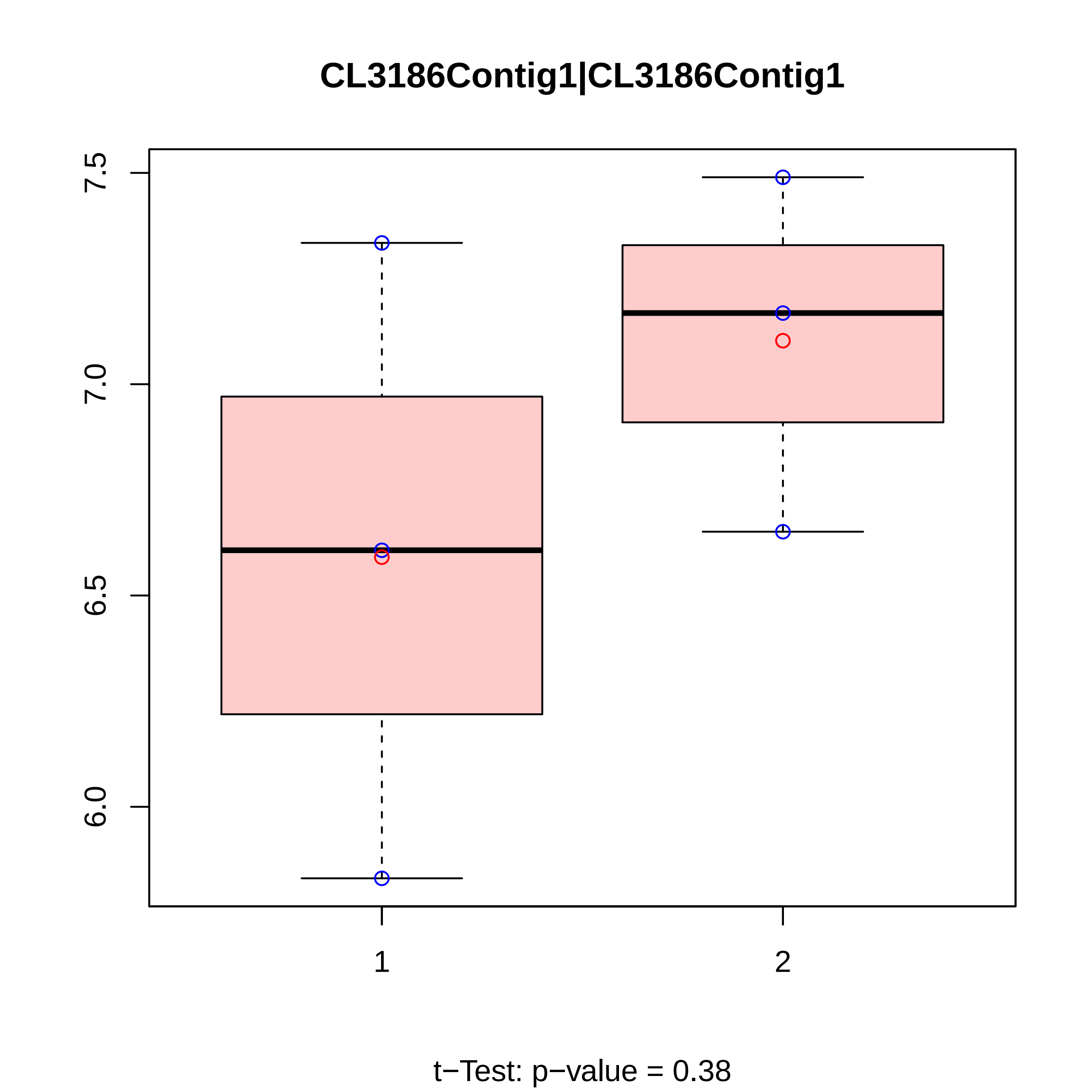
CL3186Contig1|CL3186Contig1
7.5
7.0
6.5
6.0
1
2
t−Test: p−value = 0.38

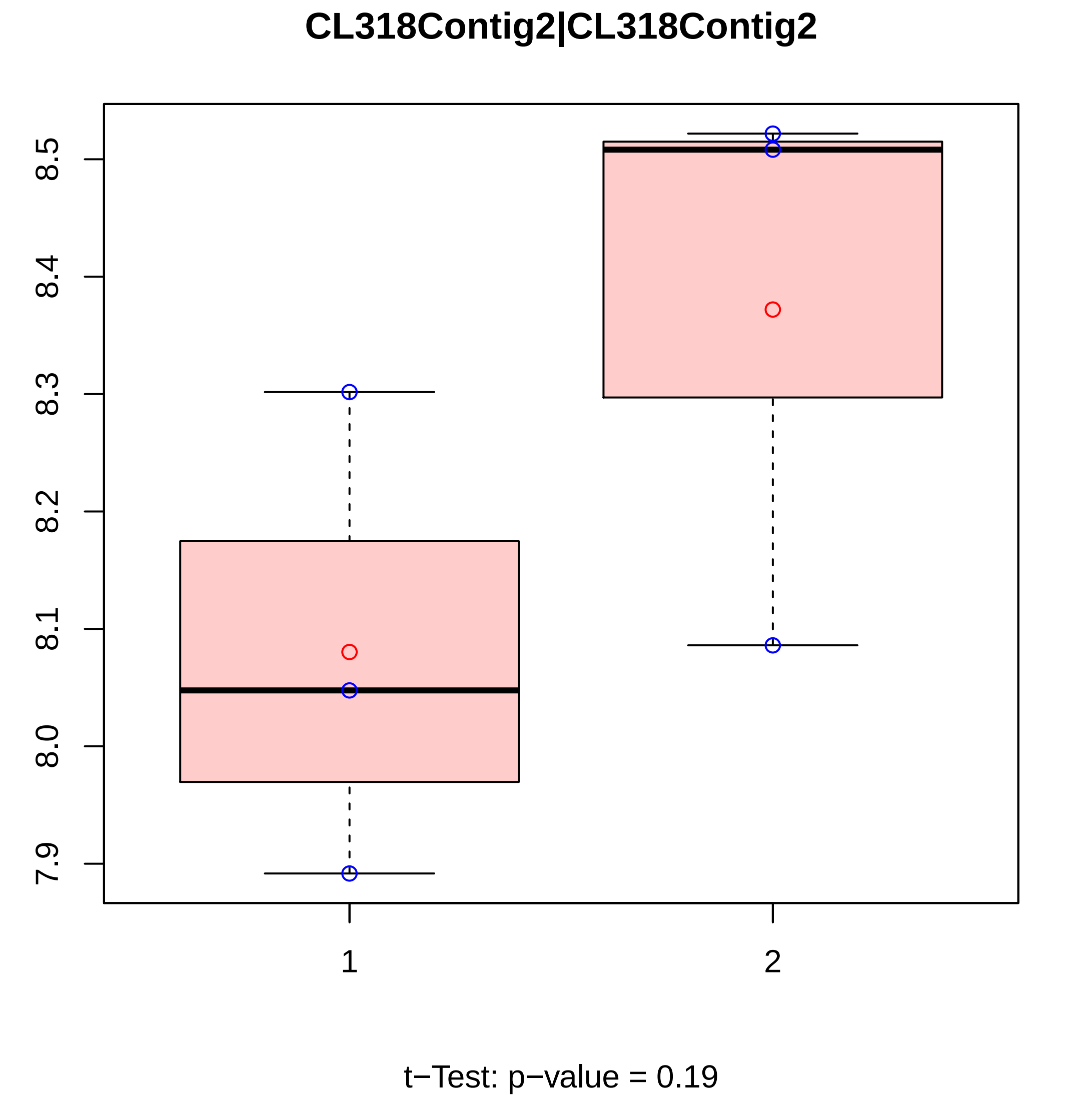
CL318Contig2|CL318Contig2
8.5
8.4
8.3
8.2
8.1
8.0
7.9
1
2
t−Test: p−value = 0.19

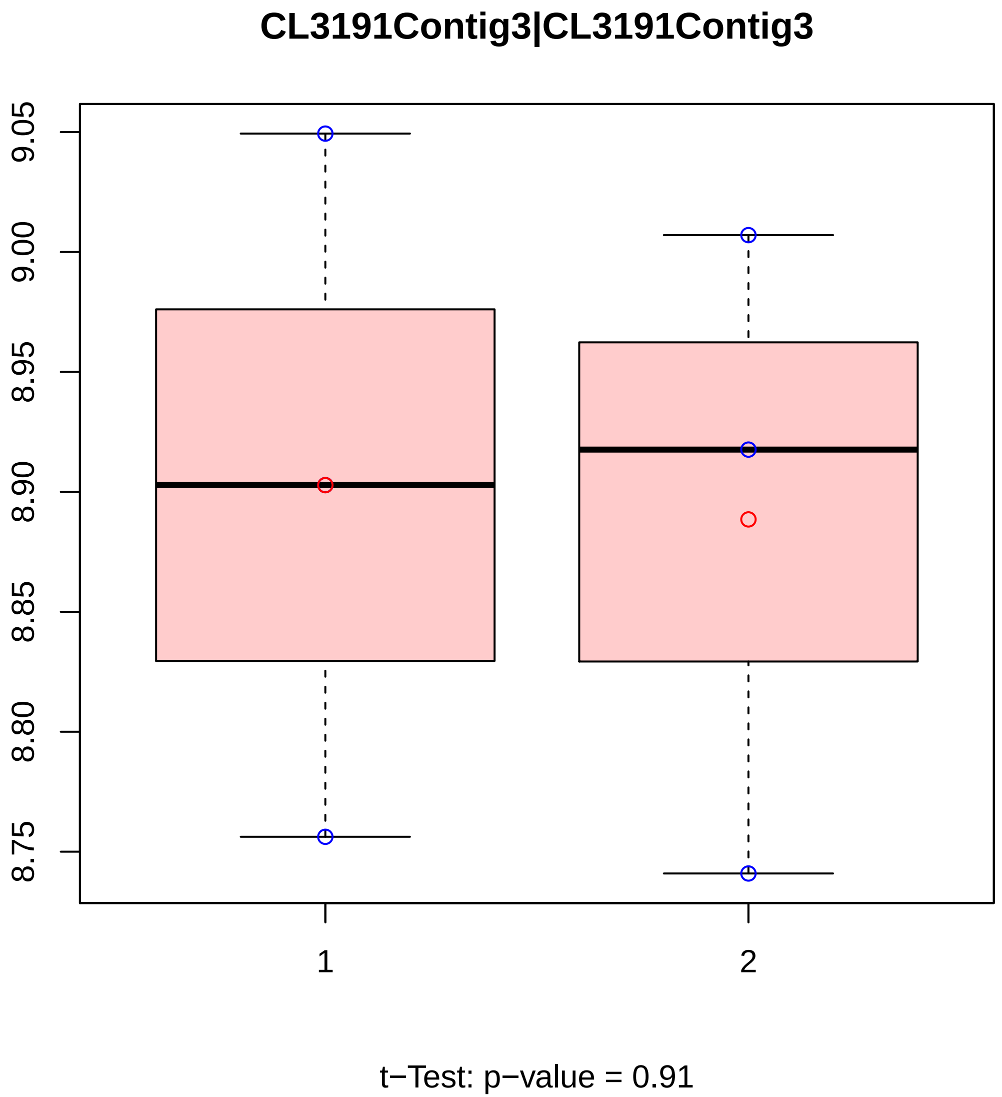

CL3191Contig3|CL3191Contig3
9.05
9.00
8.95
8.90
8.85
8.80
8.75
1
2
t−Test: p−value = 0.91

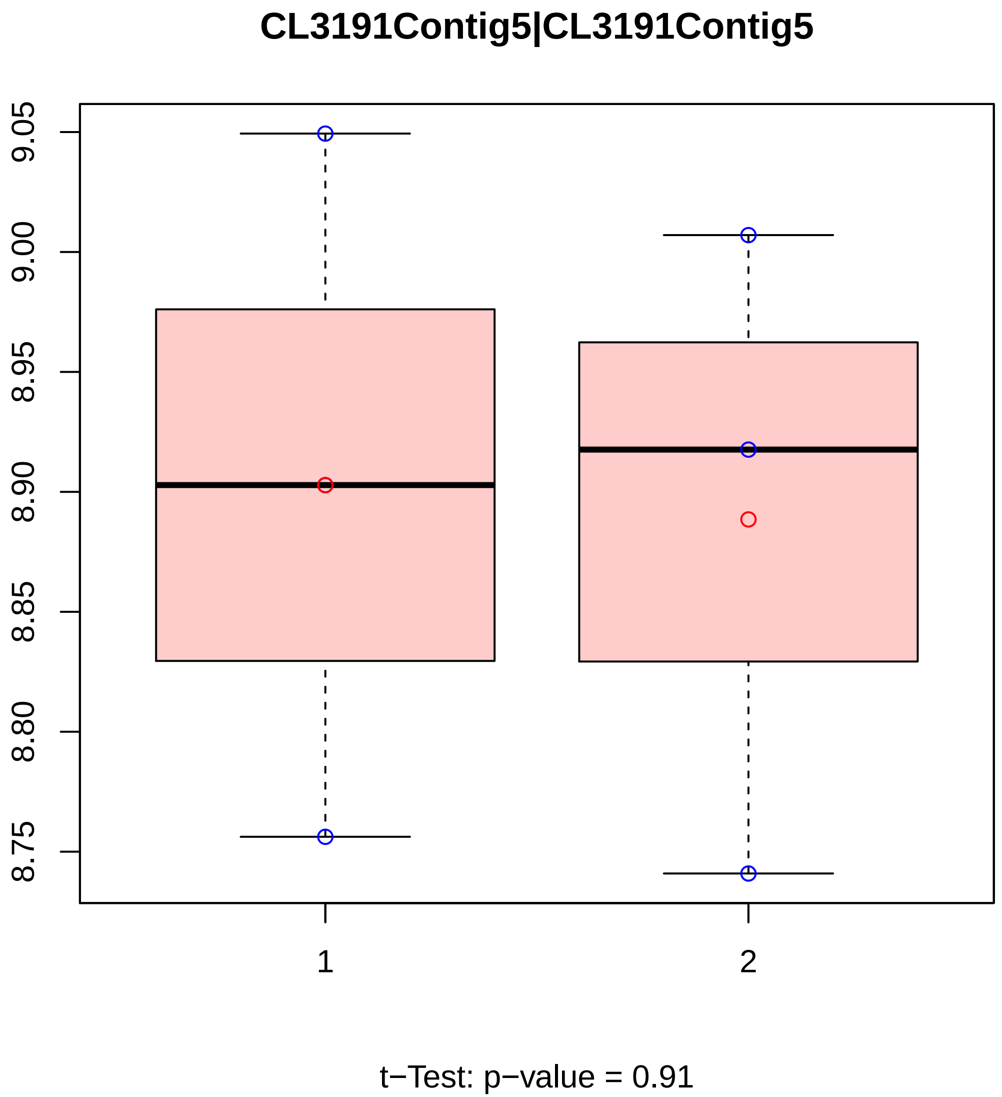

CL3191Contig5|CL3191Contig5
9.05
9.00
8.95
8.90
8.85
8.80
8.75
1
2
t−Test: p−value = 0.91

# CL3195Contig5|CL3195Contig5

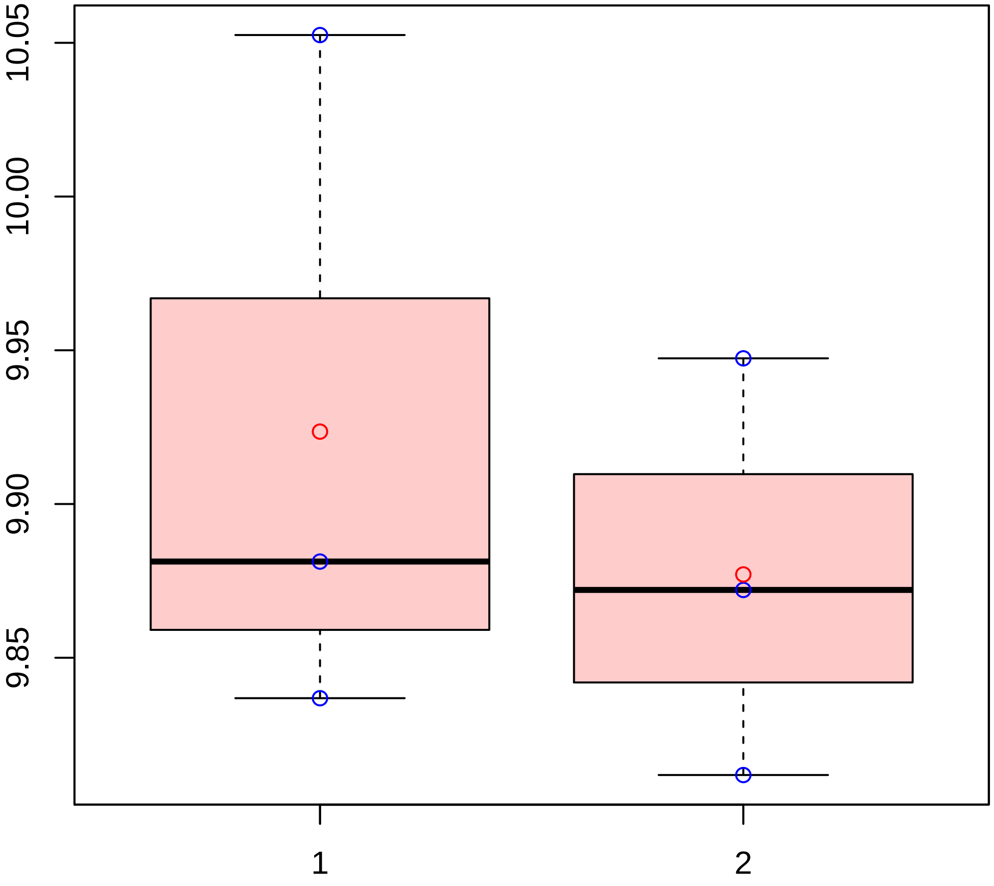


t−Test: p−value = 0.58

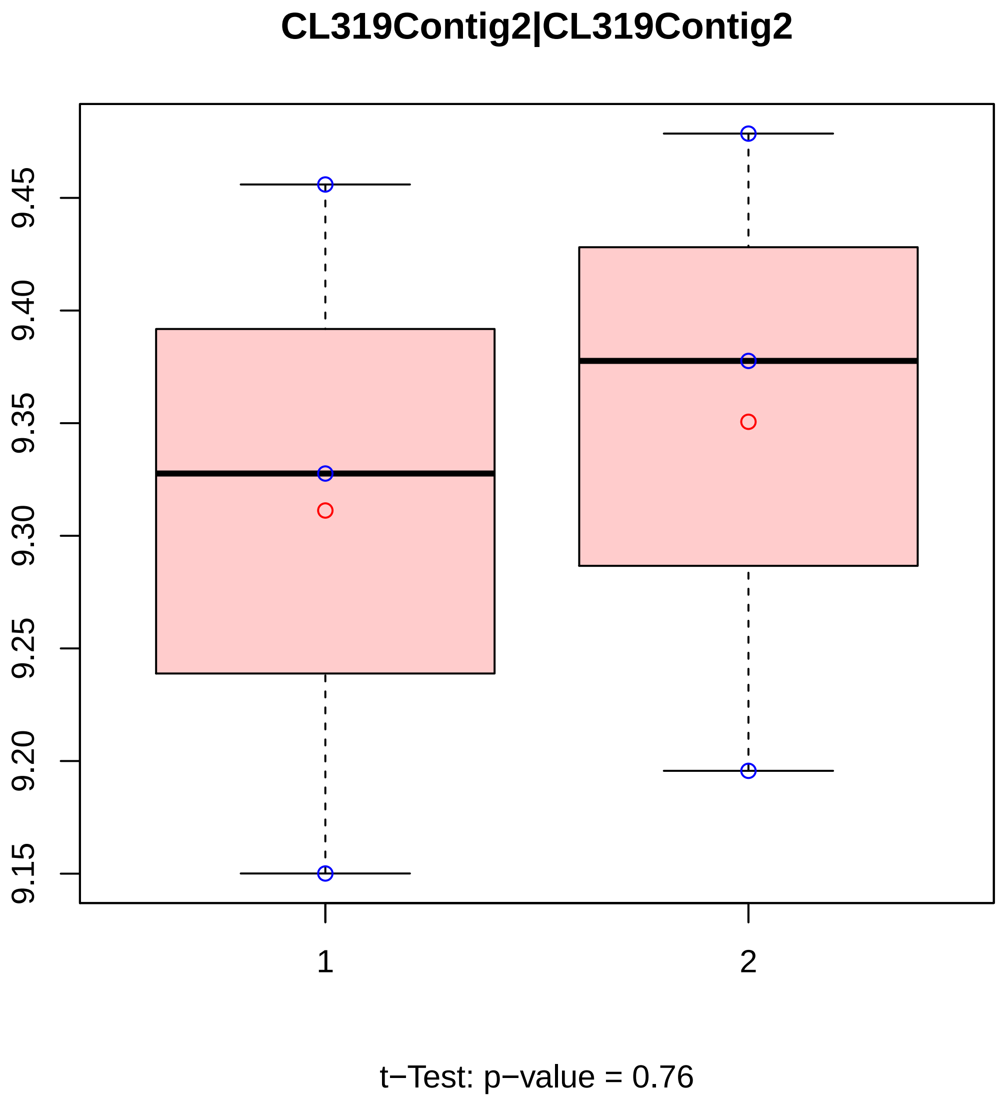
CL319Contig2|CL319Contig2
9.15
9.20
9.25
9.30
9.35
9.40
9.45
1
2
t−Test: p−value = 0.76

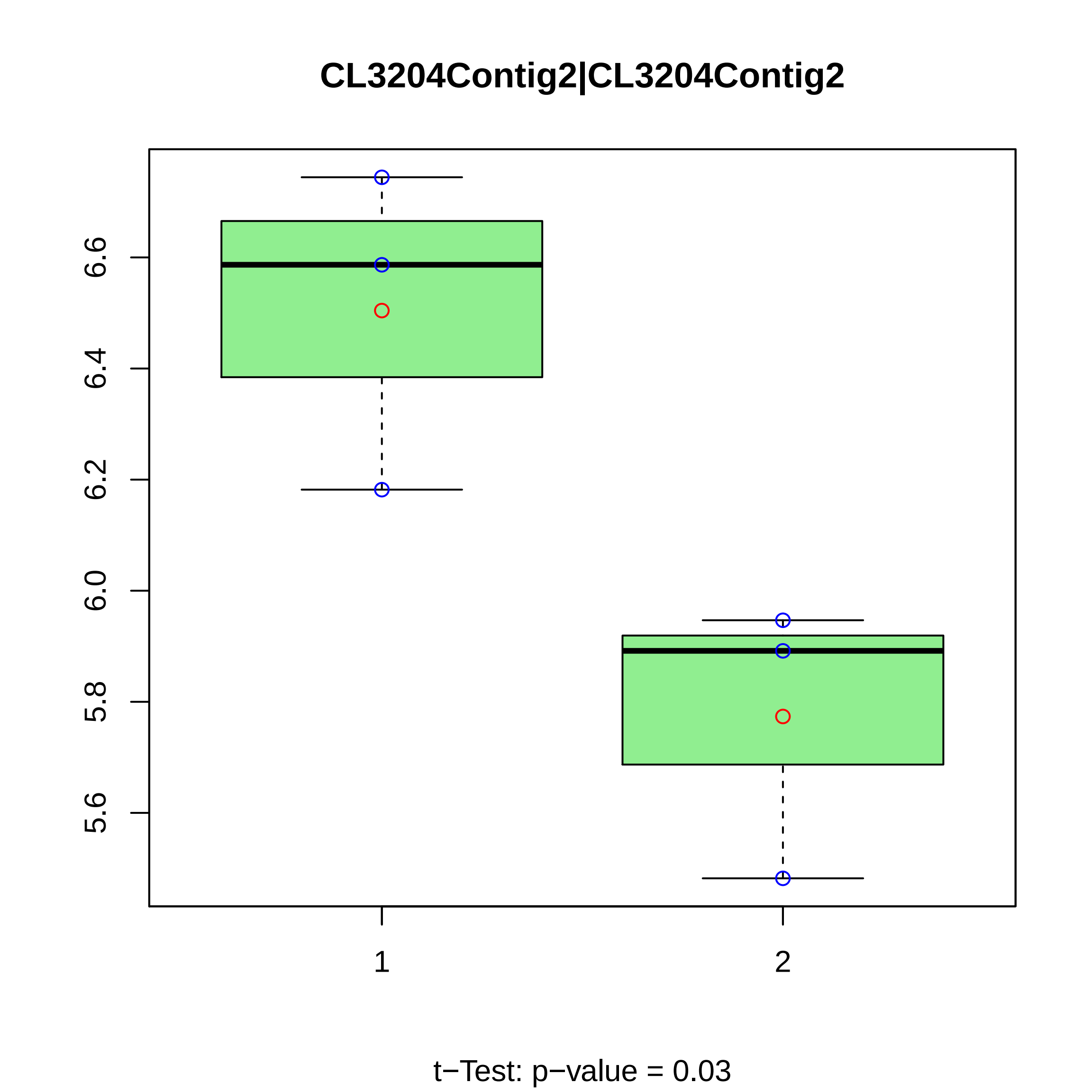
CL3204Contig2|CL3204Contig2
6.6
6.4
6.2
6.0
5.8
5.6
1
2
t−Test: p−value = 0.03

## CL3208Contig3|CL3208Contig3

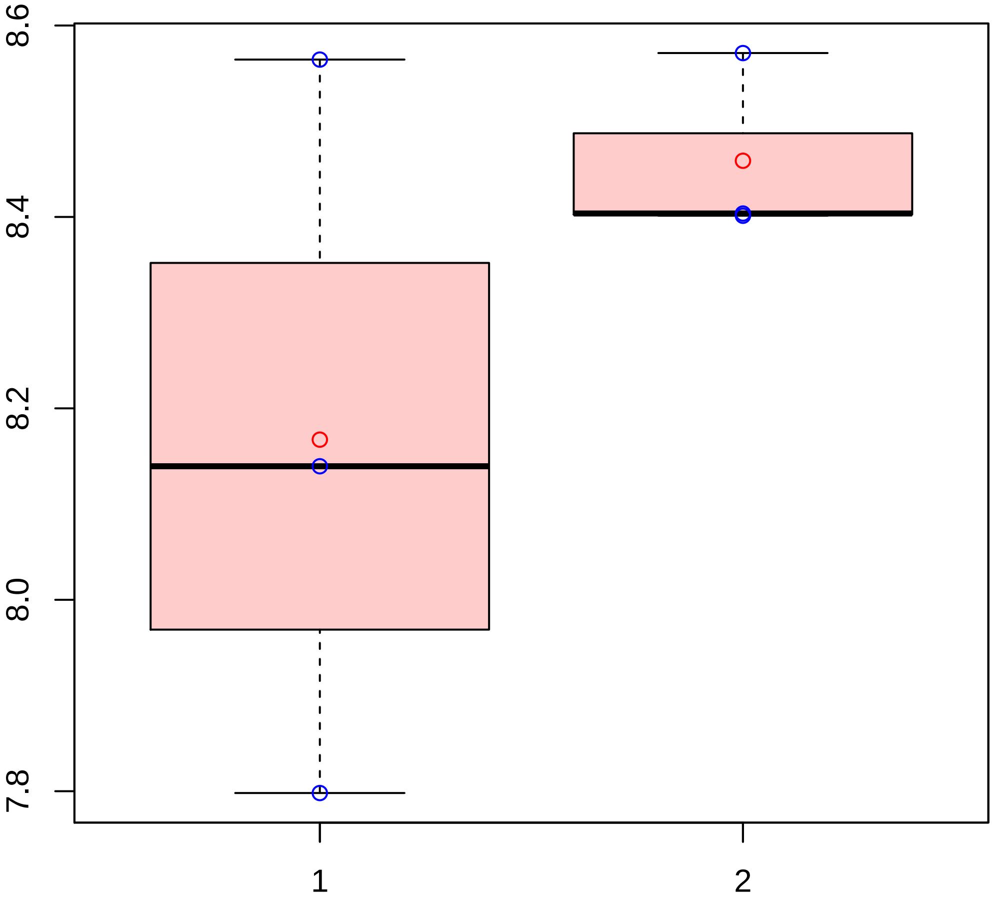


t−Test: p−value = 0.32

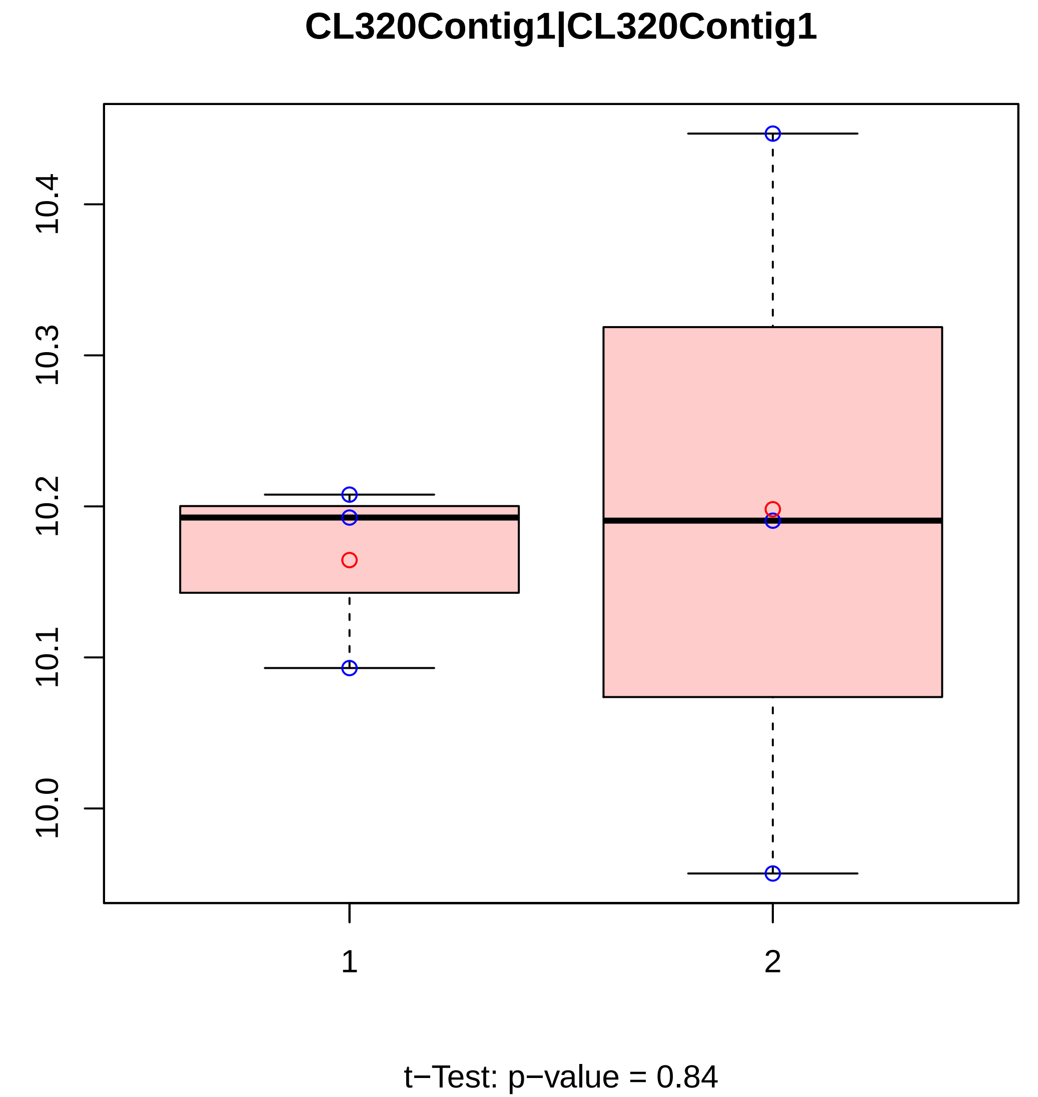
CL320Contig1|CL320Contig1
10.0
10.1
10.2
10.3
10.4
1
2
t−Test: p−value = 0.84

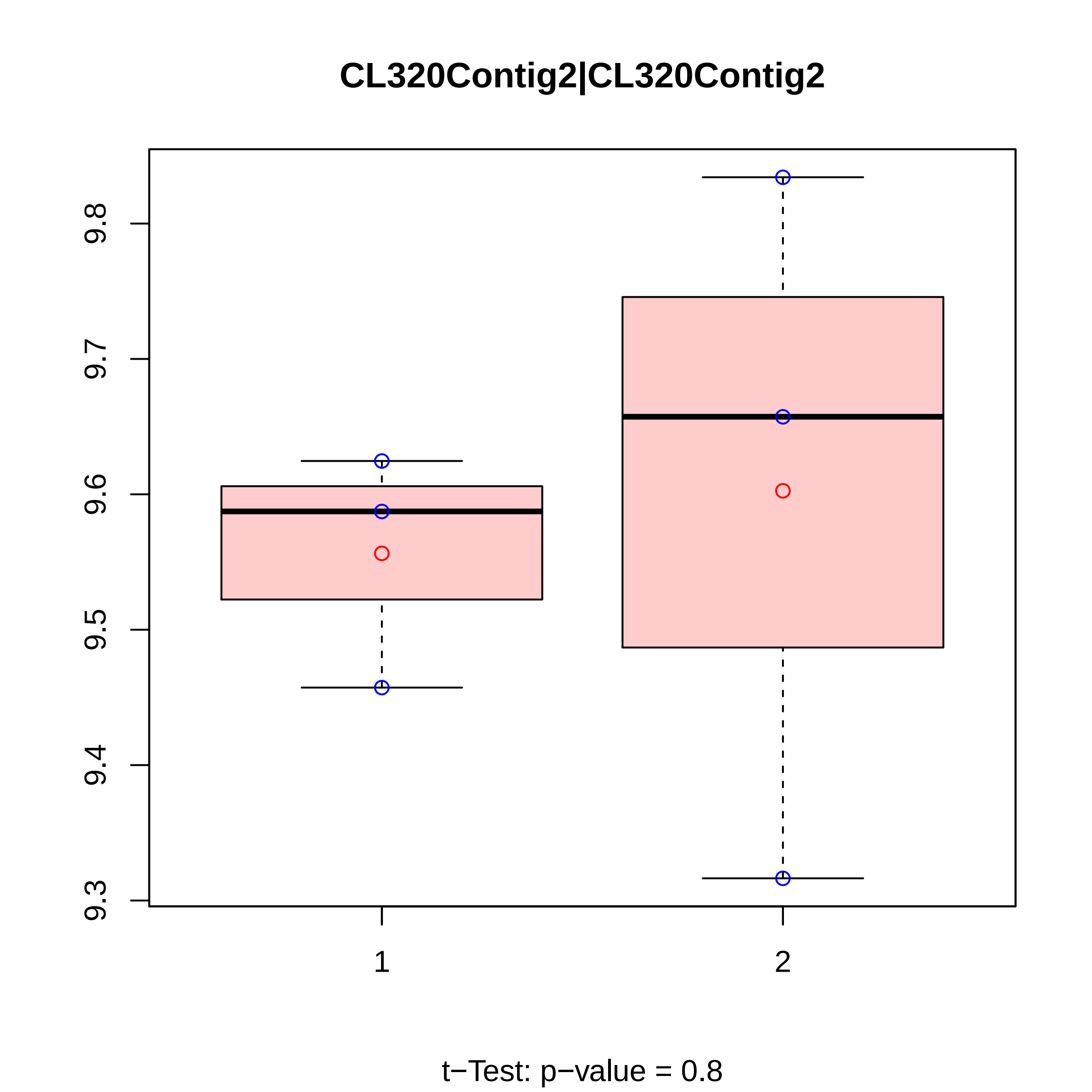

CL320Contig2|CL320Contig2
9.3
9.4
9.5
9.6
9.7
9.8
1
2
t−Test: p−value = 0.8

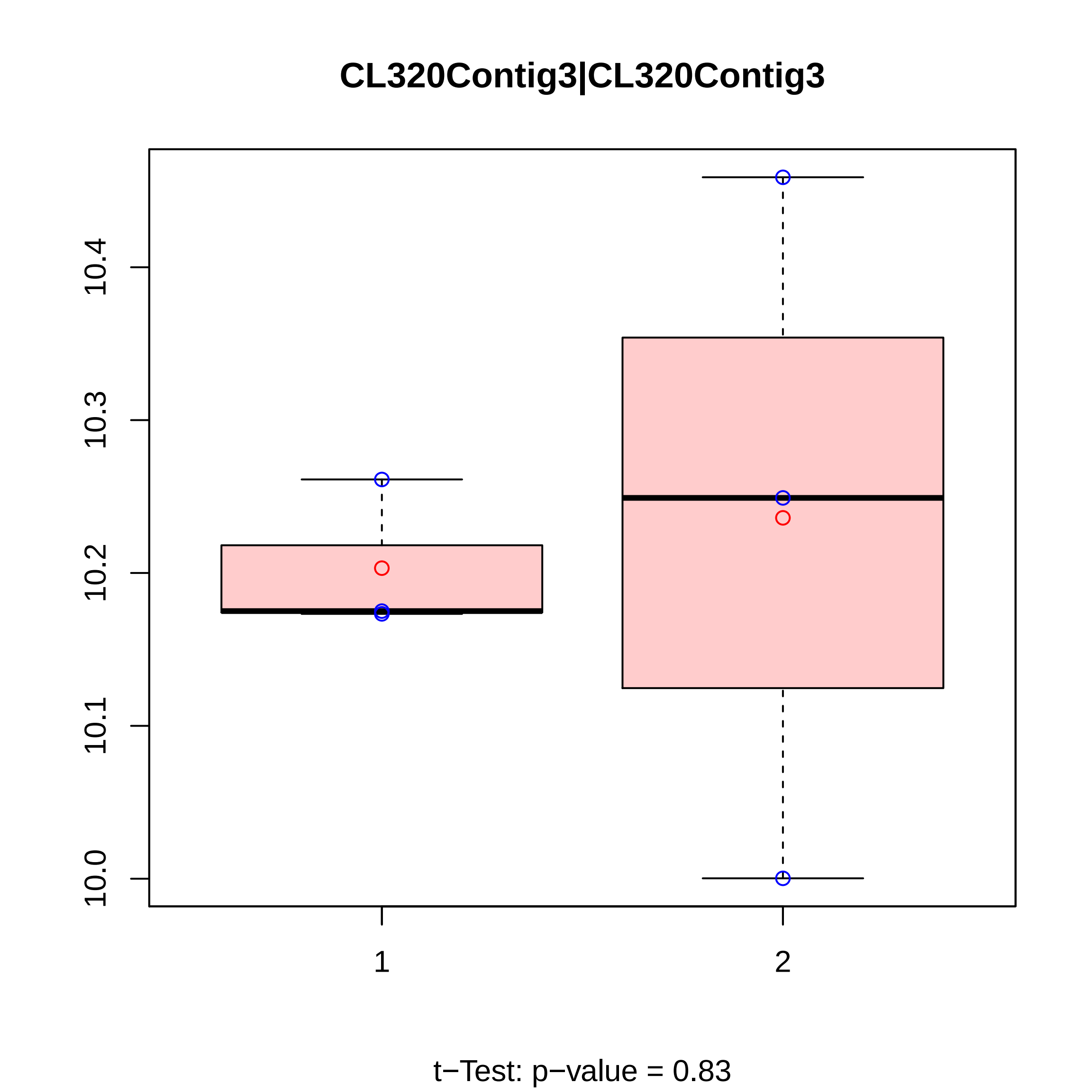
CL320Contig3|CL320Contig3
10.0
10.1
10.2
10.3
10.4
1
2
t−Test: p−value = 0.83

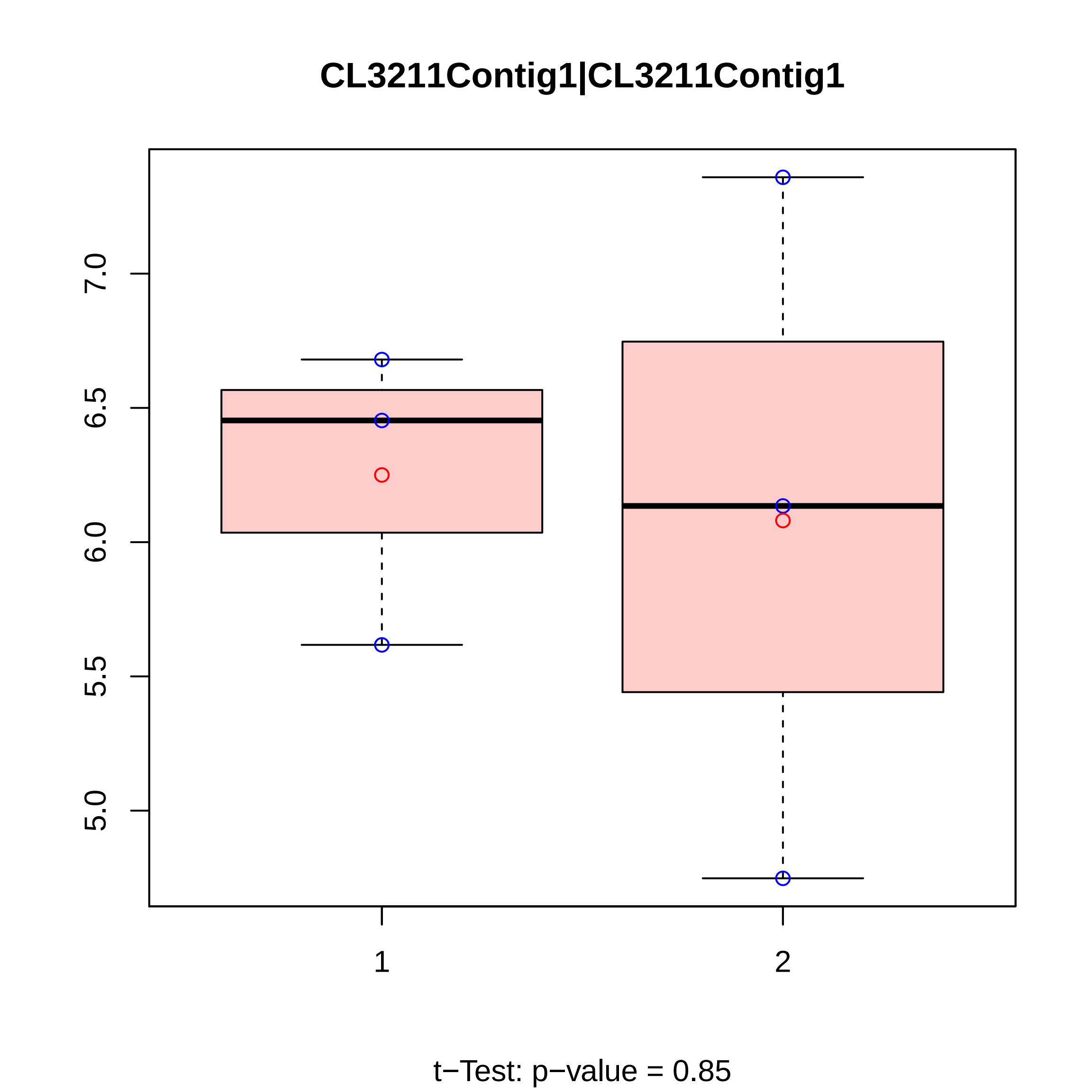

CL3211Contig1|CL3211Contig1
7.0
6.5
6.0
5.5
5.0
1
2
t−Test: p−value = 0.85

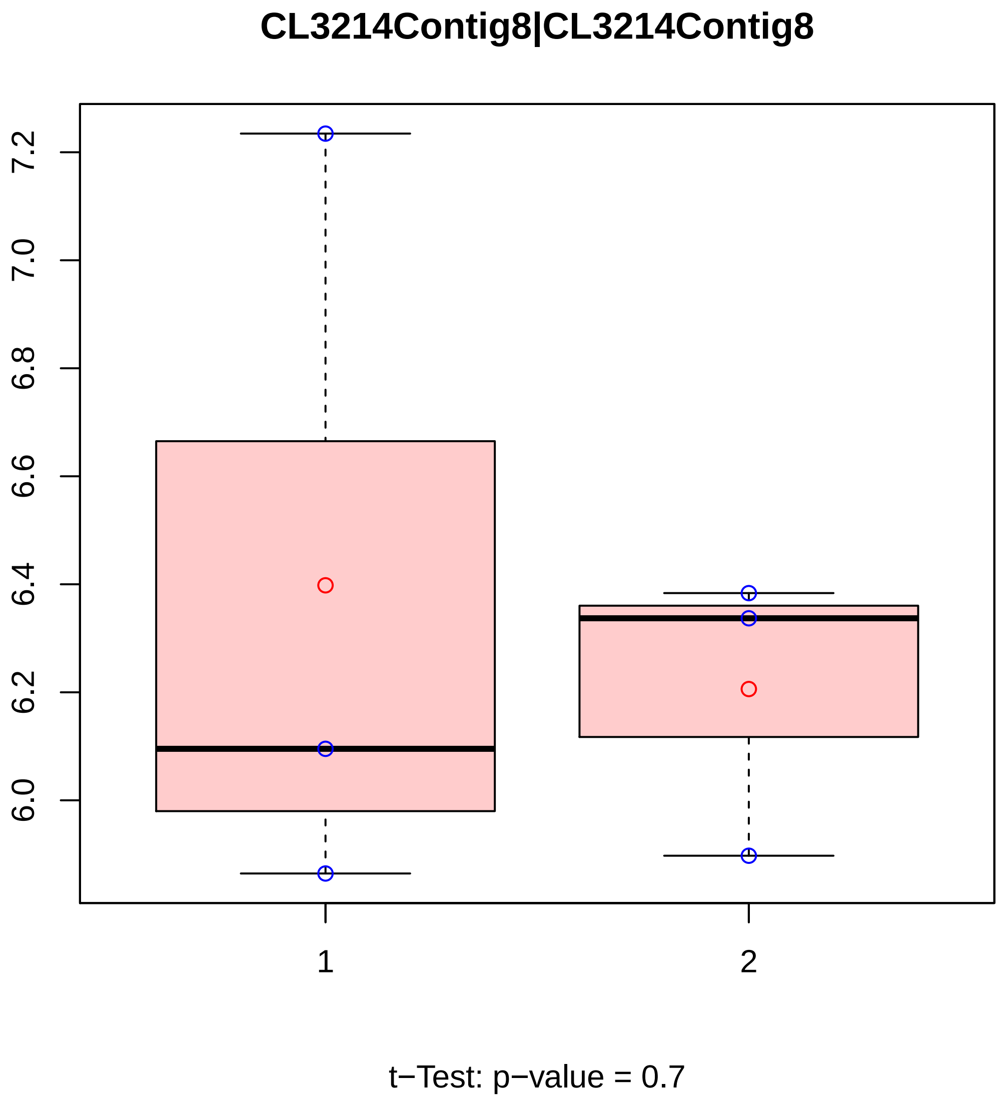

CL3214Contig8|CL3214Contig8
7.2
7.0
6.8
6.6
6.4
6.2
6.0
1
2
t−Test: p−value = 0.7

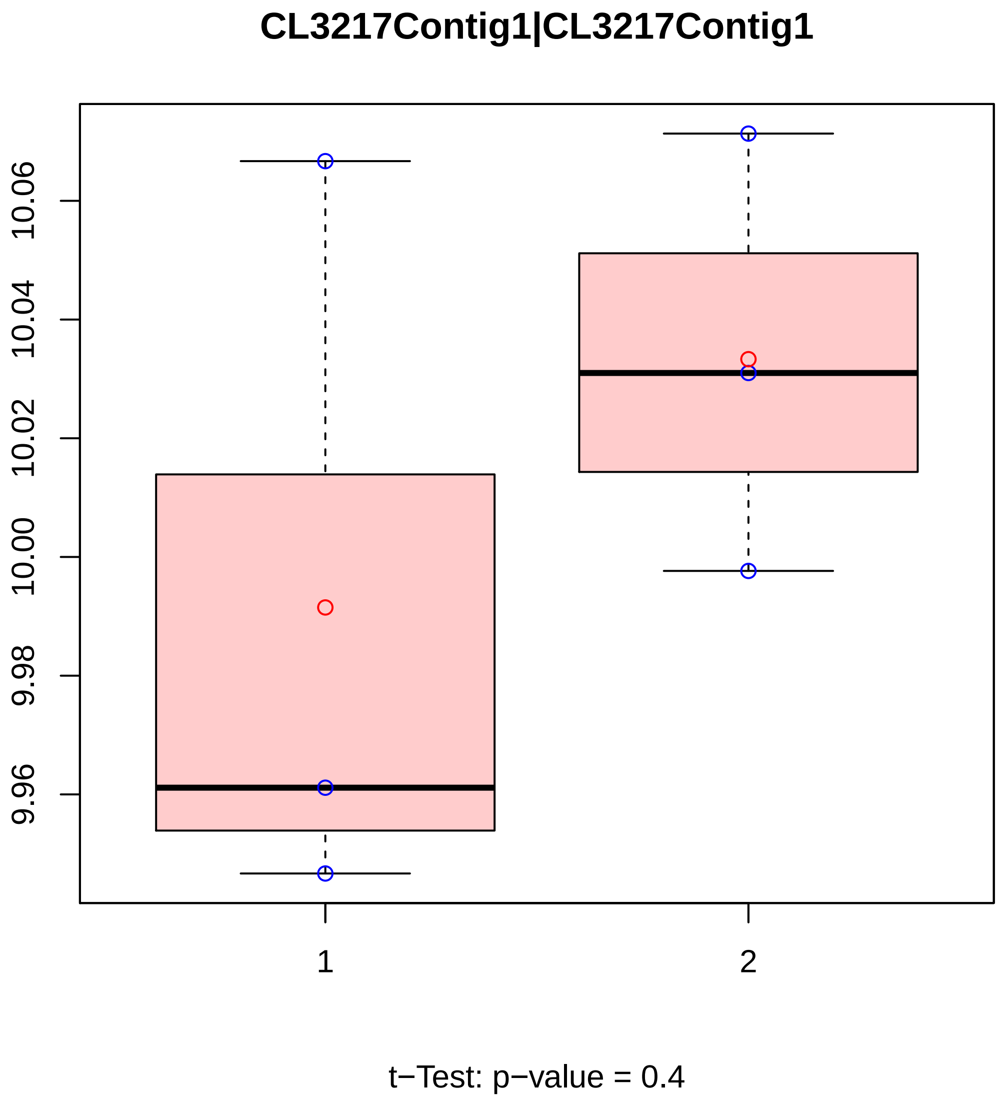

CL3217Contig1|CL3217Contig1
10.06
10.04
10.02
10.00
9.98
9.96
1
2
t−Test: p−value = 0.4

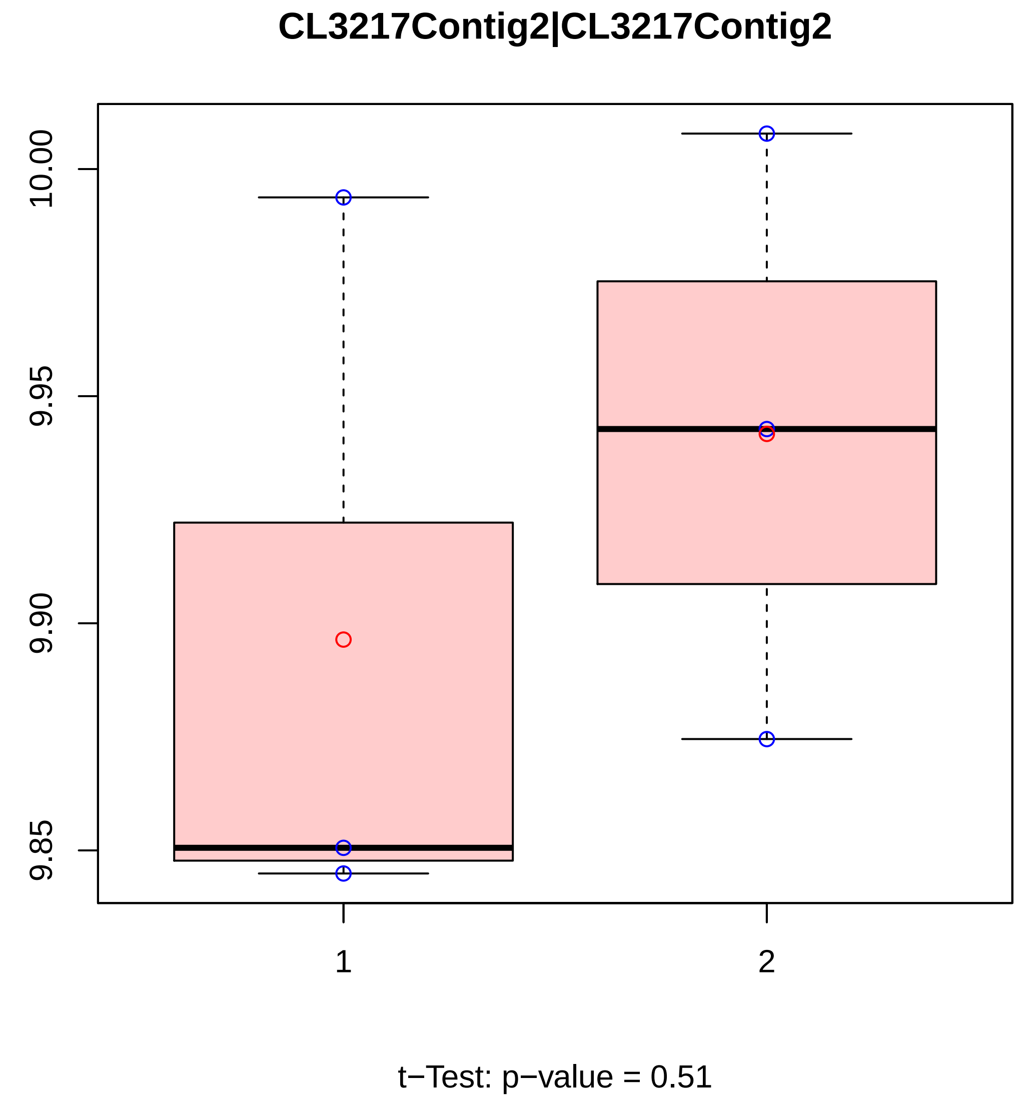
CL3217Contig2|CL3217Contig2
10.00
9.95
9.90
9.85
1
2
t−Test: p−value = 0.51

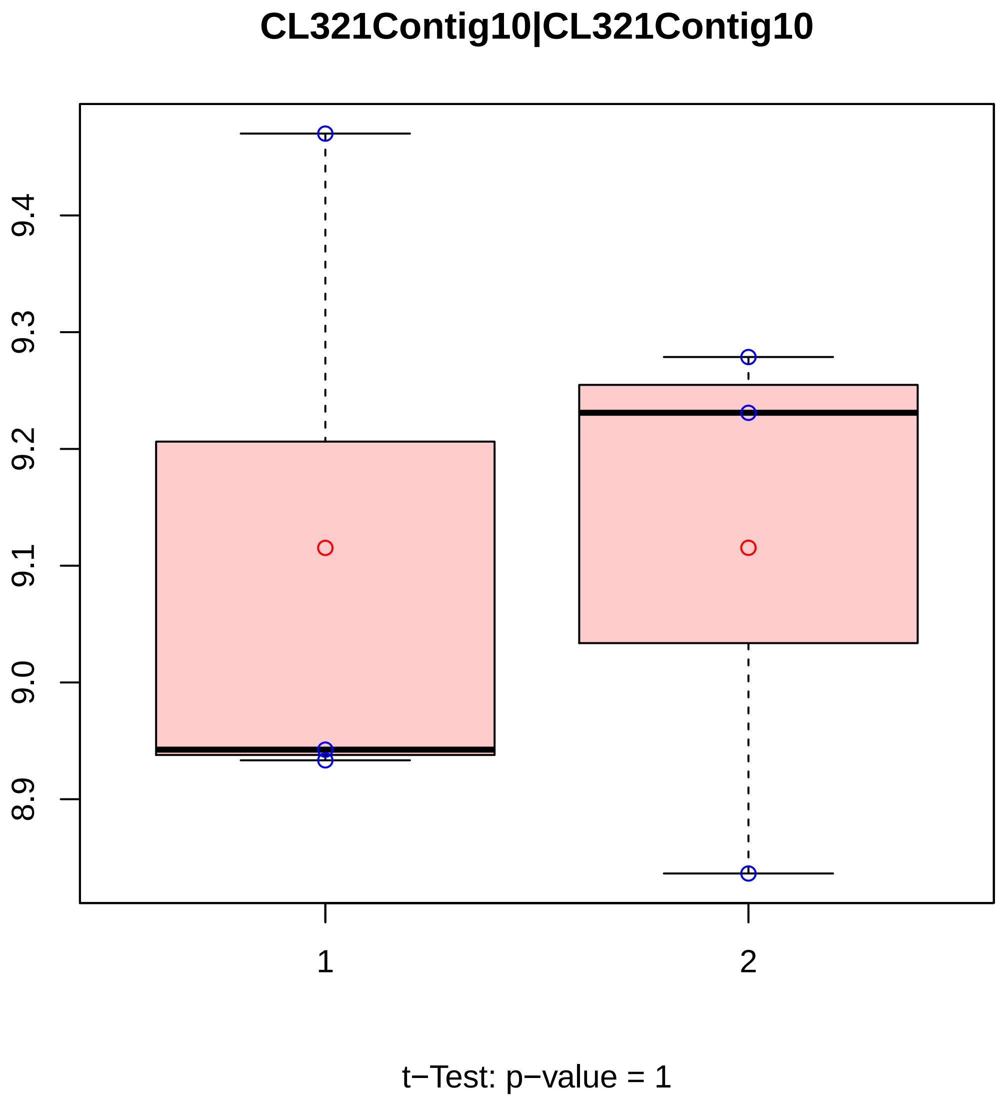

CL321Contig10|CL321Contig10
9.4
9.3
9.2
9.1
9.0
8.9
1
2
t−Test: p−value = 1

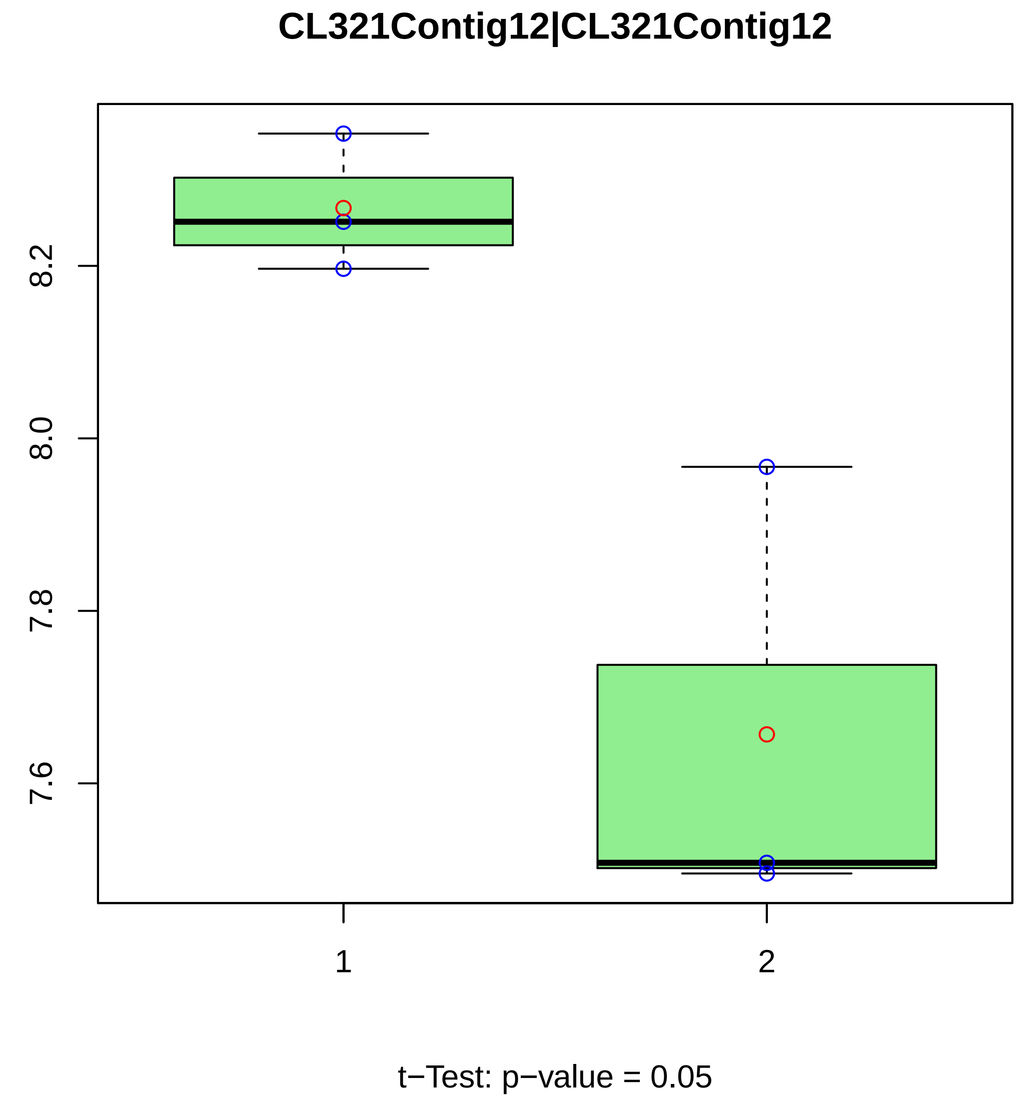
CL321Contig12|CL321Contig12
8.2
8.0
7.8
7.6
1
2
t−Test: p−value = 0.05

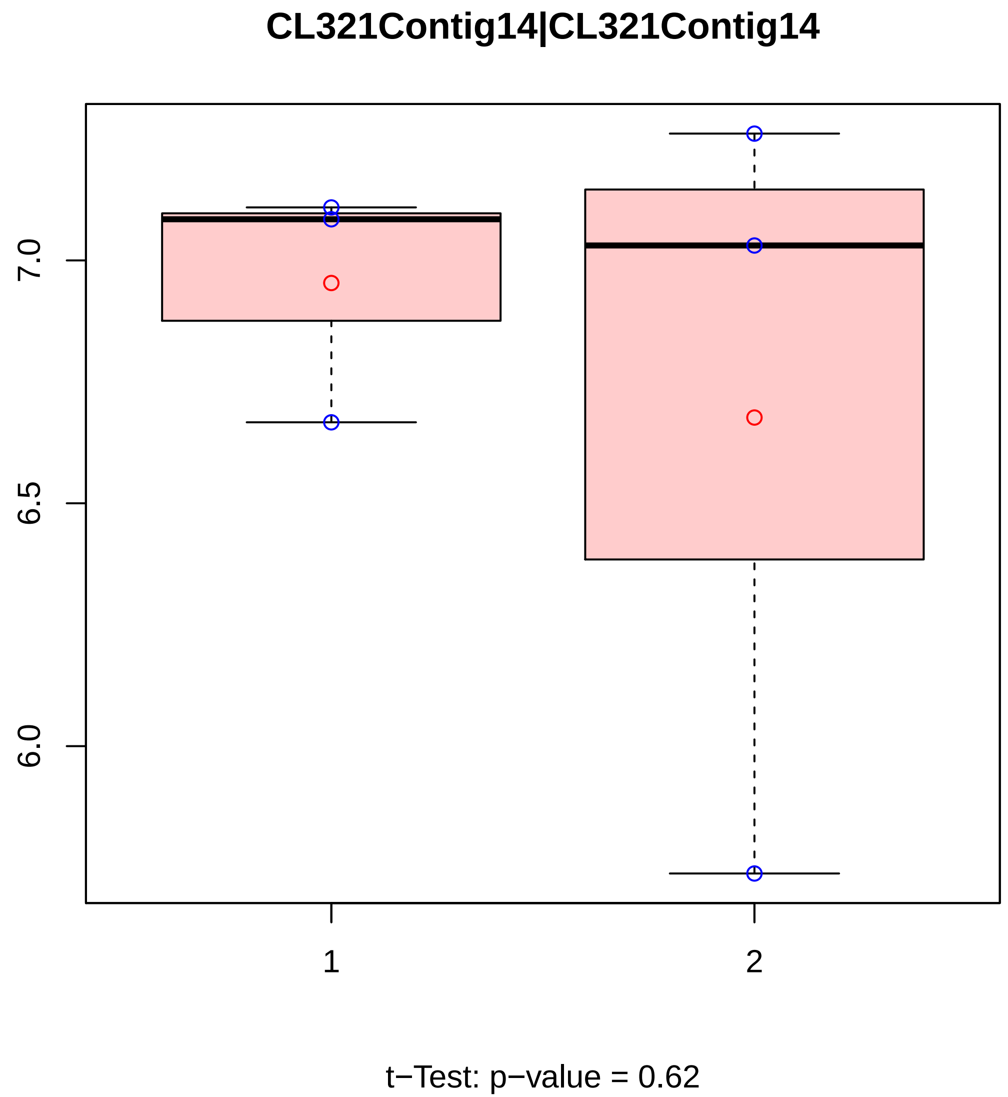
CL321Contig14|CL321Contig14
7.0
6.5
6.0
1
2
t−Test: p−value = 0.62

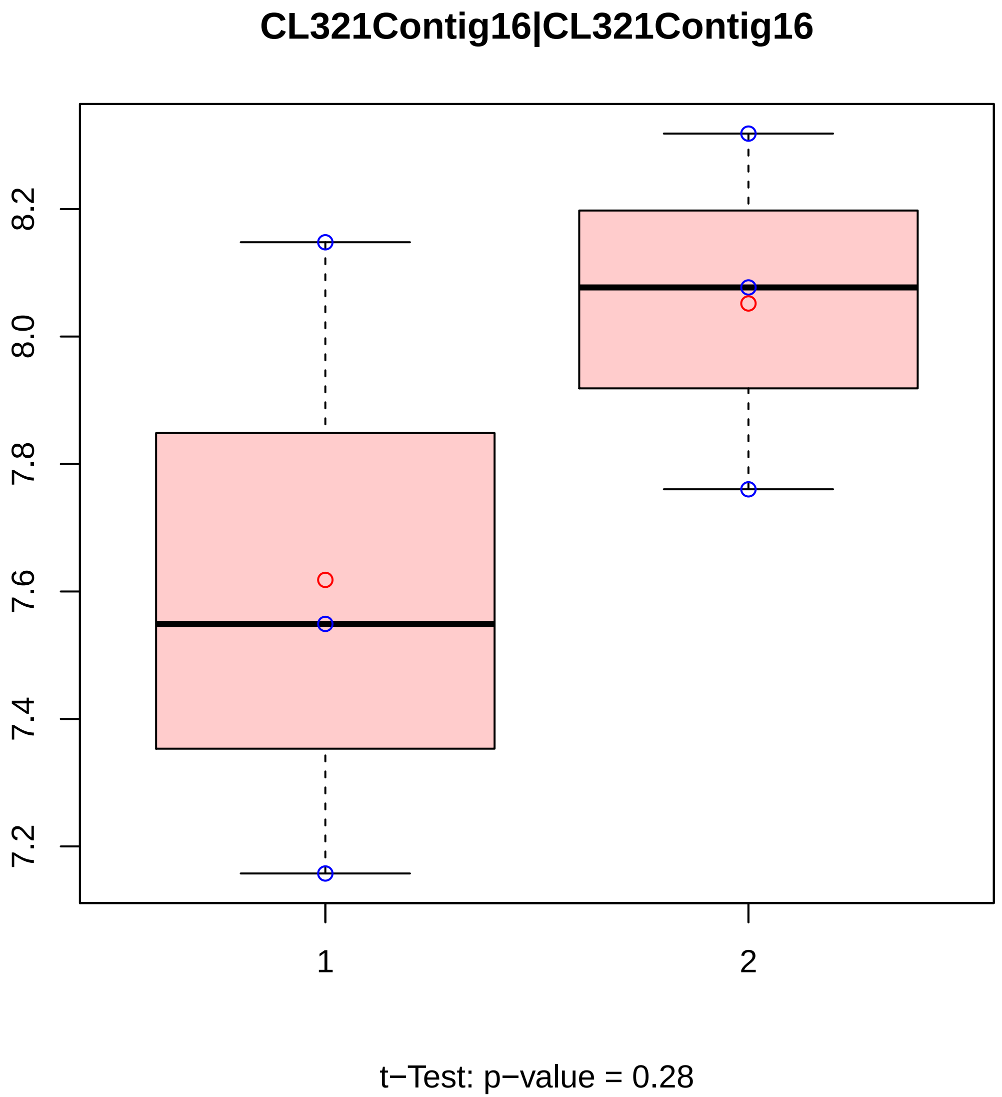
CL321Contig16|CL321Contig16
8.2
8.0
7.8
7.6
7.4
7.2
1
2
t−Test: p−value = 0.28

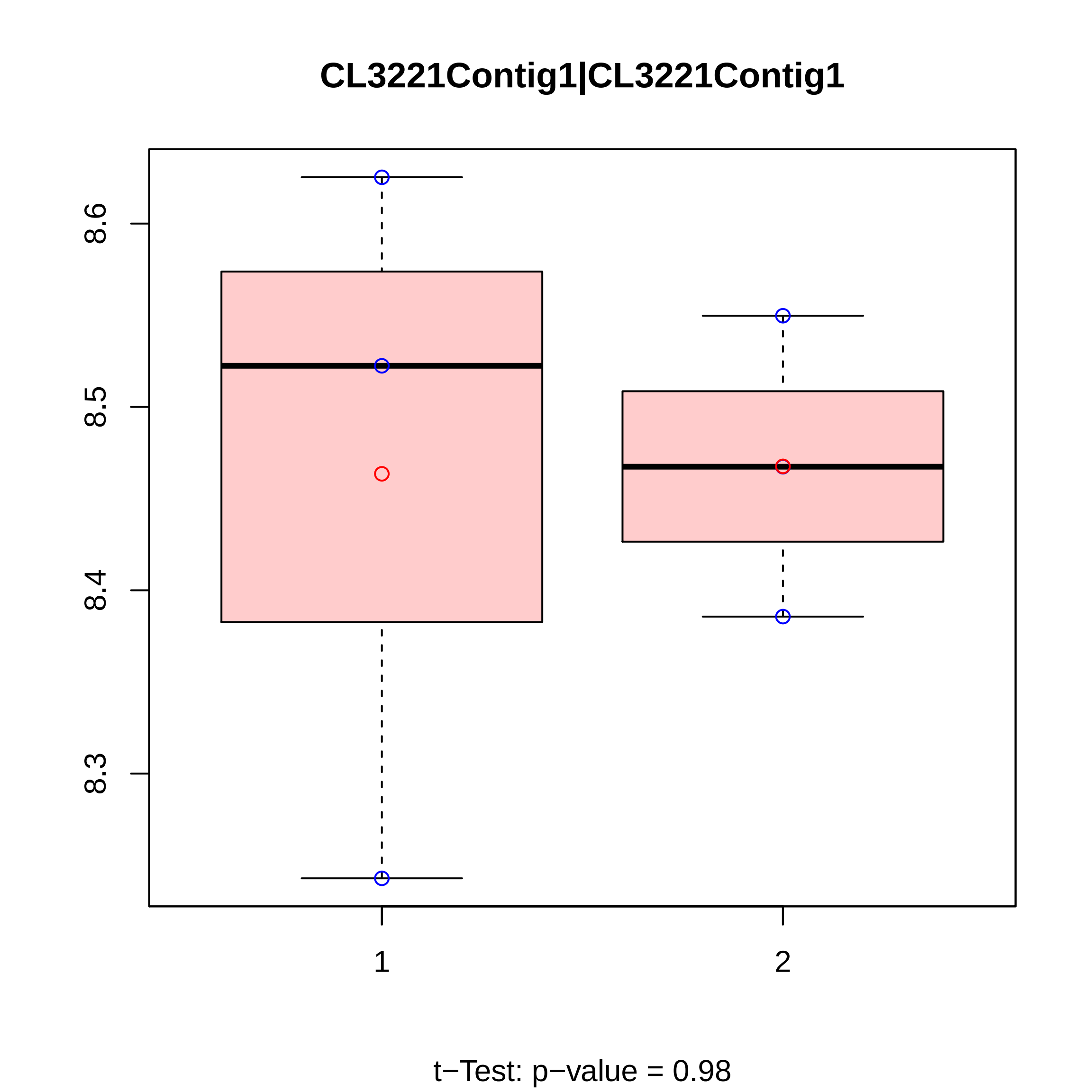

CL3221Contig1|CL3221Contig1
8.3
8.4
8.5
8.6
1
2
t−Test: p−value = 0.98

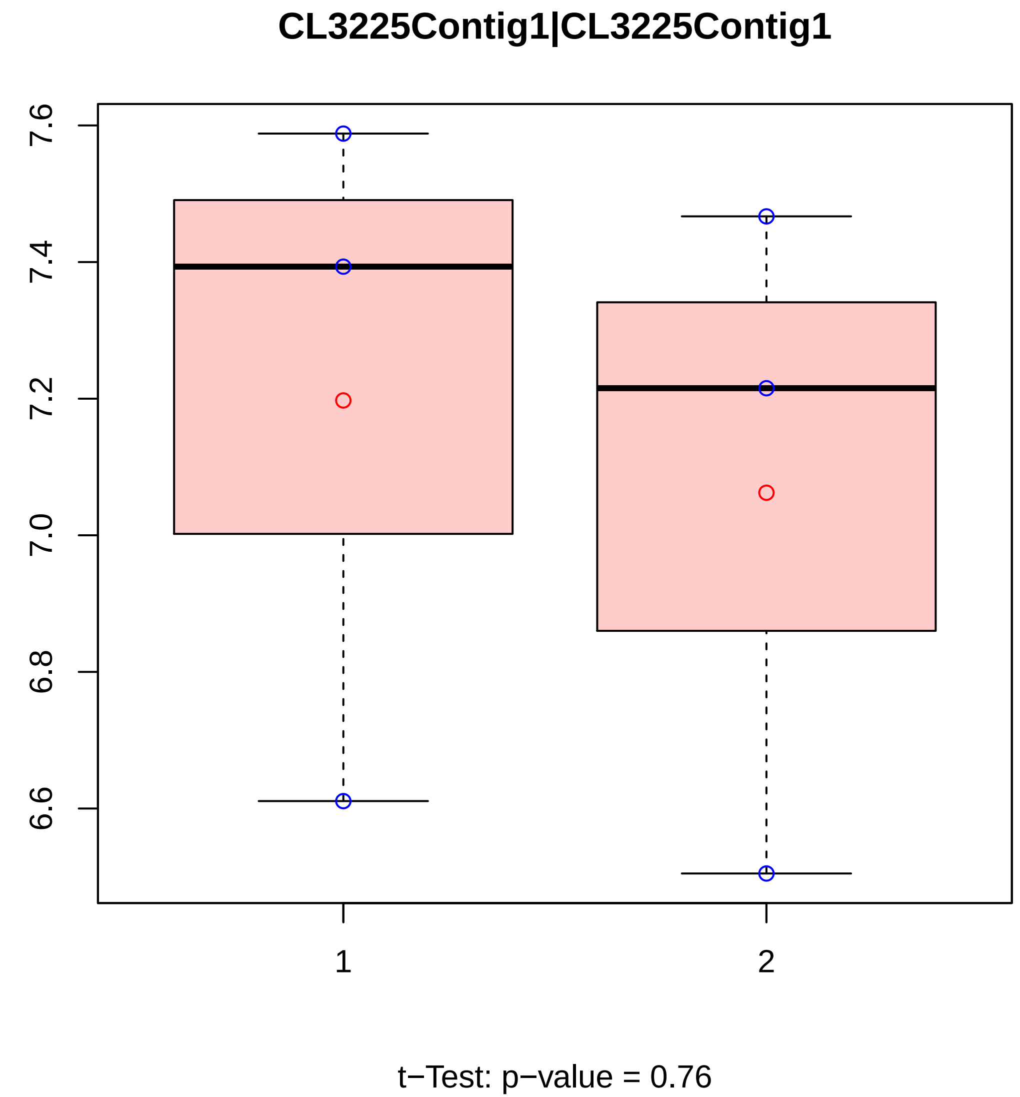
CL3225Contig1|CL3225Contig1
7.6
7.4
7.2
7.0
6.8
6.6
1
2
t−Test: p−value = 0.76

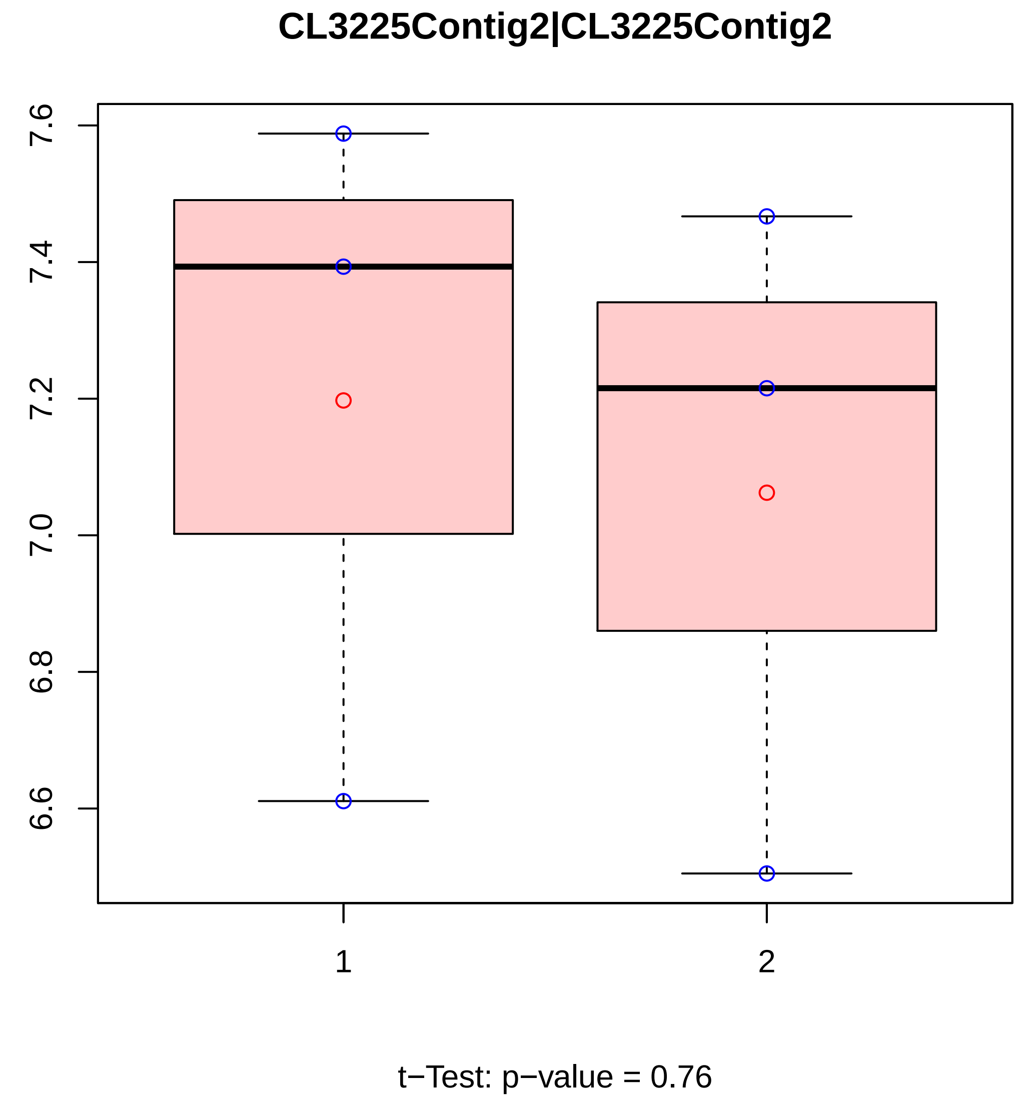
CL3225Contig2|CL3225Contig2
7.6
7.4
7.2
7.0
6.8
6.6
1
2
t−Test: p−value = 0.76

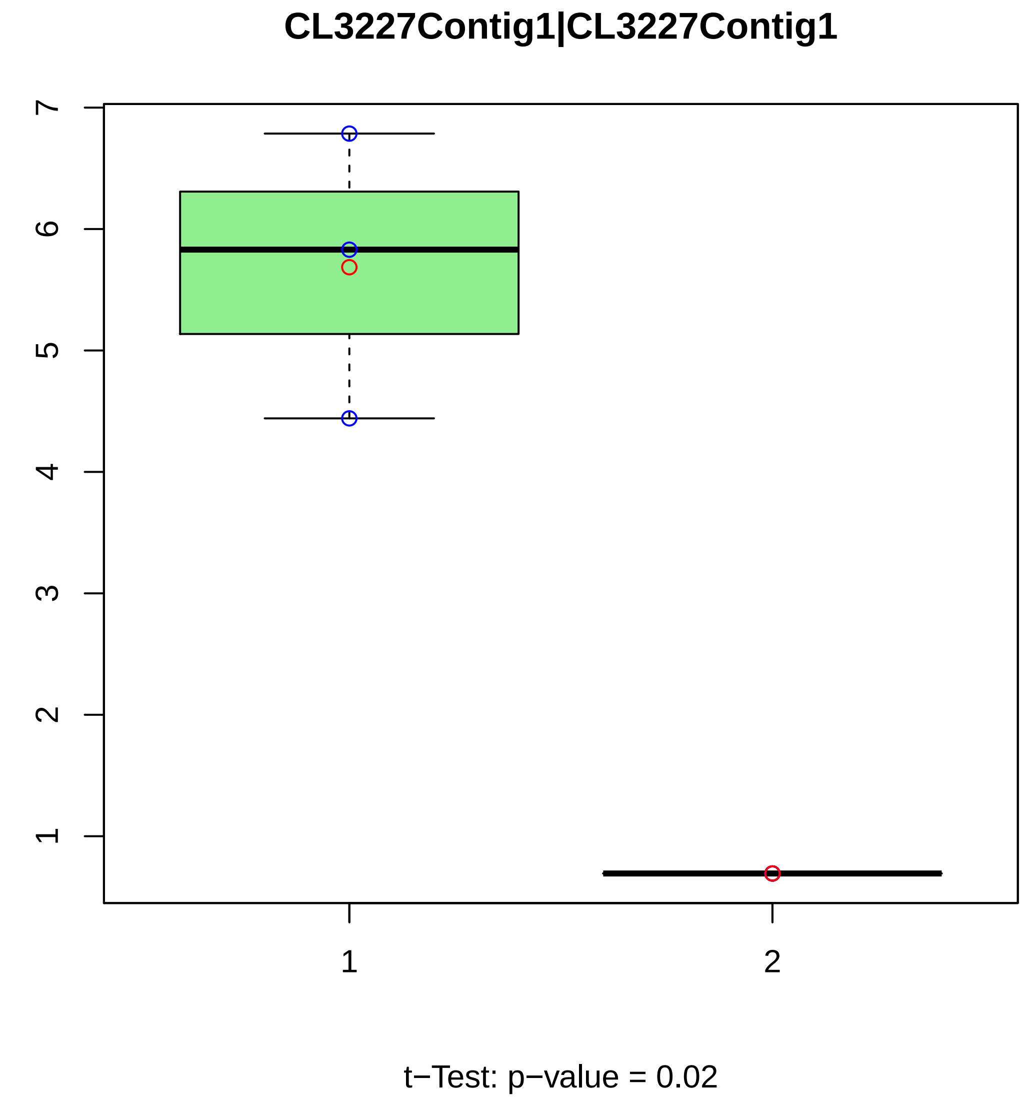
CL3227Contig1|CL3227Contig1
7
6
5
4
3
2
1
1
2
t−Test: p−value = 0.02

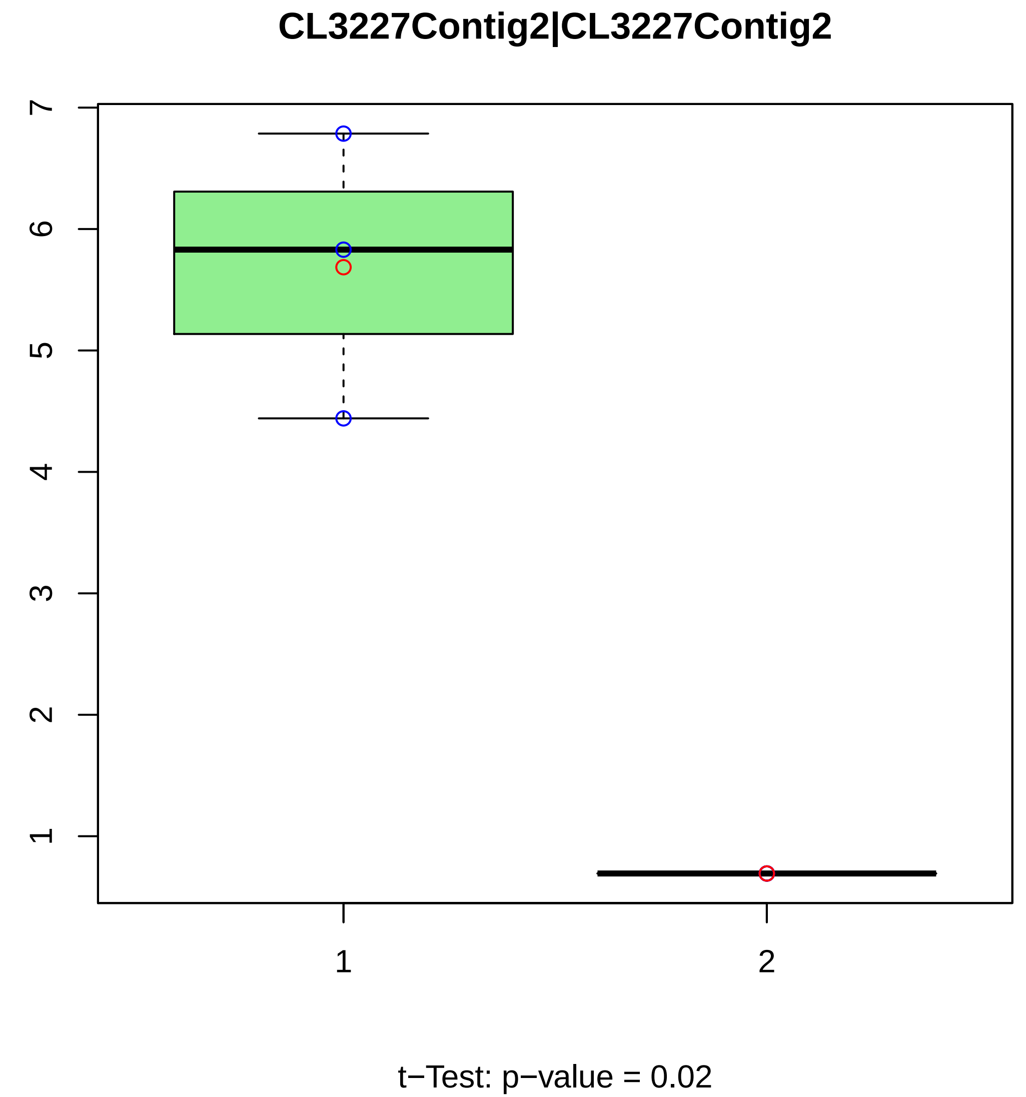
CL3227Contig2|CL3227Contig2
1
2
3
4
5
6
7
1
2
t−Test: p−value = 0.02

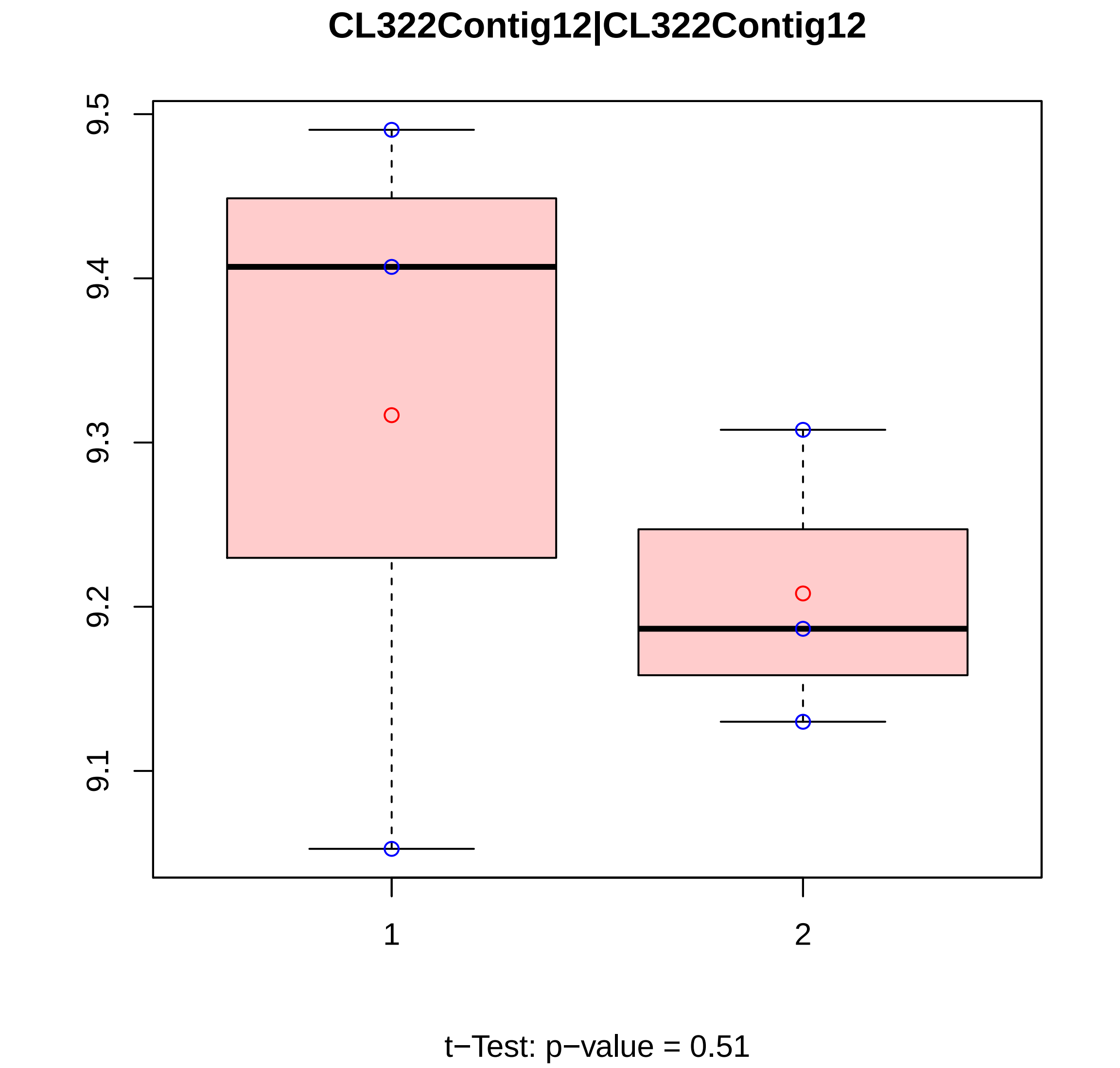
CL322Contig12|CL322Contig12
9.5
9.4
9.3
9.2
9.1
1
2
t−Test: p−value = 0.51

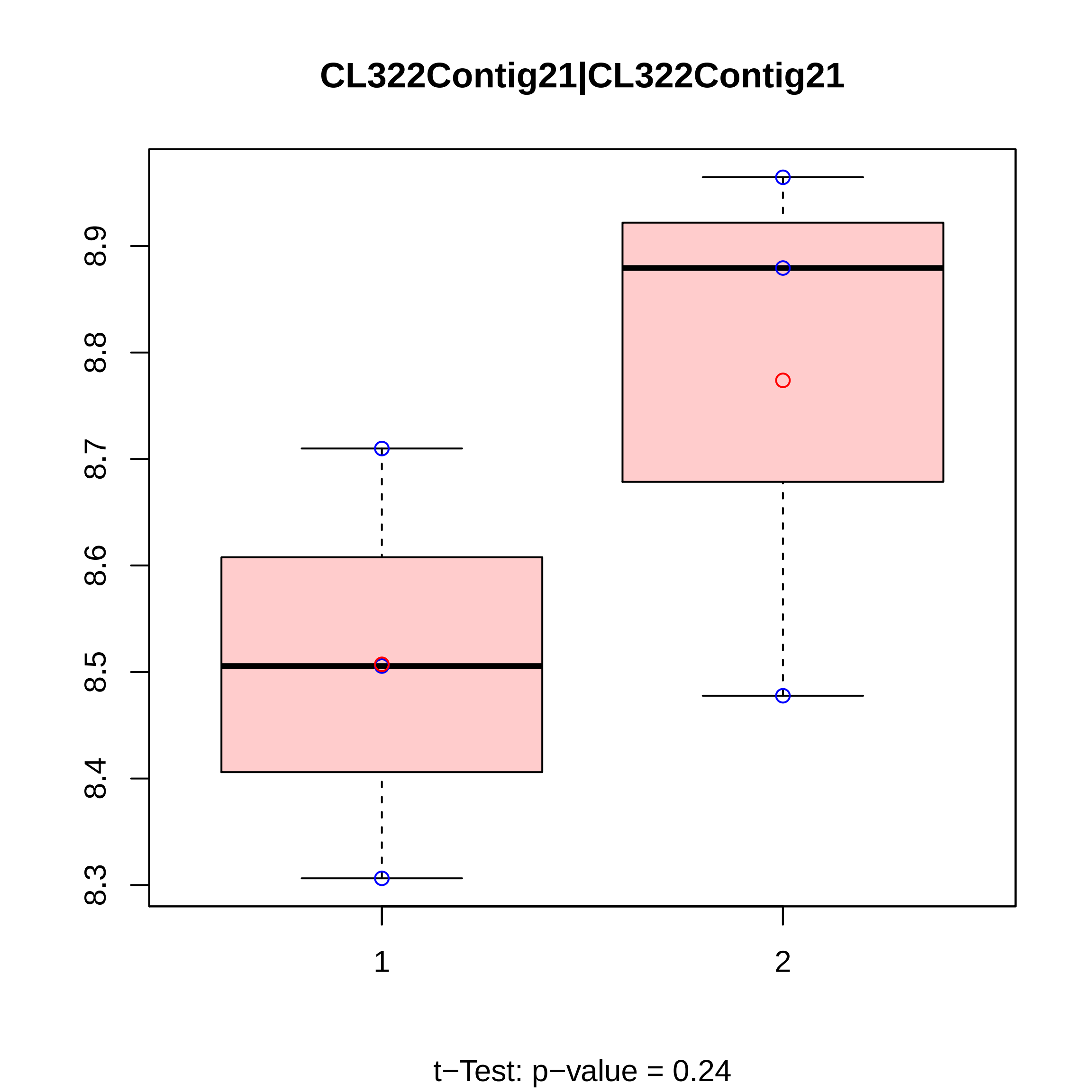

CL322Contig21|CL322Contig21
8.3
8.4
8.5
8.6
8.7
8.8
8.9
1
2
t−Test: p−value = 0.24

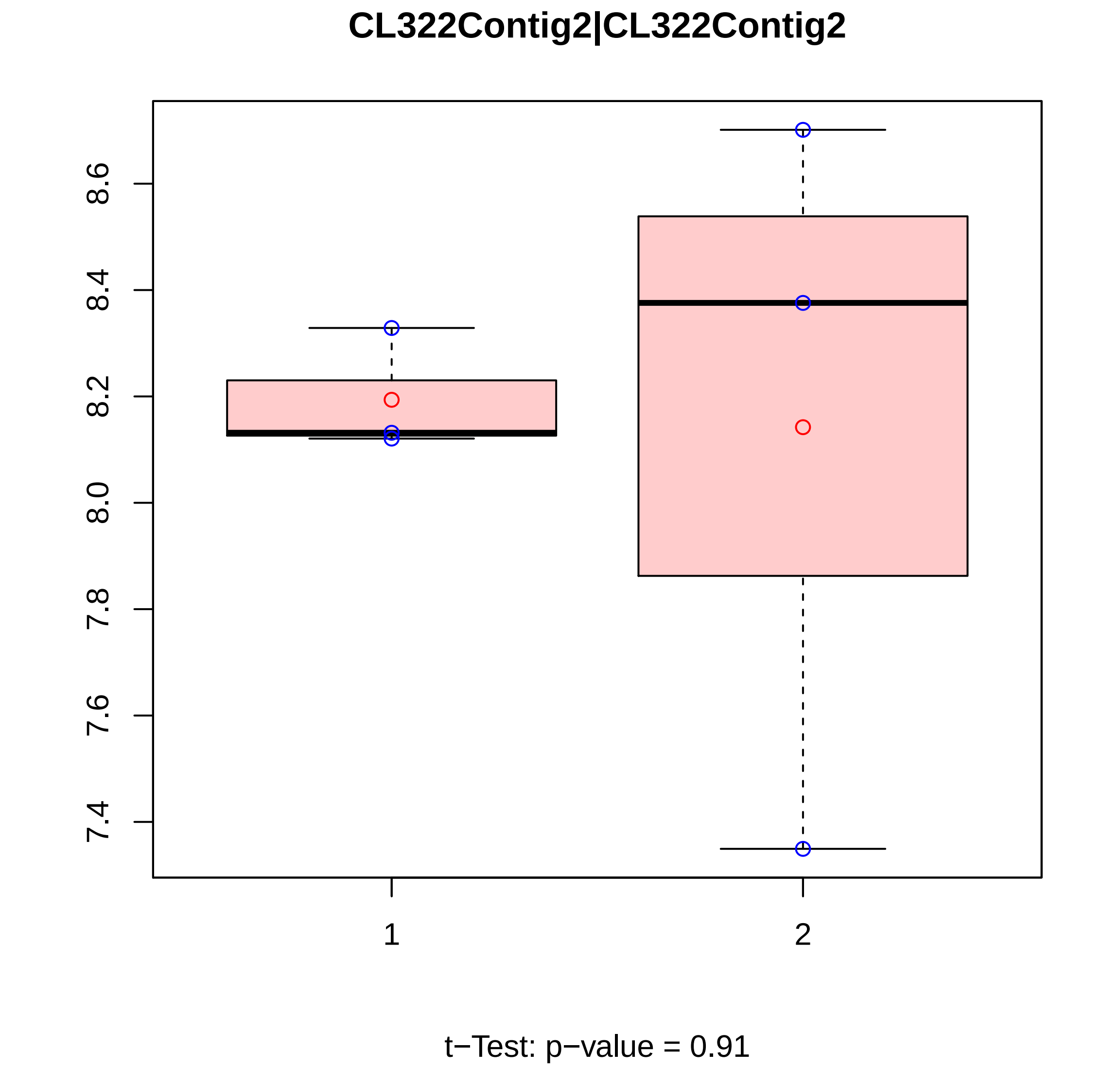
CL322Contig2|CL322Contig2
8.6
8.4
8.2
8.0
7.8
7.6
7.4
1
2
t−Test: p−value = 0.91

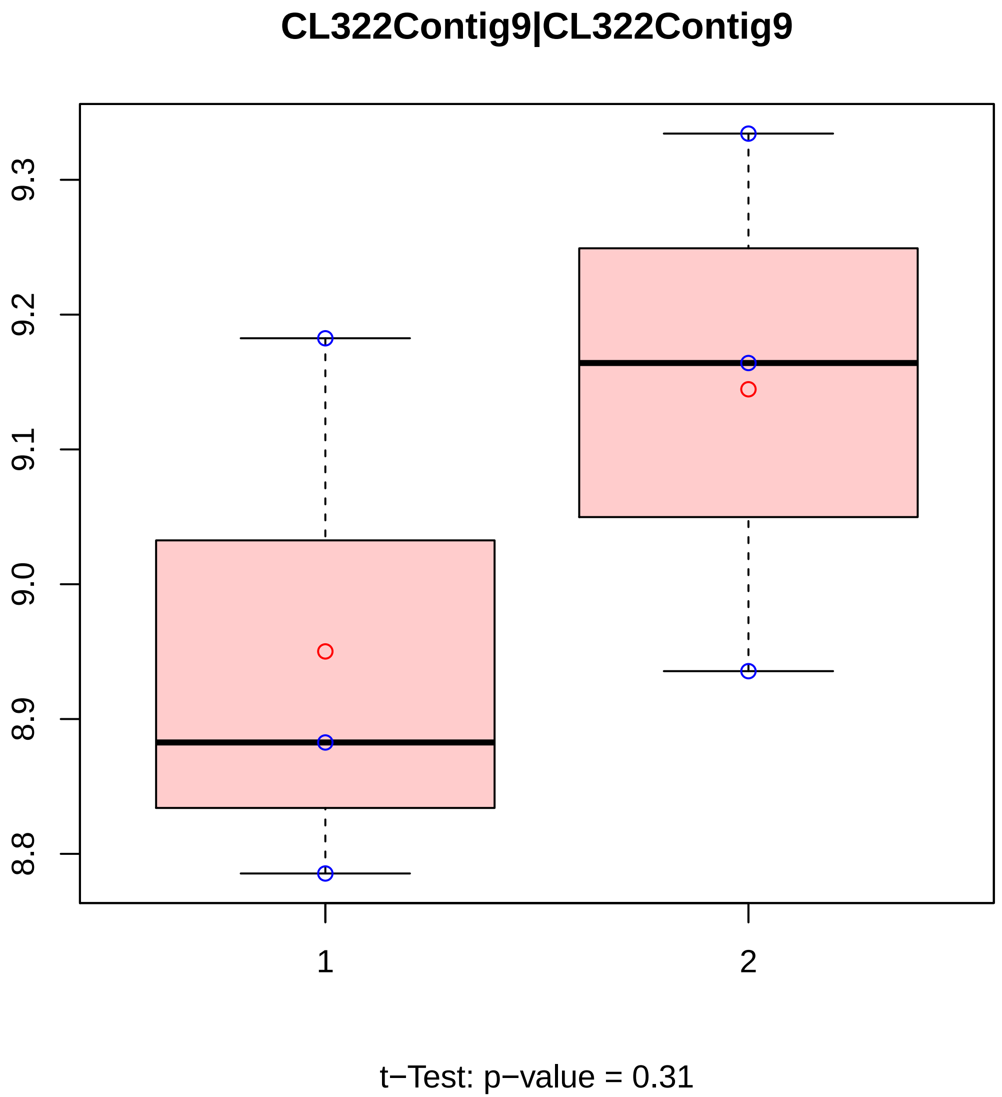
CL322Contig9|CL322Contig9
9.3
9.2
9.1
9.0
8.9
8.8
1
2
t−Test: p−value = 0.31

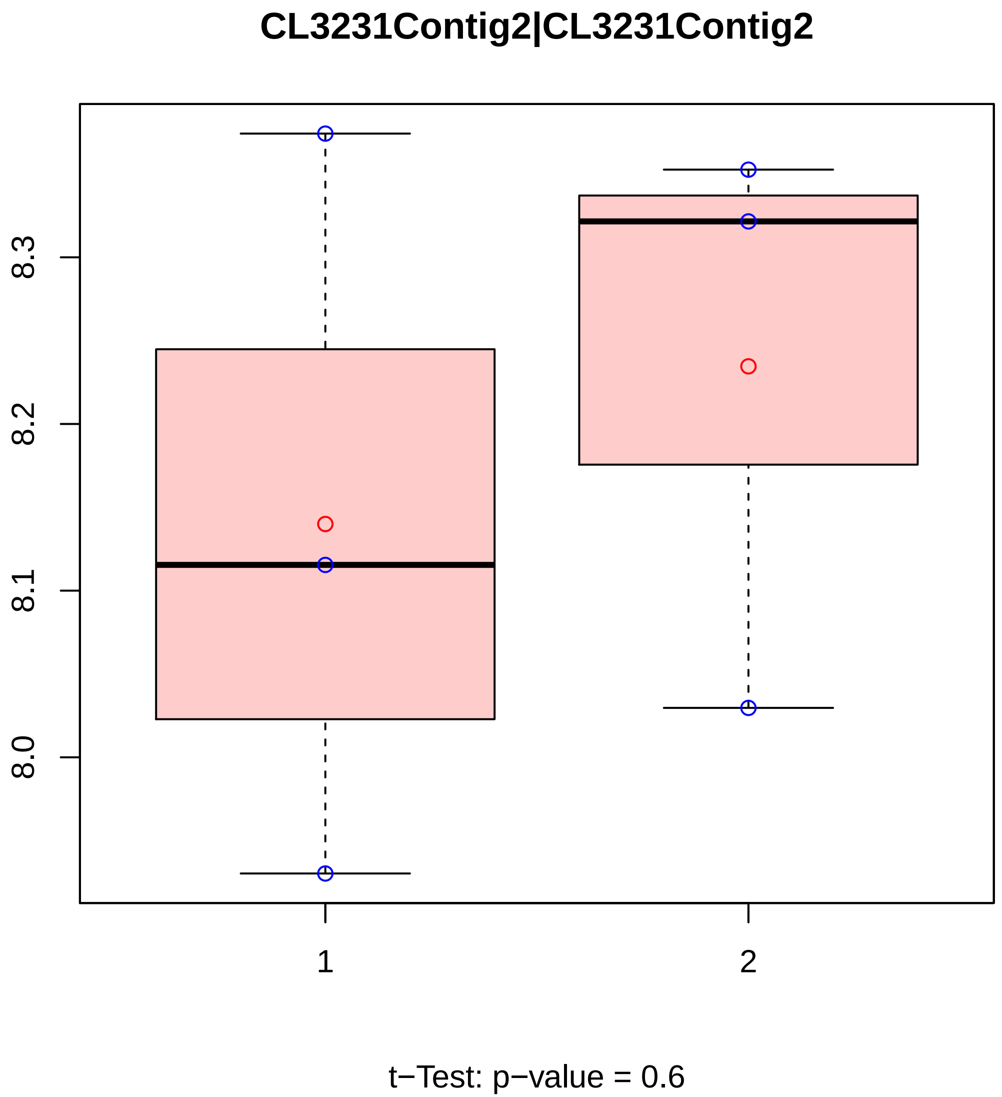

CL3231Contig2|CL3231Contig2
8.0
8.1
8.2
8.3
1
2
t−Test: p−value = 0.6

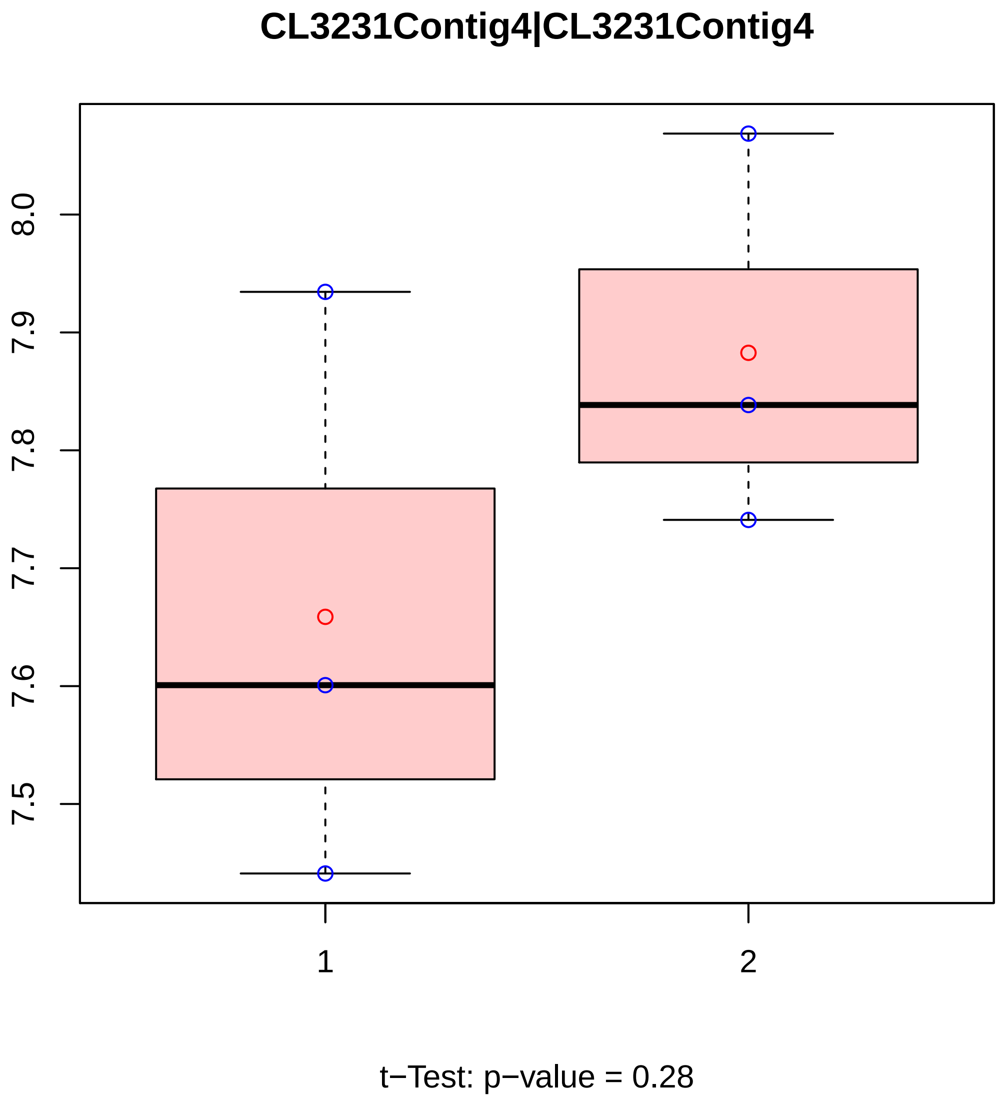

CL3231Contig4|CL3231Contig4
8.0
7.9
7.8
7.7
7.6
7.5
1
2
t−Test: p−value = 0.28

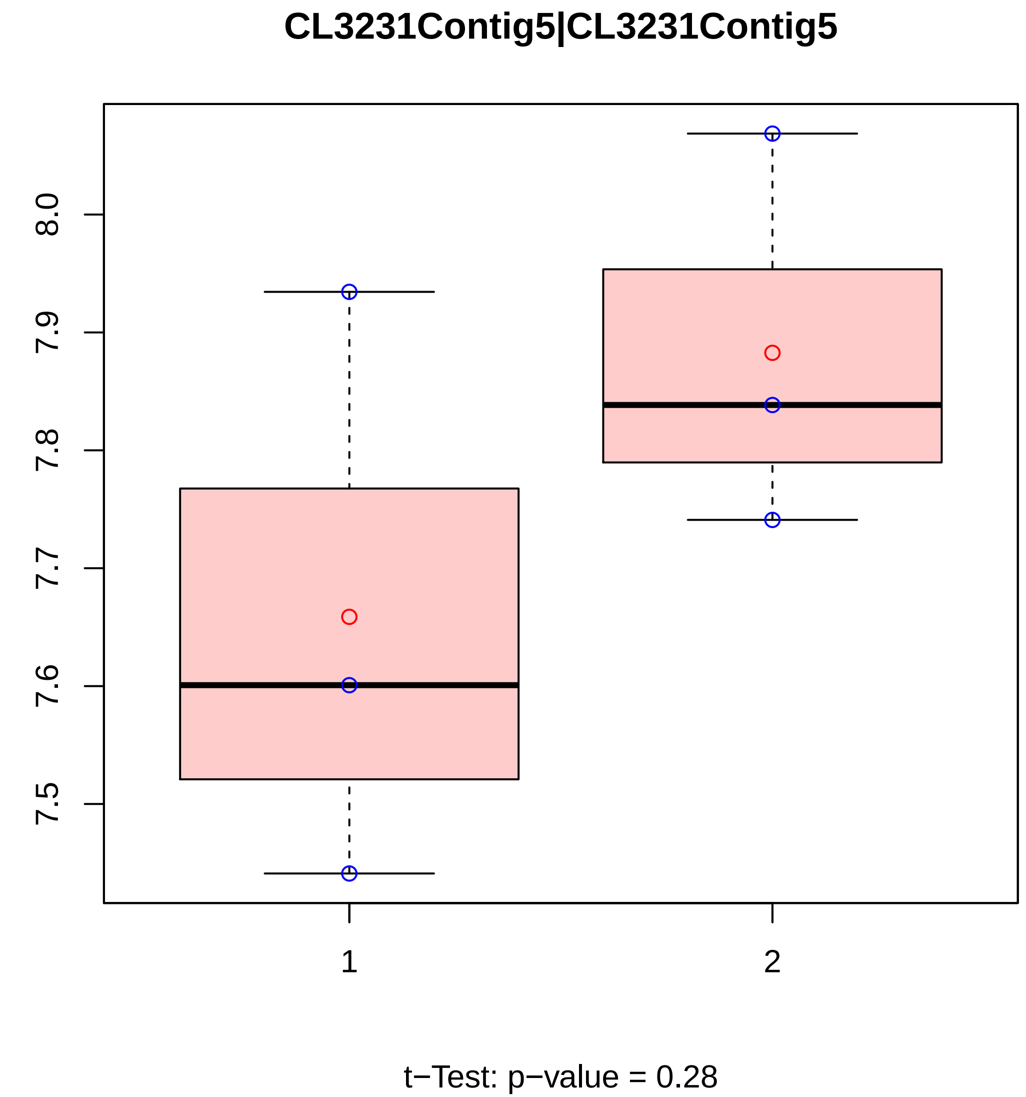
CL3231Contig5|CL3231Contig5
8.0
7.9
7.8
7.7
7.6
7.5
1
2
t−Test: p−value = 0.28

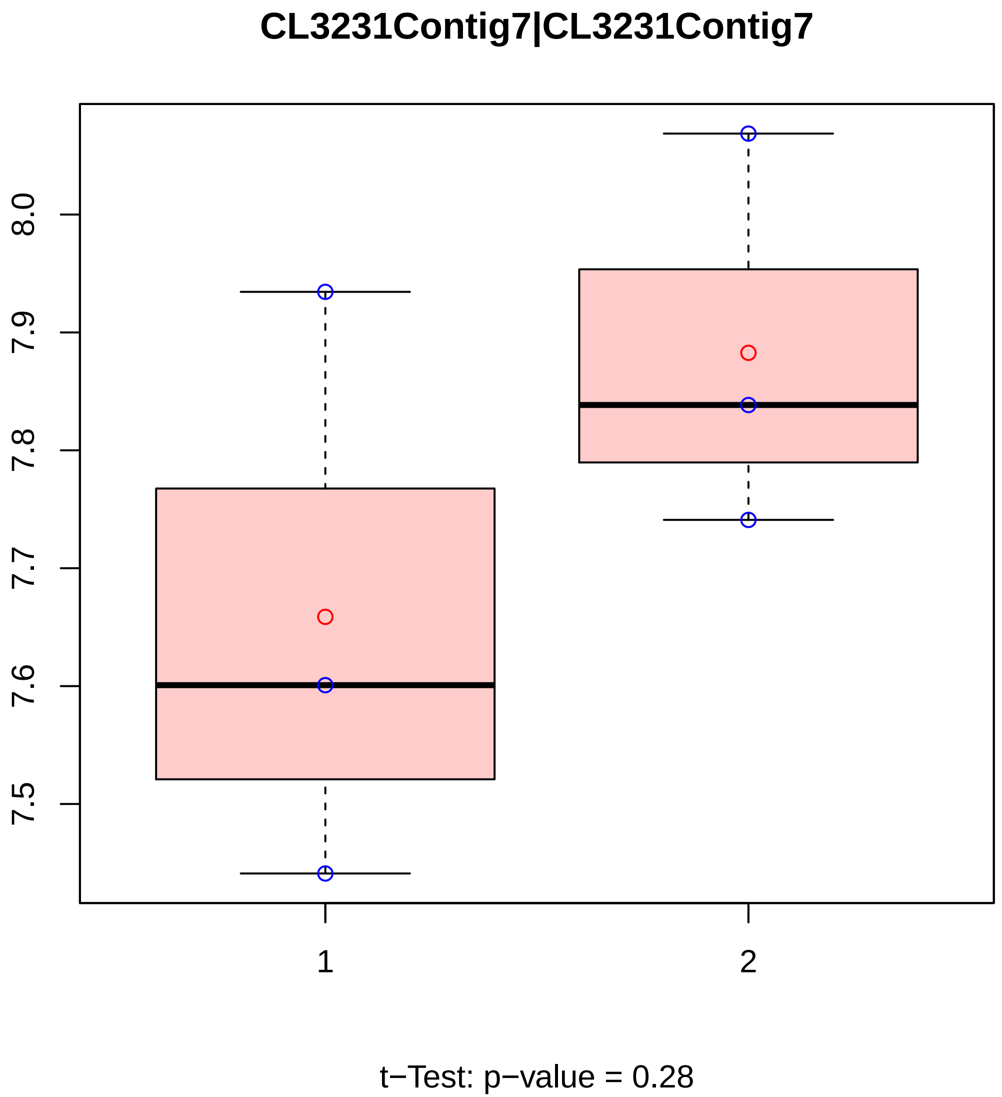
CL3231Contig7|CL3231Contig7
8.0
7.9
7.8
7.7
7.6
7.5
1
2
t−Test: p−value = 0.28

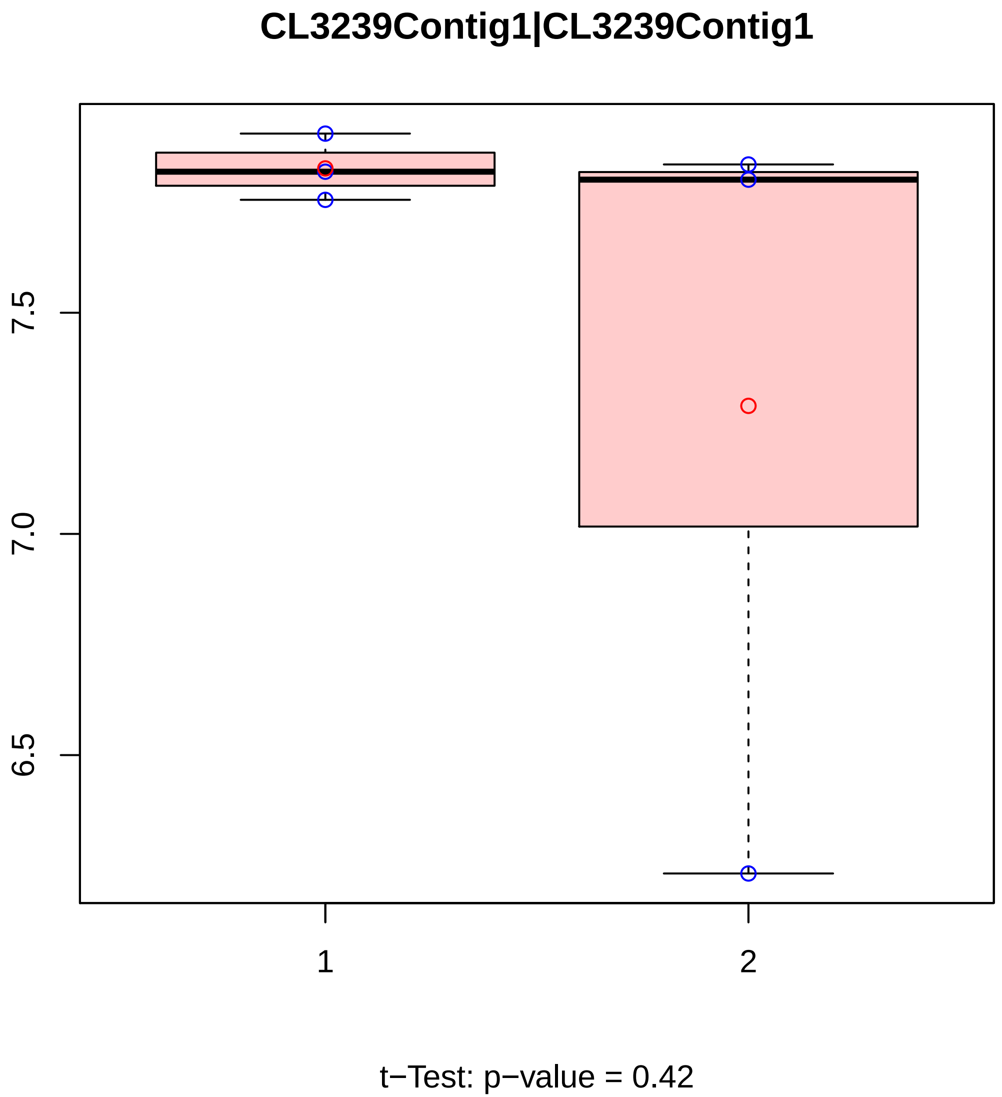
CL3239Contig1|CL3239Contig1
7.5
7.0
6.5
1
2
t−Test: p−value = 0.42

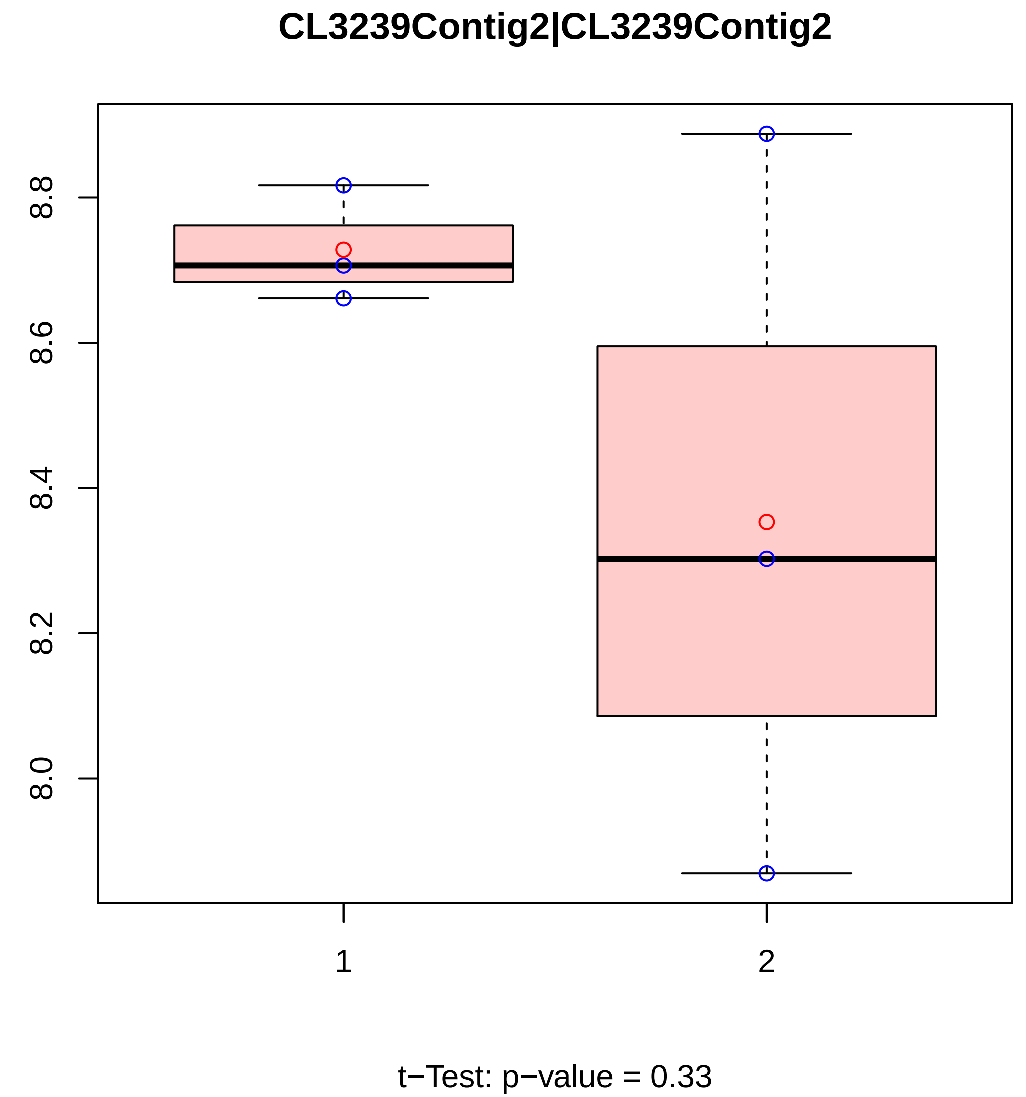
CL3239Contig2|CL3239Contig2
8.0
8.2
8.4
8.6
8.8
1
2
t−Test: p−value = 0.33

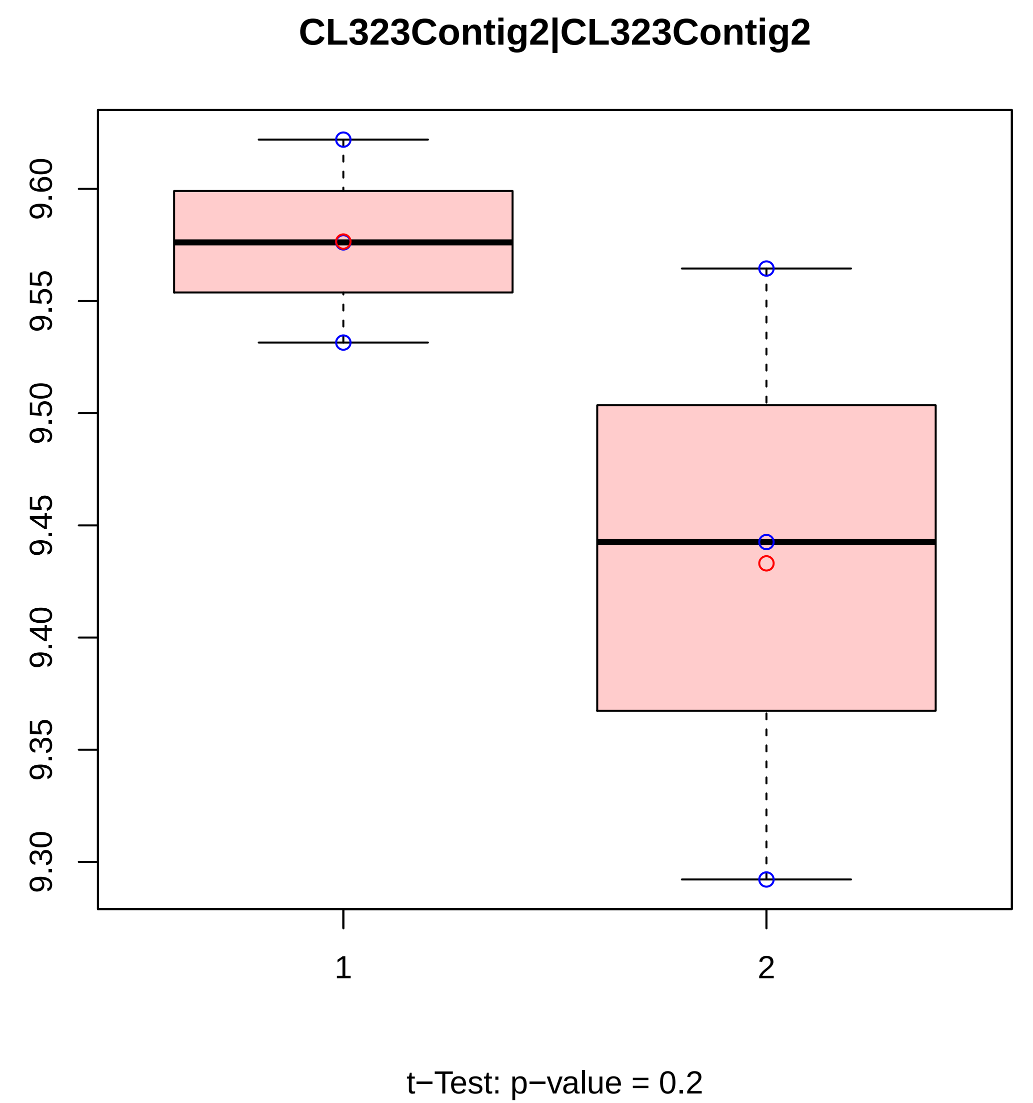
CL323Contig2|CL323Contig2
9.30
9.35
9.40
9.45
9.50
9.55
9.60
1
2
t−Test: p−value = 0.2

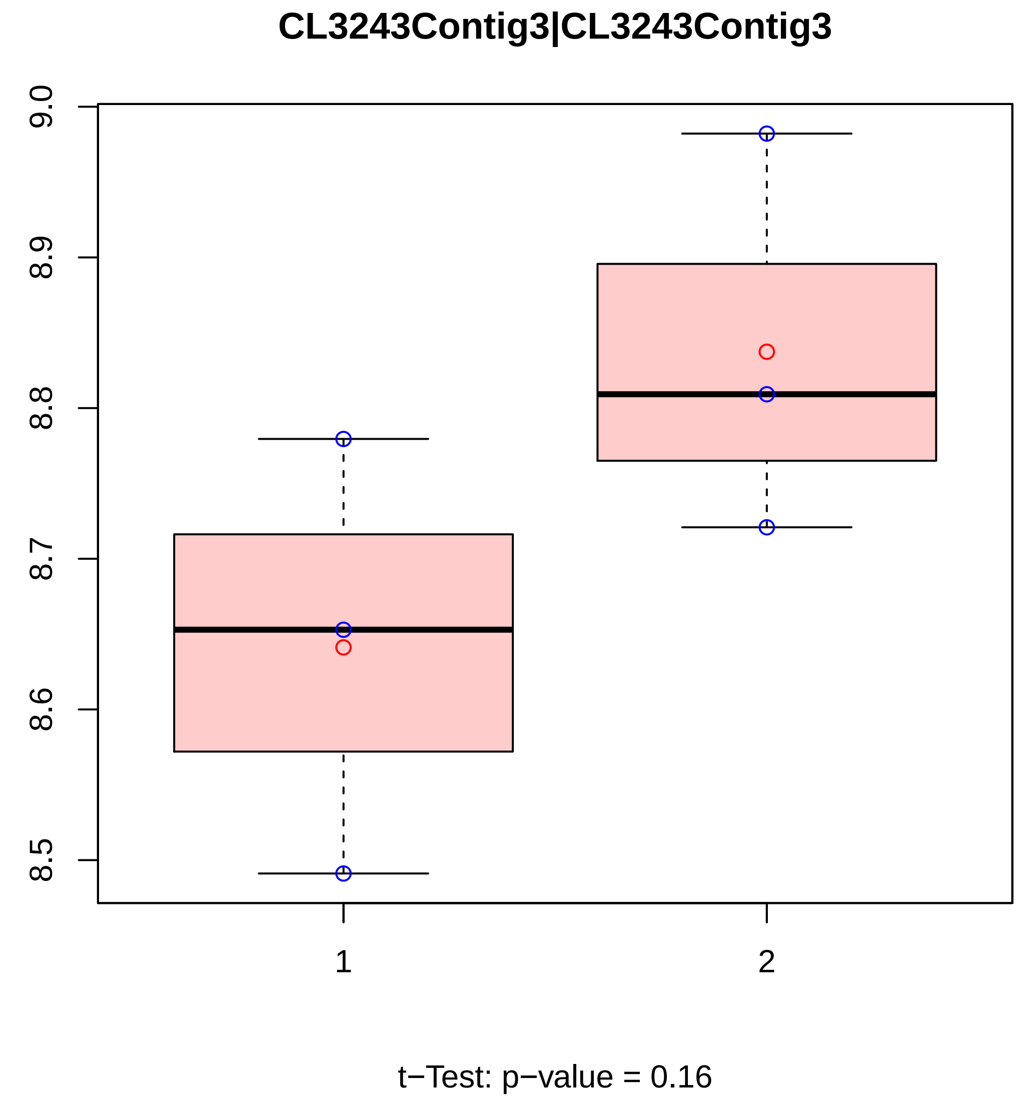

CL3243Contig3|CL3243Contig3
9.0
8.9
8.8
8.7
8.6
8.5
1
2
t−Test: p−value = 0.16

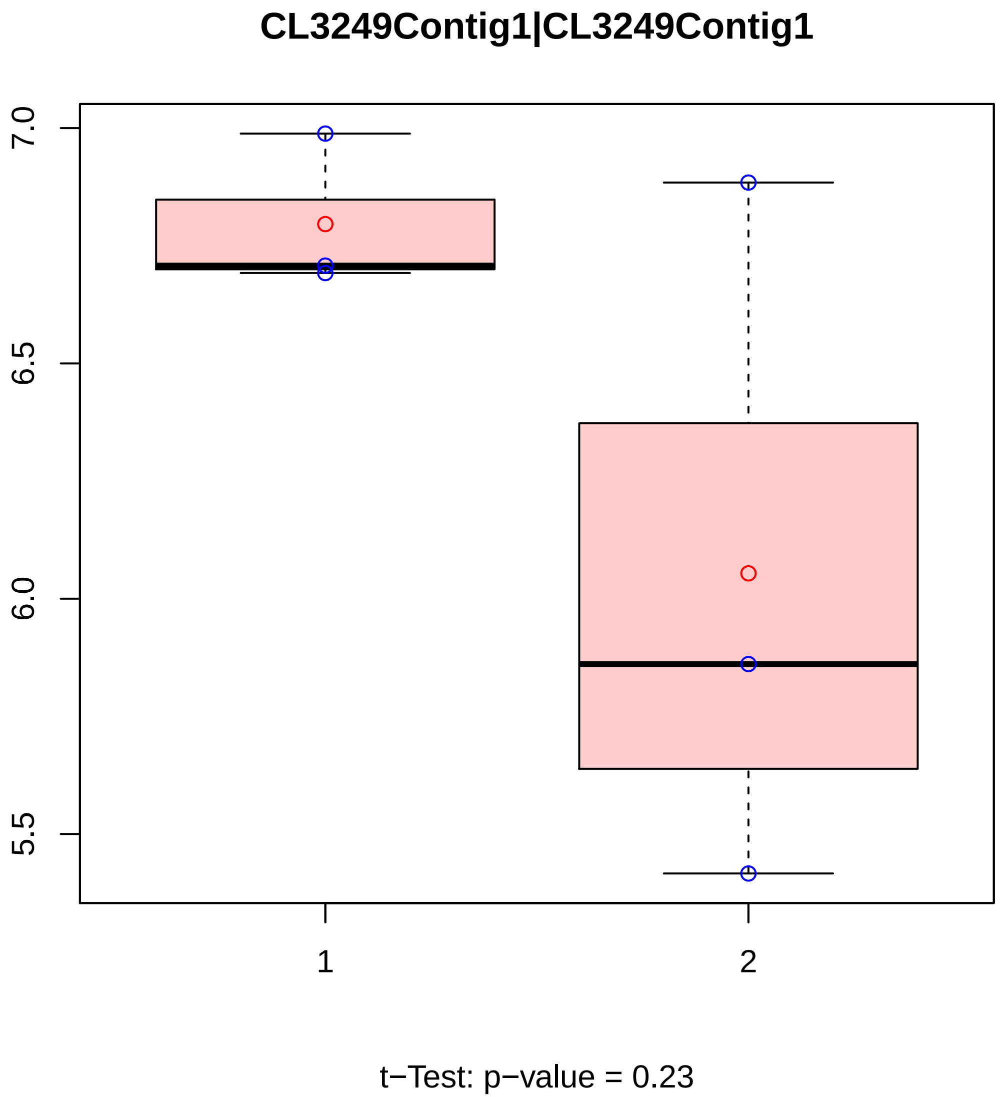
CL3249Contig1|CL3249Contig1
7.0
6.5
6.0
5.5
1
2
t−Test: p−value = 0.23

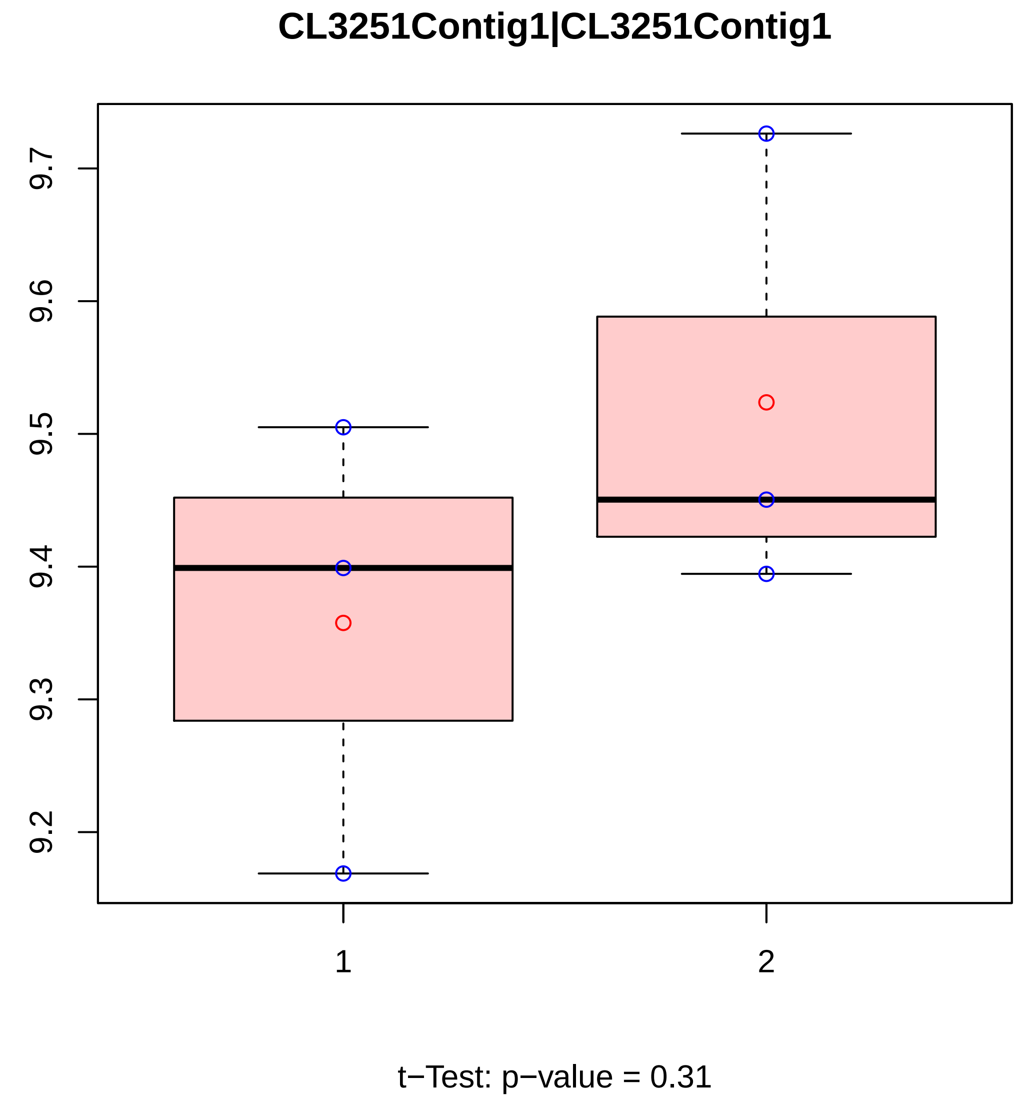
CL3251Contig1|CL3251Contig1
9.2
9.3
9.4
9.5
9.6
9.7
1
2
t−Test: p−value = 0.31

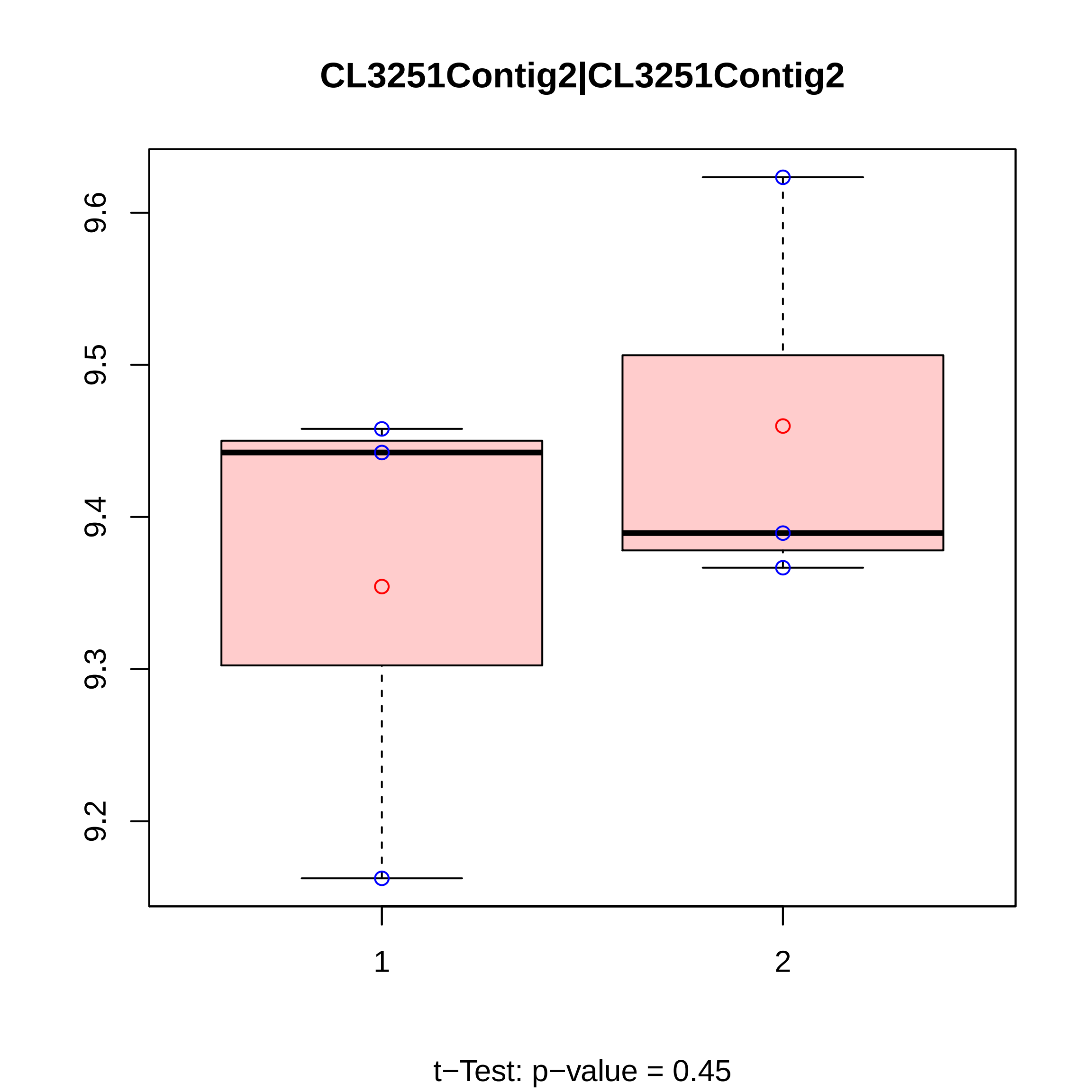
CL3251Contig2|CL3251Contig2
9.2
9.3
9.4
9.5
9.6
1
2
t−Test: p−value = 0.45

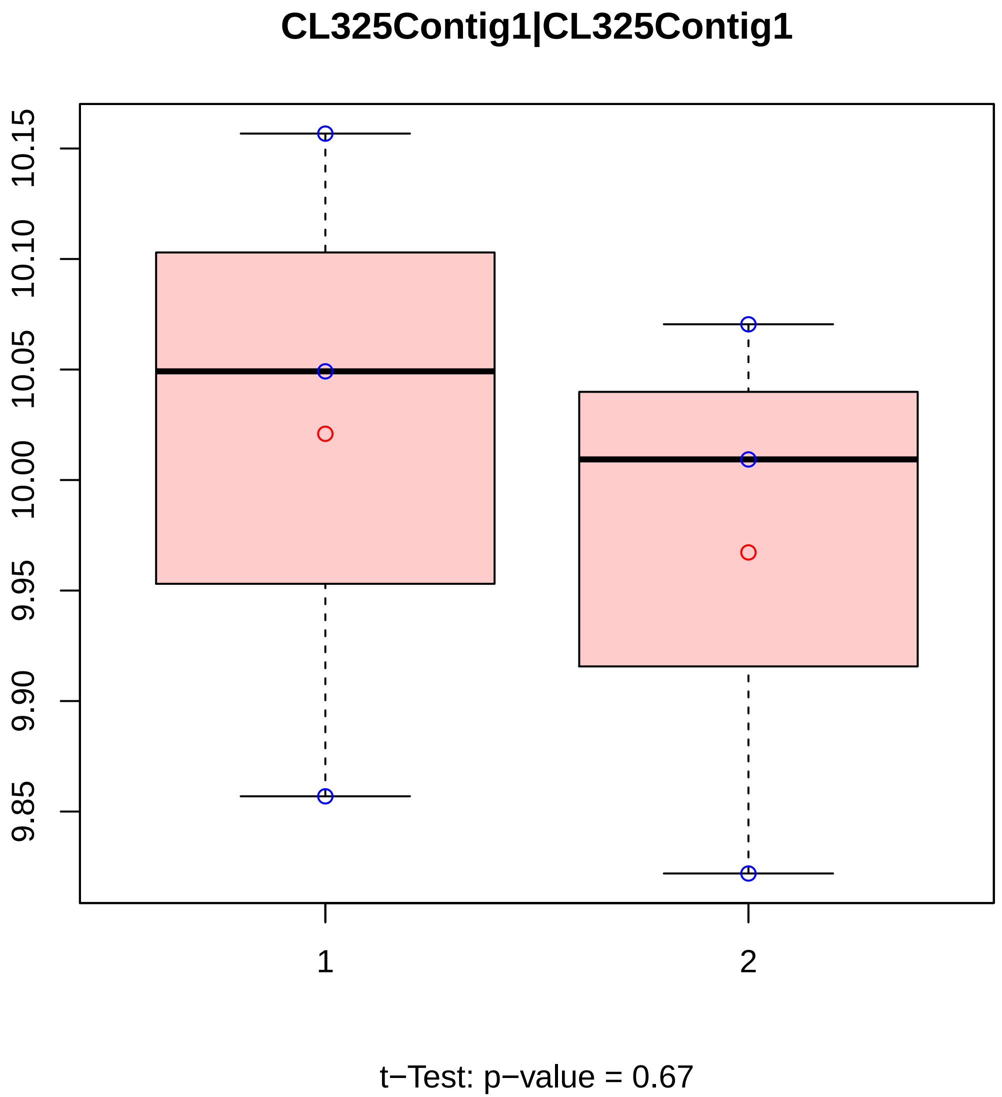

CL325Contig1|CL325Contig1
10.15
10.10
10.05
10.00
9.95
9.90
9.85
1
2
t−Test: p−value = 0.67

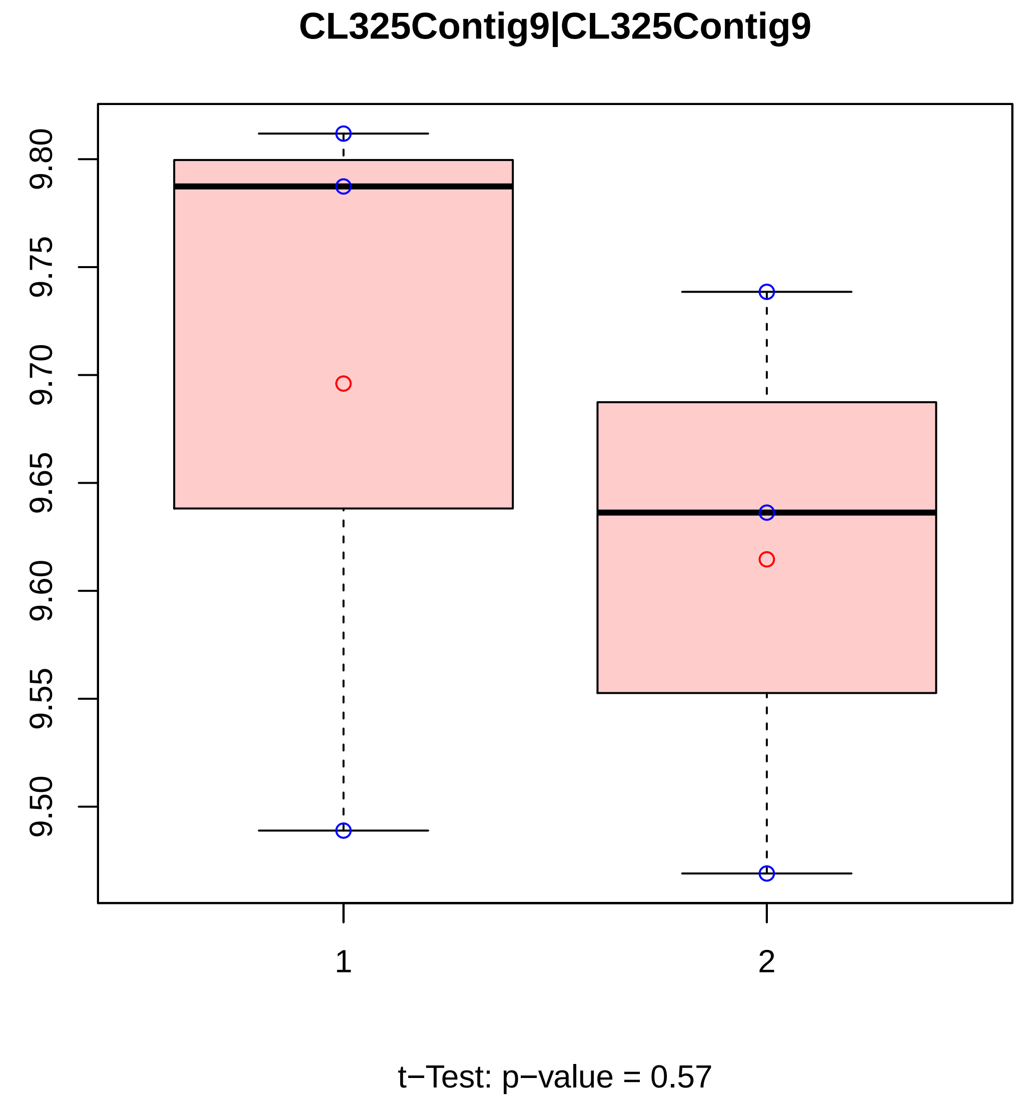
CL325Contig9|CL325Contig9
9.50
9.55
9.60
9.65
9.70
9.75
9.80
1
2
t−Test: p−value = 0.57

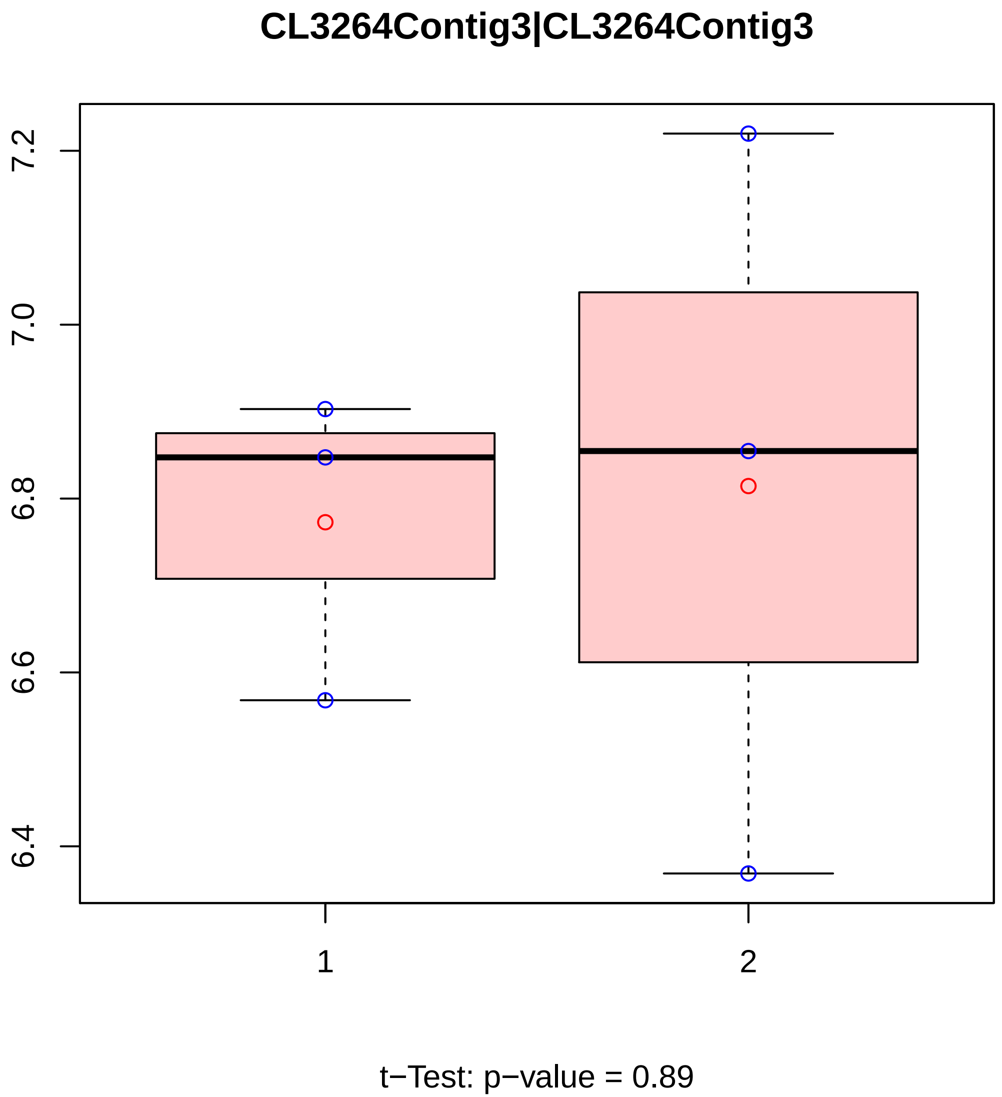

CL3264Contig3|CL3264Contig3
7.2
7.0
6.8
6.6
6.4
1
2
t−Test: p−value = 0.89

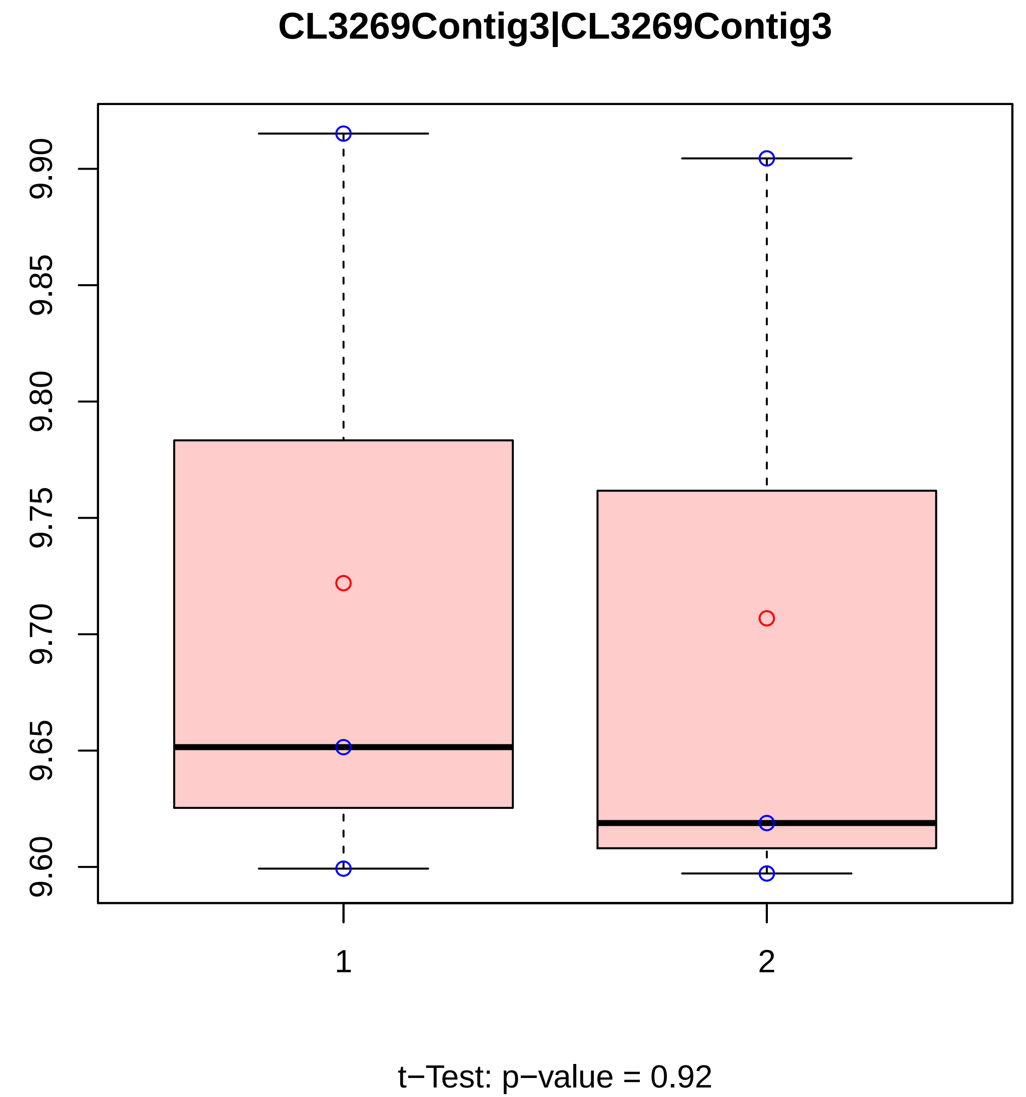
CL3269Contig3|CL3269Contig3
9.90
9.85
9.80
9.75
9.70
9.65
9.60
1
2
t−Test: p−value = 0.92

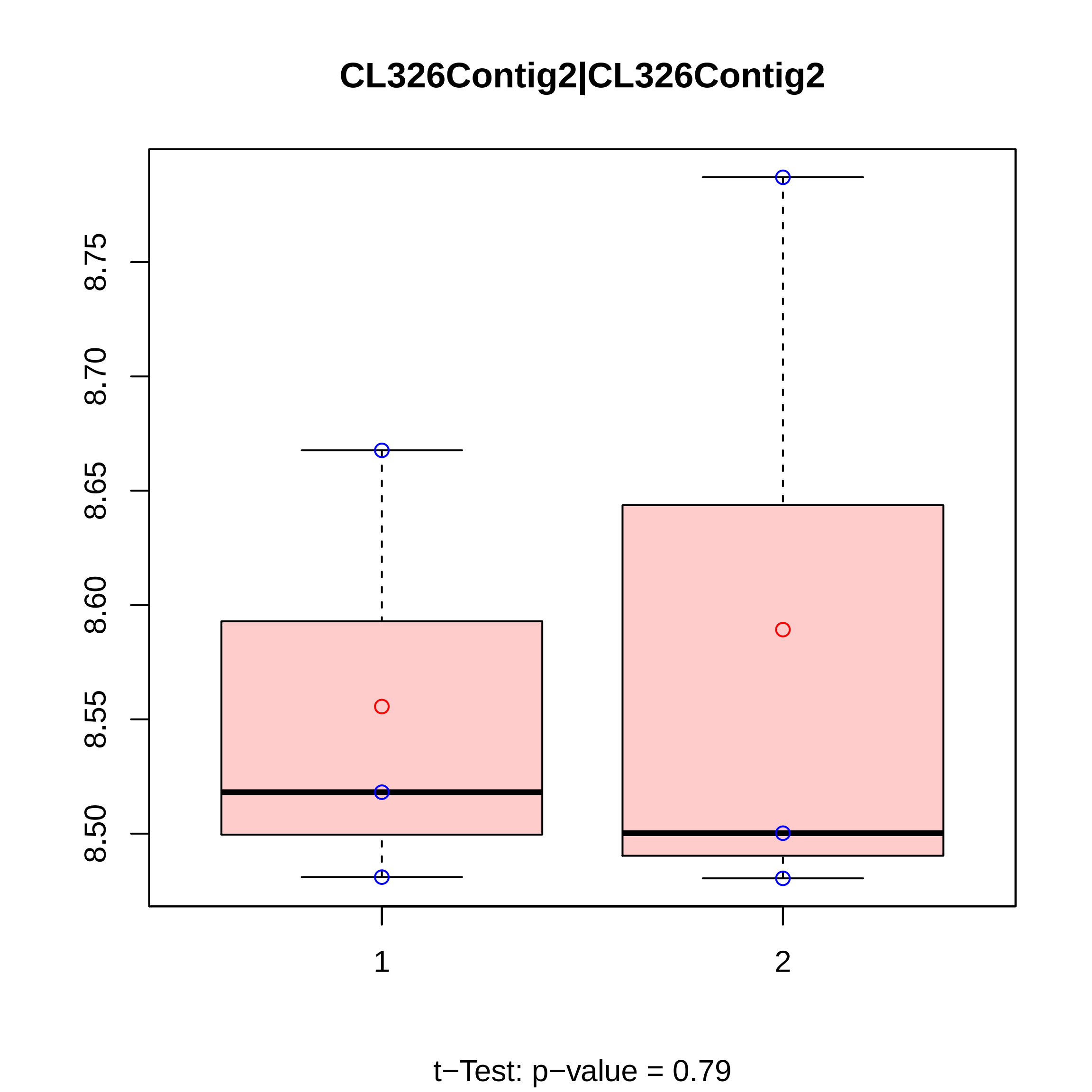

CL326Contig2|CL326Contig2
8.50
8.55
8.60
8.65
8.70
8.75
1
2
t−Test: p−value = 0.79

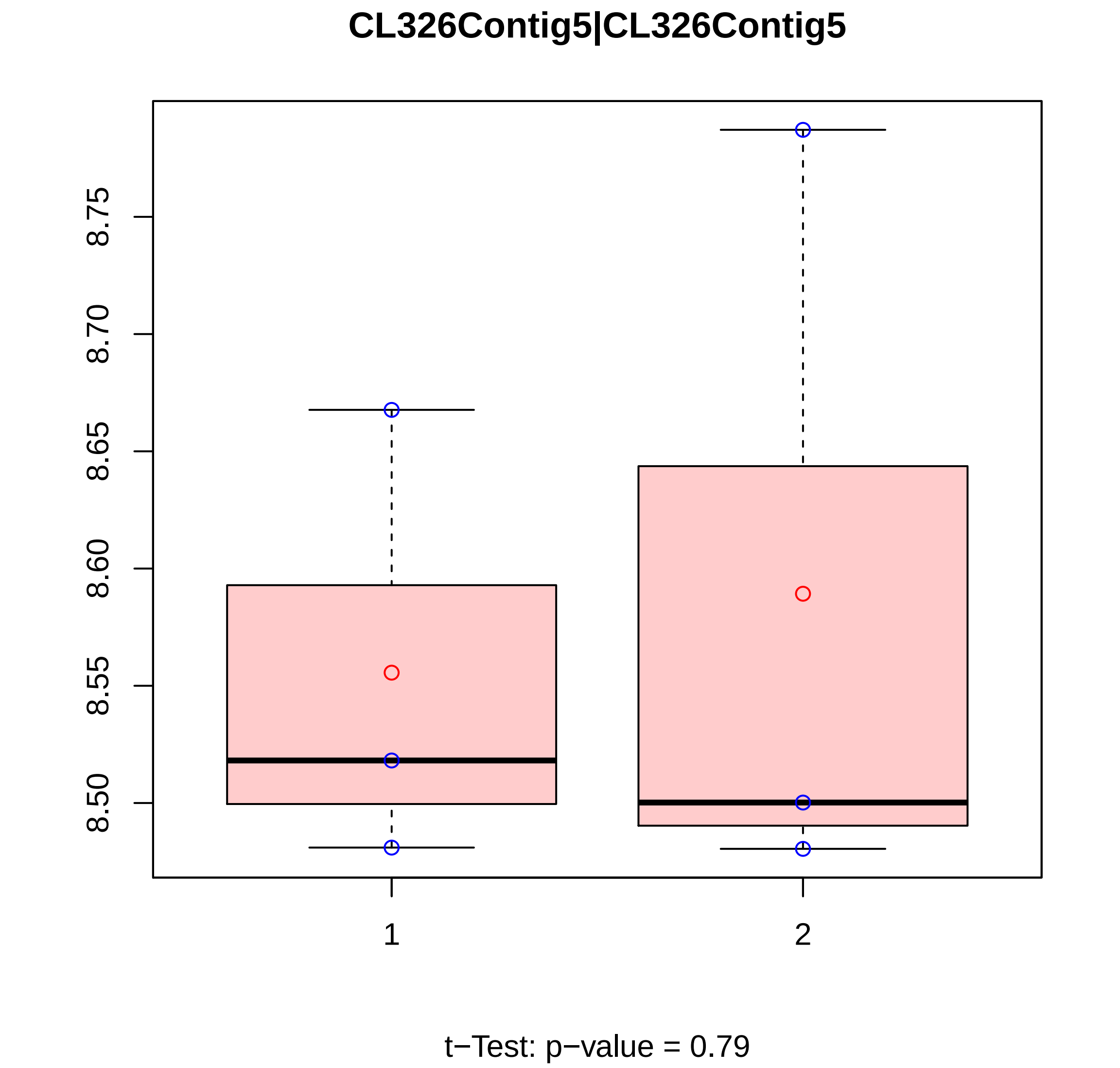

CL326Contig5|CL326Contig5
8.50
8.55
8.60
8.65
8.70
8.75
1
2
t−Test: p−value = 0.79

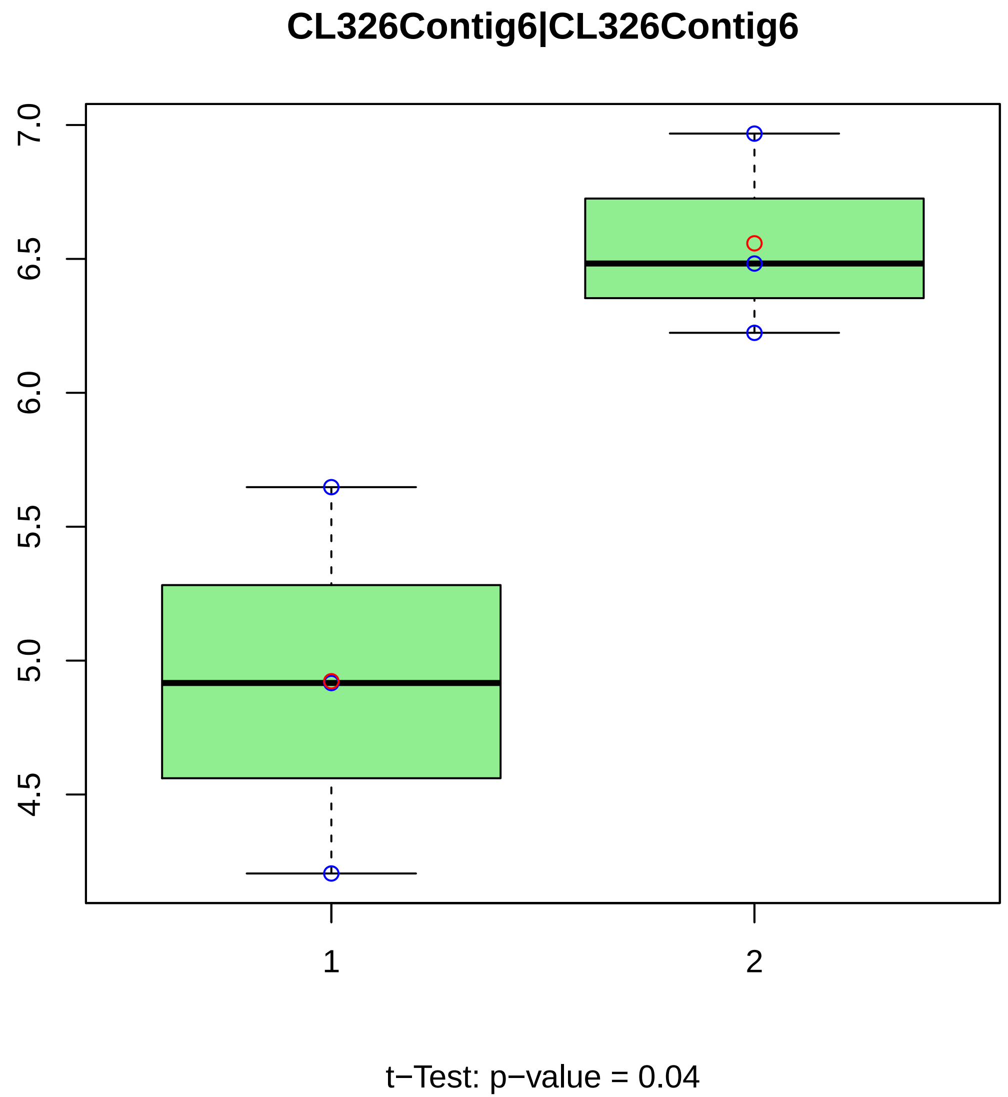
CL326Contig6|CL326Contig6
7.0
6.5
6.0
5.5
5.0
4.5
1
2
t−Test: p−value = 0.04

## CL3278Contig1|CL3278Contig1

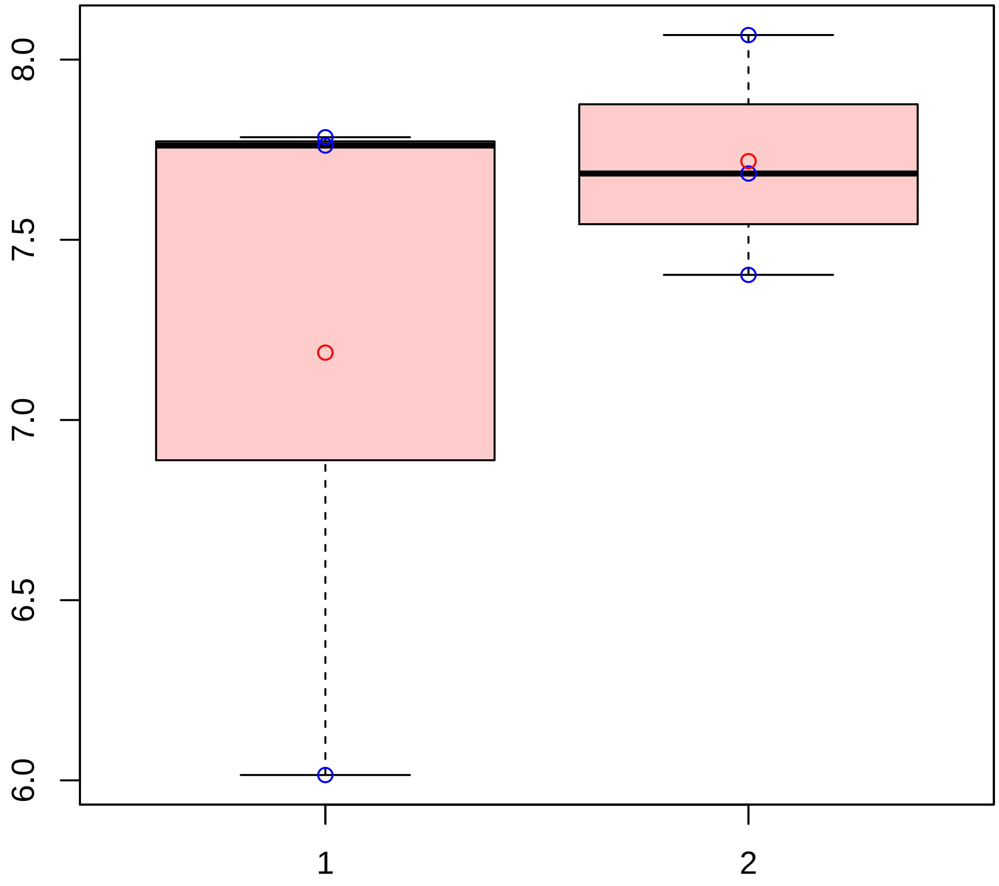


t−Test: p−value = 0.47

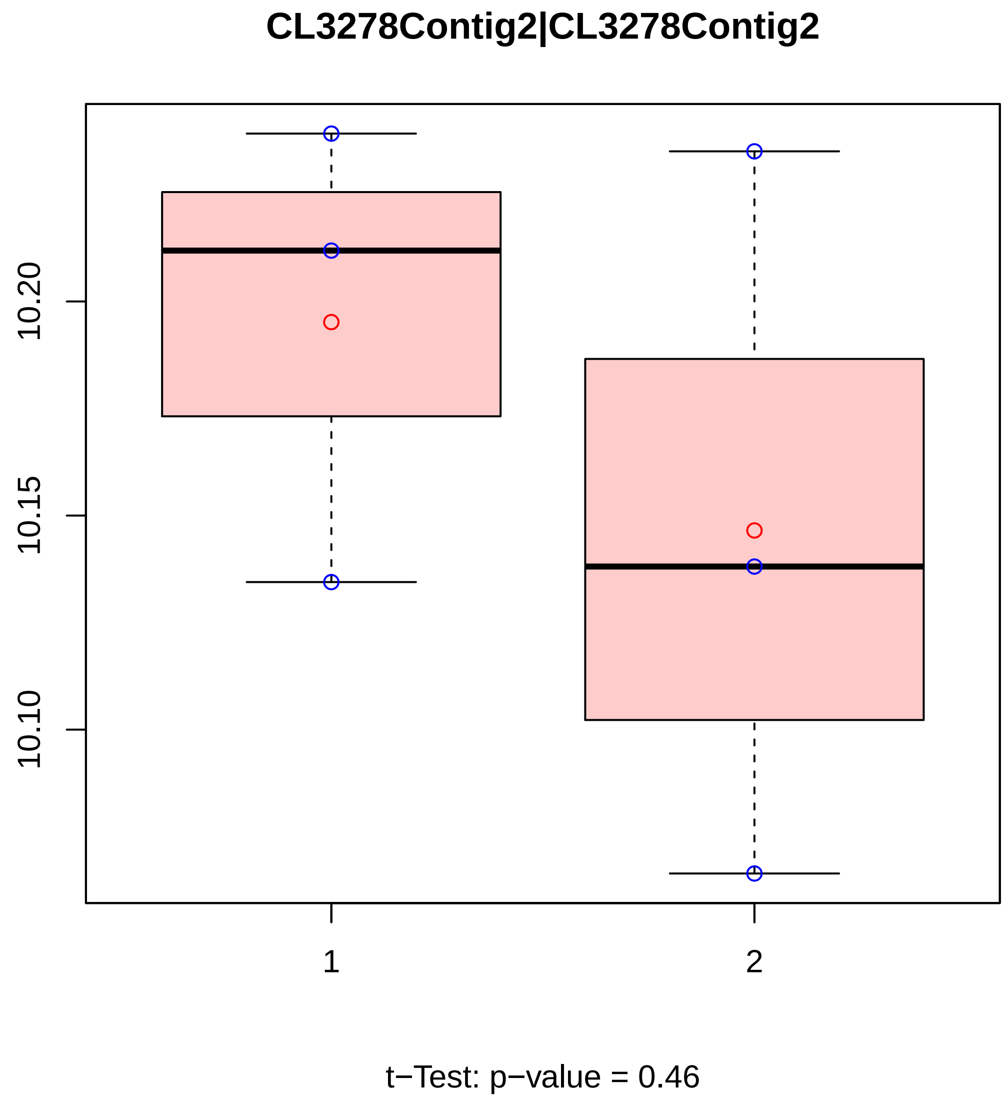
CL3278Contig2|CL3278Contig2
10.10
10.15
10.20
1
2
t−Test: p−value = 0.46

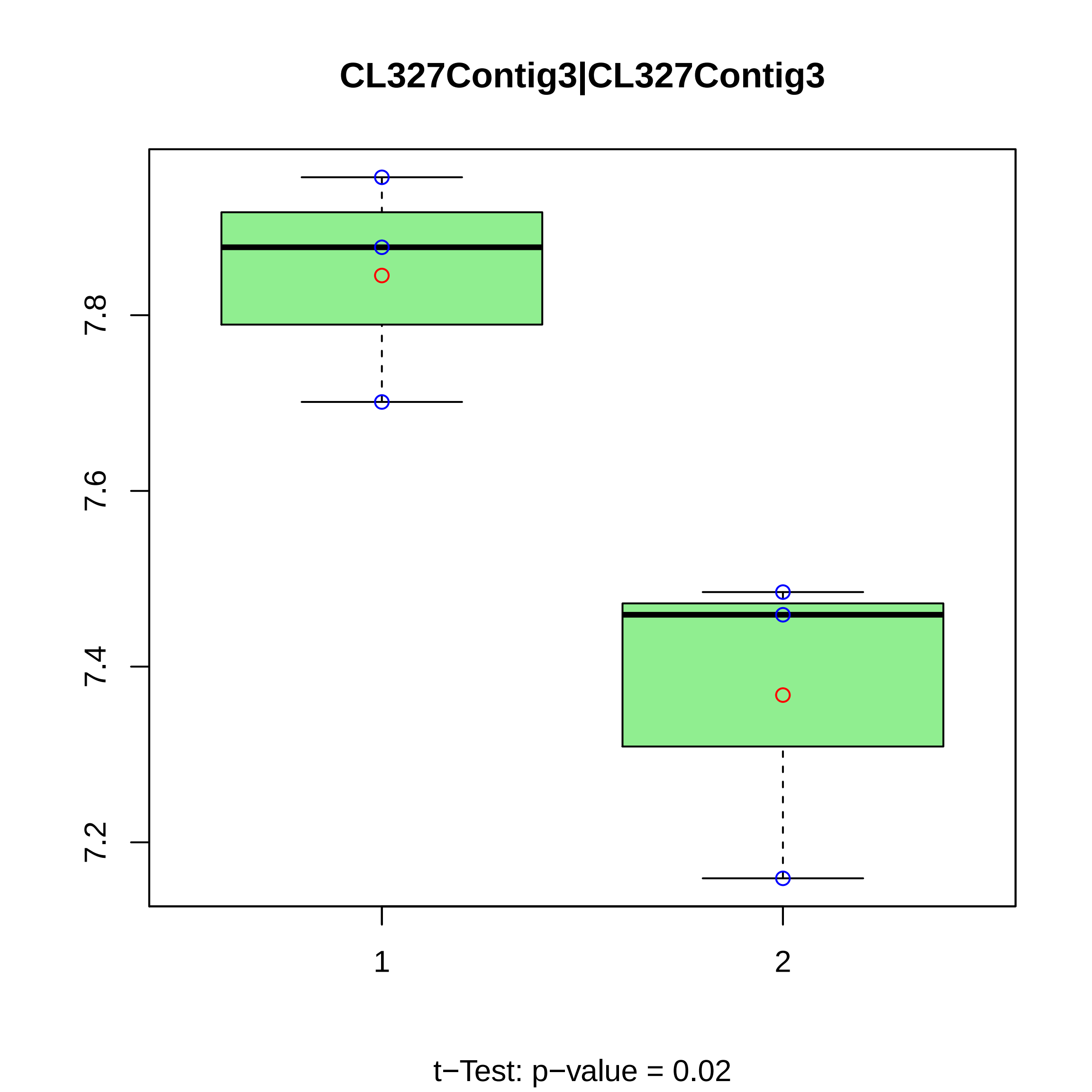
CL327Contig3|CL327Contig3
7.2
7.4
7.6
7.8
1
2
t−Test: p−value = 0.02

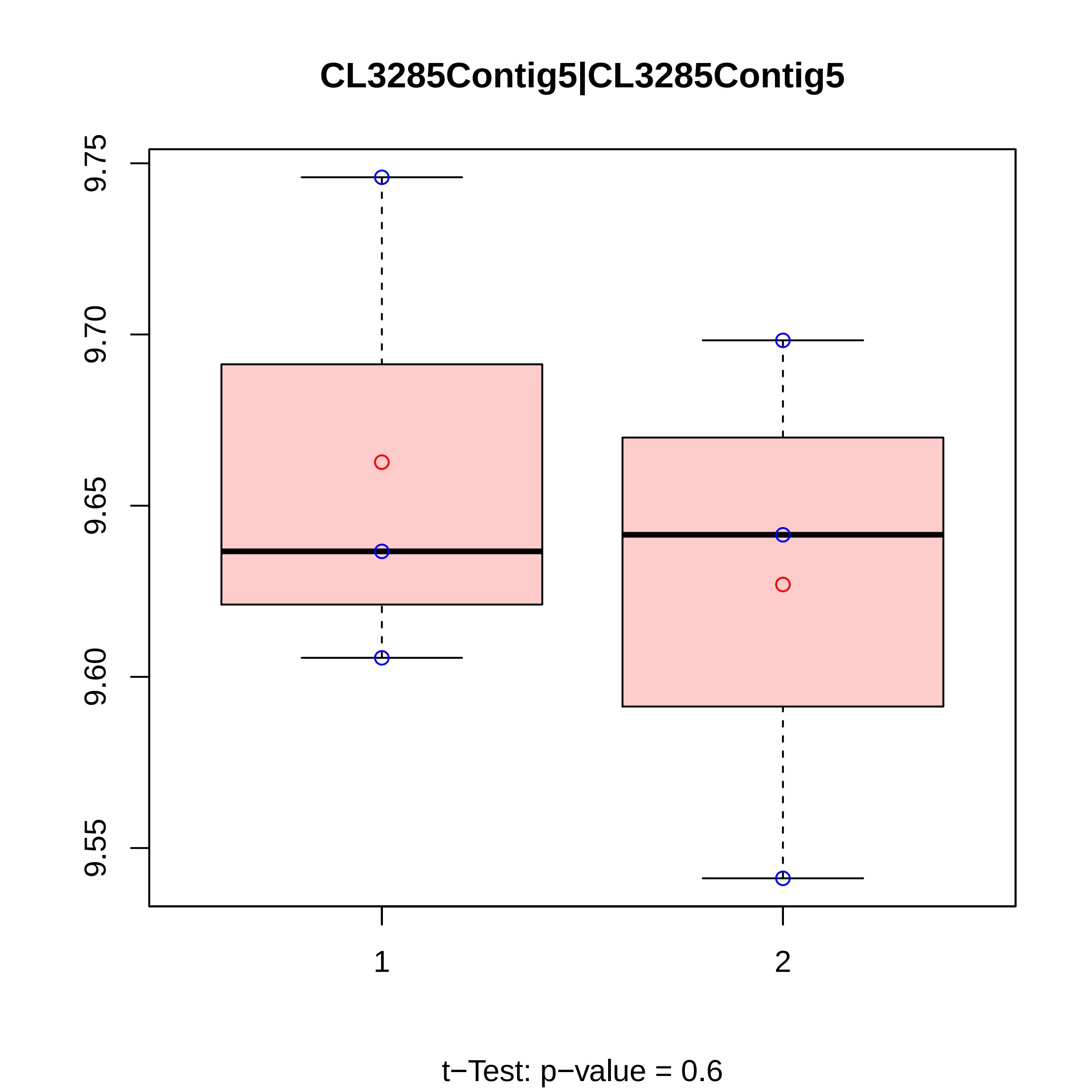
CL3285Contig5|CL3285Contig5
9.75
9.70
9.65
9.60
9.55
1
2
t−Test: p−value = 0.6

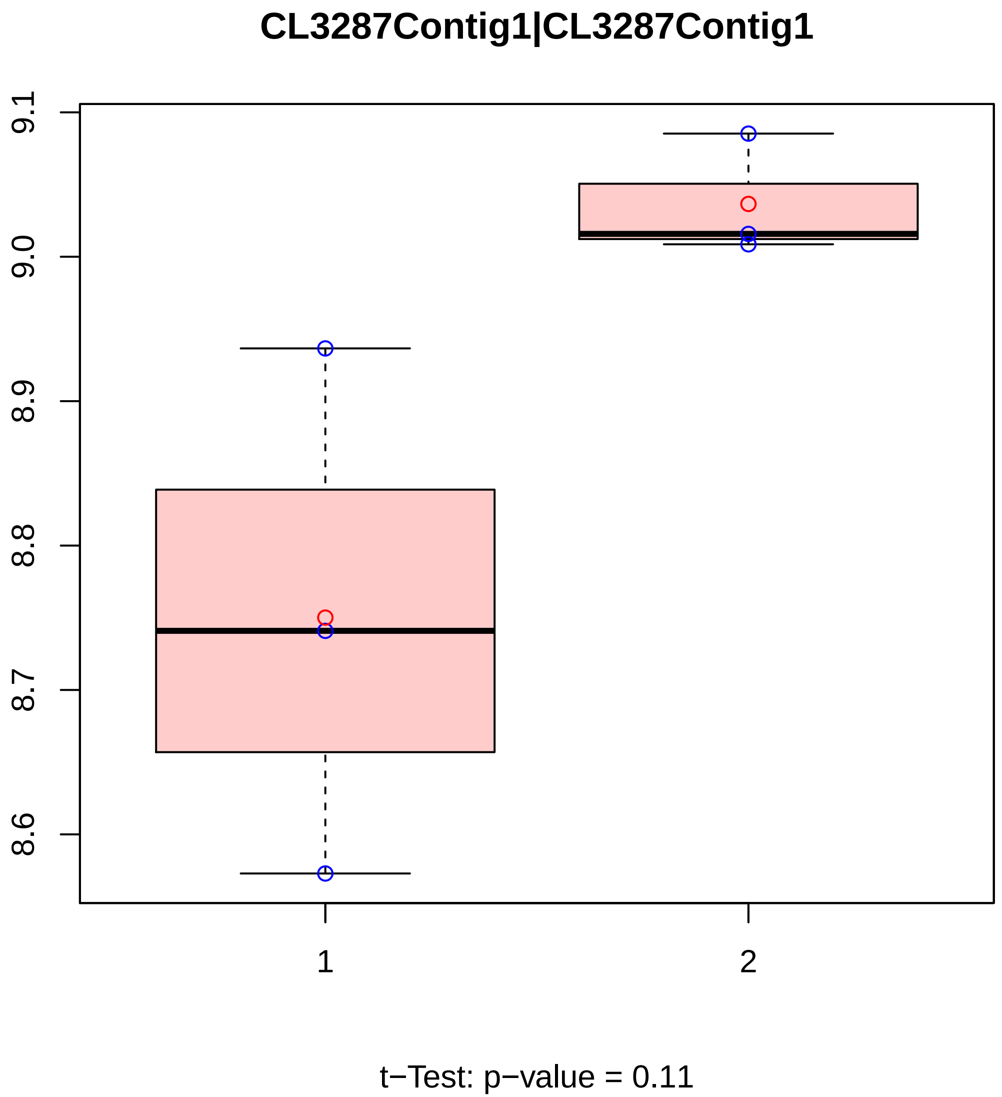
CL3287Contig1|CL3287Contig1
9.1
9.0
8.9
8.8
8.7
8.6
1
2
t−Test: p−value = 0.11

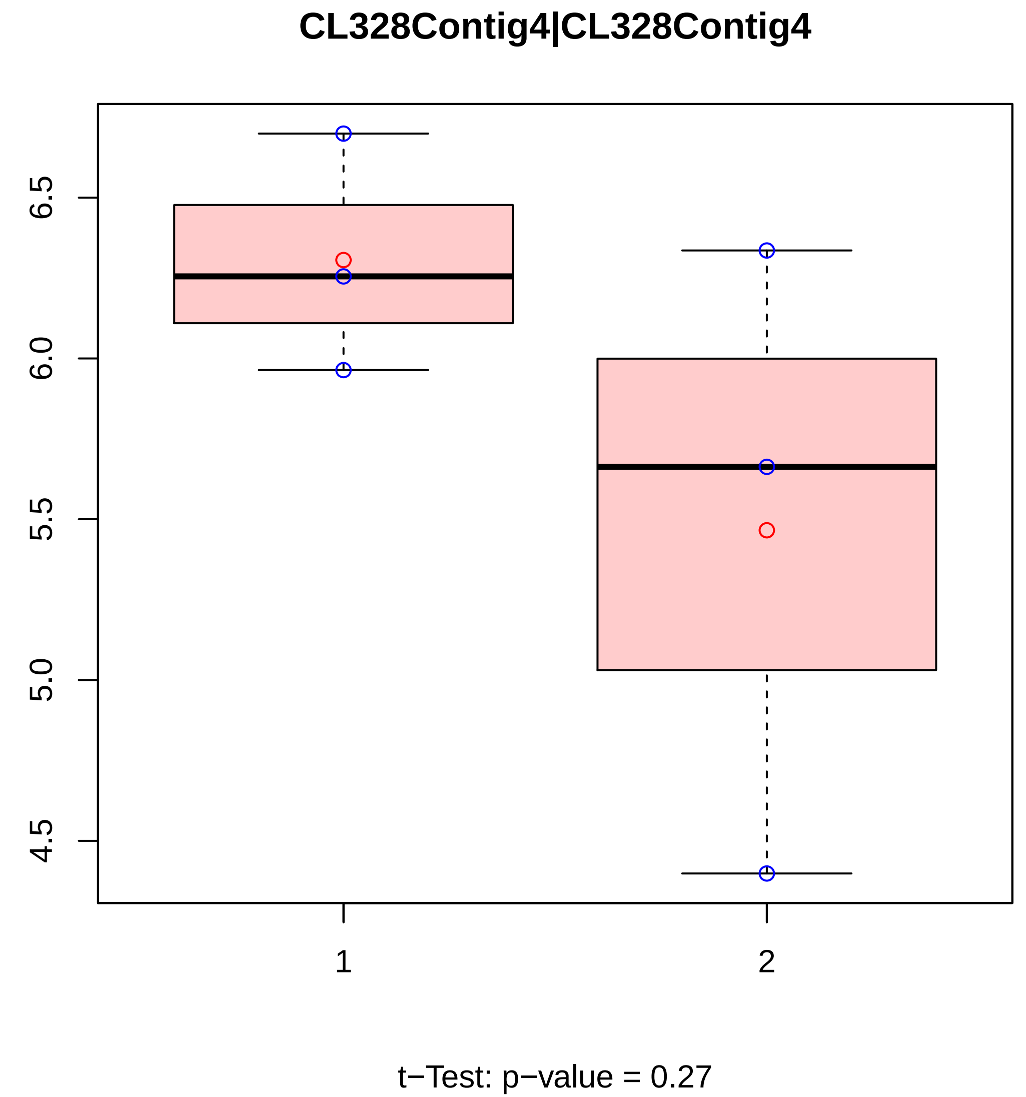
CL328Contig4|CL328Contig4
6.5
6.0
5.5
5.0
4.5
1
2
t−Test: p−value = 0.27

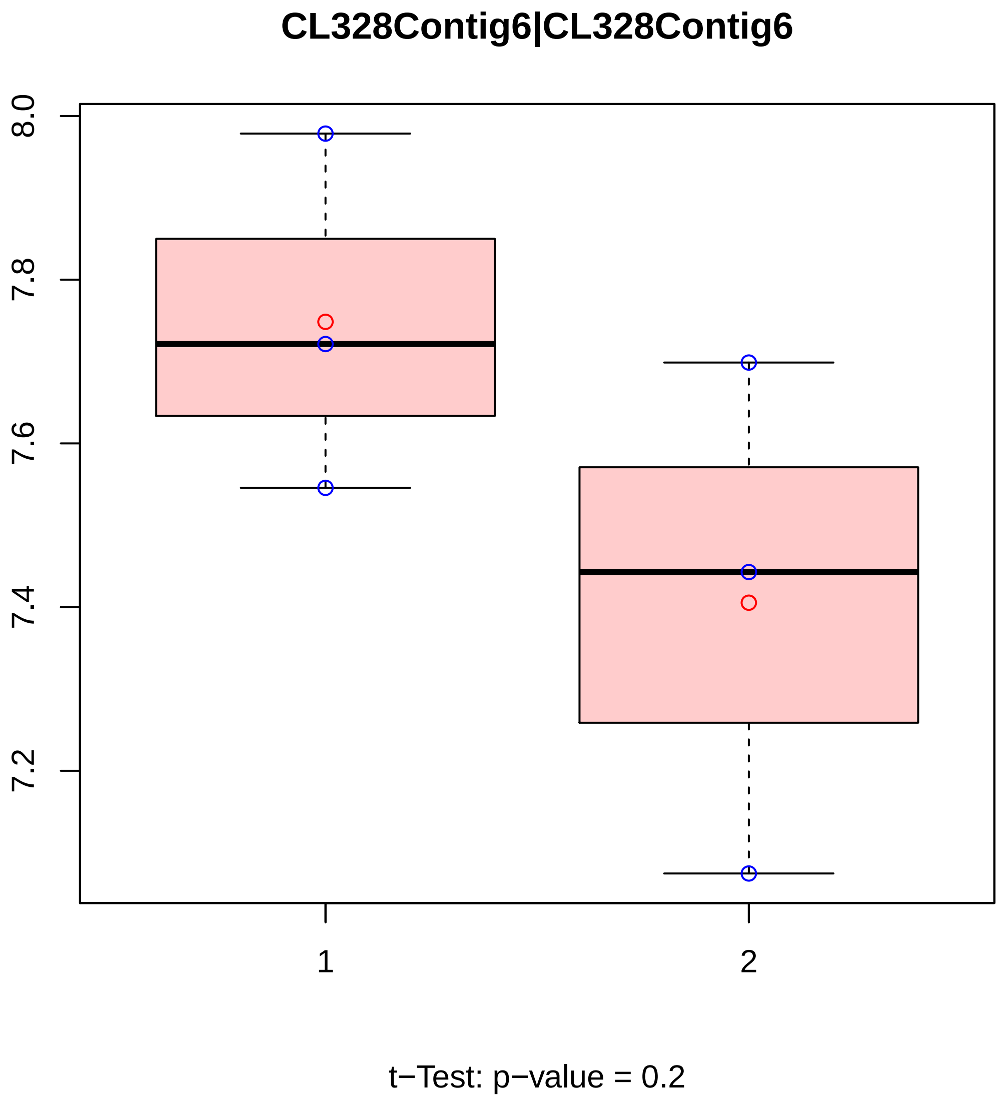
CL328Contig6|CL328Contig6
8.0
7.8
7.6
7.4
7.2
1
2
t−Test: p−value = 0.2

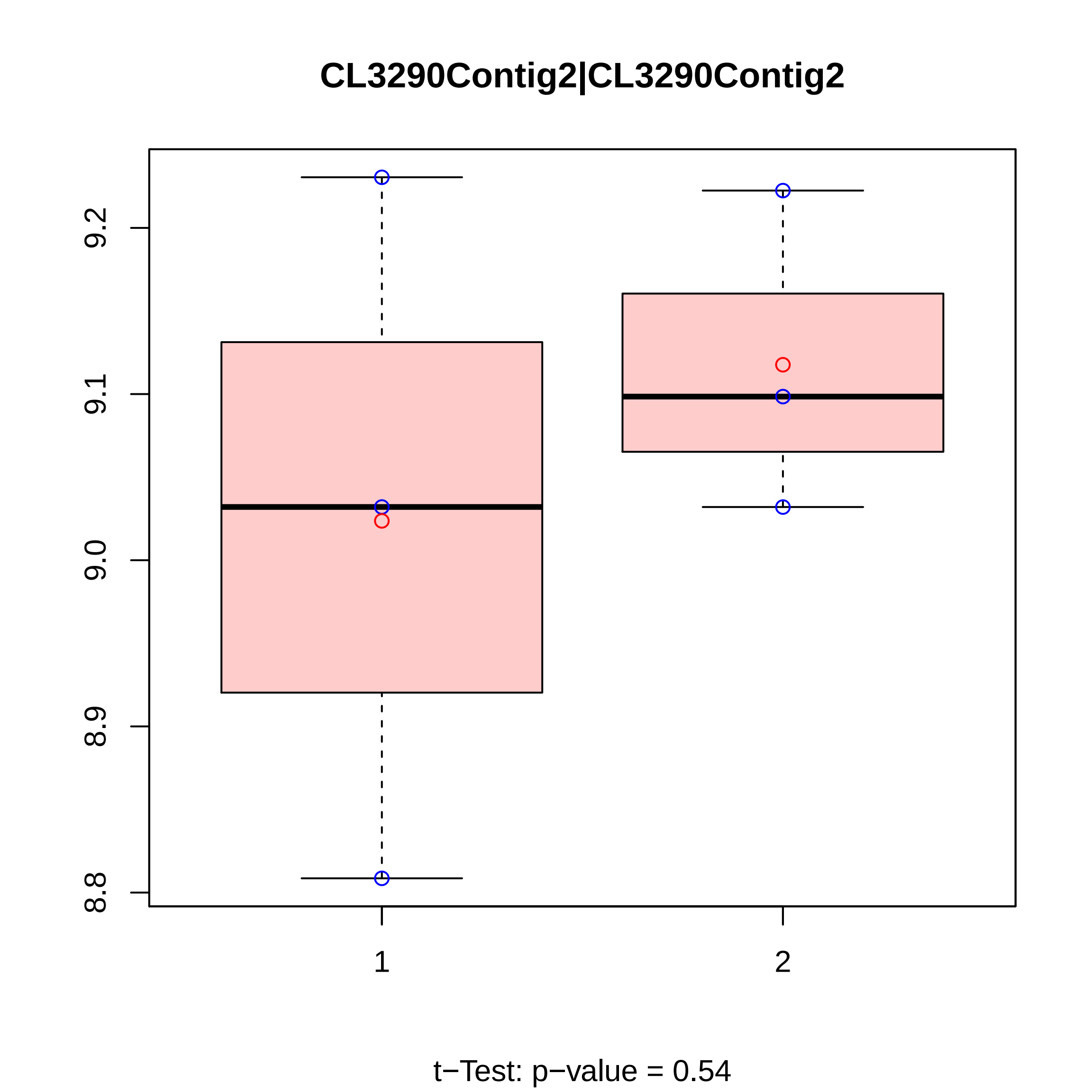
CL3290Contig2|CL3290Contig2
9.2
9.1
9.0
8.9
8.8
1
2
t−Test: p−value = 0.54

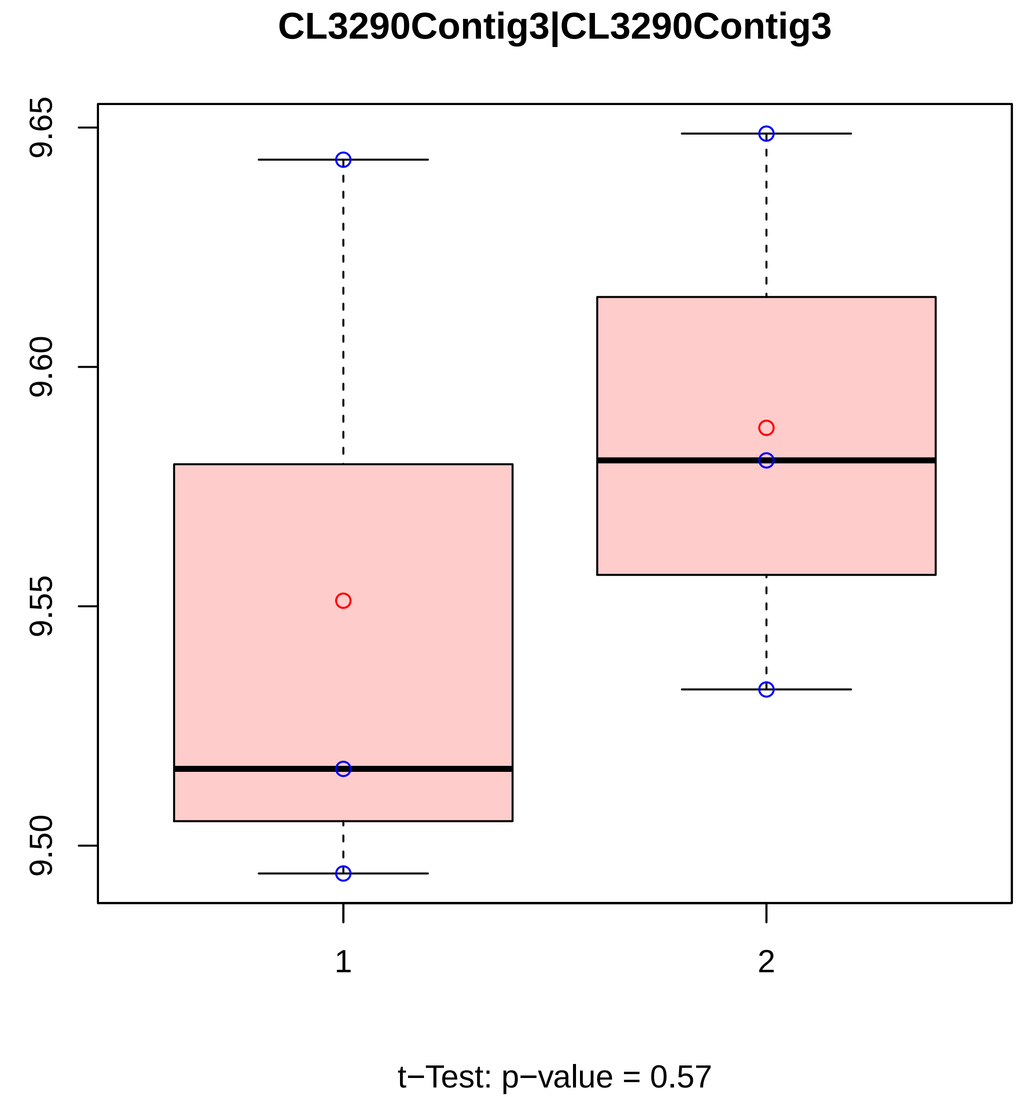
CL3290Contig3|CL3290Contig3
9.65
9.60
9.55
9.50
1
2
t−Test: p−value = 0.57

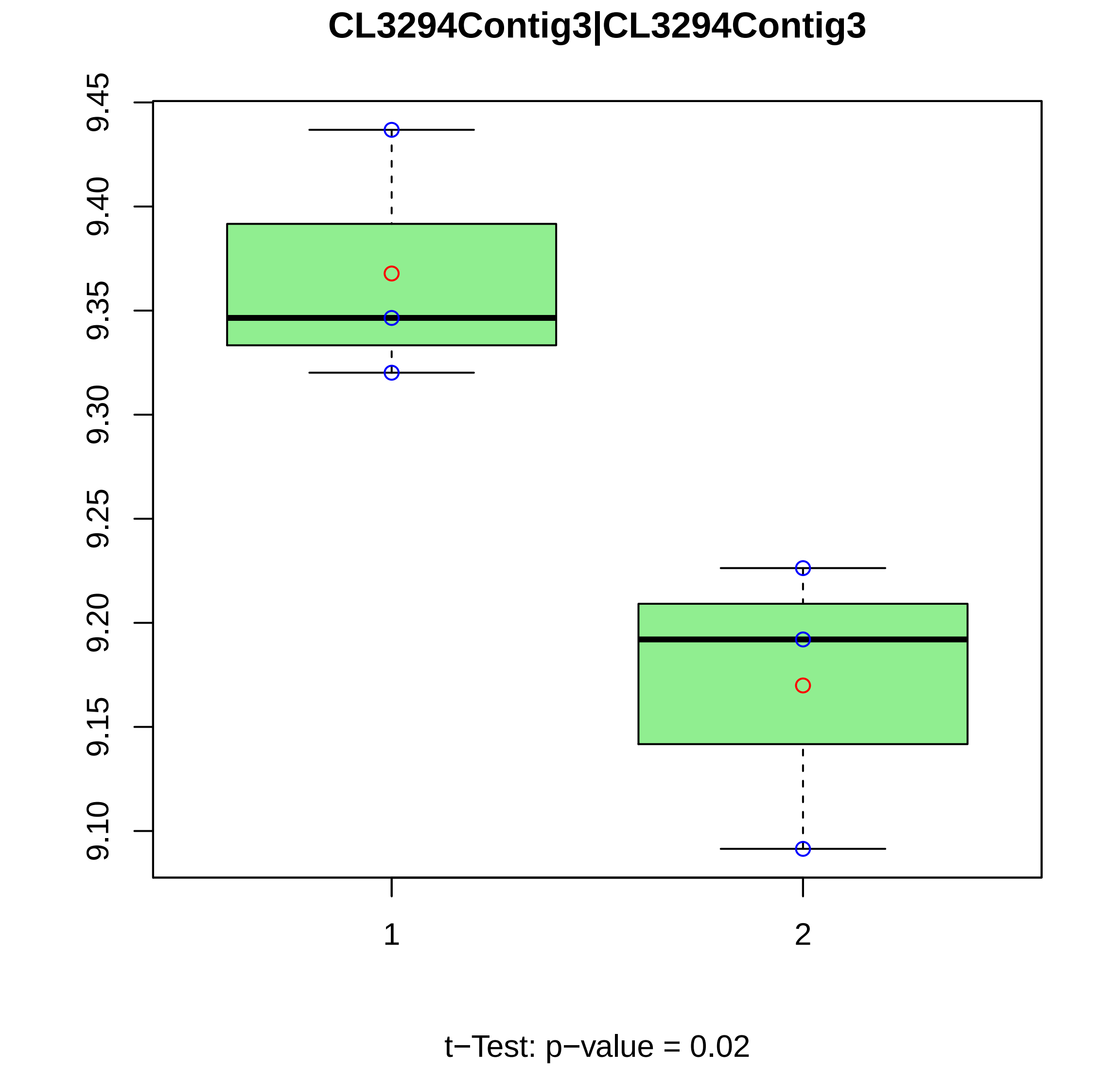
CL3294Contig3|CL3294Contig3
9.45
9.40
9.35
9.30
9.25
9.20
9.15
9.10
1
2
t−Test: p−value = 0.02

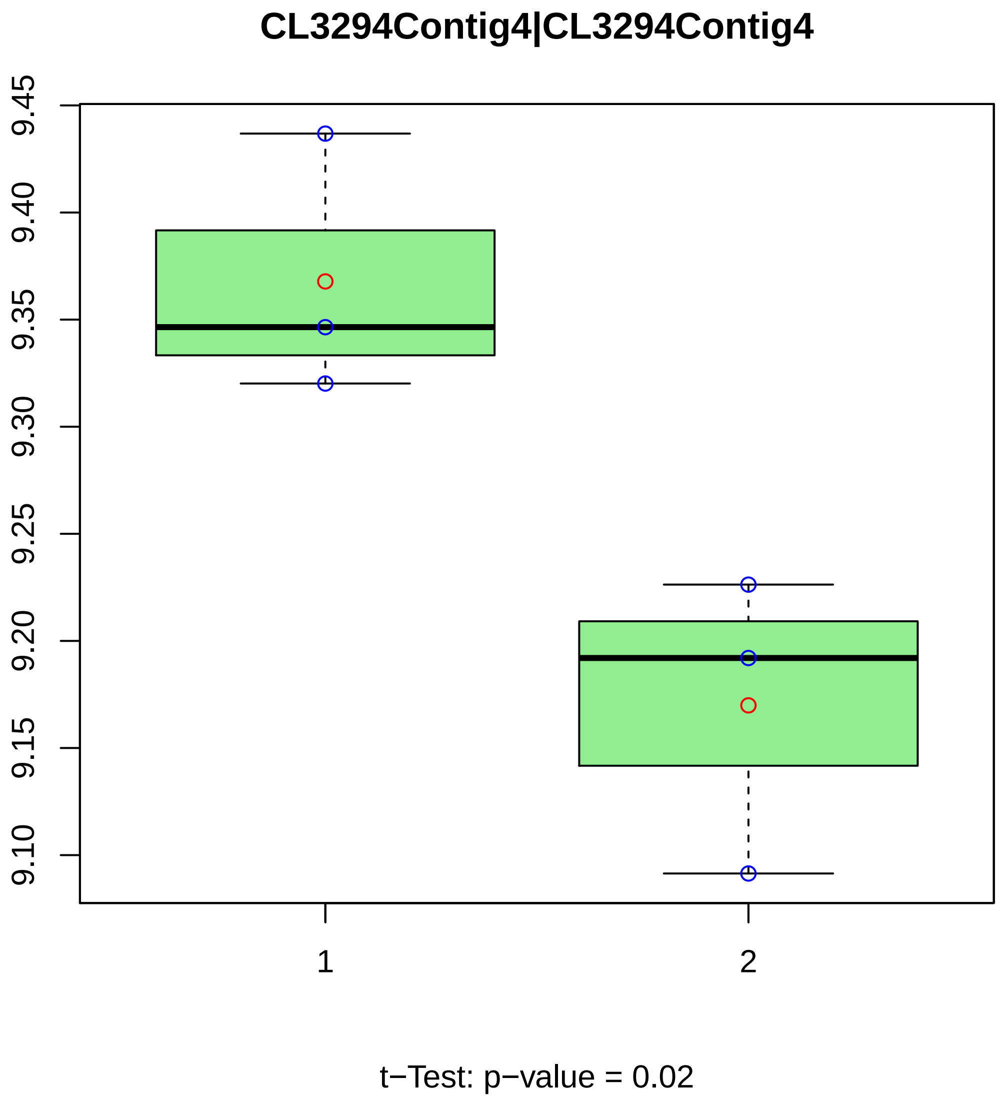
CL3294Contig4|CL3294Contig4
9.45
9.40
9.35
9.30
9.25
9.20
9.15
9.10
1
2
t−Test: p−value = 0.02

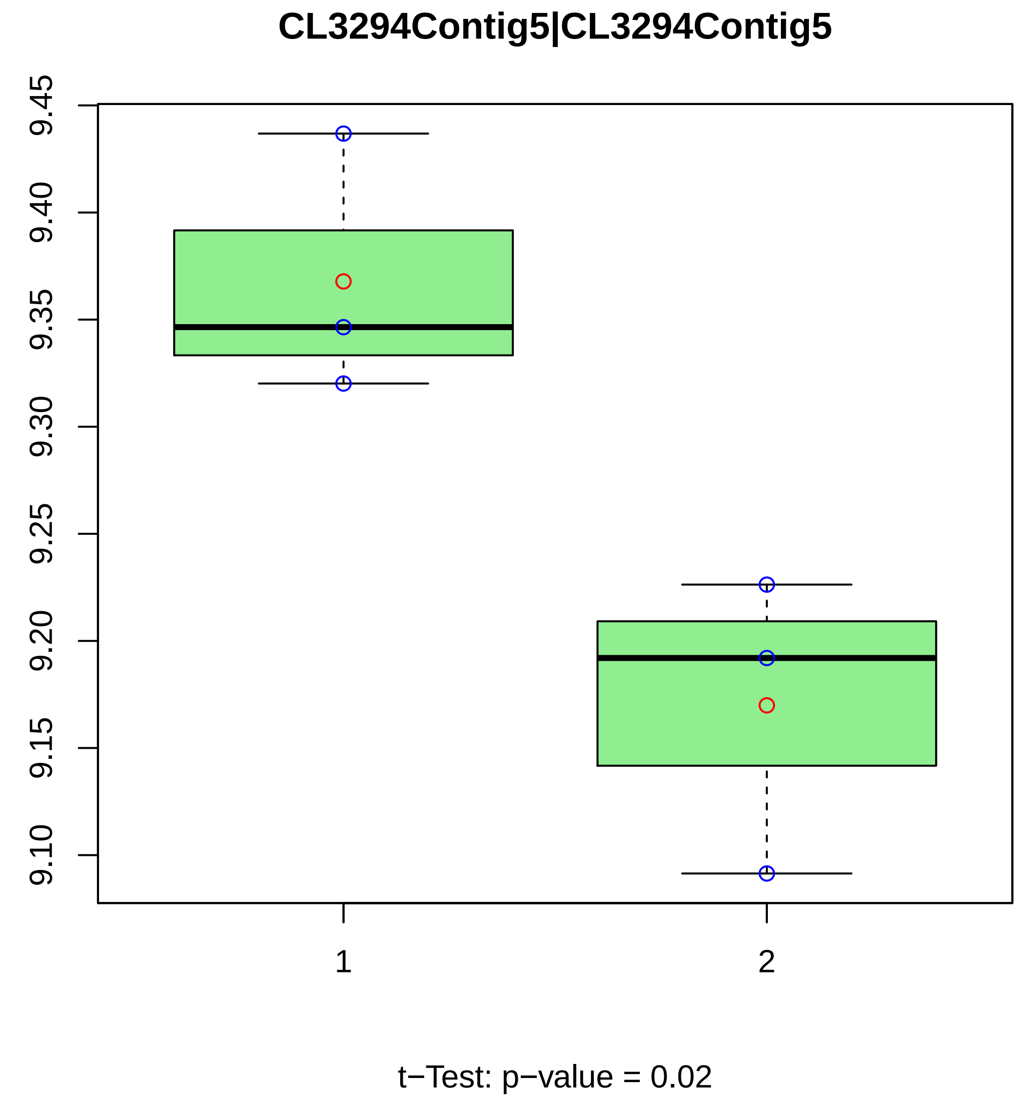

CL3294Contig5|CL3294Contig5
9.45
9.40
9.35
9.30
9.25
9.20
9.15
9.10
1
2
t−Test: p−value = 0.02

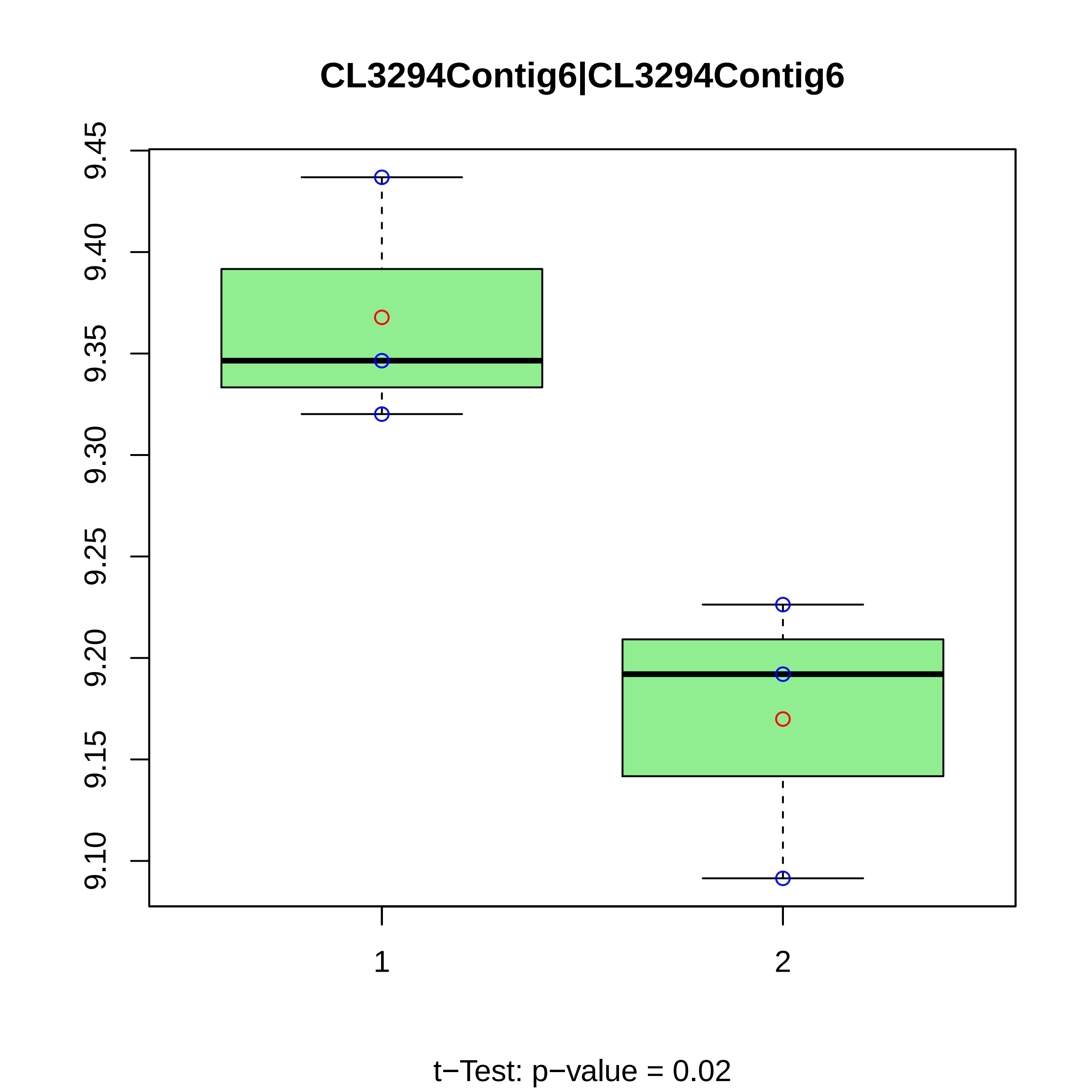

CL3294Contig6|CL3294Contig6
9.45
9.40
9.35
9.30
9.25
9.20
9.15
9.10
1
2
t−Test: p−value = 0.02

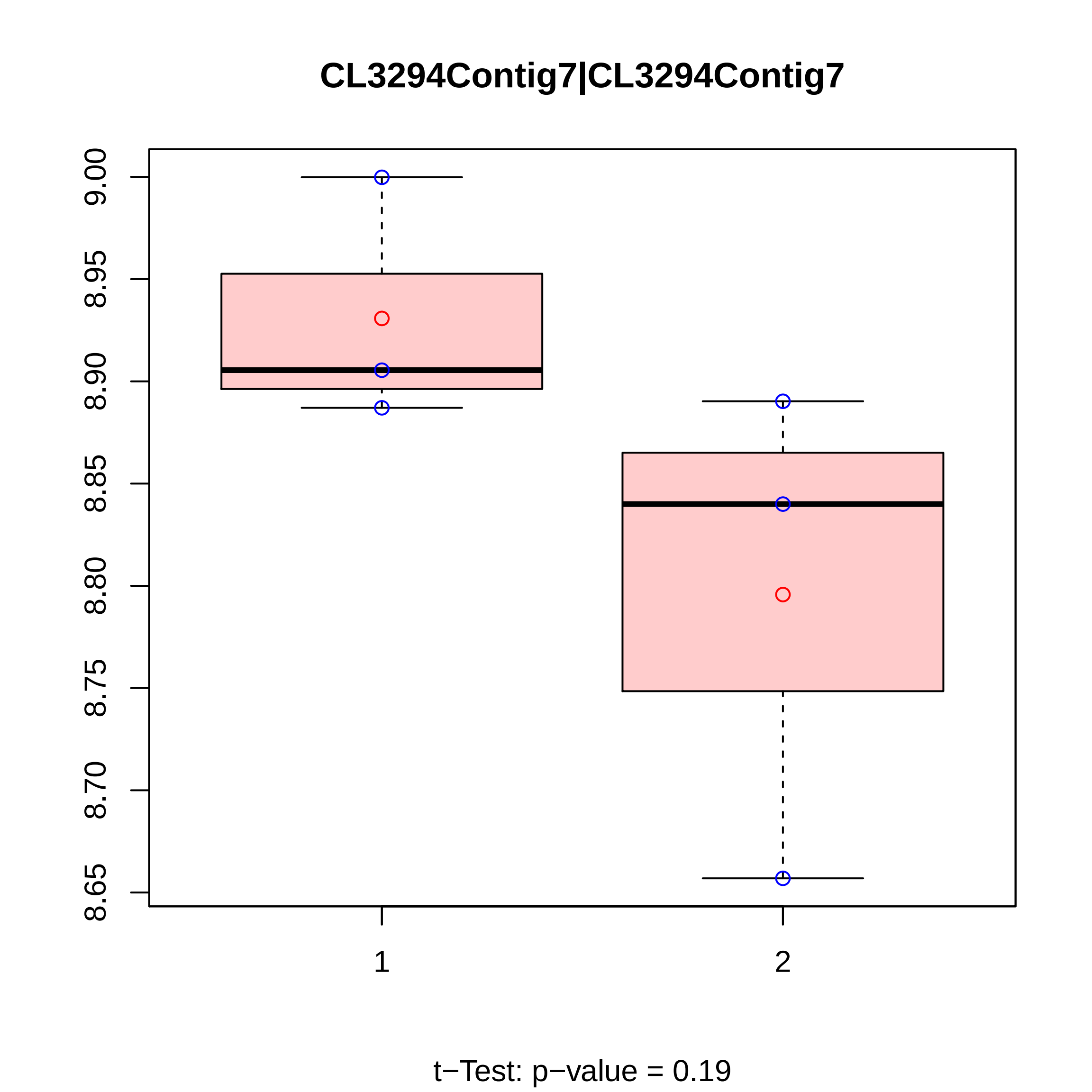
CL3294Contig7|CL3294Contig7
9.00
8.95
8.90
8.85
8.80
8.75
8.70
8.65
1
2
t−Test: p−value = 0.19

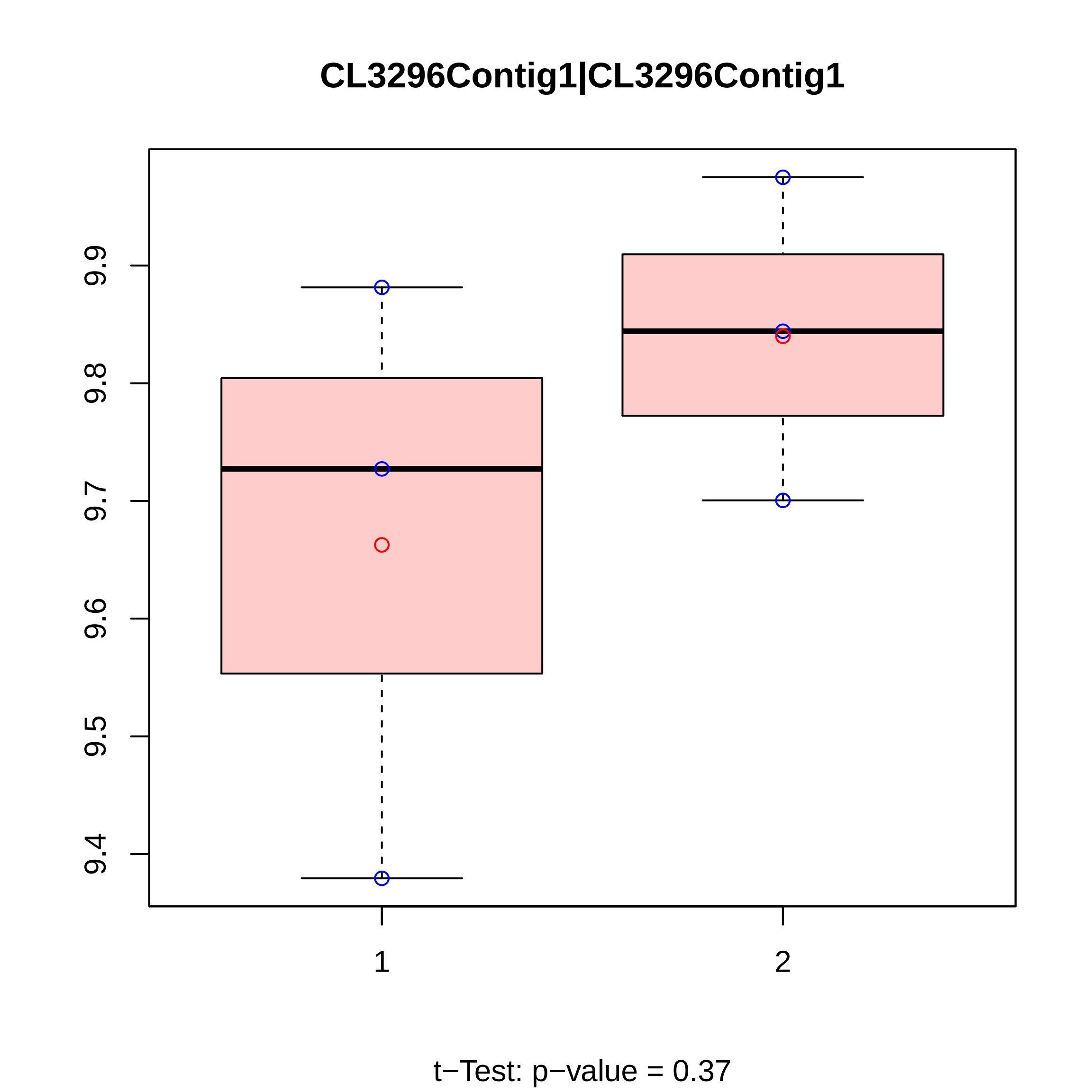
CL3296Contig1|CL3296Contig1
9.4
9.5
9.6
9.7
9.8
9.9
1
2
t−Test: p−value = 0.37

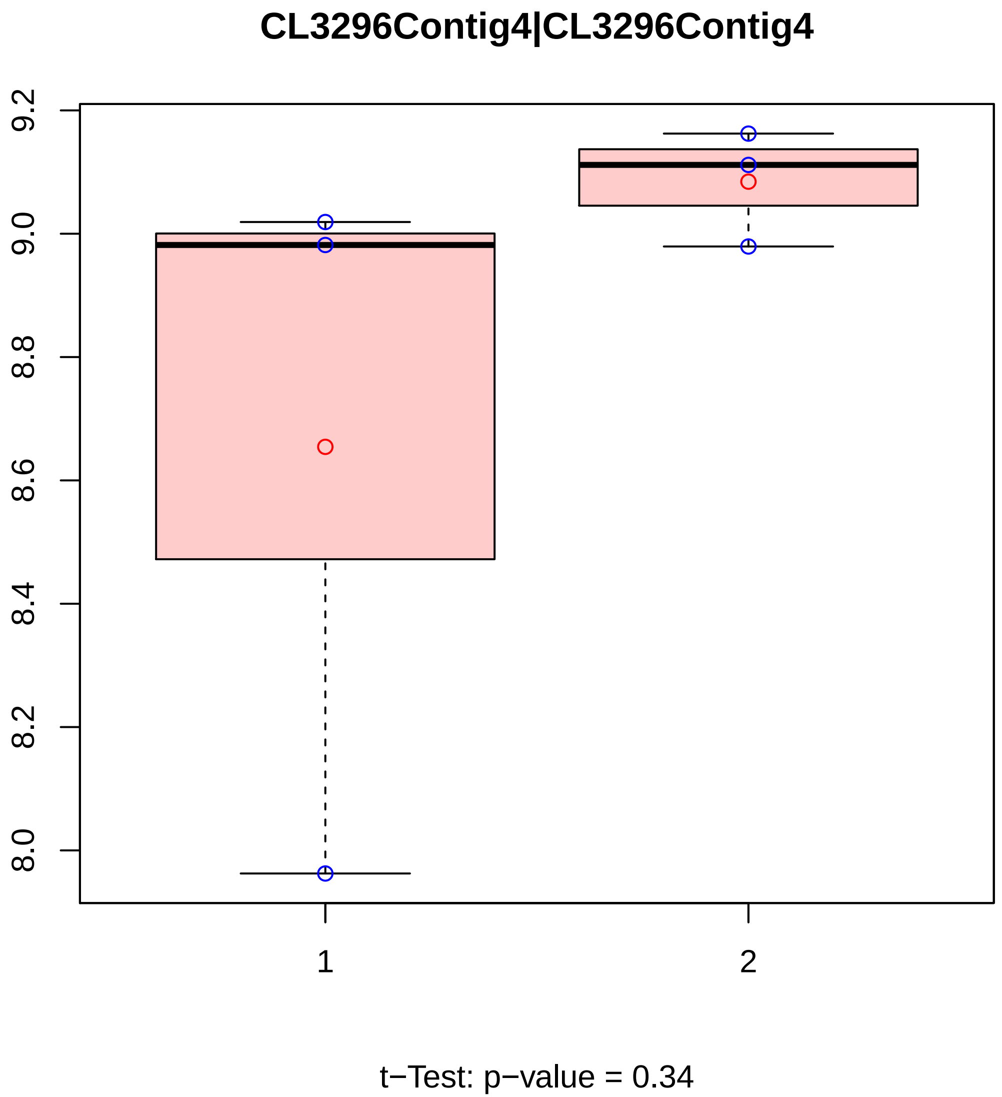

CL3296Contig4|CL3296Contig4
9.2
9.0
8.8
8.6
8.4
8.2
8.0
1
2
t−Test: p−value = 0.34

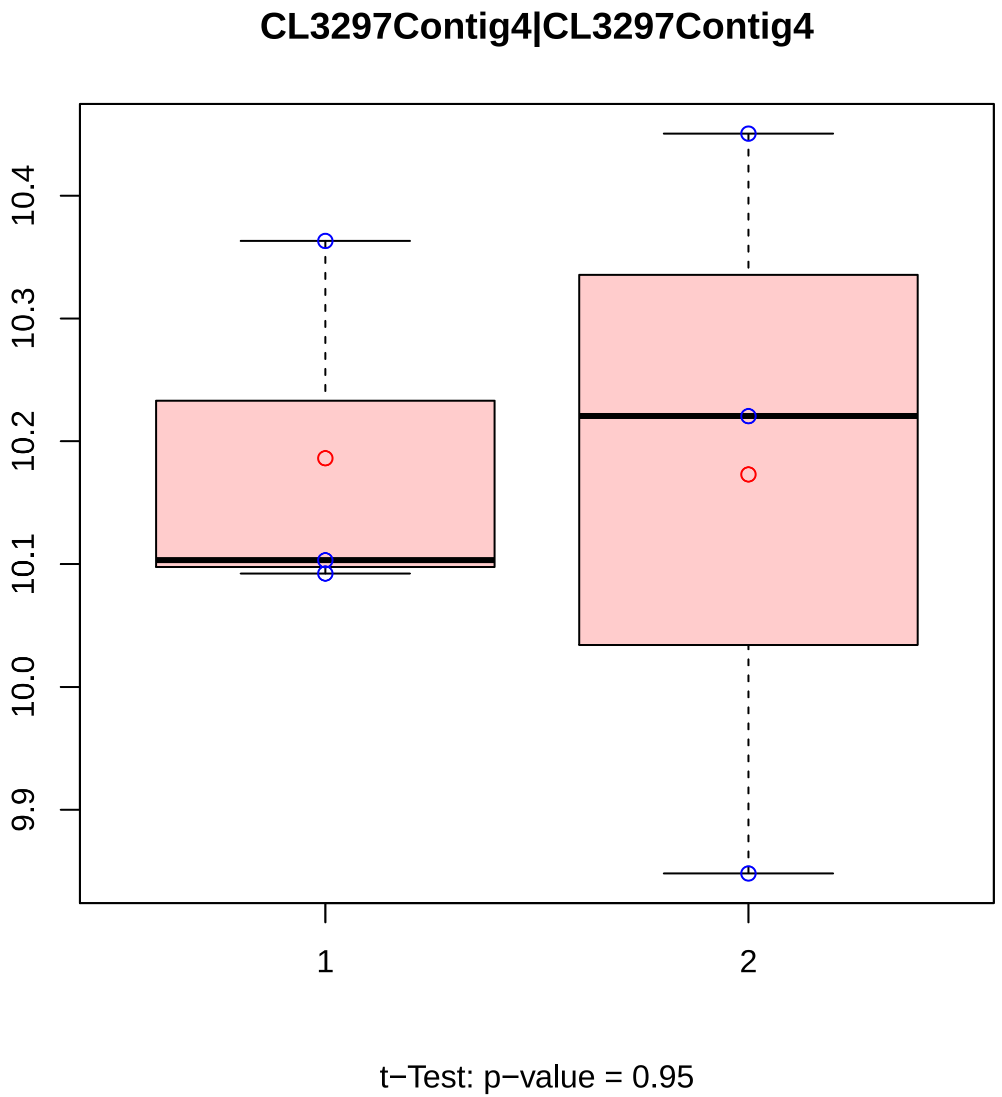
CL3297Contig4|CL3297Contig4
10.4
10.3
10.2
10.1
10.0
9.9
1
2
t−Test: p−value = 0.95

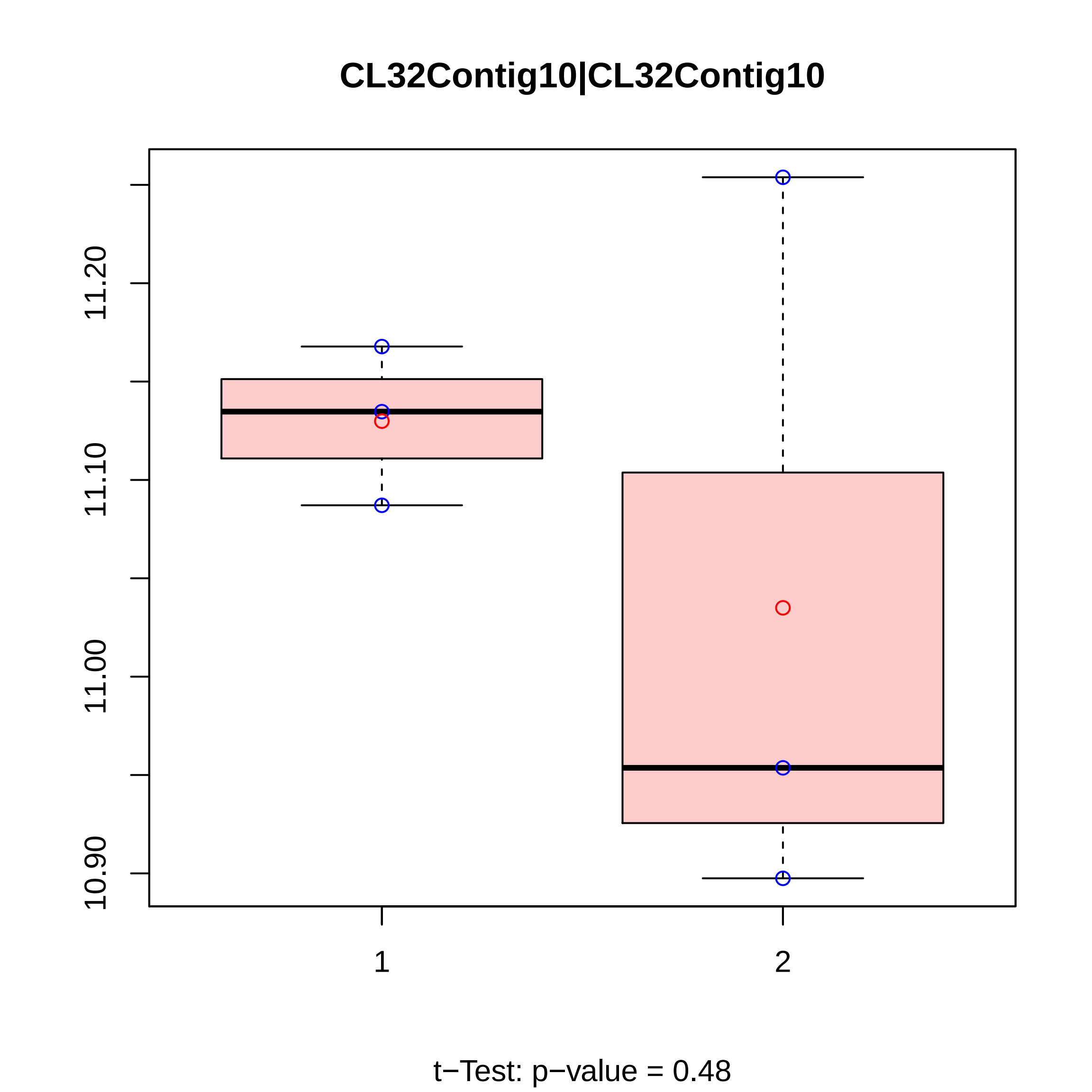

CL32Contig10|CL32Contig10
11.20
11.10
11.00
10.90
1
2
t−Test: p−value = 0.48

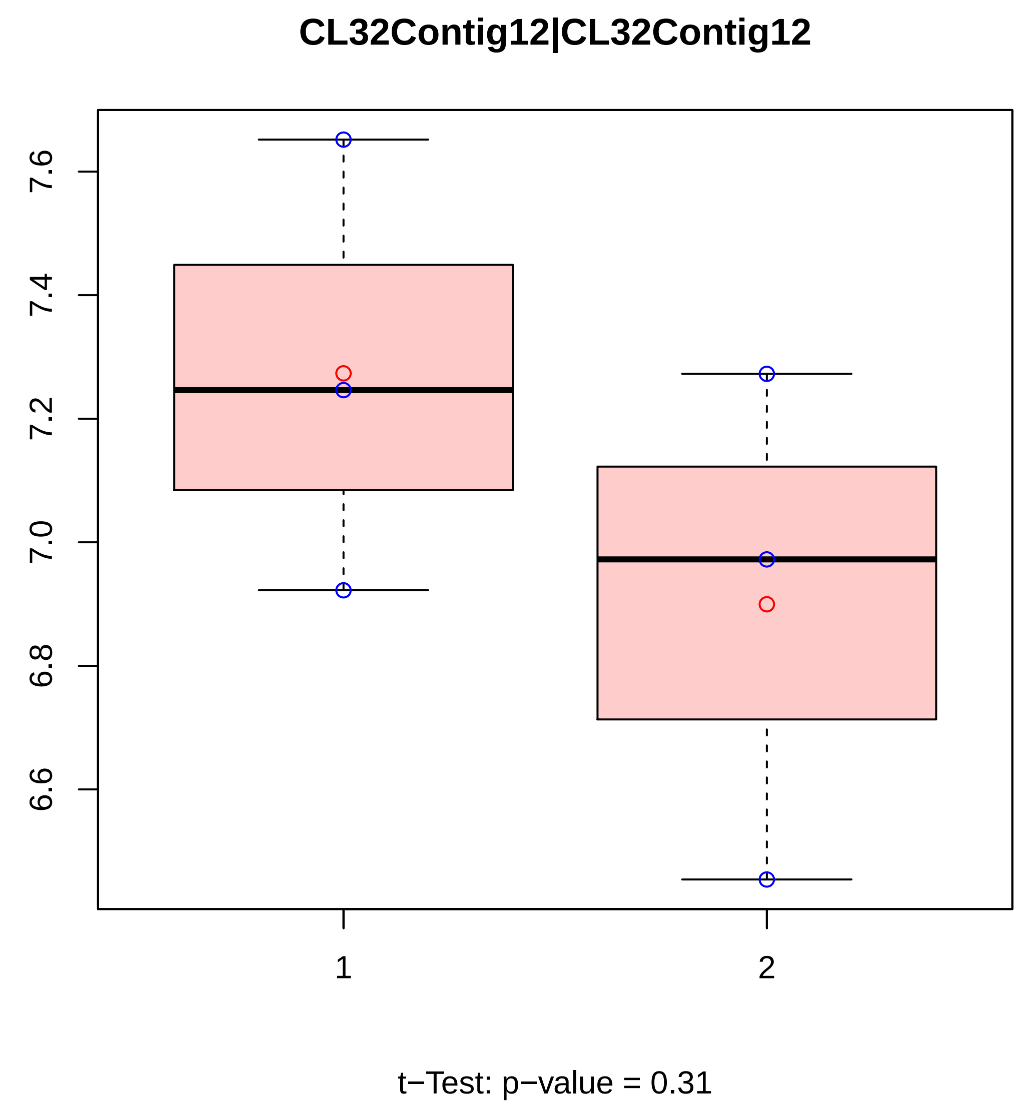

CL32Contig12|CL32Contig12
7.6
7.4
7.2
7.0
6.8
6.6
1
2
t−Test: p−value = 0.31

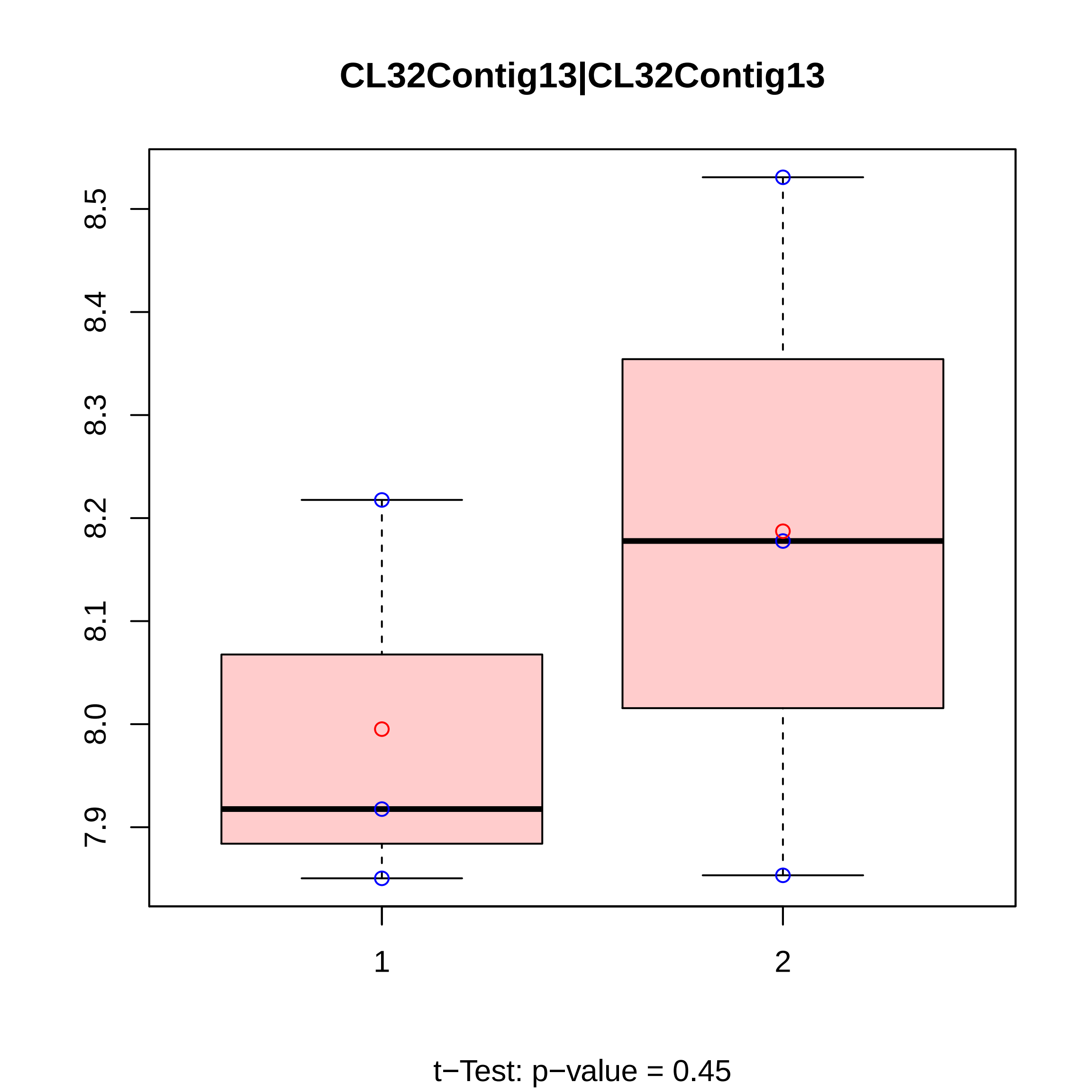

CL32Contig13|CL32Contig13
8.5
8.4
8.3
8.2
8.1
8.0
7.9
1
2
t−Test: p−value = 0.45

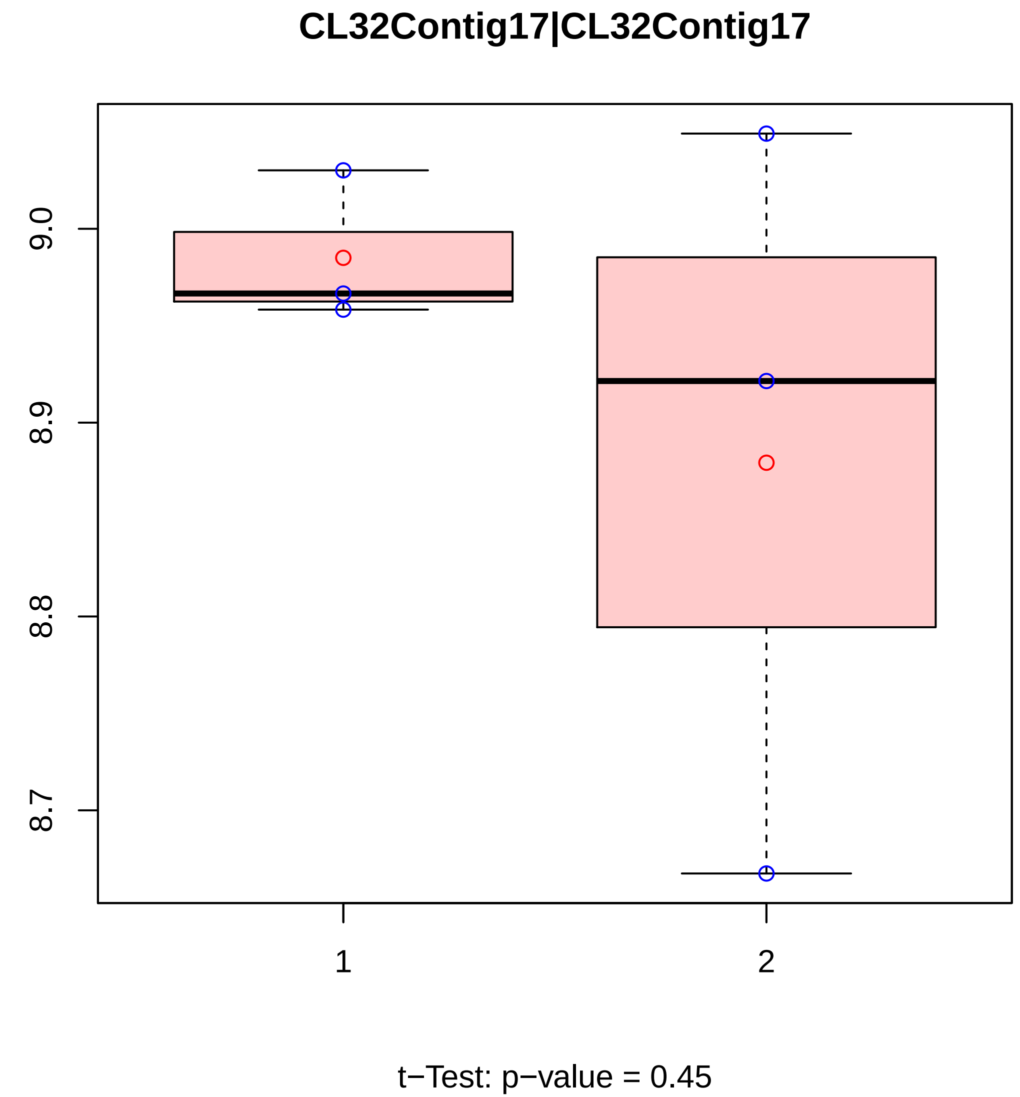
CL32Contig17|CL32Contig17
9.0
8.9
8.8
8.7
1
2
t−Test: p−value = 0.45

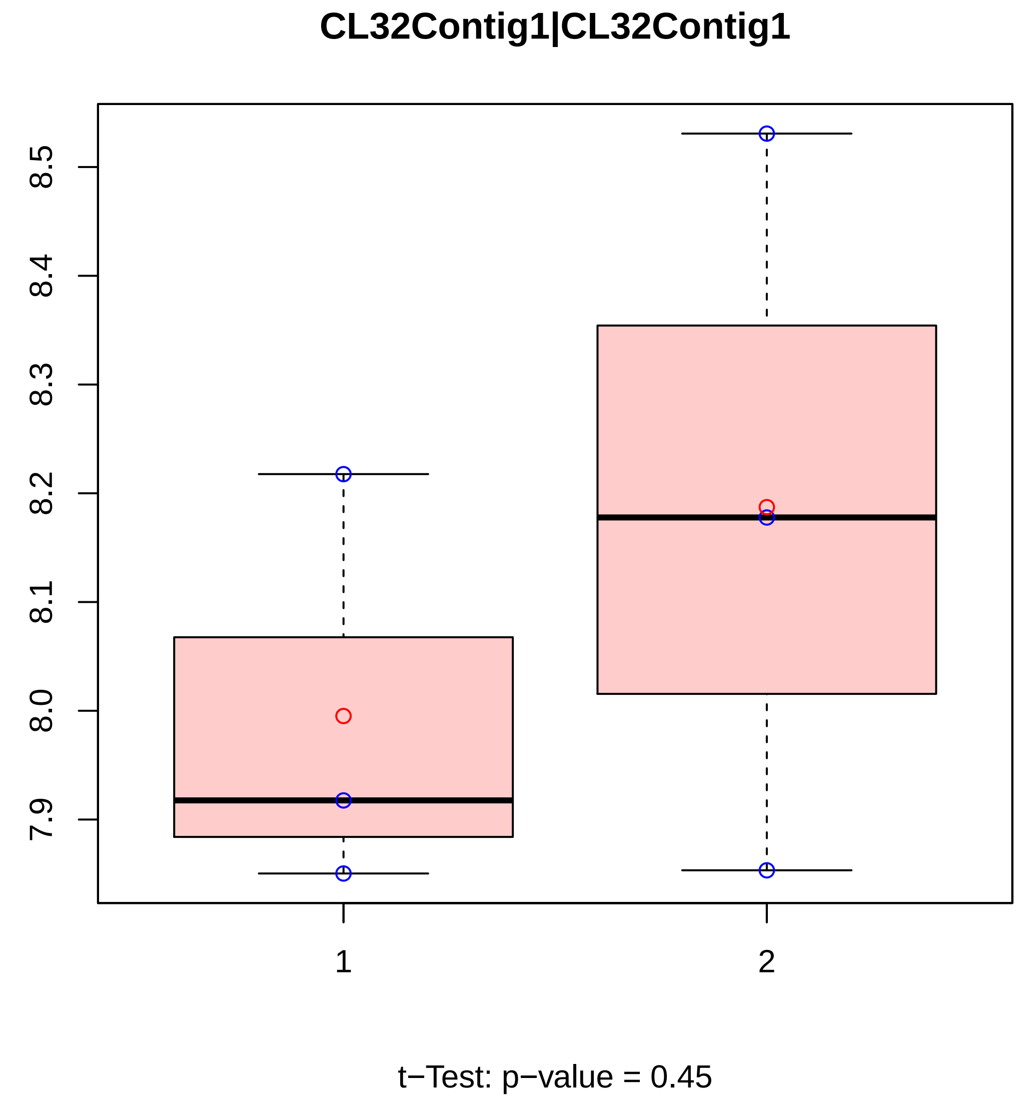

CL32Contig1|CL32Contig1
8.5
8.4
8.3
8.2
8.1
8.0
7.9
1
2
t−Test: p−value = 0.45

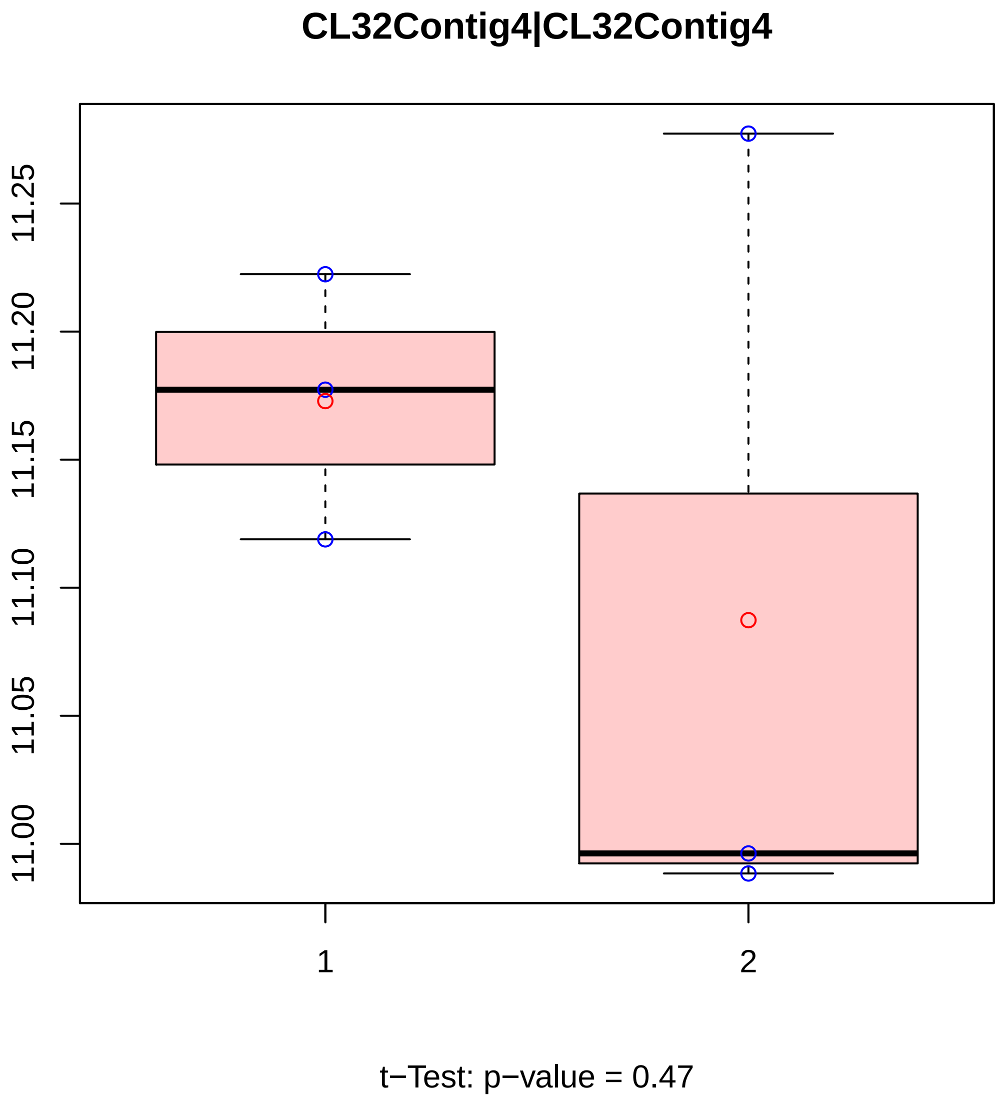

CL32Contig4|CL32Contig4
11.00
11.05
11.10
11.15
11.20
11.25
1
2
t−Test: p−value = 0.47

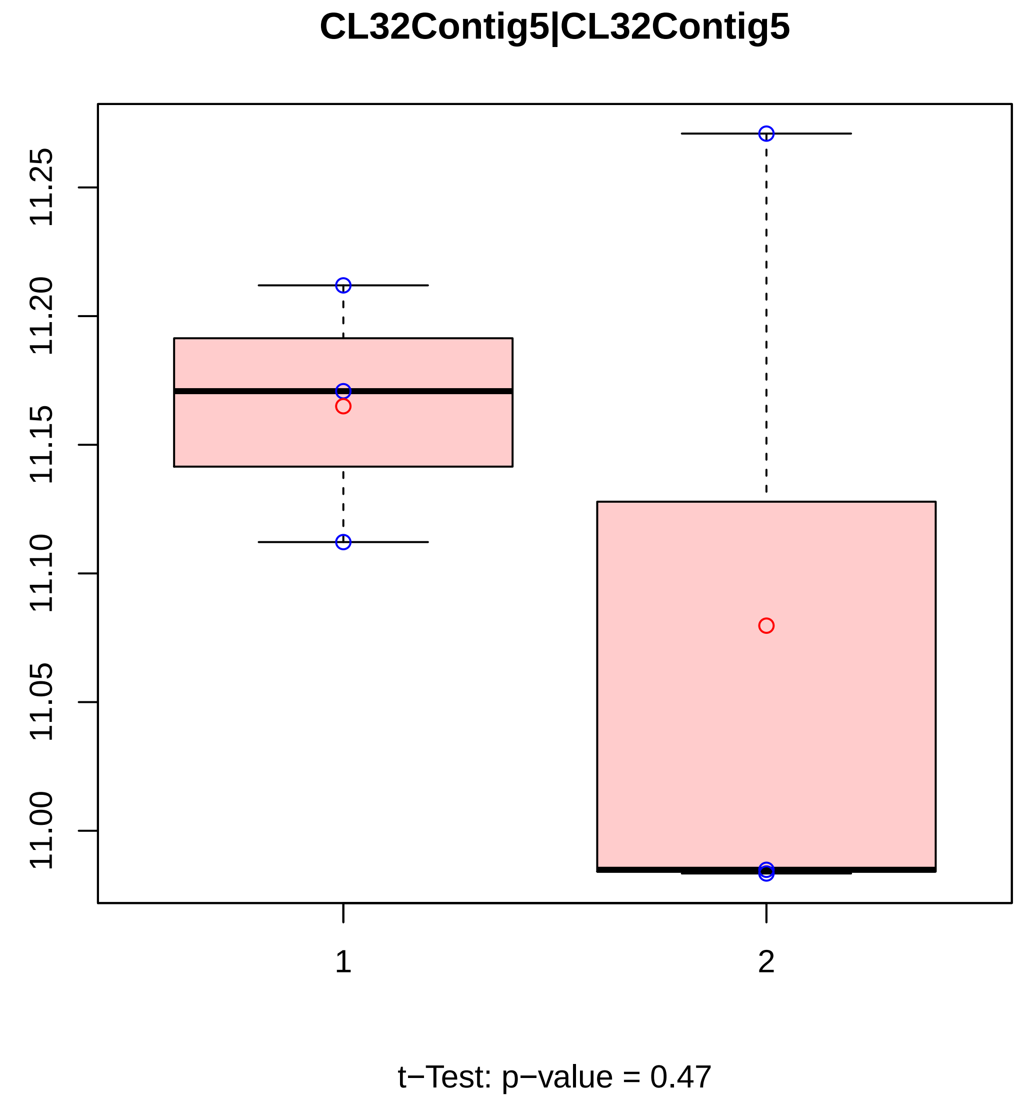
CL32Contig5|CL32Contig5
11.25
11.20
11.15
11.10
11.05
11.00
1
2
t−Test: p−value = 0.47

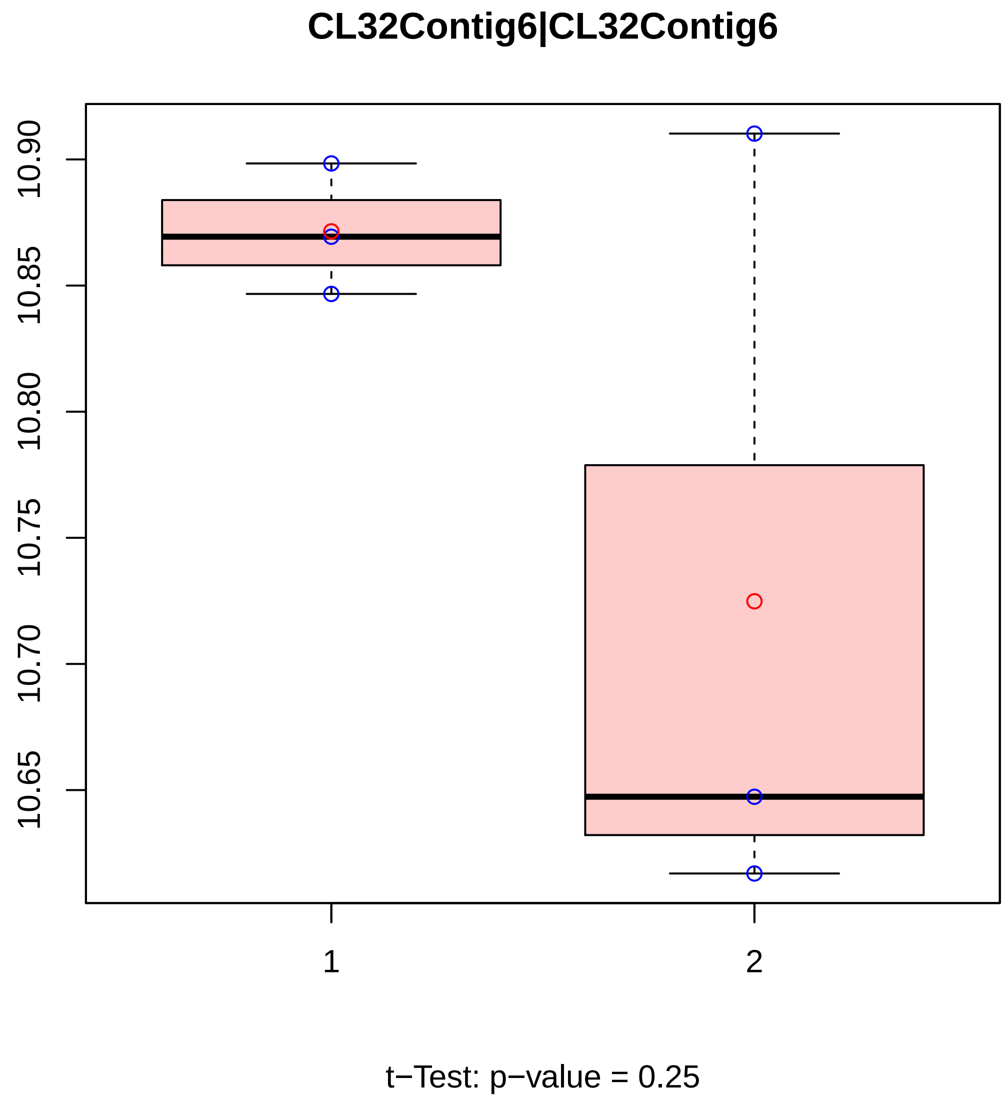
CL32Contig6|CL32Contig6
10.90
10.85
10.80
10.75
10.70
10.65
1
2
t−Test: p−value = 0.25

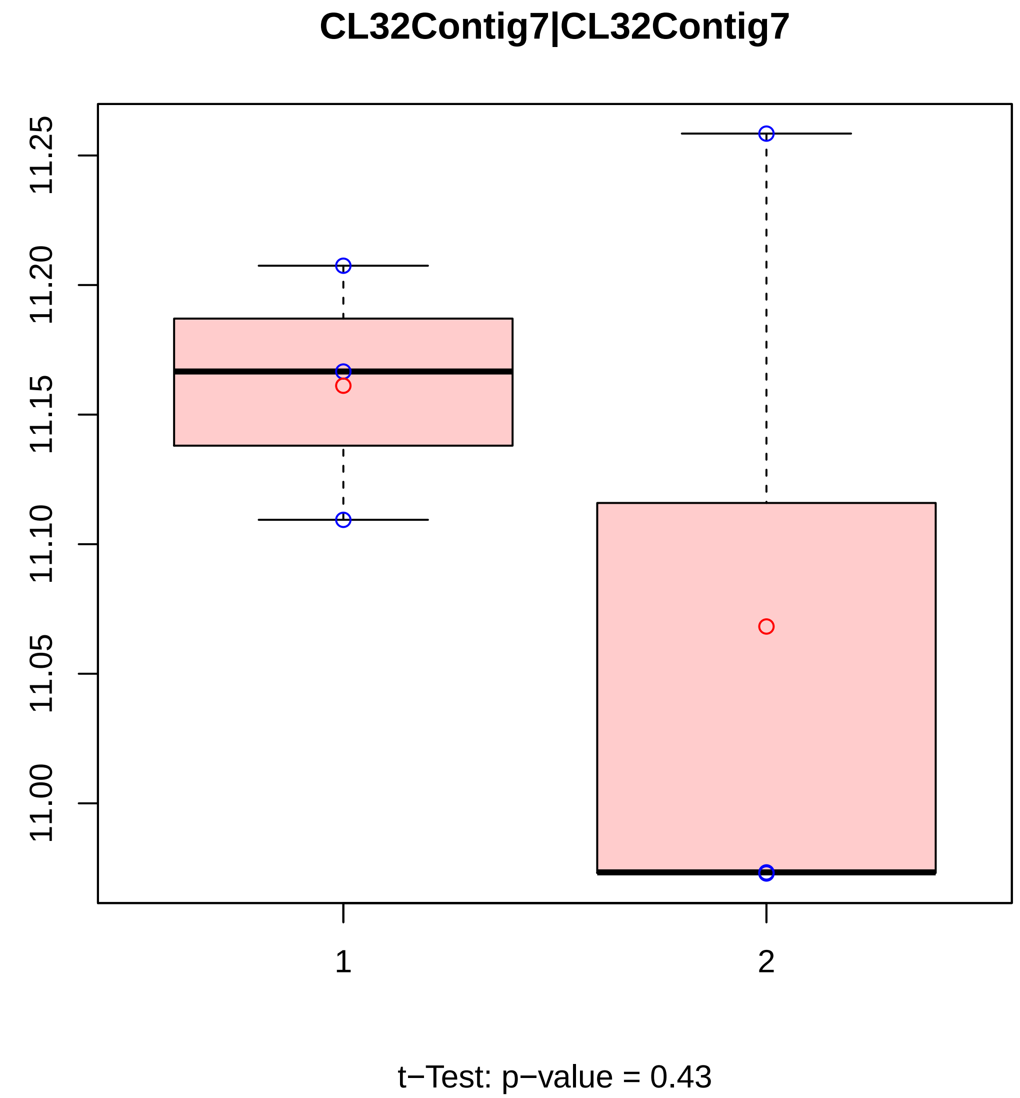
CL32Contig7|CL32Contig7
11.25
11.20
11.15
11.10
11.05
11.00
1
2
t−Test: p−value = 0.43

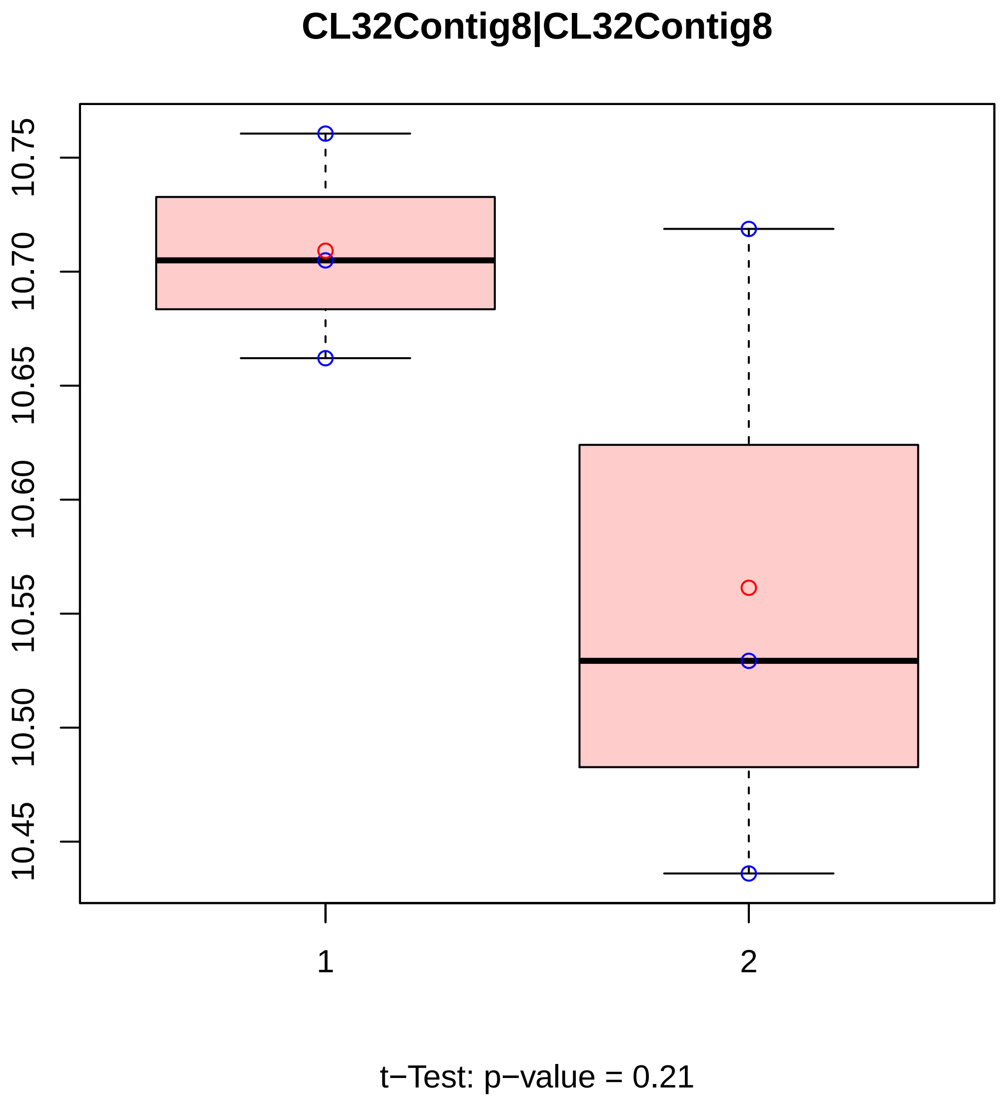

CL32Contig8|CL32Contig8
10.45
10.50
10.55
10.60
10.65
10.70
10.75
1
2
t−Test: p−value = 0.21

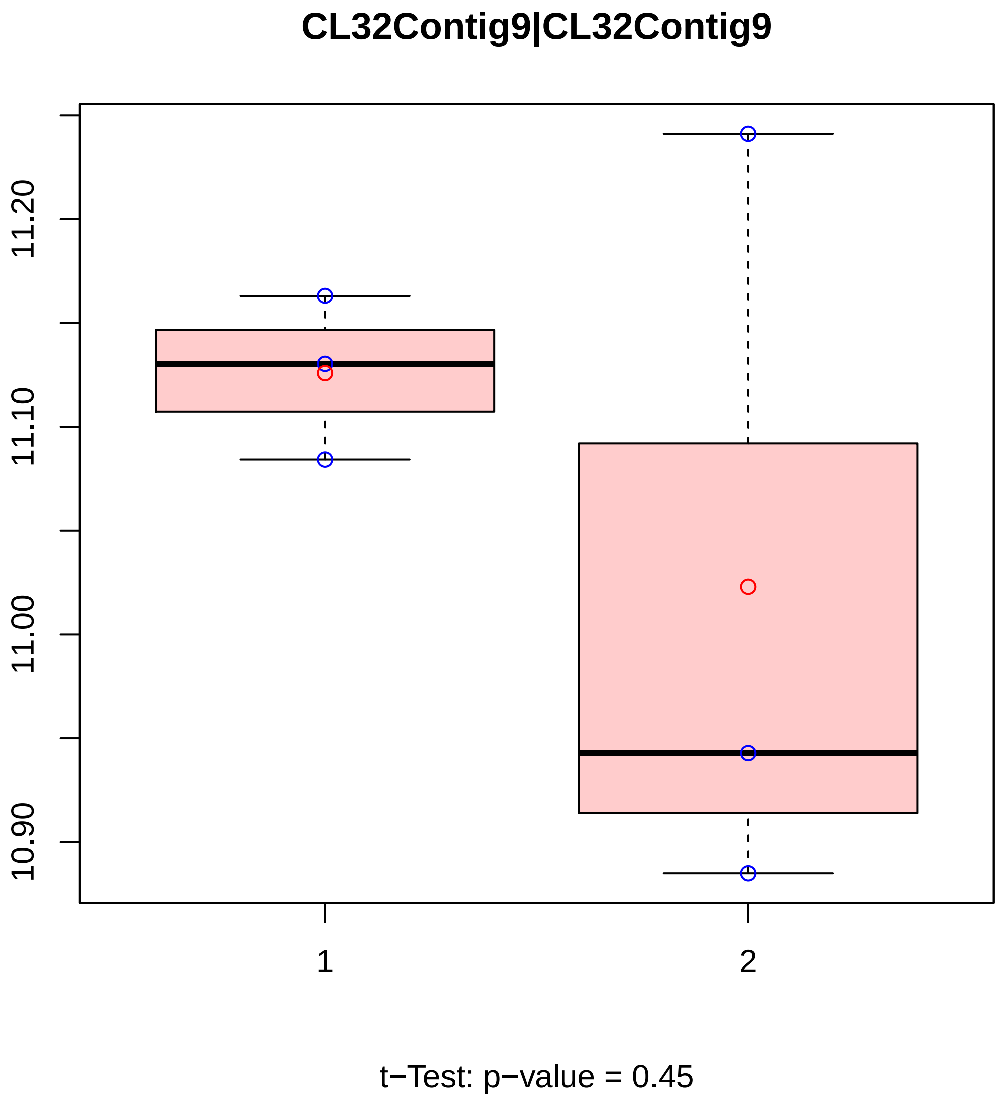
CL32Contig9|CL32Contig9
11.20
11.10
11.00
10.90
1
2
t−Test: p−value = 0.45

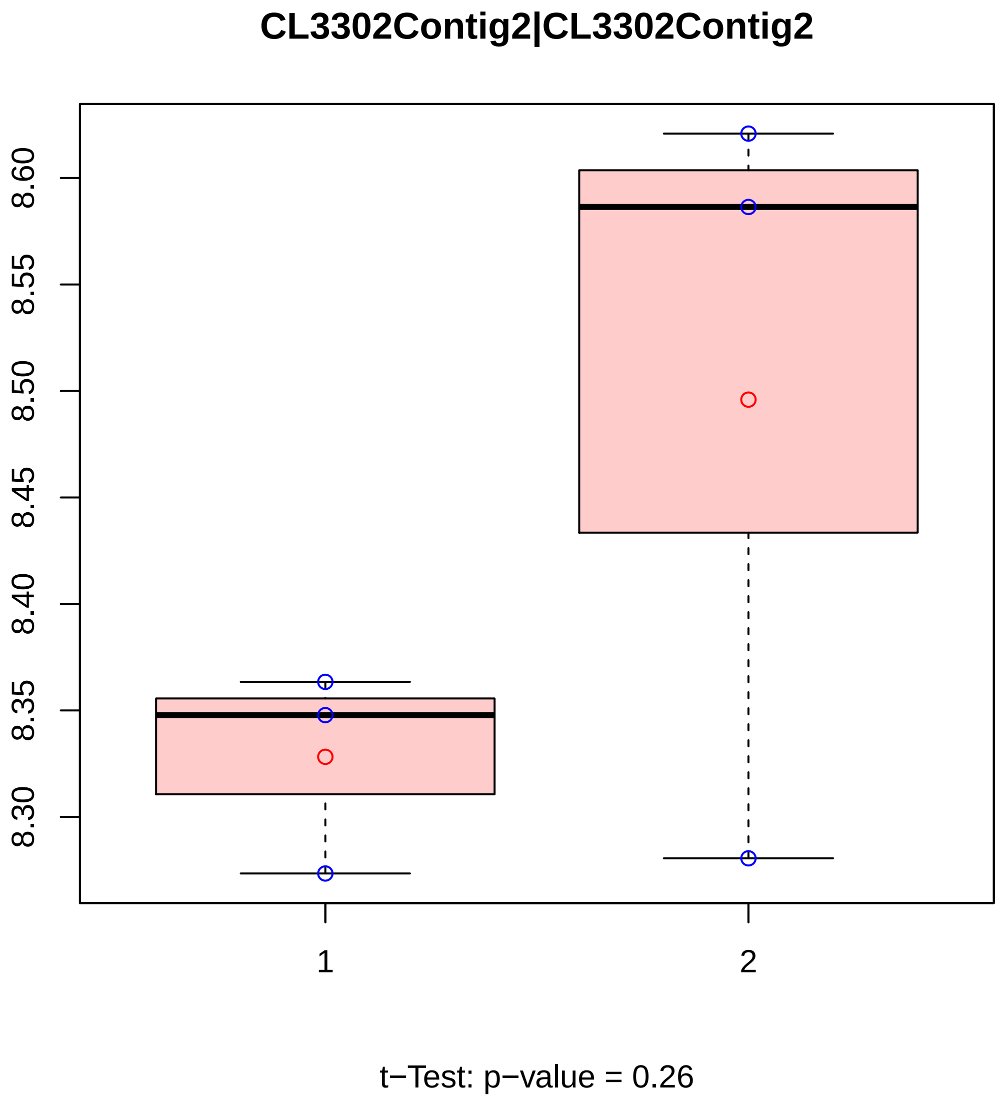

CL3302Contig2|CL3302Contig2
8.30
8.35
8.40
8.45
8.50
8.55
8.60
1
2
t−Test: p−value = 0.26

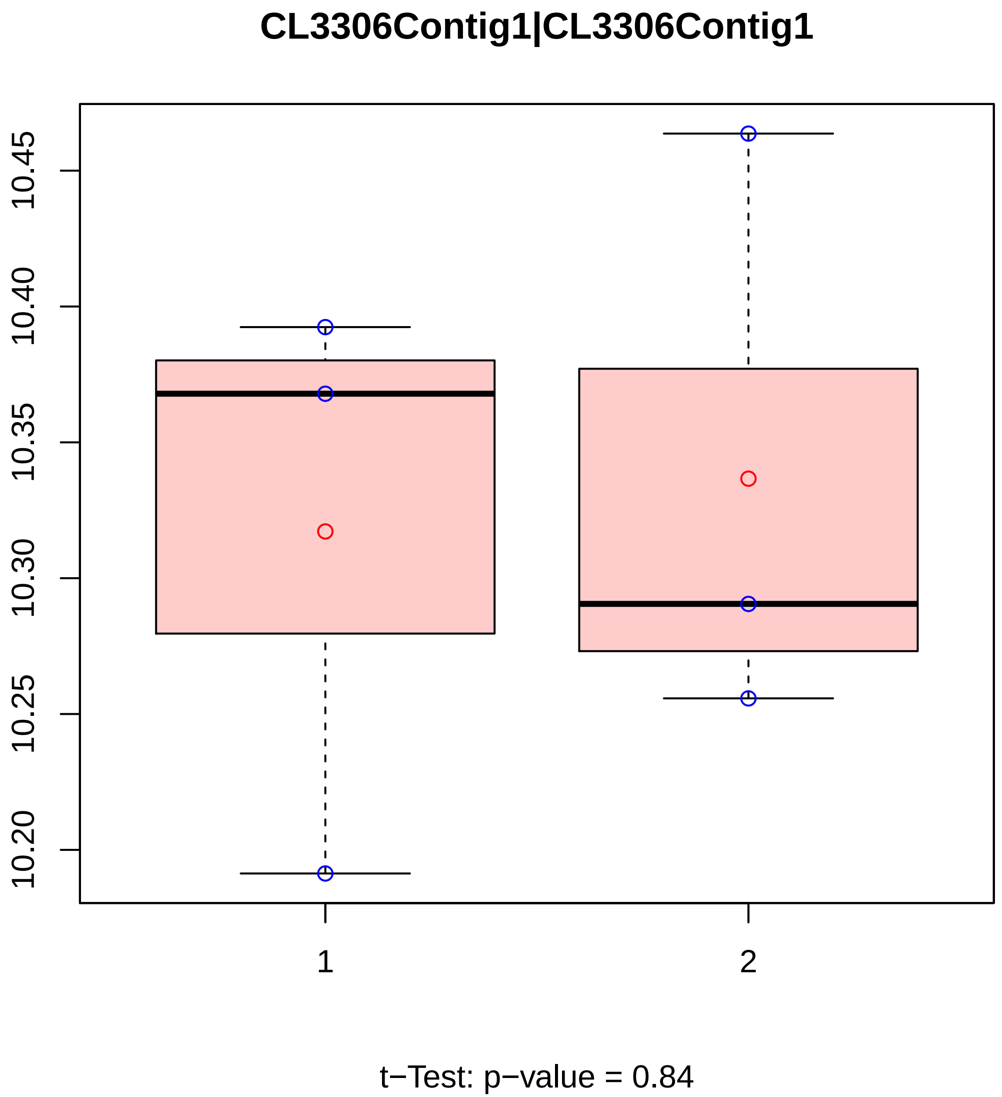

CL3306Contig1|CL3306Contig1
10.20
10.25
10.30
10.35
10.40
10.45
1
2
t−Test: p−value = 0.84

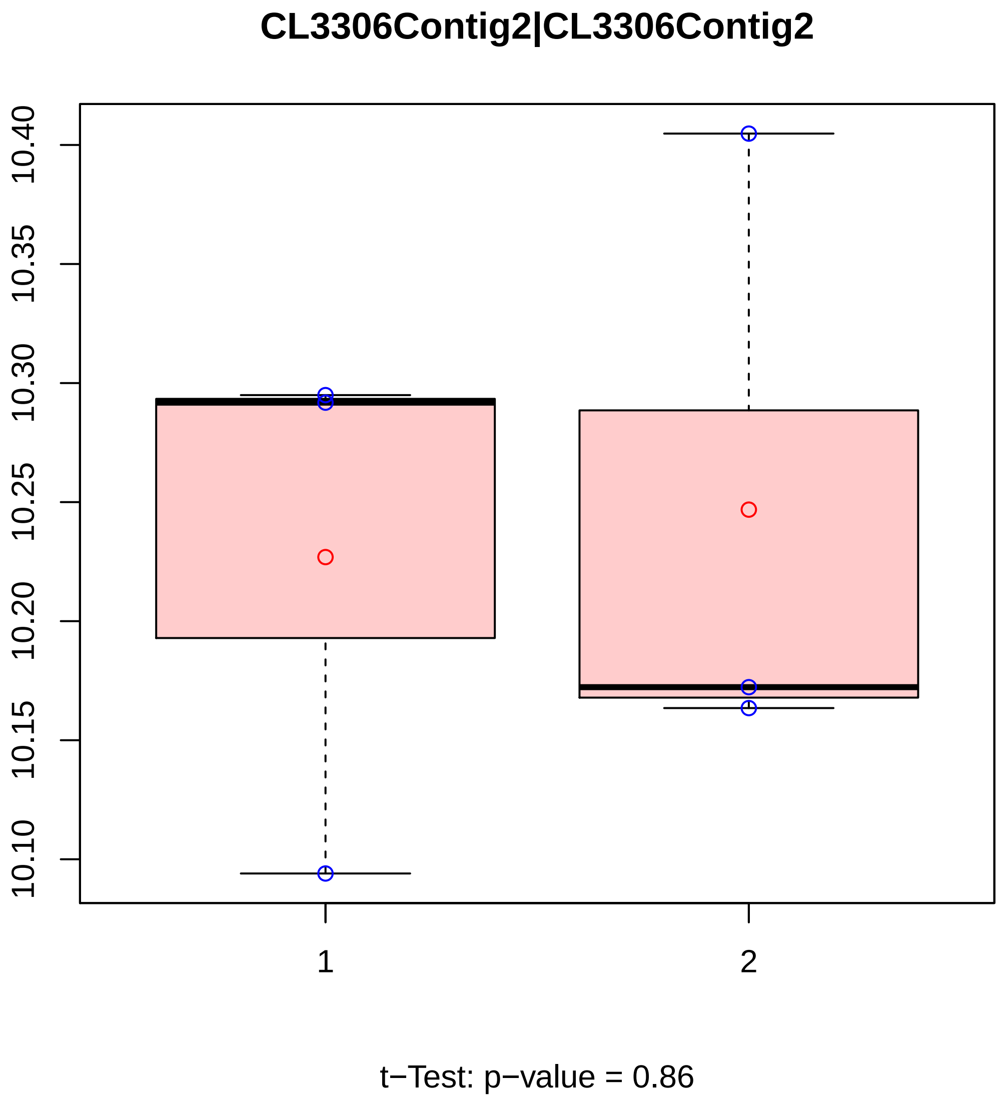

CL3306Contig2|CL3306Contig2
10.10
10.15
10.20
10.25
10.30
10.35
10.40
1
2
t−Test: p−value = 0.86

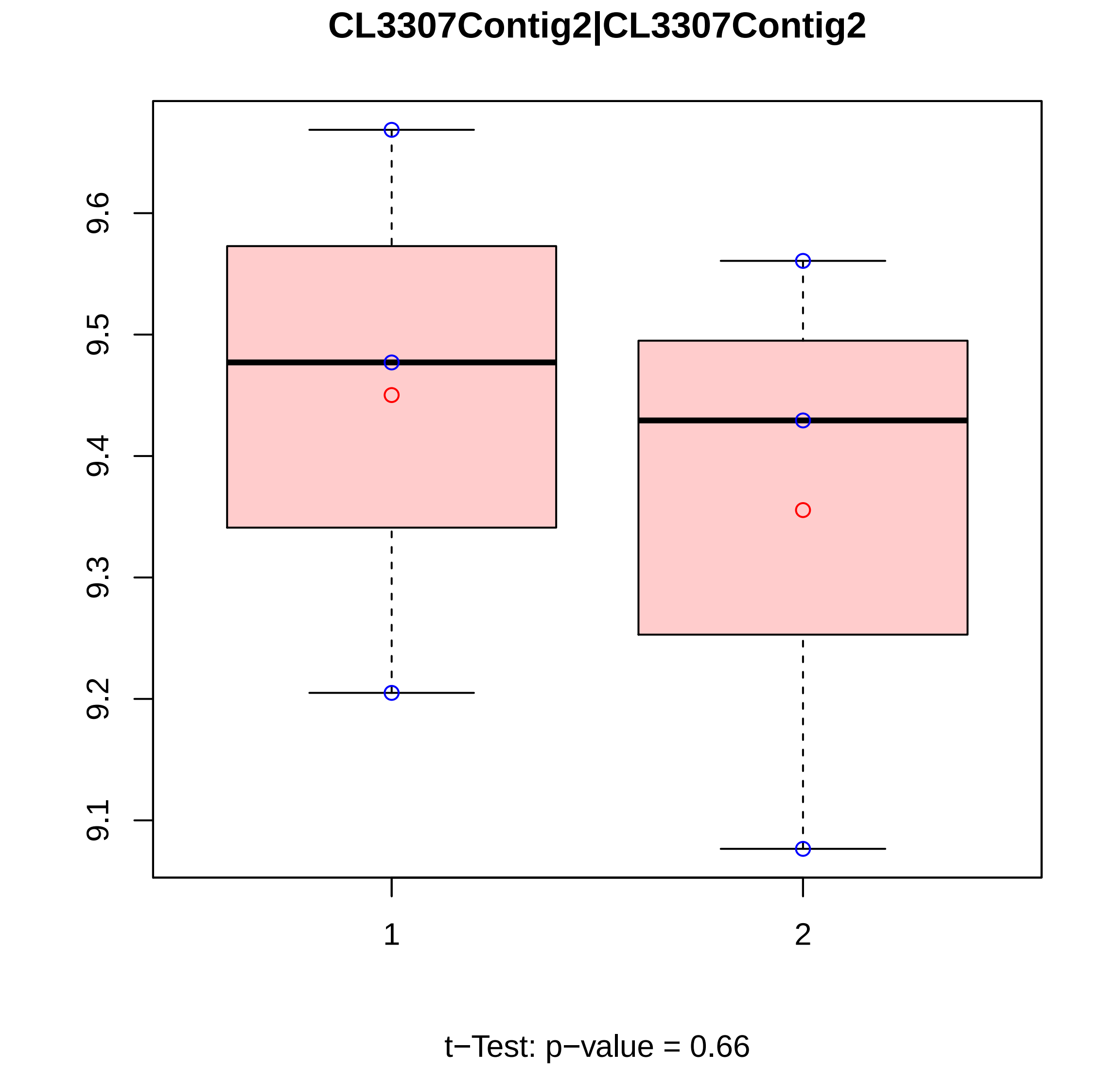
CL3307Contig2|CL3307Contig2
9.1
9.2
9.3
9.4
9.5
9.6
1
2
t−Test: p−value = 0.66

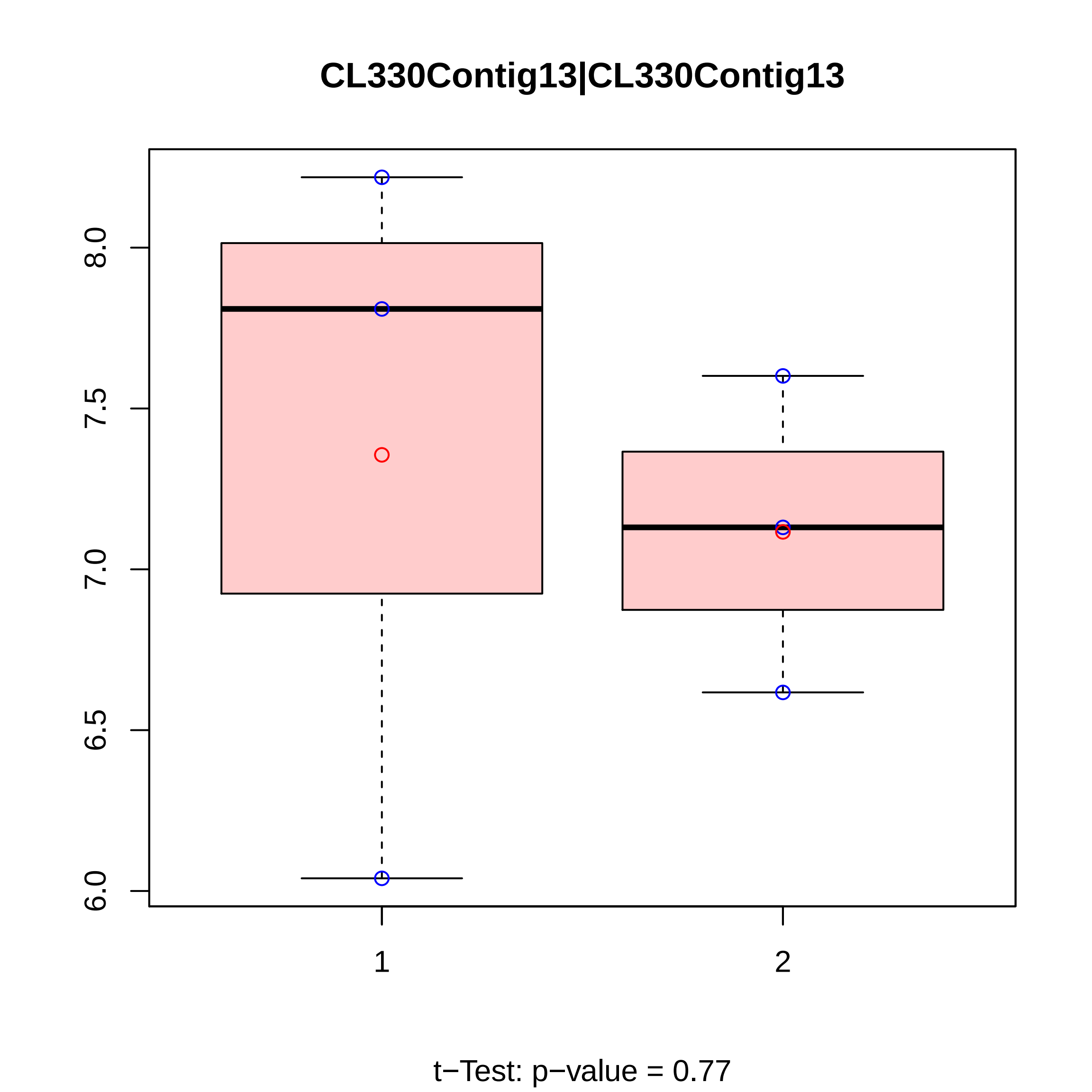

CL330Contig13|CL330Contig13
8.0
7.5
7.0
6.5
6.0
1
2
t−Test: p−value = 0.77

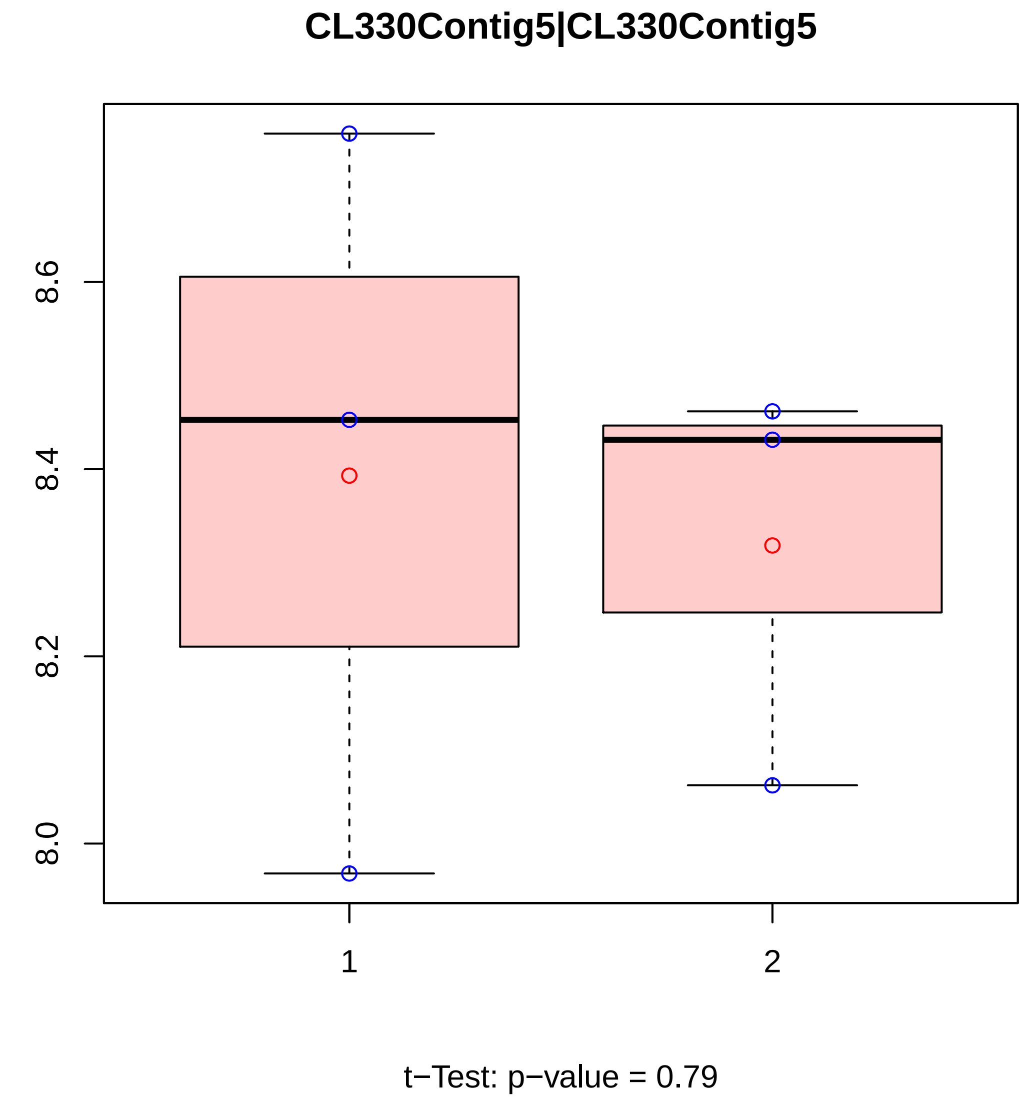
CL330Contig5|CL330Contig5
8.0
8.2
8.4
8.6
1
2
t−Test: p−value = 0.79

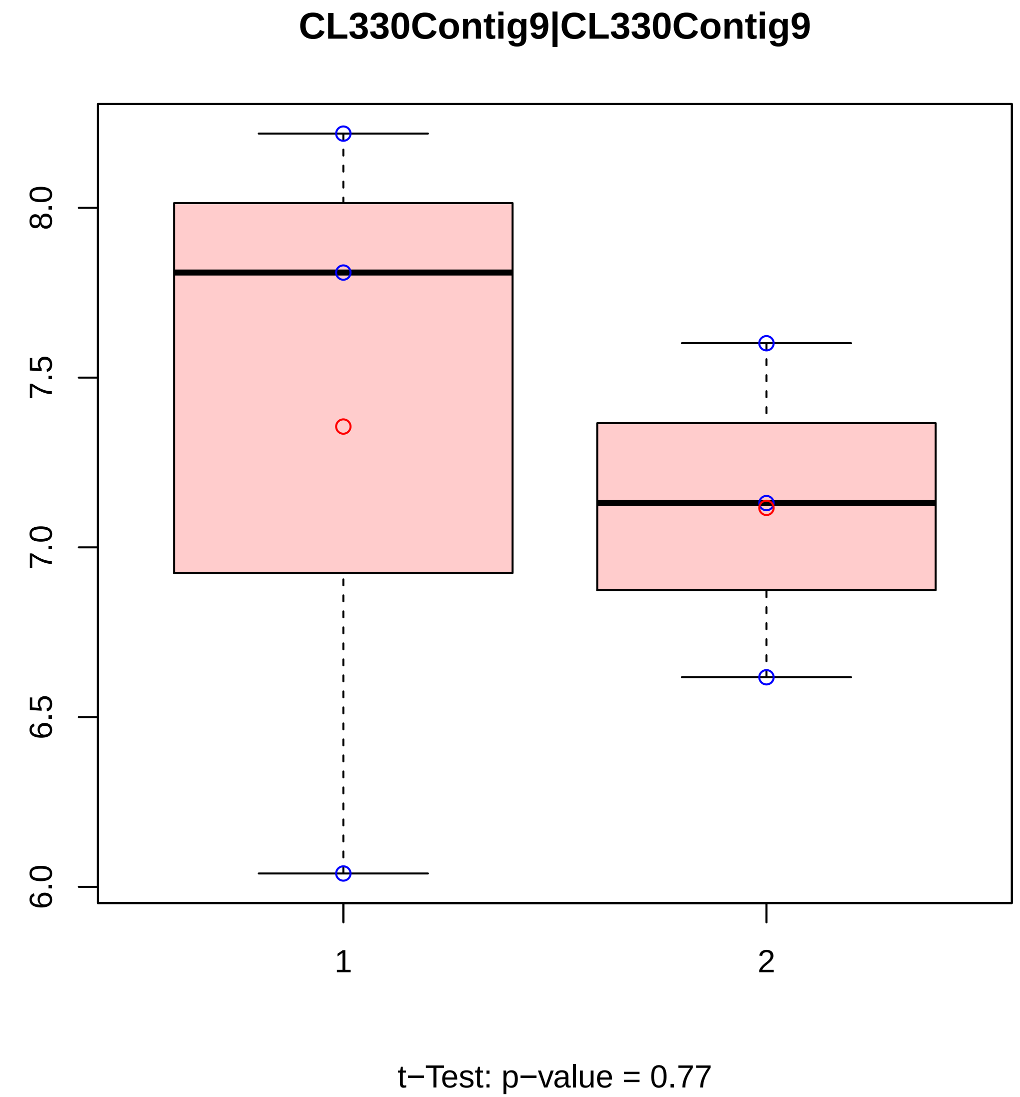

CL330Contig9|CL330Contig9
8.0
7.5
7.0
6.5
6.0
1
2
t−Test: p−value = 0.77

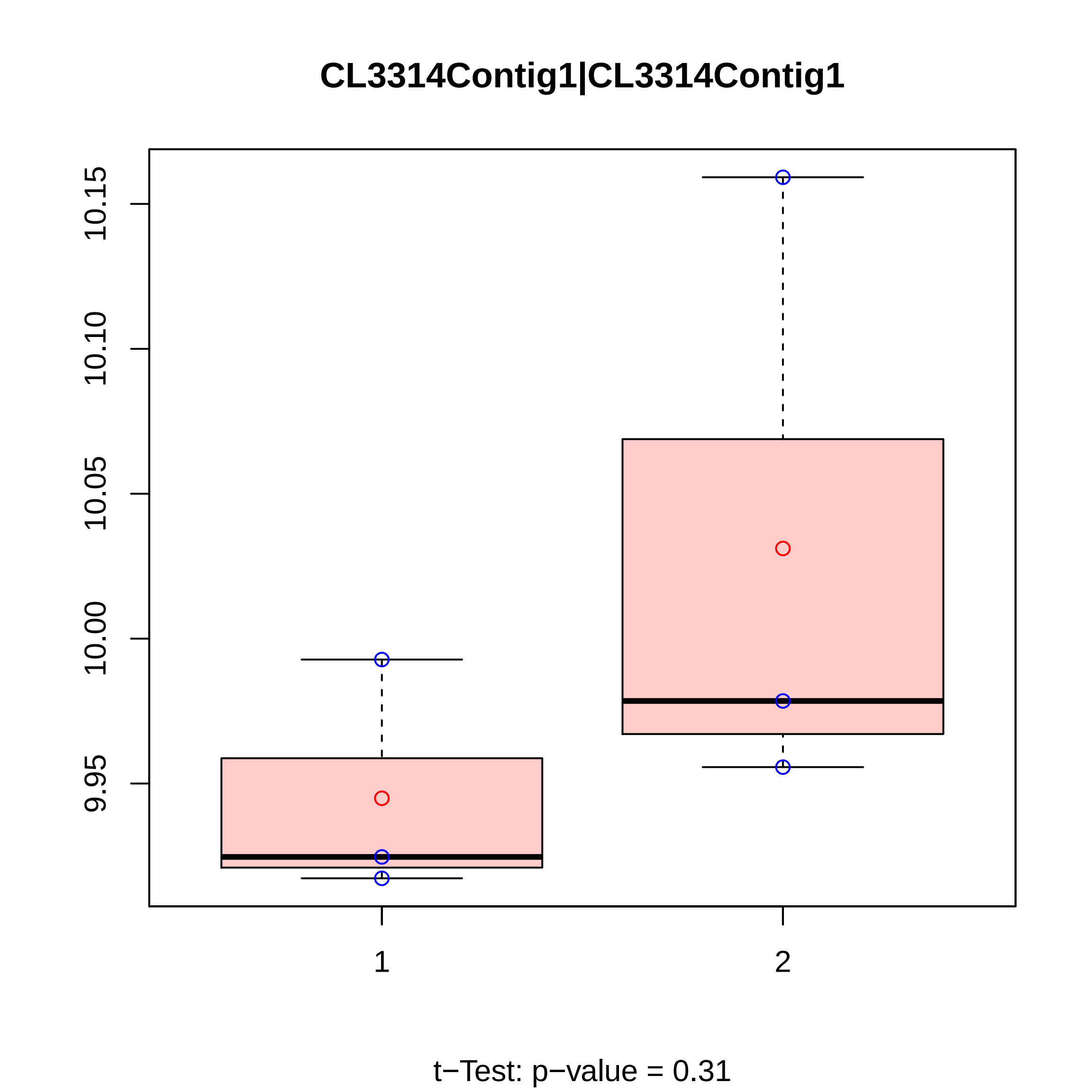
CL3314Contig1|CL3314Contig1
10.15
10.10
10.05
10.00
9.95
1
2
t−Test: p−value = 0.31

# CL3315Contig1|CL3315Contig1

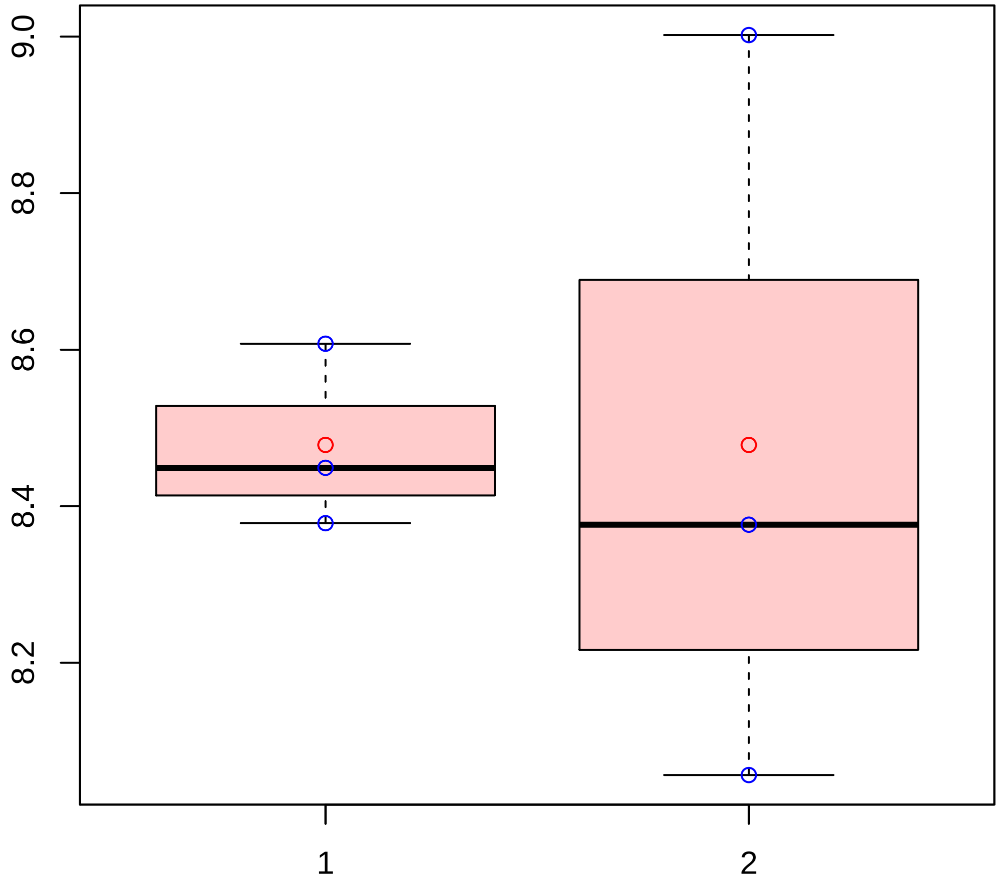


t−Test: p−value = 1

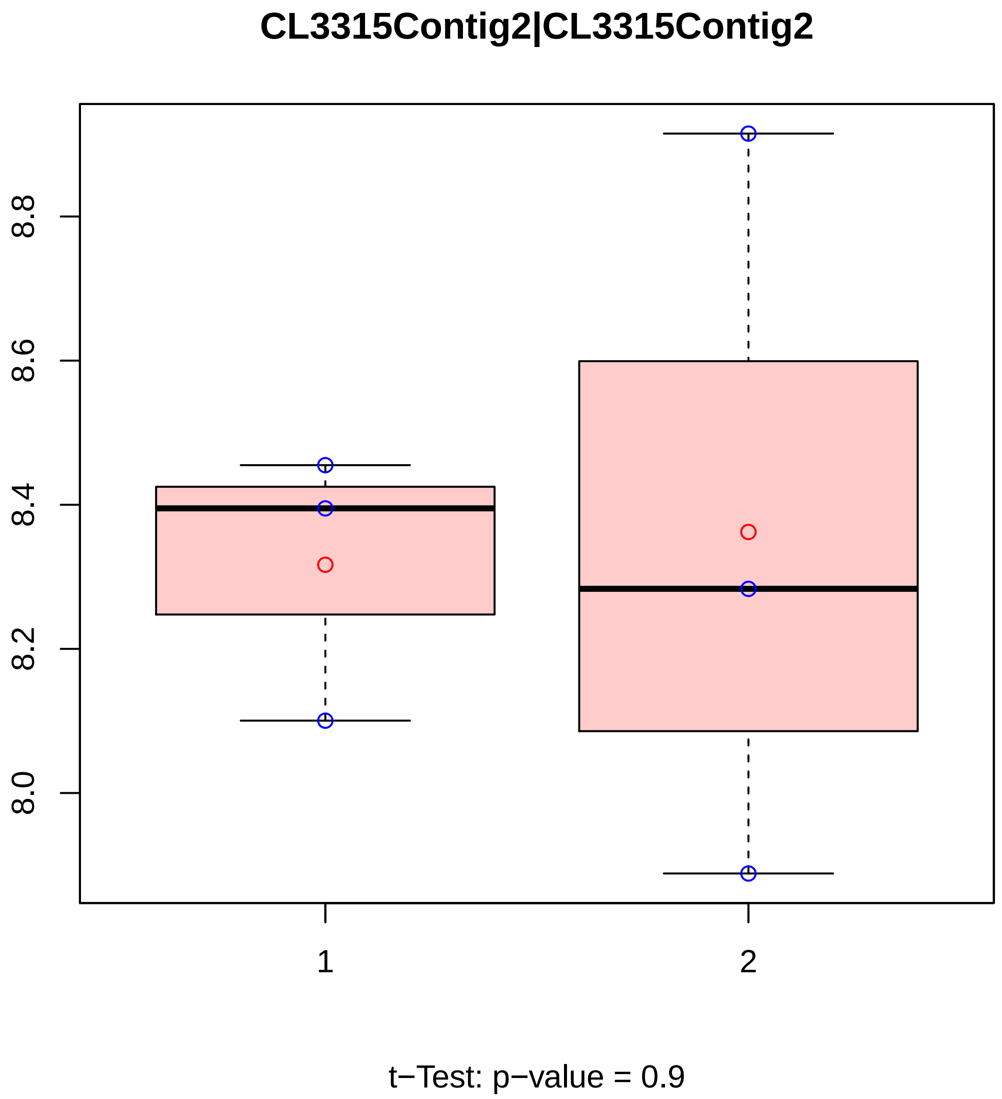

CL3315Contig2|CL3315Contig2
8.0
8.2
8.4
8.6
8.8
1
2
t−Test: p−value = 0.9

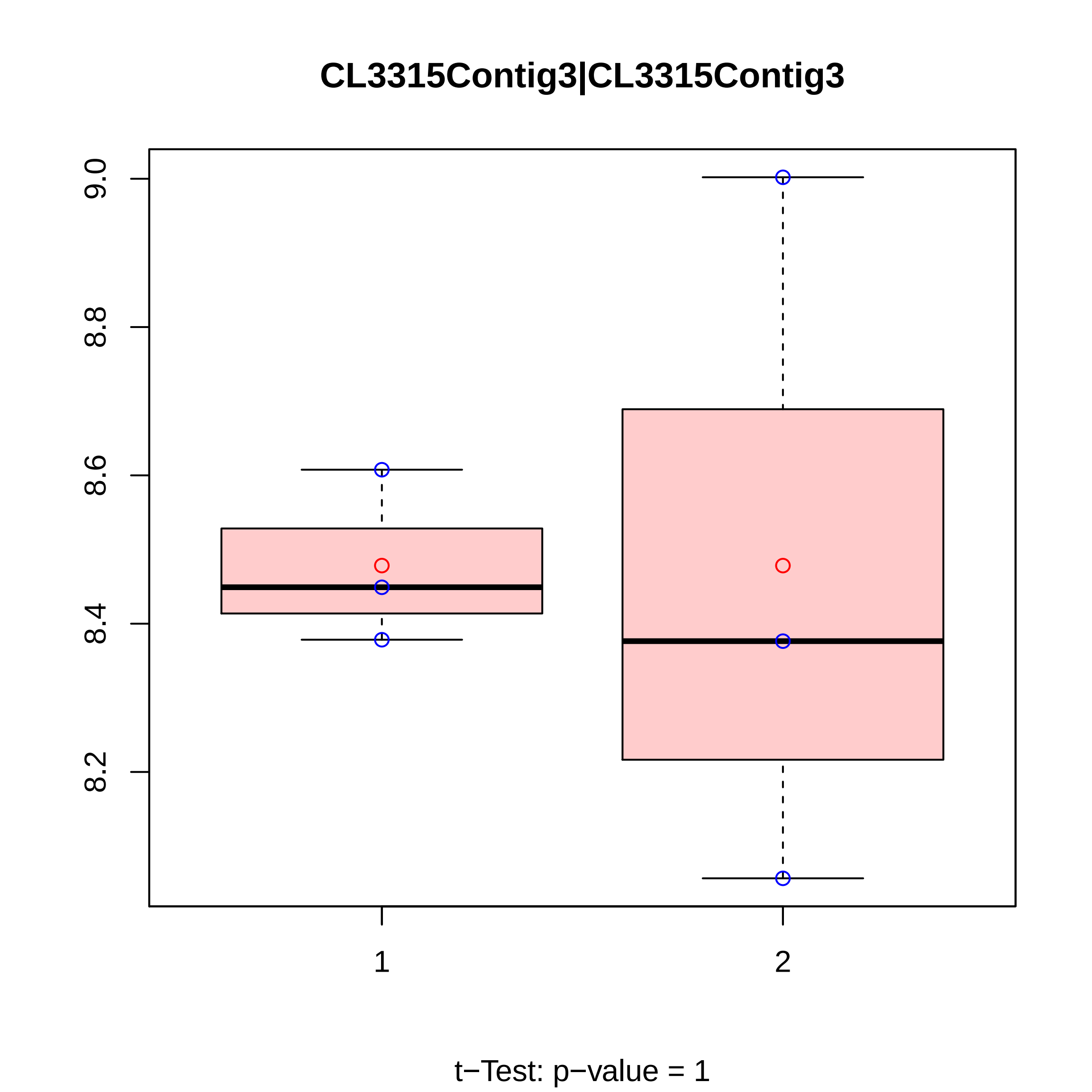

CL3315Contig3|CL3315Contig3
9.0
8.8
8.6
8.4
8.2
1
2
t−Test: p−value = 1

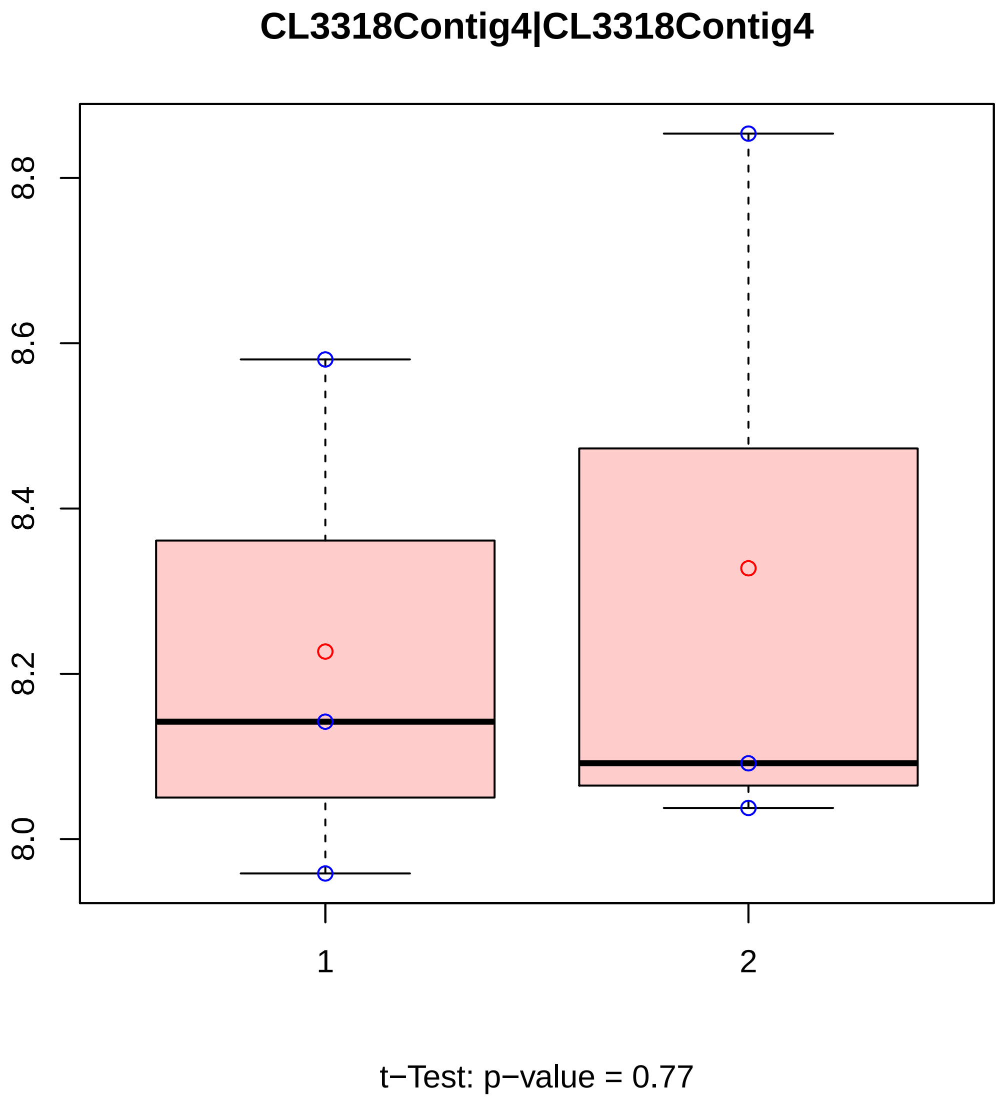
CL3318Contig4|CL3318Contig4
8.0
8.2
8.4
8.6
8.8
1
2
t−Test: p−value = 0.77

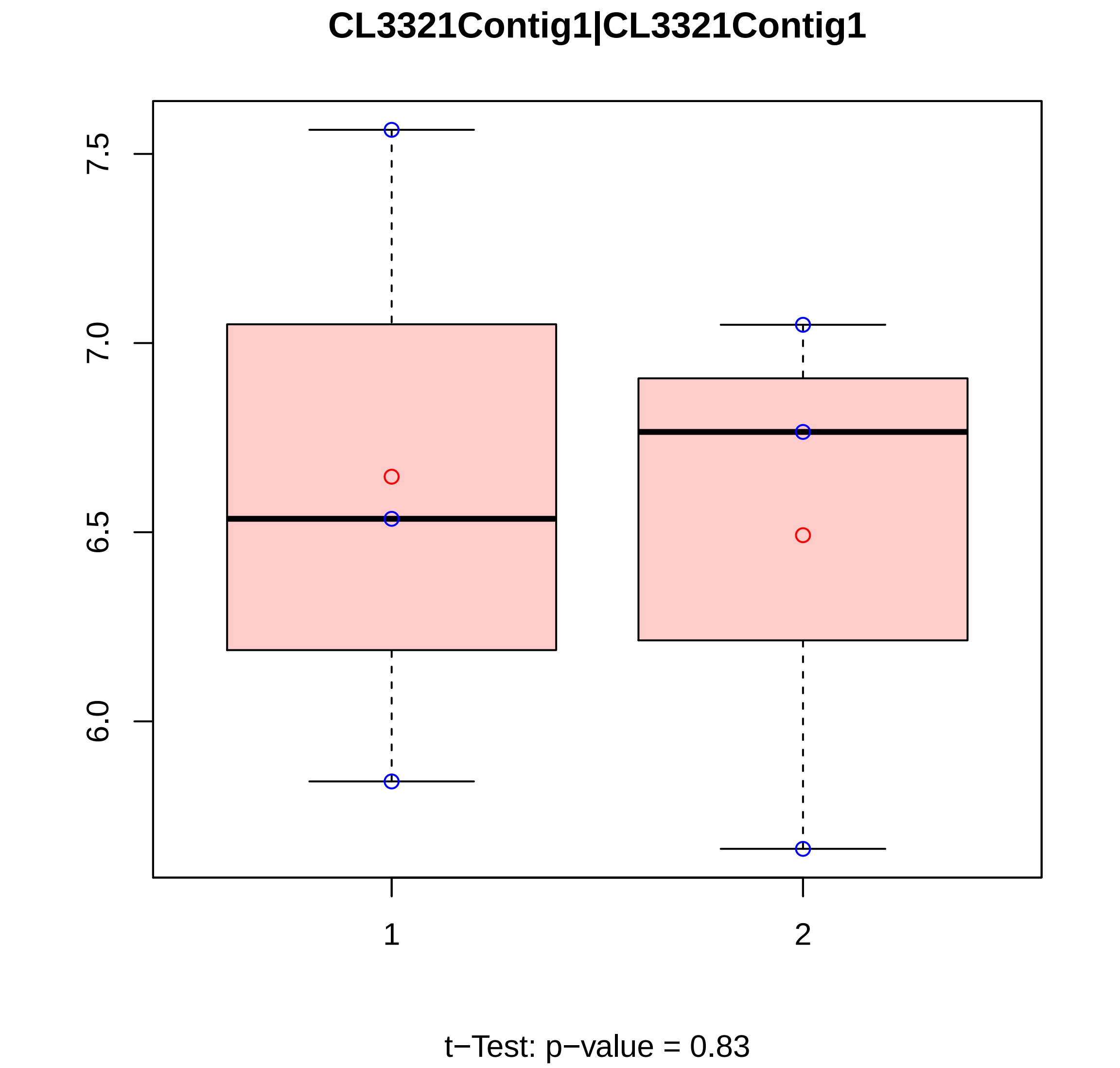
CL3321Contig1|CL3321Contig1
7.5
7.0
6.5
6.0
1
2
t−Test: p−value = 0.83

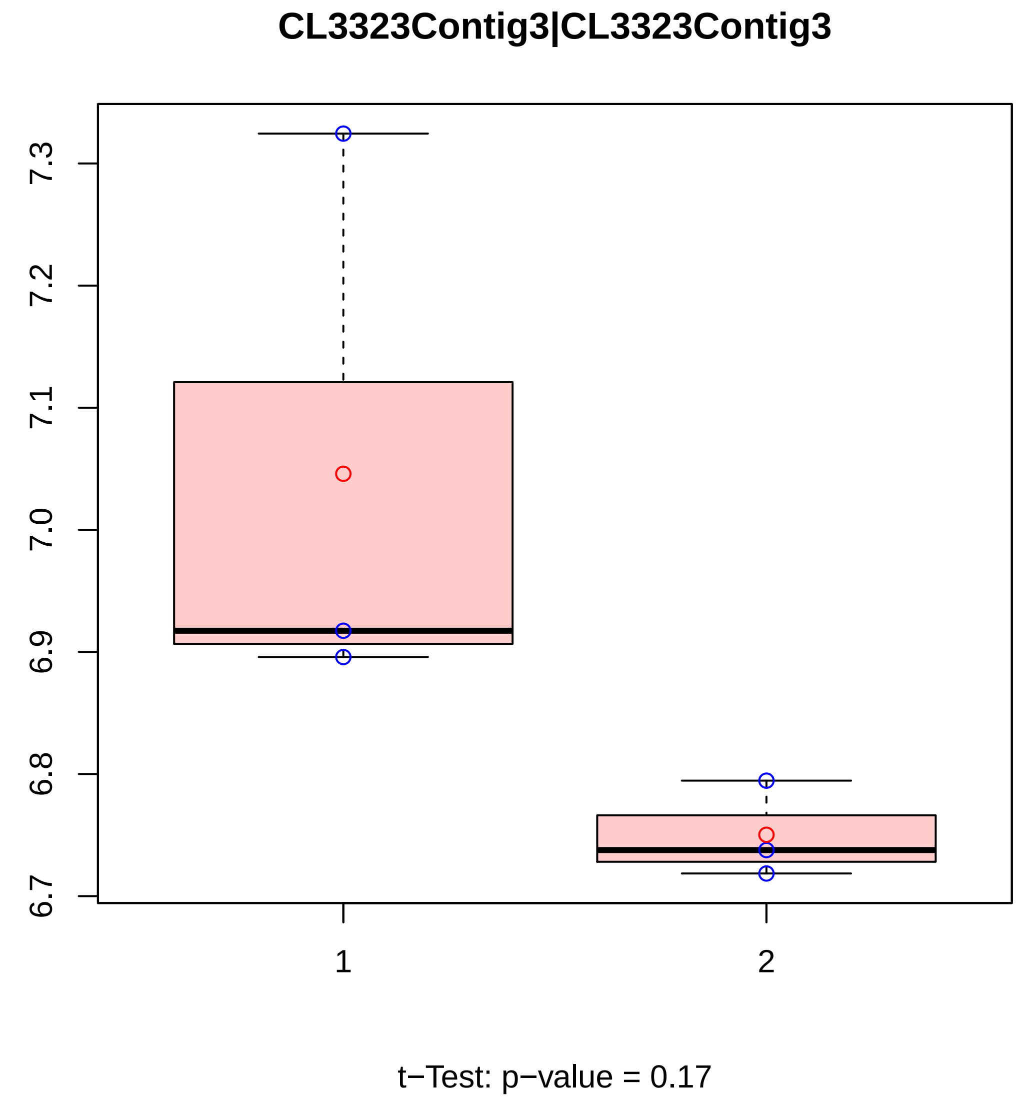
CL3323Contig3|CL3323Contig3
7.3
7.2
7.1
7.0
6.9
6.8
6.7
1
2
t−Test: p−value = 0.17

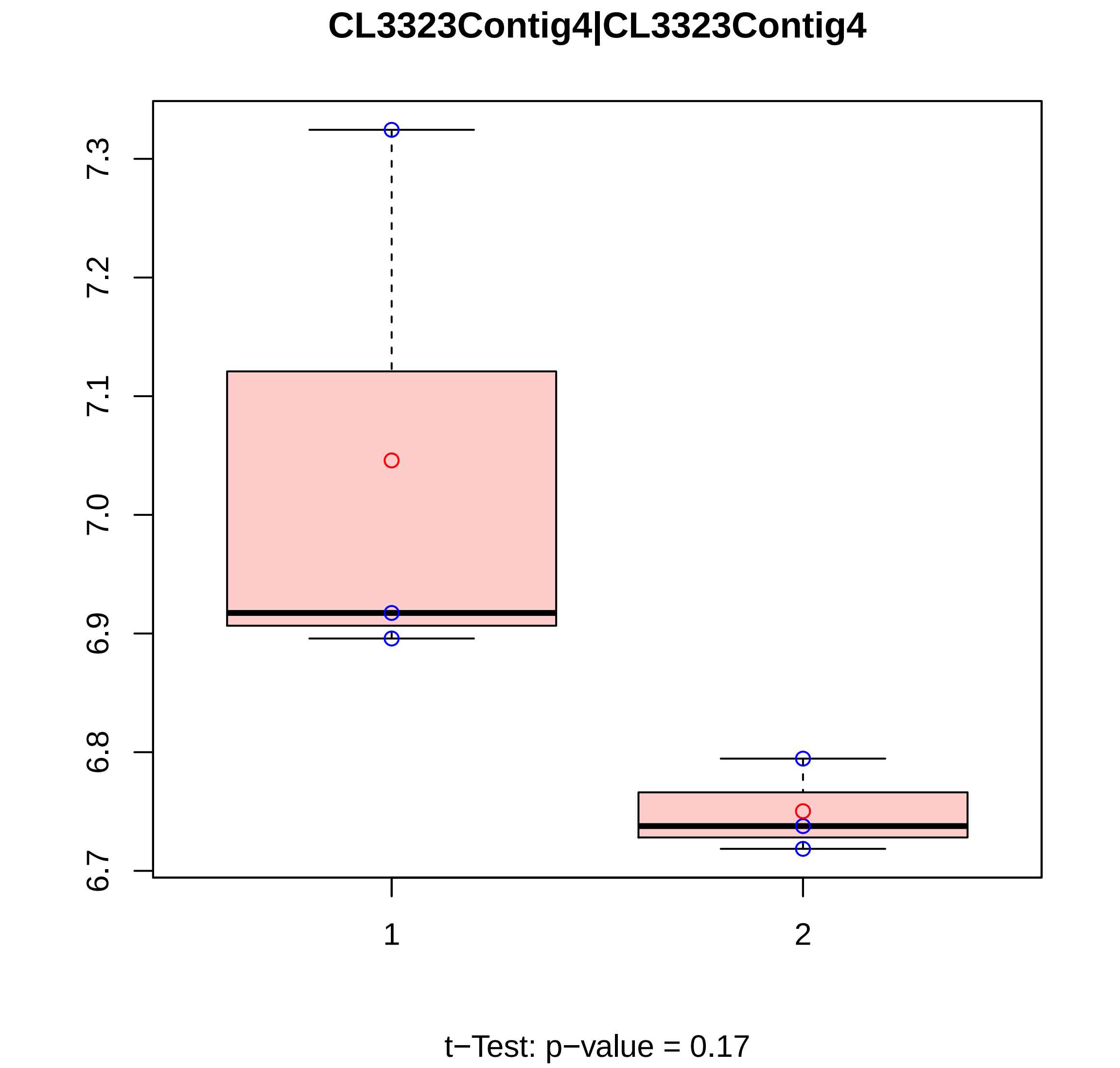
CL3323Contig4|CL3323Contig4
7.3
7.2
7.1
7.0
6.9
6.8
6.7
1
2
t−Test: p−value = 0.17

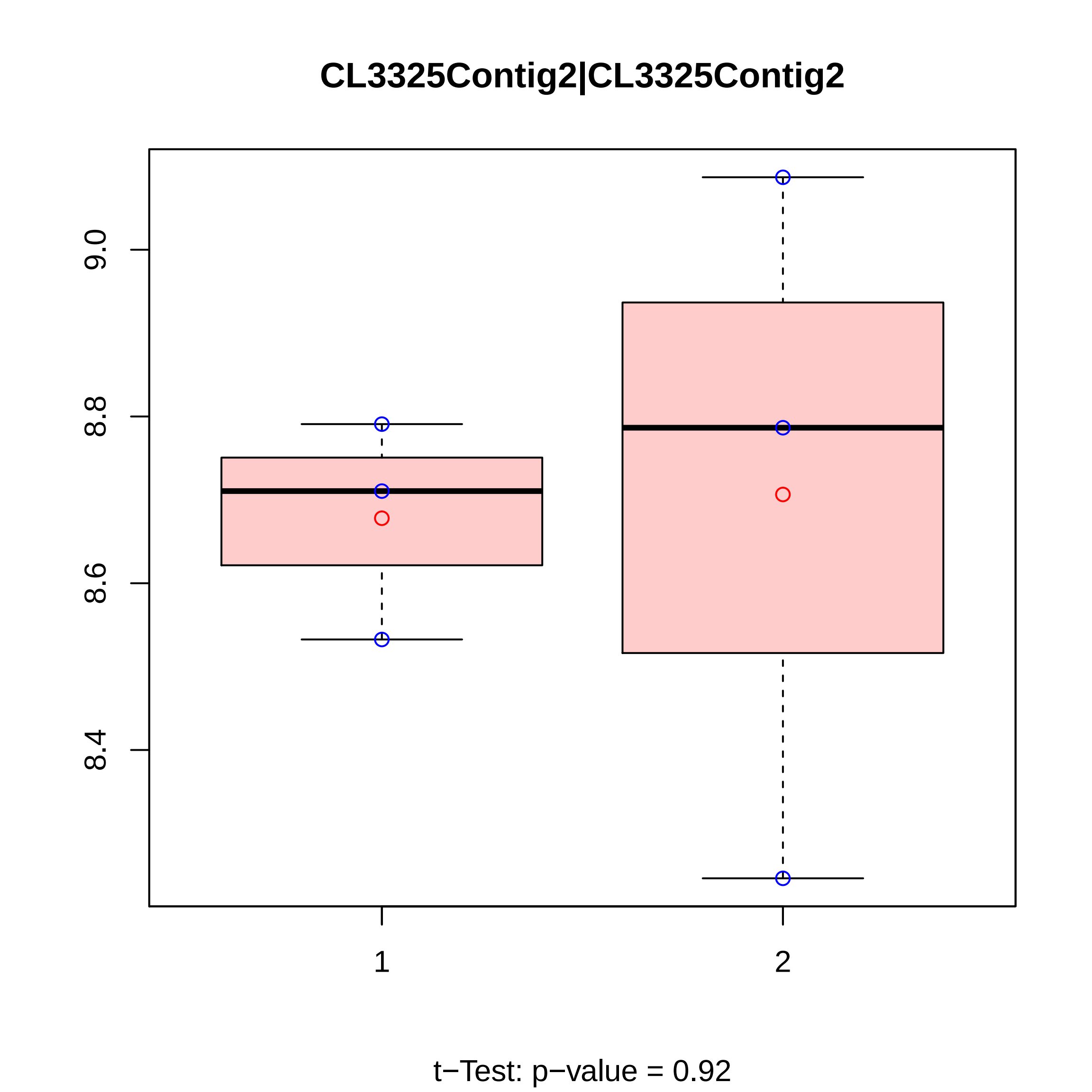
CL3325Contig2|CL3325Contig2
9.0
8.8
8.6
8.4
1
2
t−Test: p−value = 0.92

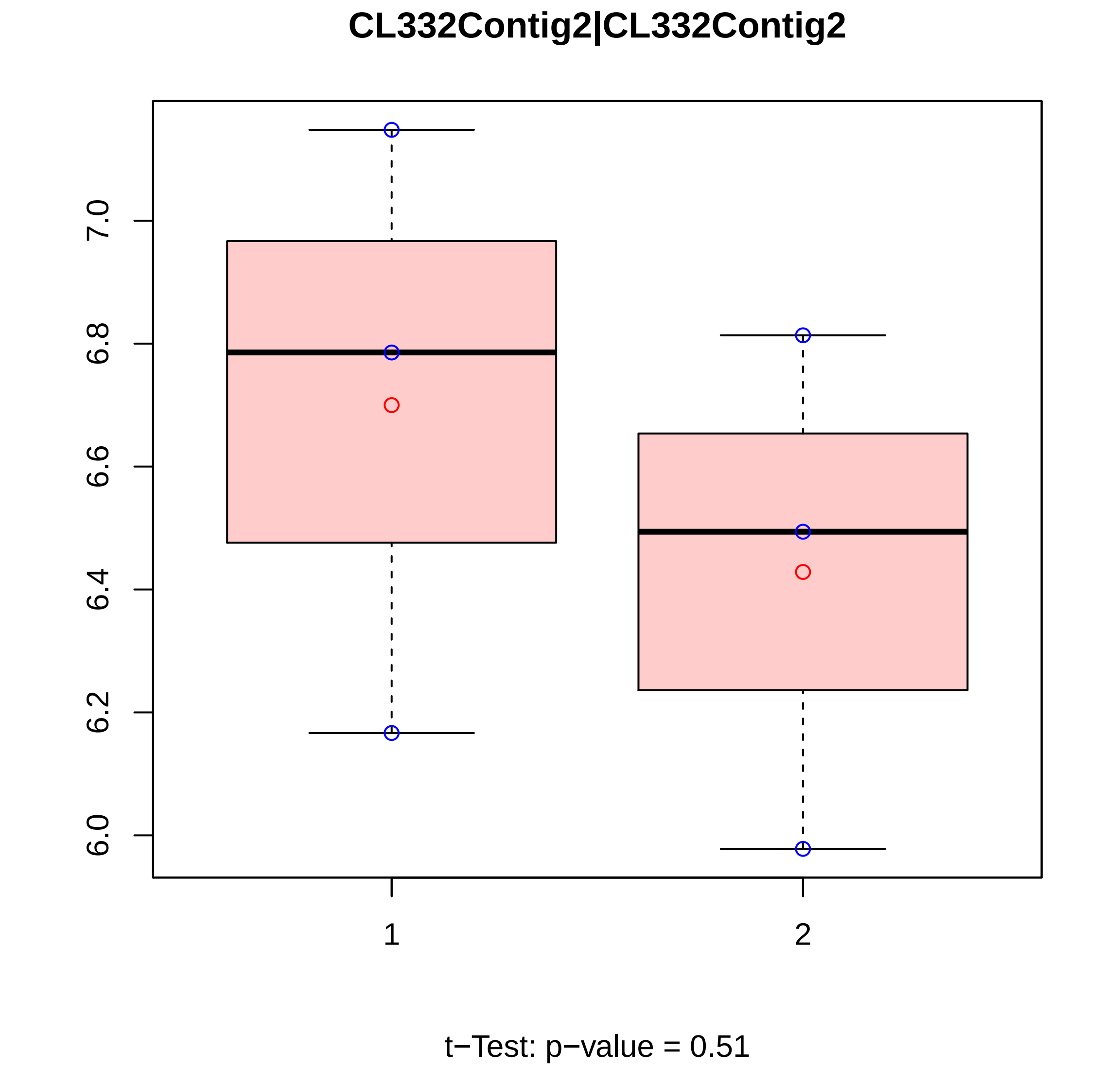

CL332Contig2|CL332Contig2
7.0
6.8
6.6
6.4
6.2
6.0
1
2
t−Test: p−value = 0.51

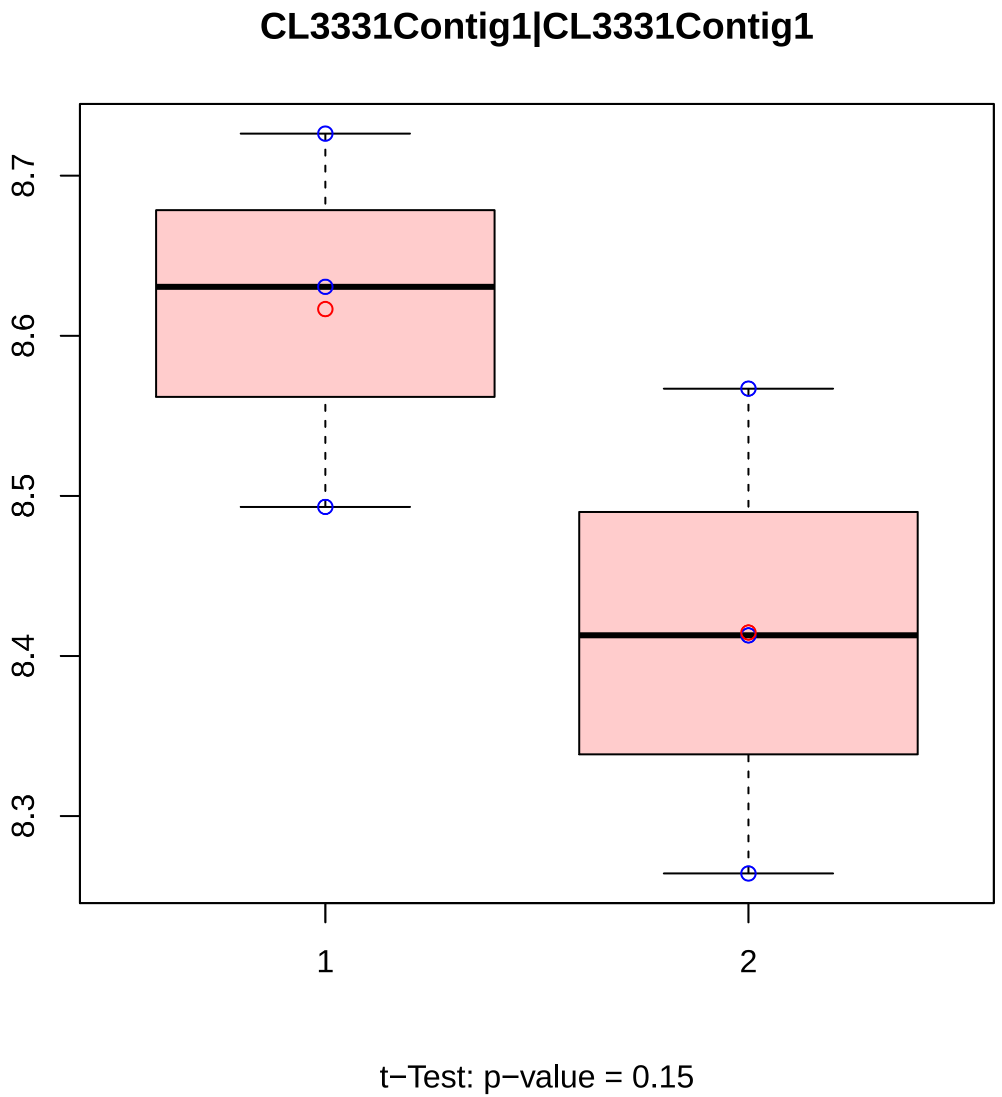
CL3331Contig1|CL3331Contig1
8.3
8.4
8.5
8.6
8.7
1
2
t−Test: p−value = 0.15

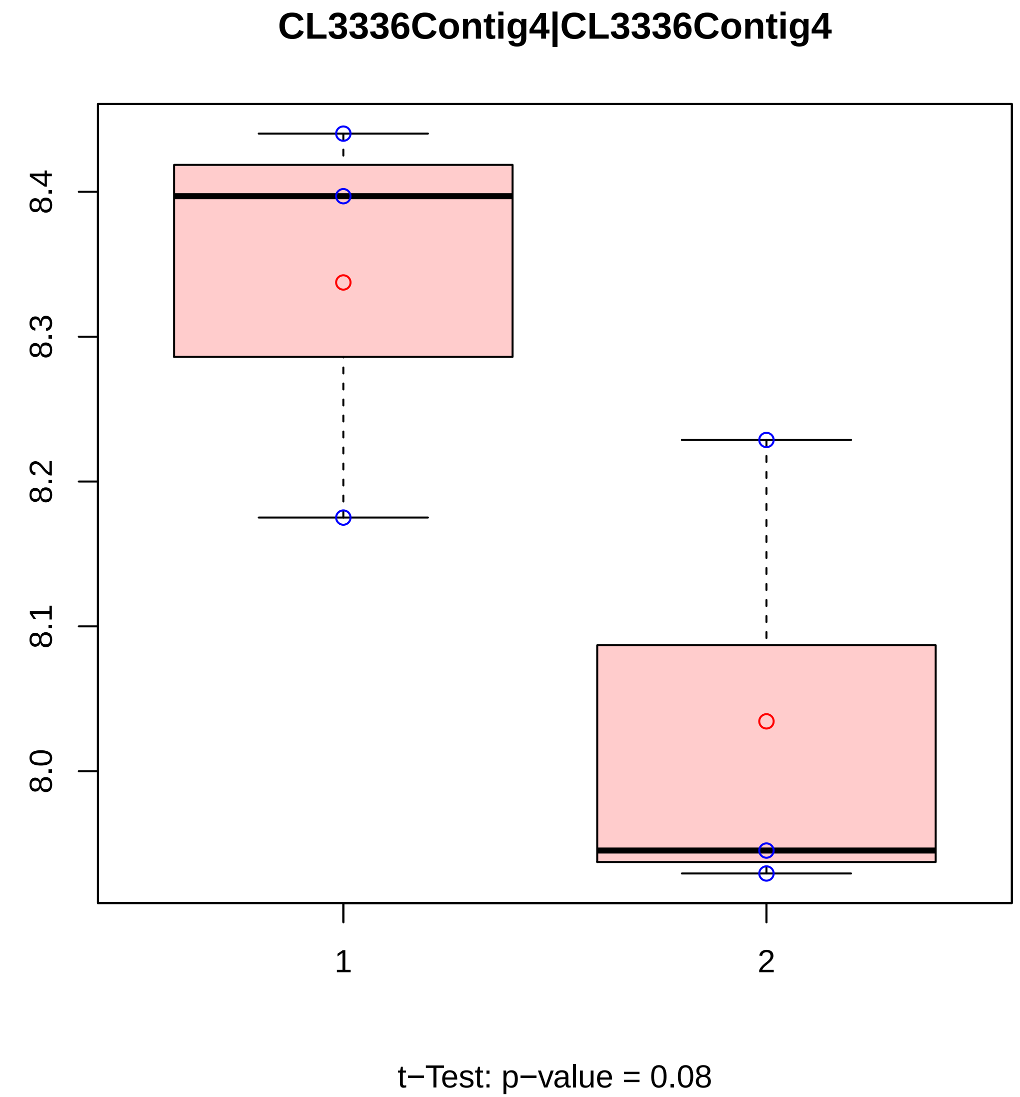
CL3336Contig4|CL3336Contig4
8.0
8.1
8.2
8.3
8.4
1
2
t−Test: p−value = 0.08

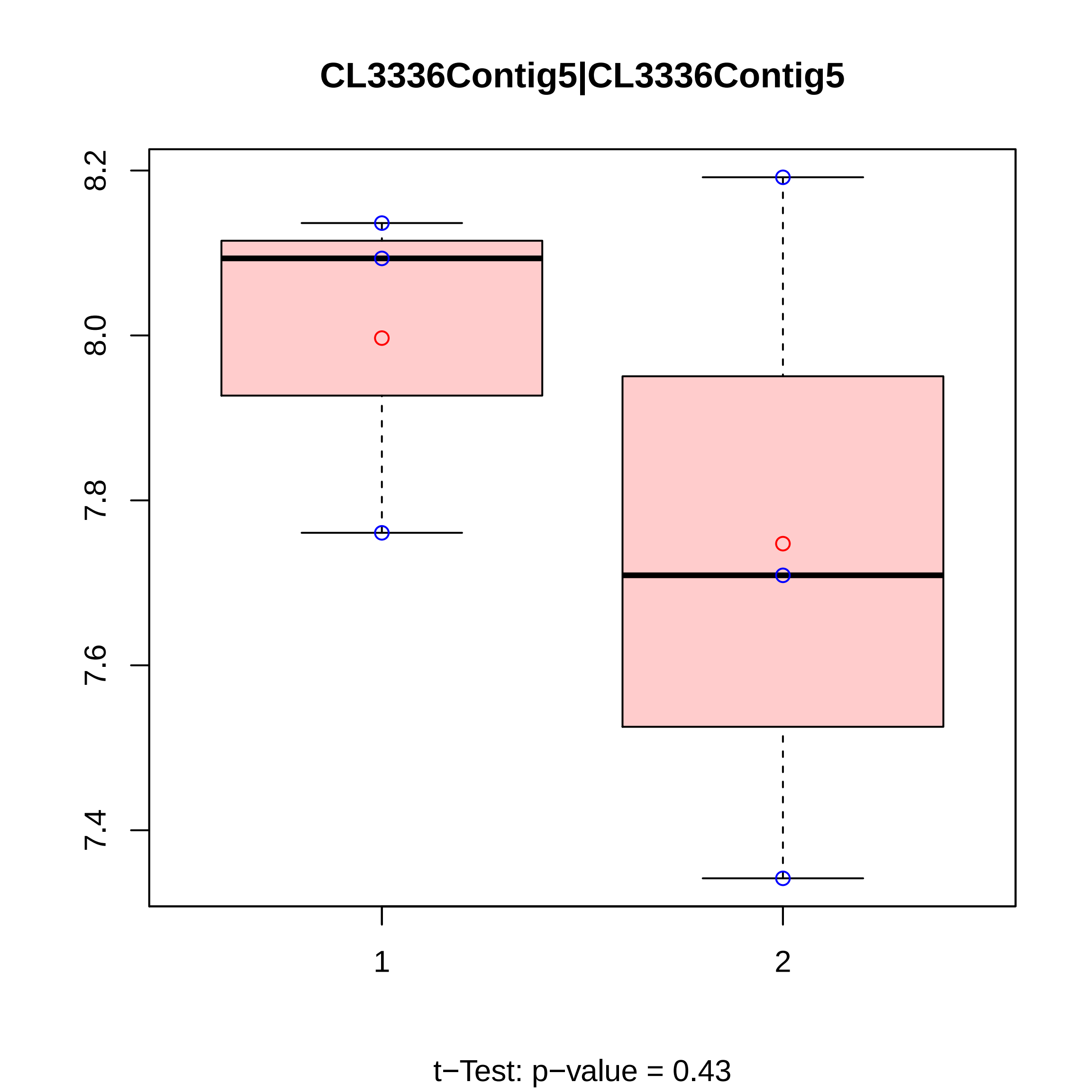
CL3336Contig5|CL3336Contig5
8.2
8.0
7.8
7.6
7.4
1
2
t−Test: p−value = 0.43

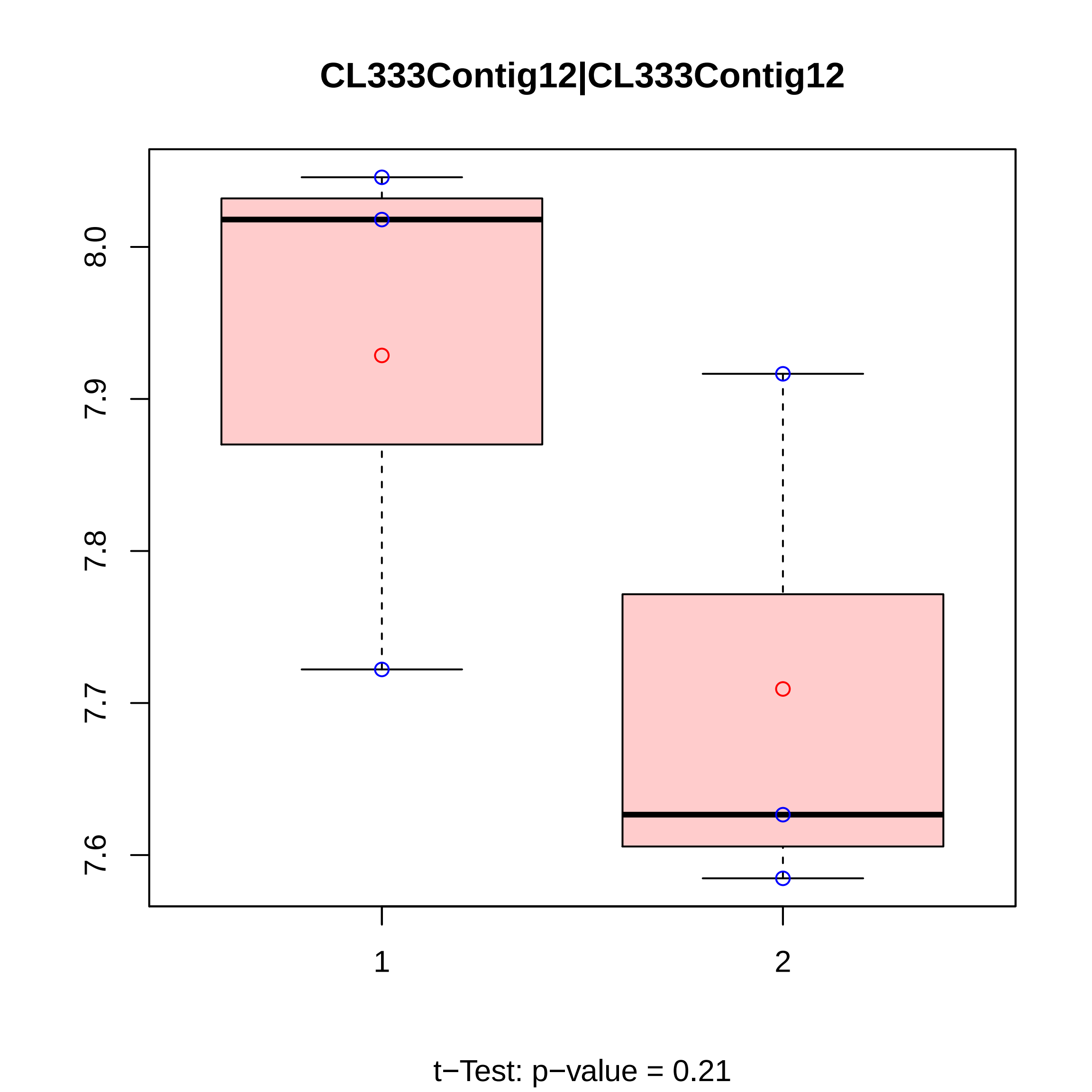

CL333Contig12|CL333Contig12
8.0
7.9
7.8
7.7
7.6
1
2
t−Test: p−value = 0.21

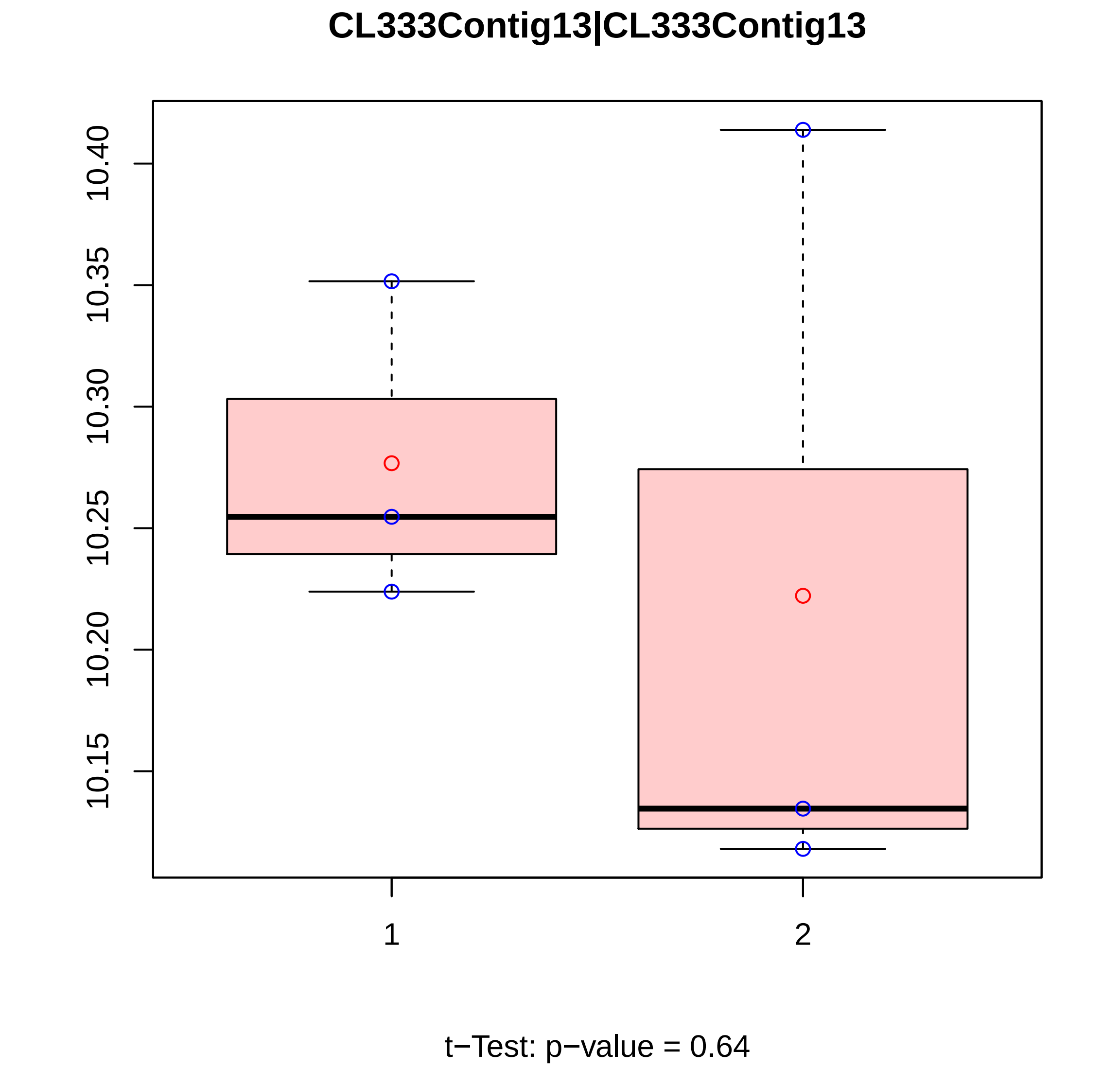

CL333Contig13|CL333Contig13
10.15
10.20
10.25
10.30
10.35
10.40
1
2
t−Test: p−value = 0.64

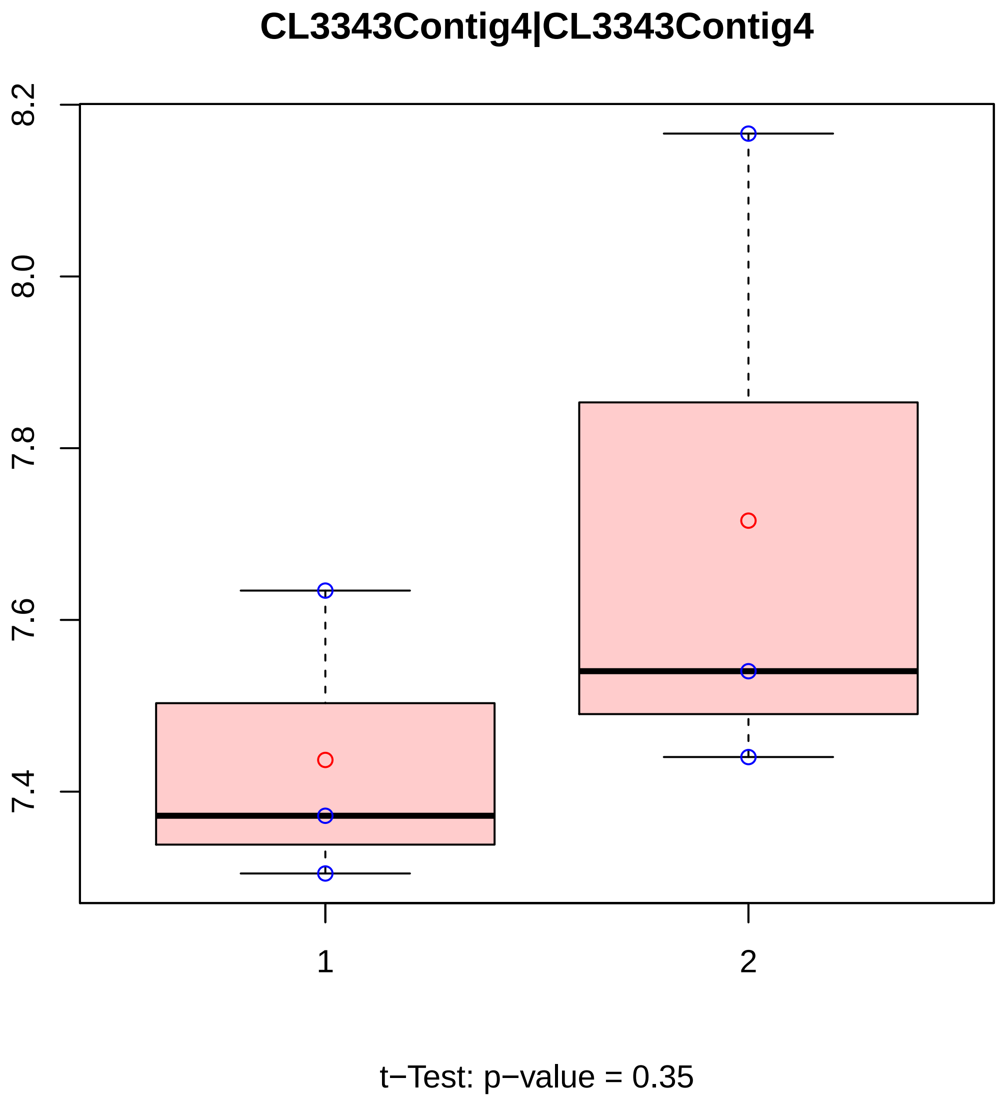
CL3343Contig4|CL3343Contig4
8.2
8.0
7.8
7.6
7.4
1
2
t−Test: p−value = 0.35

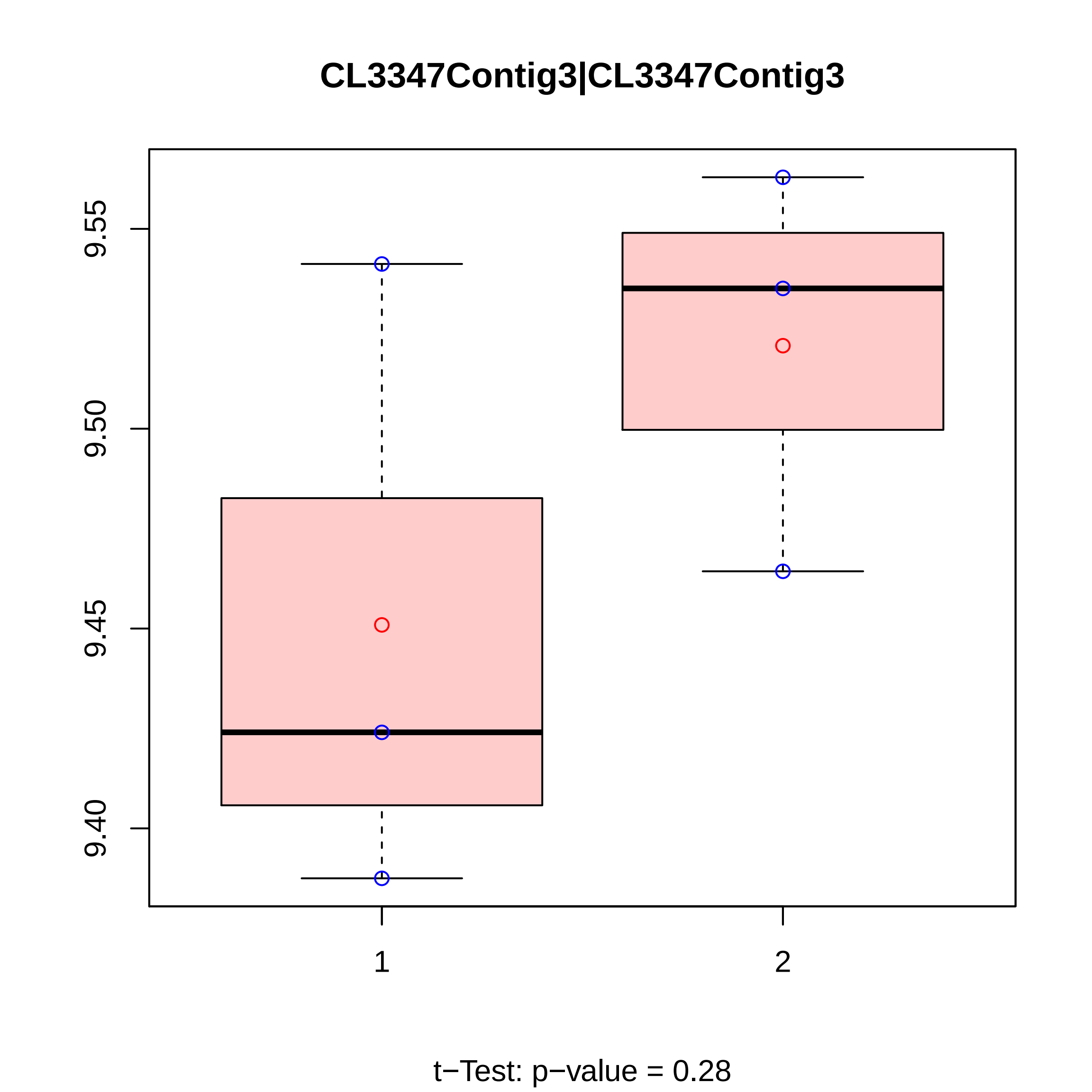

CL3347Contig3|CL3347Contig3
9.40
9.45
9.50
9.55
1
2
t−Test: p−value = 0.28

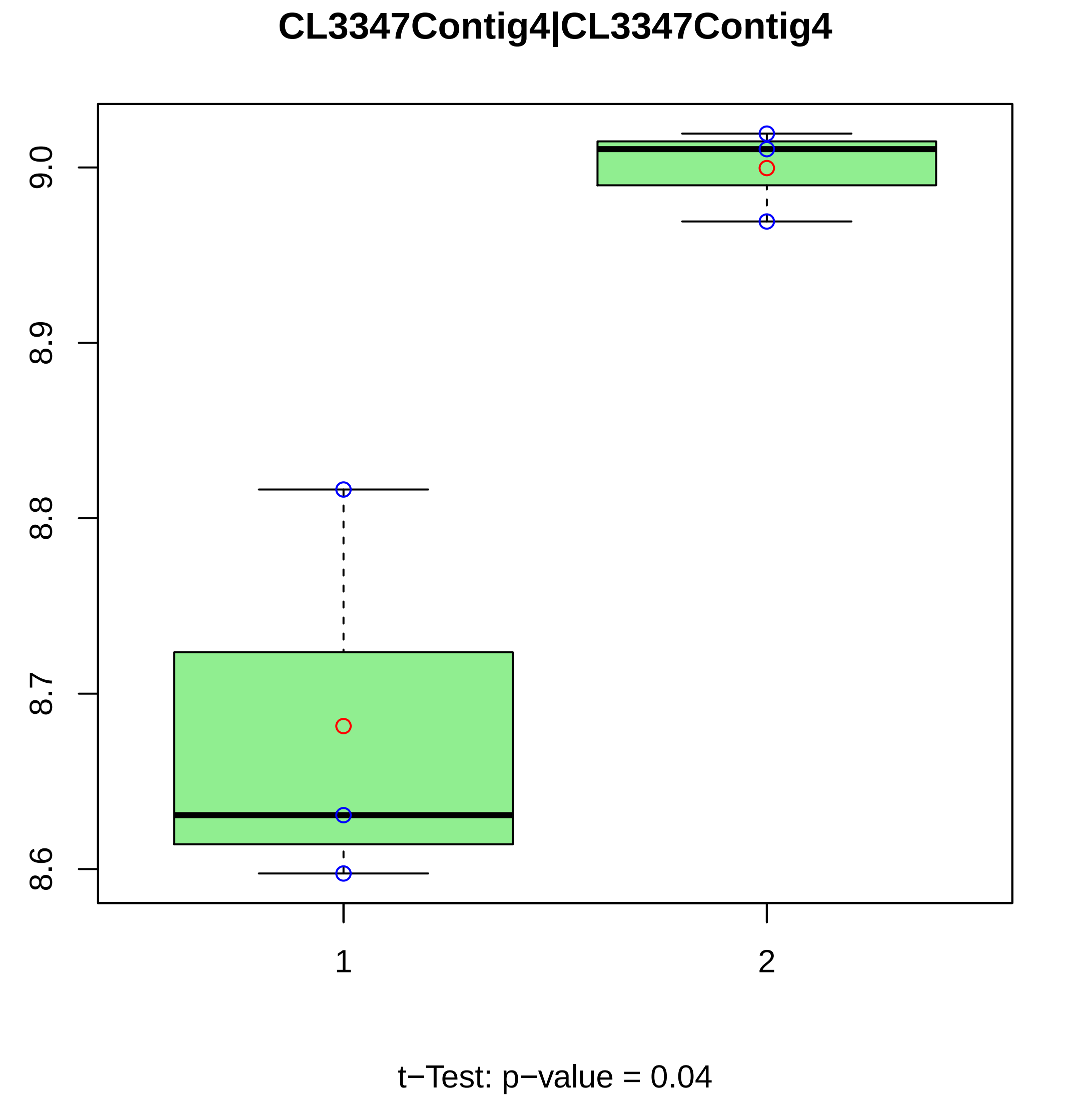
CL3347Contig4|CL3347Contig4
9.0
8.9
8.8
8.7
8.6
1
2
t−Test: p−value = 0.04

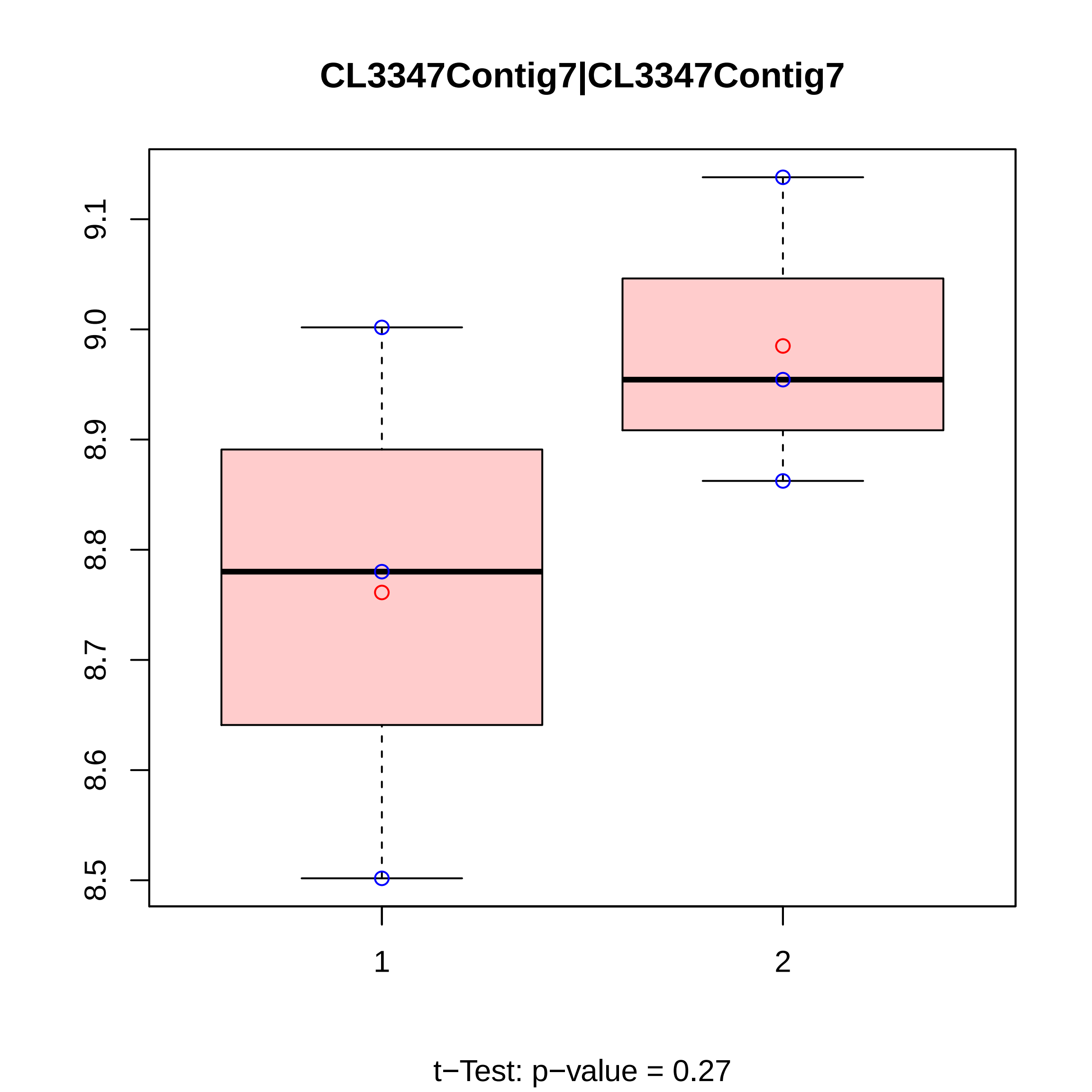

CL3347Contig7|CL3347Contig7
9.1
9.0
8.9
8.8
8.7
8.6
8.5
1
2
t−Test: p−value = 0.27

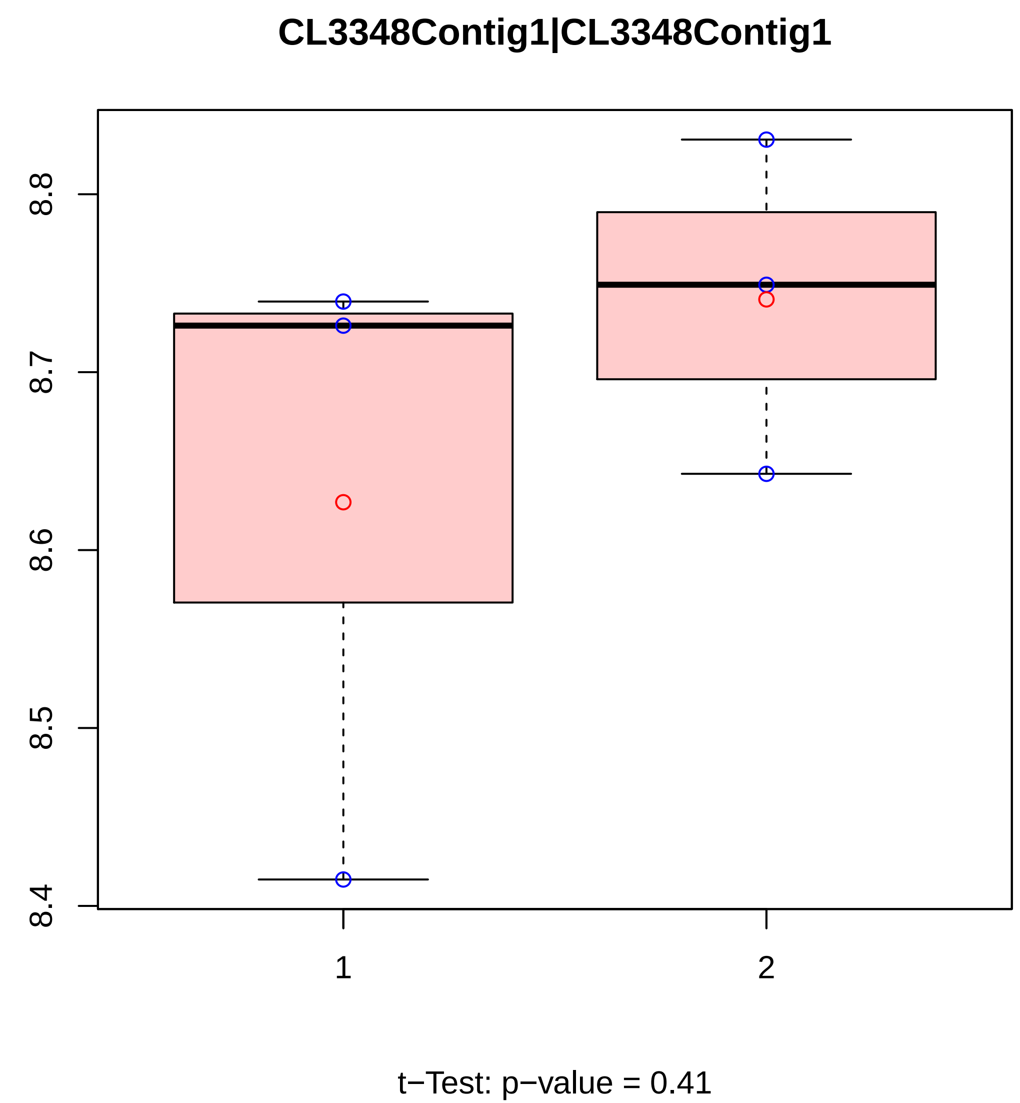

CL3348Contig1|CL3348Contig1
8.4
8.5
8.6
8.7
8.8
1
2
t−Test: p−value = 0.41

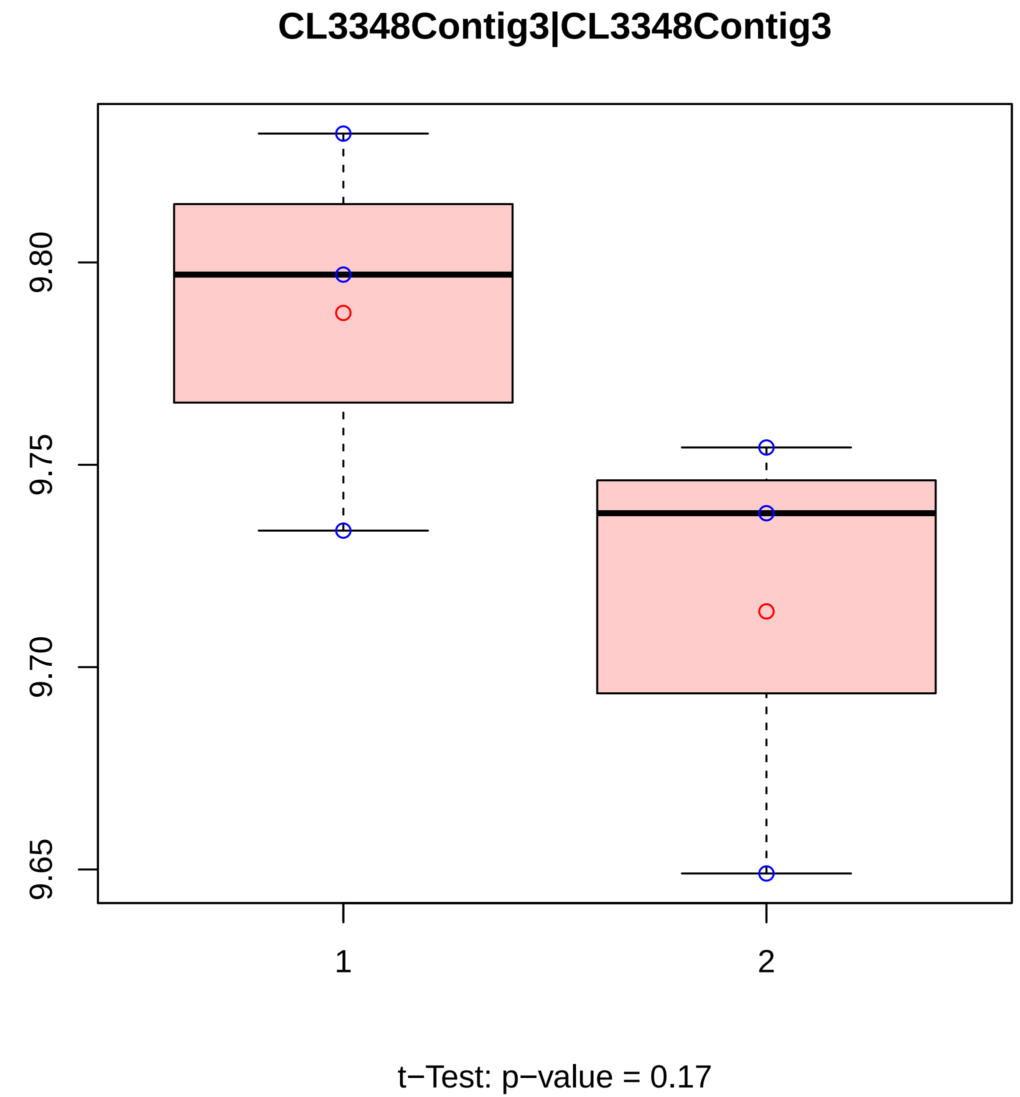

CL3348Contig3|CL3348Contig3
9.65
9.70
9.75
9.80
1
2
t−Test: p−value = 0.17

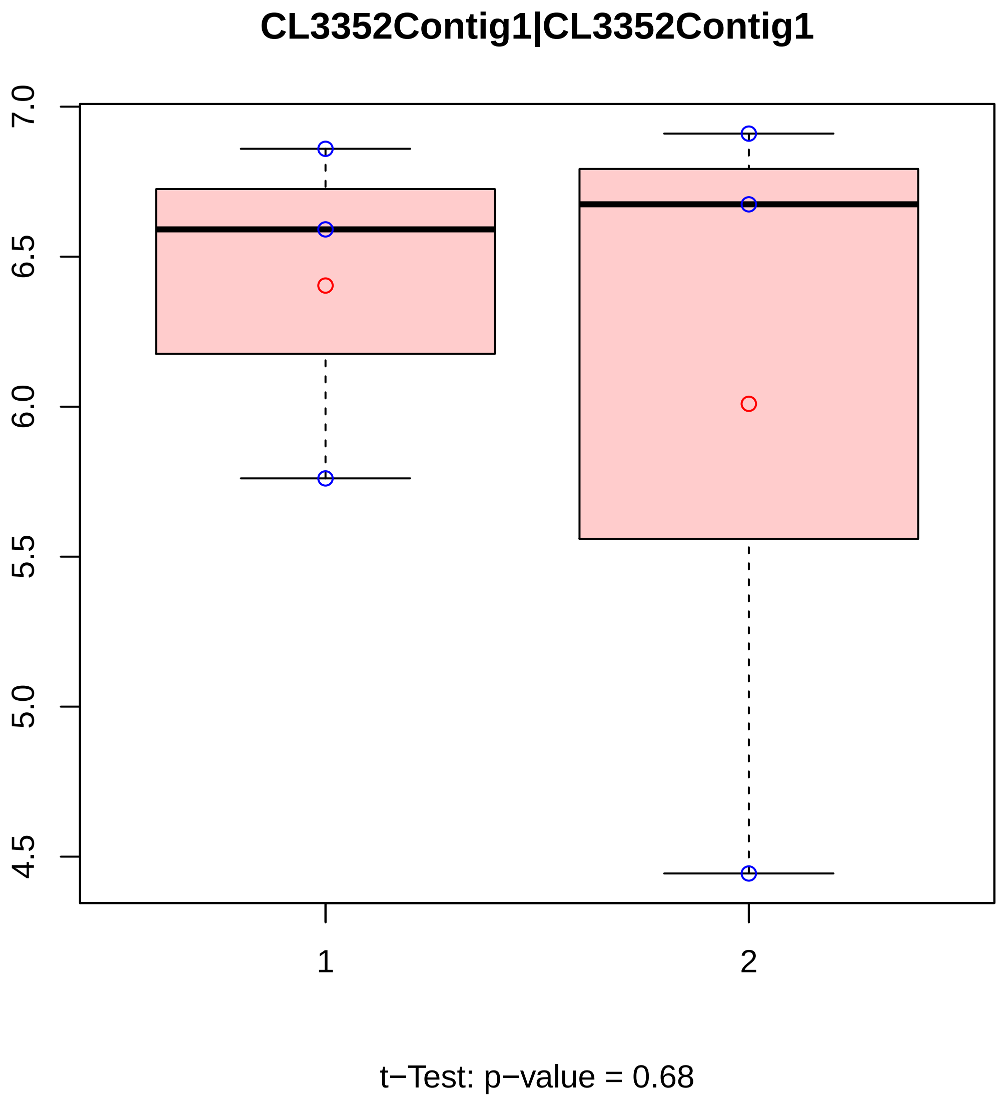
CL3352Contig1|CL3352Contig1
7.0
6.5
6.0
5.5
5.0
4.5
1
2
t−Test: p−value = 0.68

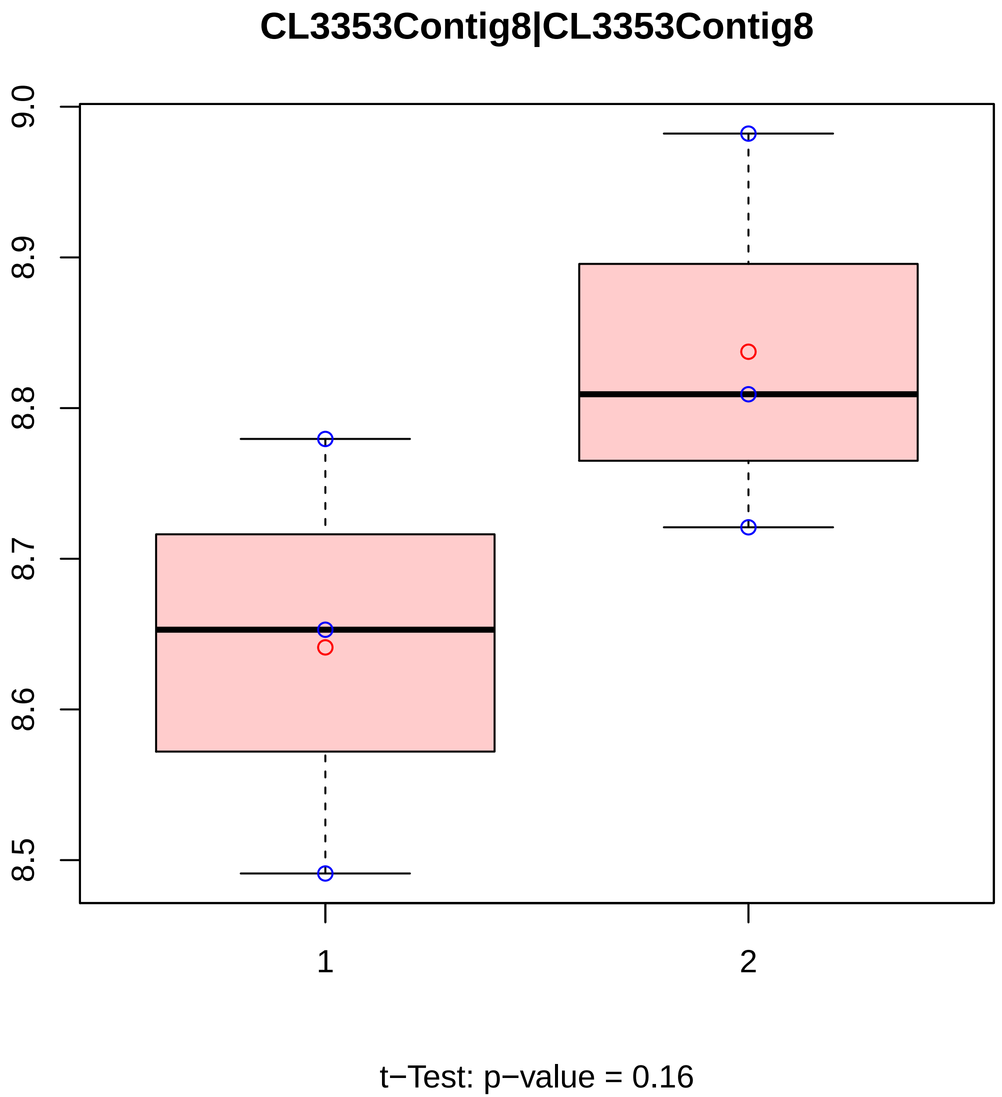
CL3353Contig8|CL3353Contig8
9.0
8.9
8.8
8.7
8.6
8.5
1
2
t−Test: p−value = 0.16

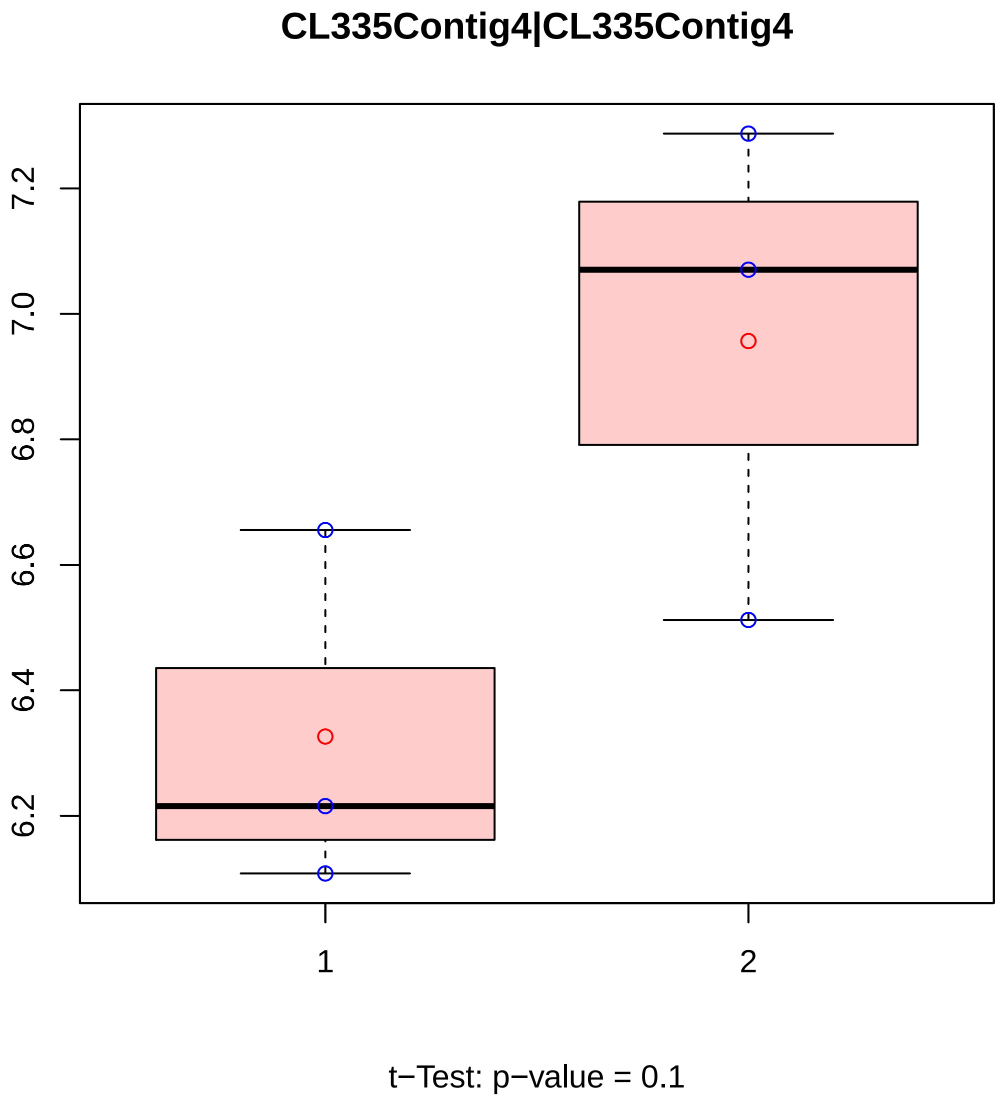
CL335Contig4|CL335Contig4
7.2
7.0
6.8
6.6
6.4
6.2
1
2
t−Test: p−value = 0.1

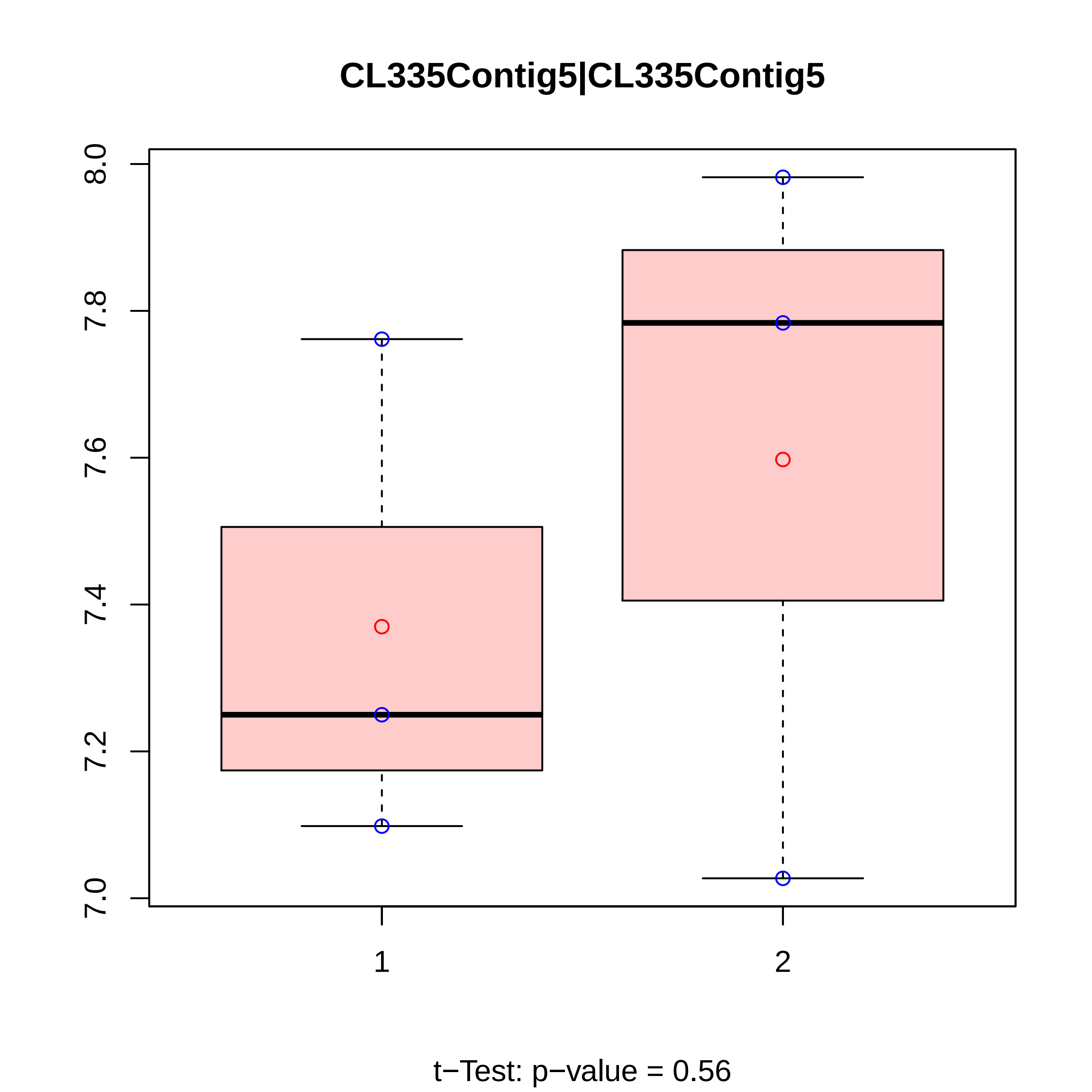
CL335Contig5|CL335Contig5
8.0
7.8
7.6
7.4
7.2
7.0
1
2
t−Test: p−value = 0.56

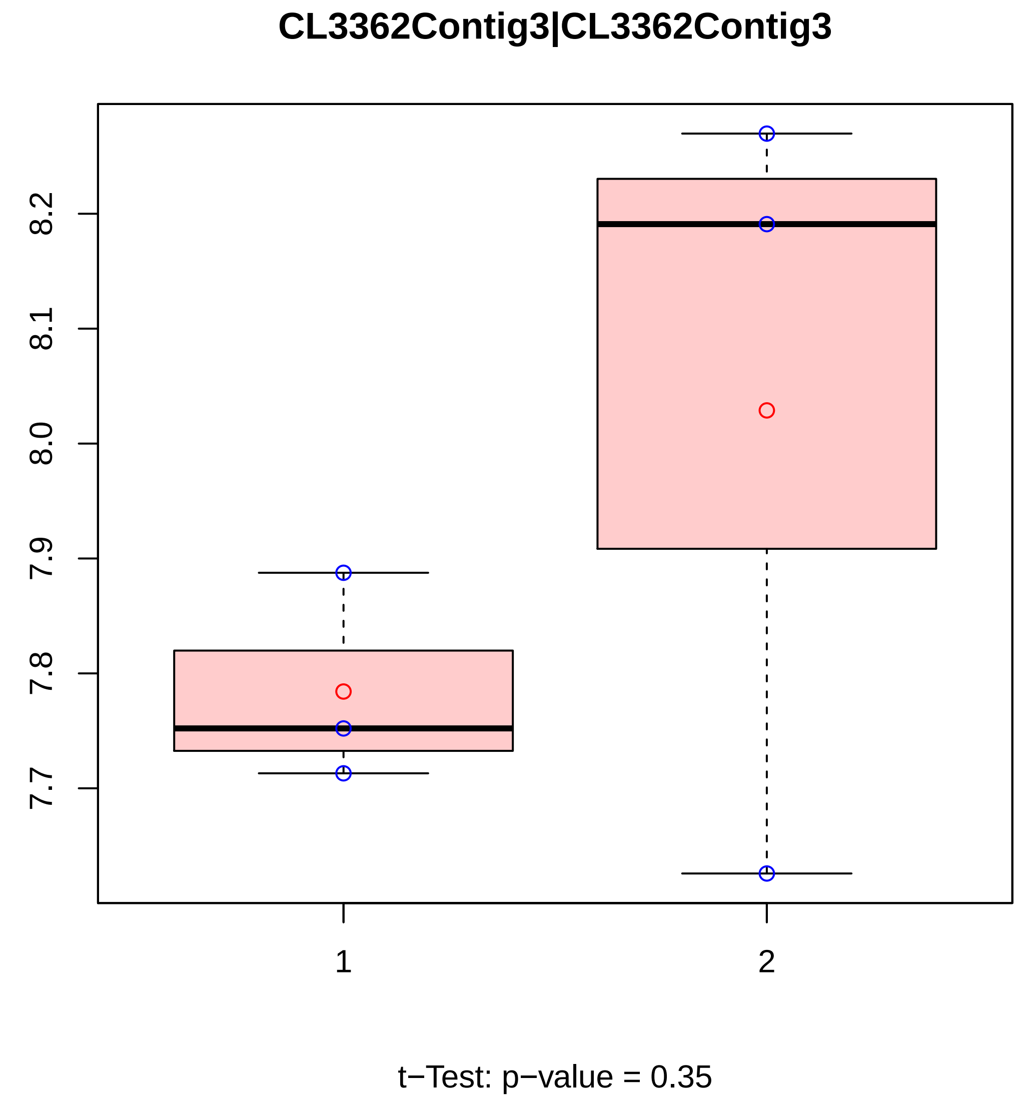
CL3362Contig3|CL3362Contig3
8.2
8.1
8.0
7.9
7.8
7.7
1
2
t−Test: p−value = 0.35

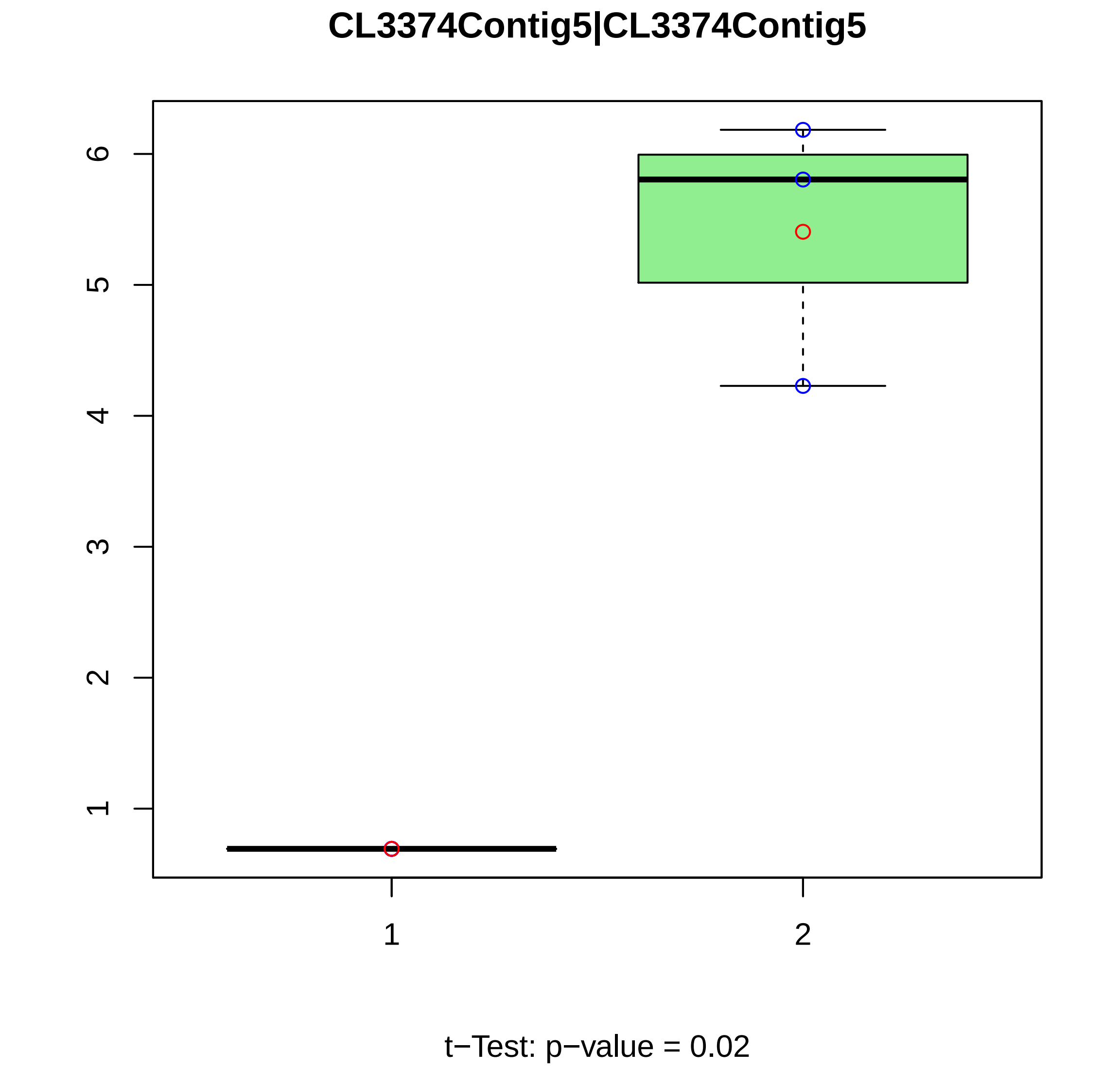

CL3374Contig5|CL3374Contig5
1
2
3
4
5
6
1
2
t−Test: p−value = 0.02

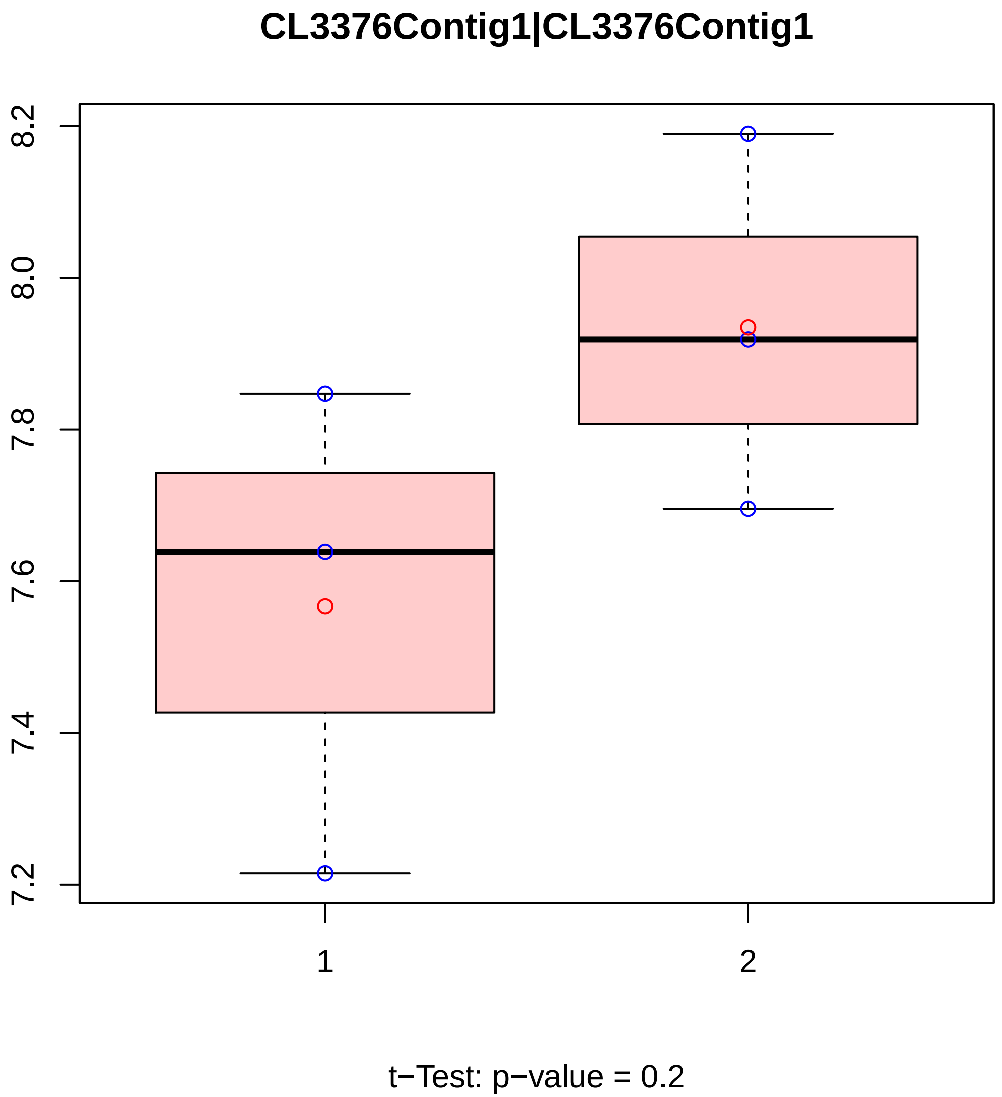
CL3376Contig1|CL3376Contig1
8.2
8.0
7.8
7.6
7.4
7.2
1
2
t−Test: p−value = 0.2

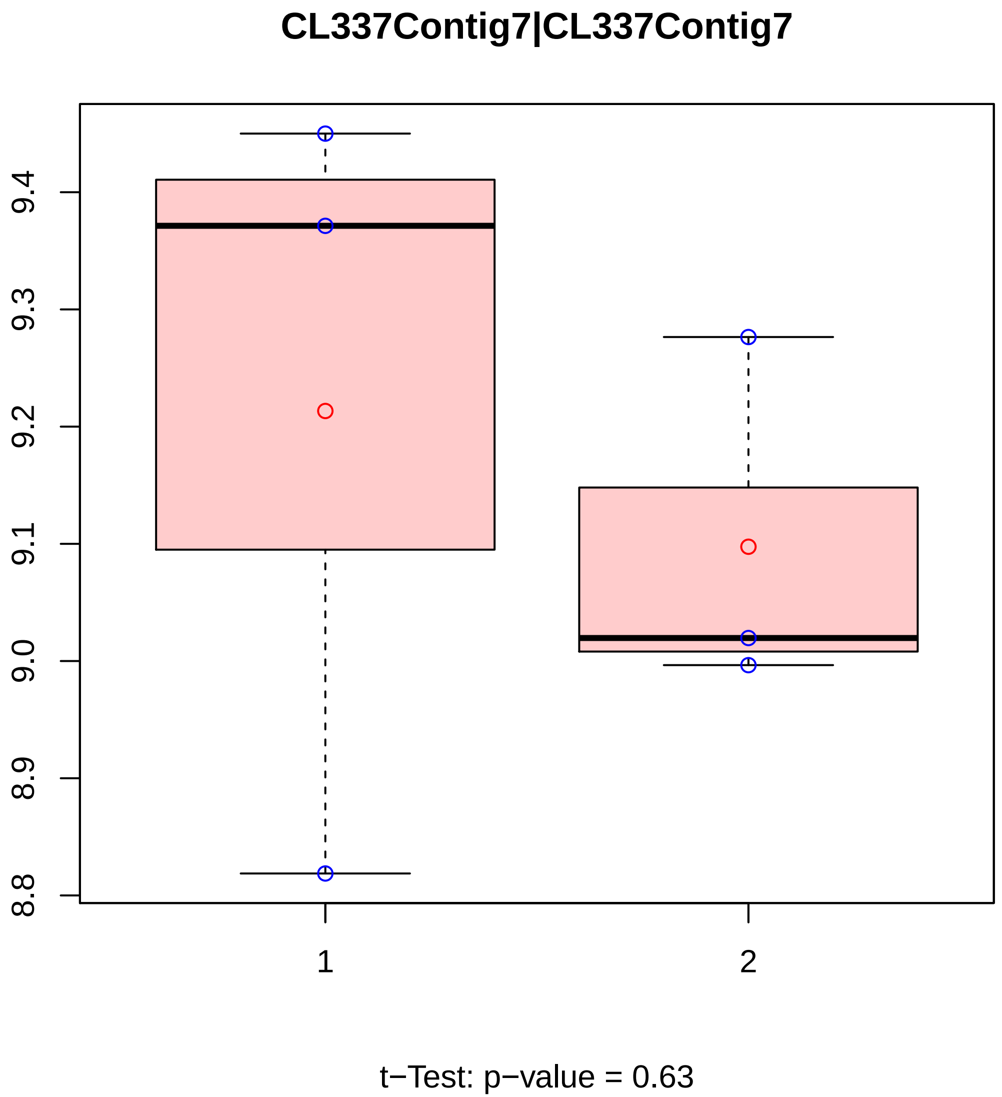
CL337Contig7|CL337Contig7
9.4
9.3
9.2
9.1
9.0
8.9
8.8
1
2
t−Test: p−value = 0.63

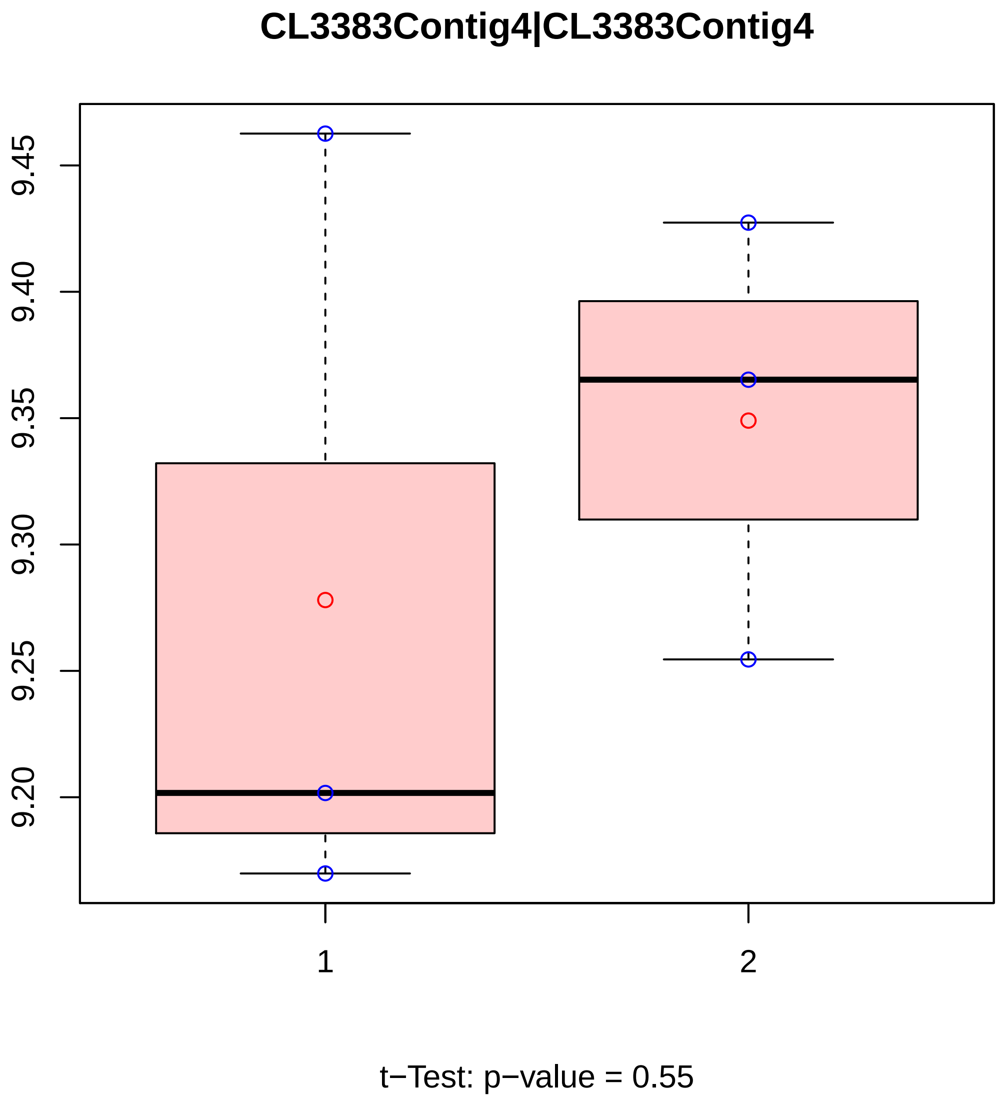

CL3383Contig4|CL3383Contig4
9.20
9.25
9.30
9.35
9.40
9.45
1
2
t−Test: p−value = 0.55

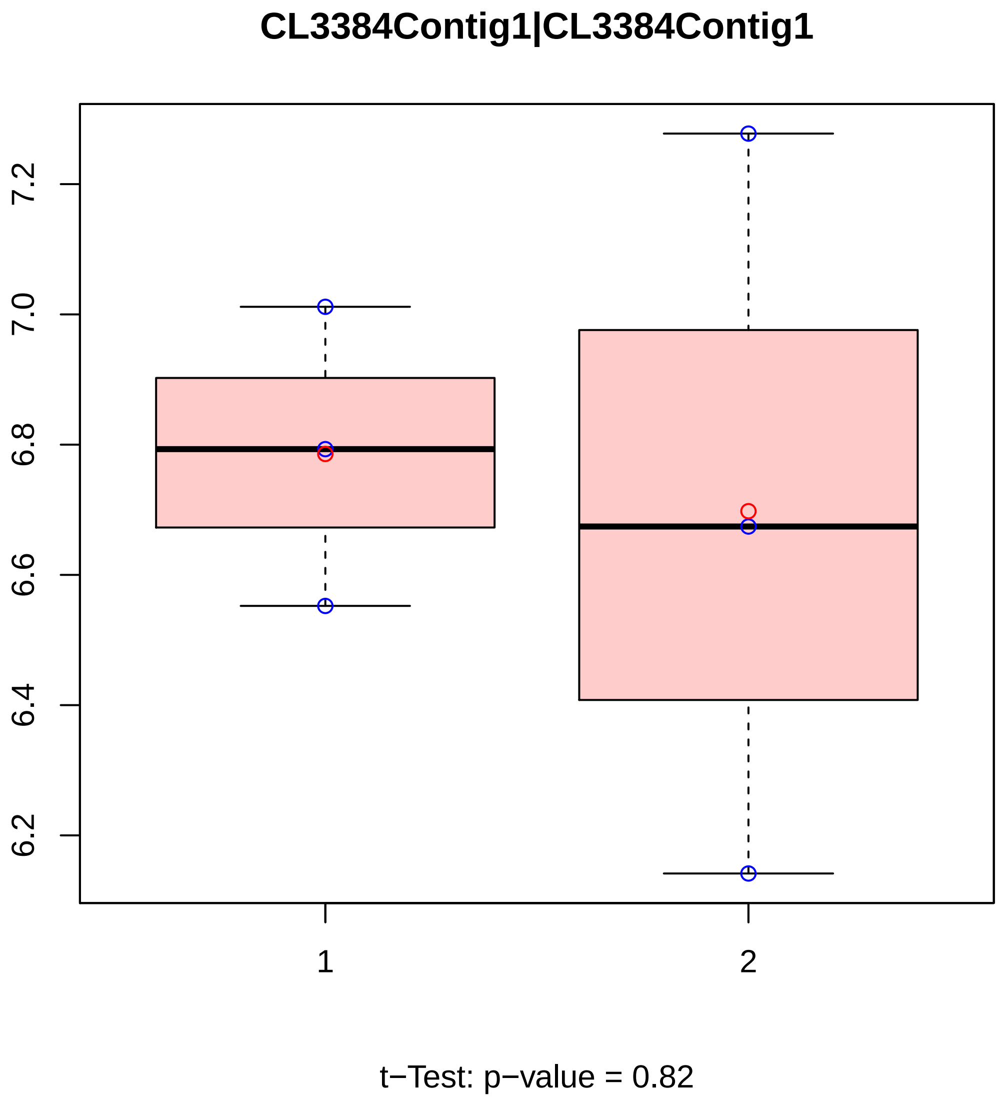
CL3384Contig1|CL3384Contig1
7.2
7.0
6.8
6.6
6.4
6.2
1
2
t−Test: p−value = 0.82

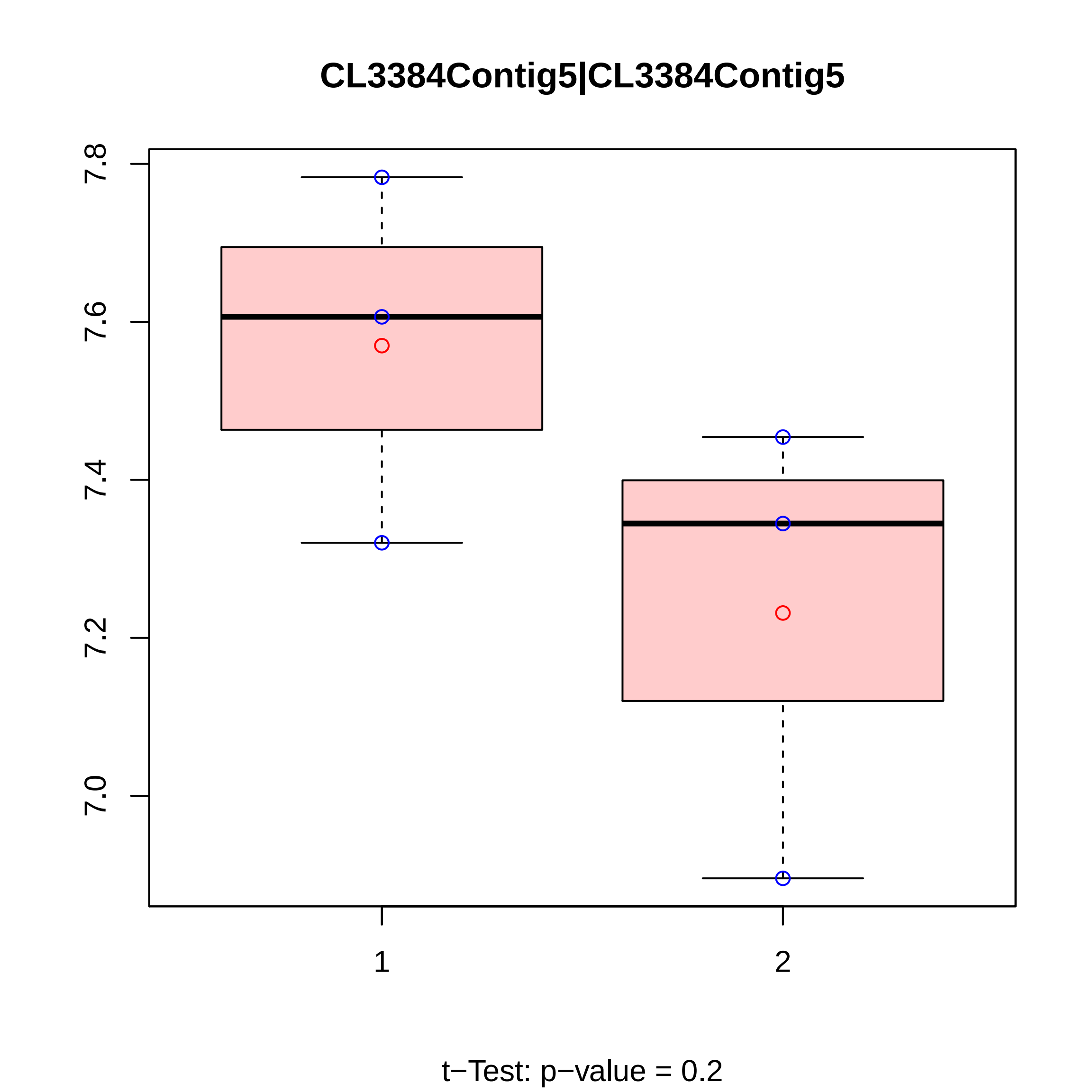
CL3384Contig5|CL3384Contig5
7.8
7.6
7.4
7.2
7.0
1
2
t−Test: p−value = 0.2

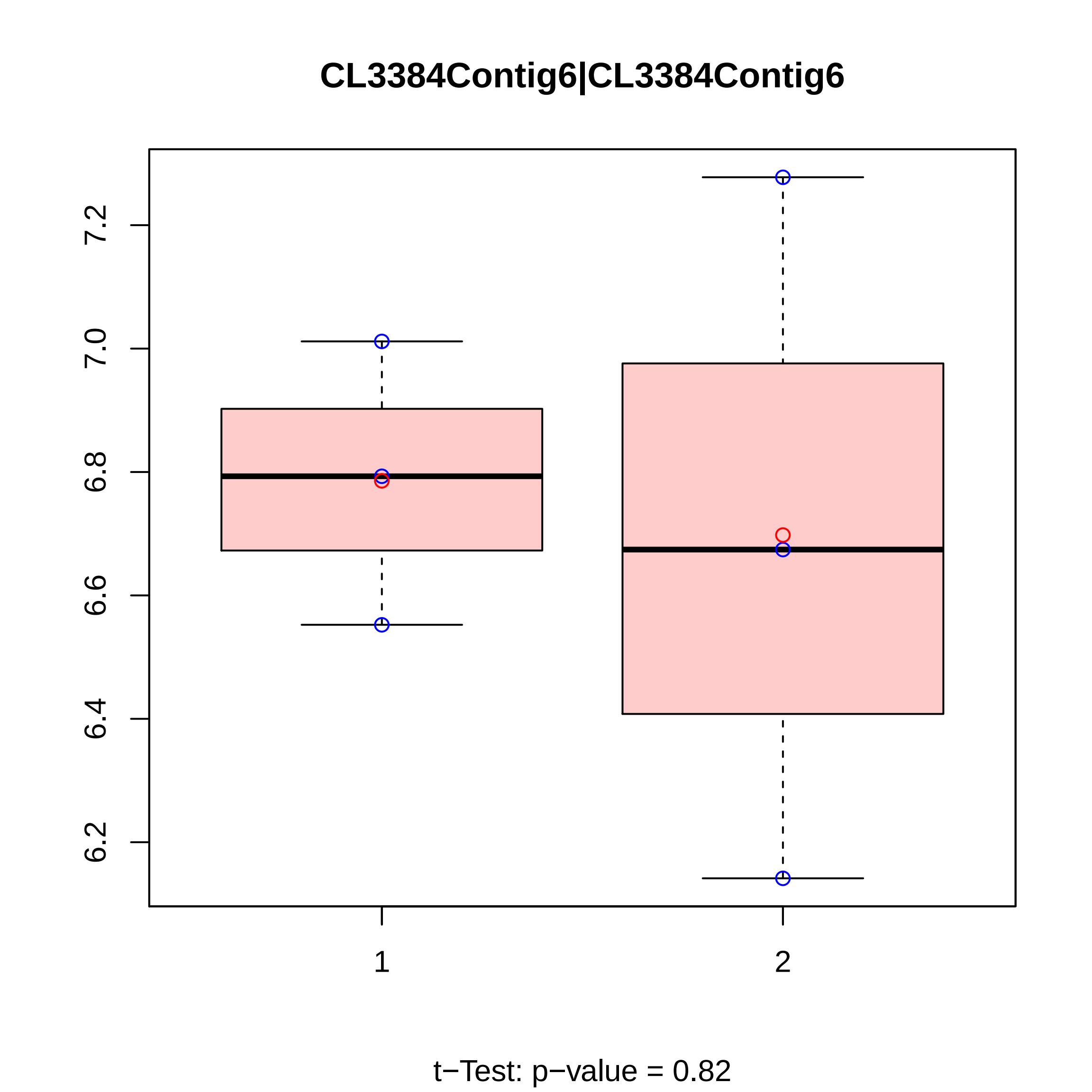

CL3384Contig6|CL3384Contig6
7.2
7.0
6.8
6.6
6.4
6.2
1
2
t−Test: p−value = 0.82

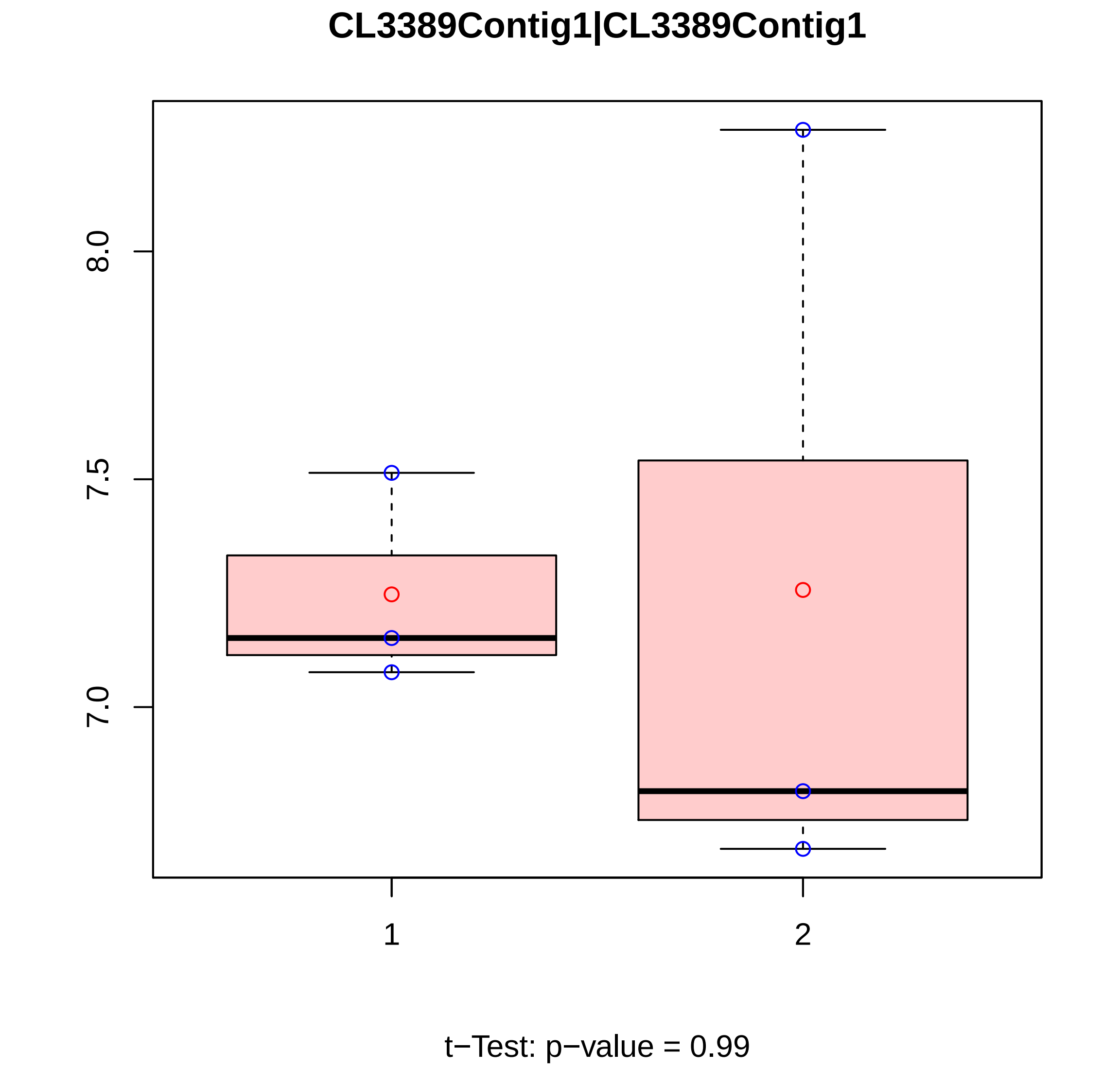
CL3389Contig1|CL3389Contig1
8.0
7.5
7.0
1
2
t−Test: p−value = 0.99

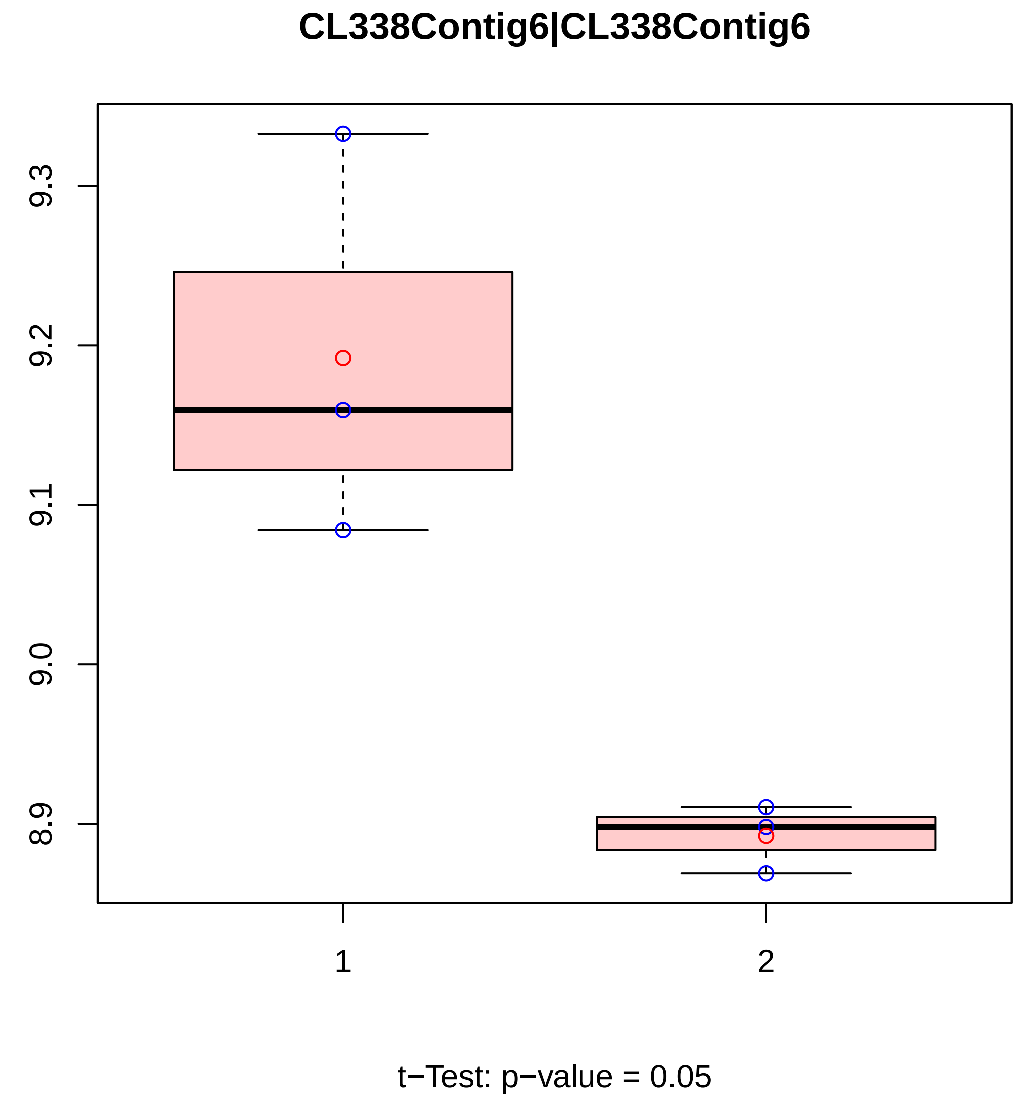

CL338Contig6|CL338Contig6
9.3
9.2
9.1
9.0
8.9
1
2
t−Test: p−value = 0.05

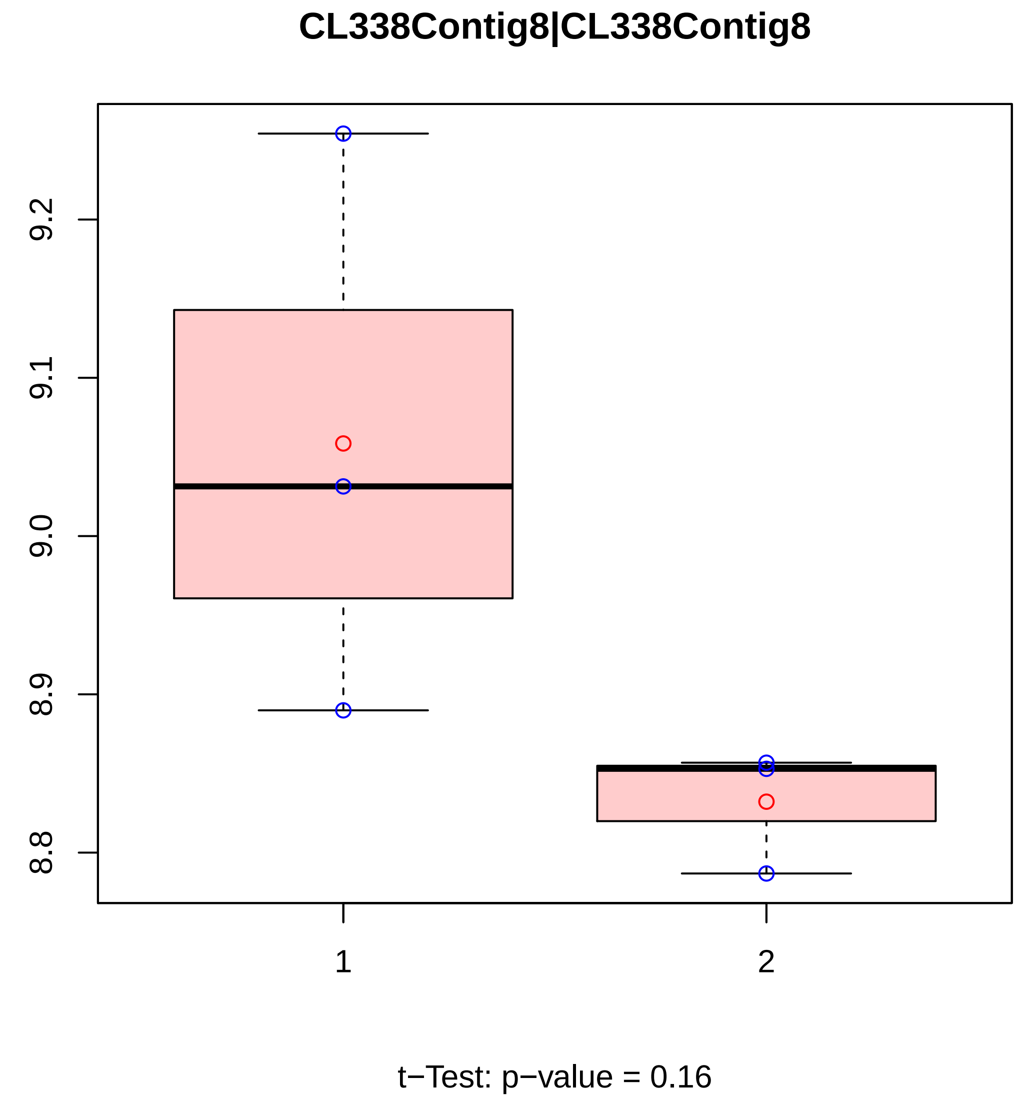

CL338Contig8|CL338Contig8
9.2
9.1
9.0
8.9
8.8
1
2
t−Test: p−value = 0.16

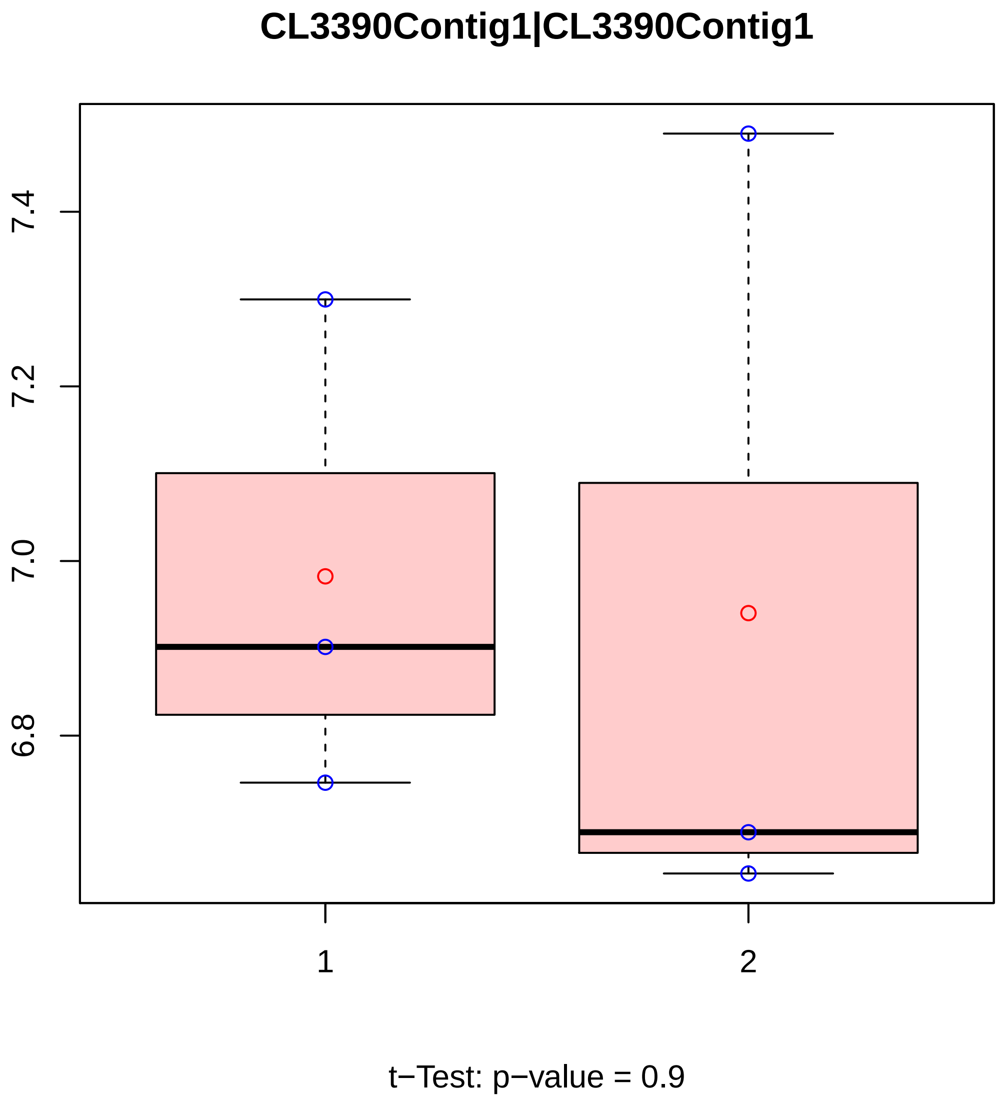

CL3390Contig1|CL3390Contig1
7.4
7.2
7.0
6.8
1
2
t−Test: p−value = 0.9

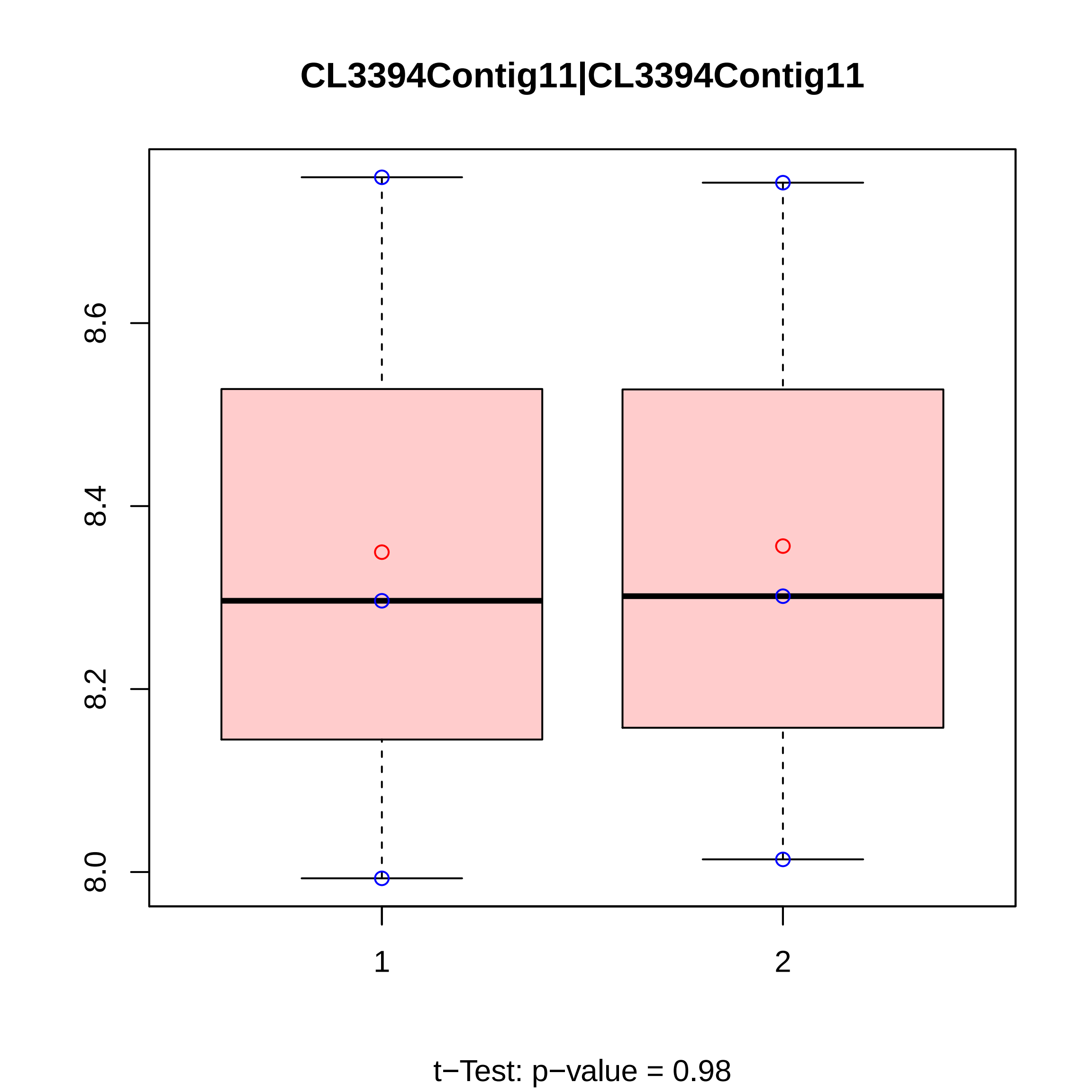
CL3394Contig11|CL3394Contig11
8.0
8.2
8.4
8.6
1
2
t−Test: p−value = 0.98

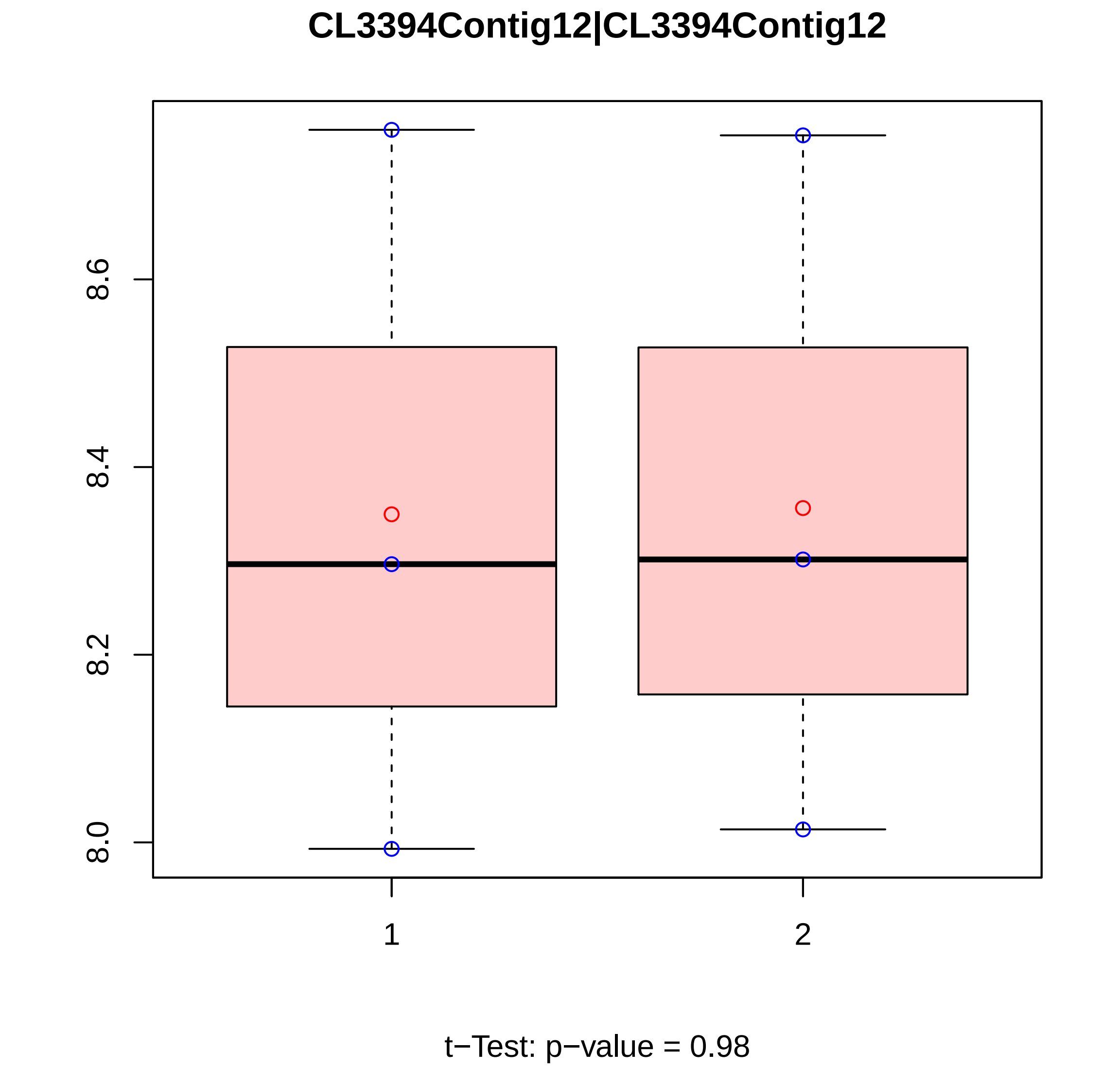

CL3394Contig12|CL3394Contig12
8.0
8.2
8.4
8.6
1
2
t−Test: p−value = 0.98

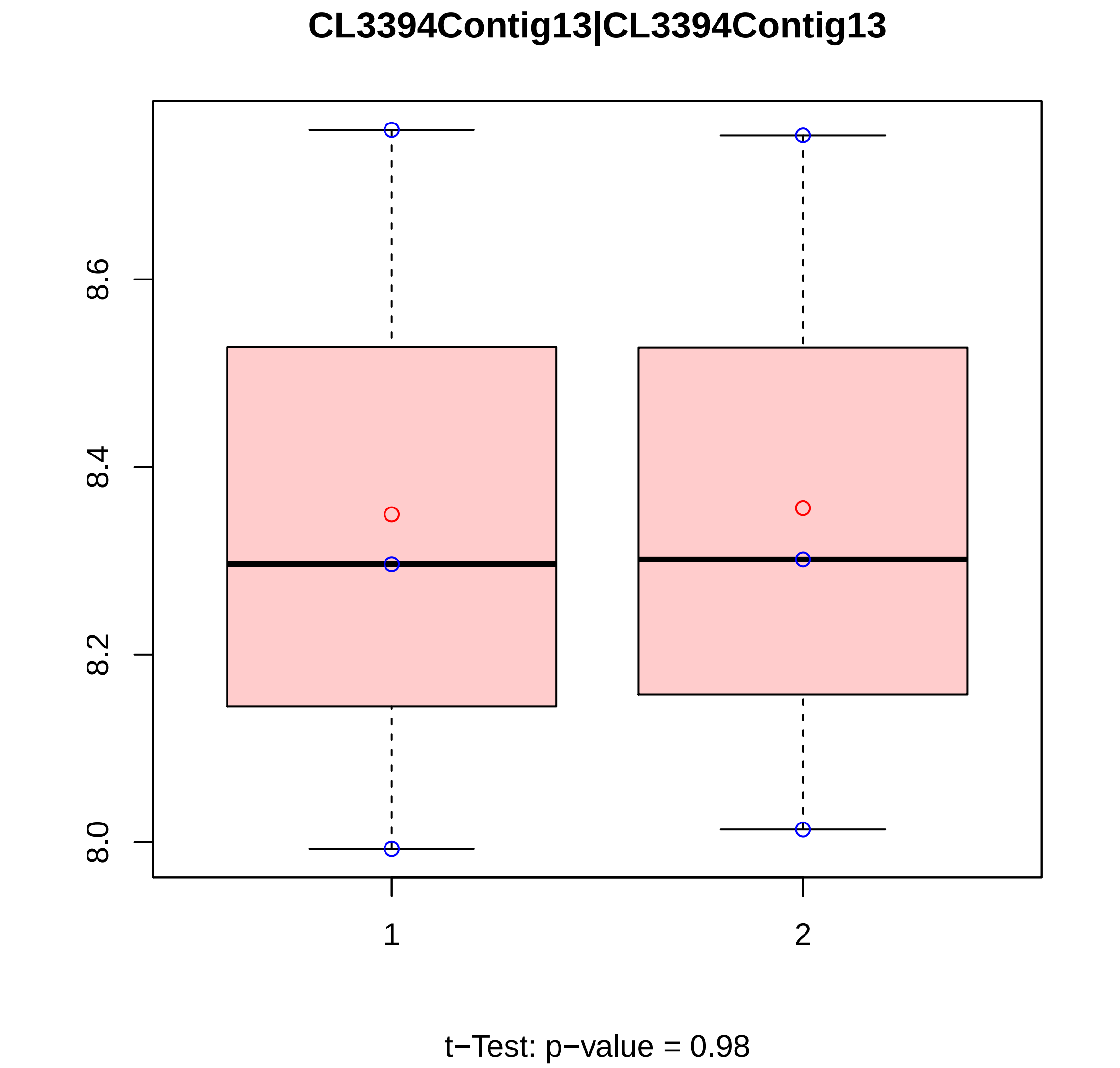
CL3394Contig13|CL3394Contig13
8.0
8.2
8.4
8.6
1
2
t−Test: p−value = 0.98

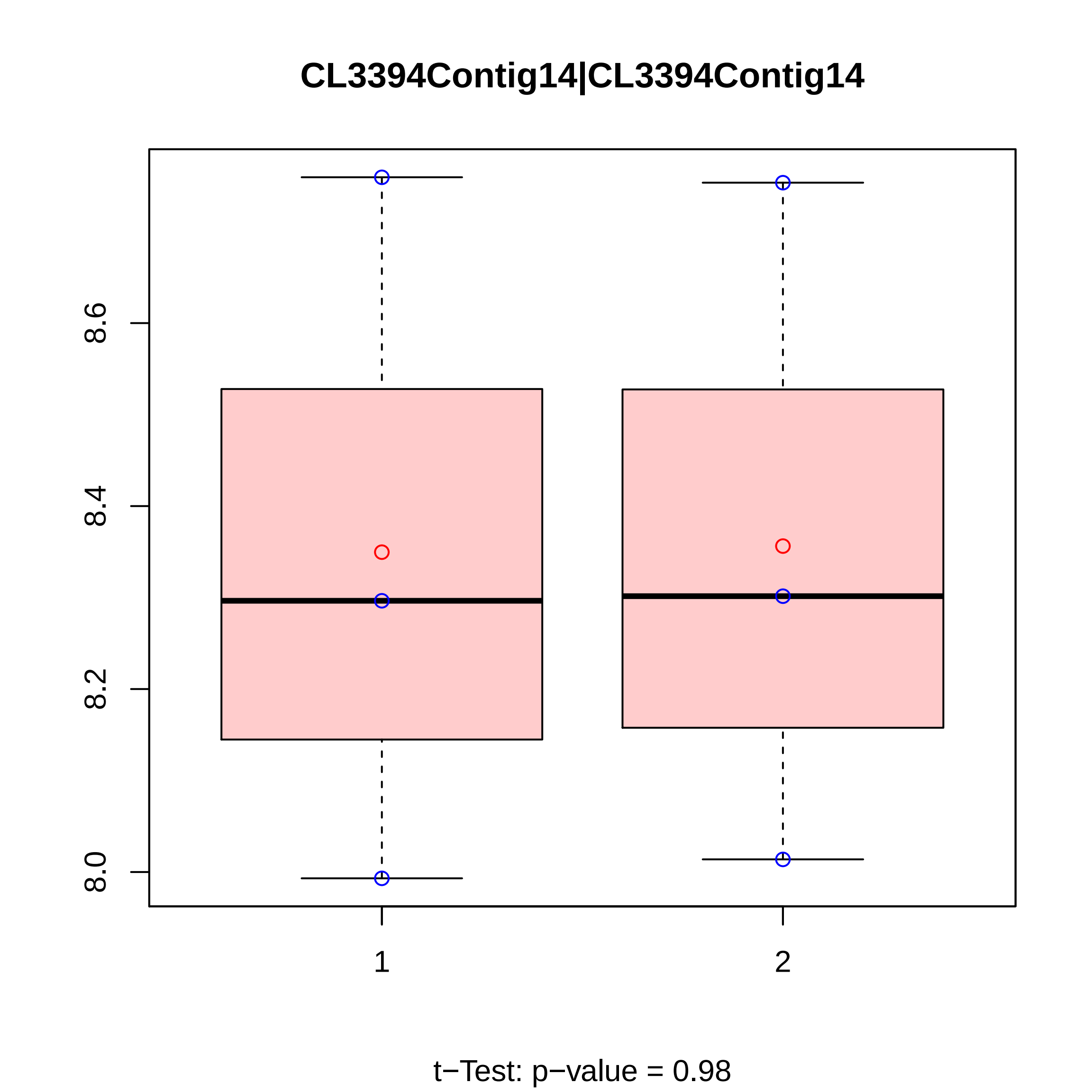

CL3394Contig14|CL3394Contig14
8.0
8.2
8.4
8.6
1
2
t−Test: p−value = 0.98

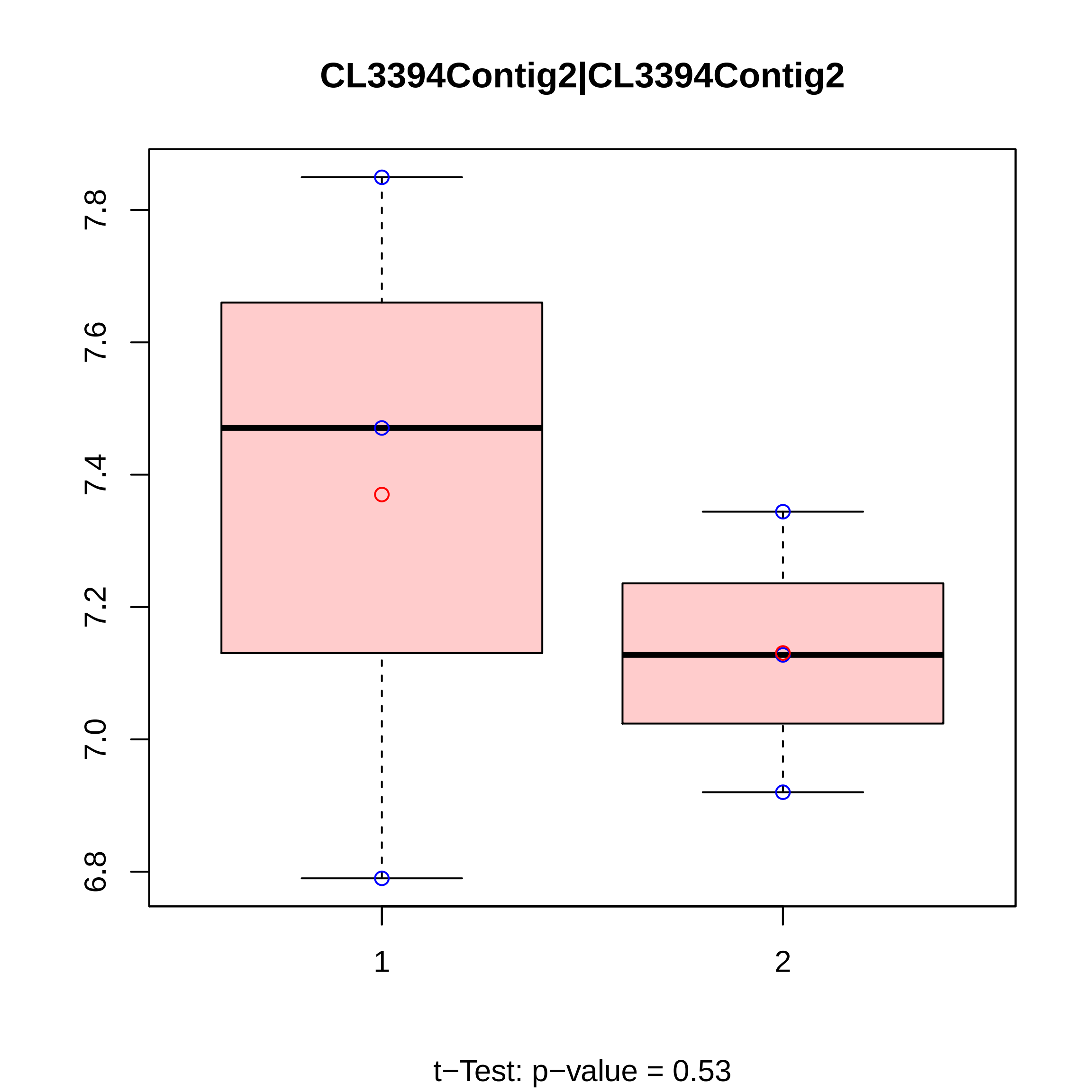

CL3394Contig2|CL3394Contig2
7.8
7.6
7.4
7.2
7.0
6.8
1
2
t−Test: p−value = 0.53

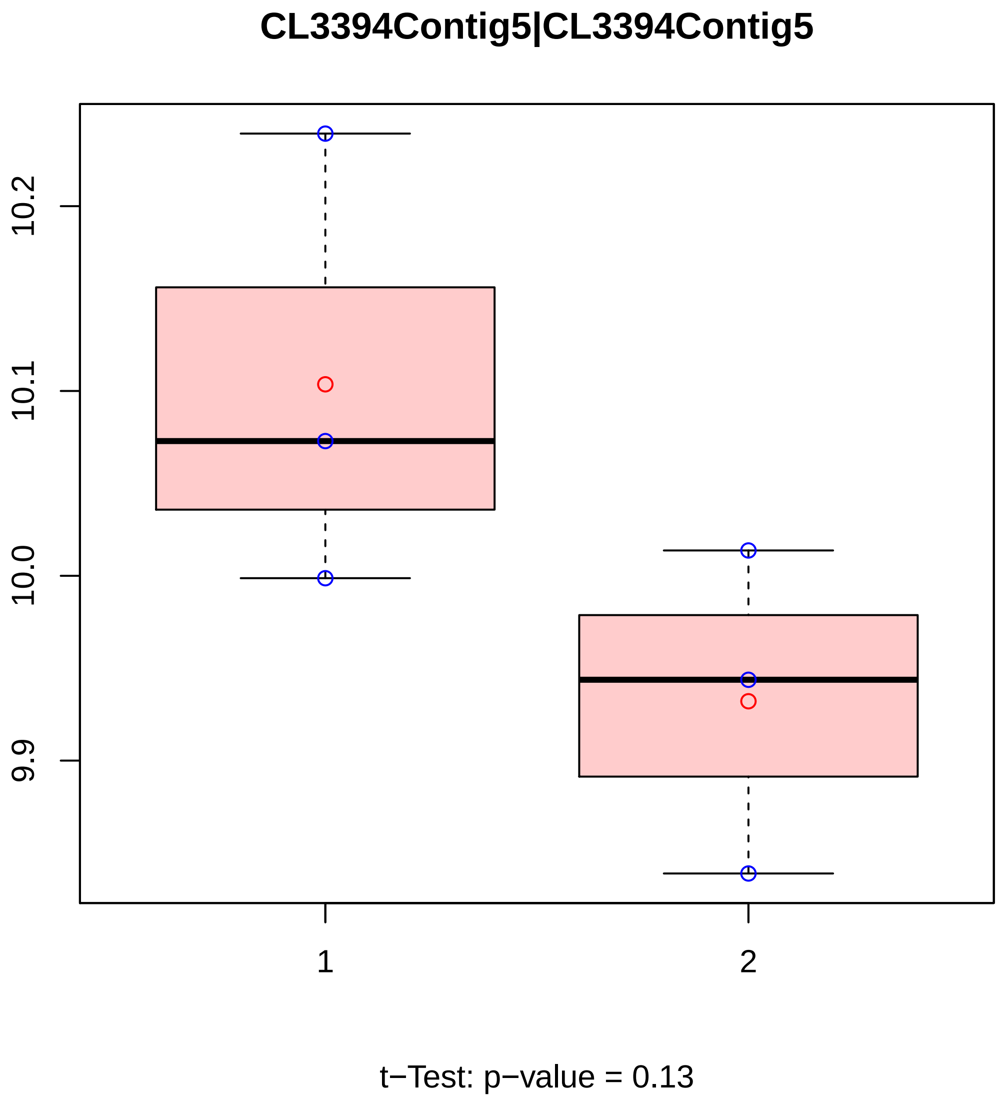
CL3394Contig5|CL3394Contig5
10.2
10.1
10.0
9.9
1
2
t−Test: p−value = 0.13

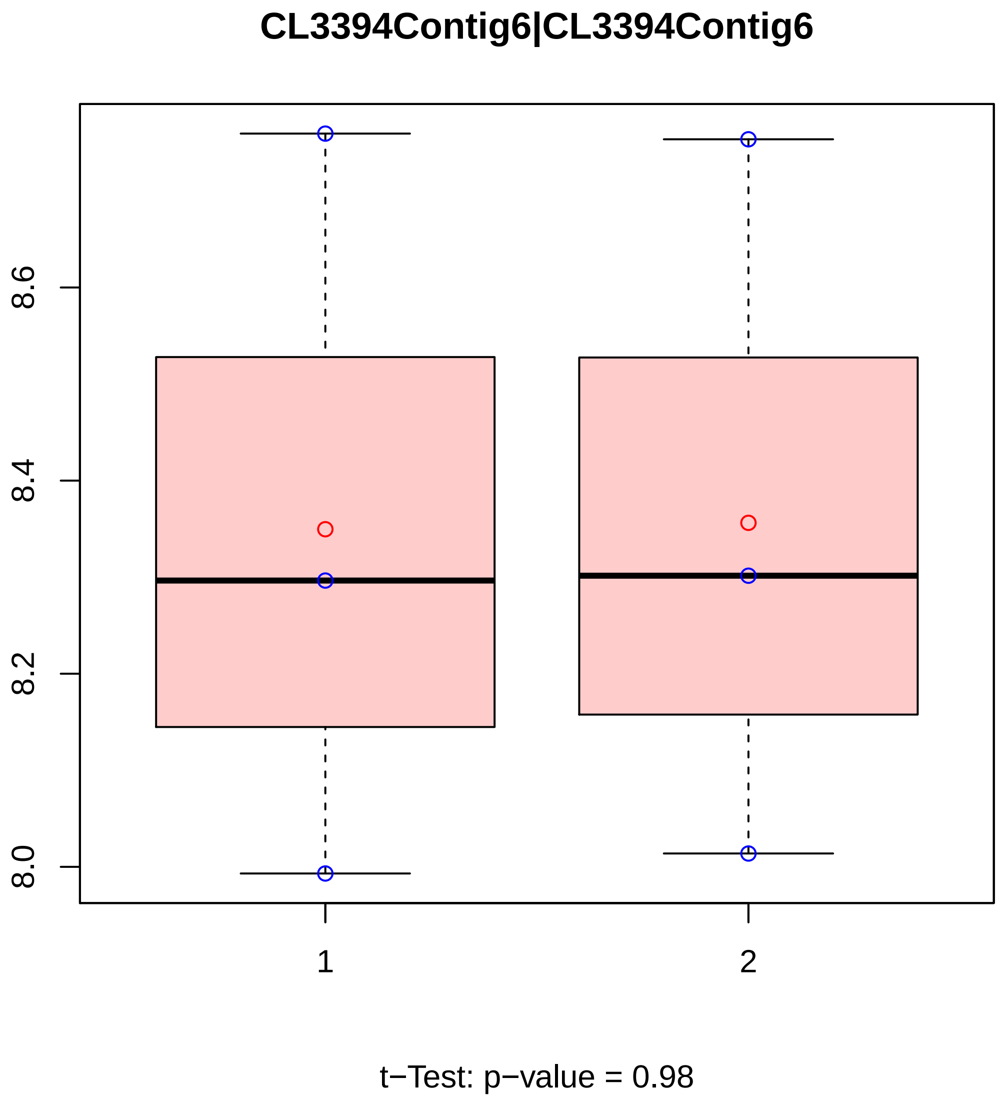
CL3394Contig6|CL3394Contig6
8.0
8.2
8.4
8.6
1
2
t−Test: p−value = 0.98

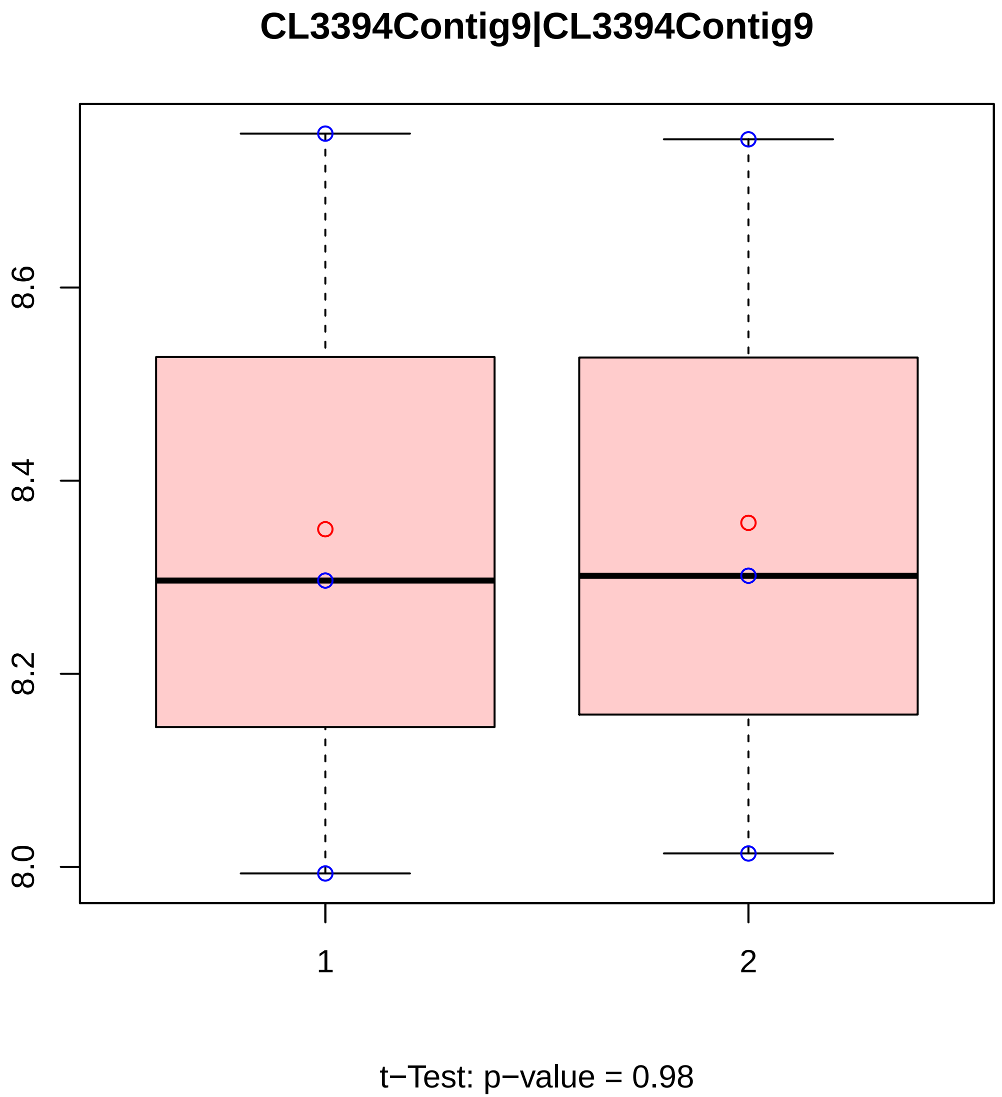

CL3394Contig9|CL3394Contig9
8.0
8.2
8.4
8.6
1
2
t−Test: p−value = 0.98

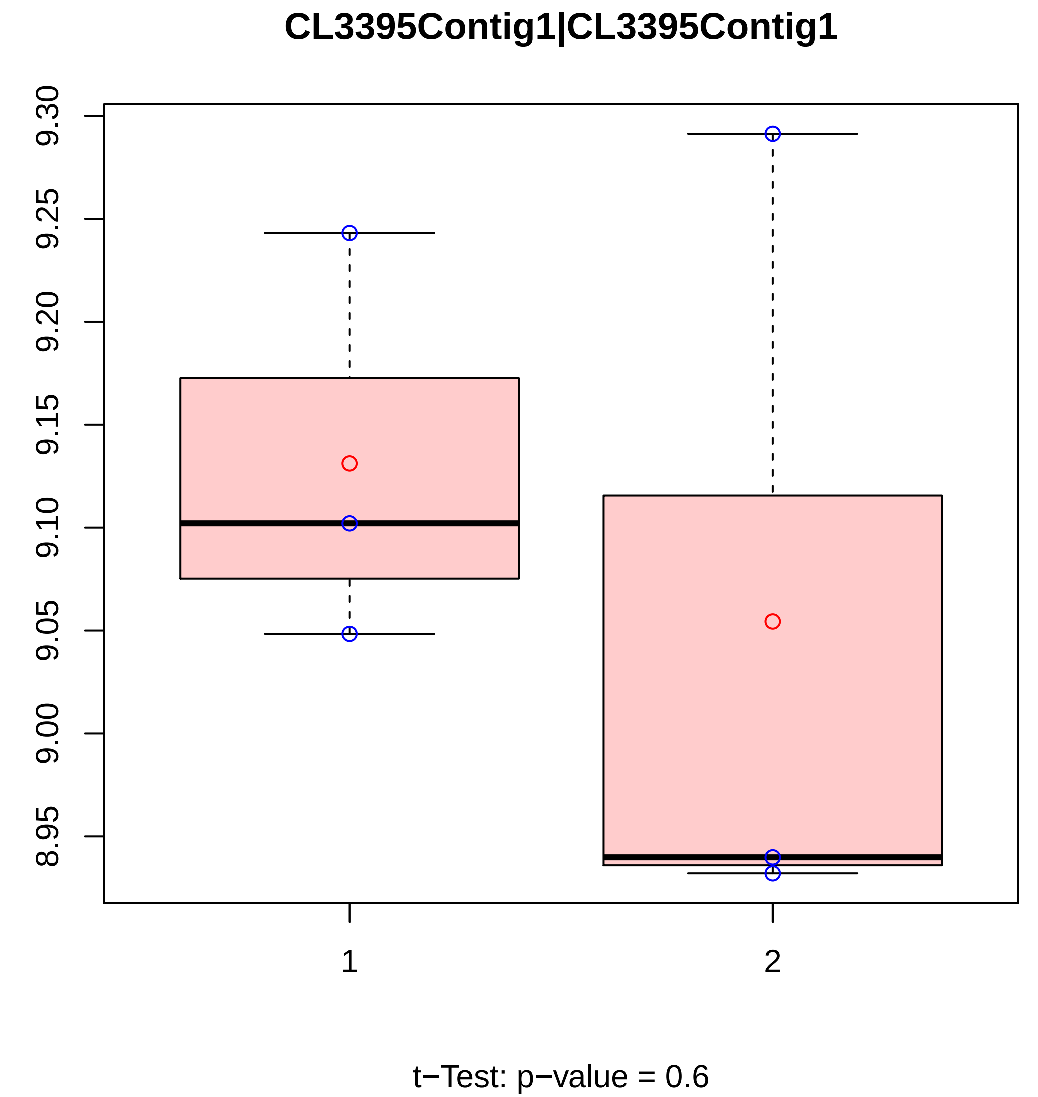
CL3395Contig1|CL3395Contig1
9.30
9.25
9.20
9.15
9.10
9.05
9.00
8.95
1
2
t−Test: p−value = 0.6

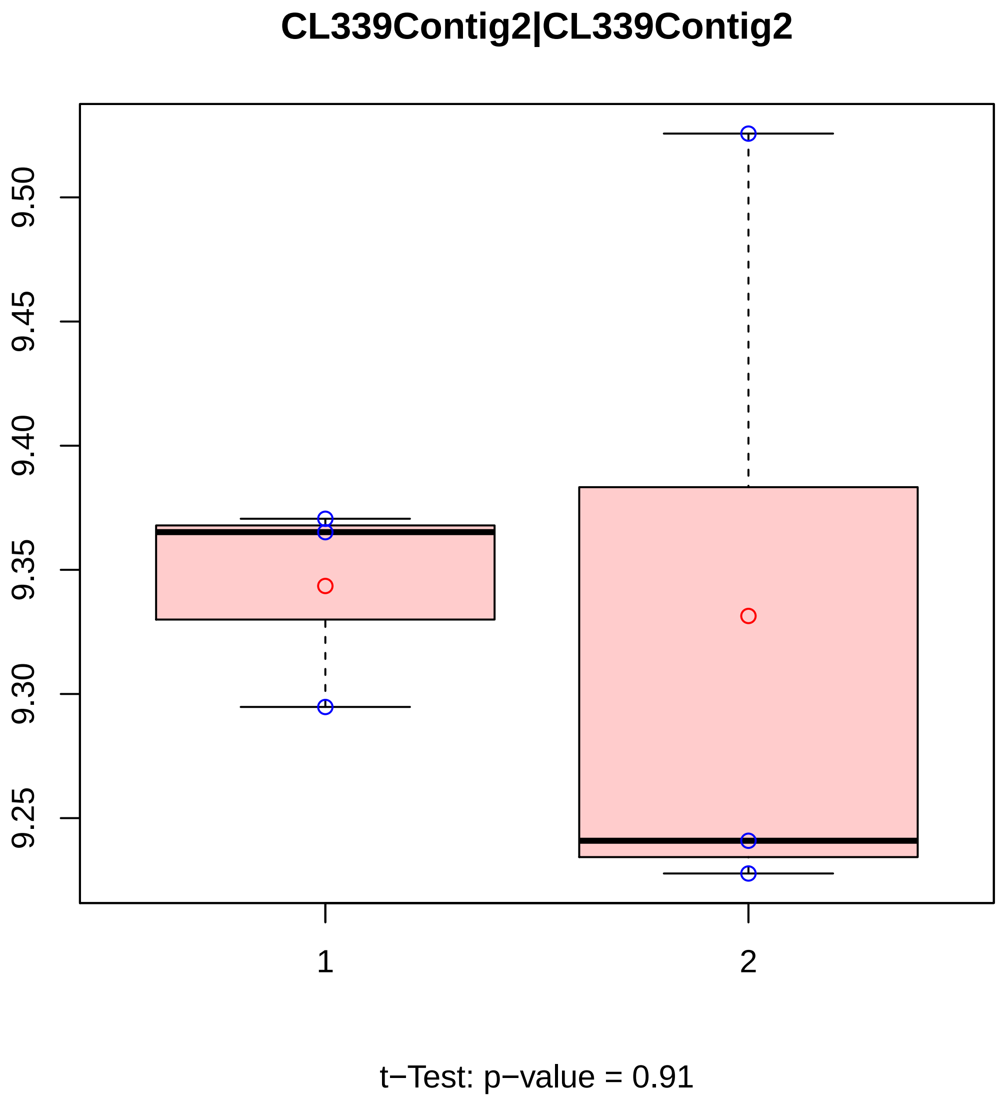

CL339Contig2|CL339Contig2
9.25
9.30
9.35
9.40
9.45
9.50
1
2
t−Test: p−value = 0.91

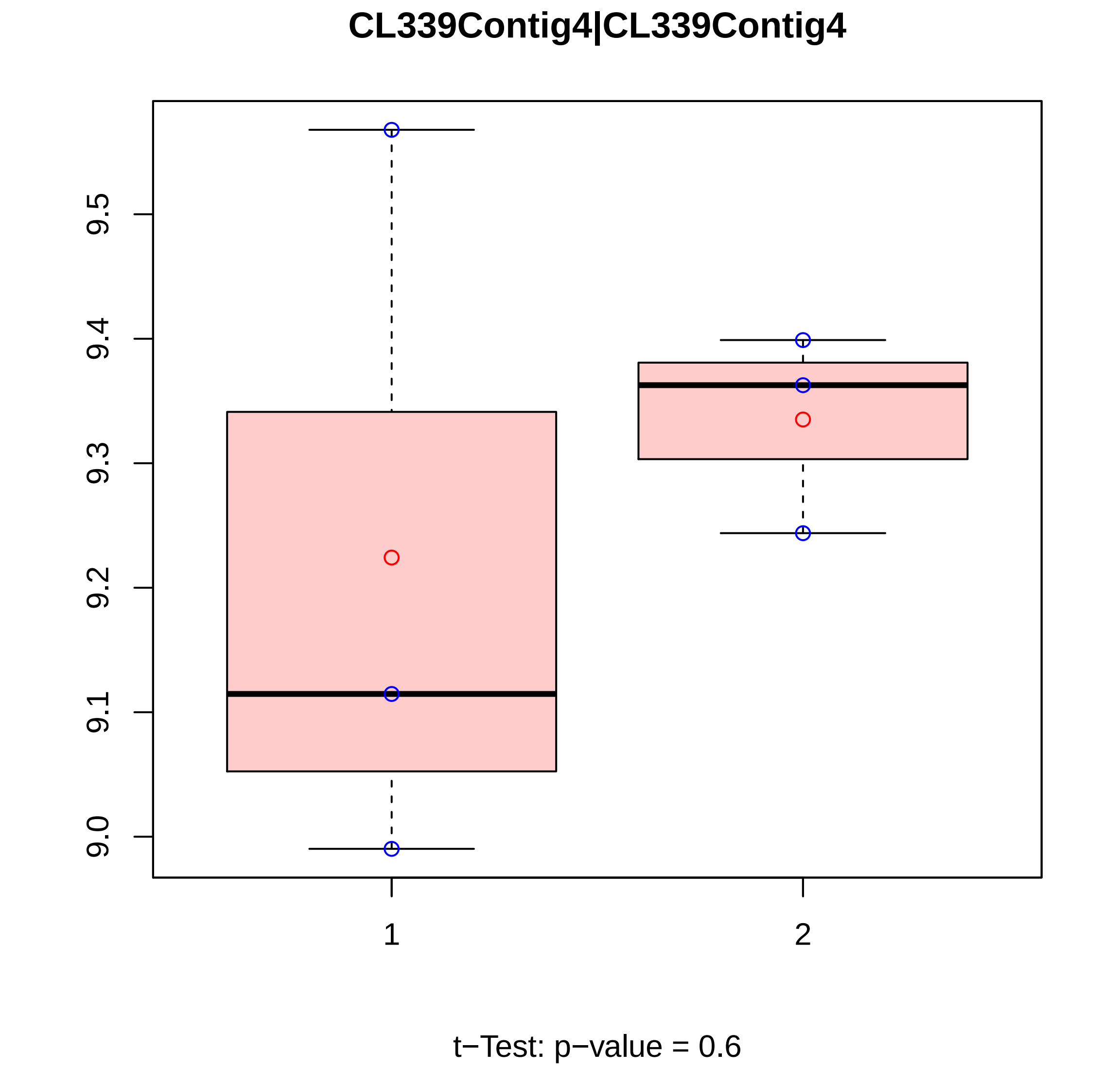
CL339Contig4|CL339Contig4
9.0
9.1
9.2
9.3
9.4
9.5
1
2
t−Test: p−value = 0.6

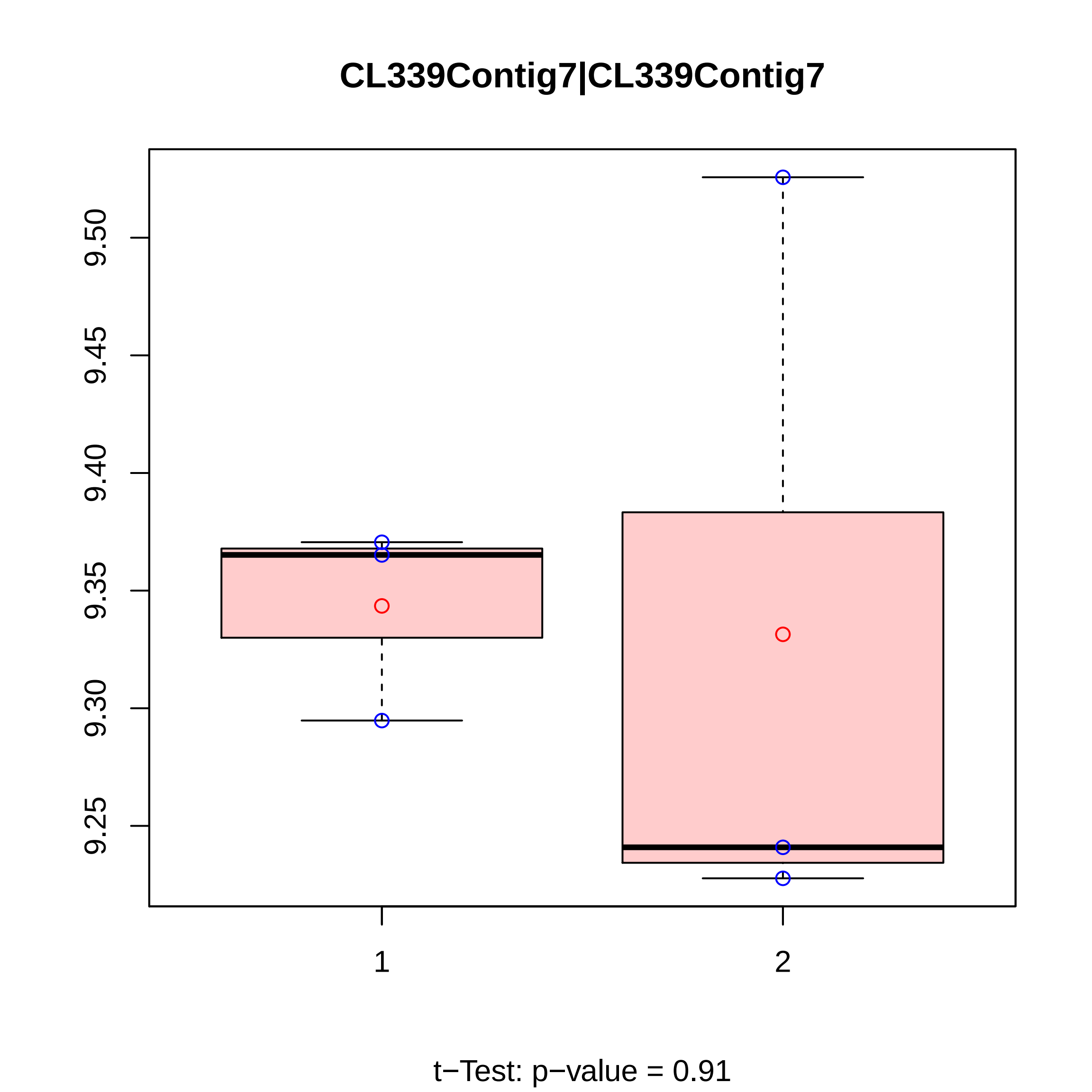

CL339Contig7|CL339Contig7
9.25
9.30
9.35
9.40
9.45
9.50
1
2
t−Test: p−value = 0.91

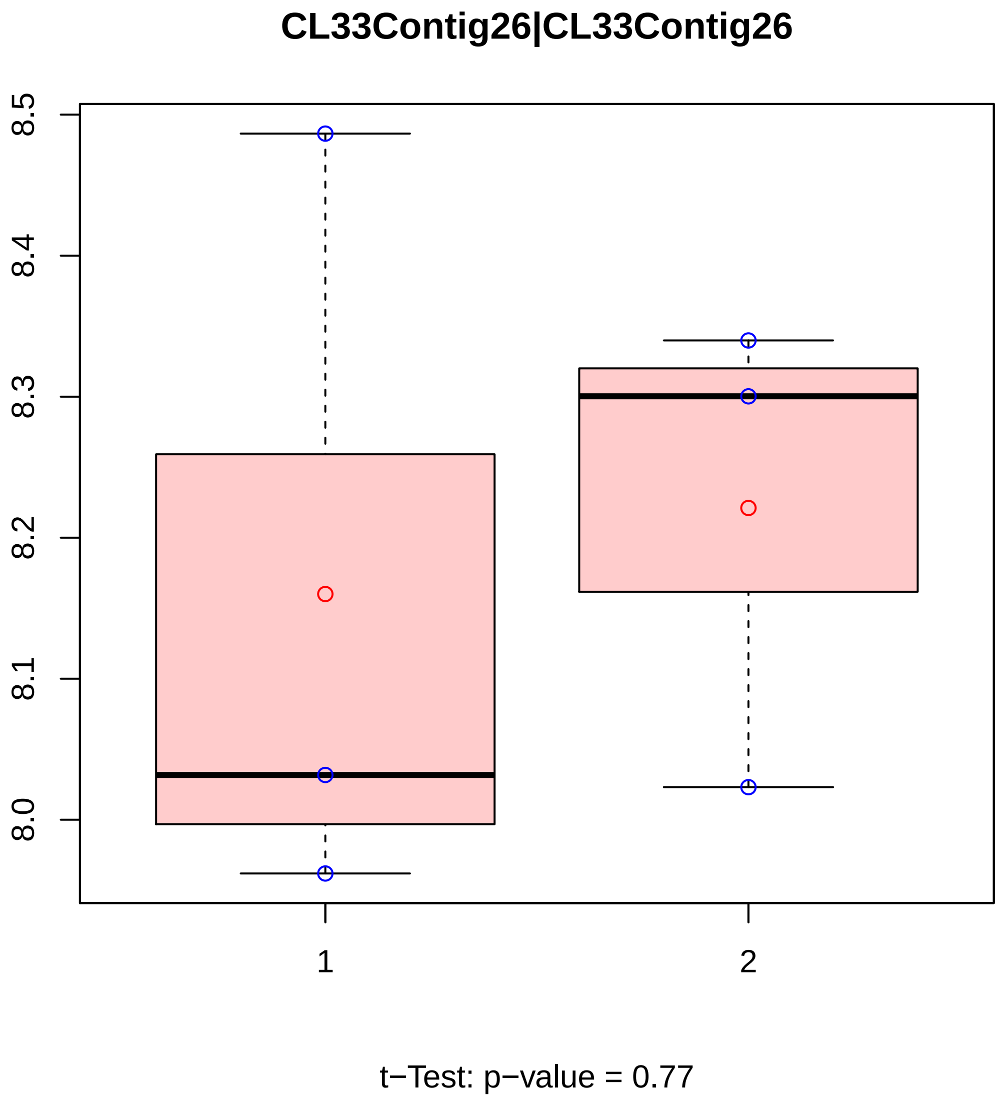
CL33Contig26|CL33Contig26
8.5
8.4
8.3
8.2
8.1
8.0
1
2
t−Test: p−value = 0.77

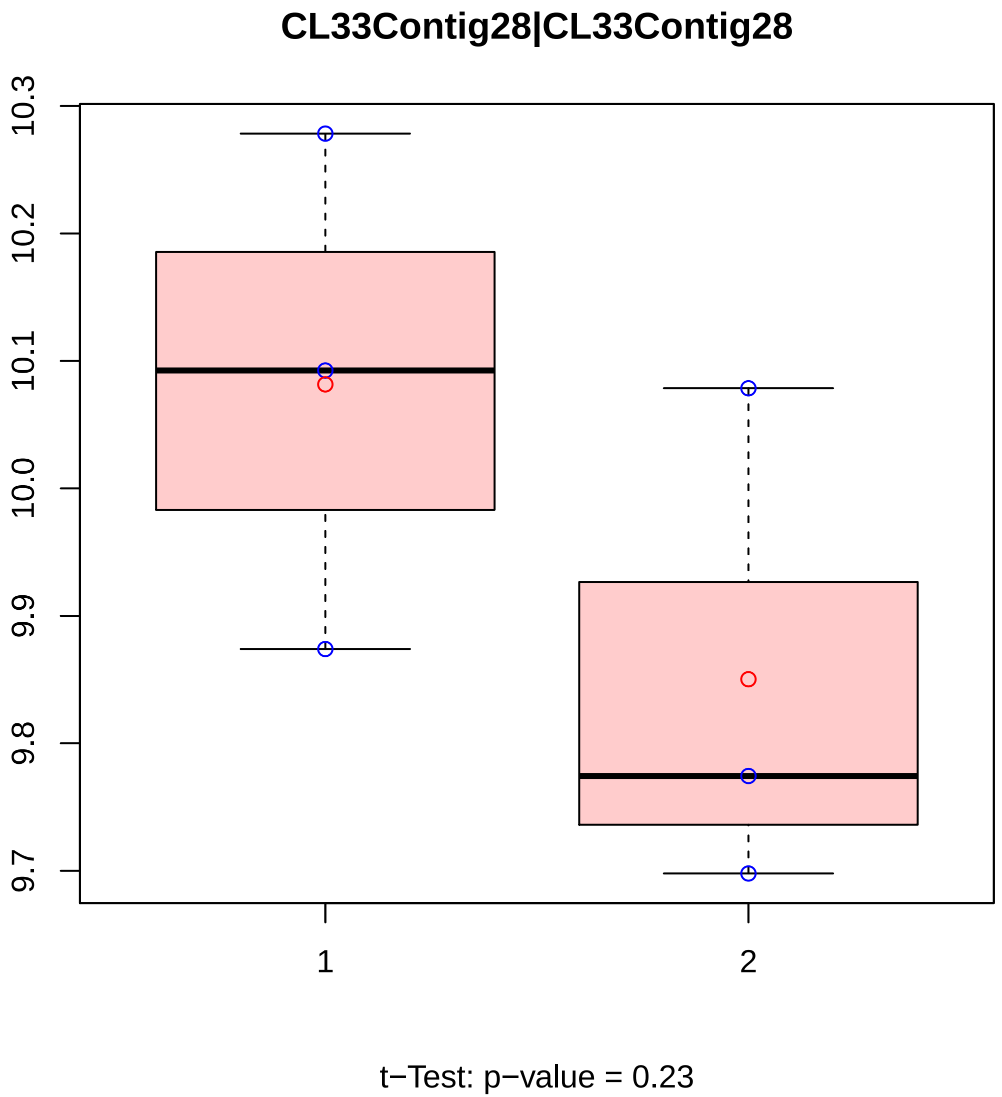

CL33Contig28|CL33Contig28
10.3
10.2
10.1
10.0
9.9
9.8
9.7
1
2
t−Test: p−value = 0.23

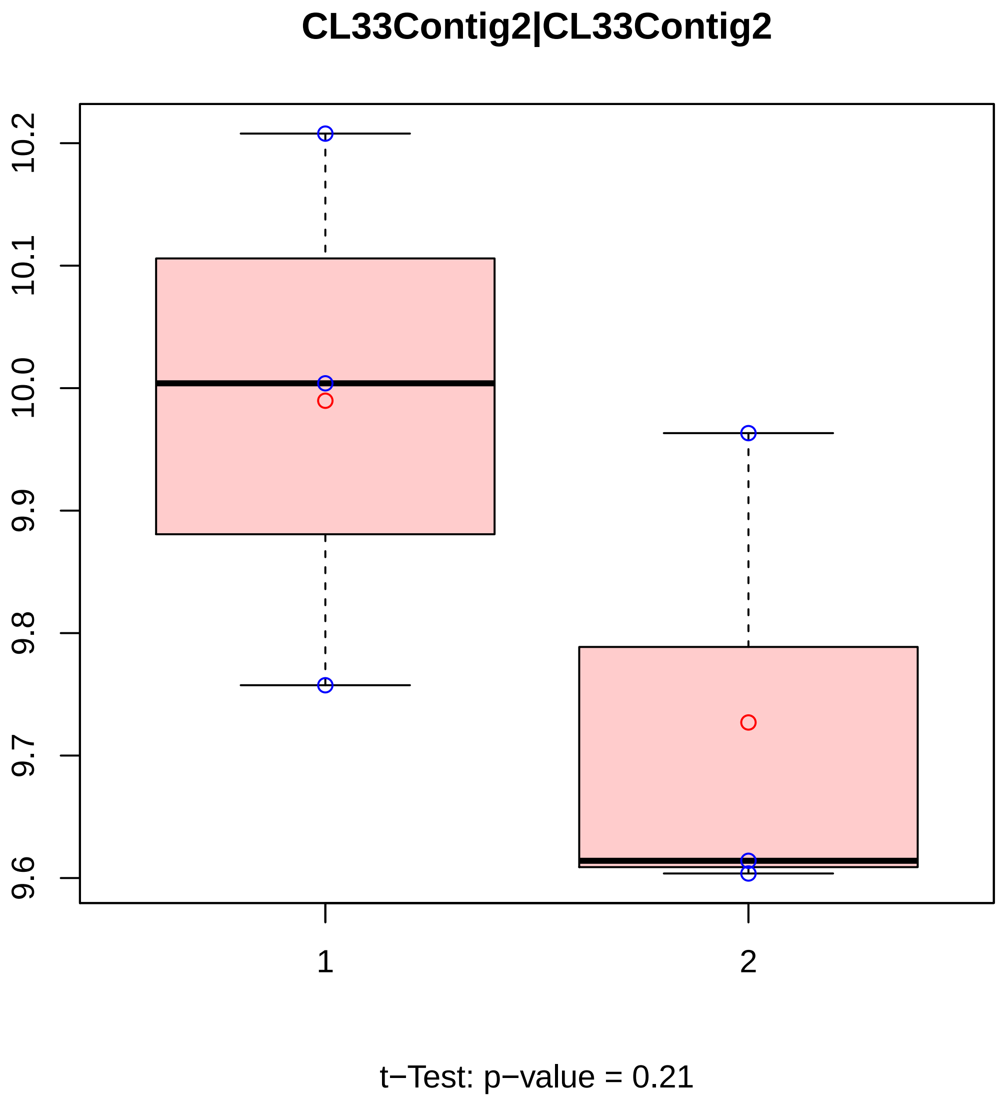

CL33Contig2|CL33Contig2
10.2
10.1
10.0
9.9
9.8
9.7
9.6
1
2
t−Test: p−value = 0.21

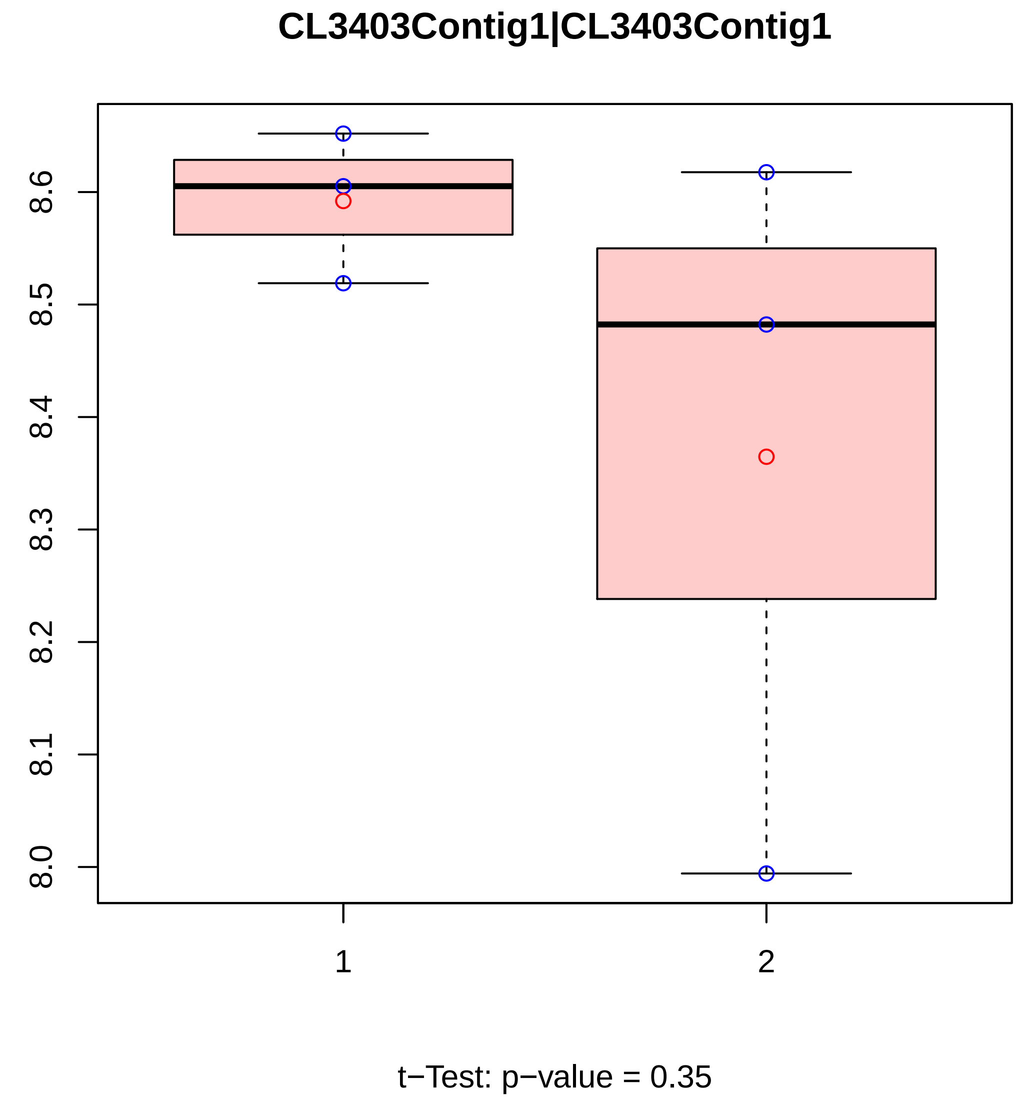
CL3403Contig1|CL3403Contig1
8.0
8.1
8.2
8.3
8.4
8.5
8.6
1
2
t−Test: p−value = 0.35

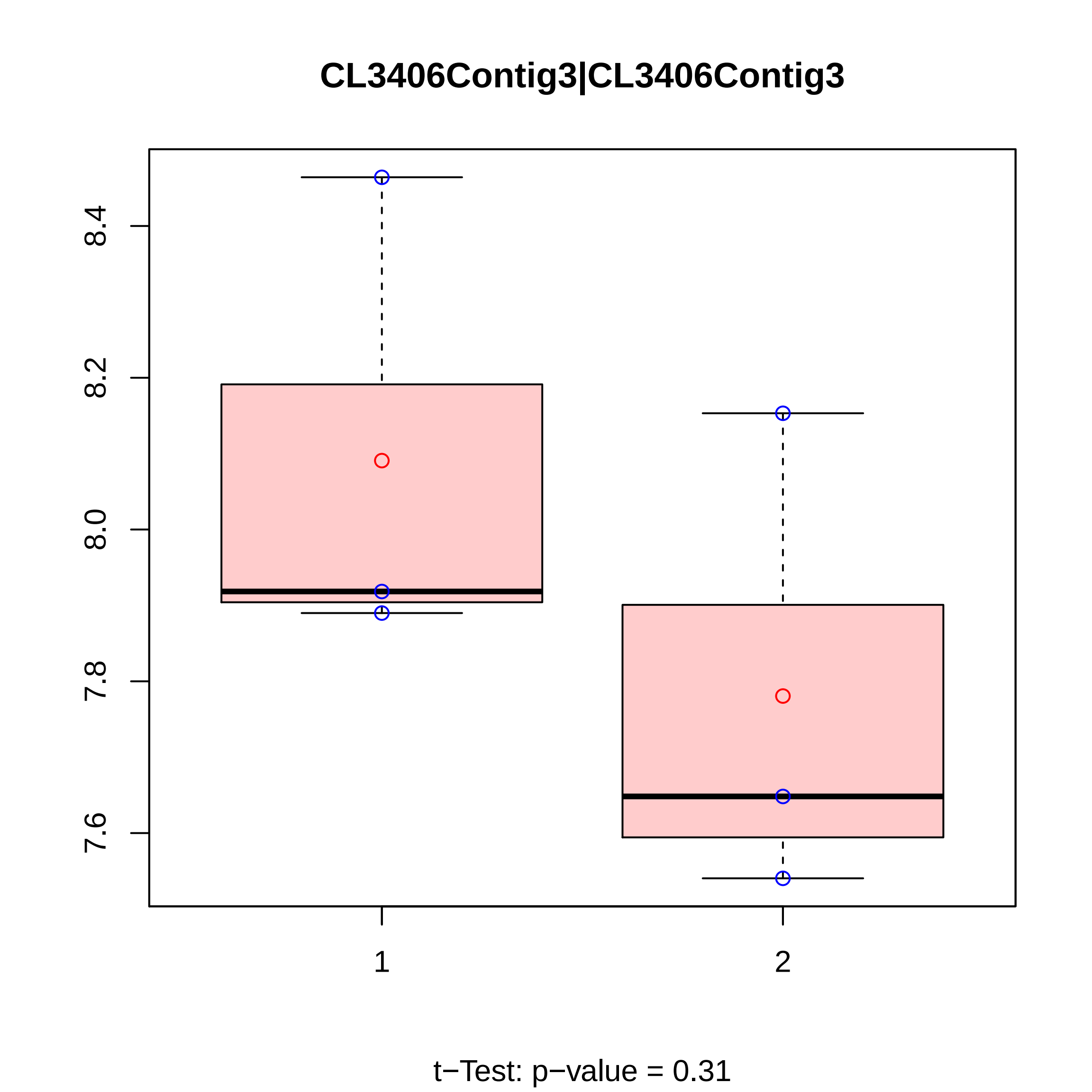

CL3406Contig3|CL3406Contig3
8.4
8.2
8.0
7.8
7.6
1
2
t−Test: p−value = 0.31

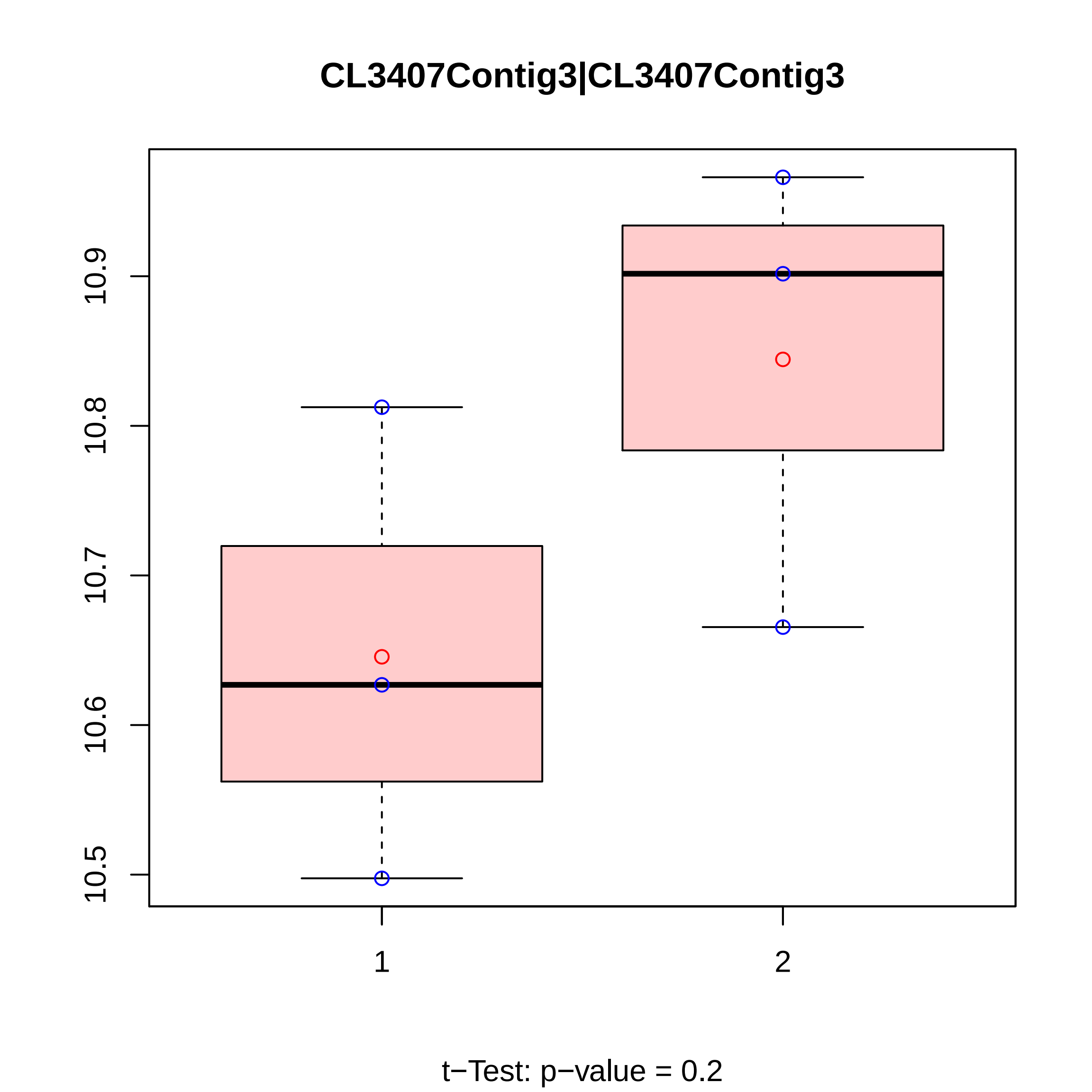
CL3407Contig3|CL3407Contig3
10.5
10.6
10.7
10.8
10.9
1
2
t−Test: p−value = 0.2

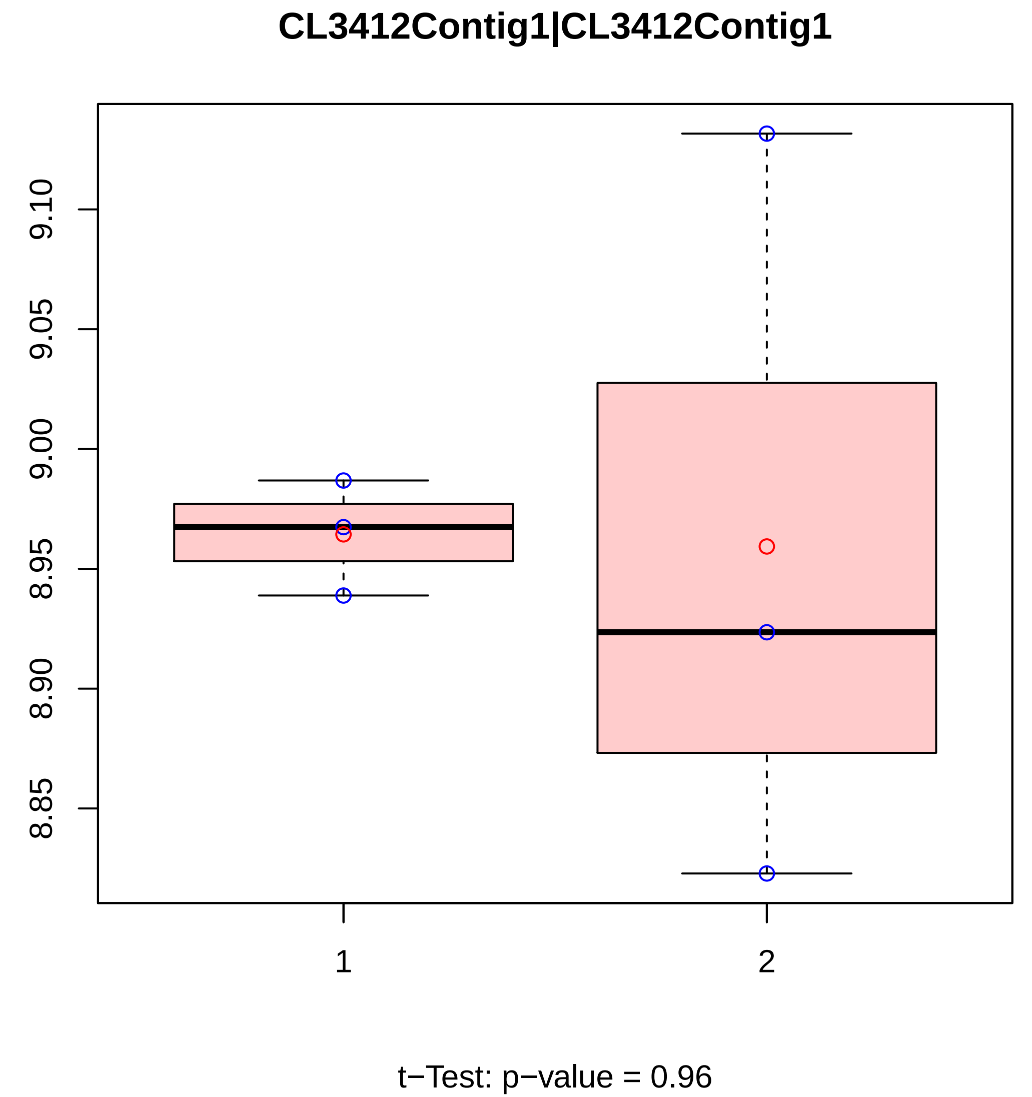
CL3412Contig1|CL3412Contig1
9.10
9.05
9.00
8.95
8.90
8.85
1
2
t−Test: p−value = 0.96

# CL3421Contig3|CL3421Contig3

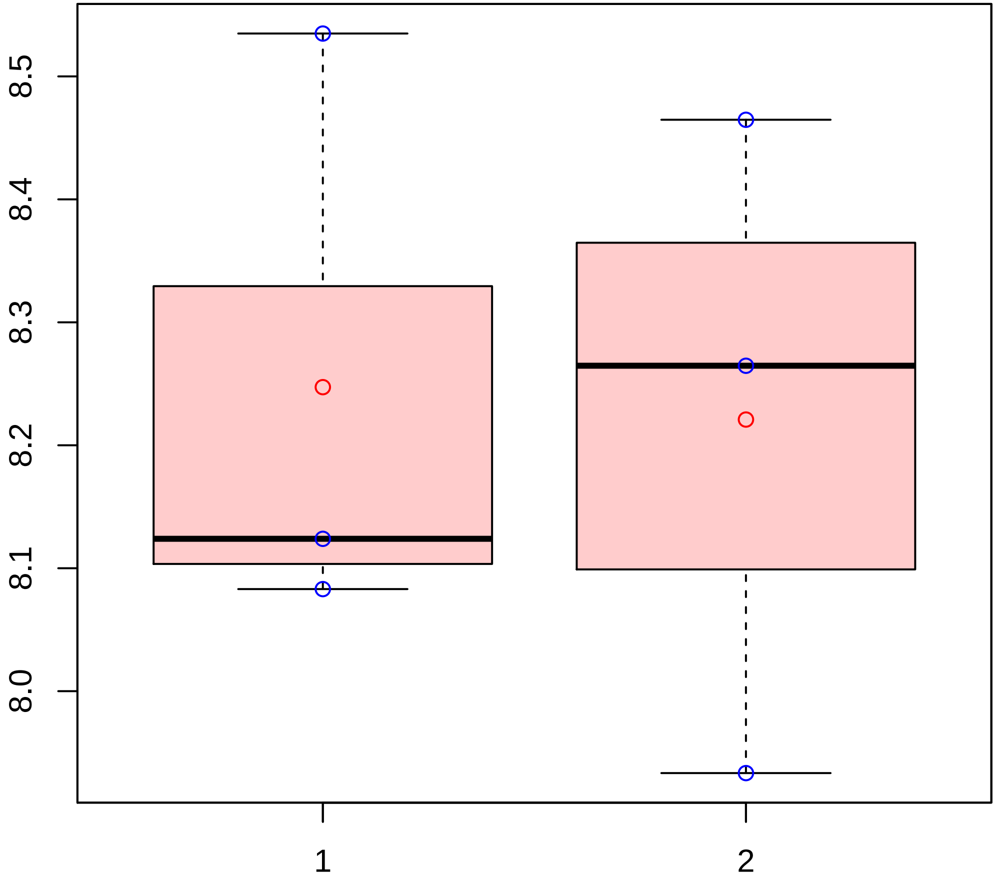


t−Test: p−value = 0.91

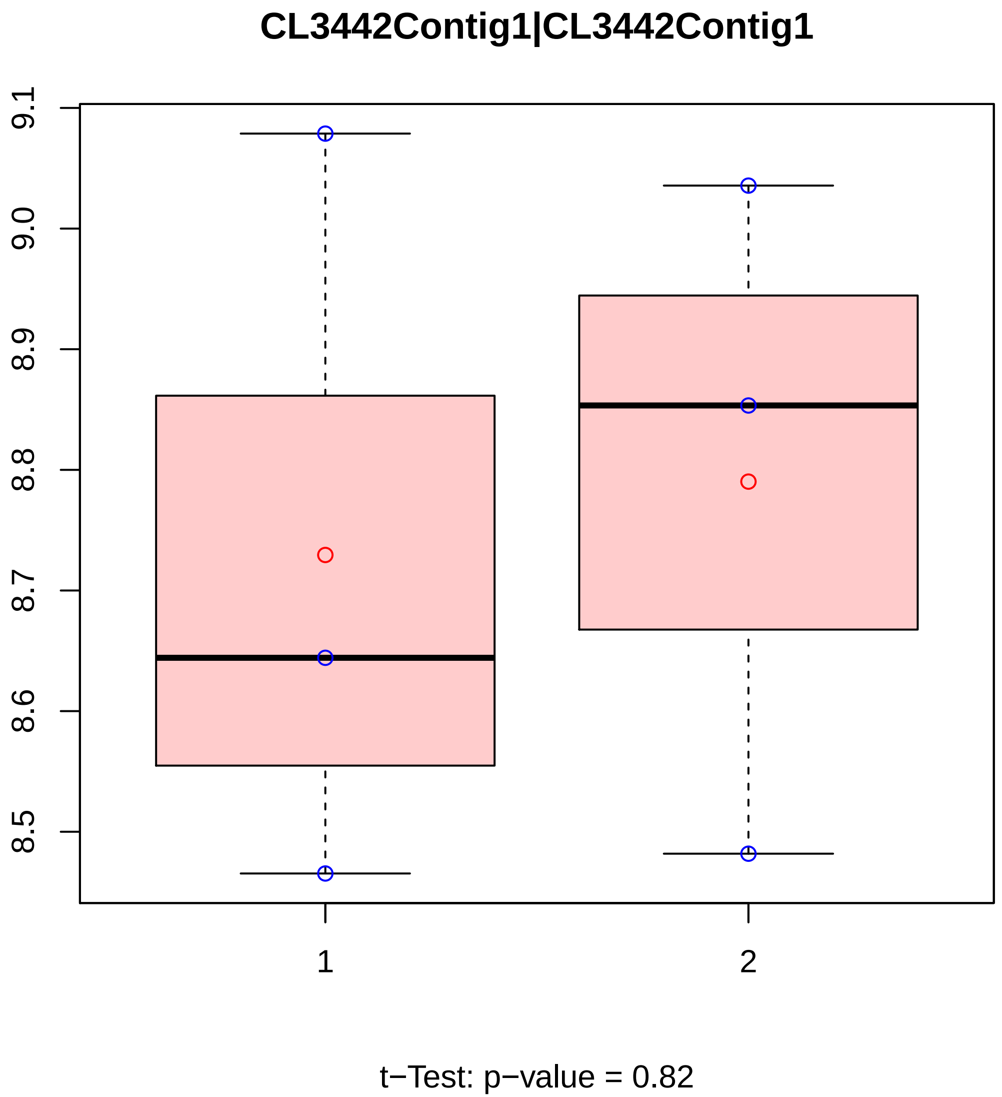
CL3442Contig1|CL3442Contig1
9.1
9.0
8.9
8.8
8.7
8.6
8.5
1
2
t−Test: p−value = 0.82

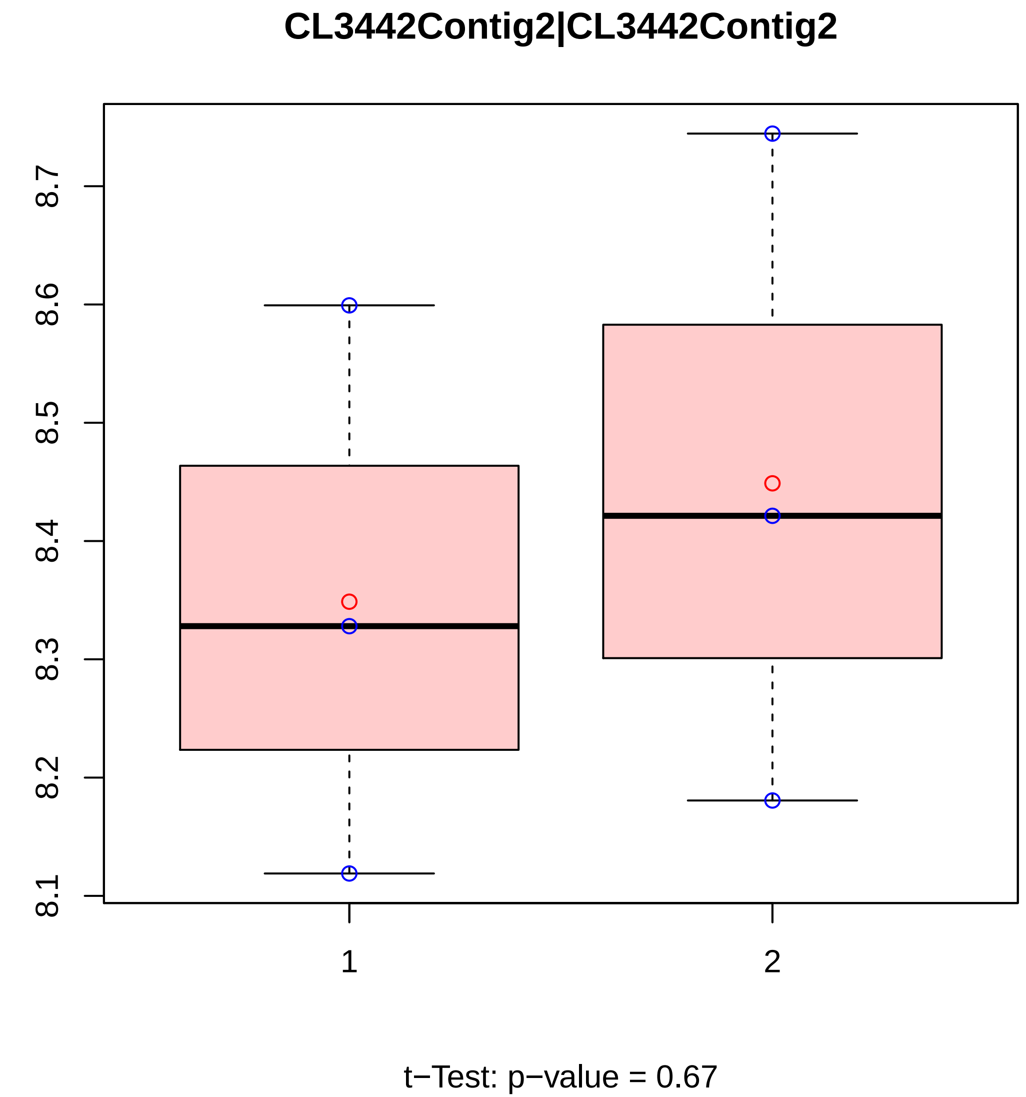
CL3442Contig2|CL3442Contig2
8.1
8.2
8.3
8.4
8.5
8.6
8.7
1
2
t−Test: p−value = 0.67

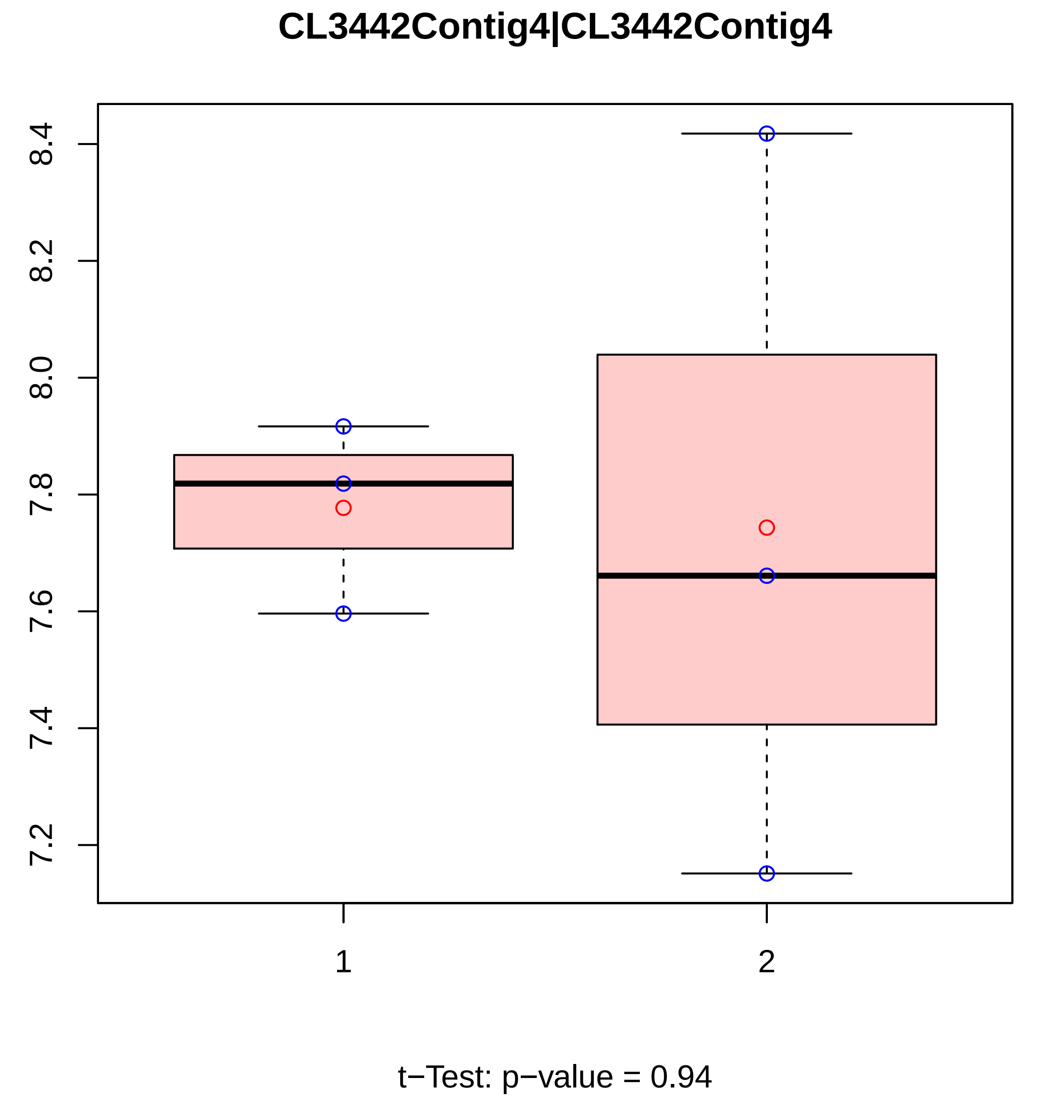

CL3442Contig4|CL3442Contig4
8.4
8.2
8.0
7.8
7.6
7.4
7.2
1
2
t−Test: p−value = 0.94

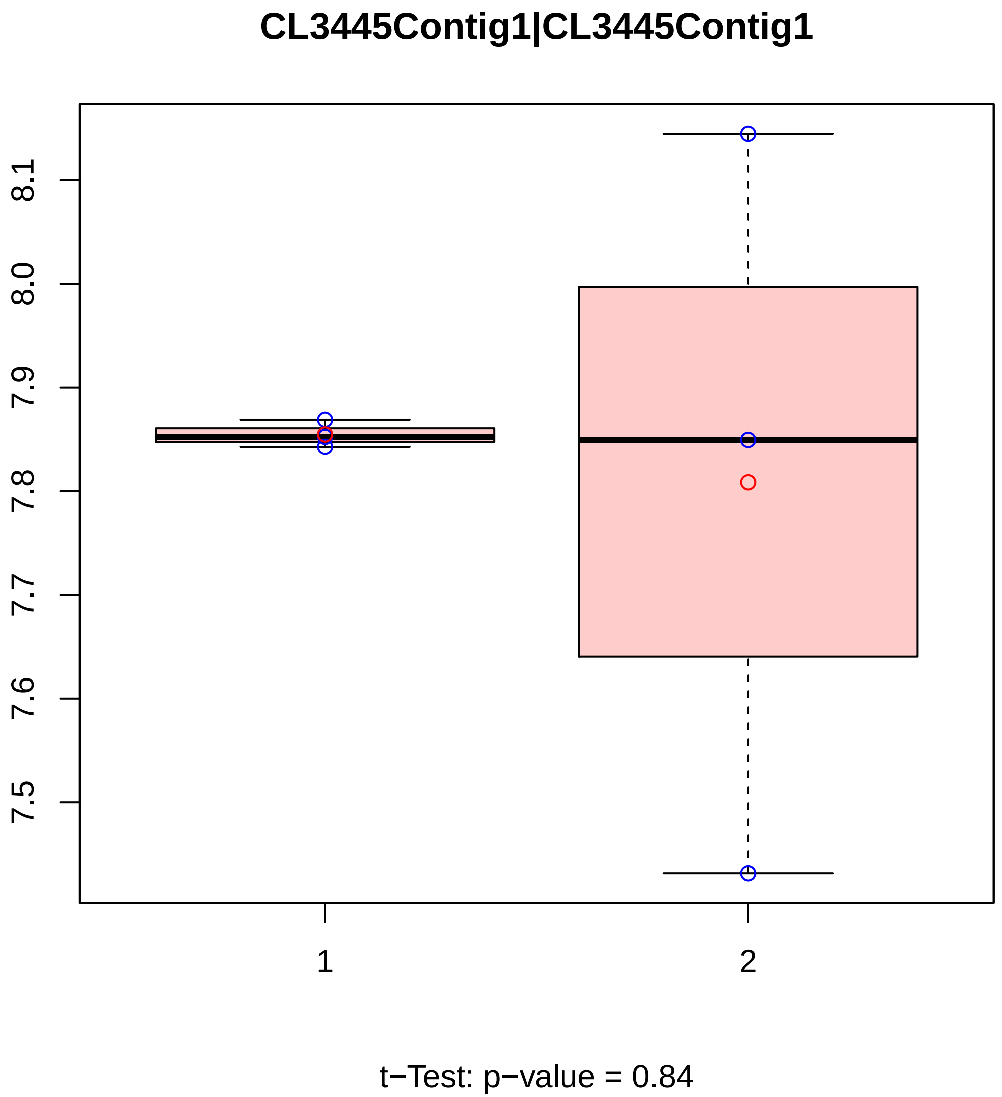
CL3445Contig1|CL3445Contig1
8.1
8.0
7.9
7.8
7.7
7.6
7.5
1
2
t−Test: p−value = 0.84

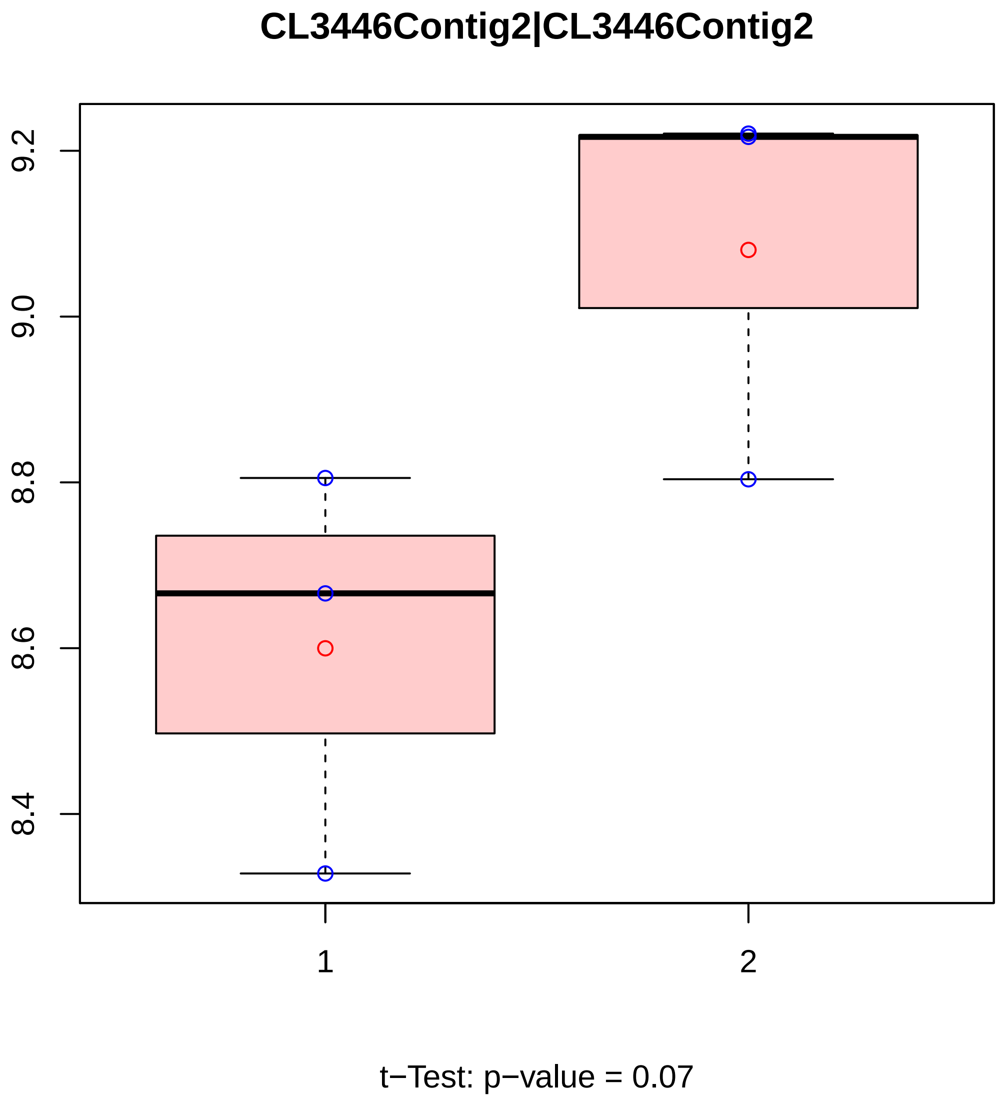
CL3446Contig2|CL3446Contig2
9.2
9.0
8.8
8.6
8.4
1
2
t−Test: p−value = 0.07

# CL3449Contig3|CL3449Contig3

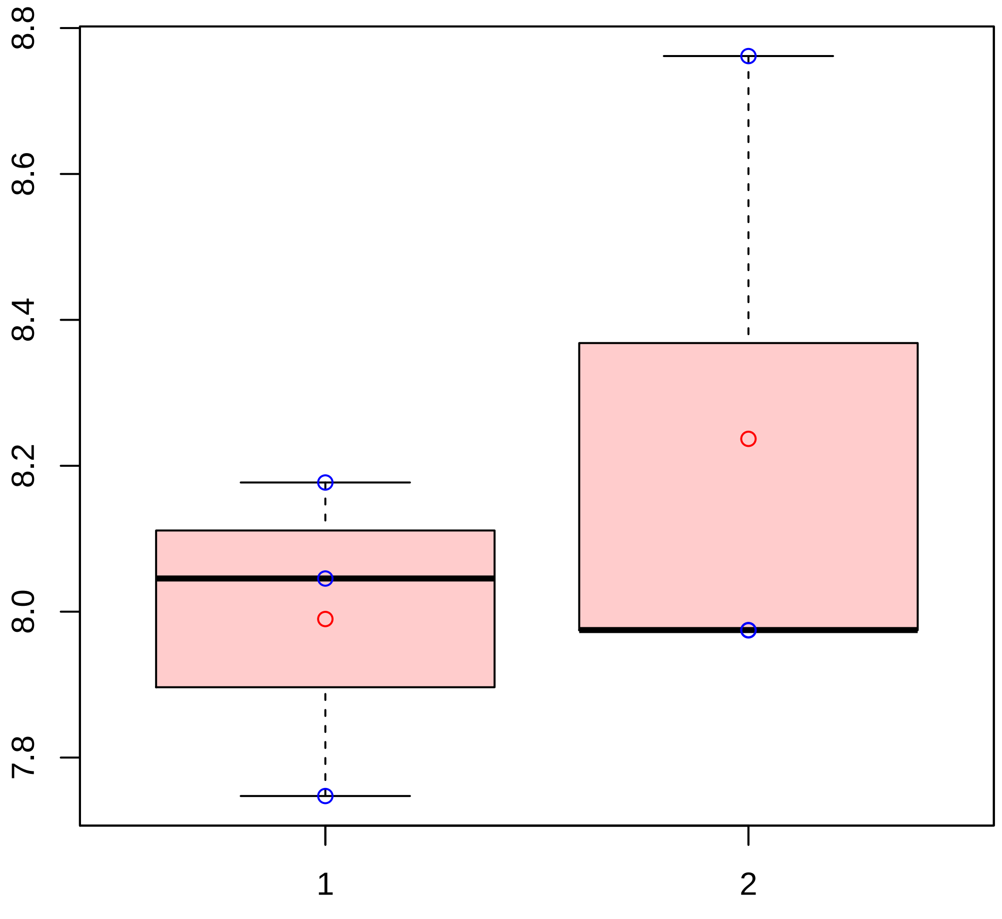


t−Test: p−value = 0.46

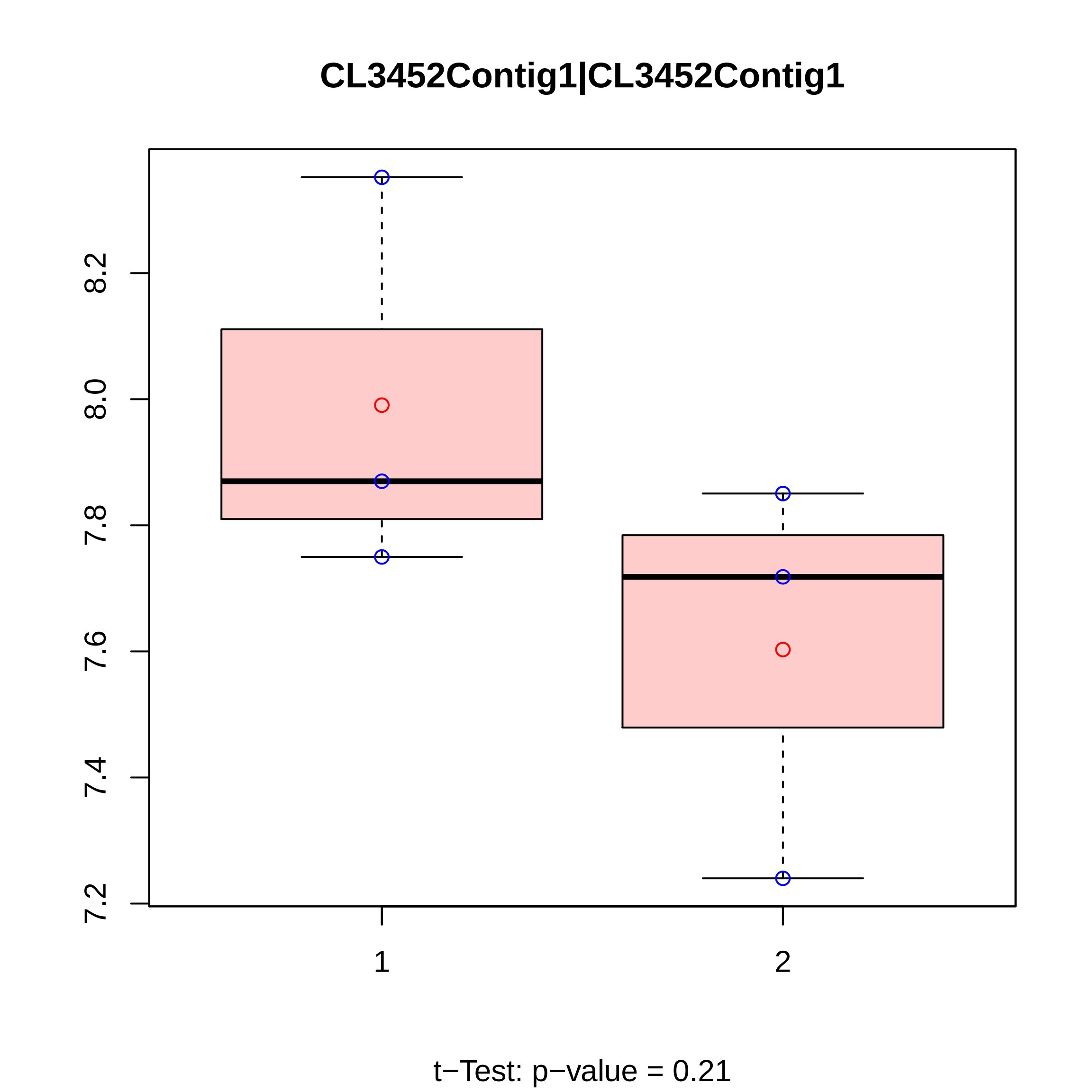

CL3452Contig1|CL3452Contig1
8.2
8.0
7.8
7.6
7.4
7.2
1
2
t−Test: p−value = 0.21

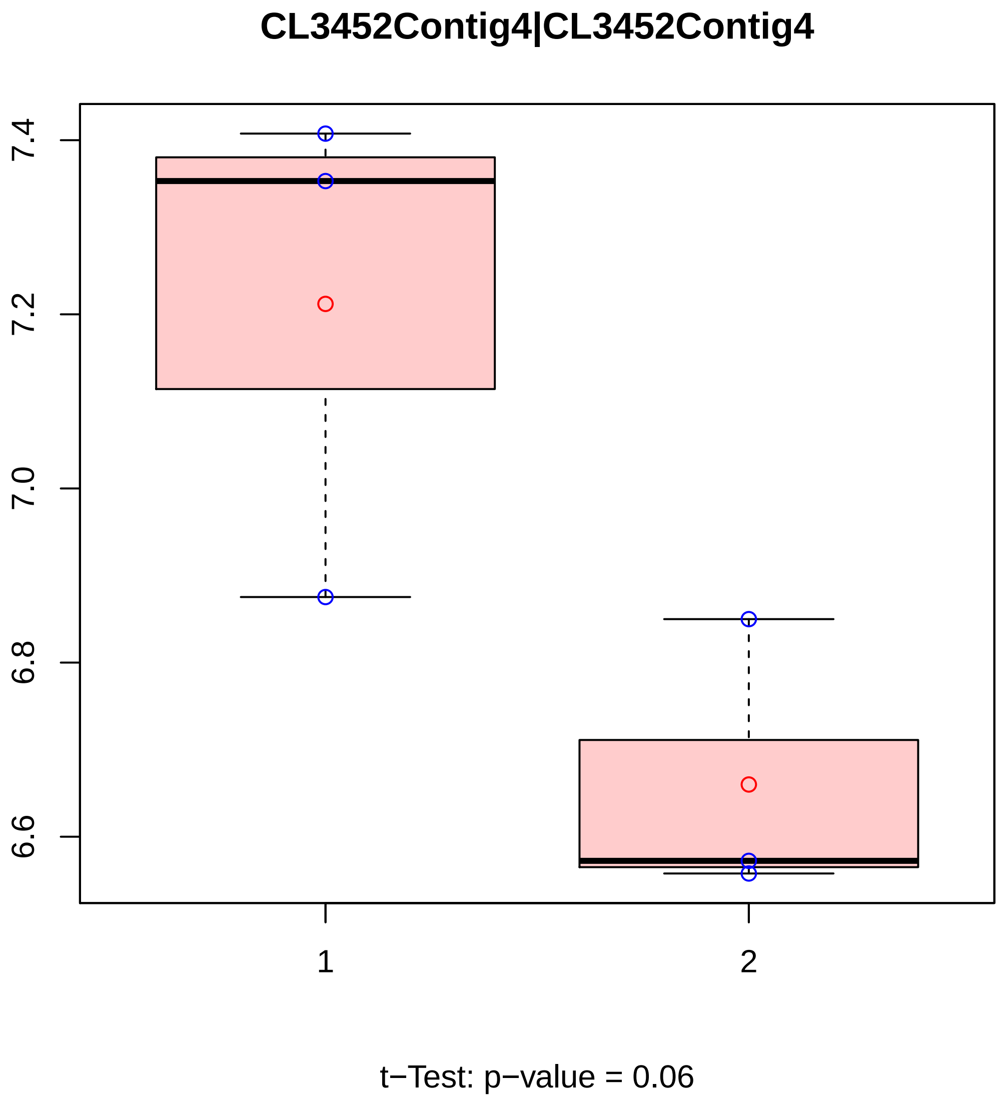

CL3452Contig4|CL3452Contig4
7.4
7.2
7.0
6.8
6.6
1
2
t−Test: p−value = 0.06

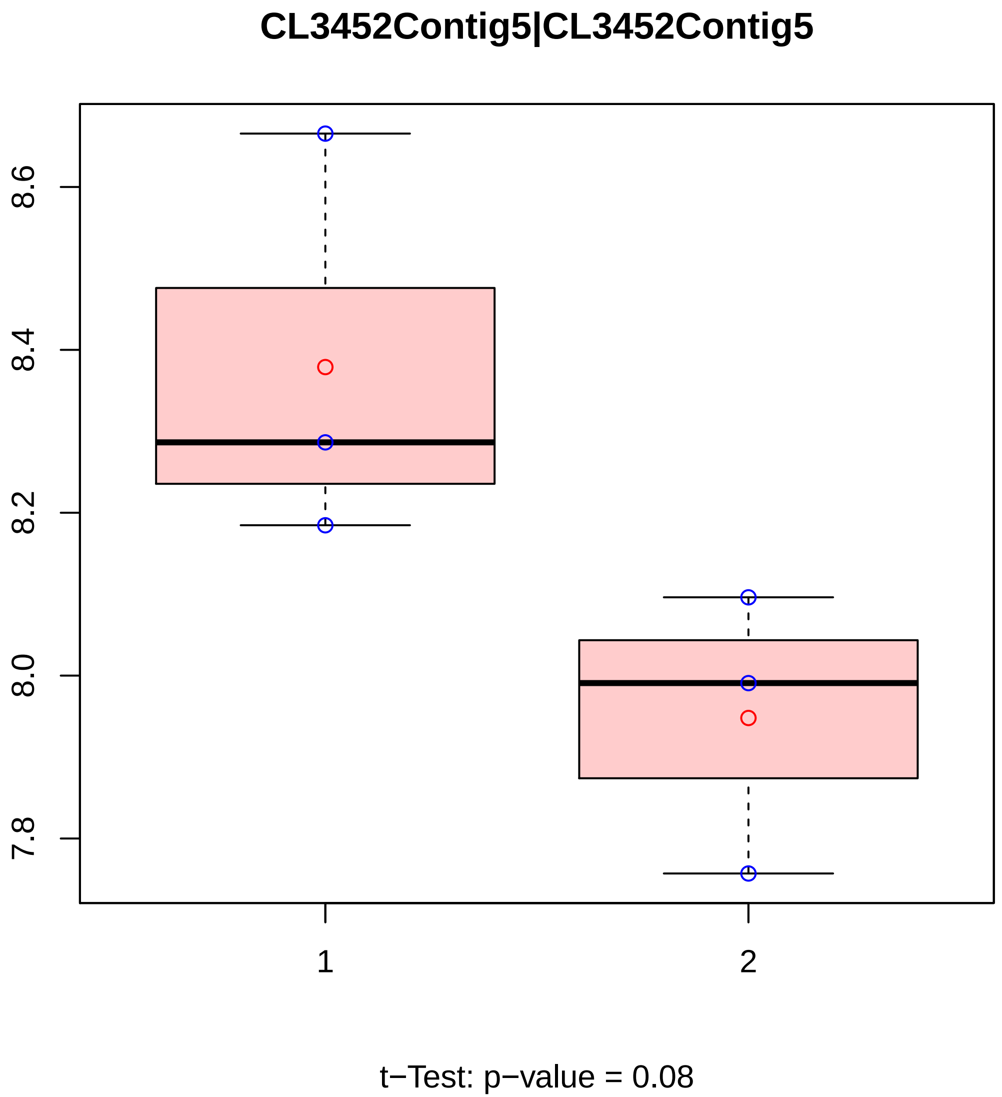

CL3452Contig5|CL3452Contig5
8.6
8.4
8.2
8.0
7.8
1
2
t−Test: p−value = 0.08

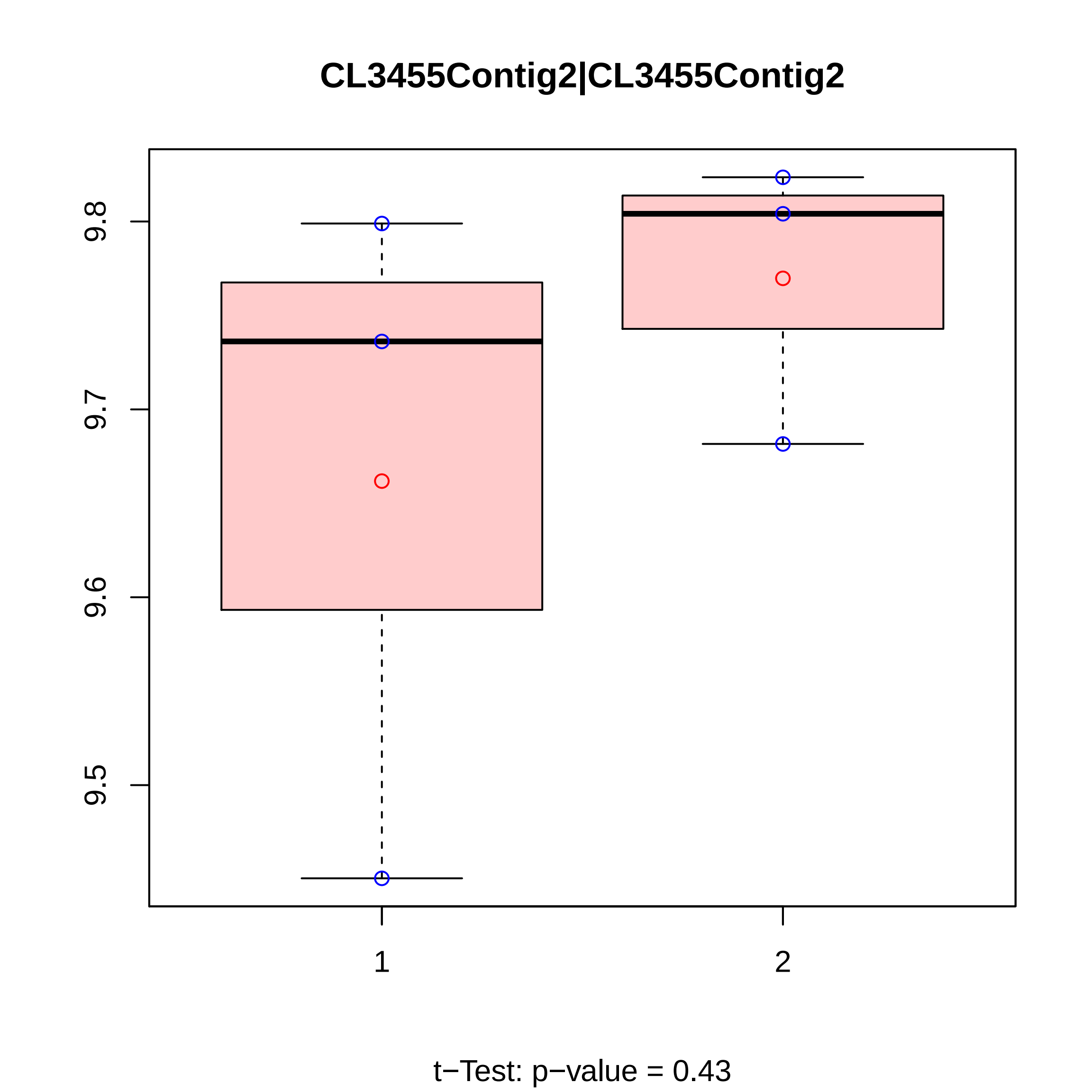
CL3455Contig2|CL3455Contig2
9.5
9.6
9.7
9.8
1
2
t−Test: p−value = 0.43

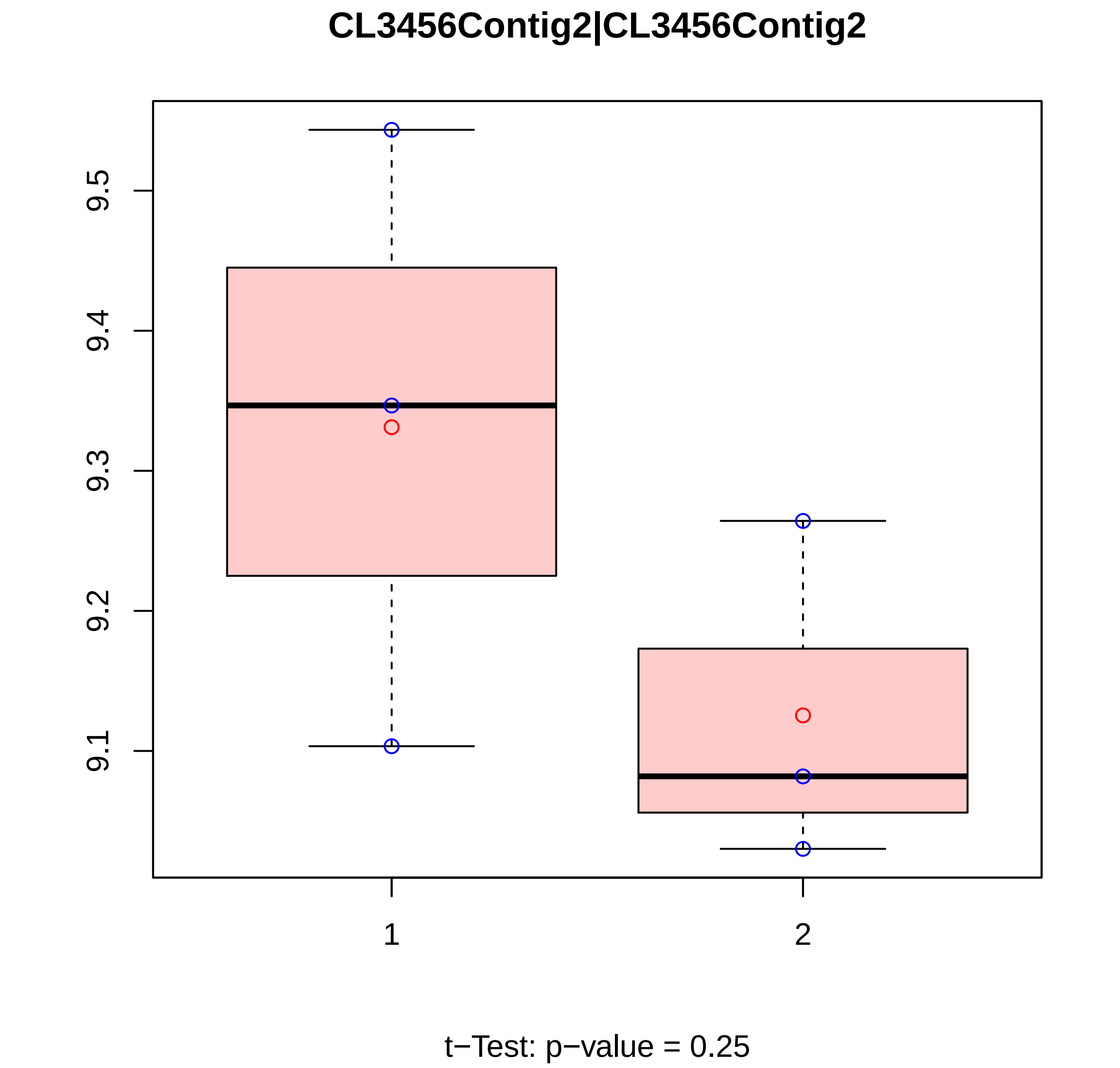
CL3456Contig2|CL3456Contig2
9.1
9.2
9.3
9.4
9.5
1
2
t−Test: p−value = 0.25

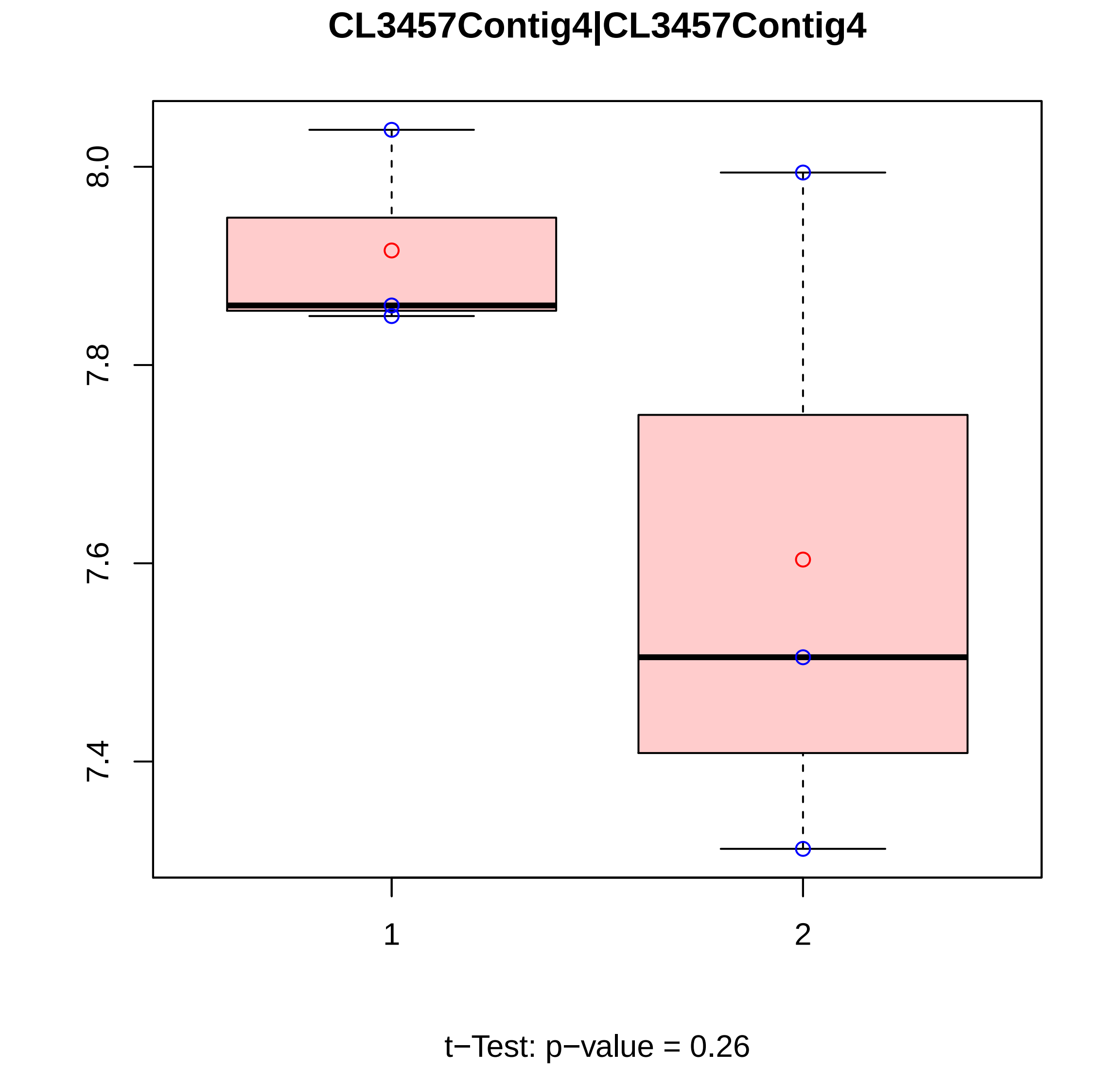
CL3457Contig4|CL3457Contig4
8.0
7.8
7.6
7.4
1
2
t−Test: p−value = 0.26

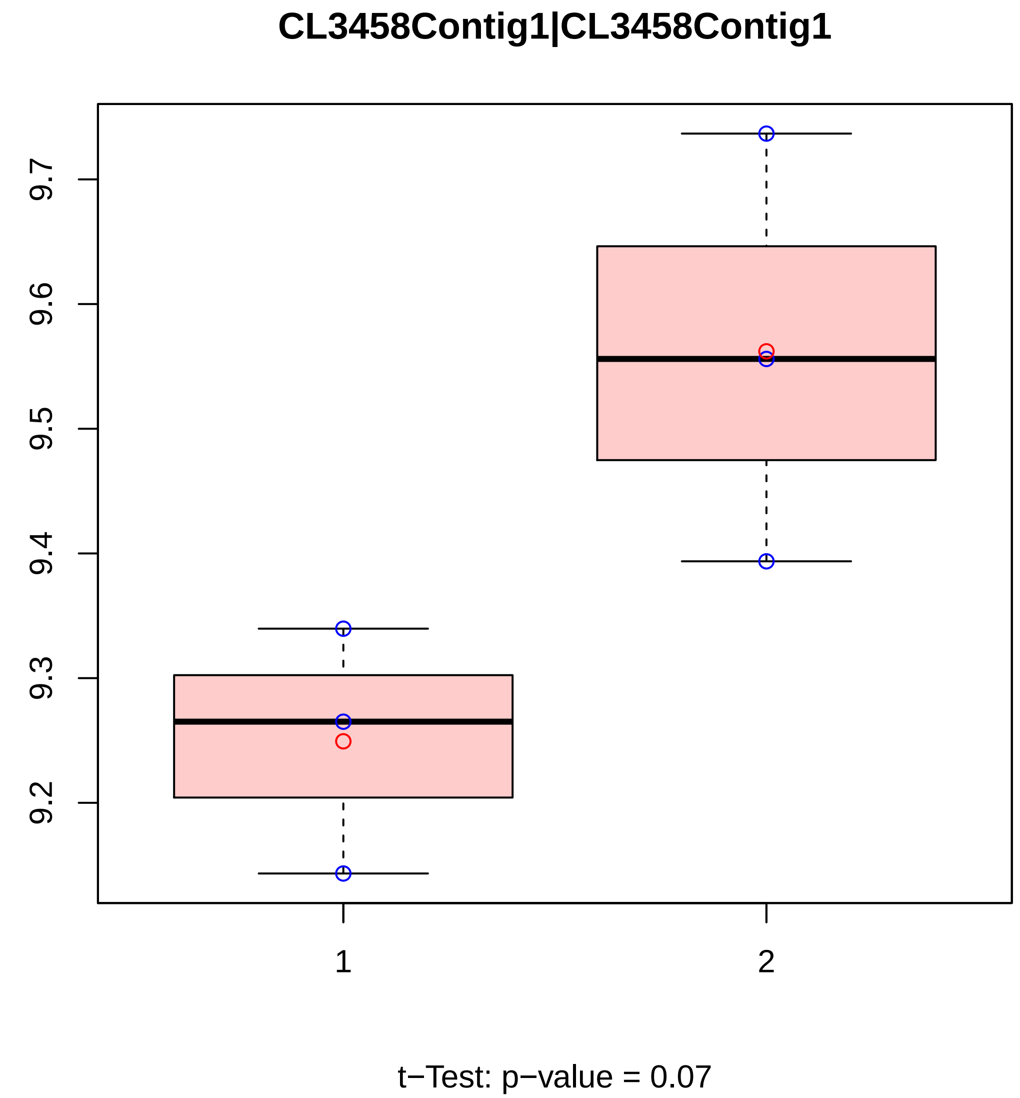
CL3458Contig1|CL3458Contig1
9.2
9.3
9.4
9.5
9.6
9.7
1
2
t−Test: p−value = 0.07

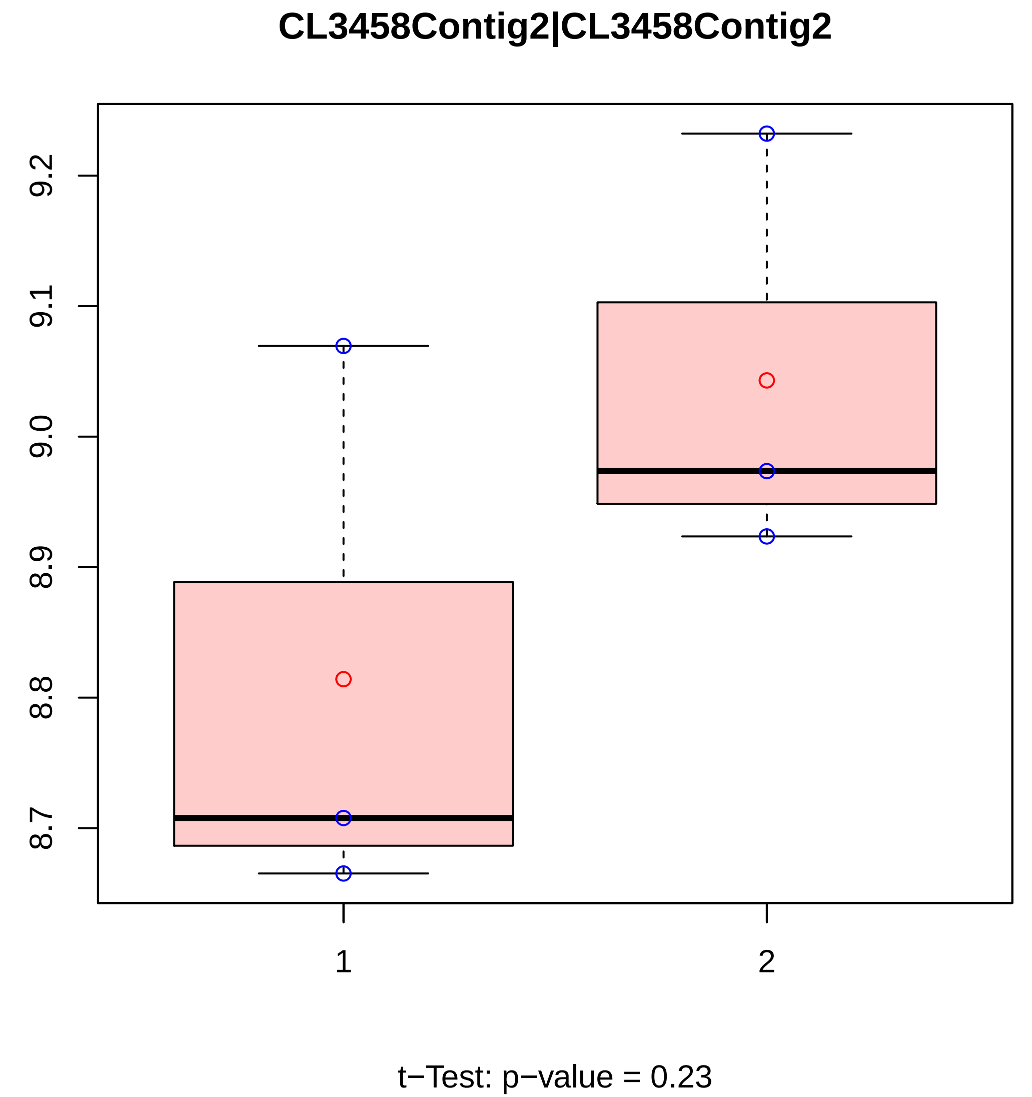

CL3458Contig2|CL3458Contig2
9.2
9.1
9.0
8.9
8.8
8.7
1
2
t−Test: p−value = 0.23

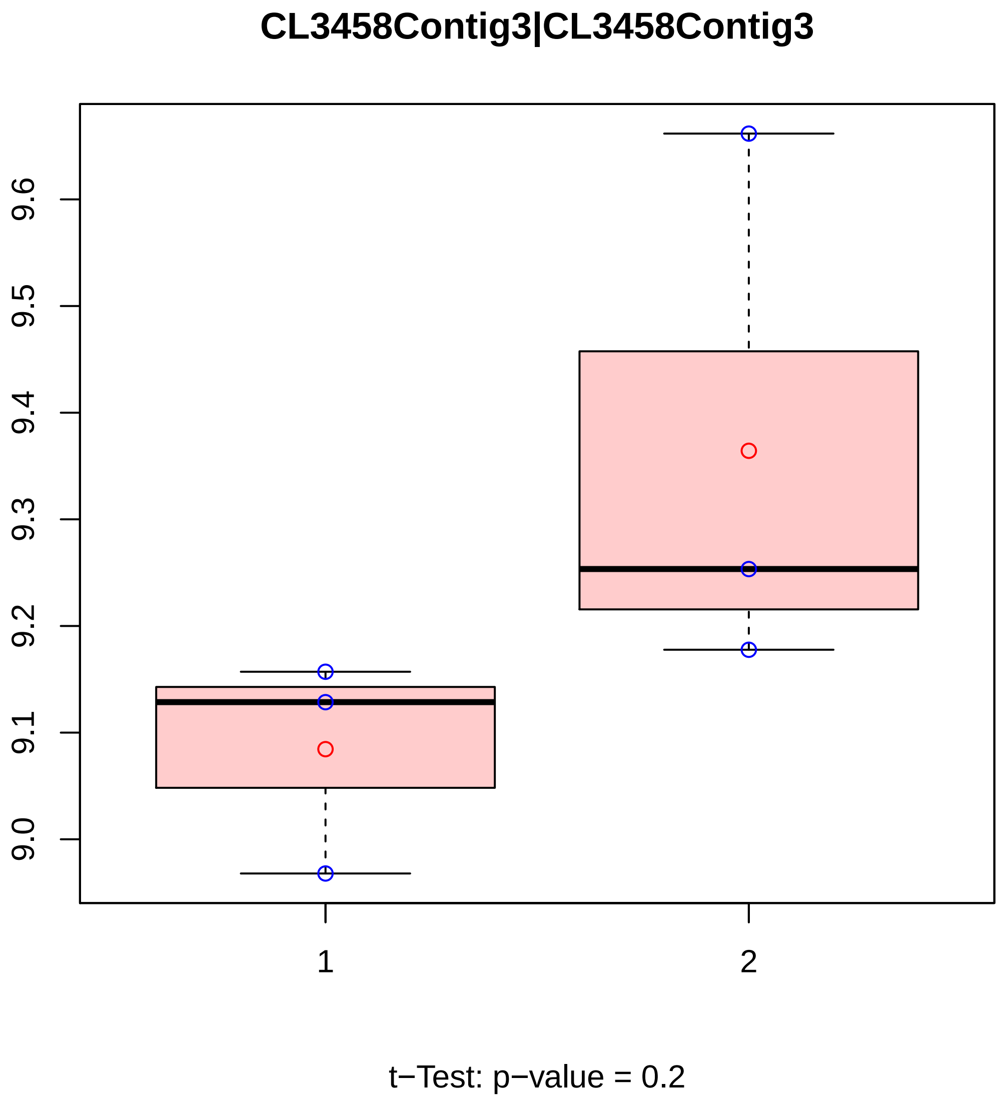

CL3458Contig3|CL3458Contig3
9.0
9.1
9.2
9.3
9.4
9.5
9.6
1
2
t−Test: p−value = 0.2

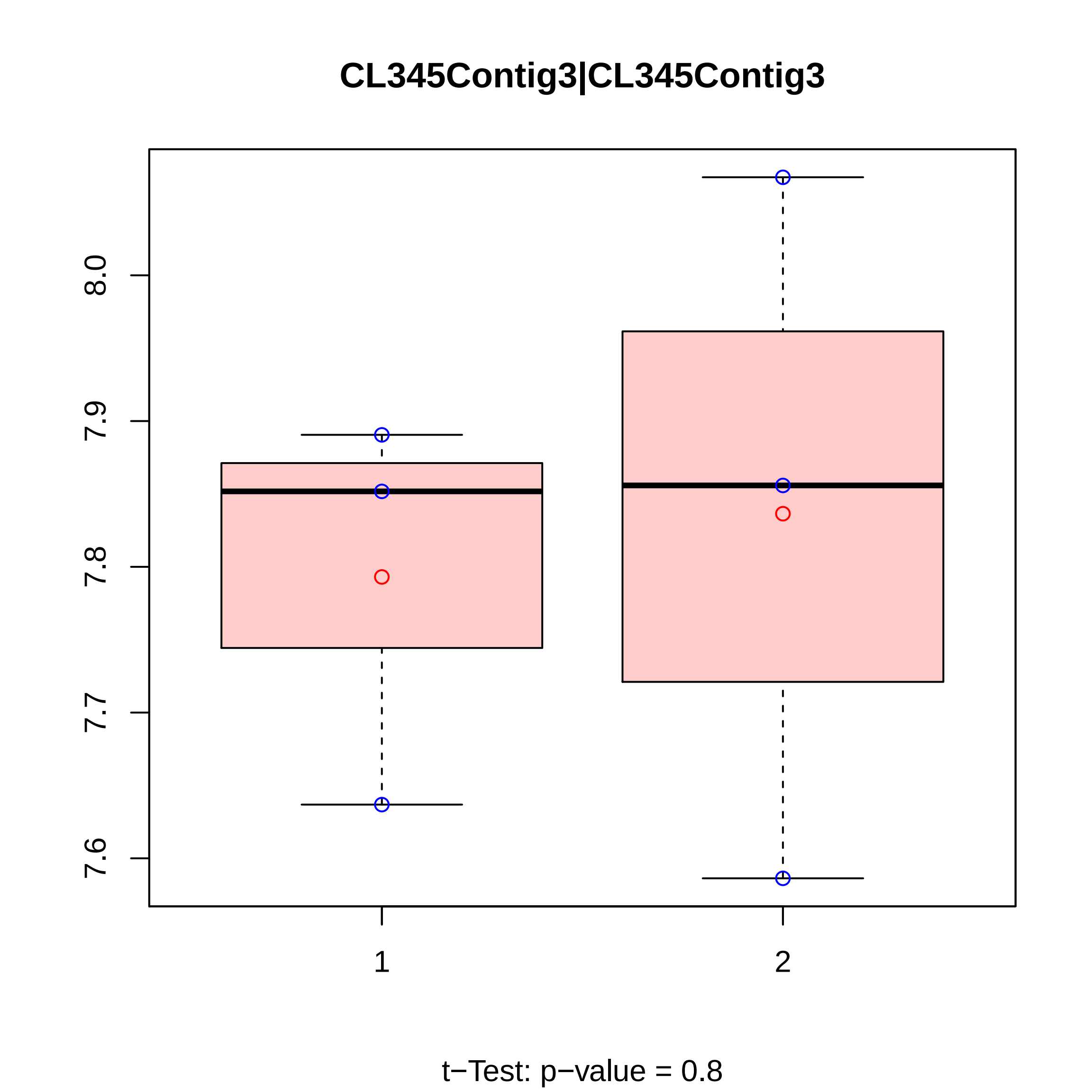

CL345Contig3|CL345Contig3
8.0
7.9
7.8
7.7
7.6
1
2
t−Test: p−value = 0.8

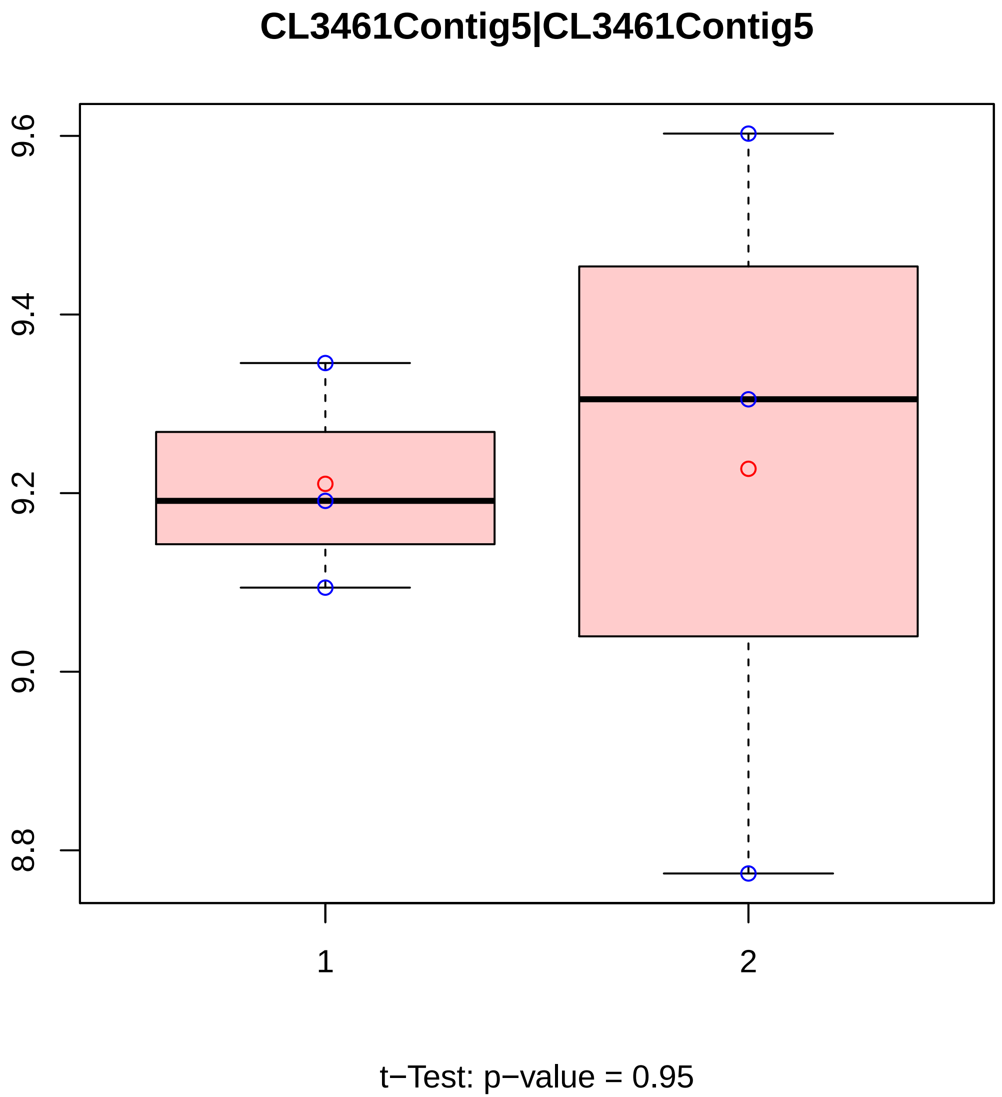
CL3461Contig5|CL3461Contig5
9.6
9.4
9.2
9.0
8.8
1
2
t−Test: p−value = 0.95

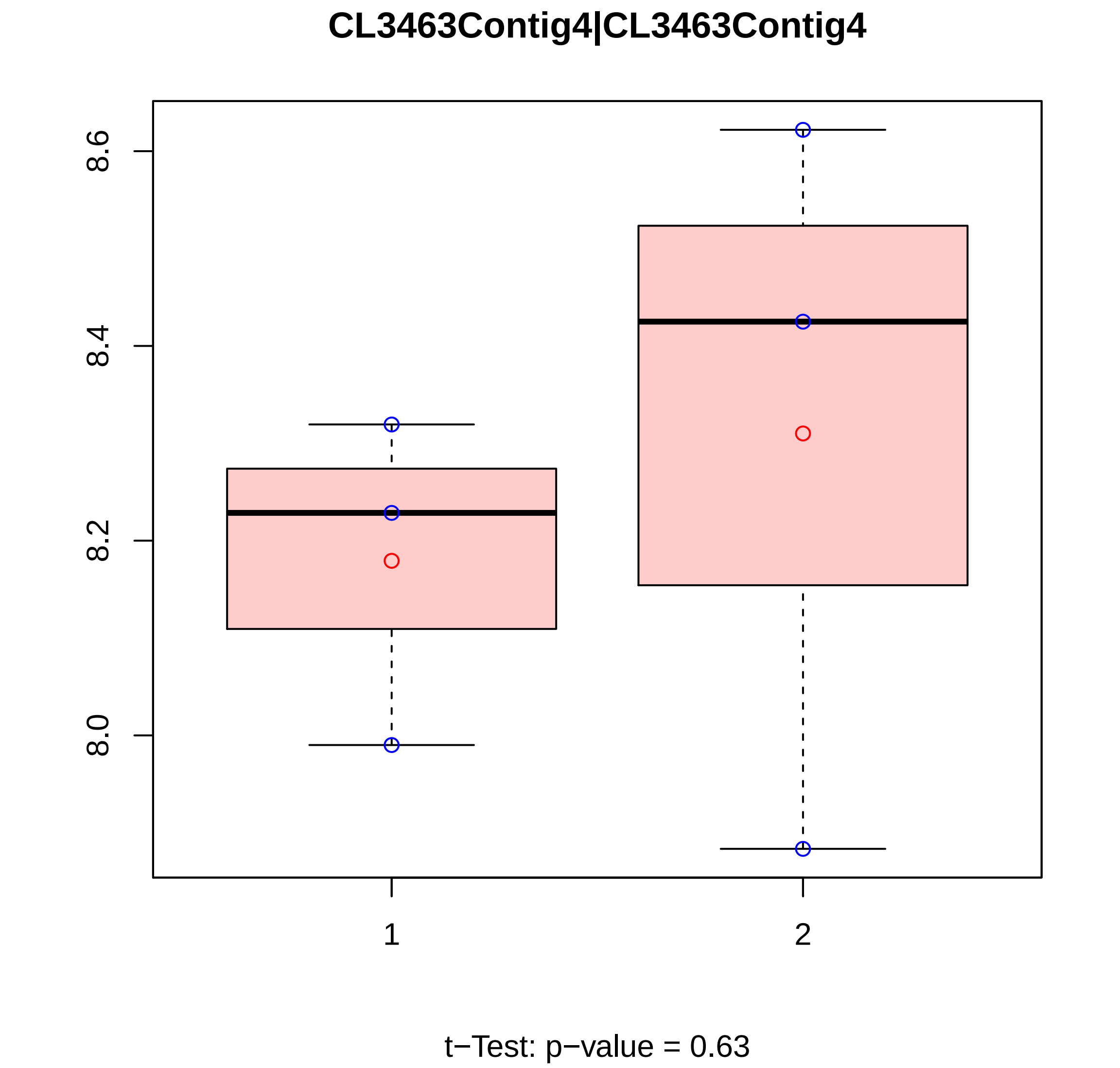
CL3463Contig4|CL3463Contig4
8.0
8.2
8.4
8.6
1
2
t−Test: p−value = 0.63

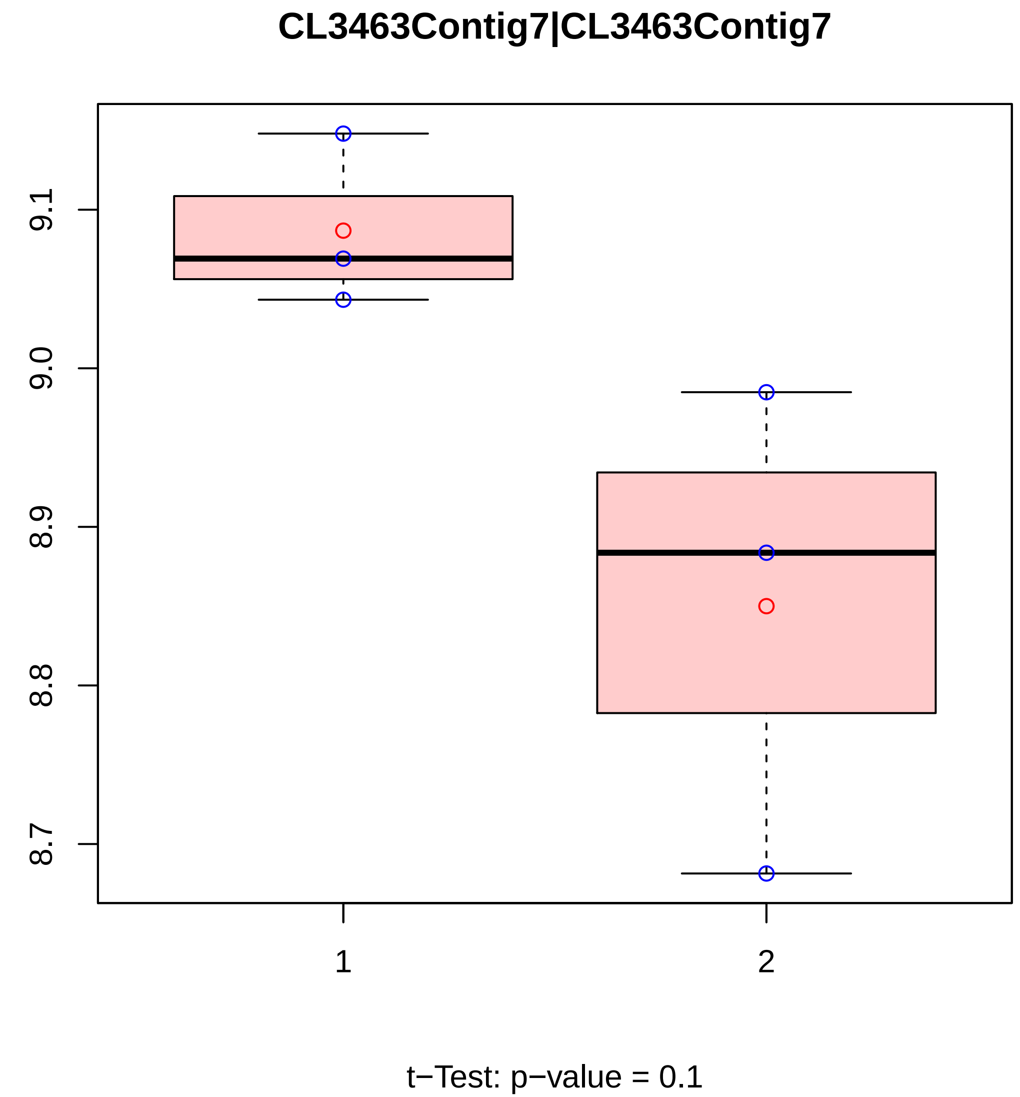
CL3463Contig7|CL3463Contig7
9.1
9.0
8.9
8.8
8.7
1
2
t−Test: p−value = 0.1

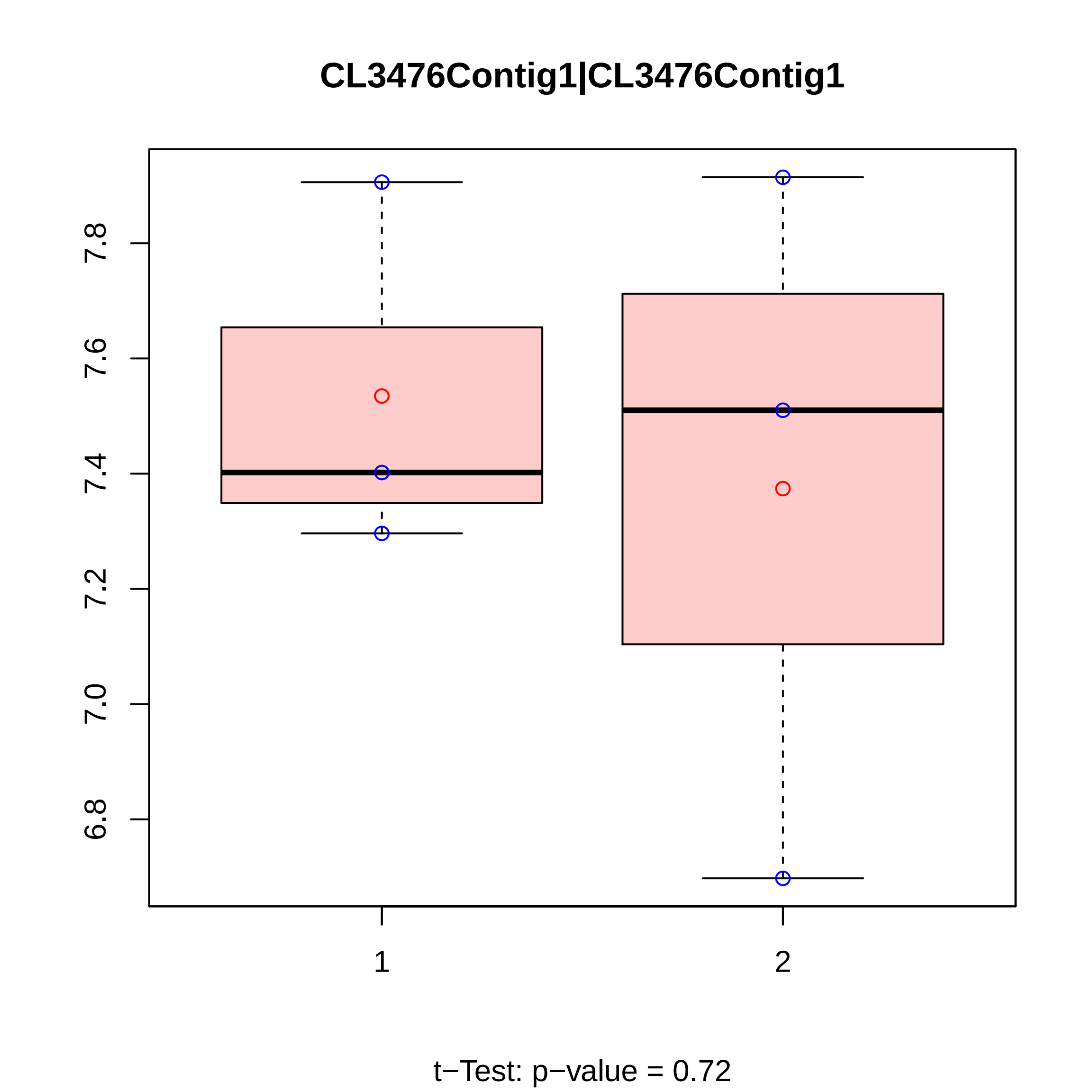

CL3476Contig1|CL3476Contig1
7.8
7.6
7.4
7.2
7.0
6.8
1
2
t−Test: p−value = 0.72

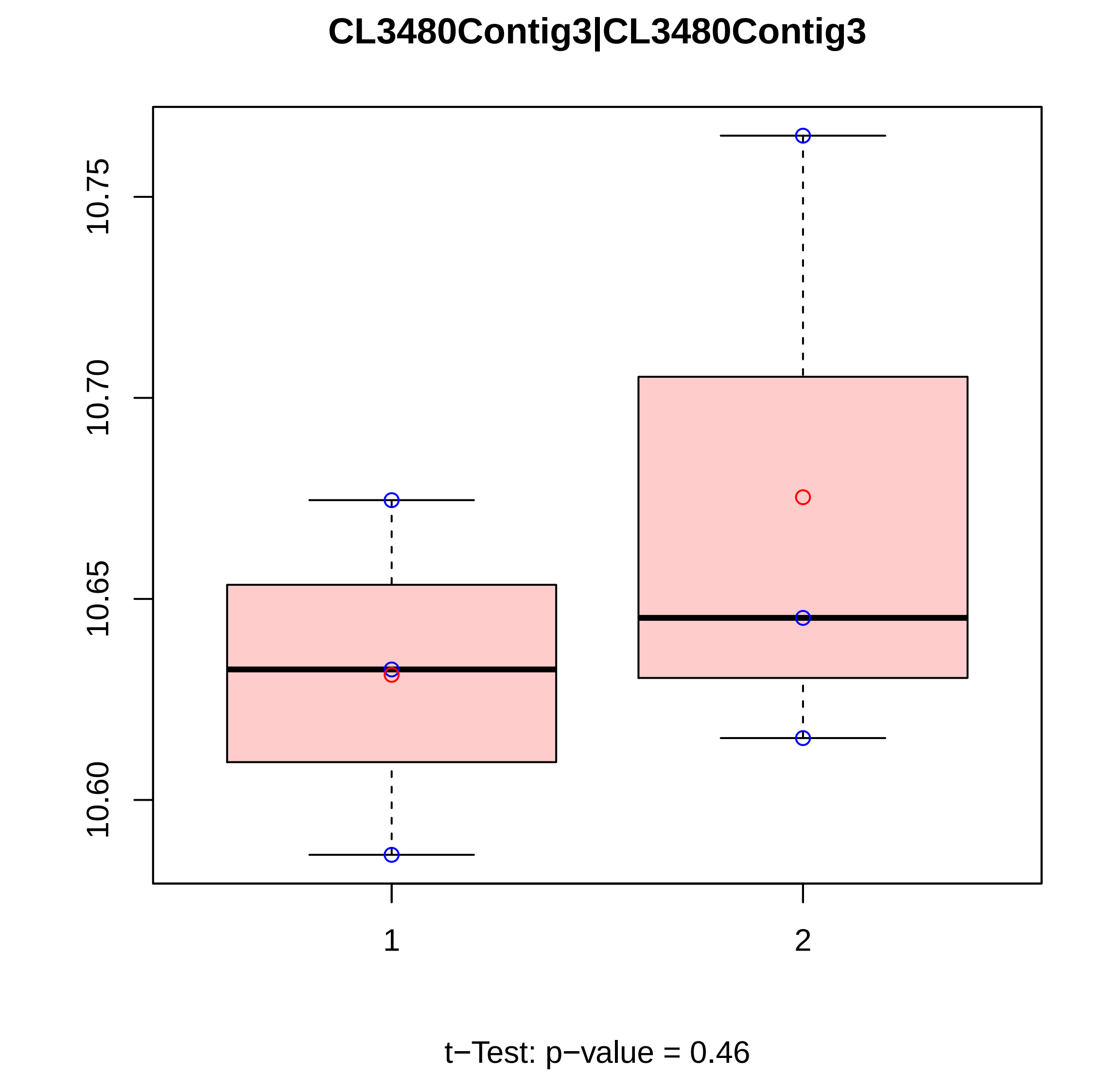
CL3480Contig3|CL3480Contig3
10.60
10.65
10.70
10.75
1
2
t−Test: p−value = 0.46

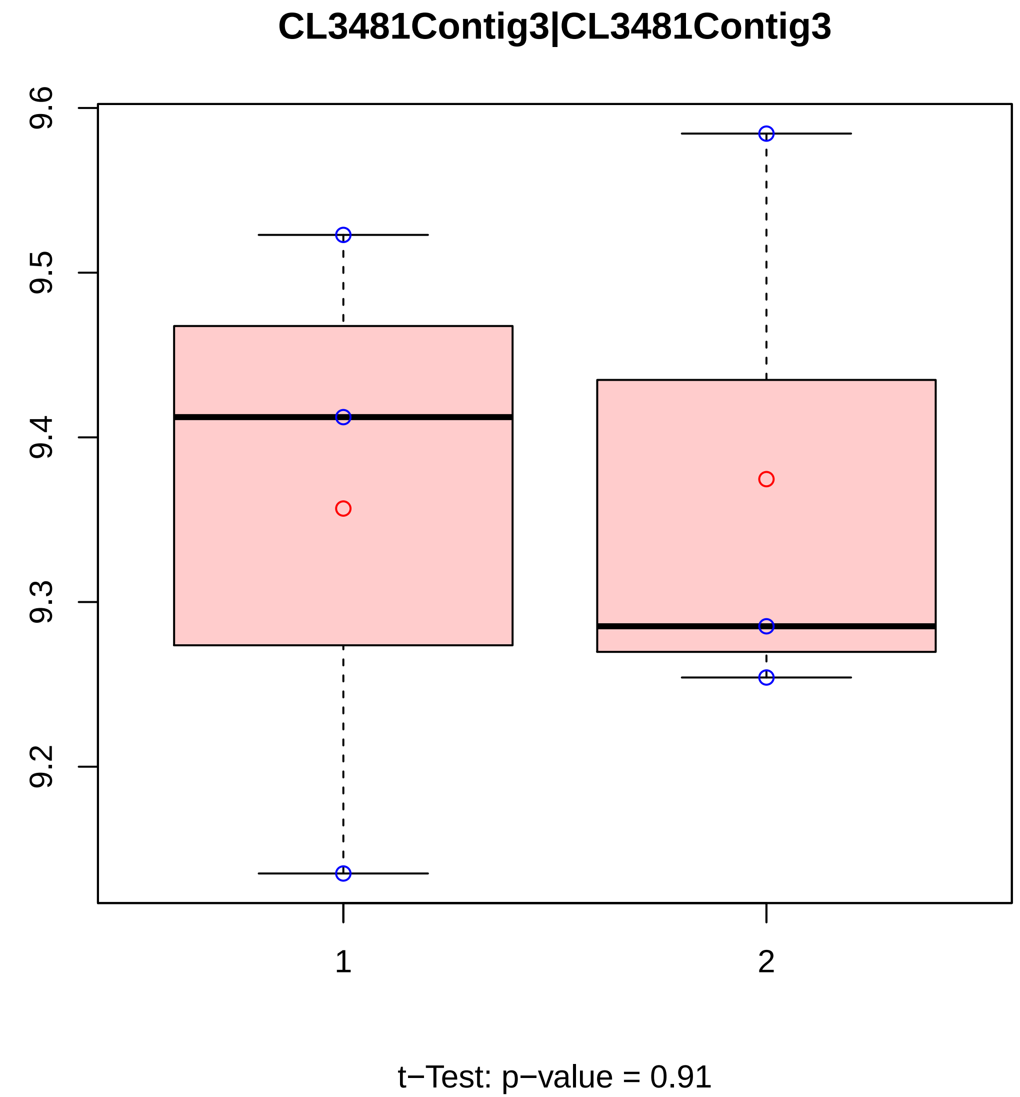
CL3481Contig3|CL3481Contig3
9.6
9.5
9.4
9.3
9.2
1
2
t−Test: p−value = 0.91

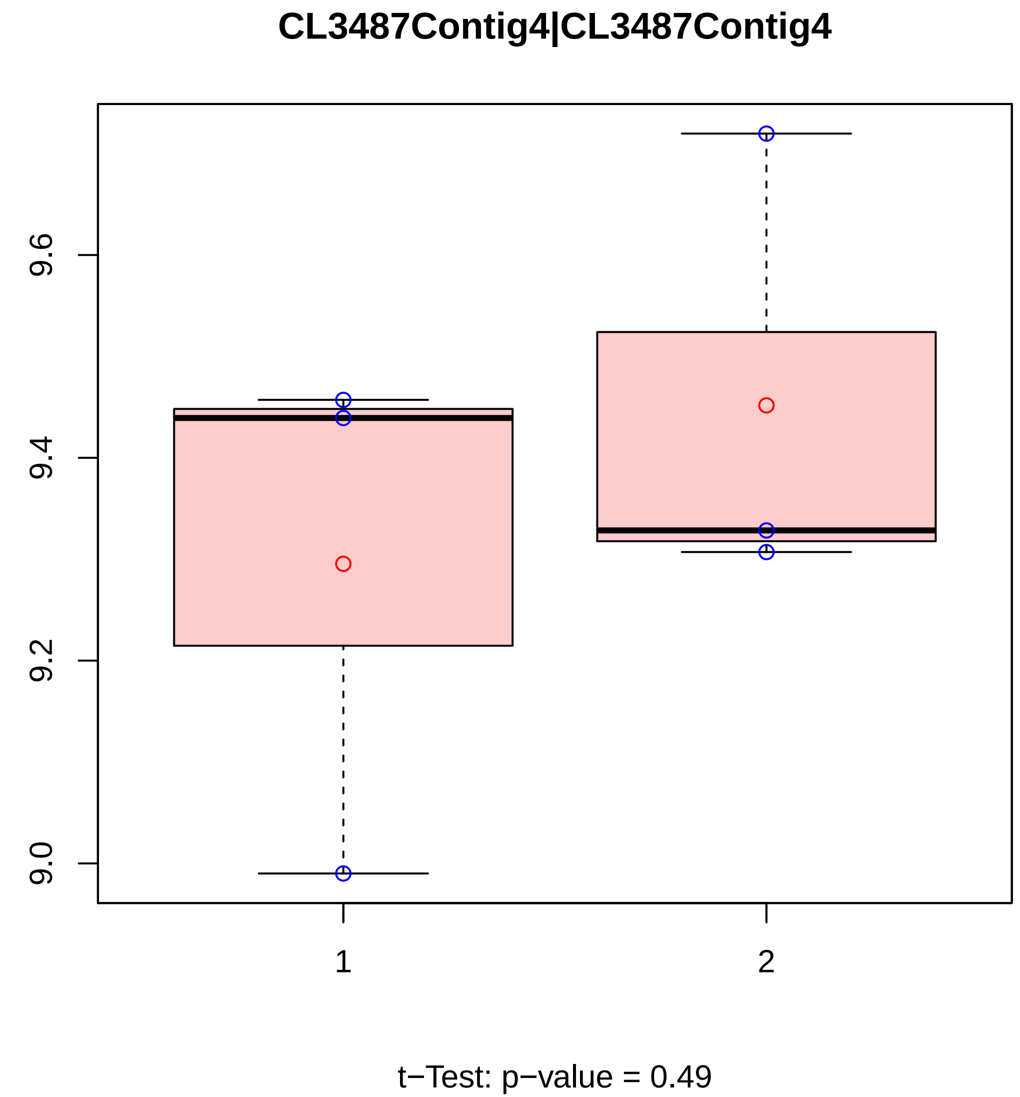

CL3487Contig4|CL3487Contig4
9.0
9.2
9.4
9.6
1
2
t−Test: p−value = 0.49

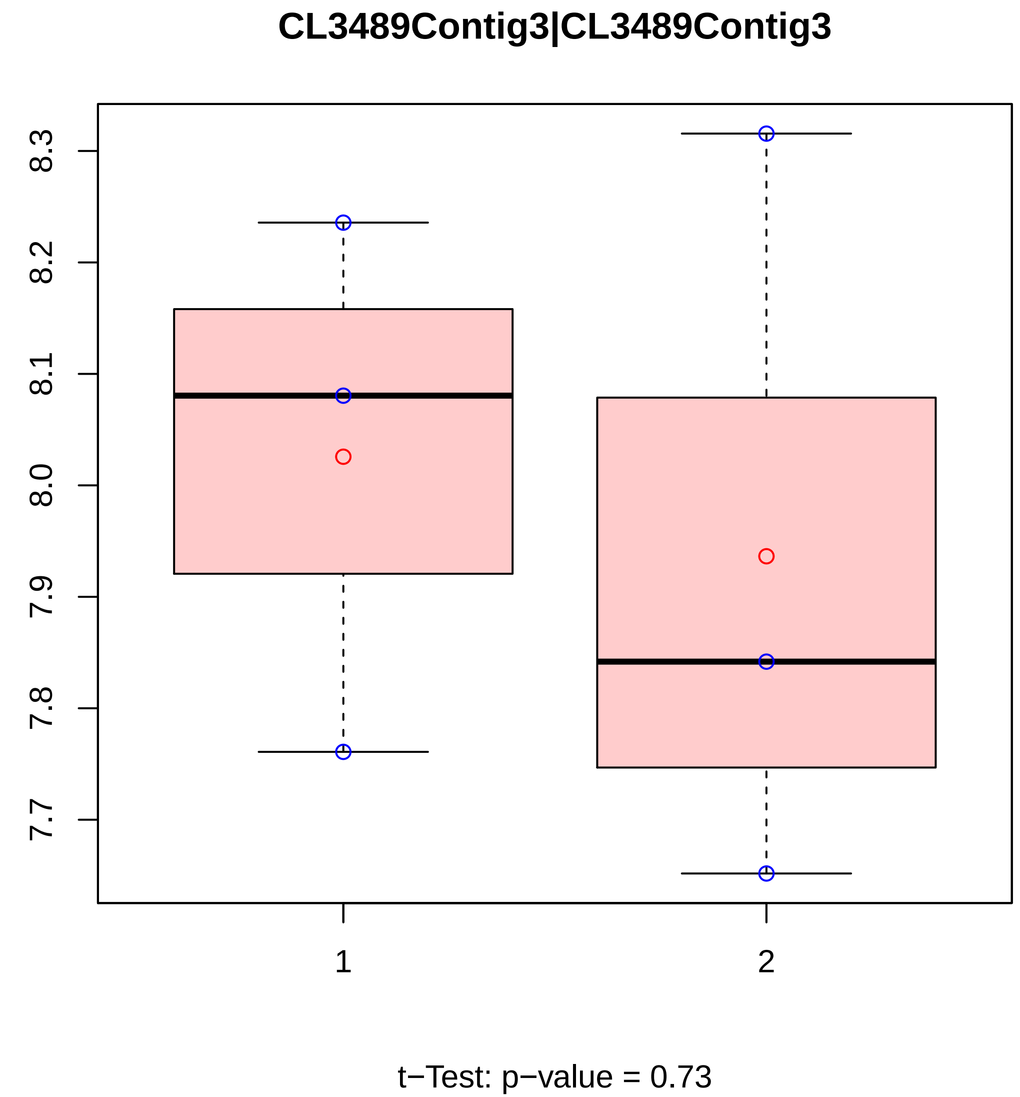
CL3489Contig3|CL3489Contig3
8.3
8.2
8.1
8.0
7.9
7.8
7.7
1
2
t−Test: p−value = 0.73

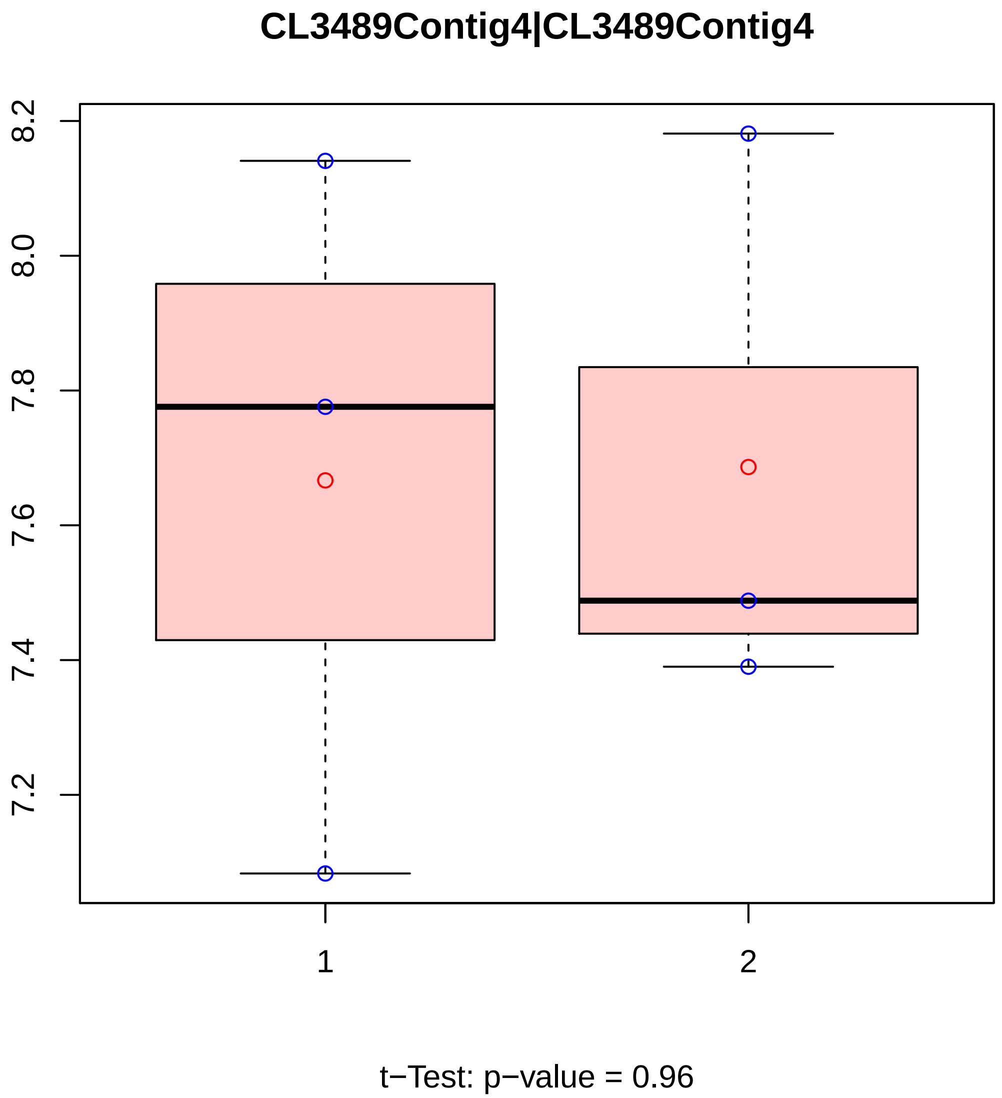
CL3489Contig4|CL3489Contig4
8.2
8.0
7.8
7.6
7.4
7.2
1
2
t−Test: p−value = 0.96

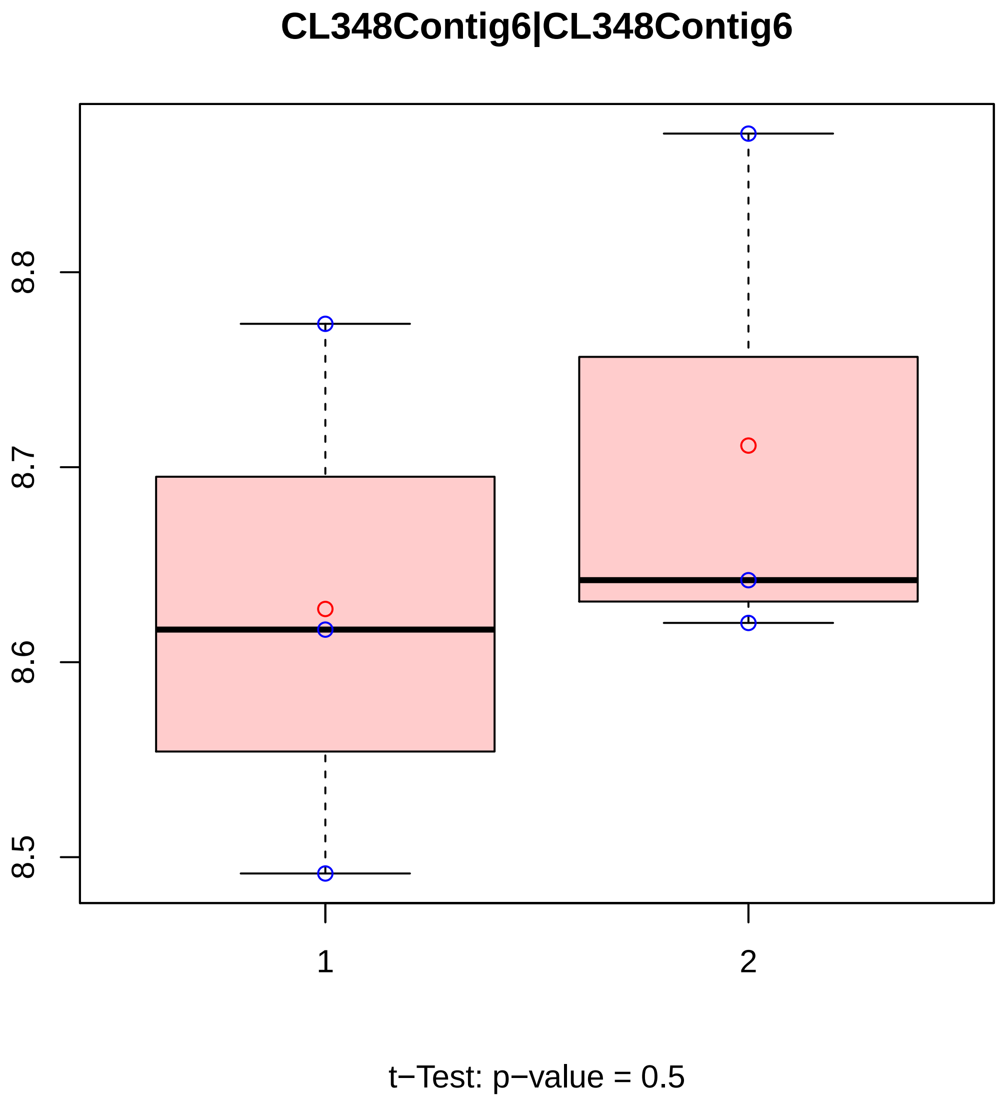
CL348Contig6|CL348Contig6
8.5
8.6
8.7
8.8
1
2
t−Test: p−value = 0.5

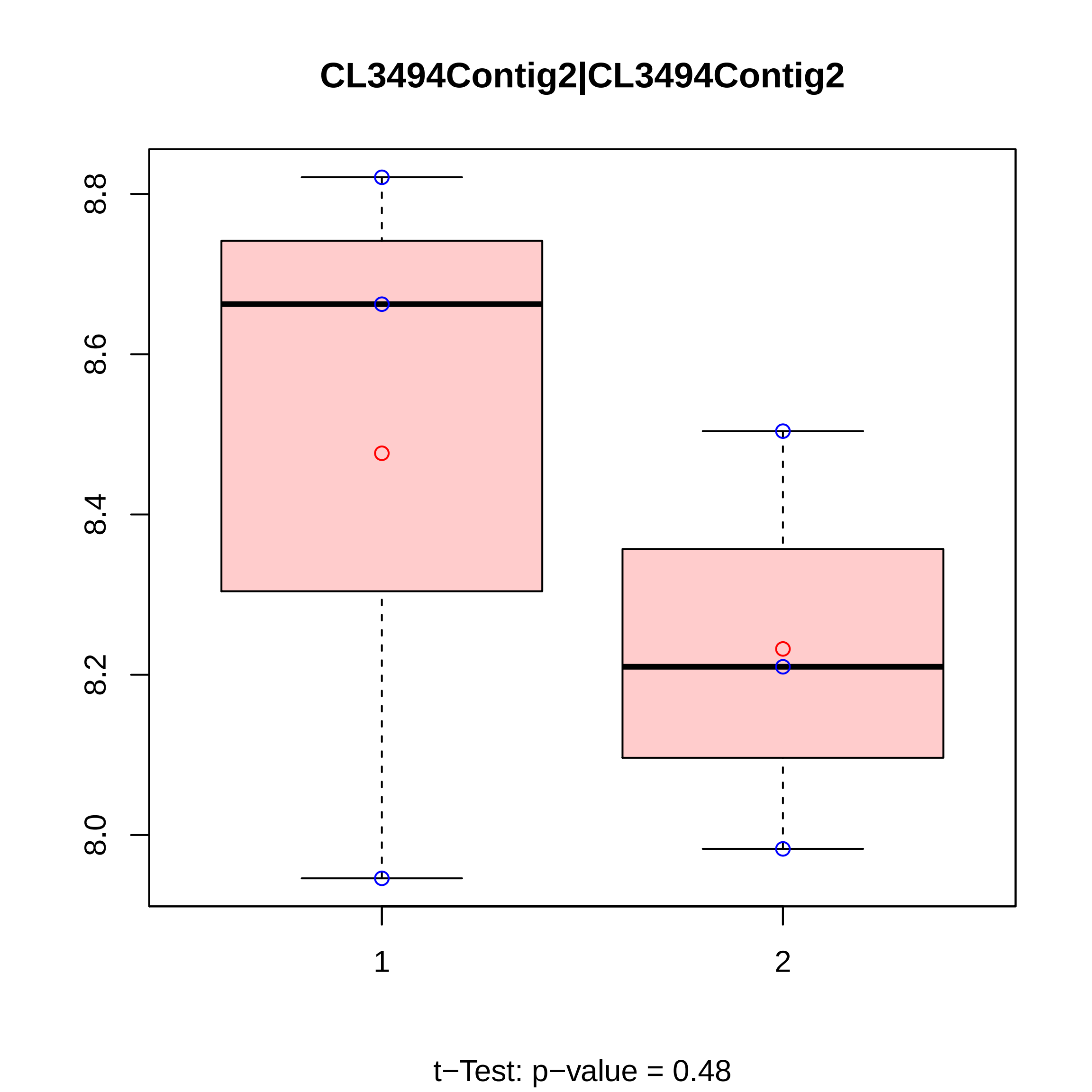
CL3494Contig2|CL3494Contig2
8.0
8.2
8.4
8.6
8.8
1
2
t−Test: p−value = 0.48

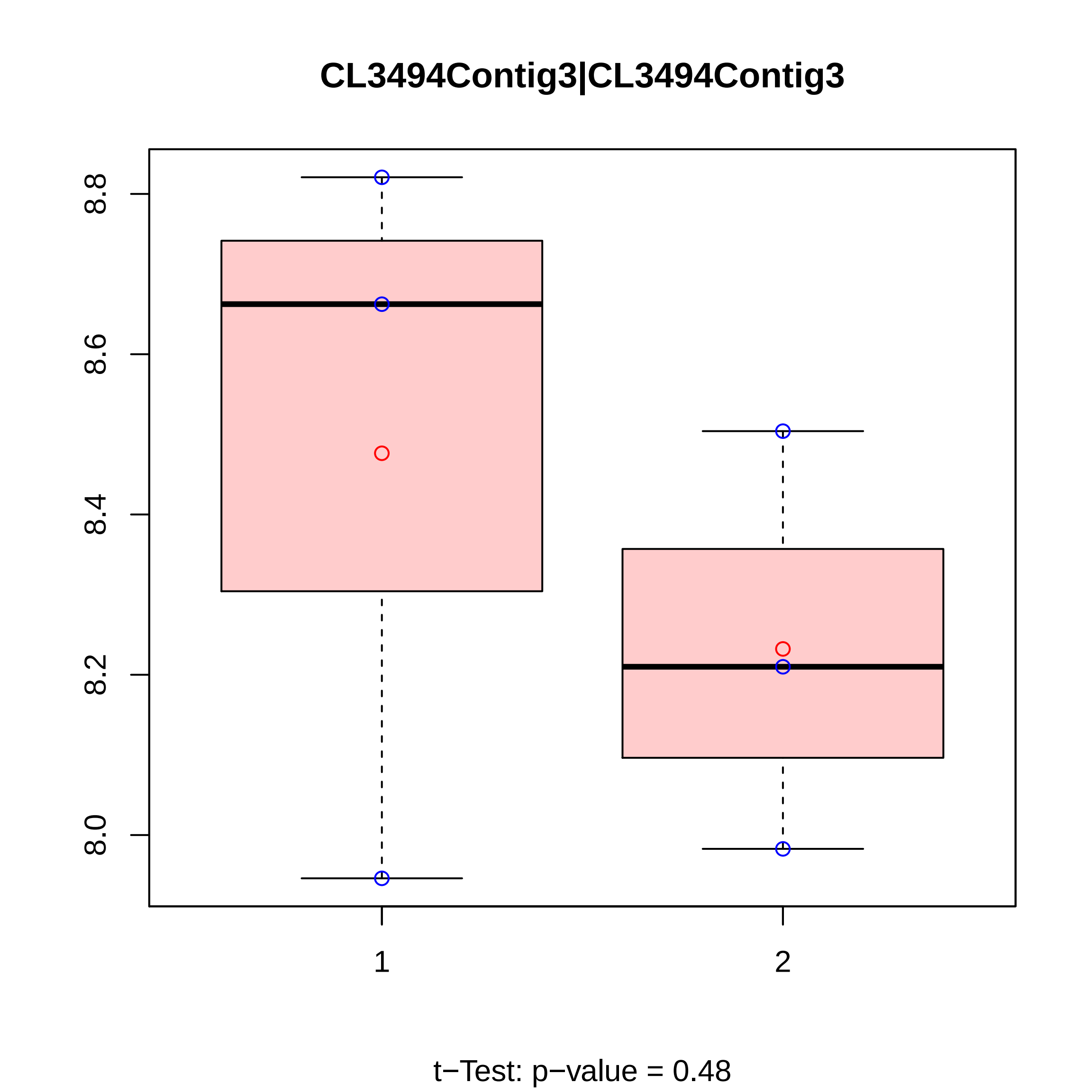

CL3494Contig3|CL3494Contig3
8.0
8.2
8.4
8.6
8.8
1
2
t−Test: p−value = 0.48

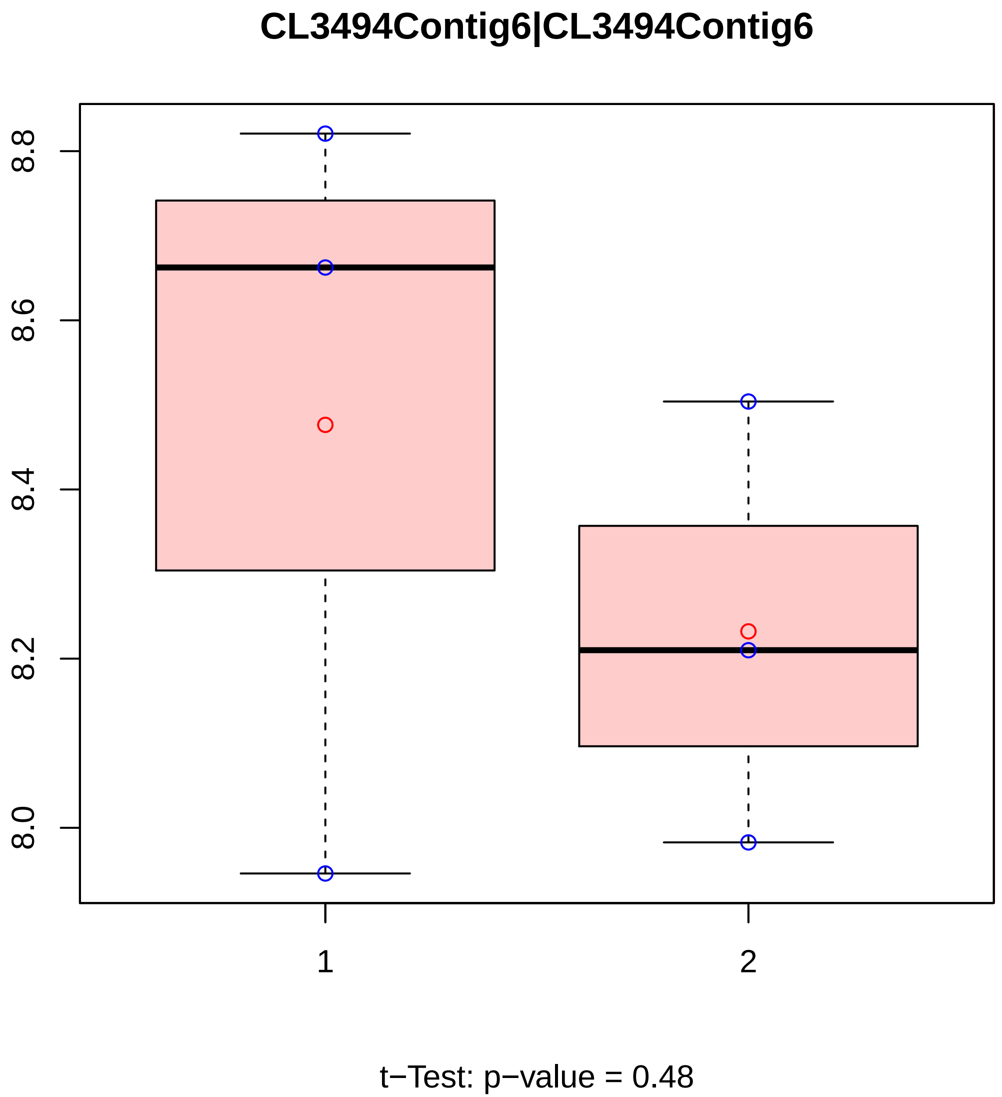
CL3494Contig6|CL3494Contig6
8.0
8.2
8.4
8.6
8.8
1
2
t−Test: p−value = 0.48

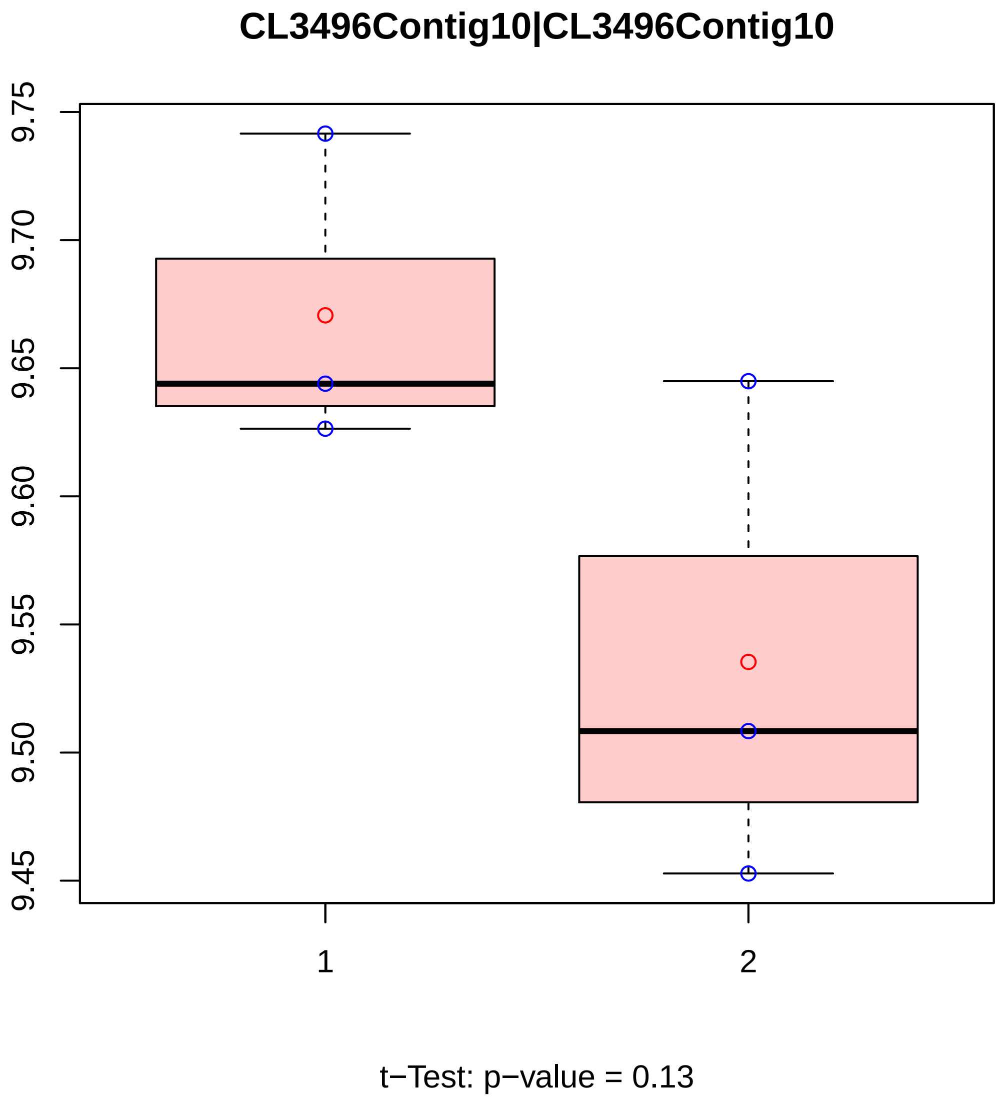

CL3496Contig10|CL3496Contig10
9.45
9.50
9.55
9.60
9.65
9.70
9.75
1
2
t−Test: p−value = 0.13

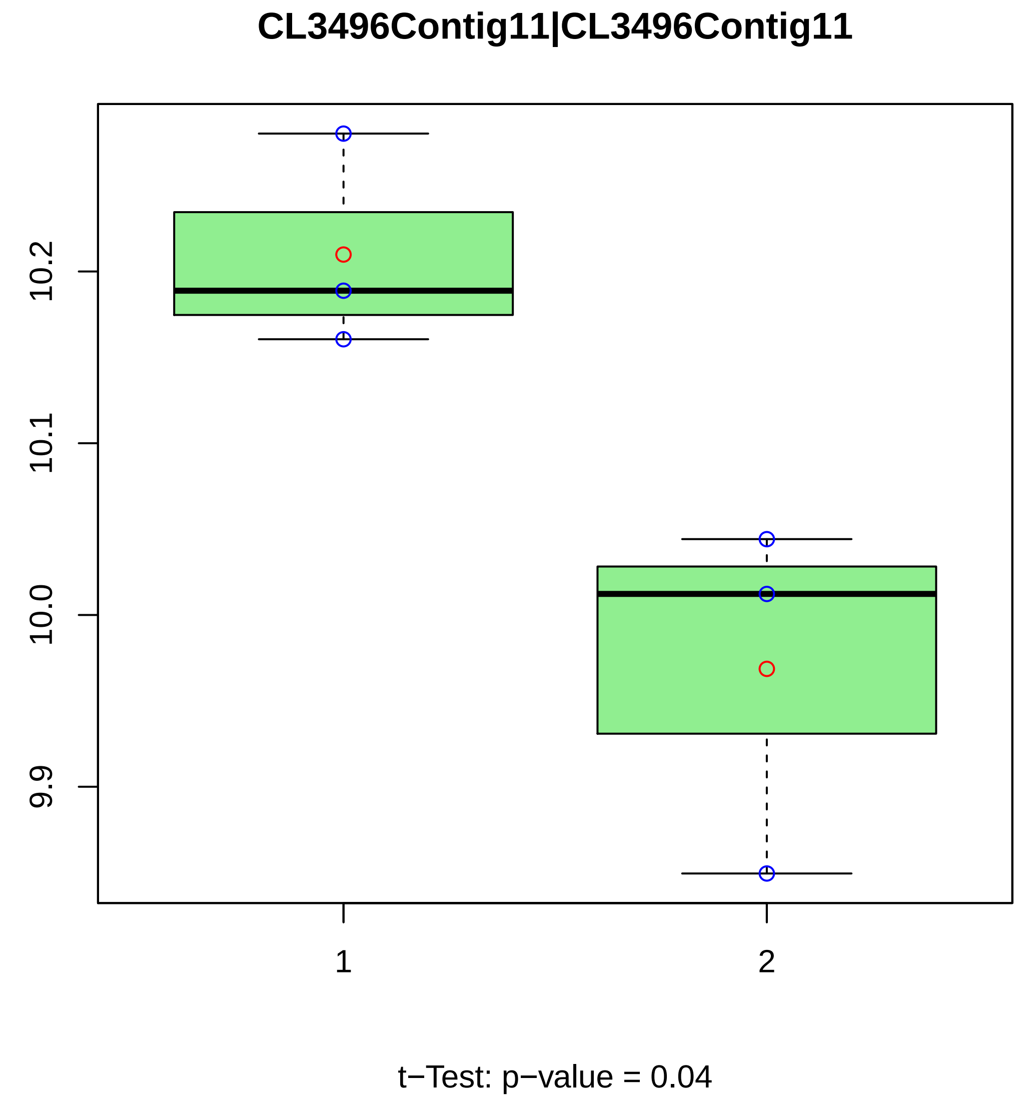
CL3496Contig11|CL3496Contig11
10.2
10.1
10.0
9.9
1
2
t−Test: p−value = 0.04

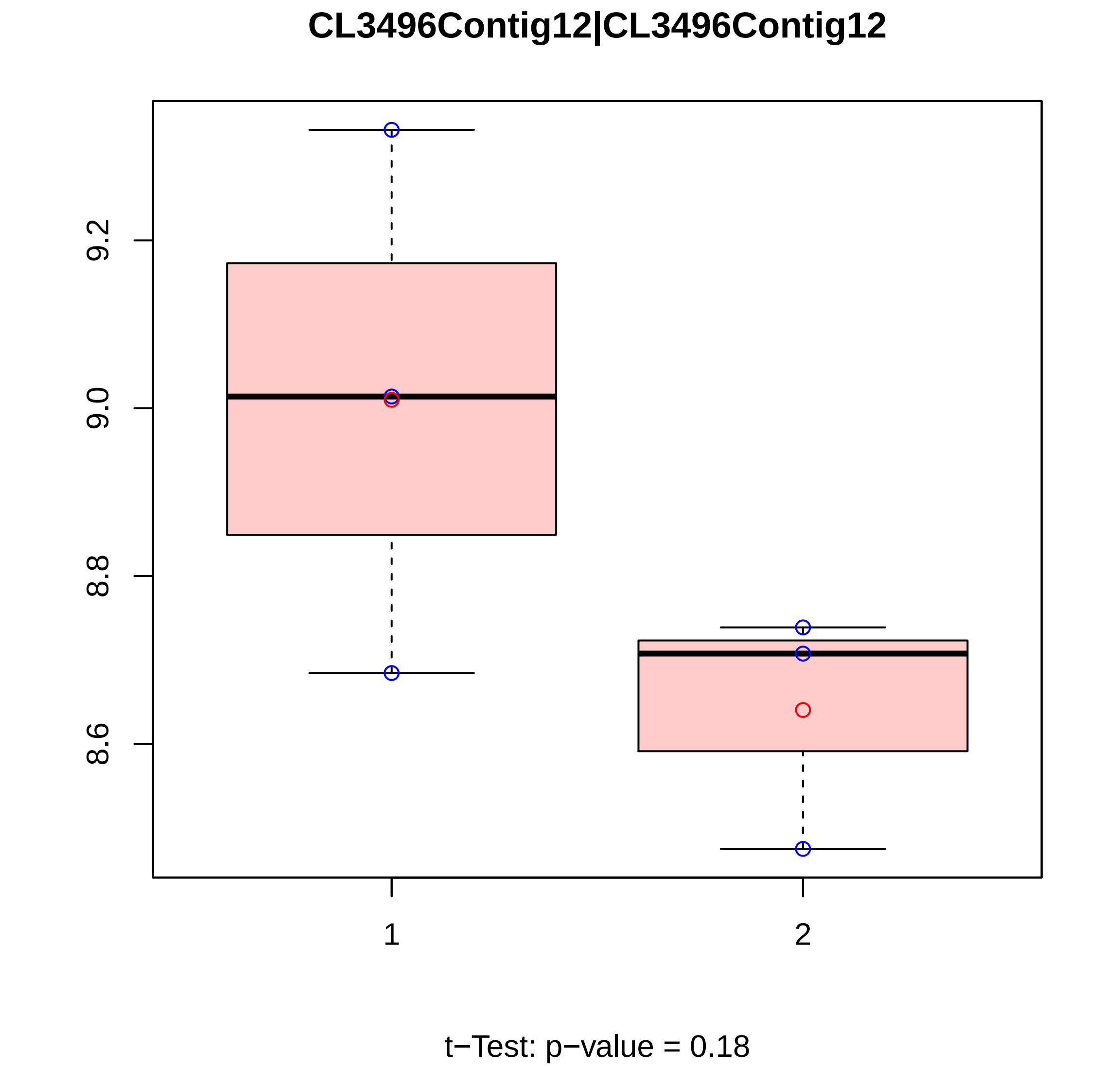
CL3496Contig12|CL3496Contig12
9.2
9.0
8.8
8.6
1
2
t−Test: p−value = 0.18

## CL3496Contig13|CL3496Contig13

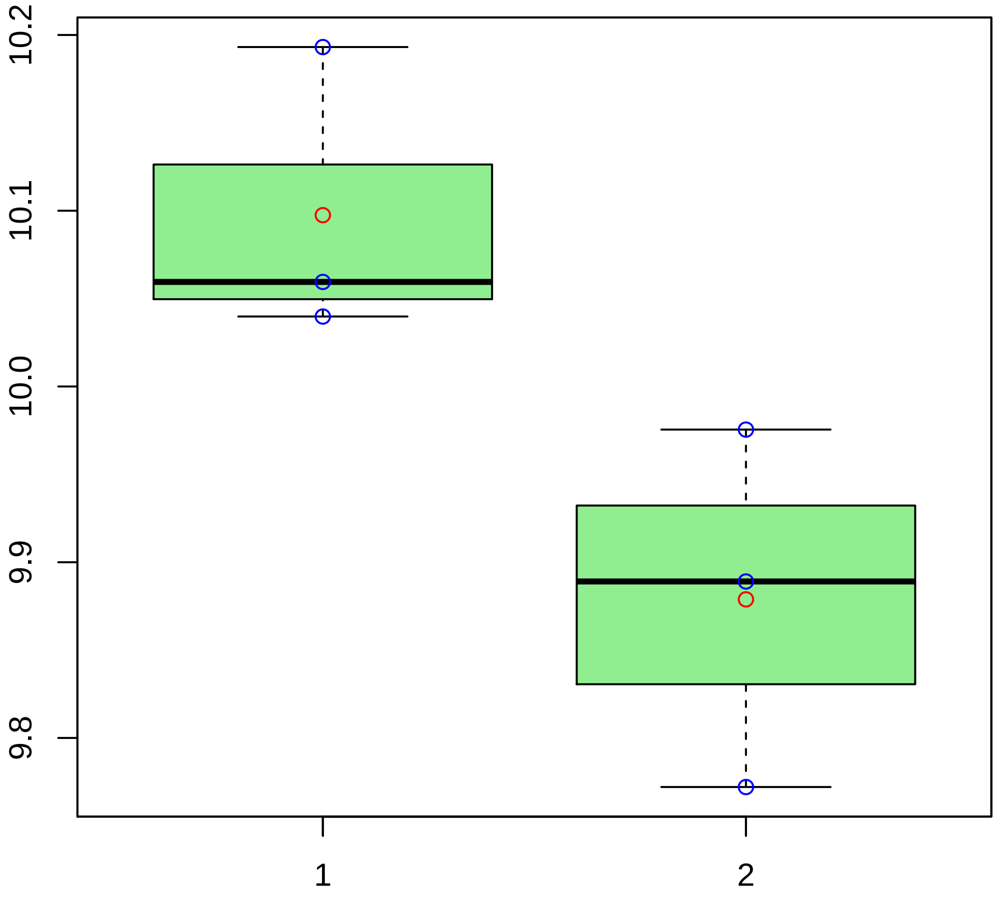


t−Test: p−value = 0.05

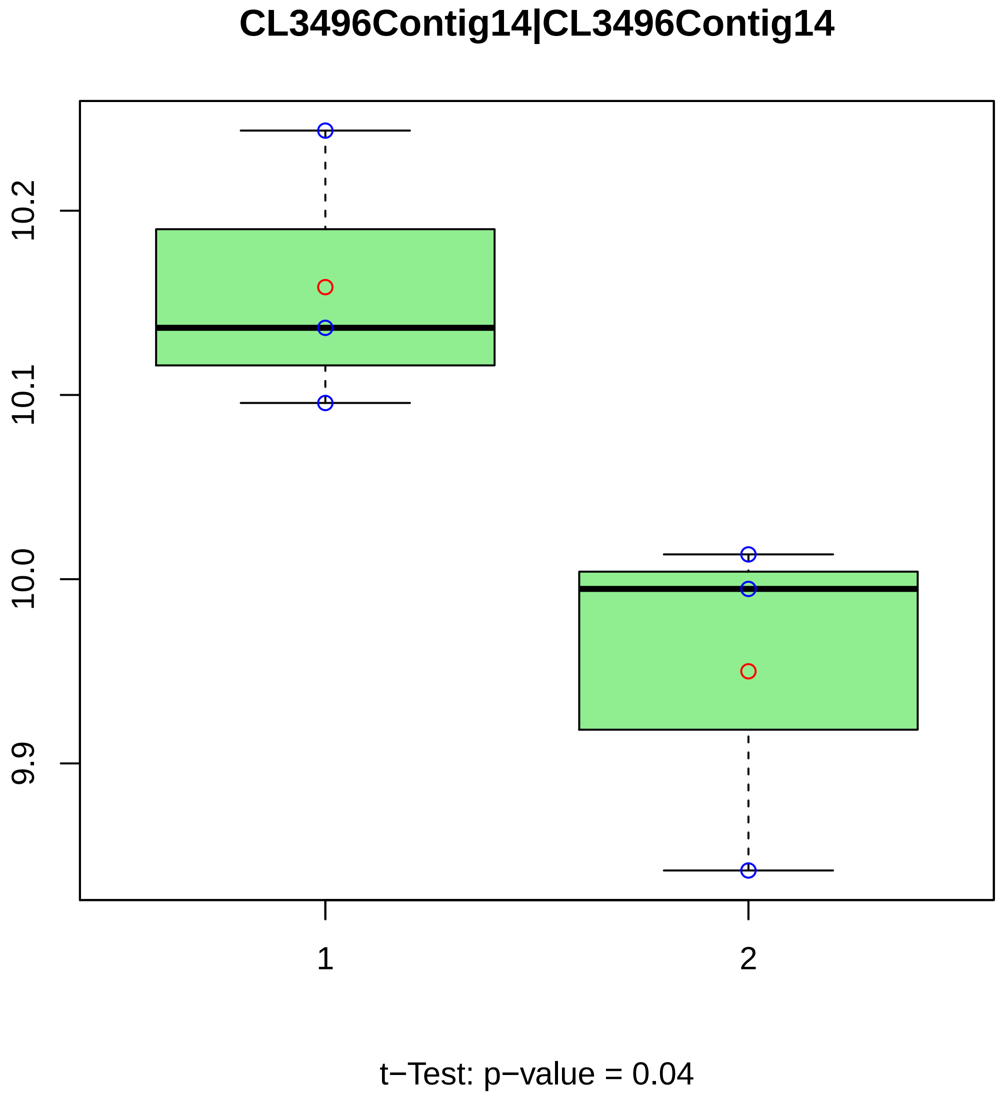

CL3496Contig14|CL3496Contig14
10.2
10.1
10.0
9.9
1
2
t−Test: p−value = 0.04

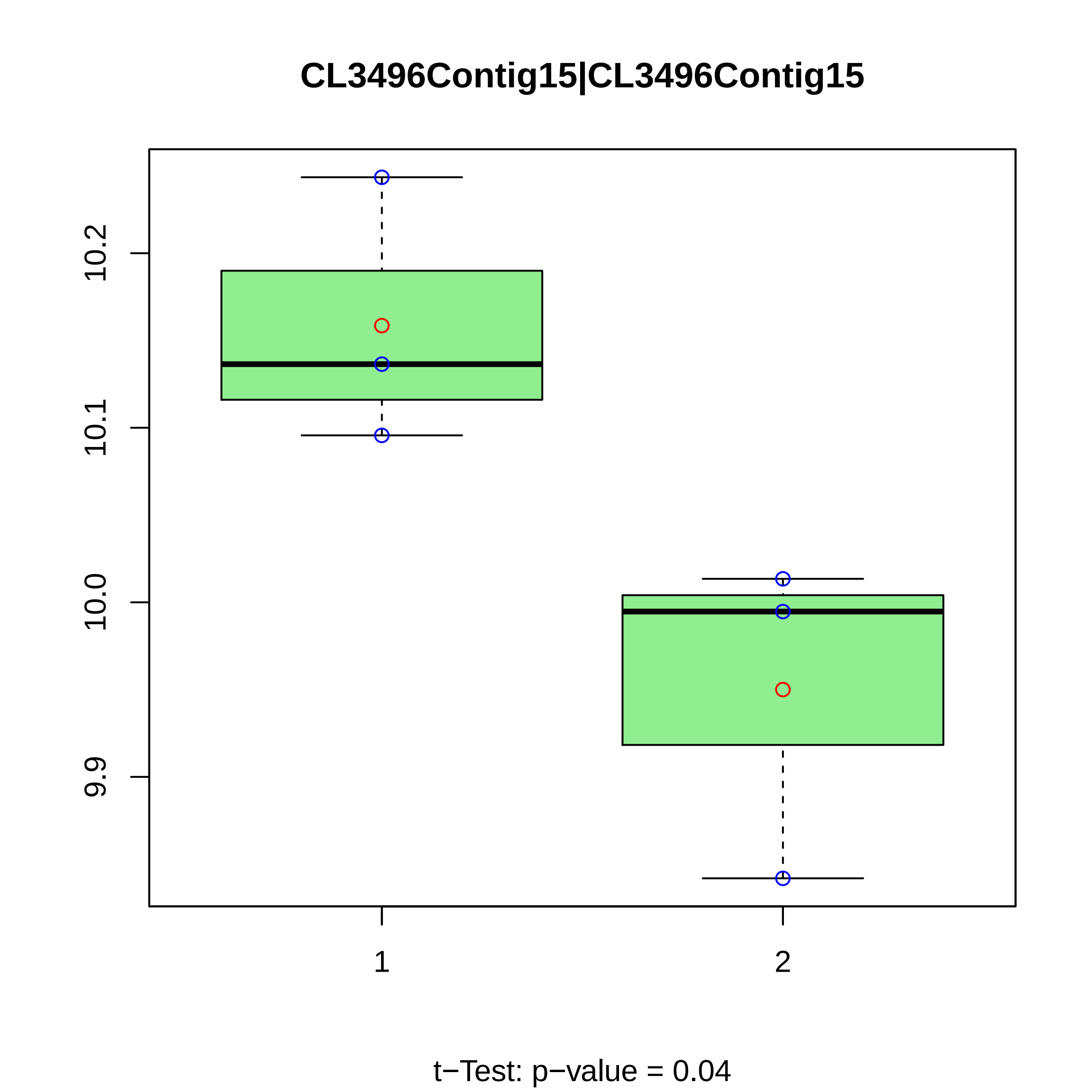
CL3496Contig15|CL3496Contig15
10.2
10.1
10.0
9.9
1
2
t−Test: p−value = 0.04

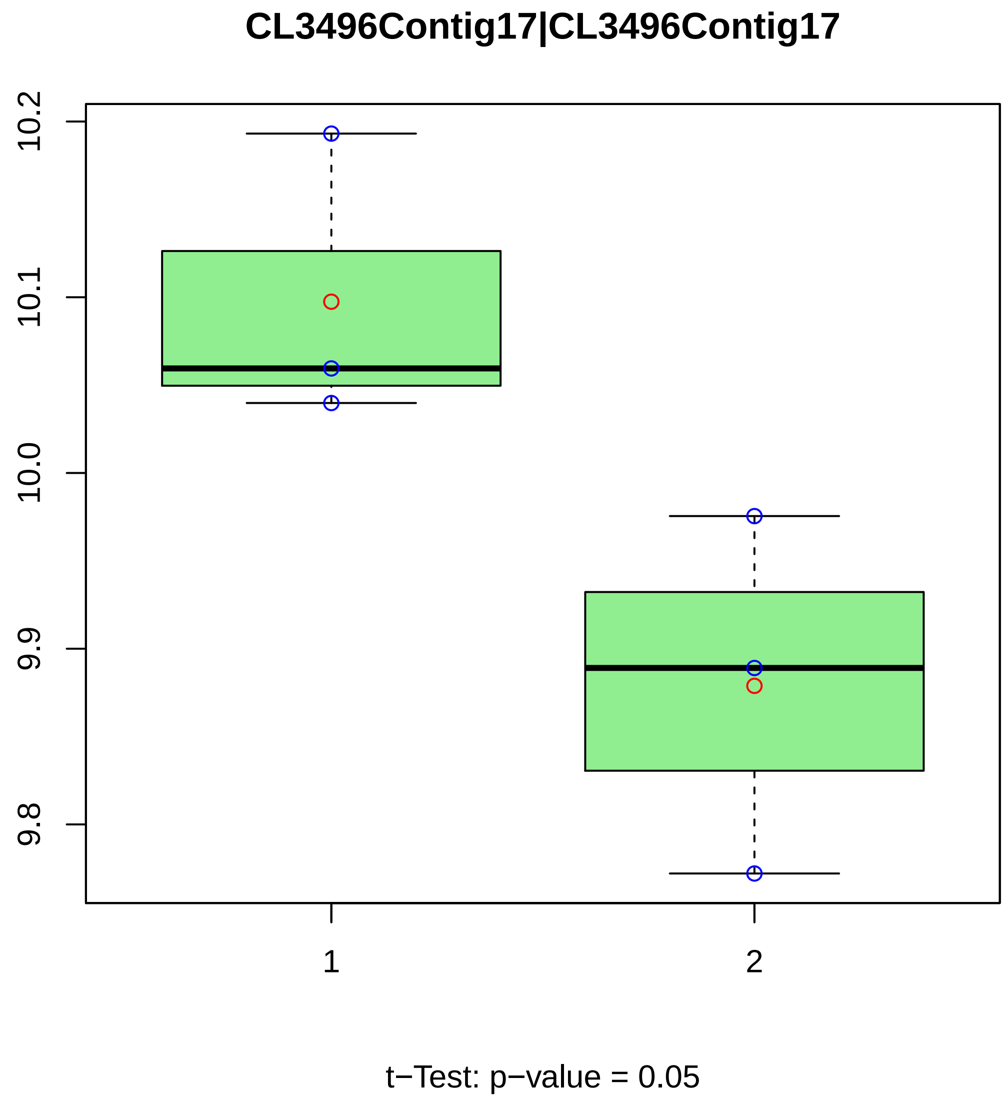

CL3496Contig17|CL3496Contig17
10.2
10.1
10.0
9.9
9.8
1
2
t−Test: p−value = 0.05

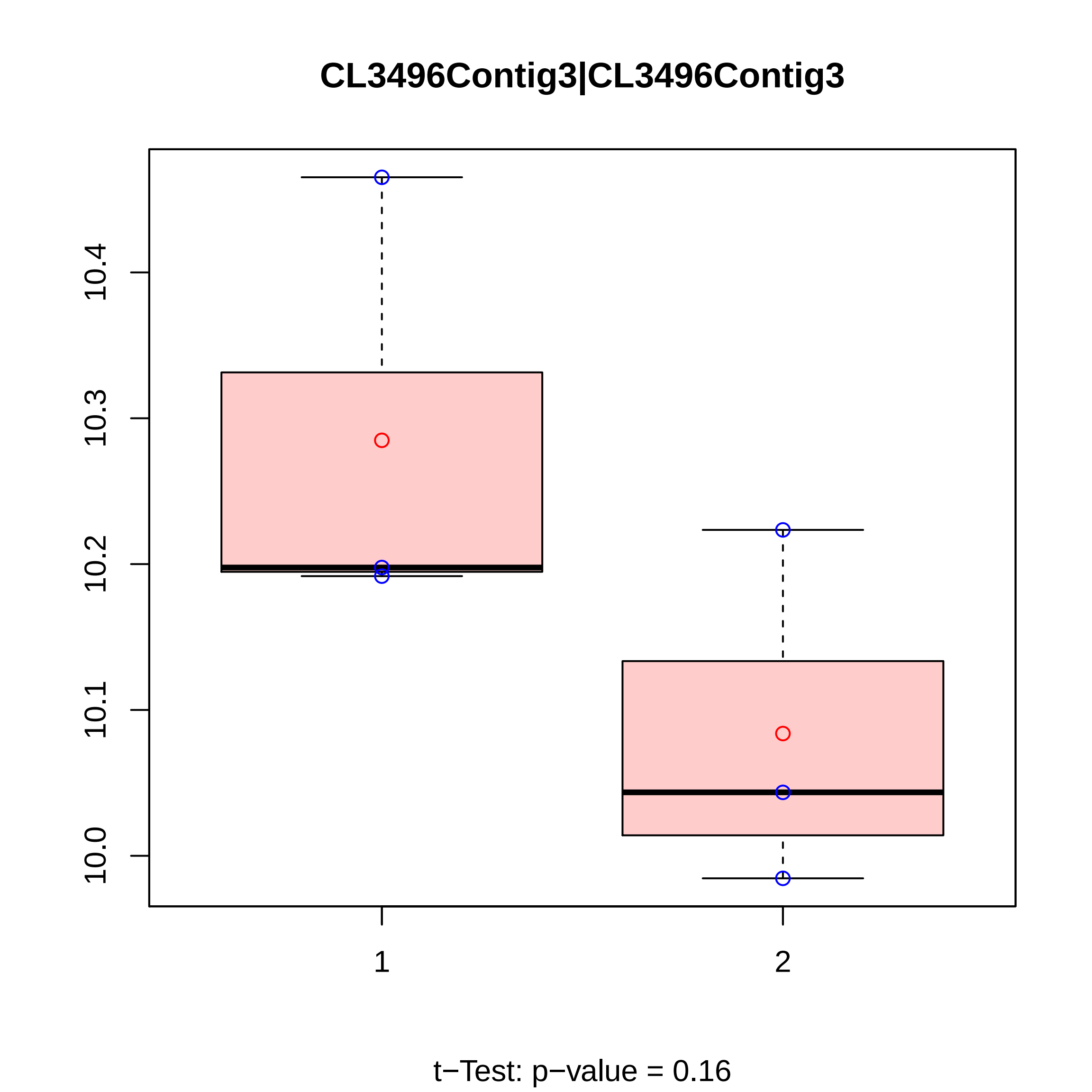
CL3496Contig3|CL3496Contig3
10.0
10.1
10.2
10.3
10.4
1
2
t−Test: p−value = 0.16

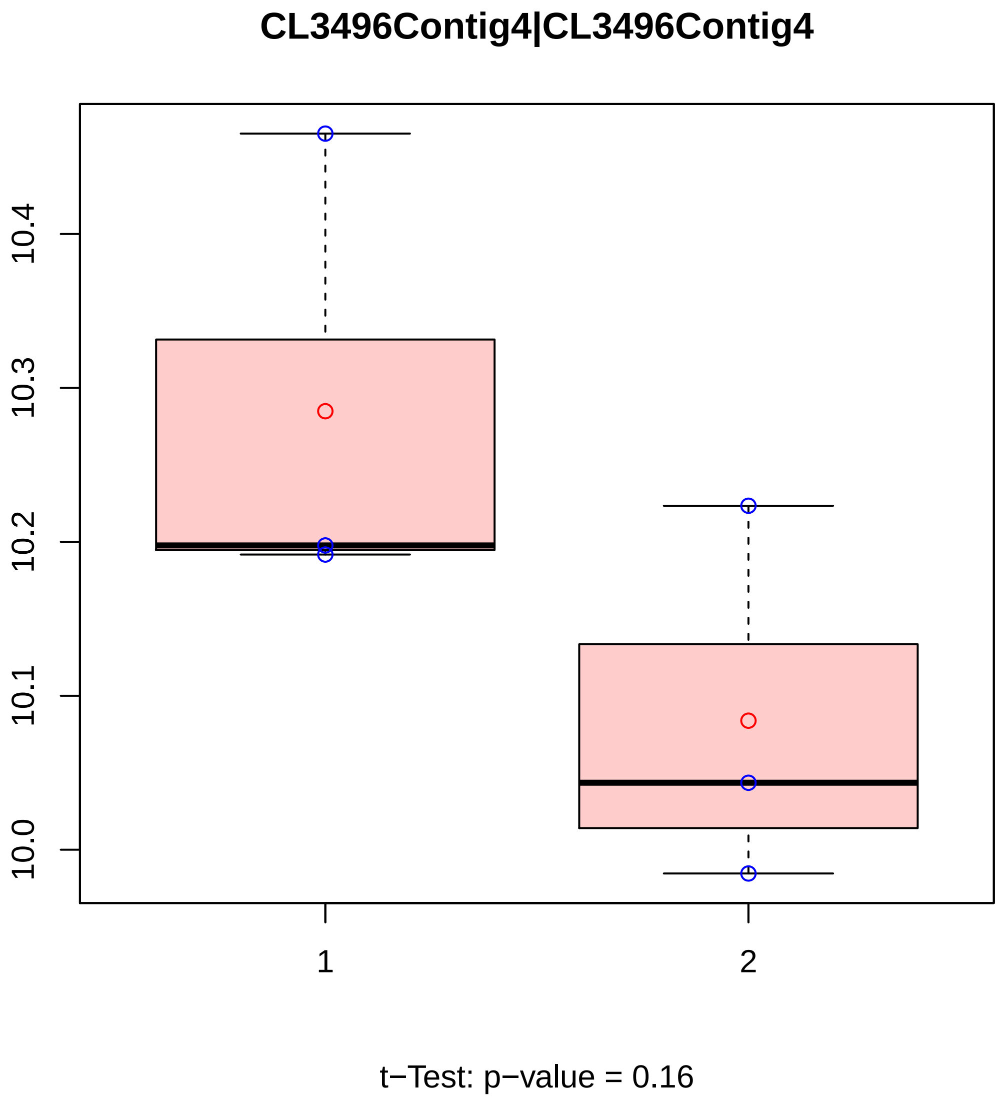
CL3496Contig4|CL3496Contig4
10.0
10.1
10.2
10.3
10.4
1
2
t−Test: p−value = 0.16

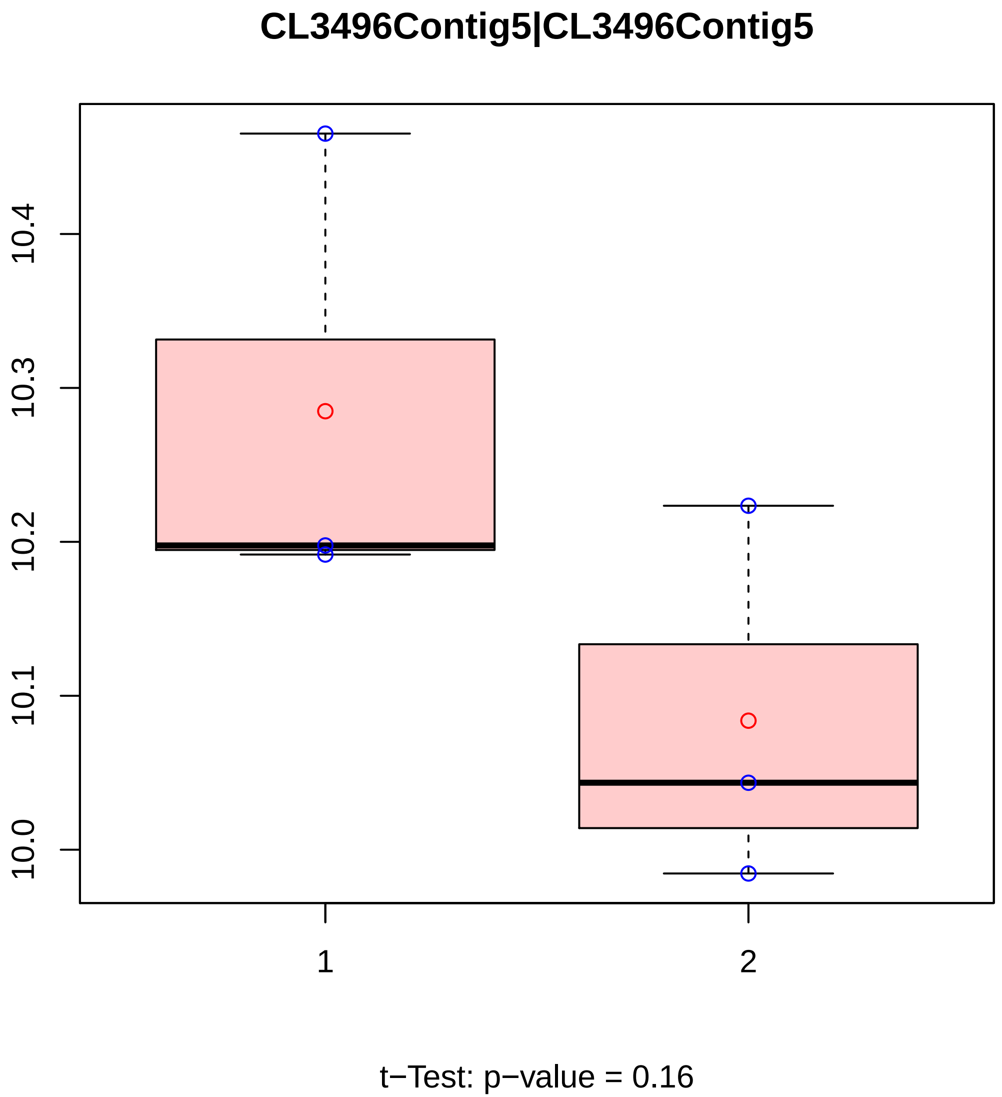
CL3496Contig5|CL3496Contig5
10.0
10.1
10.2
10.3
10.4
1
2
t−Test: p−value = 0.16

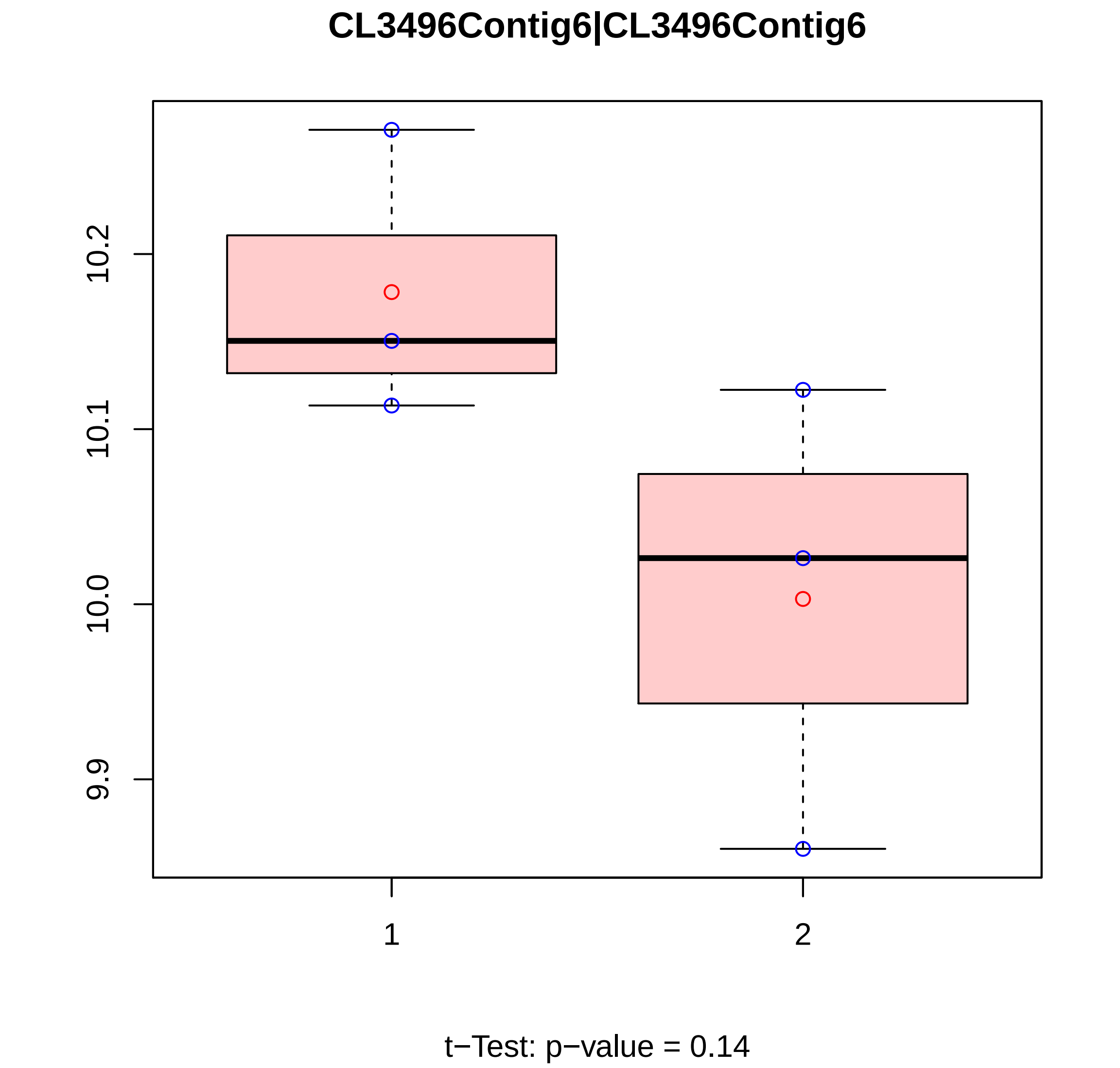

CL3496Contig6|CL3496Contig6
10.2
10.1
10.0
9.9
1
2
t−Test: p−value = 0.14

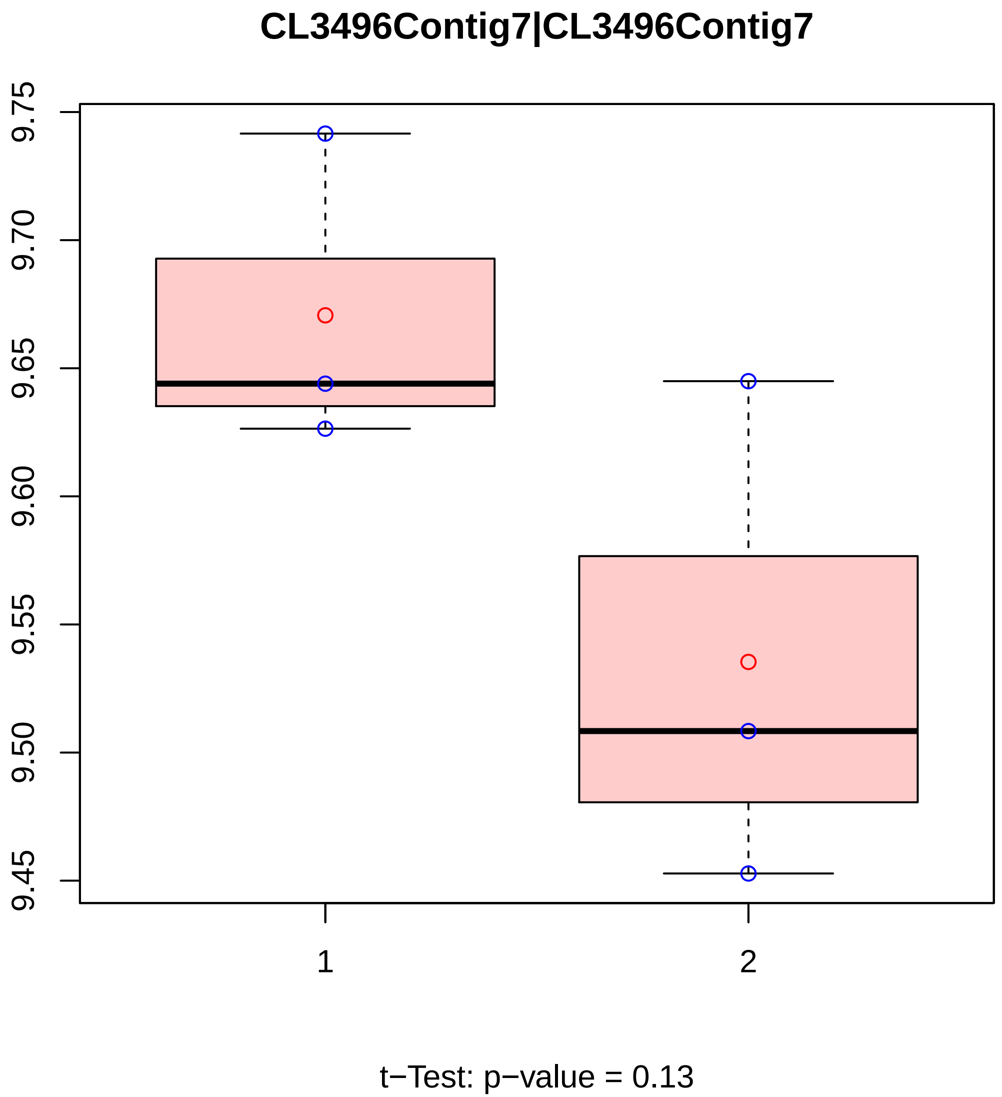
CL3496Contig7|CL3496Contig7
9.75
9.70
9.65
9.60
9.55
9.50
9.45
1
2
t−Test: p−value = 0.13

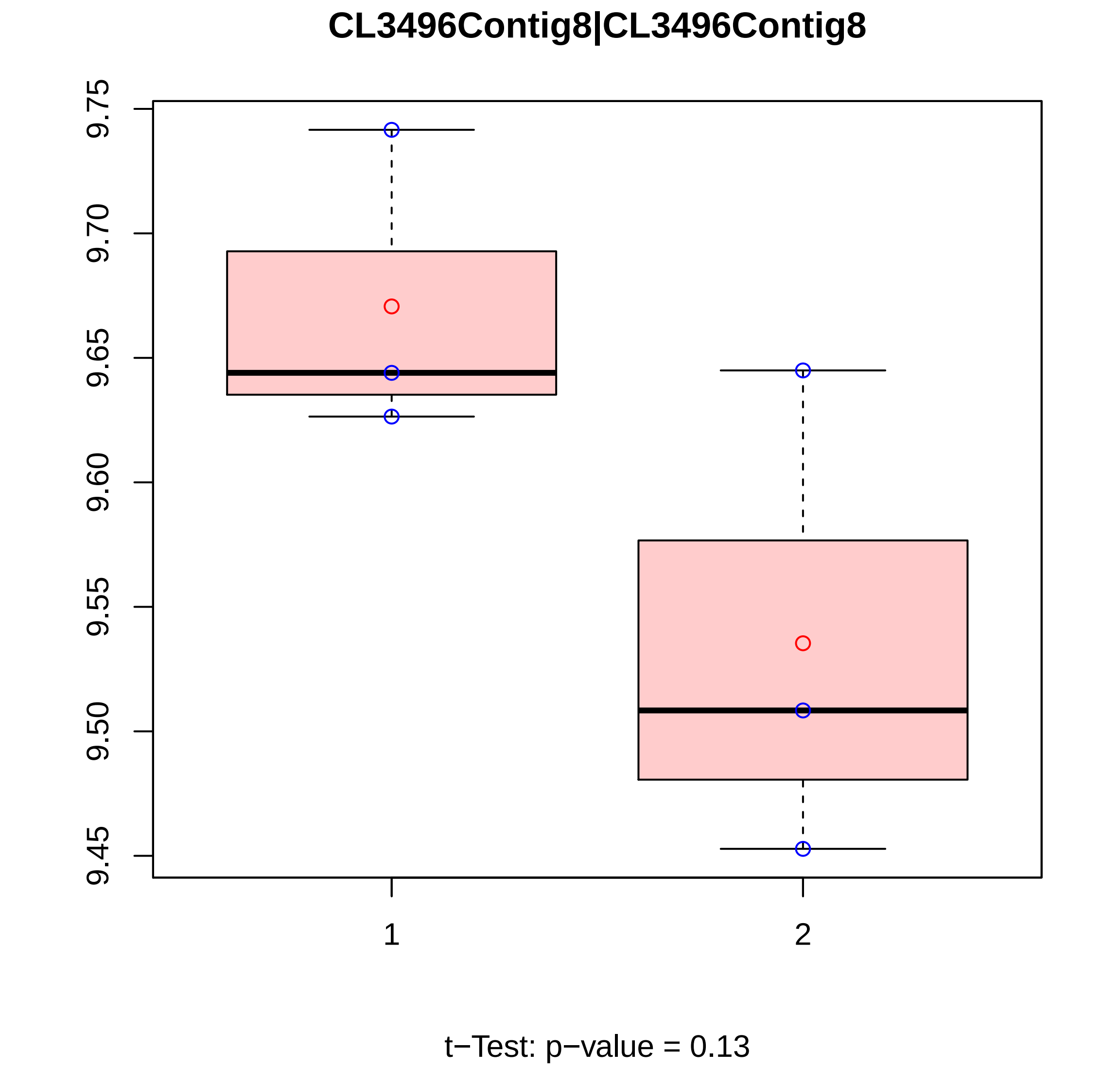

CL3496Contig8|CL3496Contig8
9.75
9.70
9.65
9.60
9.55
9.50
9.45
1
2
t−Test: p−value = 0.13

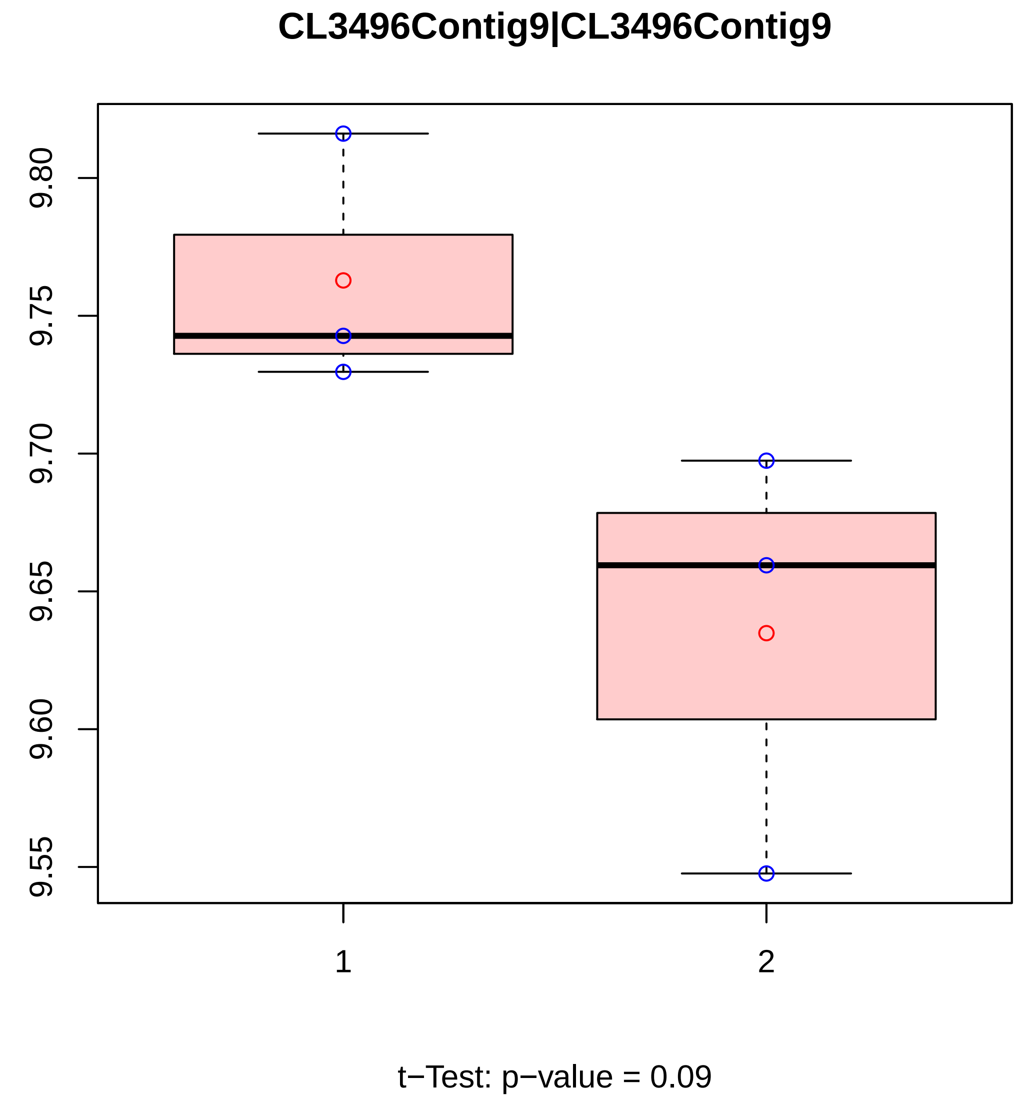
CL3496Contig9|CL3496Contig9
9.80
9.75
9.70
9.65
9.60
9.55
1
2
t−Test: p−value = 0.09

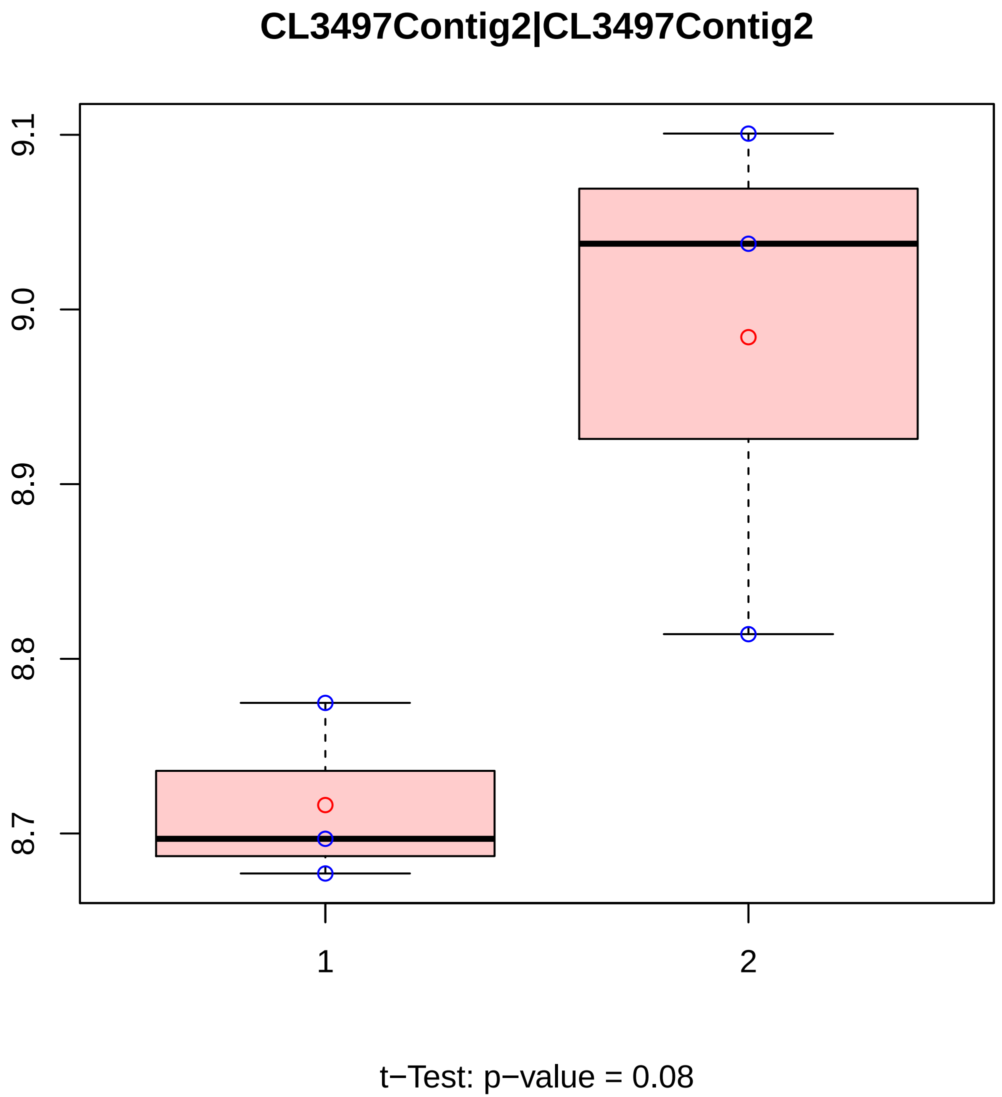

CL3497Contig2|CL3497Contig2
9.1
9.0
8.9
8.8
8.7
1
2
t−Test: p−value = 0.08

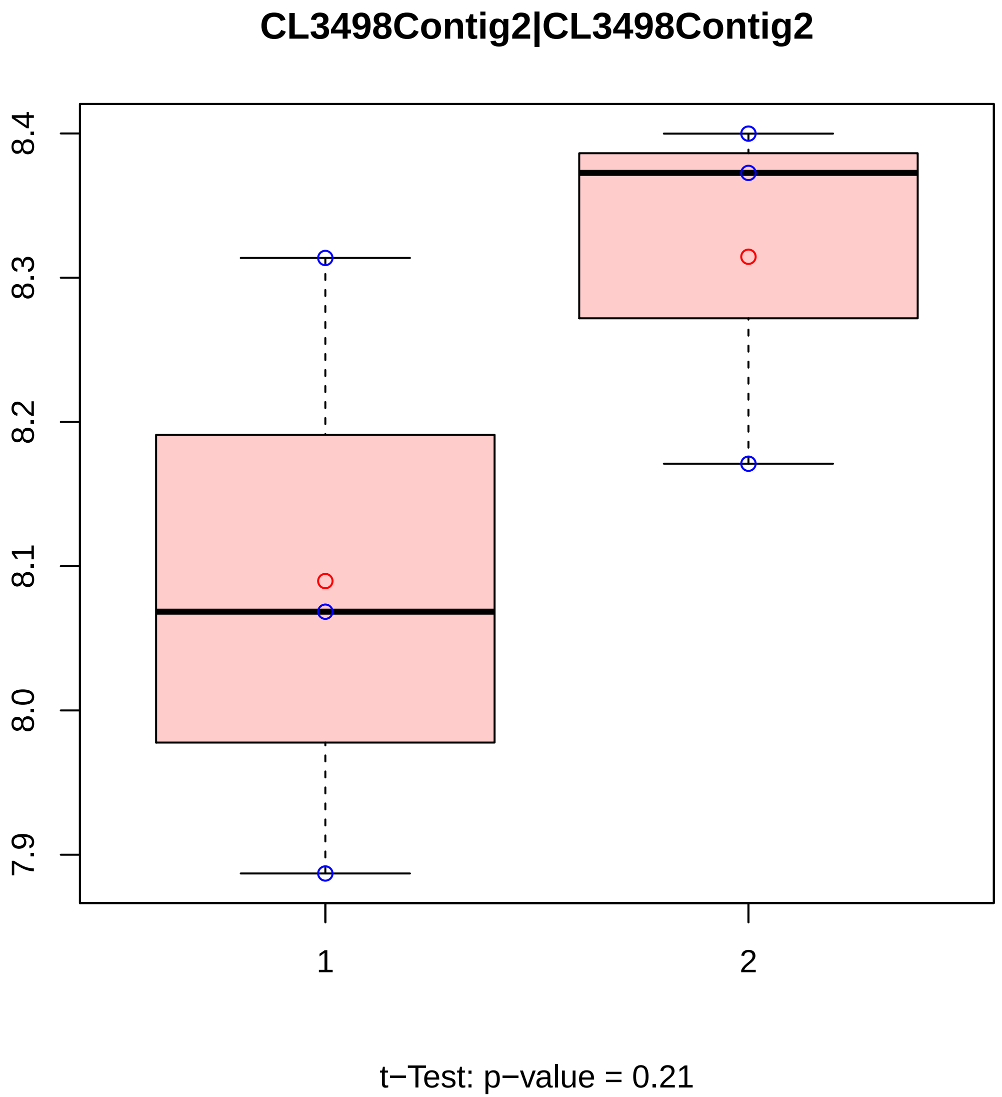
CL3498Contig2|CL3498Contig2
8.4
8.3
8.2
8.1
8.0
7.9
1
2
t−Test: p−value = 0.21

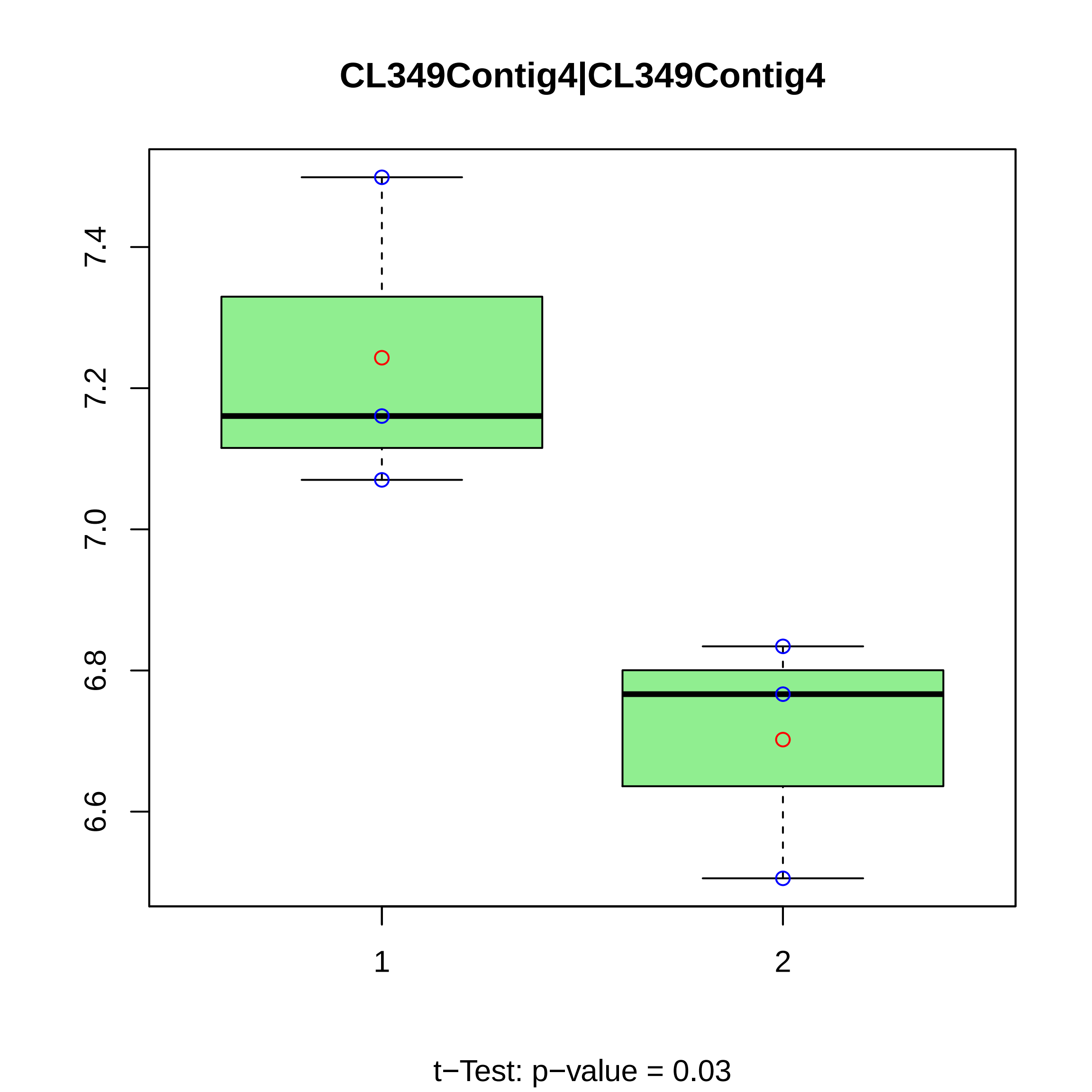
CL349Contig4|CL349Contig4
7.4
7.2
7.0
6.8
6.6
1
2
t−Test: p−value = 0.03

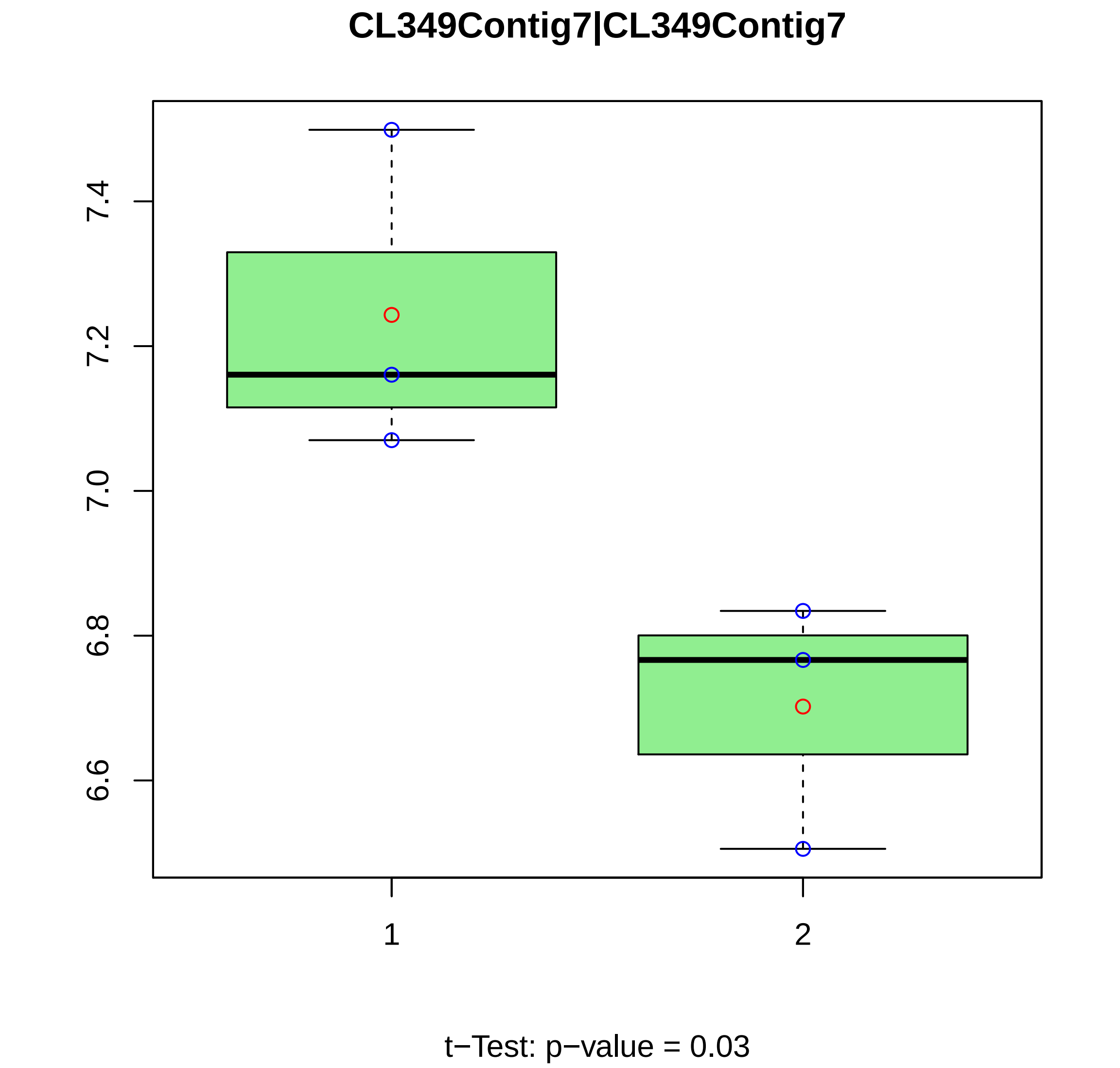

CL349Contig7|CL349Contig7
7.4
7.2
7.0
6.8
6.6
1
2
t−Test: p−value = 0.03

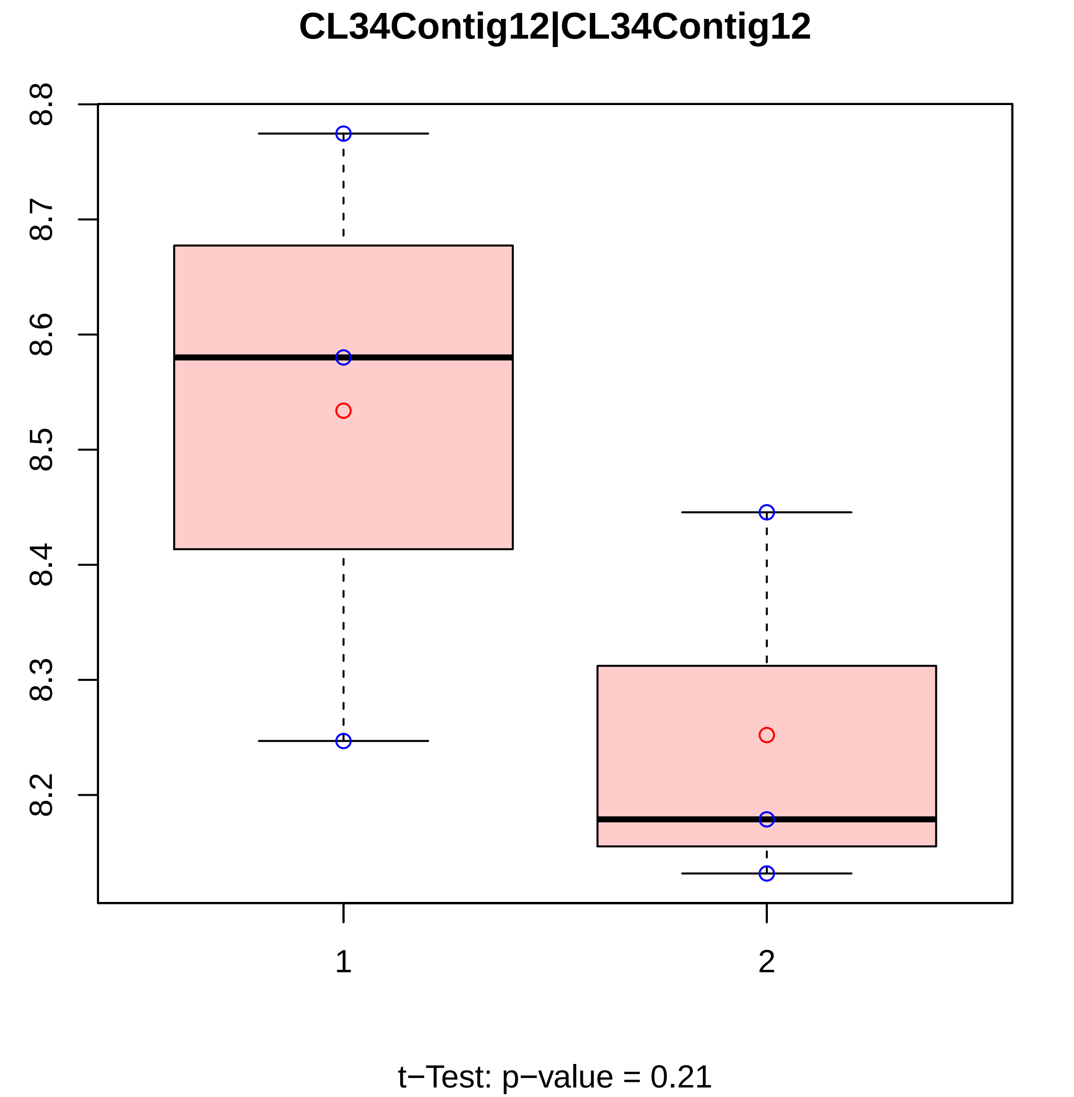

CL34Contig12|CL34Contig12
8.2
8.3
8.4
8.5
8.6
8.7
8.8
1
2
t−Test: p−value = 0.21

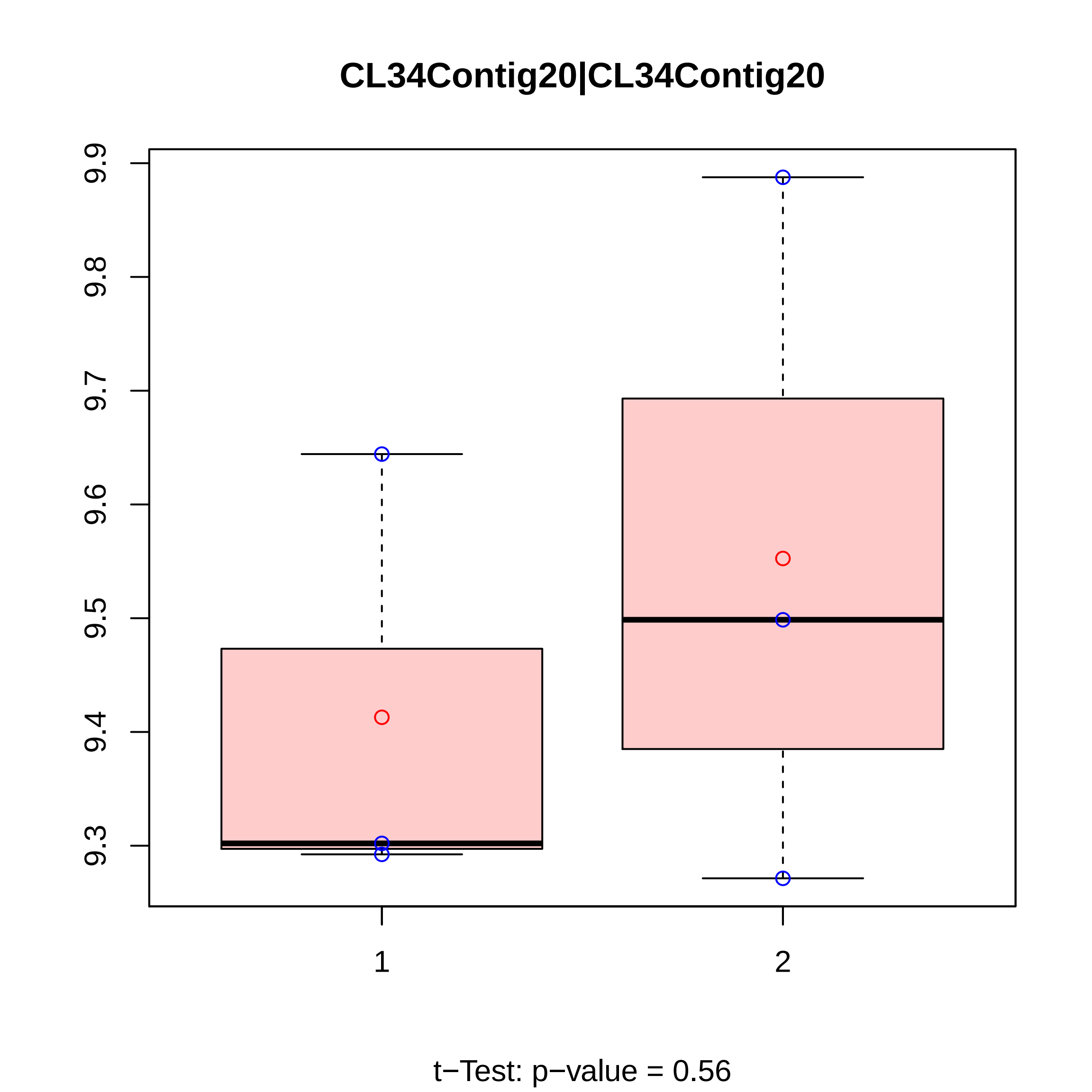
CL34Contig20|CL34Contig20
9.3
9.4
9.5
9.6
9.7
9.8
9.9
1
2
t−Test: p−value = 0.56

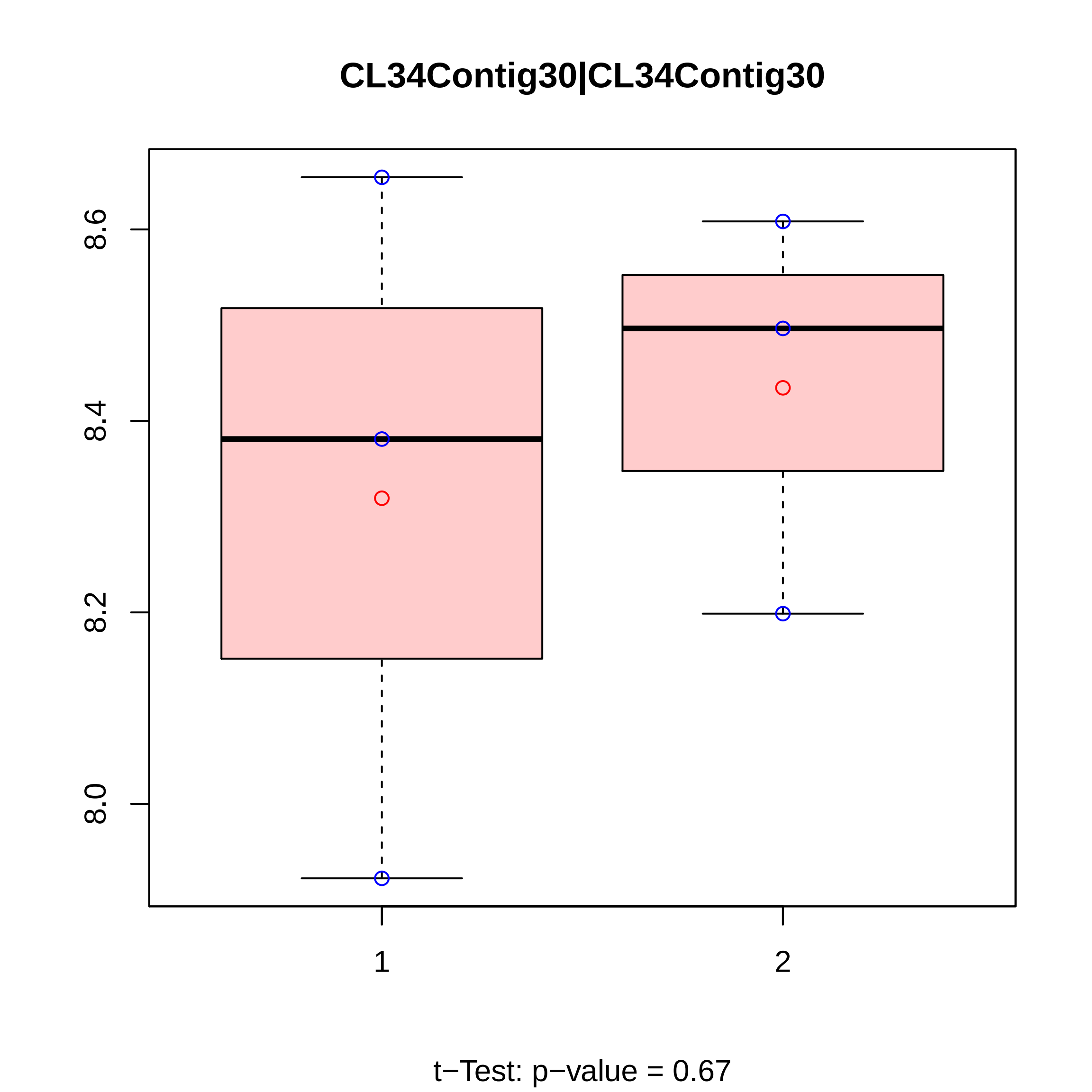
CL34Contig30|CL34Contig30
8.0
8.2
8.4
8.6
1
2
t−Test: p−value = 0.67

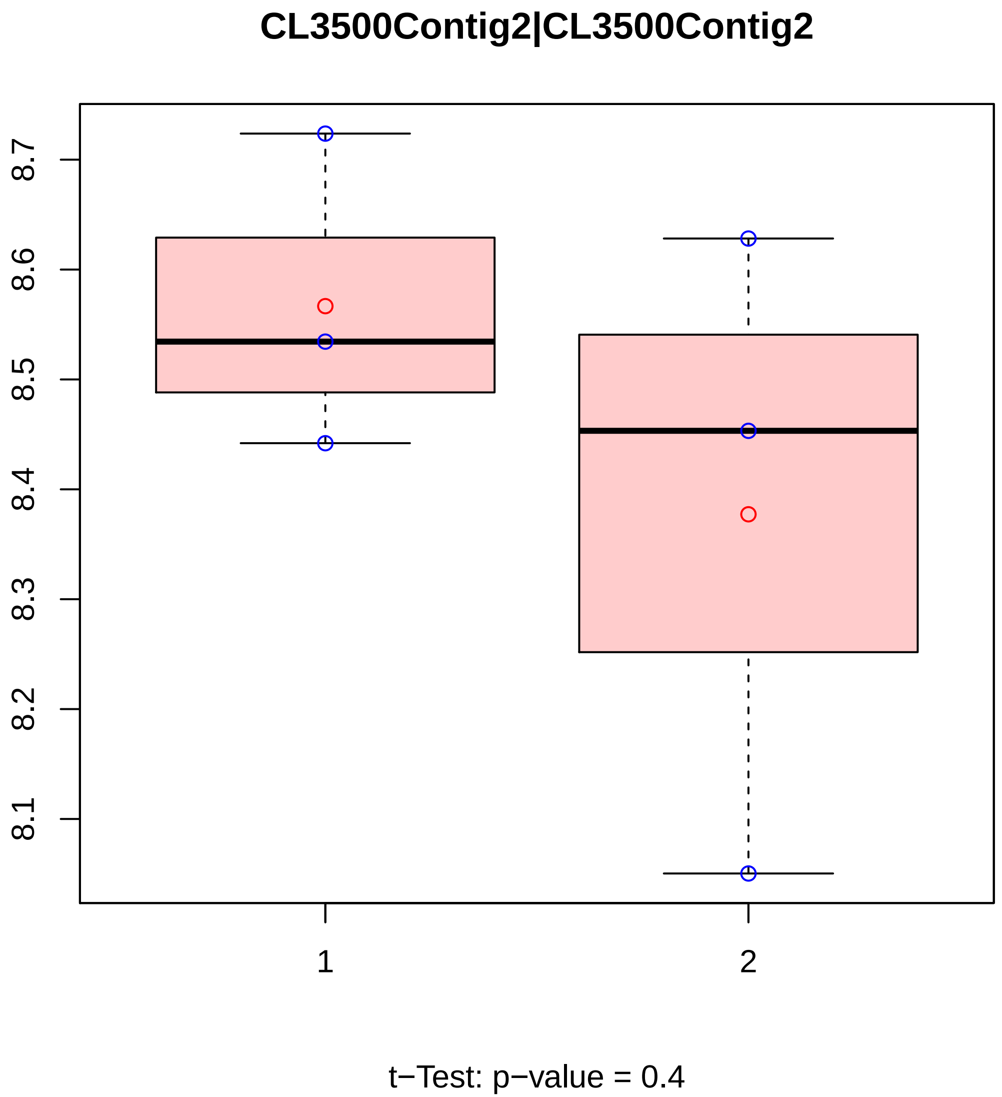

CL3500Contig2|CL3500Contig2
8.1
8.2
8.3
8.4
8.5
8.6
8.7
1
2
t−Test: p−value = 0.4

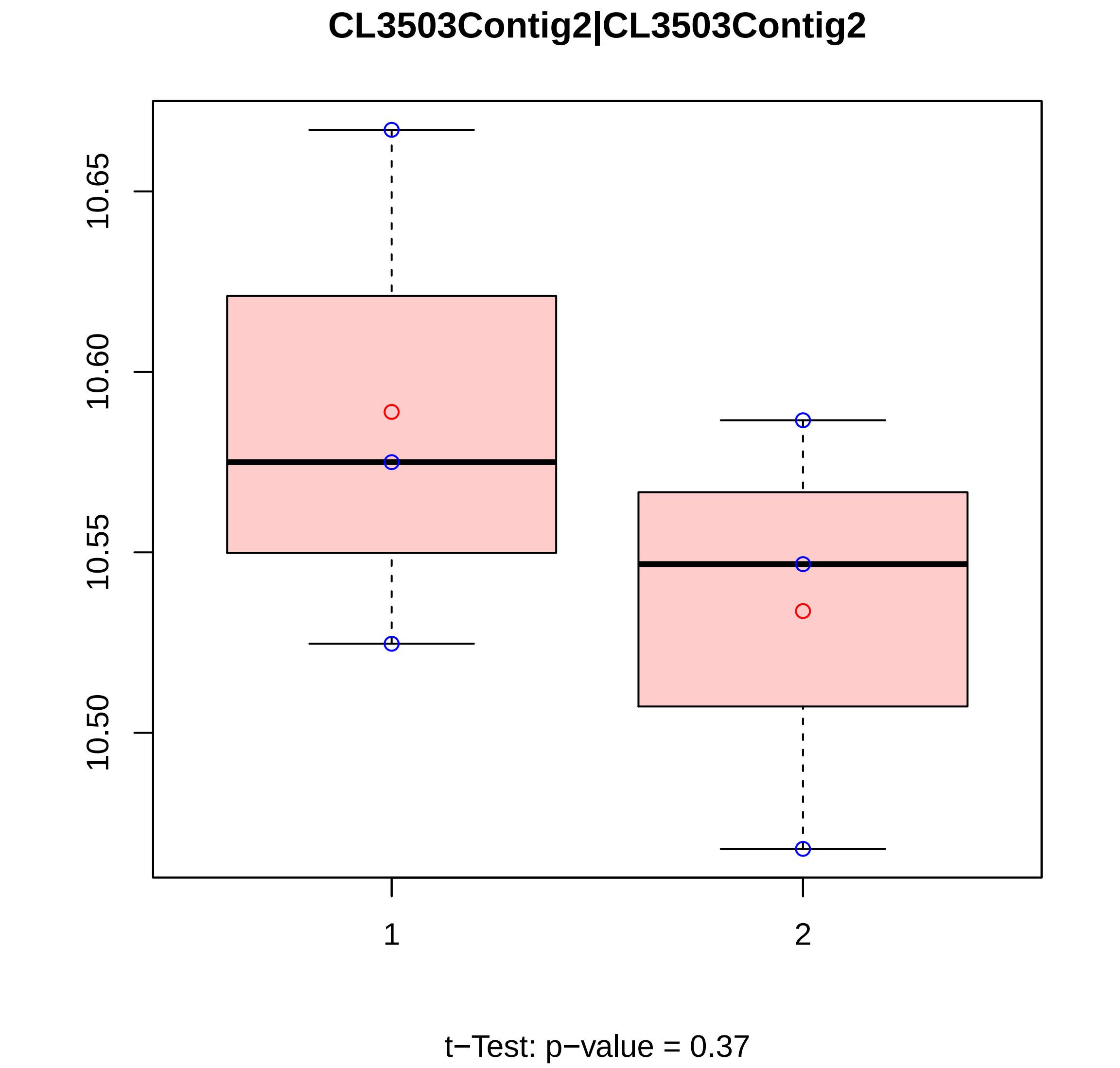
CL3503Contig2|CL3503Contig2
10.65
10.60
10.55
10.50
1
2
t−Test: p−value = 0.37

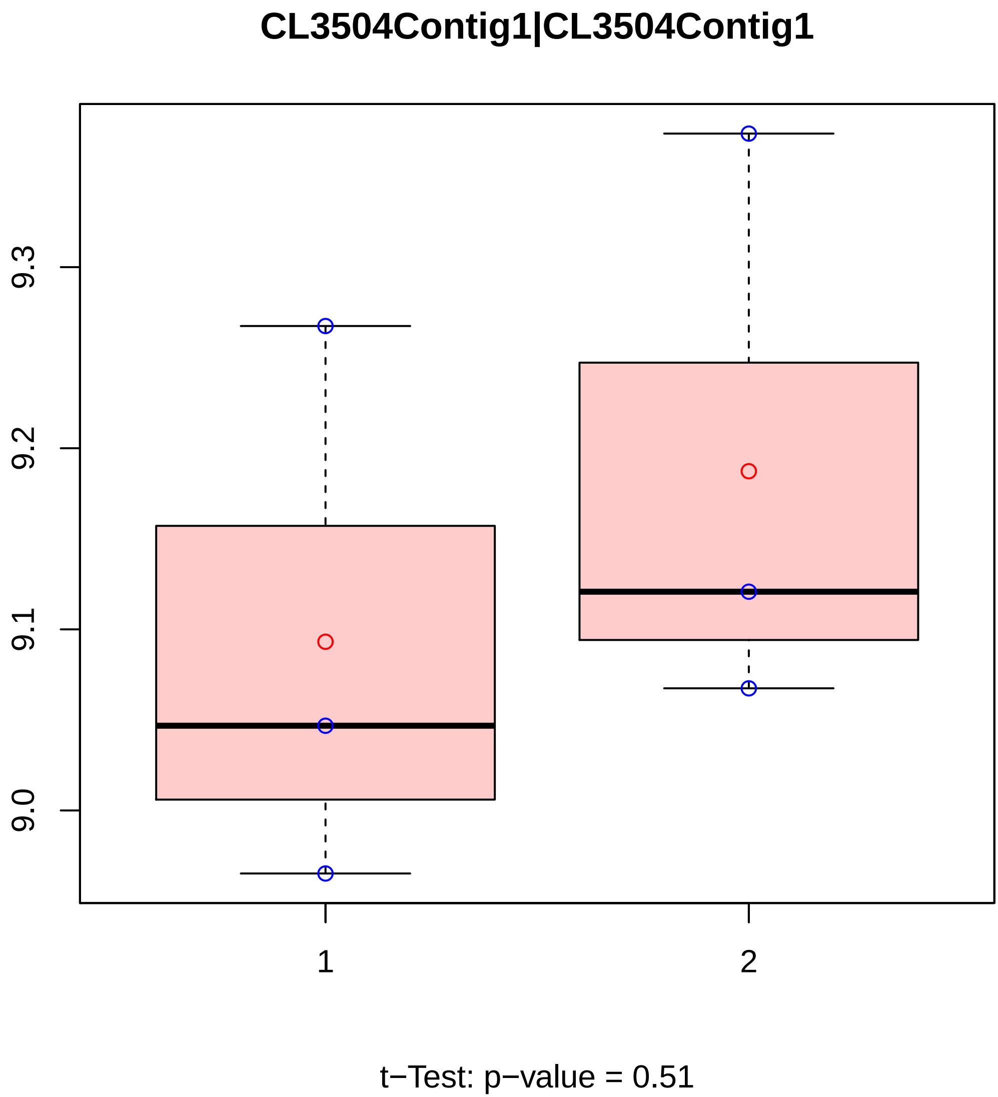
CL3504Contig1|CL3504Contig1
9.0
9.1
9.2
9.3
1
2
t−Test: p−value = 0.51

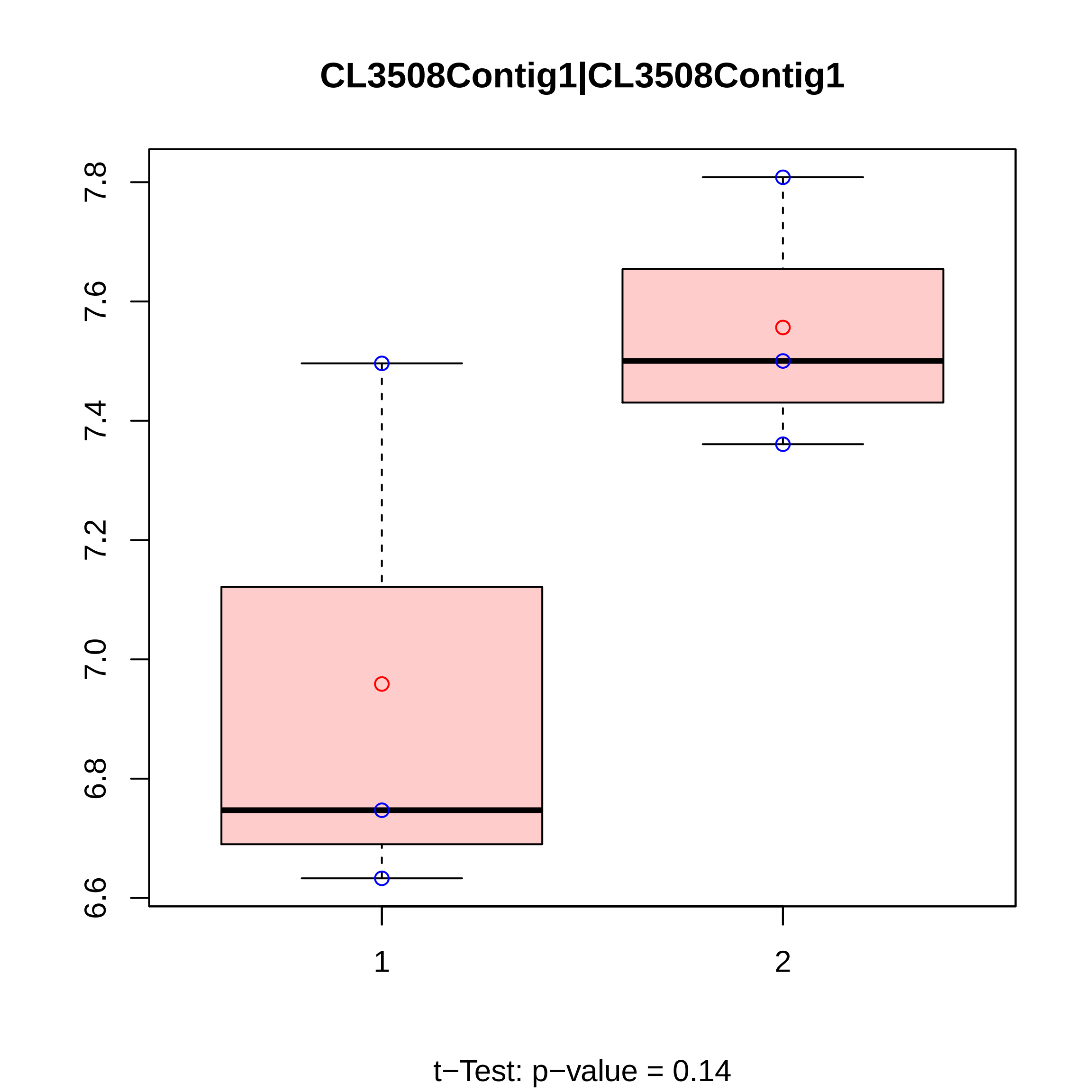
CL3508Contig1|CL3508Contig1
7.8
7.6
7.4
7.2
7.0
6.8
6.6
1
2
t−Test: p−value = 0.14

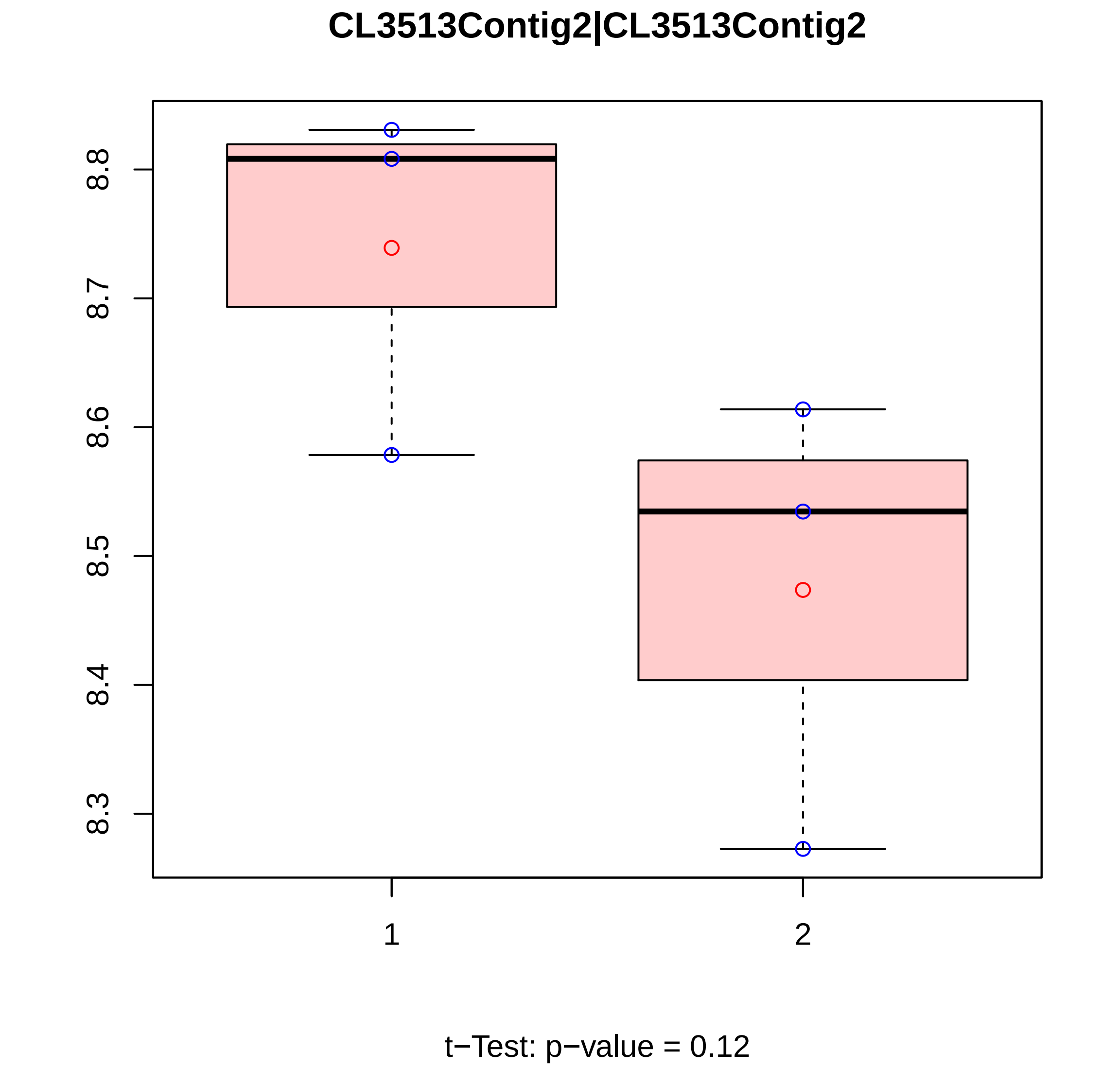

CL3513Contig2|CL3513Contig2
8.3
8.4
8.5
8.6
8.7
8.8
1
2
t−Test: p−value = 0.12

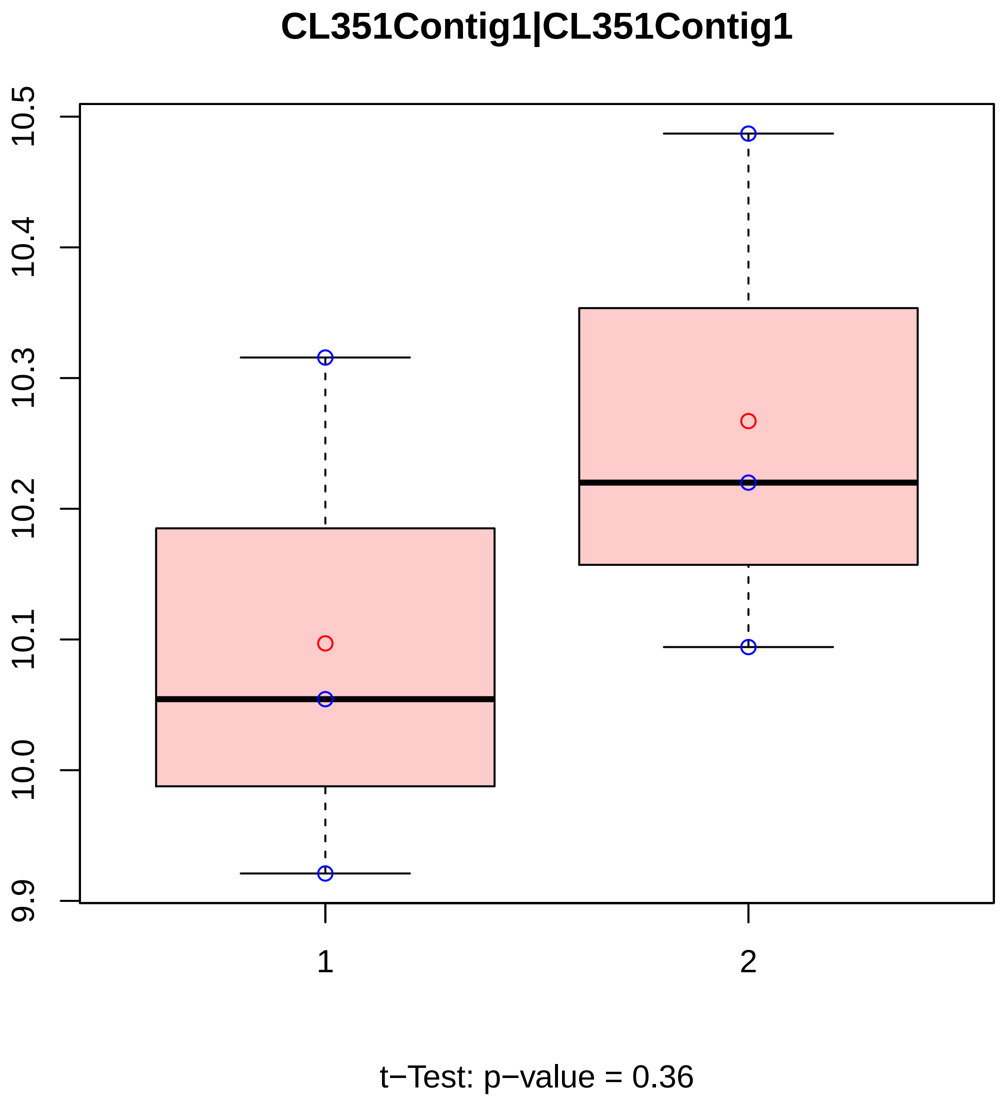

CL351Contig1|CL351Contig1
10.5
10.4
10.3
10.2
10.1
10.0
9.9
1
2
t−Test: p−value = 0.36

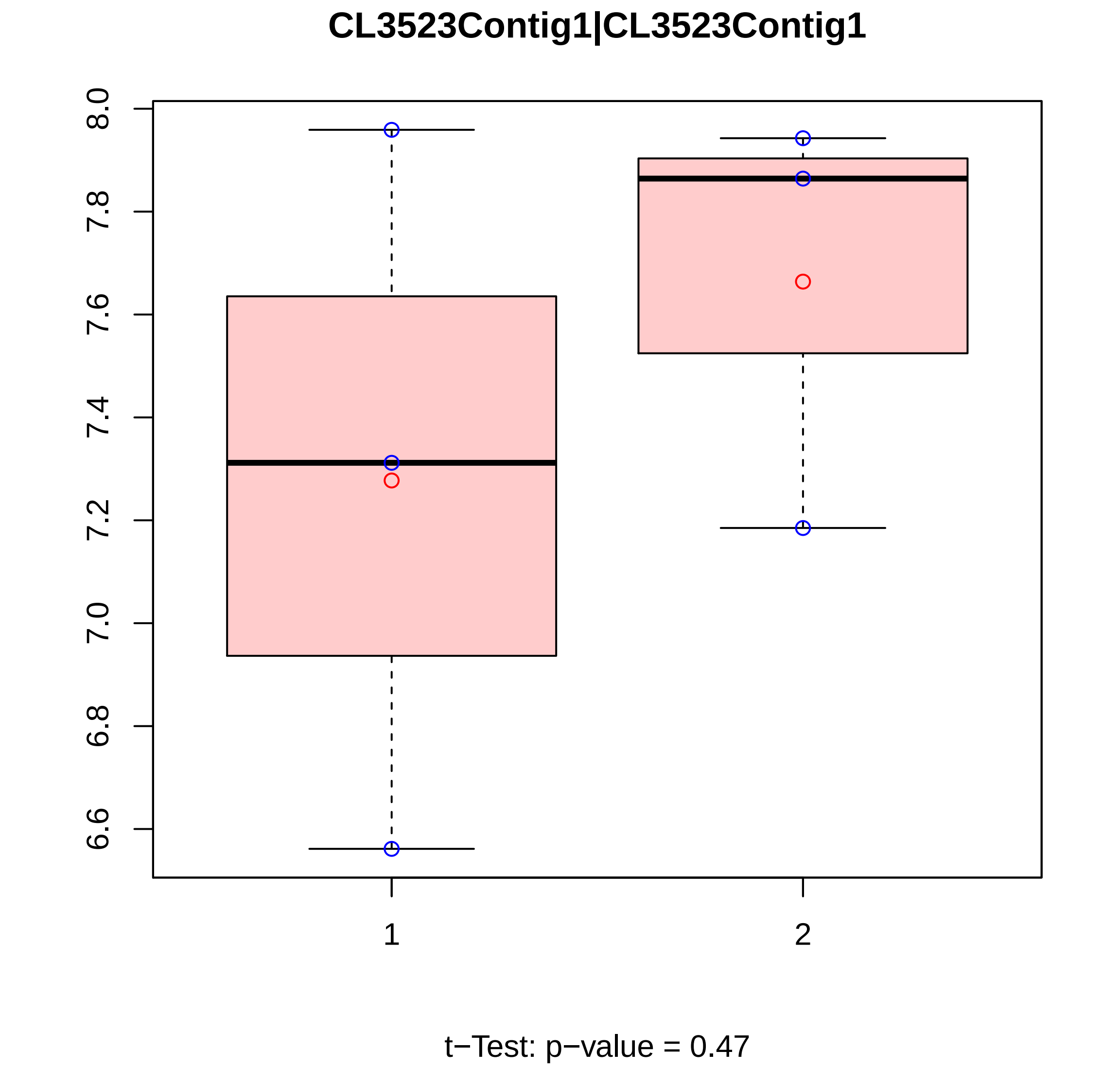
CL3523Contig1|CL3523Contig1
8.0
7.8
7.6
7.4
7.2
7.0
6.8
6.6
1
2
t−Test: p−value = 0.47

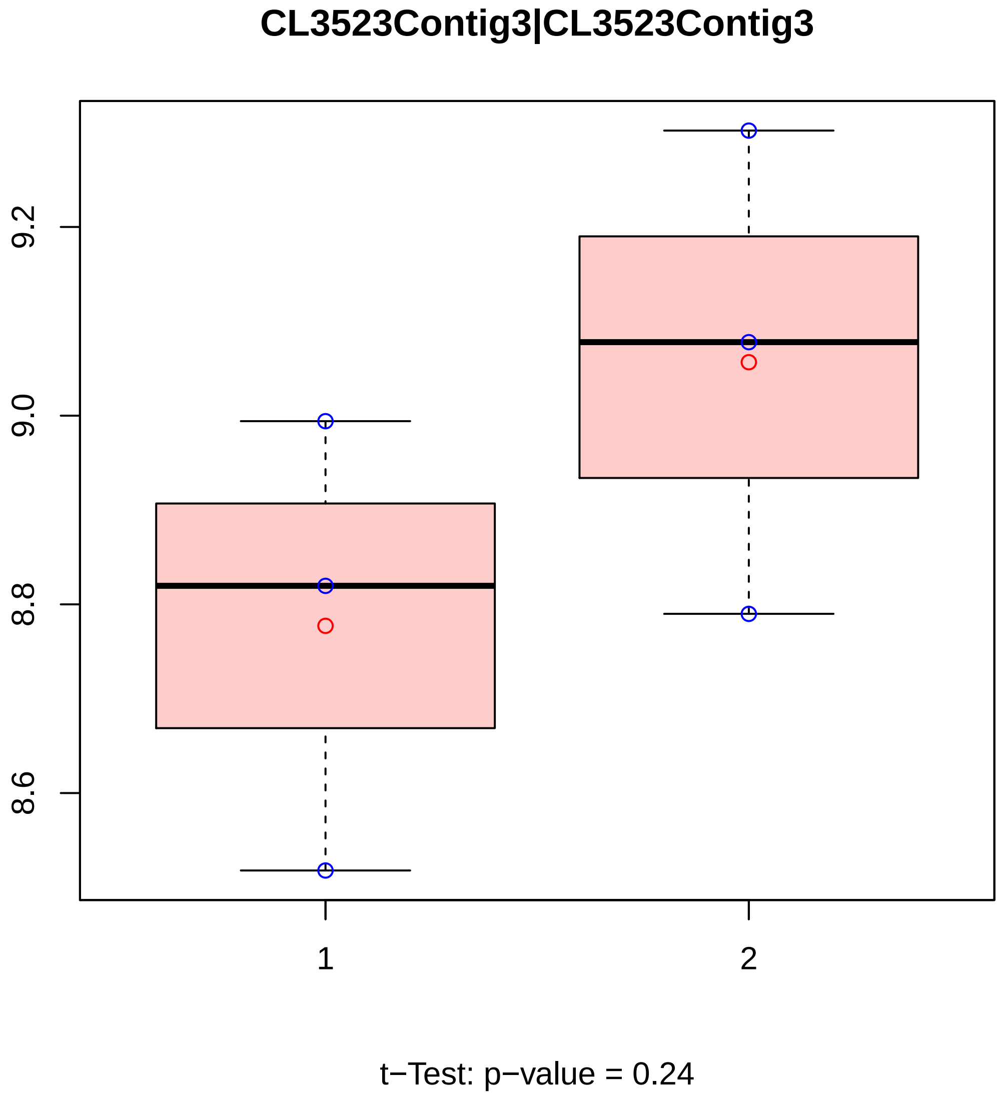

CL3523Contig3|CL3523Contig3
9.2
9.0
8.8
8.6
1
2
t−Test: p−value = 0.24

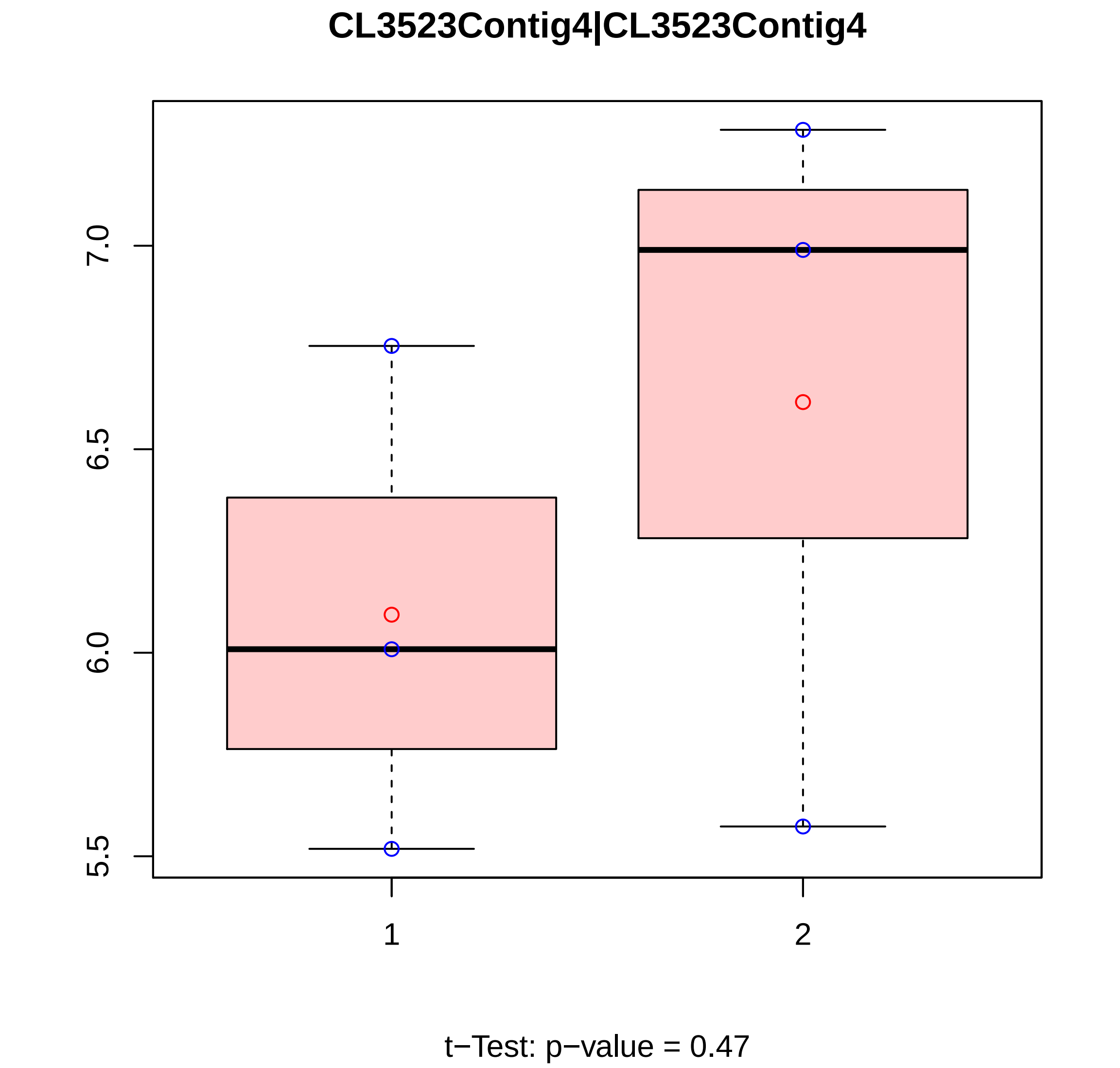

CL3523Contig4|CL3523Contig4
7.0
6.5
6.0
5.5
1
2
t−Test: p−value = 0.47

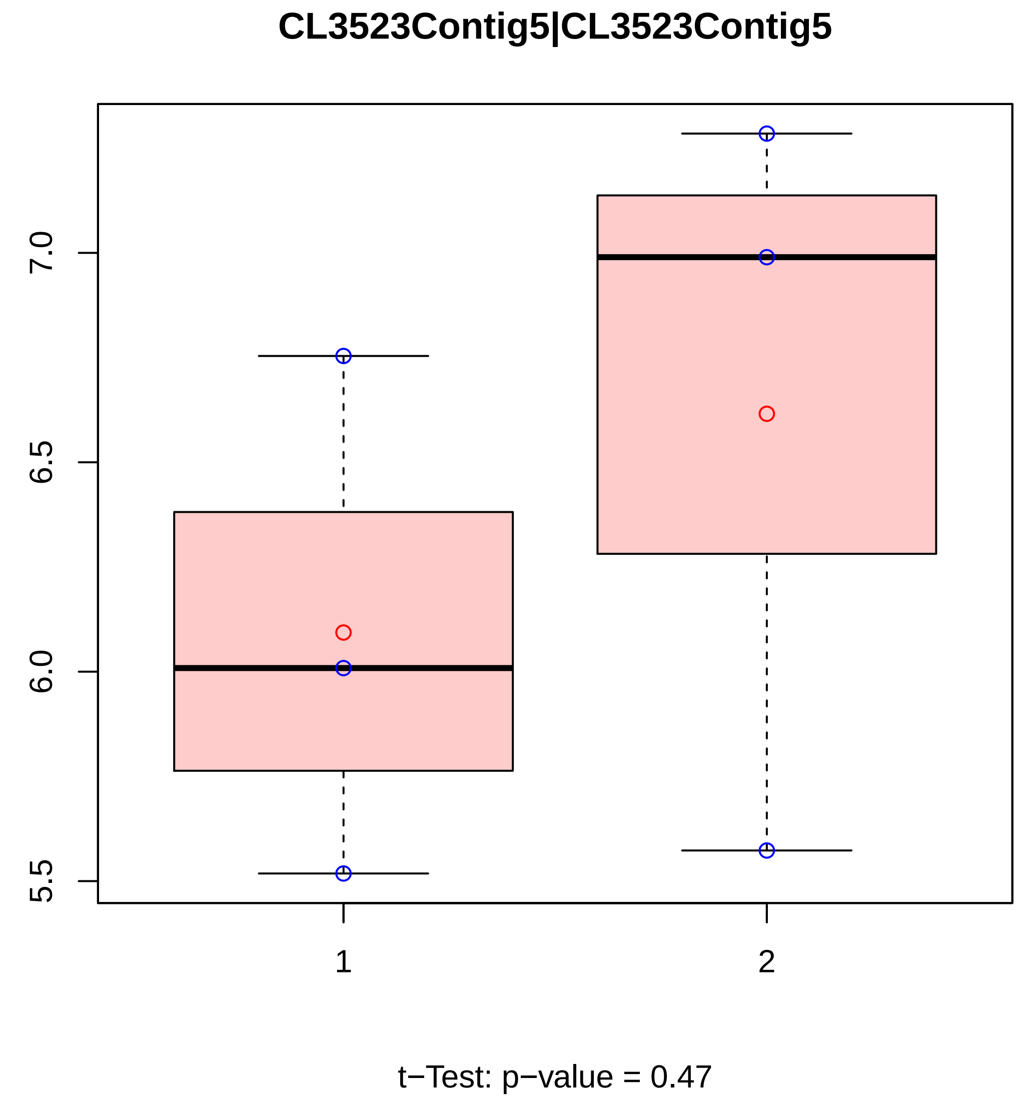
CL3523Contig5|CL3523Contig5
7.0
6.5
6.0
5.5
1
2
t−Test: p−value = 0.47

# CL3523Contig7|CL3523Contig7

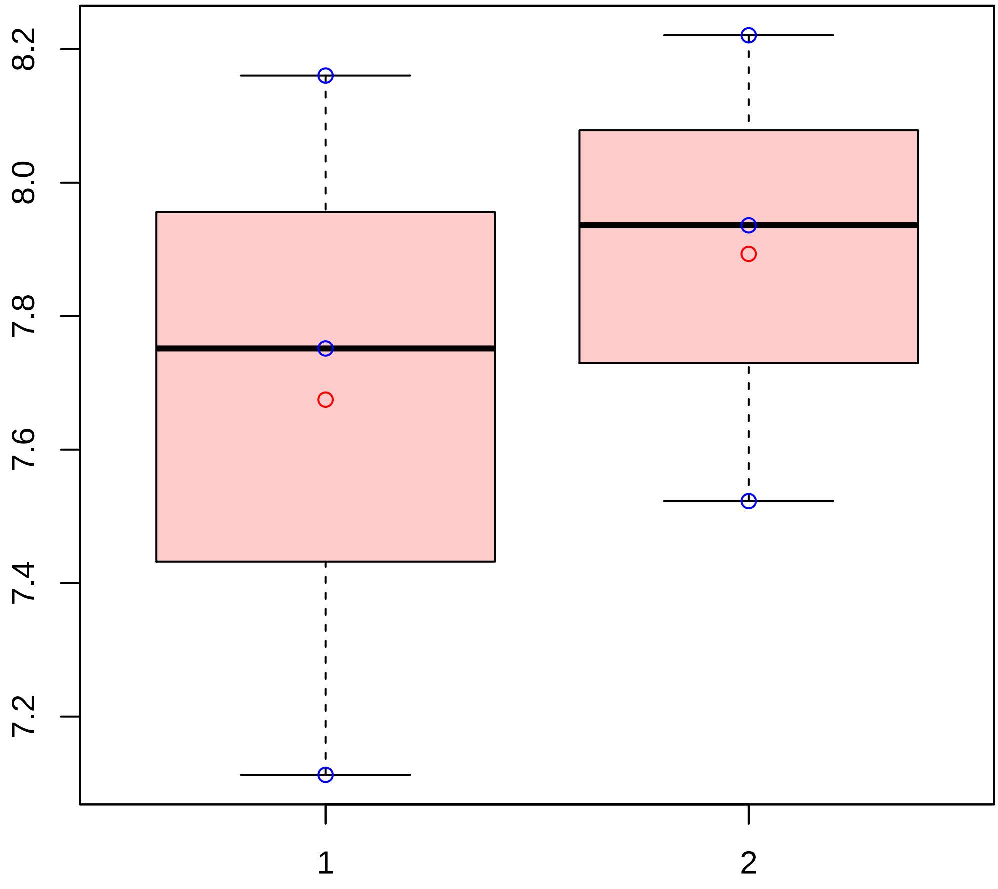


t−Test: p−value = 0.59

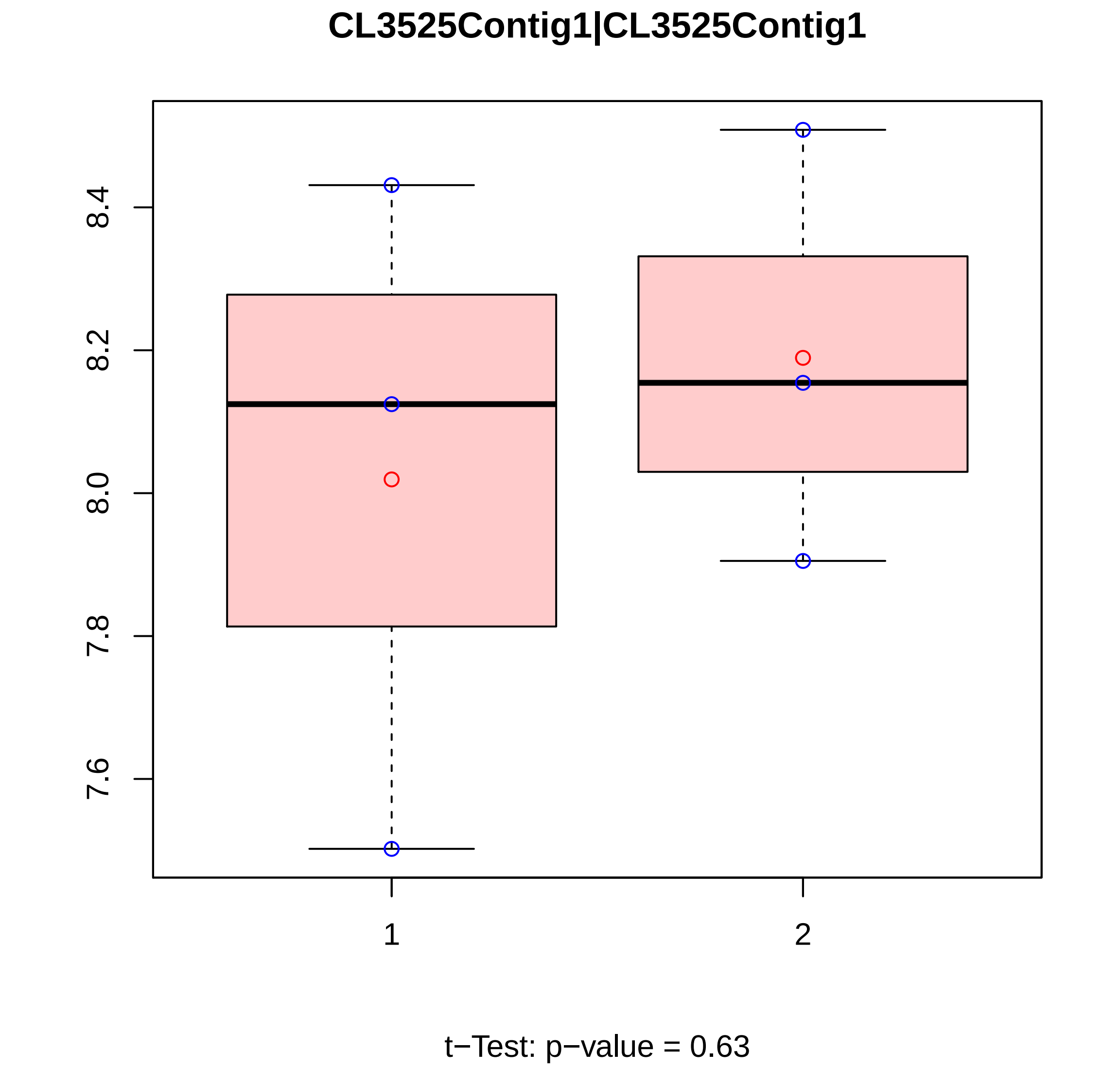
CL3525Contig1|CL3525Contig1
8.4
8.2
8.0
7.8
7.6
1
2
t−Test: p−value = 0.63

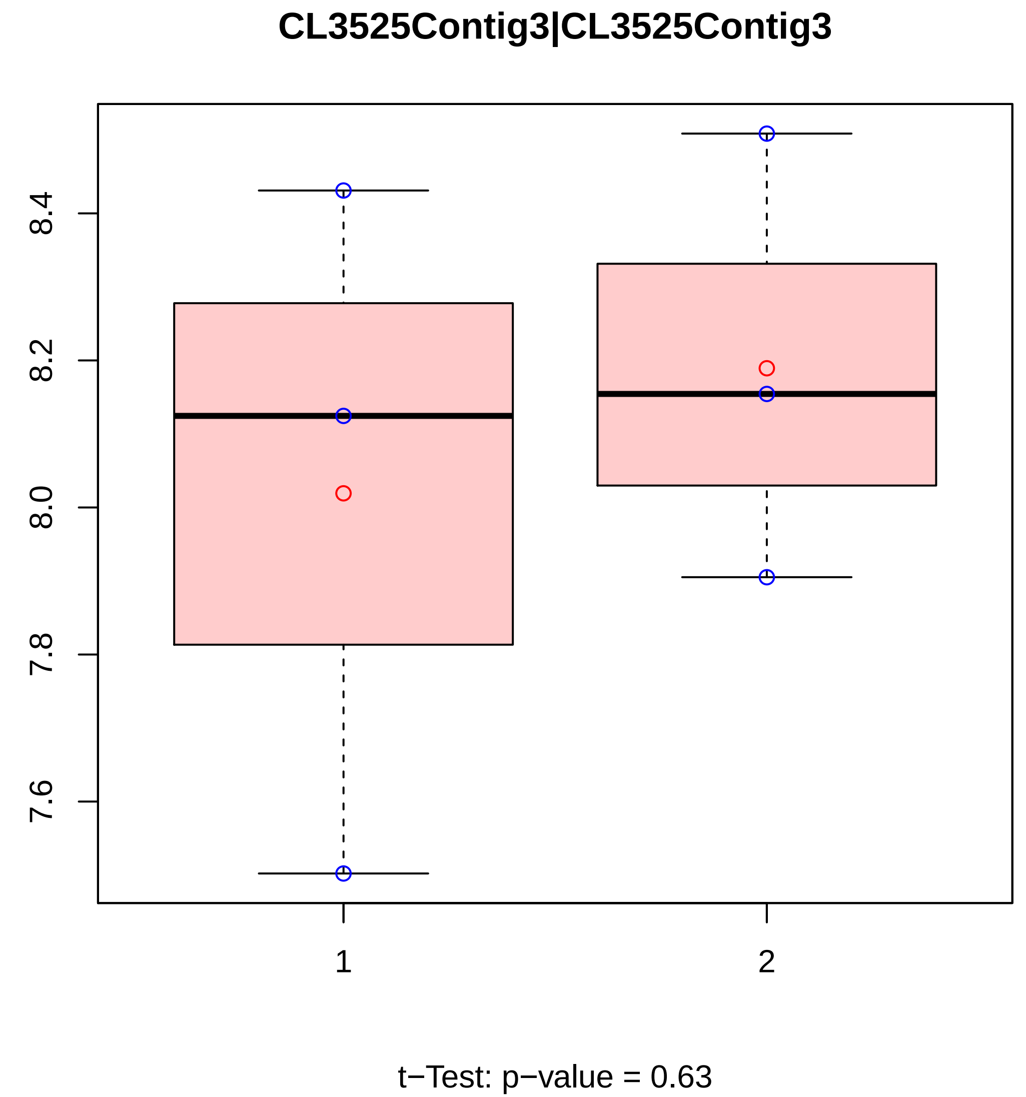
CL3525Contig3|CL3525Contig3
8.4
8.2
8.0
7.8
7.6
1
2
t−Test: p−value = 0.63

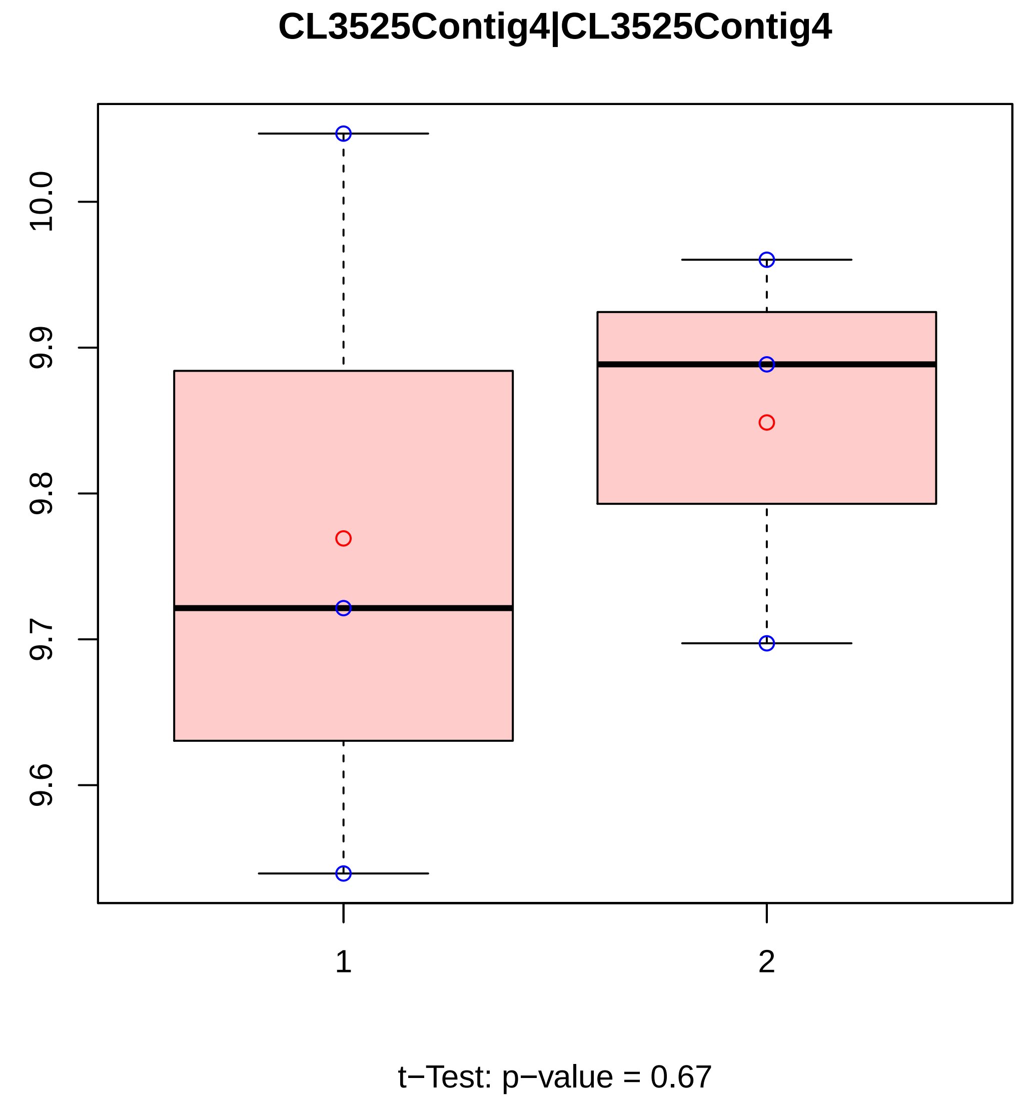
CL3525Contig4|CL3525Contig4
10.0
9.9
9.8
9.7
9.6
1
2
t−Test: p−value = 0.67

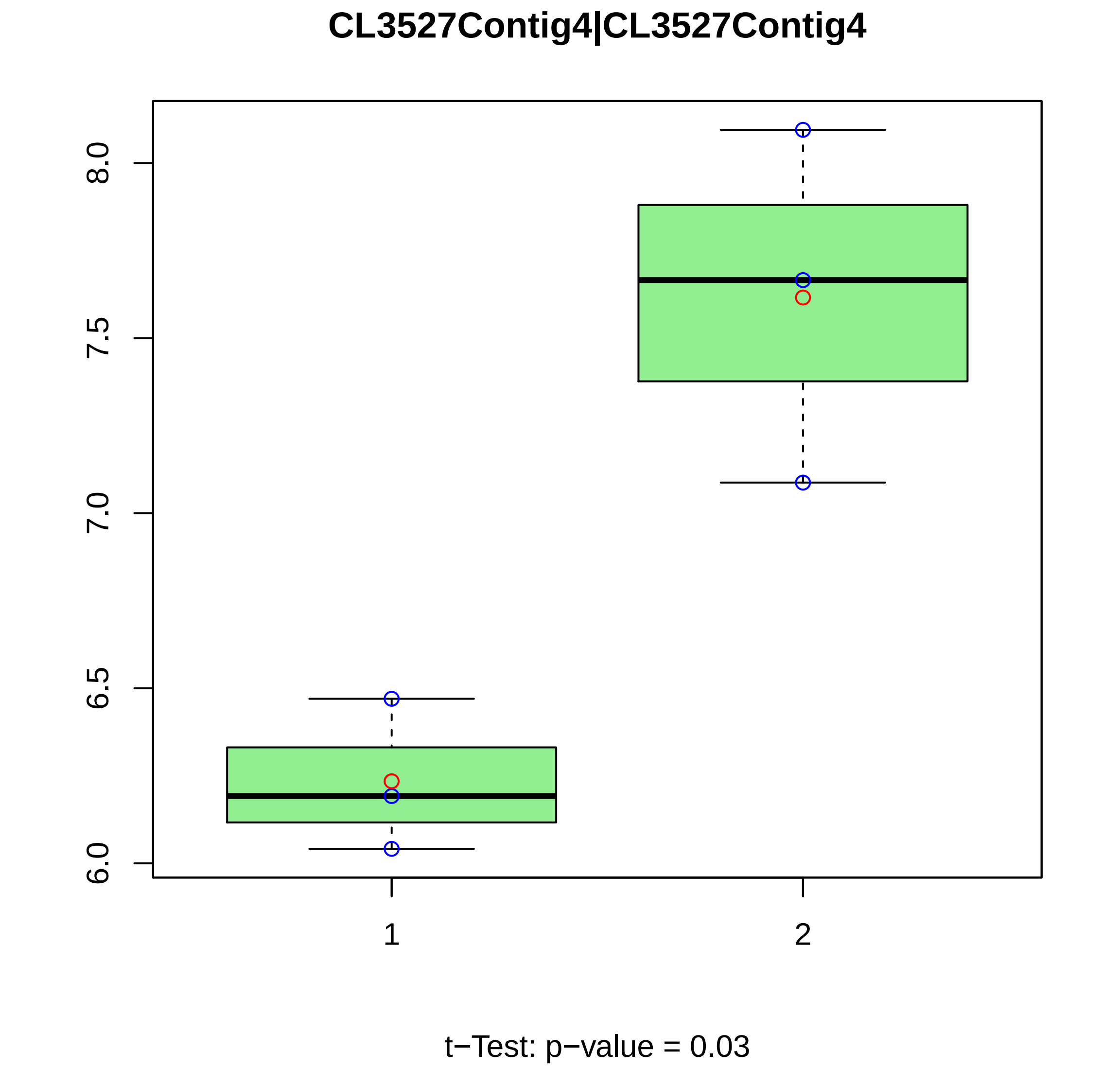

CL3527Contig4|CL3527Contig4
8.0
7.5
7.0
6.5
6.0
1
2
t−Test: p−value = 0.03

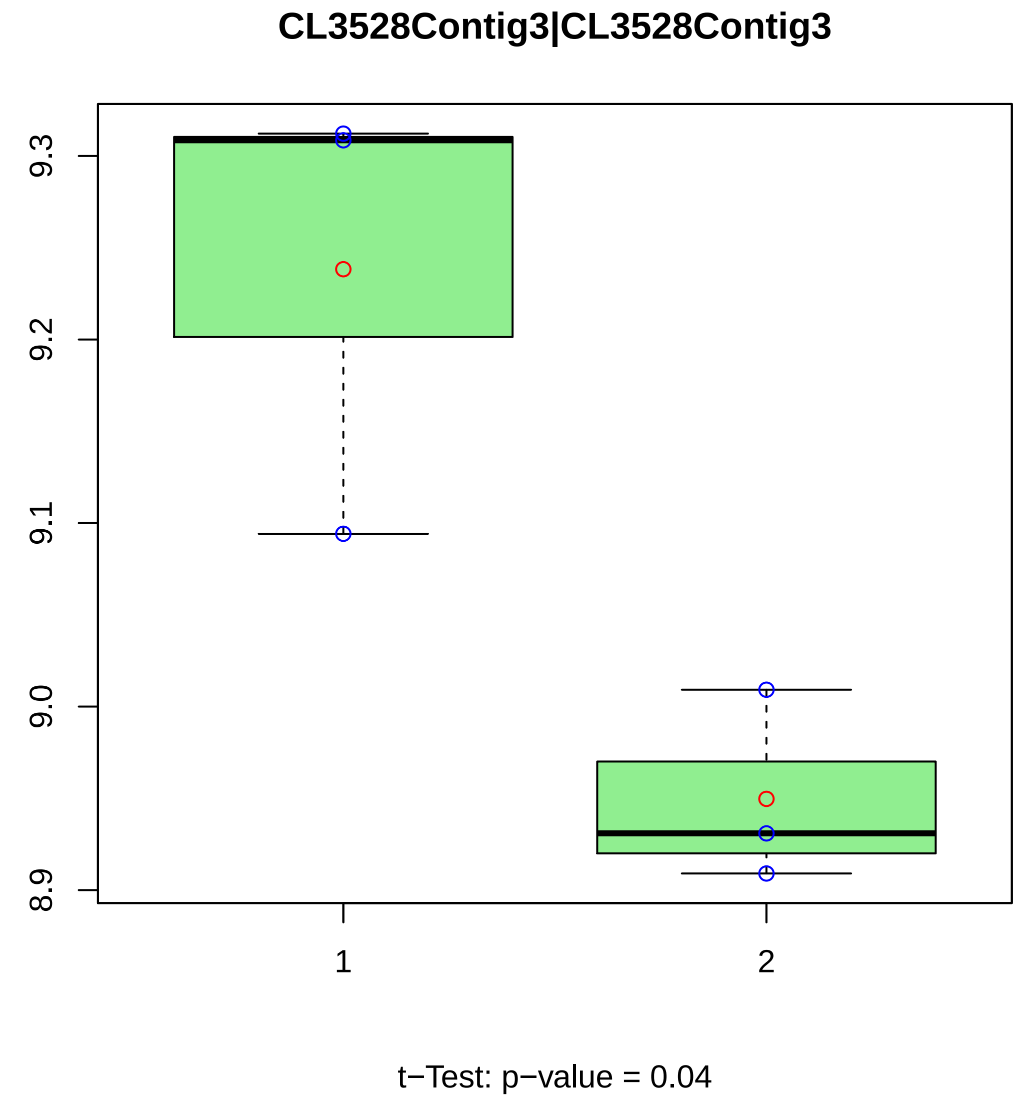

CL3528Contig3|CL3528Contig3
9.3
9.2
9.1
9.0
8.9
1
2
t−Test: p−value = 0.04

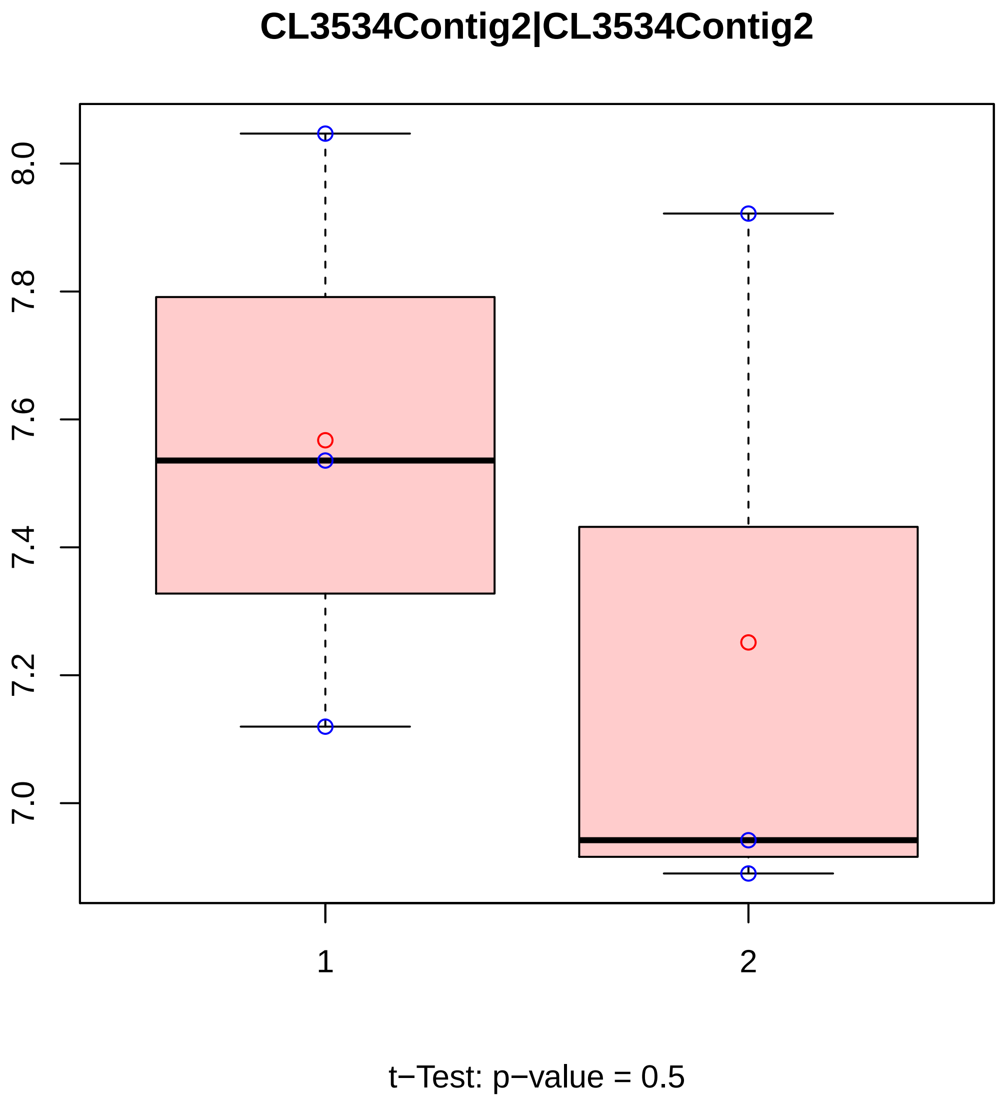
CL3534Contig2|CL3534Contig2
8.0
7.8
7.6
7.4
7.2
7.0
1
2
t−Test: p−value = 0.5

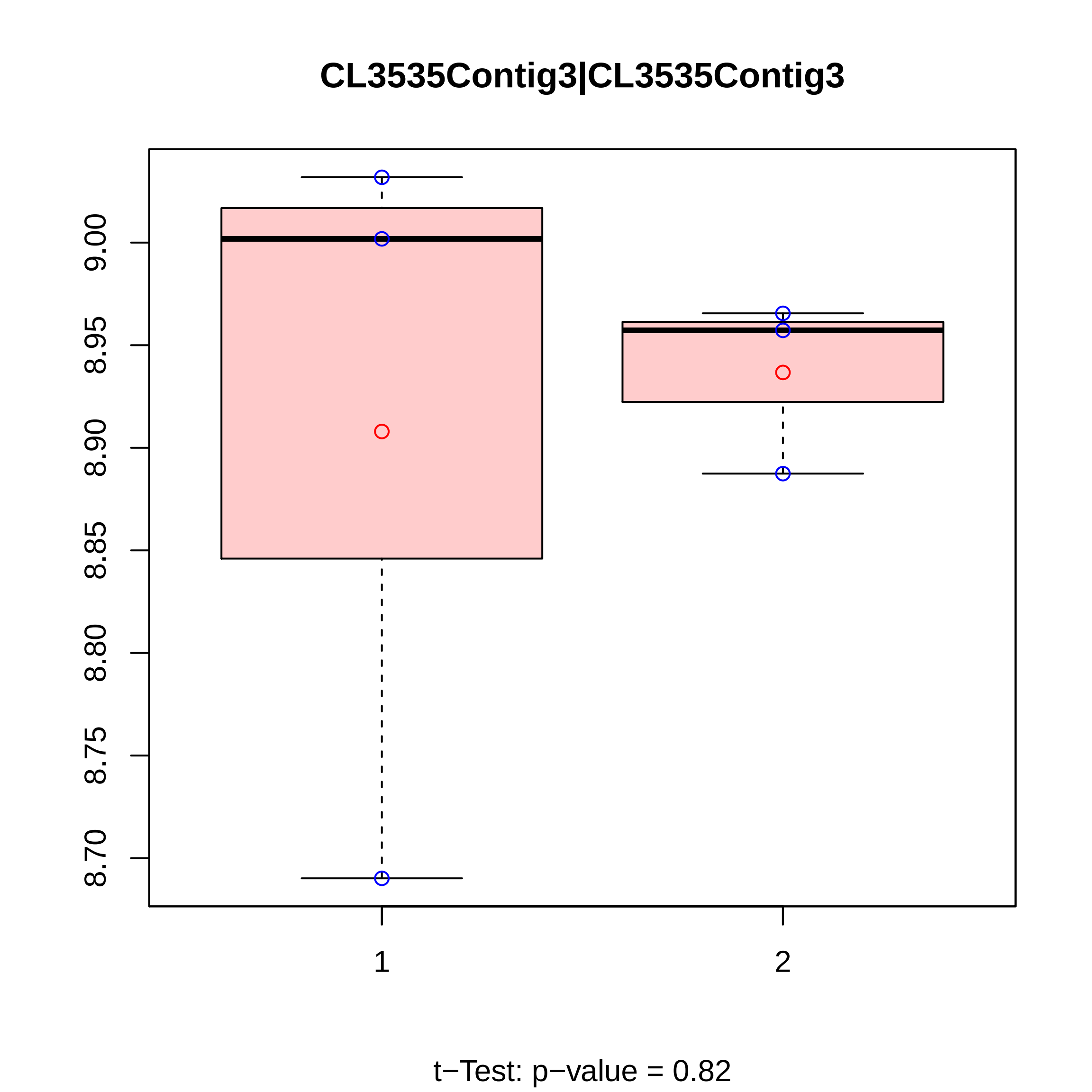

CL3535Contig3|CL3535Contig3
9.00
8.95
8.90
8.85
8.80
8.75
8.70
1
2
t−Test: p−value = 0.82

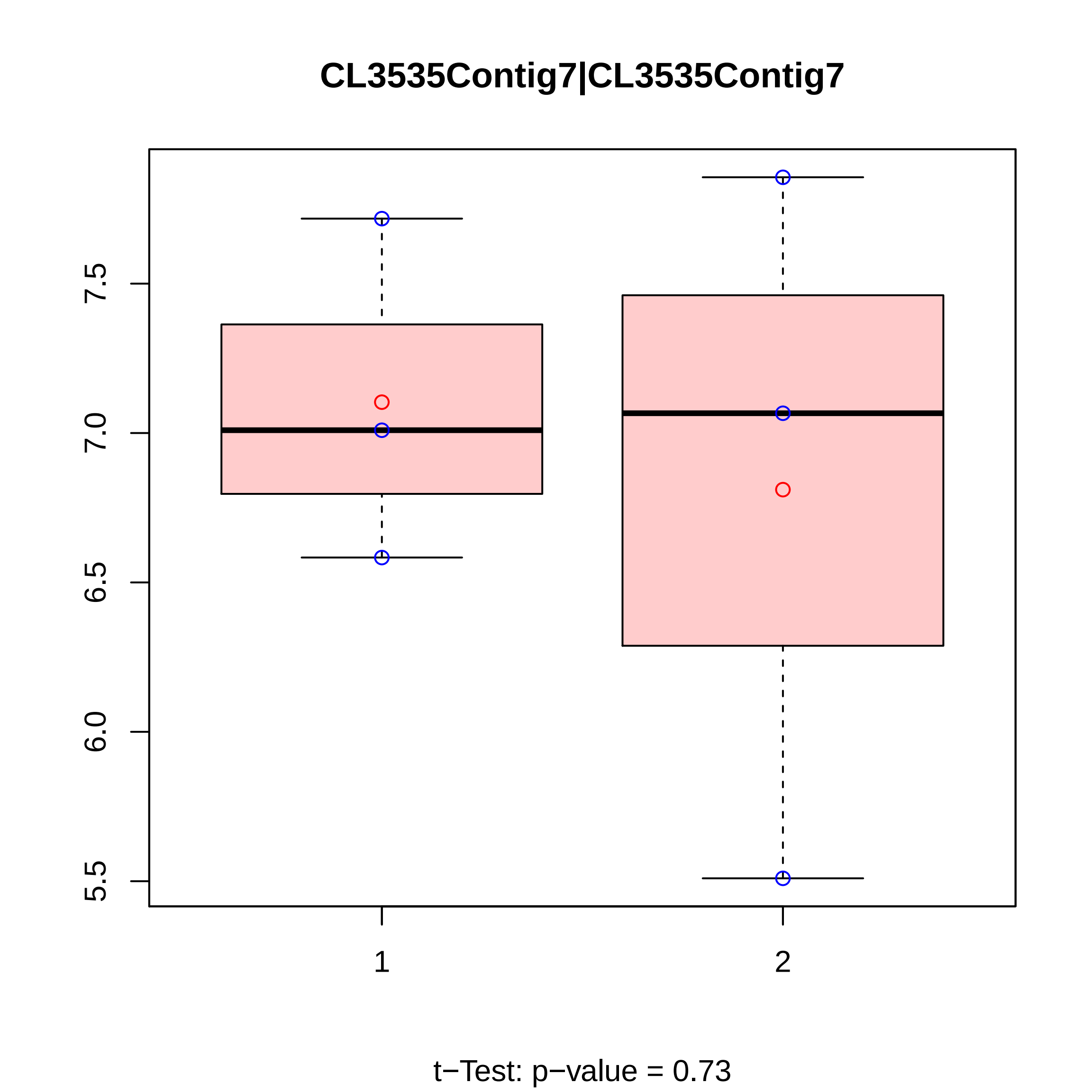
CL3535Contig7|CL3535Contig7
7.5
7.0
6.5
6.0
5.5
1
2
t−Test: p−value = 0.73

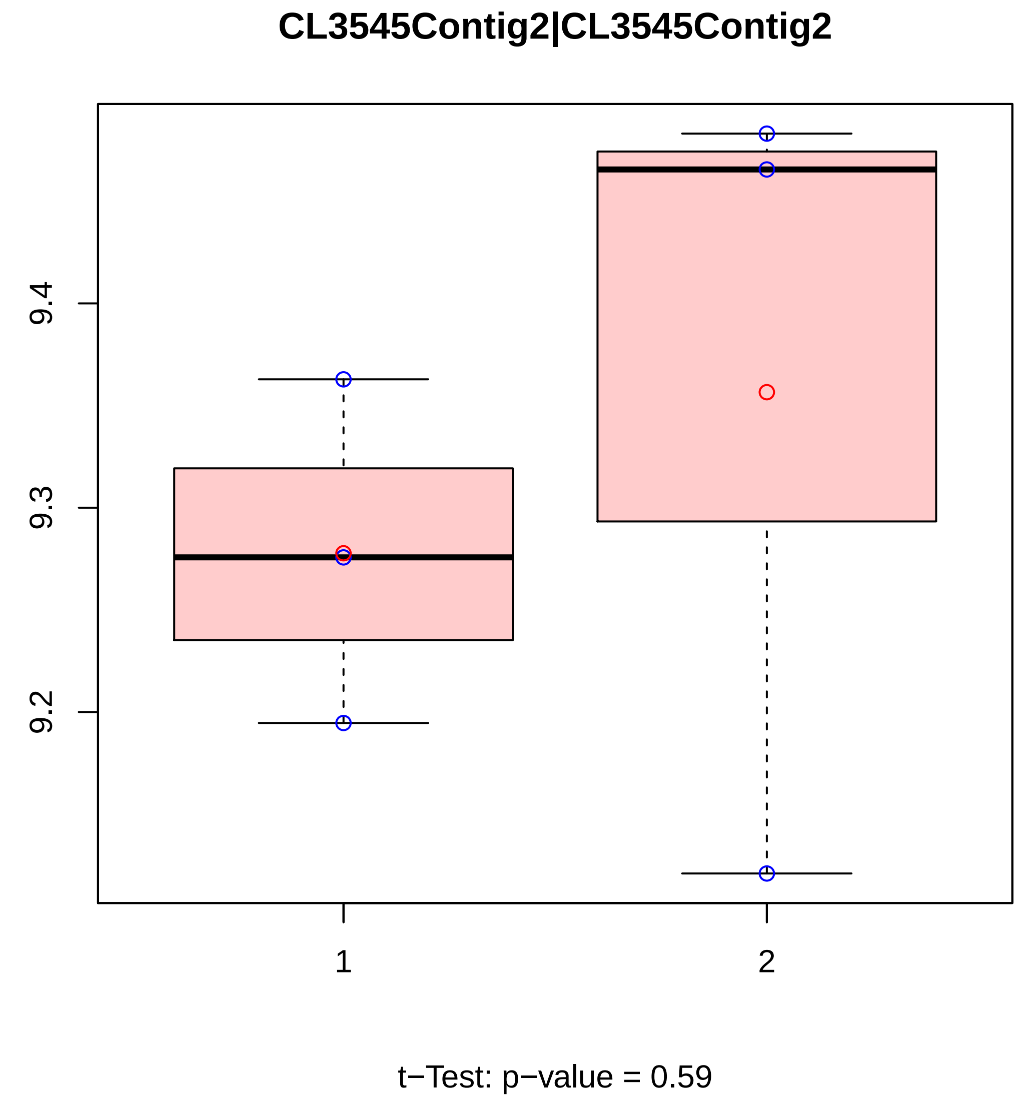
CL3545Contig2|CL3545Contig2
9.4
9.3
9.2
1
2
t−Test: p−value = 0.59

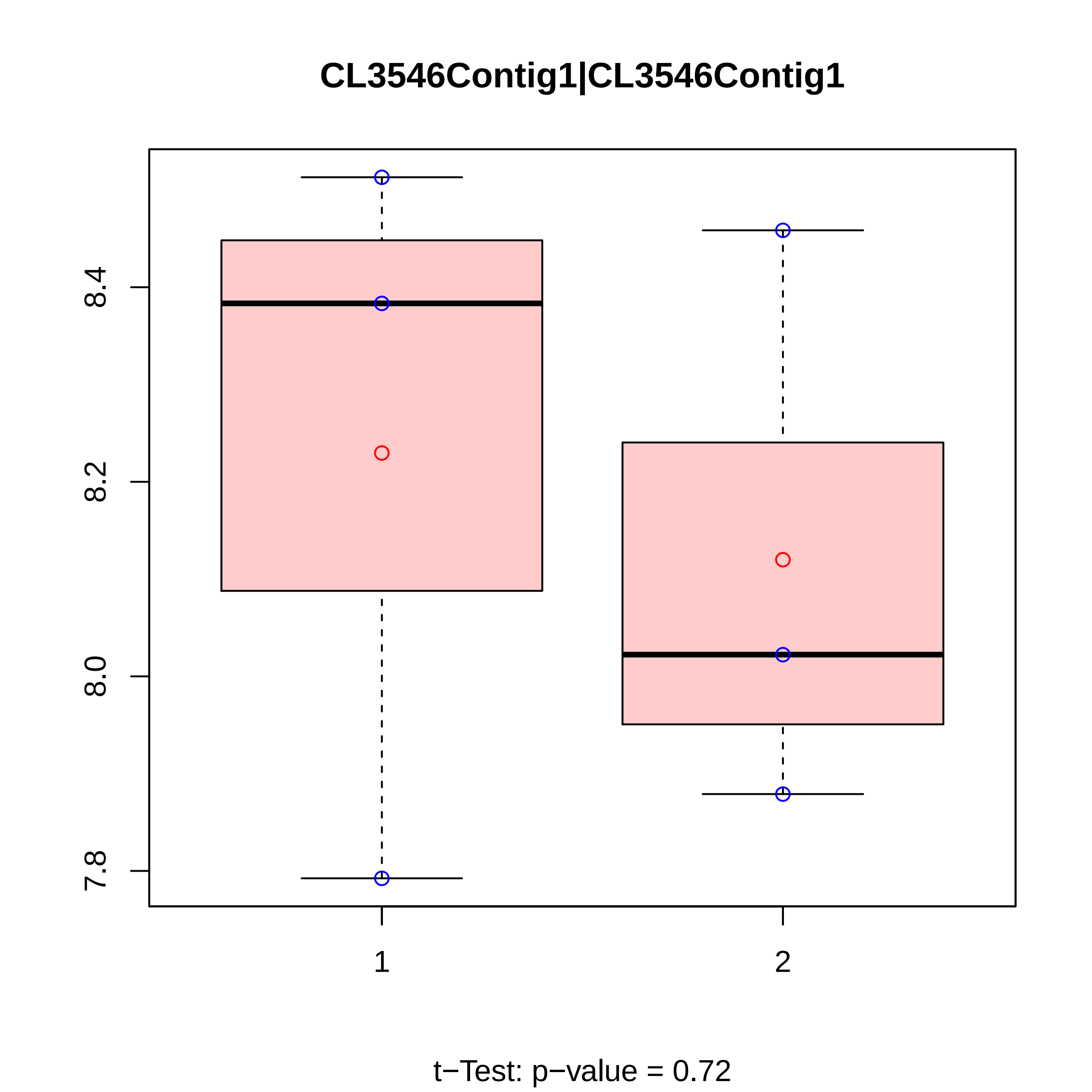
CL3546Contig1|CL3546Contig1
8.4
8.2
8.0
7.8
1
2
t−Test: p−value = 0.72

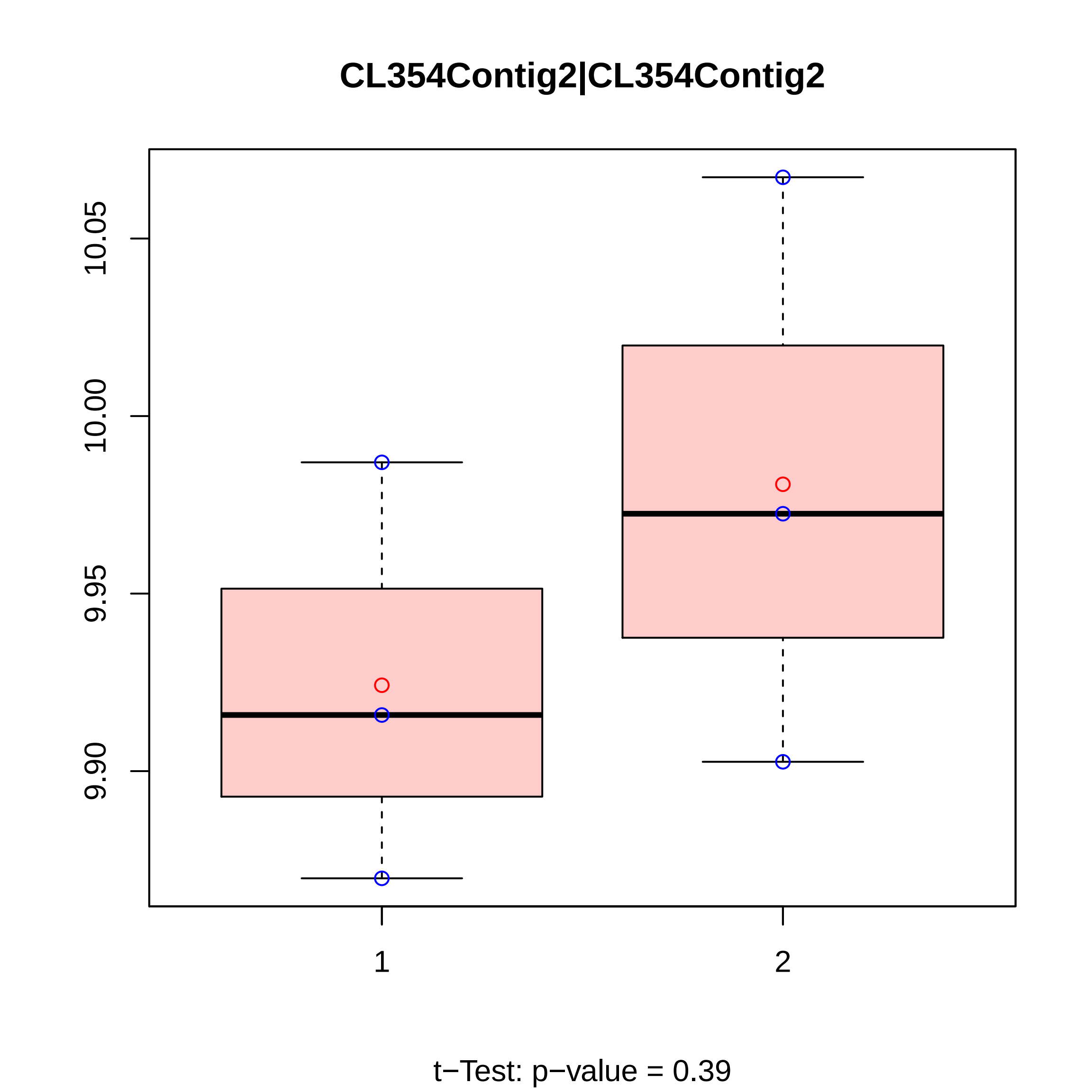
CL354Contig2|CL354Contig2
10.05
10.00
9.95
9.90
1
2
t−Test: p−value = 0.39

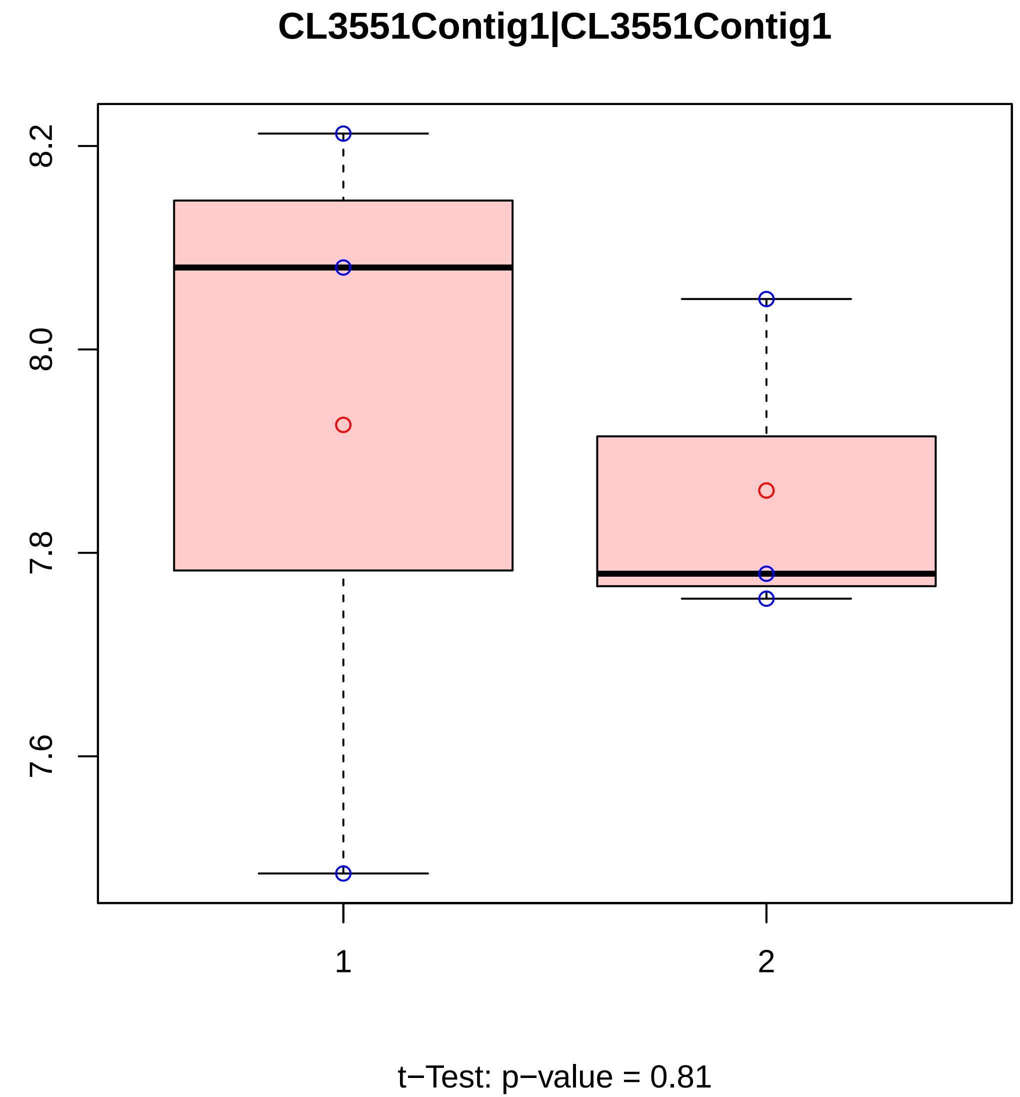
CL3551Contig1|CL3551Contig1
8.2
8.0
7.8
7.6
1
2
t−Test: p−value = 0.81

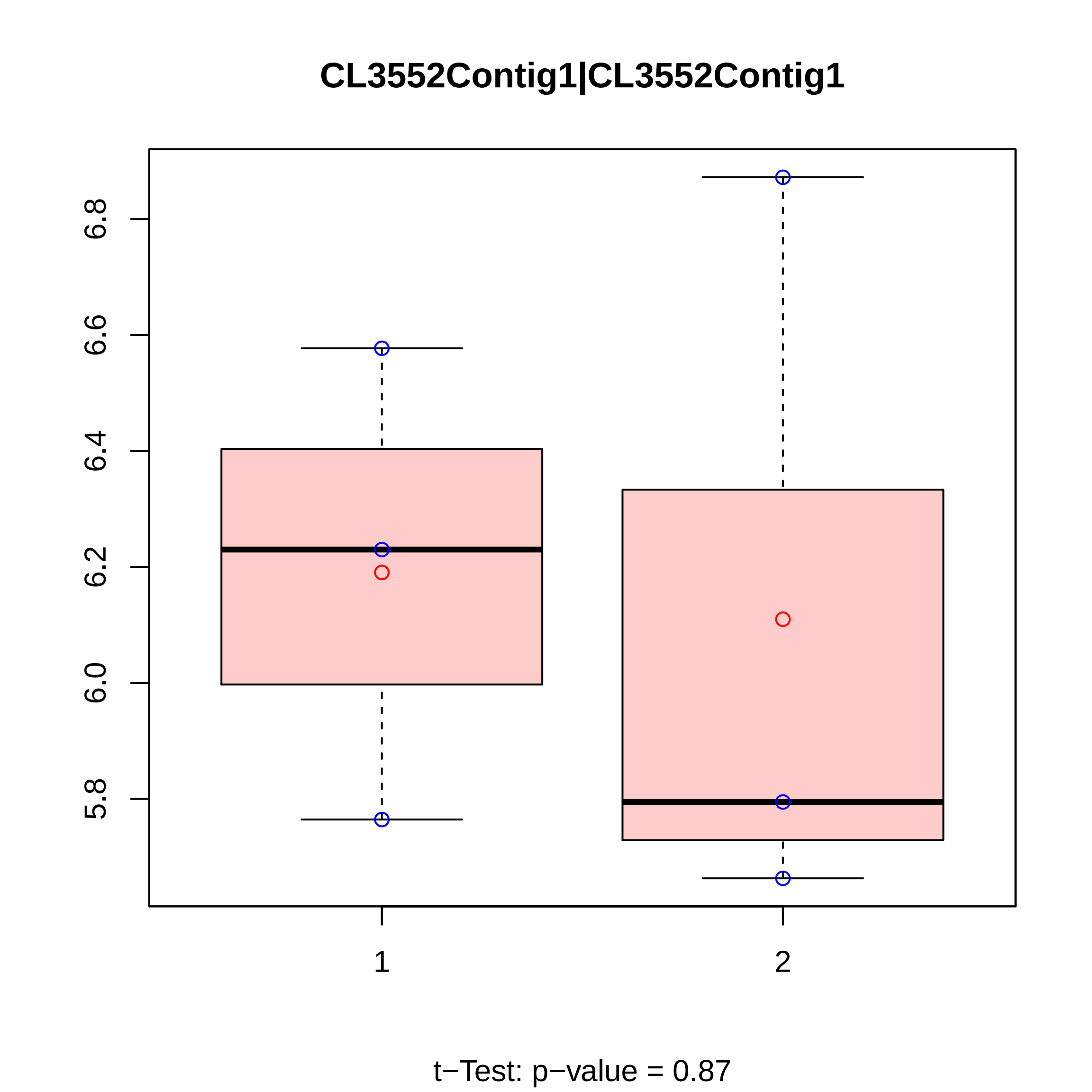
CL3552Contig1|CL3552Contig1
6.8
6.6
6.4
6.2
6.0
5.8
1
2
t−Test: p−value = 0.87

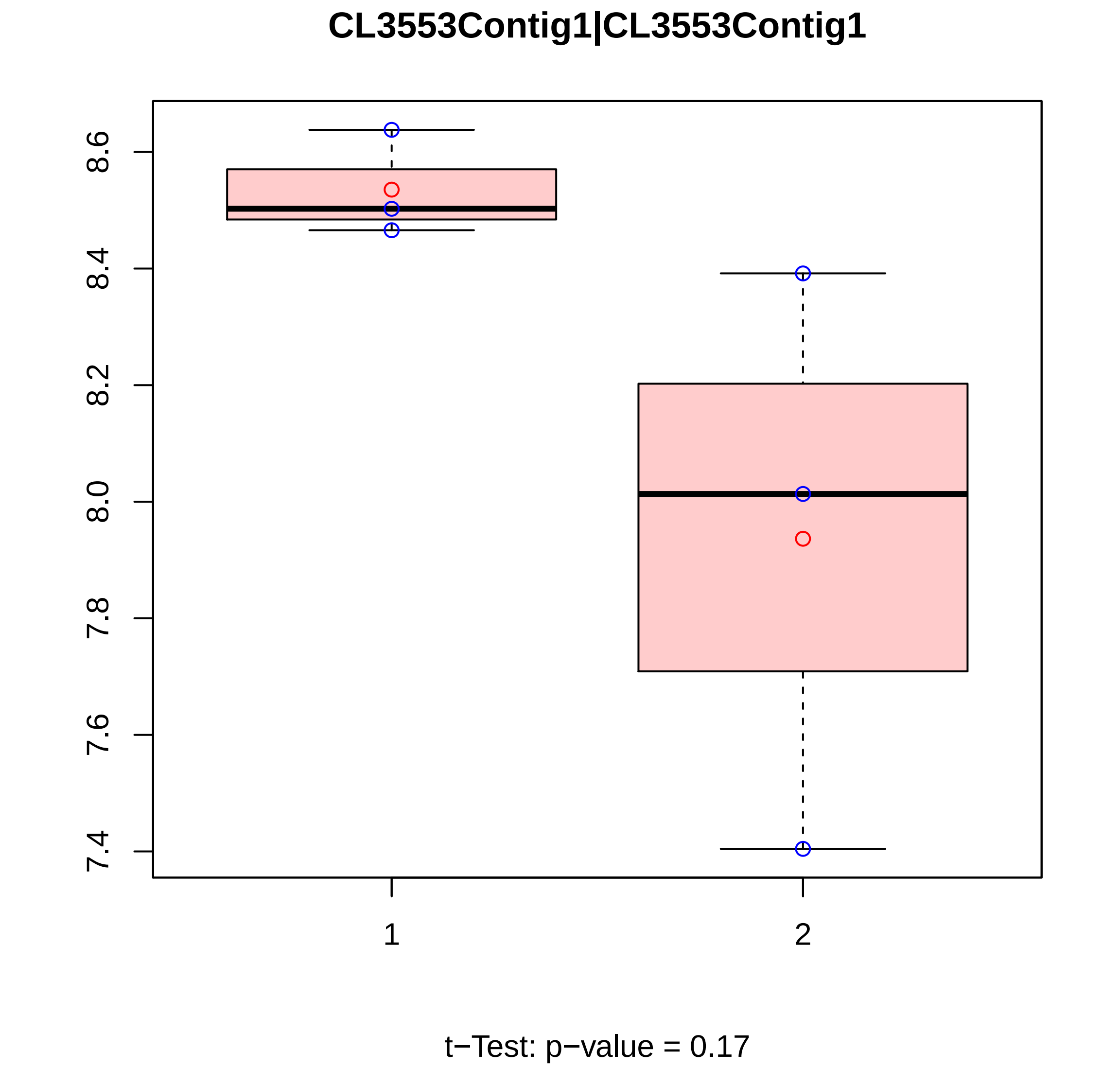
CL3553Contig1|CL3553Contig1
8.6
8.4
8.2
8.0
7.8
7.6
7.4
1
2
t−Test: p−value = 0.17

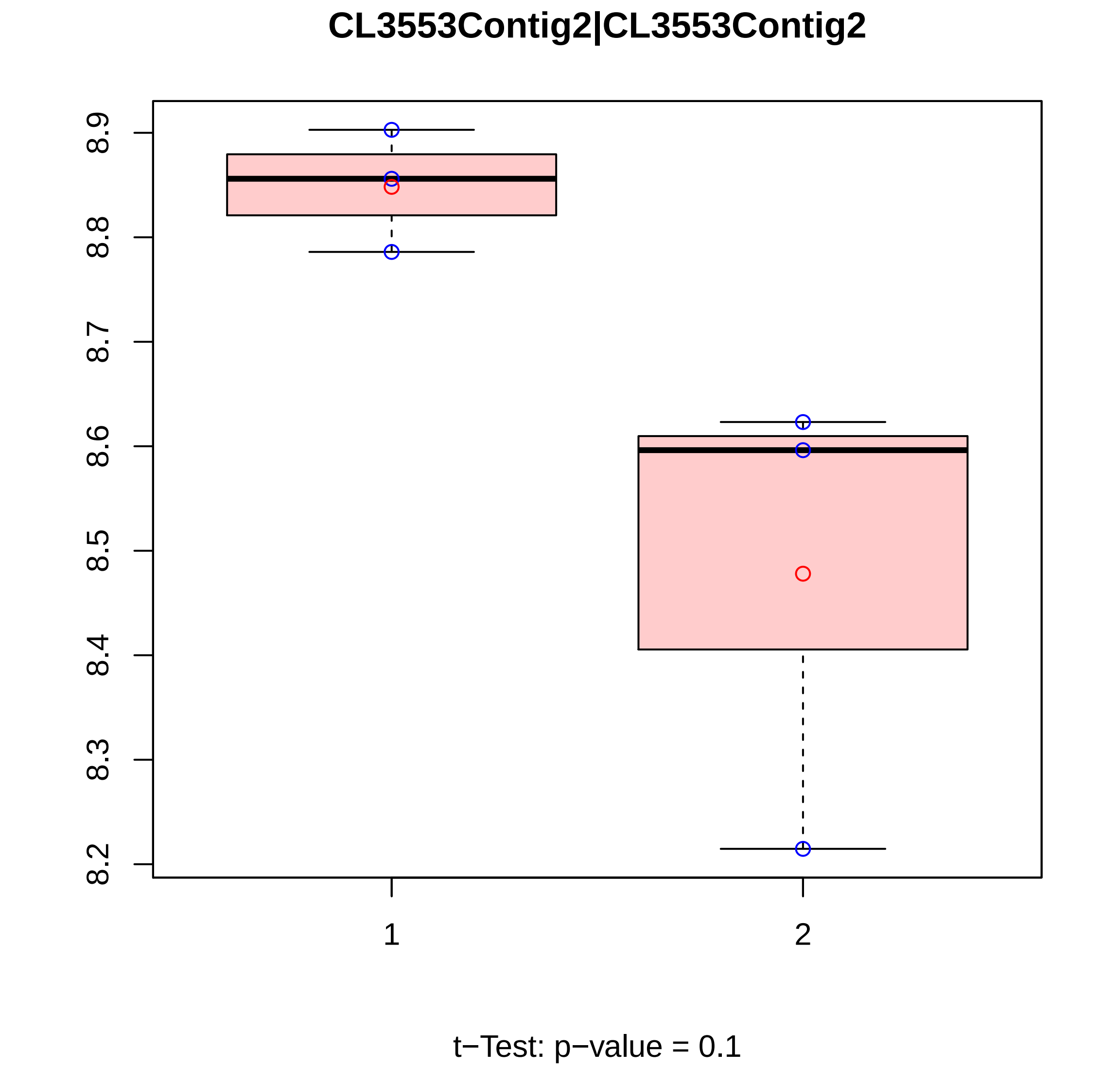

CL3553Contig2|CL3553Contig2
8.2
8.3
8.4
8.5
8.6
8.7
8.8
8.9
1
2
t−Test: p−value = 0.1

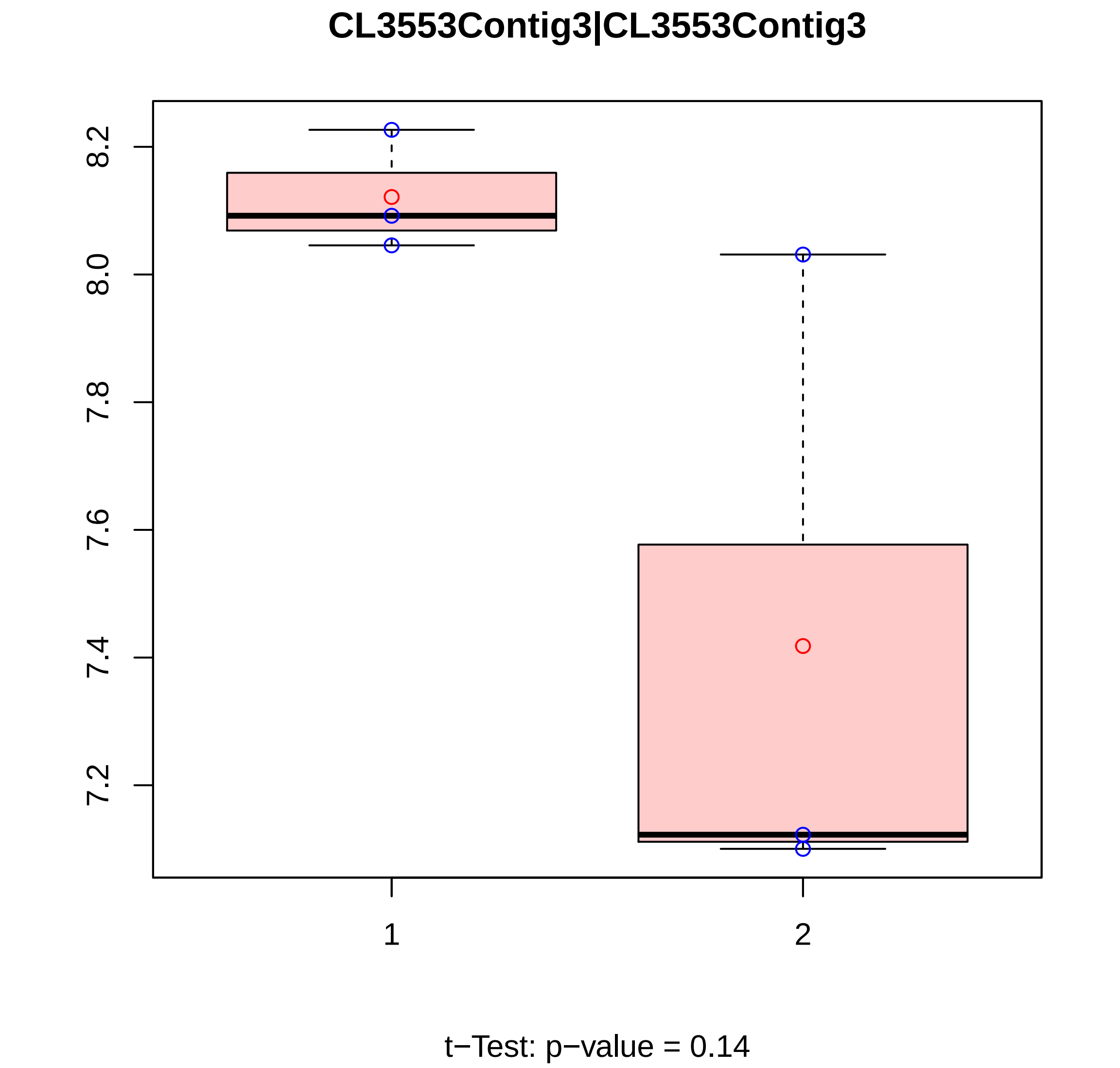
CL3553Contig3|CL3553Contig3
8.2
8.0
7.8
7.6
7.4
7.2
1
2
t−Test: p−value = 0.14

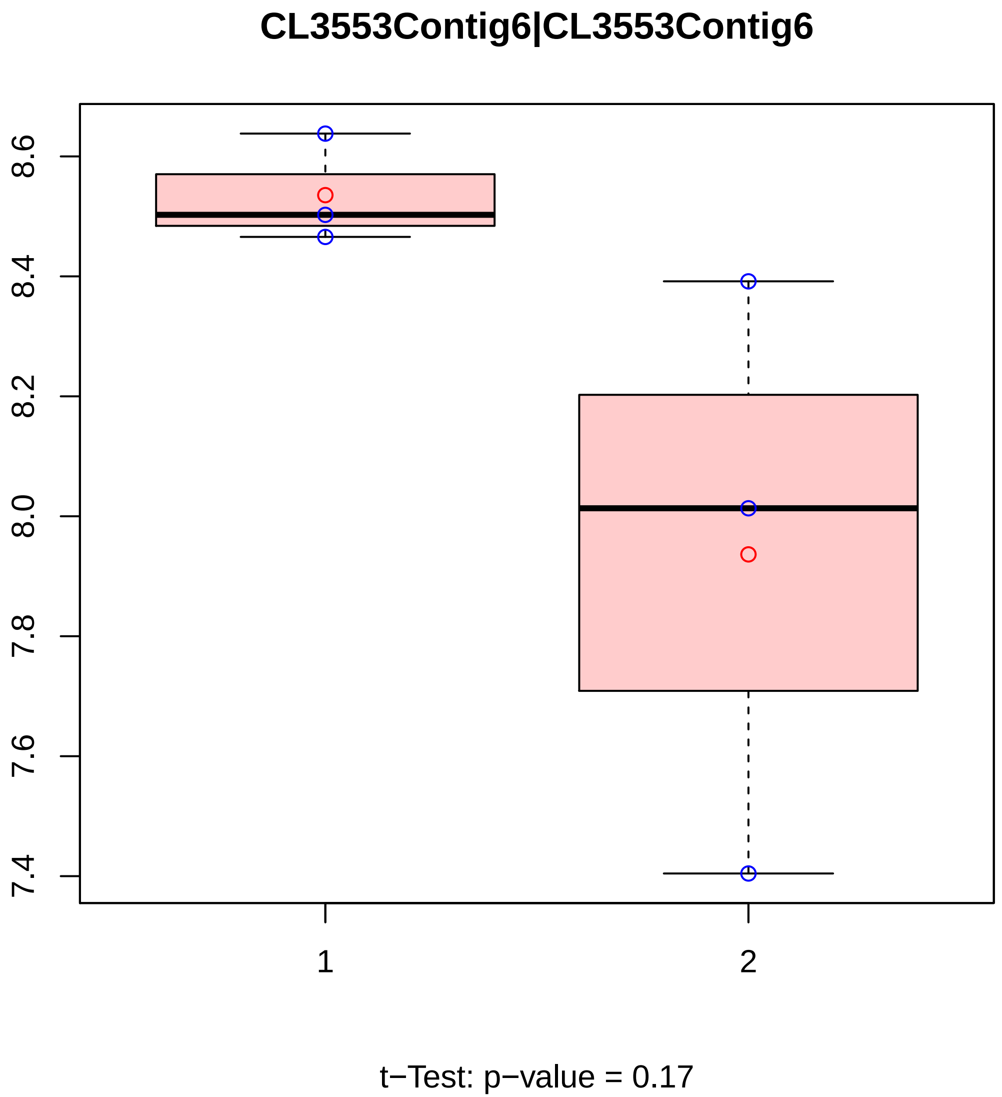
CL3553Contig6|CL3553Contig6
8.6
8.4
8.2
8.0
7.8
7.6
7.4
1
2
t−Test: p−value = 0.17

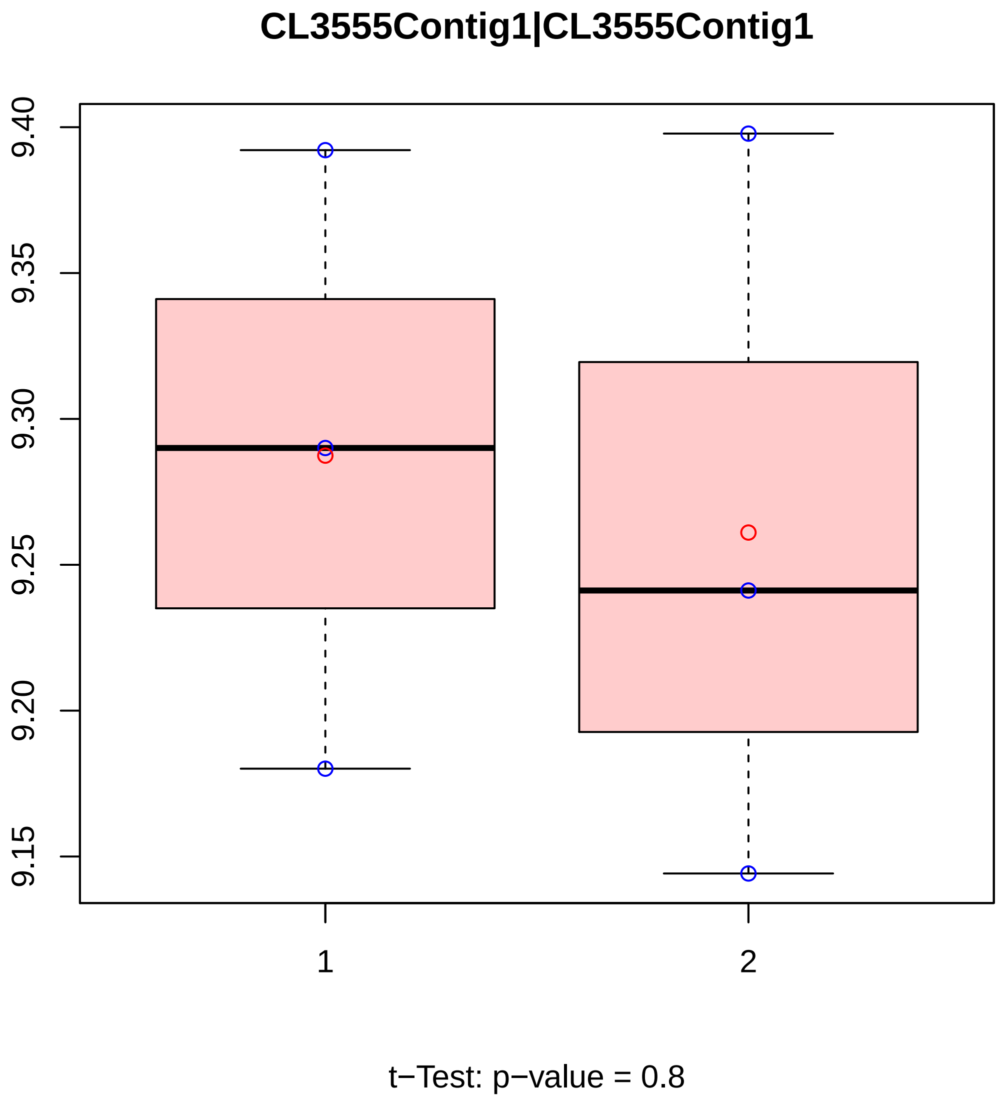

CL3555Contig1|CL3555Contig1
9.40
9.35
9.30
9.25
9.20
9.15
1
2
t−Test: p−value = 0.8

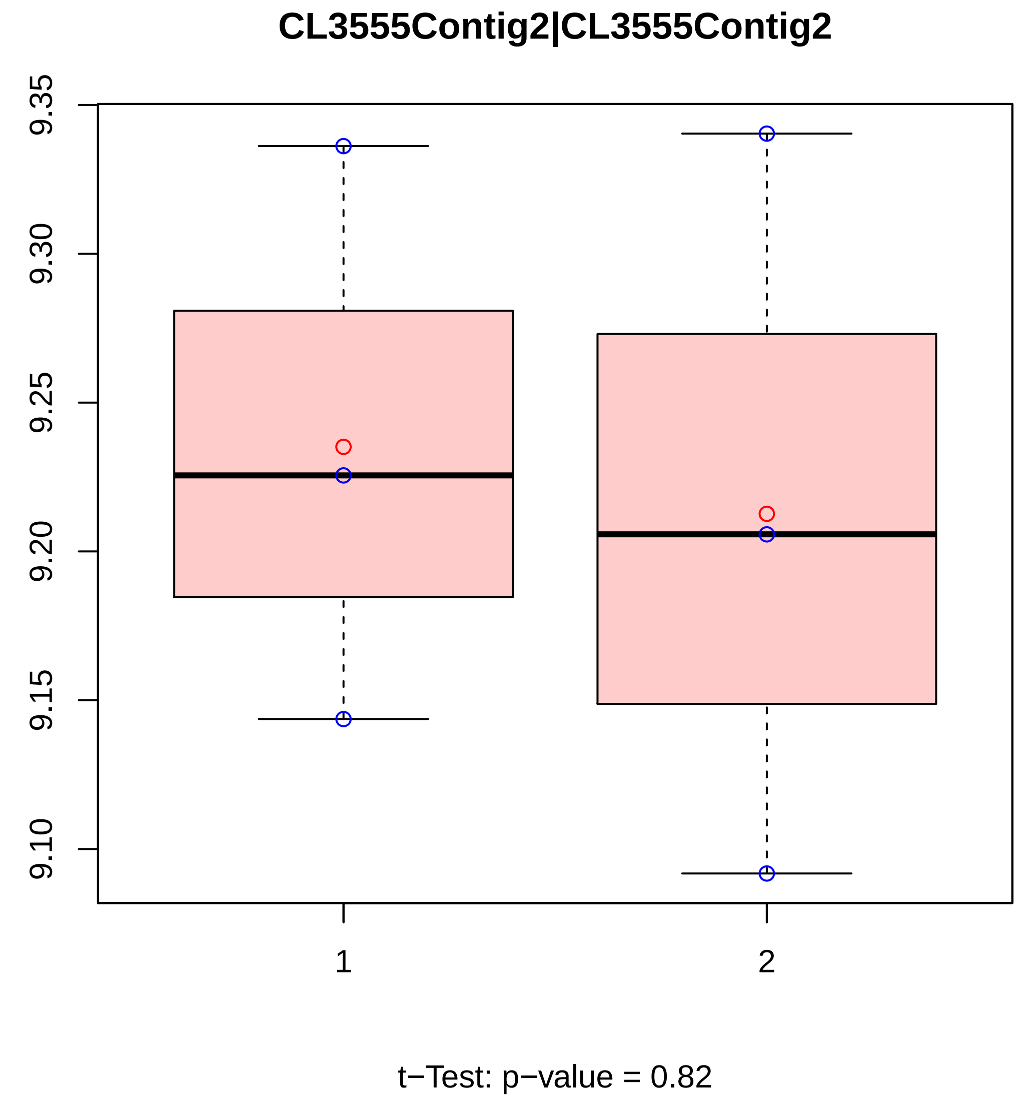
CL3555Contig2|CL3555Contig2
9.35
9.30
9.25
9.20
9.15
9.10
1
2
t−Test: p−value = 0.82

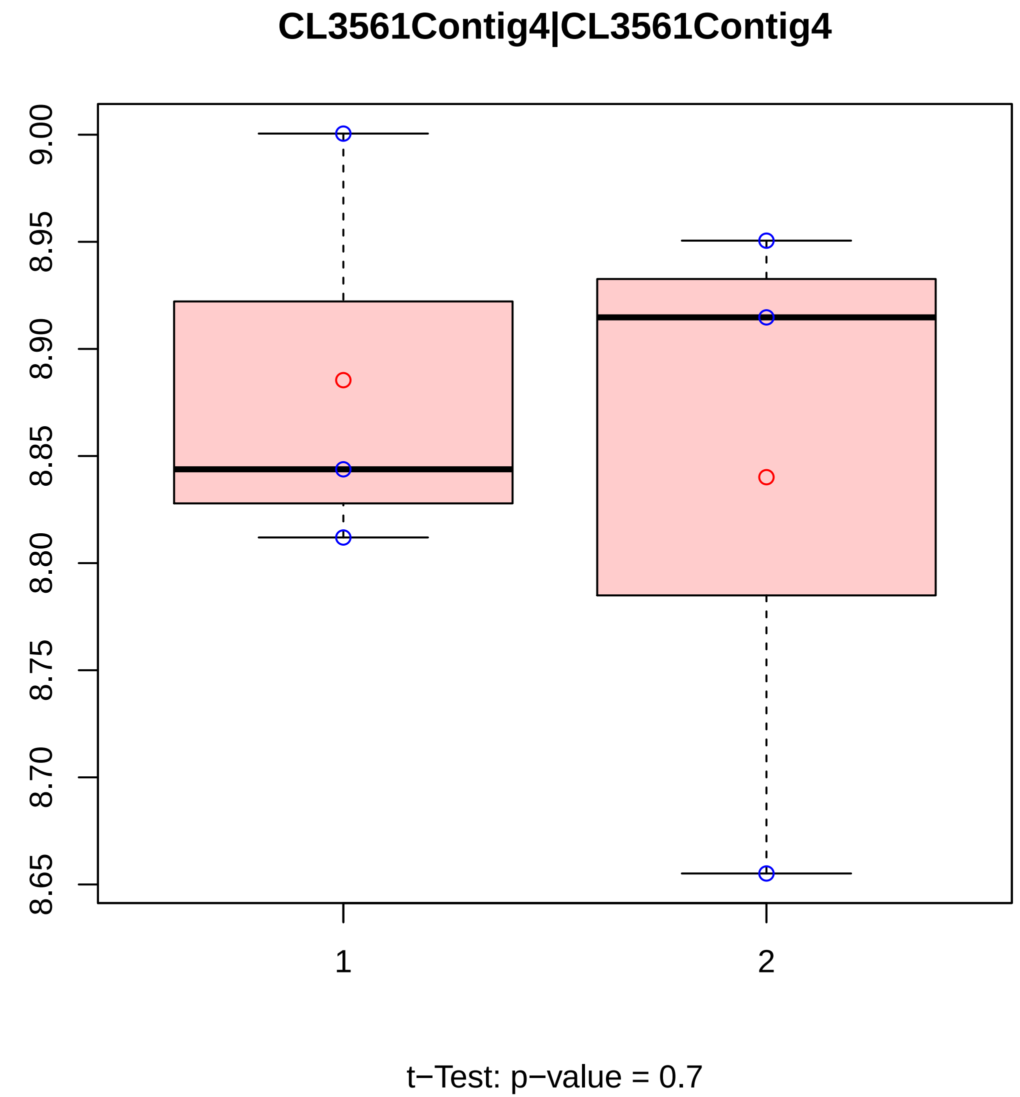

CL3561Contig4|CL3561Contig4
9.00
8.95
8.90
8.85
8.80
8.75
8.70
8.65
1
2
t−Test: p−value = 0.7

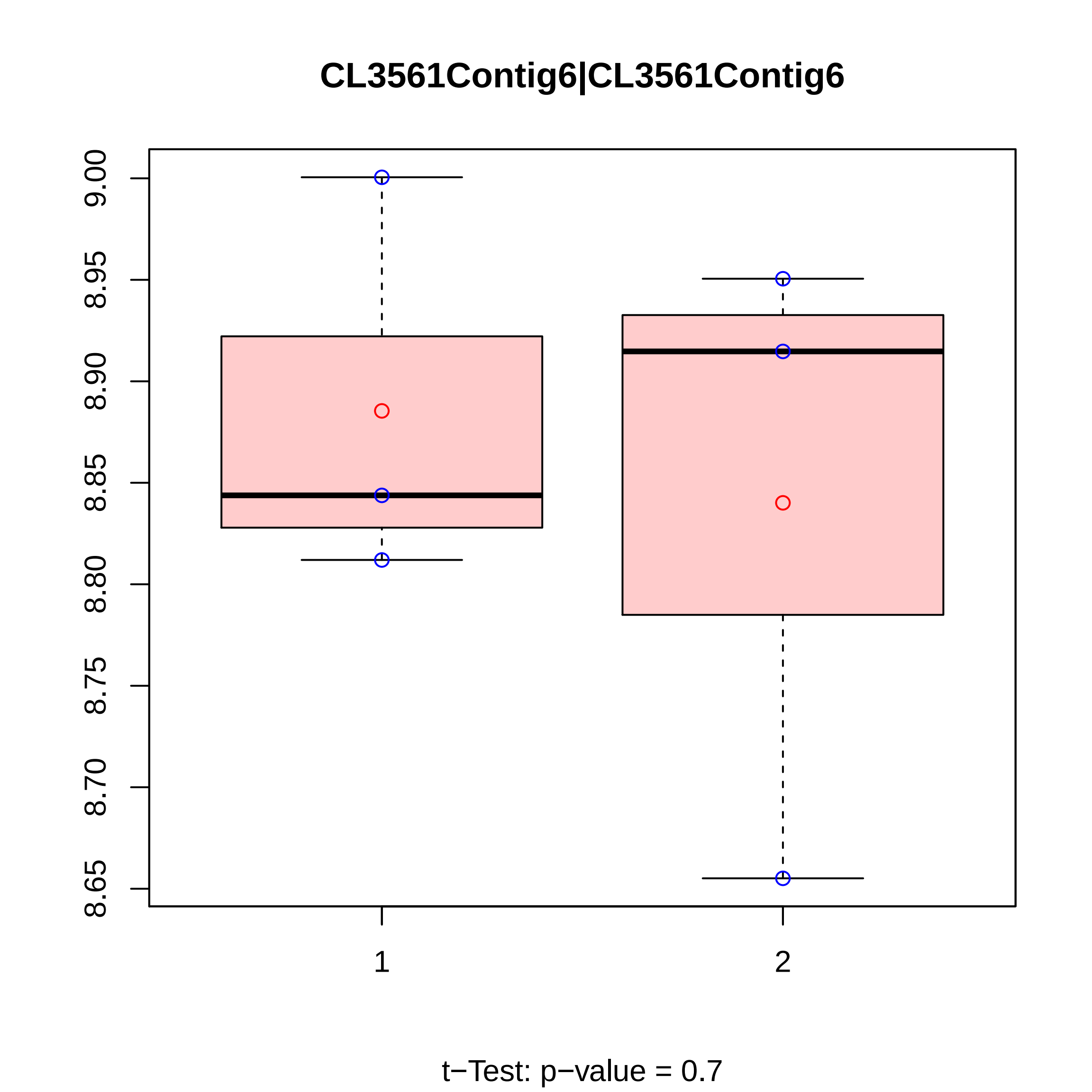

CL3561Contig6|CL3561Contig6
9.00
8.95
8.90
8.85
8.80
8.75
8.70
8.65
1
2
t−Test: p−value = 0.7

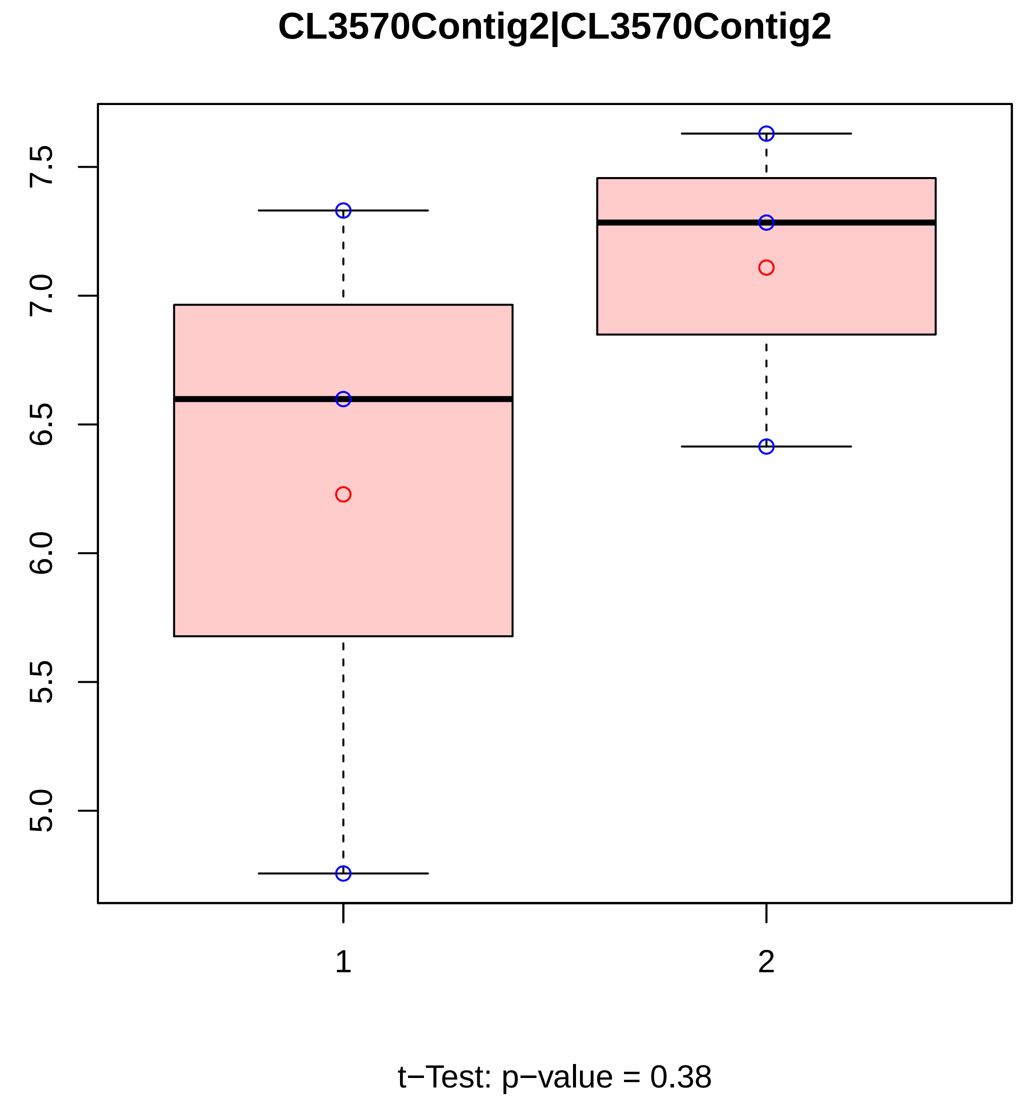
CL3570Contig2|CL3570Contig2
7.5
7.0
6.5
6.0
5.5
5.0
1
2
t−Test: p−value = 0.38

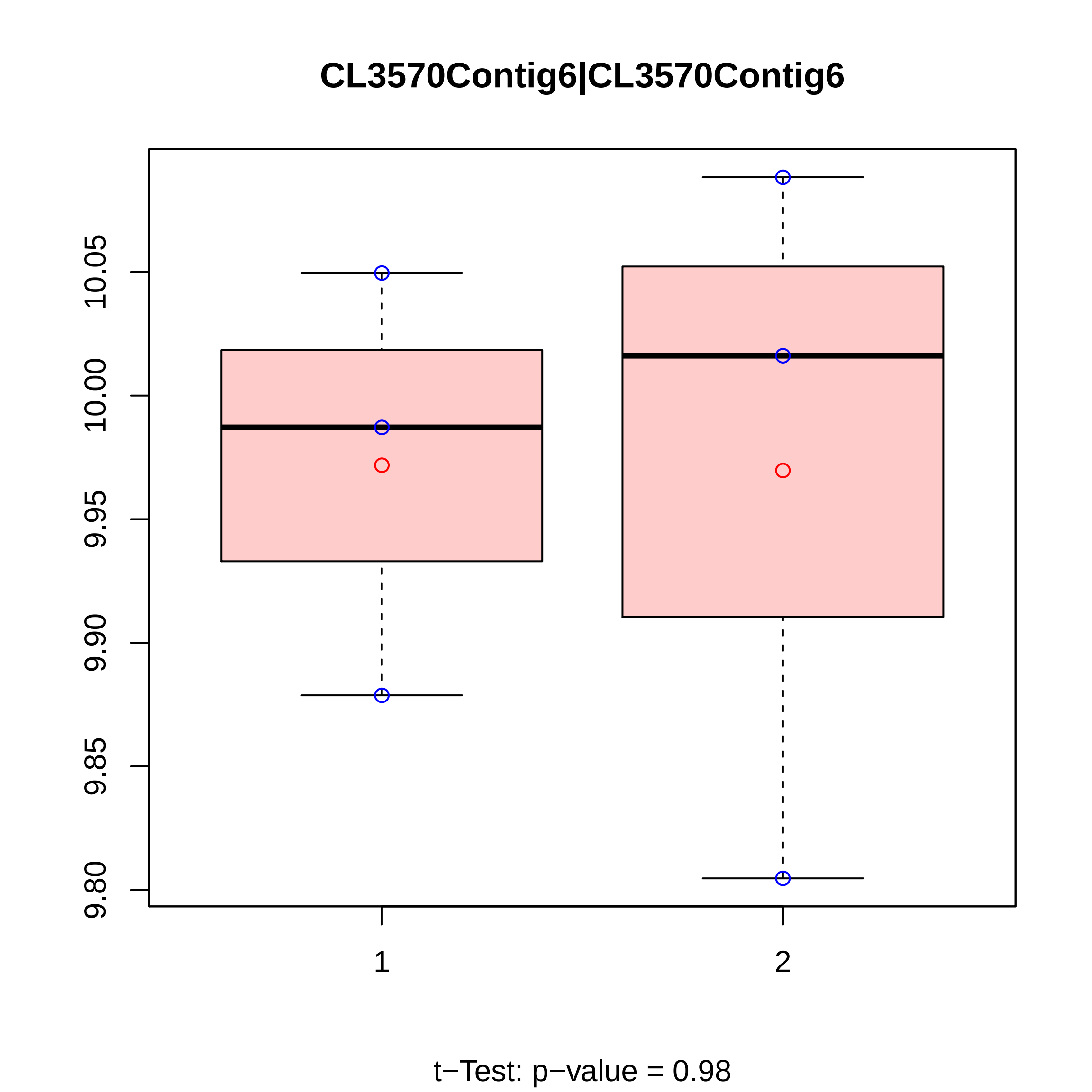
CL3570Contig6|CL3570Contig6
10.05
10.00
9.95
9.90
9.85
9.80
1
2
t−Test: p−value = 0.98

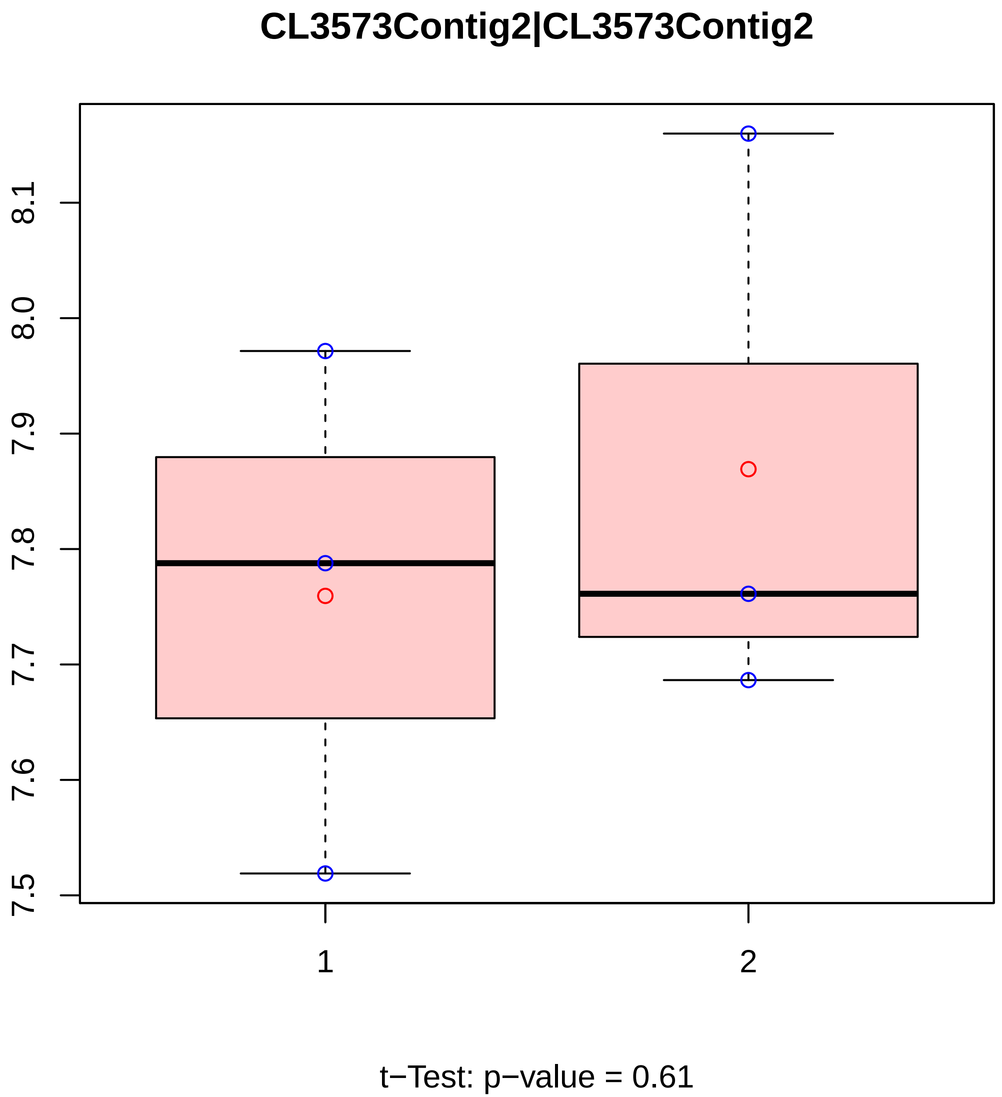

CL3573Contig2|CL3573Contig2
8.1
8.0
7.9
7.8
7.7
7.6
7.5
1
2
t−Test: p−value = 0.61

# CL3580Contig2|CL3580Contig2

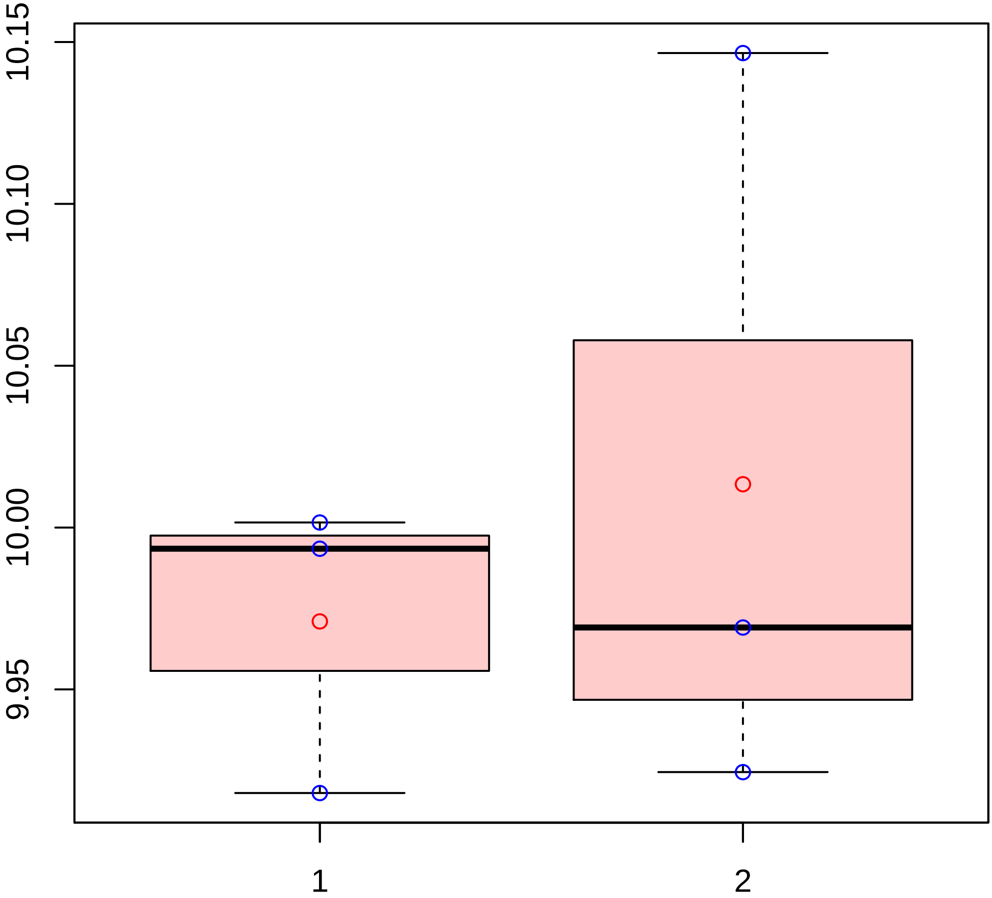


t−Test: p−value = 0.61

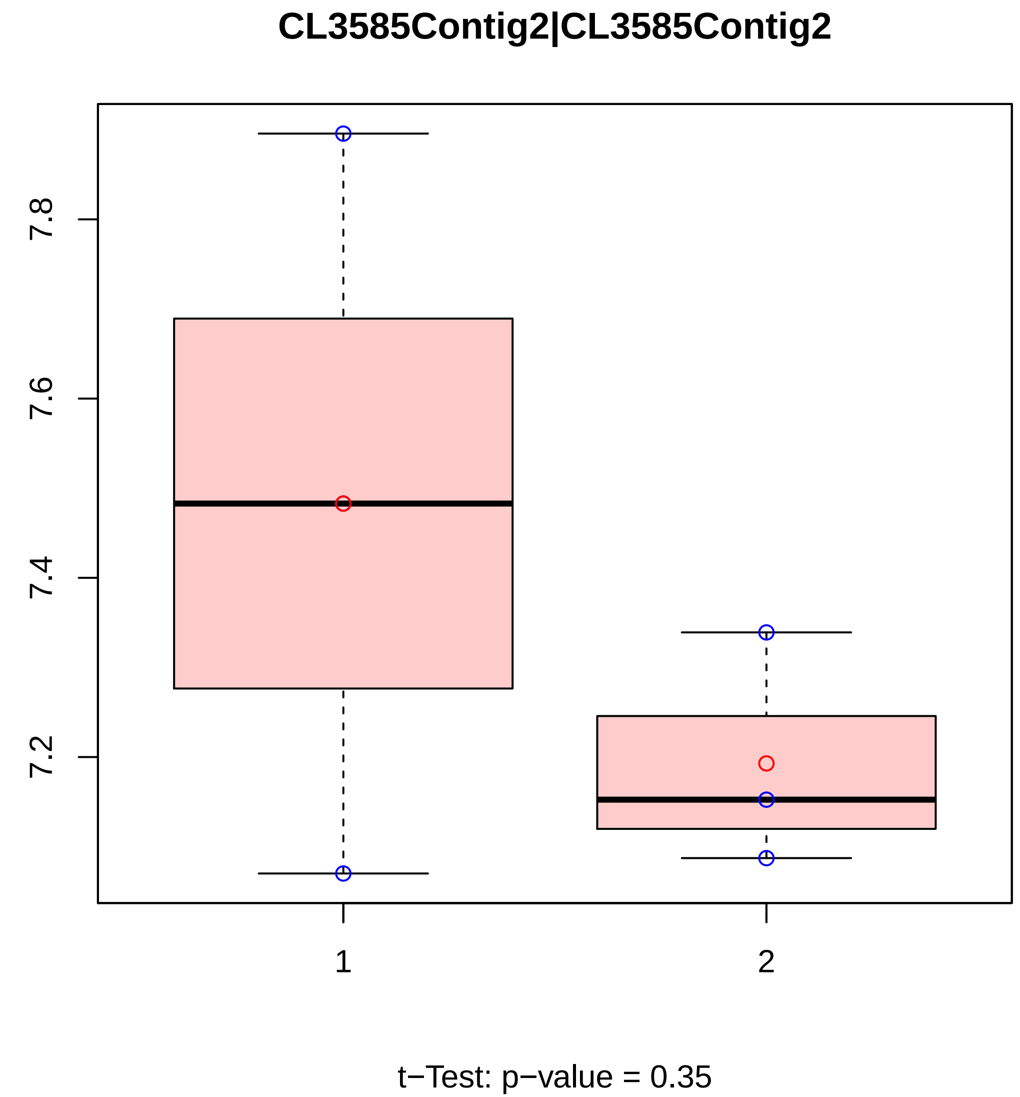

CL3585Contig2|CL3585Contig2
7.8
7.6
7.4
7.2
1
2
t−Test: p−value = 0.35

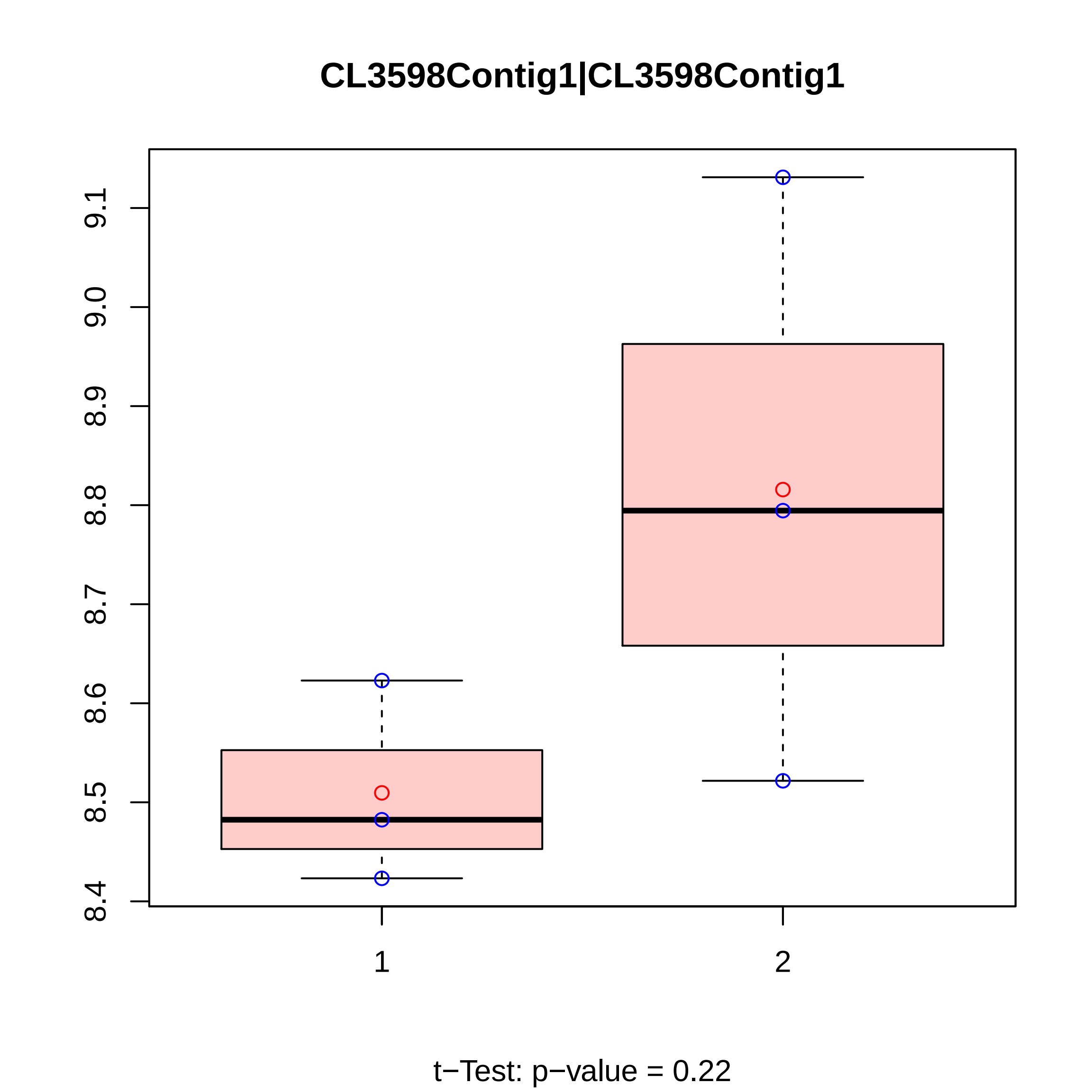
CL3598Contig1|CL3598Contig1
9.1
9.0
8.9
8.8
8.7
8.6
8.5
8.4
1
2
t−Test: p−value = 0.22

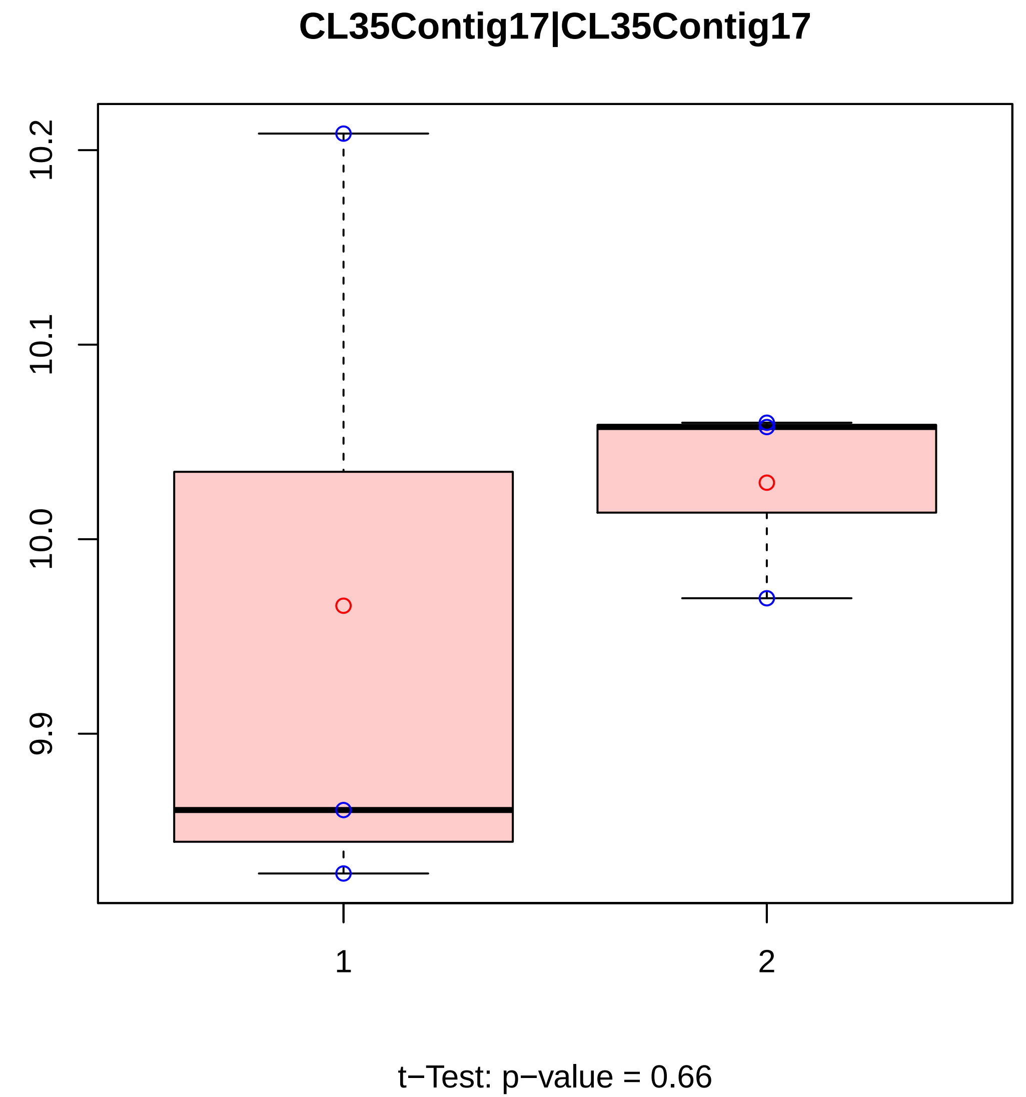
CL35Contig17|CL35Contig17
10.2
10.1
10.0
9.9
1
2
t−Test: p−value = 0.66

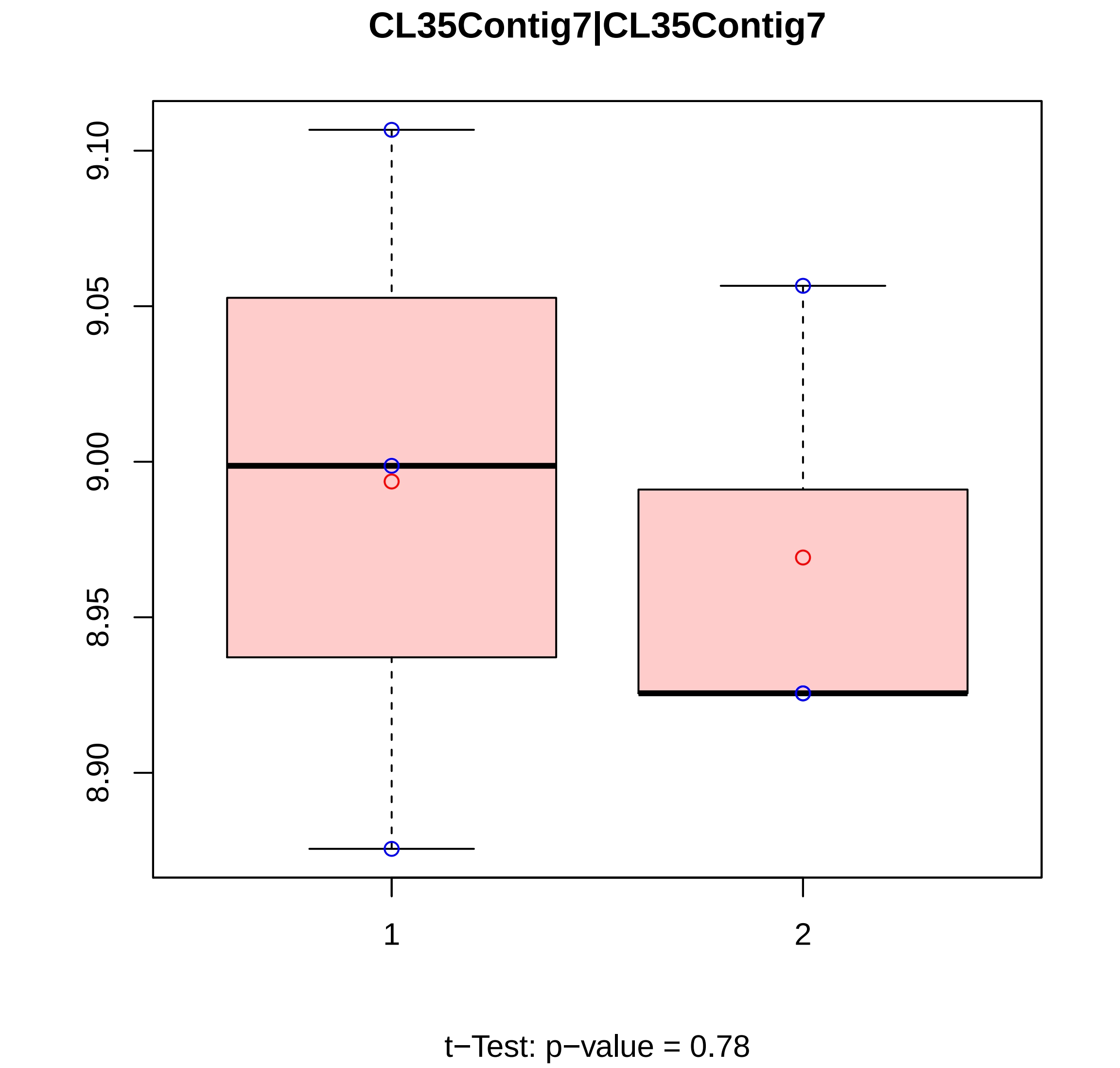

CL35Contig7|CL35Contig7
9.10
9.05
9.00
8.95
8.90
1
2
t−Test: p−value = 0.78

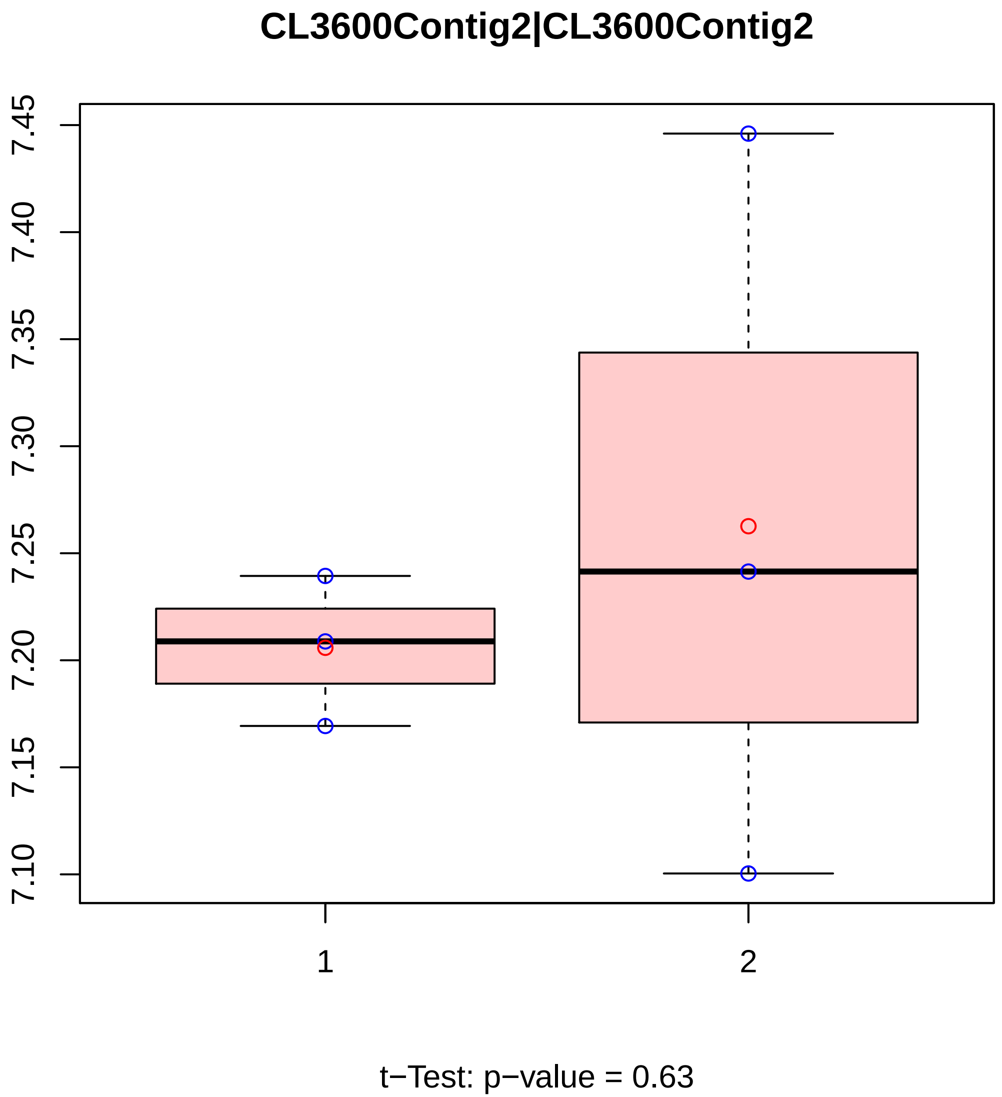
CL3600Contig2|CL3600Contig2
7.10
7.15
7.20
7.25
7.30
7.35
7.40
7.45
1
2
t−Test: p−value = 0.63

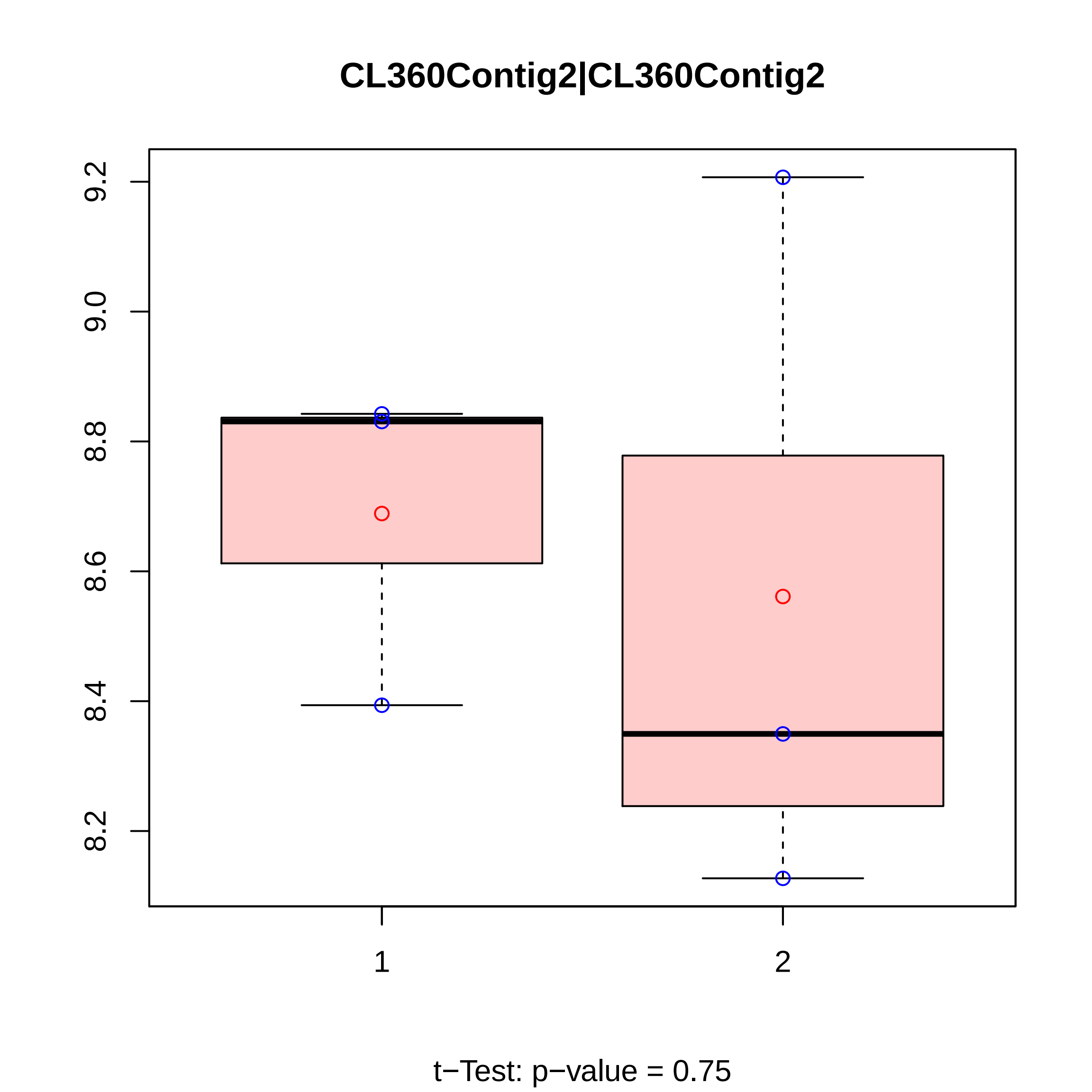

CL360Contig2|CL360Contig2
9.2
9.0
8.8
8.6
8.4
8.2
1
2
t−Test: p−value = 0.75

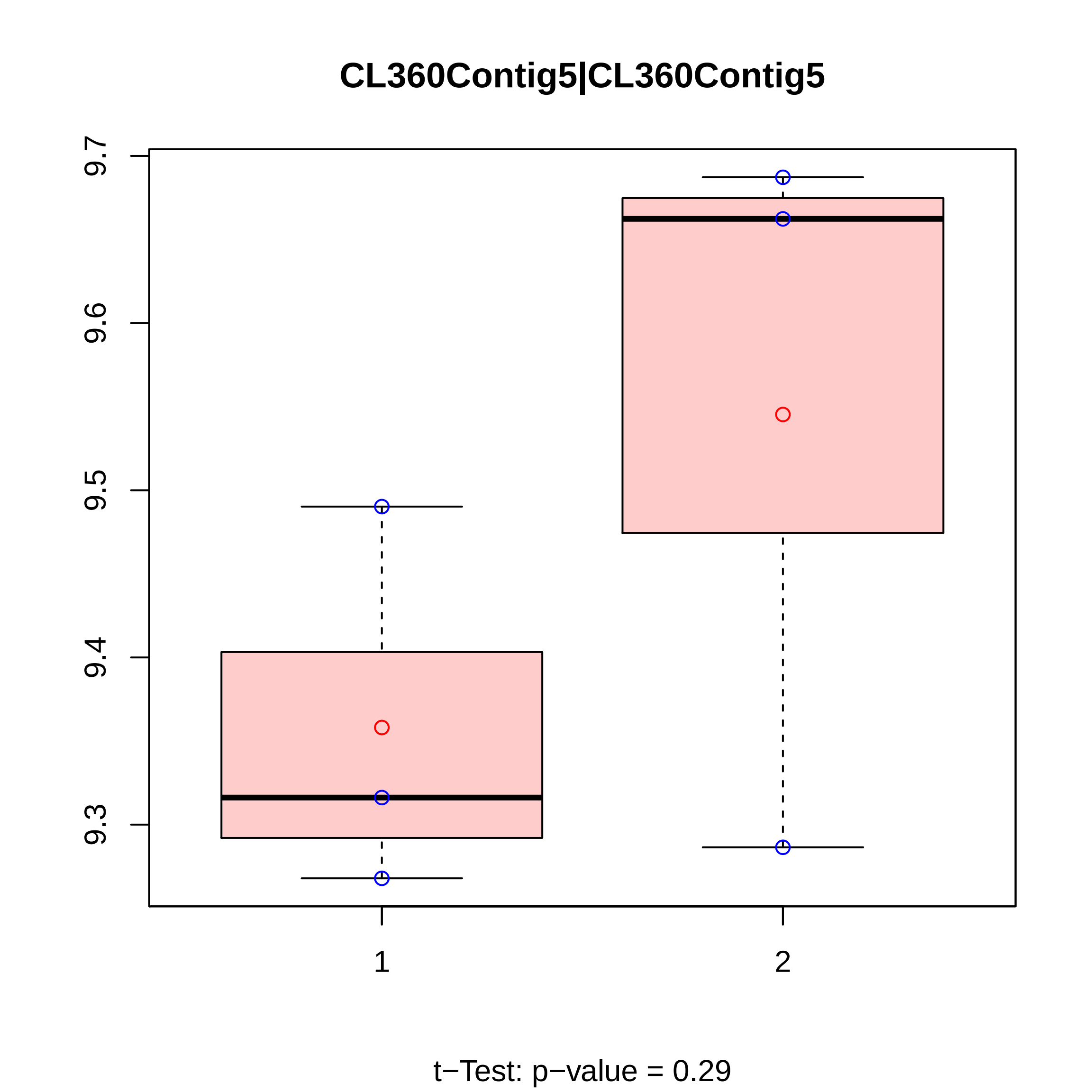

CL360Contig5|CL360Contig5
9.7
9.6
9.5
9.4
9.3
1
2
t−Test: p−value = 0.29

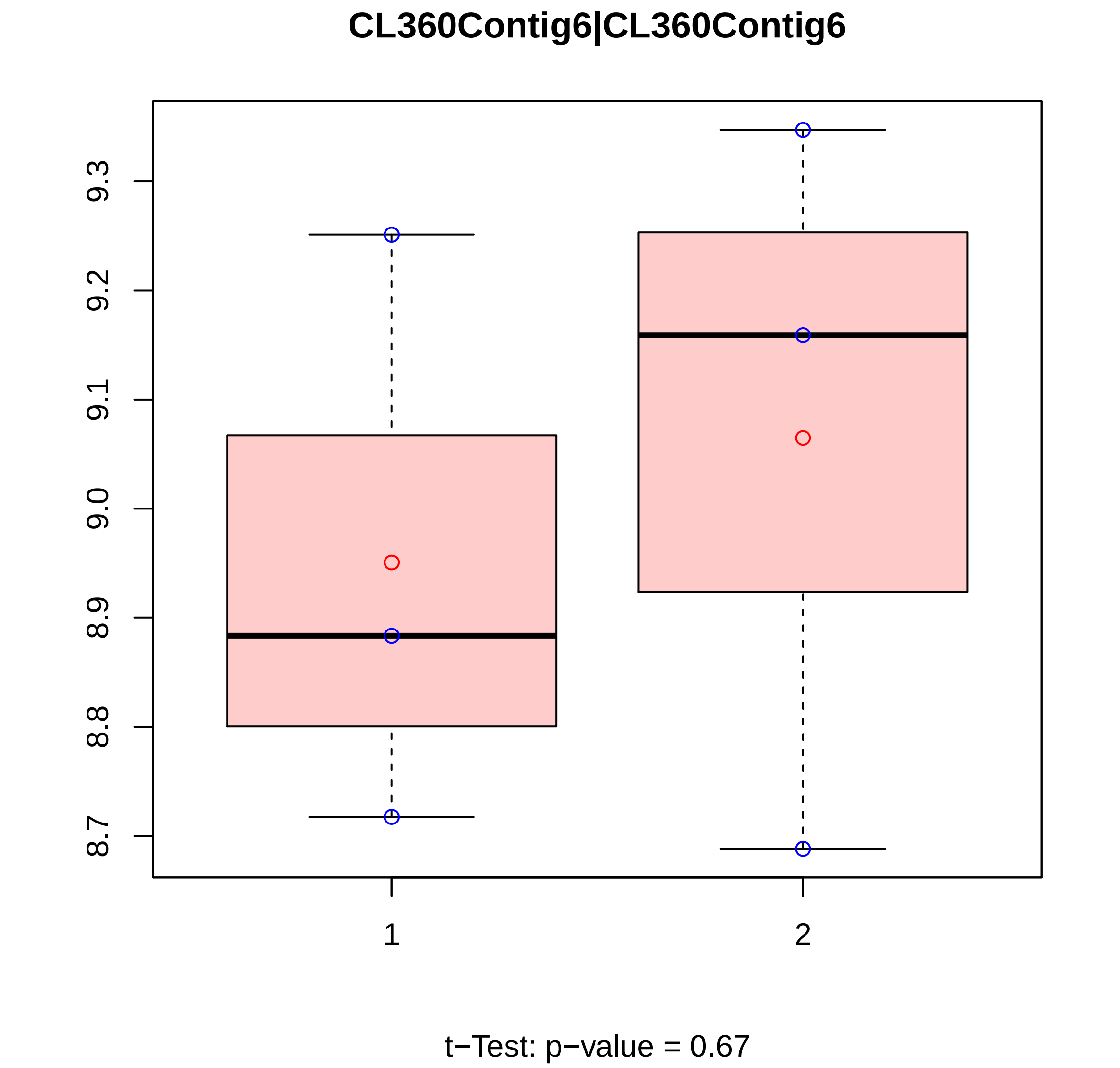

CL360Contig6|CL360Contig6
9.3
9.2
9.1
9.0
8.9
8.8
8.7
1
2
t−Test: p−value = 0.67

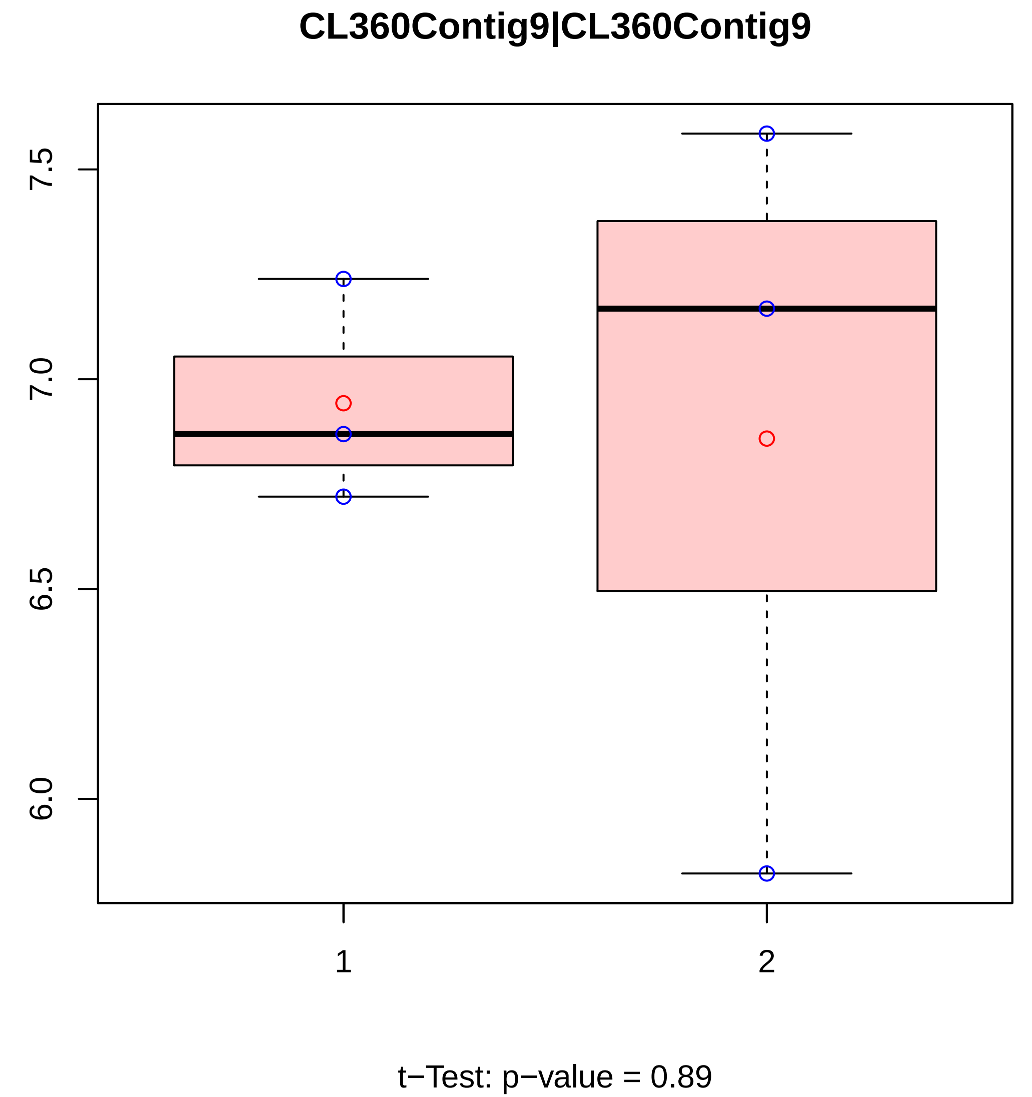
CL360Contig9|CL360Contig9
7.5
7.0
6.5
6.0
1
2
t−Test: p−value = 0.89

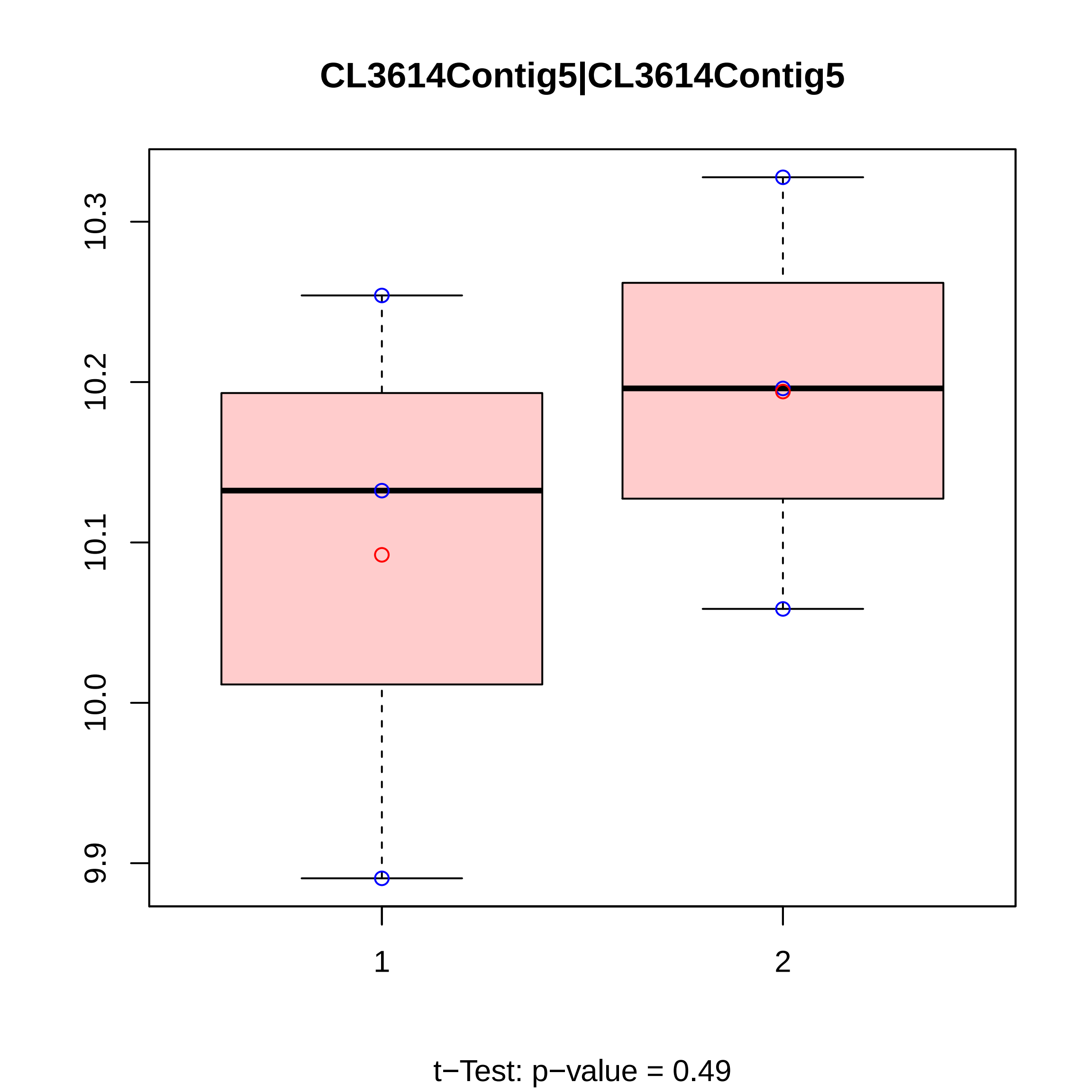
CL3614Contig5|CL3614Contig5
10.3
10.2
10.1
10.0
9.9
1
2
t−Test: p−value = 0.49

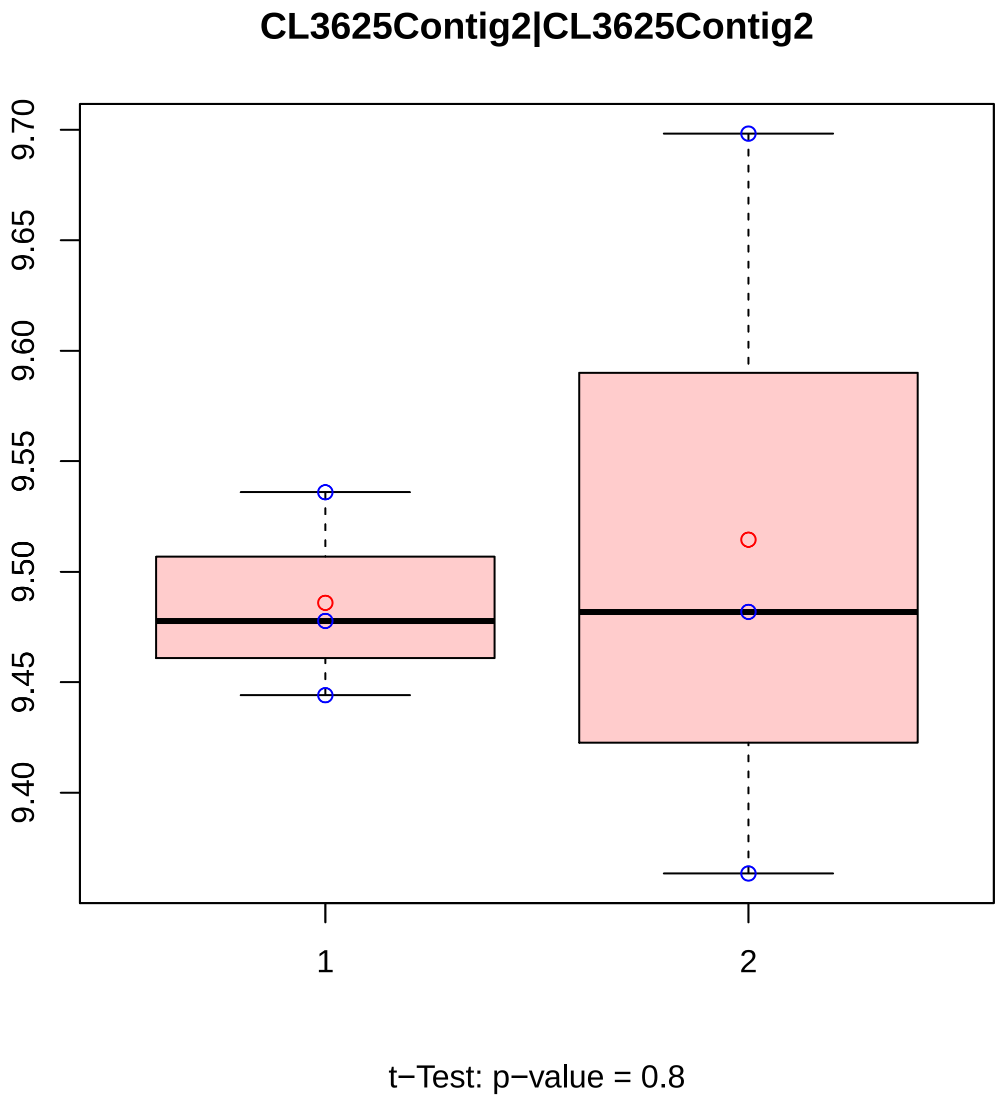
CL3625Contig2|CL3625Contig2
9.40
9.45
9.50
9.55
9.60
9.65
9.70
1
2
t−Test: p−value = 0.8

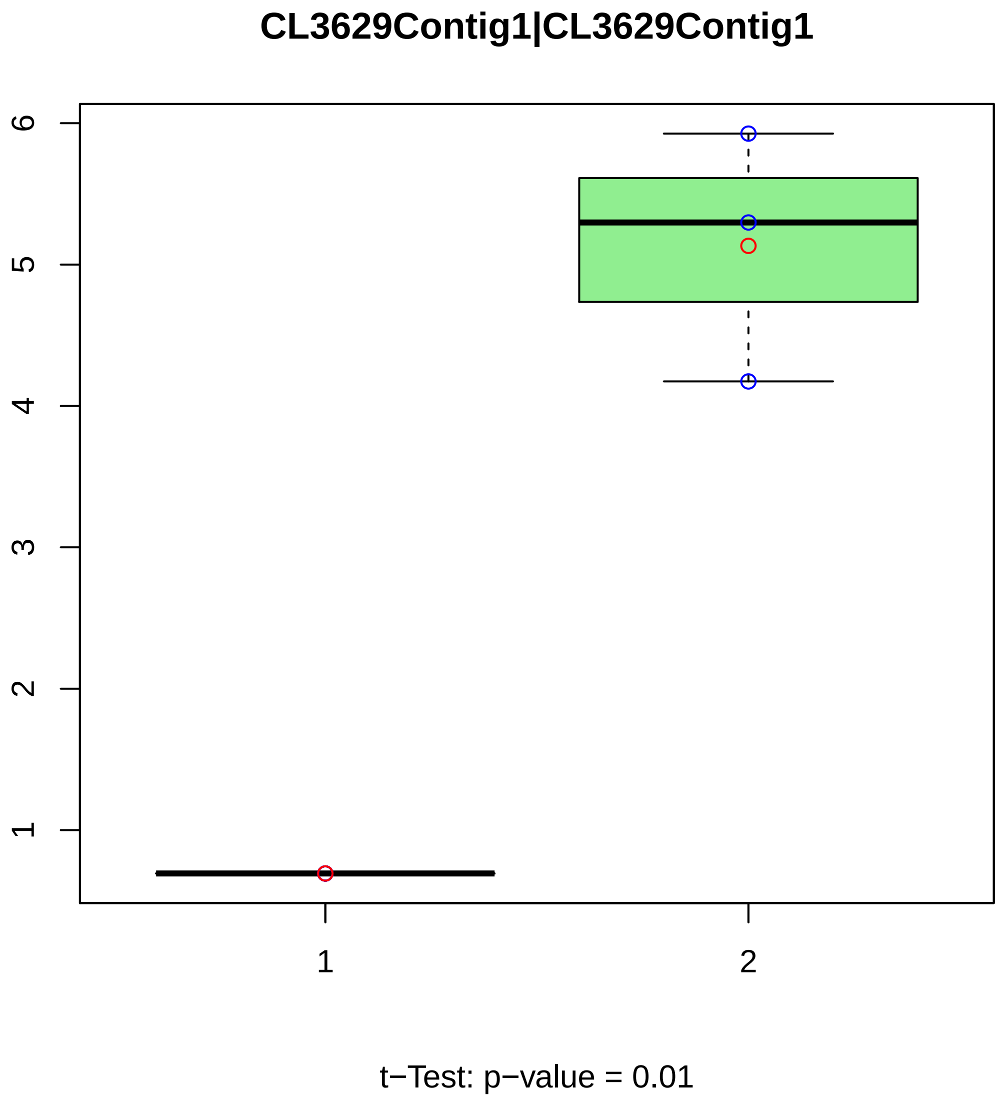

CL3629Contig1|CL3629Contig1
1
2
3
4
5
6
1
2
t−Test: p−value = 0.01

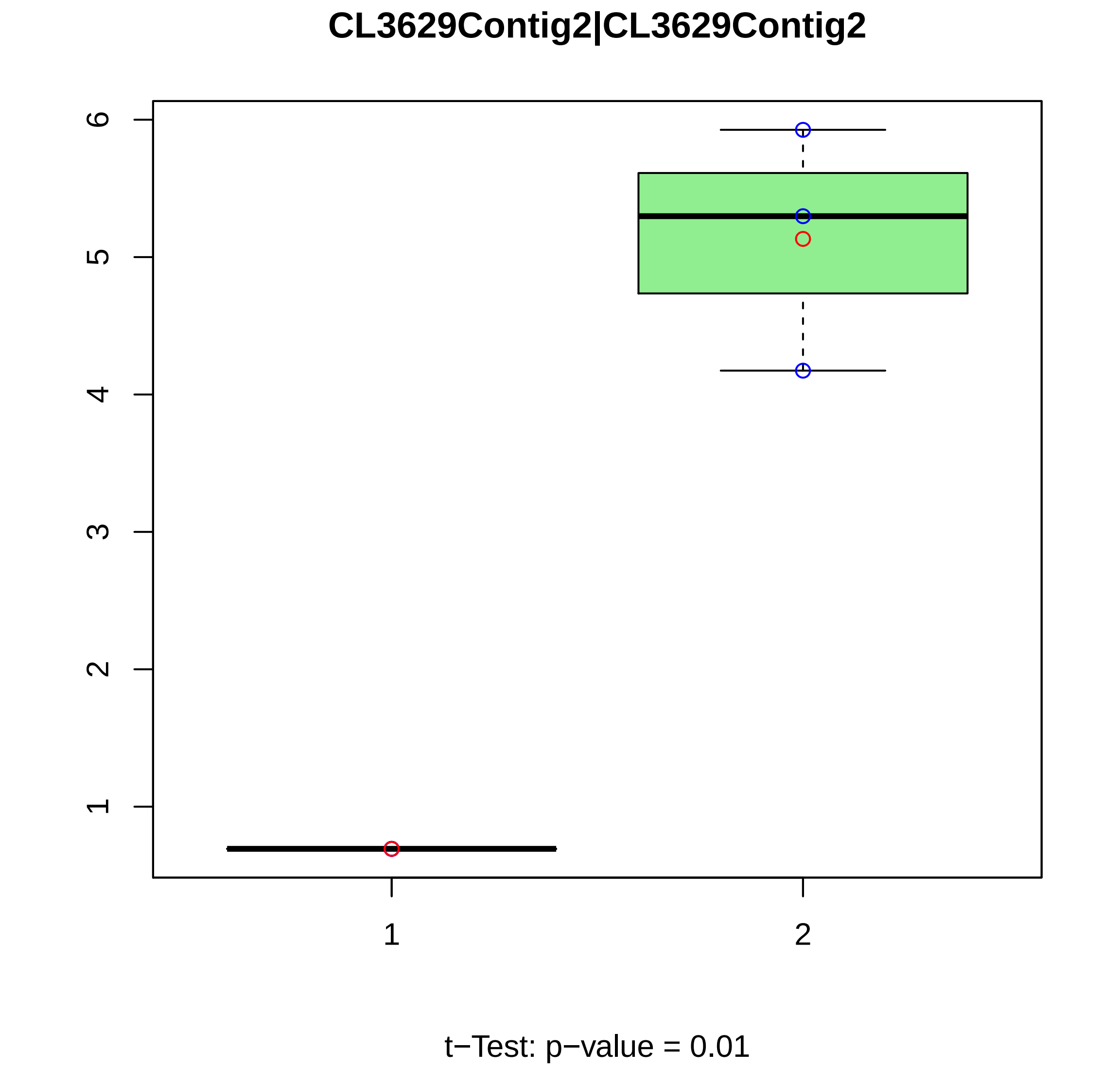
CL3629Contig2|CL3629Contig2
1
2
3
4
5
6
1
2
t−Test: p−value = 0.01

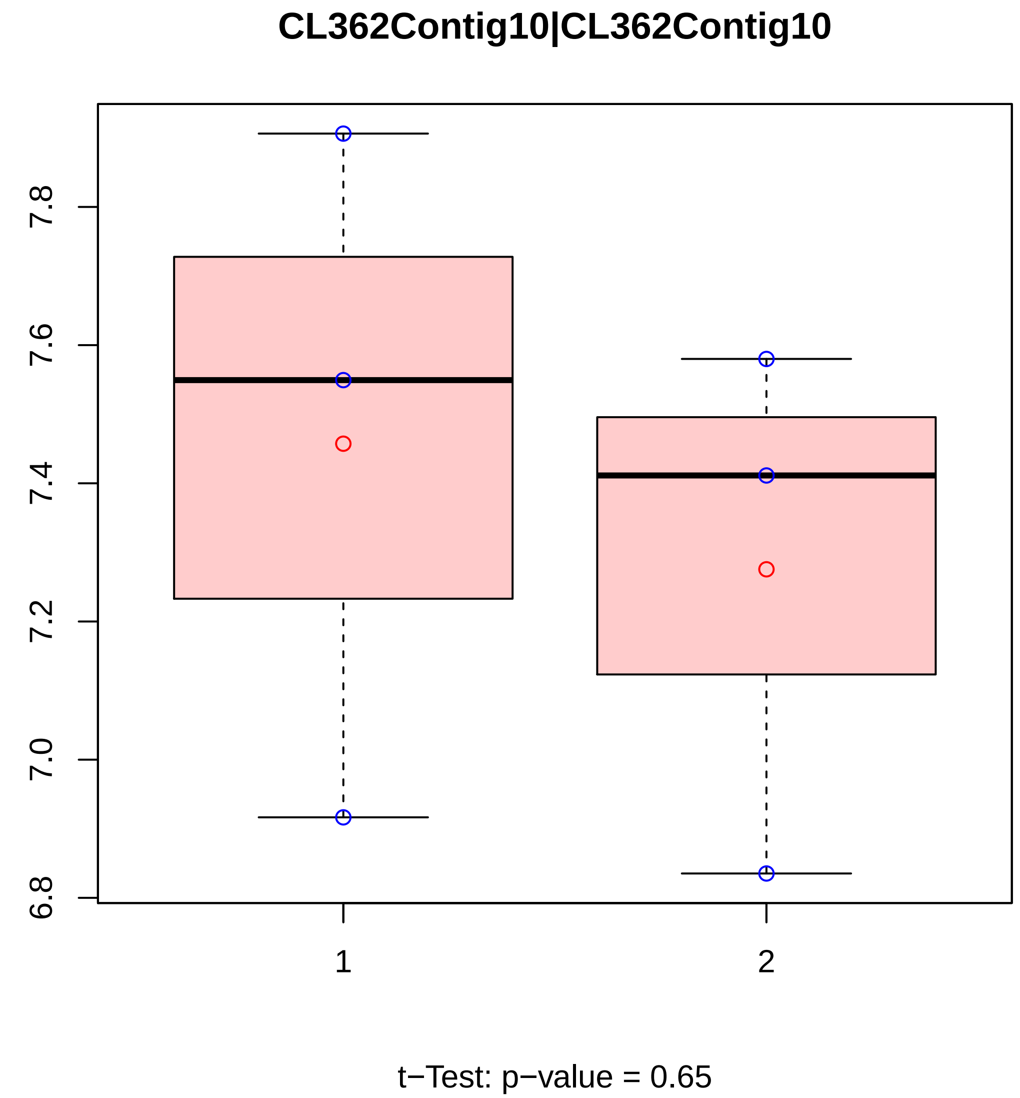
CL362Contig10|CL362Contig10
7.8
7.6
7.4
7.2
7.0
6.8
1
2
t−Test: p−value = 0.65

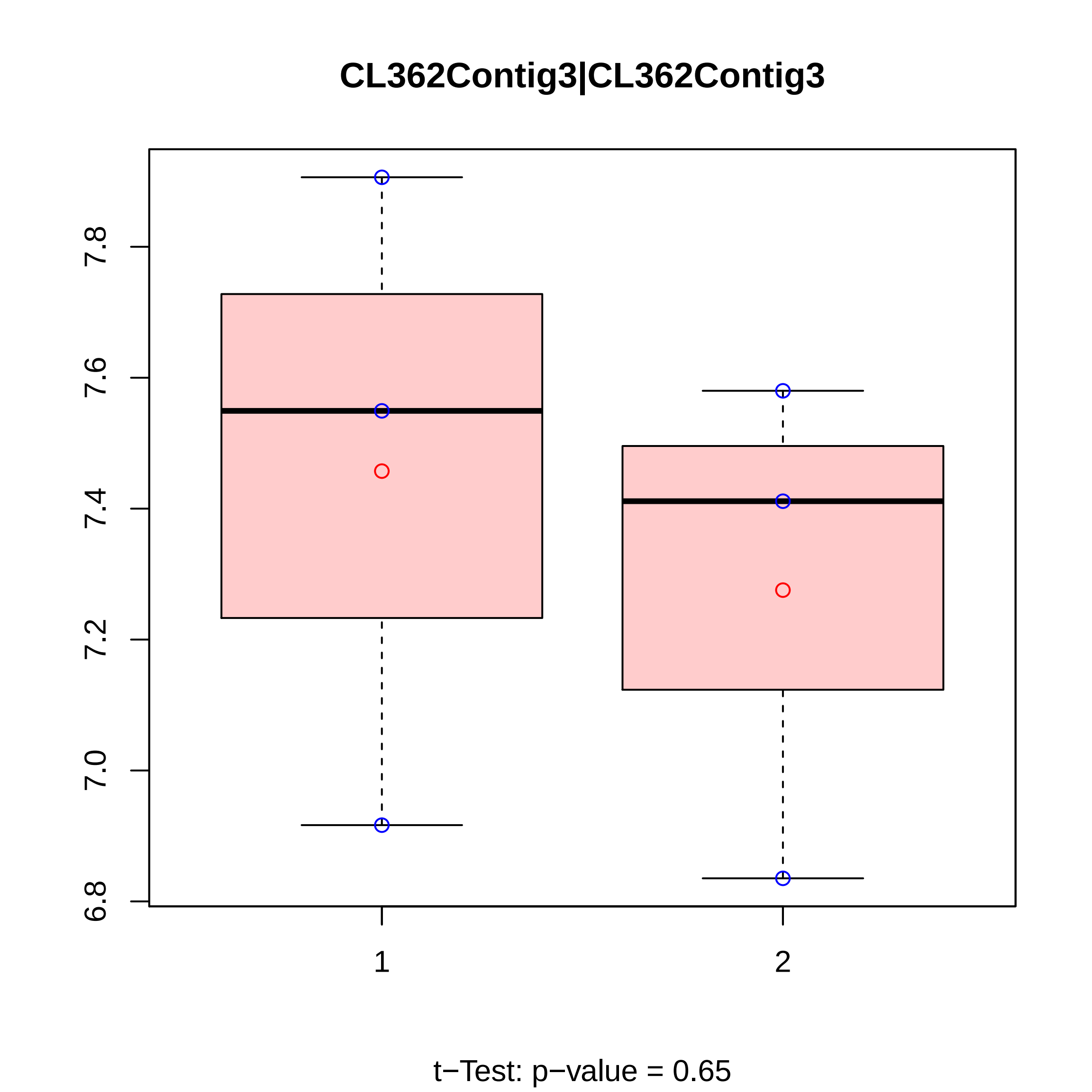

CL362Contig3|CL362Contig3
7.8
7.6
7.4
7.2
7.0
6.8
1
2
t−Test: p−value = 0.65

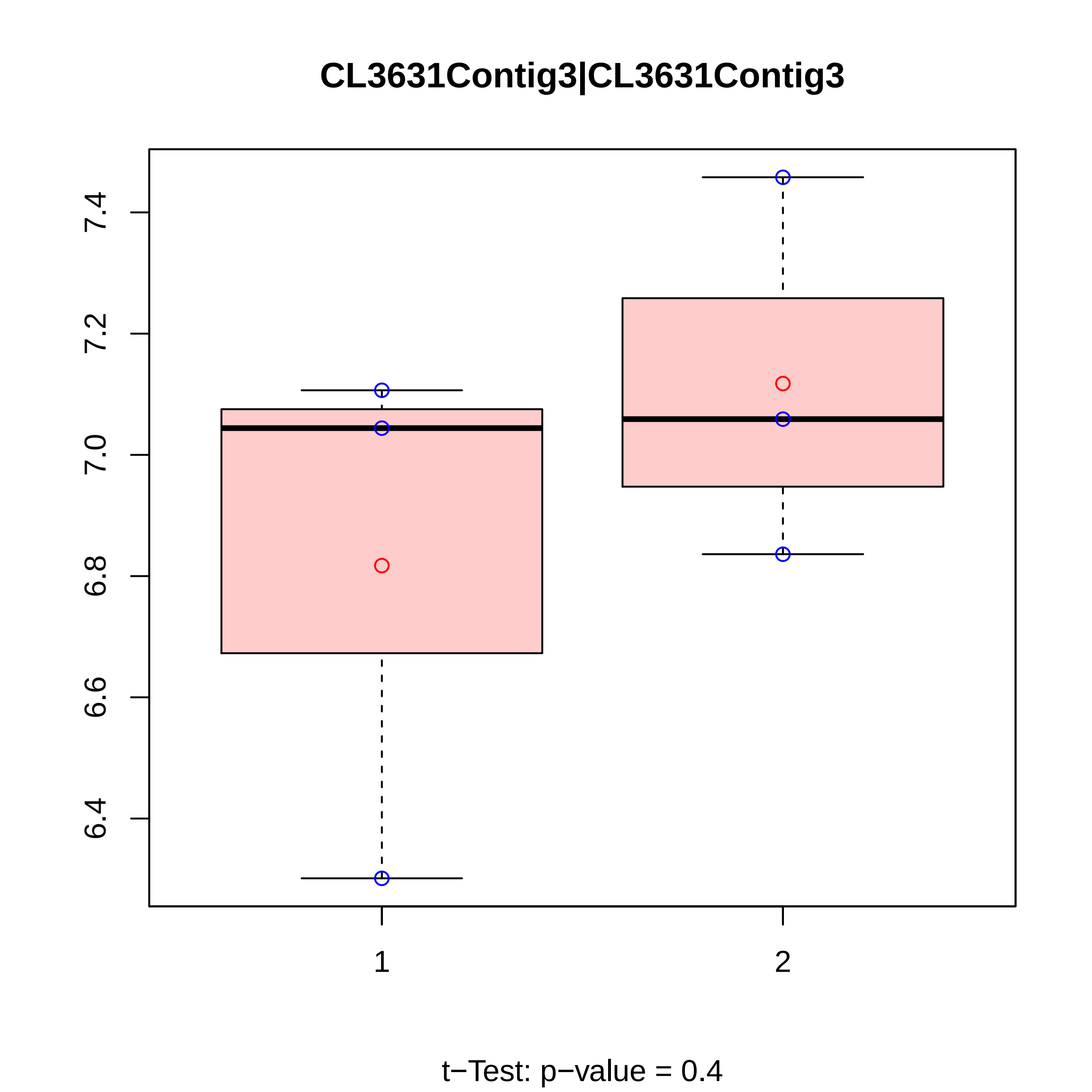

CL3631Contig3|CL3631Contig3
7.4
7.2
7.0
6.8
6.6
6.4
1
2
t−Test: p−value = 0.4

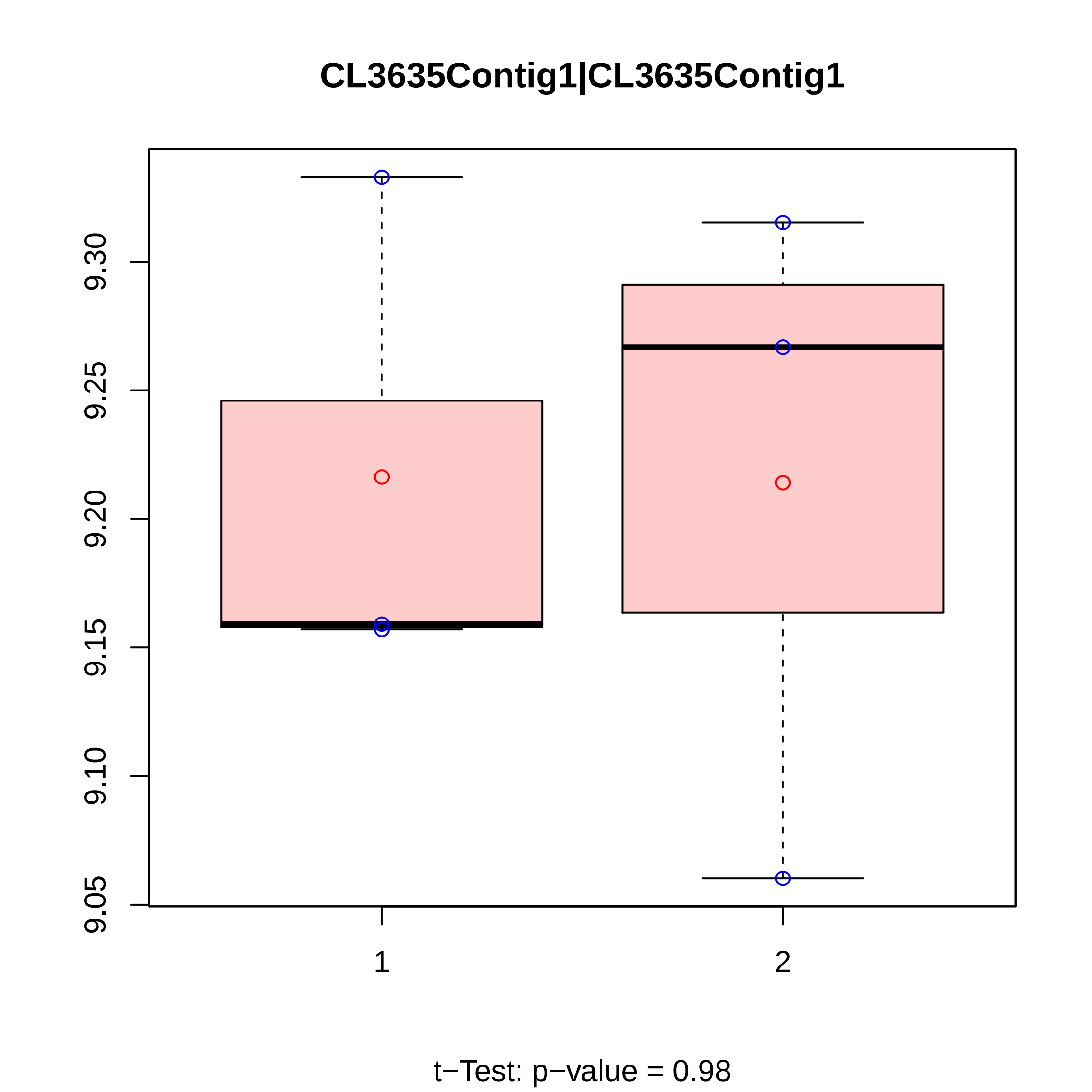
CL3635Contig1|CL3635Contig1
9.05
9.10
9.15
9.20
9.25
9.30
1
2
t−Test: p−value = 0.98

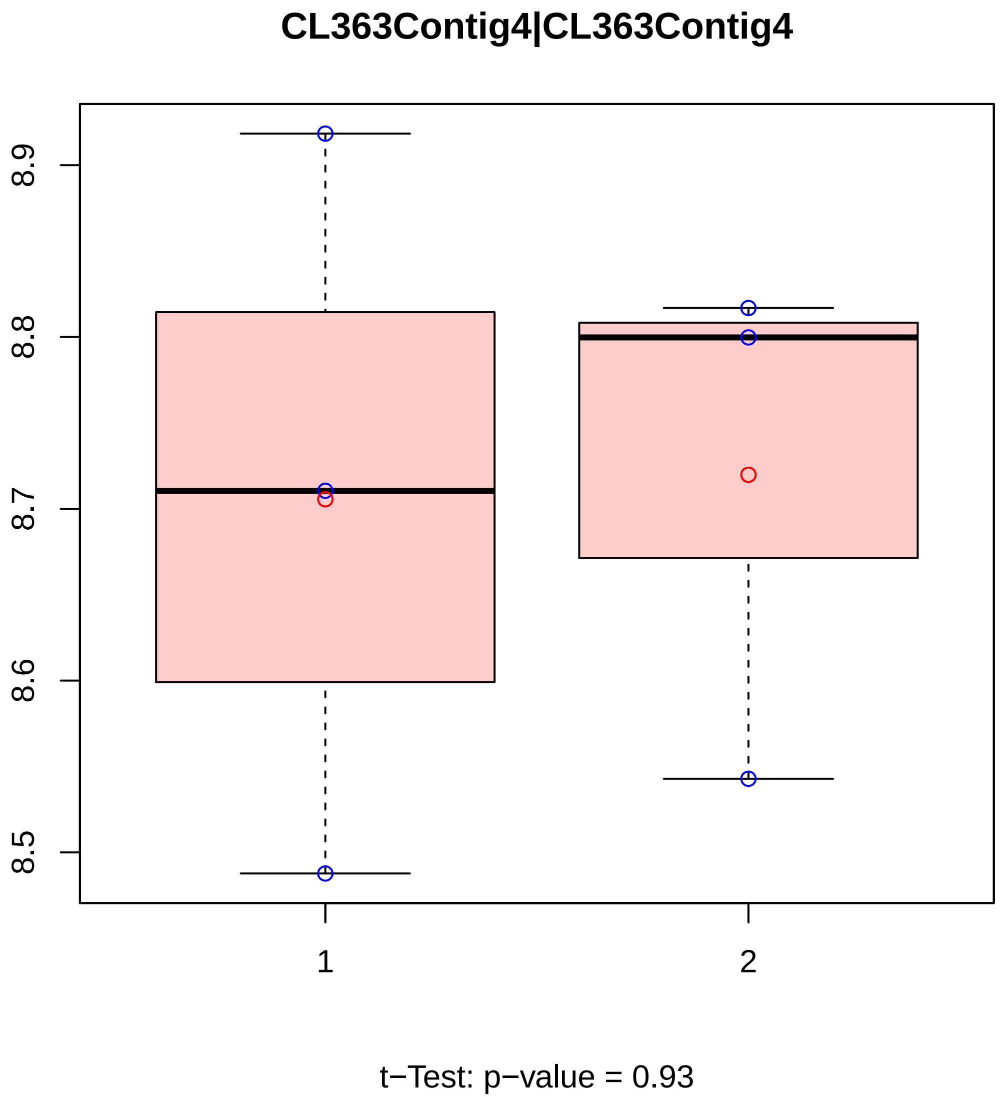
CL363Contig4|CL363Contig4
8.5
8.6
8.7
8.8
8.9
1
2
t−Test: p−value = 0.93

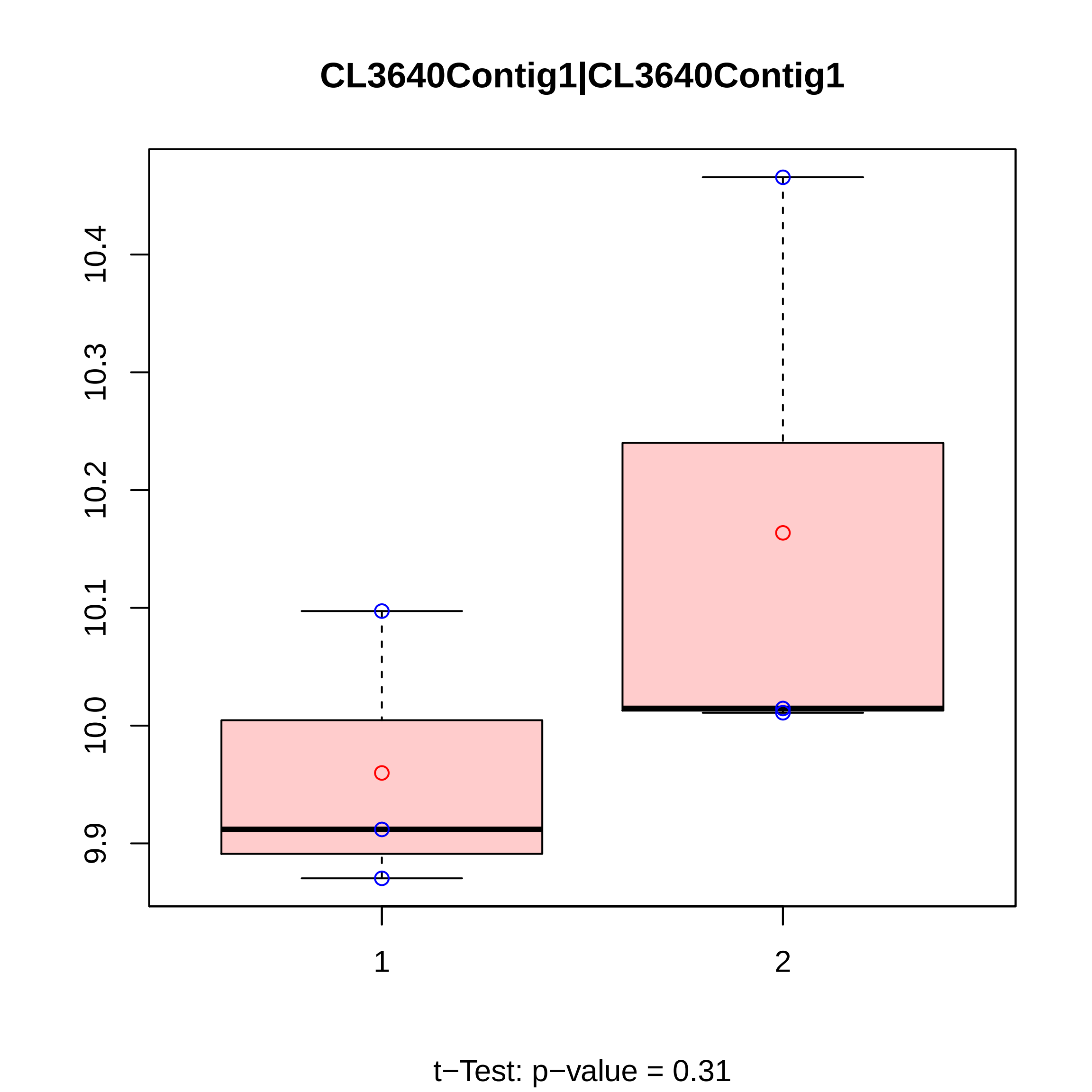
CL3640Contig1|CL3640Contig1
10.4
10.3
10.2
10.1
10.0
9.9
1
2
t−Test: p−value = 0.31

# CL3640Contig2|CL3640Contig2

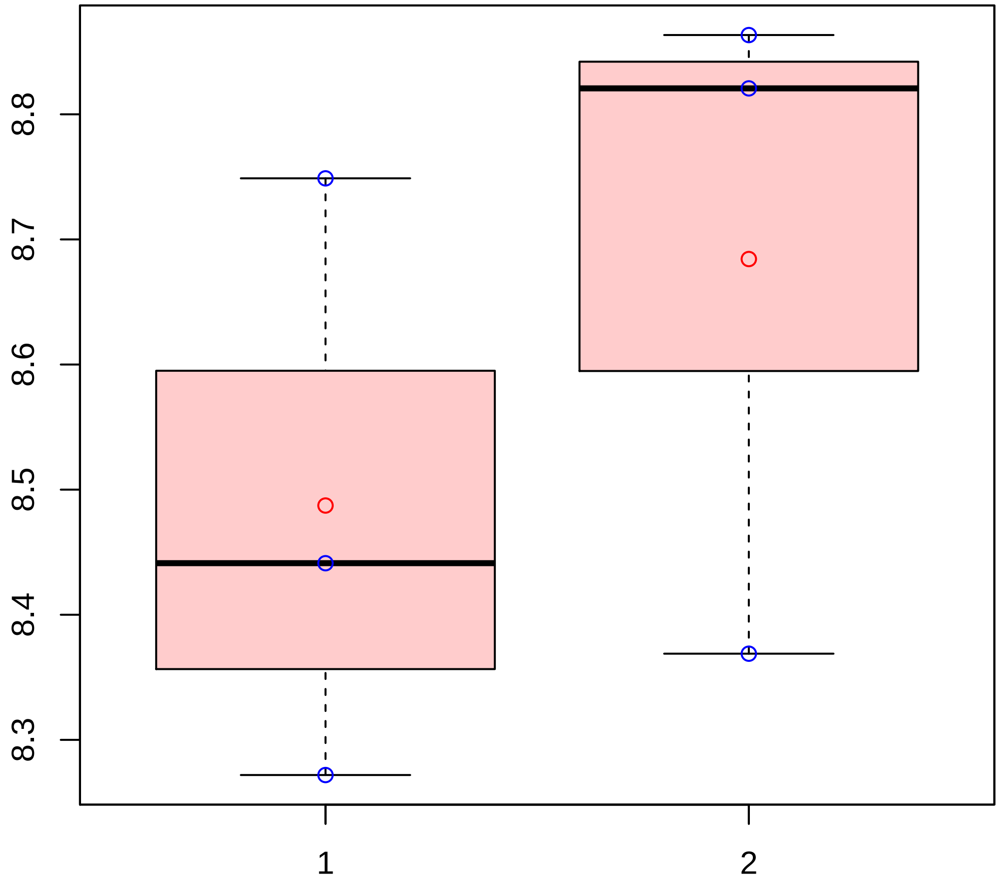


t−Test: p−value = 0.4

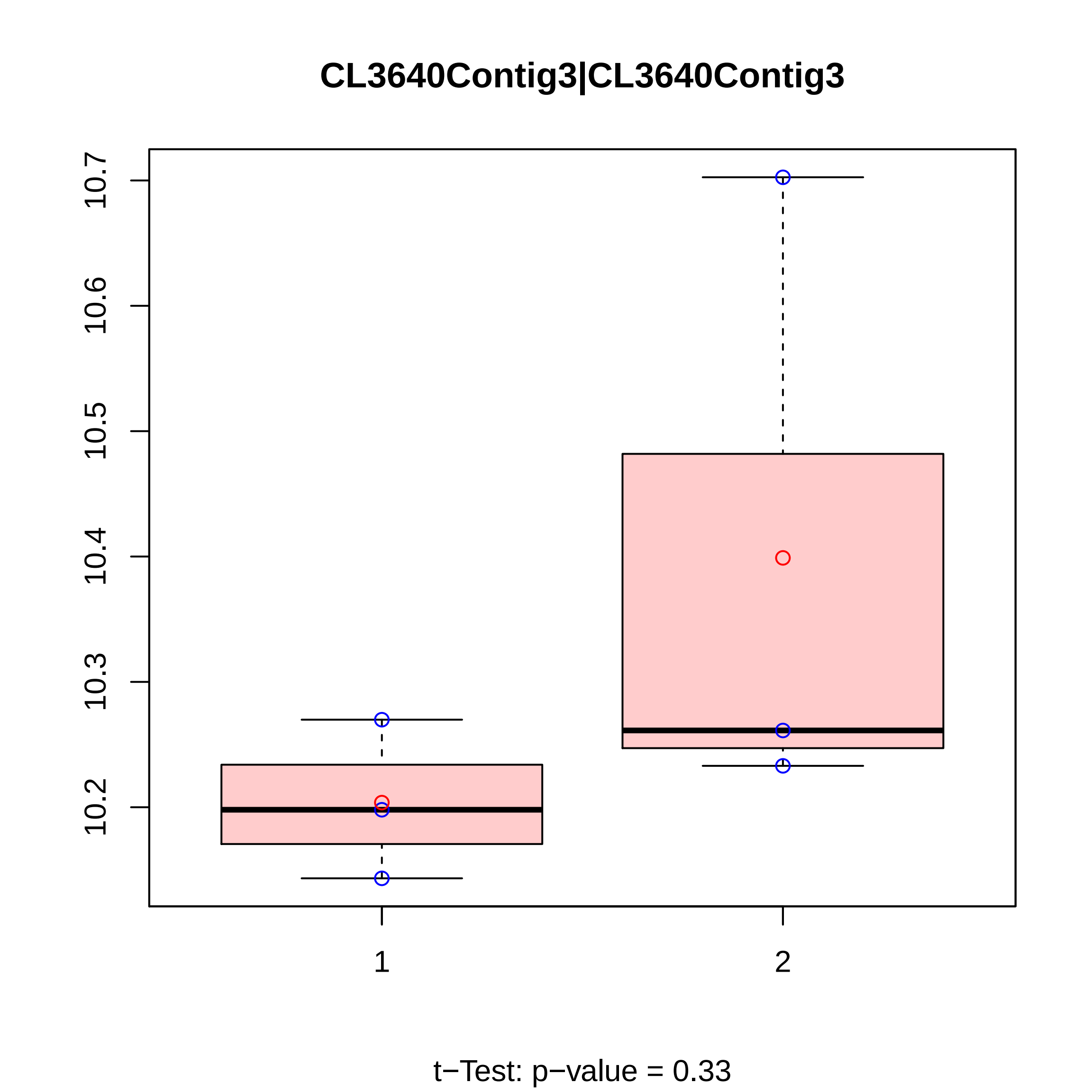

CL3640Contig3|CL3640Contig3
10.7
10.6
10.5
10.4
10.3
10.2
1
2
t−Test: p−value = 0.33

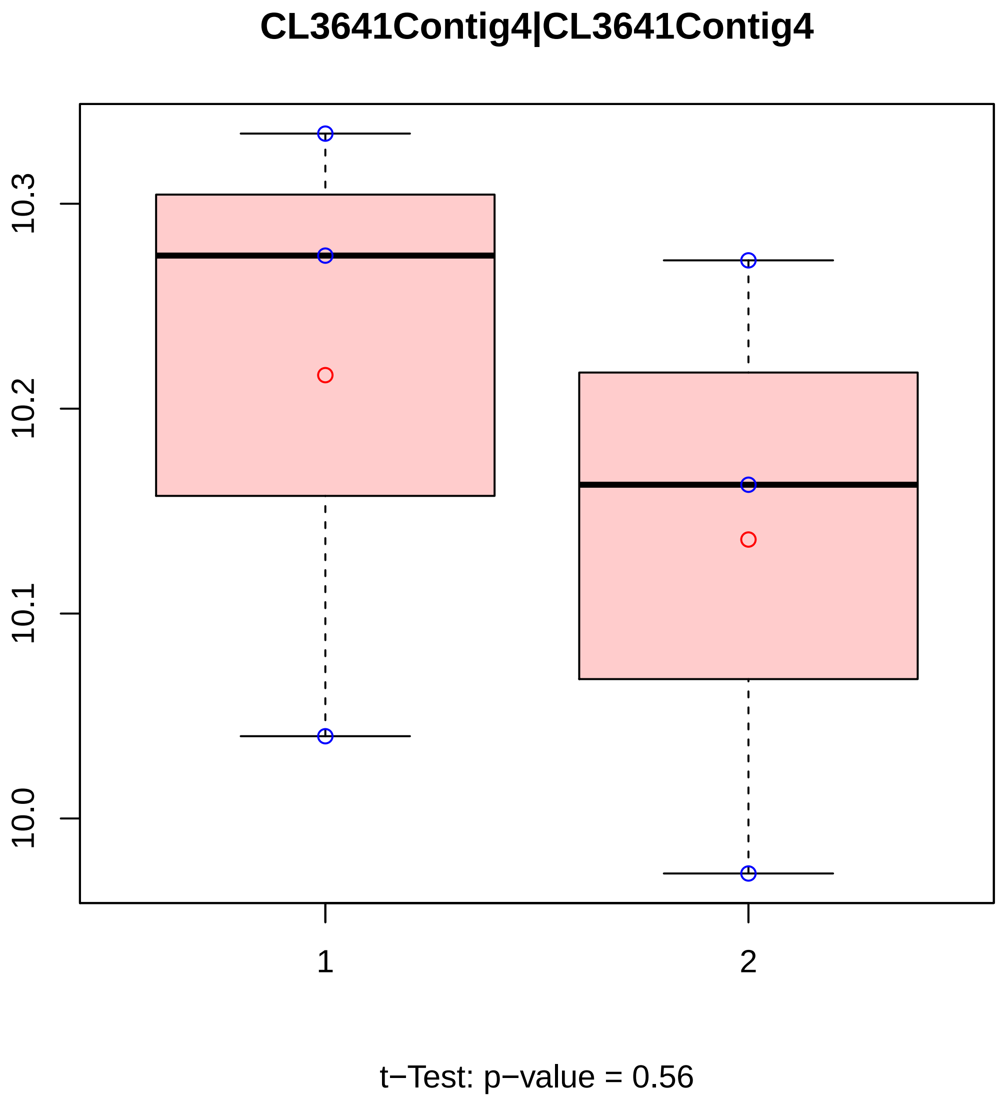
CL3641Contig4|CL3641Contig4
10.0
10.1
10.2
10.3
1
2
t−Test: p−value = 0.56

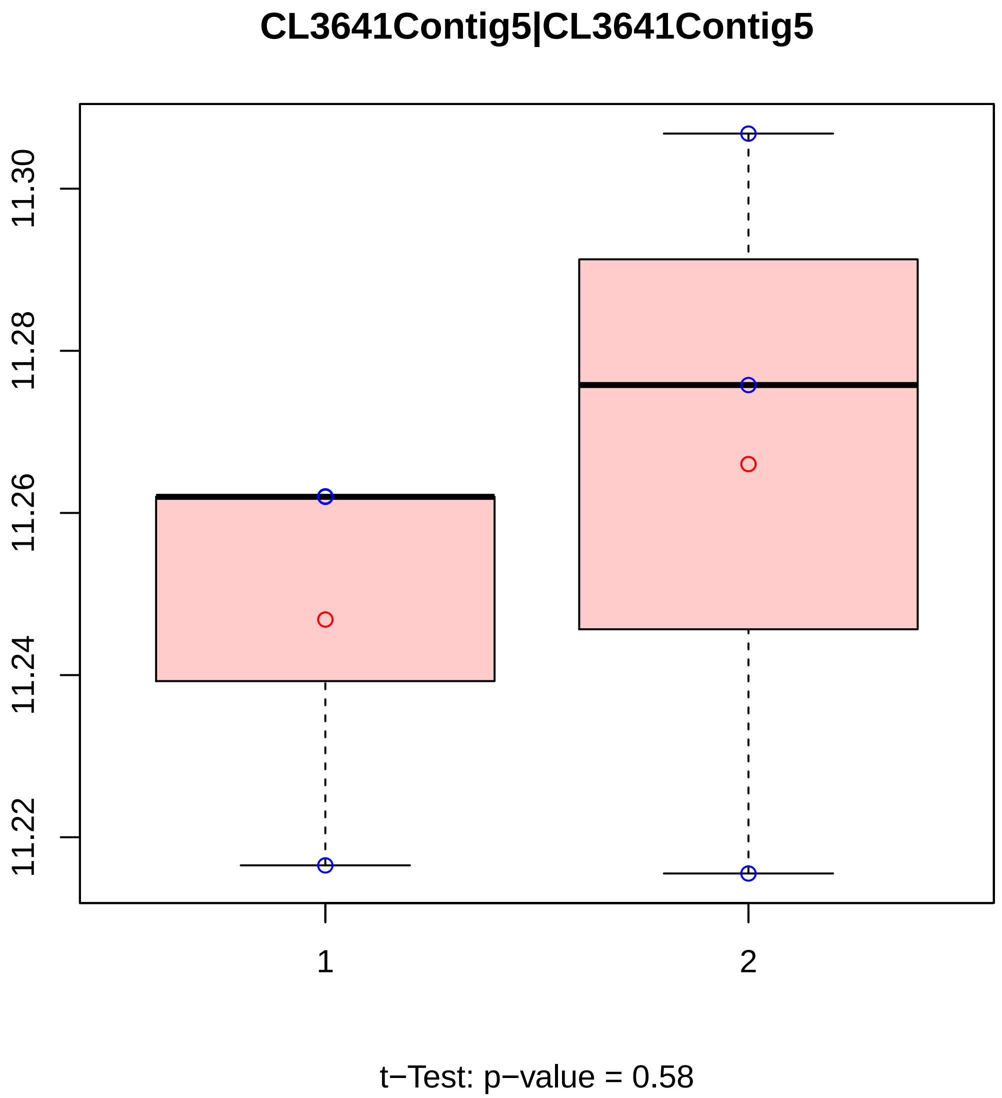

CL3641Contig5|CL3641Contig5
11.22
11.24
11.26
11.28
11.30
1
2
t−Test: p−value = 0.58

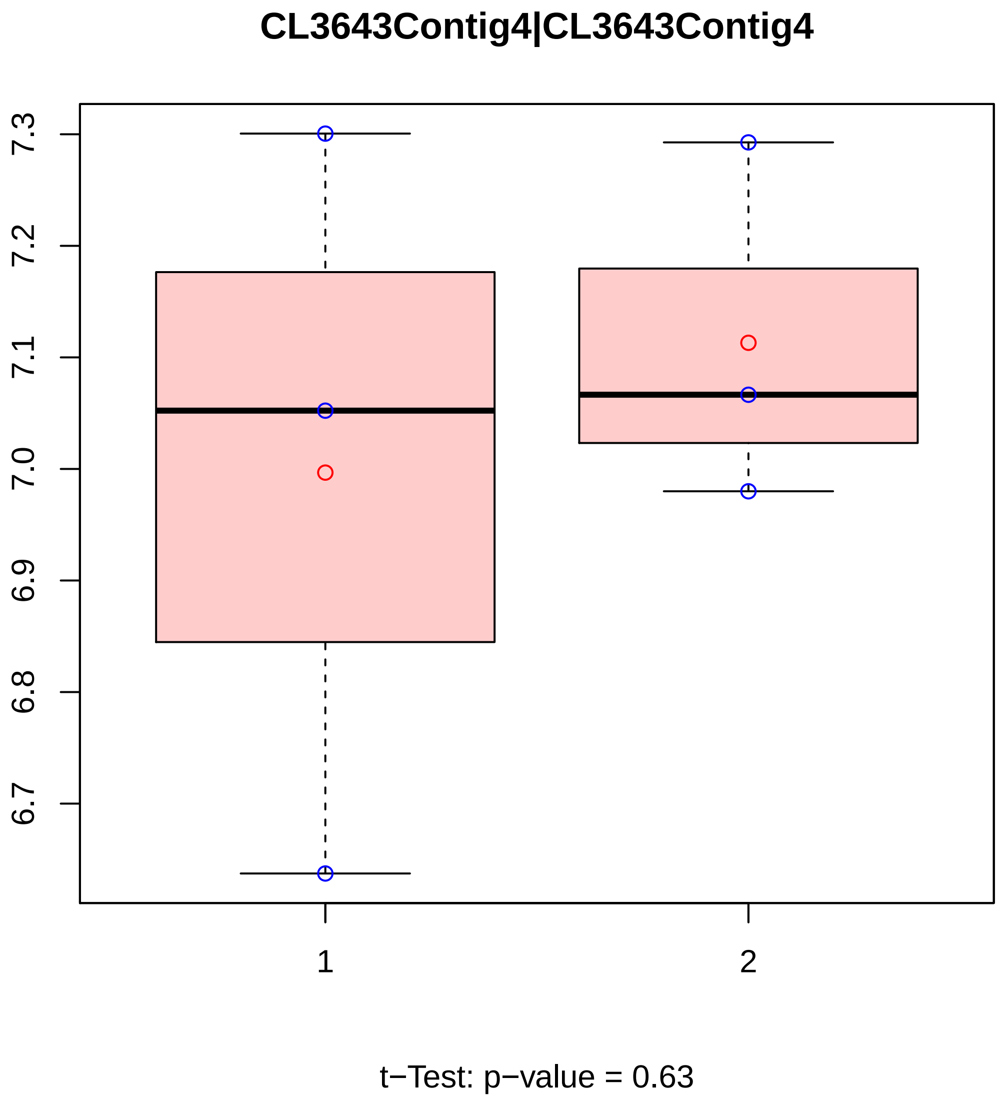

CL3643Contig4|CL3643Contig4
7.3
7.2
7.1
7.0
6.9
6.8
6.7
1
2
t−Test: p−value = 0.63

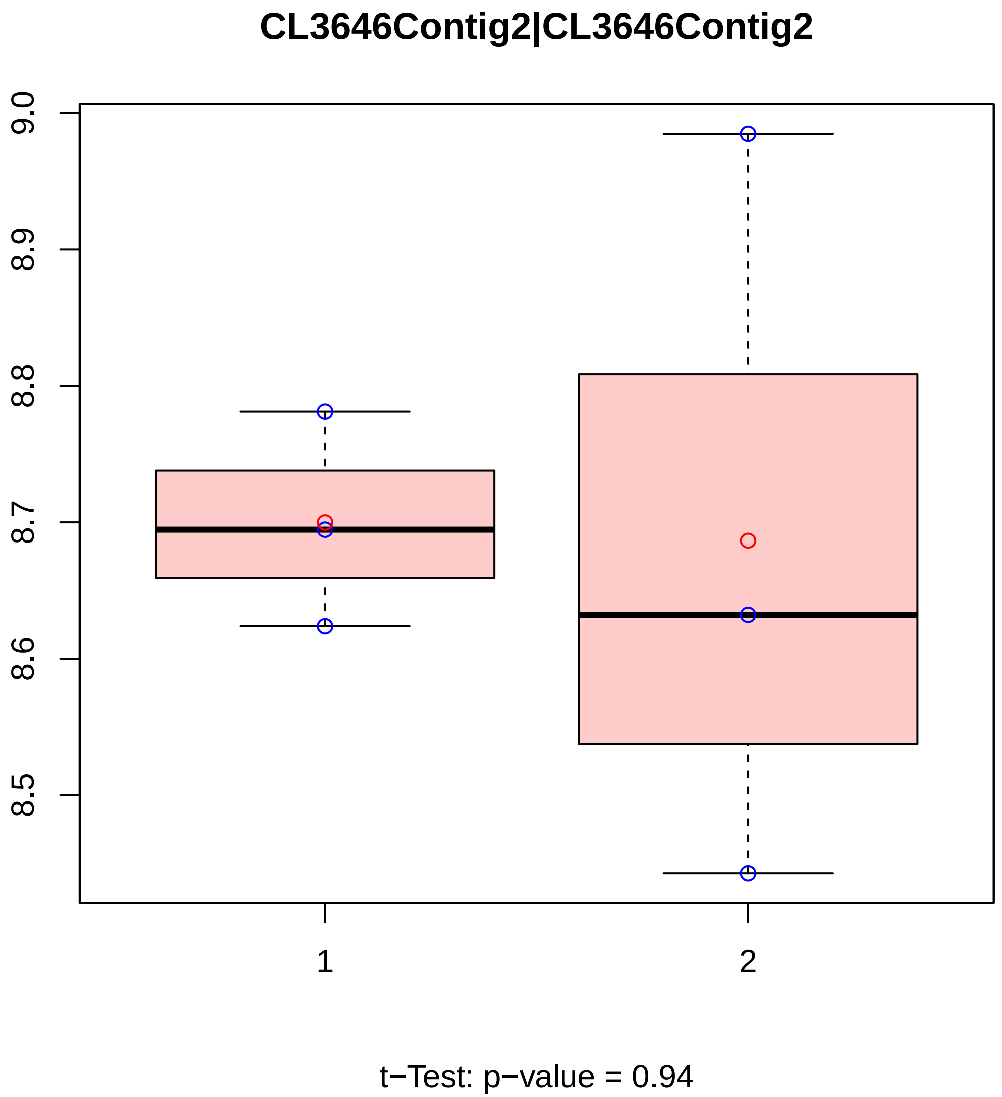
CL3646Contig2|CL3646Contig2
9.0
8.9
8.8
8.7
8.6
8.5
1
2
t−Test: p−value = 0.94

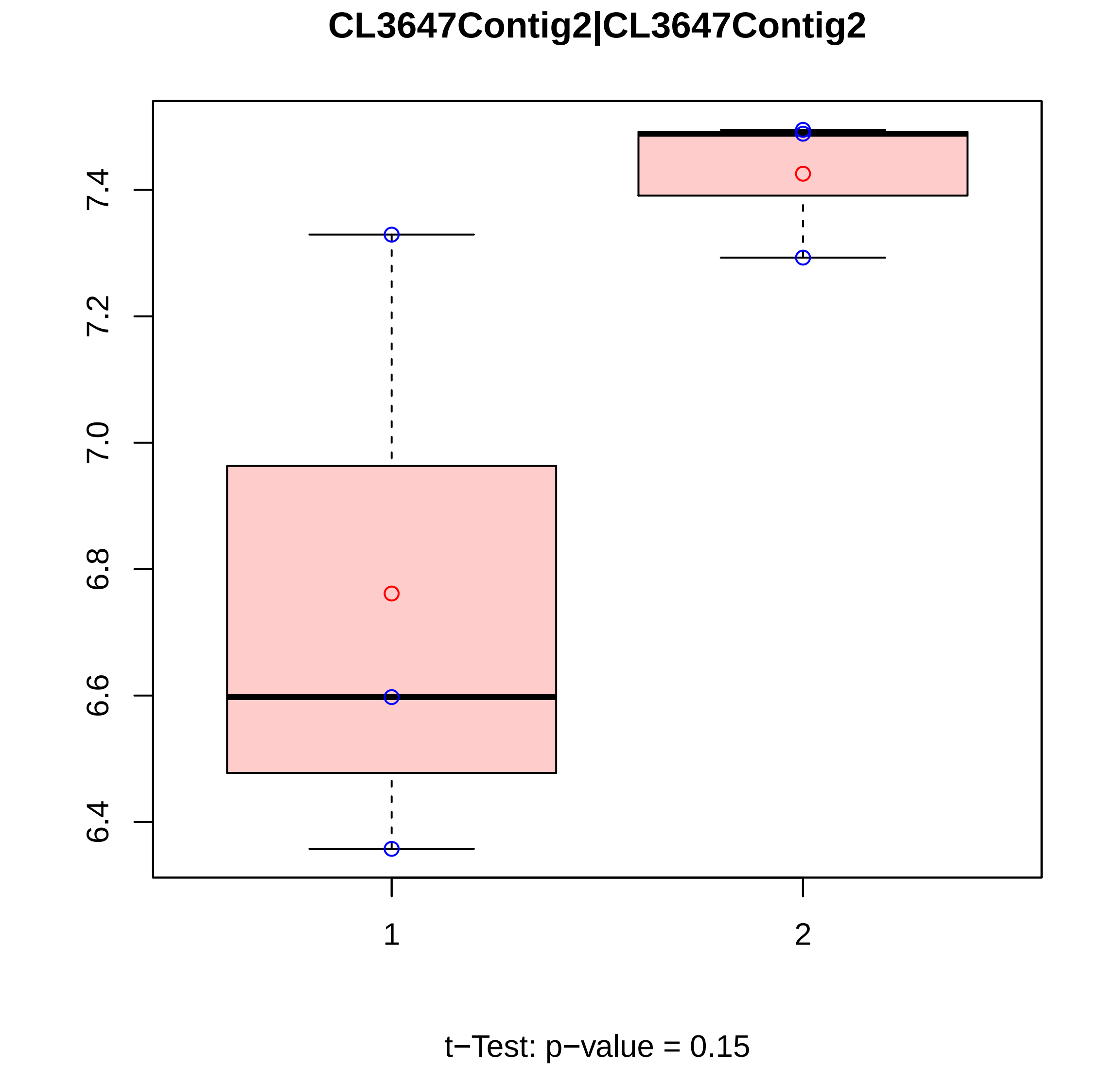
CL3647Contig2|CL3647Contig2
7.4
7.2
7.0
6.8
6.6
6.4
1
2
t−Test: p−value = 0.15

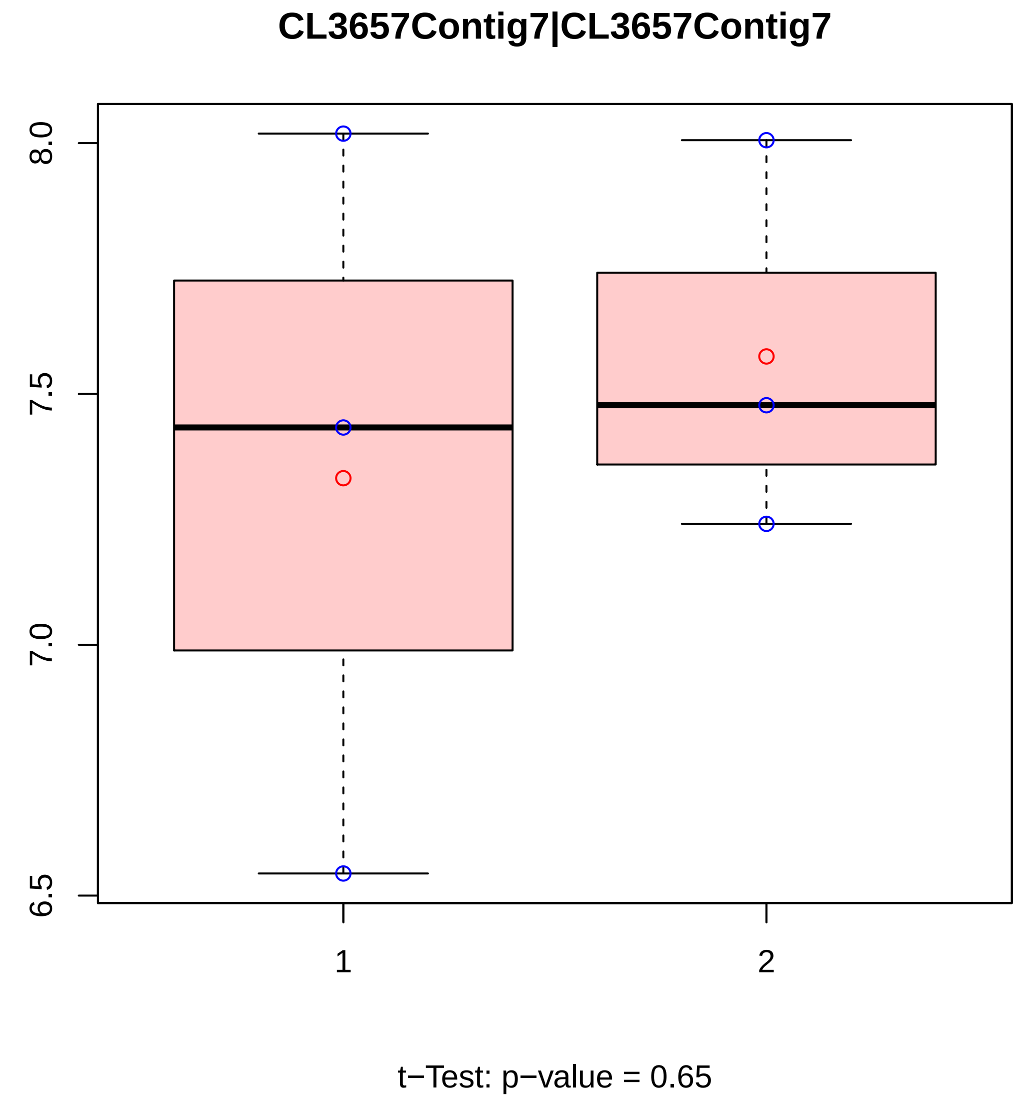
CL3657Contig7|CL3657Contig7
8.0
7.5
7.0
6.5
1
2
t−Test: p−value = 0.65

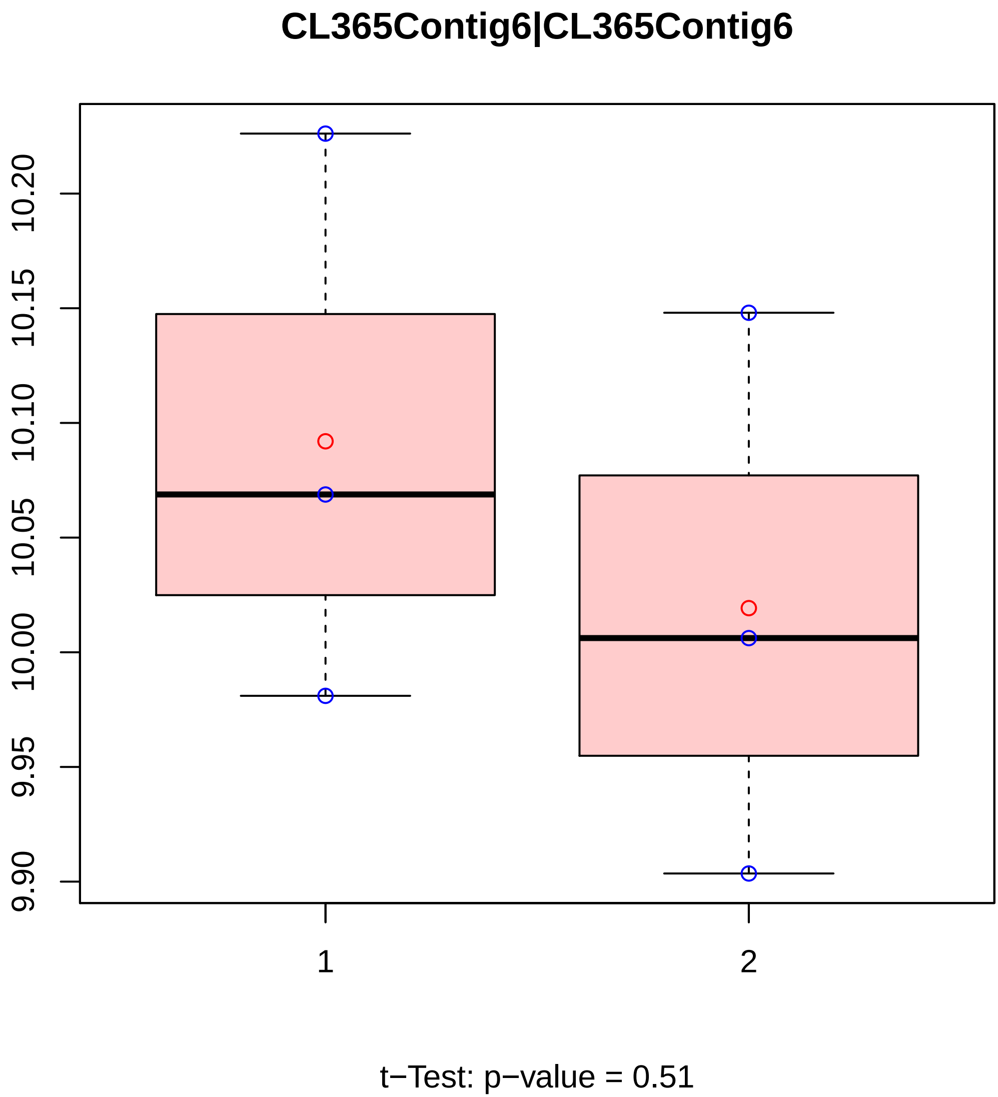

CL365Contig6|CL365Contig6
10.20
10.15
10.10
10.05
10.00
9.95
9.90
1
2
t−Test: p−value = 0.51

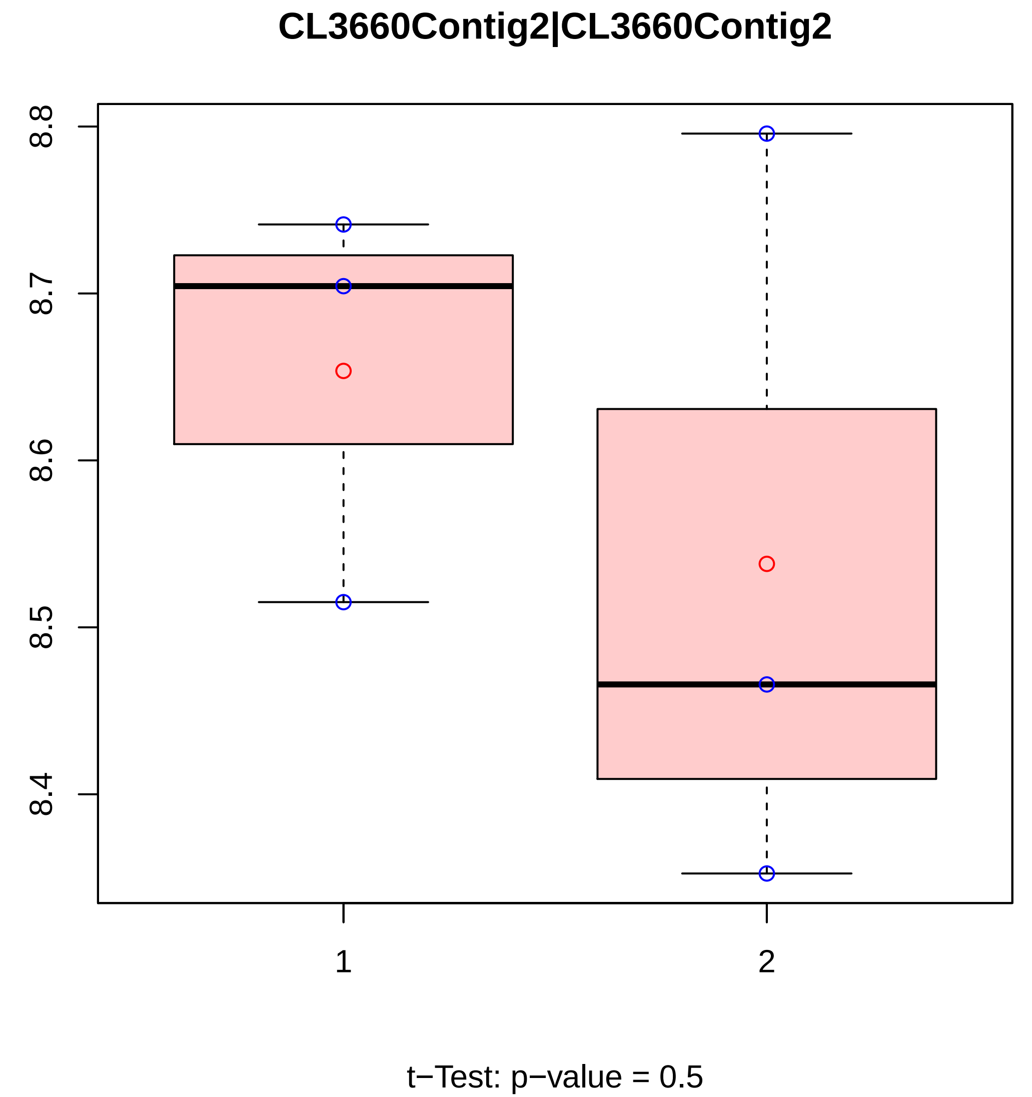

CL3660Contig2|CL3660Contig2
8.4
8.5
8.6
8.7
8.8
1
2
t−Test: p−value = 0.5

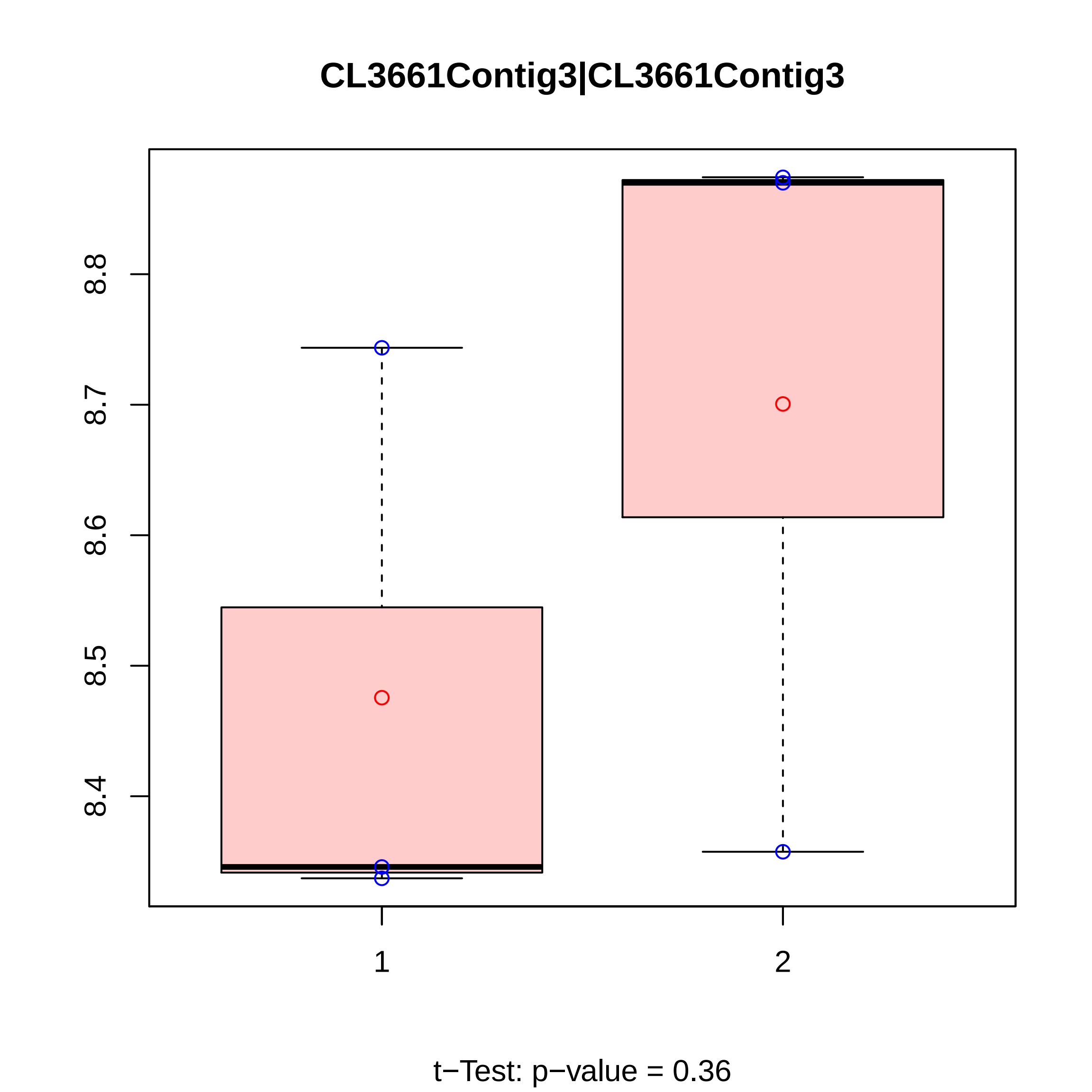
CL3661Contig3|CL3661Contig3
8.4
8.5
8.6
8.7
8.8
1
2
t−Test: p−value = 0.36

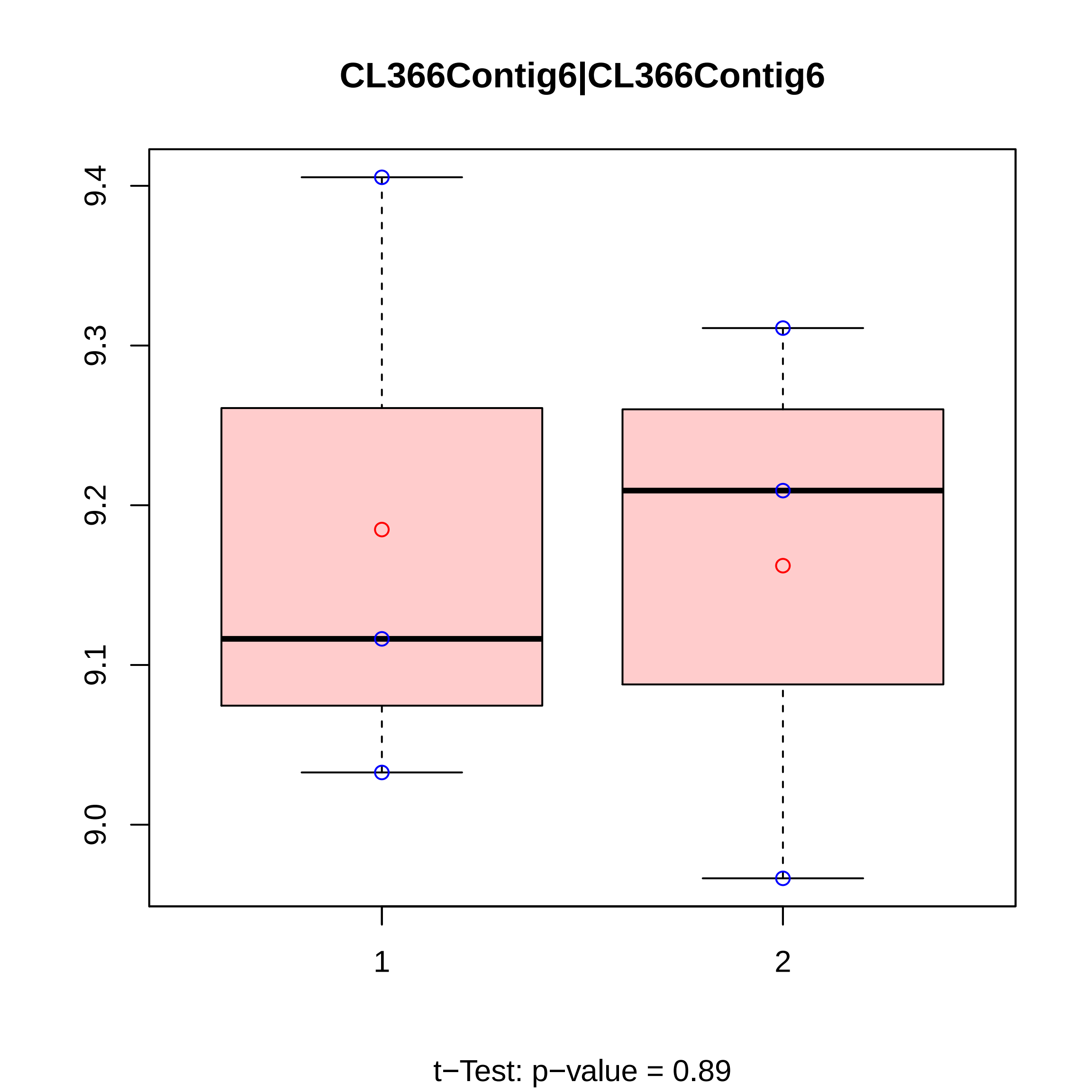
CL366Contig6|CL366Contig6
9.0
9.1
9.2
9.3
9.4
1
2
t−Test: p−value = 0.89

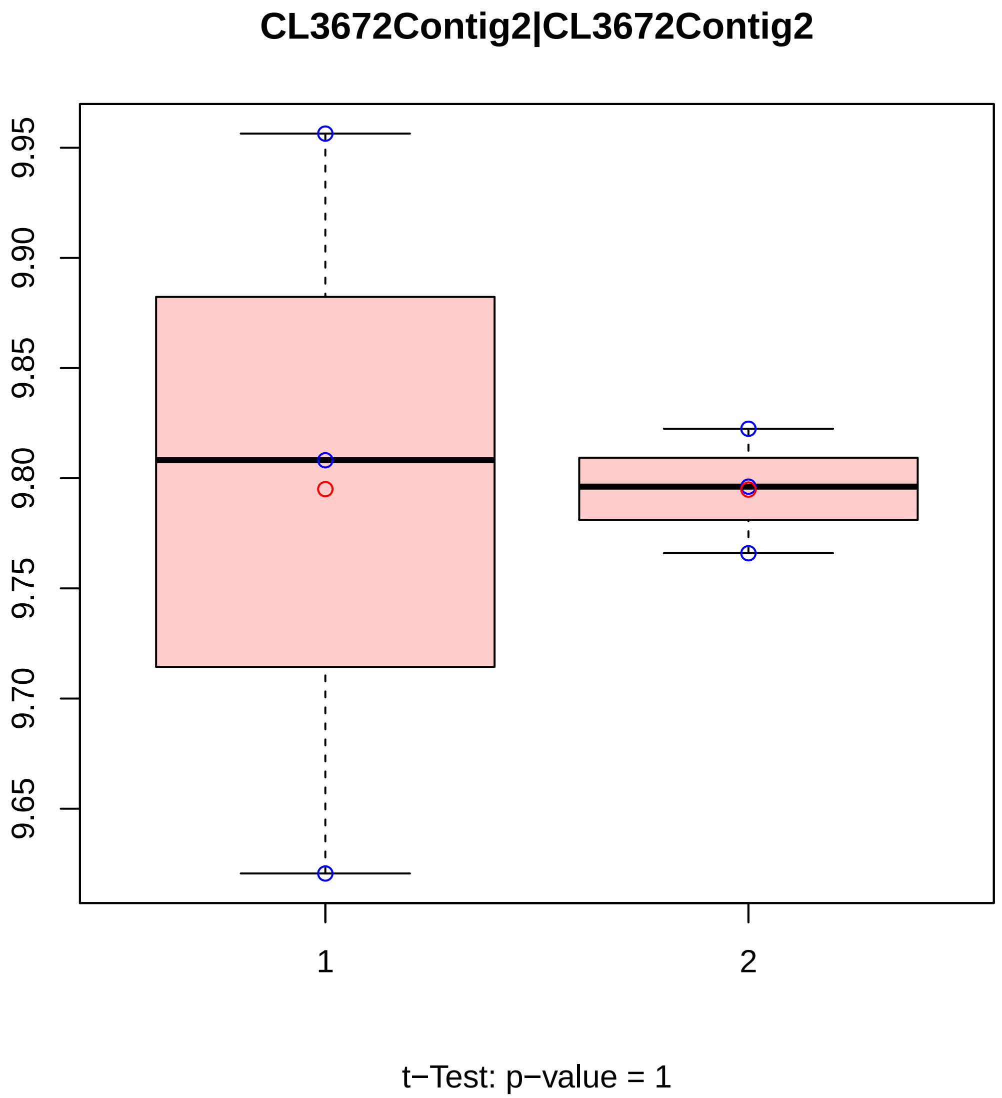

CL3672Contig2|CL3672Contig2
9.65
9.70
9.75
9.80
9.85
9.90
9.95
1
2
t−Test: p−value = 1

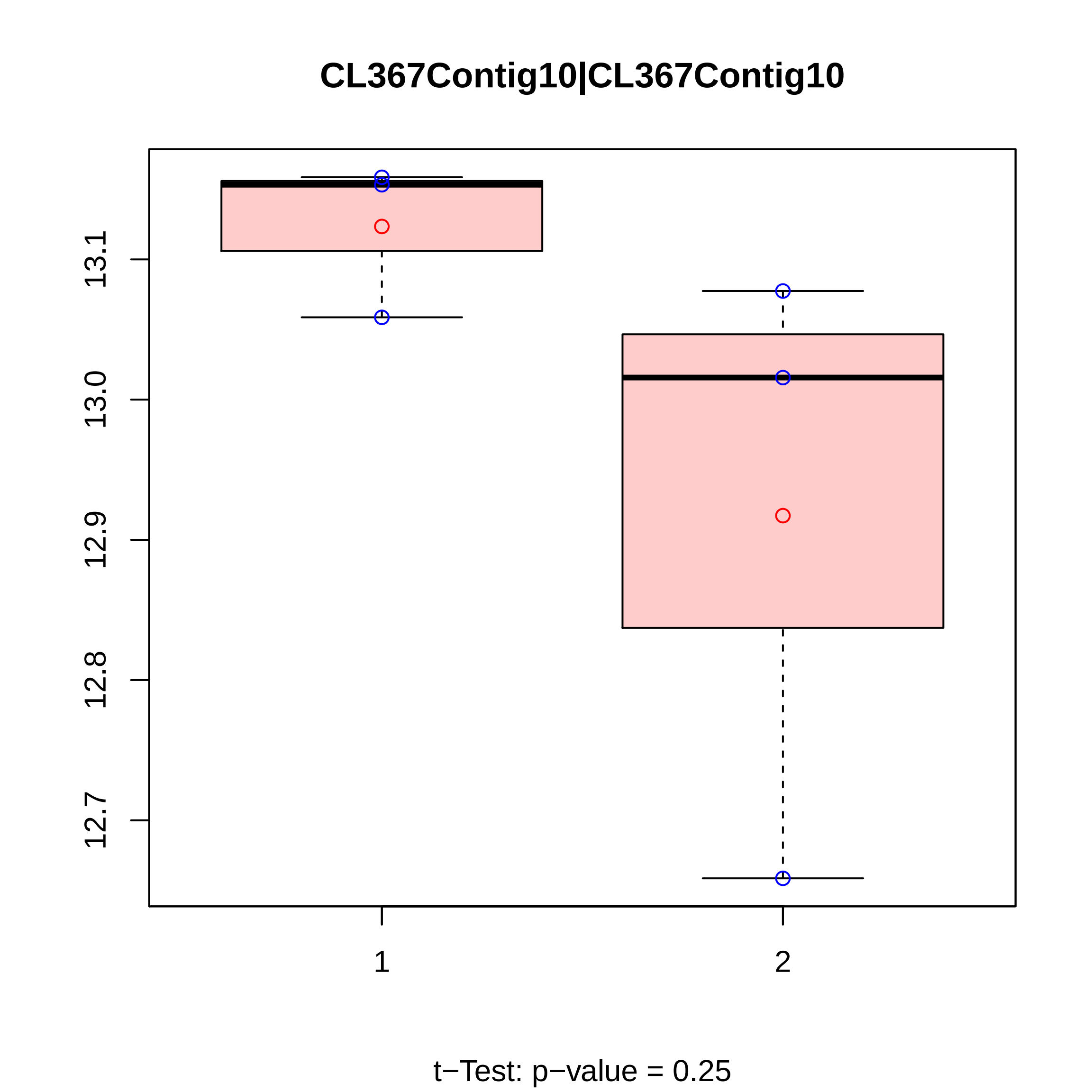

CL367Contig10|CL367Contig10
13.1
13.0
12.9
12.8
12.7
1
2
t−Test: p−value = 0.25

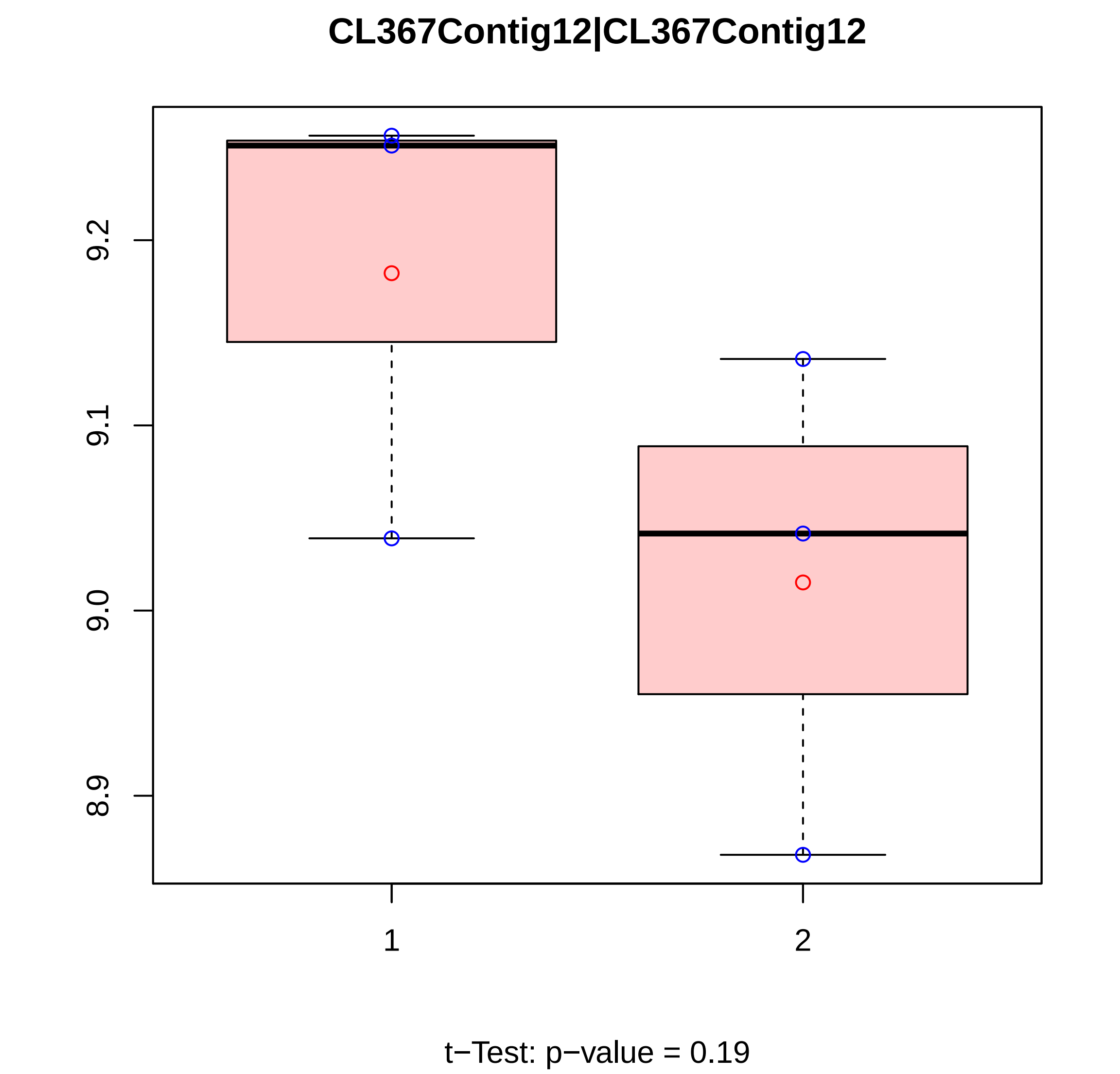

CL367Contig12|CL367Contig12
9.2
9.1
9.0
8.9
1
2
t−Test: p−value = 0.19

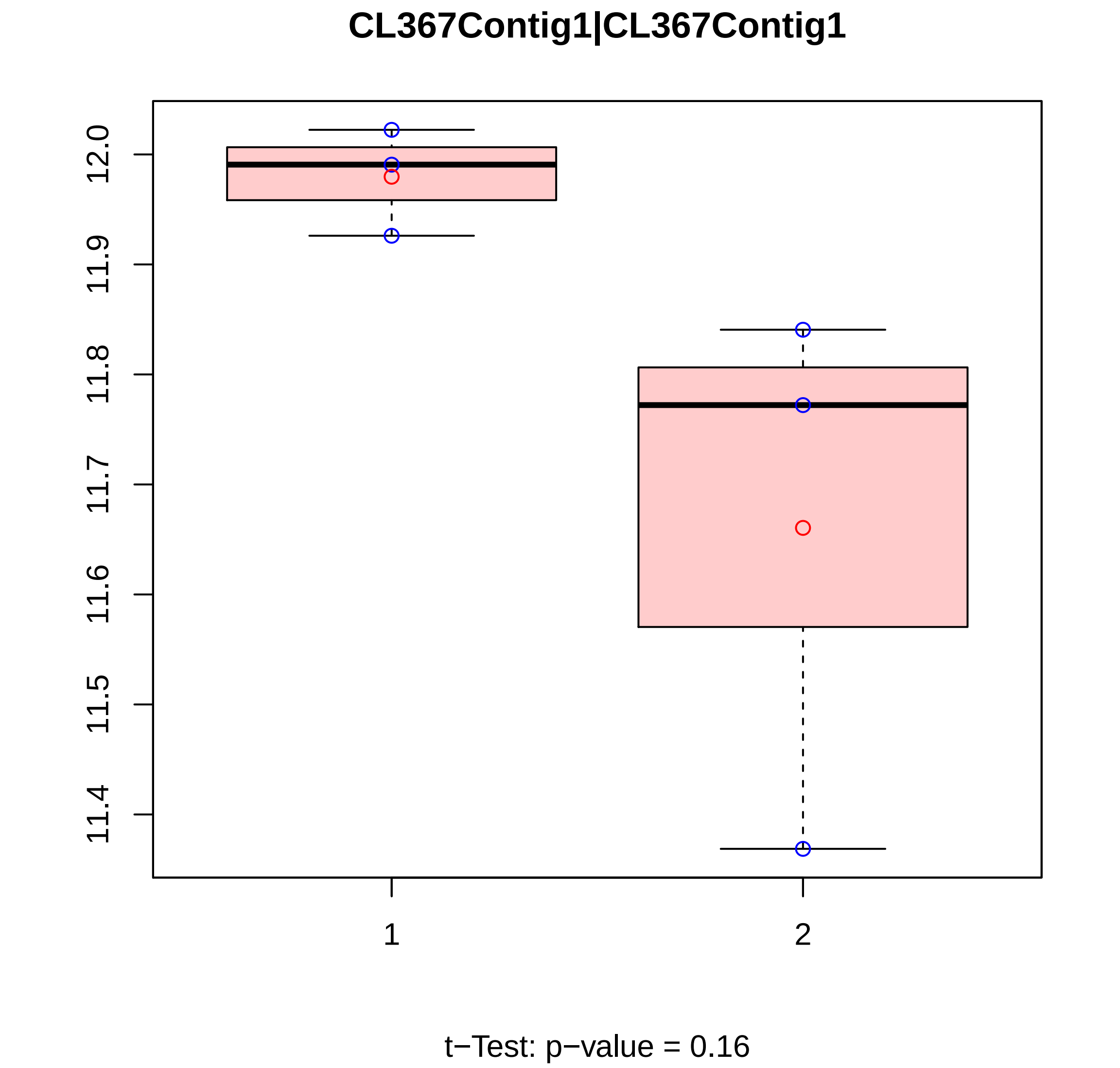
CL367Contig1|CL367Contig1
12.0
11.9
11.8
11.7
11.6
11.5
11.4
1
2
t−Test: p−value = 0.16

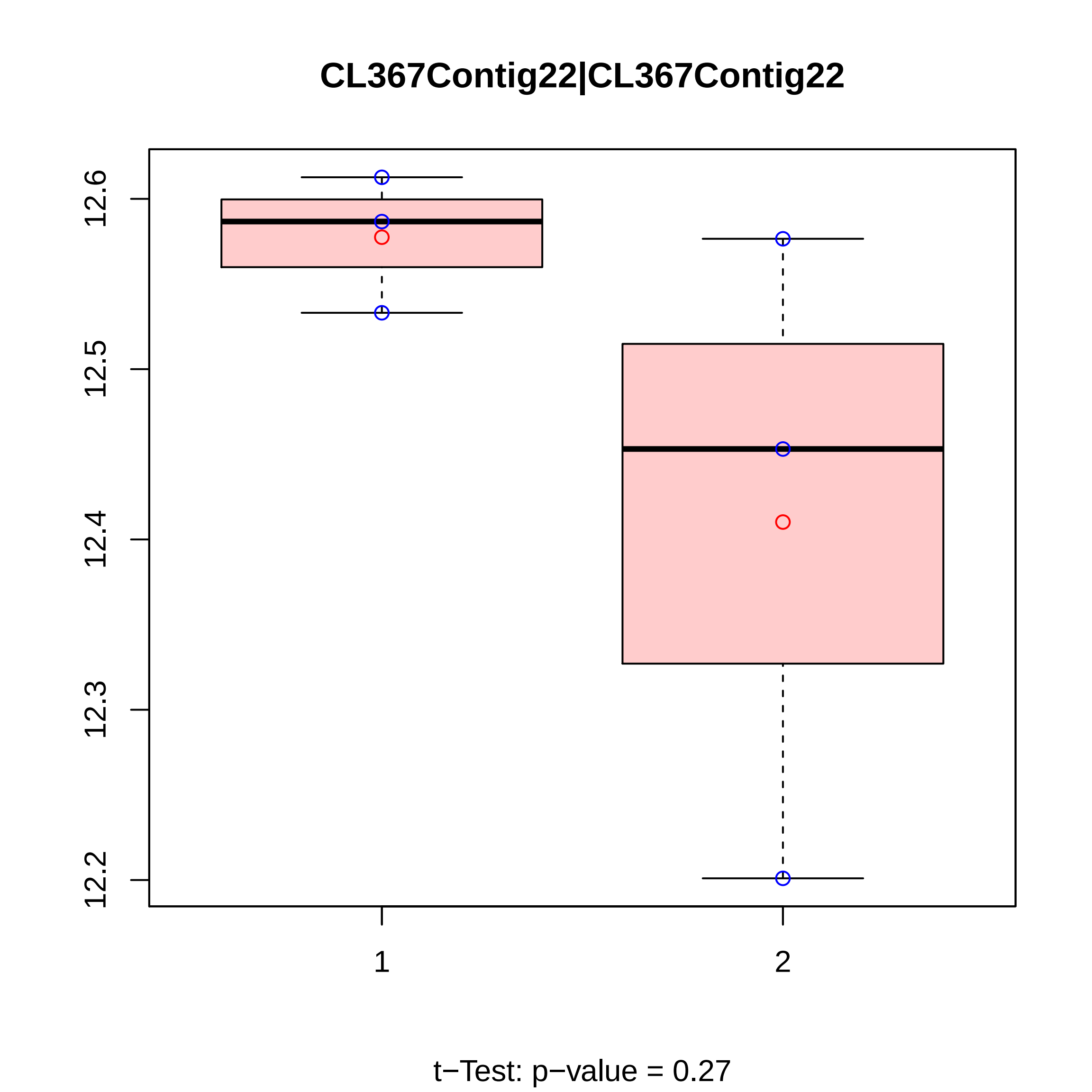

CL367Contig22|CL367Contig22
12.2
12.3
12.4
12.5
12.6
1
2
t−Test: p−value = 0.27

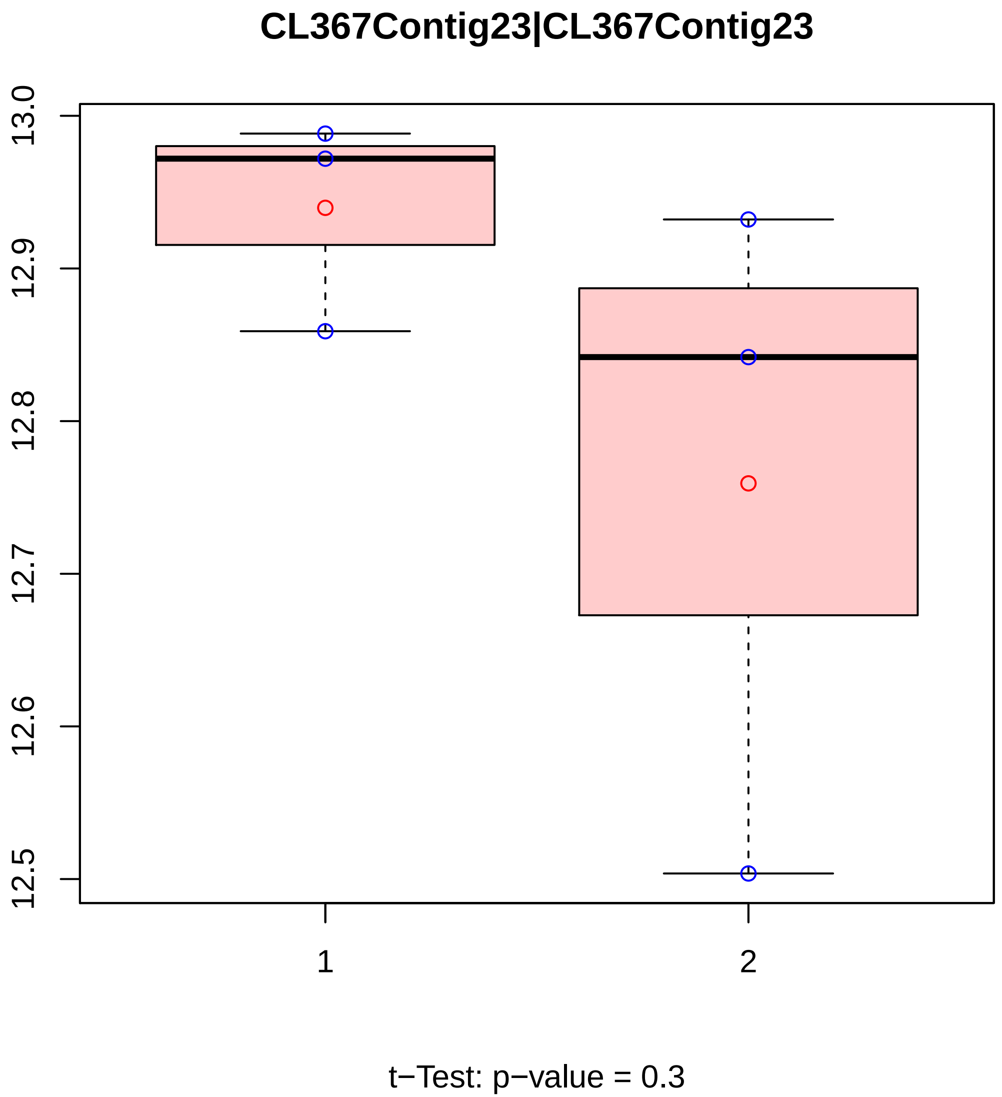
CL367Contig23|CL367Contig23
13.0
12.9
12.8
12.7
12.6
12.5
1
2
t−Test: p−value = 0.3

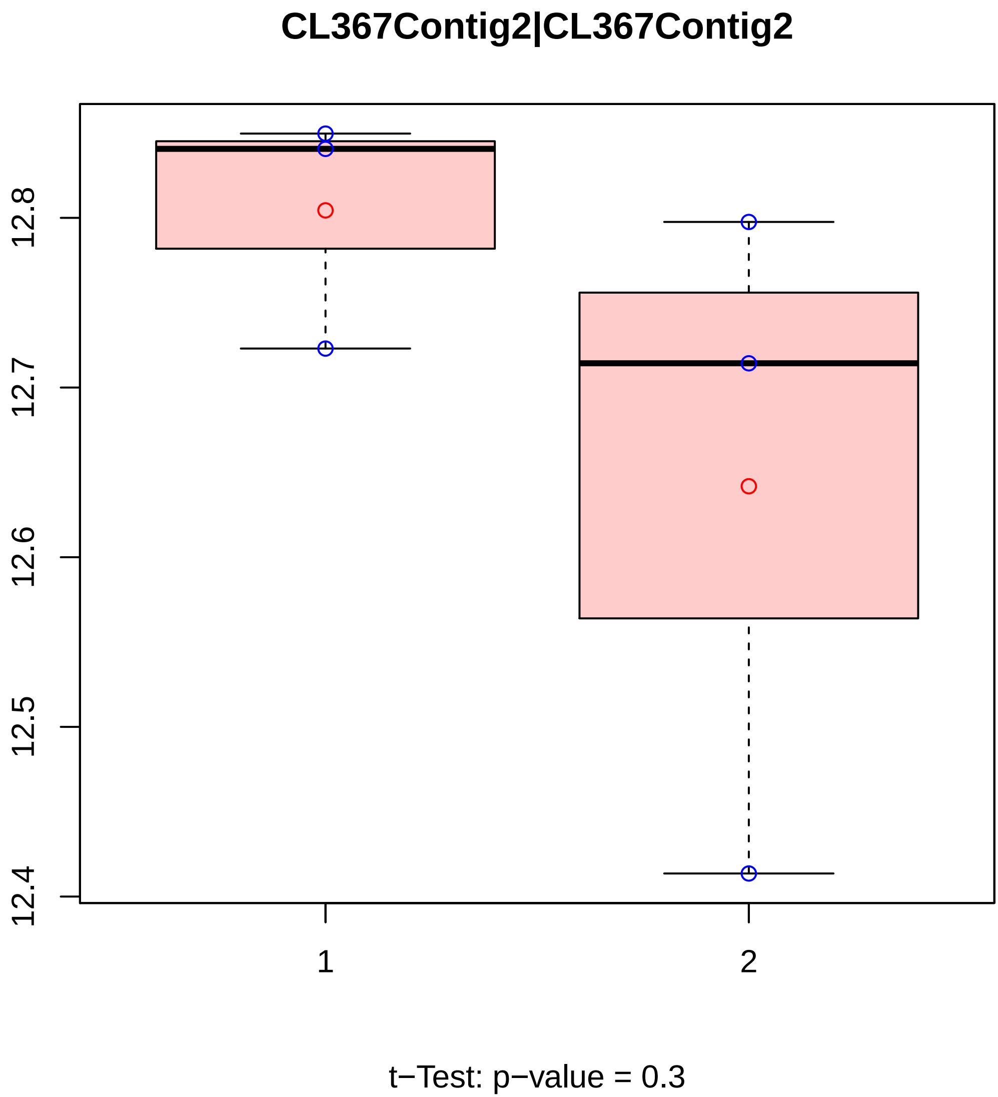
CL367Contig2|CL367Contig2
12.4
12.5
12.6
12.7
12.8
1
2
t−Test: p−value = 0.3

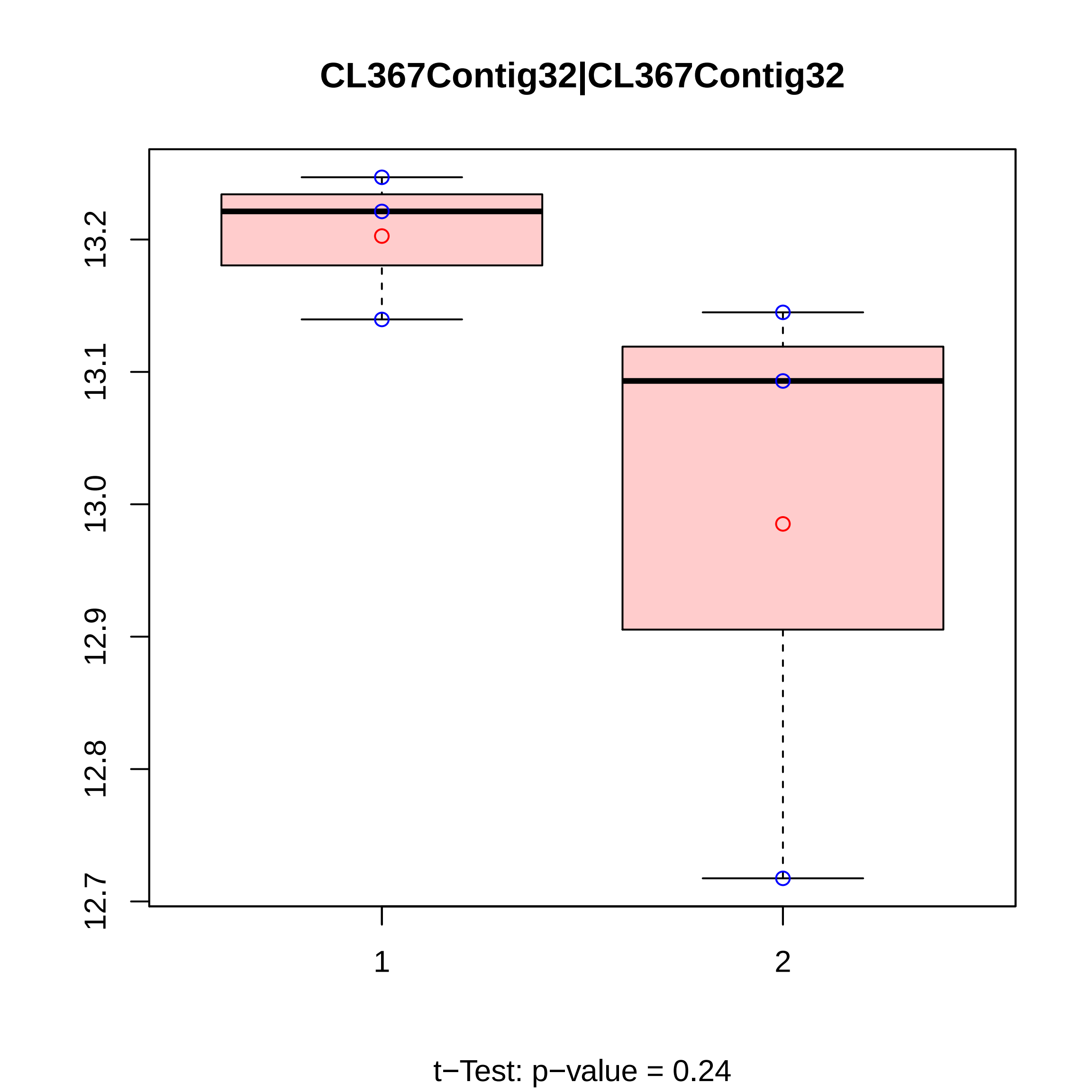

CL367Contig32|CL367Contig32
13.2
13.1
13.0
12.9
12.8
12.7
1
2
t−Test: p−value = 0.24

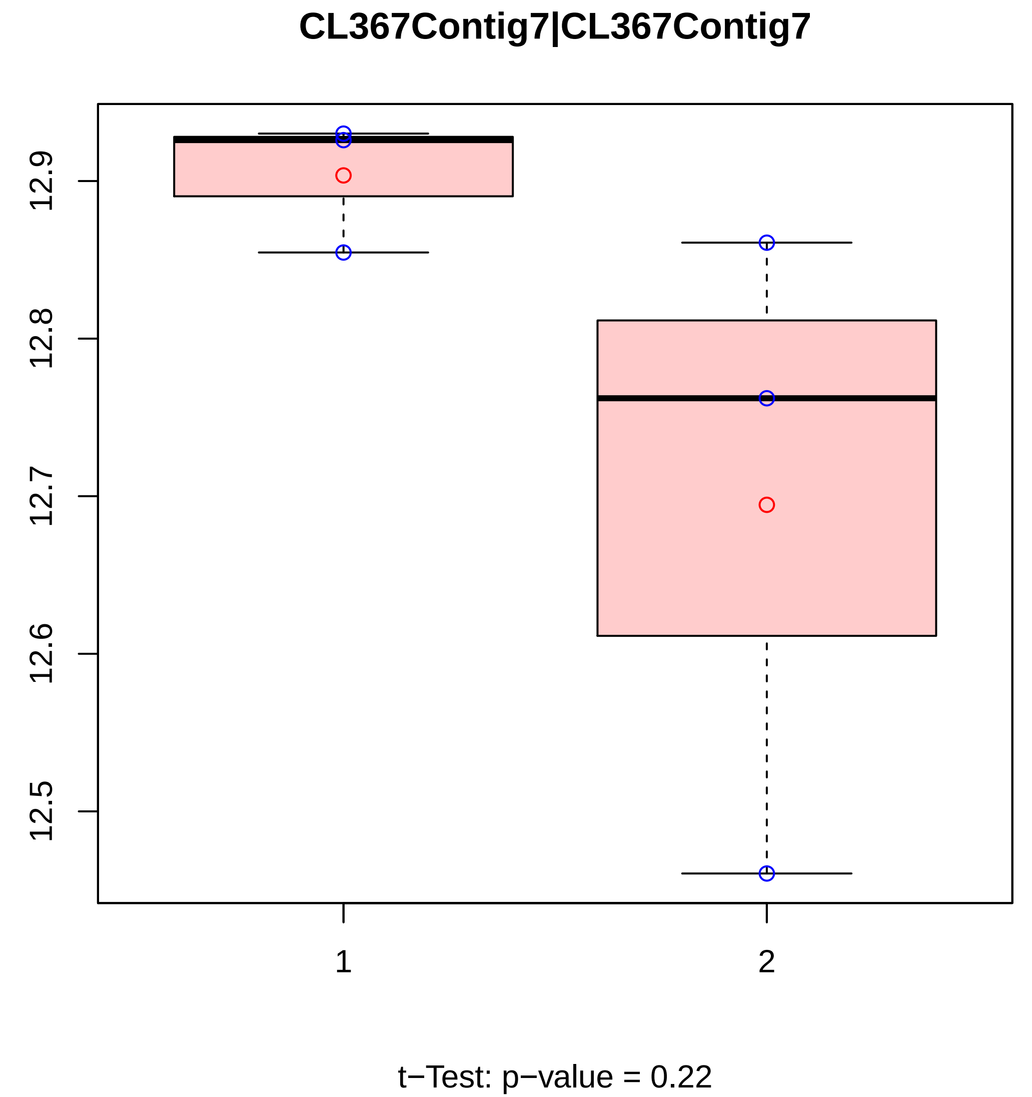
CL367Contig7|CL367Contig7
12.5
12.6
12.7
12.8
12.9
1
2
t−Test: p−value = 0.22

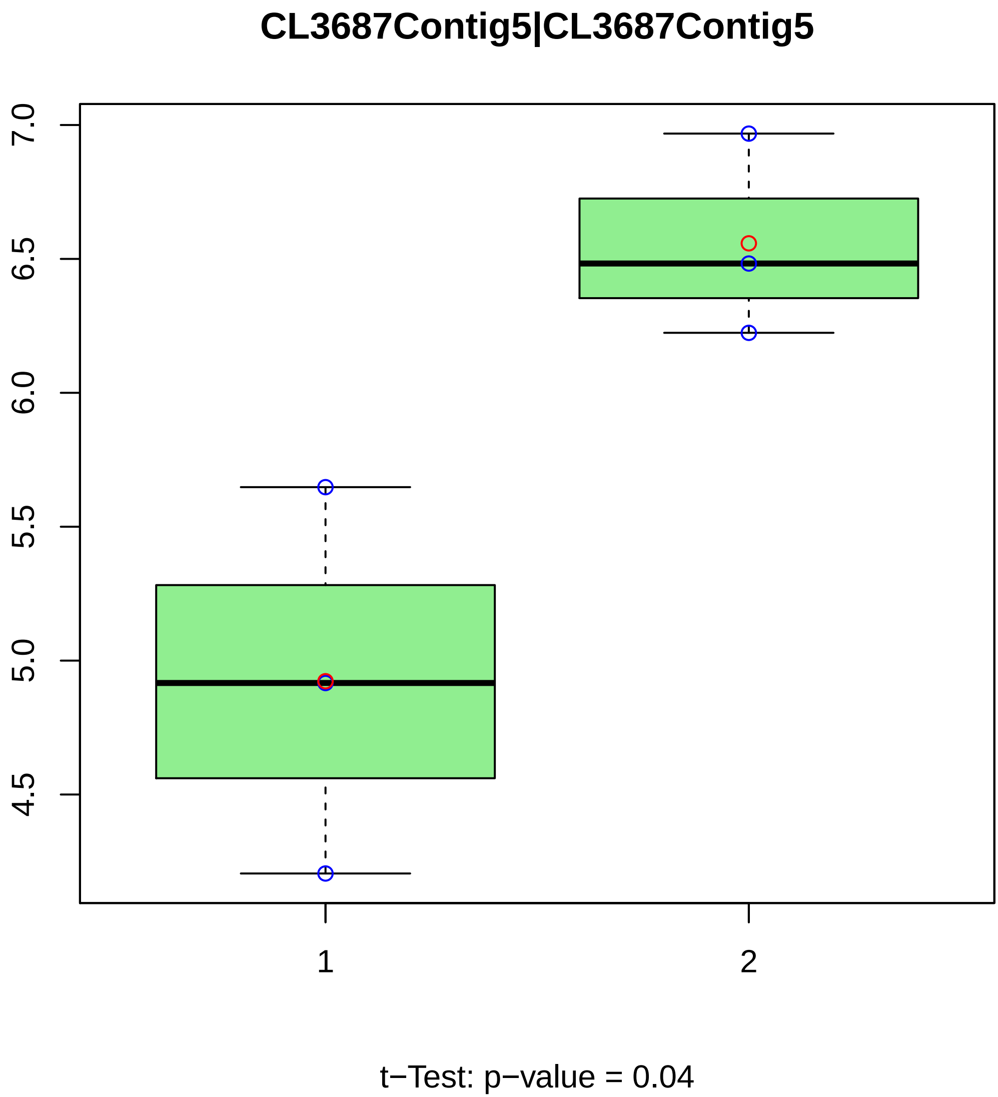

CL3687Contig5|CL3687Contig5
7.0
6.5
6.0
5.5
5.0
4.5
1
2
t−Test: p−value = 0.04

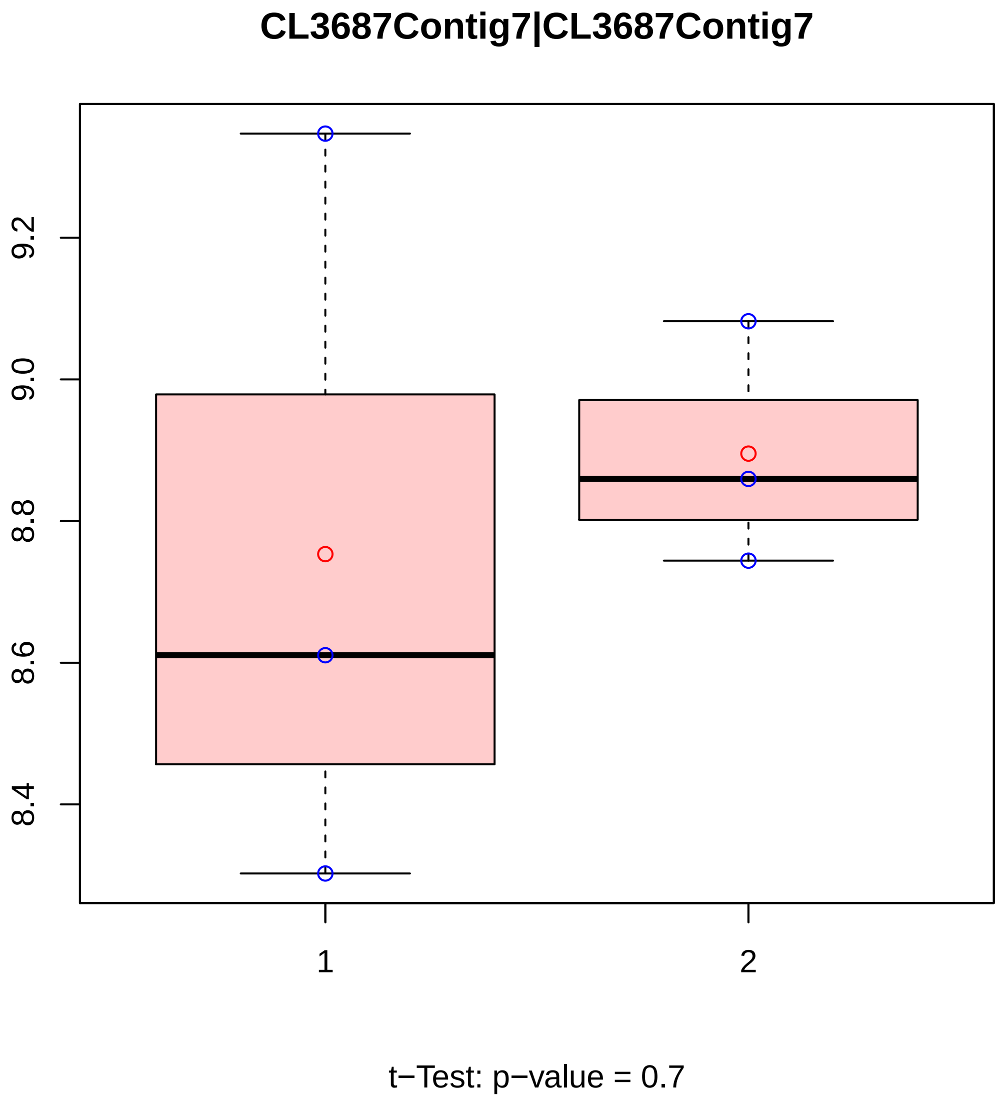

CL3687Contig7|CL3687Contig7
9.2
9.0
8.8
8.6
8.4
1
2
t−Test: p−value = 0.7

CL368Contig5|CL368Contig5

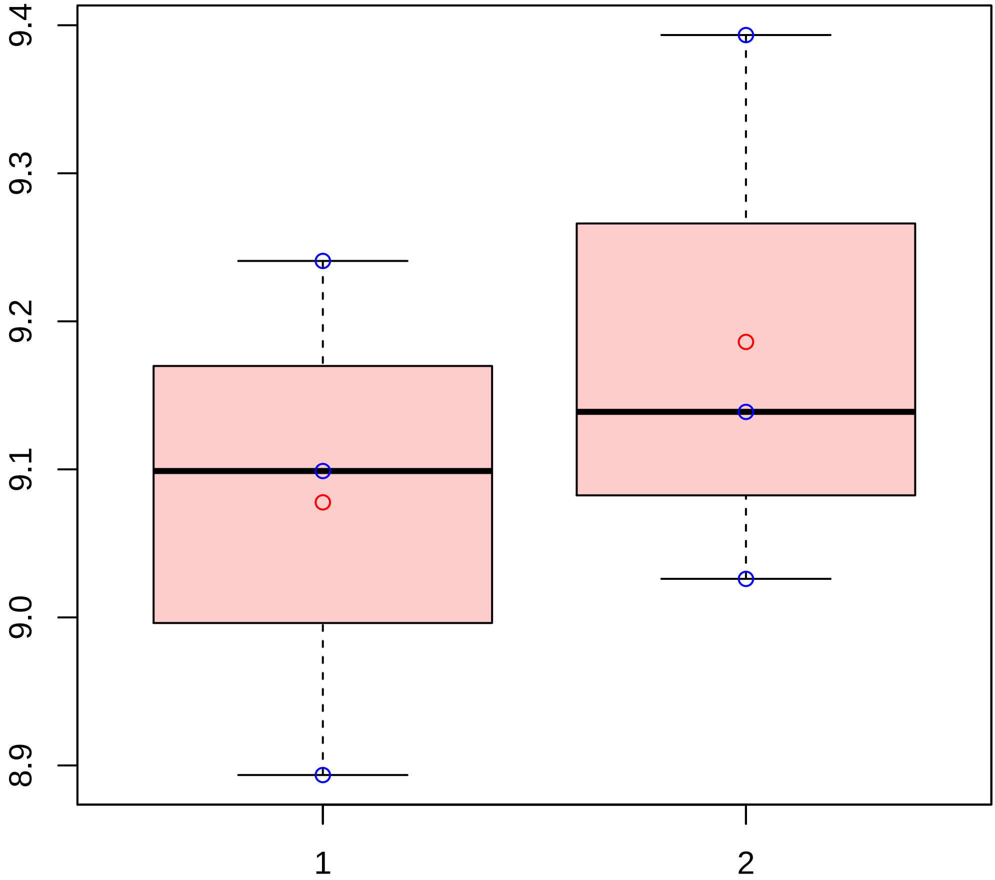


t−Test: p−value = 0.51

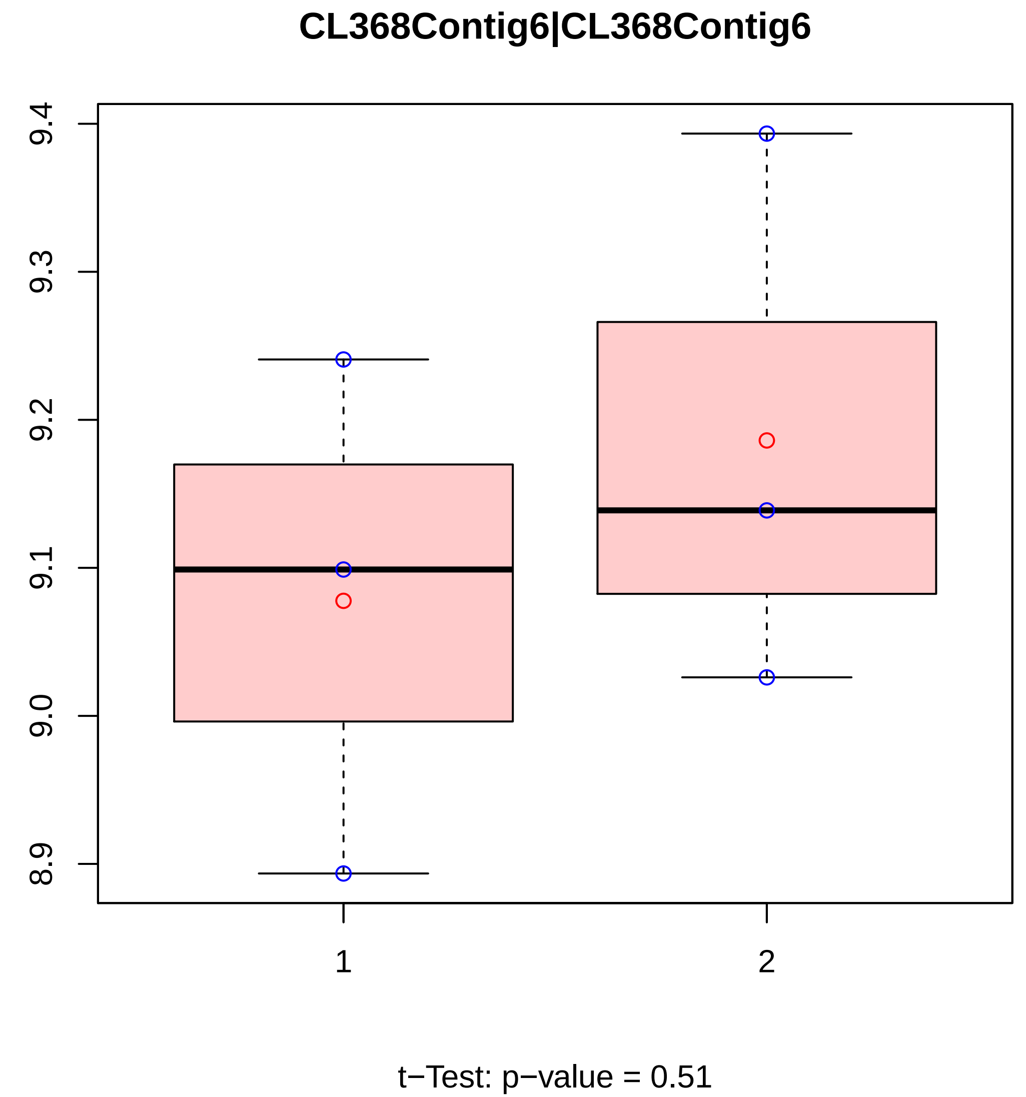
CL368Contig6|CL368Contig6
9.4
9.3
9.2
9.1
9.0
8.9
1
2
t−Test: p−value = 0.51

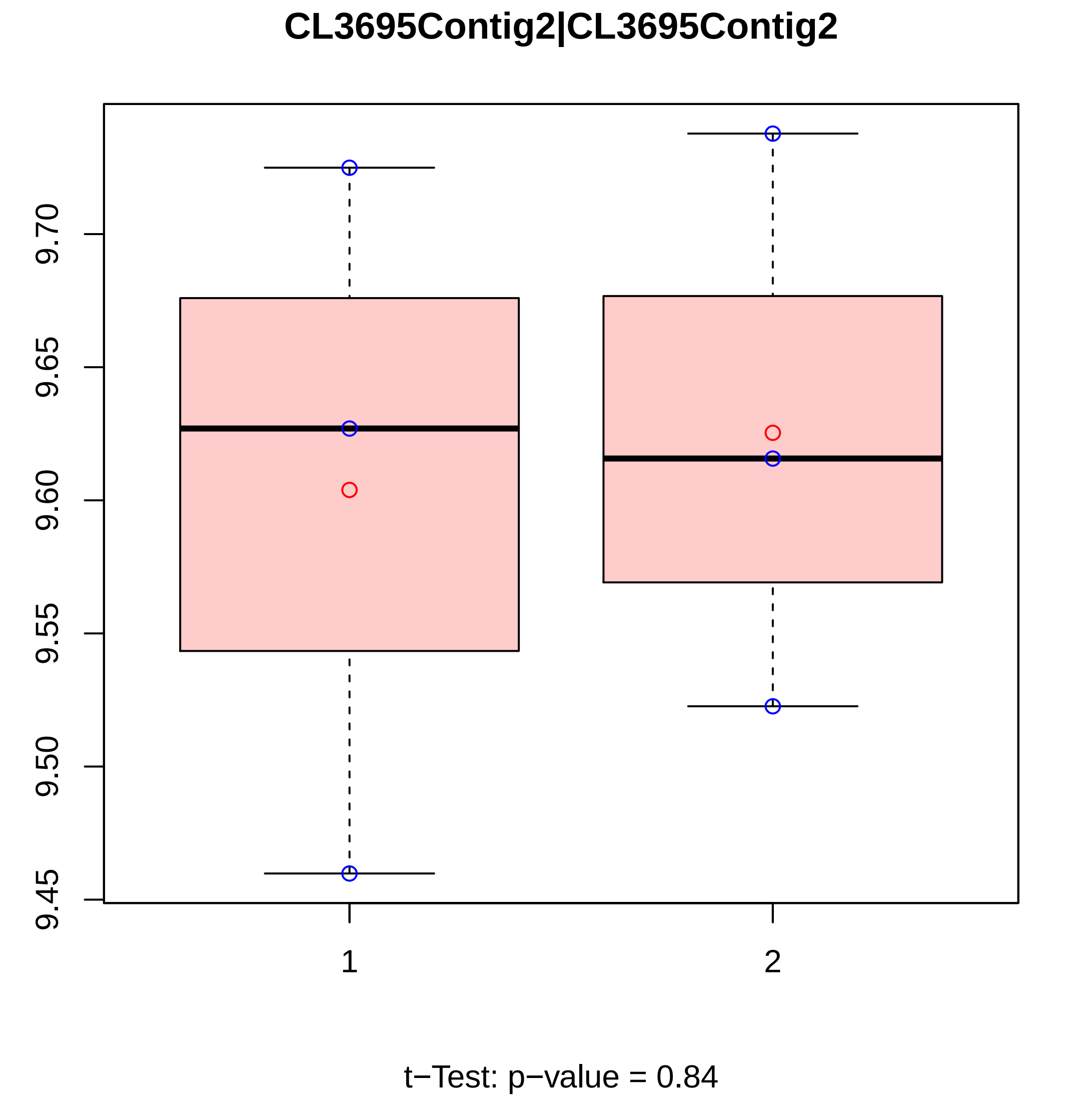
CL3695Contig2|CL3695Contig2
9.45
9.50
9.55
9.60
9.65
9.70
1
2
t−Test: p−value = 0.84

# CL3697Contig4|CL3697Contig4

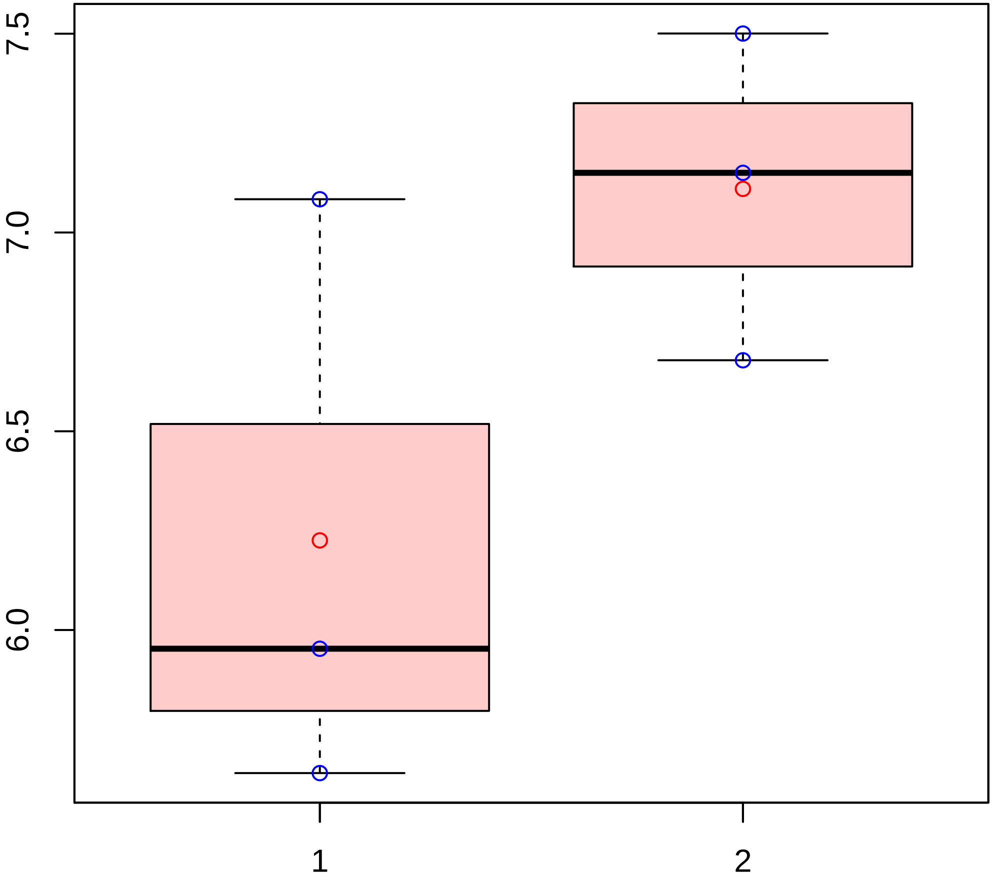


t−Test: p−value = 0.17

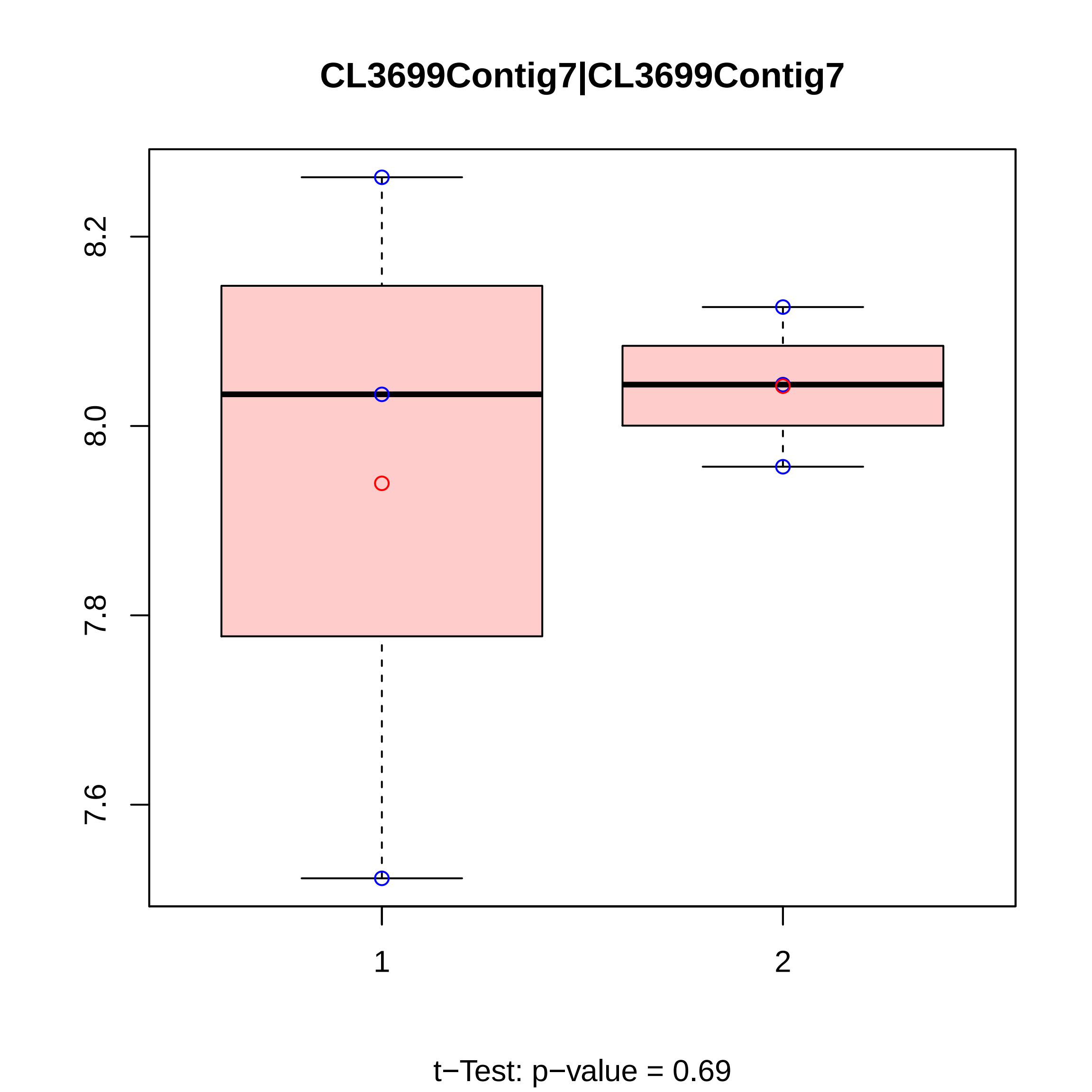

CL3699Contig7|CL3699Contig7
8.2
8.0
7.8
7.6
1
2
t−Test: p−value = 0.69

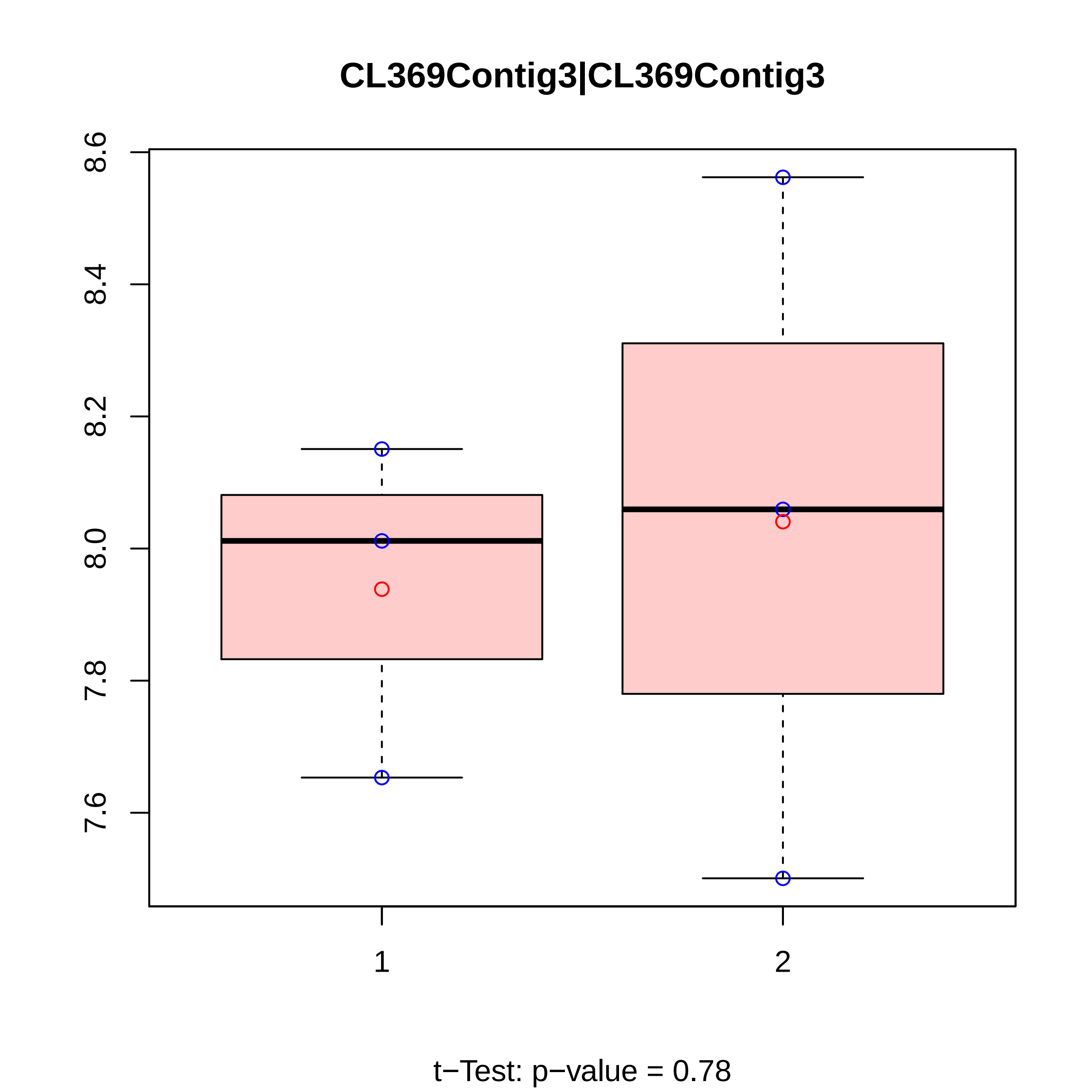

CL369Contig3|CL369Contig3
8.6
8.4
8.2
8.0
7.8
7.6
1
2
t−Test: p−value = 0.78

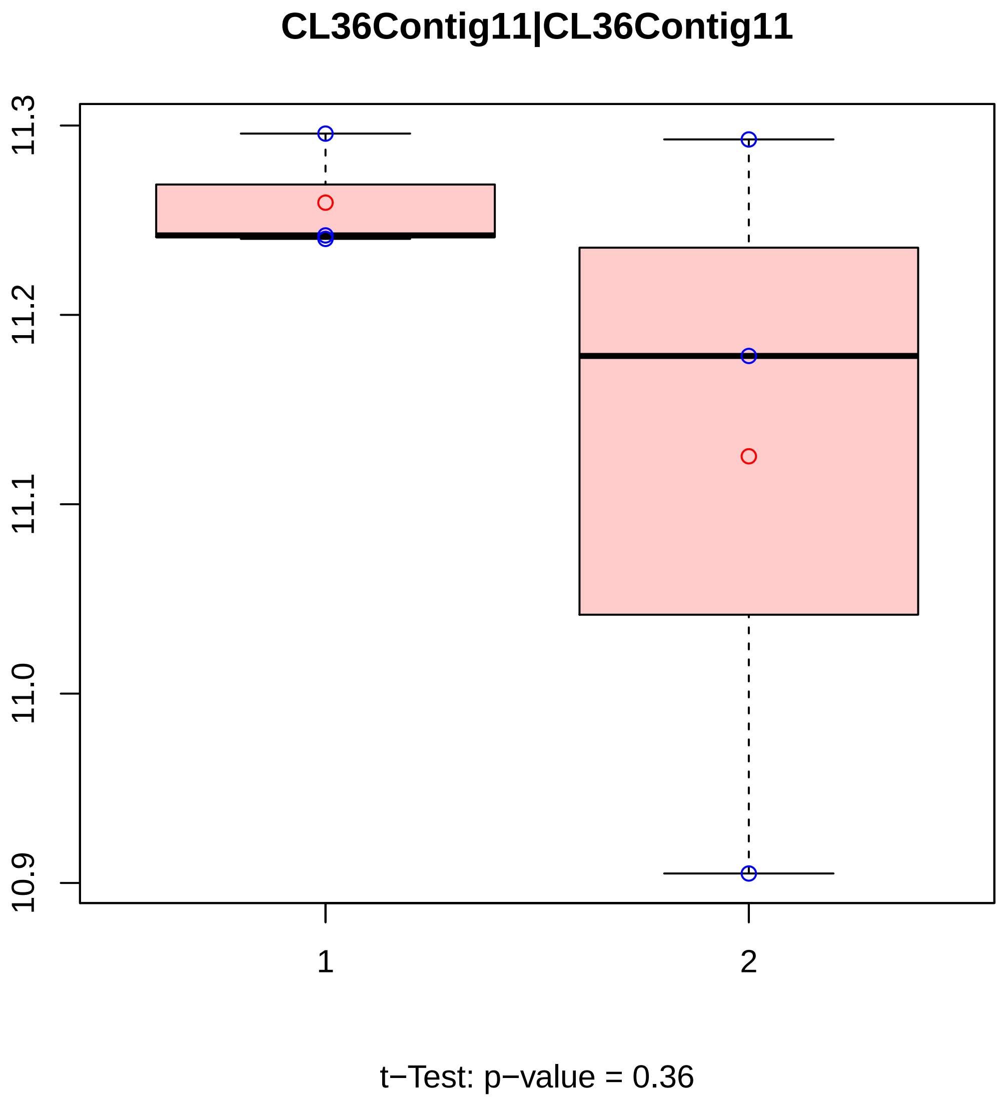
CL36Contig11|CL36Contig11
11.3
11.2
11.1
11.0
10.9
1
2
t−Test: p−value = 0.36

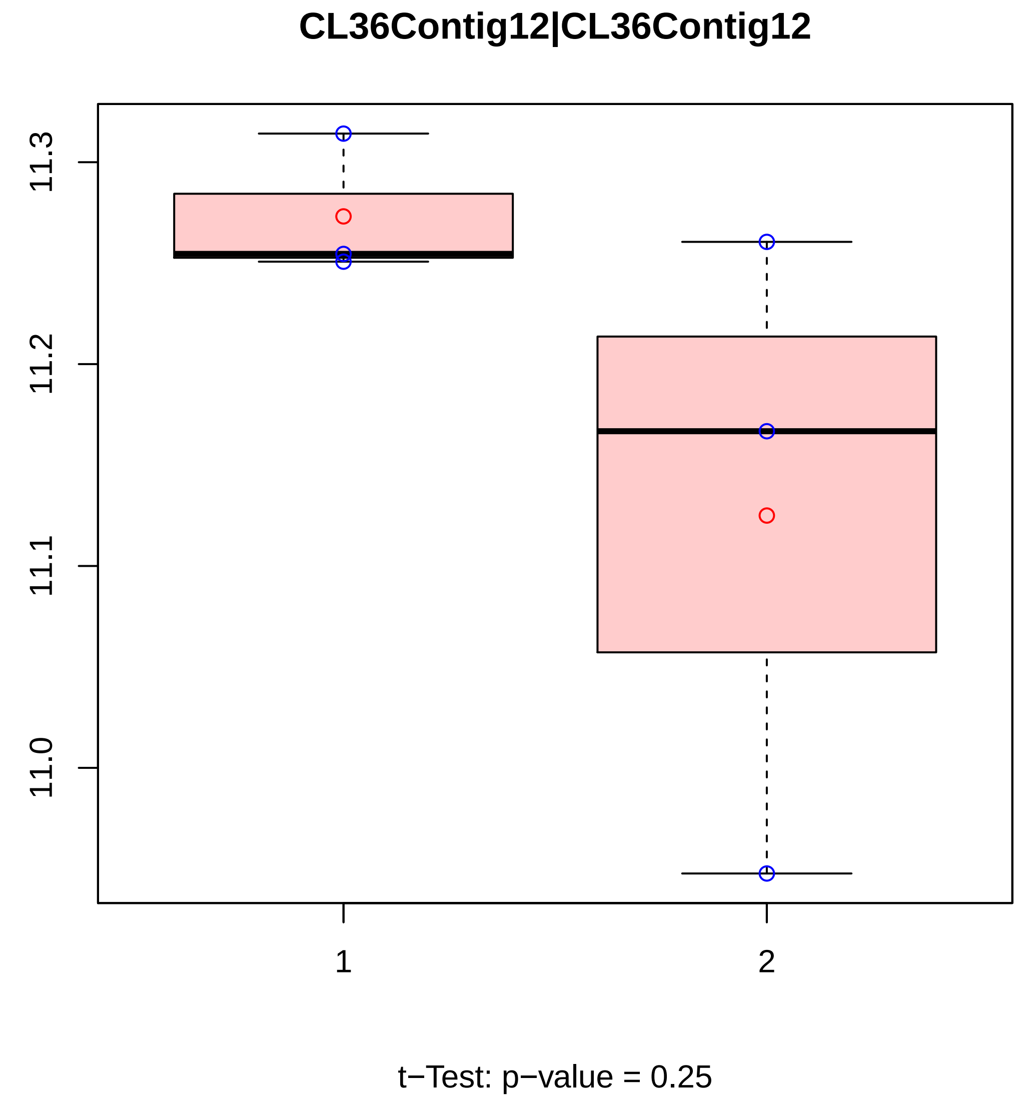
CL36Contig12|CL36Contig12
11.3
11.2
11.1
11.0
1
2
t−Test: p−value = 0.25

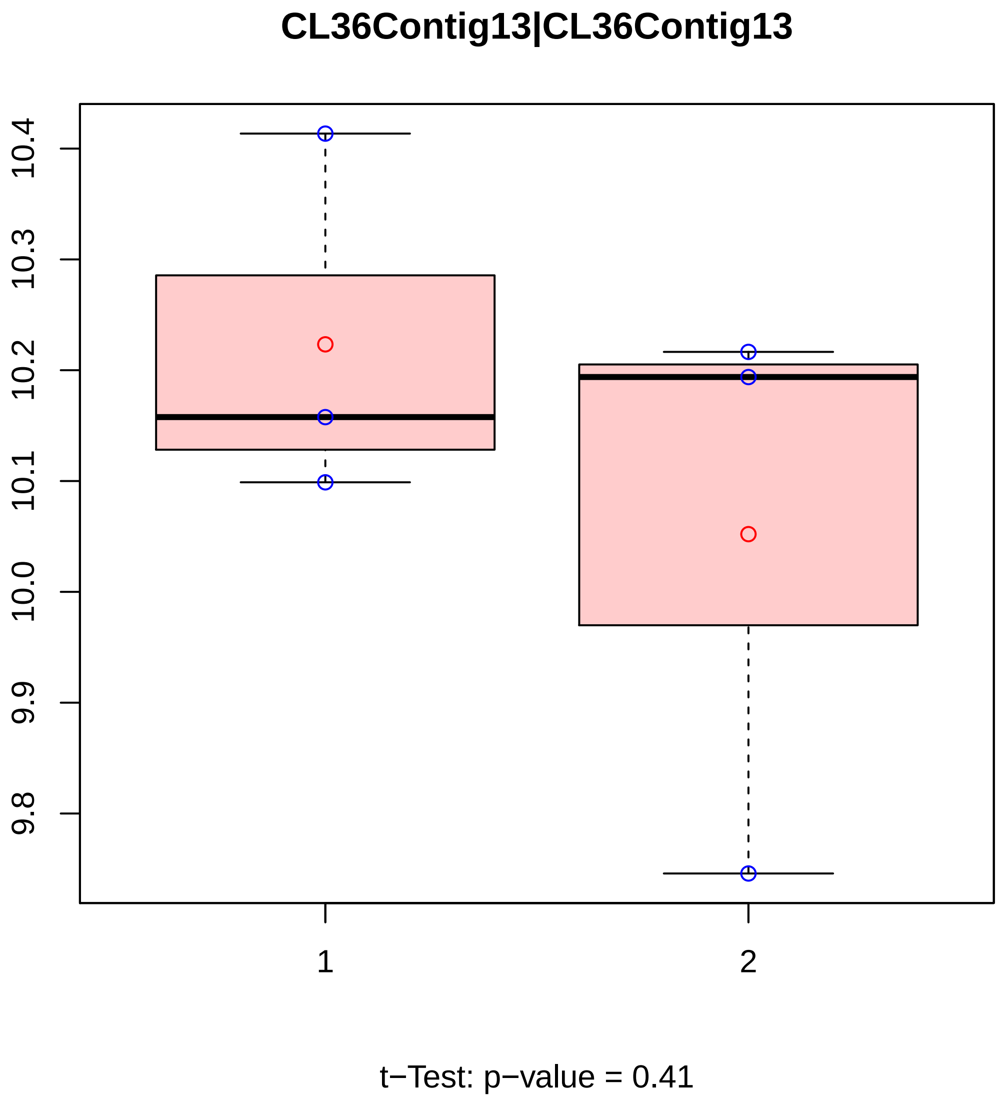
CL36Contig13|CL36Contig13
10.4
10.3
10.2
10.1
10.0
9.9
9.8
1
2
t−Test: p−value = 0.41

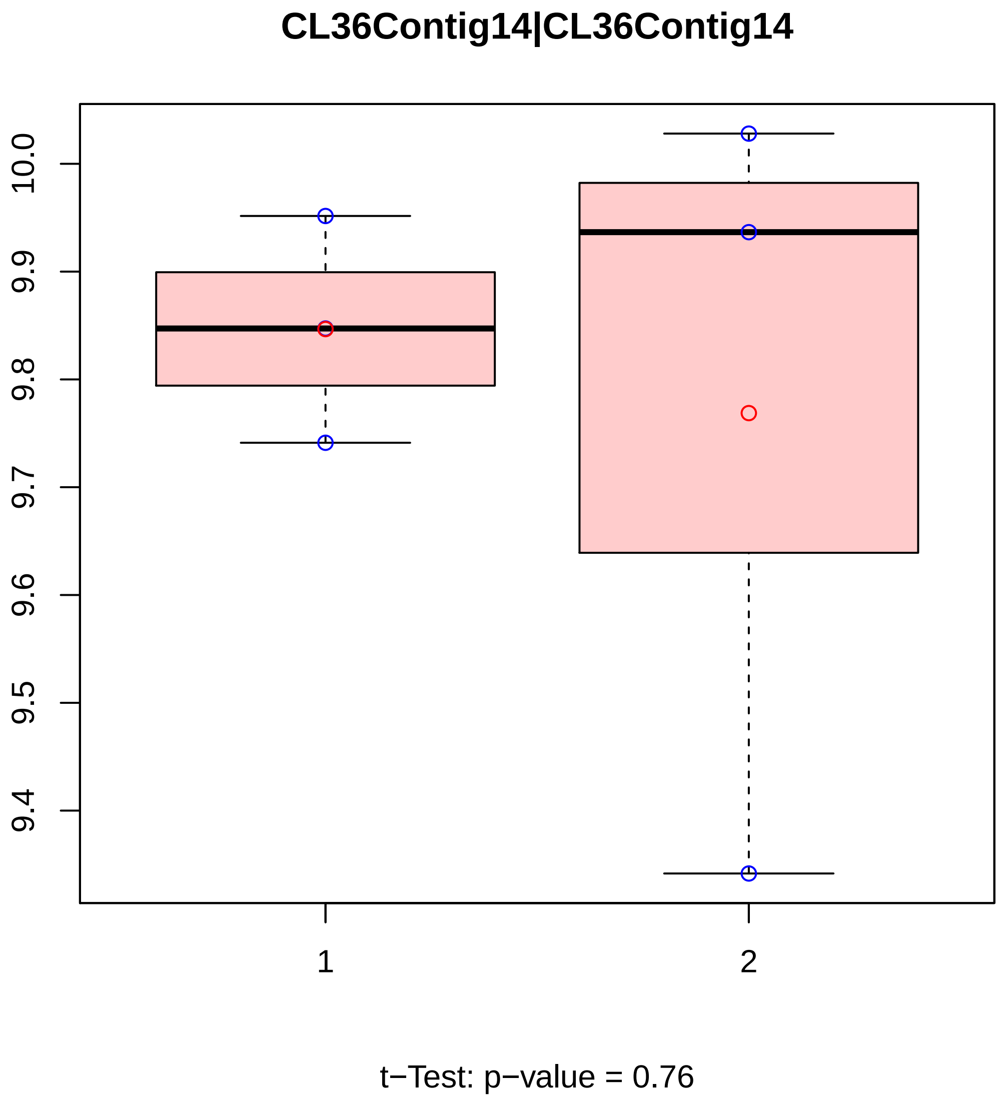
CL36Contig14|CL36Contig14
10.0
9.9
9.8
9.7
9.6
9.5
9.4
1
2
t−Test: p−value = 0.76

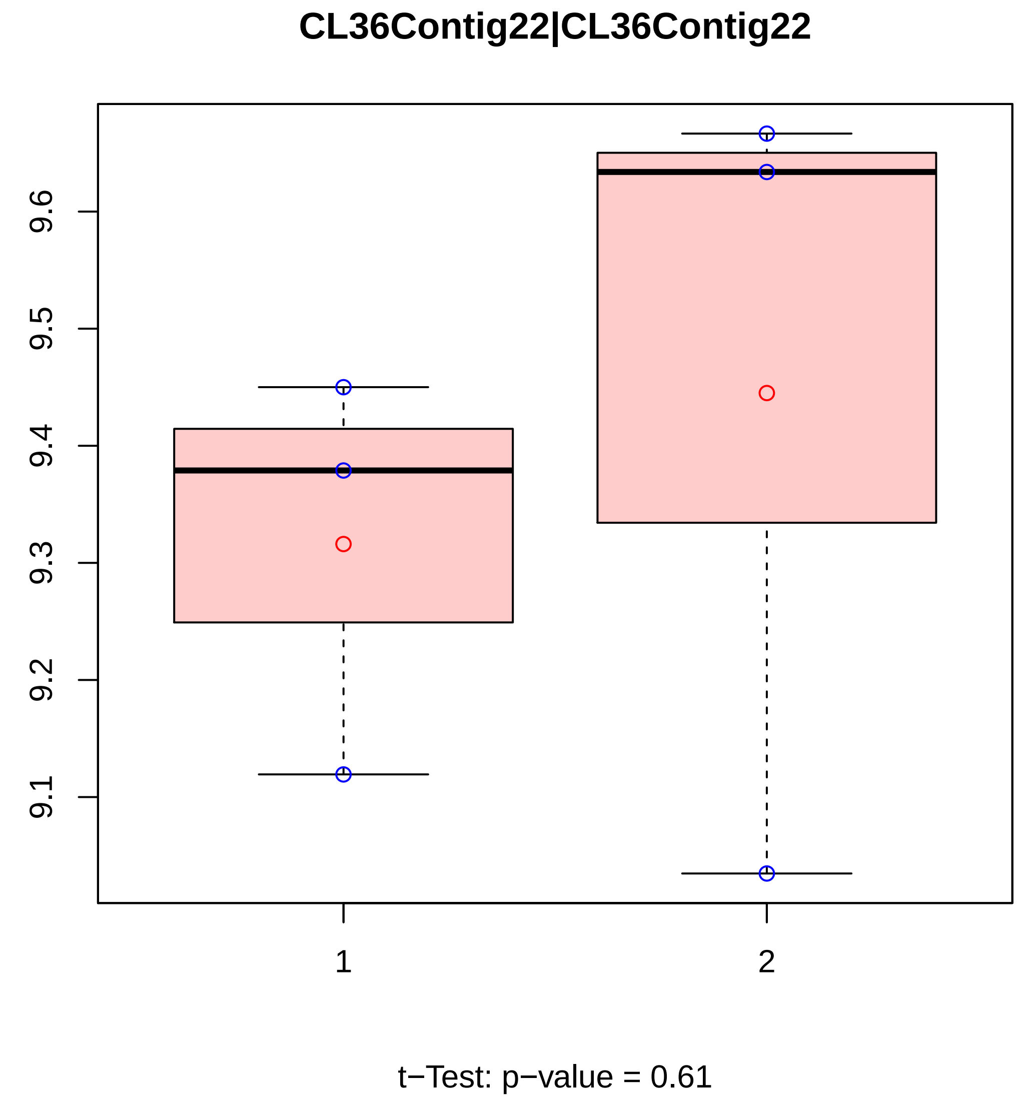
CL36Contig22|CL36Contig22
9.1
9.2
9.3
9.4
9.5
9.6
1
2
t−Test: p−value = 0.61

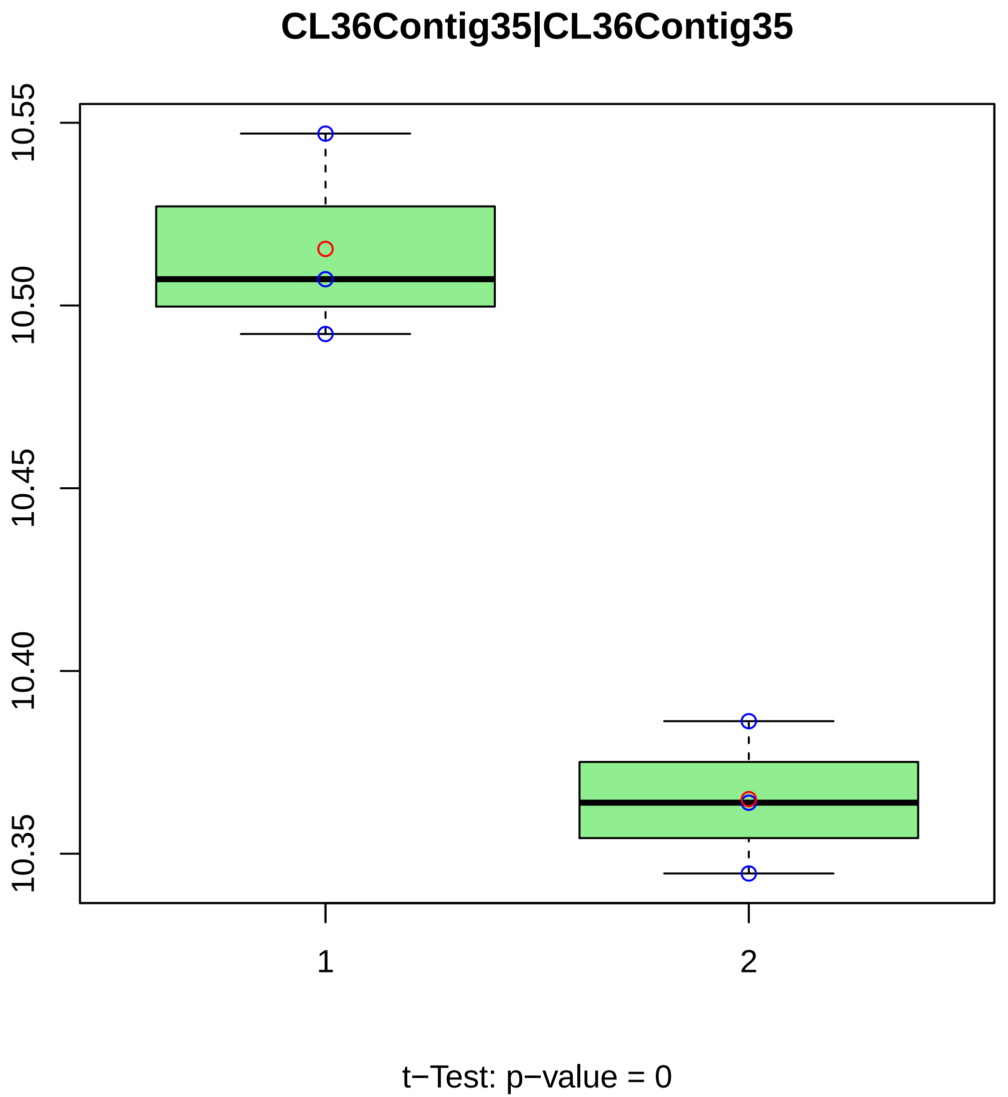

CL36Contig35|CL36Contig35
10.55
10.50
10.45
10.40
10.35
1
2
t−Test: p−value = 0

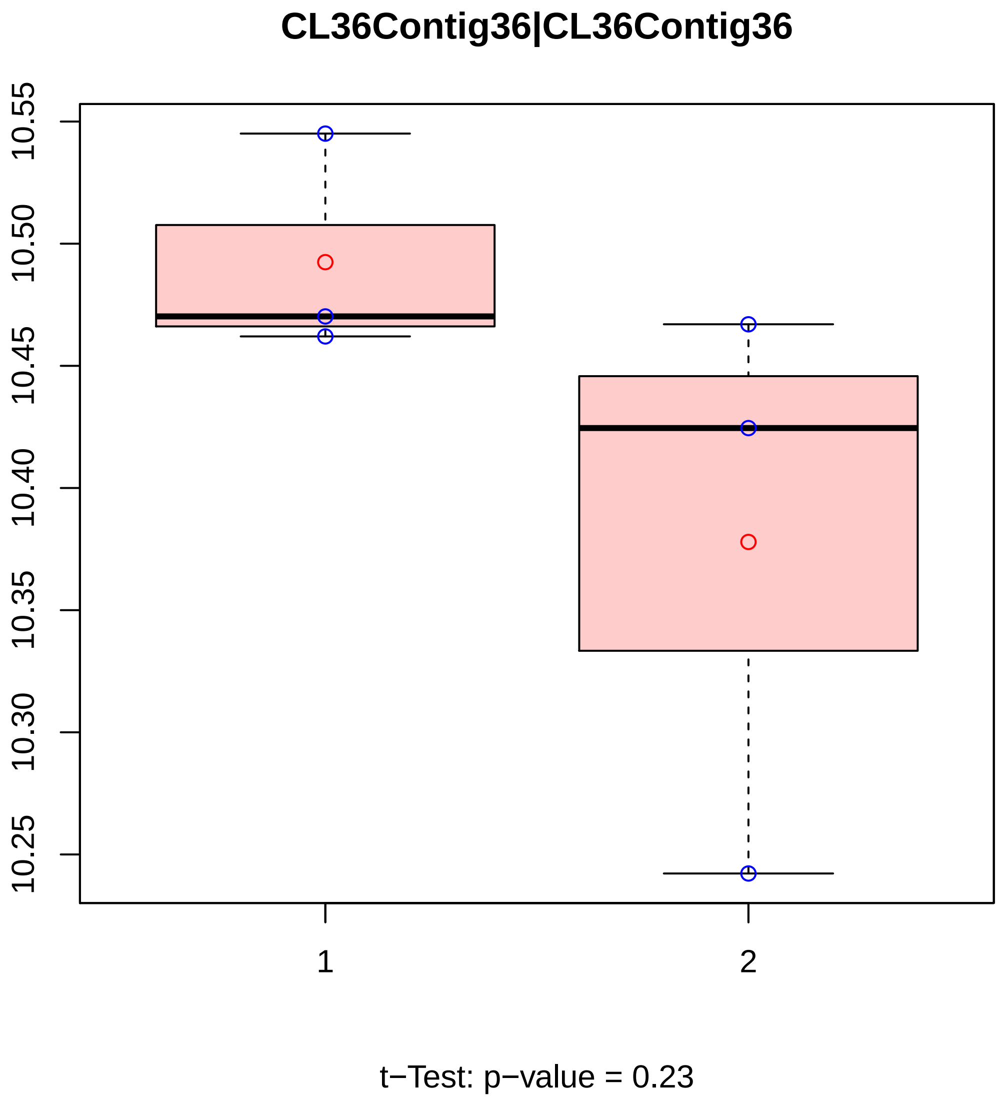
CL36Contig36|CL36Contig36
10.25
10.30
10.35
10.40
10.45
10.50
10.55
1
2
t−Test: p−value = 0.23

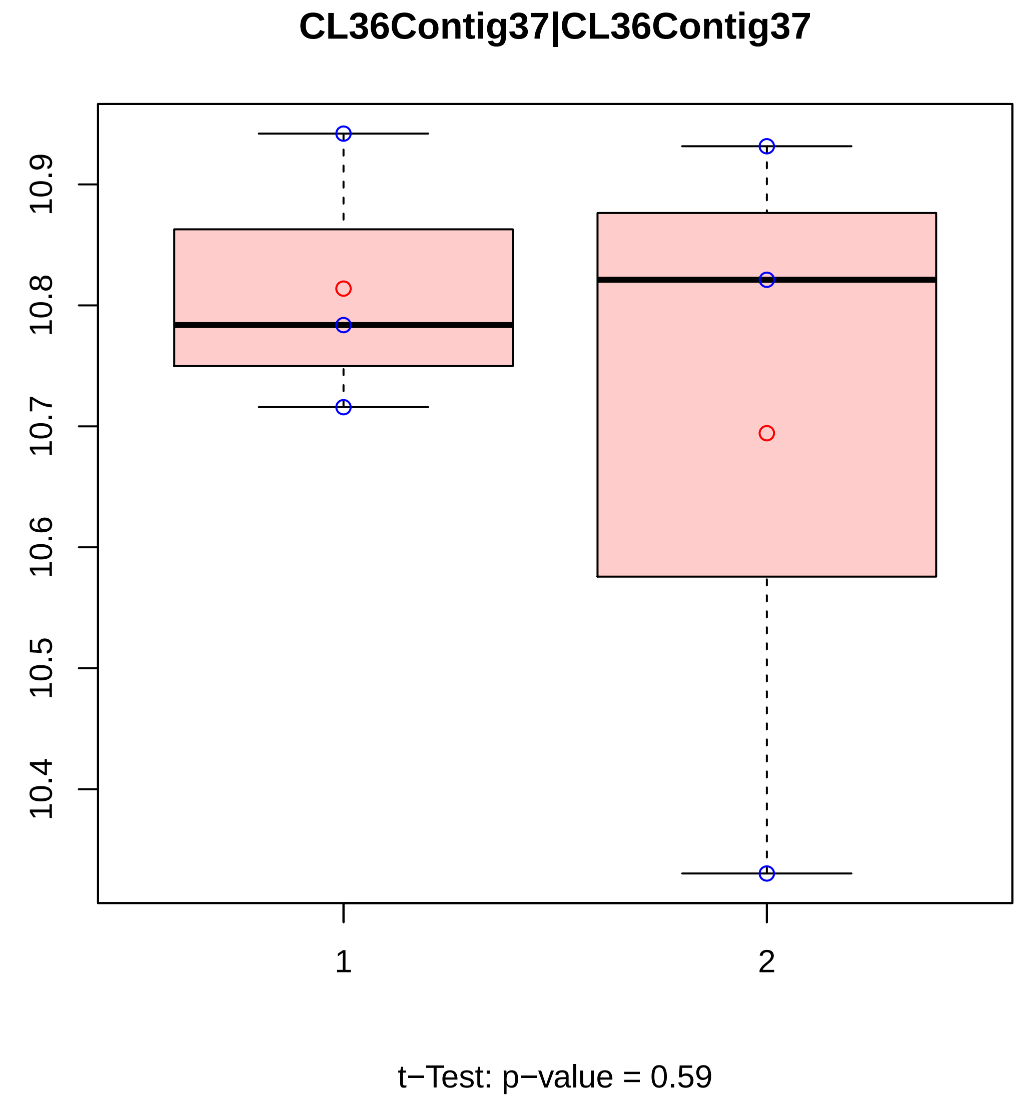

CL36Contig37|CL36Contig37
10.4
10.5
10.6
10.7
10.8
10.9
1
2
t−Test: p−value = 0.59

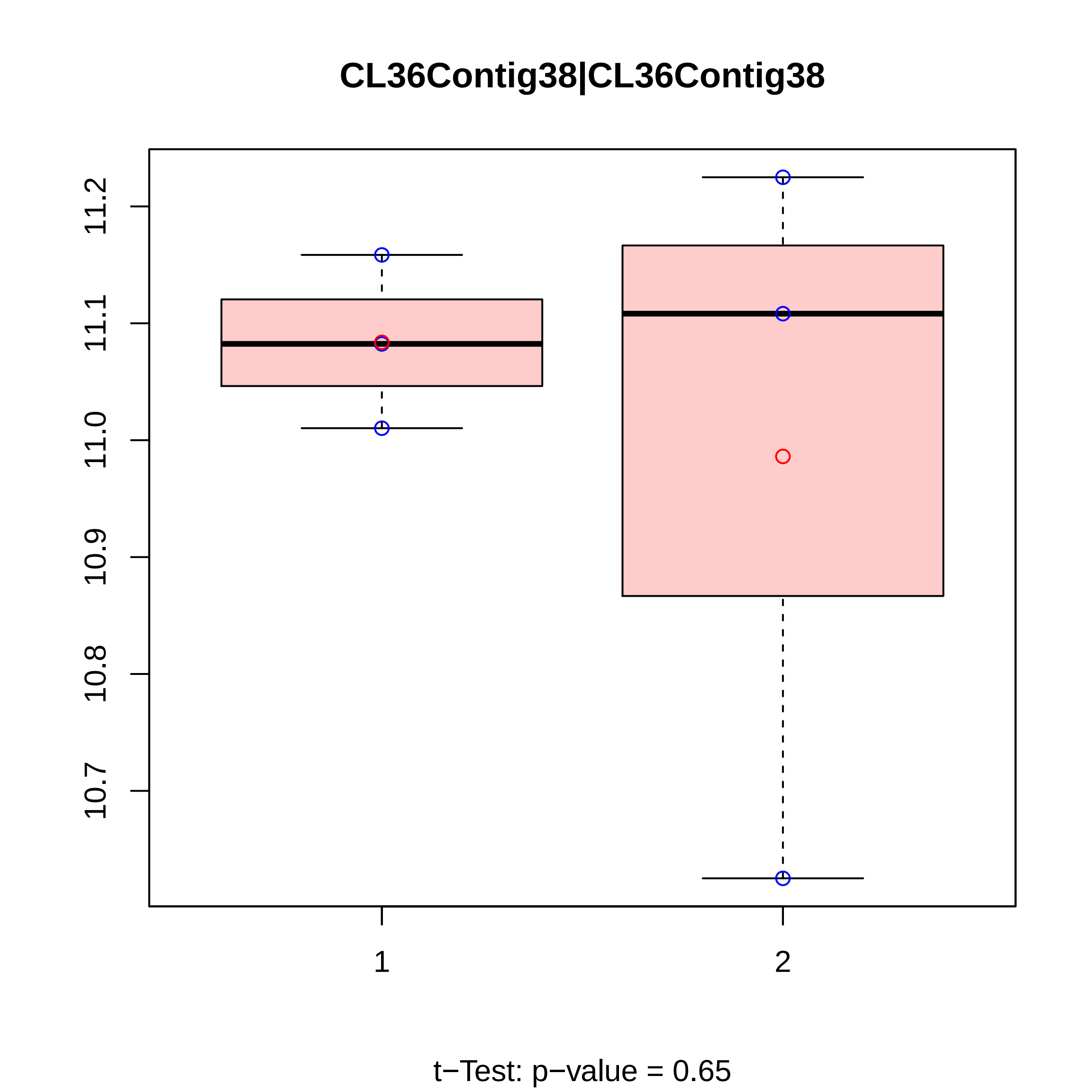

CL36Contig38|CL36Contig38
11.2
11.1
11.0
10.9
10.8
10.7
1
2
t−Test: p−value = 0.65

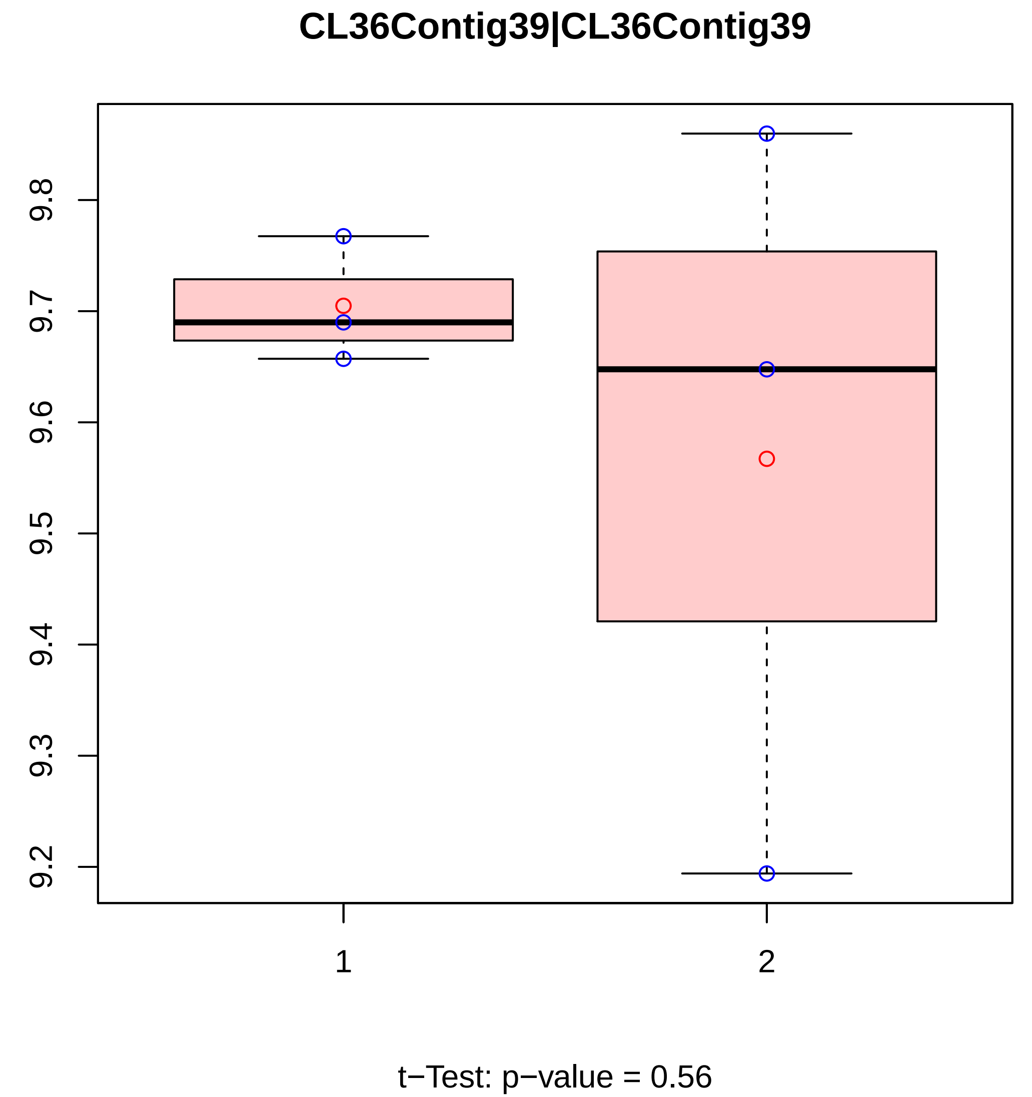
CL36Contig39|CL36Contig39
9.2
9.3
9.4
9.5
9.6
9.7
9.8
1
2
t−Test: p−value = 0.56

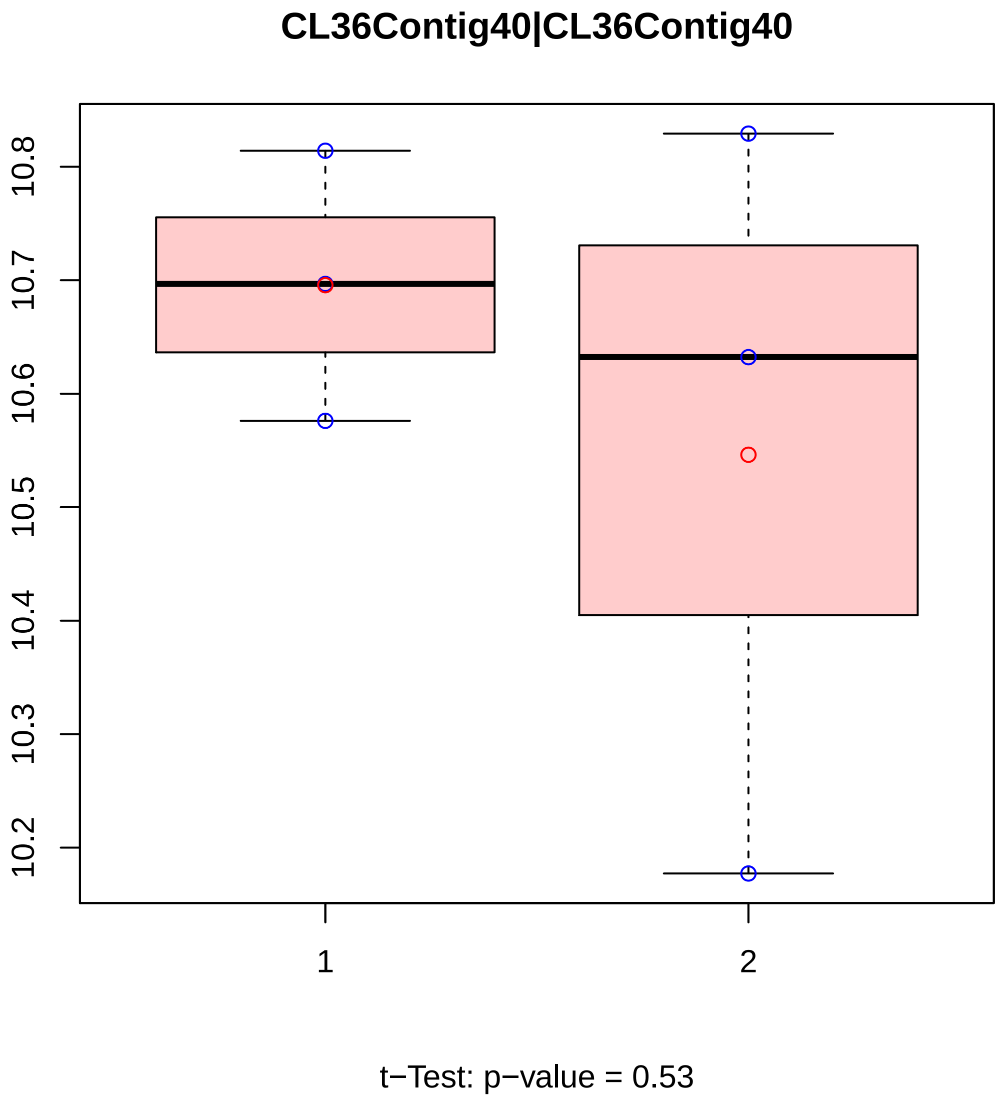
CL36Contig40|CL36Contig40
10.2
10.3
10.4
10.5
10.6
10.7
10.8
1
2
t−Test: p−value = 0.53

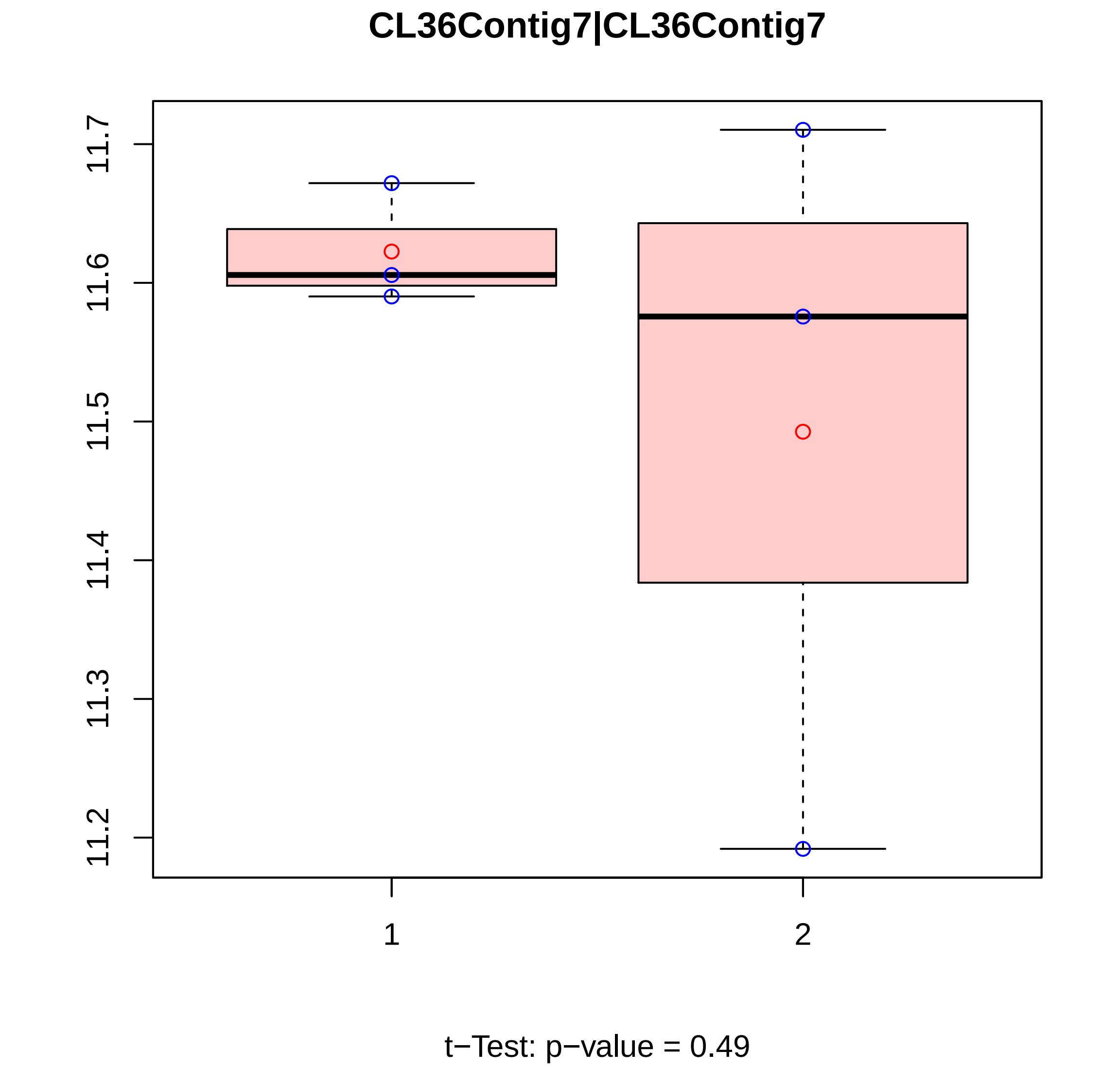
CL36Contig7|CL36Contig7
11.2
11.3
11.4
11.5
11.6
11.7
1
2
t−Test: p−value = 0.49

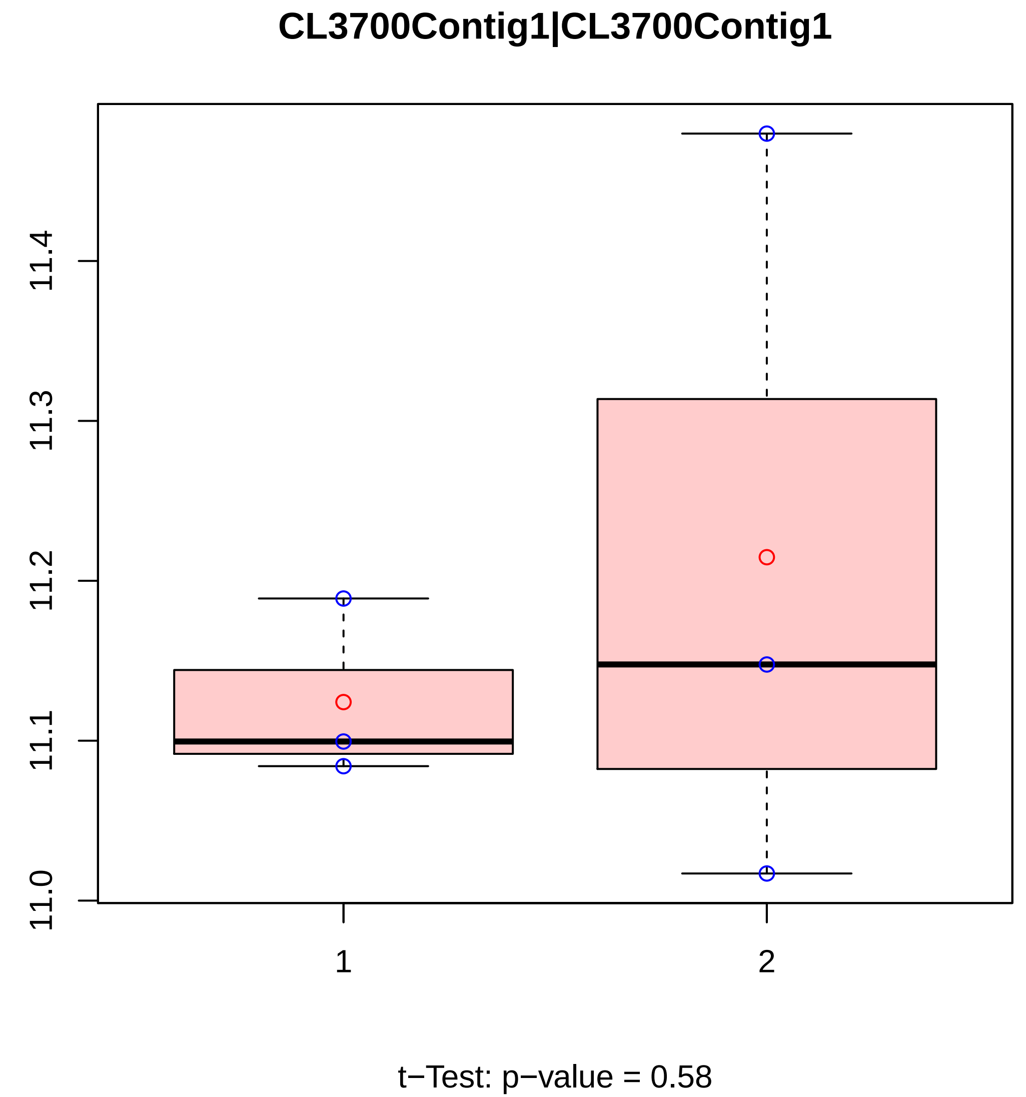

CL3700Contig1|CL3700Contig1
11.0
11.1
11.2
11.3
11.4
1
2
t−Test: p−value = 0.58

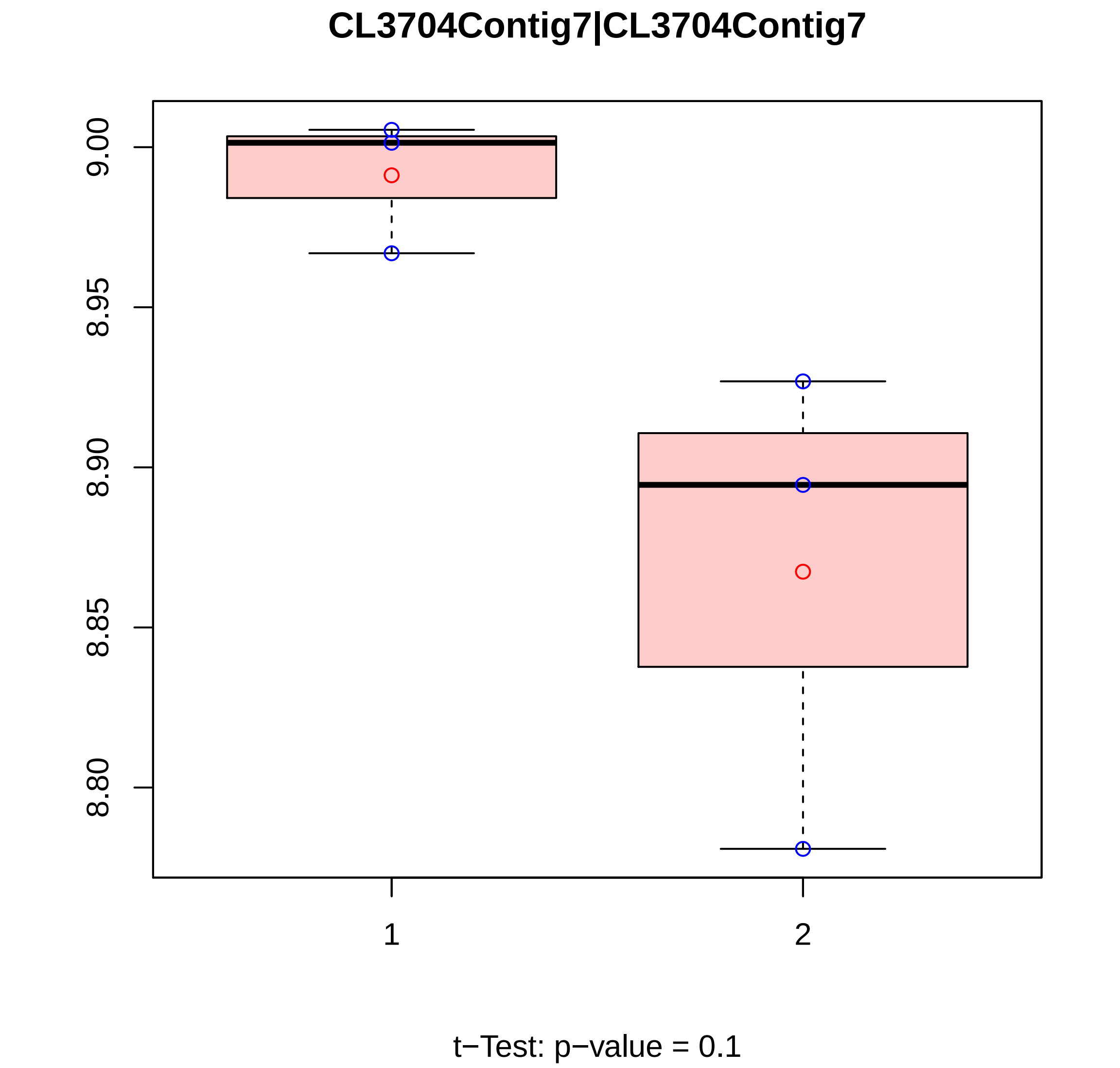
CL3704Contig7|CL3704Contig7
9.00
8.95
8.90
8.85
8.80
1
2
t−Test: p−value = 0.1

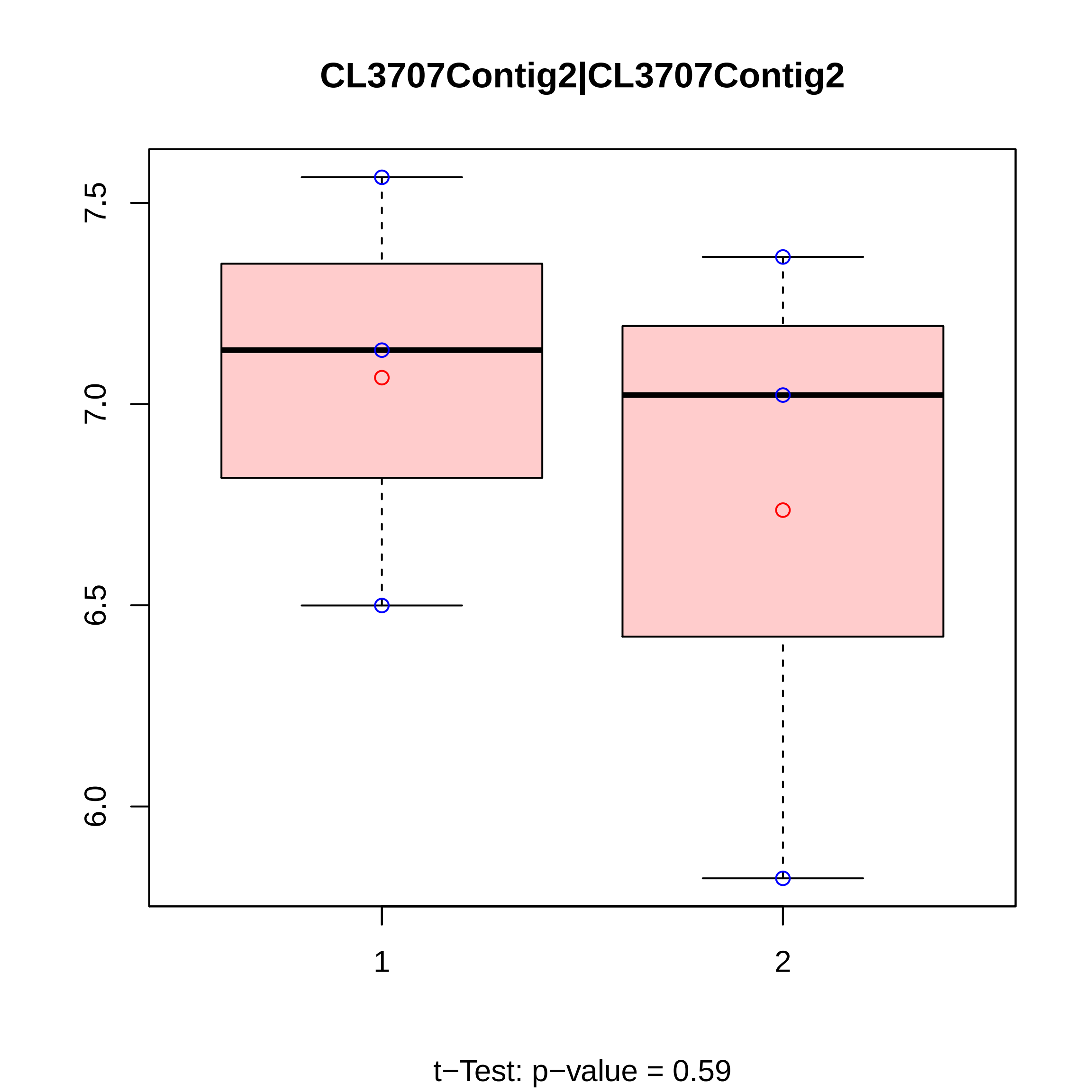
CL3707Contig2|CL3707Contig2
7.5
7.0
6.5
6.0
1
2
t−Test: p−value = 0.59

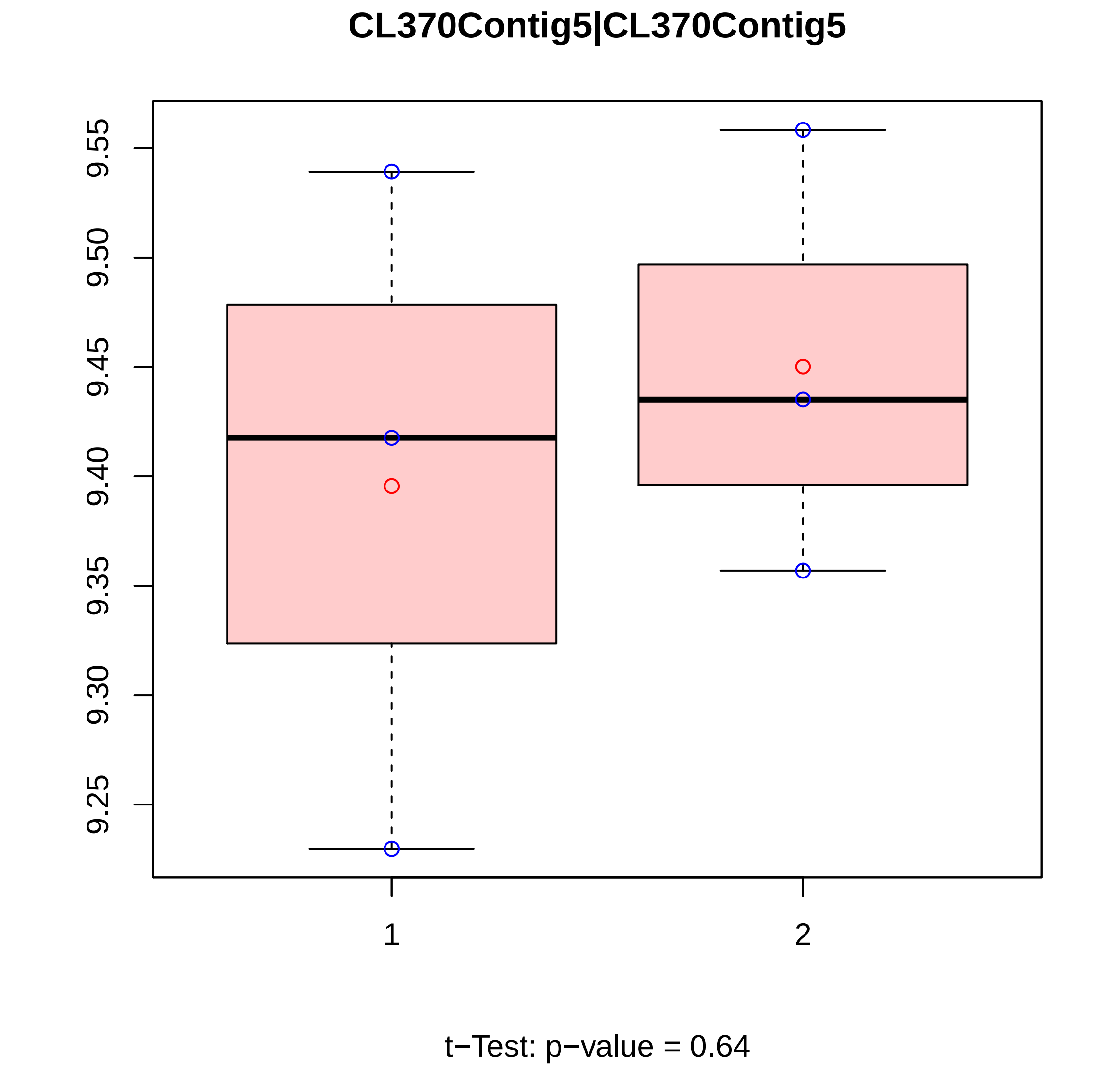

CL370Contig5|CL370Contig5
9.25
9.30
9.35
9.40
9.45
9.50
9.55
1
2
t−Test: p−value = 0.64

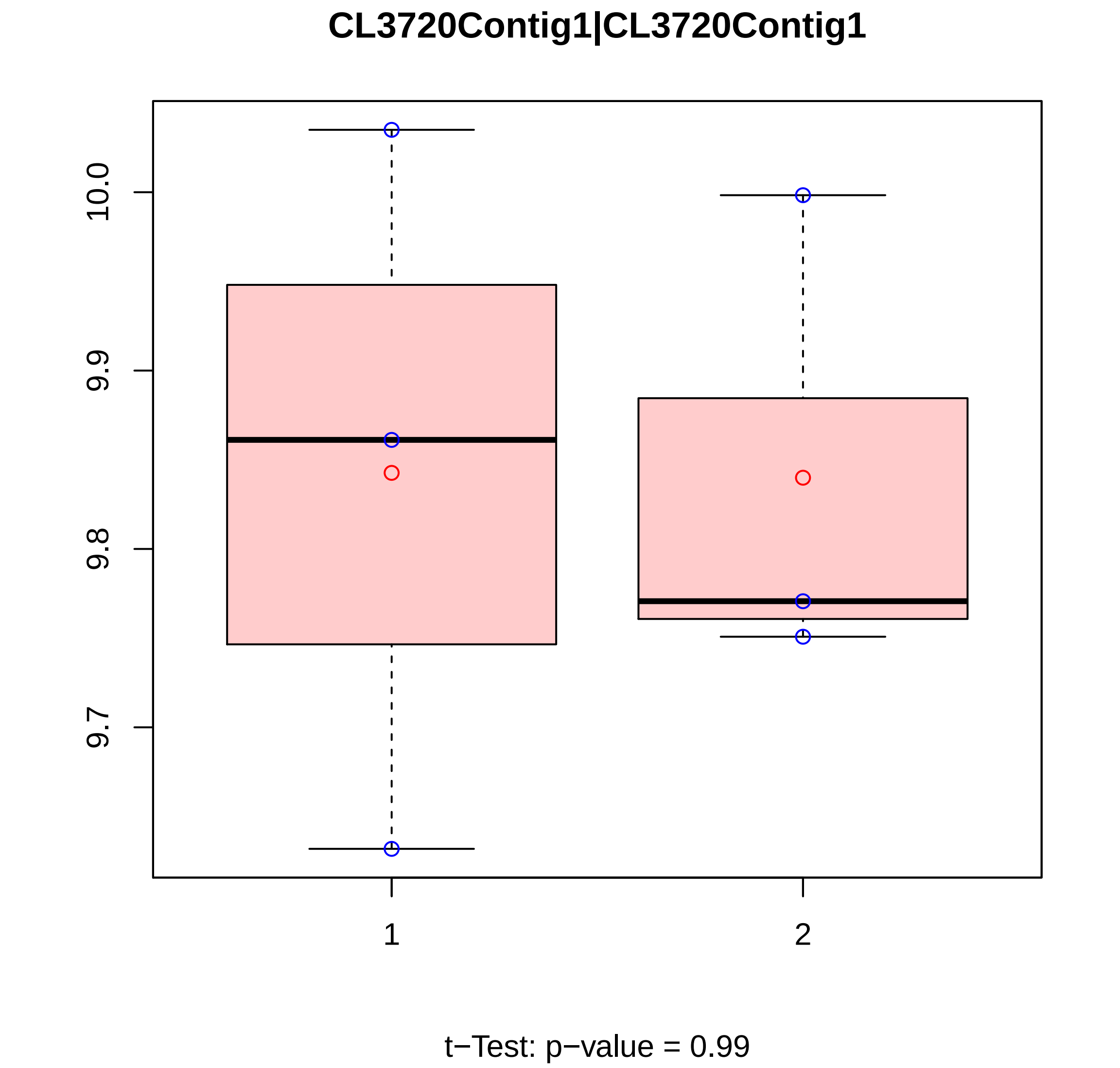
CL3720Contig1|CL3720Contig1
10.0
9.9
9.8
9.7
1
2
t−Test: p−value = 0.99

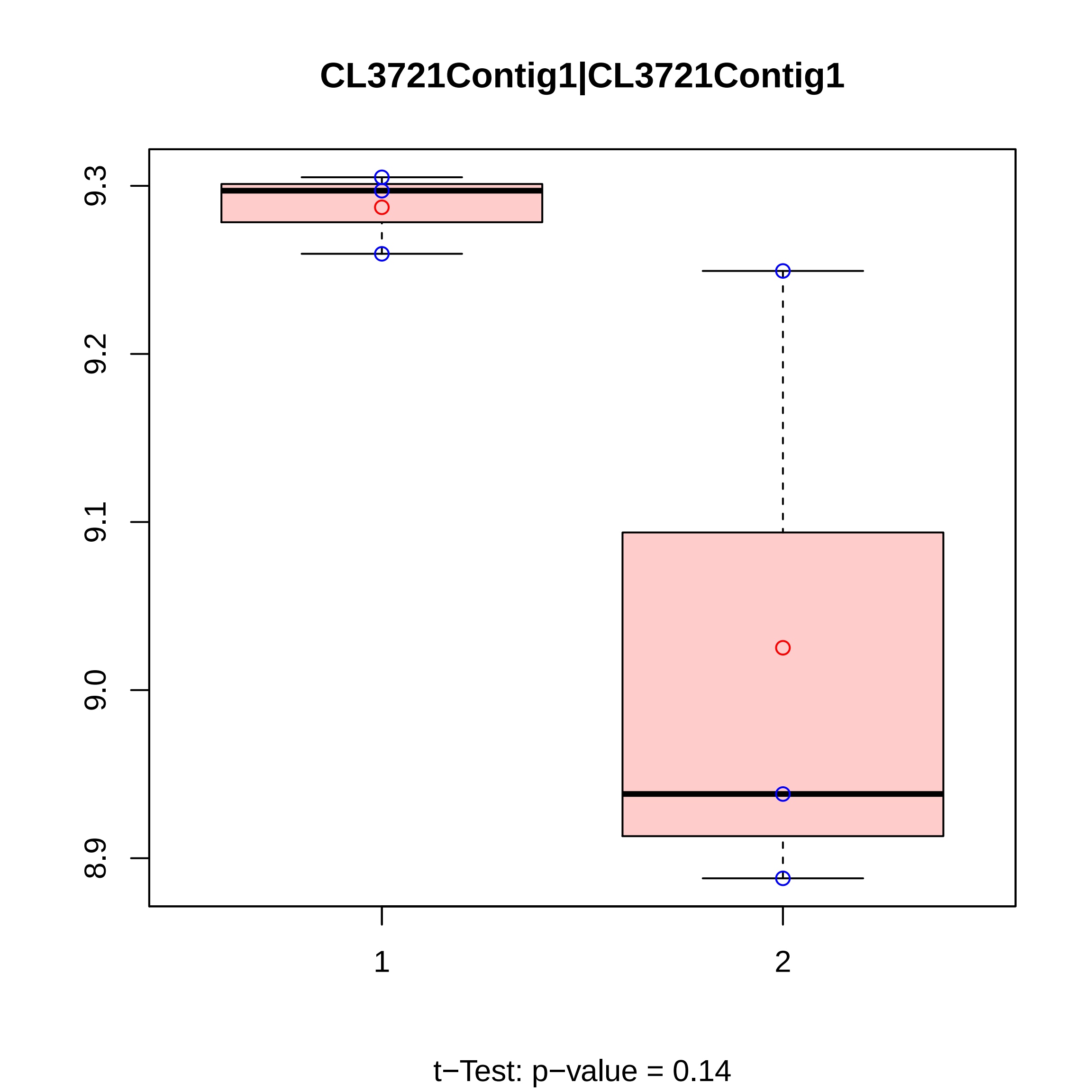

CL3721Contig1|CL3721Contig1
9.3
9.2
9.1
9.0
8.9
1
2
t−Test: p−value = 0.14

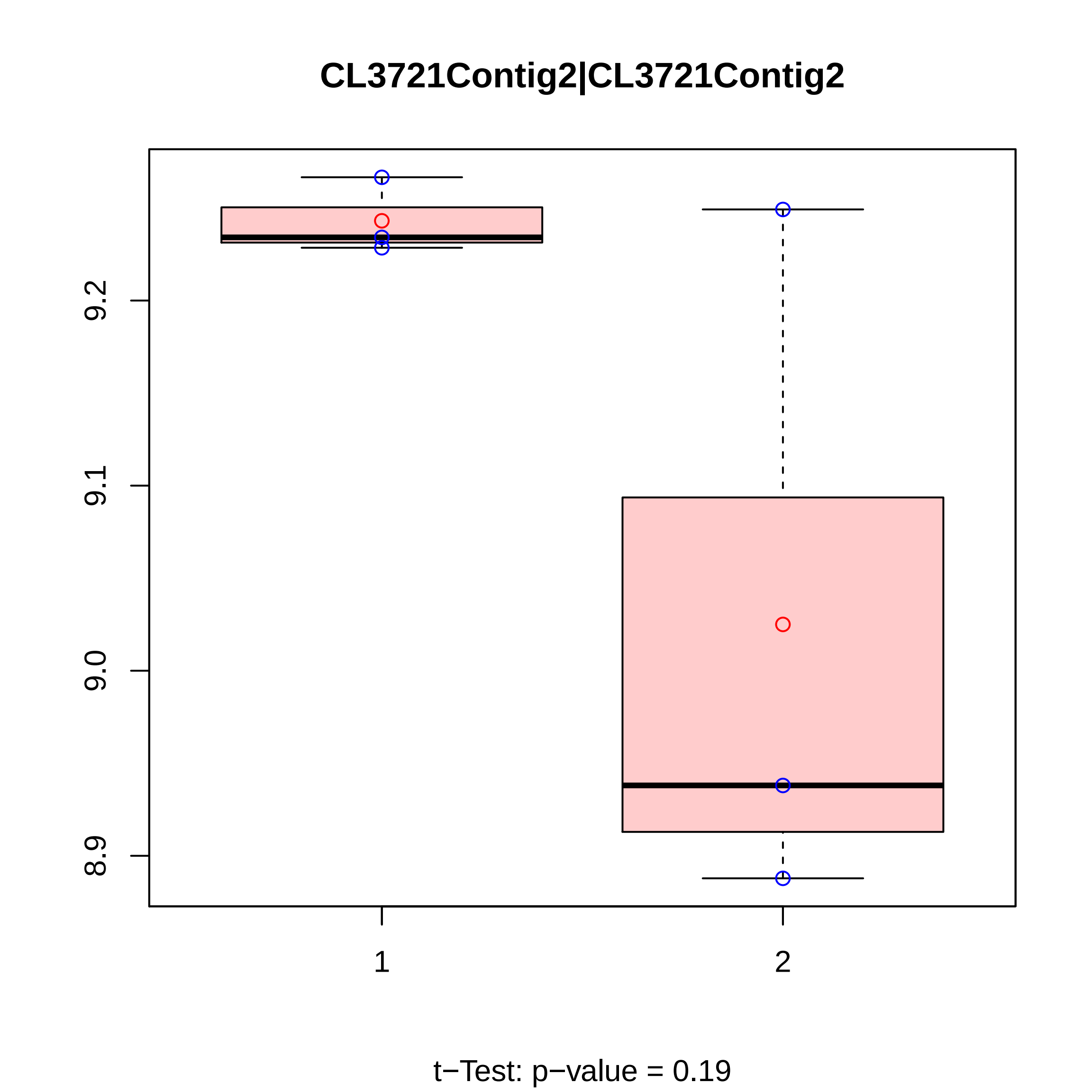
CL3721Contig2|CL3721Contig2
9.2
9.1
9.0
8.9
1
2
t−Test: p−value = 0.19

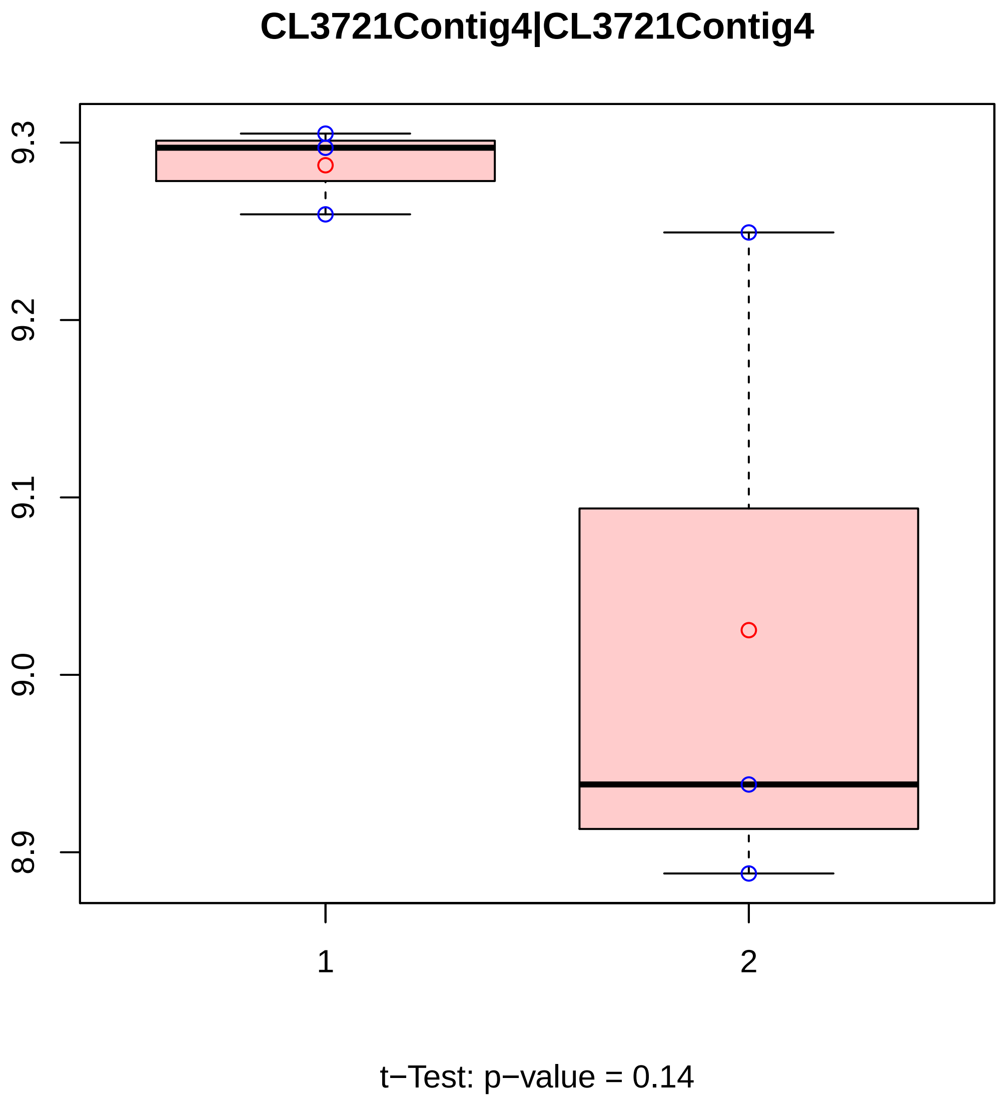
CL3721Contig4|CL3721Contig4
9.3
9.2
9.1
9.0
8.9
1
2
t−Test: p−value = 0.14

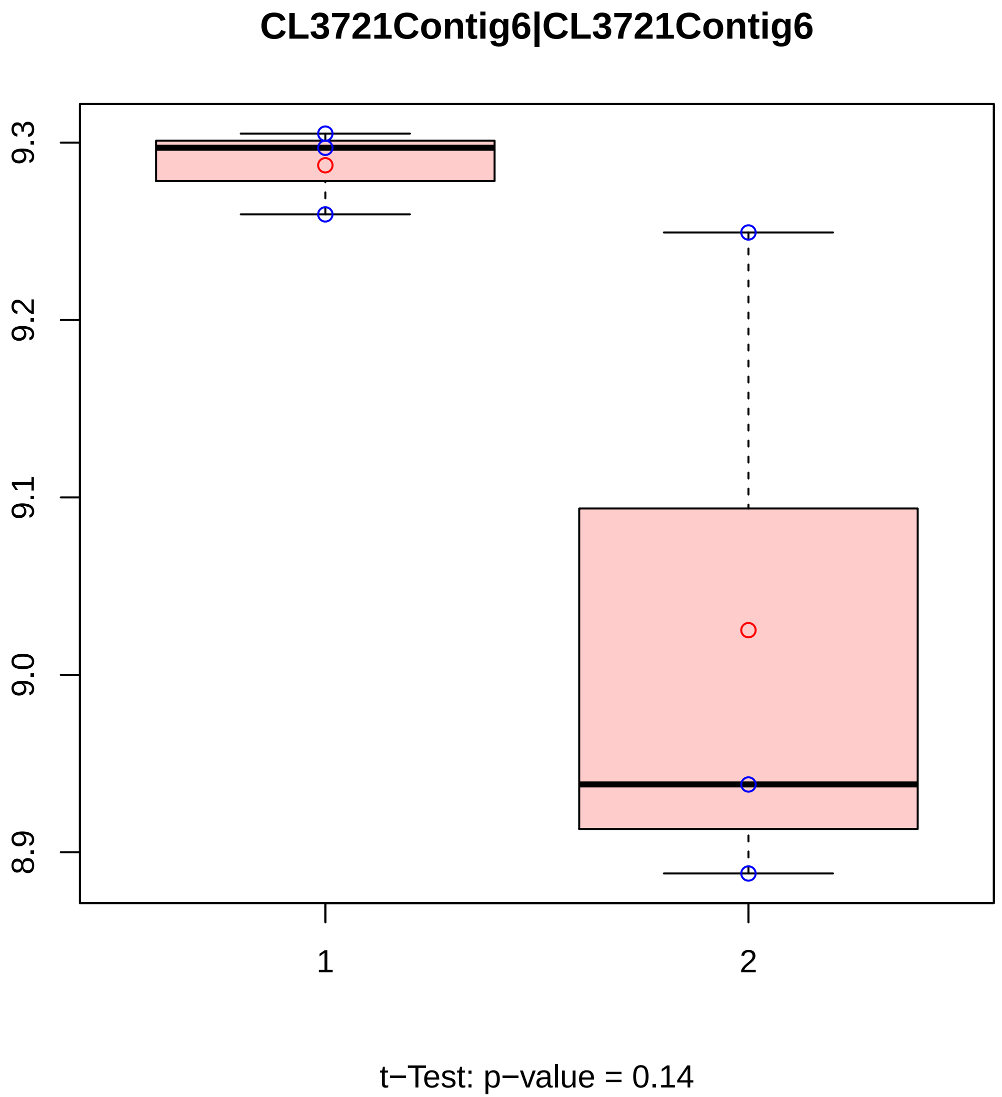
CL3721Contig6|CL3721Contig6
9.3
9.2
9.1
9.0
8.9
1
2
t−Test: p−value = 0.14

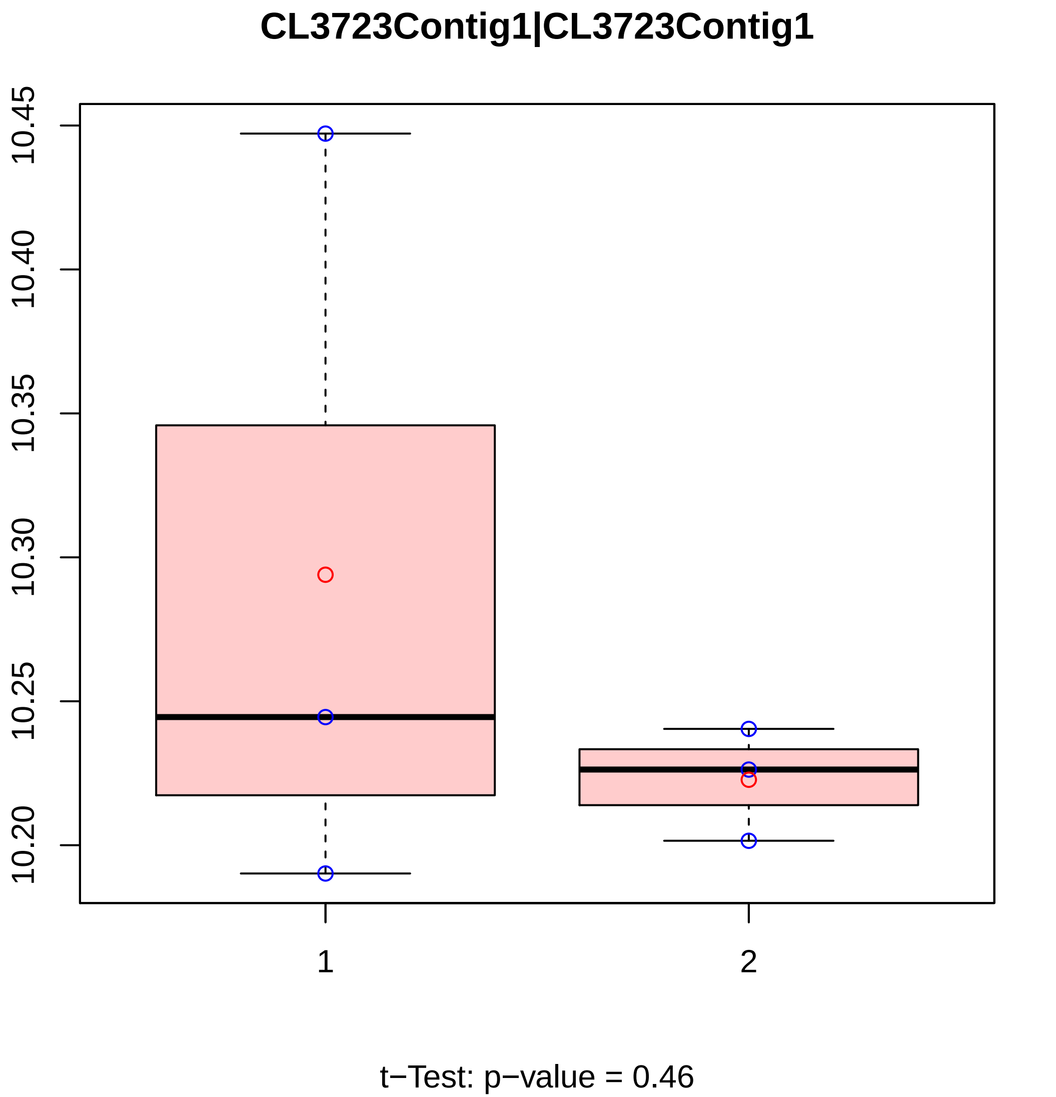

CL3723Contig1|CL3723Contig1
10.20
10.25
10.30
10.35
10.40
10.45
1
2
t−Test: p−value = 0.46

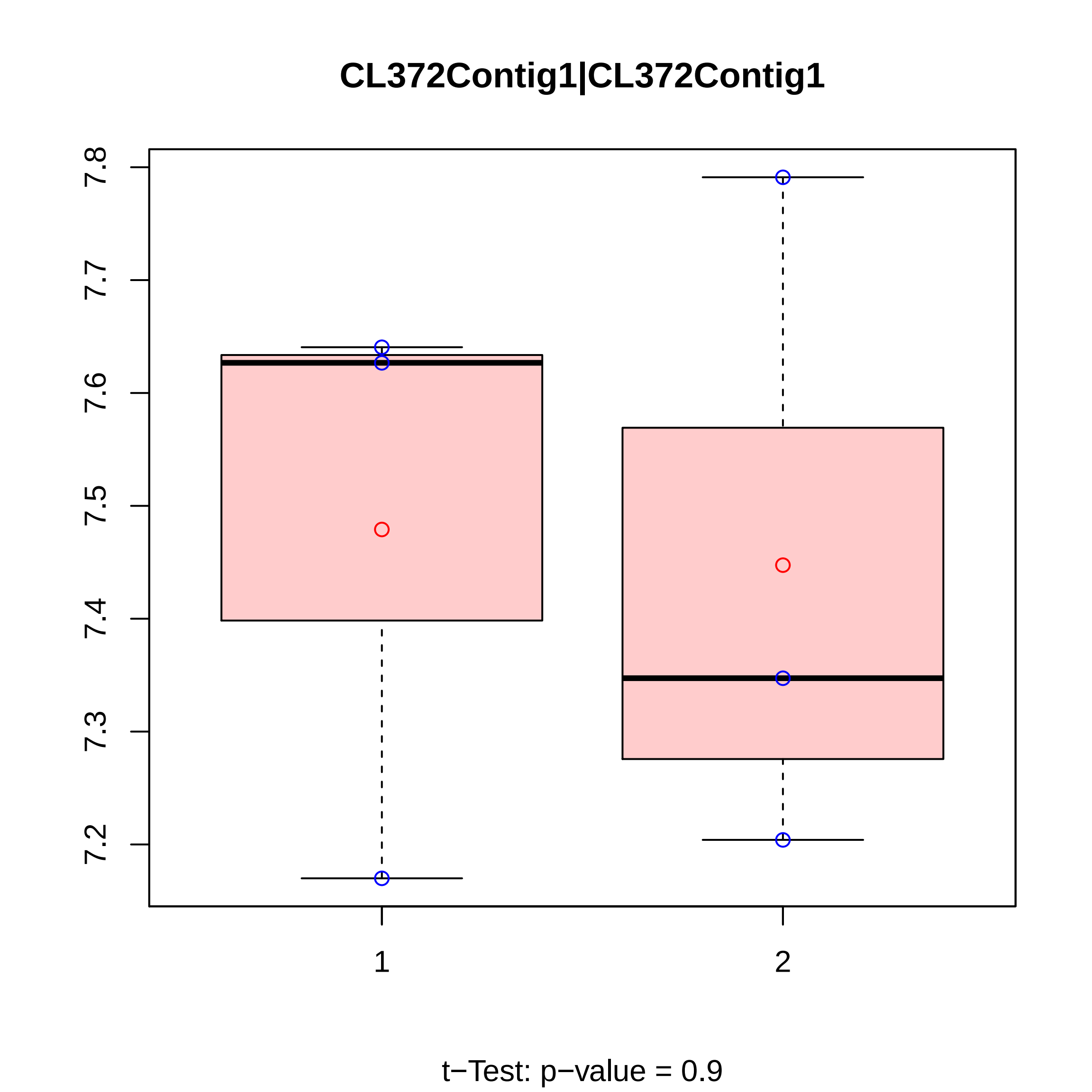

CL372Contig1|CL372Contig1
7.2
7.3
7.4
7.5
7.6
7.7
7.8
1
2
t−Test: p−value = 0.9

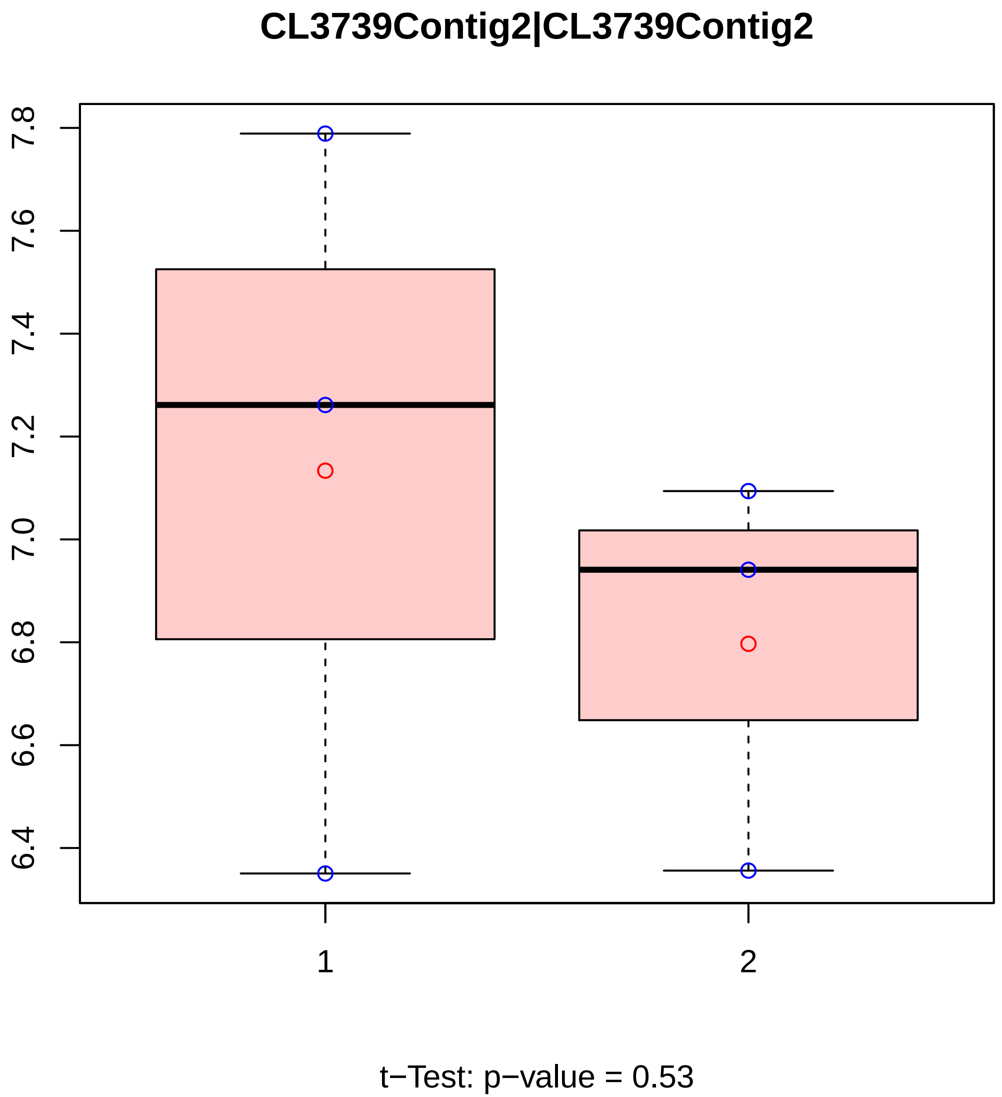

CL3739Contig2|CL3739Contig2
7.8
7.6
7.4
7.2
7.0
6.8
6.6
6.4
1
2
t−Test: p−value = 0.53

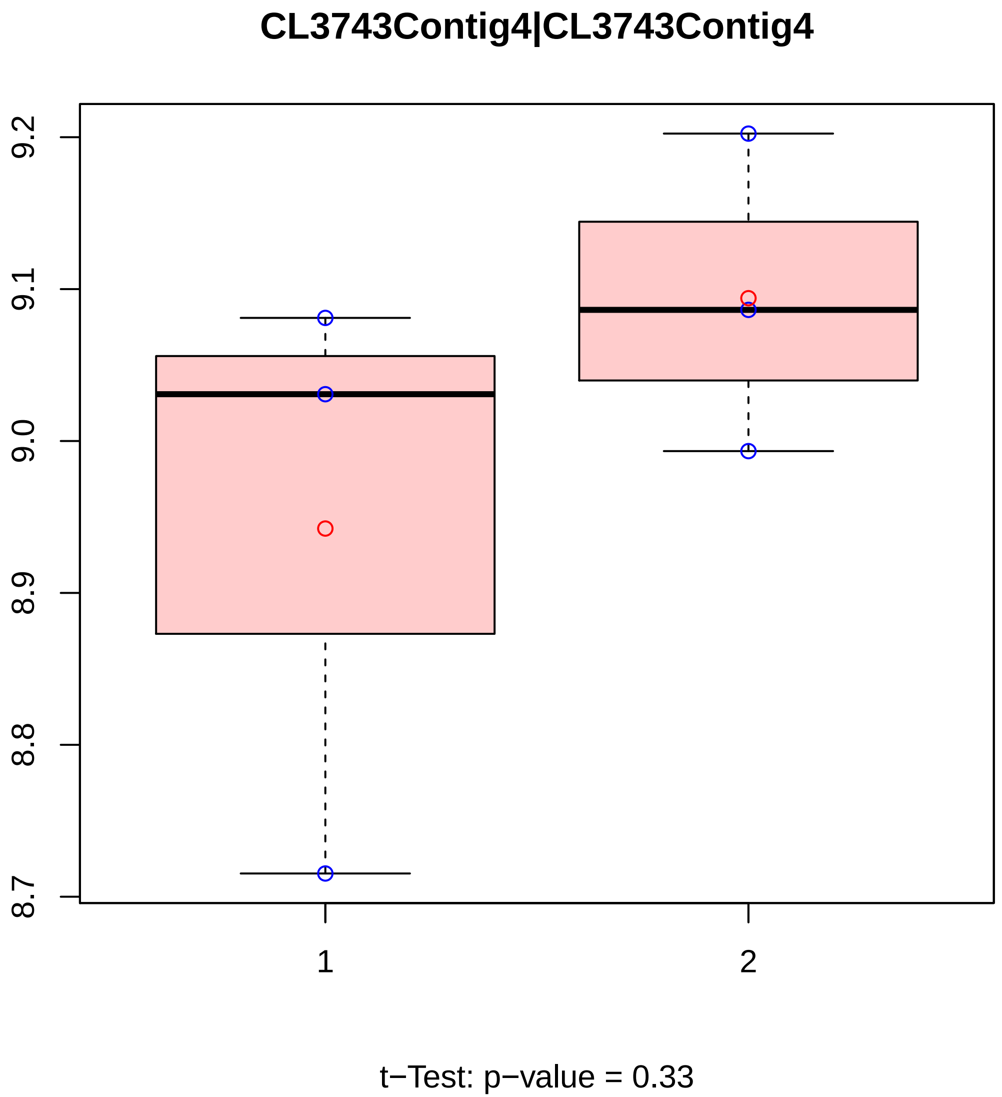

CL3743Contig4|CL3743Contig4
9.2
9.1
9.0
8.9
8.8
8.7
1
2
t−Test: p−value = 0.33

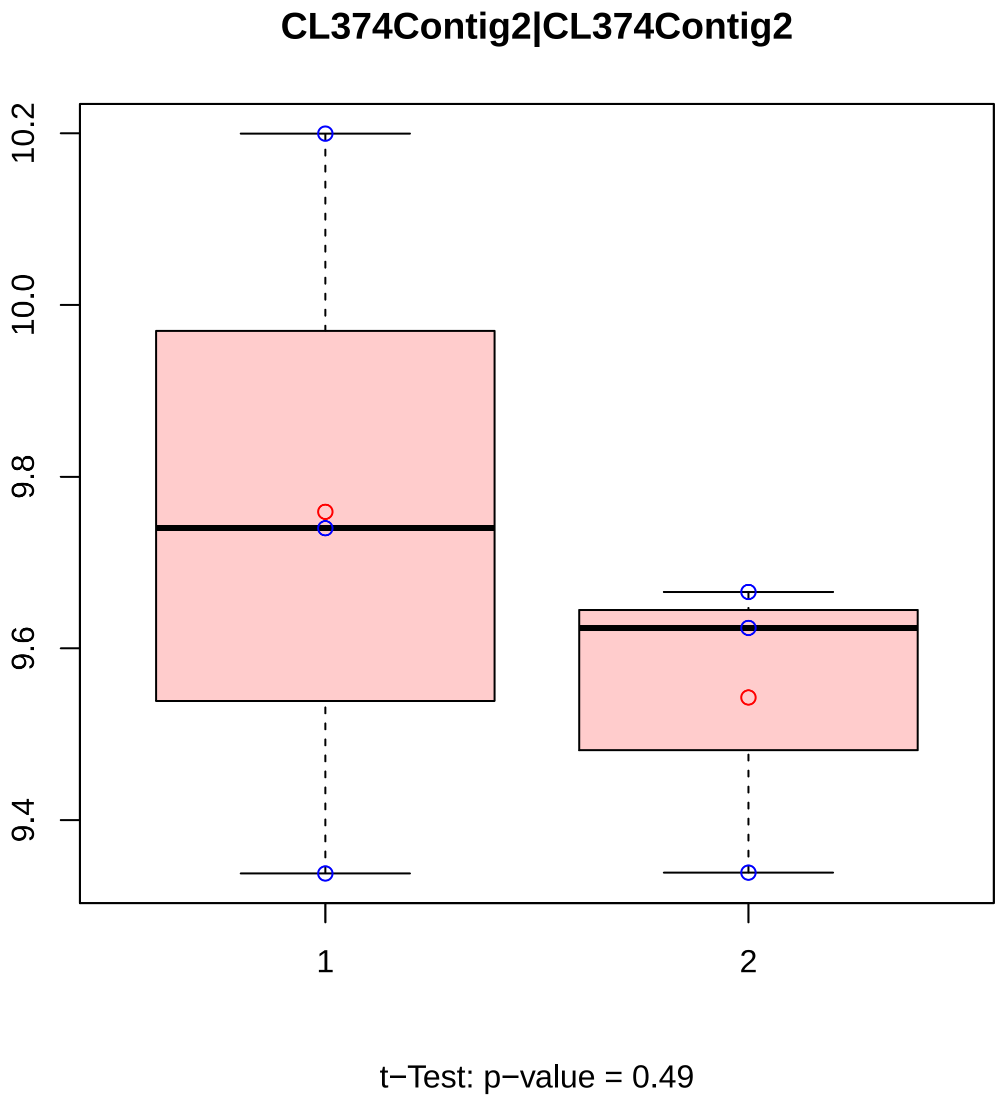

CL374Contig2|CL374Contig2
10.2
10.0
9.8
9.6
9.4
1
2
t−Test: p−value = 0.49

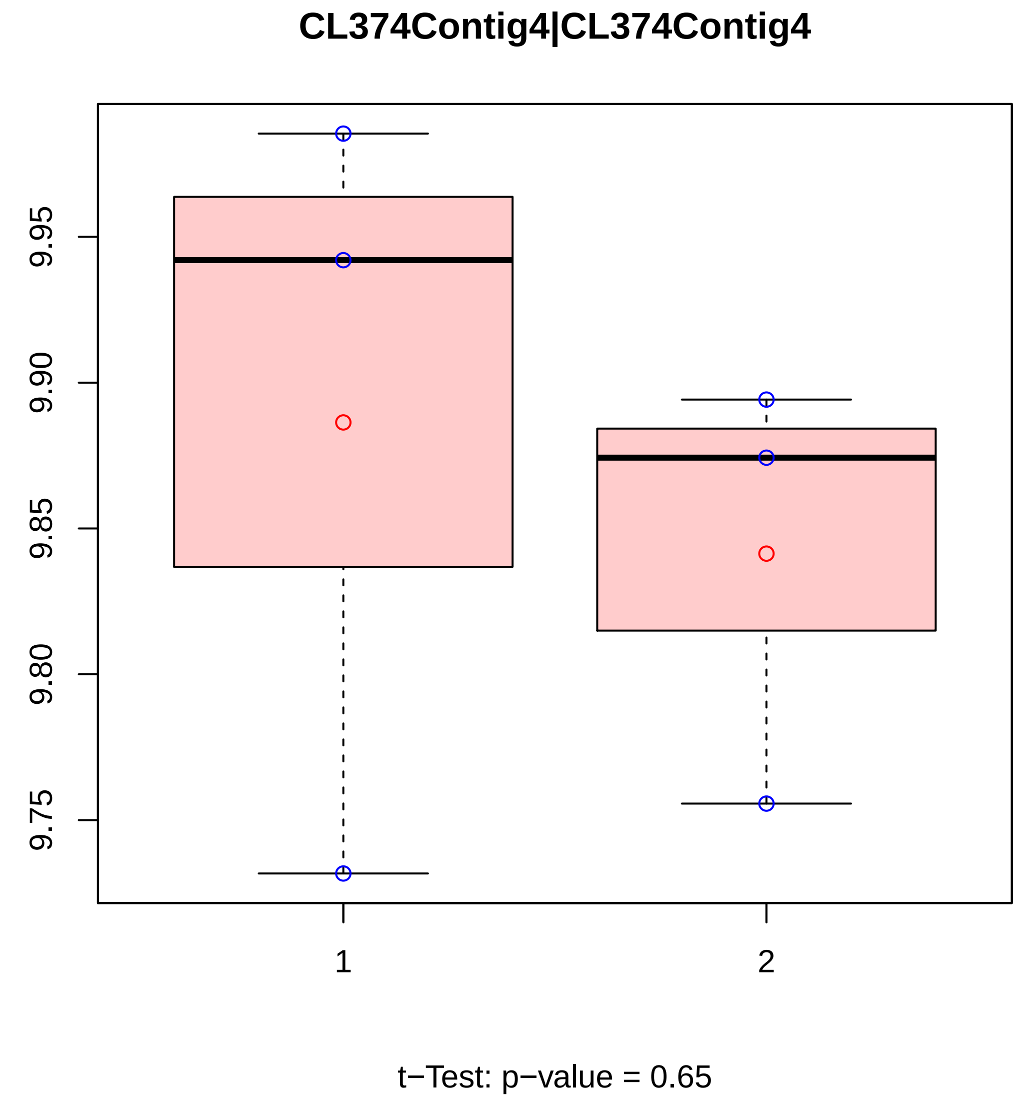
CL374Contig4|CL374Contig4
9.75
9.80
9.85
9.90
9.95
1
2
t−Test: p−value = 0.65

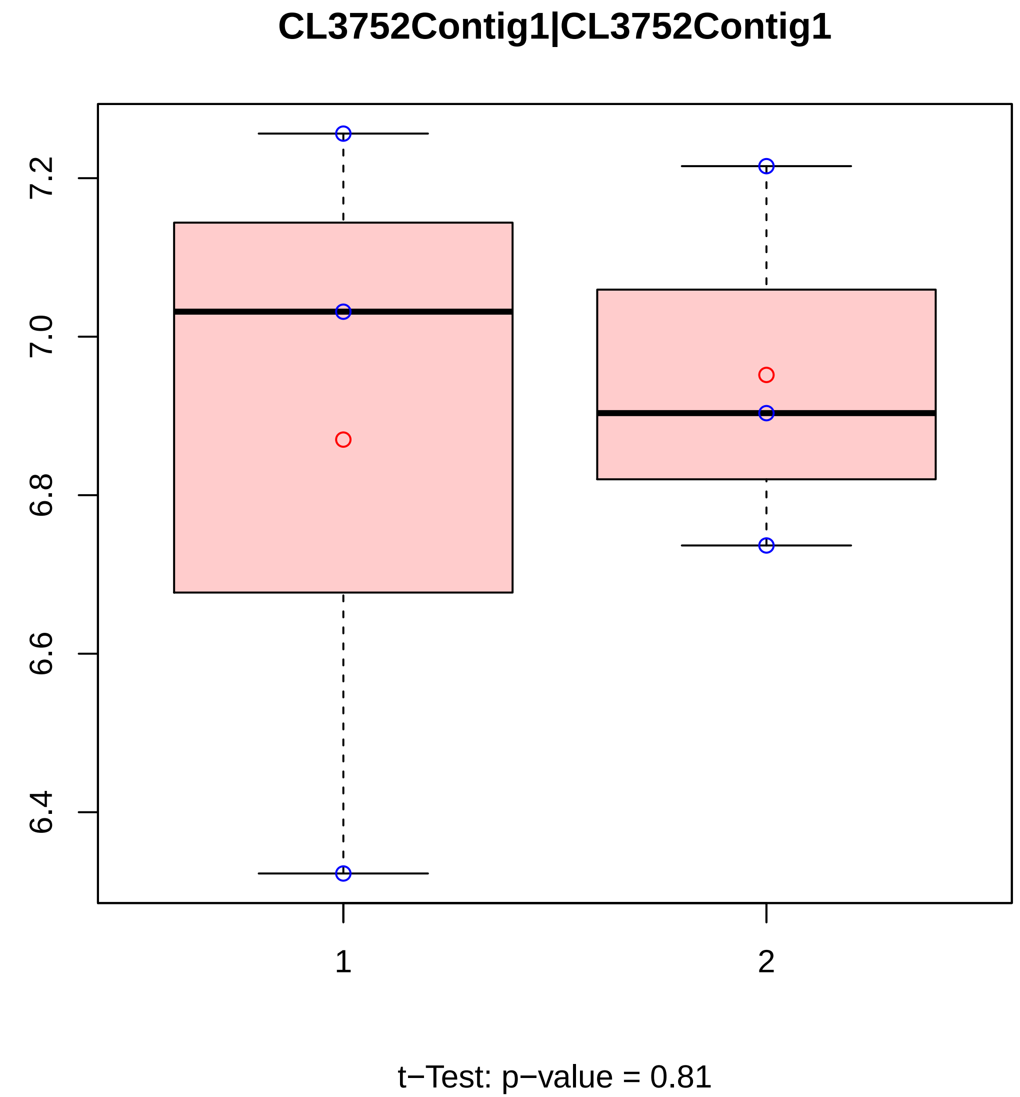
CL3752Contig1|CL3752Contig1
7.2
7.0
6.8
6.6
6.4
1
2
t−Test: p−value = 0.81

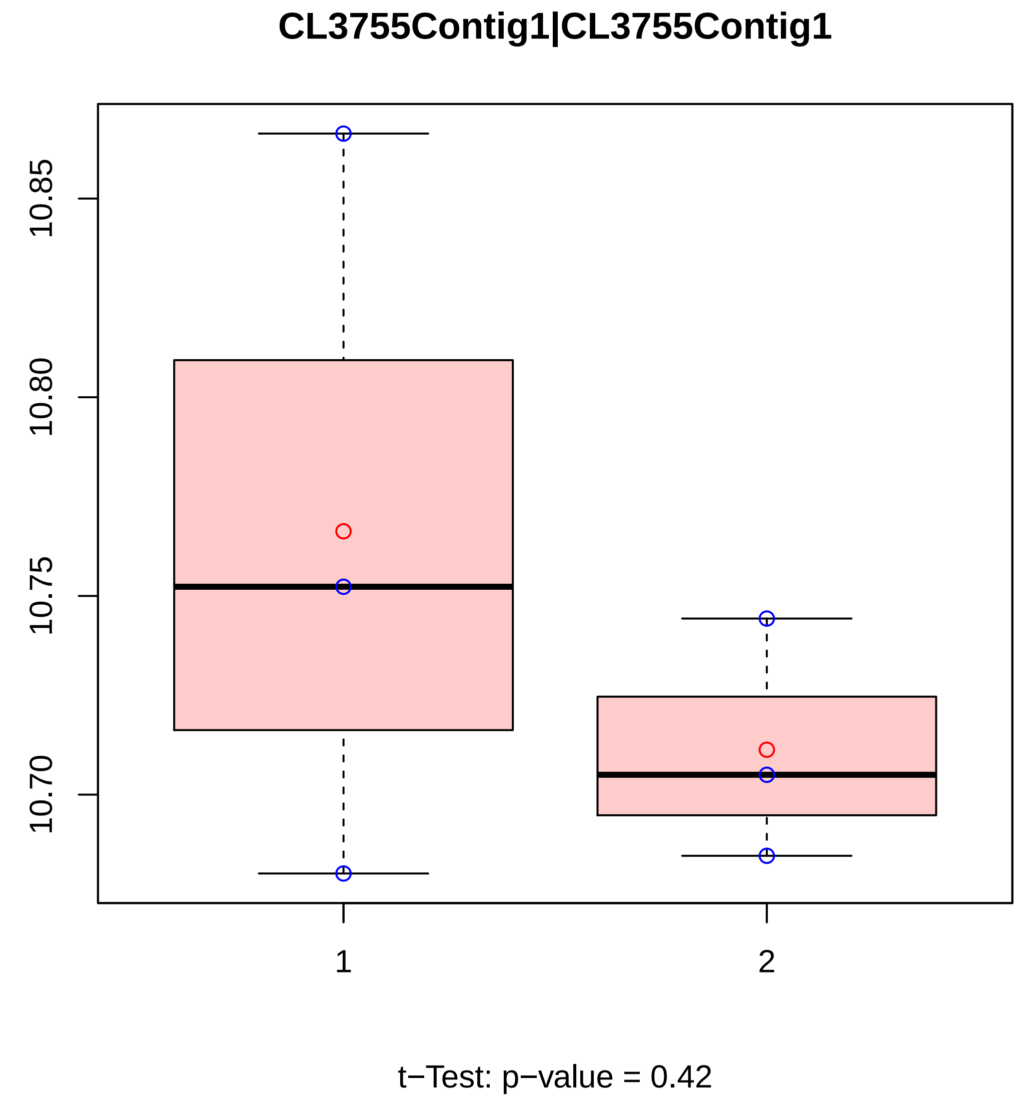

CL3755Contig1|CL3755Contig1
10.85
10.80
10.75
10.70
1
2
t−Test: p−value = 0.42

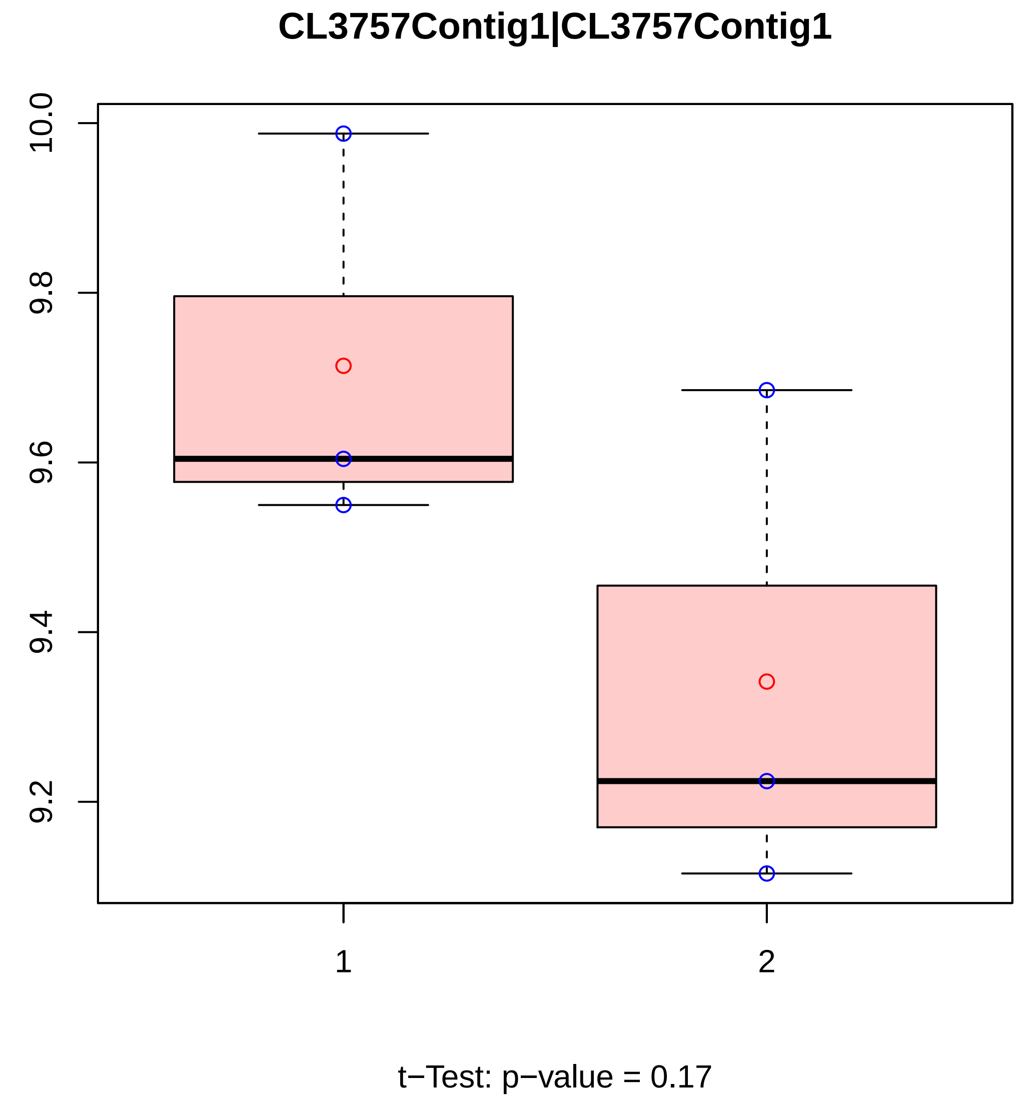

CL3757Contig1|CL3757Contig1
10.0
9.8
9.6
9.4
9.2
1
2
t−Test: p−value = 0.17

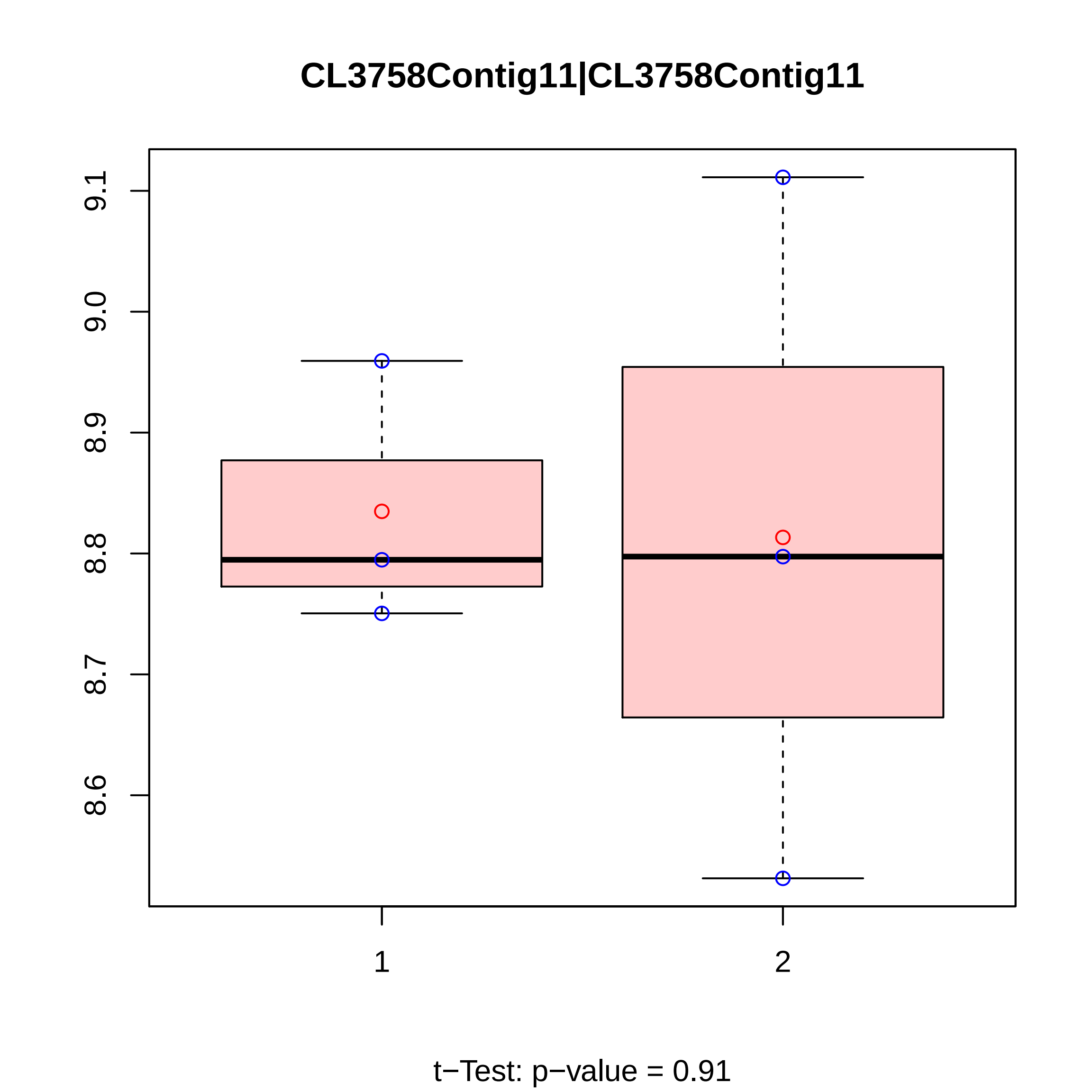
CL3758Contig11|CL3758Contig11
9.1
9.0
8.9
8.8
8.7
8.6
1
2
t−Test: p−value = 0.91

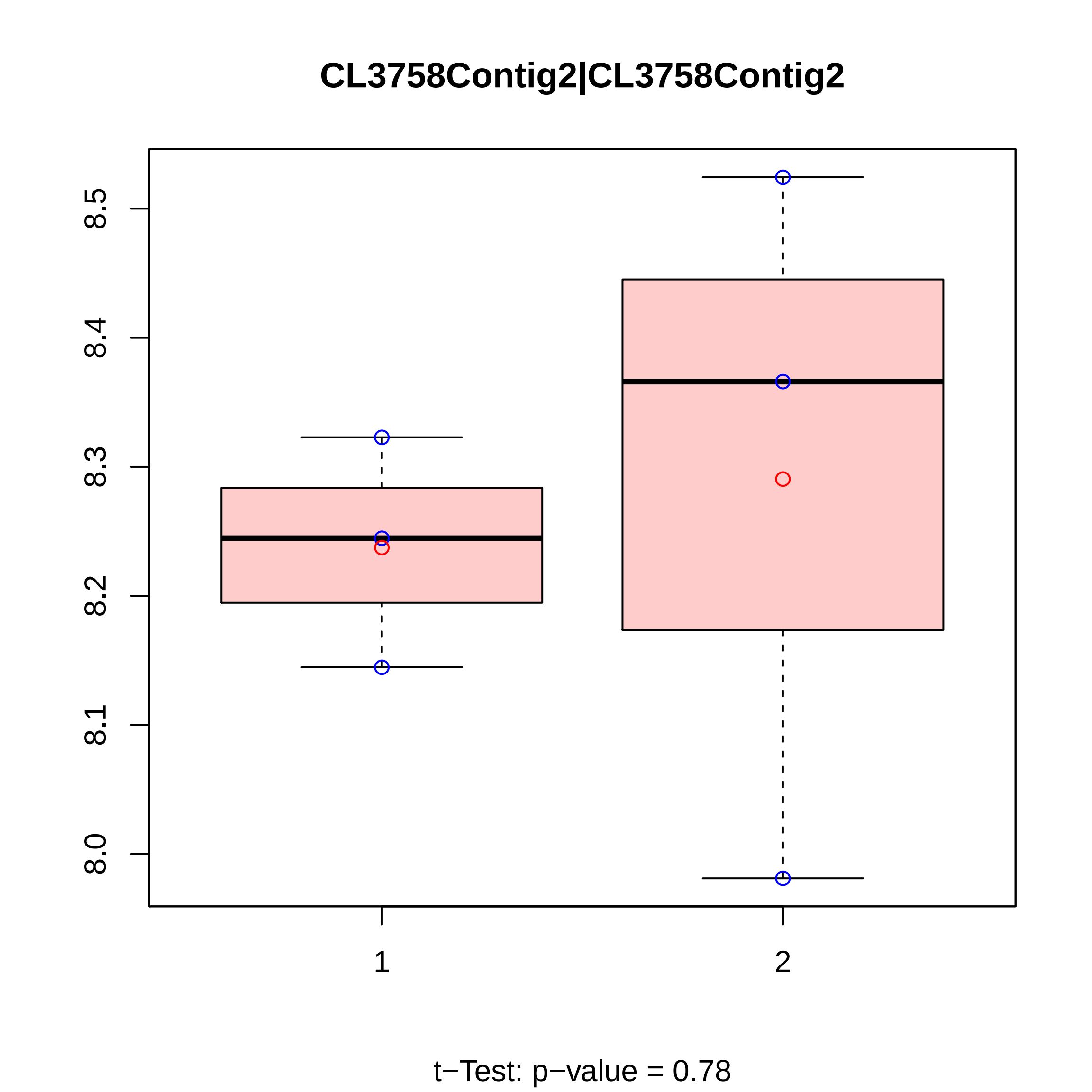
CL3758Contig2|CL3758Contig2
8.0
8.1
8.2
8.3
8.4
8.5
1
2
t−Test: p−value = 0.78

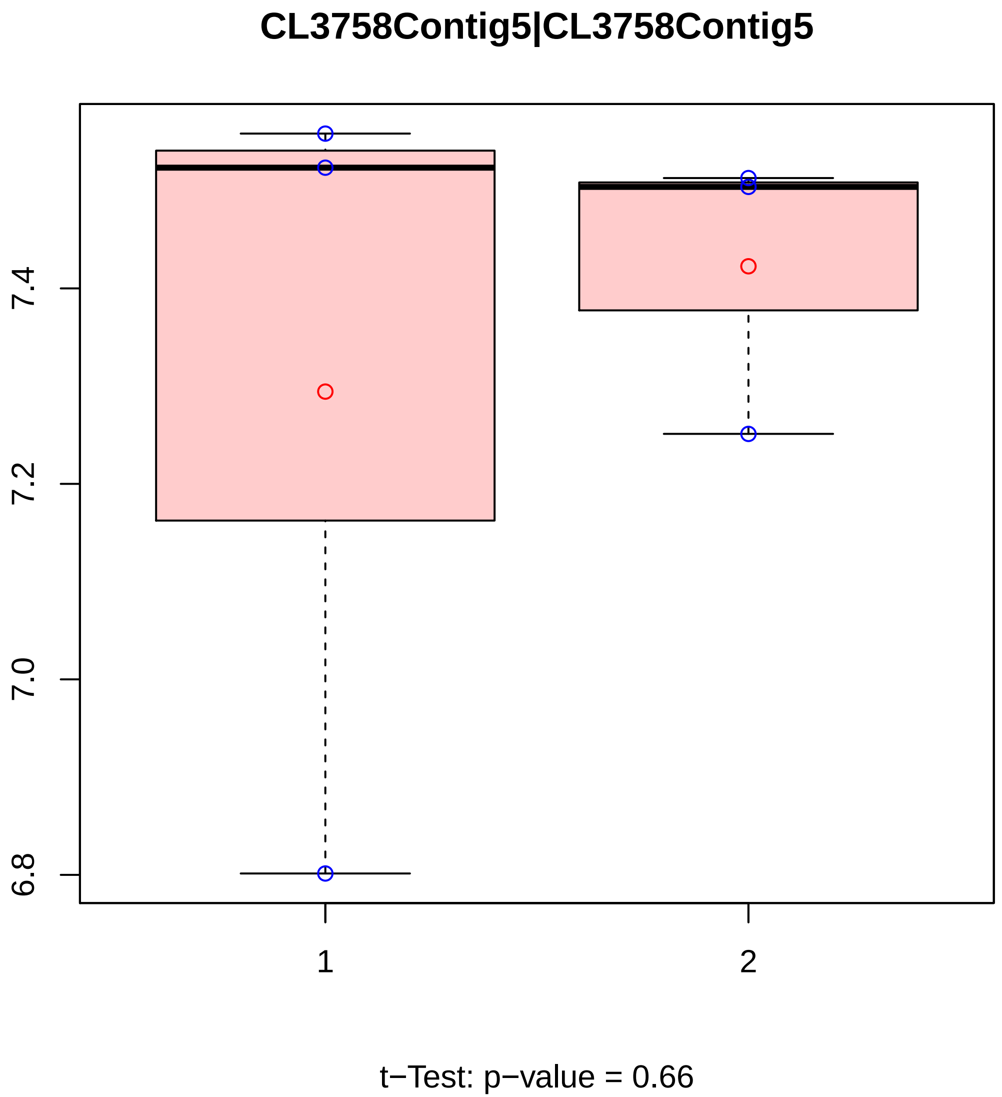
CL3758Contig5|CL3758Contig5
7.4
7.2
7.0
6.8
1
2
t−Test: p−value = 0.66

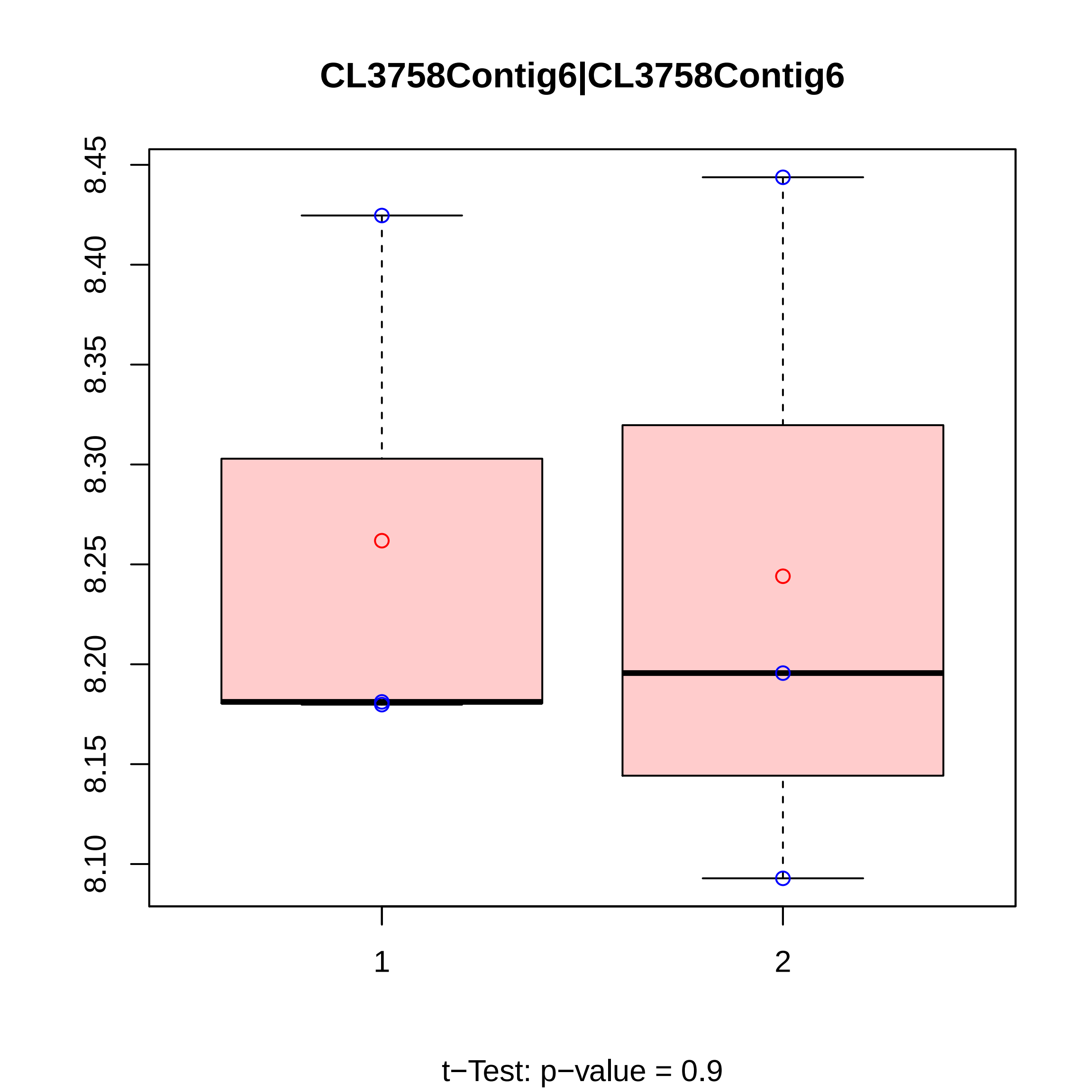
CL3758Contig6|CL3758Contig6
8.10
8.15
8.20
8.25
8.30
8.35
8.40
8.45
1
2
t−Test: p−value = 0.9

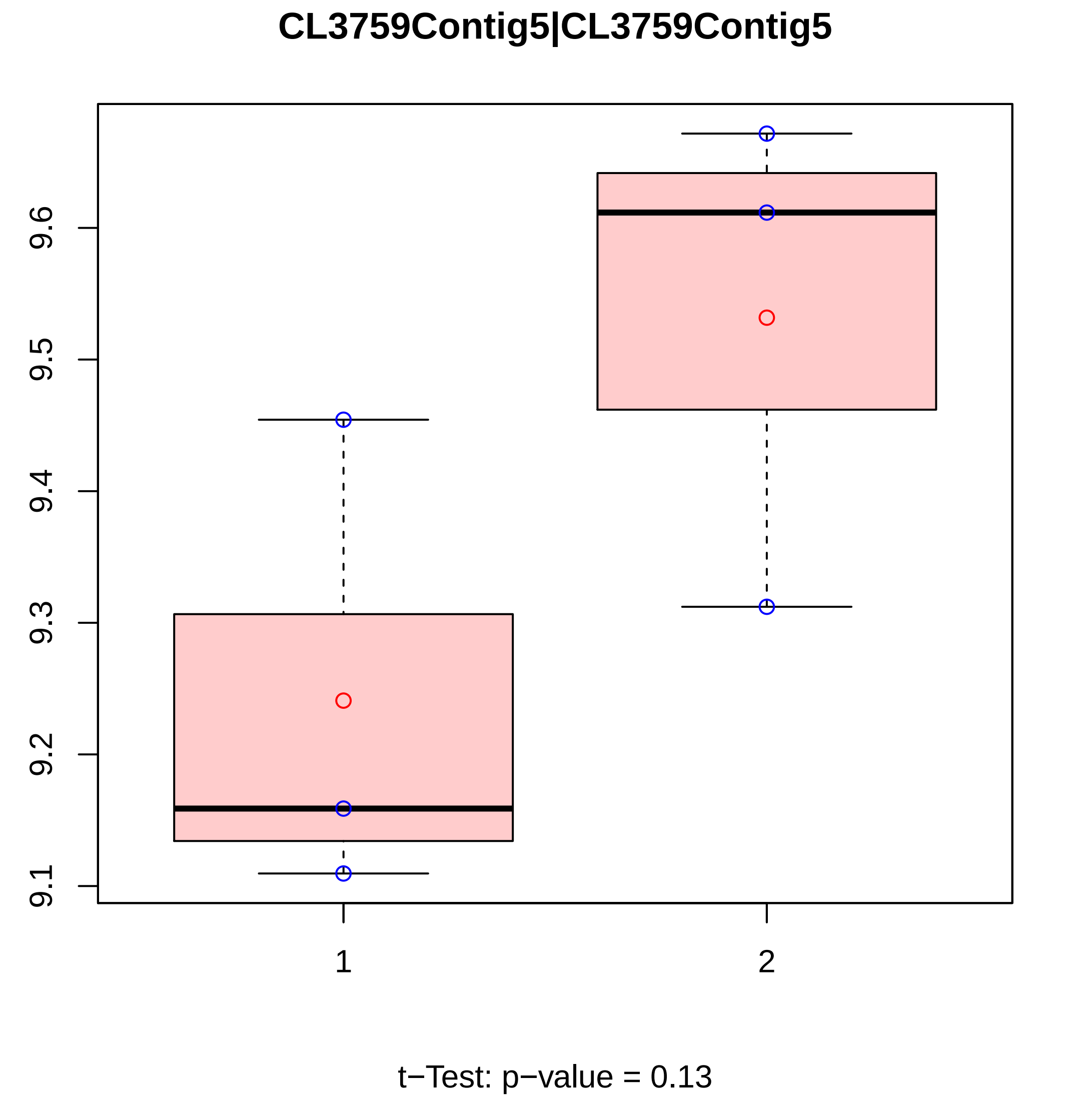
CL3759Contig5|CL3759Contig5
9.1
9.2
9.3
9.4
9.5
9.6
1
2
t−Test: p−value = 0.13

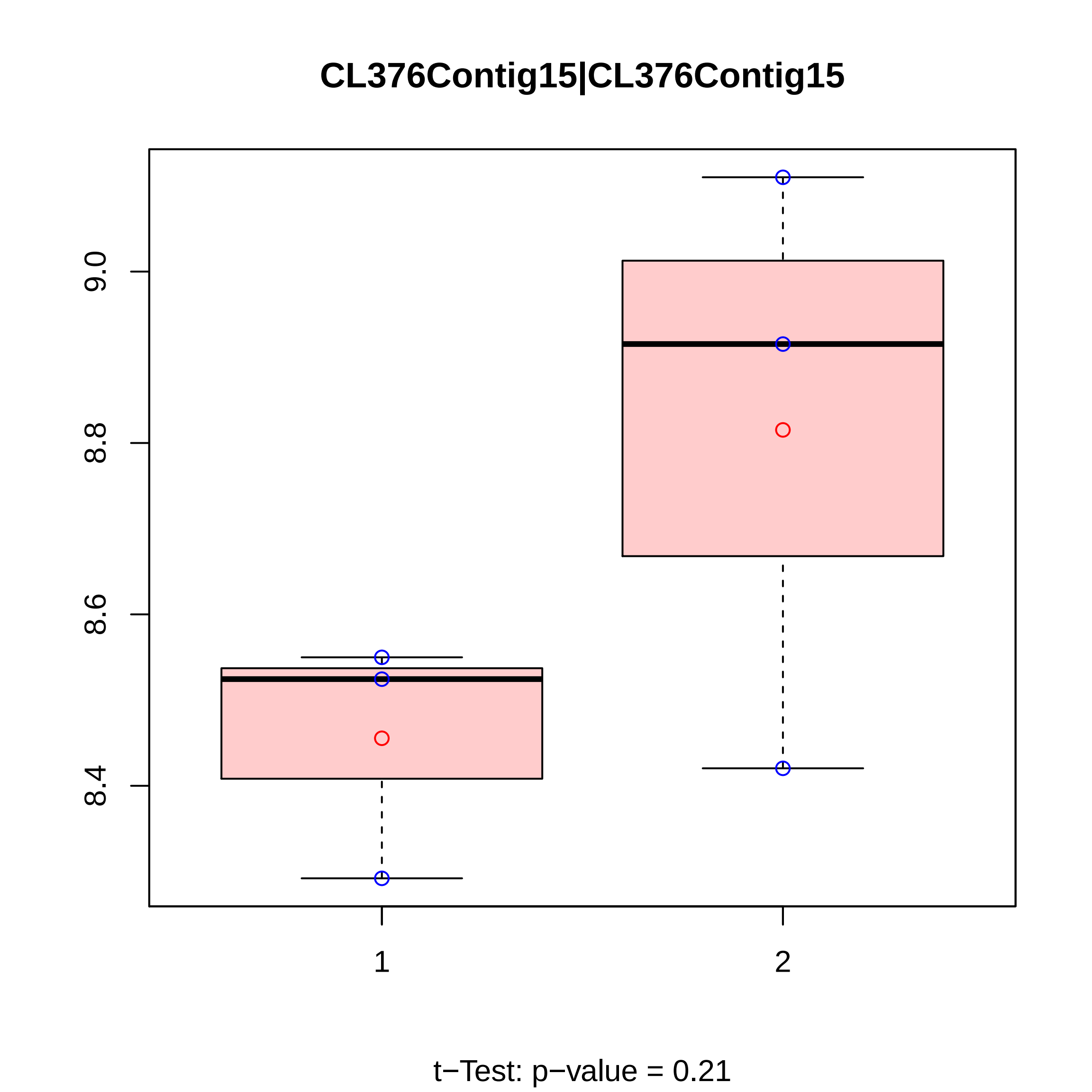
CL376Contig15|CL376Contig15
9.0
8.8
8.6
8.4
1
2
t−Test: p−value = 0.21

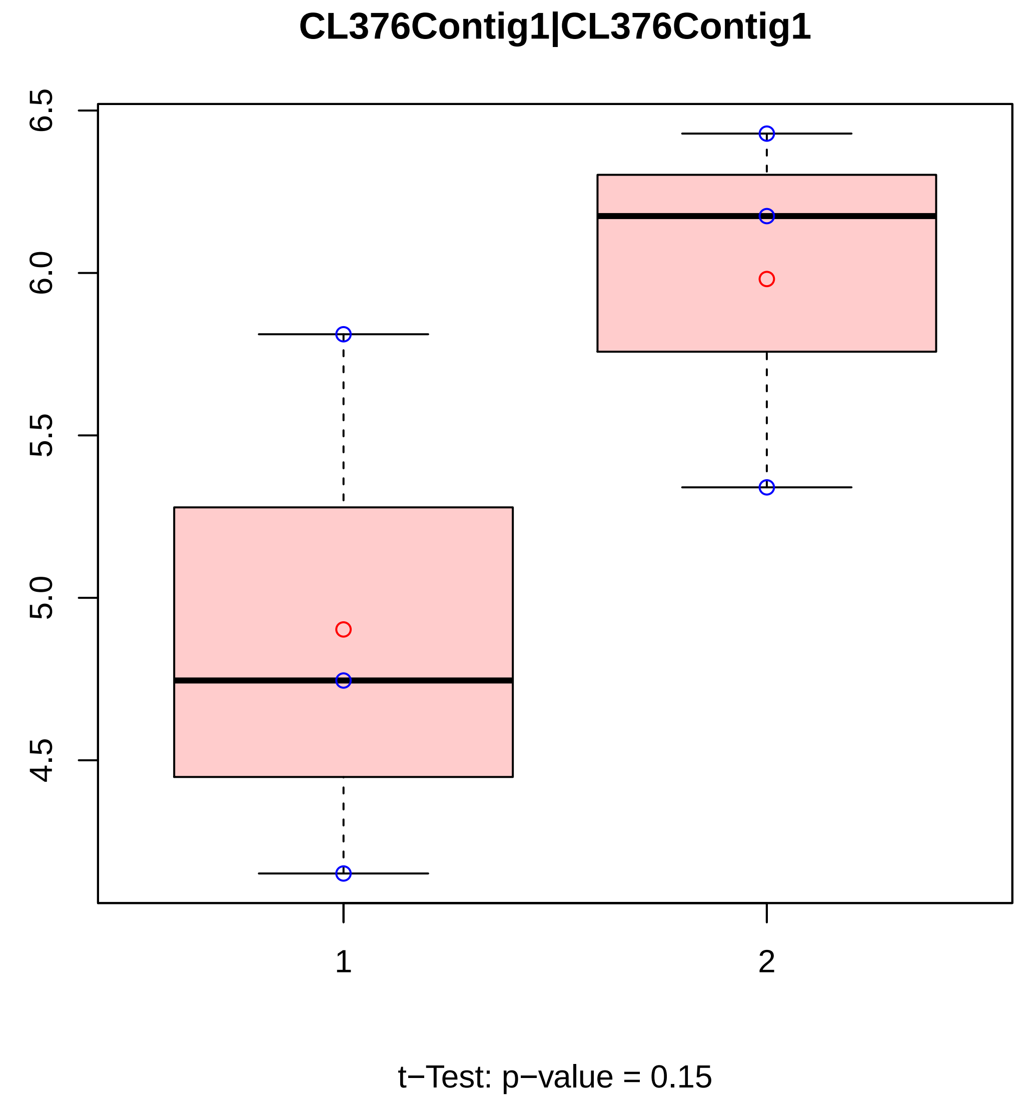

CL376Contig1|CL376Contig1
6.5
6.0
5.5
5.0
4.5
1
2
t−Test: p−value = 0.15

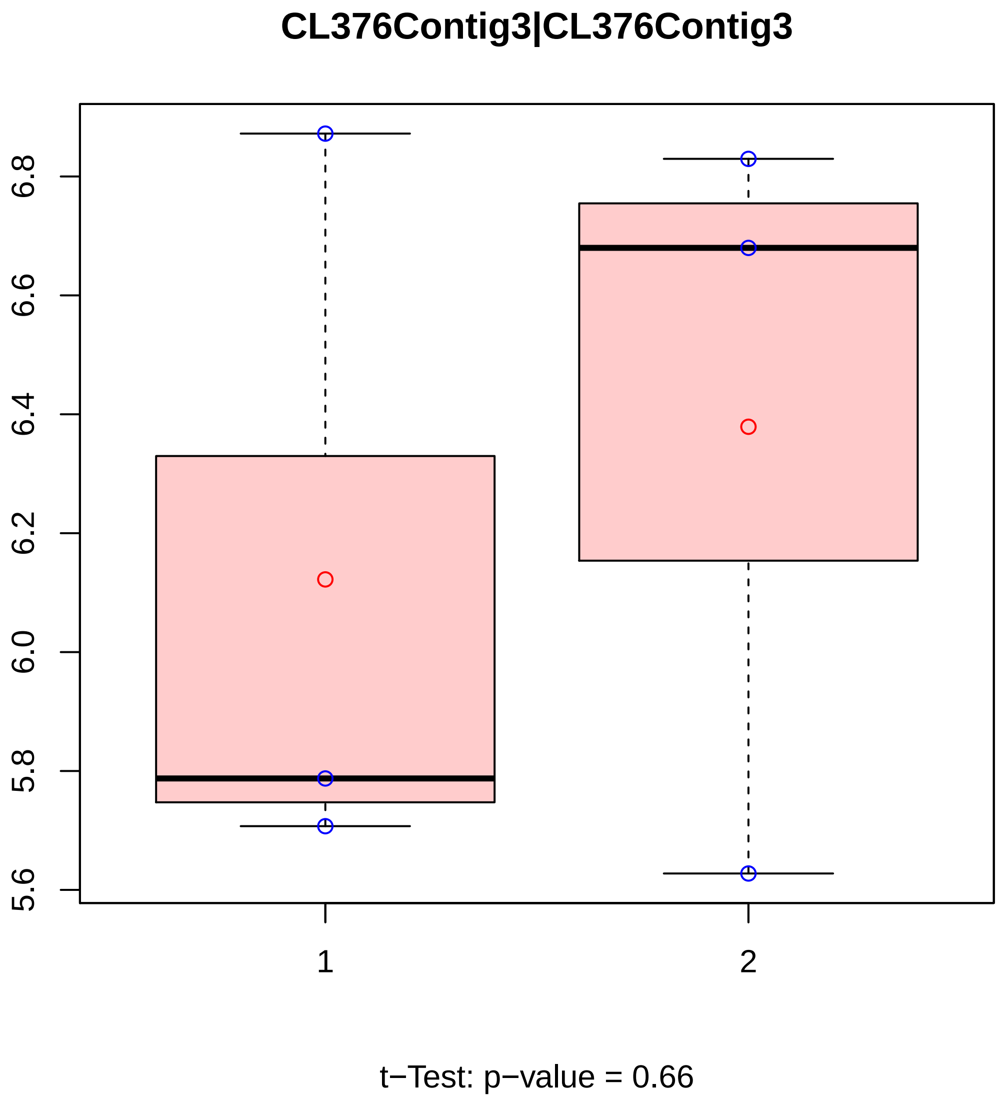

CL376Contig3|CL376Contig3
6.8
6.6
6.4
6.2
6.0
5.8
5.6
1
2
t−Test: p−value = 0.66

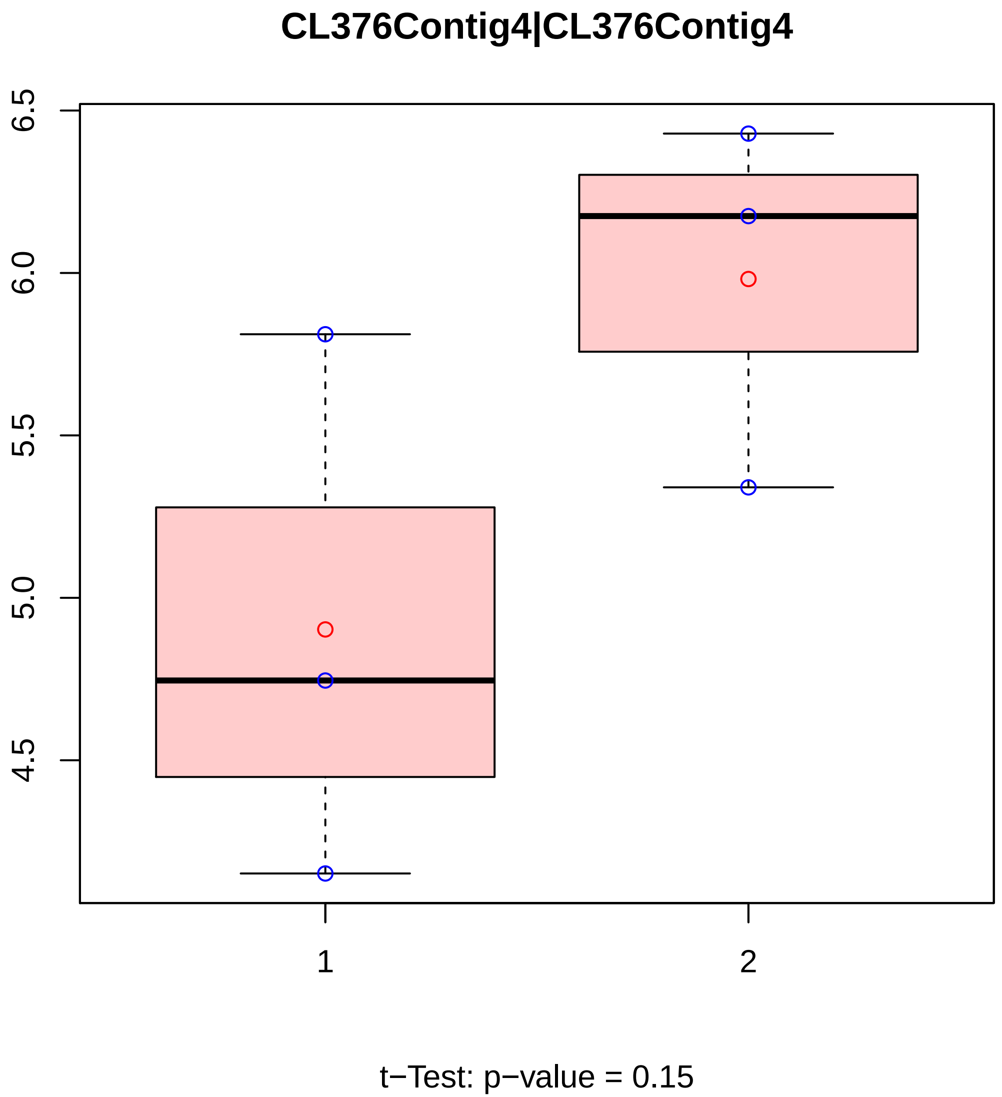
CL376Contig4|CL376Contig4
6.5
6.0
5.5
5.0
4.5
1
2
t−Test: p−value = 0.15

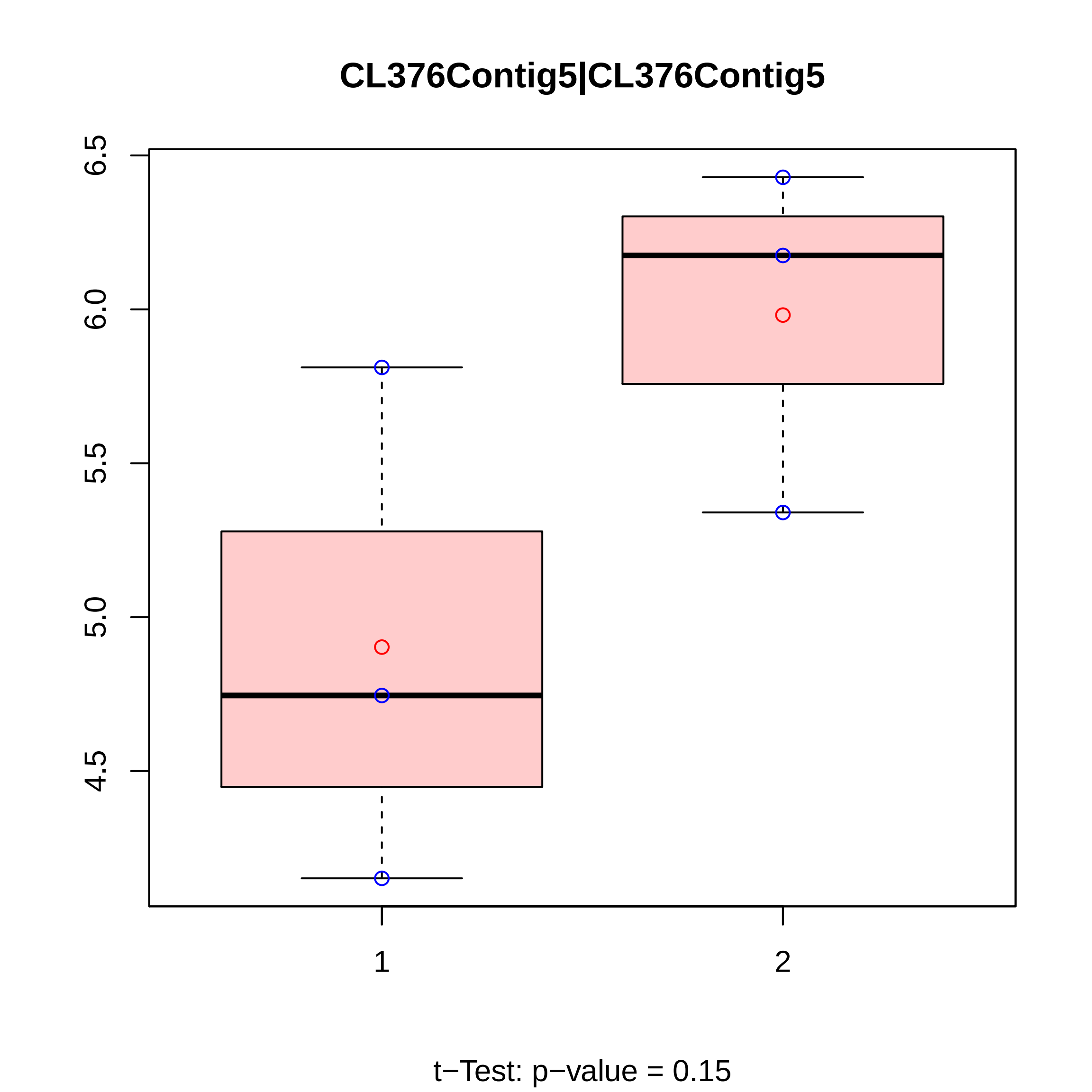
CL376Contig5|CL376Contig5
6.5
6.0
5.5
5.0
4.5
1
2
t−Test: p−value = 0.15

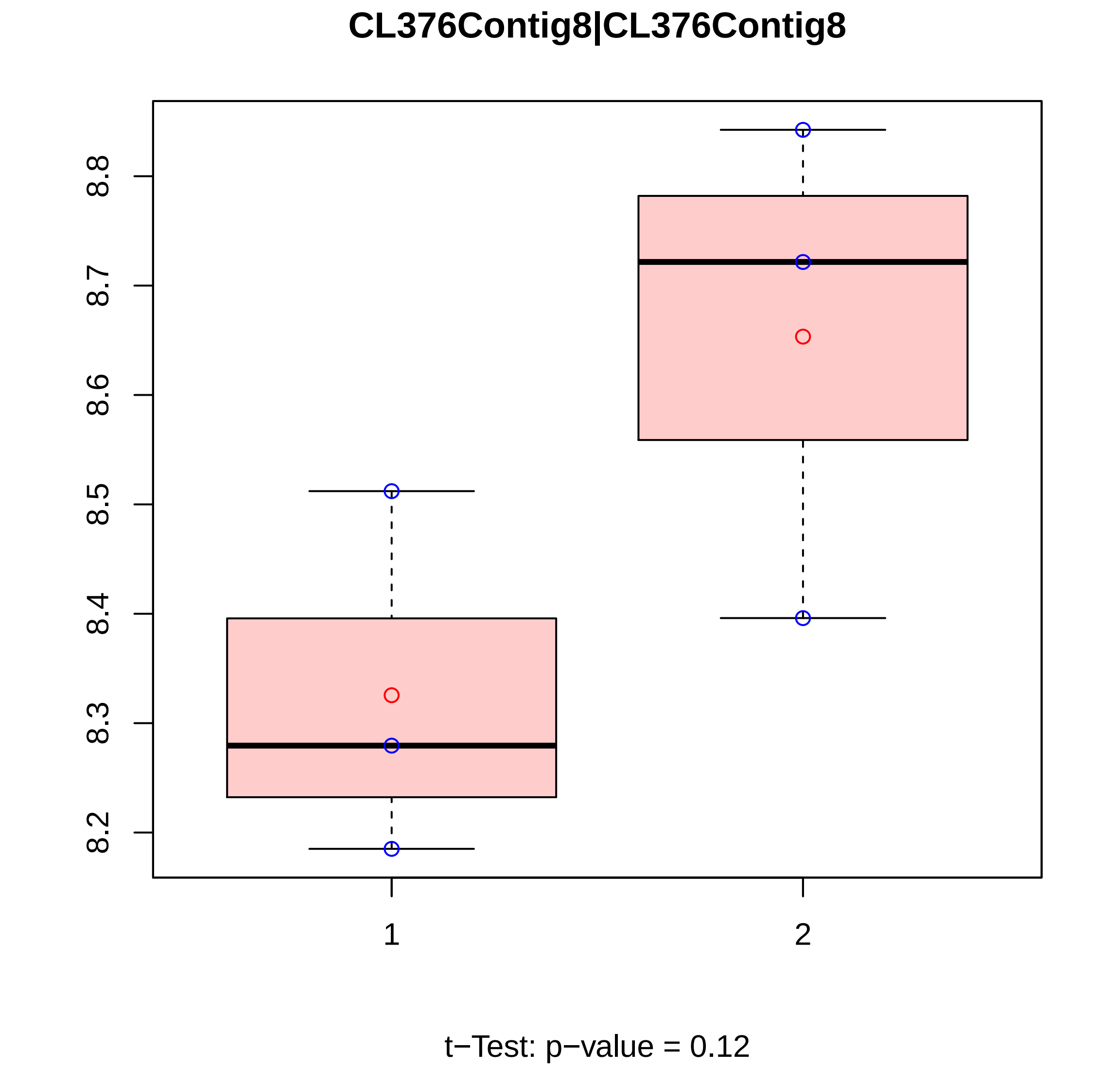

CL376Contig8|CL376Contig8
8.2
8.3
8.4
8.5
8.6
8.7
8.8
1
2
t−Test: p−value = 0.12

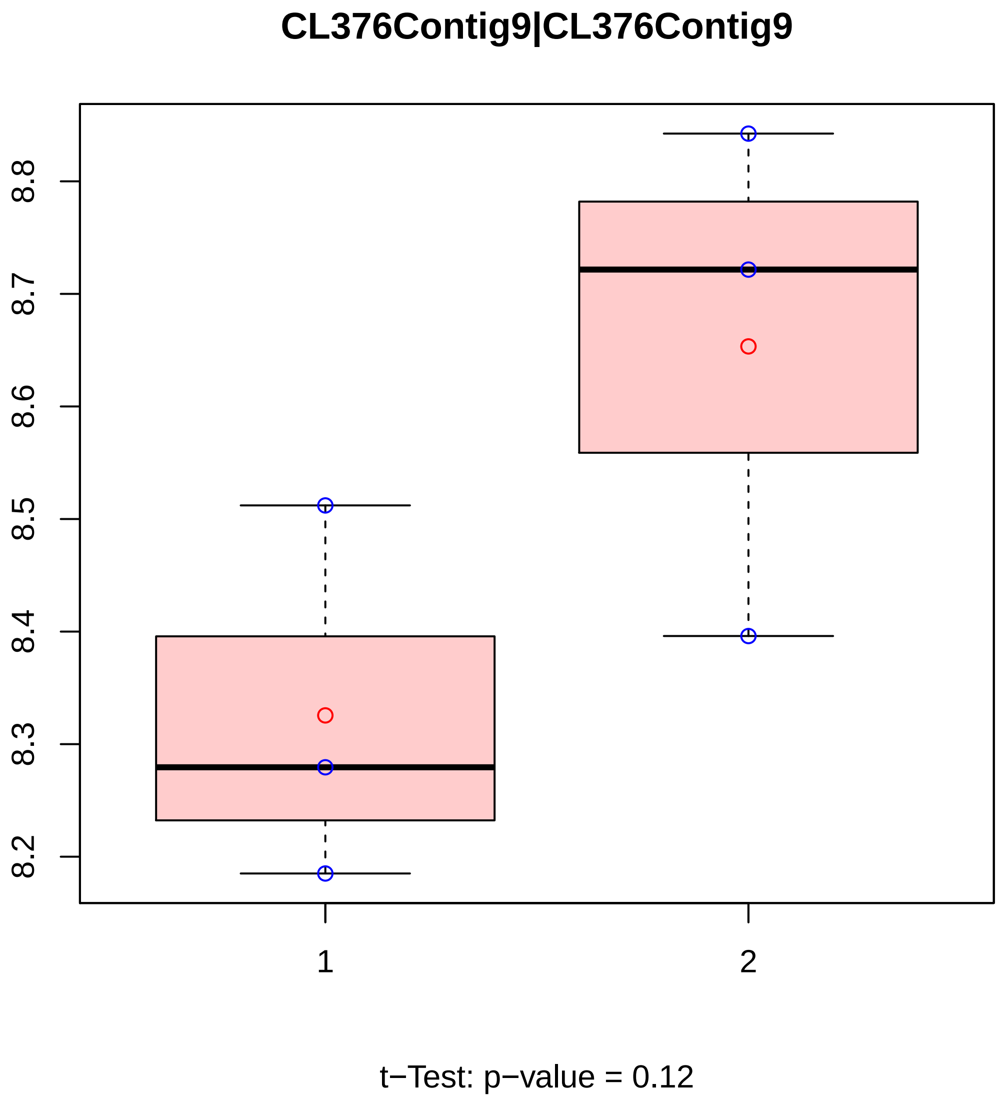
CL376Contig9|CL376Contig9
8.2
8.3
8.4
8.5
8.6
8.7
8.8
1
2
t−Test: p−value = 0.12

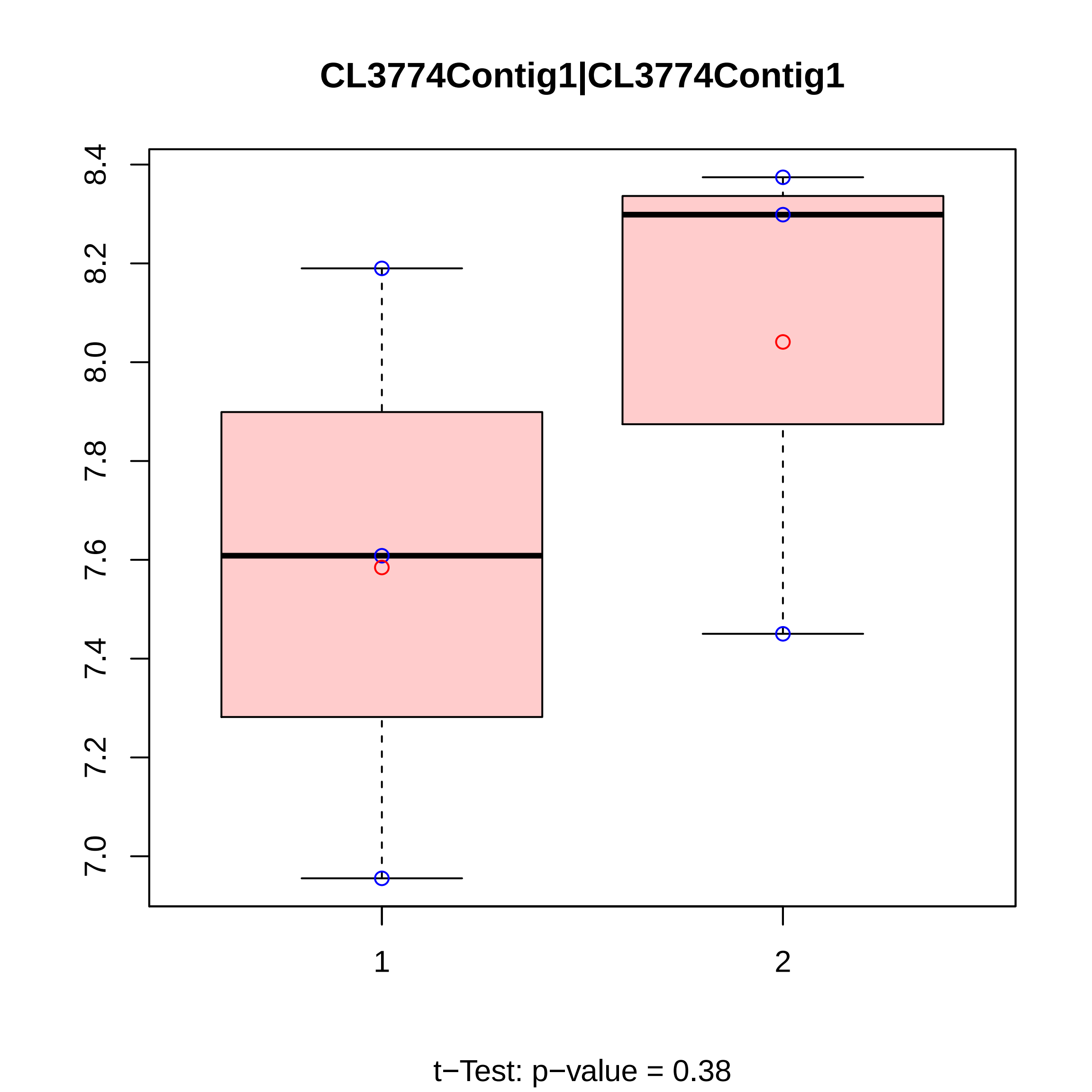

CL3774Contig1|CL3774Contig1
8.4
8.2
8.0
7.8
7.6
7.4
7.2
7.0
1
2
t−Test: p−value = 0.38

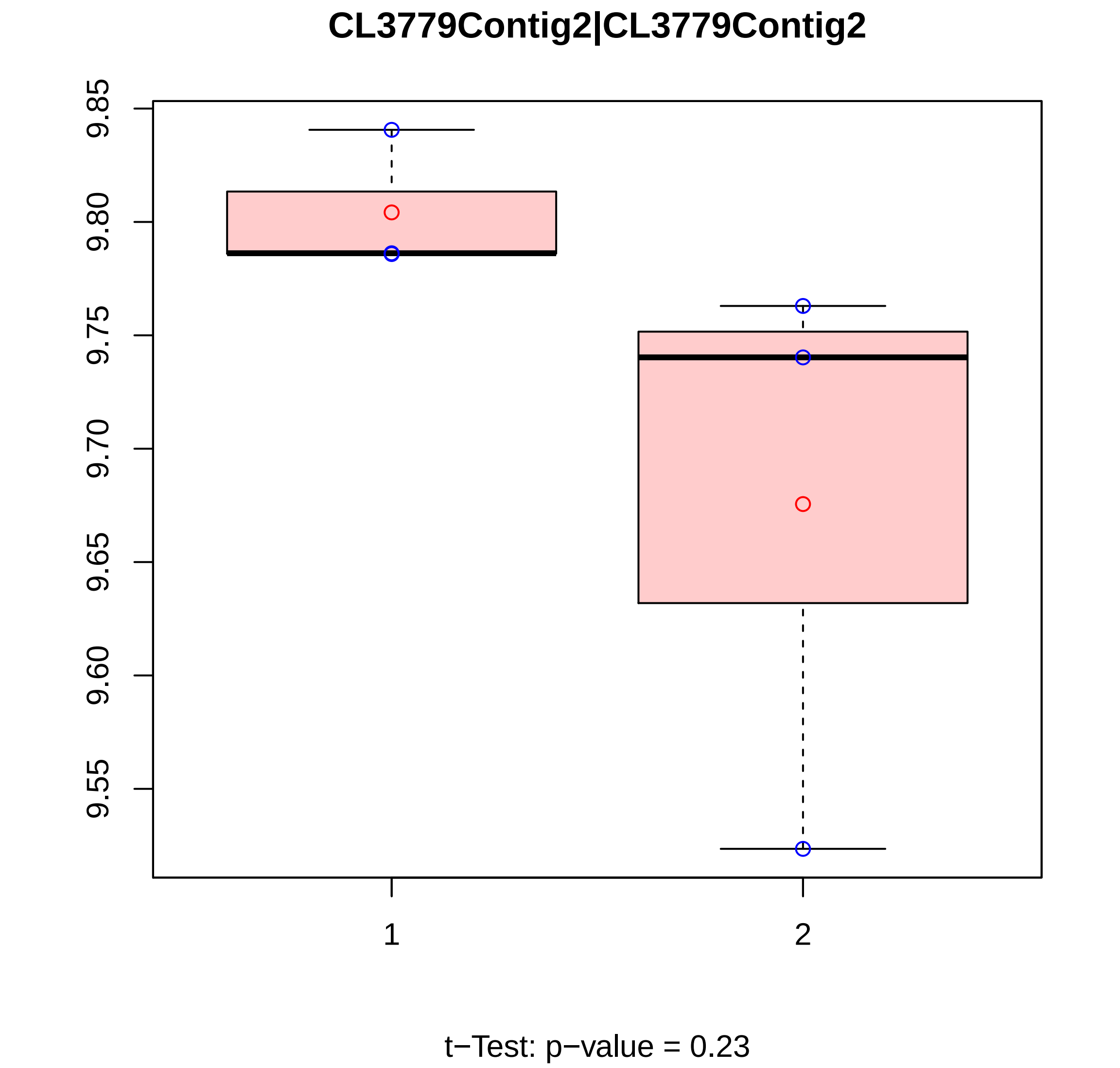

CL3779Contig2|CL3779Contig2
9.85
9.80
9.75
9.70
9.65
9.60
9.55
1
2
t−Test: p−value = 0.23

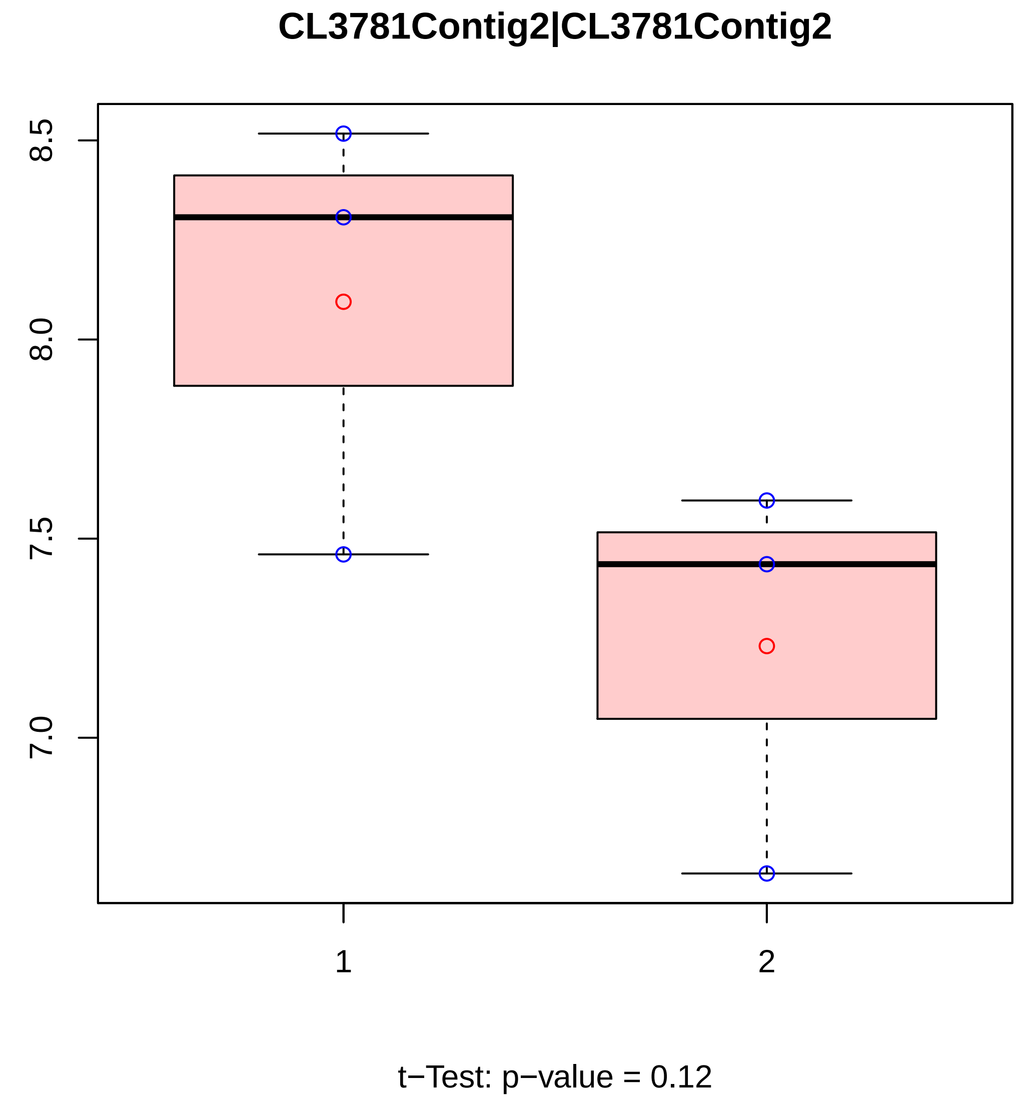
CL3781Contig2|CL3781Contig2
8.5
8.0
7.5
7.0
1
2
t−Test: p−value = 0.12

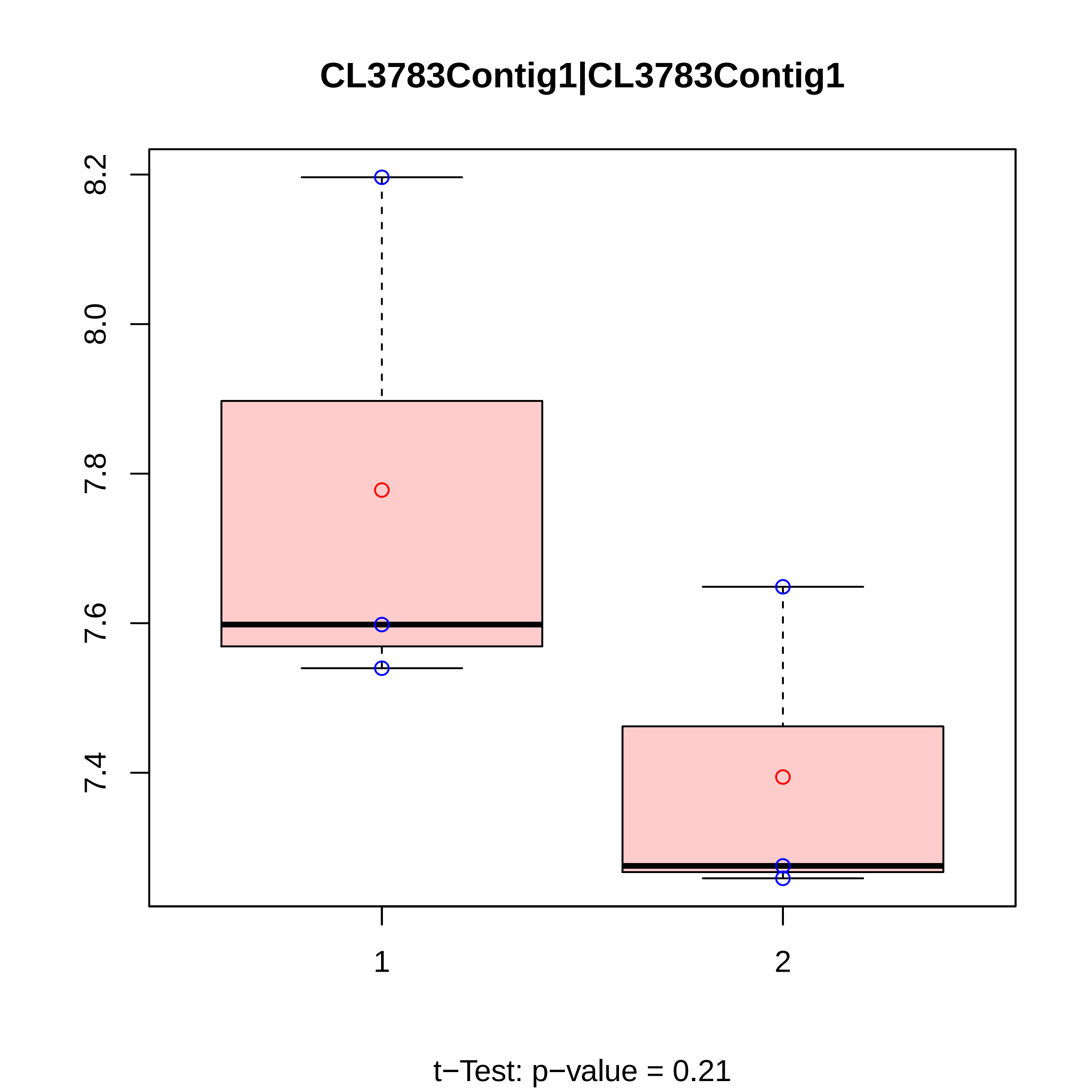
CL3783Contig1|CL3783Contig1
8.2
8.0
7.8
7.6
7.4
1
2
t−Test: p−value = 0.21

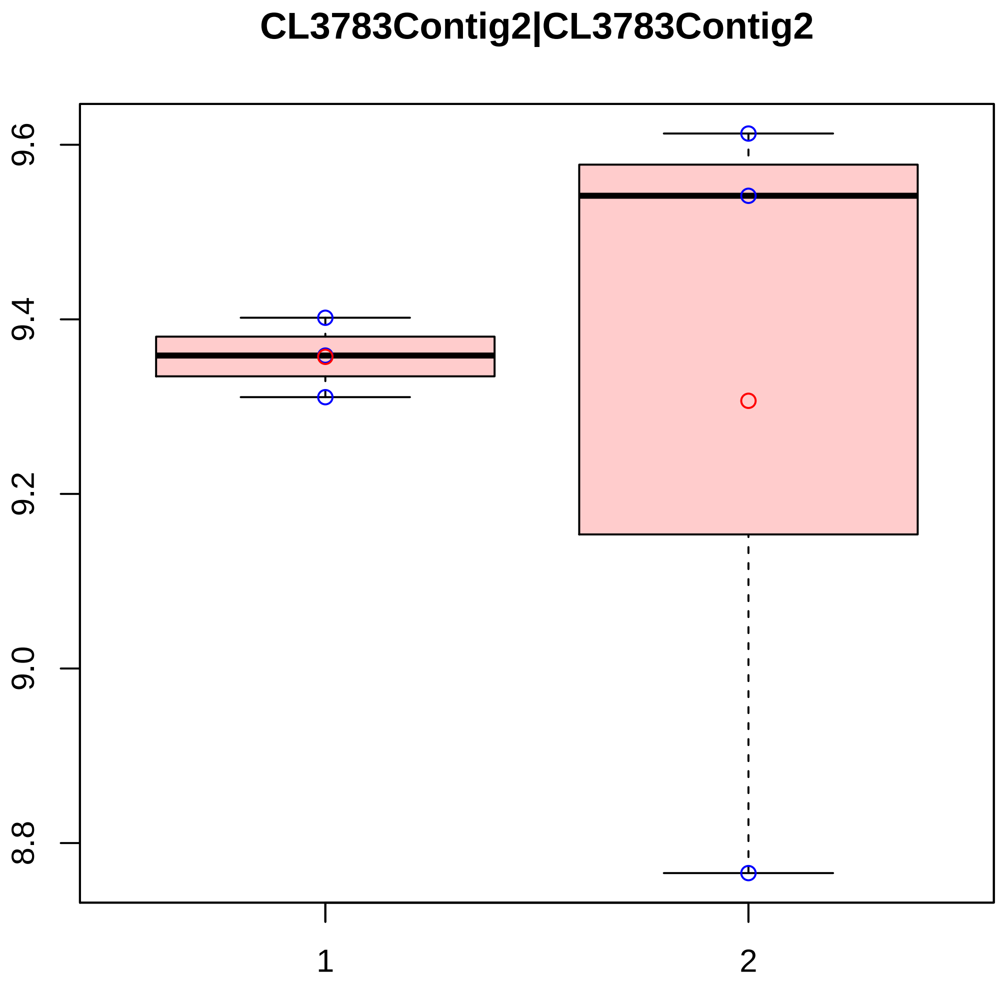

t−Test: p−value = 0.87

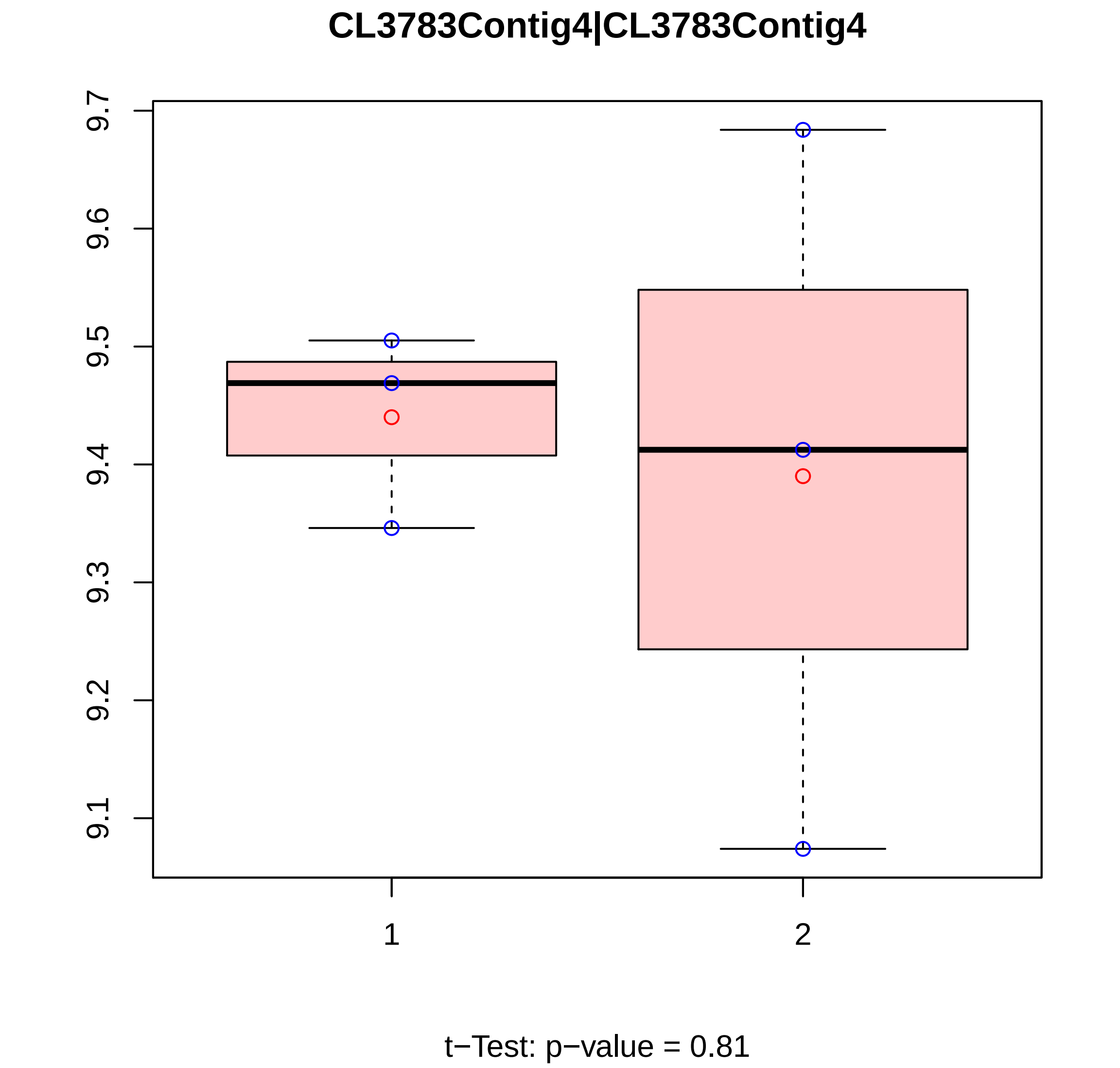
CL3783Contig4|CL3783Contig4
9.1
9.2
9.3
9.4
9.5
9.6
9.7
1
2
t−Test: p−value = 0.81

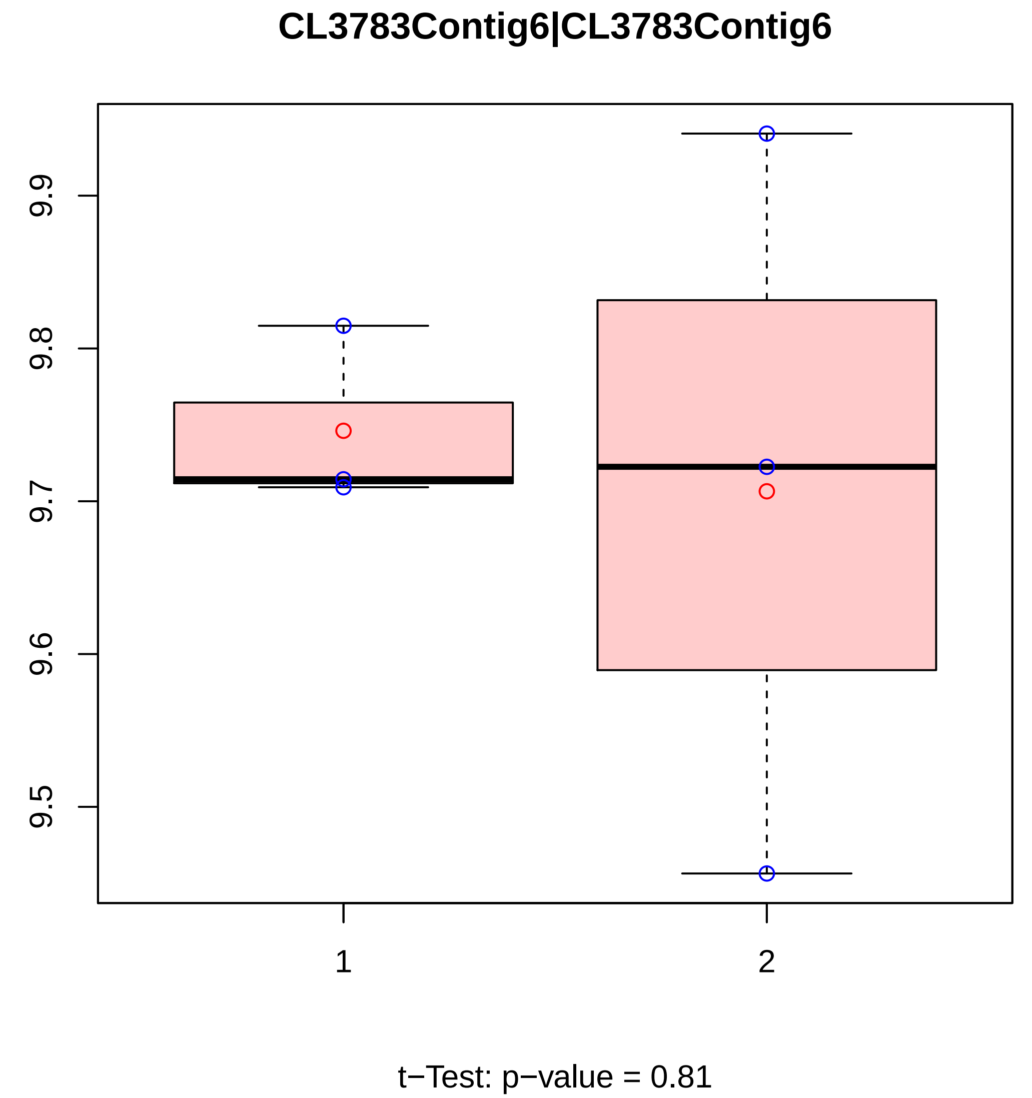
CL3783Contig6|CL3783Contig6
9.5
9.6
9.7
9.8
9.9
1
2
t−Test: p−value = 0.81

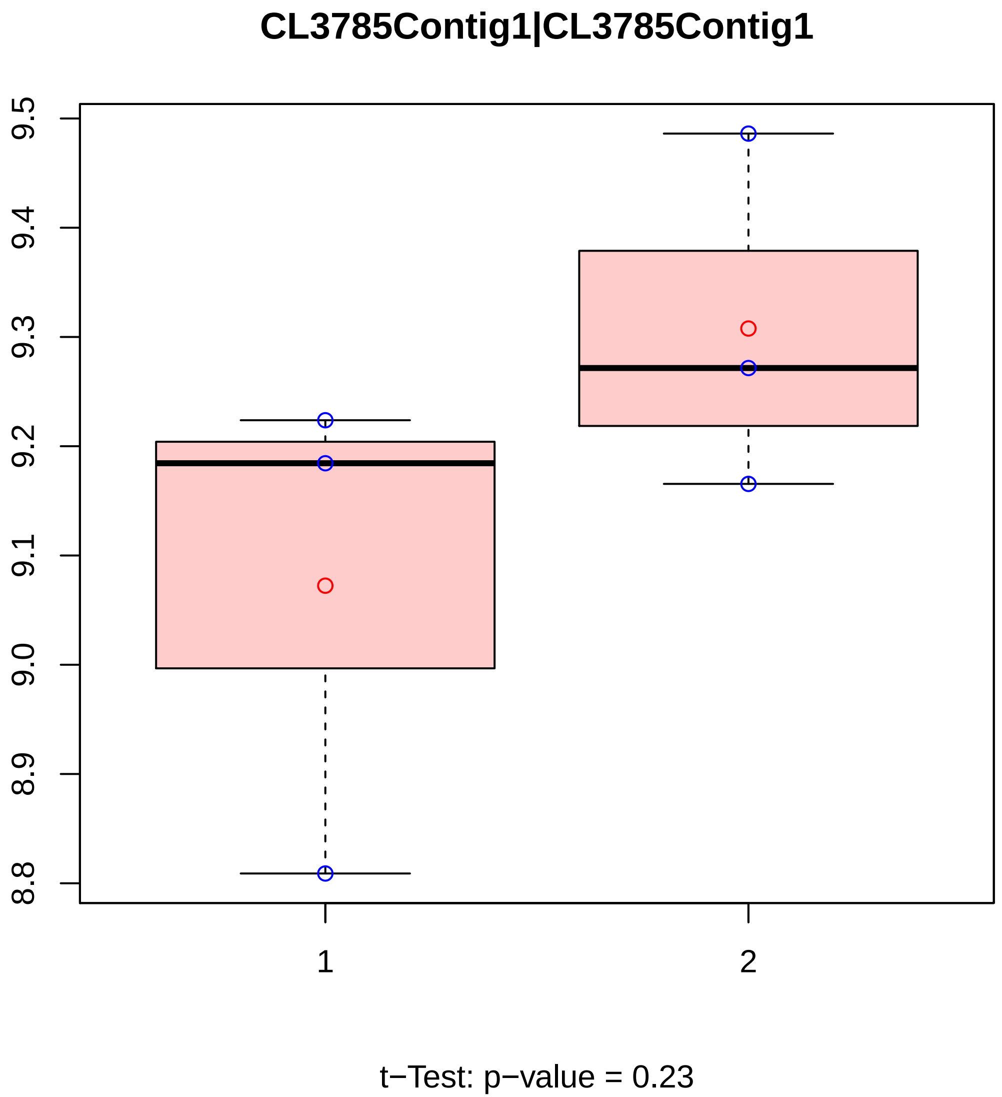

CL3785Contig1|CL3785Contig1
9.5
9.4
9.3
9.2
9.1
9.0
8.9
8.8
1
2
t−Test: p−value = 0.23

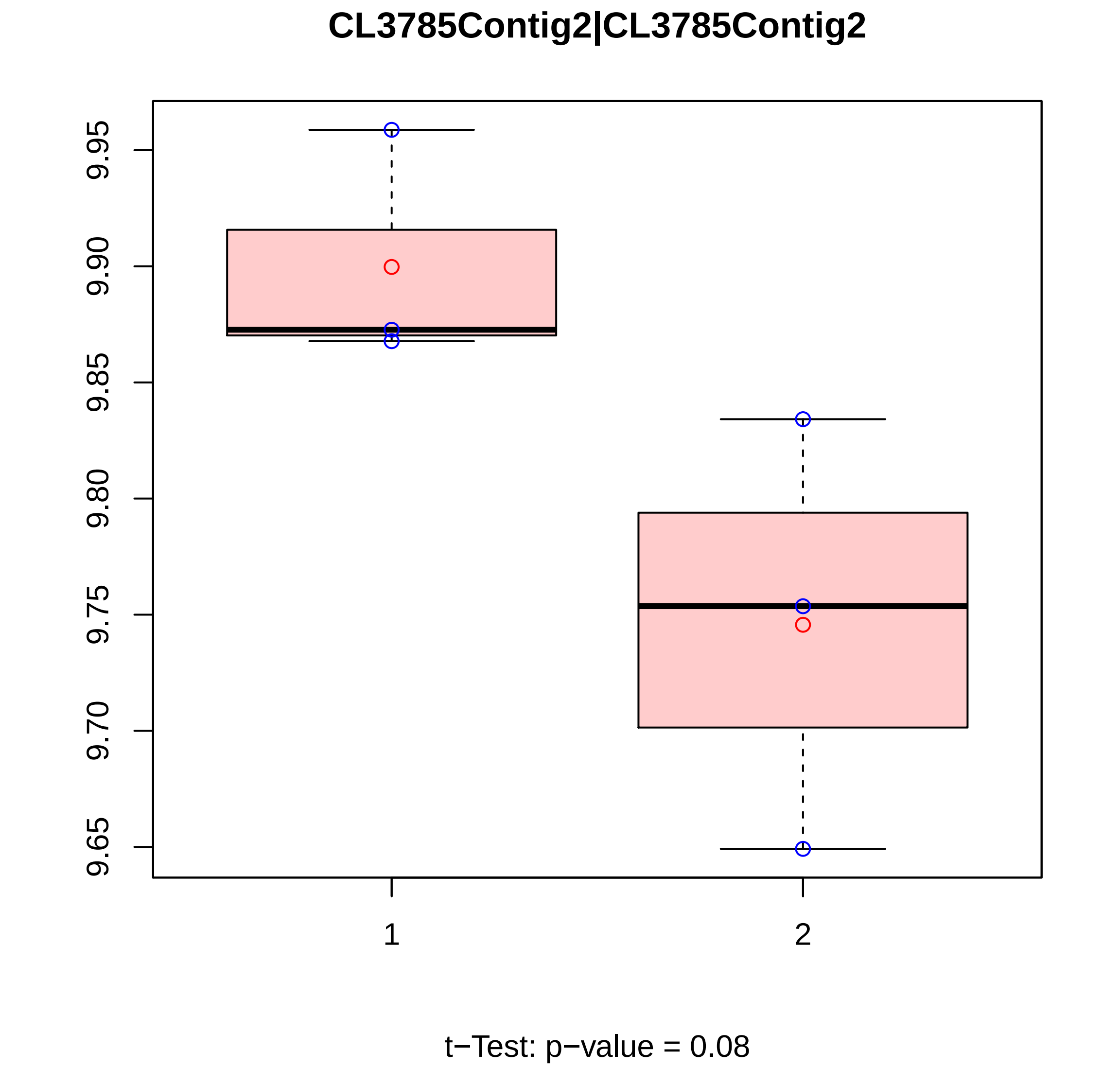

CL3785Contig2|CL3785Contig2
9.65
9.70
9.75
9.80
9.85
9.90
9.95
1
2
t−Test: p−value = 0.08

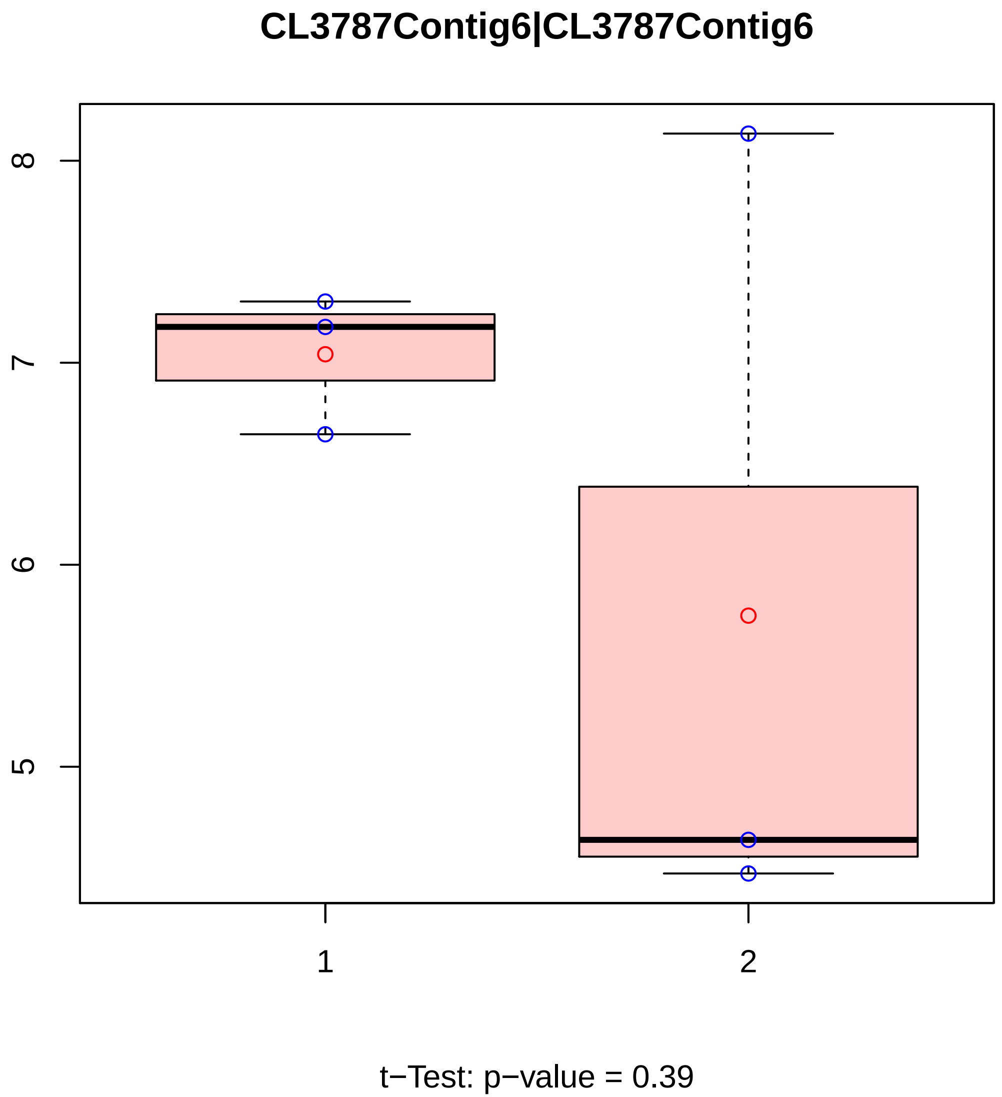

CL3787Contig6|CL3787Contig6
8
7
6
5
1
2
t−Test: p−value = 0.39

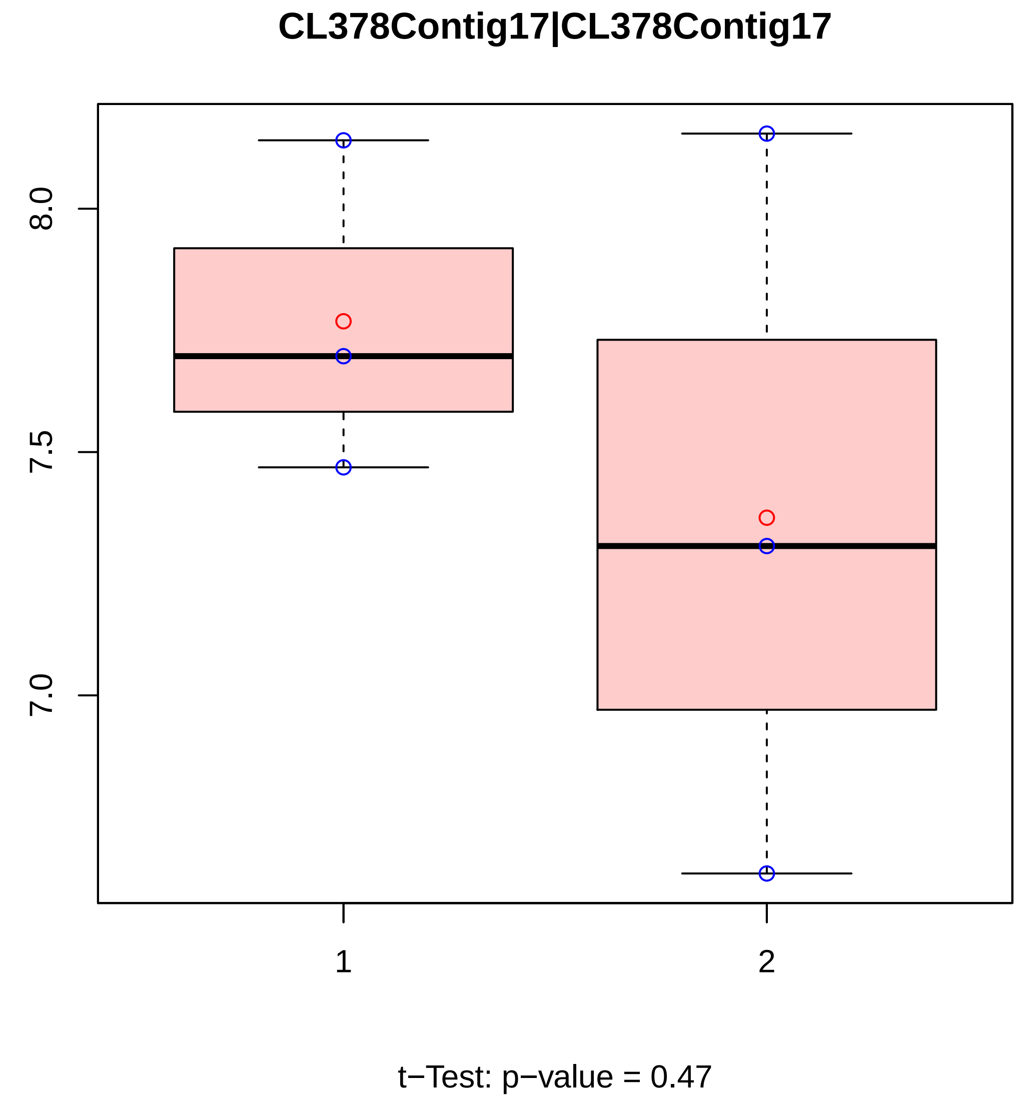
CL378Contig17|CL378Contig17
8.0
7.5
7.0
1
2
t−Test: p−value = 0.47

## CL378Contig18|CL378Contig18

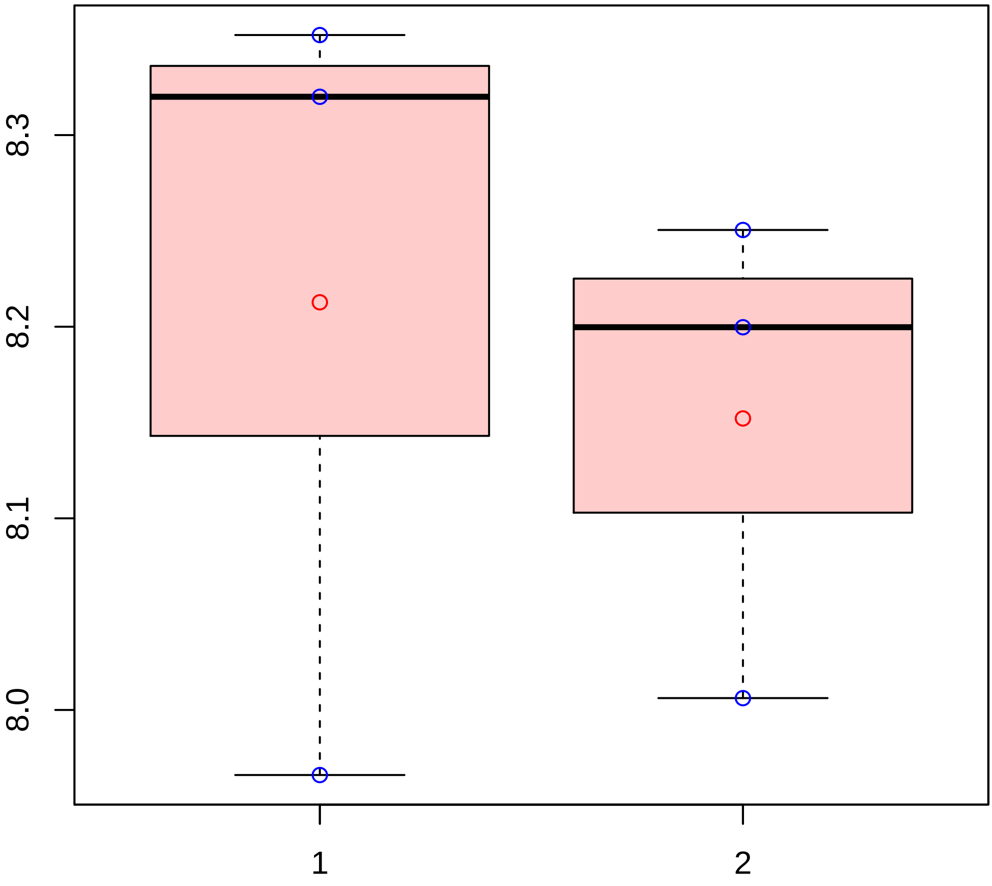


t−Test: p−value = 0.7

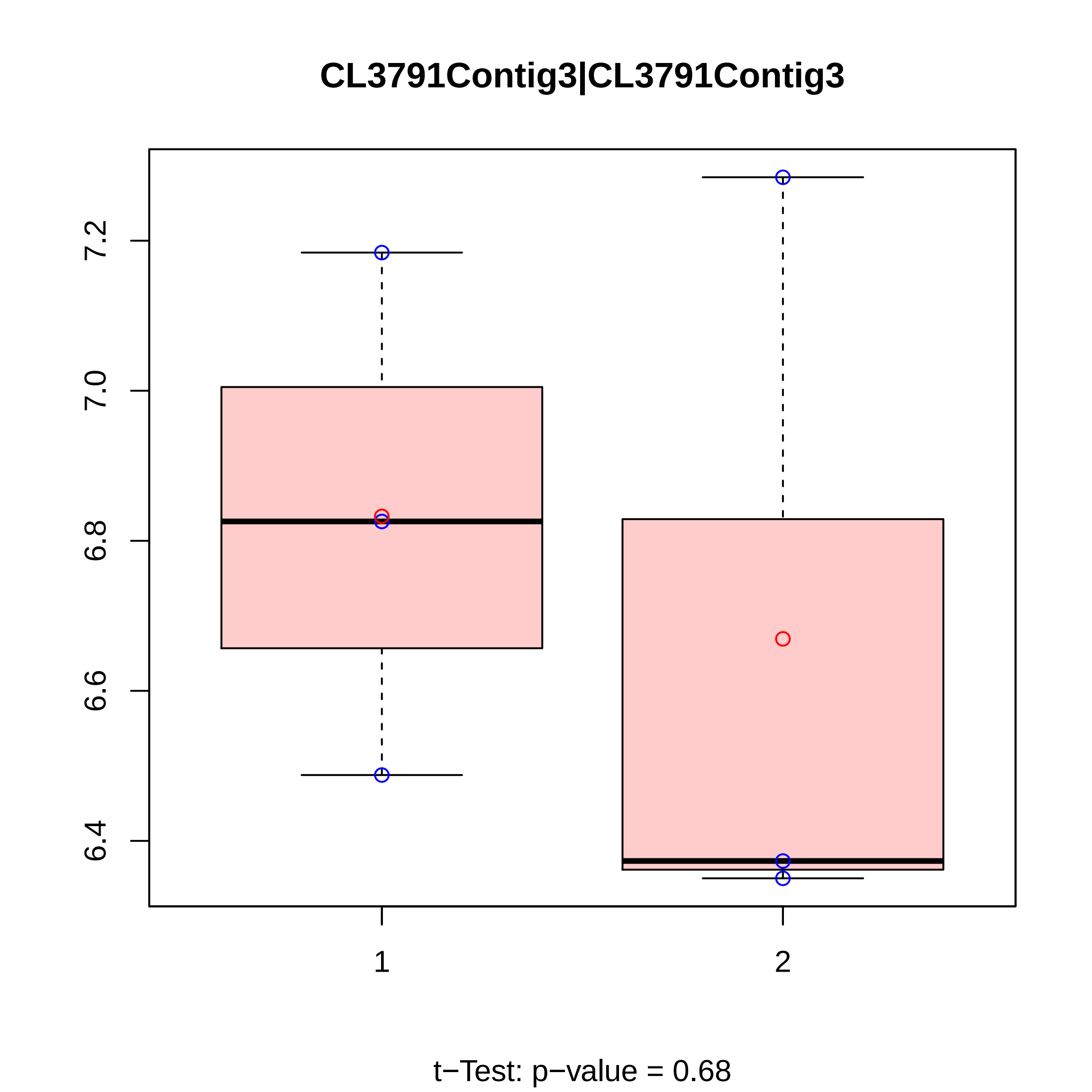
CL3791Contig3|CL3791Contig3
7.2
7.0
6.8
6.6
6.4
1
2
t−Test: p−value = 0.68

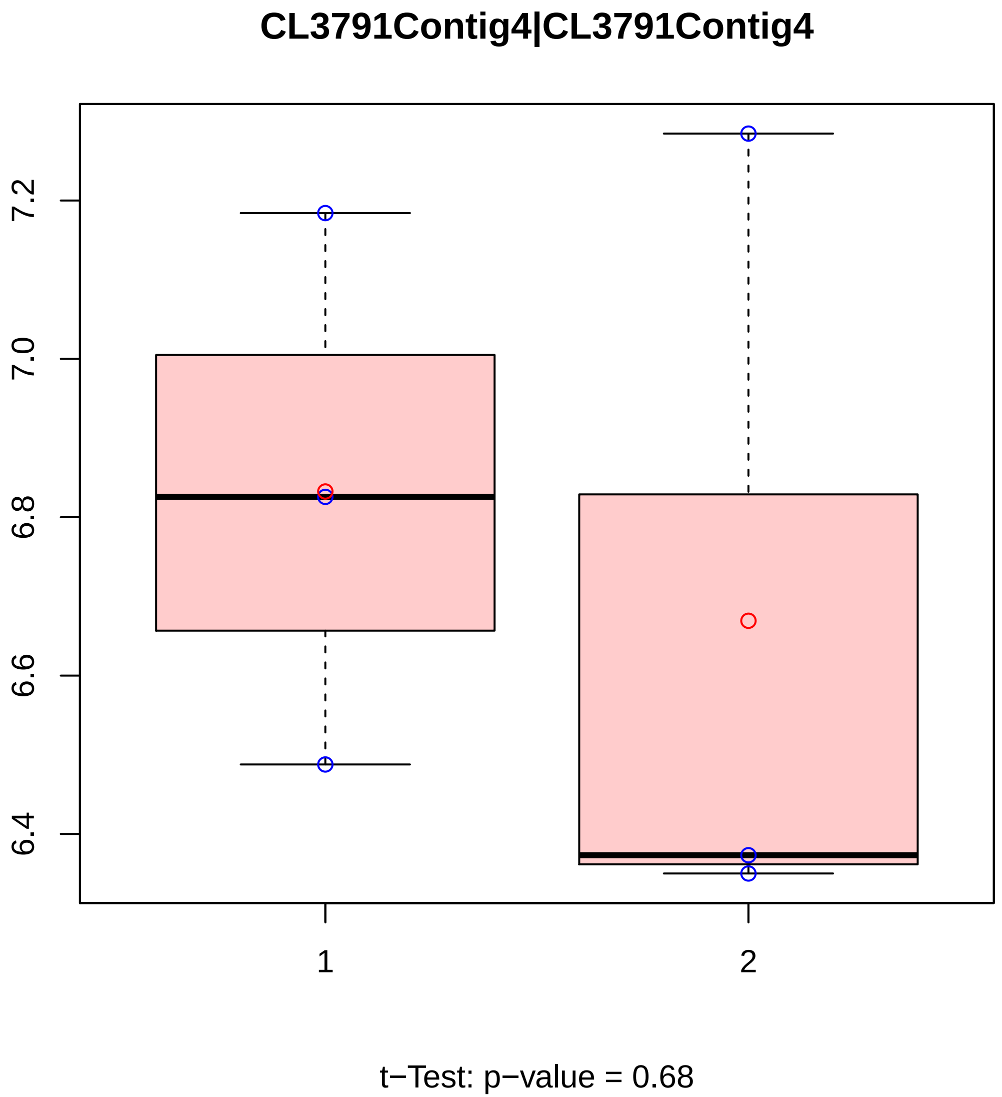
CL3791Contig4|CL3791Contig4
7.2
7.0
6.8
6.6
6.4
1
2
t−Test: p−value = 0.68

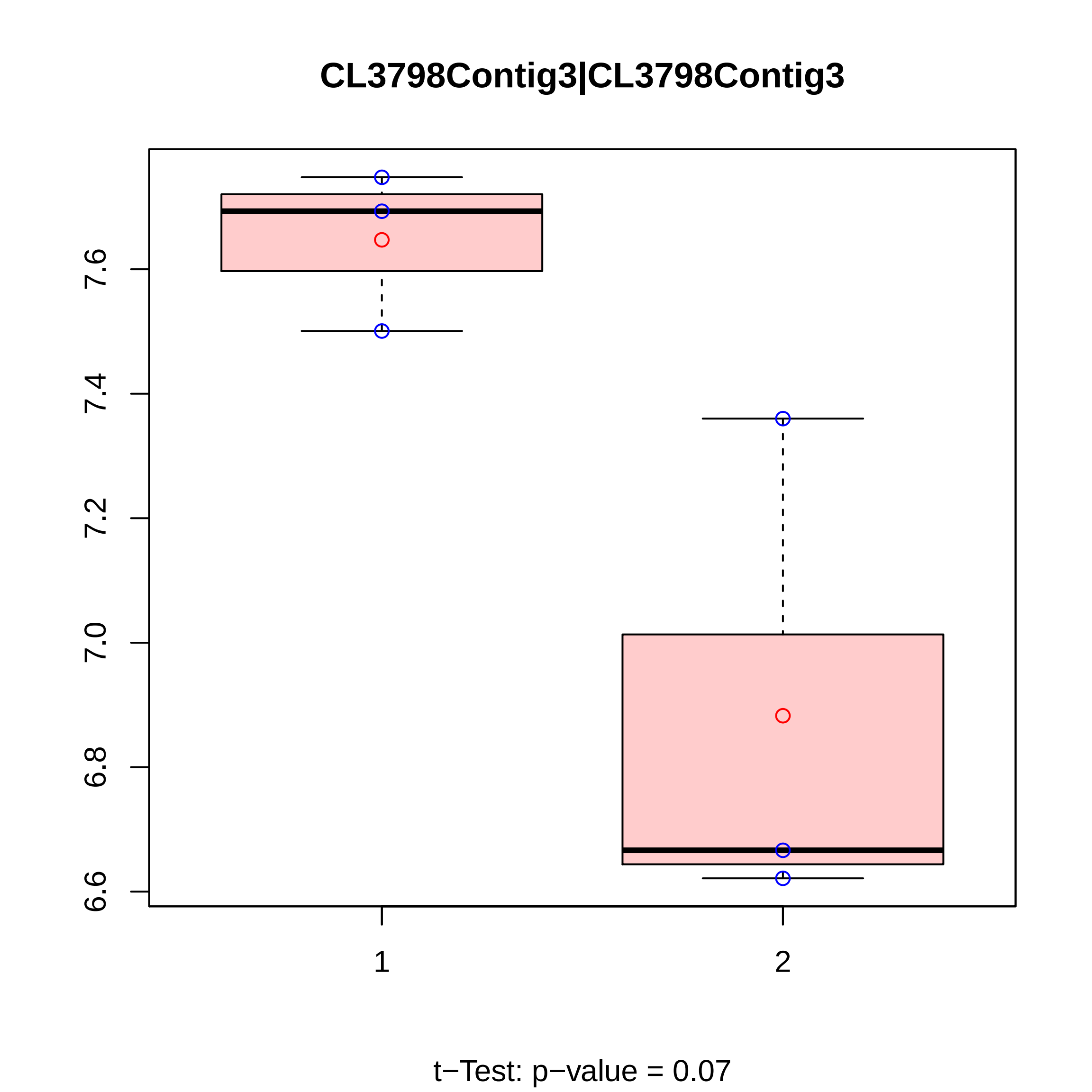

CL3798Contig3|CL3798Contig3
7.6
7.4
7.2
7.0
6.8
6.6
1
2
t−Test: p−value = 0.07

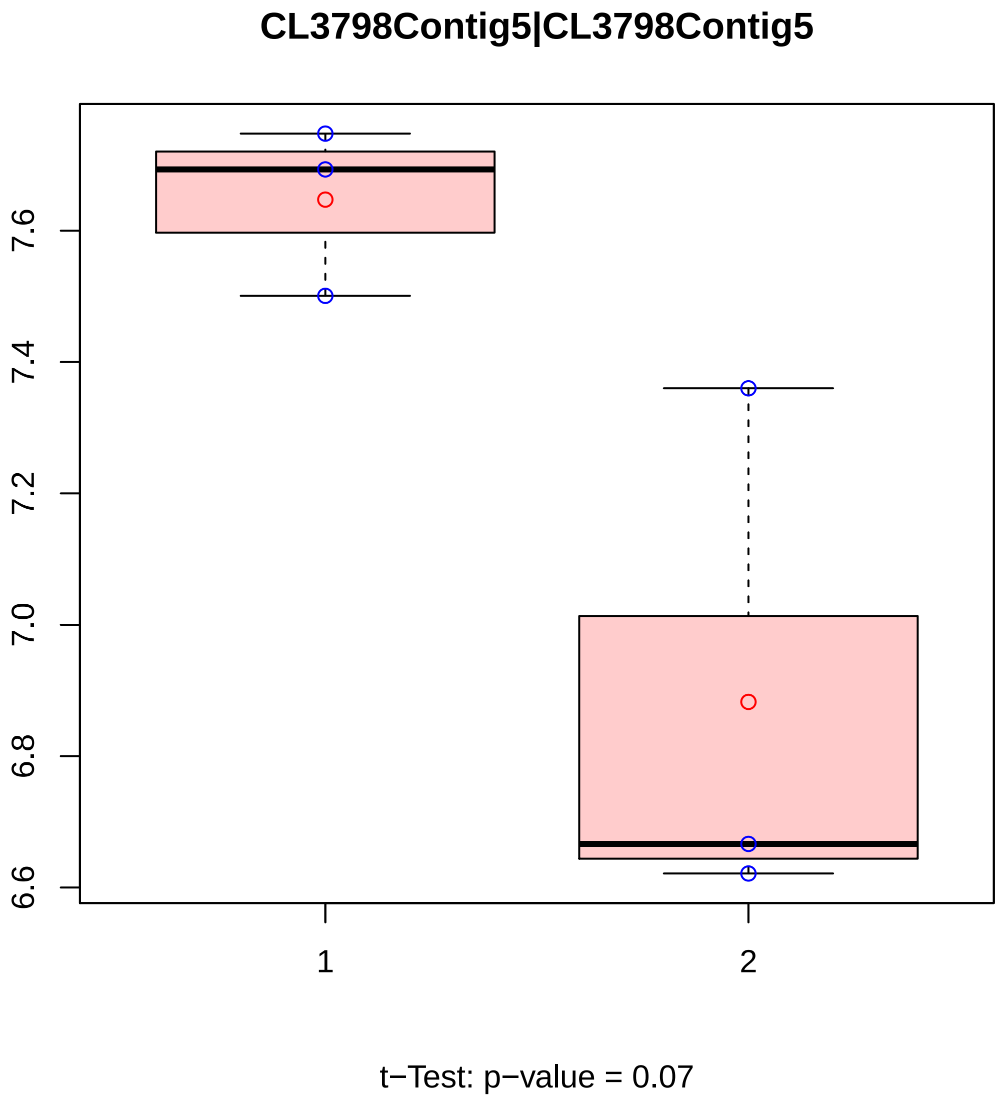

CL3798Contig5|CL3798Contig5
7.6
7.4
7.2
7.0
6.8
6.6
1
2
t−Test: p−value = 0.07

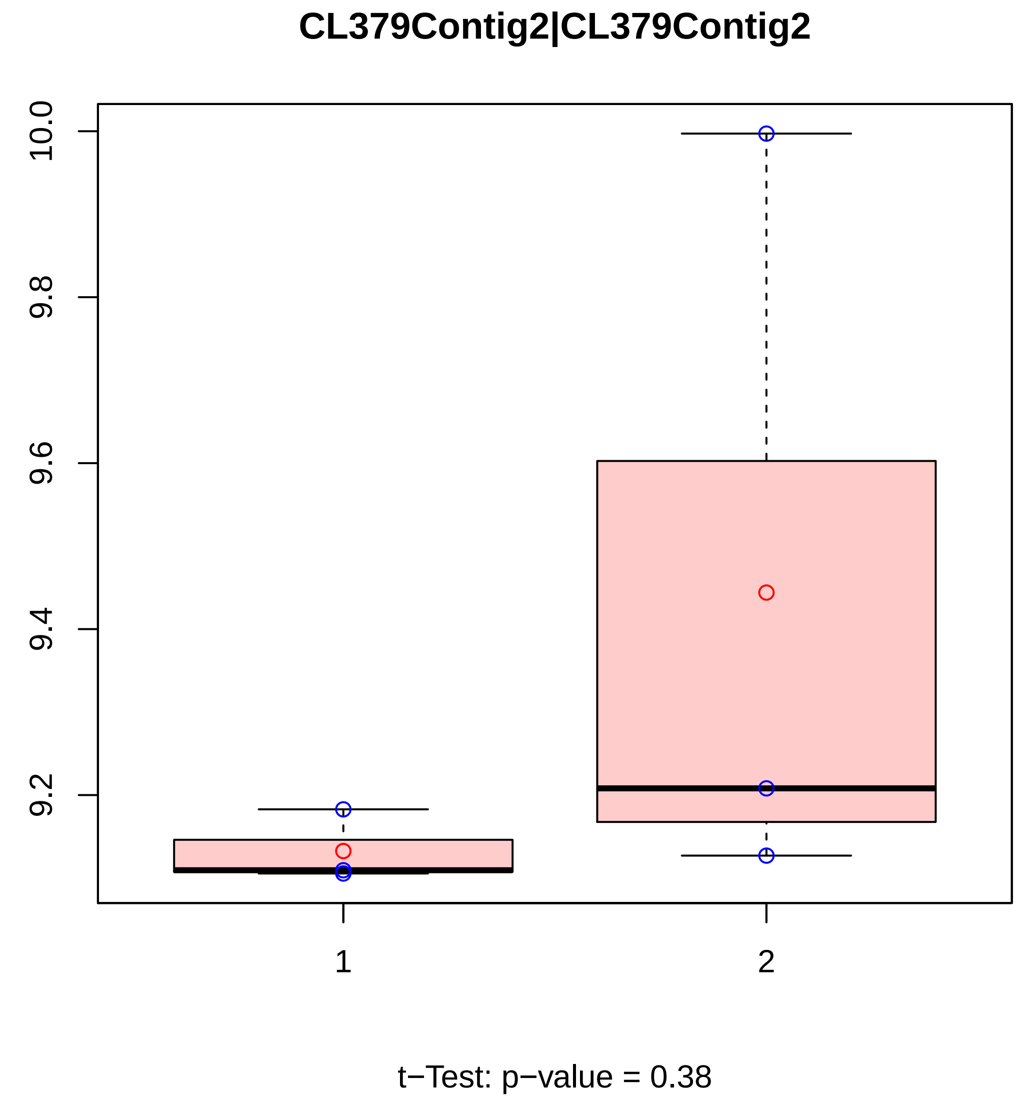

CL379Contig2|CL379Contig2
10.0
9.8
9.6
9.4
9.2
1
2
t−Test: p−value = 0.38

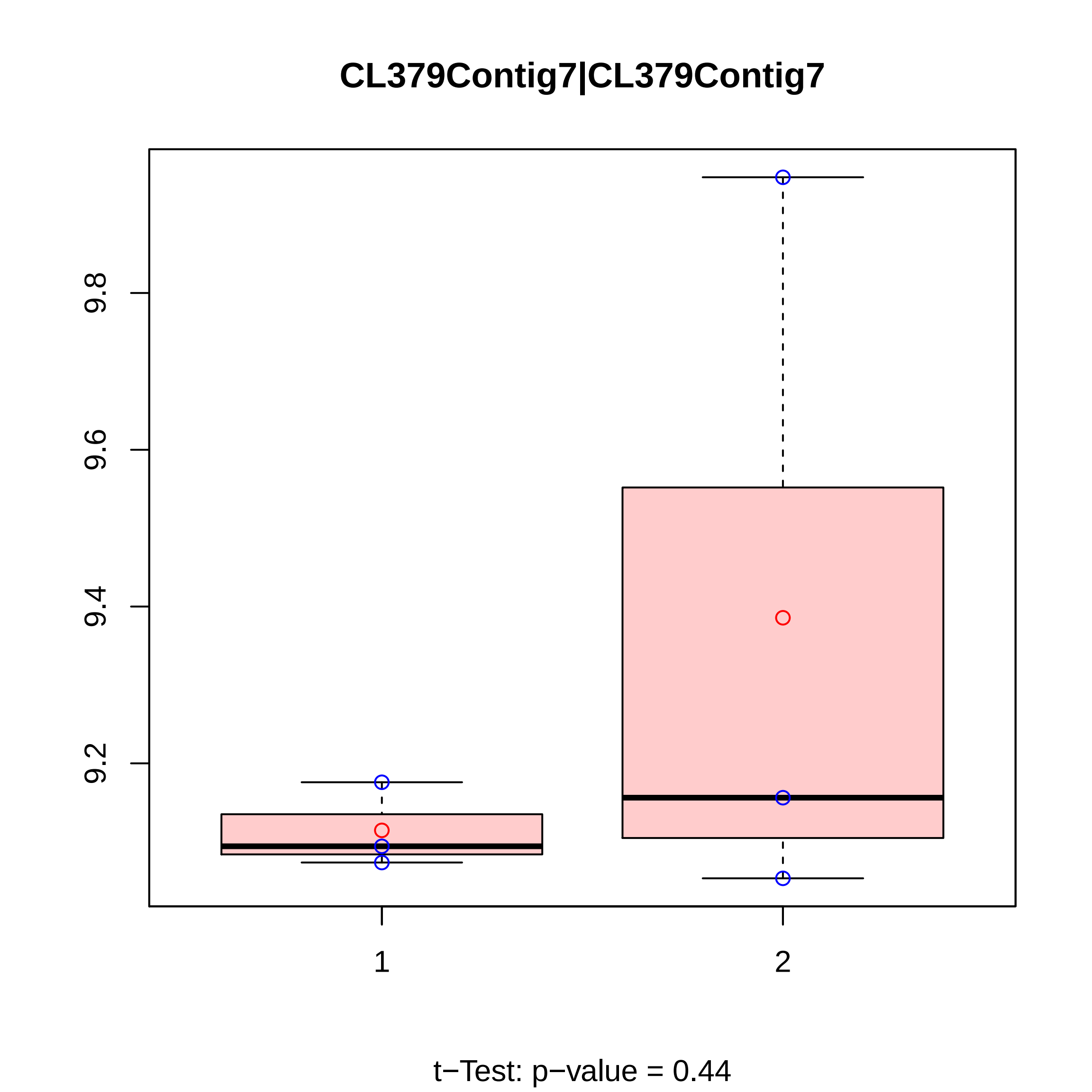

CL379Contig7|CL379Contig7
9.2
9.4
9.6
9.8
1
2
t−Test: p−value = 0.44

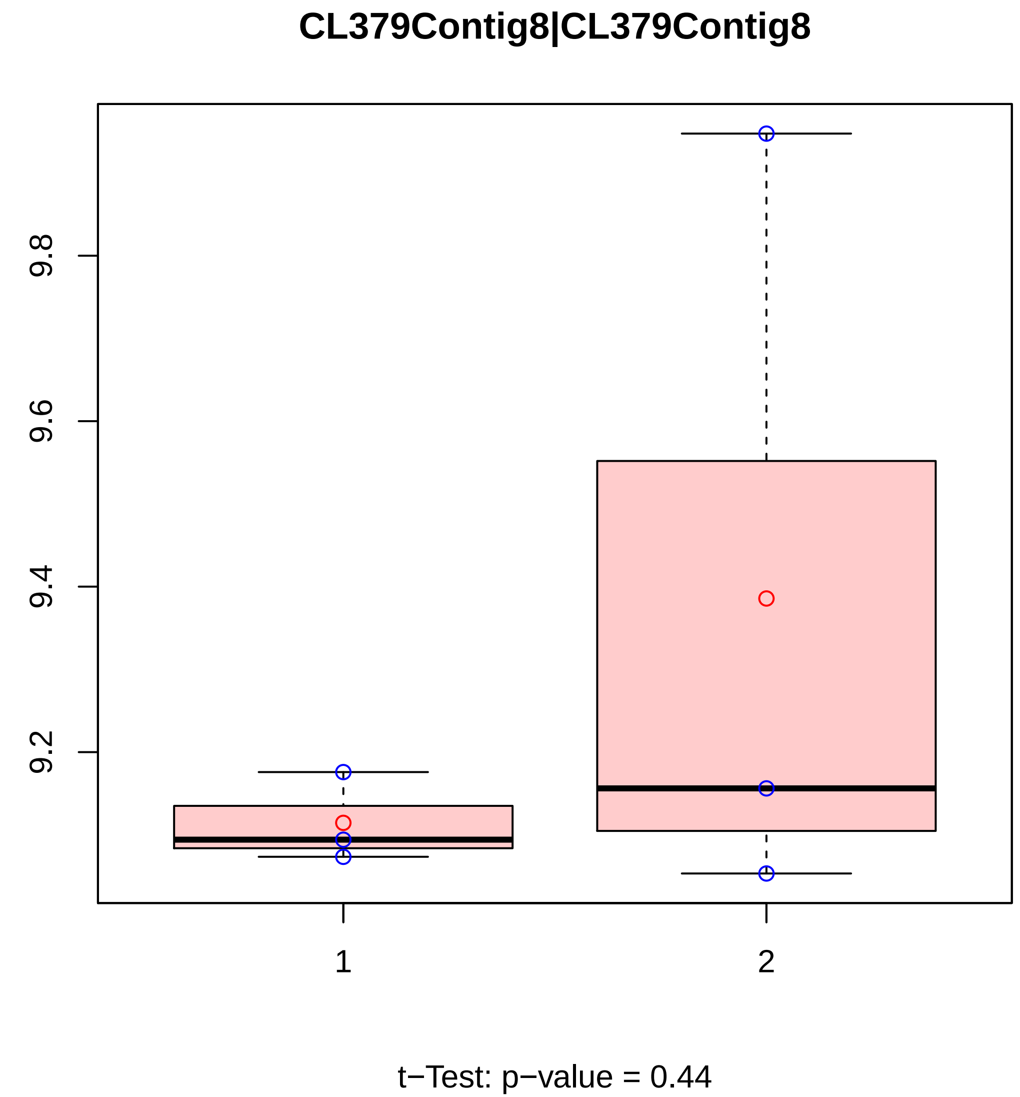
CL379Contig8|CL379Contig8
9.2
9.4
9.6
9.8
1
2
t−Test: p−value = 0.44

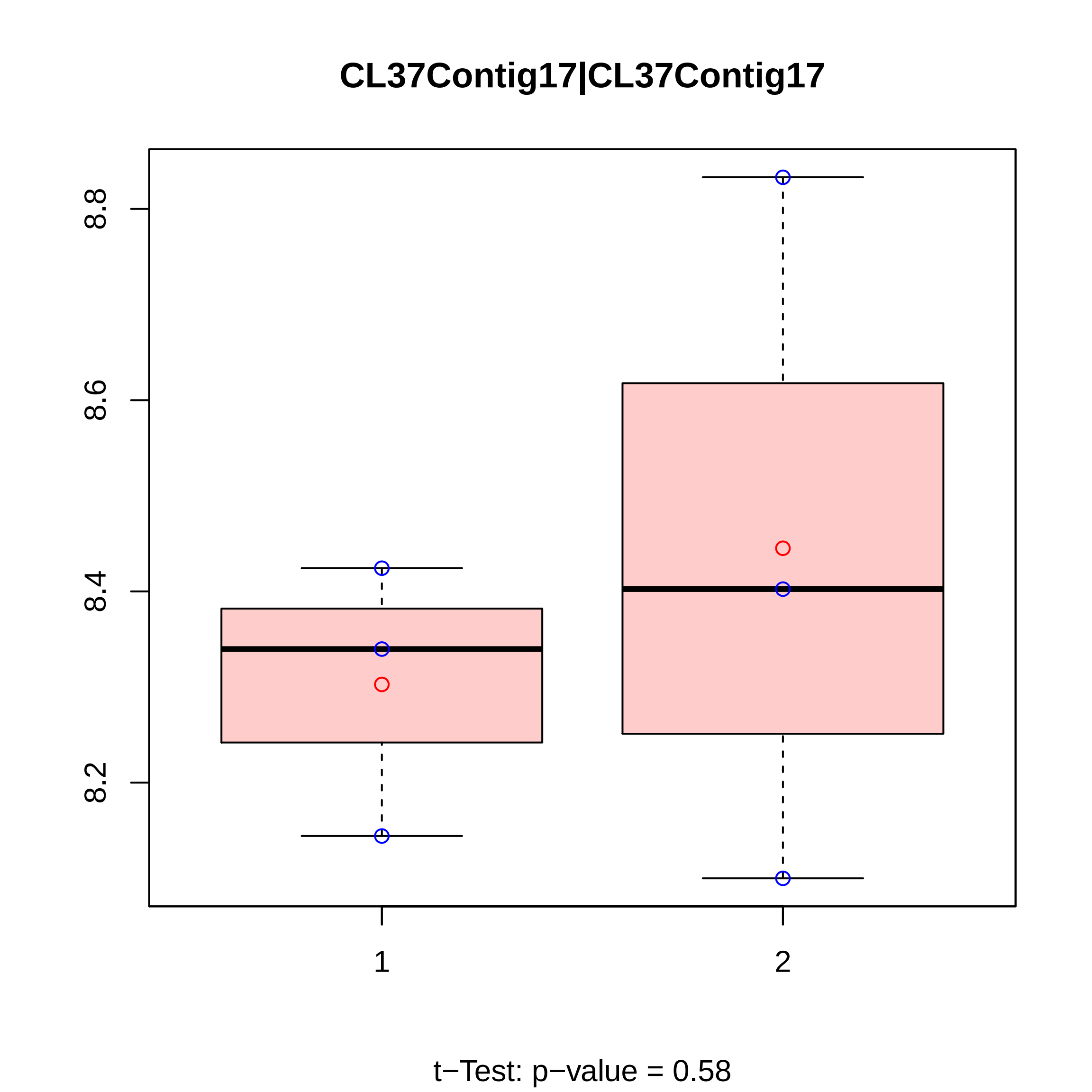
CL37Contig17|CL37Contig17
8.2
8.4
8.6
8.8
1
2
t−Test: p−value = 0.58

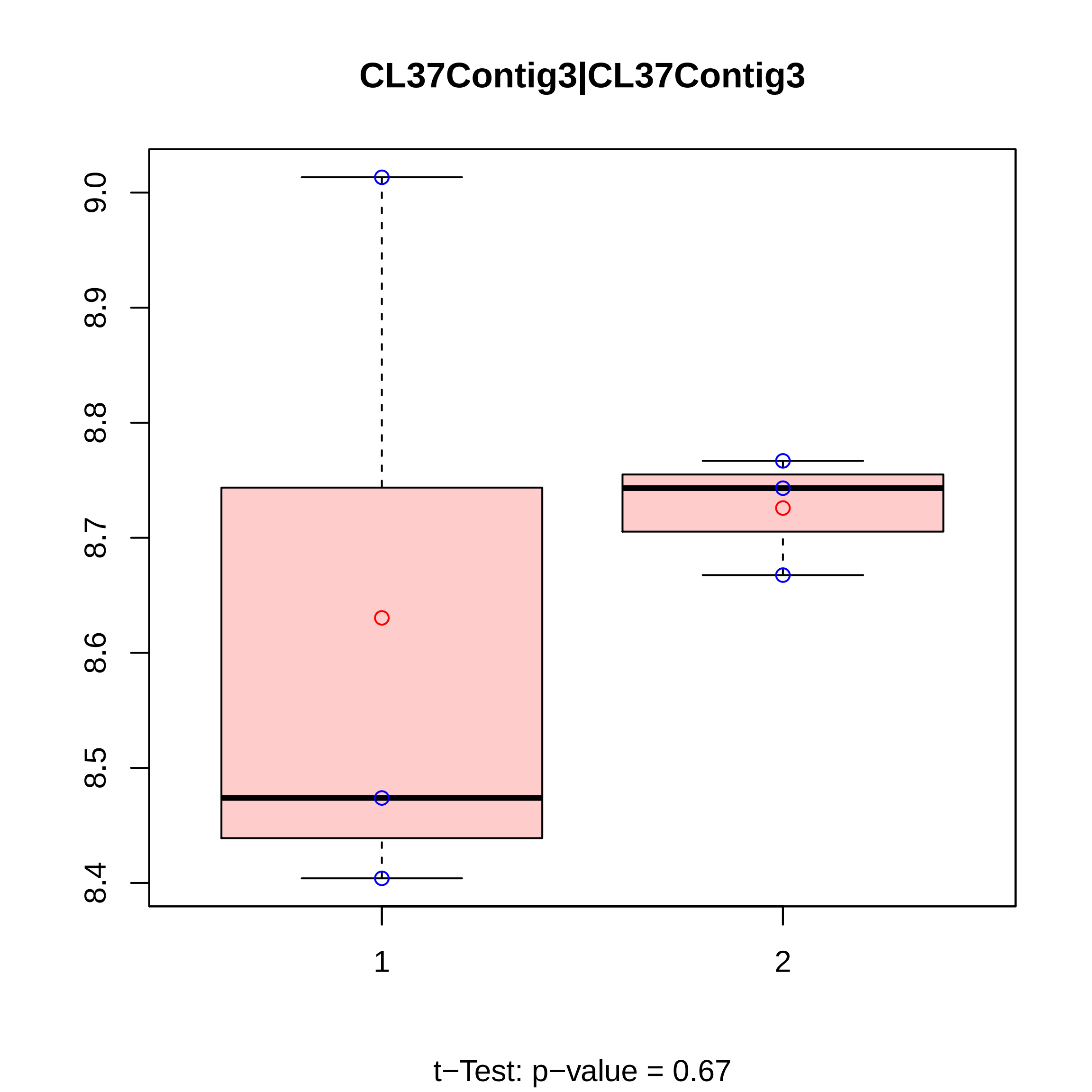
CL37Contig3|CL37Contig3
9.0
8.9
8.8
8.7
8.6
8.5
8.4
1
2
t−Test: p−value = 0.67

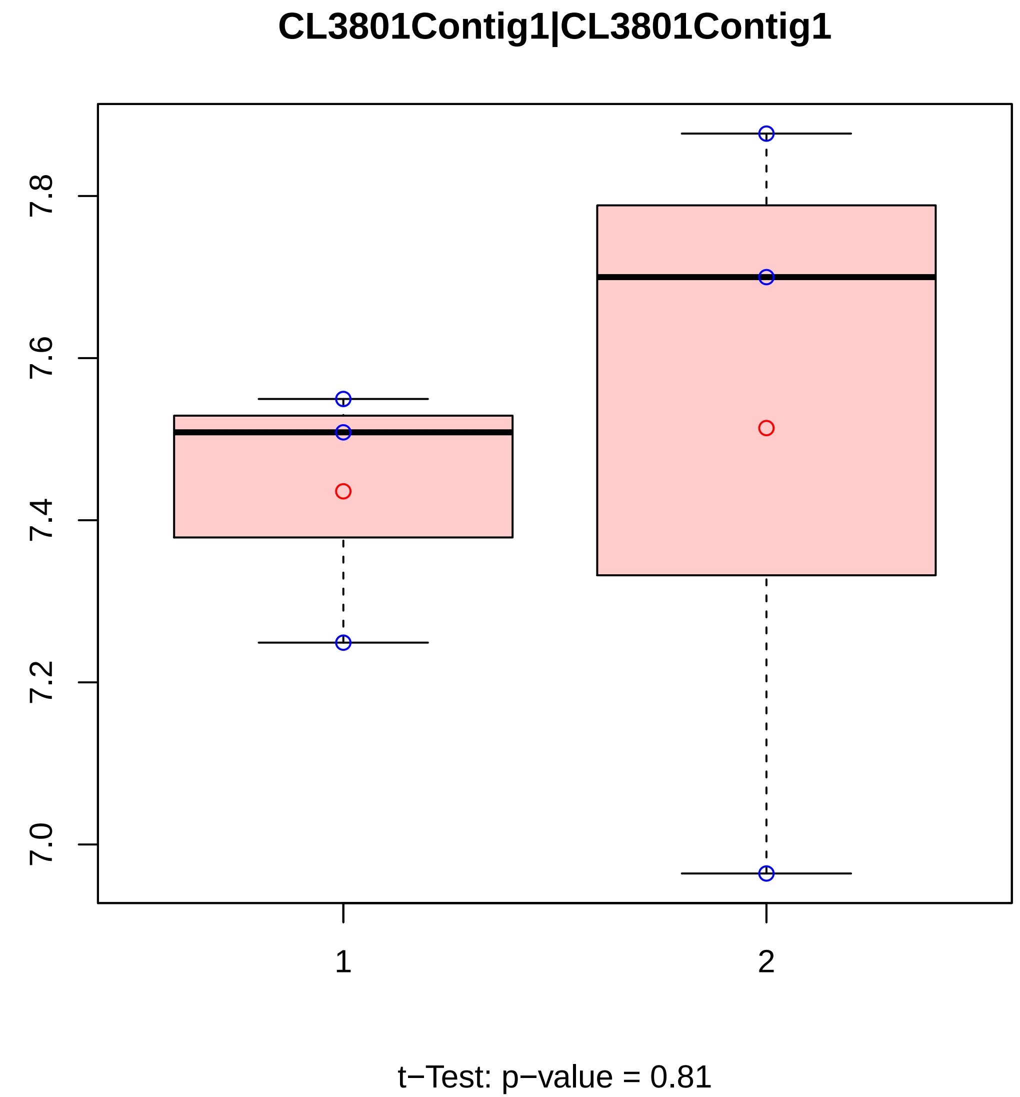
CL3801Contig1|CL3801Contig1
7.0
7.2
7.4
7.6
7.8
1
2
t−Test: p−value = 0.81

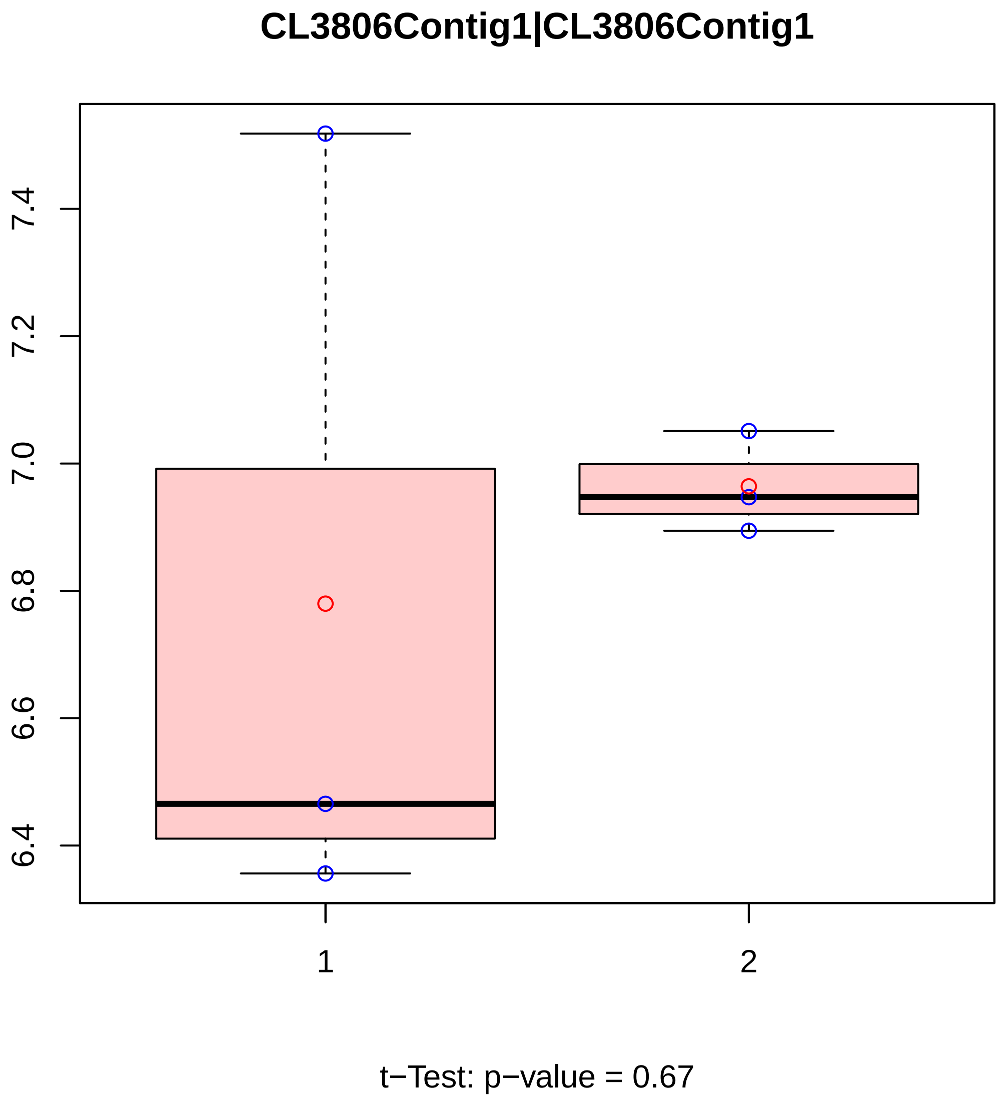
CL3806Contig1|CL3806Contig1
7.4
7.2
7.0
6.8
6.6
6.4
1
2
t−Test: p−value = 0.67

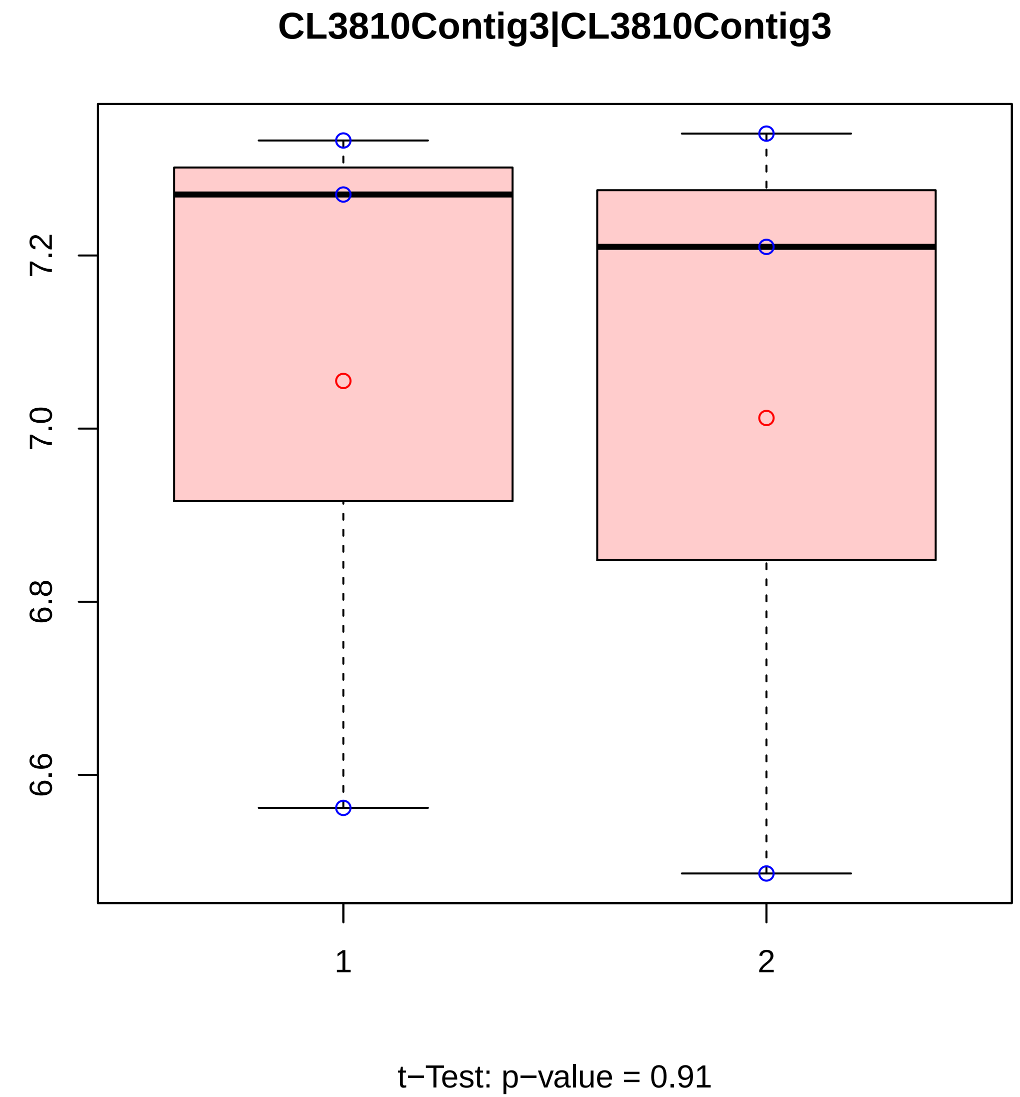
CL3810Contig3|CL3810Contig3
7.2
7.0
6.8
6.6
1
2
t−Test: p−value = 0.91

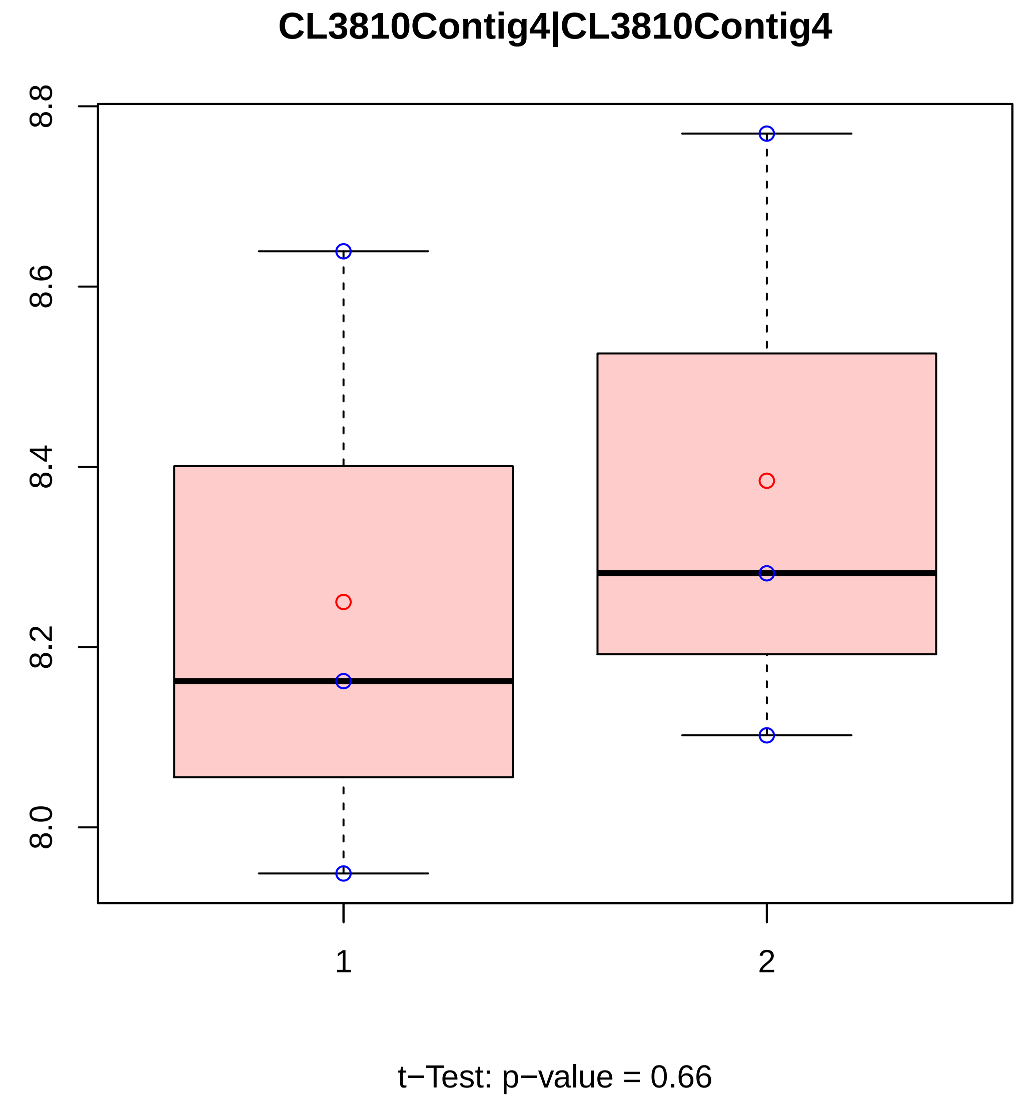
CL3810Contig4|CL3810Contig4
8.8
8.6
8.4
8.2
8.0
1
2
t−Test: p−value = 0.66

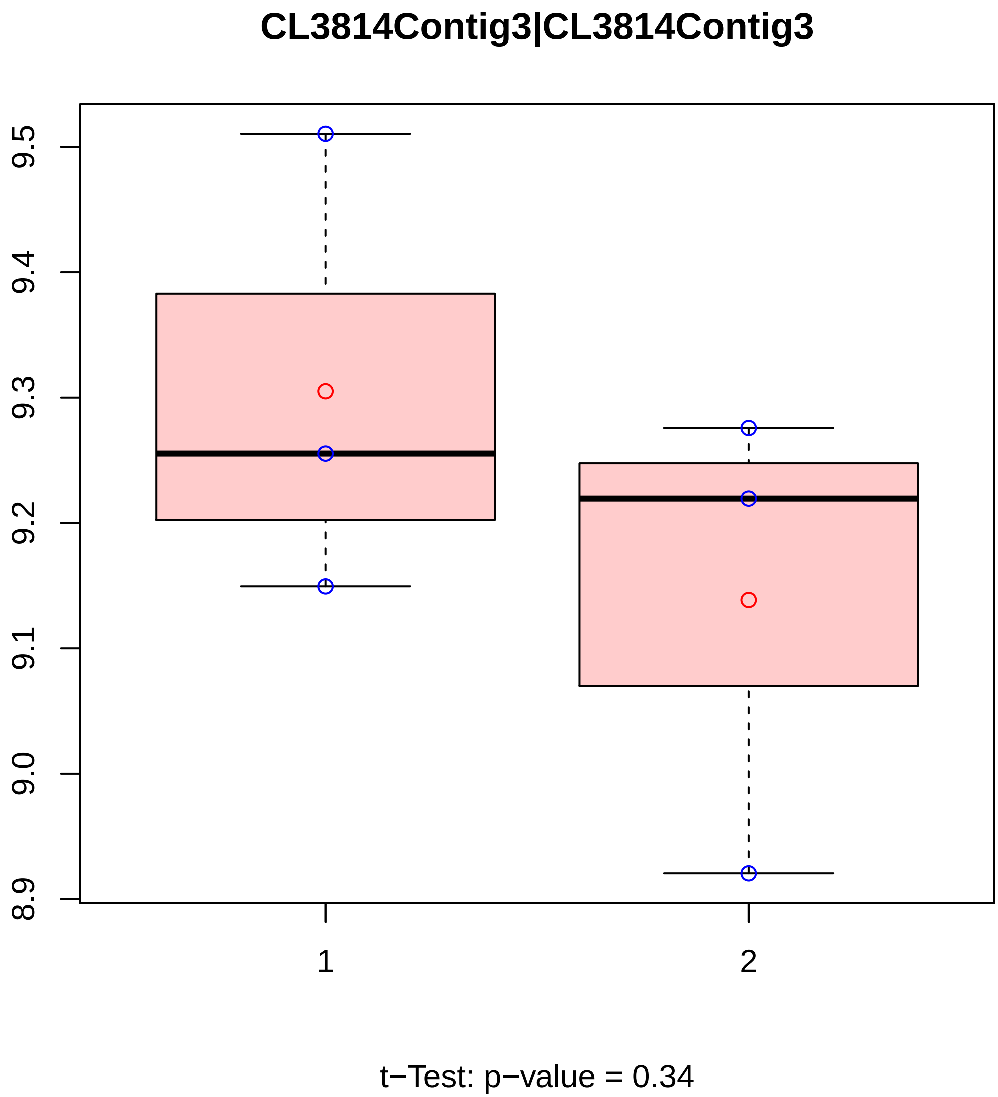
CL3814Contig3|CL3814Contig3
9.5
9.4
9.3
9.2
9.1
9.0
8.9
1
2
t−Test: p−value = 0.34

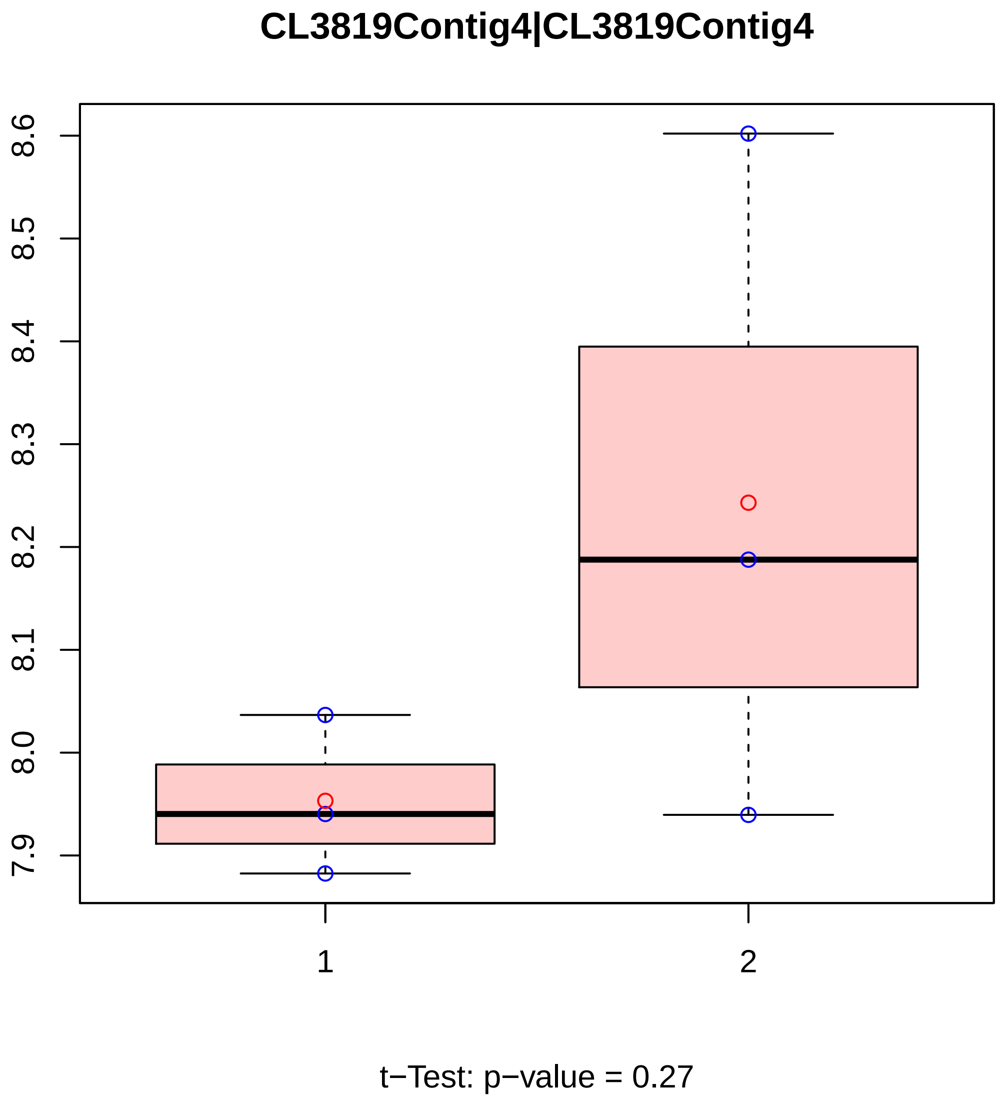
CL3819Contig4|CL3819Contig4
8.6
8.5
8.4
8.3
8.2
8.1
8.0
7.9
1
2
t−Test: p−value = 0.27

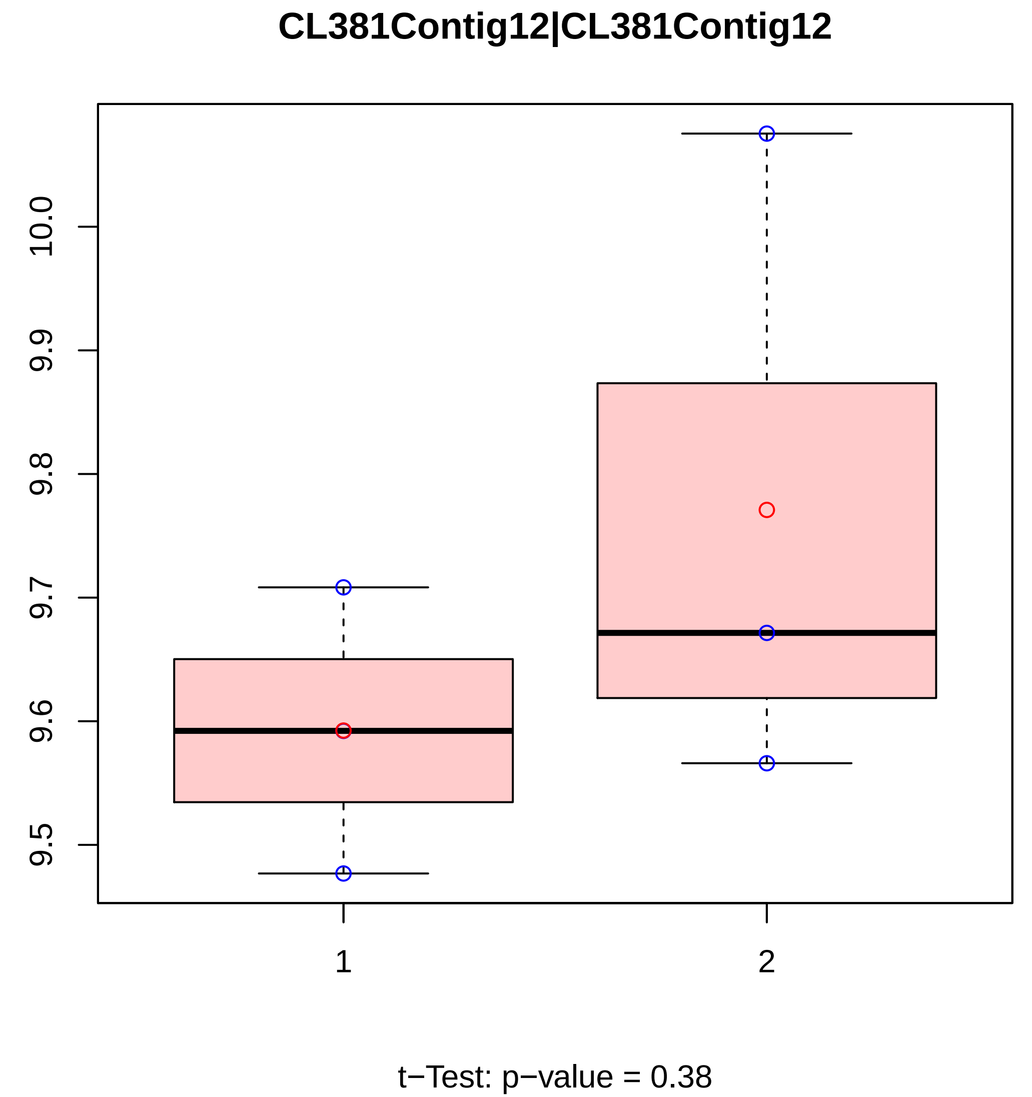
CL381Contig12|CL381Contig12
10.0
9.9
9.8
9.7
9.6
9.5
1
2
t−Test: p−value = 0.38

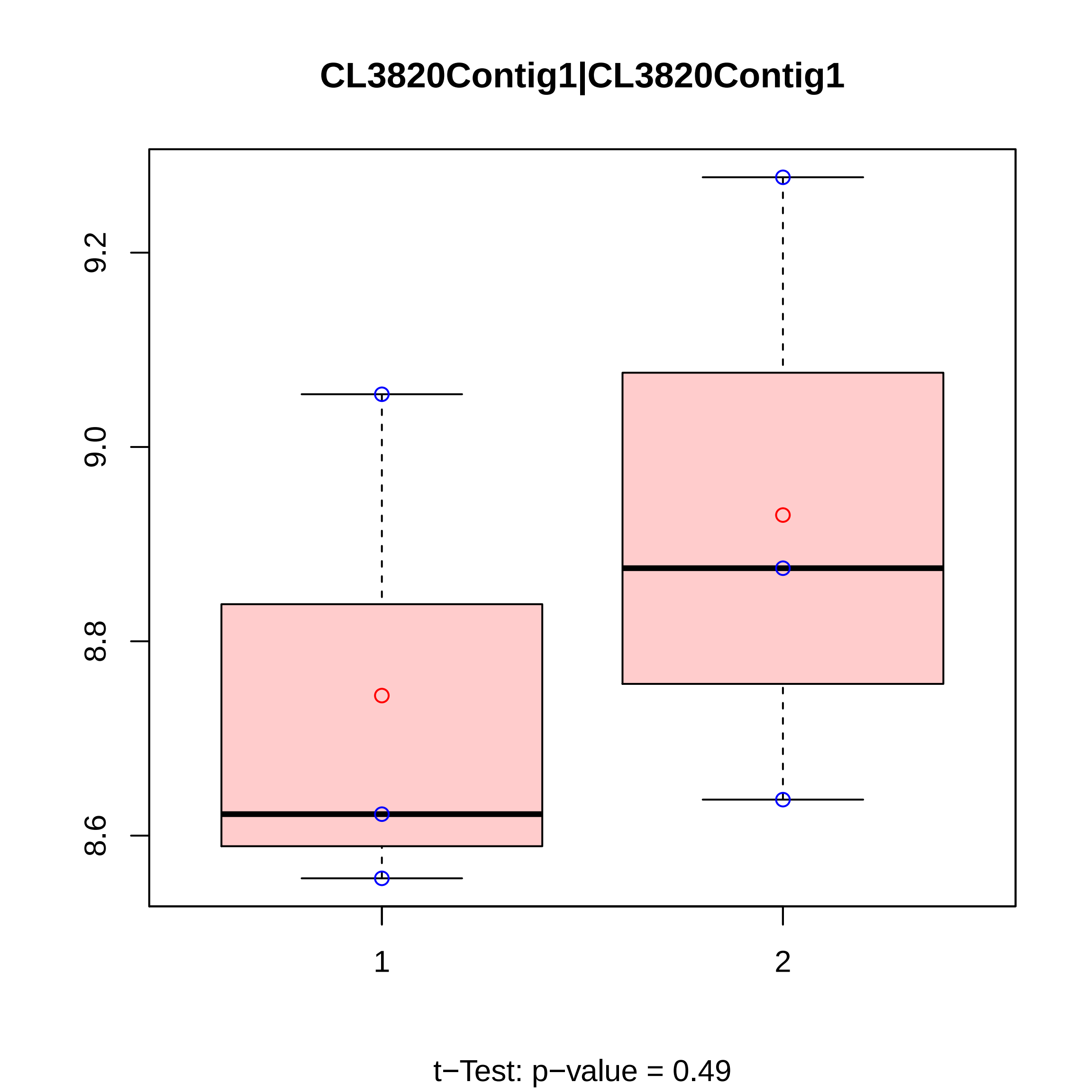

CL3820Contig1|CL3820Contig1
9.2
9.0
8.8
8.6
1
2
t−Test: p−value = 0.49

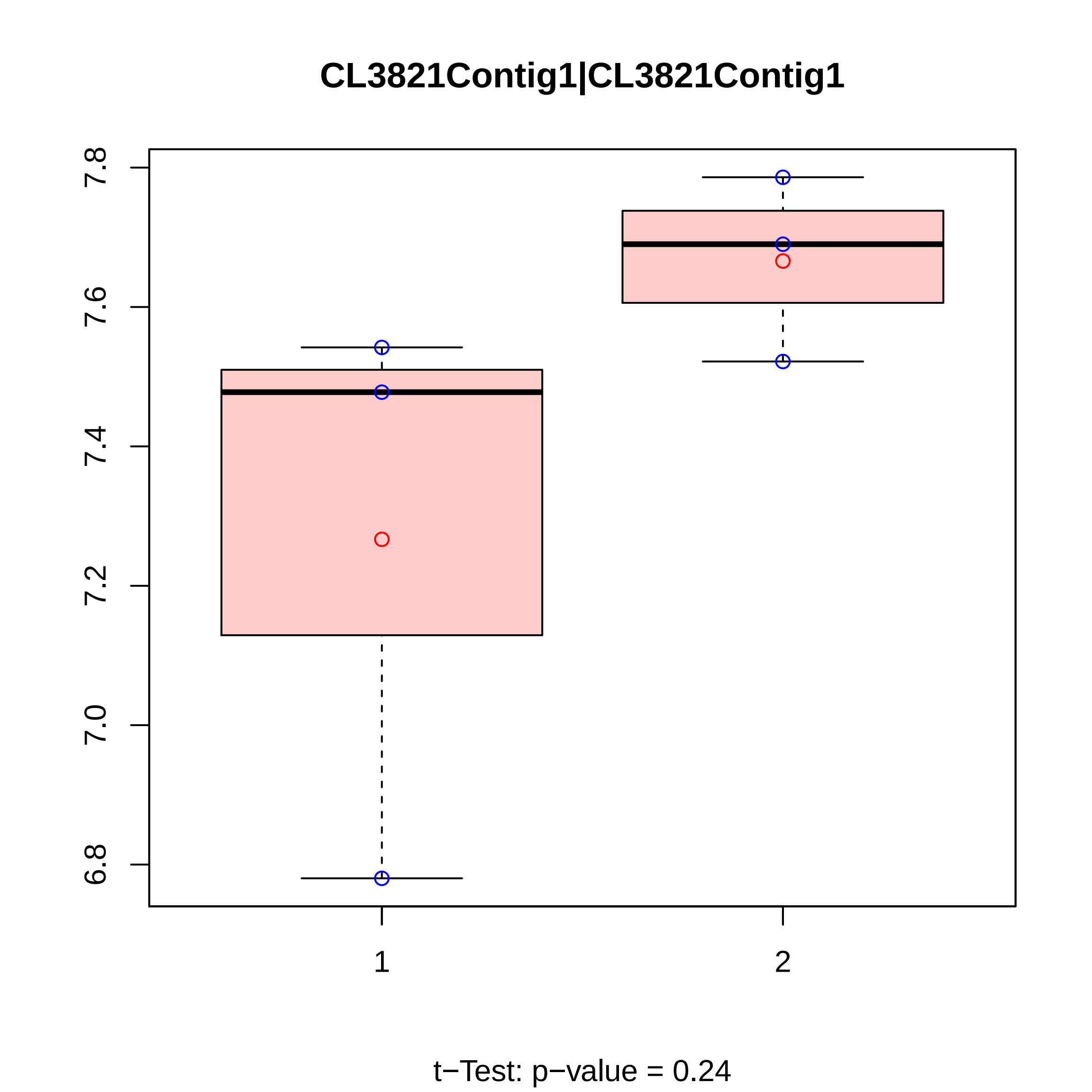

CL3821Contig1|CL3821Contig1
7.8
7.6
7.4
7.2
7.0
6.8
1
2
t−Test: p−value = 0.24

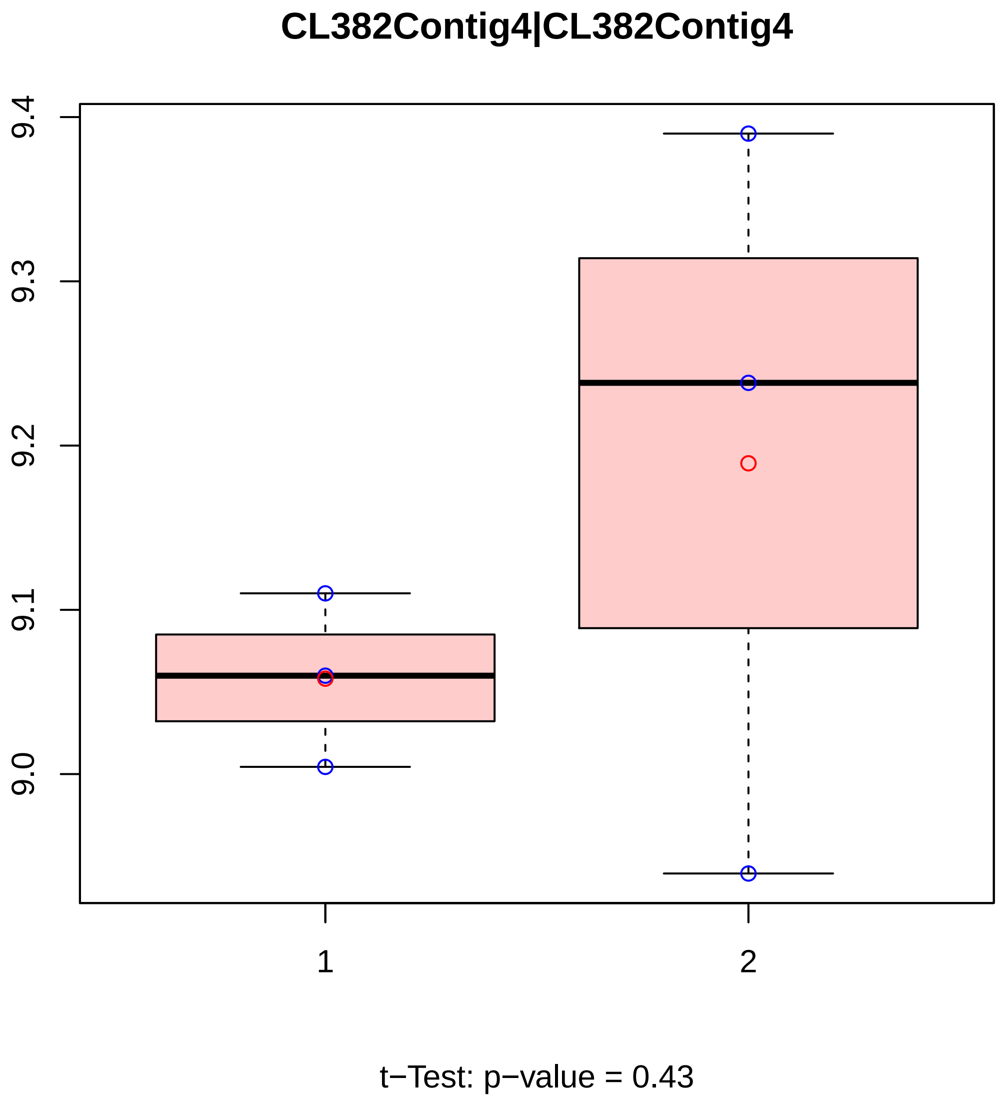
CL382Contig4|CL382Contig4
9.0
9.1
9.2
9.3
9.4
1
2
t−Test: p−value = 0.43

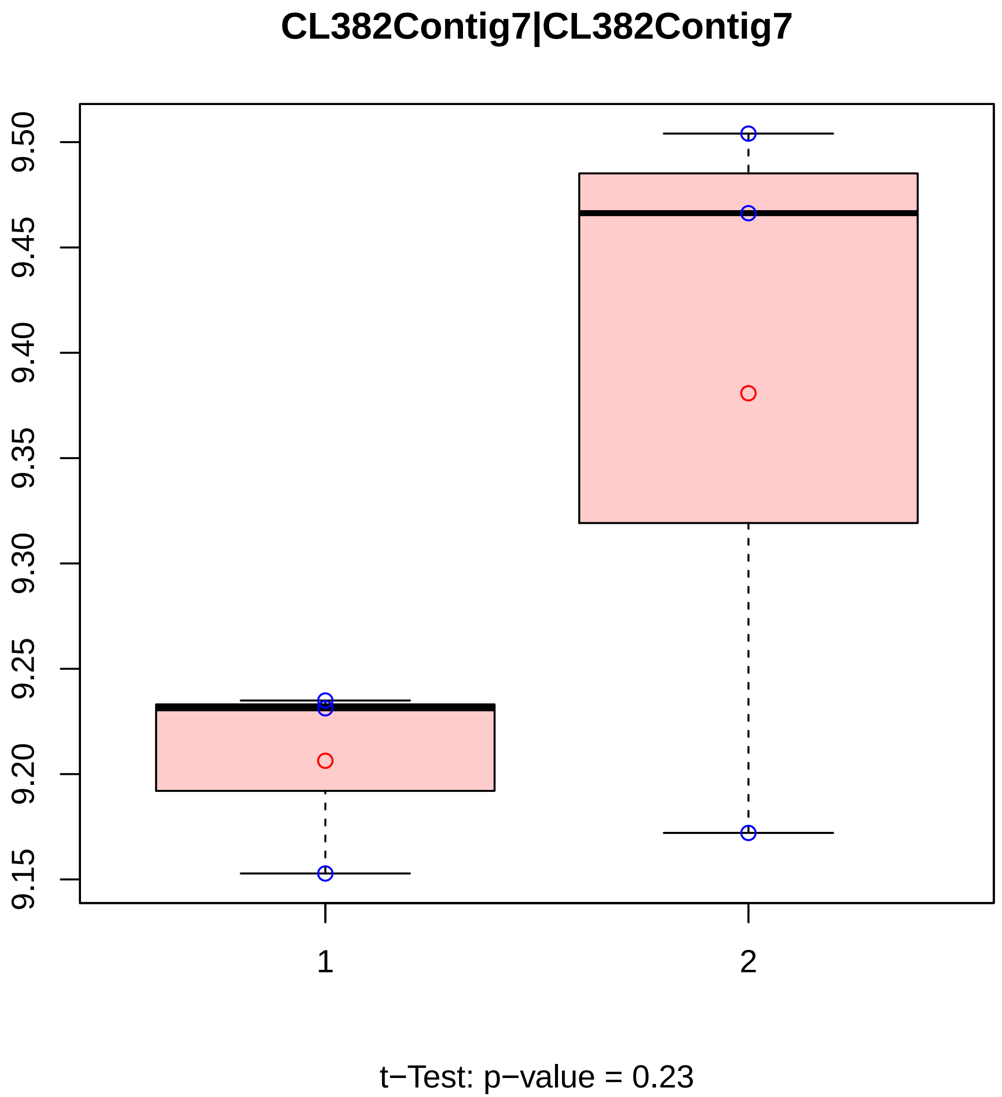

CL382Contig7|CL382Contig7
9.15
9.20
9.25
9.30
9.35
9.40
9.45
9.50
1
2
t−Test: p−value = 0.23

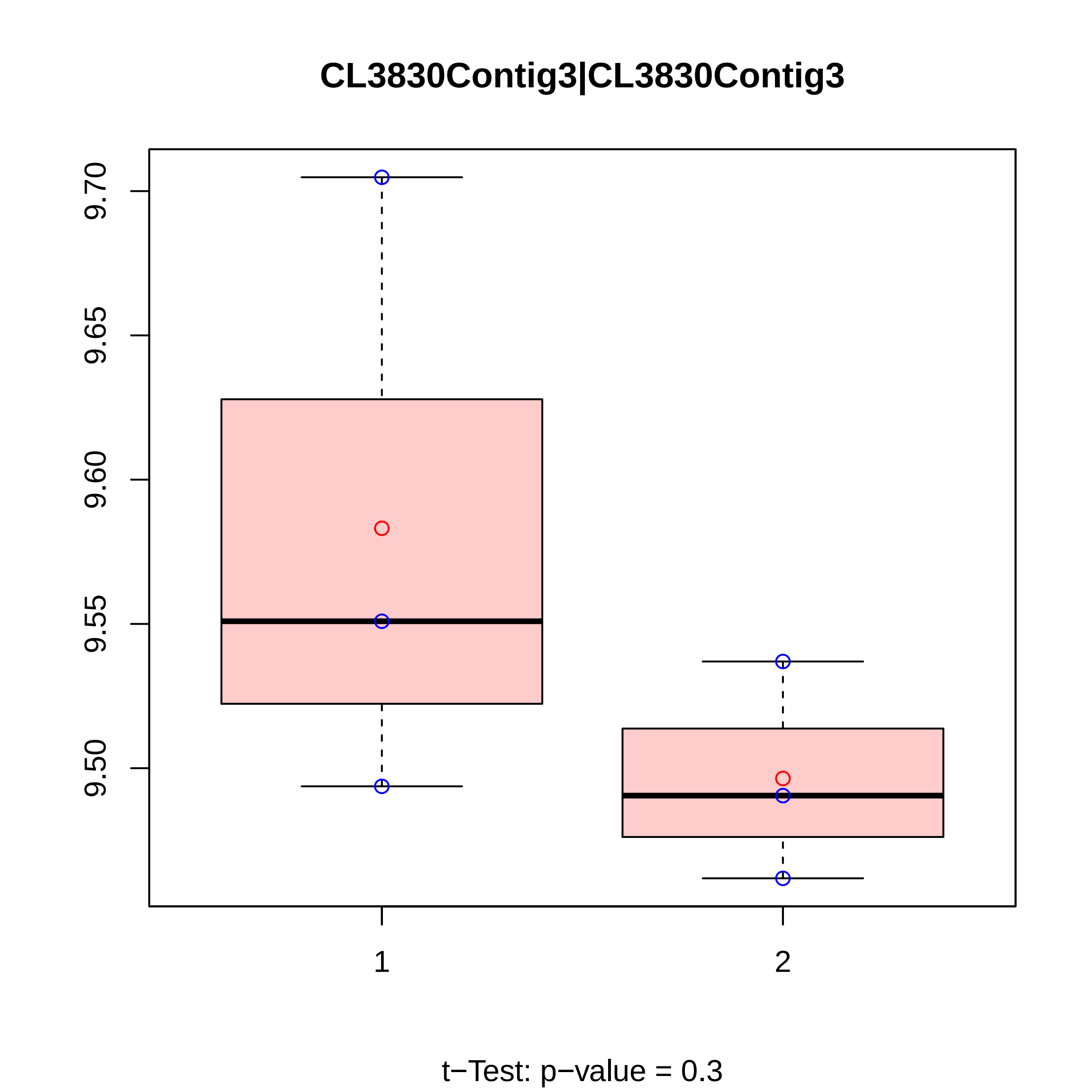

CL3830Contig3|CL3830Contig3
9.70
9.65
9.60
9.55
9.50
1
2
t−Test: p−value = 0.3

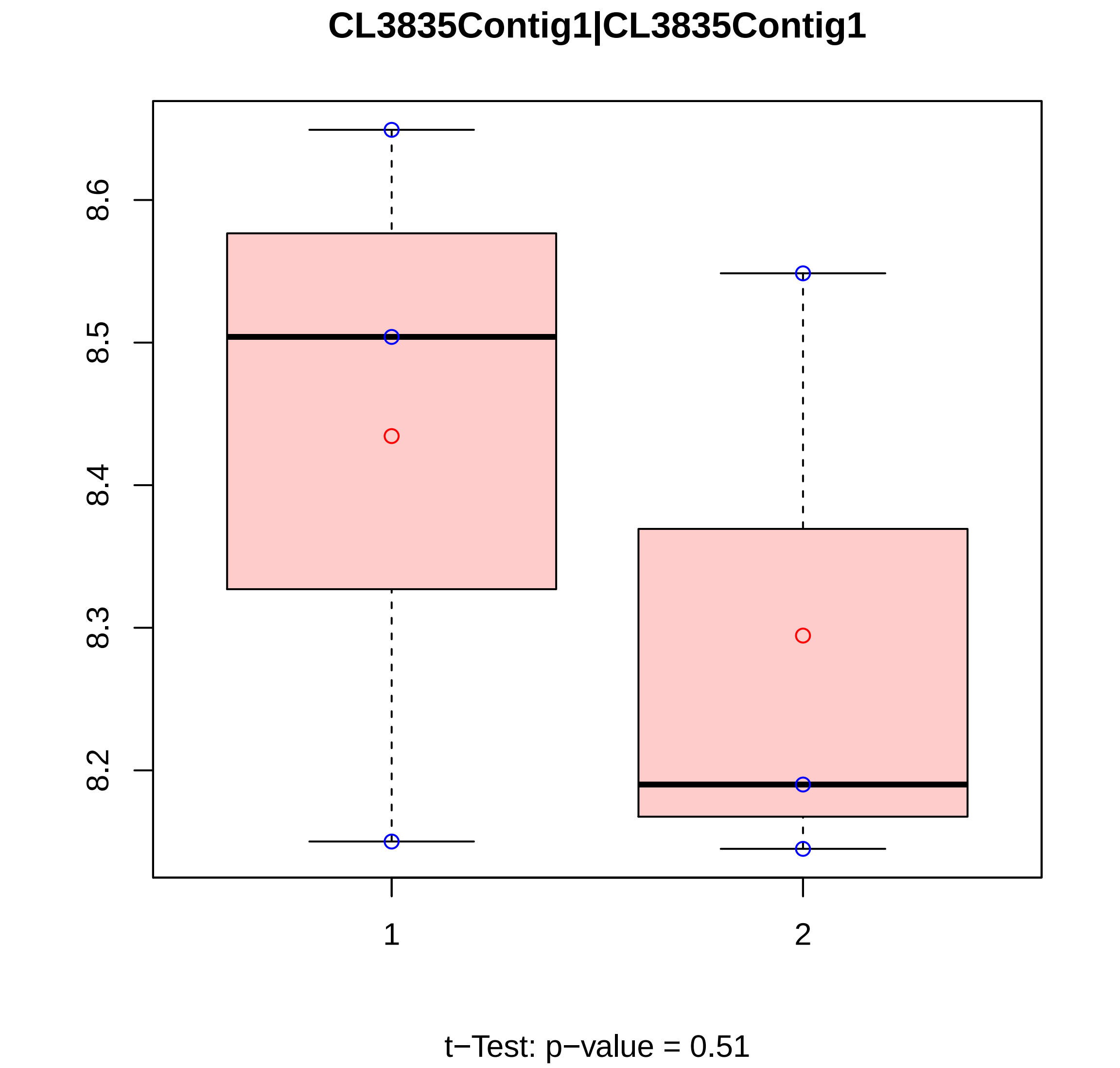

CL3835Contig1|CL3835Contig1
8.2
8.3
8.4
8.5
8.6
1
2
t−Test: p−value = 0.51

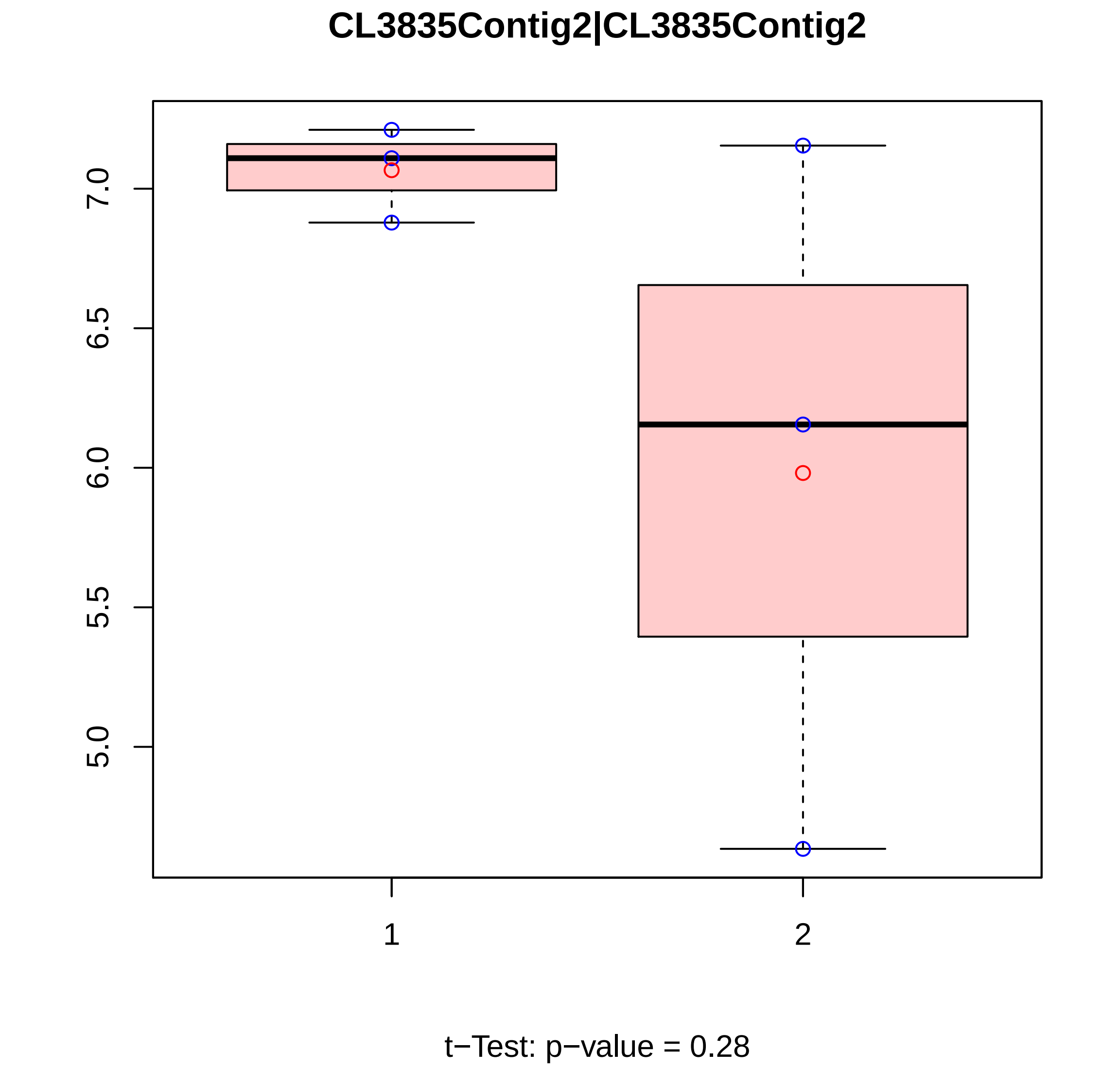
CL3835Contig2|CL3835Contig2
7.0
6.5
6.0
5.5
5.0
1
2
t−Test: p−value = 0.28

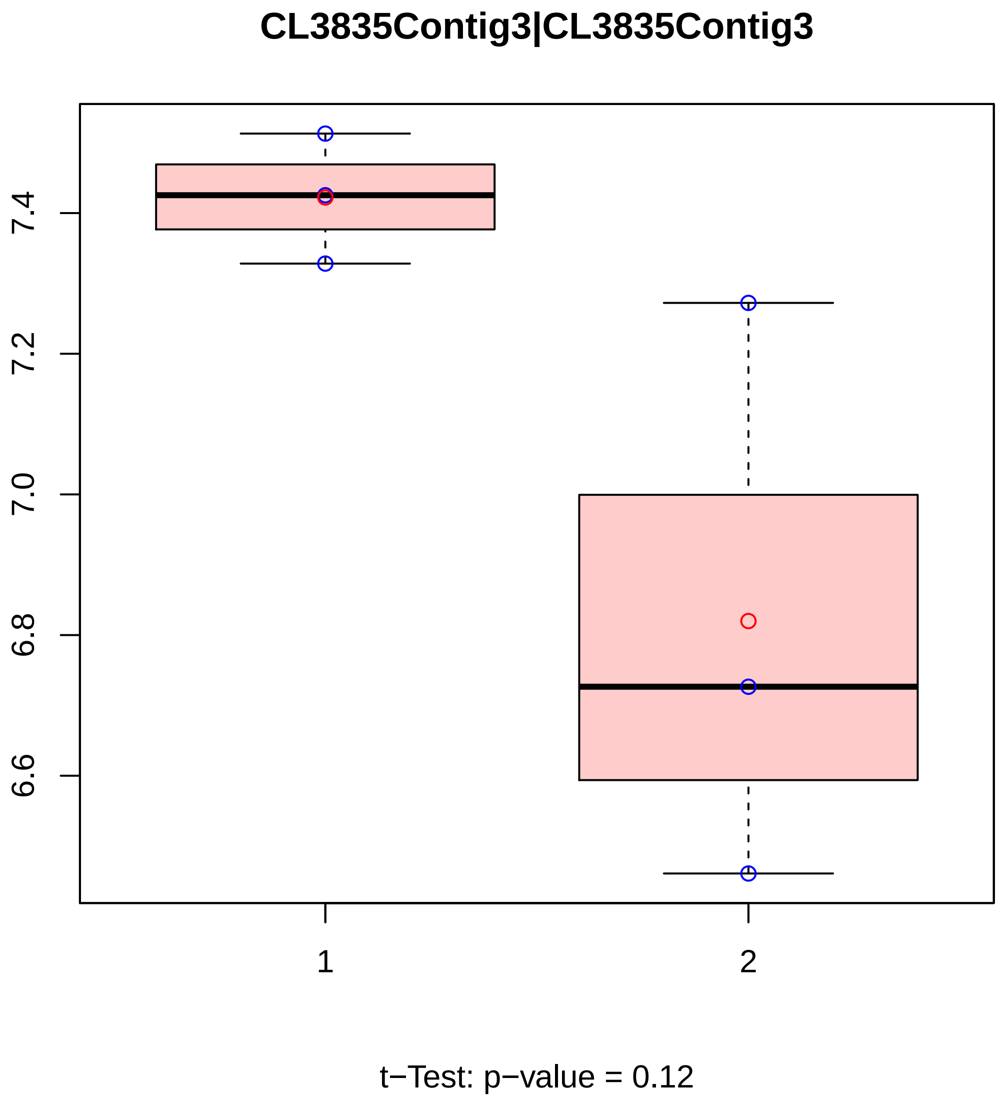
CL3835Contig3|CL3835Contig3
7.4
7.2
7.0
6.8
6.6
1
2
t−Test: p−value = 0.12

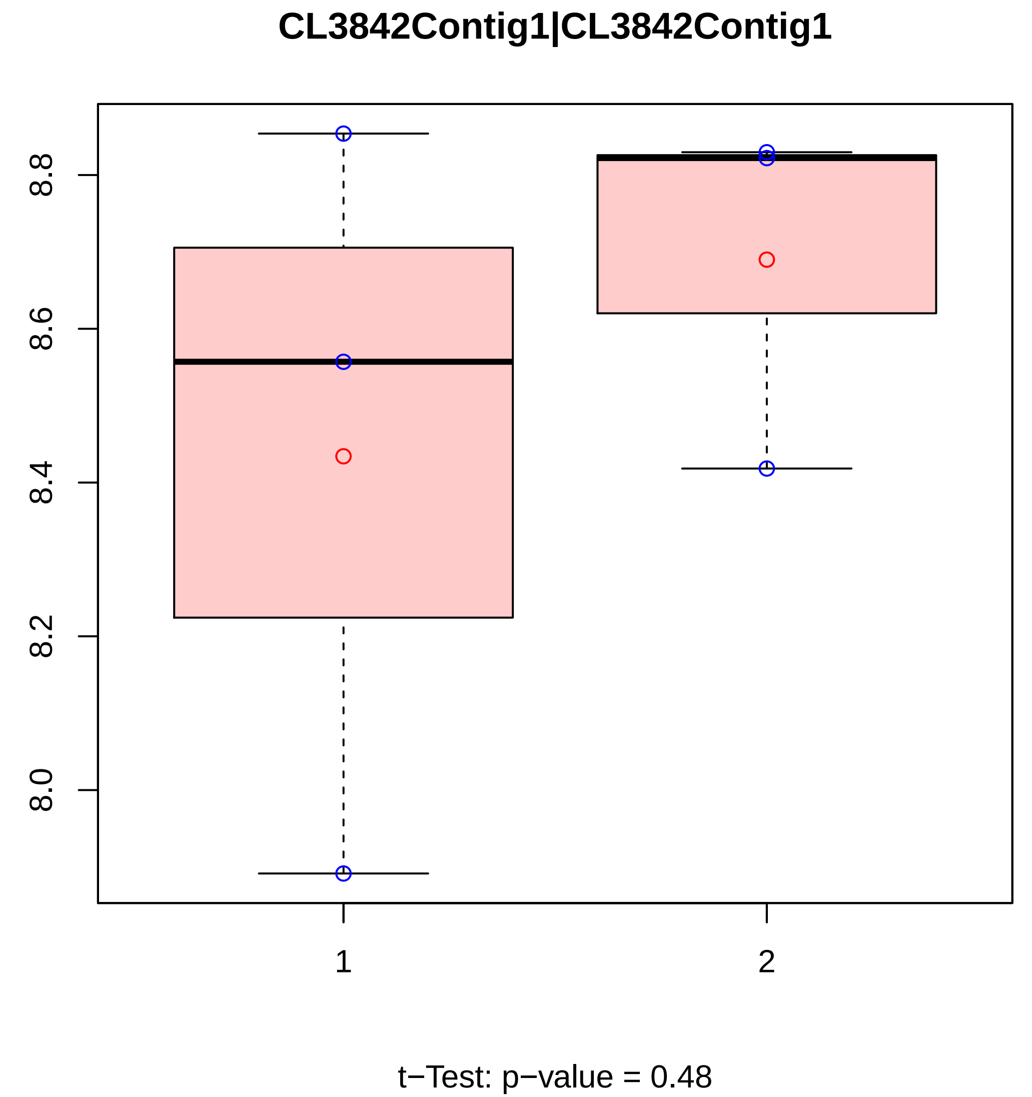

CL3842Contig1|CL3842Contig1
8.0
8.2
8.4
8.6
8.8
1
2
t−Test: p−value = 0.48

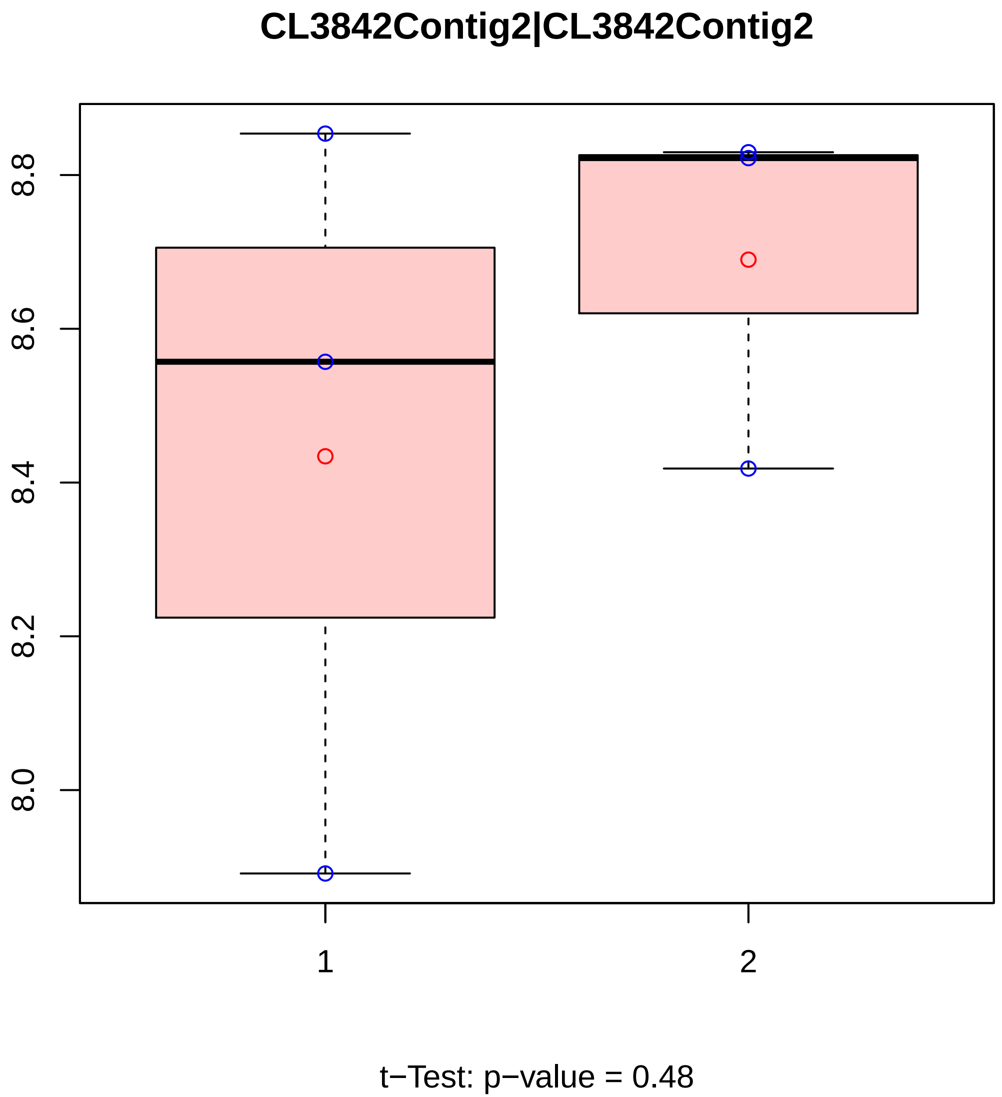
CL3842Contig2|CL3842Contig2
8.0
8.2
8.4
8.6
8.8
1
2
t−Test: p−value = 0.48

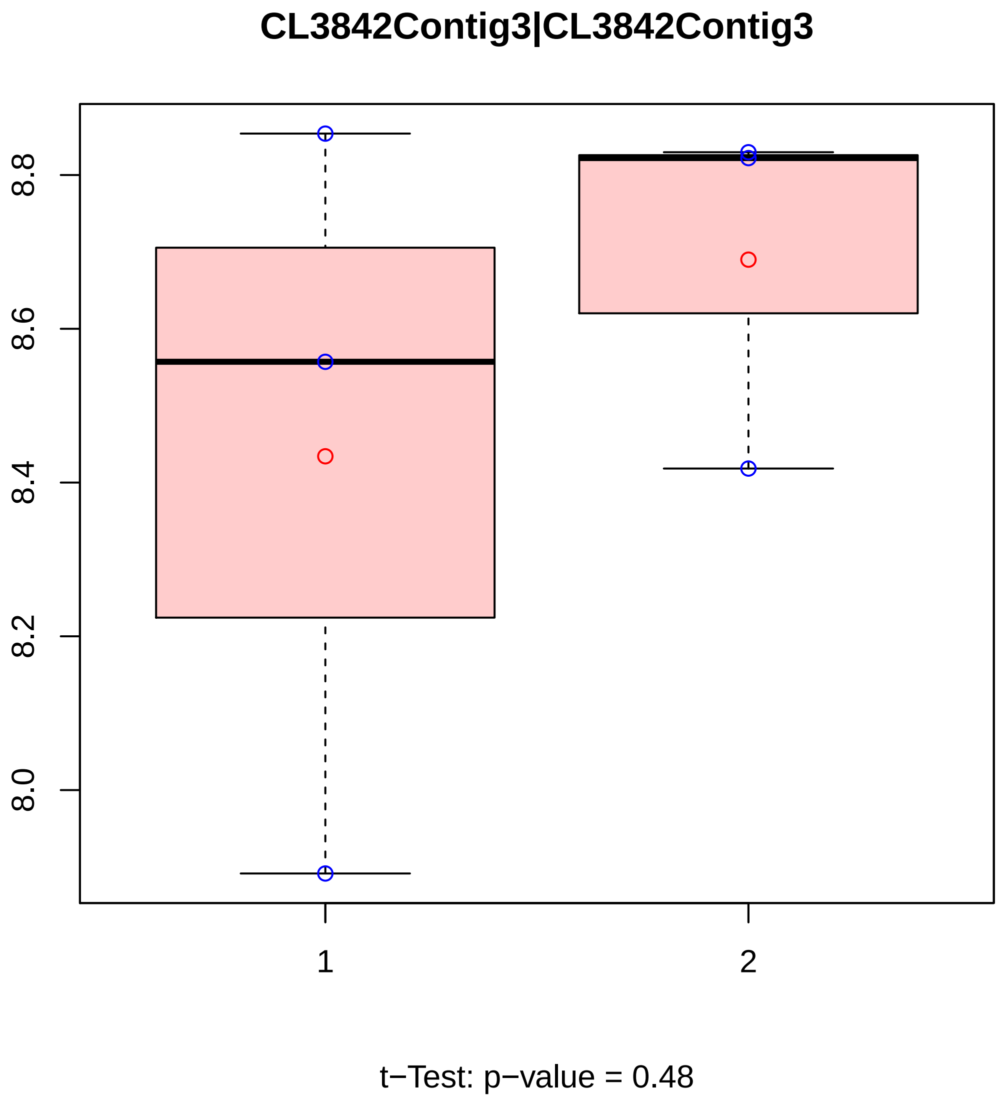

CL3842Contig3|CL3842Contig3
8.0
8.2
8.4
8.6
8.8
1
2
t−Test: p−value = 0.48

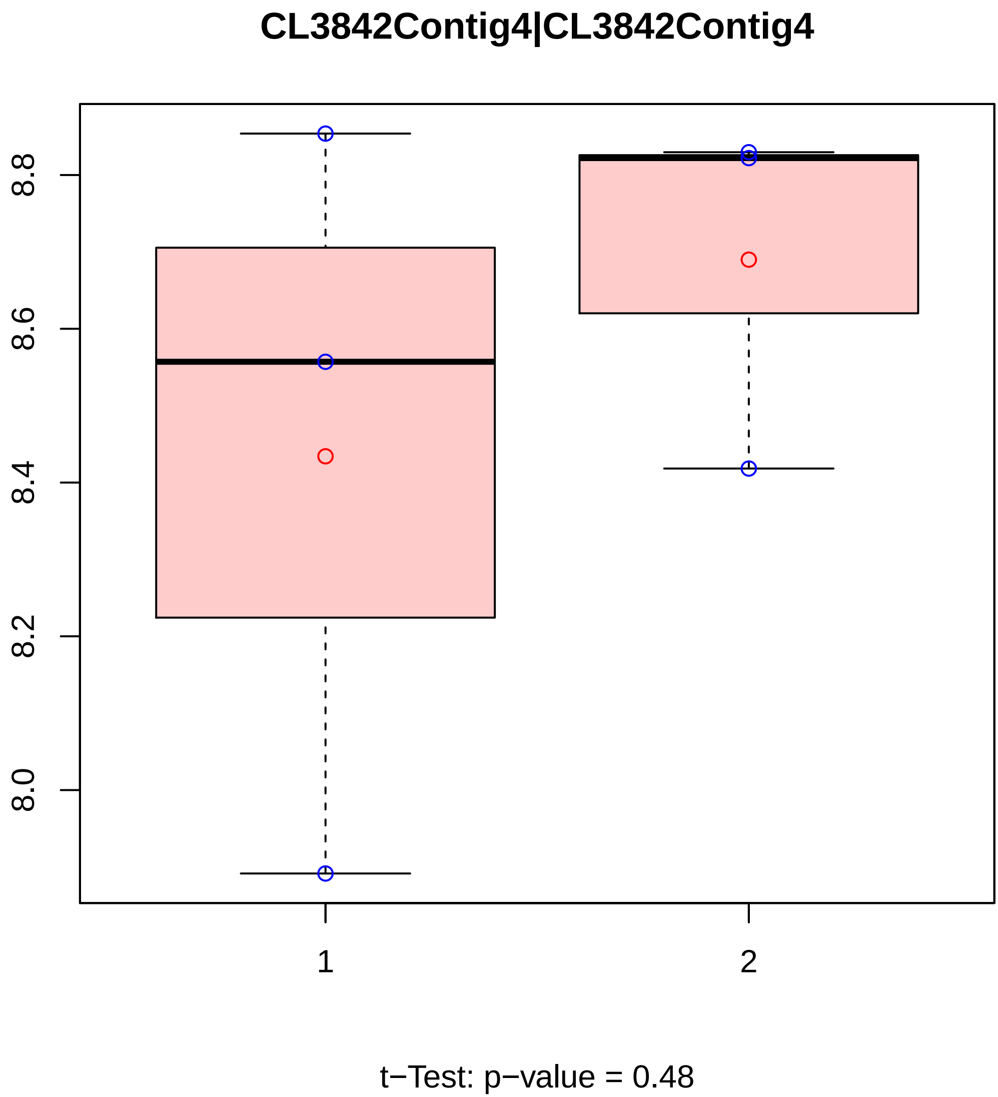
CL3842Contig4|CL3842Contig4
8.0
8.2
8.4
8.6
8.8
1
2
t−Test: p−value = 0.48

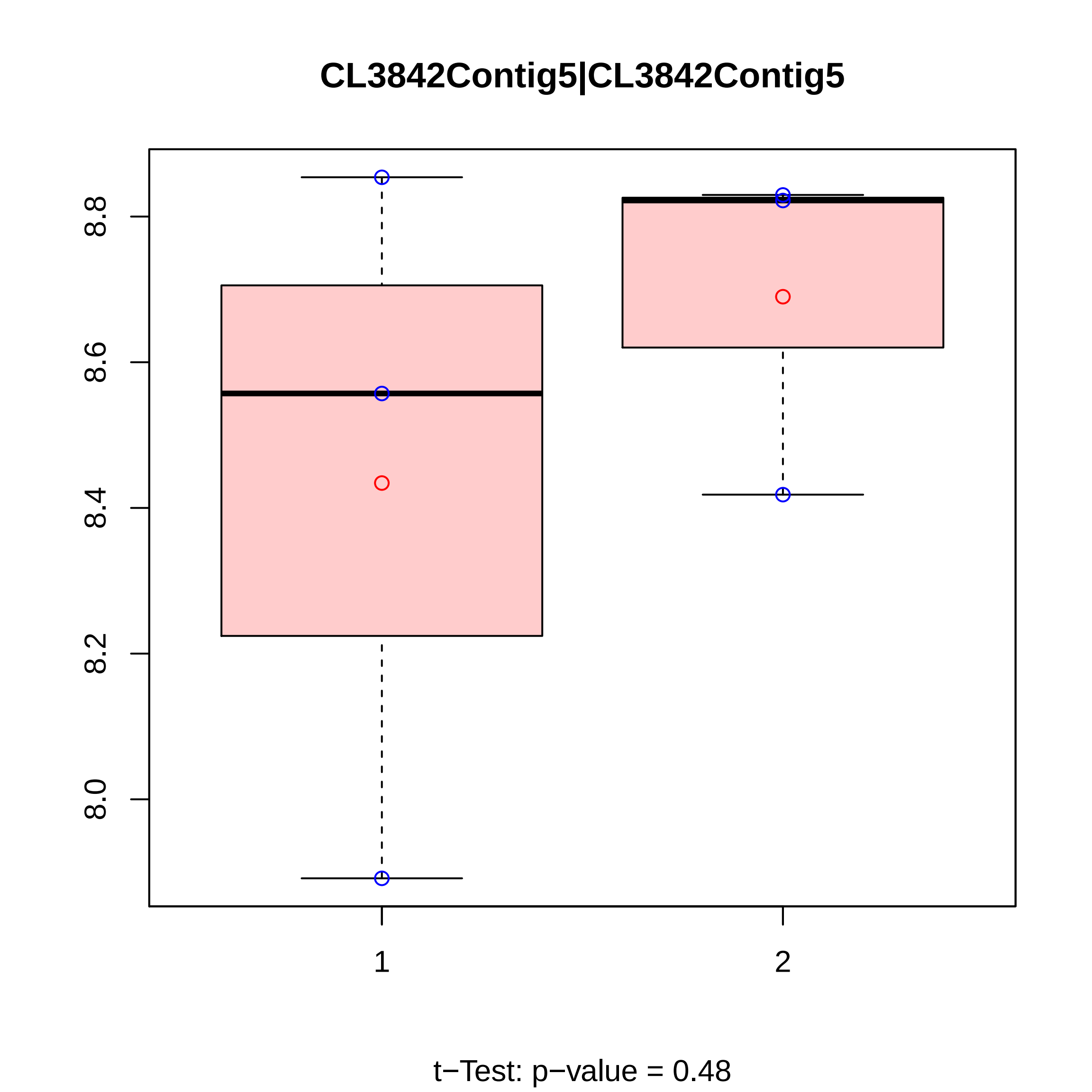

CL3842Contig5|CL3842Contig5
8.0
8.2
8.4
8.6
8.8
1
2
t−Test: p−value = 0.48

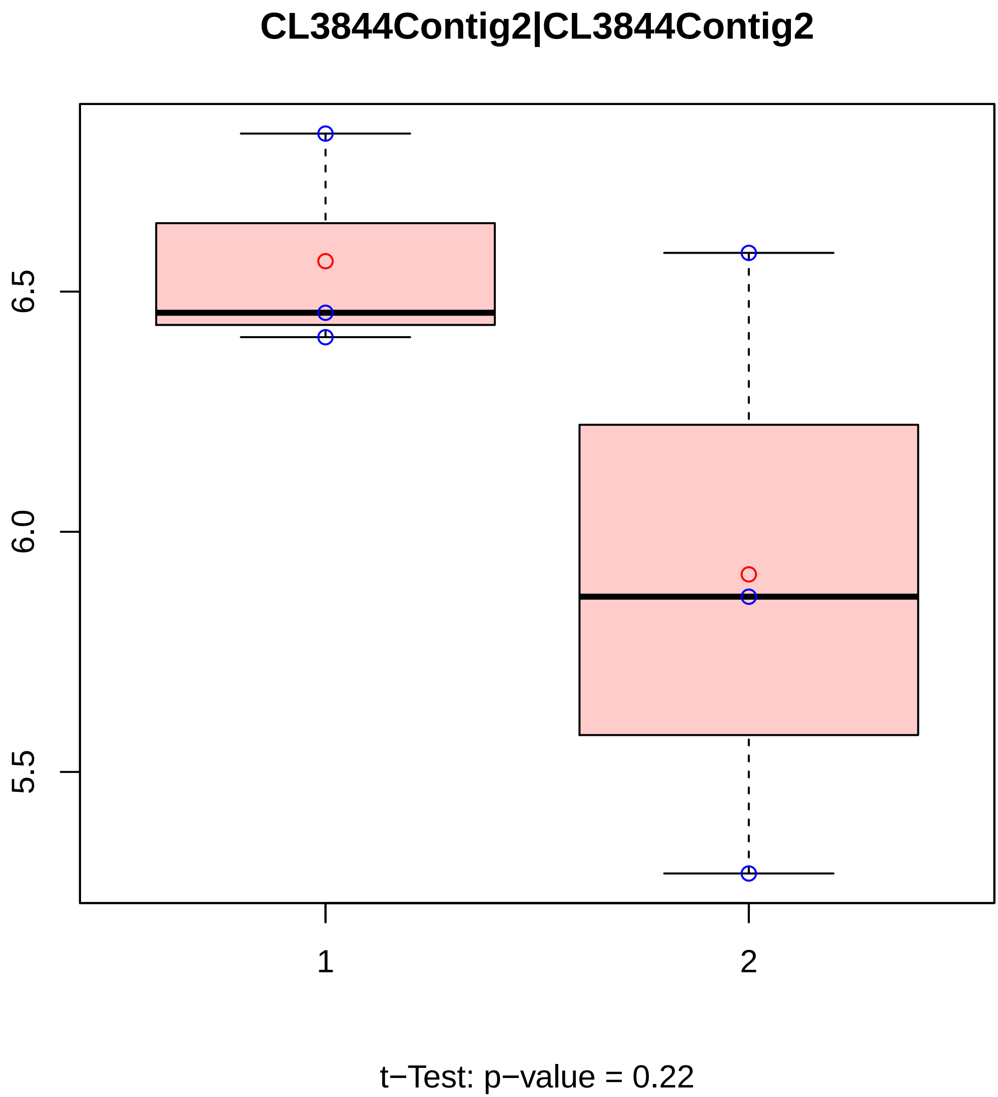
CL3844Contig2|CL3844Contig2
6.5
6.0
5.5
1
2
t−Test: p−value = 0.22

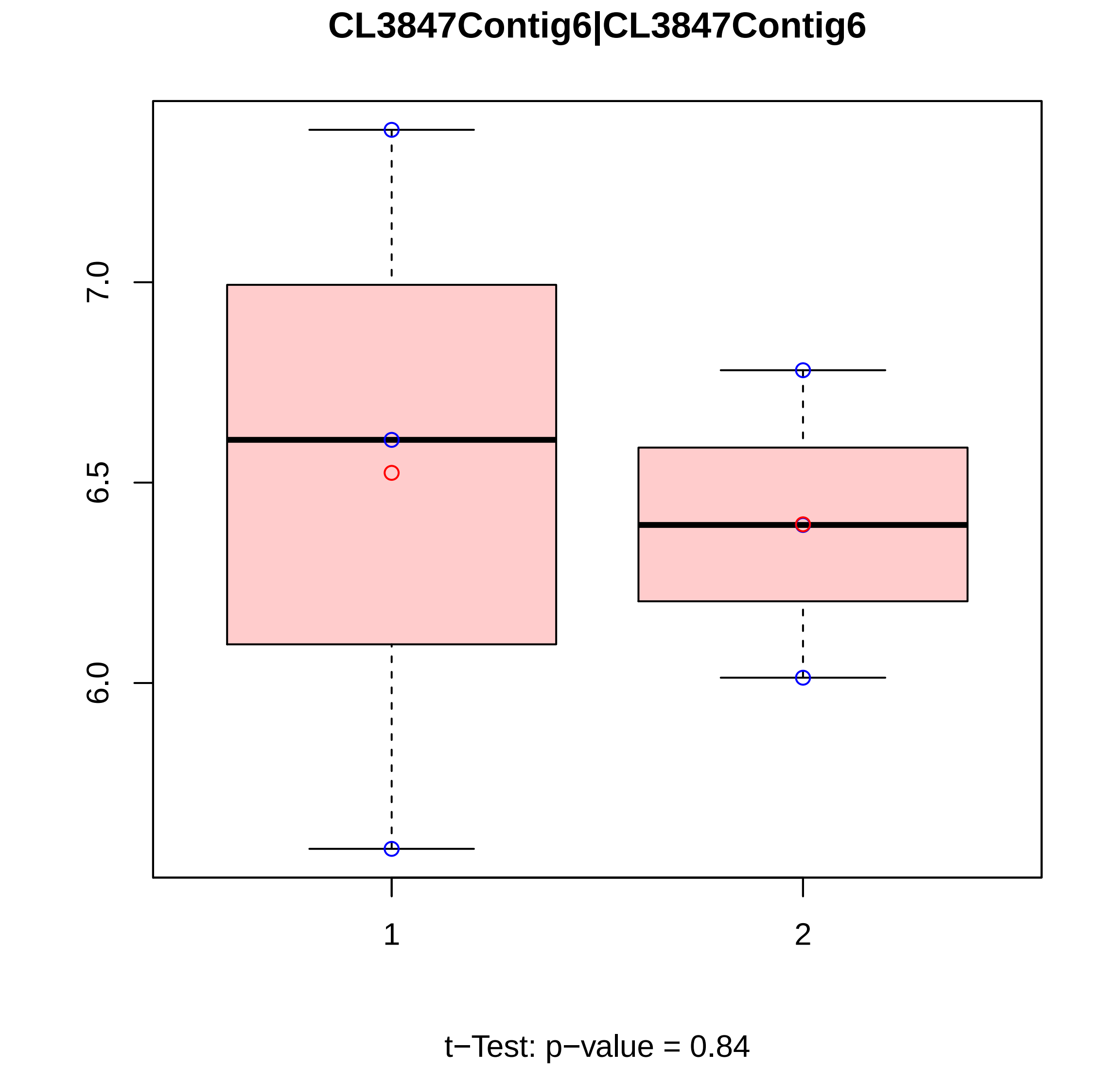

CL3847Contig6|CL3847Contig6
7.0
6.5
6.0
1
2
t−Test: p−value = 0.84

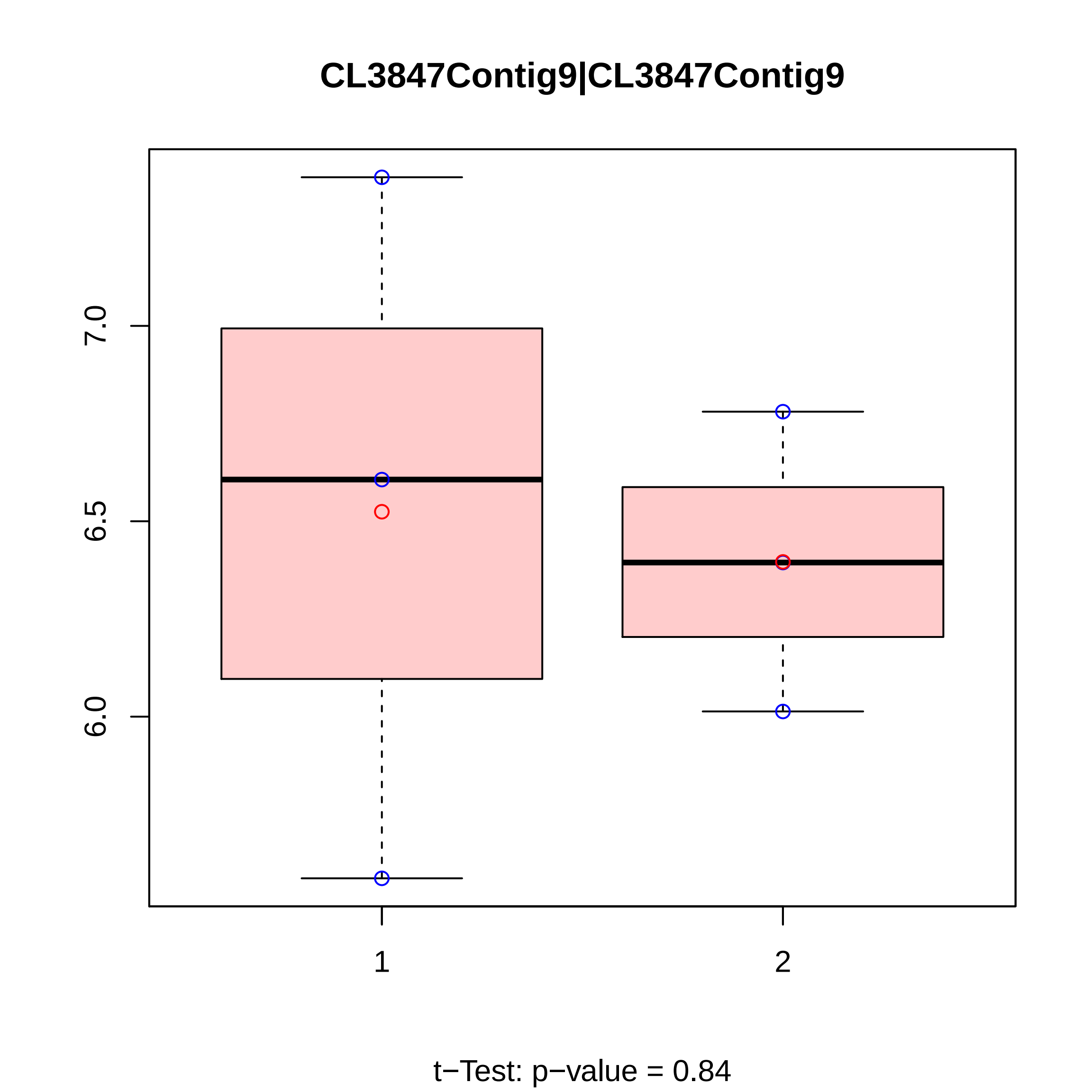
CL3847Contig9|CL3847Contig9
7.0
6.5
6.0
1
2
t−Test: p−value = 0.84

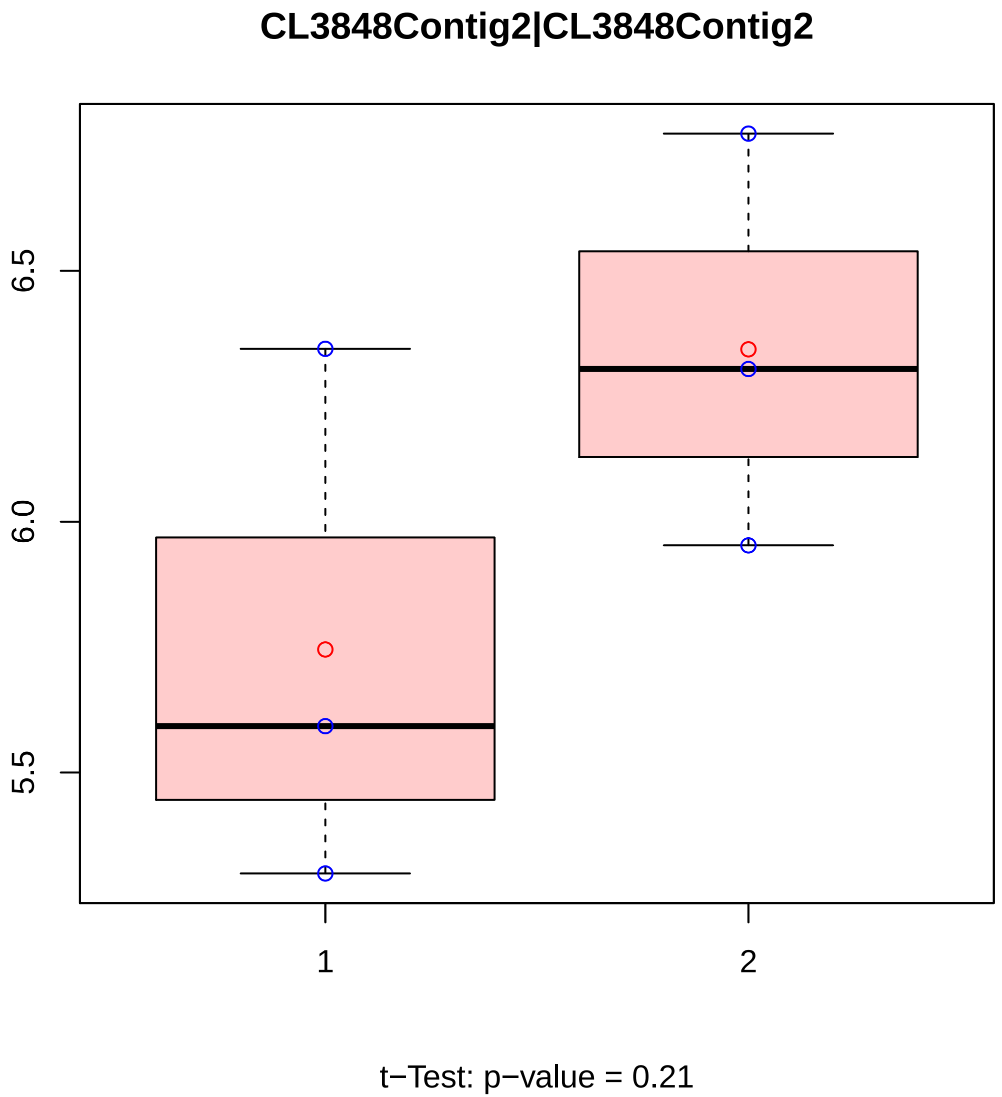
CL3848Contig2|CL3848Contig2
6.5
6.0
5.5
1
2
t−Test: p−value = 0.21

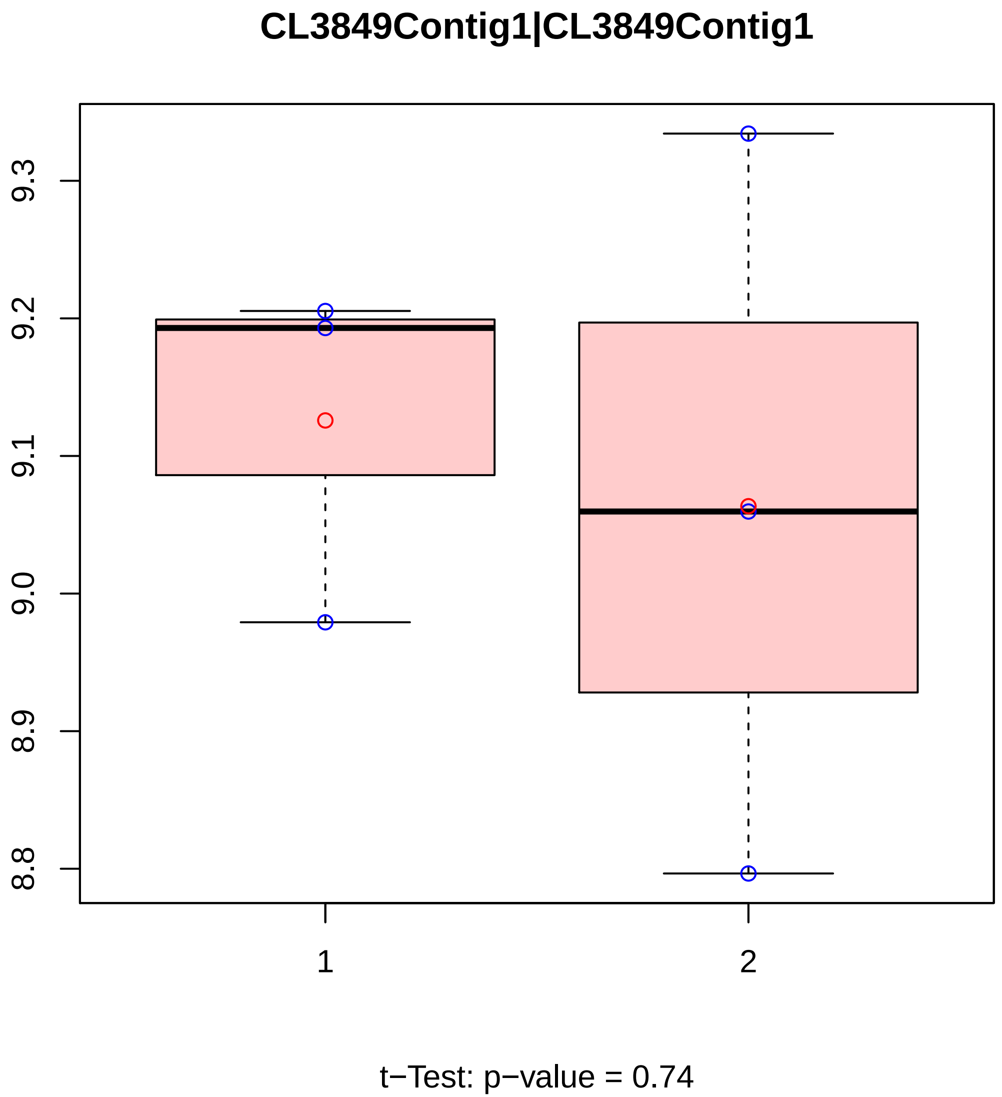
CL3849Contig1|CL3849Contig1
9.3
9.2
9.1
9.0
8.9
8.8
1
2
t−Test: p−value = 0.74

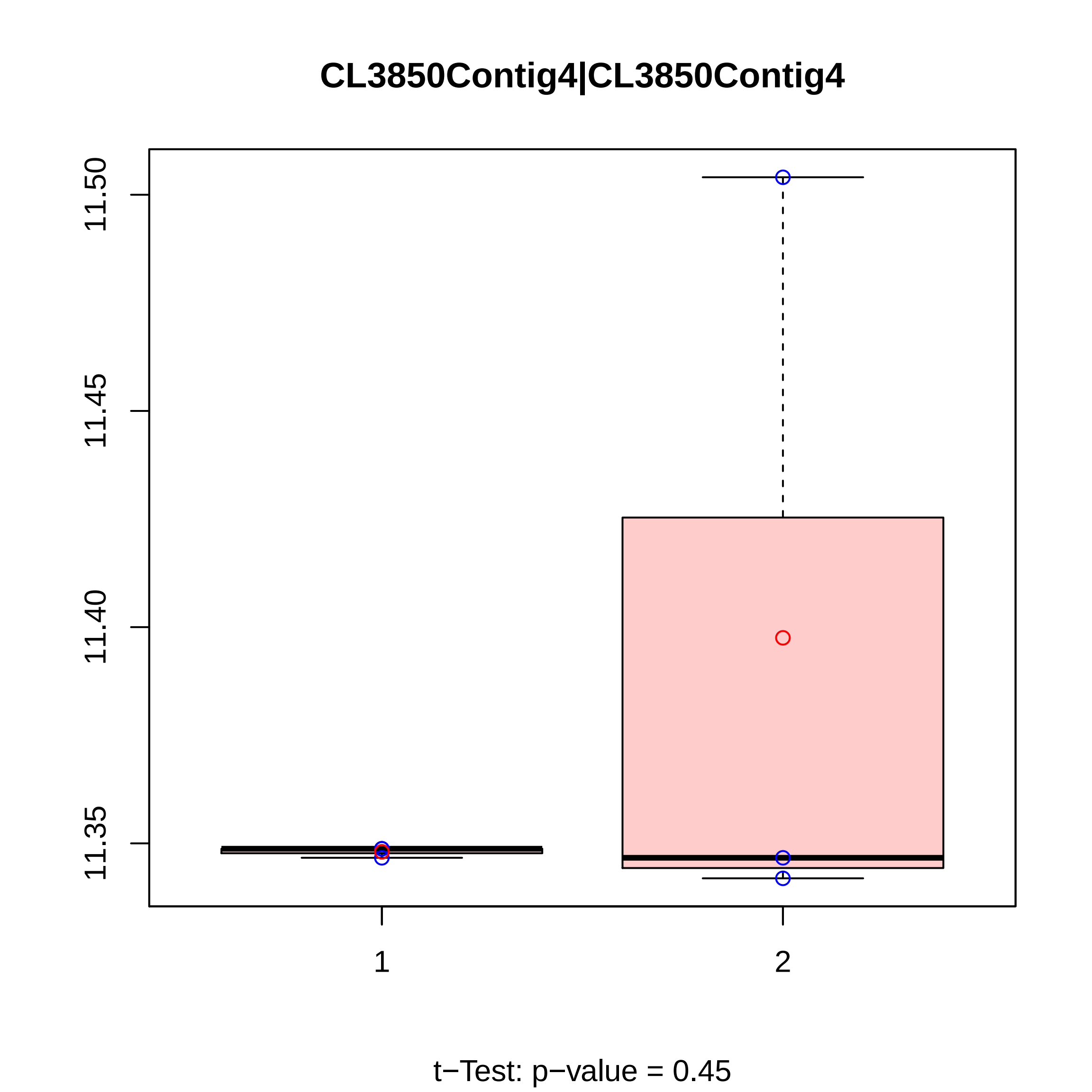

CL3850Contig4|CL3850Contig4
11.50
11.45
11.40
11.35
1
2
t−Test: p−value = 0.45

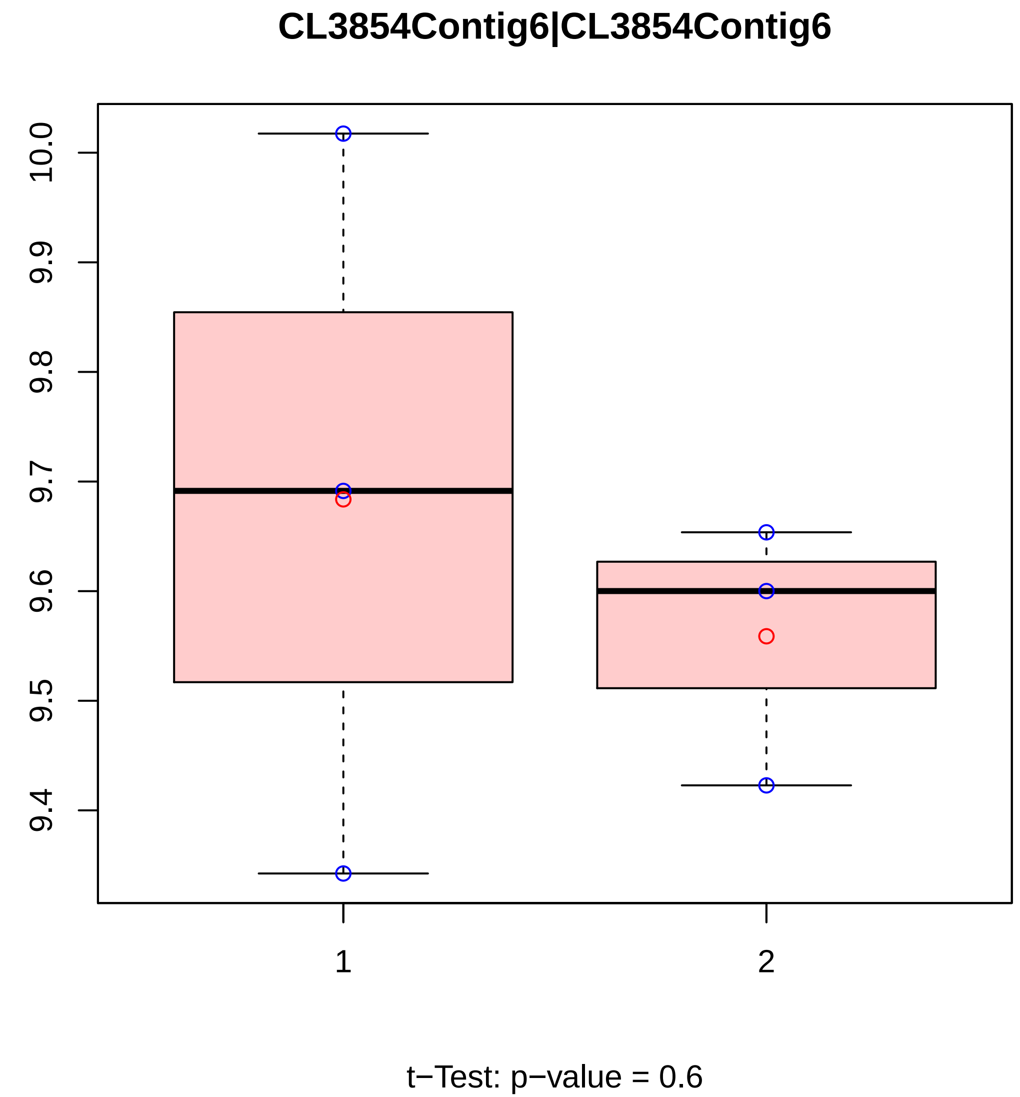
CL3854Contig6|CL3854Contig6
10.0
9.9
9.8
9.7
9.6
9.5
9.4
1
2
t−Test: p−value = 0.6

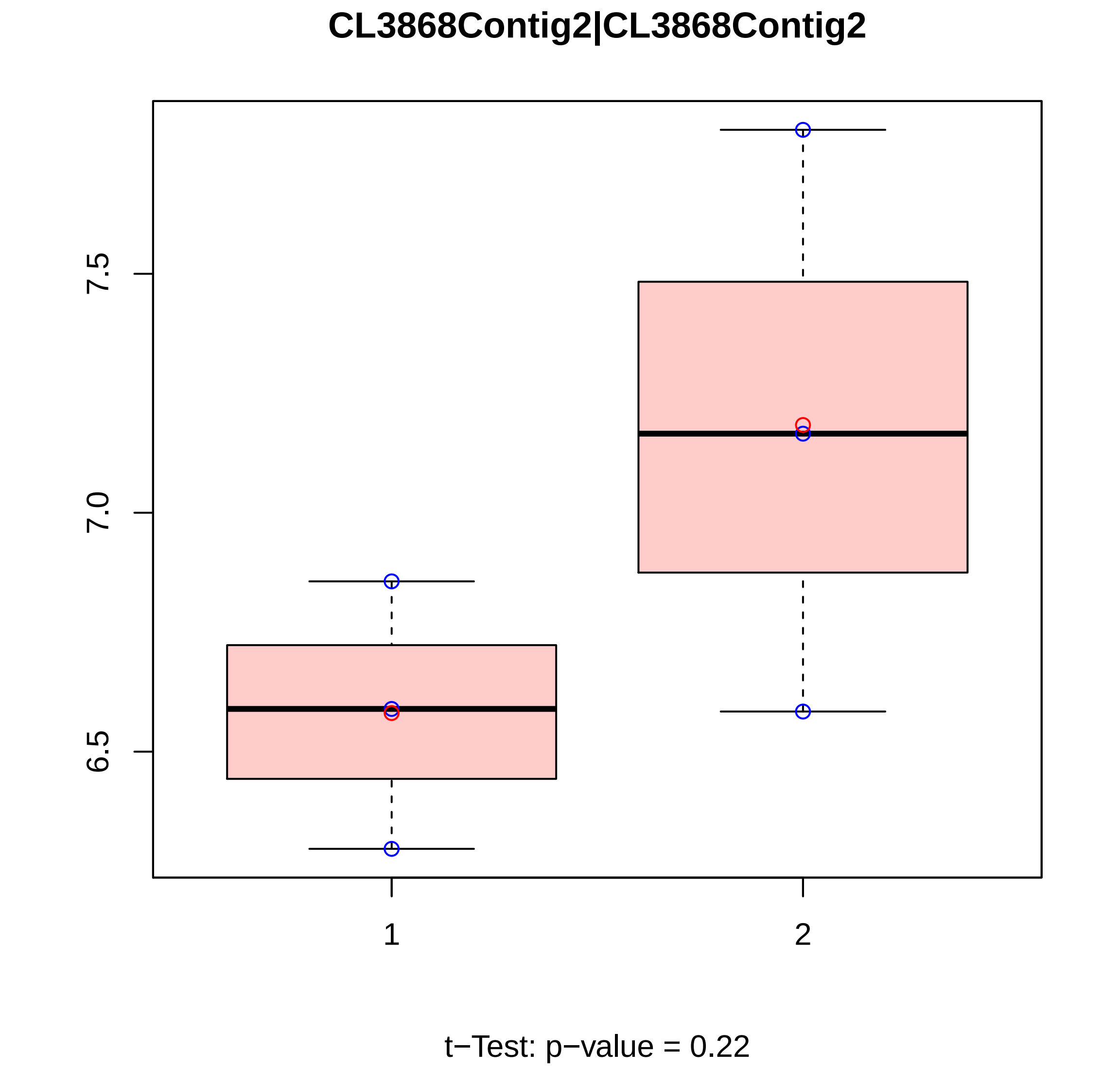
CL3868Contig2|CL3868Contig2
7.5
7.0
6.5
1
2
t−Test: p−value = 0.22

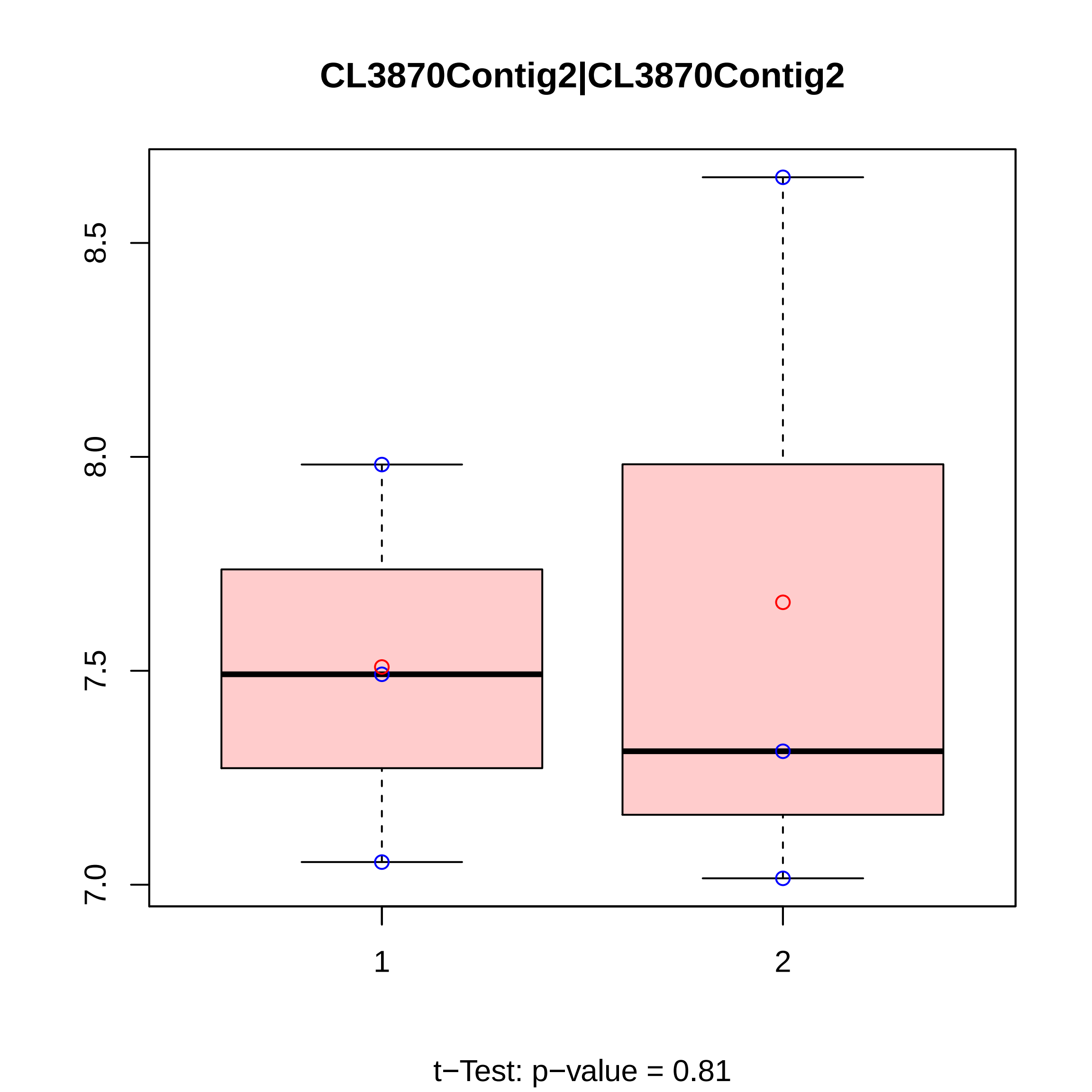
CL3870Contig2|CL3870Contig2
8.5
8.0
7.5
7.0
1
2
t−Test: p−value = 0.81

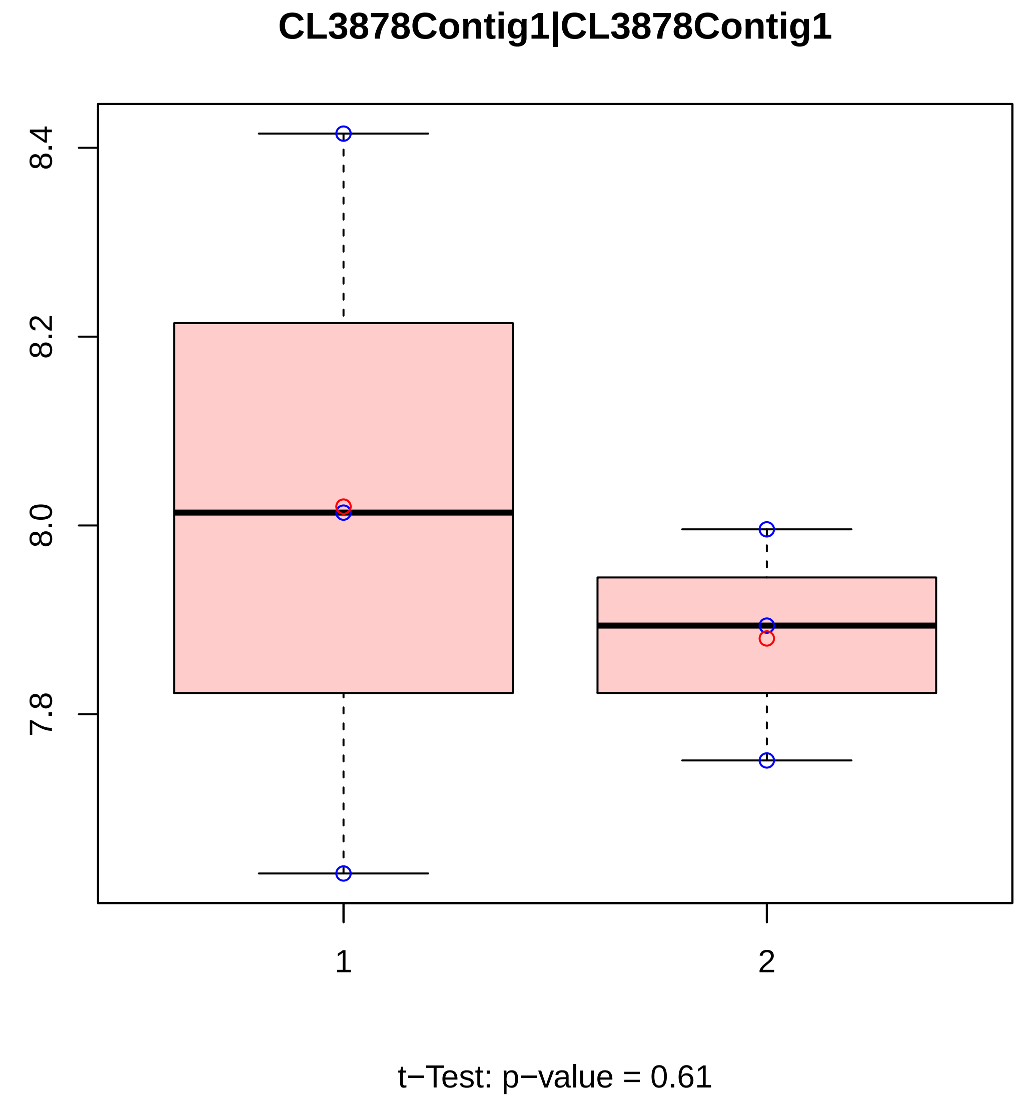

CL3878Contig1|CL3878Contig1
8.4
8.2
8.0
7.8
1
2
t−Test: p−value = 0.61

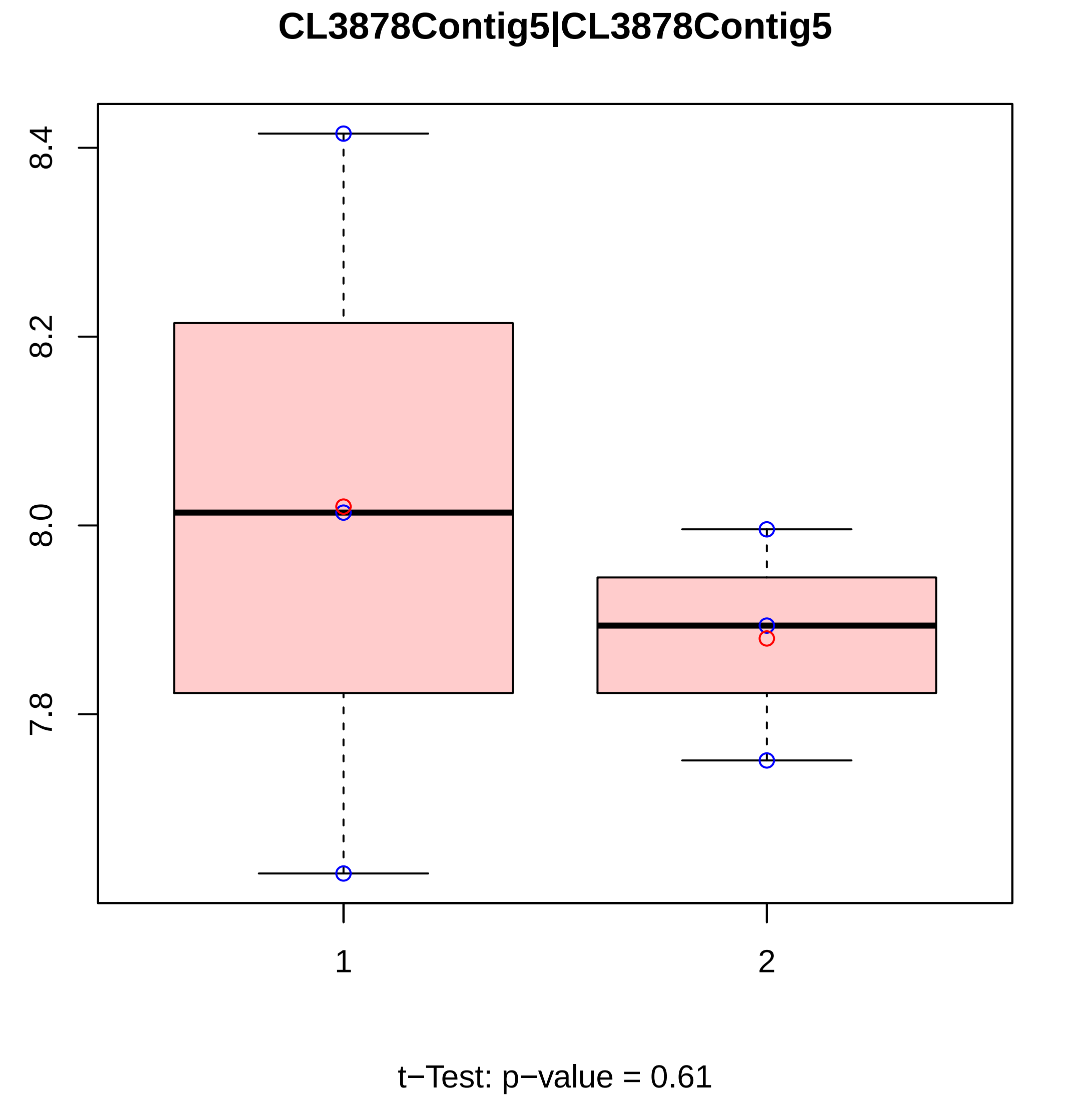
CL3878Contig5|CL3878Contig5
8.4
8.2
8.0
7.8
1
2
t−Test: p−value = 0.61

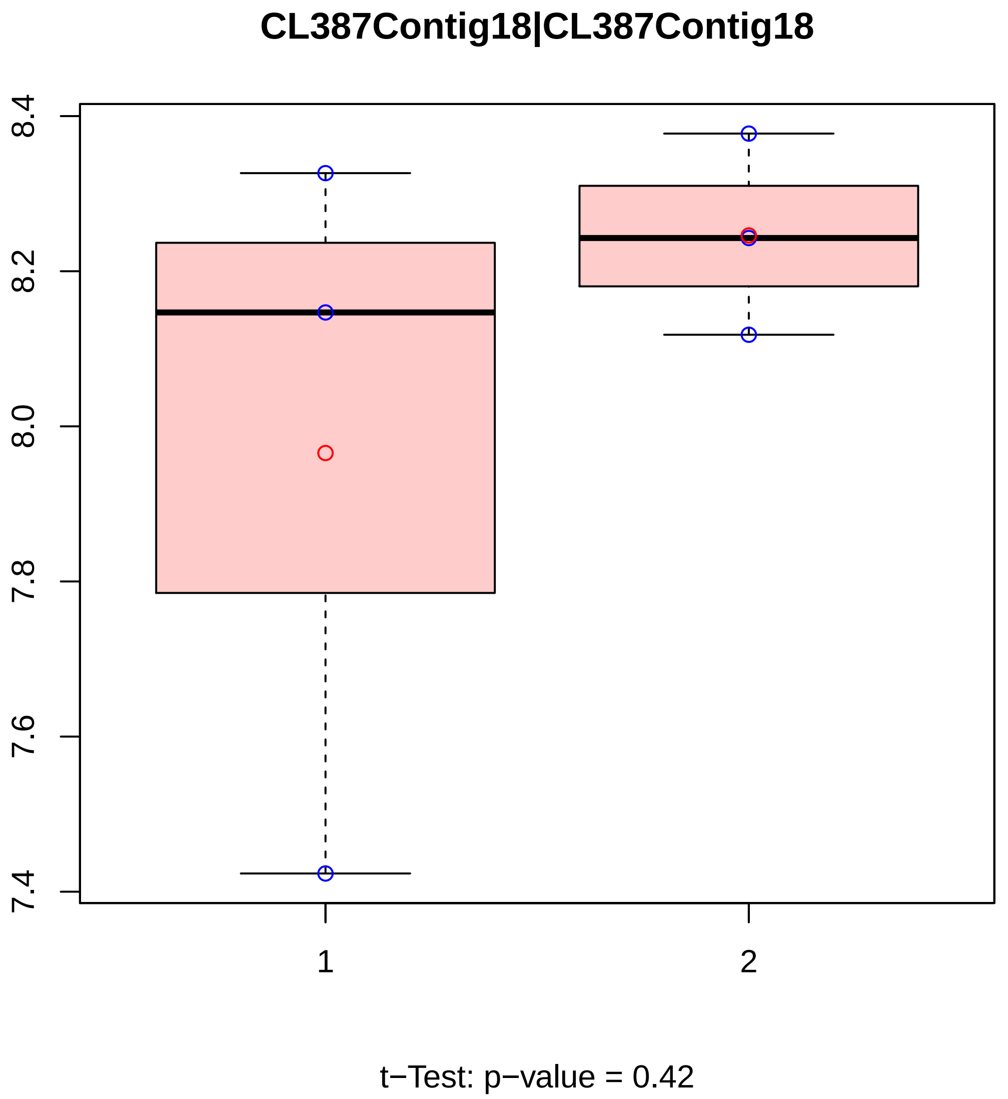
CL387Contig18|CL387Contig18
8.4
8.2
8.0
7.8
7.6
7.4
1
2
t−Test: p−value = 0.42

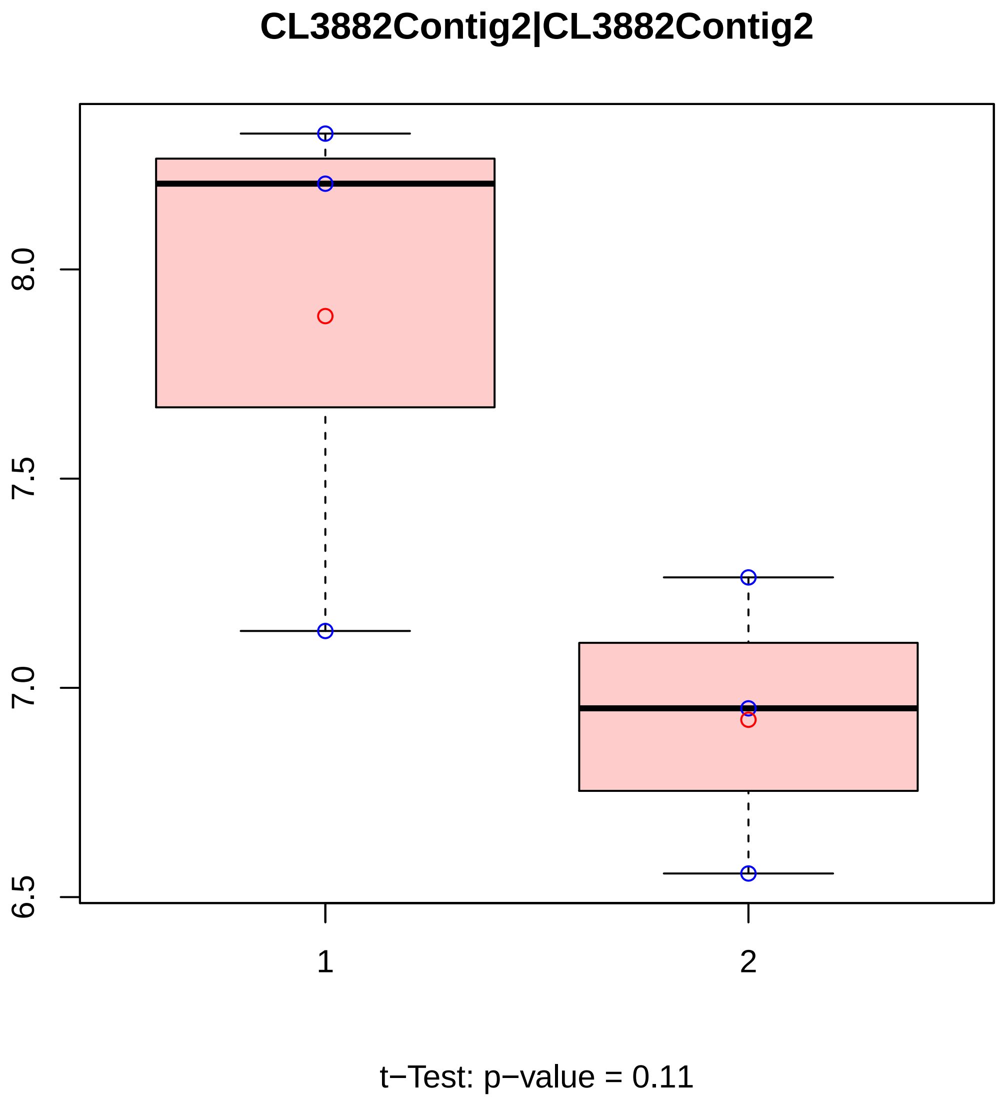

CL3882Contig2|CL3882Contig2
6.5
7.0
7.5
8.0
1
2
t−Test: p−value = 0.11

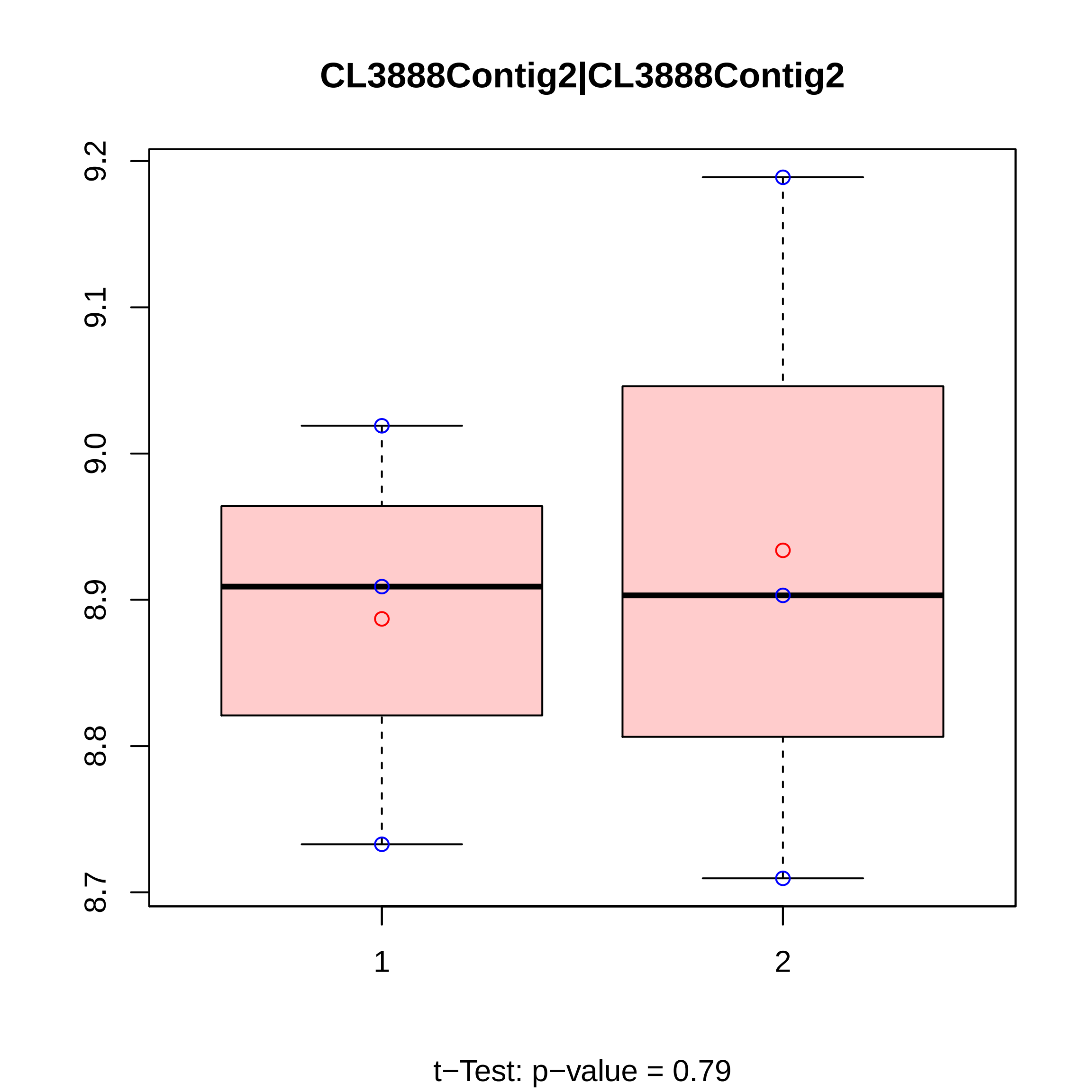

CL3888Contig2|CL3888Contig2
9.2
9.1
9.0
8.9
8.8
8.7
1
2
t−Test: p−value = 0.79

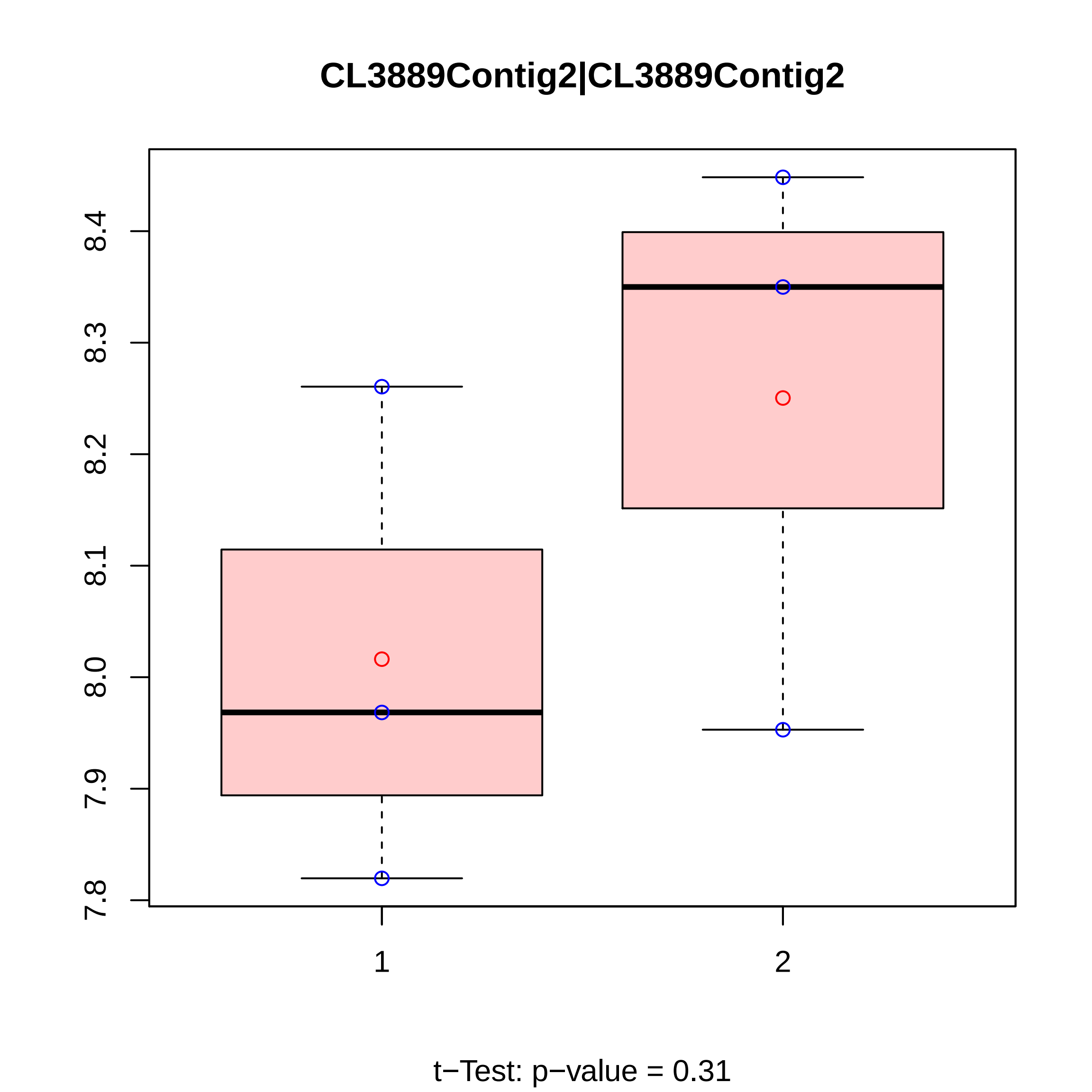
CL3889Contig2|CL3889Contig2
8.4
8.3
8.2
8.1
8.0
7.9
7.8
1
2
t−Test: p−value = 0.31

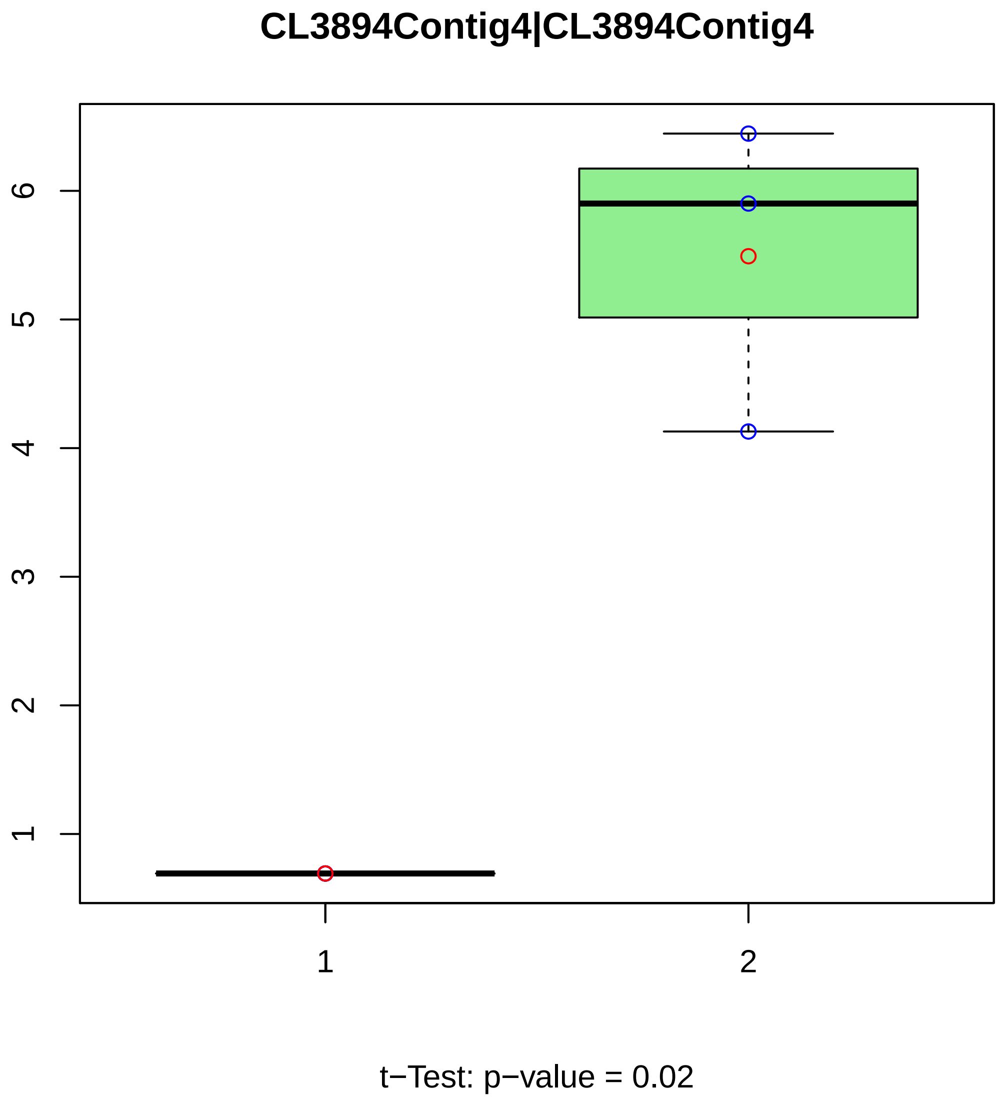

CL3894Contig4|CL3894Contig4
1
2
3
4
5
6
1
2
t−Test: p−value = 0.02

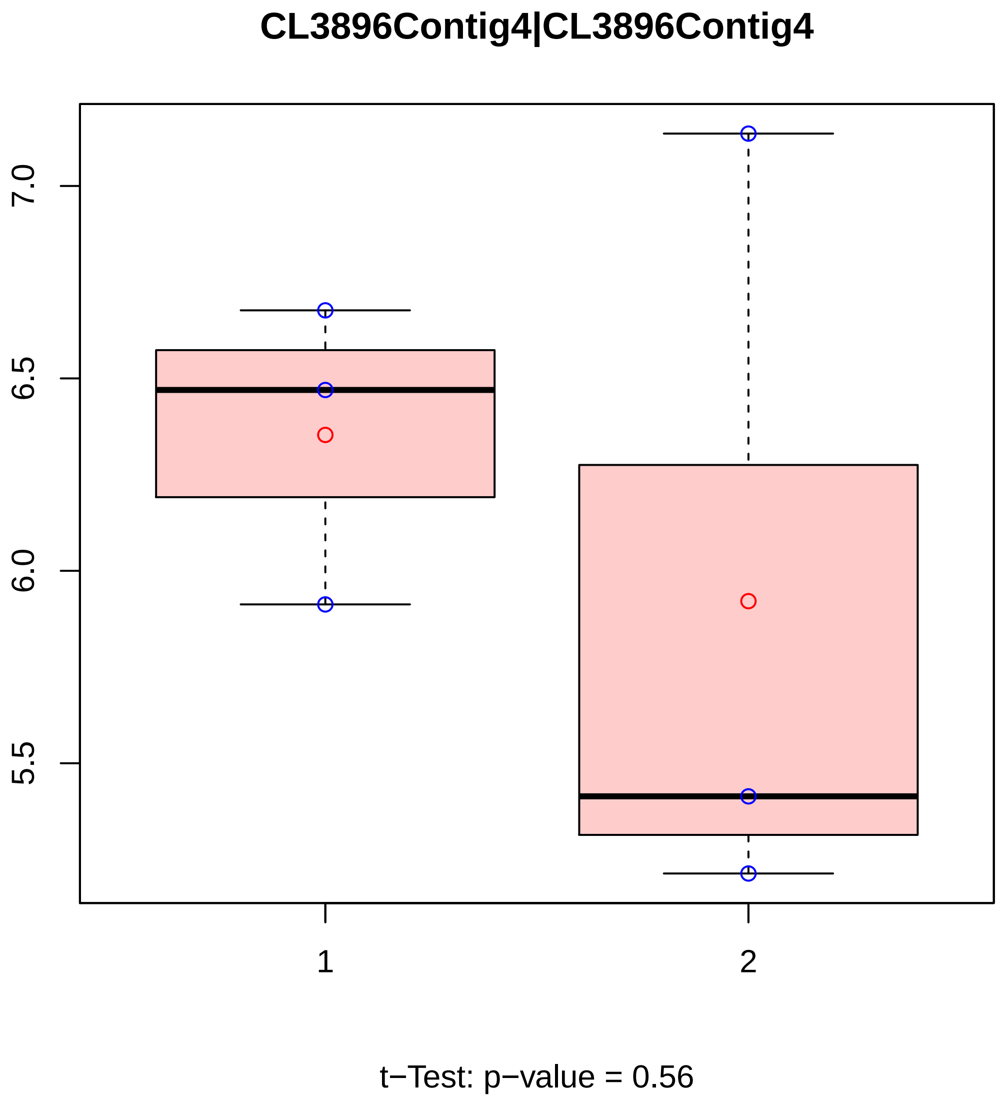

CL3896Contig4|CL3896Contig4
7.0
6.5
6.0
5.5
1
2
t−Test: p−value = 0.56

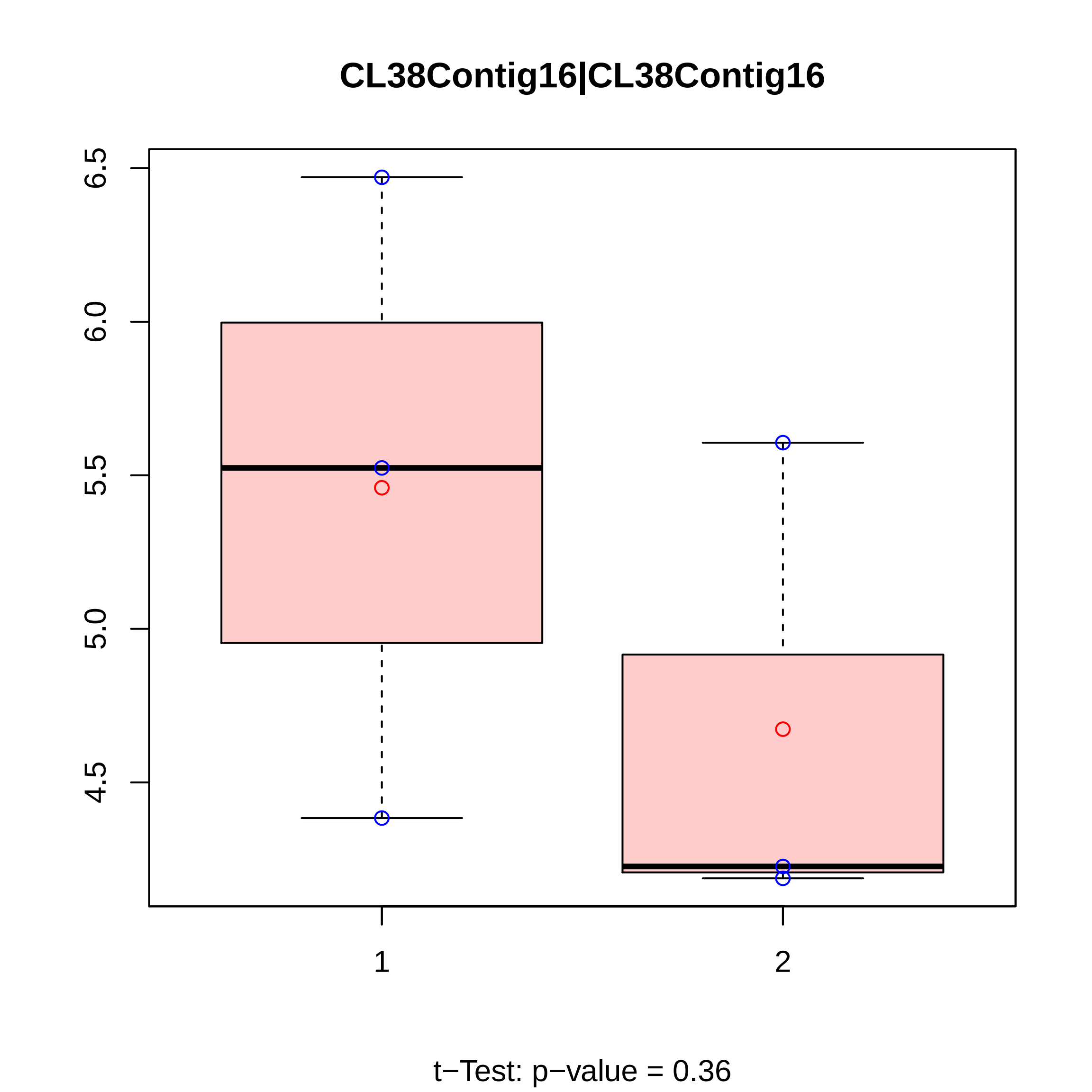
CL38Contig16|CL38Contig16
6.5
6.0
5.5
5.0
4.5
1
2
t−Test: p−value = 0.36

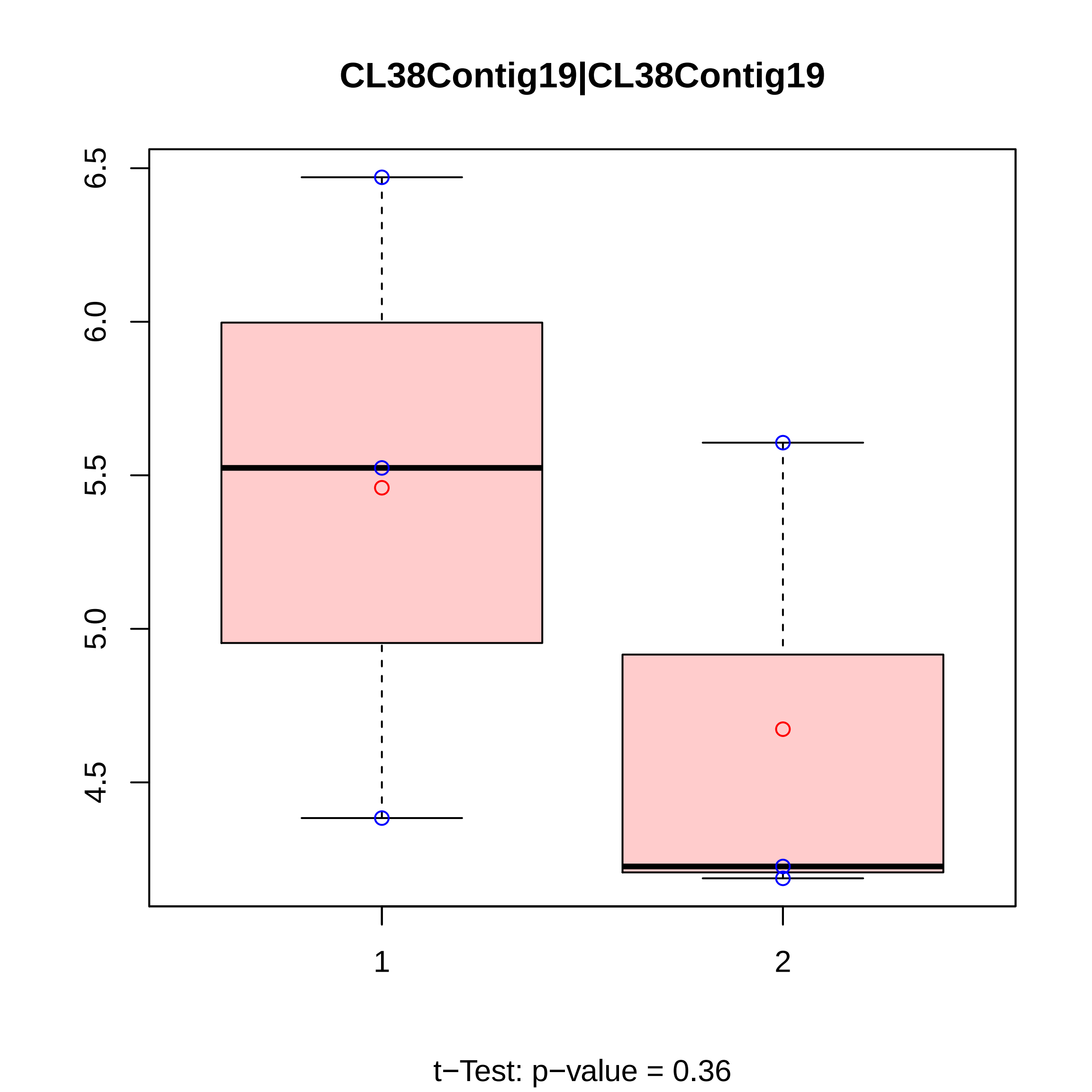
CL38Contig19|CL38Contig19
6.5
6.0
5.5
5.0
4.5
1
2
t−Test: p−value = 0.36

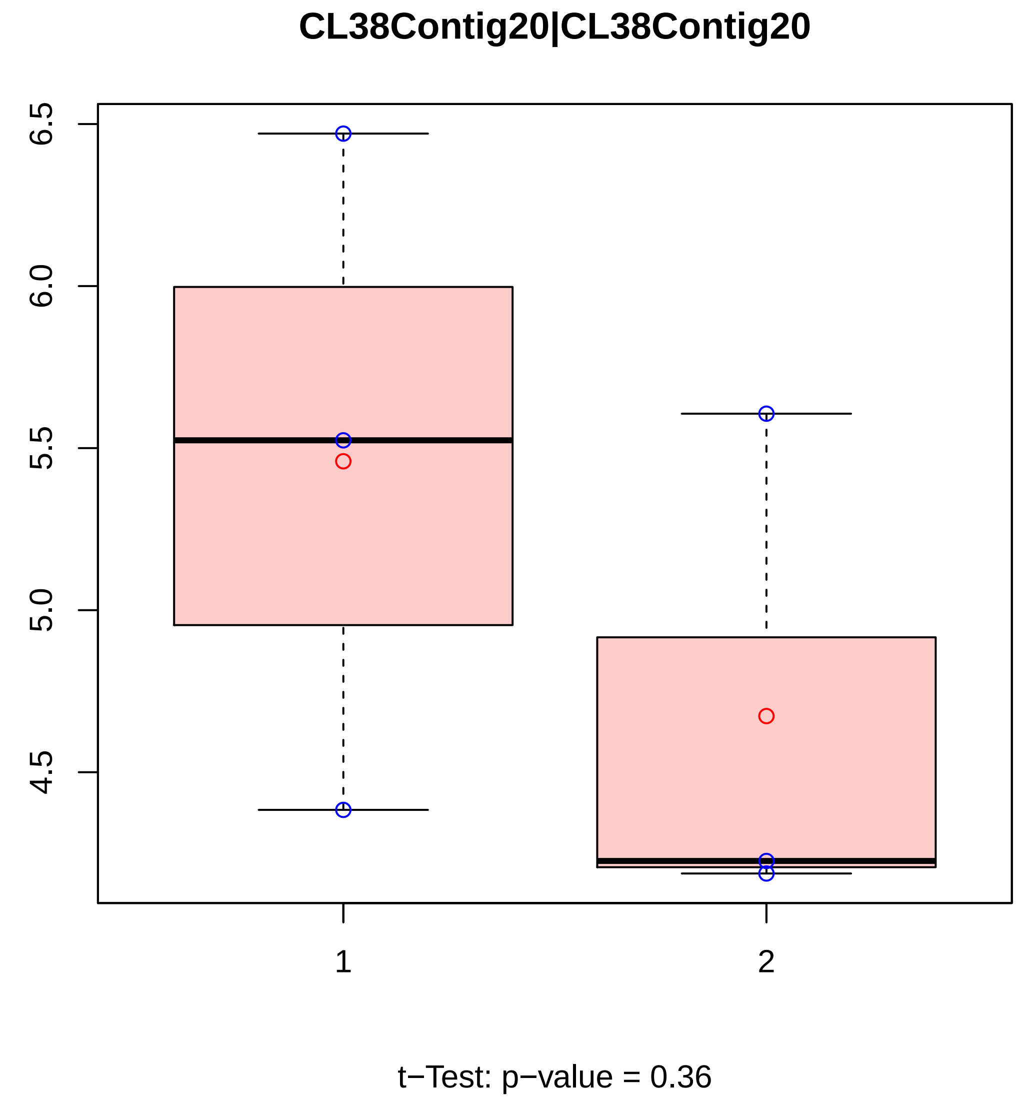
CL38Contig20|CL38Contig20
6.5
6.0
5.5
5.0
4.5
1
2
t−Test: p−value = 0.36

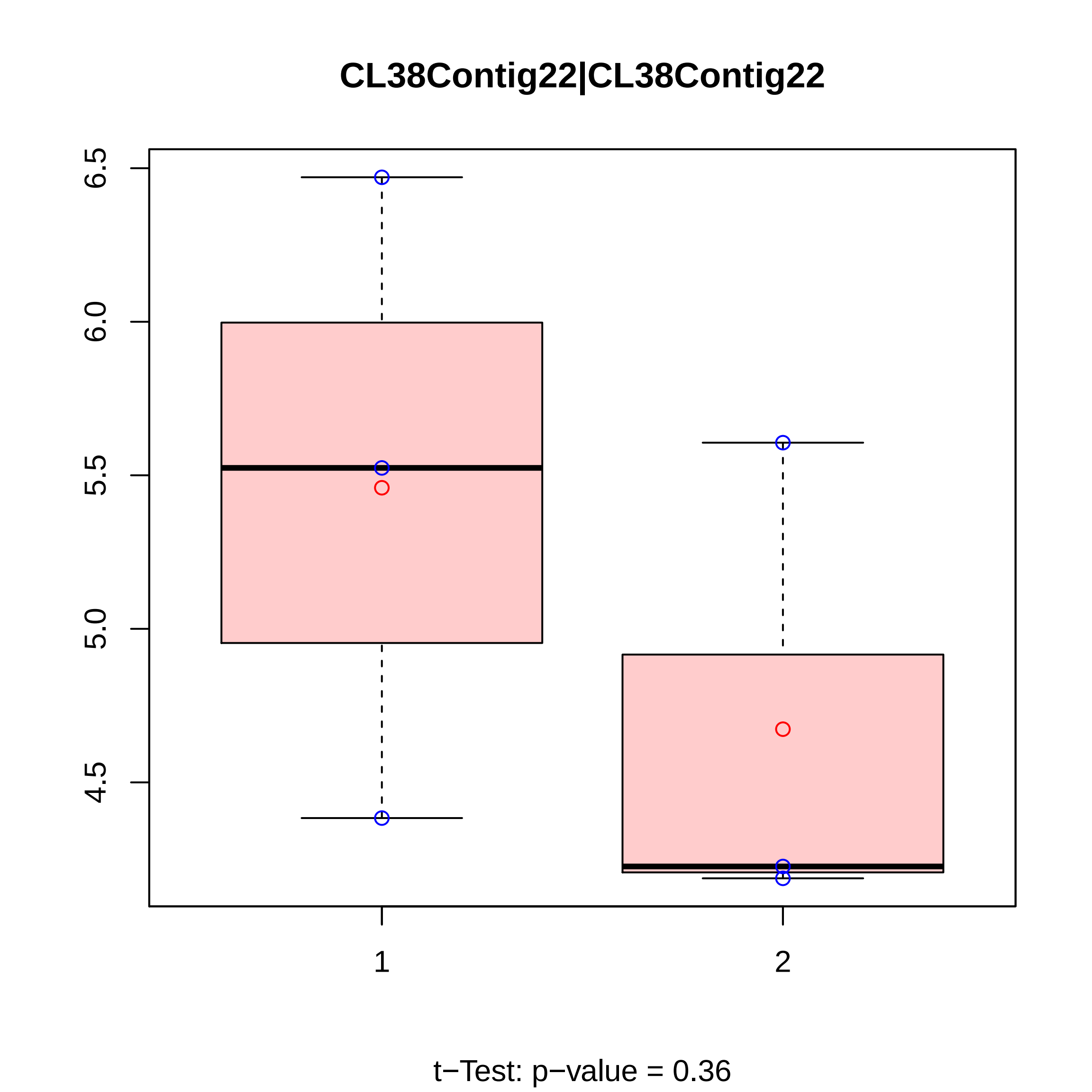
CL38Contig22|CL38Contig22
6.5
6.0
5.5
5.0
4.5
1
2
t−Test: p−value = 0.36

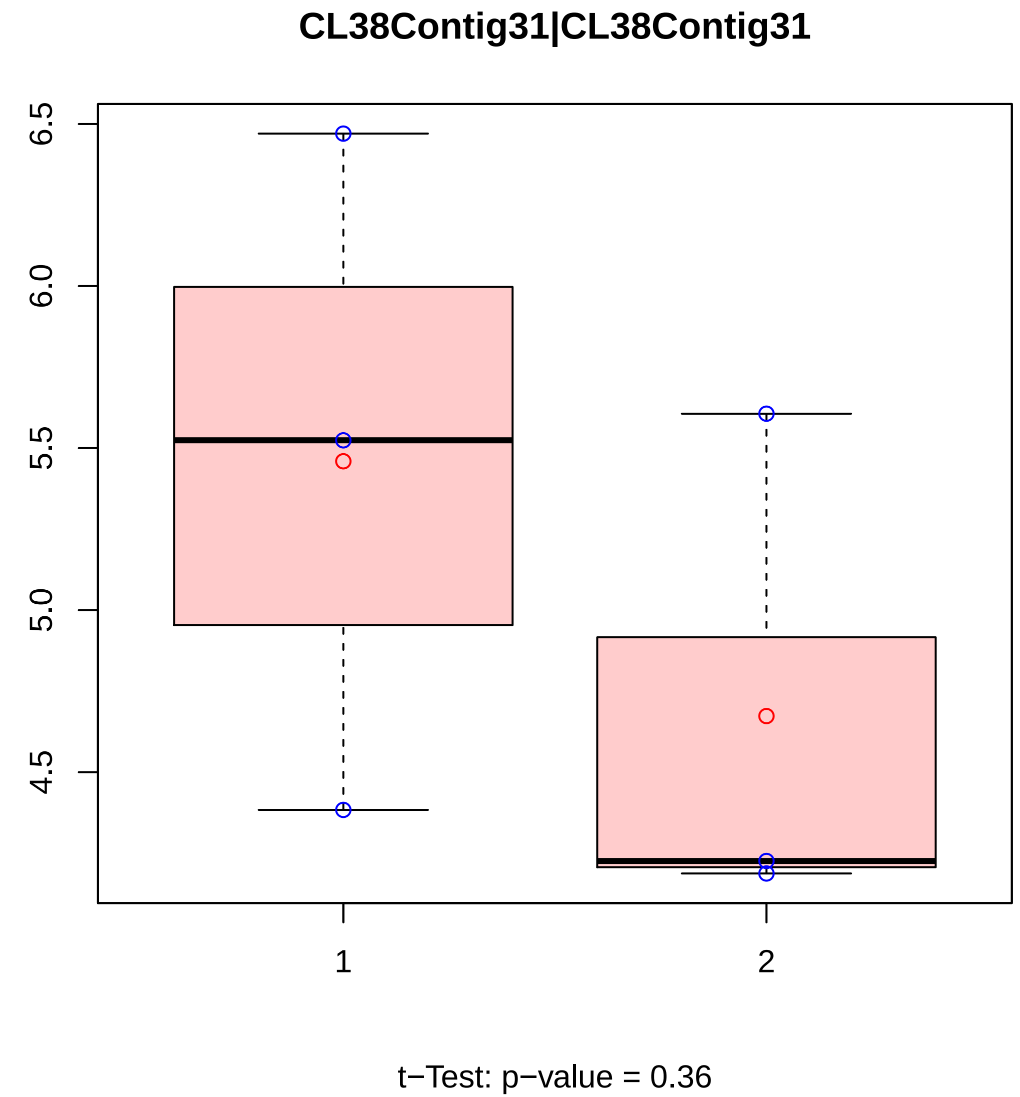

CL38Contig31|CL38Contig31
6.5
6.0
5.5
5.0
4.5
1
2
t−Test: p−value = 0.36

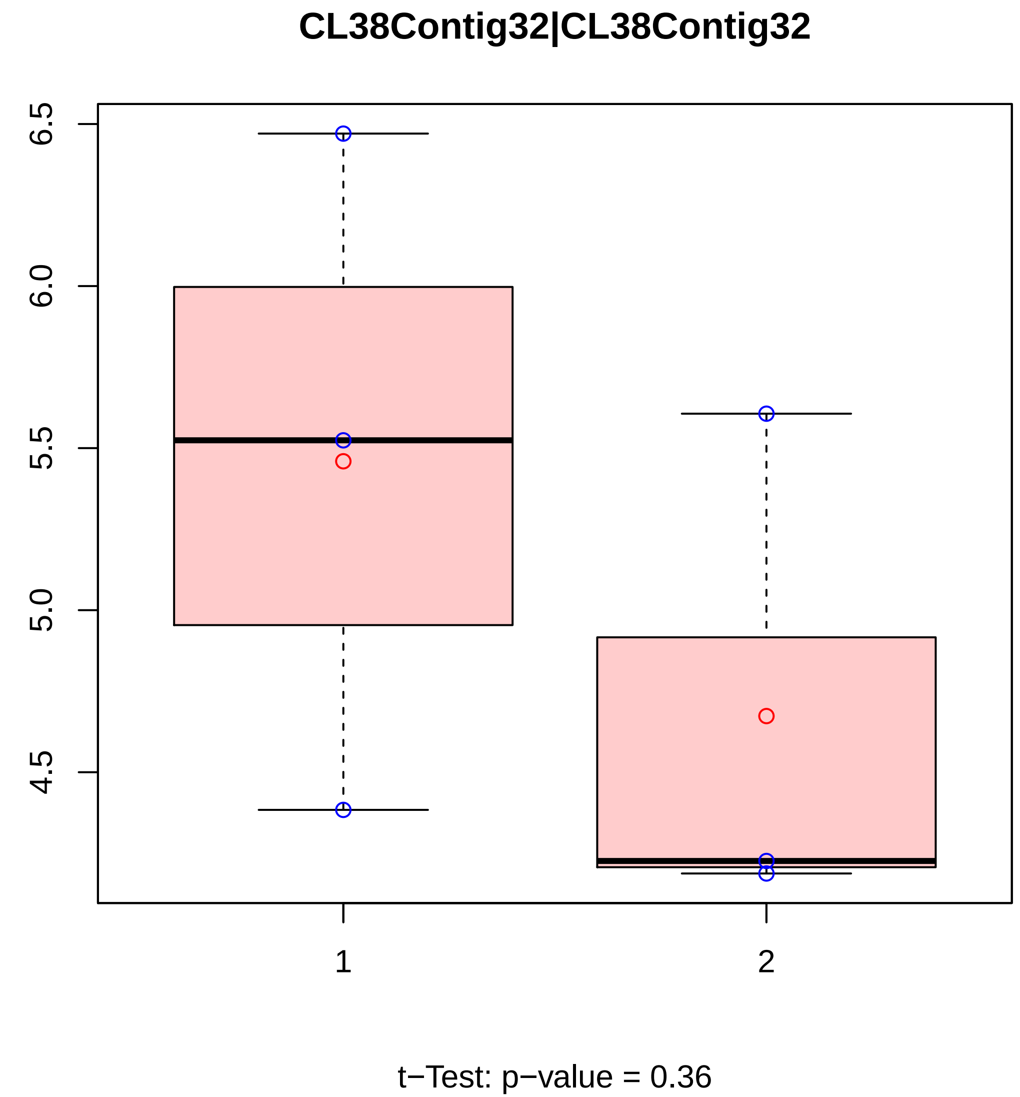
CL38Contig32|CL38Contig32
6.5
6.0
5.5
5.0
4.5
1
2
t−Test: p−value = 0.36

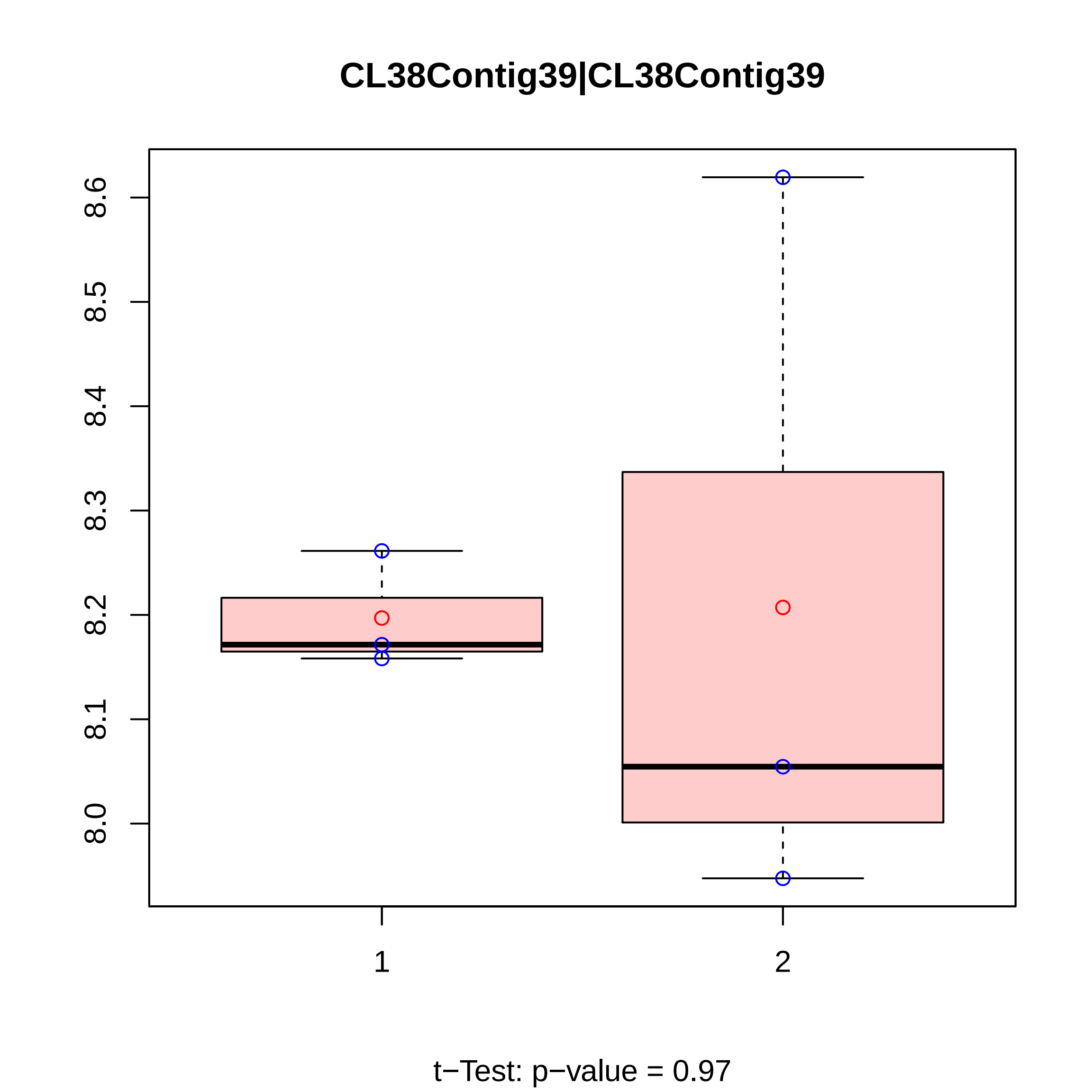
CL38Contig39|CL38Contig39
8.0
8.1
8.2
8.3
8.4
8.5
8.6
1
2
t−Test: p−value = 0.97

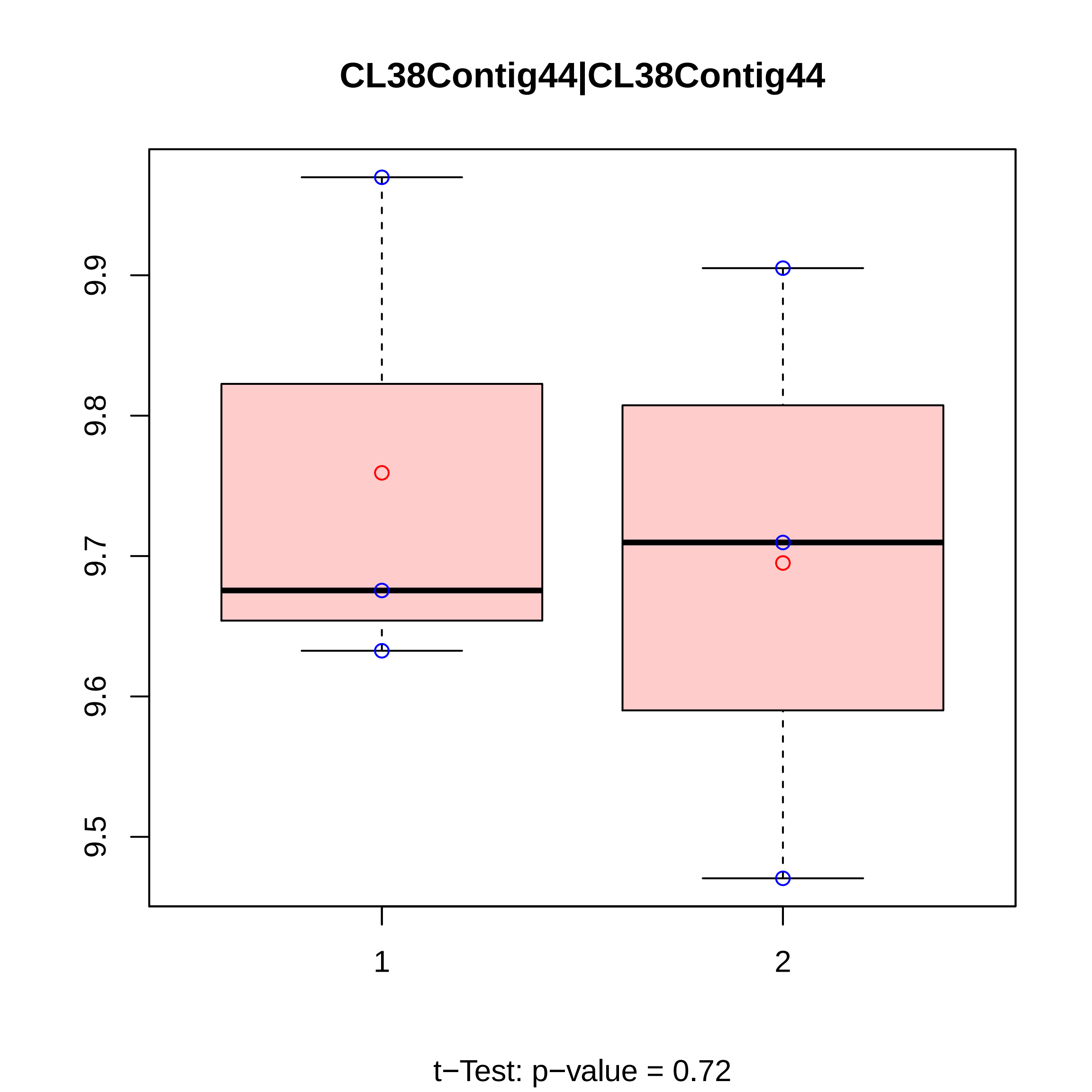
CL38Contig44|CL38Contig44
9.5
9.6
9.7
9.8
9.9
1
2
t−Test: p−value = 0.72

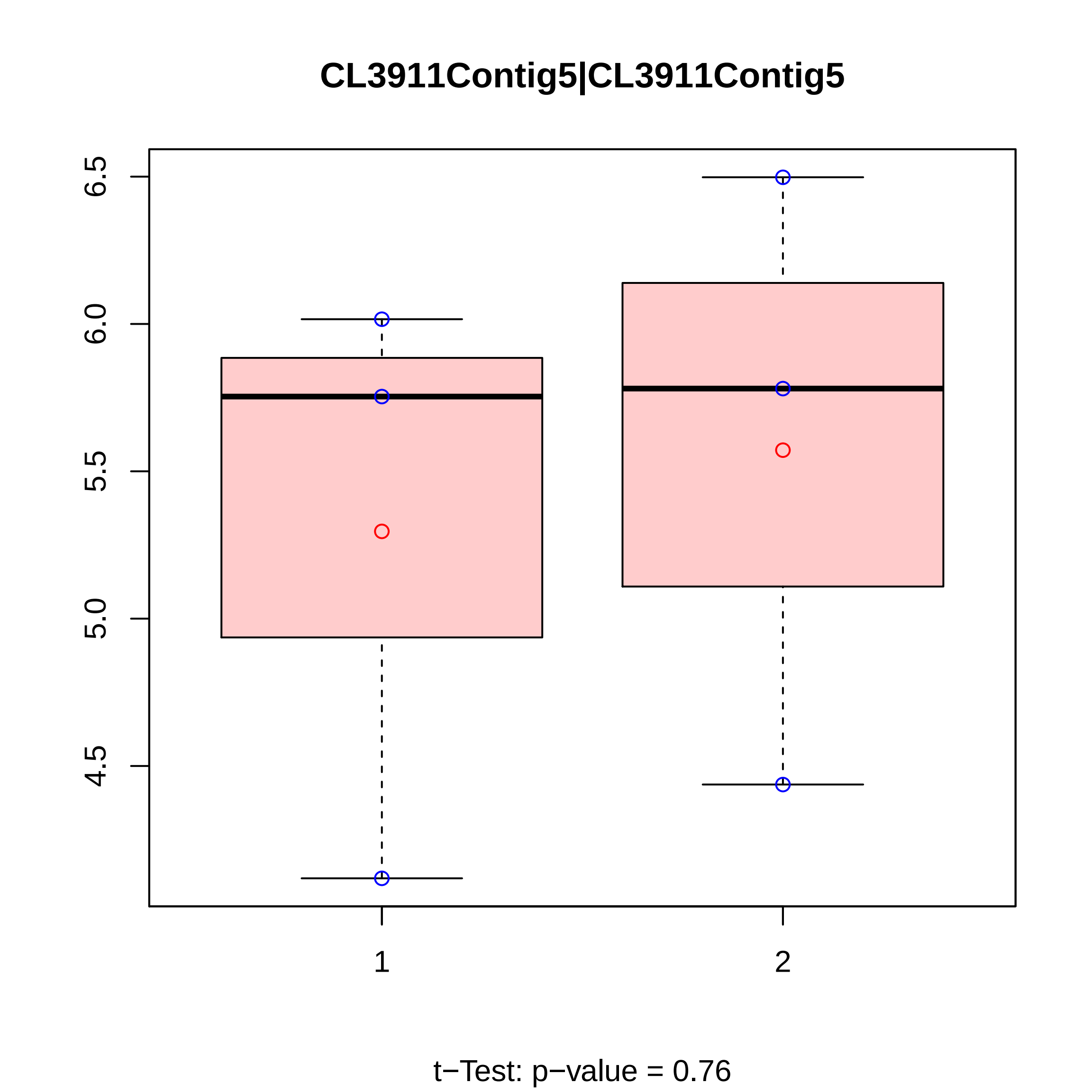
CL3911Contig5|CL3911Contig5
6.5
6.0
5.5
5.0
4.5
1
2
t−Test: p−value = 0.76

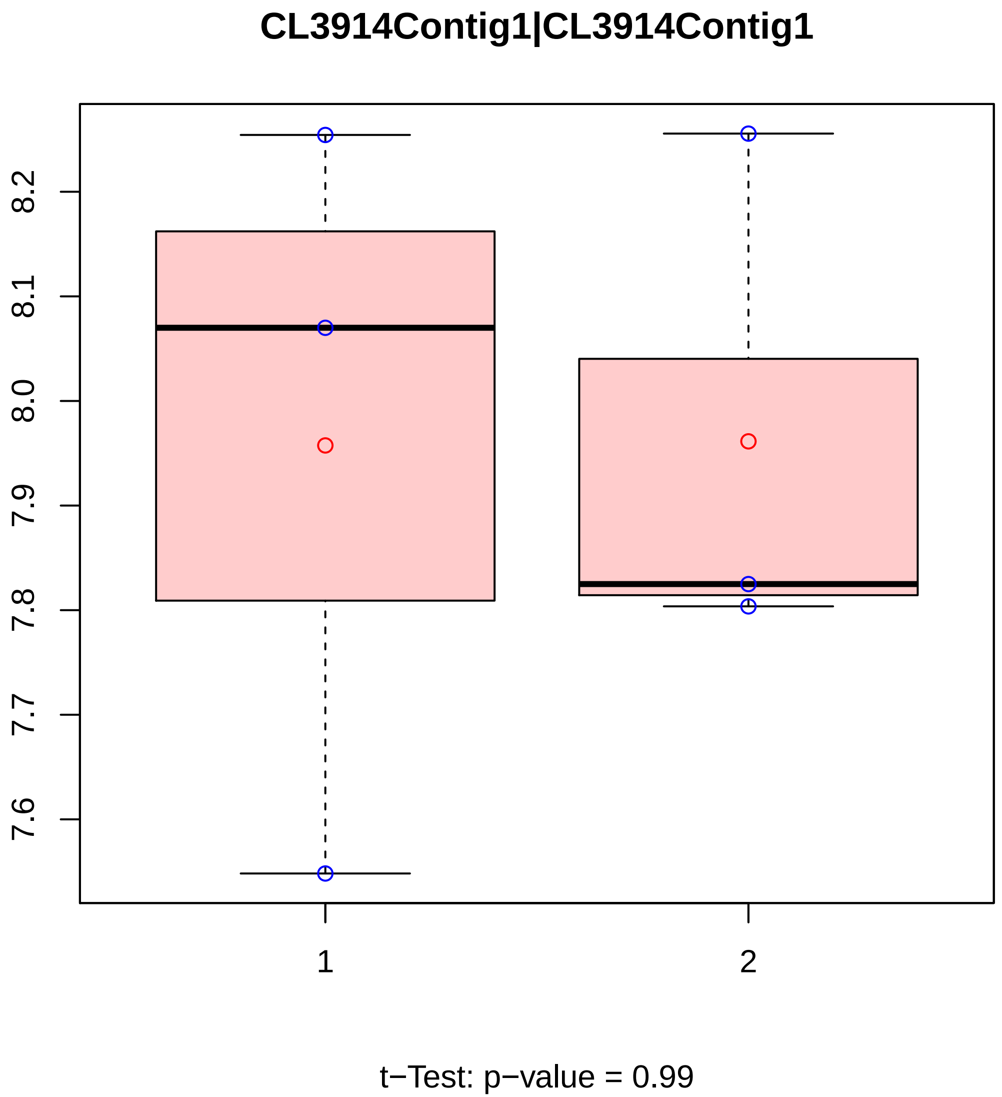
CL3914Contig1|CL3914Contig1
8.2
8.1
8.0
7.9
7.8
7.7
7.6
1
2
t−Test: p−value = 0.99

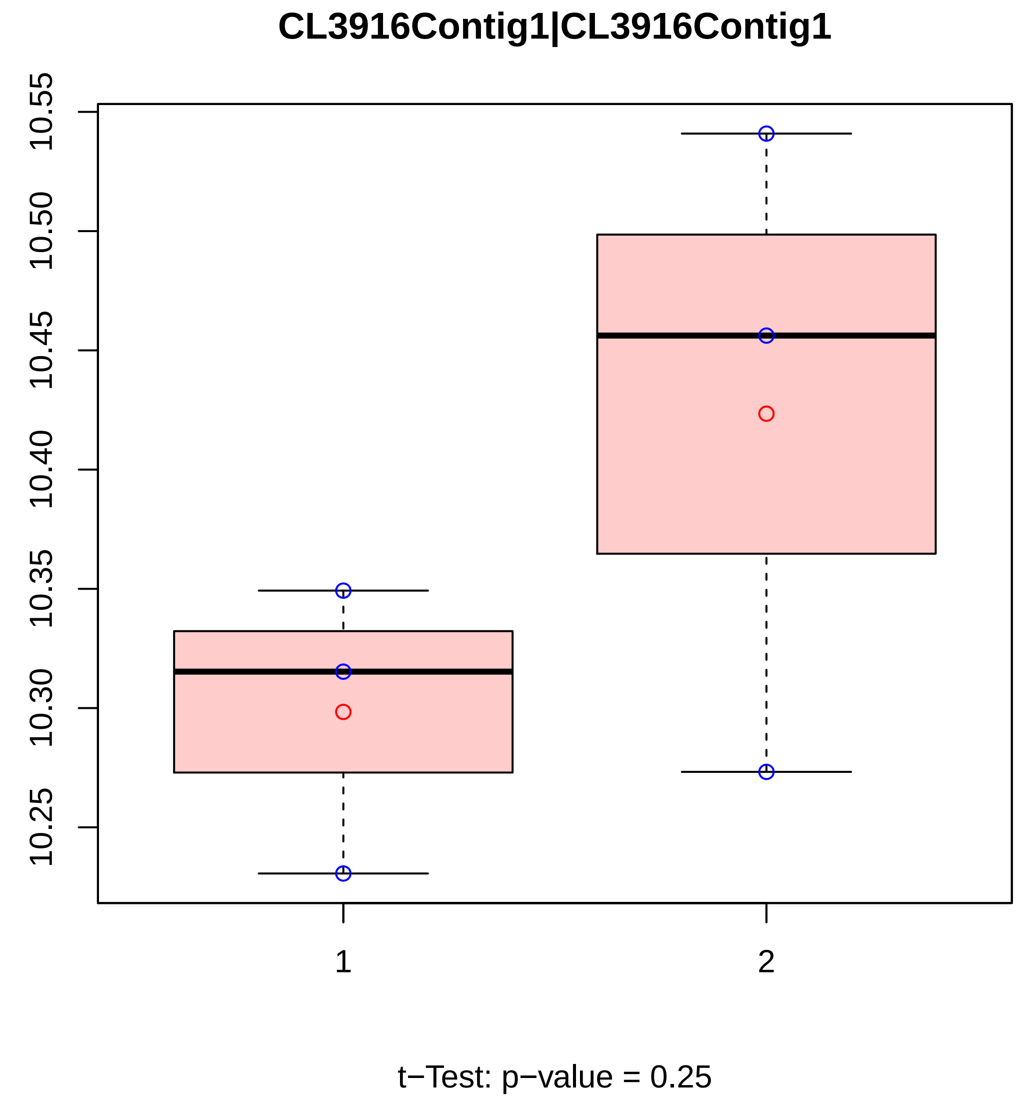

CL3916Contig1|CL3916Contig1
10.25
10.30
10.35
10.40
10.45
10.50
10.55
1
2
t−Test: p−value = 0.25

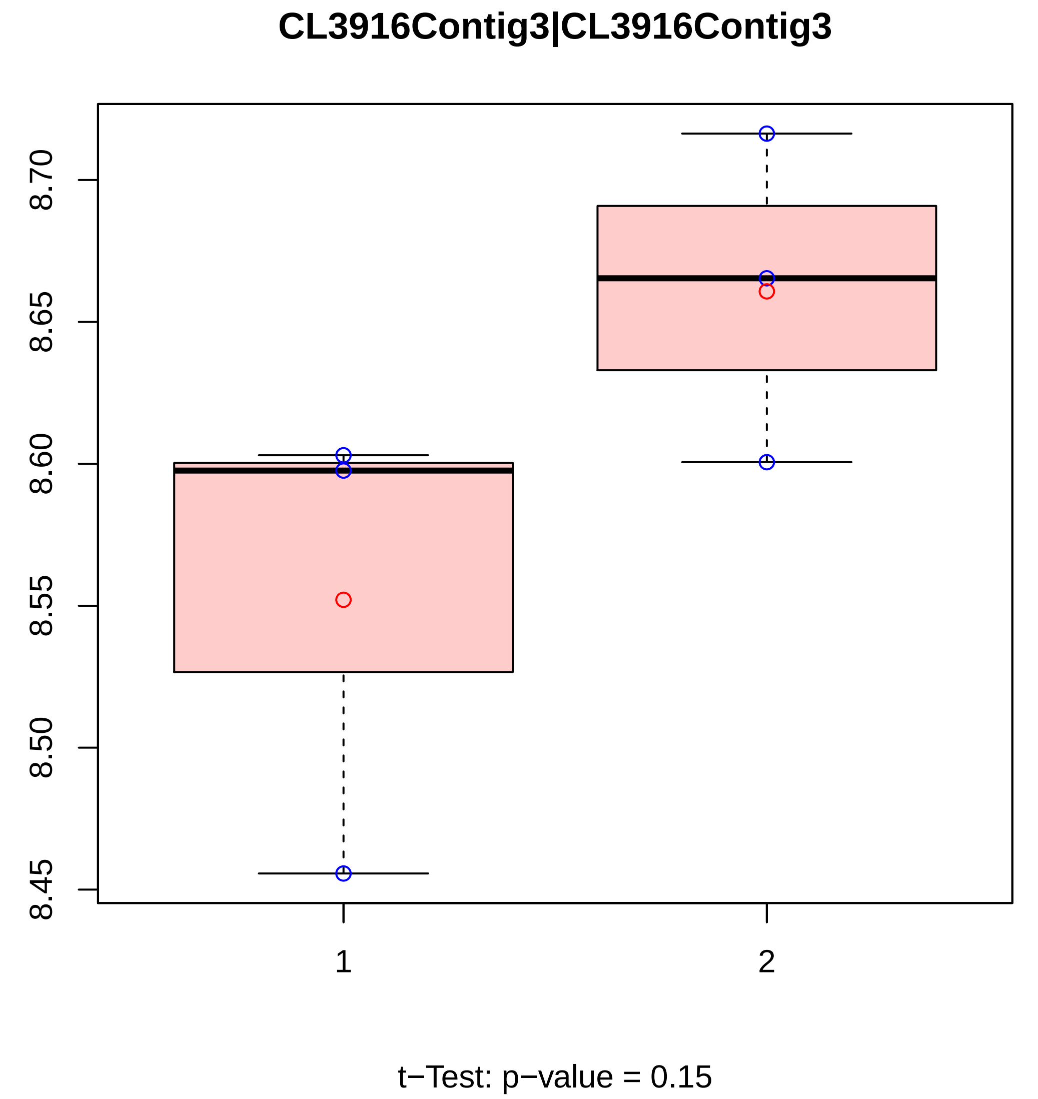

CL3916Contig3|CL3916Contig3
8.45
8.50
8.55
8.60
8.65
8.70
1
2
t−Test: p−value = 0.15

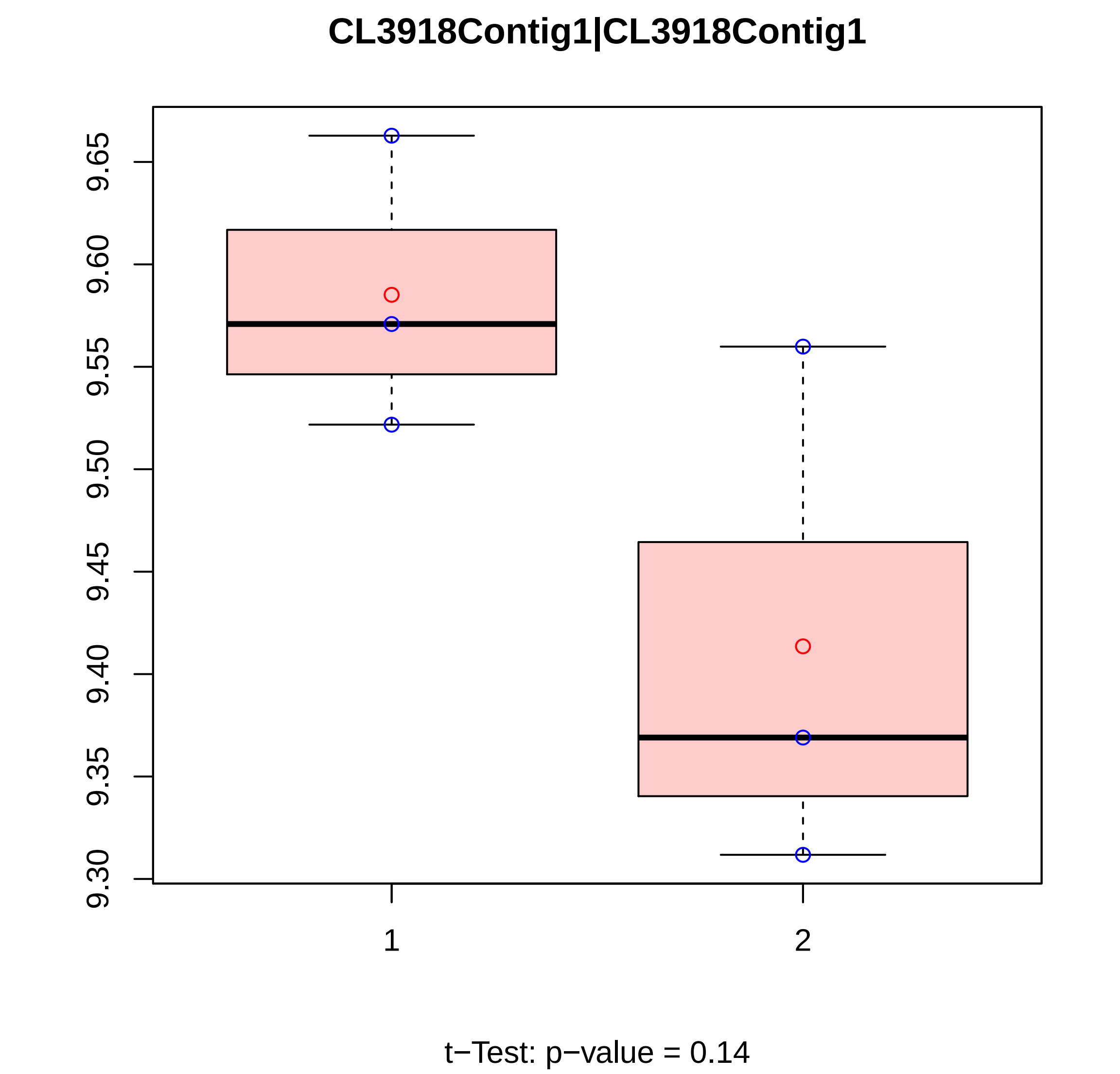
CL3918Contig1|CL3918Contig1
9.30
9.35
9.40
9.45
9.50
9.55
9.60
9.65
1
2
t−Test: p−value = 0.14

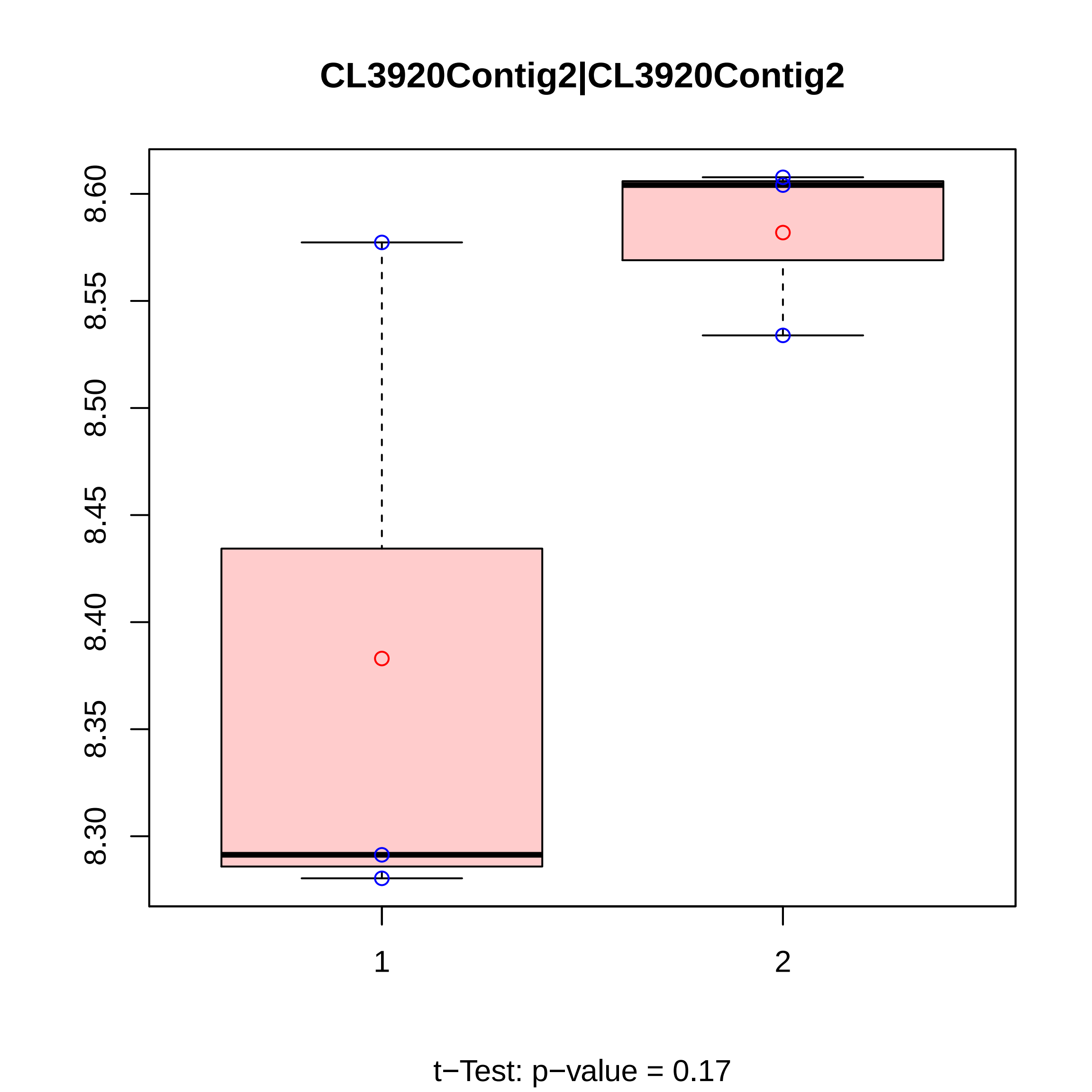

CL3920Contig2|CL3920Contig2
8.30
8.35
8.40
8.45
8.50
8.55
8.60
1
2
t−Test: p−value = 0.17

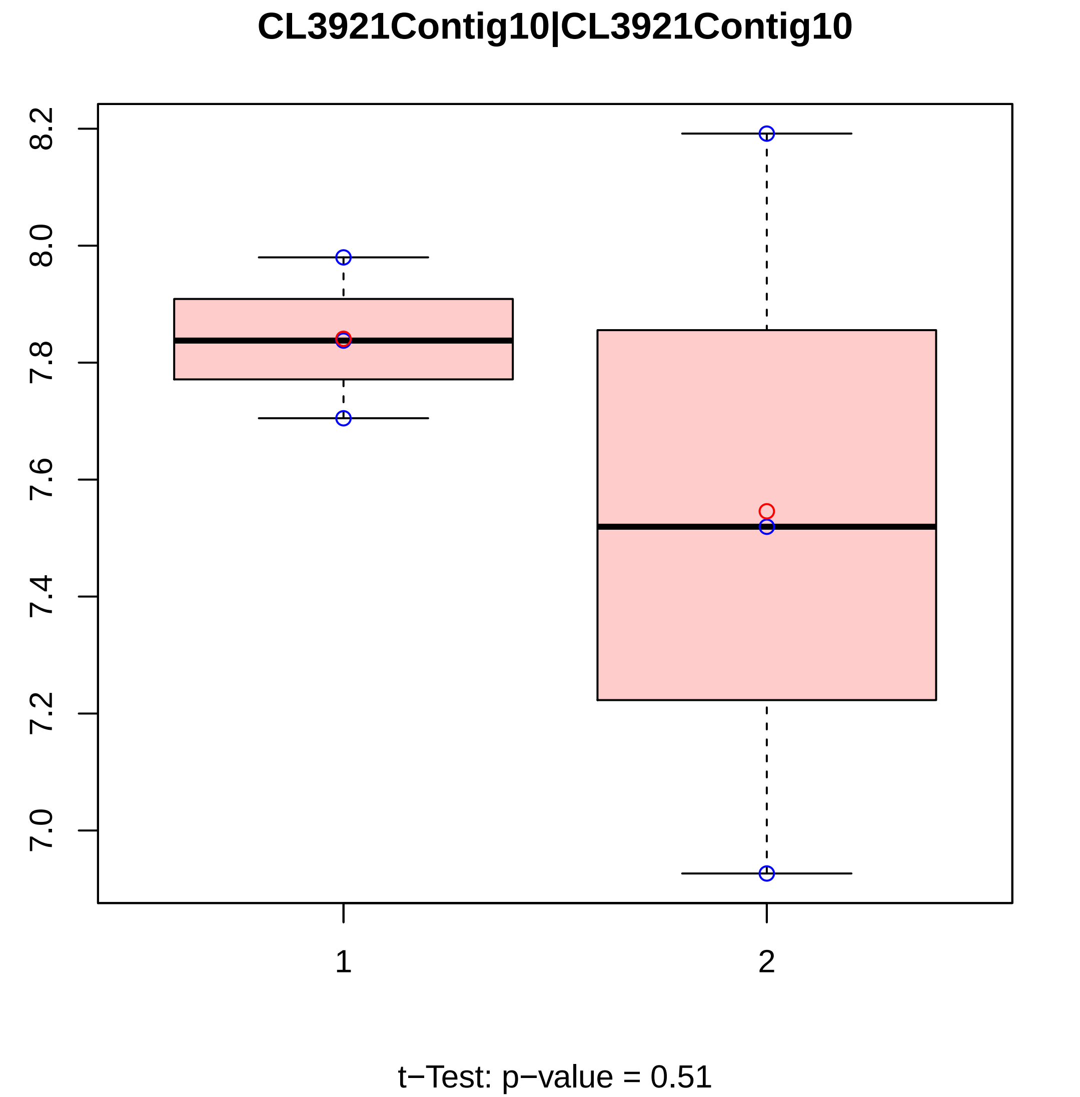
CL3921Contig10|CL3921Contig10
8.2
8.0
7.8
7.6
7.4
7.2
7.0
1
2
t−Test: p−value = 0.51

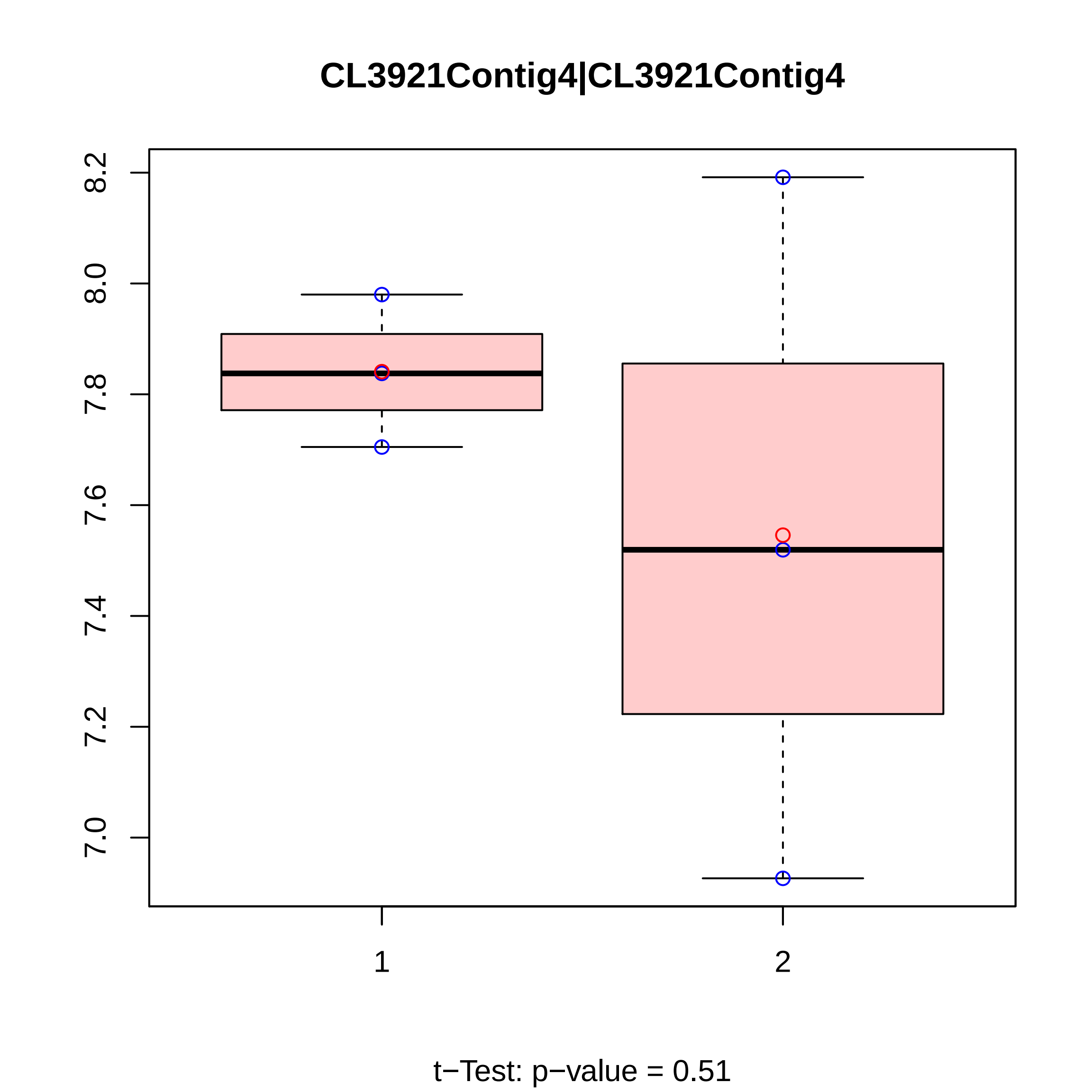

CL3921Contig4|CL3921Contig4
8.2
8.0
7.8
7.6
7.4
7.2
7.0
1
2
t−Test: p−value = 0.51

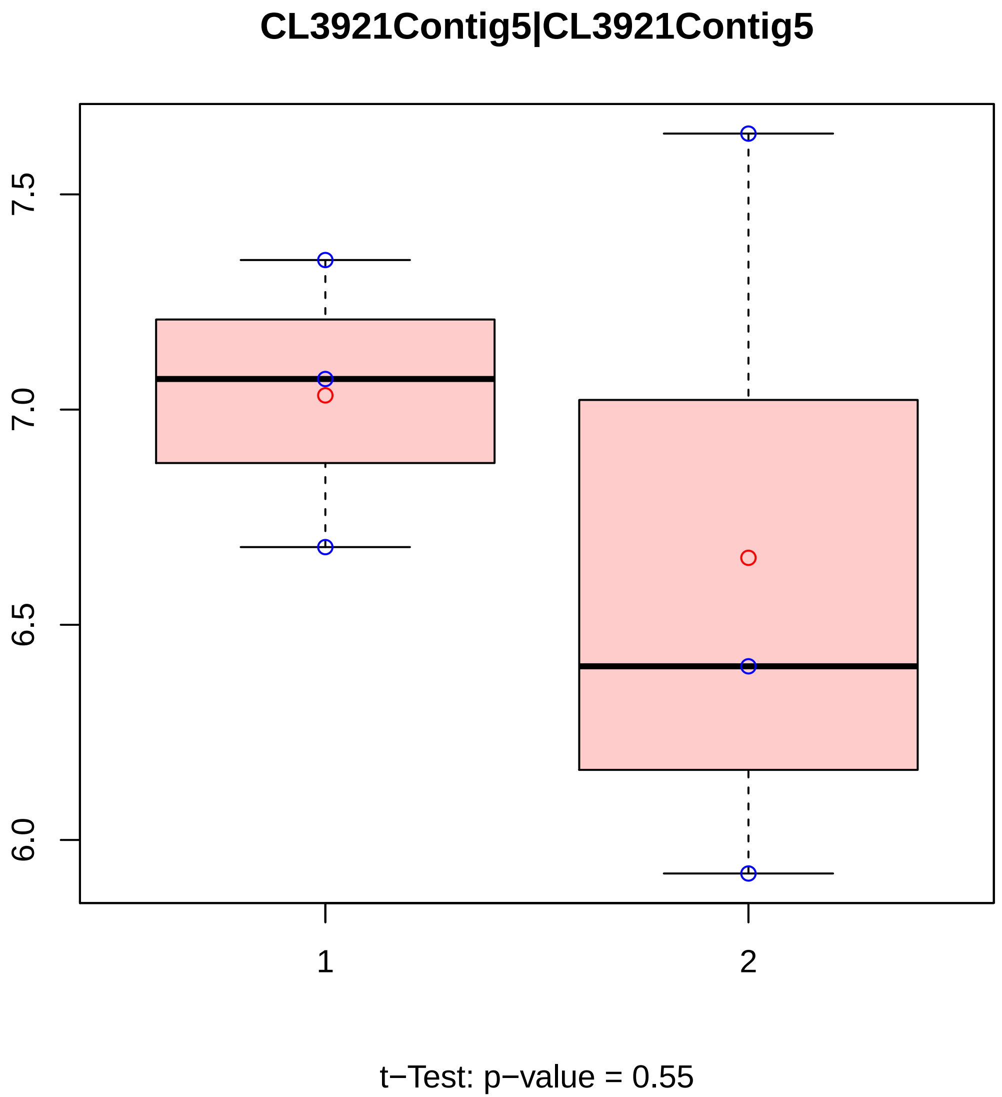
CL3921Contig5|CL3921Contig5
7.5
7.0
6.5
6.0
1
2
t−Test: p−value = 0.55

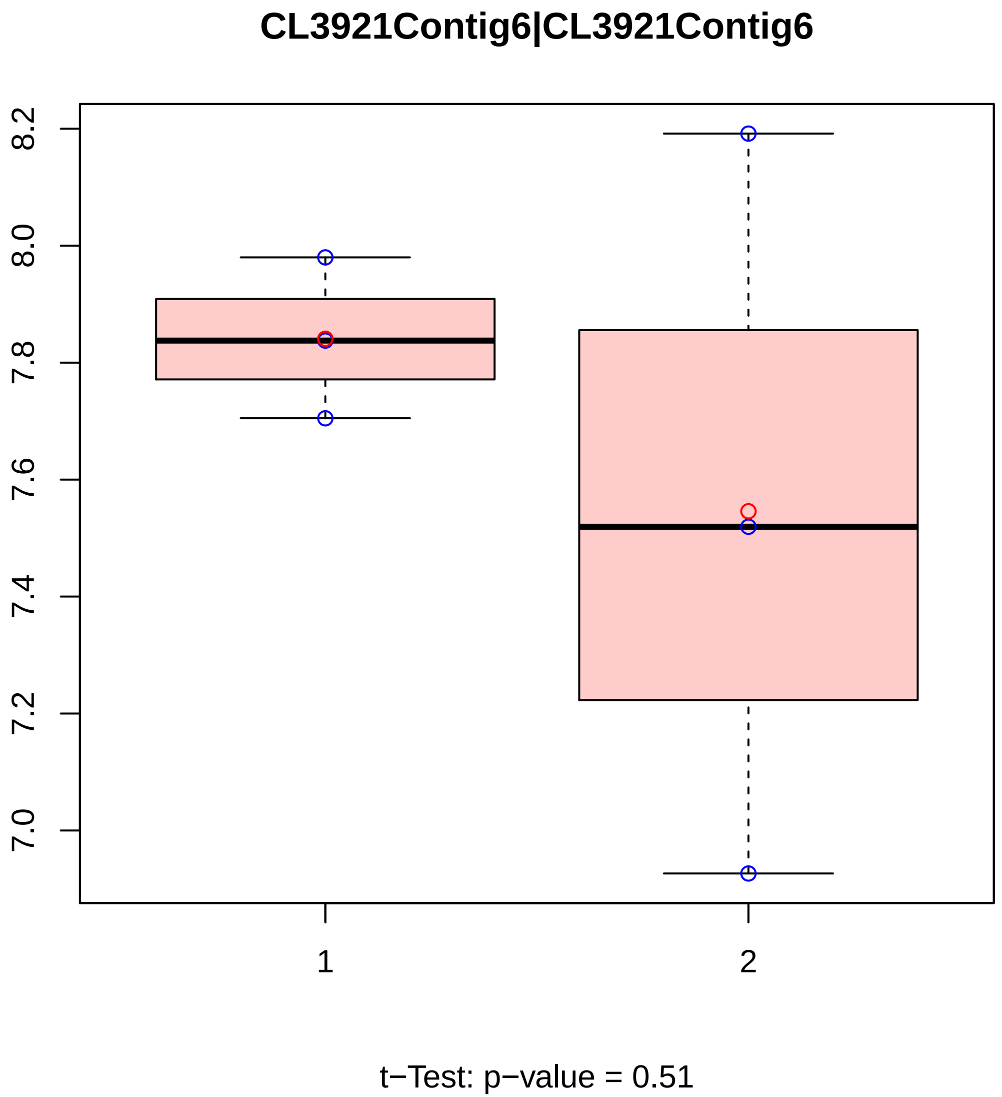

CL3921Contig6|CL3921Contig6
8.2
8.0
7.8
7.6
7.4
7.2
7.0
1
2
t−Test: p−value = 0.51

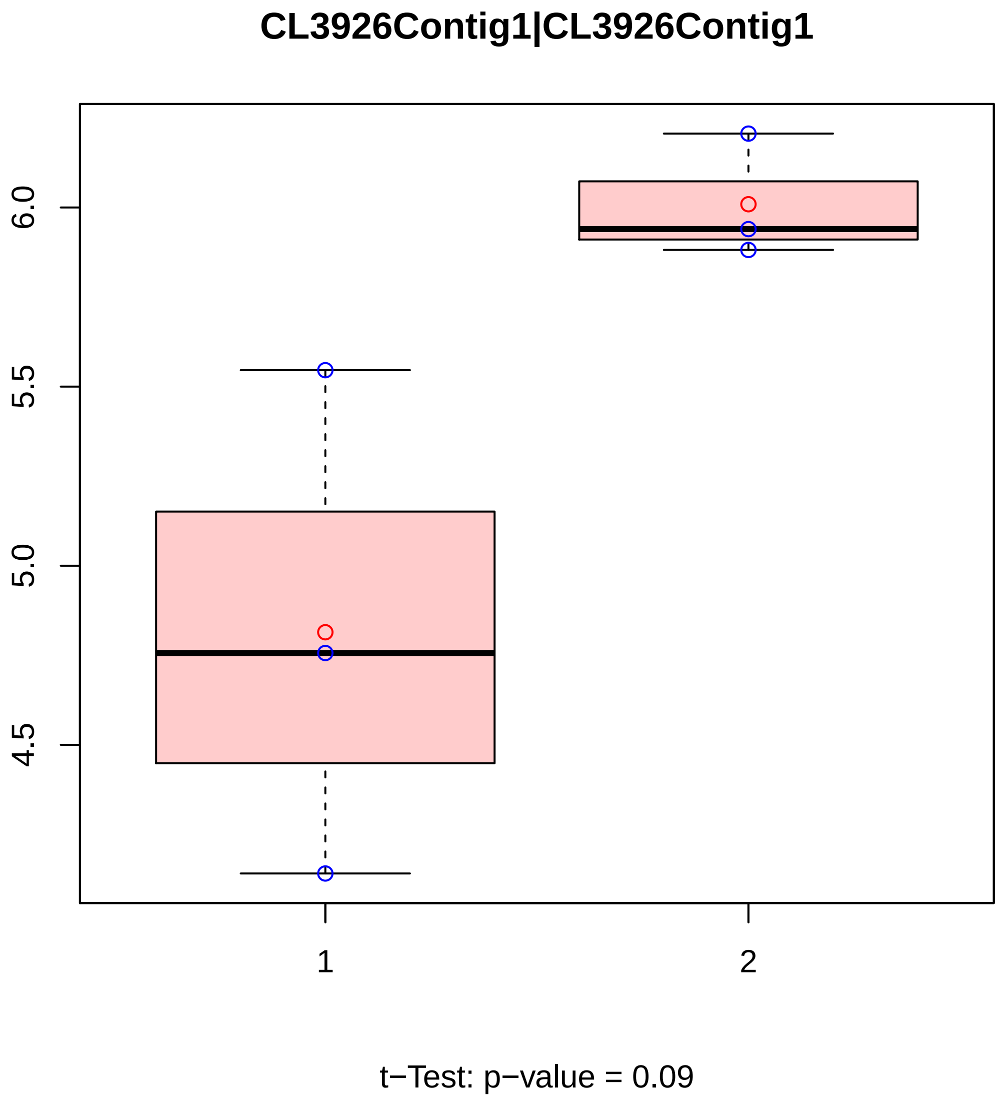

CL3926Contig1|CL3926Contig1
6.0
5.5
5.0
4.5
1
2
t−Test: p−value = 0.09

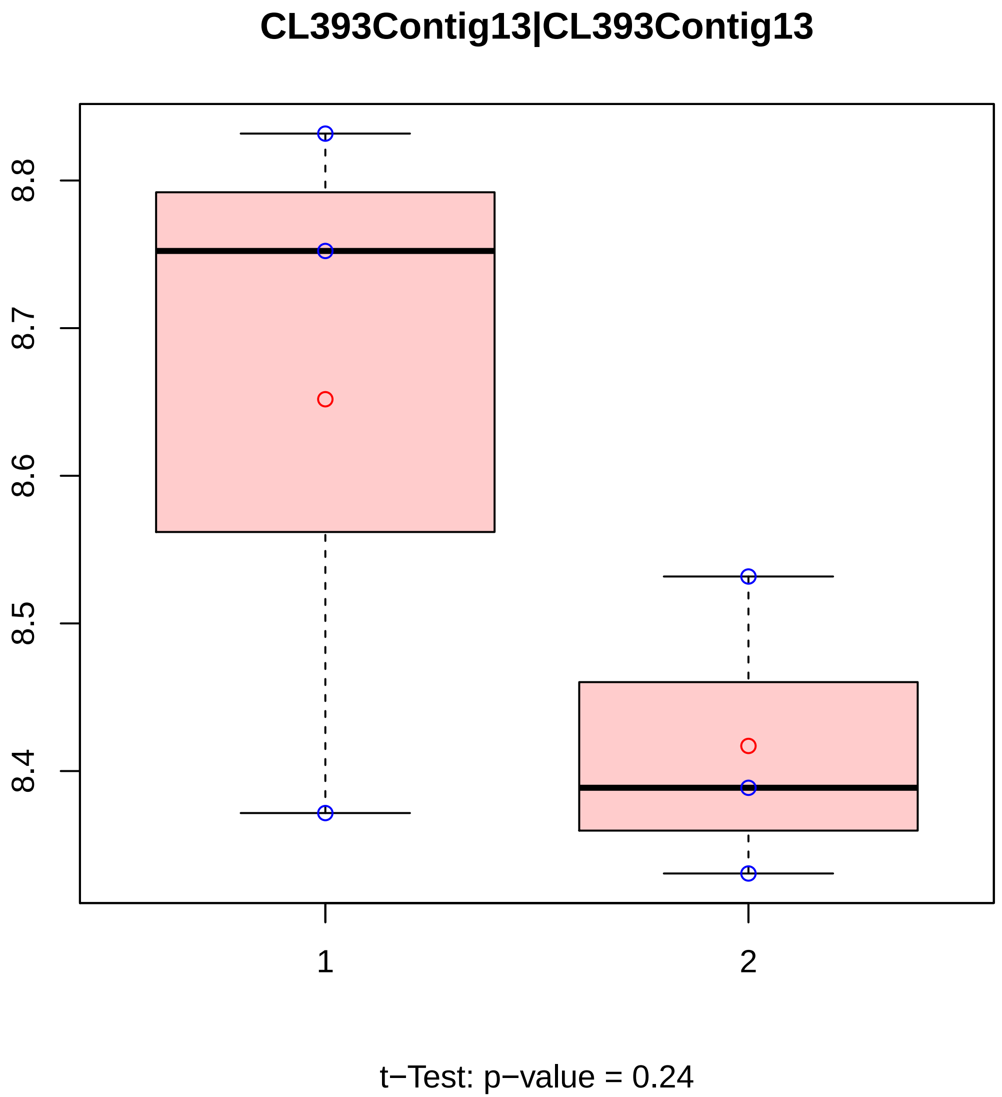
CL393Contig13|CL393Contig13
8.4
8.5
8.6
8.7
8.8
1
2
t−Test: p−value = 0.24

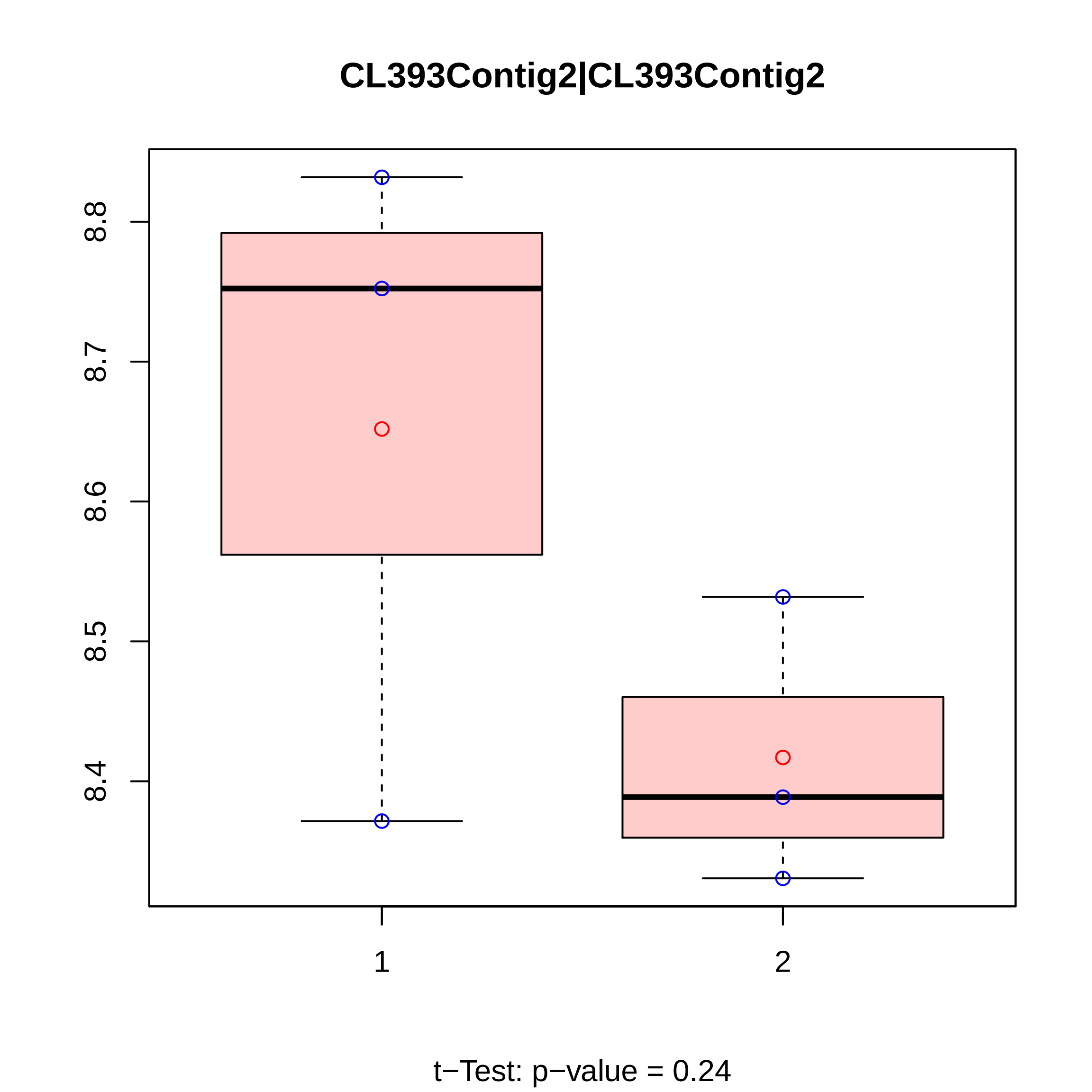
CL393Contig2|CL393Contig2
8.4
8.5
8.6
8.7
8.8
1
2
t−Test: p−value = 0.24

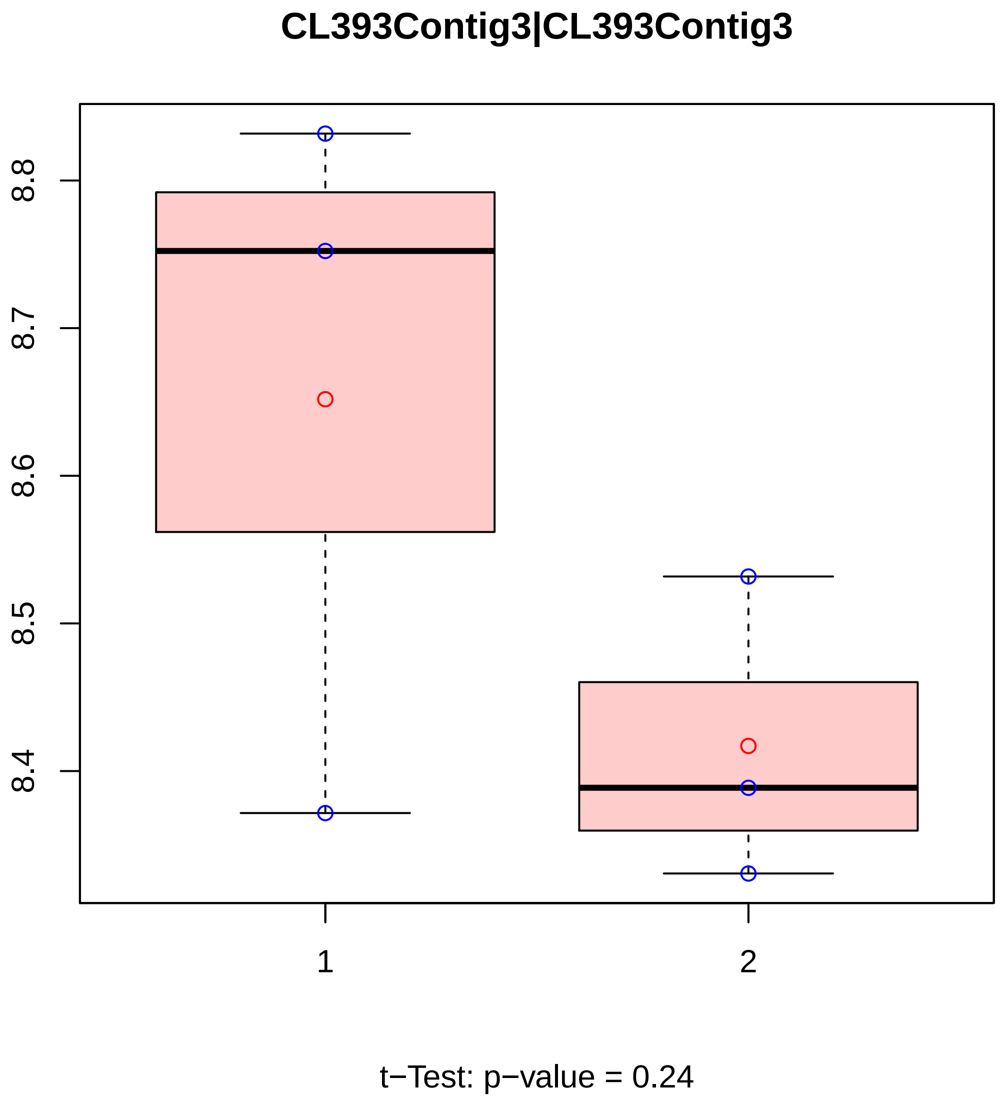

CL393Contig3|CL393Contig3
8.4
8.5
8.6
8.7
8.8
1
2
t−Test: p−value = 0.24

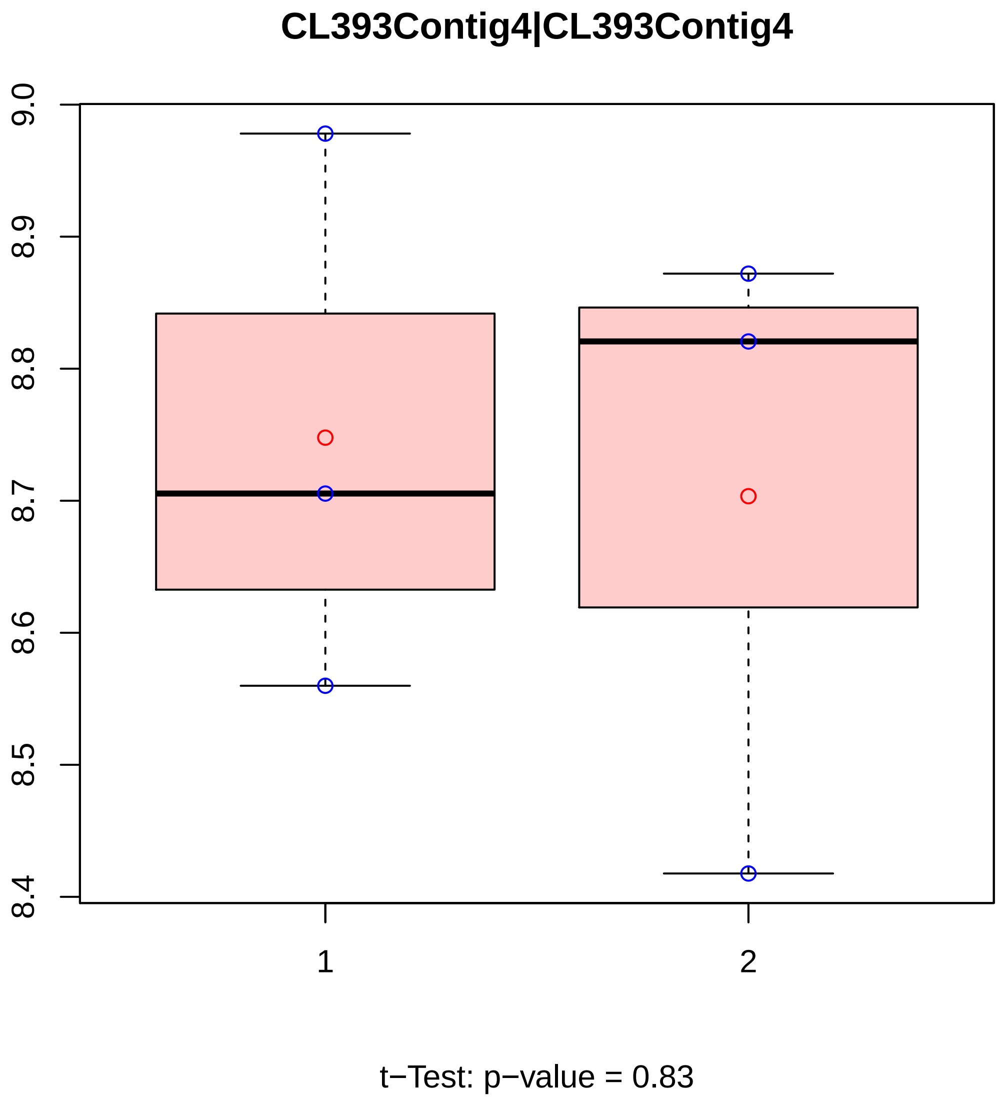
CL393Contig4|CL393Contig4
9.0
8.9
8.8
8.7
8.6
8.5
8.4
1
2
t−Test: p−value = 0.83

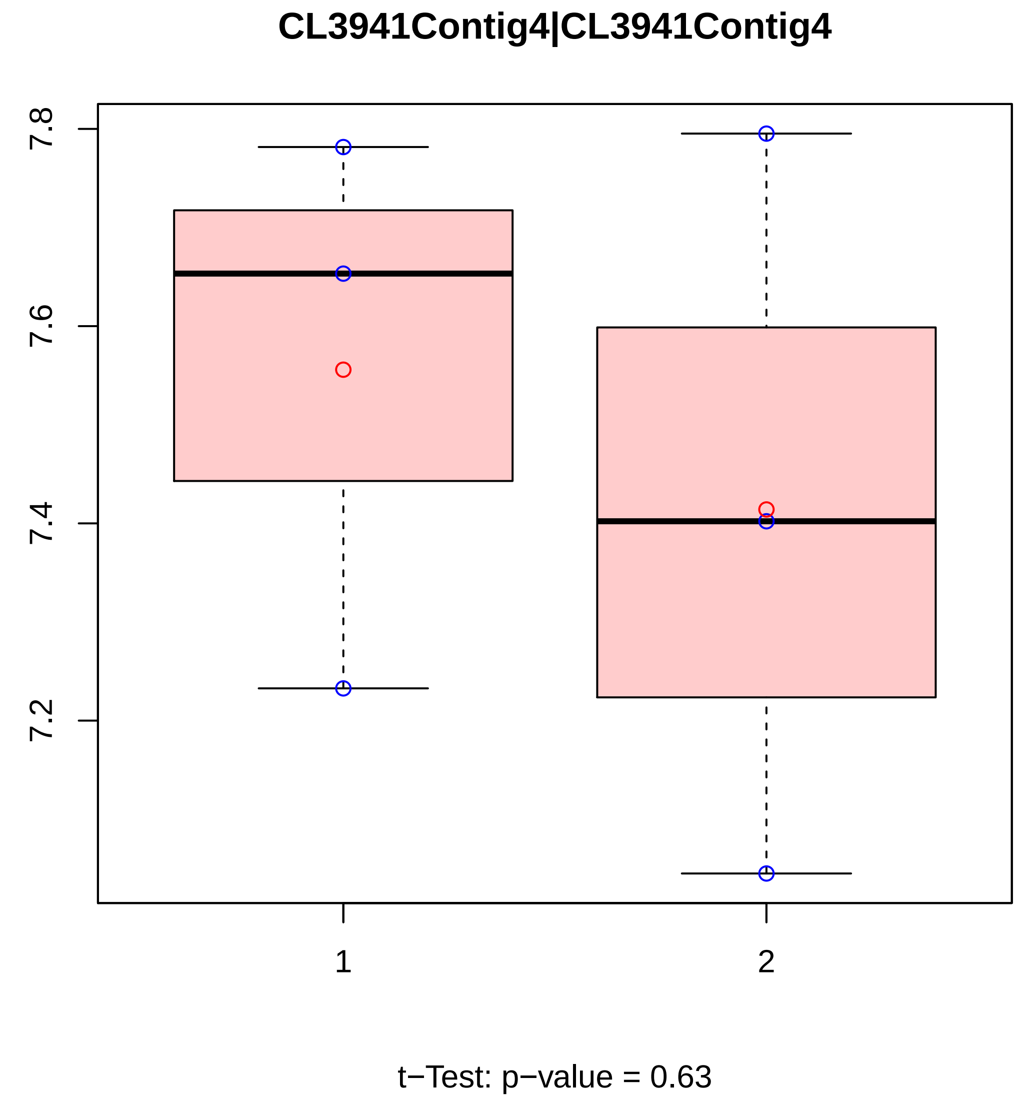
CL3941Contig4|CL3941Contig4
7.8
7.6
7.4
7.2
1
2
t−Test: p−value = 0.63

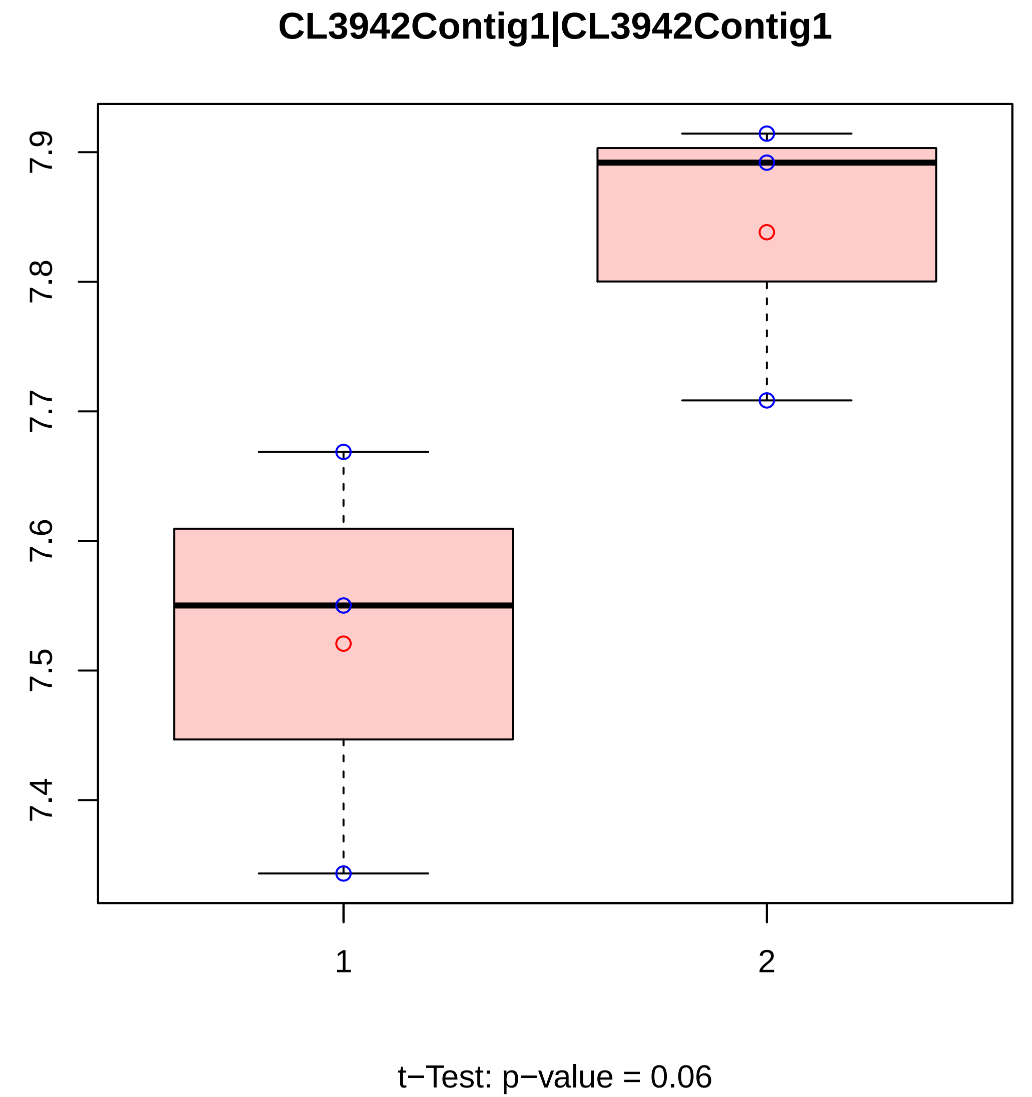
CL3942Contig1|CL3942Contig1
7.4
7.5
7.6
7.7
7.8
7.9
1
2
t−Test: p−value = 0.06

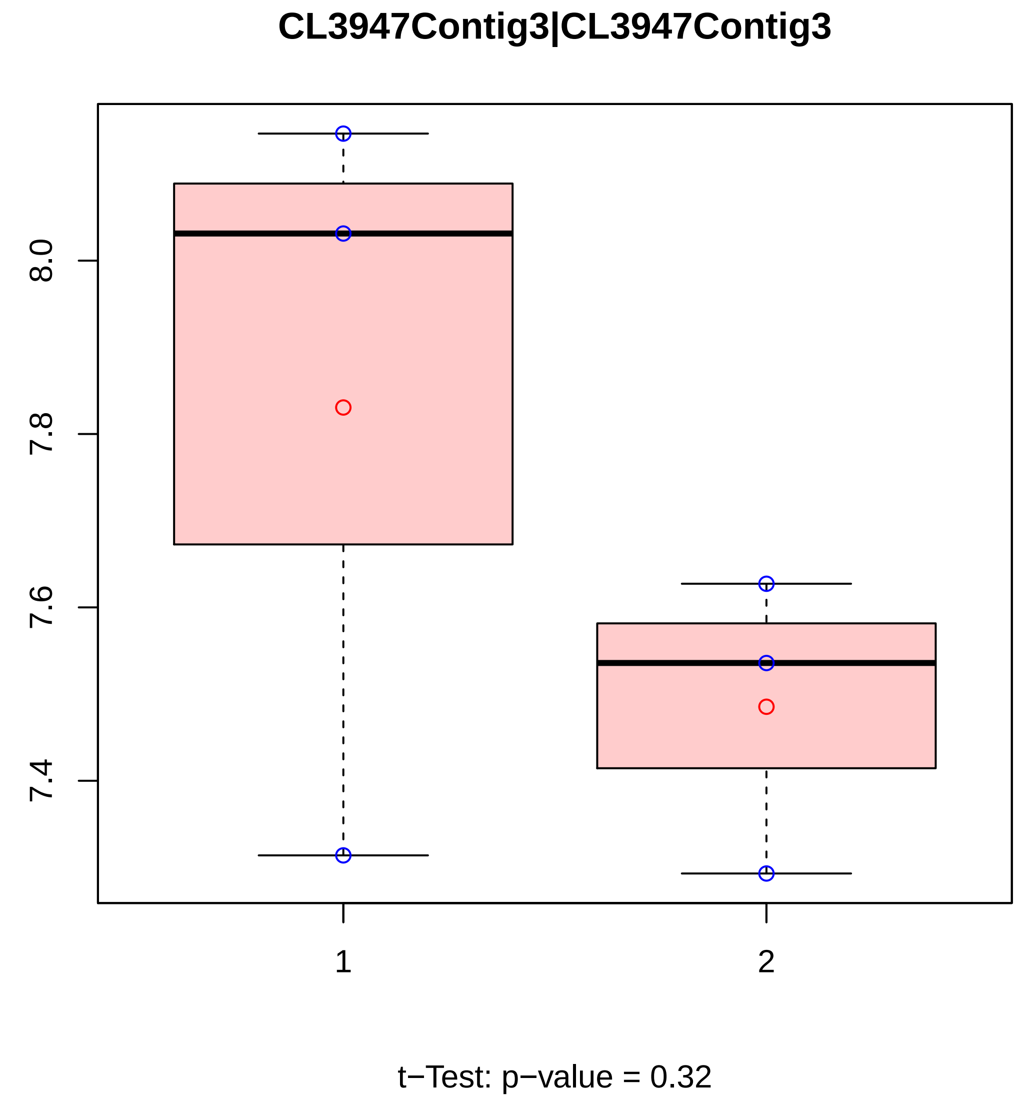
CL3947Contig3|CL3947Contig3
8.0
7.8
7.6
7.4
1
2
t−Test: p−value = 0.32

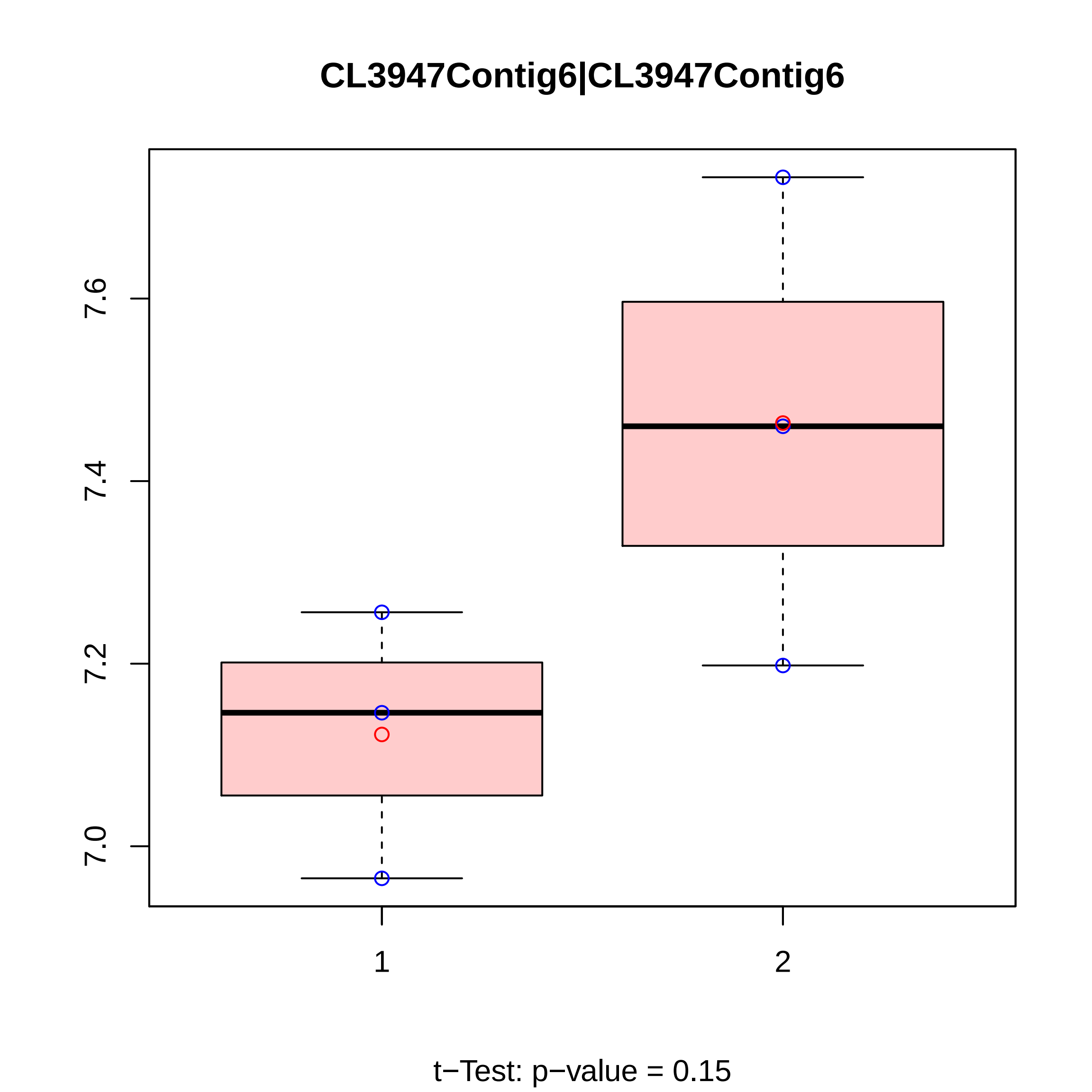

CL3947Contig6|CL3947Contig6
7.0
7.2
7.4
7.6
1
2
t−Test: p−value = 0.15

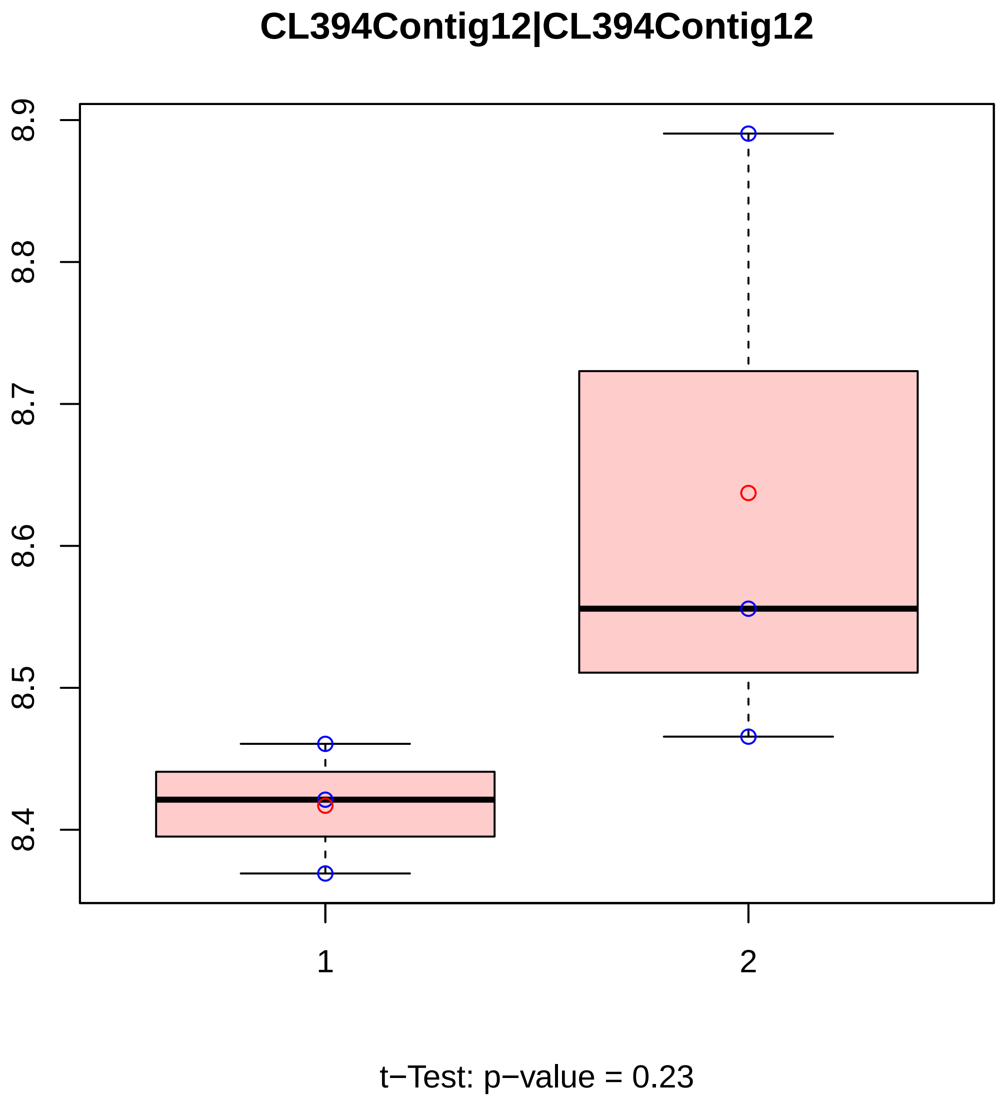

CL394Contig12|CL394Contig12
8.4
8.5
8.6
8.7
8.8
8.9
1
2
t−Test: p−value = 0.23

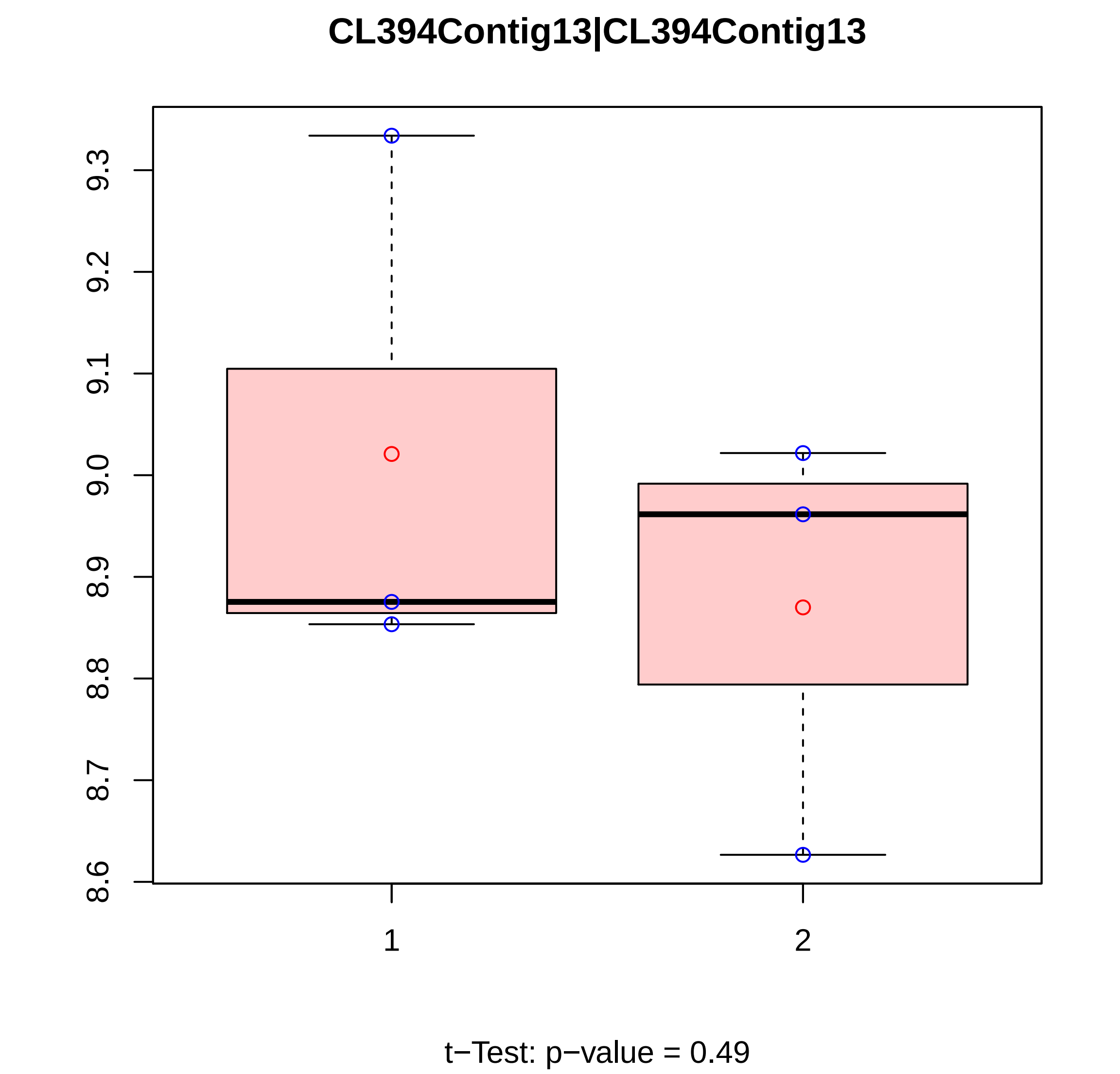

CL394Contig13|CL394Contig13
9.3
9.2
9.1
9.0
8.9
8.8
8.7
8.6
1
2
t−Test: p−value = 0.49

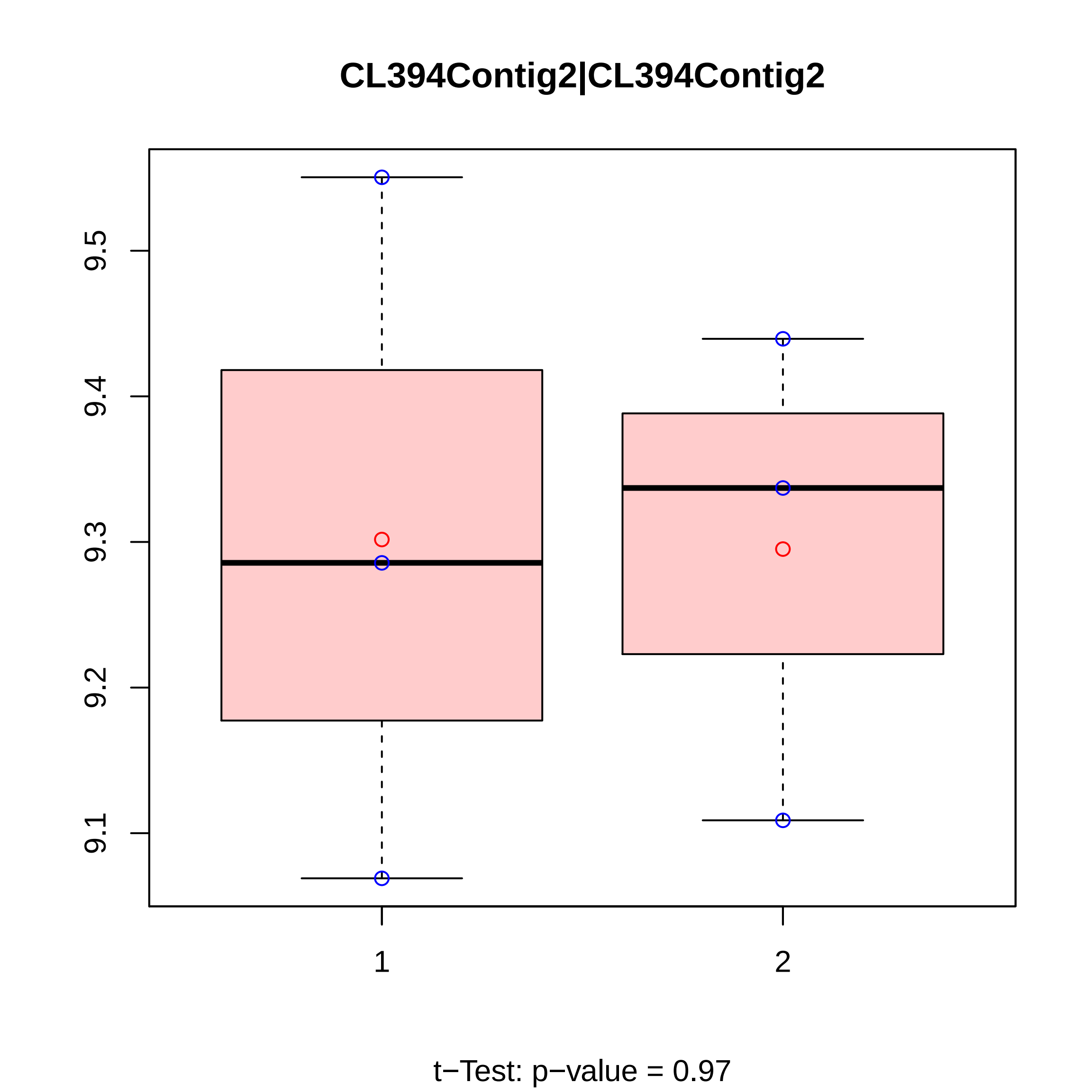
CL394Contig2|CL394Contig2
9.5
9.4
9.3
9.2
9.1
1
2
t−Test: p−value = 0.97

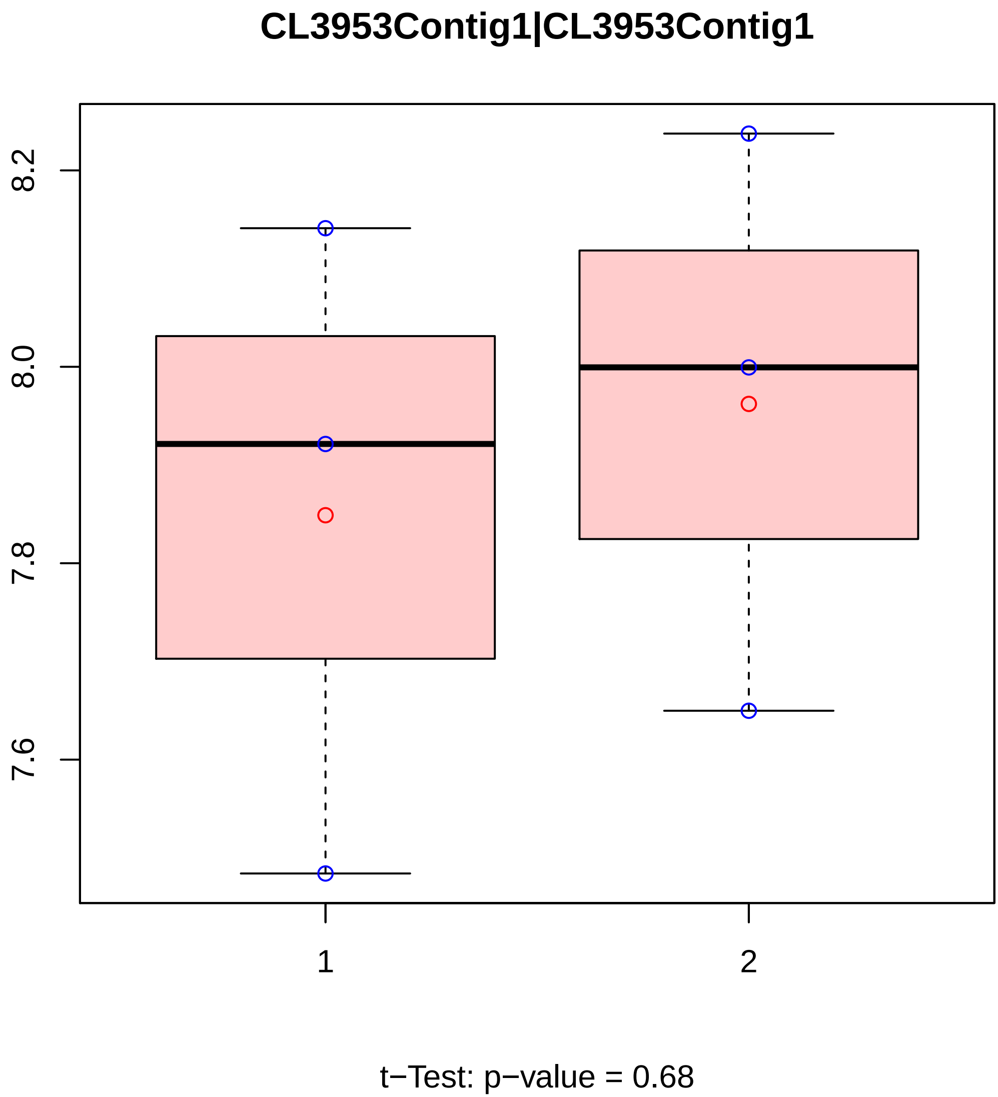
CL3953Contig1|CL3953Contig1
8.2
8.0
7.8
7.6
1
2
t−Test: p−value = 0.68

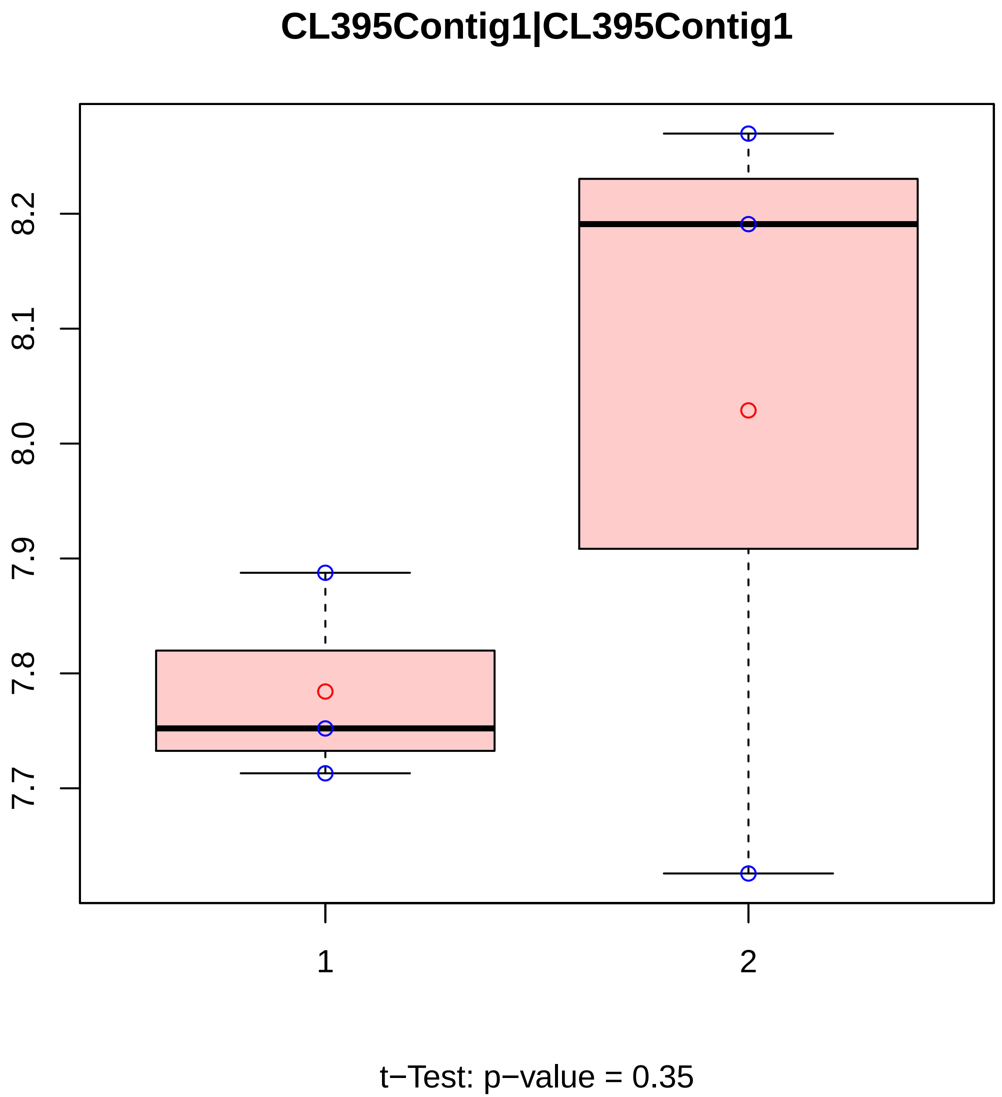

CL395Contig1|CL395Contig1
8.2
8.1
8.0
7.9
7.8
7.7
1
2
t−Test: p−value = 0.35

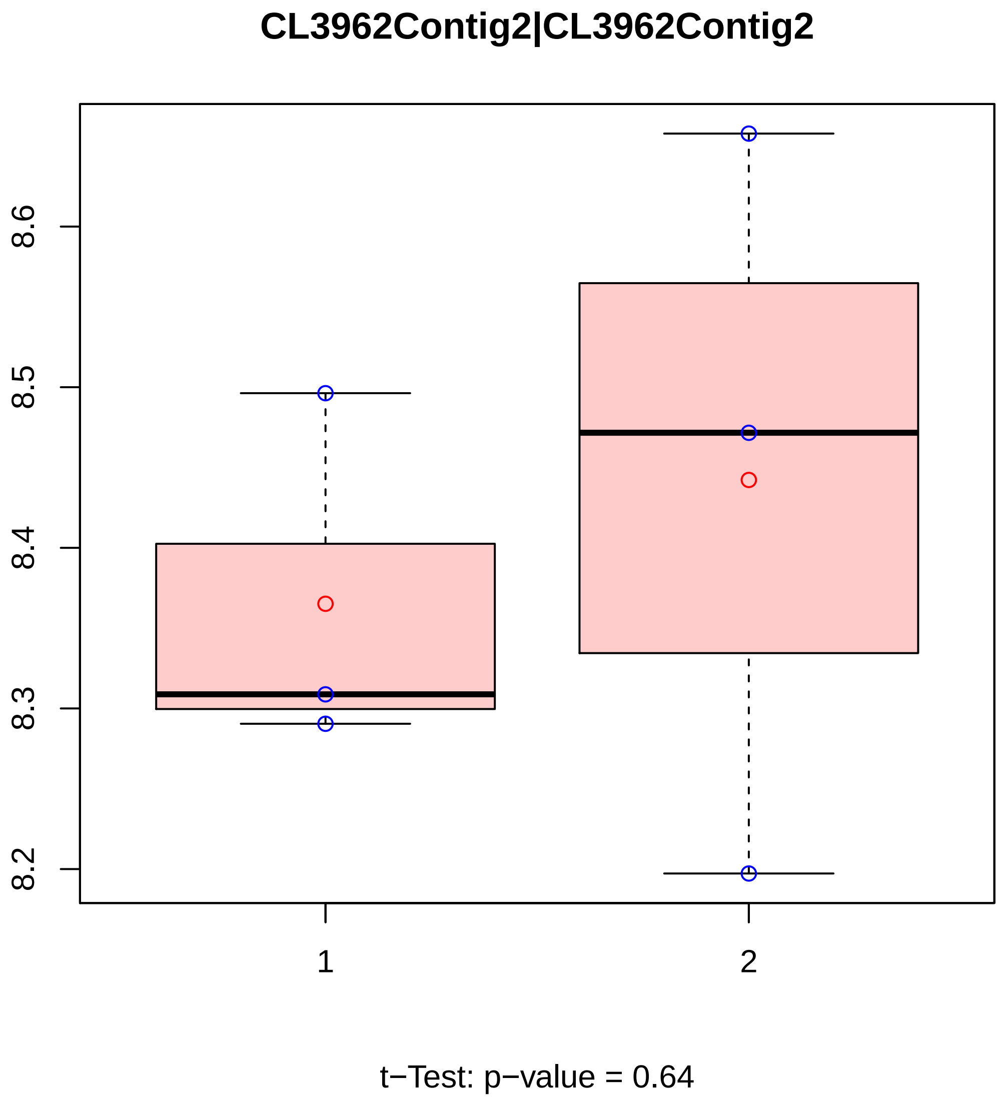
CL3962Contig2|CL3962Contig2
8.2
8.3
8.4
8.5
8.6
1
2
t−Test: p−value = 0.64

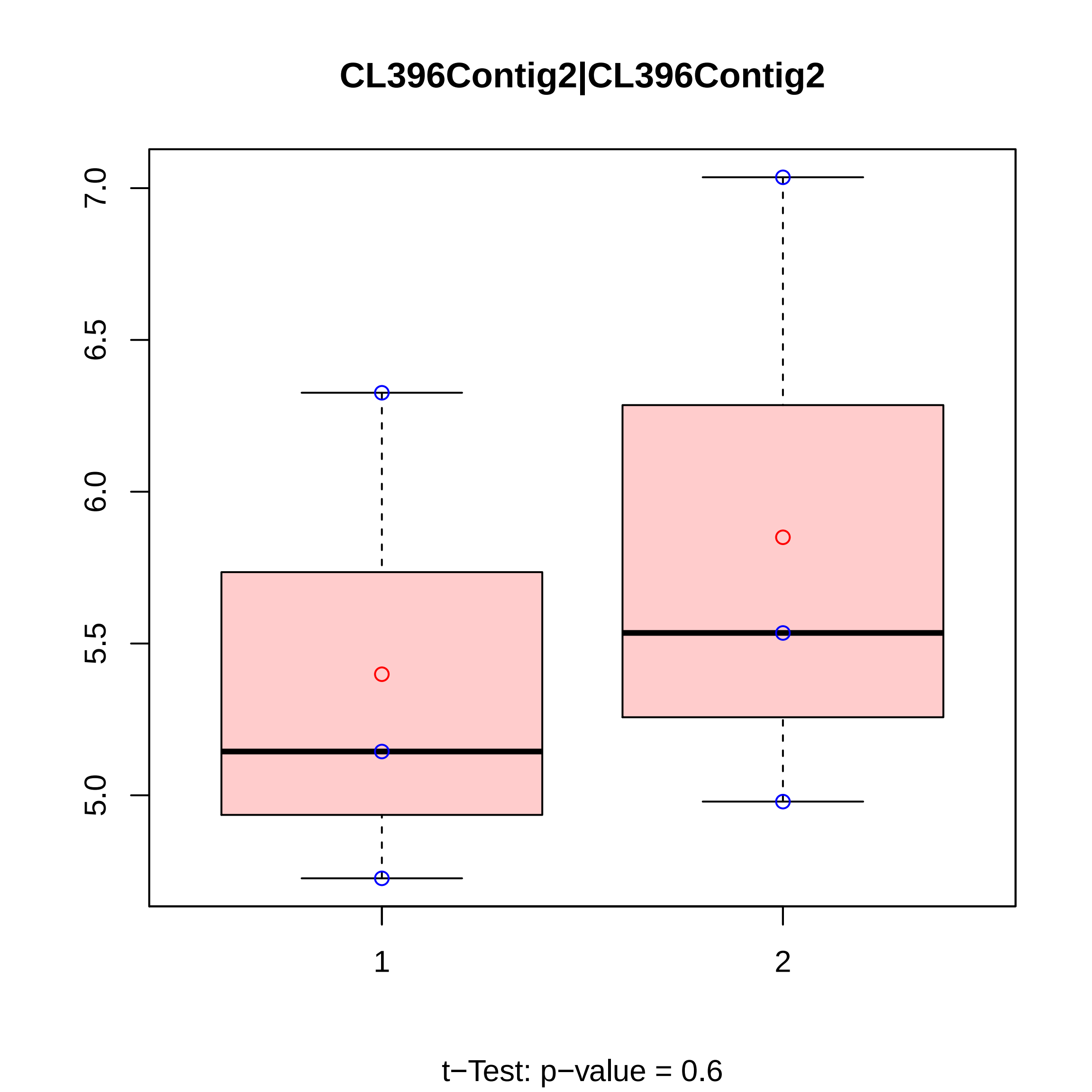
CL396Contig2|CL396Contig2
7.0
6.5
6.0
5.5
5.0
1
2
t−Test: p−value = 0.6

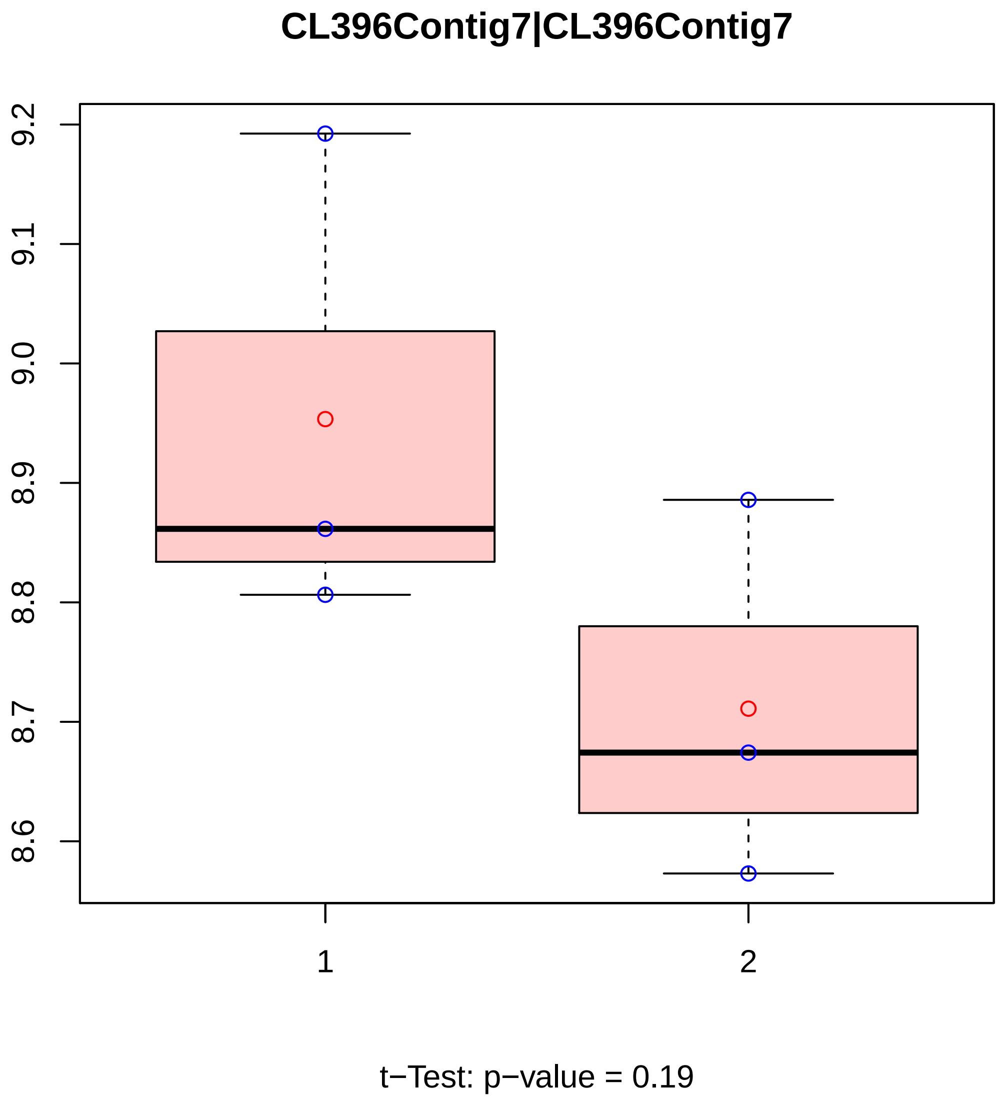

CL396Contig7|CL396Contig7
9.2
9.1
9.0
8.9
8.8
8.7
8.6
1
2
t−Test: p−value = 0.19

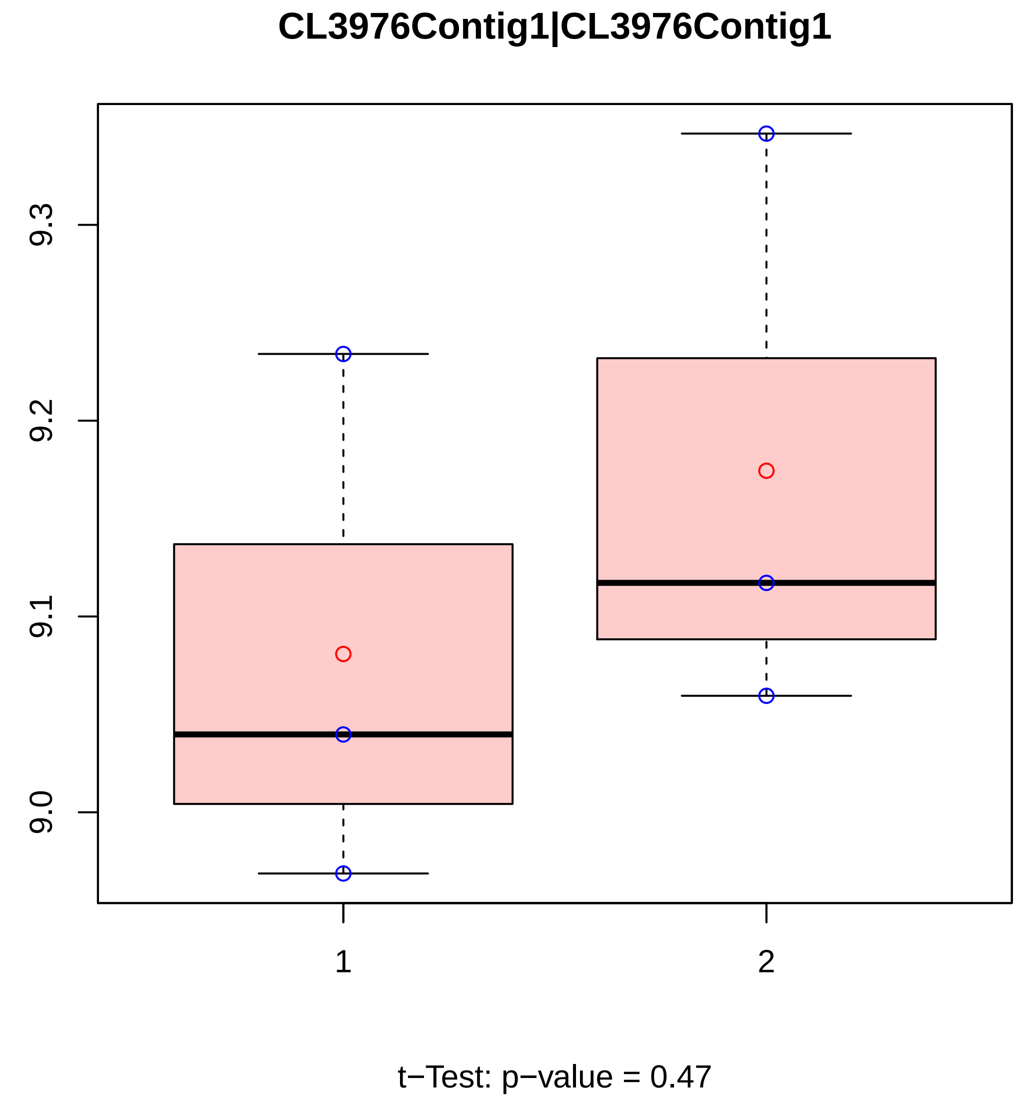

CL3976Contig1|CL3976Contig1
9.0
9.1
9.2
9.3
1
2
t−Test: p−value = 0.47

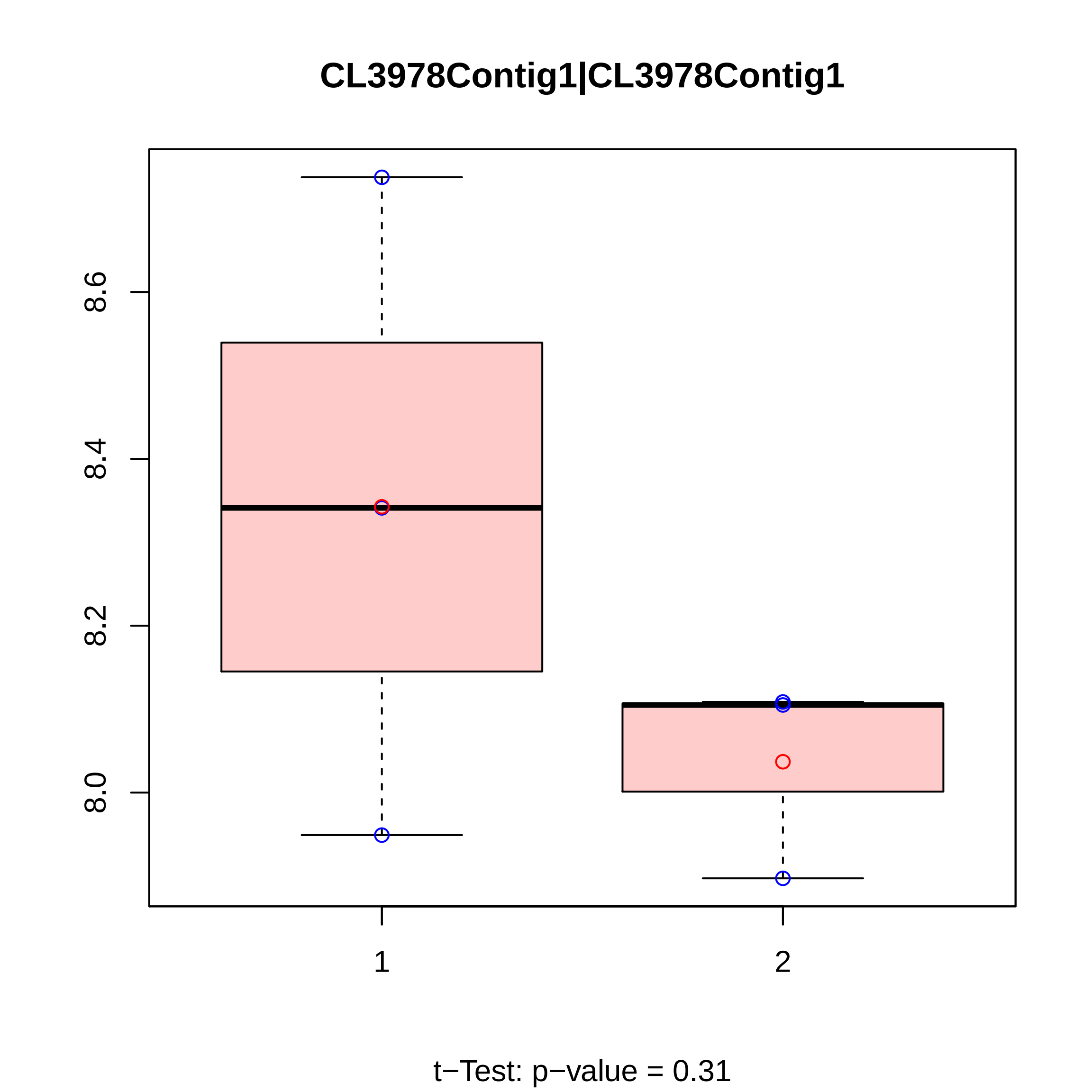
CL3978Contig1|CL3978Contig1
8.0
8.2
8.4
8.6
1
2
t−Test: p−value = 0.31

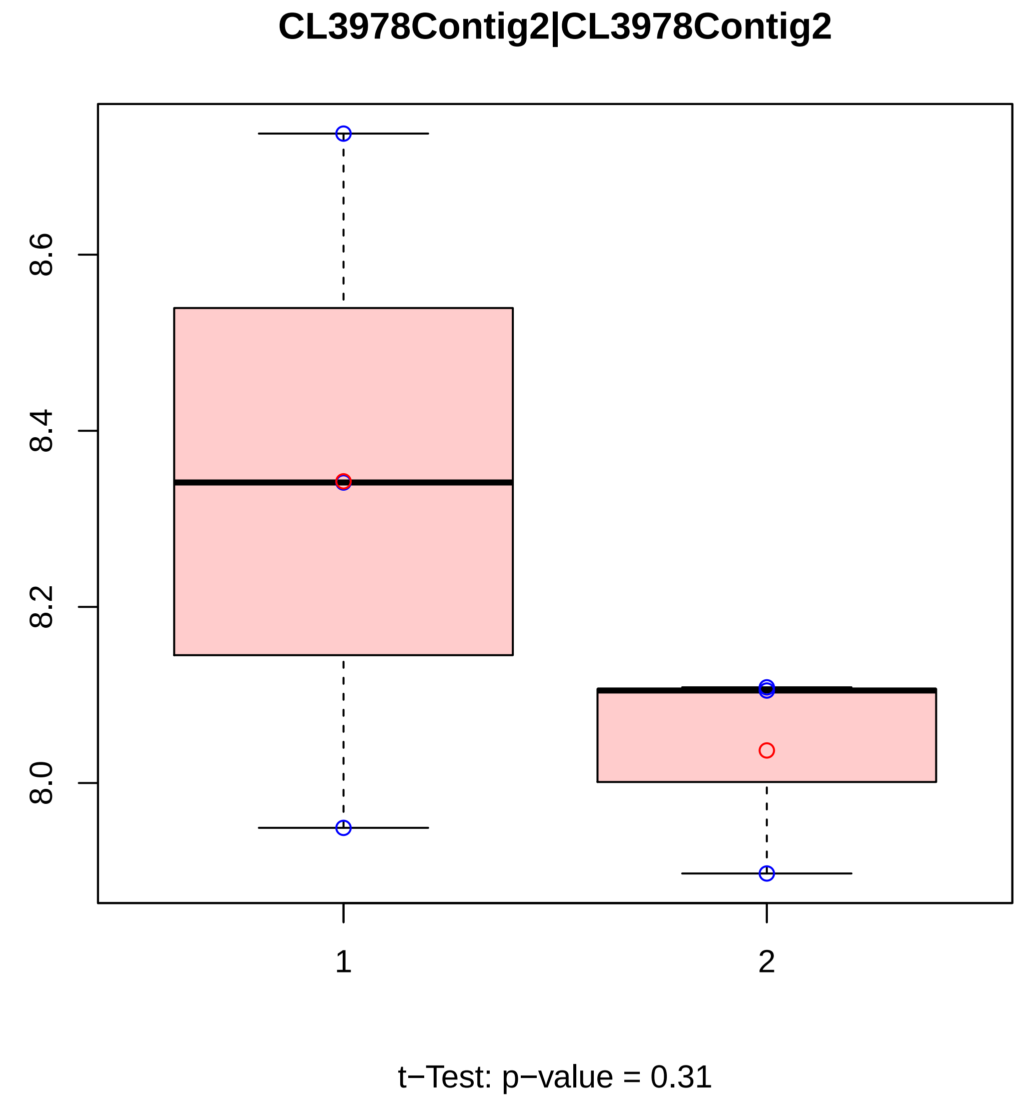

CL3978Contig2|CL3978Contig2
8.0
8.2
8.4
8.6
1
2
t−Test: p−value = 0.31

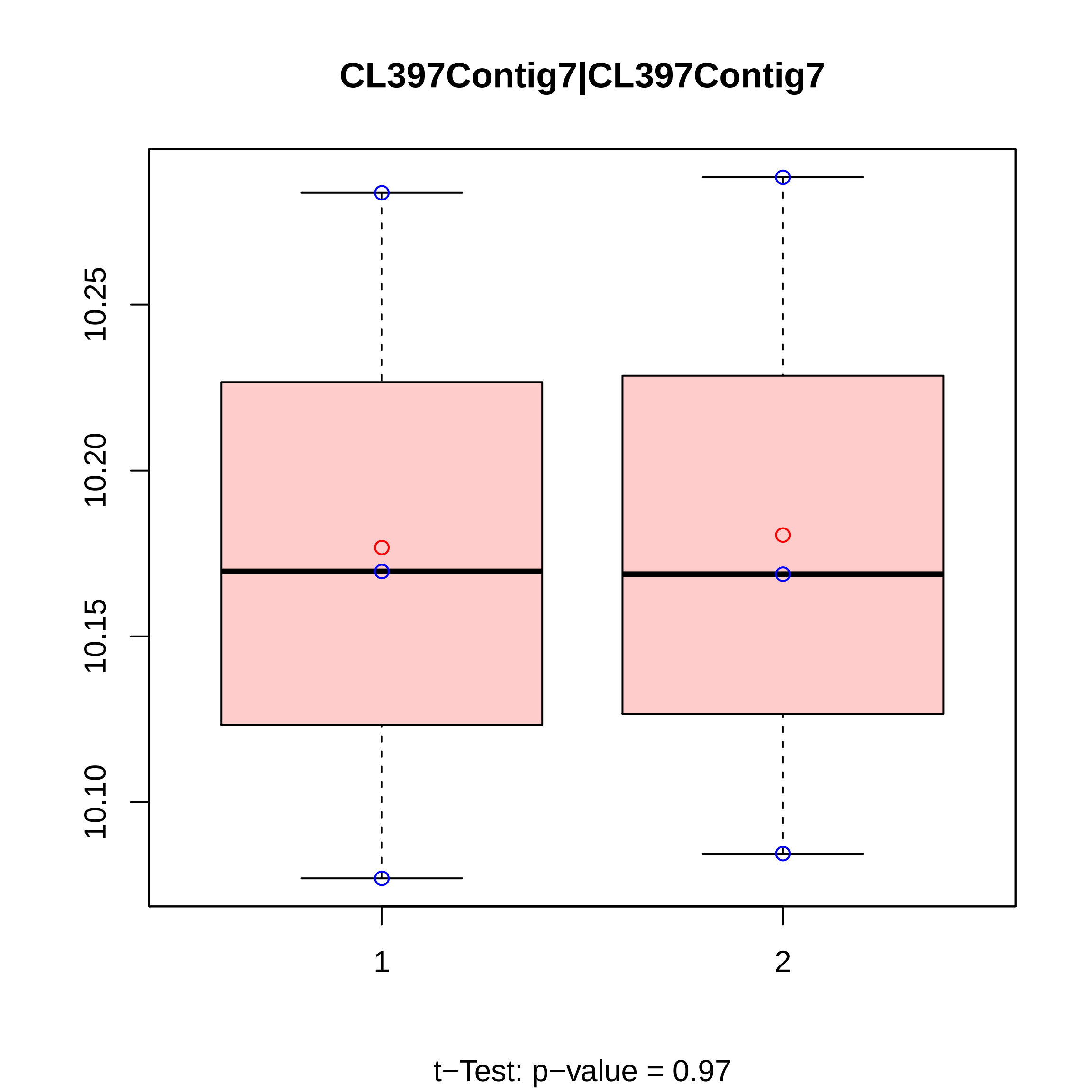

CL397Contig7|CL397Contig7
10.10
10.15
10.20
10.25
1
2
t−Test: p−value = 0.97

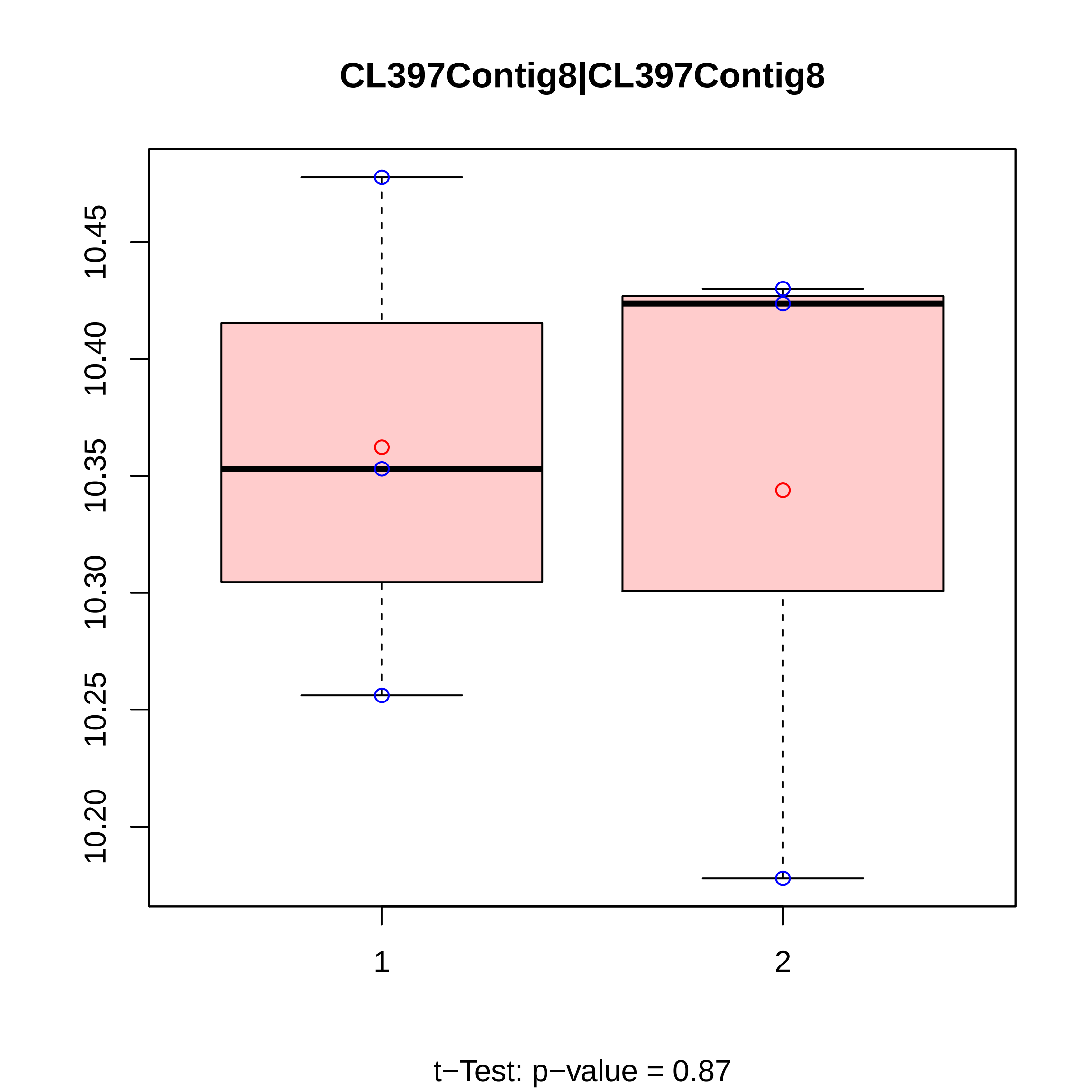
CL397Contig8|CL397Contig8
10.20
10.25
10.30
10.35
10.40
10.45
1
2
t−Test: p−value = 0.87

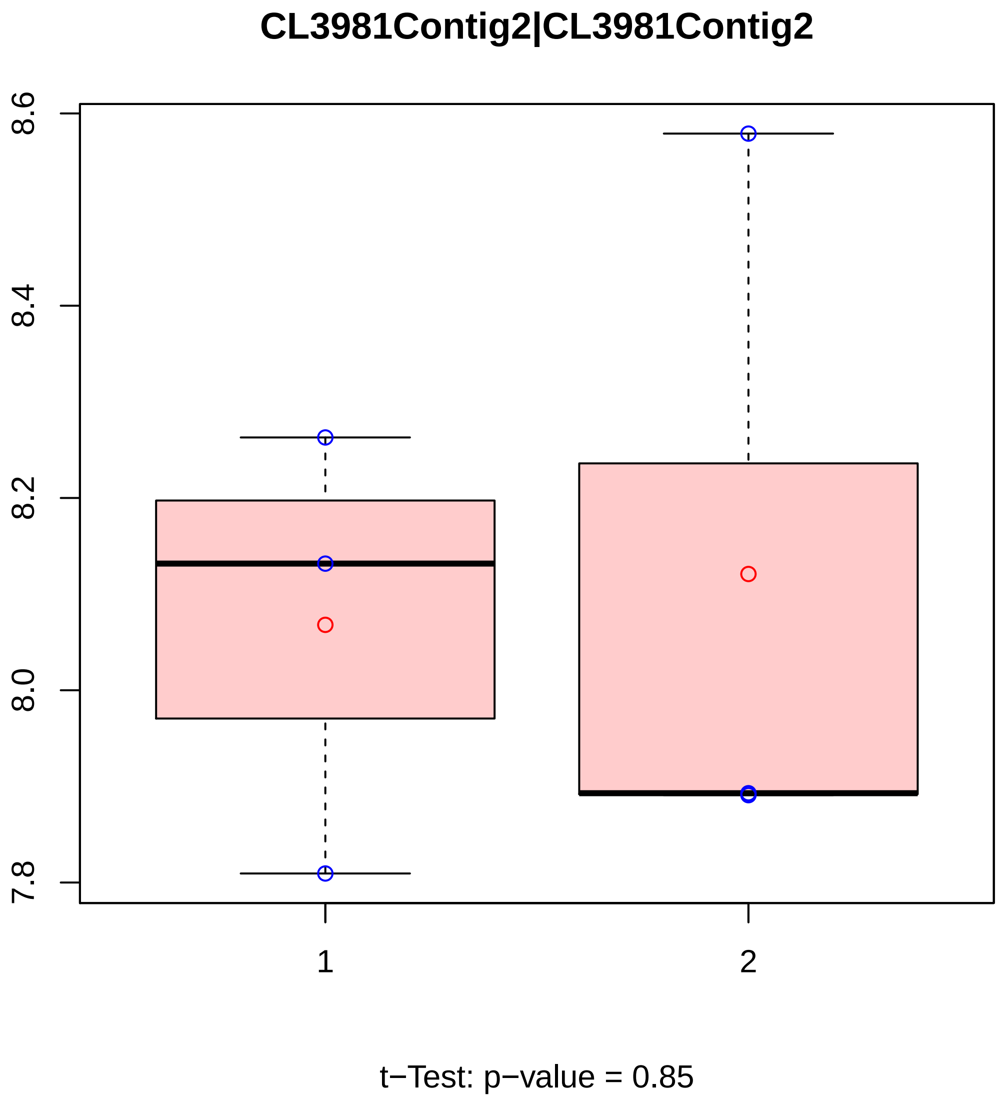
CL3981Contig2|CL3981Contig2
8.6
8.4
8.2
8.0
7.8
1
2
t−Test: p−value = 0.85

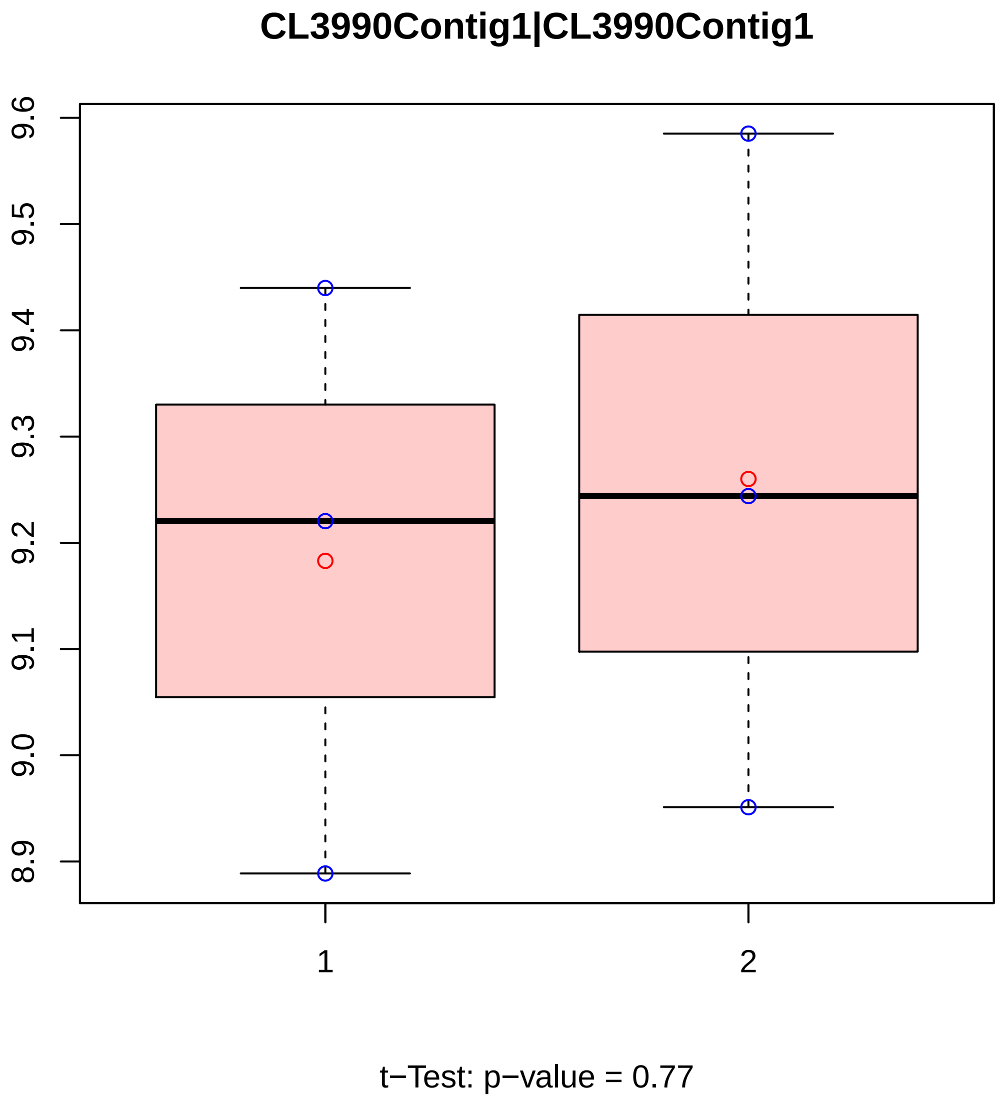

CL3990Contig1|CL3990Contig1
9.6
9.5
9.4
9.3
9.2
9.1
9.0
8.9
1
2
t−Test: p−value = 0.77

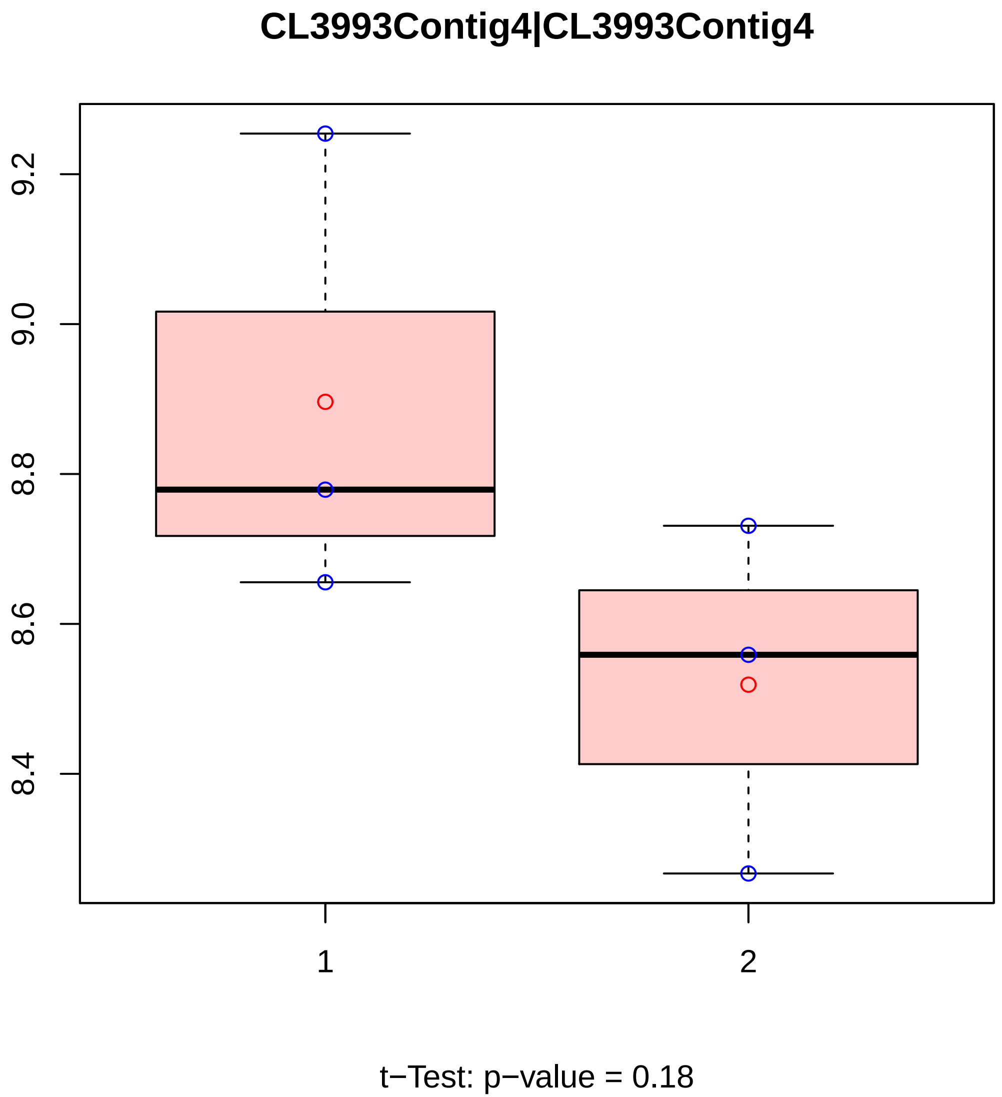

CL3993Contig4|CL3993Contig4
9.2
9.0
8.8
8.6
8.4
1
2
t−Test: p−value = 0.18

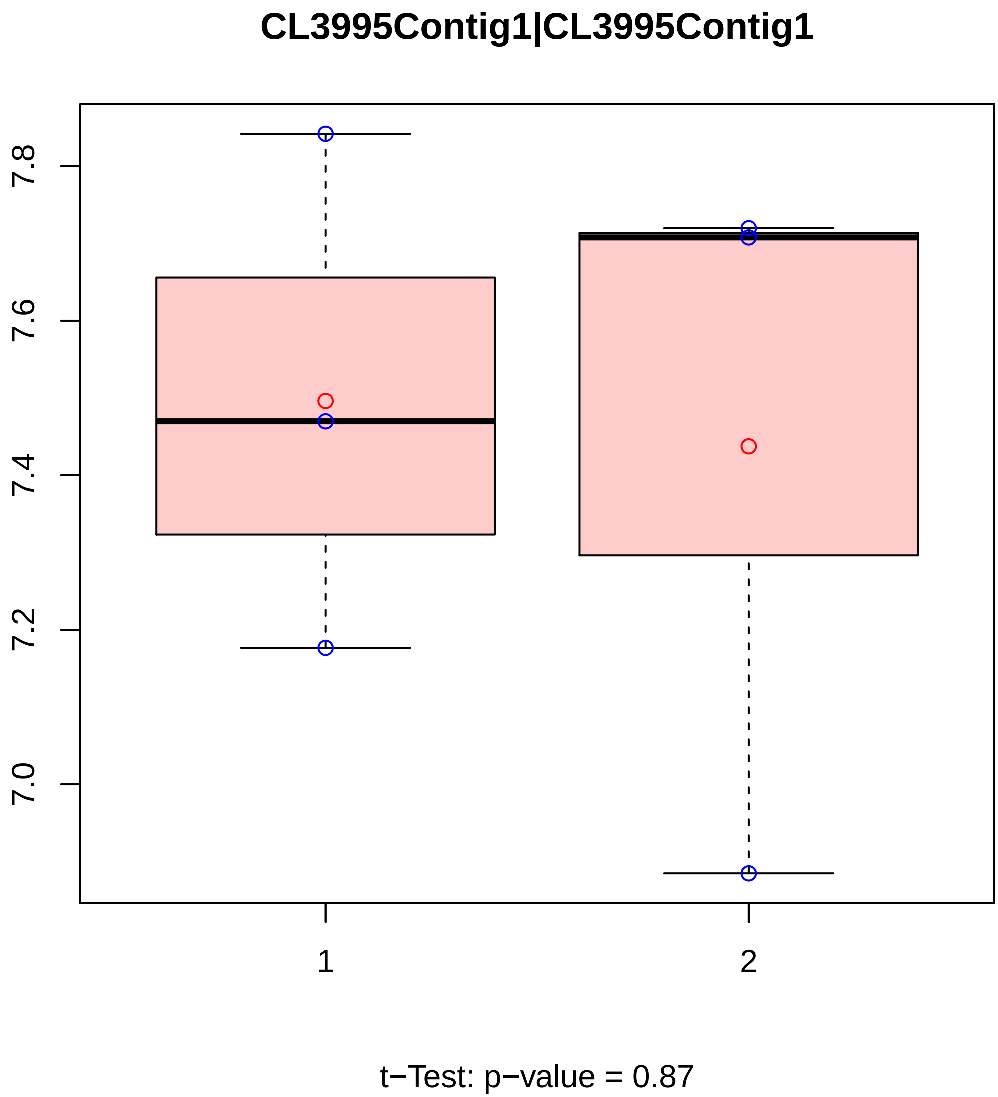

CL3995Contig1|CL3995Contig1
7.0
7.2
7.4
7.6
7.8
1
2
t−Test: p−value = 0.87

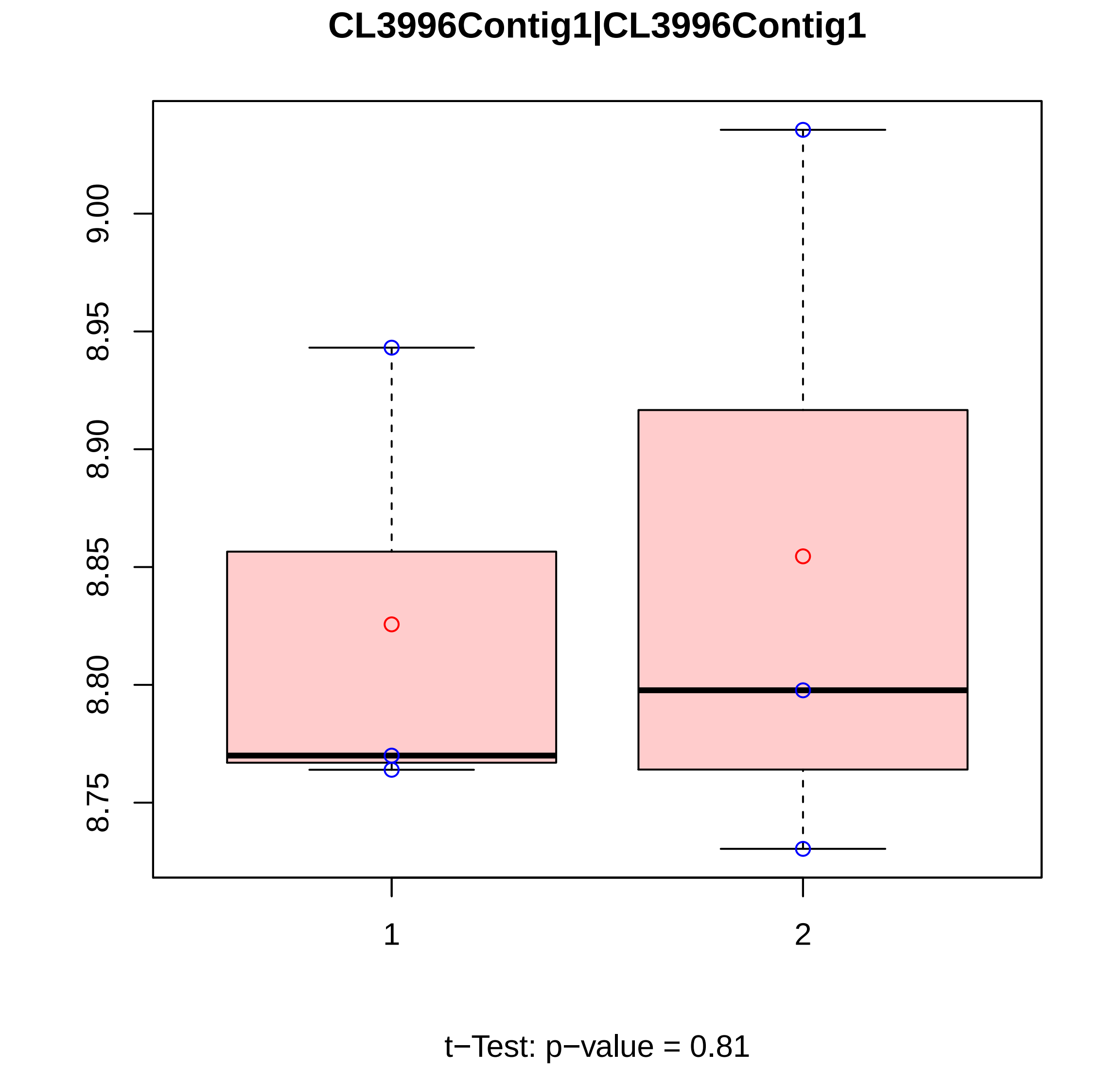
CL3996Contig1|CL3996Contig1
9.00
8.95
8.90
8.85
8.80
8.75
1
2
t−Test: p−value = 0.81

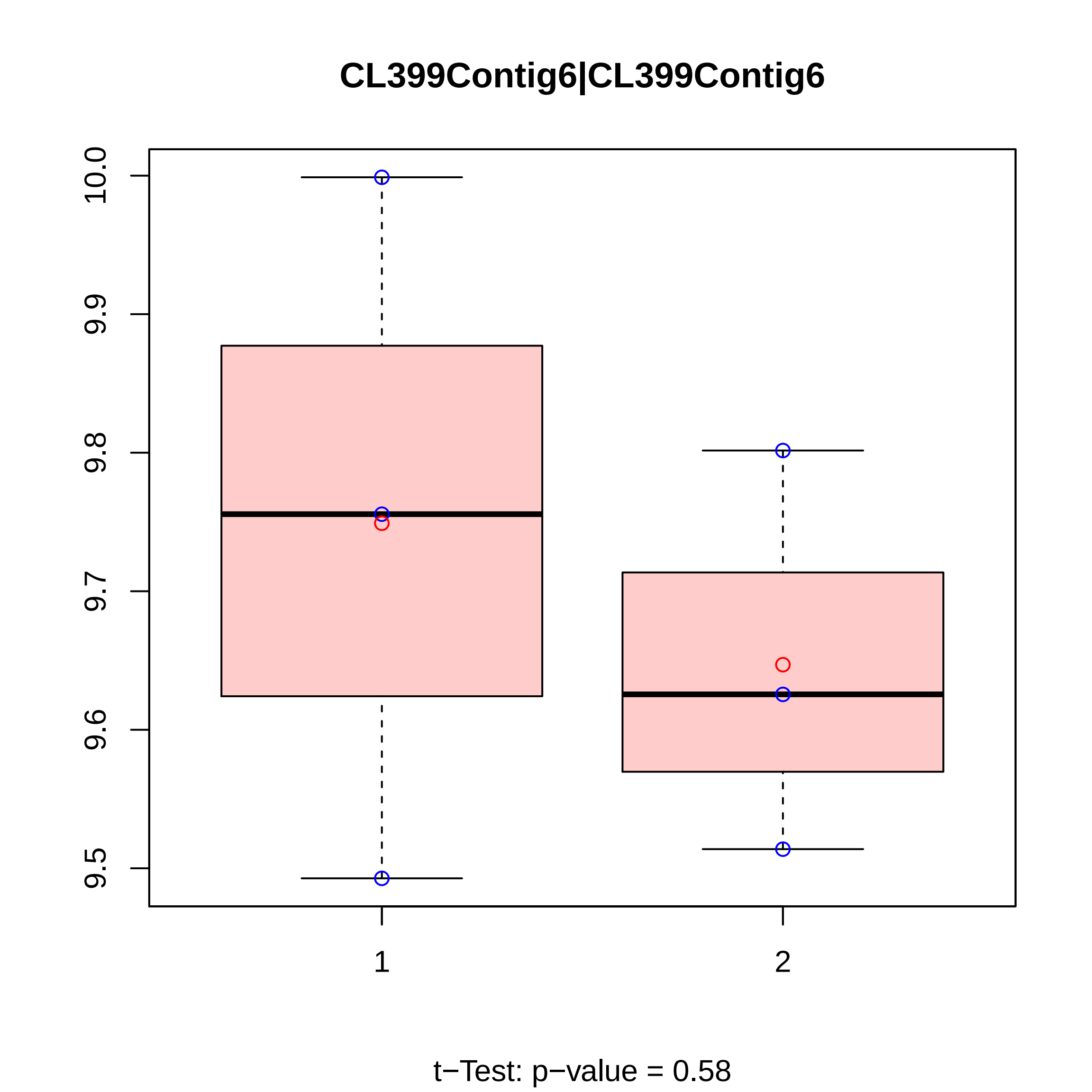
CL399Contig6|CL399Contig6
10.0
9.9
9.8
9.7
9.6
9.5
1
2
t−Test: p−value = 0.58

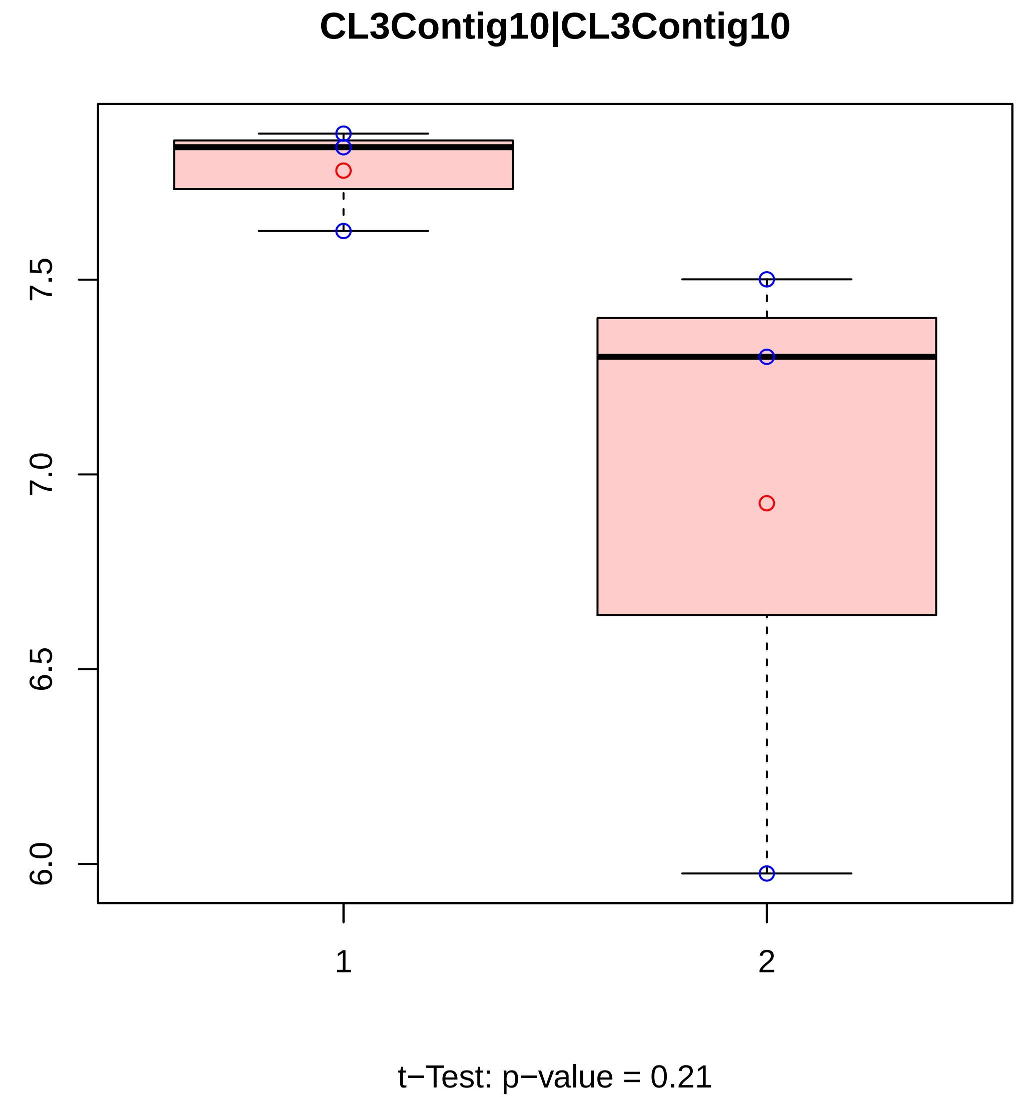
CL3Contig10|CL3Contig10
7.5
7.0
6.5
6.0
1
2
t−Test: p−value = 0.21

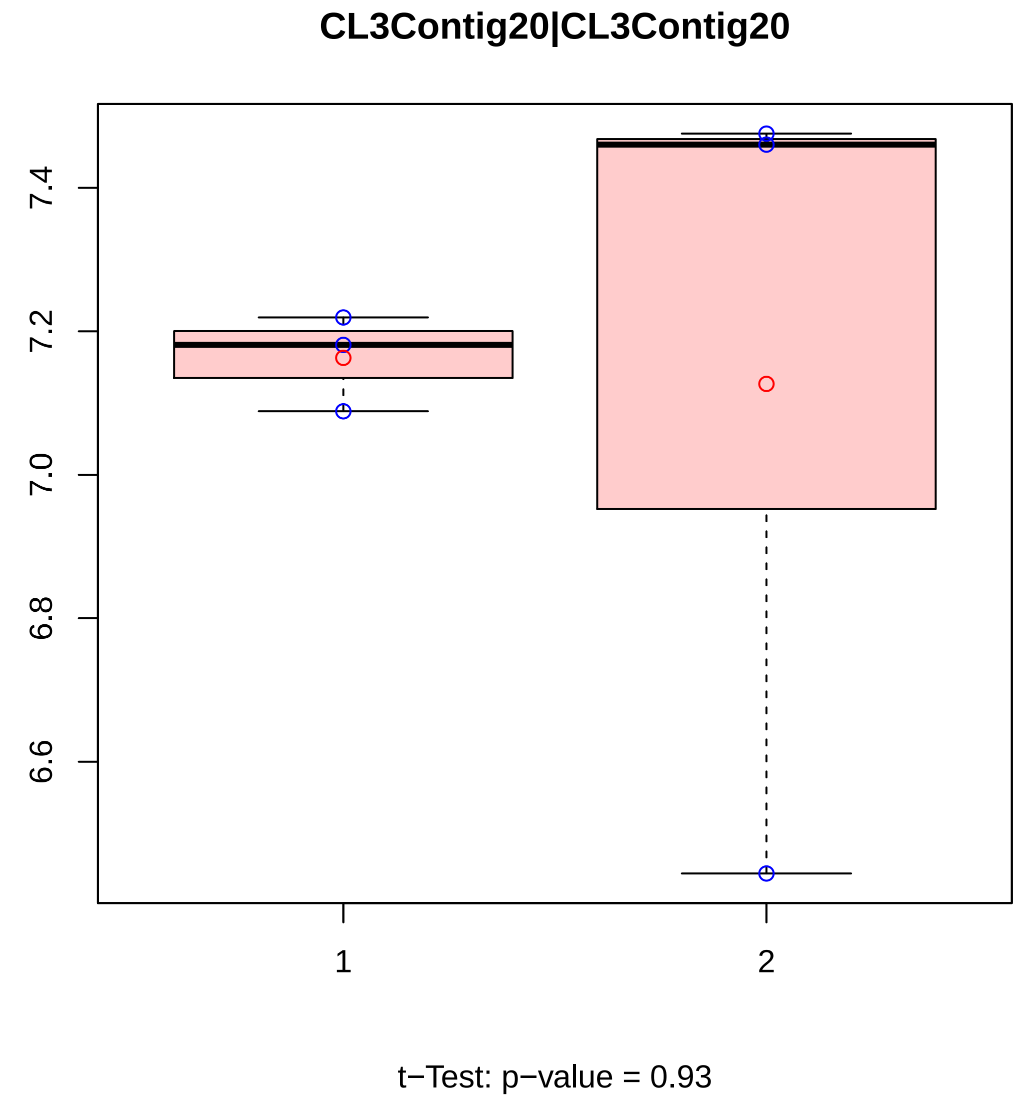
CL3Contig20|CL3Contig20
7.4
7.2
7.0
6.8
6.6
1
2
t−Test: p−value = 0.93

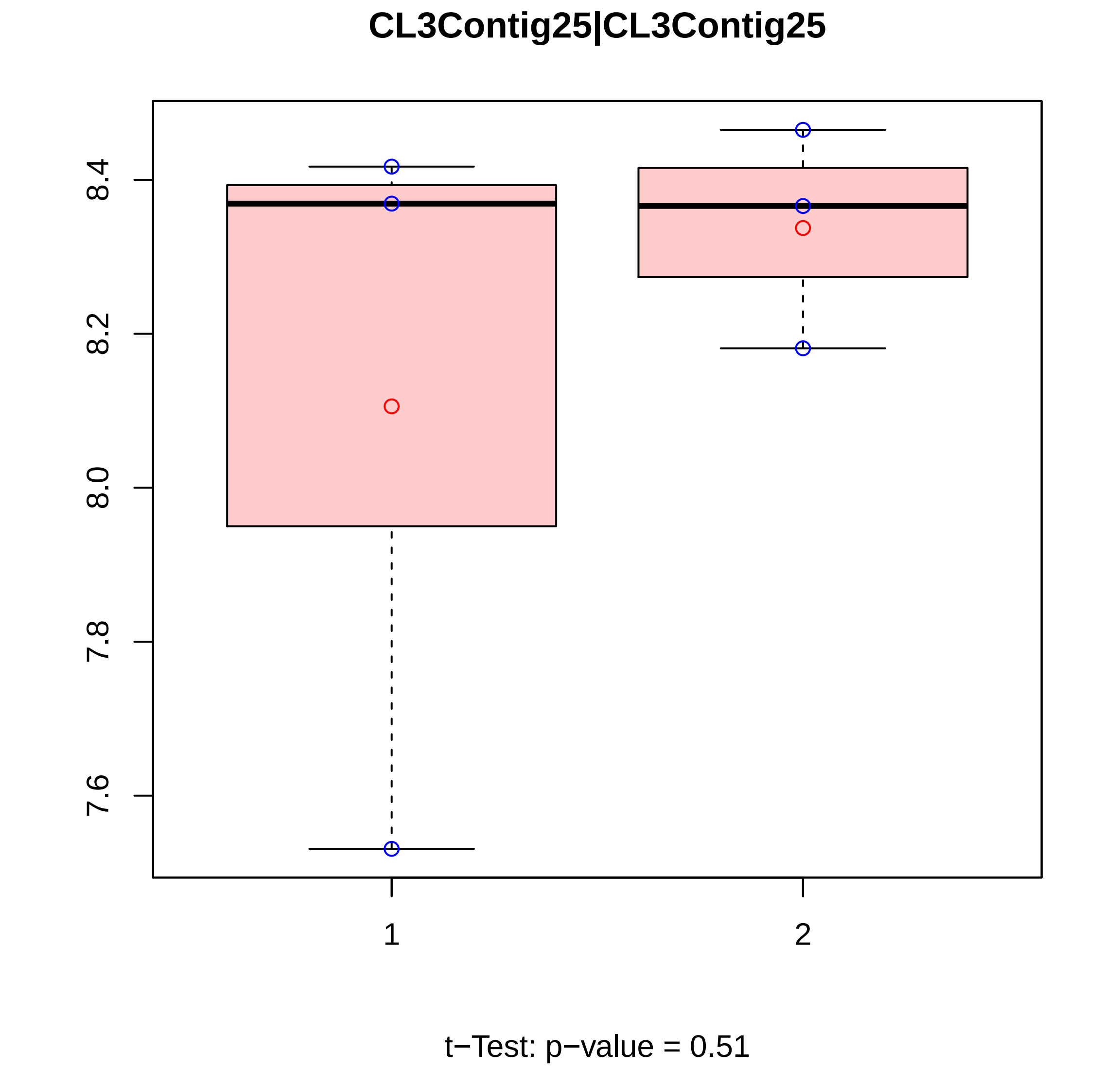

CL3Contig25|CL3Contig25
8.4
8.2
8.0
7.8
7.6
1
2
t−Test: p−value = 0.51

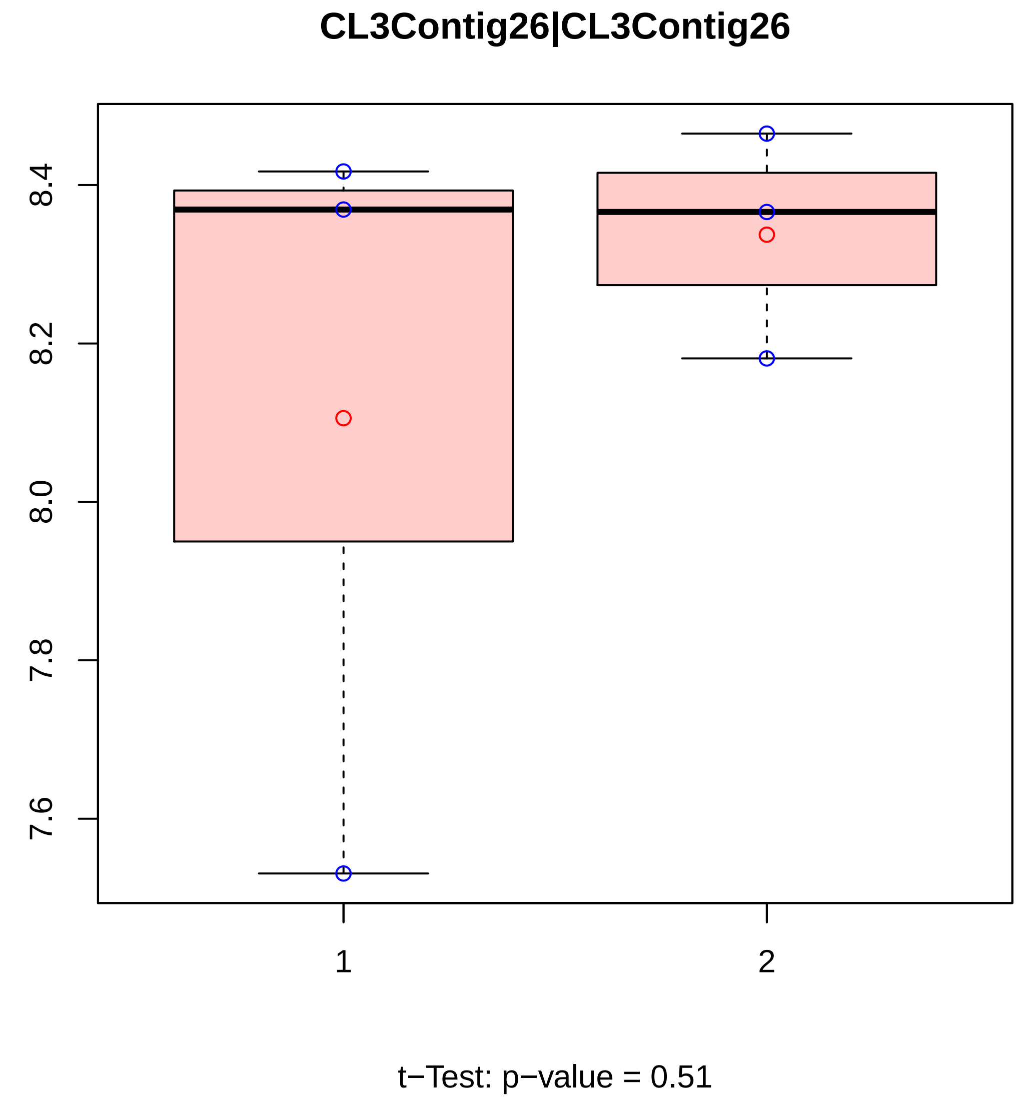

CL3Contig26|CL3Contig26
8.4
8.2
8.0
7.8
7.6
1
2
t−Test: p−value = 0.51

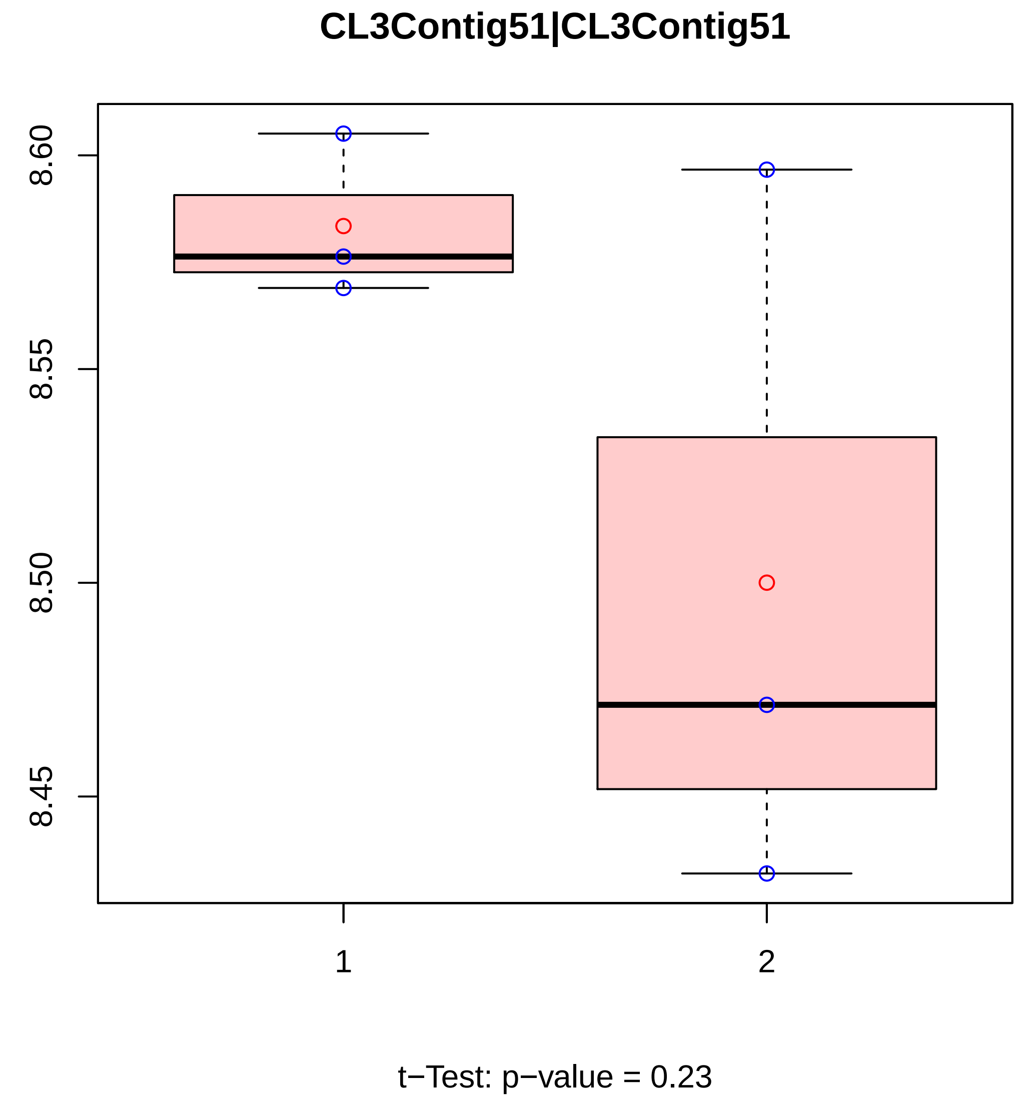
CL3Contig51|CL3Contig51
8.60
8.55
8.50
8.45
1
2
t−Test: p−value = 0.23

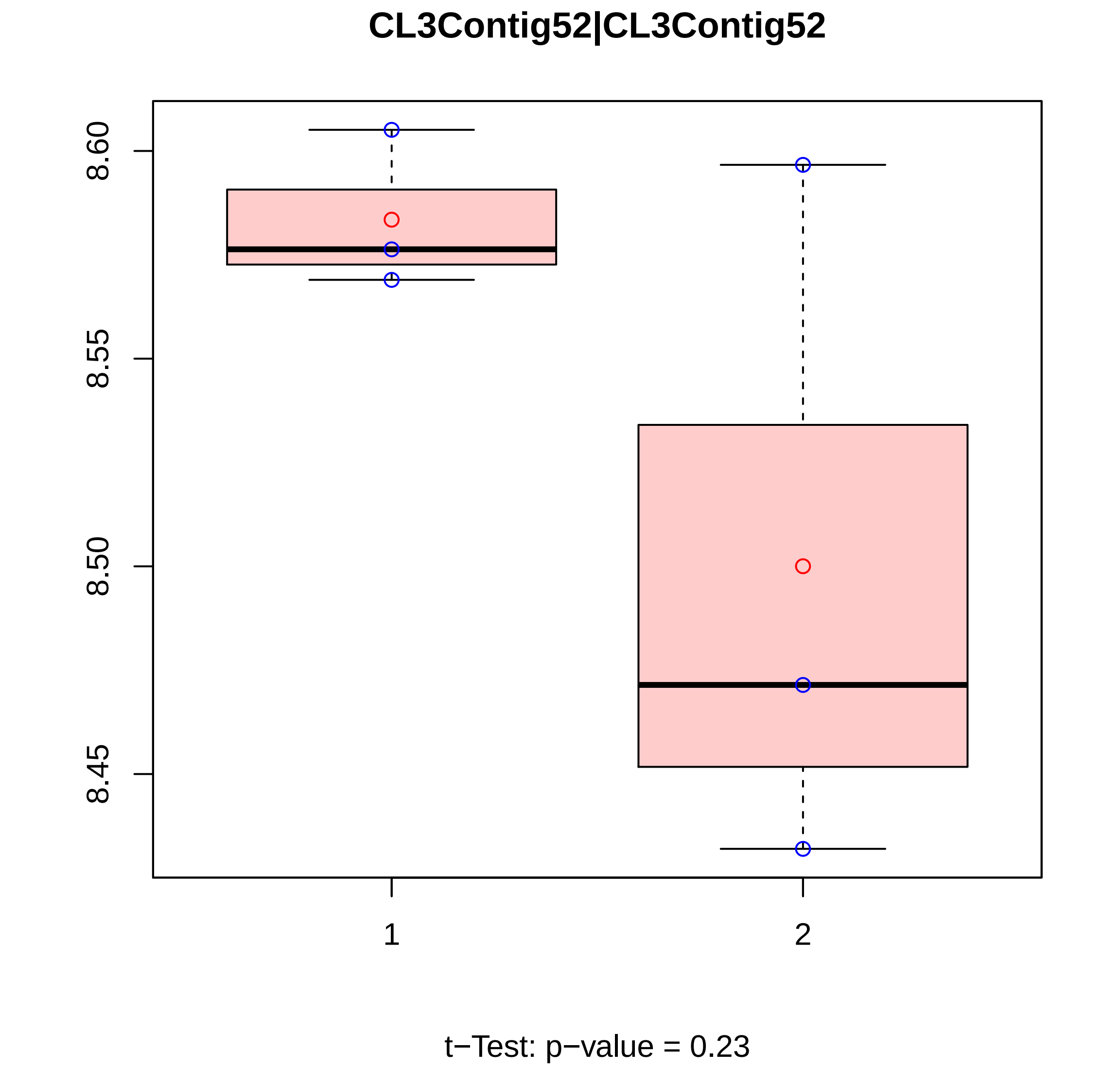

CL3Contig52|CL3Contig52
8.60
8.55
8.50
8.45
1
2
t−Test: p−value = 0.23

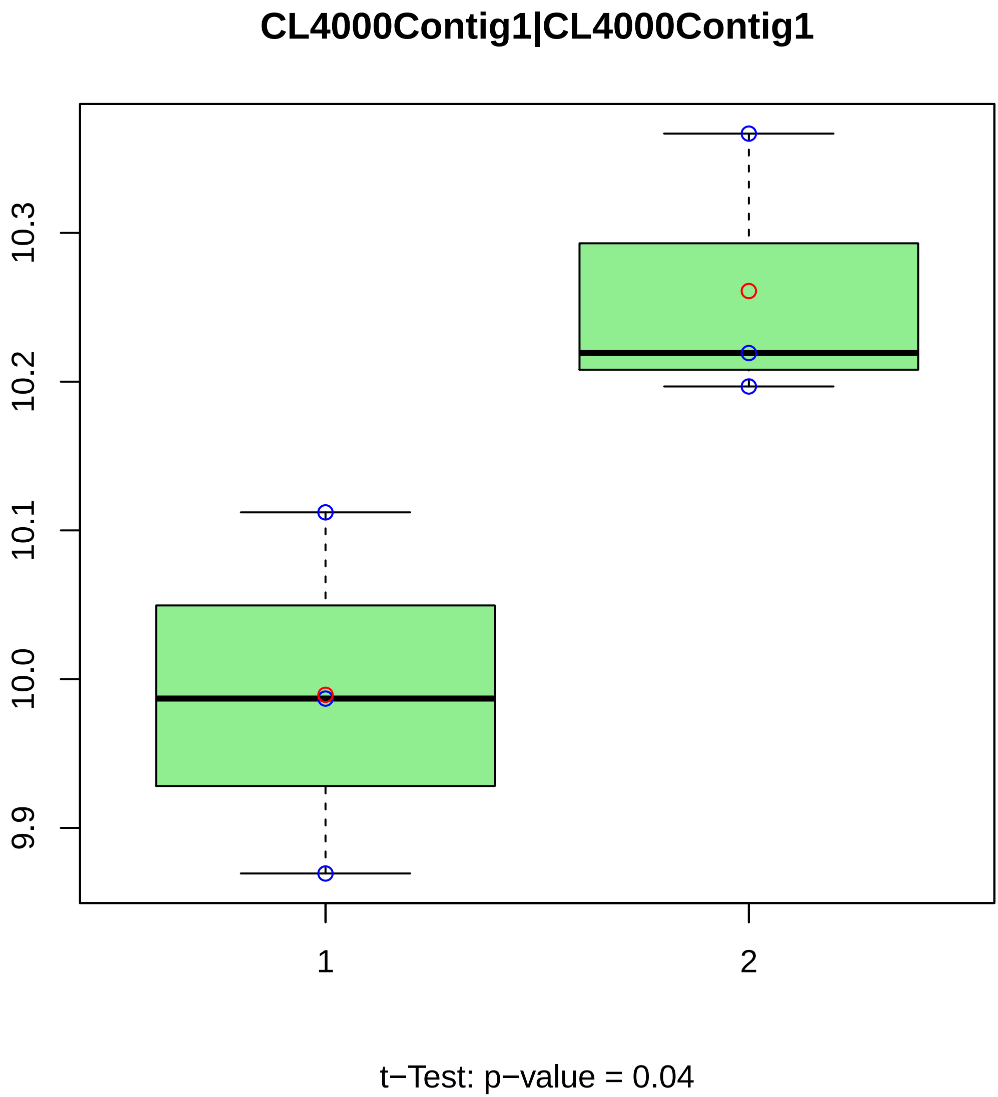
CL4000Contig1|CL4000Contig1
10.3
10.2
10.1
10.0
9.9
1
2
t−Test: p−value = 0.04

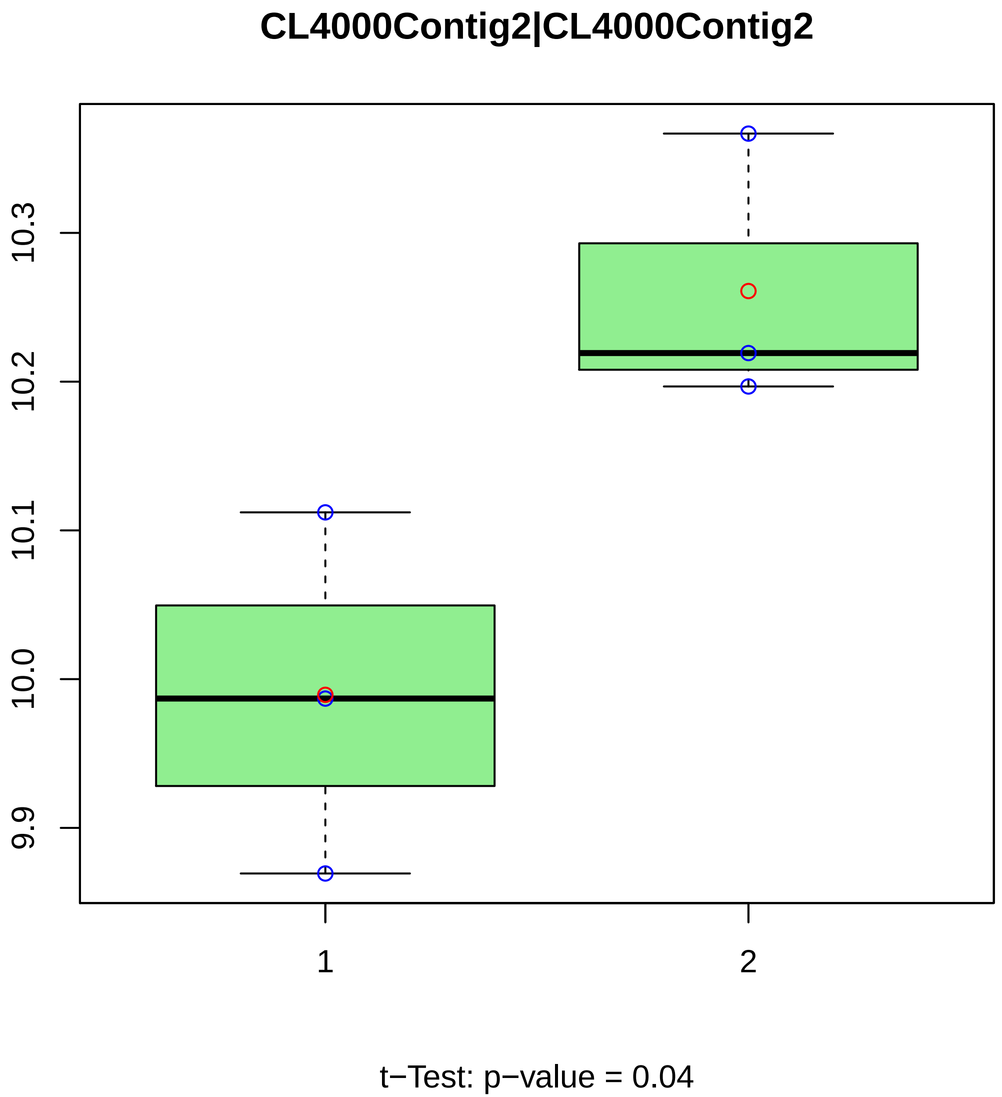

CL4000Contig2|CL4000Contig2
10.3
10.2
10.1
10.0
9.9
1
2
t−Test: p−value = 0.04

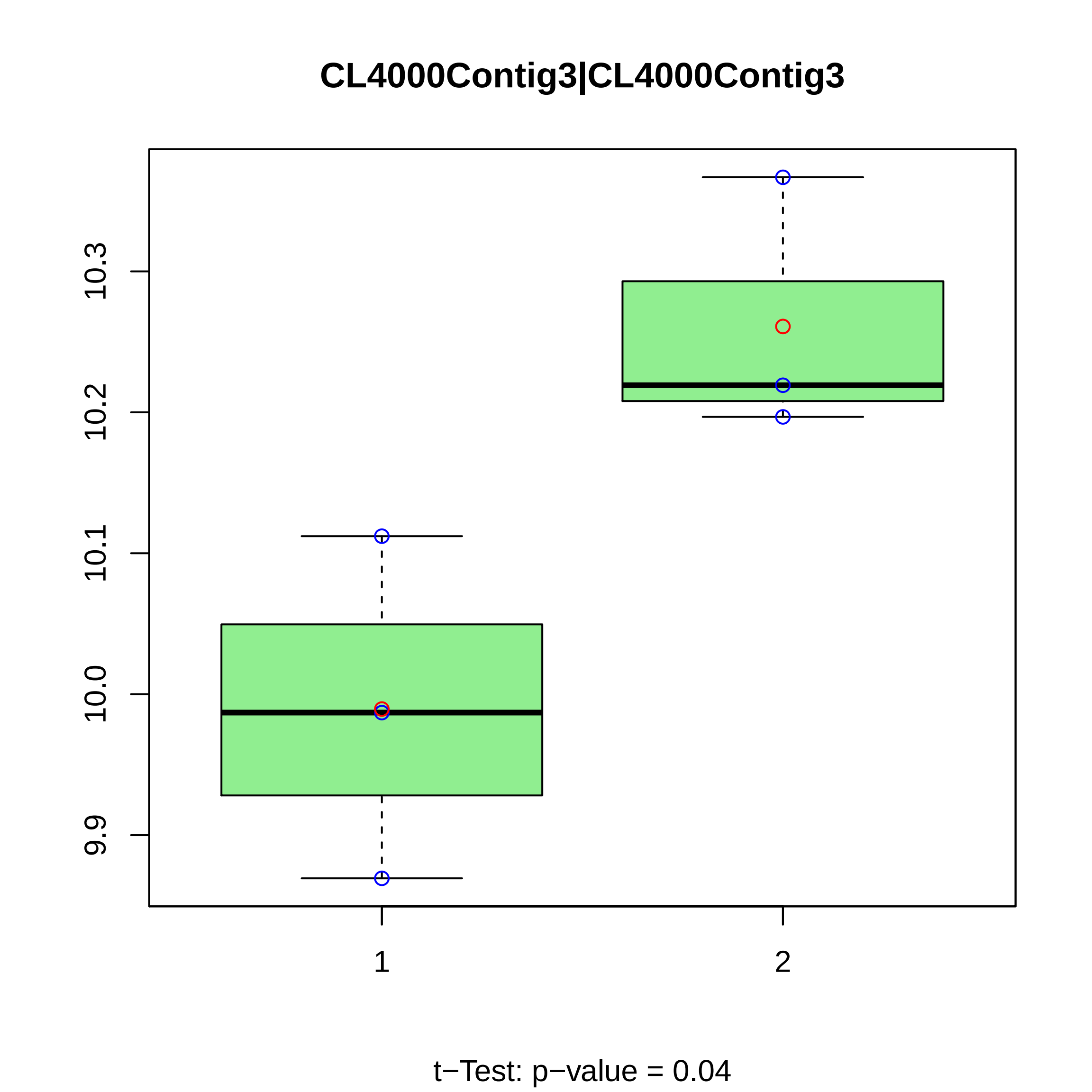
CL4000Contig3|CL4000Contig3
10.3
10.2
10.1
10.0
9.9
1
2
t−Test: p−value = 0.04

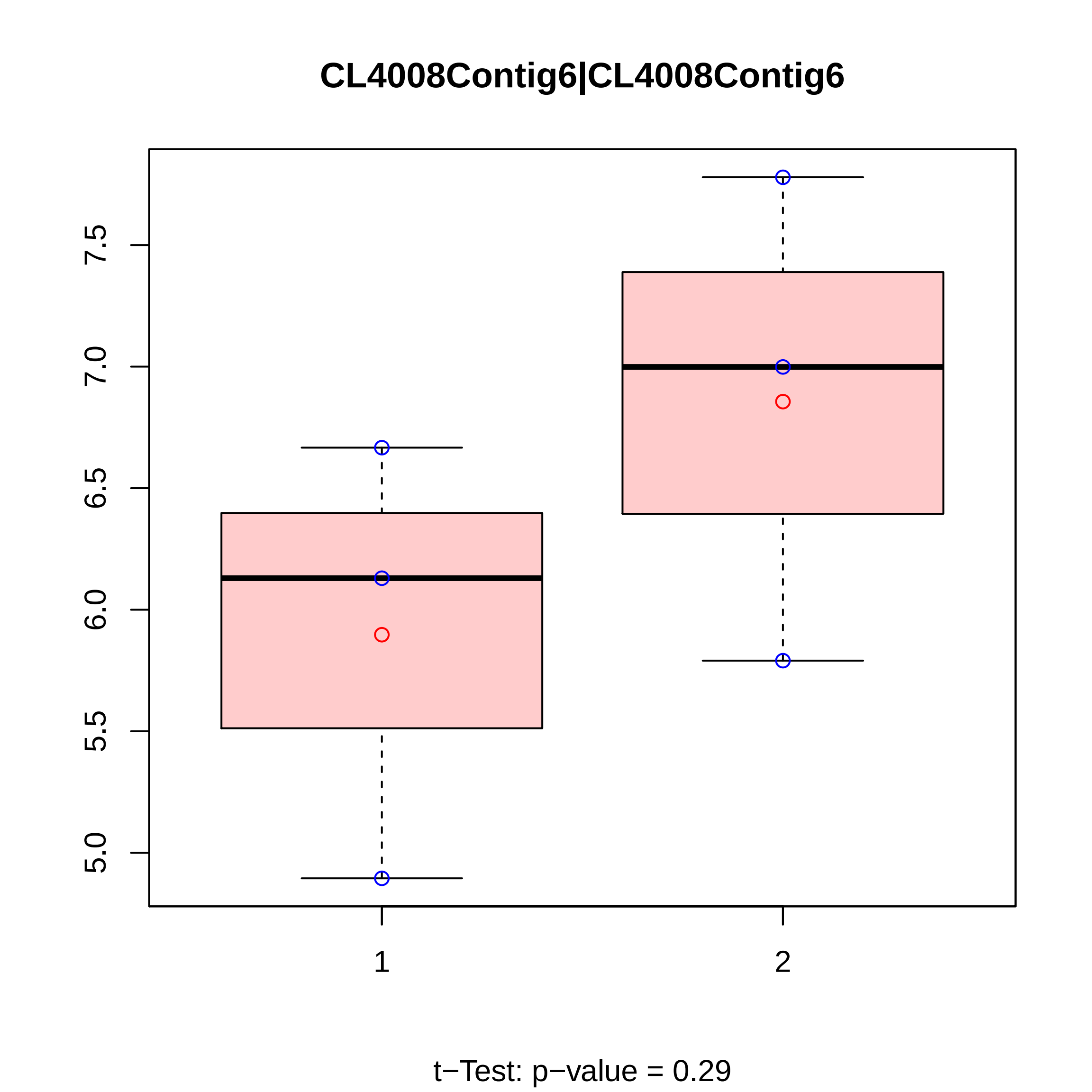

CL4008Contig6|CL4008Contig6
7.5
7.0
6.5
6.0
5.5
5.0
1
2
t−Test: p−value = 0.29

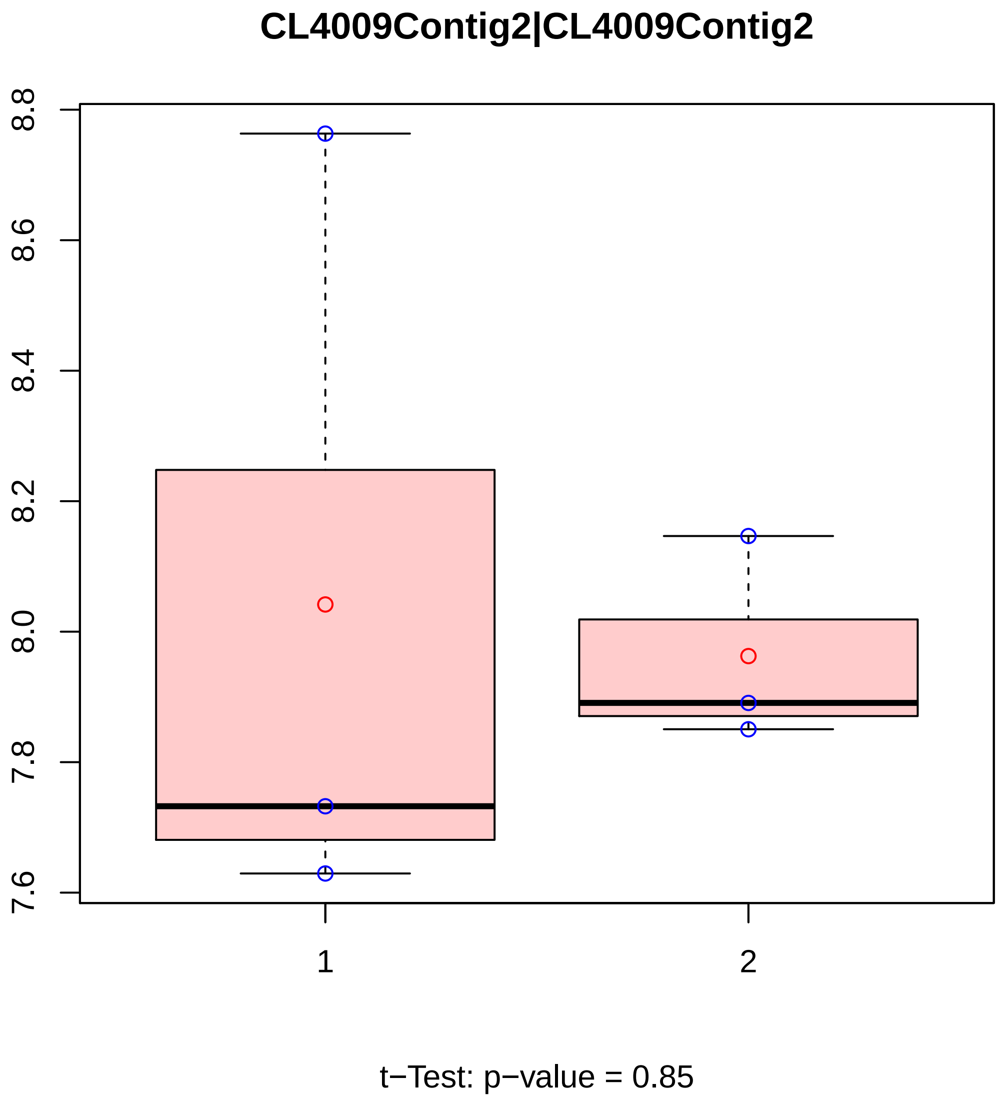

CL4009Contig2|CL4009Contig2
8.8
8.6
8.4
8.2
8.0
7.8
7.6
1
2
t−Test: p−value = 0.85

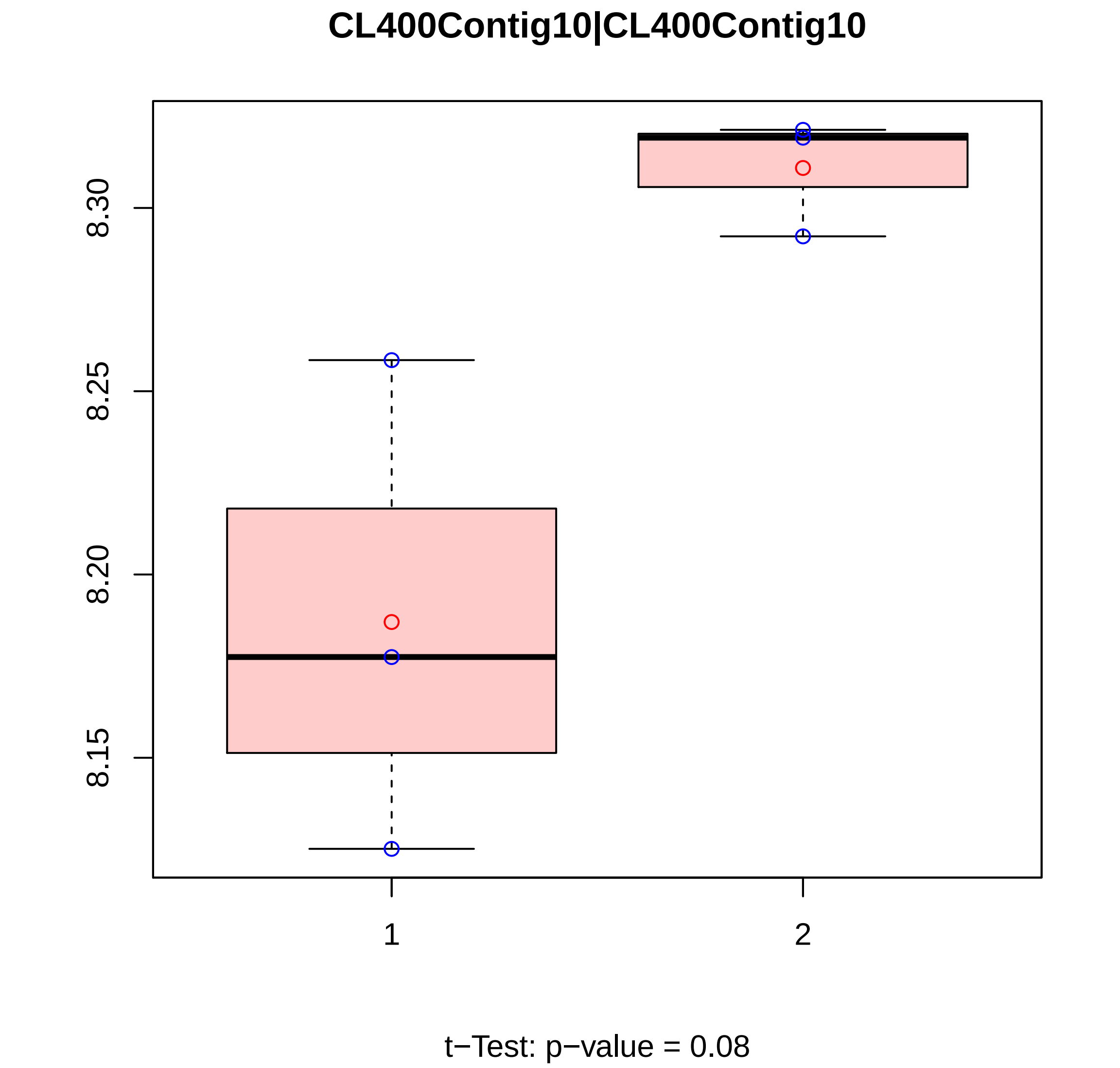

CL400Contig10|CL400Contig10
8.15
8.20
8.25
8.30
1
2
t−Test: p−value = 0.08

# CL400Contig11|CL400Contig11

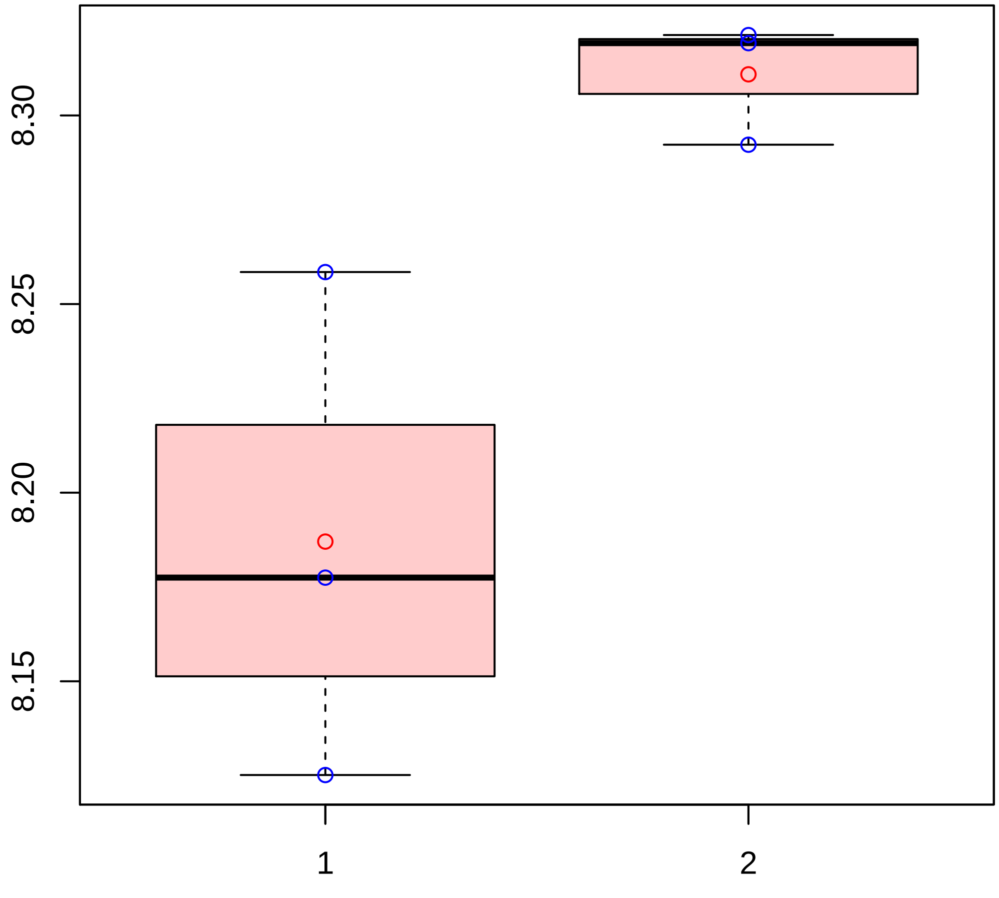


t−Test: p−value = 0.08

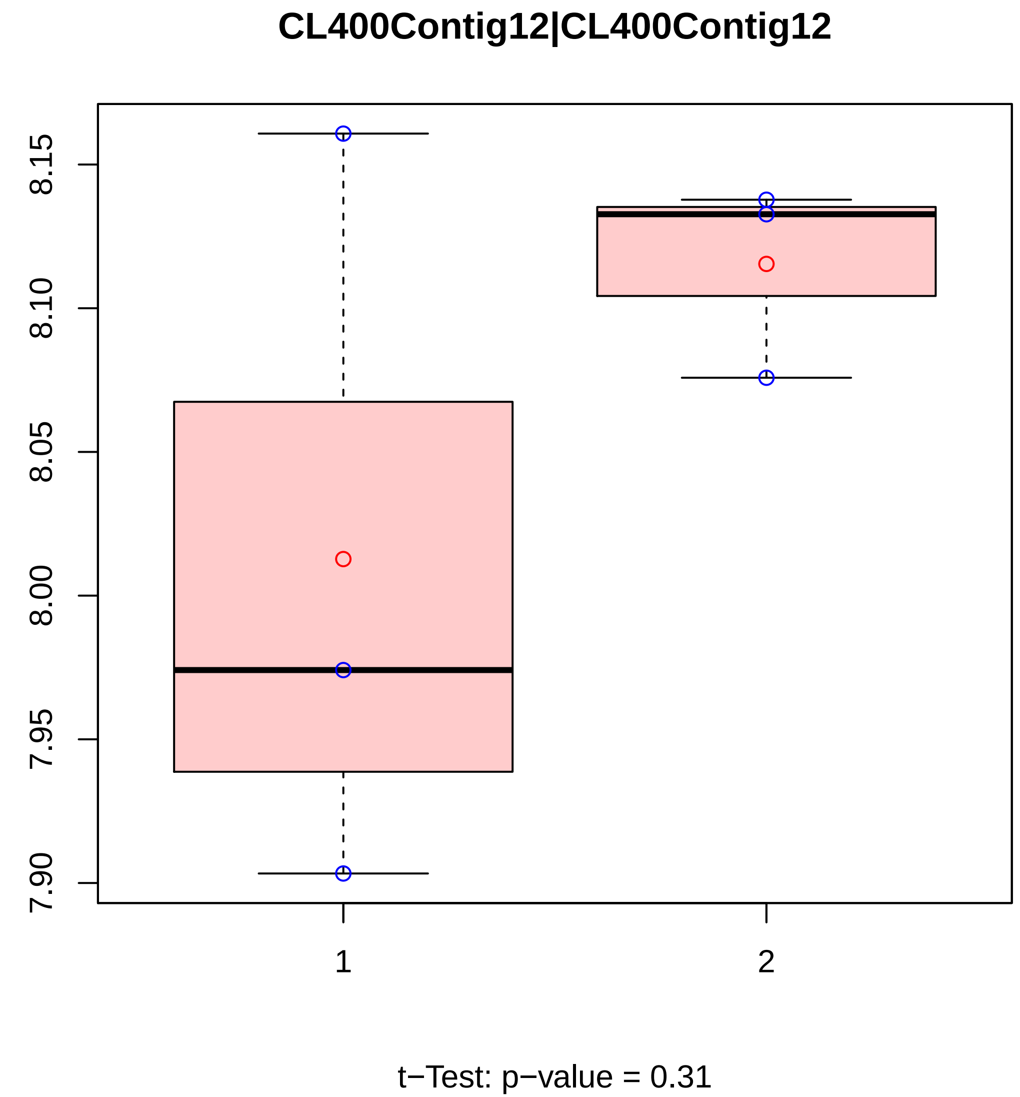

CL400Contig12|CL400Contig12
8.15
8.10
8.05
8.00
7.95
7.90
1
2
t−Test: p−value = 0.31

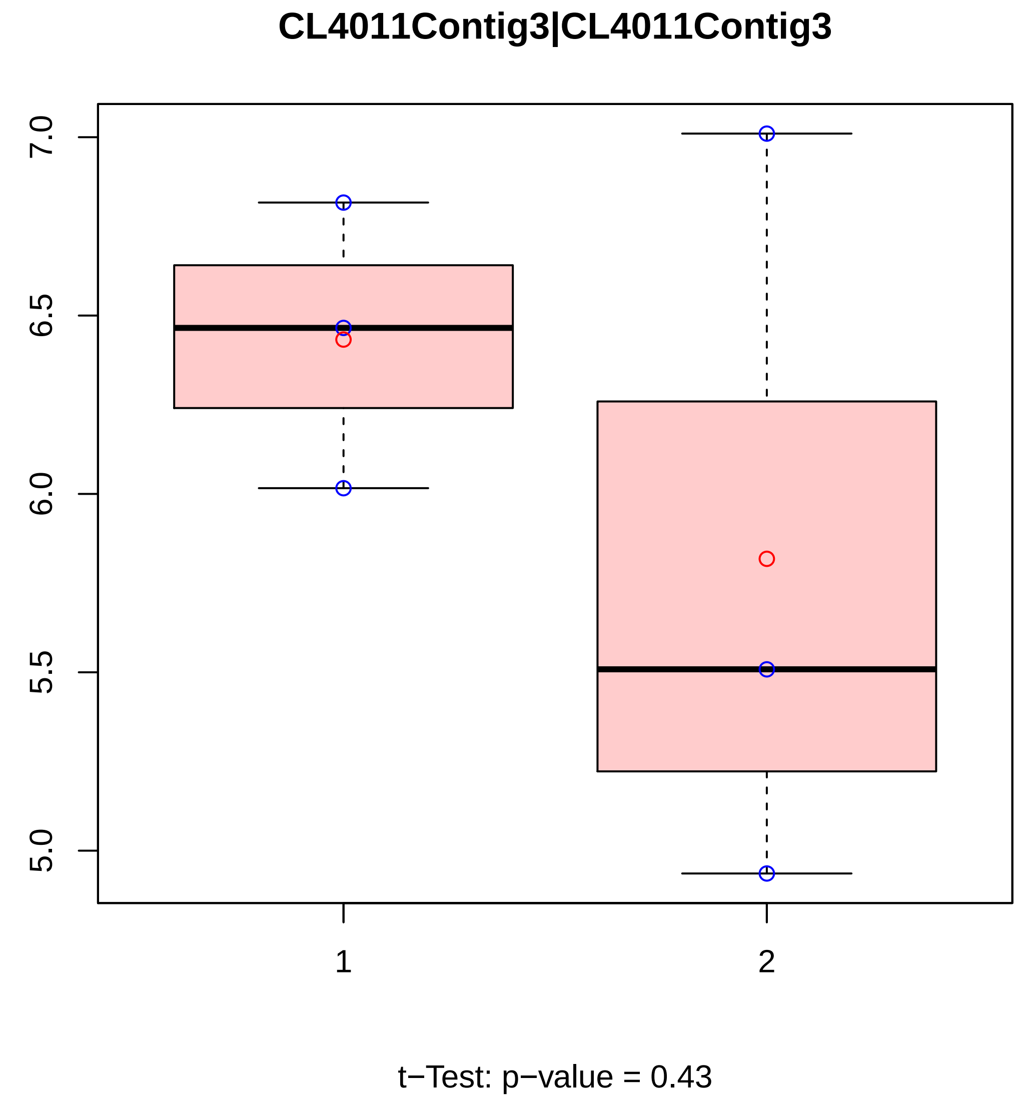
CL4011Contig3|CL4011Contig3
7.0
6.5
6.0
5.5
5.0
1
2
t−Test: p−value = 0.43

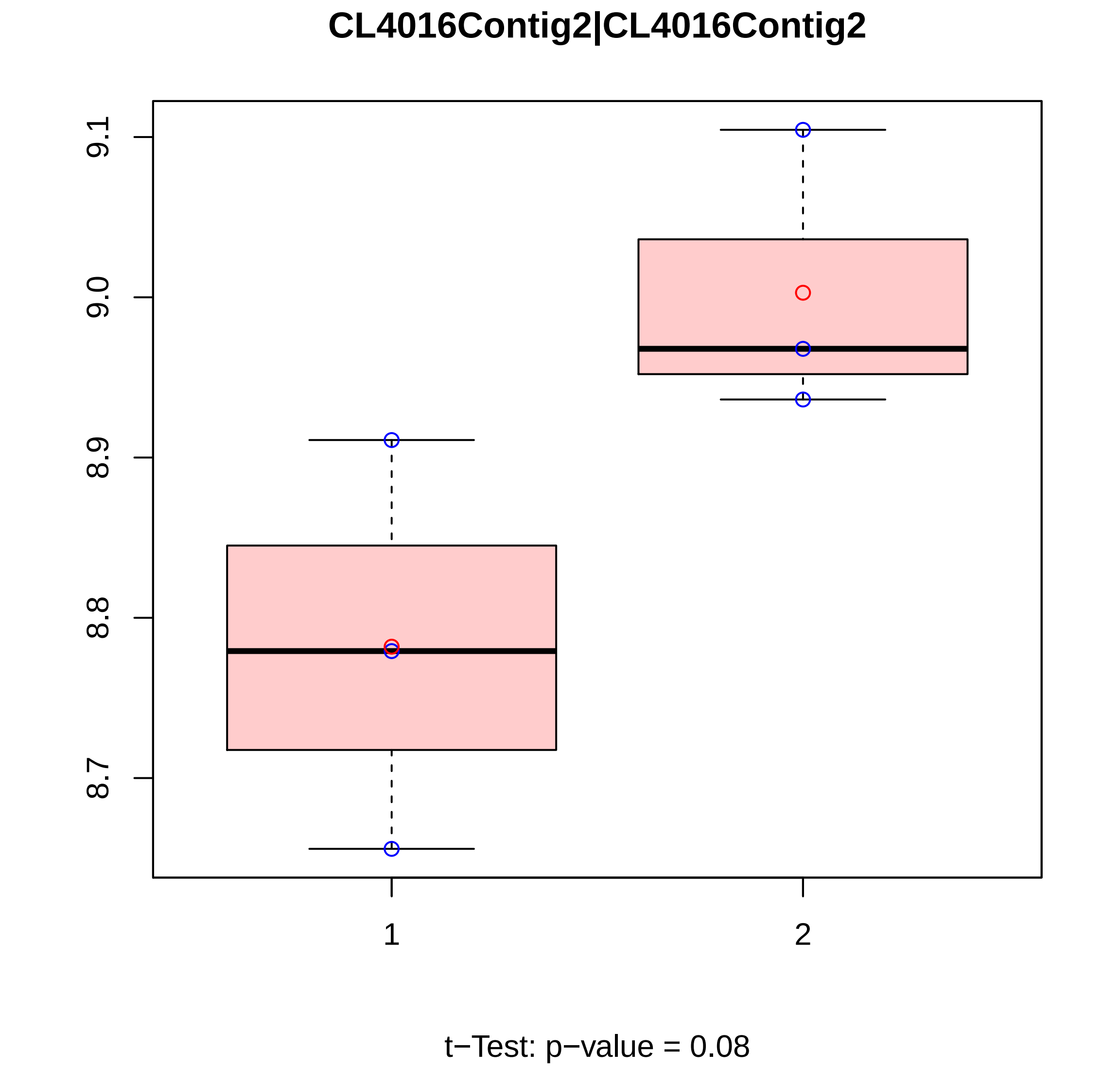
CL4016Contig2|CL4016Contig2
9.1
9.0
8.9
8.8
8.7
1
2
t−Test: p−value = 0.08

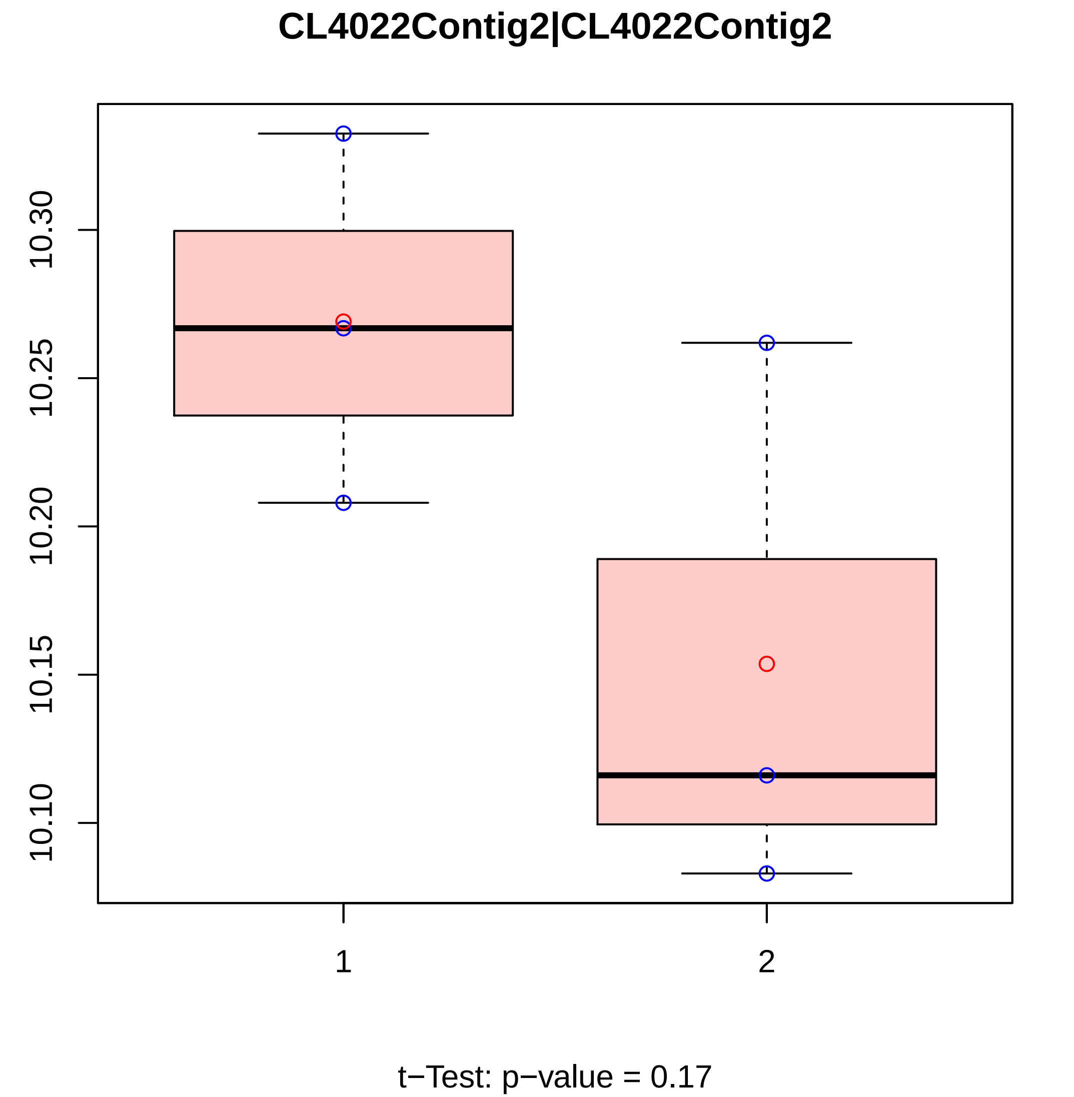
CL4022Contig2|CL4022Contig2
10.10
10.15
10.20
10.25
10.30
1
2
t−Test: p−value = 0.17

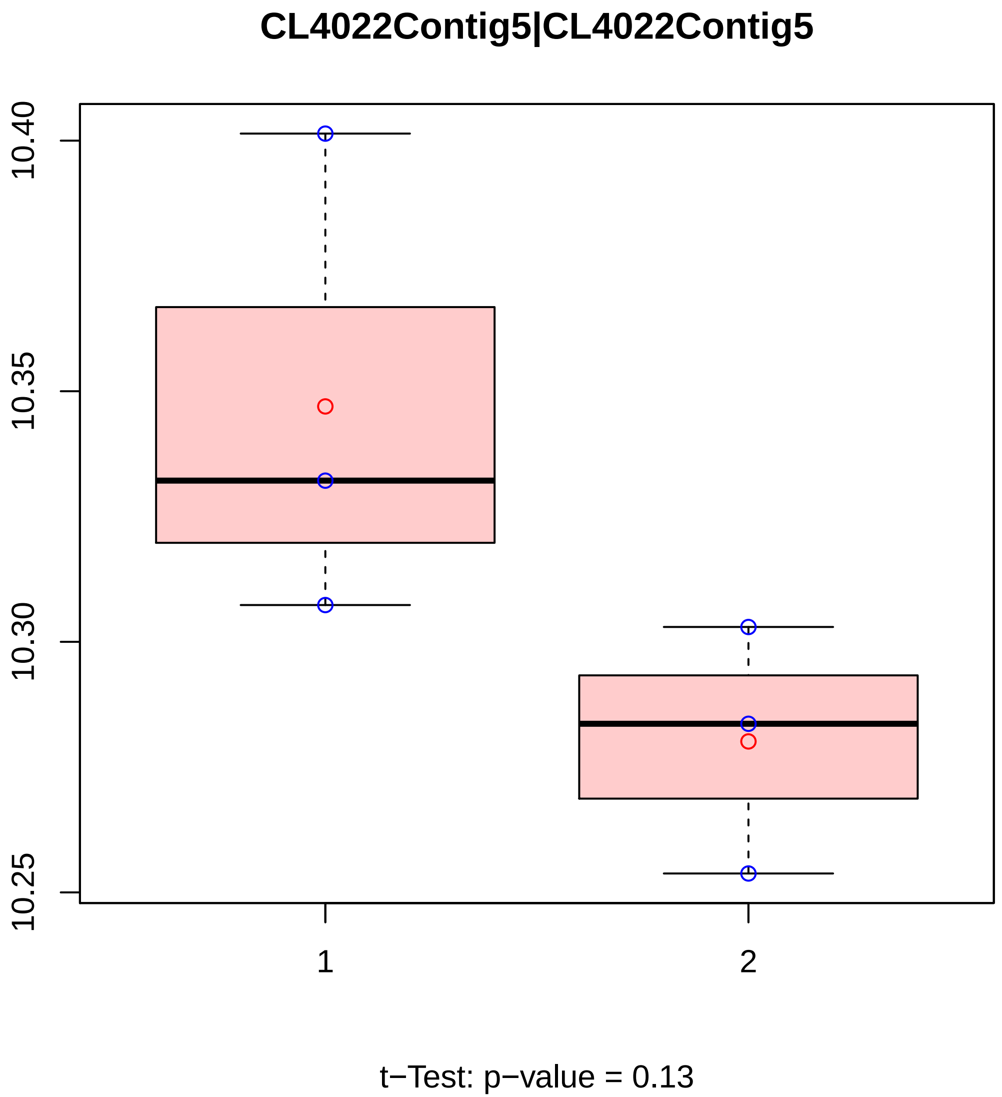
CL4022Contig5|CL4022Contig5
10.40
10.35
10.30
10.25
1
2
t−Test: p−value = 0.13

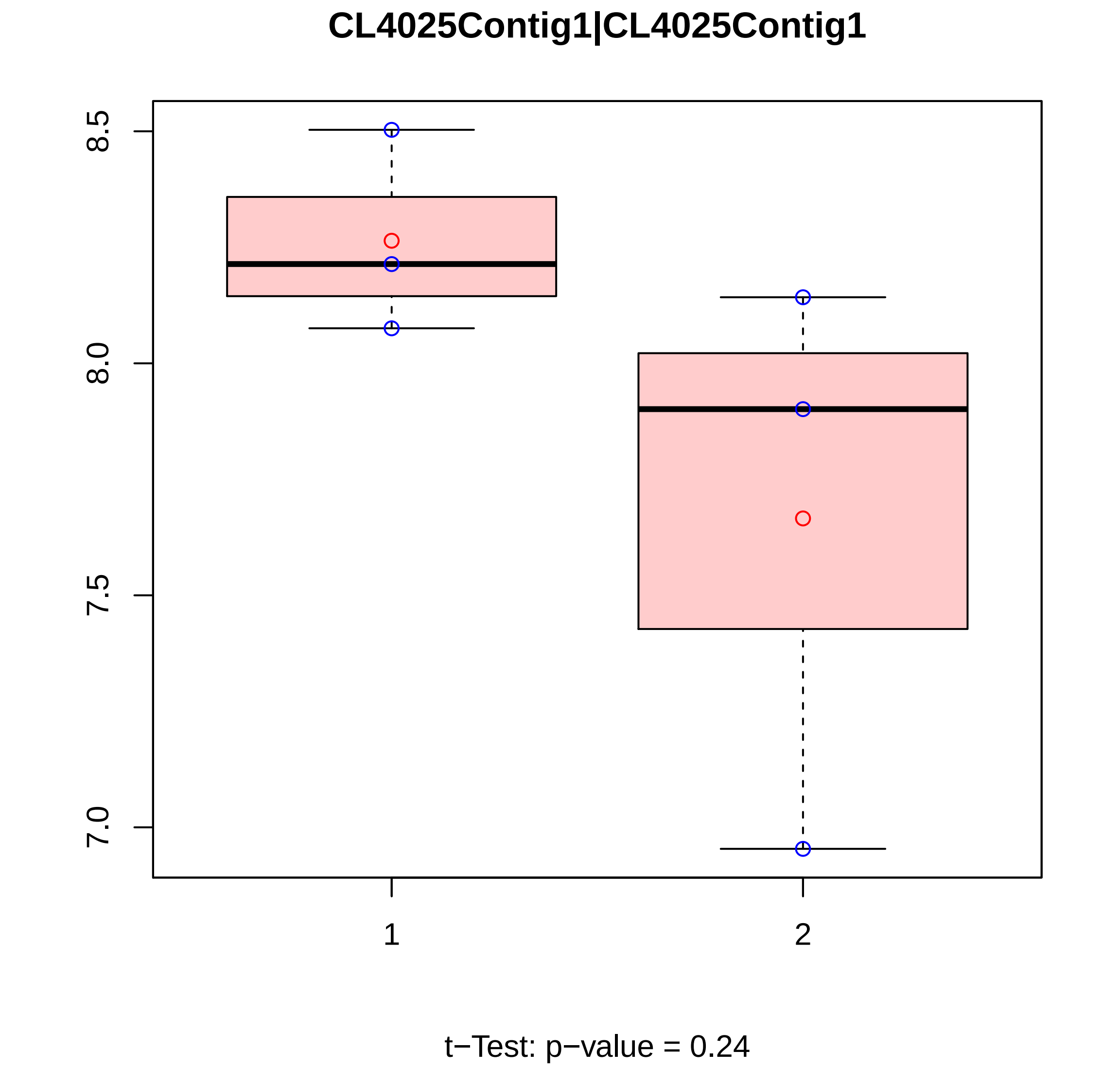
CL4025Contig1|CL4025Contig1
8.5
8.0
7.5
7.0
1
2
t−Test: p−value = 0.24

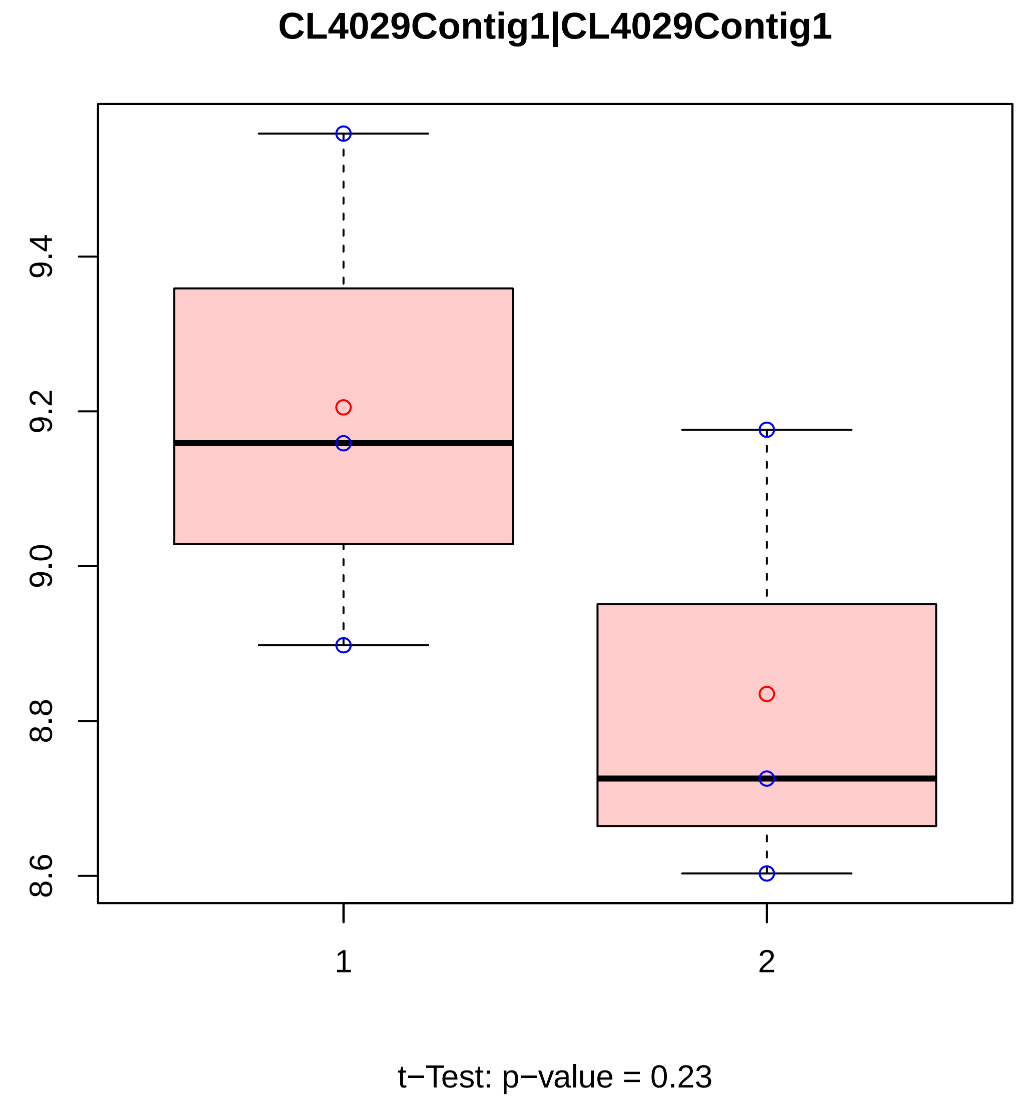

CL4029Contig1|CL4029Contig1
9.4
9.2
9.0
8.8
8.6
1
2
t−Test: p−value = 0.23

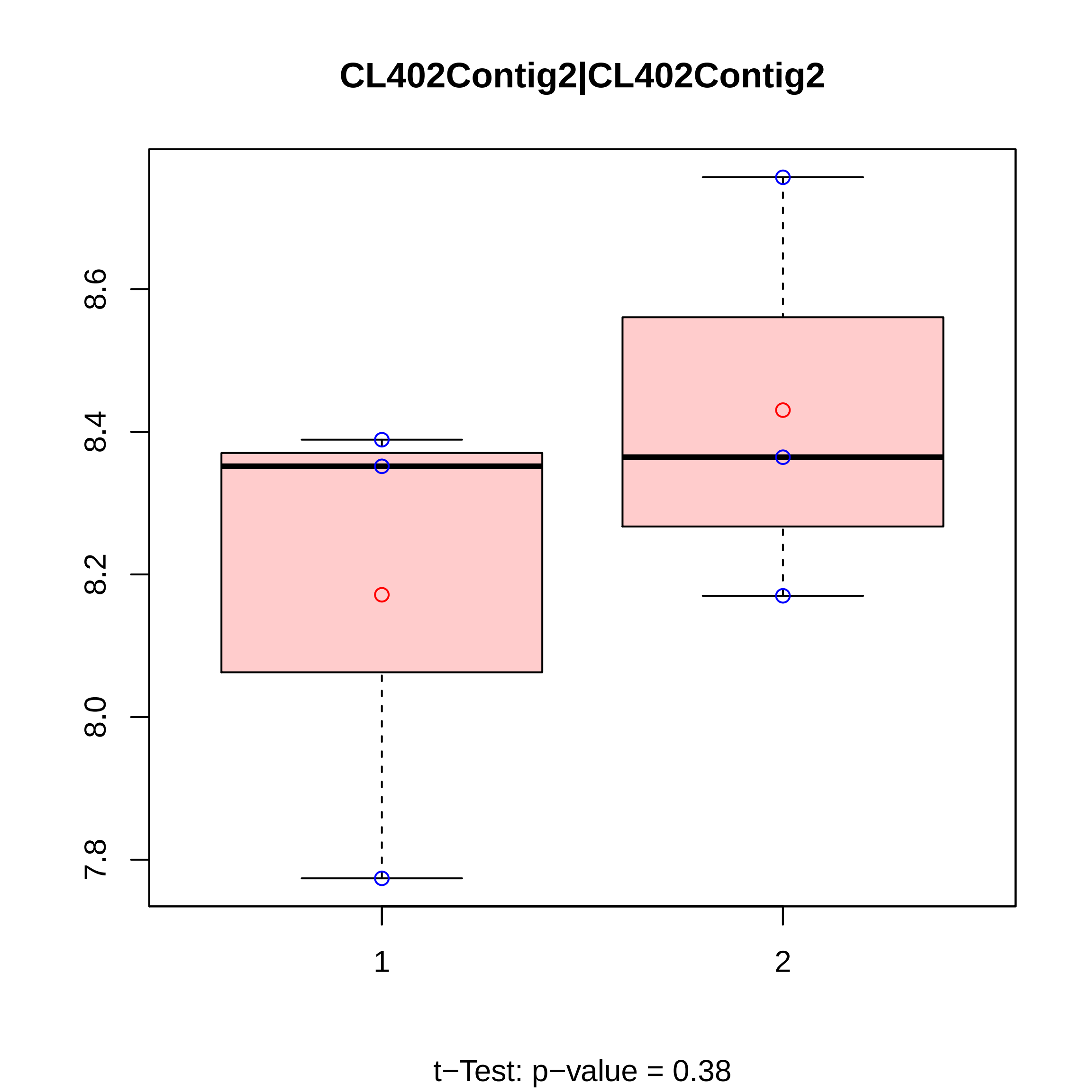
CL402Contig2|CL402Contig2
8.6
8.4
8.2
8.0
7.8
1
2
t−Test: p−value = 0.38

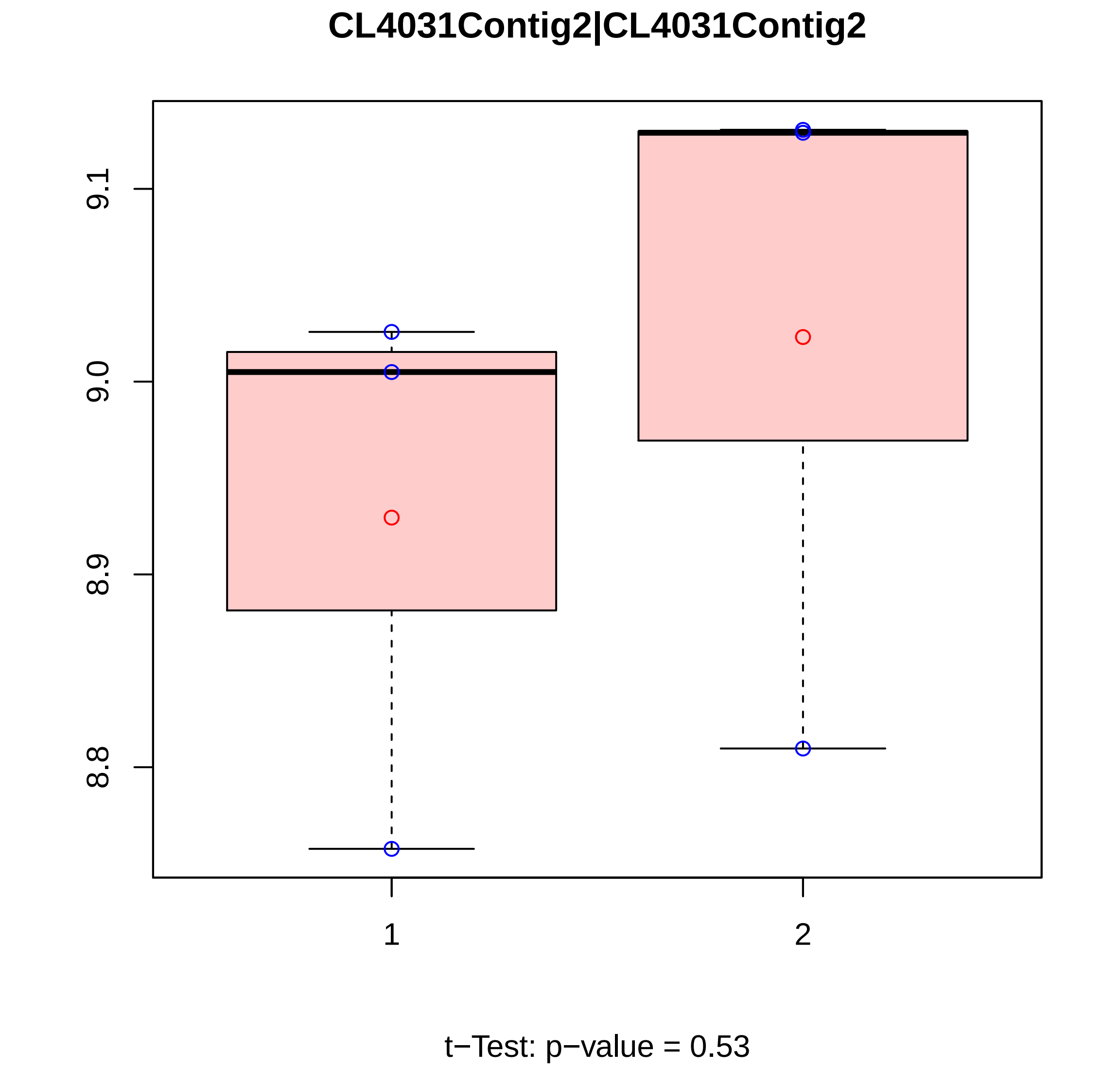
CL4031Contig2|CL4031Contig2
9.1
9.0
8.9
8.8
1
2
t−Test: p−value = 0.53

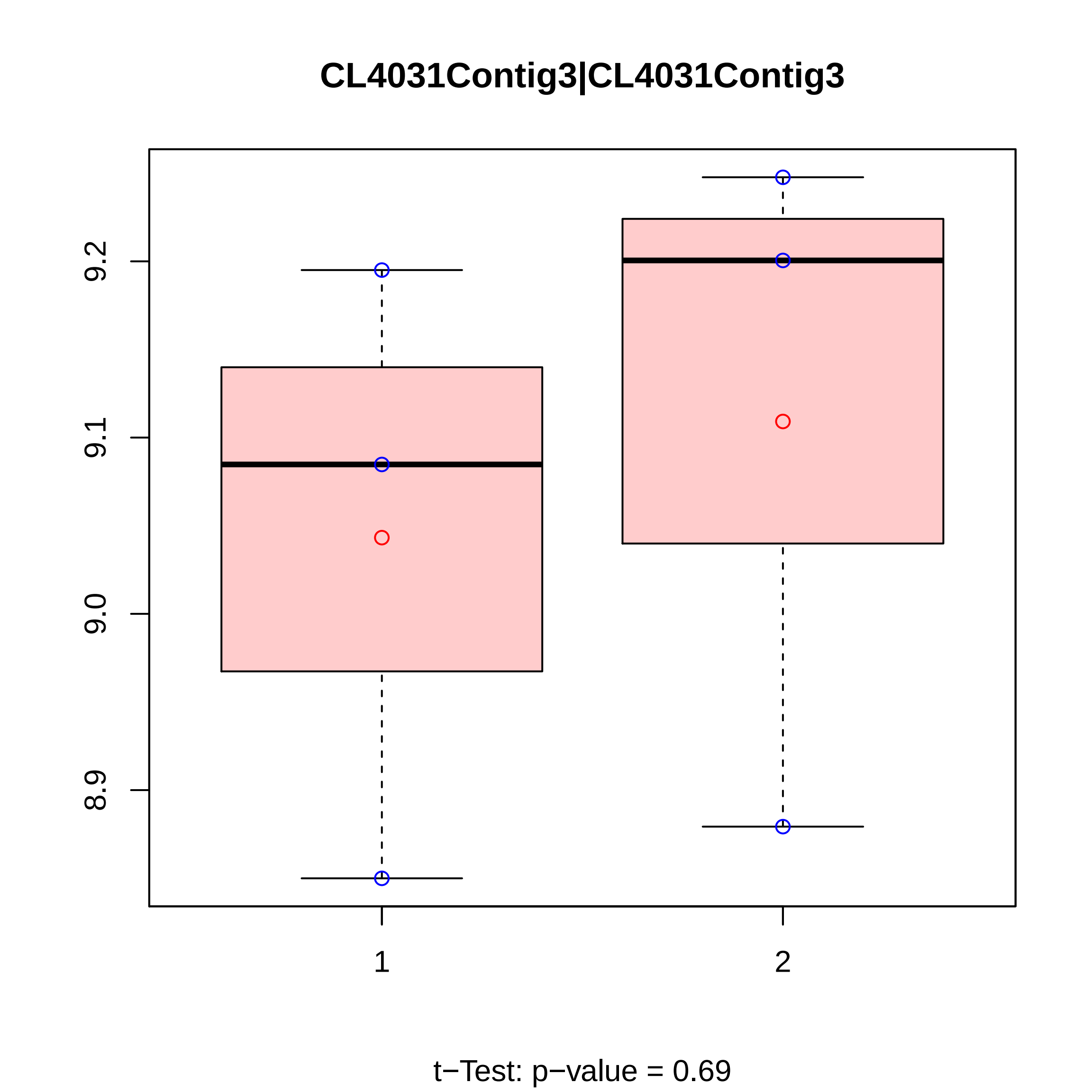

CL4031Contig3|CL4031Contig3
9.2
9.1
9.0
8.9
1
2
t−Test: p−value = 0.69

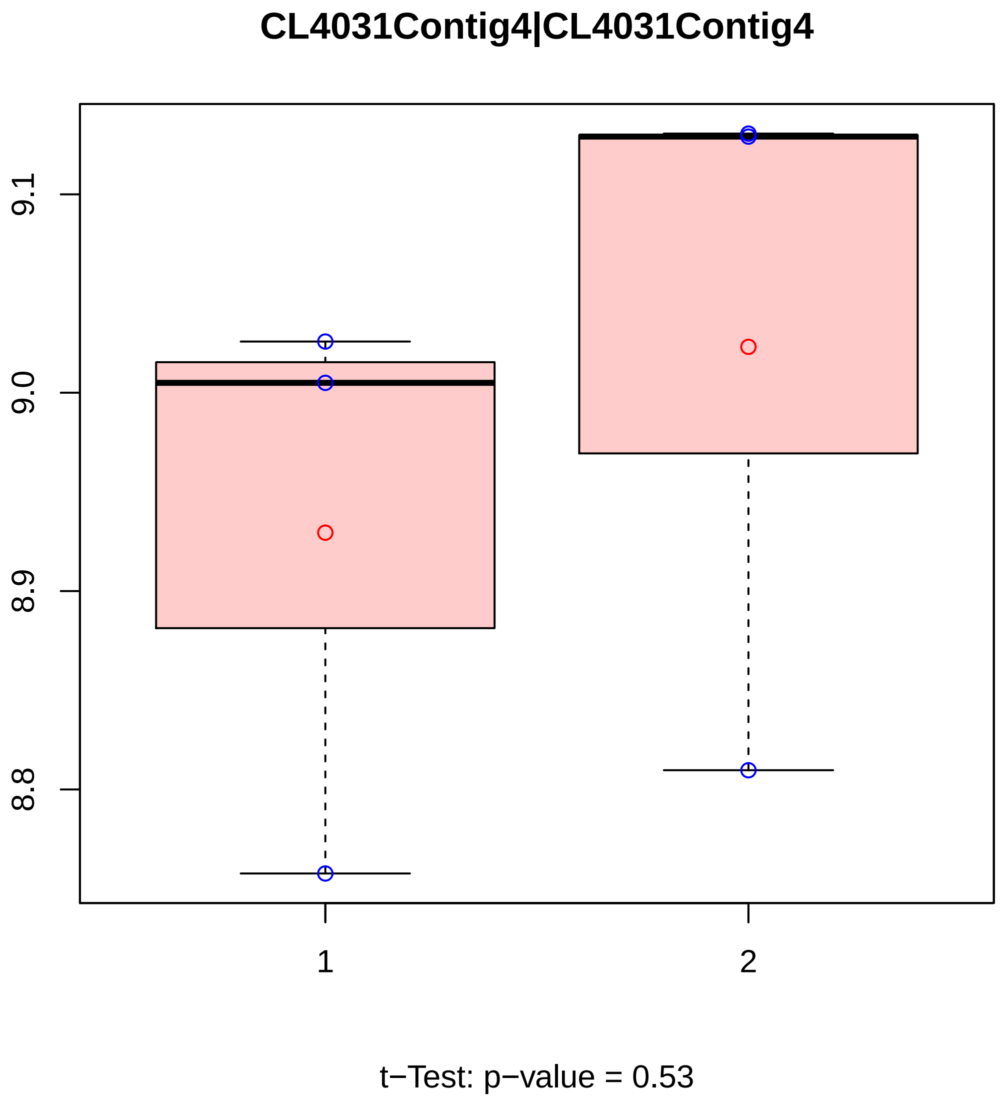

CL4031Contig4|CL4031Contig4
9.1
9.0
8.9
8.8
1
2
t−Test: p−value = 0.53

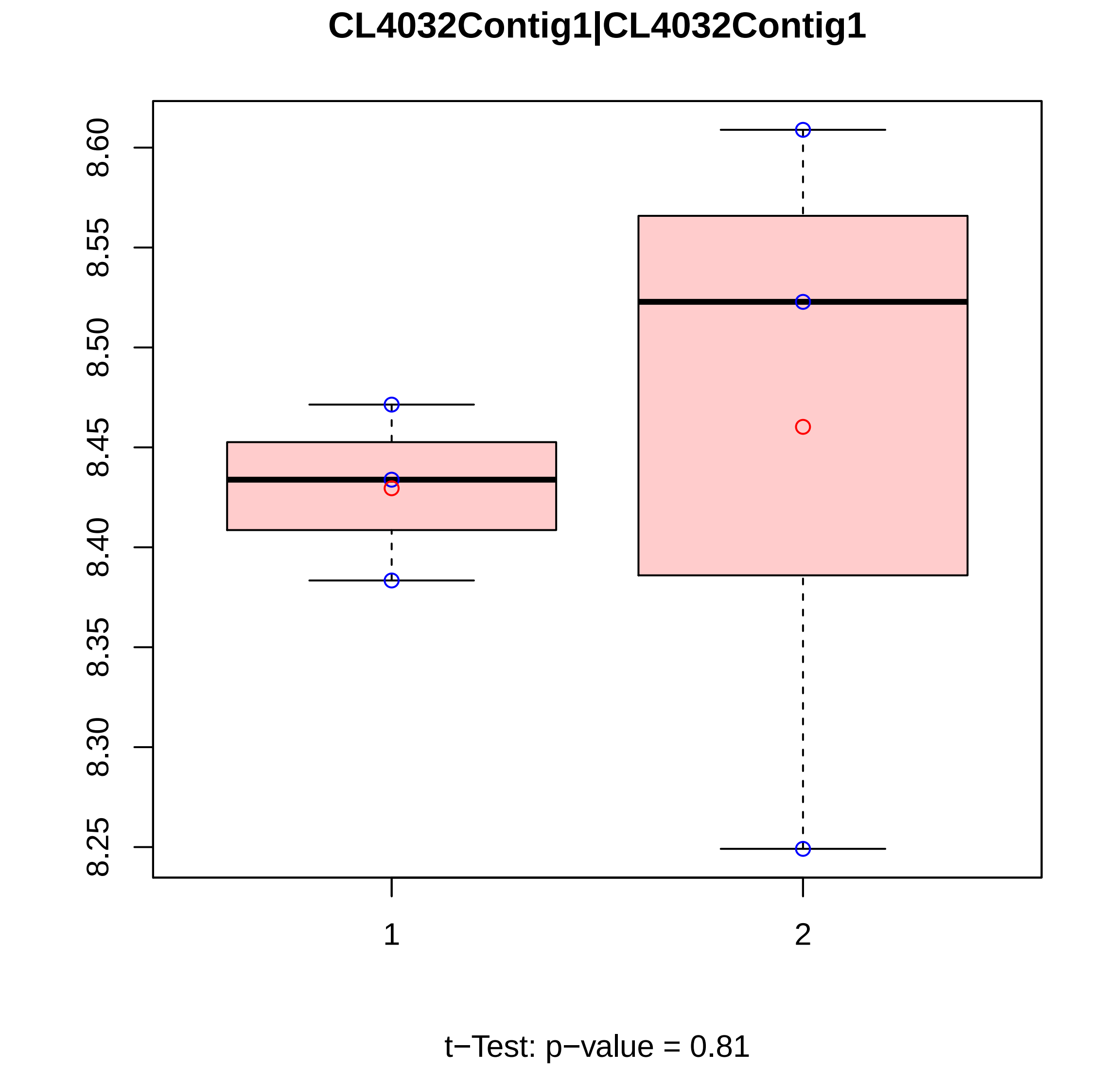

CL4032Contig1|CL4032Contig1
8.60
8.55
8.50
8.45
8.40
8.35
8.30
8.25
1
2
t−Test: p−value = 0.81

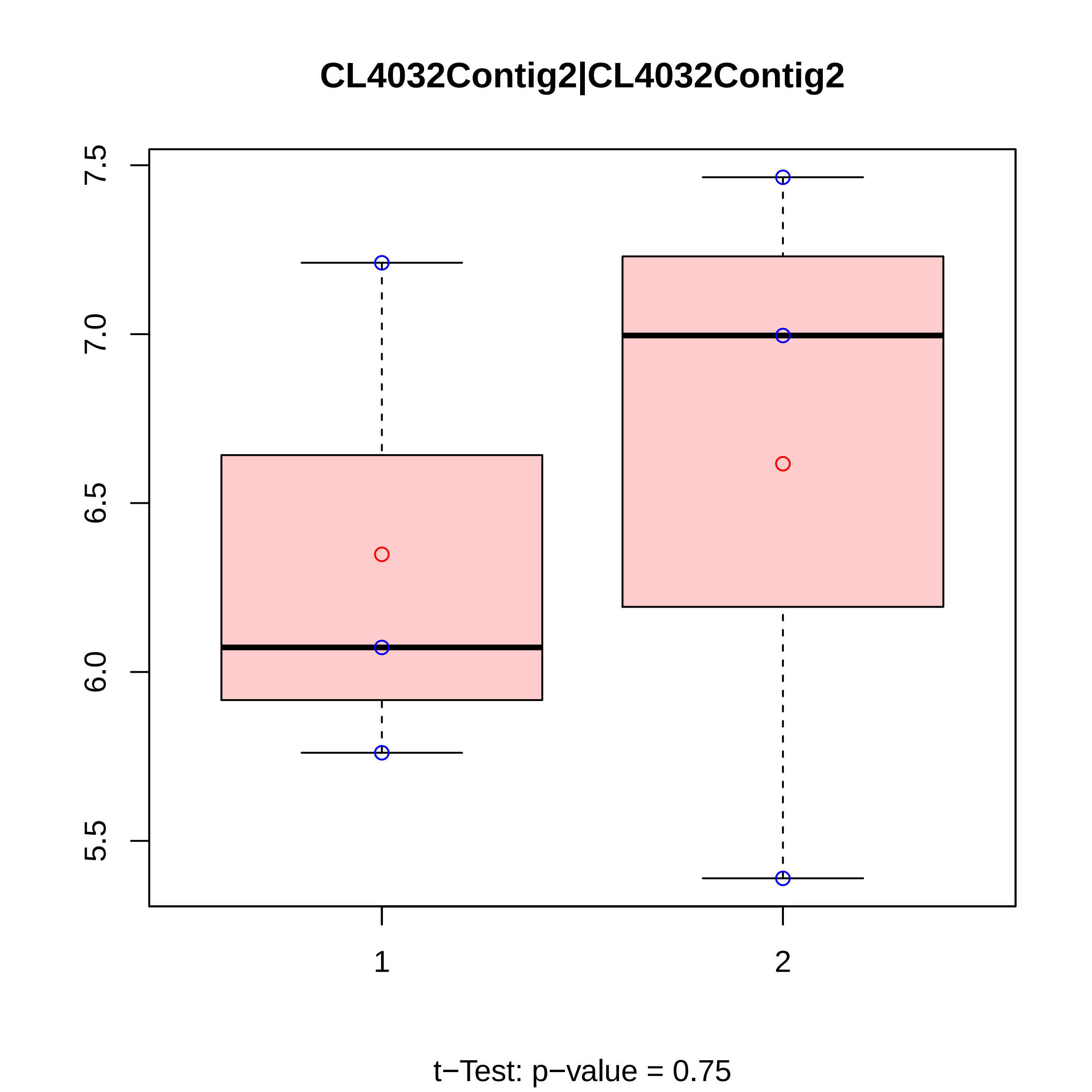
CL4032Contig2|CL4032Contig2
7.5
7.0
6.5
6.0
5.5
1
2
t−Test: p−value = 0.75

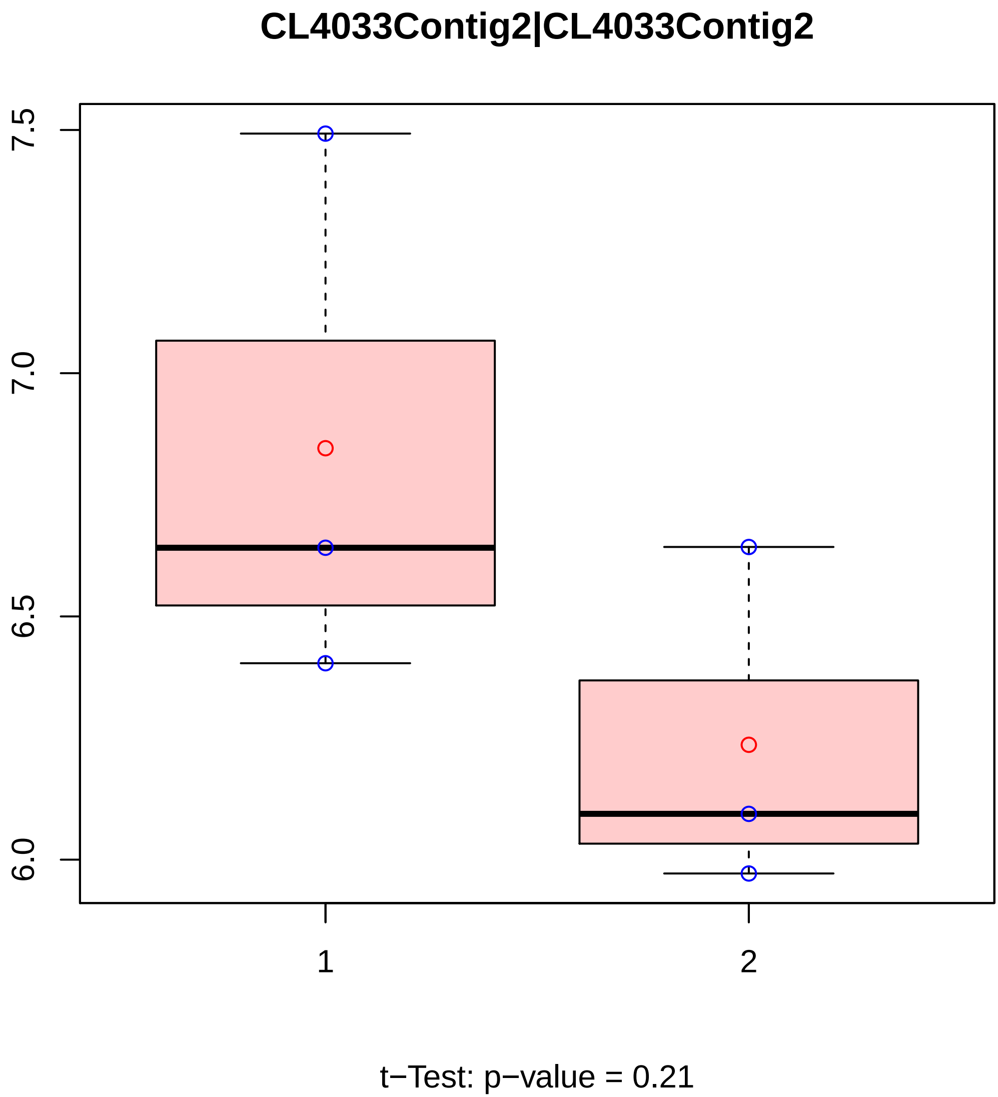
CL4033Contig2|CL4033Contig2
7.5
7.0
6.5
6.0
1
2
t−Test: p−value = 0.21

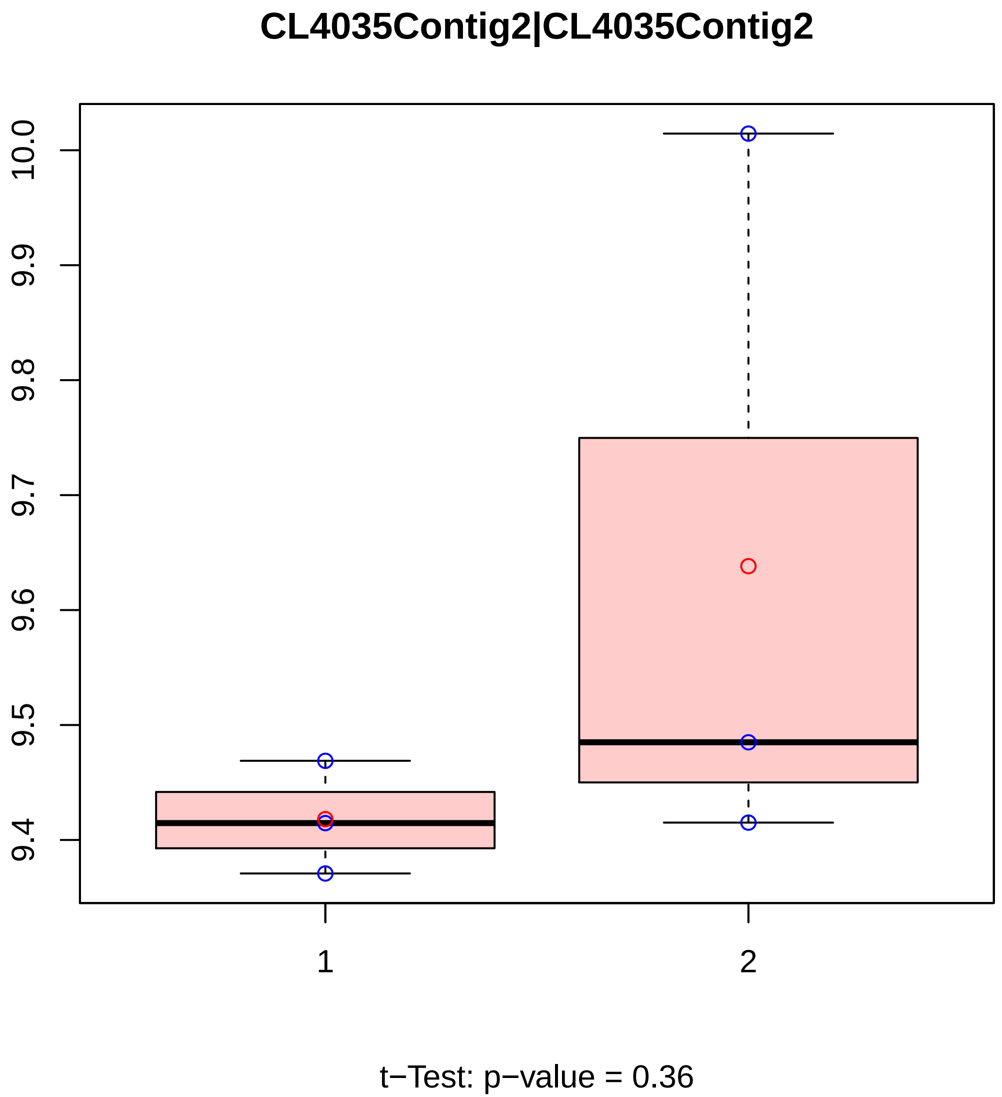
CL4035Contig2|CL4035Contig2
10.0
9.9
9.8
9.7
9.6
9.5
9.4
1
2
t−Test: p−value = 0.36

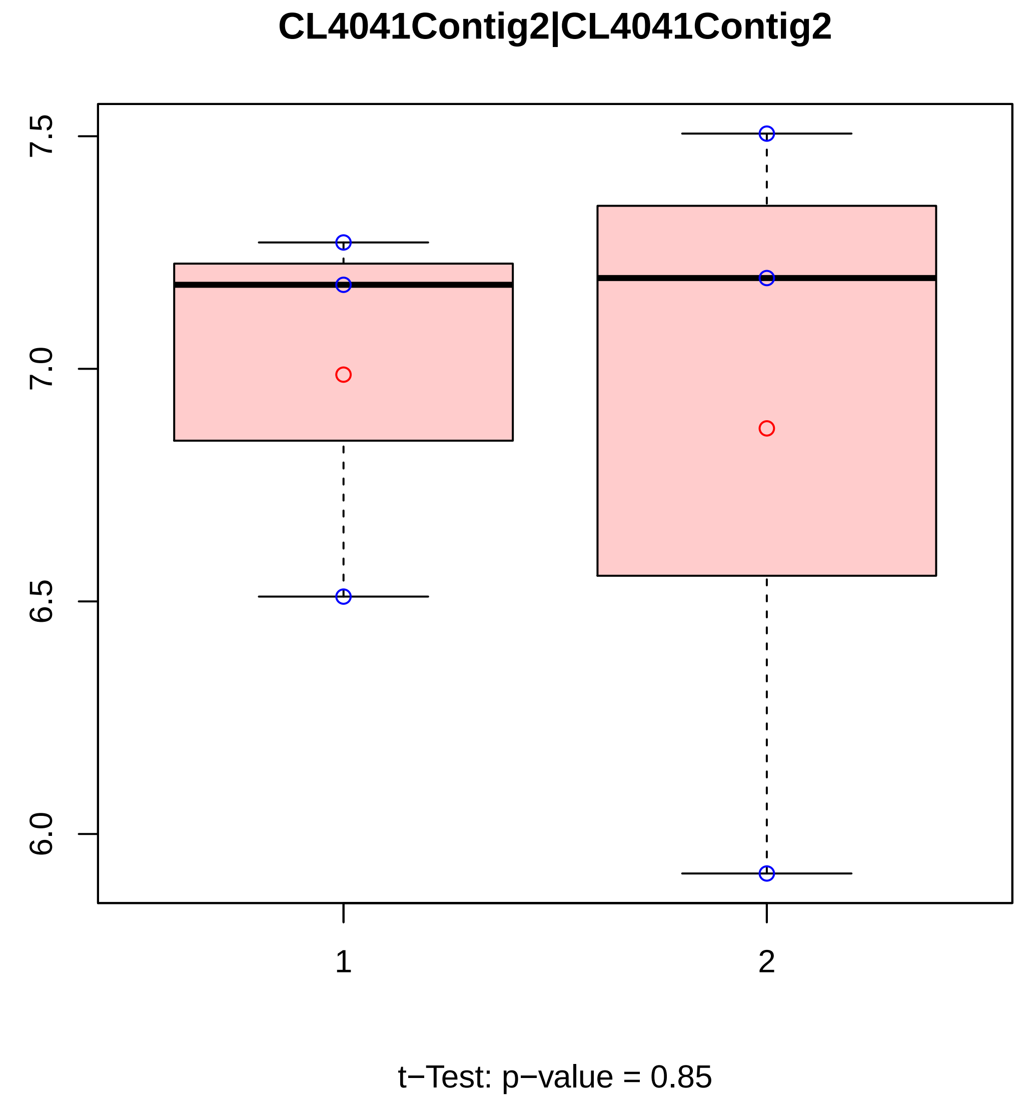

CL4041Contig2|CL4041Contig2
7.5
7.0
6.5
6.0
1
2
t−Test: p−value = 0.85

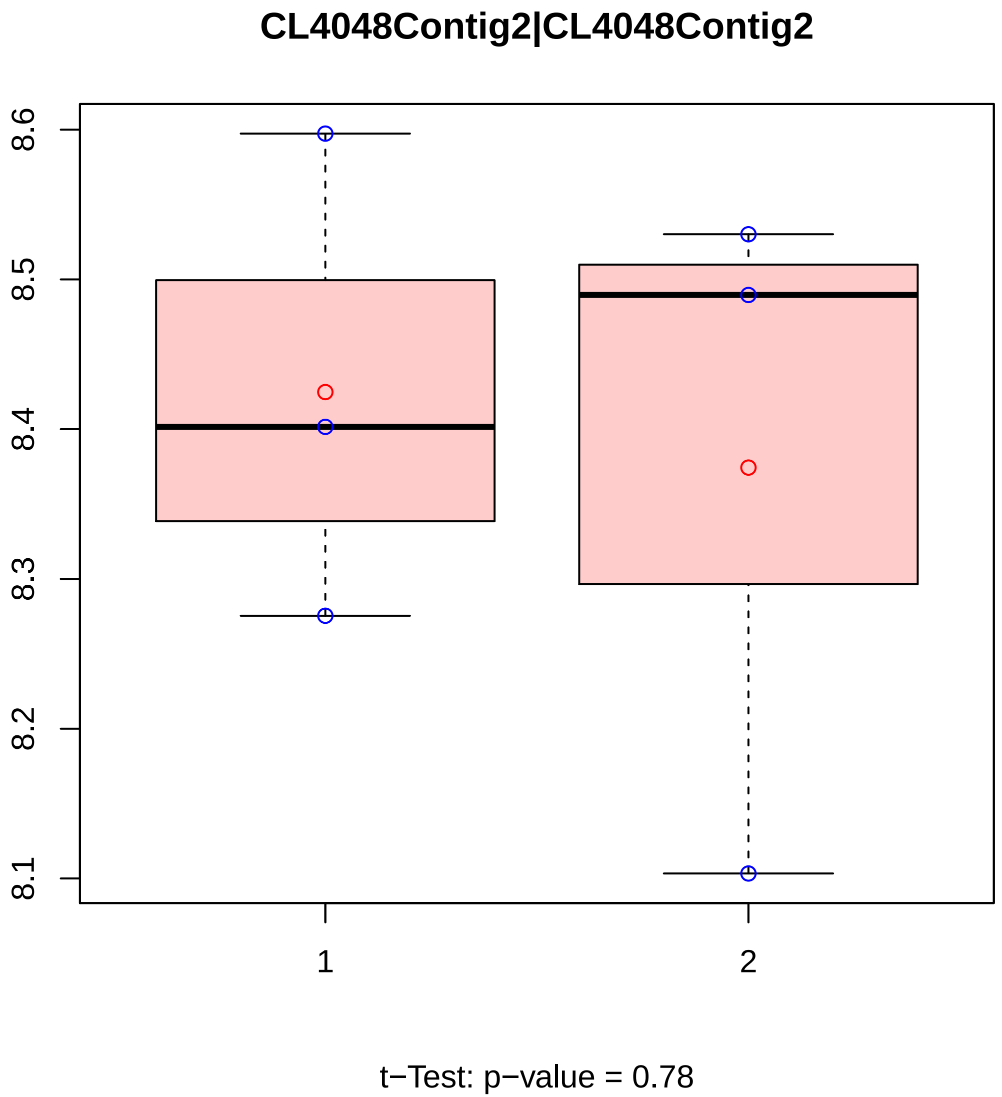

CL4048Contig2|CL4048Contig2
8.1
8.2
8.3
8.4
8.5
8.6
1
2
t−Test: p−value = 0.78

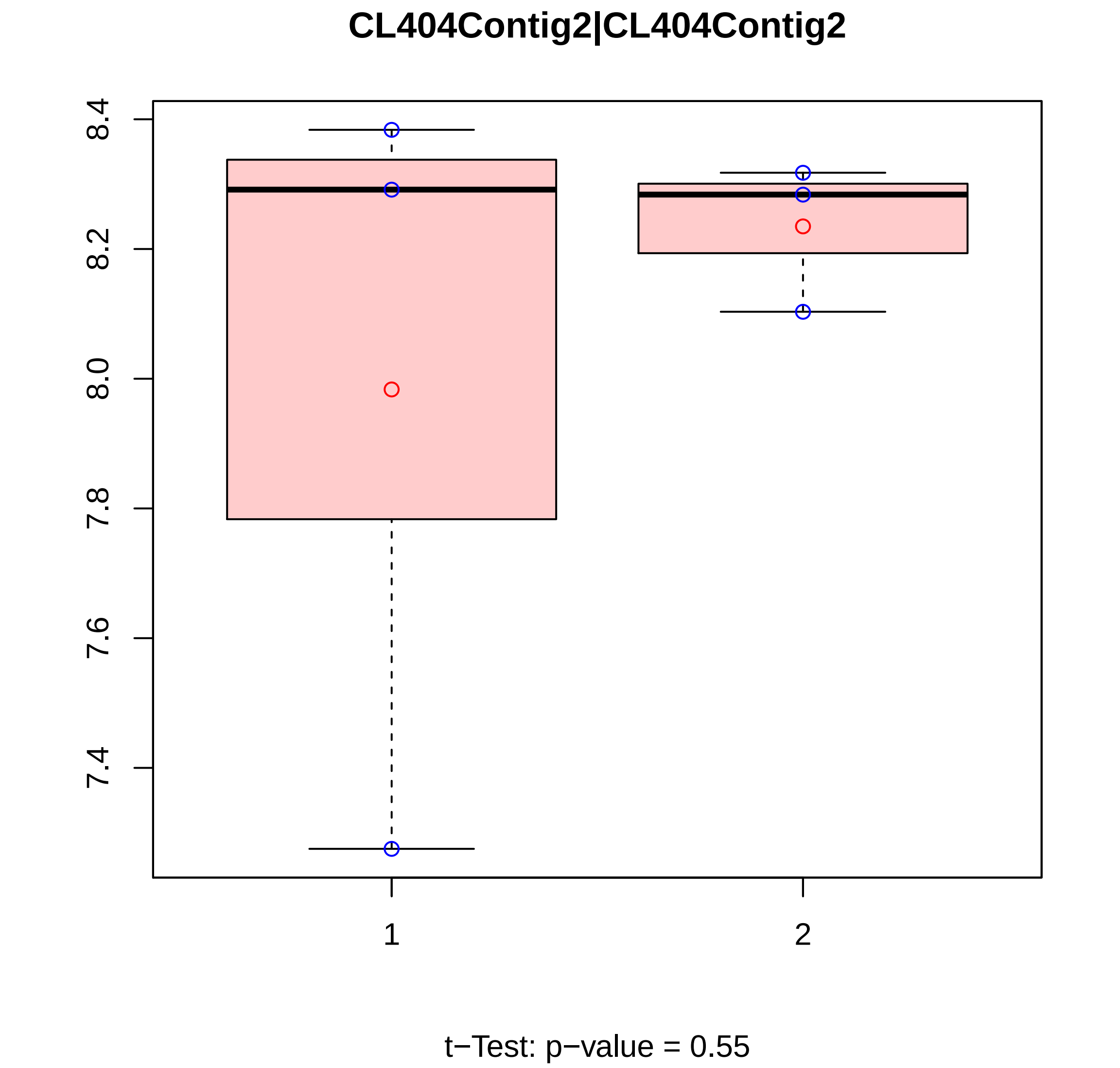
CL404Contig2|CL404Contig2
8.4
8.2
8.0
7.8
7.6
7.4
1
2
t−Test: p−value = 0.55

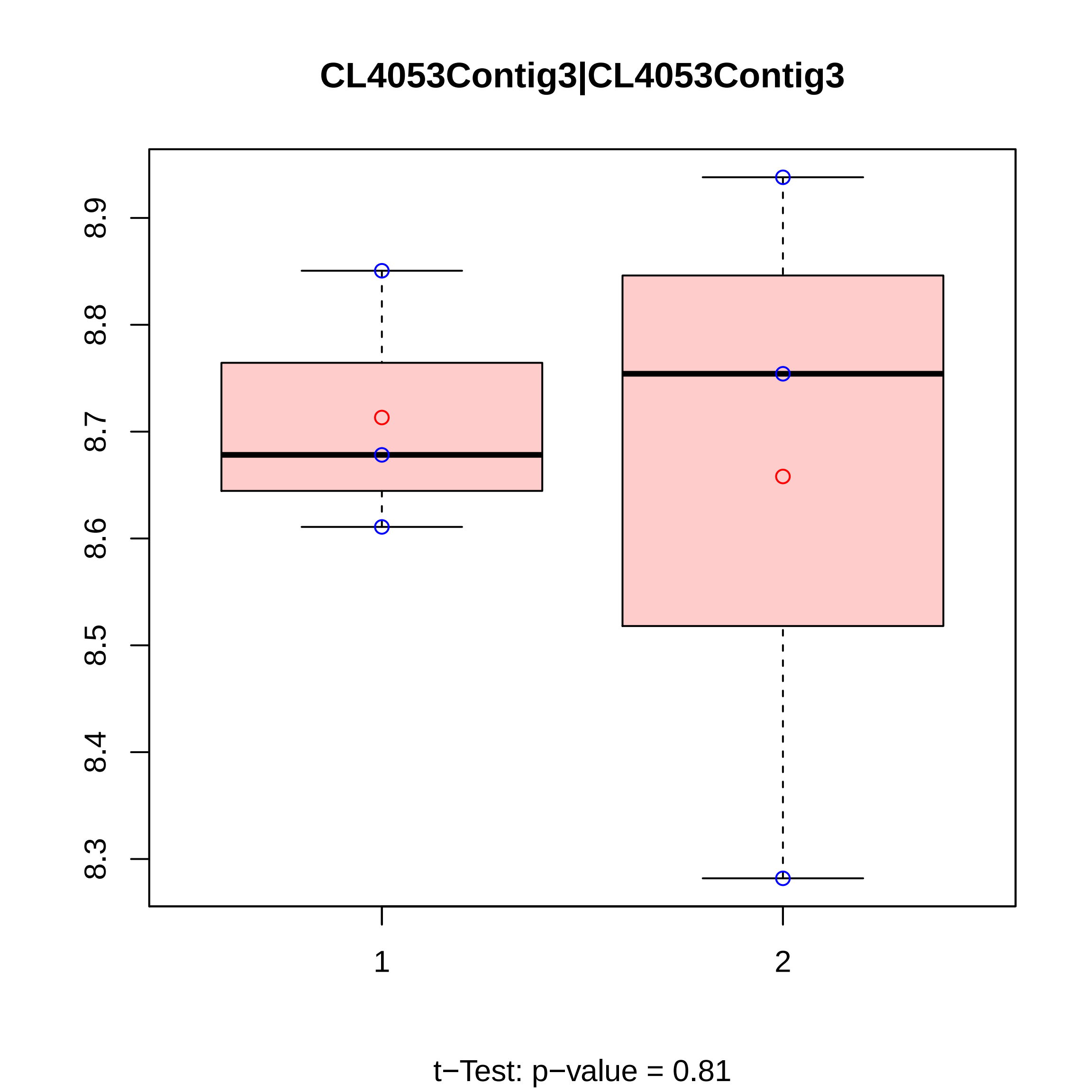
CL4053Contig3|CL4053Contig3
8.3
8.4
8.5
8.6
8.7
8.8
8.9
1
2
t−Test: p−value = 0.81

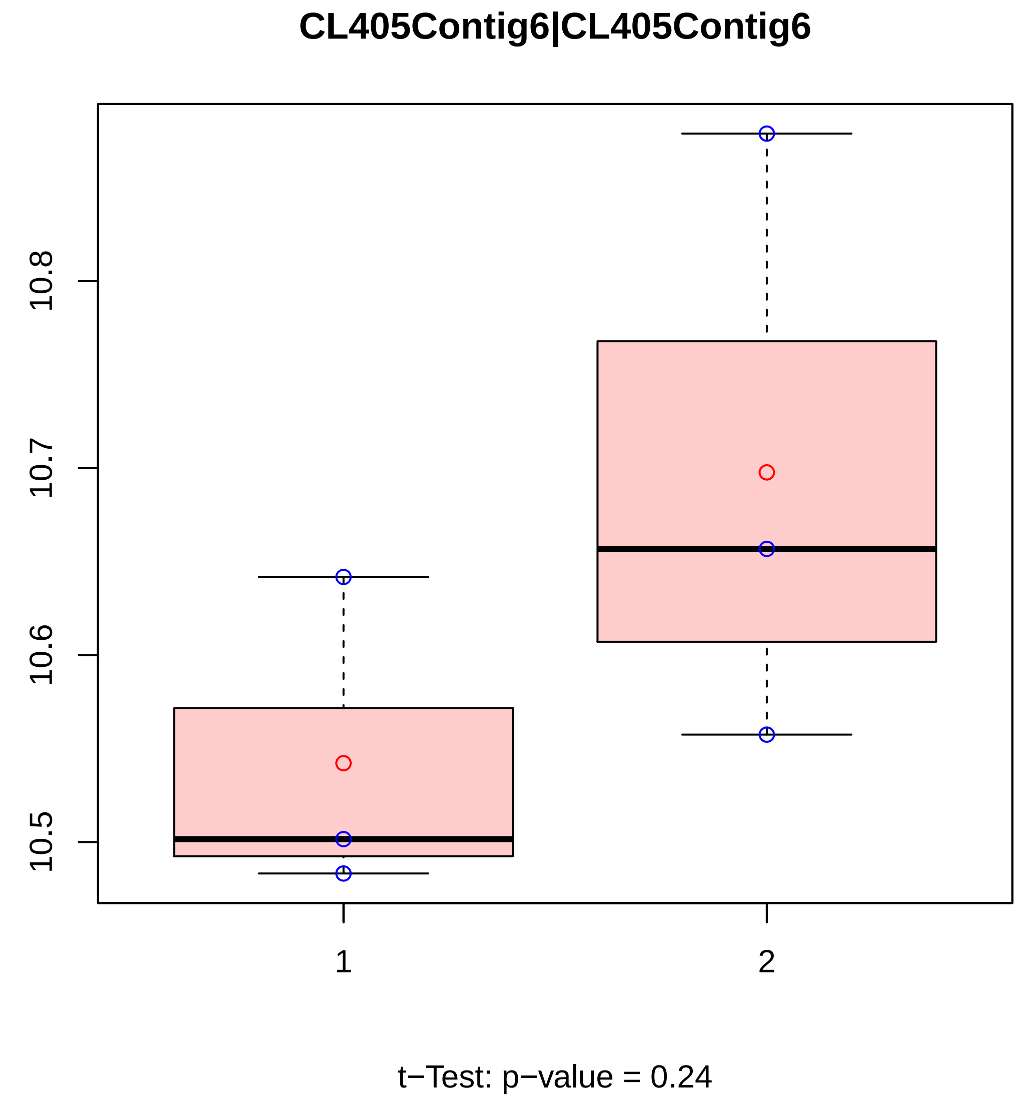
CL405Contig6|CL405Contig6
10.5
10.6
10.7
10.8
1
2
t−Test: p−value = 0.24

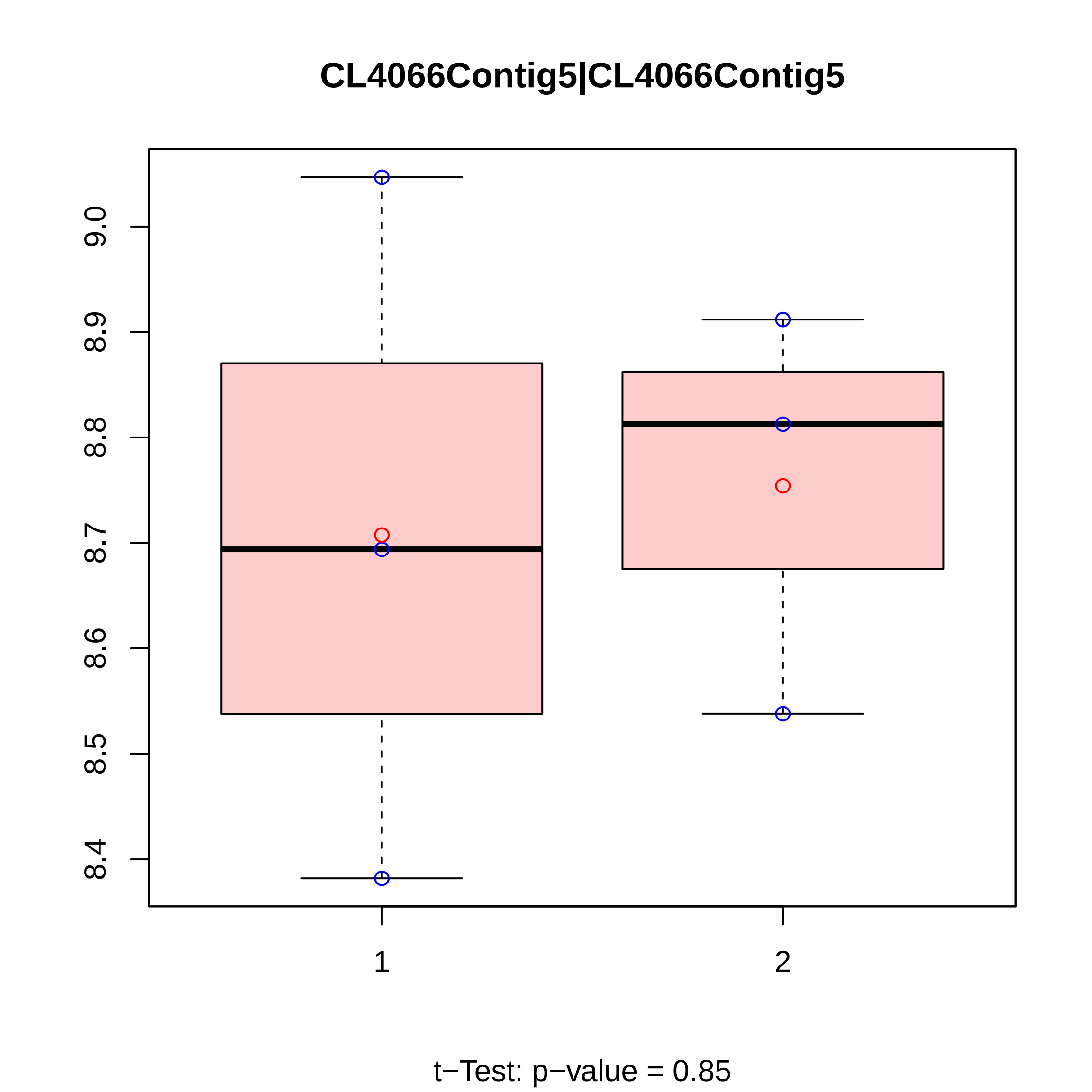

CL4066Contig5|CL4066Contig5
9.0
8.9
8.8
8.7
8.6
8.5
8.4
1
2
t−Test: p−value = 0.85

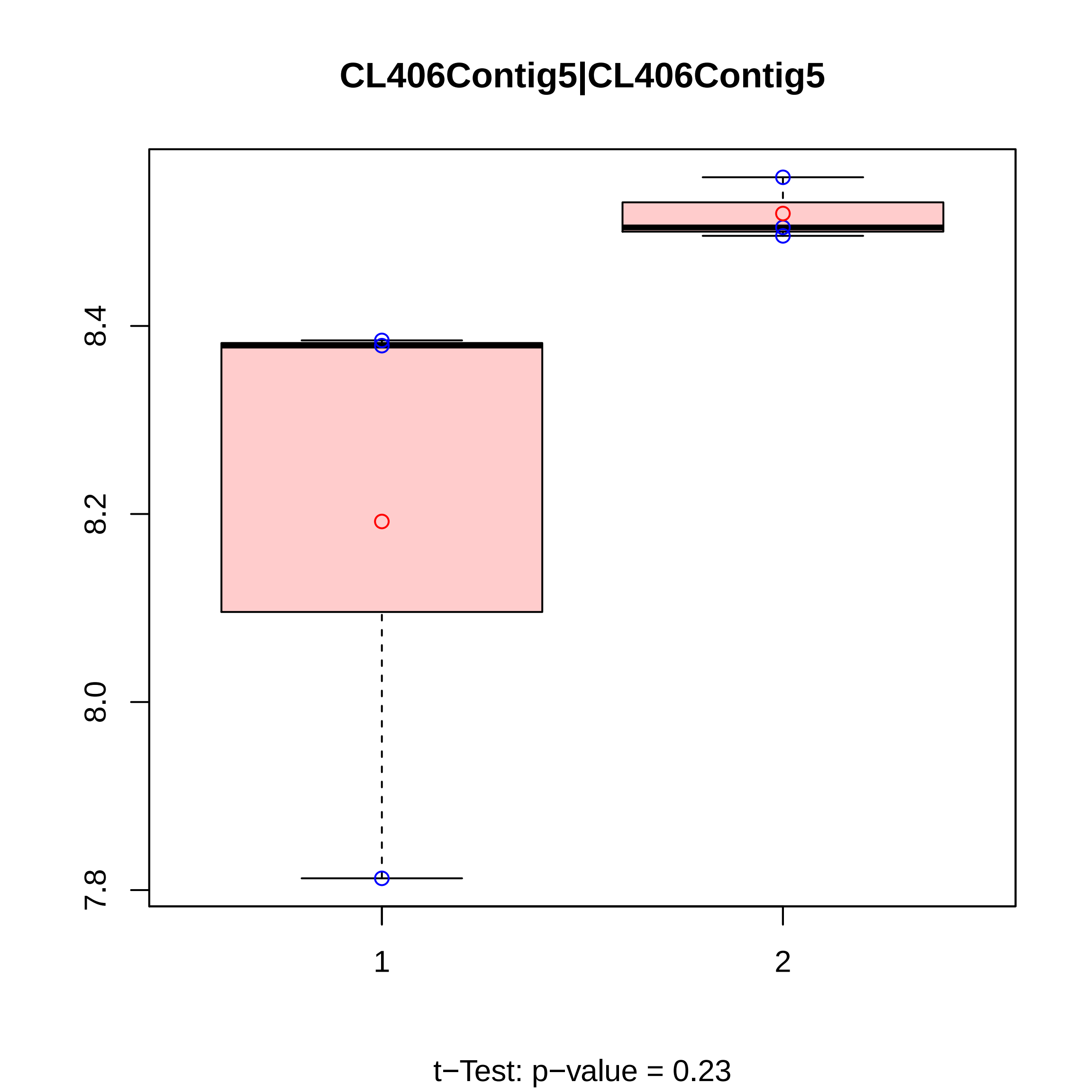
CL406Contig5|CL406Contig5
8.4
8.2
8.0
7.8
1
2
t−Test: p−value = 0.23

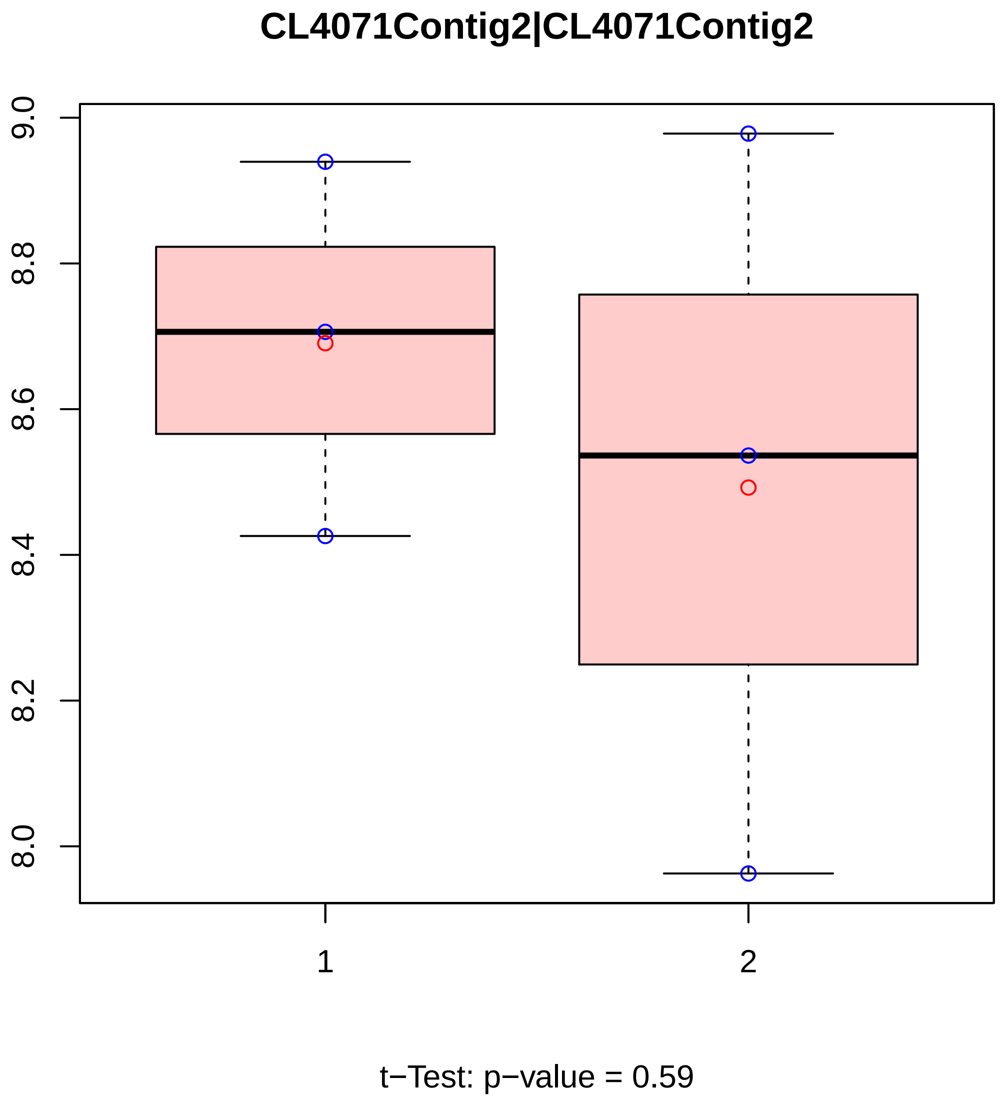
CL4071Contig2|CL4071Contig2
9.0
8.8
8.6
8.4
8.2
8.0
1
2
t−Test: p−value = 0.59

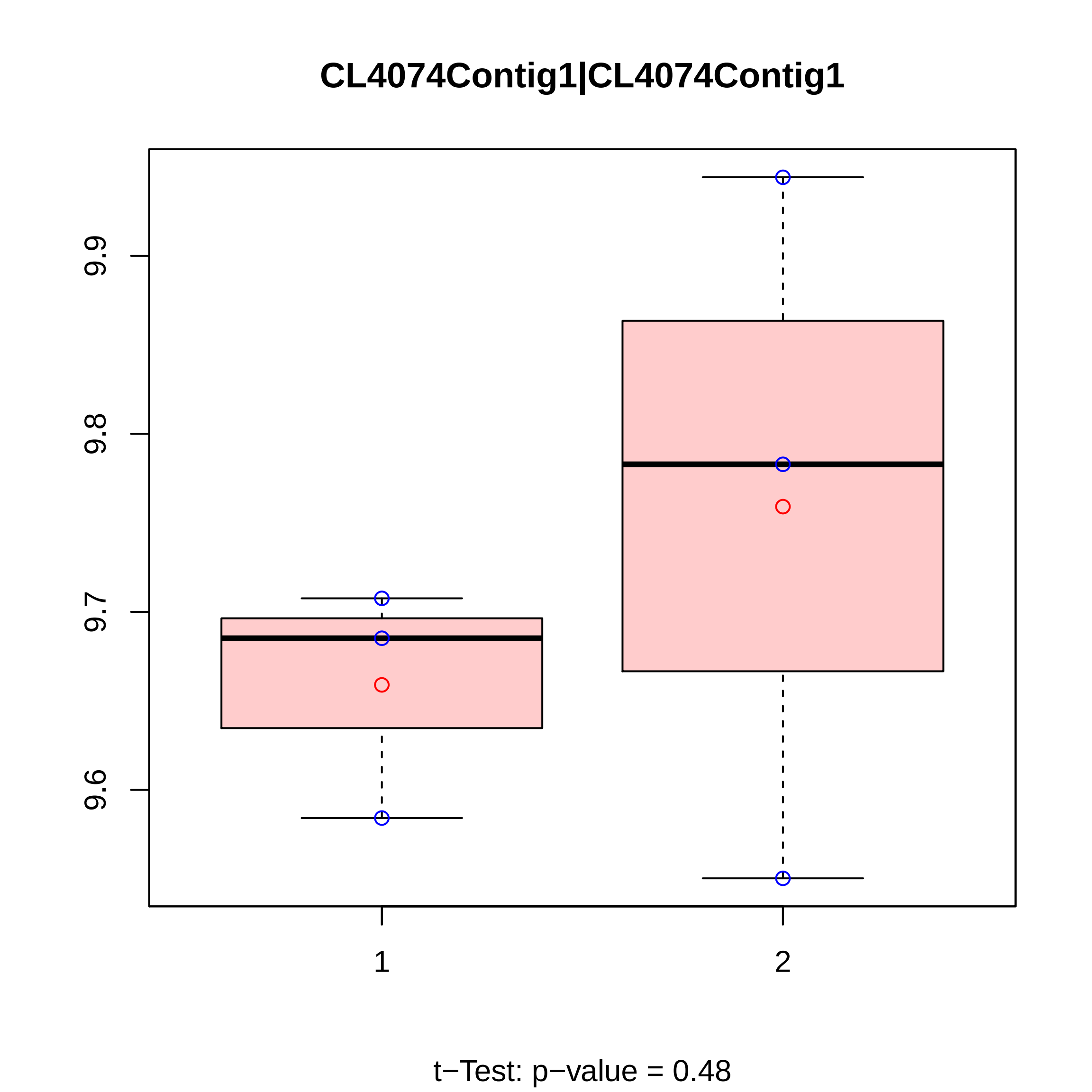

CL4074Contig1|CL4074Contig1
9.6
9.7
9.8
9.9
1
2
t−Test: p−value = 0.48

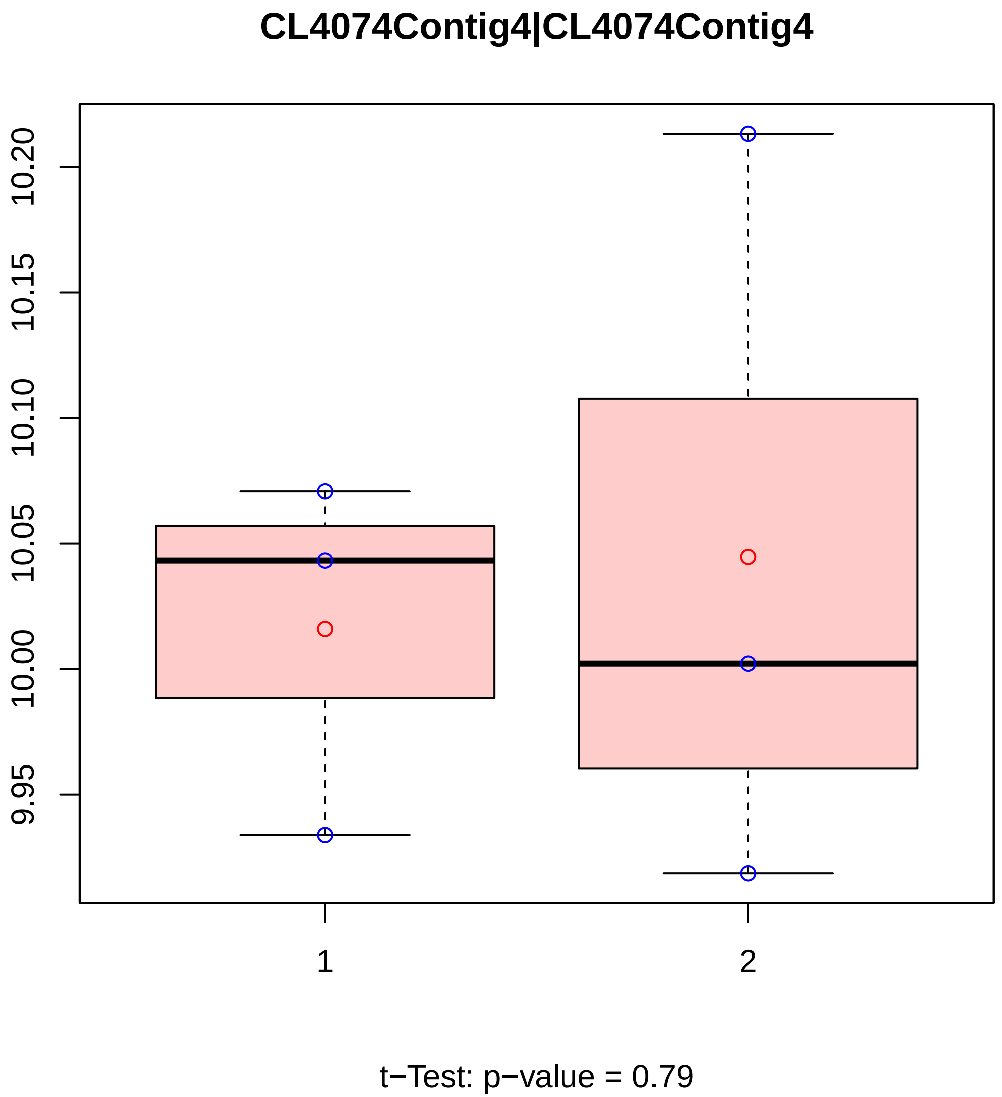
CL4074Contig4|CL4074Contig4
10.20
10.15
10.10
10.05
10.00
9.95
1
2
t−Test: p−value = 0.79

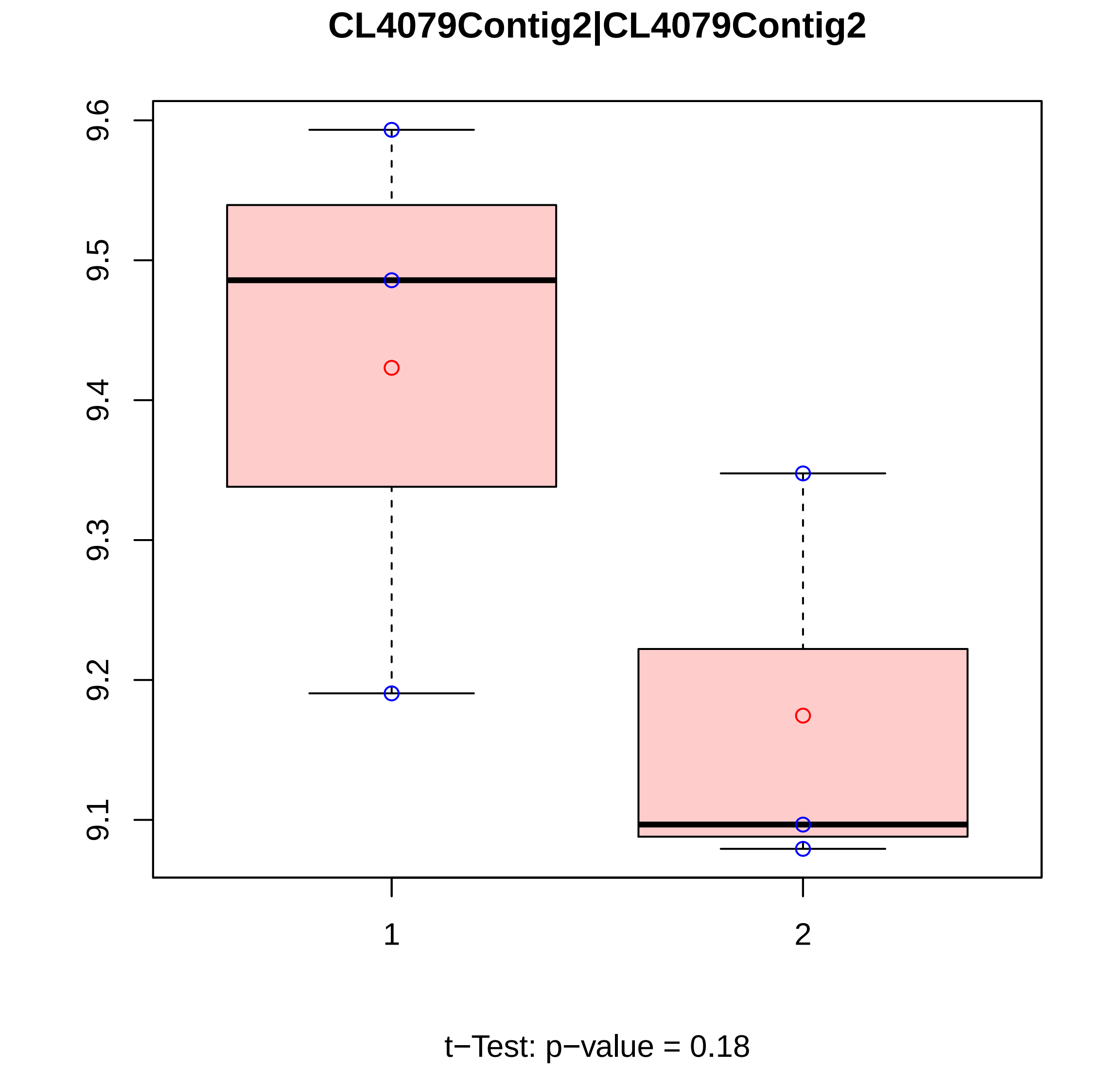

CL4079Contig2|CL4079Contig2
9.1
9.2
9.3
9.4
9.5
9.6
1
2
t−Test: p−value = 0.18

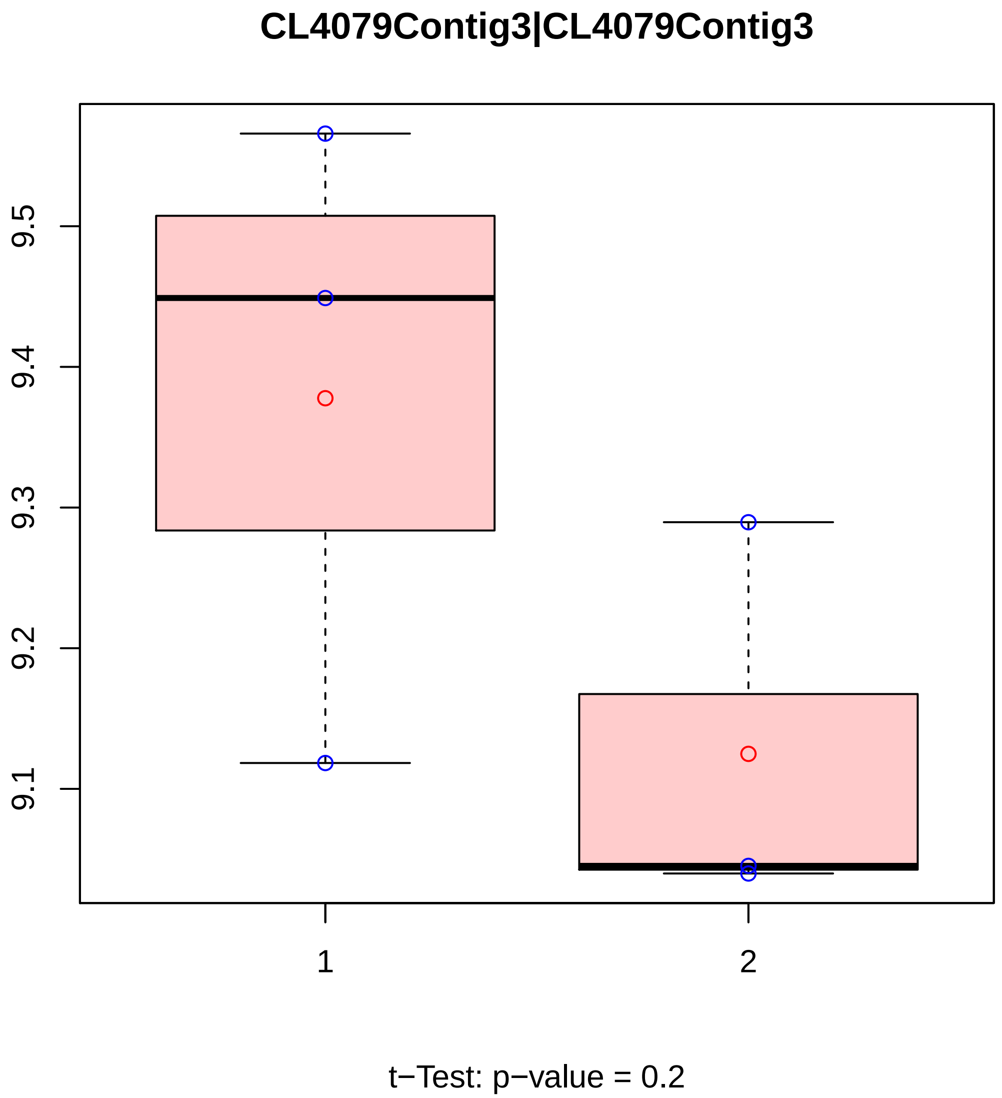

CL4079Contig3|CL4079Contig3
9.1
9.2
9.3
9.4
9.5
1
2
t−Test: p−value = 0.2

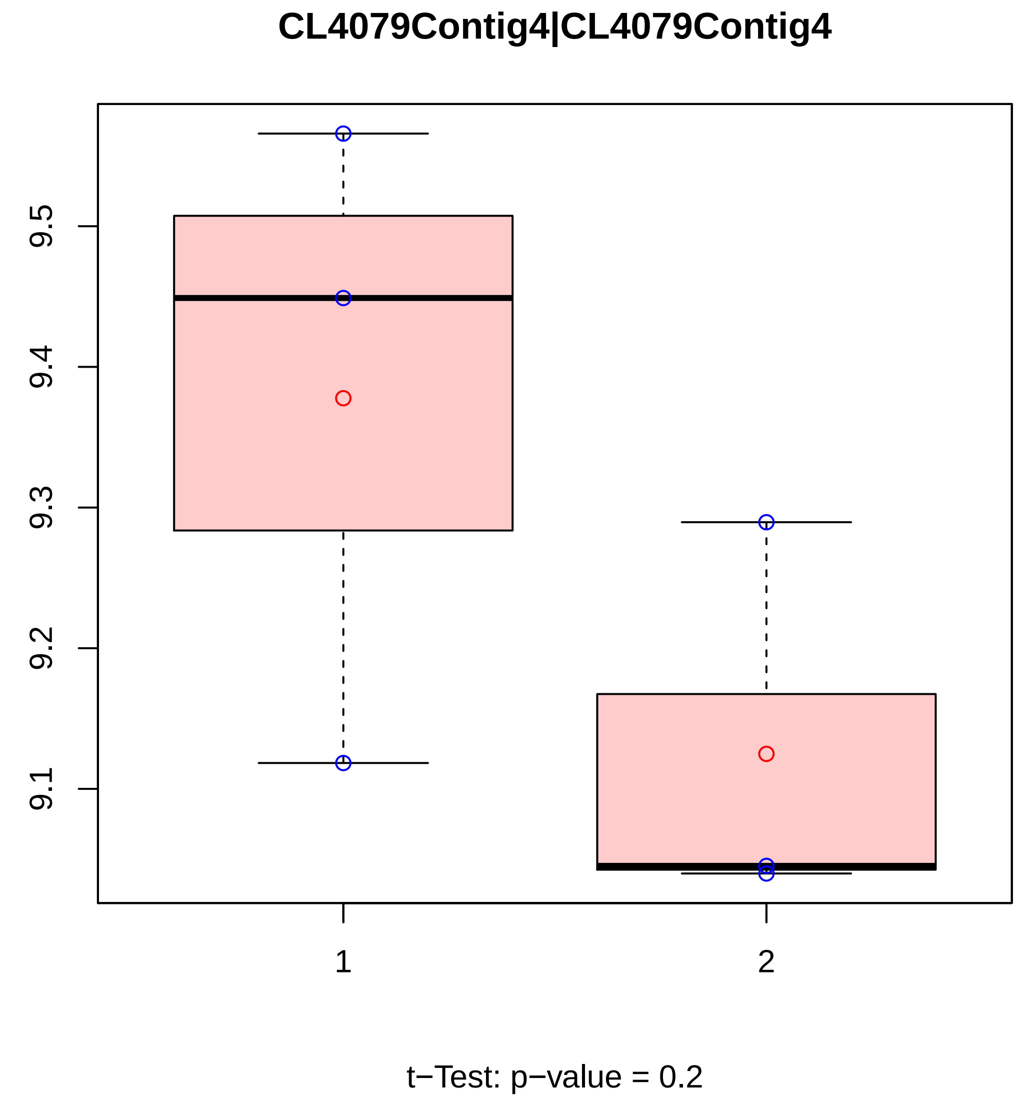
CL4079Contig4|CL4079Contig4
9.5
9.4
9.3
9.2
9.1
1
2
t−Test: p−value = 0.2

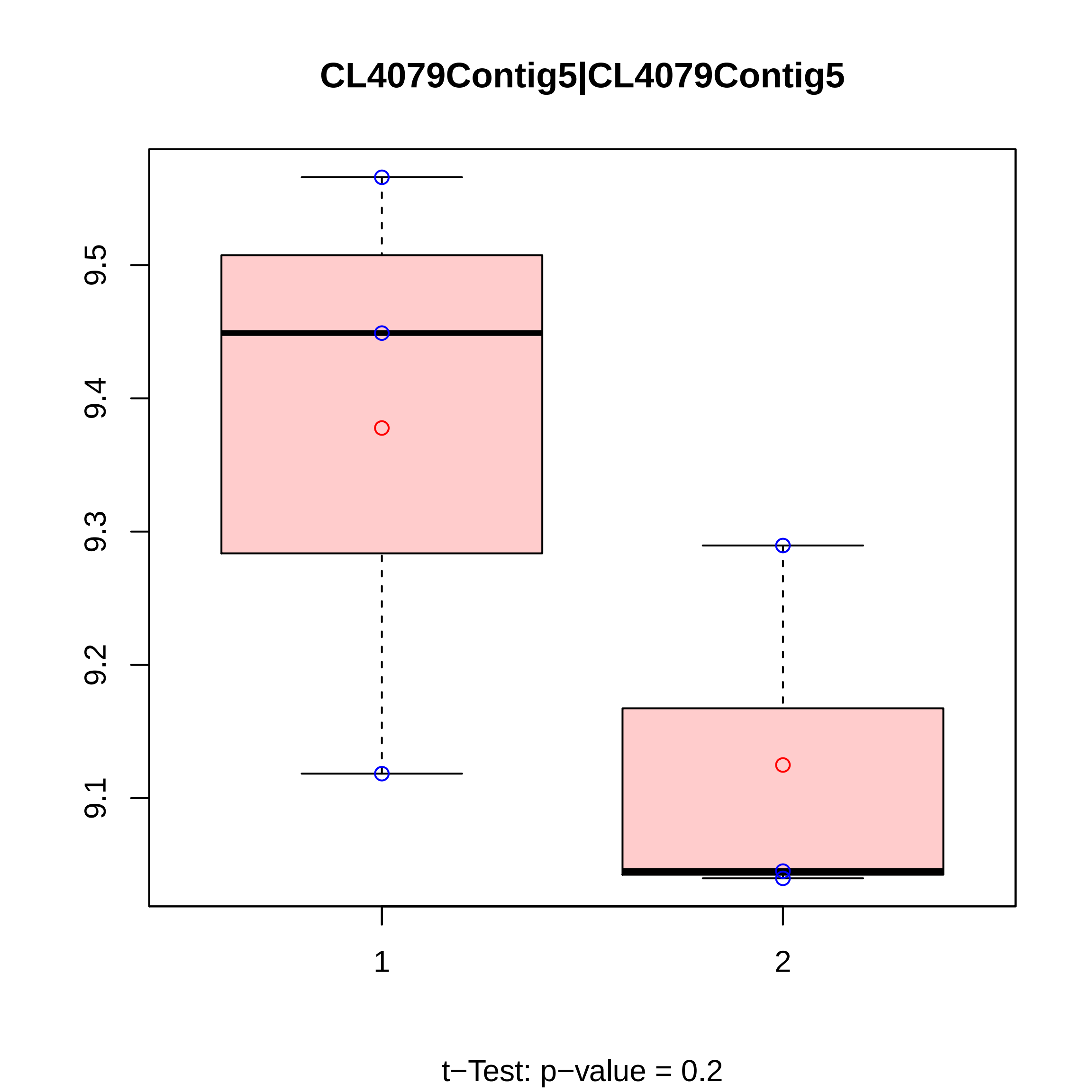
CL4079Contig5|CL4079Contig5
9.1
9.2
9.3
9.4
9.5
1
2
t−Test: p−value = 0.2

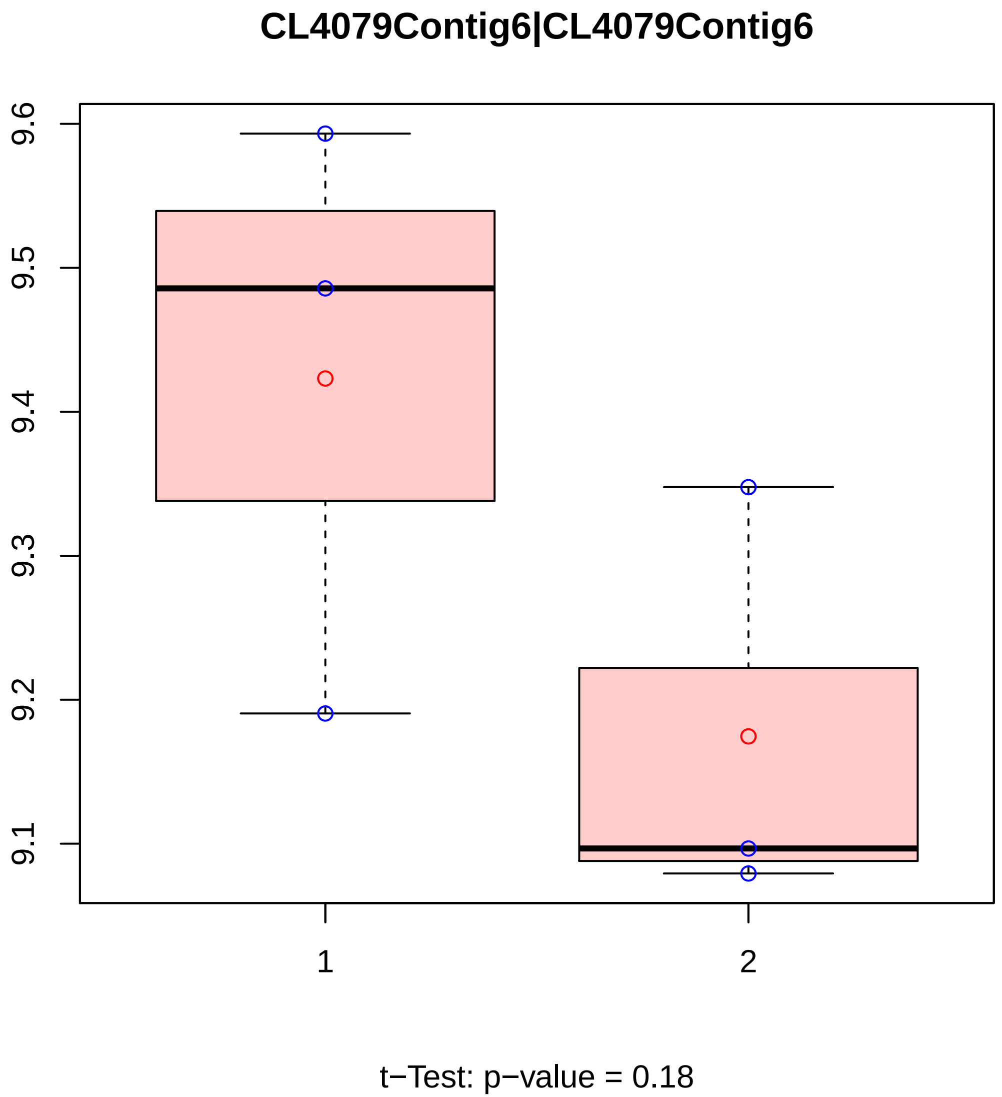

CL4079Contig6|CL4079Contig6
9.6
9.5
9.4
9.3
9.2
9.1
1
2
t−Test: p−value = 0.18

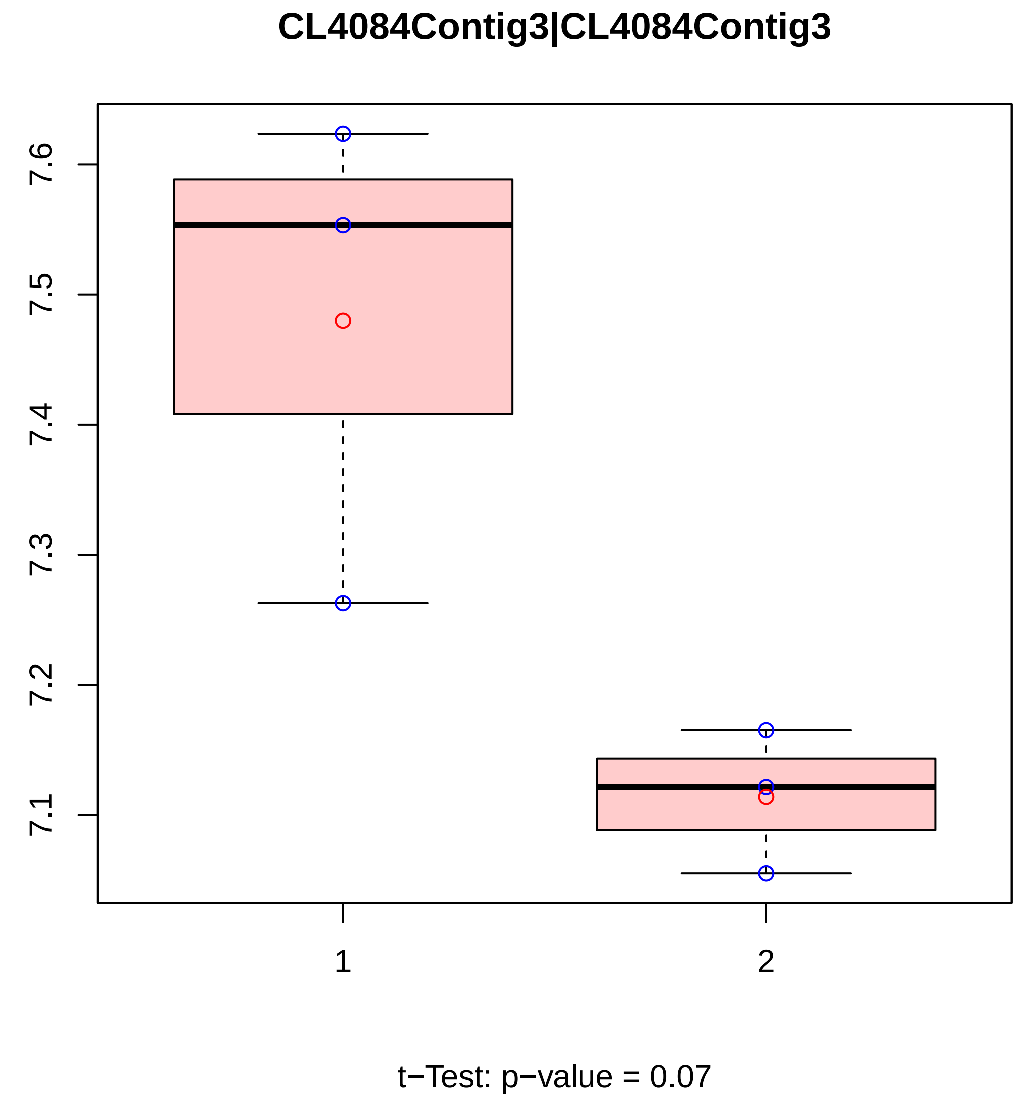
CL4084Contig3|CL4084Contig3
7.1
7.2
7.3
7.4
7.5
7.6
1
2
t−Test: p−value = 0.07

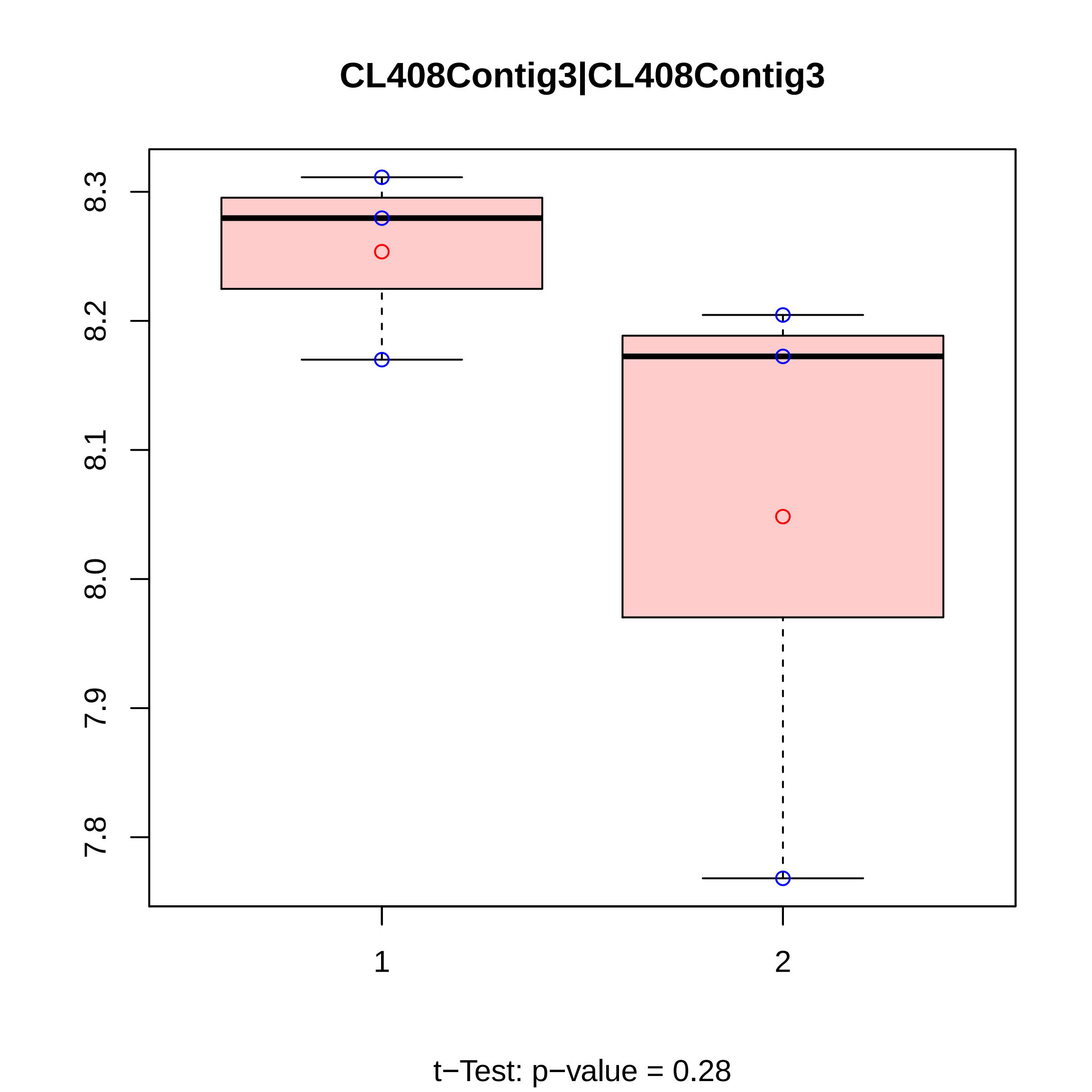
CL408Contig3|CL408Contig3
8.3
8.2
8.1
8.0
7.9
7.8
1
2
t−Test: p−value = 0.28

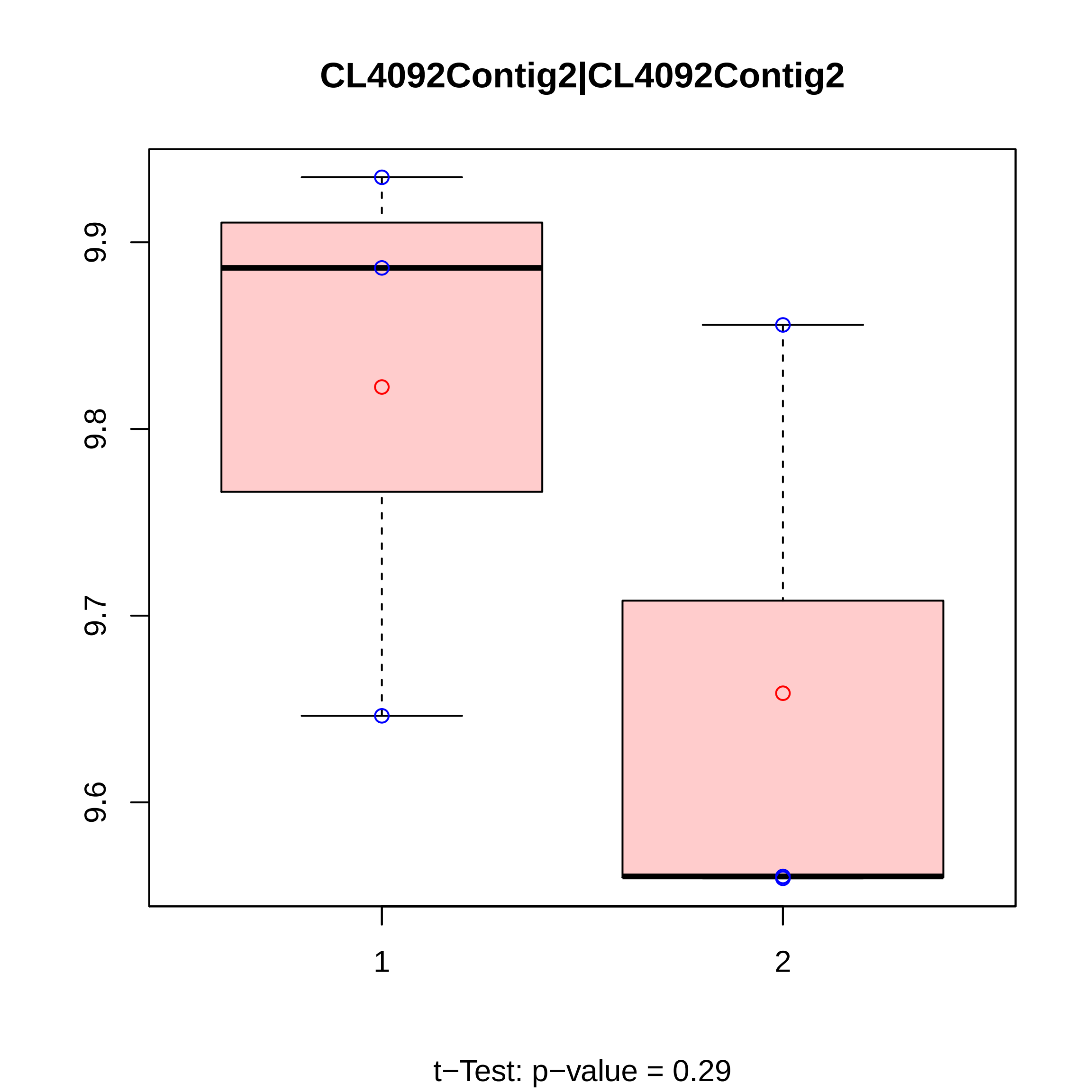
CL4092Contig2|CL4092Contig2
9.9
9.8
9.7
9.6
1
2
t−Test: p−value = 0.29

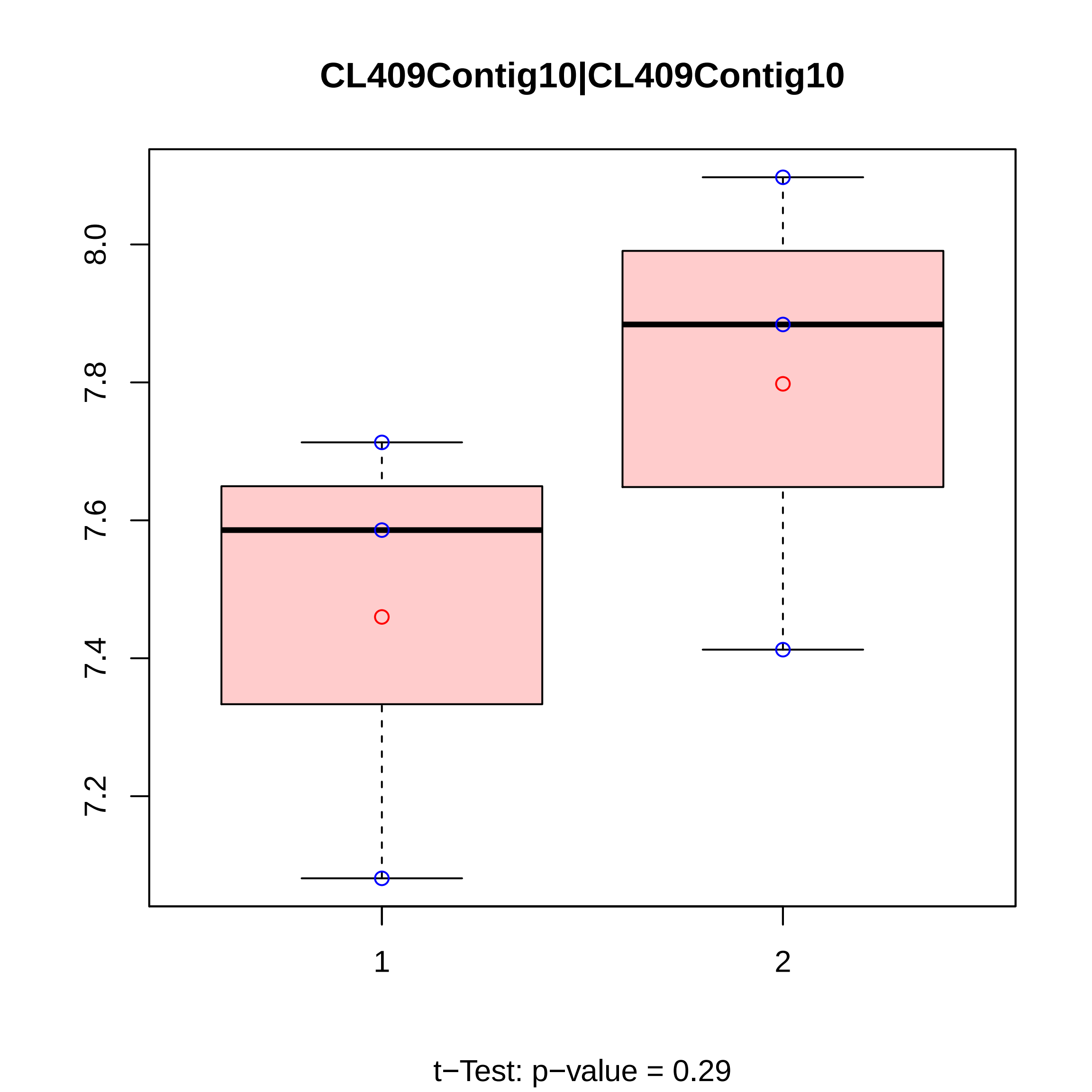
CL409Contig10|CL409Contig10
8.0
7.8
7.6
7.4
7.2
1
2
t−Test: p−value = 0.29

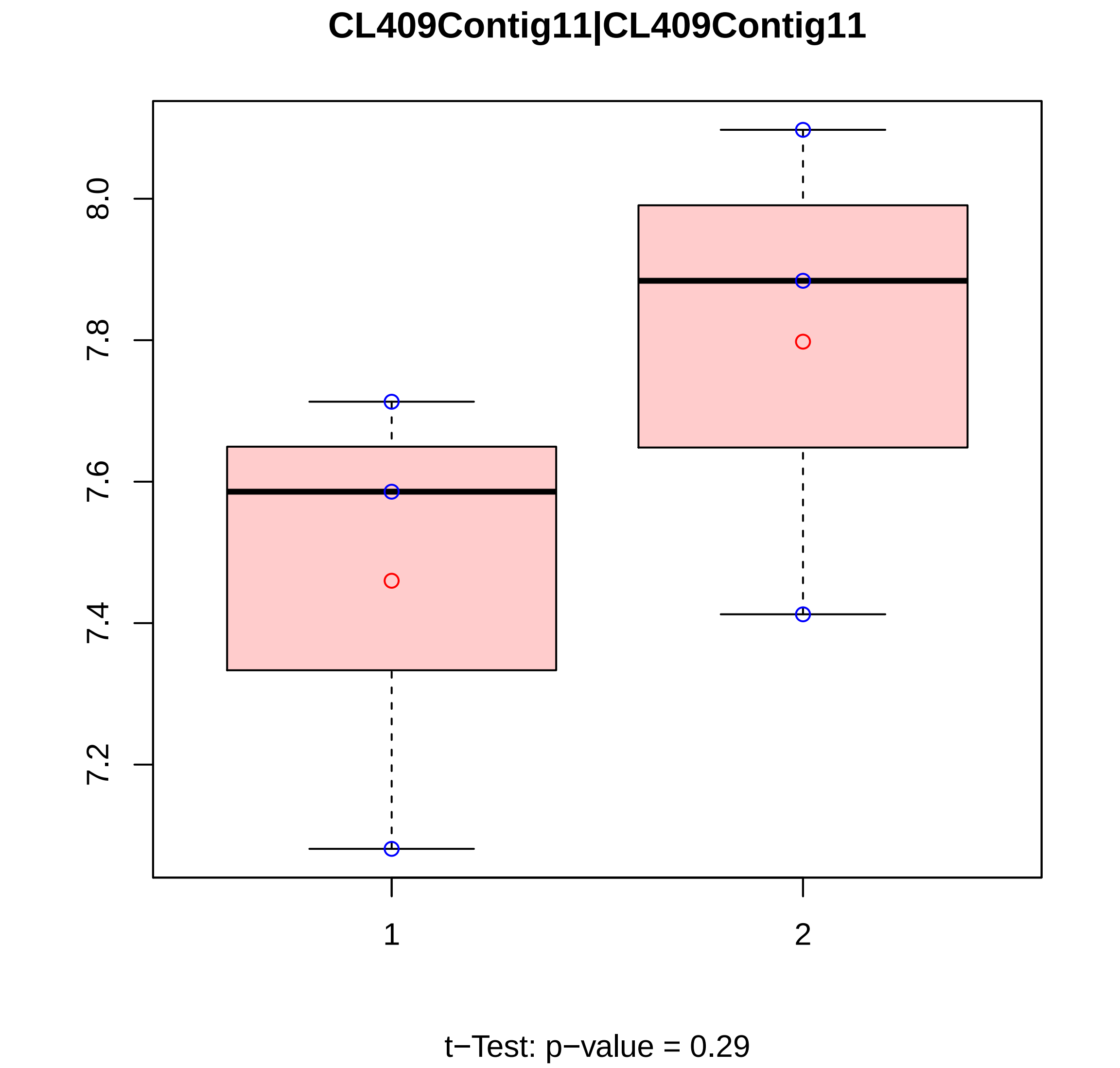
CL409Contig11|CL409Contig11
8.0
7.8
7.6
7.4
7.2
1
2
t−Test: p−value = 0.29

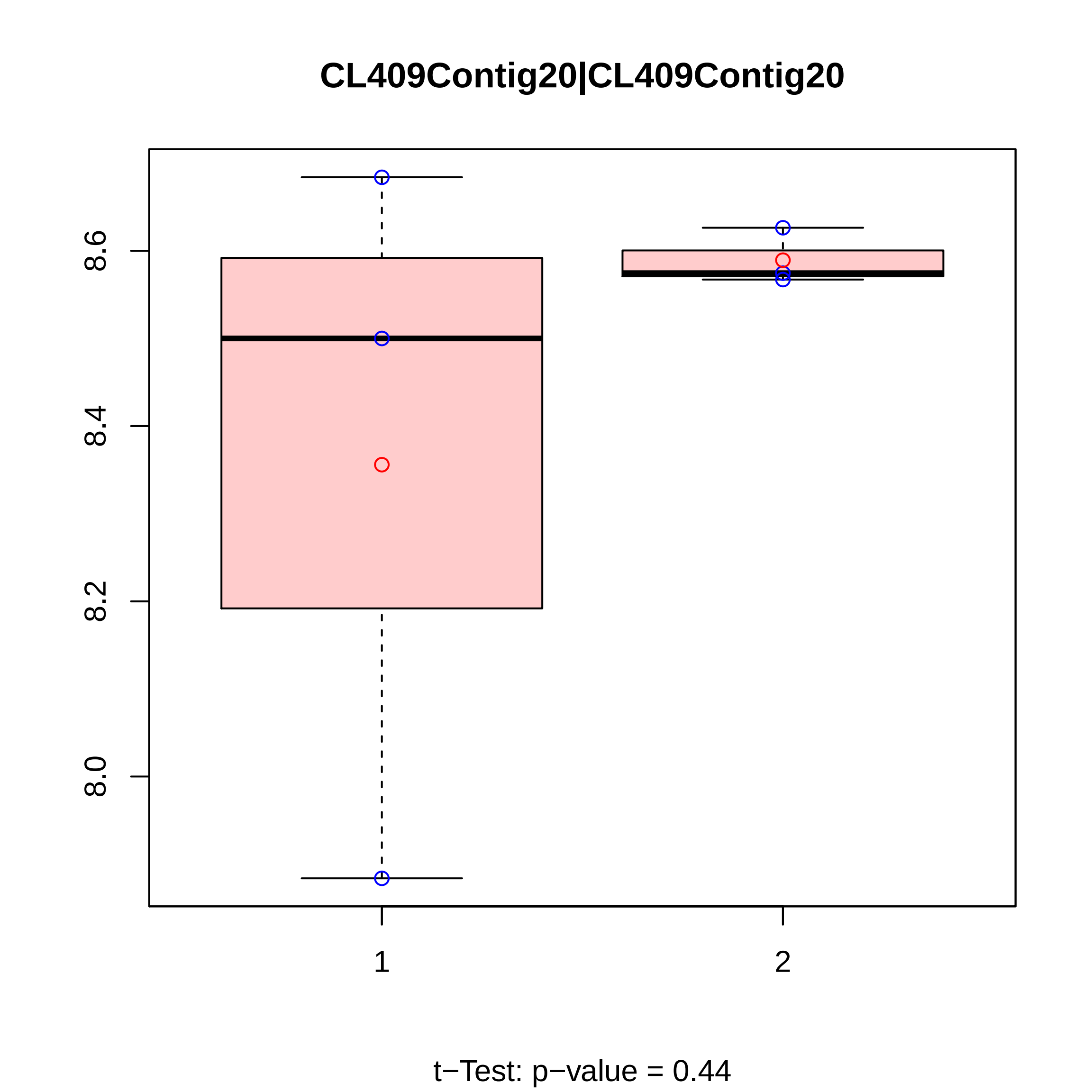
CL409Contig20|CL409Contig20
8.0
8.2
8.4
8.6
1
2
t−Test: p−value = 0.44

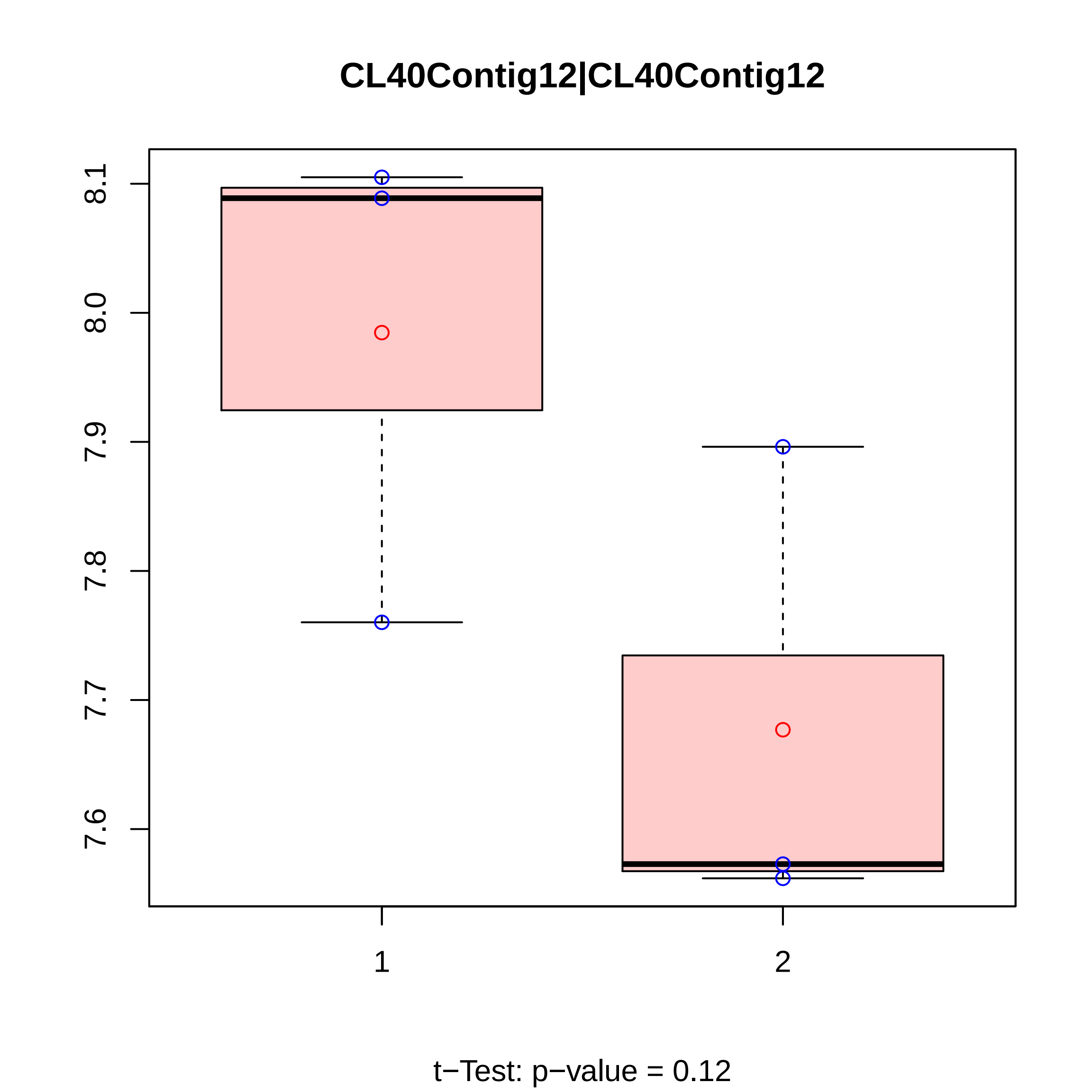
CL40Contig12|CL40Contig12
8.1
8.0
7.9
7.8
7.7
7.6
1
2
t−Test: p−value = 0.12

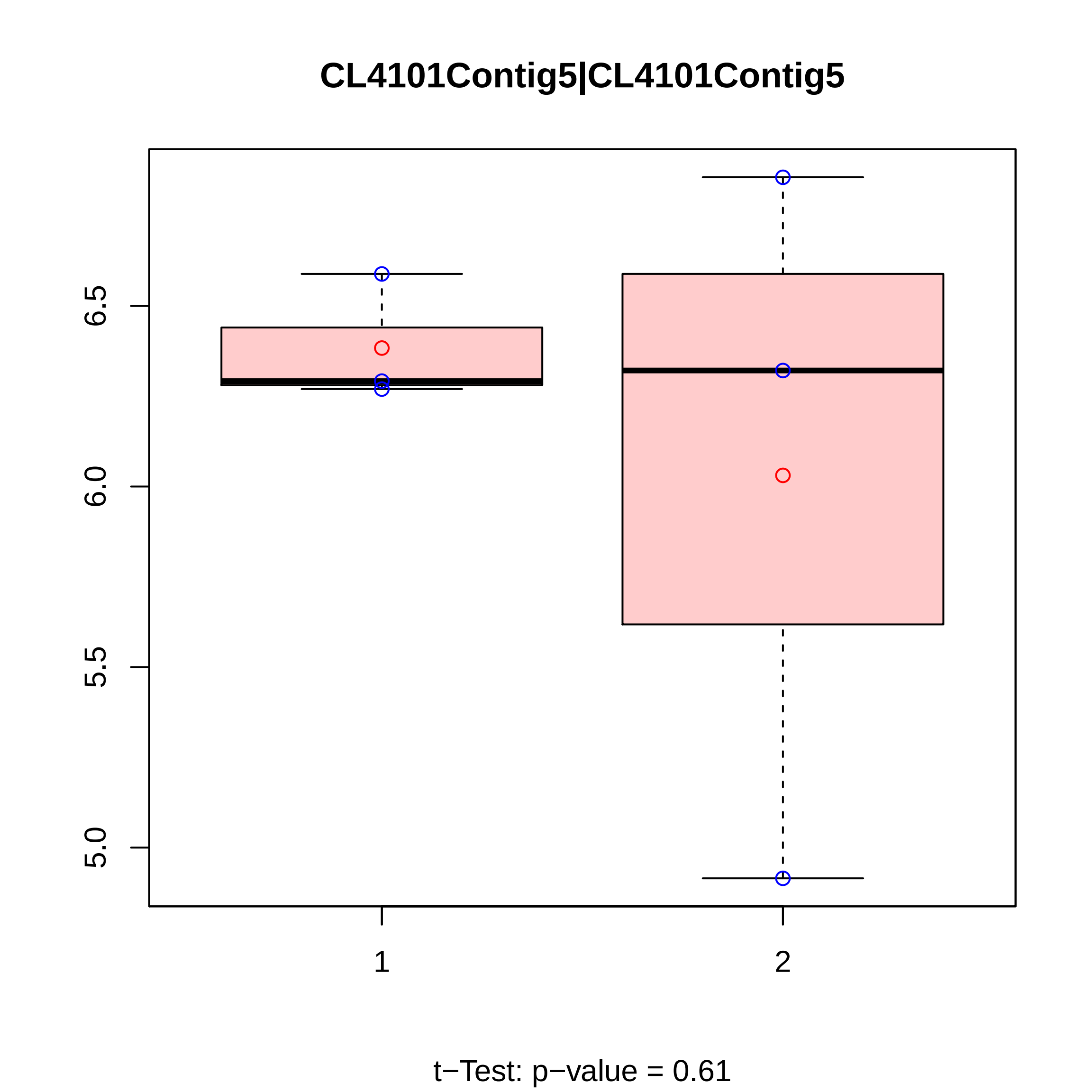
CL4101Contig5|CL4101Contig5
6.5
6.0
5.5
5.0
1
2
t−Test: p−value = 0.61

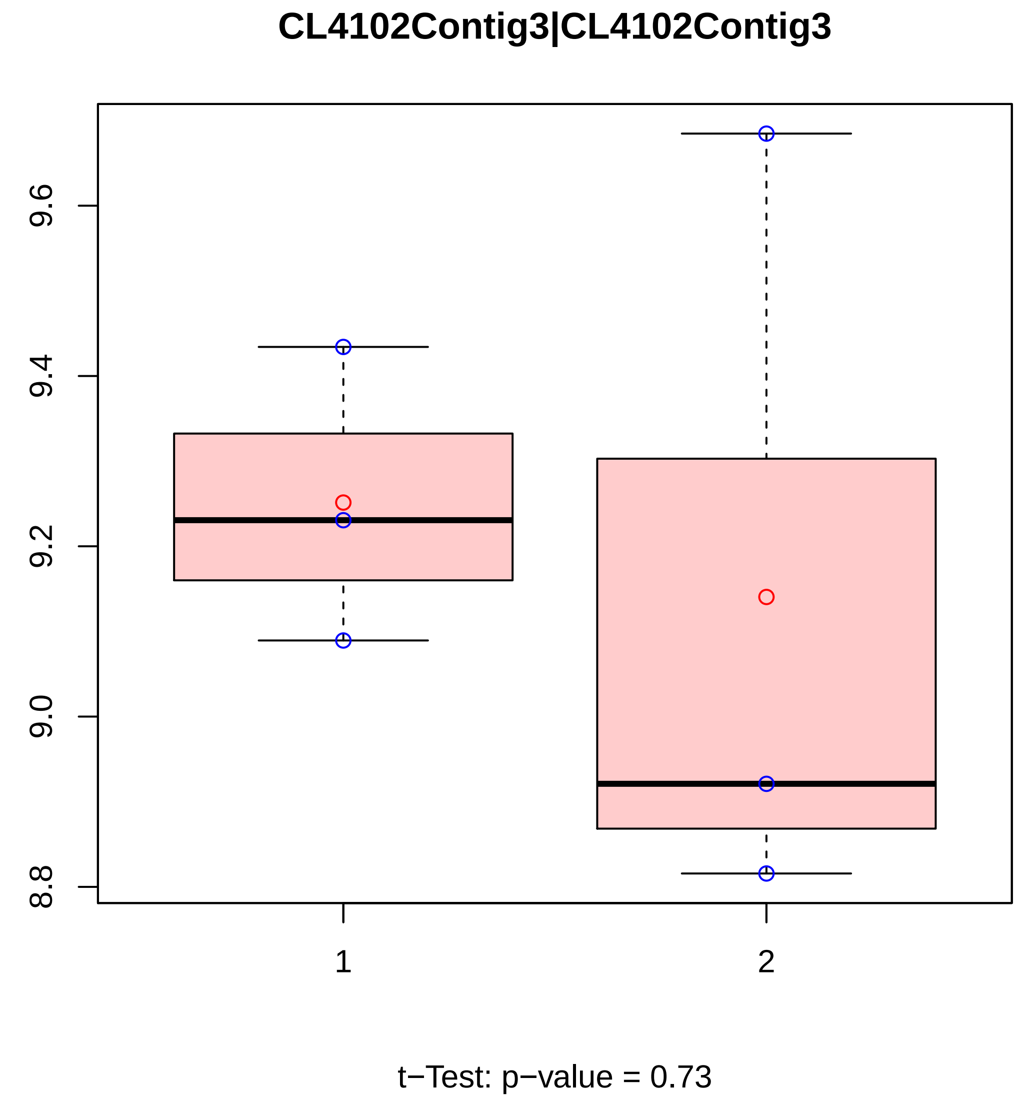
CL4102Contig3|CL4102Contig3
9.6
9.4
9.2
9.0
8.8
1
2
t−Test: p−value = 0.73

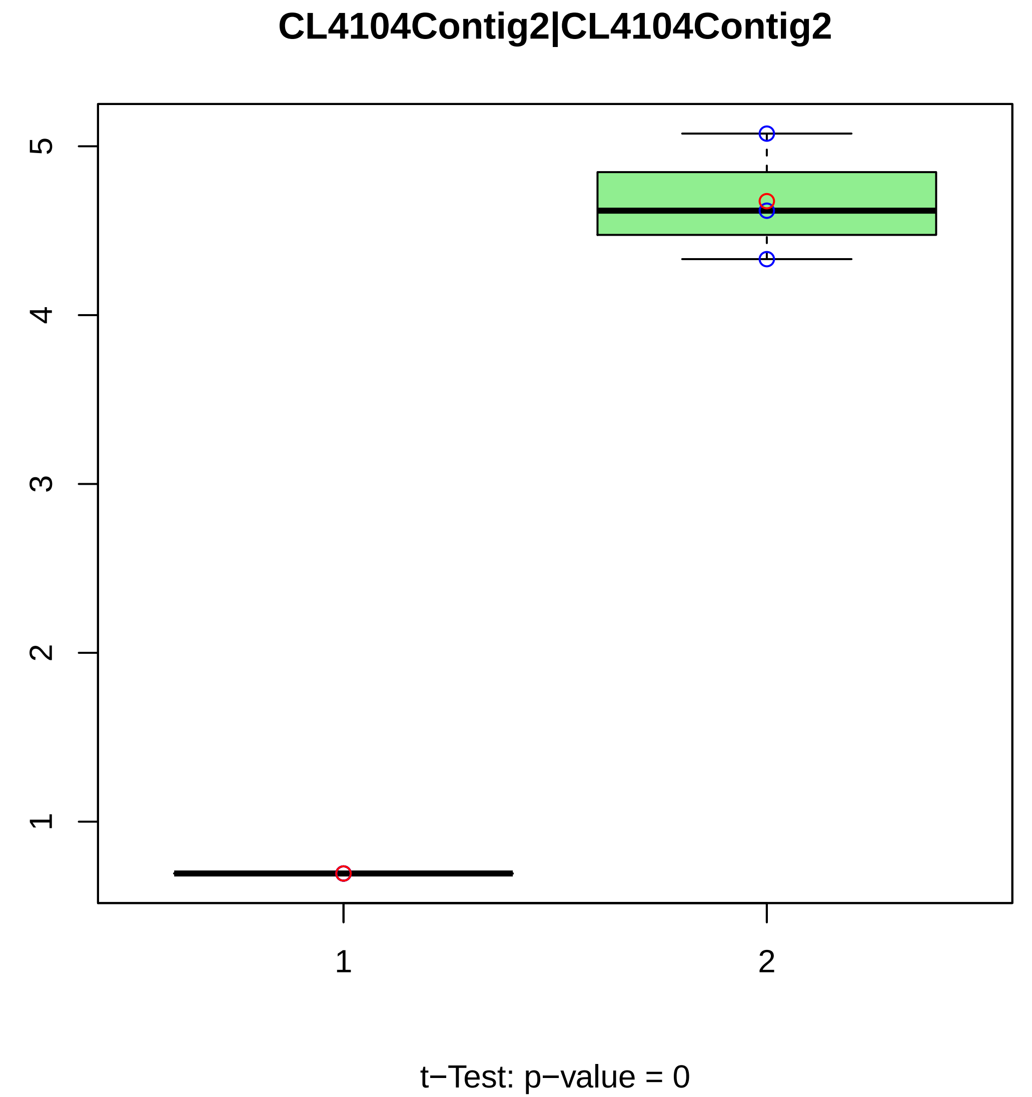
CL4104Contig2|CL4104Contig2
1
2
3
4
5
1
2
t−Test: p−value = 0

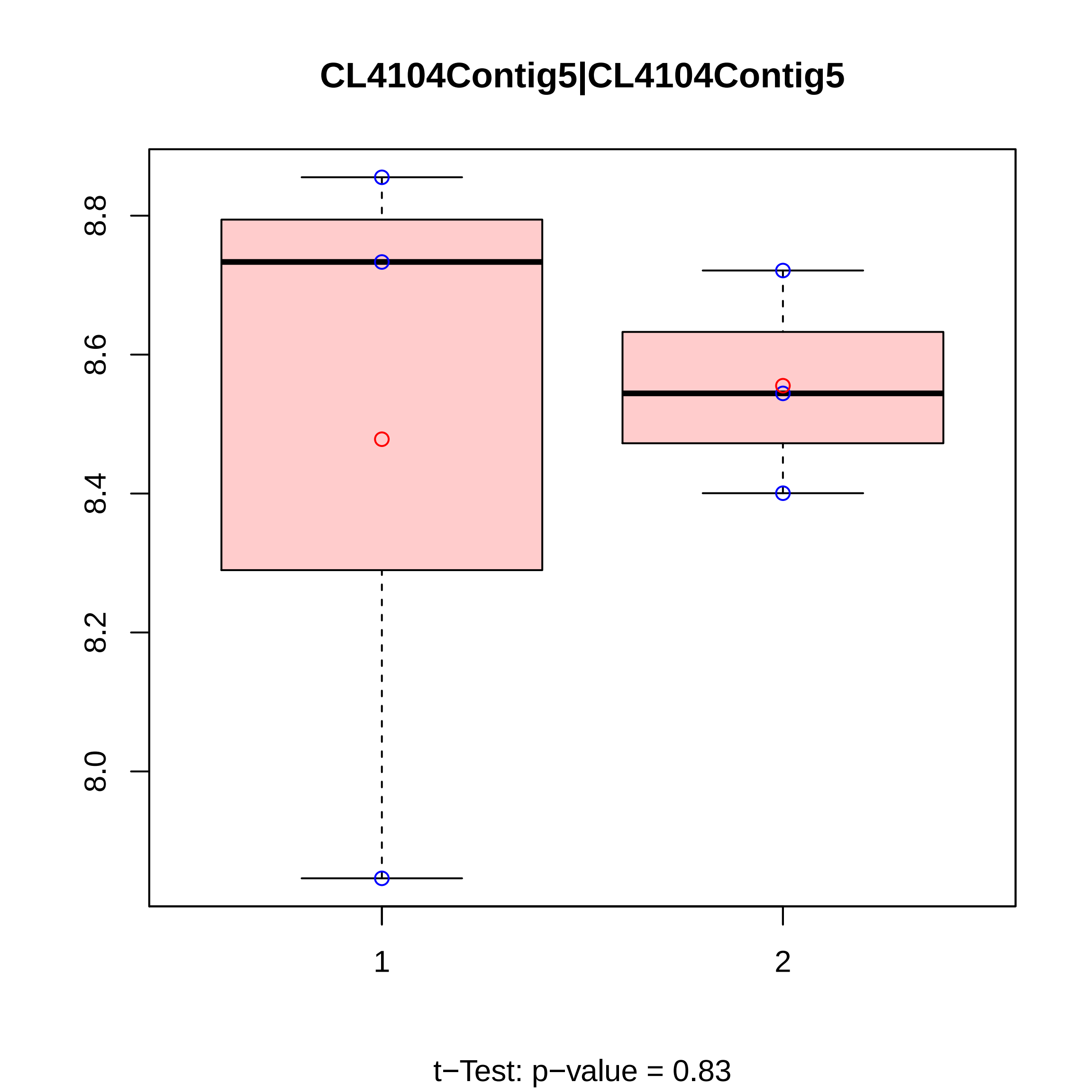
CL4104Contig5|CL4104Contig5
8.0
8.2
8.4
8.6
8.8
1
2
t−Test: p−value = 0.83

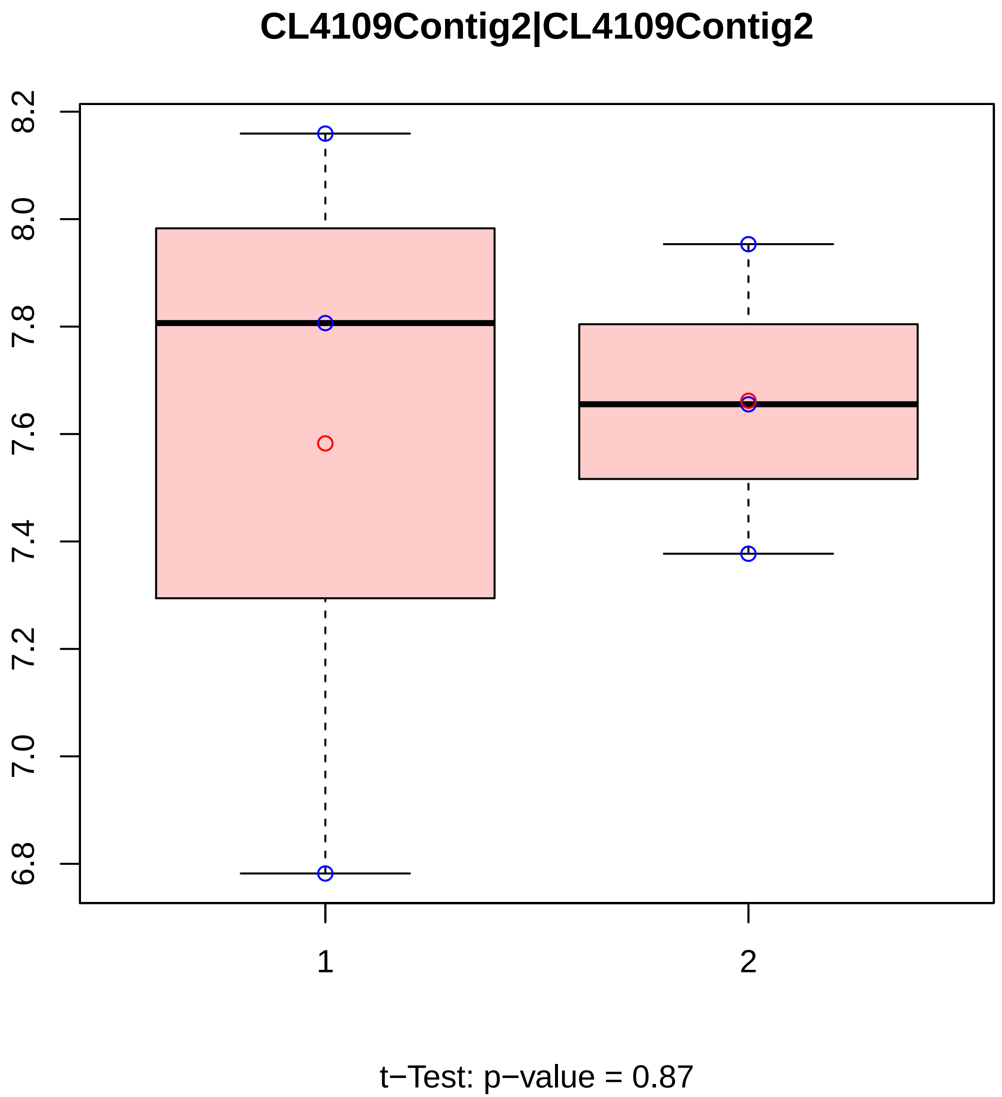
CL4109Contig2|CL4109Contig2
8.2
8.0
7.8
7.6
7.4
7.2
7.0
6.8
1
2
t−Test: p−value = 0.87

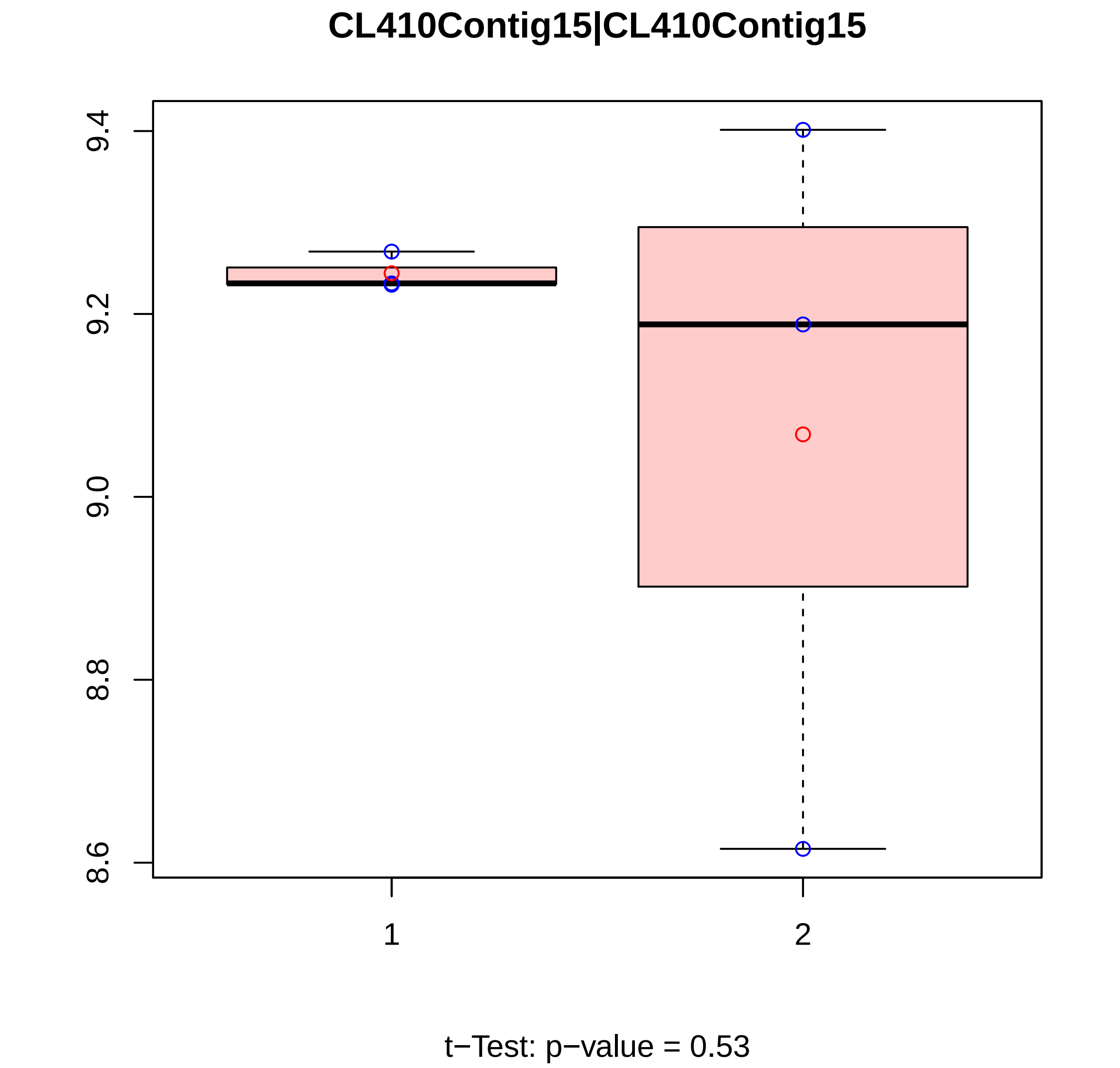
CL410Contig15|CL410Contig15
9.4
9.2
9.0
8.8
8.6
1
2
t−Test: p−value = 0.53

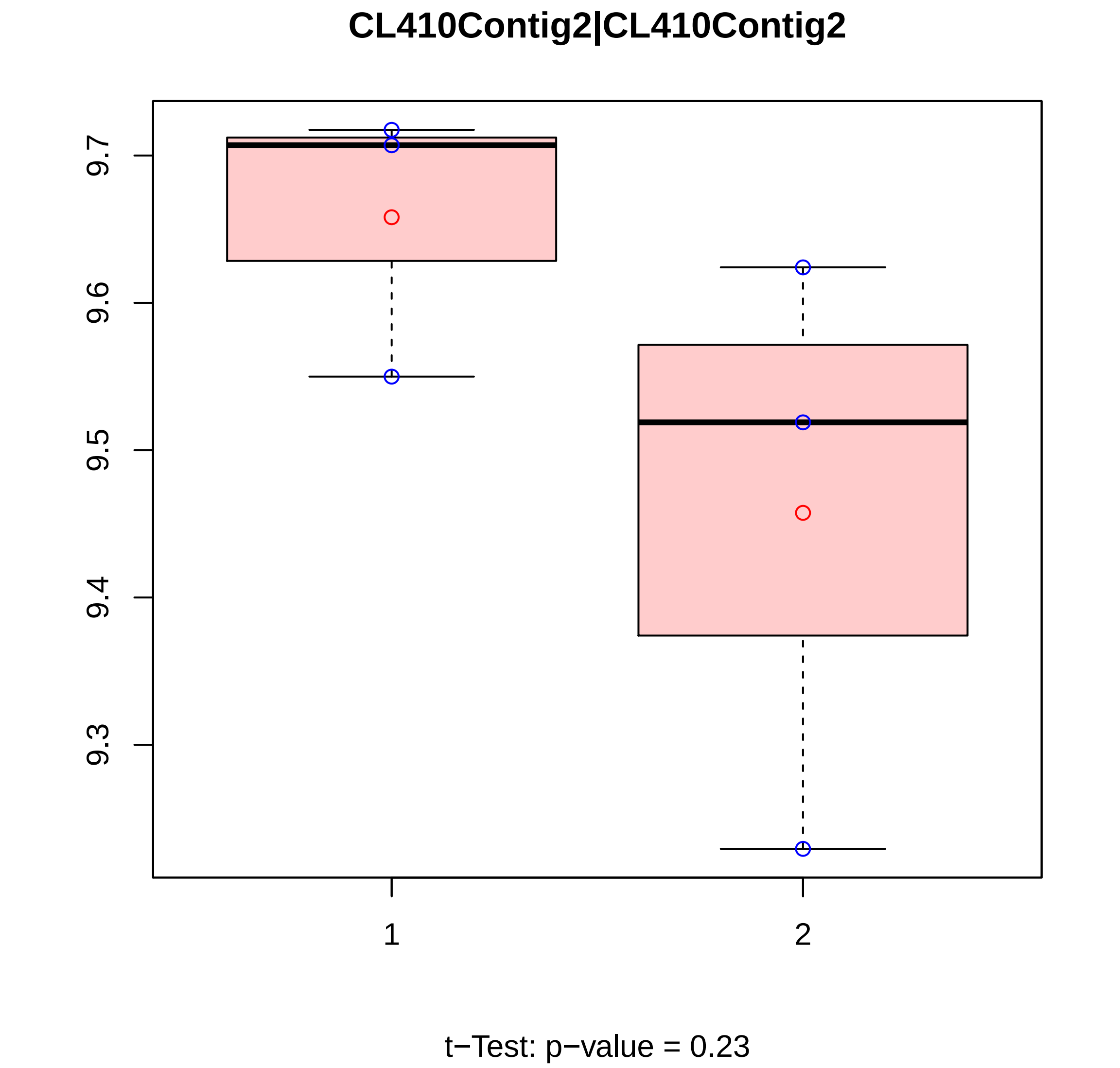
CL410Contig2|CL410Contig2
9.3
9.4
9.5
9.6
9.7
1
2
t−Test: p−value = 0.23

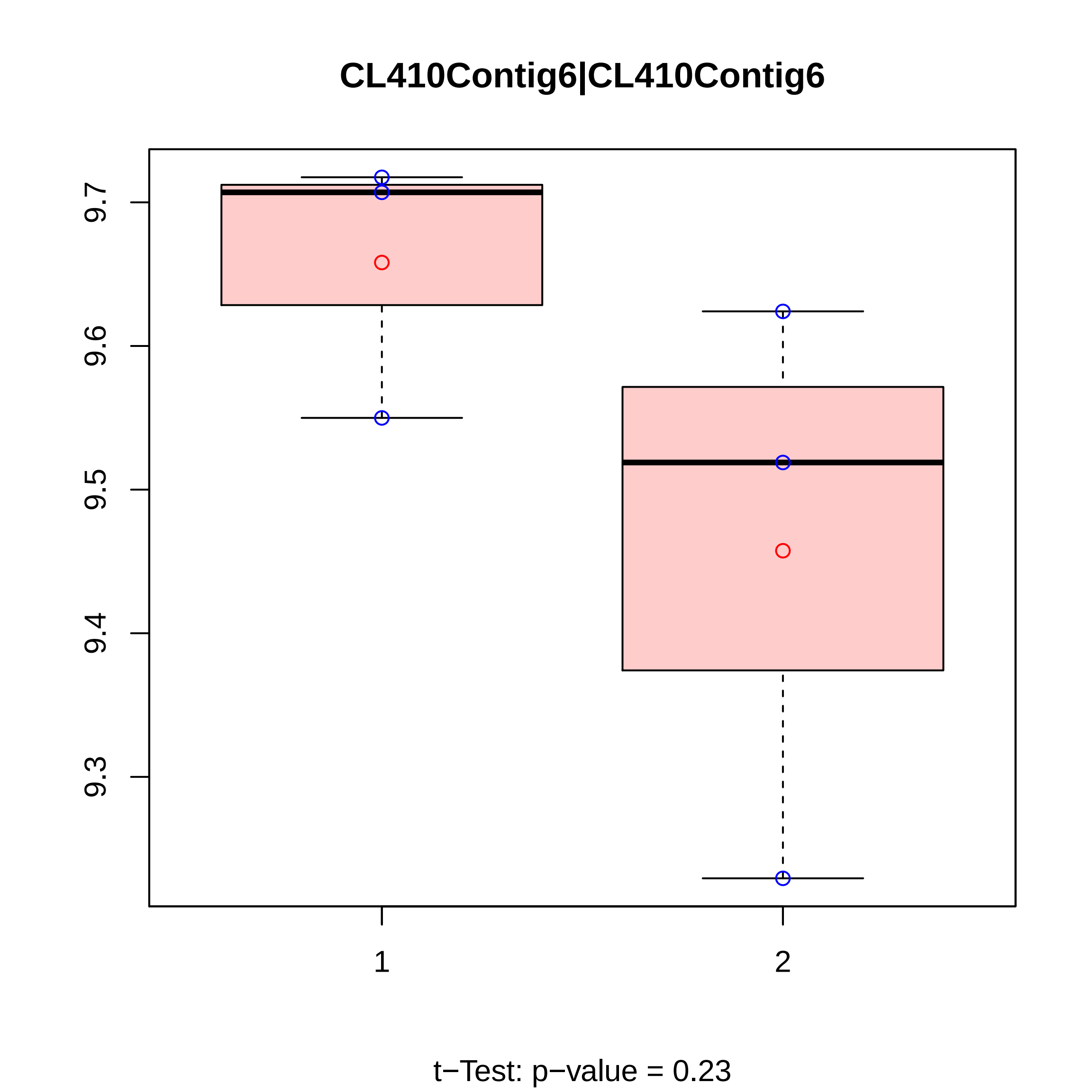

CL410Contig6|CL410Contig6
9.3
9.4
9.5
9.6
9.7
1
2
t−Test: p−value = 0.23

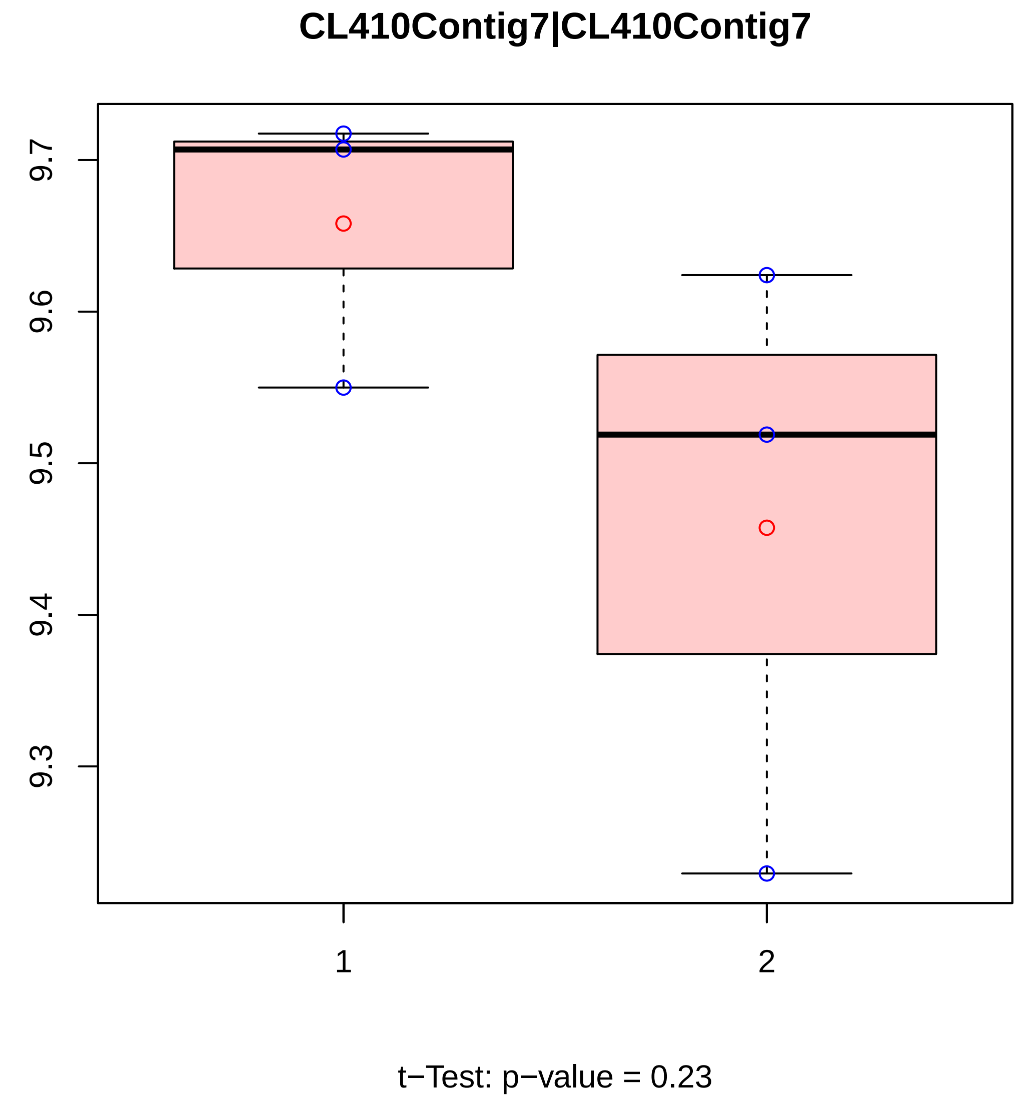
CL410Contig7|CL410Contig7
9.3
9.4
9.5
9.6
9.7
1
2
t−Test: p−value = 0.23

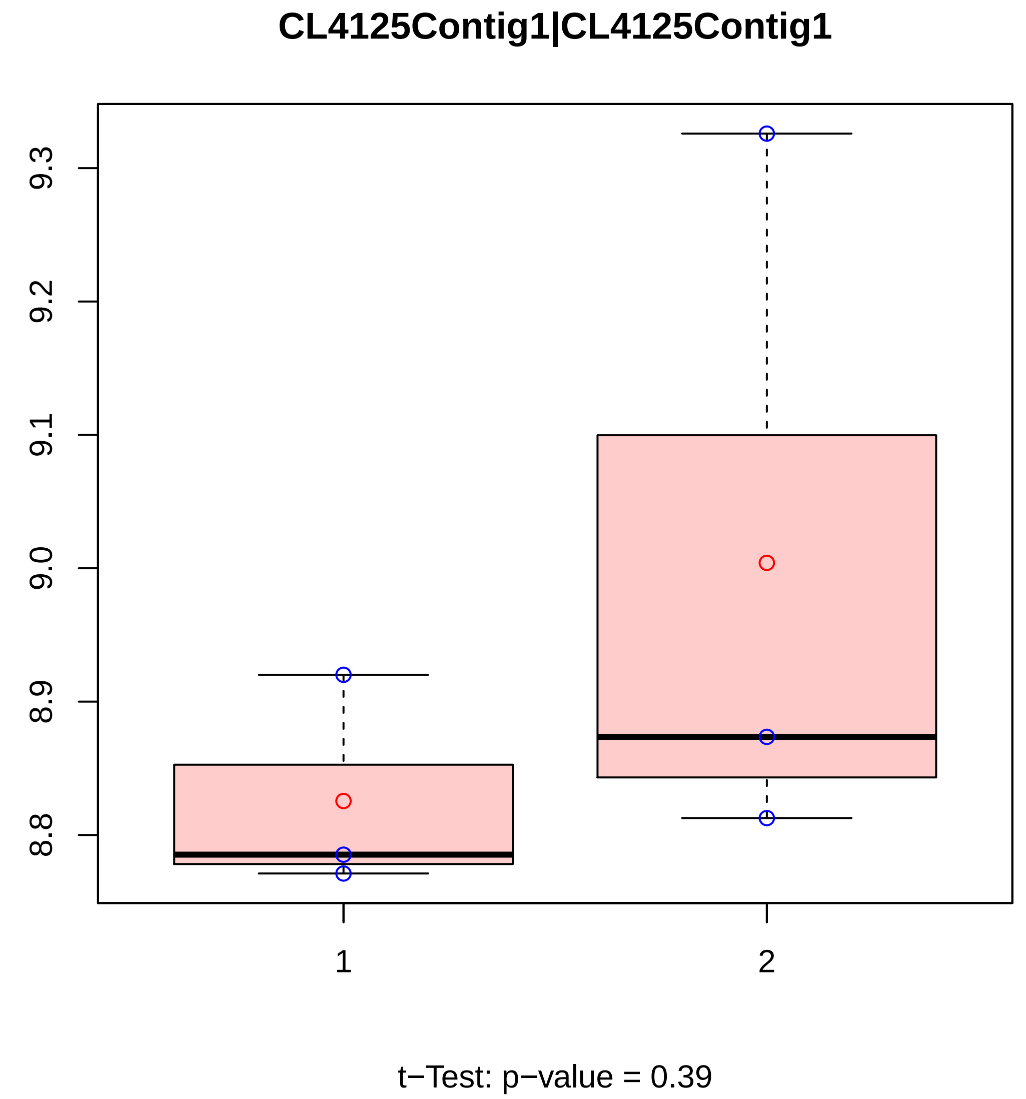

CL4125Contig1|CL4125Contig1
9.3
9.2
9.1
9.0
8.9
8.8
1
2
t−Test: p−value = 0.39

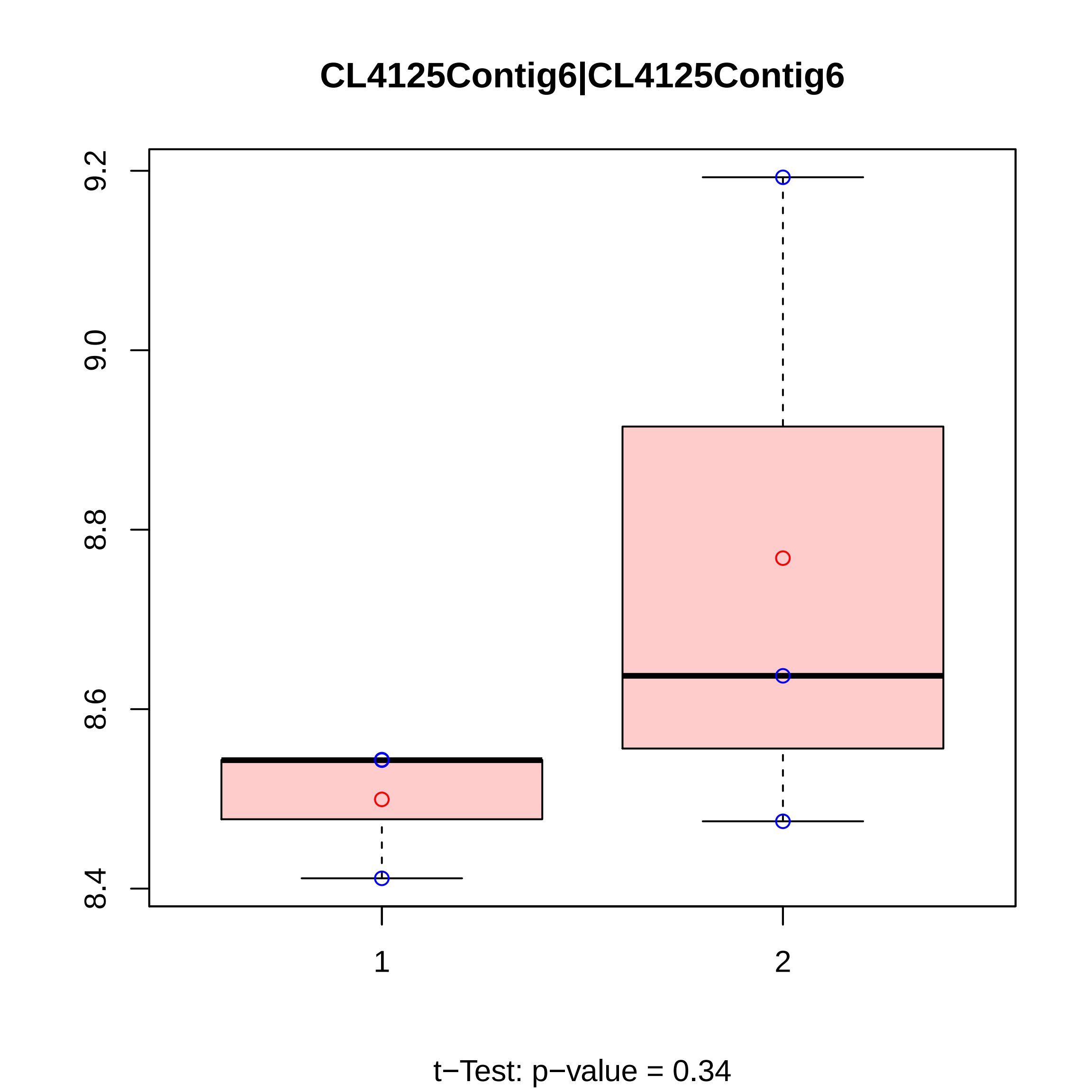

CL4125Contig6|CL4125Contig6
9.2
9.0
8.8
8.6
8.4
1
2
t−Test: p−value = 0.34

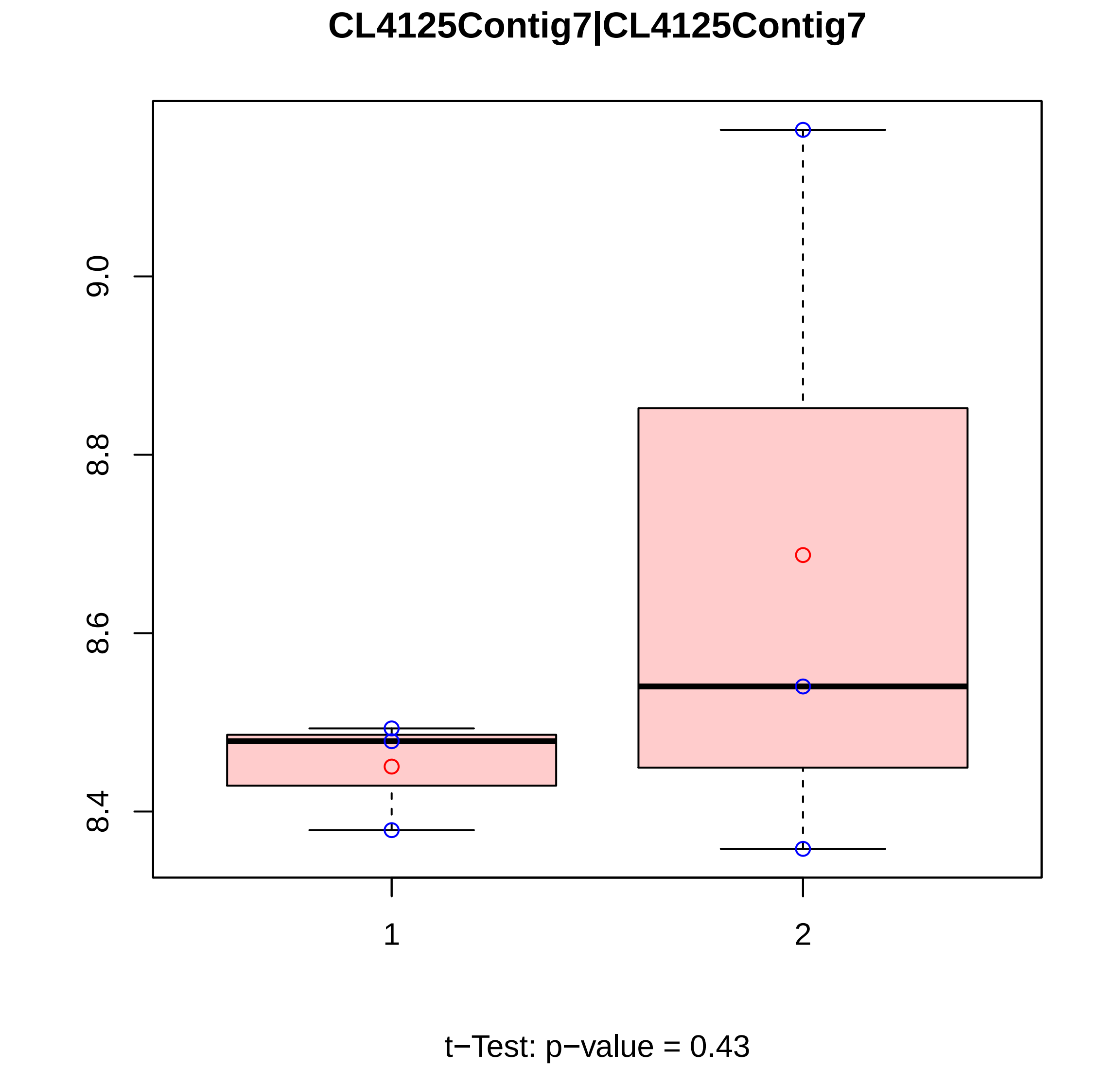
CL4125Contig7|CL4125Contig7
9.0
8.8
8.6
8.4
1
2
t−Test: p−value = 0.43

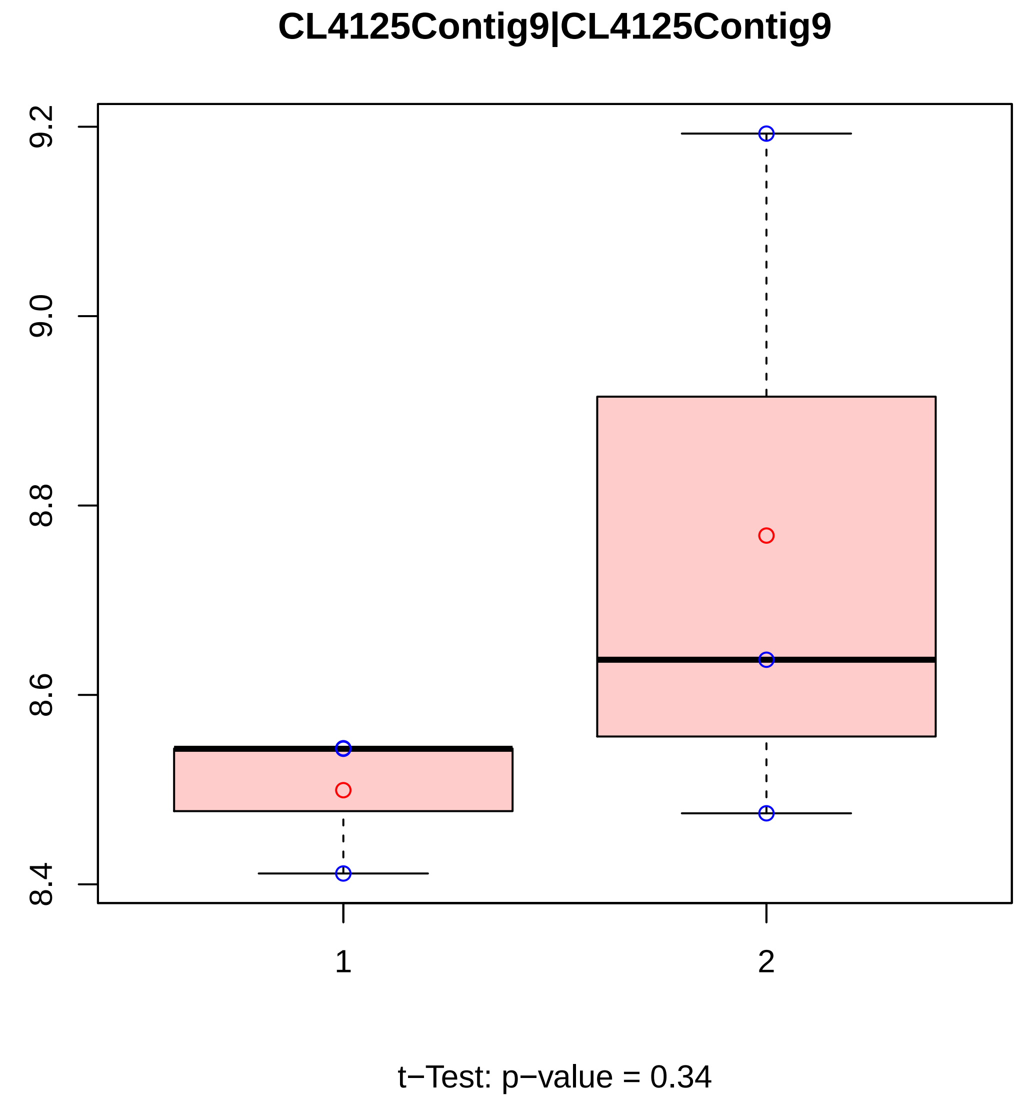

CL4125Contig9|CL4125Contig9
9.2
9.0
8.8
8.6
8.4
1
2
t−Test: p−value = 0.34

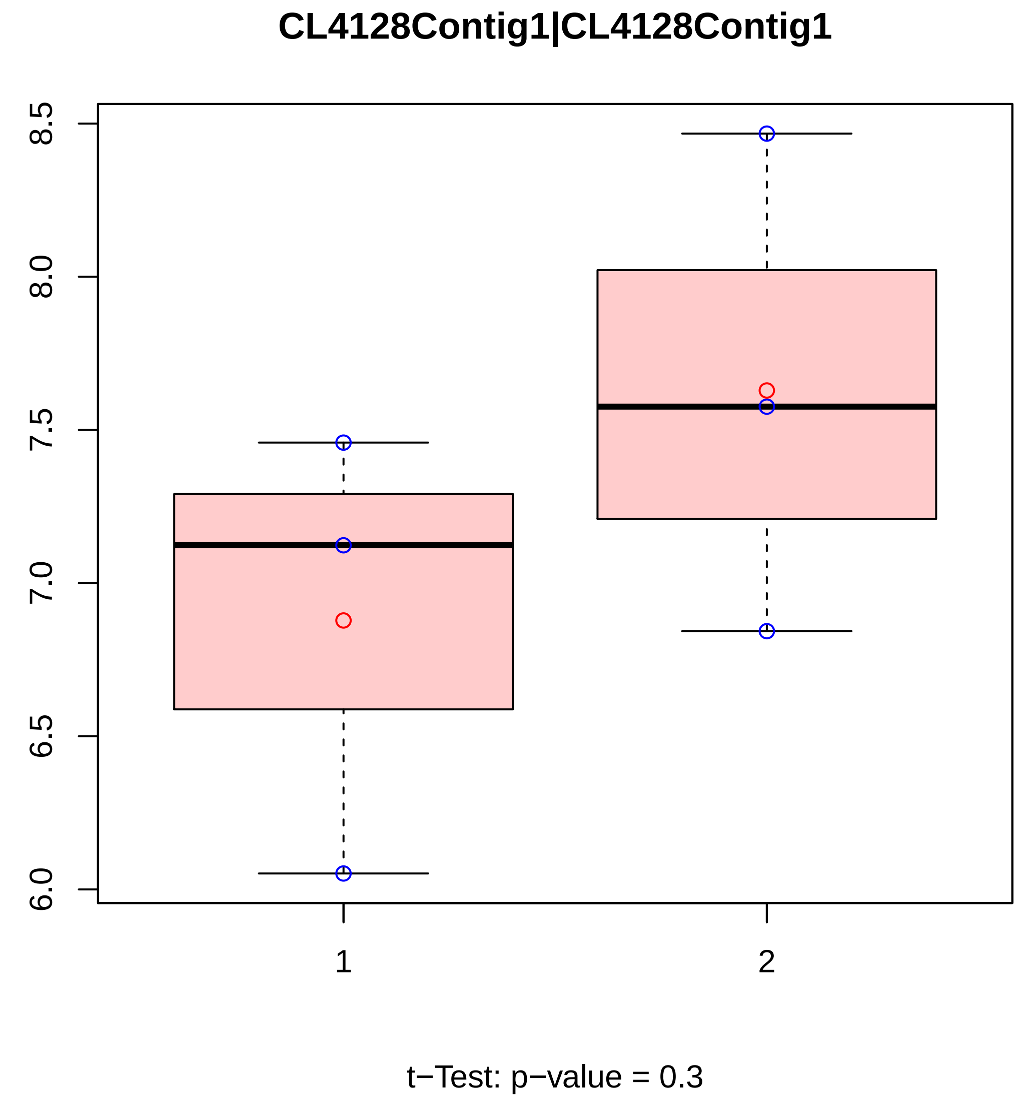
CL4128Contig1|CL4128Contig1
8.5
8.0
7.5
7.0
6.5
6.0
1
2
t−Test: p−value = 0.3

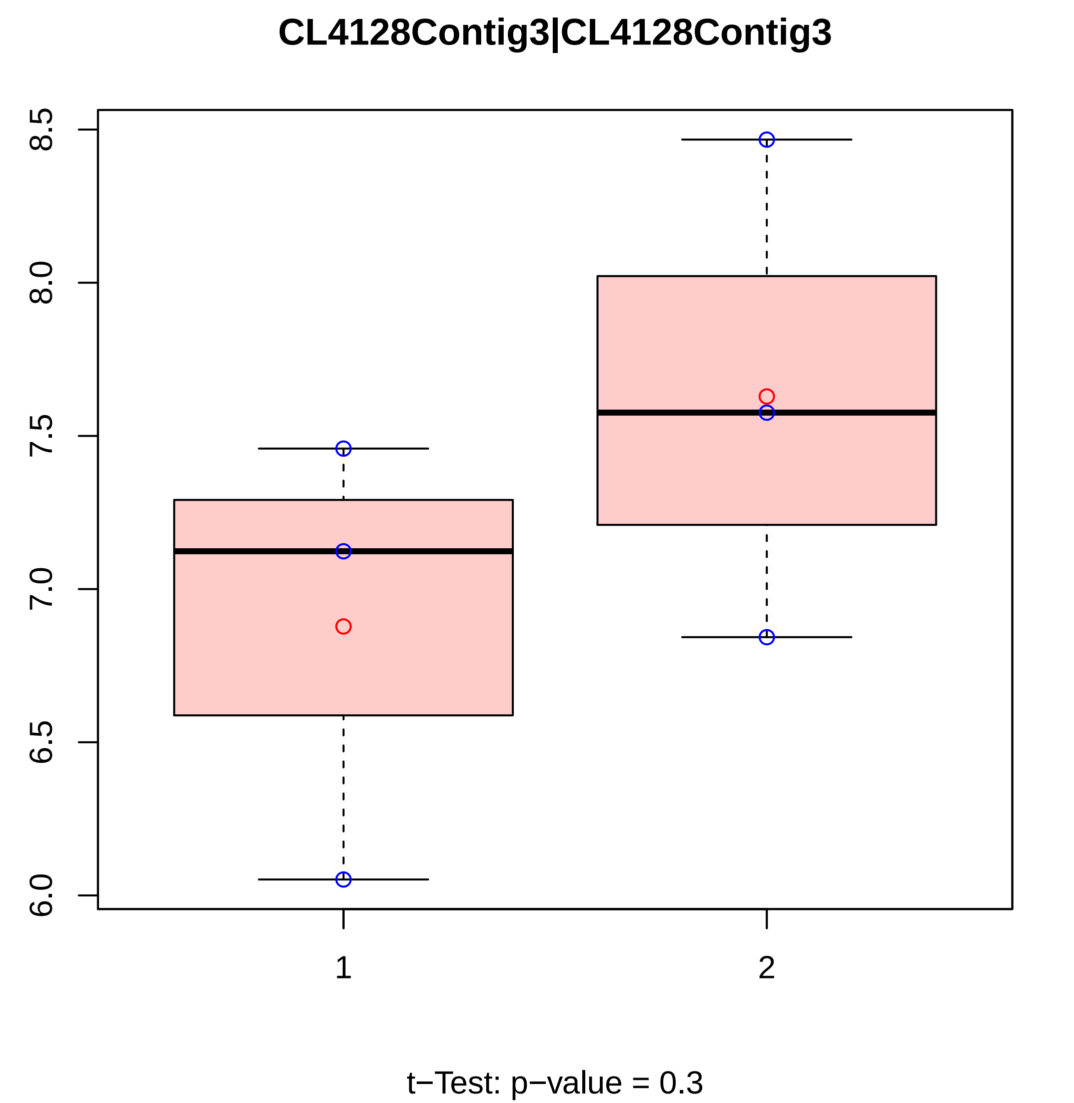
CL4128Contig3|CL4128Contig3
8.5
8.0
7.5
7.0
6.5
6.0
1
2
t−Test: p−value = 0.3

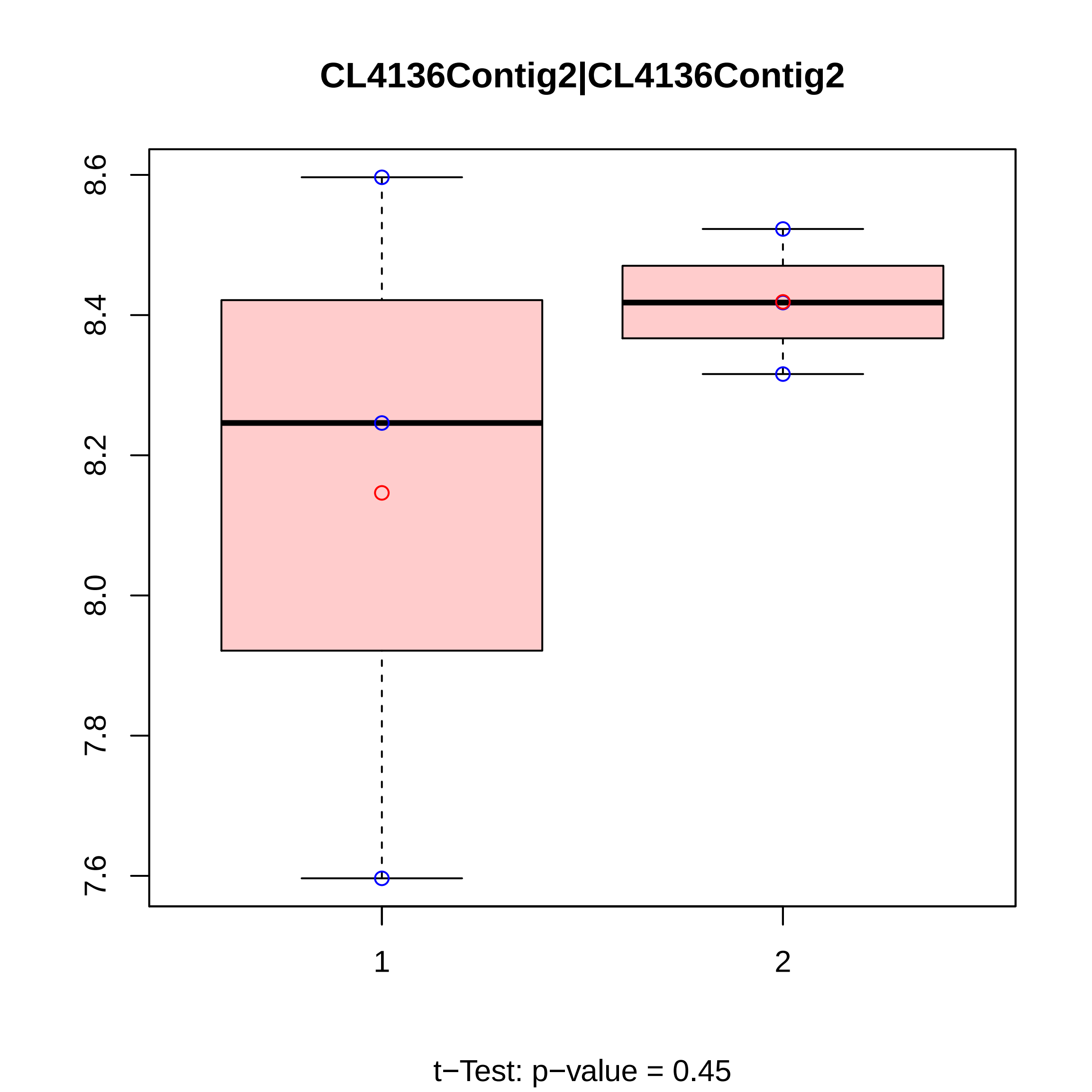
CL4136Contig2|CL4136Contig2
8.6
8.4
8.2
8.0
7.8
7.6
1
2
t−Test: p−value = 0.45

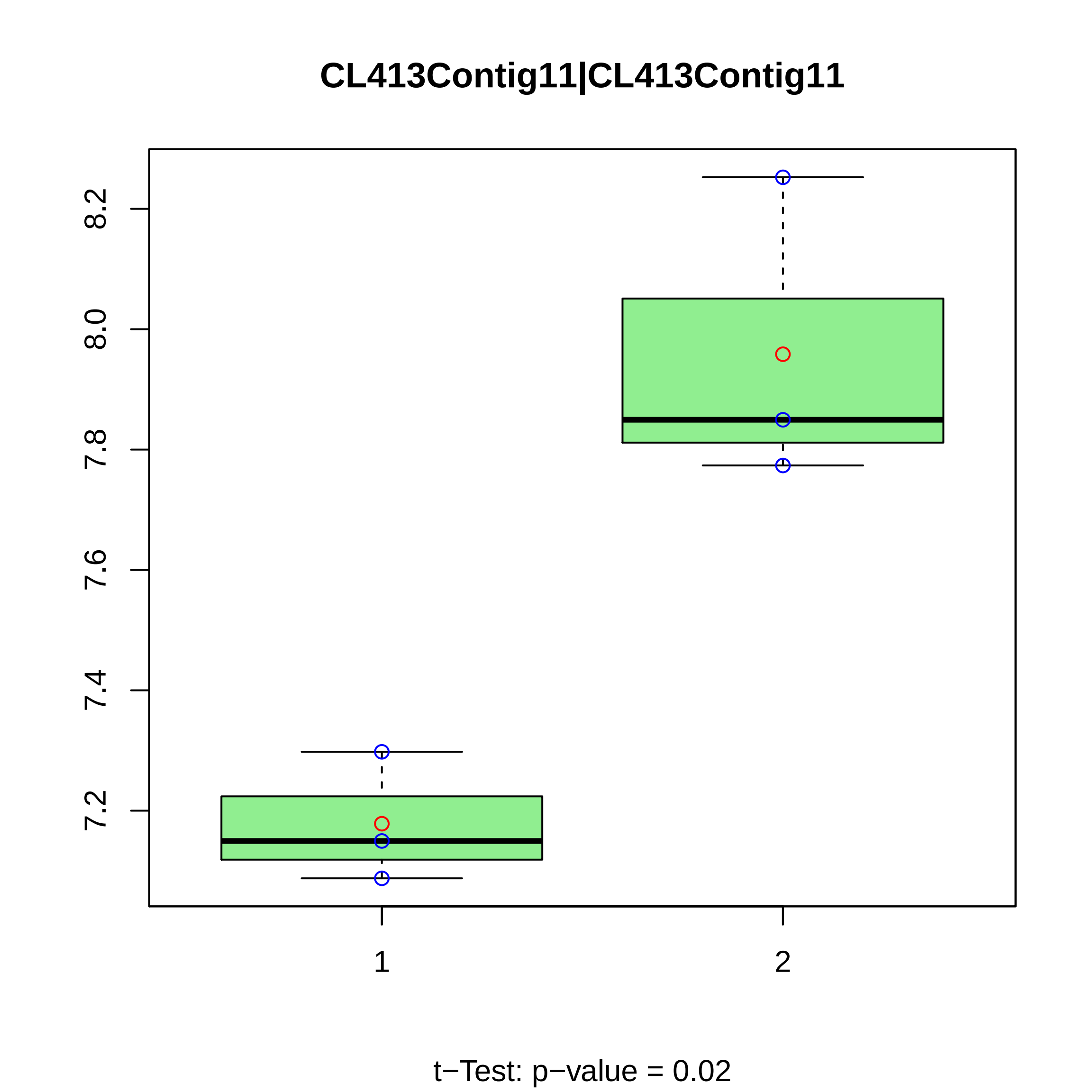
CL413Contig11|CL413Contig11
8.2
8.0
7.8
7.6
7.4
7.2
1
2
t−Test: p−value = 0.02

# CL413Contig9|CL413Contig9

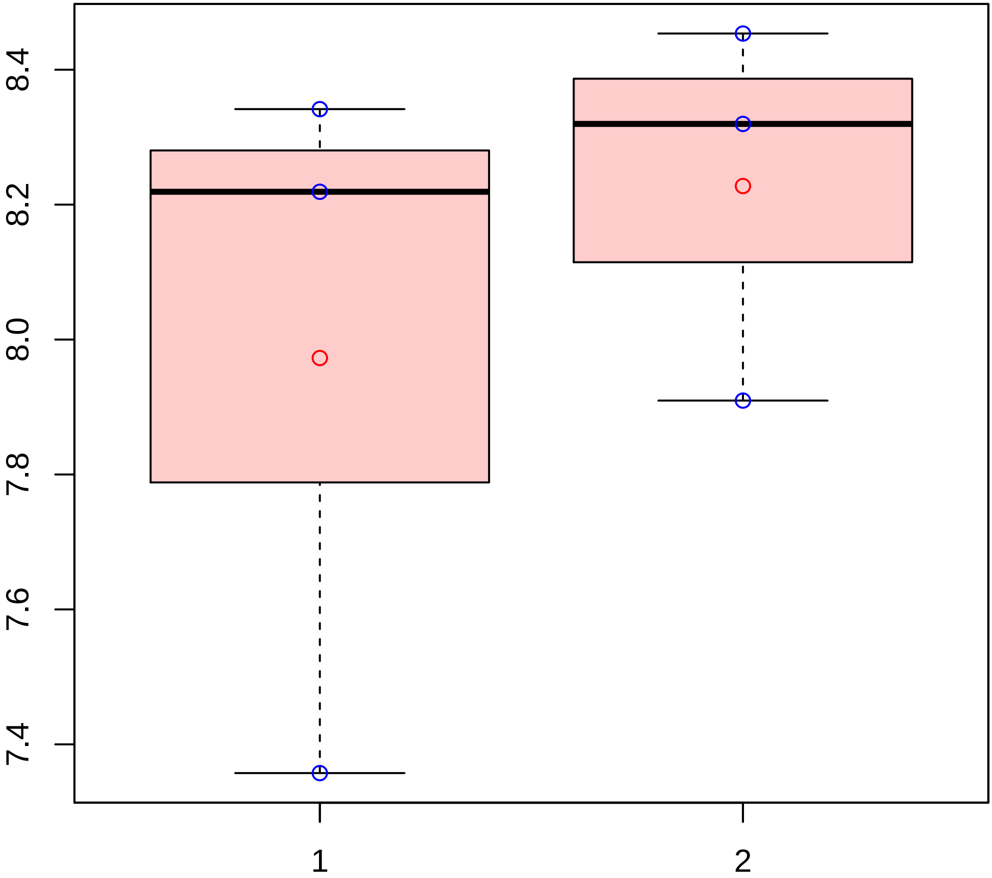


t−Test: p−value = 0.52

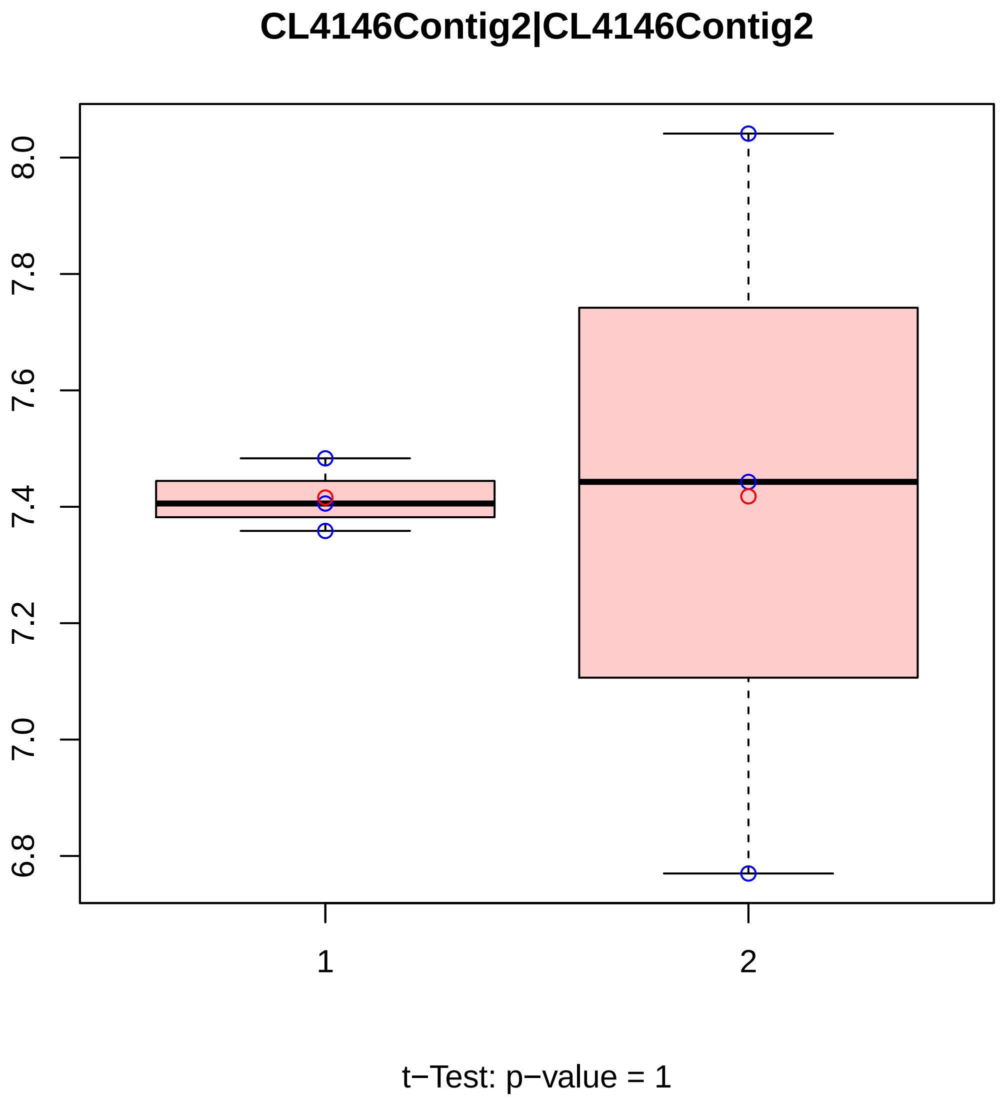

CL4146Contig2|CL4146Contig2
8.0
7.8
7.6
7.4
7.2
7.0
6.8
1
2
t−Test: p−value = 1

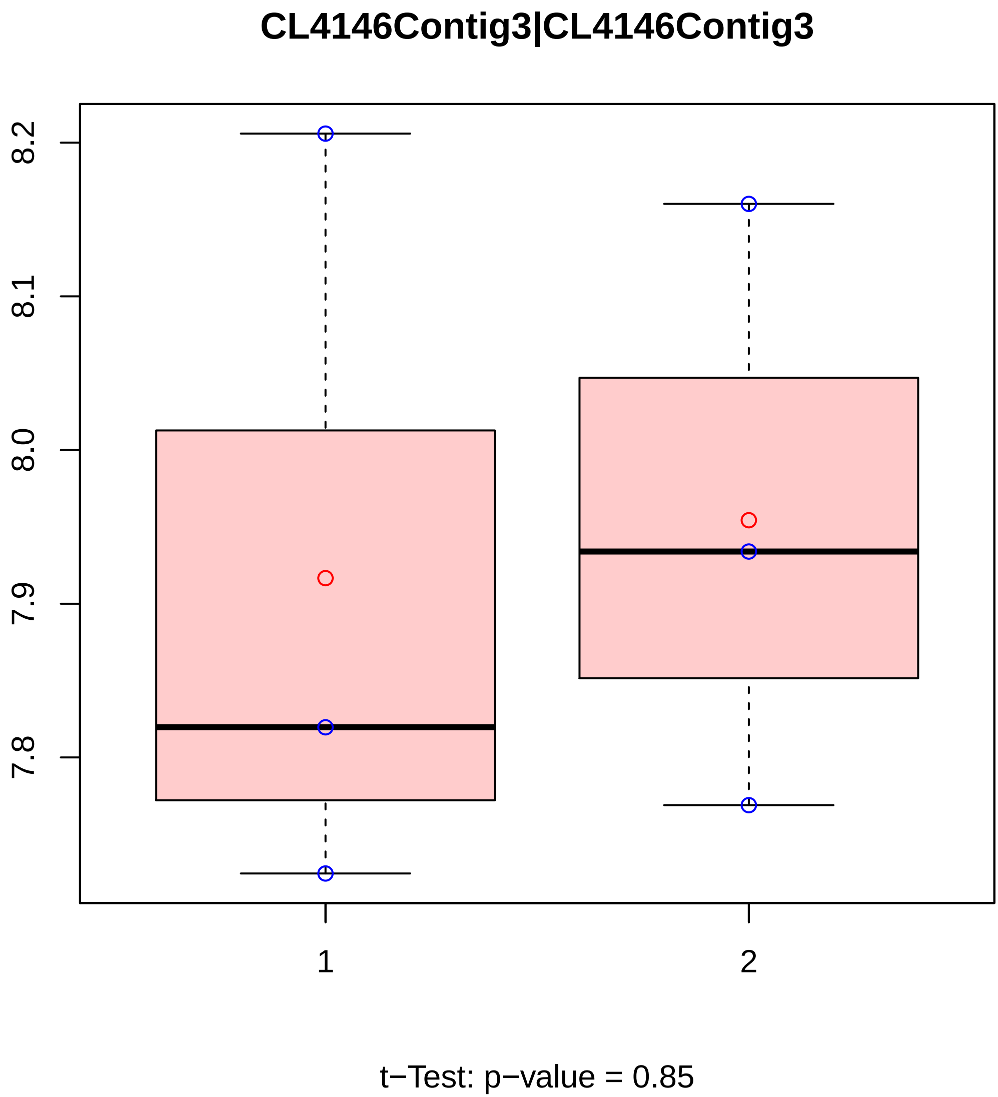
CL4146Contig3|CL4146Contig3
8.2
8.1
8.0
7.9
7.8
1
2
t−Test: p−value = 0.85

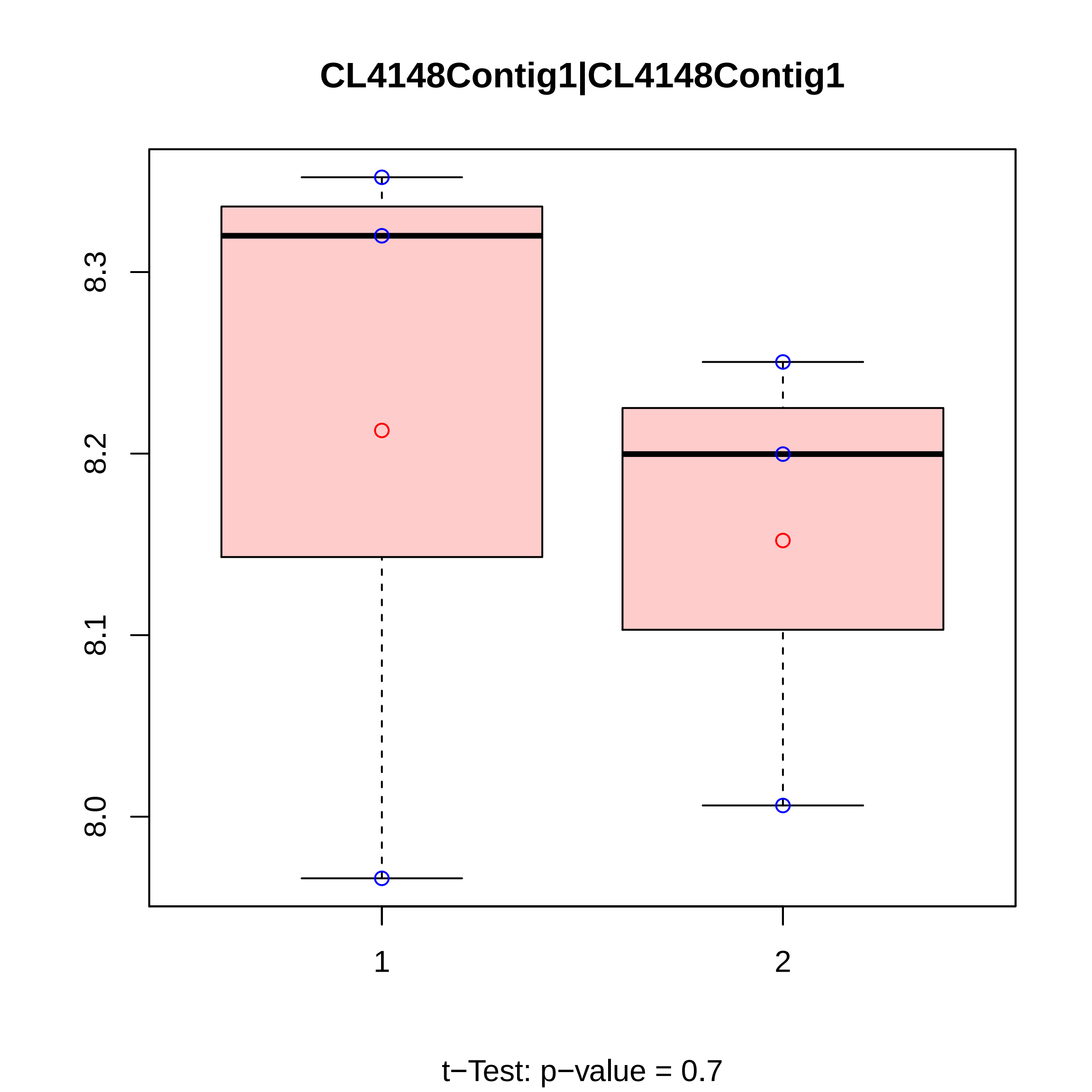
CL4148Contig1|CL4148Contig1
8.0
8.1
8.2
8.3
1
2
t−Test: p−value = 0.7

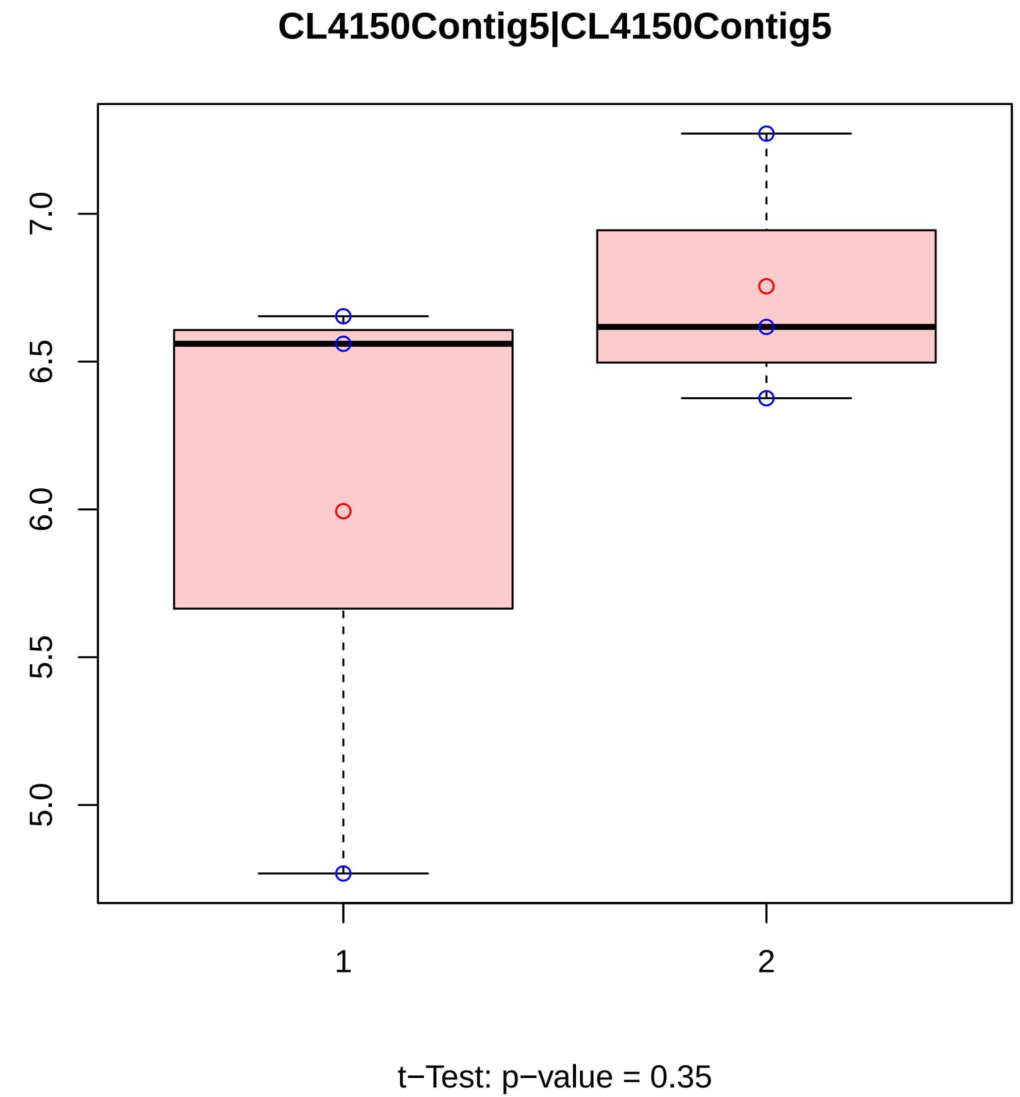
CL4150Contig5|CL4150Contig5
7.0
6.5
6.0
5.5
5.0
1
2
t−Test: p−value = 0.35

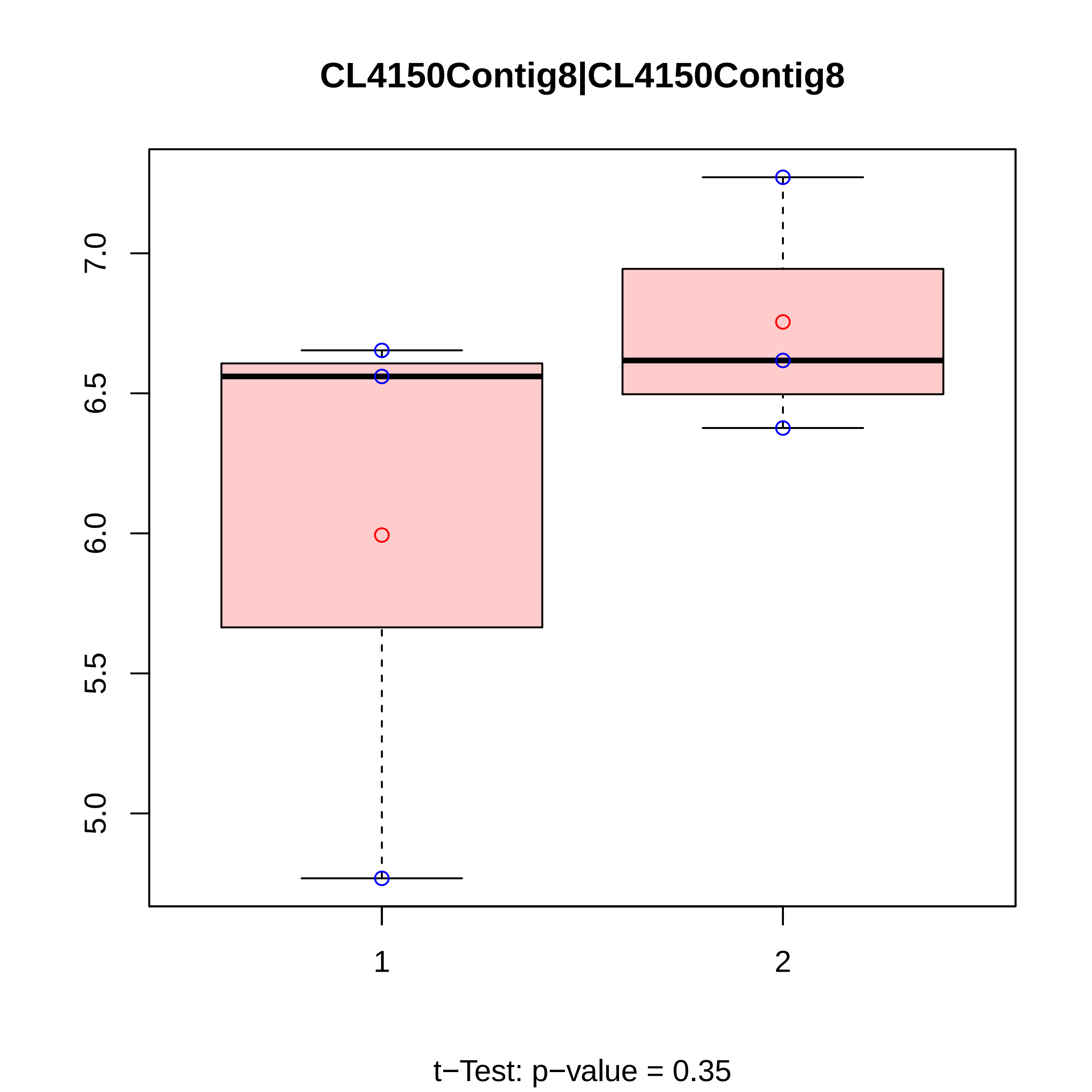

CL4150Contig8|CL4150Contig8
7.0
6.5
6.0
5.5
5.0
1
2
t−Test: p−value = 0.35

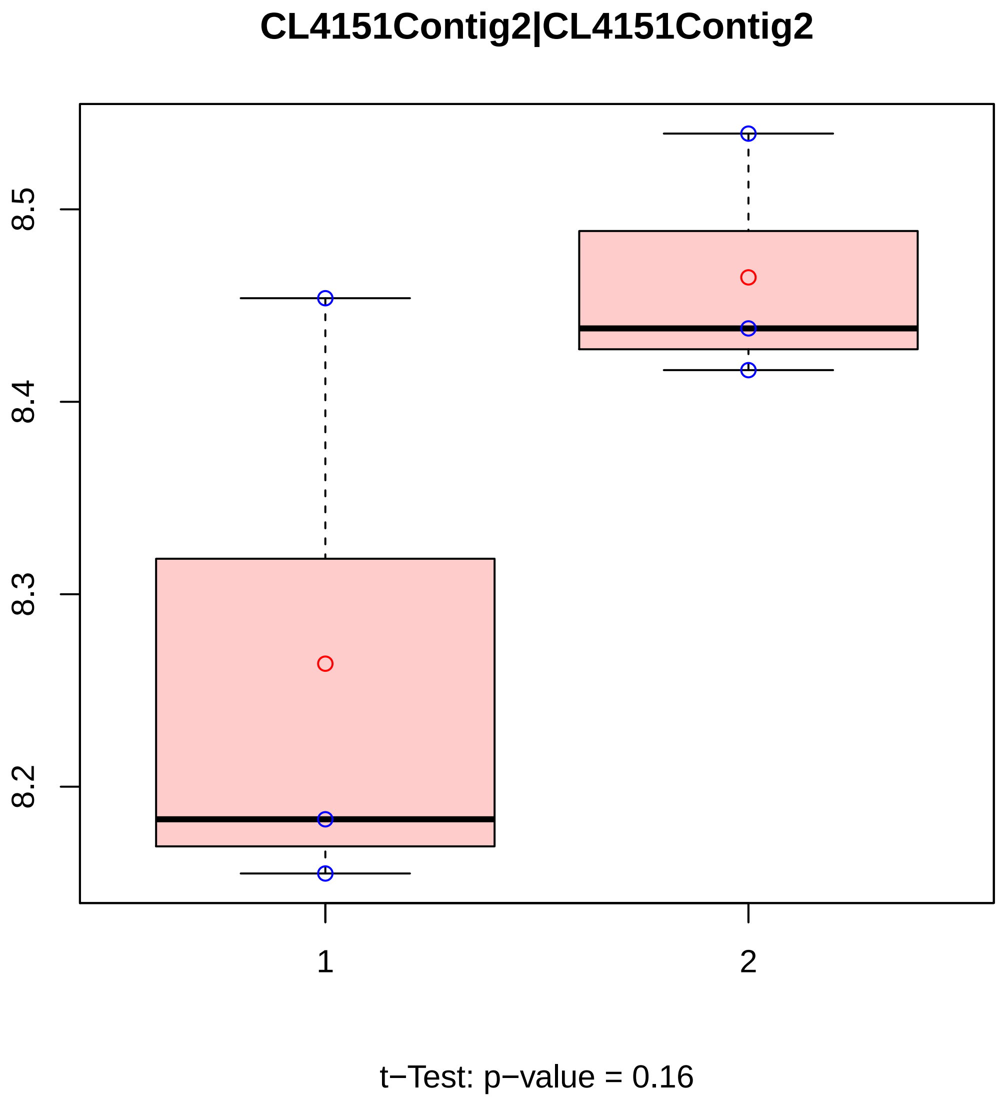
CL4151Contig2|CL4151Contig2
8.2
8.3
8.4
8.5
1
2
t−Test: p−value = 0.16

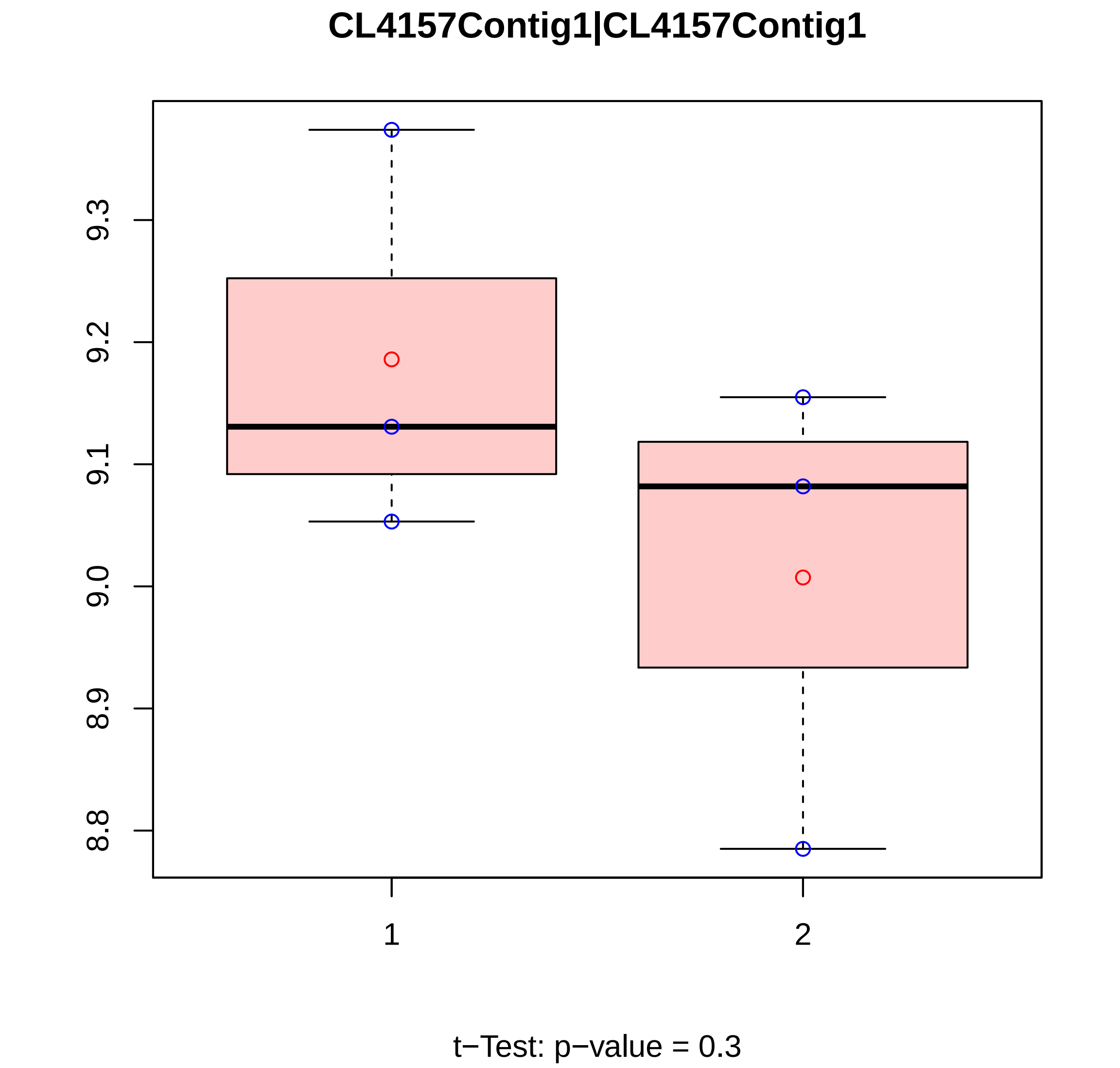
CL4157Contig1|CL4157Contig1
9.3
9.2
9.1
9.0
8.9
8.8
1
2
t−Test: p−value = 0.3

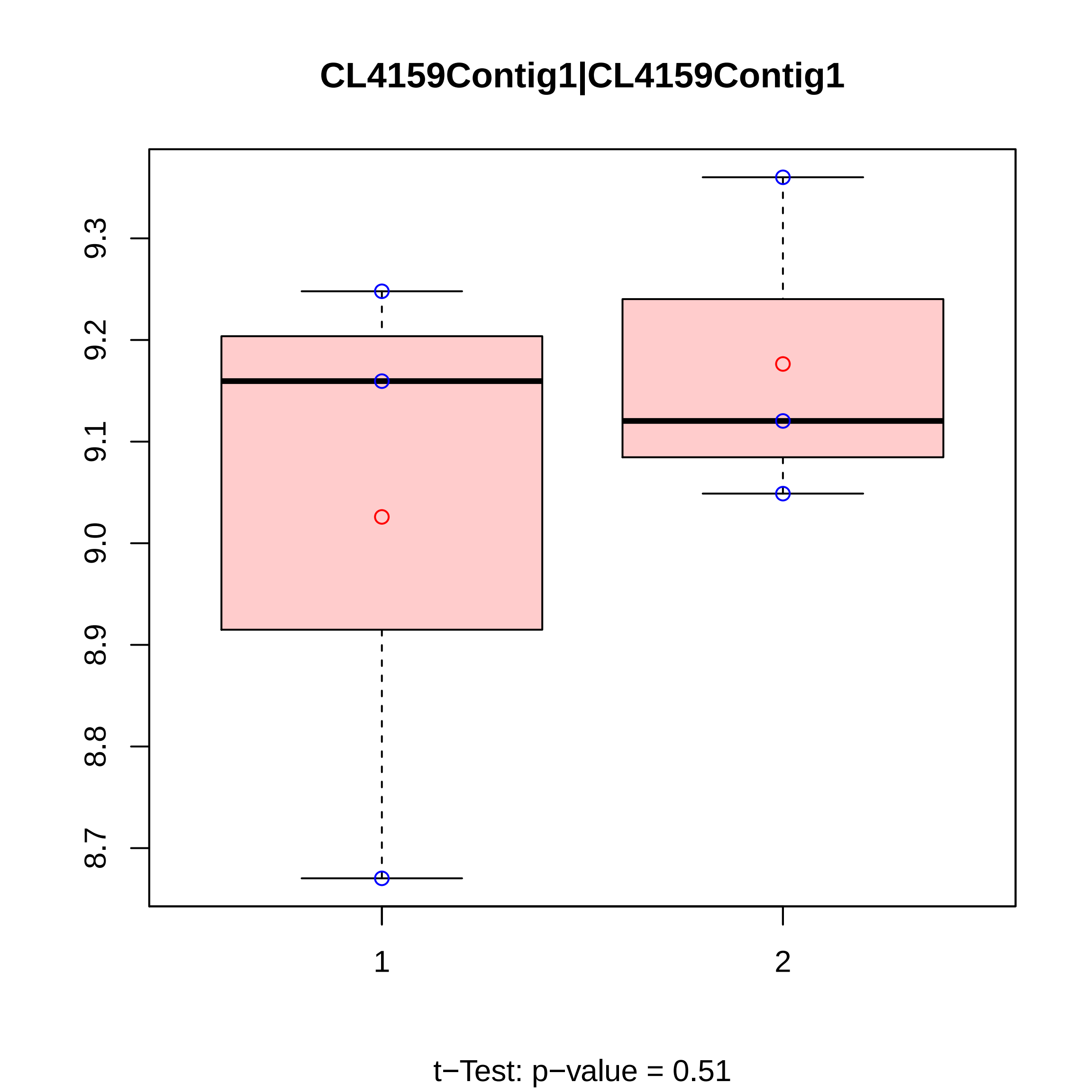

CL4159Contig1|CL4159Contig1
9.3
9.2
9.1
9.0
8.9
8.8
8.7
1
2
t−Test: p−value = 0.51

## CL415Contig1|CL415Contig1

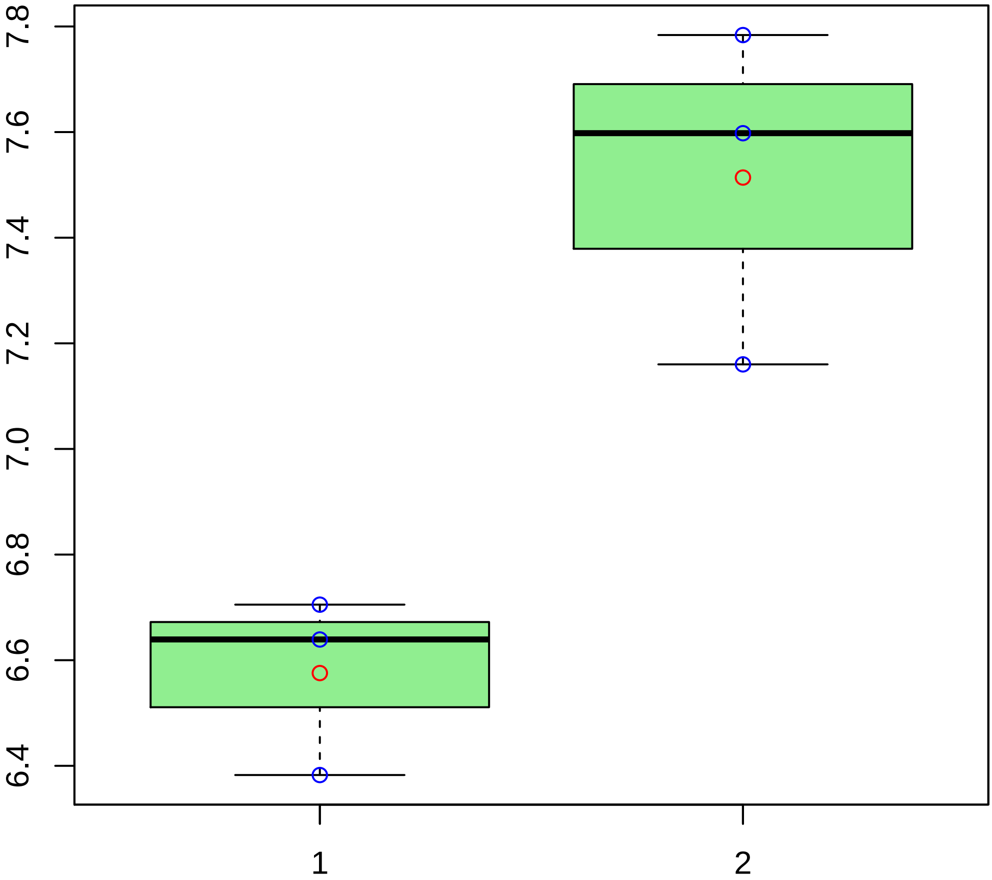


t−Test: p−value = 0.02

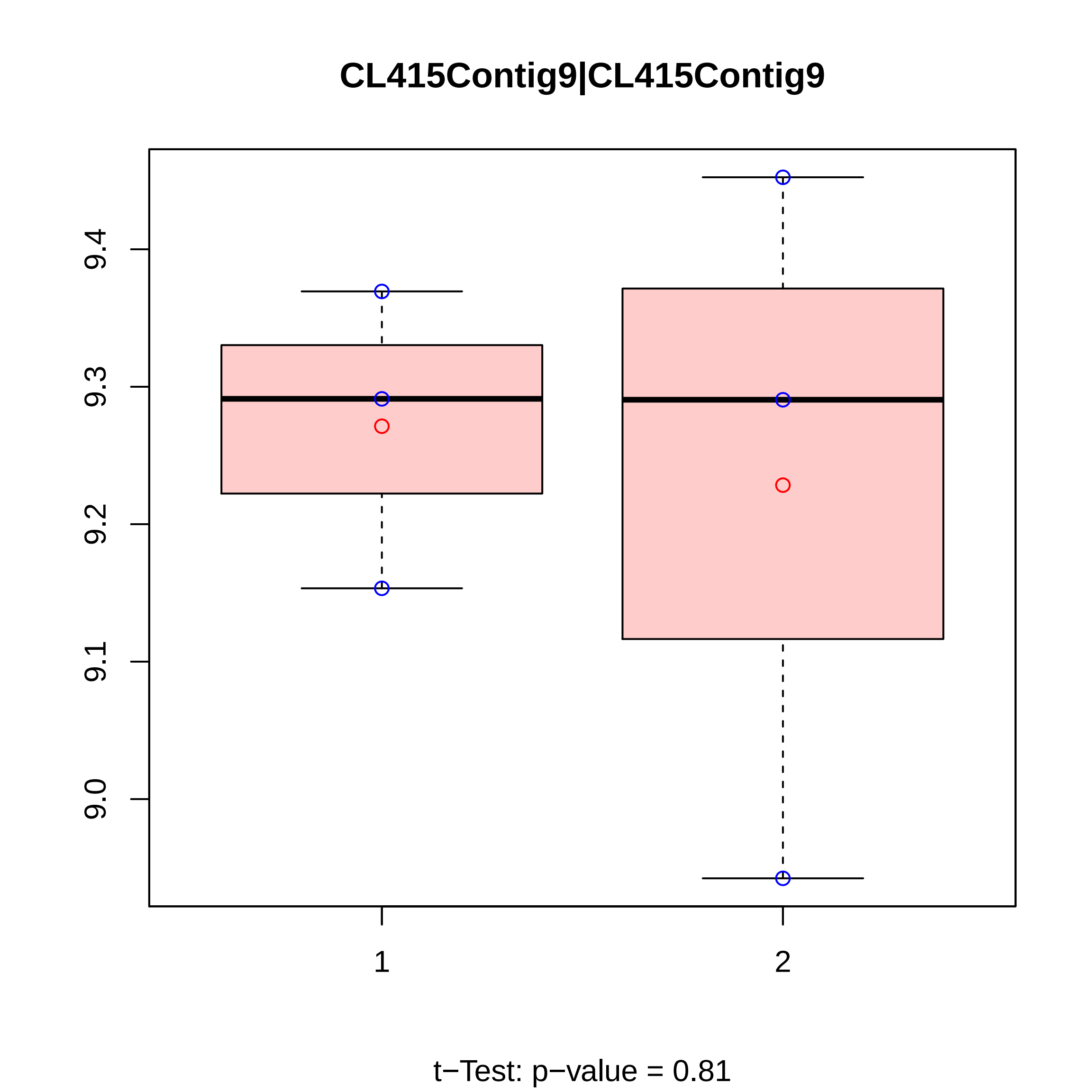
CL415Contig9|CL415Contig9
9.0
9.1
9.2
9.3
9.4
1
2
t−Test: p−value = 0.81

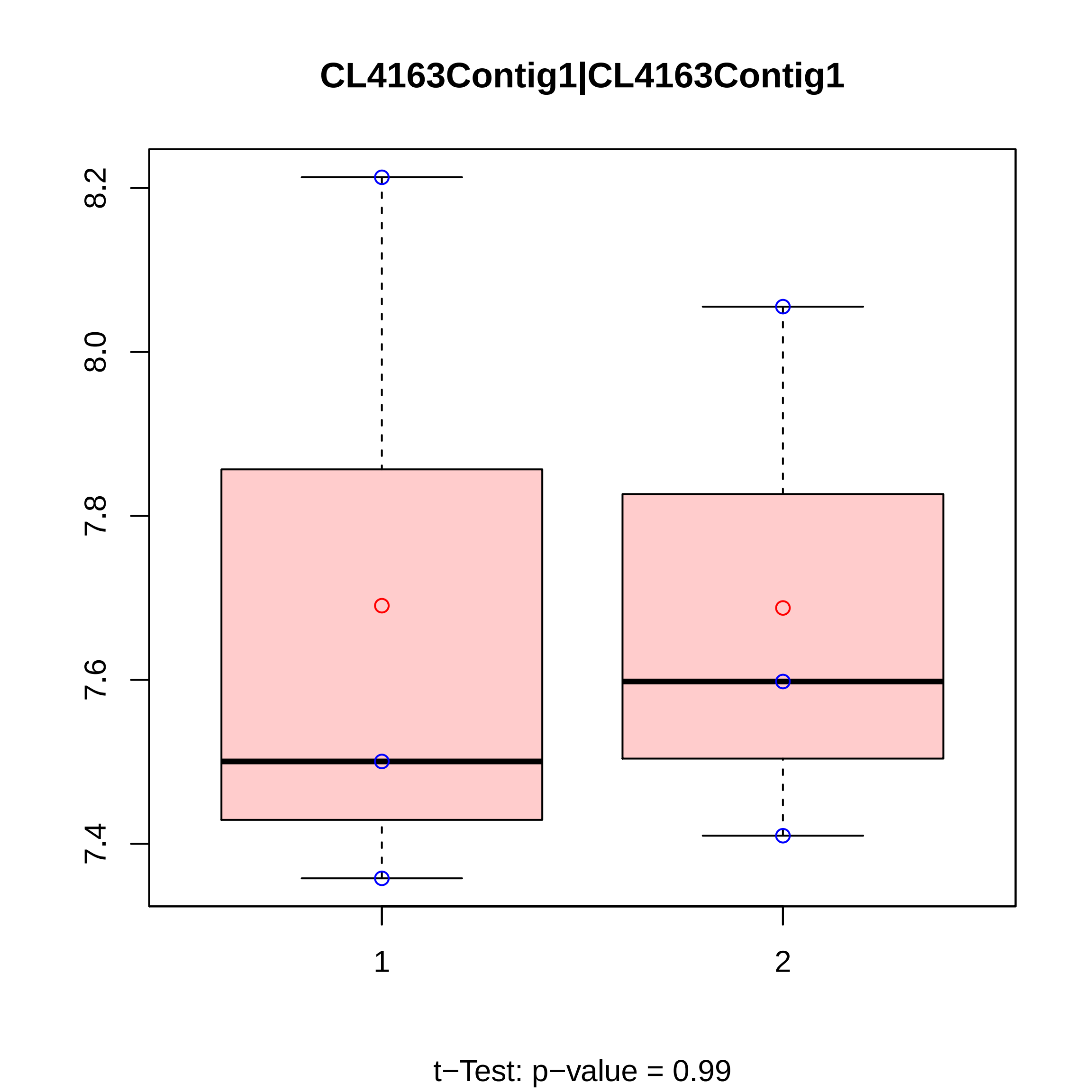

CL4163Contig1|CL4163Contig1
8.2
8.0
7.8
7.6
7.4
1
2
t−Test: p−value = 0.99

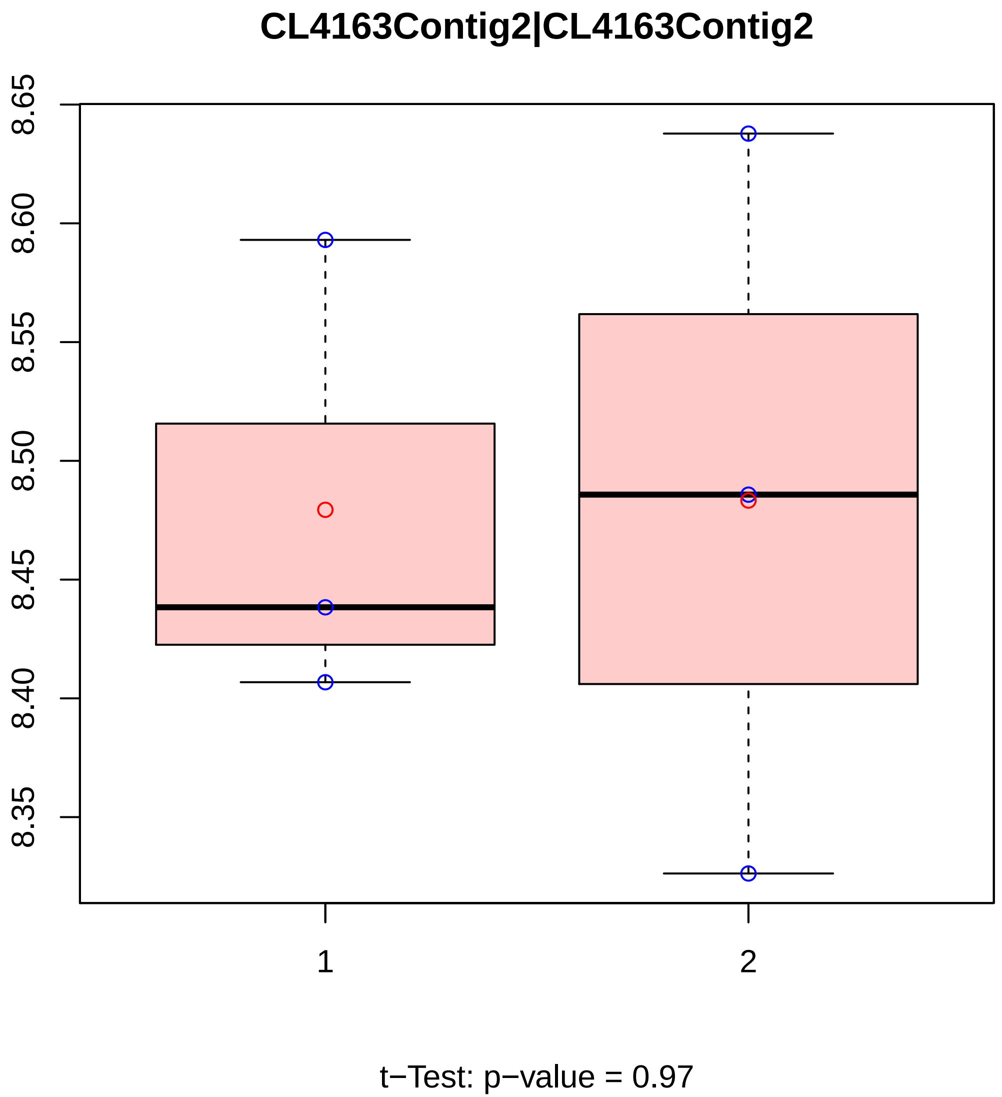
CL4163Contig2|CL4163Contig2
8.35
8.40
8.45
8.50
8.55
8.60
8.65
1
2
t−Test: p−value = 0.97

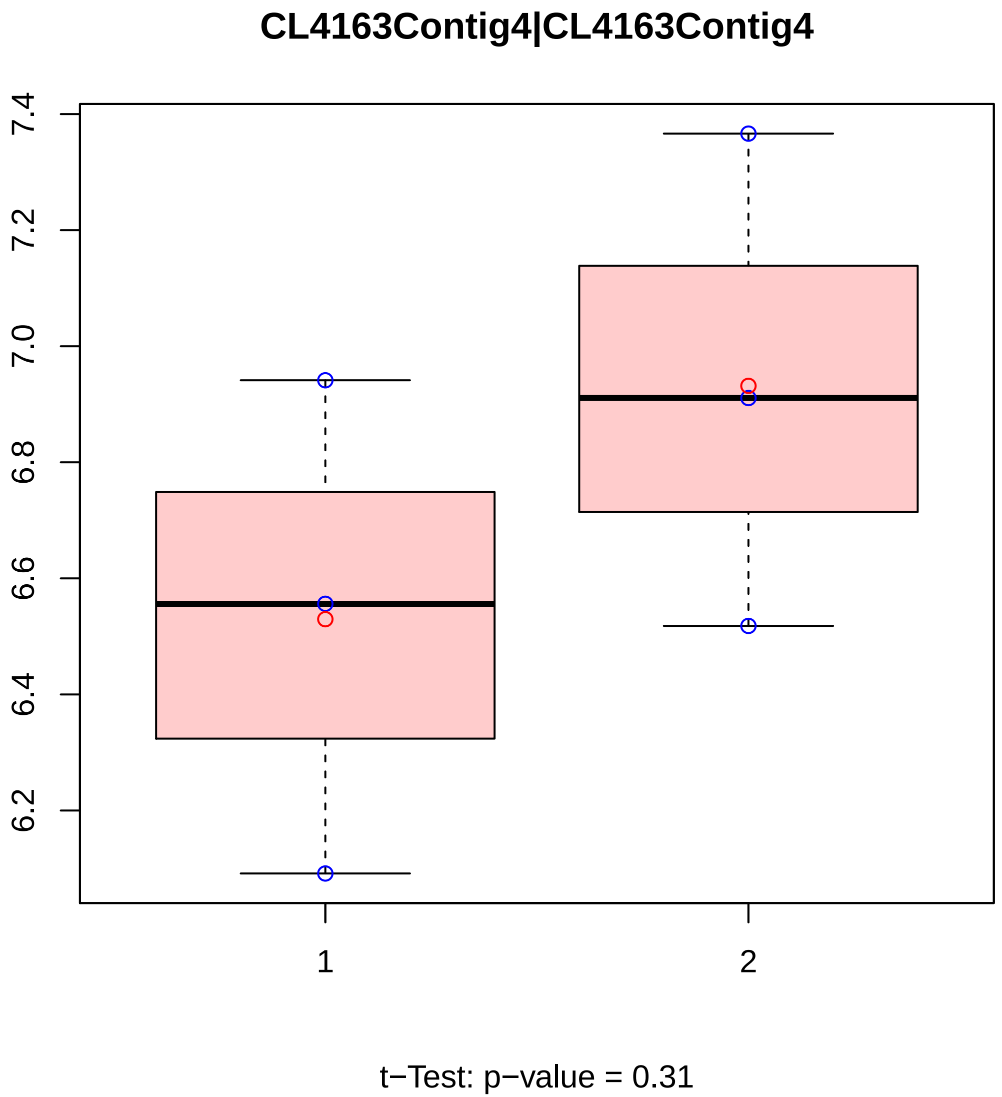

CL4163Contig4|CL4163Contig4
7.4
7.2
7.0
6.8
6.6
6.4
6.2
1
2
t−Test: p−value = 0.31

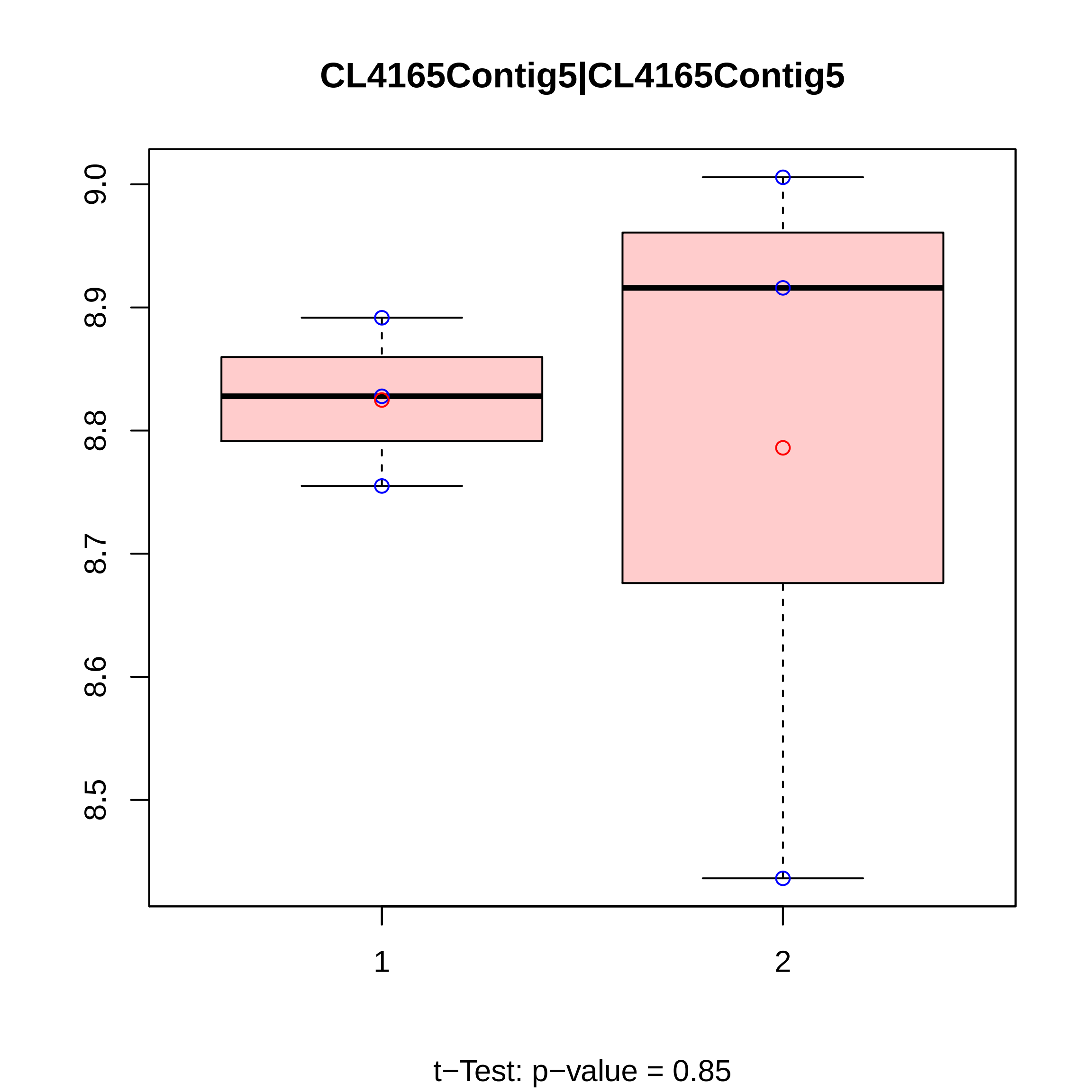
CL4165Contig5|CL4165Contig5
9.0
8.9
8.8
8.7
8.6
8.5
1
2
t−Test: p−value = 0.85

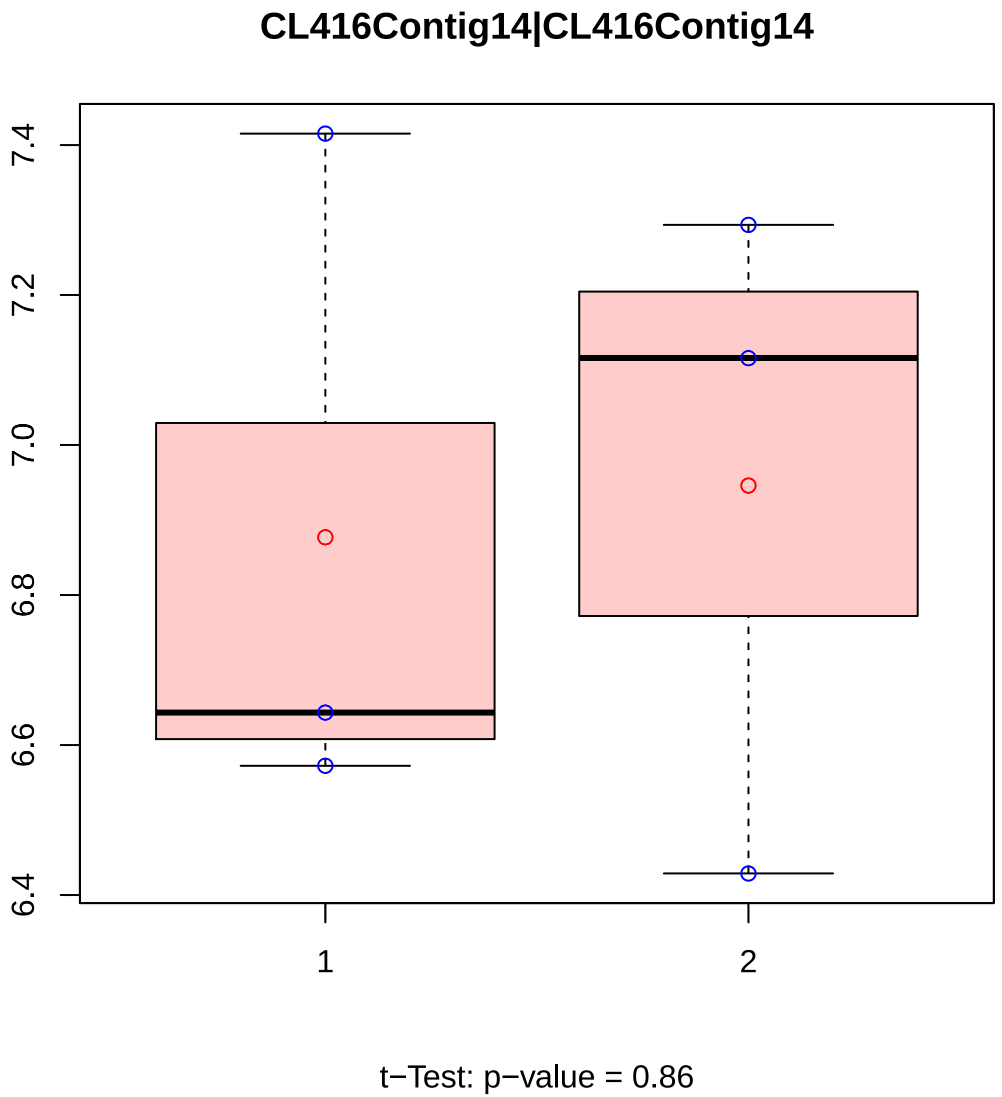

CL416Contig14|CL416Contig14
7.4
7.2
7.0
6.8
6.6
6.4
1
2
t−Test: p−value = 0.86

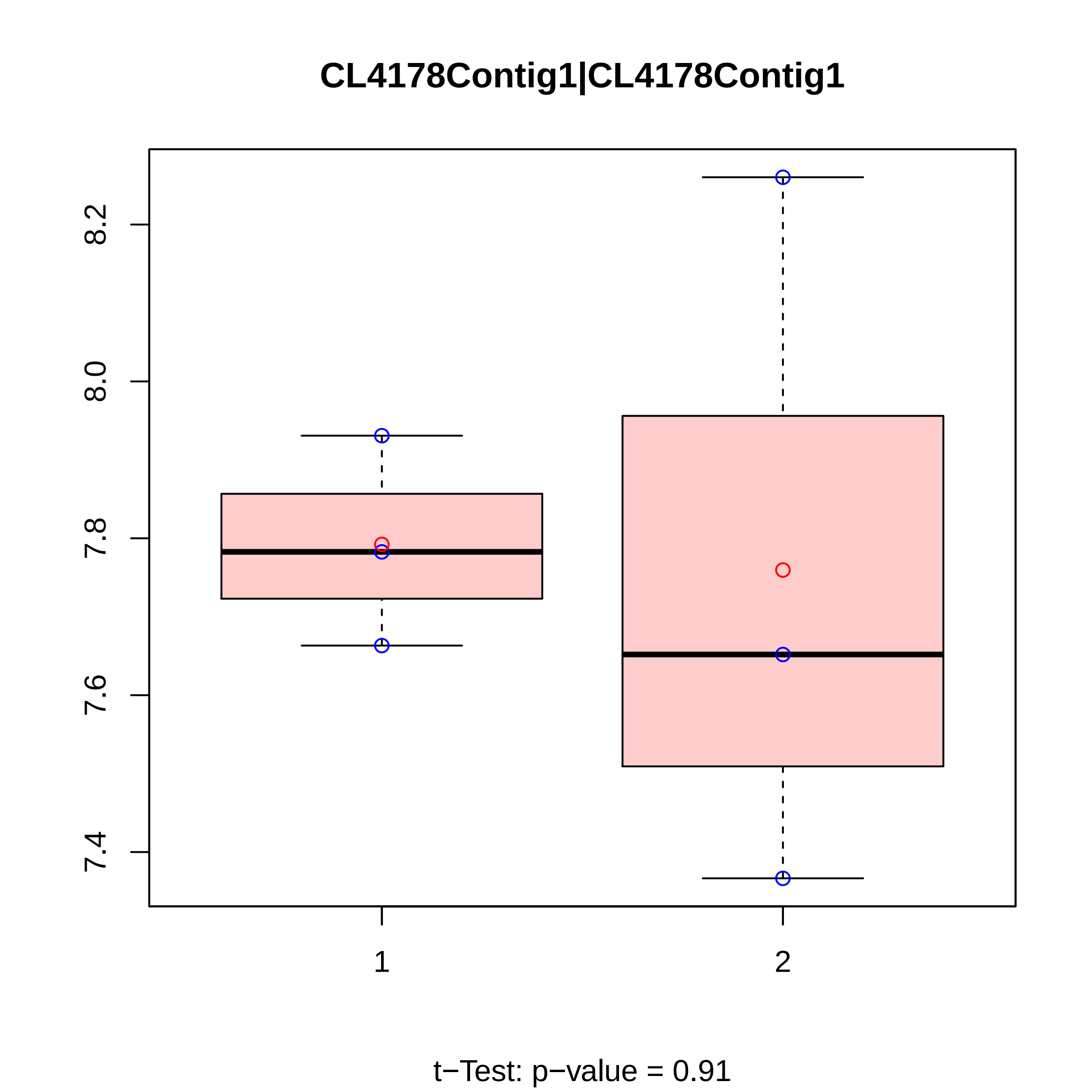

CL4178Contig1|CL4178Contig1
8.2
8.0
7.8
7.6
7.4
1
2
t−Test: p−value = 0.91

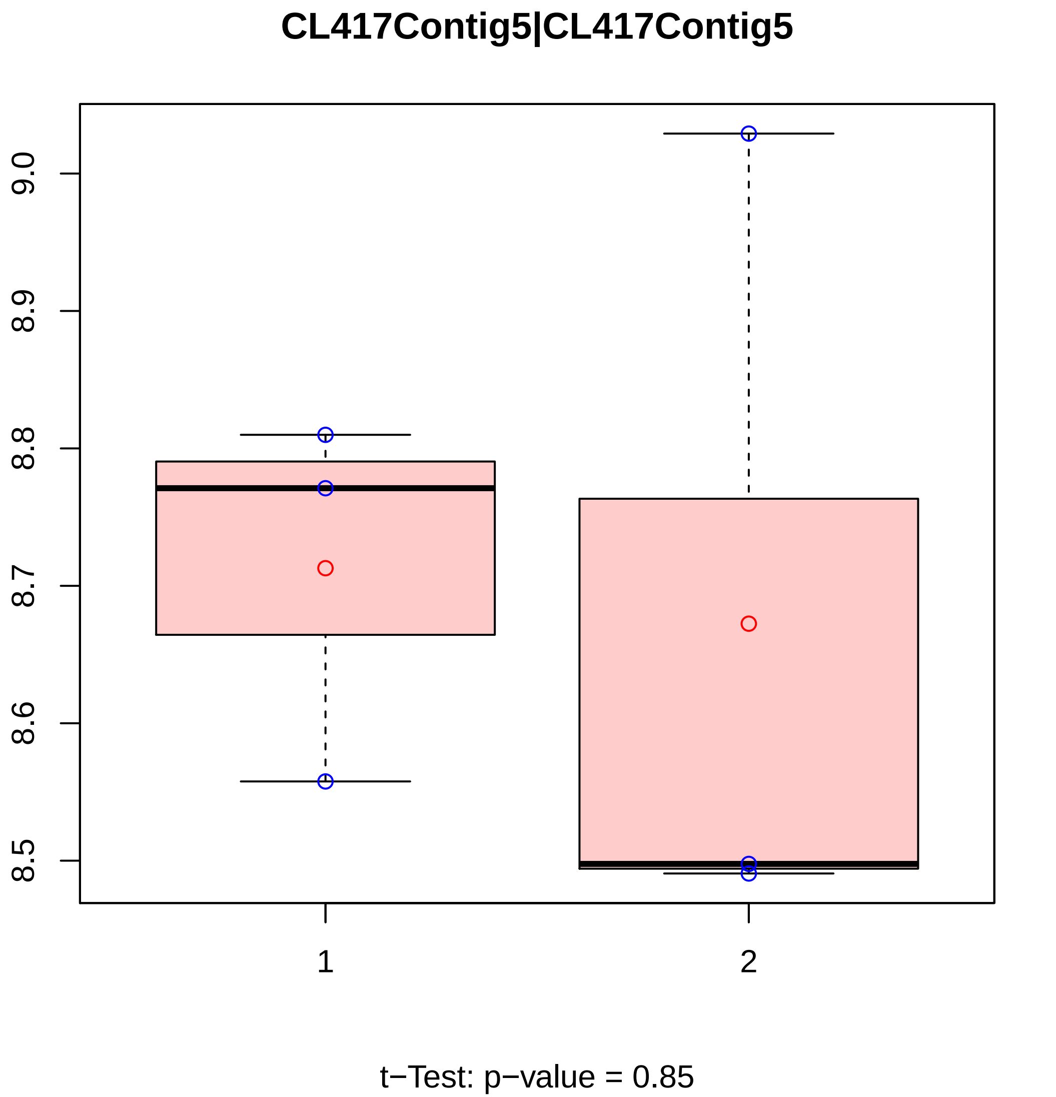

CL417Contig5|CL417Contig5
9.0
8.9
8.8
8.7
8.6
8.5
1
2
t−Test: p−value = 0.85

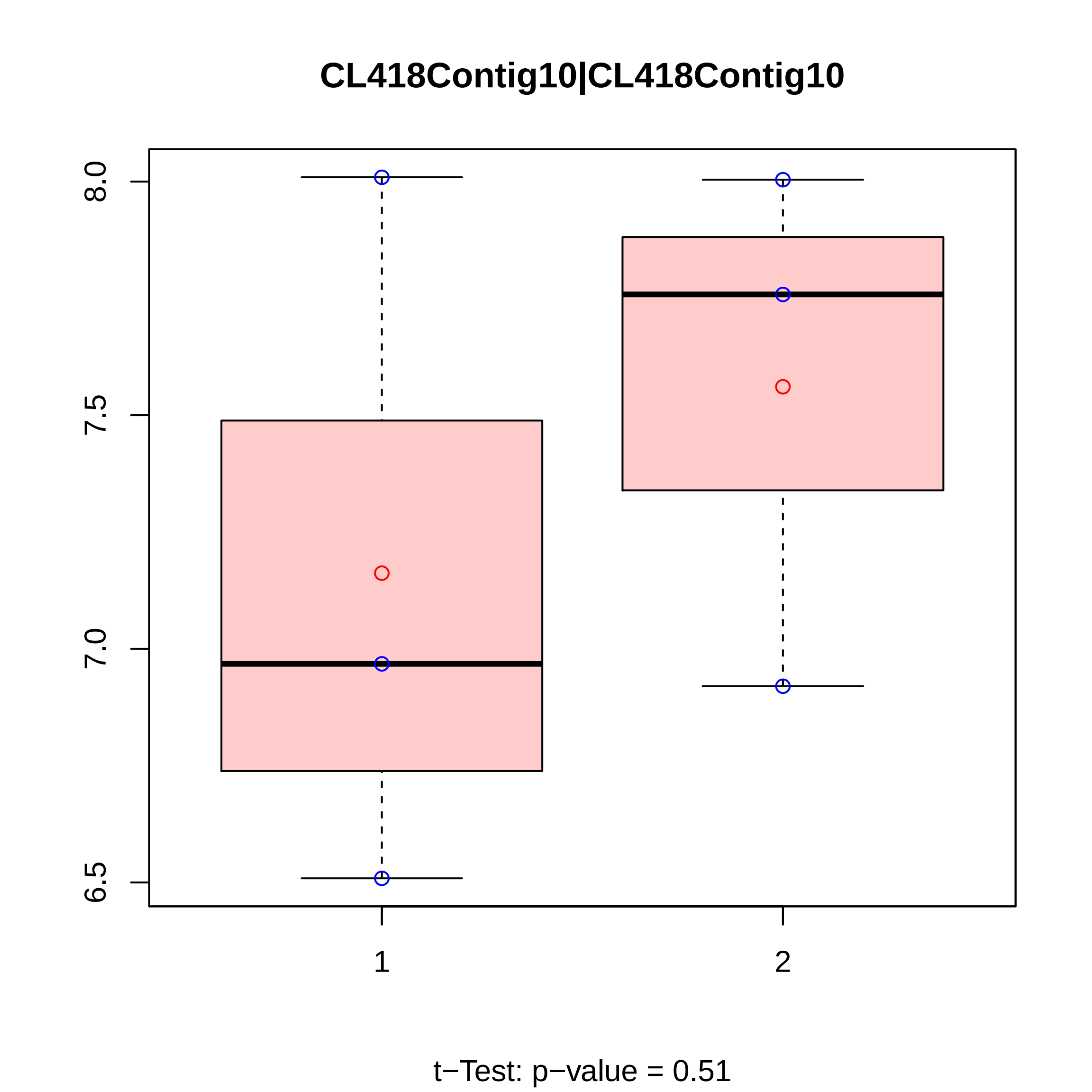

CL418Contig10|CL418Contig10
8.0
7.5
7.0
6.5
1
2
t−Test: p−value = 0.51

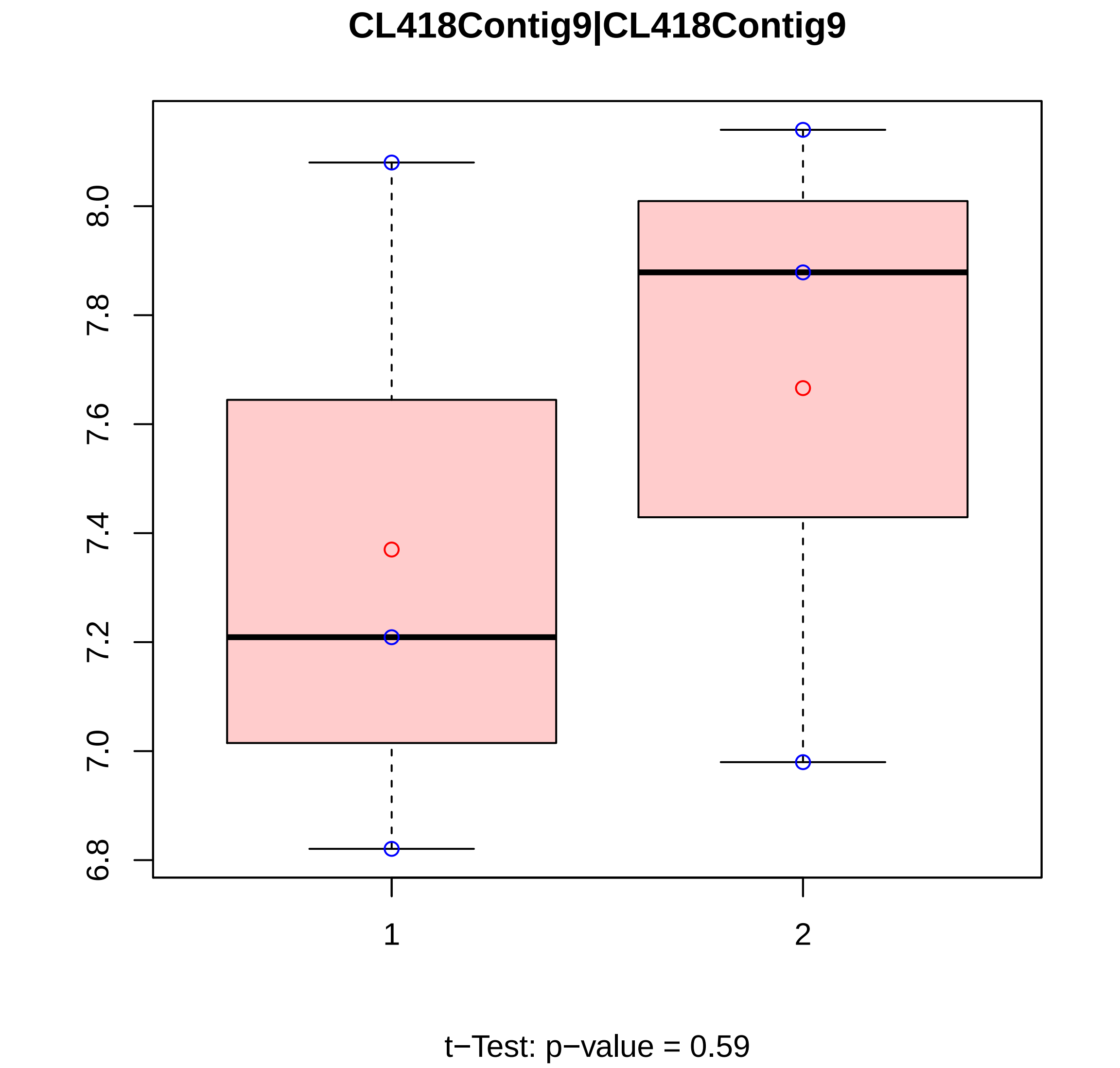

CL418Contig9|CL418Contig9
8.0
7.8
7.6
7.4
7.2
7.0
6.8
1
2
t−Test: p−value = 0.59

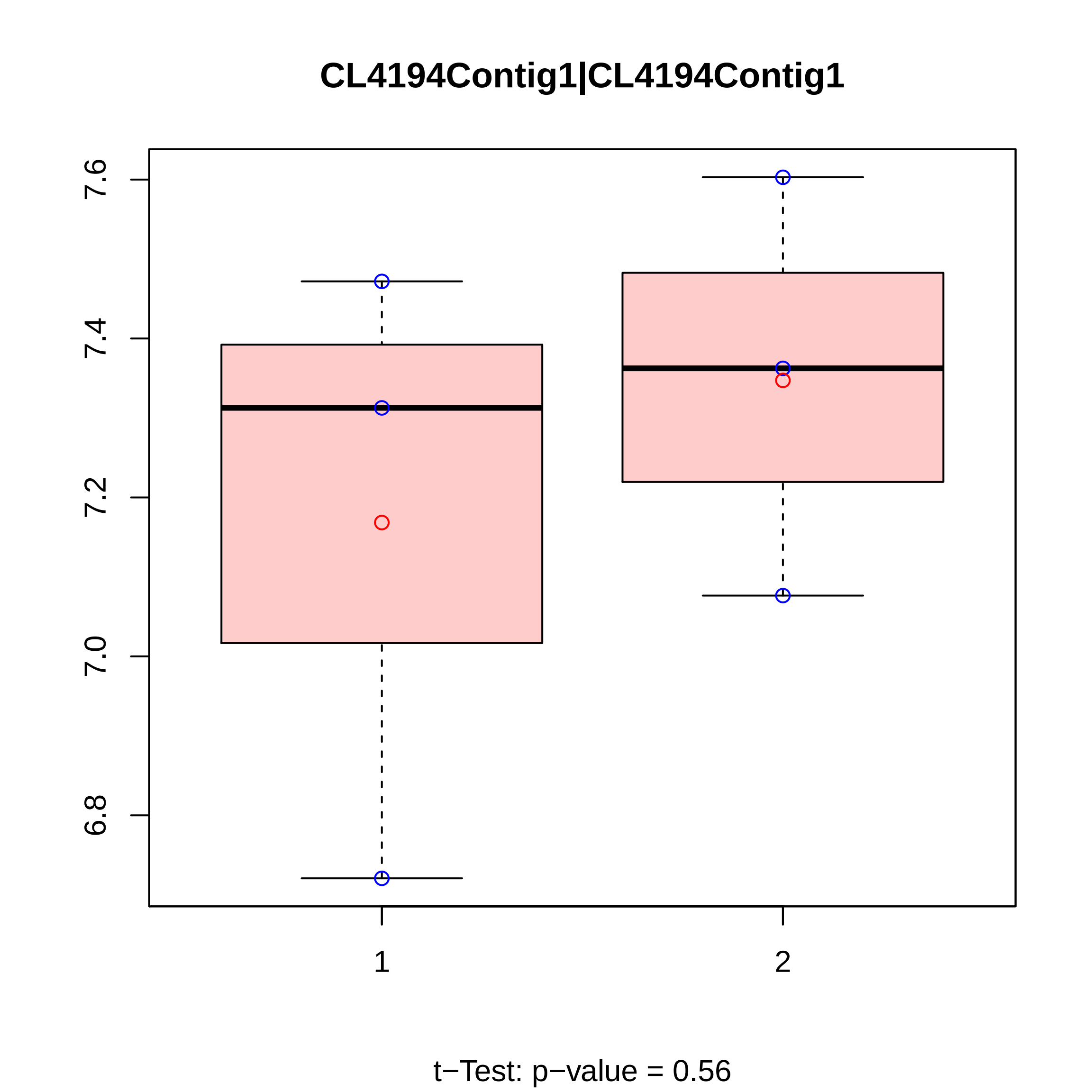
CL4194Contig1|CL4194Contig1
7.6
7.4
7.2
7.0
6.8
1
2
t−Test: p−value = 0.56

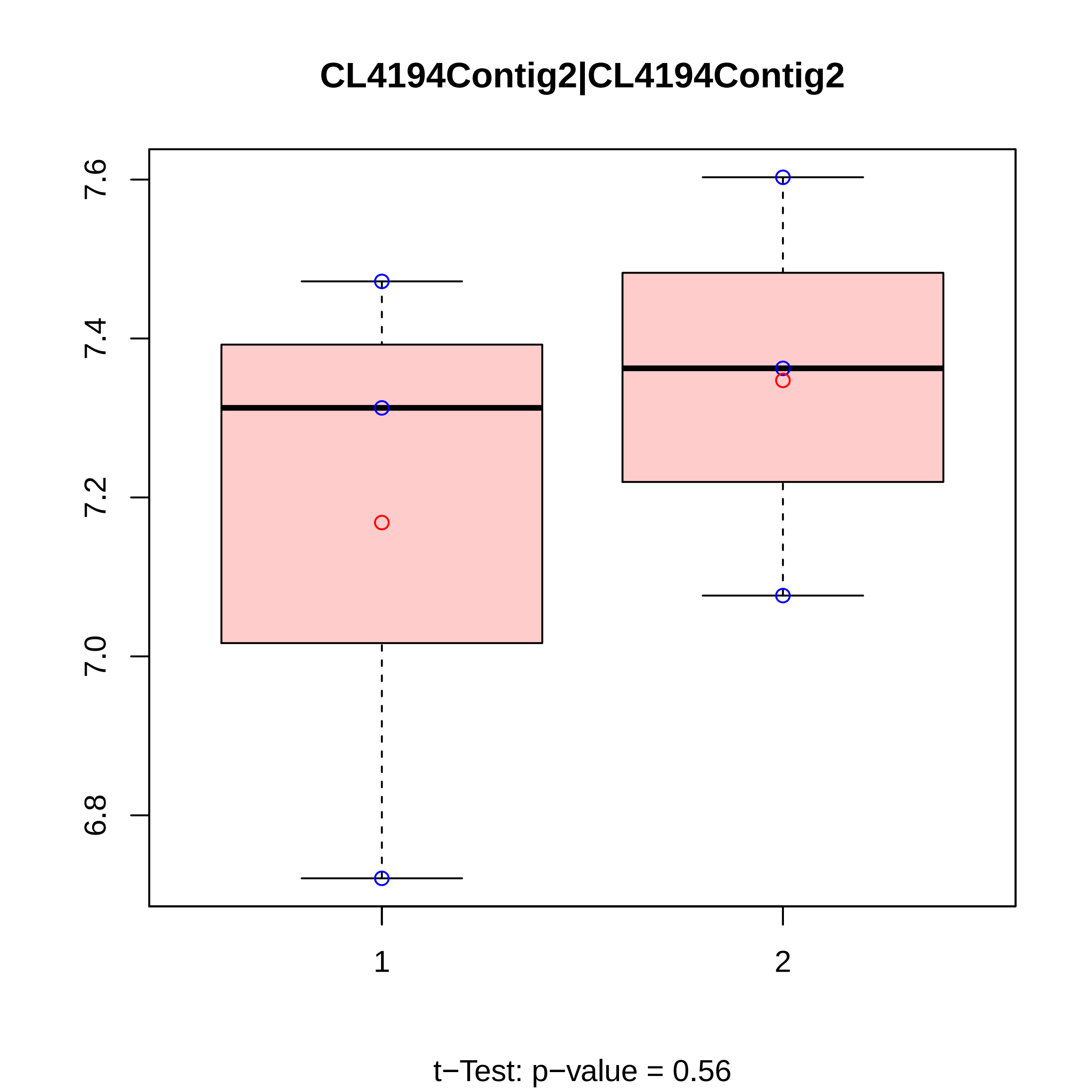
CL4194Contig2|CL4194Contig2
7.6
7.4
7.2
7.0
6.8
1
2
t−Test: p−value = 0.56

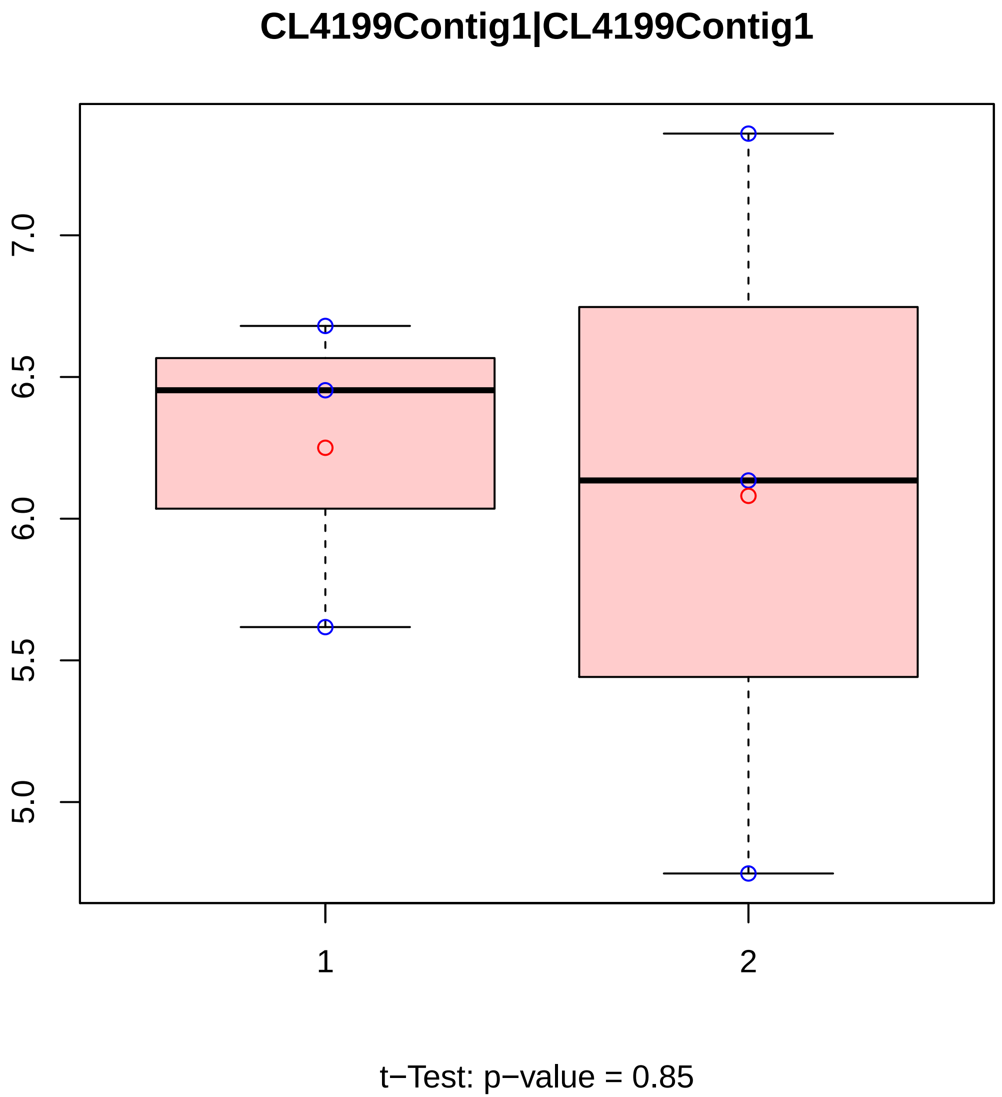

CL4199Contig1|CL4199Contig1
7.0
6.5
6.0
5.5
5.0
1
2
t−Test: p−value = 0.85

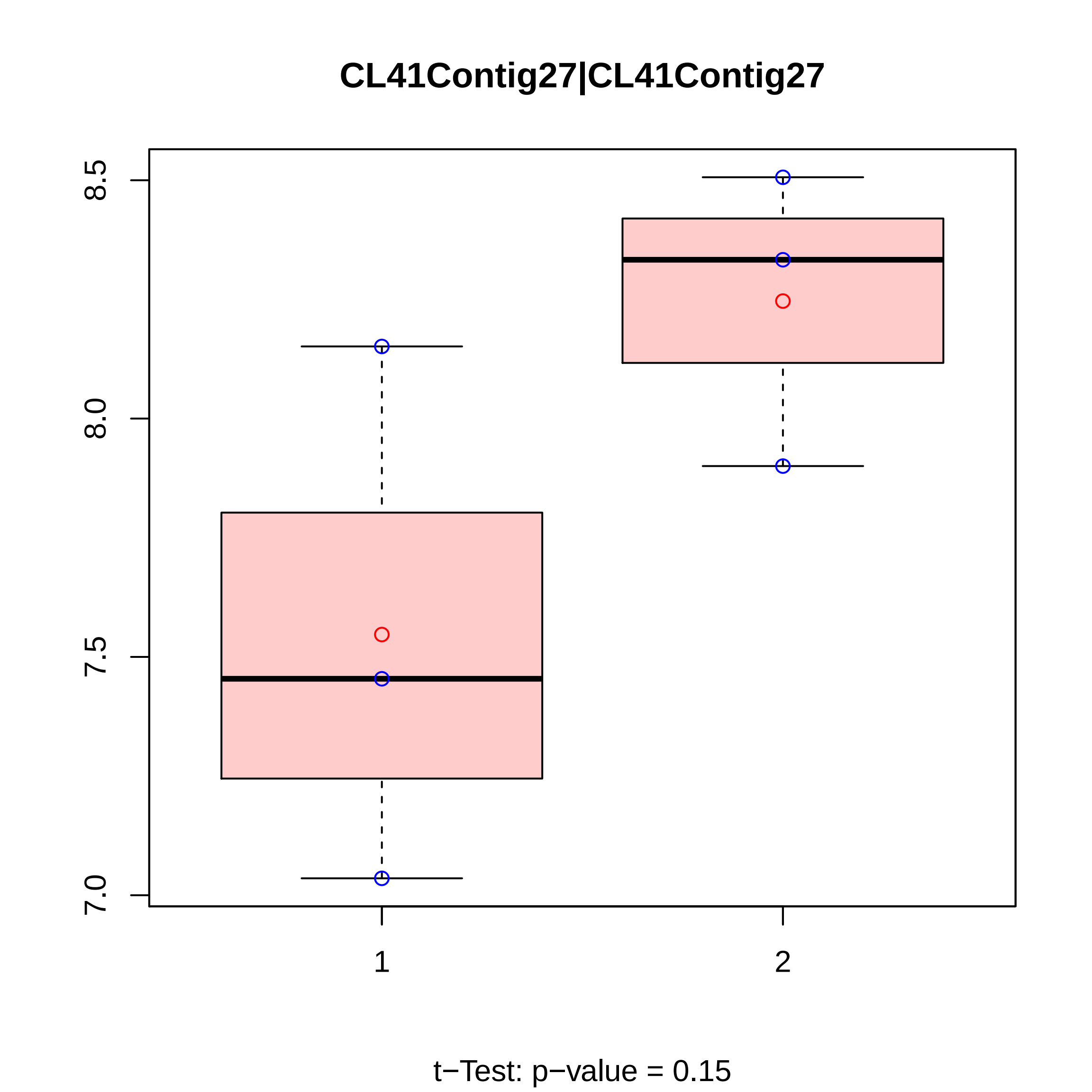

CL41Contig27|CL41Contig27
8.5
8.0
7.5
7.0
1
2
t−Test: p−value = 0.15

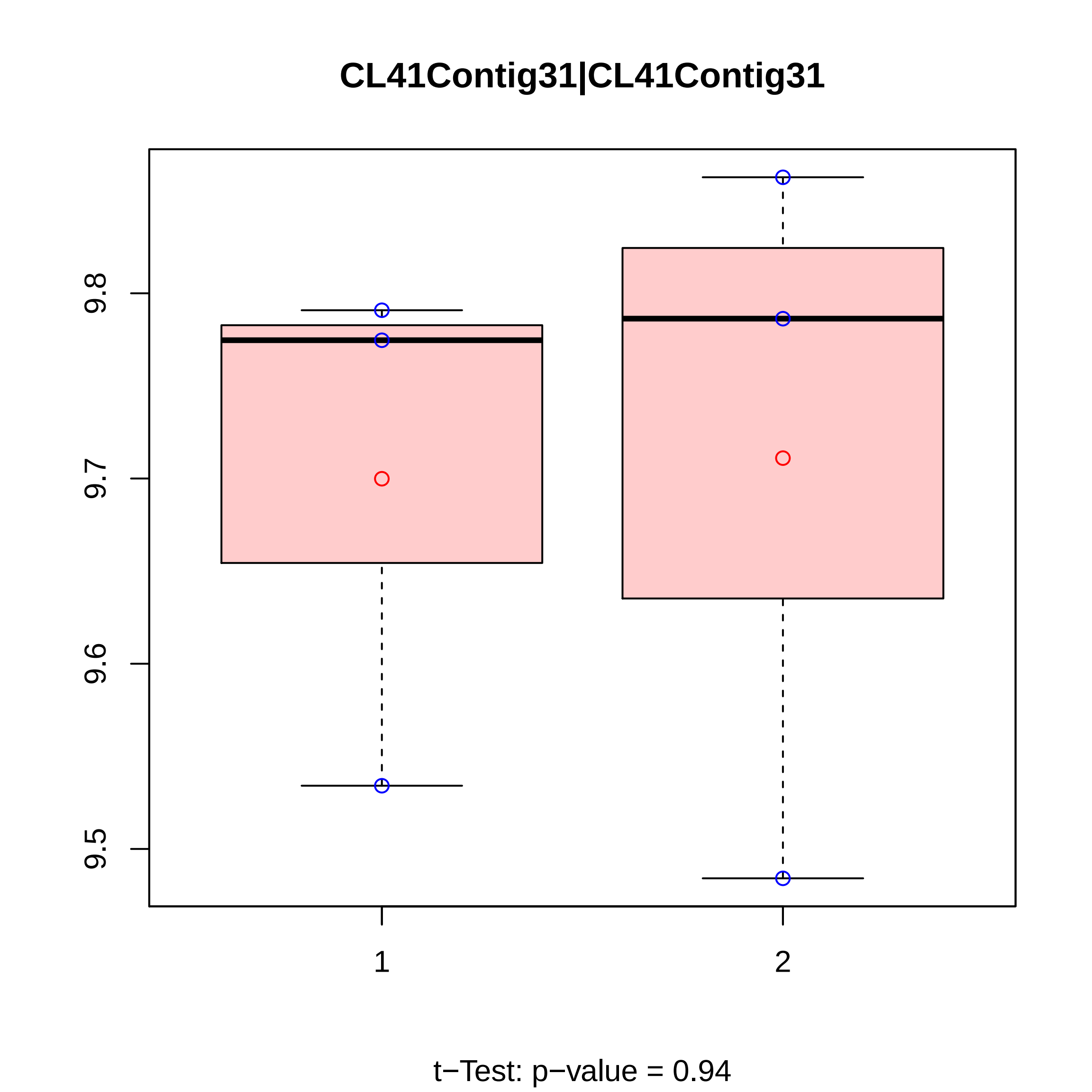

CL41Contig31|CL41Contig31
9.5
9.6
9.7
9.8
1
2
t−Test: p−value = 0.94

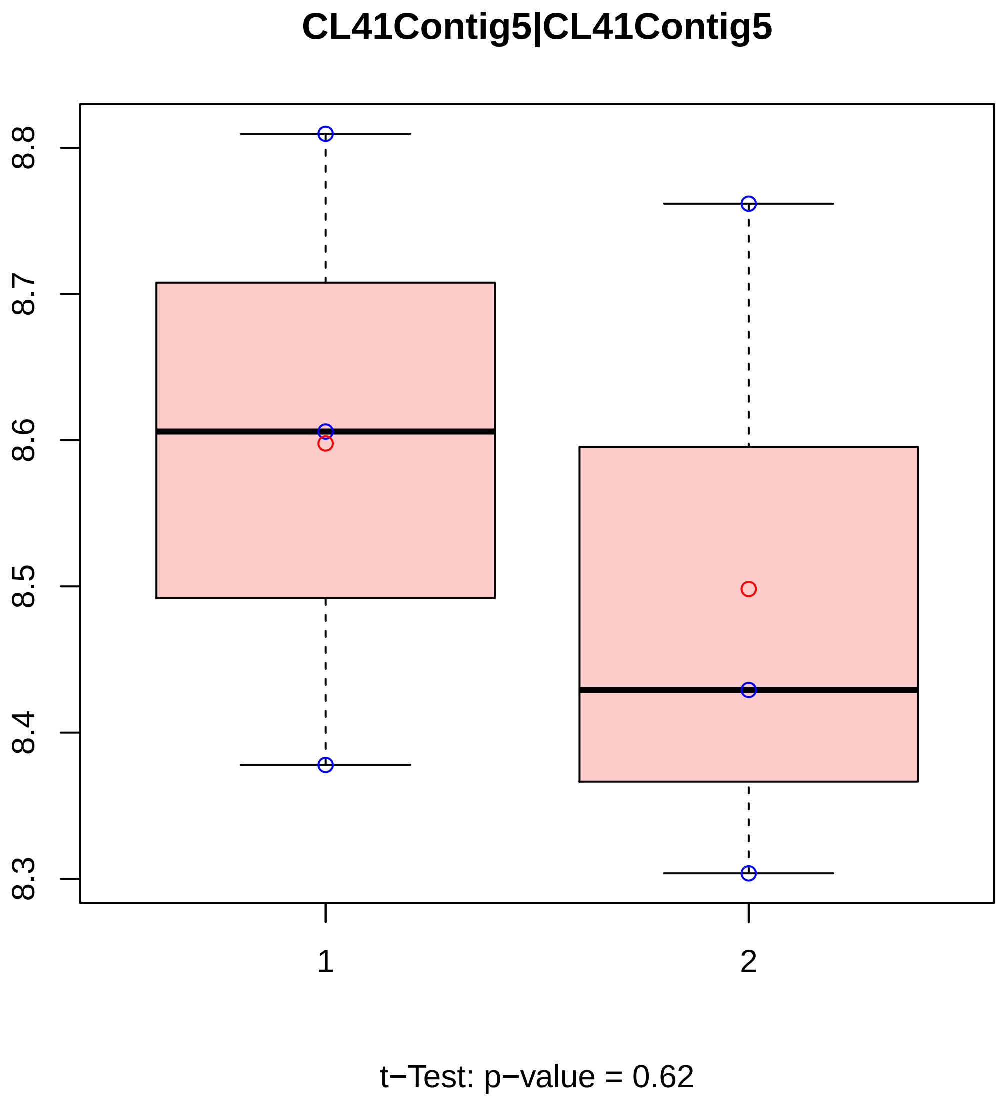
CL41Contig5|CL41Contig5
8.8
8.7
8.6
8.5
8.4
8.3
1
2
t−Test: p−value = 0.62

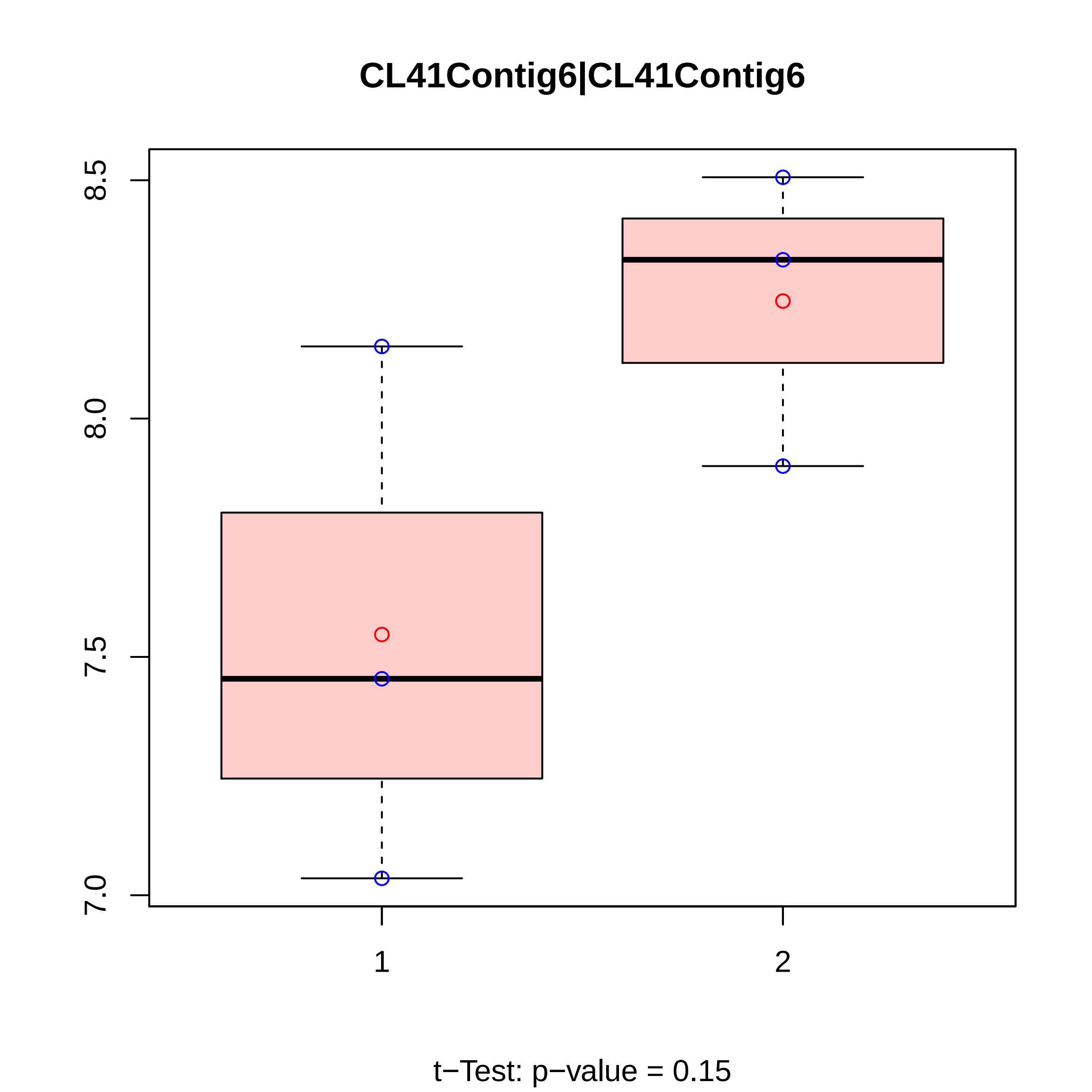
CL41Contig6|CL41Contig6
8.5
8.0
7.5
7.0
1
2
t−Test: p−value = 0.15

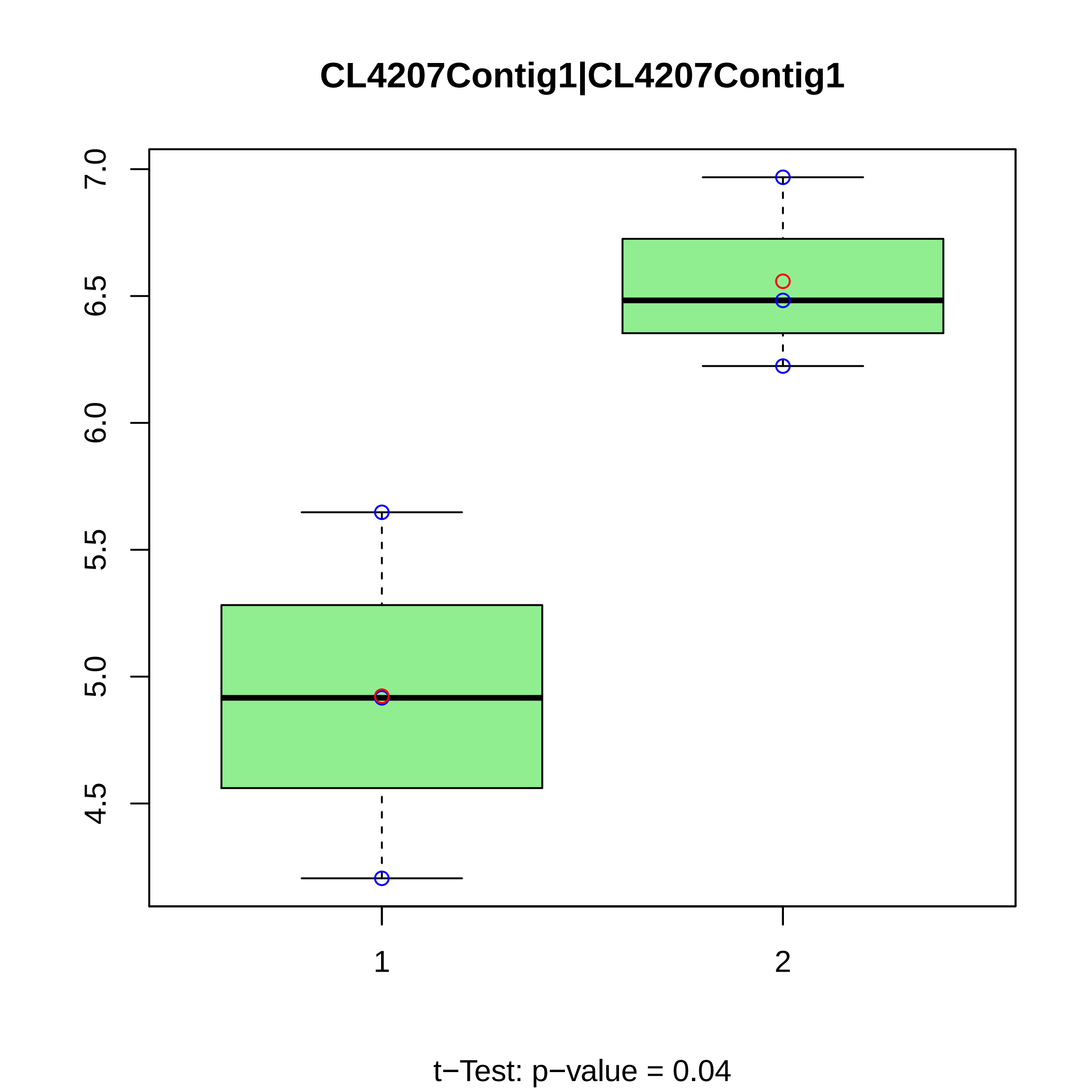
CL4207Contig1|CL4207Contig1
7.0
6.5
6.0
5.5
5.0
4.5
1
2
t−Test: p−value = 0.04

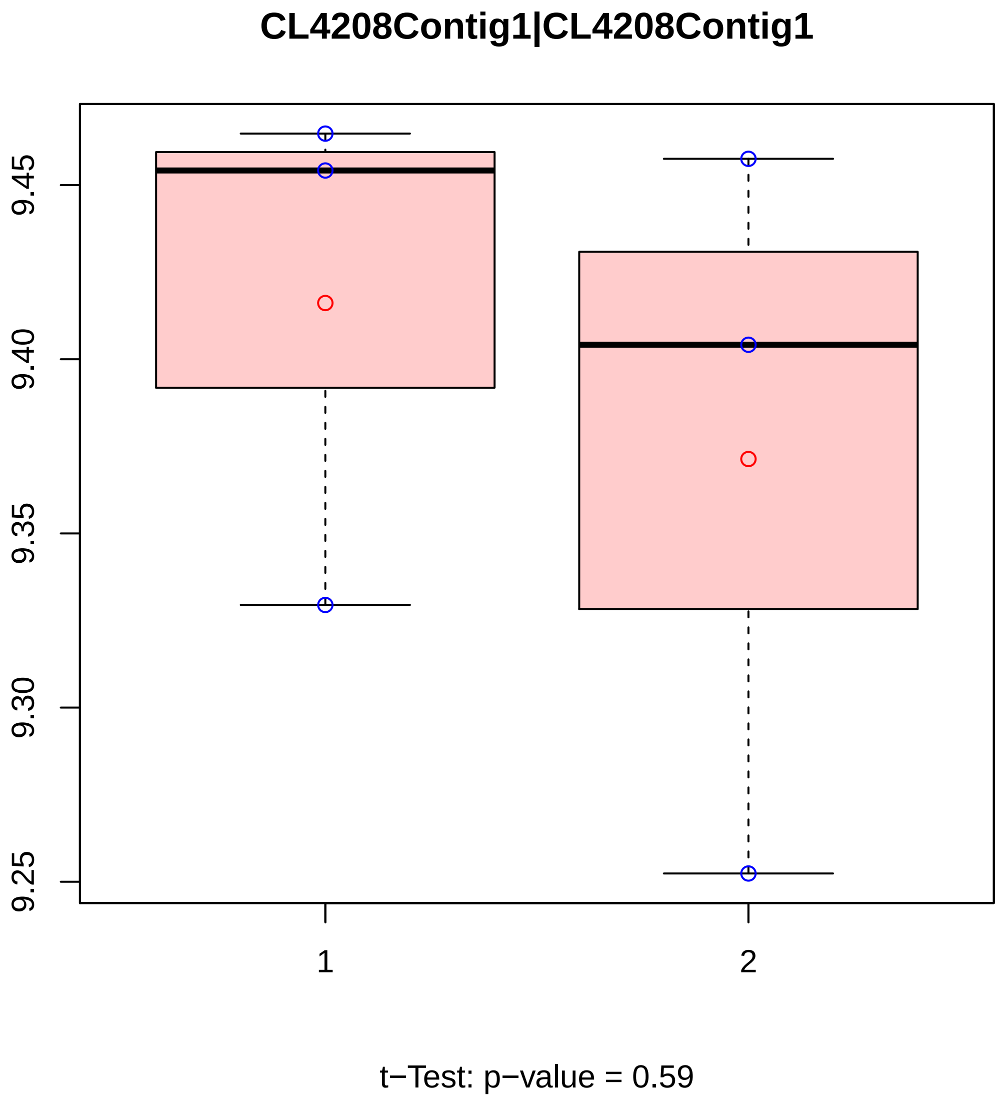
CL4208Contig1|CL4208Contig1
9.45
9.40
9.35
9.30
9.25
1
2
t−Test: p−value = 0.59

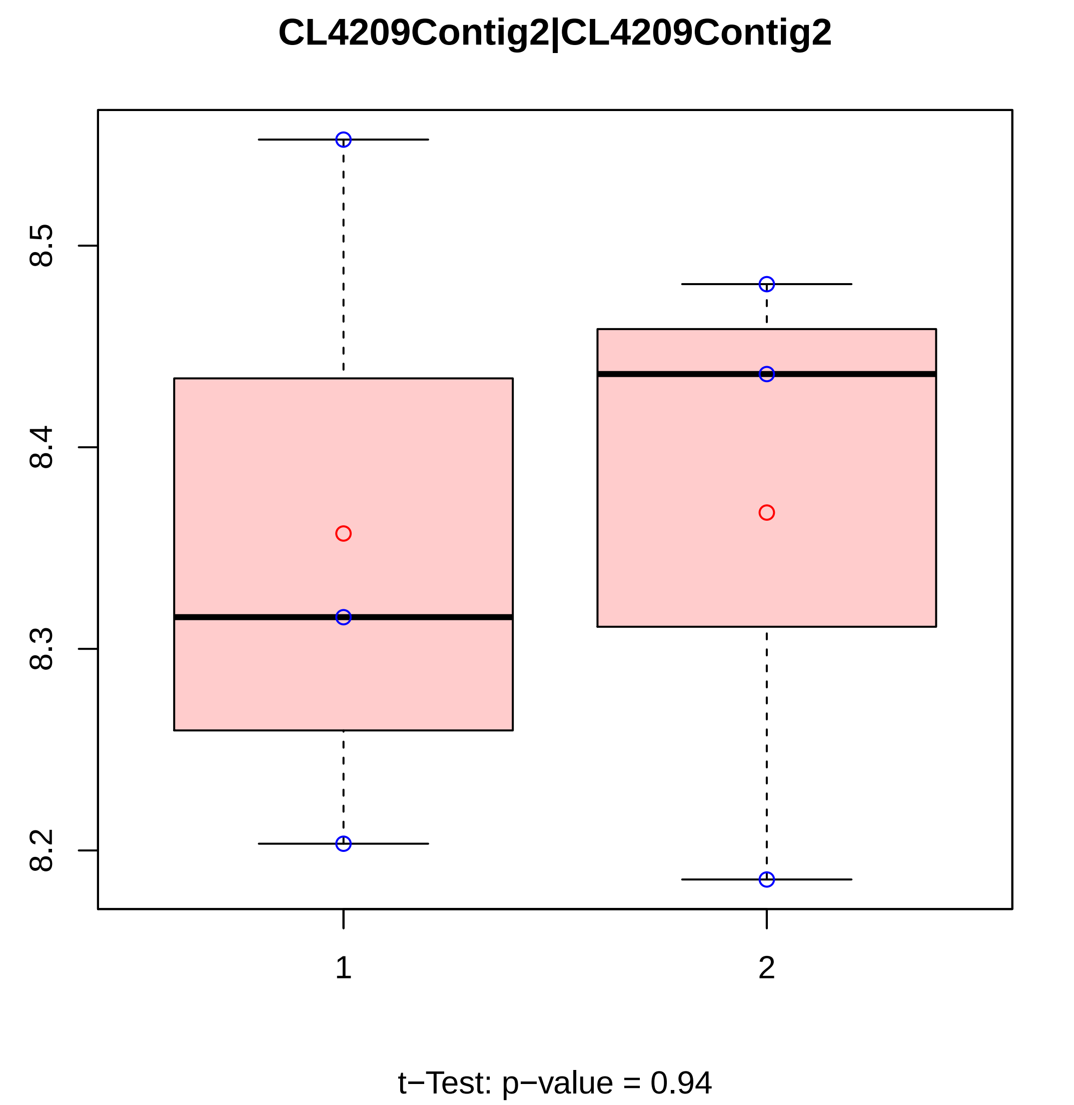

CL4209Contig2|CL4209Contig2
8.2
8.3
8.4
8.5
1
2
t−Test: p−value = 0.94

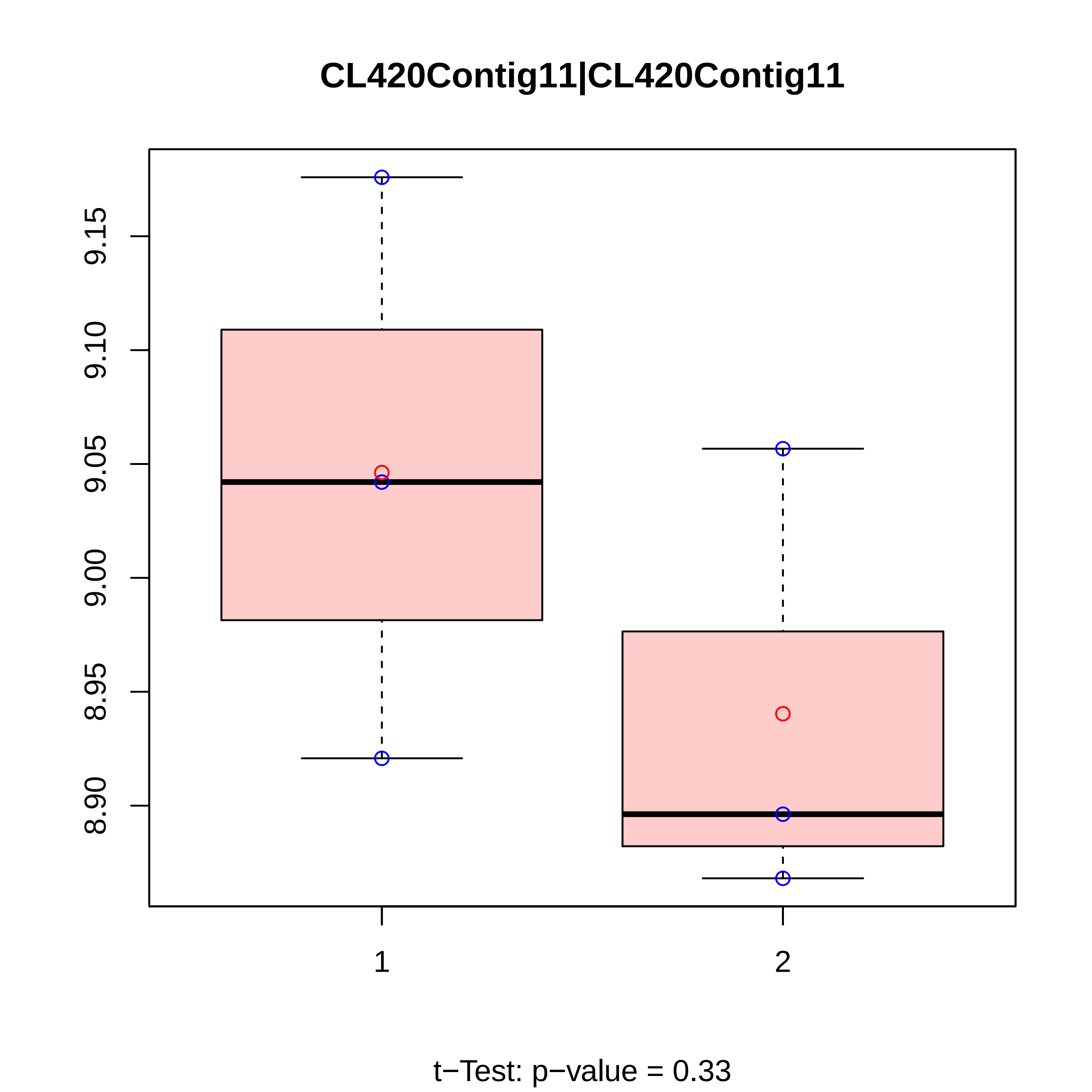

CL420Contig11|CL420Contig11
9.15
9.10
9.05
9.00
8.95
8.90
1
2
t−Test: p−value = 0.33

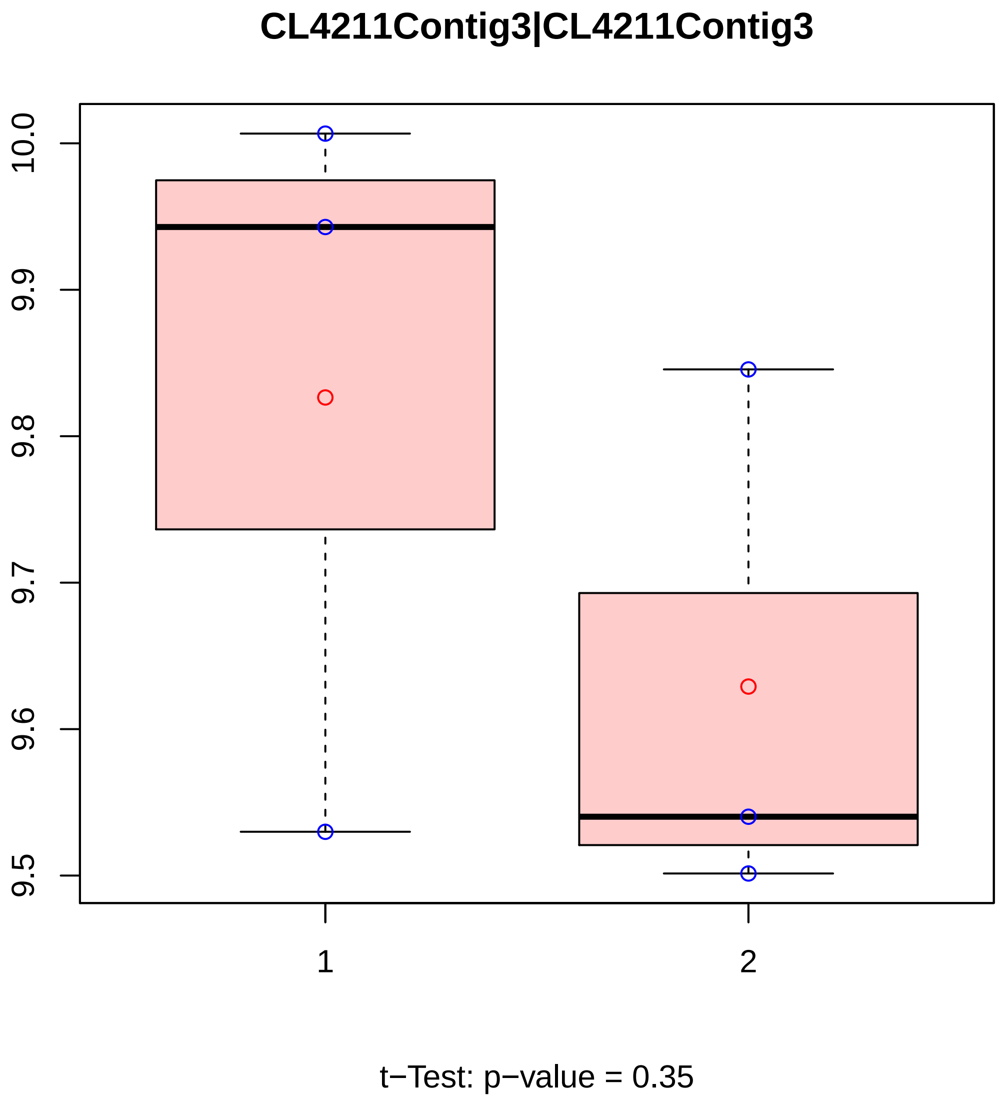

CL4211Contig3|CL4211Contig3
10.0
9.9
9.8
9.7
9.6
9.5
1
2
t−Test: p−value = 0.35

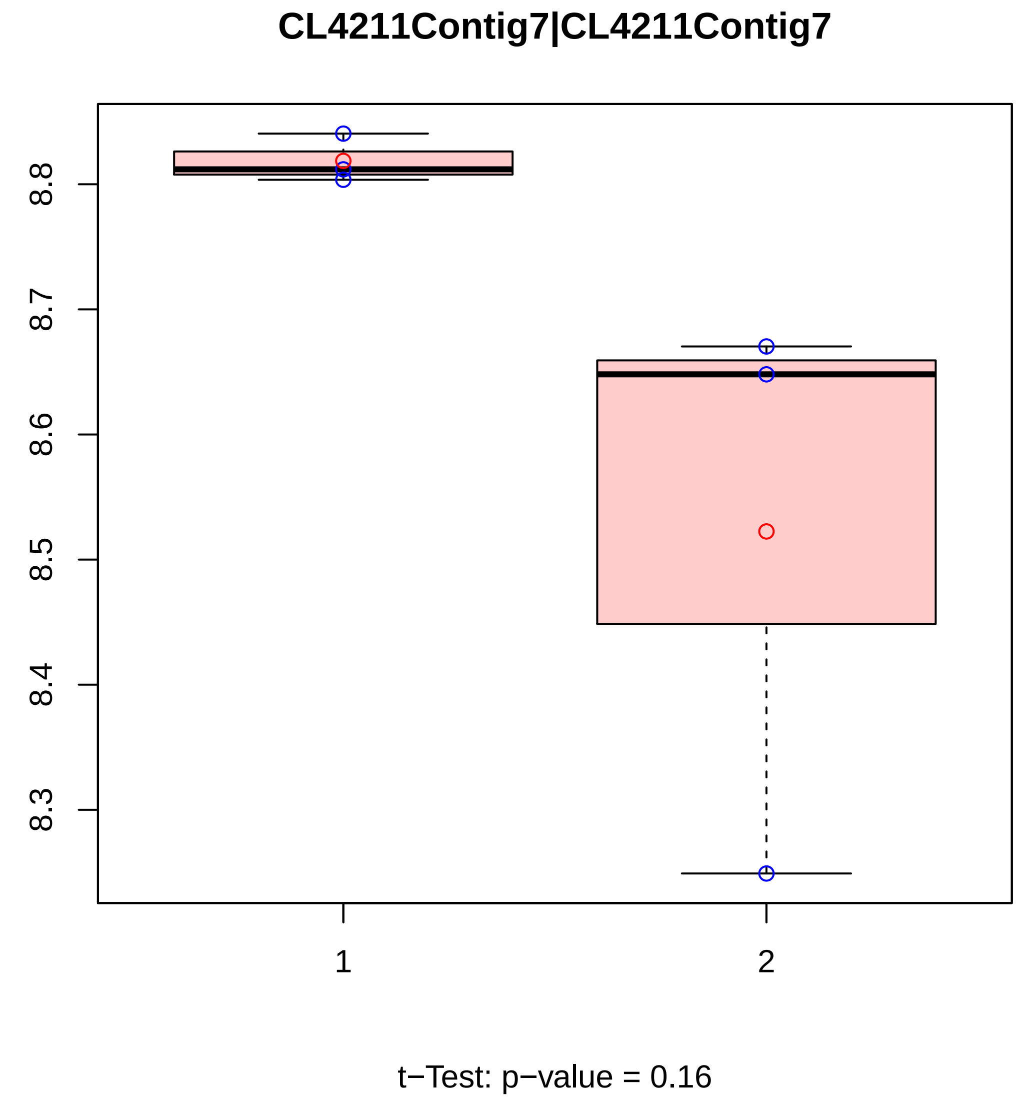

CL4211Contig7|CL4211Contig7
8.3
8.4
8.5
8.6
8.7
8.8
1
2
t−Test: p−value = 0.16

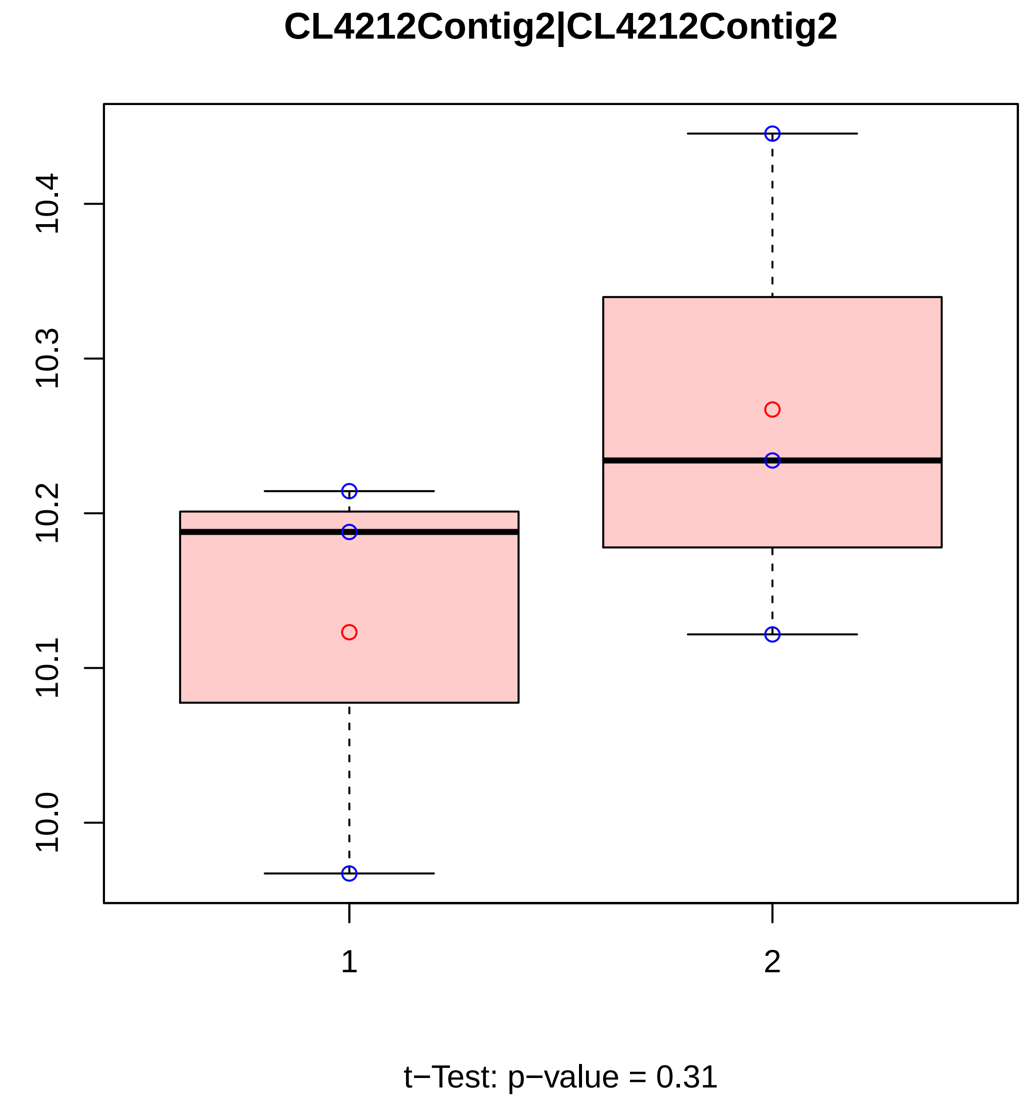
CL4212Contig2|CL4212Contig2
10.0
10.1
10.2
10.3
10.4
1
2
t−Test: p−value = 0.31

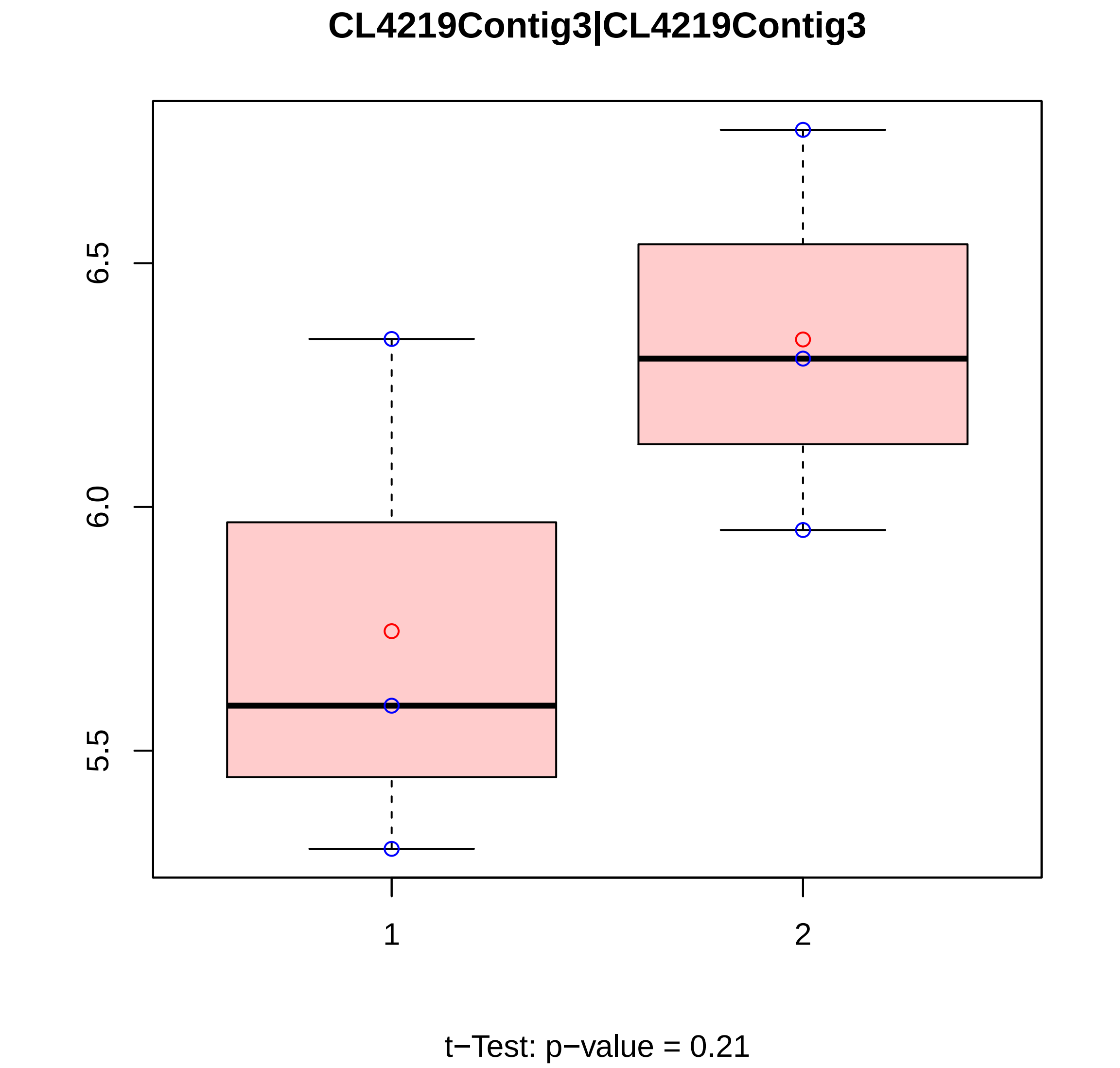
CL4219Contig3|CL4219Contig3
6.5
6.0
5.5
1
2
t−Test: p−value = 0.21

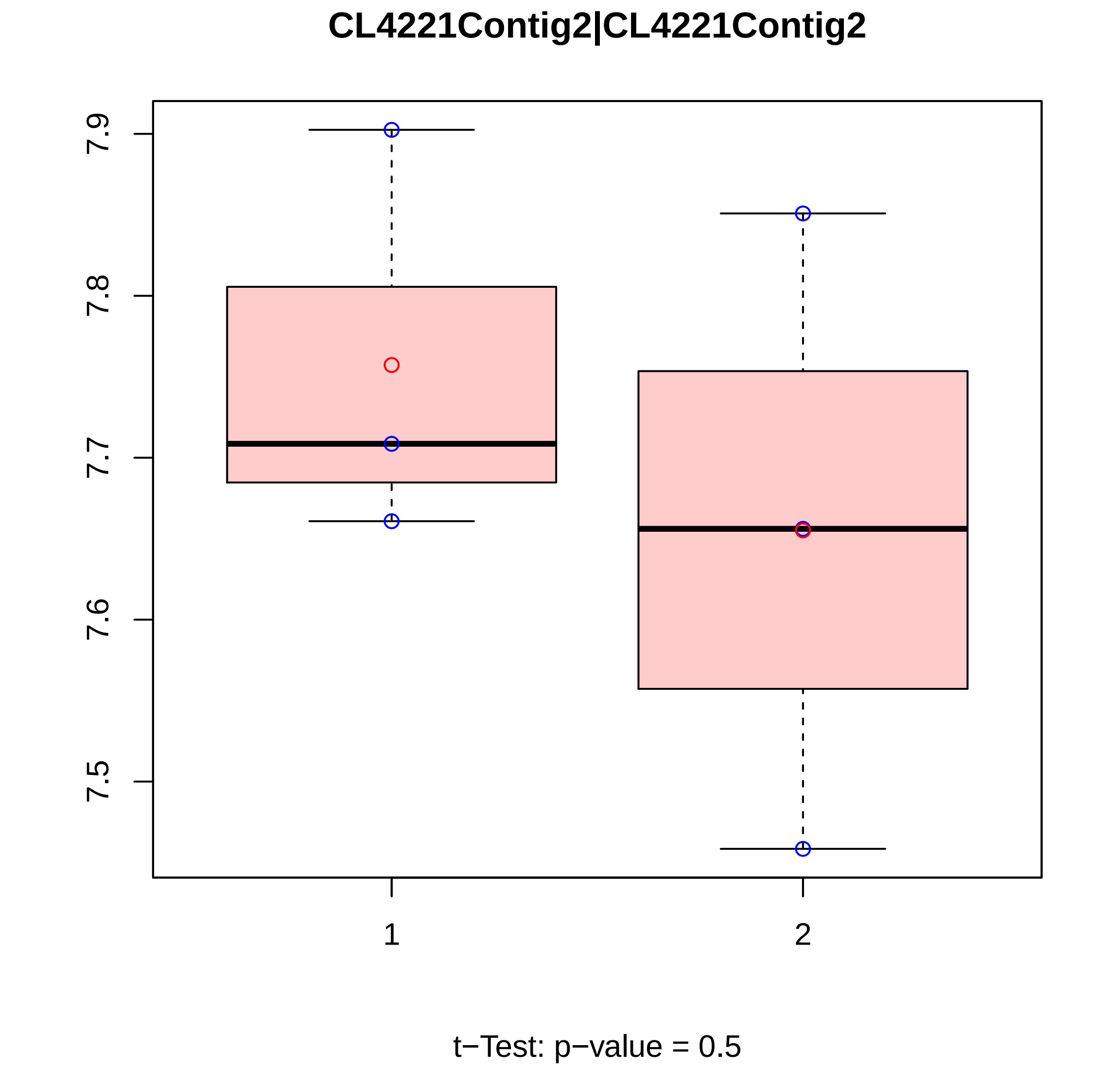
CL4221Contig2|CL4221Contig2
7.5
7.6
7.7
7.8
7.9
1
2
t−Test: p−value = 0.5

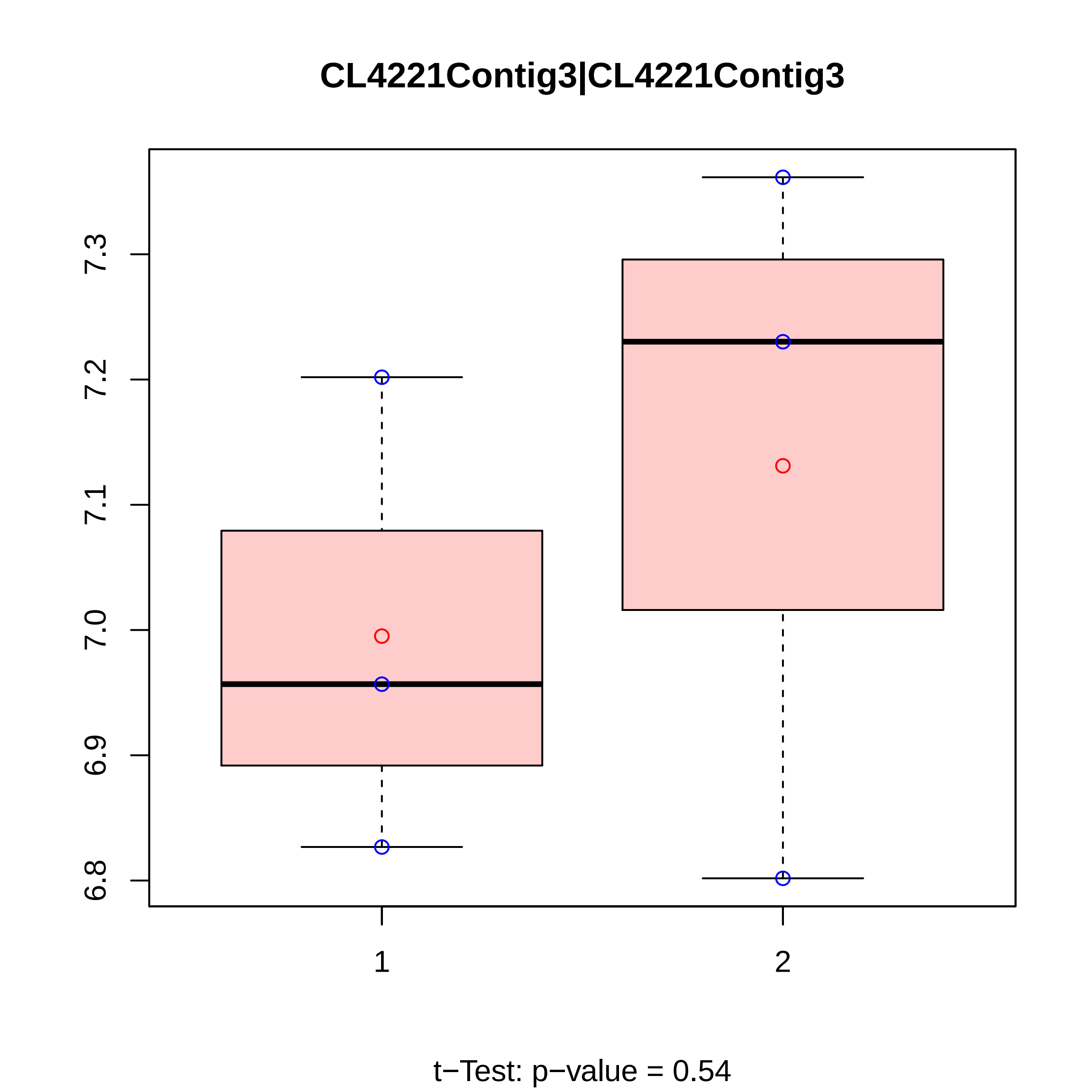

CL4221Contig3|CL4221Contig3
6.8
6.9
7.0
7.1
7.2
7.3
1
2
t−Test: p−value = 0.54

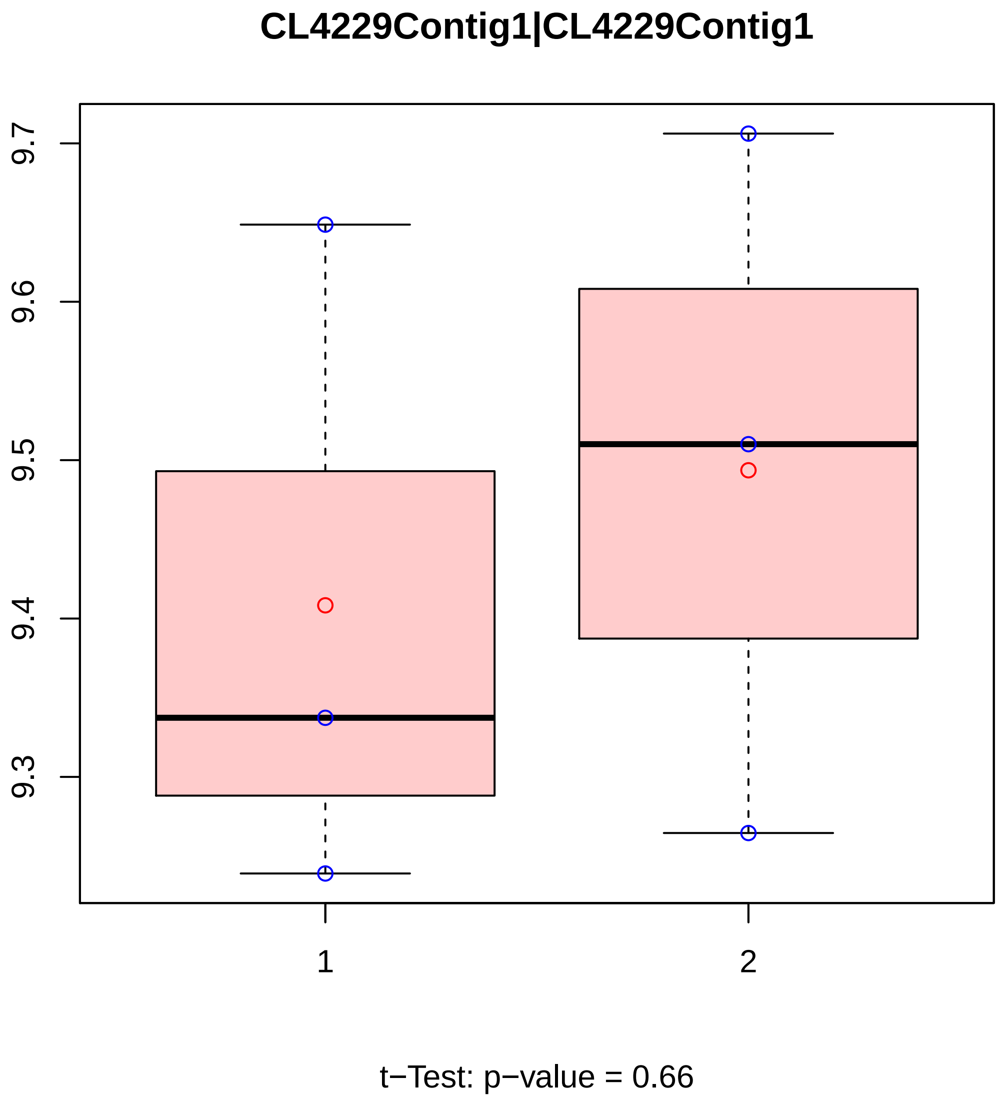
CL4229Contig1|CL4229Contig1
9.3
9.4
9.5
9.6
9.7
1
2
t−Test: p−value = 0.66

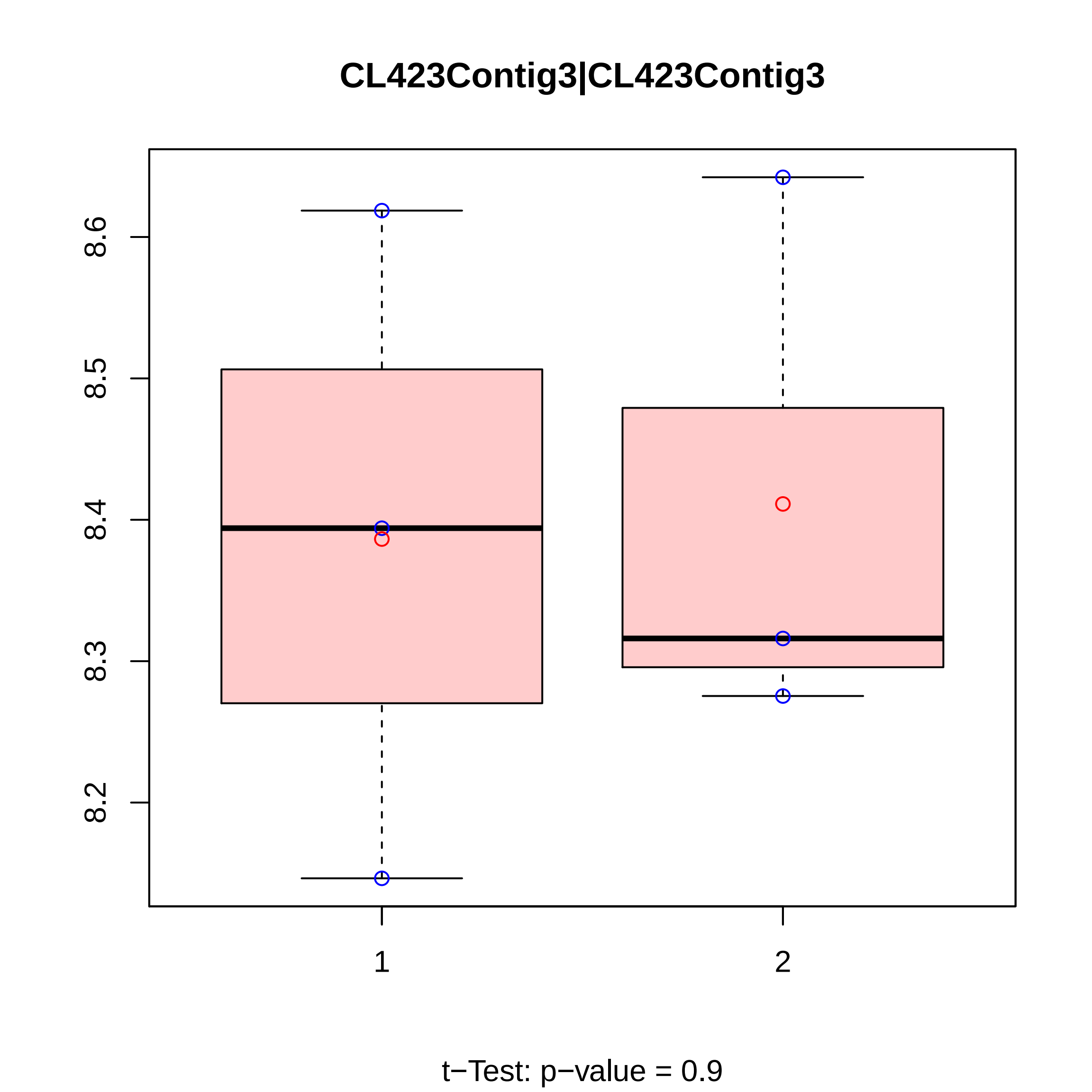

CL423Contig3|CL423Contig3
8.2
8.3
8.4
8.5
8.6
1
2
t−Test: p−value = 0.9

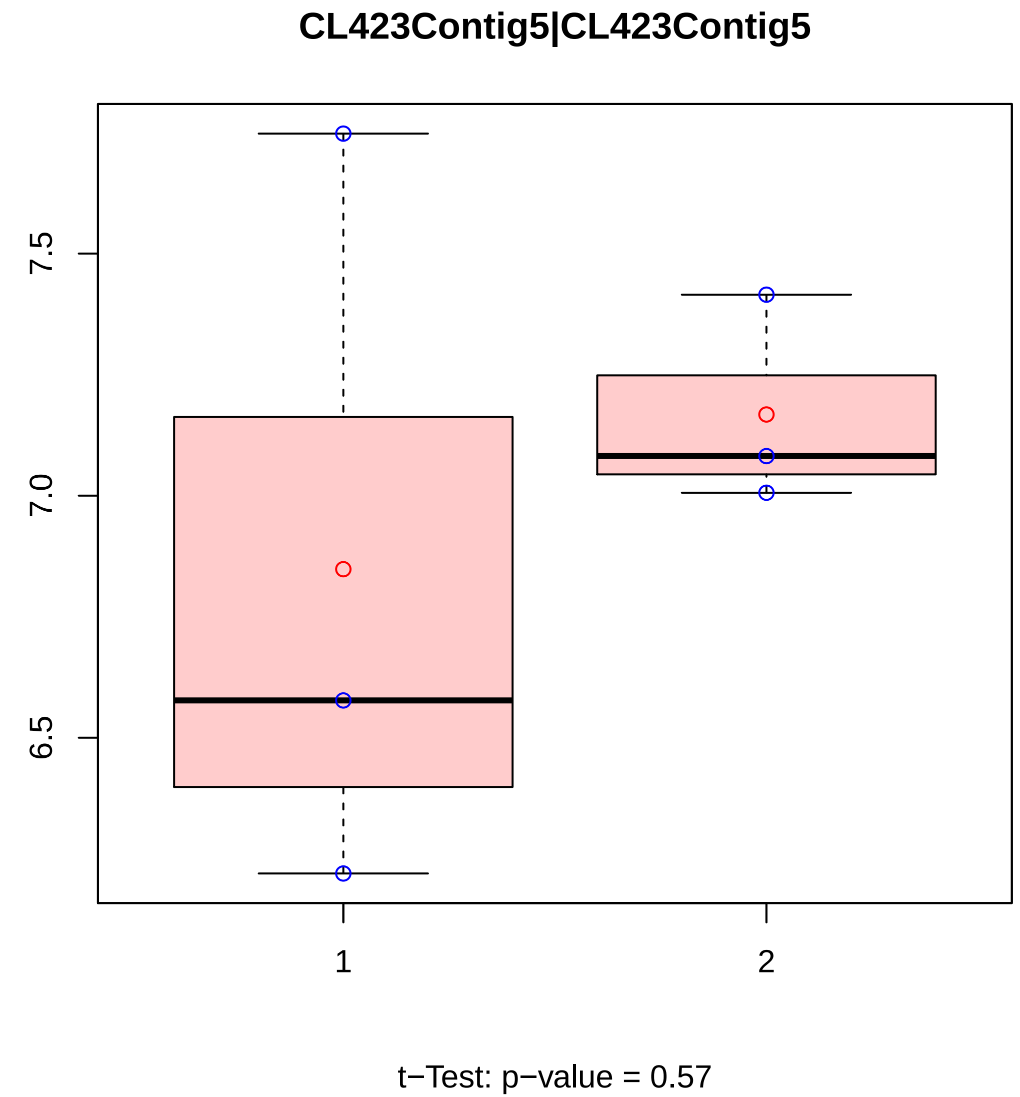
CL423Contig5|CL423Contig5
7.5
7.0
6.5
1
2
t−Test: p−value = 0.57

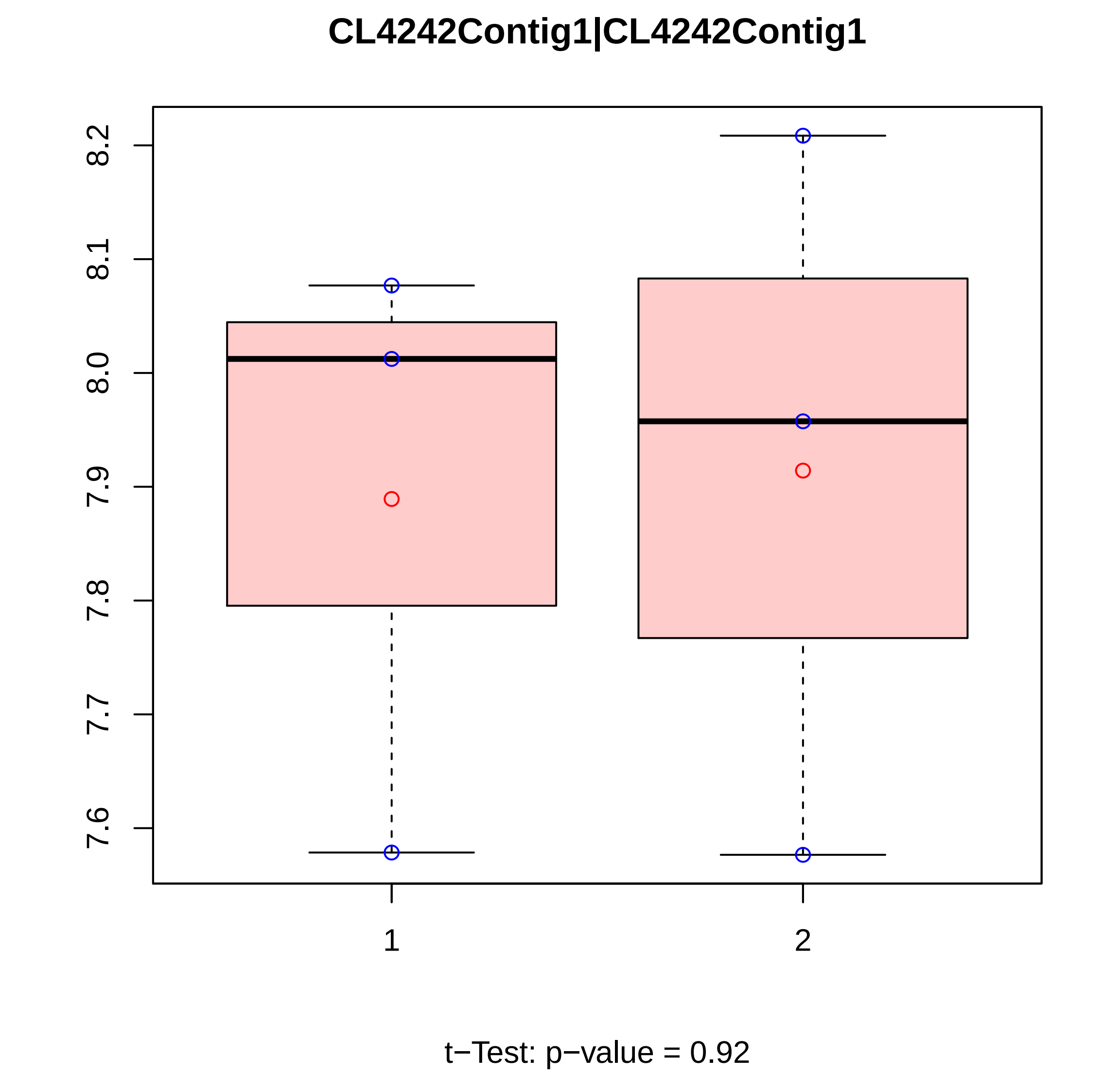
CL4242Contig1|CL4242Contig1
8.2
8.1
8.0
7.9
7.8
7.7
7.6
1
2
t−Test: p−value = 0.92

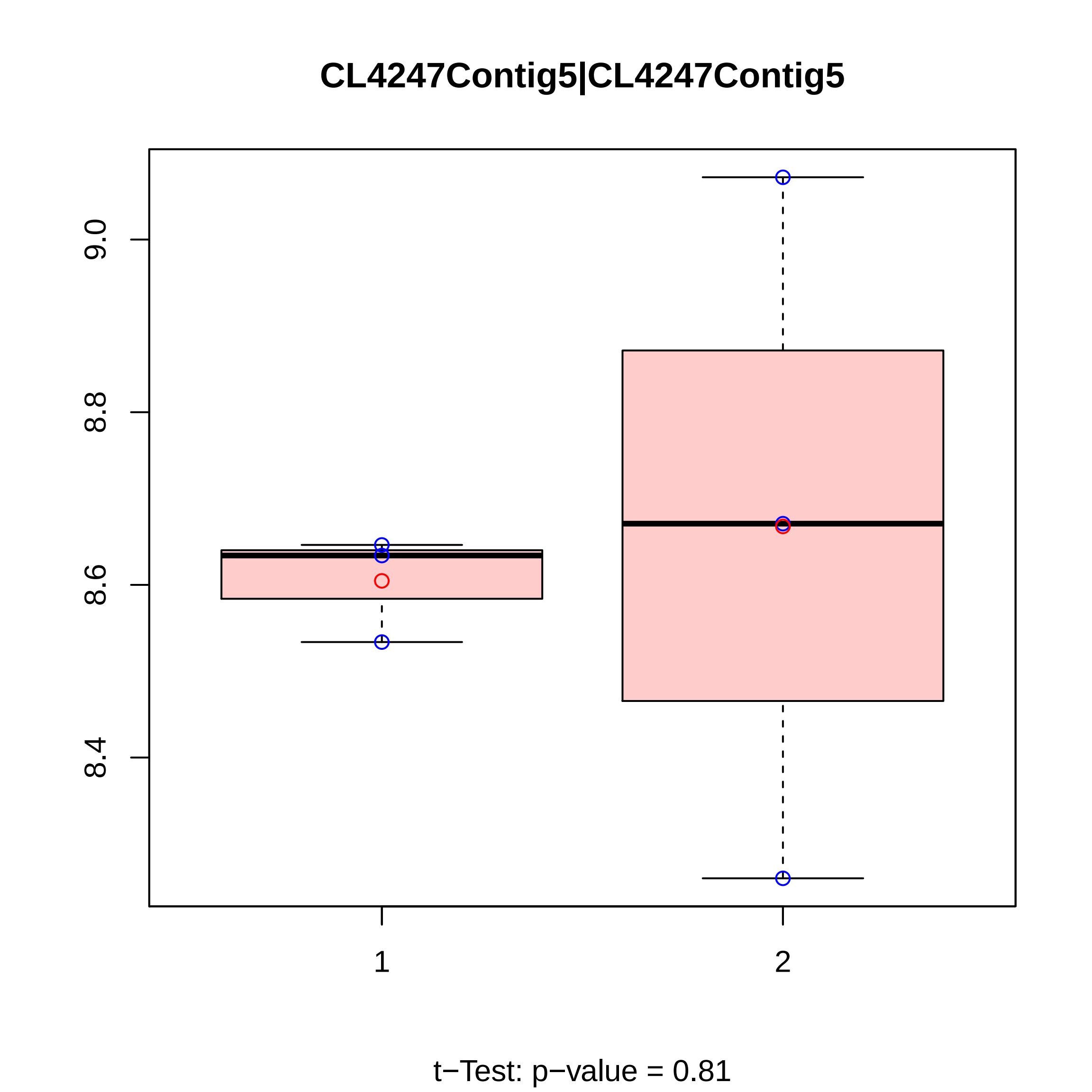
CL4247Contig5|CL4247Contig5
9.0
8.8
8.6
8.4
1
2
t−Test: p−value = 0.81

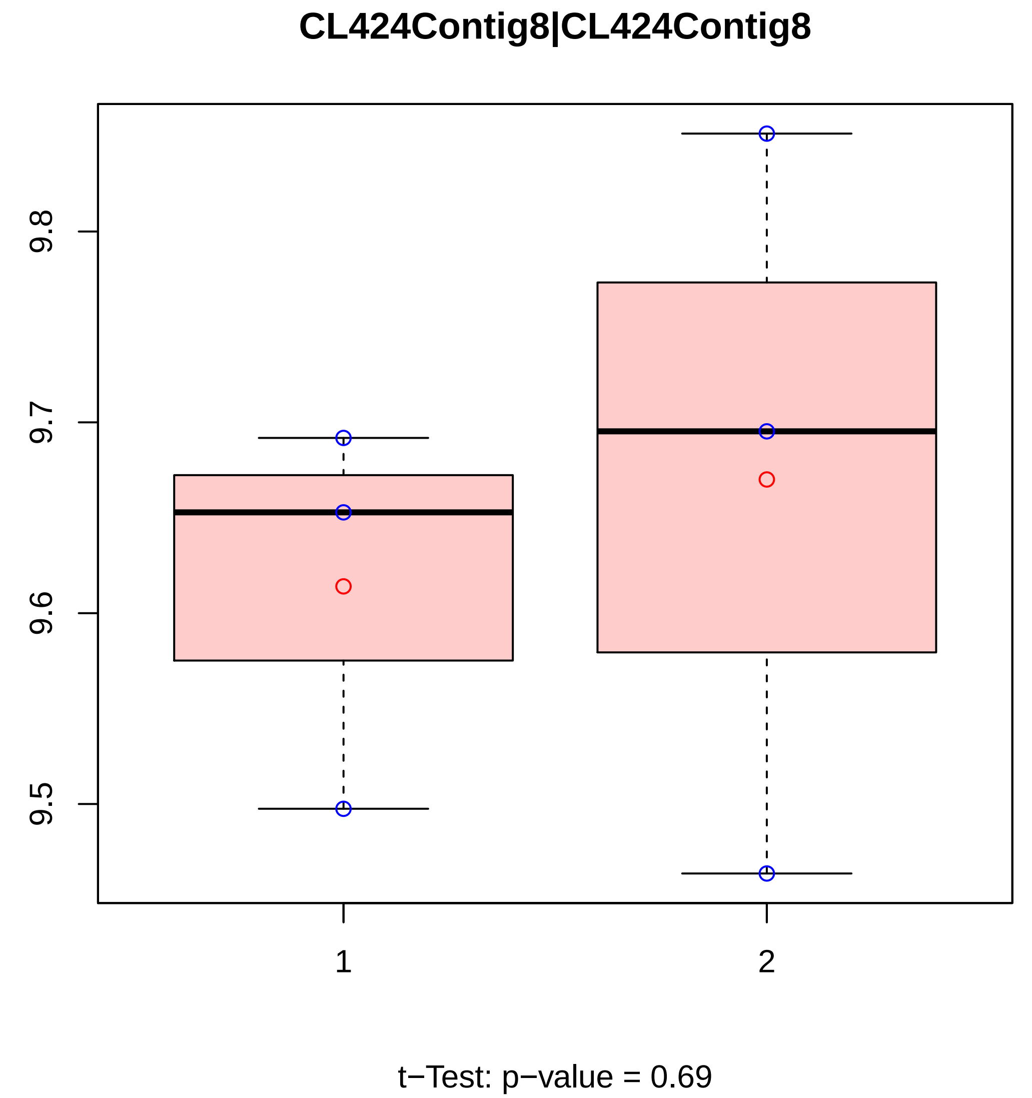

CL424Contig8|CL424Contig8
9.5
9.6
9.7
9.8
1
2
t−Test: p−value = 0.69

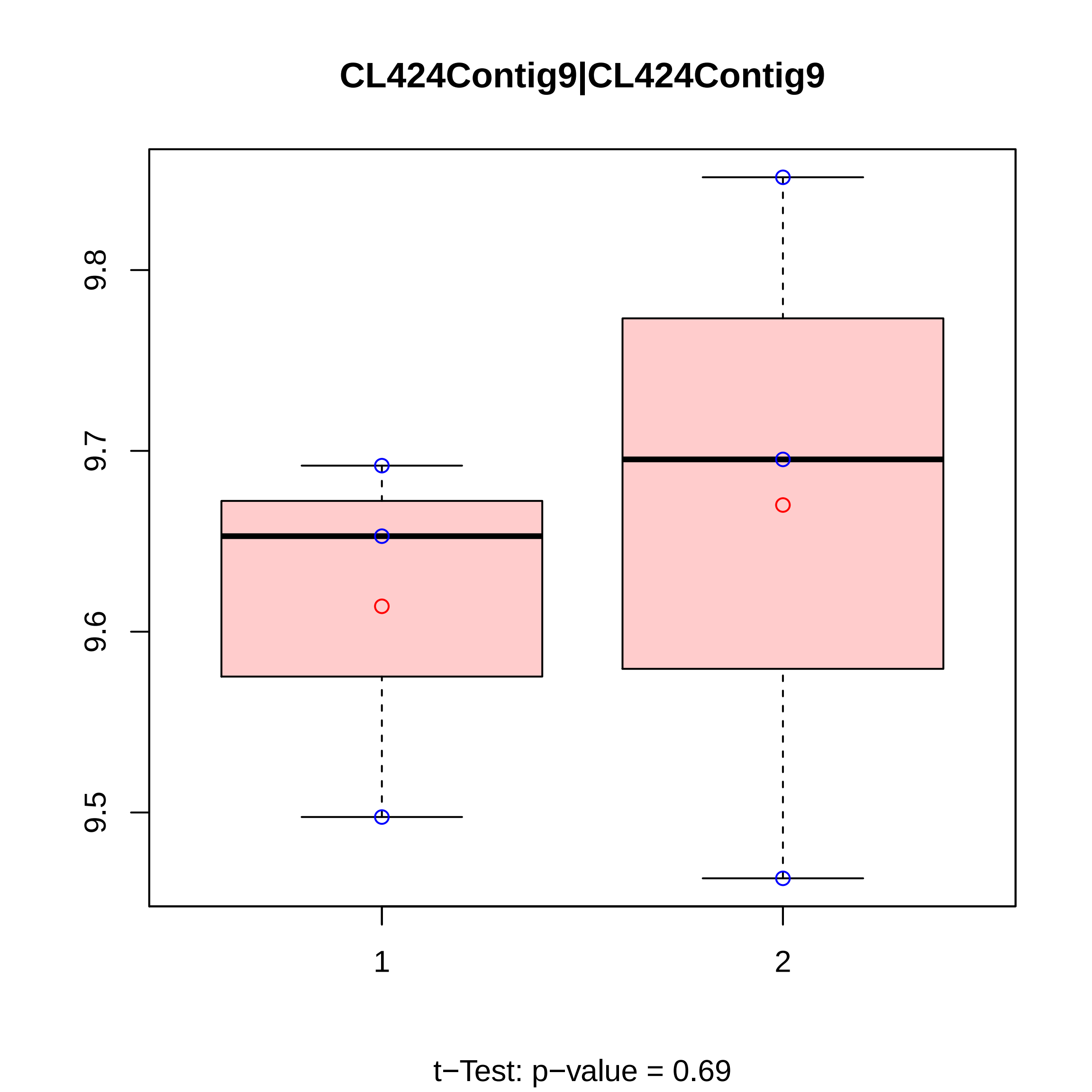
CL424Contig9|CL424Contig9
9.5
9.6
9.7
9.8
1
2
t−Test: p−value = 0.69

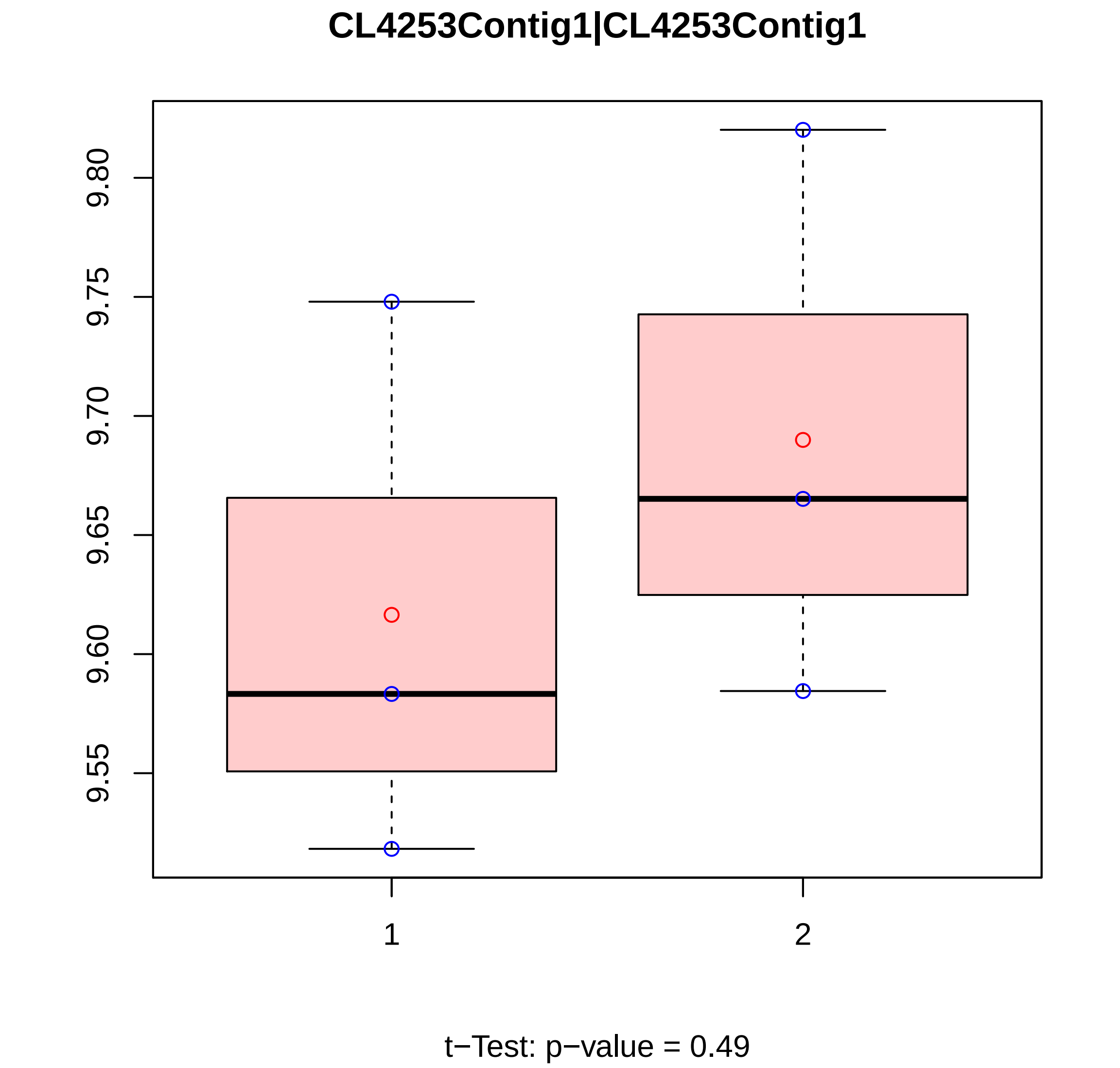

CL4253Contig1|CL4253Contig1
9.55
9.60
9.65
9.70
9.75
9.80
1
2
t−Test: p−value = 0.49

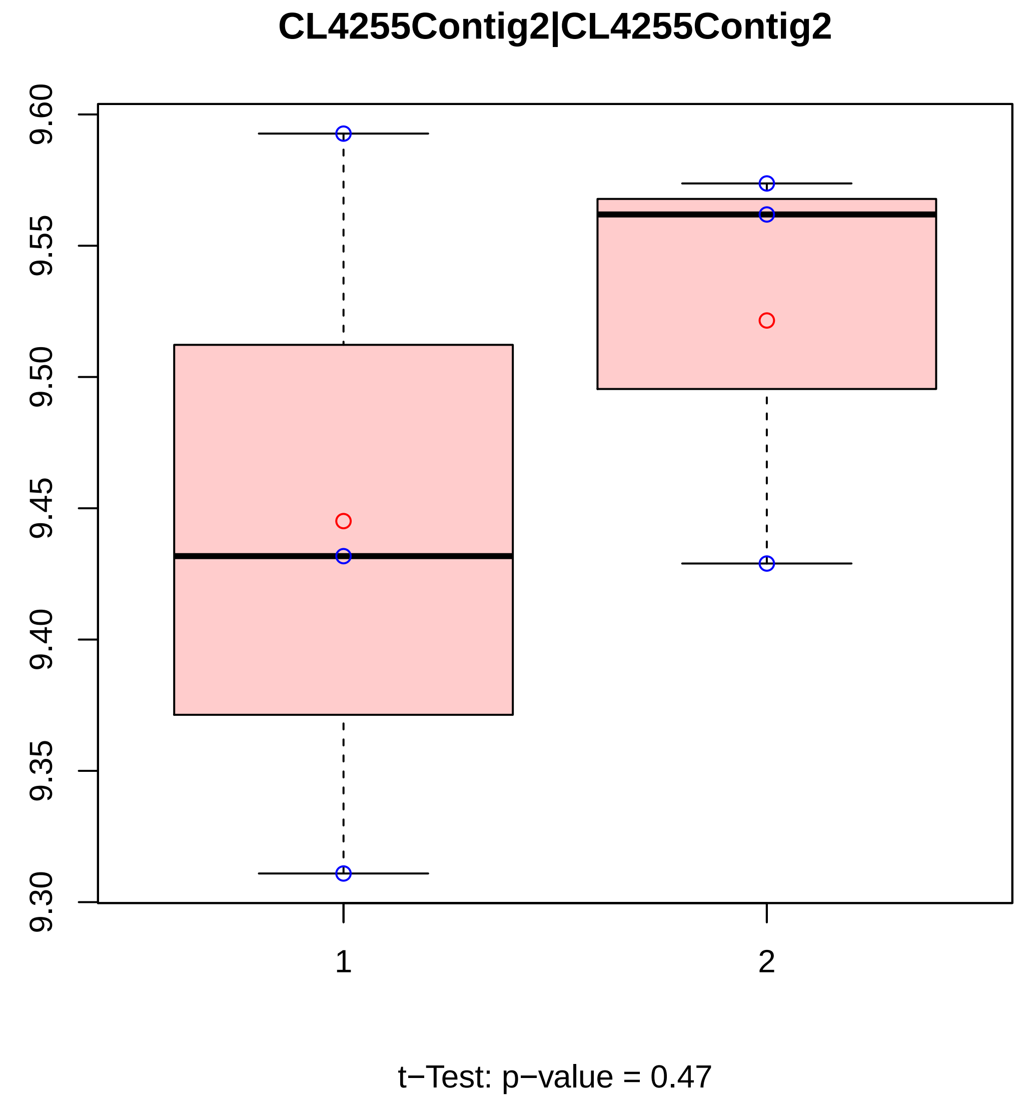

CL4255Contig2|CL4255Contig2
9.30
9.35
9.40
9.45
9.50
9.55
9.60
1
2
t−Test: p−value = 0.47

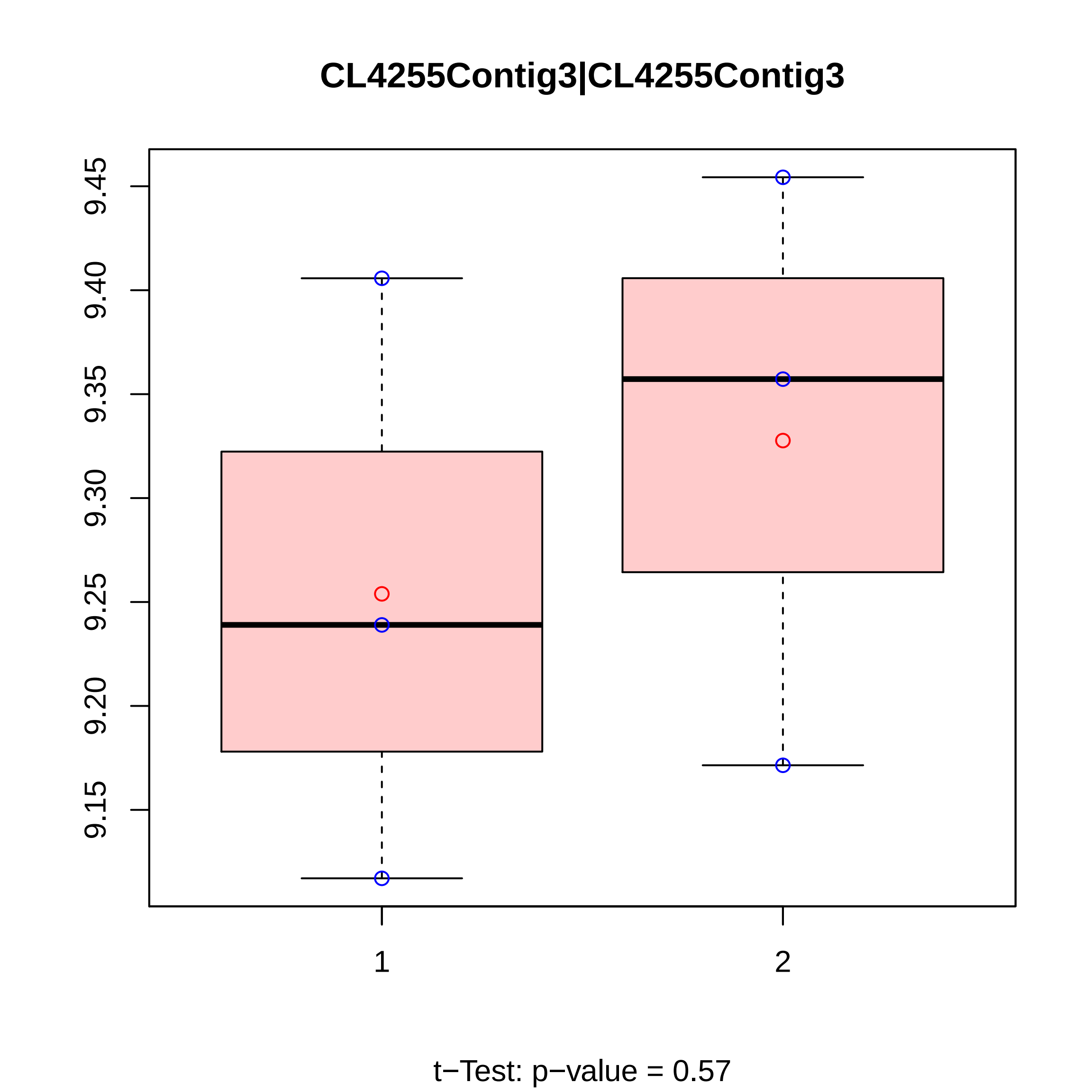
CL4255Contig3|CL4255Contig3
9.45
9.40
9.35
9.30
9.25
9.20
9.15
1
2
t−Test: p−value = 0.57

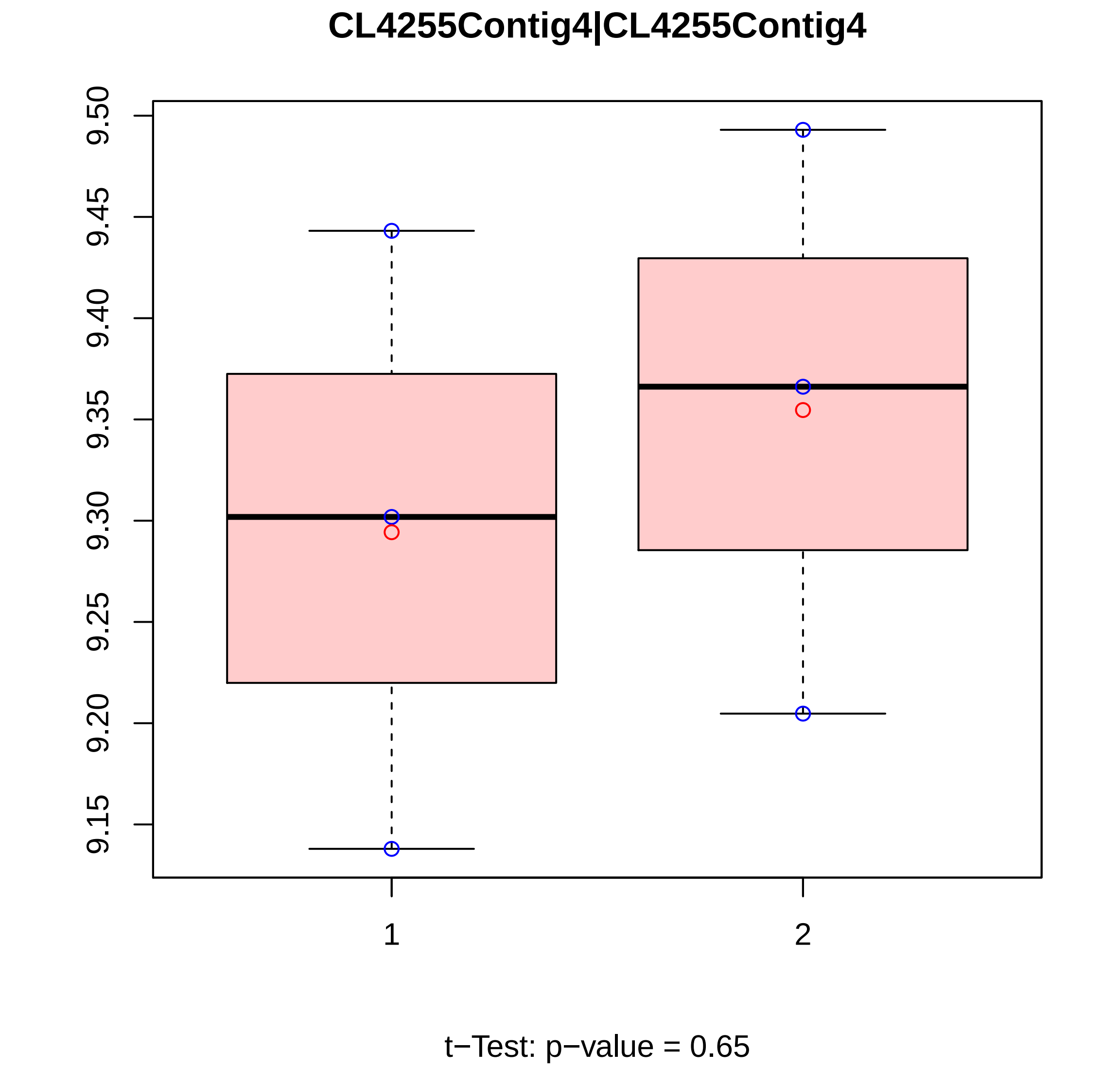
CL4255Contig4|CL4255Contig4
9.15
9.20
9.25
9.30
9.35
9.40
9.45
9.50
1
2
t−Test: p−value = 0.65

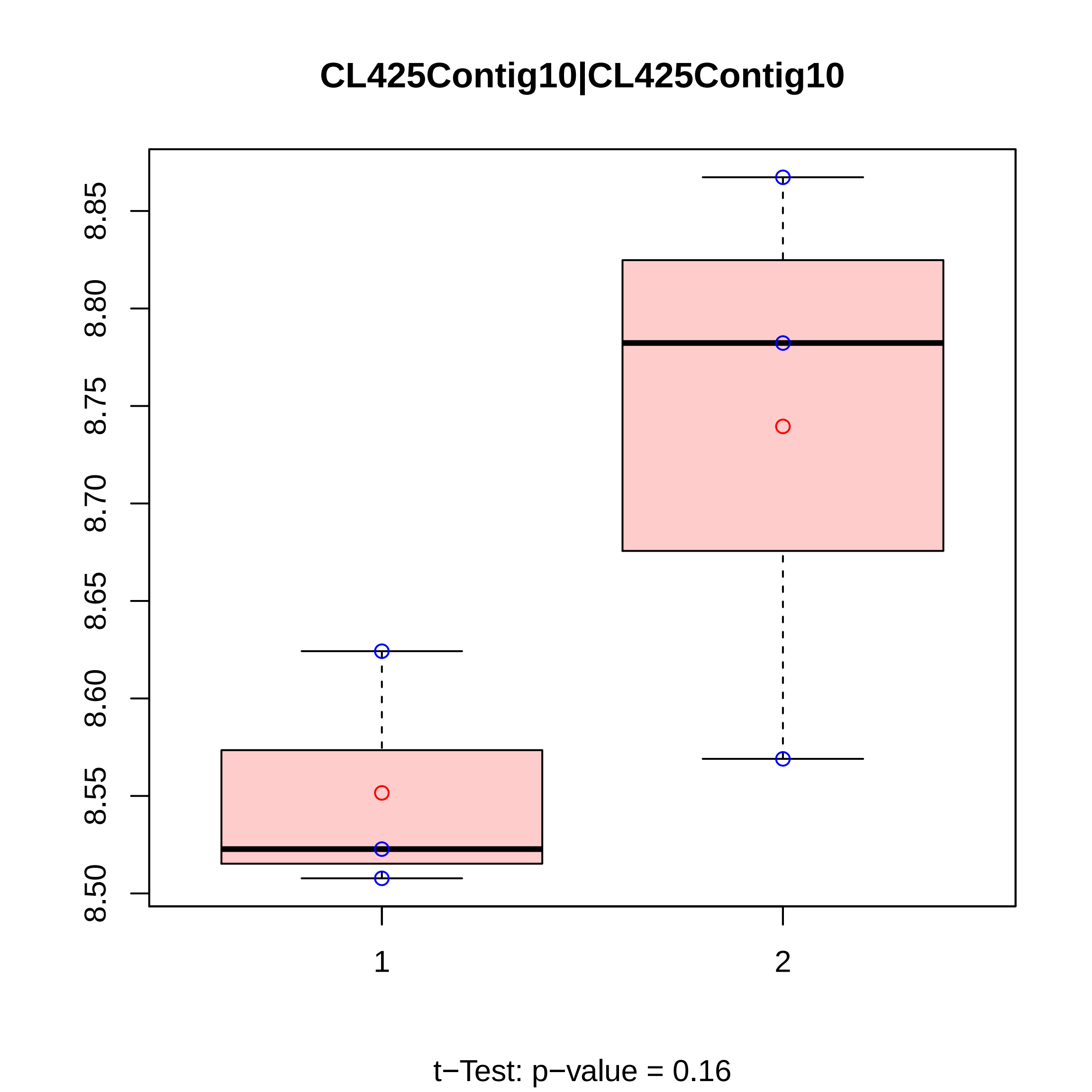

CL425Contig10|CL425Contig10
8.50
8.55
8.60
8.65
8.70
8.75
8.80
8.85
1
2
t−Test: p−value = 0.16

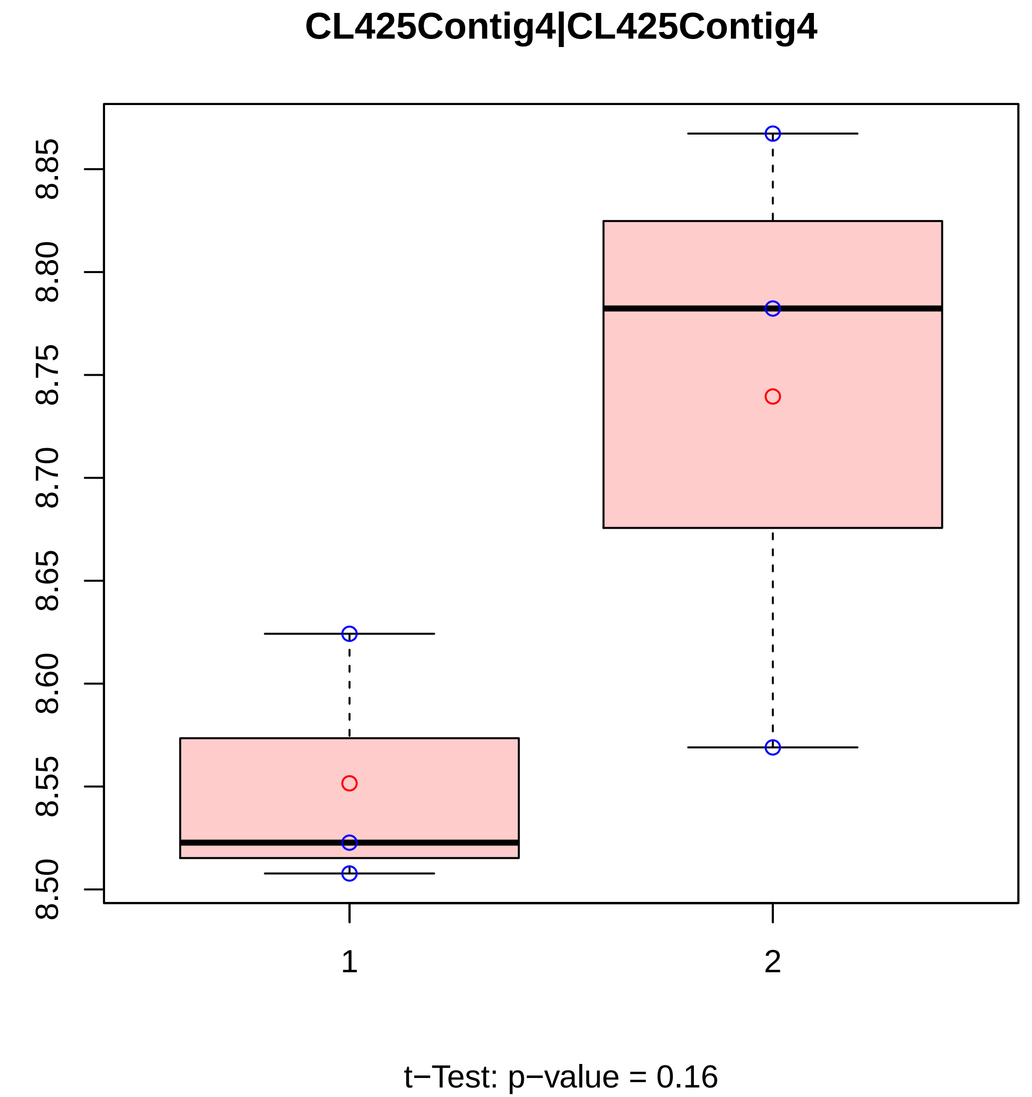
CL425Contig4|CL425Contig4
8.50
8.55
8.60
8.65
8.70
8.75
8.80
8.85
1
2
t−Test: p−value = 0.16

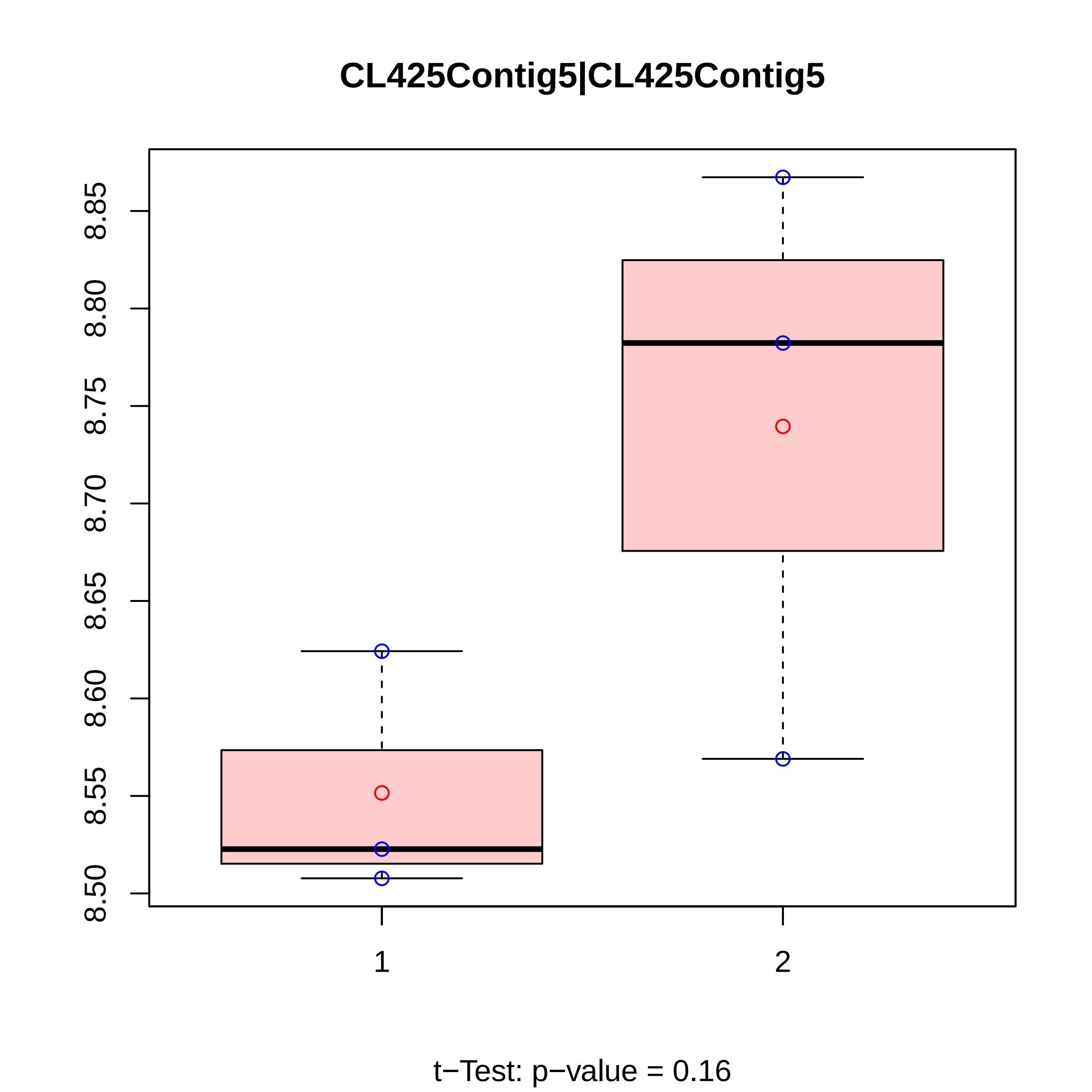

CL425Contig5|CL425Contig5
8.50
8.55
8.60
8.65
8.70
8.75
8.80
8.85
1
2
t−Test: p−value = 0.16

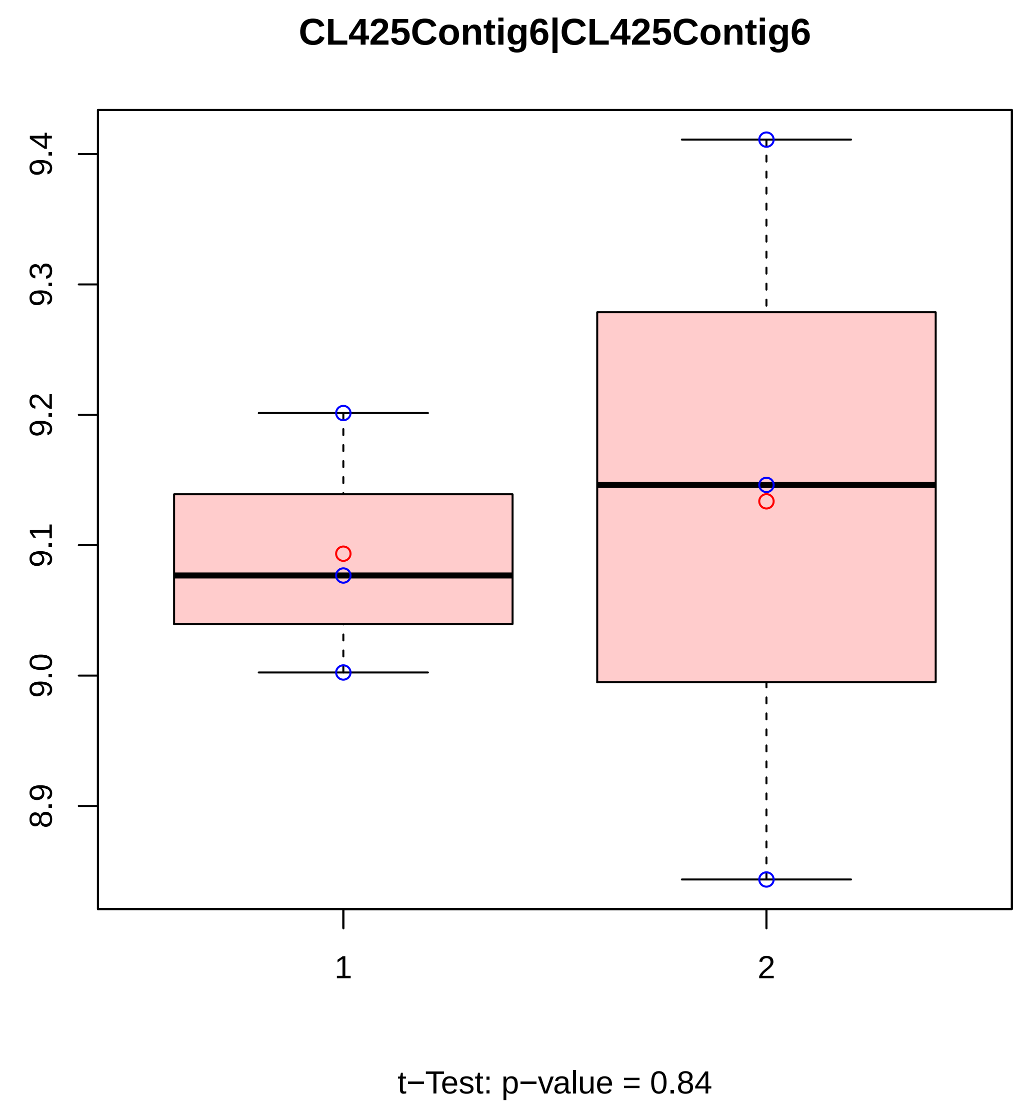
CL425Contig6|CL425Contig6
9.4
9.3
9.2
9.1
9.0
8.9
1
2
t−Test: p−value = 0.84

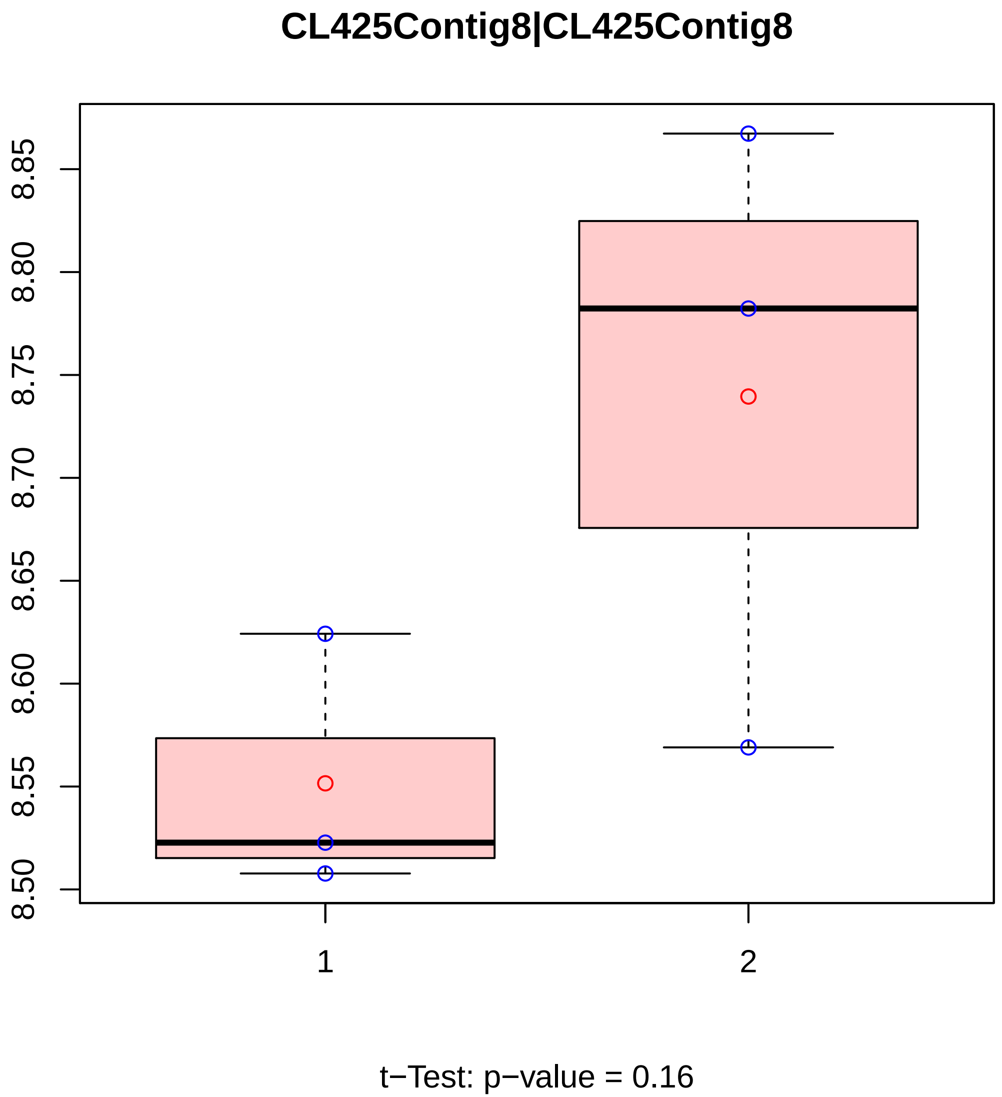
CL425Contig8|CL425Contig8
8.50
8.55
8.60
8.65
8.70
8.75
8.80
8.85
1
2
t−Test: p−value = 0.16

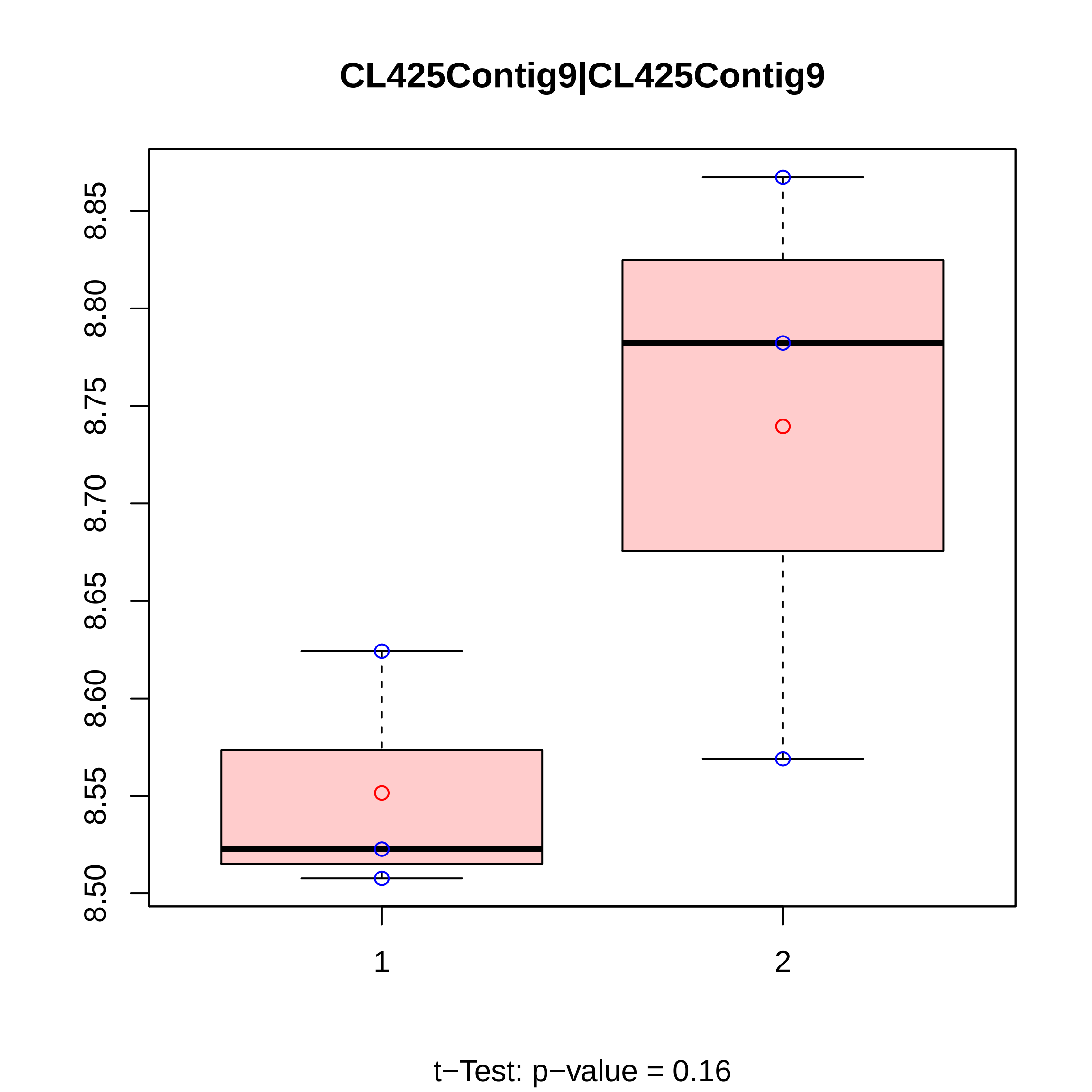
CL425Contig9|CL425Contig9
8.50
8.55
8.60
8.65
8.70
8.75
8.80
8.85
1
2
t−Test: p−value = 0.16

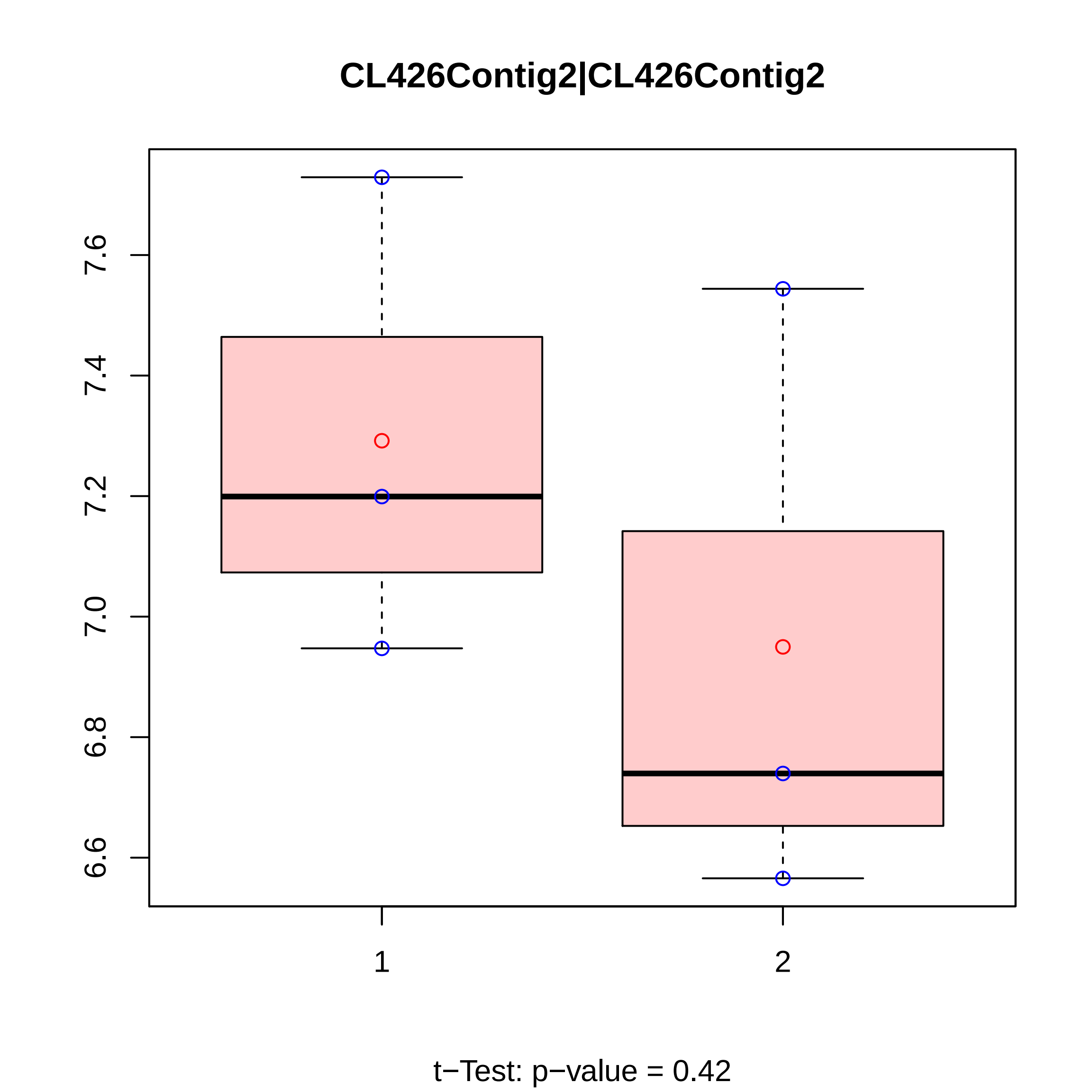
CL426Contig2|CL426Contig2
7.6
7.4
7.2
7.0
6.8
6.6
1
2
t−Test: p−value = 0.42

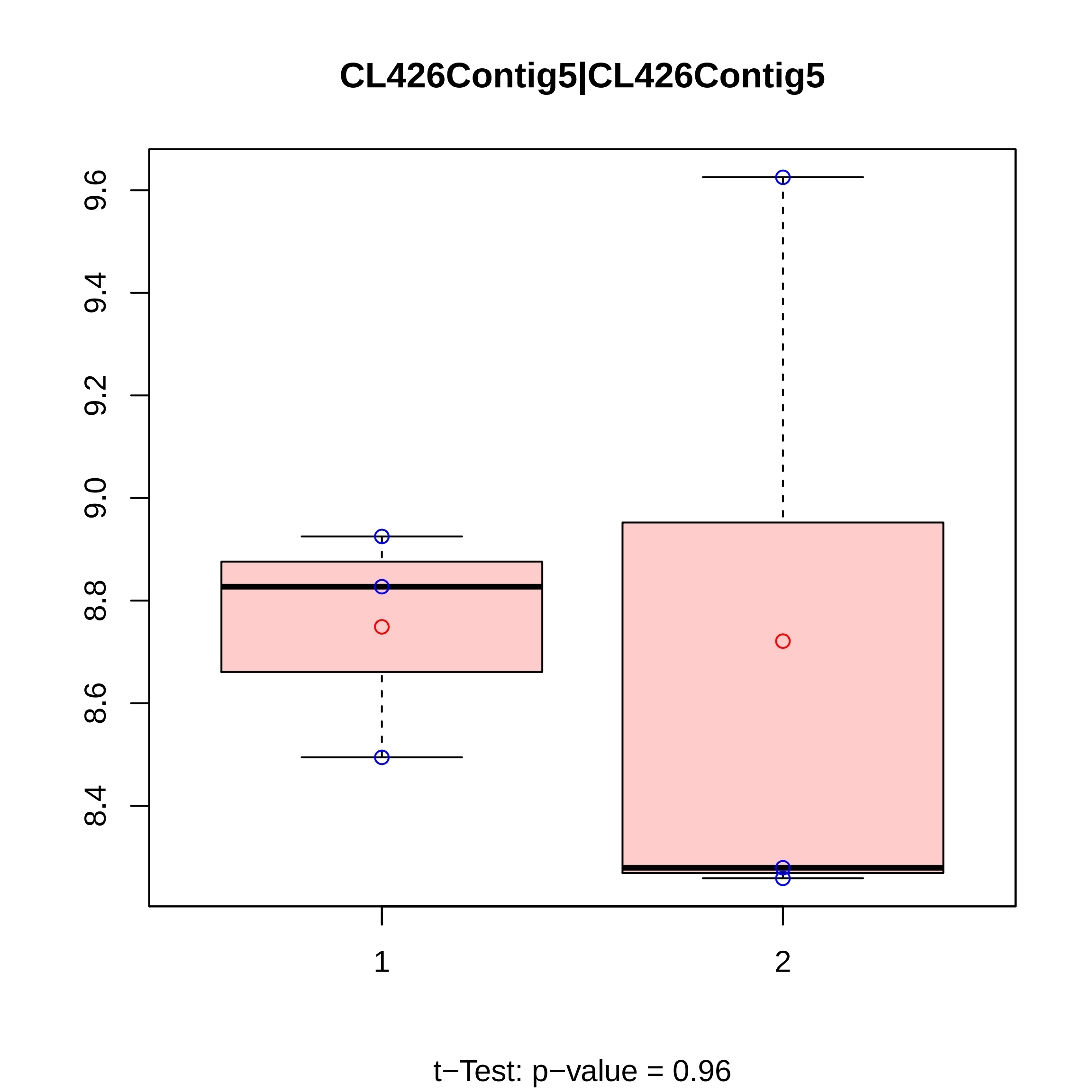

CL426Contig5|CL426Contig5
9.6
9.4
9.2
9.0
8.8
8.6
8.4
1
2
t−Test: p−value = 0.96

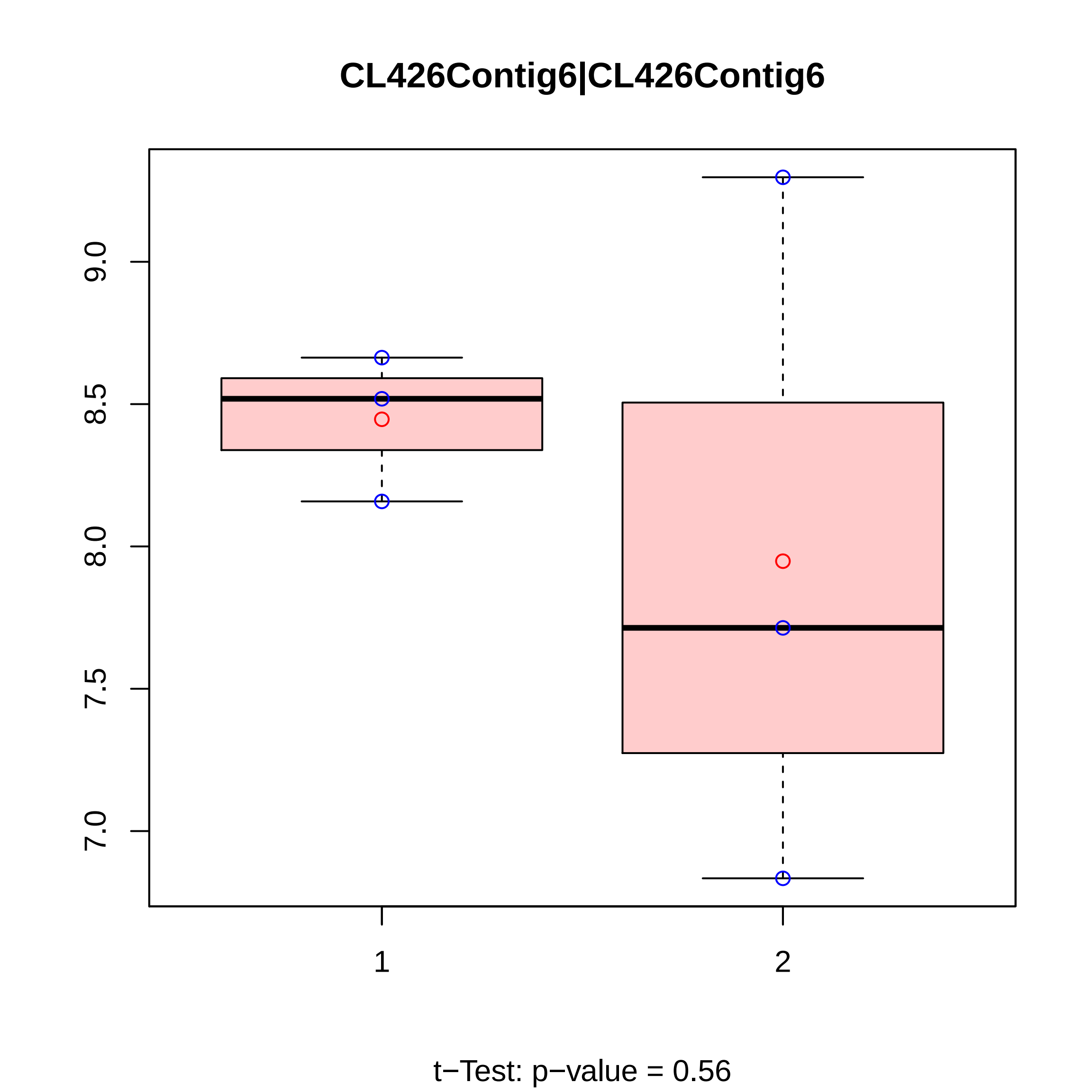

CL426Contig6|CL426Contig6
9.0
8.5
8.0
7.5
7.0
1
2
t−Test: p−value = 0.56

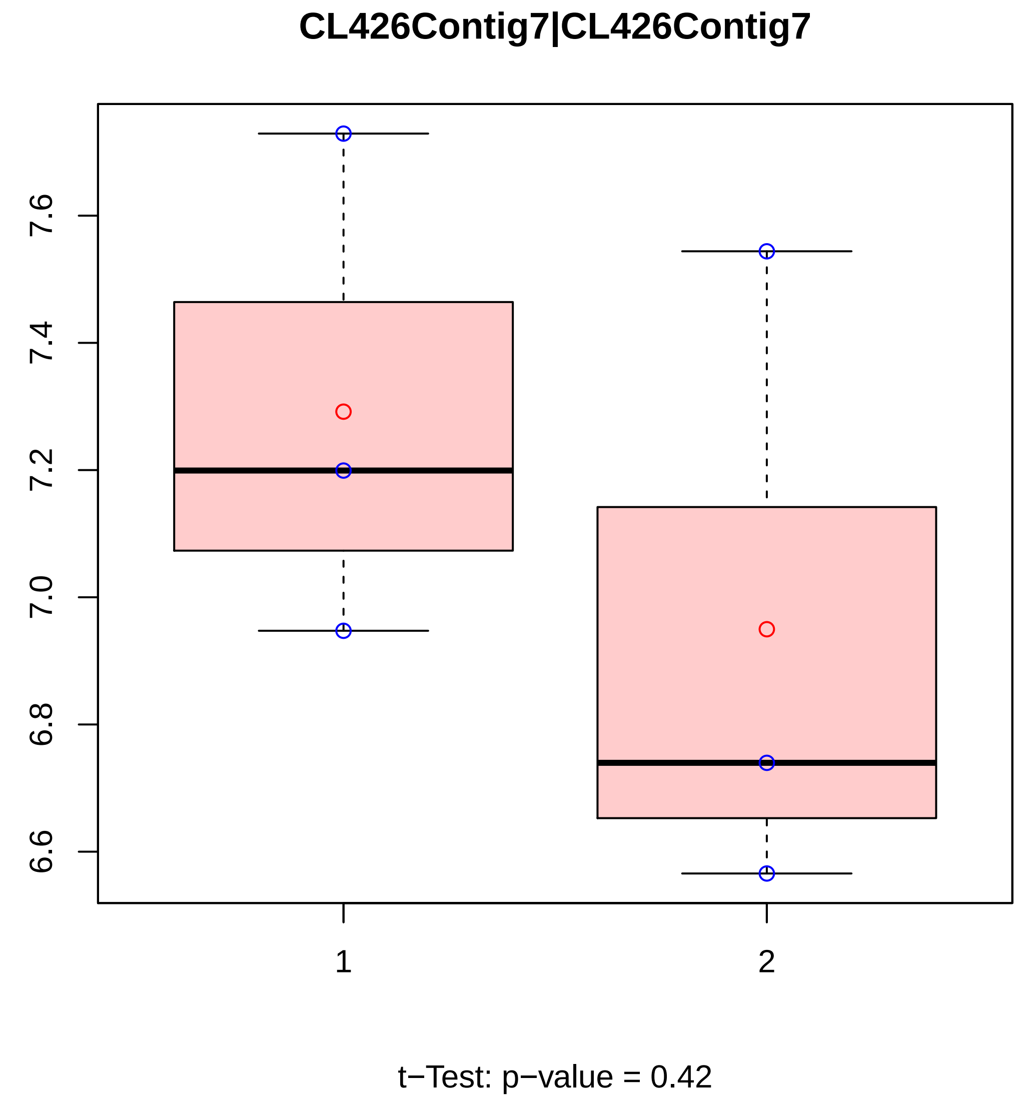
CL426Contig7|CL426Contig7
7.6
7.4
7.2
7.0
6.8
6.6
1
2
t−Test: p−value = 0.42

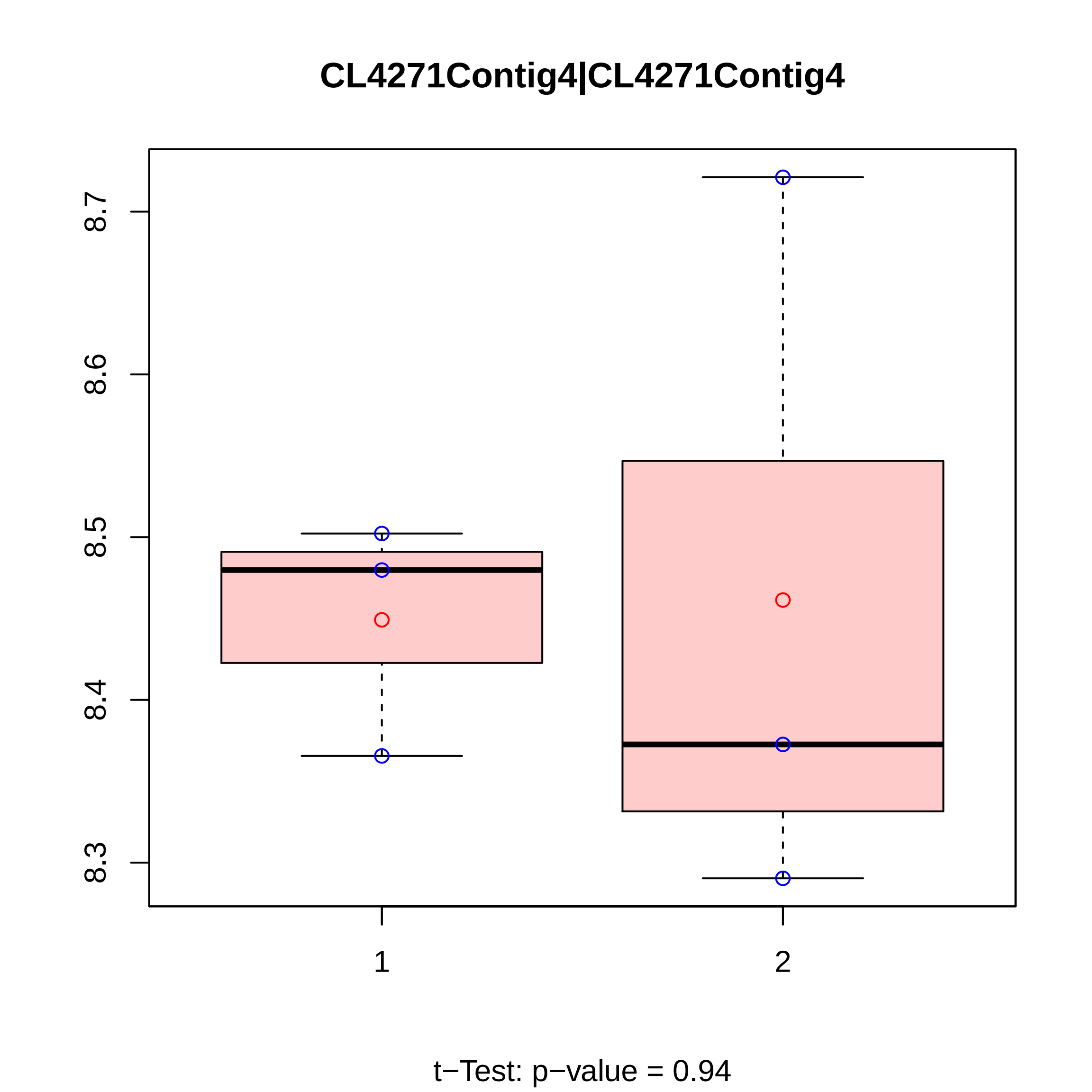

CL4271Contig4|CL4271Contig4
8.3
8.4
8.5
8.6
8.7
1
2
t−Test: p−value = 0.94

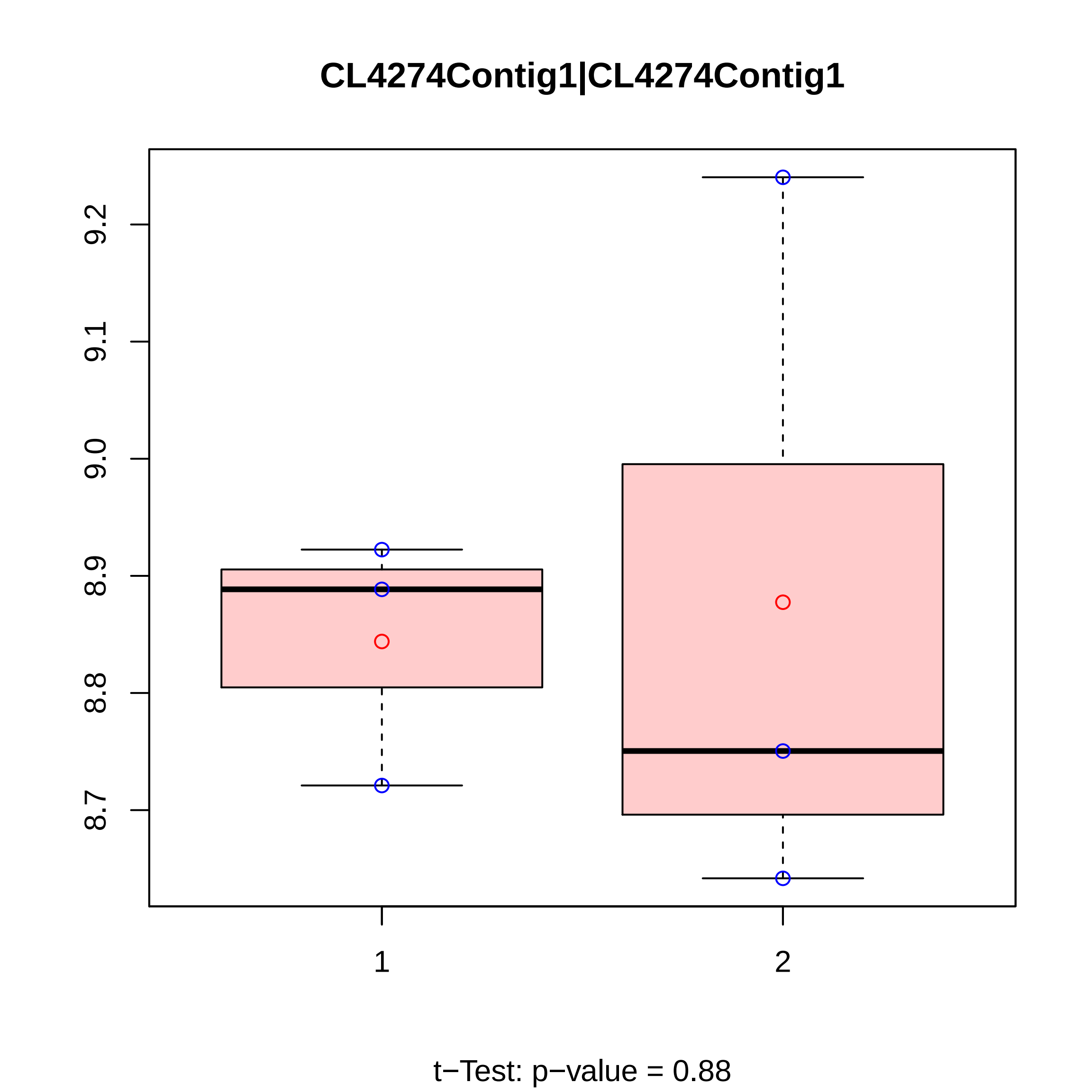

CL4274Contig1|CL4274Contig1
9.2
9.1
9.0
8.9
8.8
8.7
1
2
t−Test: p−value = 0.88

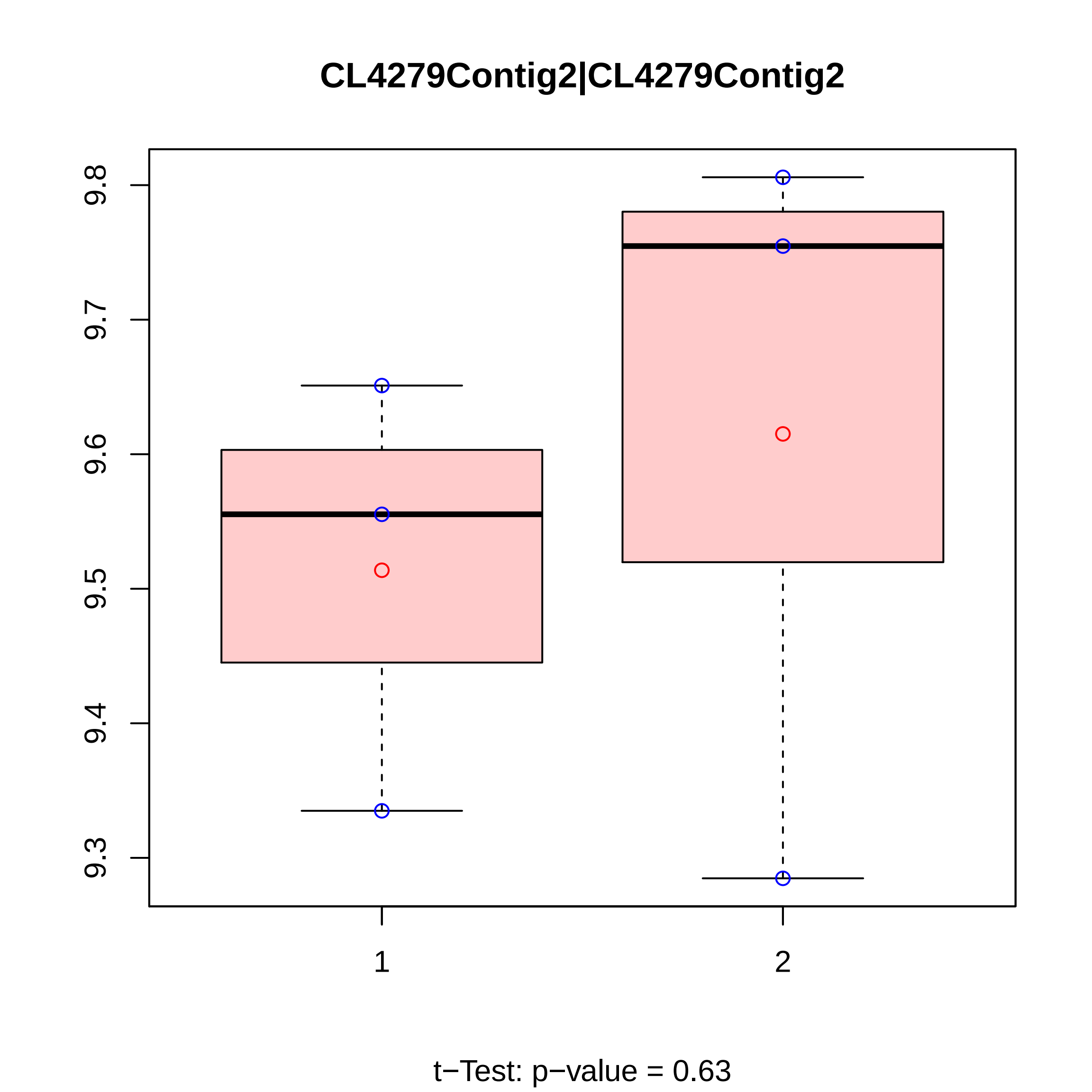

CL4279Contig2|CL4279Contig2
9.3
9.4
9.5
9.6
9.7
9.8
1
2
t−Test: p−value = 0.63

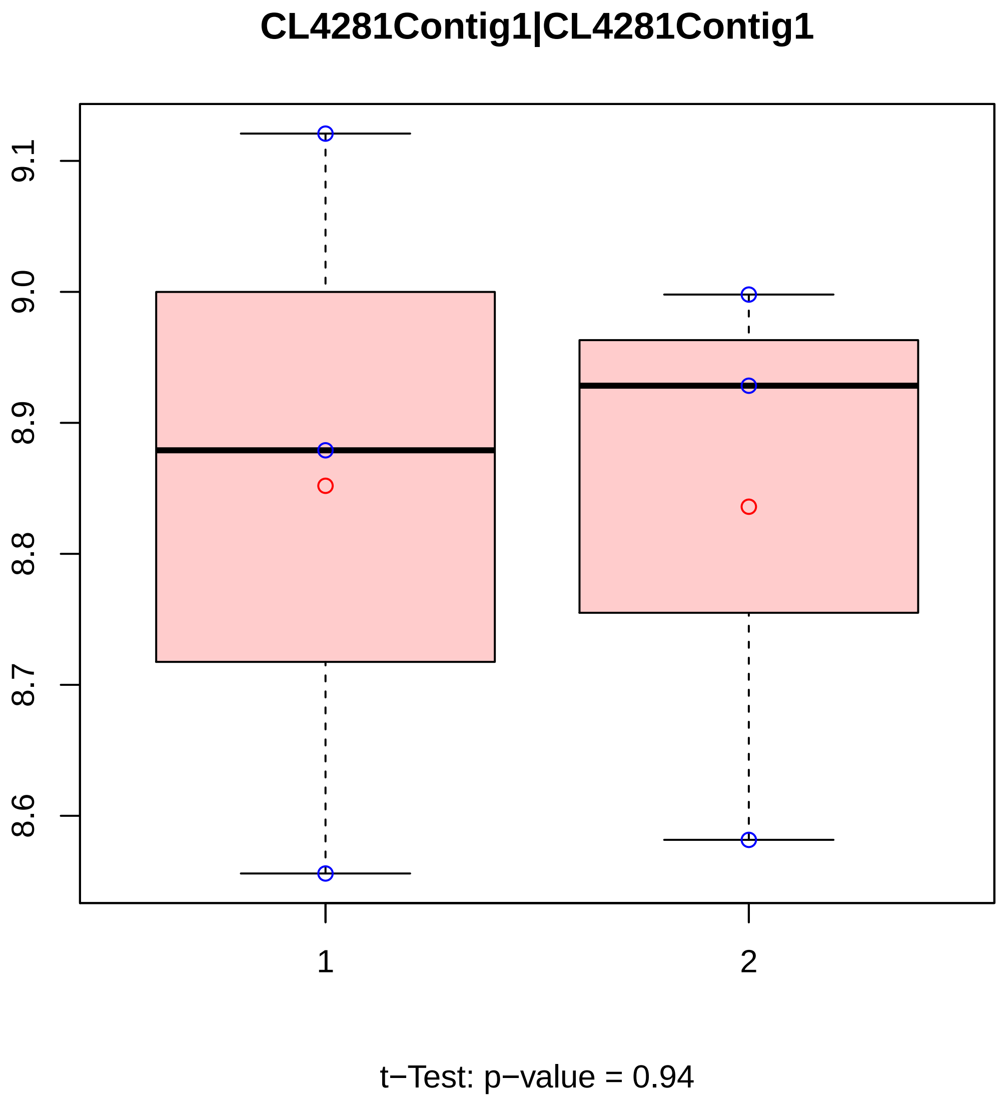
CL4281Contig1|CL4281Contig1
9.1
9.0
8.9
8.8
8.7
8.6
1
2
t−Test: p−value = 0.94

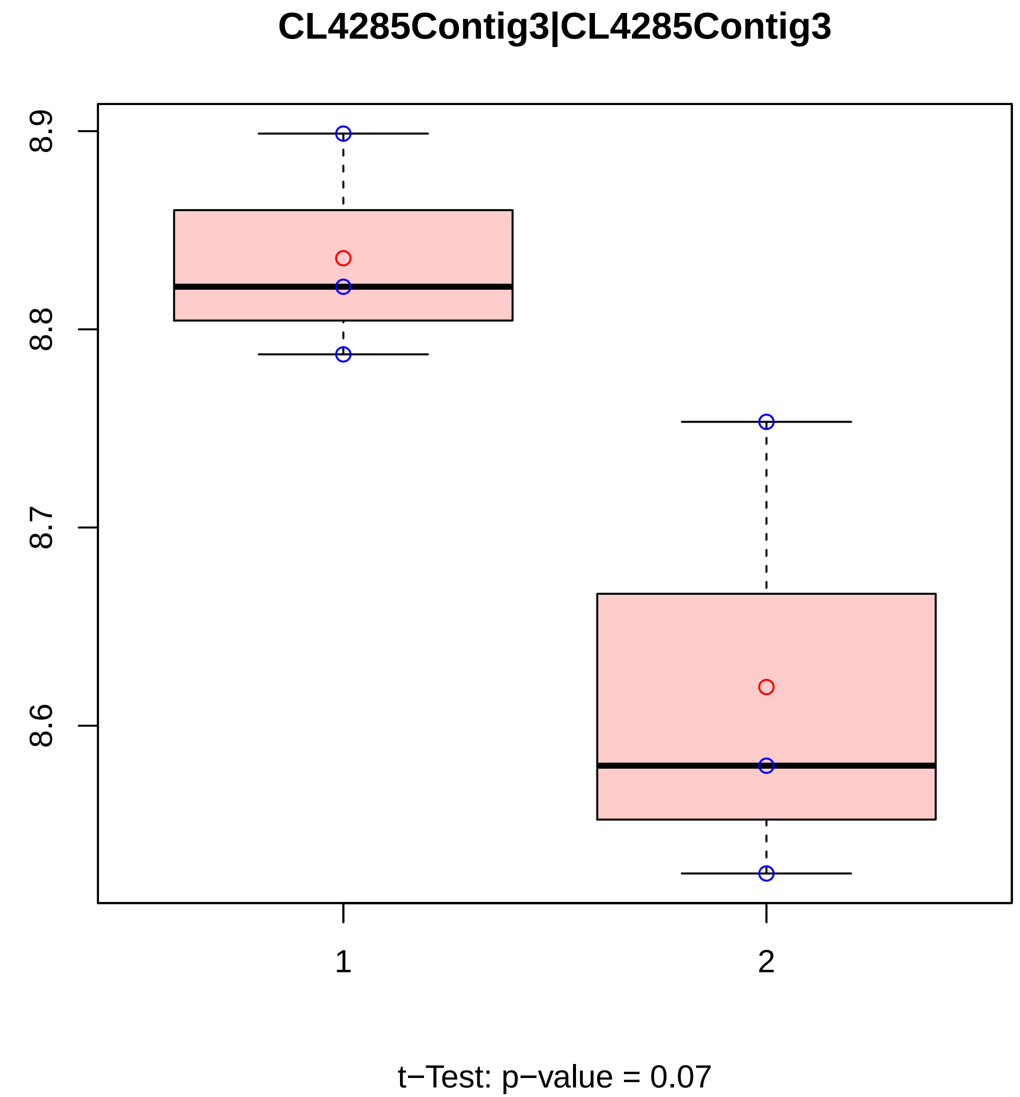
CL4285Contig3|CL4285Contig3
8.9
8.8
8.7
8.6
1
2
t−Test: p−value = 0.07

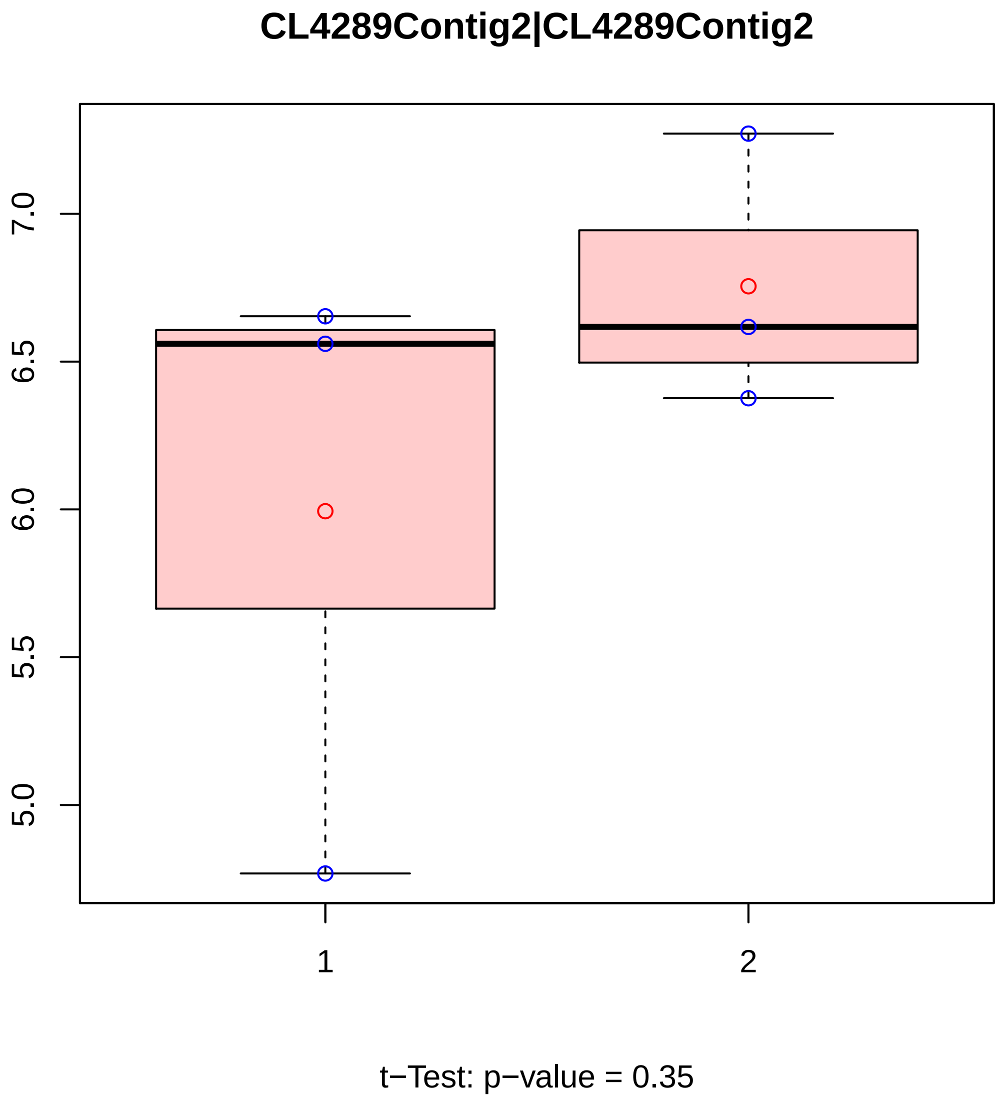

CL4289Contig2|CL4289Contig2
5.0
5.5
6.0
6.5
7.0
1
2
t−Test: p−value = 0.35

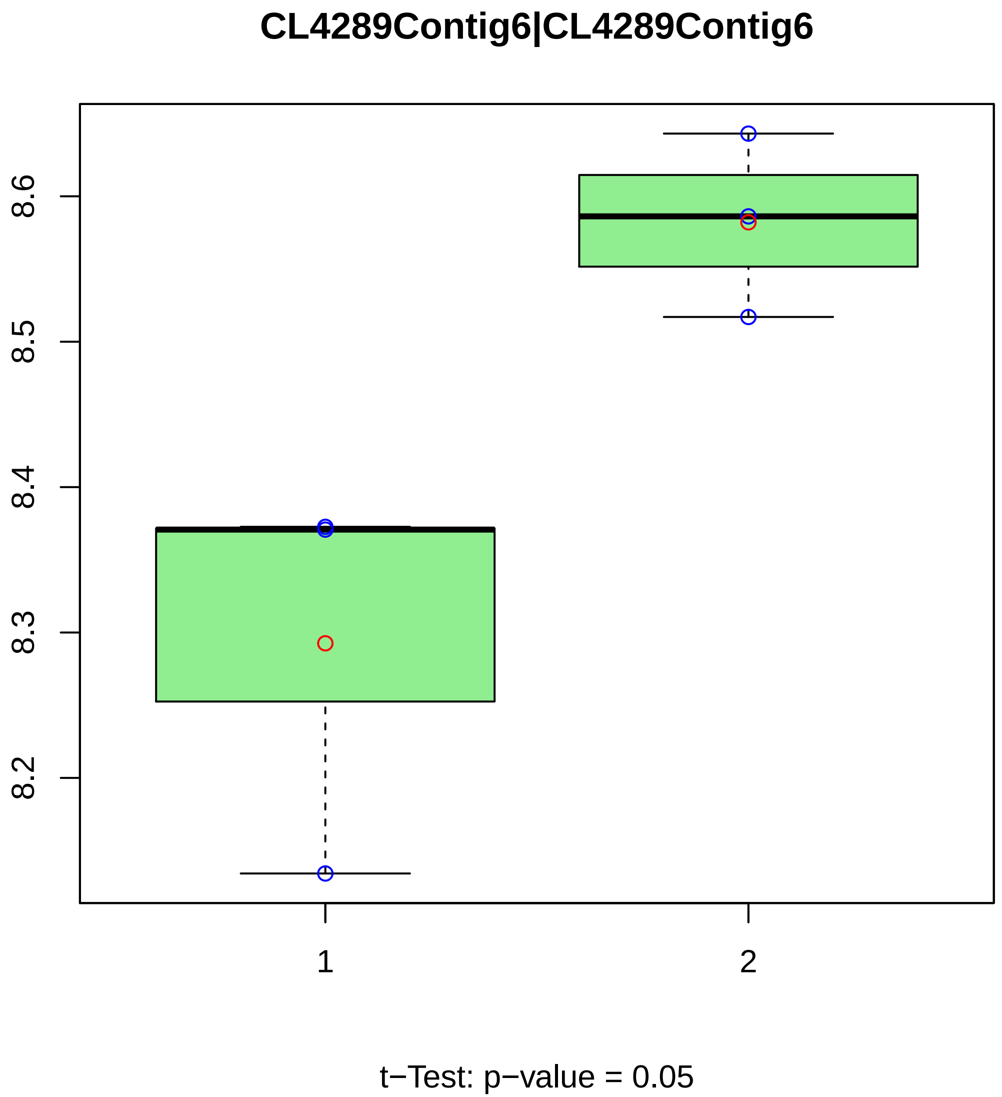

CL4289Contig6|CL4289Contig6
8.2
8.3
8.4
8.5
8.6
1
2
t−Test: p−value = 0.05

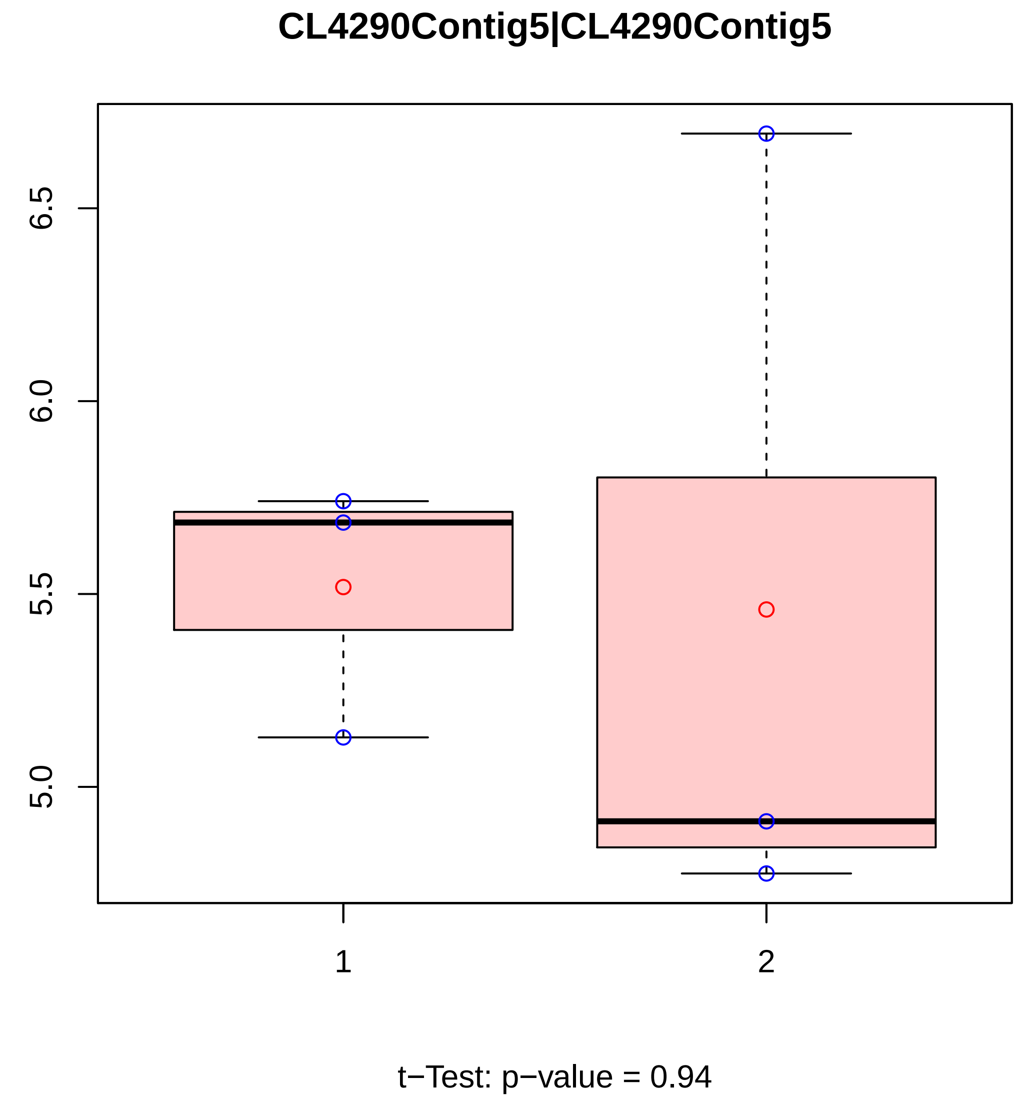

CL4290Contig5|CL4290Contig5
6.5
6.0
5.5
5.0
1
2
t−Test: p−value = 0.94

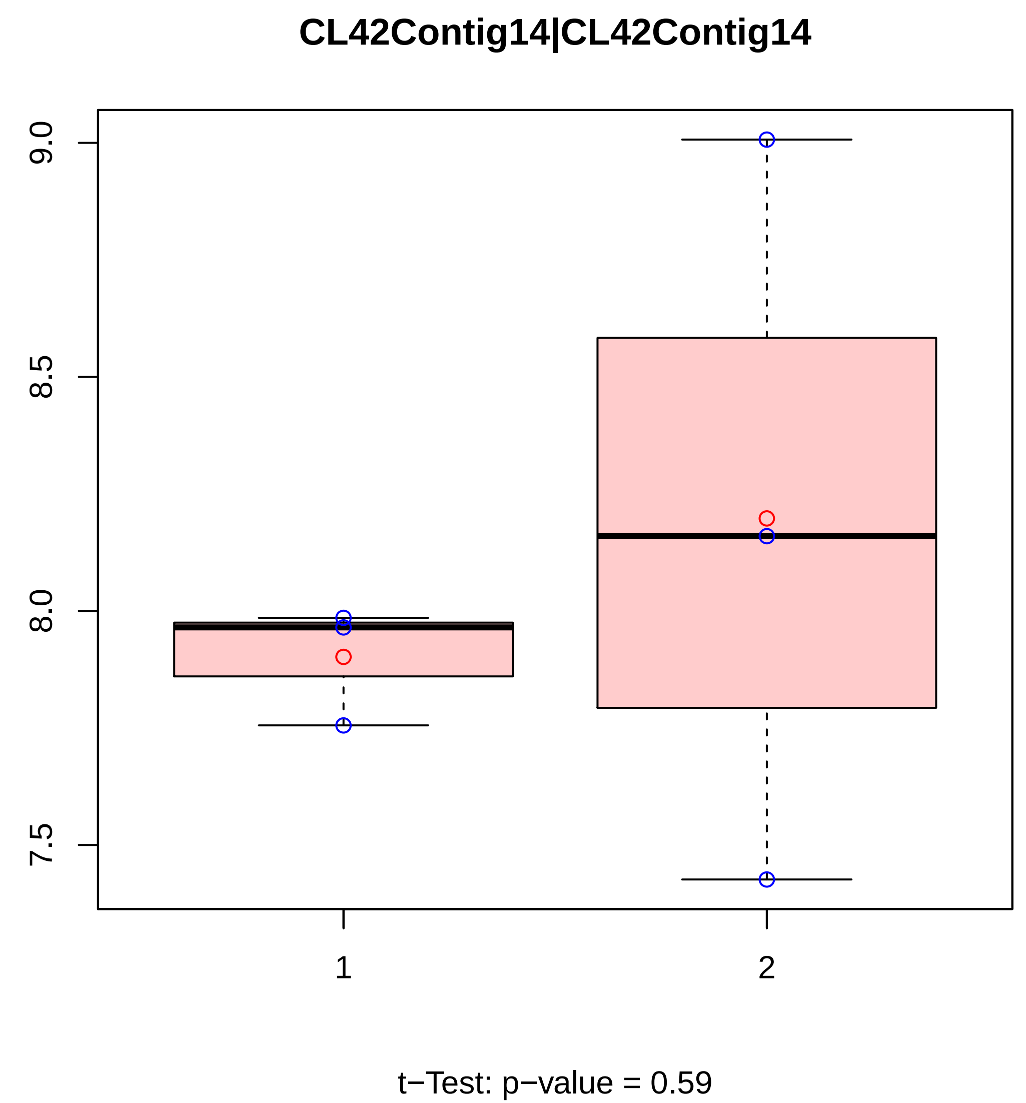

CL42Contig14|CL42Contig14
9.0
8.5
8.0
7.5
1
2
t−Test: p−value = 0.59

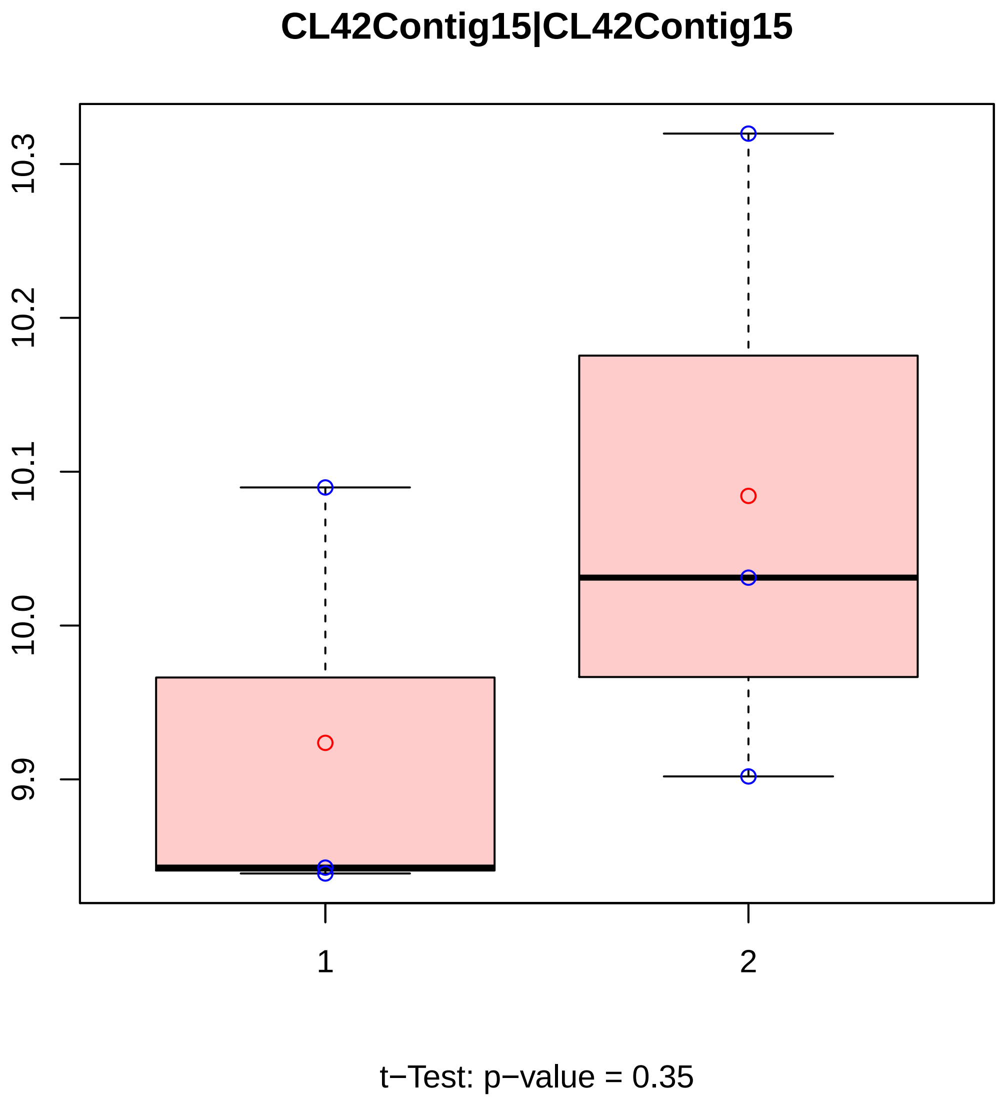
CL42Contig15|CL42Contig15
10.3
10.2
10.1
10.0
9.9
1
2
t−Test: p−value = 0.35

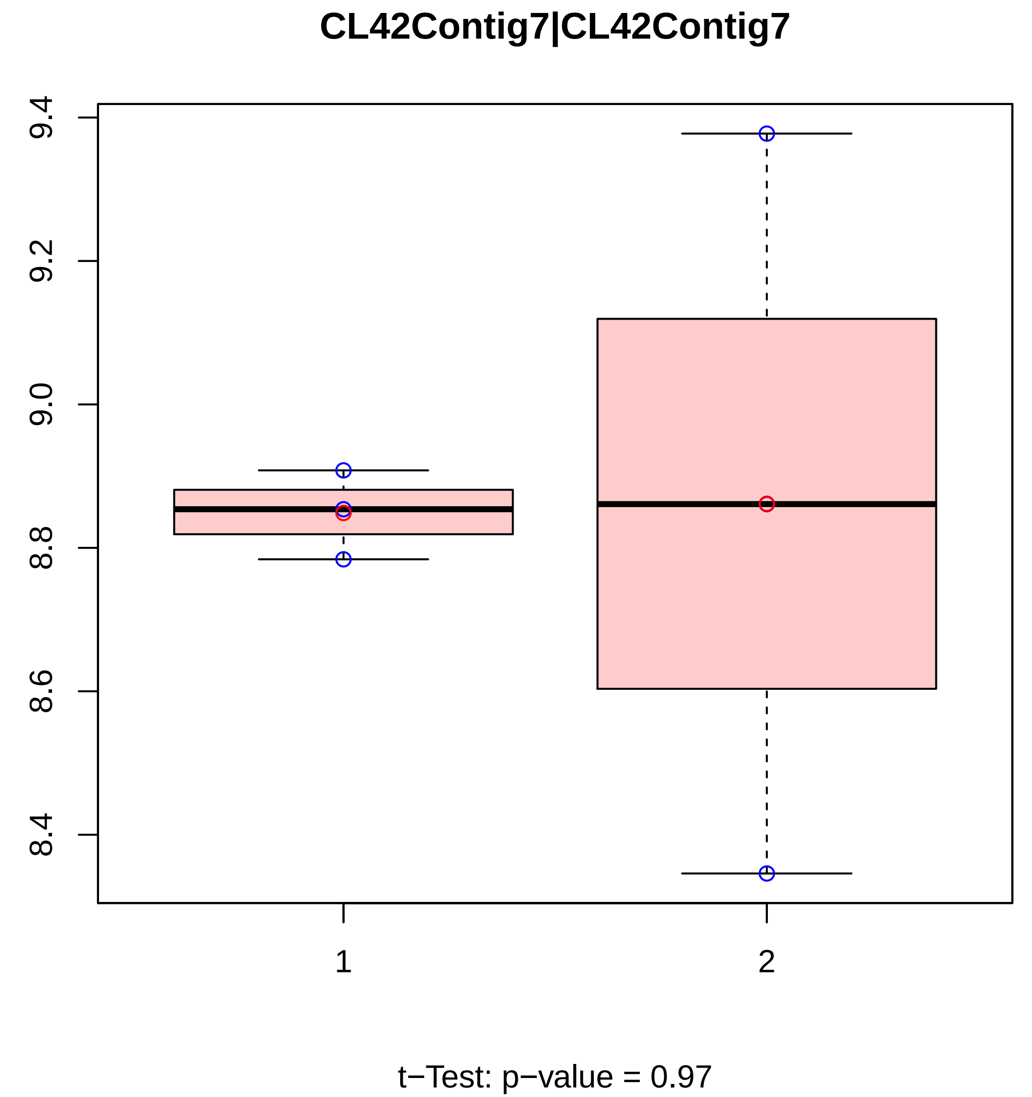
CL42Contig7|CL42Contig7
9.4
9.2
9.0
8.8
8.6
8.4
1
2
t−Test: p−value = 0.97

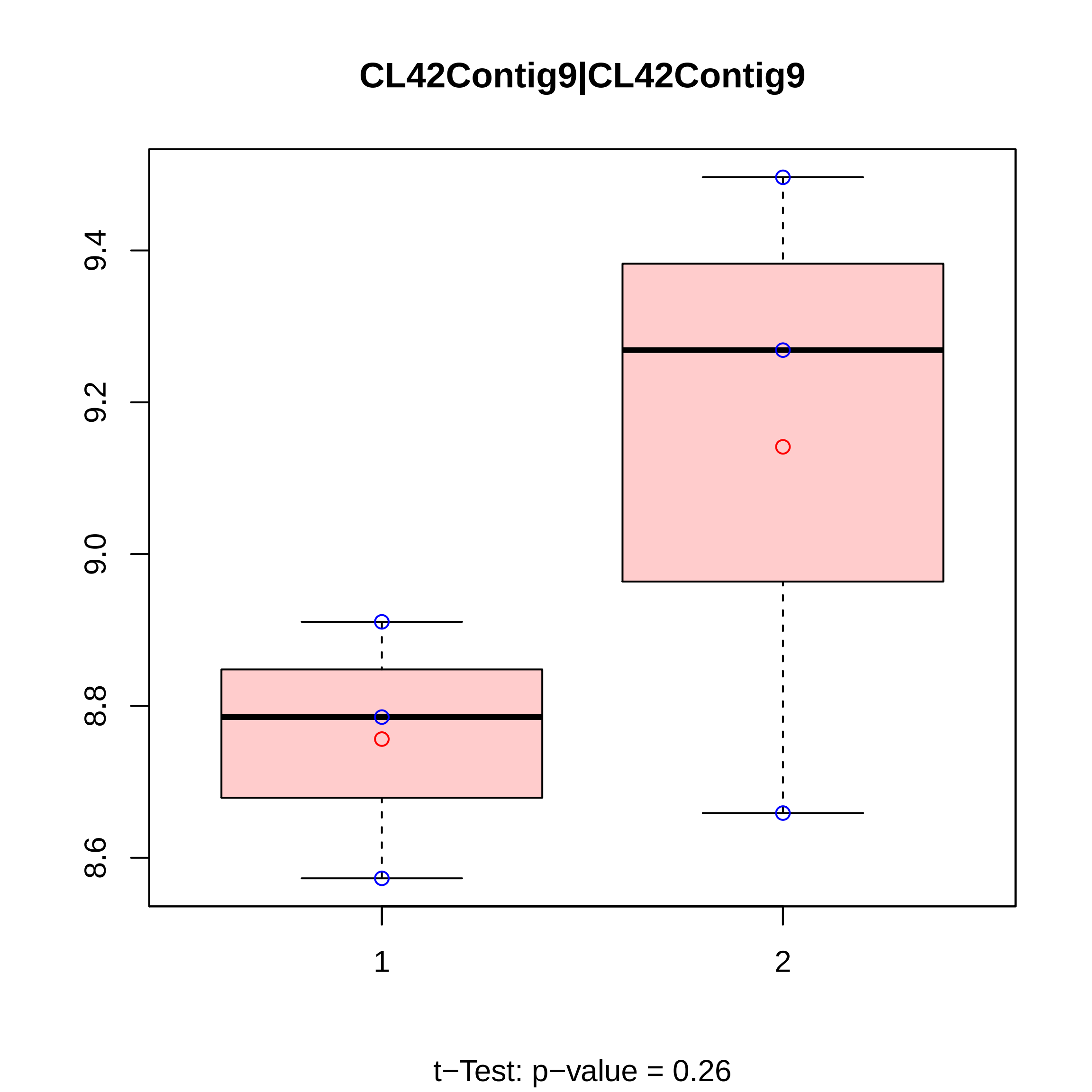

CL42Contig9|CL42Contig9
9.4
9.2
9.0
8.8
8.6
1
2
t−Test: p−value = 0.26

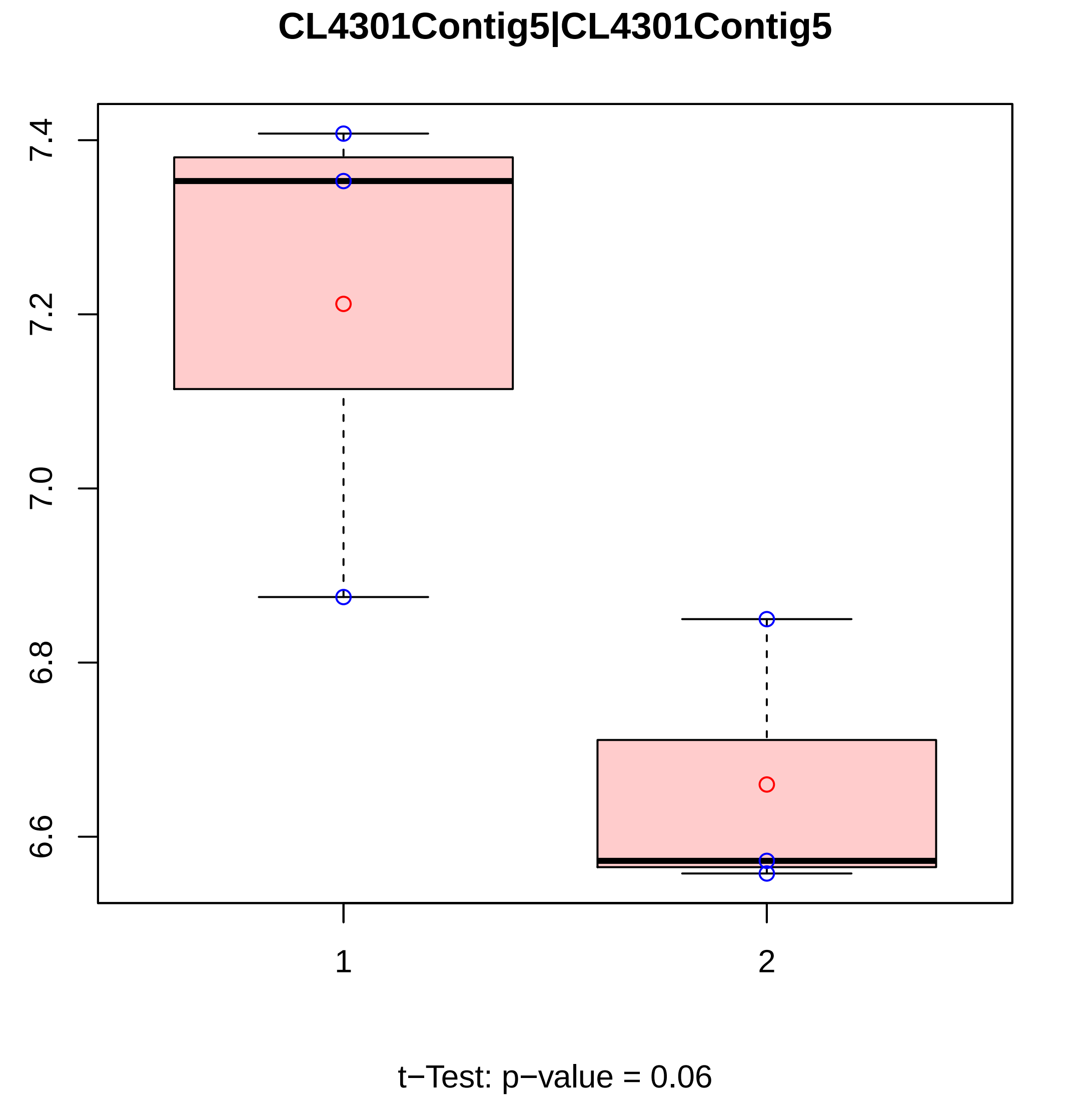
CL4301Contig5|CL4301Contig5
7.4
7.2
7.0
6.8
6.6
1
2
t−Test: p−value = 0.06

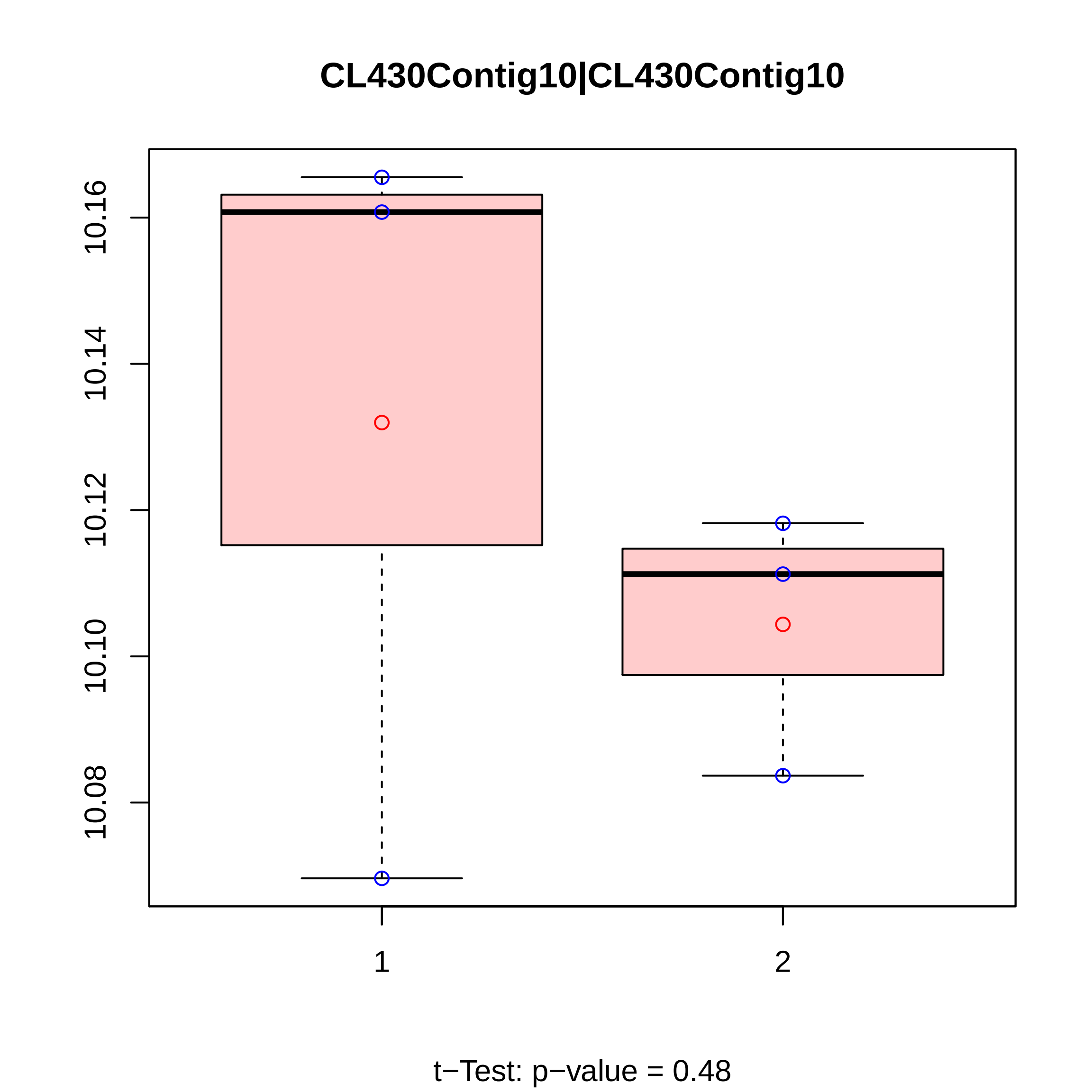

CL430Contig10|CL430Contig10
10.08
10.10
10.12
10.14
10.16
1
2
t−Test: p−value = 0.48

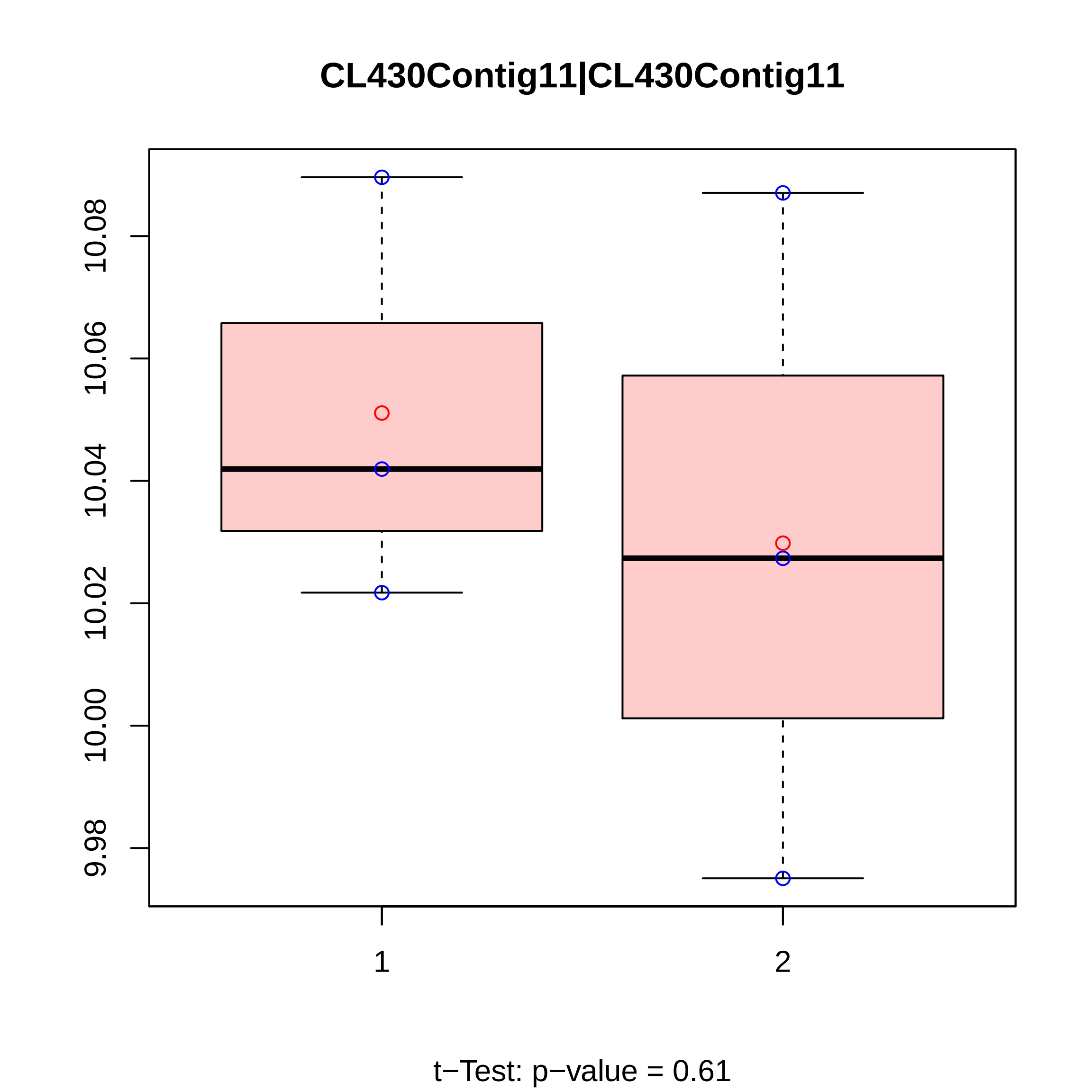
CL430Contig11|CL430Contig11
10.08
10.06
10.04
10.02
10.00
9.98
1
2
t−Test: p−value = 0.61

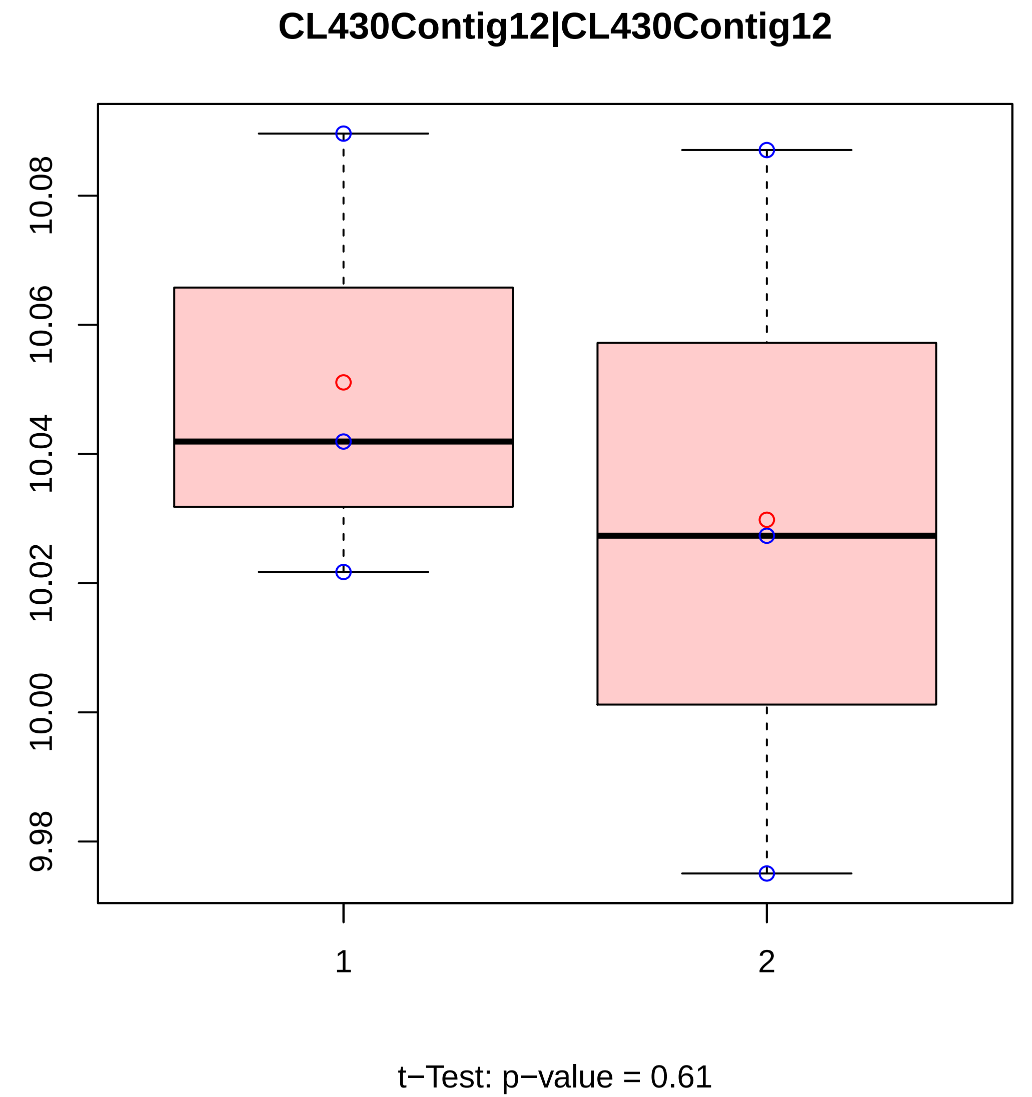

CL430Contig12|CL430Contig12
10.08
10.06
10.04
10.02
10.00
9.98
1
2
t−Test: p−value = 0.61

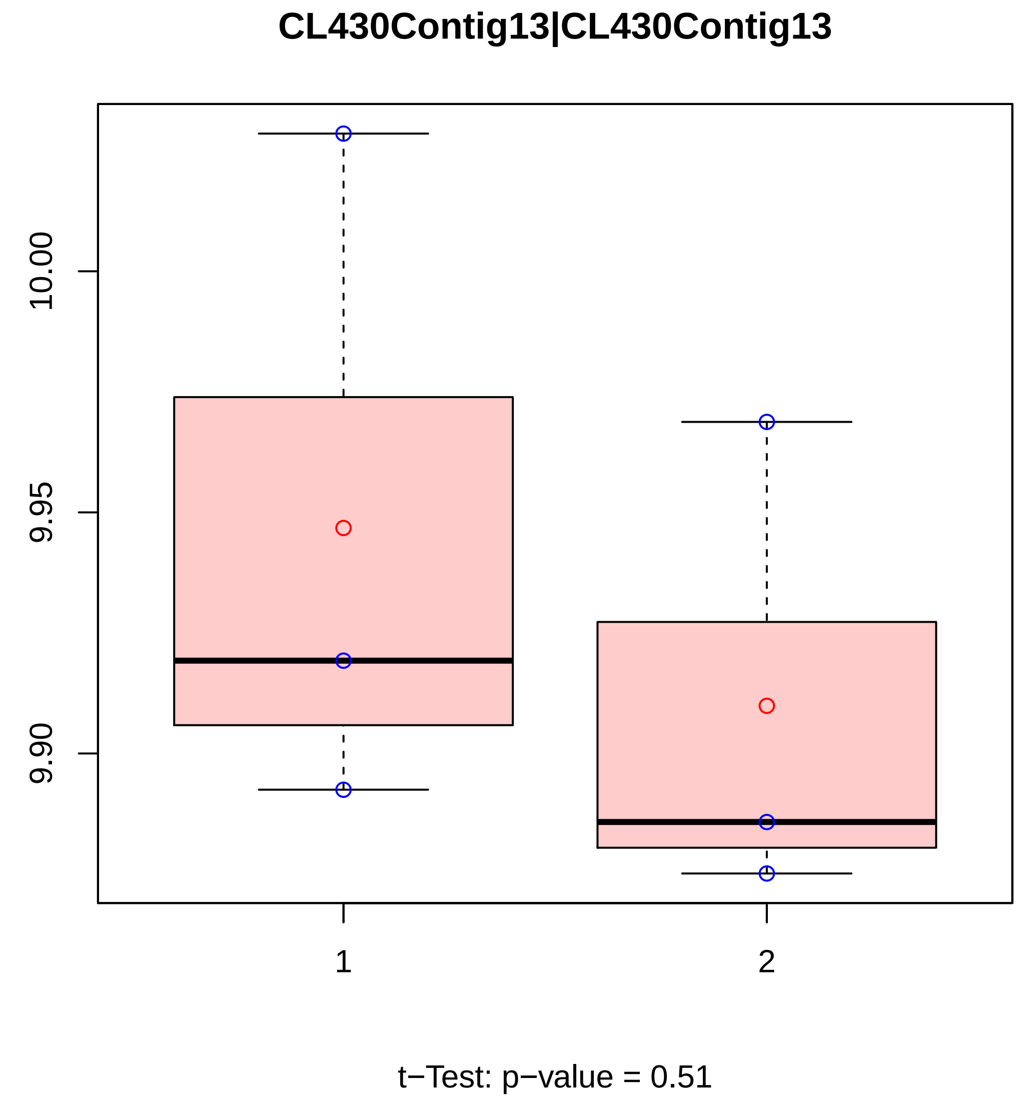
CL430Contig13|CL430Contig13
10.00
9.95
9.90
1
2
t−Test: p−value = 0.51

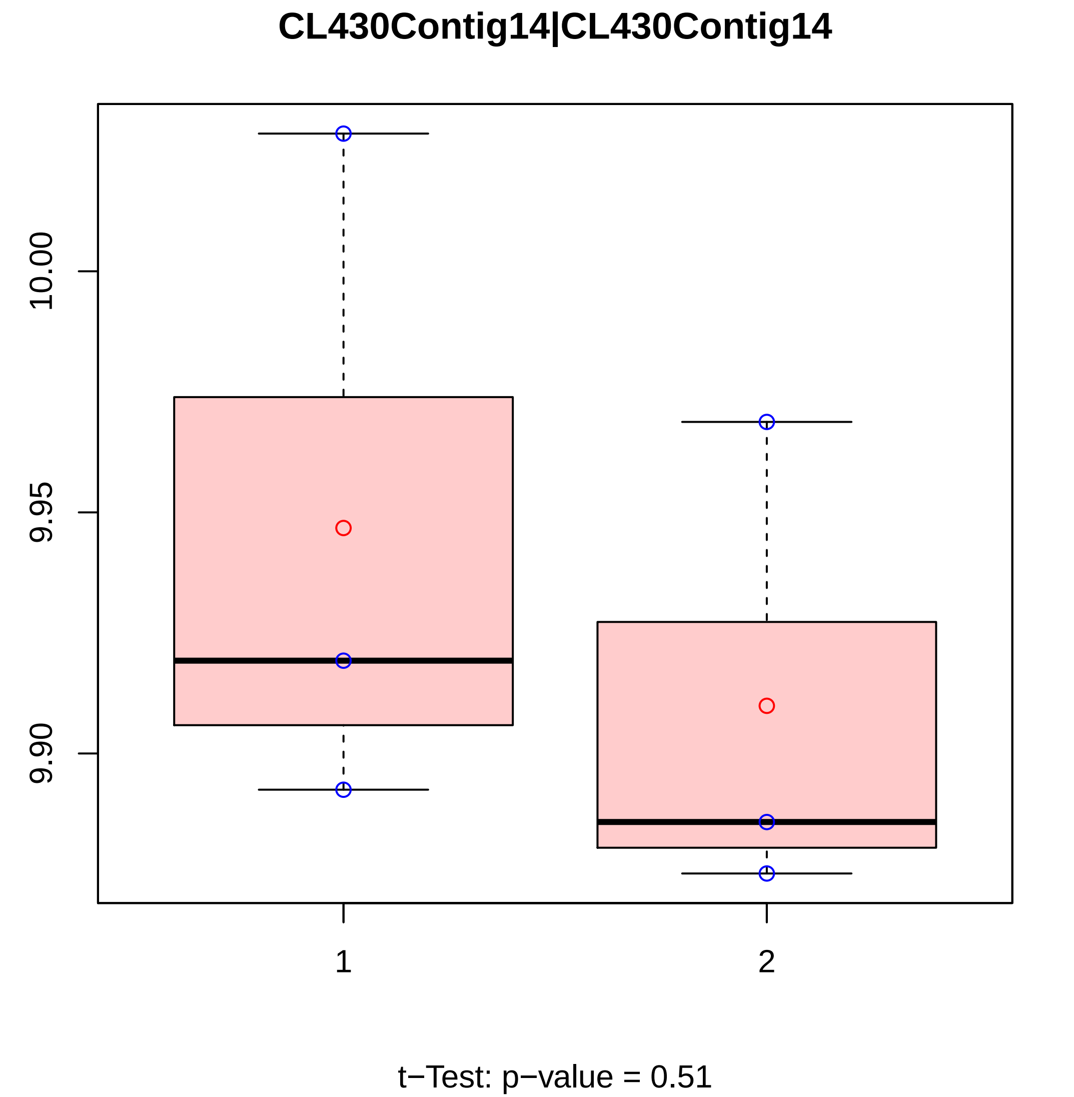
CL430Contig14|CL430Contig14
10.00
9.95
9.90
1
2
t−Test: p−value = 0.51

# CL430Contig2|CL430Contig2

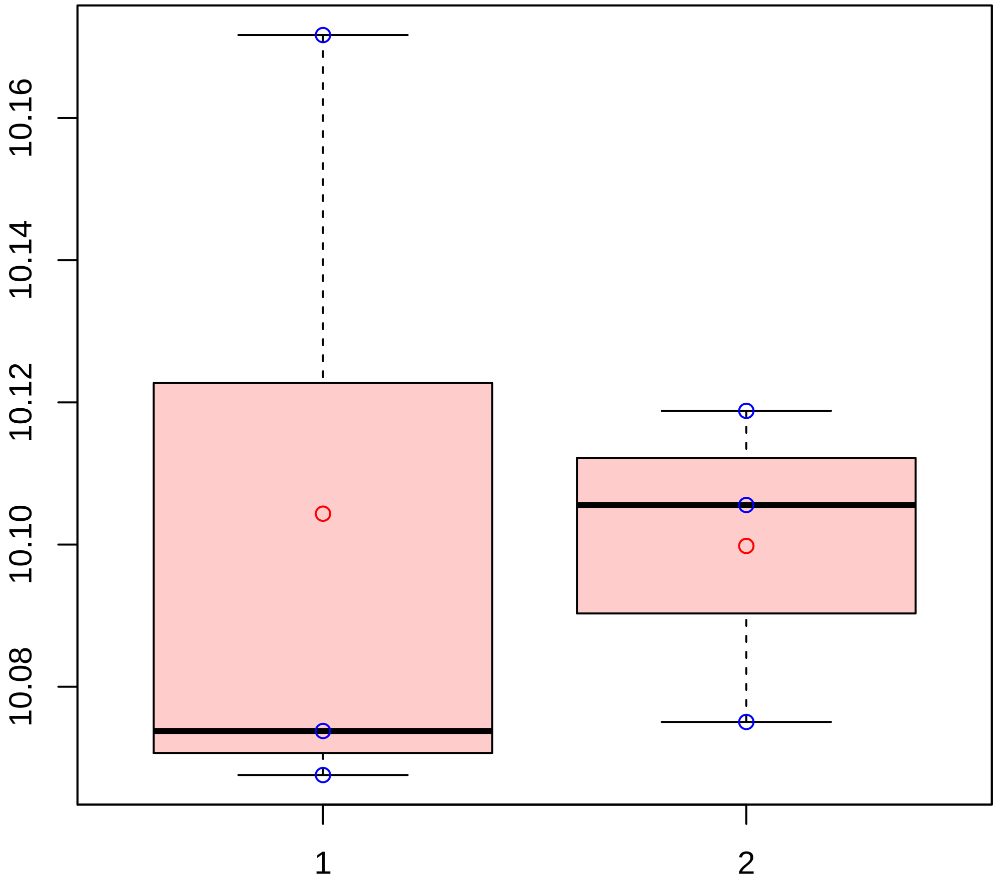


t−Test: p−value = 0.91

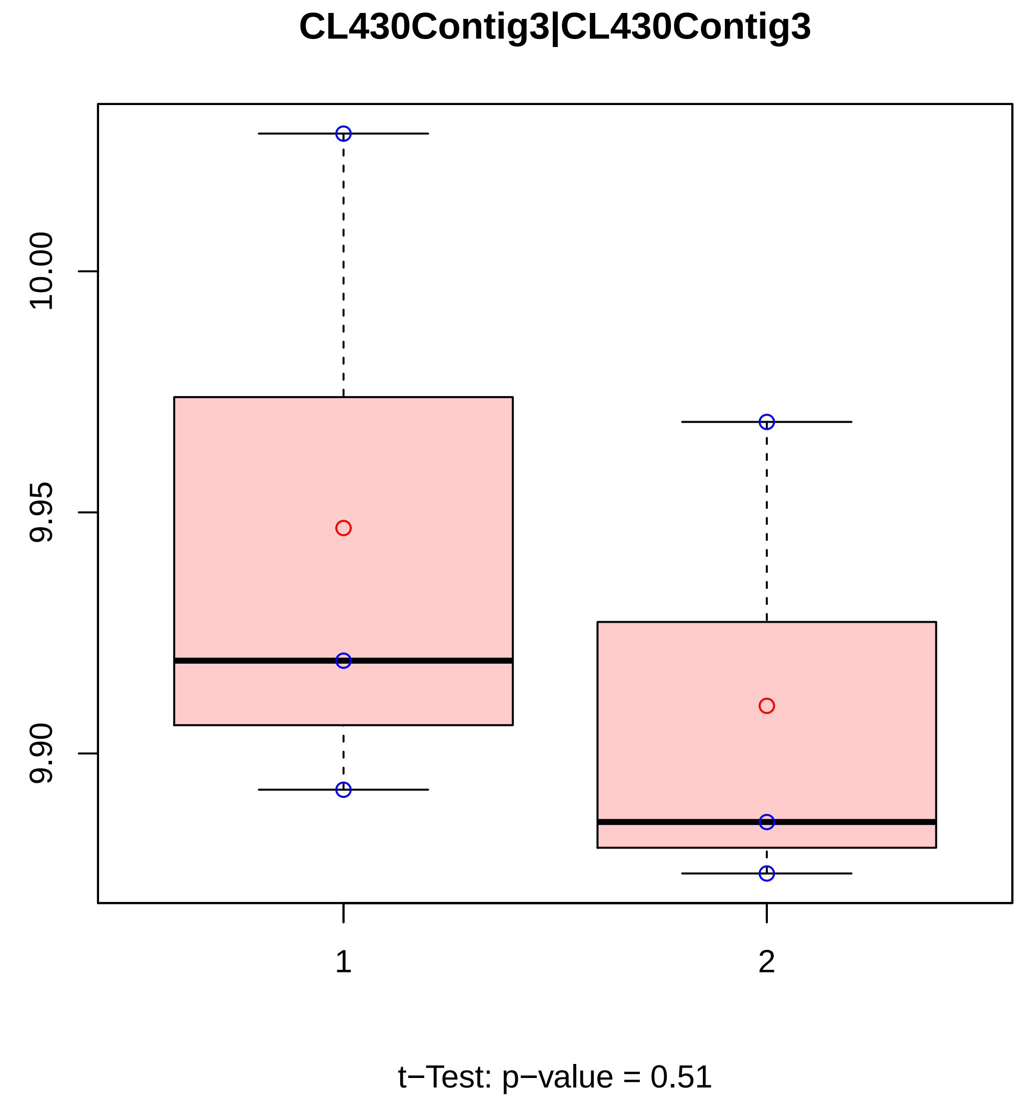
CL430Contig3|CL430Contig3
10.00
9.95
9.90
1
2
t−Test: p−value = 0.51

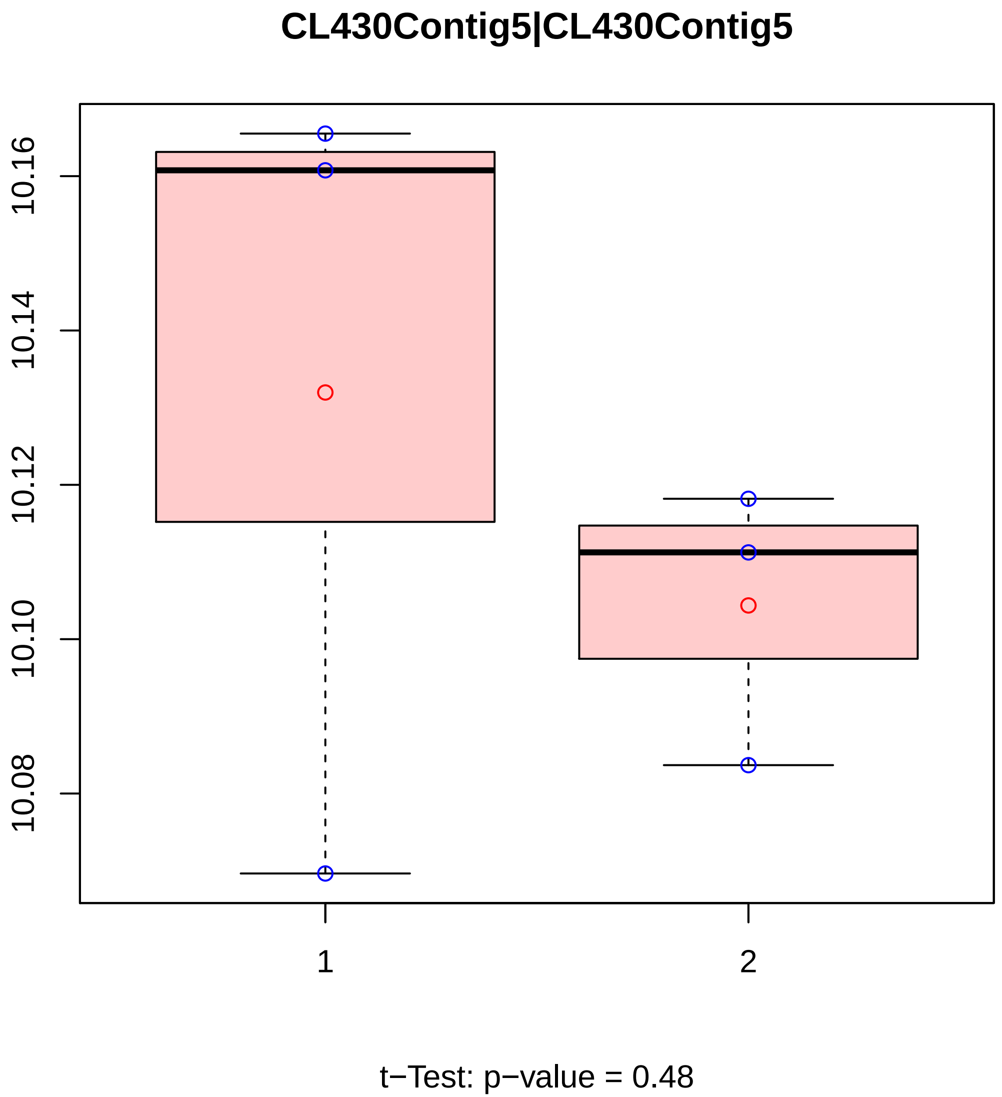

CL430Contig5|CL430Contig5
10.16
10.14
10.12
10.10
10.08
1
2
t−Test: p−value = 0.48

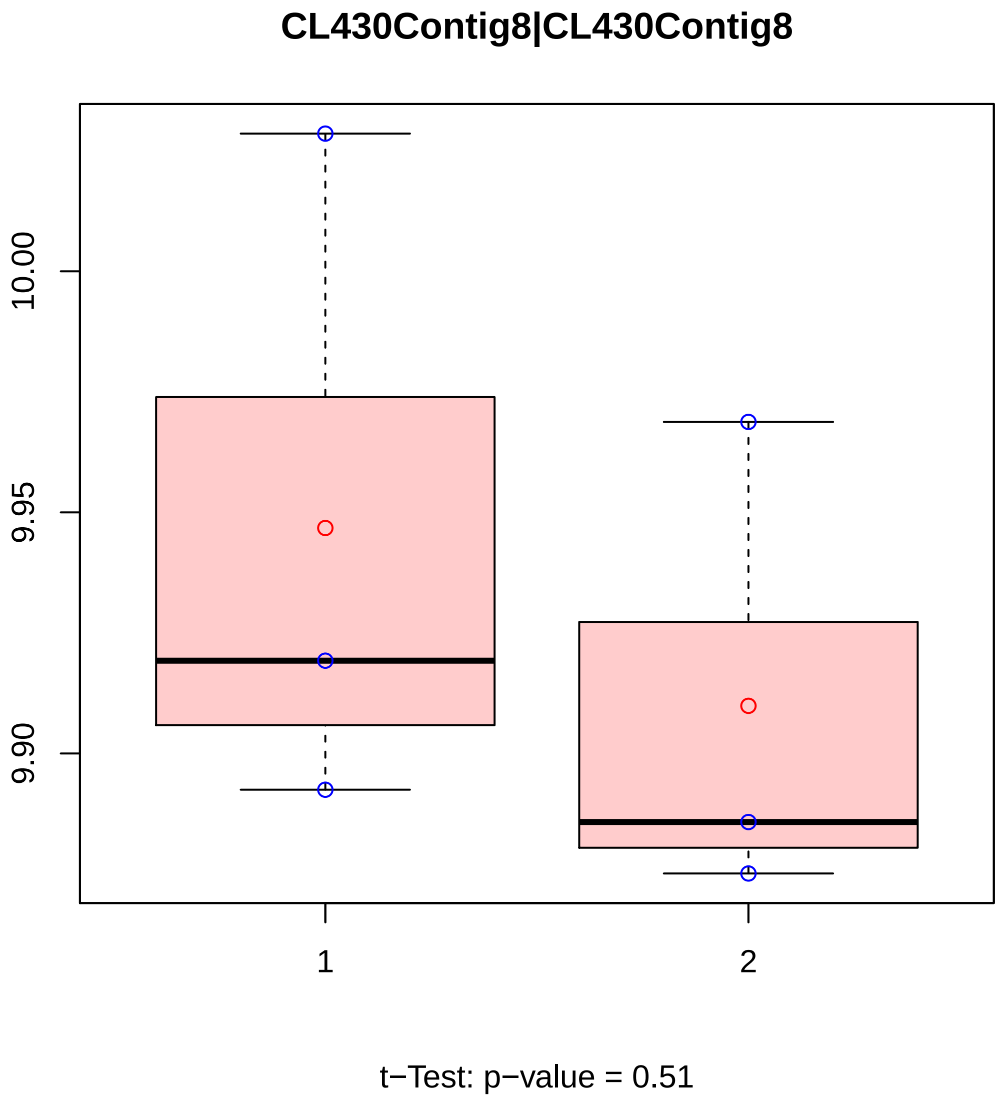
CL430Contig8|CL430Contig8
10.00
9.95
9.90
1
2
t−Test: p−value = 0.51

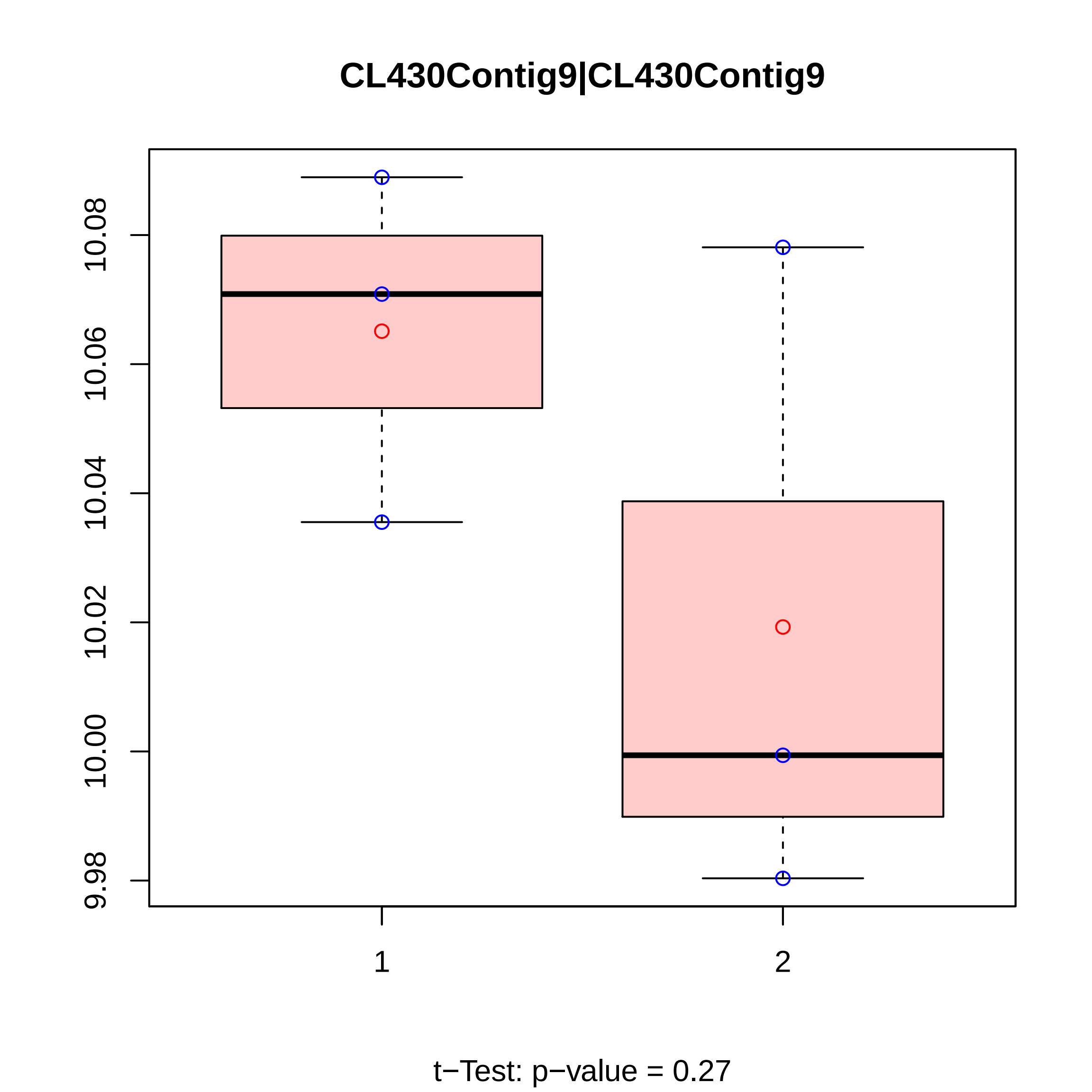

CL430Contig9|CL430Contig9
10.08
10.06
10.04
10.02
10.00
9.98
1
2
t−Test: p−value = 0.27

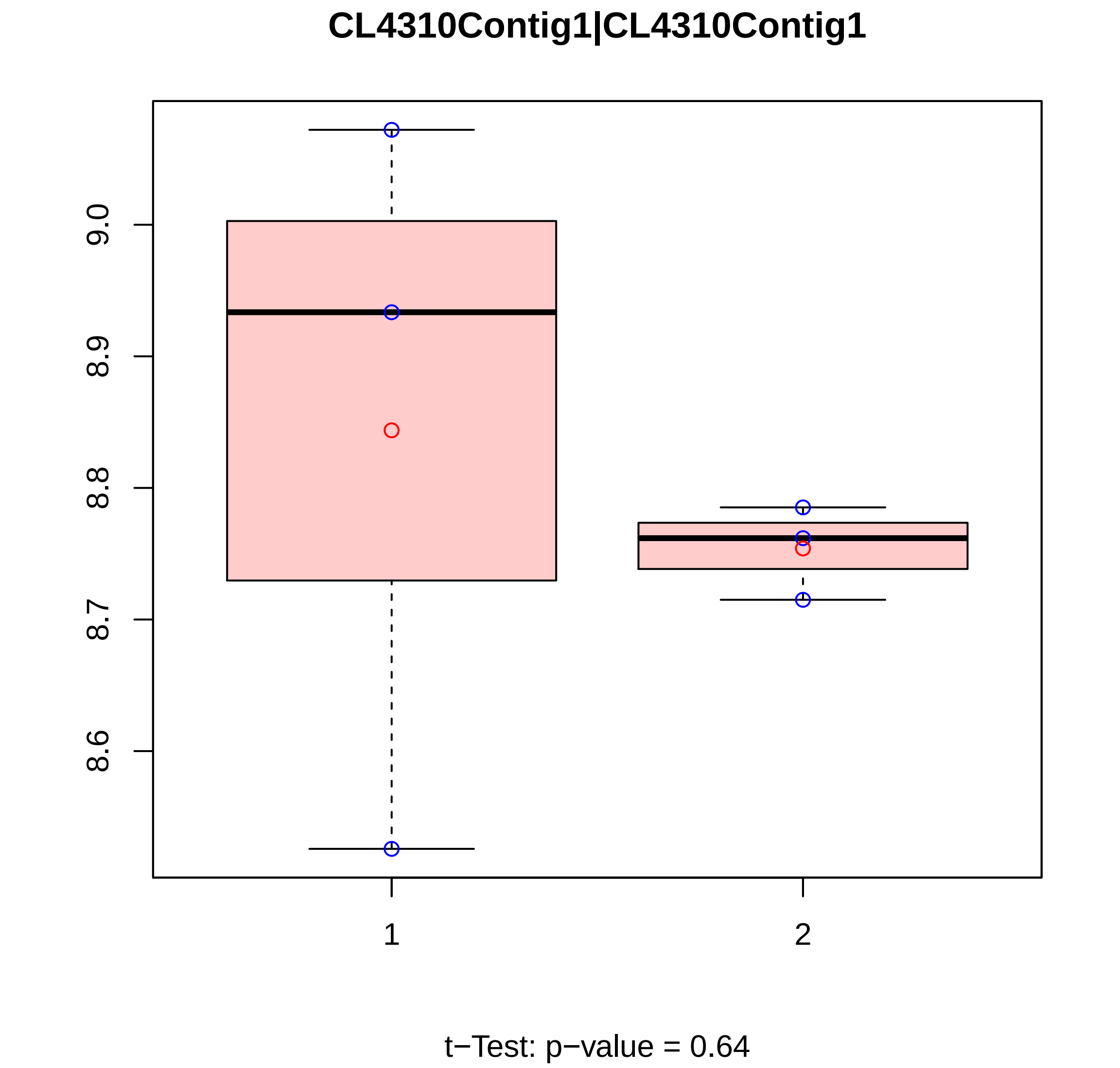
CL4310Contig1|CL4310Contig1
9.0
8.9
8.8
8.7
8.6
1
2
t−Test: p−value = 0.64

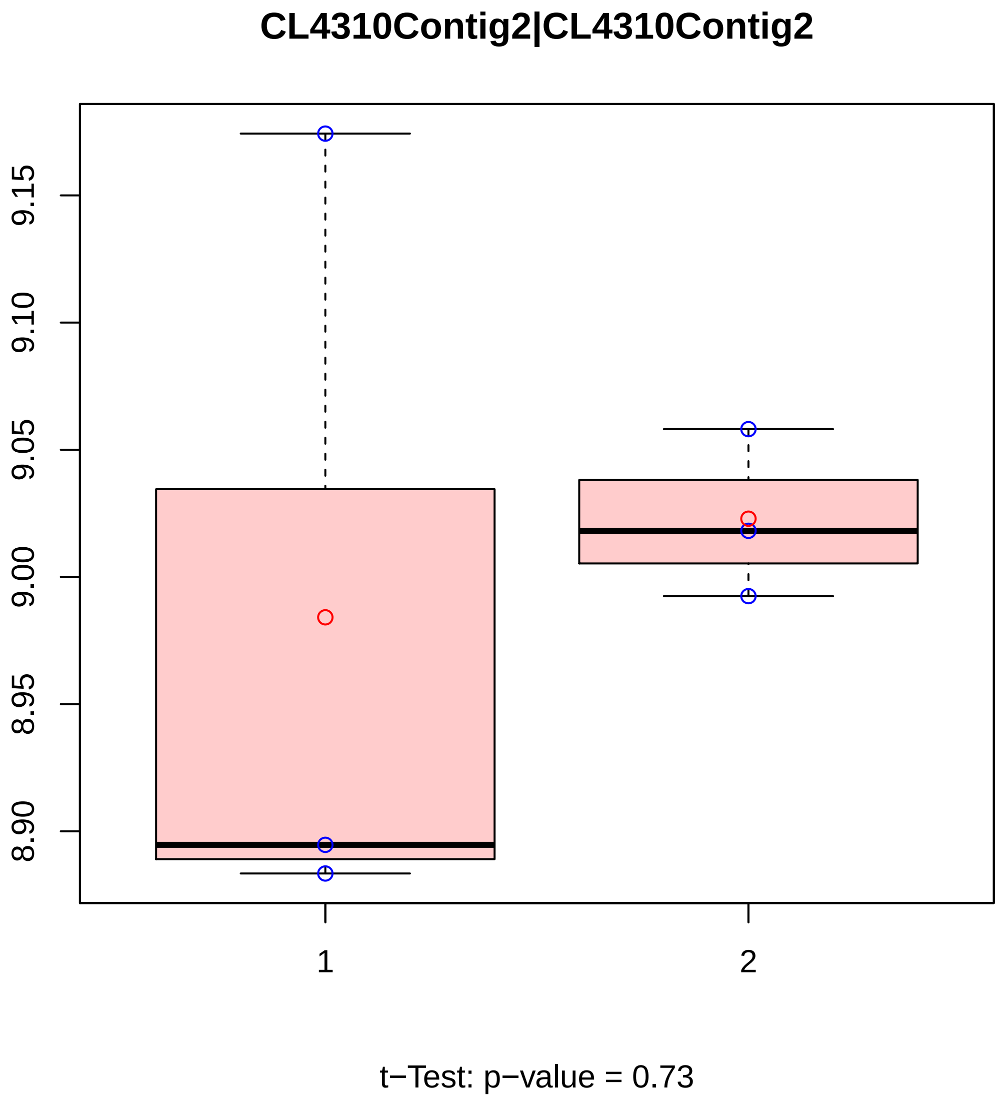
CL4310Contig2|CL4310Contig2
9.15
9.10
9.05
9.00
8.95
8.90
1
2
t−Test: p−value = 0.73

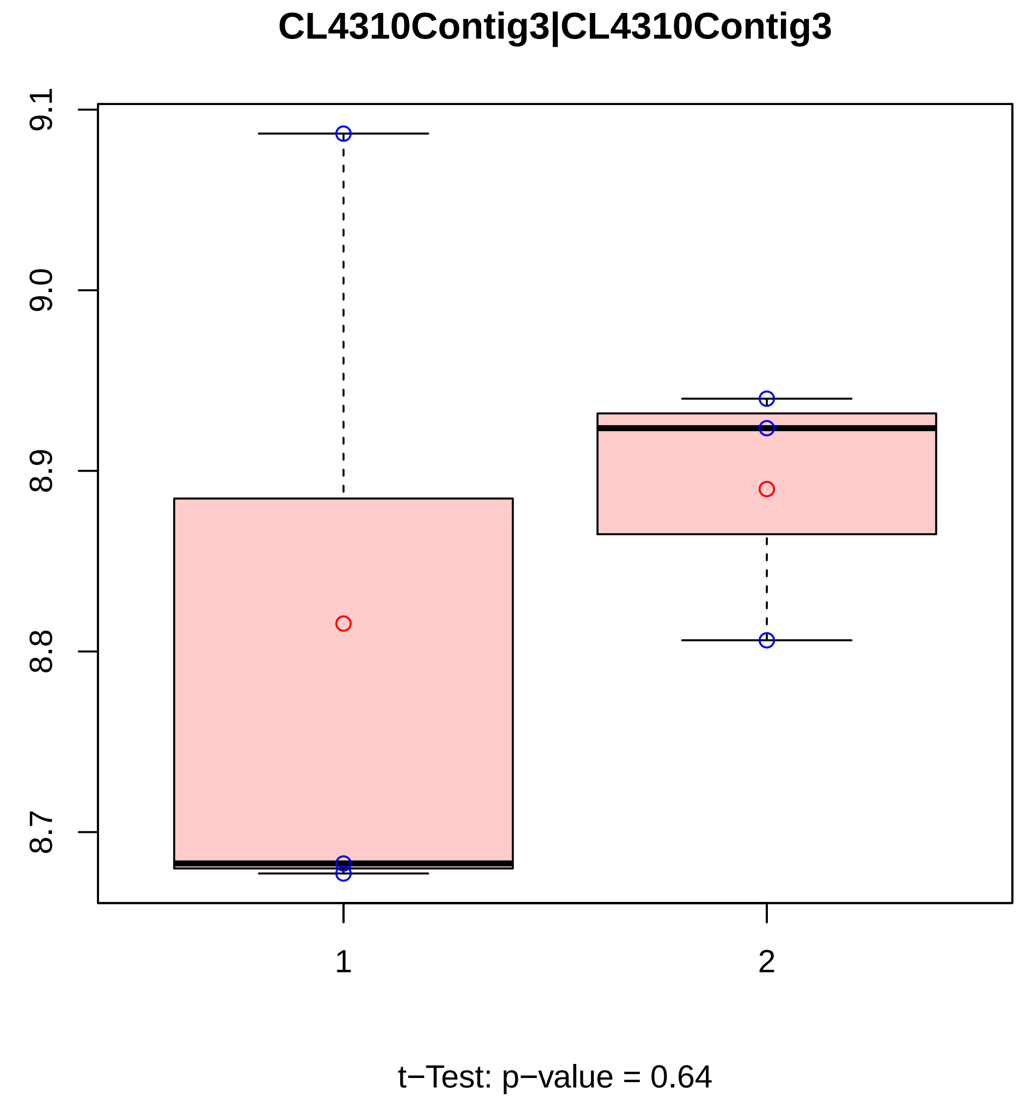

CL4310Contig3|CL4310Contig3
9.1
9.0
8.9
8.8
8.7
1
2
t−Test: p−value = 0.64

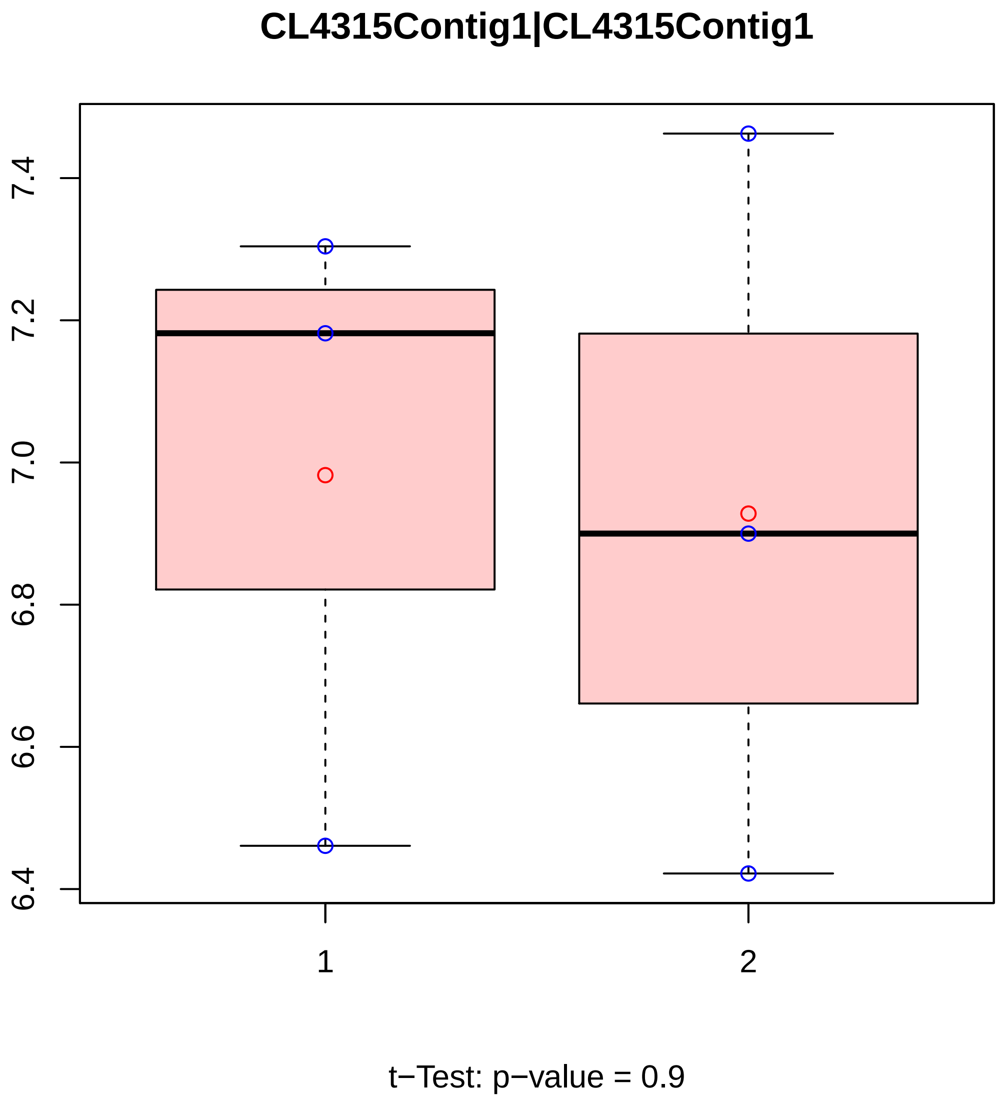
CL4315Contig1|CL4315Contig1
6.4
6.6
6.8
7.0
7.2
7.4
1
2
t−Test: p−value = 0.9

# CL4321Contig2|CL4321Contig2

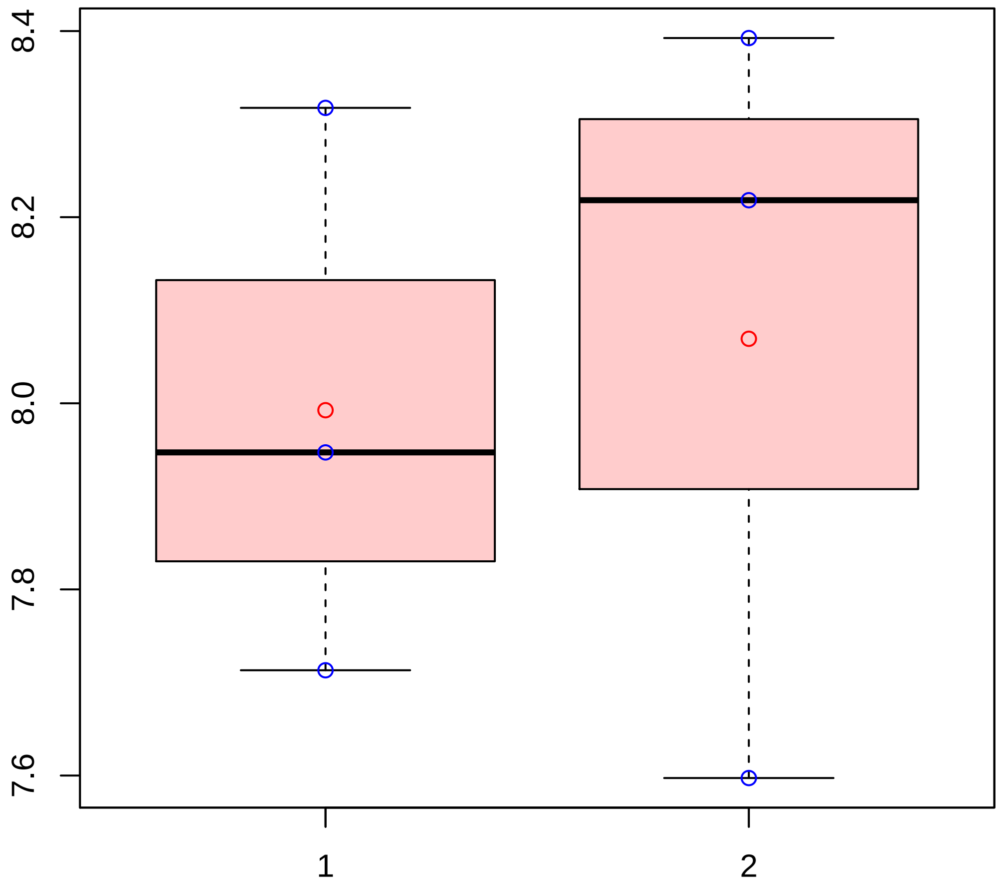


t−Test: p−value = 0.81

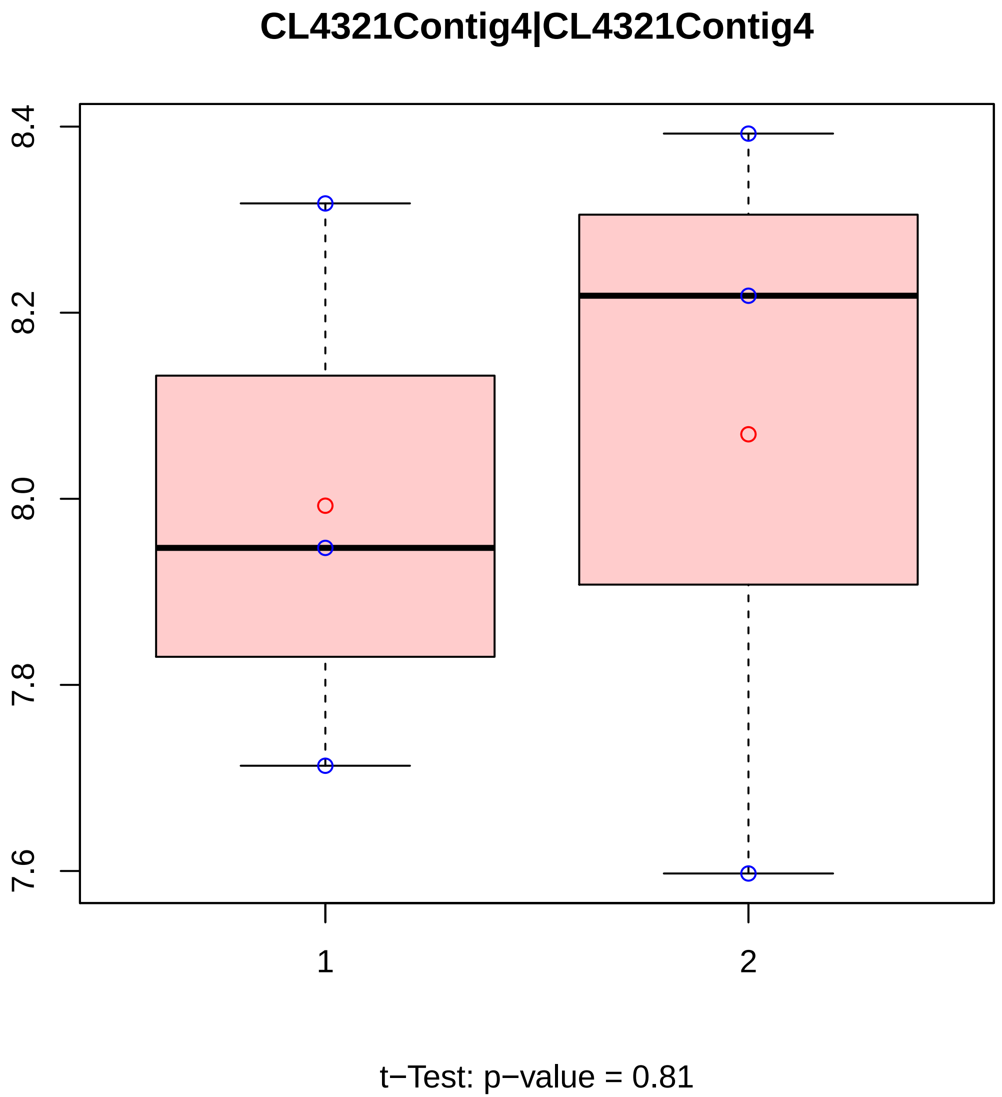
CL4321Contig4|CL4321Contig4
8.4
8.2
8.0
7.8
7.6
1
2
t−Test: p−value = 0.81

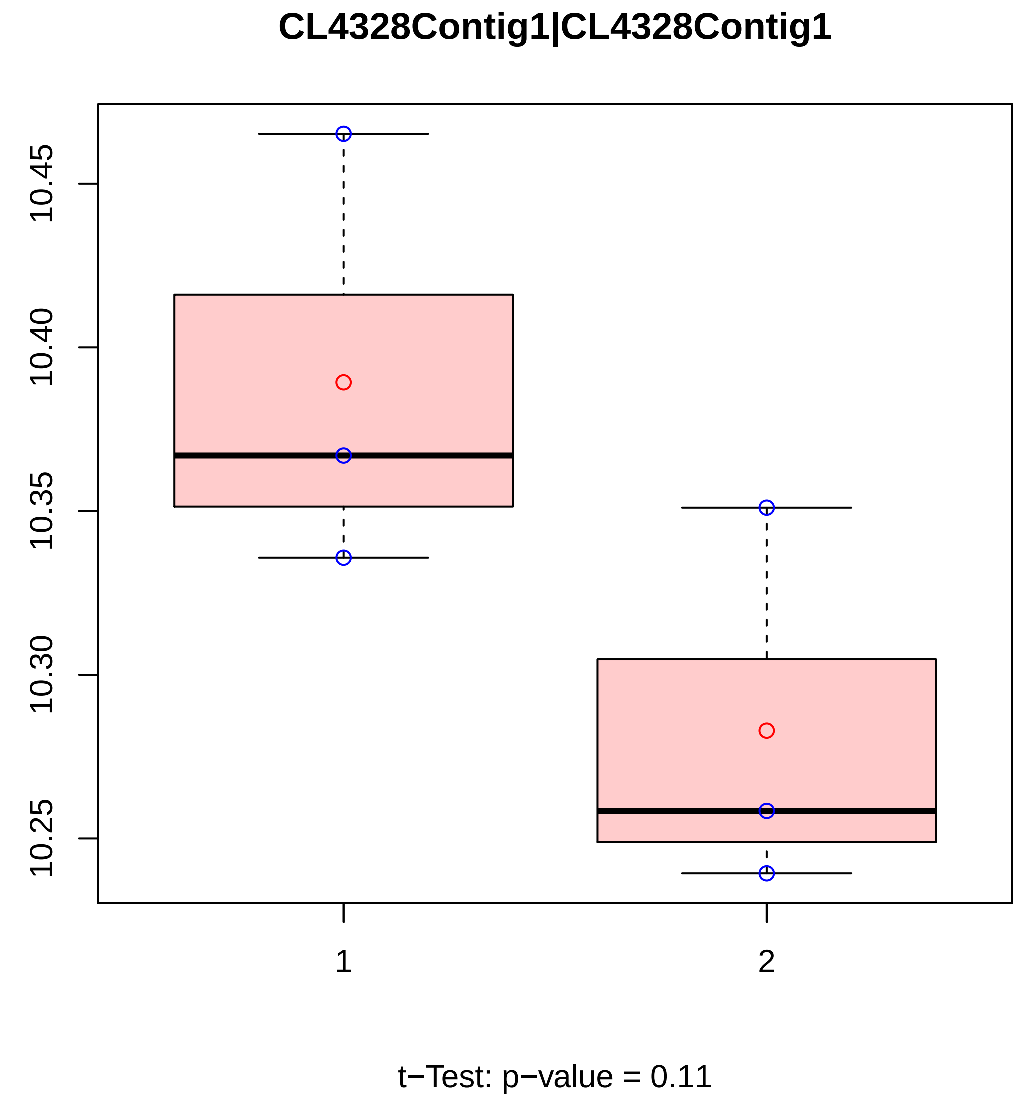
CL4328Contig1|CL4328Contig1
10.45
10.40
10.35
10.30
10.25
1
2
t−Test: p−value = 0.11

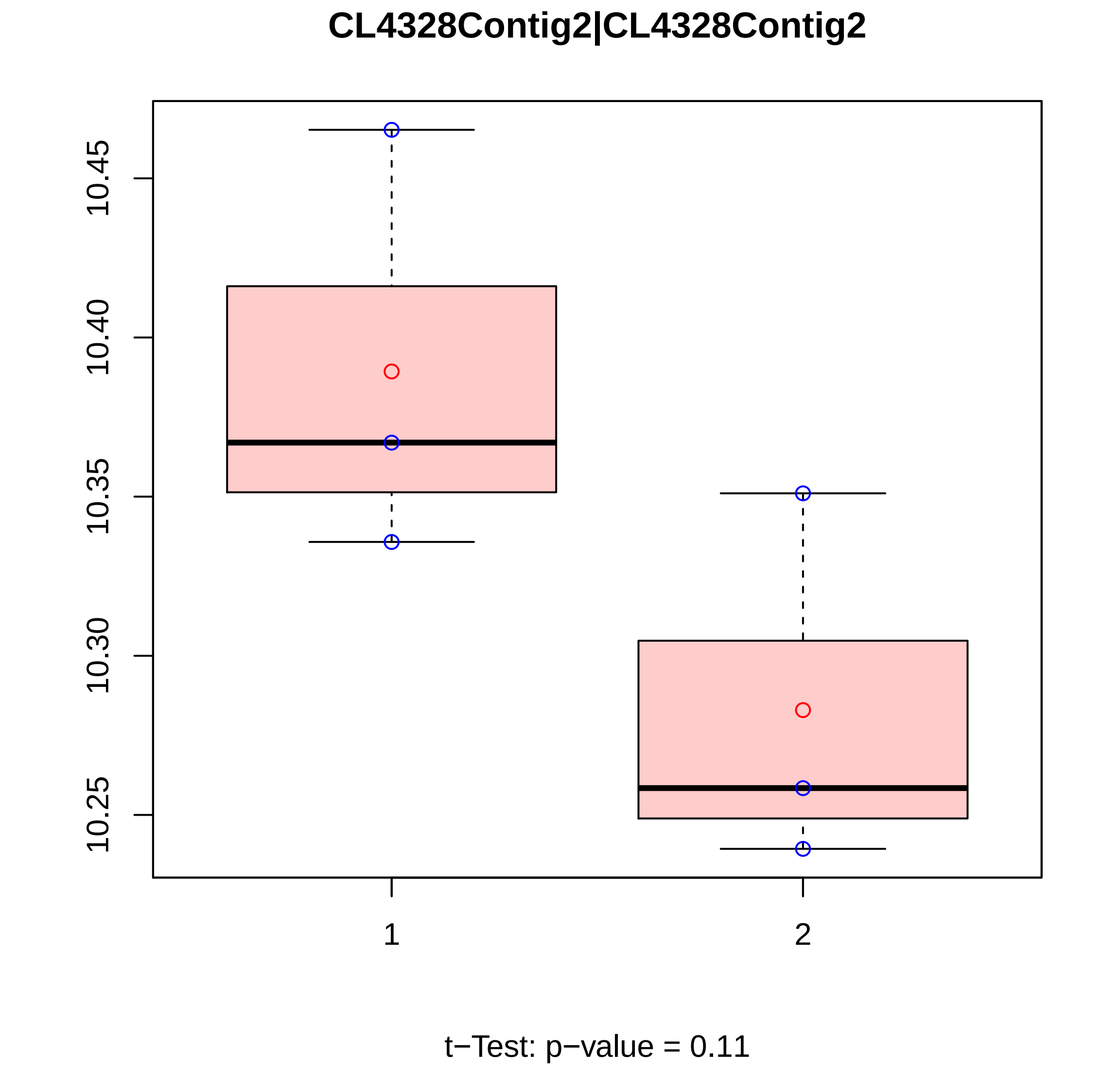
CL4328Contig2|CL4328Contig2
10.45
10.40
10.35
10.30
10.25
1
2
t−Test: p−value = 0.11

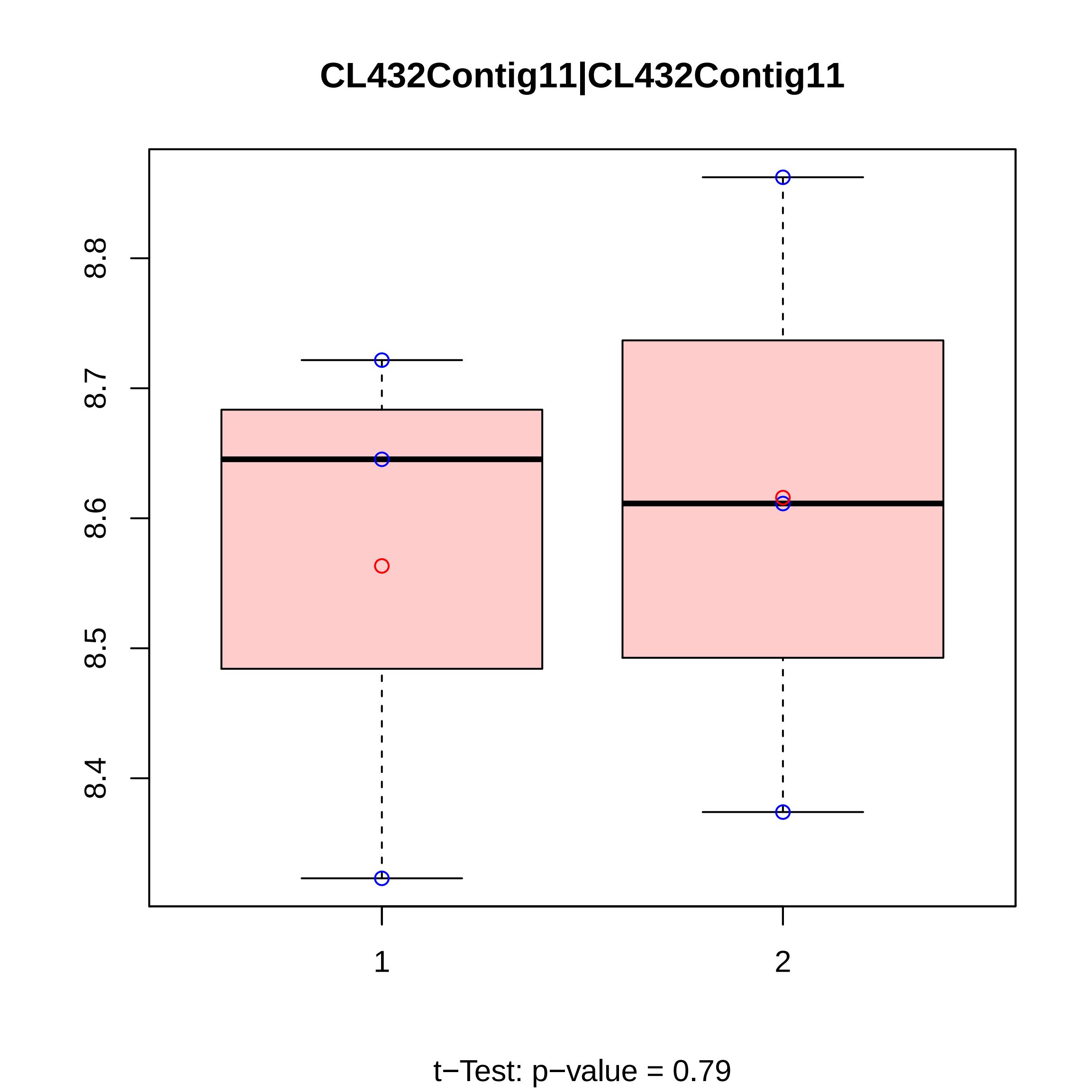

CL432Contig11|CL432Contig11
8.4
8.5
8.6
8.7
8.8
1
2
t−Test: p−value = 0.79

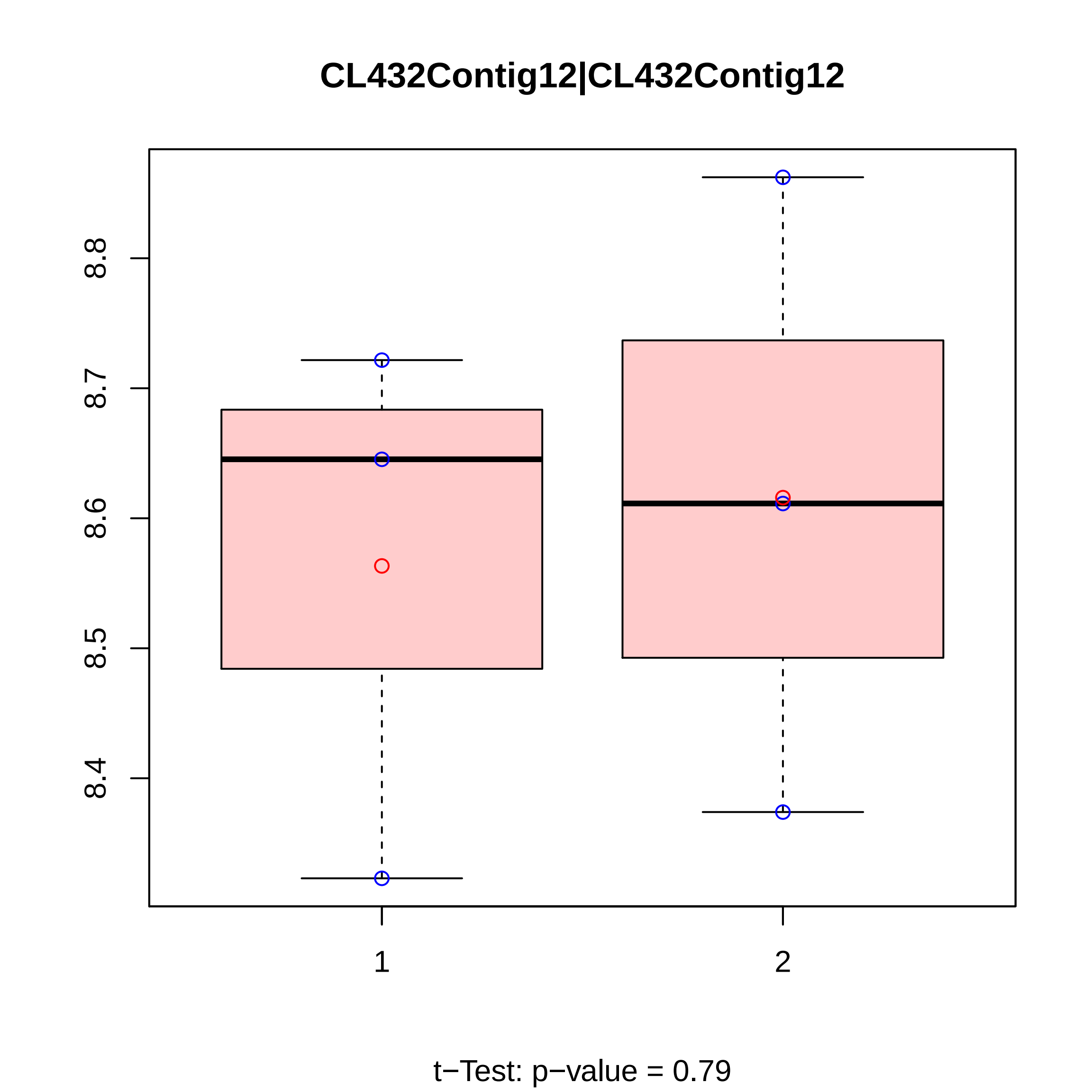
CL432Contig12|CL432Contig12
8.4
8.5
8.6
8.7
8.8
1
2
t−Test: p−value = 0.79

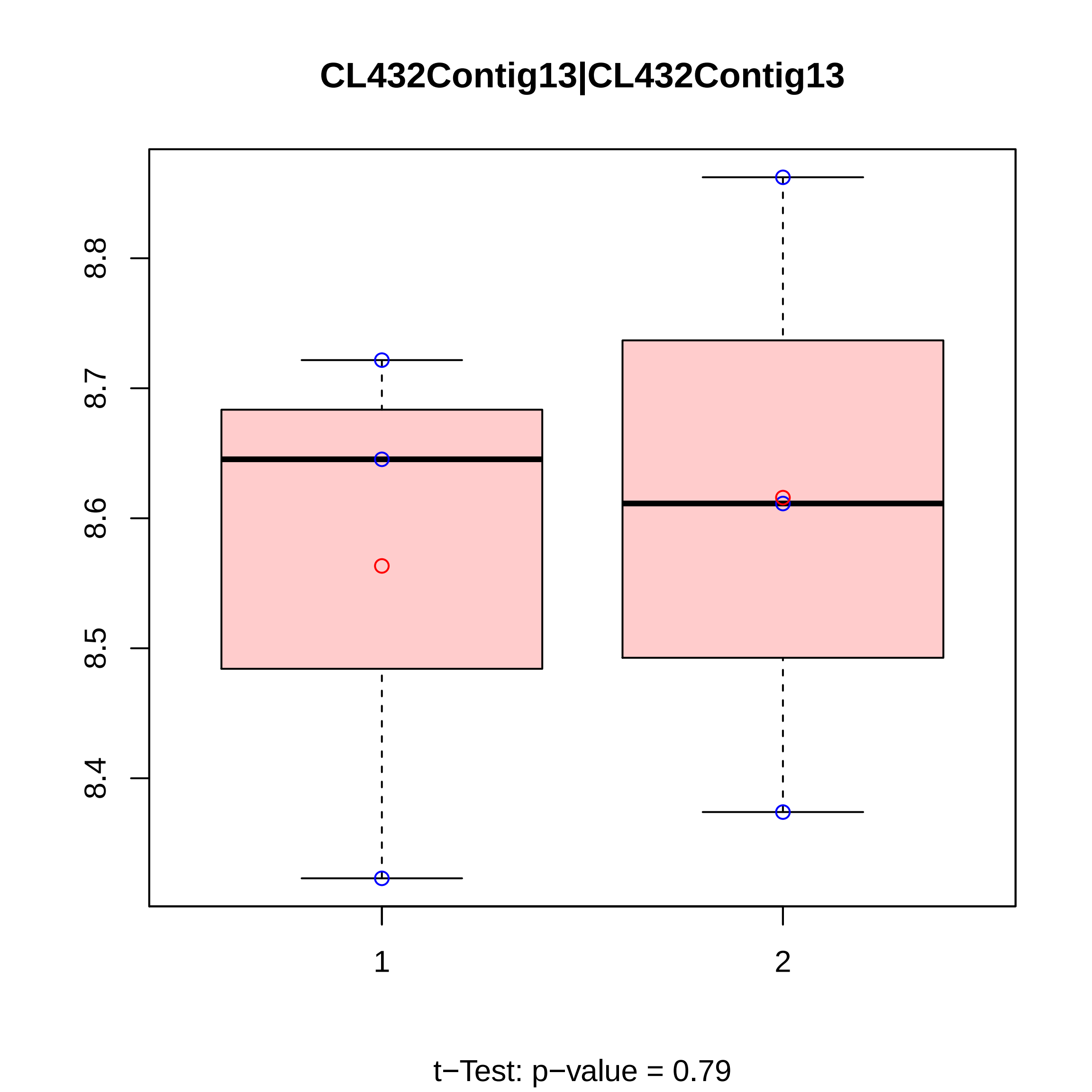
CL432Contig13|CL432Contig13
8.4
8.5
8.6
8.7
8.8
1
2
t−Test: p−value = 0.79

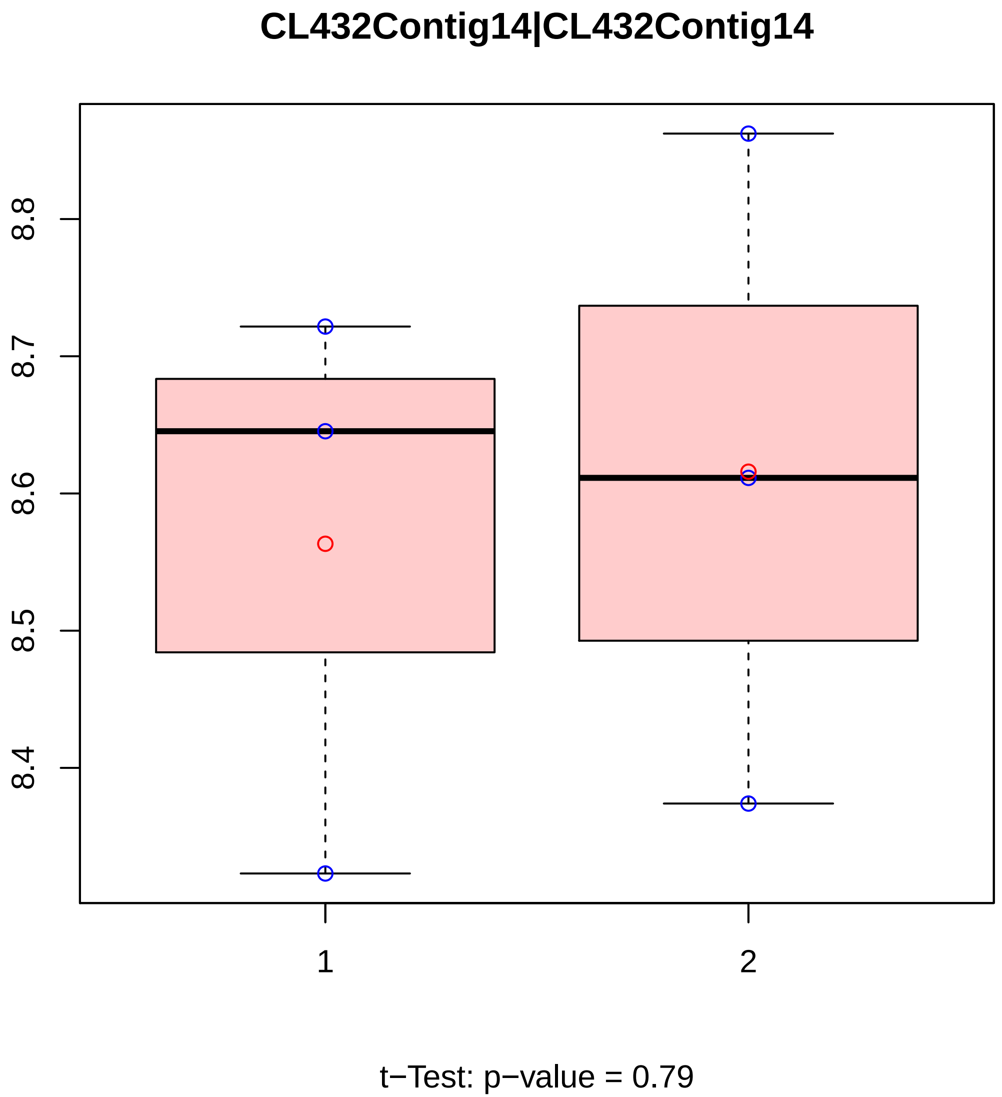

CL432Contig14|CL432Contig14
8.4
8.5
8.6
8.7
8.8
1
2
t−Test: p−value = 0.79

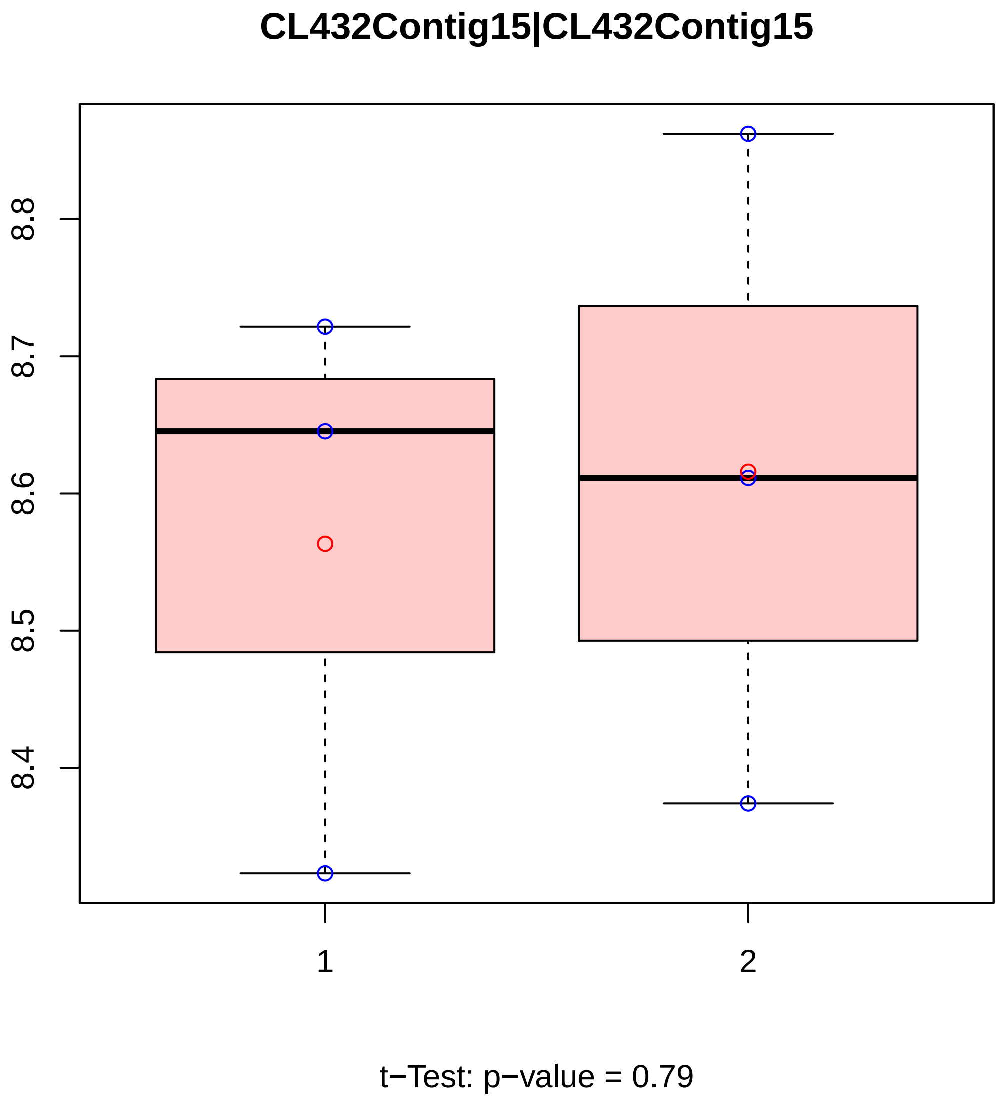
CL432Contig15|CL432Contig15
8.4
8.5
8.6
8.7
8.8
1
2
t−Test: p−value = 0.79

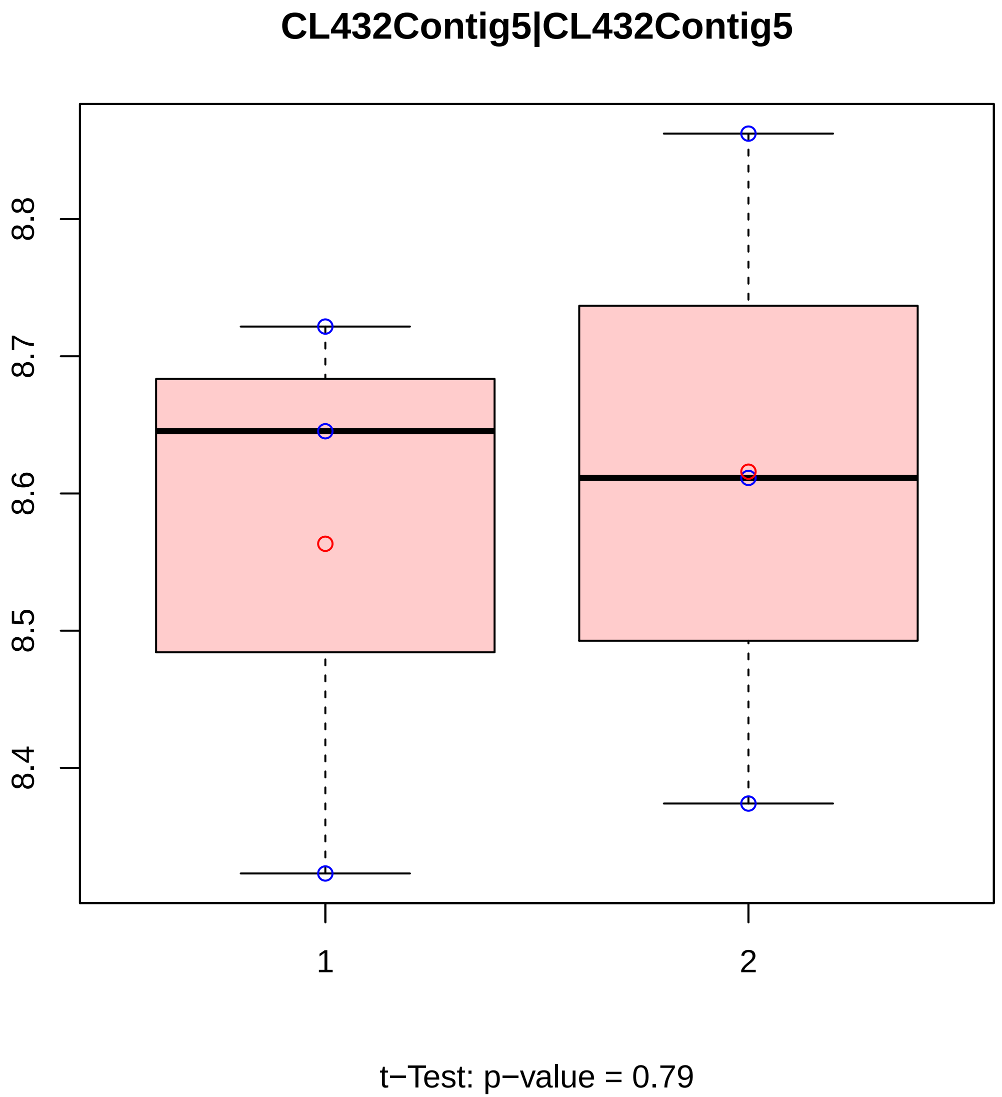
CL432Contig5|CL432Contig5
8.4
8.5
8.6
8.7
8.8
1
2
t−Test: p−value = 0.79

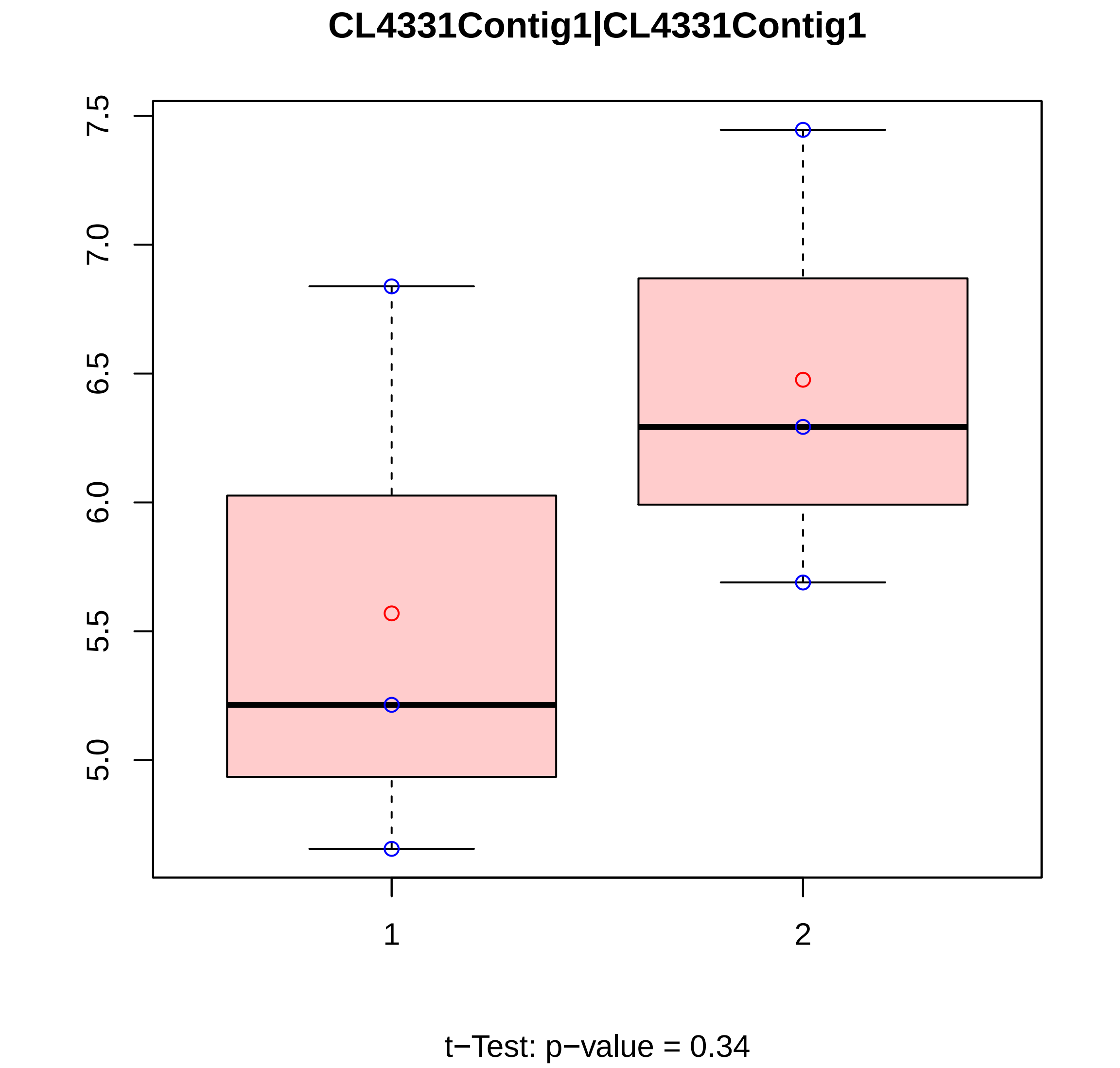
CL4331Contig1|CL4331Contig1
7.5
7.0
6.5
6.0
5.5
5.0
1
2
t−Test: p−value = 0.34

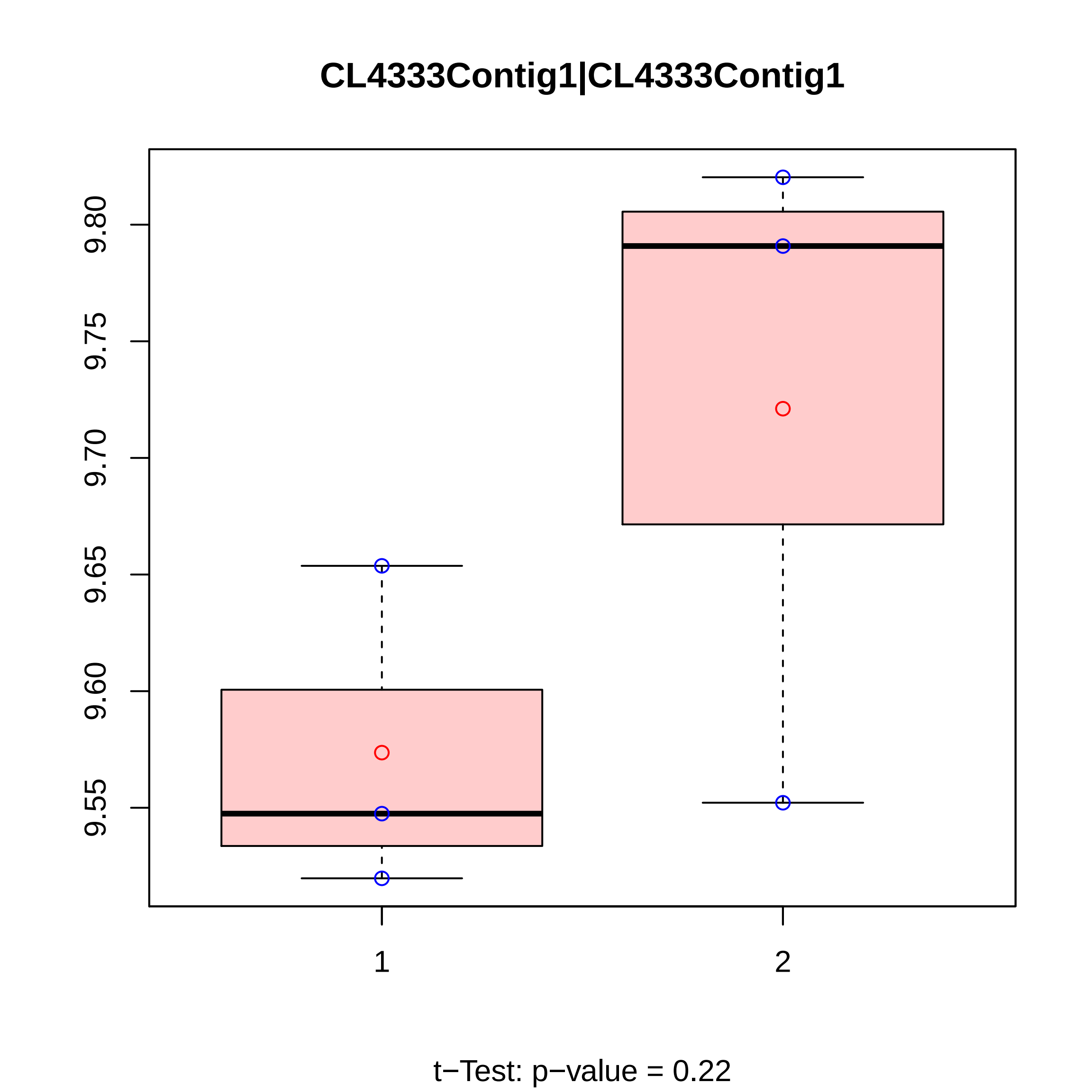
CL4333Contig1|CL4333Contig1
9.55
9.60
9.65
9.70
9.75
9.80
1
2
t−Test: p−value = 0.22

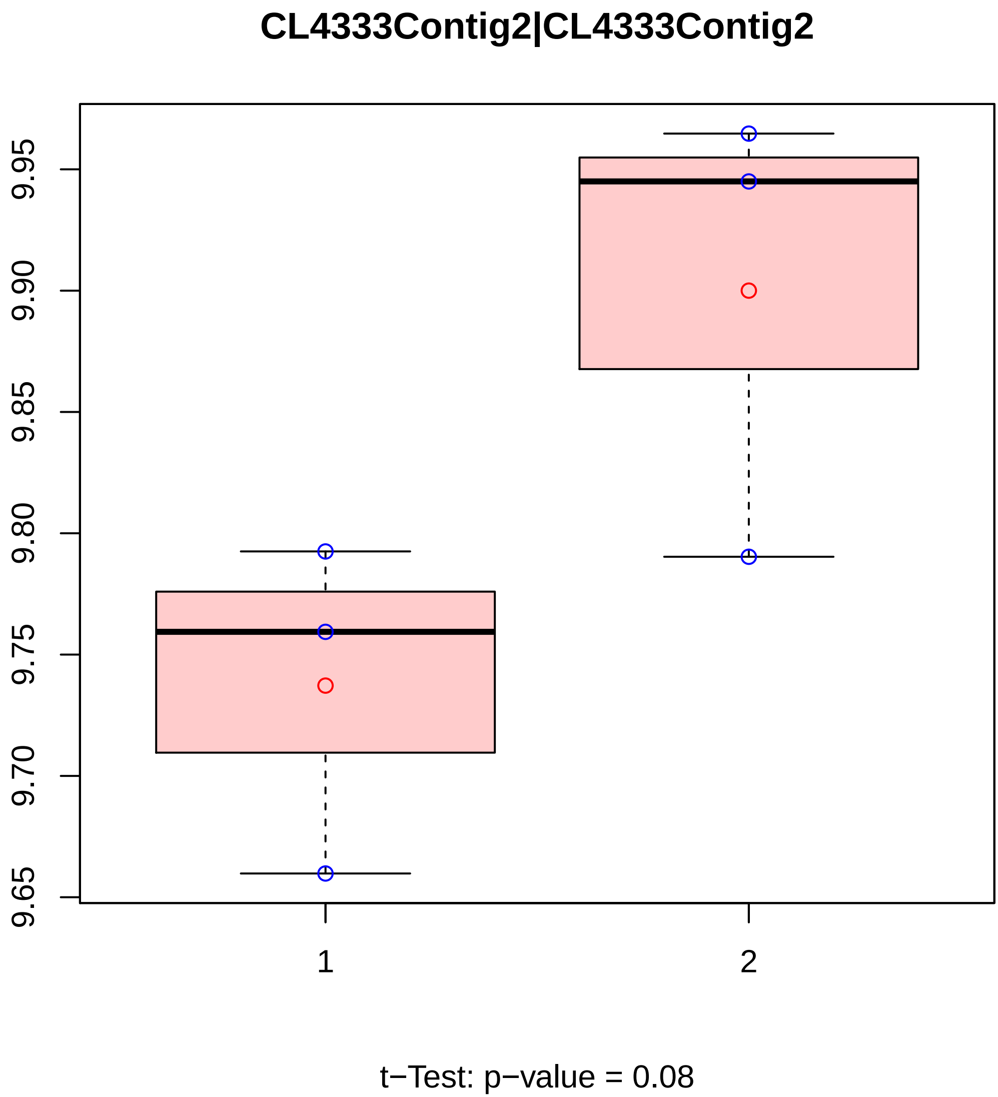
CL4333Contig2|CL4333Contig2
9.65
9.70
9.75
9.80
9.85
9.90
9.95
1
2
t−Test: p−value = 0.08

## CL433Contig1|CL433Contig1

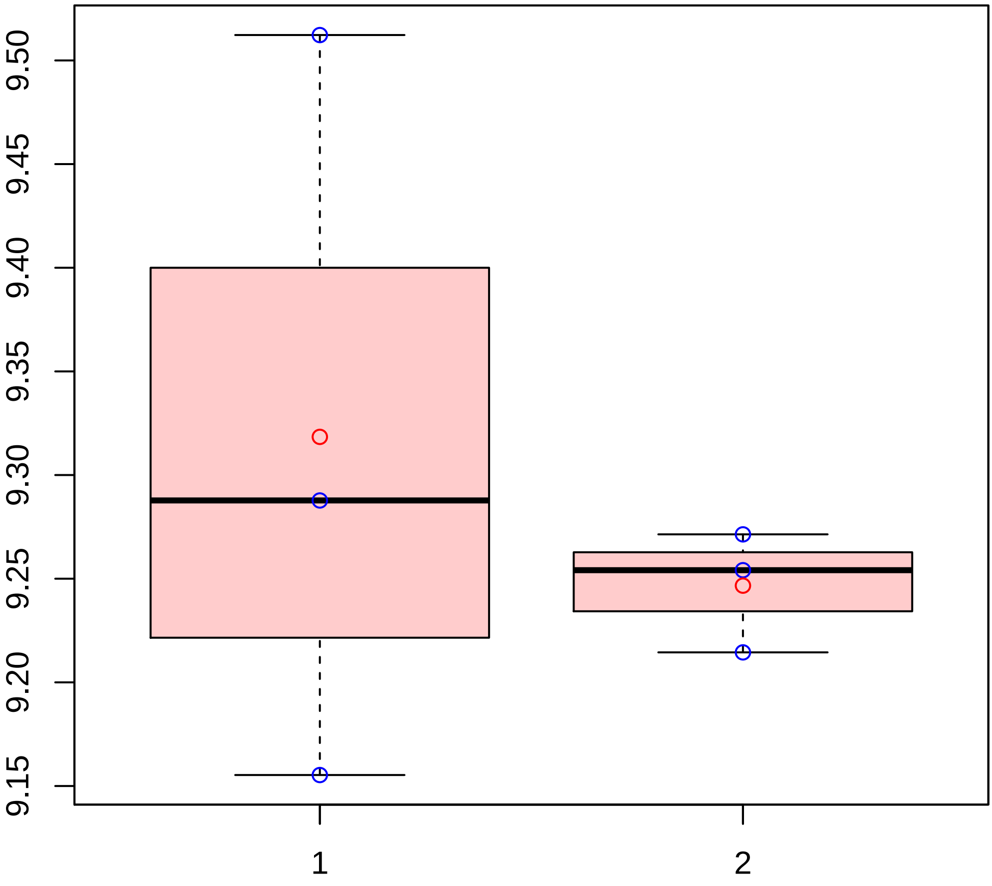


t−Test: p−value = 0.56

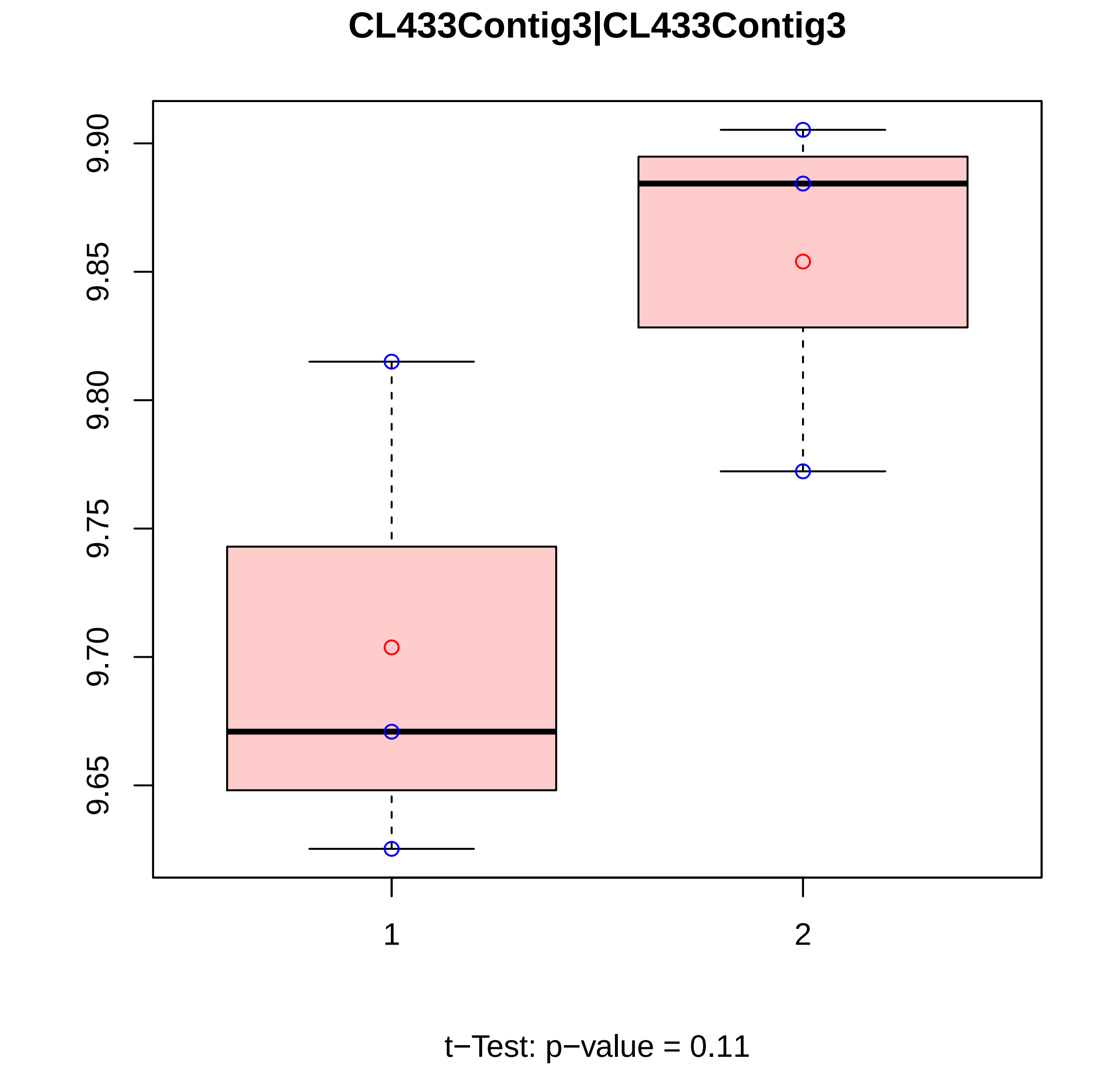
CL433Contig3|CL433Contig3
9.65
9.70
9.75
9.80
9.85
9.90
1
2
t−Test: p−value = 0.11

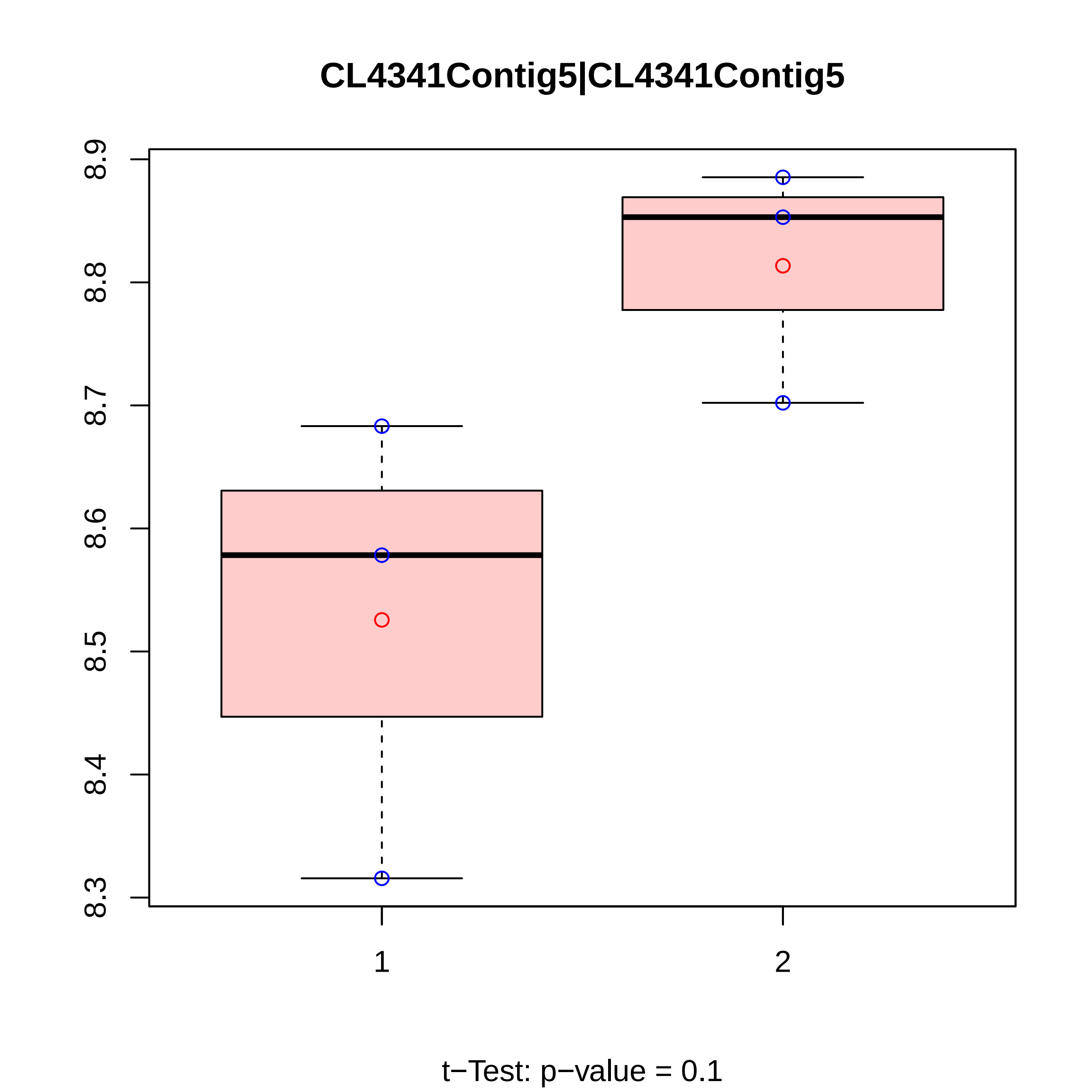

CL4341Contig5|CL4341Contig5
8.3
8.4
8.5
8.6
8.7
8.8
8.9
1
2
t−Test: p−value = 0.1

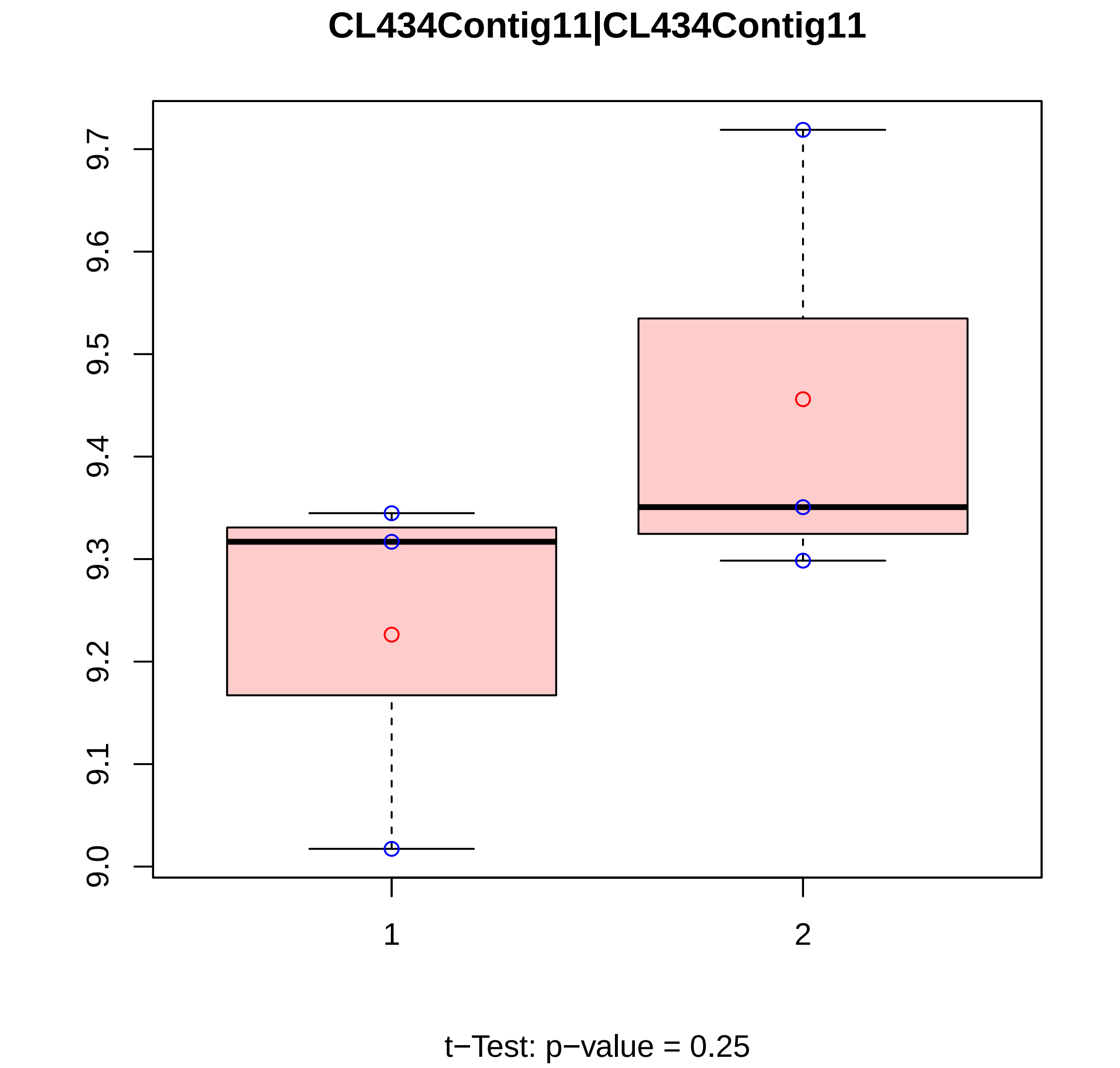

CL434Contig11|CL434Contig11
9.0
9.1
9.2
9.3
9.4
9.5
9.6
9.7
1
2
t−Test: p−value = 0.25

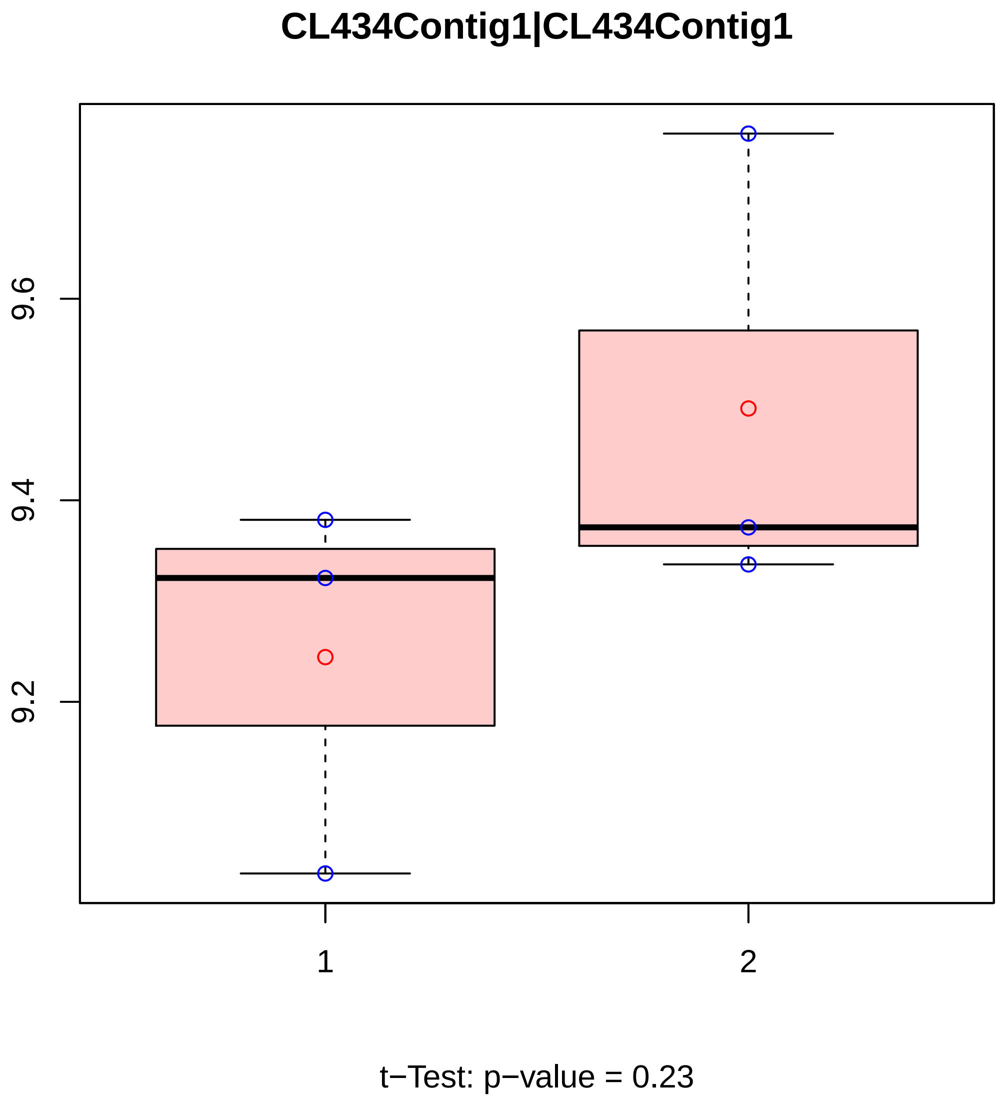
CL434Contig1|CL434Contig1
9.2
9.4
9.6
1
2
t−Test: p−value = 0.23

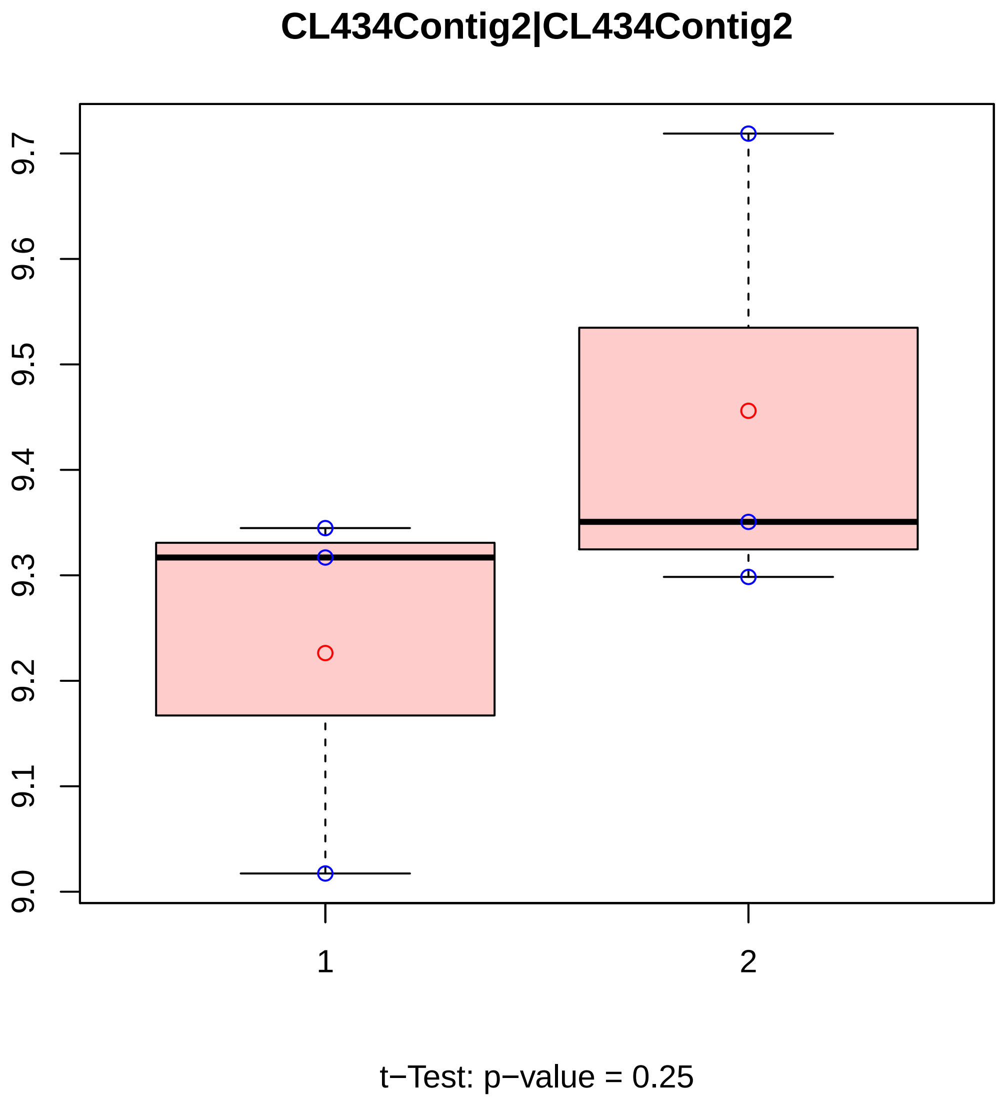

CL434Contig2|CL434Contig2
9.0
9.1
9.2
9.3
9.4
9.5
9.6
9.7
1
2
t−Test: p−value = 0.25

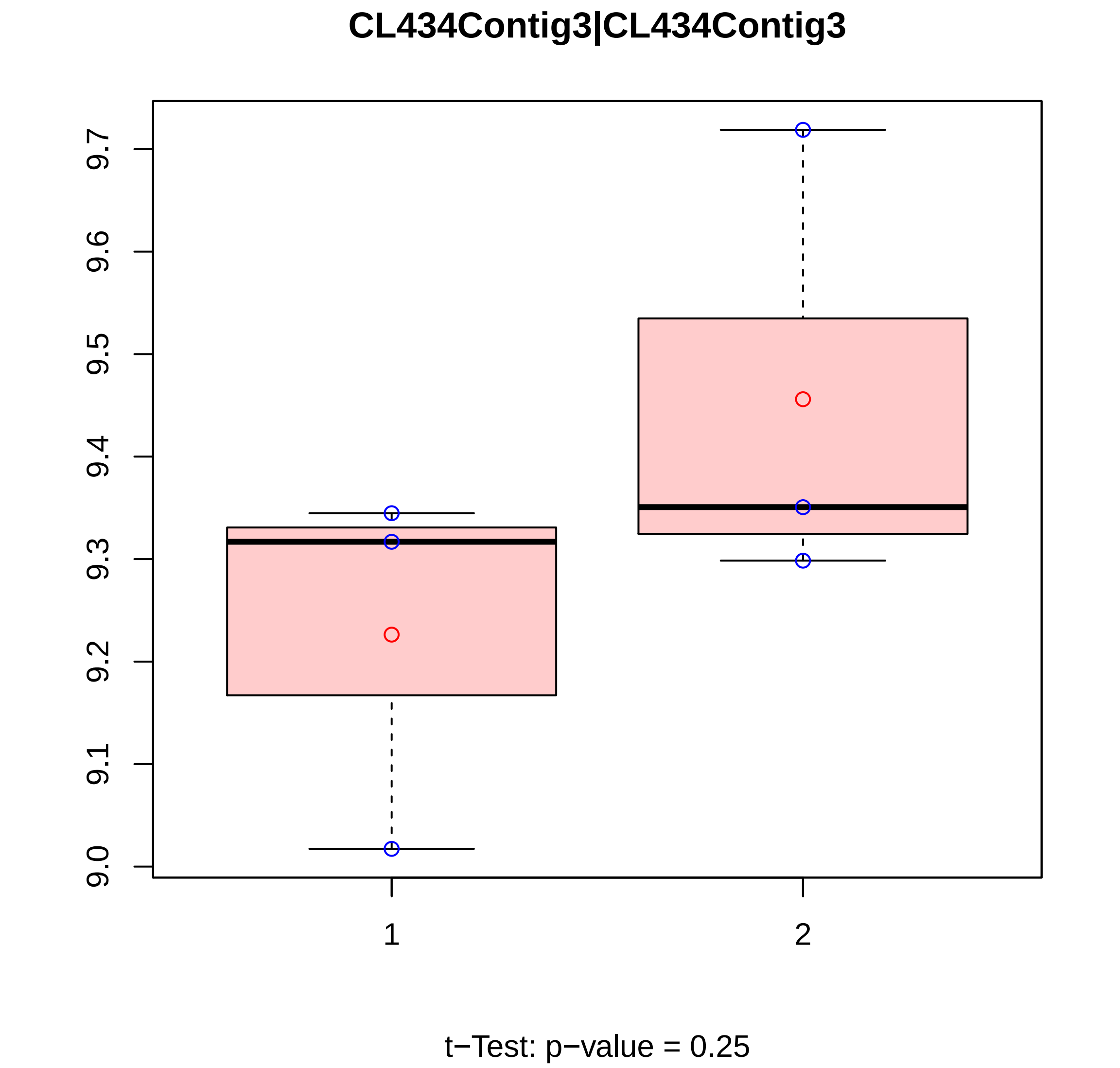

CL434Contig3|CL434Contig3
9.0
9.1
9.2
9.3
9.4
9.5
9.6
9.7
1
2
t−Test: p−value = 0.25

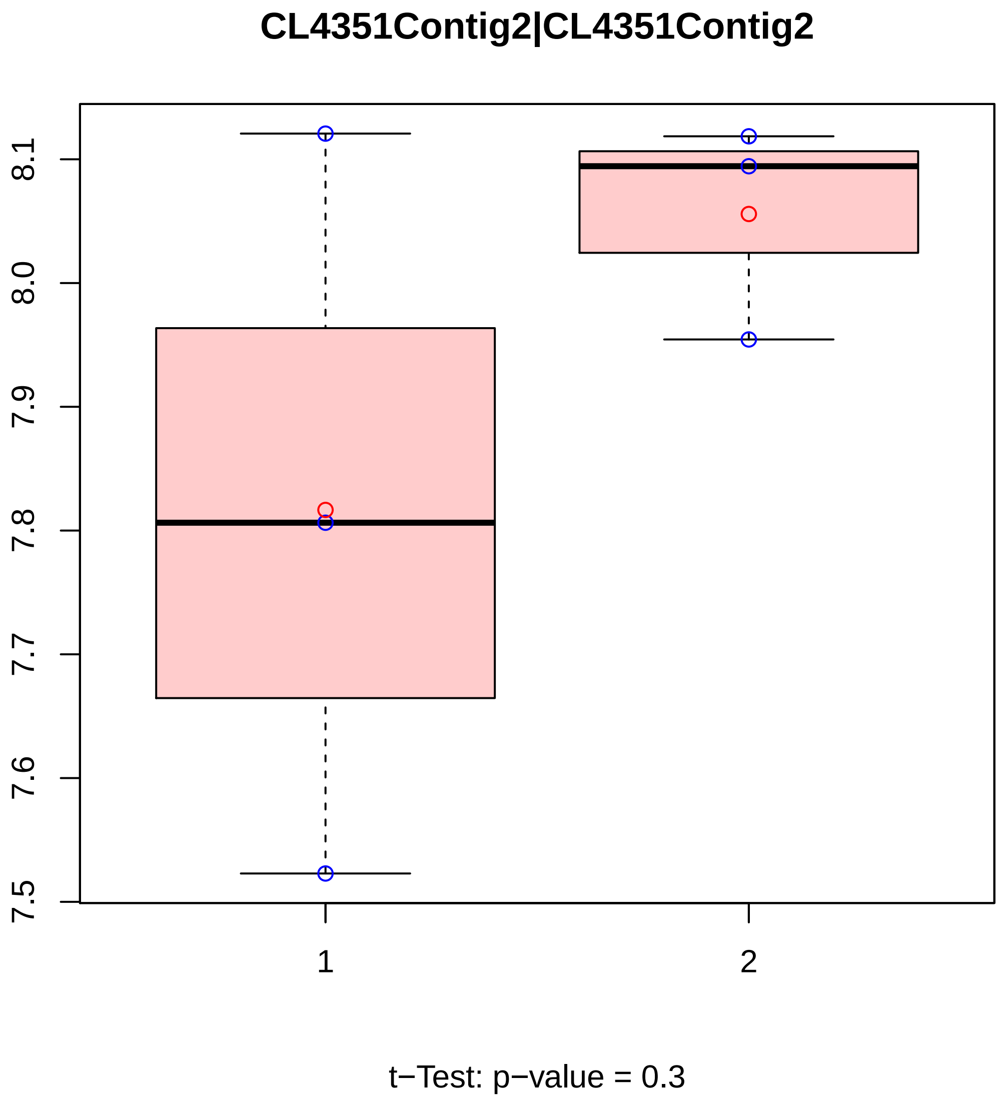

CL4351Contig2|CL4351Contig2
8.1
8.0
7.9
7.8
7.7
7.6
7.5
1
2
t−Test: p−value = 0.3

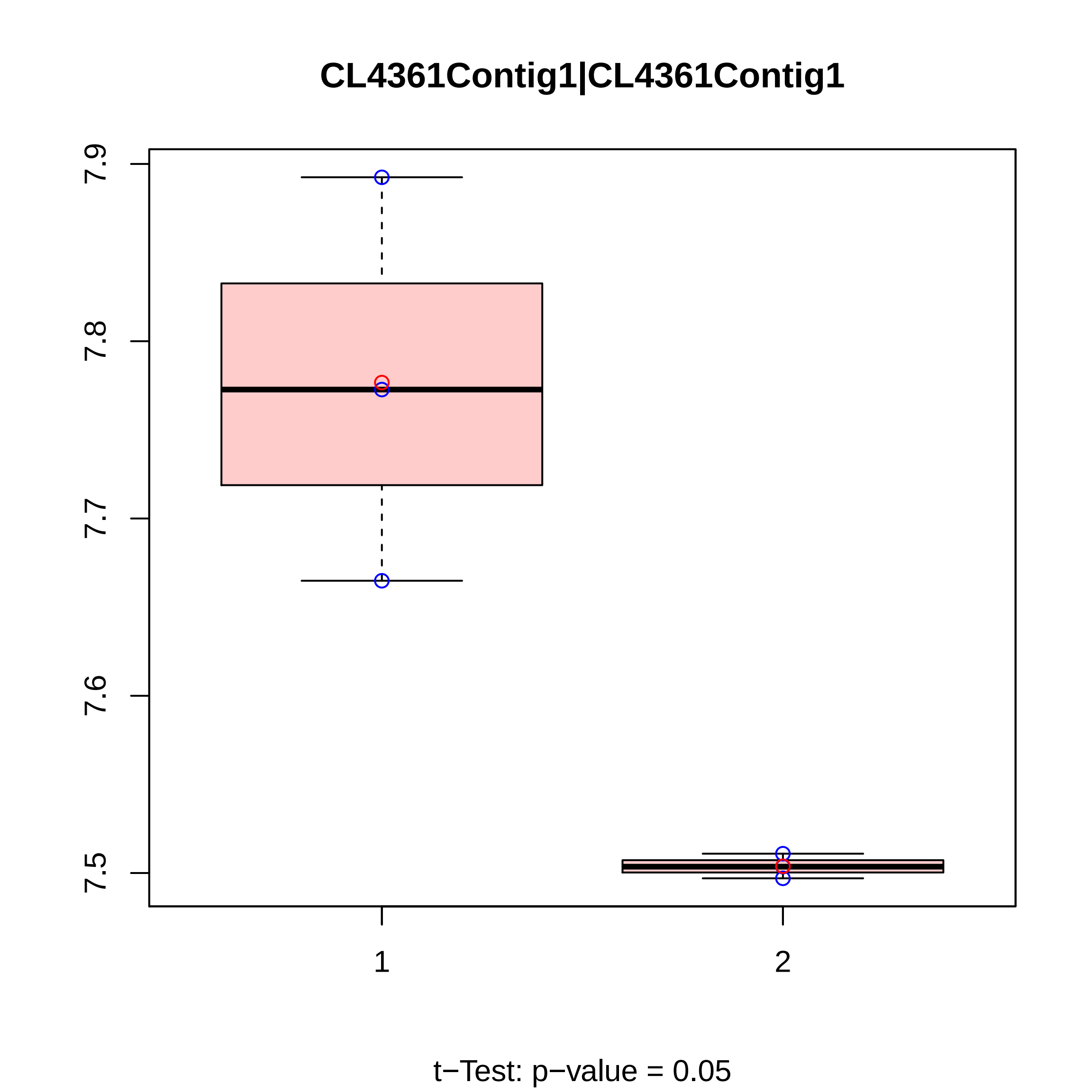
CL4361Contig1|CL4361Contig1
7.9
7.8
7.7
7.6
7.5
1
2
t−Test: p−value = 0.05

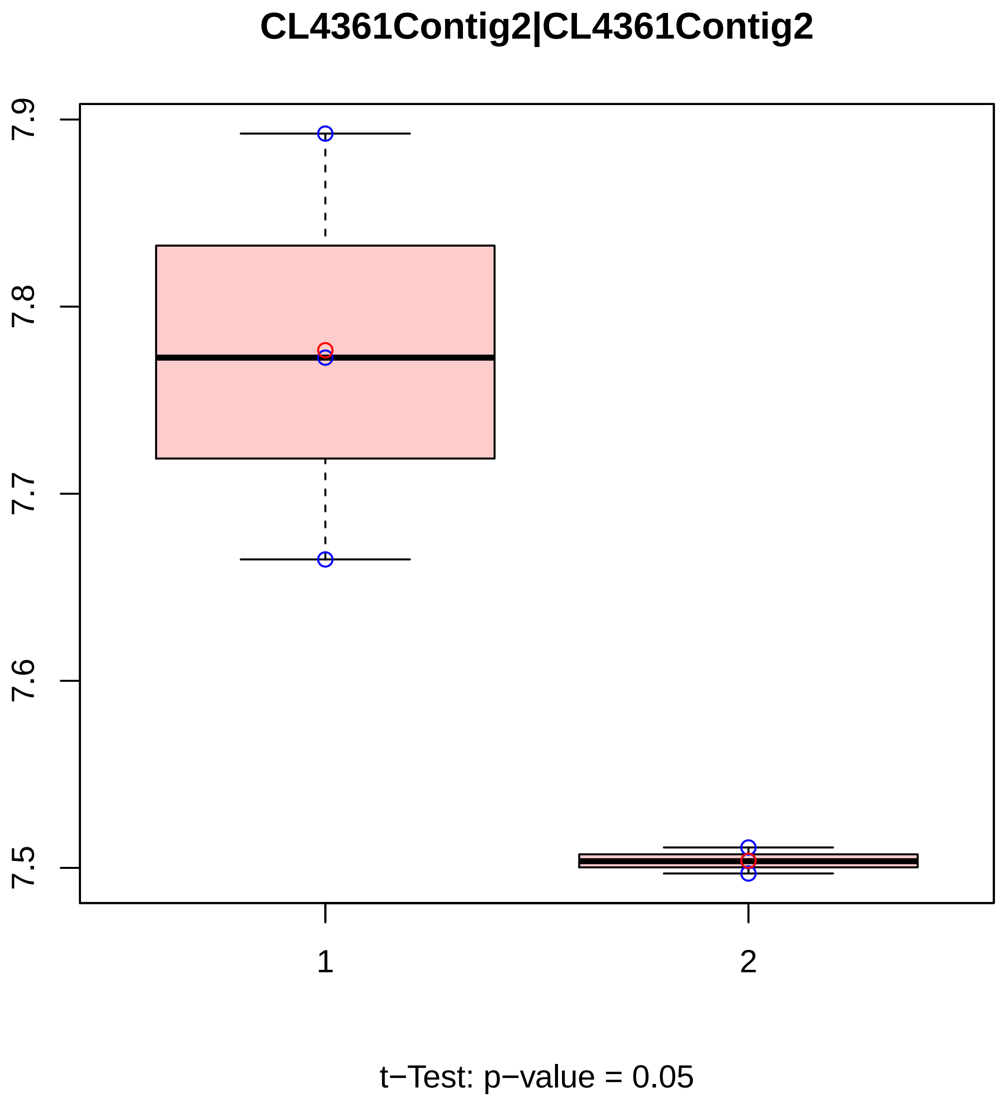

CL4361Contig2|CL4361Contig2
7.9
7.8
7.7
7.6
7.5
1
2
t−Test: p−value = 0.05

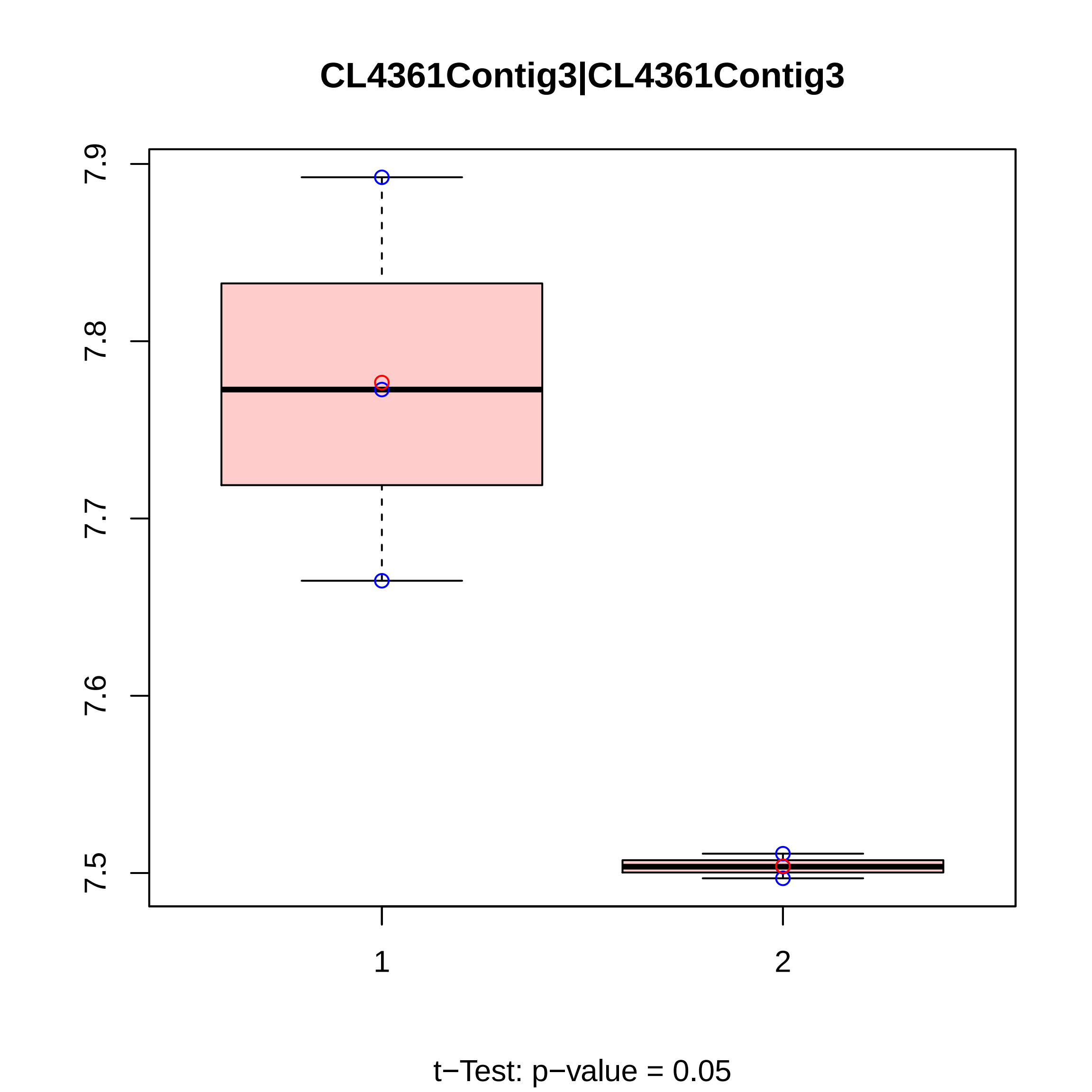

CL4361Contig3|CL4361Contig3
7.5
7.6
7.7
7.8
7.9
1
2
t−Test: p−value = 0.05

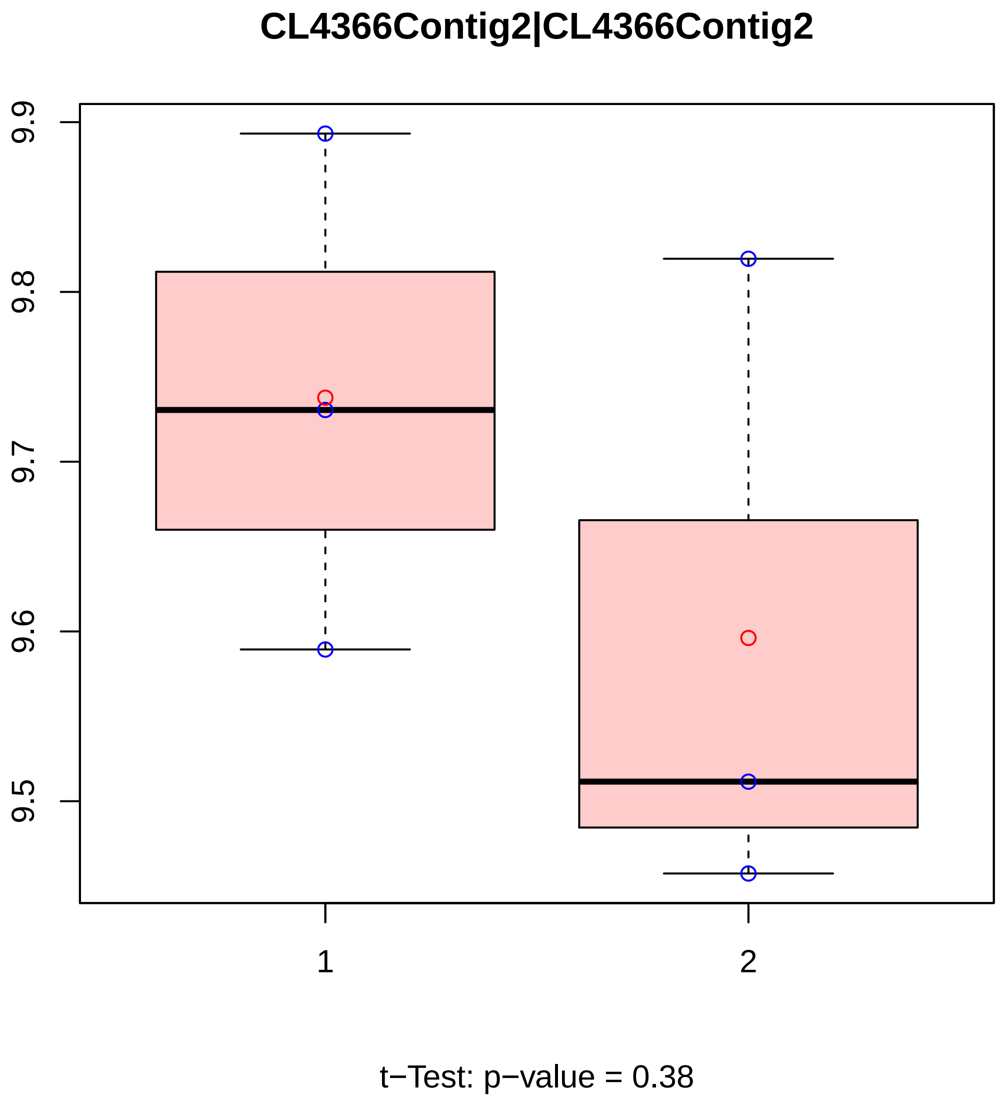

CL4366Contig2|CL4366Contig2
9.9
9.8
9.7
9.6
9.5
1
2
t−Test: p−value = 0.38

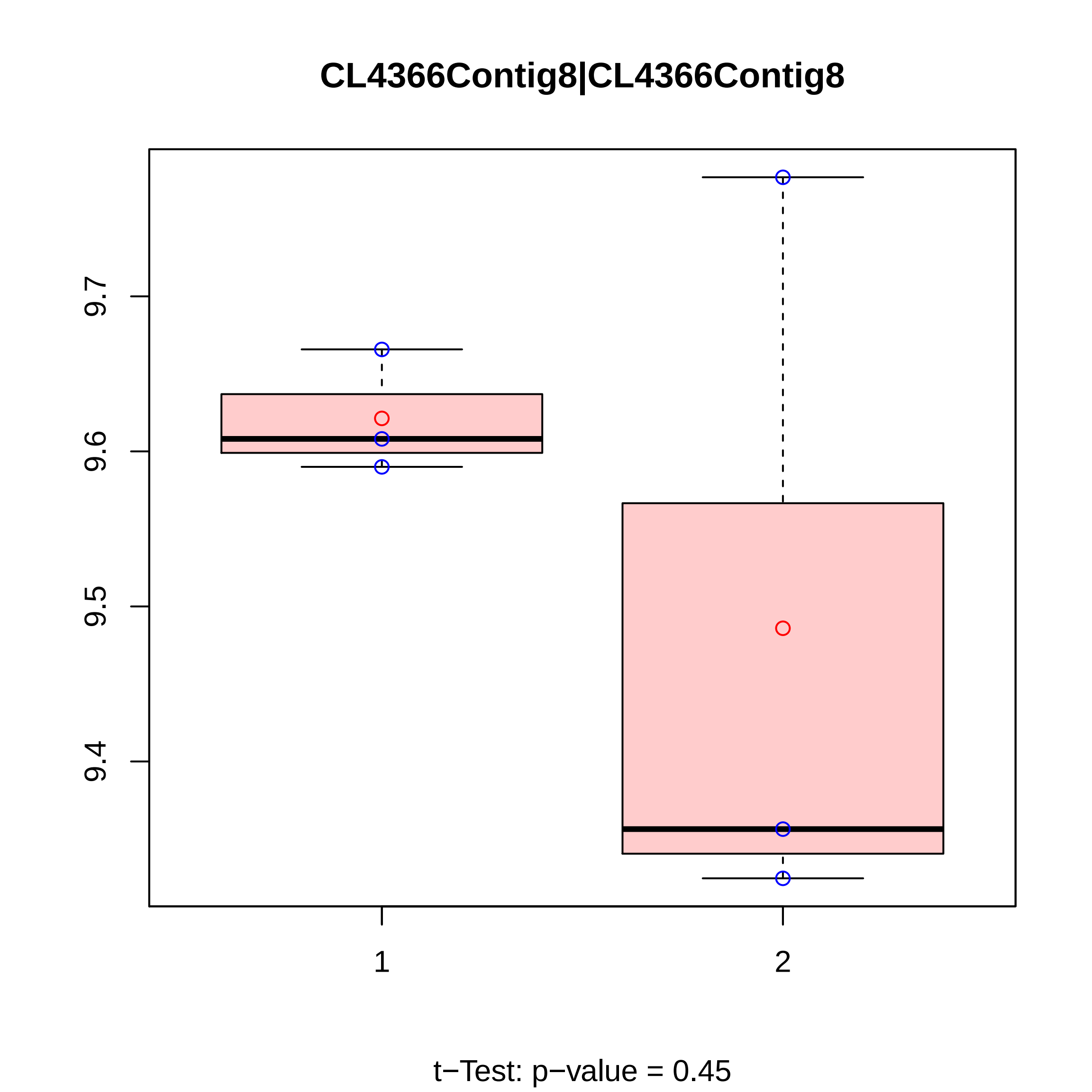
CL4366Contig8|CL4366Contig8
9.7
9.6
9.5
9.4
1
2
t−Test: p−value = 0.45

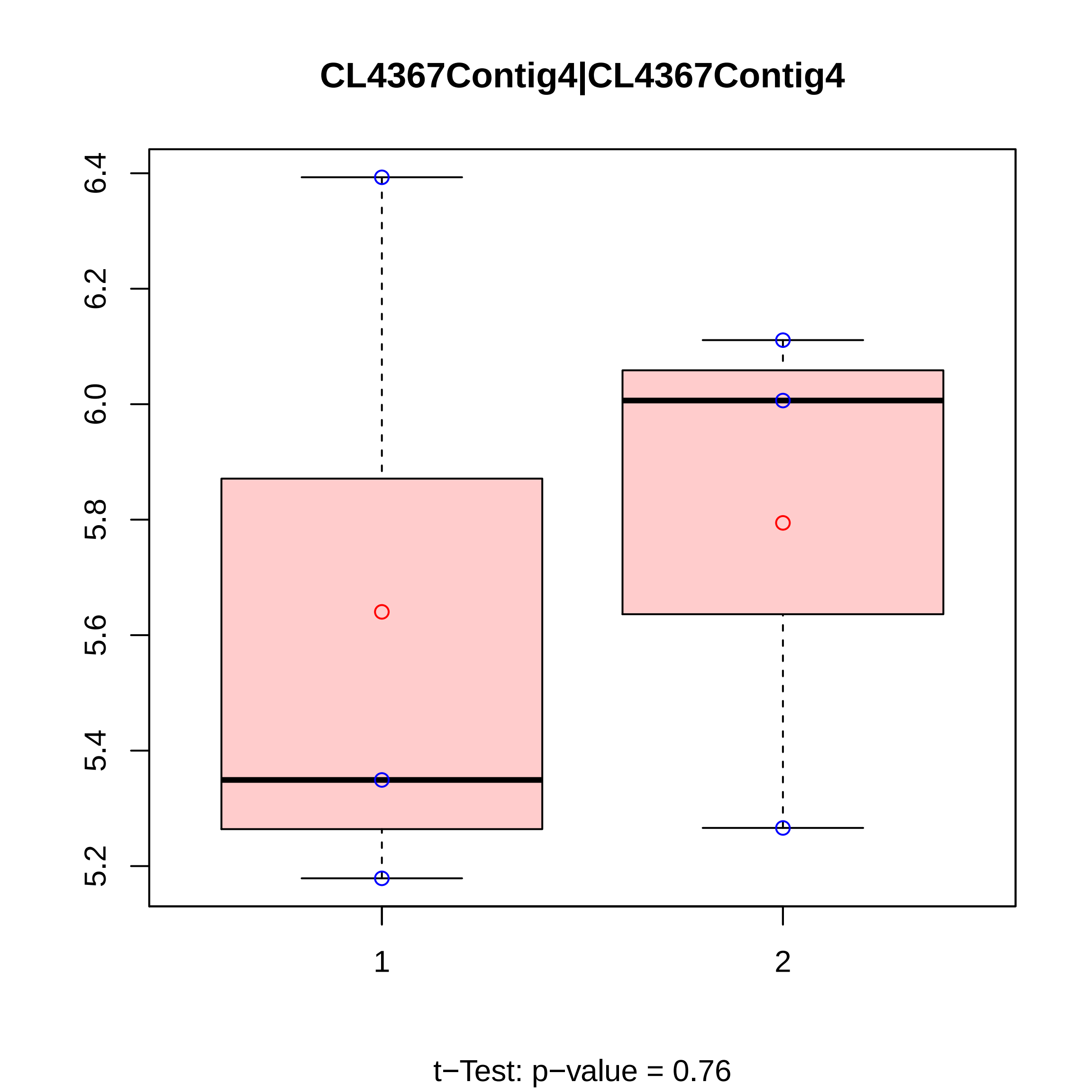

CL4367Contig4|CL4367Contig4
6.4
6.2
6.0
5.8
5.6
5.4
5.2
1
2
t−Test: p−value = 0.76

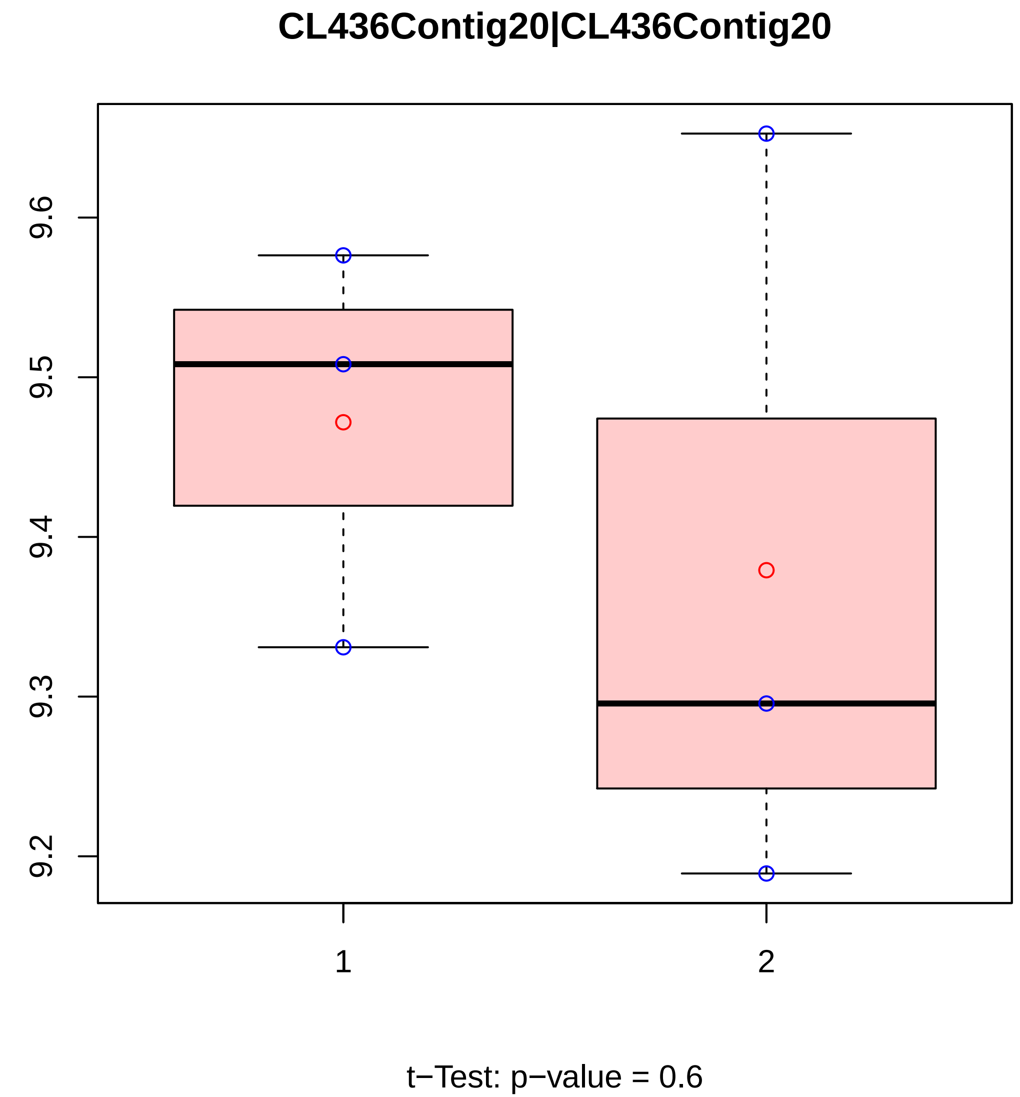
CL436Contig20|CL436Contig20
9.2
9.3
9.4
9.5
9.6
1
2
t−Test: p−value = 0.6

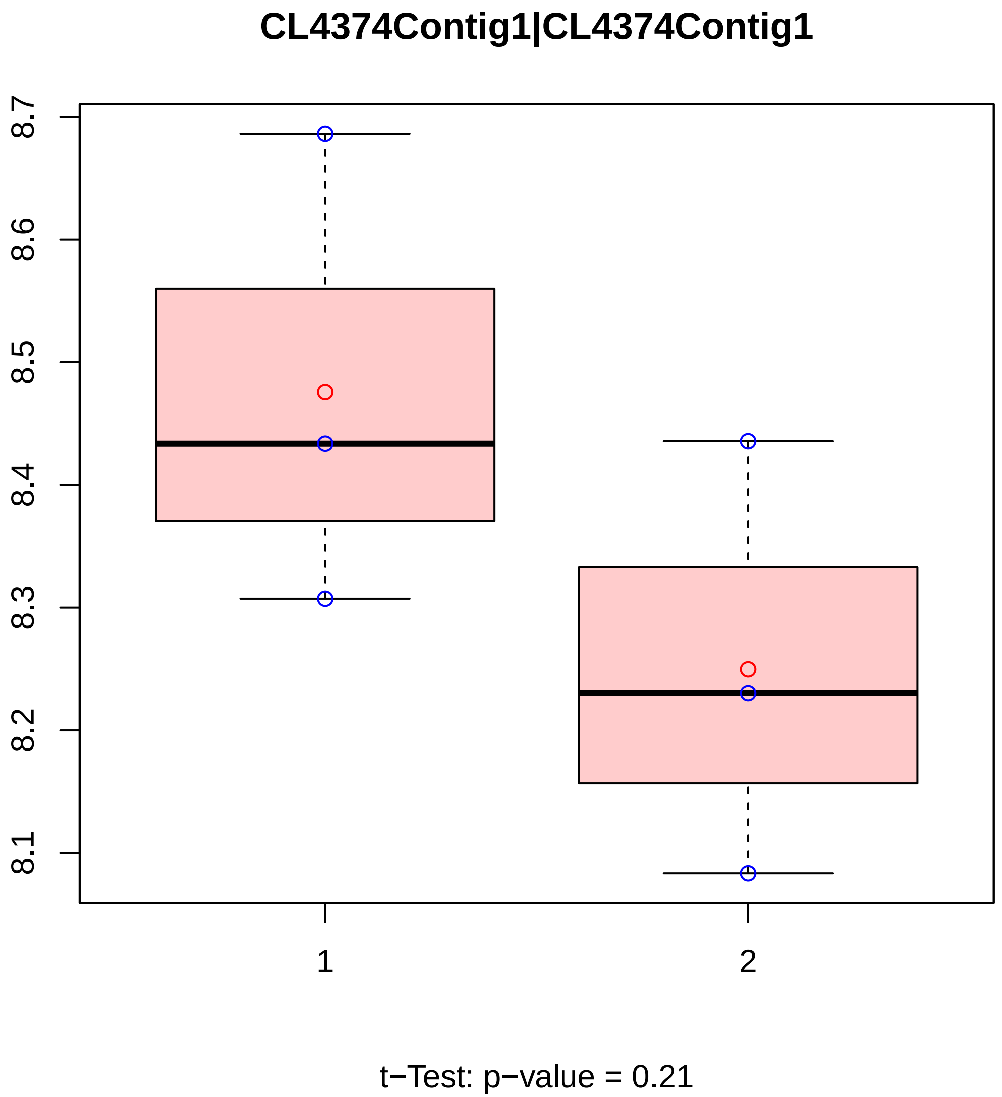

CL4374Contig1|CL4374Contig1
8.7
8.6
8.5
8.4
8.3
8.2
8.1
1
2
t−Test: p−value = 0.21

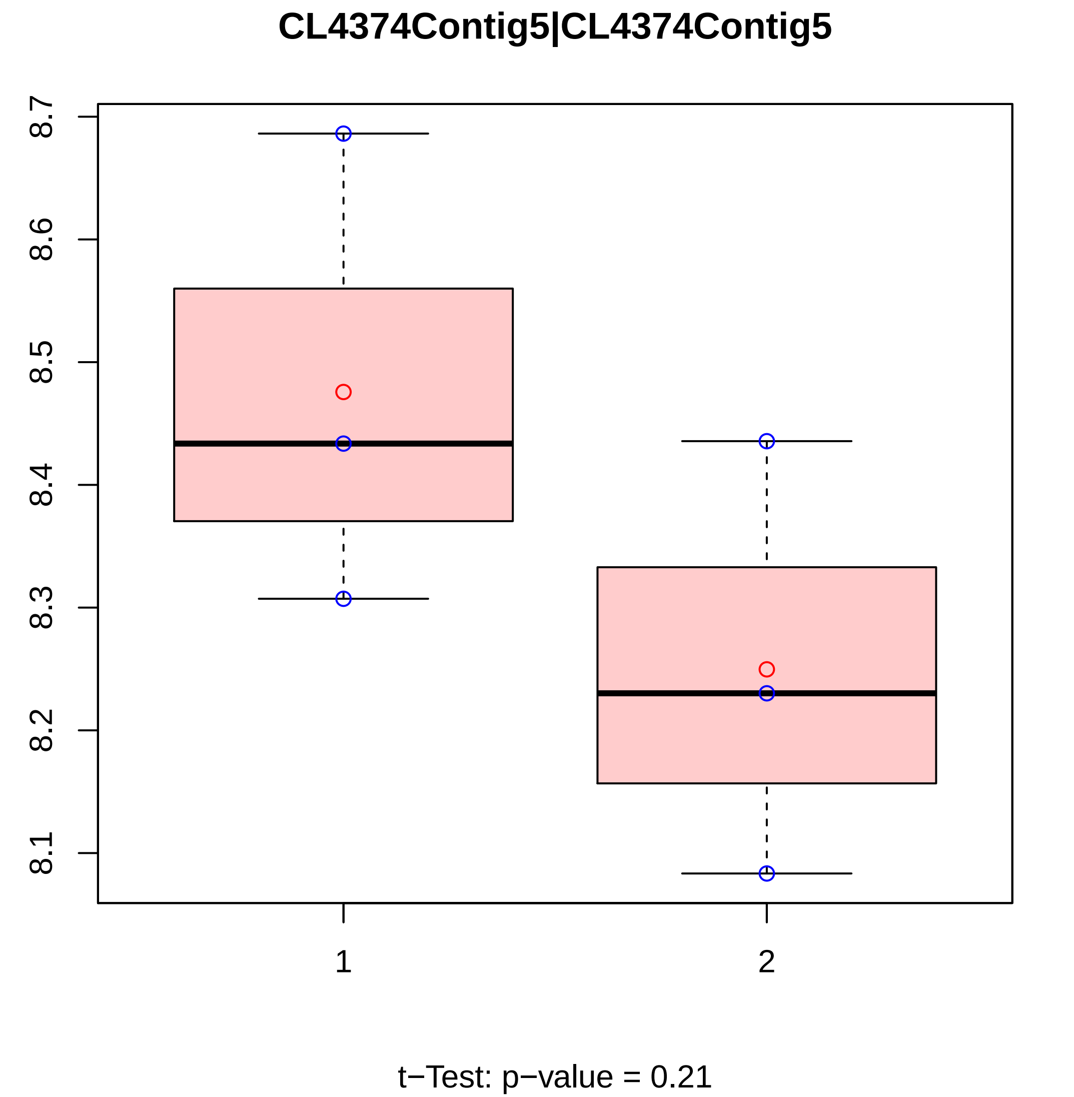
CL4374Contig5|CL4374Contig5
8.1
8.2
8.3
8.4
8.5
8.6
8.7
1
2
t−Test: p−value = 0.21

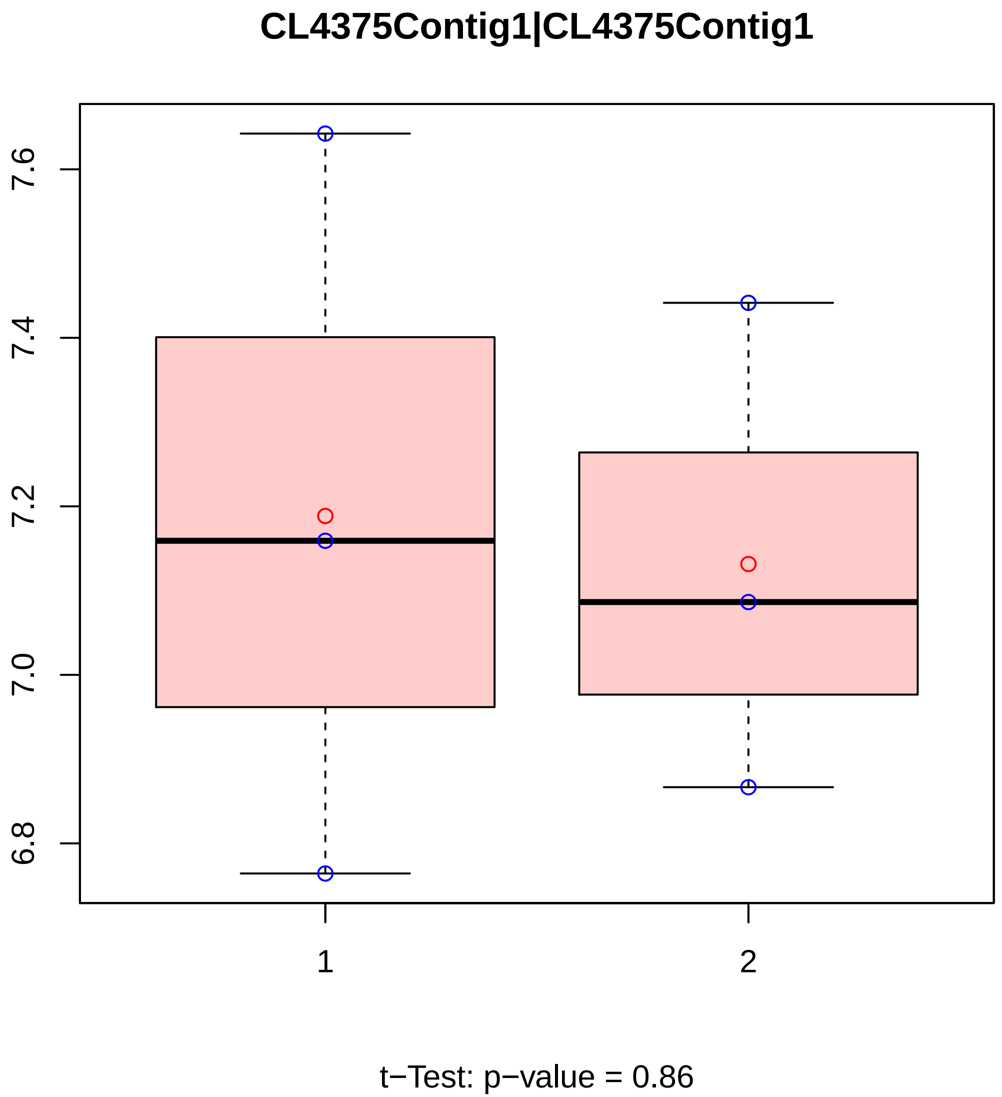
CL4375Contig1|CL4375Contig1
7.6
7.4
7.2
7.0
6.8
1
2
t−Test: p−value = 0.86

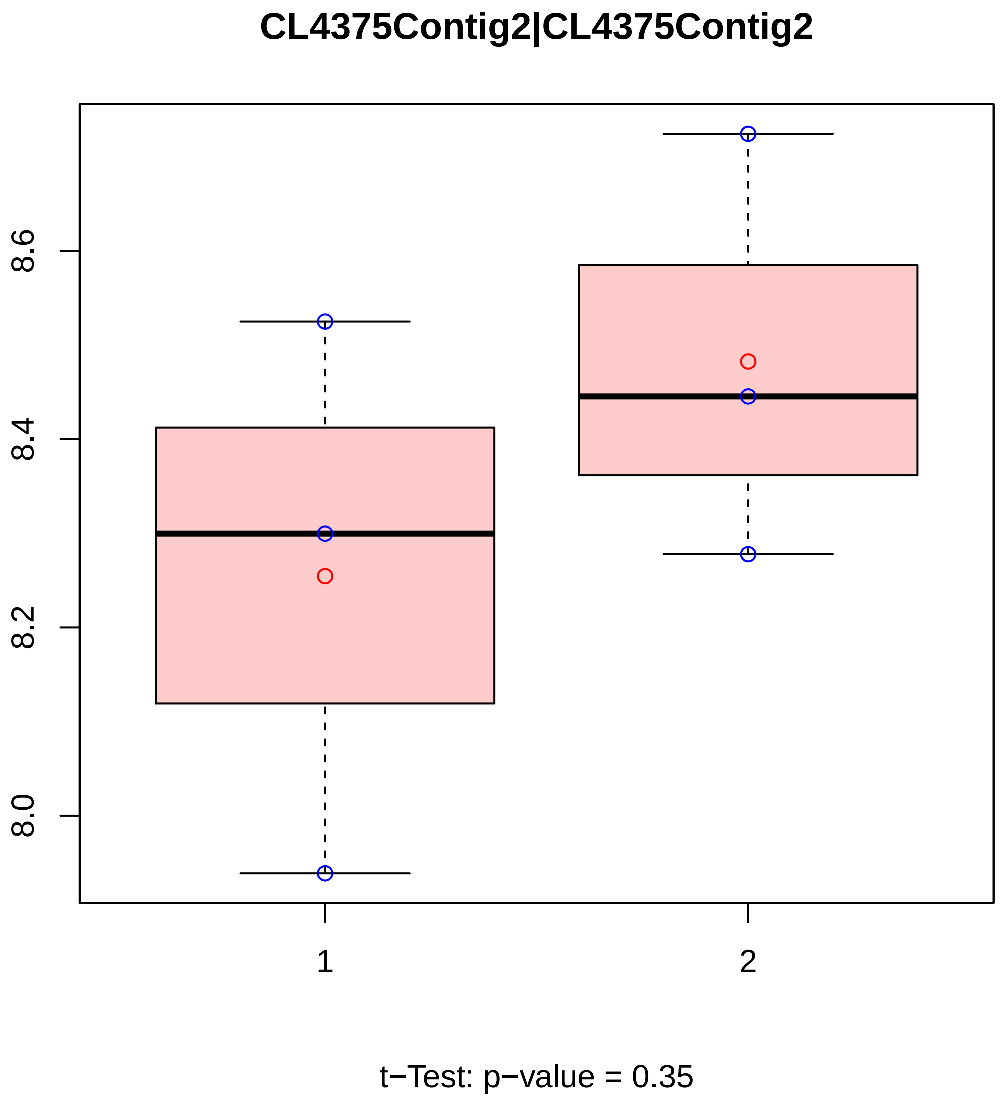
CL4375Contig2|CL4375Contig2
8.0
8.2
8.4
8.6
1
2
t−Test: p−value = 0.35

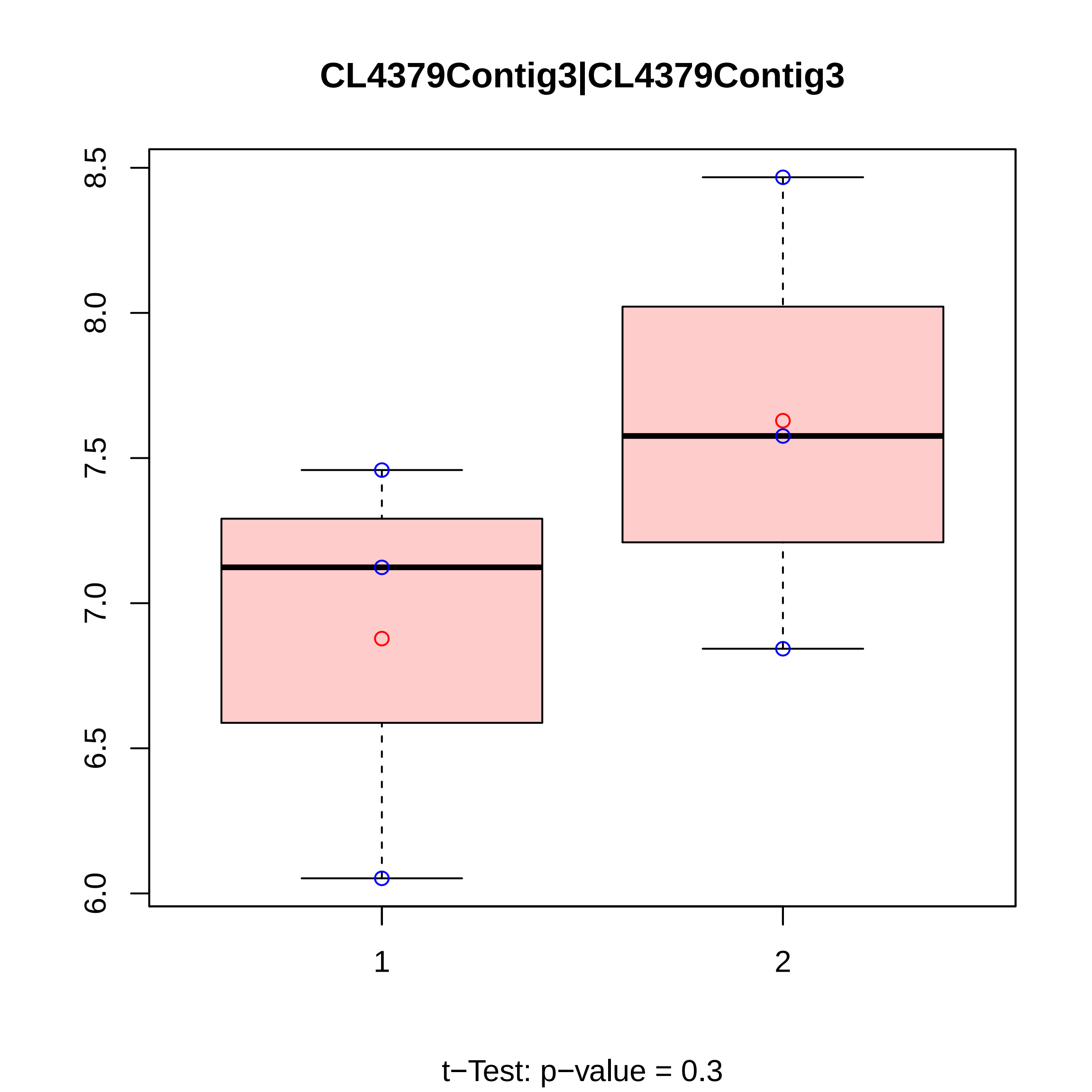
CL4379Contig3|CL4379Contig3
8.5
8.0
7.5
7.0
6.5
6.0
1
2
t−Test: p−value = 0.3

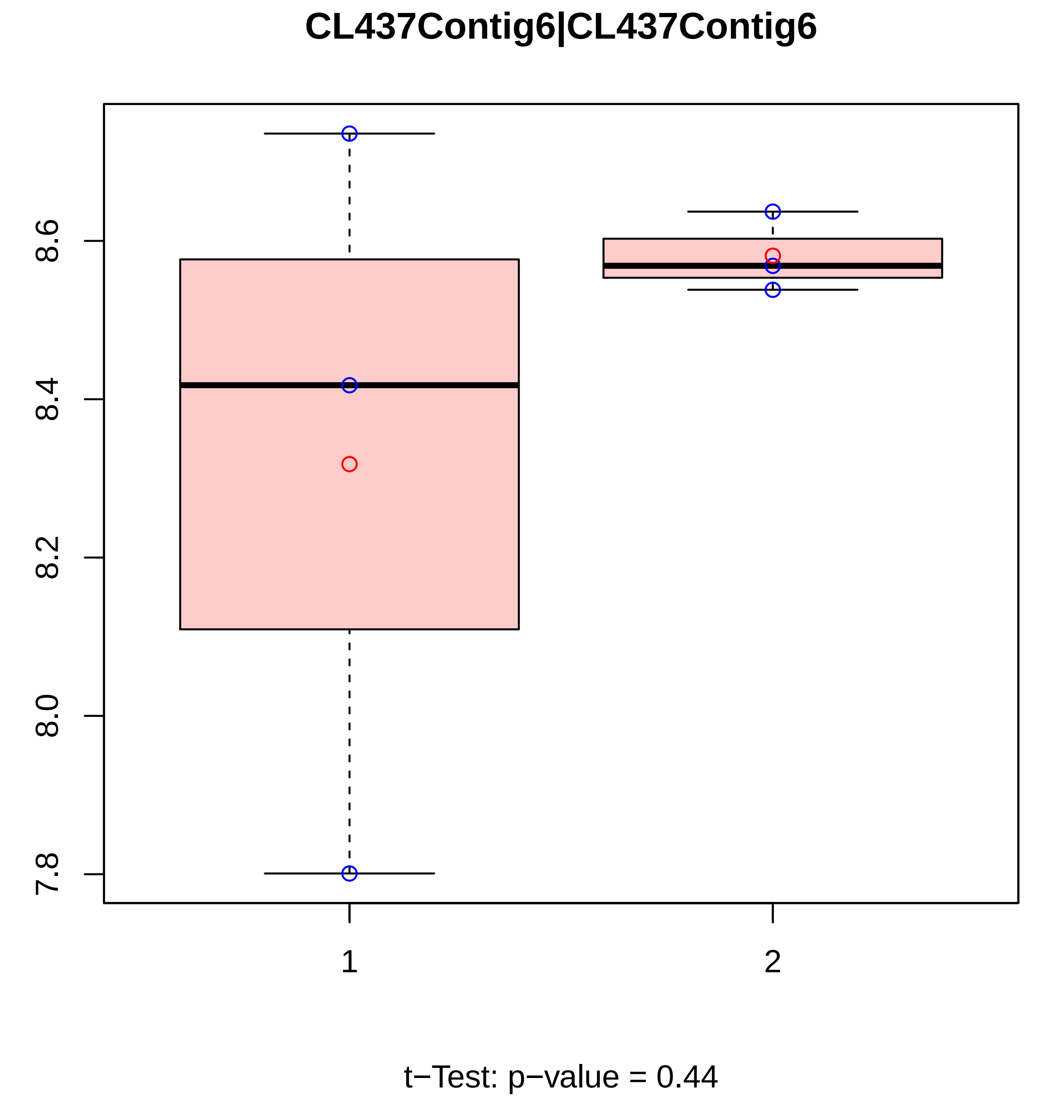
CL437Contig6|CL437Contig6
8.6
8.4
8.2
8.0
7.8
1
2
t−Test: p−value = 0.44

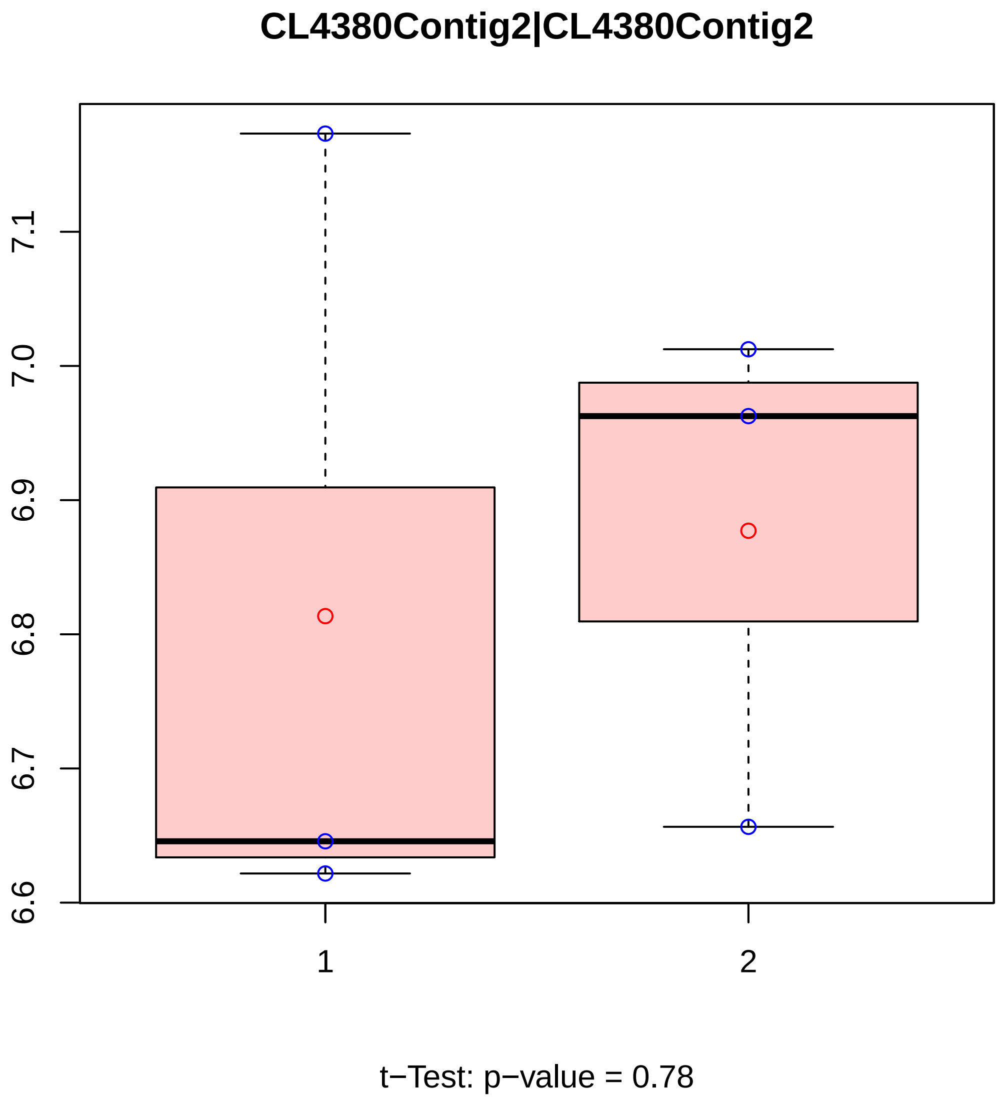

CL4380Contig2|CL4380Contig2
7.1
7.0
6.9
6.8
6.7
6.6
1
2
t−Test: p−value = 0.78

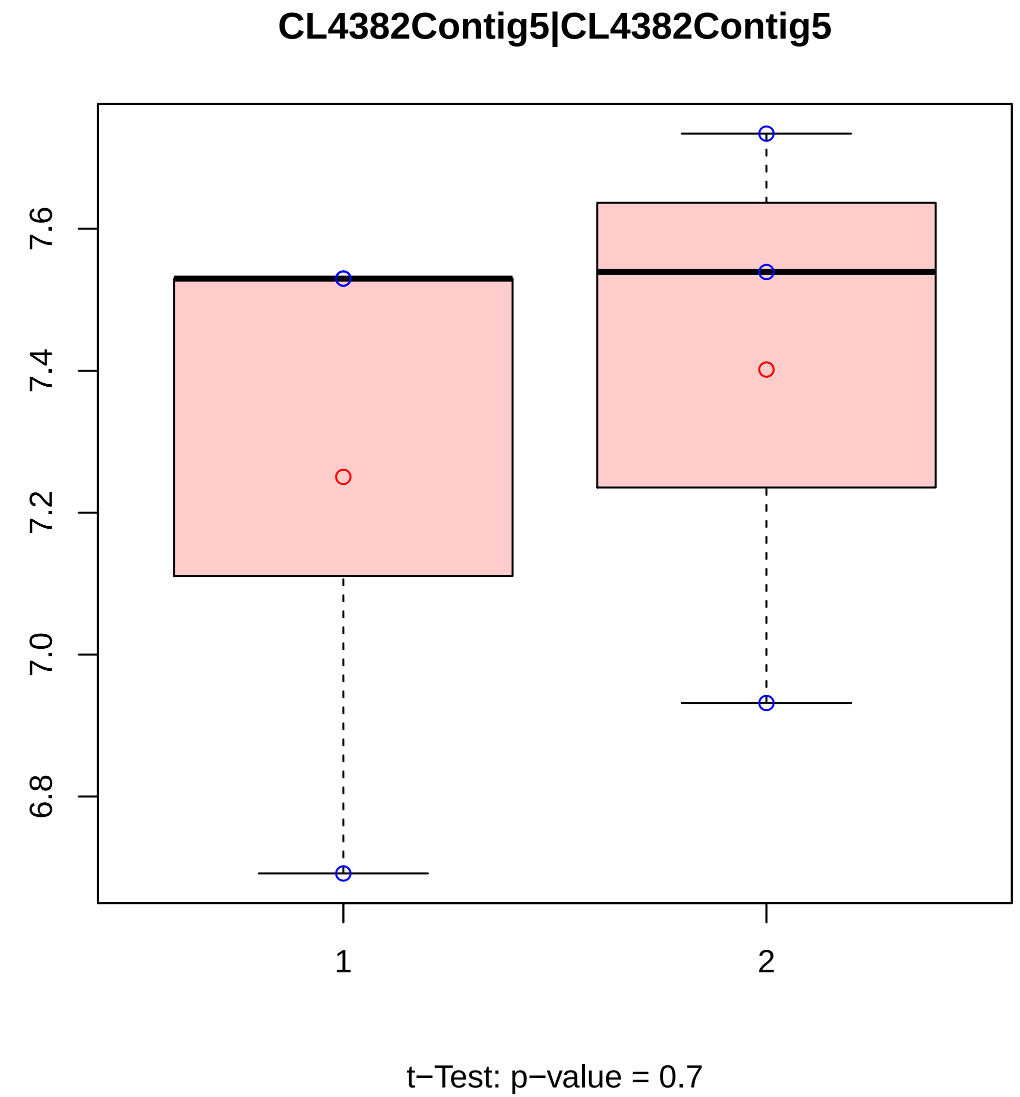

CL4382Contig5|CL4382Contig5
7.6
7.4
7.2
7.0
6.8
1
2
t−Test: p−value = 0.7

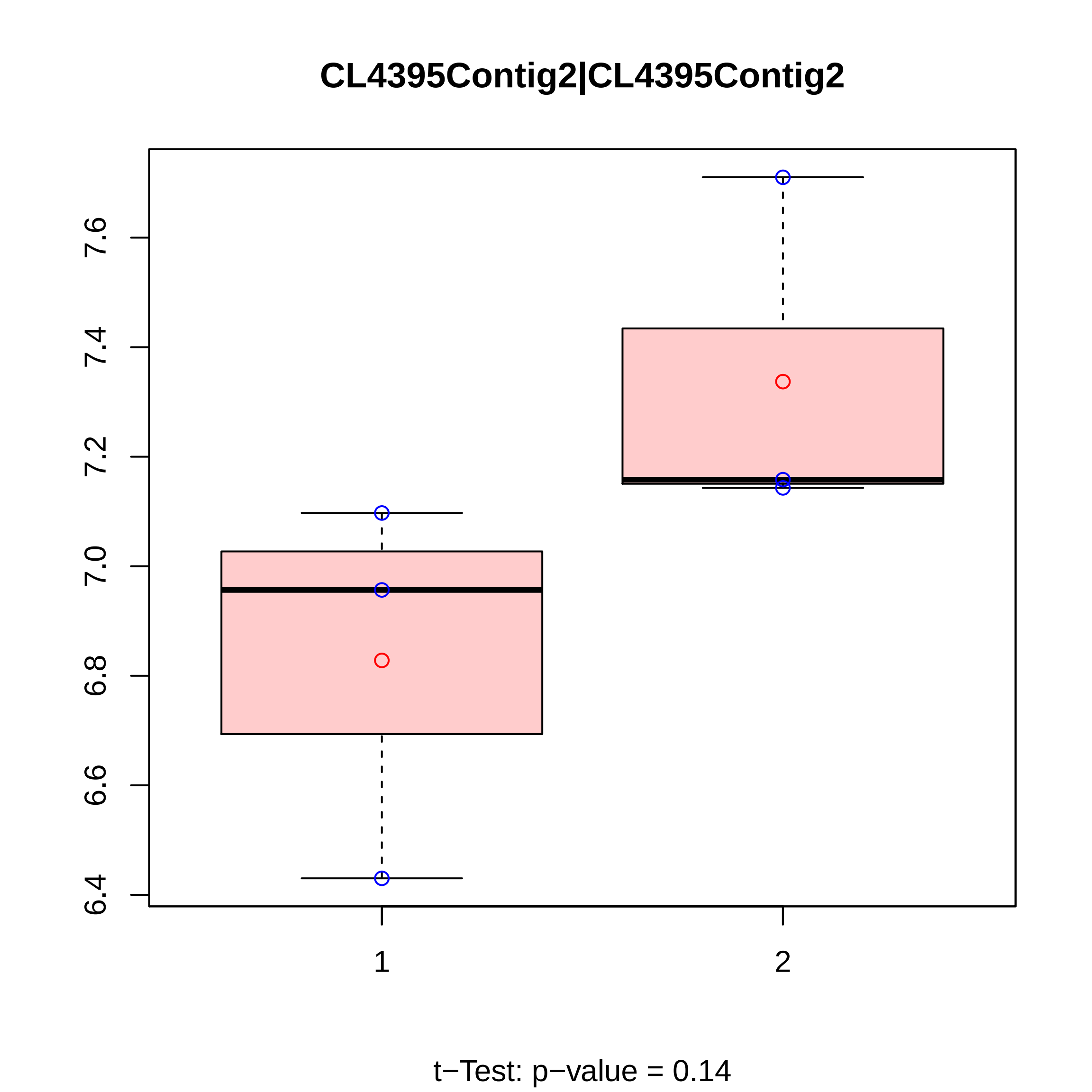
CL4395Contig2|CL4395Contig2
6.4
6.6
6.8
7.0
7.2
7.4
7.6
1
2
t−Test: p−value = 0.14

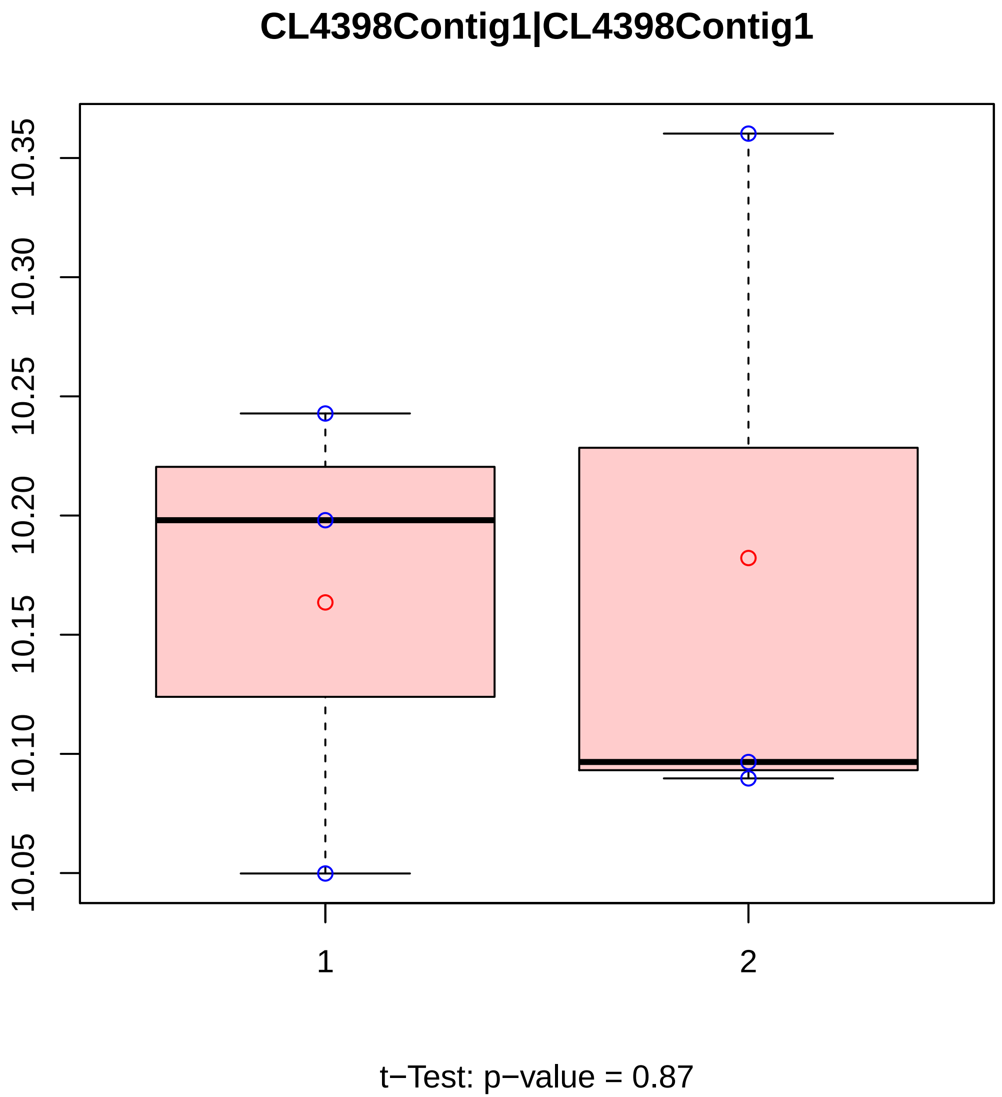

CL4398Contig1|CL4398Contig1
10.05
10.10
10.15
10.20
10.25
10.30
10.35
1
2
t−Test: p−value = 0.87

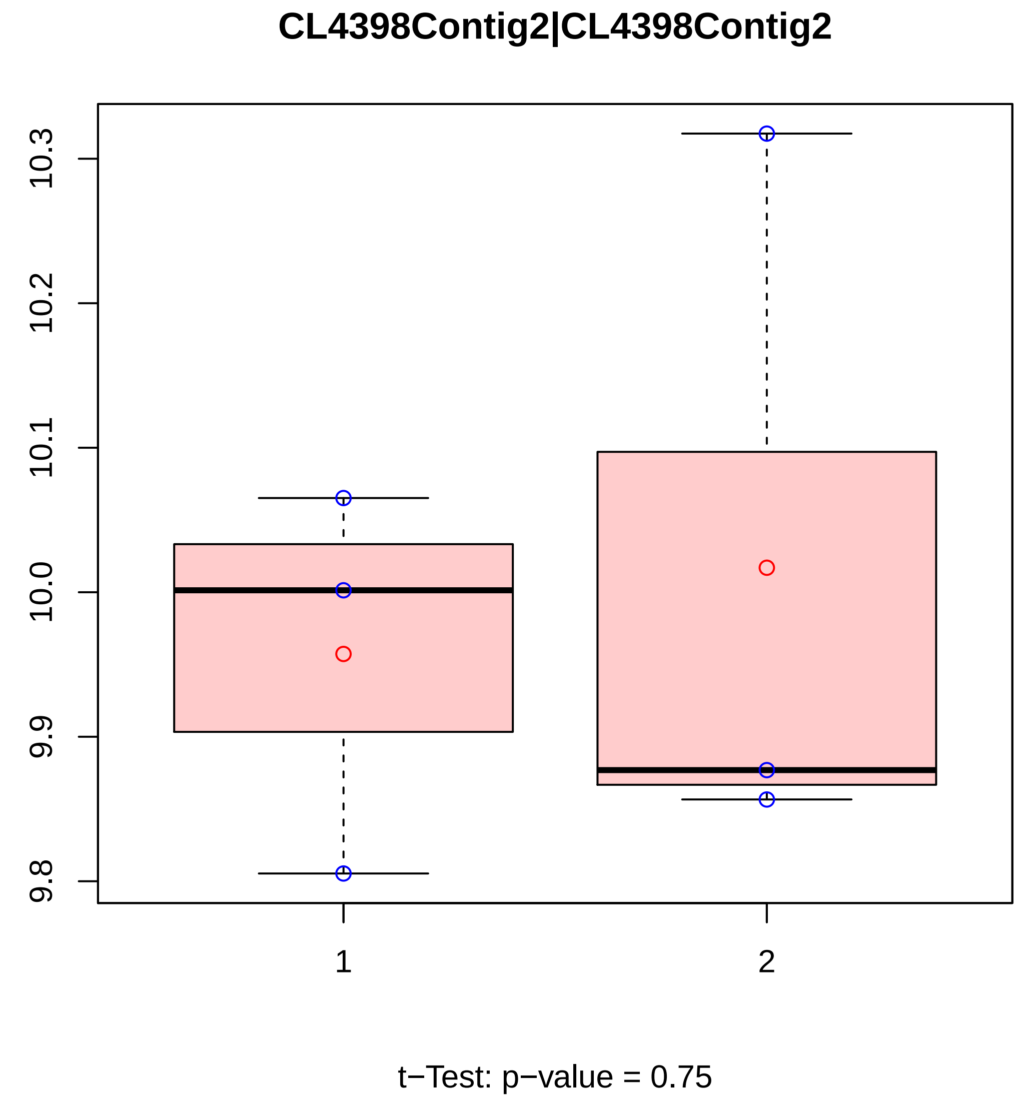
CL4398Contig2|CL4398Contig2
10.3
10.2
10.1
10.0
9.9
9.8
1
2
t−Test: p−value = 0.75

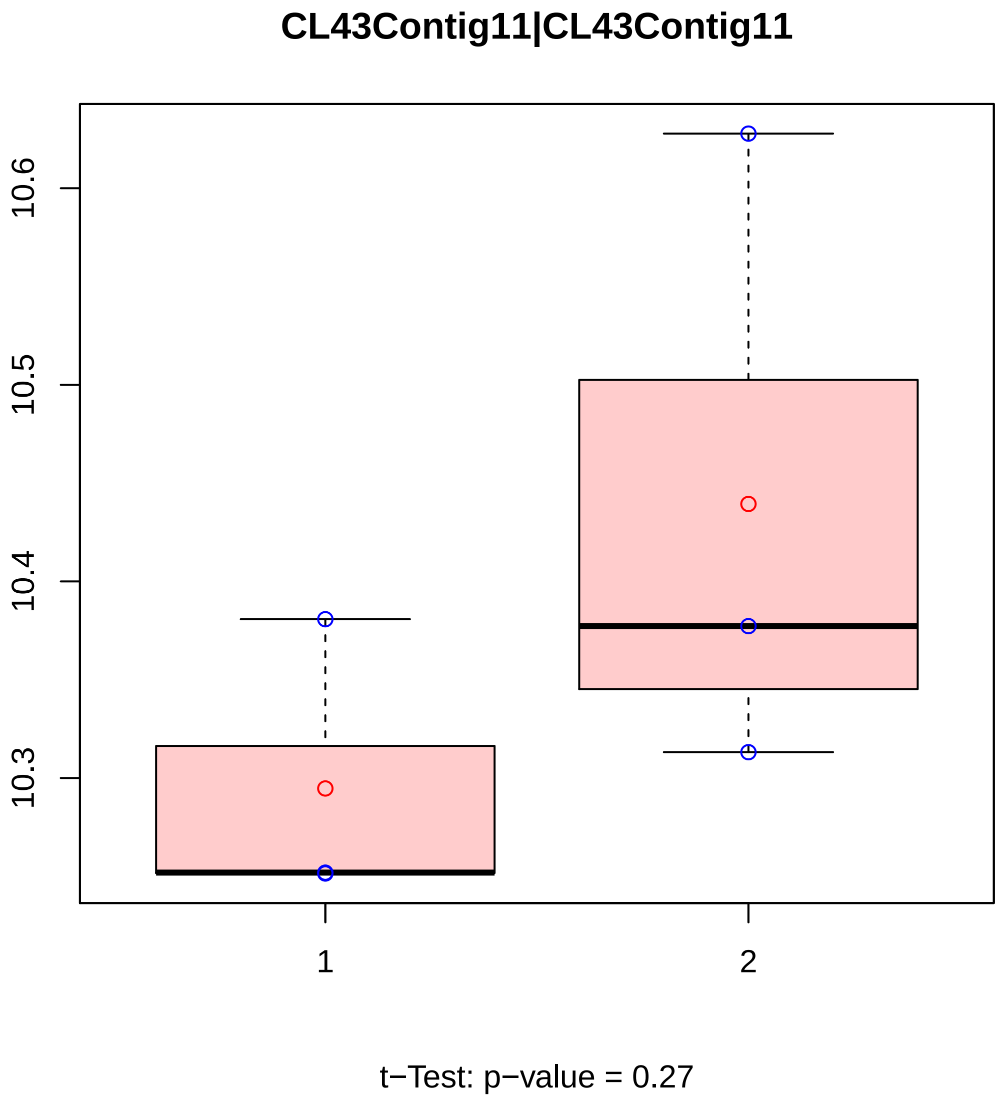

CL43Contig11|CL43Contig11
10.3
10.4
10.5
10.6
1
2
t−Test: p−value = 0.27

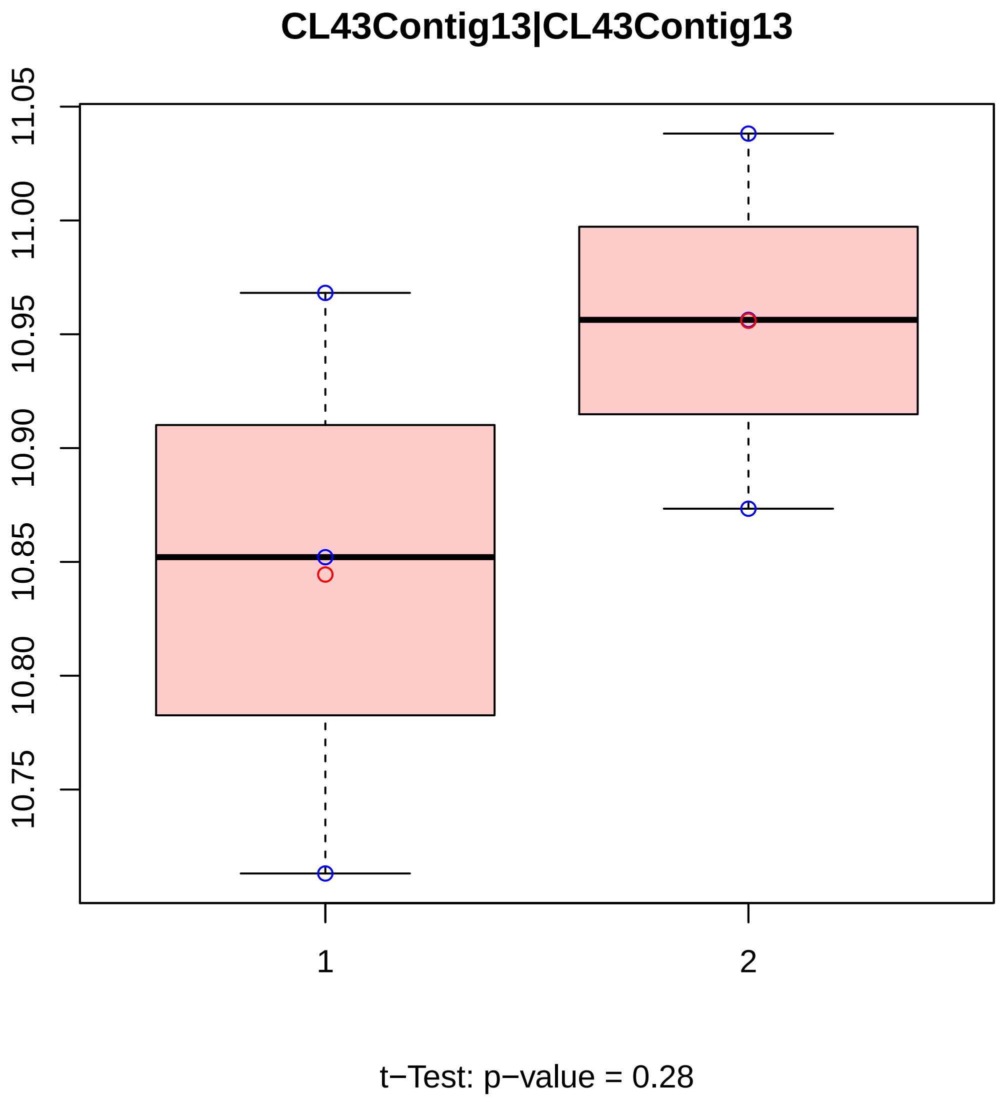

CL43Contig13|CL43Contig13
11.05
11.00
10.95
10.90
10.85
10.80
10.75
1
2
t−Test: p−value = 0.28

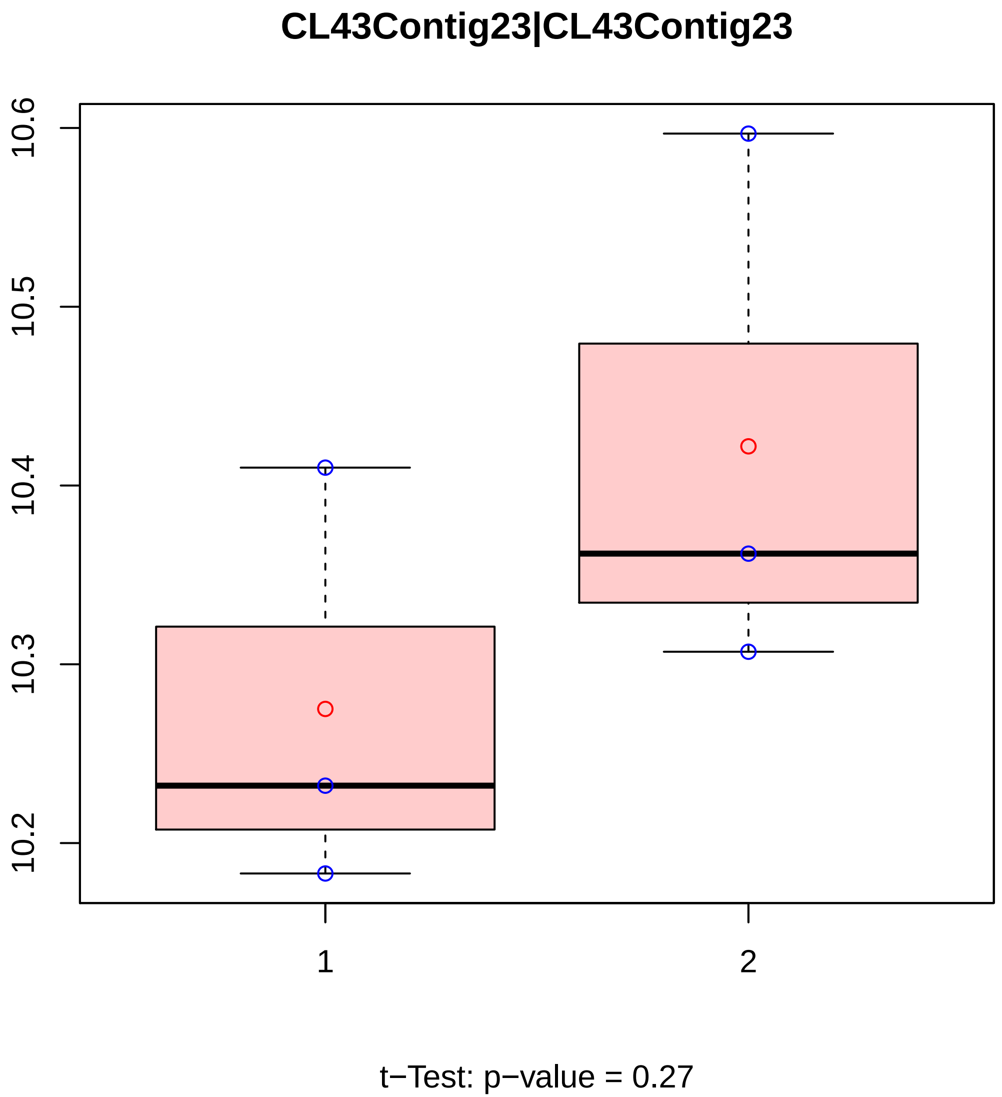
CL43Contig23|CL43Contig23
10.6
10.5
10.4
10.3
10.2
1
2
t−Test: p−value = 0.27

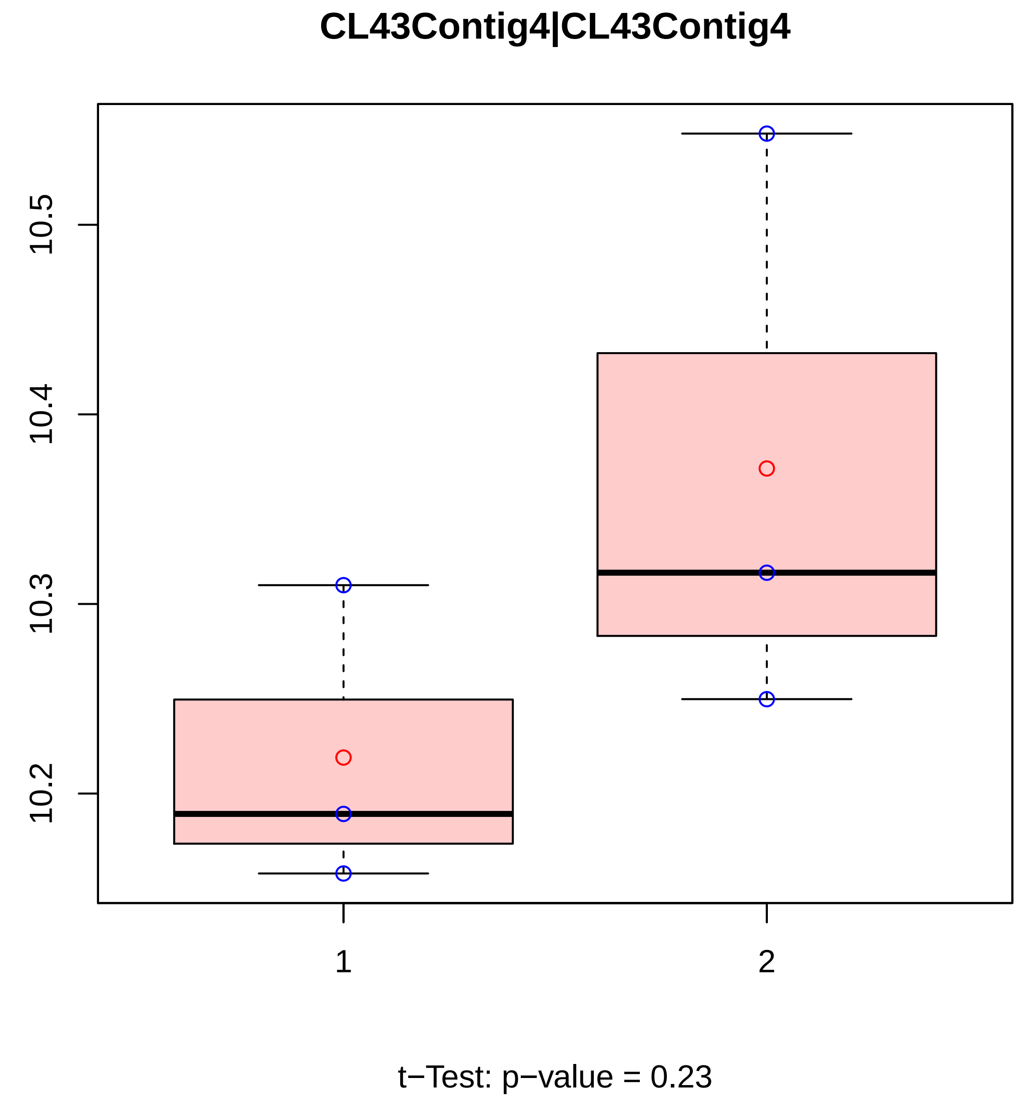
CL43Contig4|CL43Contig4
10.2
10.3
10.4
10.5
1
2
t−Test: p−value = 0.23

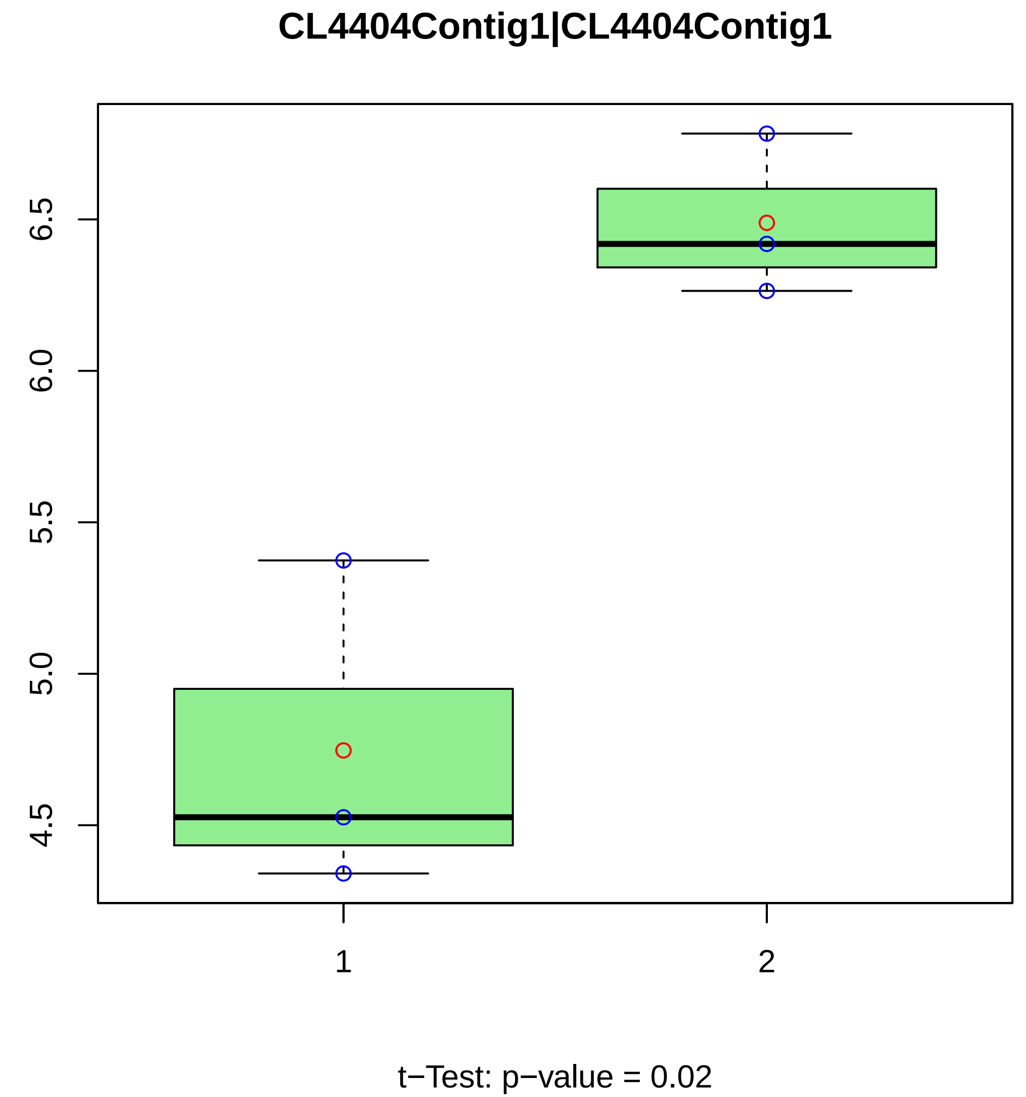
CL4404Contig1|CL4404Contig1
6.5
6.0
5.5
5.0
4.5
1
2
t−Test: p−value = 0.02

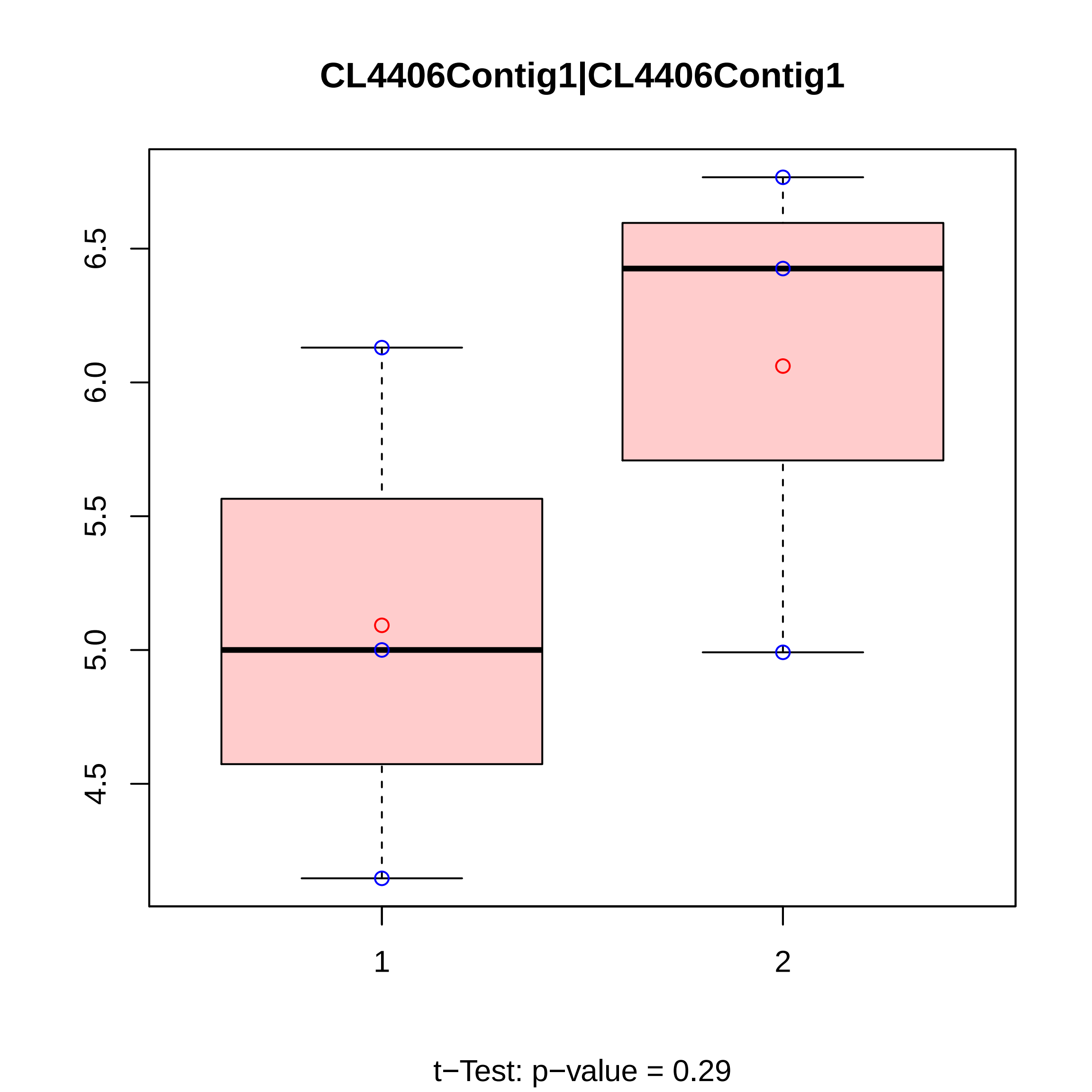

CL4406Contig1|CL4406Contig1
6.5
6.0
5.5
5.0
4.5
1
2
t−Test: p−value = 0.29

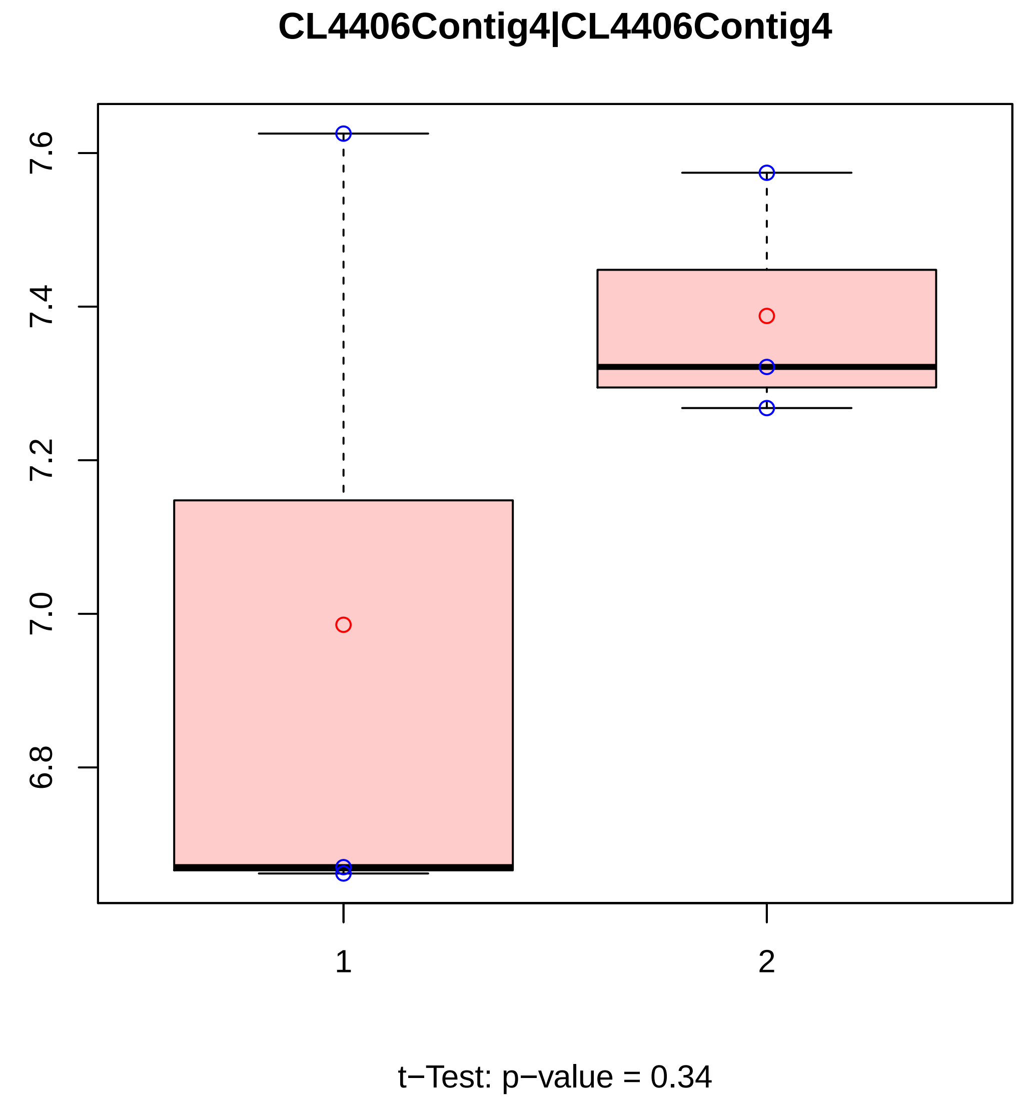
CL4406Contig4|CL4406Contig4
7.6
7.4
7.2
7.0
6.8
1
2
t−Test: p−value = 0.34

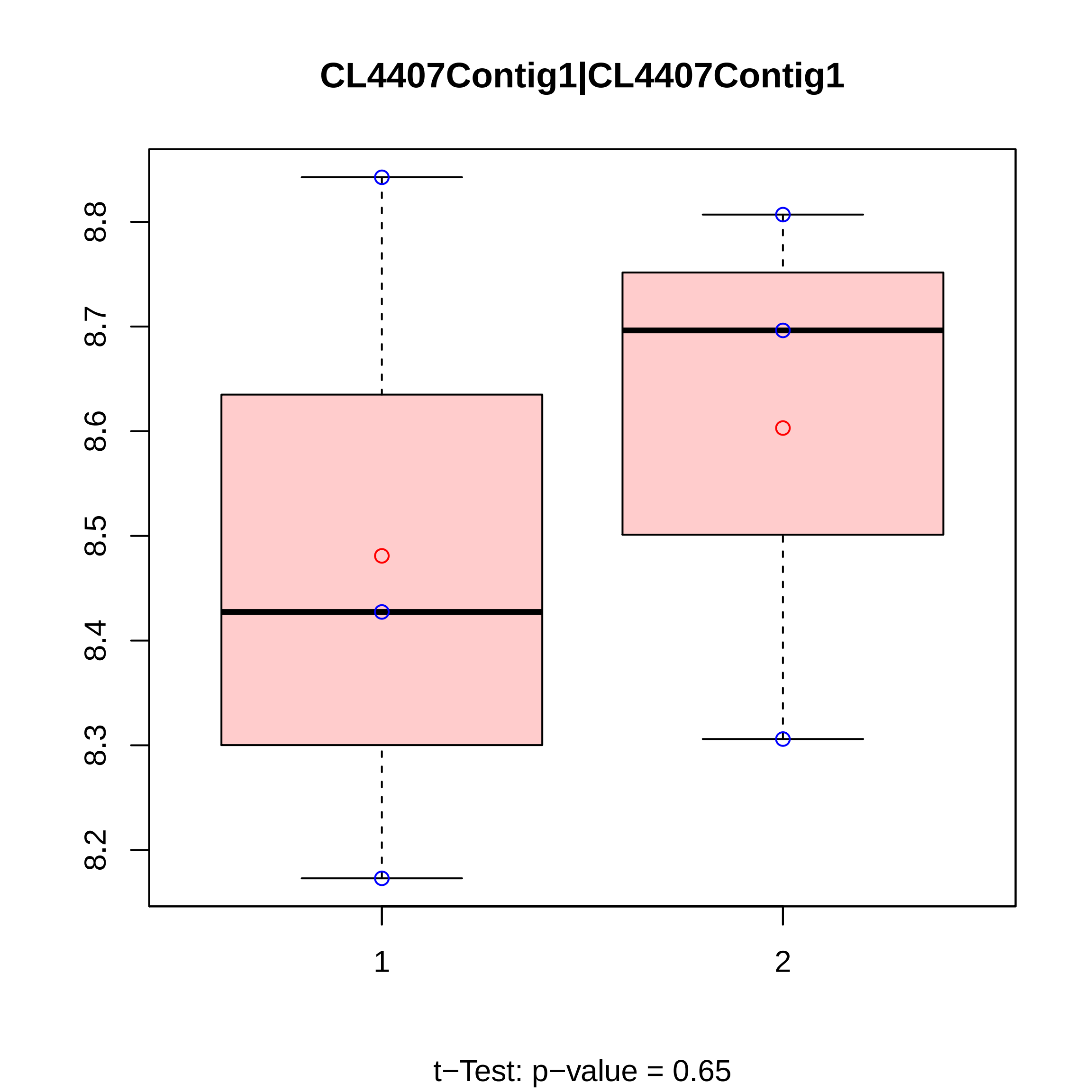

CL4407Contig1|CL4407Contig1
8.2
8.3
8.4
8.5
8.6
8.7
8.8
1
2
t−Test: p−value = 0.65

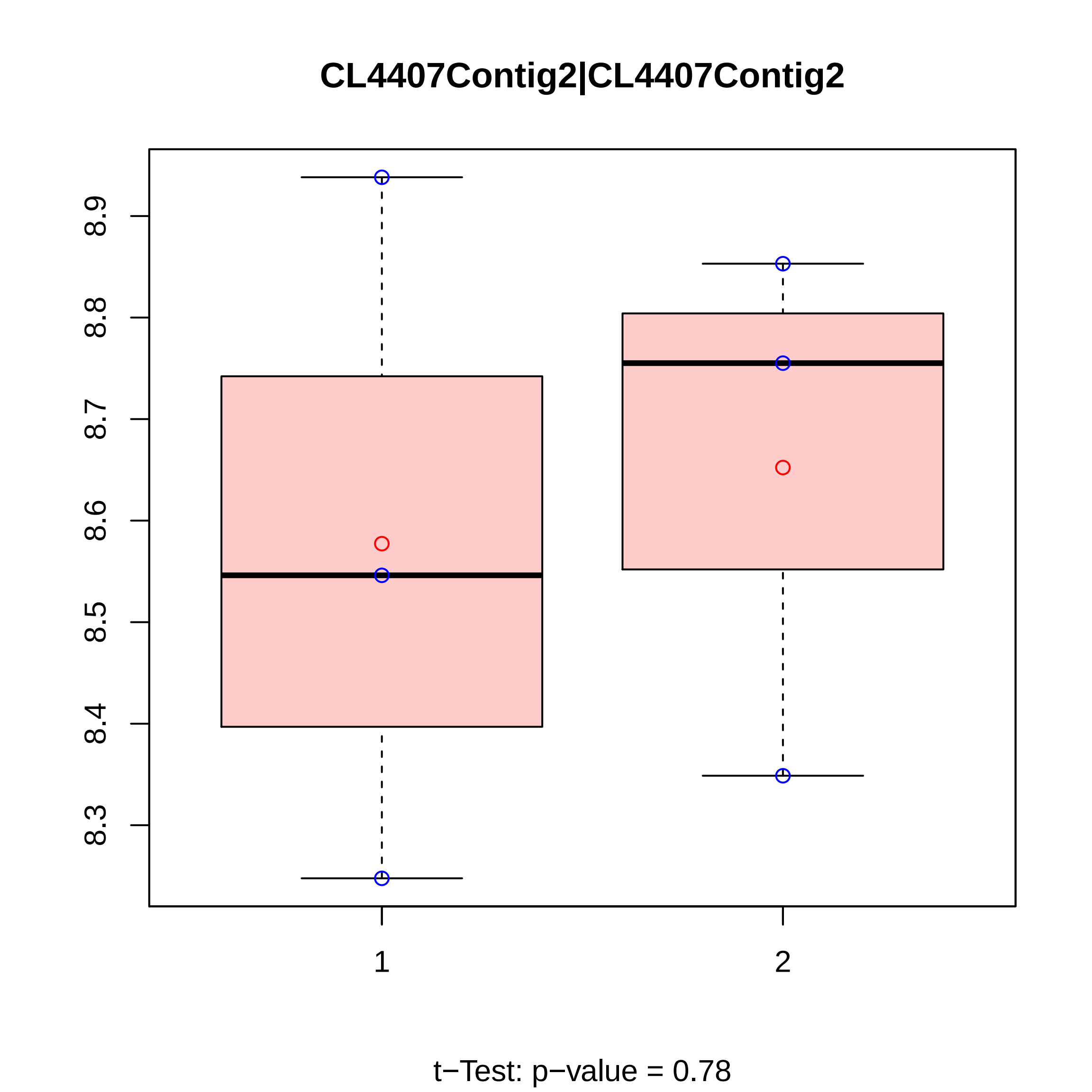

CL4407Contig2|CL4407Contig2
8.3
8.4
8.5
8.6
8.7
8.8
8.9
1
2
t−Test: p−value = 0.78

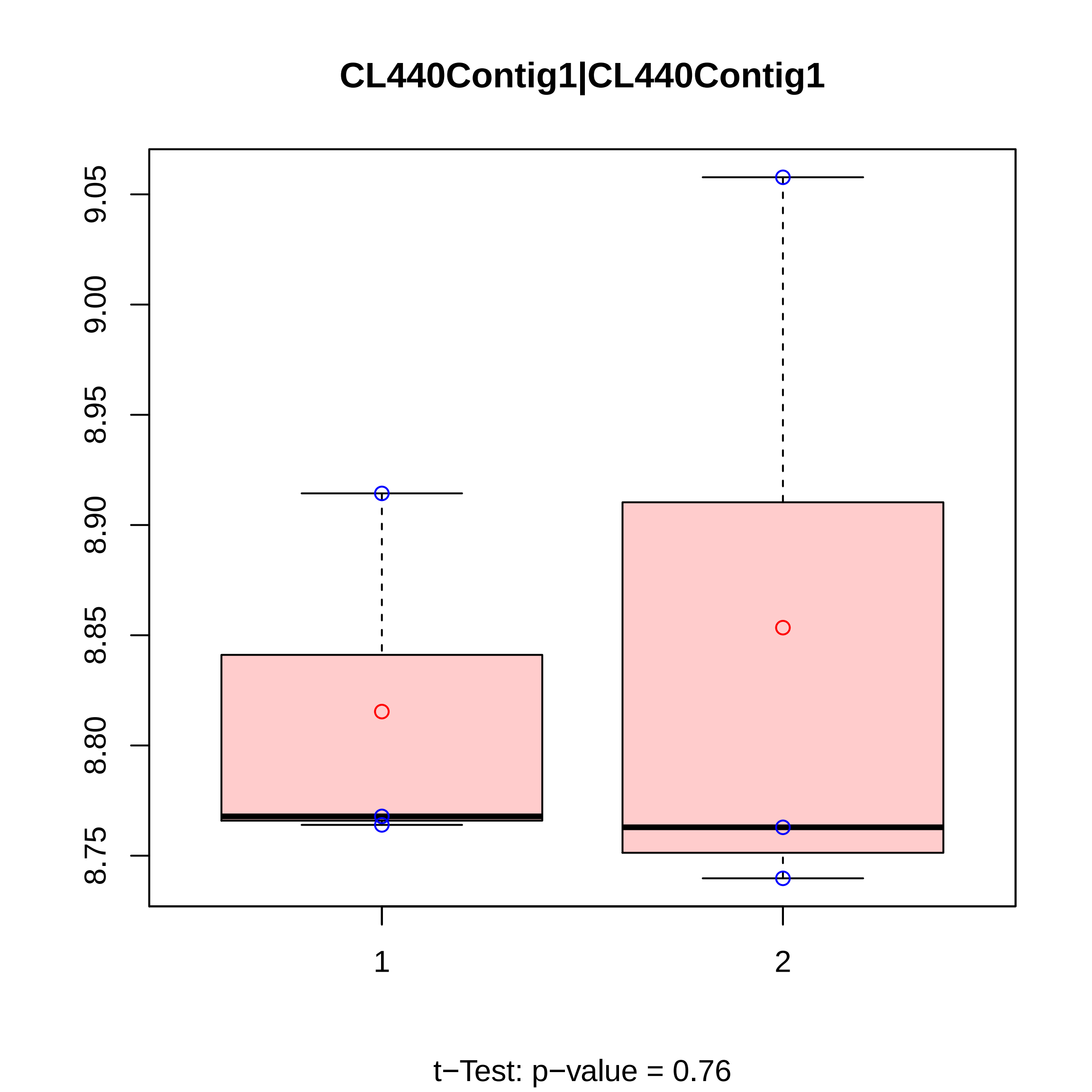

CL440Contig1|CL440Contig1
9.05
9.00
8.95
8.90
8.85
8.80
8.75
1
2
t−Test: p−value = 0.76

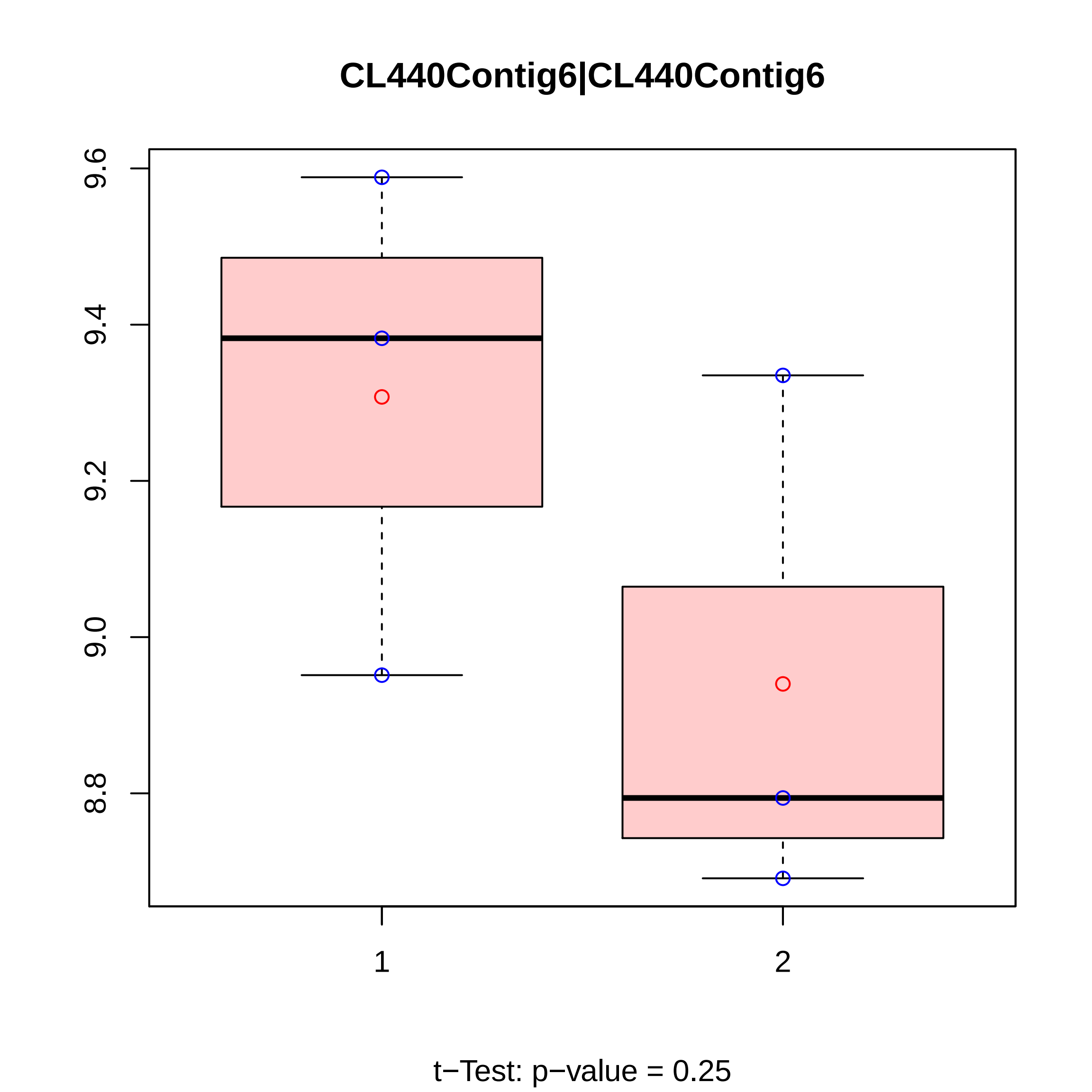

CL440Contig6|CL440Contig6
9.6
9.4
9.2
9.0
8.8
1
2
t−Test: p−value = 0.25

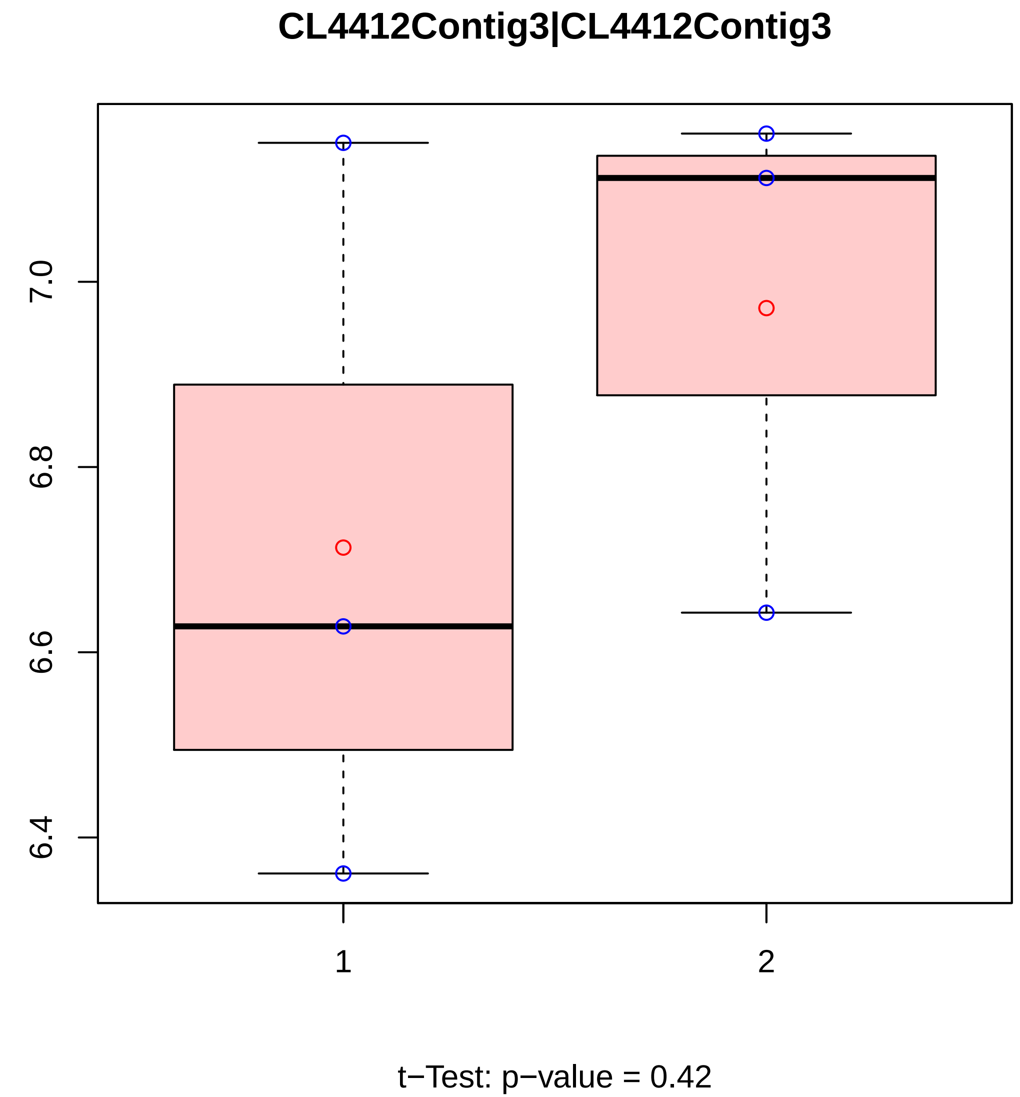

CL4412Contig3|CL4412Contig3
7.0
6.8
6.6
6.4
1
2
t−Test: p−value = 0.42

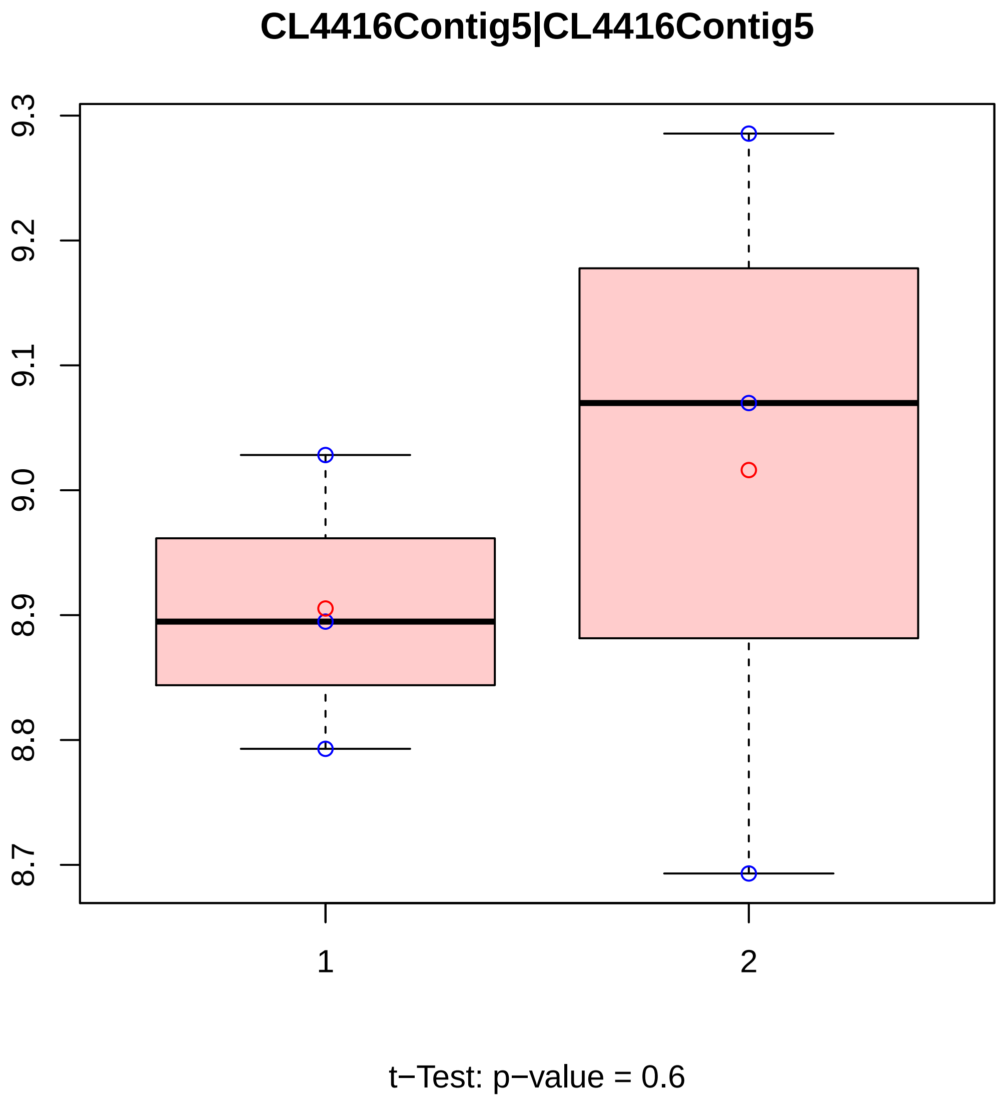

CL4416Contig5|CL4416Contig5
9.3
9.2
9.1
9.0
8.9
8.8
8.7
1
2
t−Test: p−value = 0.6

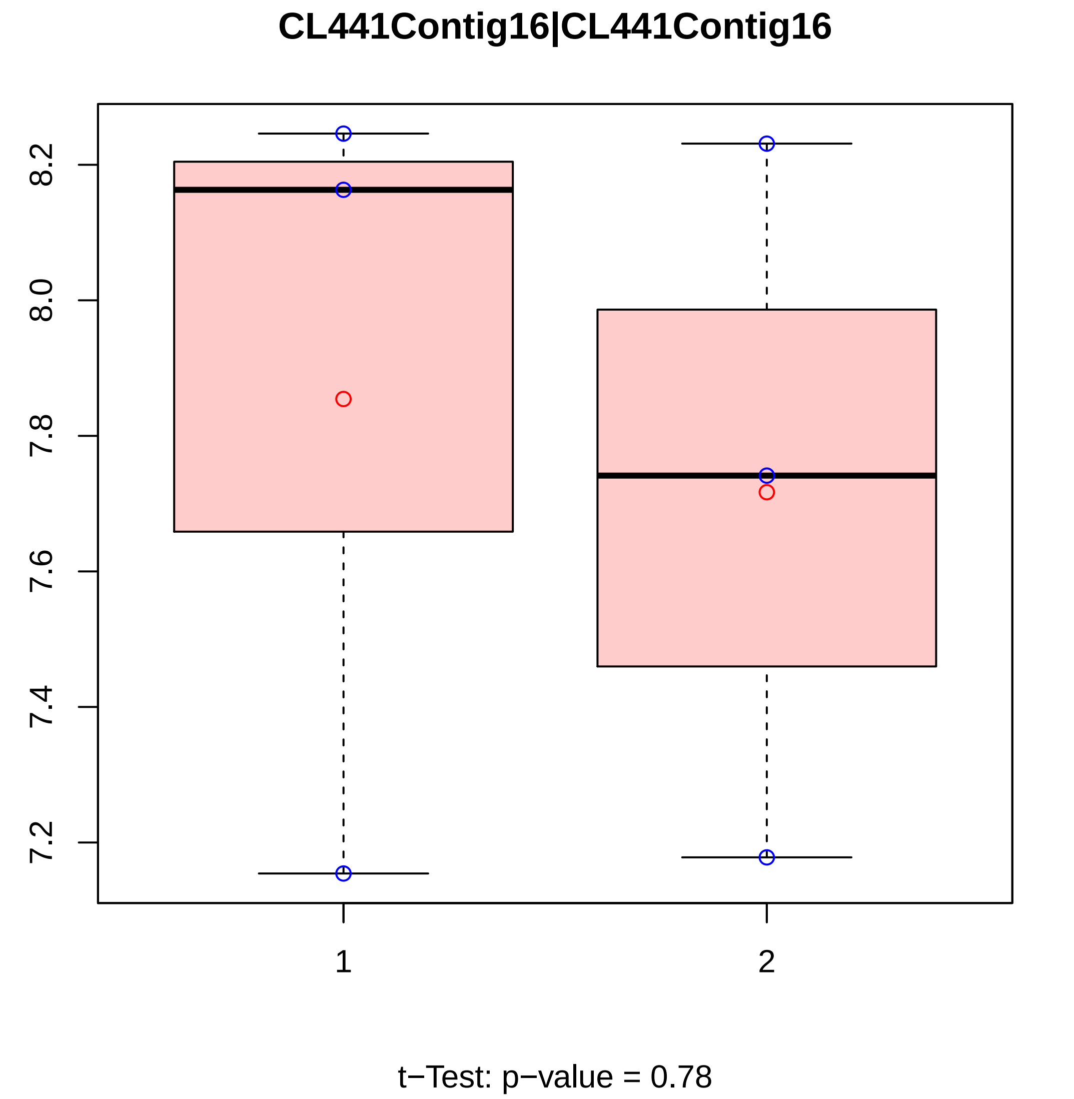
CL441Contig16|CL441Contig16
8.2
8.0
7.8
7.6
7.4
7.2
1
2
t−Test: p−value = 0.78

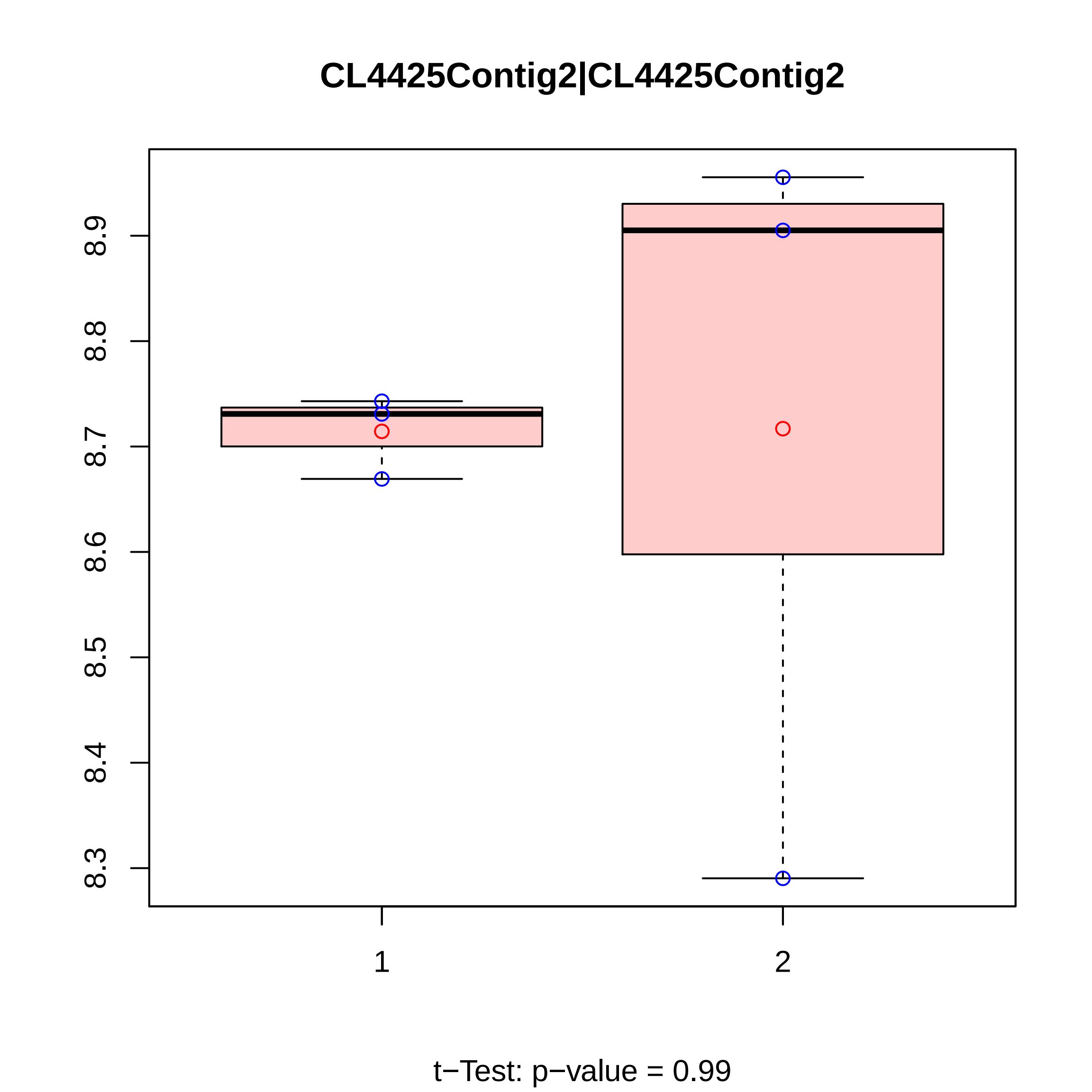
CL4425Contig2|CL4425Contig2
8.3
8.4
8.5
8.6
8.7
8.8
8.9
1
2
t−Test: p−value = 0.99

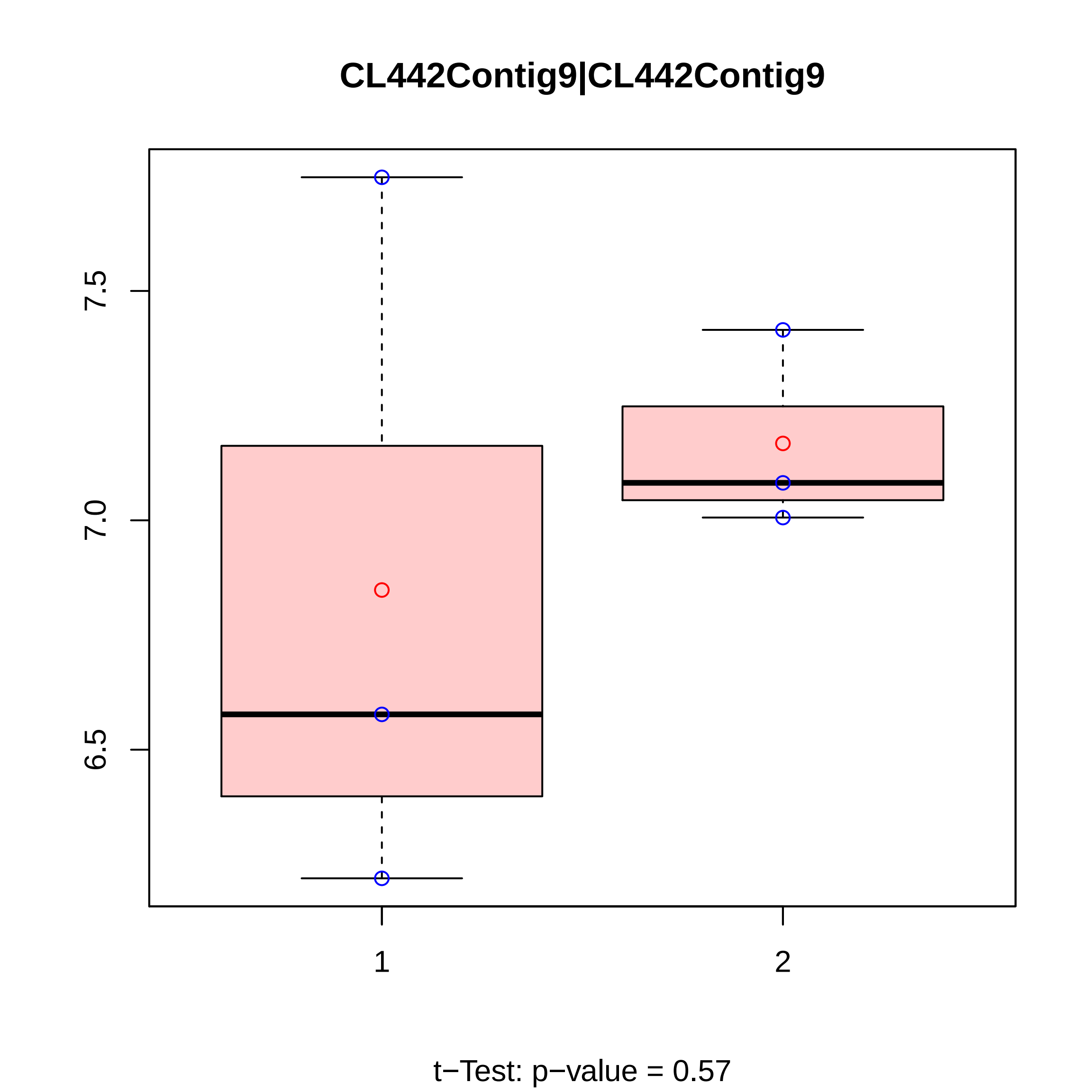
CL442Contig9|CL442Contig9
7.5
7.0
6.5
1
2
t−Test: p−value = 0.57

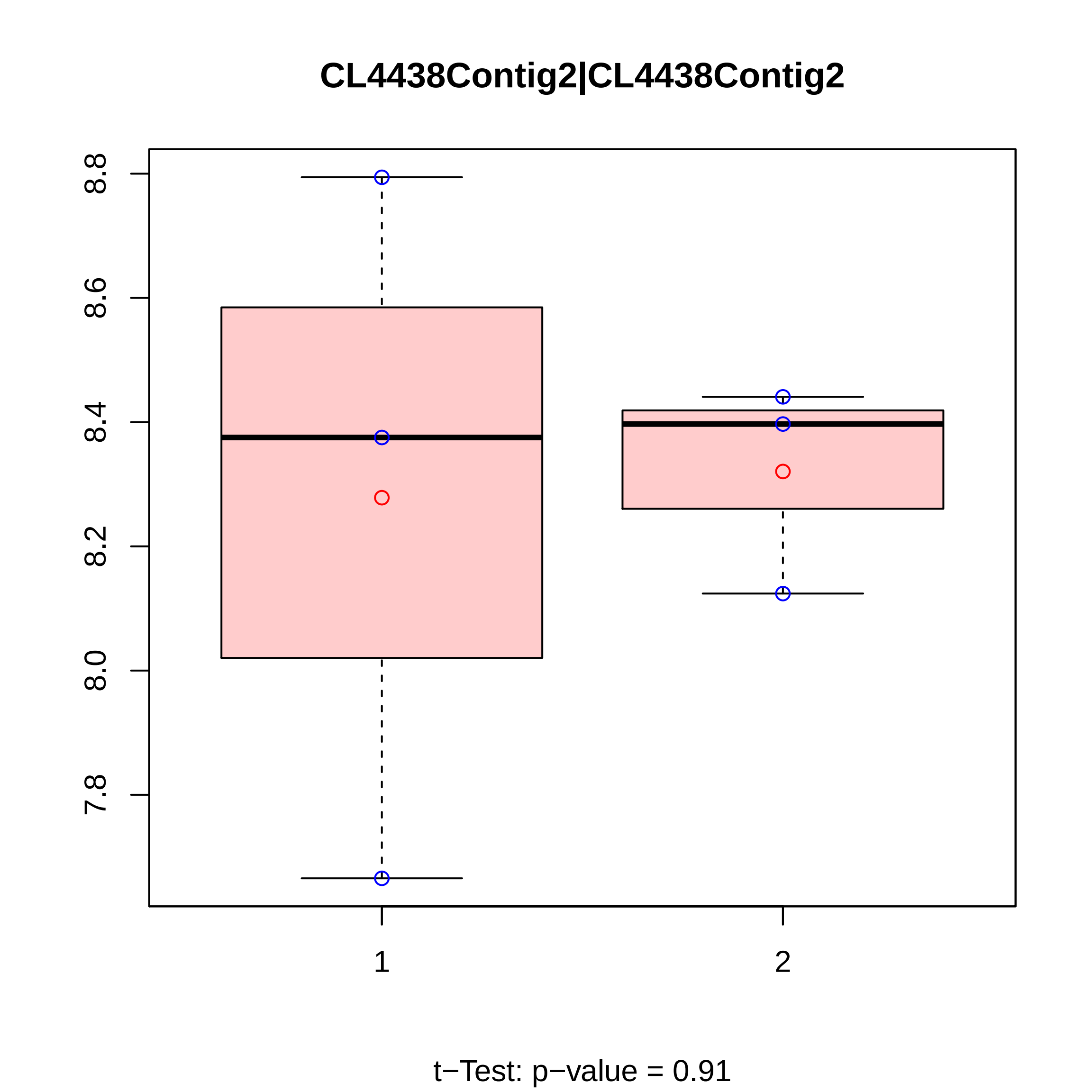

CL4438Contig2|CL4438Contig2
8.8
8.6
8.4
8.2
8.0
7.8
1
2
t−Test: p−value = 0.91

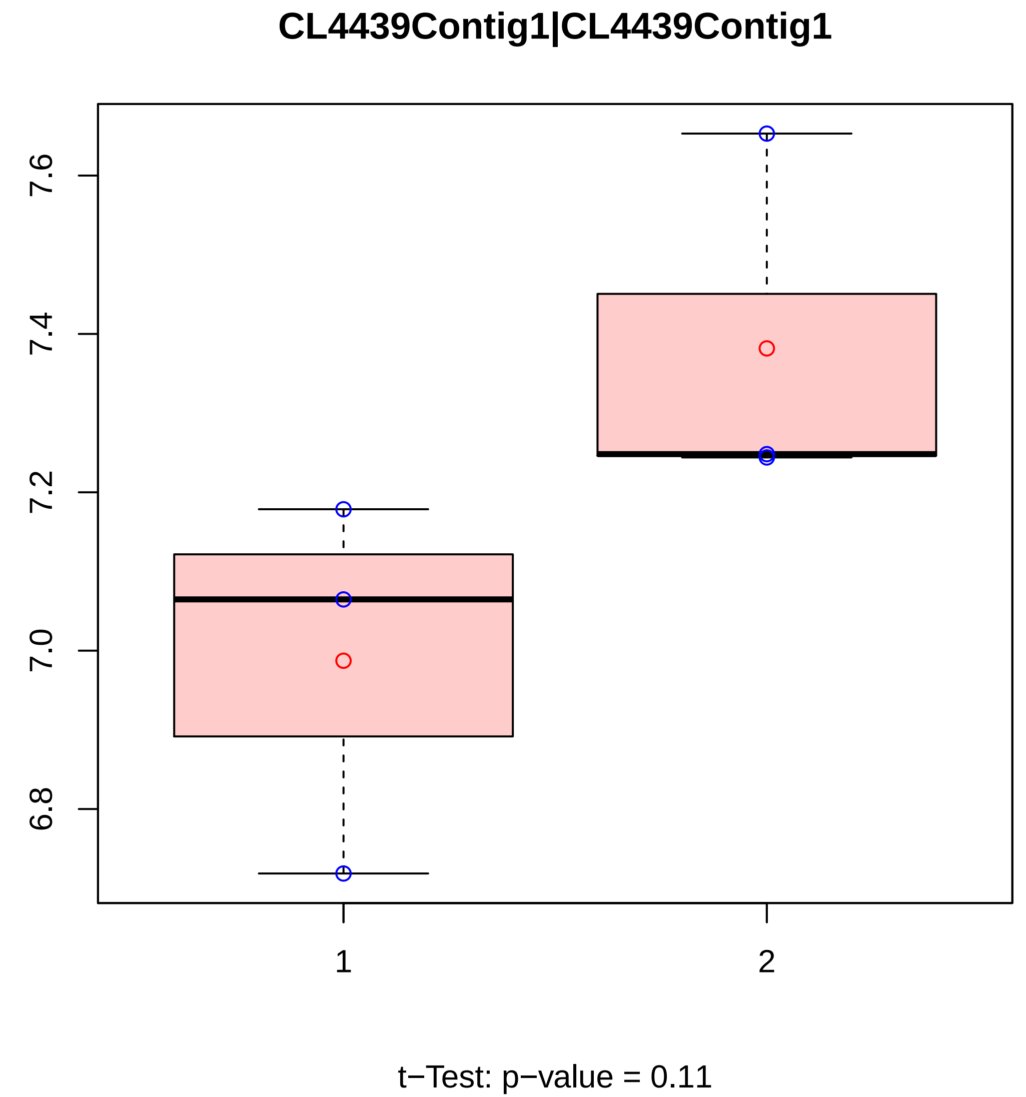
CL4439Contig1|CL4439Contig1
7.6
7.4
7.2
7.0
6.8
1
2
t−Test: p−value = 0.11

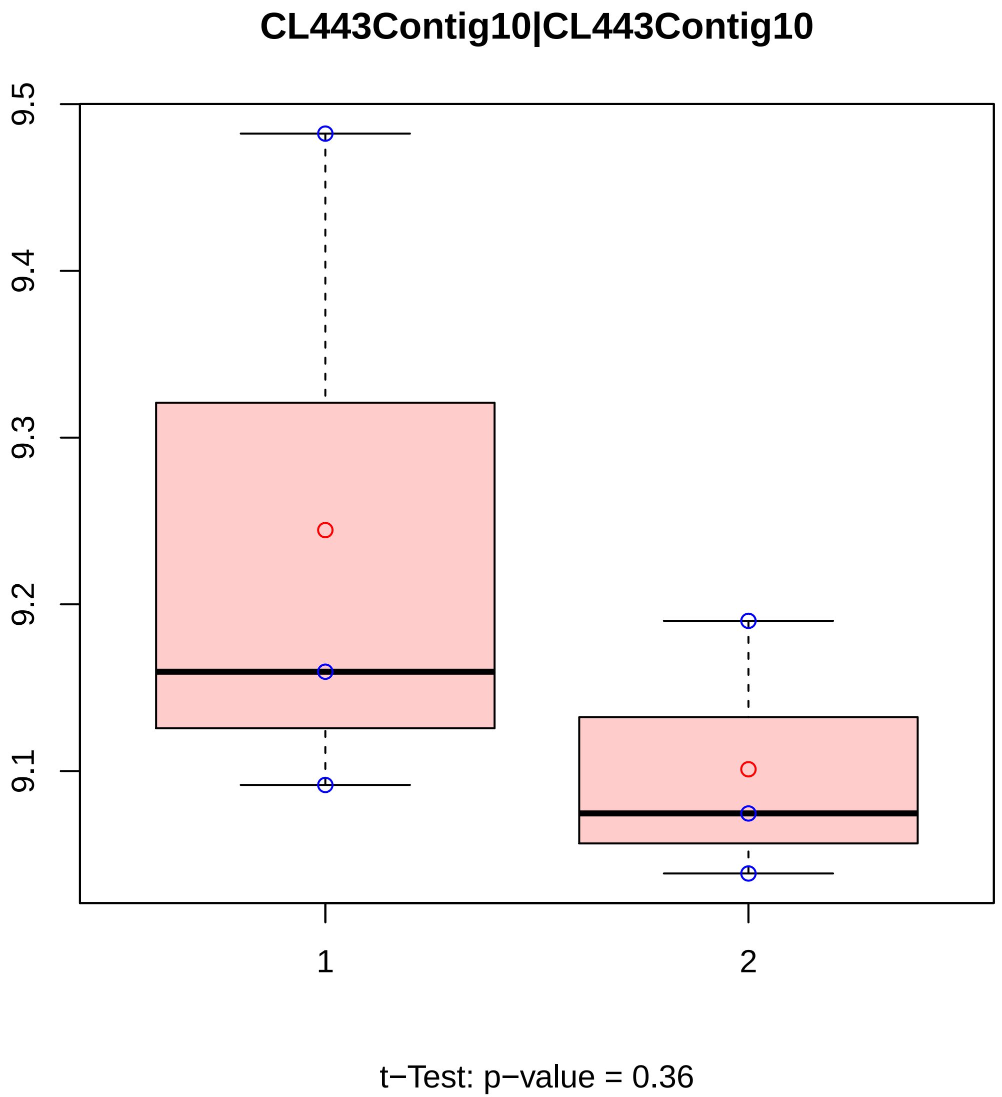
CL443Contig10|CL443Contig10
9.5
9.4
9.3
9.2
9.1
1
2
t−Test: p−value = 0.36

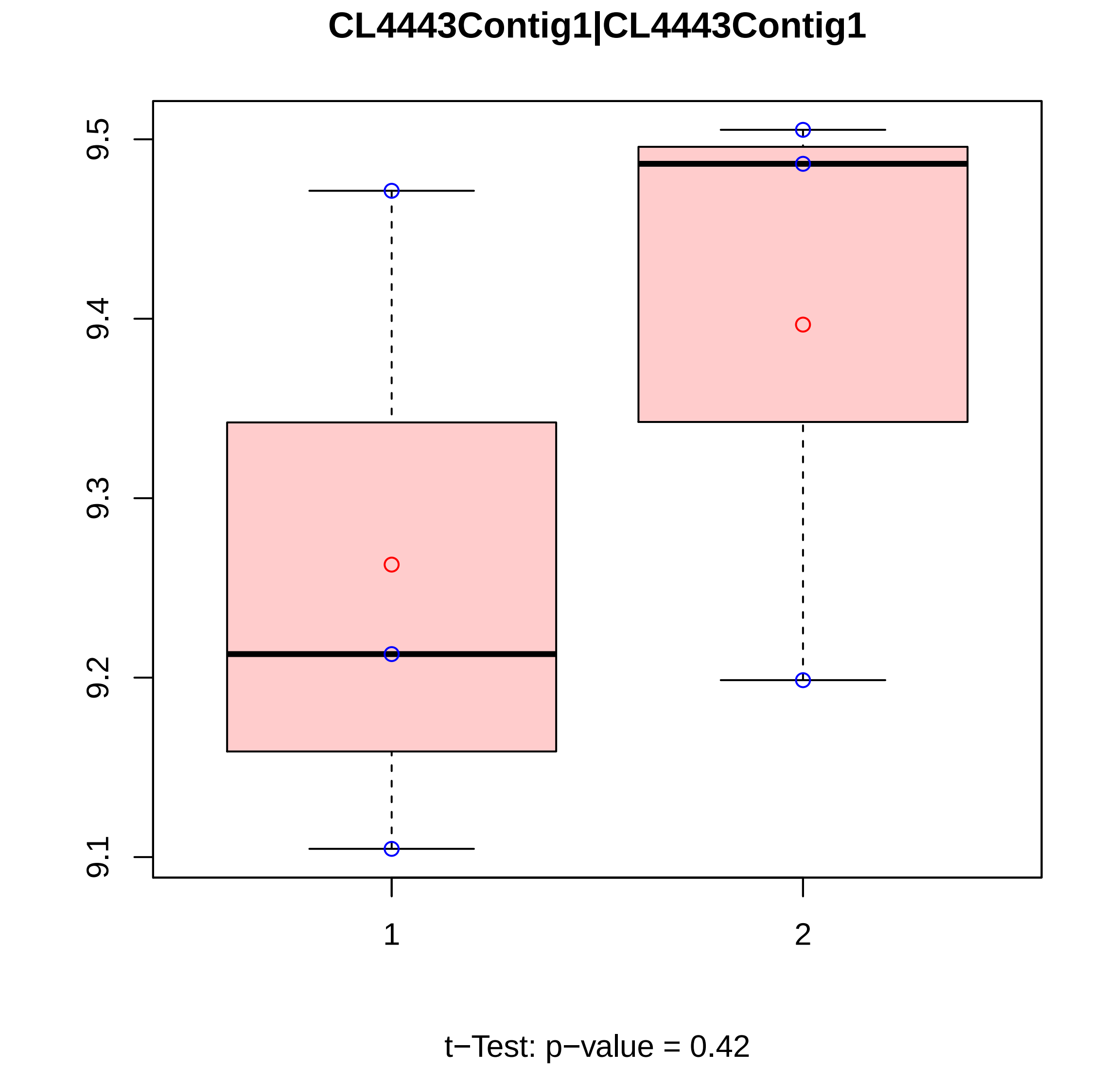

CL4443Contig1|CL4443Contig1
9.1
9.2
9.3
9.4
9.5
1
2
t−Test: p−value = 0.42

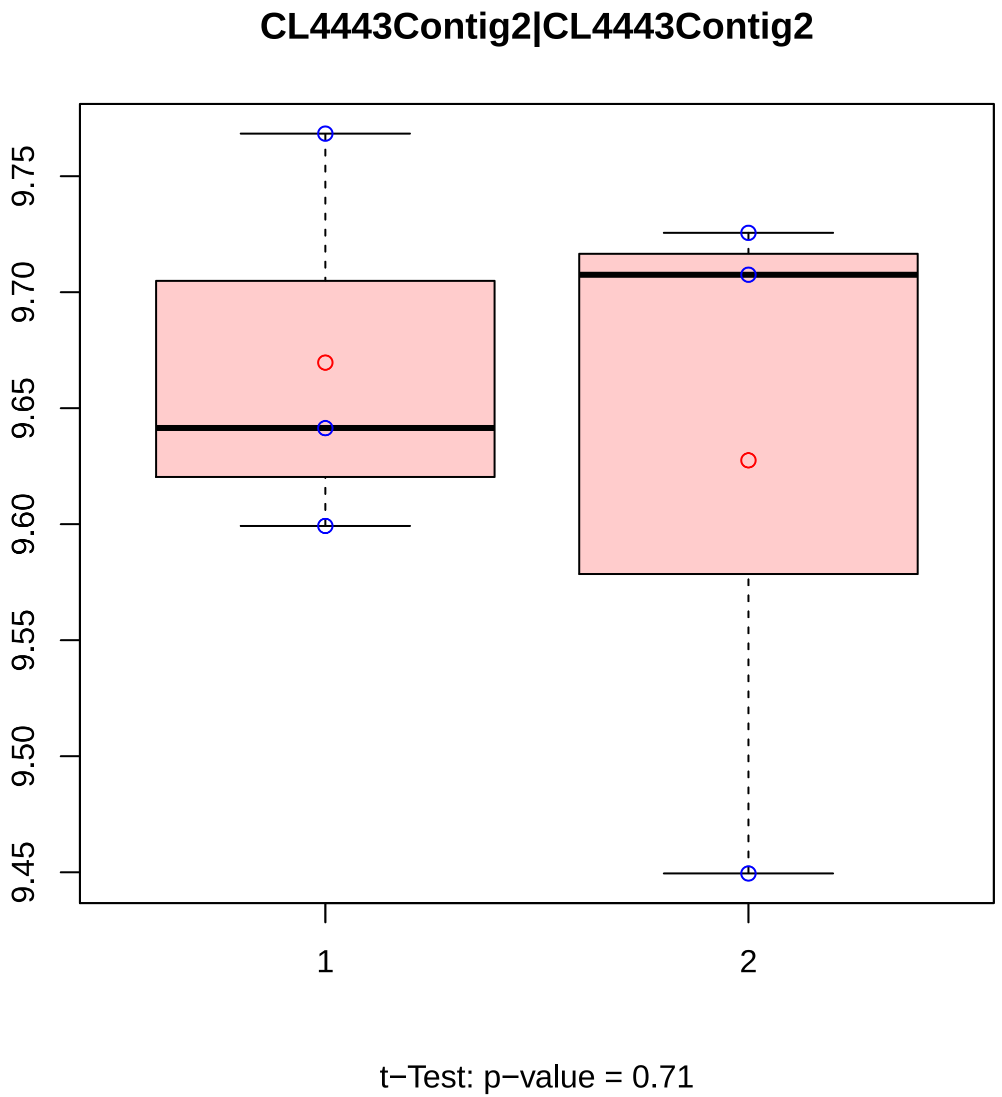

CL4443Contig2|CL4443Contig2
9.45
9.50
9.55
9.60
9.65
9.70
9.75
1
2
t−Test: p−value = 0.71

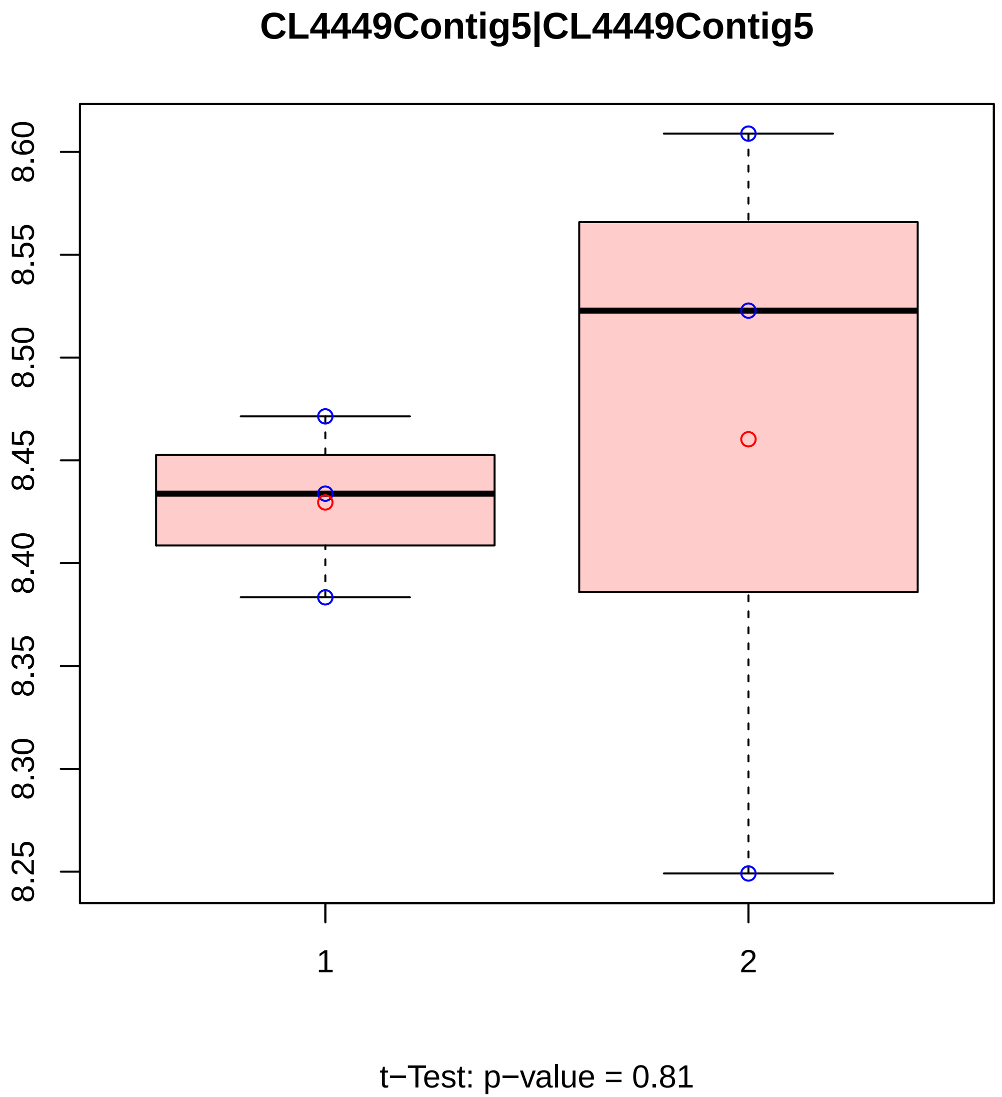

CL4449Contig5|CL4449Contig5
8.25
8.30
8.35
8.40
8.45
8.50
8.55
8.60
1
2
t−Test: p−value = 0.81

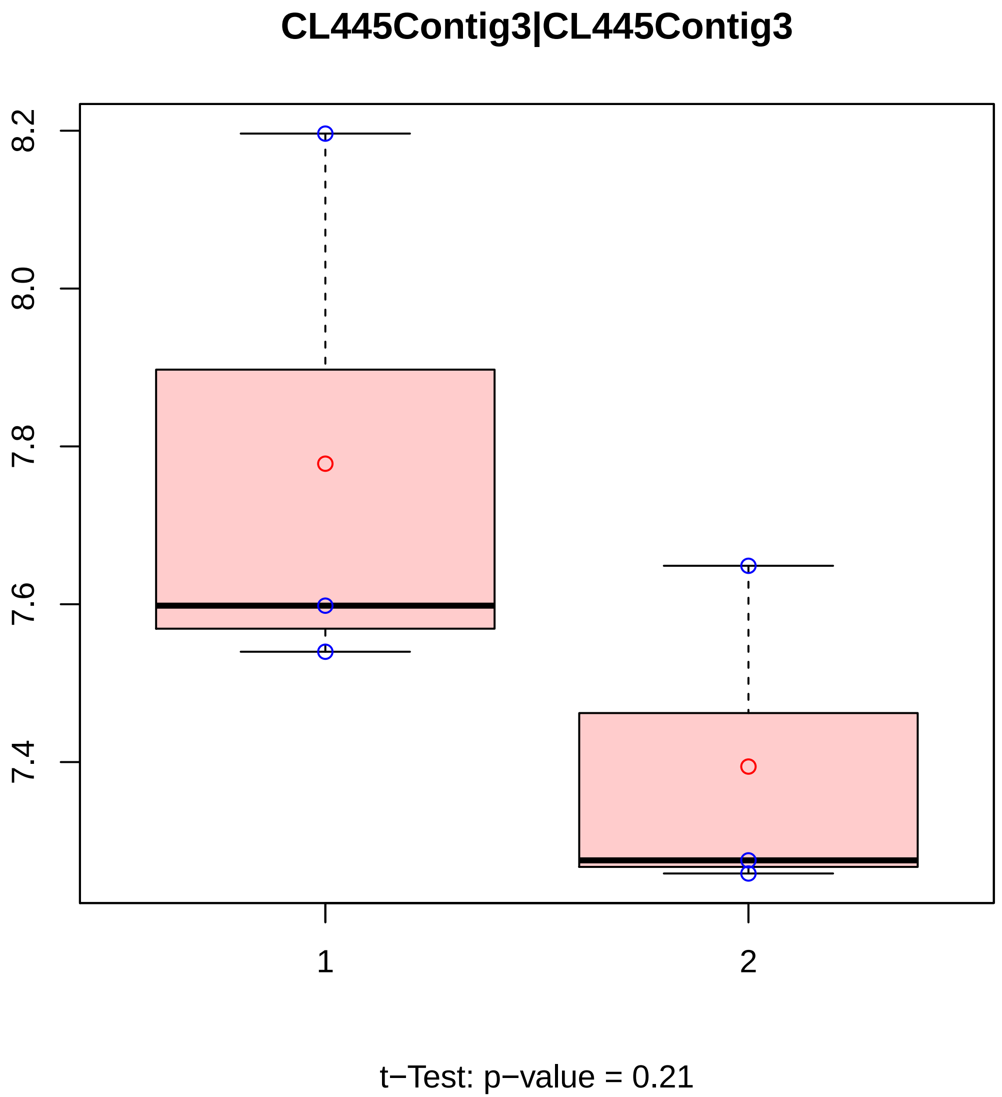
CL445Contig3|CL445Contig3
8.2
8.0
7.8
7.6
7.4
1
2
t−Test: p−value = 0.21

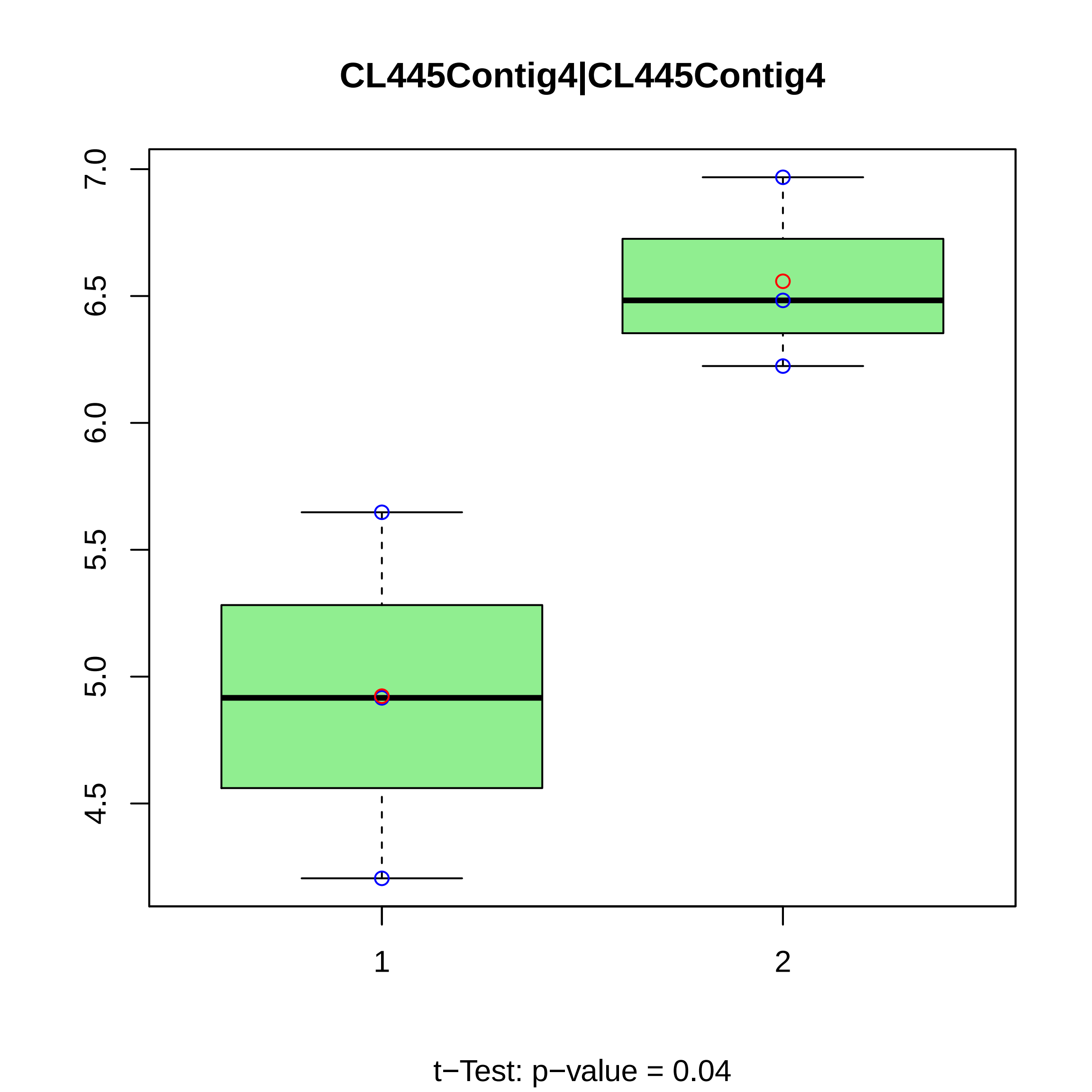
CL445Contig4|CL445Contig4
7.0
6.5
6.0
5.5
5.0
4.5
1
2
t−Test: p−value = 0.04

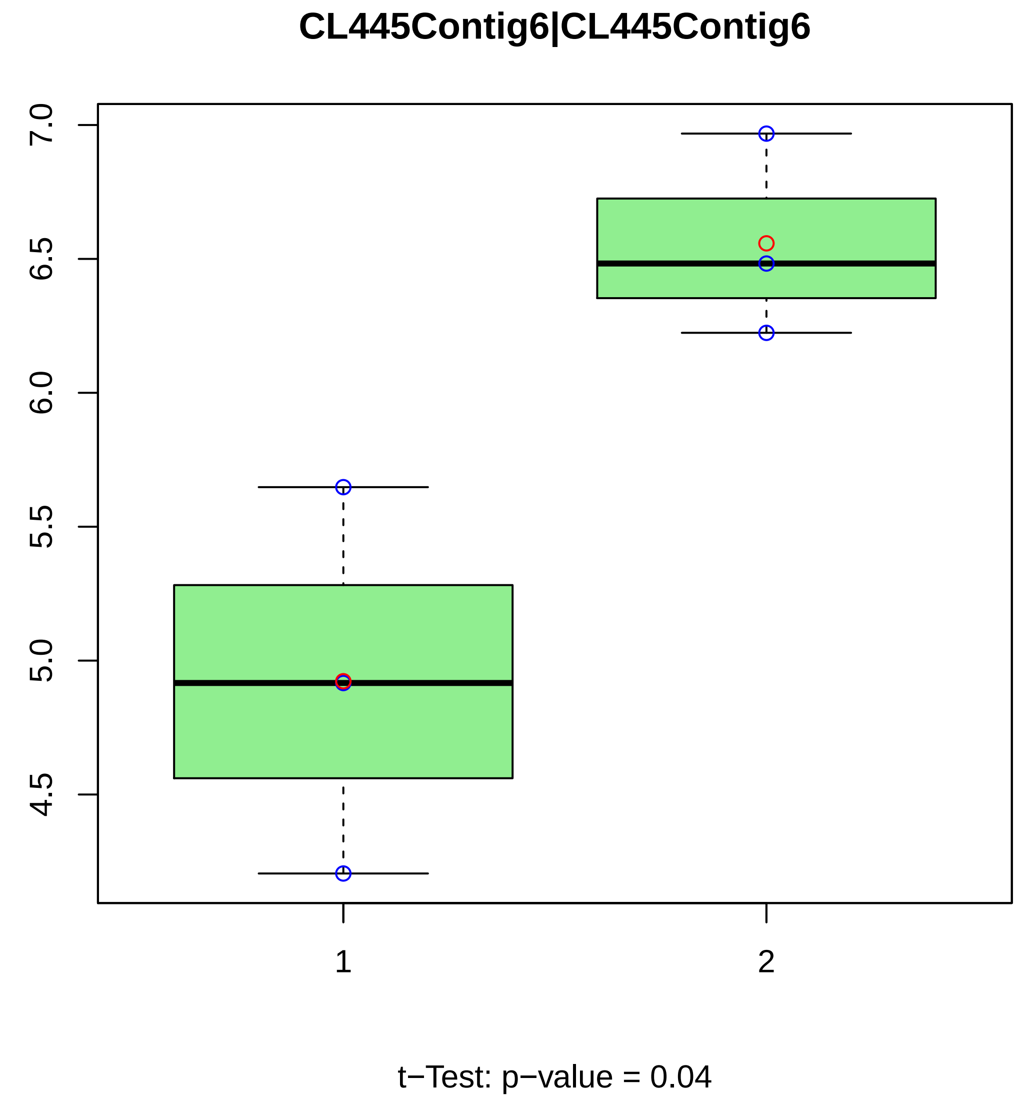
CL445Contig6|CL445Contig6
7.0
6.5
6.0
5.5
5.0
4.5
1
2
t−Test: p−value = 0.04

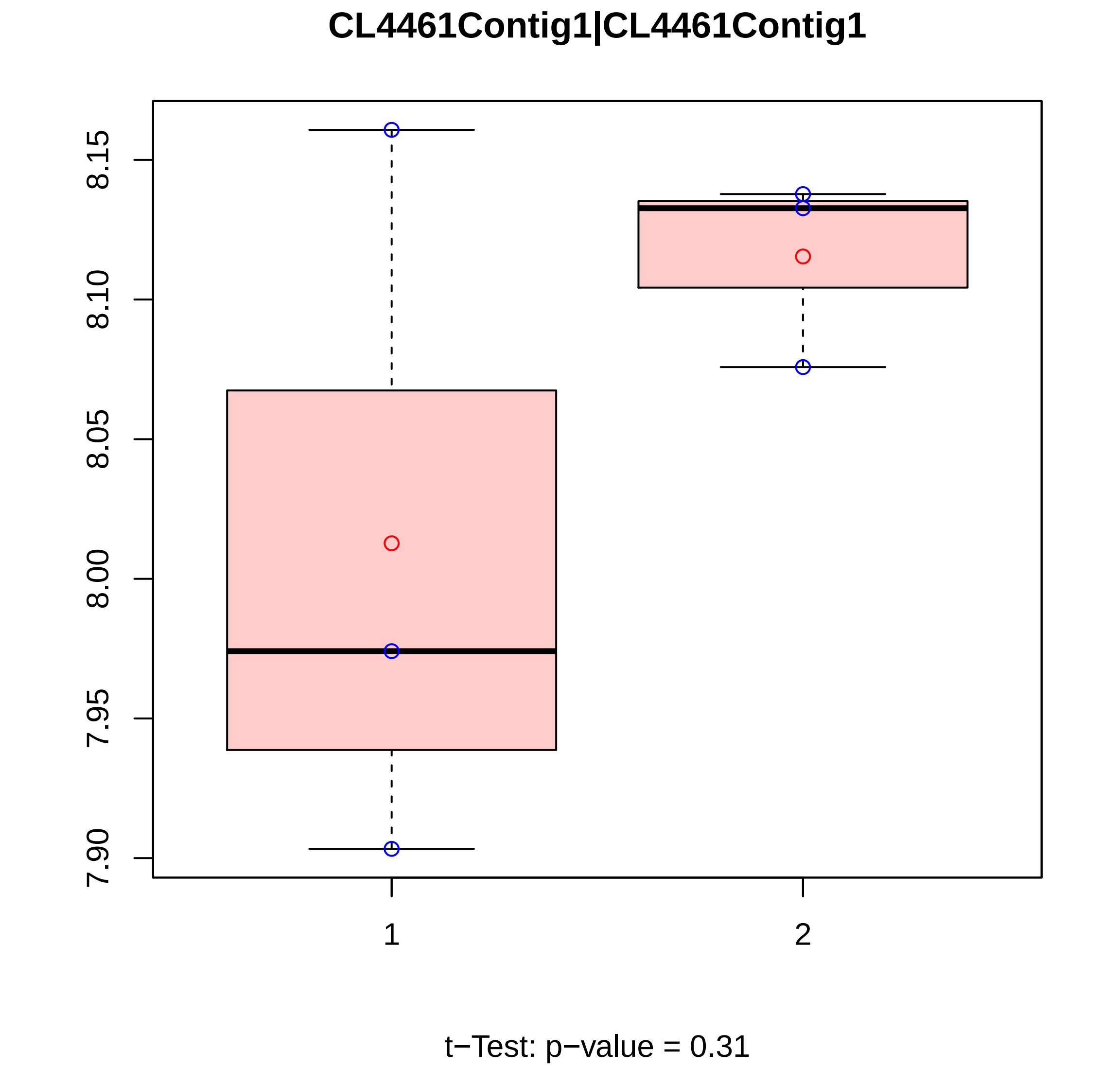
CL4461Contig1|CL4461Contig1
8.15
8.10
8.05
8.00
7.95
7.90
1
2
t−Test: p−value = 0.31

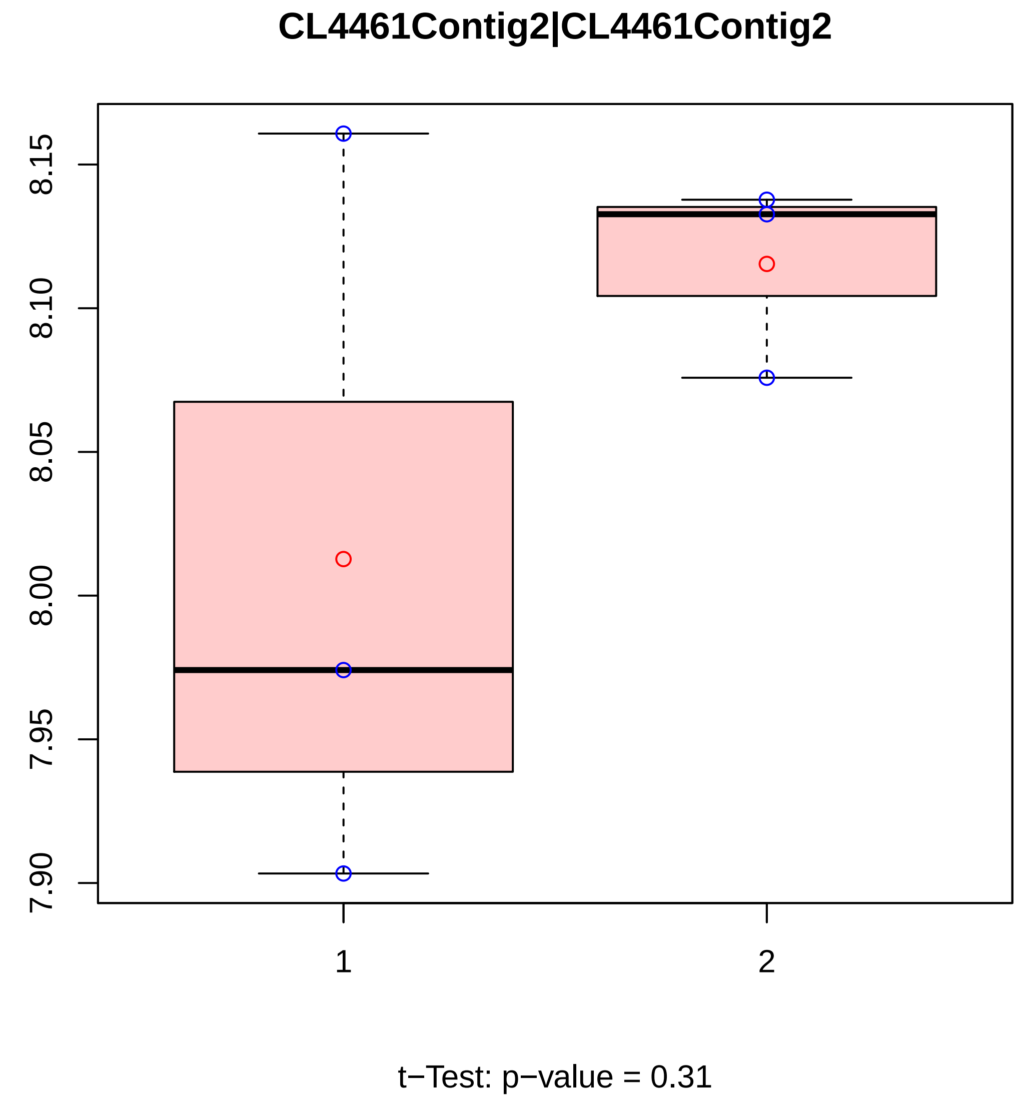
CL4461Contig2|CL4461Contig2
8.15
8.10
8.05
8.00
7.95
7.90
1
2
t−Test: p−value = 0.31

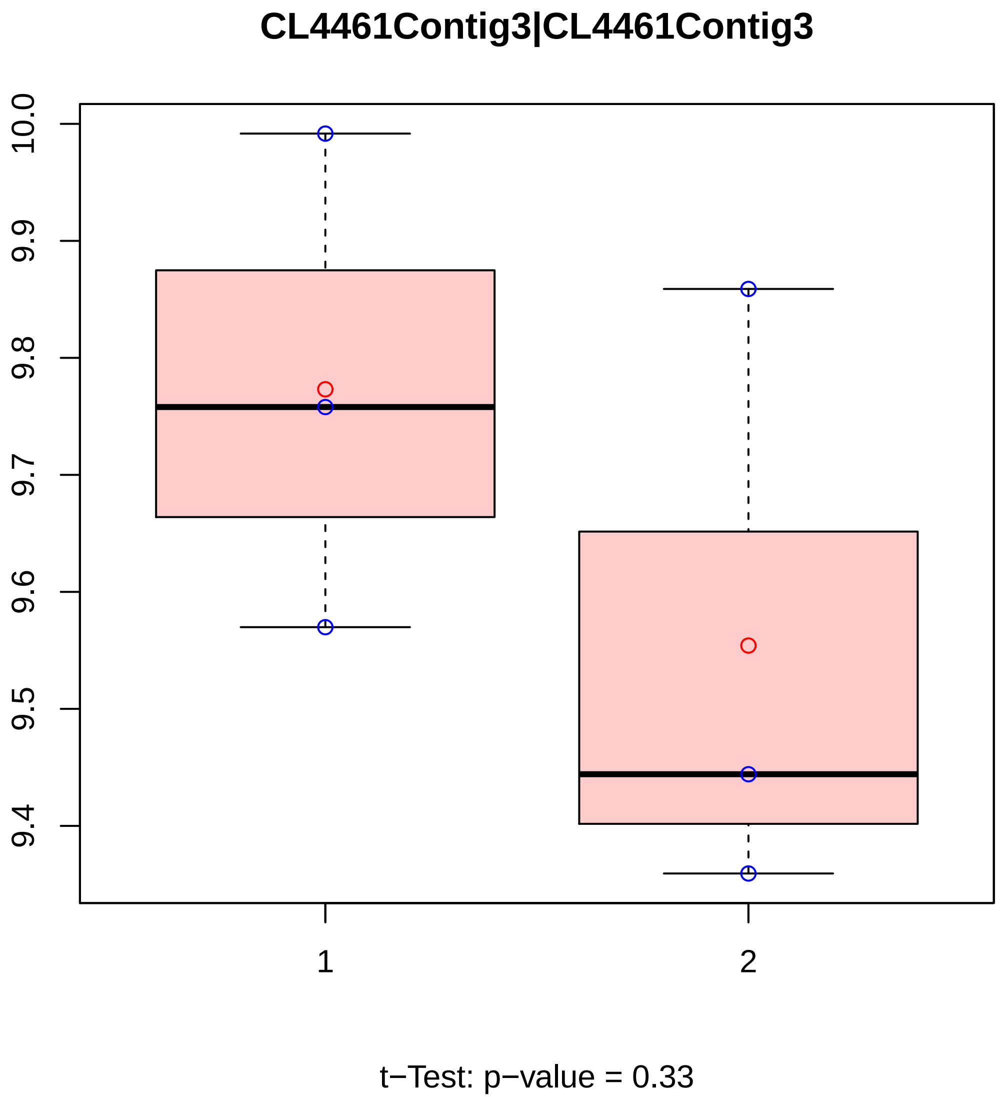

CL4461Contig3|CL4461Contig3
10.0
9.9
9.8
9.7
9.6
9.5
9.4
1
2
t−Test: p−value = 0.33

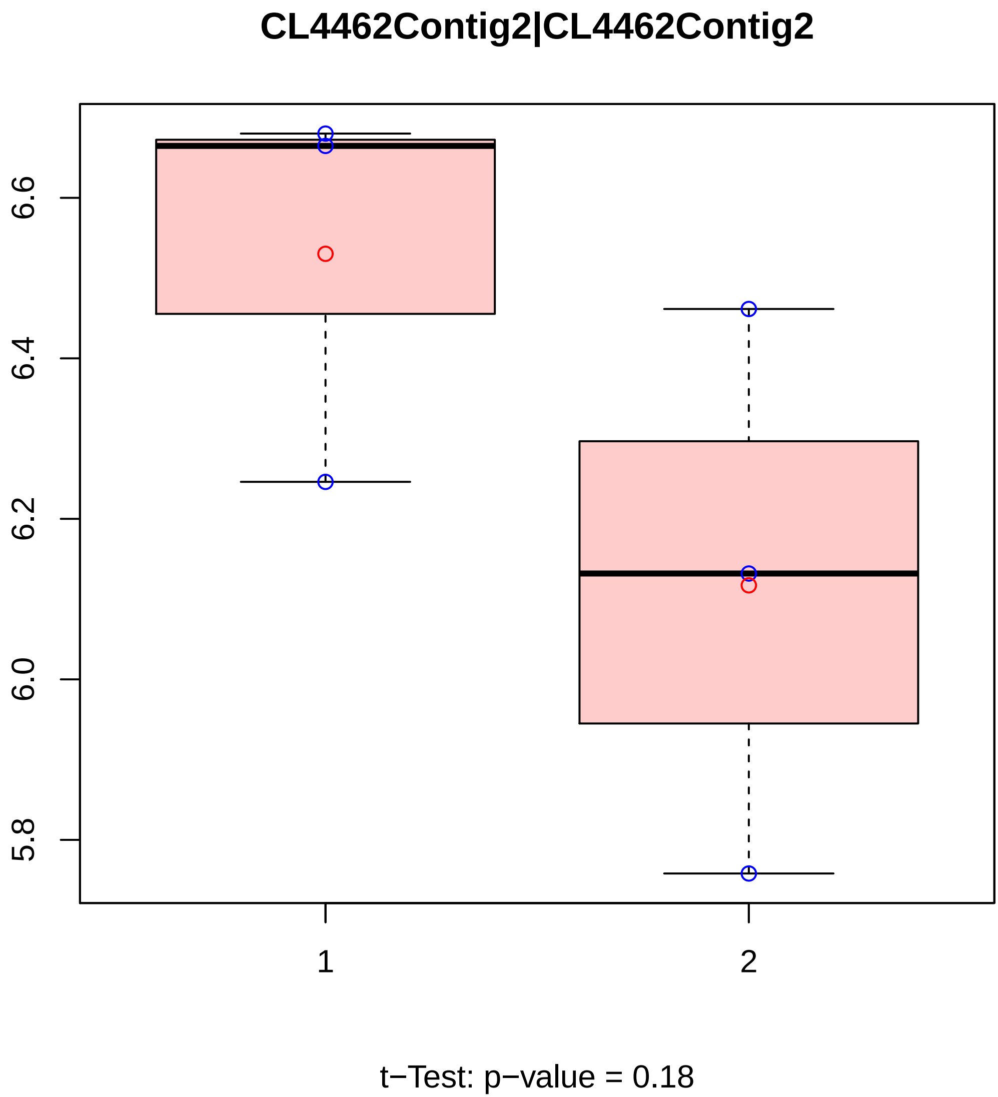
CL4462Contig2|CL4462Contig2
6.6
6.4
6.2
6.0
5.8
1
2
t−Test: p−value = 0.18

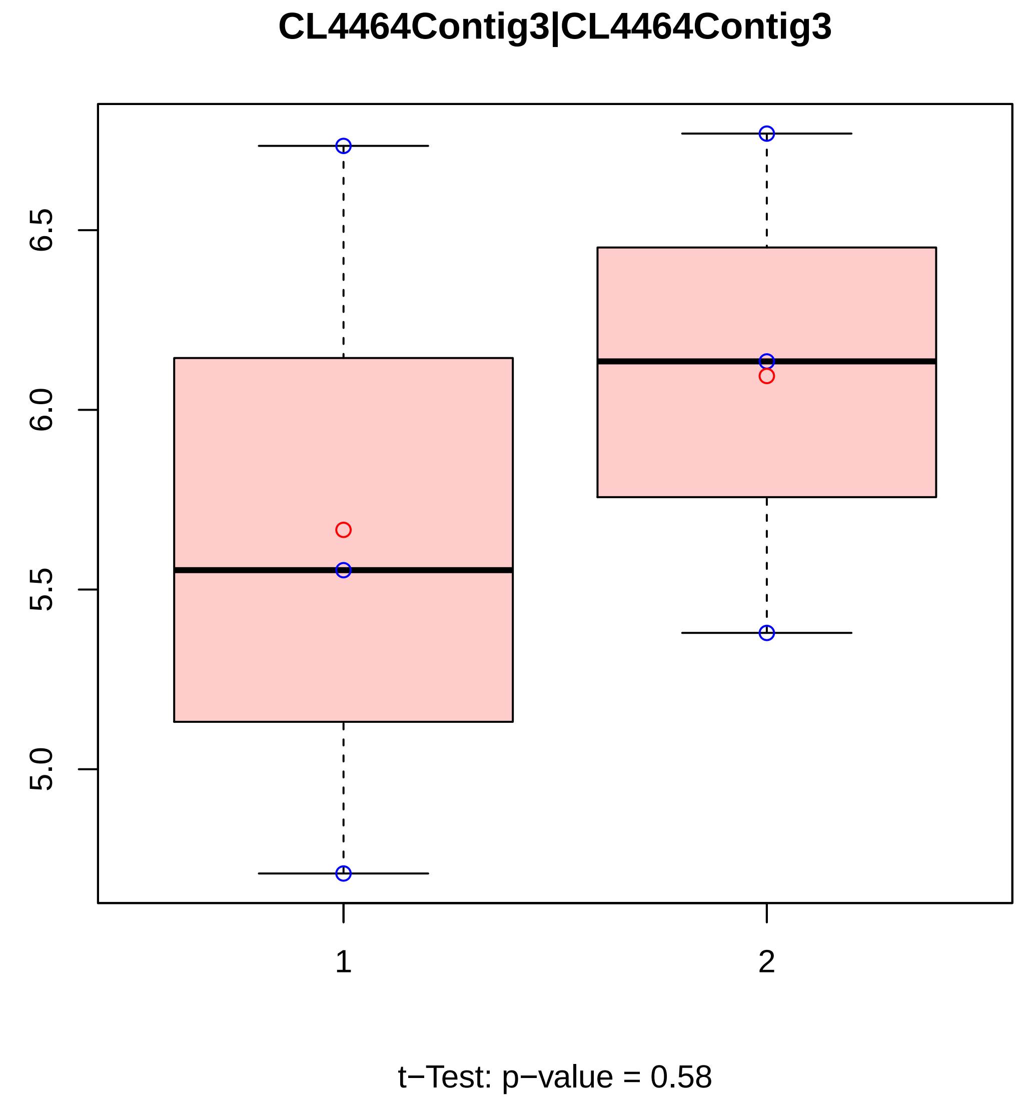
CL4464Contig3|CL4464Contig3
6.5
6.0
5.5
5.0
1
2
t−Test: p−value = 0.58

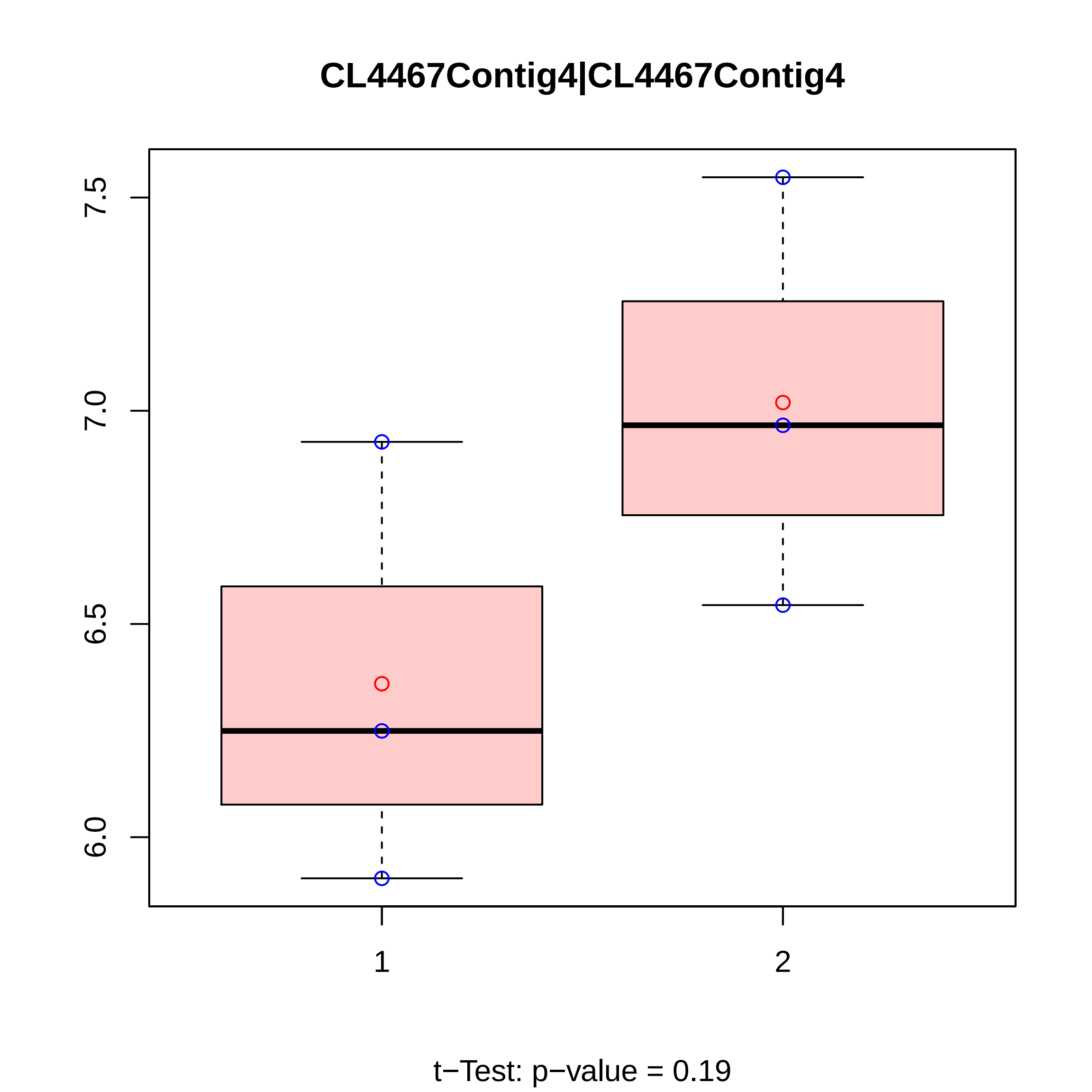

CL4467Contig4|CL4467Contig4
7.5
7.0
6.5
6.0
1
2
t−Test: p−value = 0.19

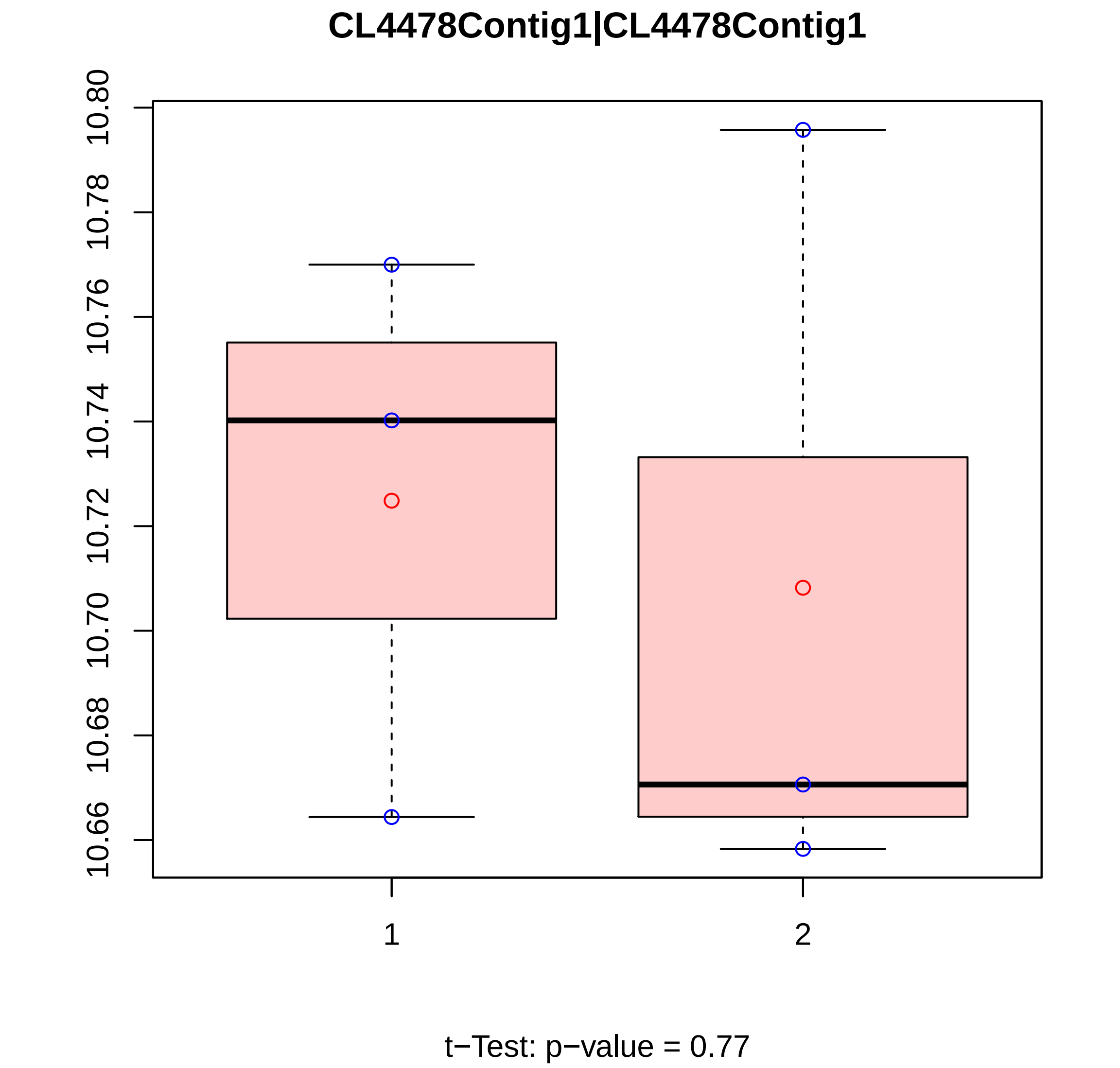
CL4478Contig1|CL4478Contig1
10.66
10.68
10.70
10.72
10.74
10.76
10.78
10.80
1
2
t−Test: p−value = 0.77

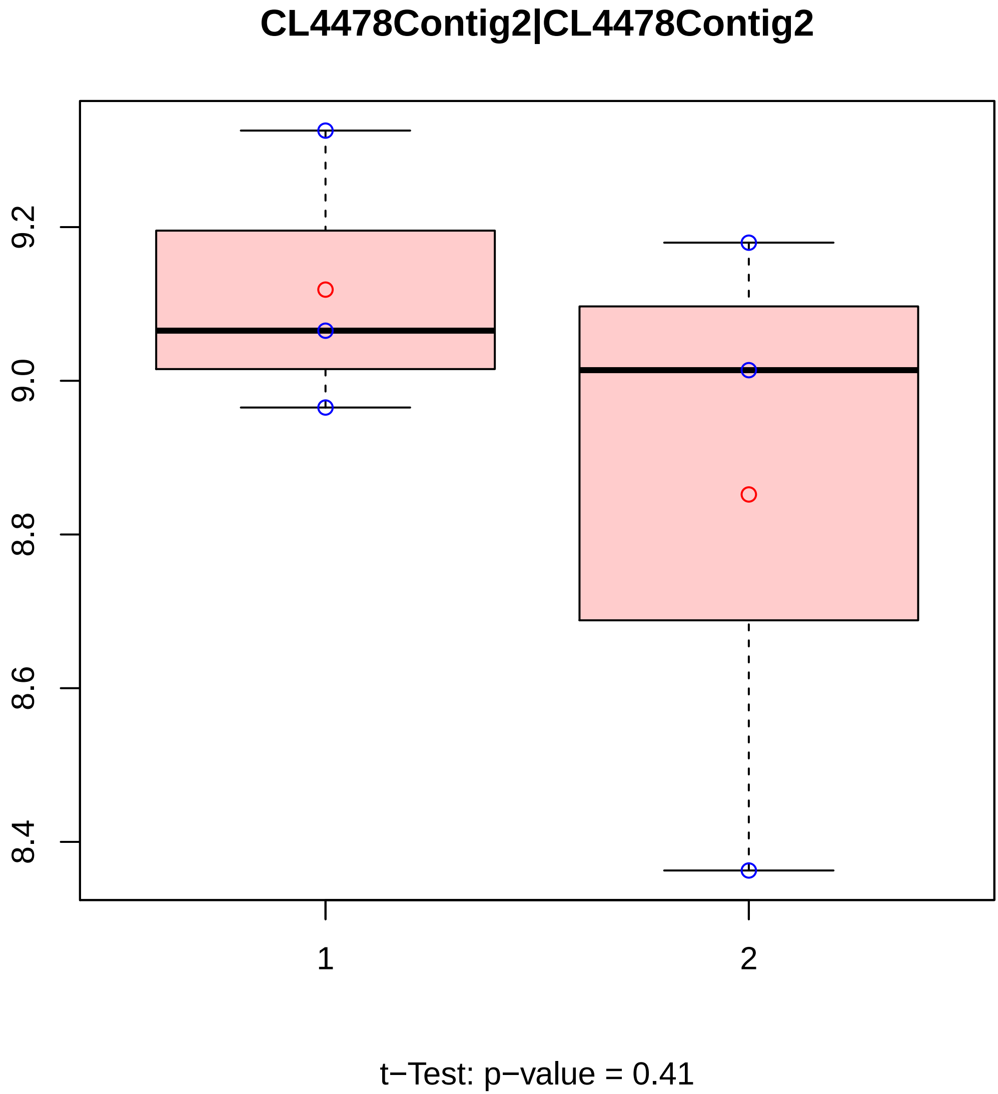
CL4478Contig2|CL4478Contig2
9.2
9.0
8.8
8.6
8.4
1
2
t−Test: p−value = 0.41

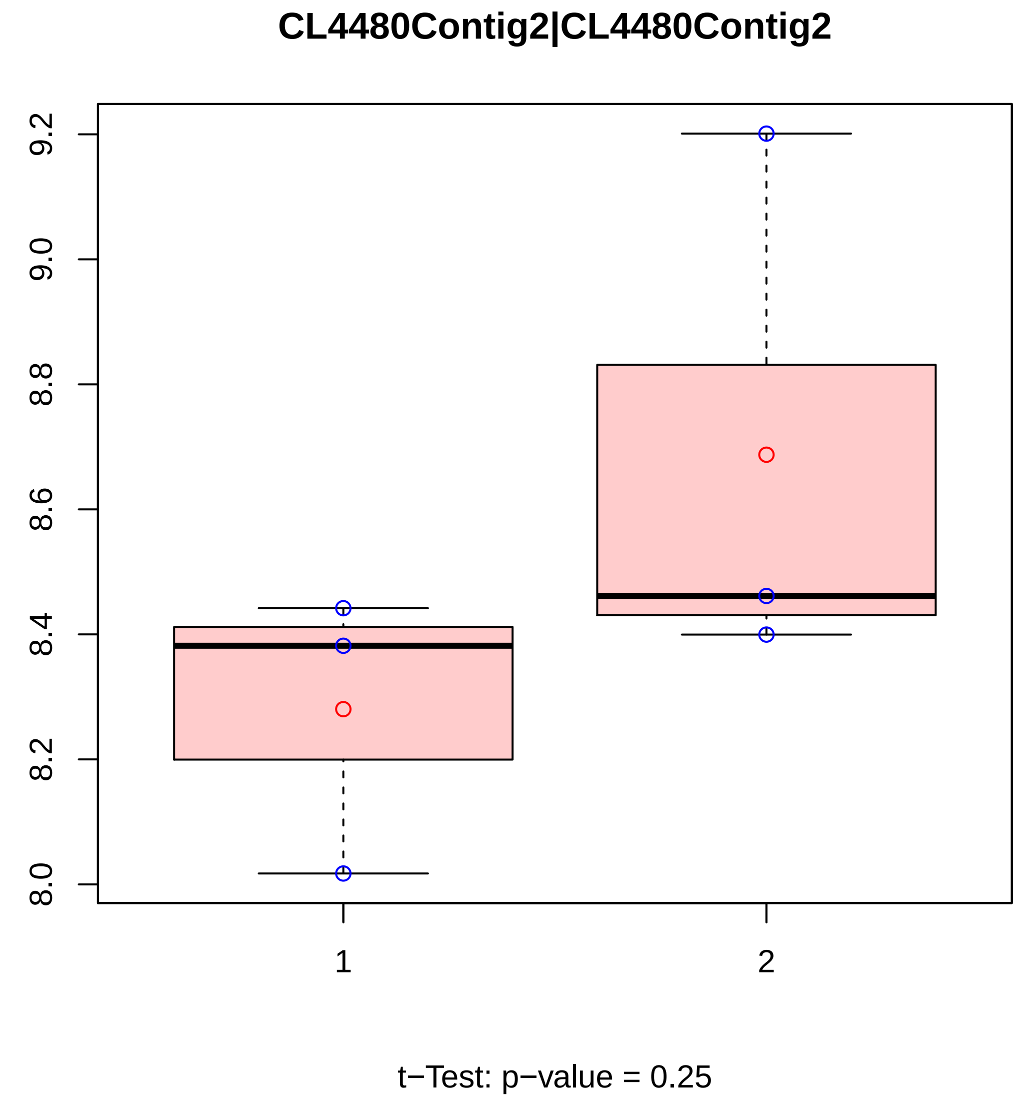

CL4480Contig2|CL4480Contig2
9.2
9.0
8.8
8.6
8.4
8.2
8.0
1
2
t−Test: p−value = 0.25

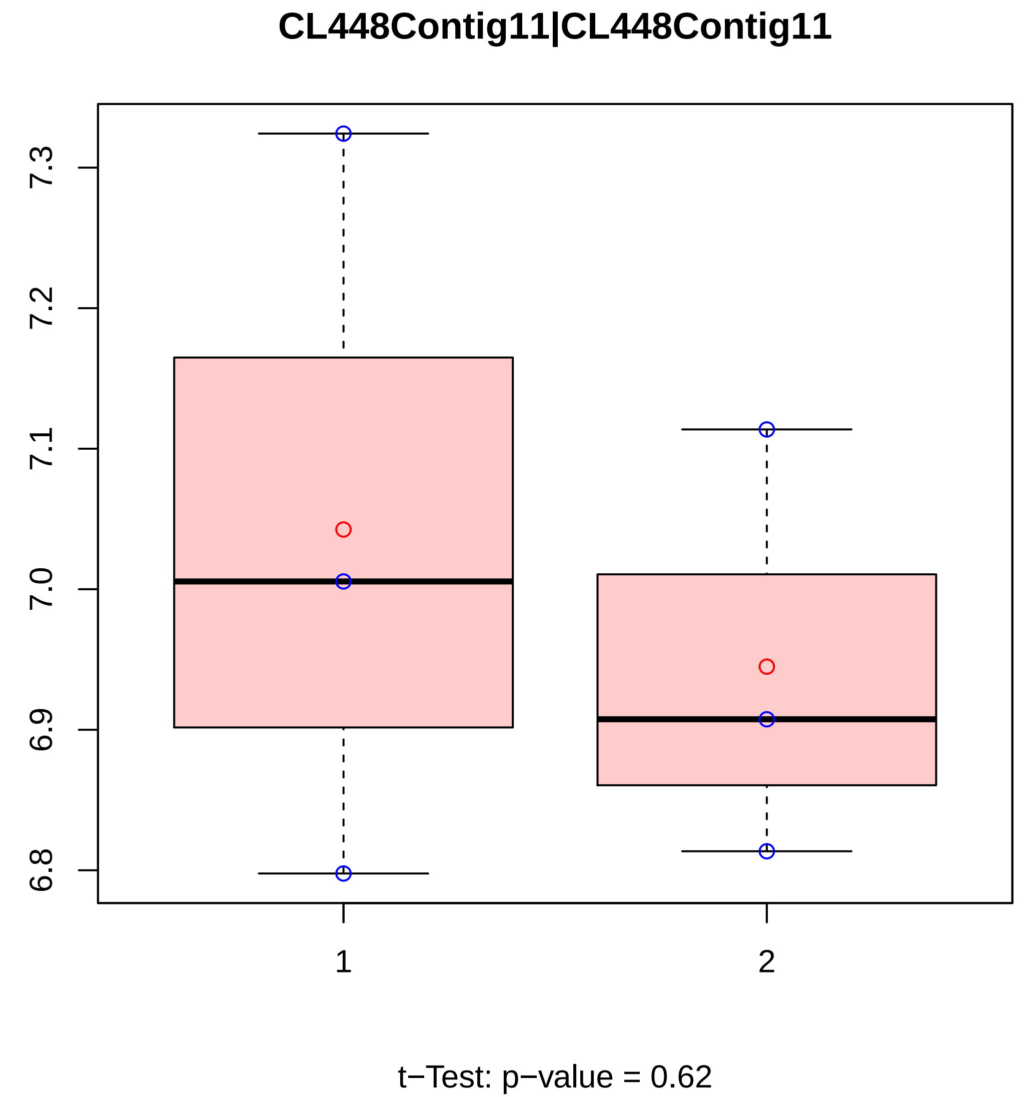

CL448Contig11|CL448Contig11
7.3
7.2
7.1
7.0
6.9
6.8
1
2
t−Test: p−value = 0.62

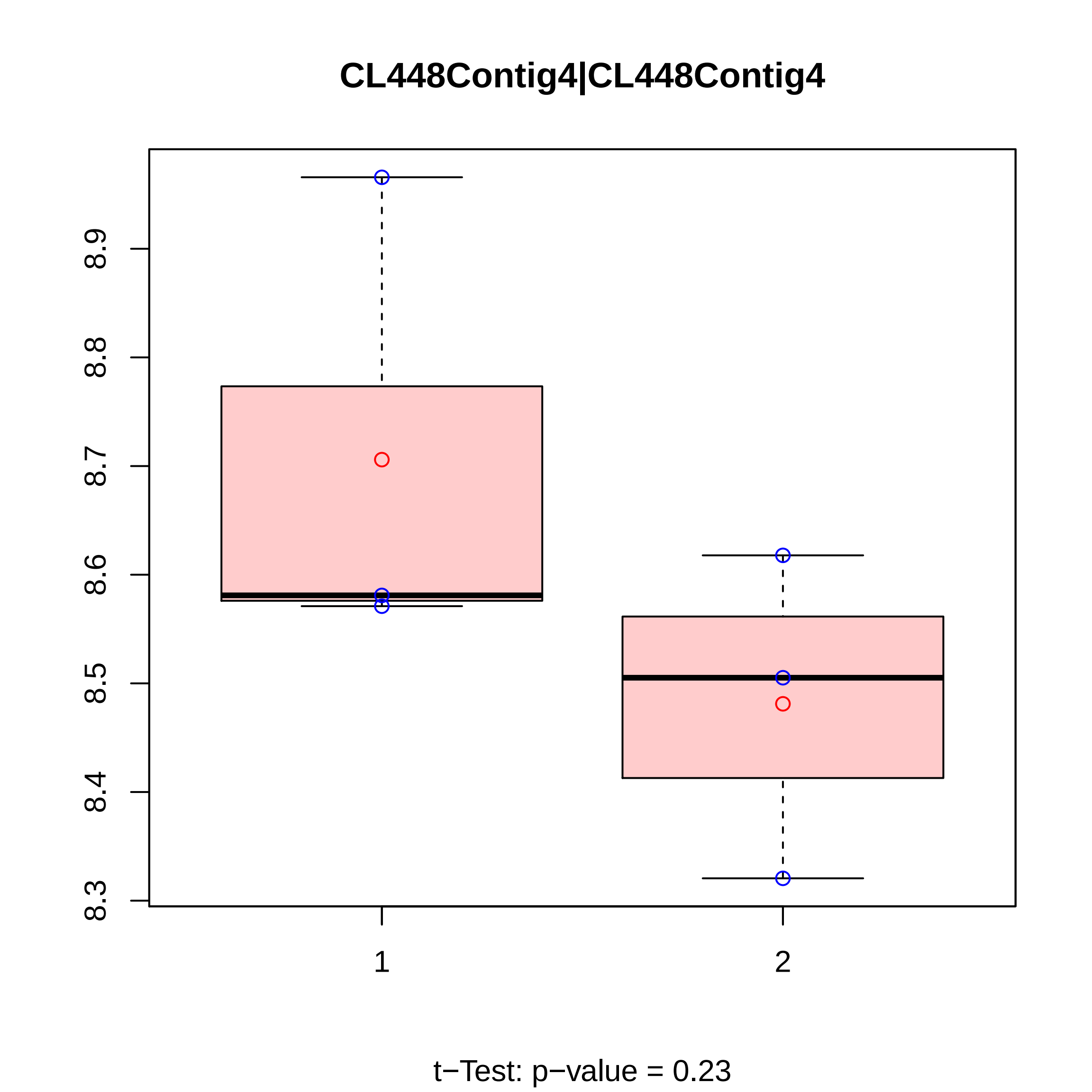
CL448Contig4|CL448Contig4
8.3
8.4
8.5
8.6
8.7
8.8
8.9
1
2
t−Test: p−value = 0.23

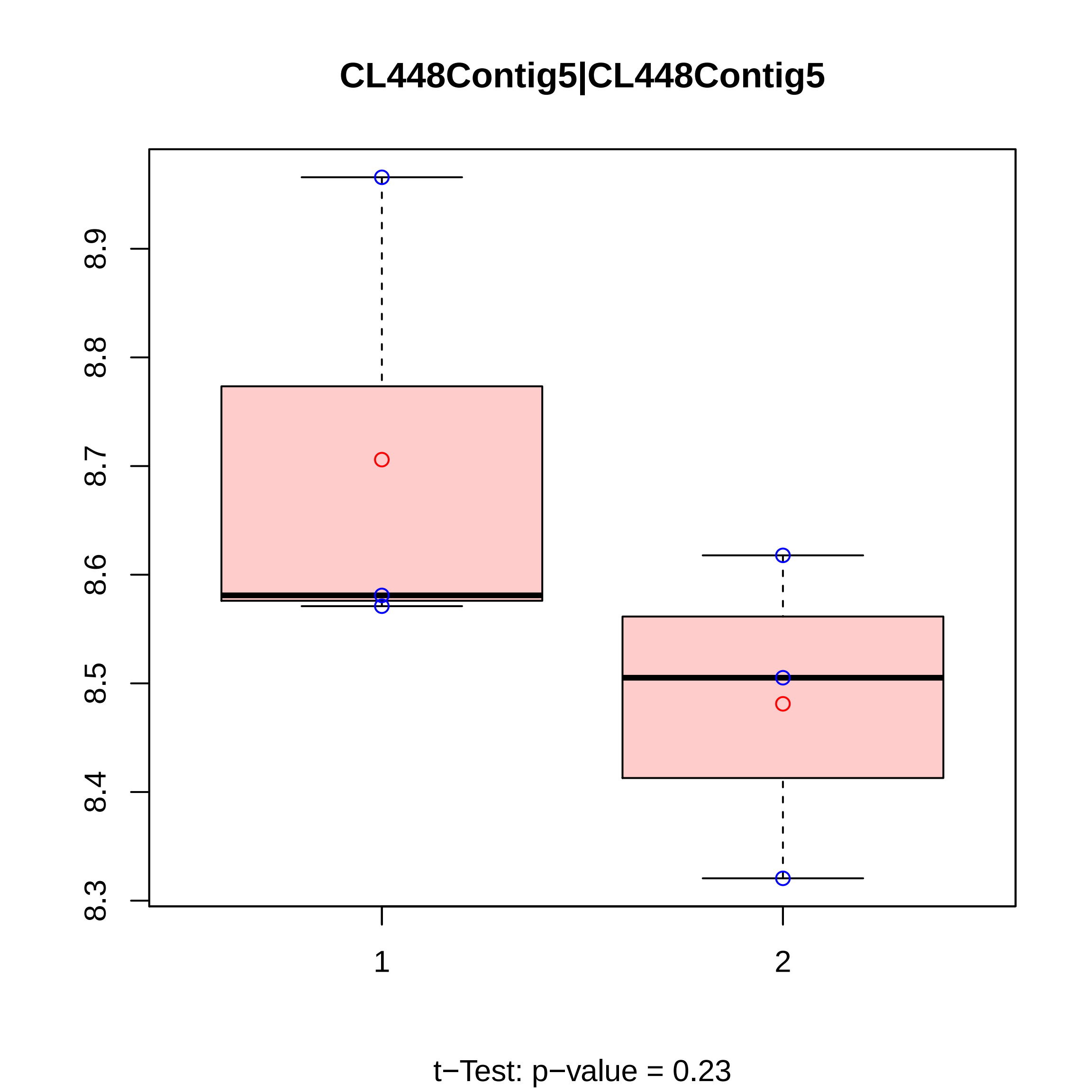
CL448Contig5|CL448Contig5
8.3
8.4
8.5
8.6
8.7
8.8
8.9
1
2
t−Test: p−value = 0.23

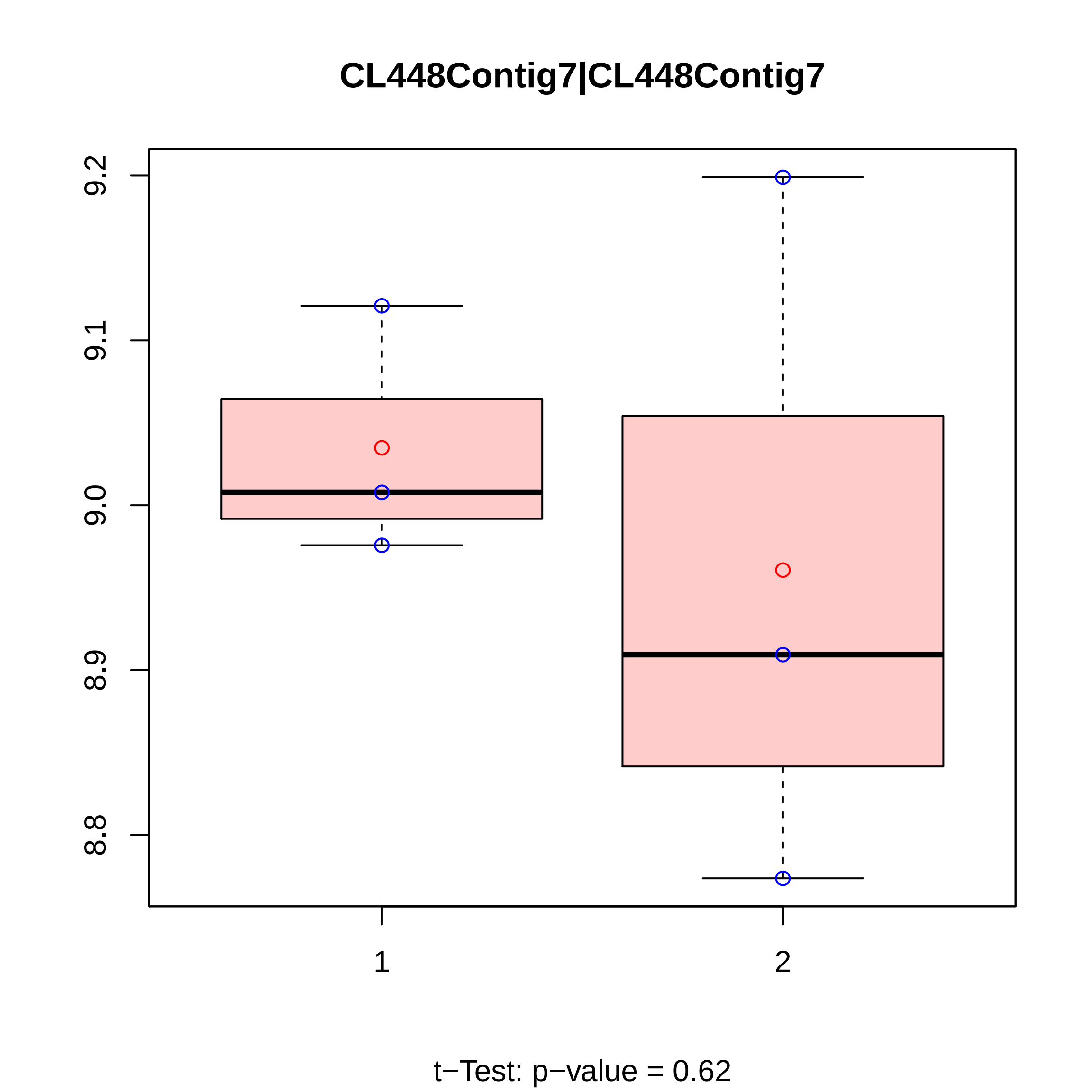
CL448Contig7|CL448Contig7
9.2
9.1
9.0
8.9
8.8
1
2
t−Test: p−value = 0.62

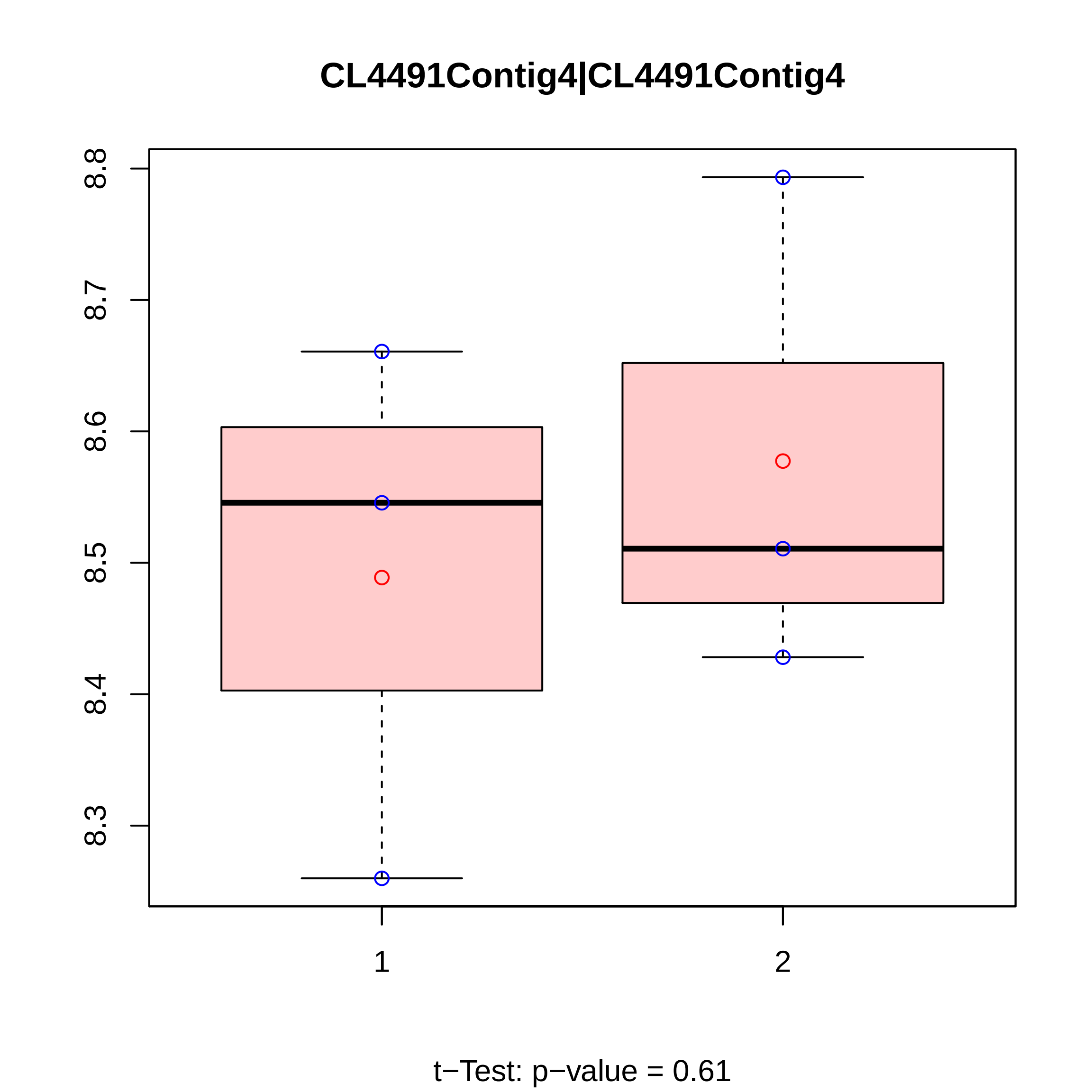
CL4491Contig4|CL4491Contig4
8.3
8.4
8.5
8.6
8.7
8.8
1
2
t−Test: p−value = 0.61

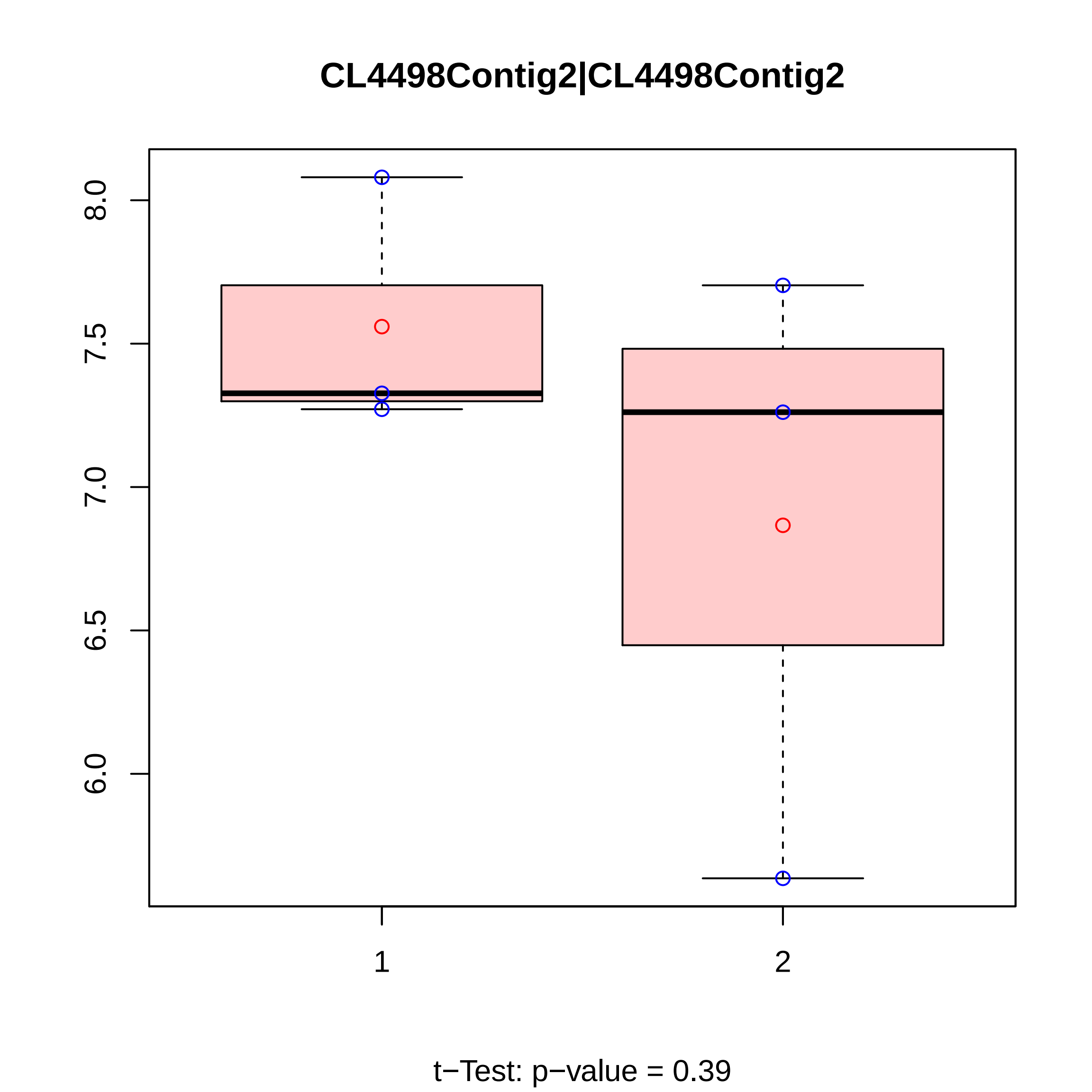

CL4498Contig2|CL4498Contig2
8.0
7.5
7.0
6.5
6.0
1
2
t−Test: p−value = 0.39

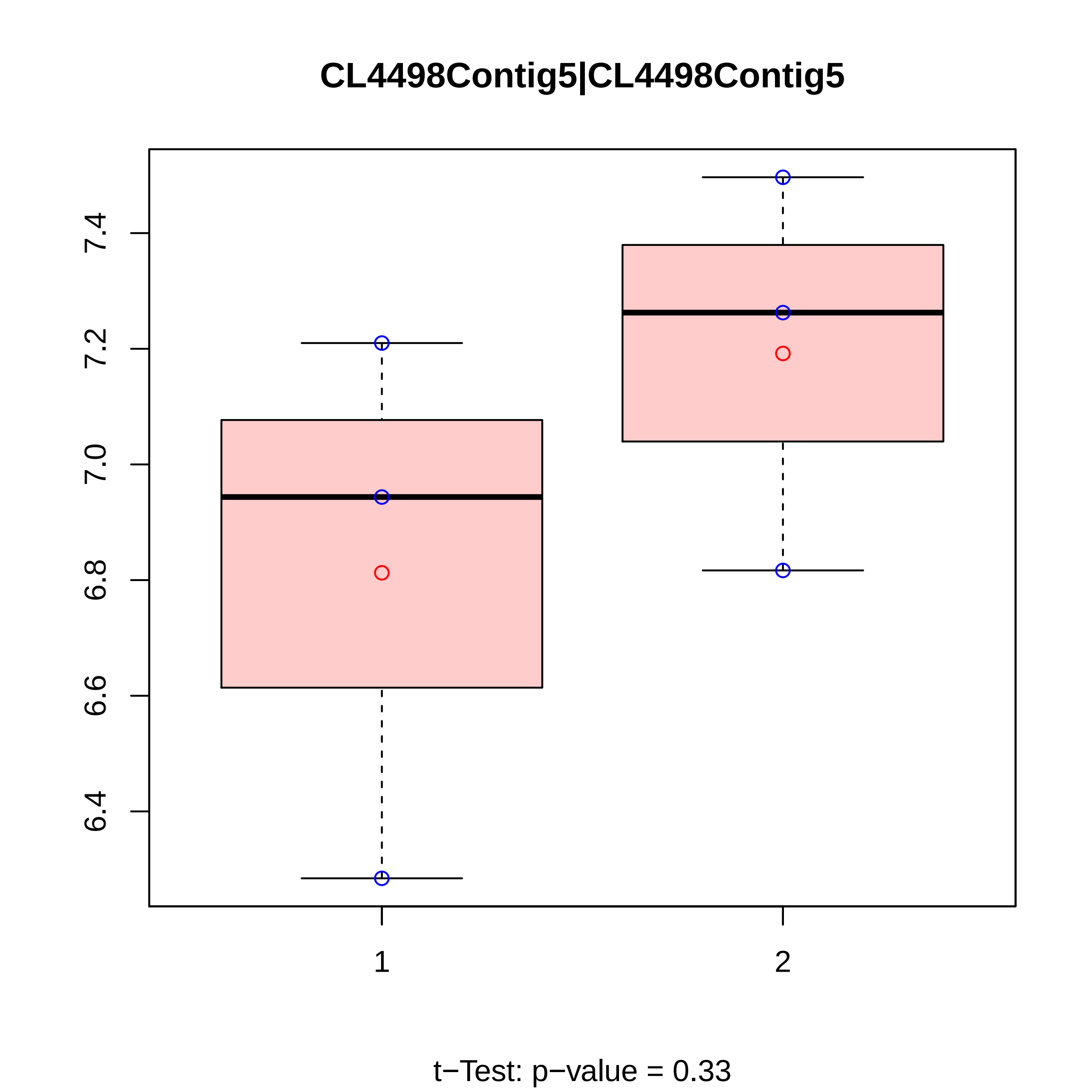

CL4498Contig5|CL4498Contig5
6.4
6.6
6.8
7.0
7.2
7.4
1
2
t−Test: p−value = 0.33

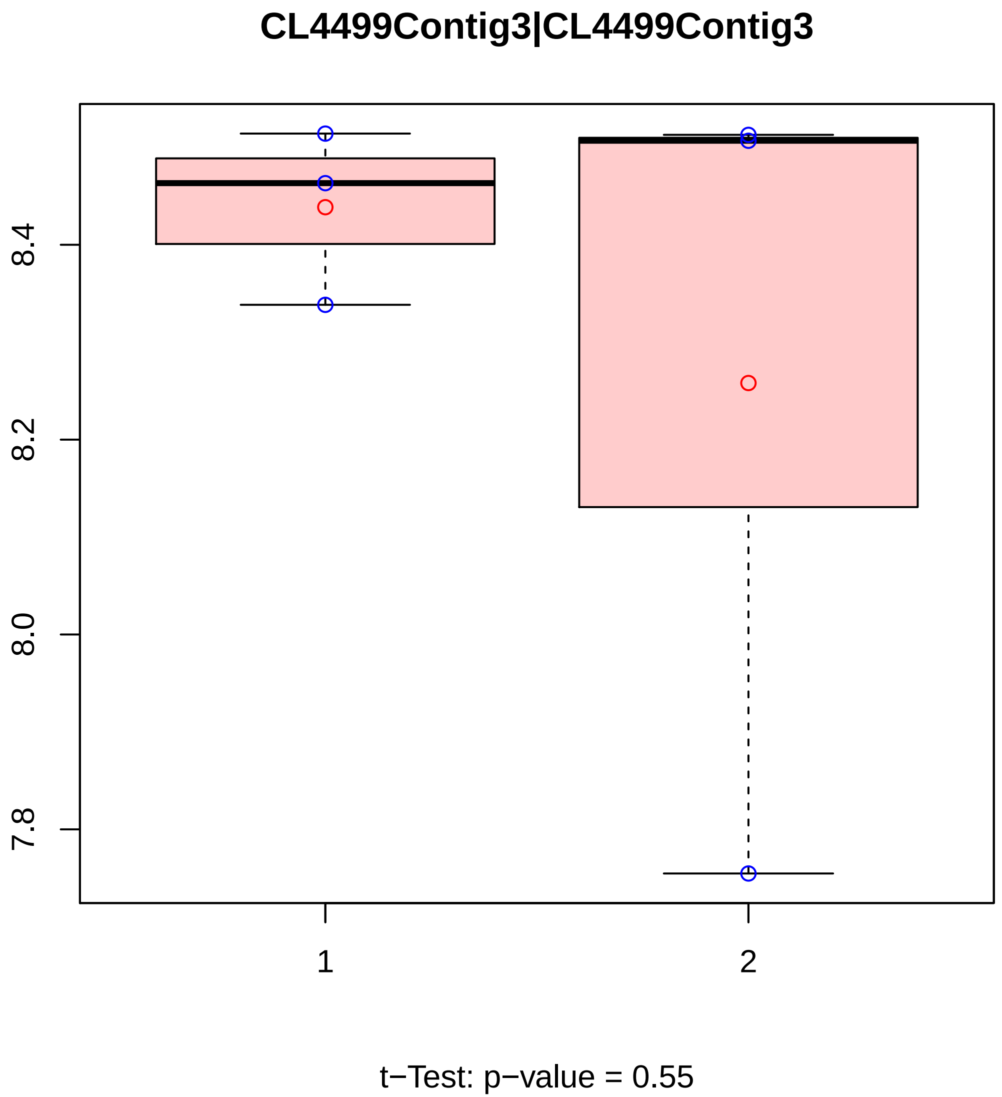
CL4499Contig3|CL4499Contig3
8.4
8.2
8.0
7.8
1
2
t−Test: p−value = 0.55

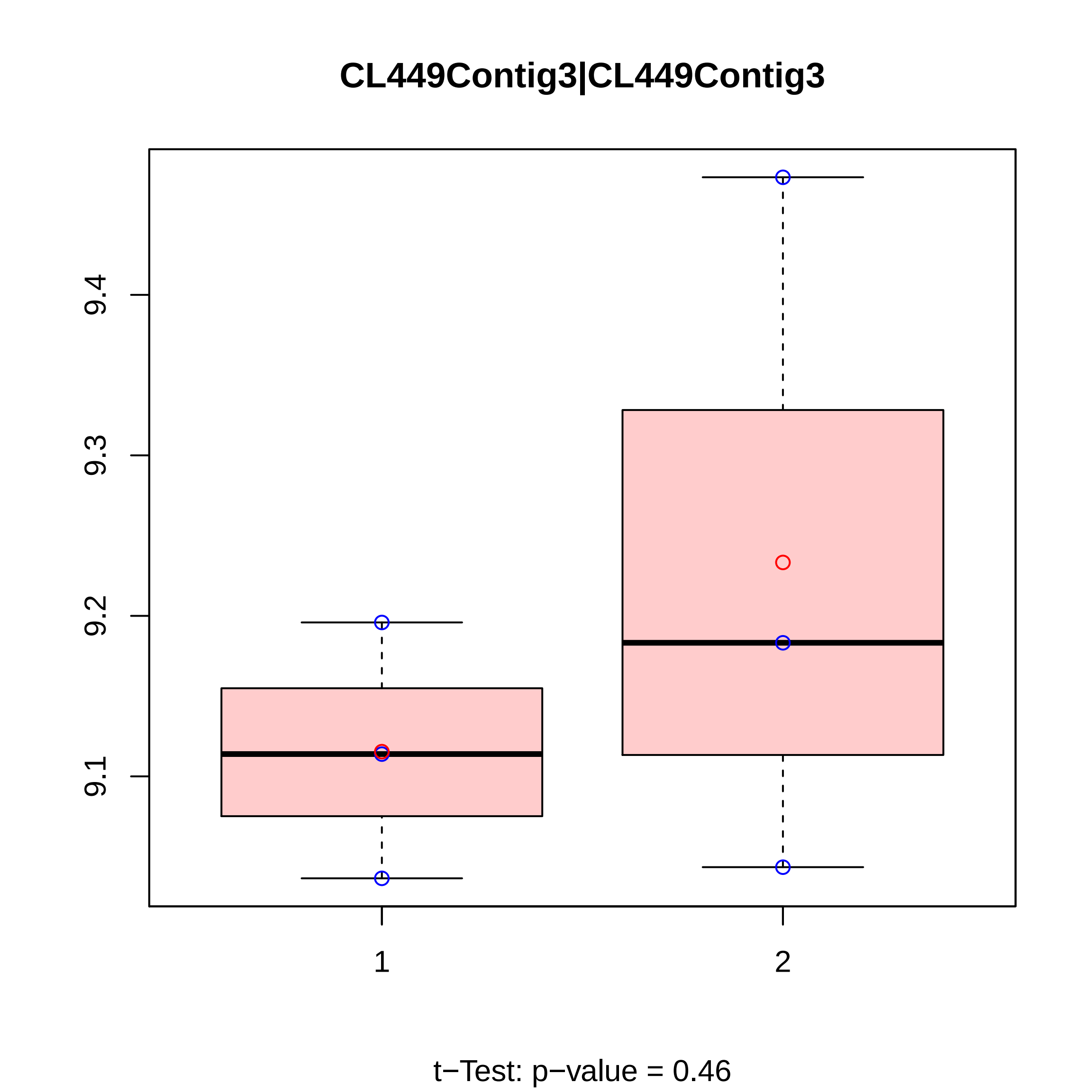

CL449Contig3|CL449Contig3
9.1
9.2
9.3
9.4
1
2
t−Test: p−value = 0.46

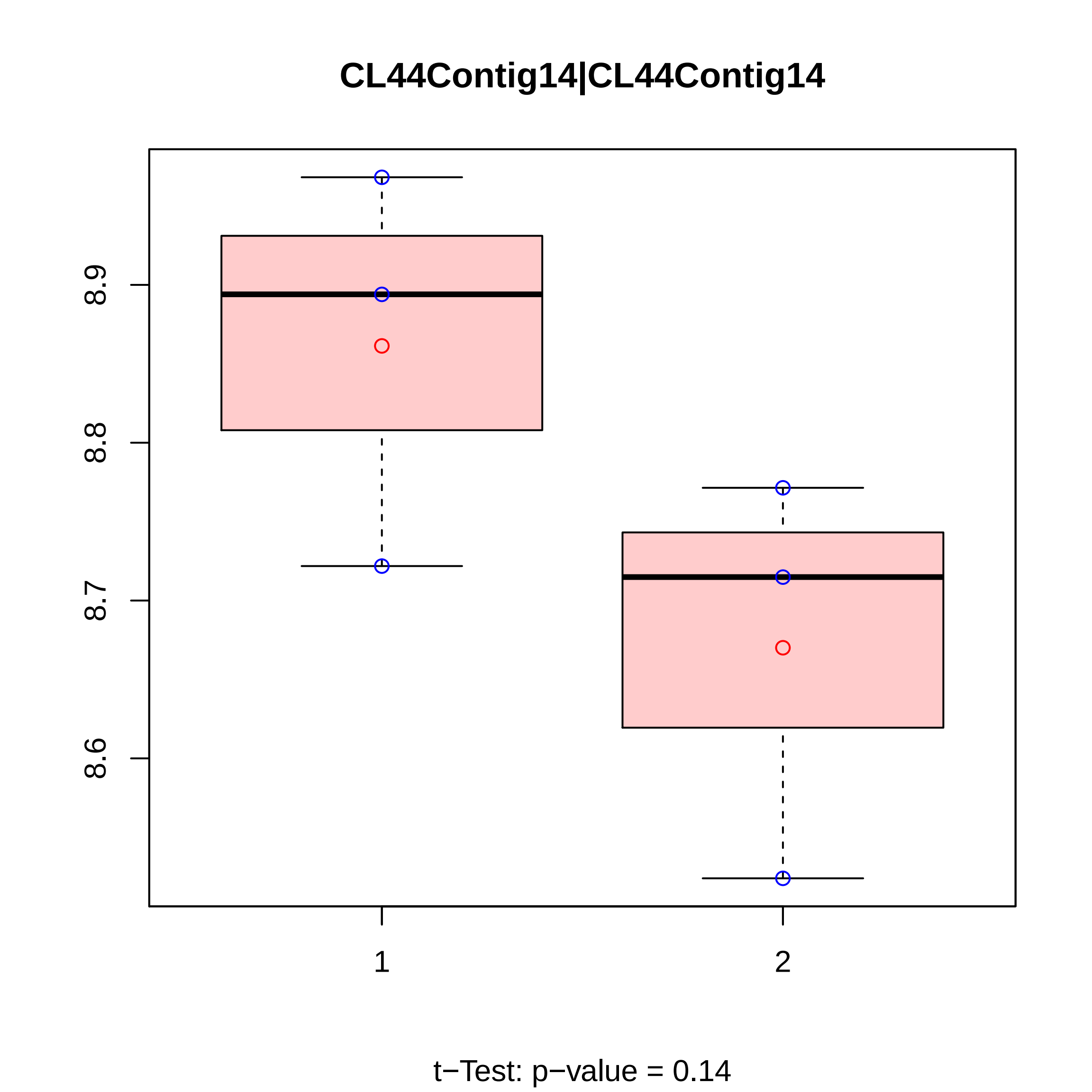

CL44Contig14|CL44Contig14
8.6
8.7
8.8
8.9
1
2
t−Test: p−value = 0.14

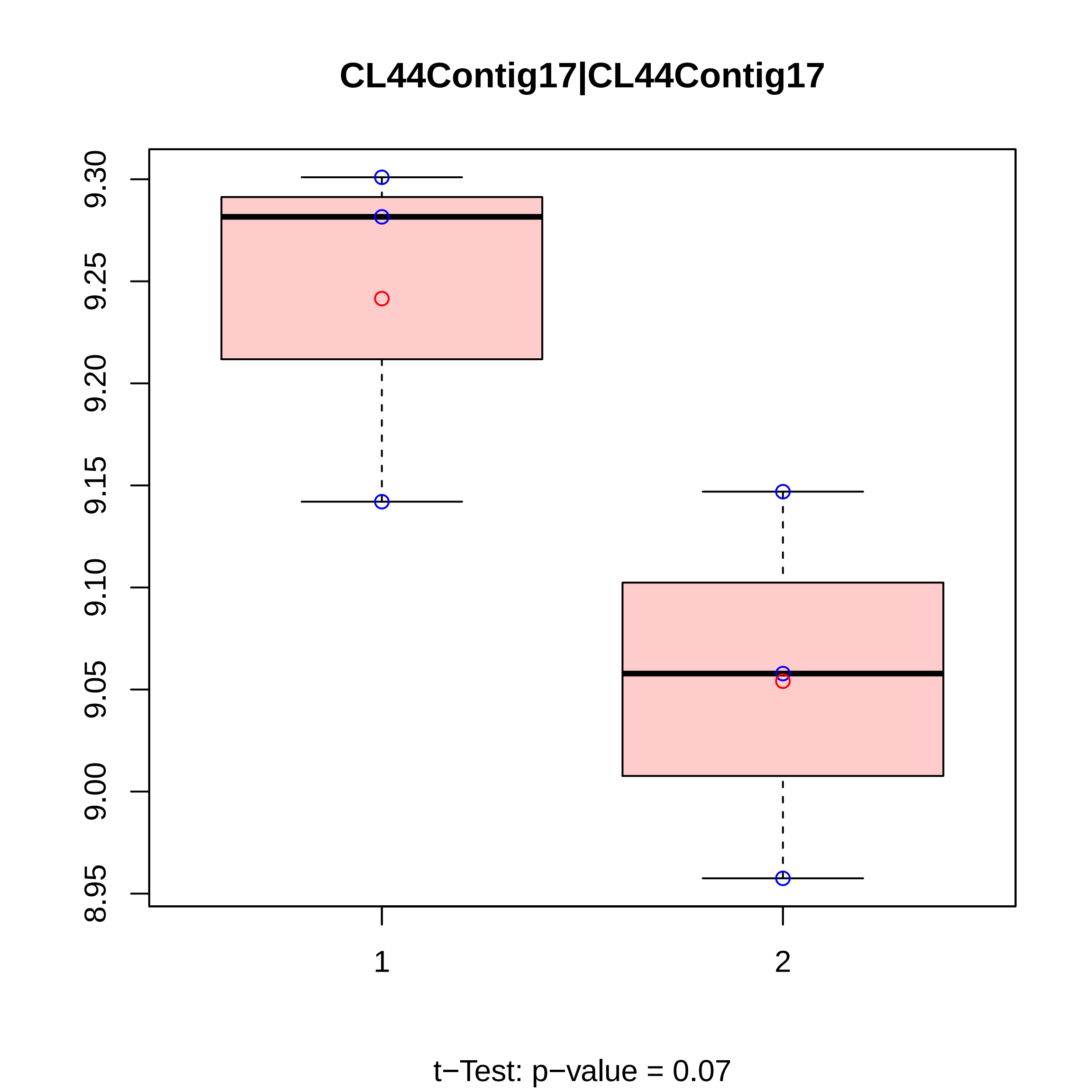
CL44Contig17|CL44Contig17
9.30
9.25
9.20
9.15
9.10
9.05
9.00
8.95
1
2
t−Test: p−value = 0.07

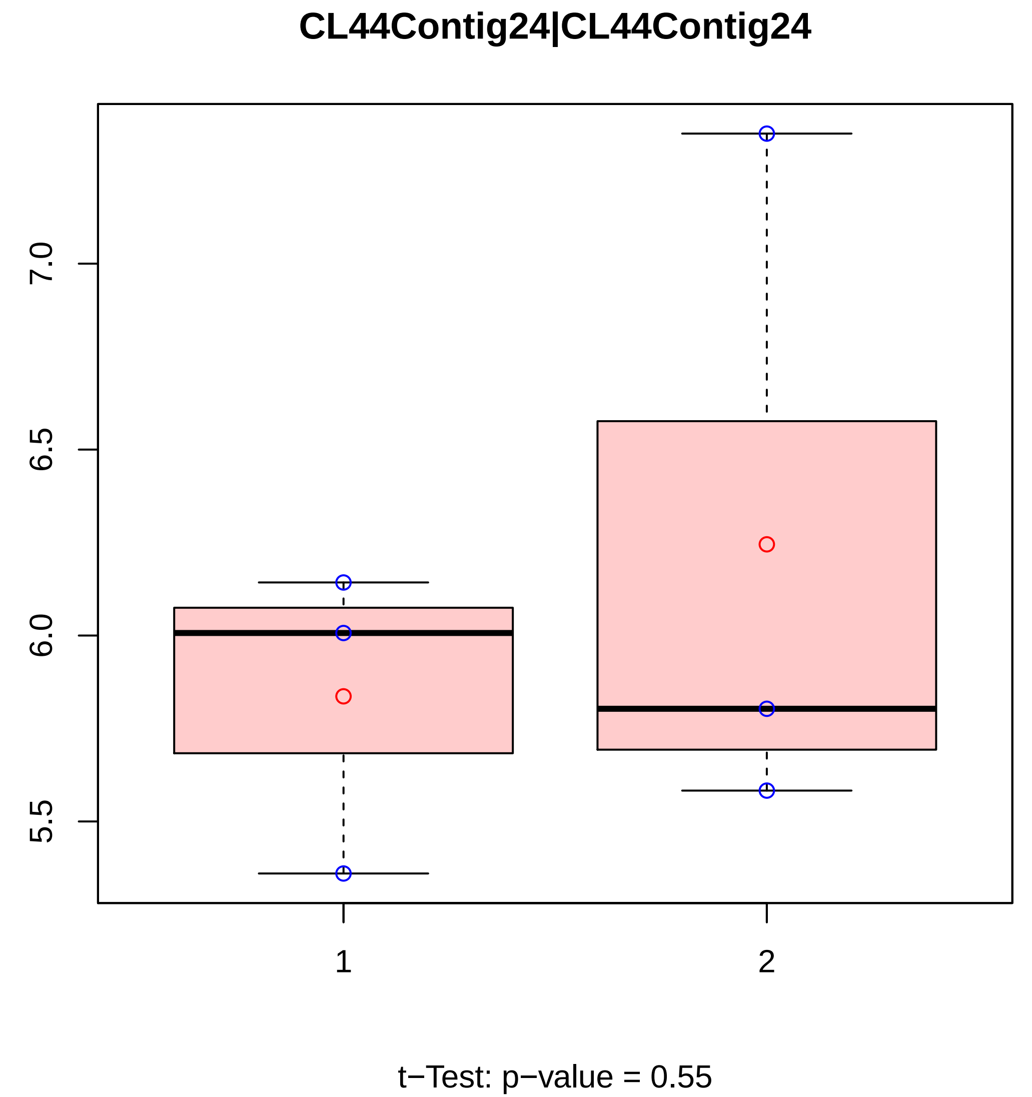
CL44Contig24|CL44Contig24
7.0
6.5
6.0
5.5
1
2
t−Test: p−value = 0.55

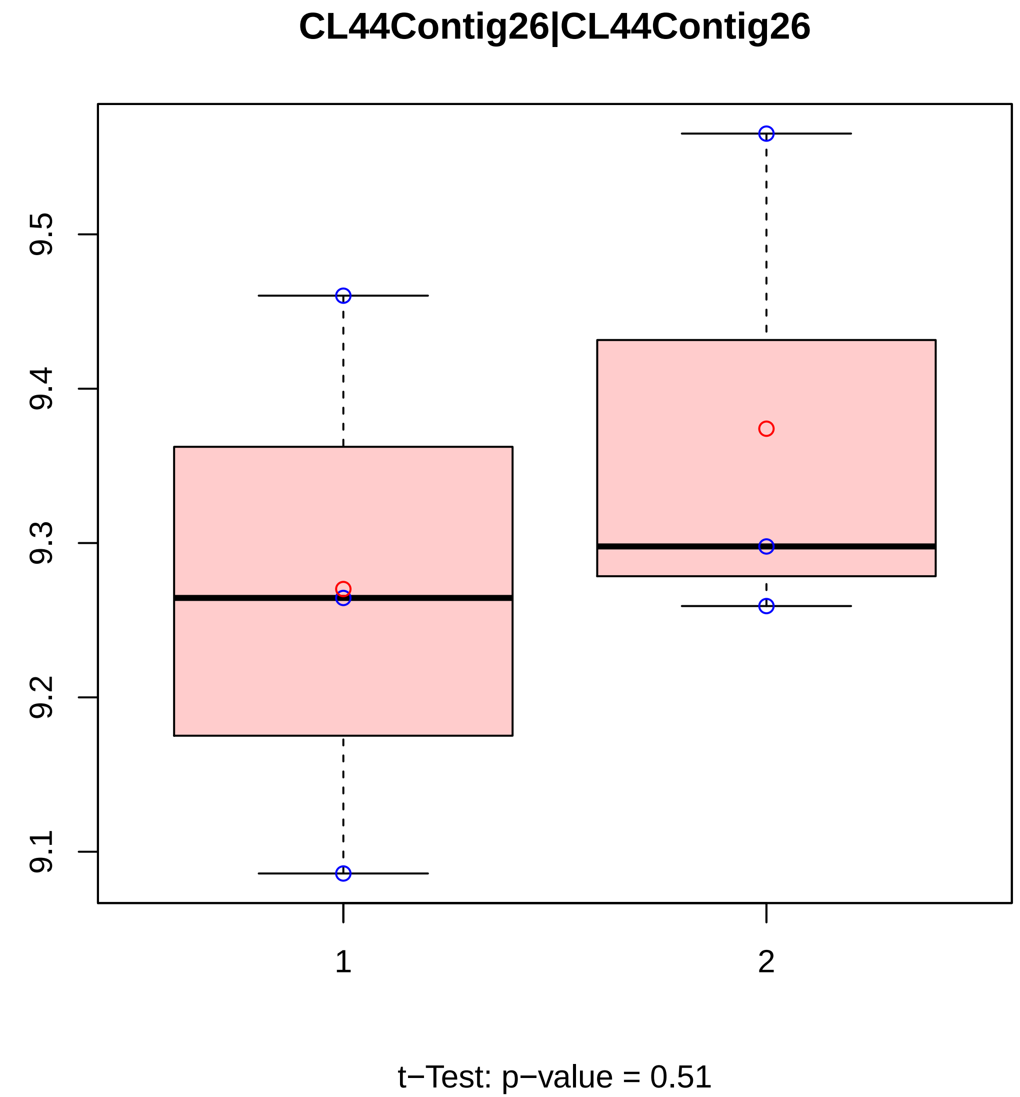
CL44Contig26|CL44Contig26
9.1
9.2
9.3
9.4
9.5
1
2
t−Test: p−value = 0.51

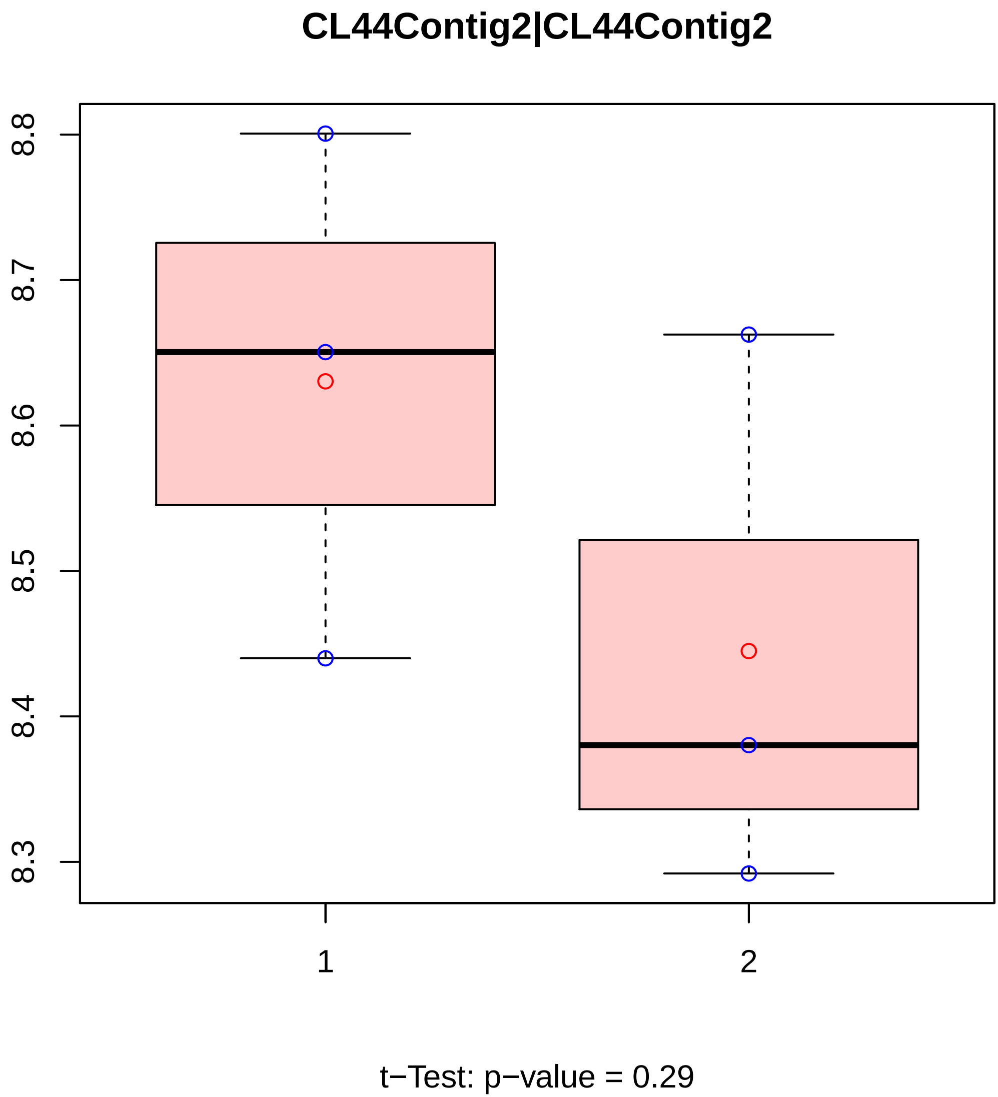

CL44Contig2|CL44Contig2
8.8
8.7
8.6
8.5
8.4
8.3
1
2
t−Test: p−value = 0.29

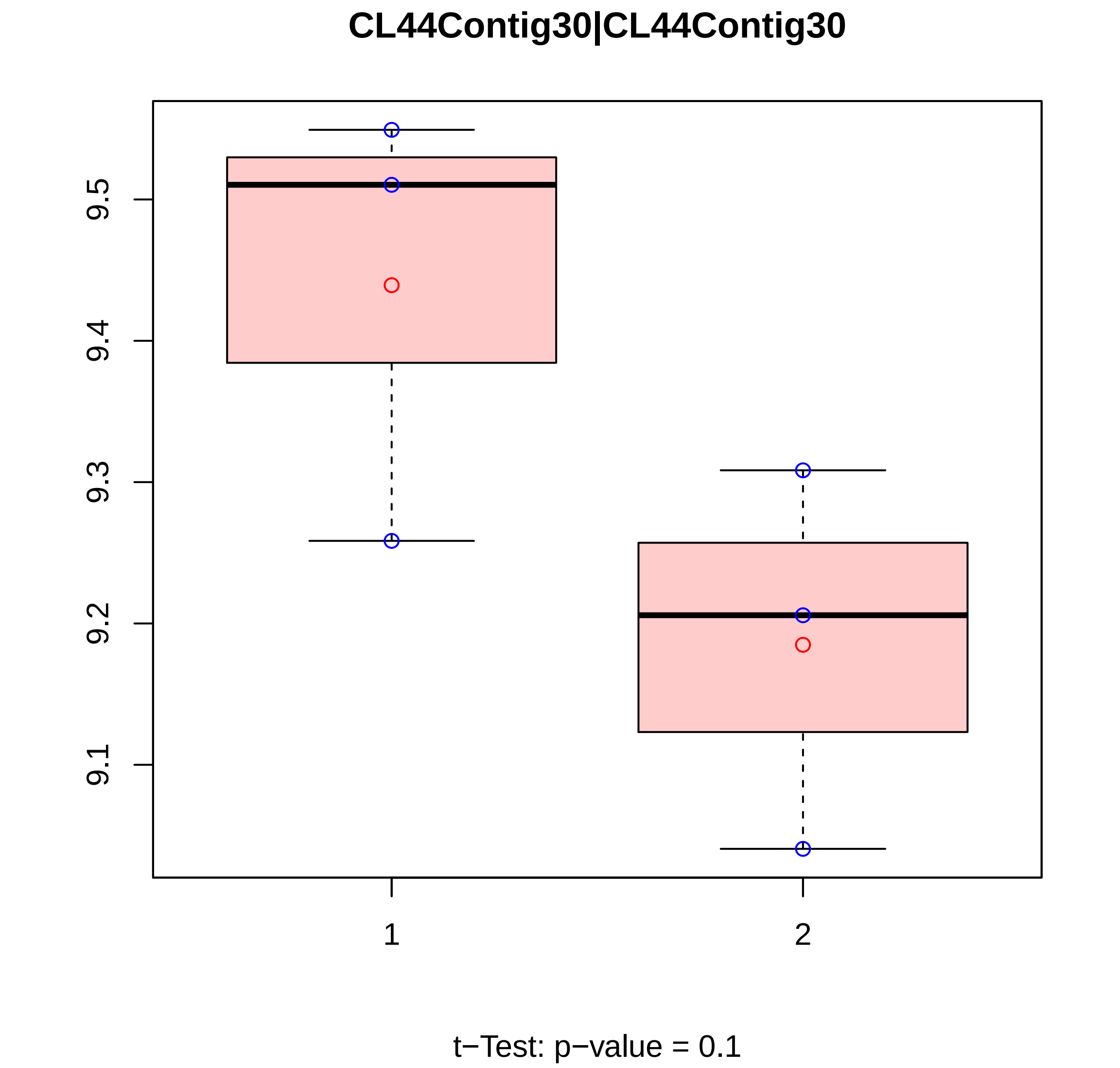
CL44Contig30|CL44Contig30
9.1
9.2
9.3
9.4
9.5
1
2
t−Test: p−value = 0.1

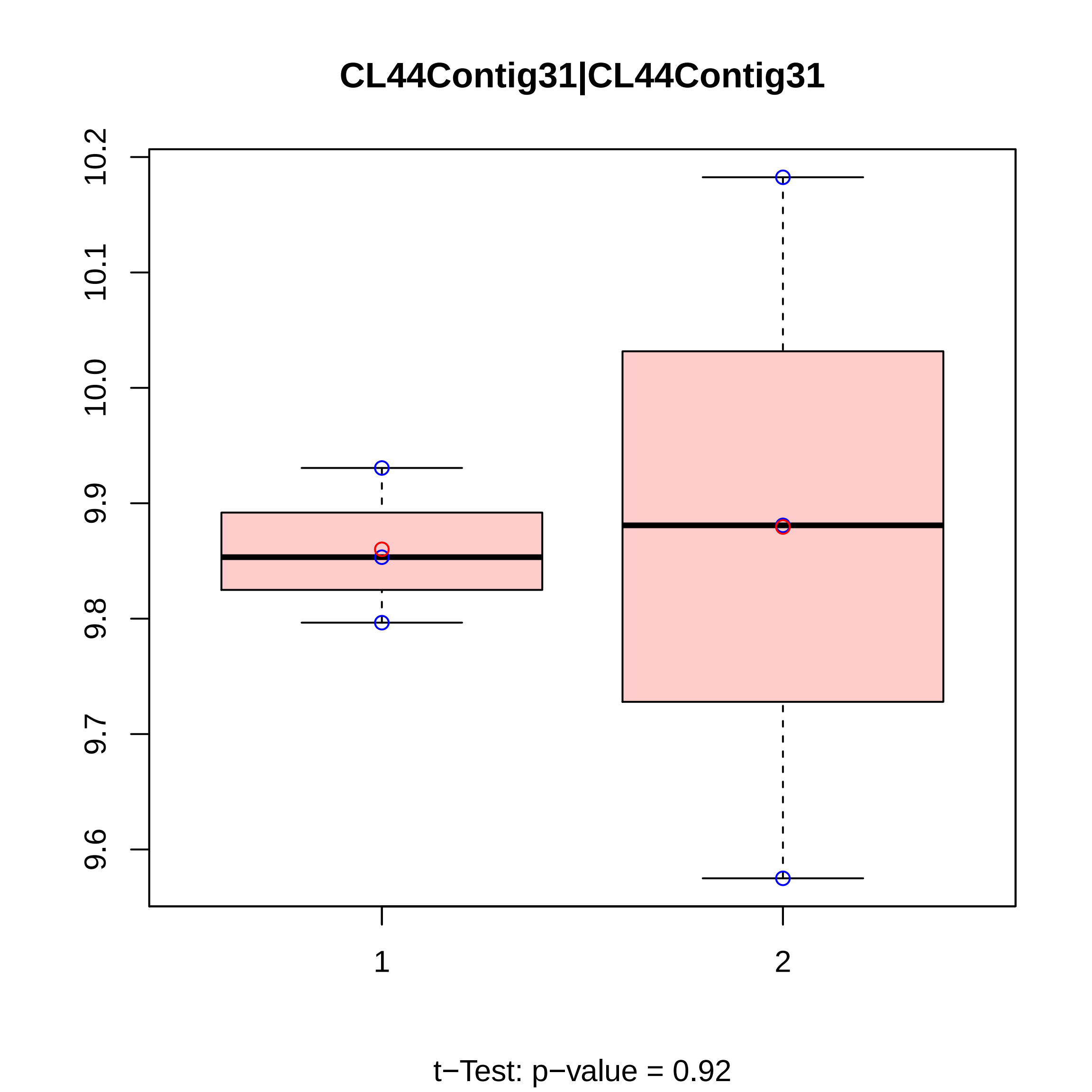

CL44Contig31|CL44Contig31
10.2
10.1
10.0
9.9
9.8
9.7
9.6
1
2
t−Test: p−value = 0.92

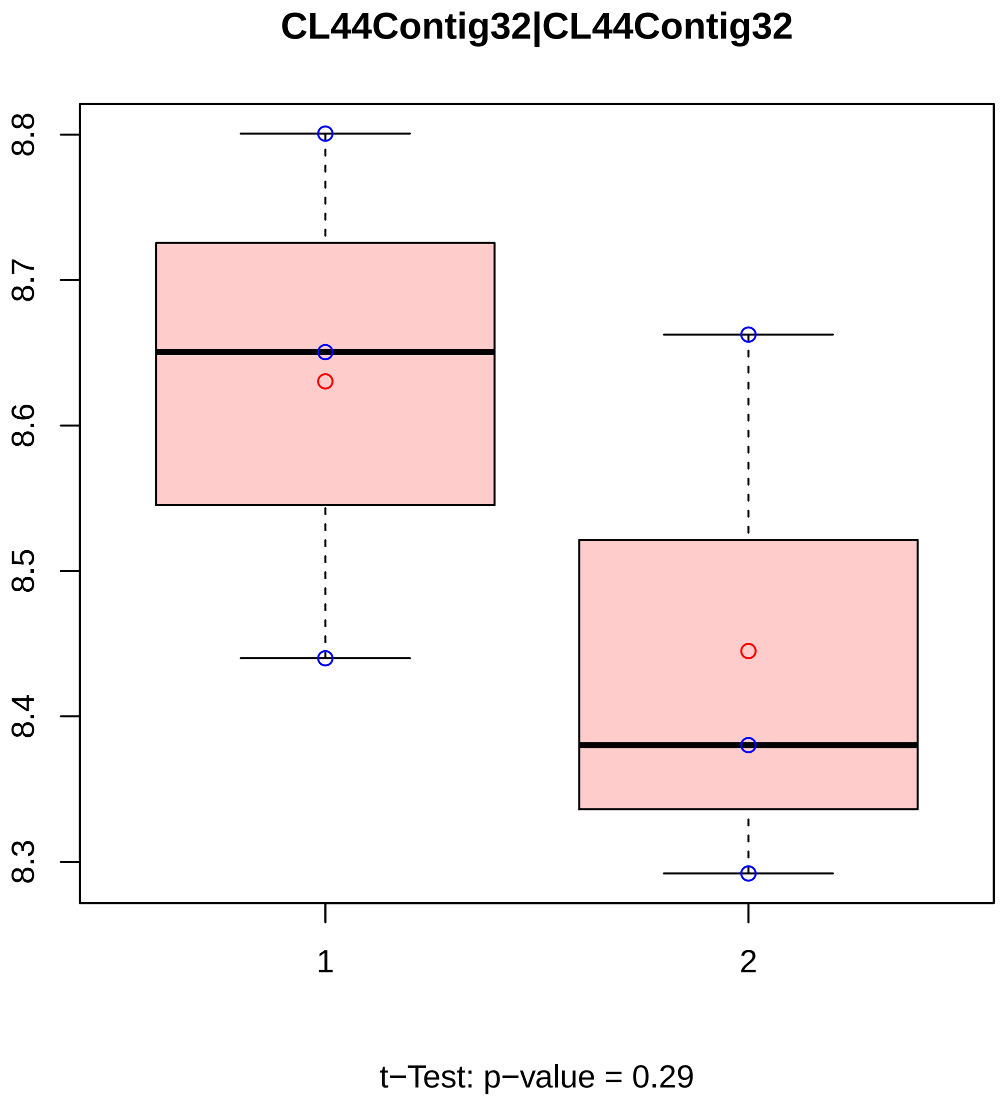
CL44Contig32|CL44Contig32
8.3
8.4
8.5
8.6
8.7
8.8
1
2
t−Test: p−value = 0.29

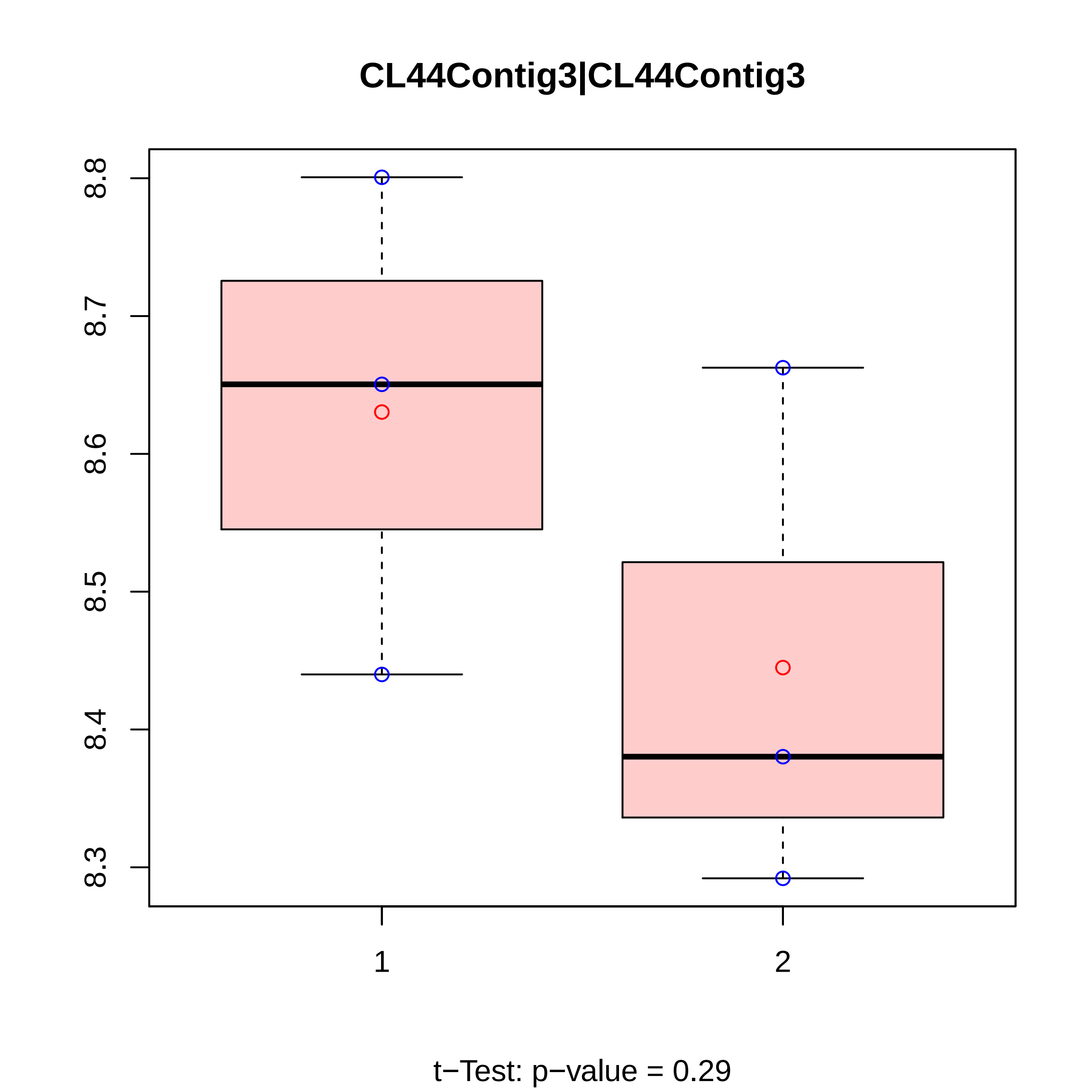
CL44Contig3|CL44Contig3
8.8
8.7
8.6
8.5
8.4
8.3
1
2
t−Test: p−value = 0.29

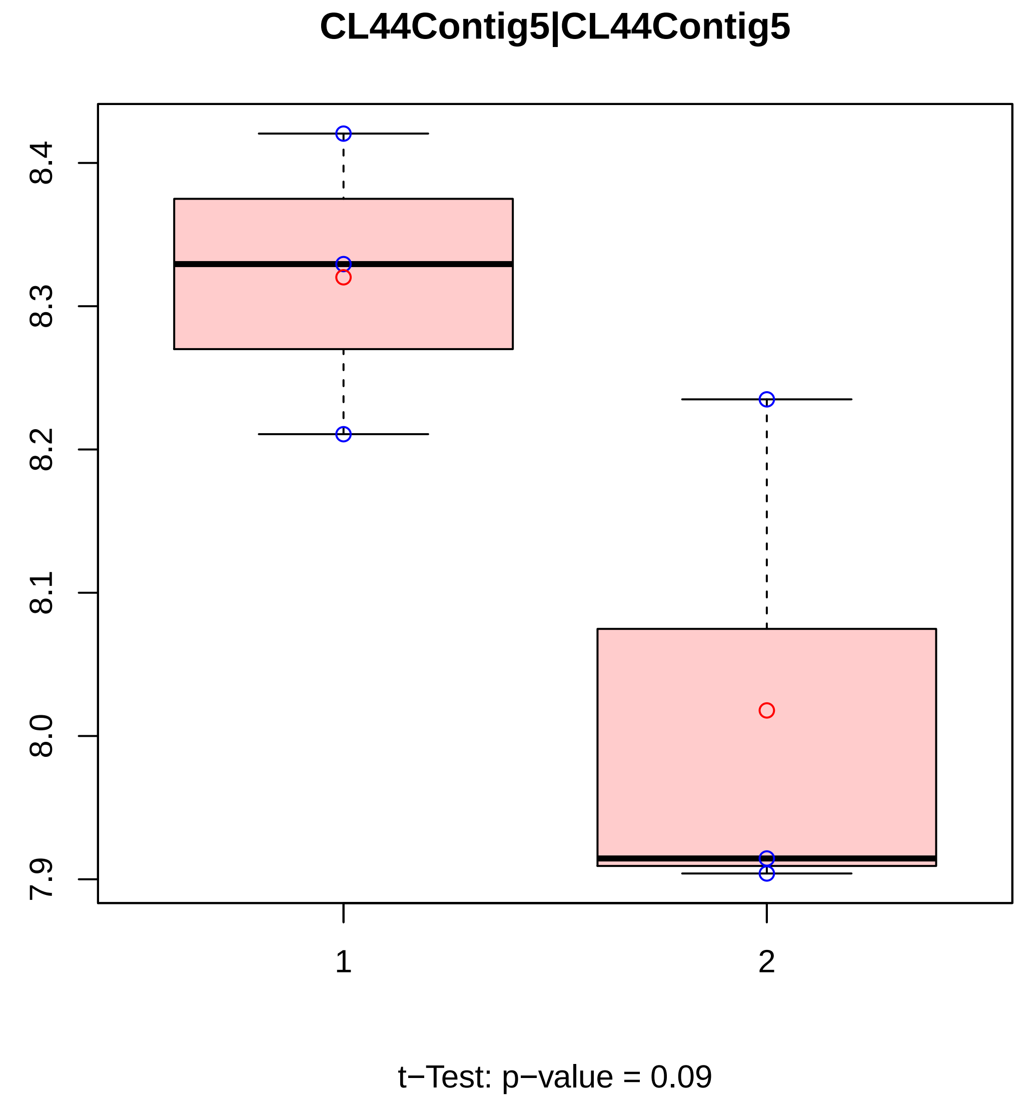
CL44Contig5|CL44Contig5
8.4
8.3
8.2
8.1
8.0
7.9
1
2
t−Test: p−value = 0.09

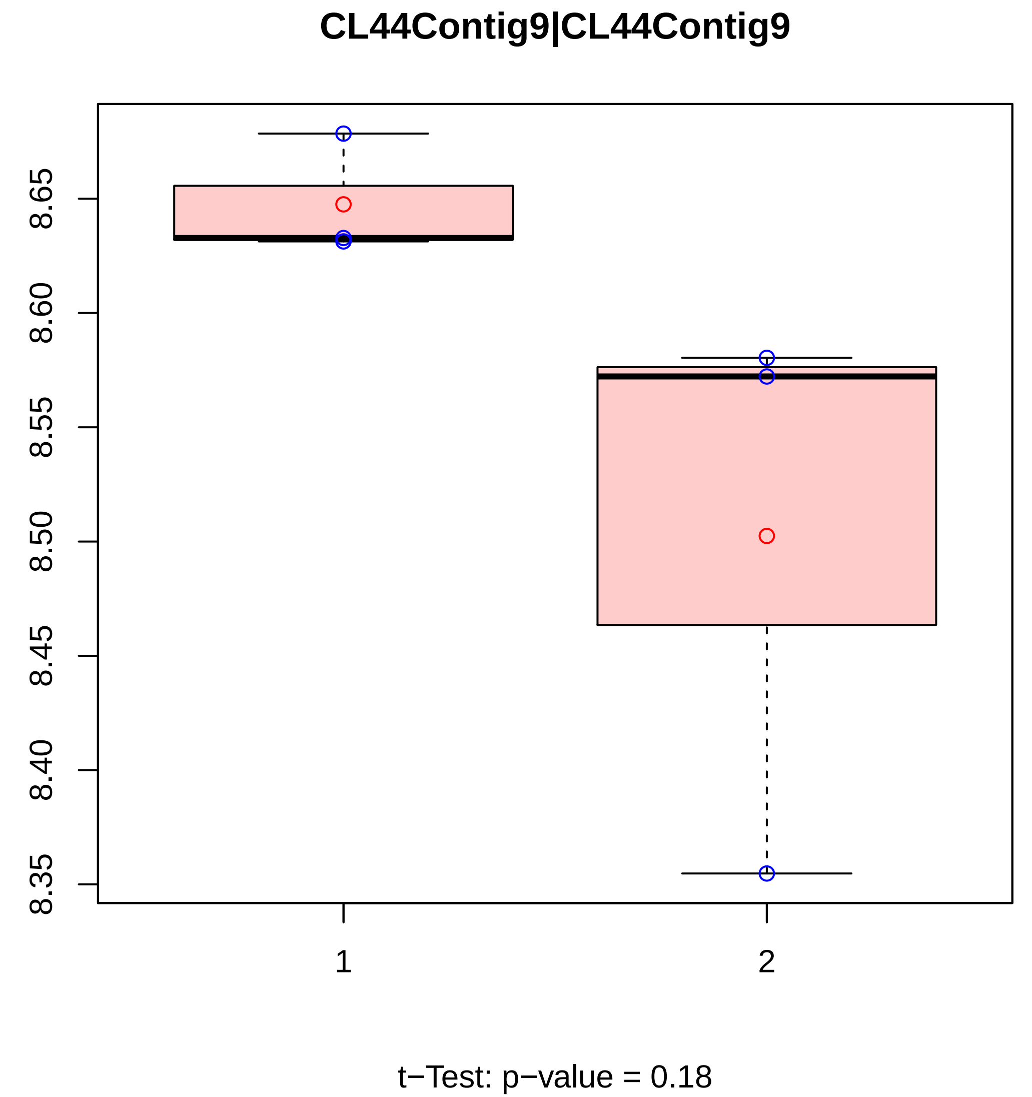
CL44Contig9|CL44Contig9
8.35
8.40
8.45
8.50
8.55
8.60
8.65
1
2
t−Test: p−value = 0.18

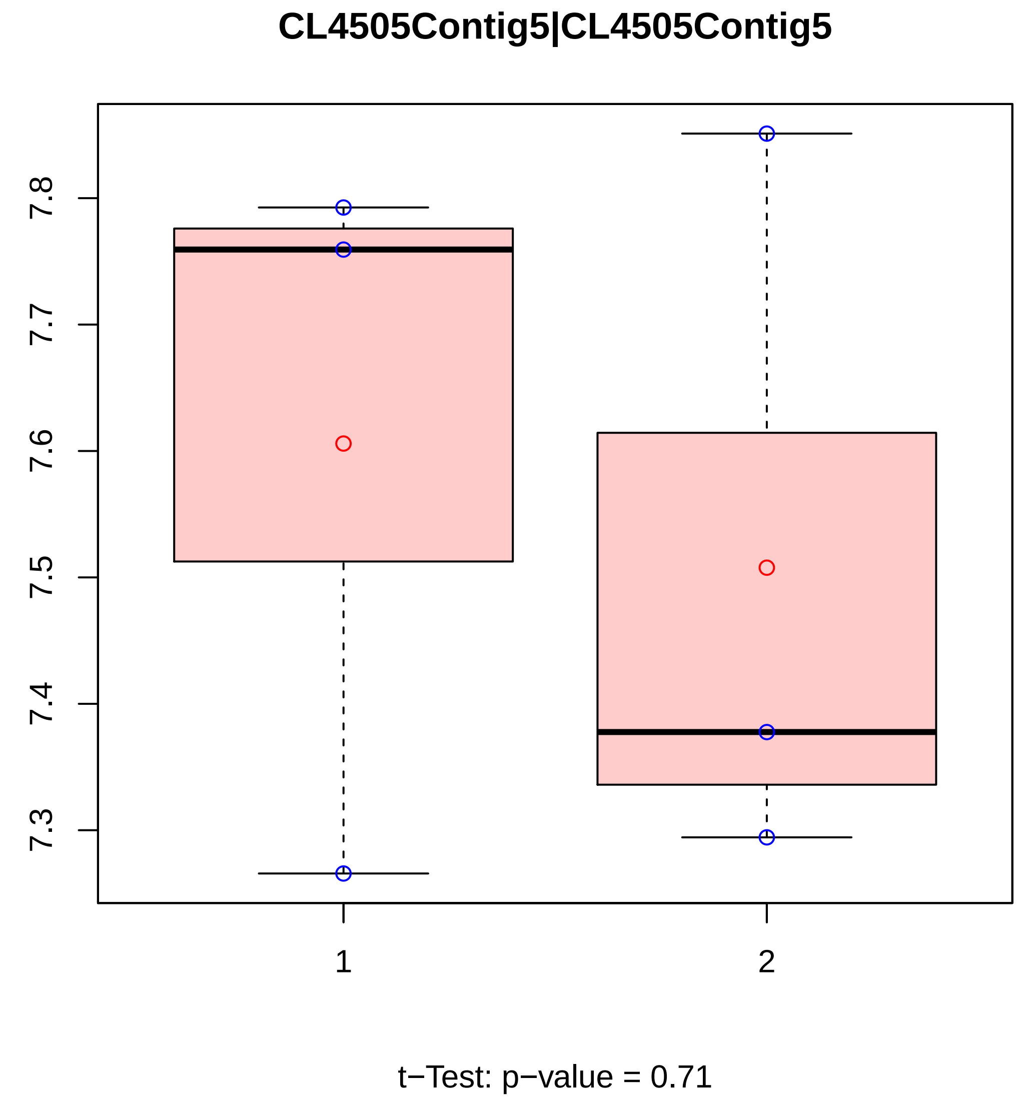

CL4505Contig5|CL4505Contig5
7.3
7.4
7.5
7.6
7.7
7.8
1
2
t−Test: p−value = 0.71

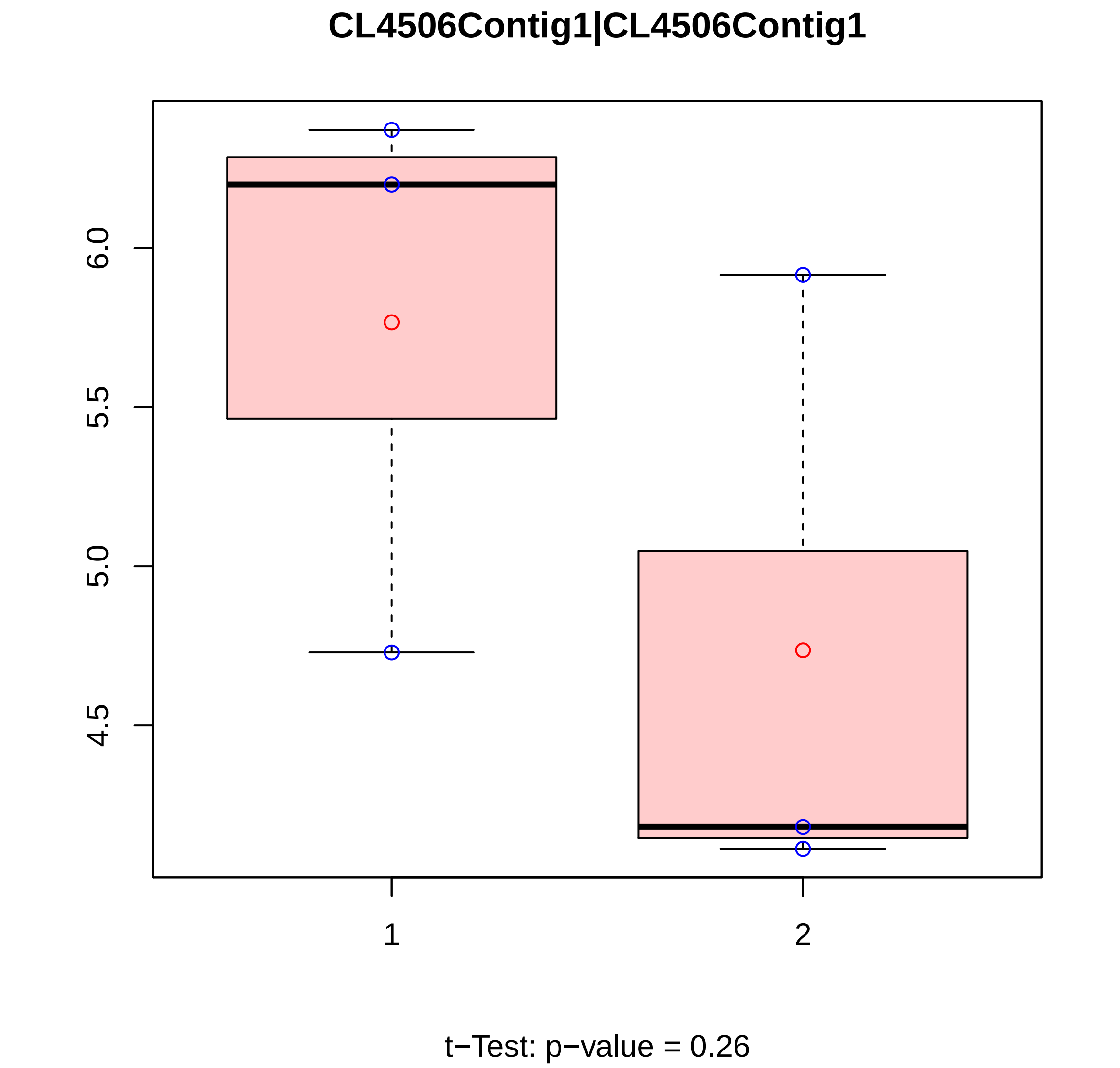
CL4506Contig1|CL4506Contig1
6.0
5.5
5.0
4.5
1
2
t−Test: p−value = 0.26

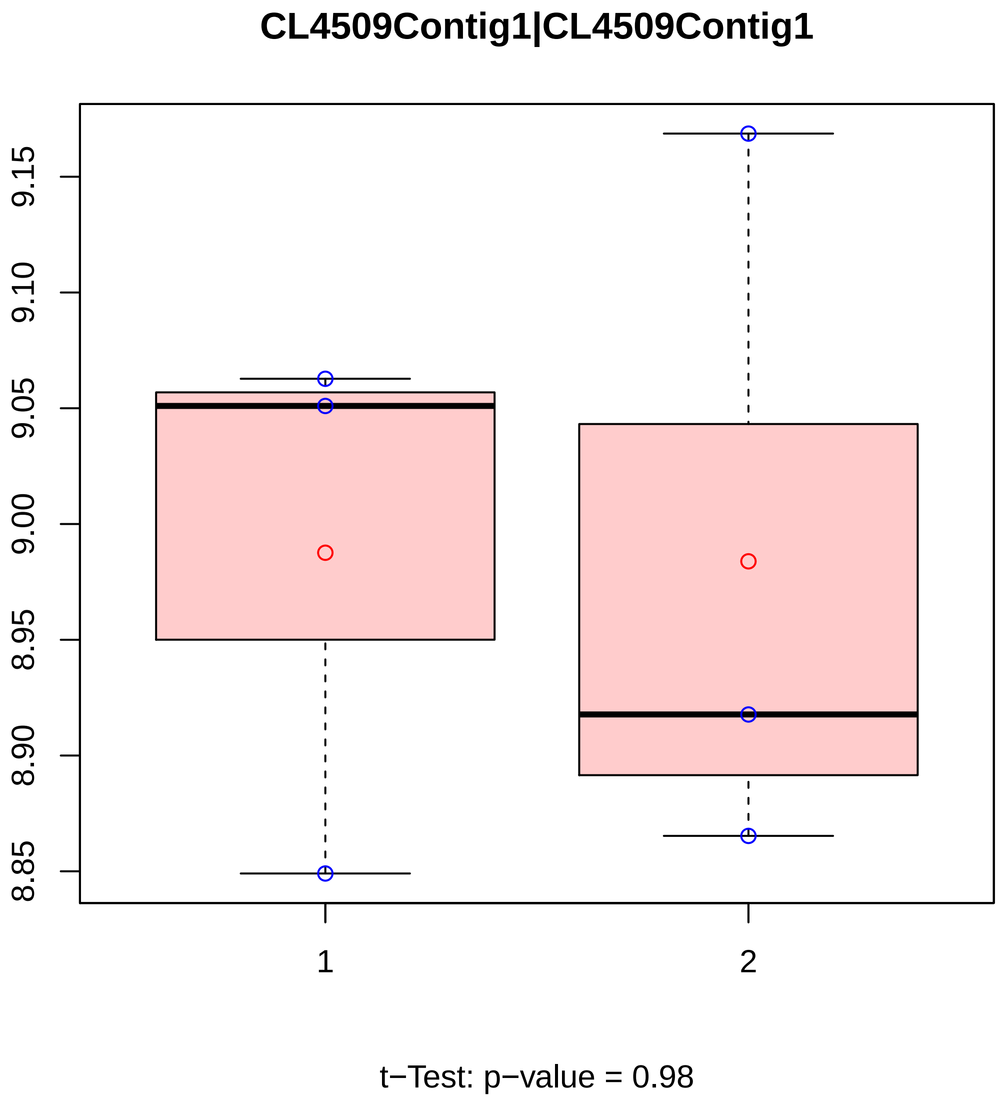
CL4509Contig1|CL4509Contig1
9.15
9.10
9.05
9.00
8.95
8.90
8.85
1
2
t−Test: p−value = 0.98

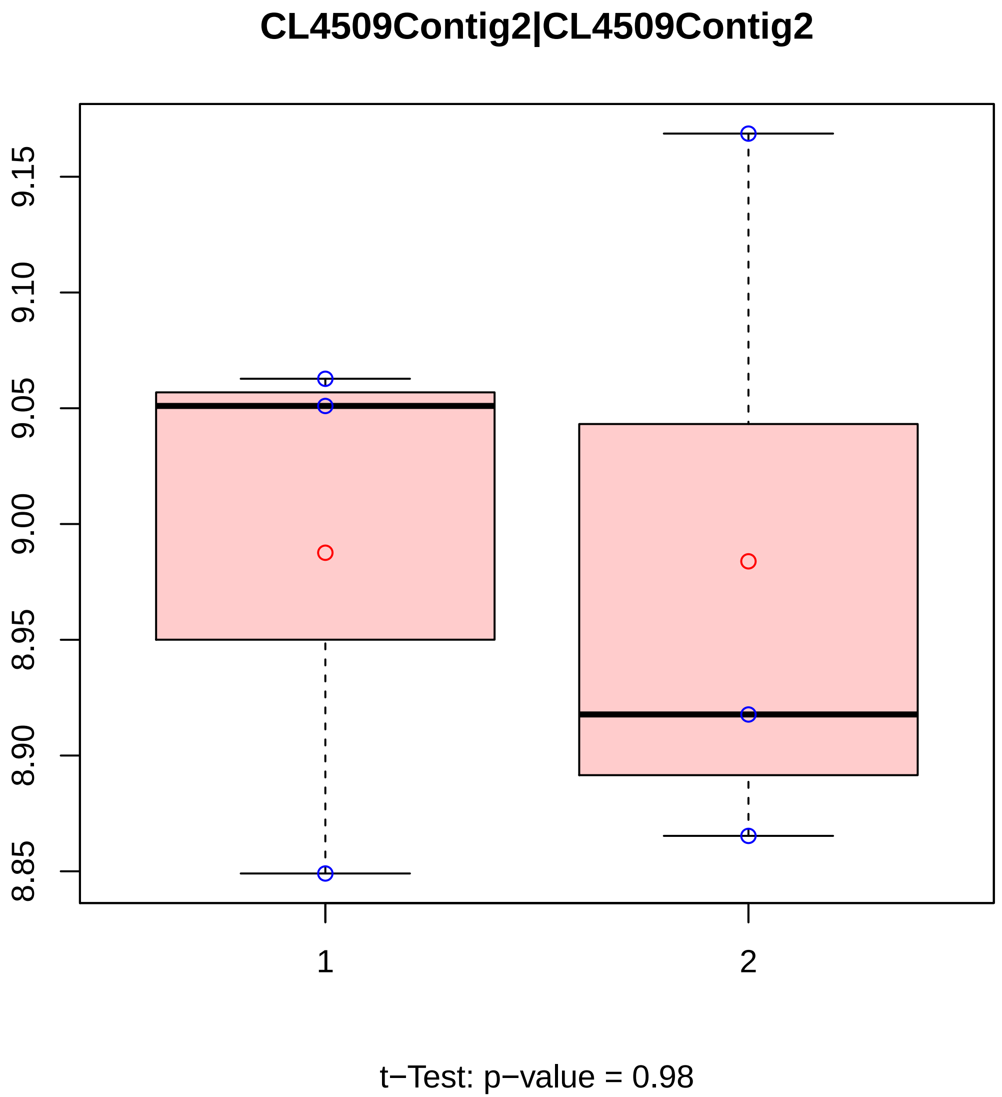

CL4509Contig2|CL4509Contig2
9.15
9.10
9.05
9.00
8.95
8.90
8.85
1
2
t−Test: p−value = 0.98

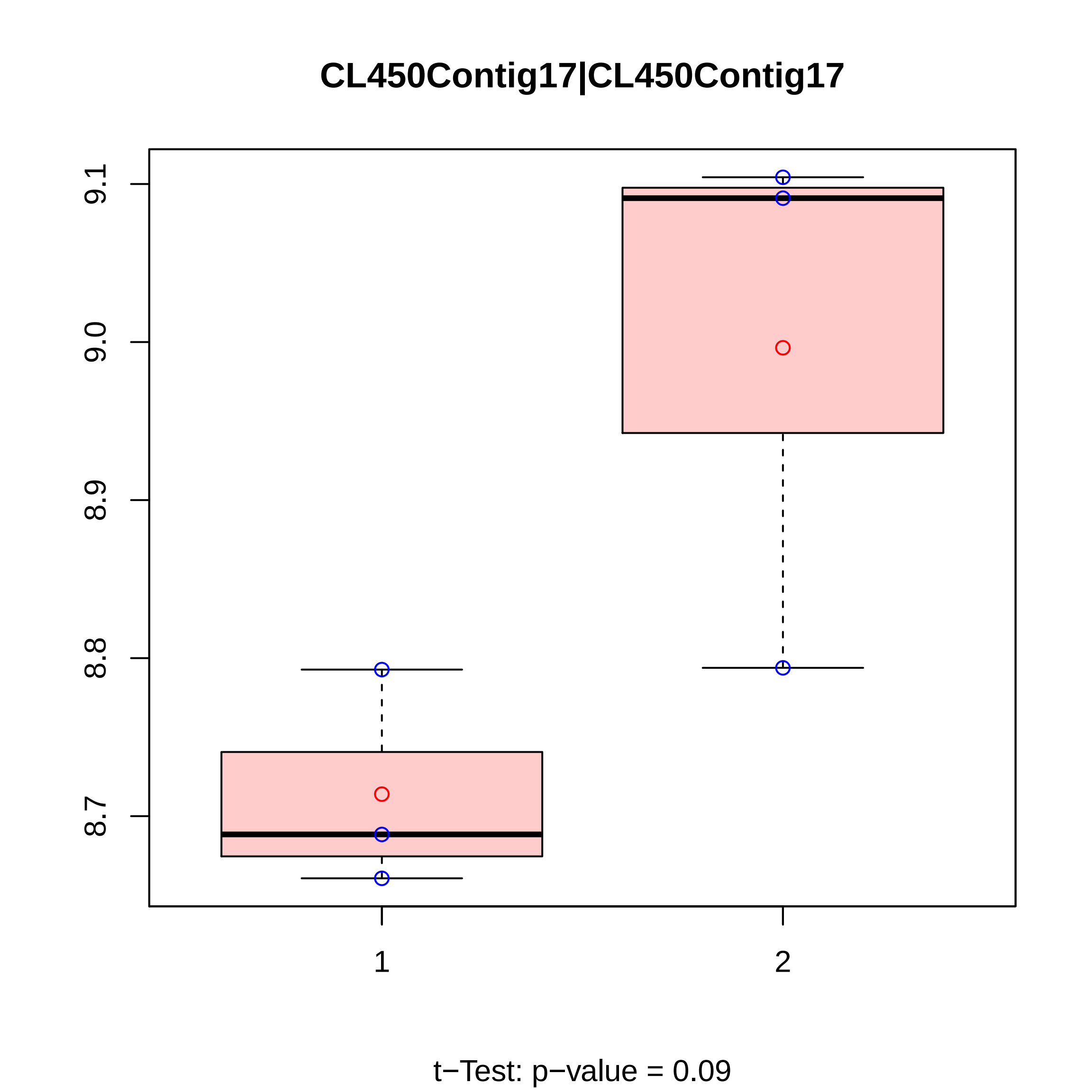

CL450Contig17|CL450Contig17
9.1
9.0
8.9
8.8
8.7
1
2
t−Test: p−value = 0.09

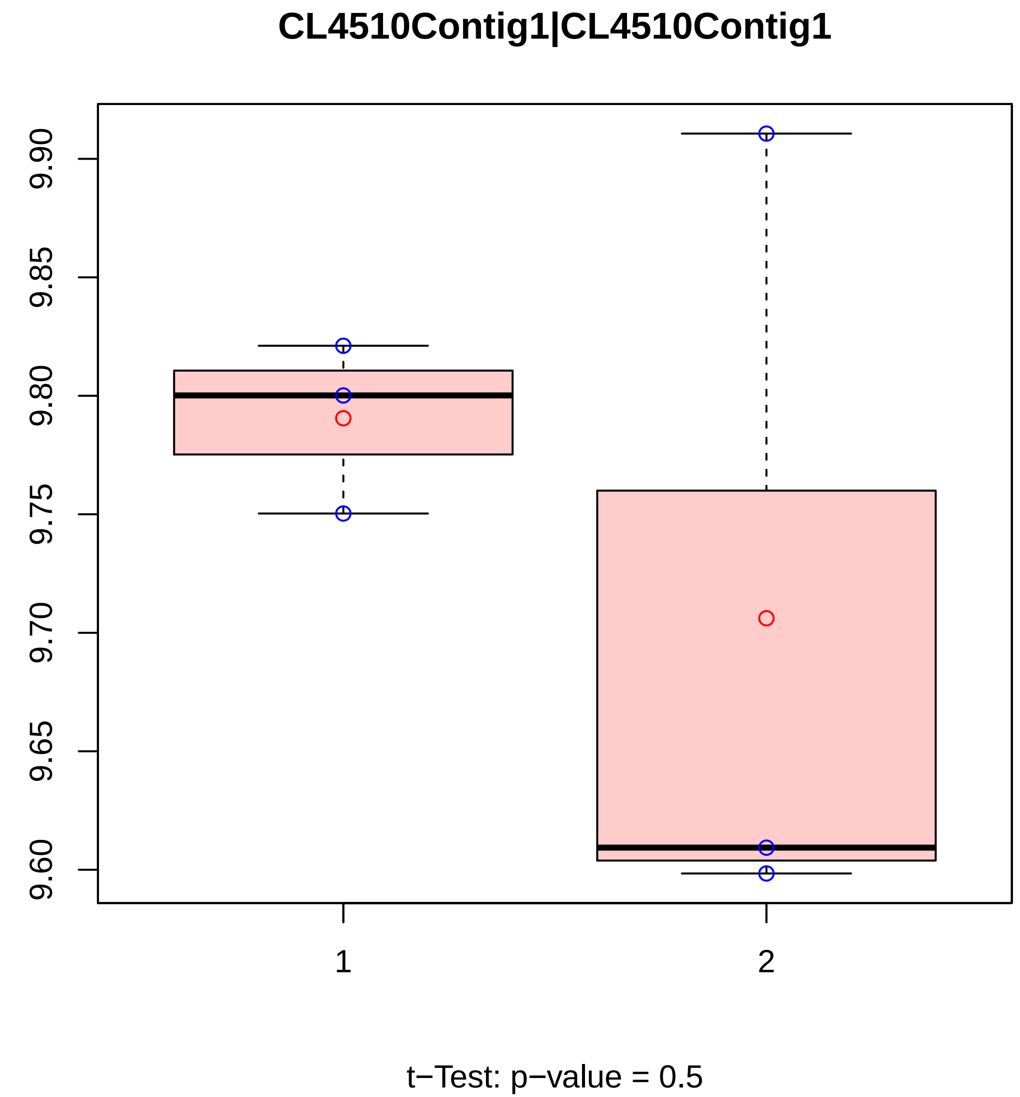
CL4510Contig1|CL4510Contig1
9.90
9.85
9.80
9.75
9.70
9.65
9.60
1
2
t−Test: p−value = 0.5

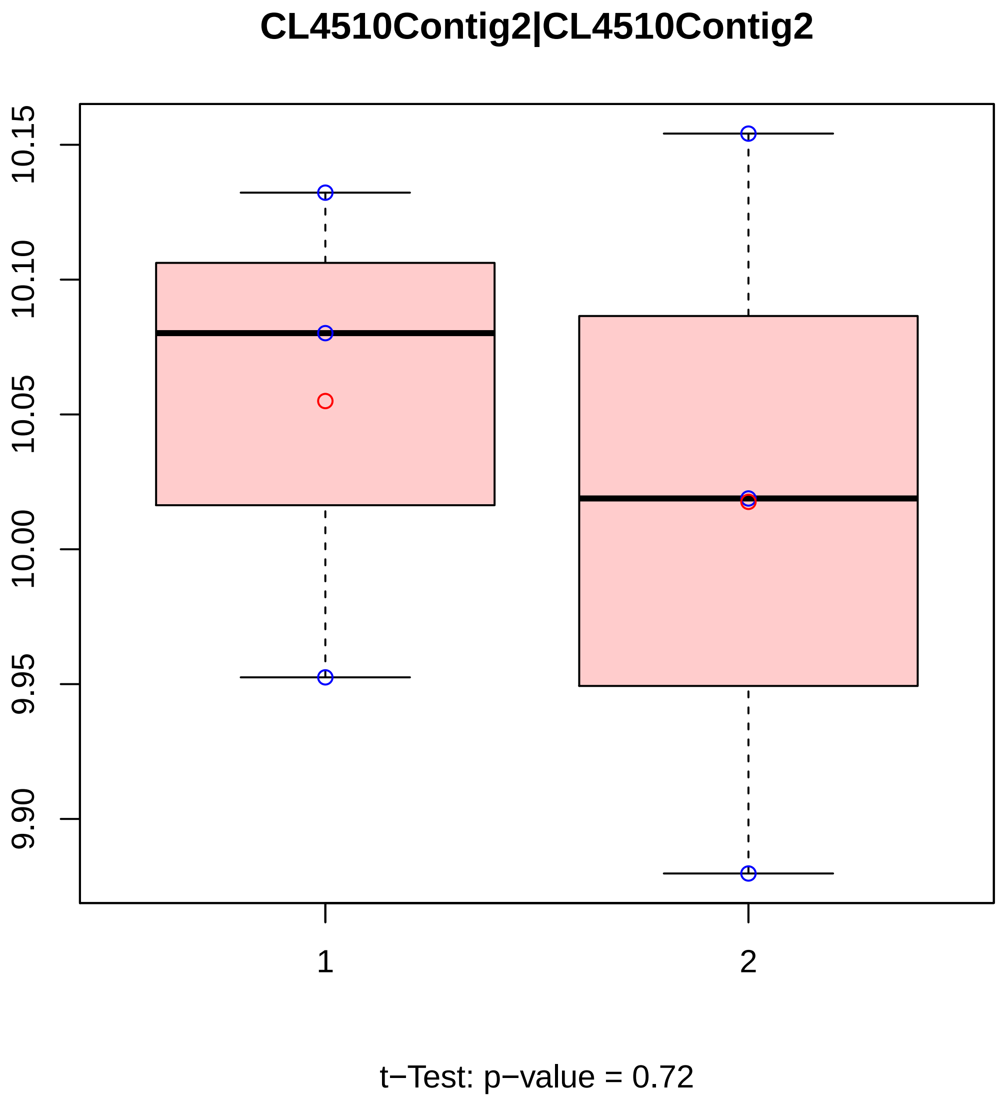
CL4510Contig2|CL4510Contig2
10.15
10.10
10.05
10.00
9.95
9.90
1
2
t−Test: p−value = 0.72

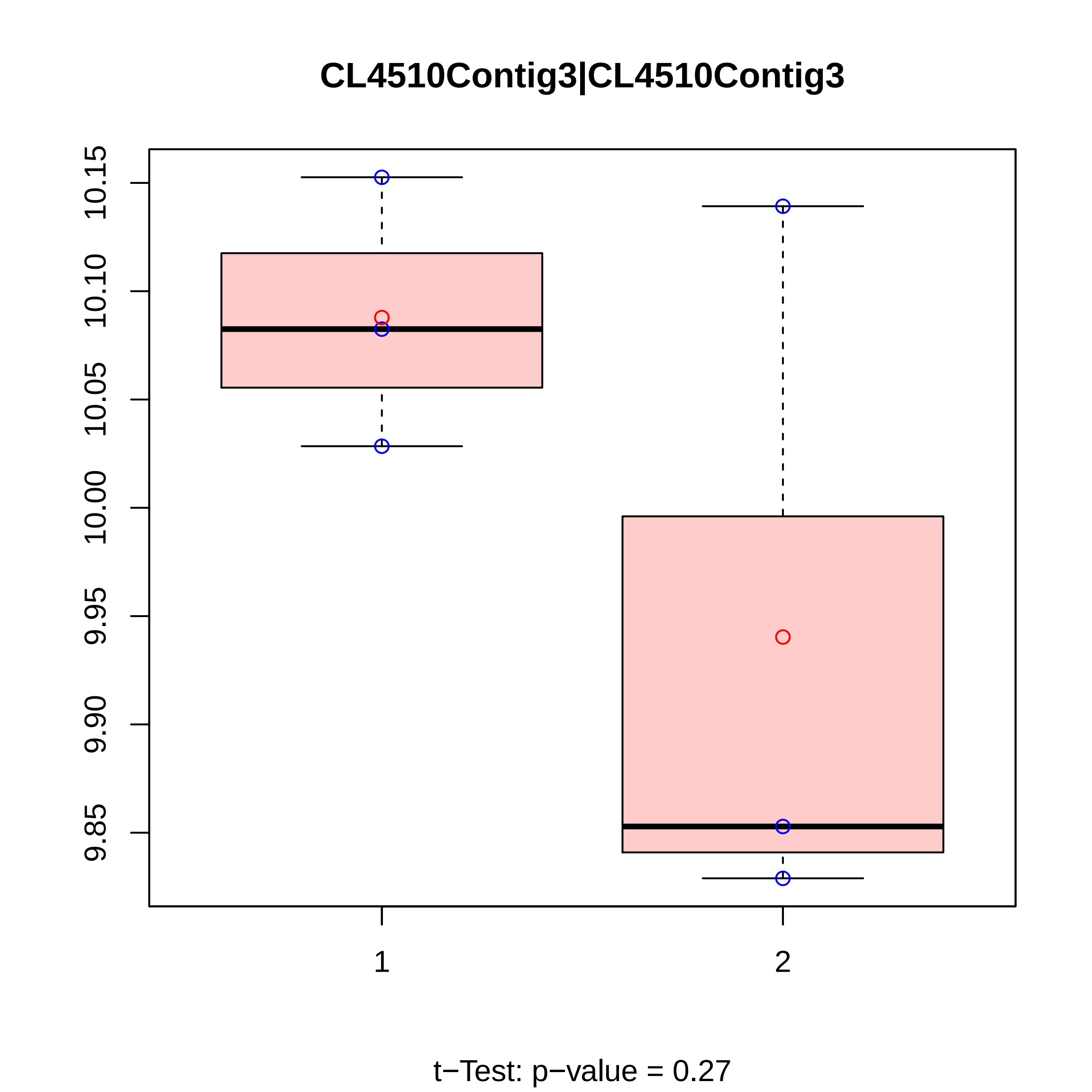
CL4510Contig3|CL4510Contig3
10.15
10.10
10.05
10.00
9.95
9.90
9.85
1
2
t−Test: p−value = 0.27

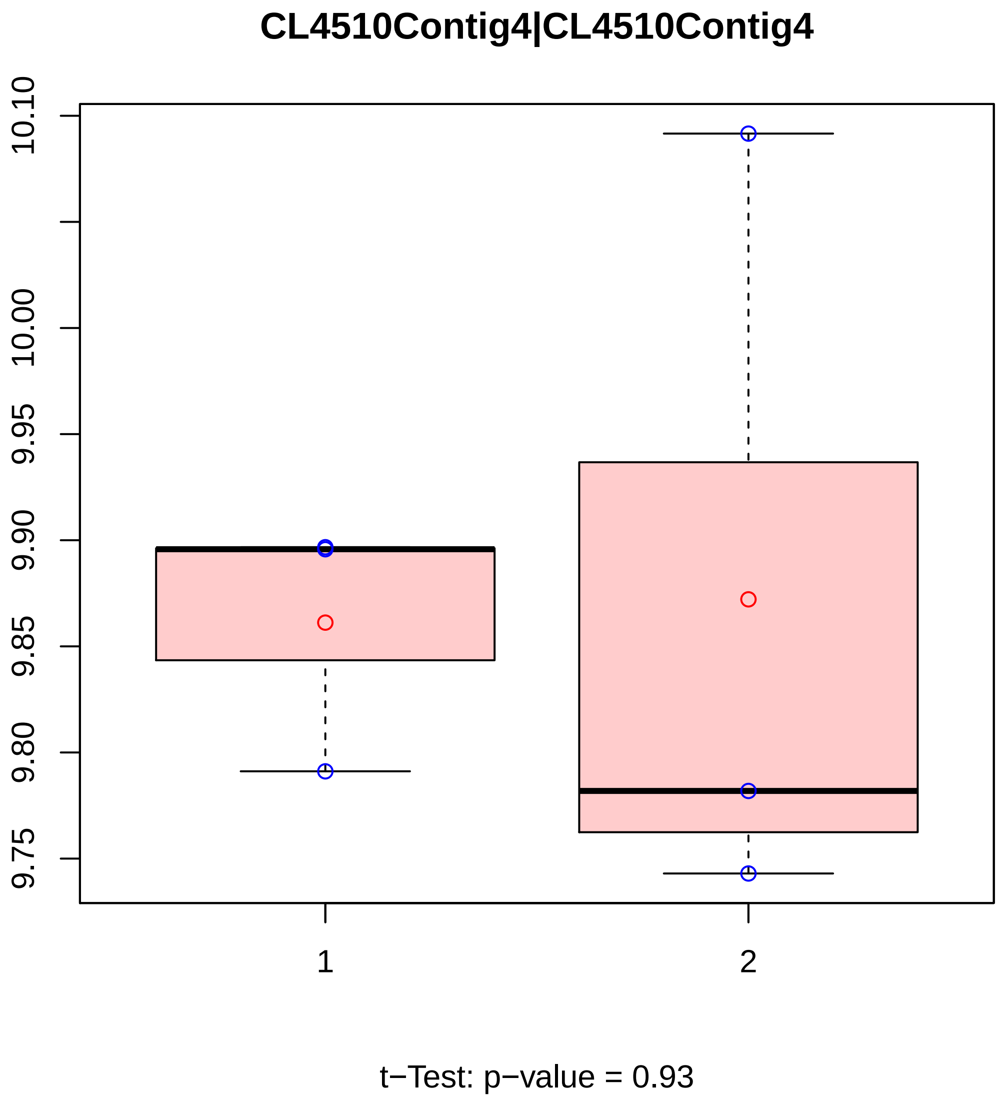

CL4510Contig4|CL4510Contig4
10.10
10.00
9.95
9.90
9.85
9.80
9.75
1
2
t−Test: p−value = 0.93

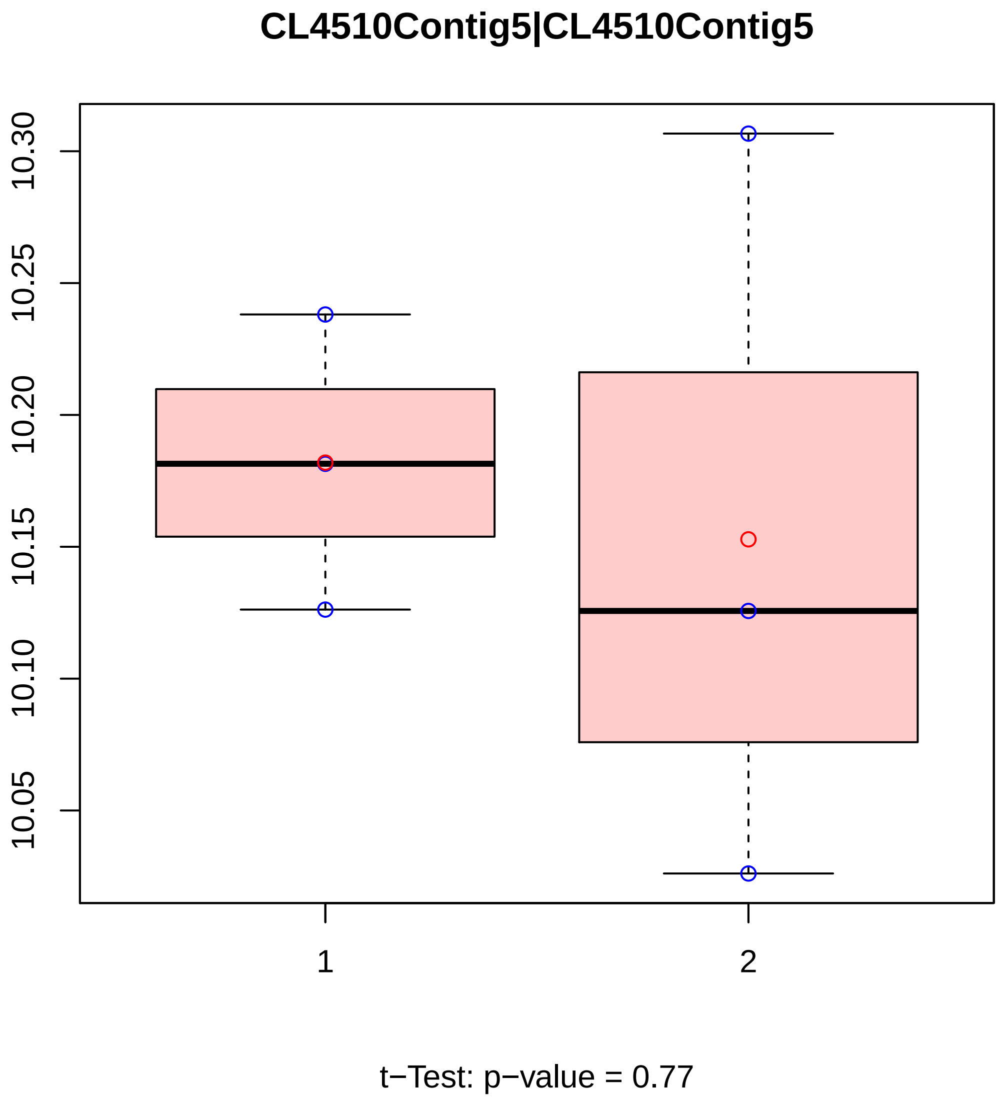
CL4510Contig5|CL4510Contig5
10.05
10.10
10.15
10.20
10.25
10.30
1
2
t−Test: p−value = 0.77

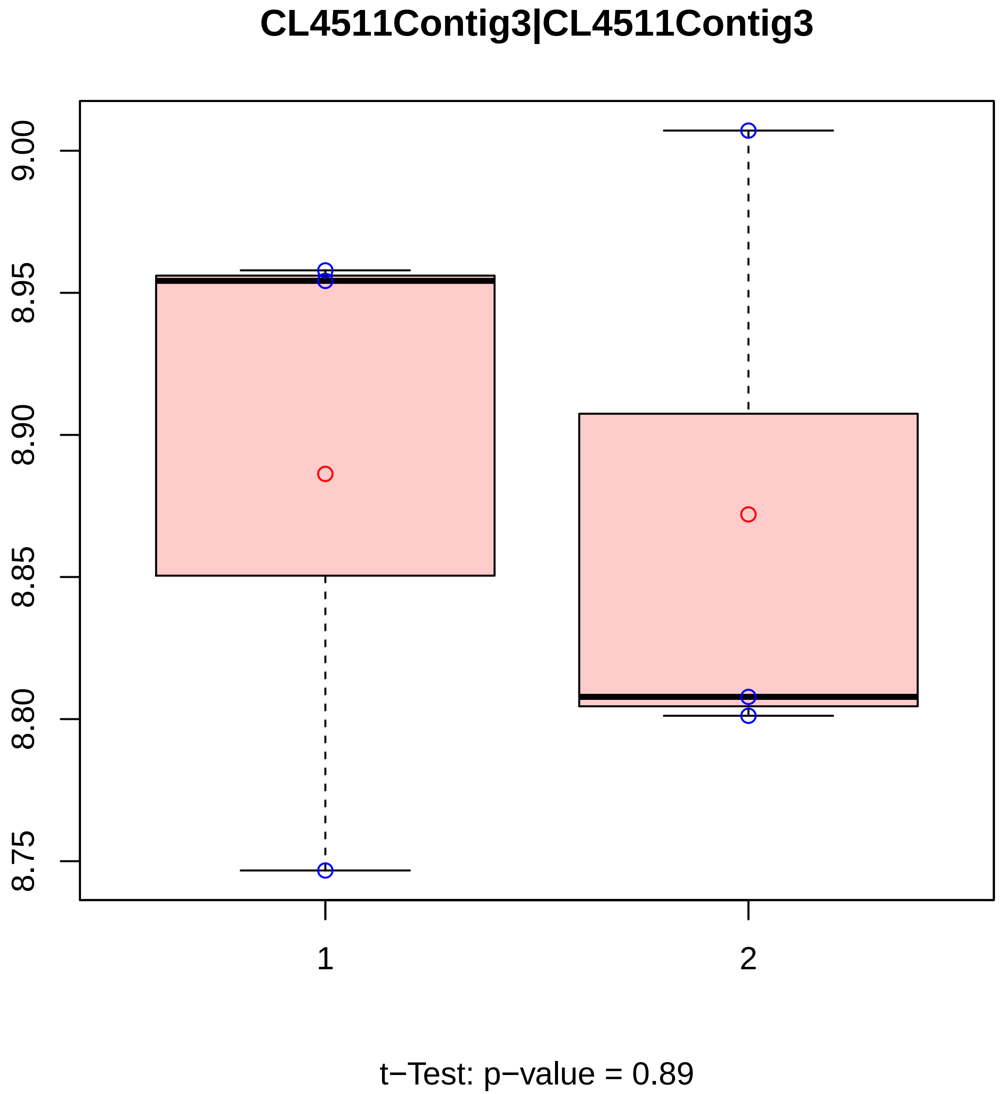
CL4511Contig3|CL4511Contig3
9.00
8.95
8.90
8.85
8.80
8.75
1
2
t−Test: p−value = 0.89

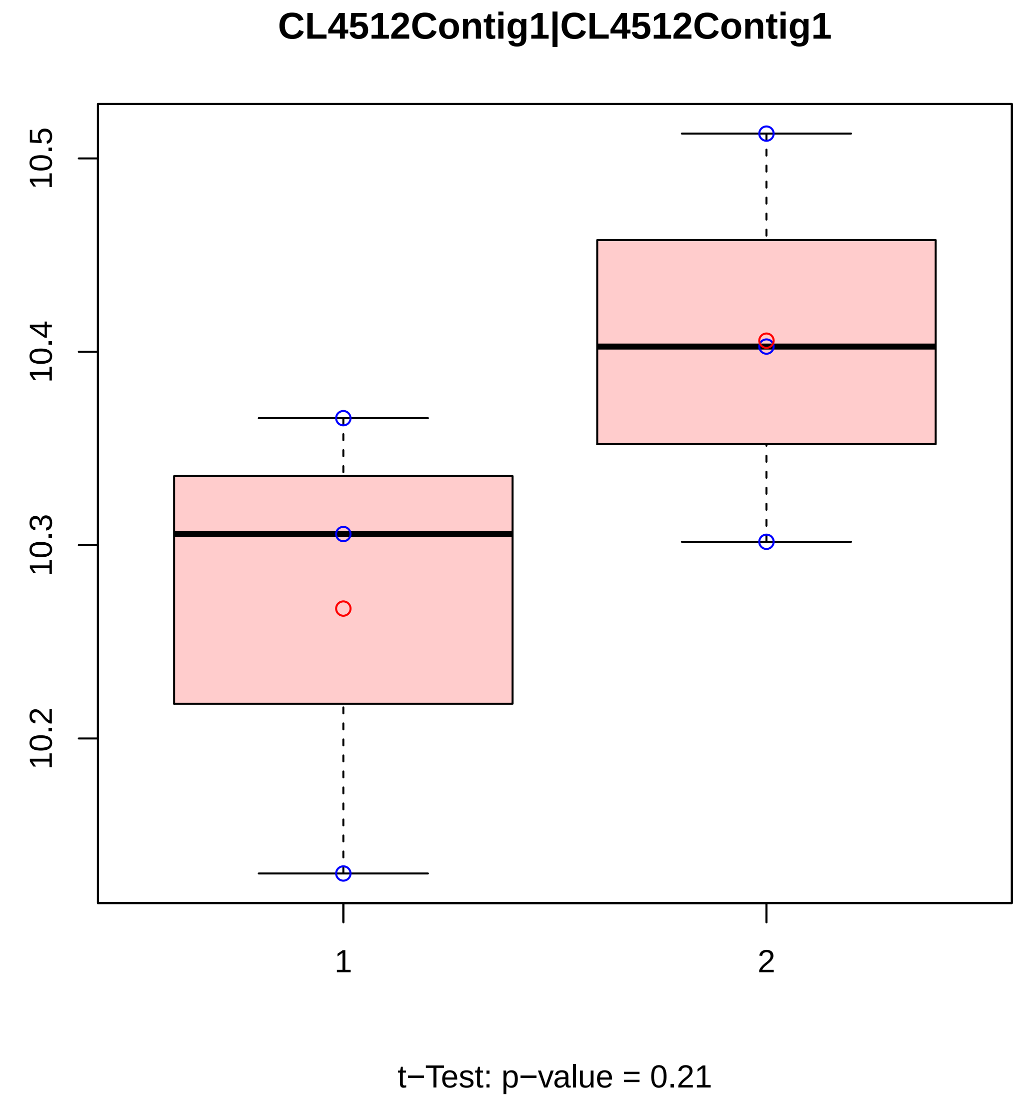
CL4512Contig1|CL4512Contig1
10.5
10.4
10.3
10.2
1
2
t−Test: p−value = 0.21

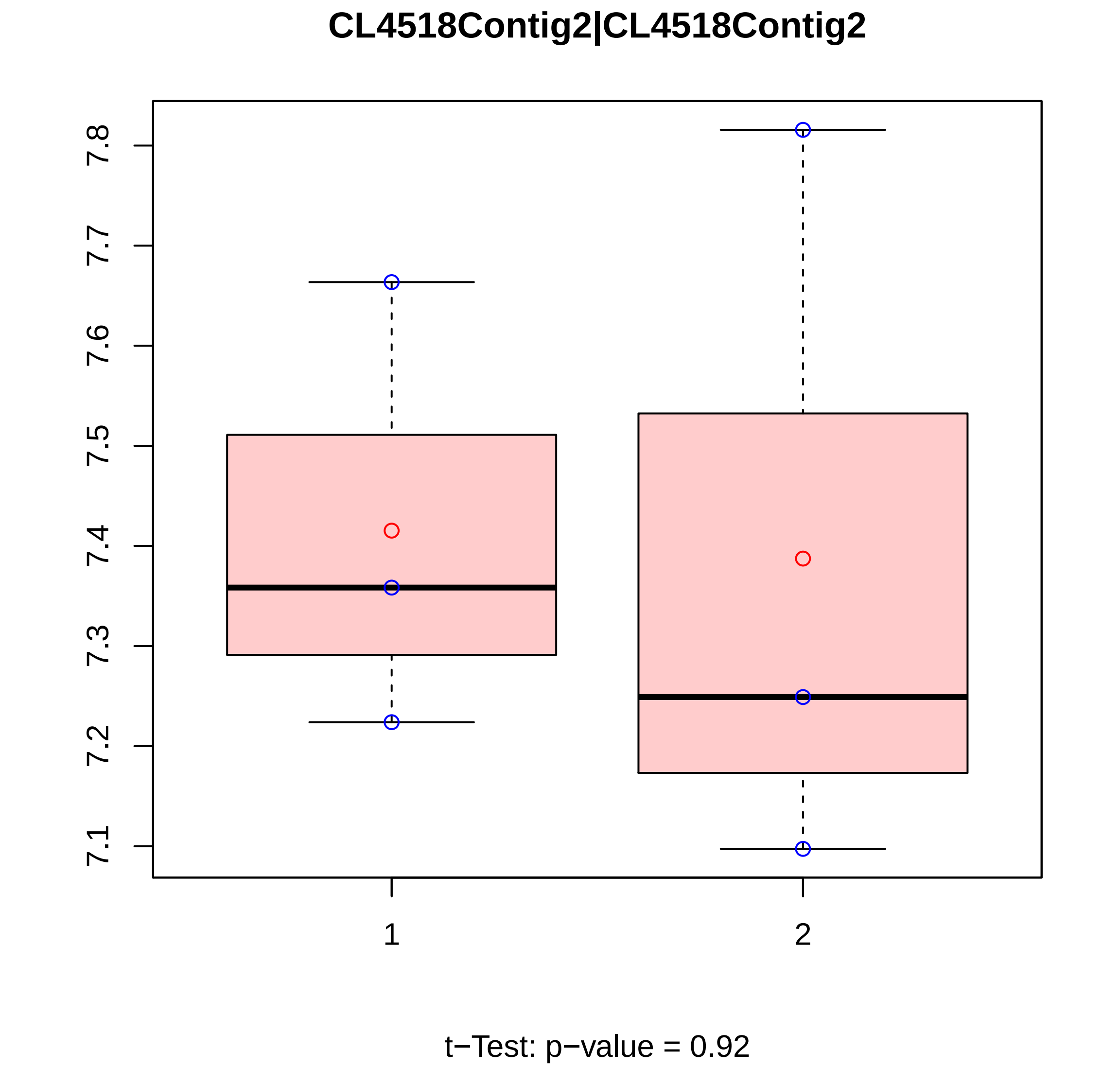
CL4518Contig2|CL4518Contig2
7.1
7.2
7.3
7.4
7.5
7.6
7.7
7.8
1
2
t−Test: p−value = 0.92

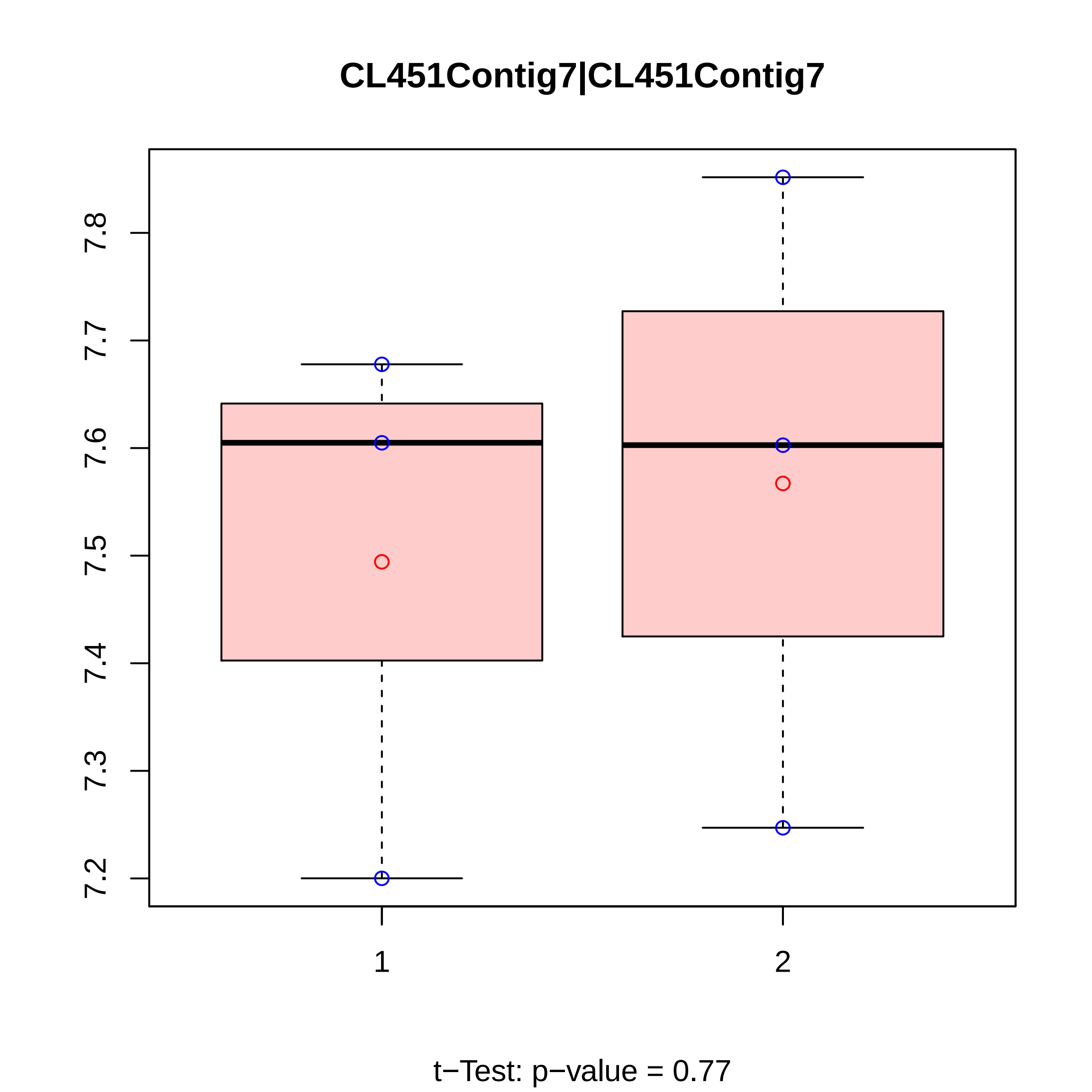
CL451Contig7|CL451Contig7
7.2
7.3
7.4
7.5
7.6
7.7
7.8
1
2
t−Test: p−value = 0.77

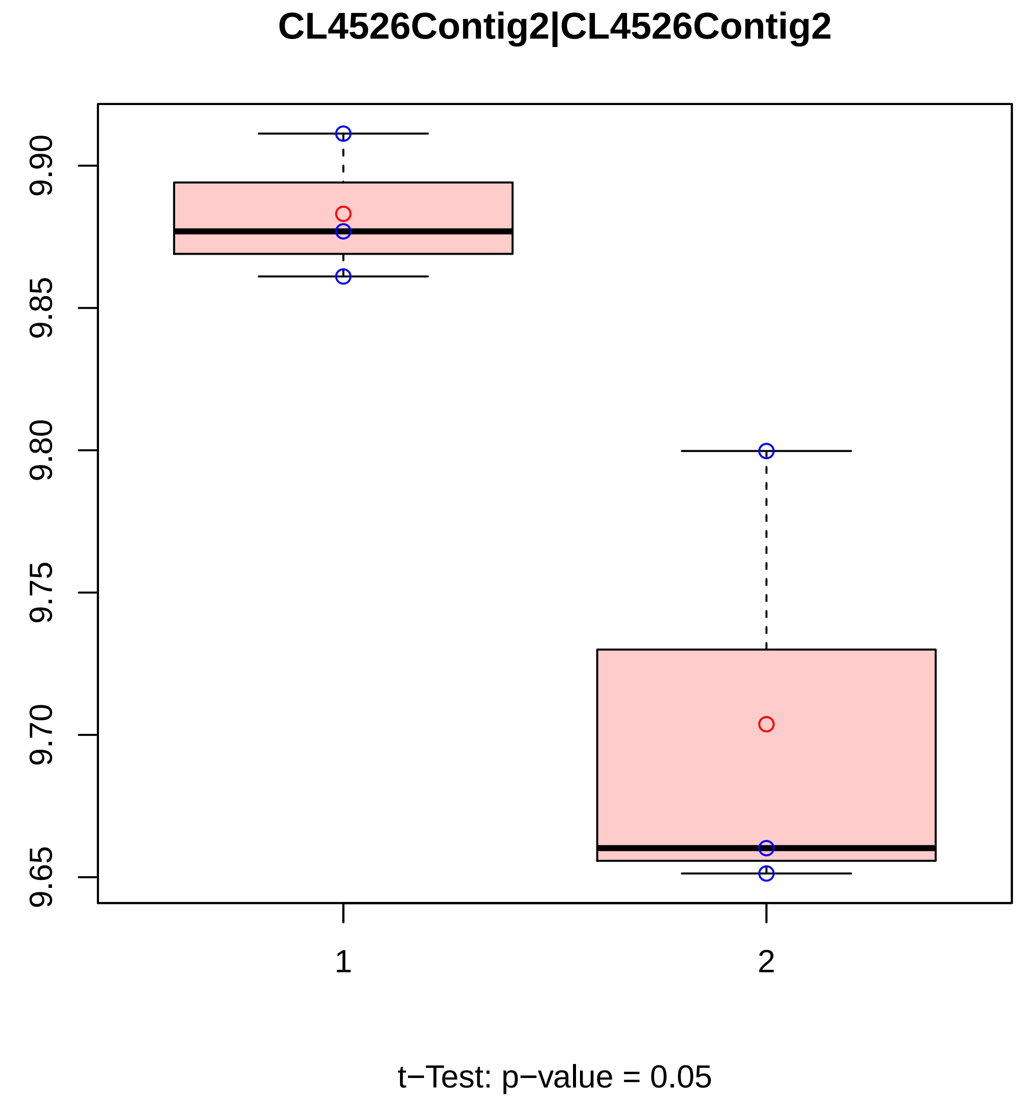
CL4526Contig2|CL4526Contig2
9.65
9.70
9.75
9.80
9.85
9.90
1
2
t−Test: p−value = 0.05

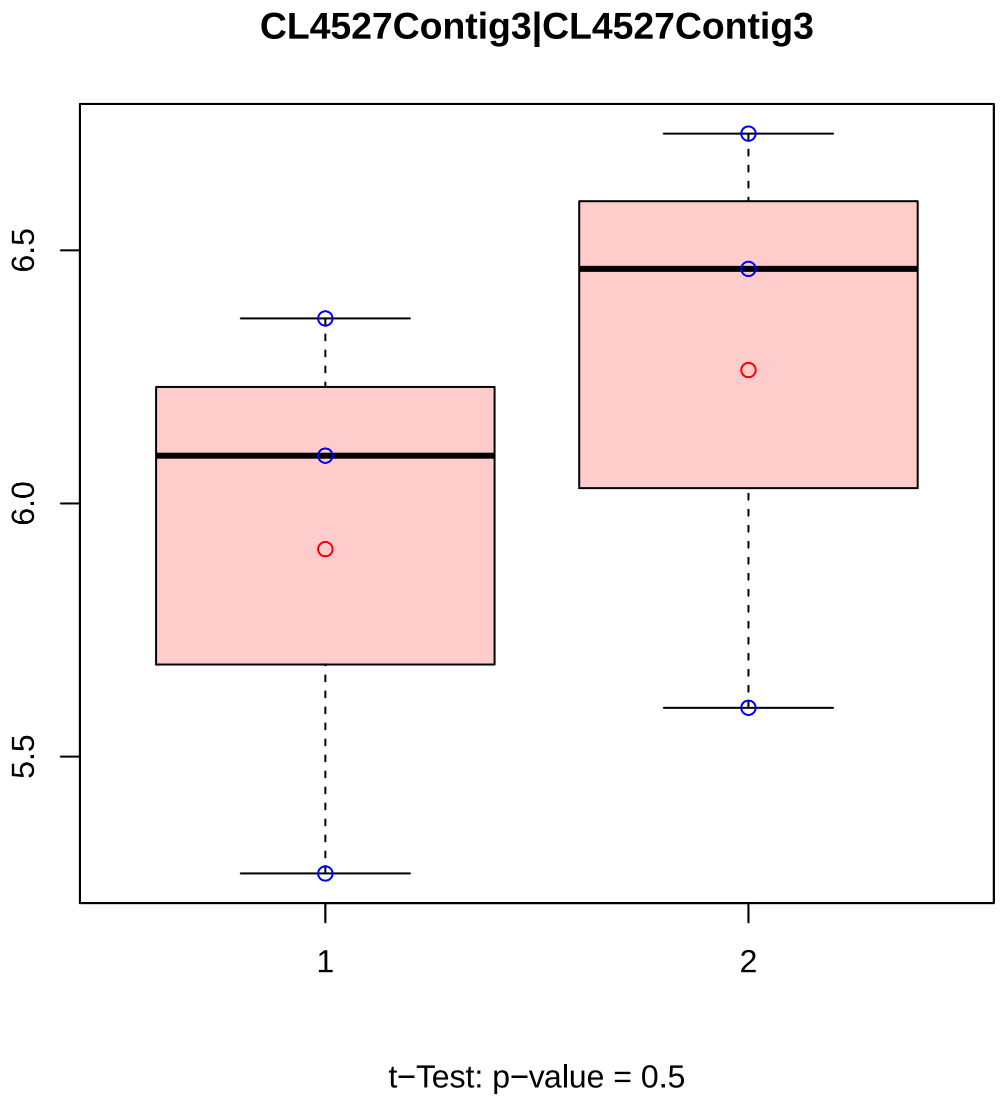
CL4527Contig3|CL4527Contig3
6.5
6.0
5.5
1
2
t−Test: p−value = 0.5

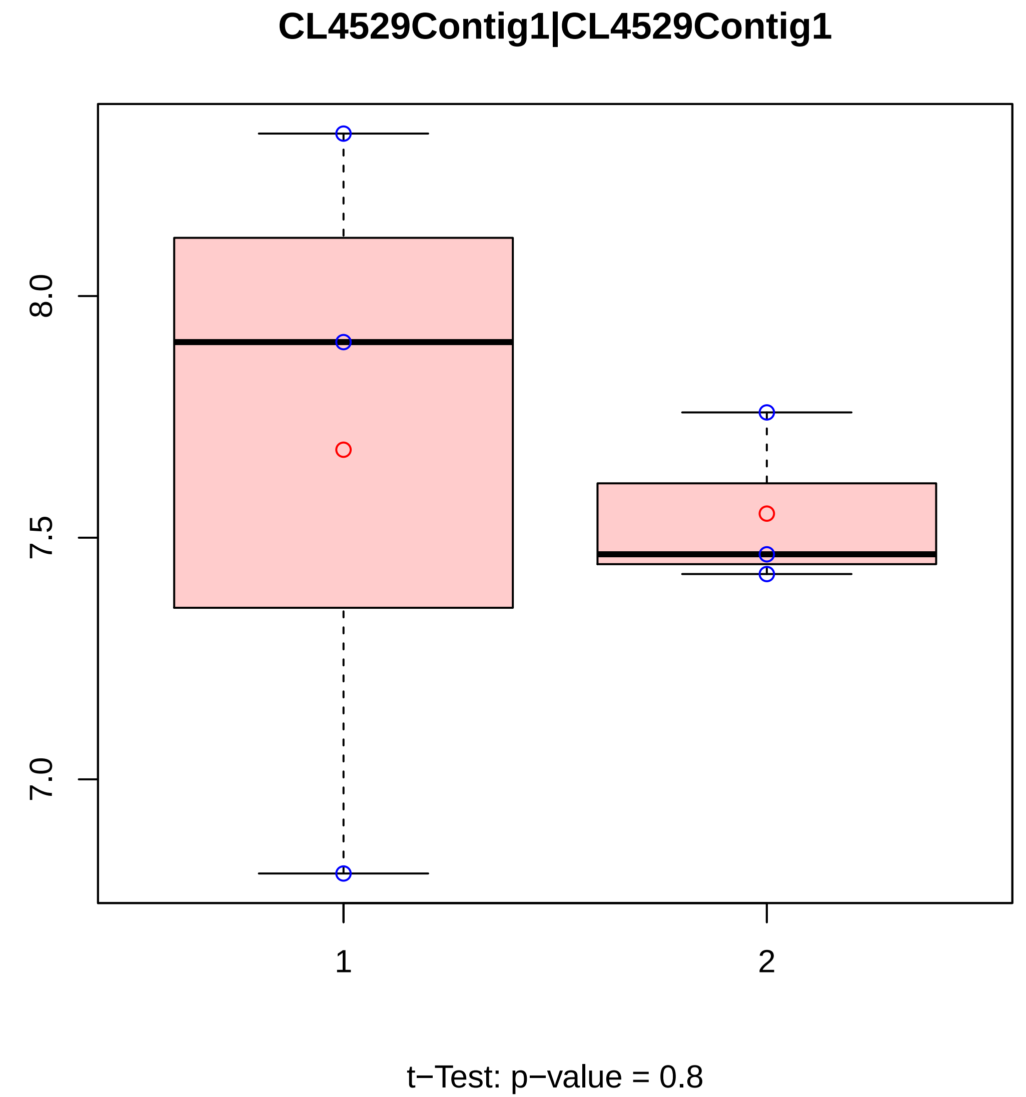

CL4529Contig1|CL4529Contig1
8.0
7.5
7.0
1
2
t−Test: p−value = 0.8

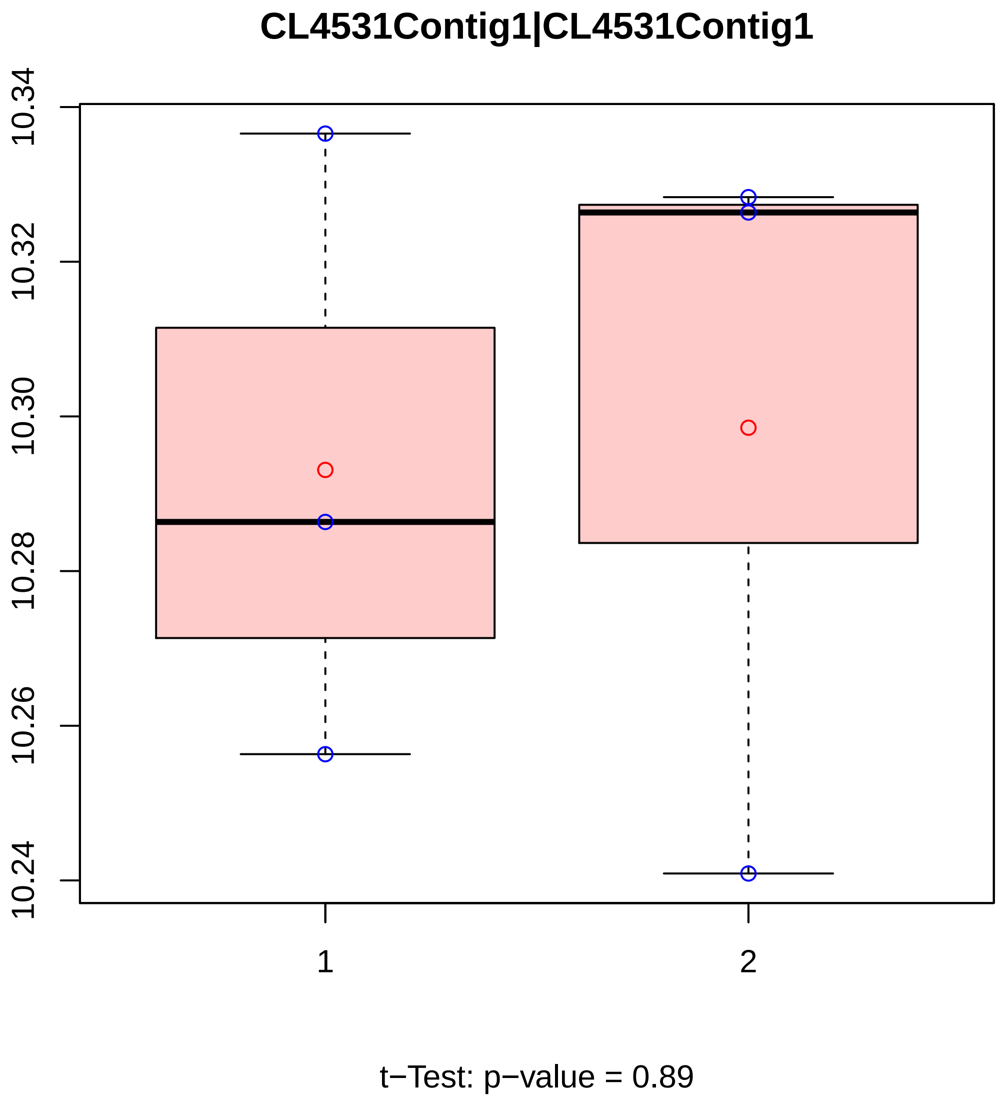

CL4531Contig1|CL4531Contig1
10.34
10.32
10.30
10.28
10.26
10.24
1
2
t−Test: p−value = 0.89

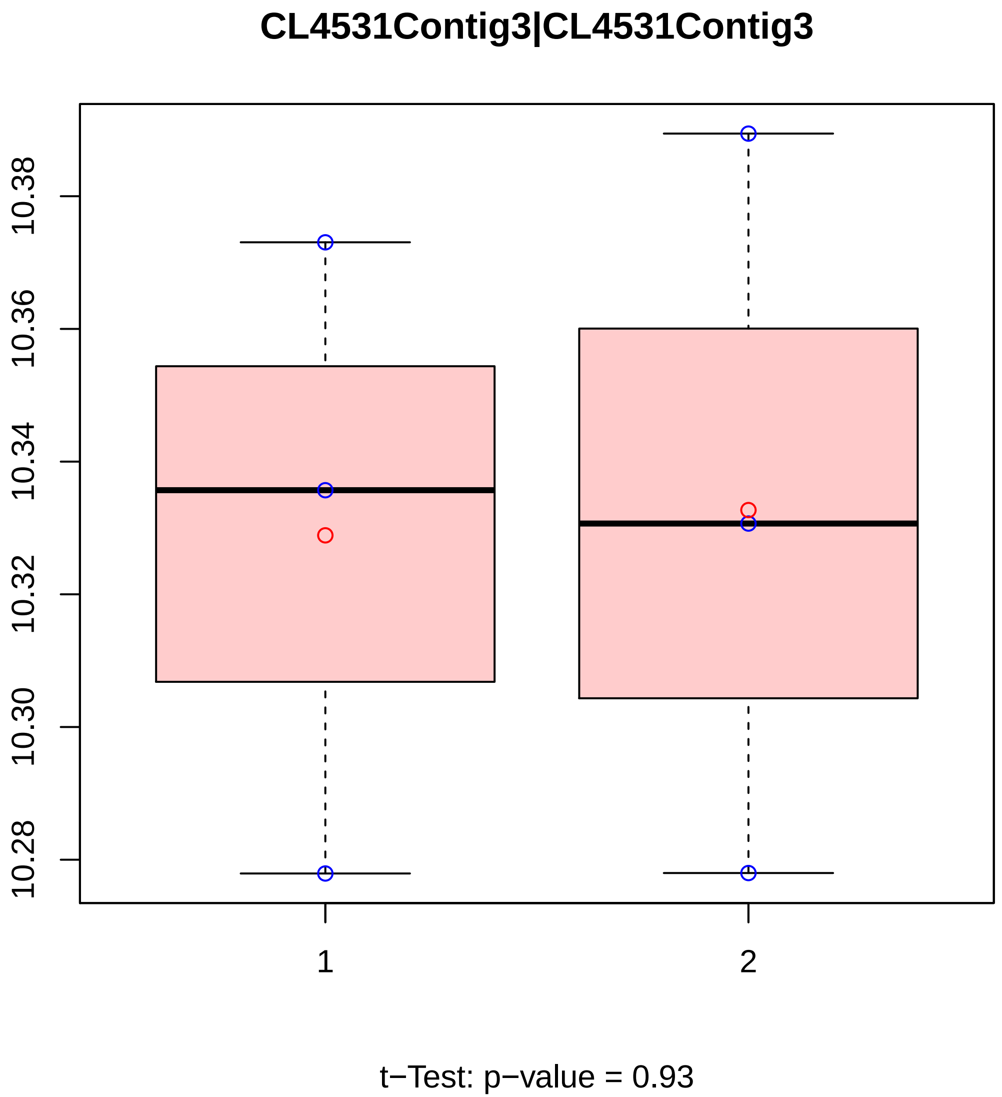
CL4531Contig3|CL4531Contig3
10.38
10.36
10.34
10.32
10.30
10.28
1
2
t−Test: p−value = 0.93

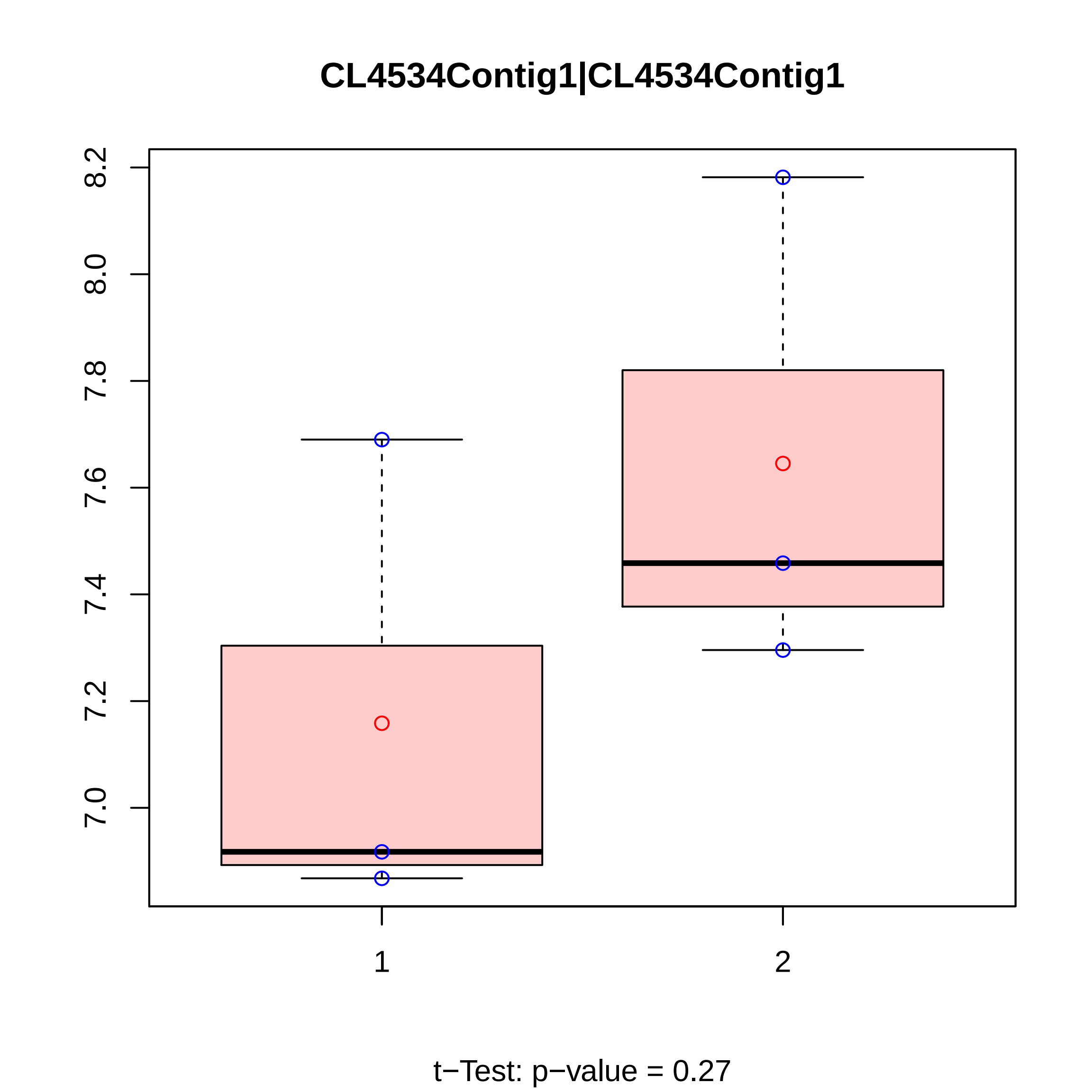

CL4534Contig1|CL4534Contig1
8.2
8.0
7.8
7.6
7.4
7.2
7.0
1
2
t−Test: p−value = 0.27

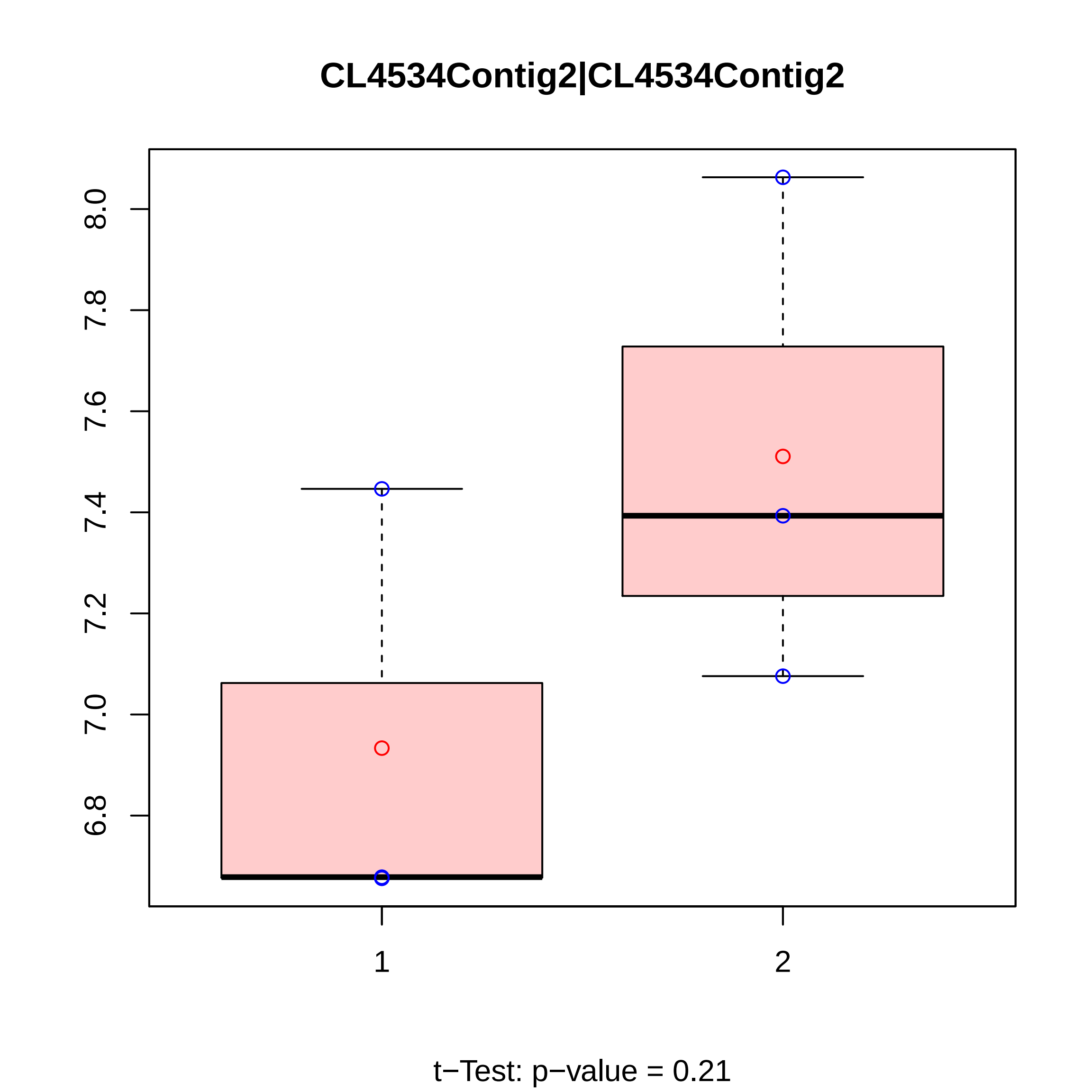

CL4534Contig2|CL4534Contig2
8.0
7.8
7.6
7.4
7.2
7.0
6.8
1
2
t−Test: p−value = 0.21

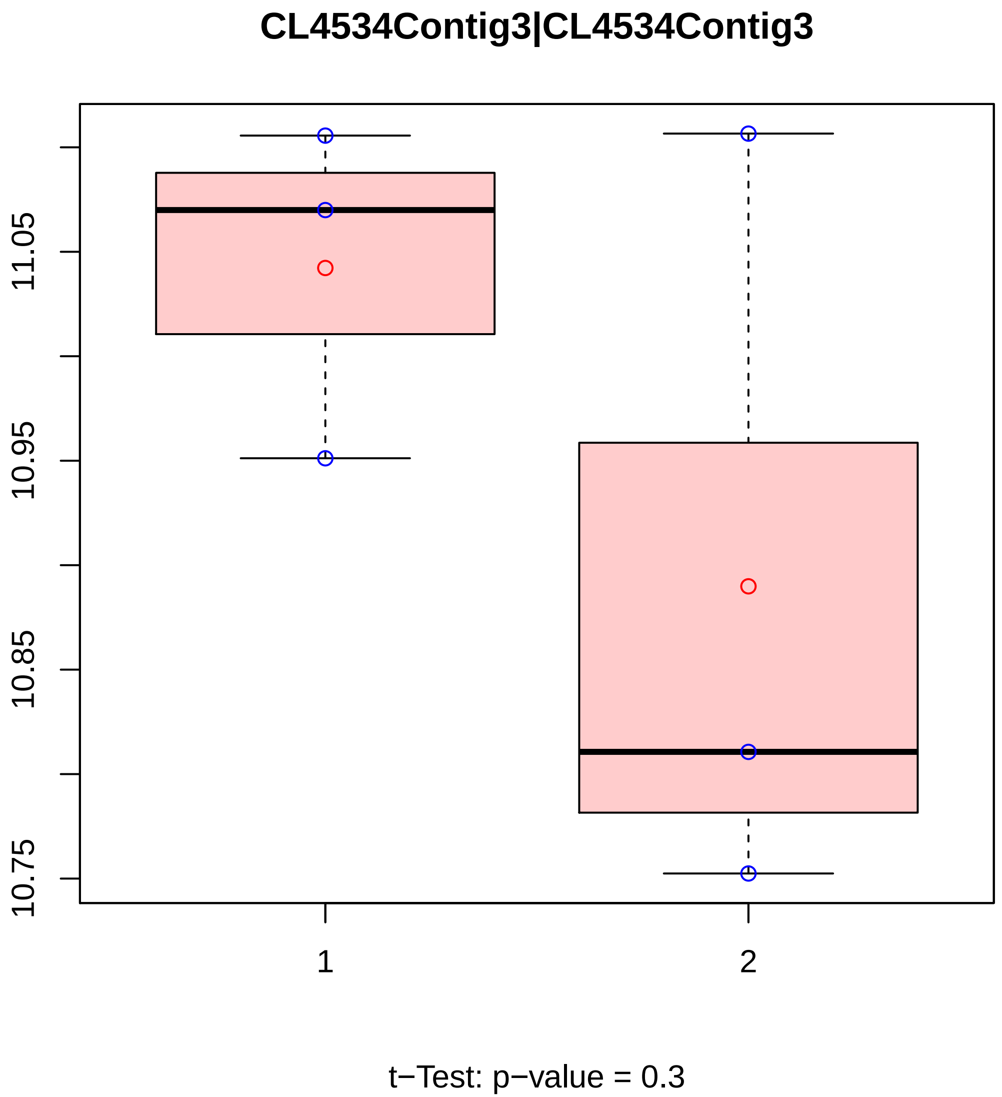
CL4534Contig3|CL4534Contig3
11.05
10.95
10.85
10.75
1
2
t−Test: p−value = 0.3

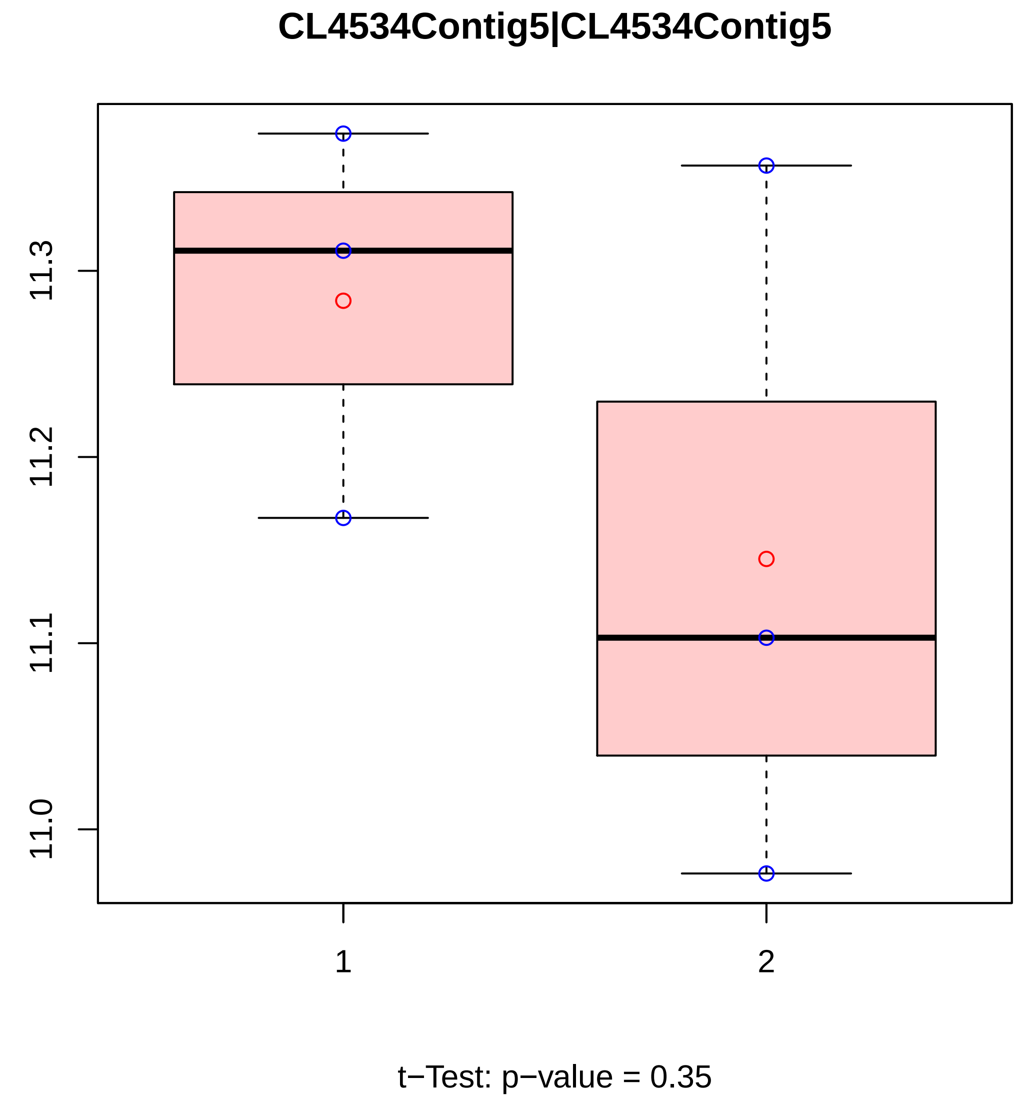
CL4534Contig5|CL4534Contig5
11.0
11.1
11.2
11.3
1
2
t−Test: p−value = 0.35

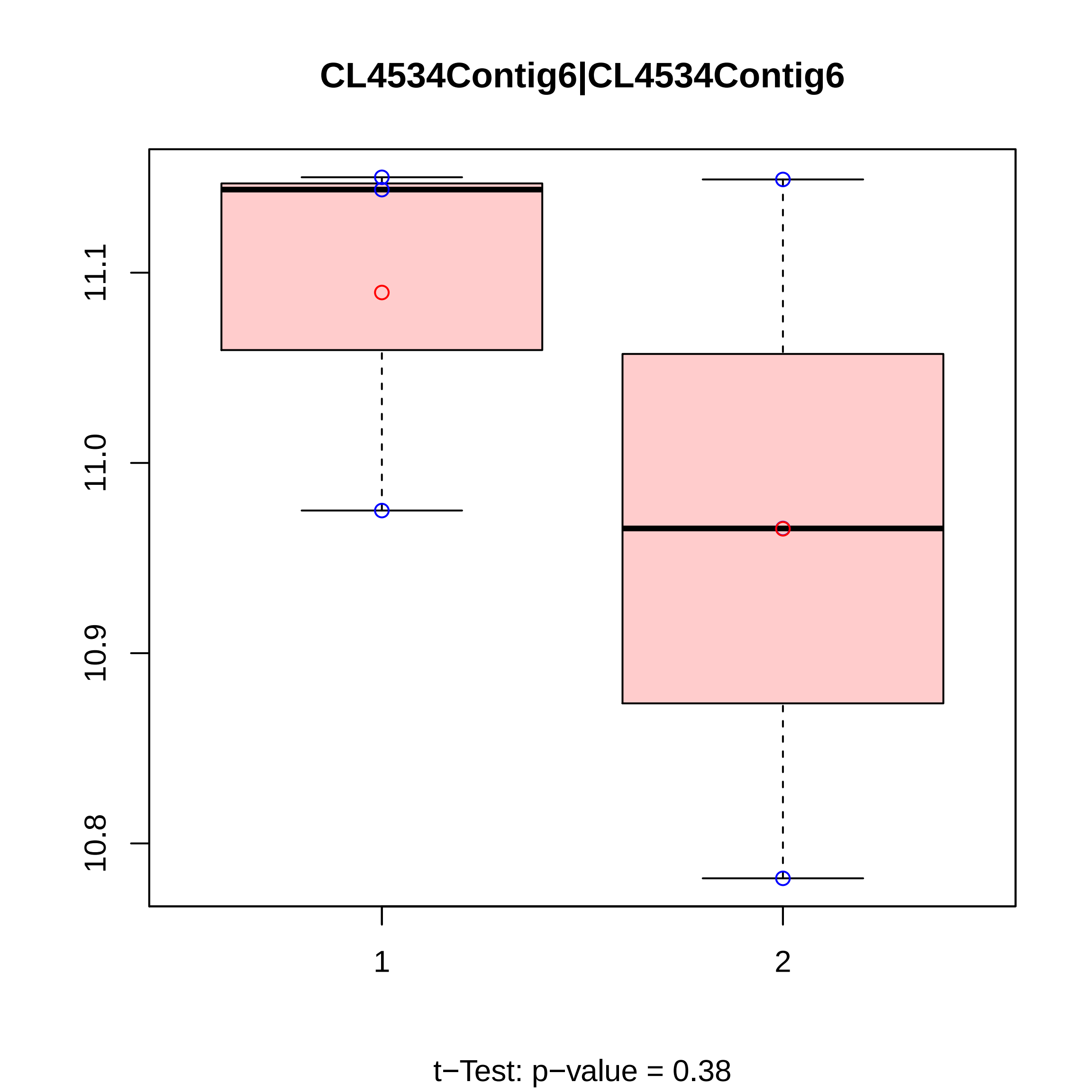
CL4534Contig6|CL4534Contig6
11.1
11.0
10.9
10.8
1
2
t−Test: p−value = 0.38

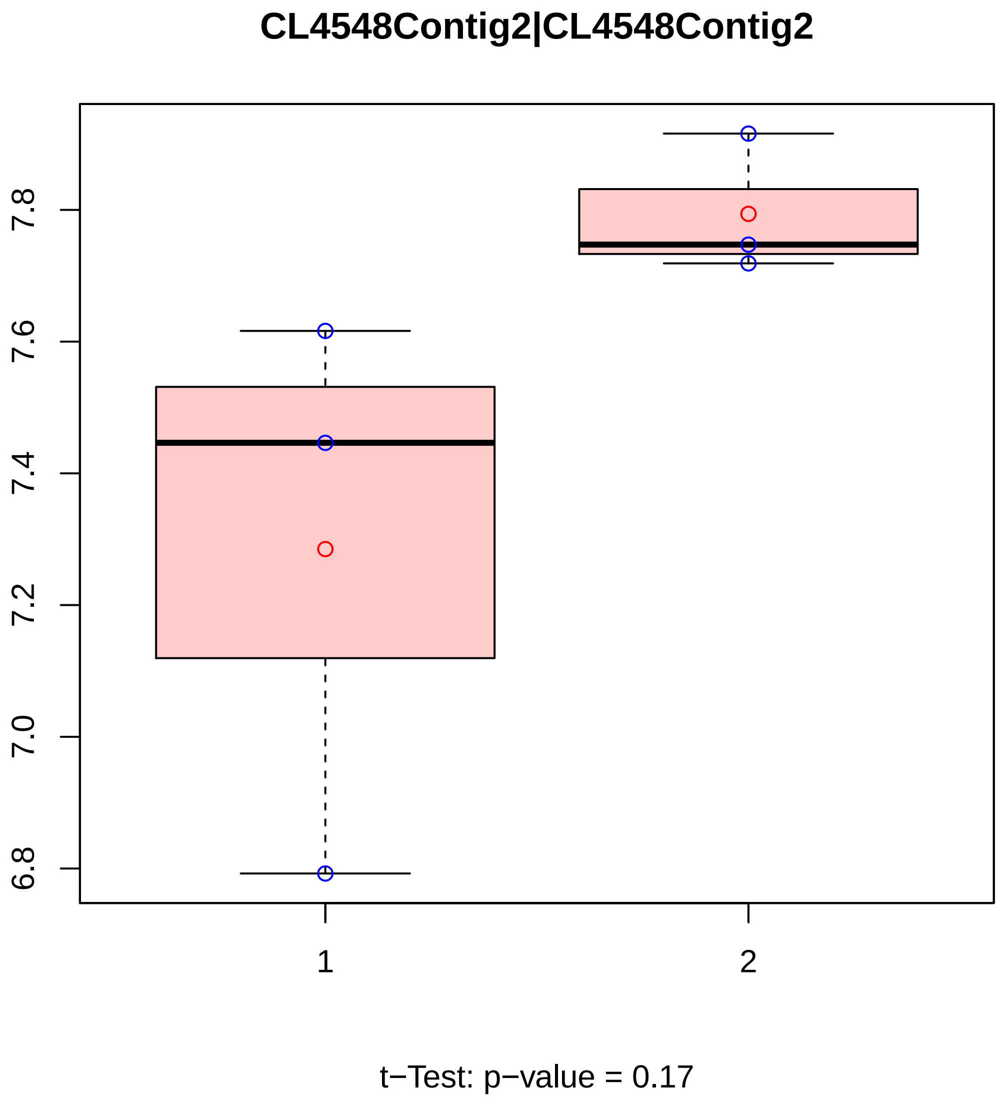
CL4548Contig2|CL4548Contig2
7.8
7.6
7.4
7.2
7.0
6.8
1
2
t−Test: p−value = 0.17

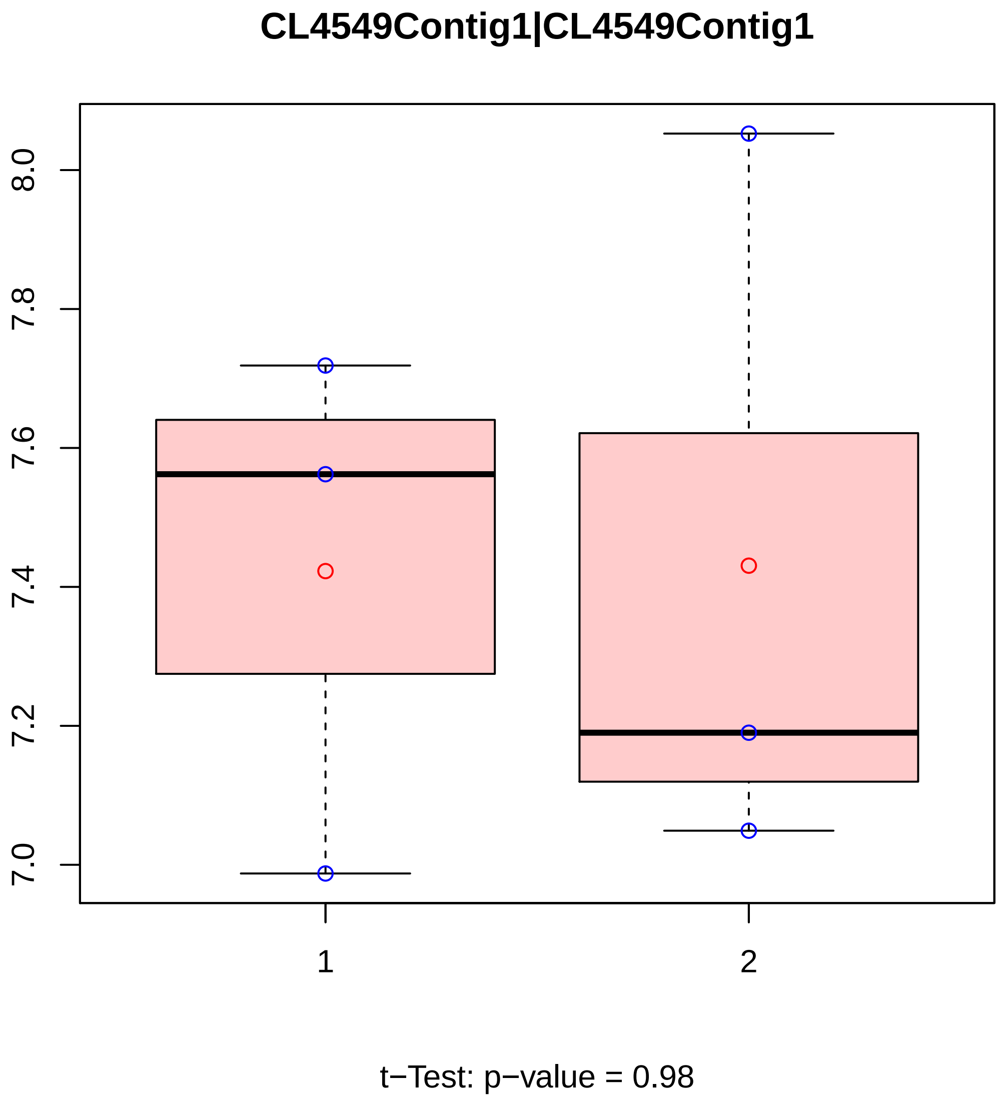

CL4549Contig1|CL4549Contig1
7.0
7.2
7.4
7.6
7.8
8.0
1
2
t−Test: p−value = 0.98

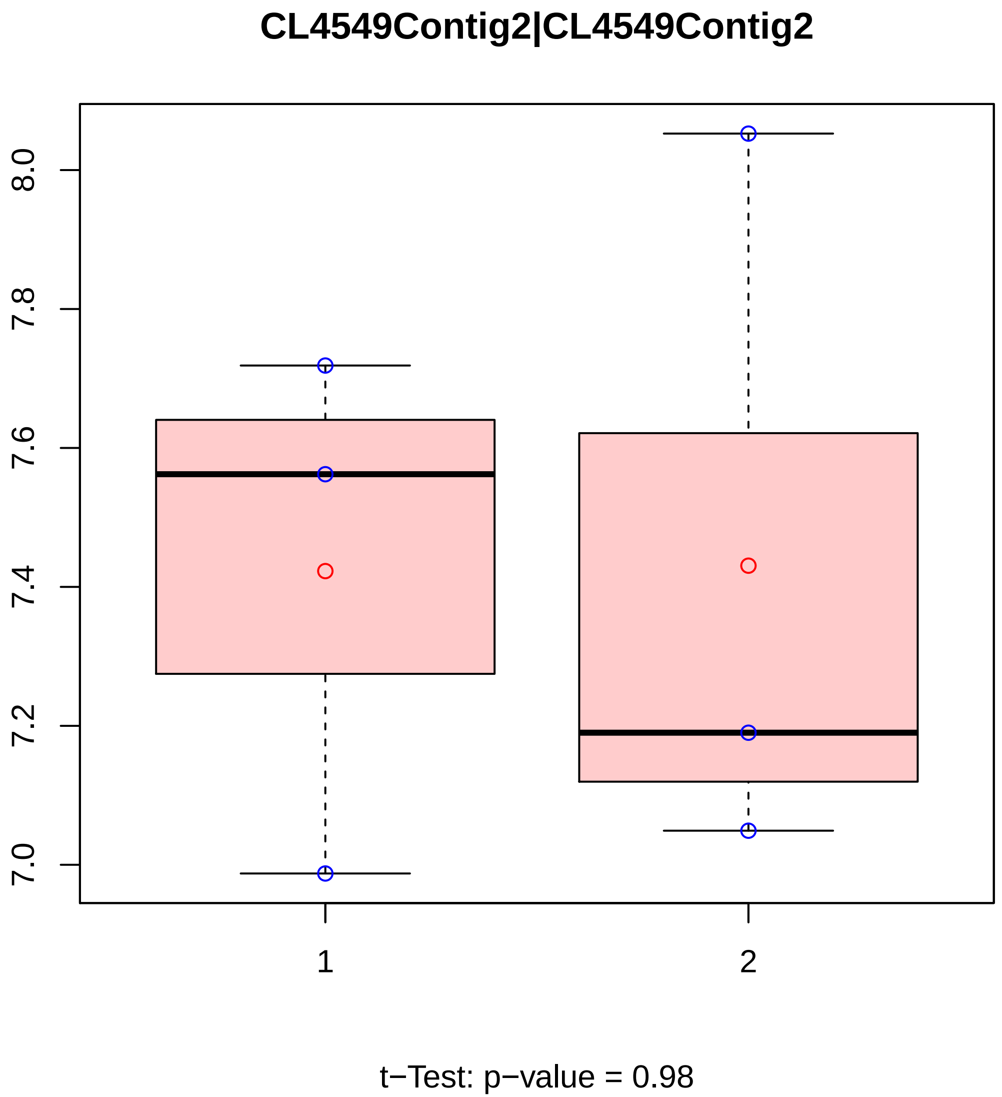

CL4549Contig2|CL4549Contig2
8.0
7.8
7.6
7.4
7.2
7.0
1
2
t−Test: p−value = 0.98

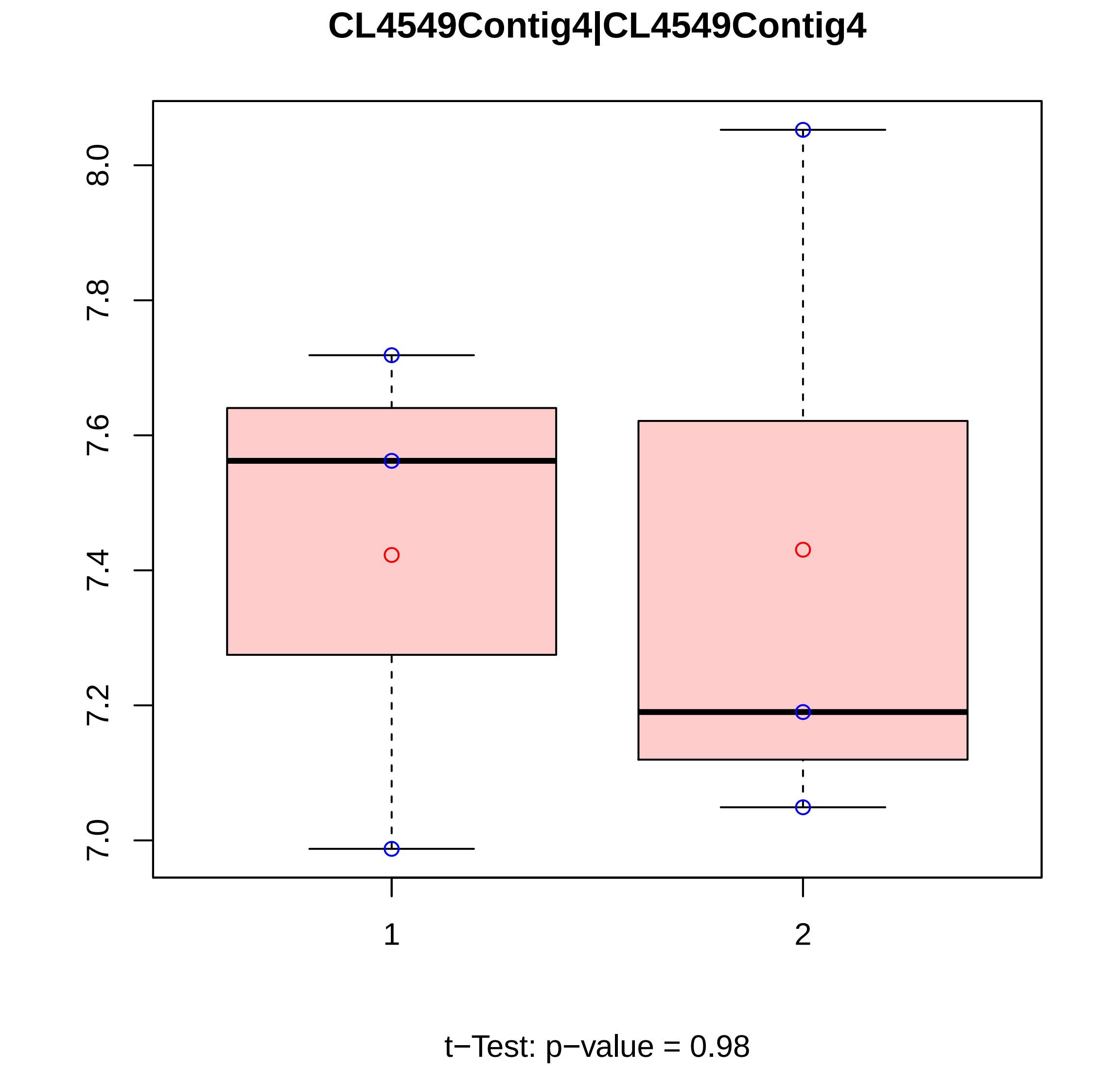
CL4549Contig4|CL4549Contig4
7.0
7.2
7.4
7.6
7.8
8.0
1
2
t−Test: p−value = 0.98

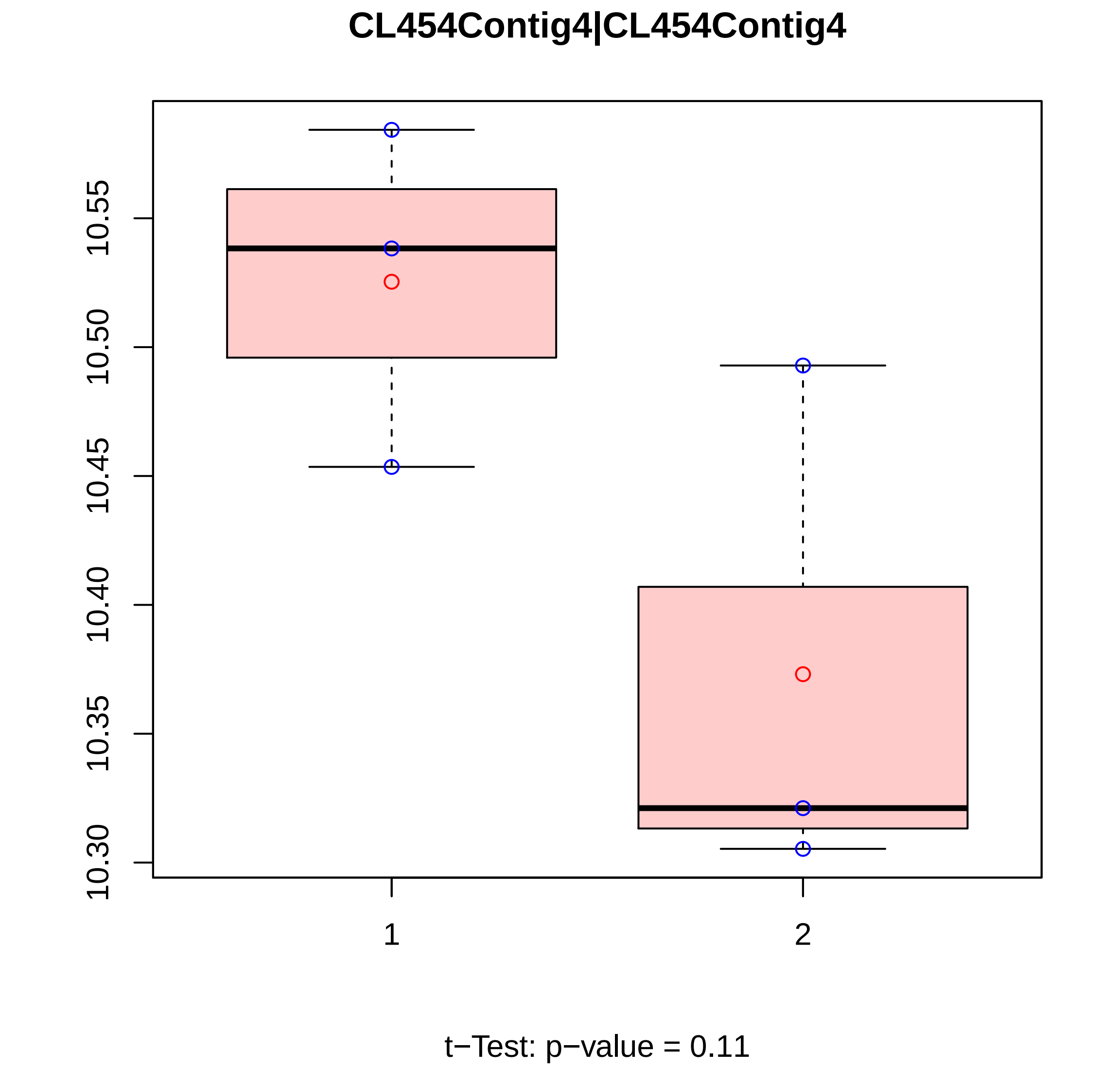
CL454Contig4|CL454Contig4
10.30
10.35
10.40
10.45
10.50
10.55
1
2
t−Test: p−value = 0.11

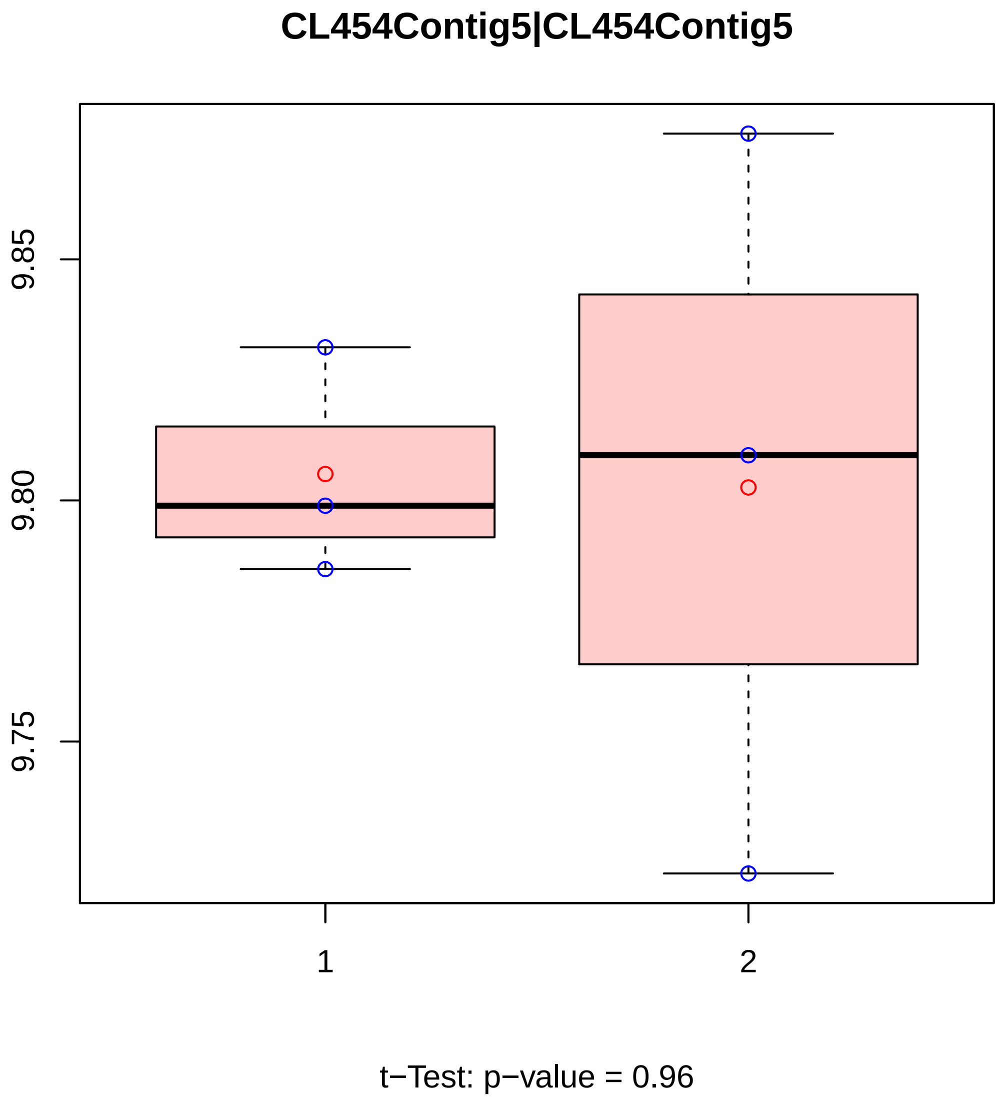
CL454Contig5|CL454Contig5
9.85
9.80
9.75
1
2
t−Test: p−value = 0.96

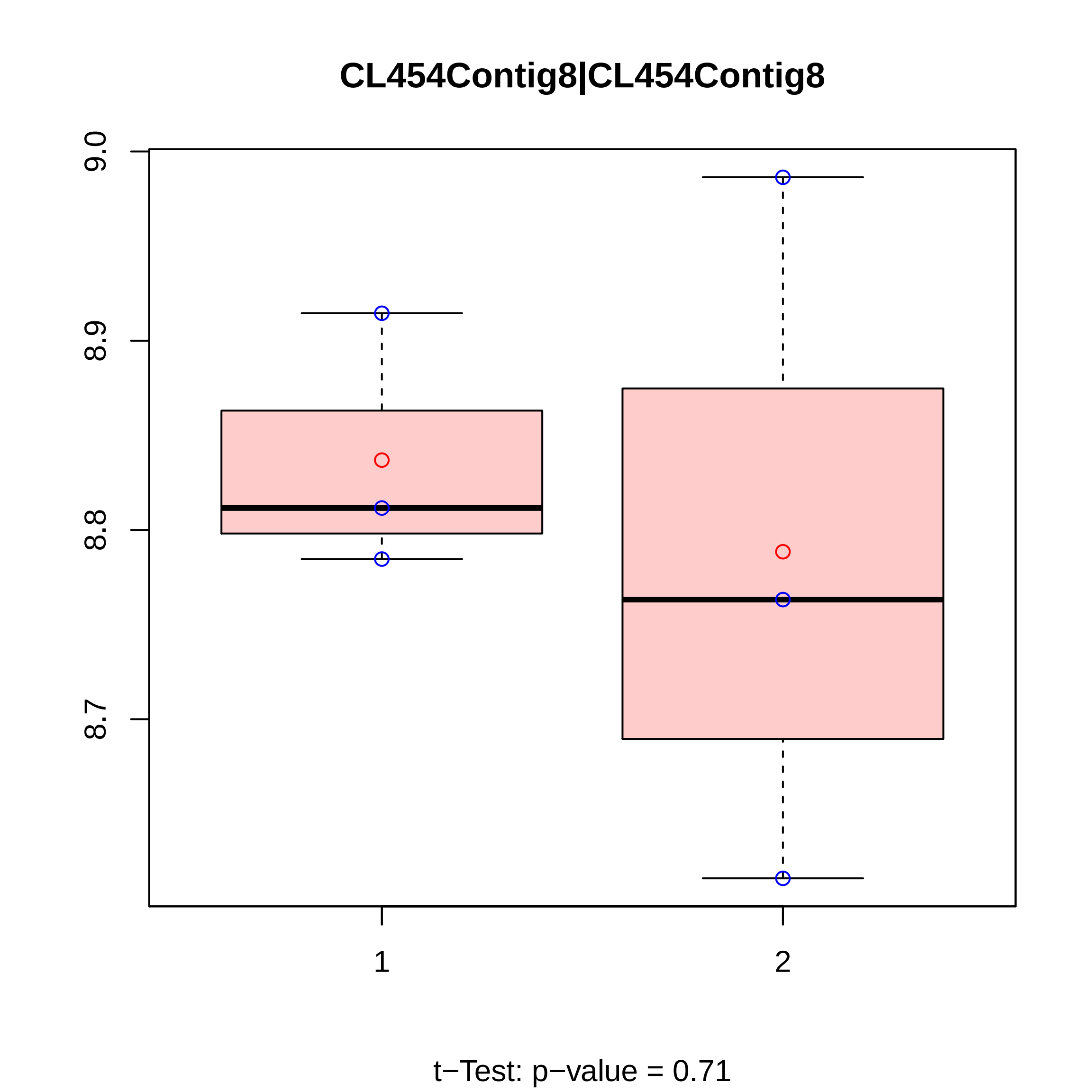

CL454Contig8|CL454Contig8
9.0
8.9
8.8
8.7
1
2
t−Test: p−value = 0.71

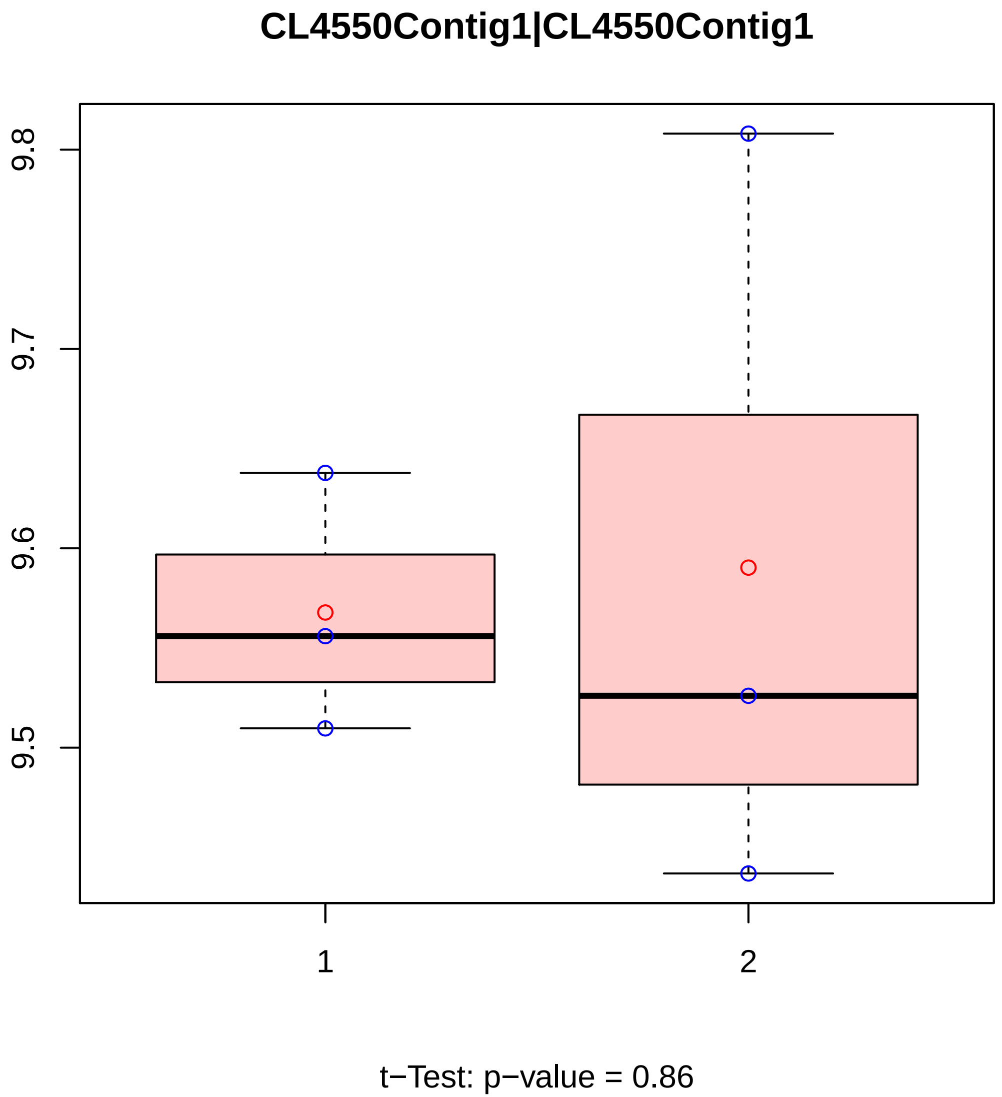

CL4550Contig1|CL4550Contig1
9.8
9.7
9.6
9.5
1
2
t−Test: p−value = 0.86

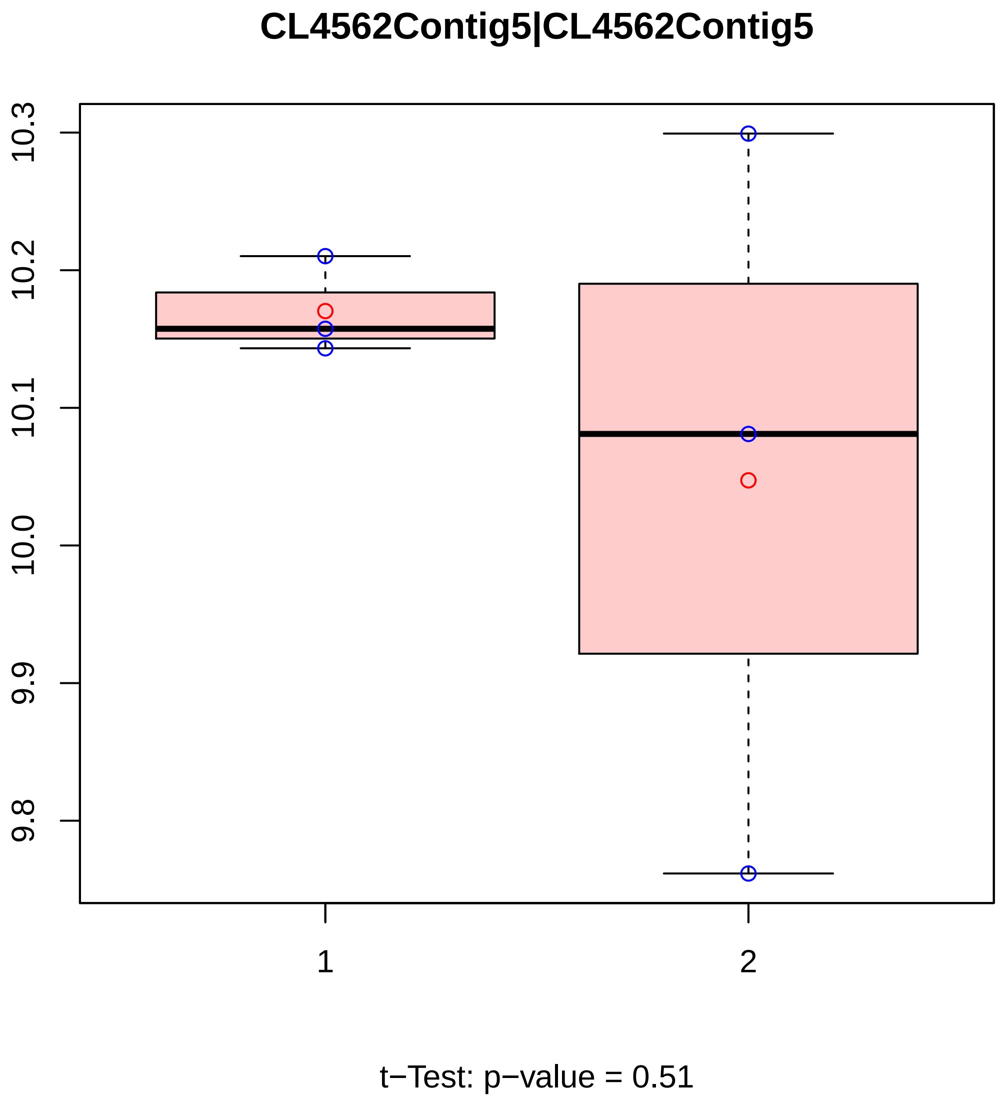
CL4562Contig5|CL4562Contig5
10.3
10.2
10.1
10.0
9.9
9.8
1
2
t−Test: p−value = 0.51

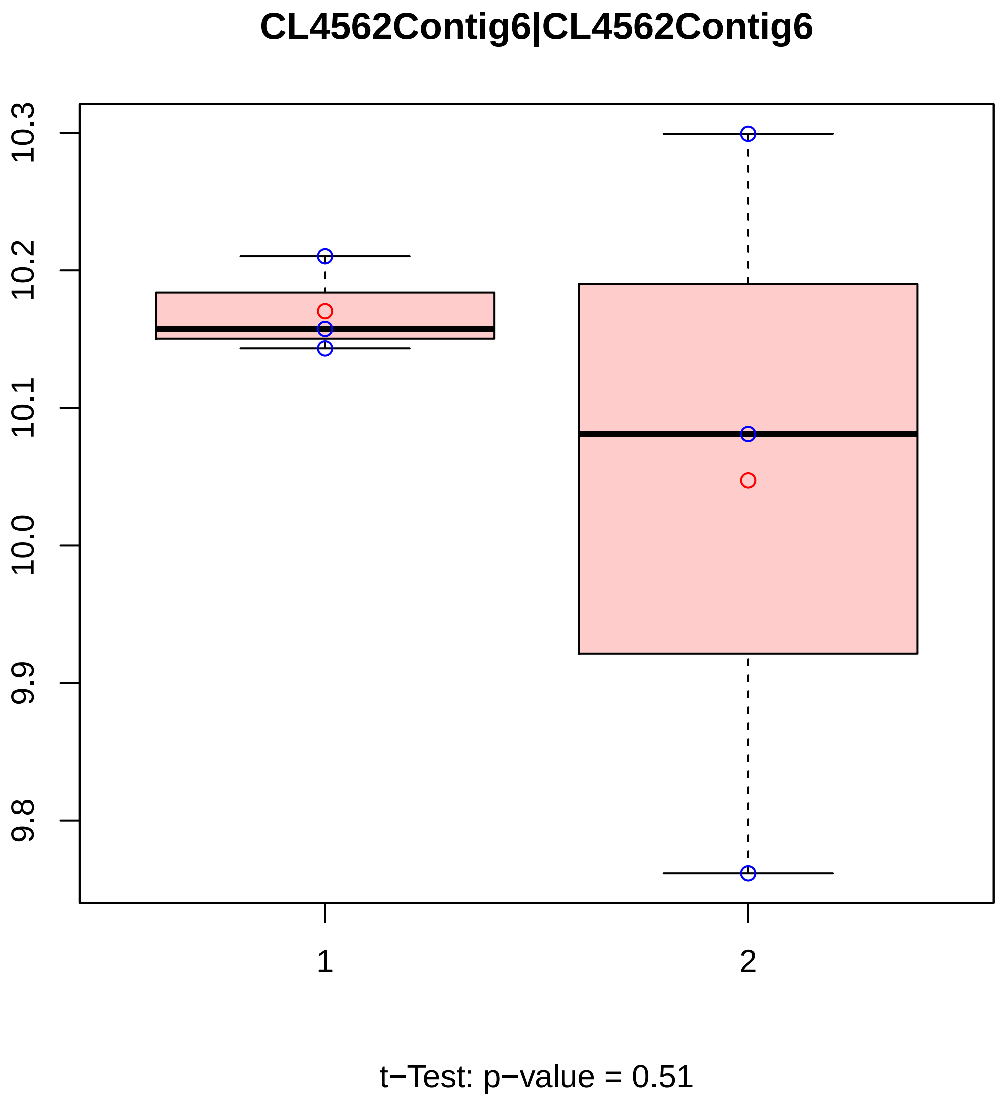
CL4562Contig6|CL4562Contig6
10.3
10.2
10.1
10.0
9.9
9.8
1
2
t−Test: p−value = 0.51

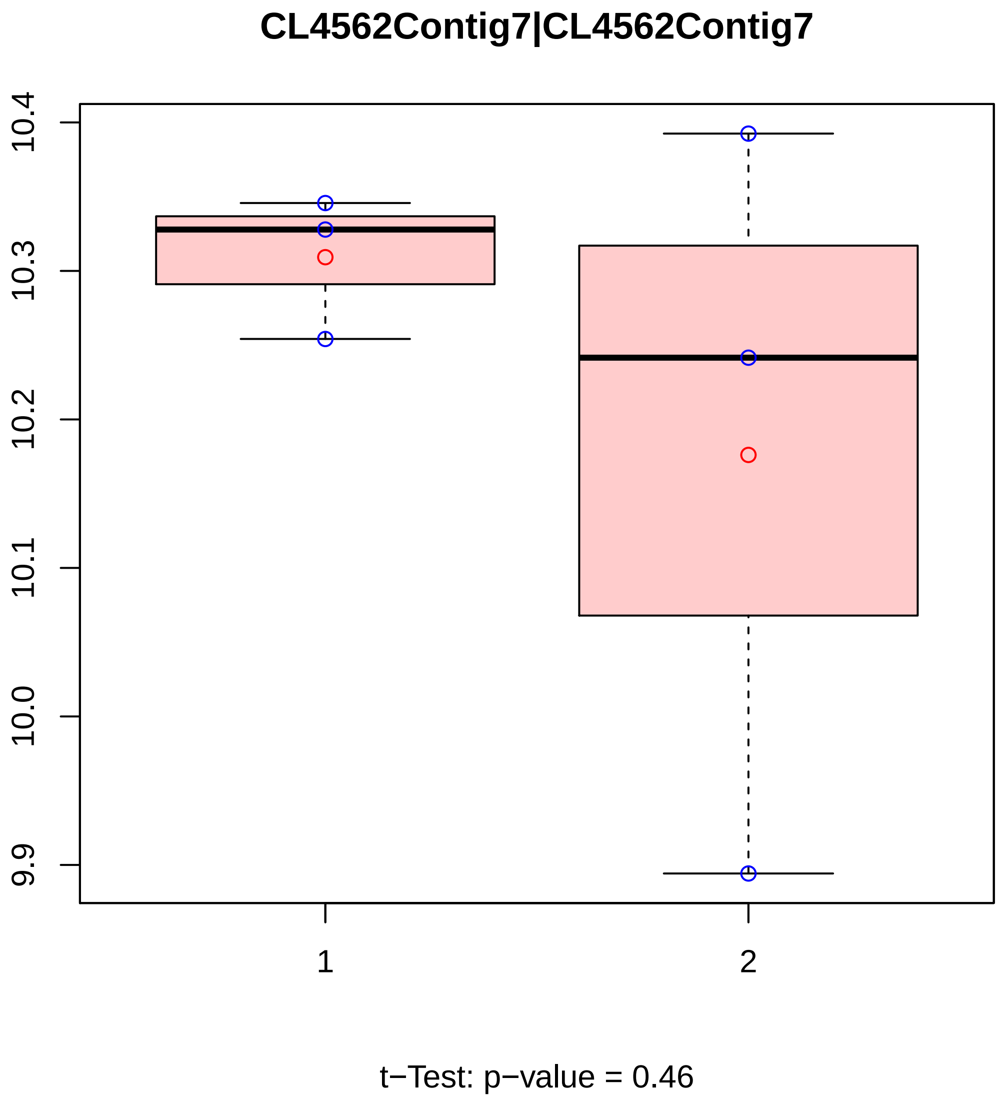

CL4562Contig7|CL4562Contig7
10.4
10.3
10.2
10.1
10.0
9.9
1
2
t−Test: p−value = 0.46

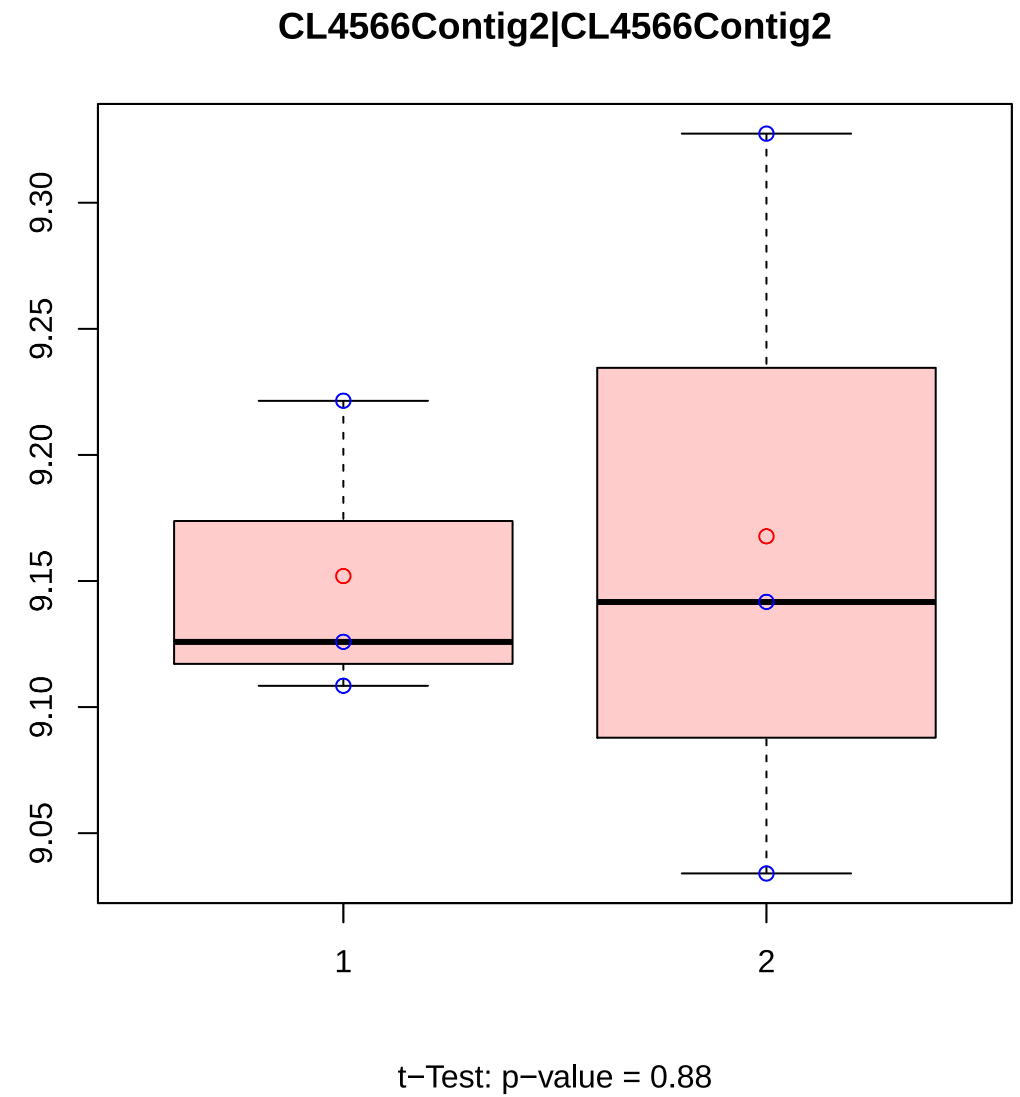
CL4566Contig2|CL4566Contig2
9.05
9.10
9.15
9.20
9.25
9.30
1
2
t−Test: p−value = 0.88

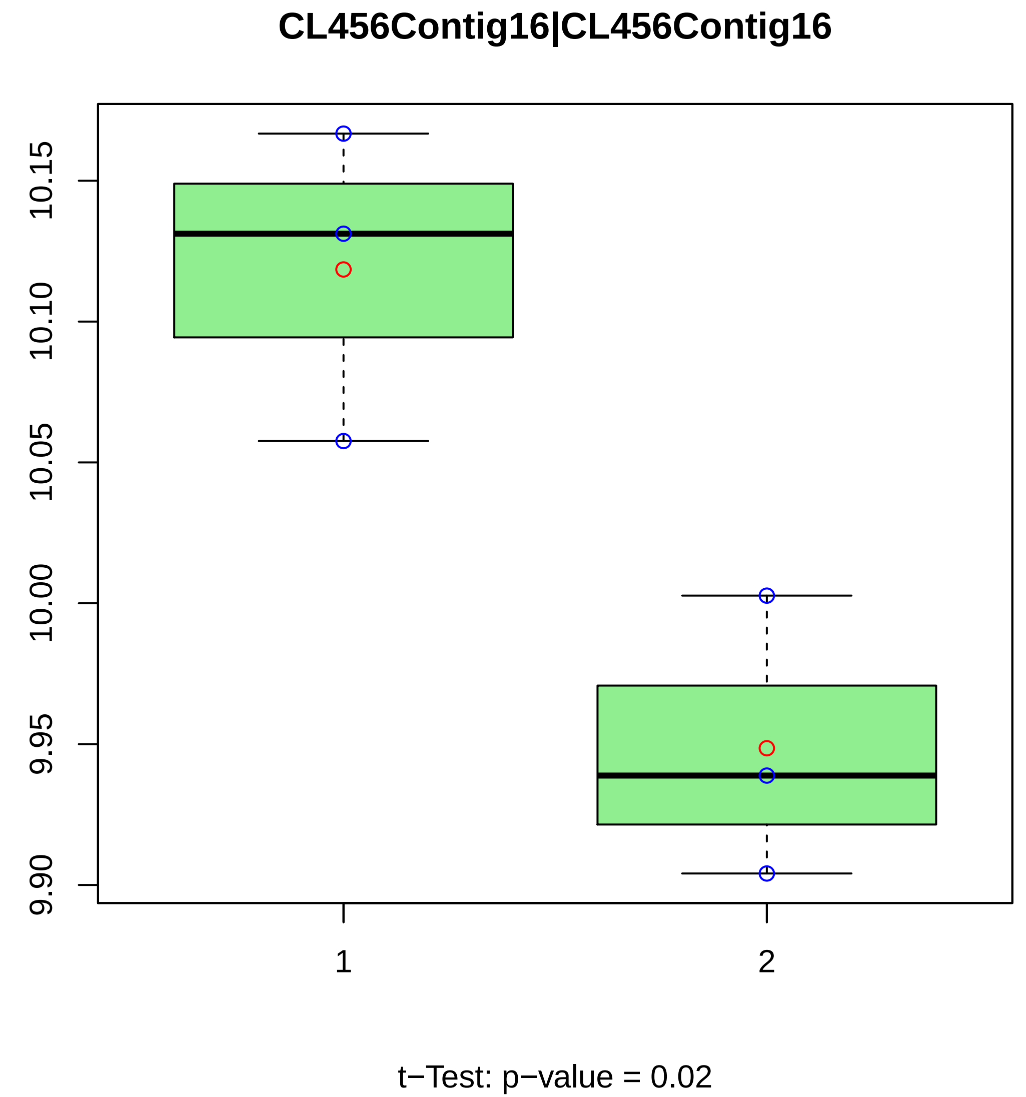

CL456Contig16|CL456Contig16
10.15
10.10
10.05
10.00
9.95
9.90
1
2
t−Test: p−value = 0.02

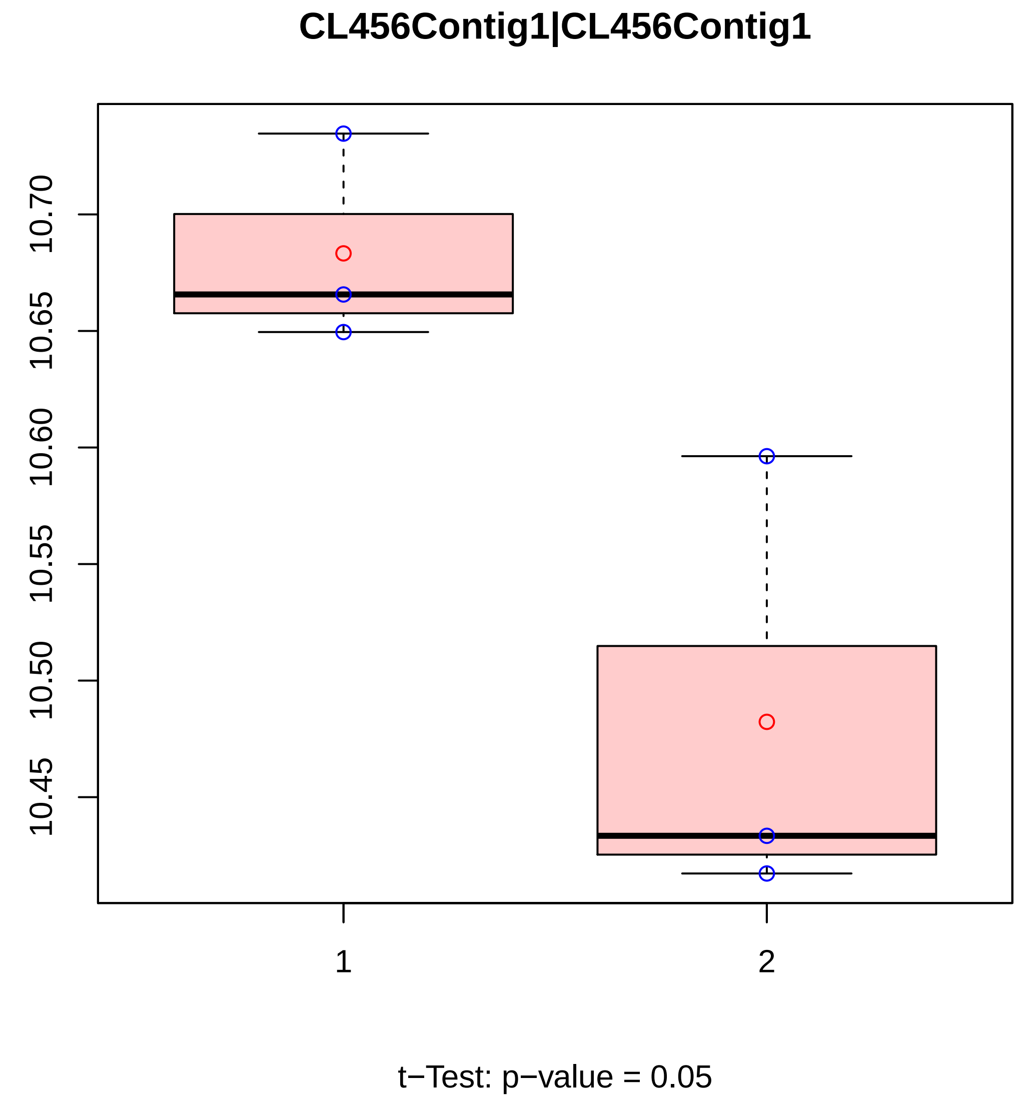
CL456Contig1|CL456Contig1
10.70
10.65
10.60
10.55
10.50
10.45
1
2
t−Test: p−value = 0.05

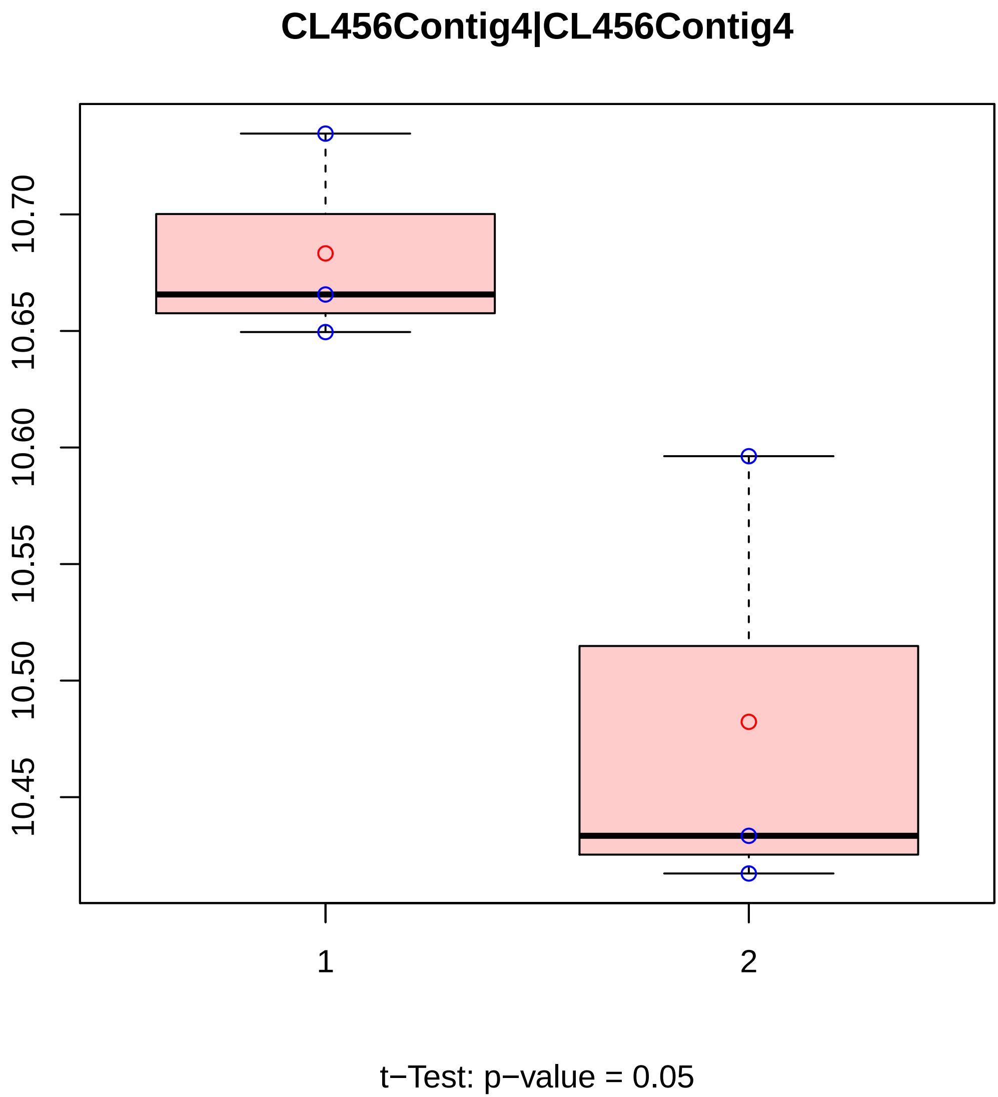
CL456Contig4|CL456Contig4
10.70
10.65
10.60
10.55
10.50
10.45
1
2
t−Test: p−value = 0.05

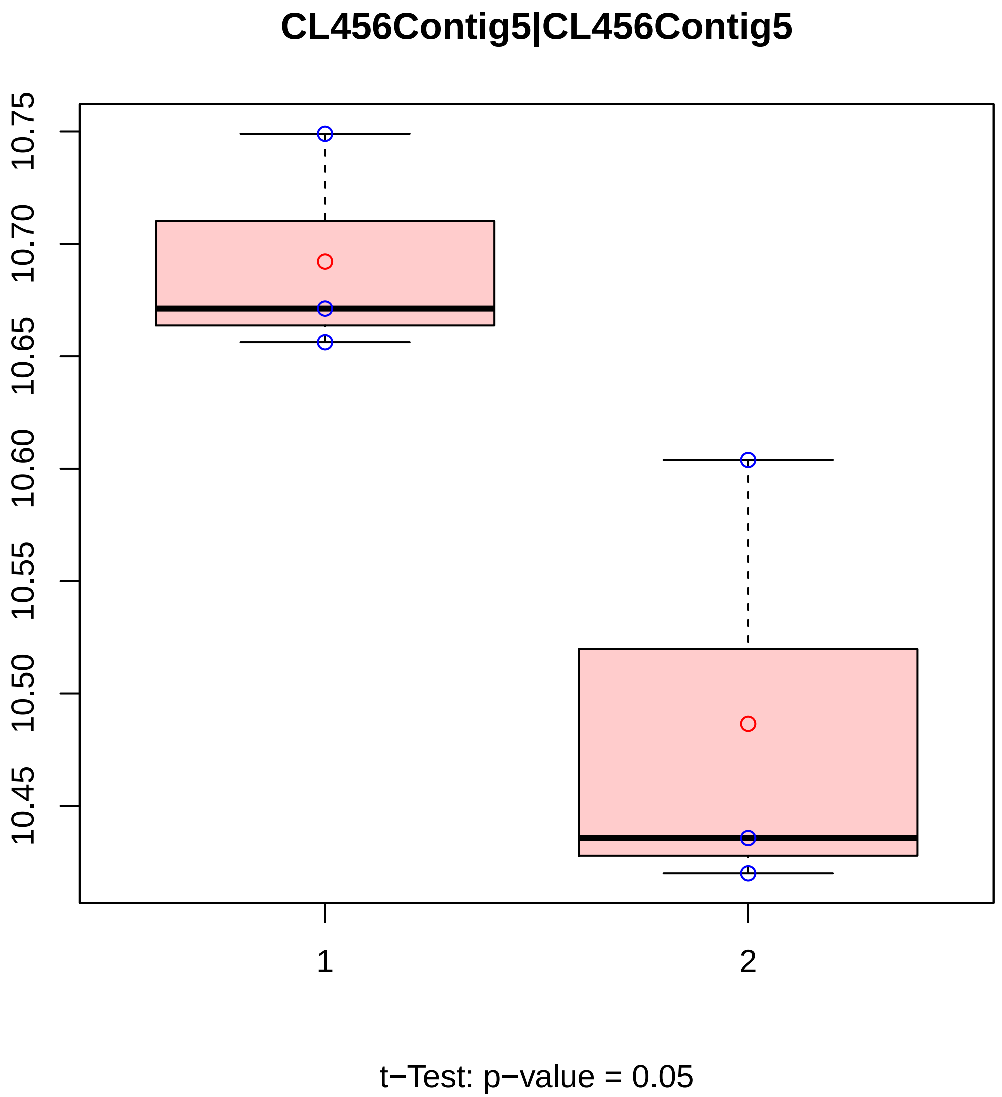
CL456Contig5|CL456Contig5
10.75
10.70
10.65
10.60
10.55
10.50
10.45
1
2
t−Test: p−value = 0.05

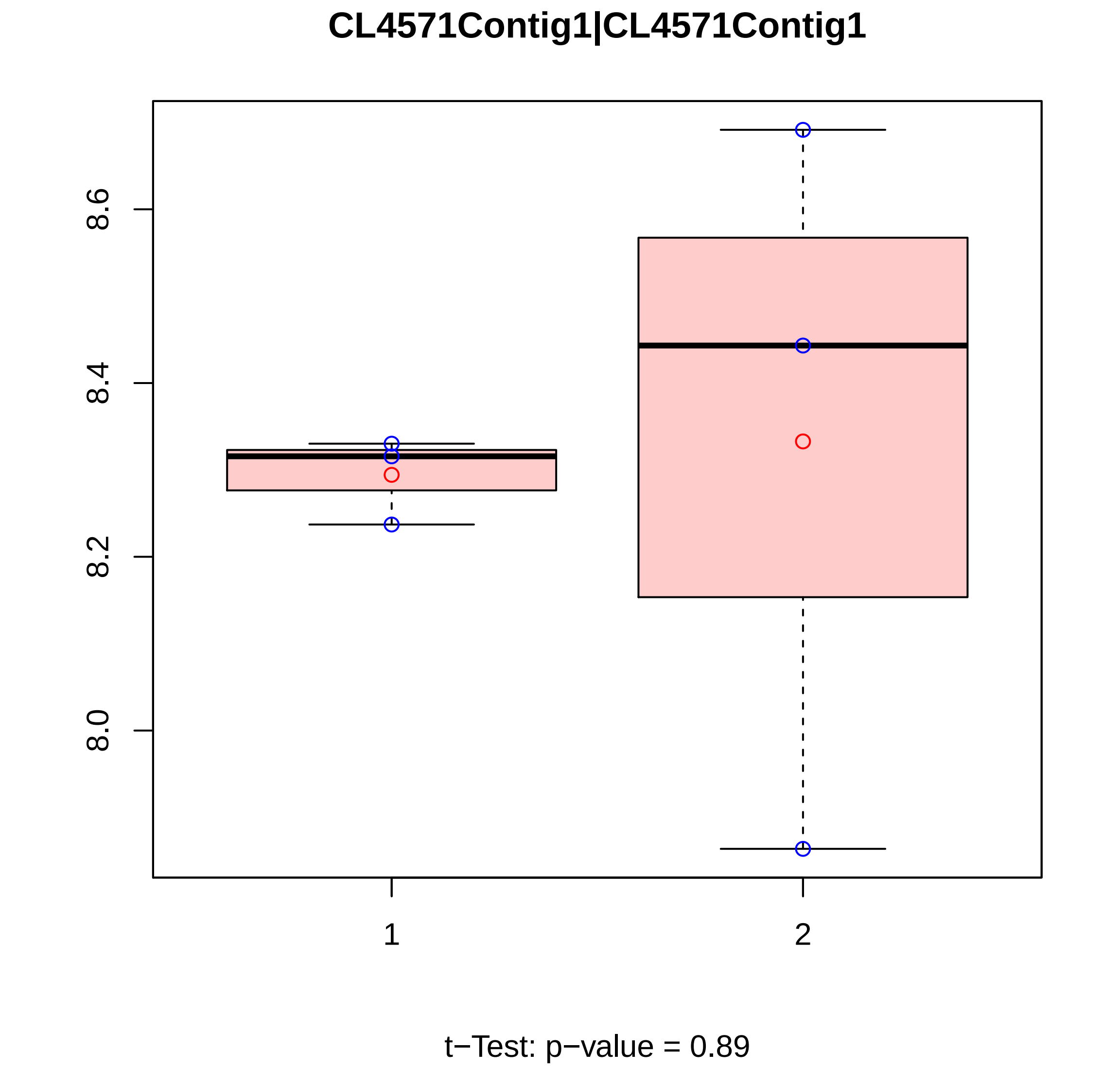
CL4571Contig1|CL4571Contig1
8.0
8.2
8.4
8.6
1
2
t−Test: p−value = 0.89

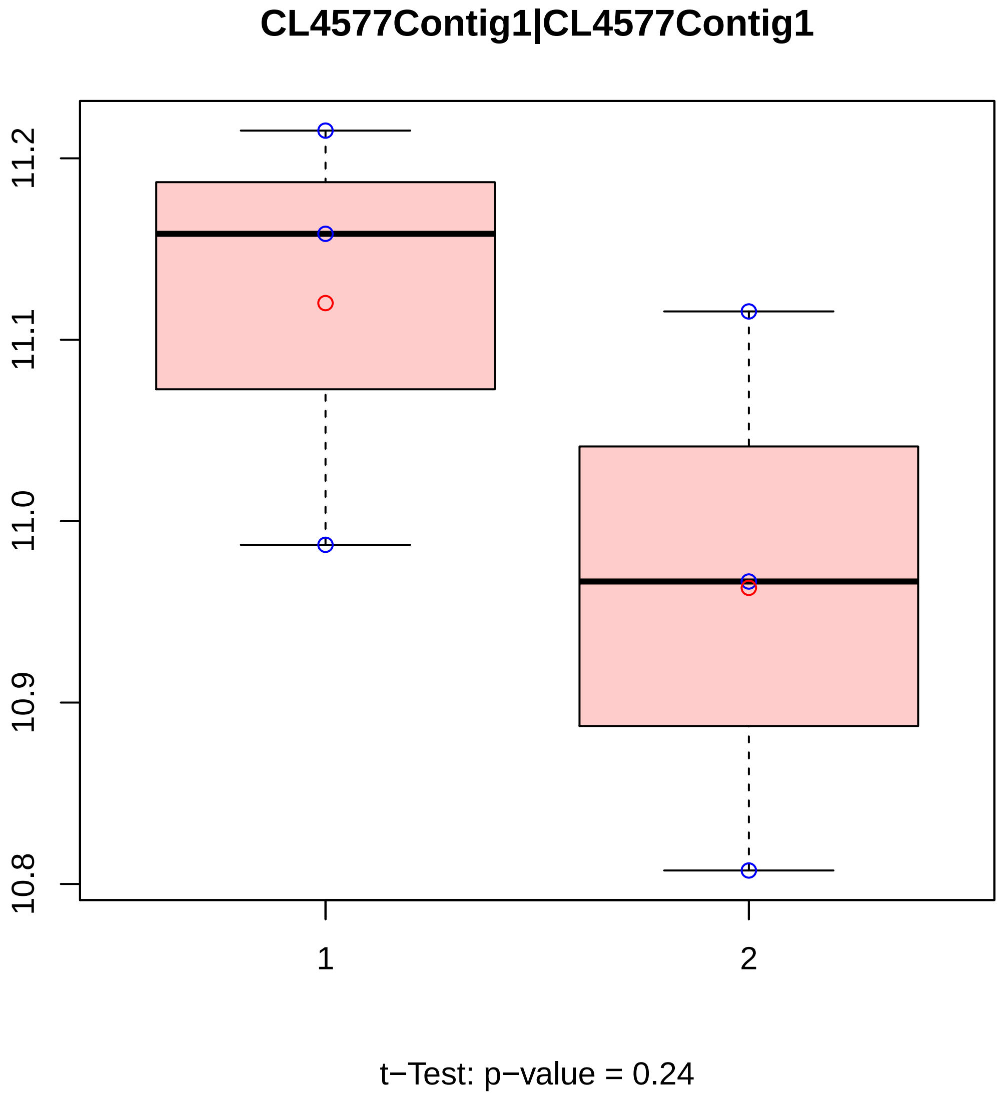

CL4577Contig1|CL4577Contig1
11.2
11.1
11.0
10.9
10.8
1
2
t−Test: p−value = 0.24

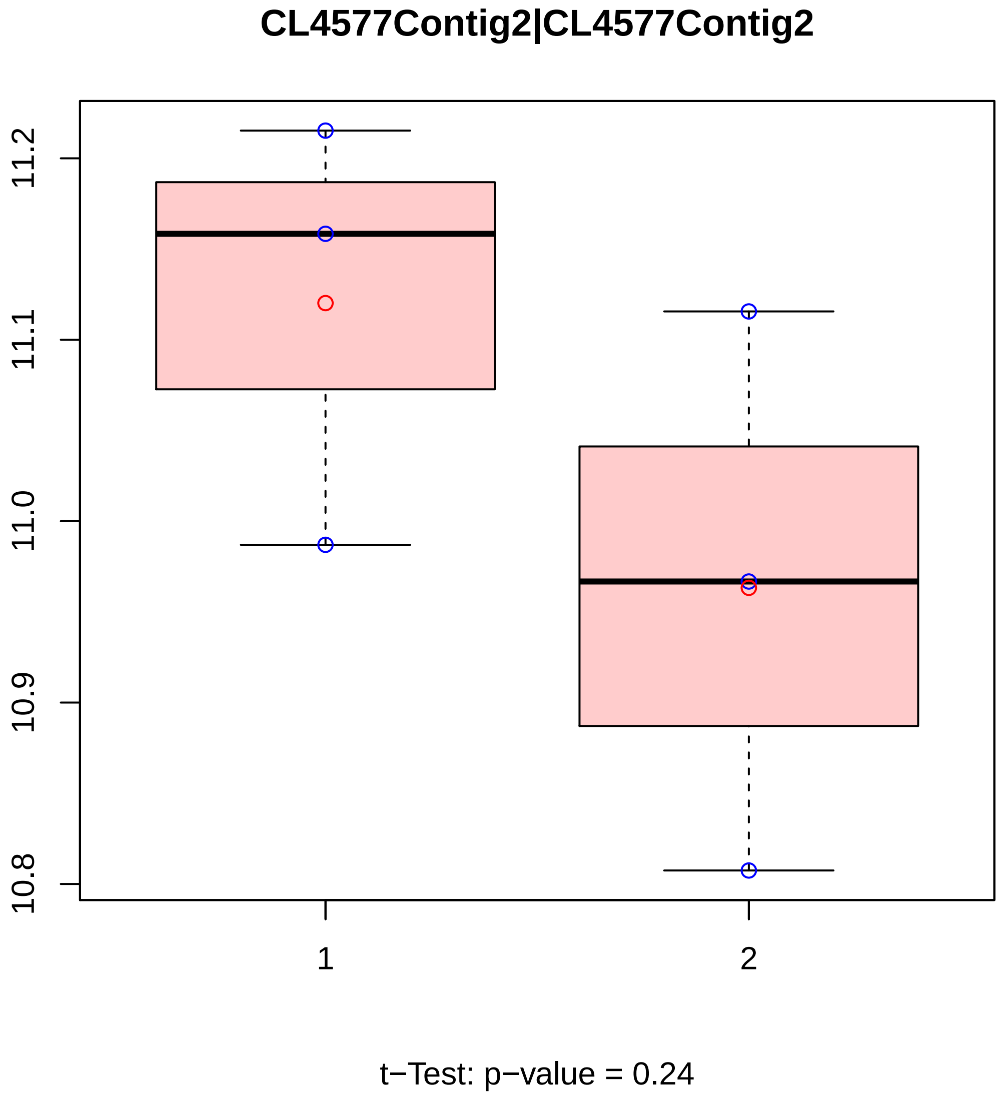

CL4577Contig2|CL4577Contig2
11.2
11.1
11.0
10.9
10.8
1
2
t−Test: p−value = 0.24

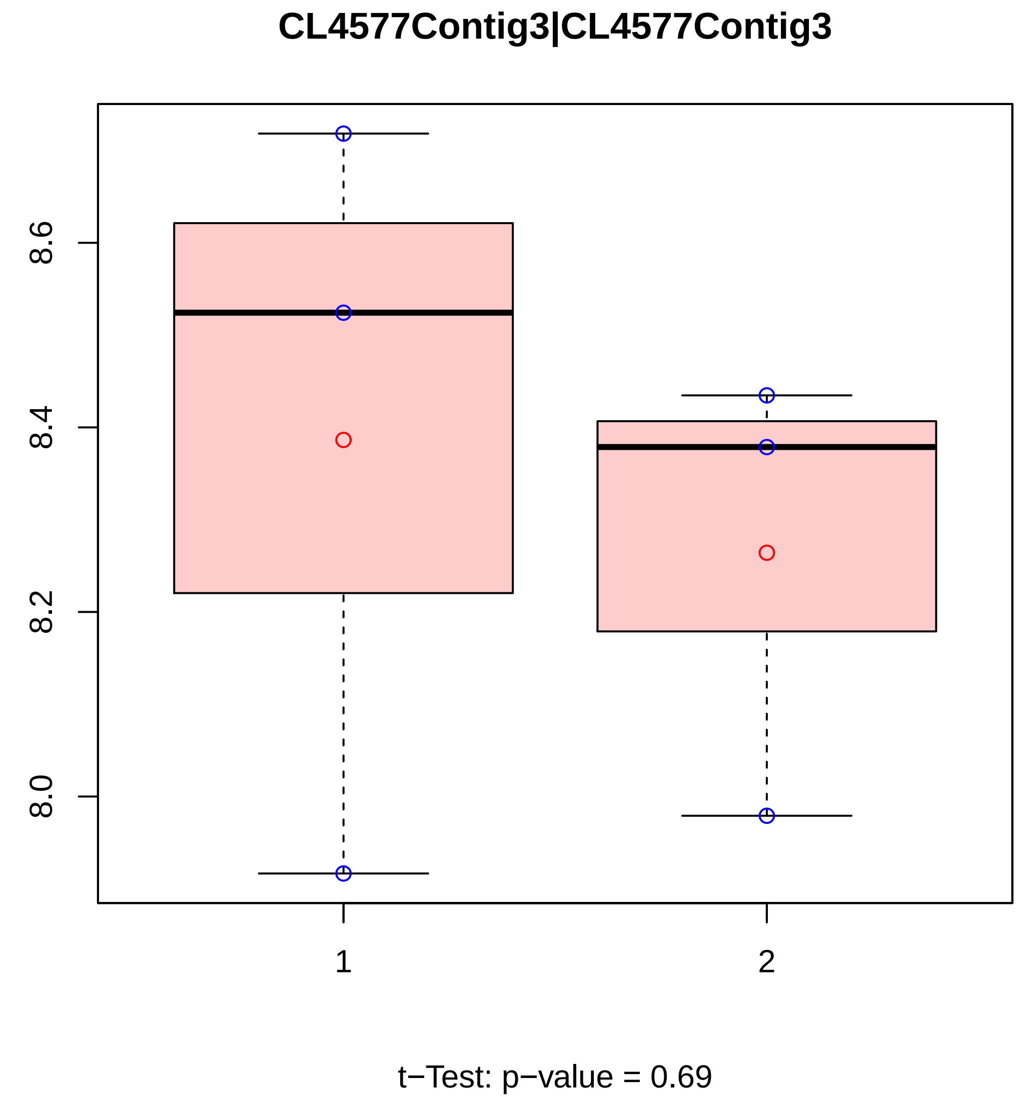

CL4577Contig3|CL4577Contig3
8.0
8.2
8.4
8.6
1
2
t−Test: p−value = 0.69

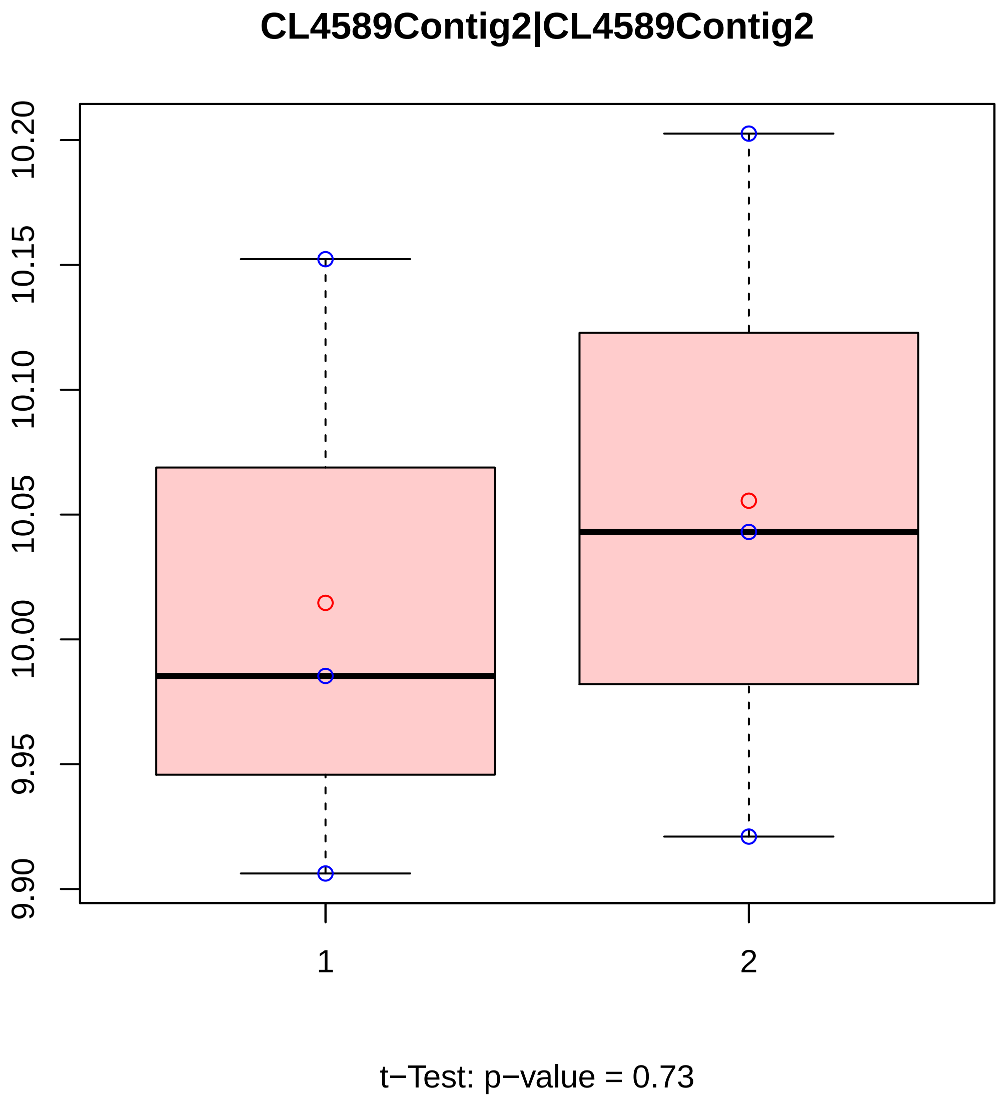

CL4589Contig2|CL4589Contig2
10.20
10.15
10.10
10.05
10.00
9.95
9.90
1
2
t−Test: p−value = 0.73

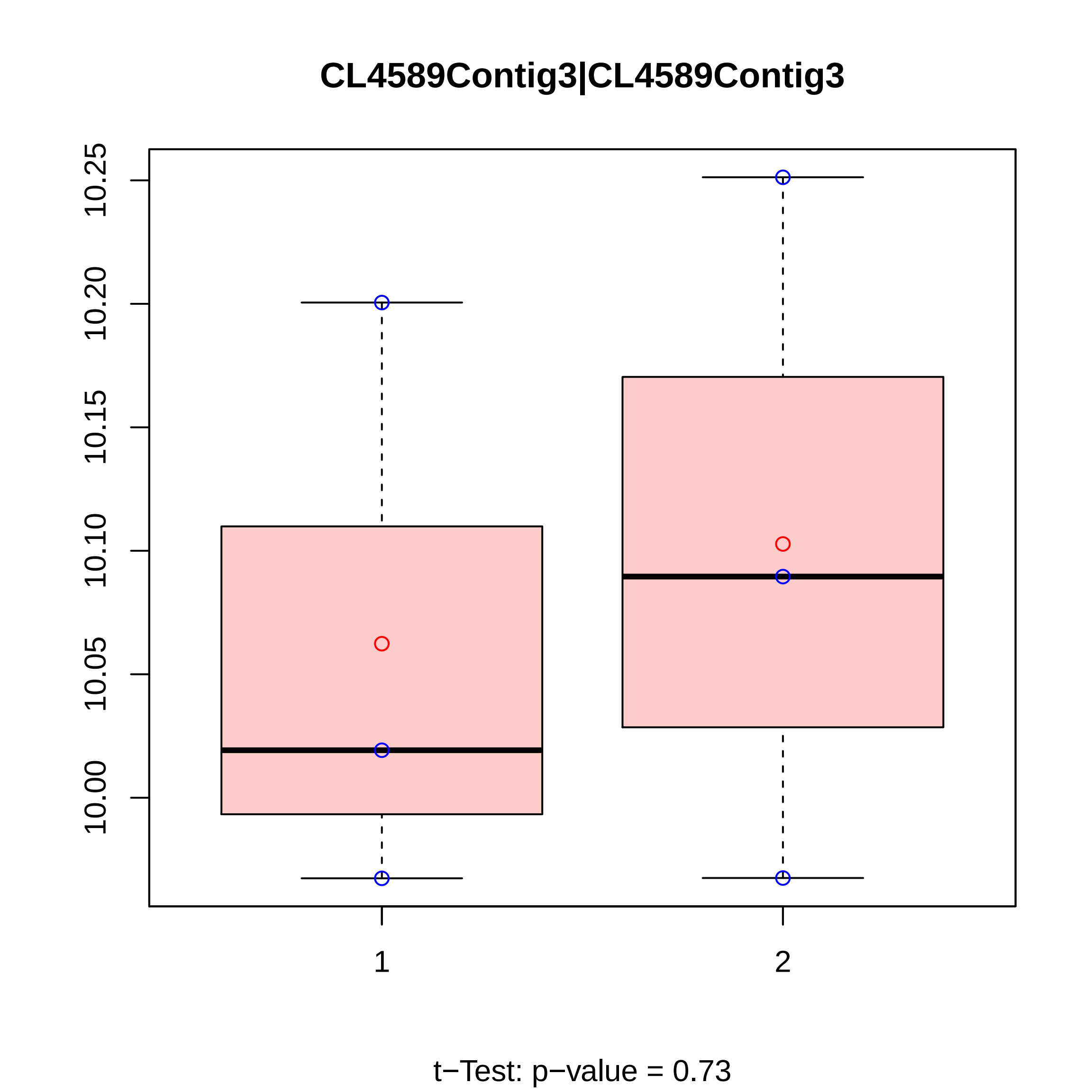
CL4589Contig3|CL4589Contig3
10.00
10.05
10.10
10.15
10.20
10.25
1
2
t−Test: p−value = 0.73

# CL4591Contig2|CL4591Contig2

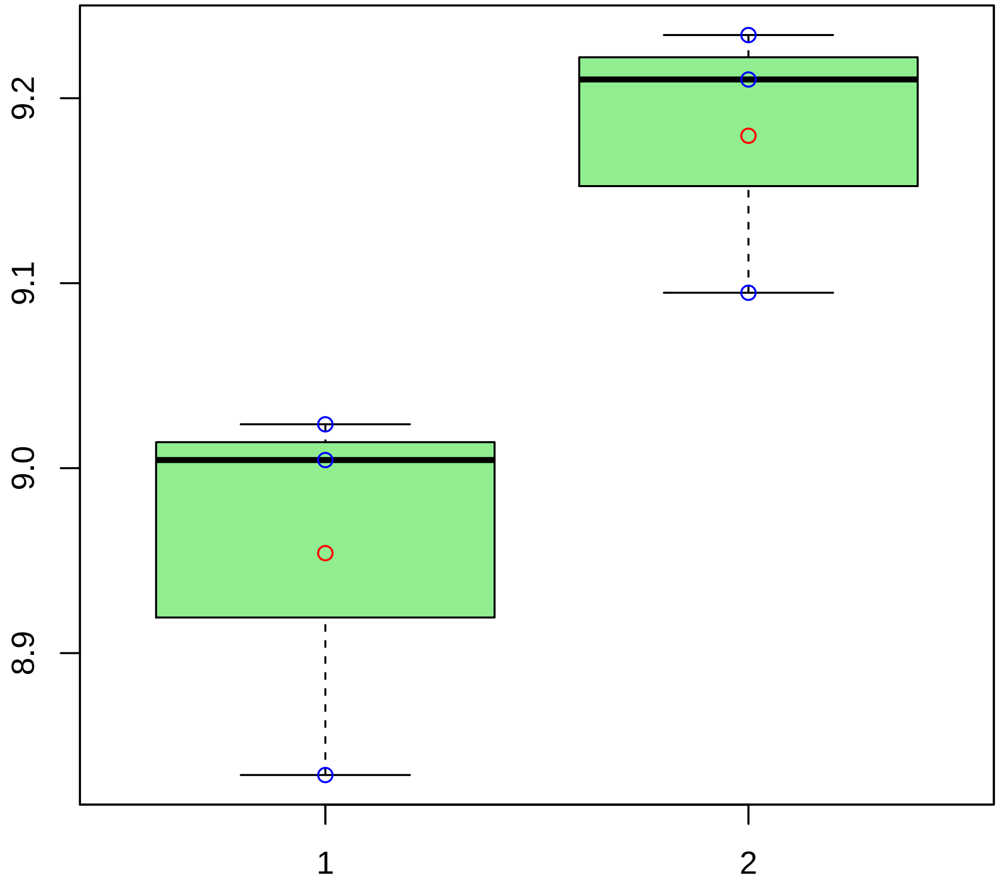


t−Test: p−value = 0.04

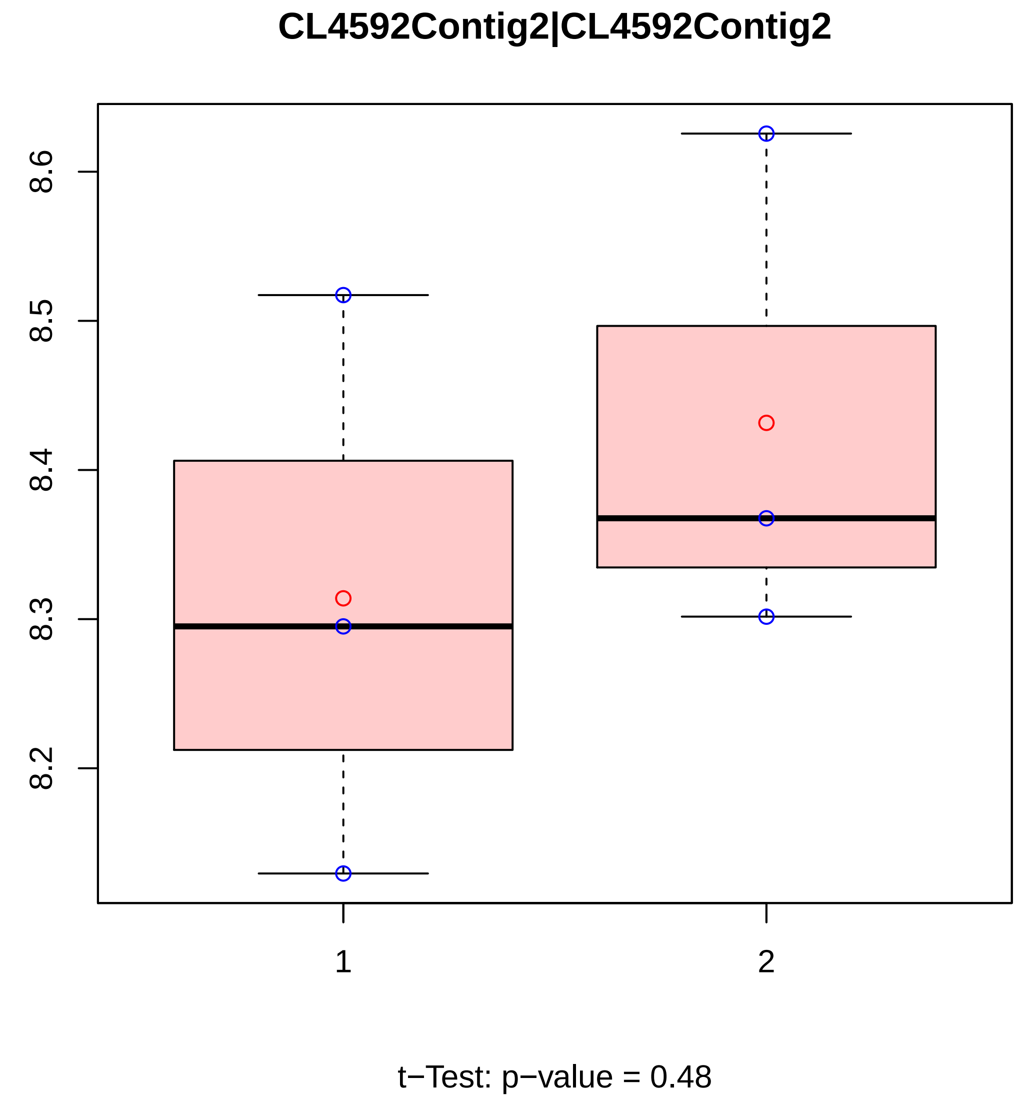
CL4592Contig2|CL4592Contig2
8.2
8.3
8.4
8.5
8.6
1
2
t−Test: p−value = 0.48

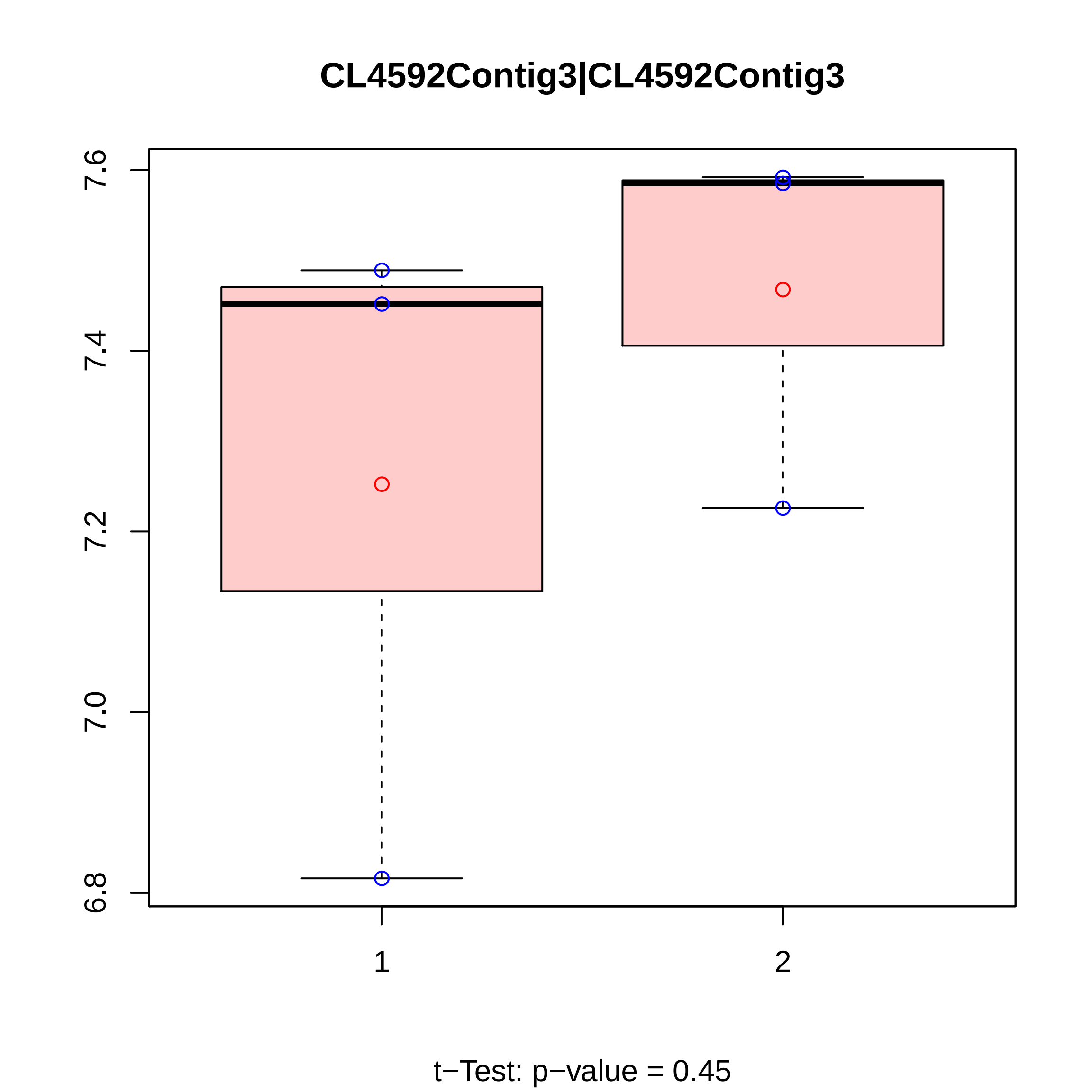
CL4592Contig3|CL4592Contig3
7.6
7.4
7.2
7.0
6.8
1
2
t−Test: p−value = 0.45

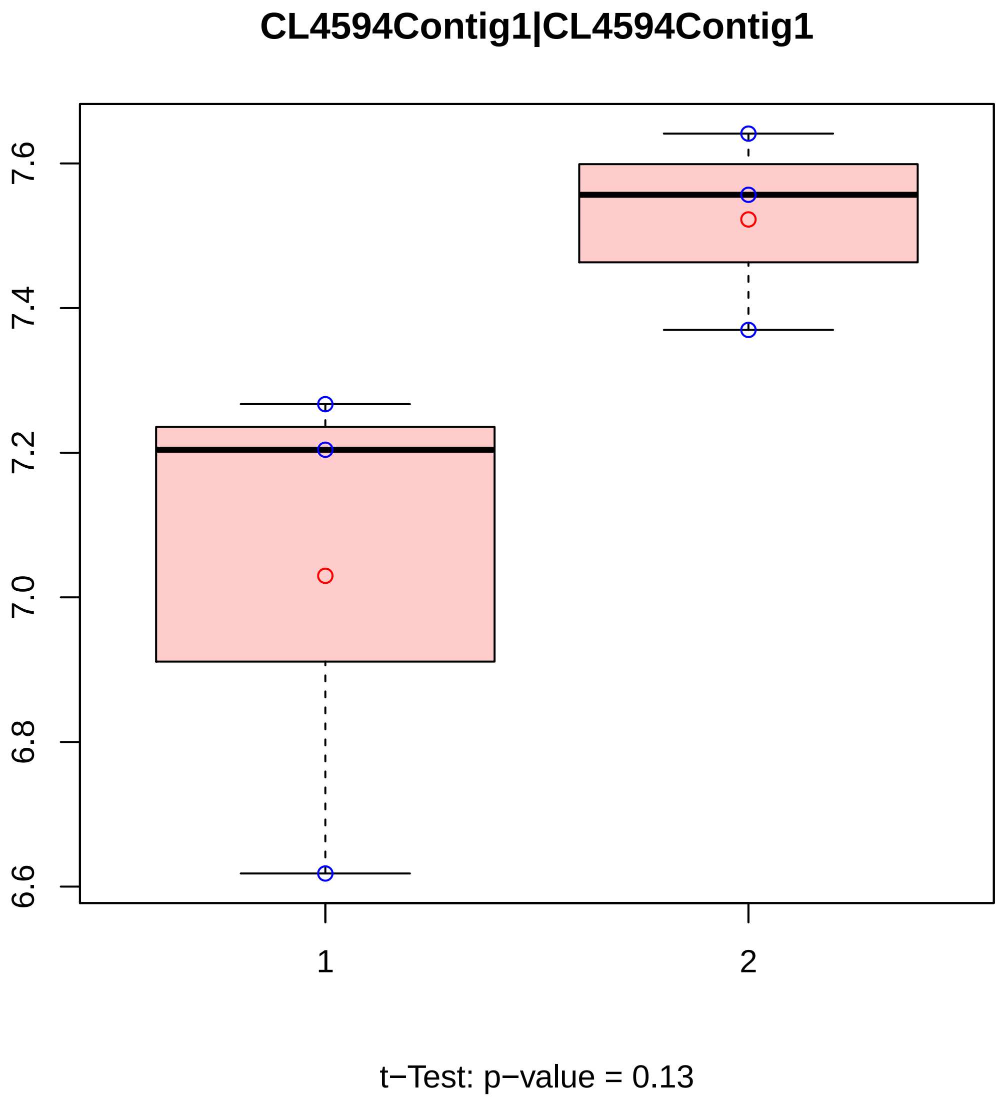
CL4594Contig1|CL4594Contig1
6.6
6.8
7.0
7.2
7.4
7.6
1
2
t−Test: p−value = 0.13

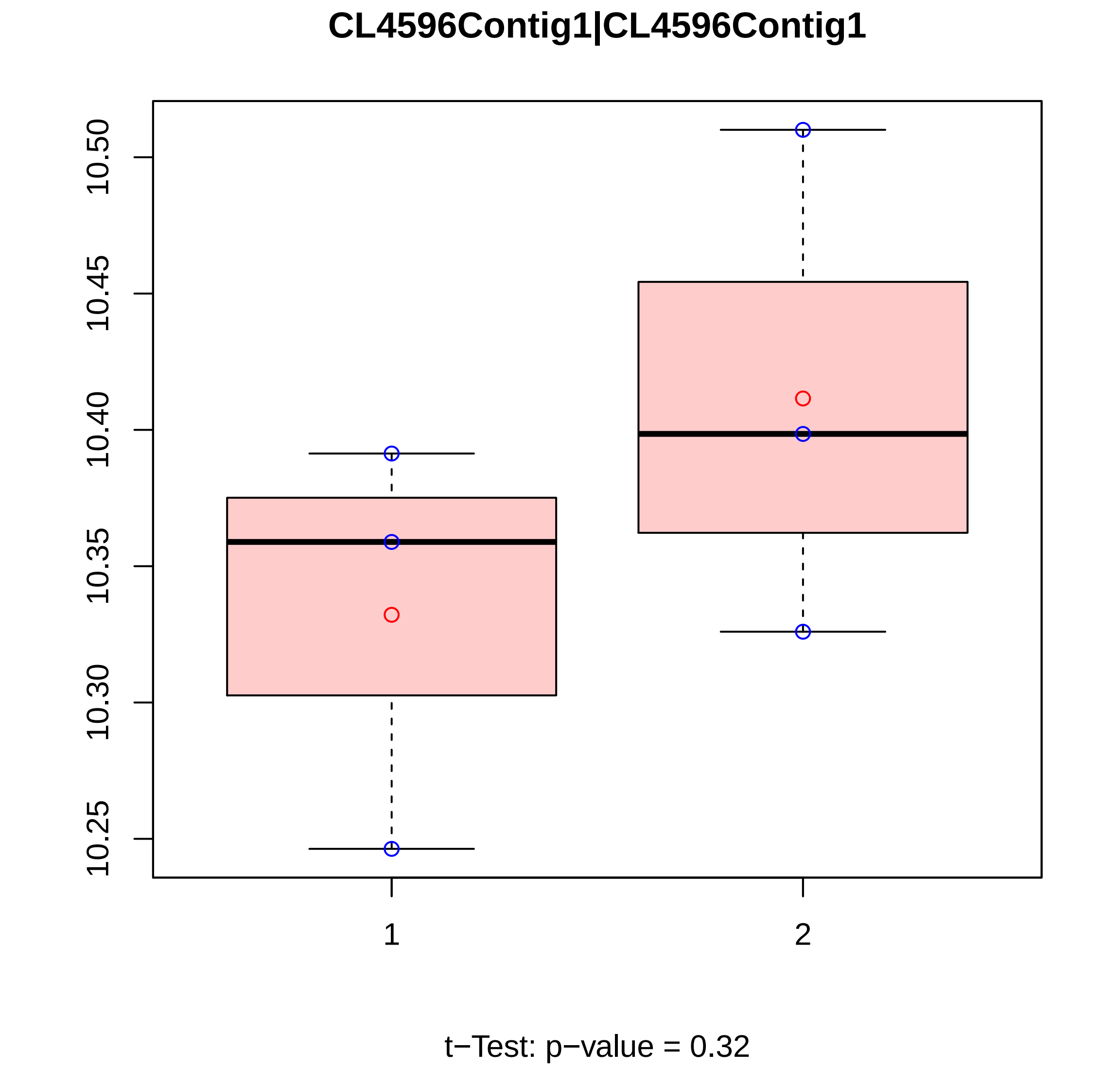

CL4596Contig1|CL4596Contig1
10.25
10.30
10.35
10.40
10.45
10.50
1
2
t−Test: p−value = 0.32

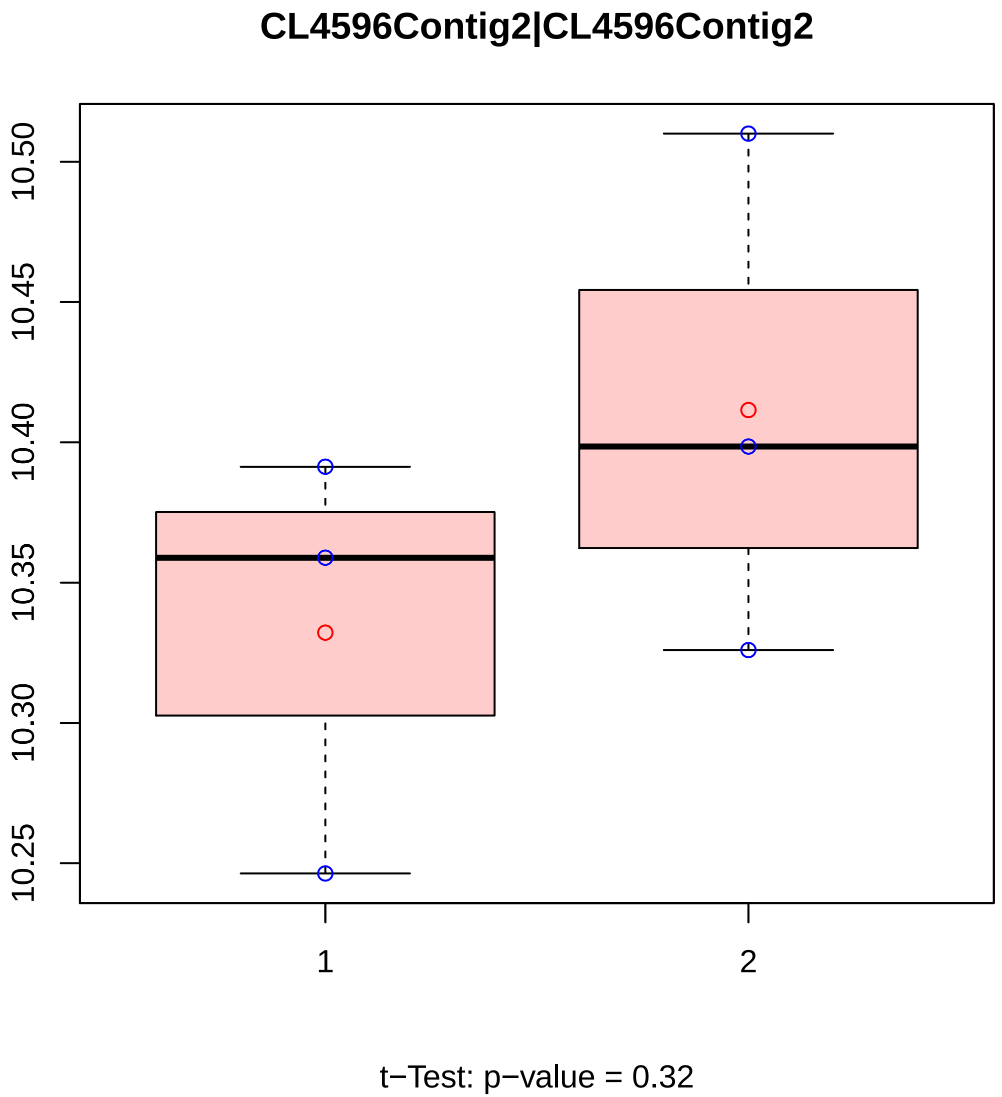

CL4596Contig2|CL4596Contig2
10.25
10.30
10.35
10.40
10.45
10.50
1
2
t−Test: p−value = 0.32

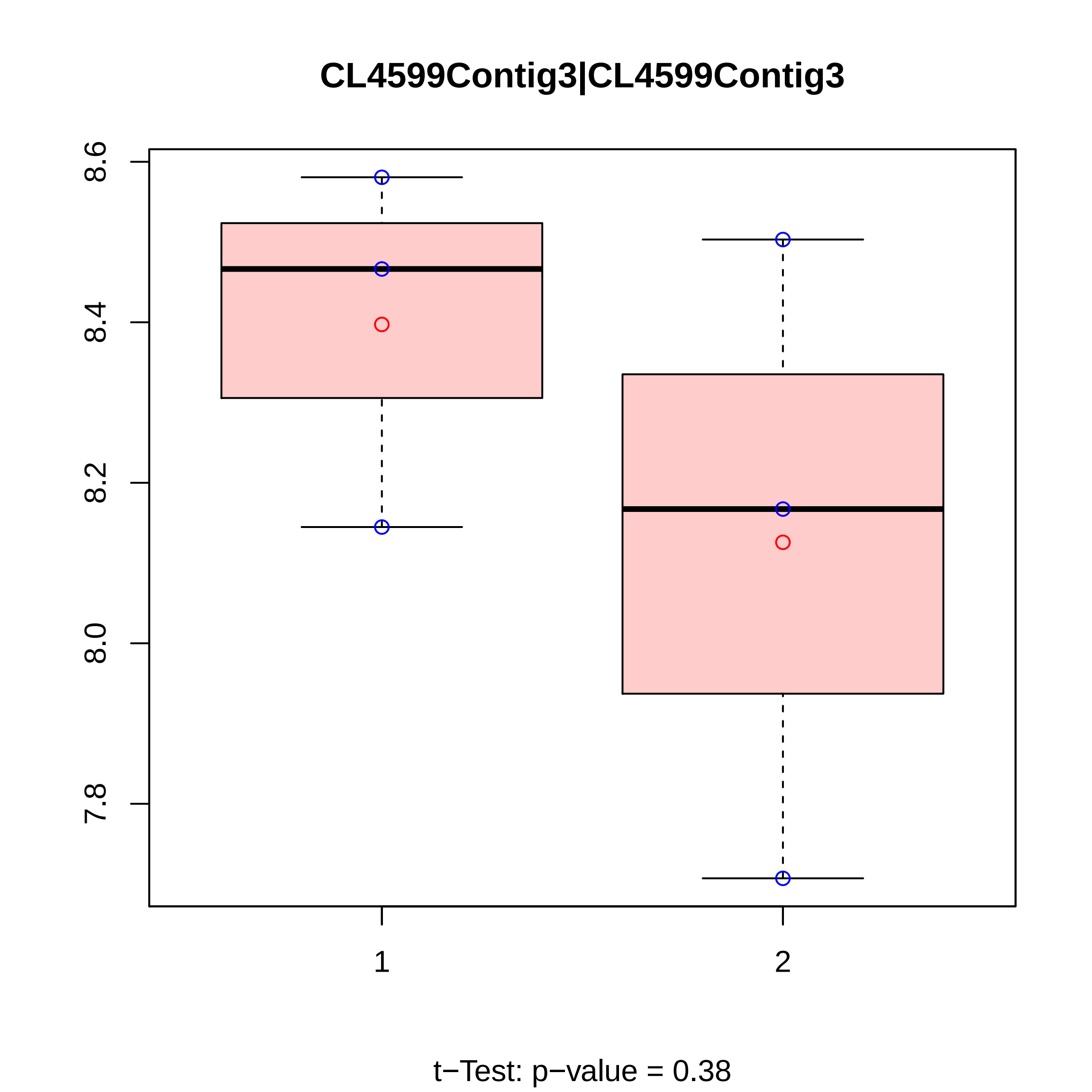
CL4599Contig3|CL4599Contig3
8.6
8.4
8.2
8.0
7.8
1
2
t−Test: p−value = 0.38

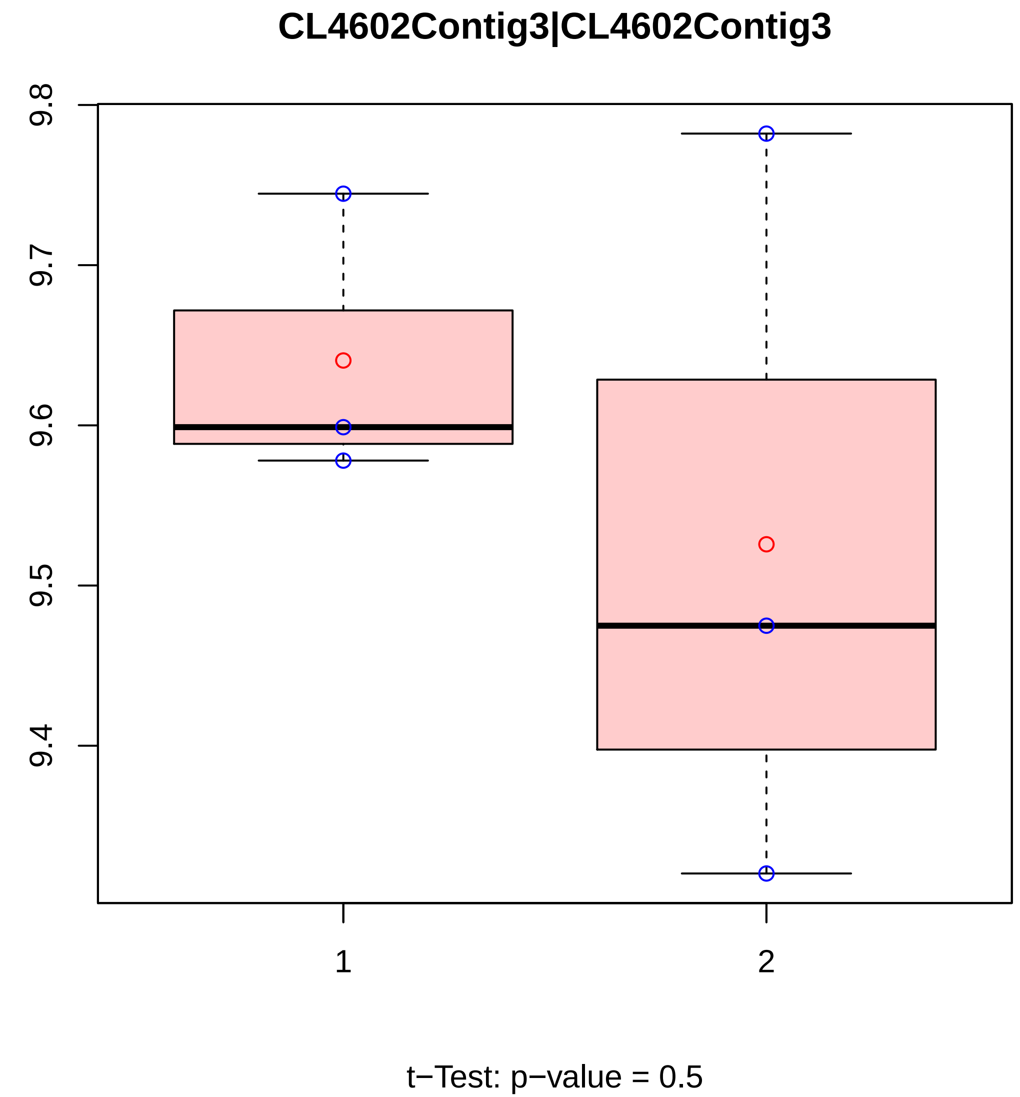
CL4602Contig3|CL4602Contig3
9.8
9.7
9.6
9.5
9.4
1
2
t−Test: p−value = 0.5

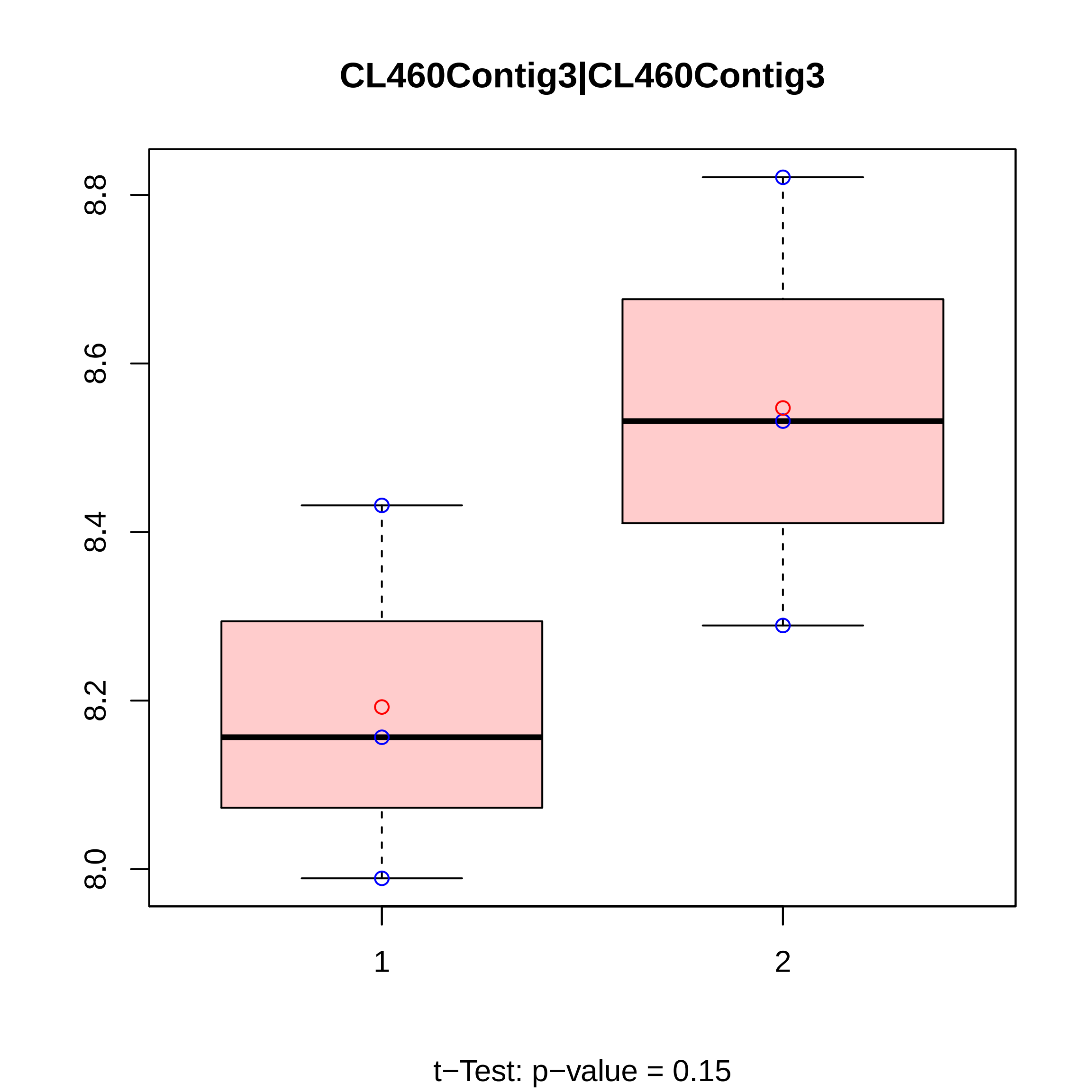

CL460Contig3|CL460Contig3
8.0
8.2
8.4
8.6
8.8
1
2
t−Test: p−value = 0.15

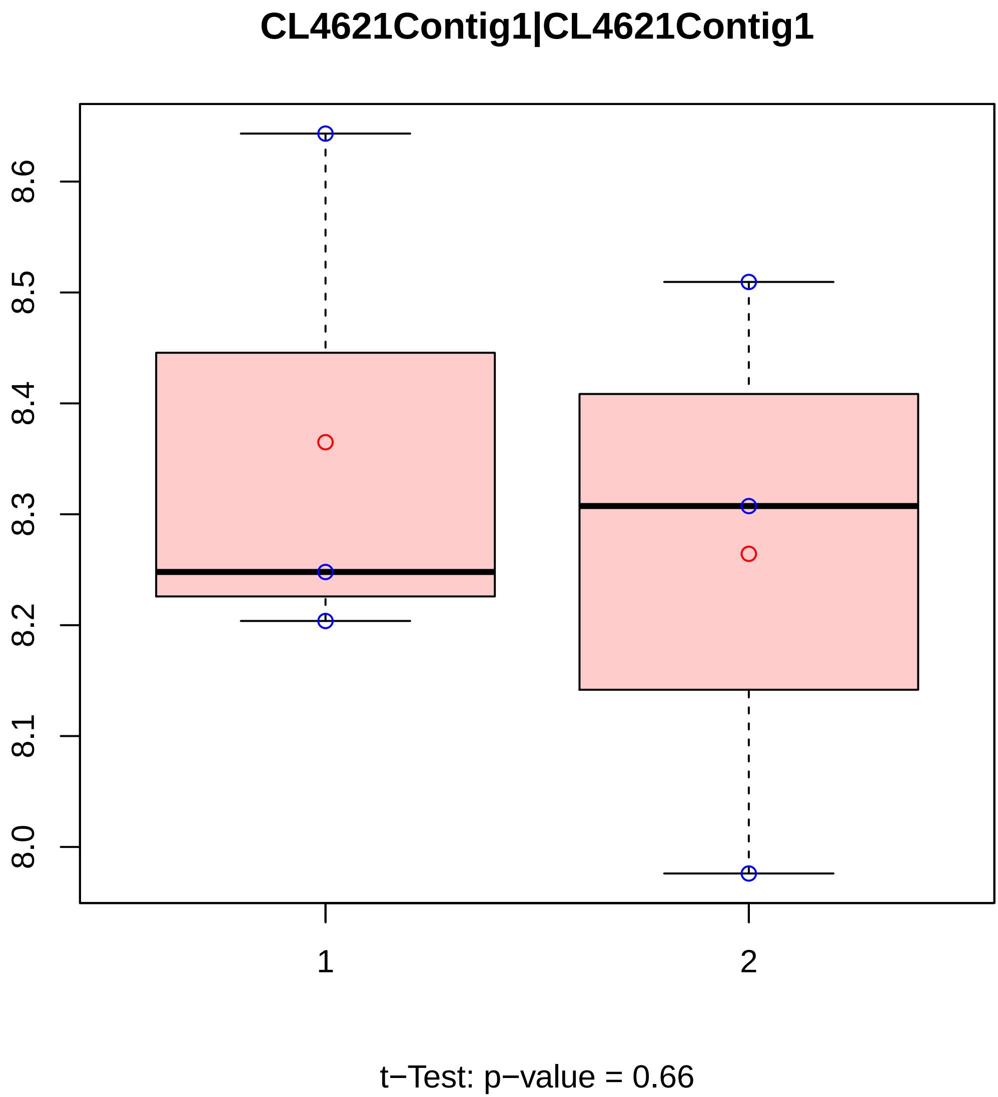
CL4621Contig1|CL4621Contig1
8.0
8.1
8.2
8.3
8.4
8.5
8.6
1
2
t−Test: p−value = 0.66

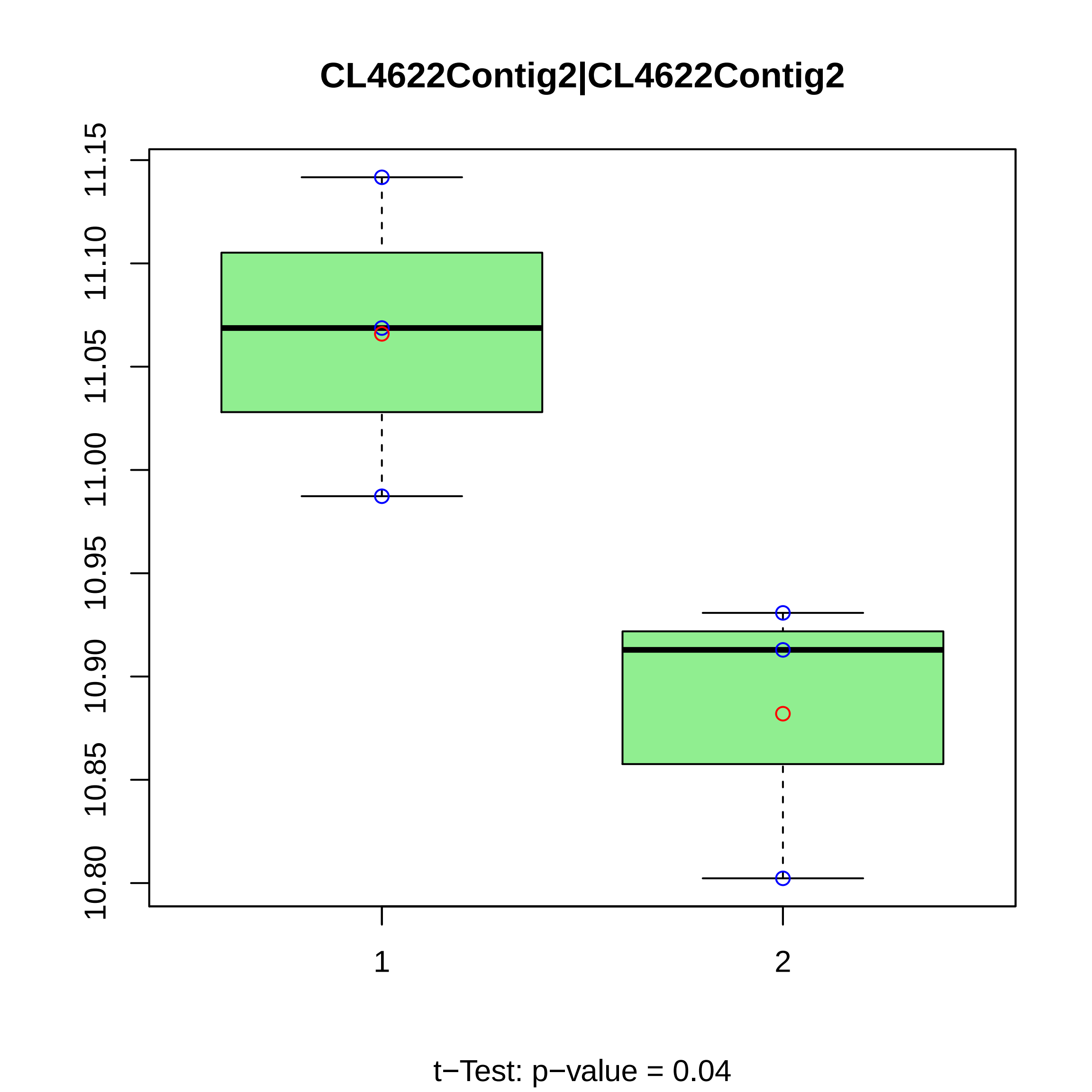
CL4622Contig2|CL4622Contig2
11.15
11.10
11.05
11.00
10.95
10.90
10.85
10.80
1
2
t−Test: p−value = 0.04

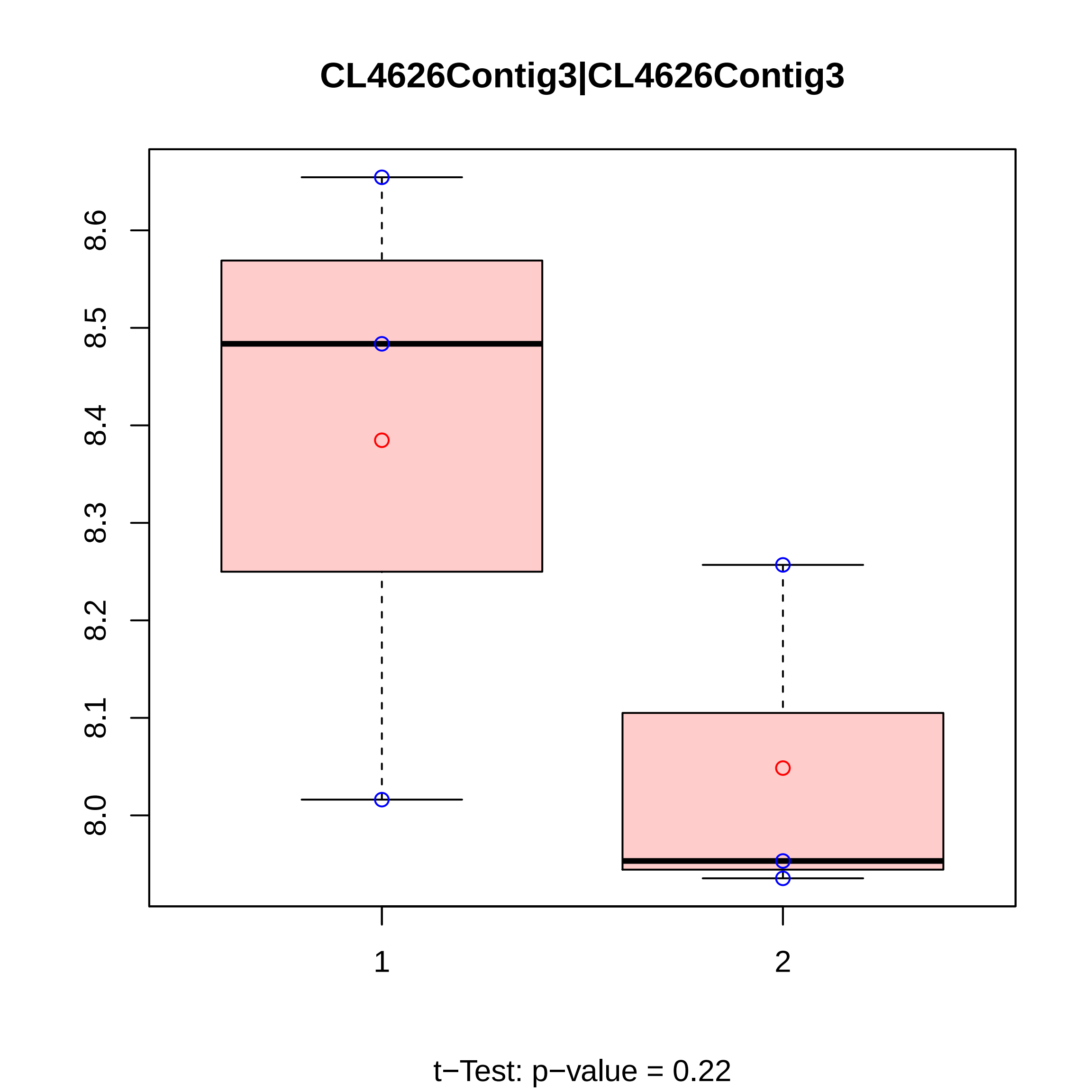
CL4626Contig3|CL4626Contig3
8.0
8.1
8.2
8.3
8.4
8.5
8.6
1
2
t−Test: p−value = 0.22

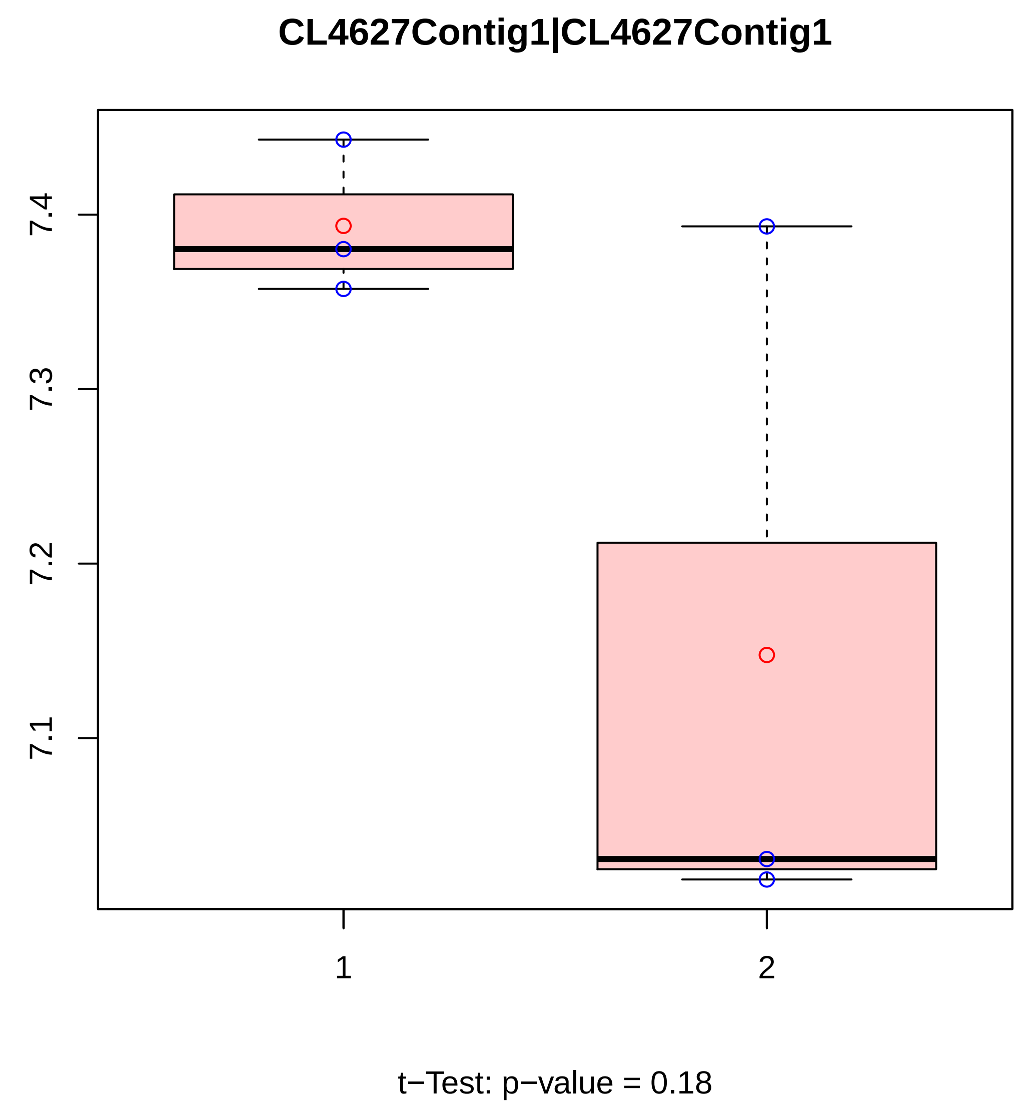

CL4627Contig1|CL4627Contig1
7.4
7.3
7.2
7.1
1
2
t−Test: p−value = 0.18

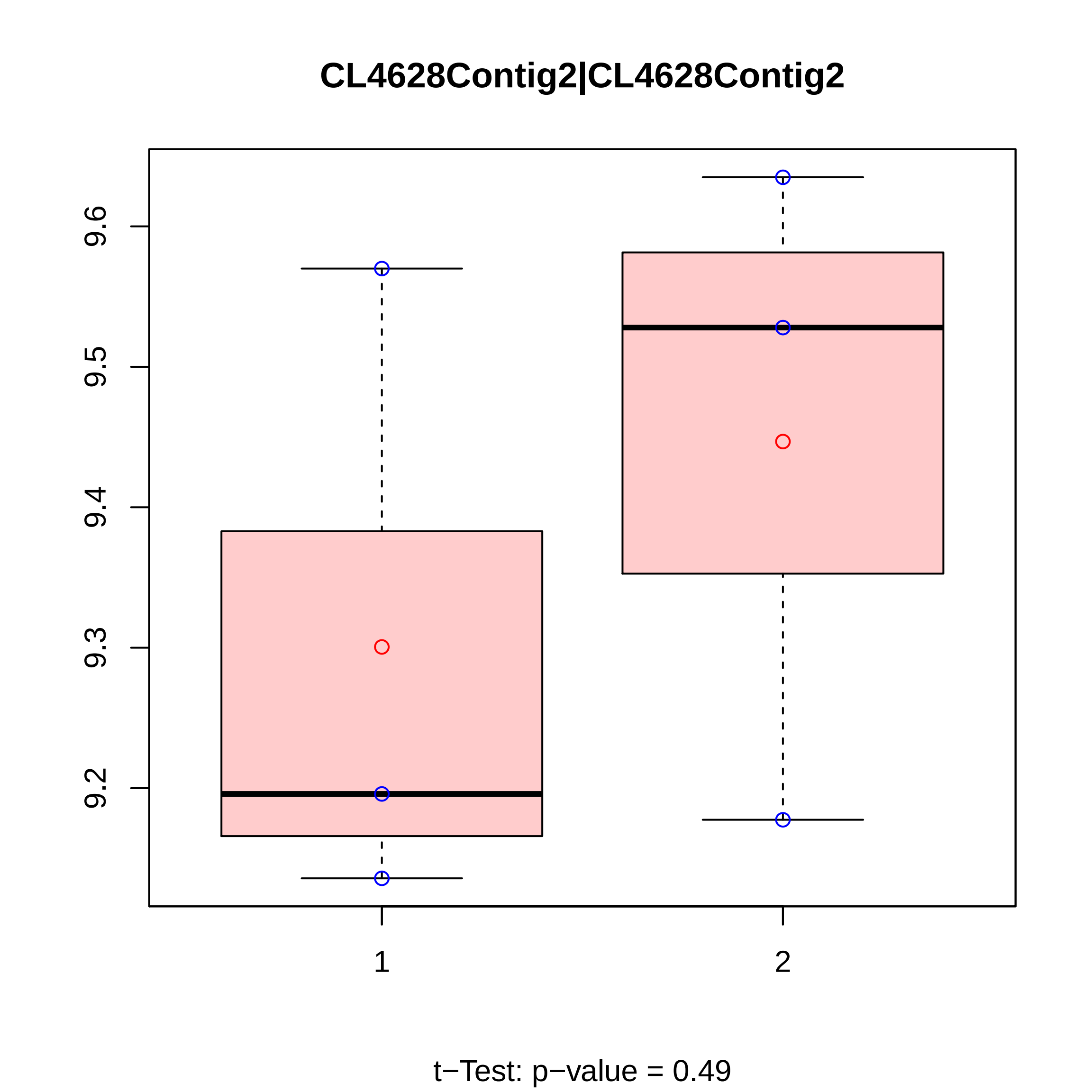
CL4628Contig2|CL4628Contig2
9.2
9.3
9.4
9.5
9.6
1
2
t−Test: p−value = 0.49

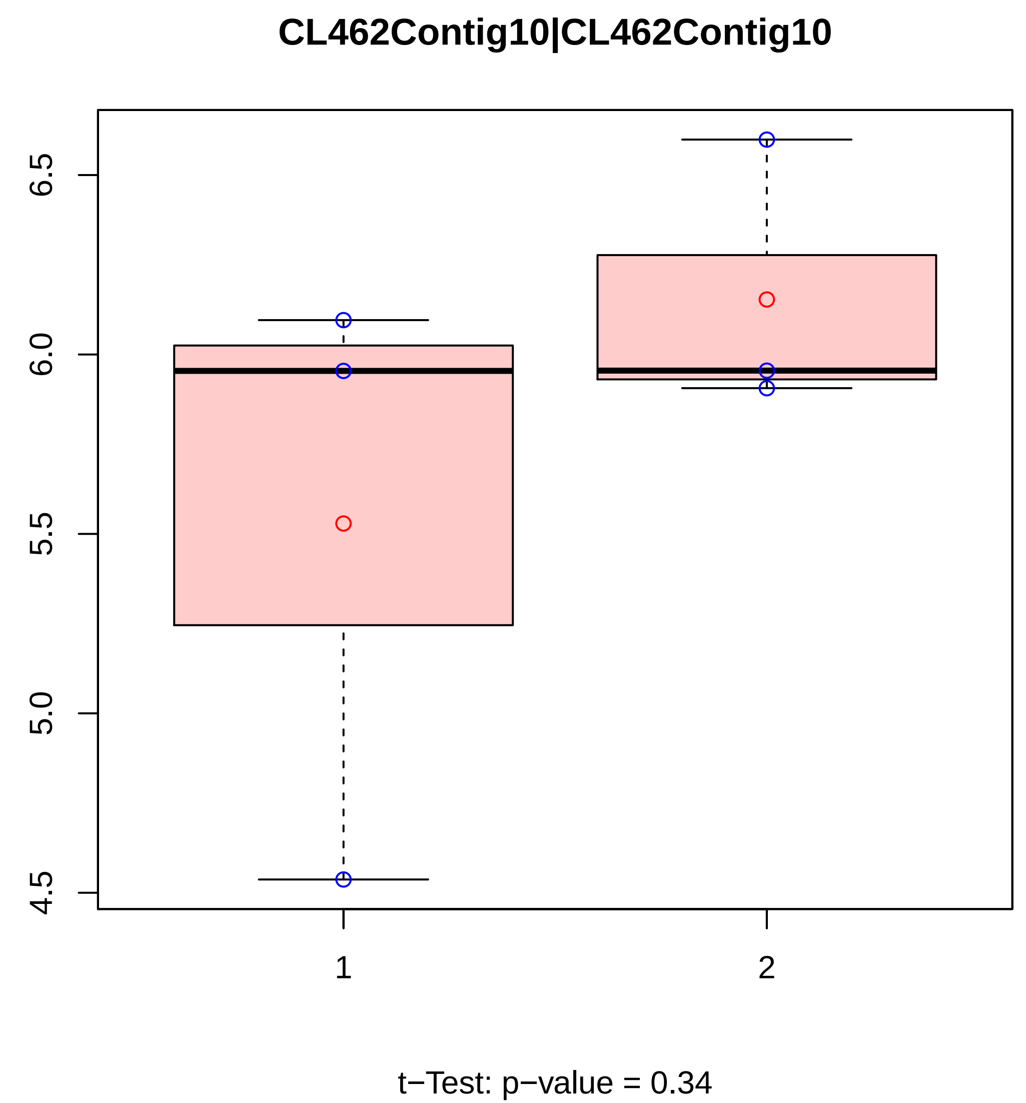
CL462Contig10|CL462Contig10
6.5
6.0
5.5
5.0
4.5
1
2
t−Test: p−value = 0.34

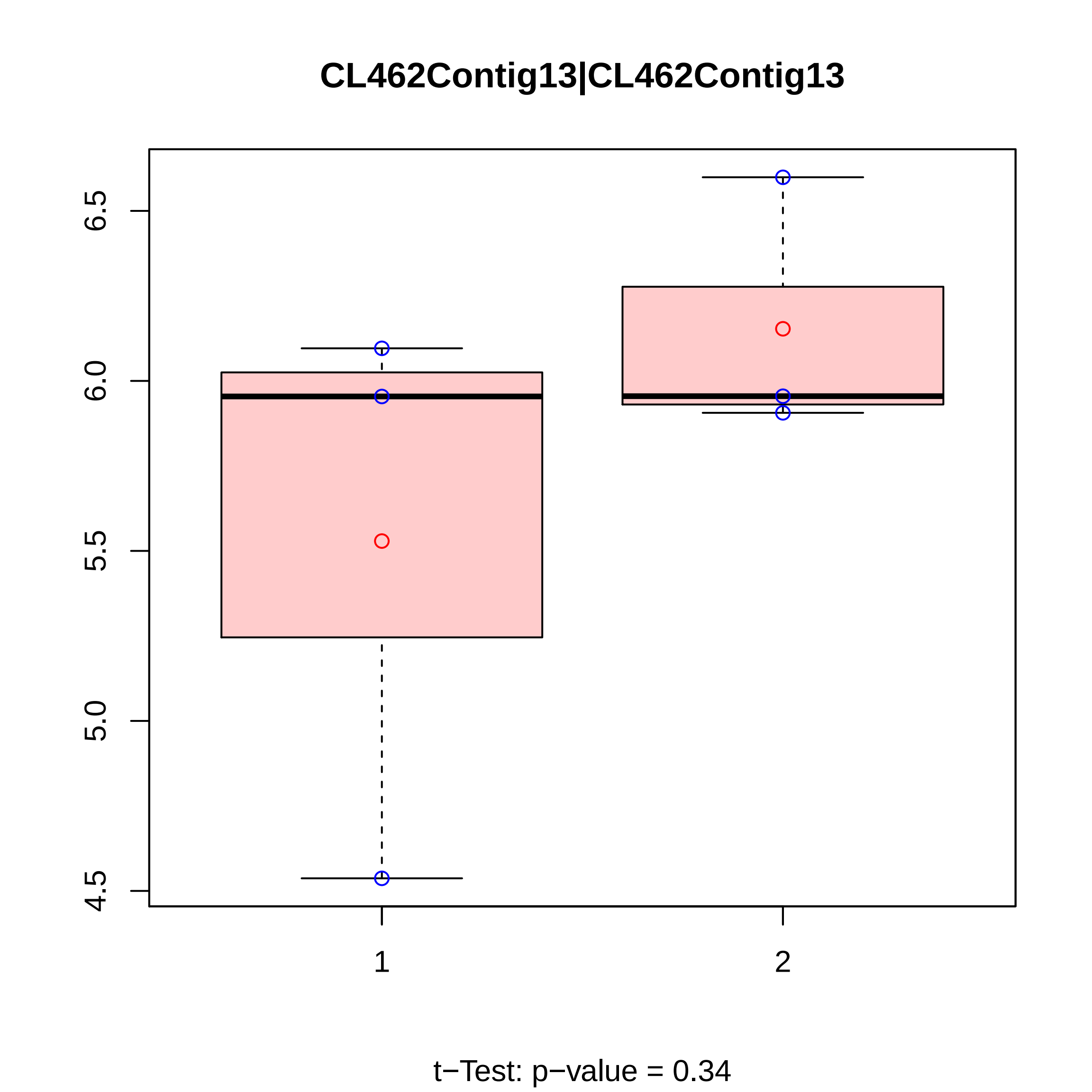

CL462Contig13|CL462Contig13
6.5
6.0
5.5
5.0
4.5
1
2
t−Test: p−value = 0.34

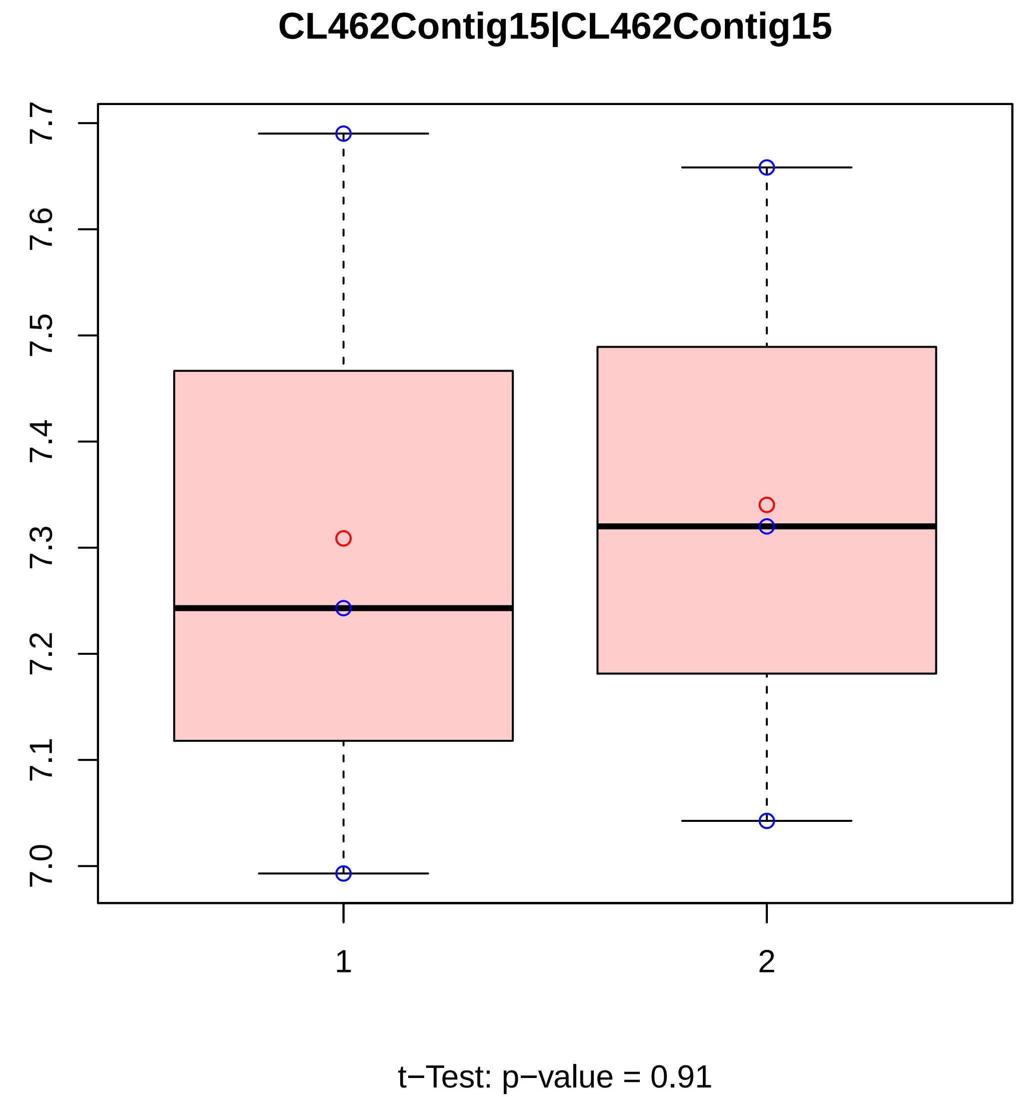
CL462Contig15|CL462Contig15
7.0
7.1
7.2
7.3
7.4
7.5
7.6
7.7
1
2
t−Test: p−value = 0.91

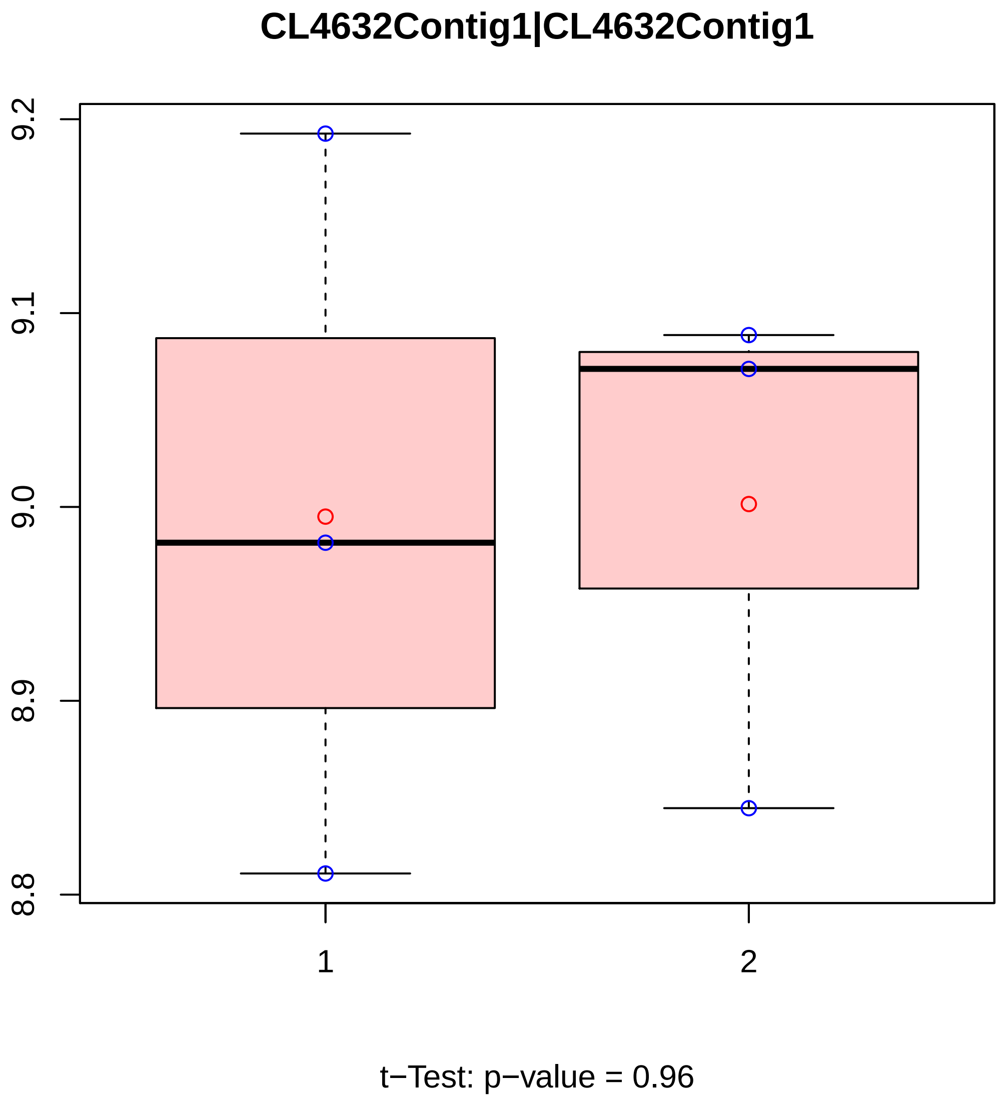

CL4632Contig1|CL4632Contig1
9.2
9.1
9.0
8.9
8.8
1
2
t−Test: p−value = 0.96

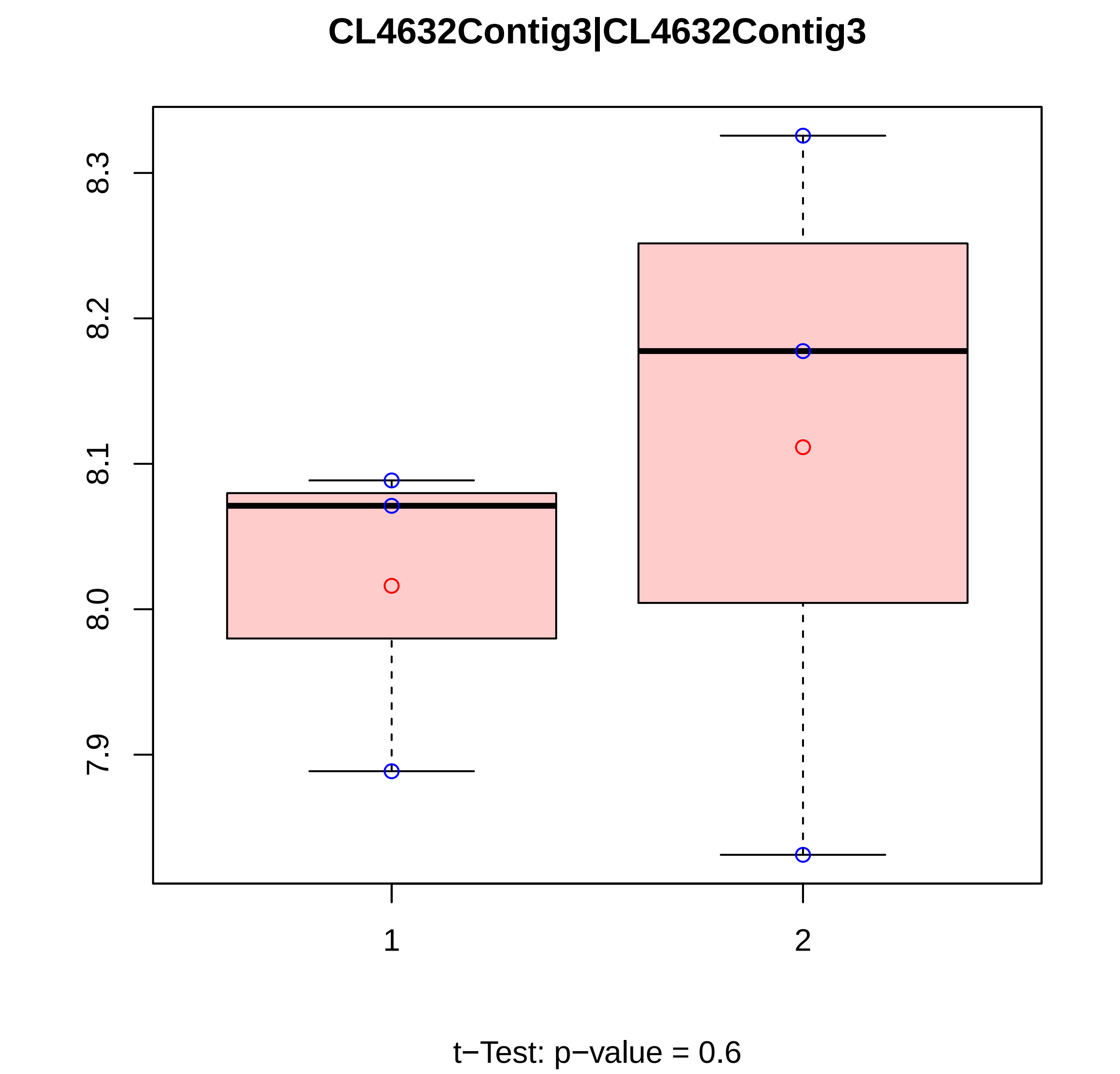

CL4632Contig3|CL4632Contig3
8.3
8.2
8.1
8.0
7.9
1
2
t−Test: p−value = 0.6

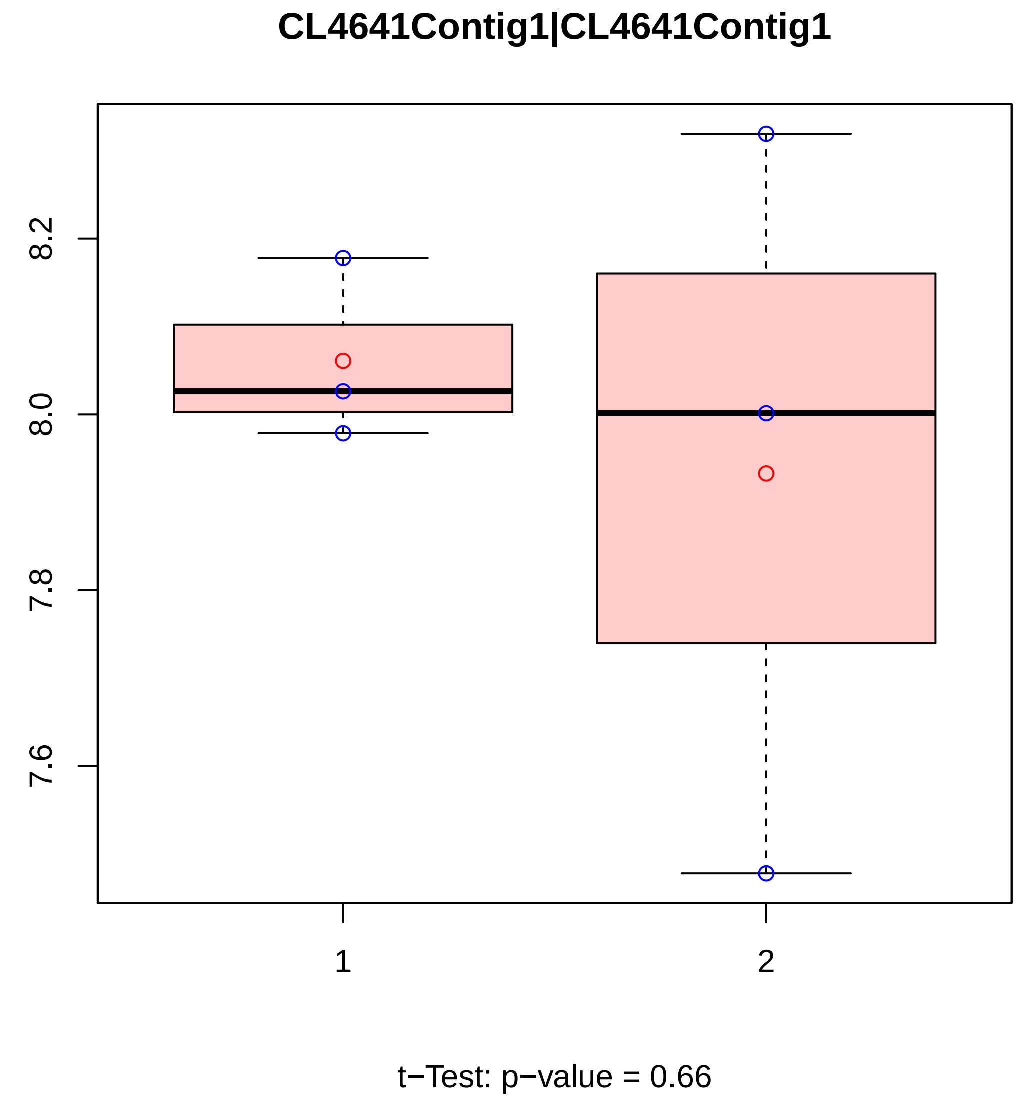
CL4641Contig1|CL4641Contig1
8.2
8.0
7.8
7.6
1
2
t−Test: p−value = 0.66

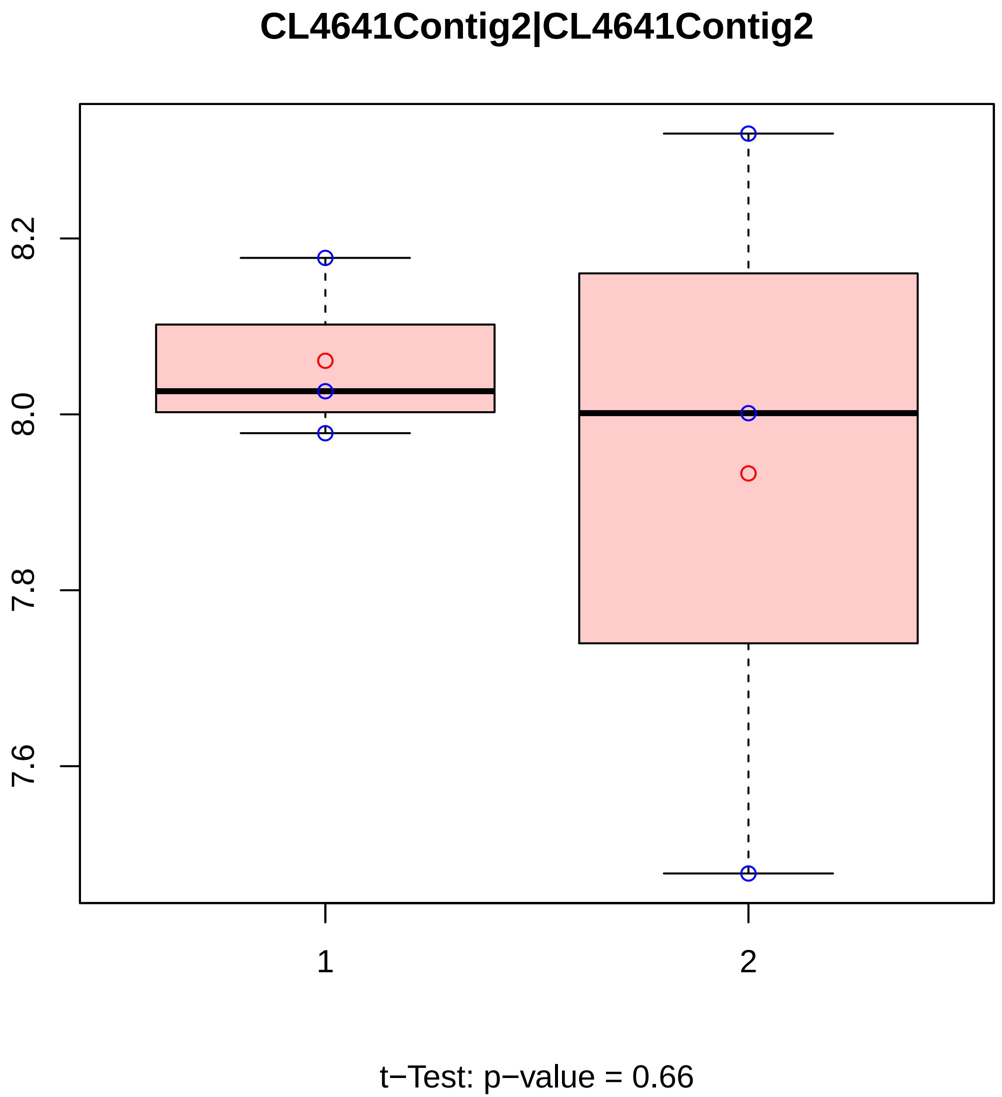
CL4641Contig2|CL4641Contig2
8.2
8.0
7.8
7.6
1
2
t−Test: p−value = 0.66

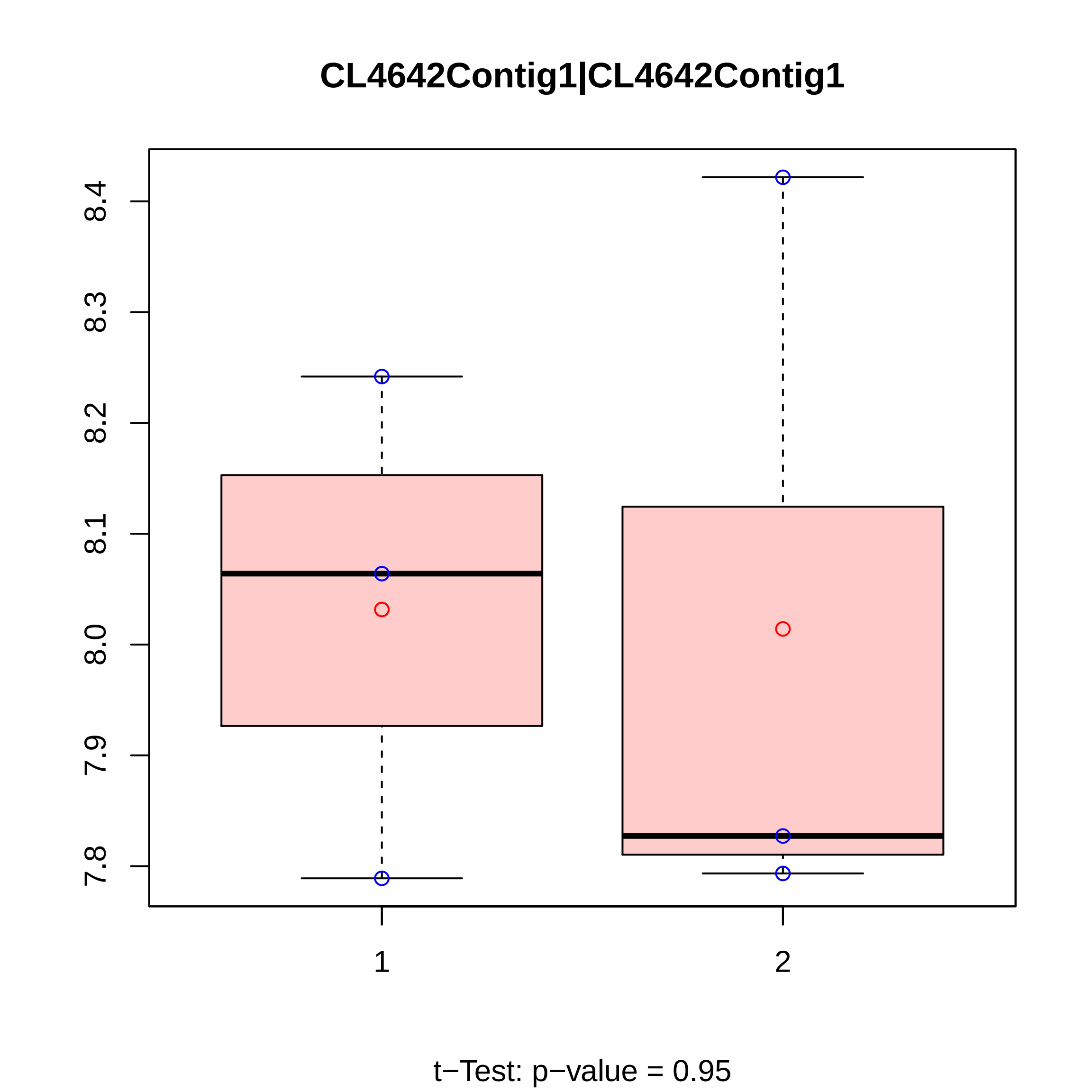
CL4642Contig1|CL4642Contig1
8.4
8.3
8.2
8.1
8.0
7.9
7.8
1
2
t−Test: p−value = 0.95

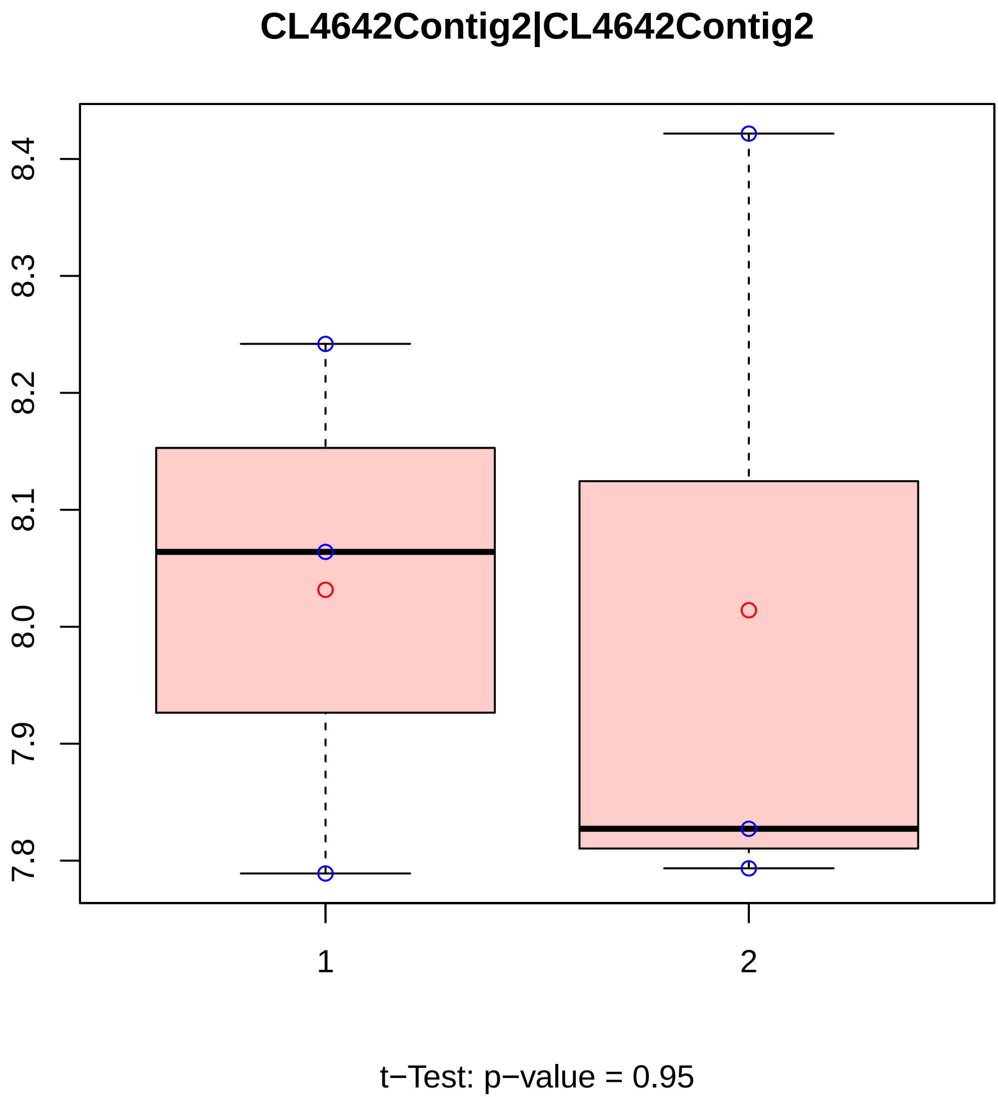

CL4642Contig2|CL4642Contig2
8.4
8.3
8.2
8.1
8.0
7.9
7.8
1
2
t−Test: p−value = 0.95

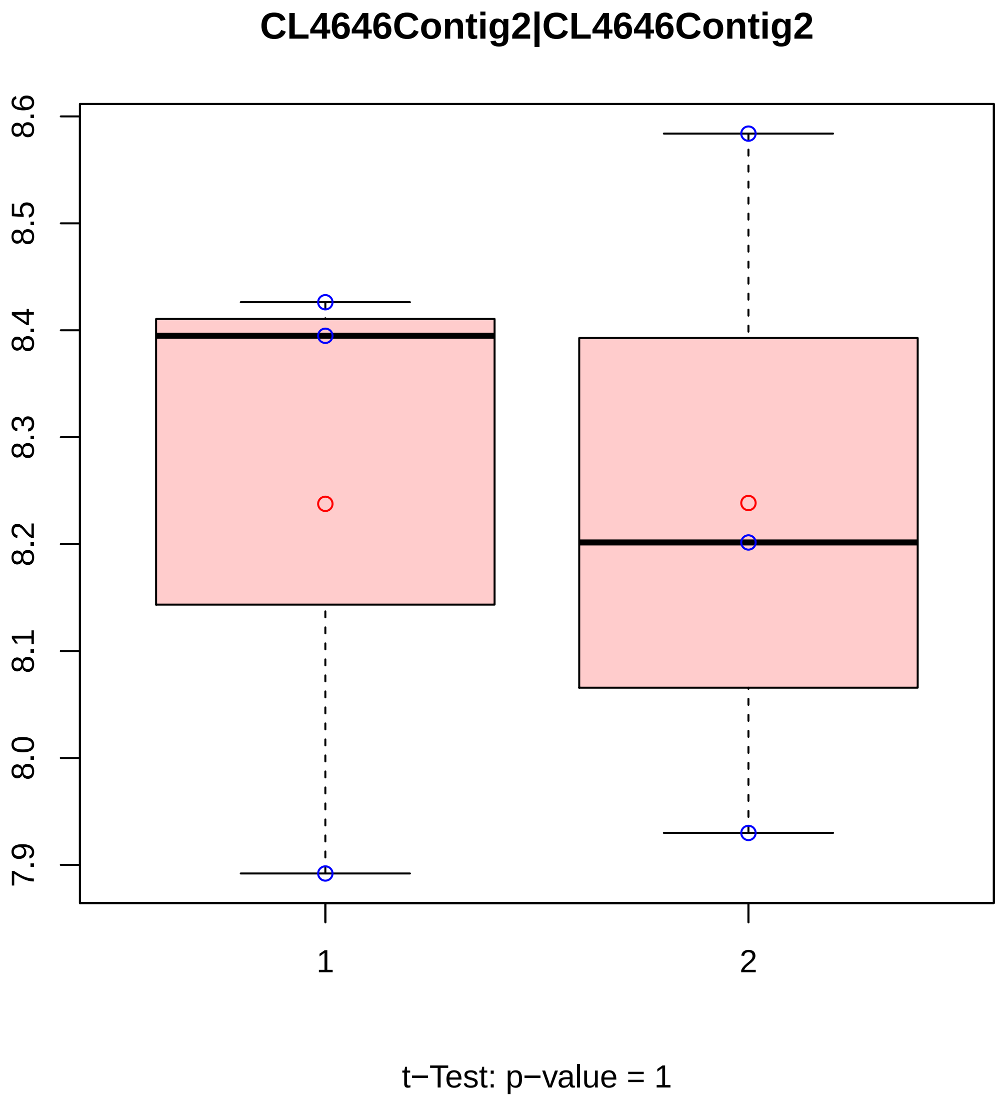

CL4646Contig2|CL4646Contig2
8.6
8.5
8.4
8.3
8.2
8.1
8.0
7.9
1
2
t−Test: p−value = 1

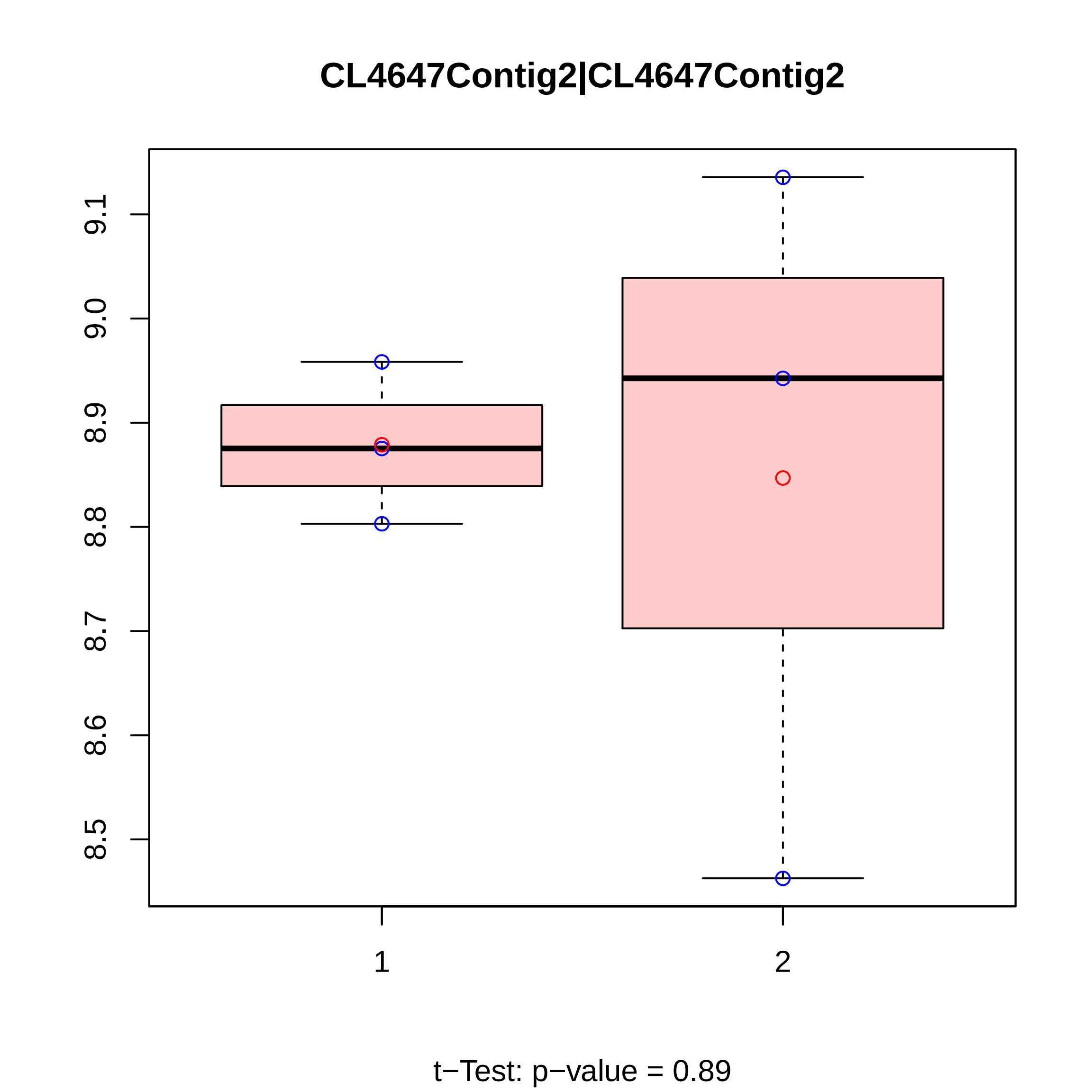
CL4647Contig2|CL4647Contig2
9.1
9.0
8.9
8.8
8.7
8.6
8.5
1
2
t−Test: p−value = 0.89

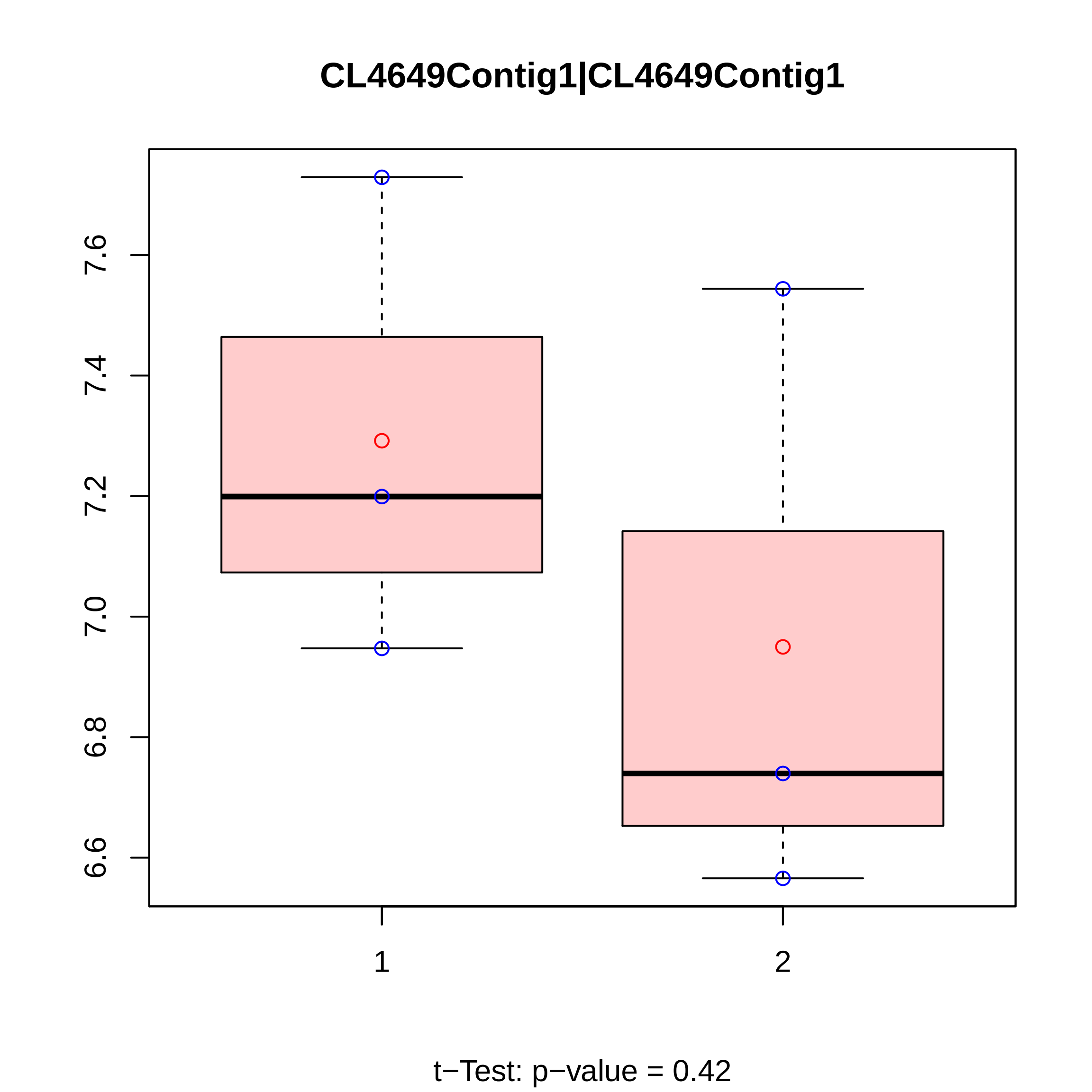

CL4649Contig1|CL4649Contig1
7.6
7.4
7.2
7.0
6.8
6.6
1
2
t−Test: p−value = 0.42

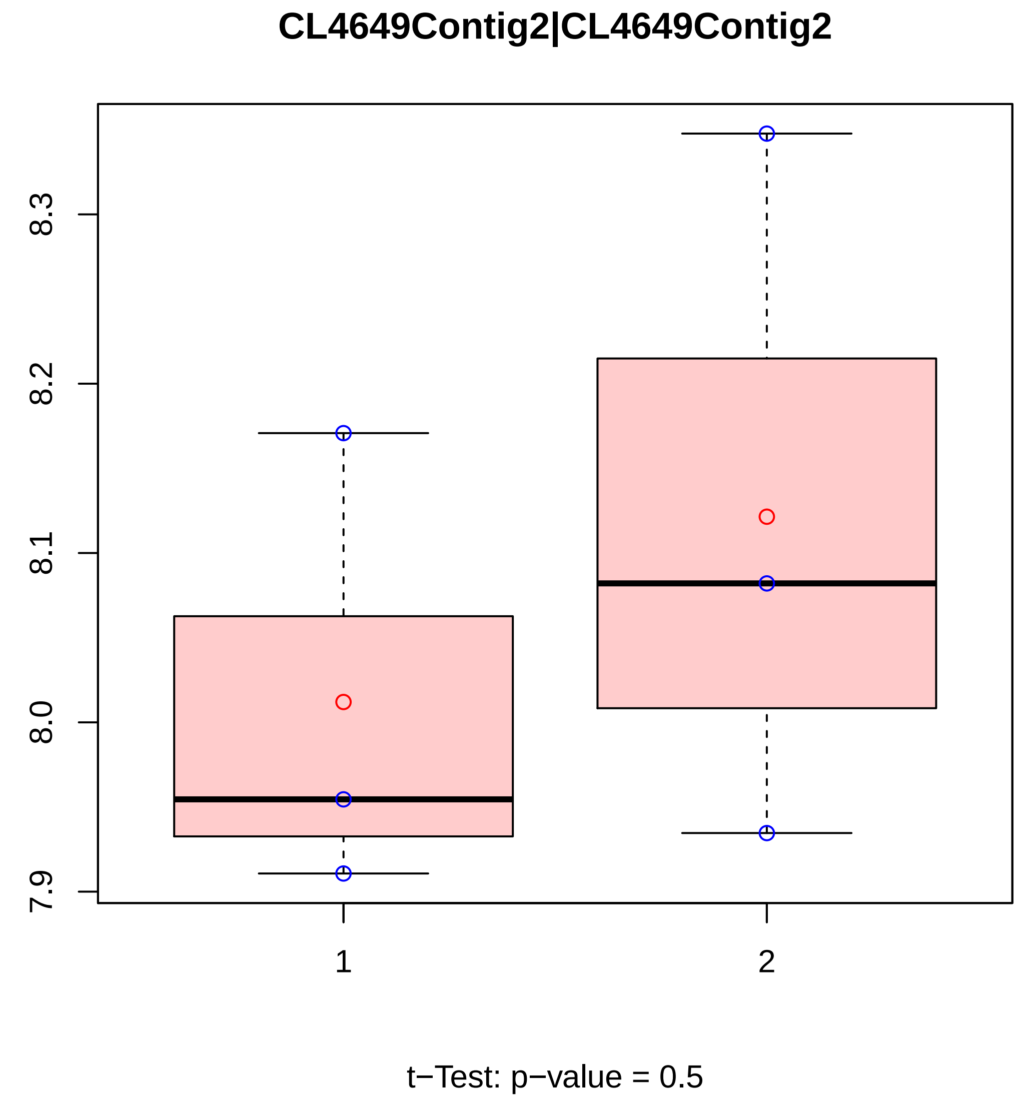
CL4649Contig2|CL4649Contig2
8.3
8.2
8.1
8.0
7.9
1
2
t−Test: p−value = 0.5

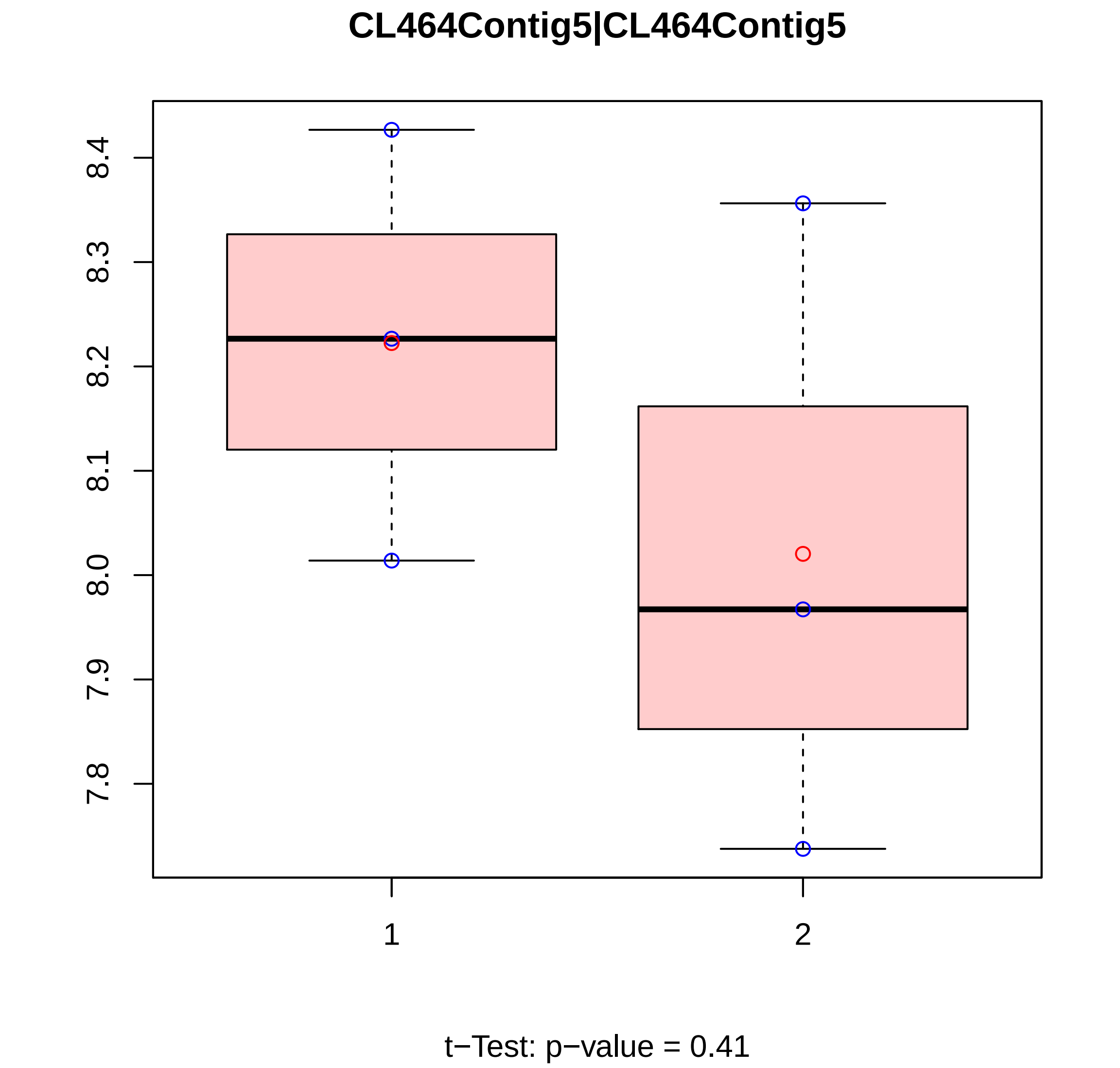
CL464Contig5|CL464Contig5
8.4
8.3
8.2
8.1
8.0
7.9
7.8
1
2
t−Test: p−value = 0.41

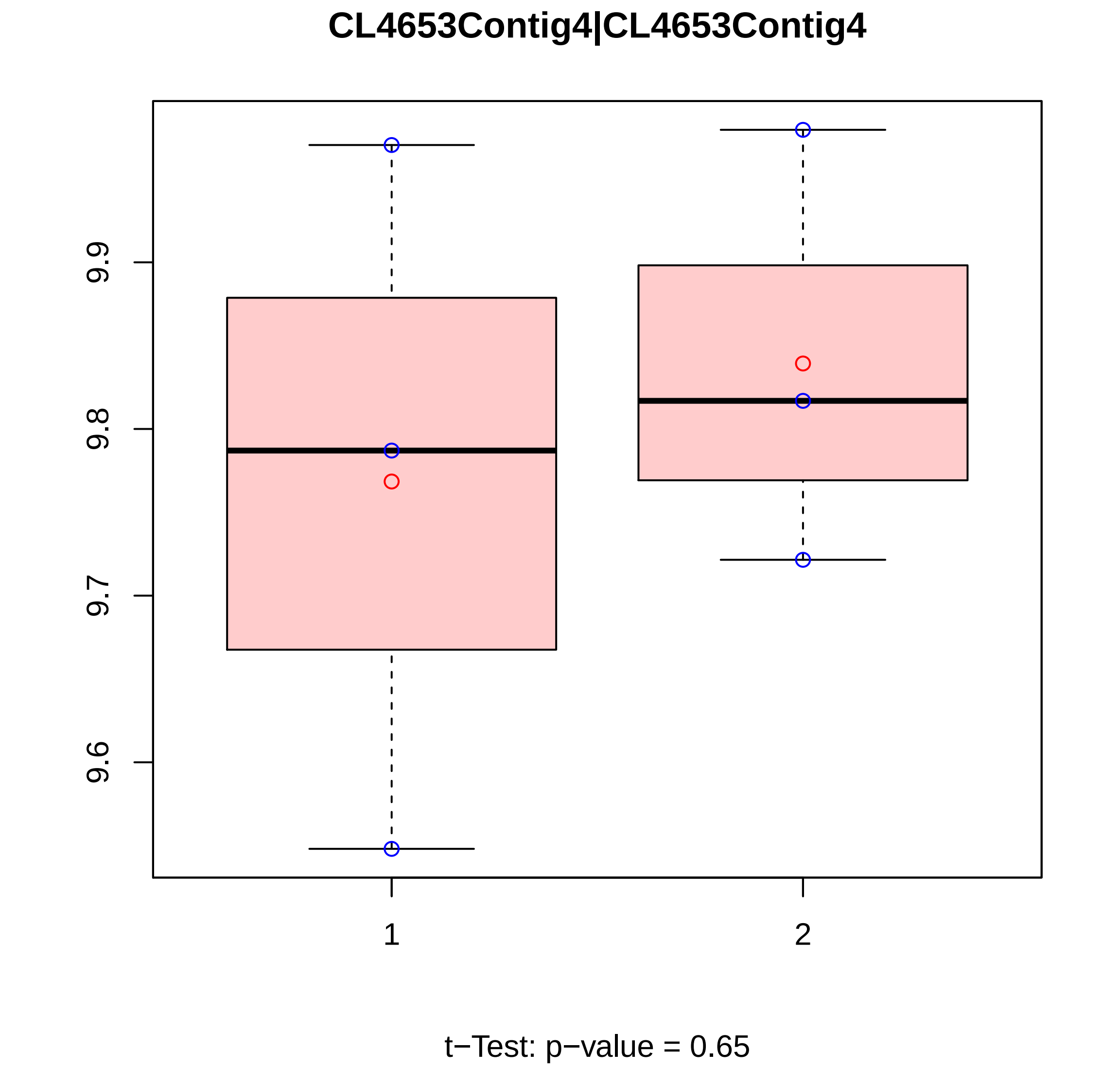

CL4653Contig4|CL4653Contig4
9.6
9.7
9.8
9.9
1
2
t−Test: p−value = 0.65

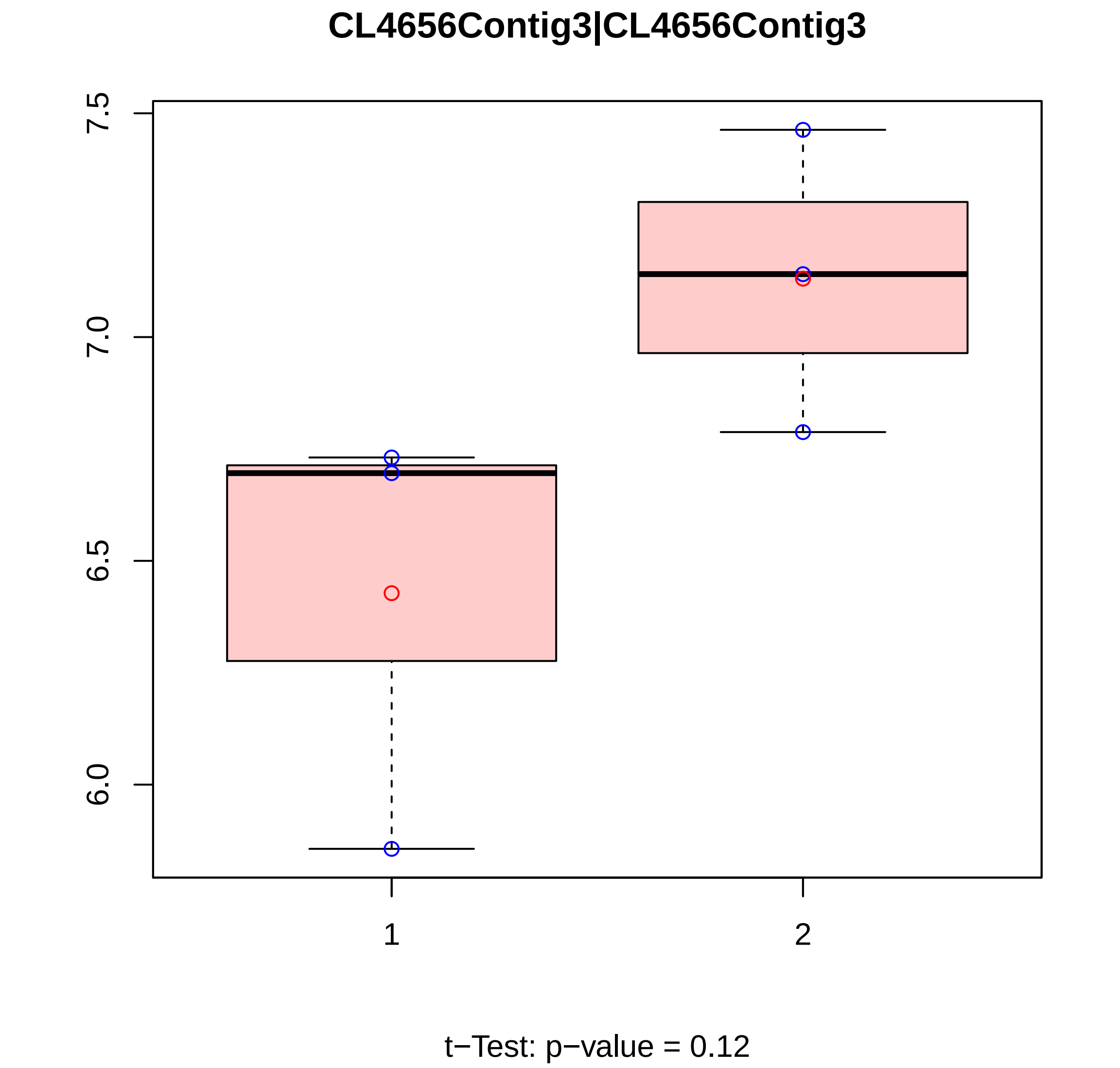
CL4656Contig3|CL4656Contig3
7.5
7.0
6.5
6.0
1
2
t−Test: p−value = 0.12

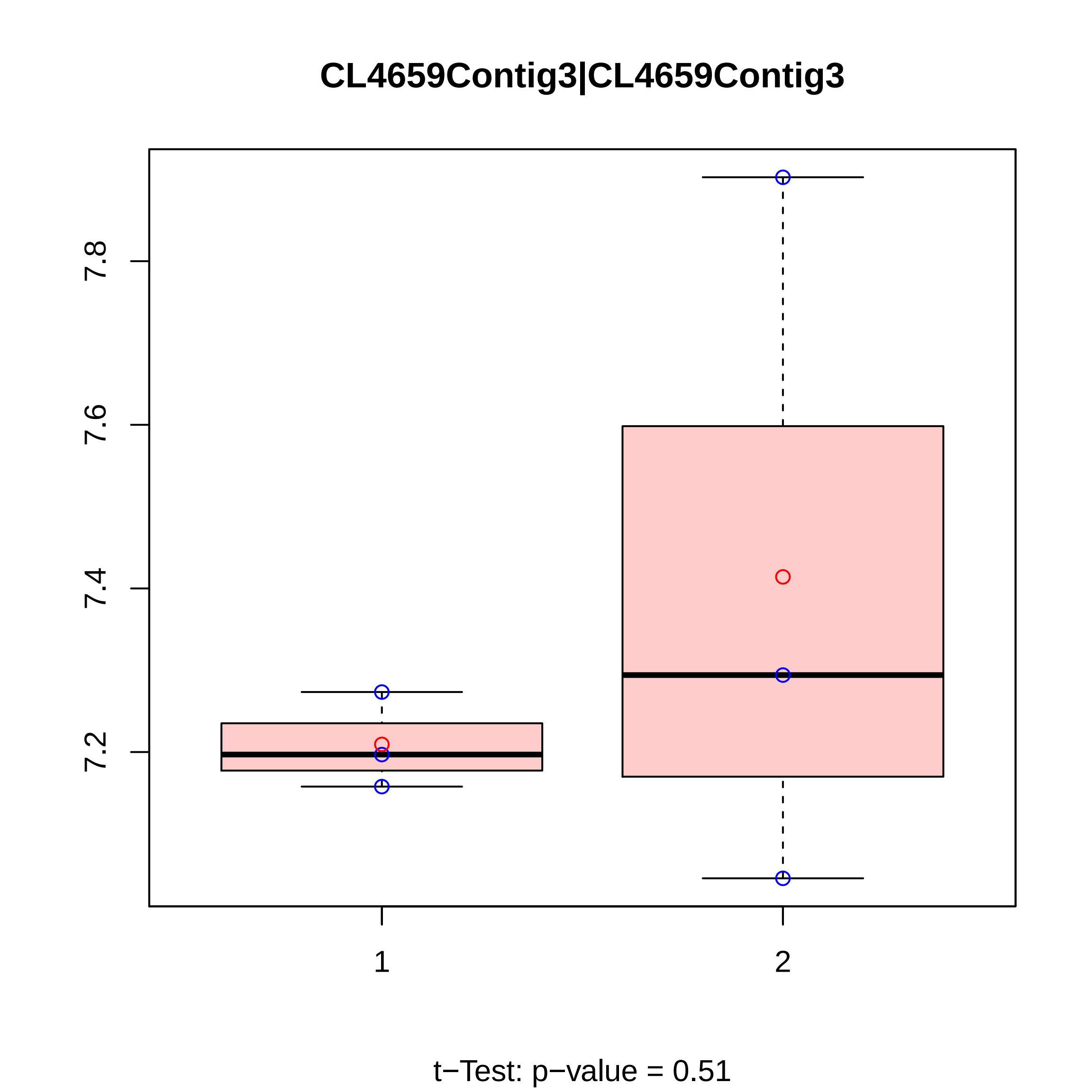
CL4659Contig3|CL4659Contig3
7.2
7.4
7.6
7.8
1
2
t−Test: p−value = 0.51

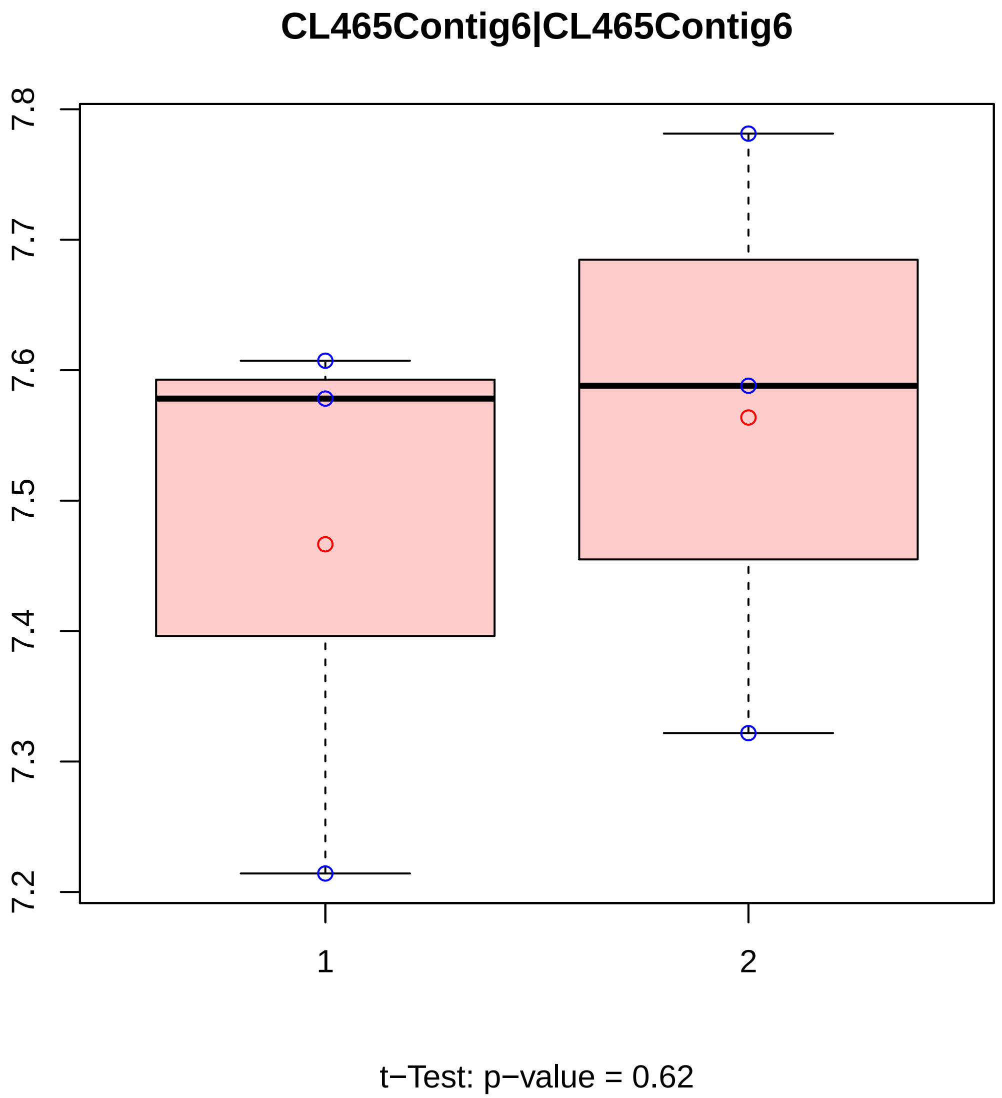

CL465Contig6|CL465Contig6
7.2
7.3
7.4
7.5
7.6
7.7
7.8
1
2
t−Test: p−value = 0.62

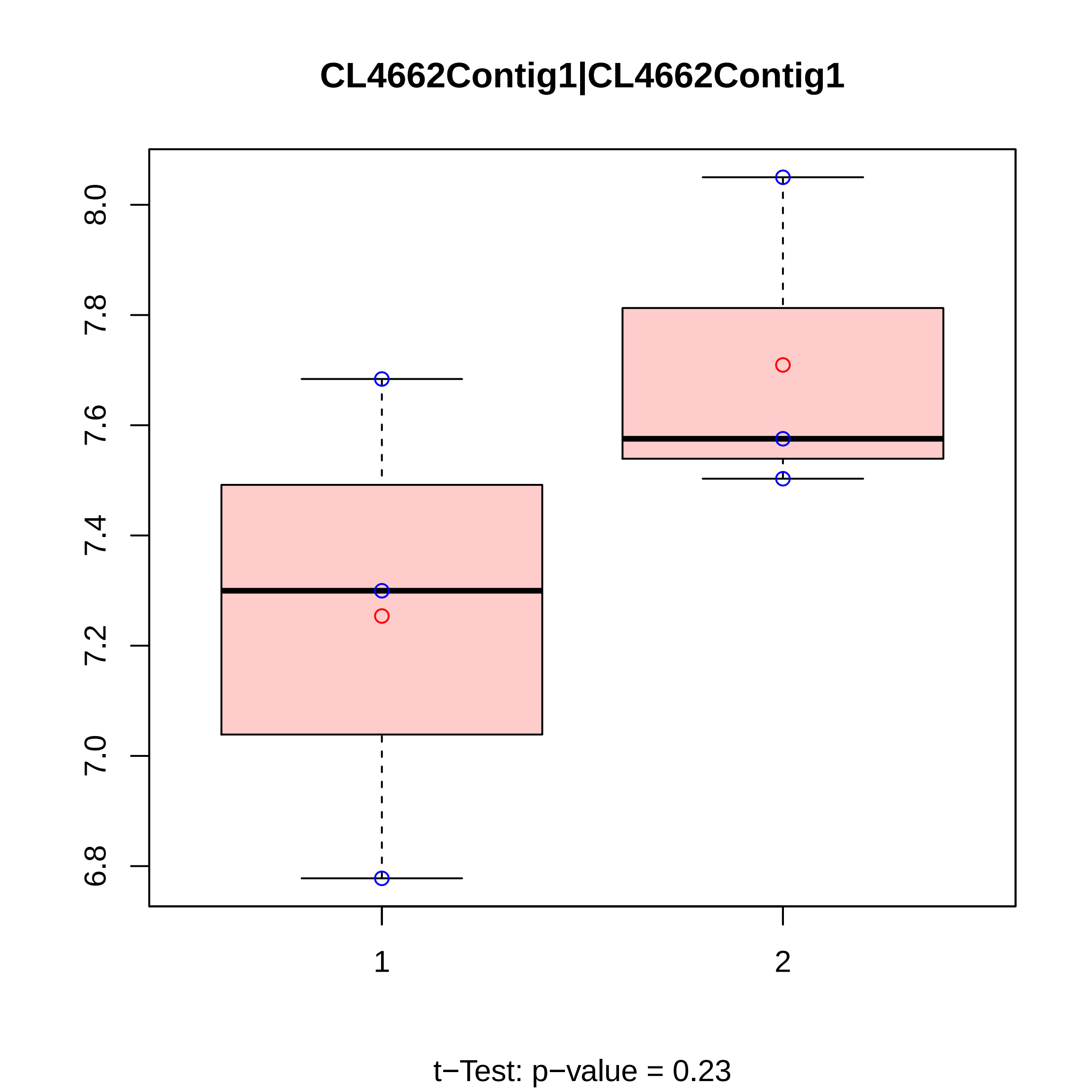
CL4662Contig1|CL4662Contig1
8.0
7.8
7.6
7.4
7.2
7.0
6.8
1
2
t−Test: p−value = 0.23

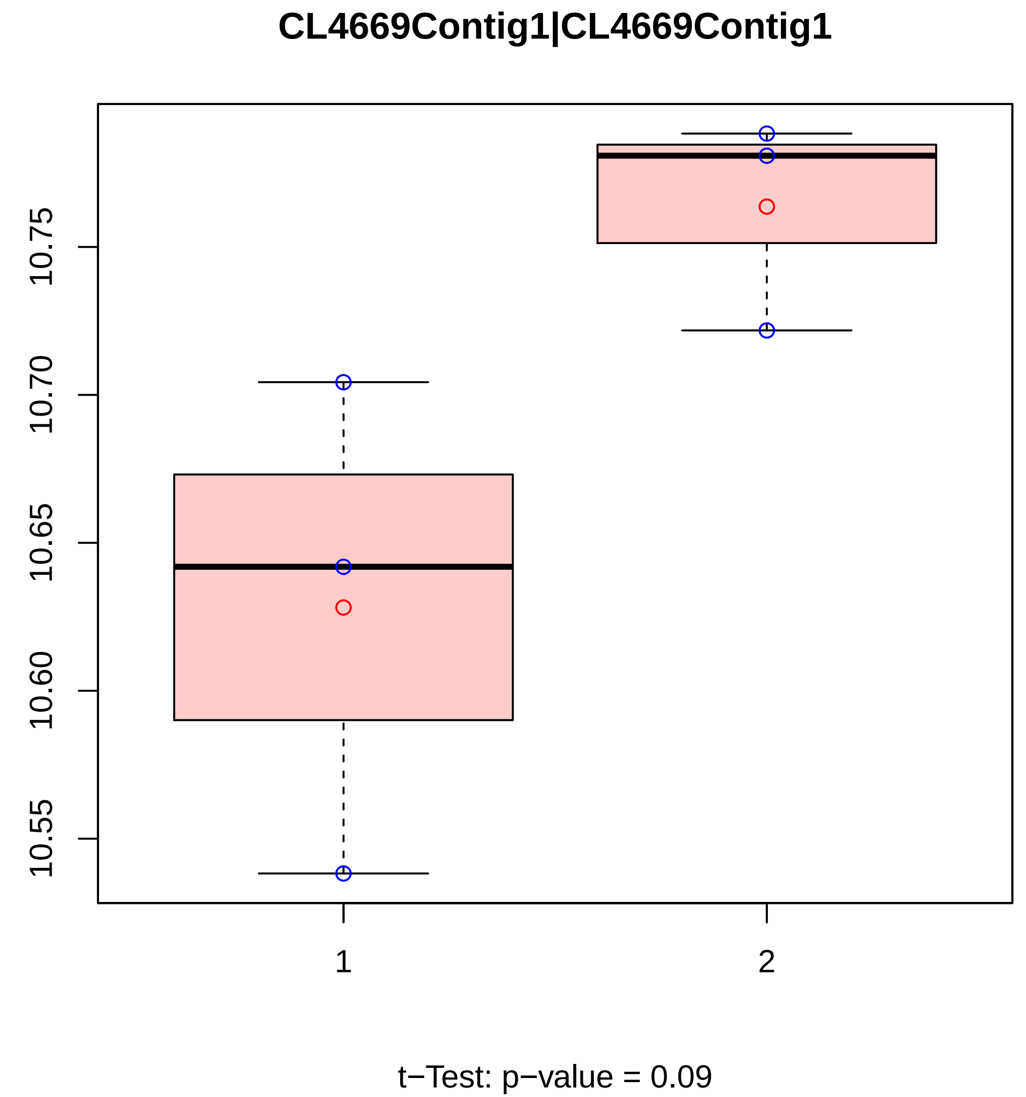
CL4669Contig1|CL4669Contig1
10.55
10.60
10.65
10.70
10.75
1
2
t−Test: p−value = 0.09

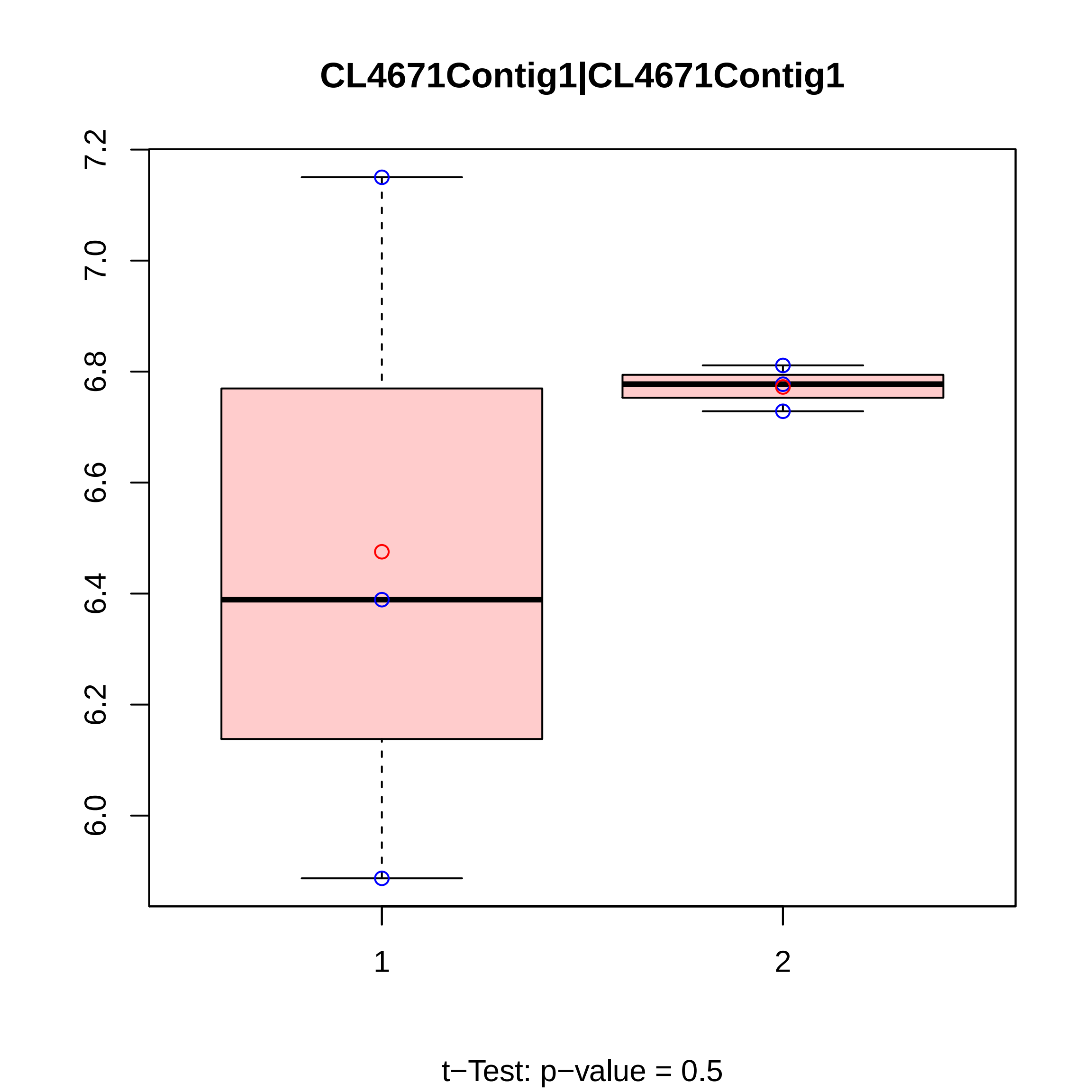
CL4671Contig1|CL4671Contig1
7.2
7.0
6.8
6.6
6.4
6.2
6.0
1
2
t−Test: p−value = 0.5

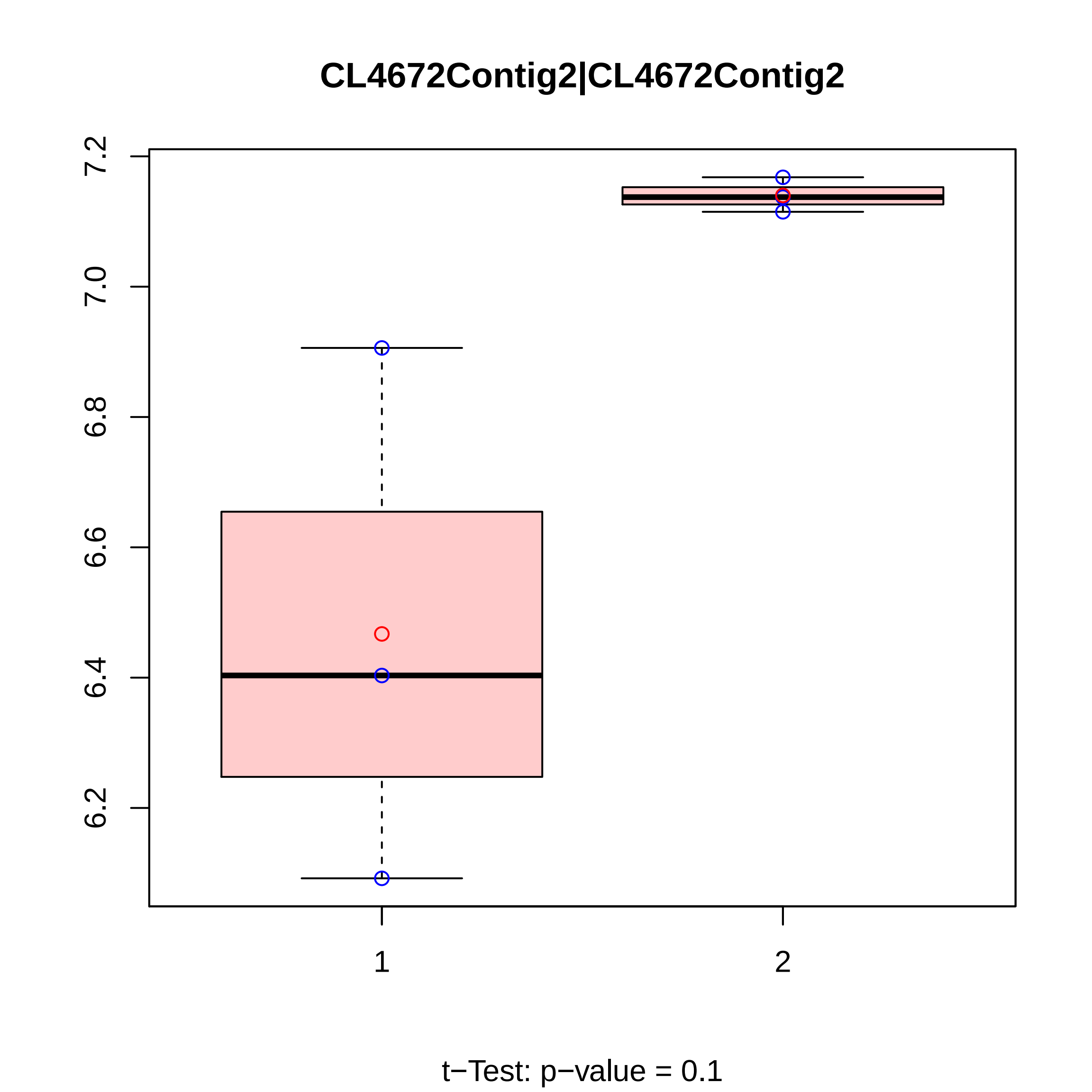
CL4672Contig2|CL4672Contig2
7.2
7.0
6.8
6.6
6.4
6.2
1
2
t−Test: p−value = 0.1

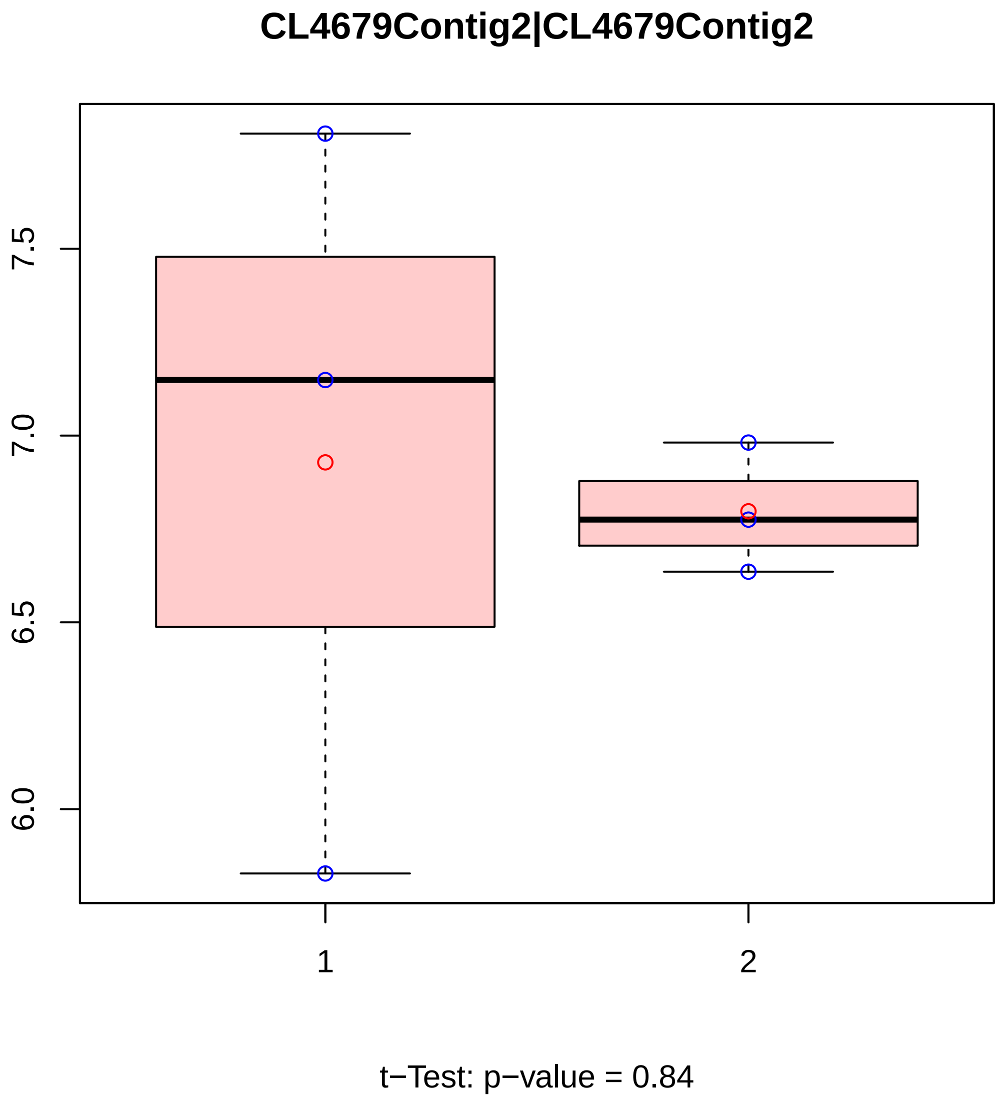
CL4679Contig2|CL4679Contig2
7.5
7.0
6.5
6.0
1
2
t−Test: p−value = 0.84

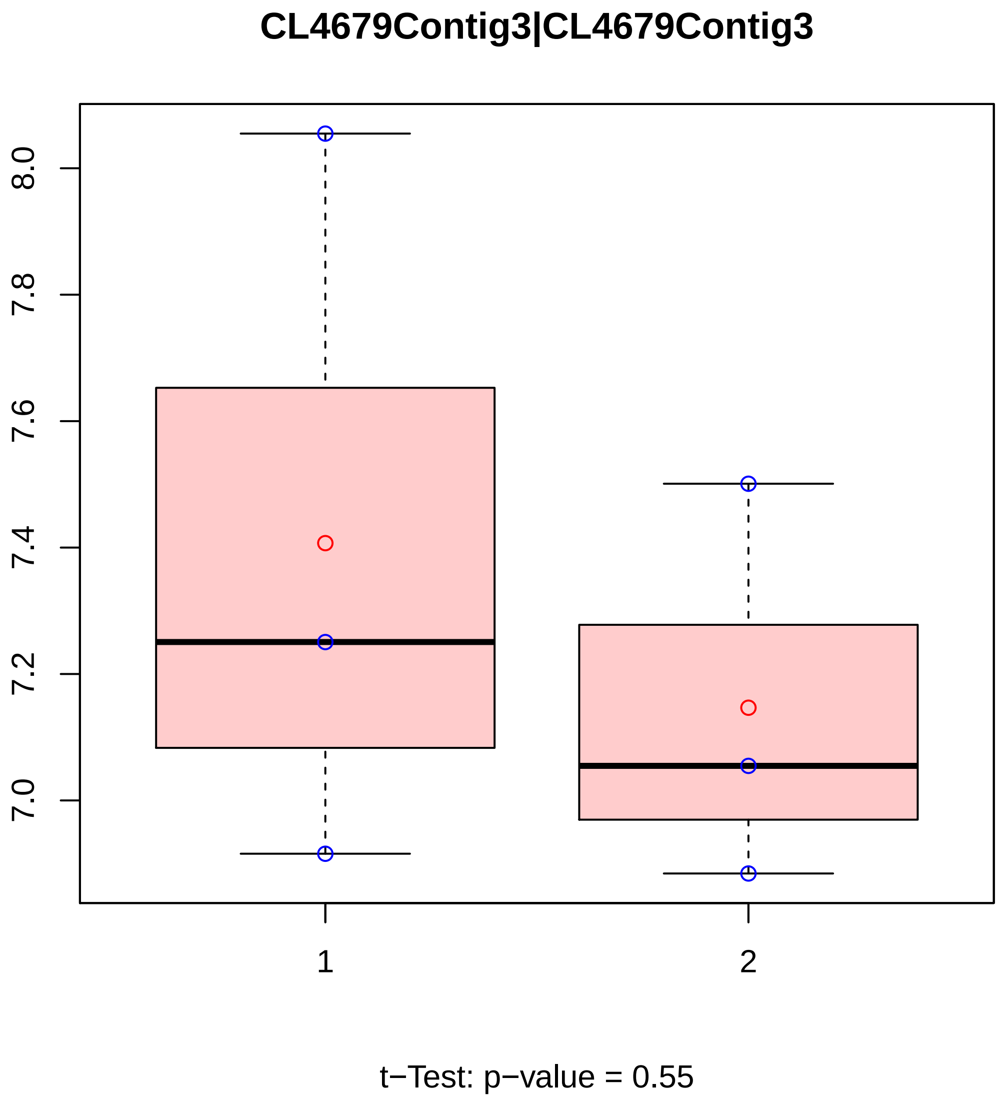
CL4679Contig3|CL4679Contig3
8.0
7.8
7.6
7.4
7.2
7.0
1
2
t−Test: p−value = 0.55

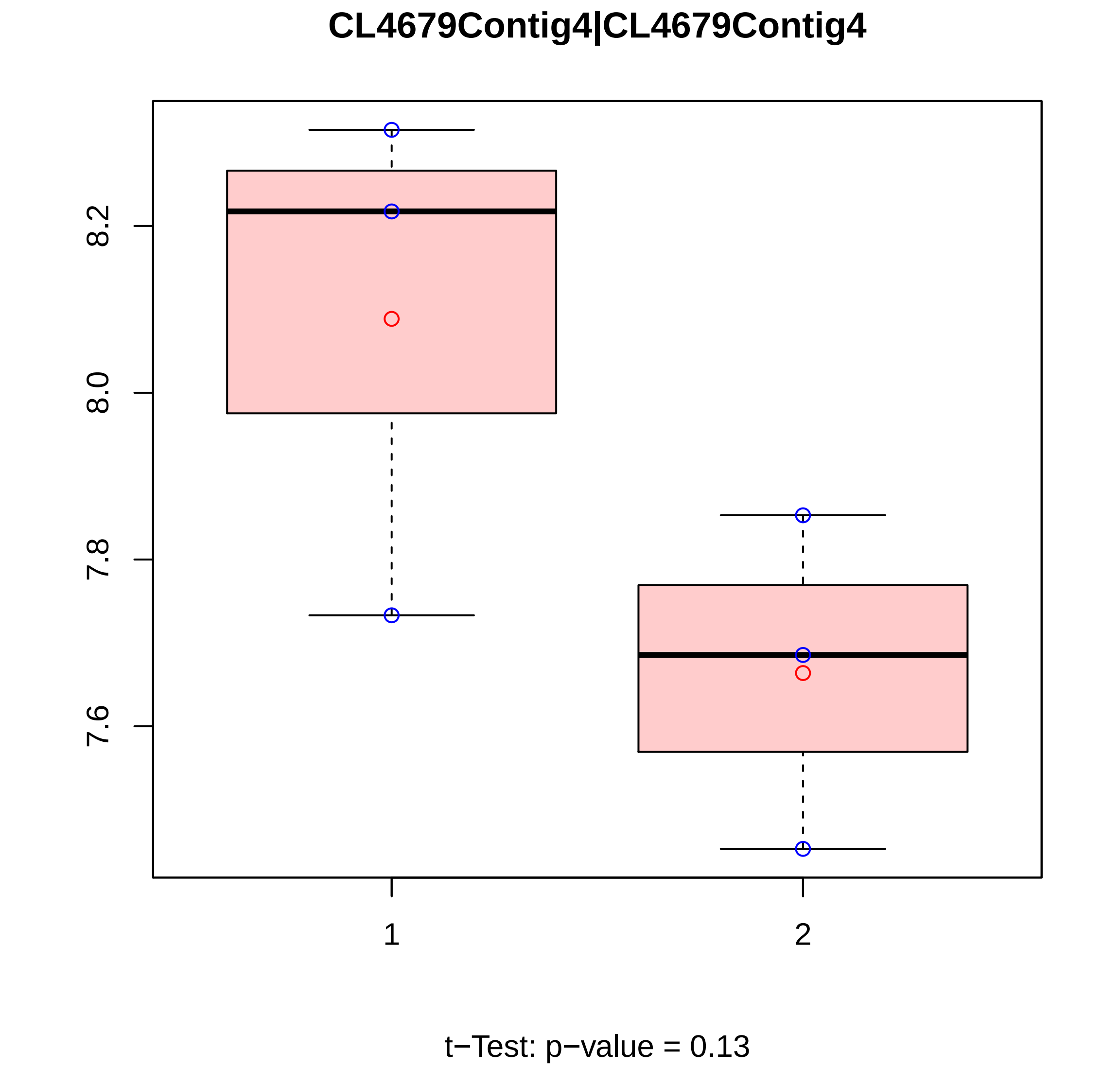

CL4679Contig4|CL4679Contig4
8.2
8.0
7.8
7.6
1
2
t−Test: p−value = 0.13

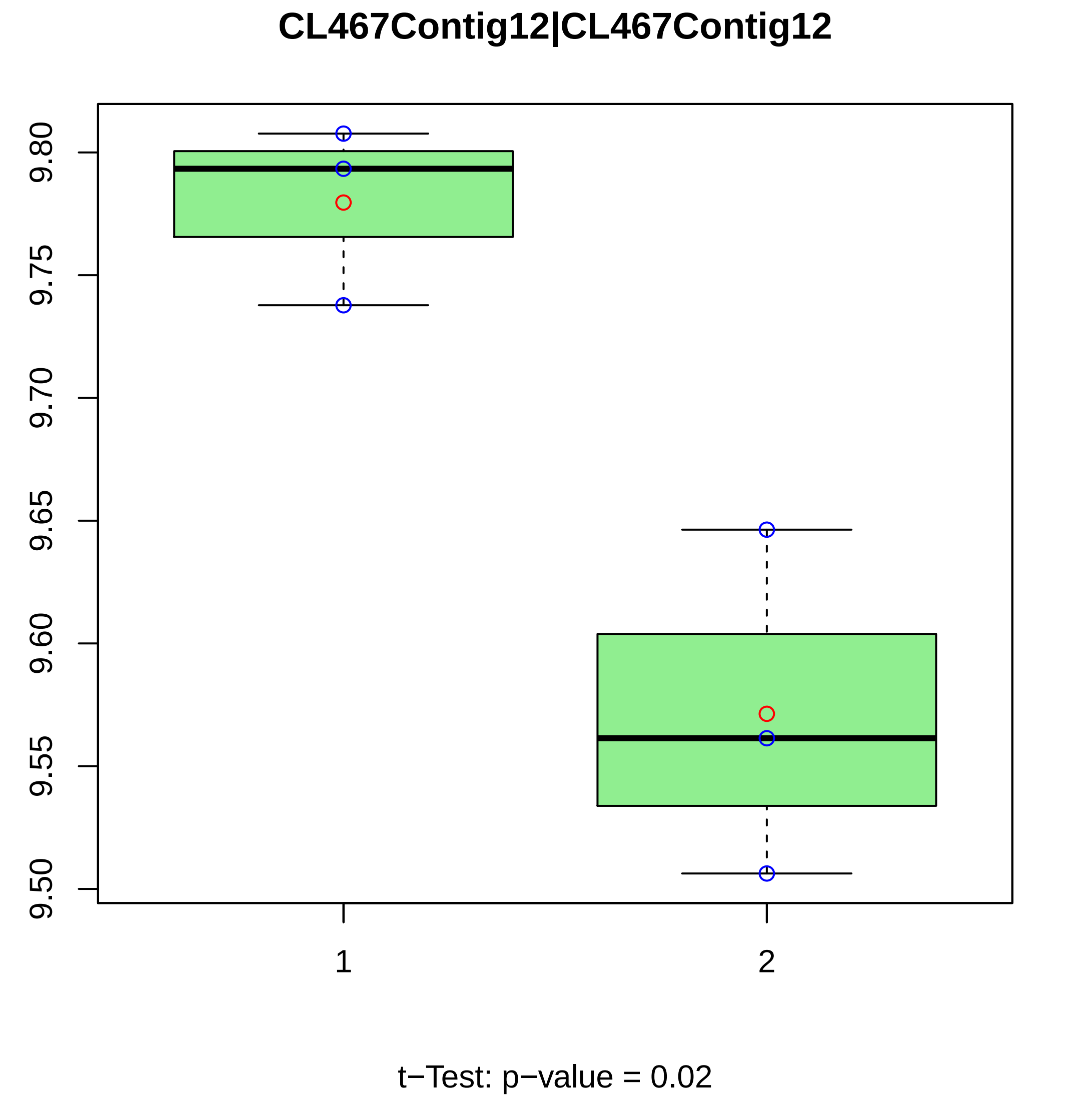
CL467Contig12|CL467Contig12
9.80
9.75
9.70
9.65
9.60
9.55
9.50
1
2
t−Test: p−value = 0.02

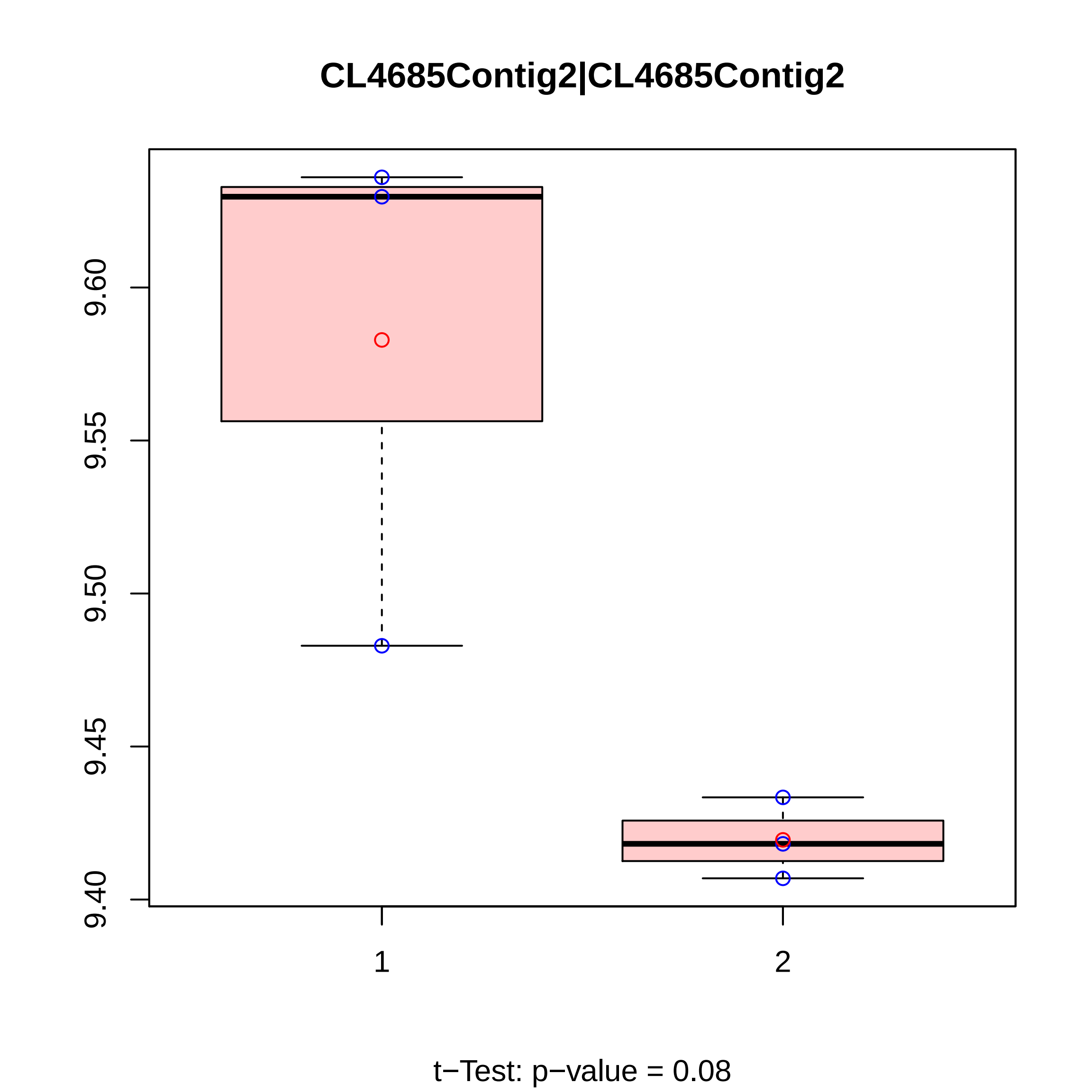

CL4685Contig2|CL4685Contig2
9.40
9.45
9.50
9.55
9.60
1
2
t−Test: p−value = 0.08

## CL4685Contig3|CL4685Contig3

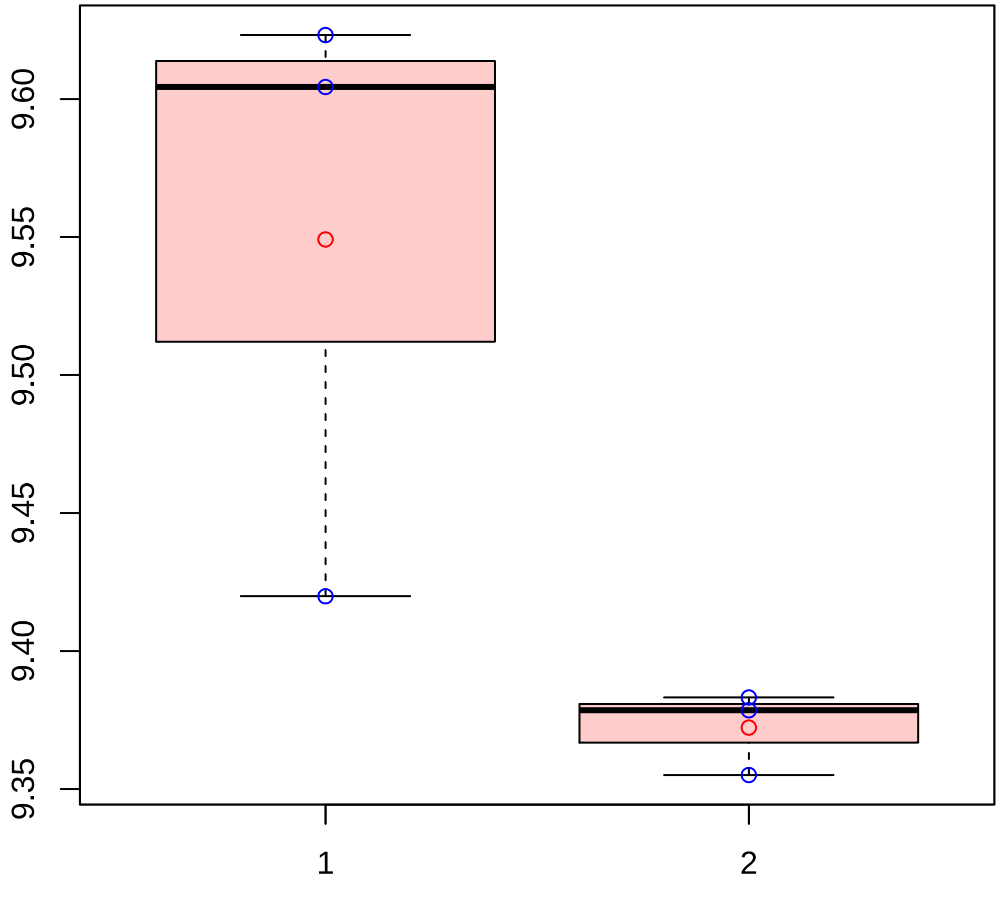


t−Test: p−value = 0.11

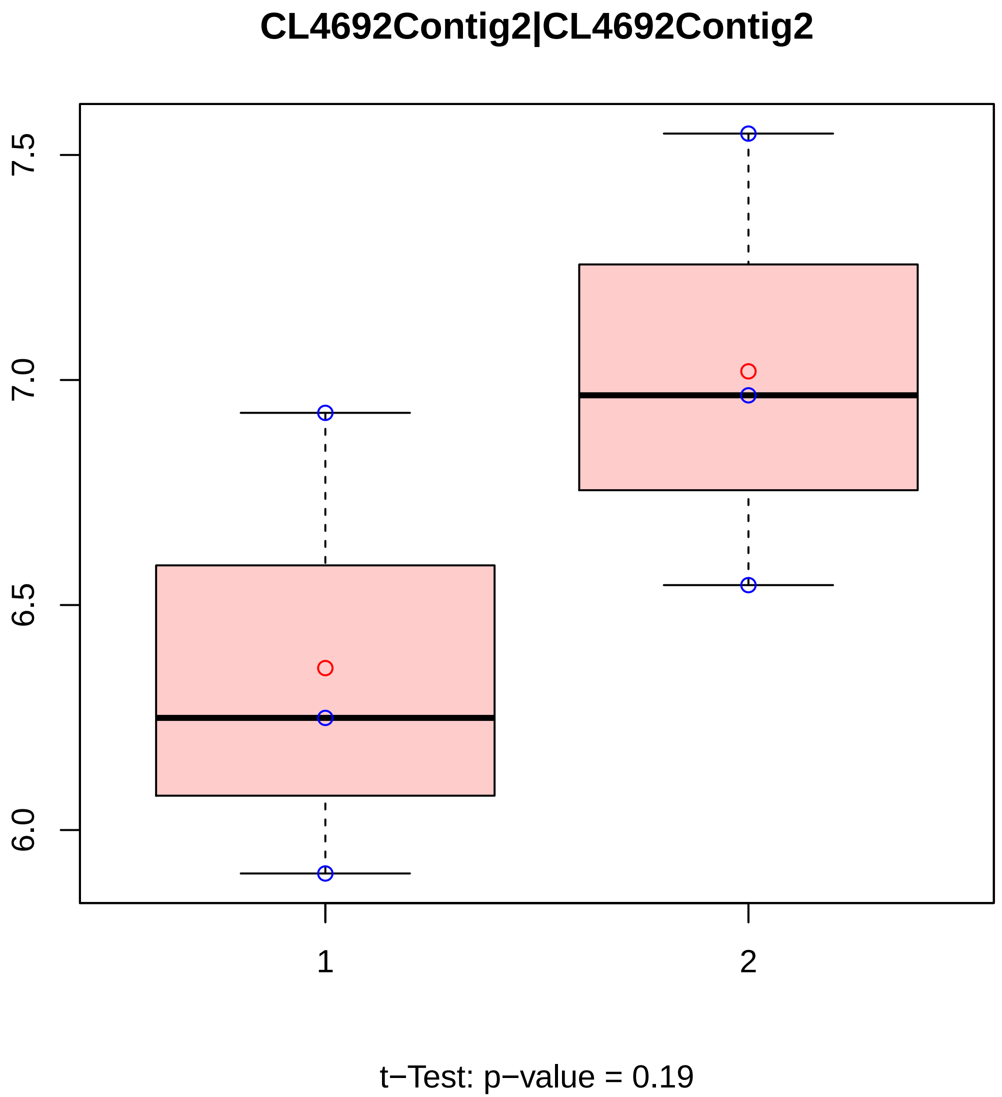

CL4692Contig2|CL4692Contig2
7.5
7.0
6.5
6.0
1
2
t−Test: p−value = 0.19

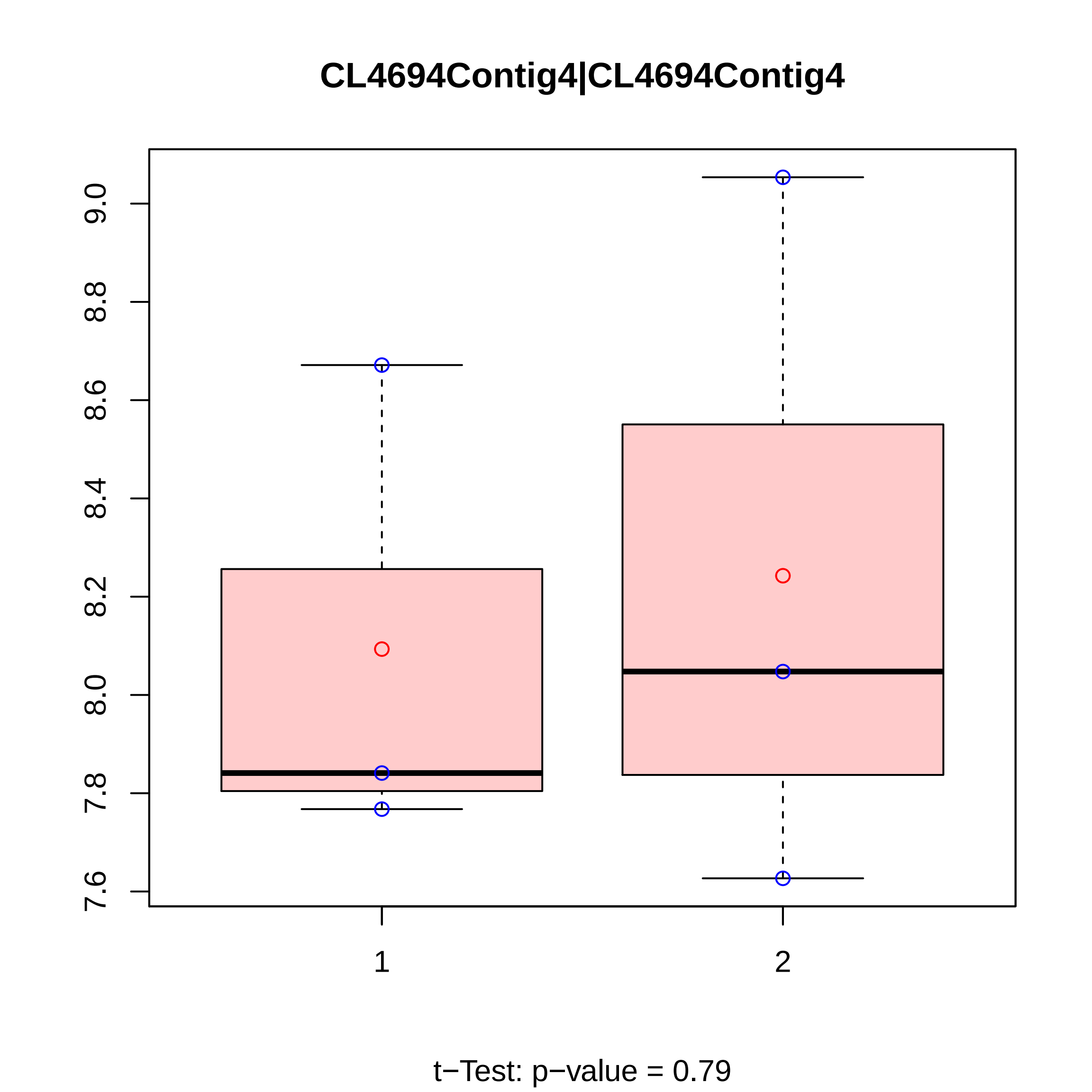
CL4694Contig4|CL4694Contig4
9.0
8.8
8.6
8.4
8.2
8.0
7.8
7.6
1
2
t−Test: p−value = 0.79

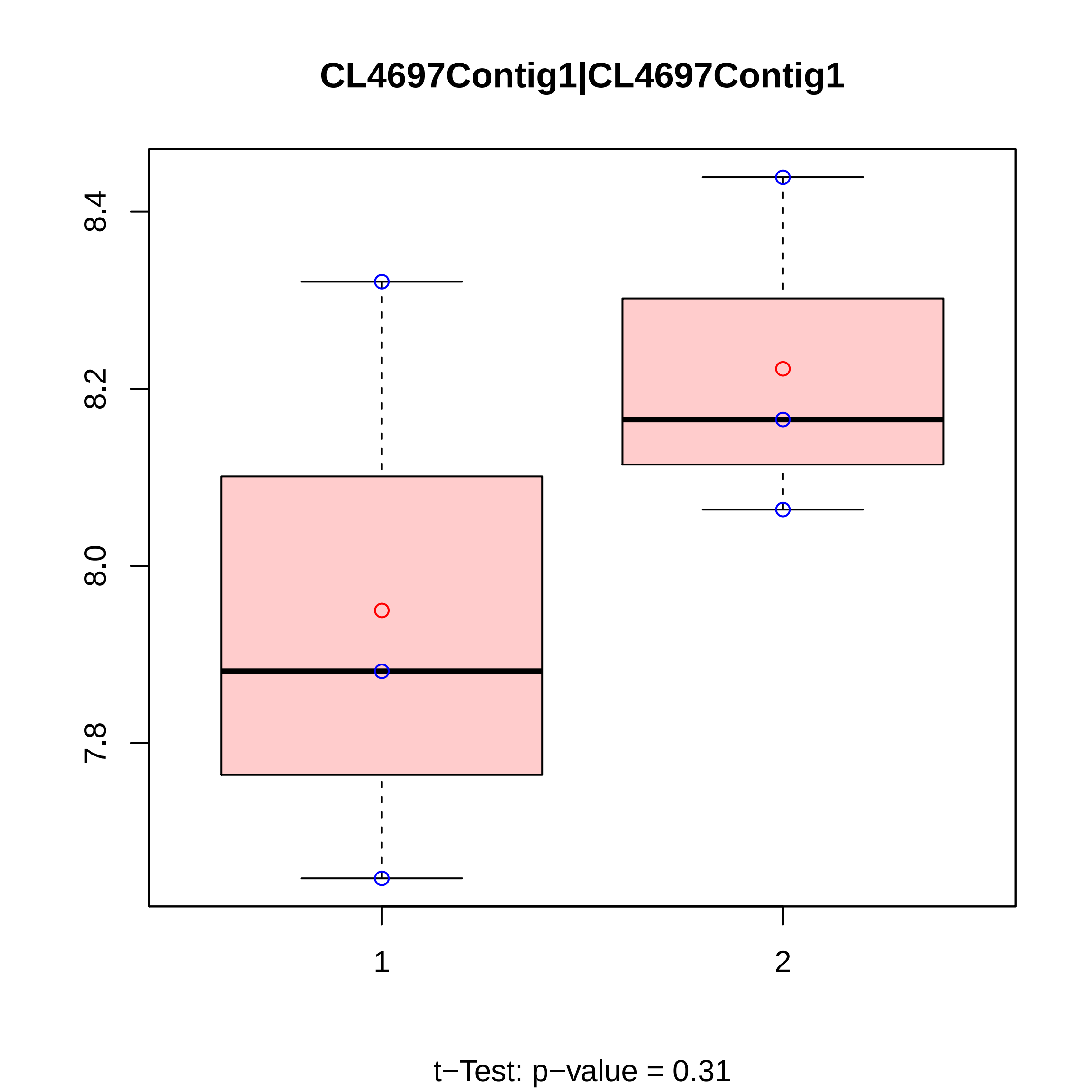

CL4697Contig1|CL4697Contig1
8.4
8.2
8.0
7.8
1
2
t−Test: p−value = 0.31

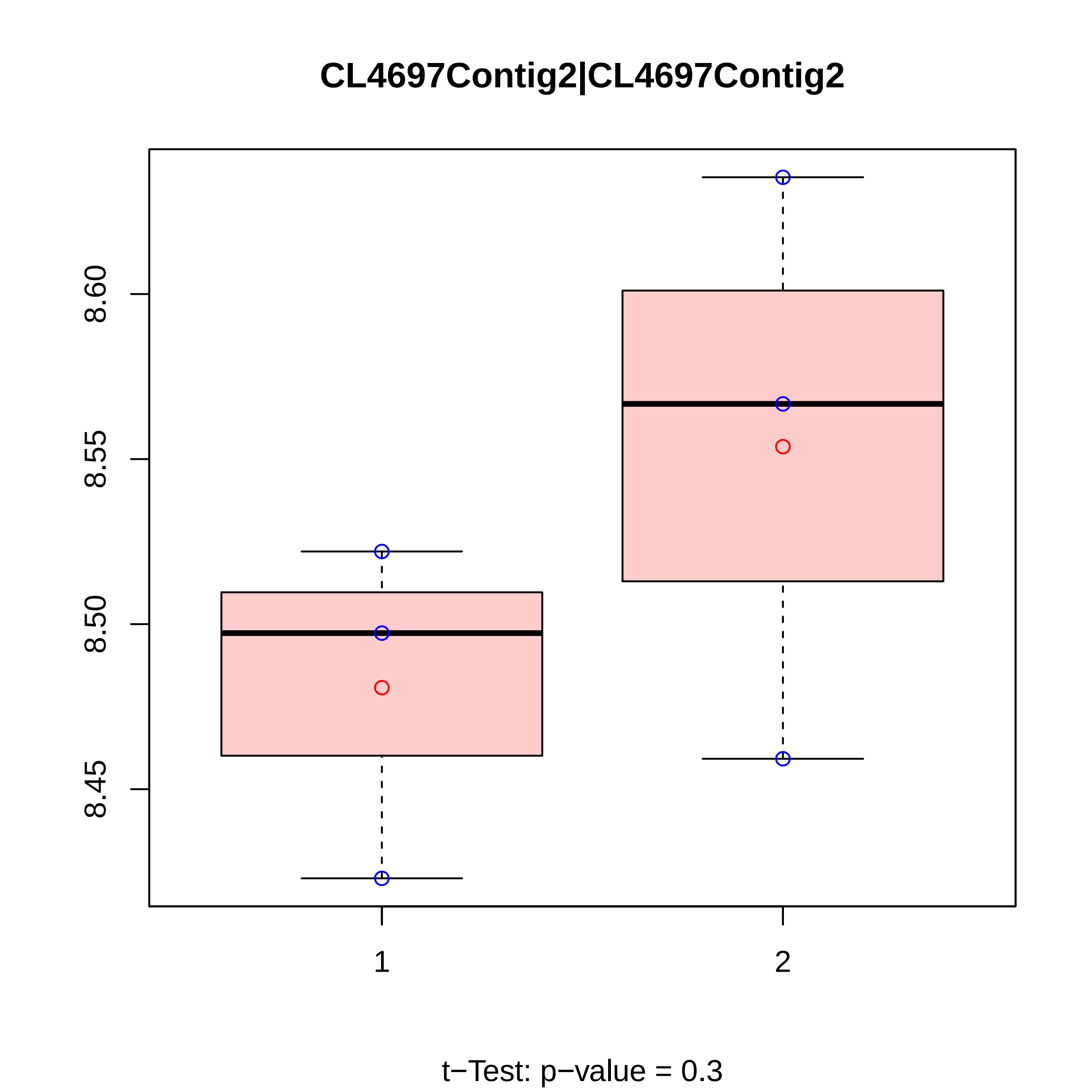
CL4697Contig2|CL4697Contig2
8.45
8.50
8.55
8.60
1
2
t−Test: p−value = 0.3

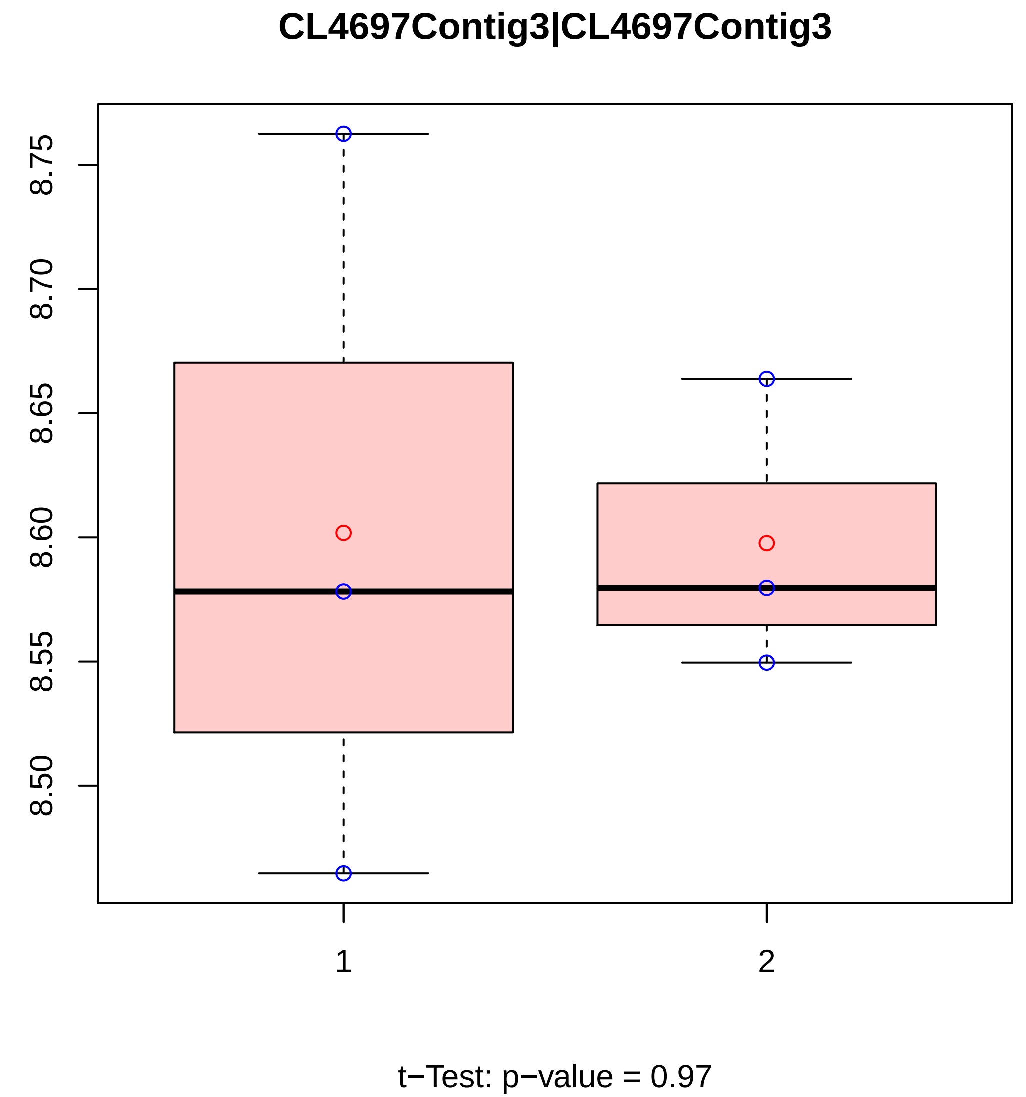

CL4697Contig3|CL4697Contig3
8.50
8.55
8.60
8.65
8.70
8.75
1
2
t−Test: p−value = 0.97

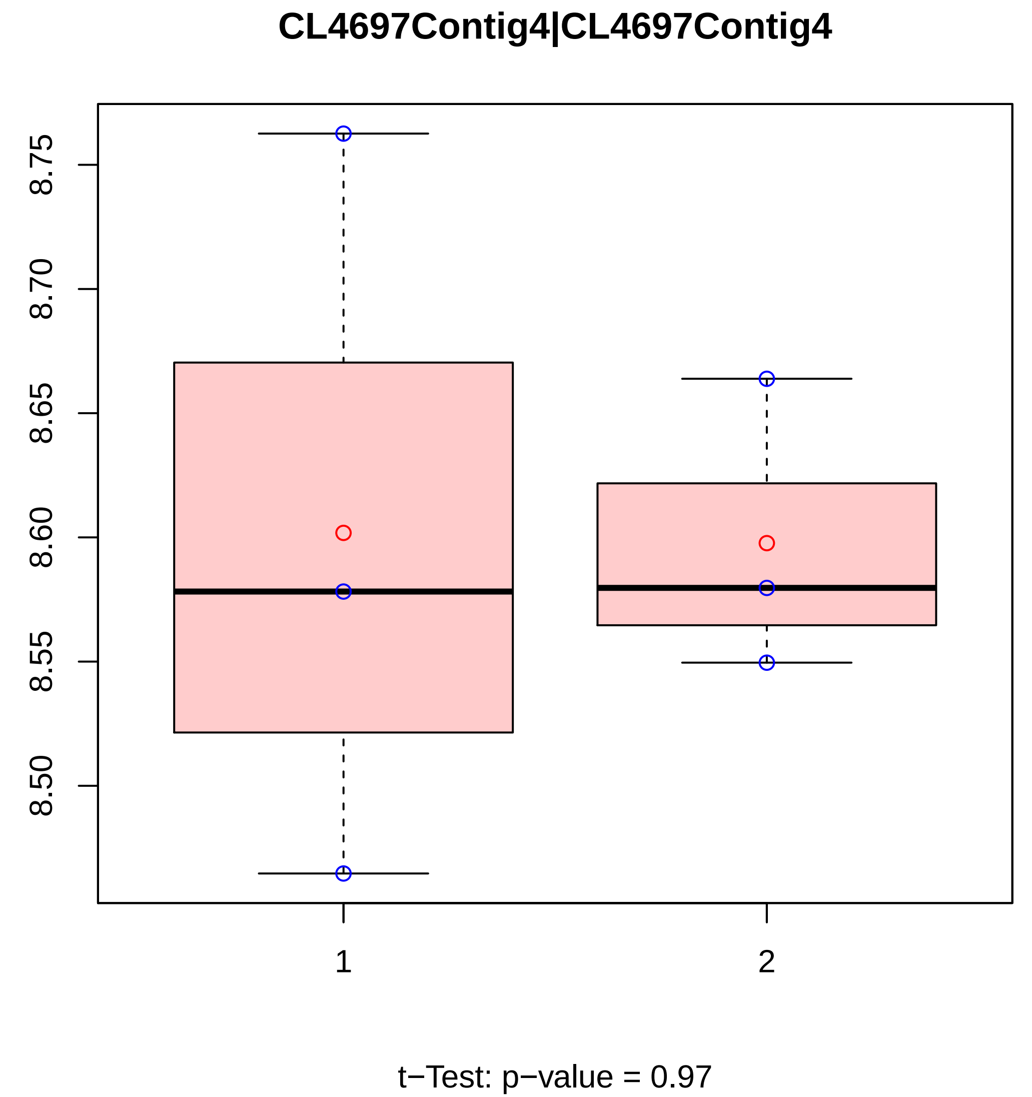
CL4697Contig4|CL4697Contig4
8.50
8.55
8.60
8.65
8.70
8.75
1
2
t−Test: p−value = 0.97

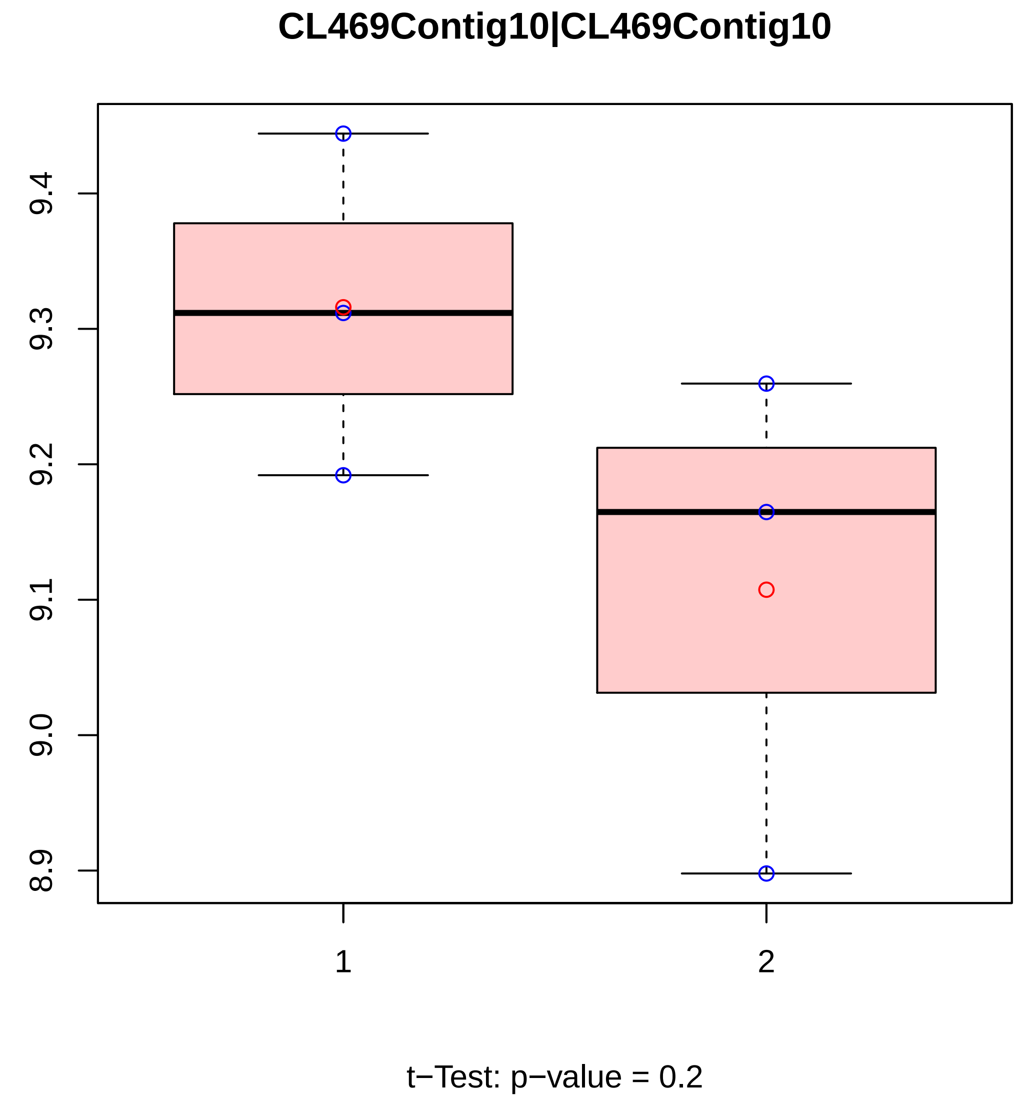
CL469Contig10|CL469Contig10
9.4
9.3
9.2
9.1
9.0
8.9
1
2
t−Test: p−value = 0.2

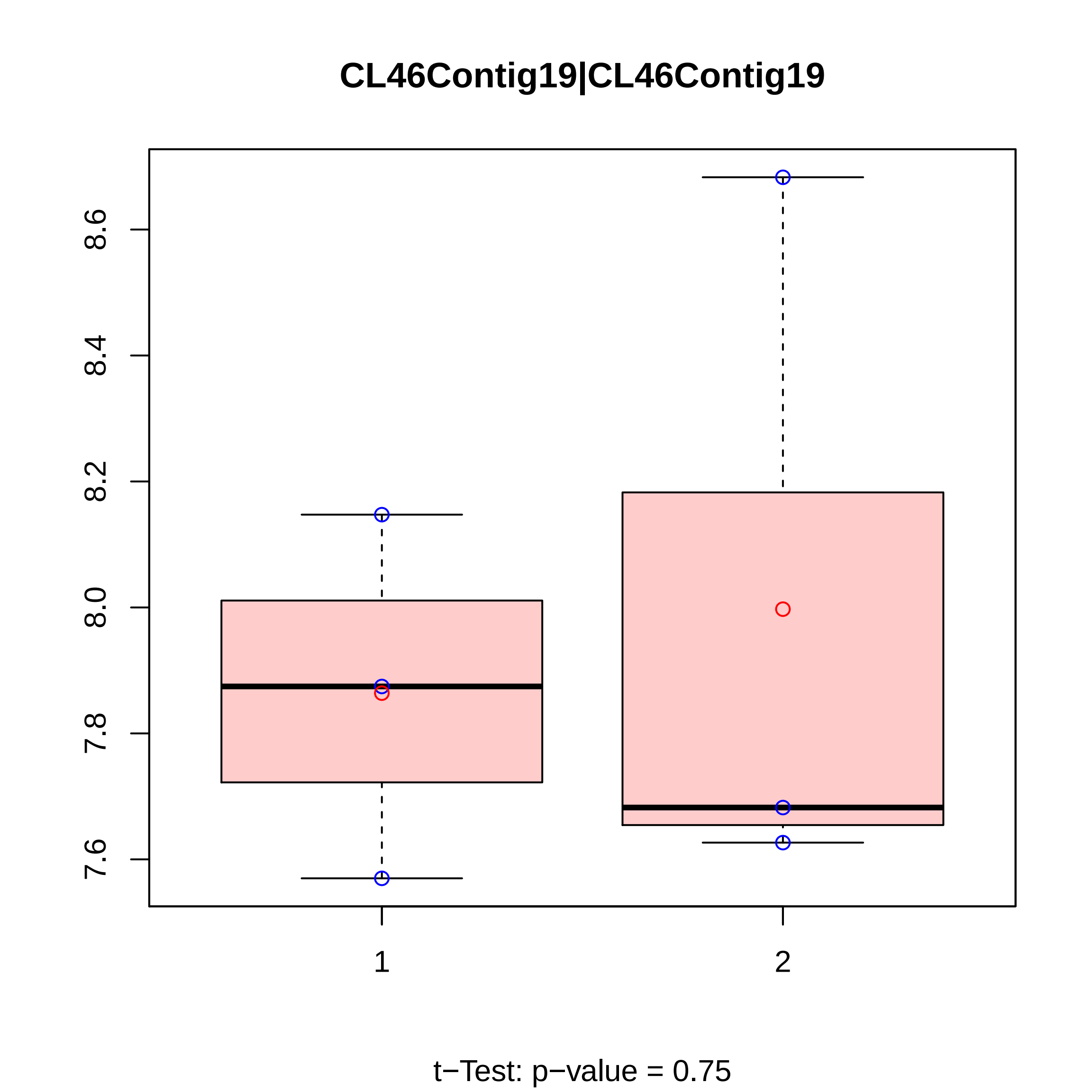
CL46Contig19|CL46Contig19
8.6
8.4
8.2
8.0
7.8
7.6
1
2
t−Test: p−value = 0.75

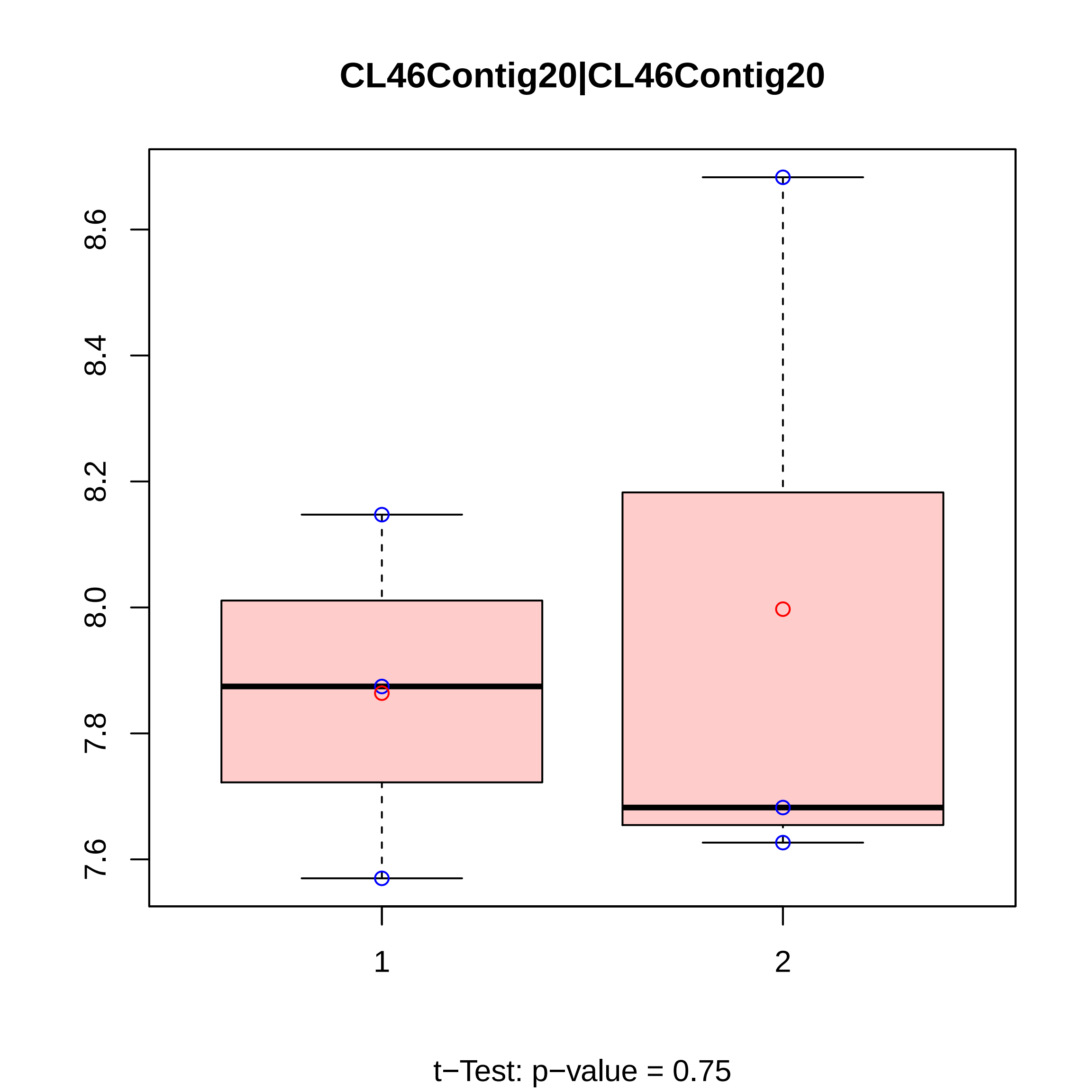
CL46Contig20|CL46Contig20
8.6
8.4
8.2
8.0
7.8
7.6
1
2
t−Test: p−value = 0.75

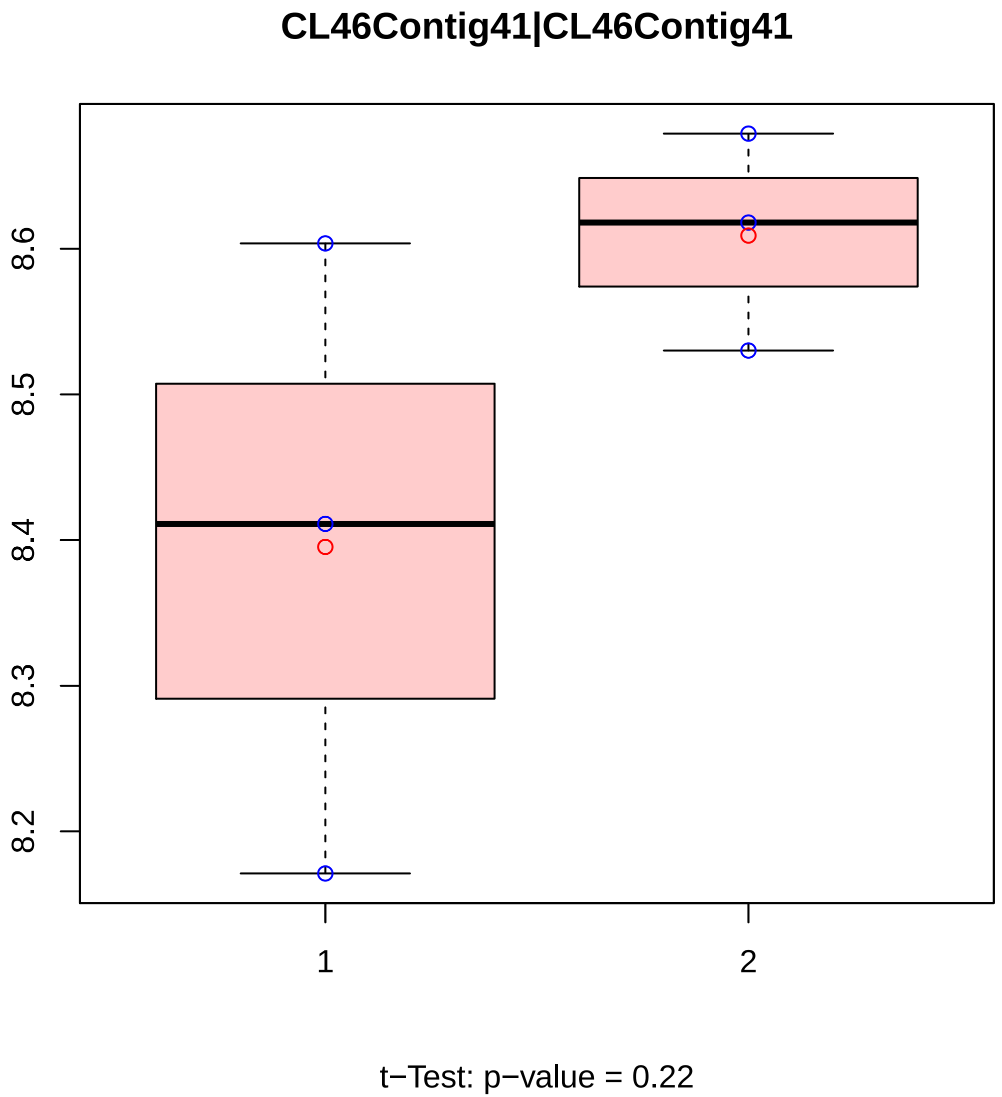
CL46Contig41|CL46Contig41
8.2
8.3
8.4
8.5
8.6
1
2
t−Test: p−value = 0.22

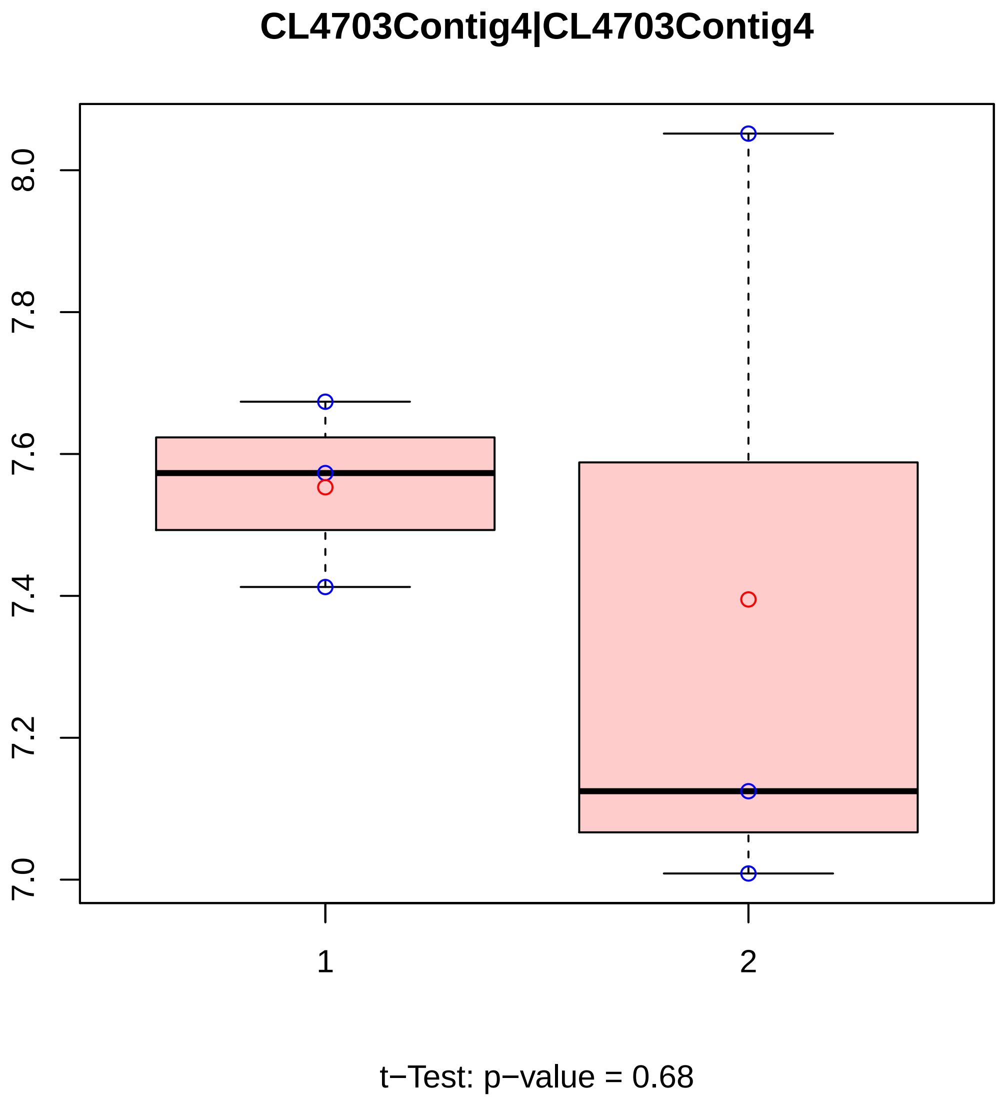
CL4703Contig4|CL4703Contig4
8.0
7.8
7.6
7.4
7.2
7.0
1
2
t−Test: p−value = 0.68

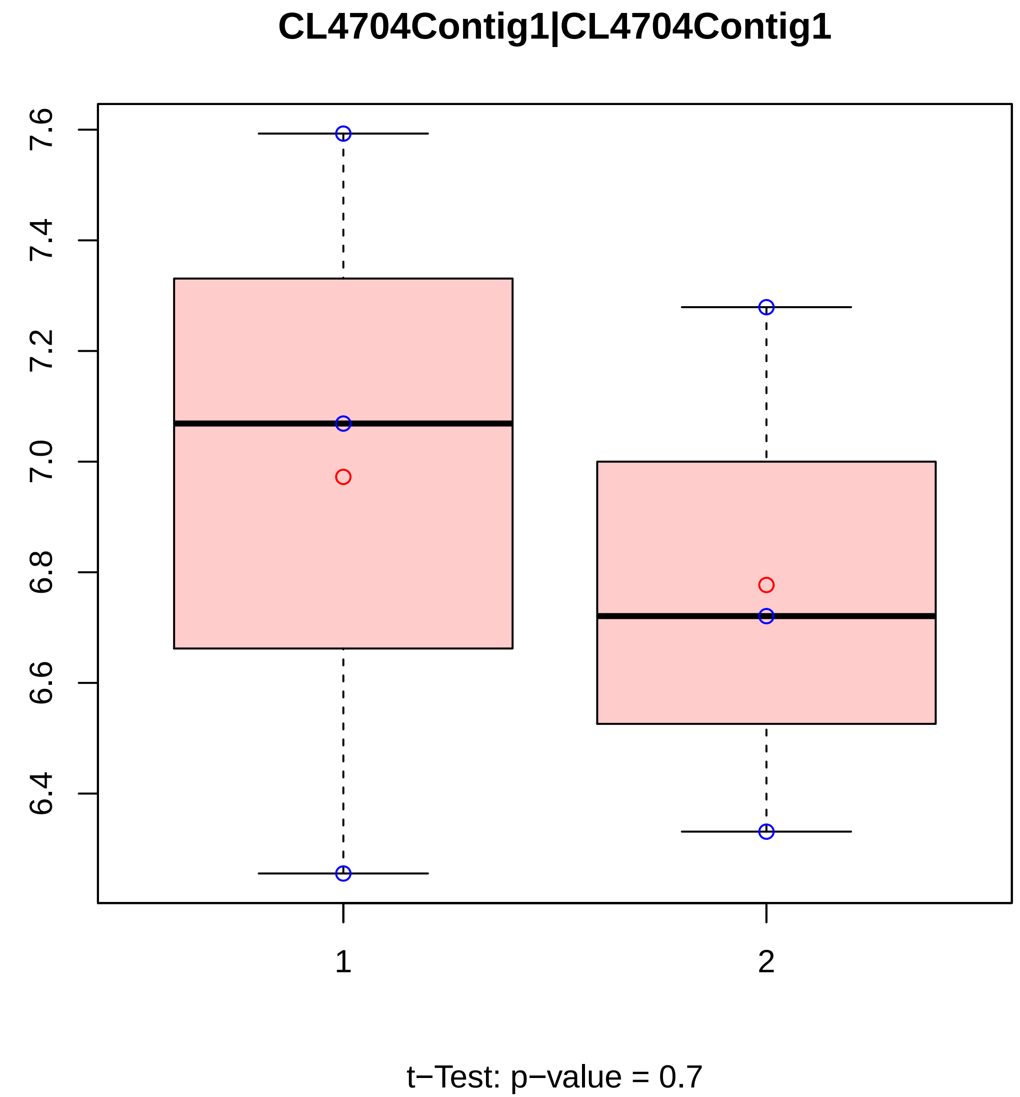
CL4704Contig1|CL4704Contig1
7.6
7.4
7.2
7.0
6.8
6.6
6.4
1
2
t−Test: p−value = 0.7

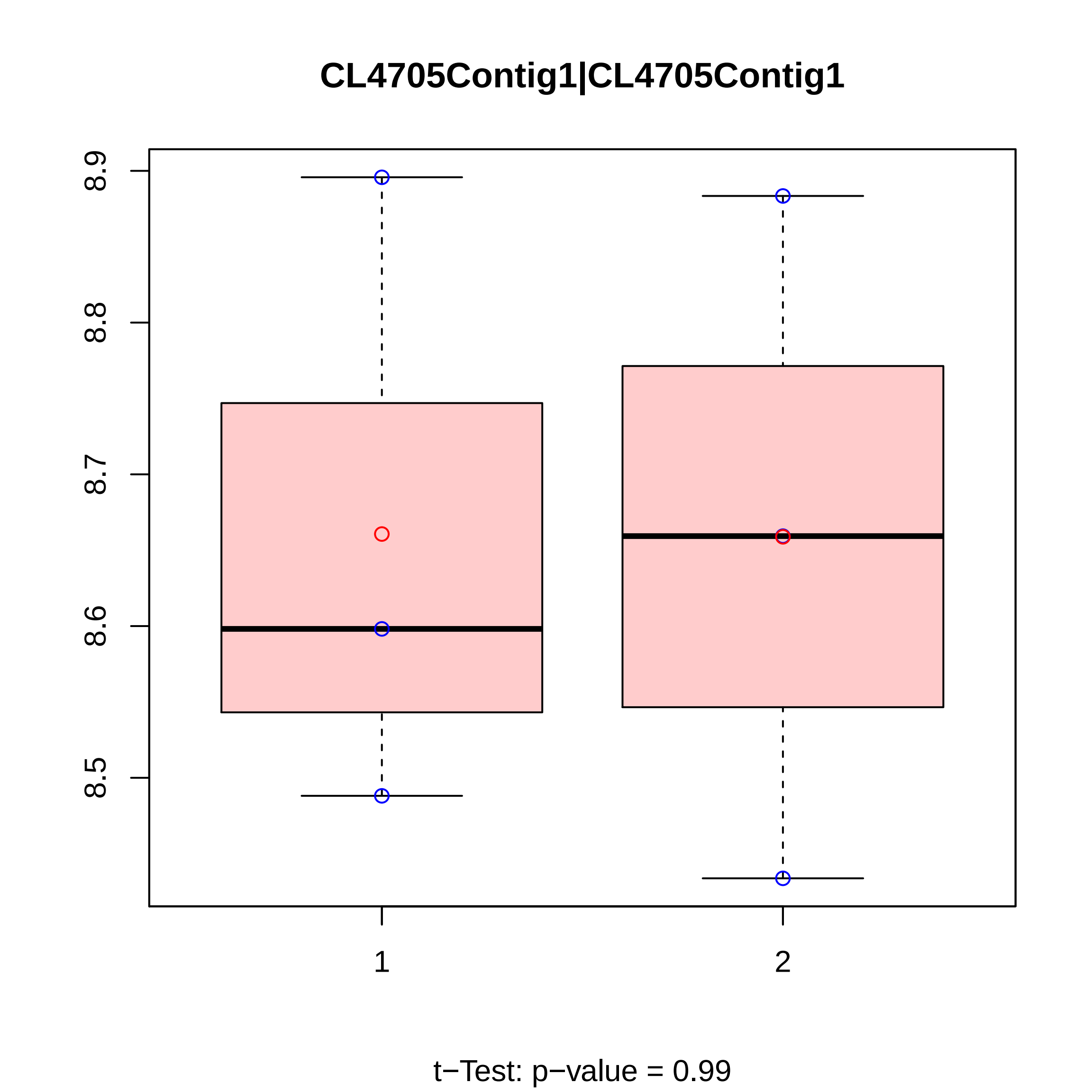
CL4705Contig1|CL4705Contig1
8.9
8.8
8.7
8.6
8.5
1
2
t−Test: p−value = 0.99

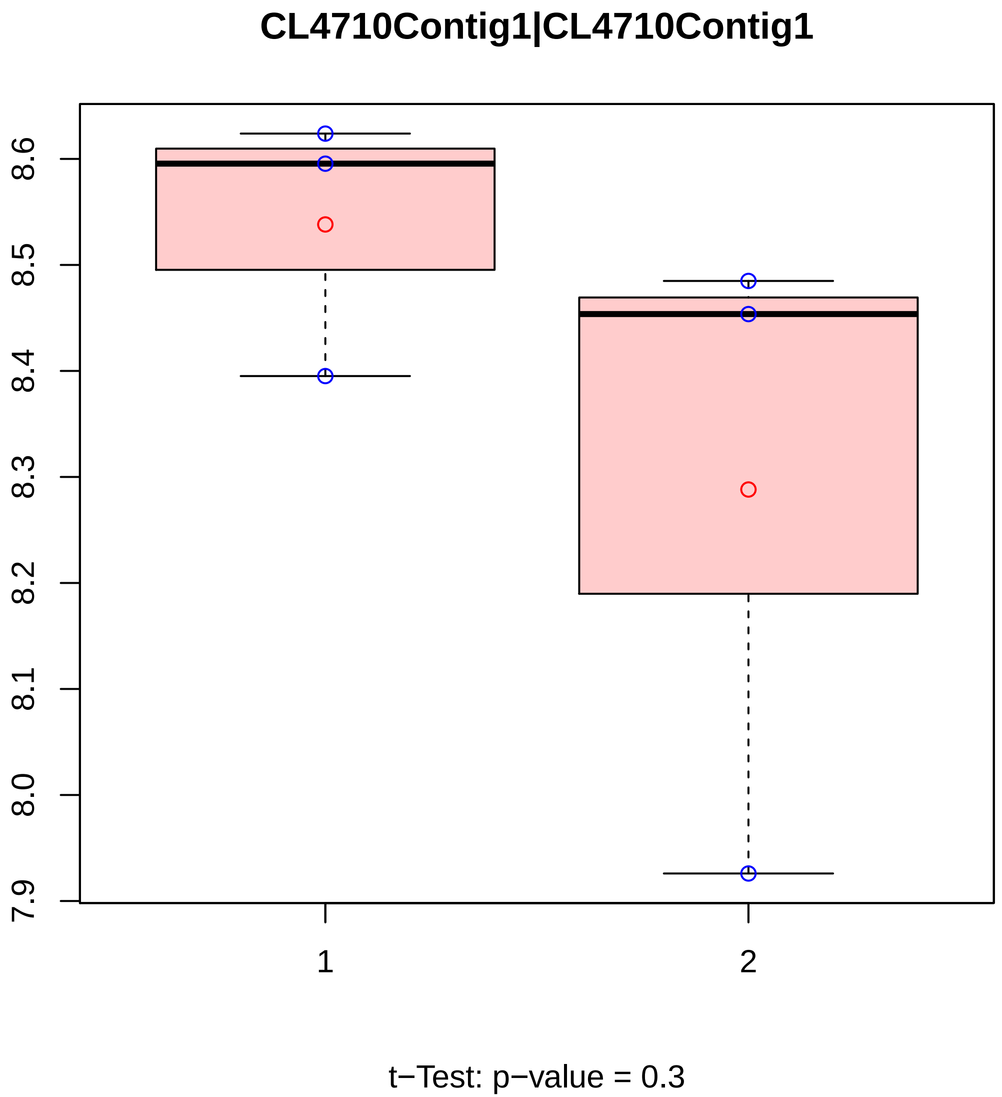

CL4710Contig1|CL4710Contig1
8.6
8.5
8.4
8.3
8.2
8.1
8.0
7.9
1
2
t−Test: p−value = 0.3

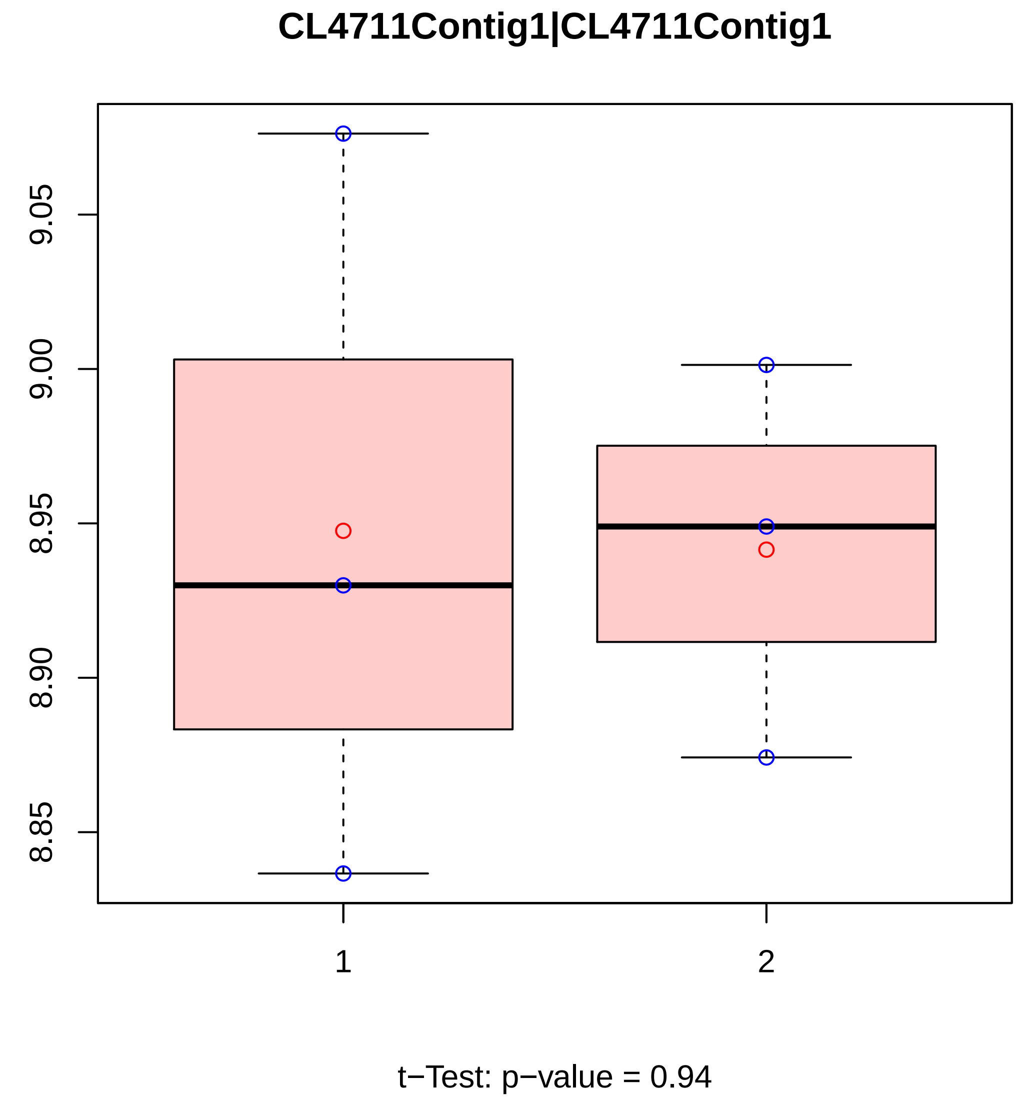
CL4711Contig1|CL4711Contig1
9.05
9.00
8.95
8.90
8.85
1
2
t−Test: p−value = 0.94

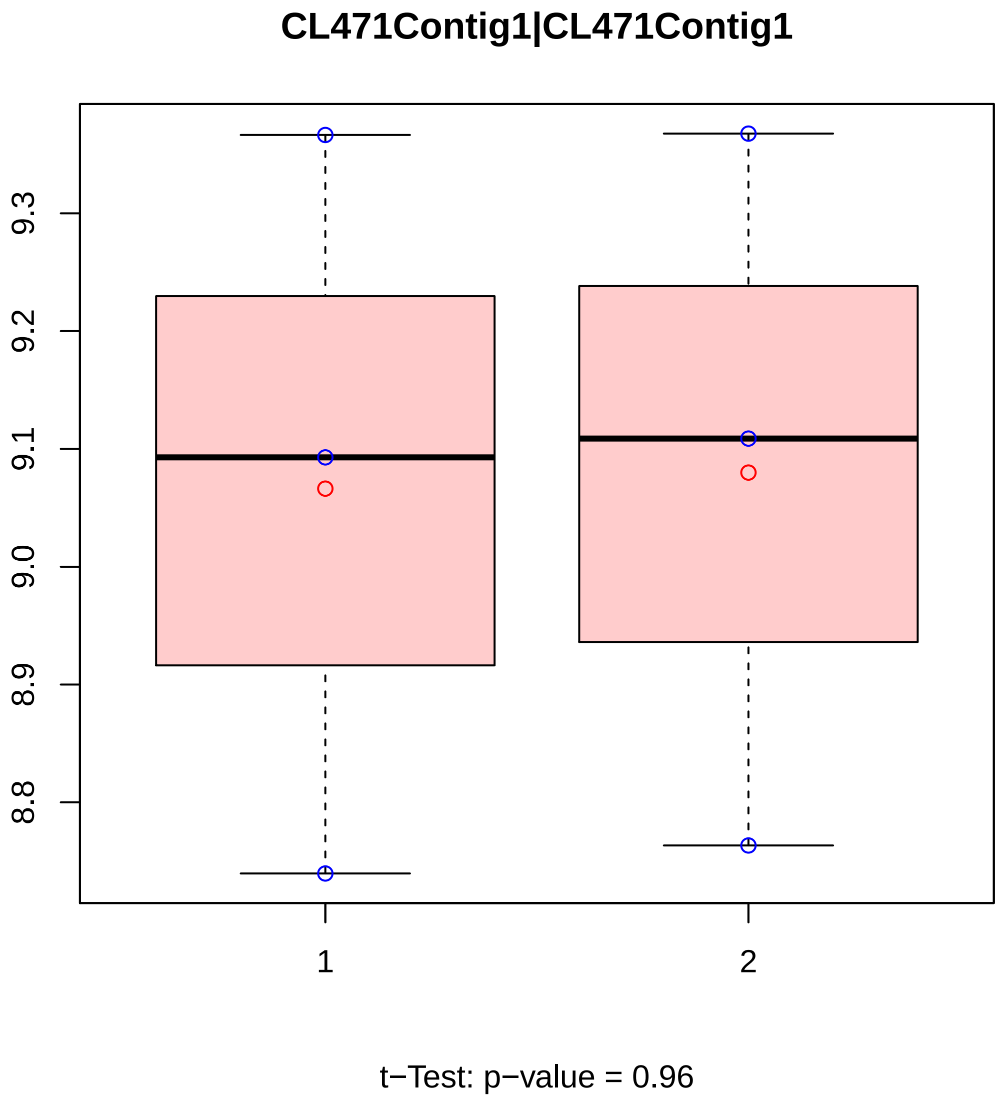

CL471Contig1|CL471Contig1
9.3
9.2
9.1
9.0
8.9
8.8
1
2
t−Test: p−value = 0.96

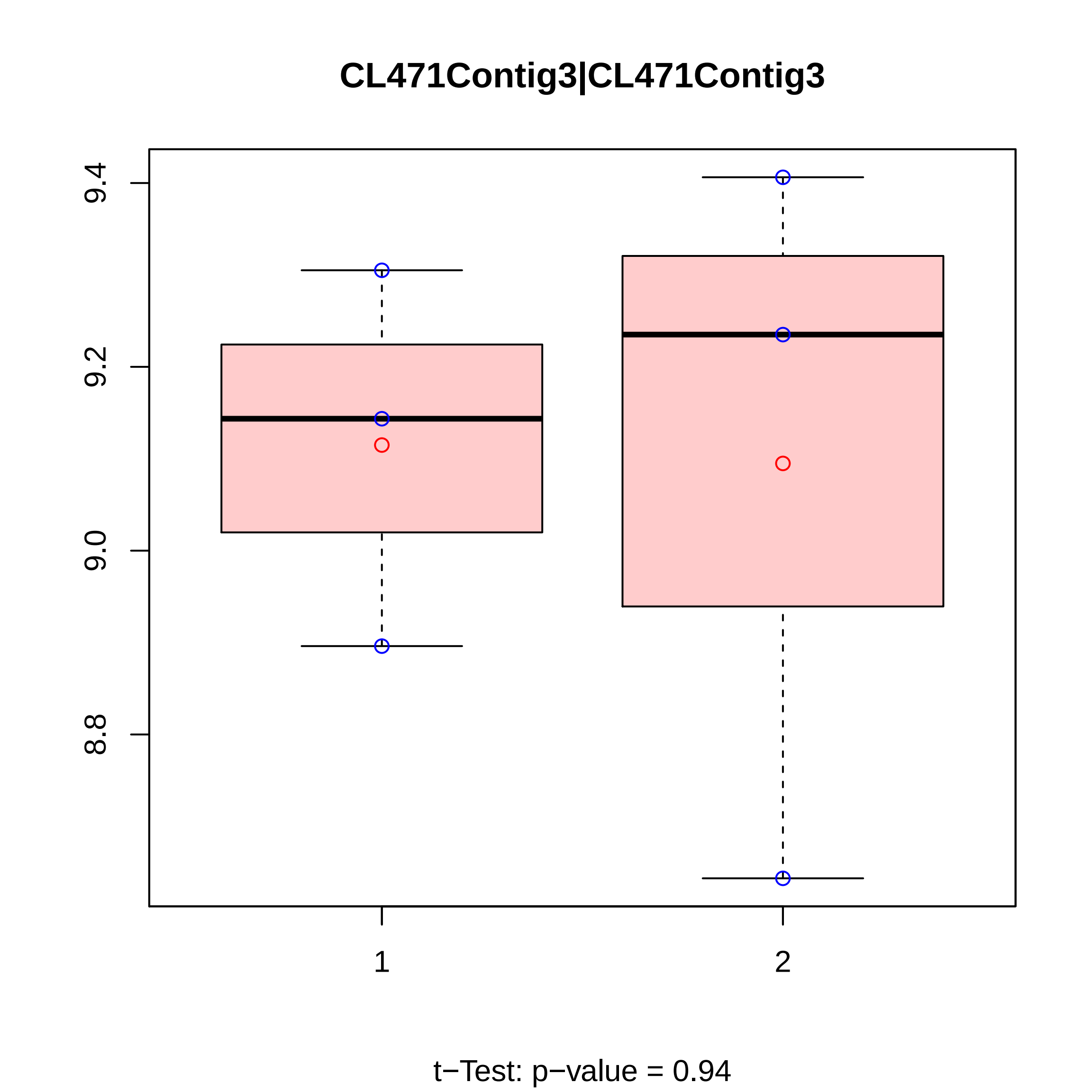
CL471Contig3|CL471Contig3
9.4
9.2
9.0
8.8
1
2
t−Test: p−value = 0.94

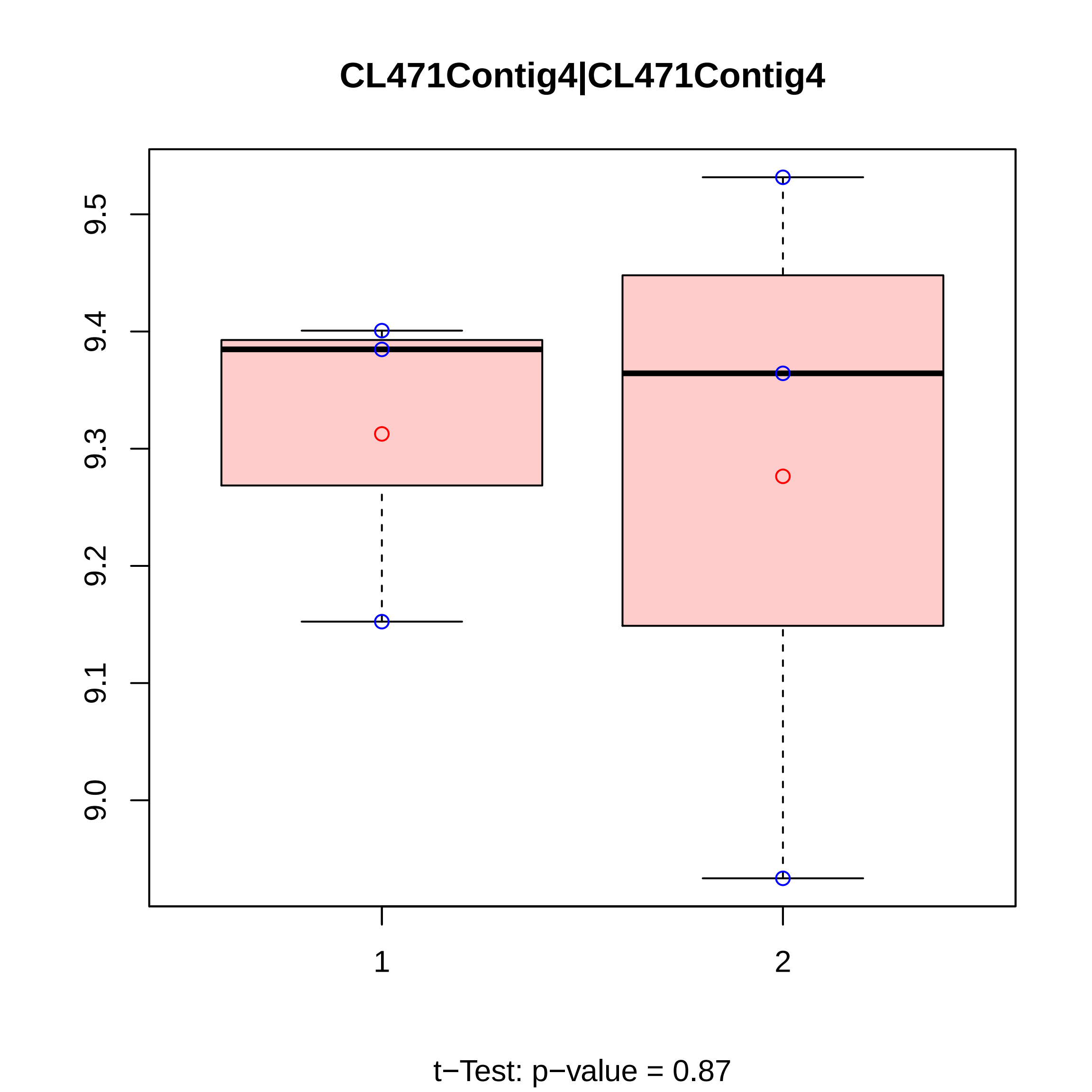
CL471Contig4|CL471Contig4
9.0
9.1
9.2
9.3
9.4
9.5
1
2
t−Test: p−value = 0.87

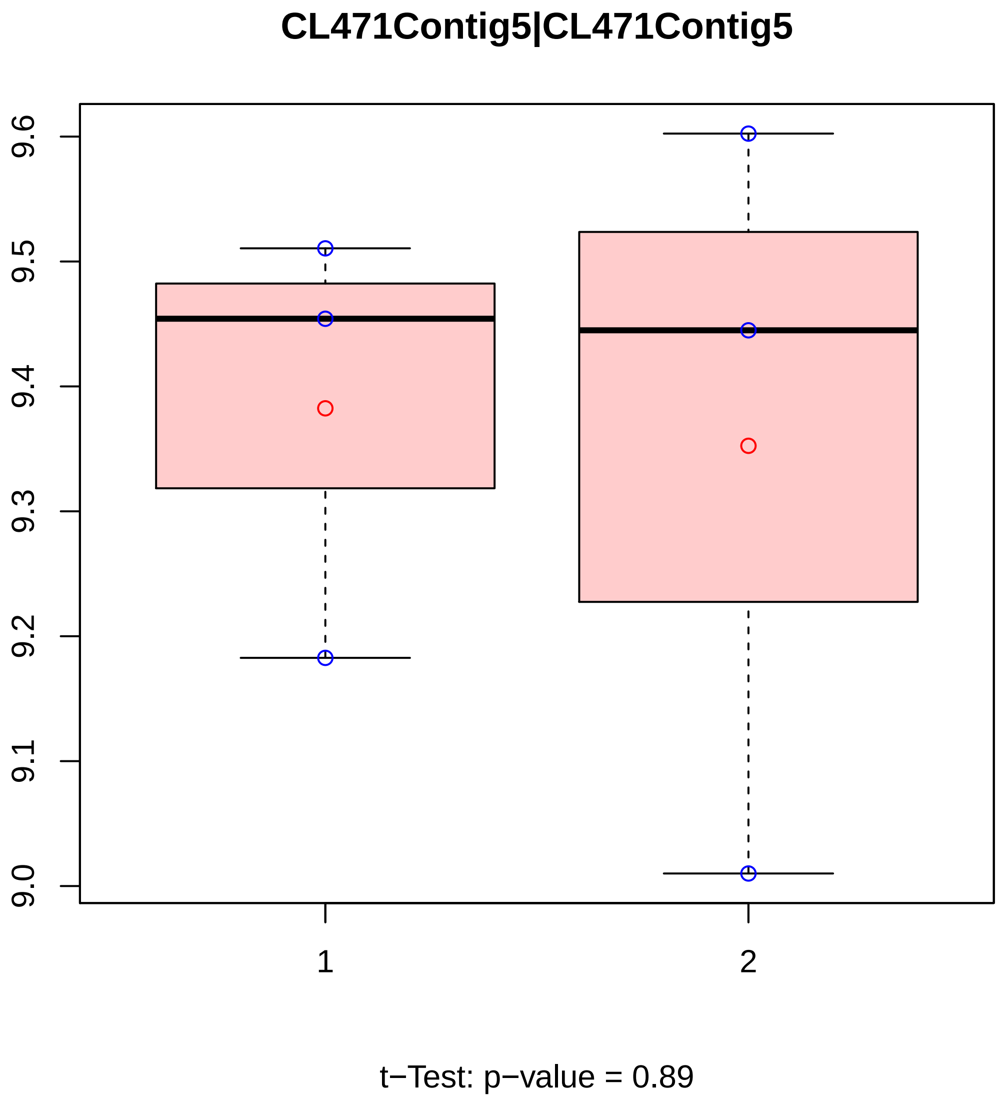

CL471Contig5|CL471Contig5
9.0
9.1
9.2
9.3
9.4
9.5
9.6
1
2
t−Test: p−value = 0.89

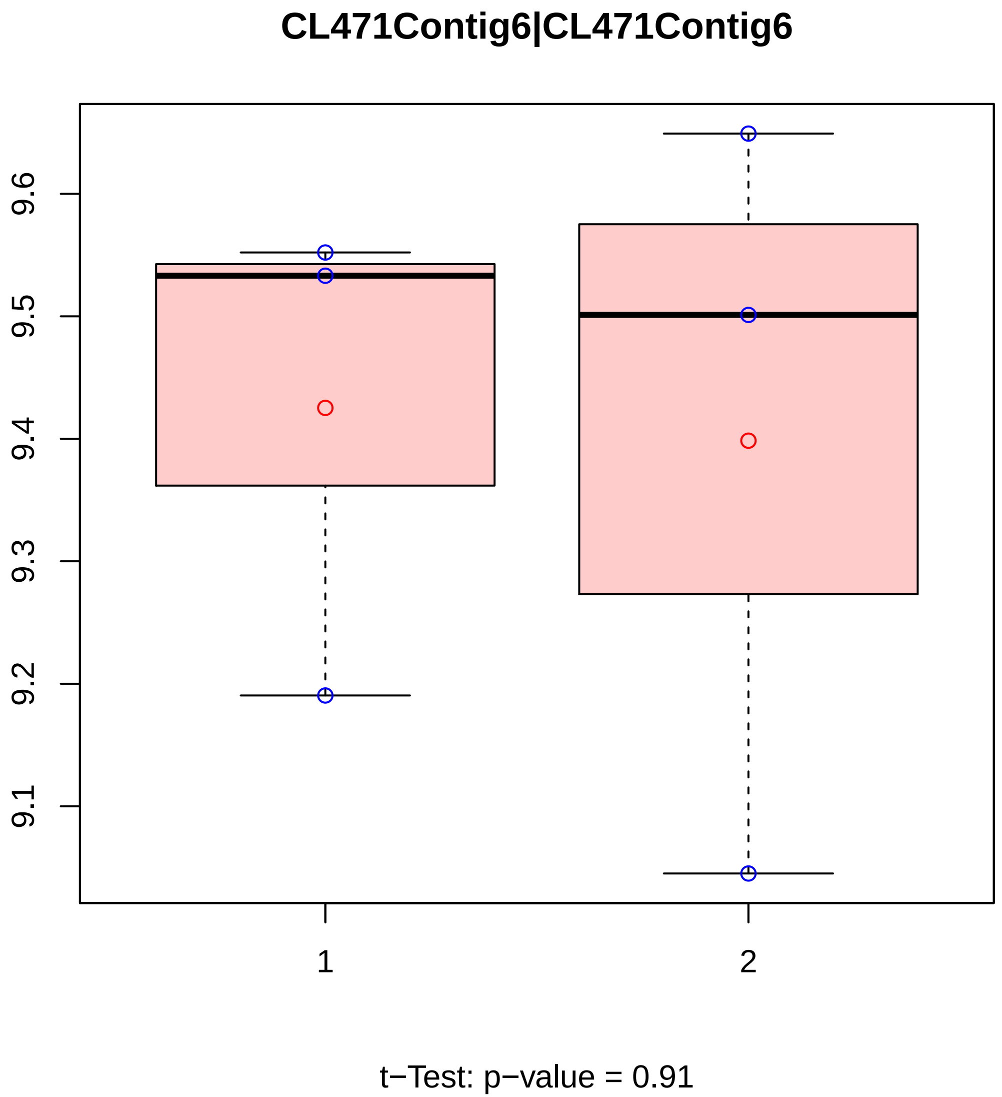
CL471Contig6|CL471Contig6
9.1
9.2
9.3
9.4
9.5
9.6
1
2
t−Test: p−value = 0.91

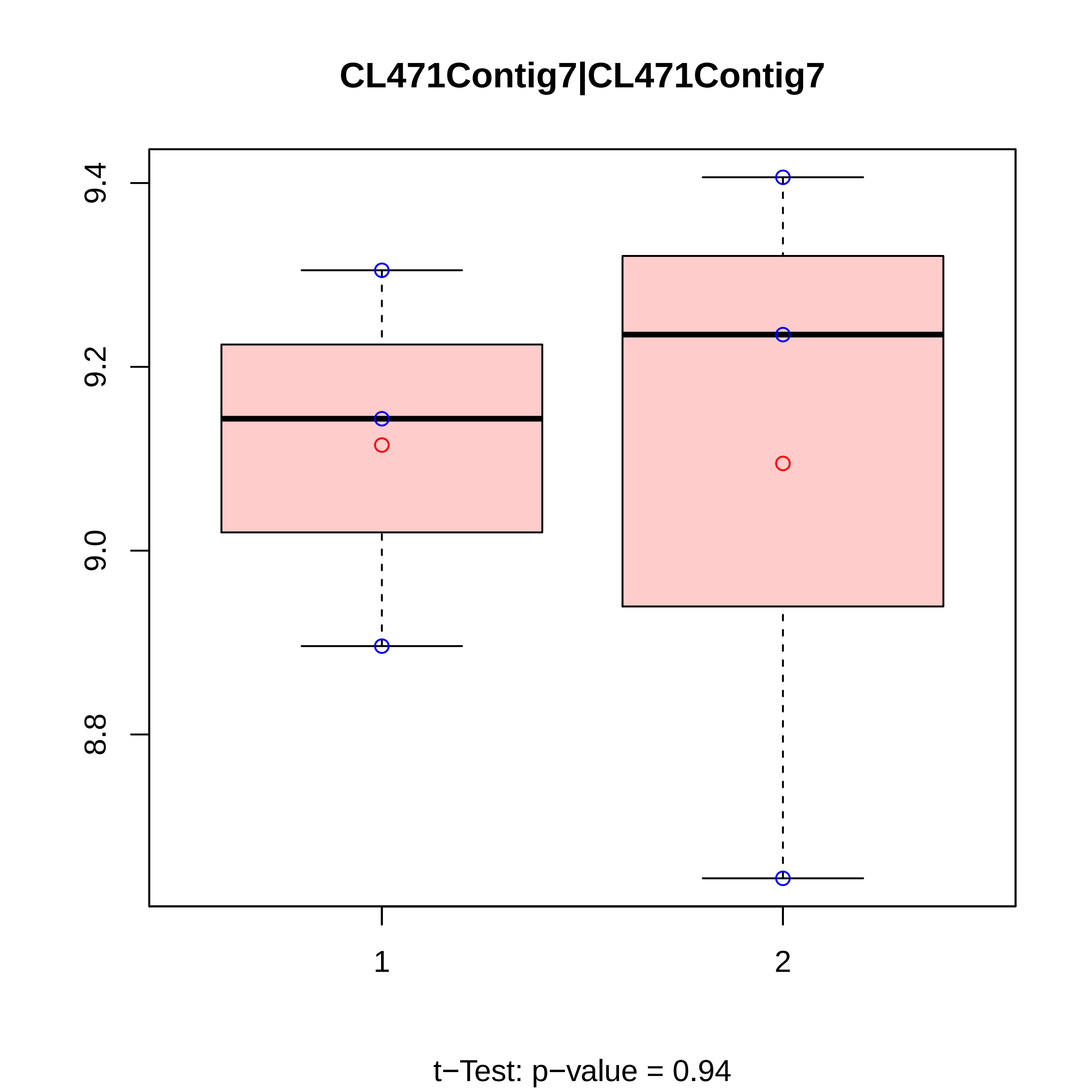

CL471Contig7|CL471Contig7
9.4
9.2
9.0
8.8
1
2
t−Test: p−value = 0.94

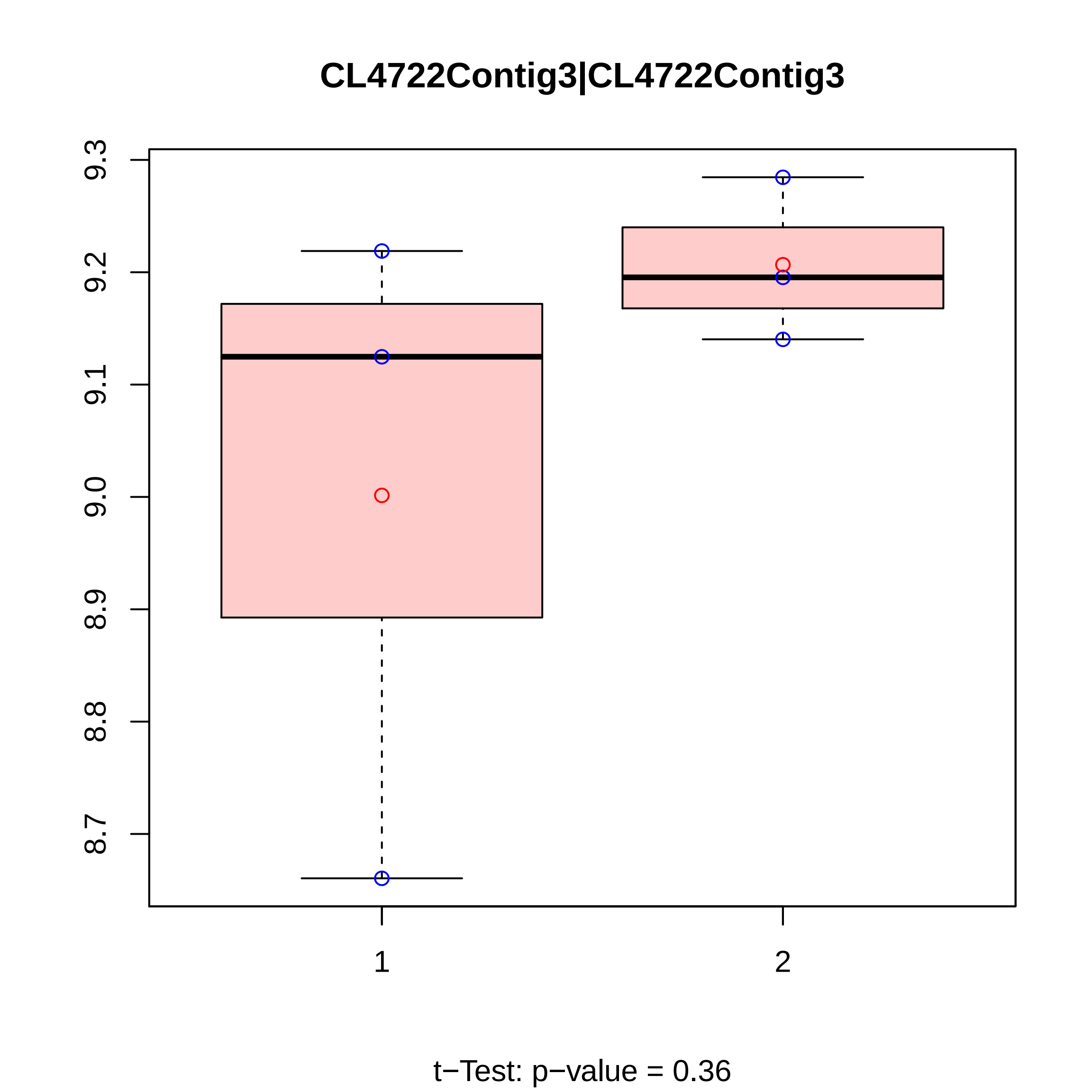
CL4722Contig3|CL4722Contig3
9.3
9.2
9.1
9.0
8.9
8.8
8.7
1
2
t−Test: p−value = 0.36

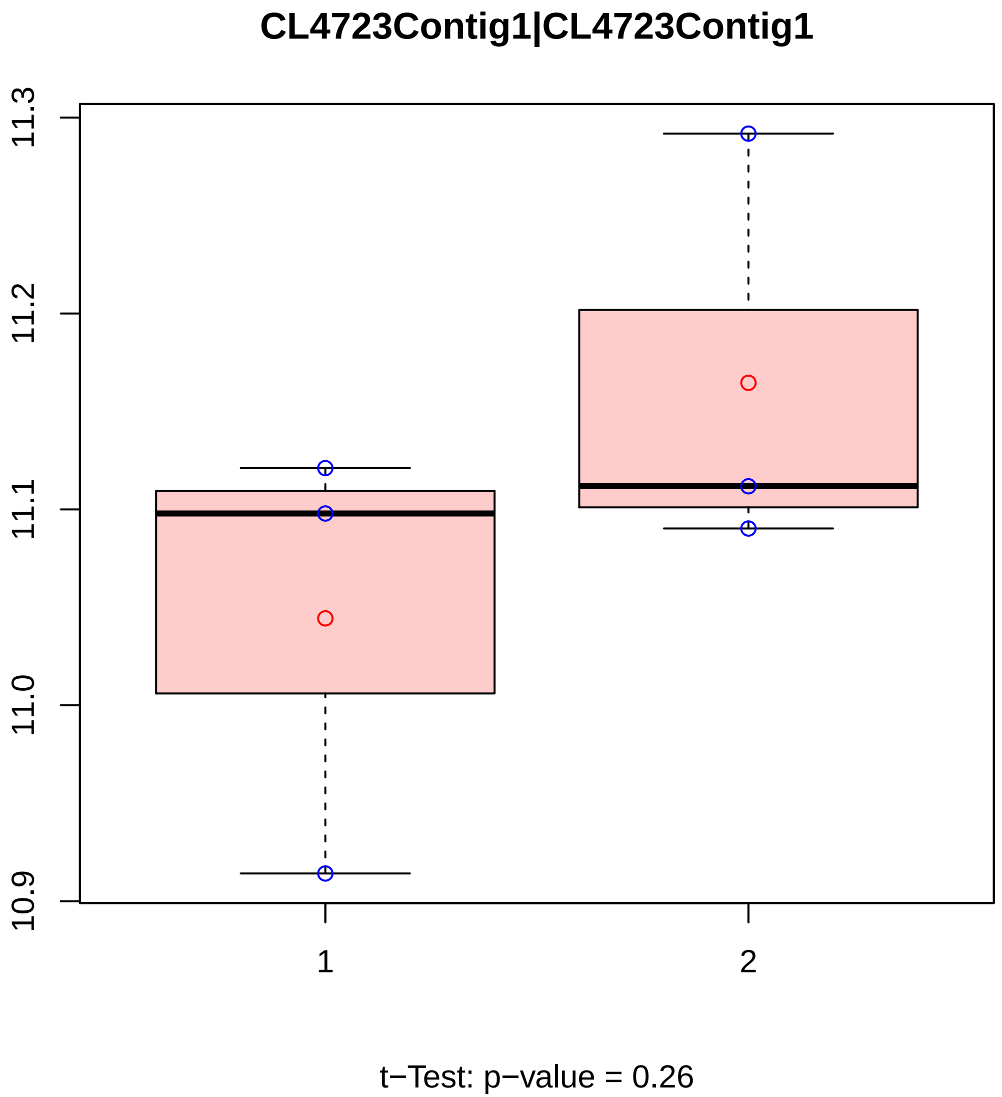

CL4723Contig1|CL4723Contig1
11.3
11.2
11.1
11.0
10.9
1
2
t−Test: p−value = 0.26

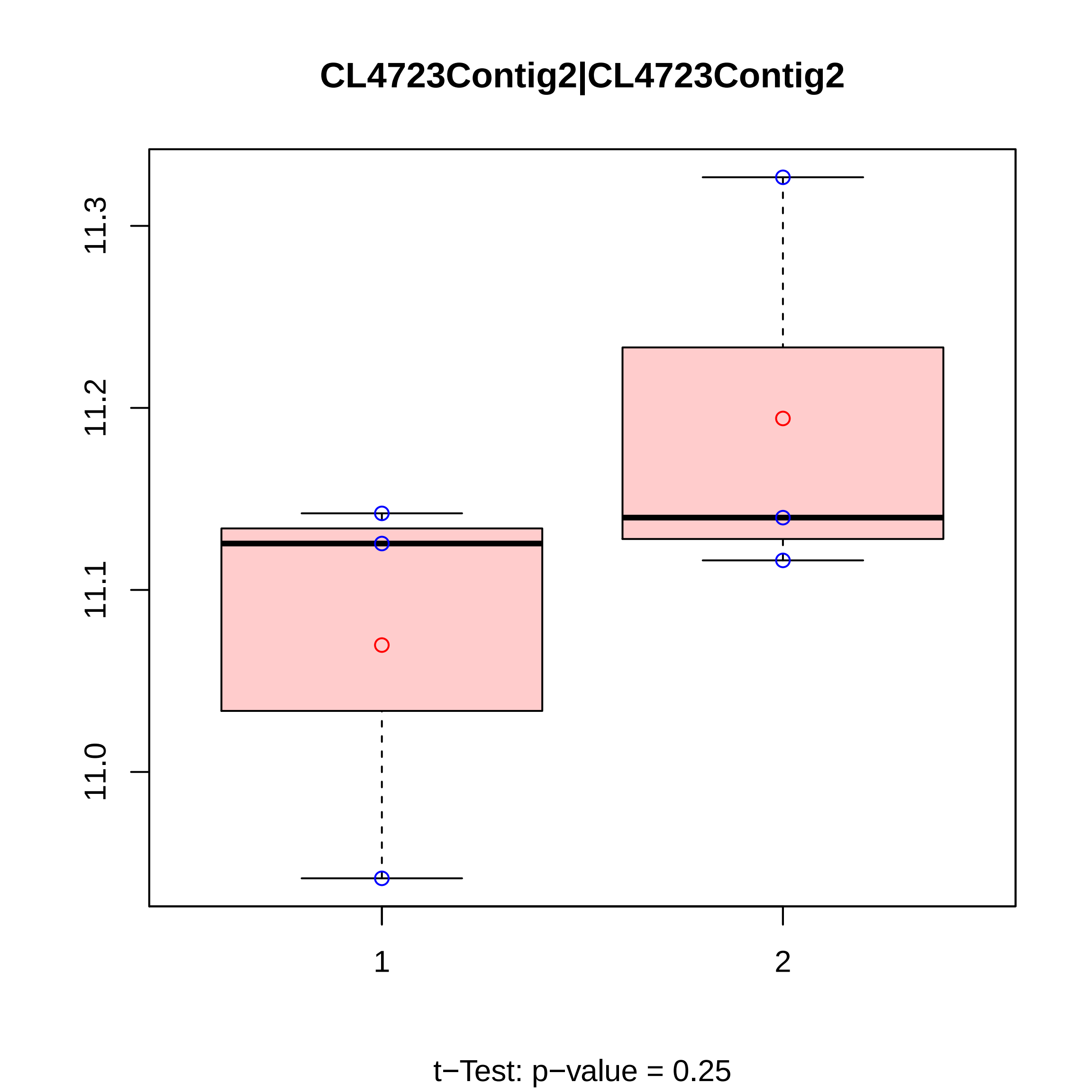
CL4723Contig2|CL4723Contig2
11.0
11.1
11.2
11.3
1
2
t−Test: p−value = 0.25

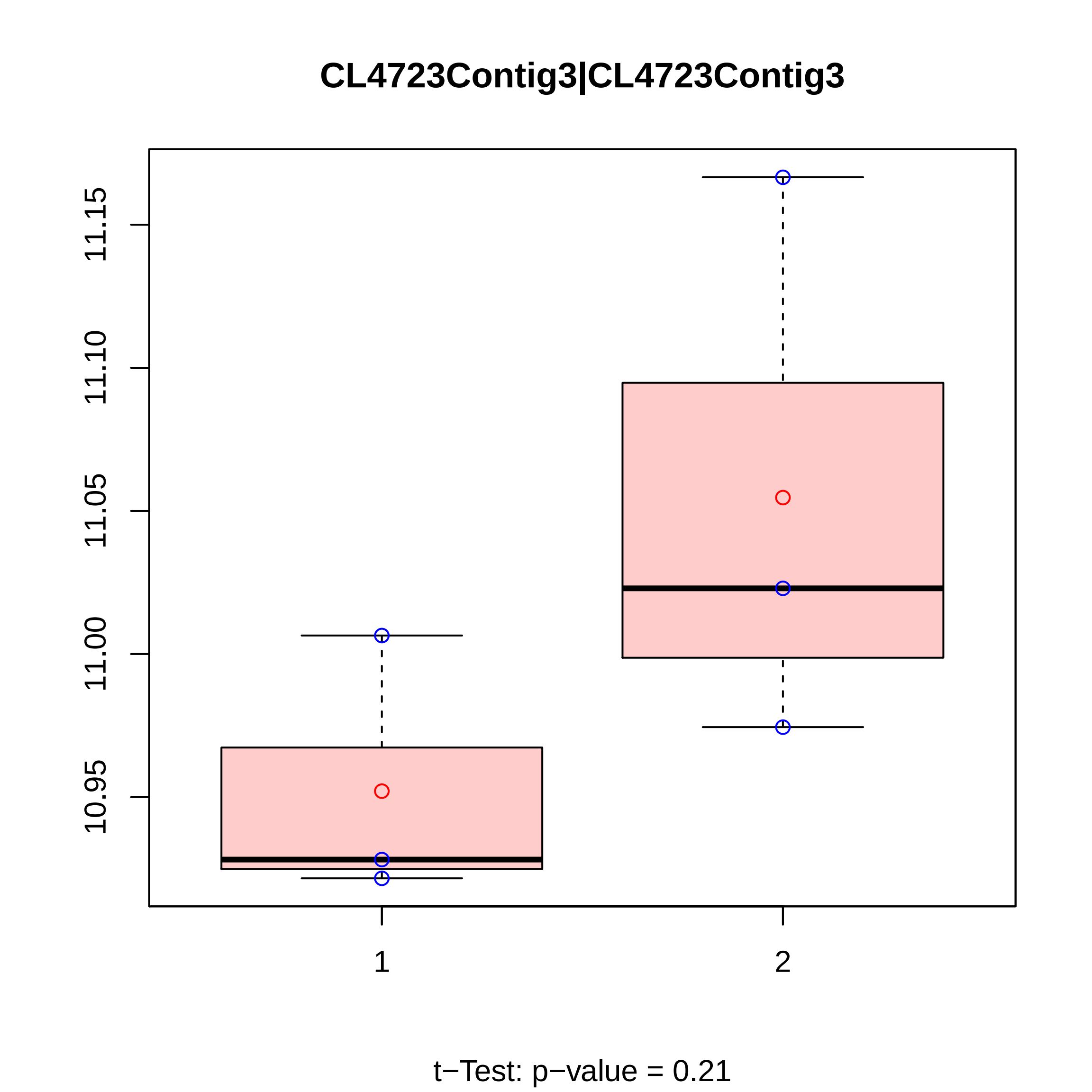
CL4723Contig3|CL4723Contig3
11.15
11.10
11.05
11.00
10.95
1
2
t−Test: p−value = 0.21

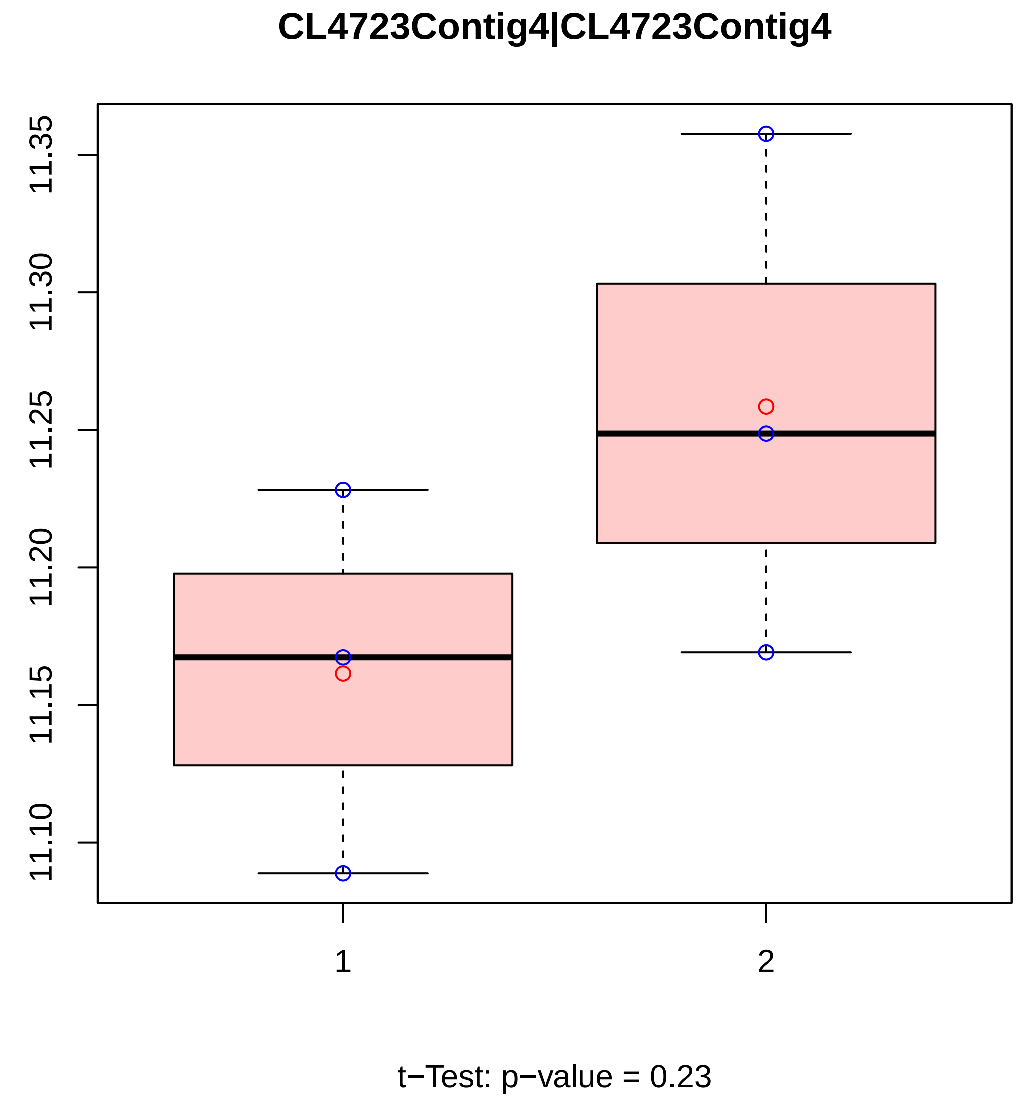
CL4723Contig4|CL4723Contig4
11.10
11.15
11.20
11.25
11.30
11.35
1
2
t−Test: p−value = 0.23

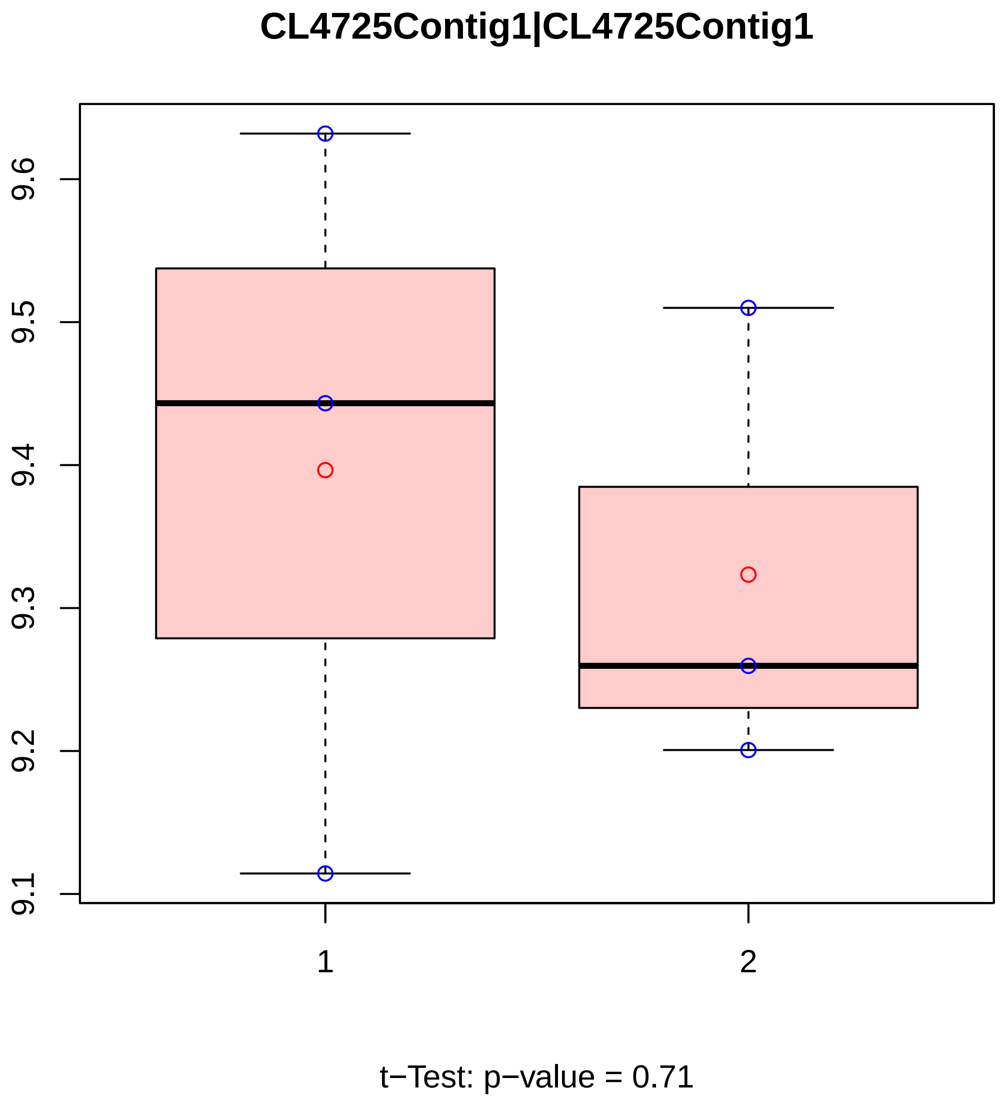
CL4725Contig1|CL4725Contig1
9.1
9.2
9.3
9.4
9.5
9.6
1
2
t−Test: p−value = 0.71

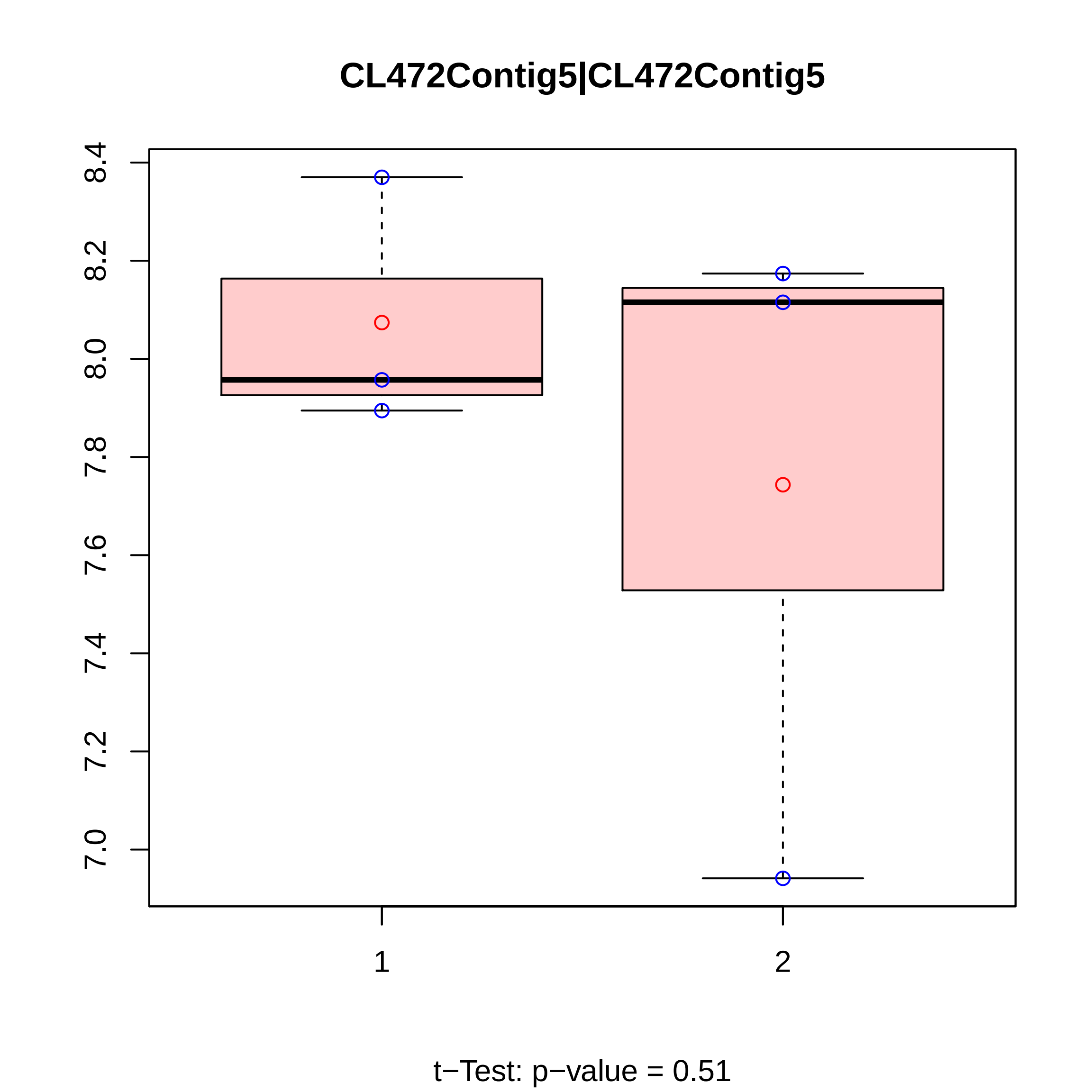
CL472Contig5|CL472Contig5
8.4
8.2
8.0
7.8
7.6
7.4
7.2
7.0
1
2
t−Test: p−value = 0.51

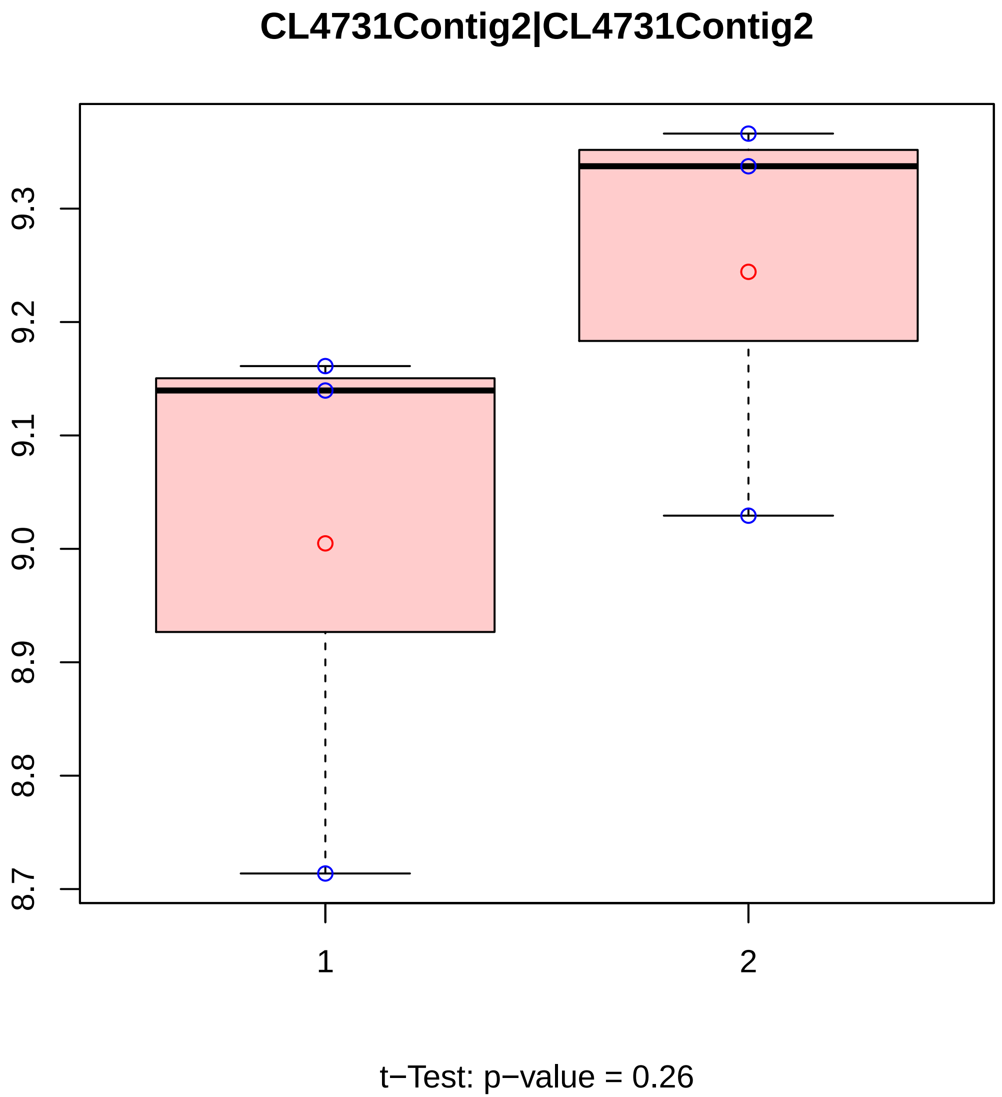

CL4731Contig2|CL4731Contig2
9.3
9.2
9.1
9.0
8.9
8.8
8.7
1
2
t−Test: p−value = 0.26

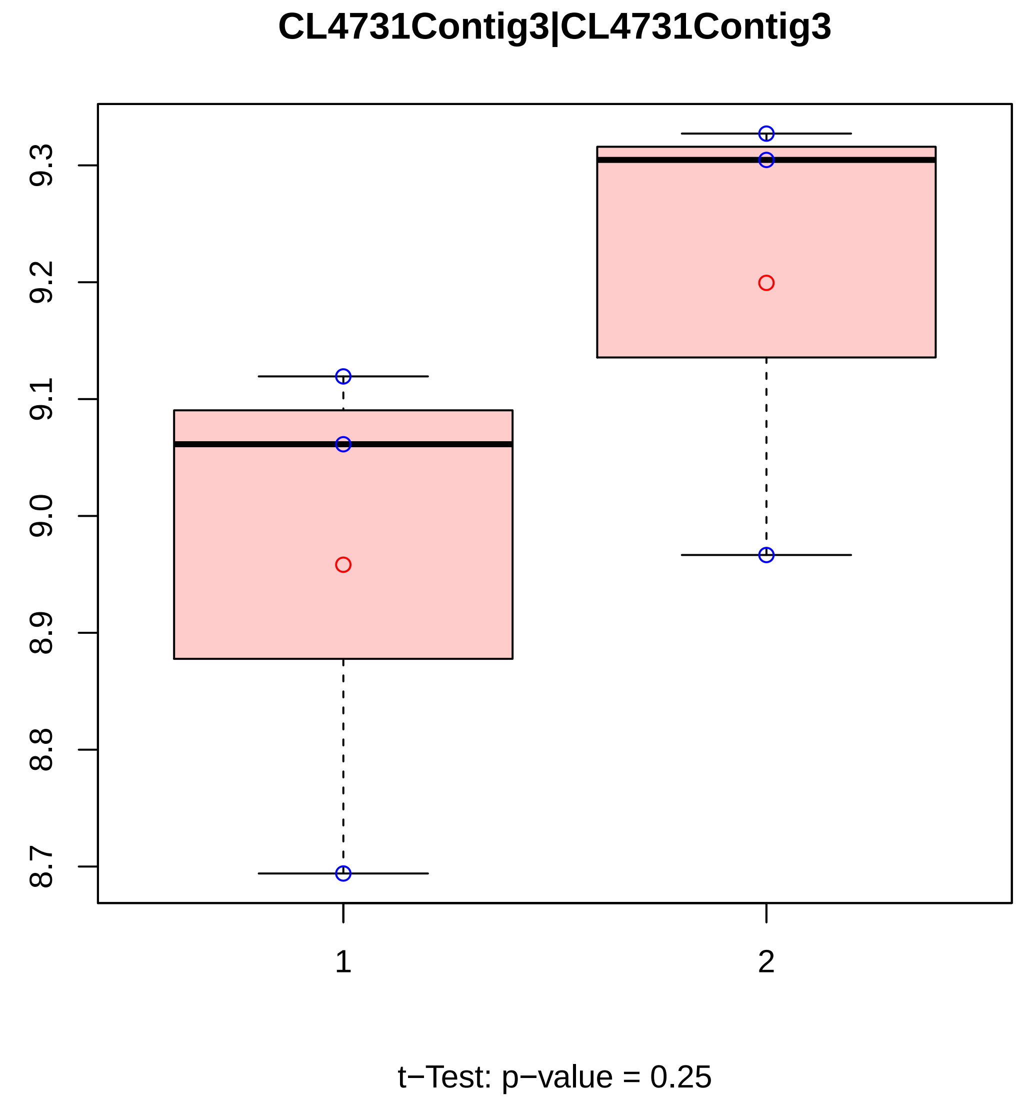
CL4731Contig3|CL4731Contig3
9.3
9.2
9.1
9.0
8.9
8.8
8.7
1
2
t−Test: p−value = 0.25

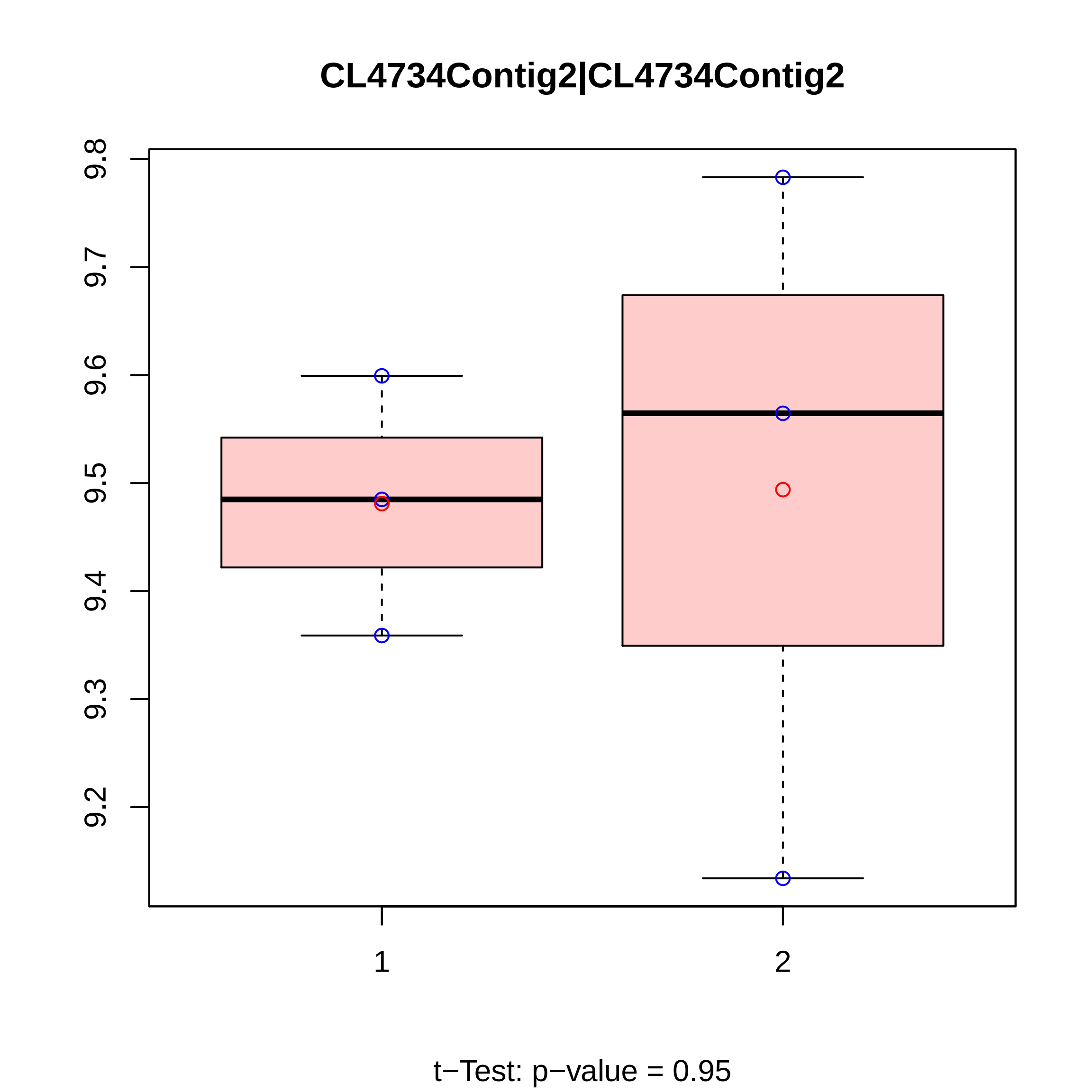
CL4734Contig2|CL4734Contig2
9.8
9.7
9.6
9.5
9.4
9.3
9.2
1
2
t−Test: p−value = 0.95

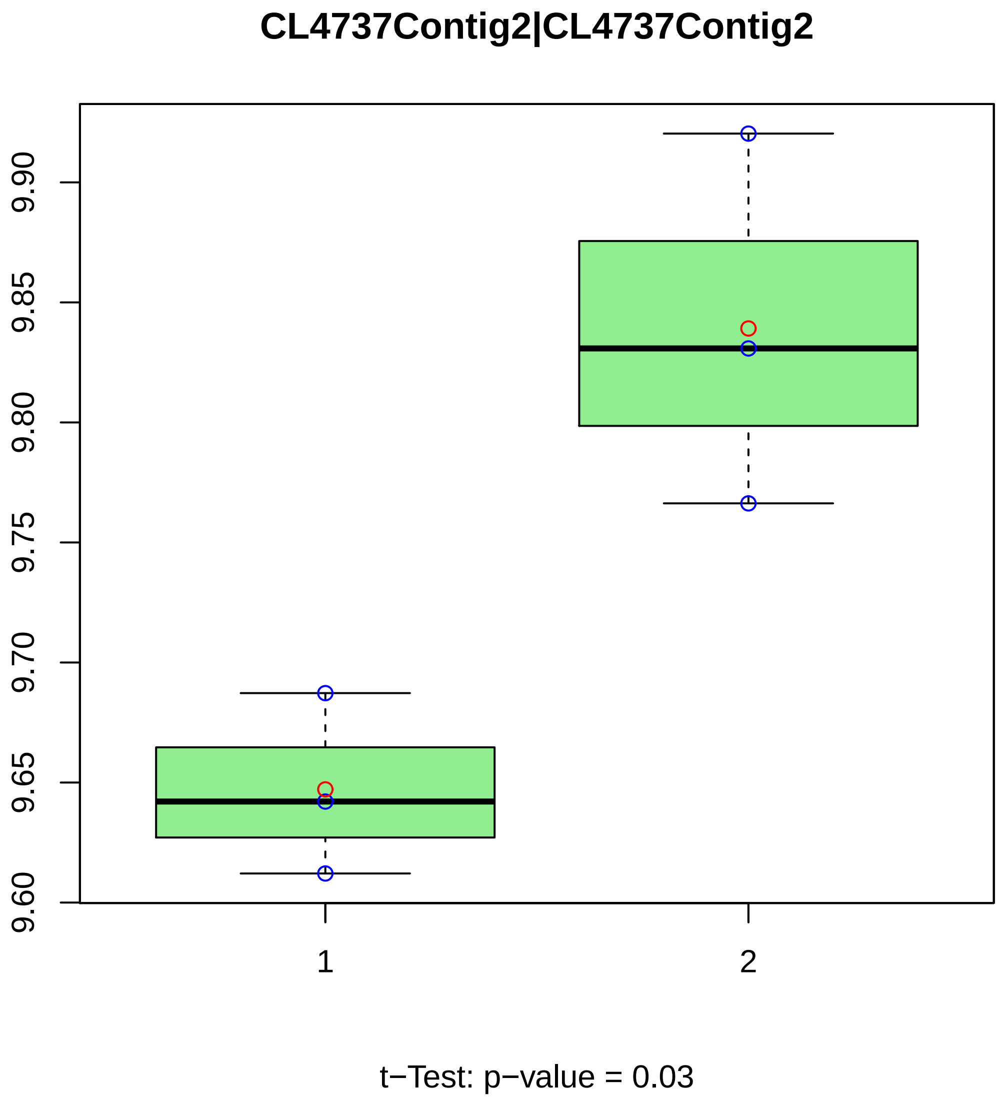
CL4737Contig2|CL4737Contig2
9.60
9.65
9.70
9.75
9.80
9.85
9.90
1
2
t−Test: p−value = 0.03

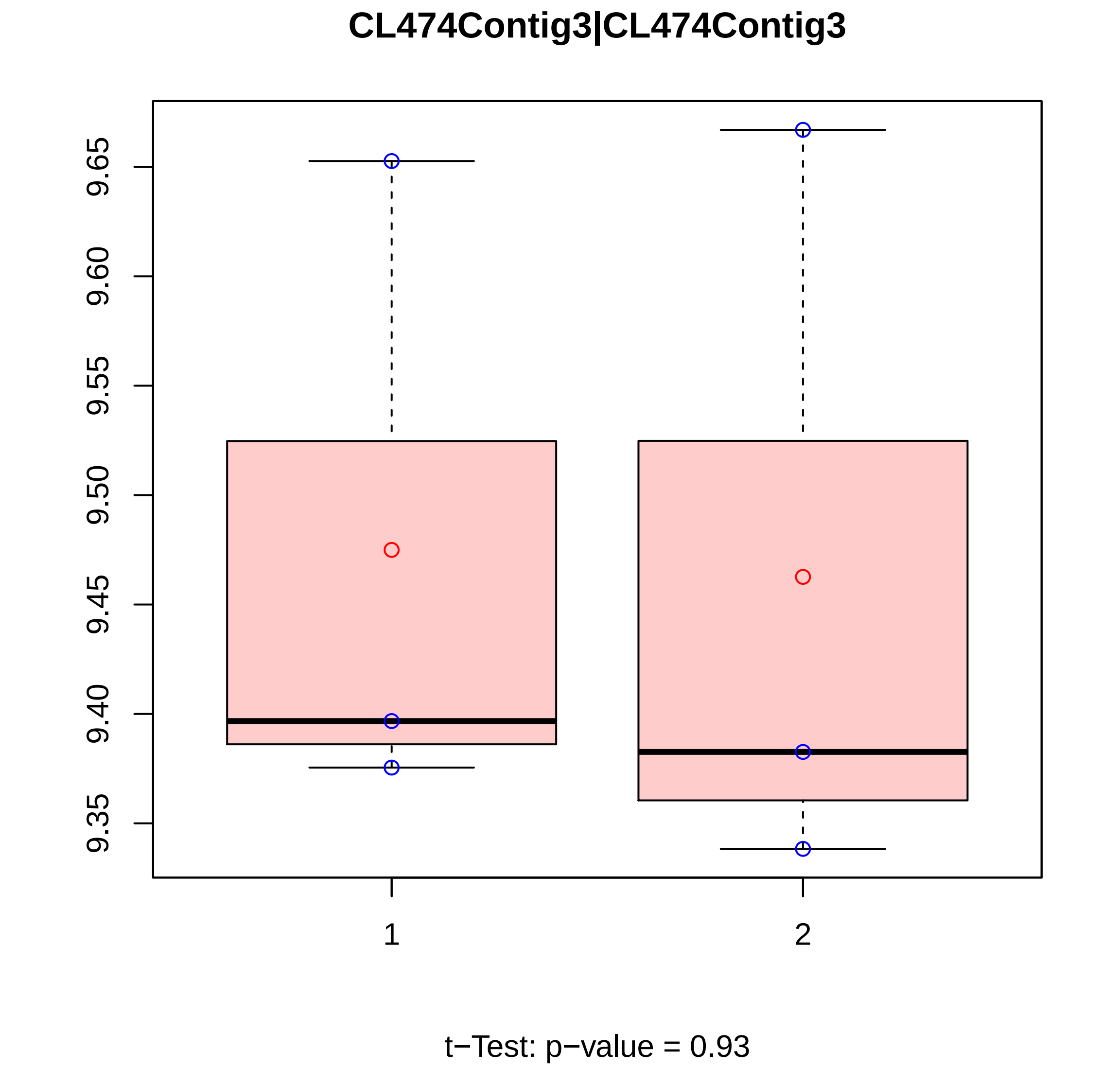
CL474Contig3|CL474Contig3
9.35
9.40
9.45
9.50
9.55
9.60
9.65
1
2
t−Test: p−value = 0.93

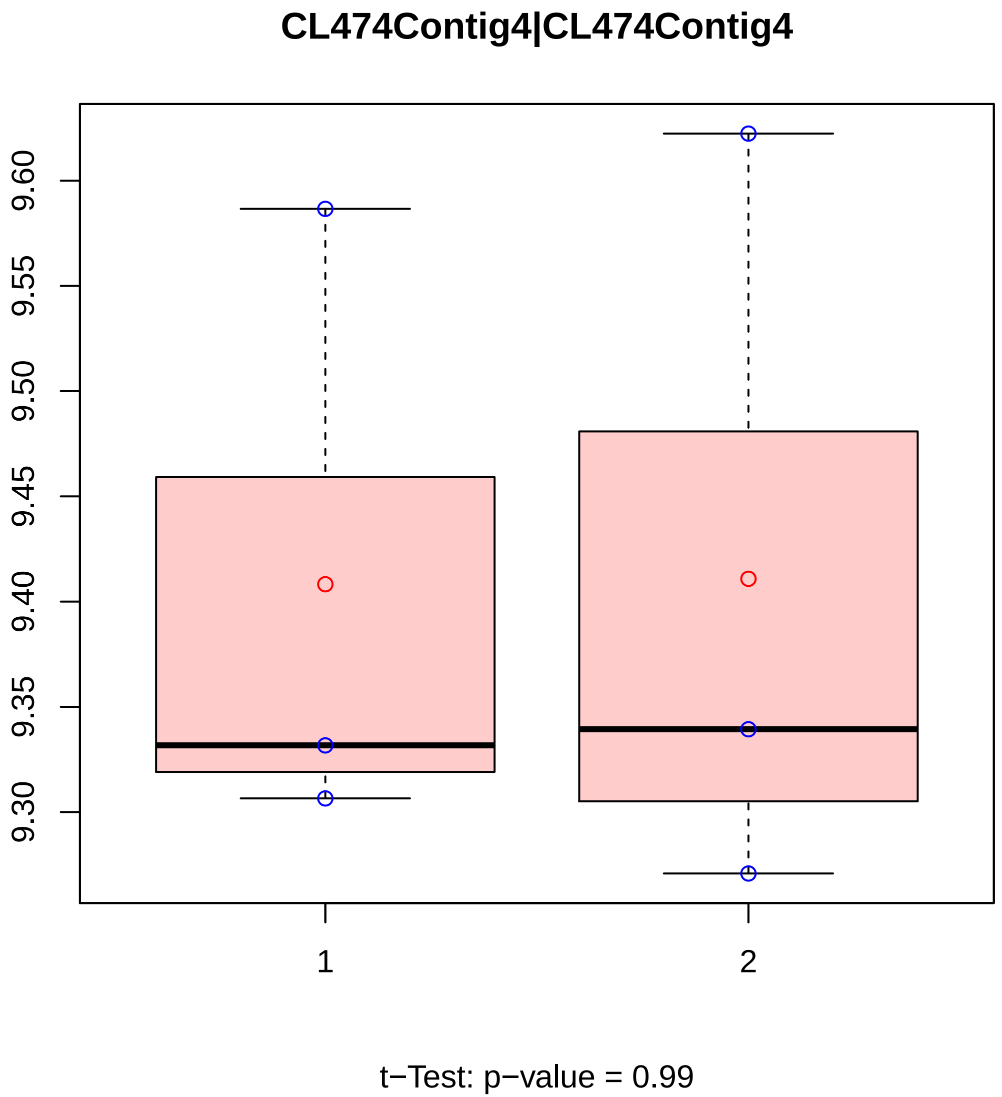

CL474Contig4|CL474Contig4
9.30
9.35
9.40
9.45
9.50
9.55
9.60
1
2
t−Test: p−value = 0.99

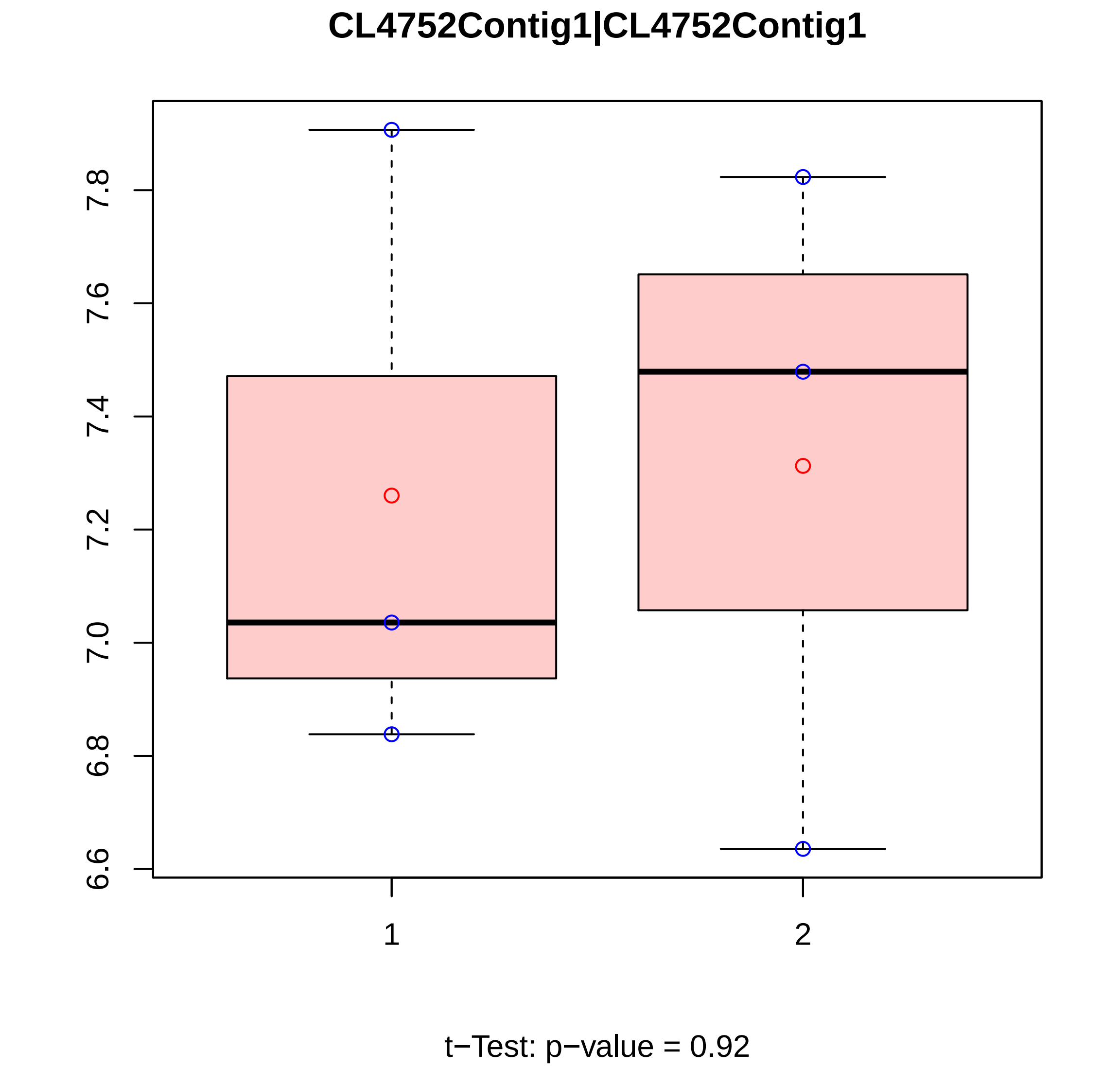
CL4752Contig1|CL4752Contig1
6.6
6.8
7.0
7.2
7.4
7.6
7.8
1
2
t−Test: p−value = 0.92

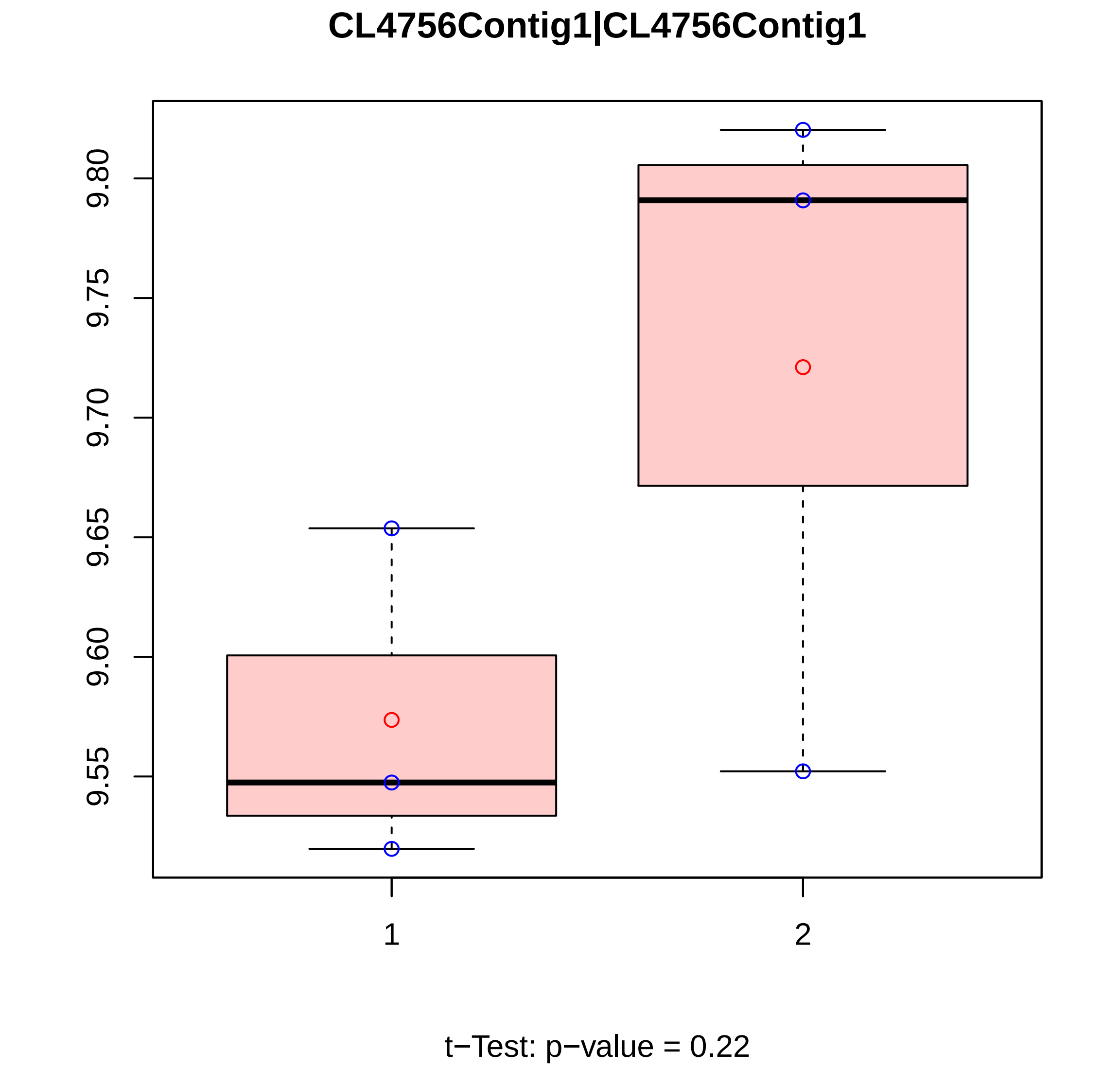
CL4756Contig1|CL4756Contig1
9.55
9.60
9.65
9.70
9.75
9.80
1
2
t−Test: p−value = 0.22

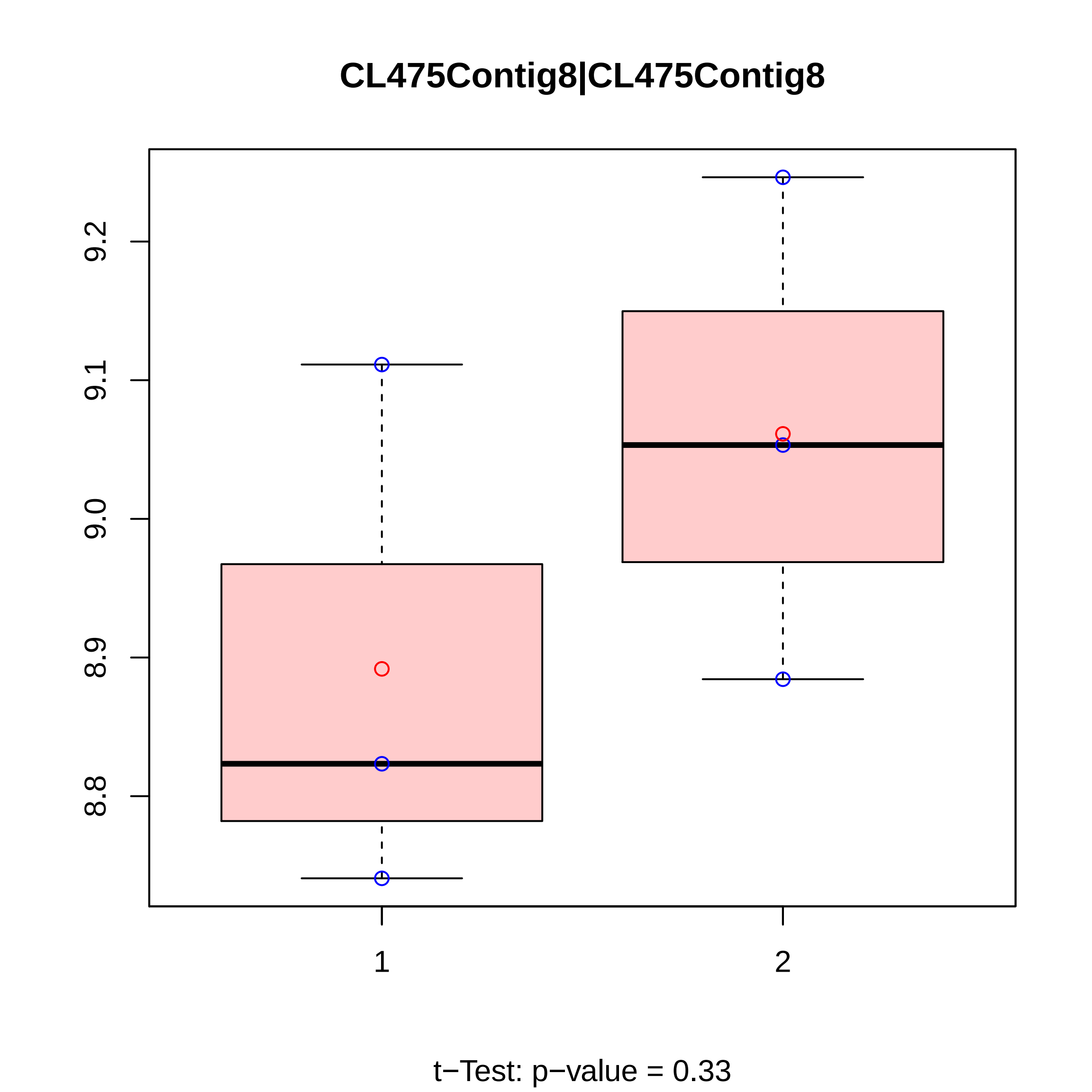

CL475Contig8|CL475Contig8
9.2
9.1
9.0
8.9
8.8
1
2
t−Test: p−value = 0.33

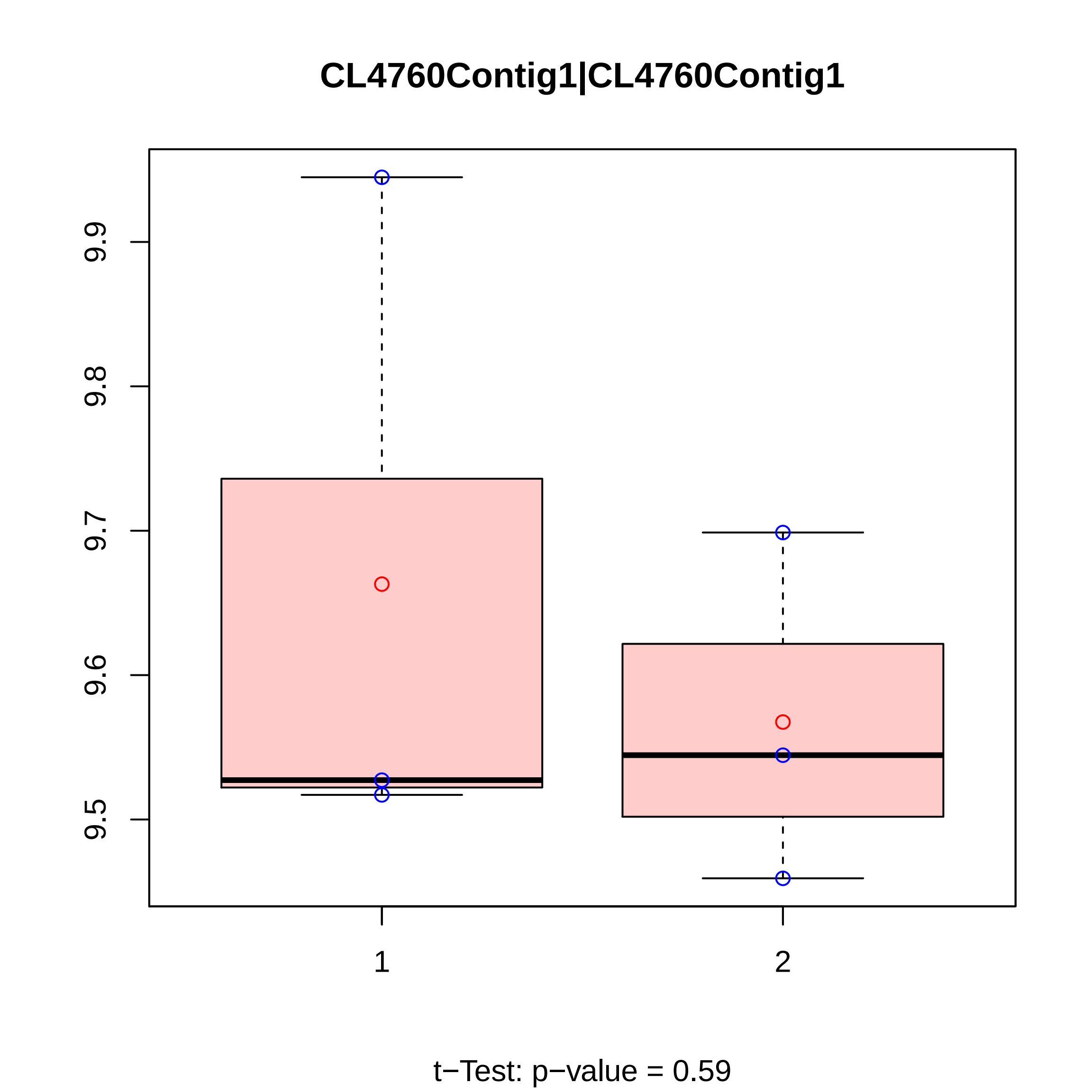
CL4760Contig1|CL4760Contig1
9.5
9.6
9.7
9.8
9.9
1
2
t−Test: p−value = 0.59

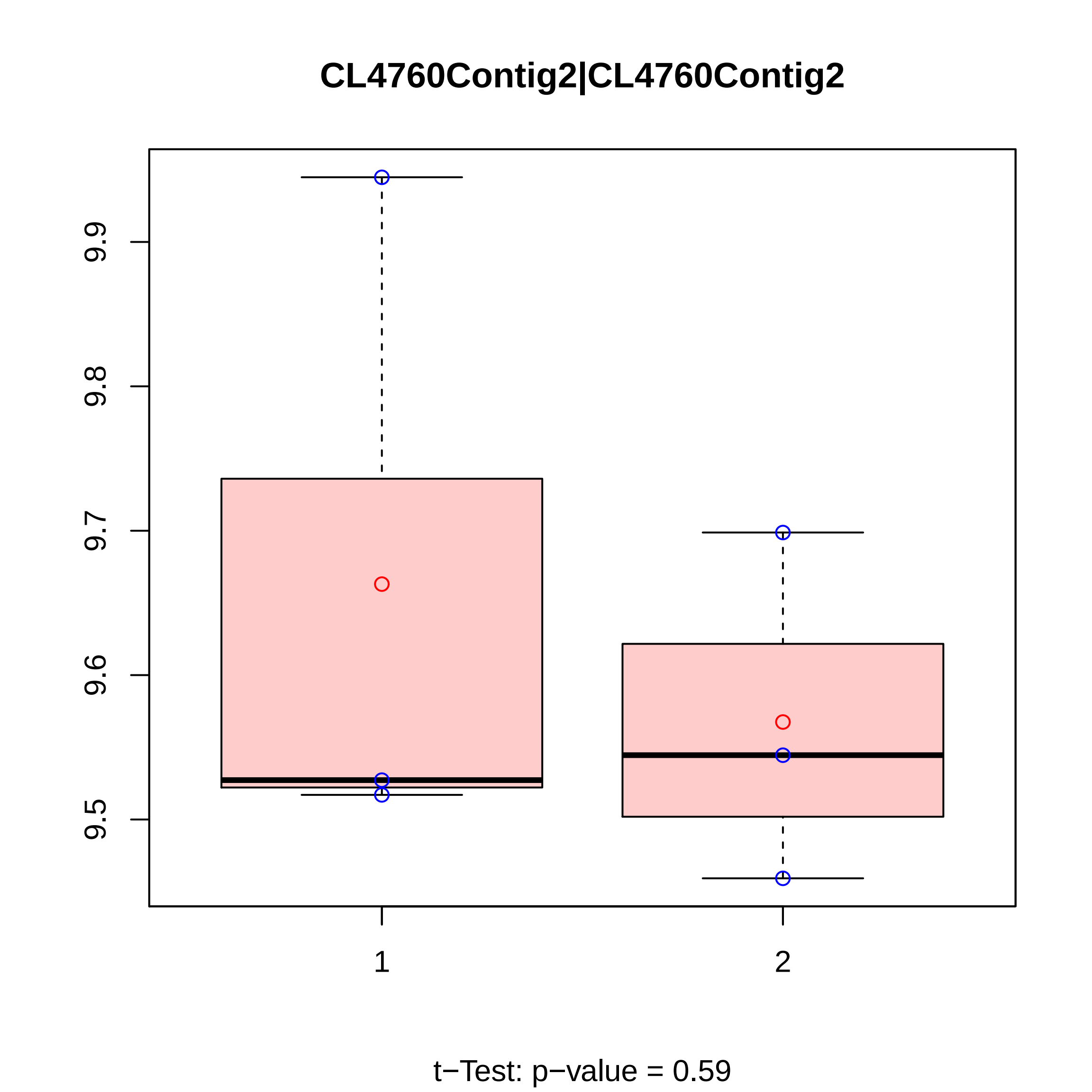

CL4760Contig2|CL4760Contig2
9.5
9.6
9.7
9.8
9.9
1
2
t−Test: p−value = 0.59

# CL4761Contig4|CL4761Contig4

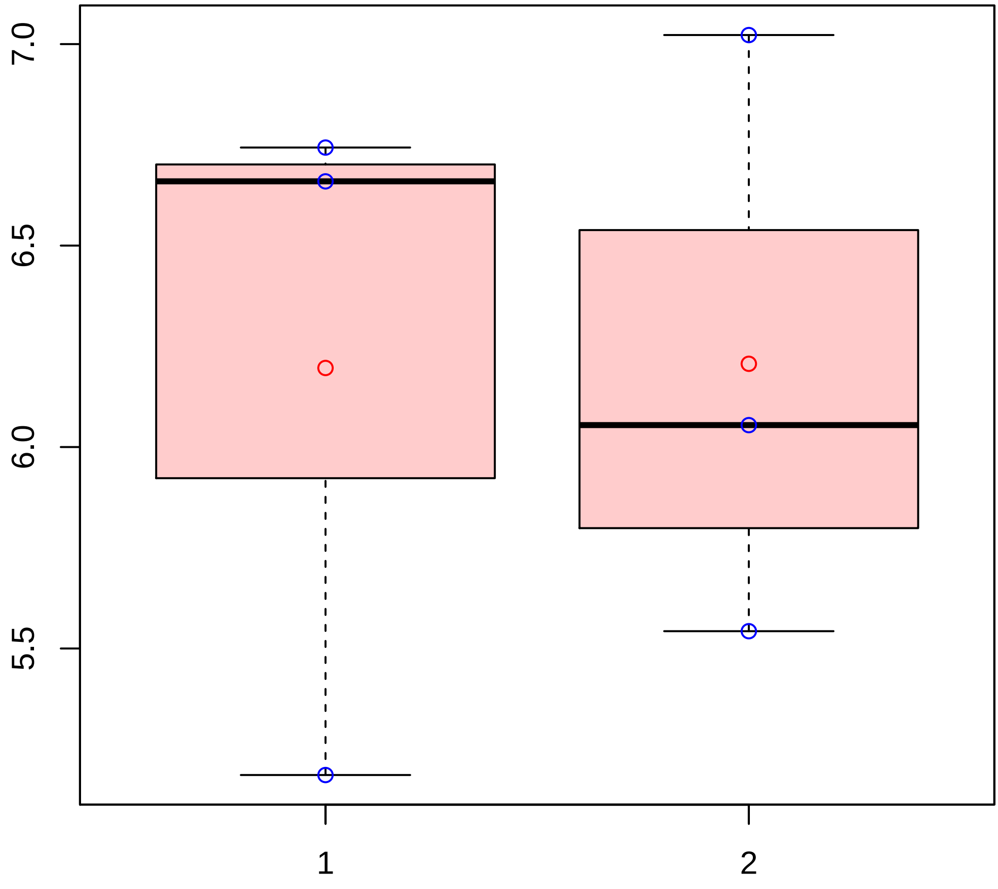


t−Test: p−value = 0.99

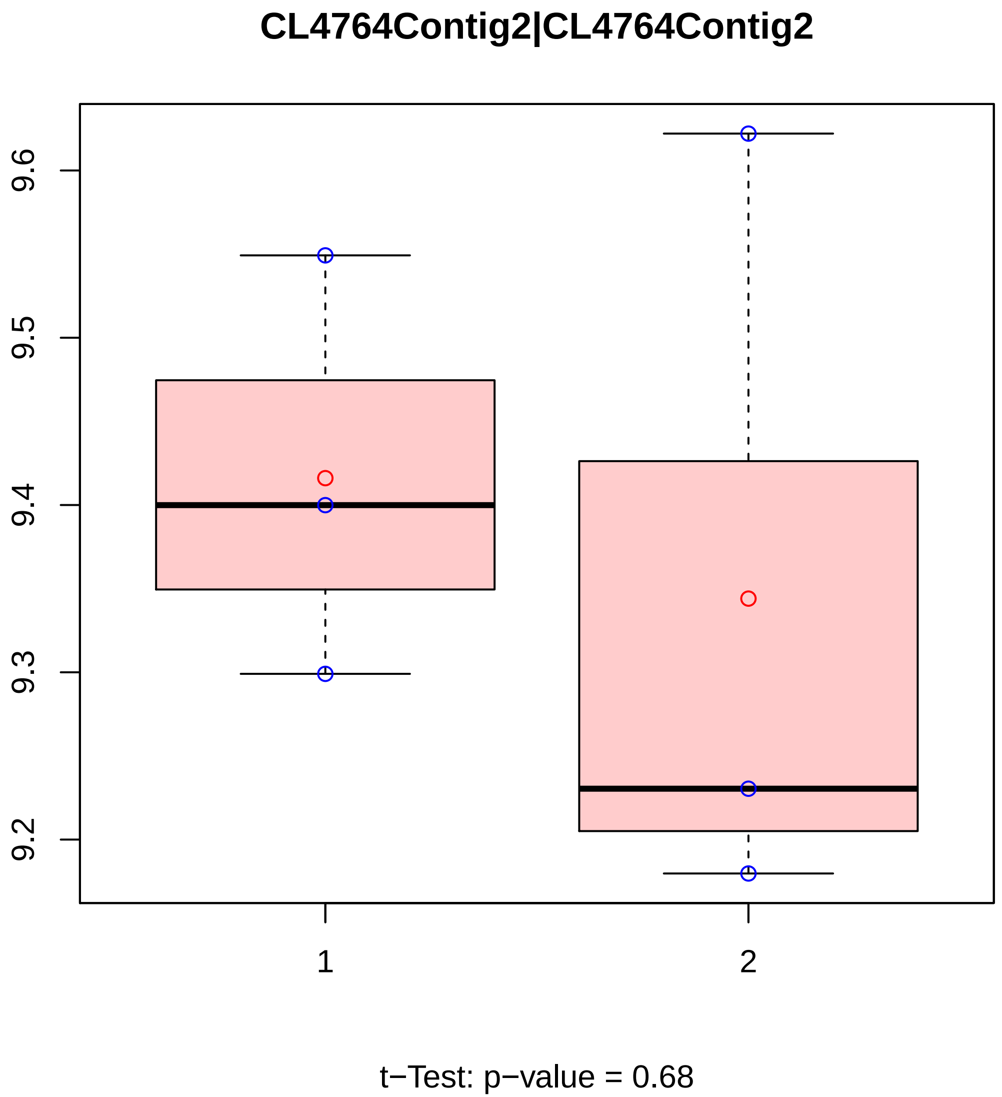

CL4764Contig2|CL4764Contig2
9.6
9.5
9.4
9.3
9.2
1
2
t−Test: p−value = 0.68

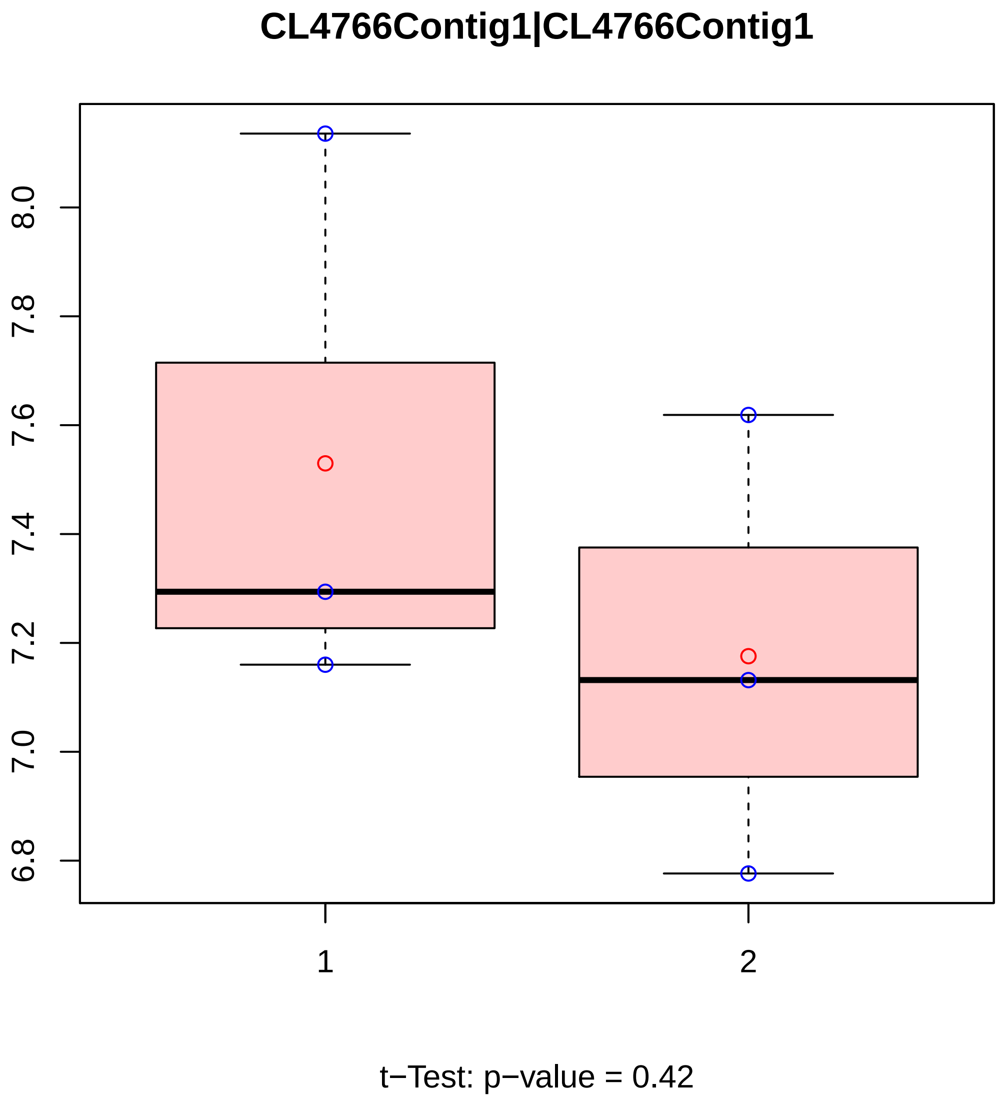
CL4766Contig1|CL4766Contig1
8.0
7.8
7.6
7.4
7.2
7.0
6.8
1
2
t−Test: p−value = 0.42

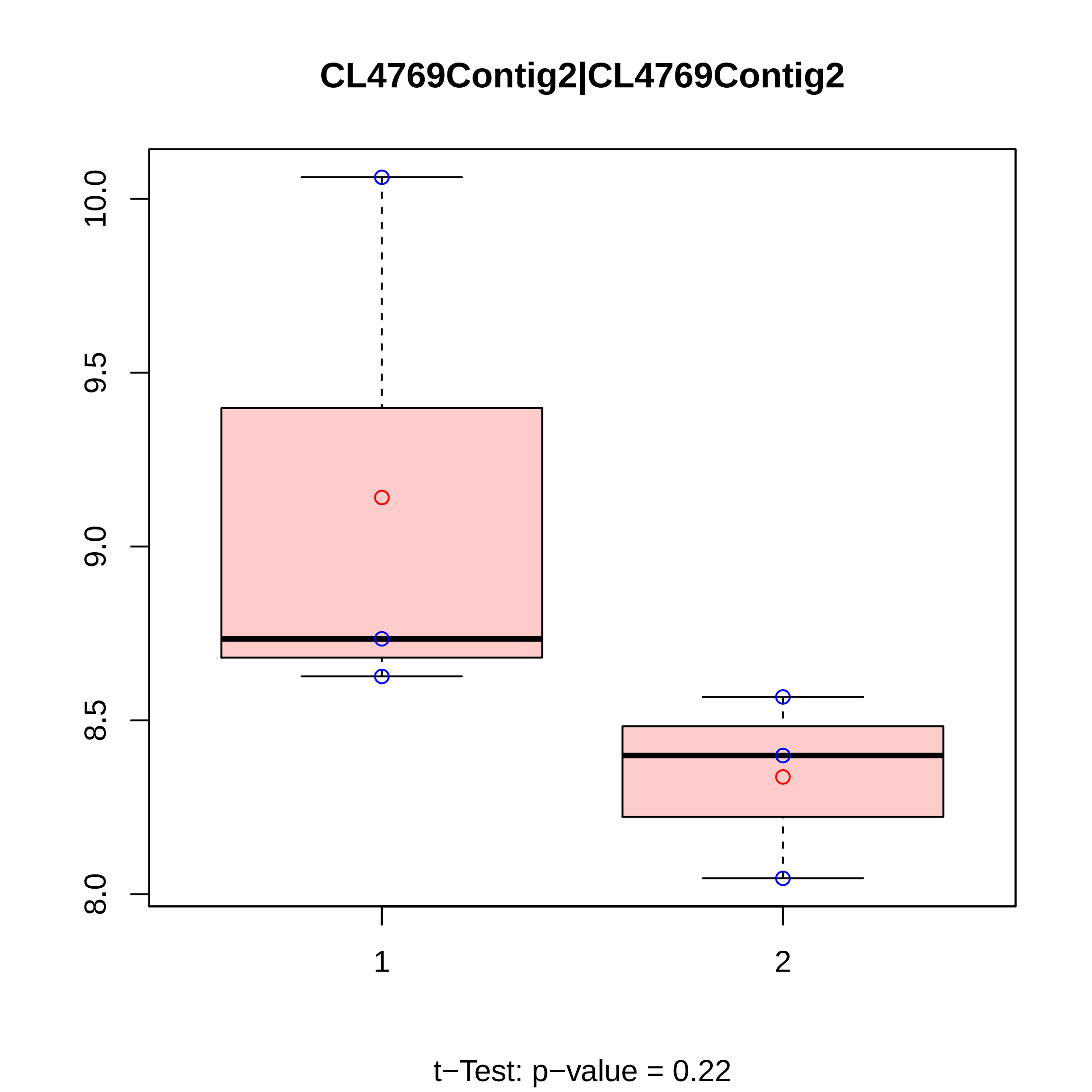
CL4769Contig2|CL4769Contig2
10.0
9.5
9.0
8.5
8.0
1
2
t−Test: p−value = 0.22

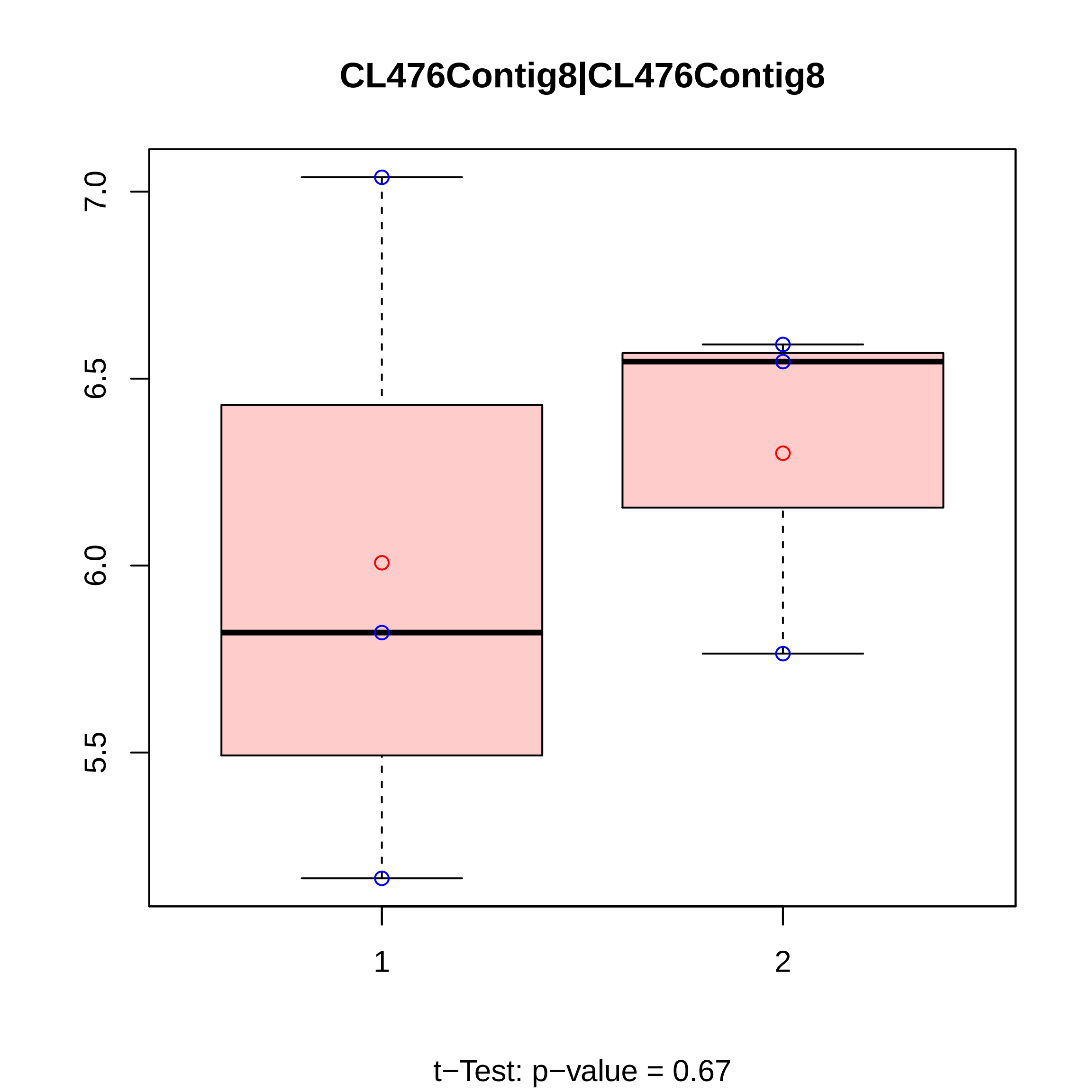
CL476Contig8|CL476Contig8
7.0
6.5
6.0
5.5
1
2
t−Test: p−value = 0.67

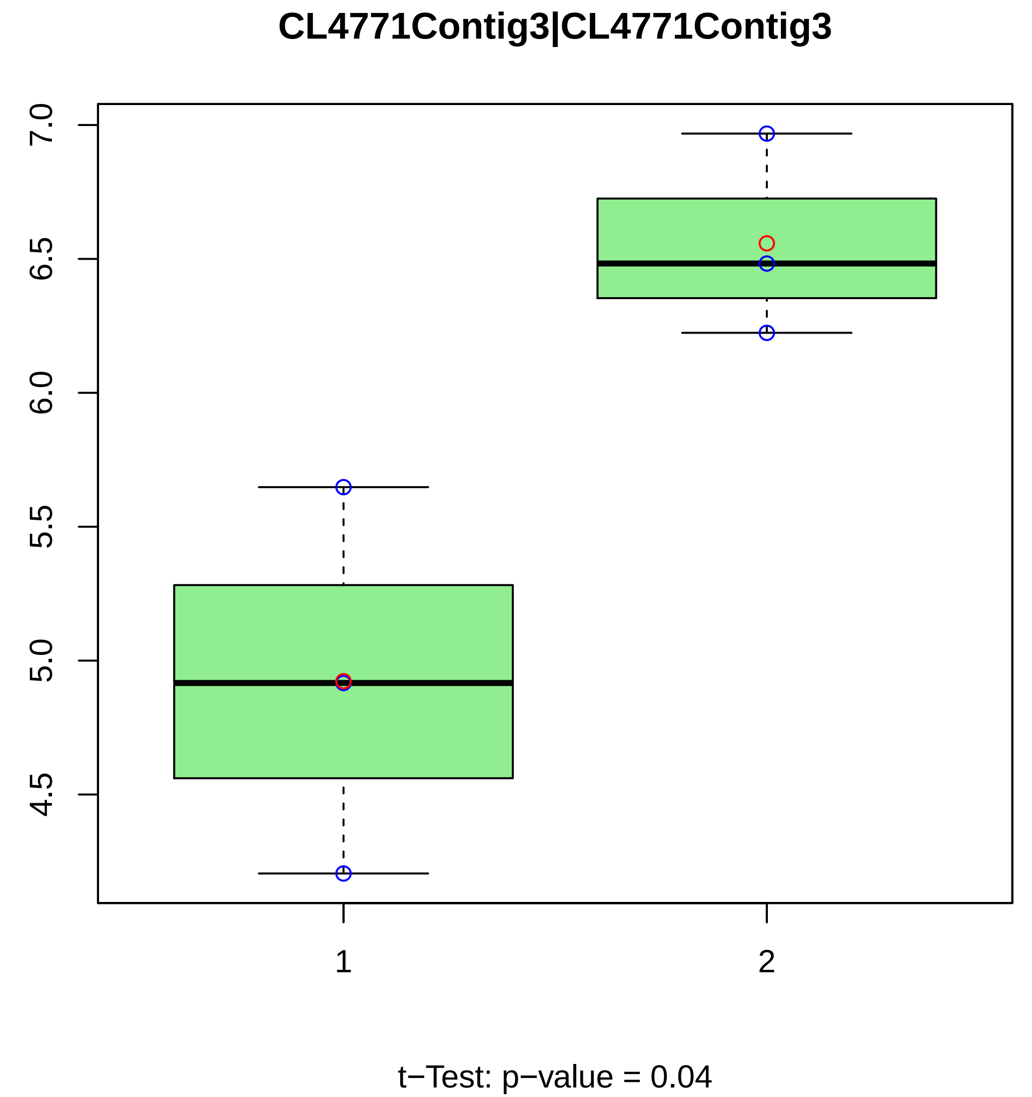
CL4771Contig3|CL4771Contig3
7.0
6.5
6.0
5.5
5.0
4.5
1
2
t−Test: p−value = 0.04

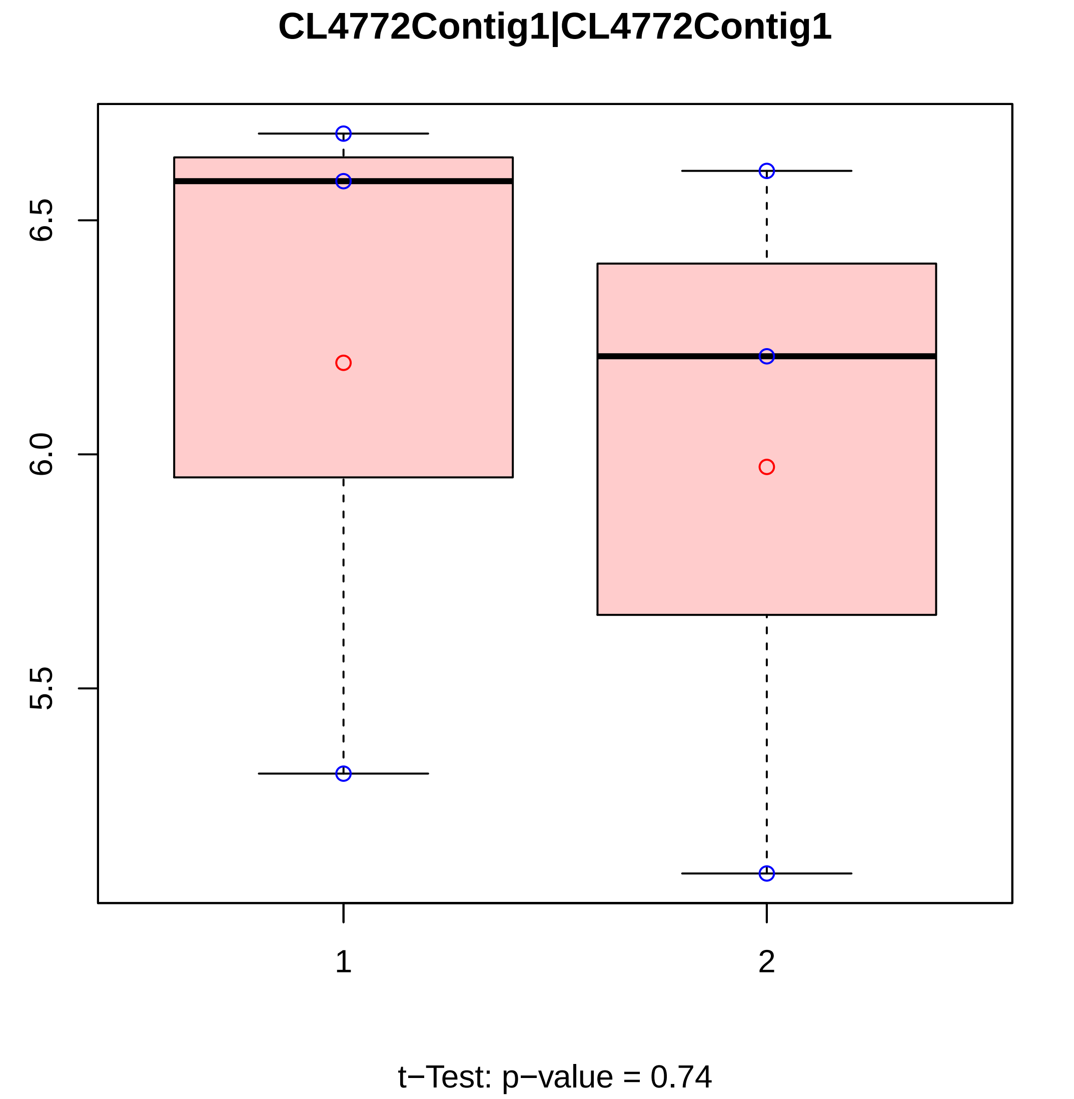
CL4772Contig1|CL4772Contig1
6.5
6.0
5.5
1
2
t−Test: p−value = 0.74

# CL477Contig1|CL477Contig1

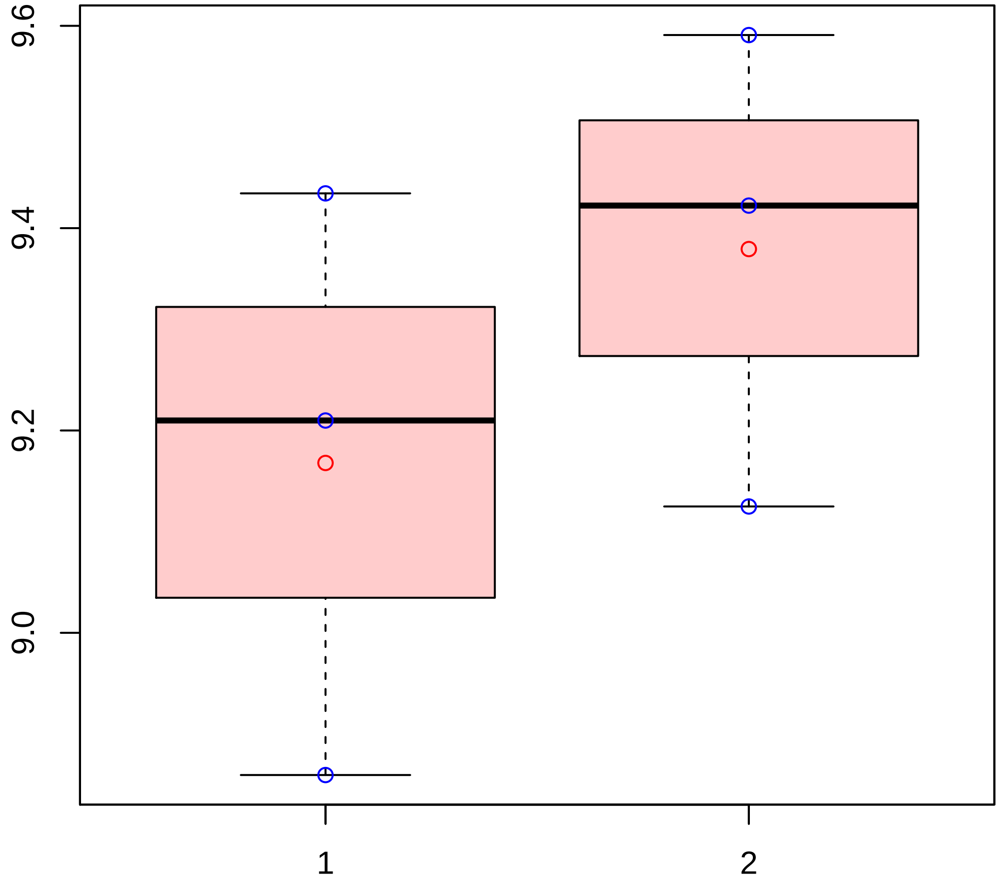


t−Test: p−value = 0.38

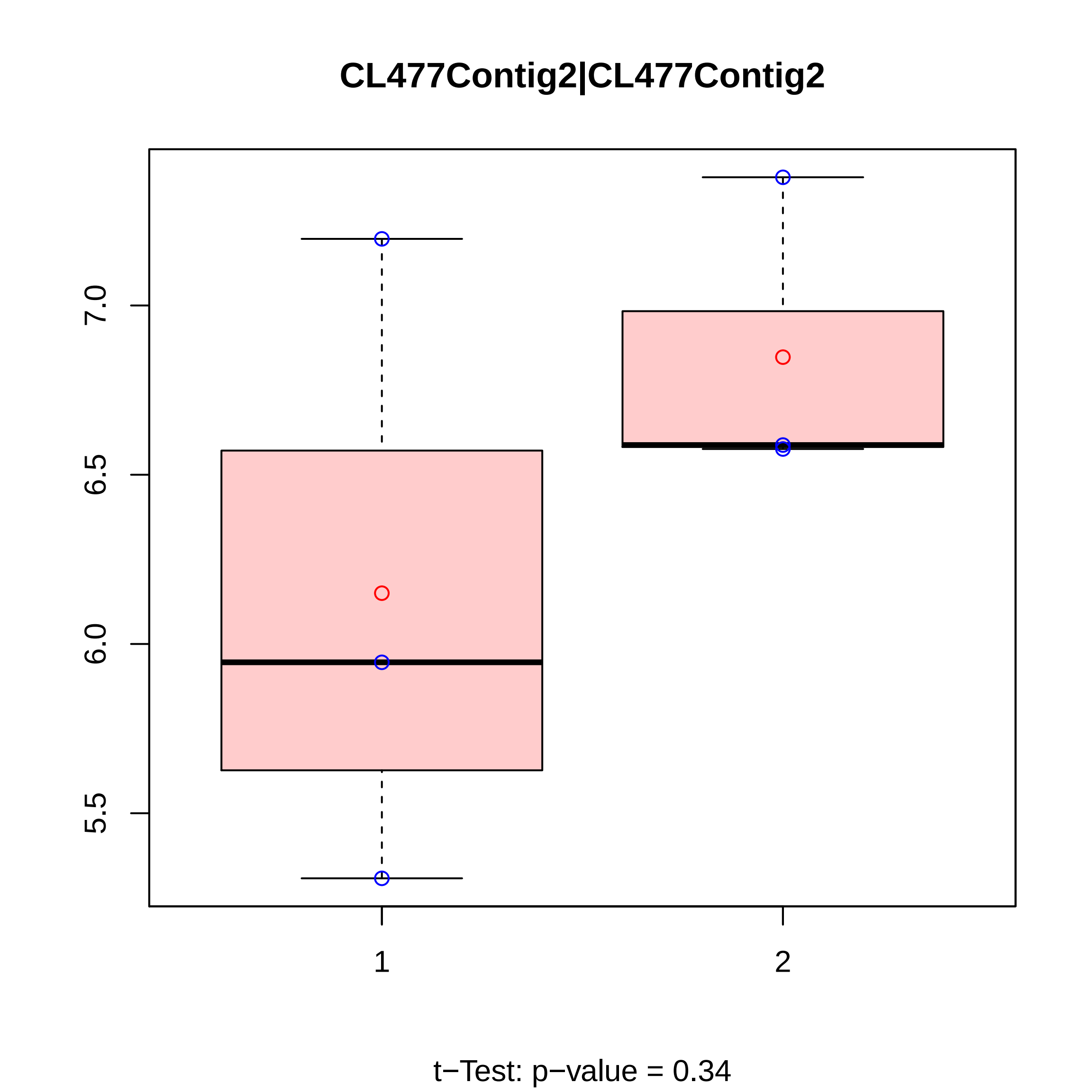
CL477Contig2|CL477Contig2
7.0
6.5
6.0
5.5
1
2
t−Test: p−value = 0.34

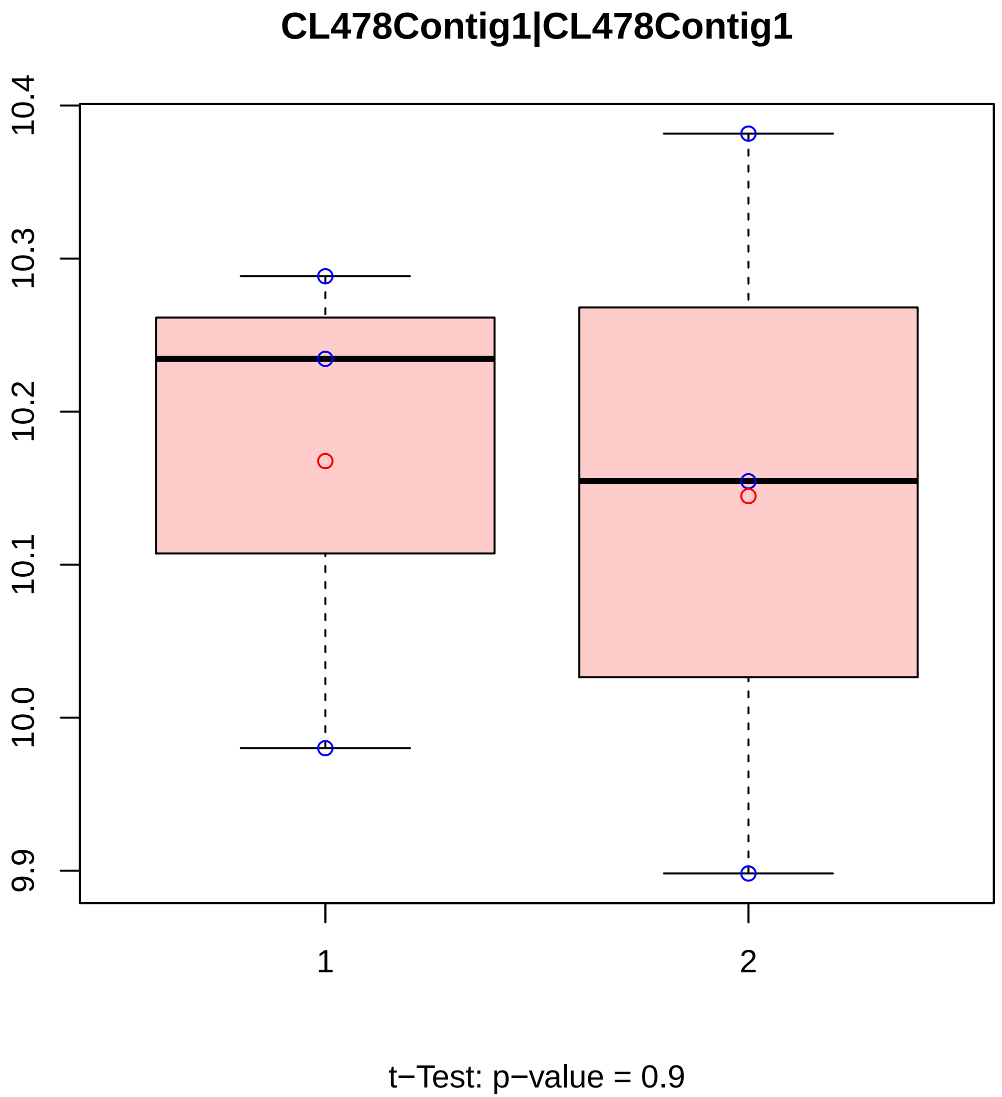
CL478Contig1|CL478Contig1
10.4
10.3
10.2
10.1
10.0
9.9
1
2
t−Test: p−value = 0.9

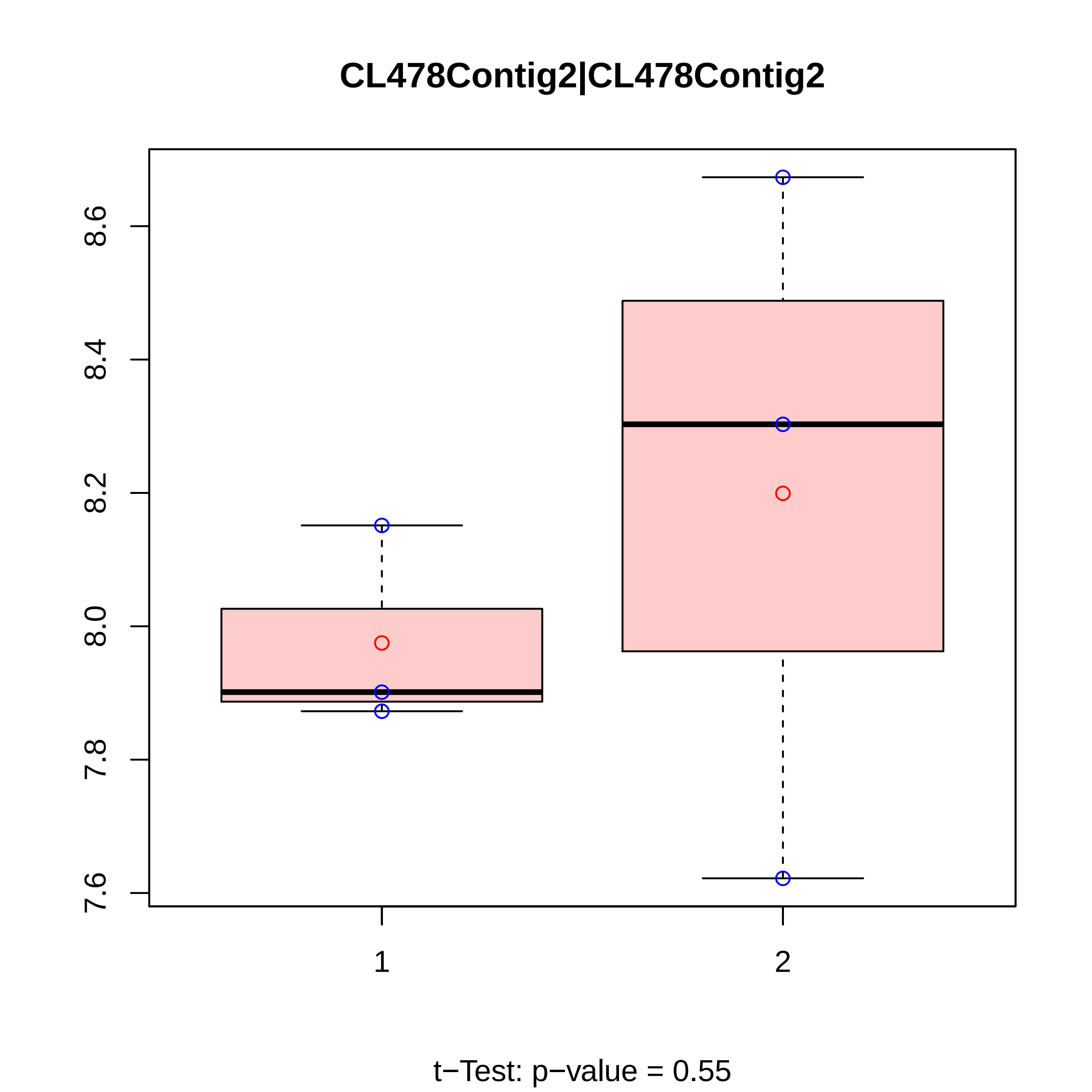

CL478Contig2|CL478Contig2
8.6
8.4
8.2
8.0
7.8
7.6
1
2
t−Test: p−value = 0.55

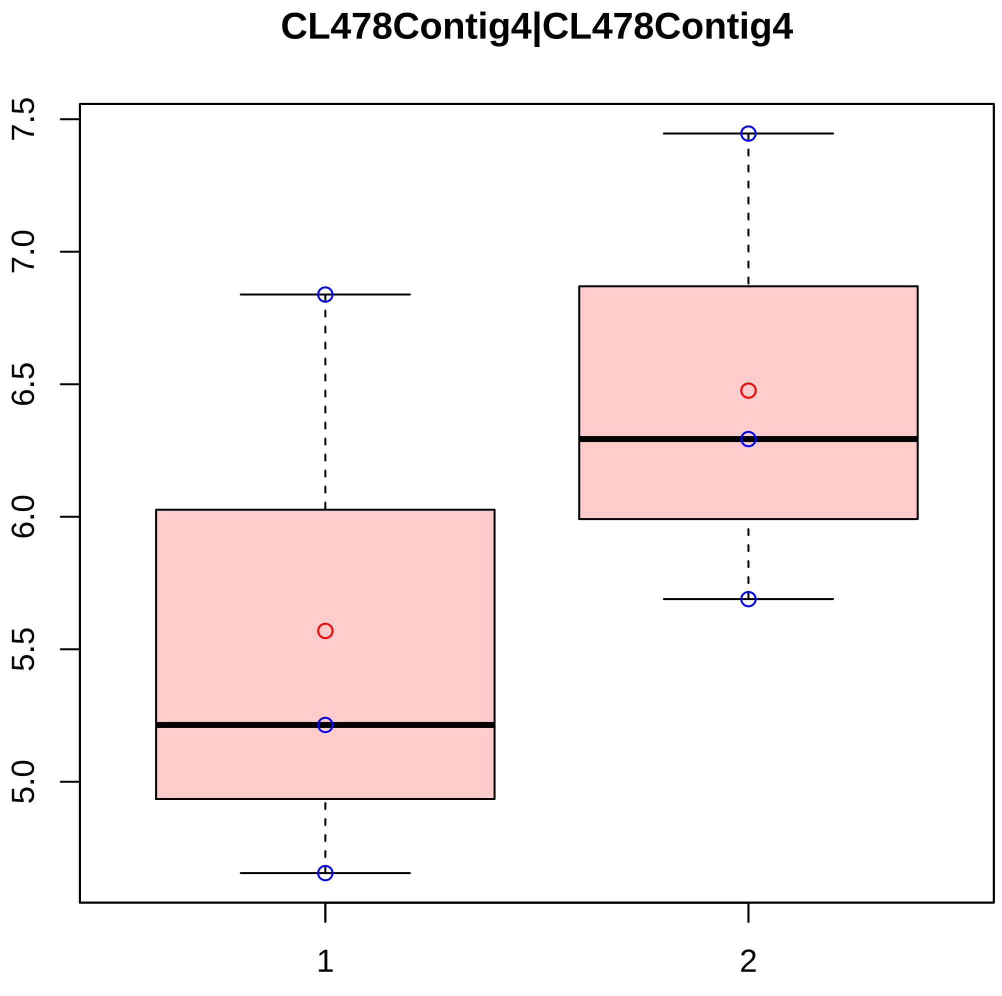

t−Test: p−value = 0.34

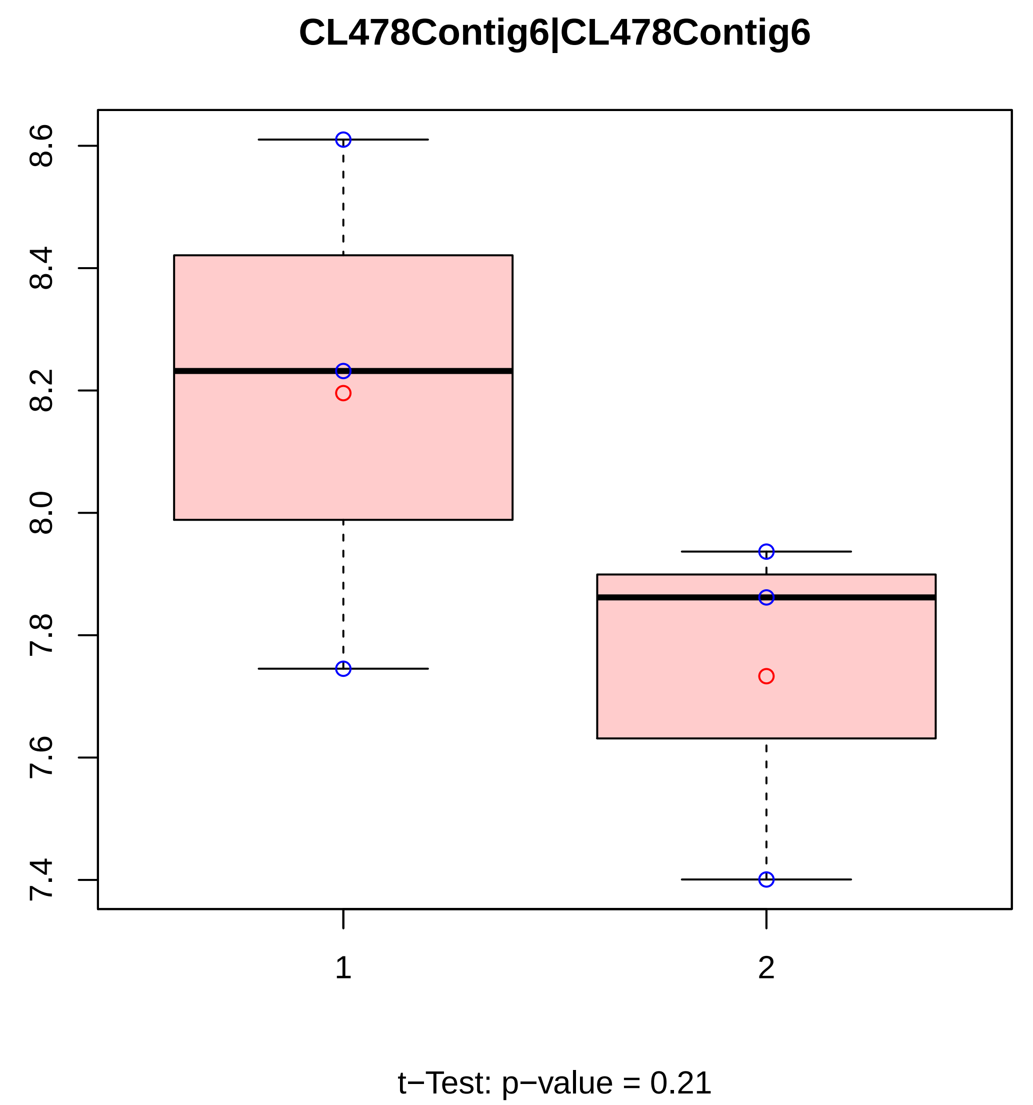
CL478Contig6|CL478Contig6
8.6
8.4
8.2
8.0
7.8
7.6
7.4
1
2
t−Test: p−value = 0.21

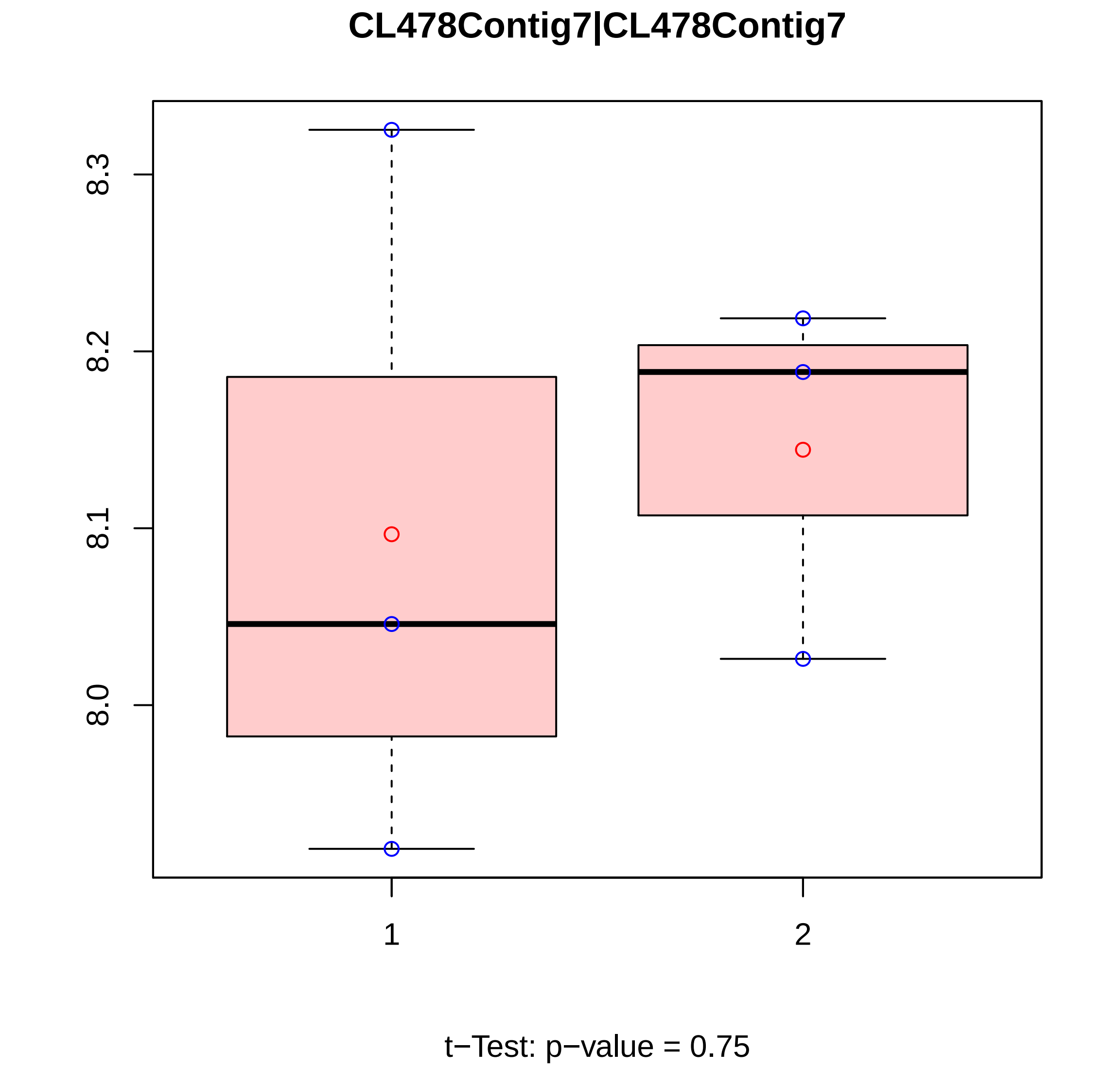

CL478Contig7|CL478Contig7
8.0
8.1
8.2
8.3
1
2
t−Test: p−value = 0.75

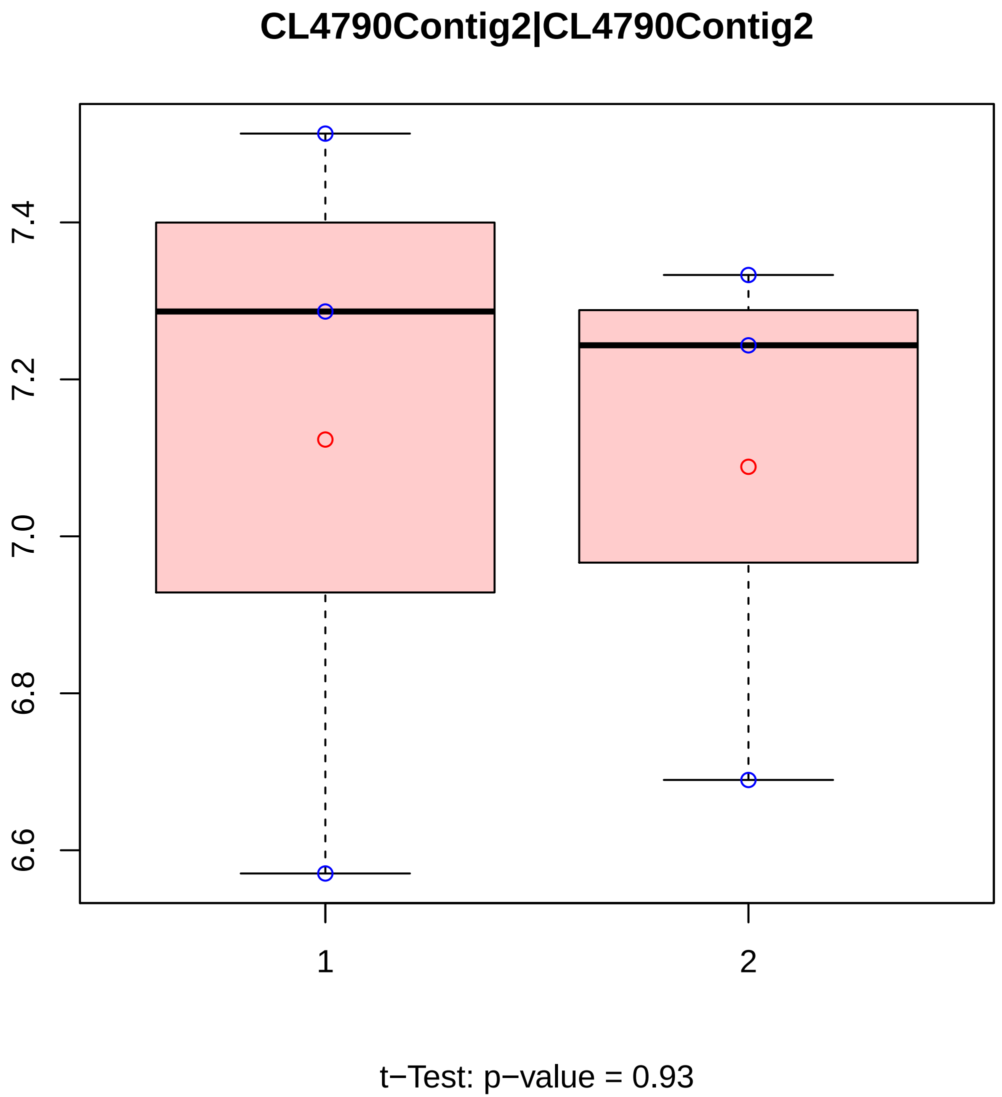

CL4790Contig2|CL4790Contig2
7.4
7.2
7.0
6.8
6.6
1
2
t−Test: p−value = 0.93

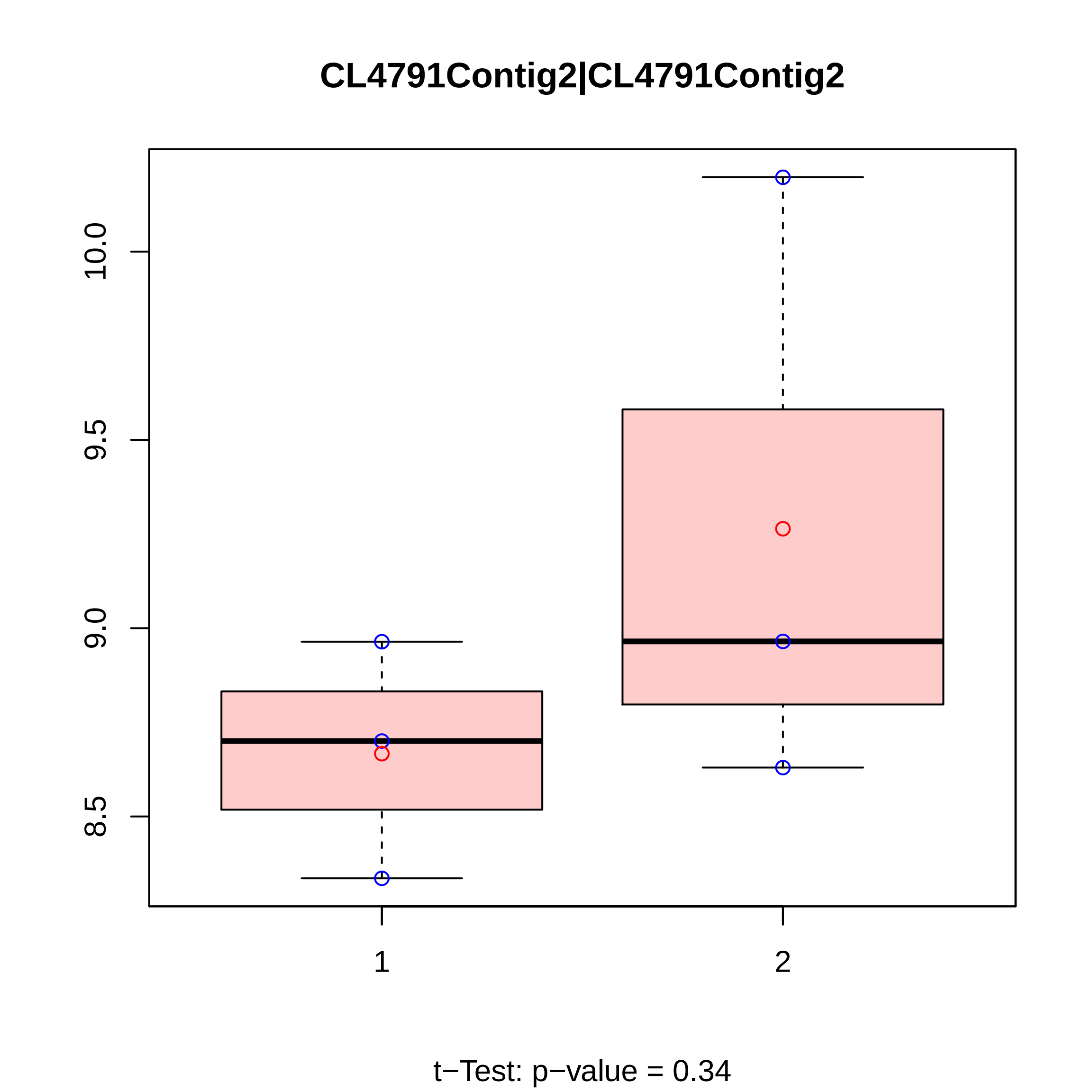

CL4791Contig2|CL4791Contig2
10.0
9.5
9.0
8.5
1
2
t−Test: p−value = 0.34

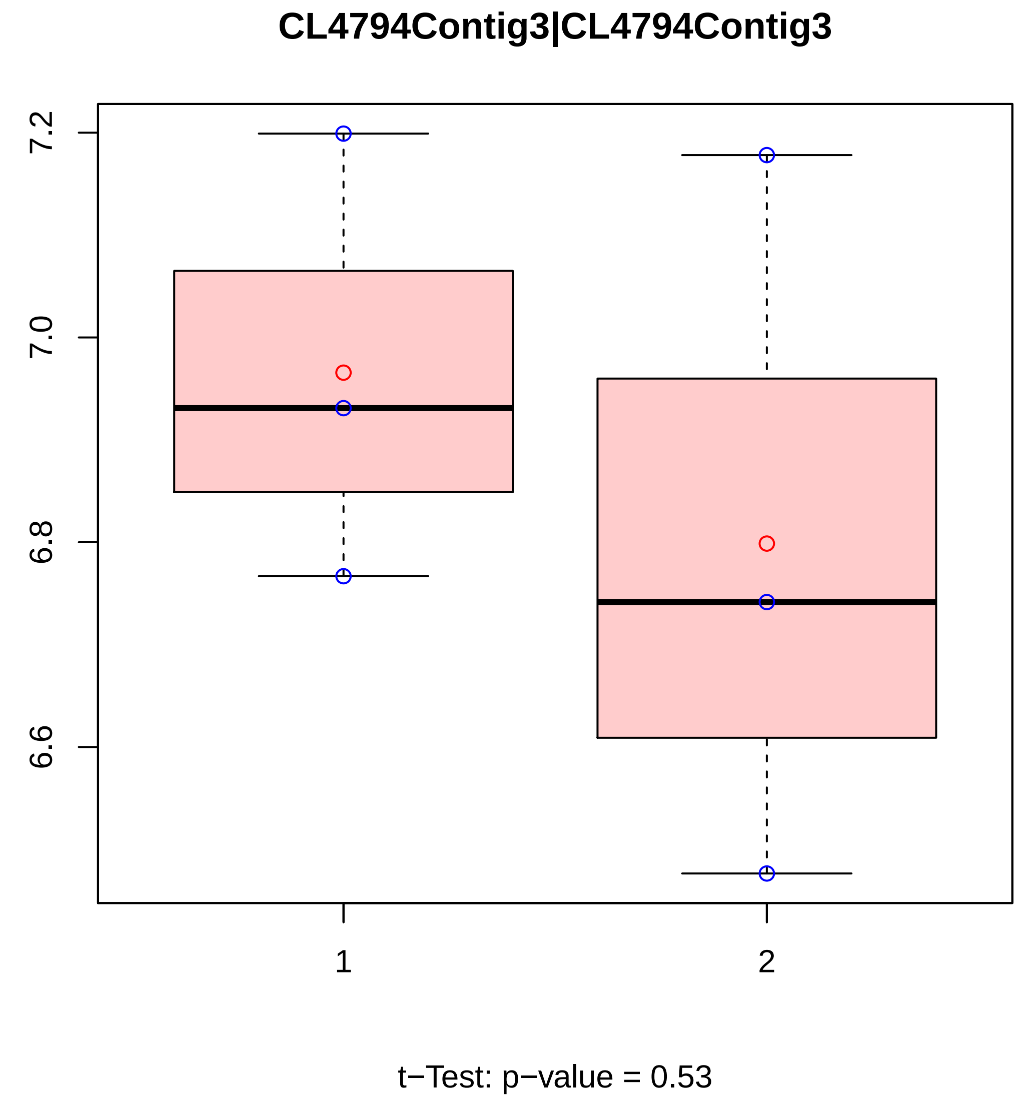

CL4794Contig3|CL4794Contig3
7.2
7.0
6.8
6.6
1
2
t−Test: p−value = 0.53

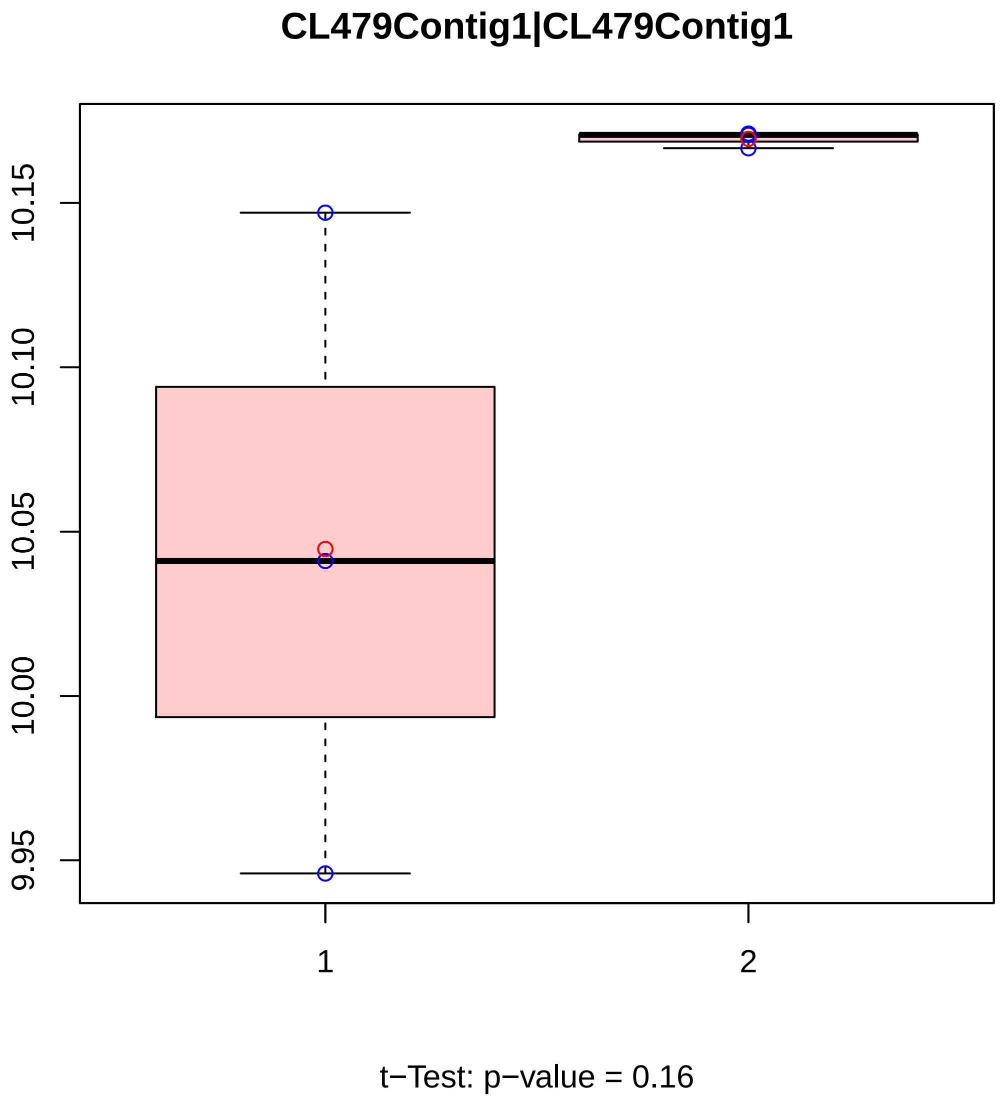

CL479Contig1|CL479Contig1
10.15
10.10
10.05
10.00
9.95
1
2
t−Test: p−value = 0.16

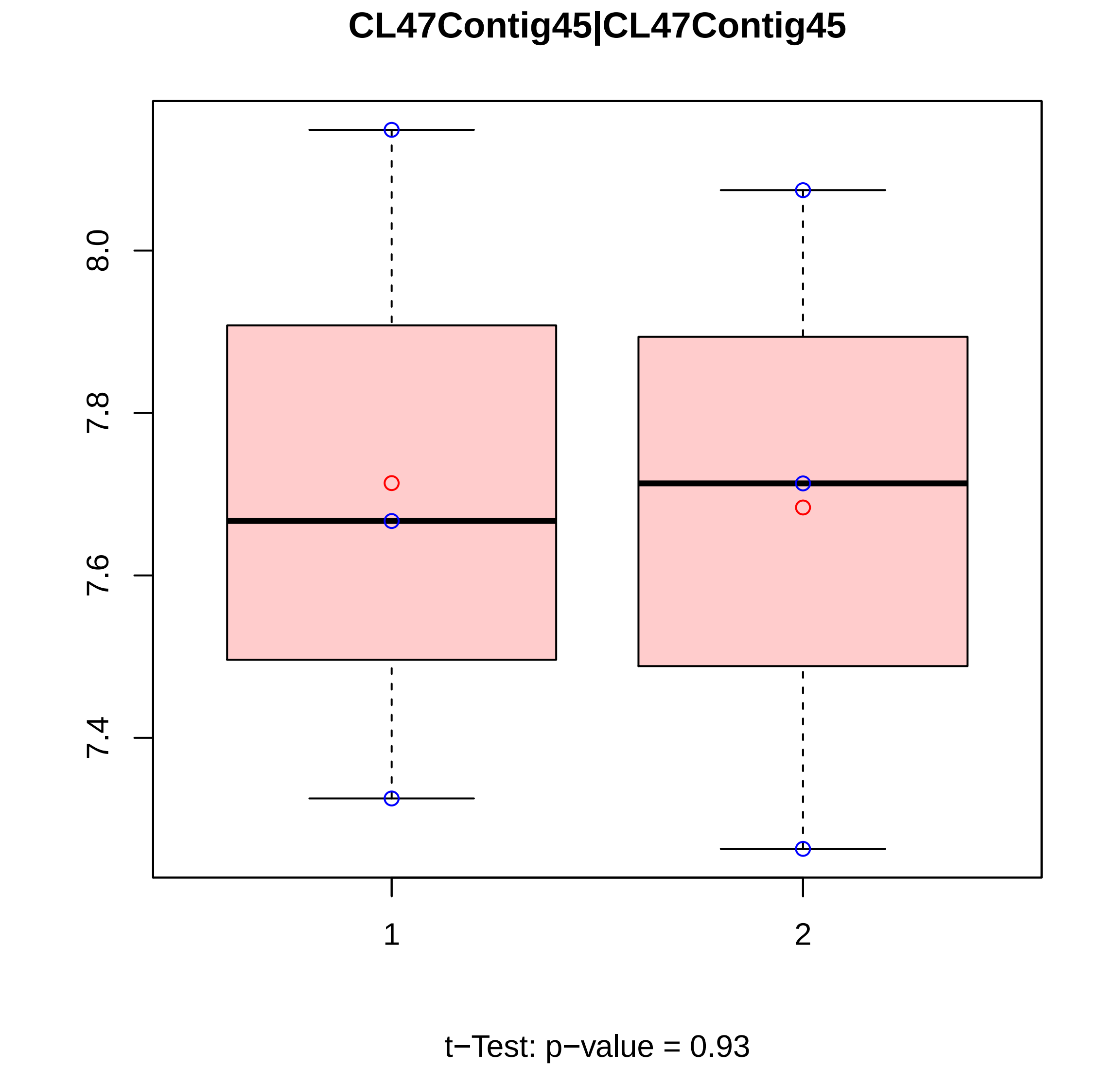

CL47Contig45|CL47Contig45
8.0
7.8
7.6
7.4
1
2
t−Test: p−value = 0.93

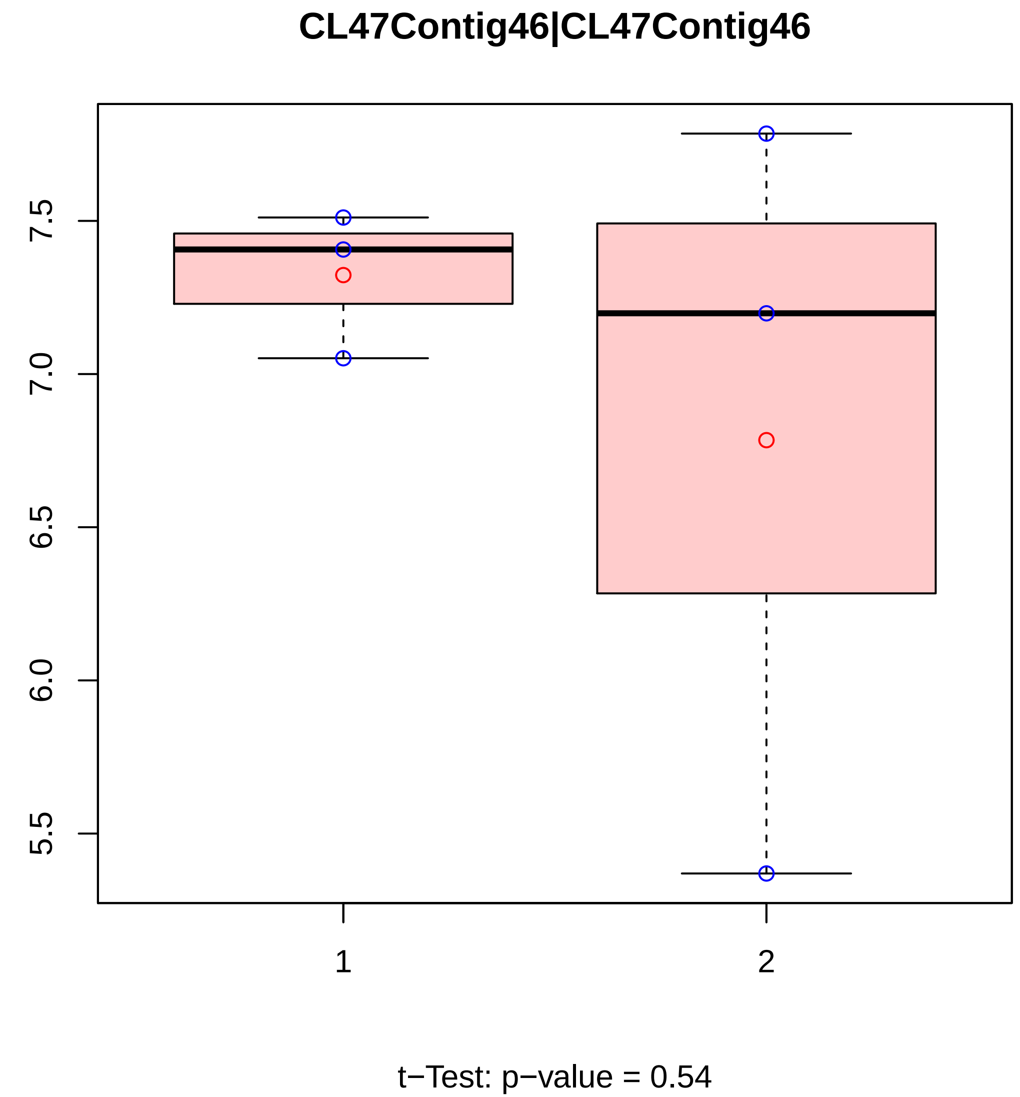
CL47Contig46|CL47Contig46
7.5
7.0
6.5
6.0
5.5
1
2
t−Test: p−value = 0.54

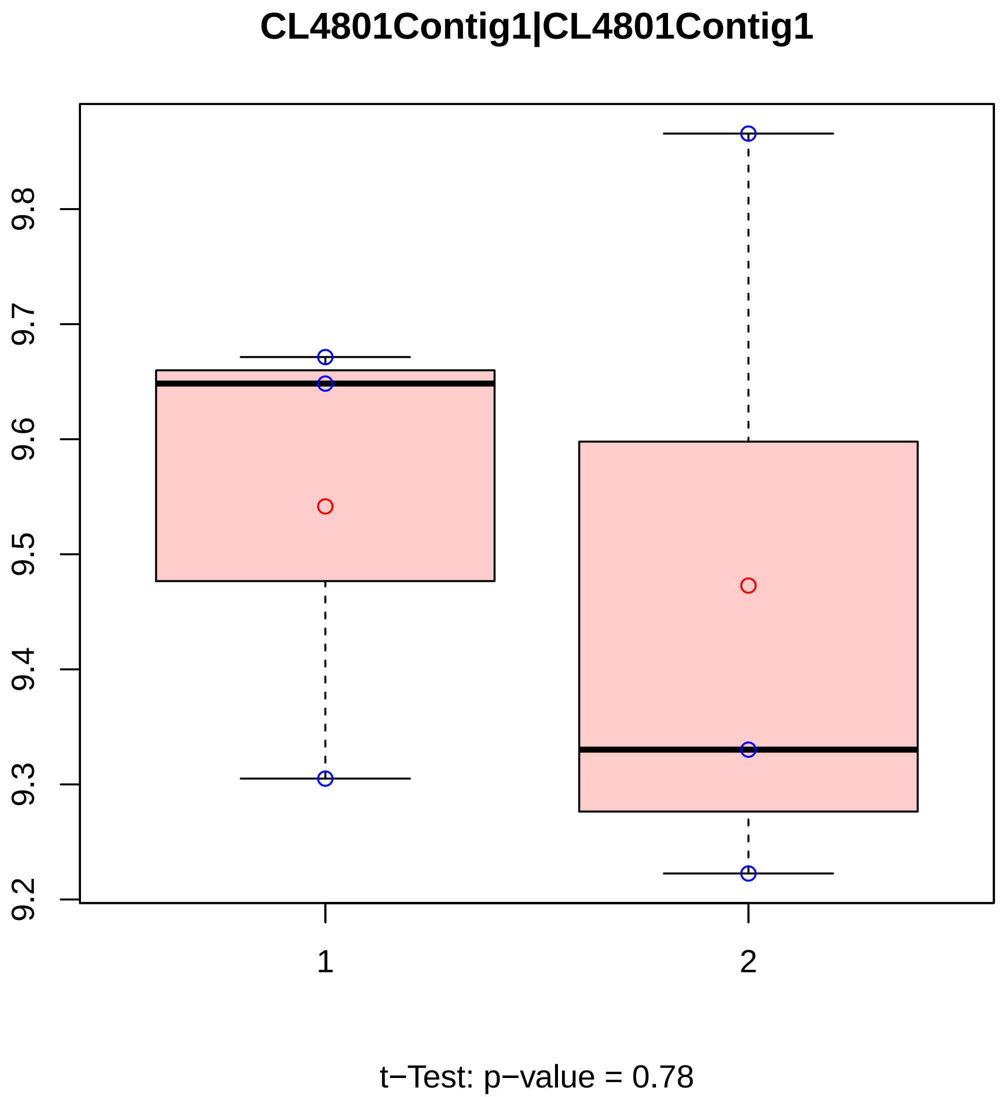

CL4801Contig1|CL4801Contig1
9.2
9.3
9.4
9.5
9.6
9.7
9.8
1
2
t−Test: p−value = 0.78

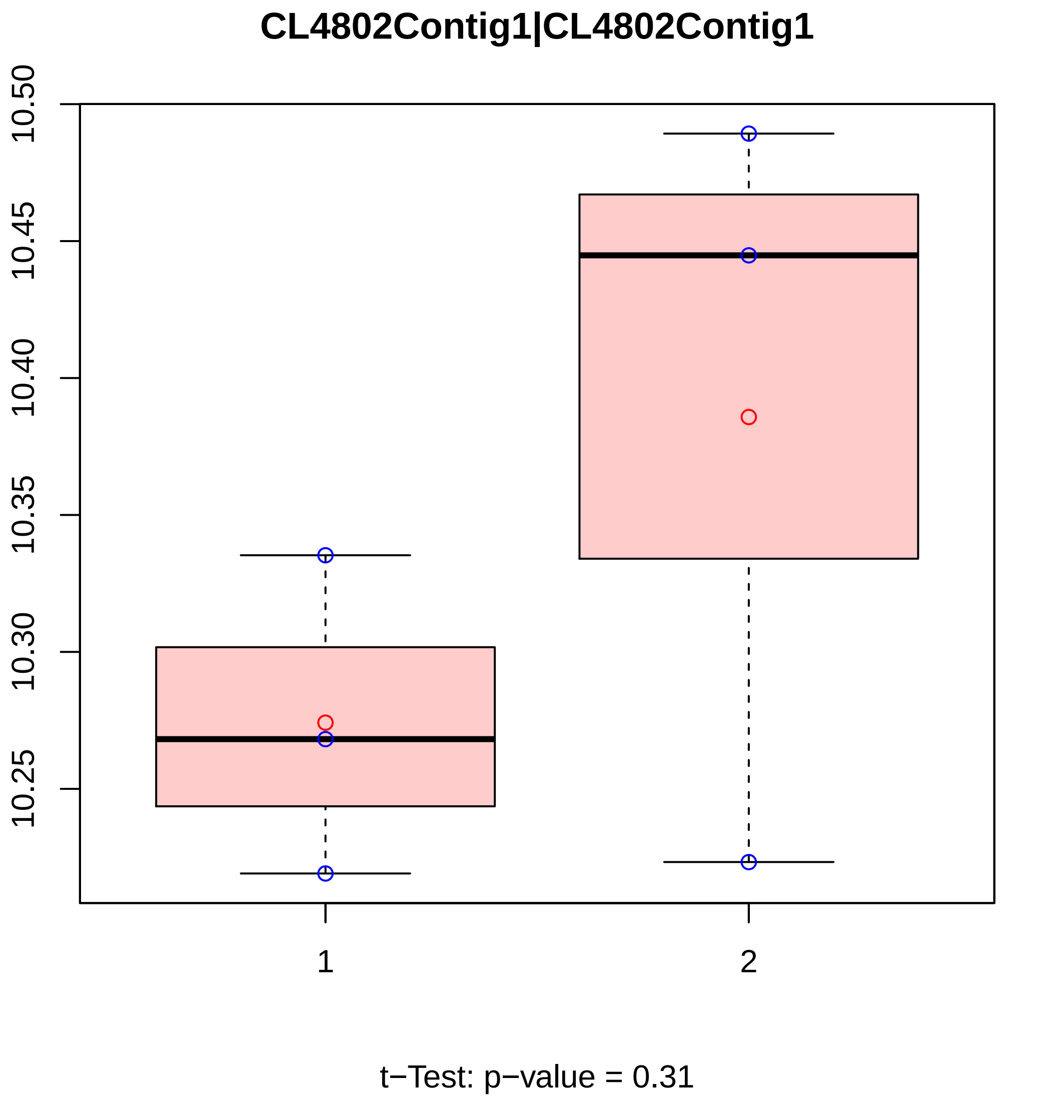
CL4802Contig1|CL4802Contig1
10.25
10.30
10.35
10.40
10.45
10.50
1
2
t−Test: p−value = 0.31

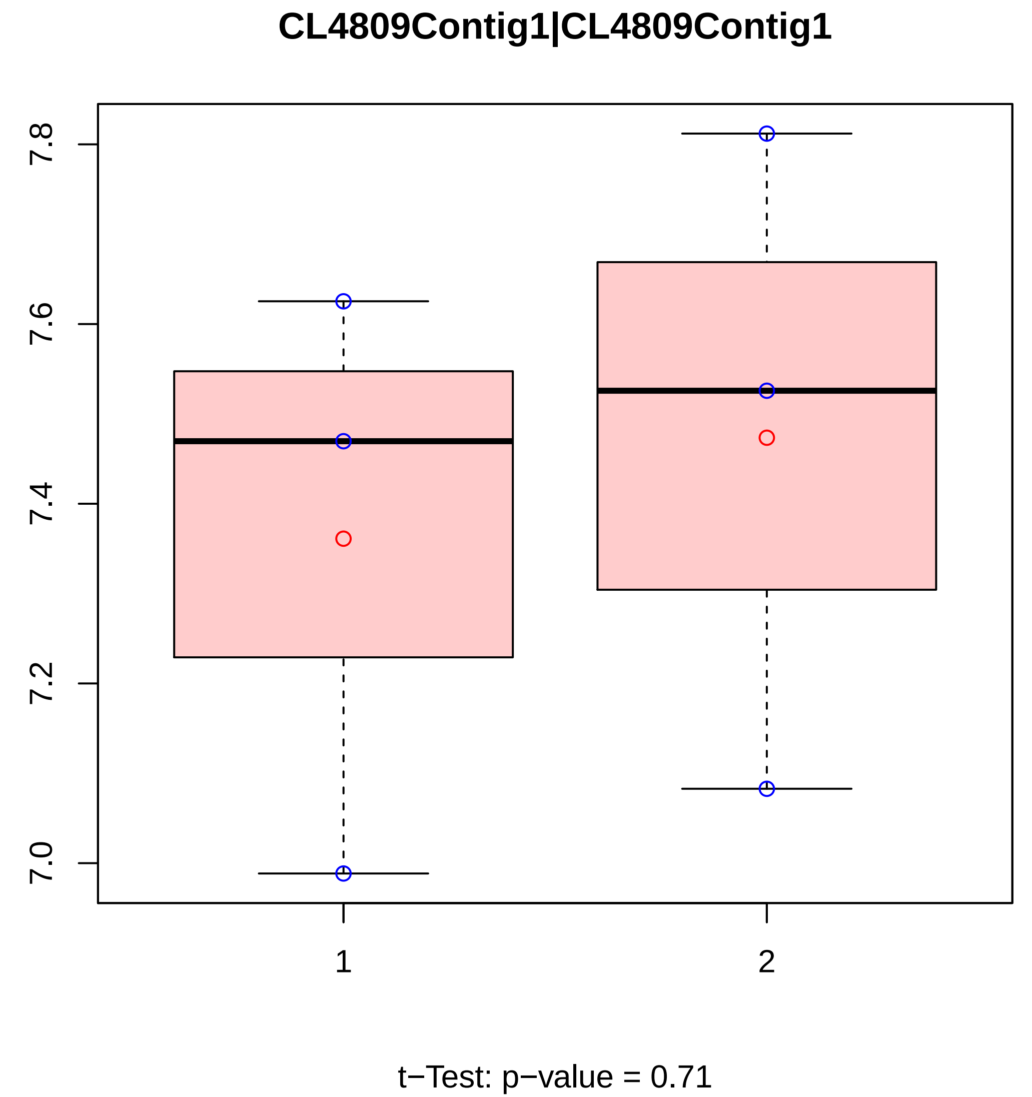

CL4809Contig1|CL4809Contig1
7.0
7.2
7.4
7.6
7.8
1
2
t−Test: p−value = 0.71

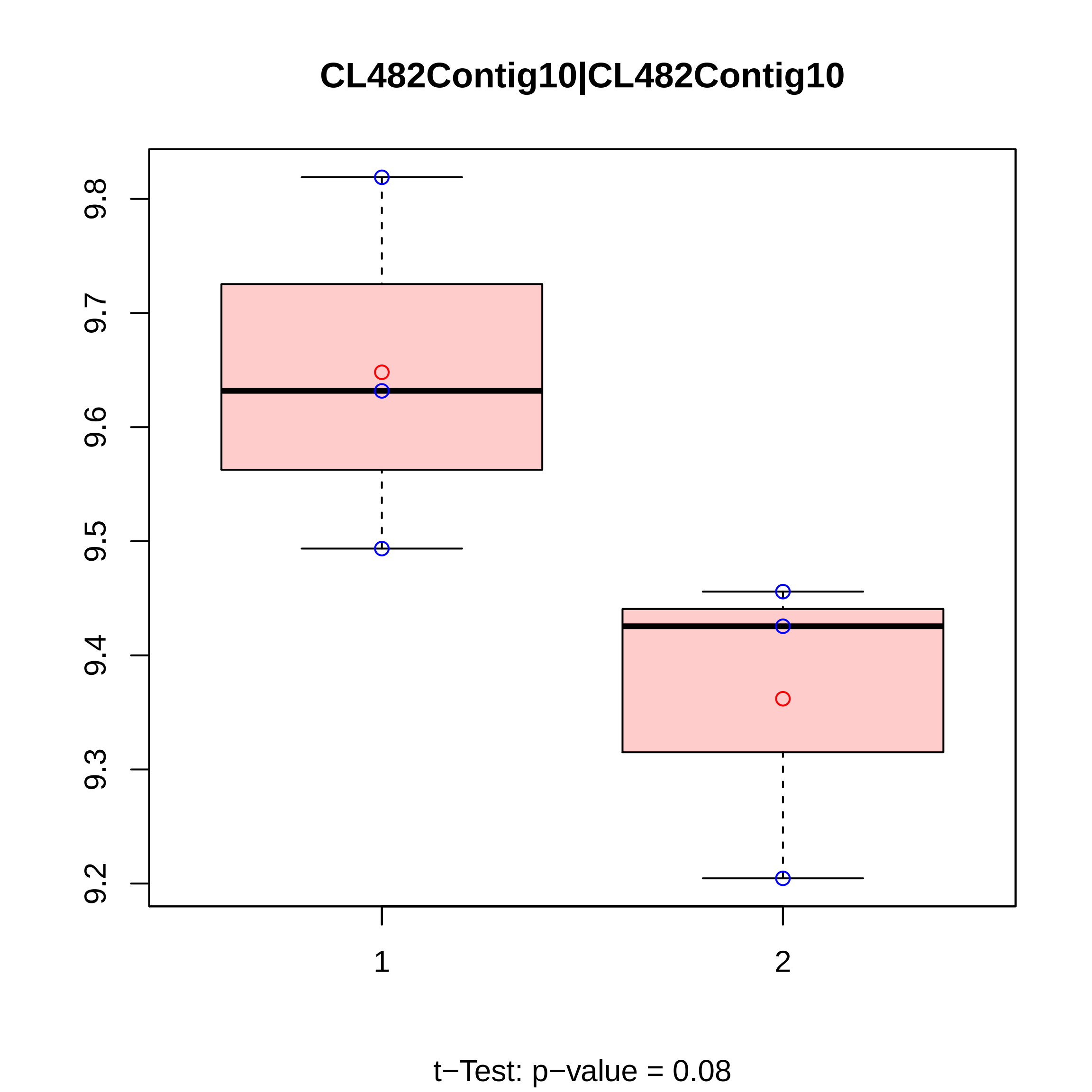
CL482Contig10|CL482Contig10
9.2
9.3
9.4
9.5
9.6
9.7
9.8
1
2
t−Test: p−value = 0.08

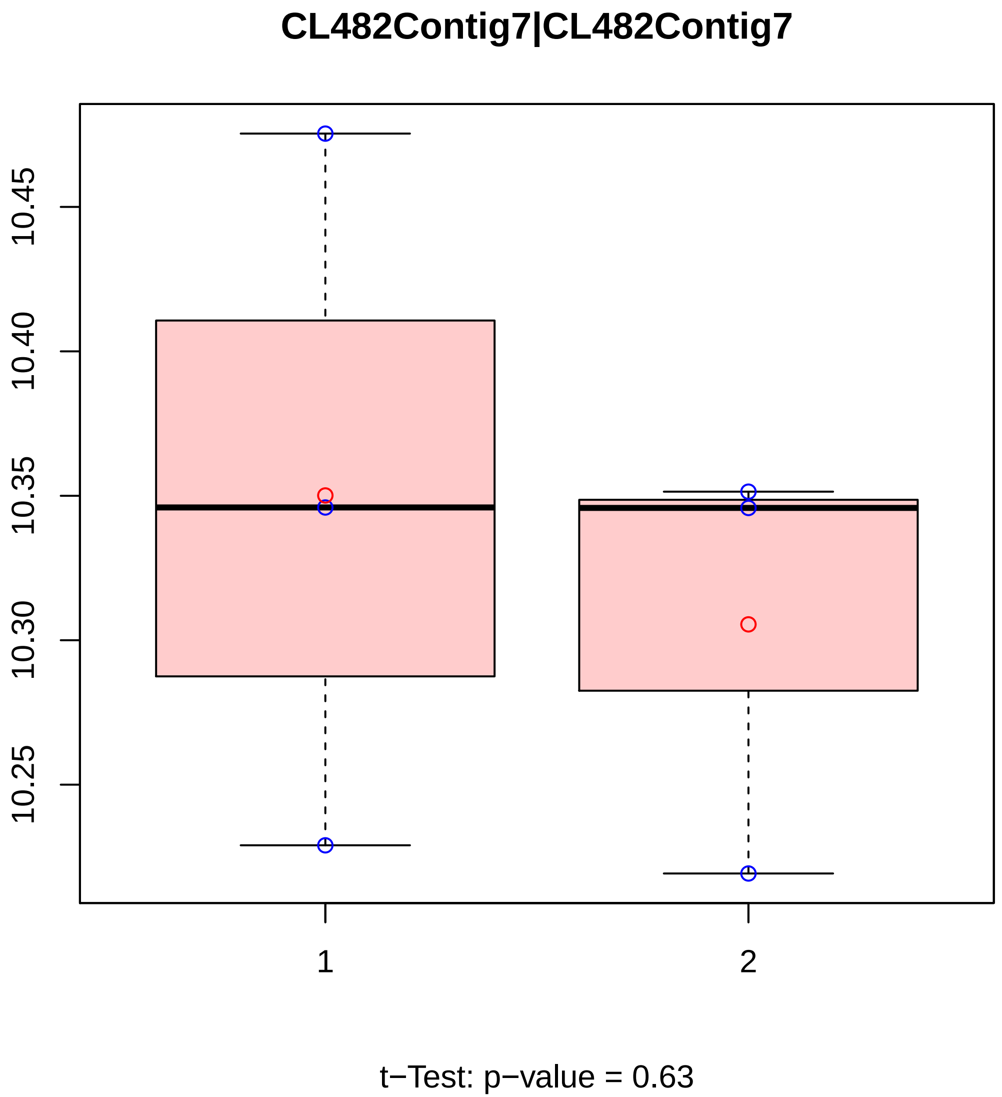

CL482Contig7|CL482Contig7
10.25
10.30
10.35
10.40
10.45
1
2
t−Test: p−value = 0.63

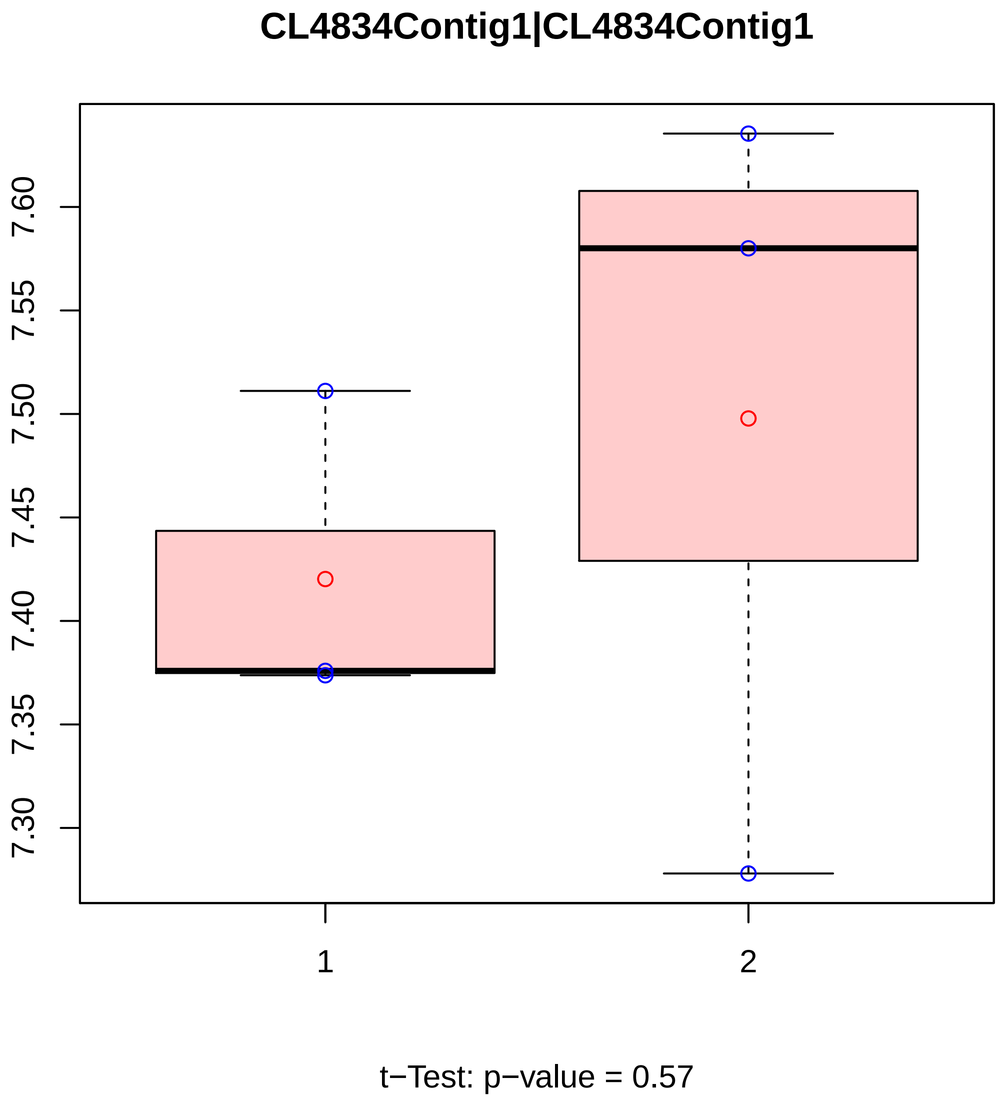
CL4834Contig1|CL4834Contig1
7.30
7.35
7.40
7.45
7.50
7.55
7.60
1
2
t−Test: p−value = 0.57

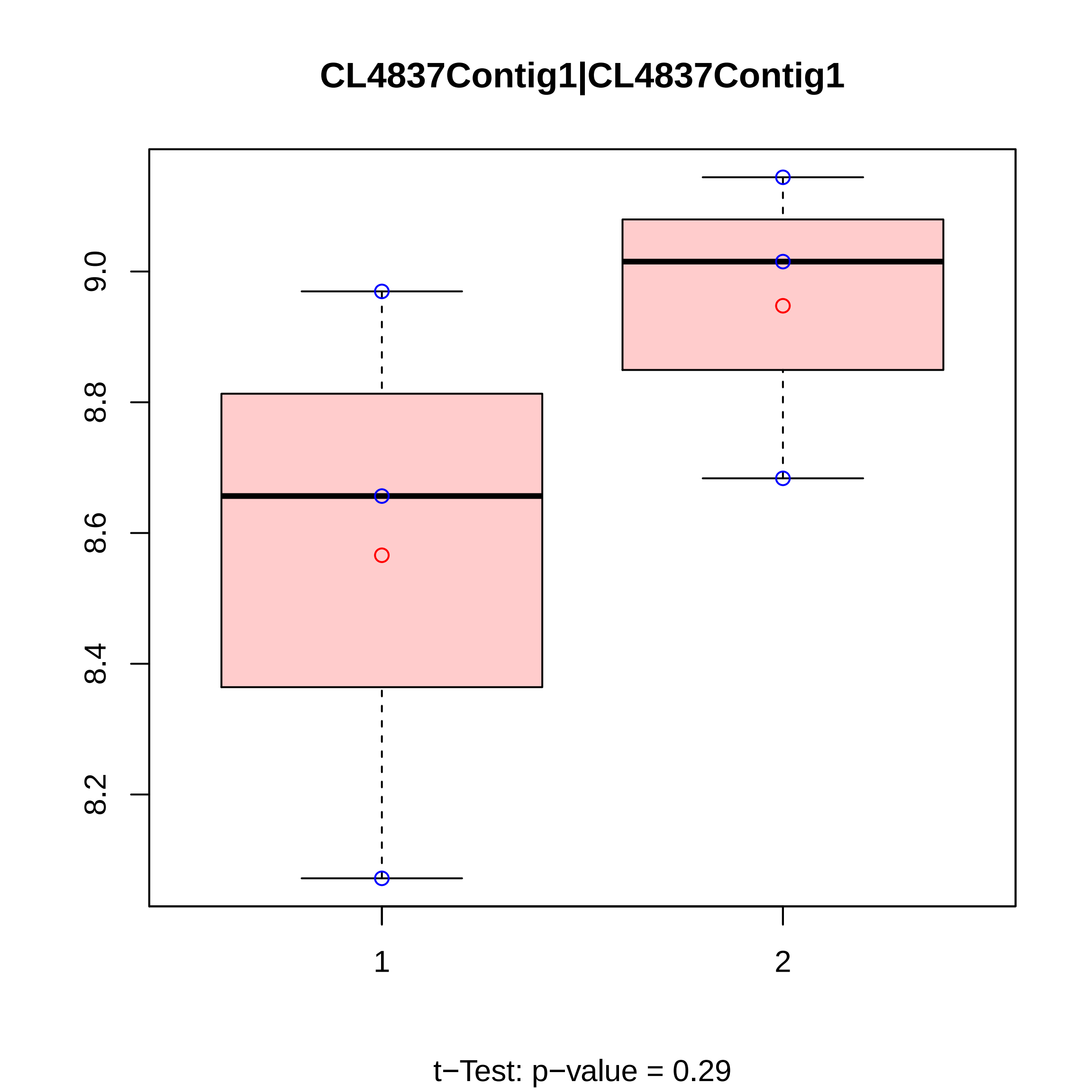
CL4837Contig1|CL4837Contig1
9.0
8.8
8.6
8.4
8.2
1
2
t−Test: p−value = 0.29

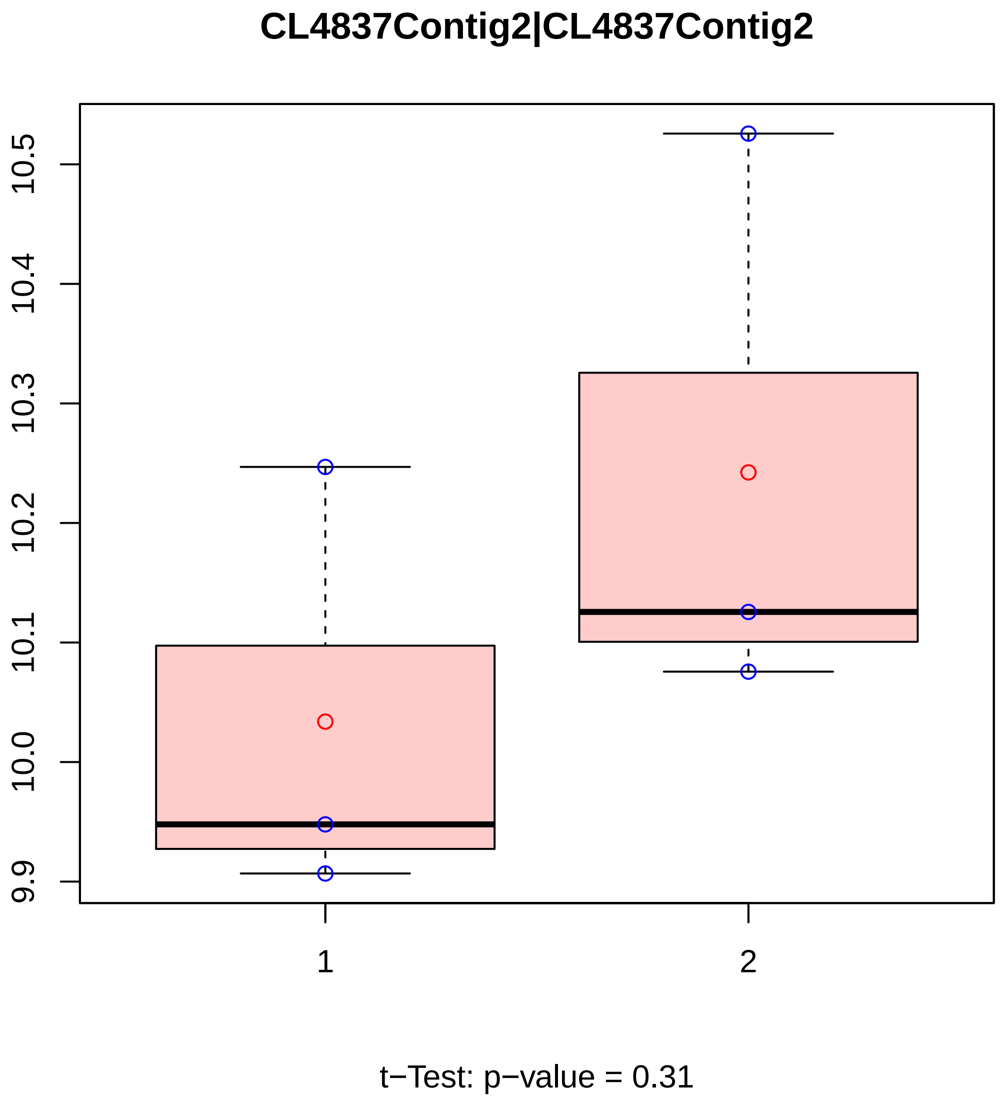
CL4837Contig2|CL4837Contig2
10.5
10.4
10.3
10.2
10.1
10.0
9.9
1
2
t−Test: p−value = 0.31

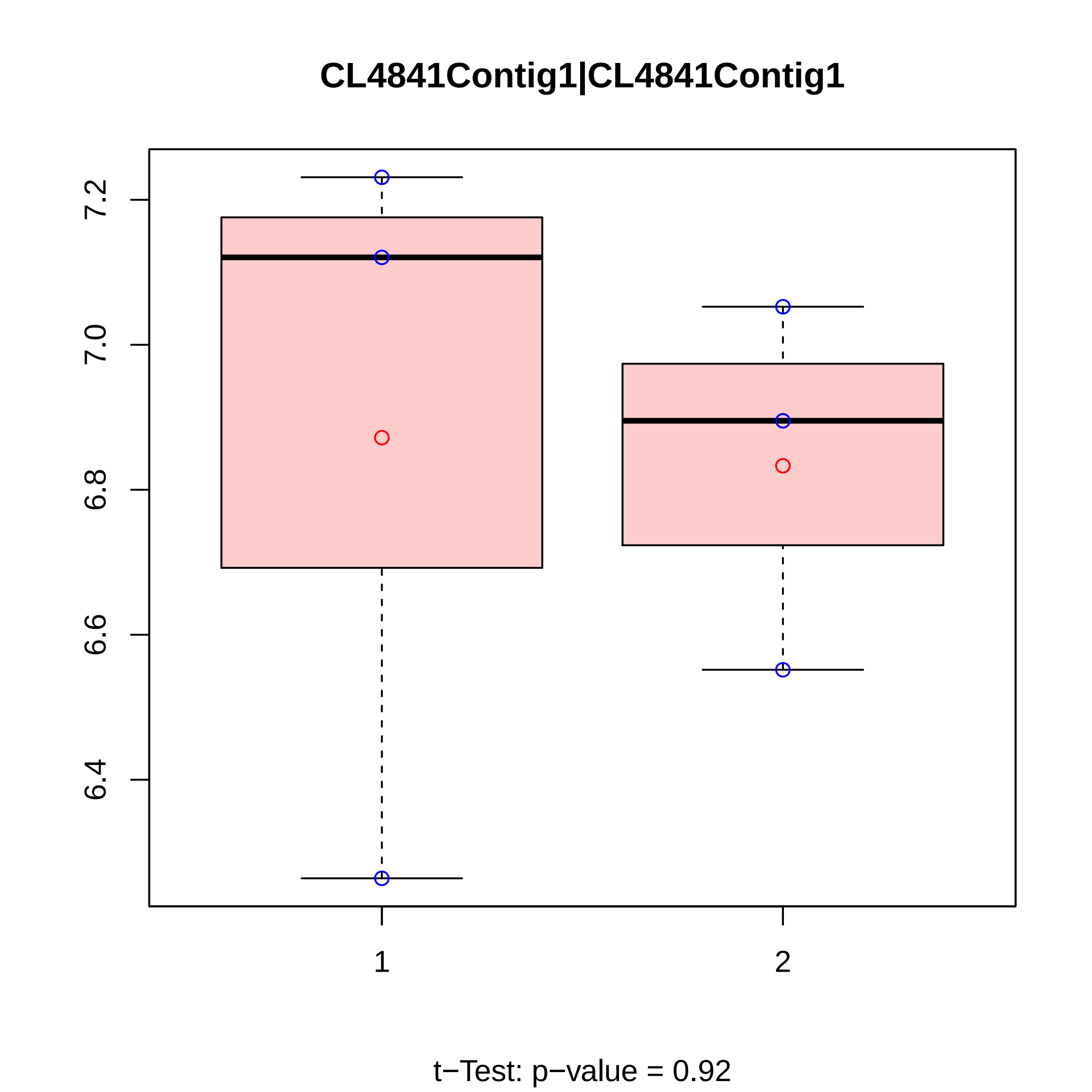
CL4841Contig1|CL4841Contig1
7.2
7.0
6.8
6.6
6.4
1
2
t−Test: p−value = 0.92

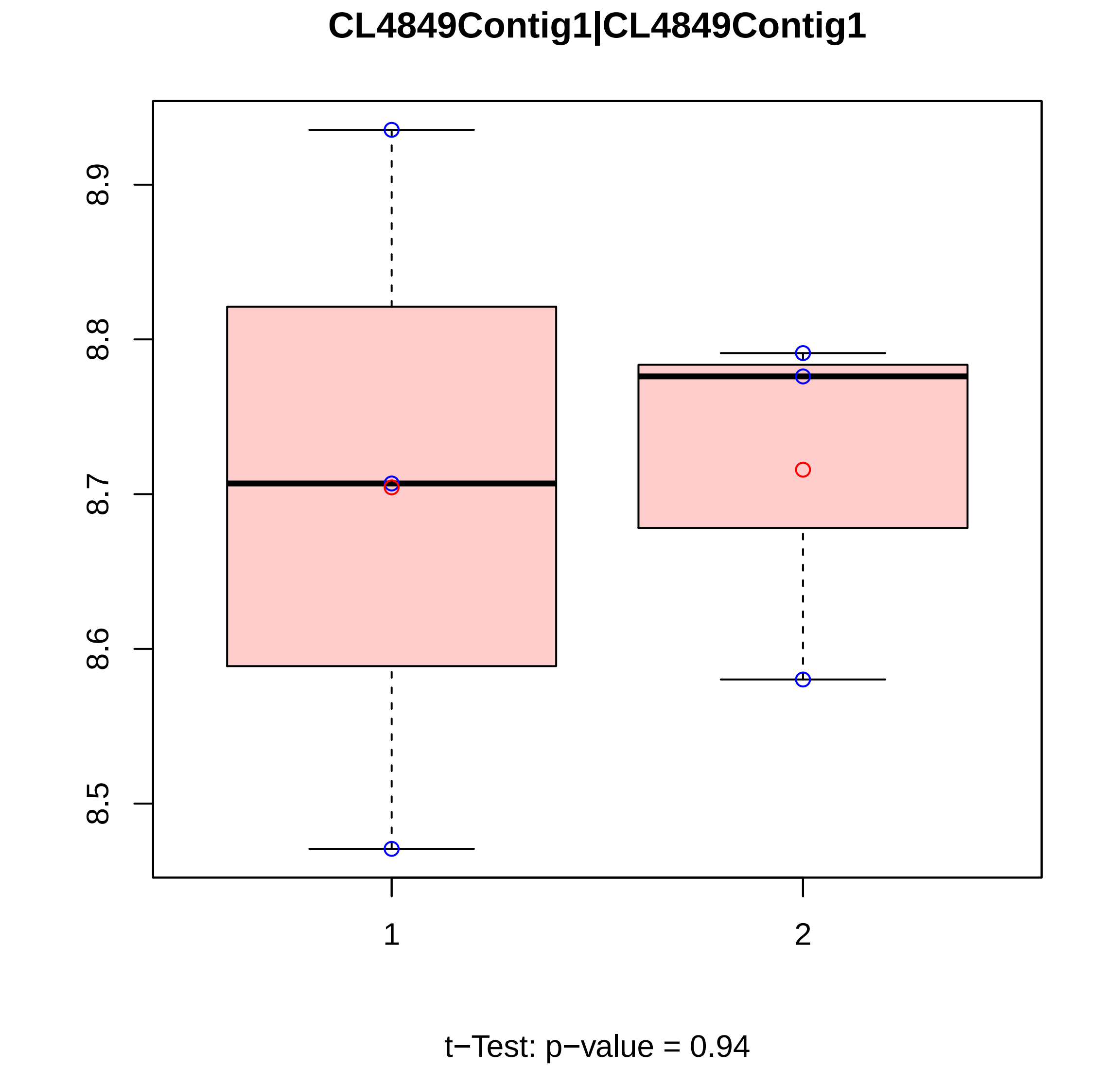

CL4849Contig1|CL4849Contig1
8.5
8.6
8.7
8.8
8.9
1
2
t−Test: p−value = 0.94

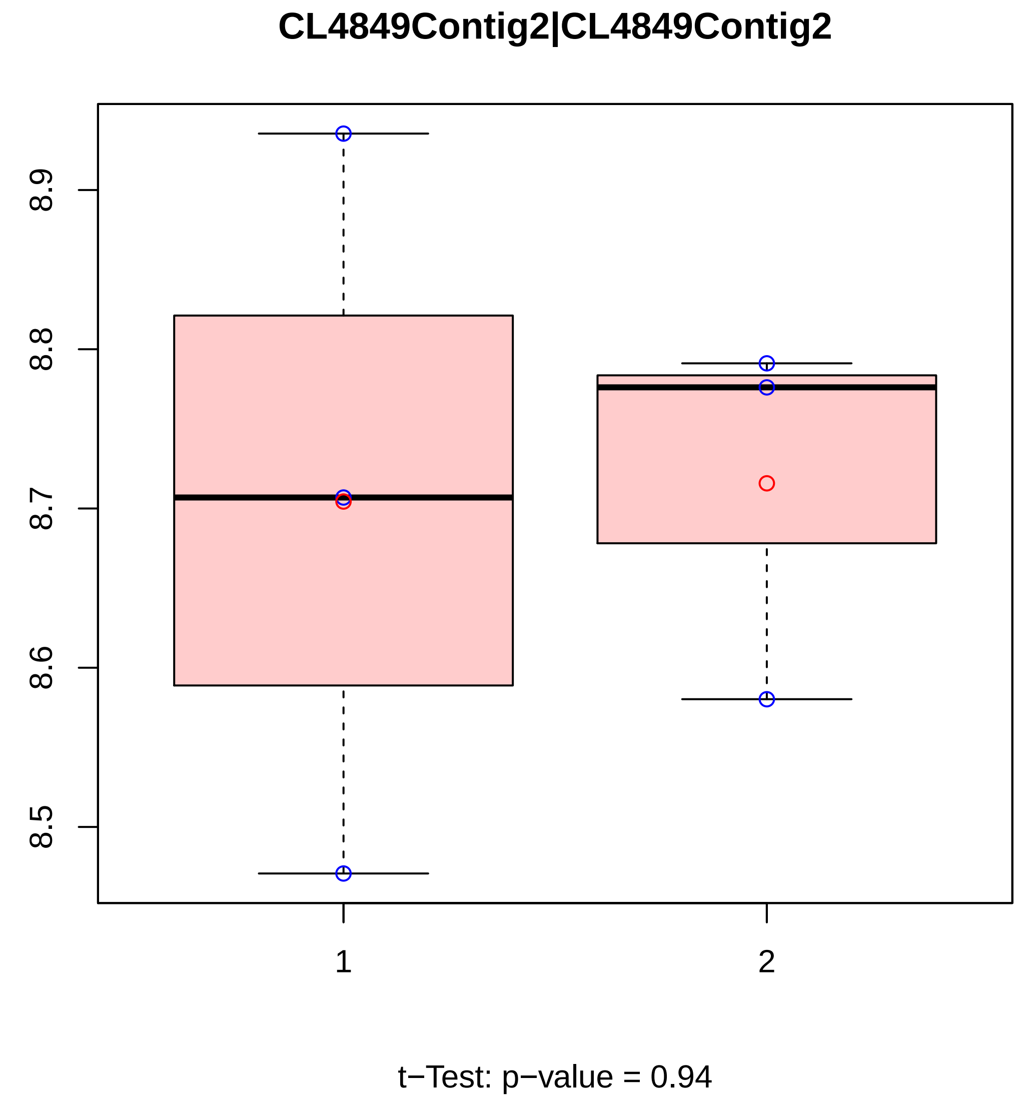
CL4849Contig2|CL4849Contig2
8.5
8.6
8.7
8.8
8.9
1
2
t−Test: p−value = 0.94

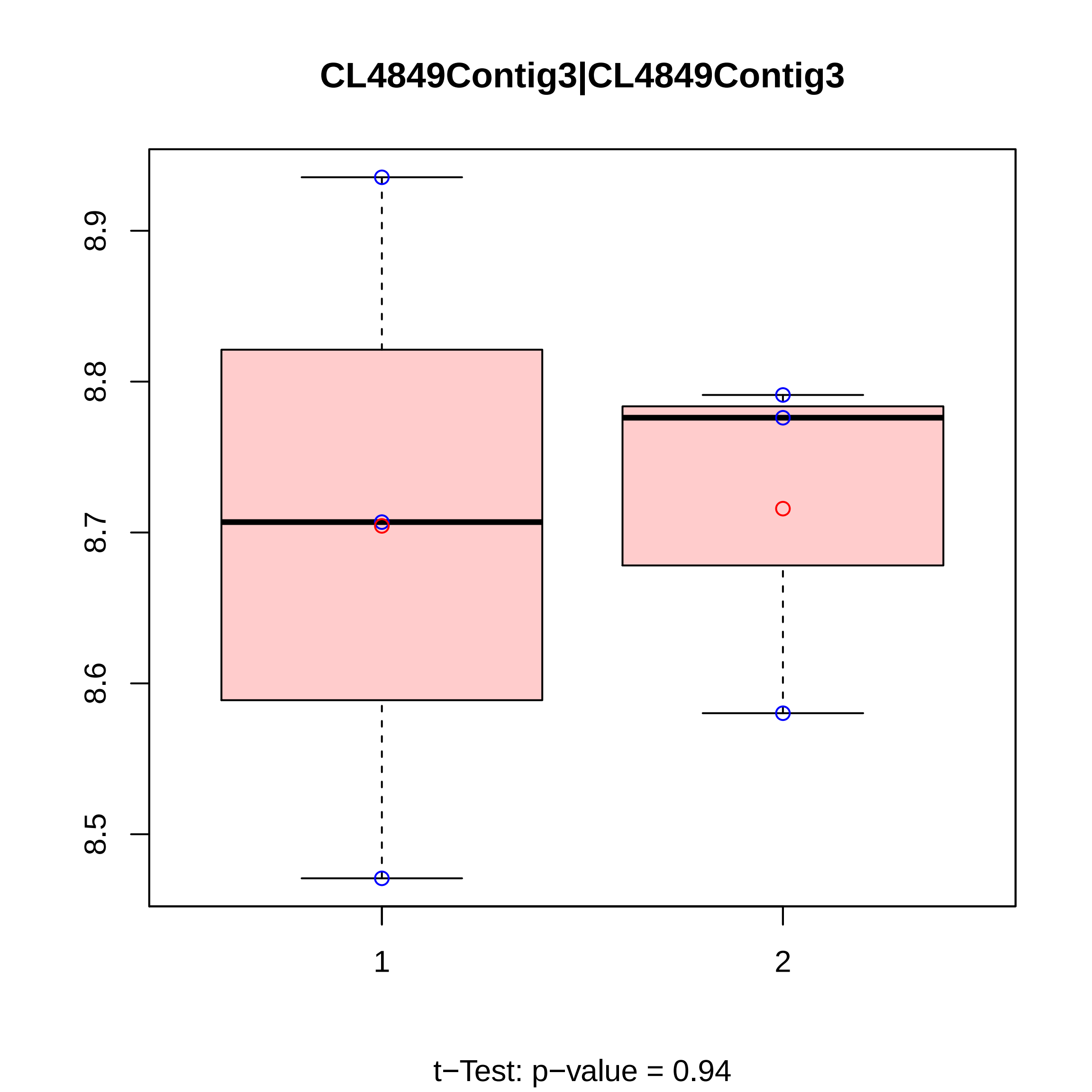
CL4849Contig3|CL4849Contig3
8.5
8.6
8.7
8.8
8.9
1
2
t−Test: p−value = 0.94

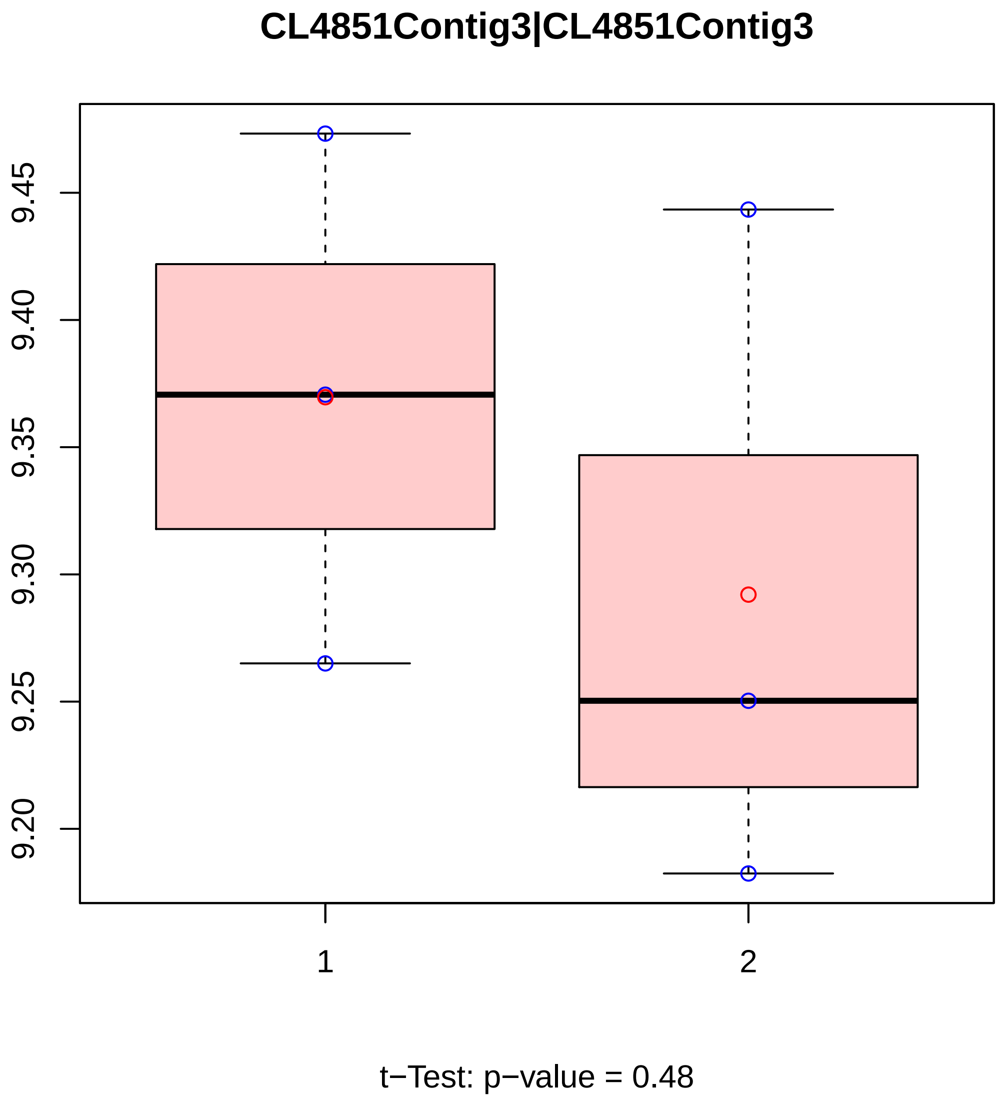
CL4851Contig3|CL4851Contig3
9.45
9.40
9.35
9.30
9.25
9.20
1
2
t−Test: p−value = 0.48

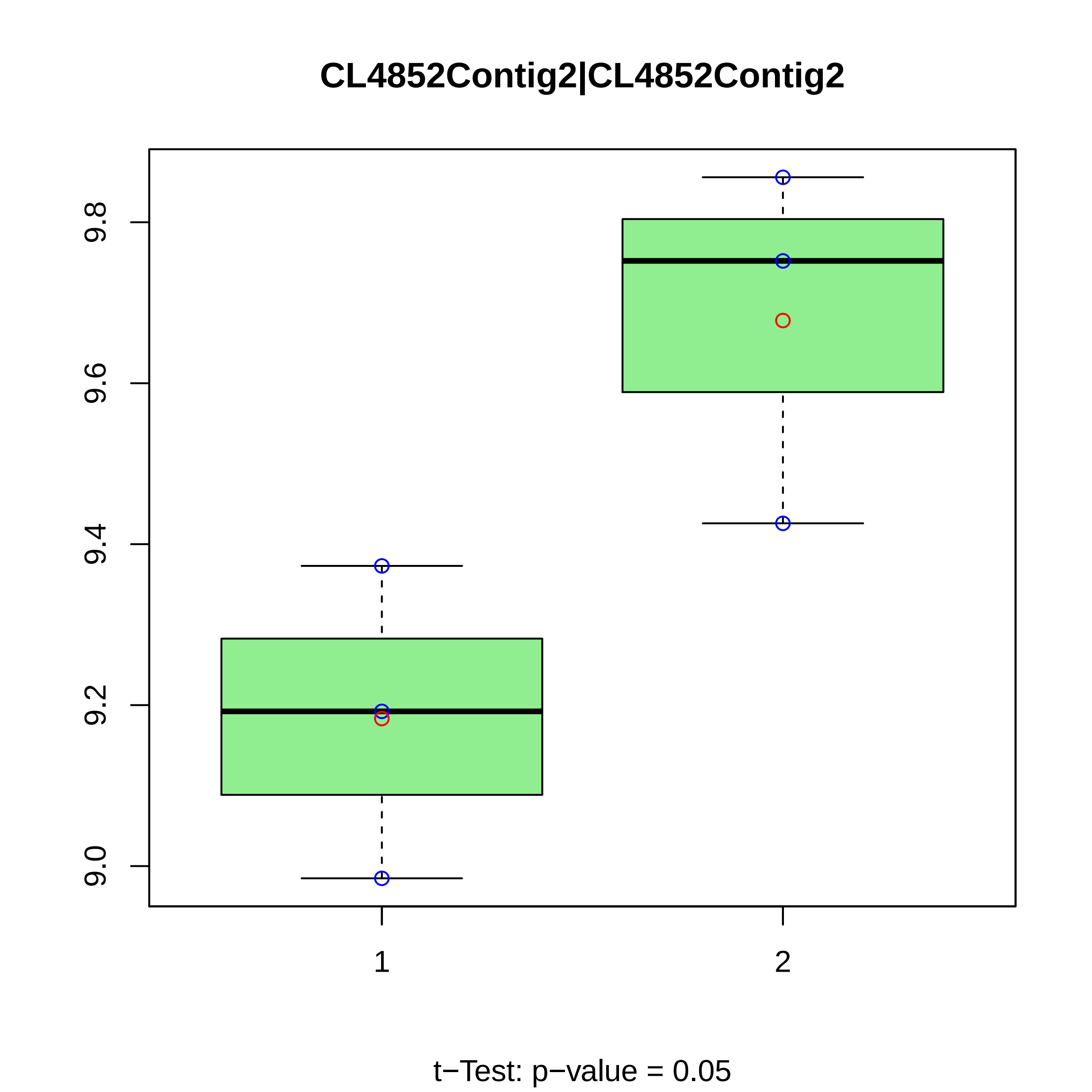
CL4852Contig2|CL4852Contig2
9.0
9.2
9.4
9.6
9.8
1
2
t−Test: p−value = 0.05

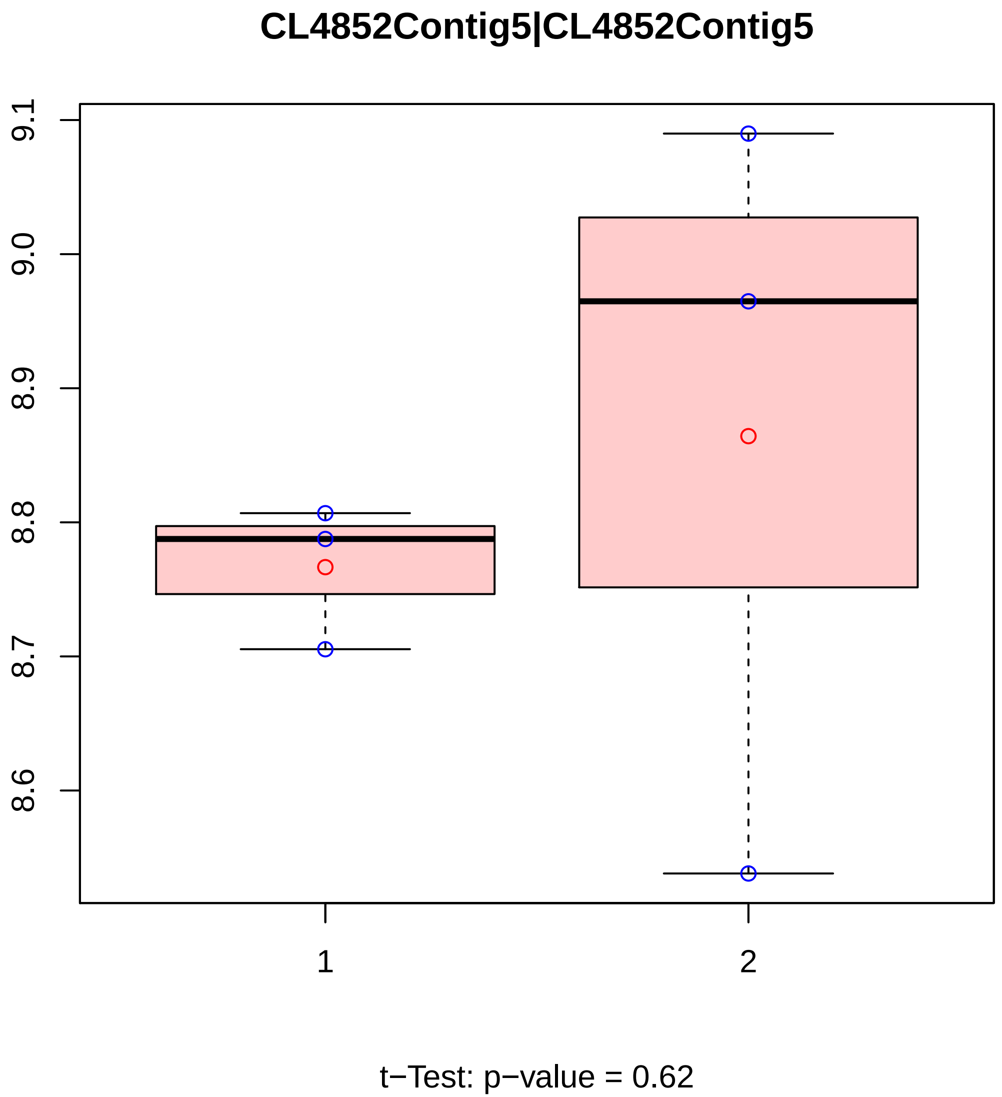
CL4852Contig5|CL4852Contig5
9.1
9.0
8.9
8.8
8.7
8.6
1
2
t−Test: p−value = 0.62

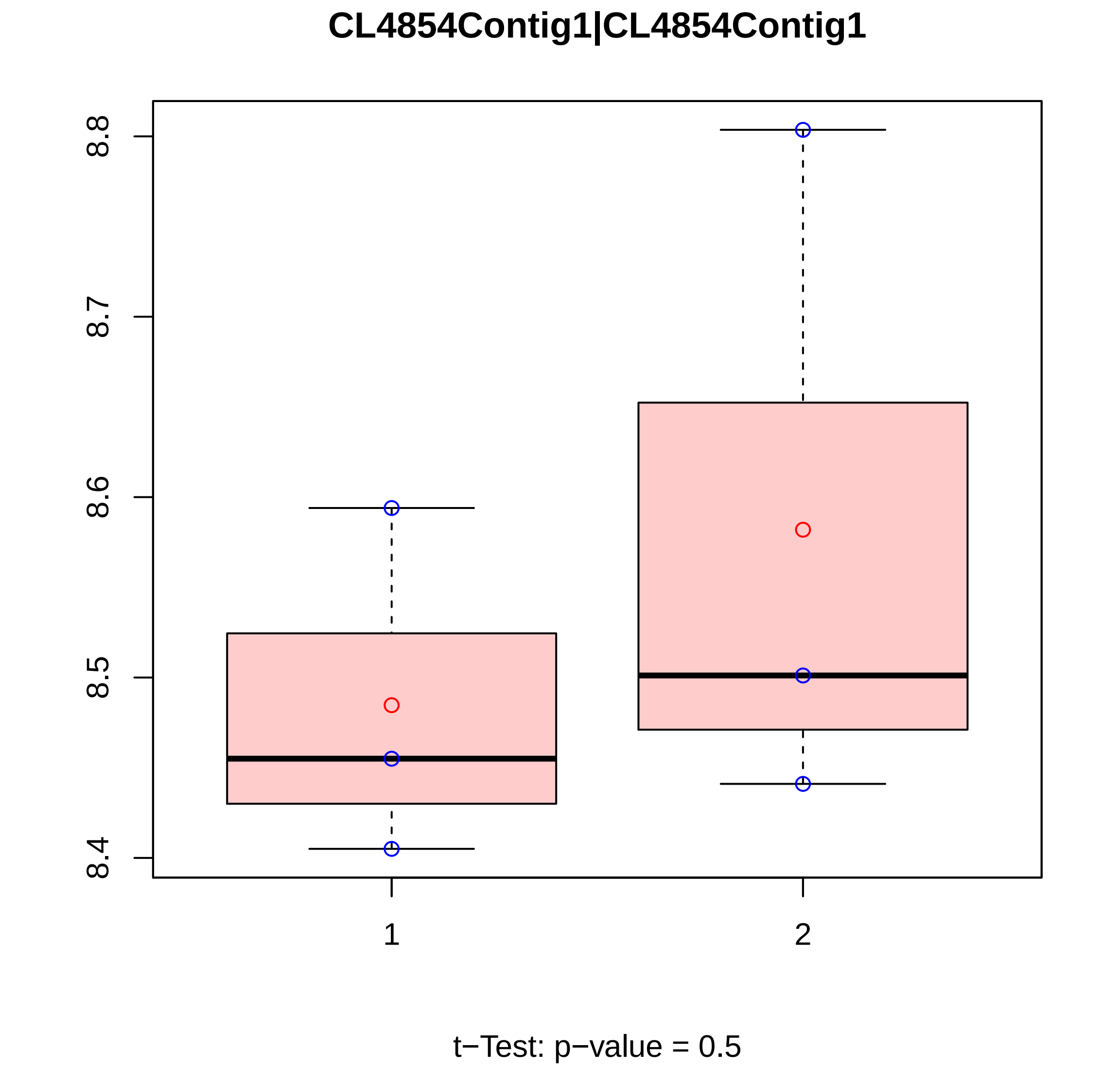

CL4854Contig1|CL4854Contig1
8.4
8.5
8.6
8.7
8.8
1
2
t−Test: p−value = 0.5

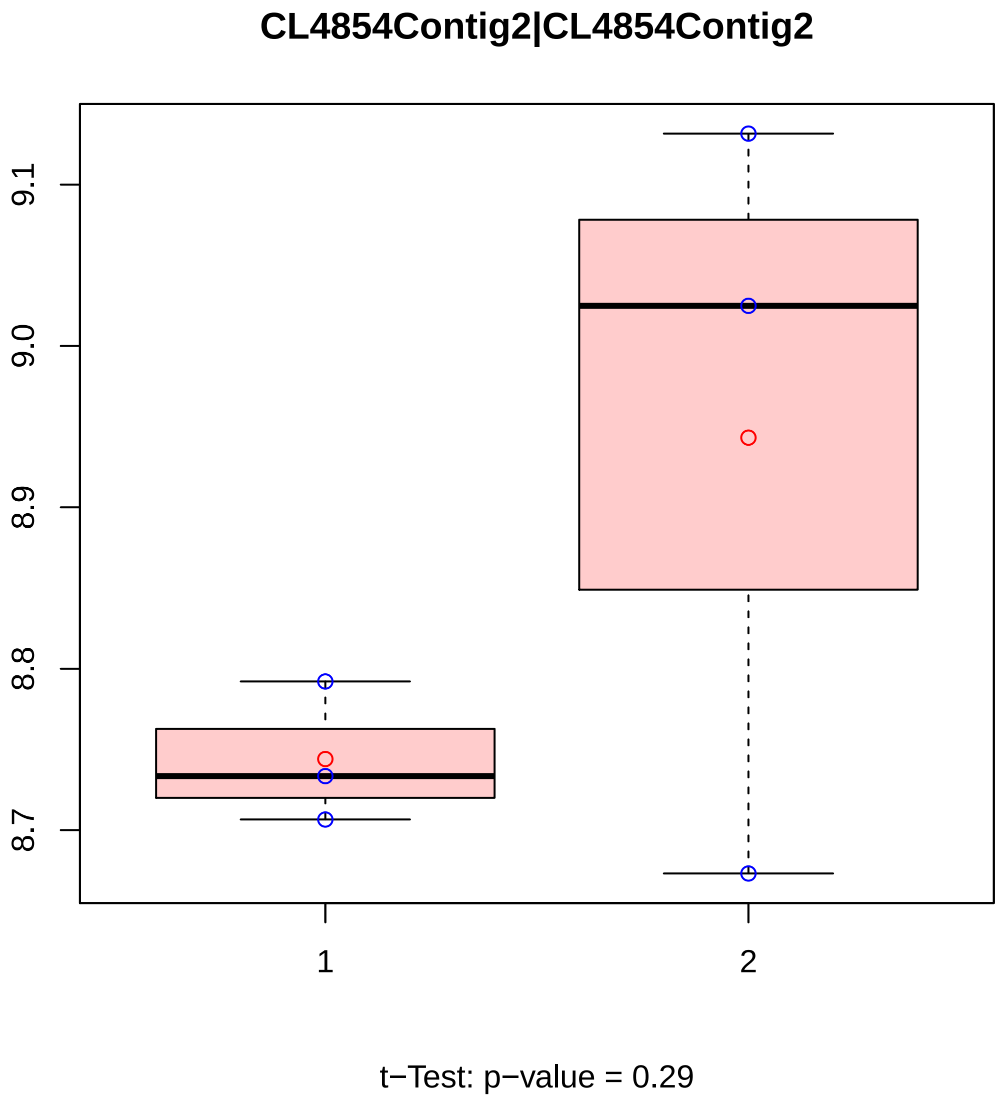

CL4854Contig2|CL4854Contig2
9.1
9.0
8.9
8.8
8.7
1
2
t−Test: p−value = 0.29

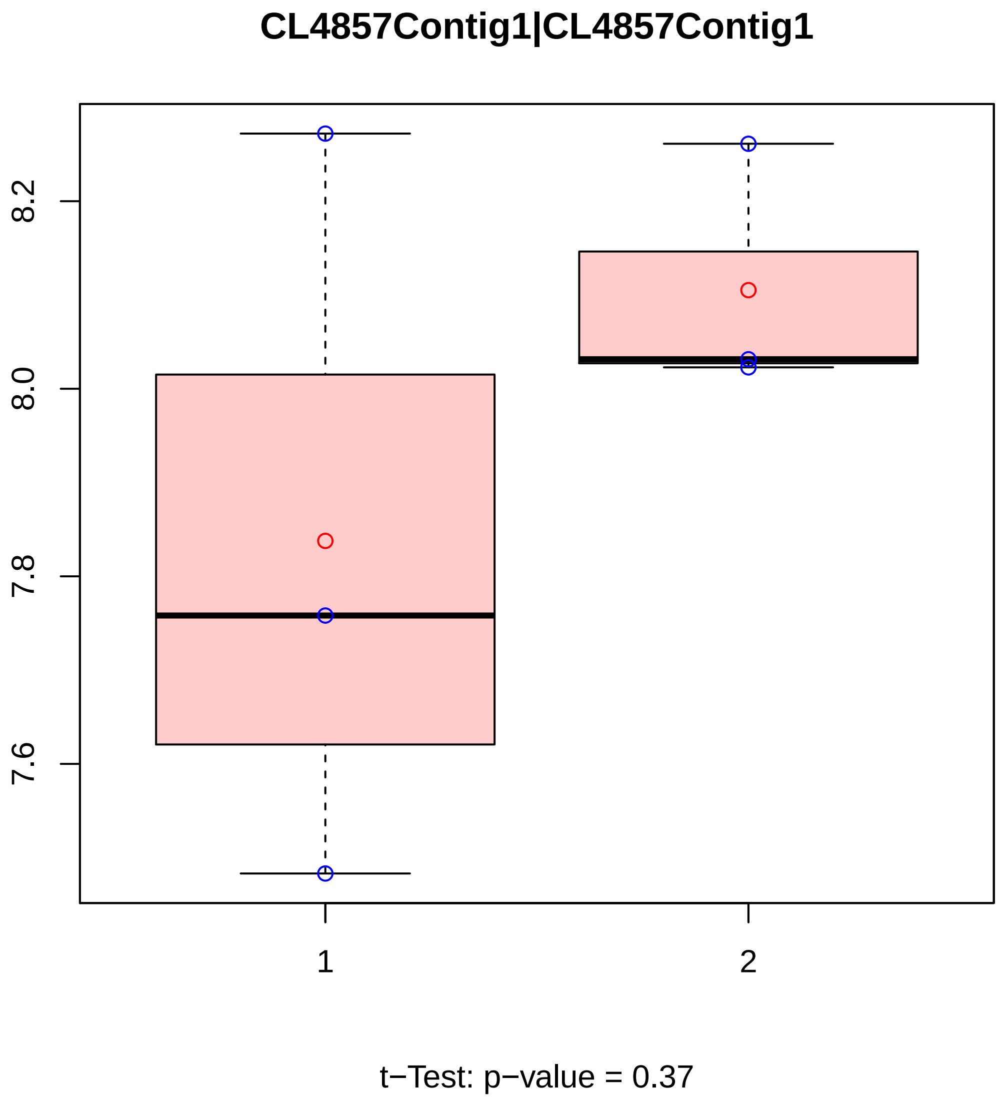

CL4857Contig1|CL4857Contig1
8.2
8.0
7.8
7.6
1
2
t−Test: p−value = 0.37

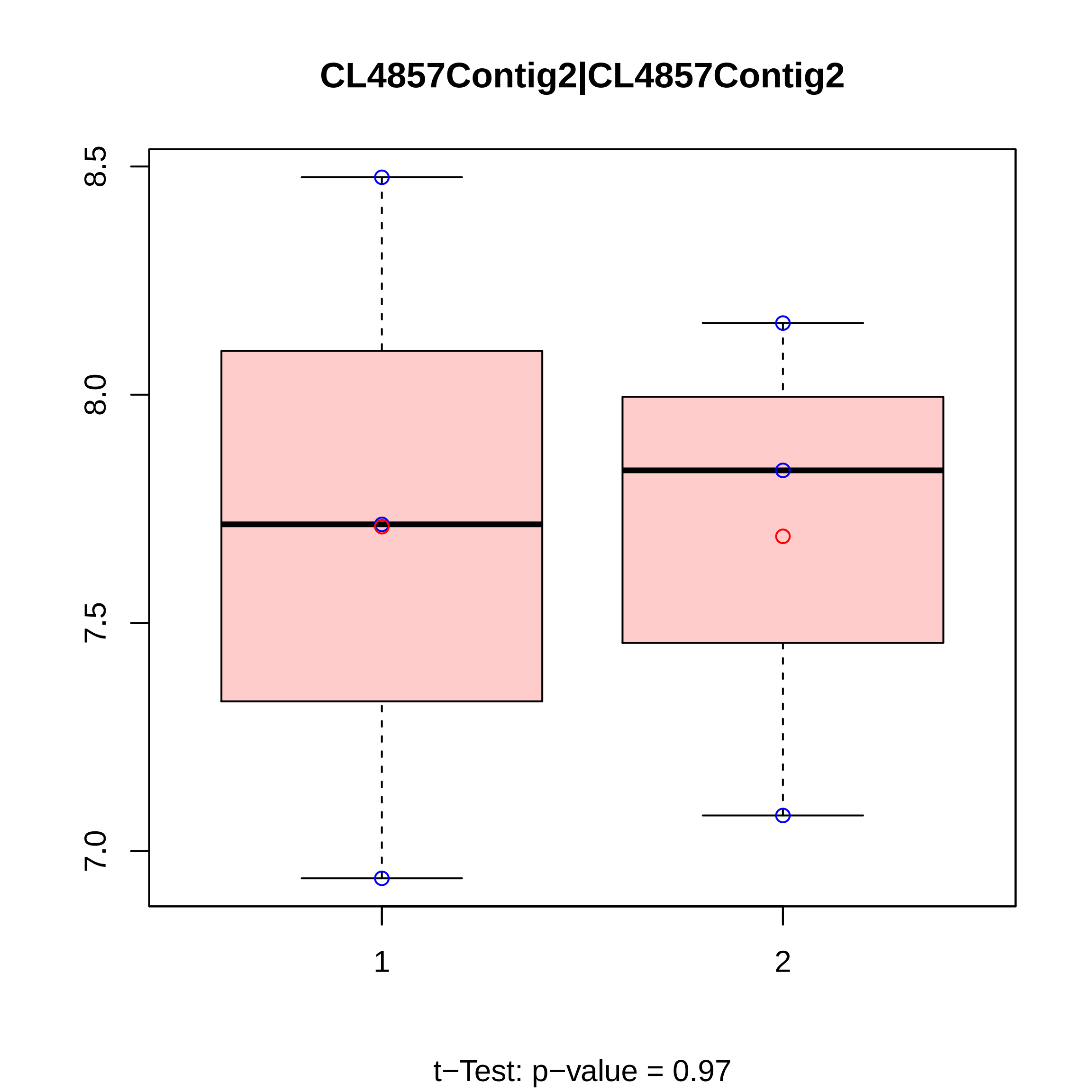

CL4857Contig2|CL4857Contig2
8.5
8.0
7.5
7.0
1
2
t−Test: p−value = 0.97

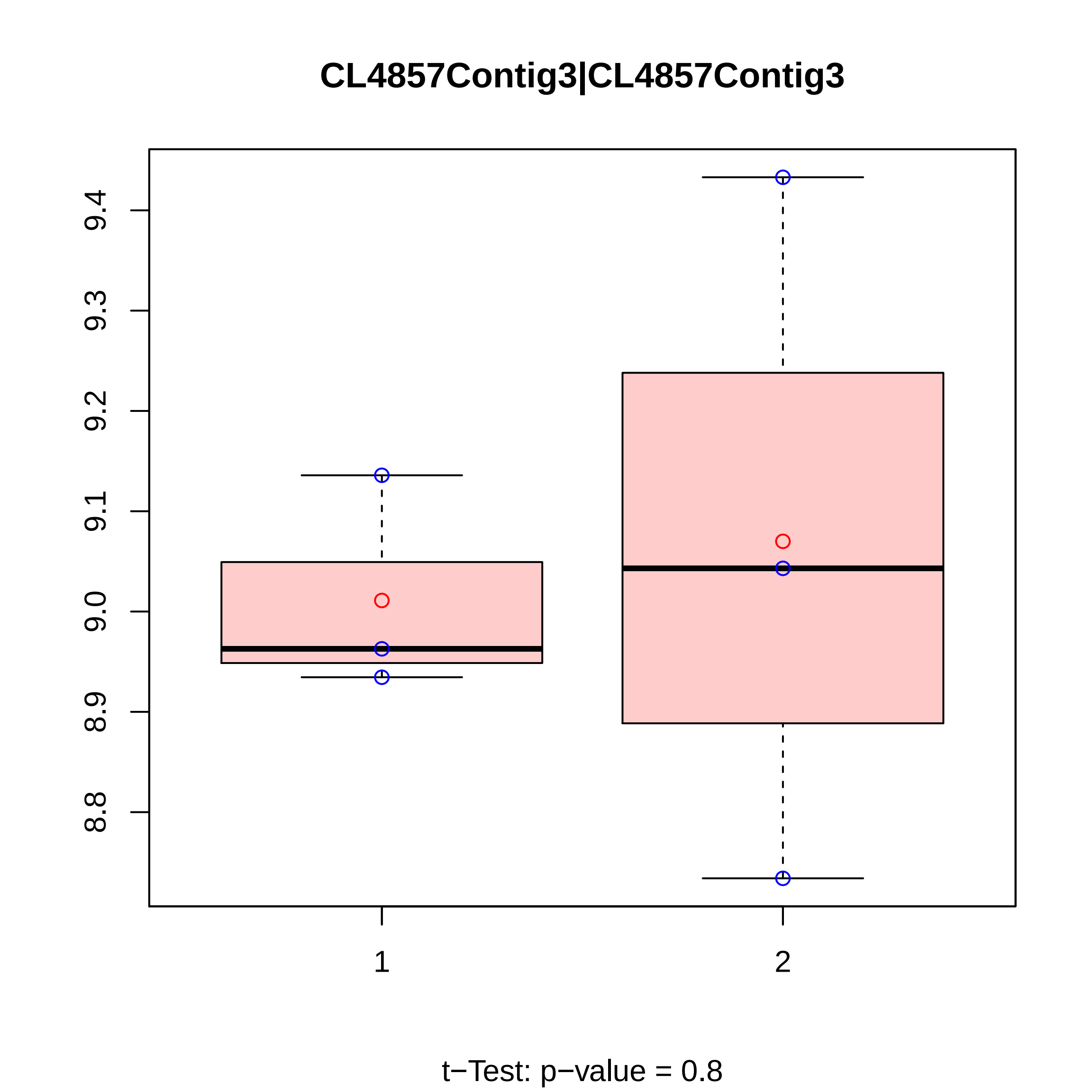

CL4857Contig3|CL4857Contig3
9.4
9.3
9.2
9.1
9.0
8.9
8.8
1
2
t−Test: p−value = 0.8

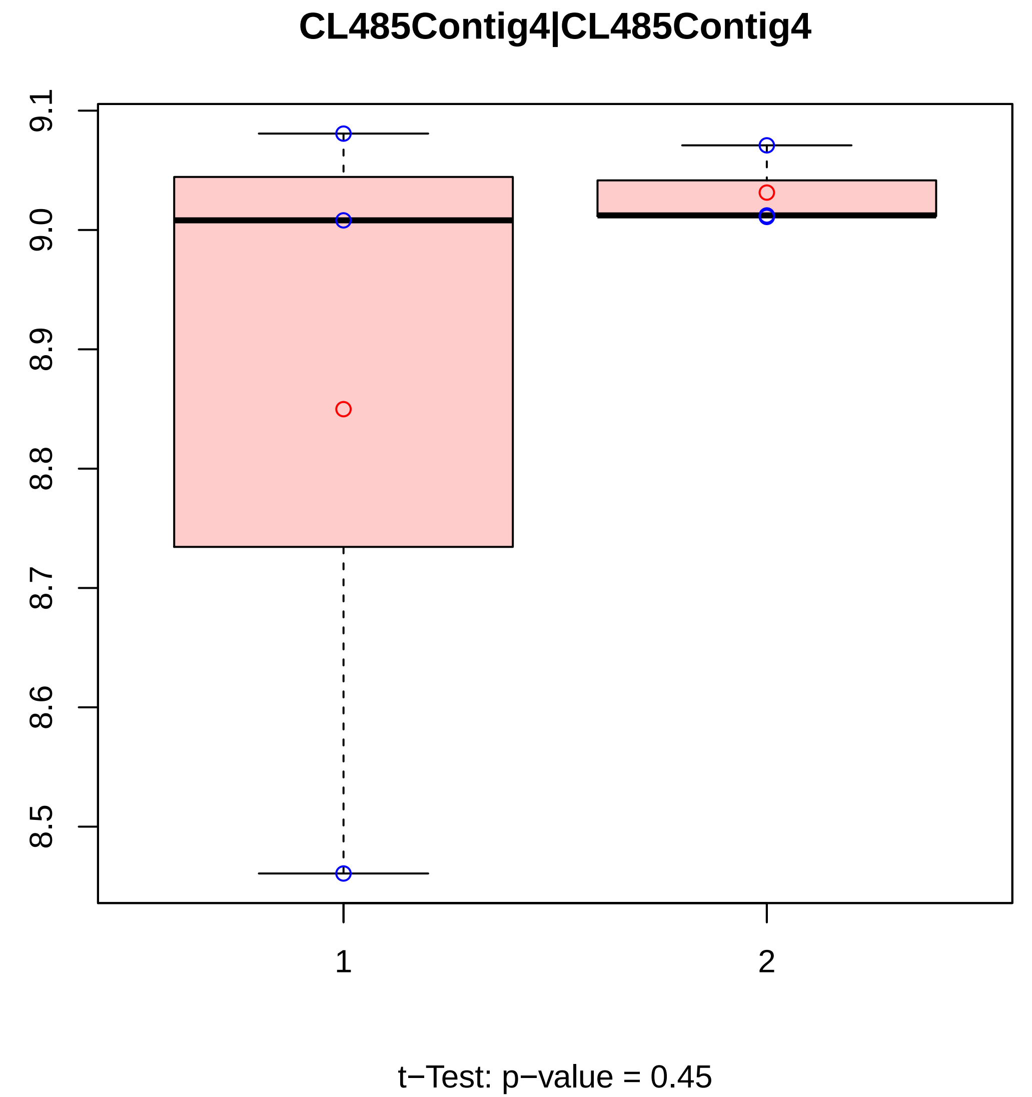

CL485Contig4|CL485Contig4
9.1
9.0
8.9
8.8
8.7
8.6
8.5
1
2
t−Test: p−value = 0.45

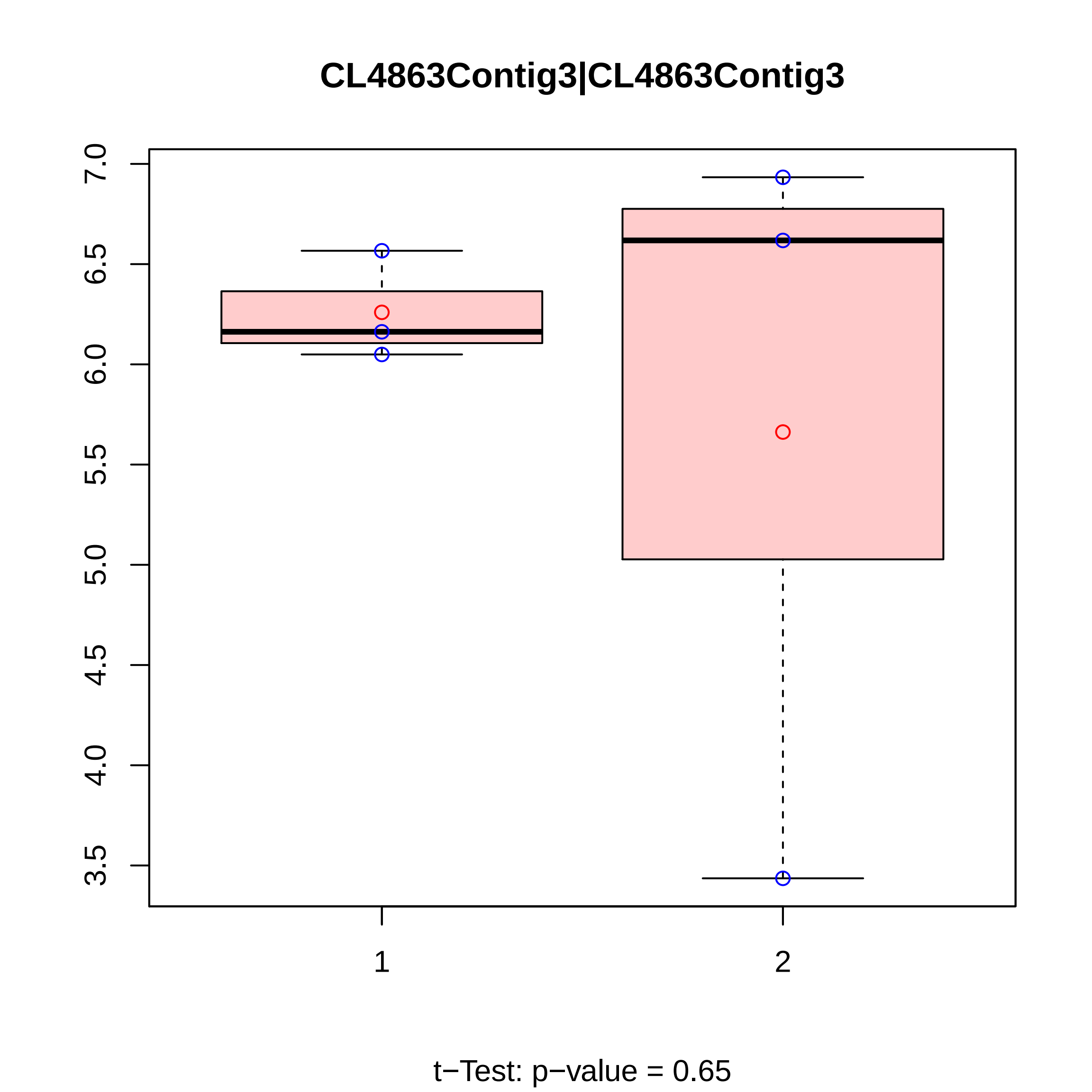
CL4863Contig3|CL4863Contig3
7.0
6.5
6.0
5.5
5.0
4.5
4.0
3.5
1
2
t−Test: p−value = 0.65

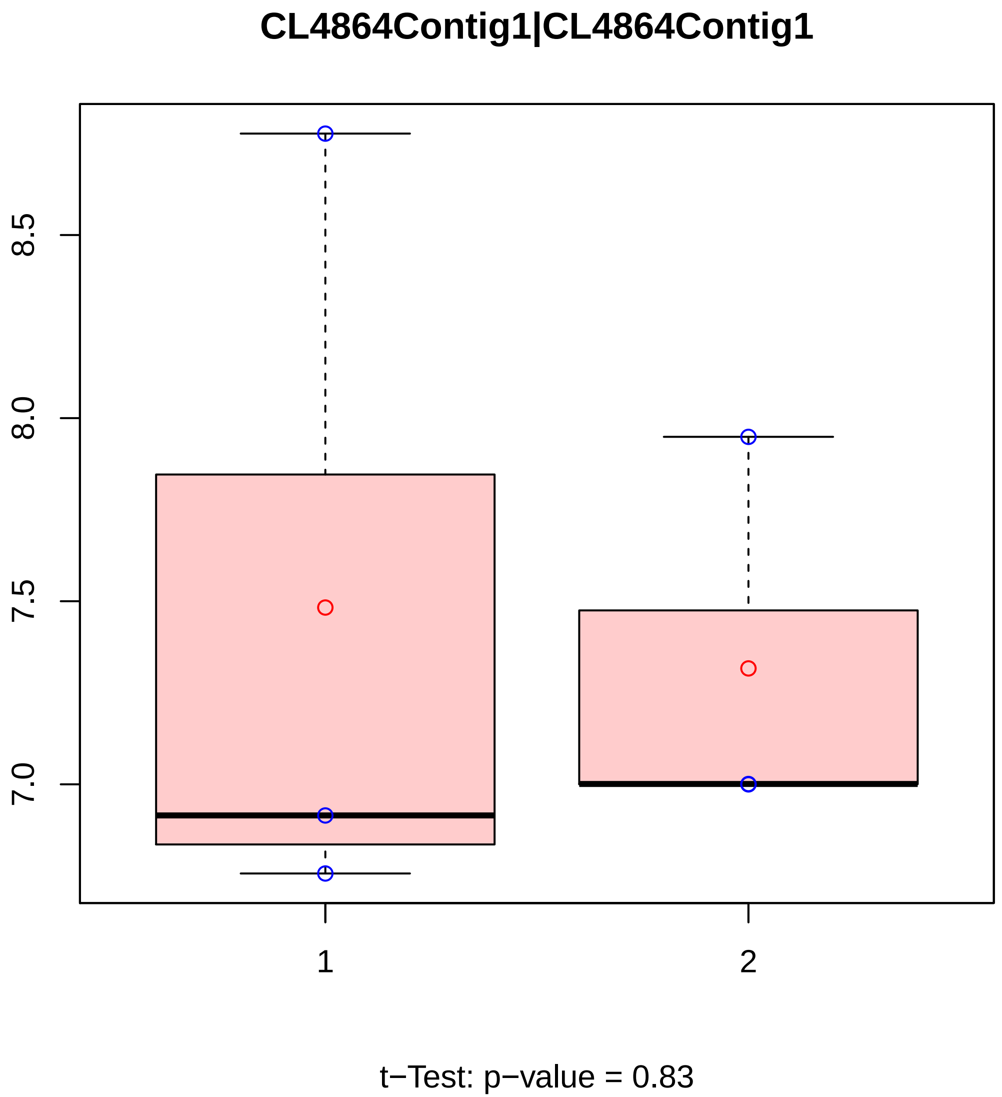
CL4864Contig1|CL4864Contig1
8.5
8.0
7.5
7.0
1
2
t−Test: p−value = 0.83

# CL4867Contig1|CL4867Contig1

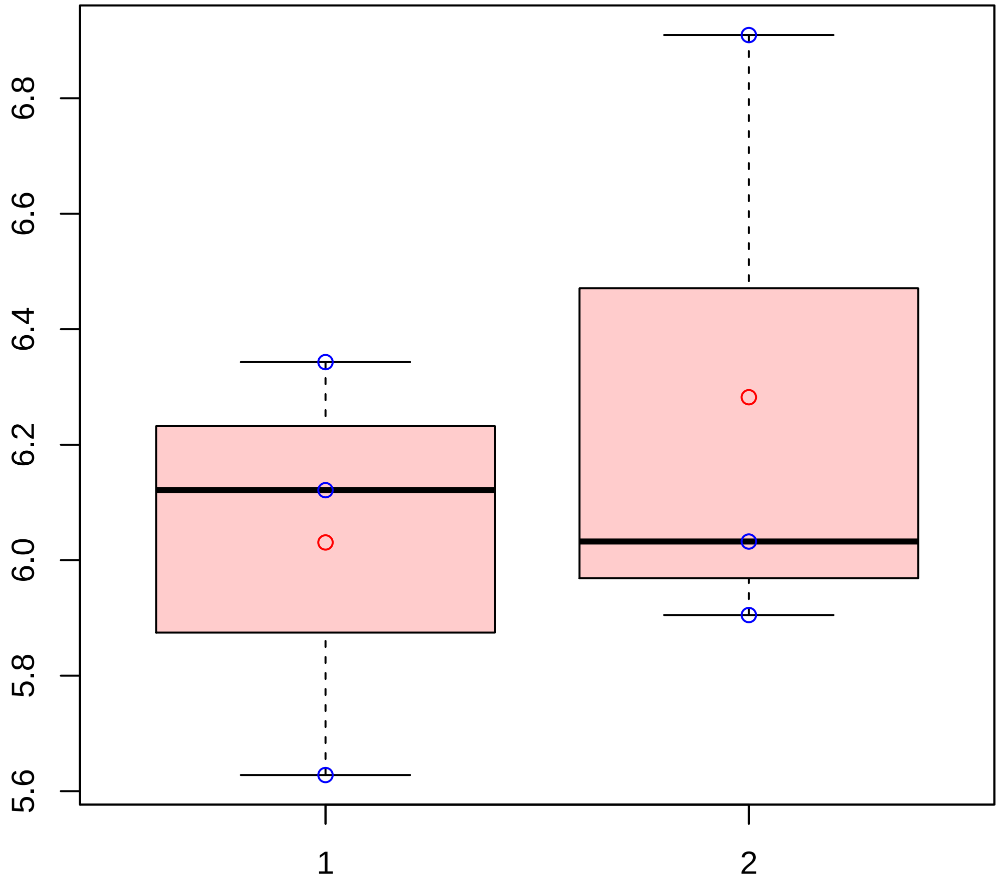


t−Test: p−value = 0.55

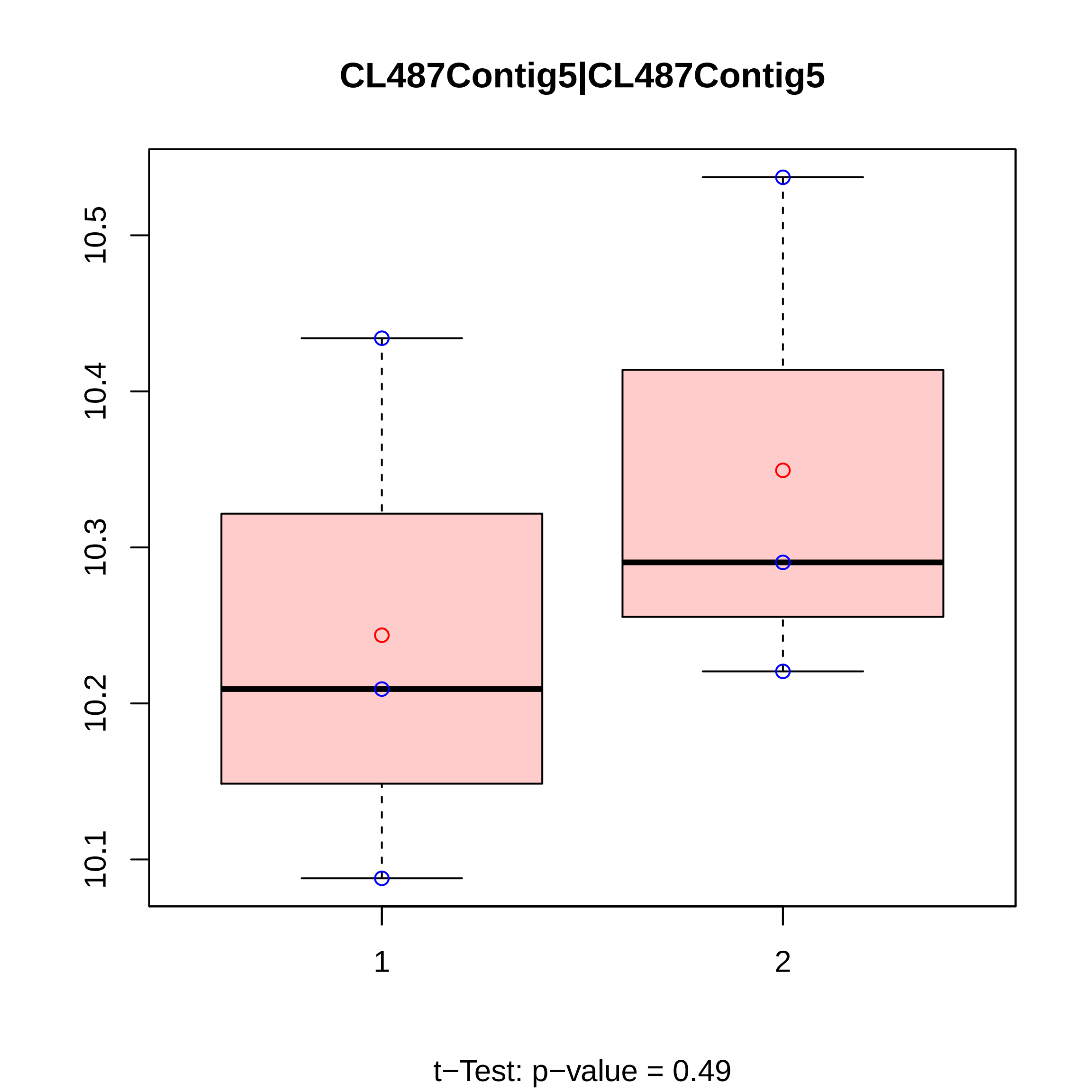

CL487Contig5|CL487Contig5
10.1
10.2
10.3
10.4
10.5
1
2
t−Test: p−value = 0.49

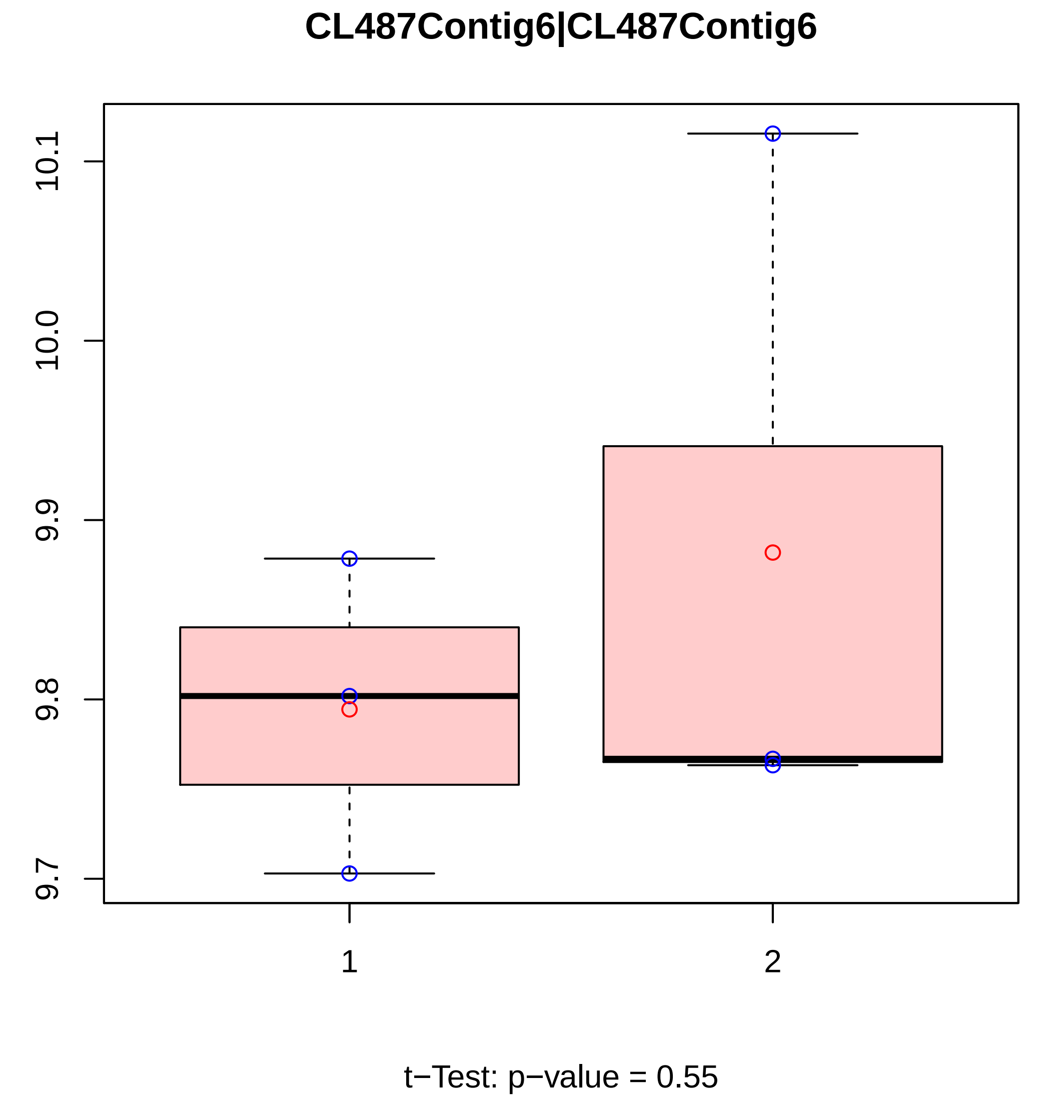
CL487Contig6|CL487Contig6
10.1
10.0
9.9
9.8
9.7
1
2
t−Test: p−value = 0.55

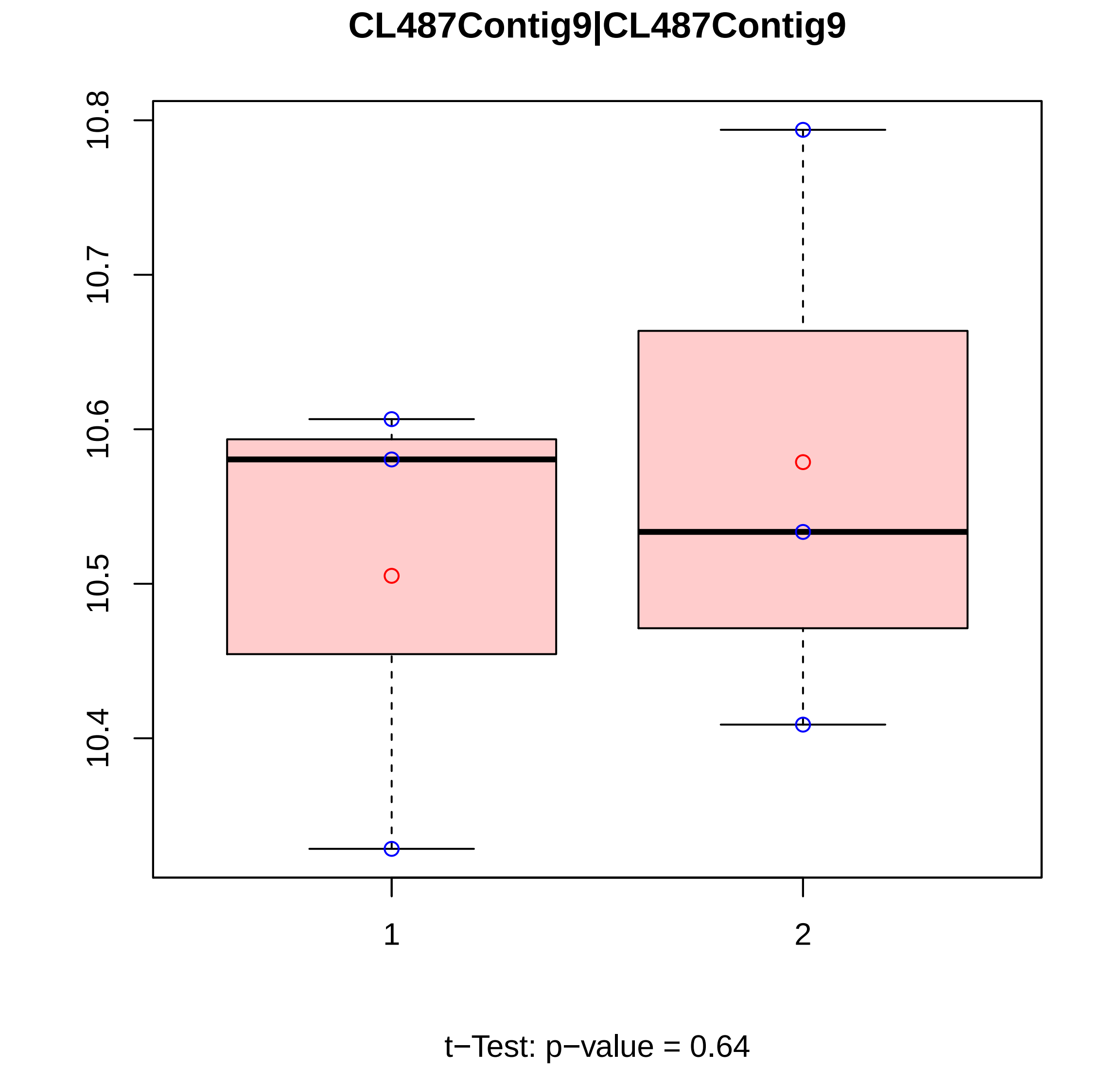

CL487Contig9|CL487Contig9
10.8
10.7
10.6
10.5
10.4
1
2
t−Test: p−value = 0.64

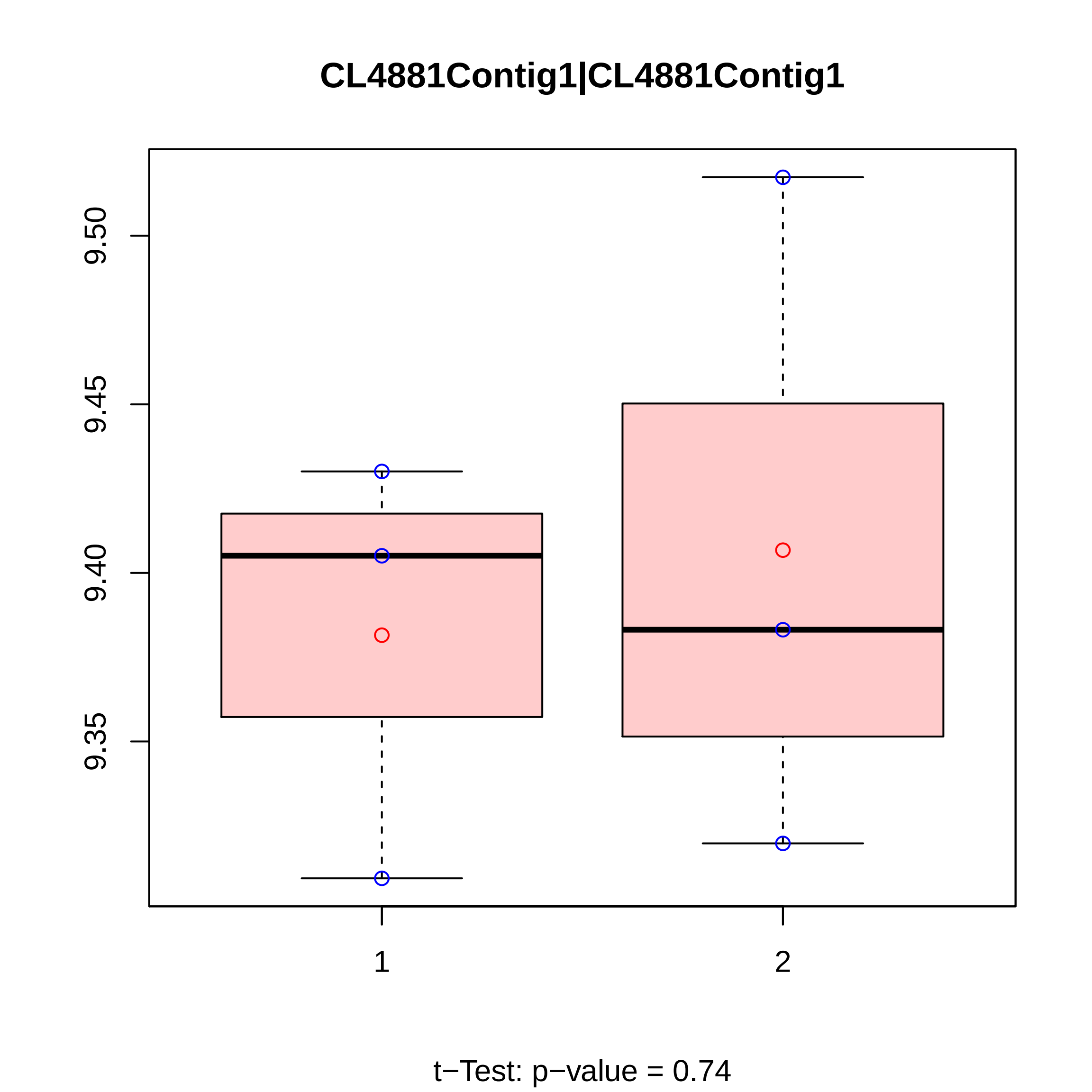

CL4881Contig1|CL4881Contig1
9.35
9.40
9.45
9.50
1
2
t−Test: p−value = 0.74

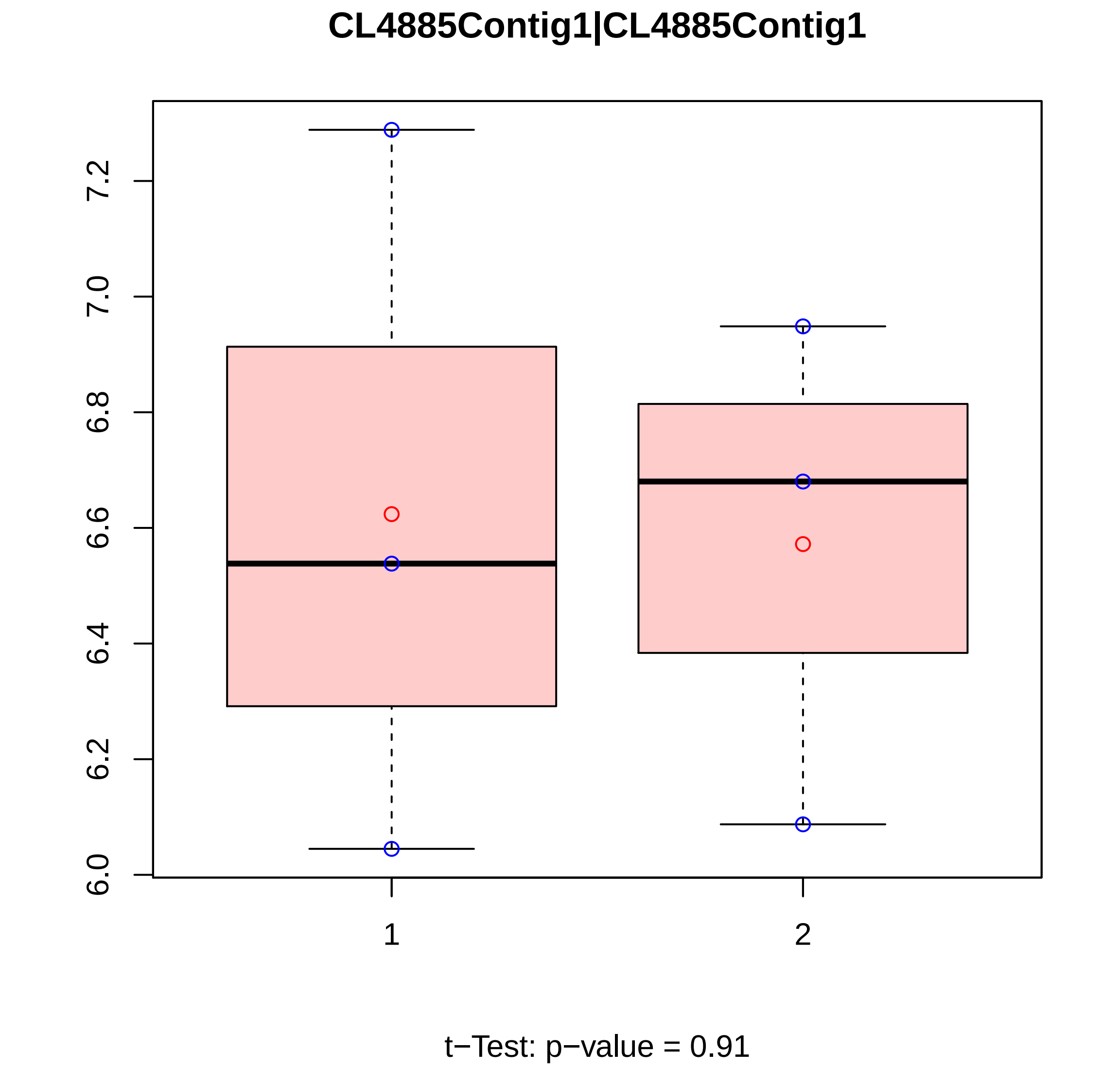

CL4885Contig1|CL4885Contig1
6.0
6.2
6.4
6.6
6.8
7.0
7.2
1
2
t−Test: p−value = 0.91

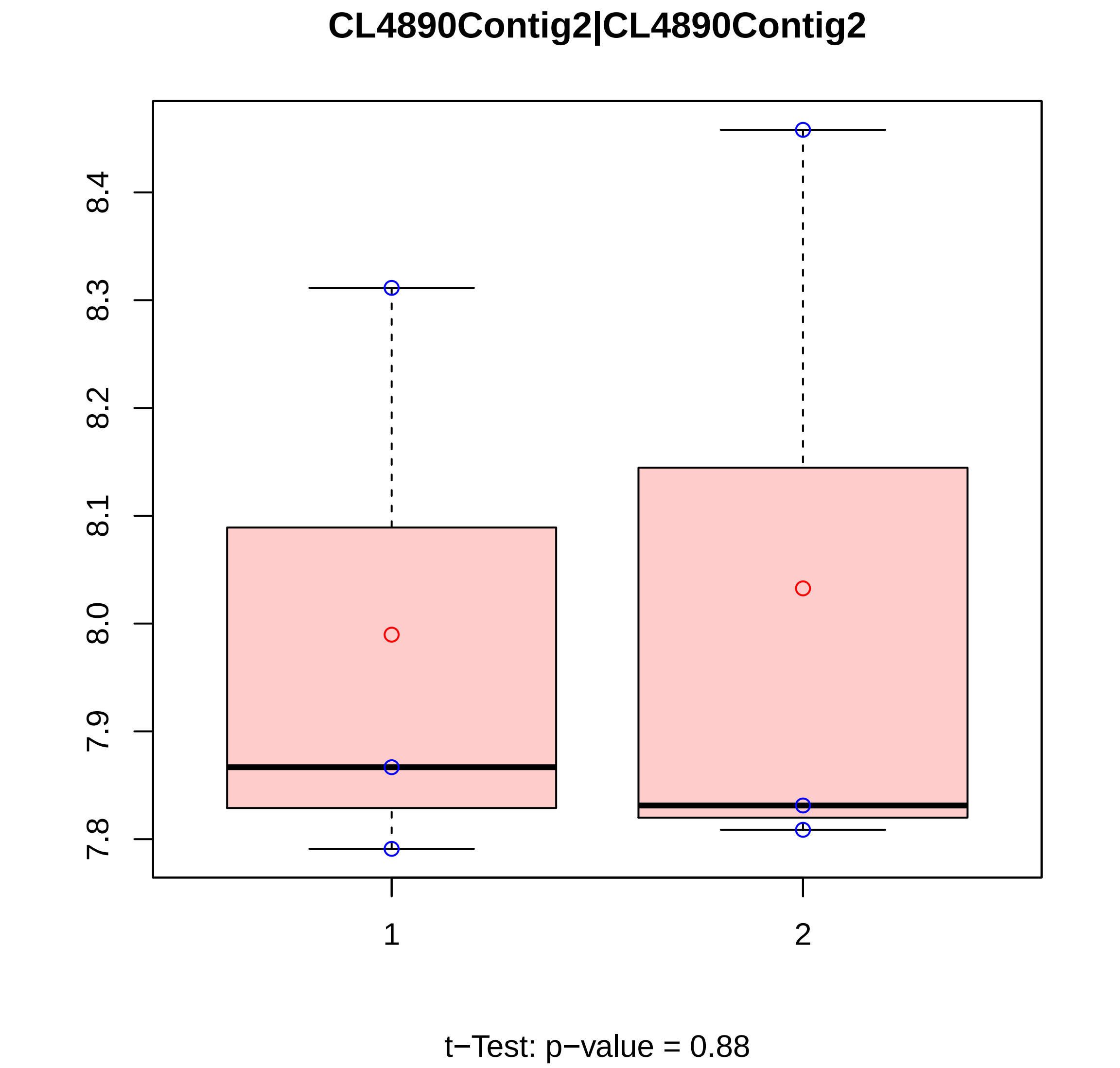
CL4890Contig2|CL4890Contig2
8.4
8.3
8.2
8.1
8.0
7.9
7.8
1
2
t−Test: p−value = 0.88

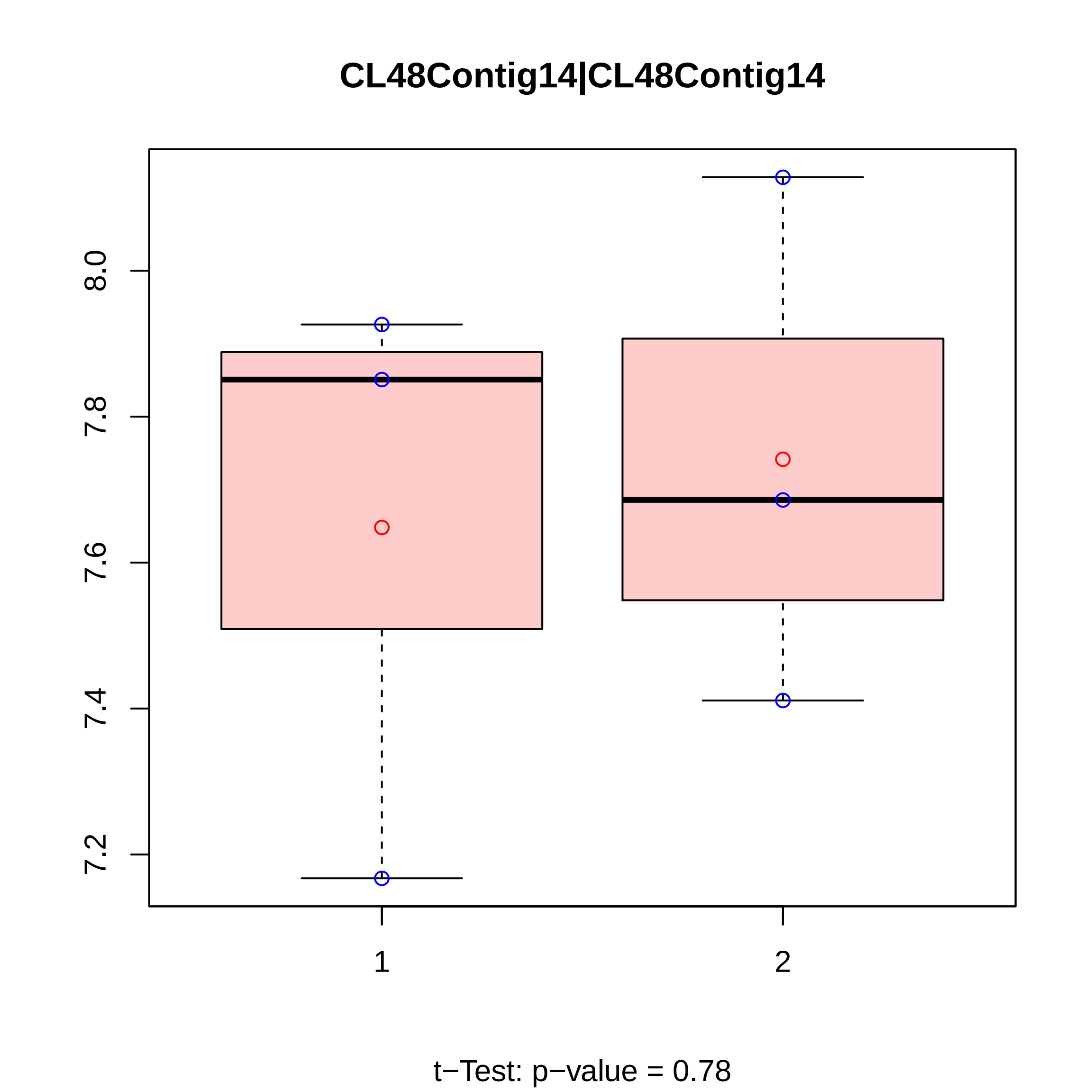

CL48Contig14|CL48Contig14
8.0
7.8
7.6
7.4
7.2
1
2
t−Test: p−value = 0.78

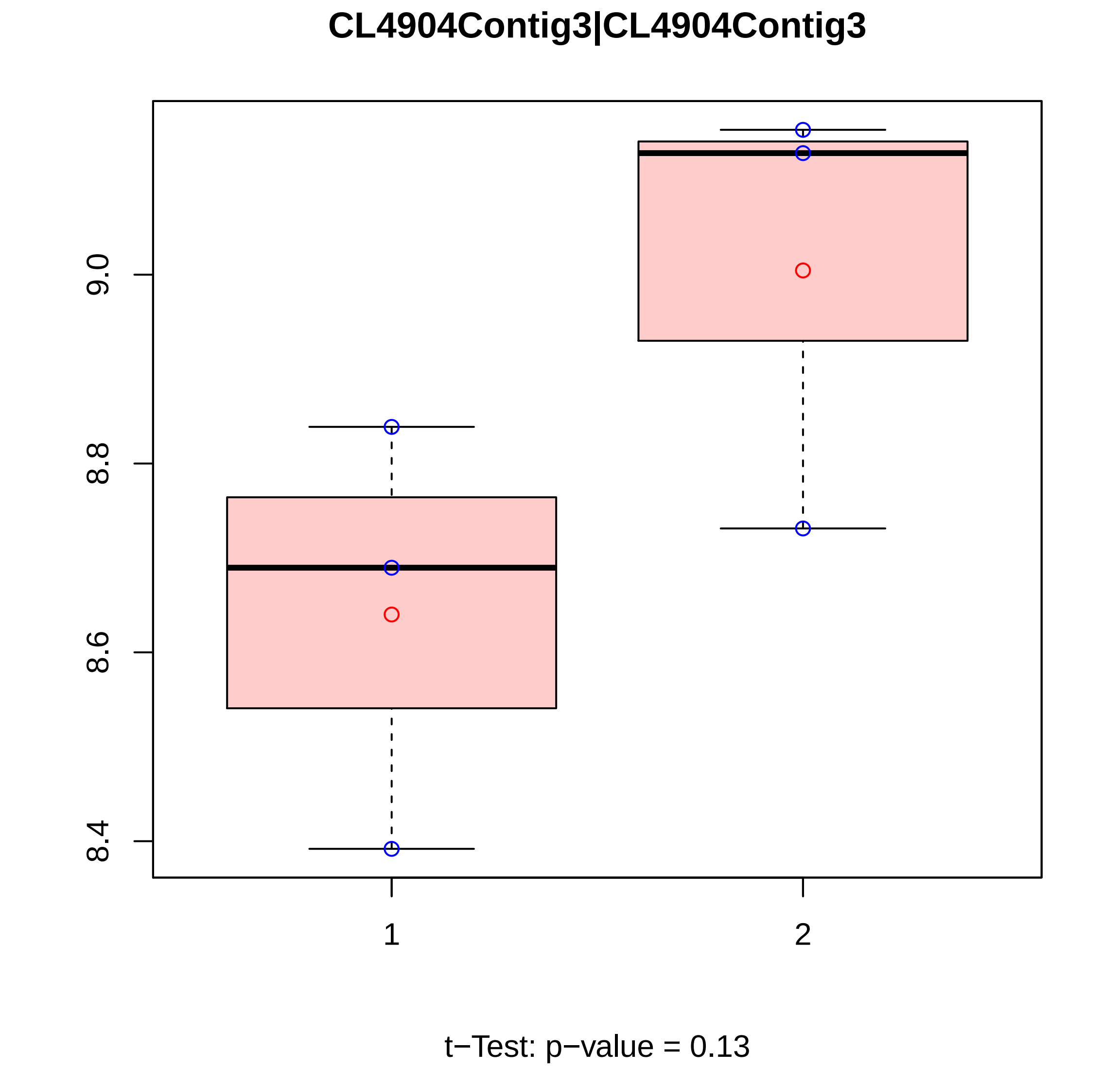
CL4904Contig3|CL4904Contig3
9.0
8.8
8.6
8.4
1
2
t−Test: p−value = 0.13

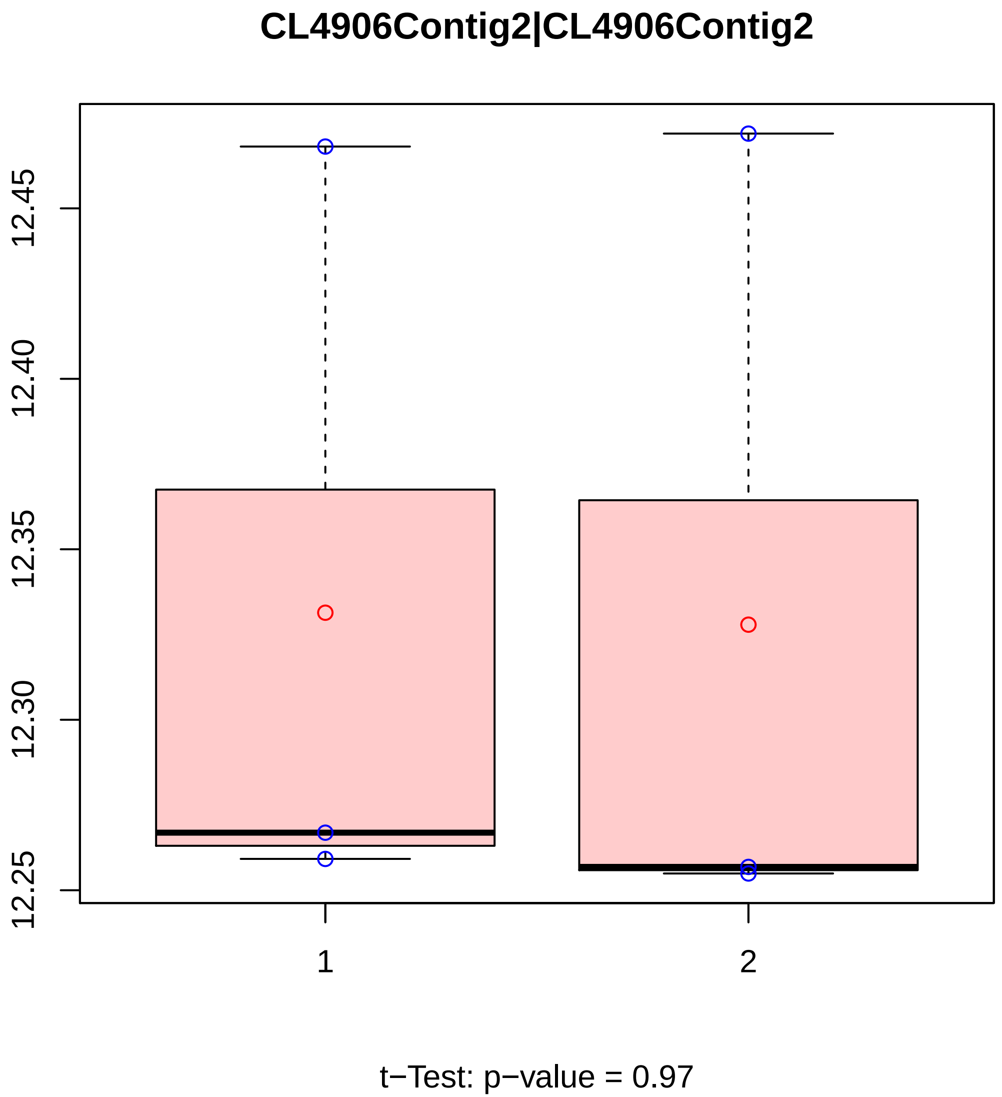

CL4906Contig2|CL4906Contig2
12.25
12.30
12.35
12.40
12.45
1
2
t−Test: p−value = 0.97

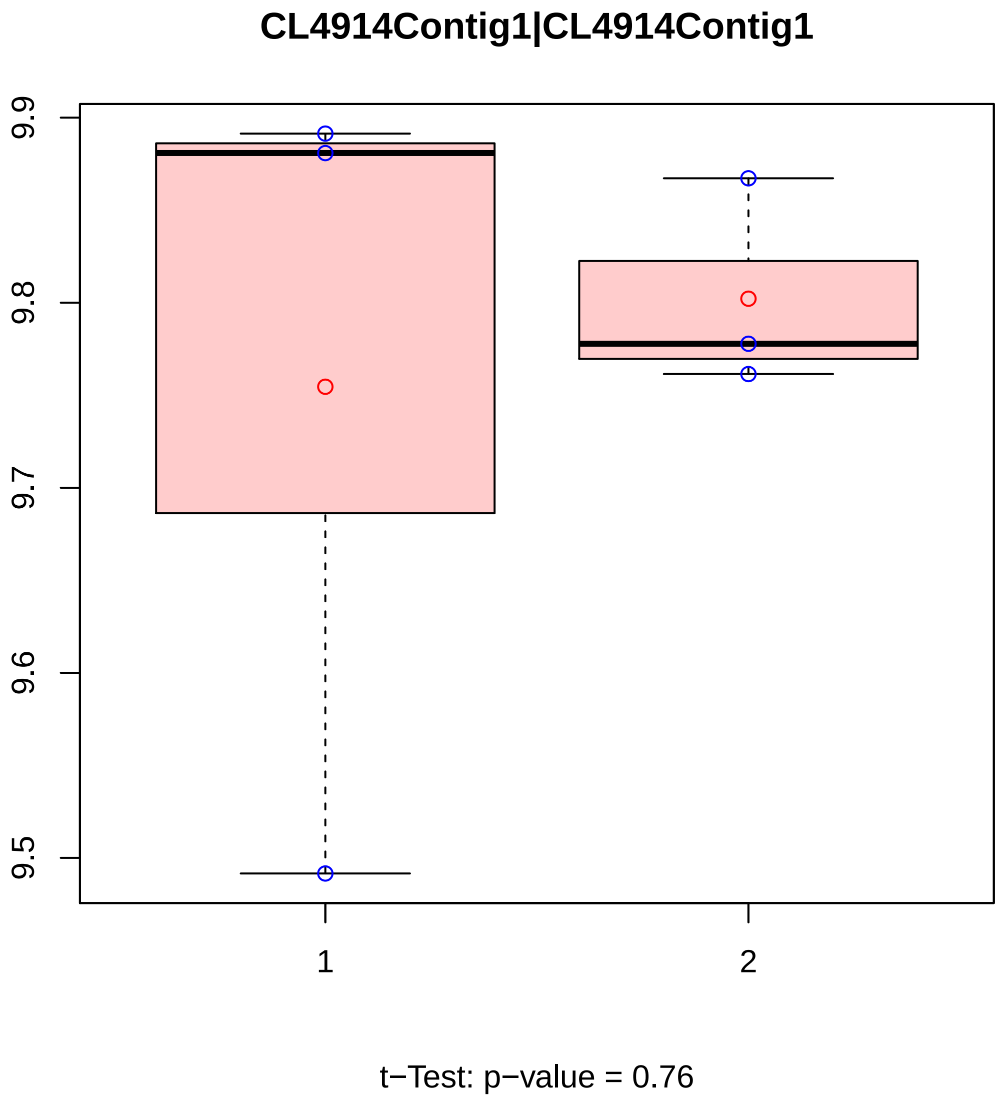
CL4914Contig1|CL4914Contig1
9.9
9.8
9.7
9.6
9.5
1
2
t−Test: p−value = 0.76

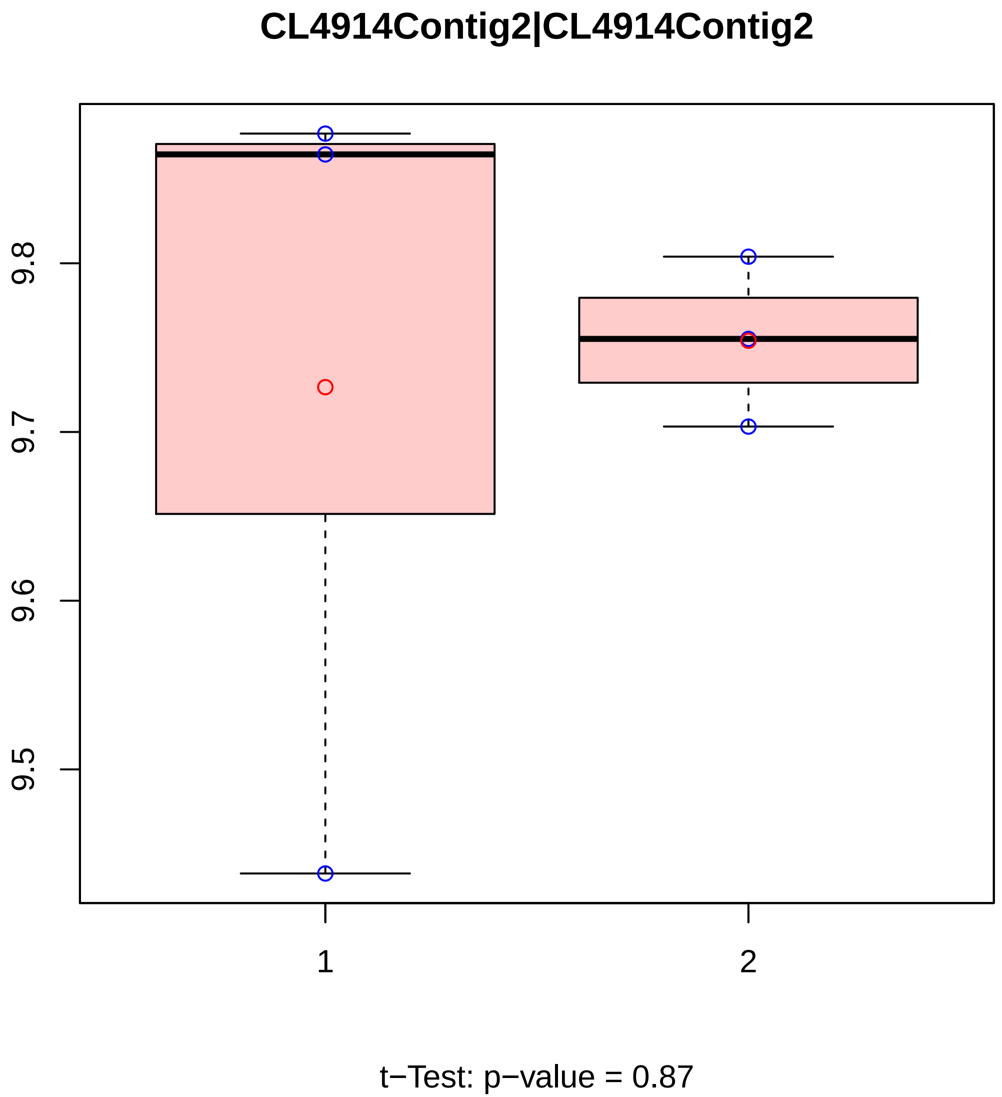

CL4914Contig2|CL4914Contig2
9.5
9.6
9.7
9.8
1
2
t−Test: p−value = 0.87

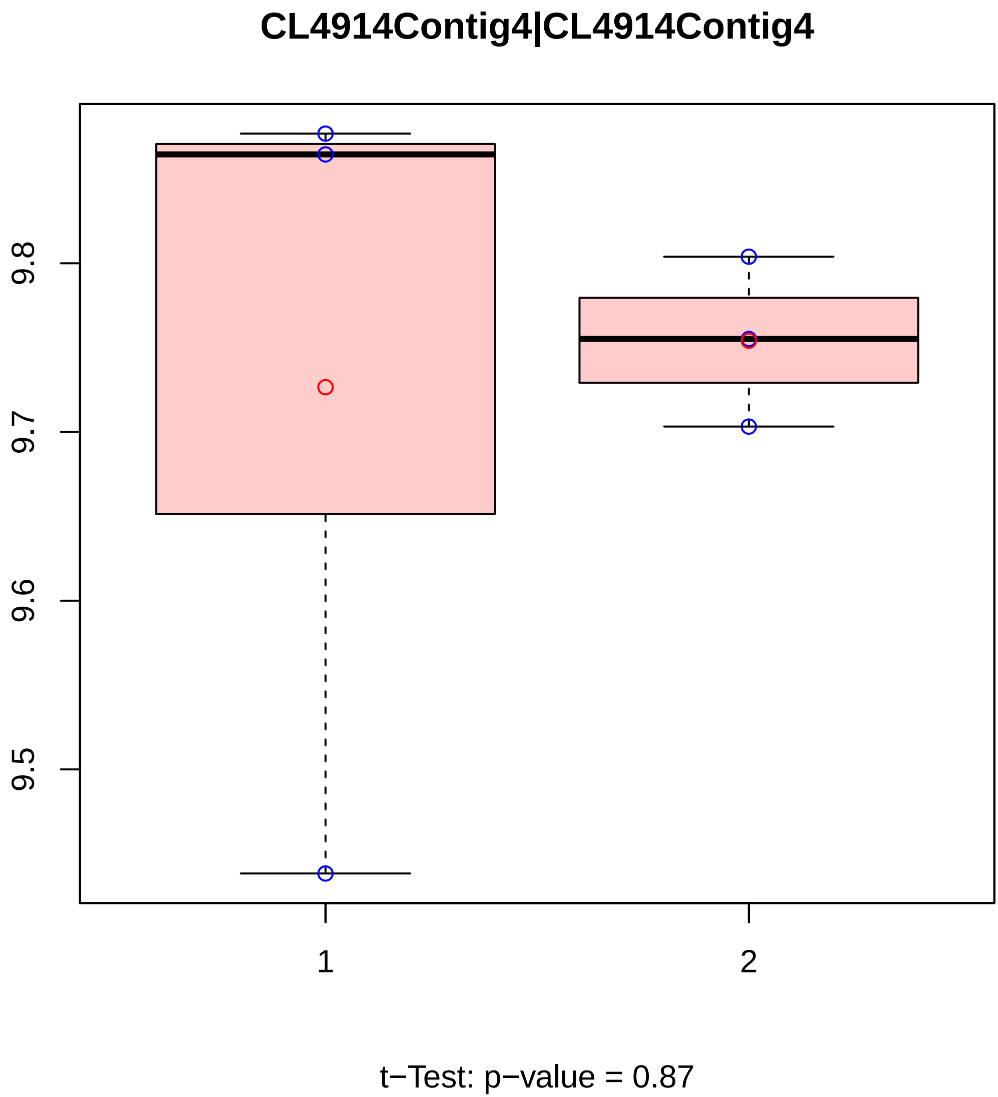
CL4914Contig4|CL4914Contig4
9.8
9.7
9.6
9.5
1
2
t−Test: p−value = 0.87

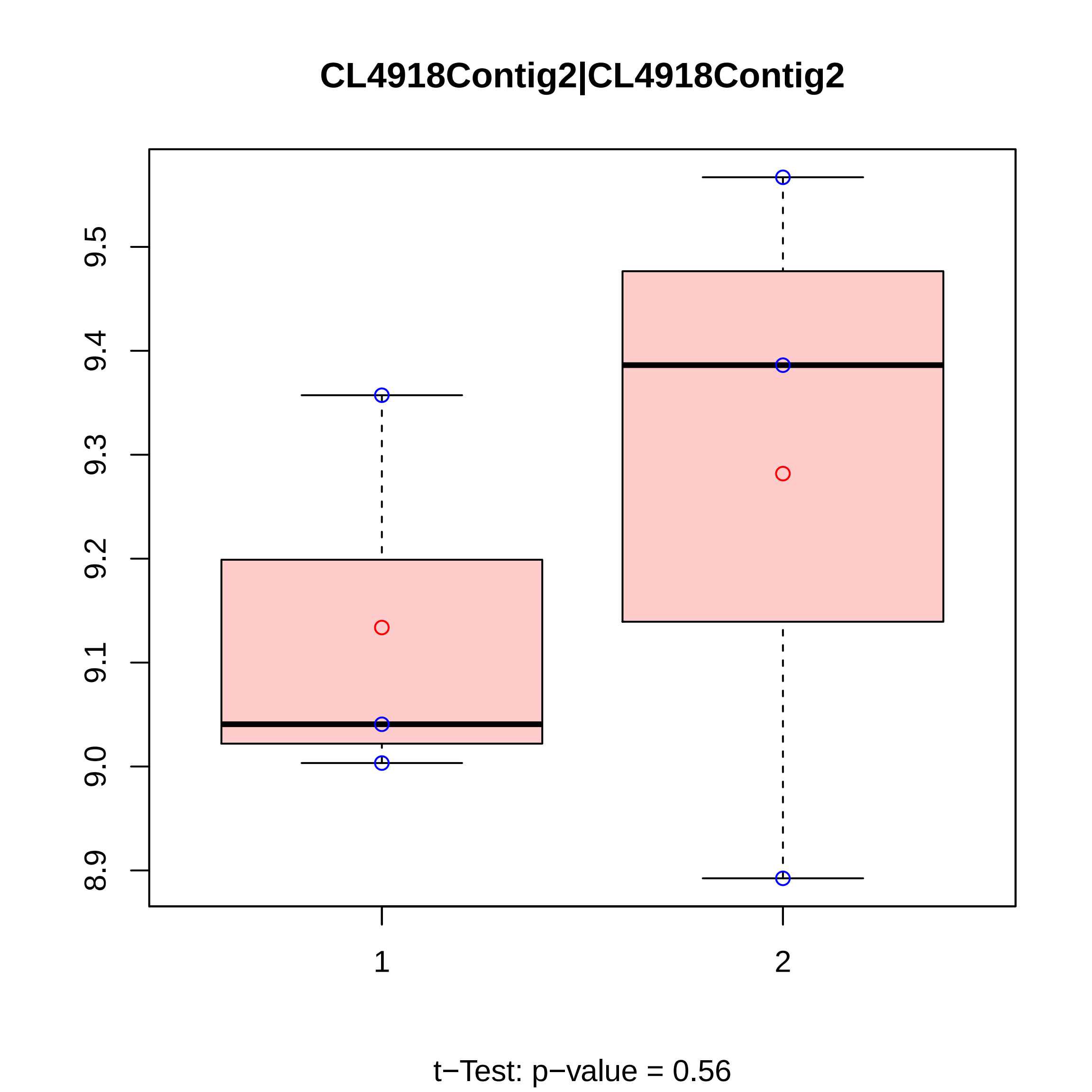

CL4918Contig2|CL4918Contig2
9.5
9.4
9.3
9.2
9.1
9.0
8.9
1
2
t−Test: p−value = 0.56

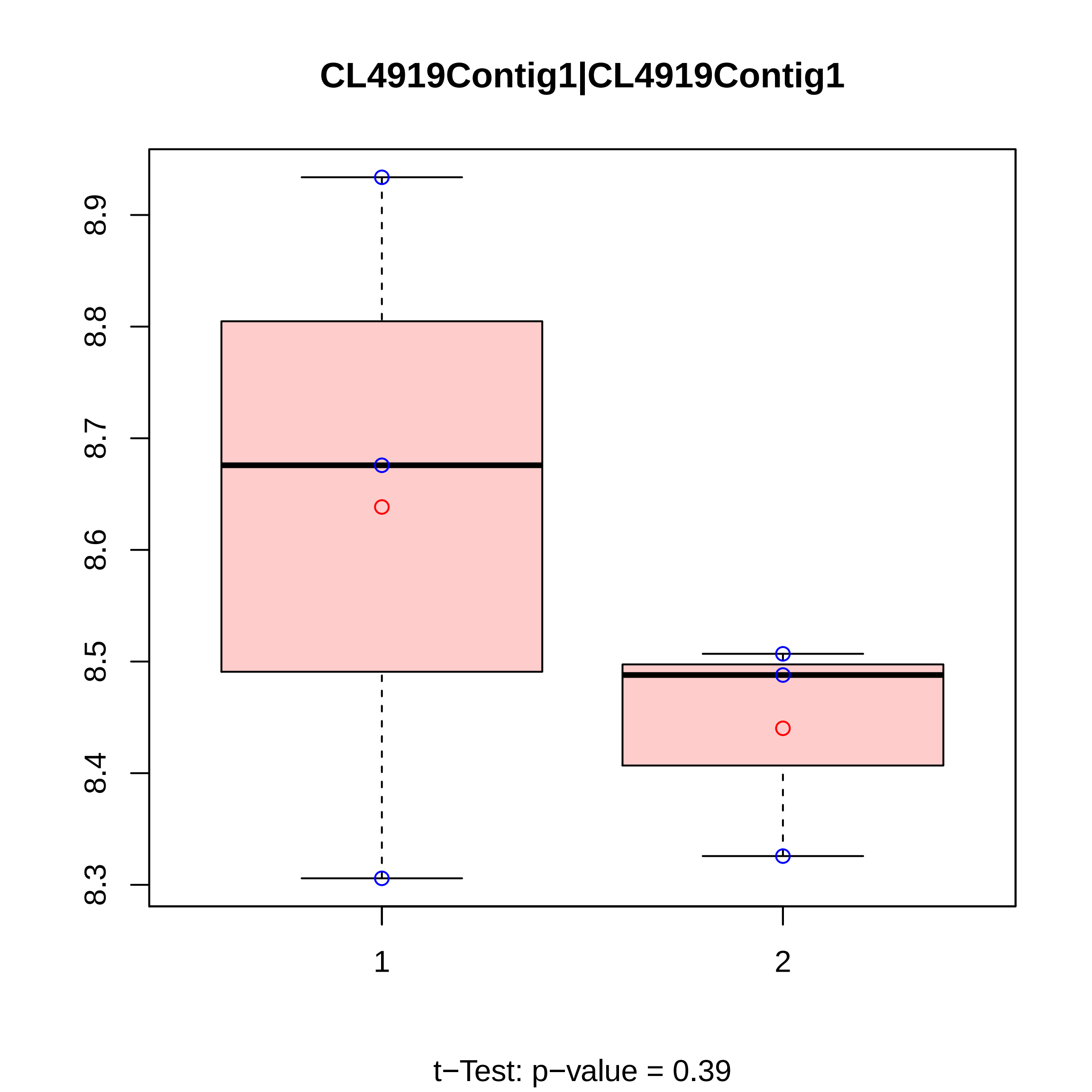
CL4919Contig1|CL4919Contig1
8.3
8.4
8.5
8.6
8.7
8.8
8.9
1
2
t−Test: p−value = 0.39

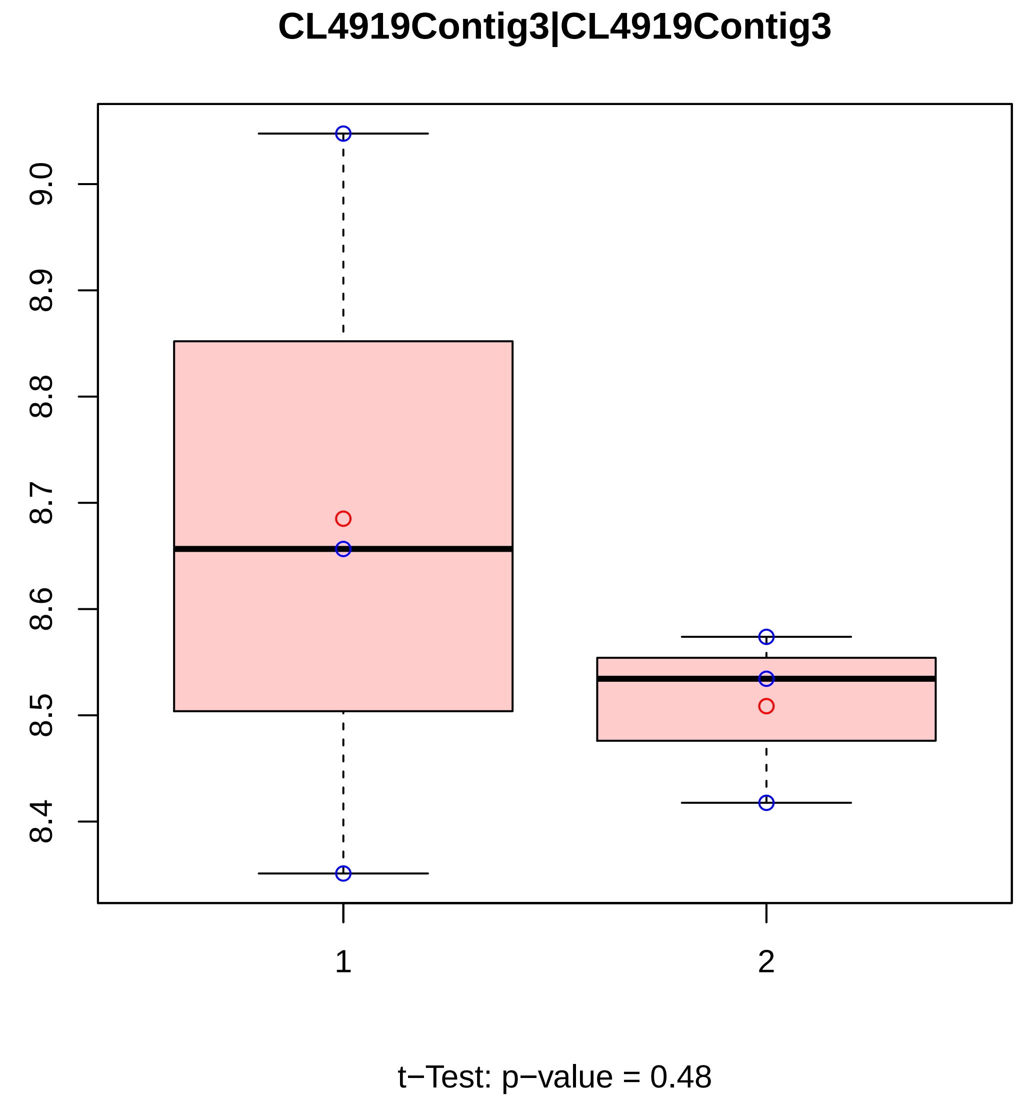
CL4919Contig3|CL4919Contig3
9.0
8.9
8.8
8.7
8.6
8.5
8.4
1
2
t−Test: p−value = 0.48

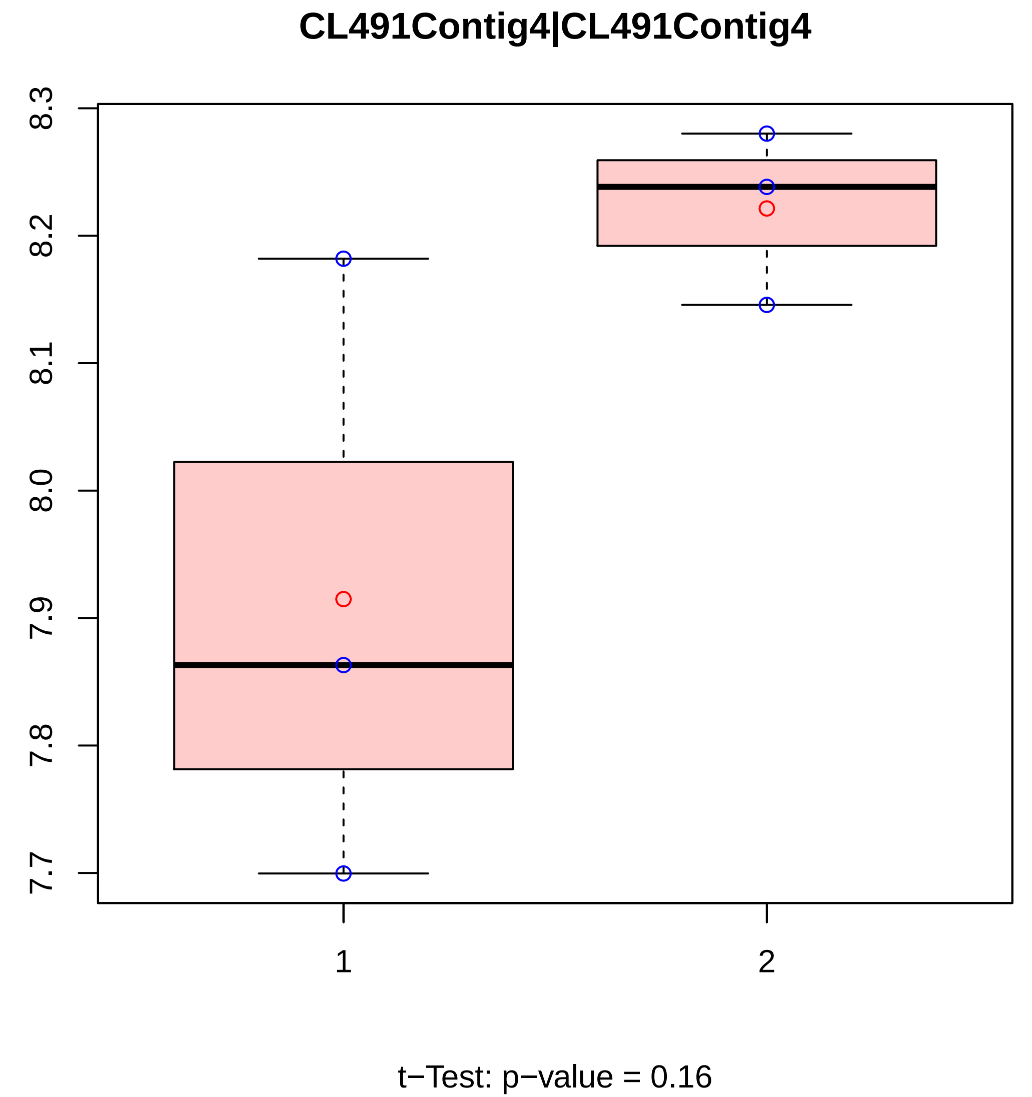
CL491Contig4|CL491Contig4
8.3
8.2
8.1
8.0
7.9
7.8
7.7
1
2
t−Test: p−value = 0.16

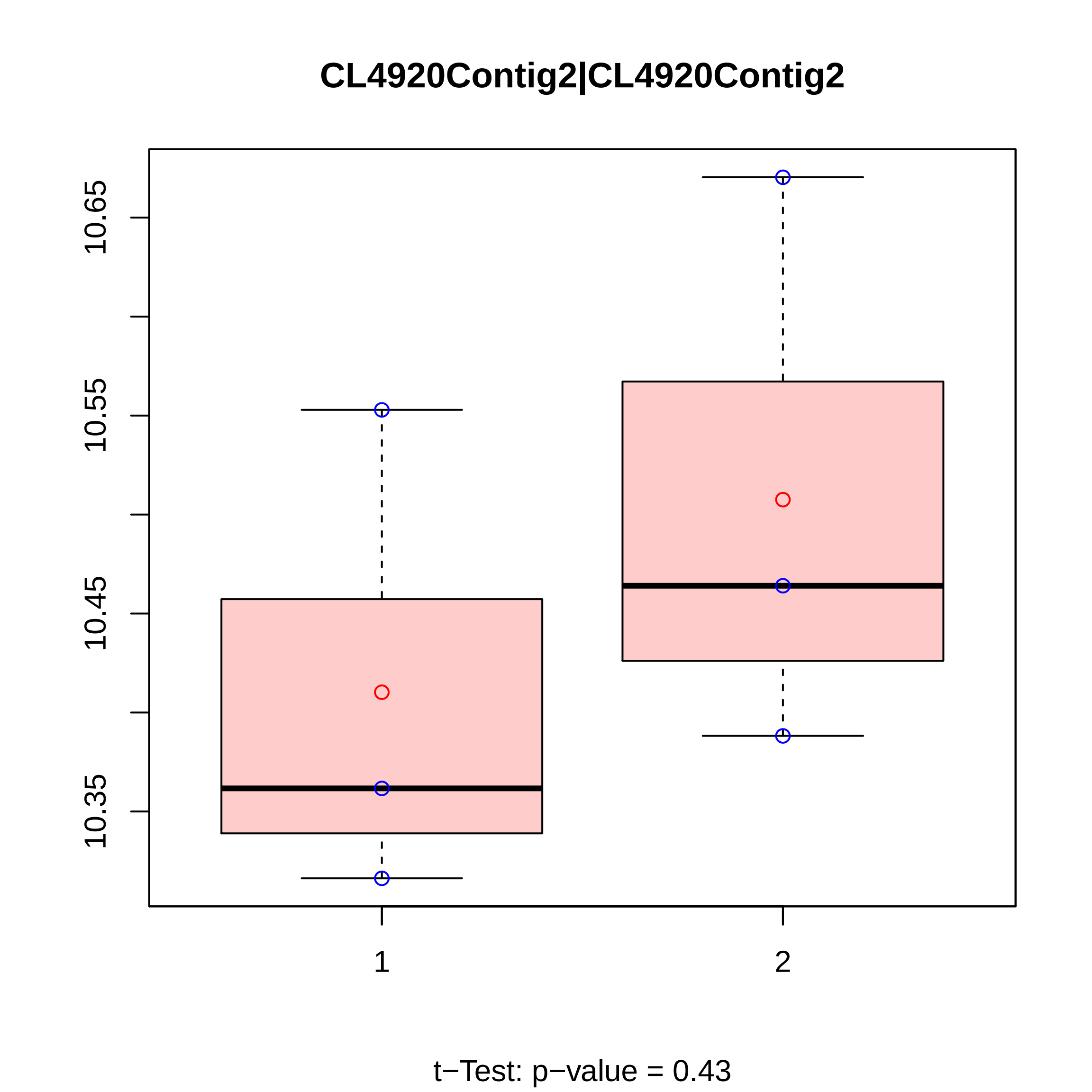

CL4920Contig2|CL4920Contig2
10.35
10.45
10.55
10.65
1
2
t−Test: p−value = 0.43

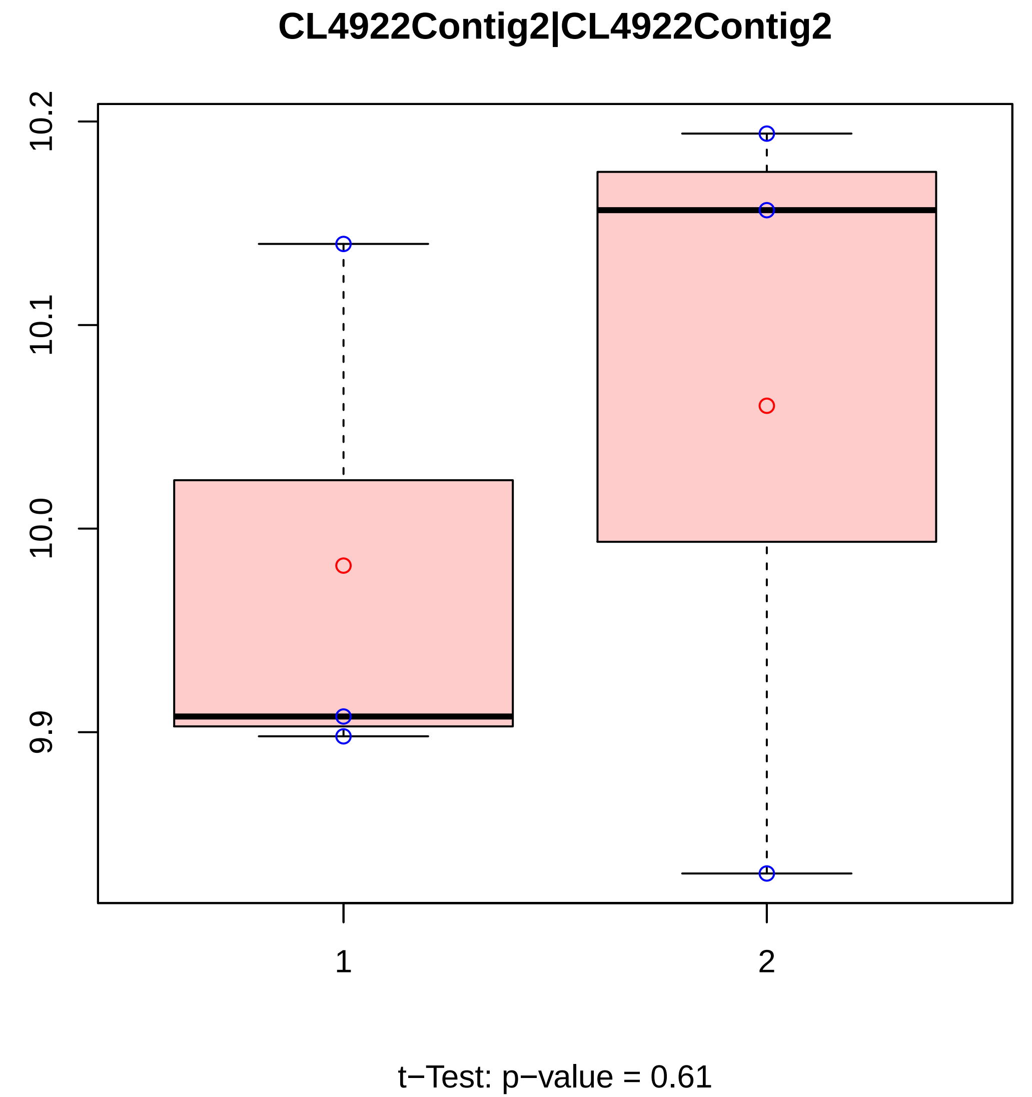
CL4922Contig2|CL4922Contig2
10.2
10.1
10.0
9.9
1
2
t−Test: p−value = 0.61

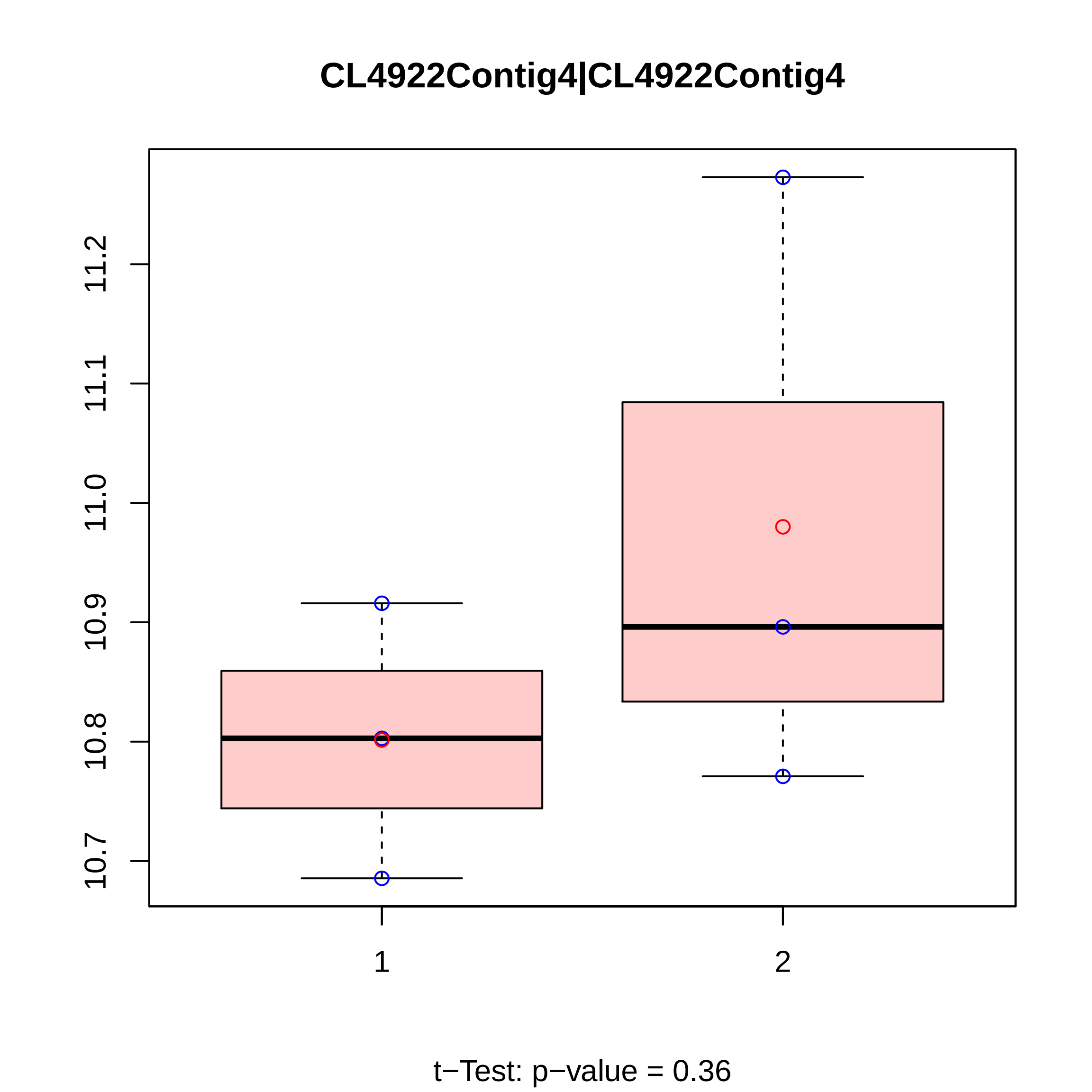

CL4922Contig4|CL4922Contig4
11.2
11.1
11.0
10.9
10.8
10.7
1
2
t−Test: p−value = 0.36

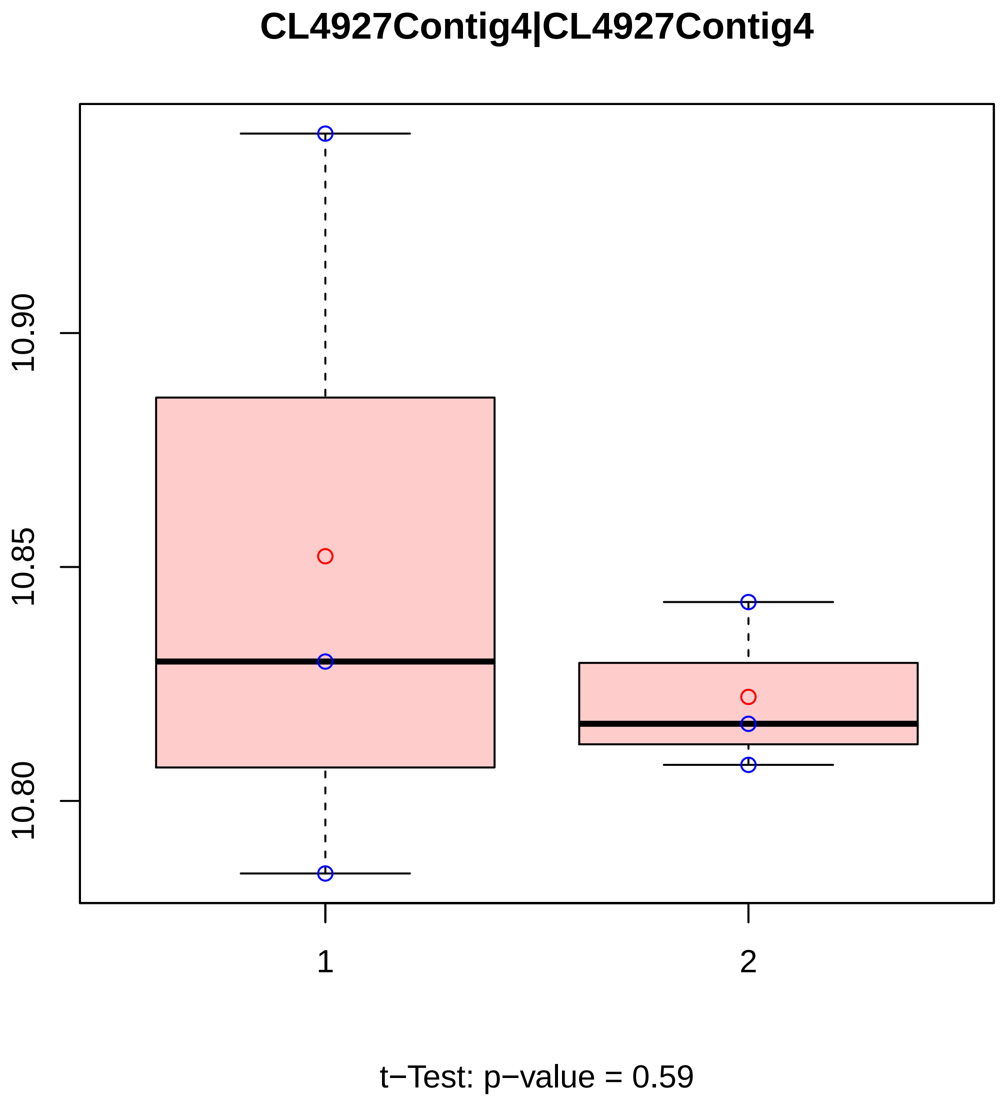
CL4927Contig4|CL4927Contig4
10.80
10.85
10.90
1
2
t−Test: p−value = 0.59

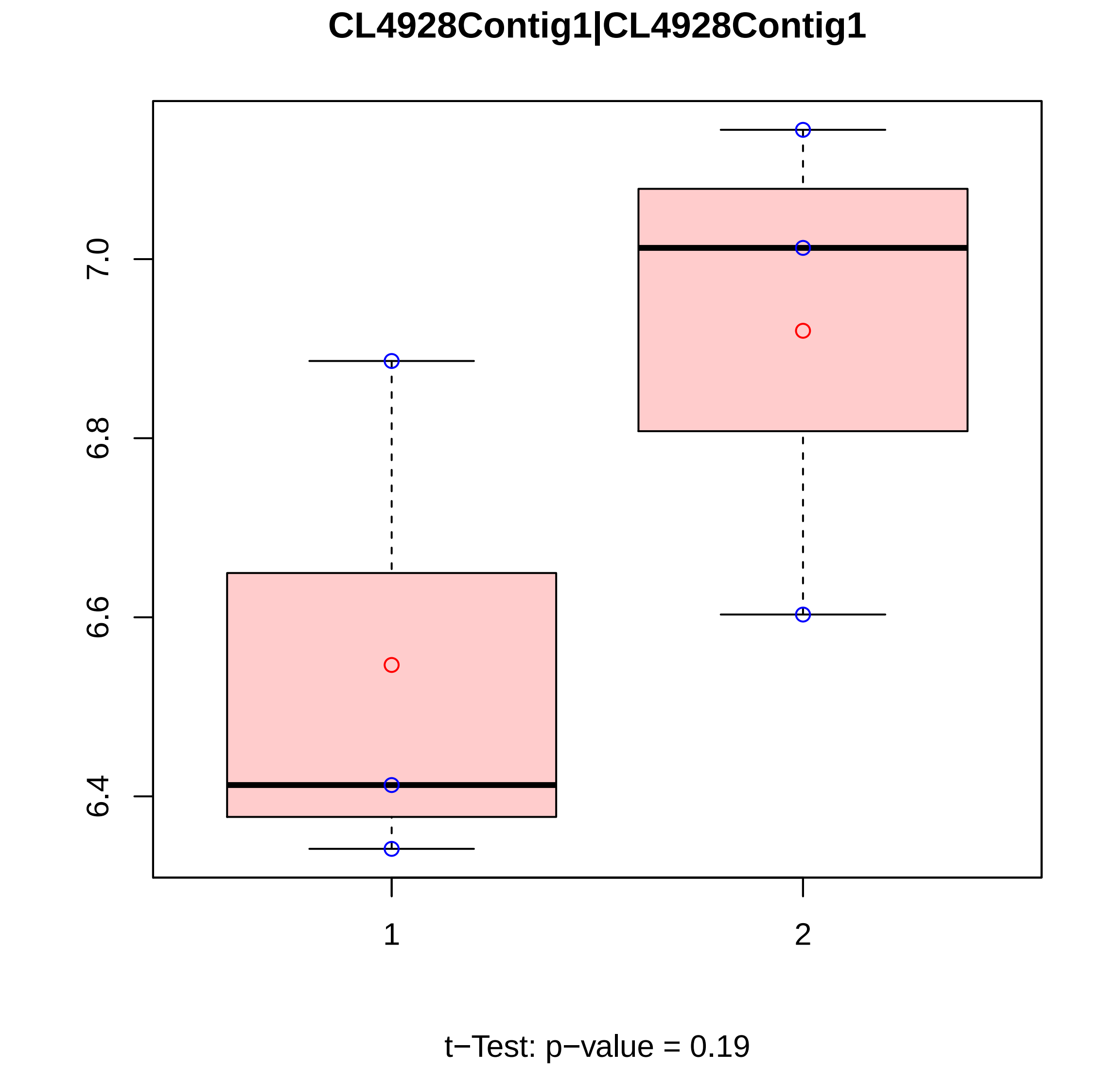

CL4928Contig1|CL4928Contig1
7.0
6.8
6.6
6.4
1
2
t−Test: p−value = 0.19

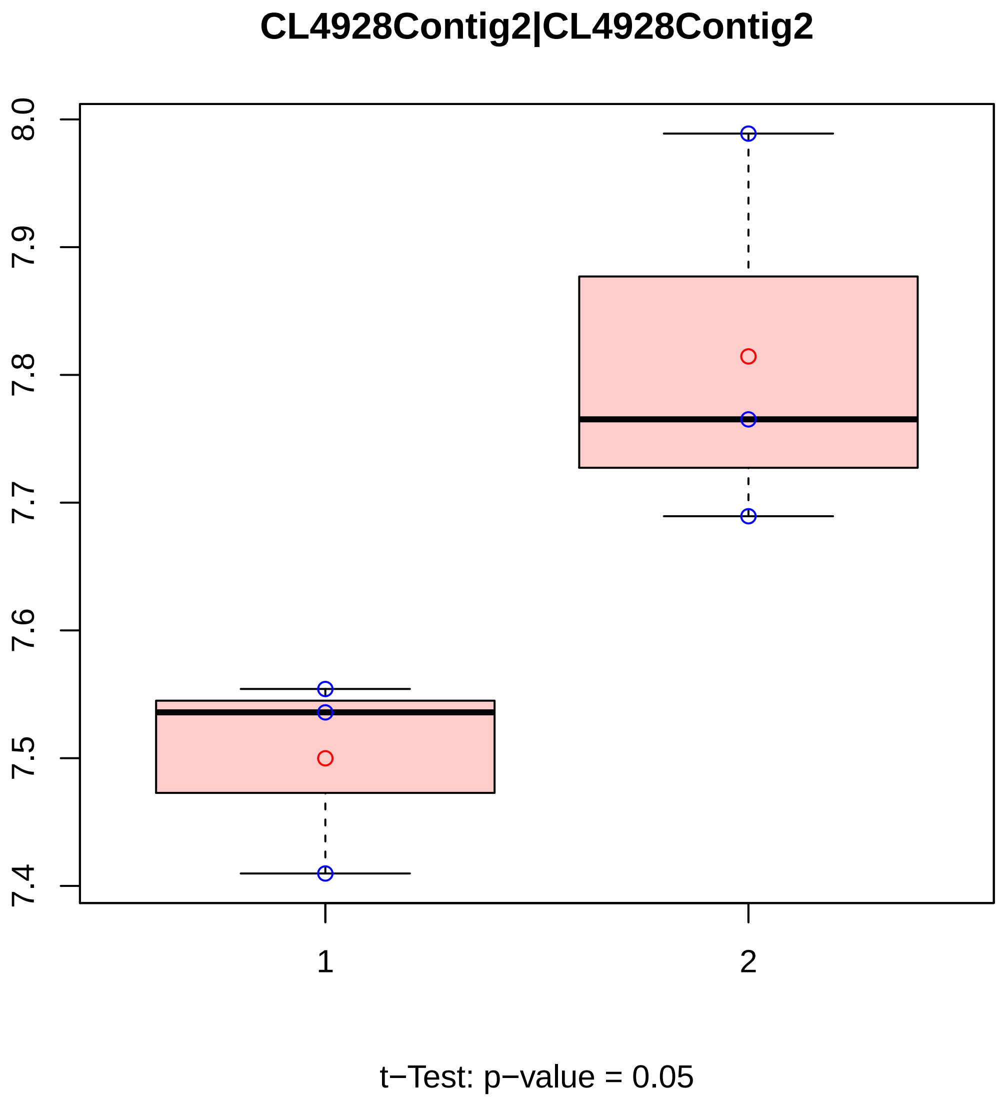
CL4928Contig2|CL4928Contig2
8.0
7.9
7.8
7.7
7.6
7.5
7.4
1
2
t−Test: p−value = 0.05

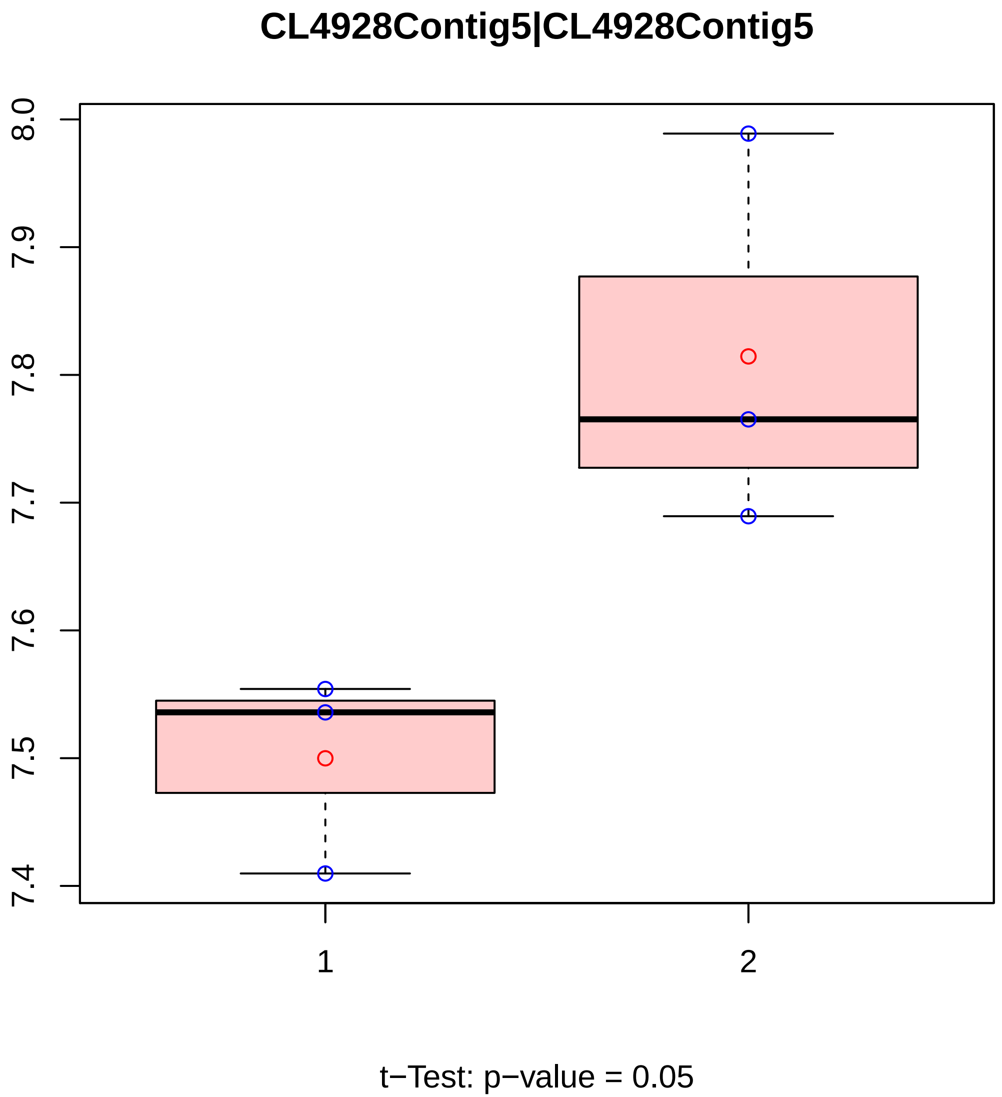
CL4928Contig5|CL4928Contig5
8.0
7.9
7.8
7.7
7.6
7.5
7.4
1
2
t−Test: p−value = 0.05

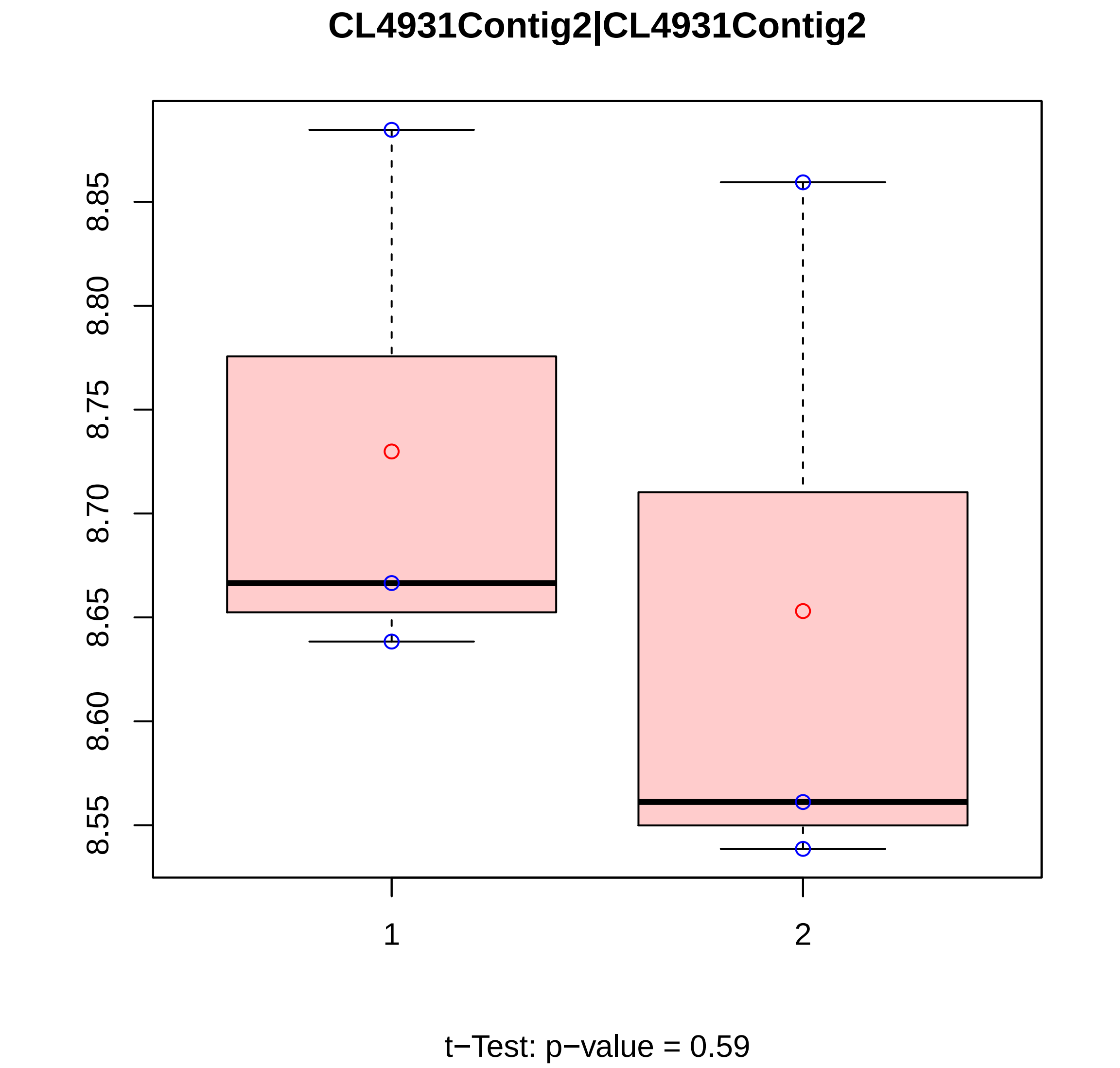

CL4931Contig2|CL4931Contig2
8.55
8.60
8.65
8.70
8.75
8.80
8.85
1
2
t−Test: p−value = 0.59

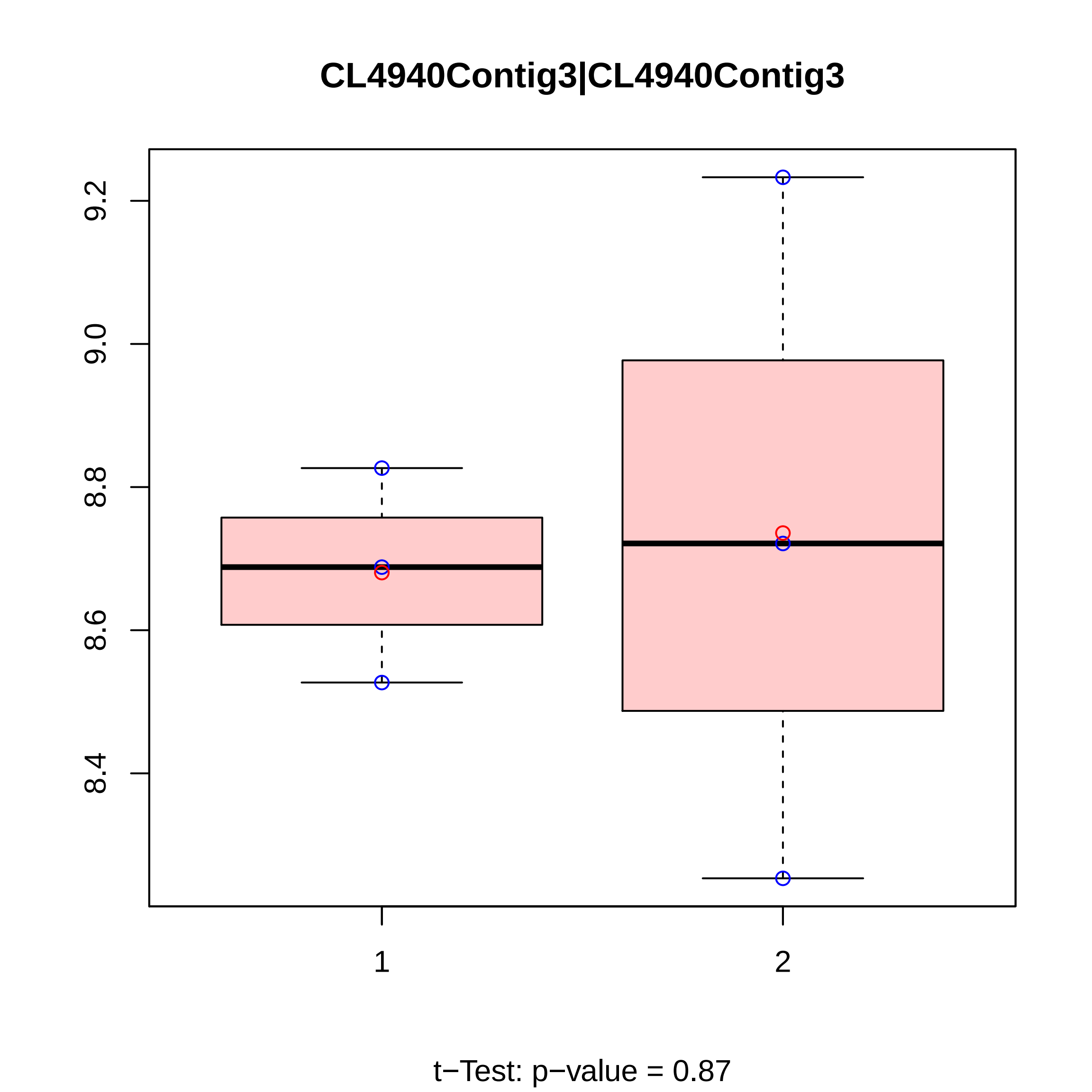

CL4940Contig3|CL4940Contig3
9.2
9.0
8.8
8.6
8.4
1
2
t−Test: p−value = 0.87

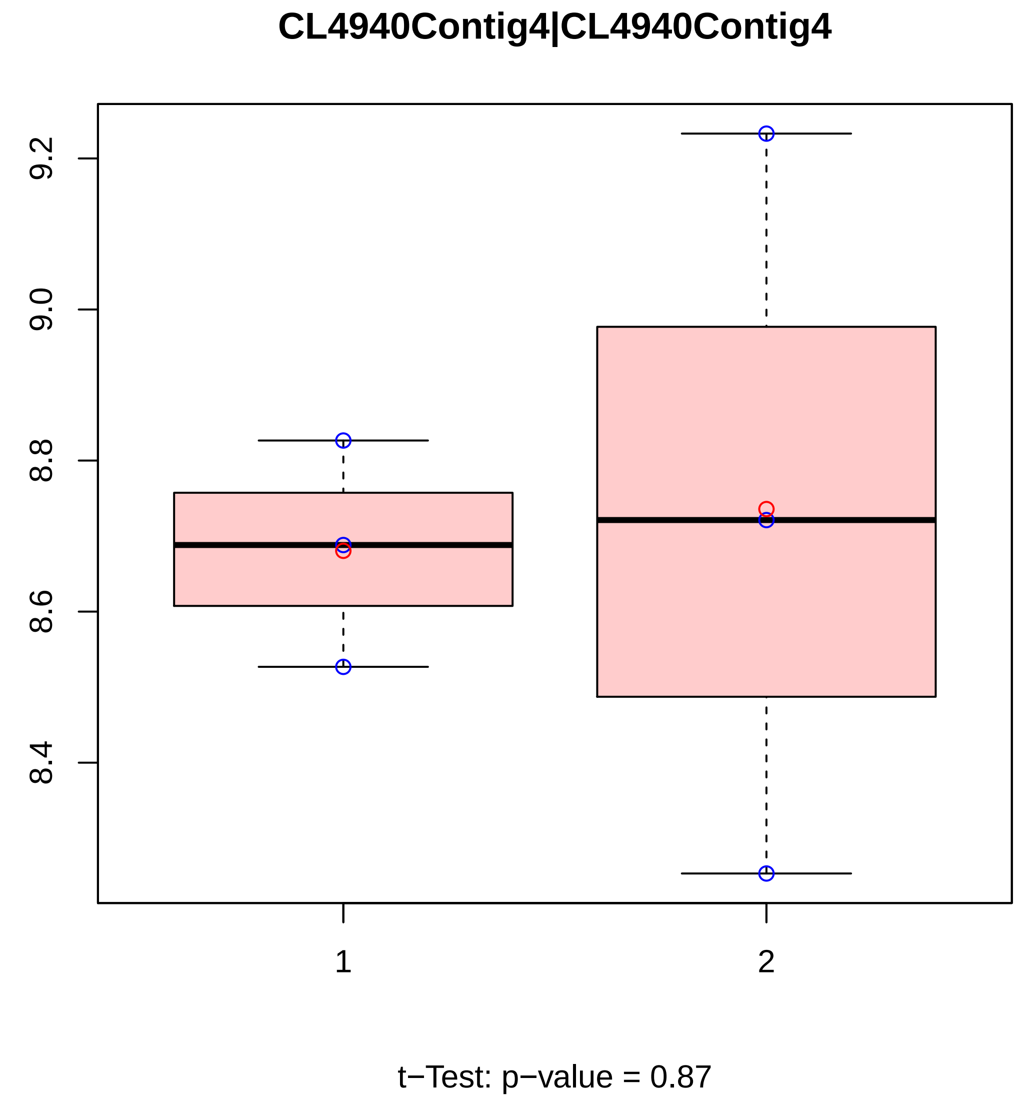
CL4940Contig4|CL4940Contig4
9.2
9.0
8.8
8.6
8.4
1
2
t−Test: p−value = 0.87

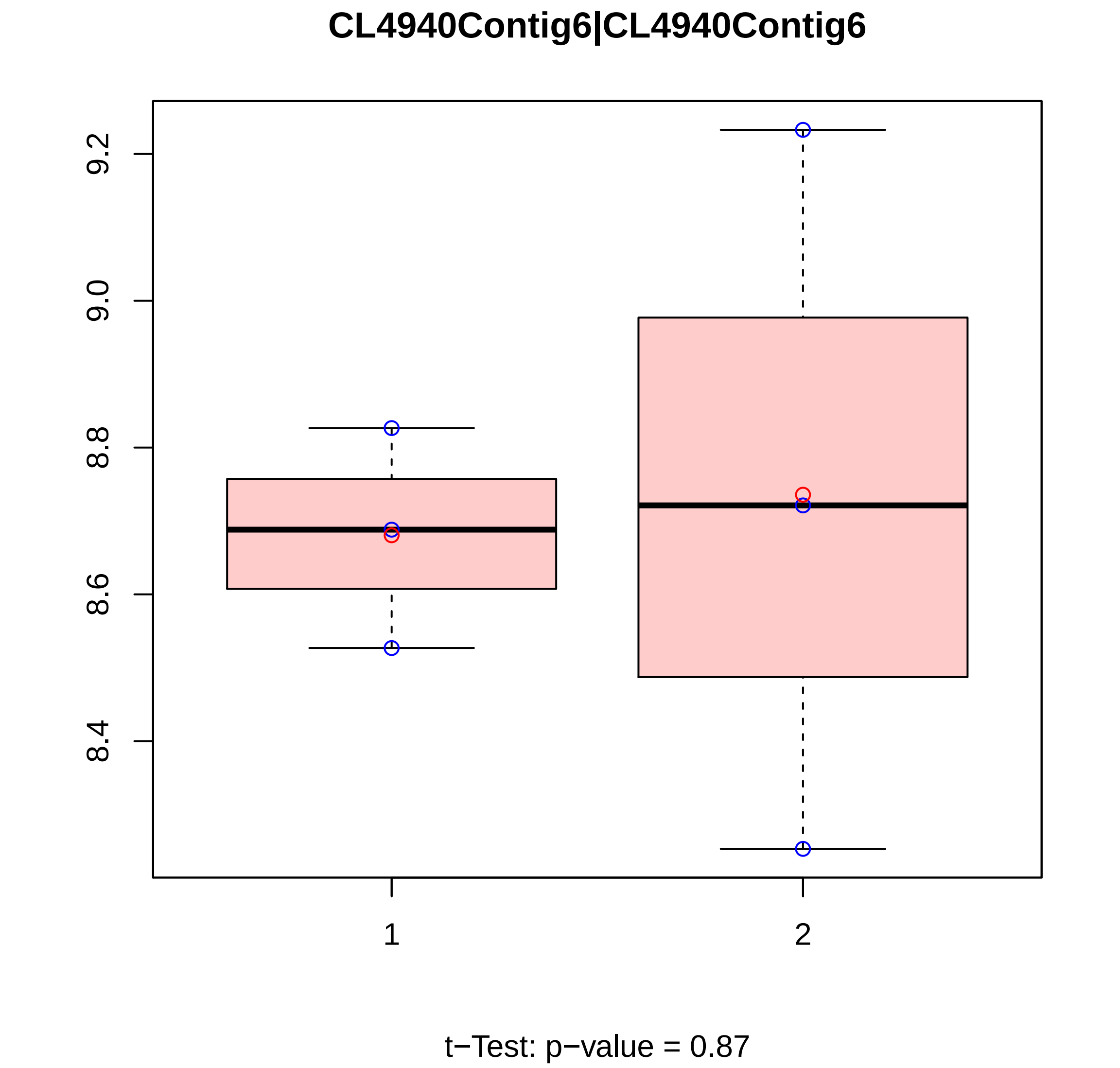
CL4940Contig6|CL4940Contig6
9.2
9.0
8.8
8.6
8.4
1
2
t−Test: p−value = 0.87

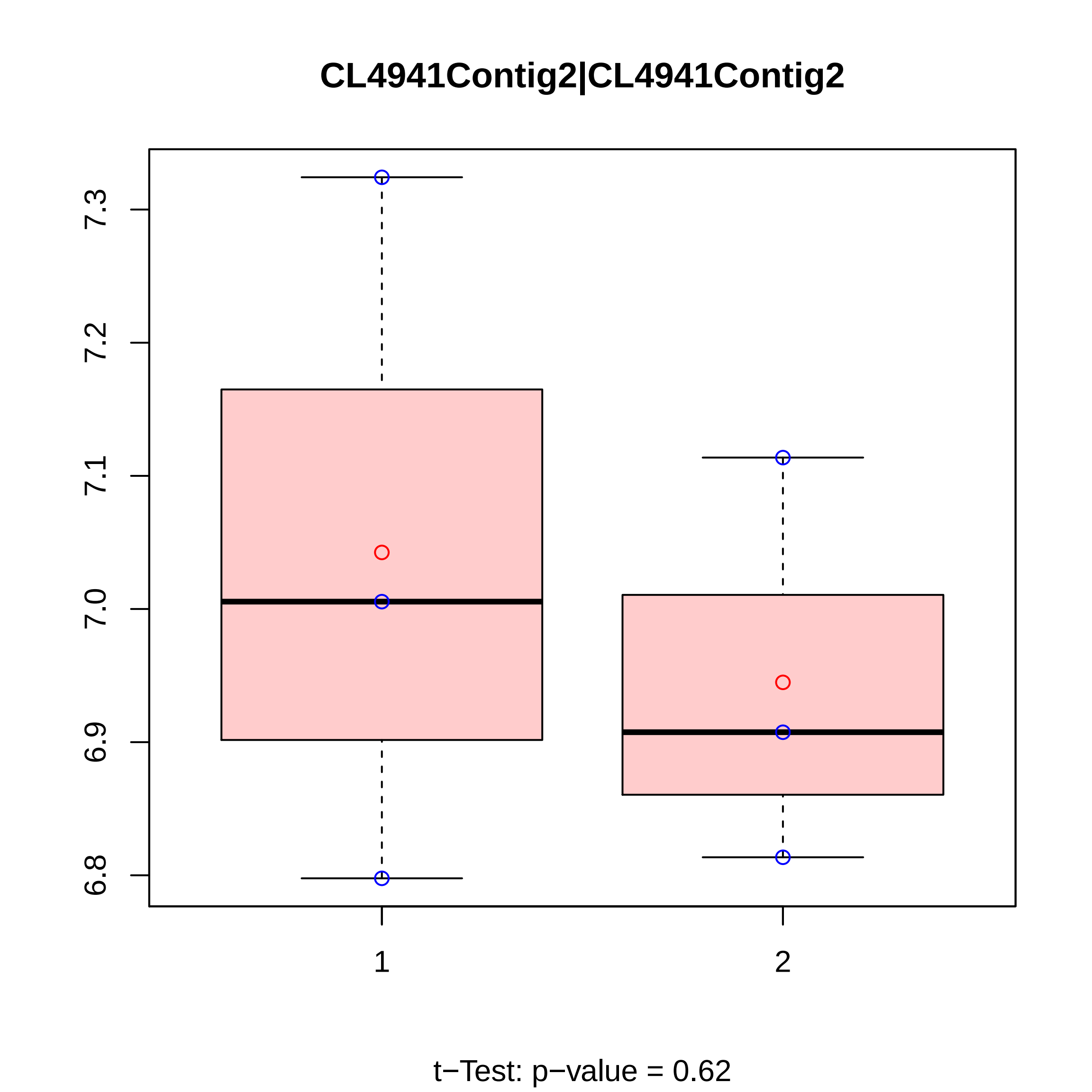
CL4941Contig2|CL4941Contig2
7.3
7.2
7.1
7.0
6.9
6.8
1
2
t−Test: p−value = 0.62

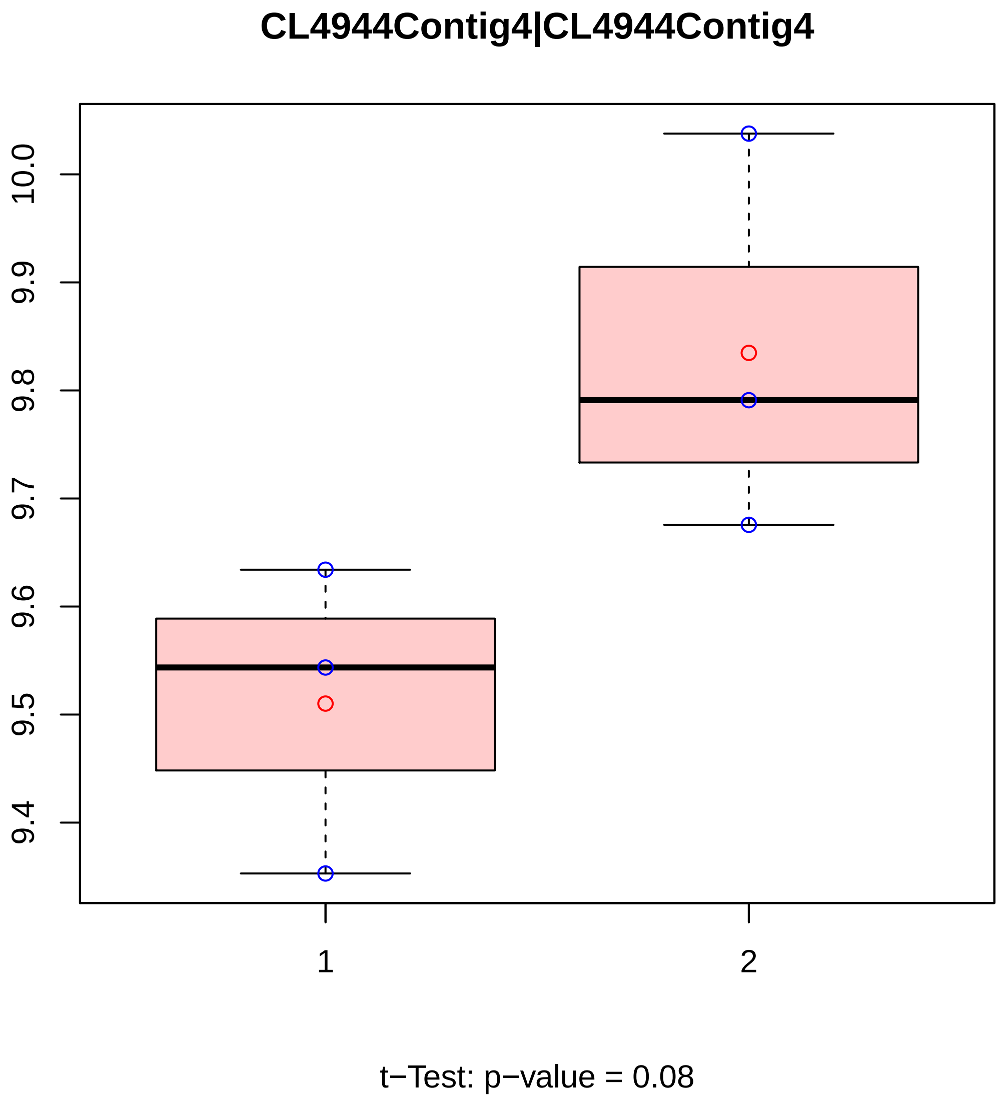
CL4944Contig4|CL4944Contig4
10.0
9.9
9.8
9.7
9.6
9.5
9.4
1
2
t−Test: p−value = 0.08

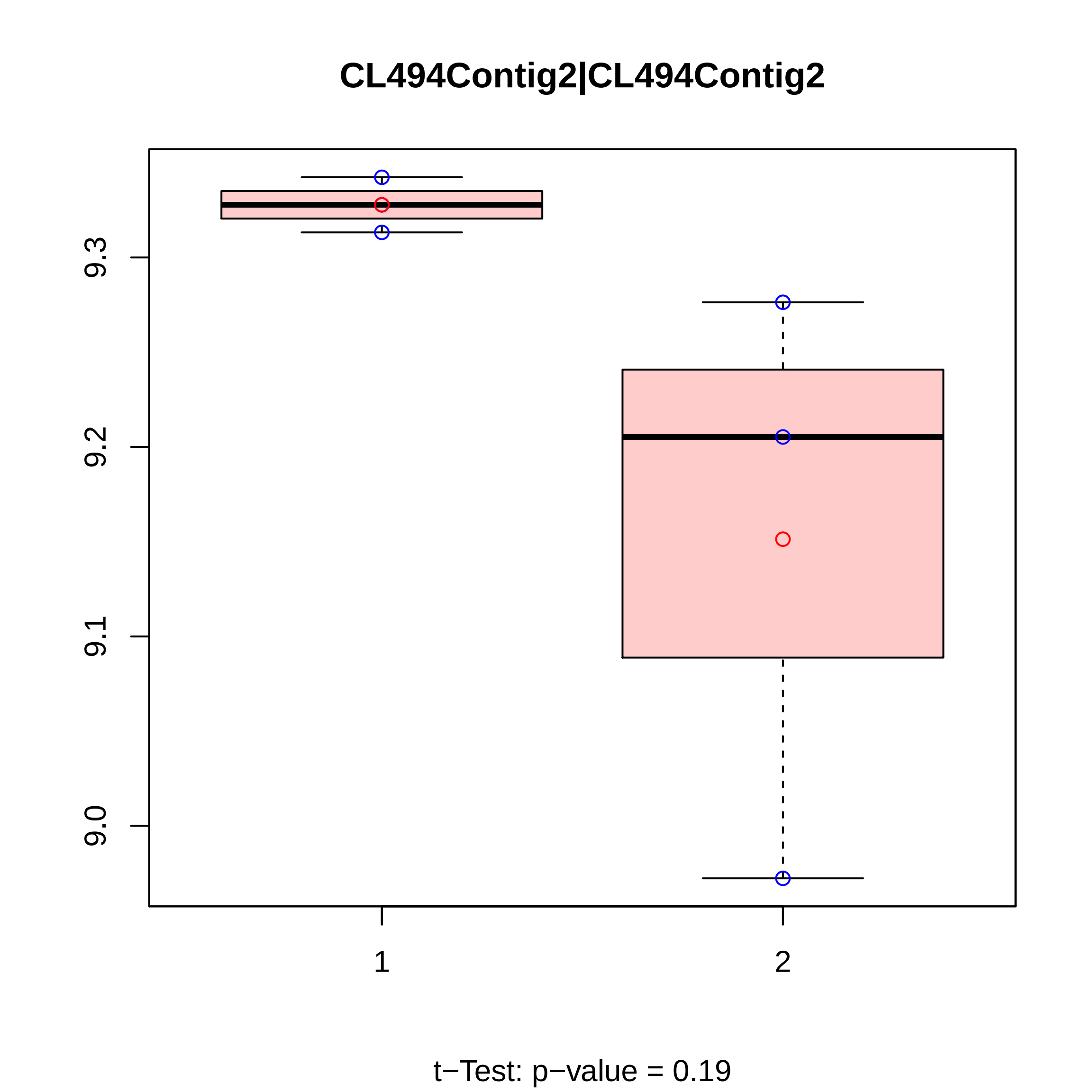

CL494Contig2|CL494Contig2
9.0
9.1
9.2
9.3
1
2
t−Test: p−value = 0.19

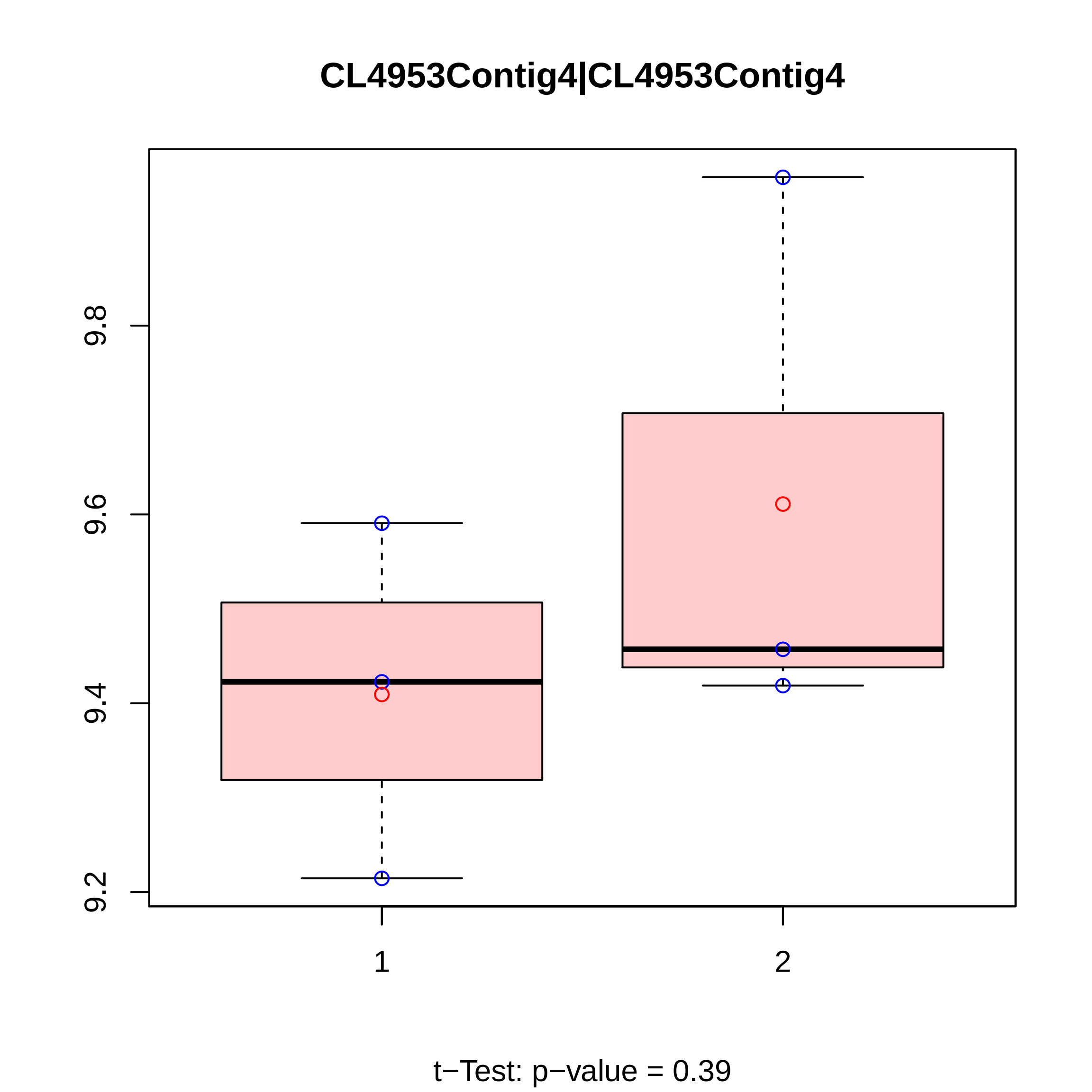
CL4953Contig4|CL4953Contig4
9.2
9.4
9.6
9.8
1
2
t−Test: p−value = 0.39

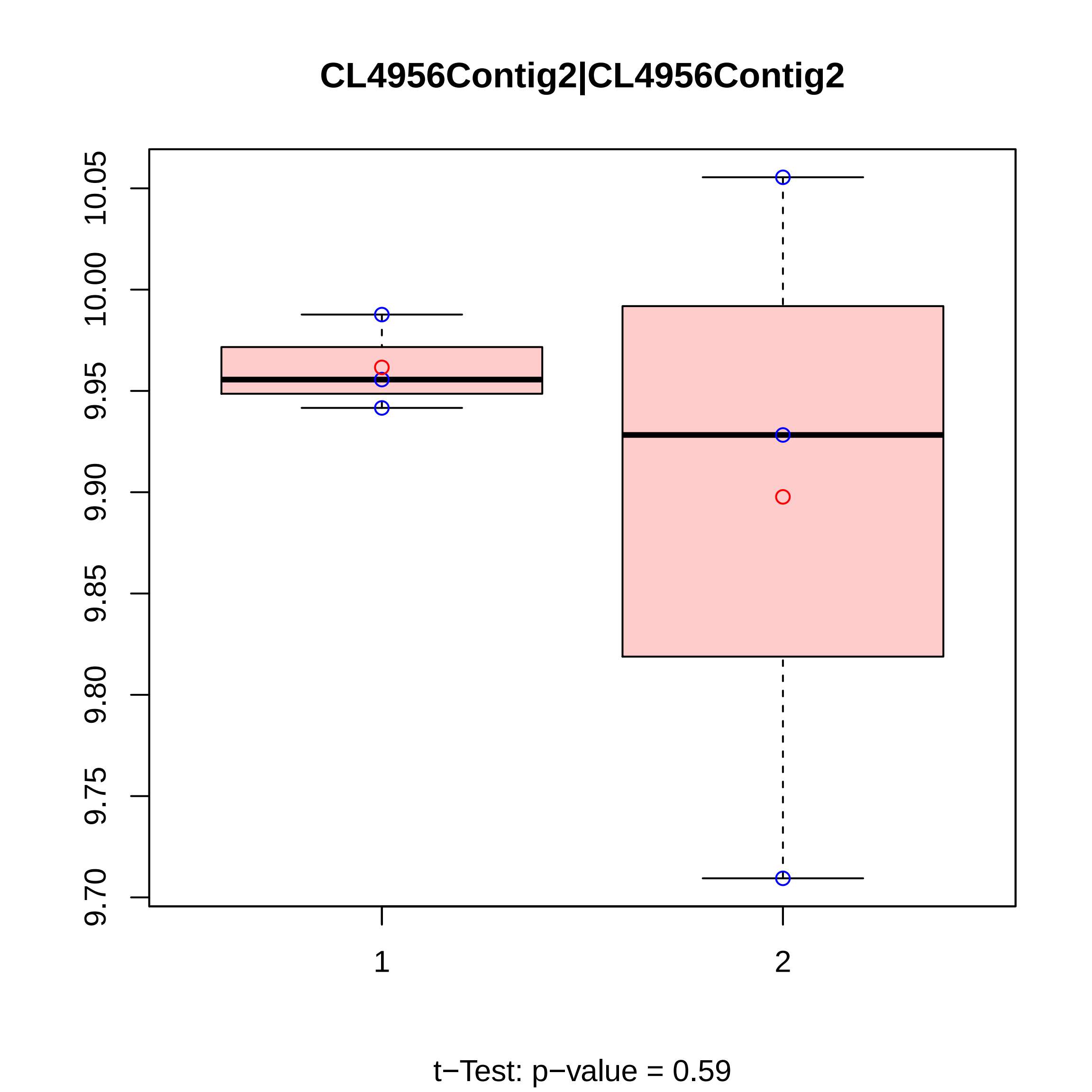

CL4956Contig2|CL4956Contig2
10.05
10.00
9.95
9.90
9.85
9.80
9.75
9.70
1
2
t−Test: p−value = 0.59

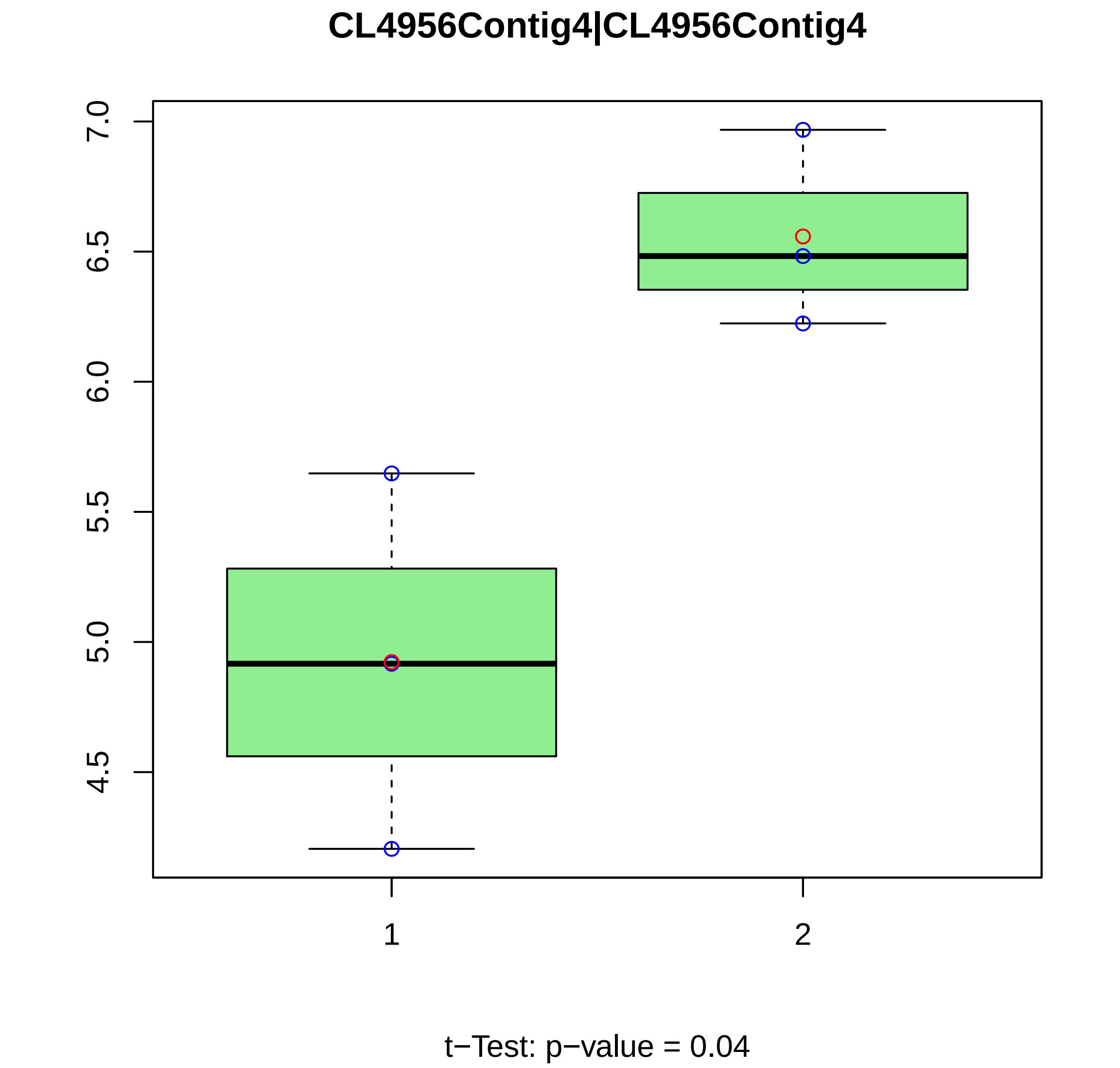

CL4956Contig4|CL4956Contig4
7.0
6.5
6.0
5.5
5.0
4.5
1
2
t−Test: p−value = 0.04

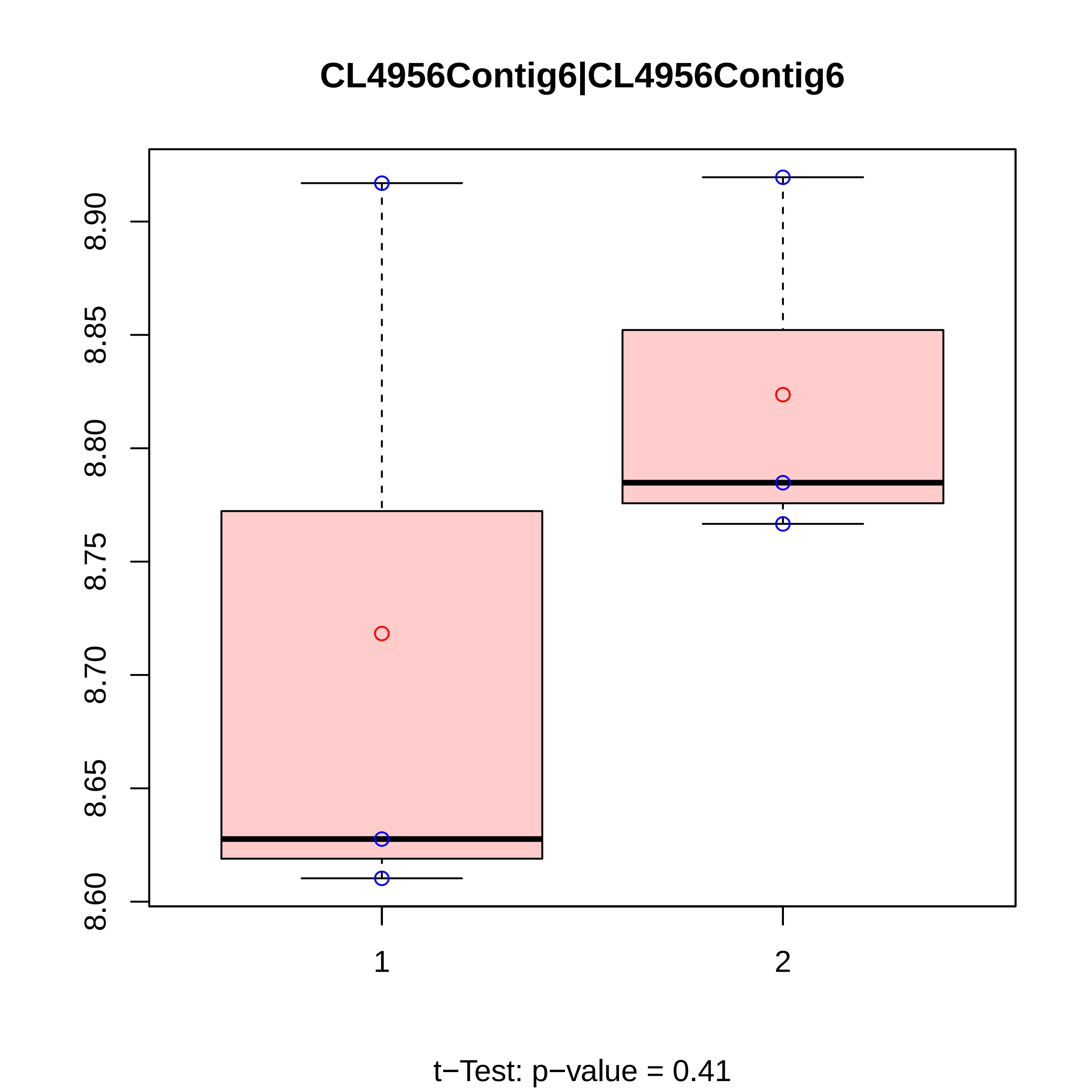
CL4956Contig6|CL4956Contig6
8.60
8.65
8.70
8.75
8.80
8.85
8.90
1
2
t−Test: p−value = 0.41

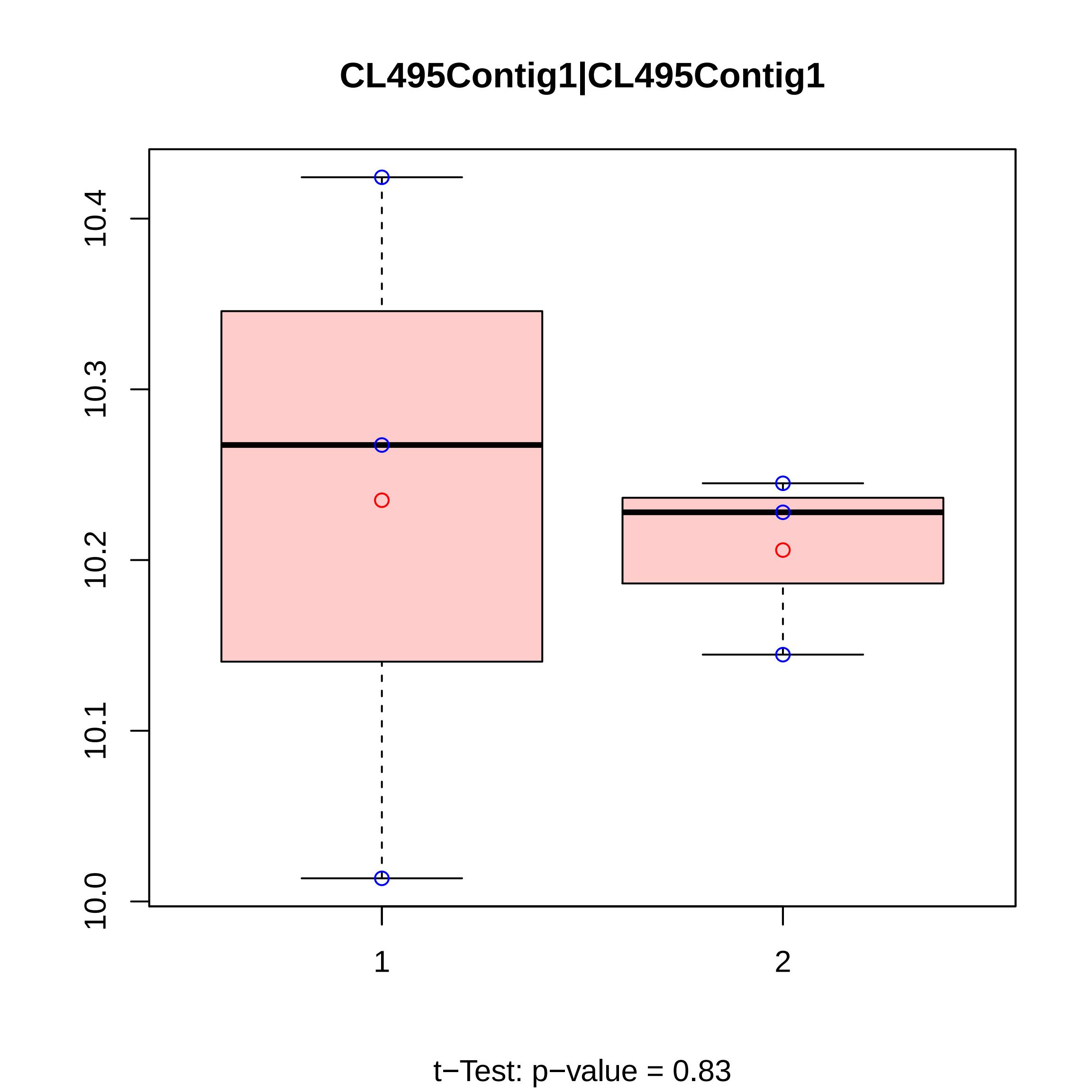

CL495Contig1|CL495Contig1
10.0
10.1
10.2
10.3
10.4
1
2
t−Test: p−value = 0.83

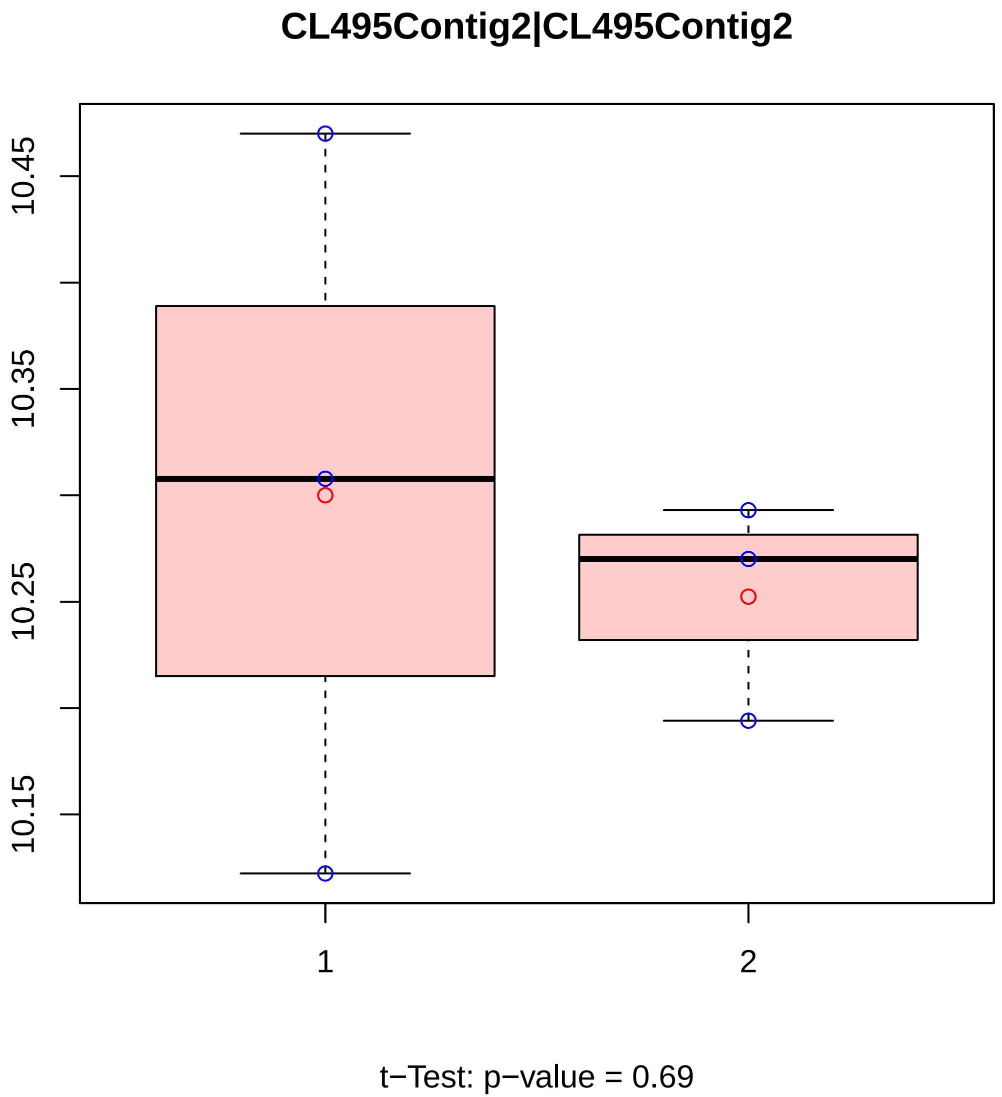

CL495Contig2|CL495Contig2
10.45
10.35
10.25
10.15
1
2
t−Test: p−value = 0.69

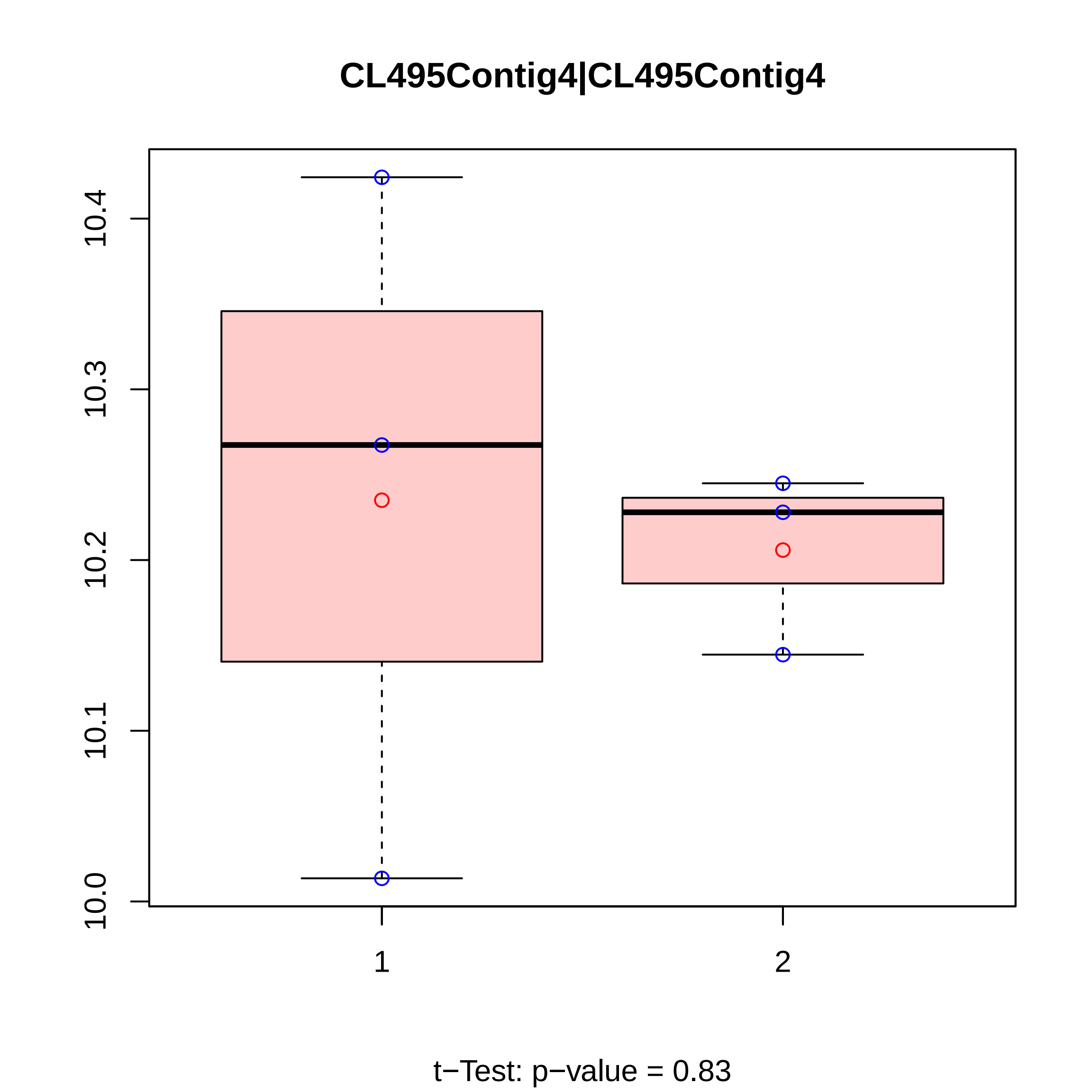

CL495Contig4|CL495Contig4
10.0
10.1
10.2
10.3
10.4
1
2
t−Test: p−value = 0.83

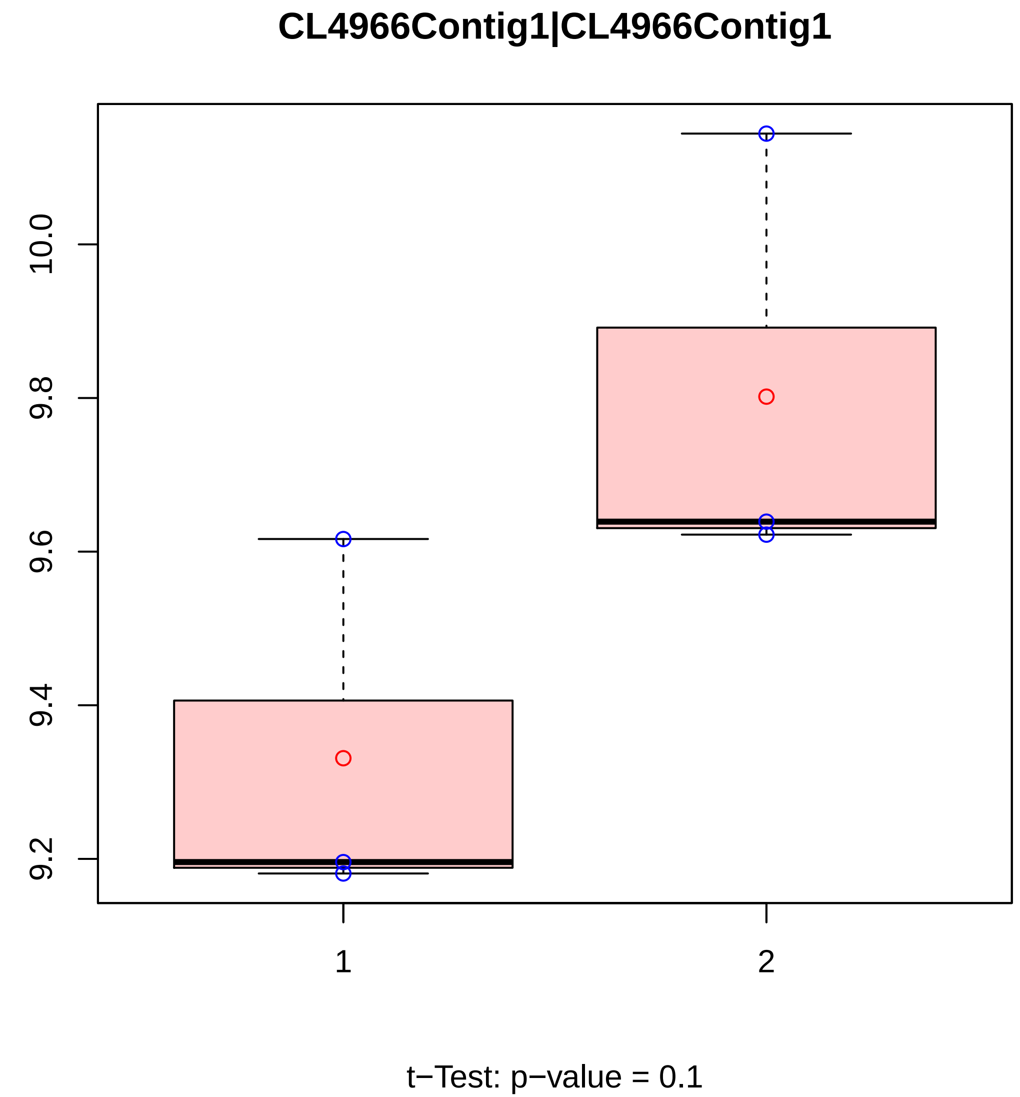

CL4966Contig1|CL4966Contig1
10.0
9.8
9.6
9.4
9.2
1
2
t−Test: p−value = 0.1

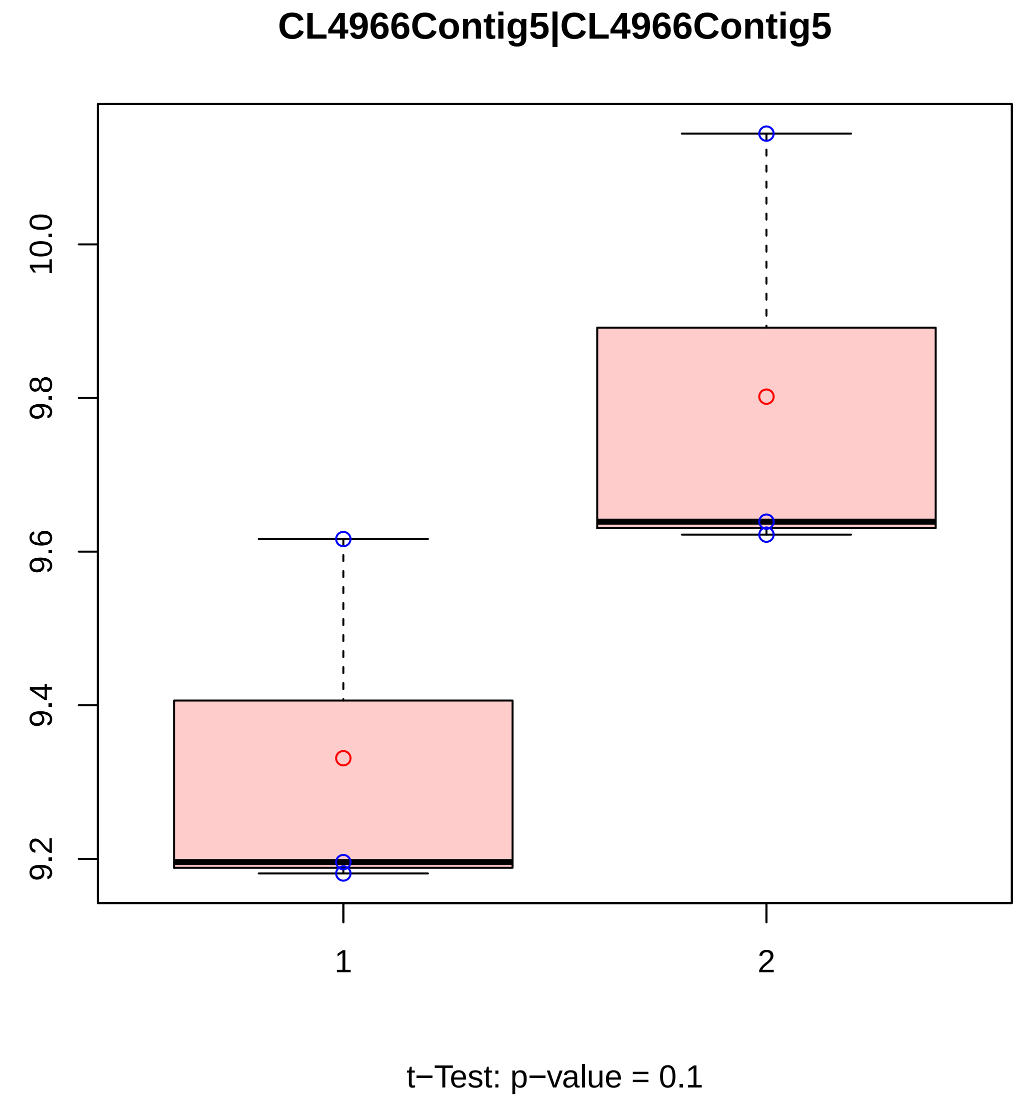
CL4966Contig5|CL4966Contig5
10.0
9.8
9.6
9.4
9.2
1
2
t−Test: p−value = 0.1

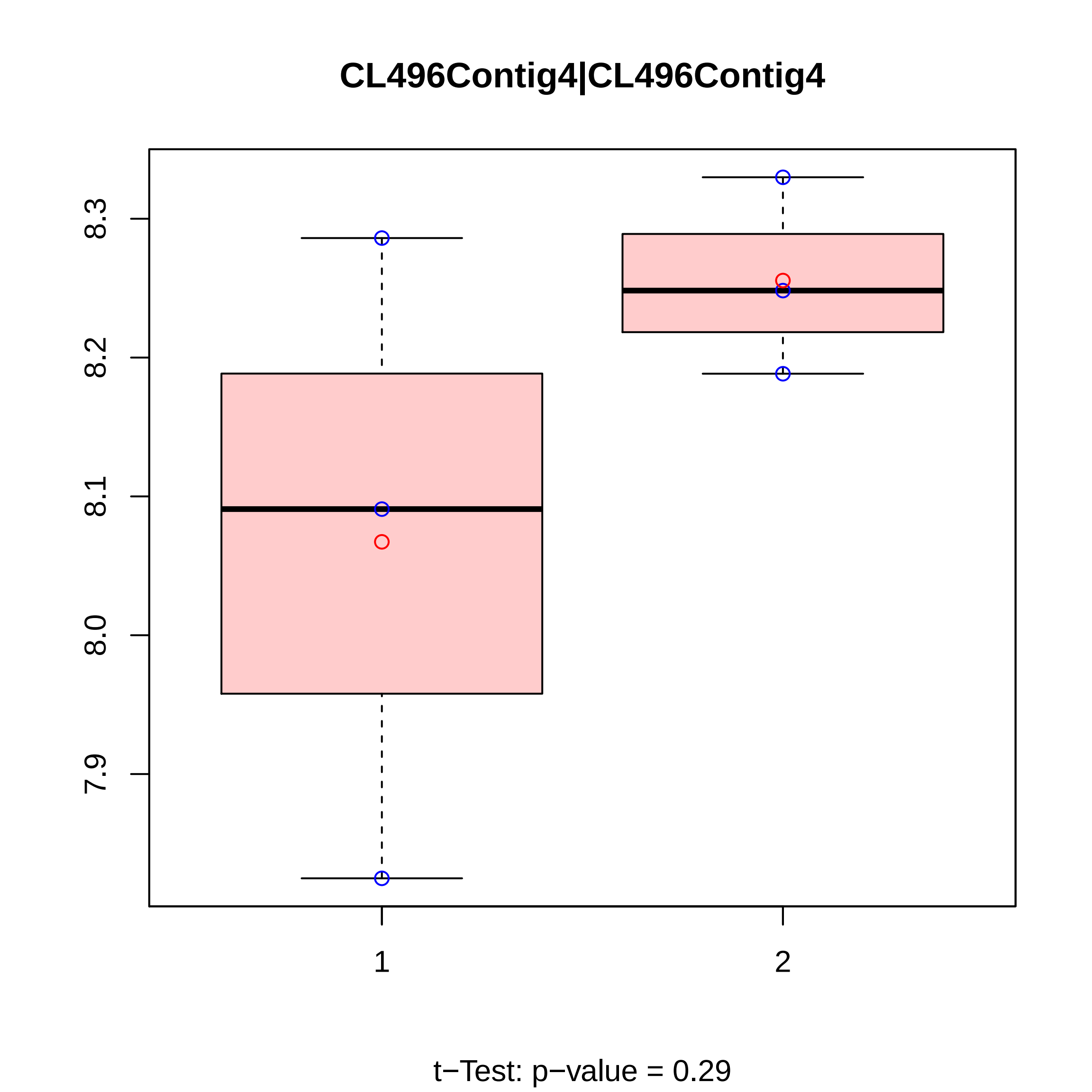
CL496Contig4|CL496Contig4
8.3
8.2
8.1
8.0
7.9
1
2
t−Test: p−value = 0.29

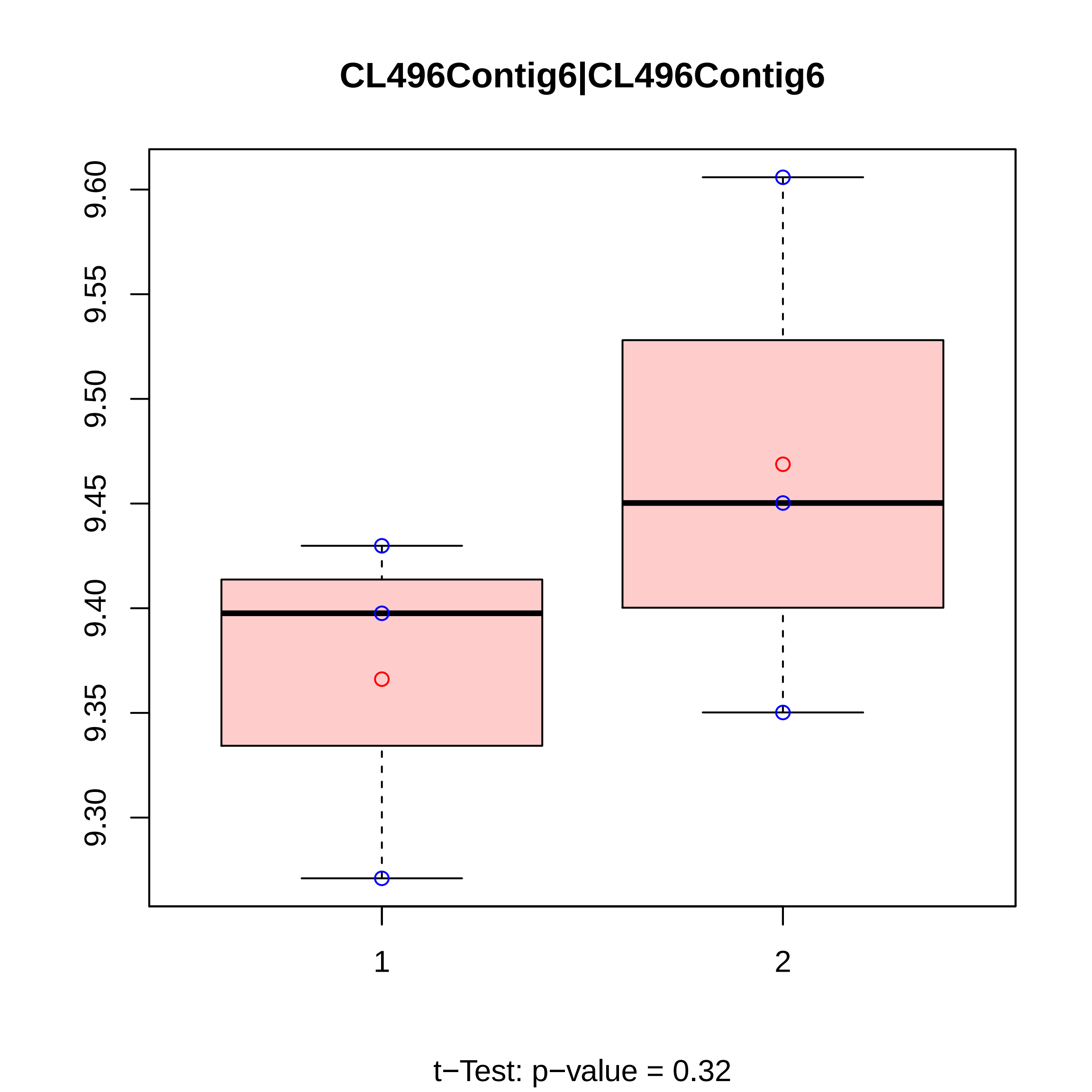

CL496Contig6|CL496Contig6
9.30
9.35
9.40
9.45
9.50
9.55
9.60
1
2
t−Test: p−value = 0.32

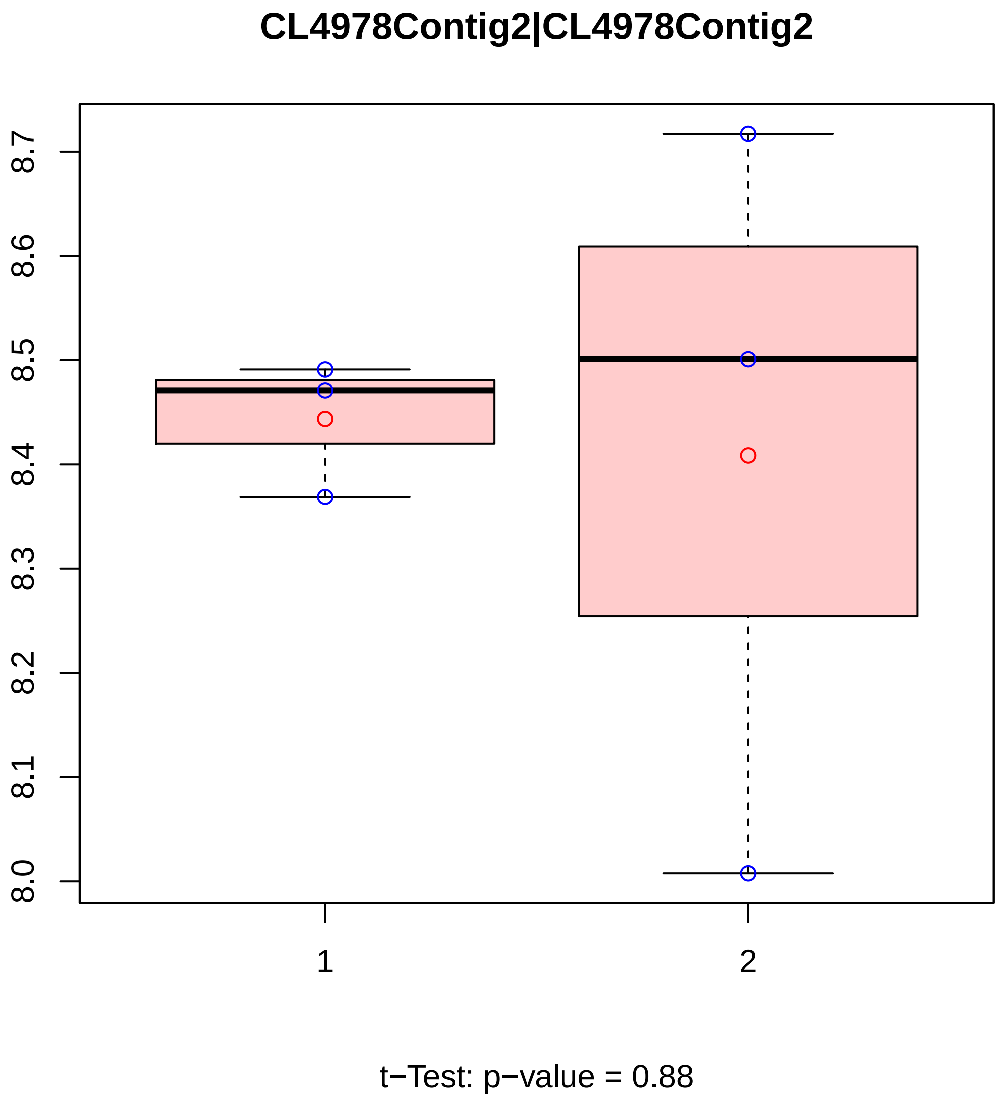
CL4978Contig2|CL4978Contig2
8.0
8.1
8.2
8.3
8.4
8.5
8.6
8.7
1
2
t−Test: p−value = 0.88

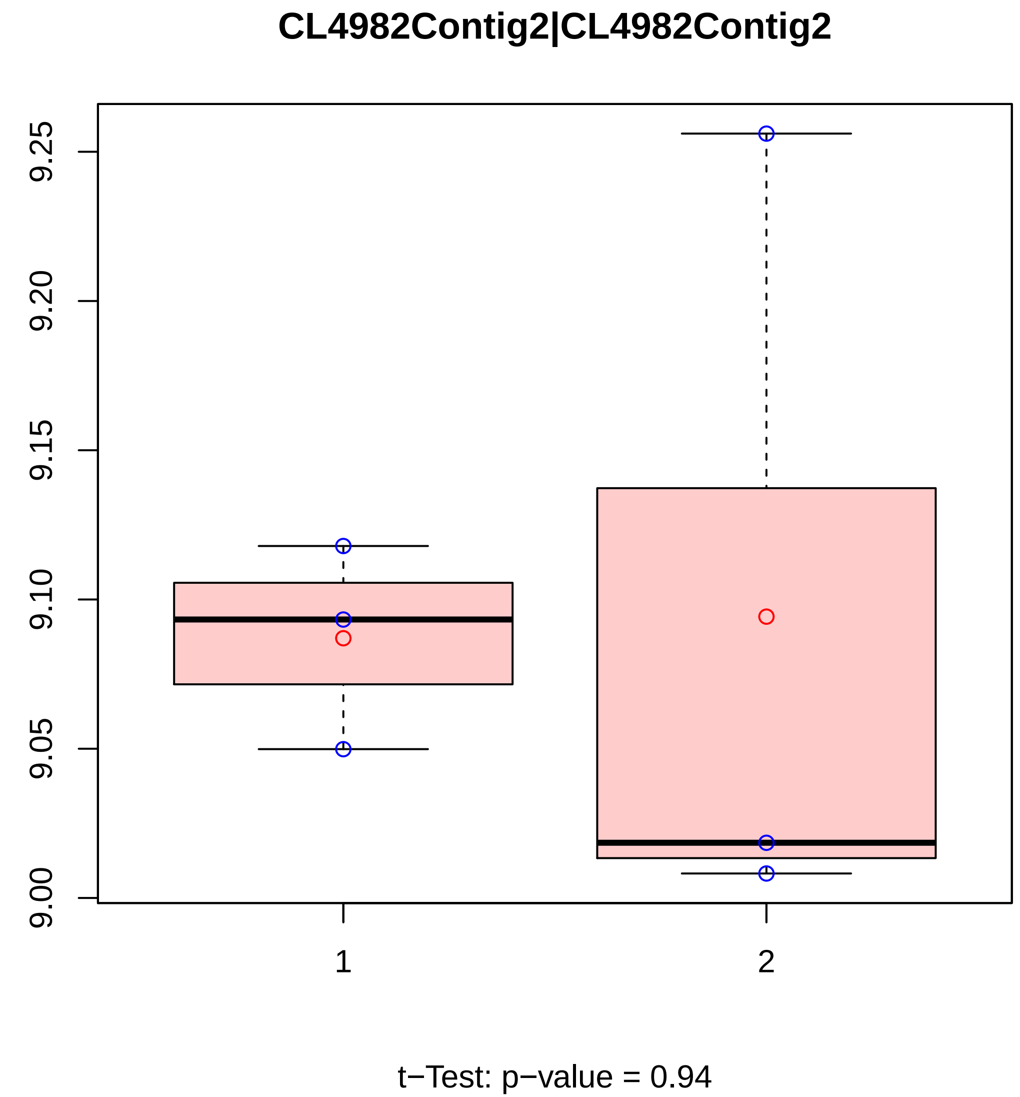

CL4982Contig2|CL4982Contig2
9.00
9.05
9.10
9.15
9.20
9.25
1
2
t−Test: p−value = 0.94

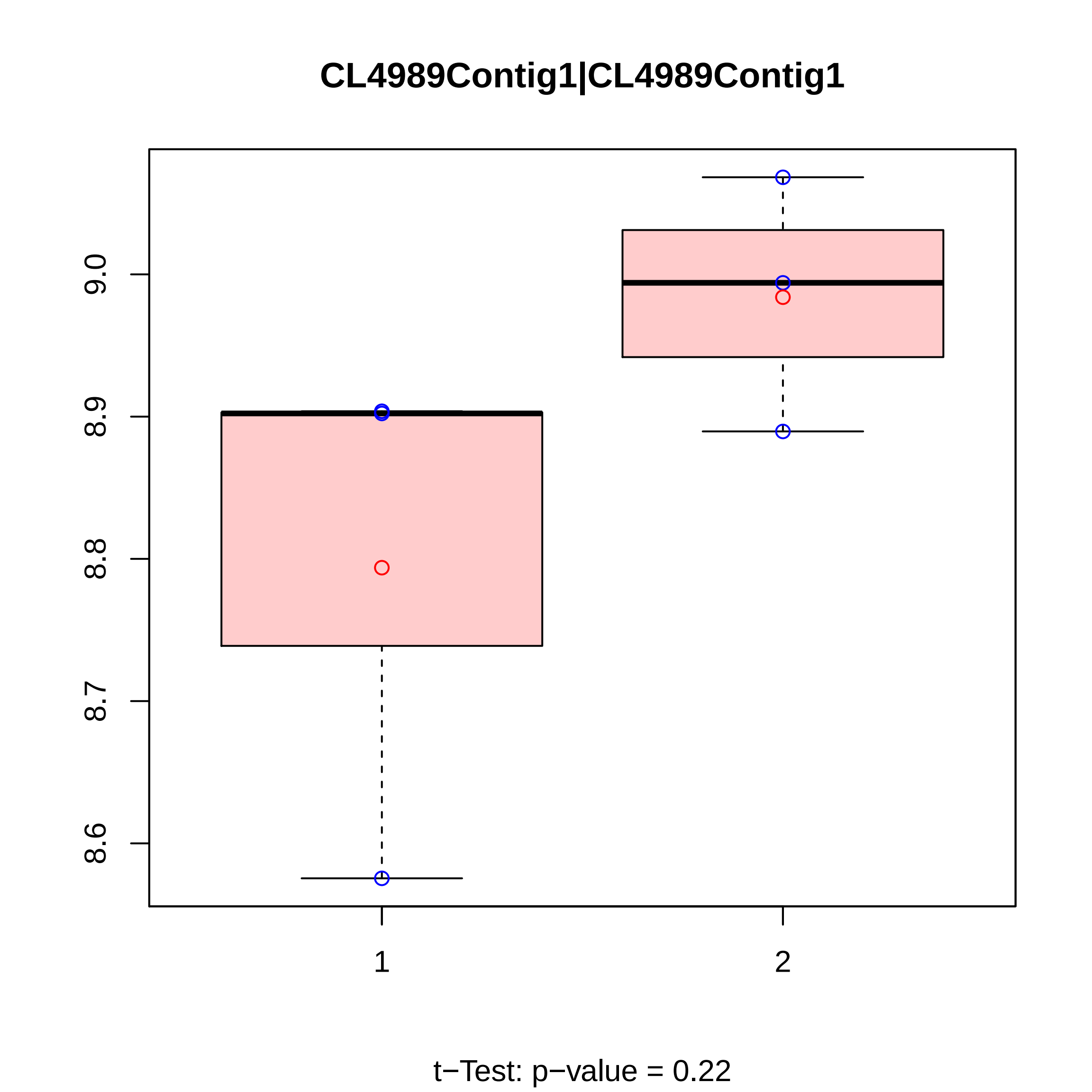

CL4989Contig1|CL4989Contig1
9.0
8.9
8.8
8.7
8.6
1
2
t−Test: p−value = 0.22

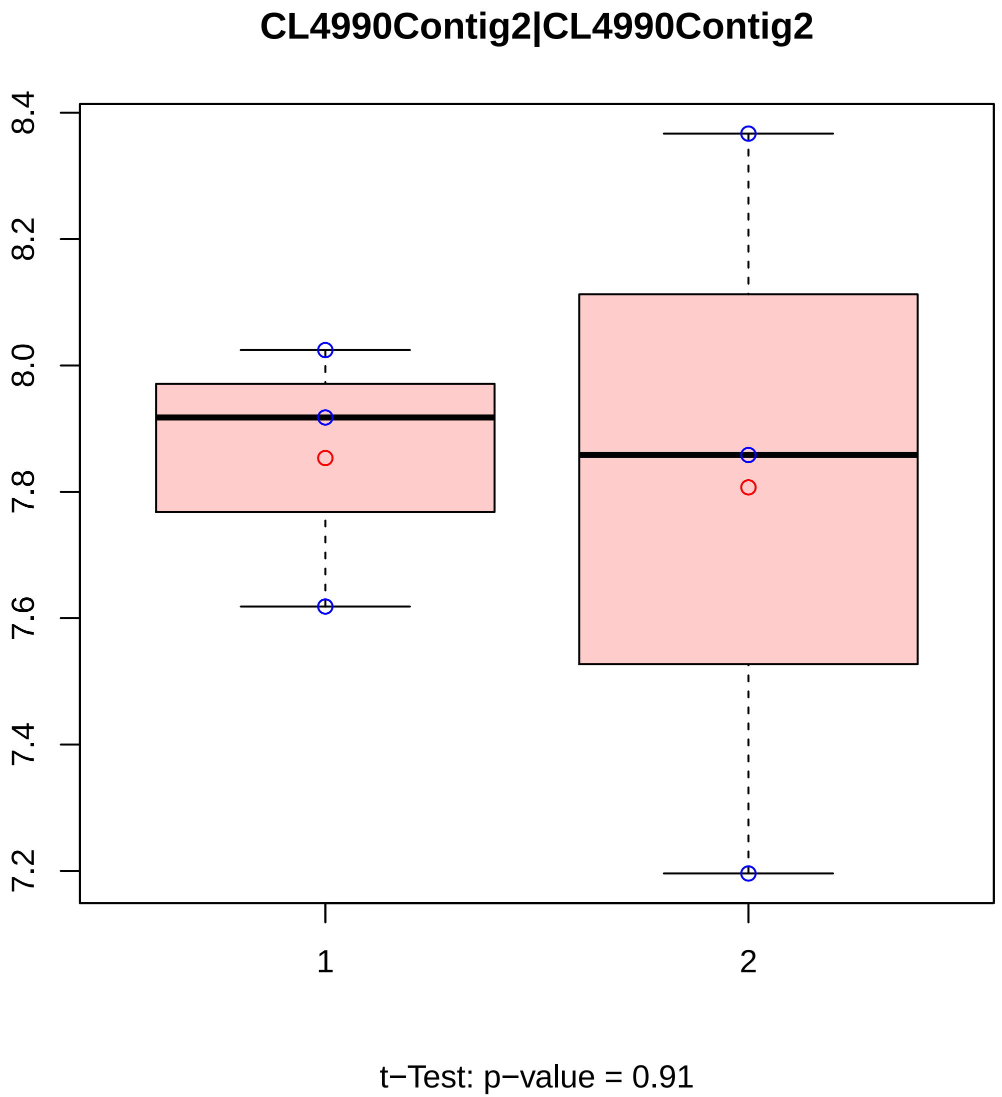
CL4990Contig2|CL4990Contig2
8.4
8.2
8.0
7.8
7.6
7.4
7.2
1
2
t−Test: p−value = 0.91

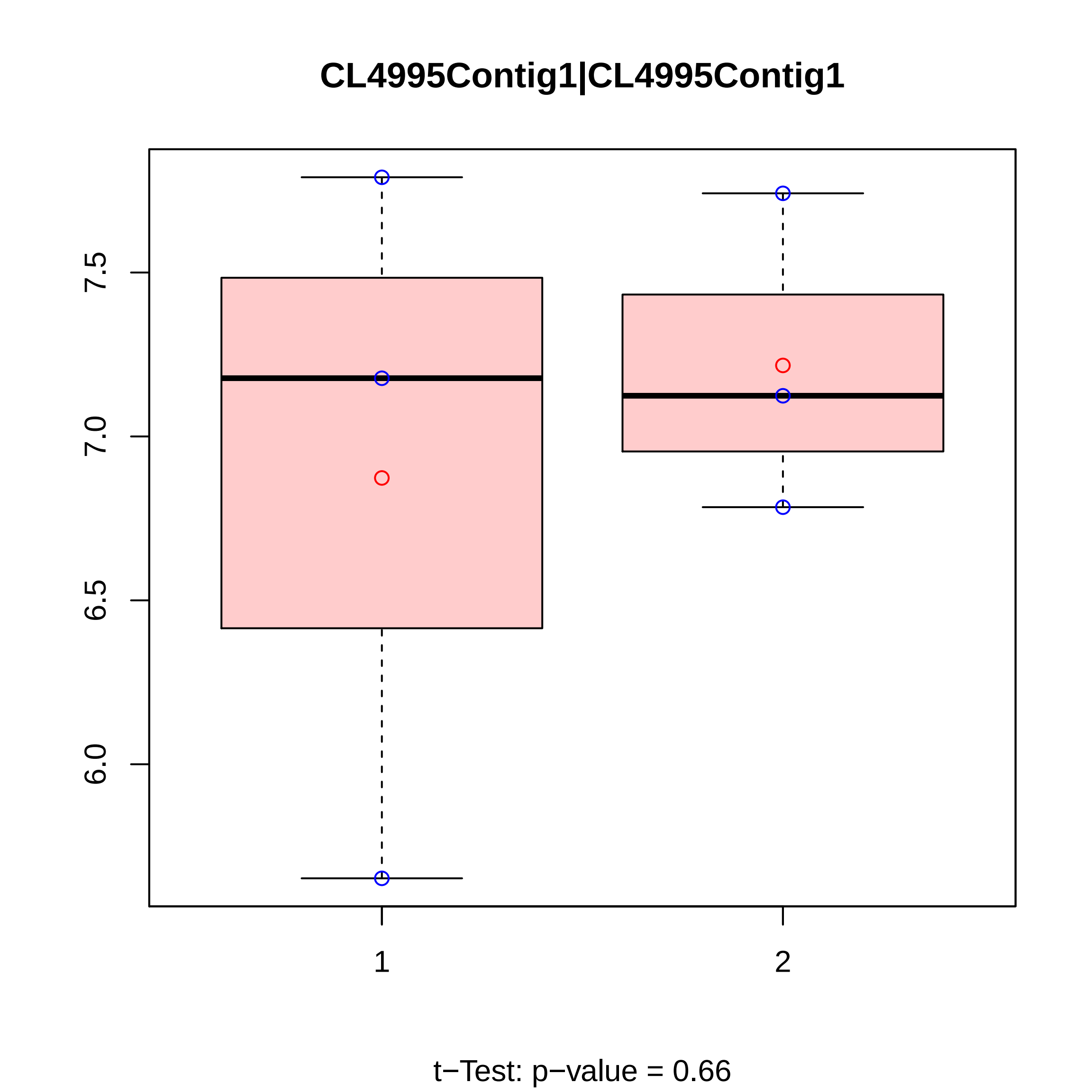

CL4995Contig1|CL4995Contig1
7.5
7.0
6.5
6.0
1
2
t−Test: p−value = 0.66

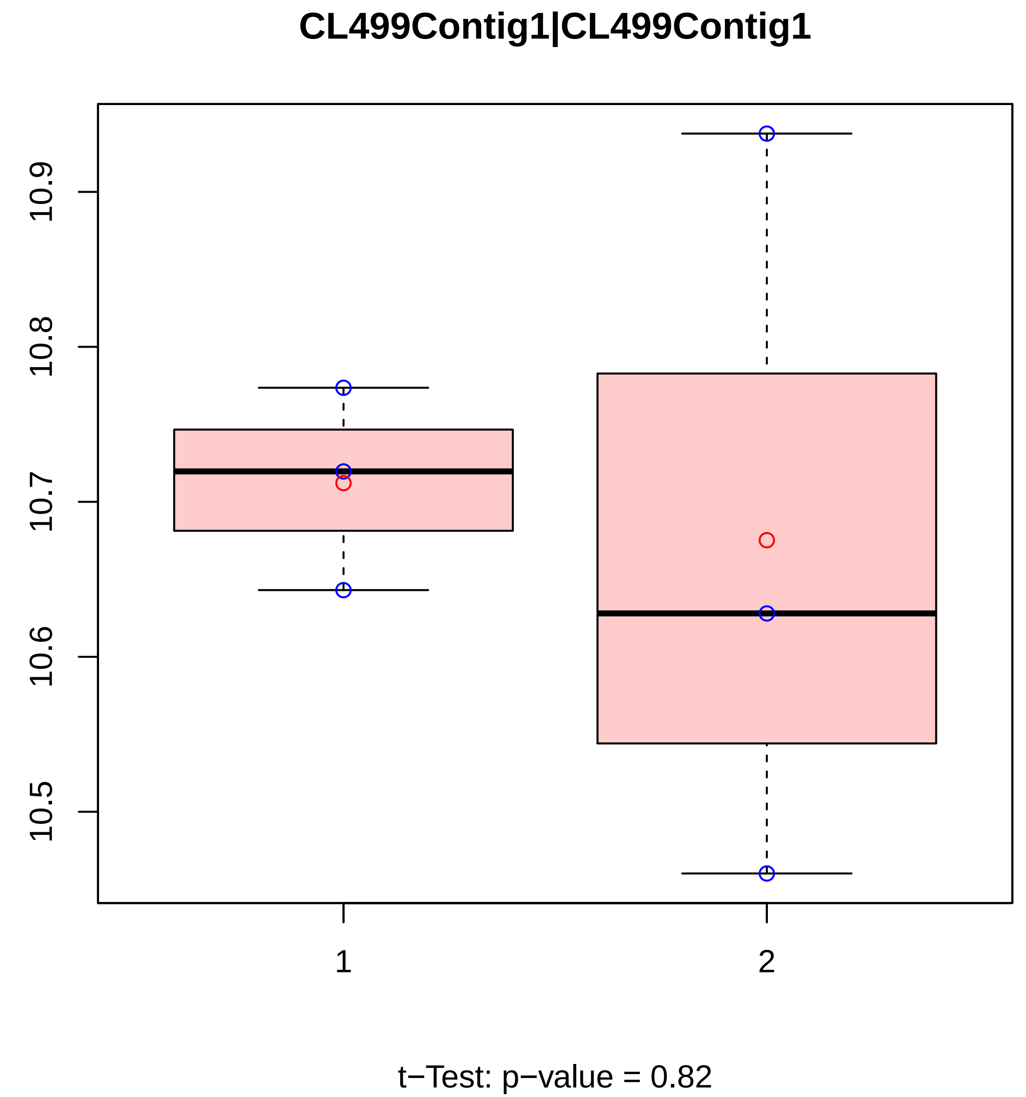
CL499Contig1|CL499Contig1
10.5
10.6
10.7
10.8
10.9
1
2
t−Test: p−value = 0.82

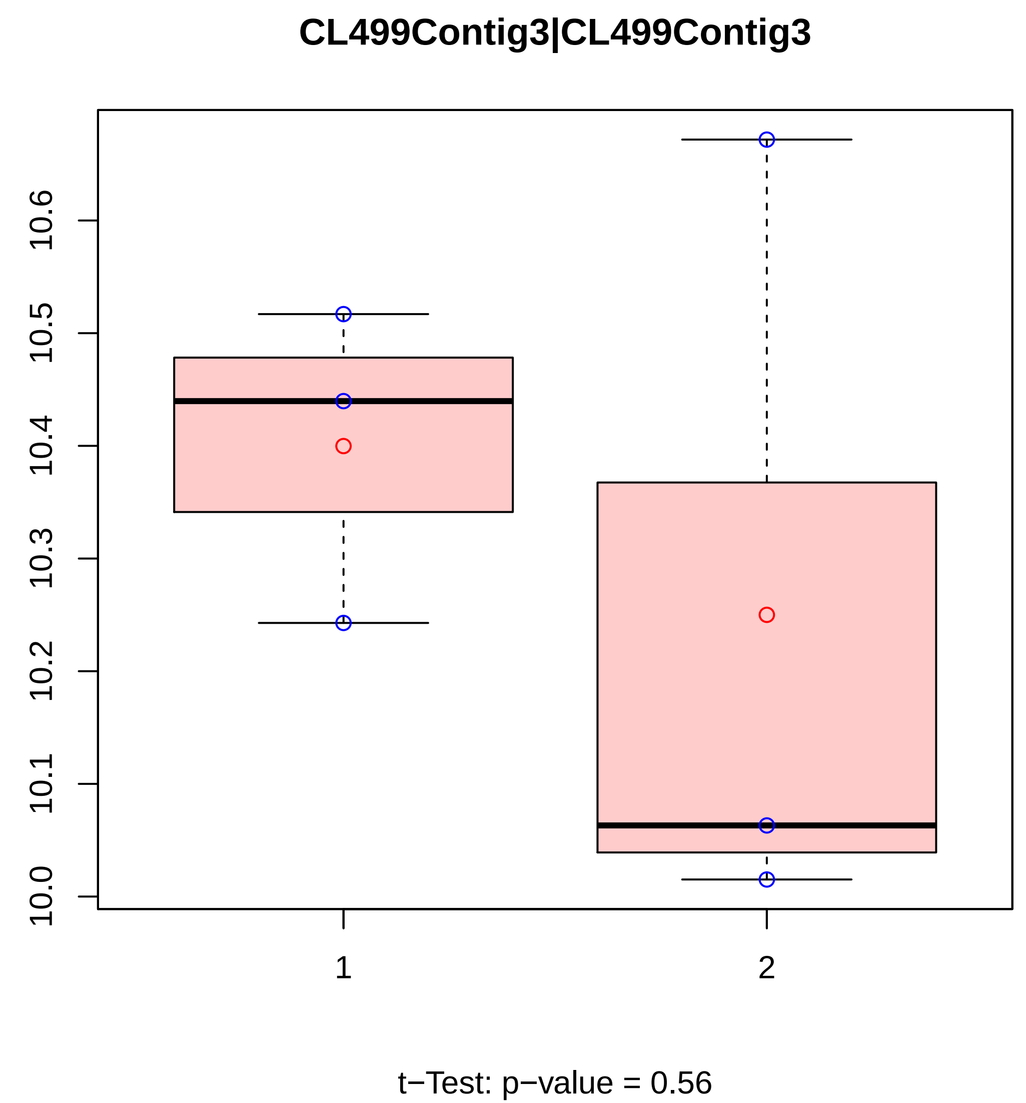
CL499Contig3|CL499Contig3
10.0
10.1
10.2
10.3
10.4
10.5
10.6
1
2
t−Test: p−value = 0.56

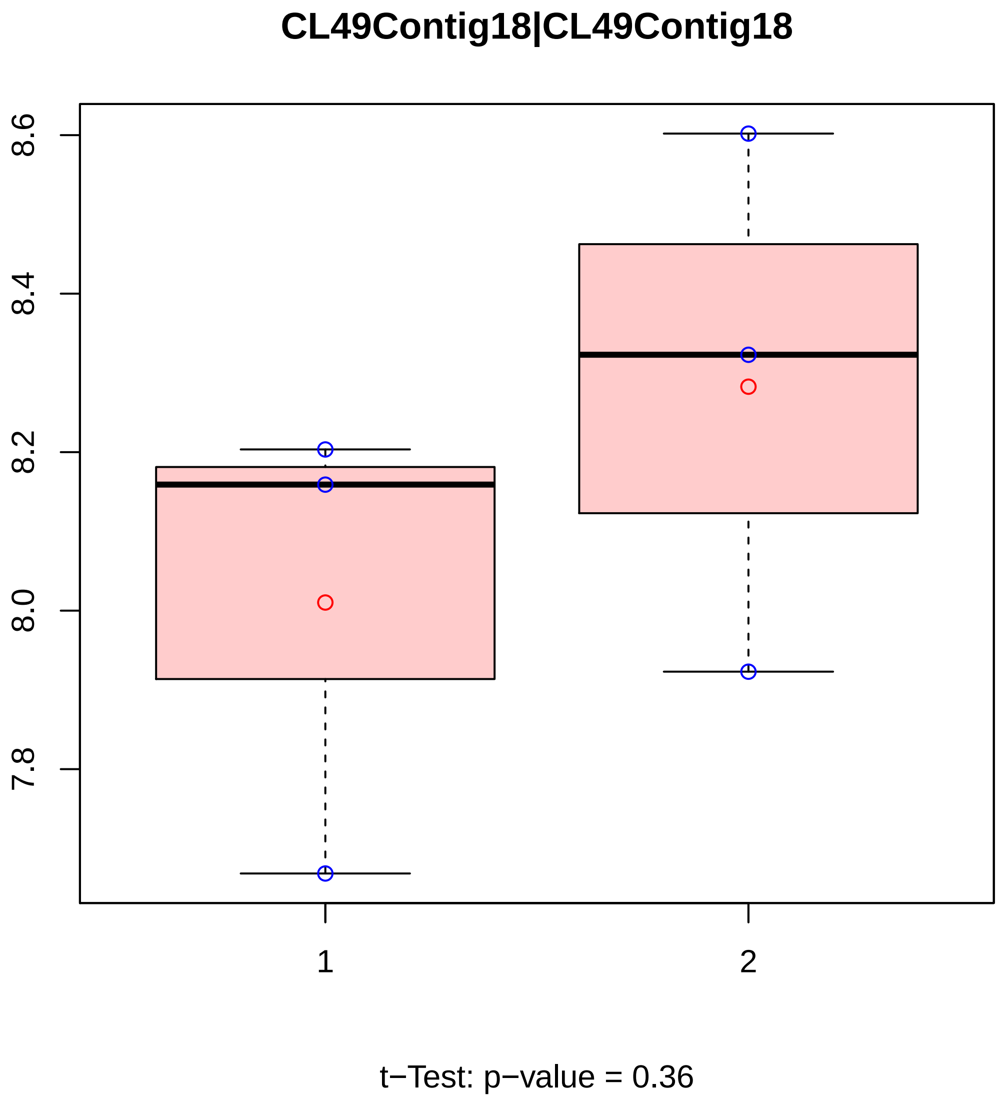
CL49Contig18|CL49Contig18
8.6
8.4
8.2
8.0
7.8
1
2
t−Test: p−value = 0.36

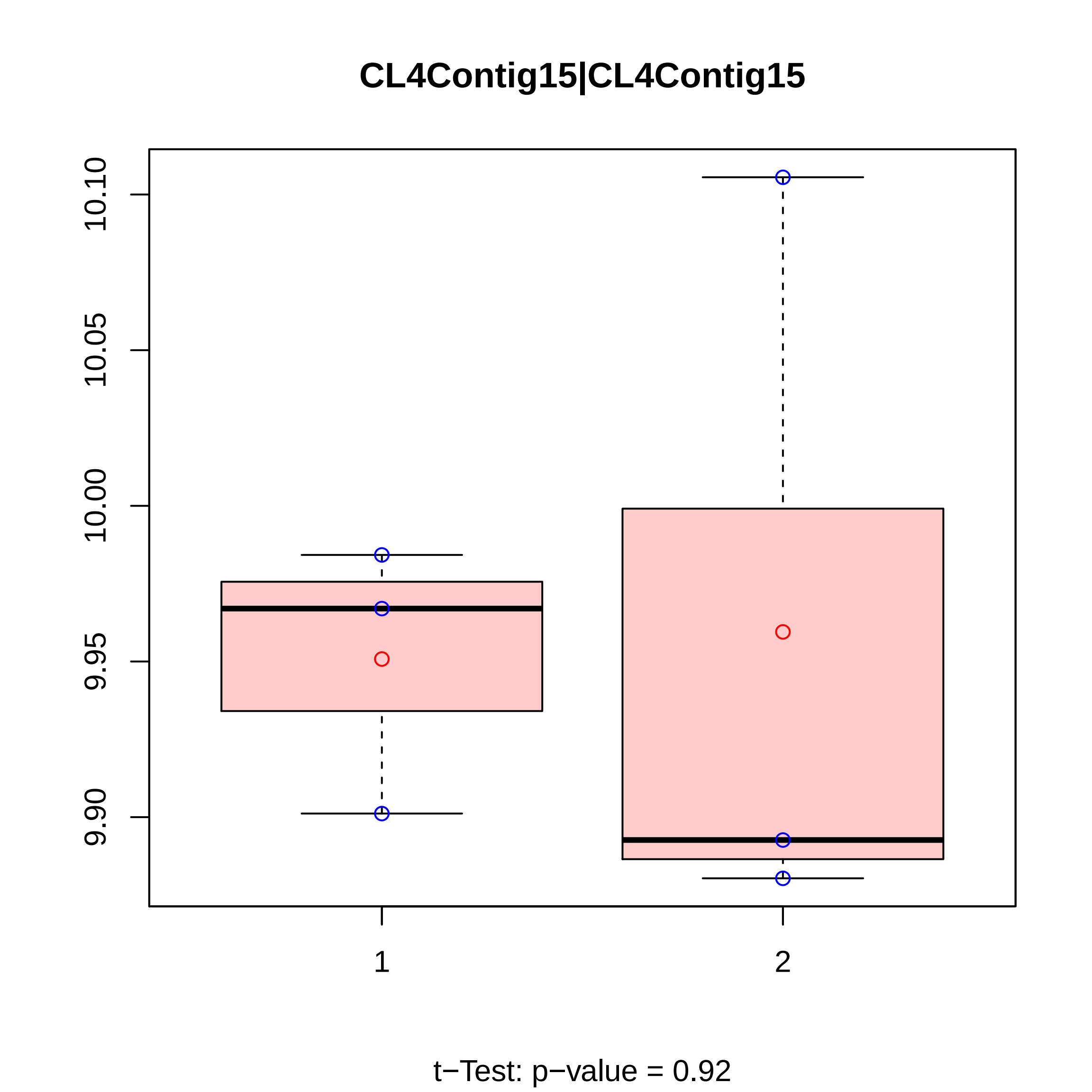

CL4Contig15|CL4Contig15
10.10
10.05
10.00
9.95
9.90
1
2
t−Test: p−value = 0.92

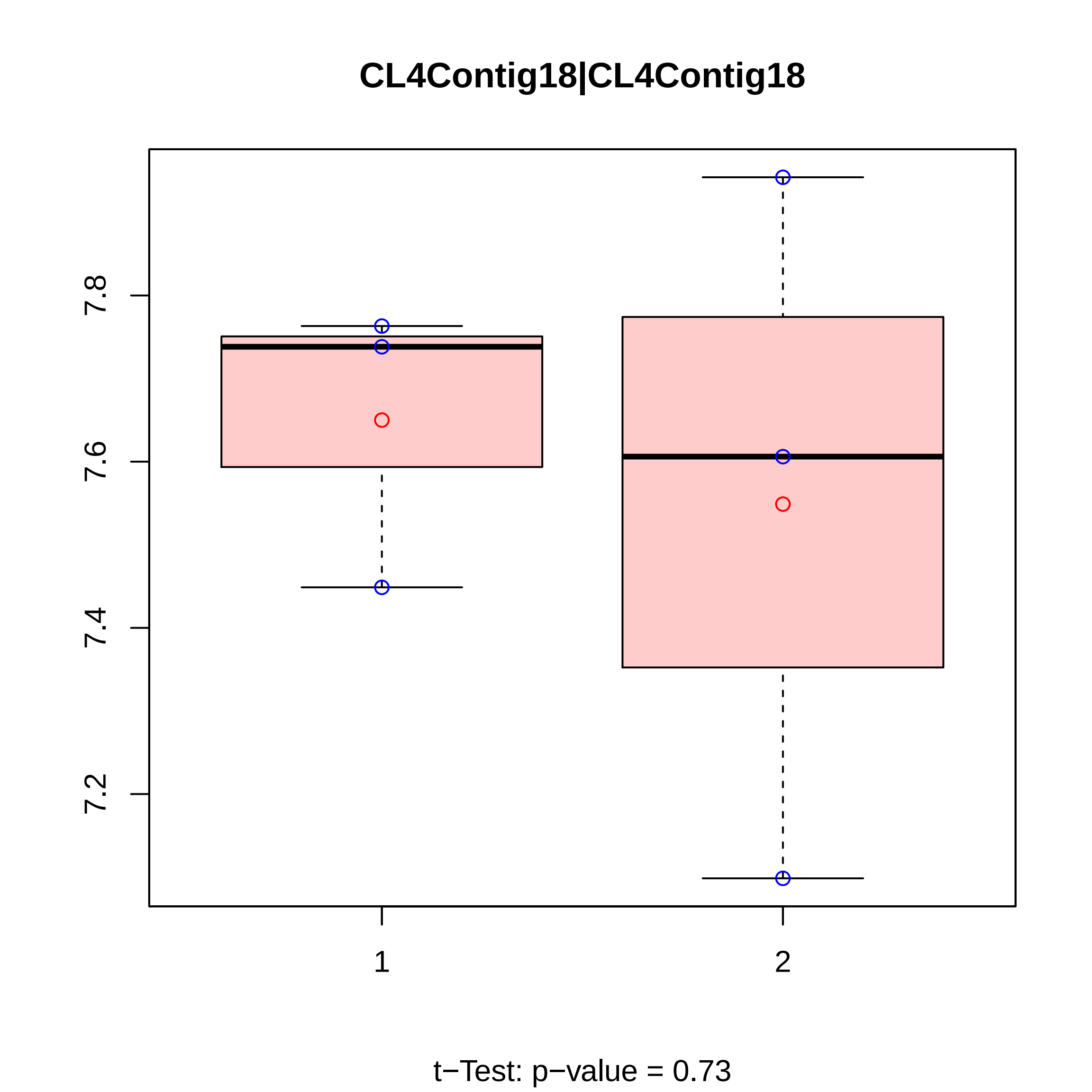
CL4Contig18|CL4Contig18
7.2
7.4
7.6
7.8
1
2
t−Test: p−value = 0.73

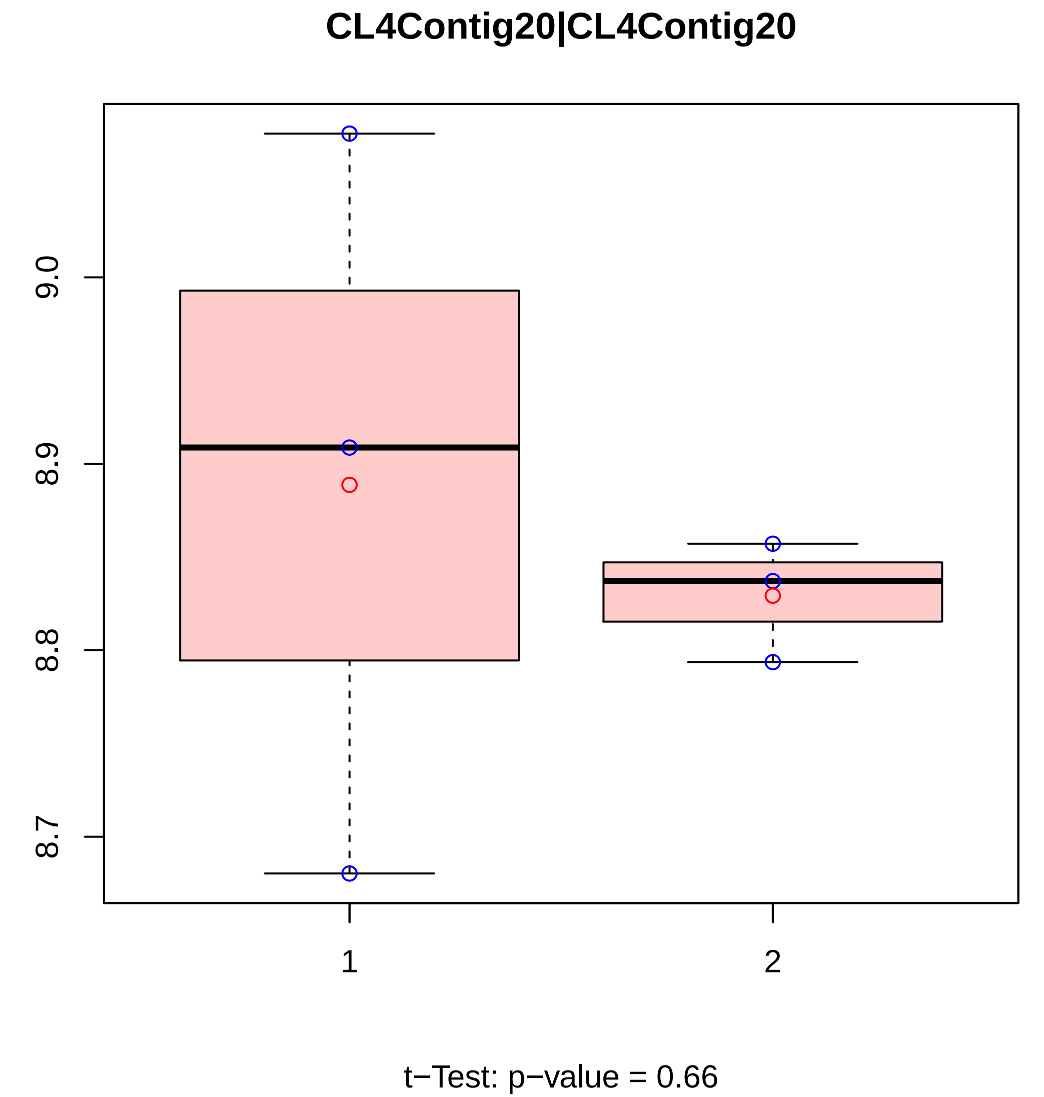
CL4Contig20|CL4Contig20
9.0
8.9
8.8
8.7
1
2
t−Test: p−value = 0.66

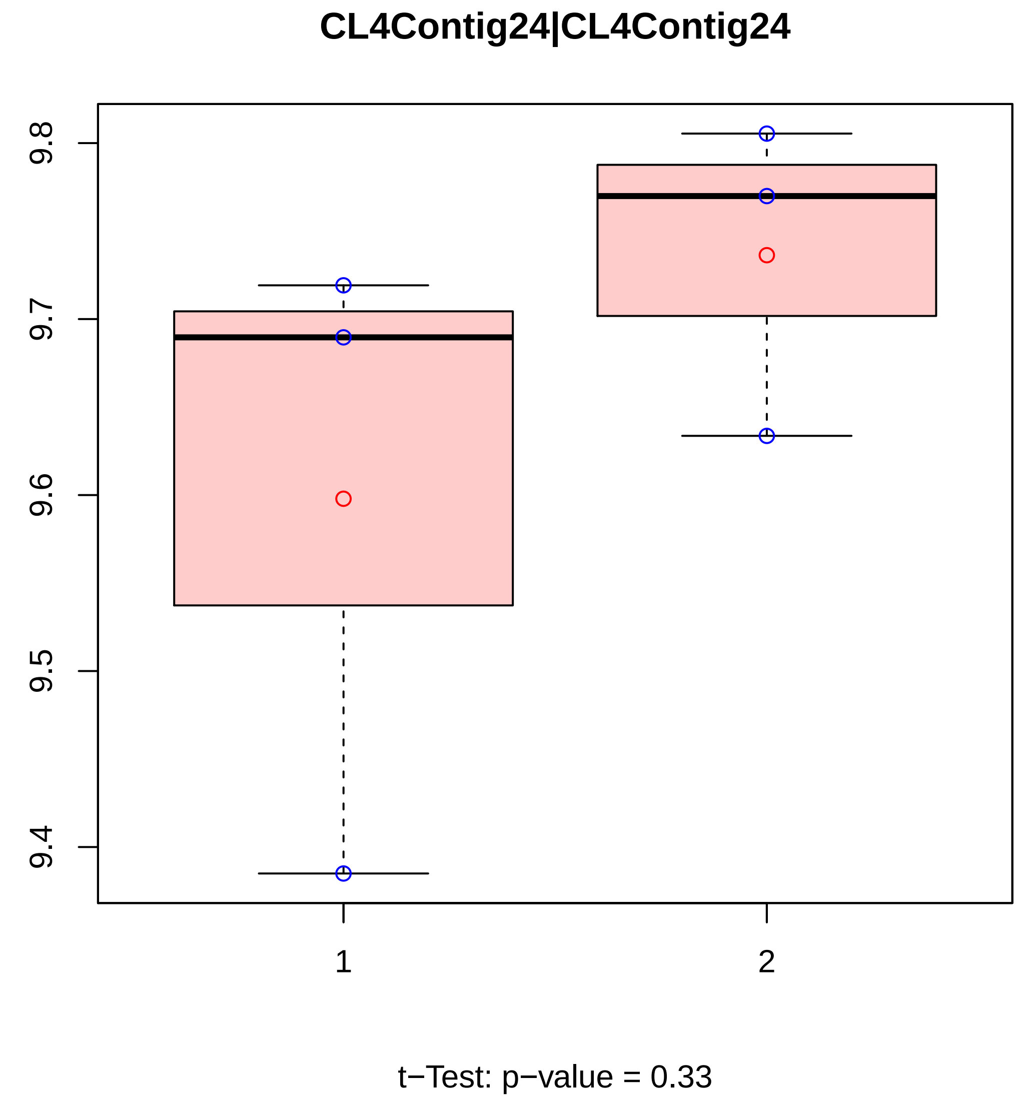
CL4Contig24|CL4Contig24
9.4
9.5
9.6
9.7
9.8
1
2
t−Test: p−value = 0.33

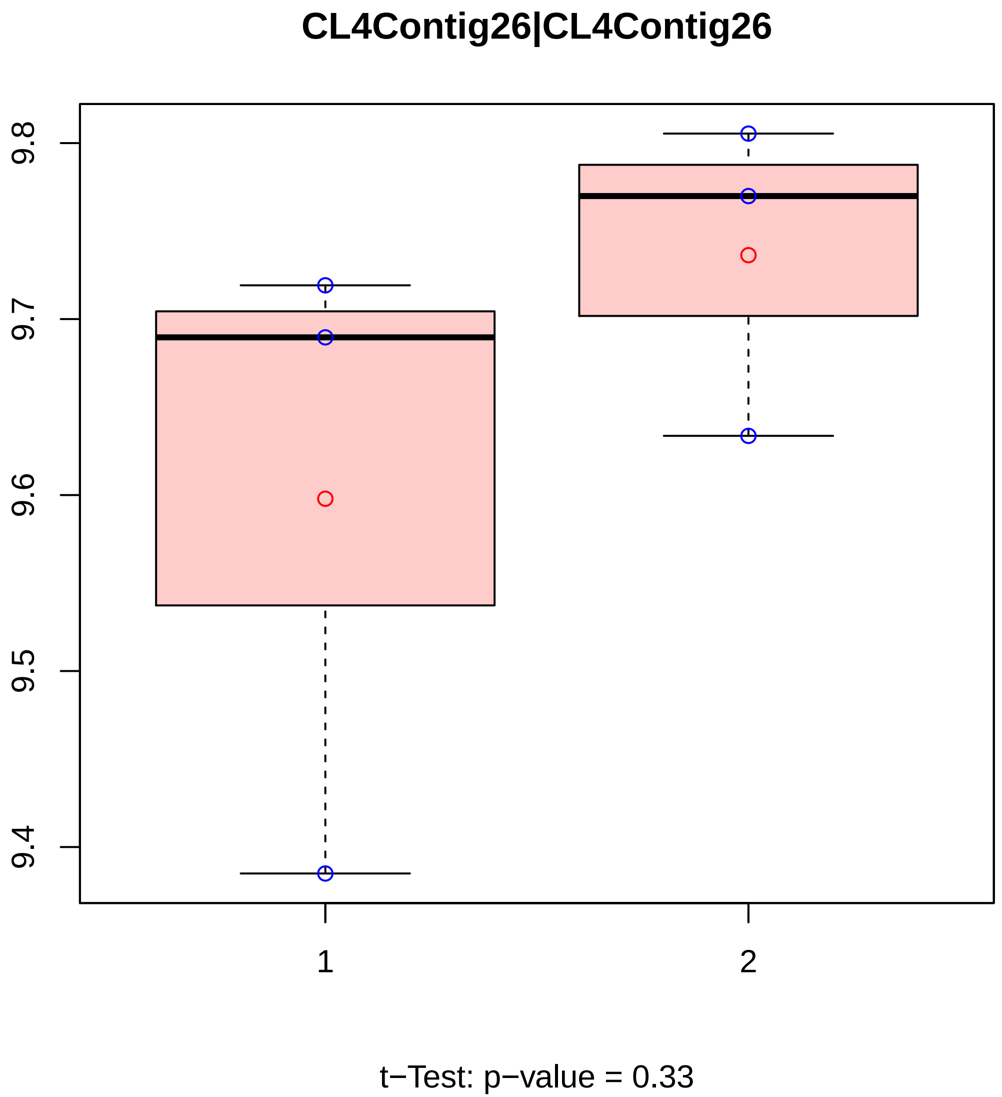
CL4Contig26|CL4Contig26
9.4
9.5
9.6
9.7
9.8
1
2
t−Test: p−value = 0.33

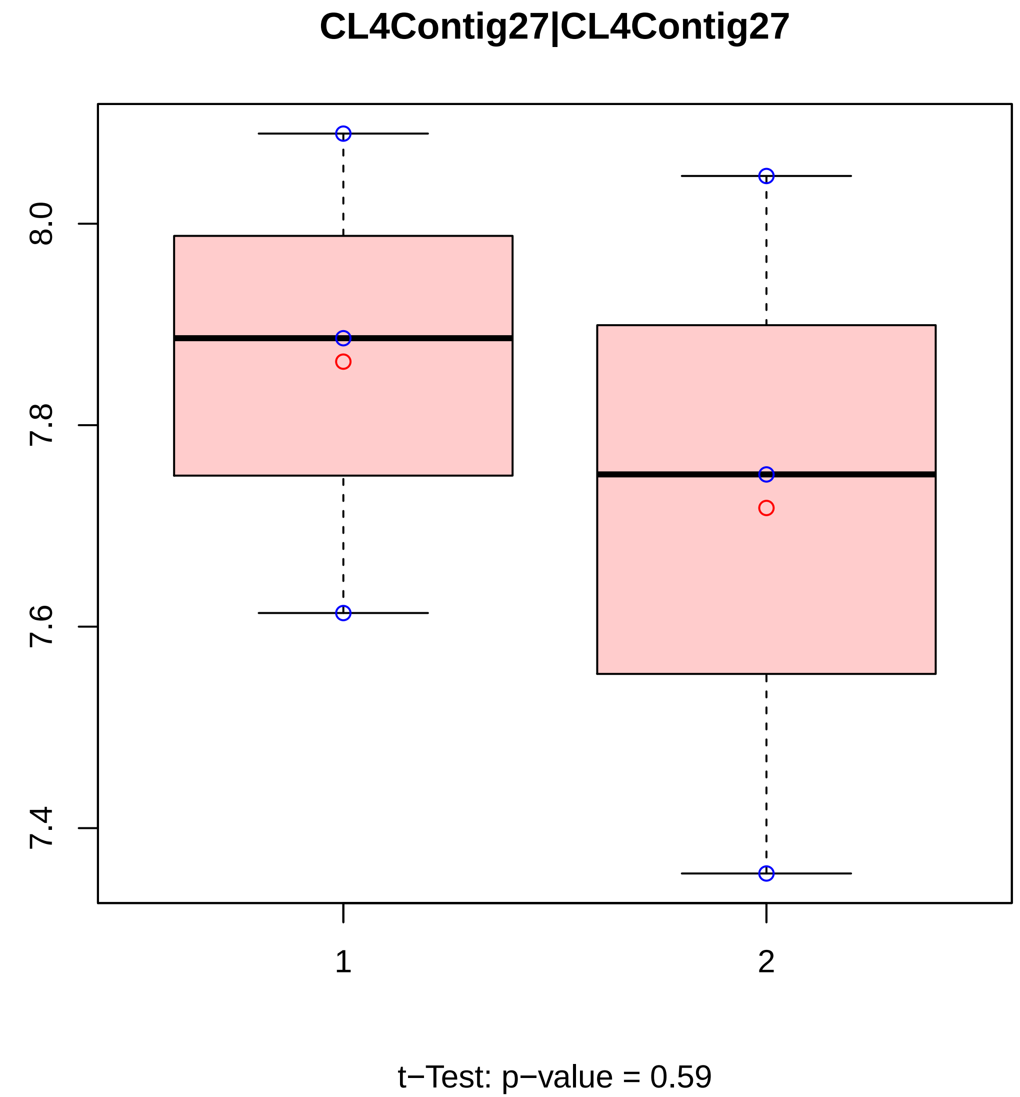

CL4Contig27|CL4Contig27
8.0
7.8
7.6
7.4
1
2
t−Test: p−value = 0.59

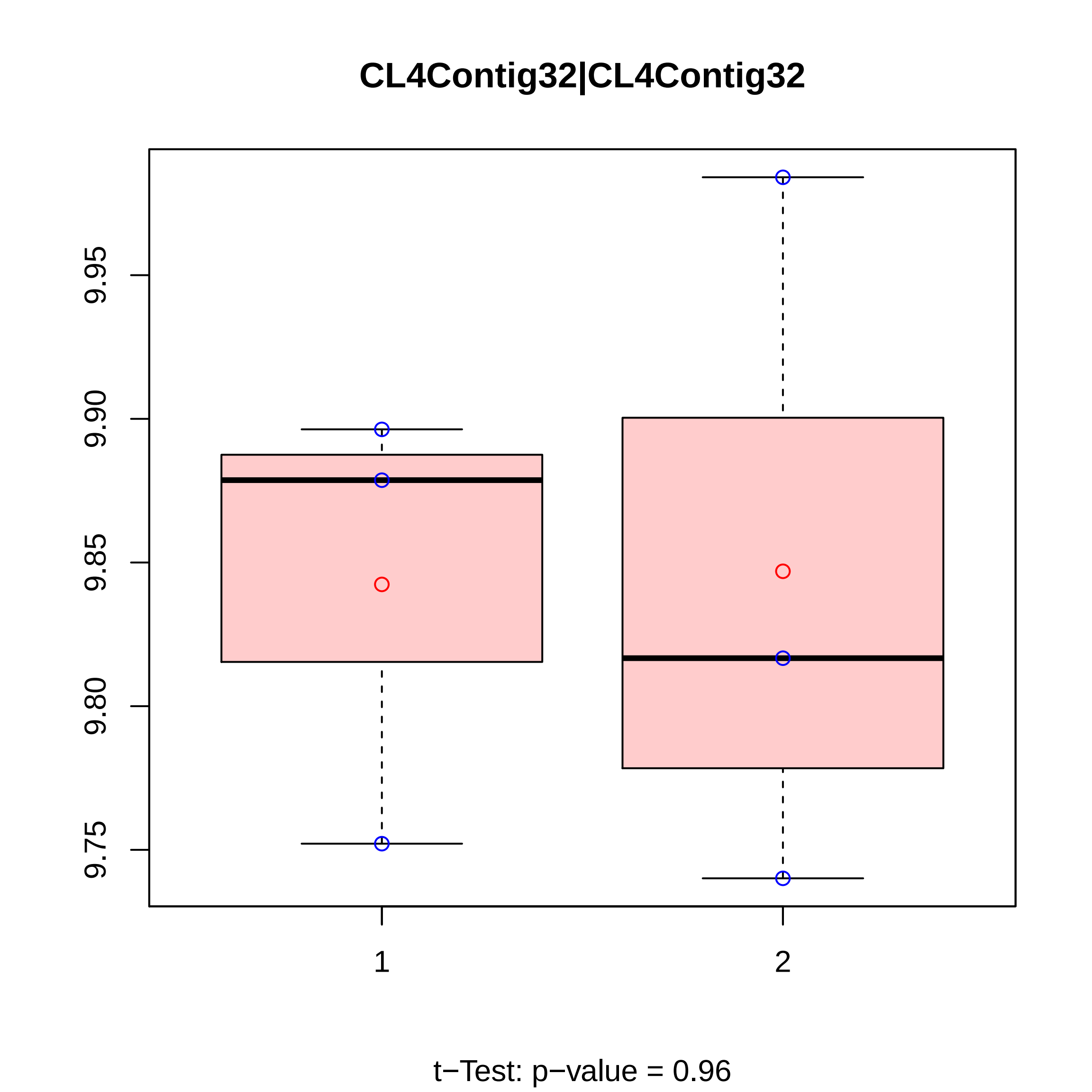

CL4Contig32|CL4Contig32
9.75
9.80
9.85
9.90
9.95
1
2
t−Test: p−value = 0.96

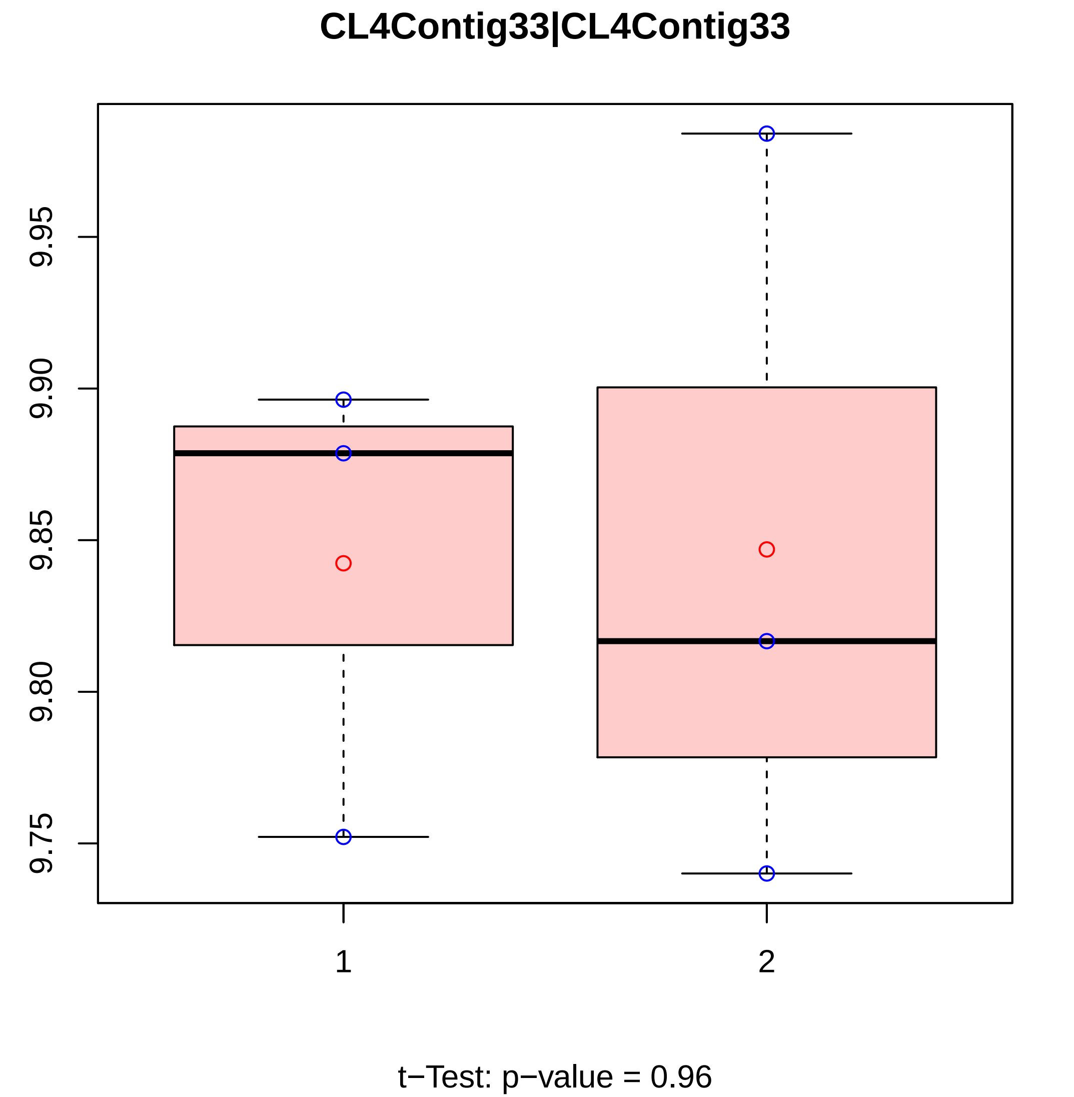
CL4Contig33|CL4Contig33
9.75
9.80
9.85
9.90
9.95
1
2
t−Test: p−value = 0.96

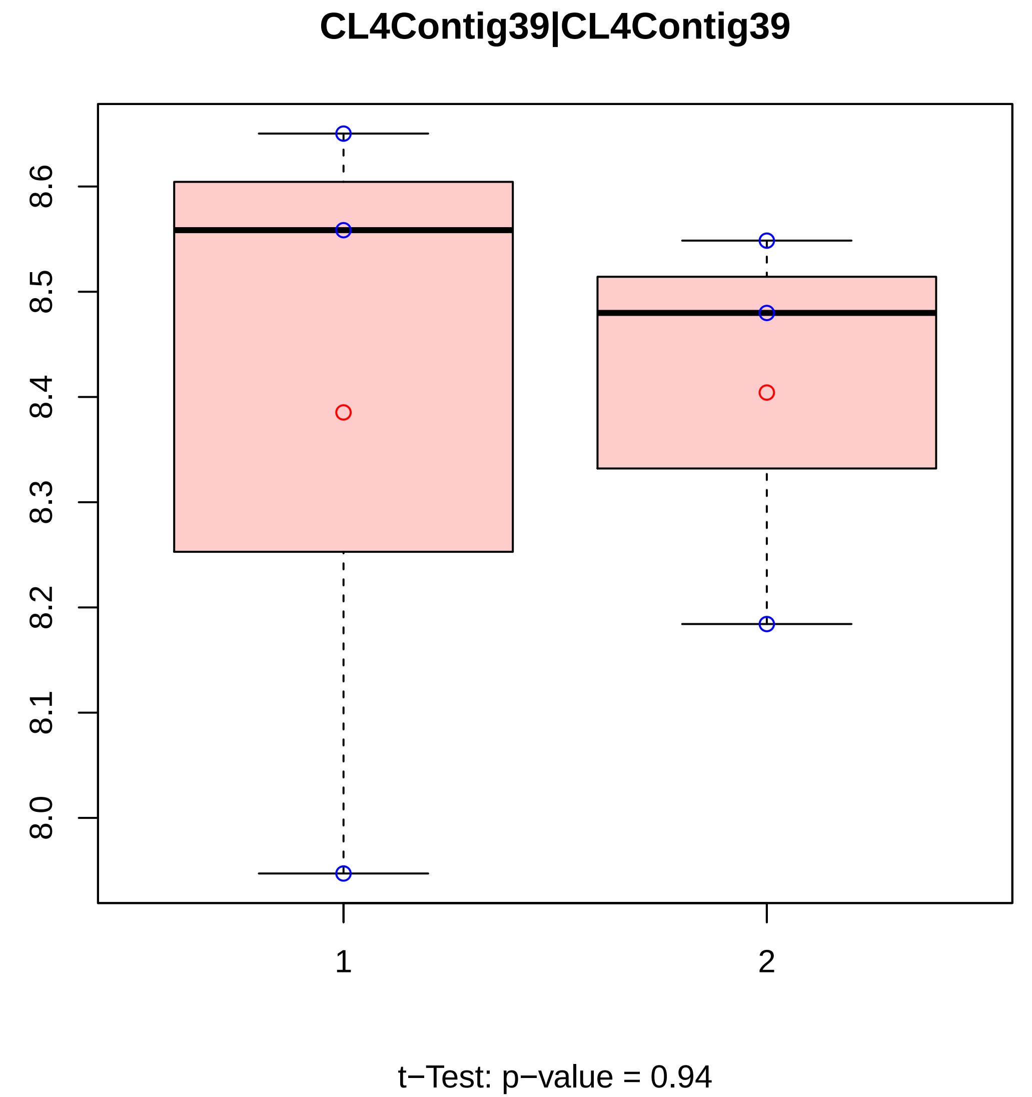
CL4Contig39|CL4Contig39
8.0
8.1
8.2
8.3
8.4
8.5
8.6
1
2
t−Test: p−value = 0.94

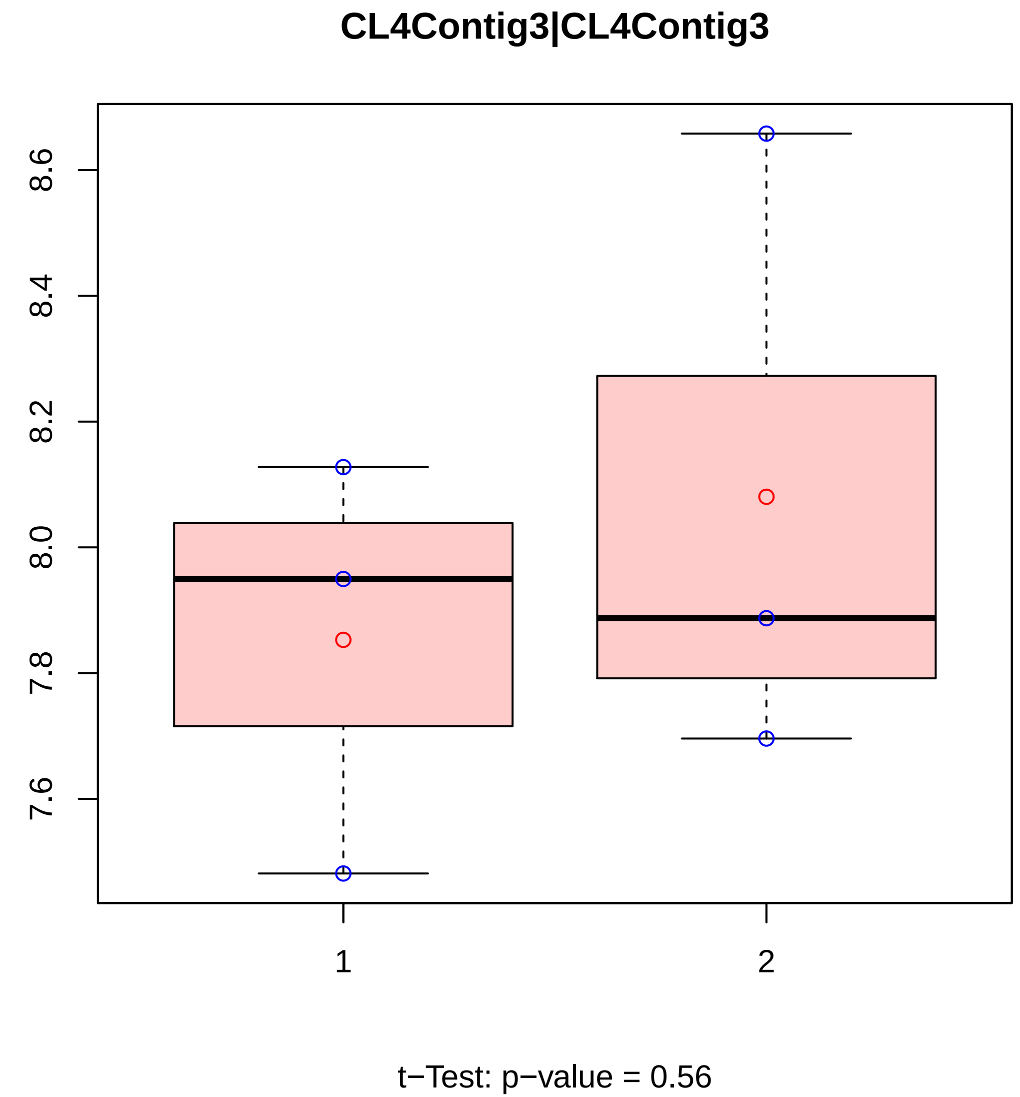

CL4Contig3|CL4Contig3
8.6
8.4
8.2
8.0
7.8
7.6
1
2
t−Test: p−value = 0.56

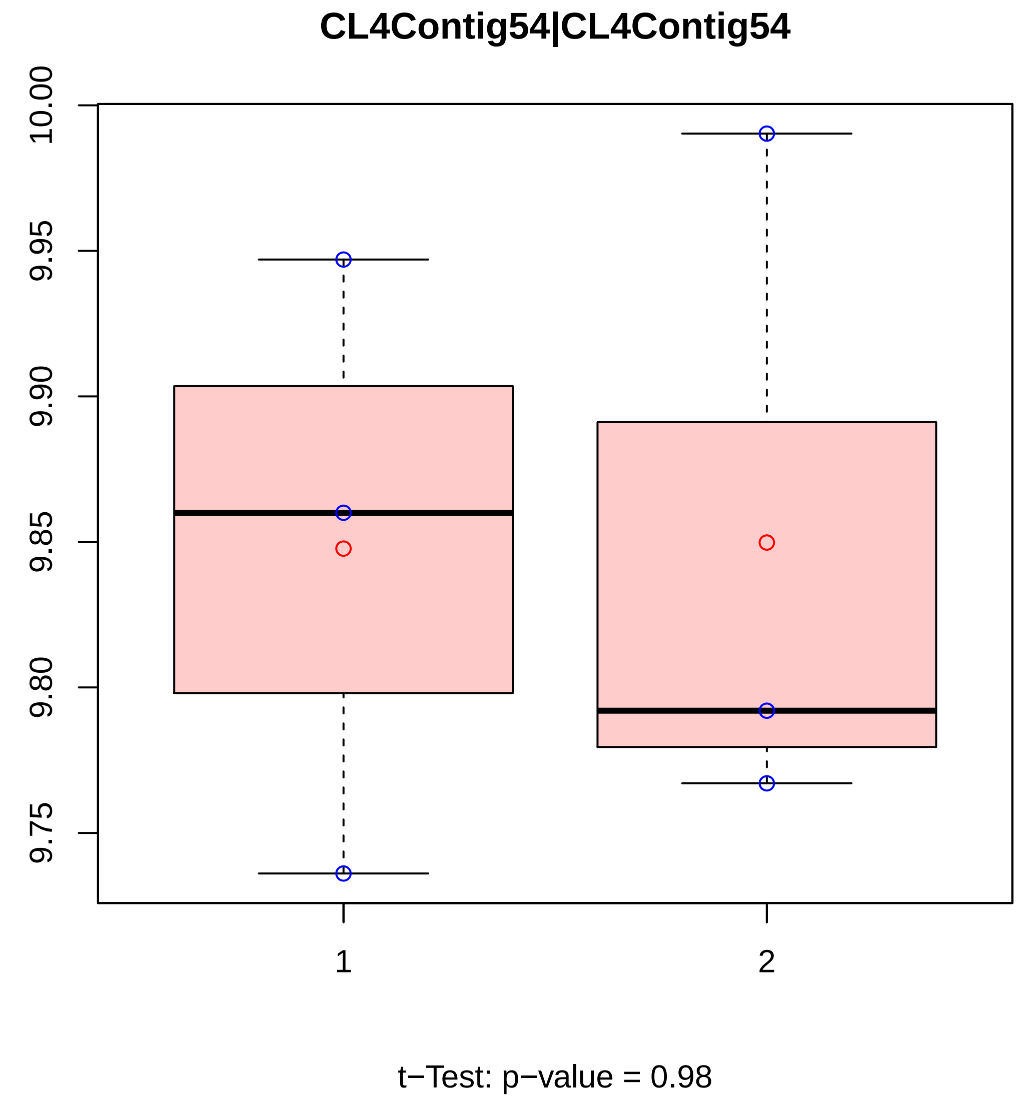
CL4Contig54|CL4Contig54
10.00
9.95
9.90
9.85
9.80
9.75
1
2
t−Test: p−value = 0.98

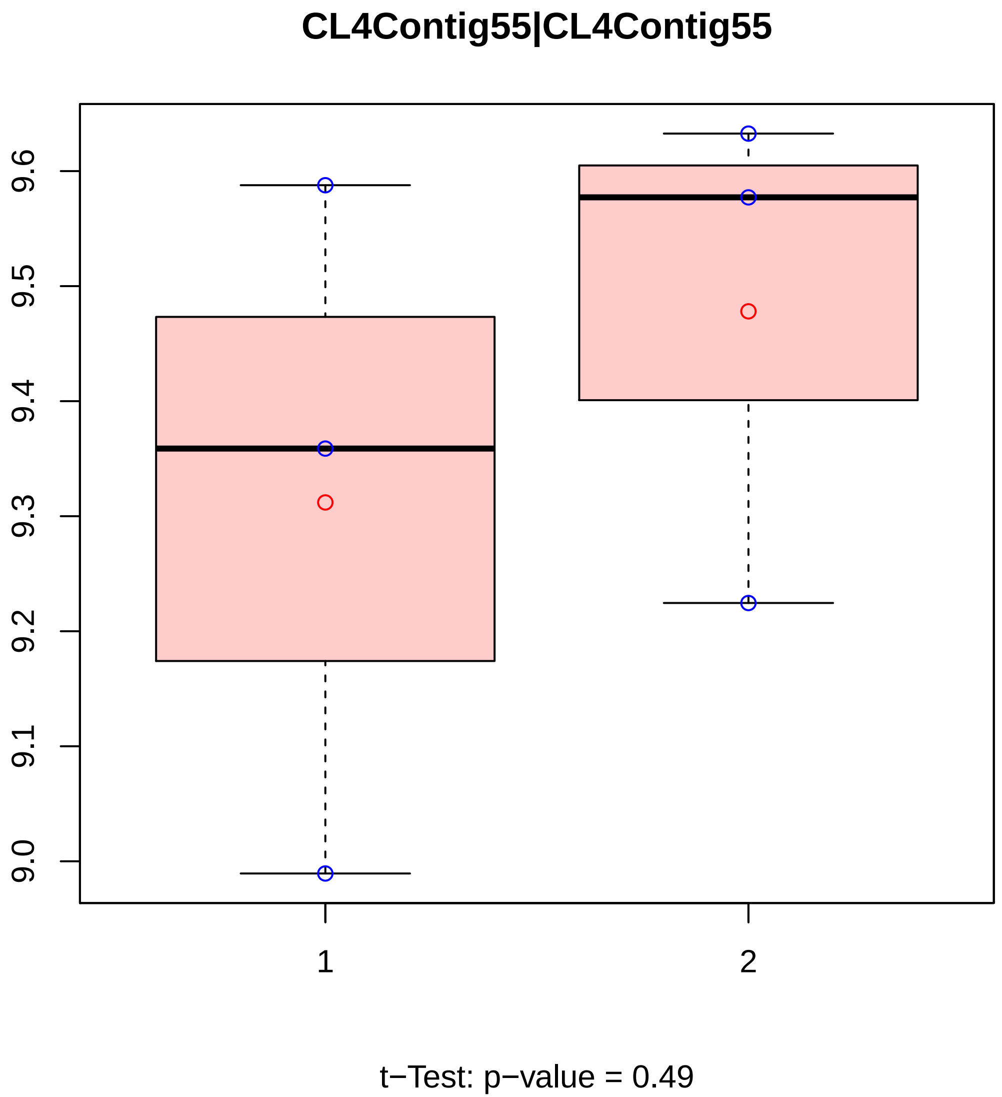
CL4Contig55|CL4Contig55
9.0
9.1
9.2
9.3
9.4
9.5
9.6
1
2
t−Test: p−value = 0.49

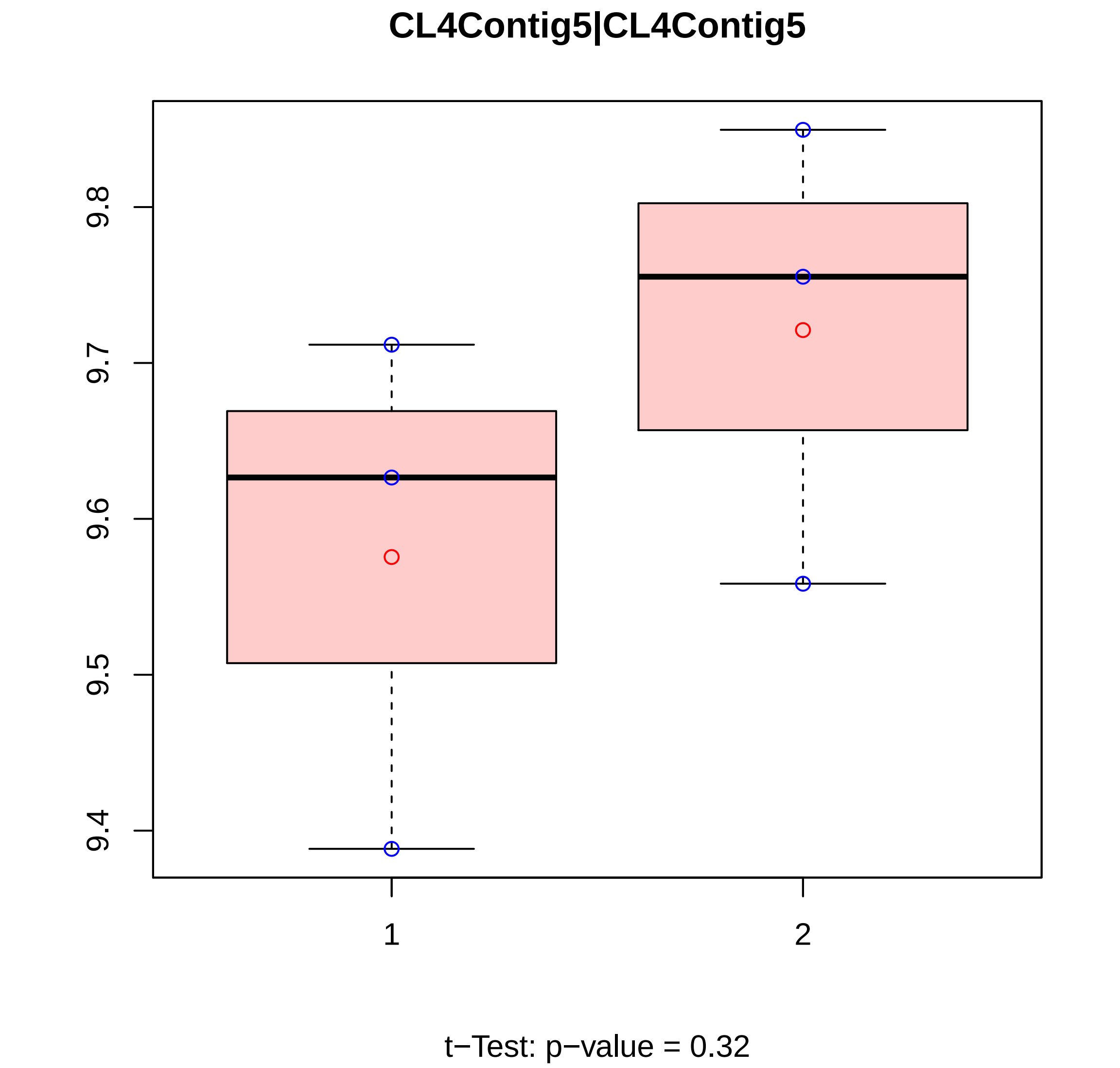
CL4Contig5|CL4Contig5
9.4
9.5
9.6
9.7
9.8
1
2
t−Test: p−value = 0.32

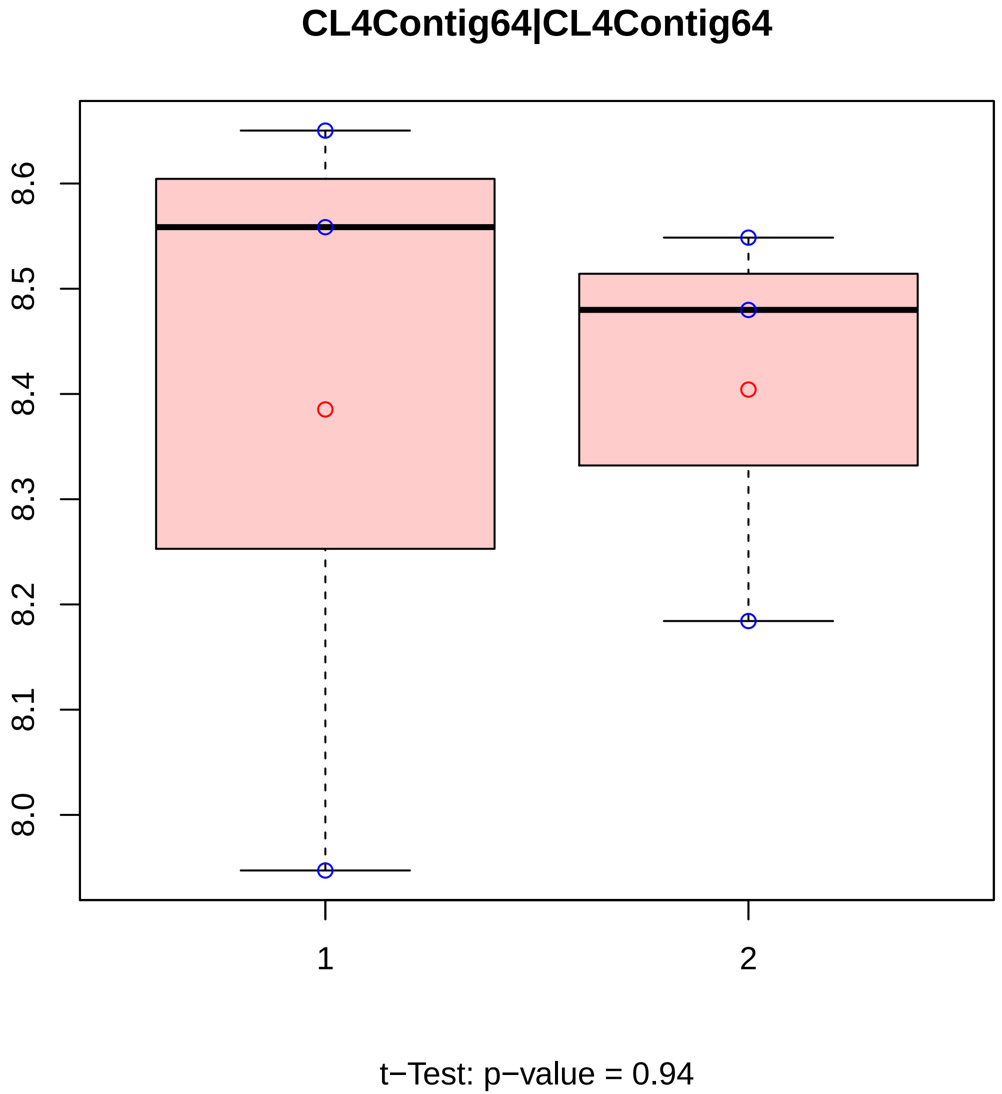
CL4Contig64|CL4Contig64
8.0
8.1
8.2
8.3
8.4
8.5
8.6
1
2
t−Test: p−value = 0.94

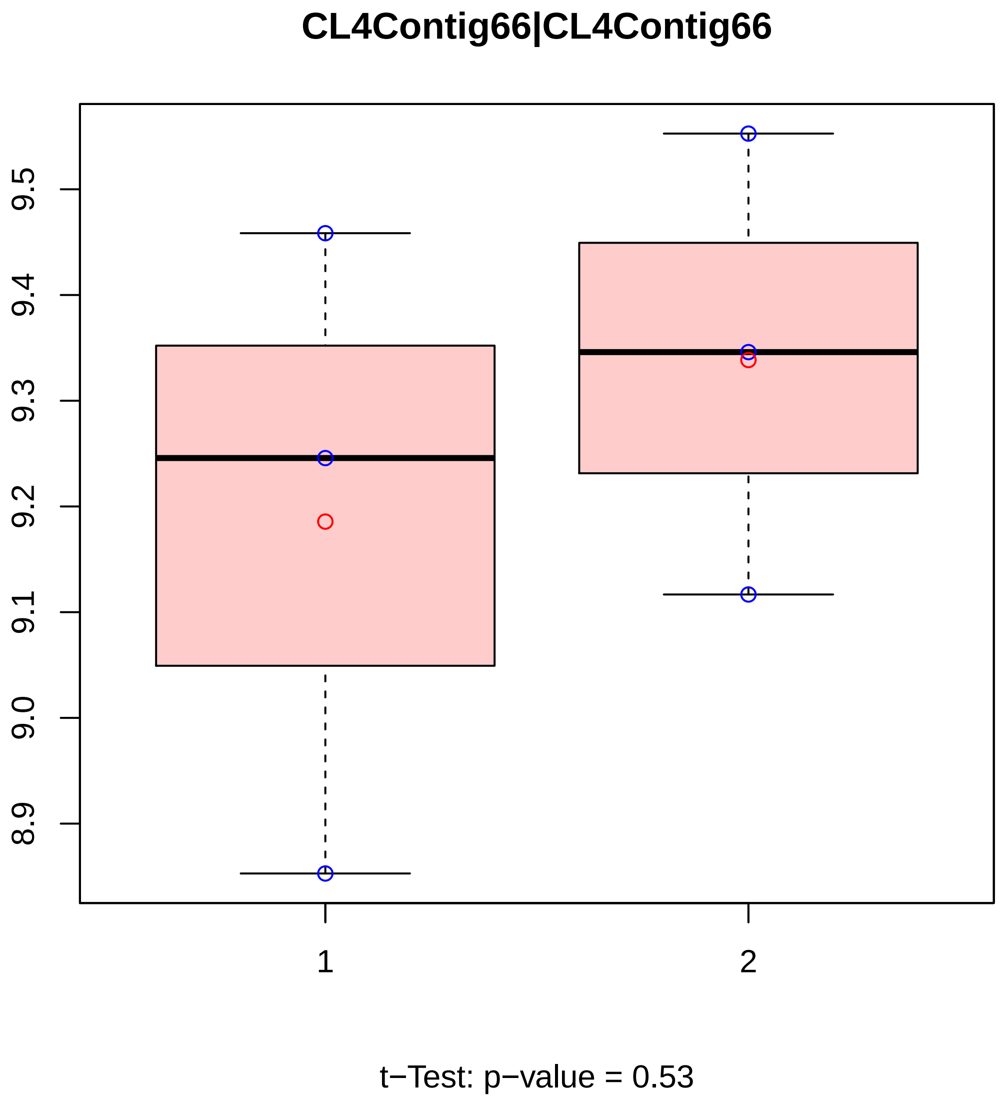
CL4Contig66|CL4Contig66
9.5
9.4
9.3
9.2
9.1
9.0
8.9
1
2
t−Test: p−value = 0.53

# CL4Contig67|CL4Contig67

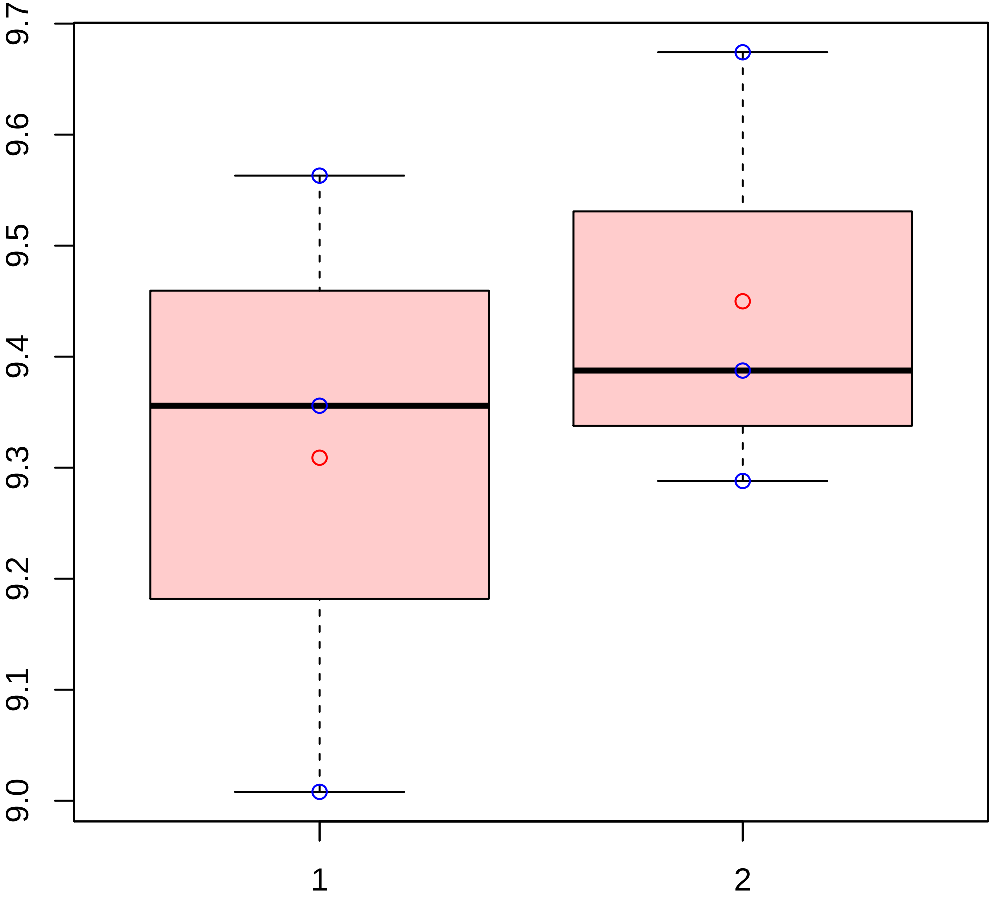


t−Test: p−value = 0.52

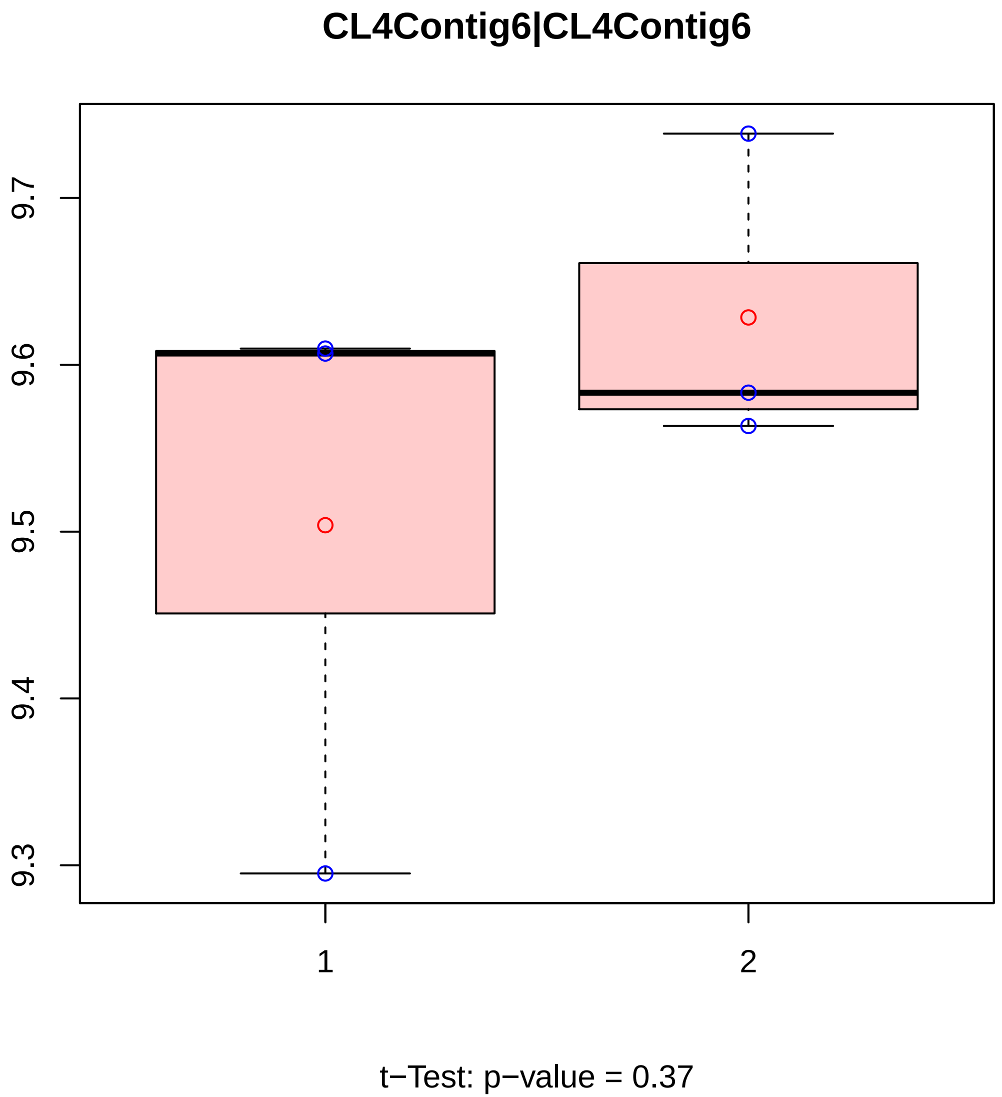
CL4Contig6|CL4Contig6
9.3
9.4
9.5
9.6
9.7
1
2
t−Test: p−value = 0.37

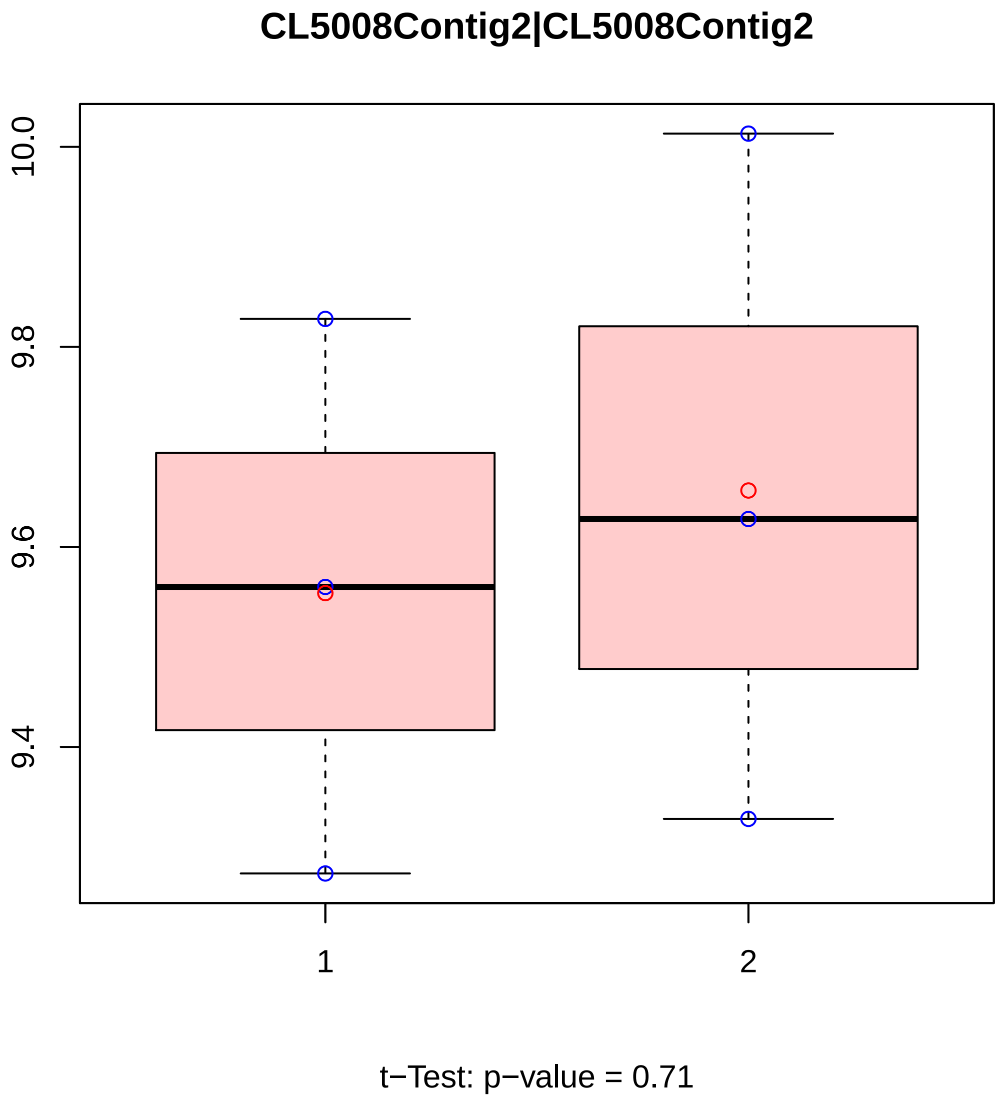
CL5008Contig2|CL5008Contig2
10.0
9.8
9.6
9.4
1
2
t−Test: p−value = 0.71

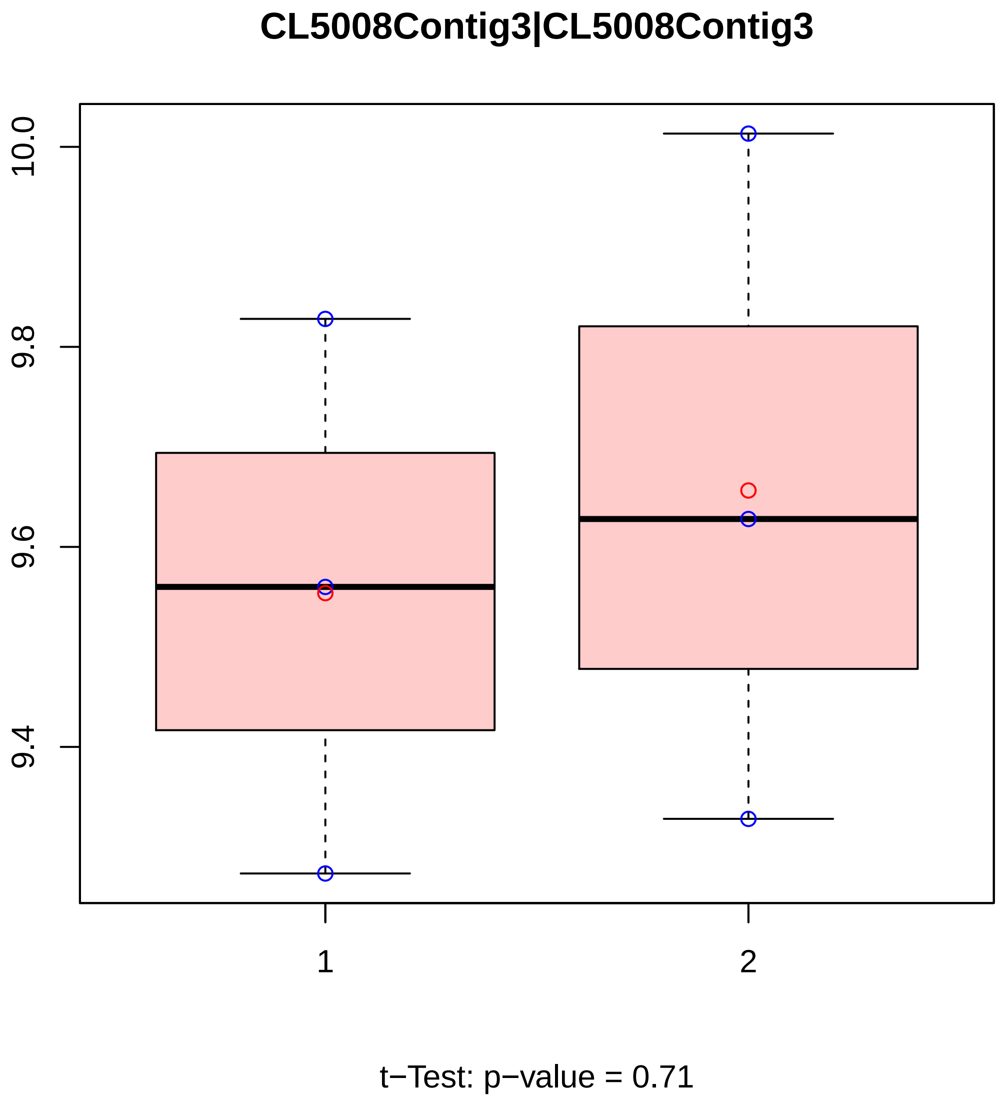

CL5008Contig3|CL5008Contig3
10.0
9.8
9.6
9.4
1
2
t−Test: p−value = 0.71

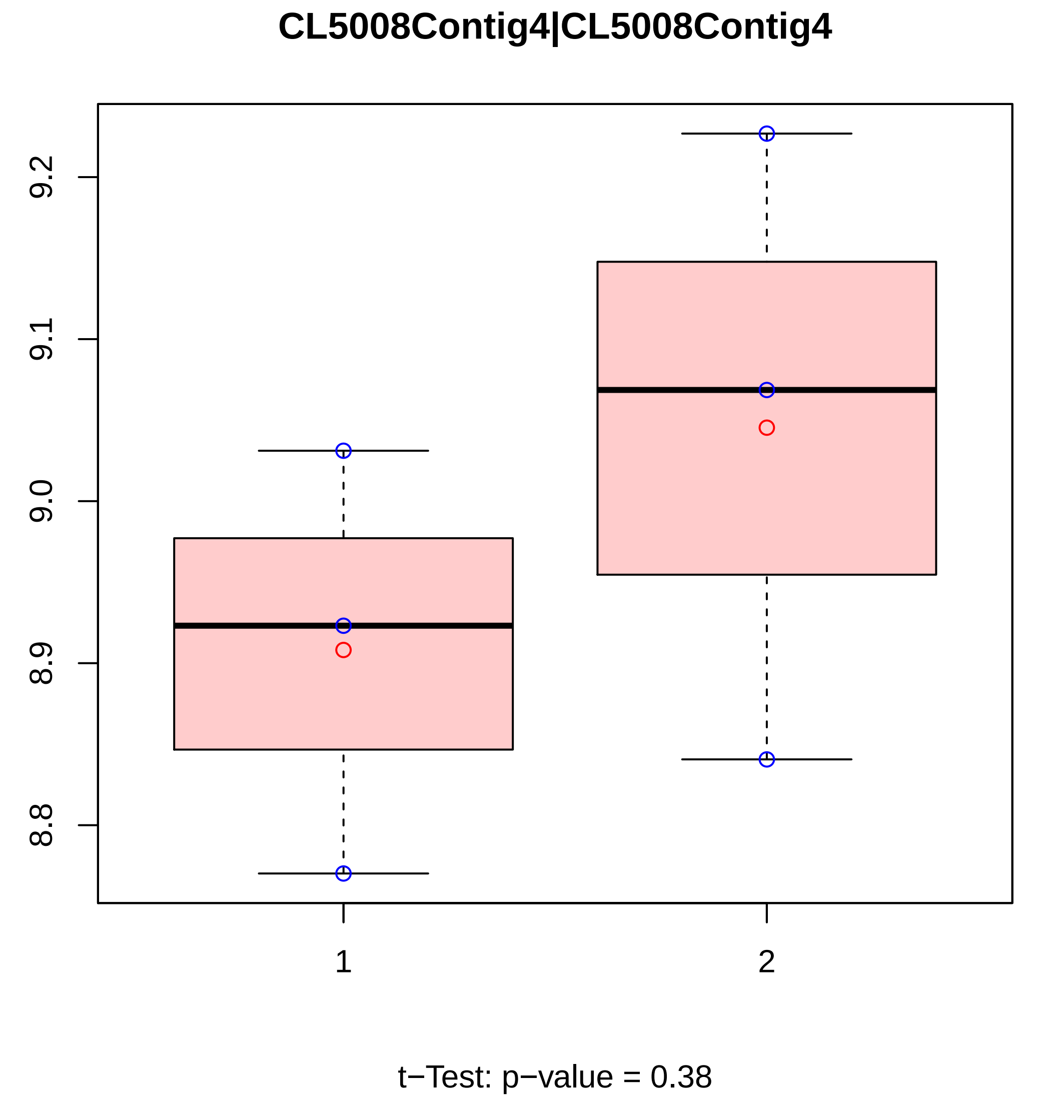

CL5008Contig4|CL5008Contig4
9.2
9.1
9.0
8.9
8.8
1
2
t−Test: p−value = 0.38

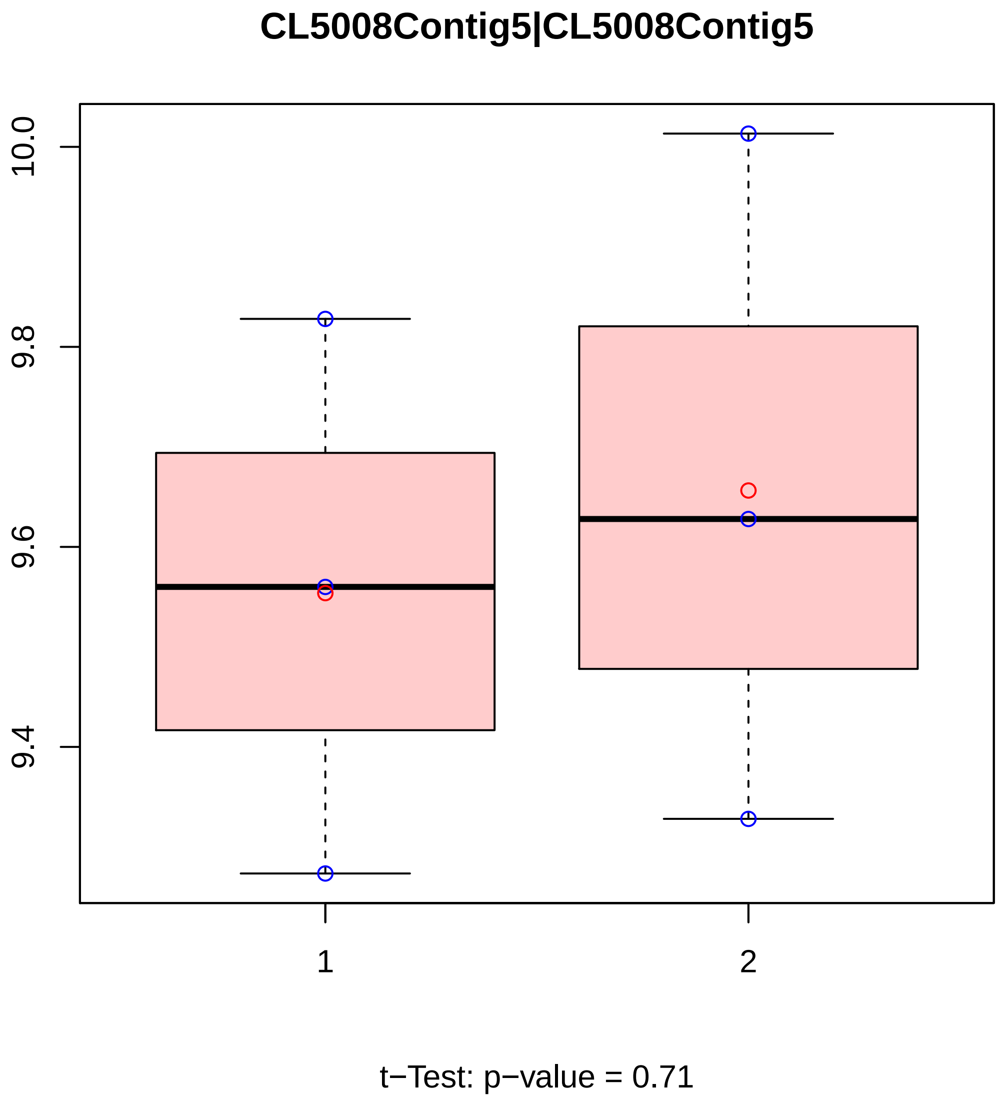
CL5008Contig5|CL5008Contig5
10.0
9.8
9.6
9.4
1
2
t−Test: p−value = 0.71

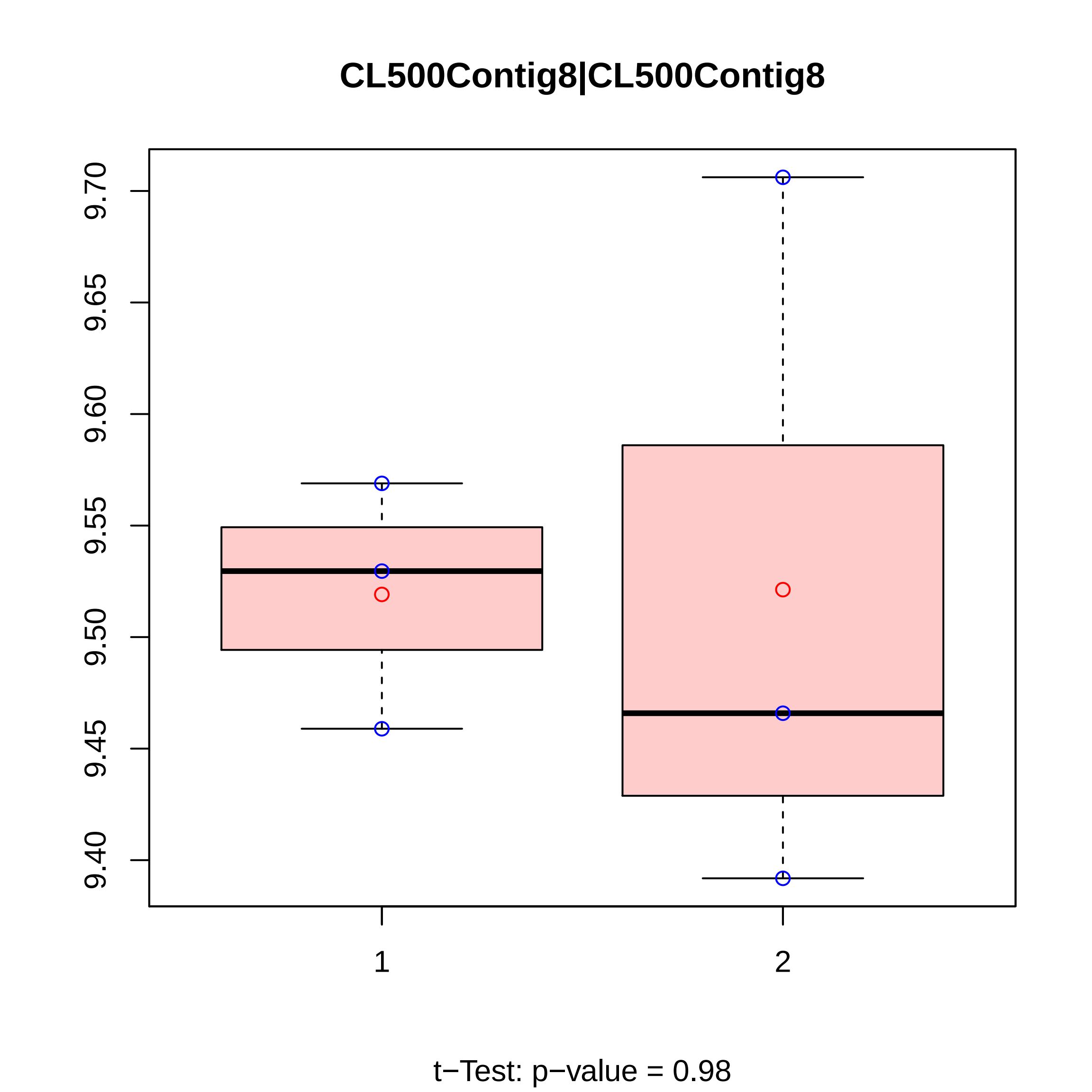

CL500Contig8|CL500Contig8
9.40
9.45
9.50
9.55
9.60
9.65
9.70
1
2
t−Test: p−value = 0.98

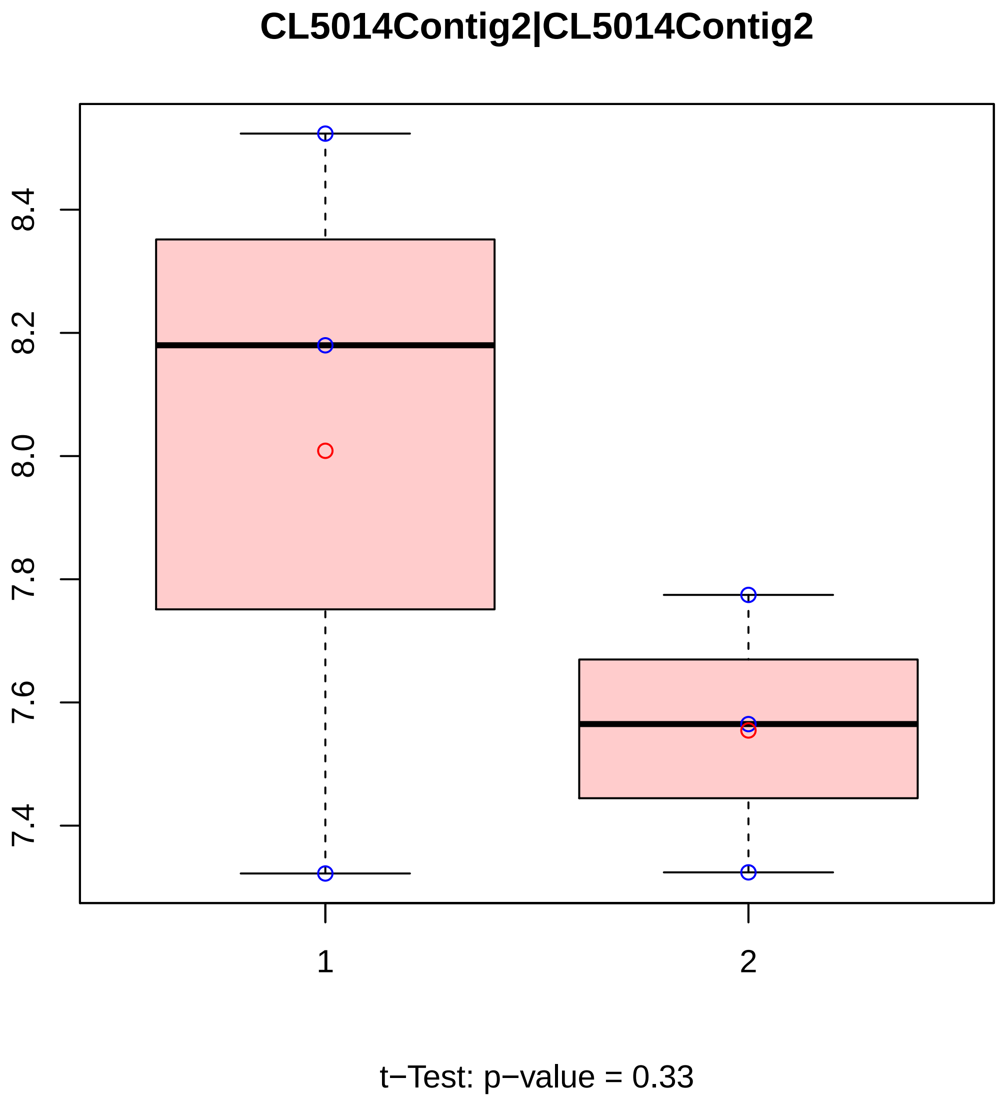

CL5014Contig2|CL5014Contig2
8.4
8.2
8.0
7.8
7.6
7.4
1
2
t−Test: p−value = 0.33

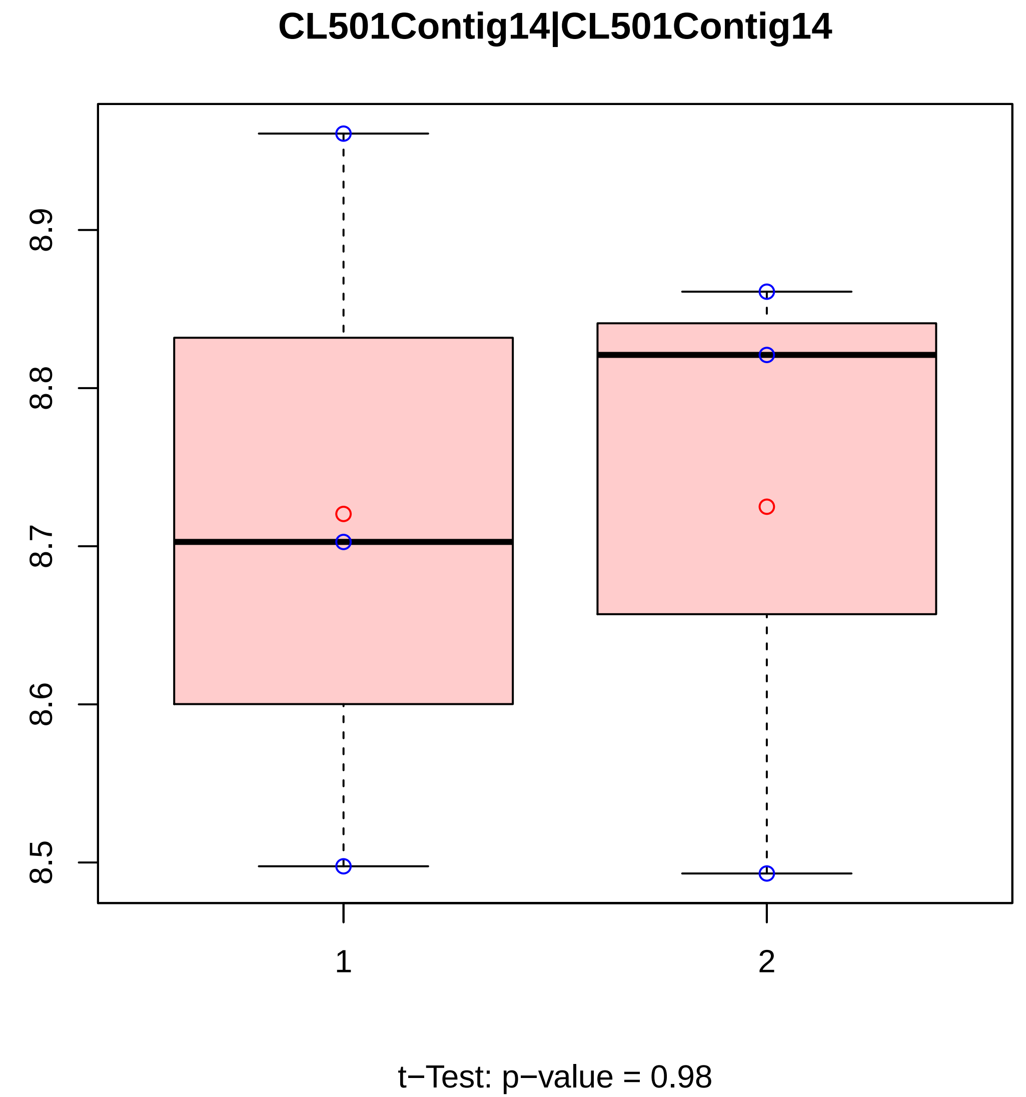

CL501Contig14|CL501Contig14
8.5
8.6
8.7
8.8
8.9
1
2
t−Test: p−value = 0.98

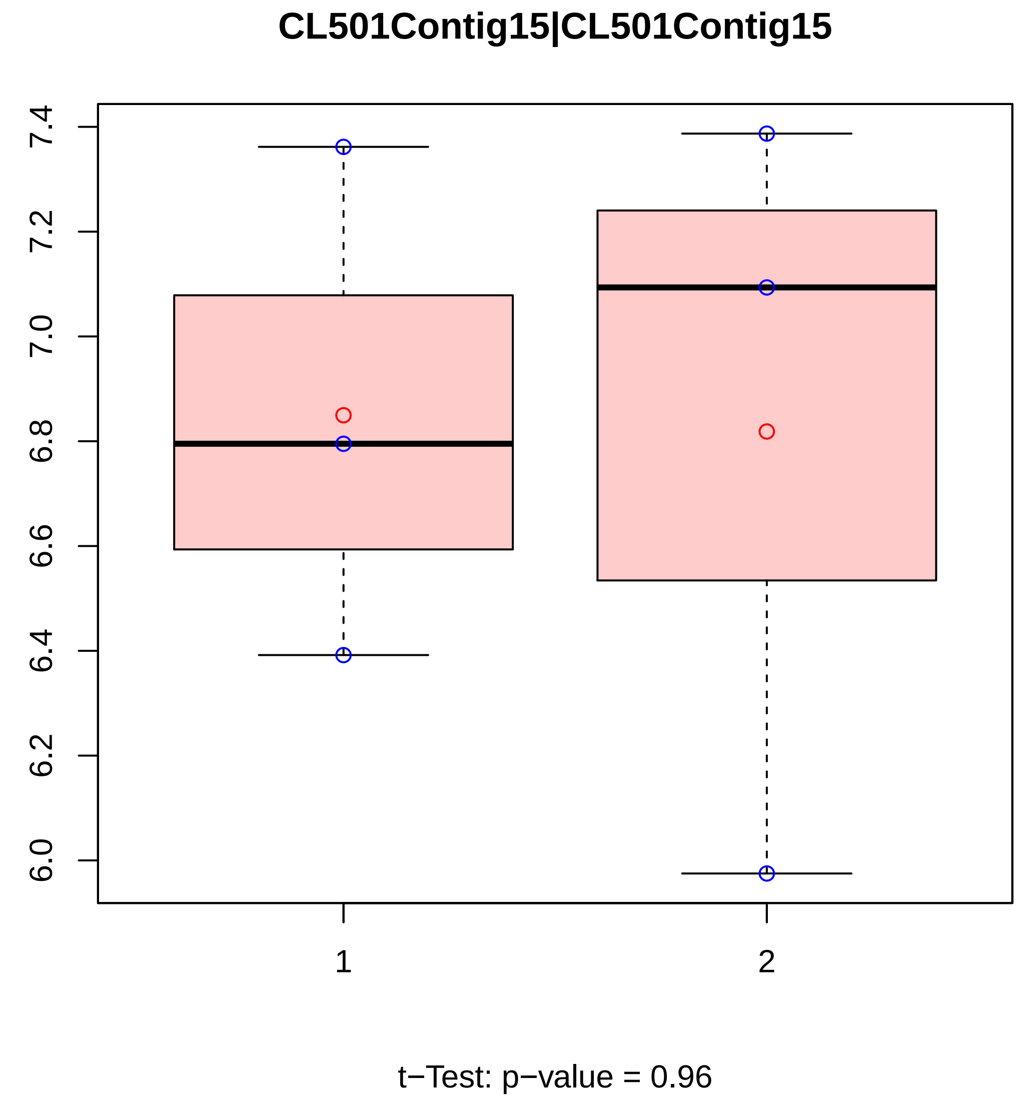
CL501Contig15|CL501Contig15
7.4
7.2
7.0
6.8
6.6
6.4
6.2
6.0
1
2
t−Test: p−value = 0.96

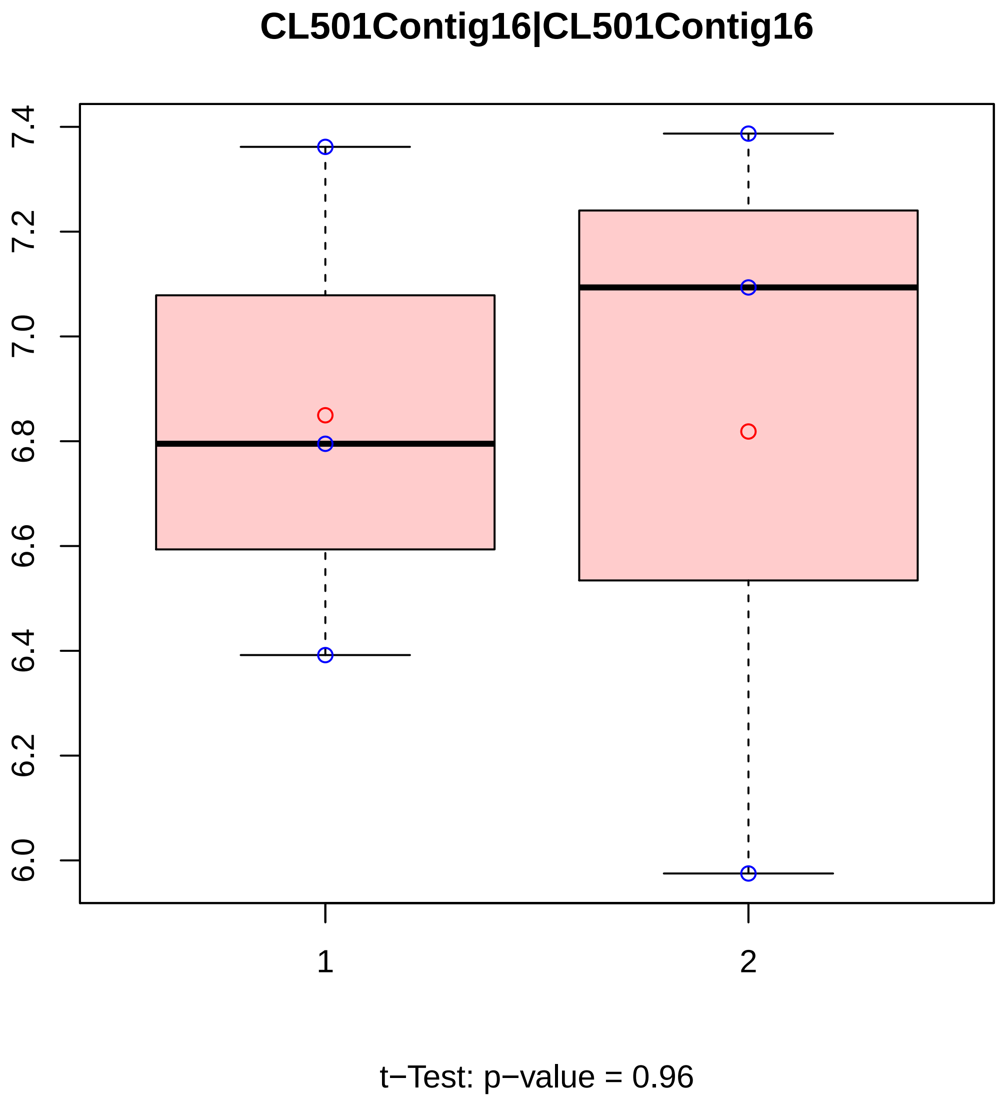
CL501Contig16|CL501Contig16
7.4
7.2
7.0
6.8
6.6
6.4
6.2
6.0
1
2
t−Test: p−value = 0.96

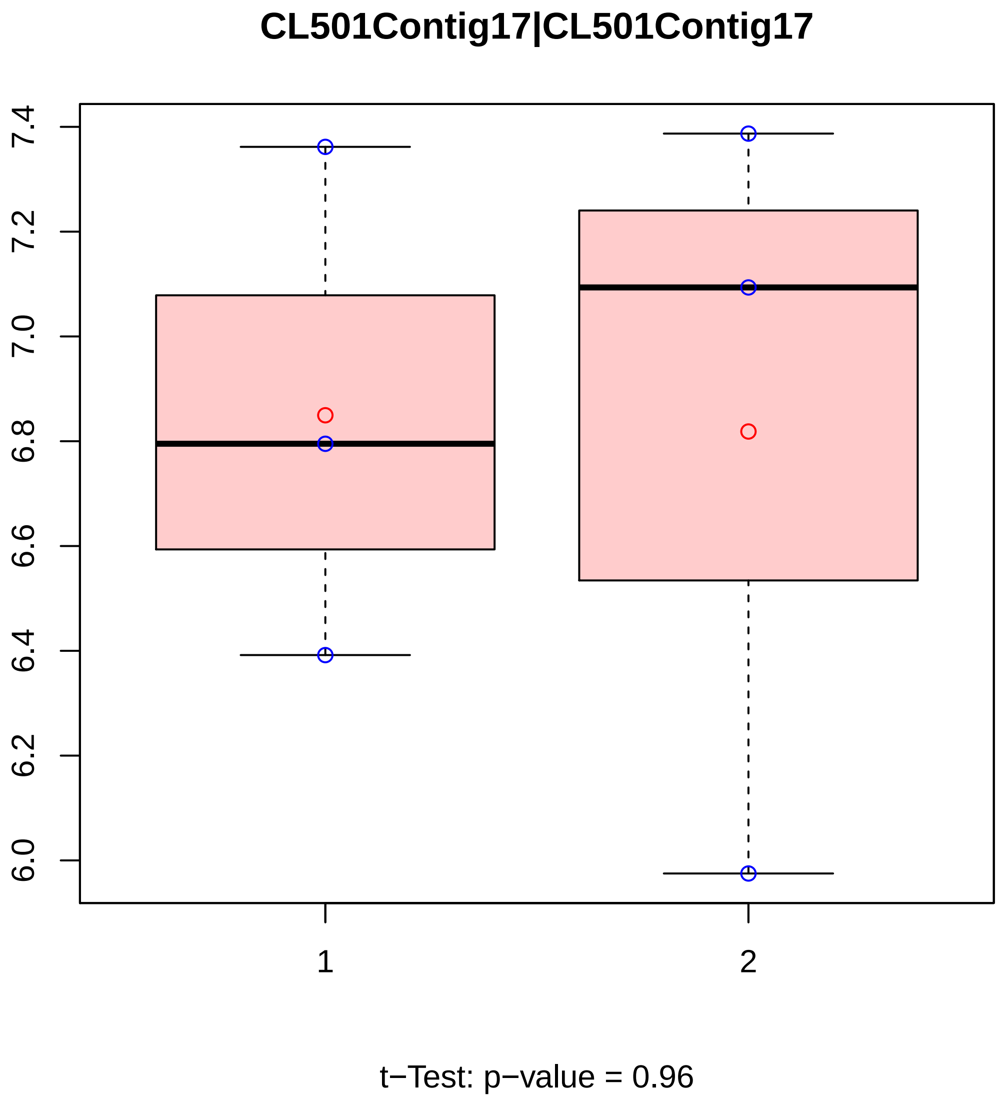

CL501Contig17|CL501Contig17
7.4
7.2
7.0
6.8
6.6
6.4
6.2
6.0
1
2
t−Test: p−value = 0.96

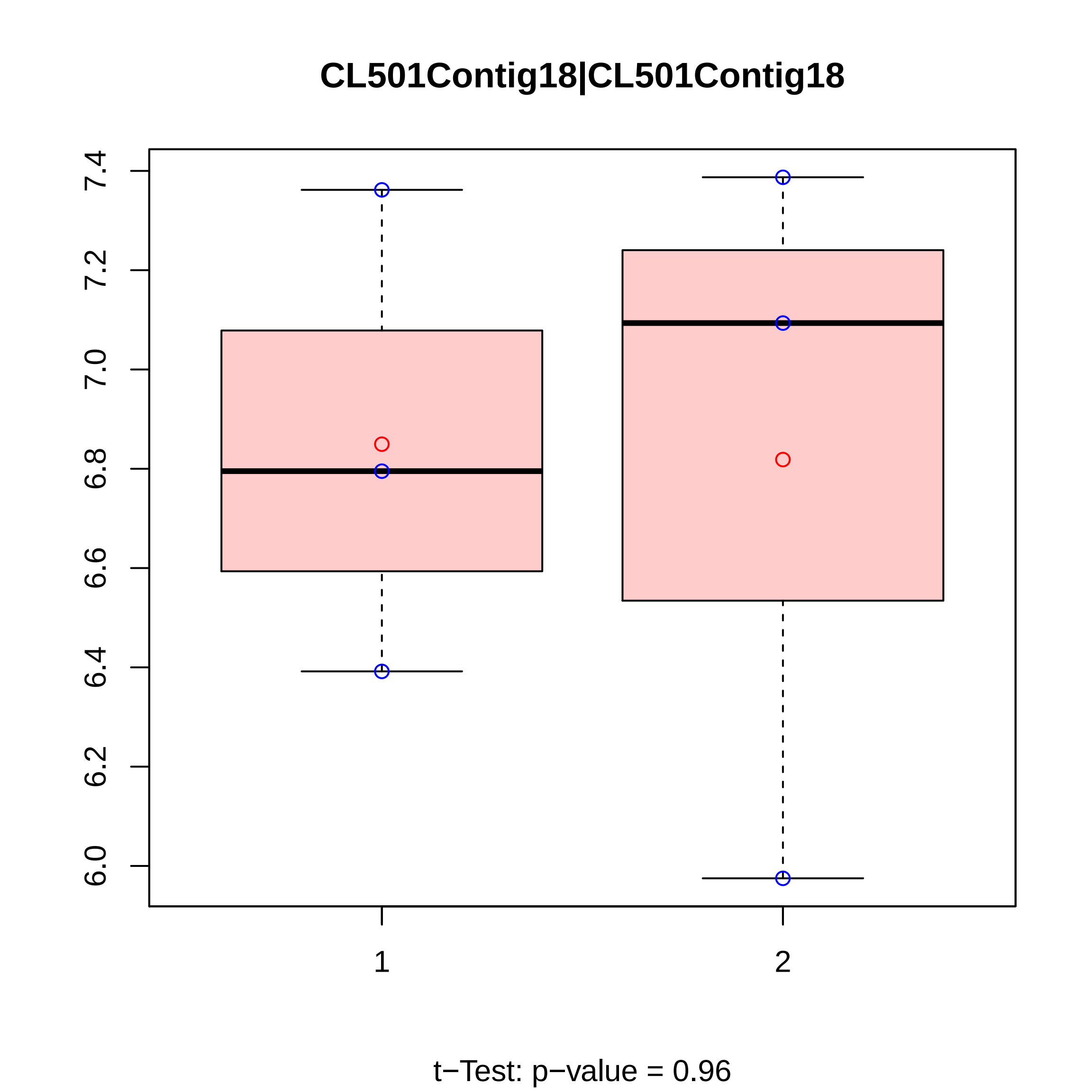
CL501Contig18|CL501Contig18
7.4
7.2
7.0
6.8
6.6
6.4
6.2
6.0
1
2
t−Test: p−value = 0.96

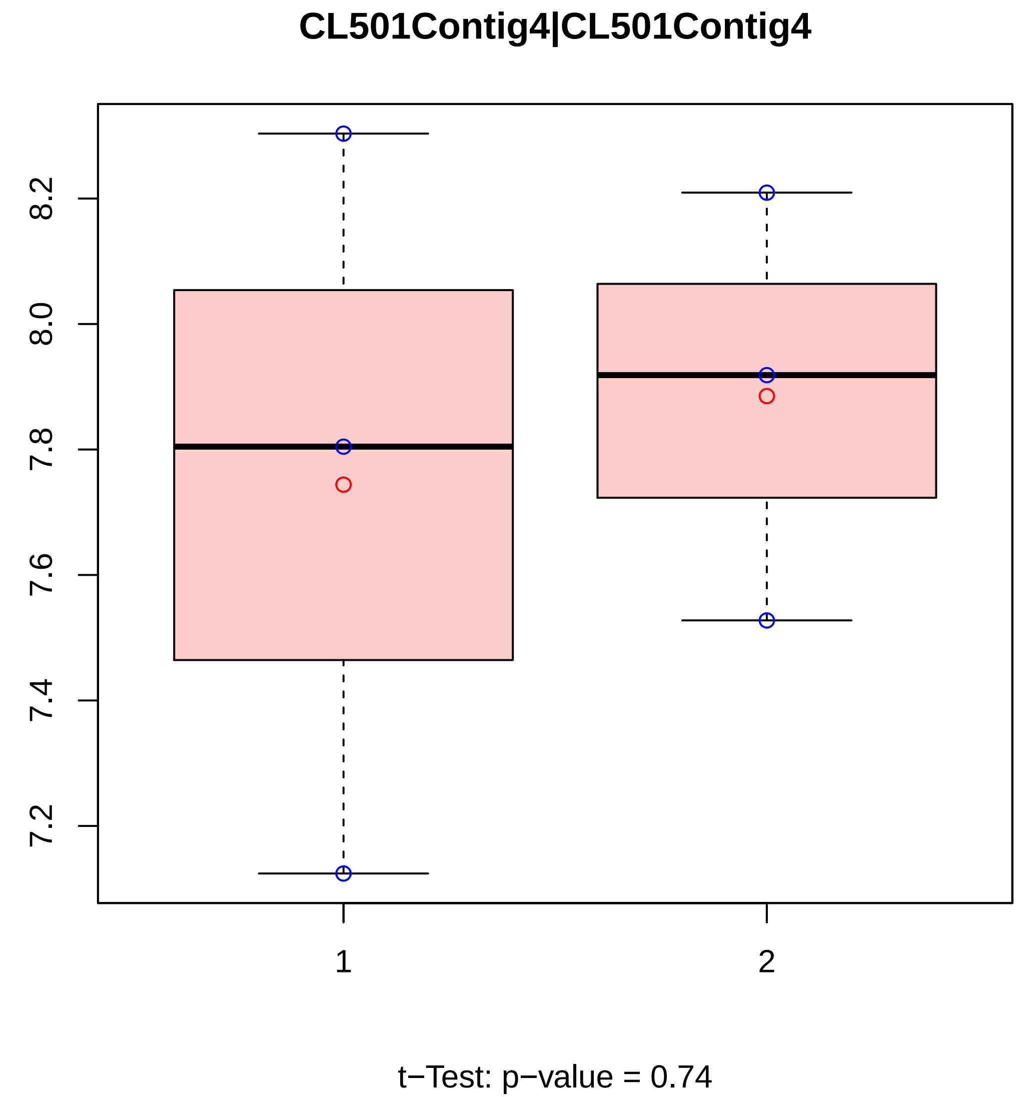
CL501Contig4|CL501Contig4
8.2
8.0
7.8
7.6
7.4
7.2
1
2
t−Test: p−value = 0.74

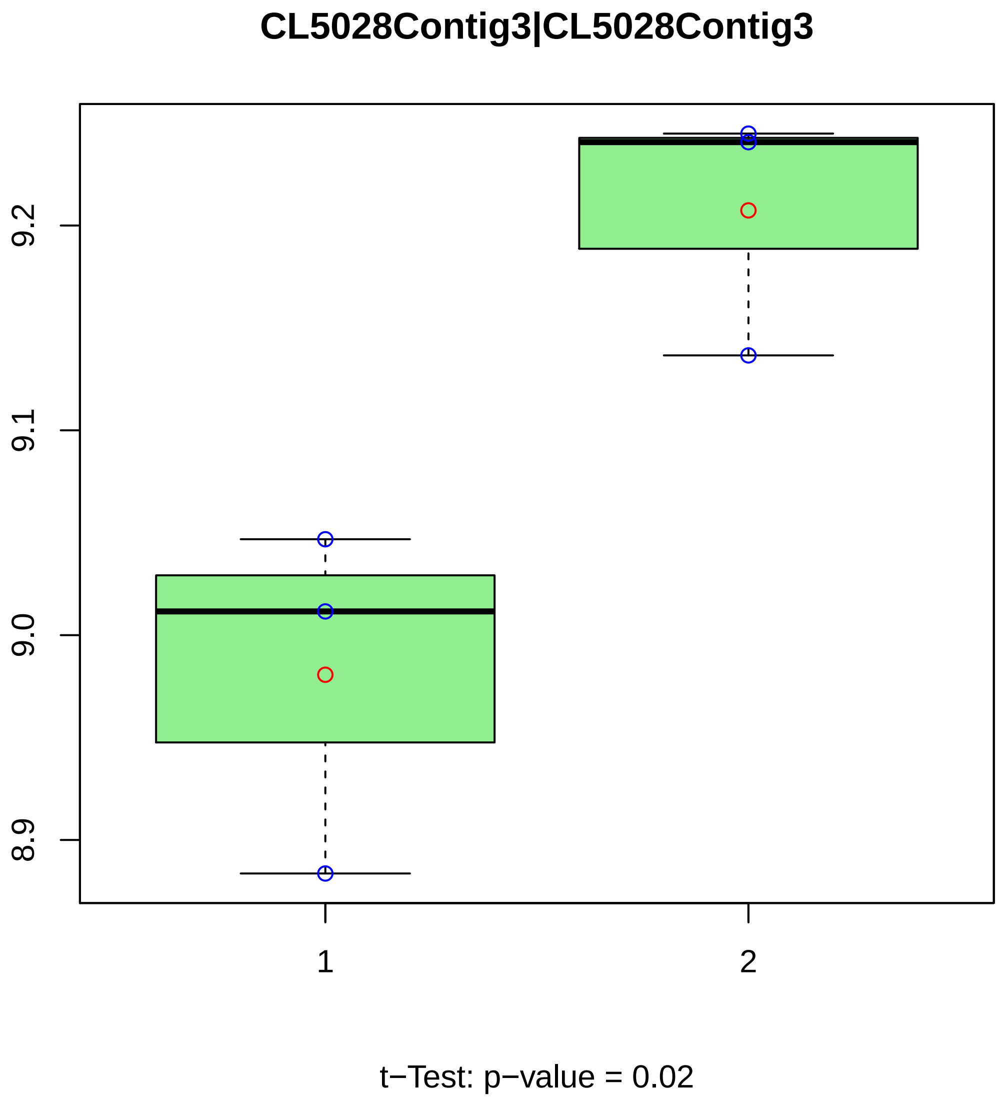
CL5028Contig3|CL5028Contig3
9.2
9.1
9.0
8.9
1
2
t−Test: p−value = 0.02

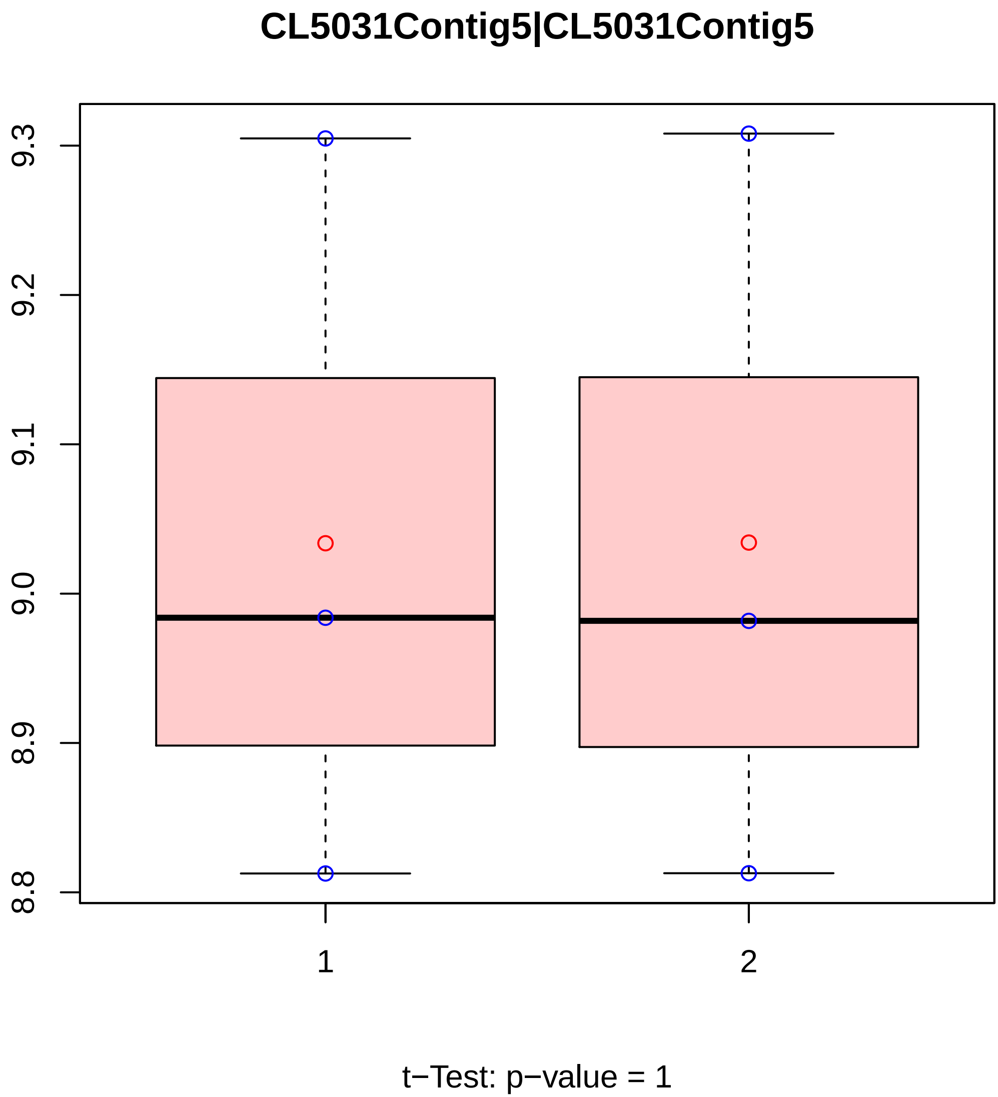
CL5031Contig5|CL5031Contig5
9.3
9.2
9.1
9.0
8.9
8.8
1
2
t−Test: p−value = 1

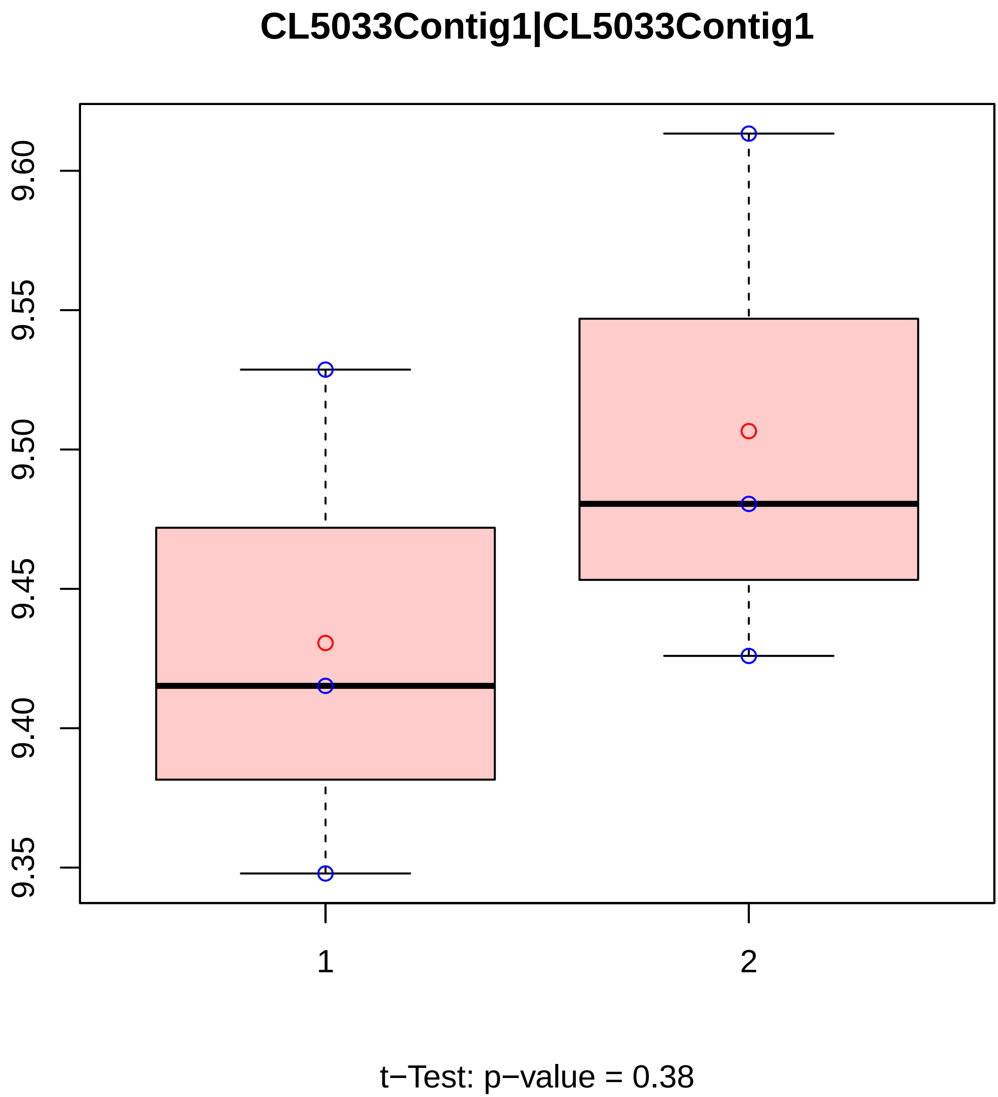
CL5033Contig1|CL5033Contig1
9.35
9.40
9.45
9.50
9.55
9.60
1
2
t−Test: p−value = 0.38

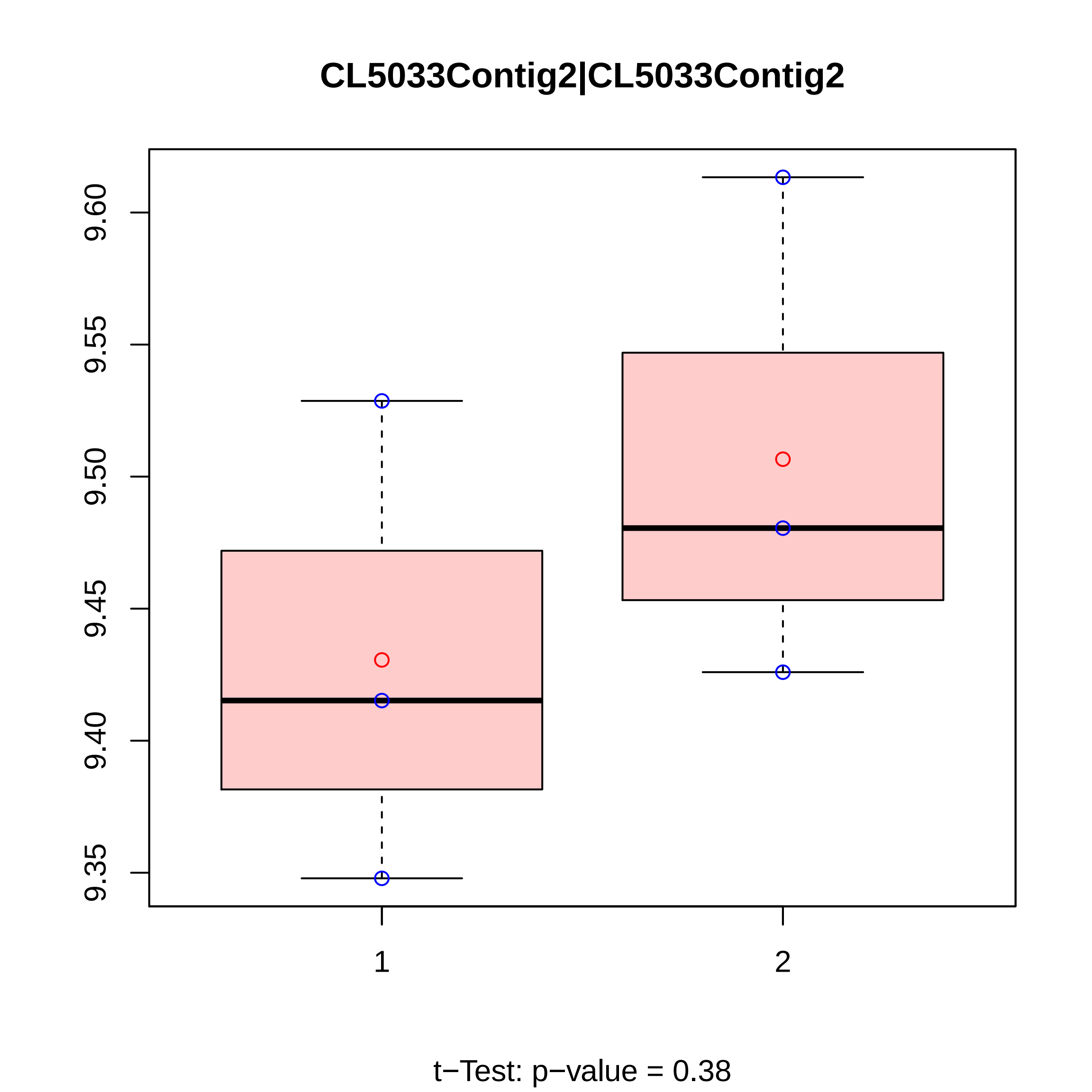
CL5033Contig2|CL5033Contig2
9.35
9.40
9.45
9.50
9.55
9.60
1
2
t−Test: p−value = 0.38

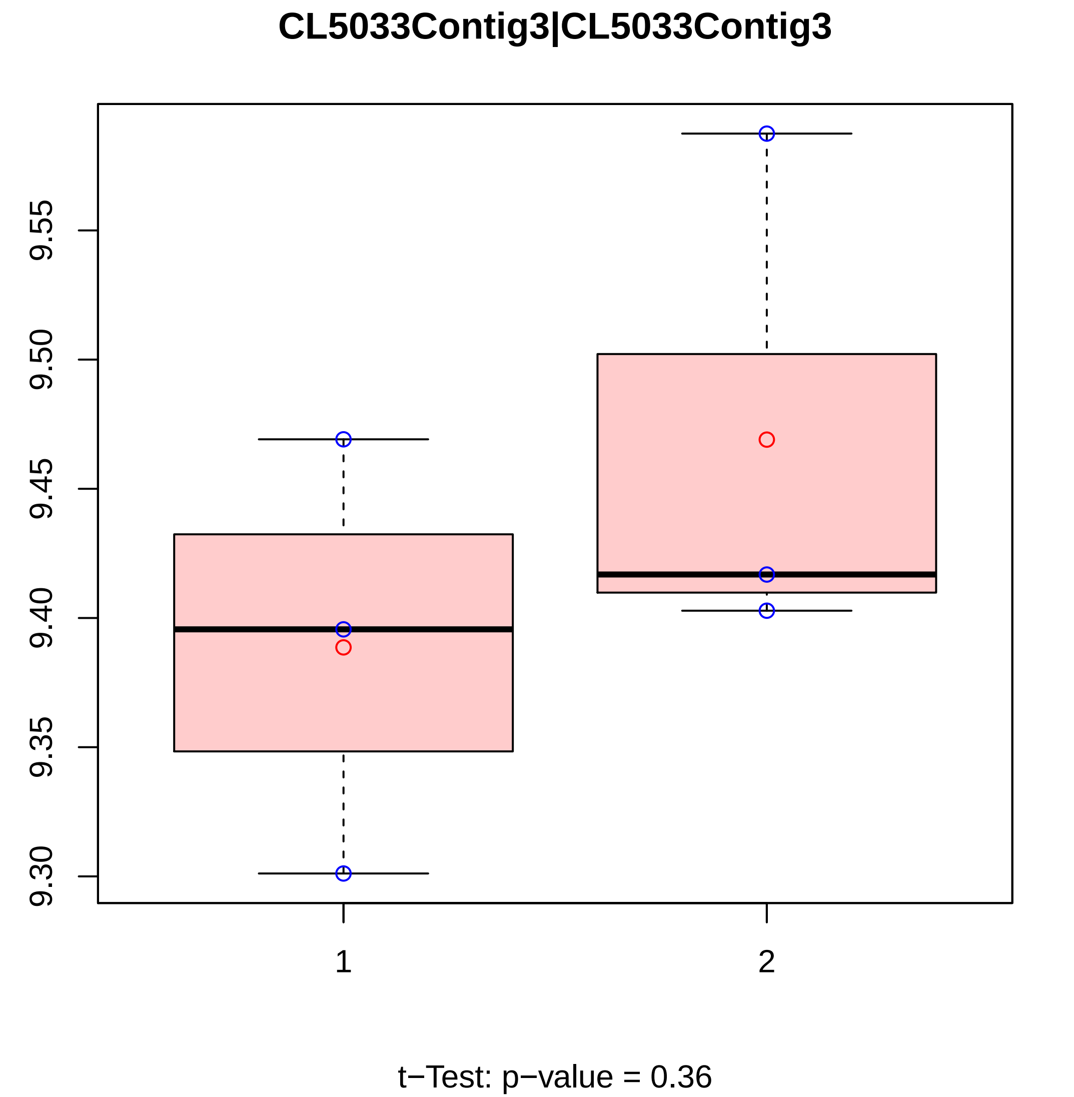
CL5033Contig3|CL5033Contig3
9.30
9.35
9.40
9.45
9.50
9.55
1
2
t−Test: p−value = 0.36

# CL5039Contig2|CL5039Contig2

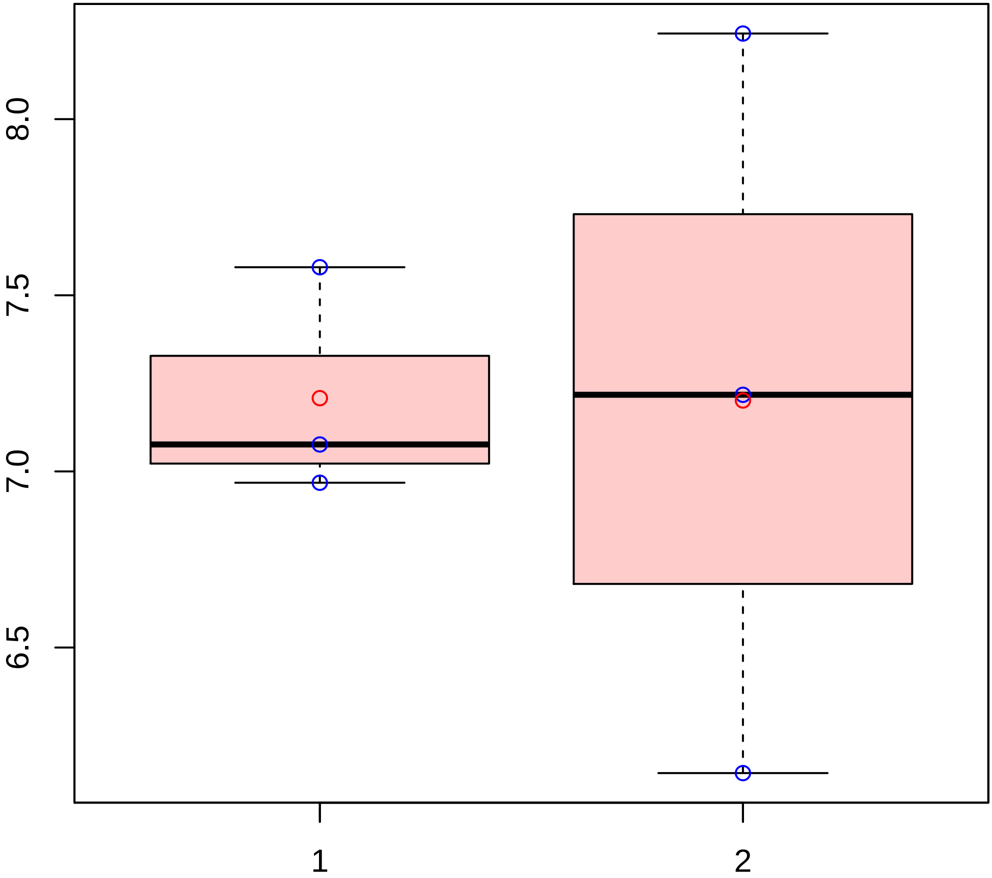


t−Test: p−value = 0.99

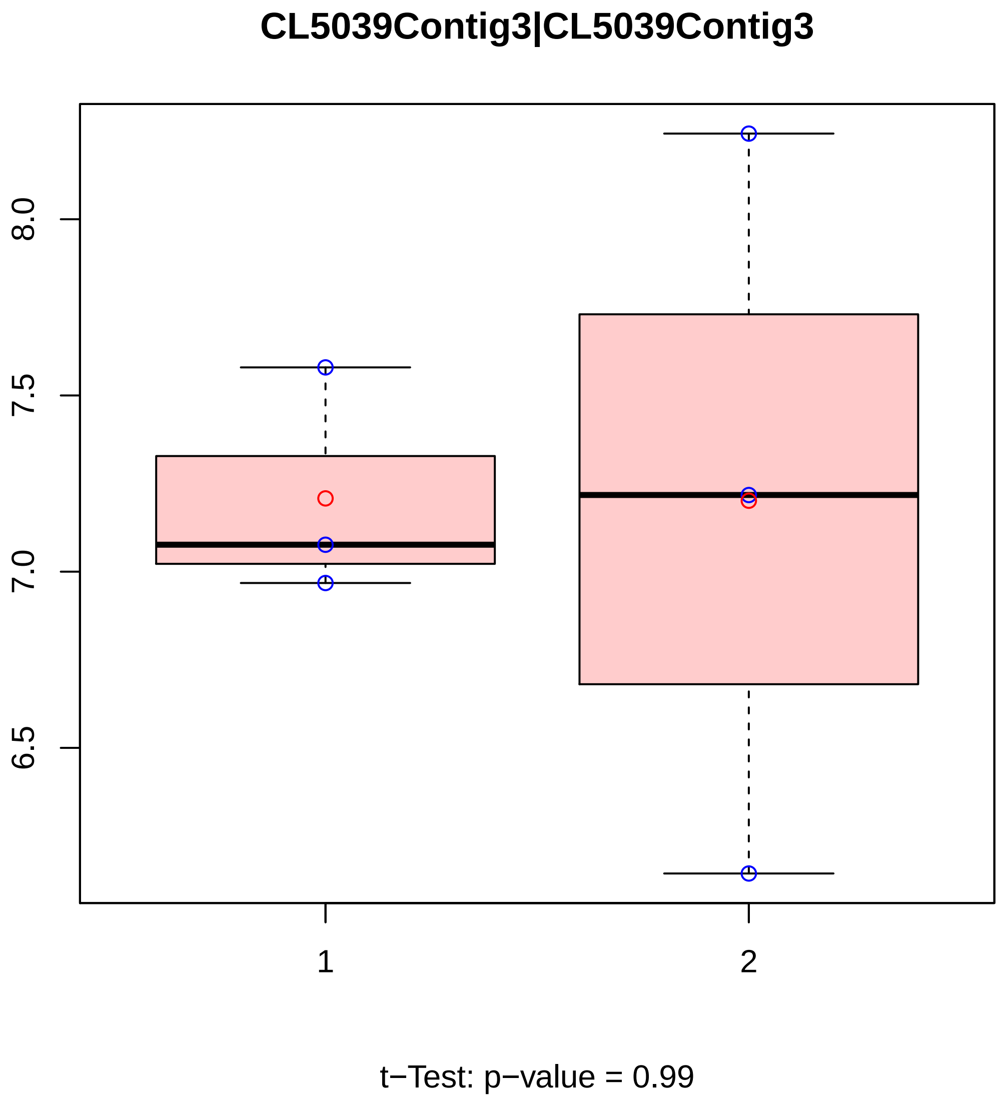
CL5039Contig3|CL5039Contig3
8.0
7.5
7.0
6.5
1
2
t−Test: p−value = 0.99

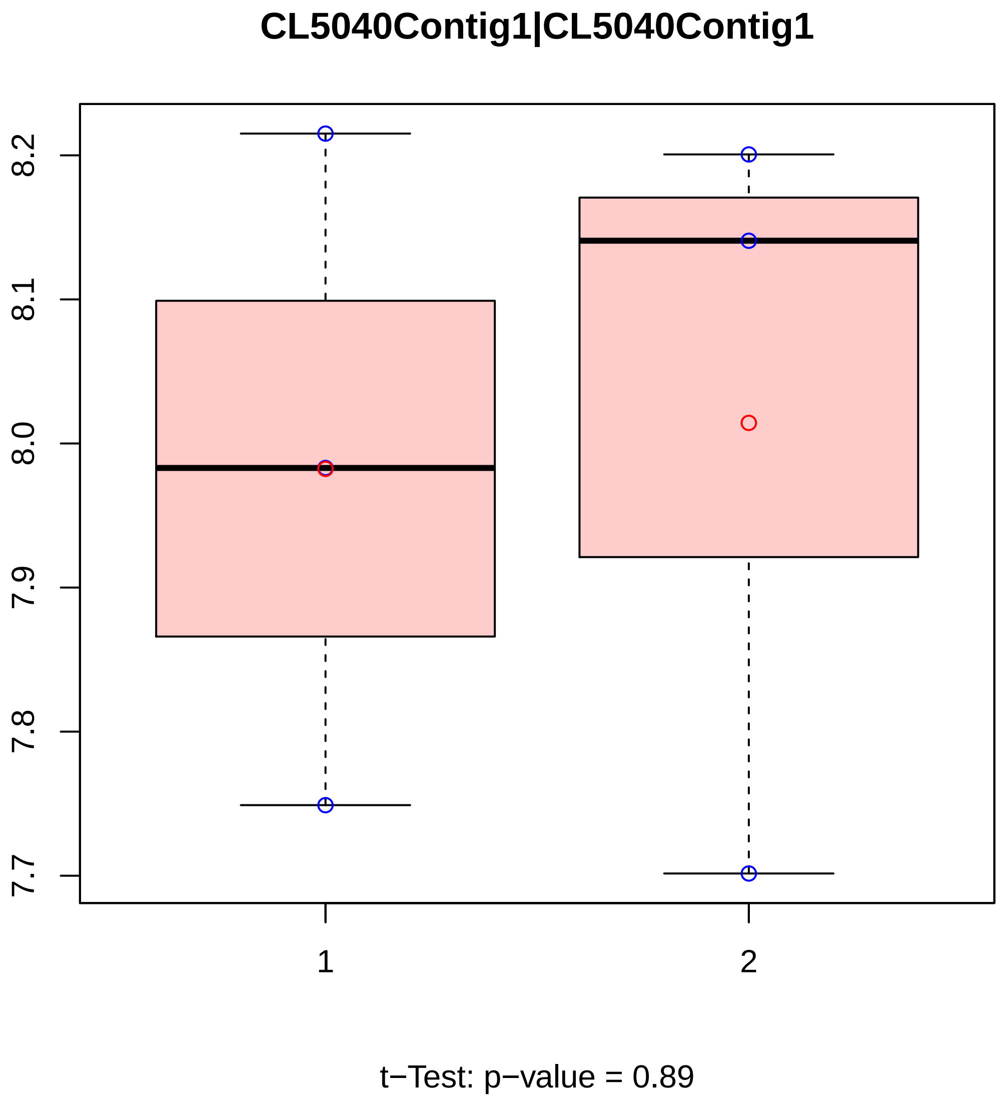
CL5040Contig1|CL5040Contig1
8.2
8.1
8.0
7.9
7.8
7.7
1
2
t−Test: p−value = 0.89

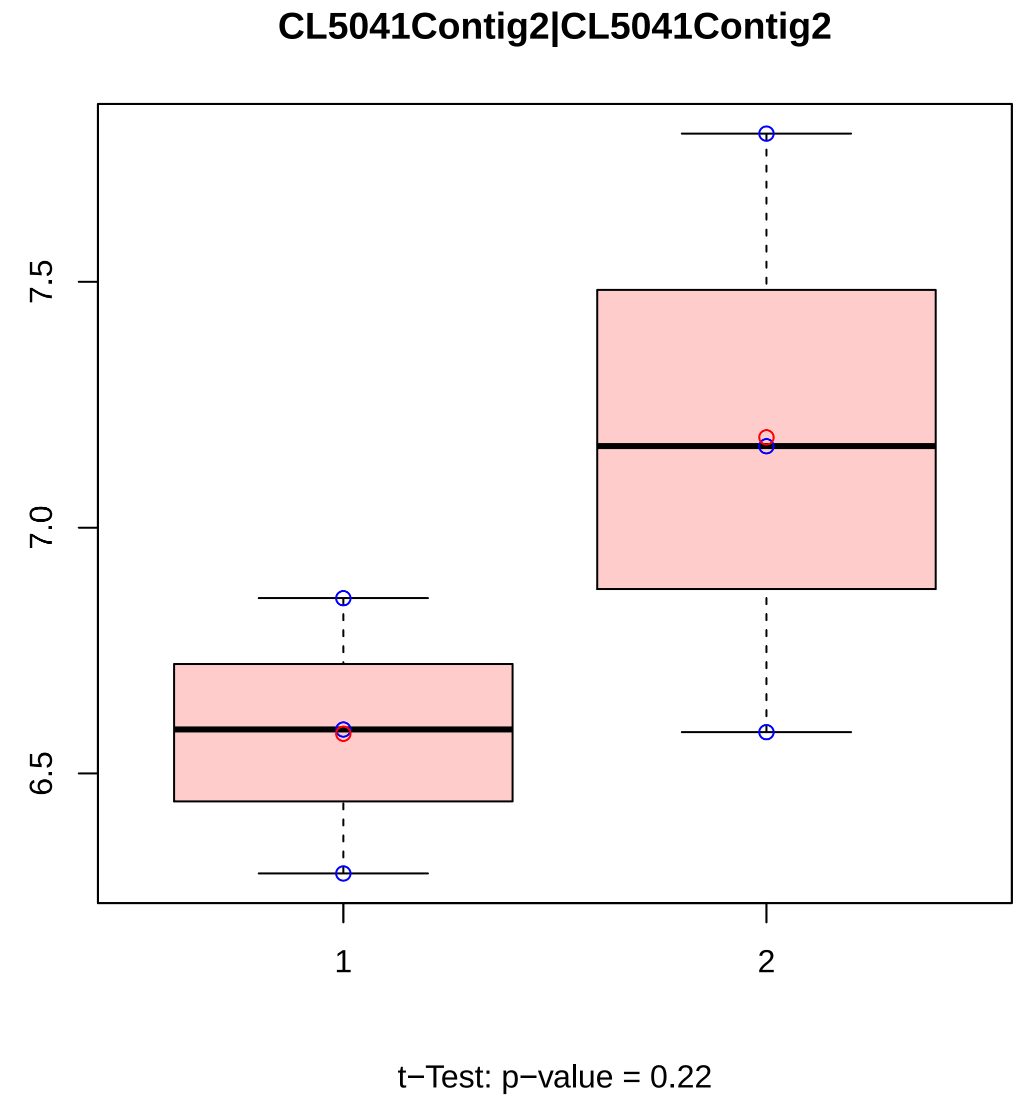
CL5041Contig2|CL5041Contig2
7.5
7.0
6.5
1
2
t−Test: p−value = 0.22

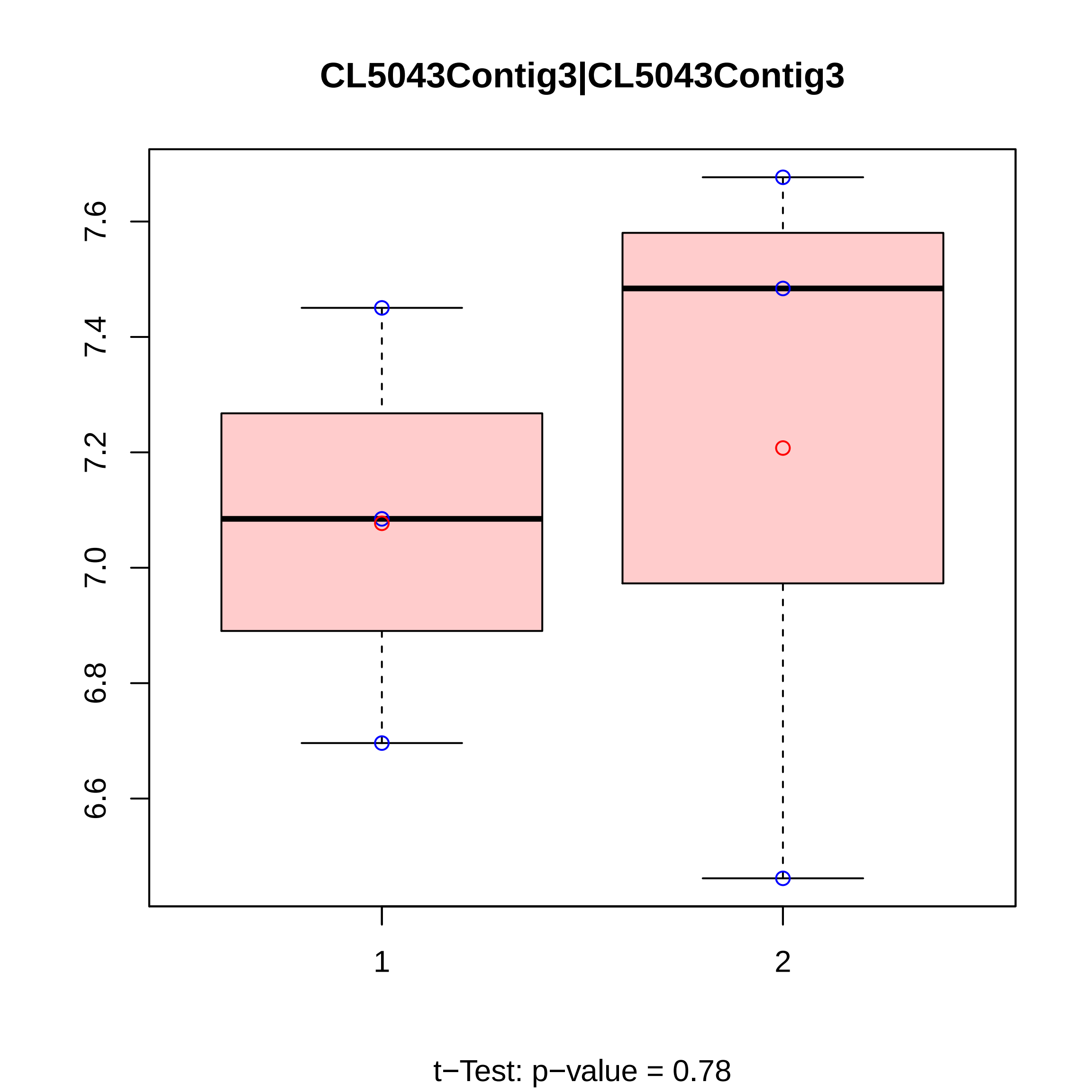

CL5043Contig3|CL5043Contig3
7.6
7.4
7.2
7.0
6.8
6.6
1
2
t−Test: p−value = 0.78

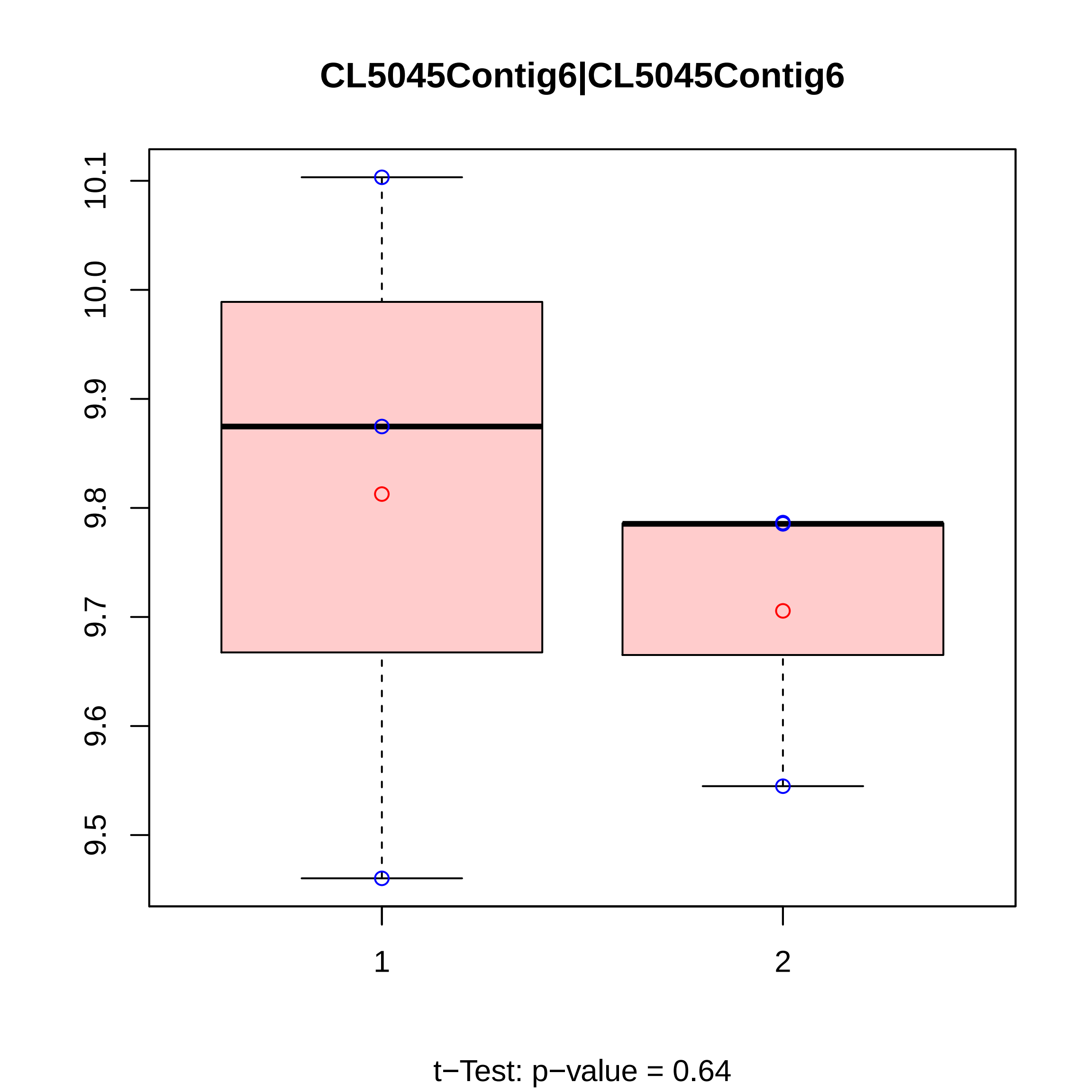
CL5045Contig6|CL5045Contig6
10.1
10.0
9.9
9.8
9.7
9.6
9.5
1
2
t−Test: p−value = 0.64

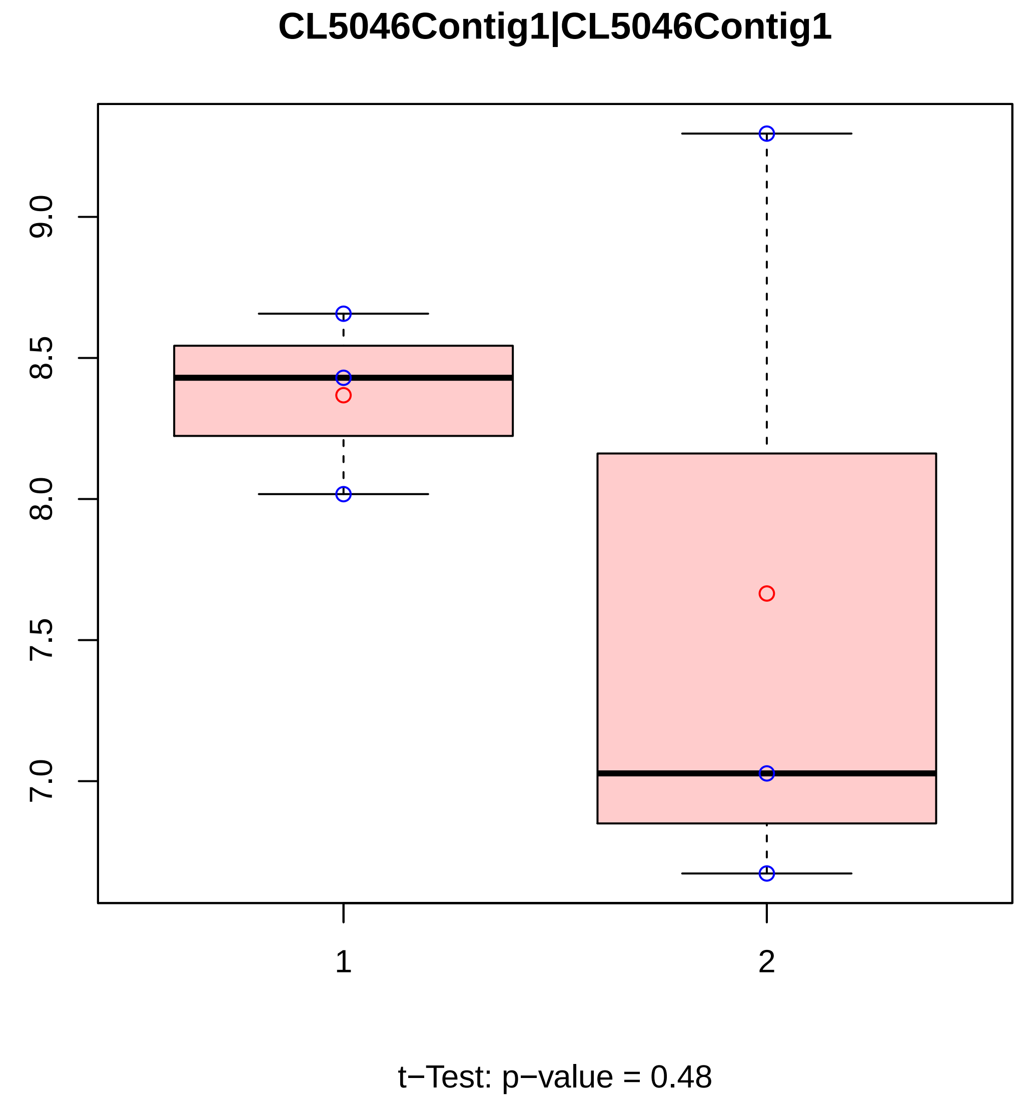
CL5046Contig1|CL5046Contig1
9.0
8.5
8.0
7.5
7.0
1
2
t−Test: p−value = 0.48

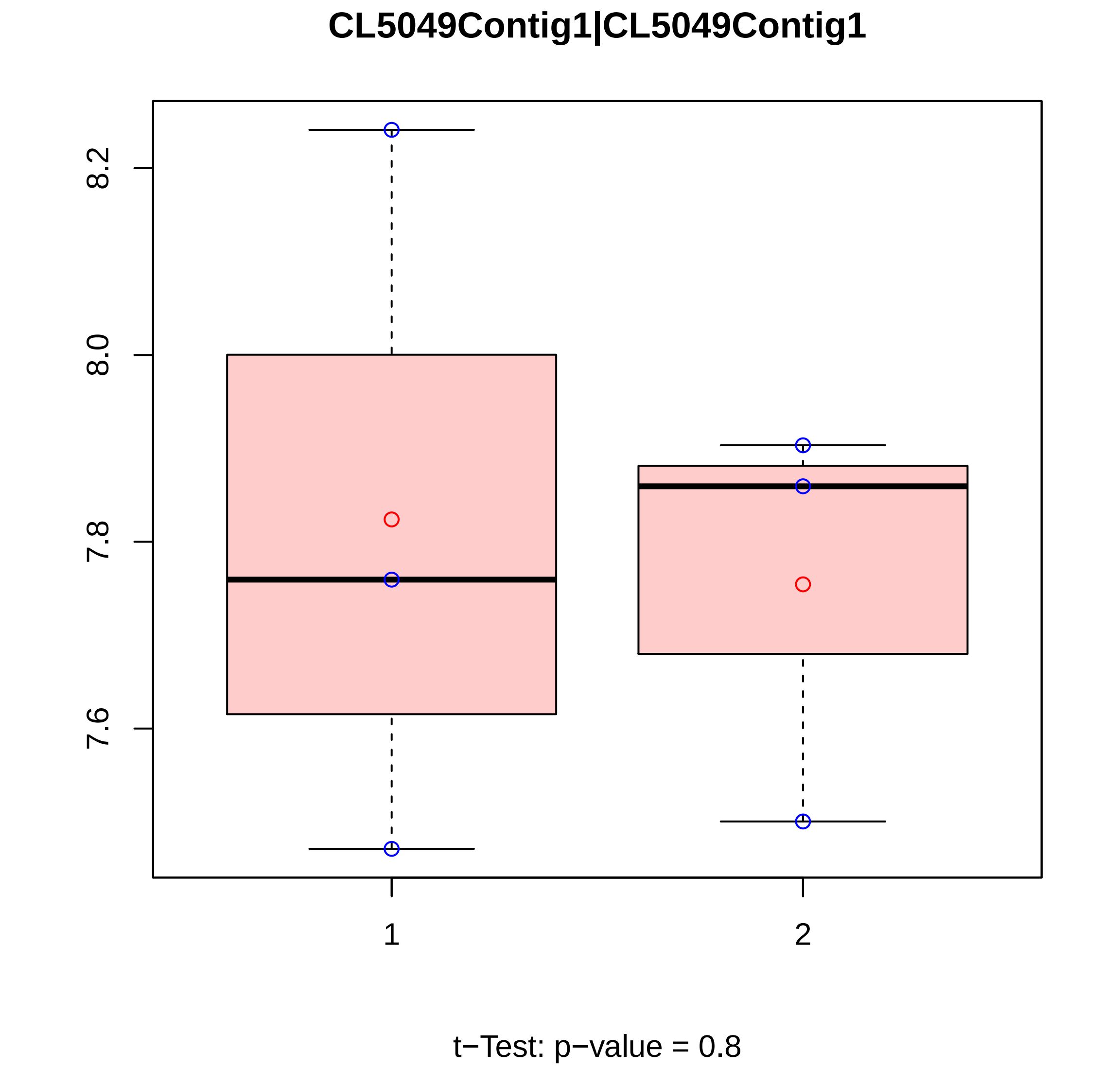
CL5049Contig1|CL5049Contig1
8.2
8.0
7.8
7.6
1
2
t−Test: p−value = 0.8

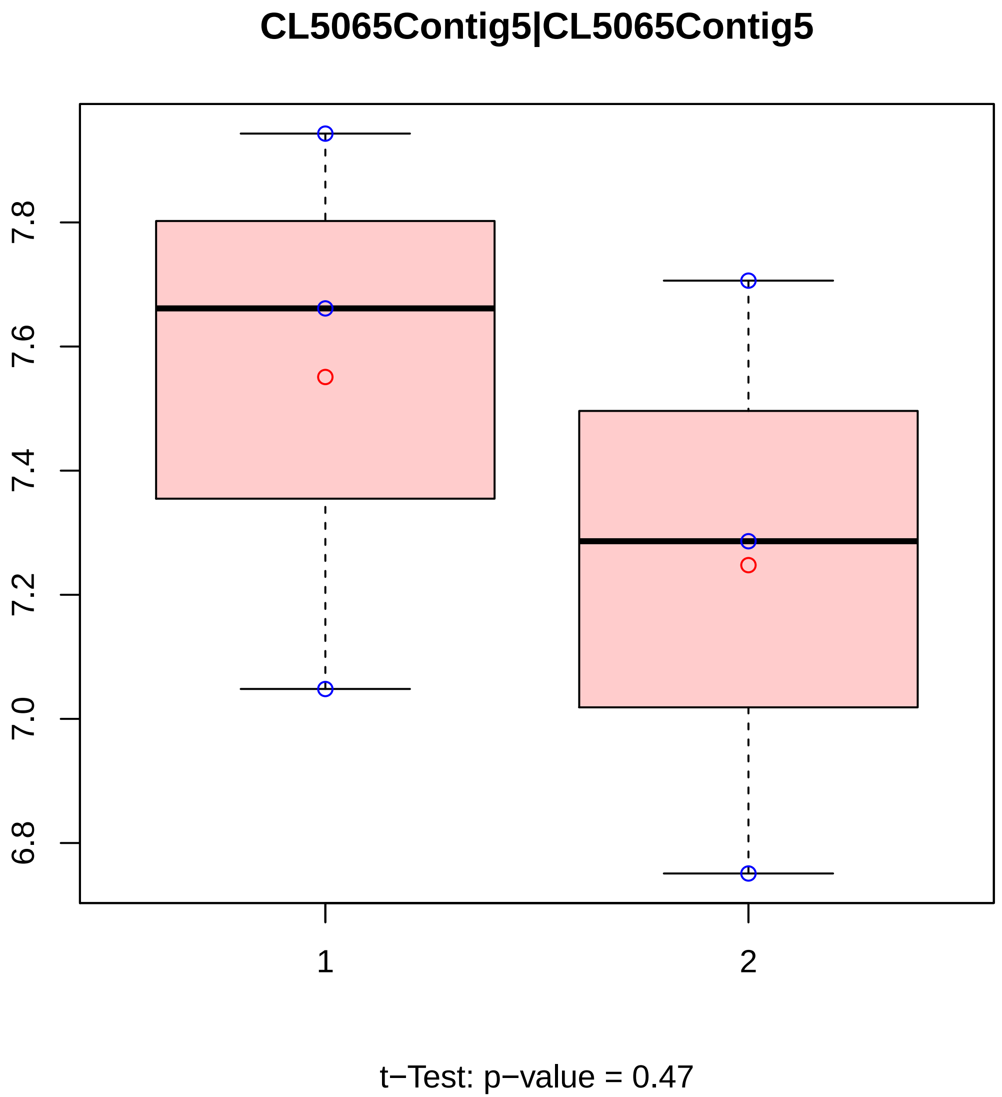
CL5065Contig5|CL5065Contig5
7.8
7.6
7.4
7.2
7.0
6.8
1
2
t−Test: p−value = 0.47

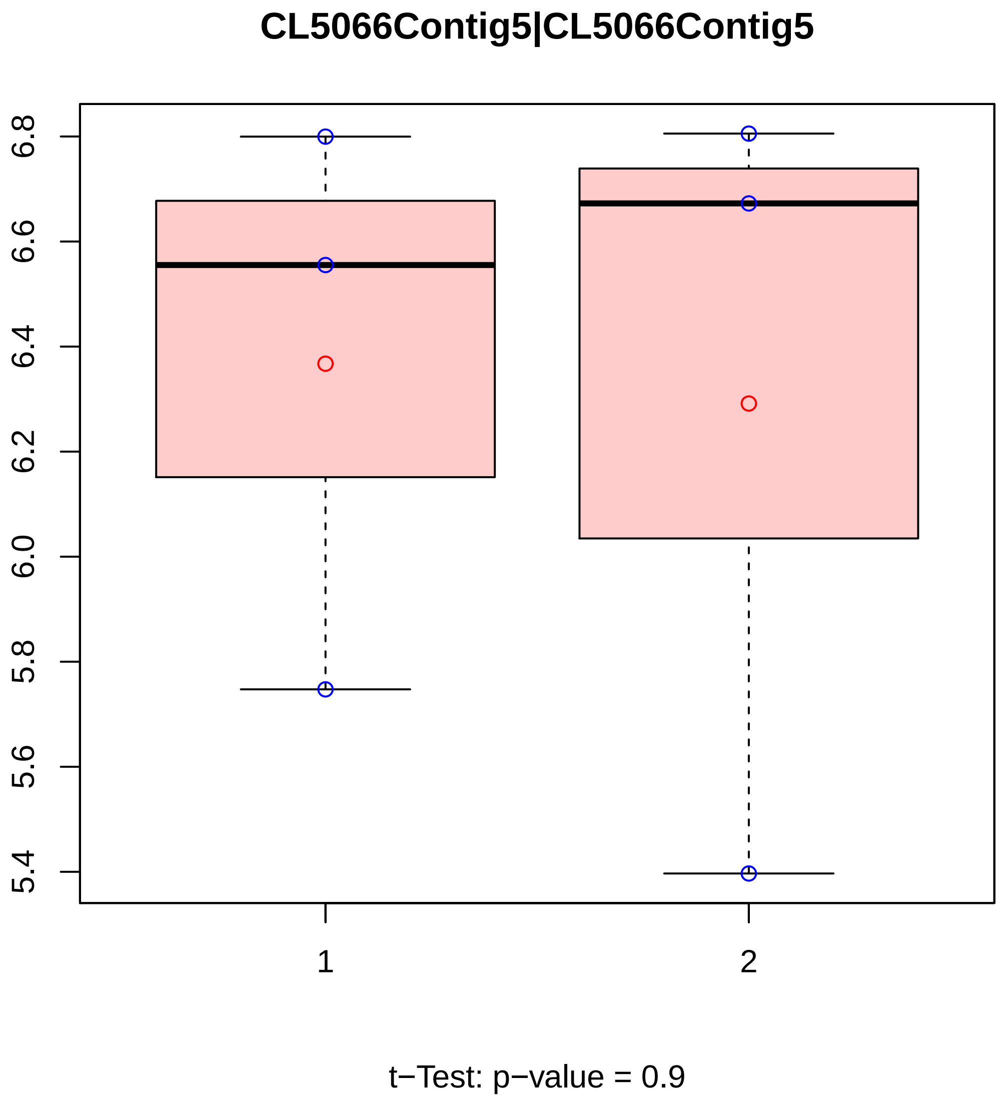
CL5066Contig5|CL5066Contig5
6.8
6.6
6.4
6.2
6.0
5.8
5.6
5.4
1
2
t−Test: p−value = 0.9

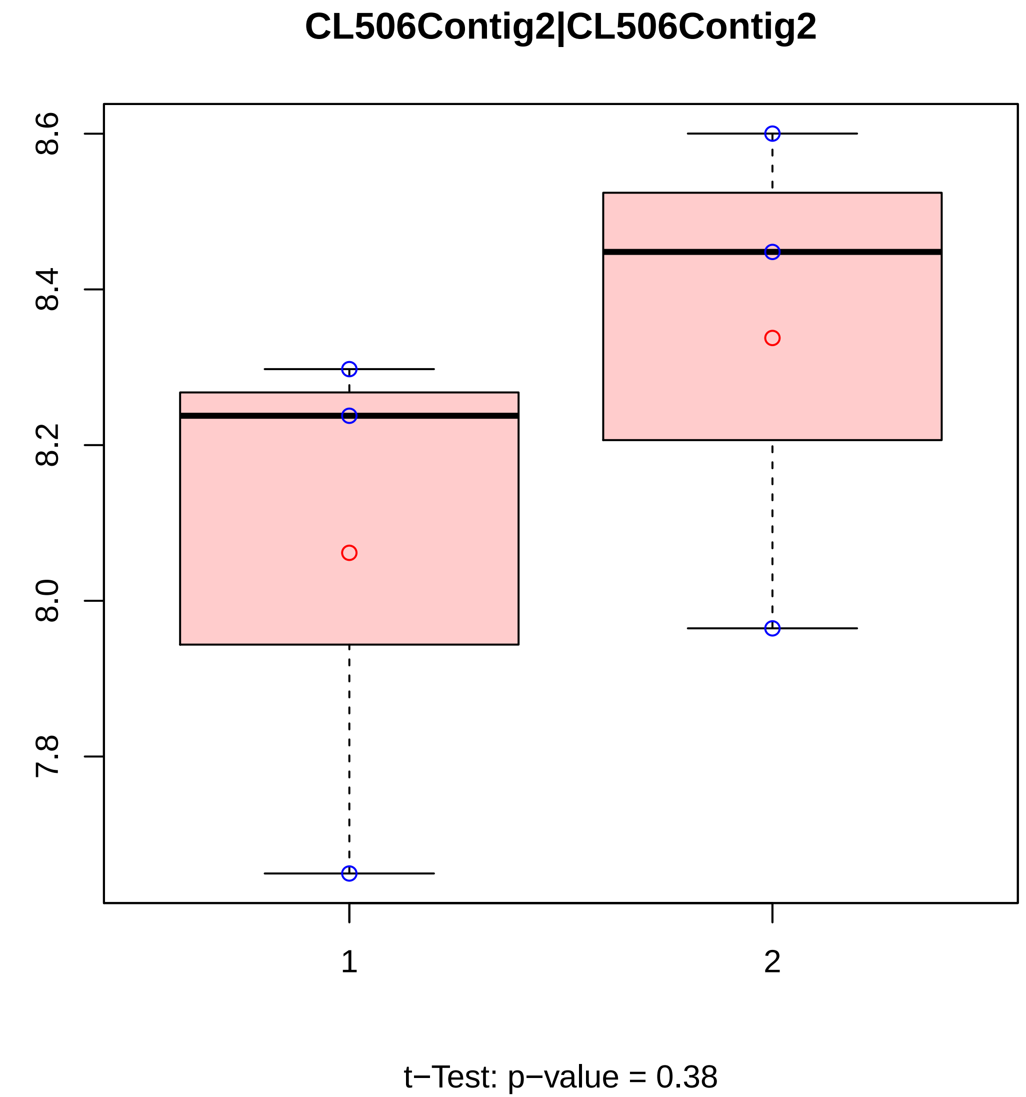

CL506Contig2|CL506Contig2
8.6
8.4
8.2
8.0
7.8
1
2
t−Test: p−value = 0.38

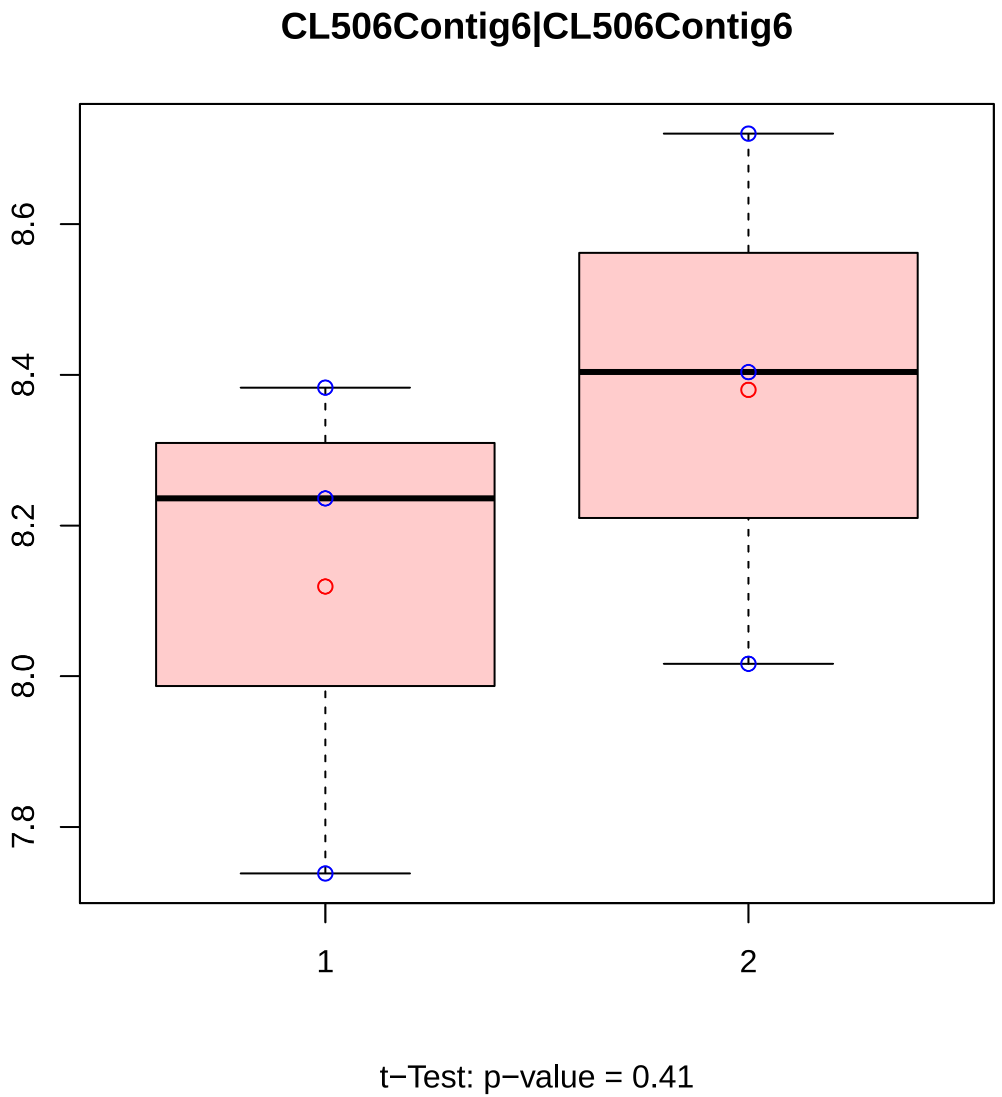
CL506Contig6|CL506Contig6
8.6
8.4
8.2
8.0
7.8
1
2
t−Test: p−value = 0.41

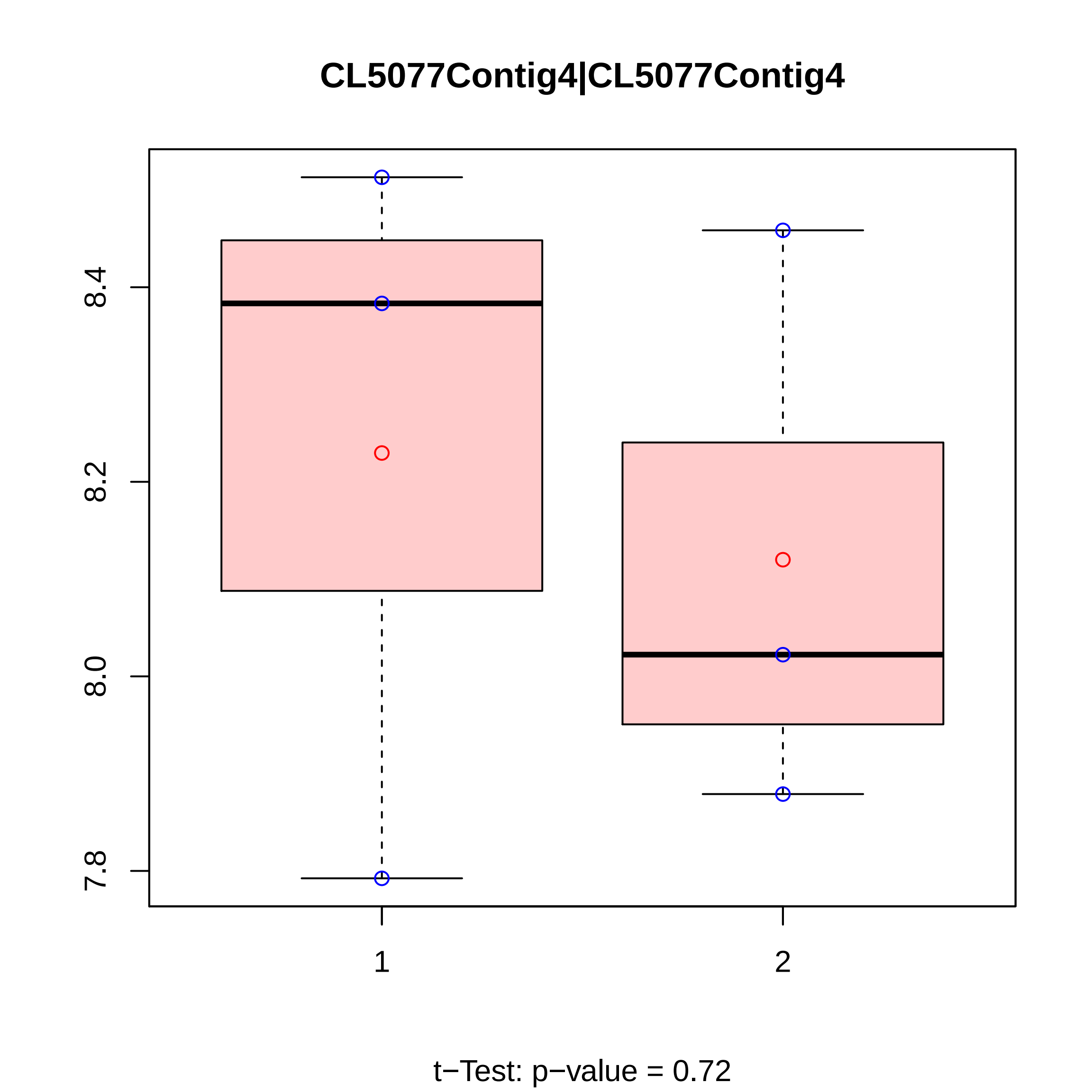
CL5077Contig4|CL5077Contig4
8.4
8.2
8.0
7.8
1
2
t−Test: p−value = 0.72

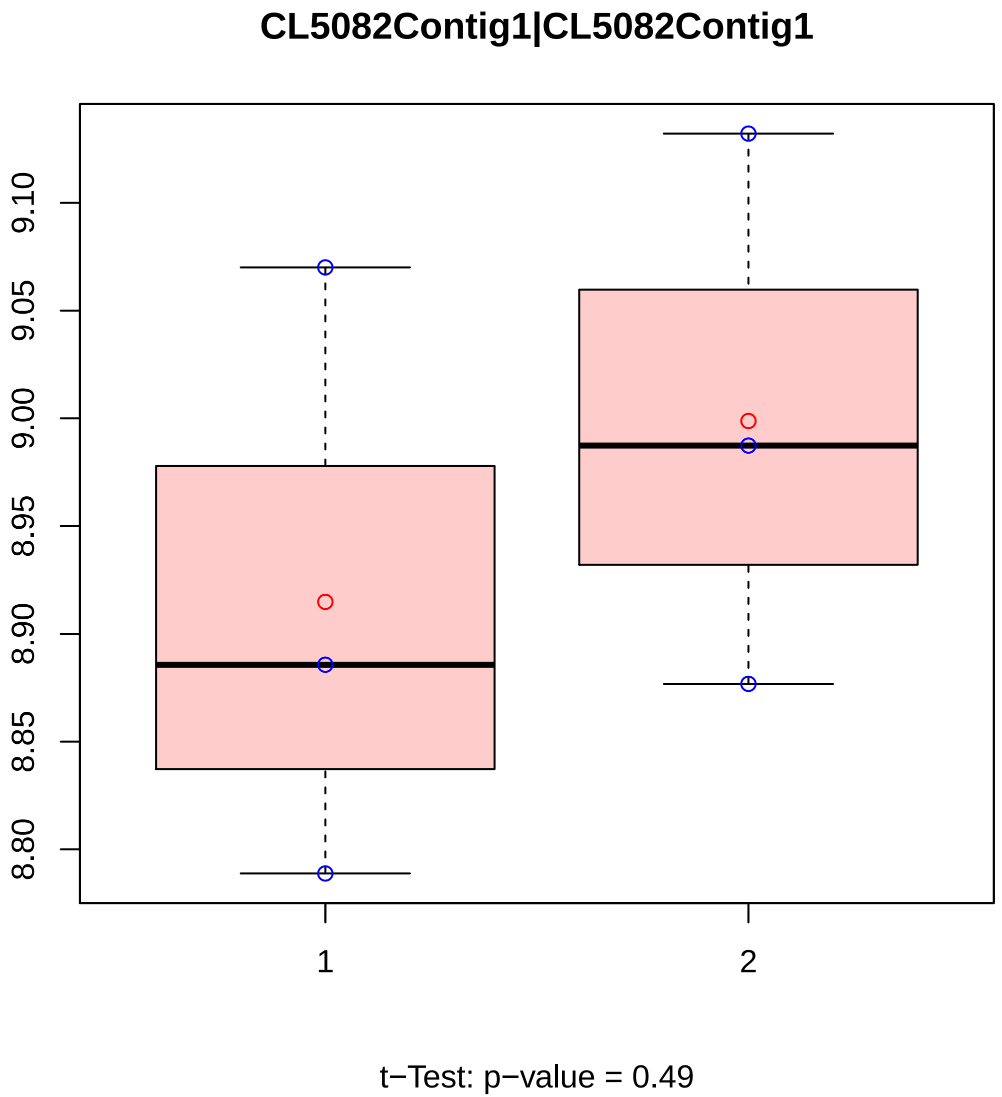
CL5082Contig1|CL5082Contig1
9.10
9.05
9.00
8.95
8.90
8.85
8.80
1
2
t−Test: p−value = 0.49

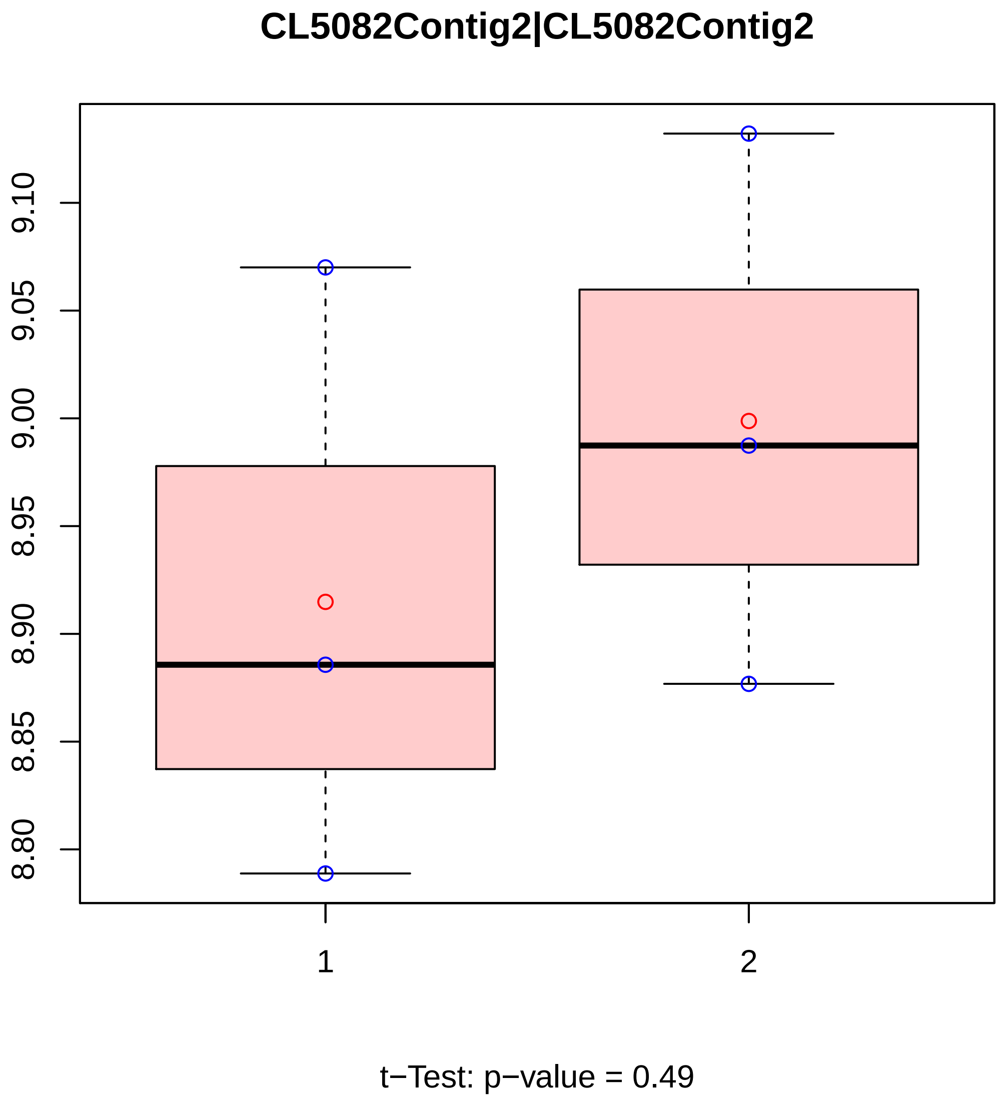

CL5082Contig2|CL5082Contig2
9.10
9.05
9.00
8.95
8.90
8.85
8.80
1
2
t−Test: p−value = 0.49

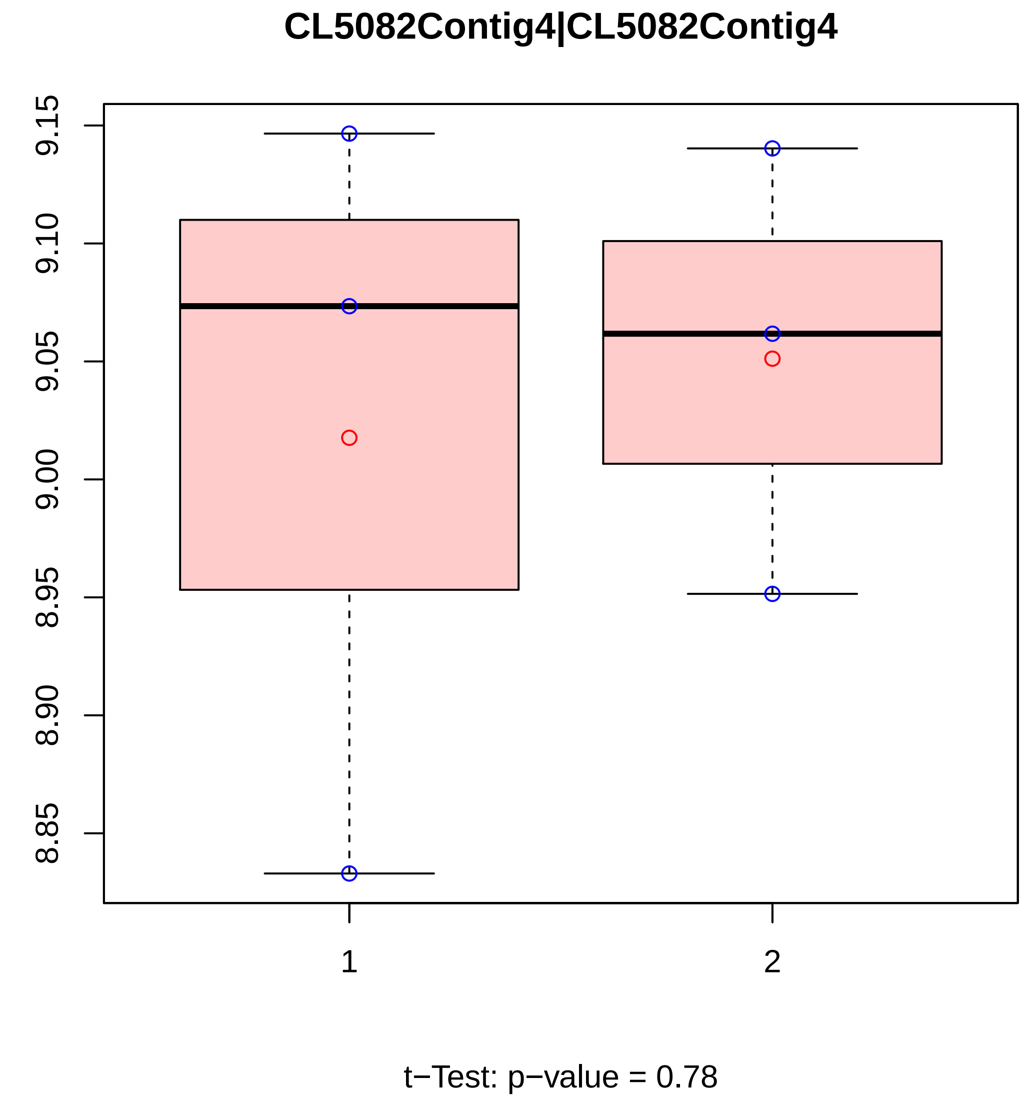
CL5082Contig4|CL5082Contig4
9.15
9.10
9.05
9.00
8.95
8.90
8.85
1
2
t−Test: p−value = 0.78

# CL5082Contig5|CL5082Contig5

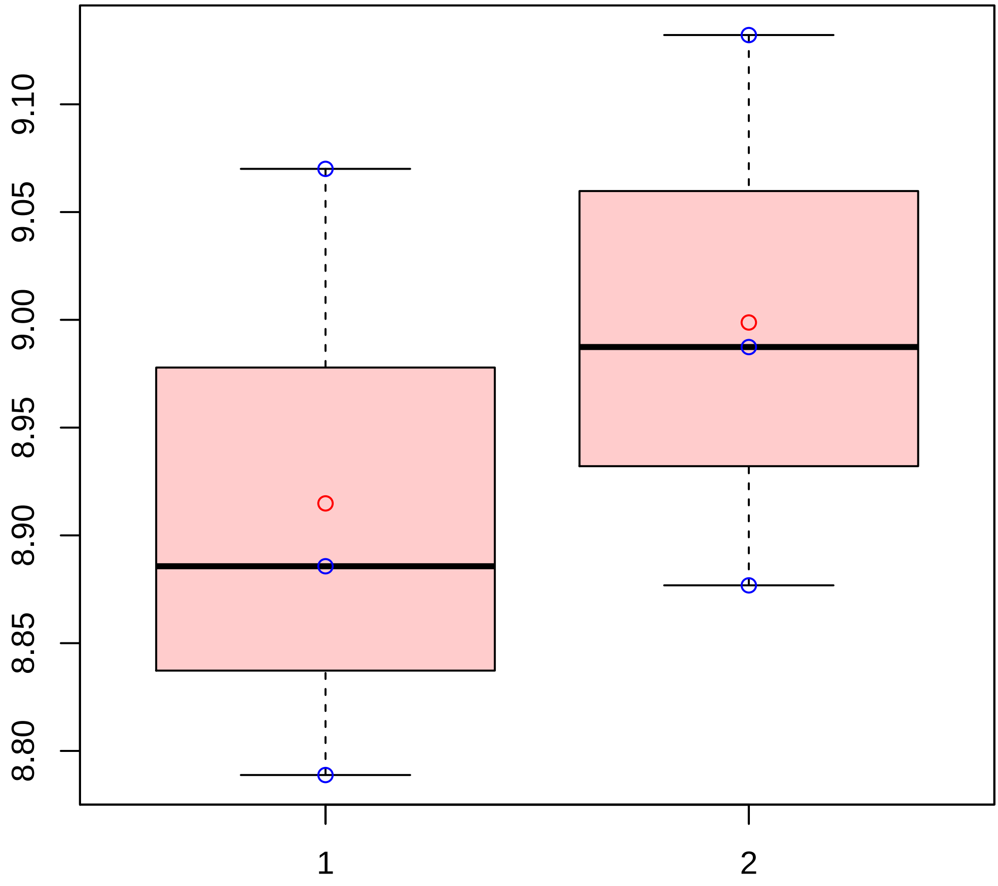


t−Test: p−value = 0.49

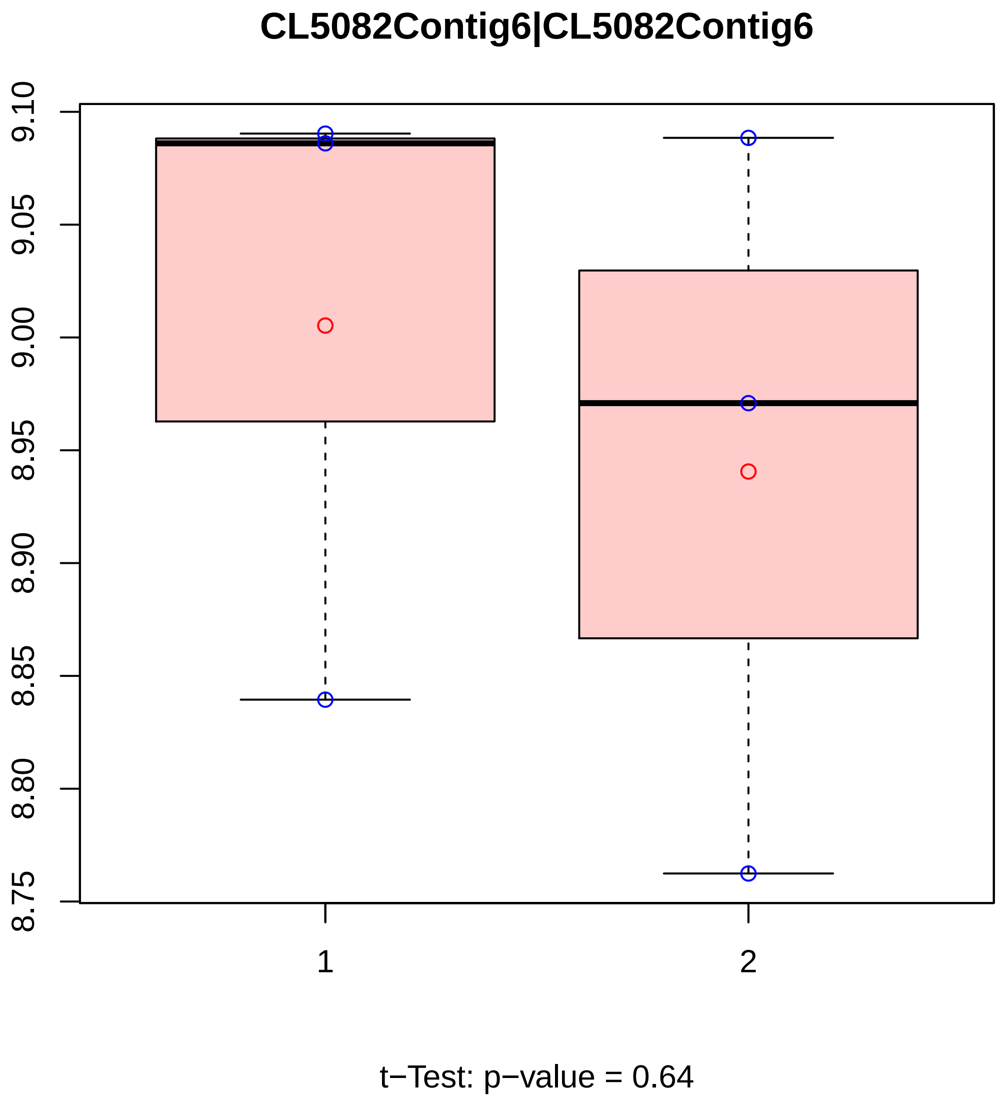

CL5082Contig6|CL5082Contig6
9.10
9.05
9.00
8.95
8.90
8.85
8.80
8.75
1
2
t−Test: p−value = 0.64

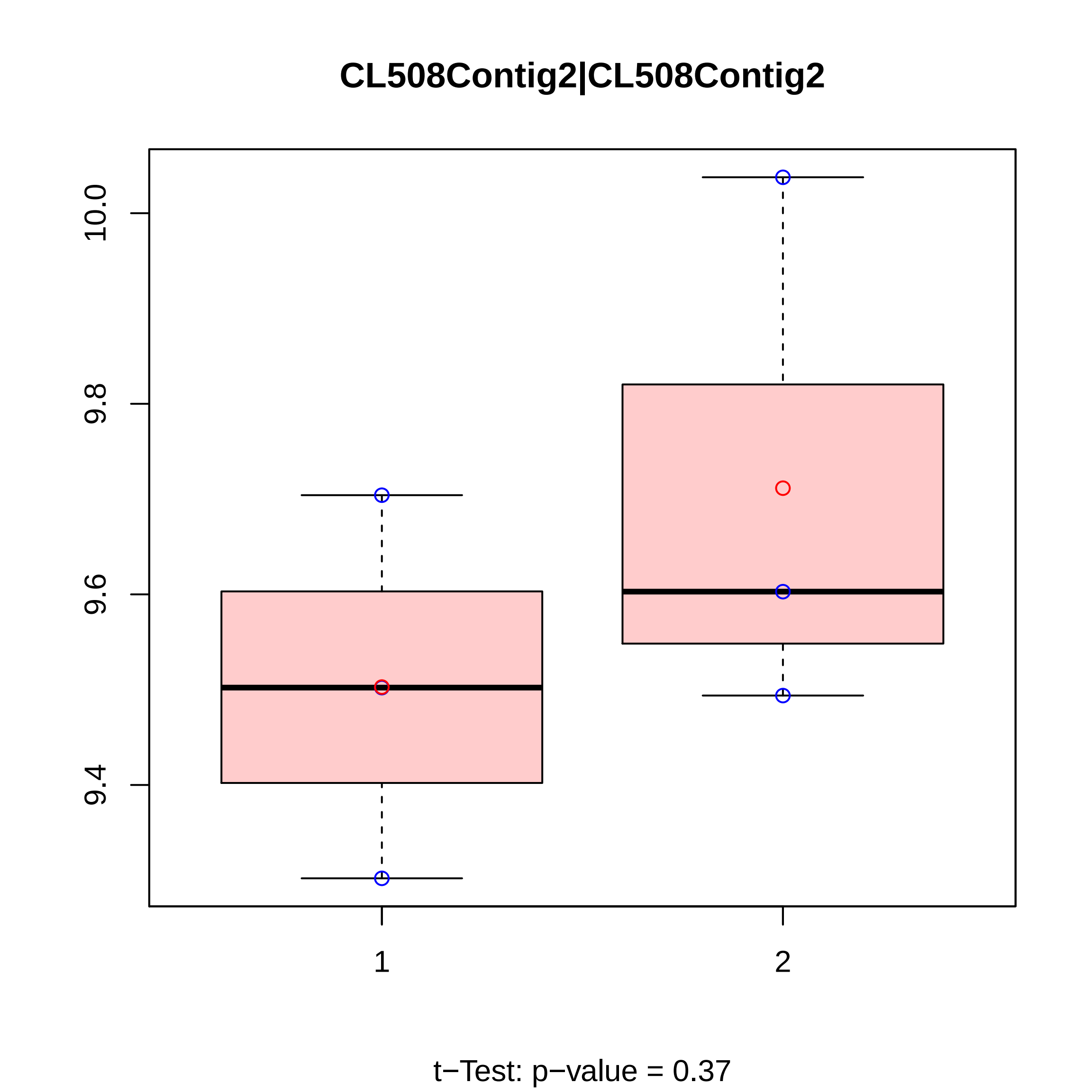
CL508Contig2|CL508Contig2
10.0
9.8
9.6
9.4
1
2
t−Test: p−value = 0.37

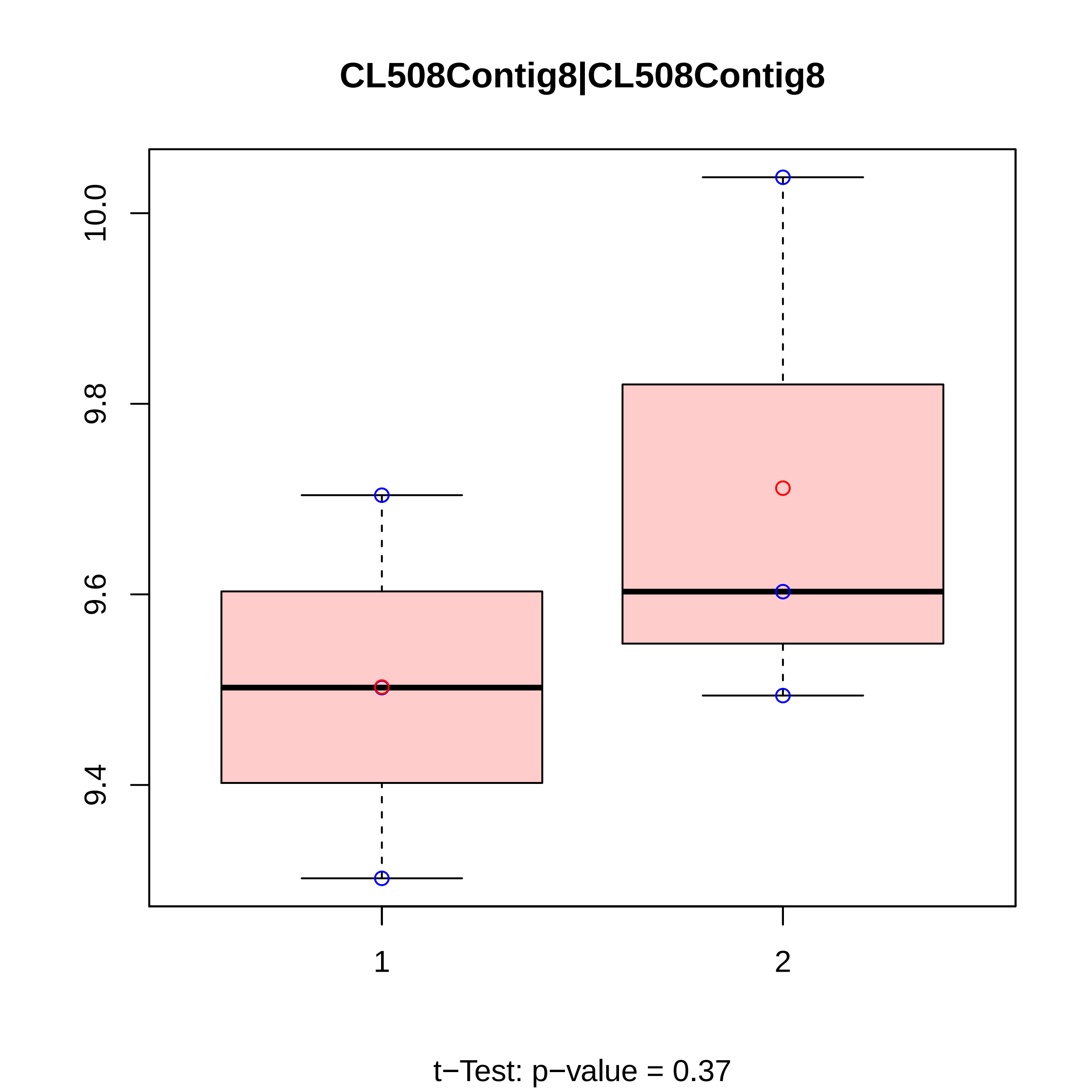
CL508Contig8|CL508Contig8
10.0
9.8
9.6
9.4
1
2
t−Test: p−value = 0.37

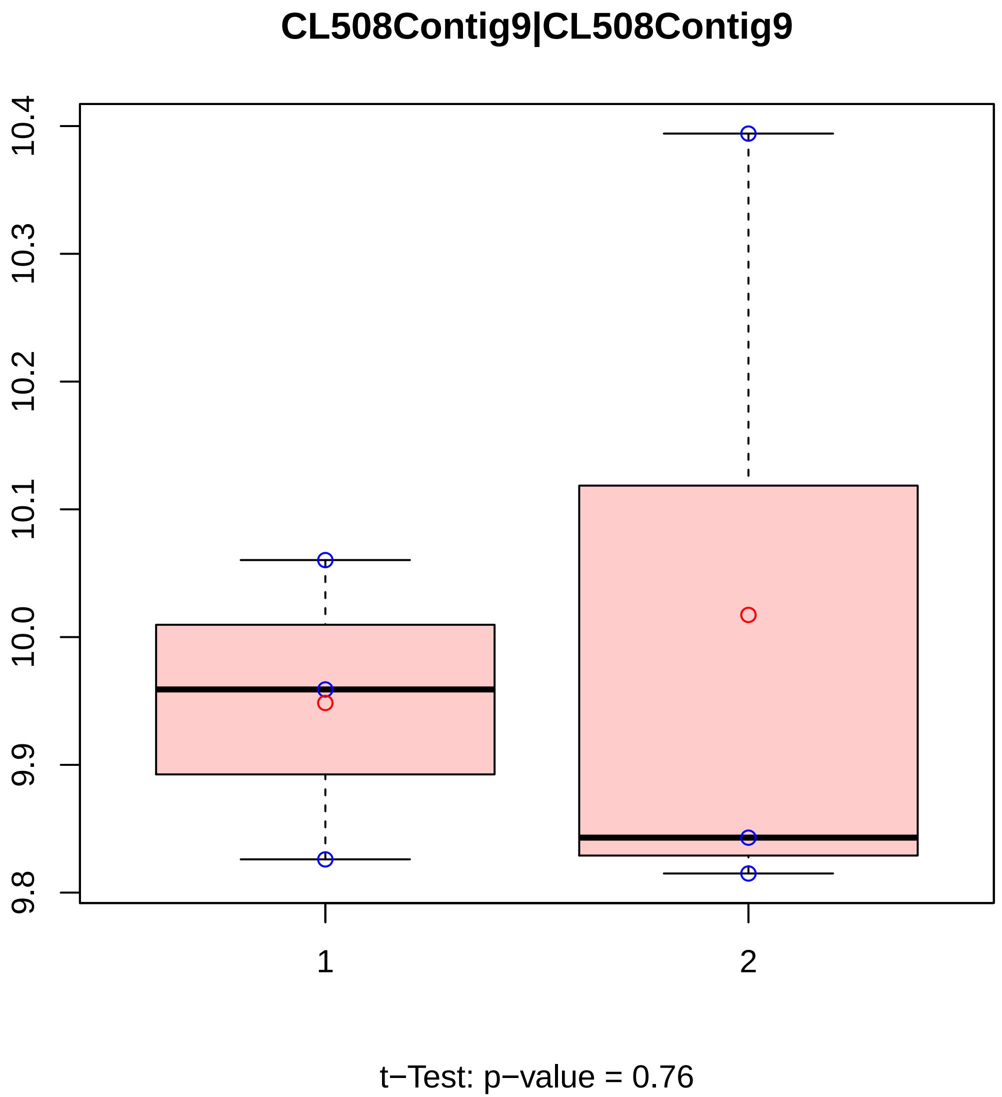
CL508Contig9|CL508Contig9
10.4
10.3
10.2
10.1
10.0
9.9
9.8
1
2
t−Test: p−value = 0.76

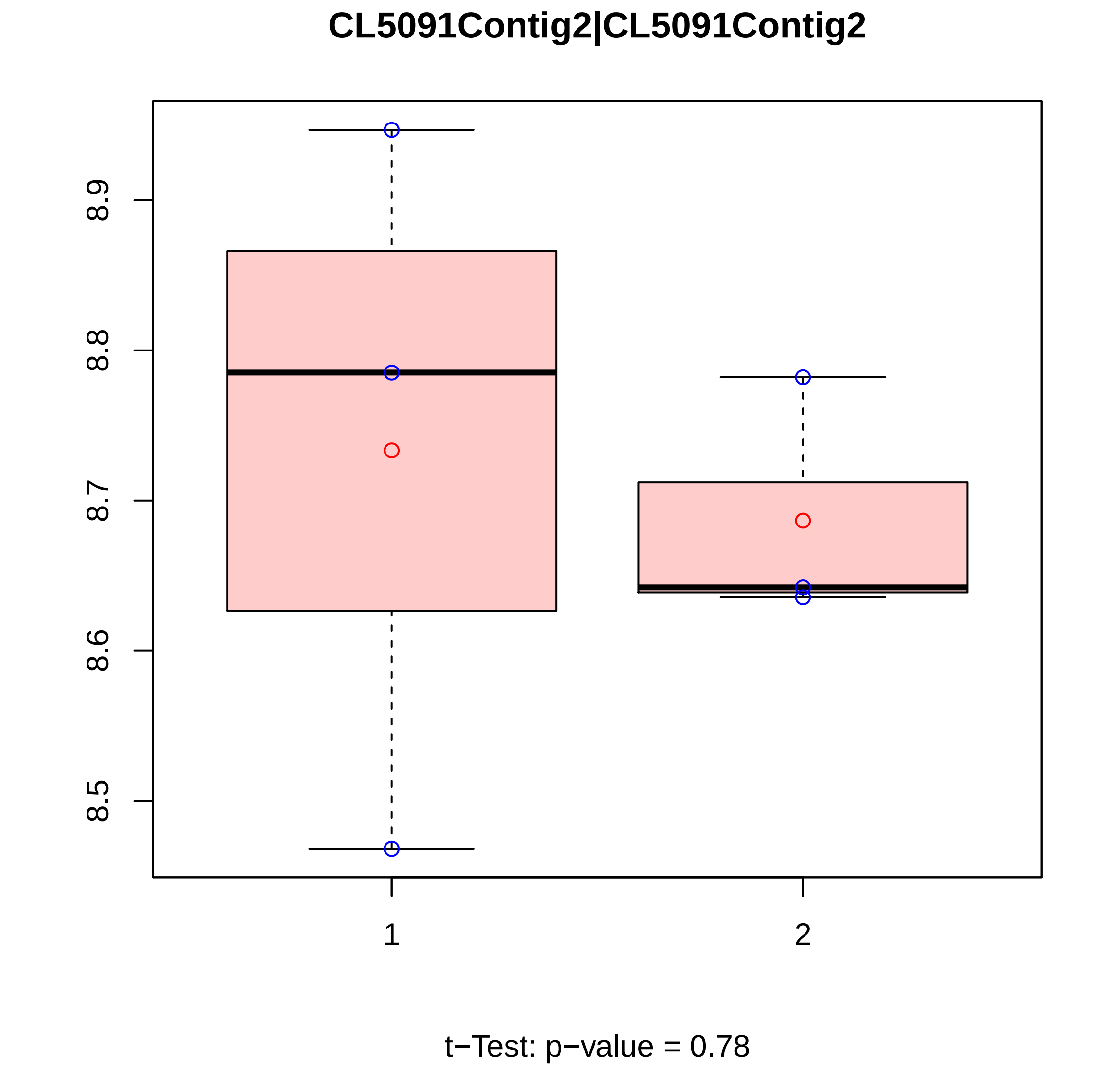

CL5091Contig2|CL5091Contig2
8.9
8.8
8.7
8.6
8.5
1
2
t−Test: p−value = 0.78

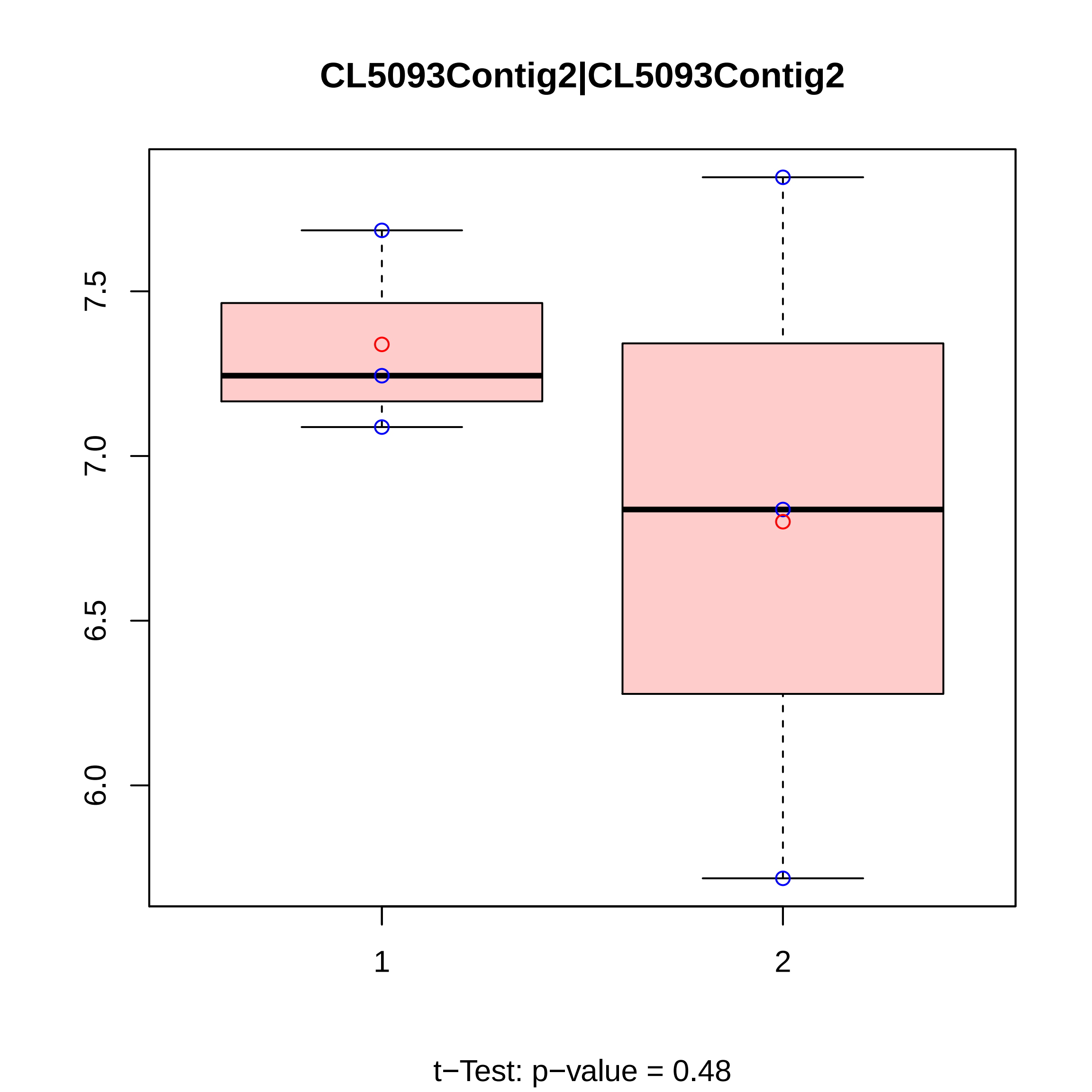

CL5093Contig2|CL5093Contig2
7.5
7.0
6.5
6.0
1
2
t−Test: p−value = 0.48

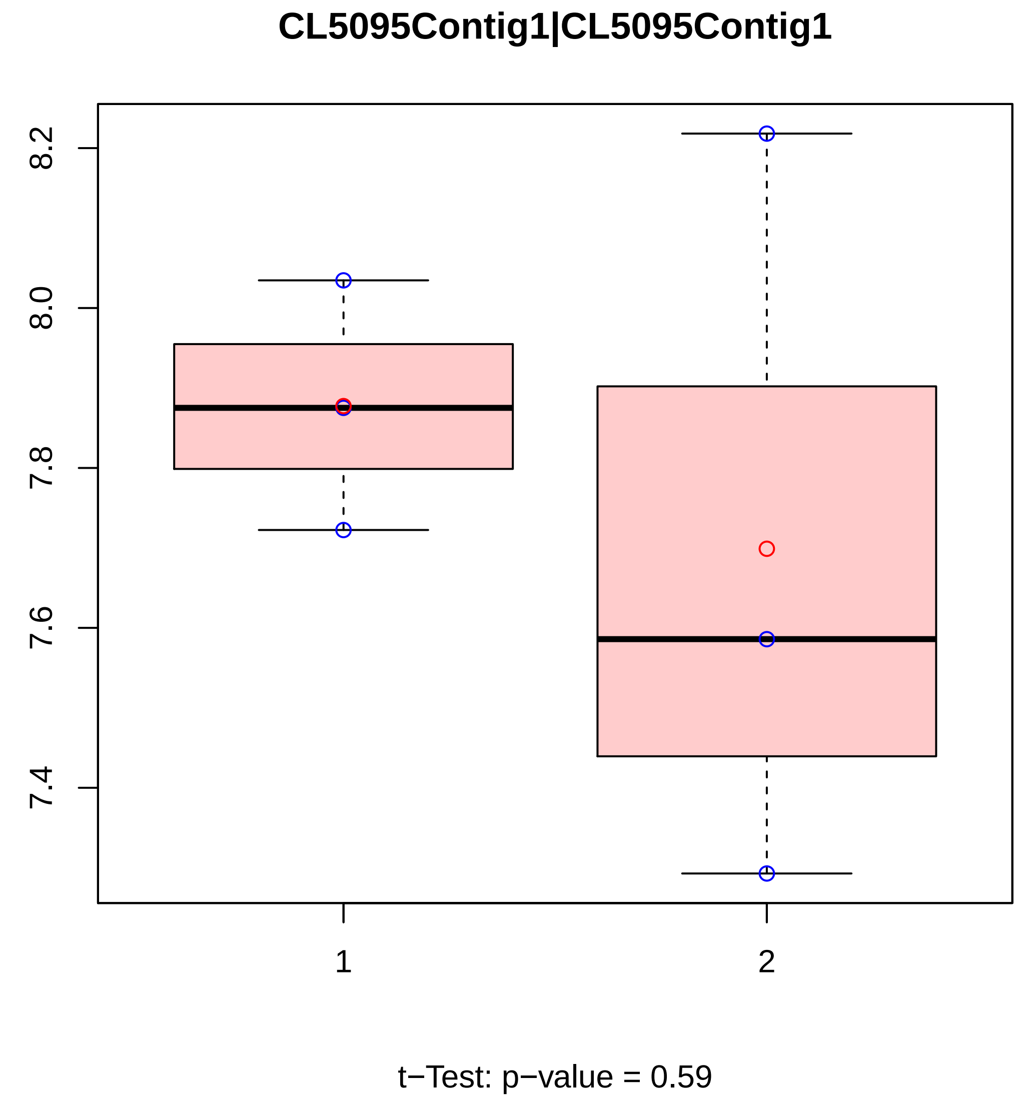

CL5095Contig1|CL5095Contig1
8.2
8.0
7.8
7.6
7.4
1
2
t−Test: p−value = 0.59

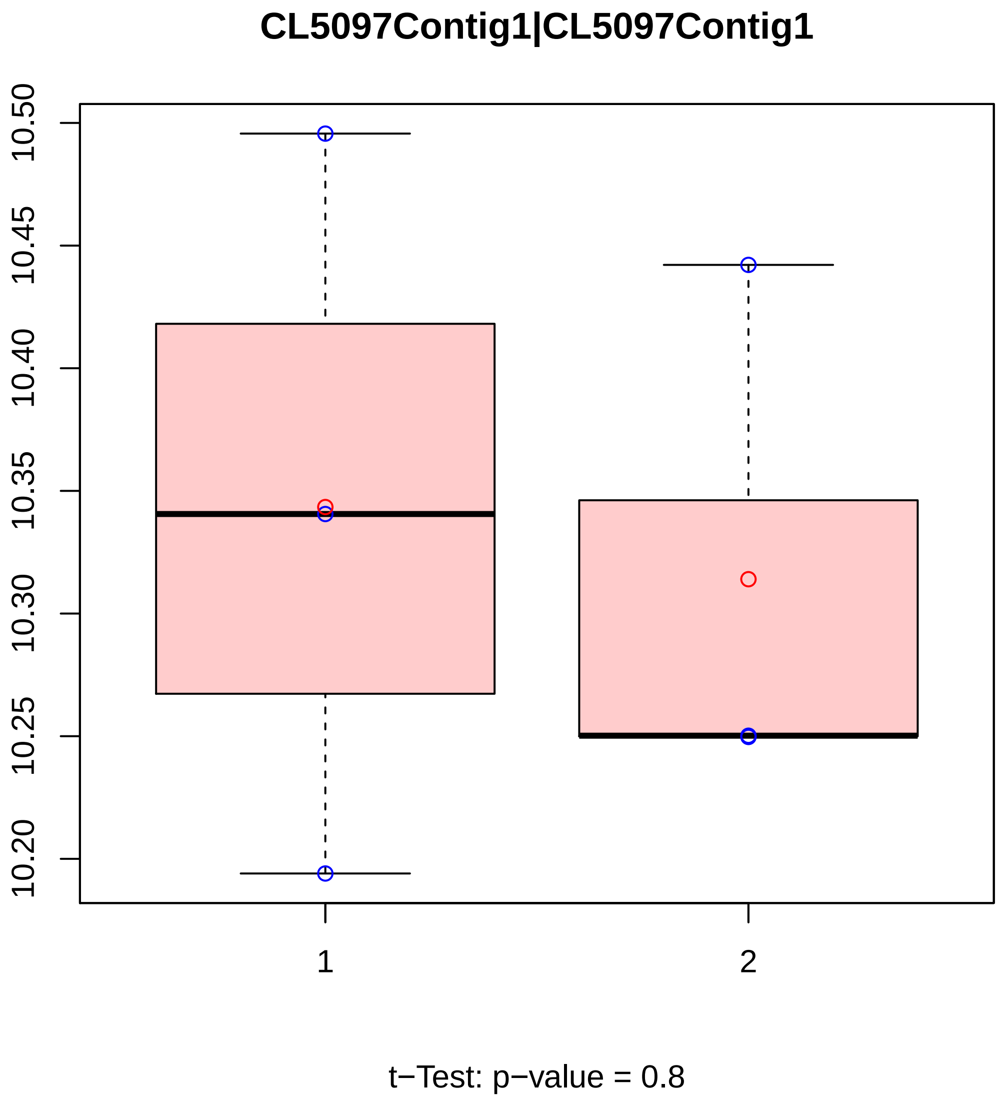
CL5097Contig1|CL5097Contig1
10.50
10.45
10.40
10.35
10.30
10.25
10.20
1
2
t−Test: p−value = 0.8

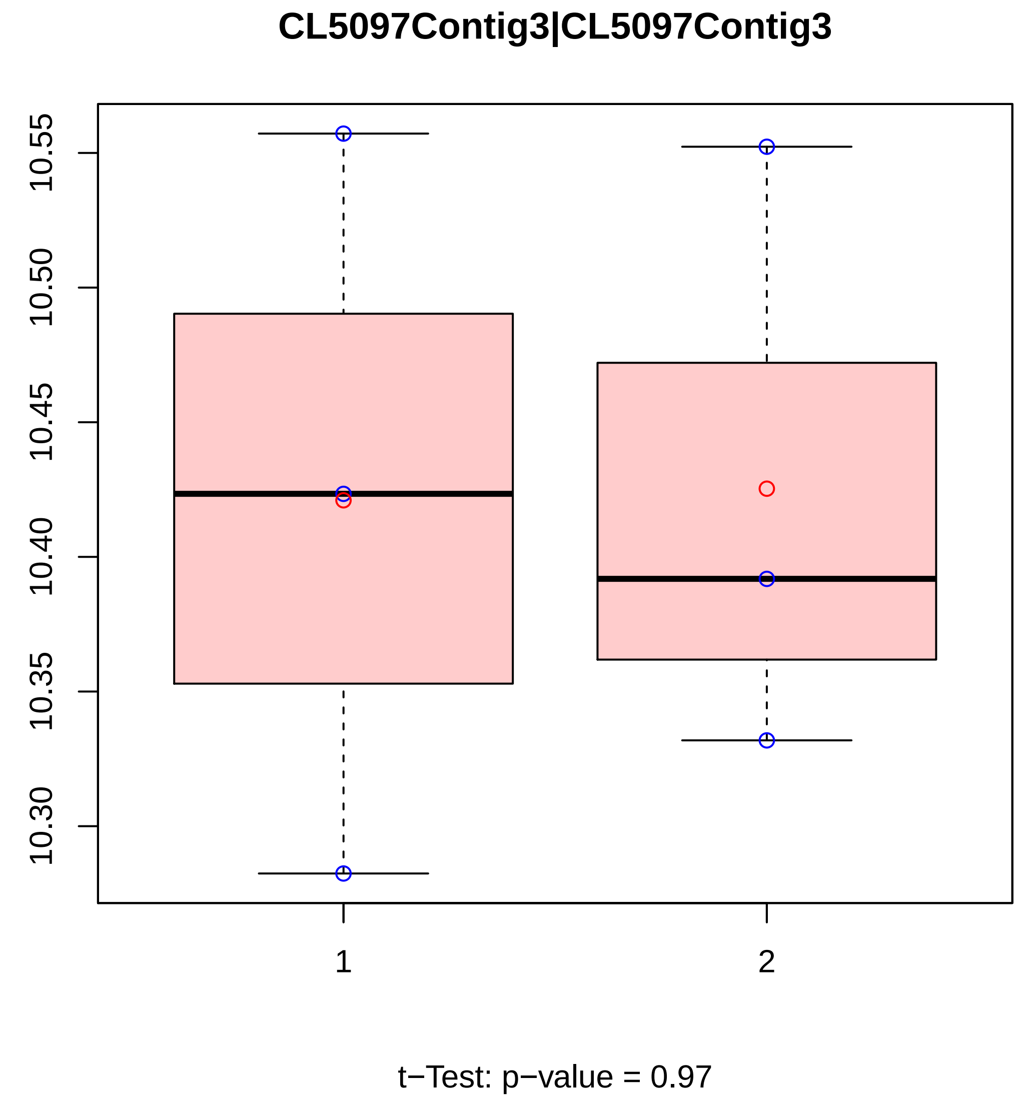
CL5097Contig3|CL5097Contig3
10.30
10.35
10.40
10.45
10.50
10.55
1
2
t−Test: p−value = 0.97

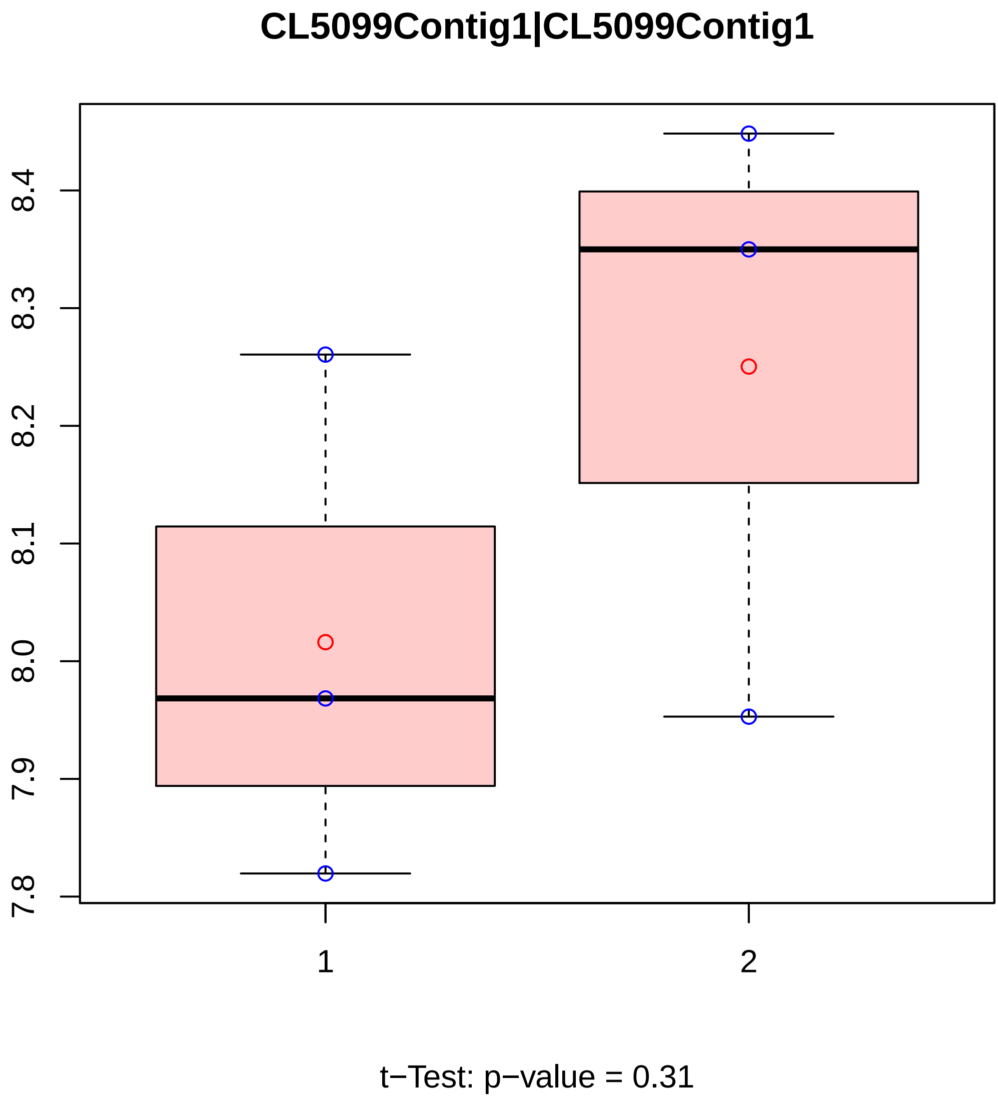
CL5099Contig1|CL5099Contig1
8.4
8.3
8.2
8.1
8.0
7.9
7.8
1
2
t−Test: p−value = 0.31

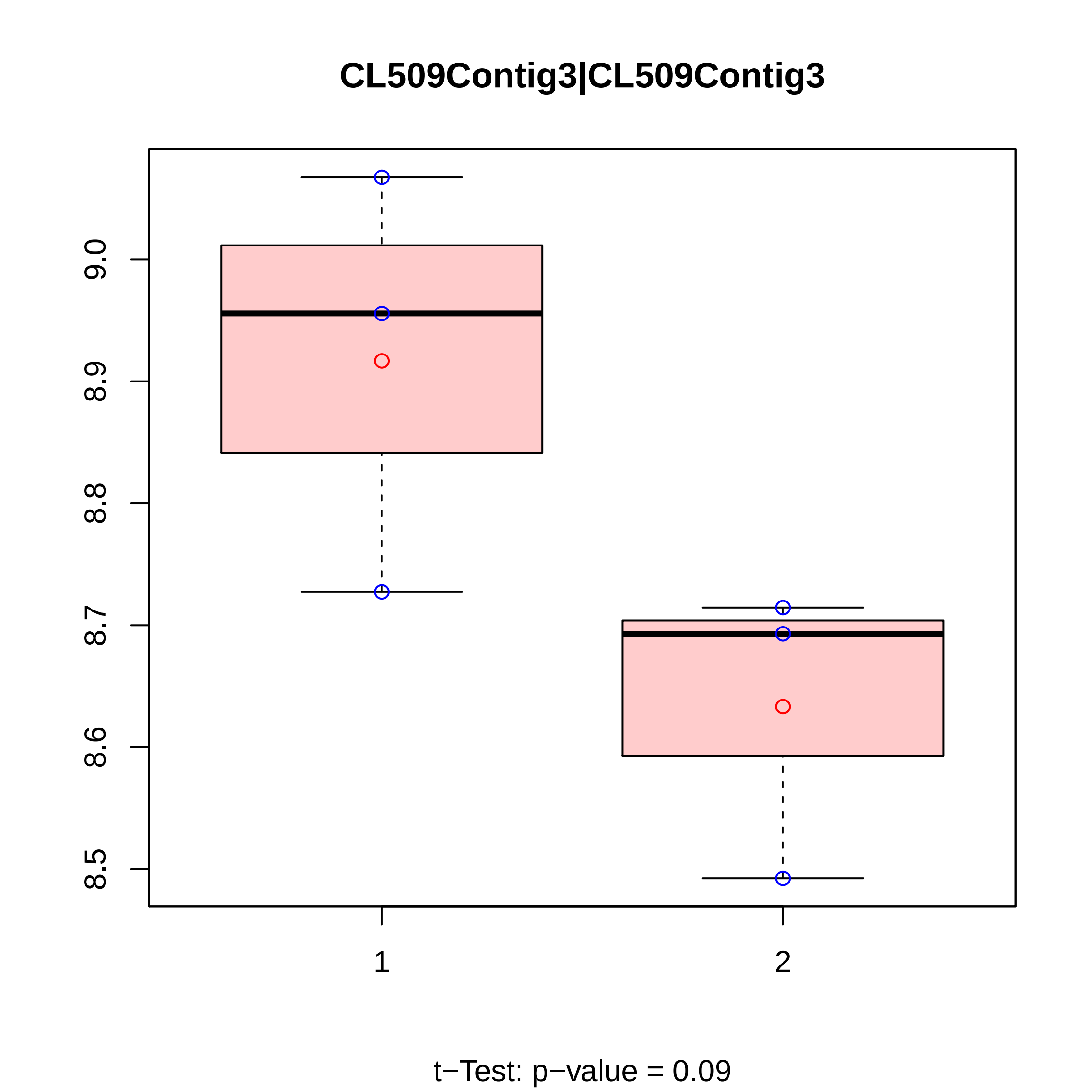

CL509Contig3|CL509Contig3
9.0
8.9
8.8
8.7
8.6
8.5
1
2
t−Test: p−value = 0.09

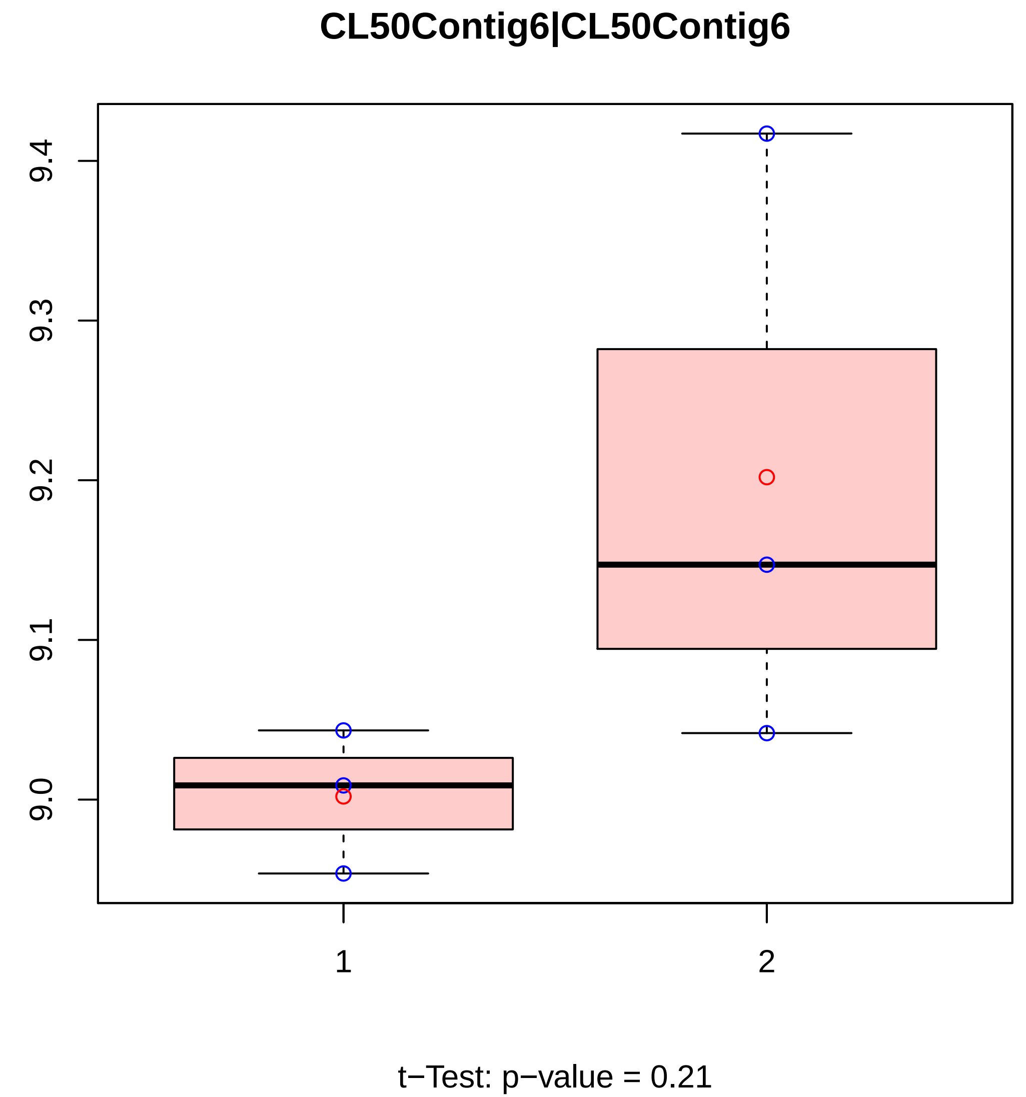
CL50Contig6|CL50Contig6
9.0
9.1
9.2
9.3
9.4
1
2
t−Test: p−value = 0.21

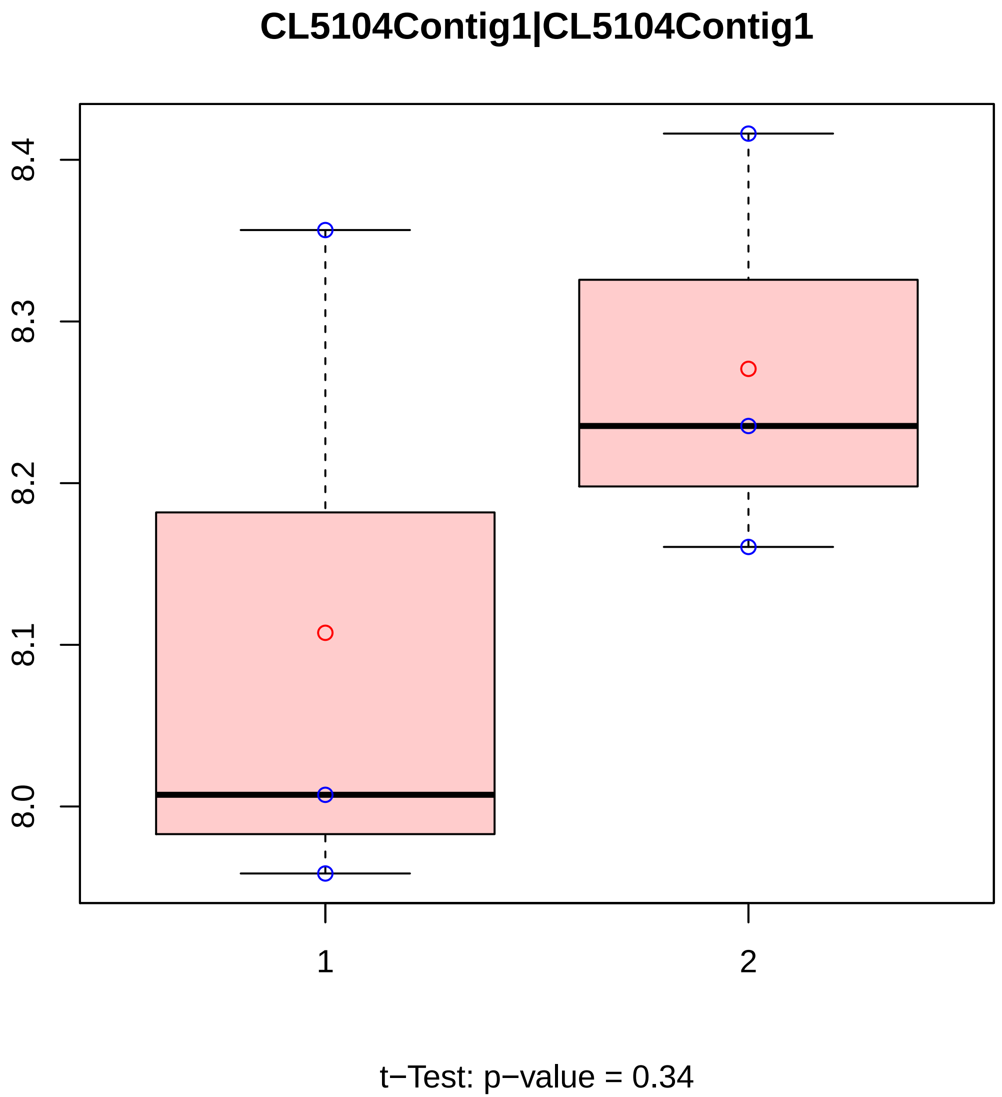

CL5104Contig1|CL5104Contig1
8.0
8.1
8.2
8.3
8.4
1
2
t−Test: p−value = 0.34

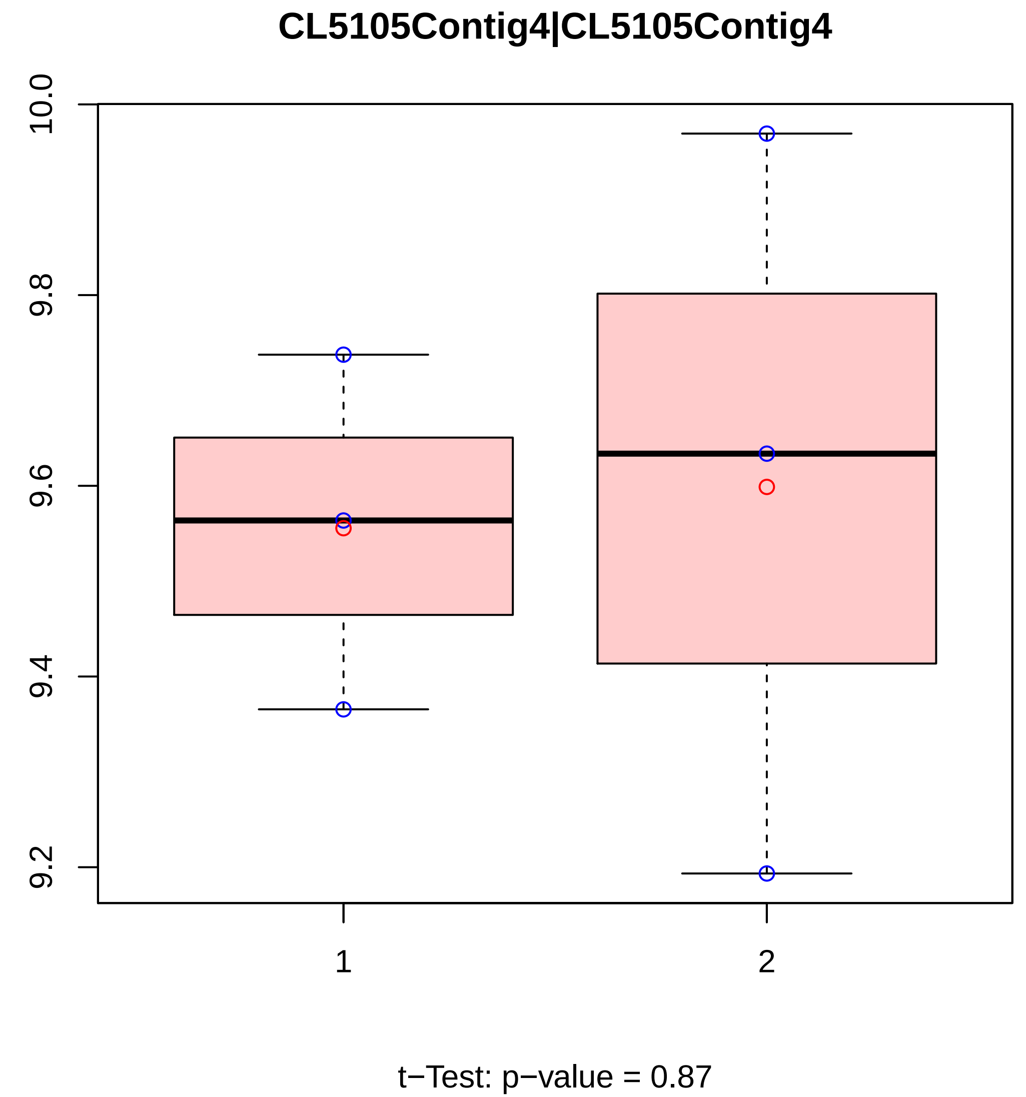

CL5105Contig4|CL5105Contig4
10.0
9.8
9.6
9.4
9.2
1
2
t−Test: p−value = 0.87

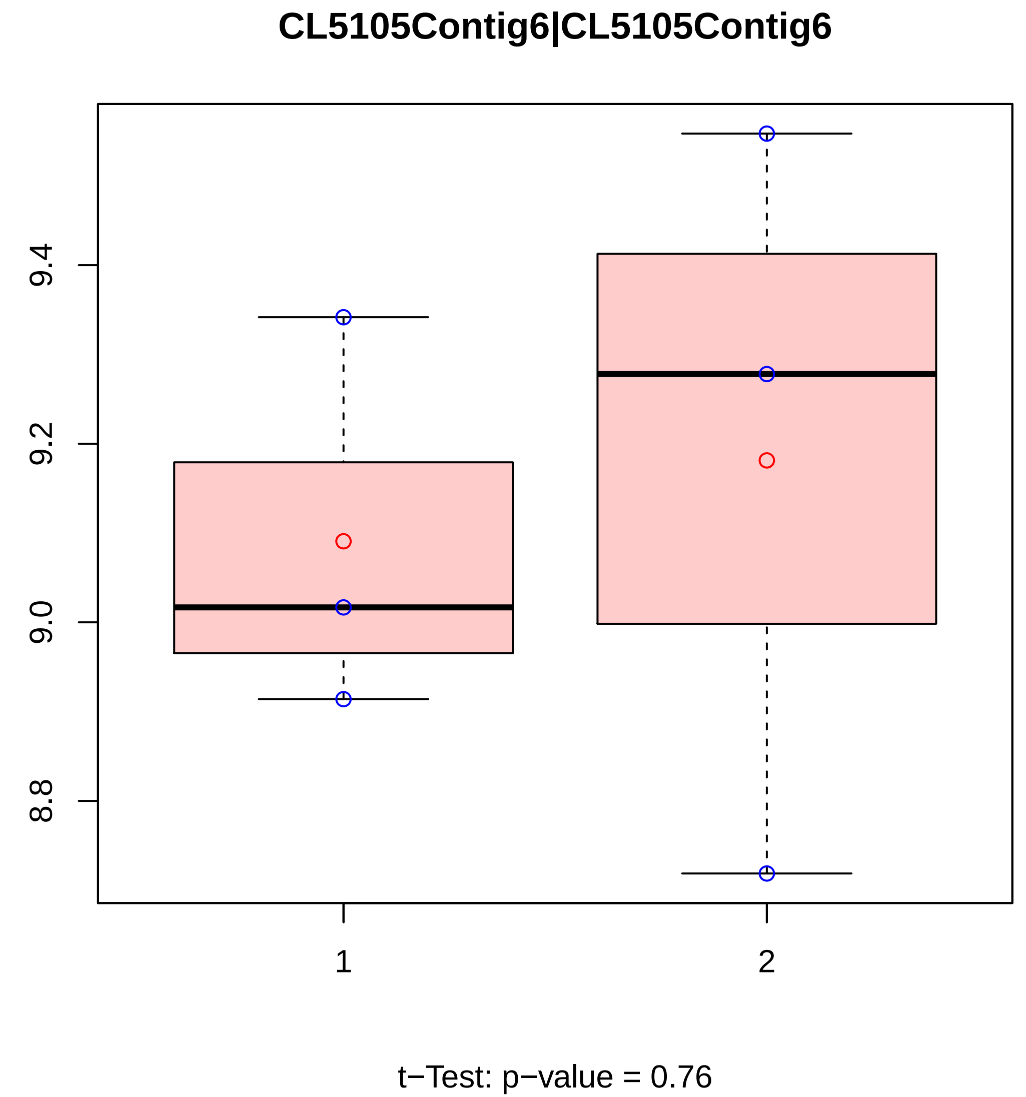
CL5105Contig6|CL5105Contig6
9.4
9.2
9.0
8.8
1
2
t−Test: p−value = 0.76

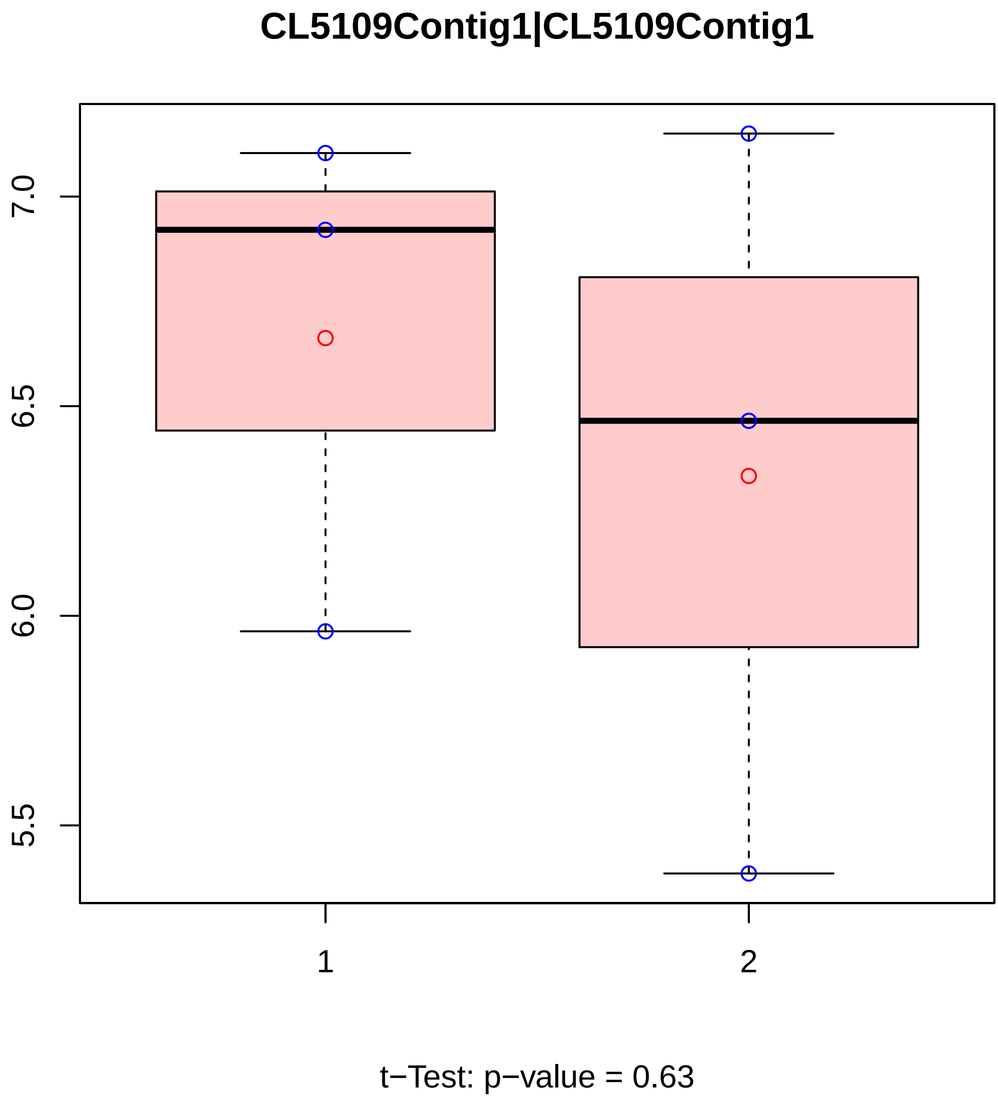
CL5109Contig1|CL5109Contig1
7.0
6.5
6.0
5.5
1
2
t−Test: p−value = 0.63

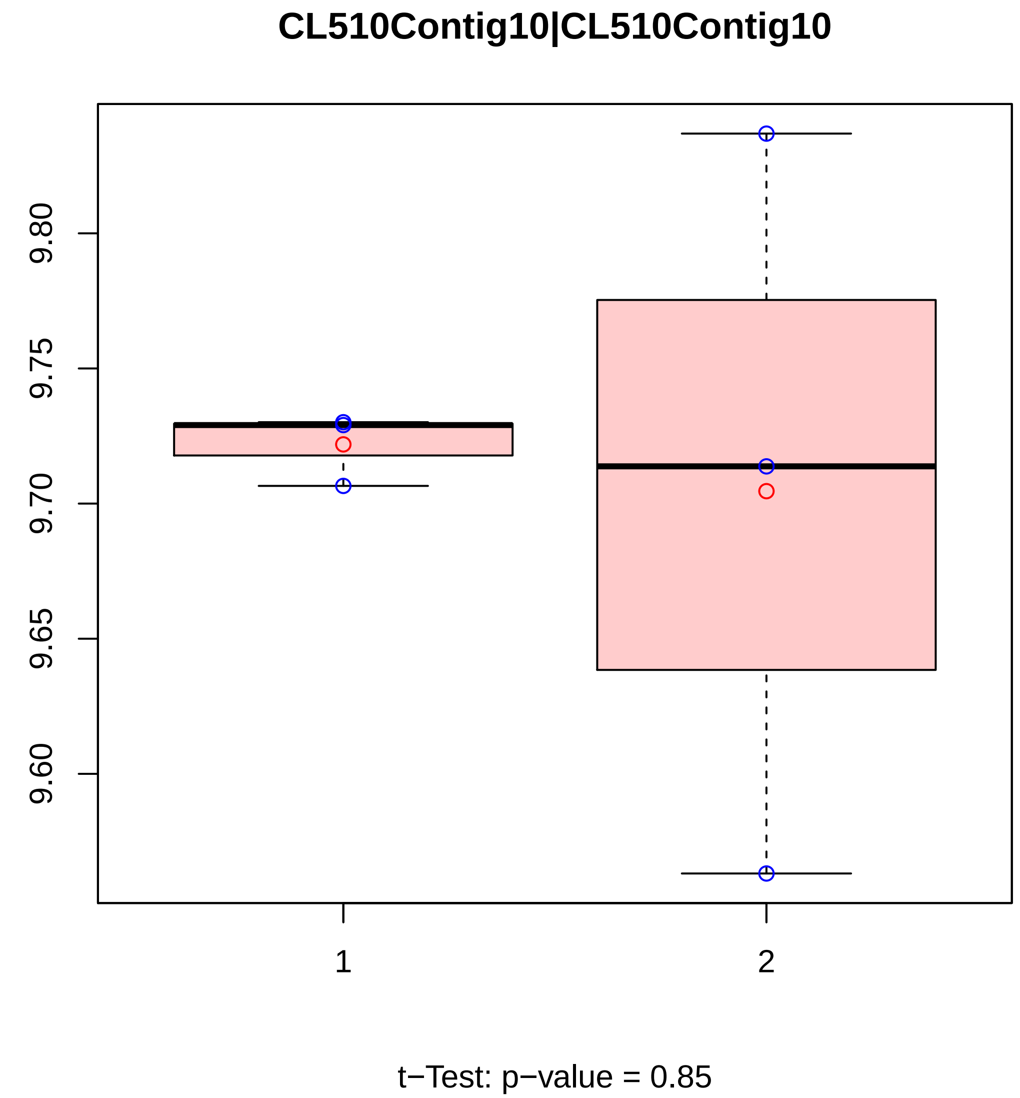
CL510Contig10|CL510Contig10
9.60
9.65
9.70
9.75
9.80
1
2
t−Test: p−value = 0.85

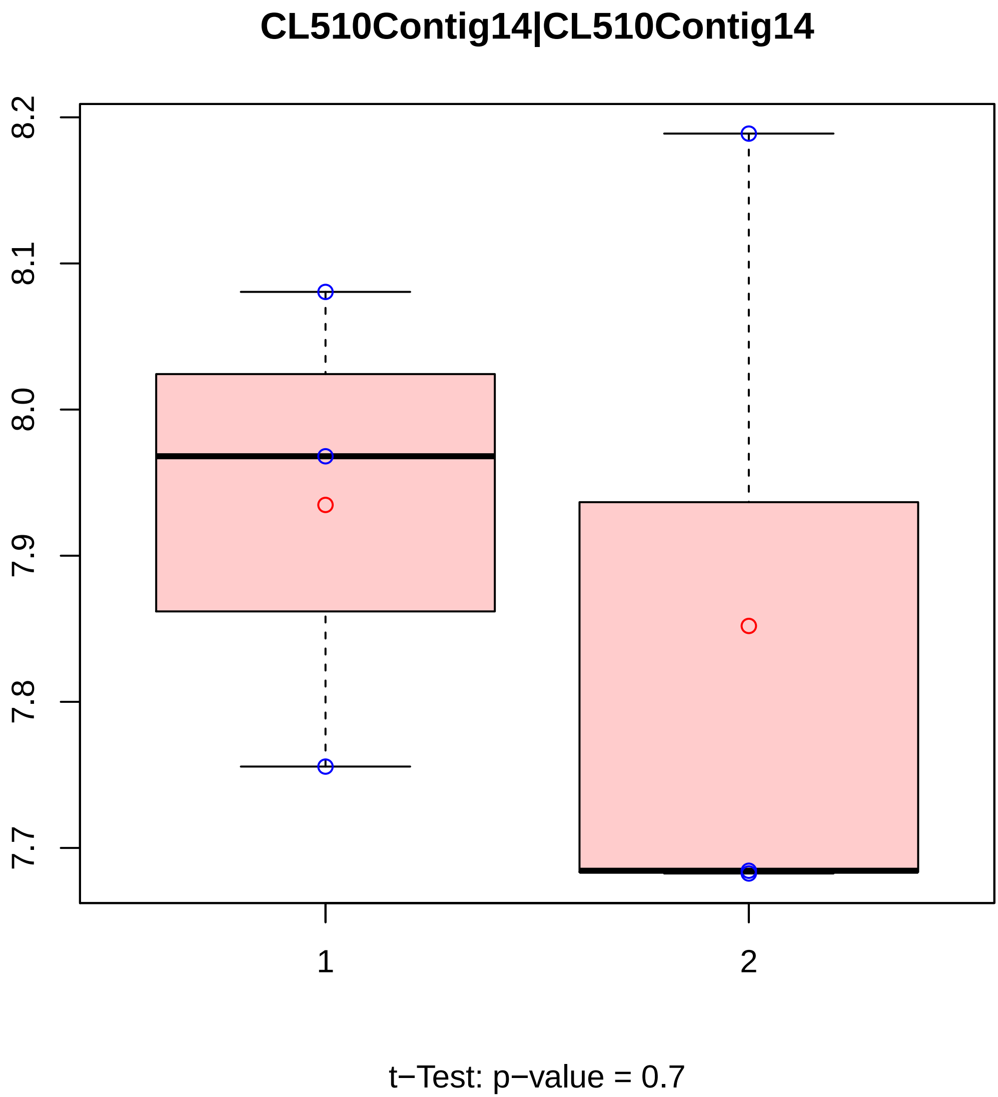
CL510Contig14|CL510Contig14
8.2
8.1
8.0
7.9
7.8
7.7
1
2
t−Test: p−value = 0.7

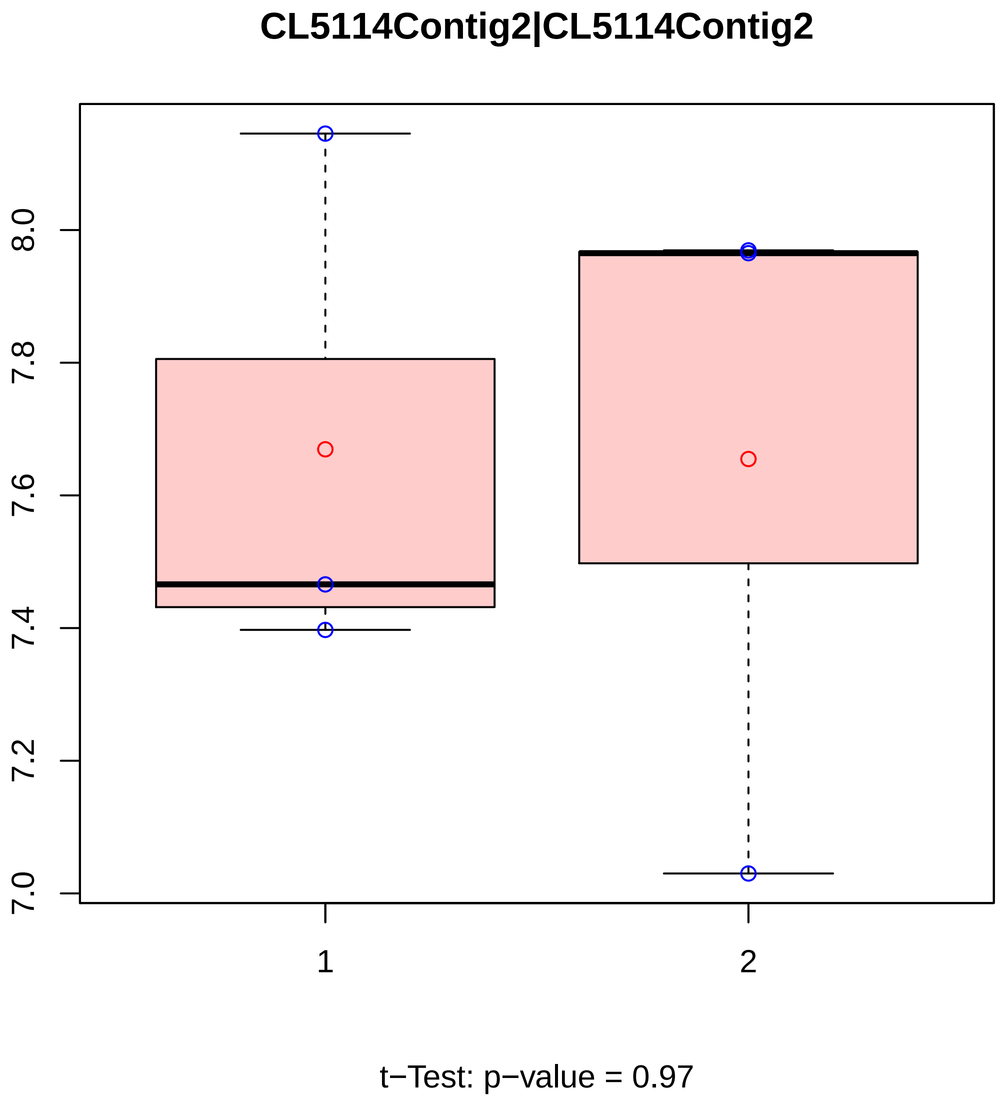

CL5114Contig2|CL5114Contig2
8.0
7.8
7.6
7.4
7.2
7.0
1
2
t−Test: p−value = 0.97

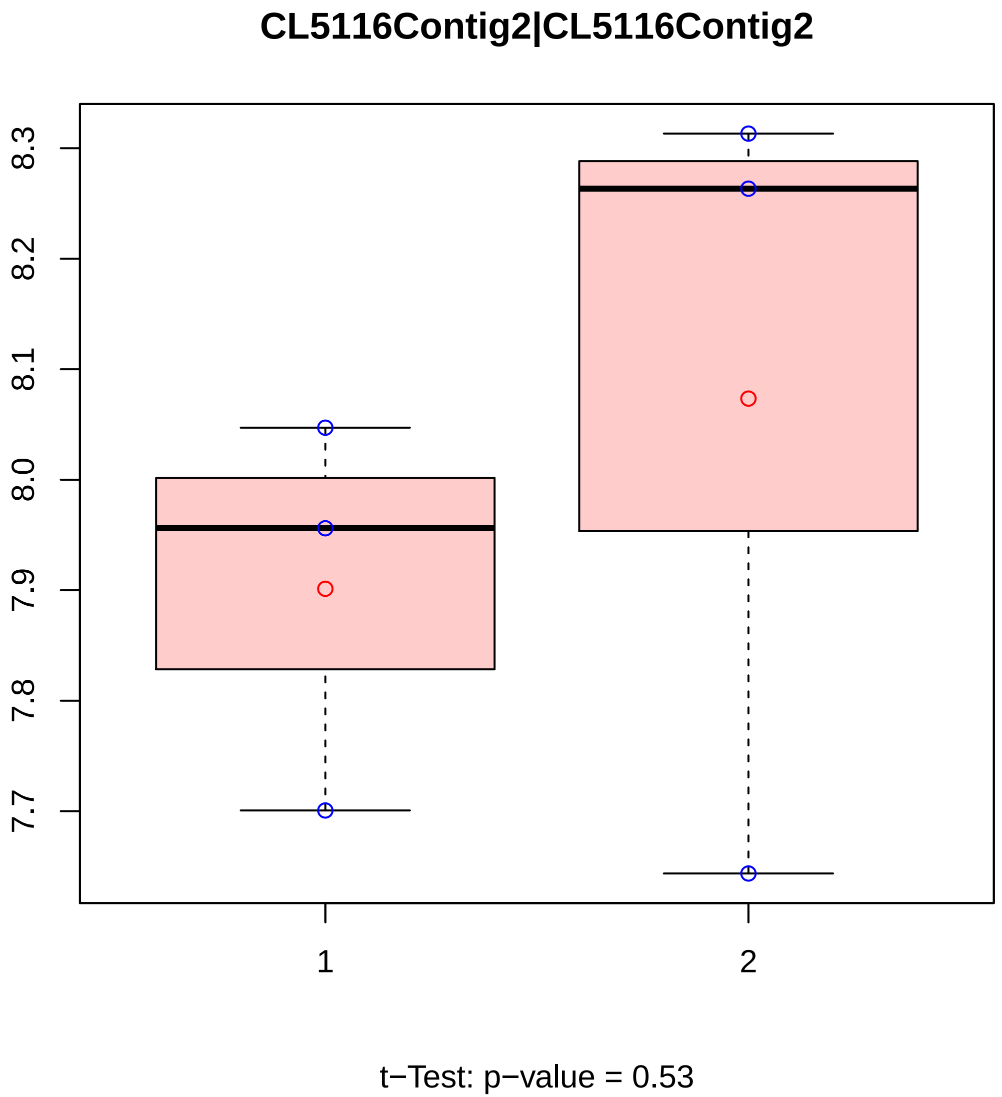
CL5116Contig2|CL5116Contig2
8.3
8.2
8.1
8.0
7.9
7.8
7.7
1
2
t−Test: p−value = 0.53

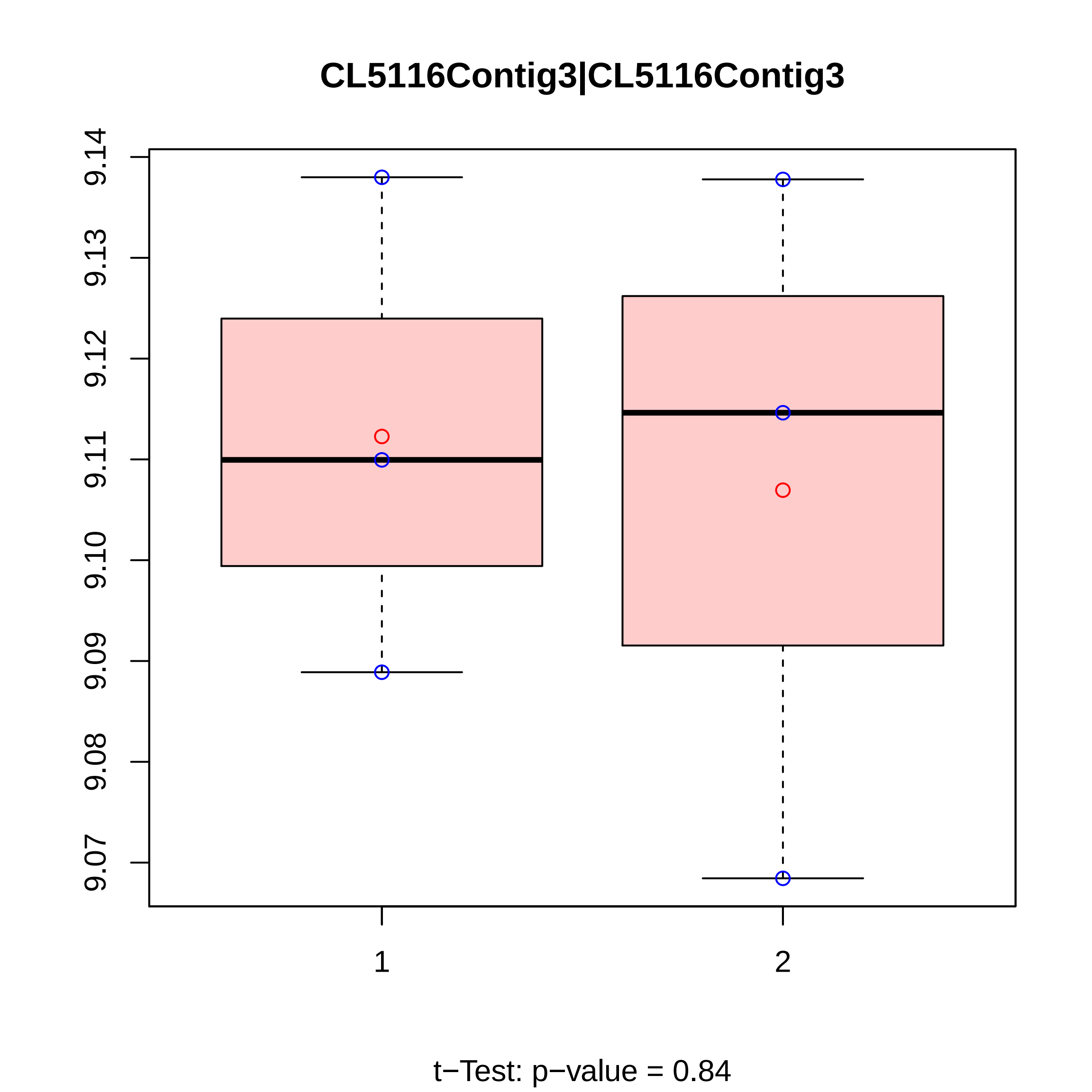

CL5116Contig3|CL5116Contig3
9.14
9.13
9.12
9.11
9.10
9.09
9.08
9.07
1
2
t−Test: p−value = 0.84

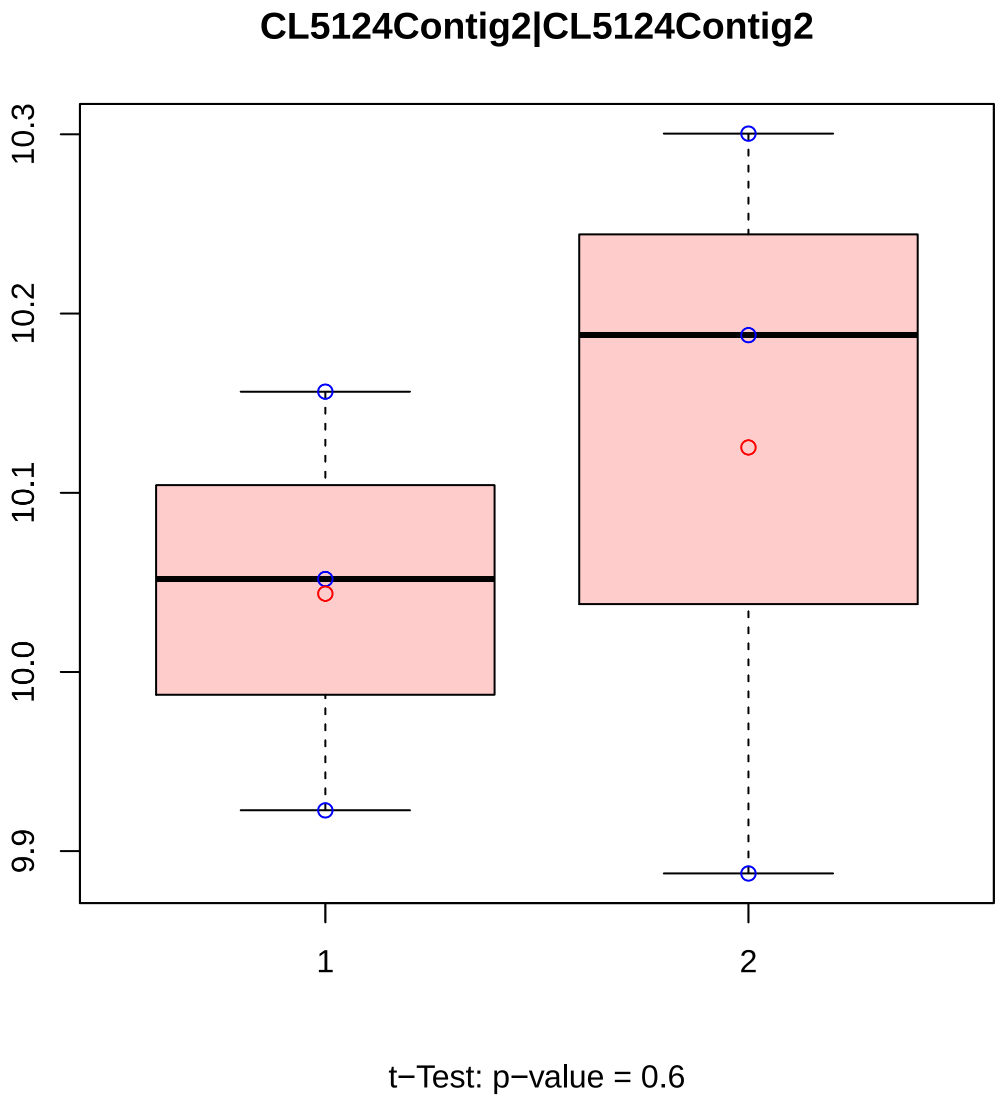

CL5124Contig2|CL5124Contig2
10.3
10.2
10.1
10.0
9.9
1
2
t−Test: p−value = 0.6

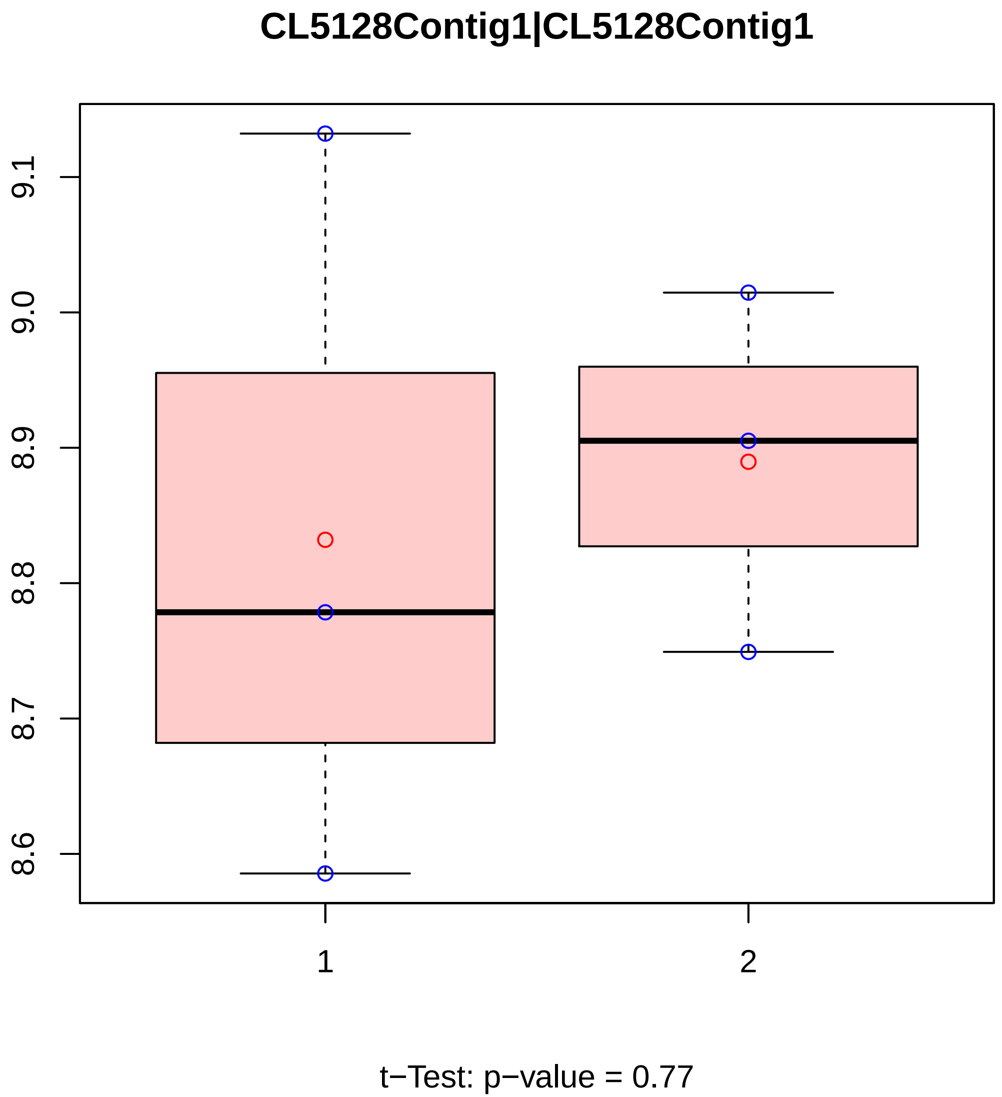
CL5128Contig1|CL5128Contig1
9.1
9.0
8.9
8.8
8.7
8.6
1
2
t−Test: p−value = 0.77

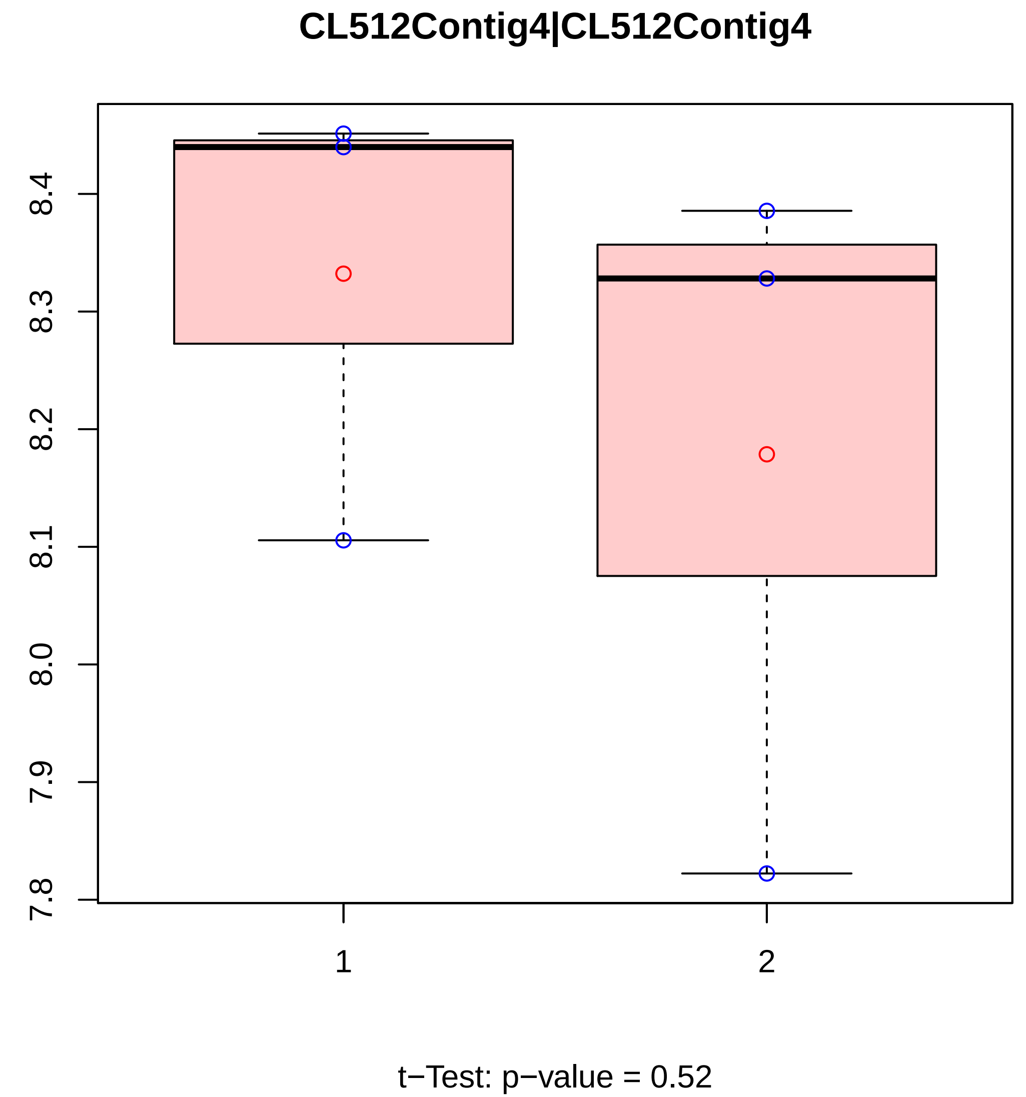
CL512Contig4|CL512Contig4
8.4
8.3
8.2
8.1
8.0
7.9
7.8
1
2
t−Test: p−value = 0.52

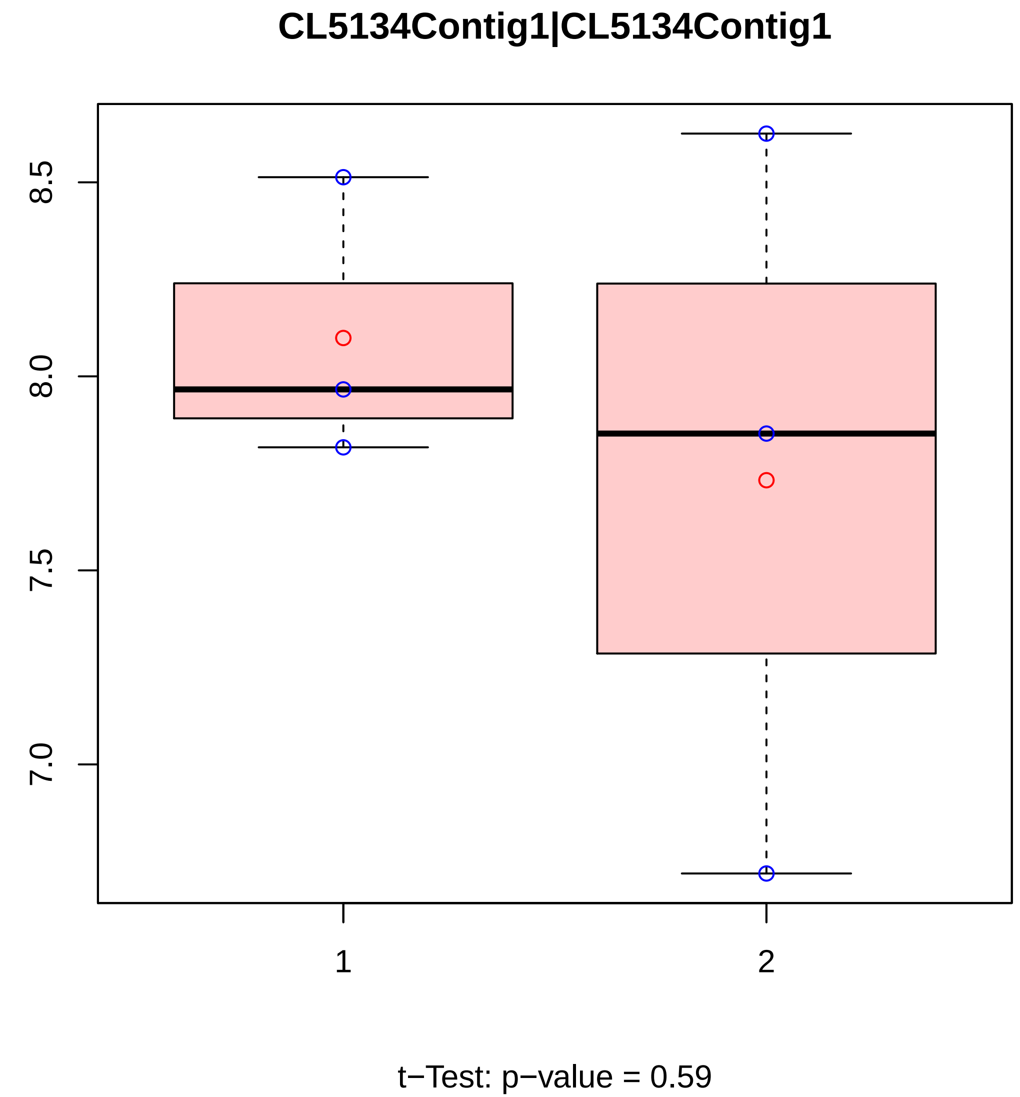
CL5134Contig1|CL5134Contig1
8.5
8.0
7.5
7.0
1
2
t−Test: p−value = 0.59

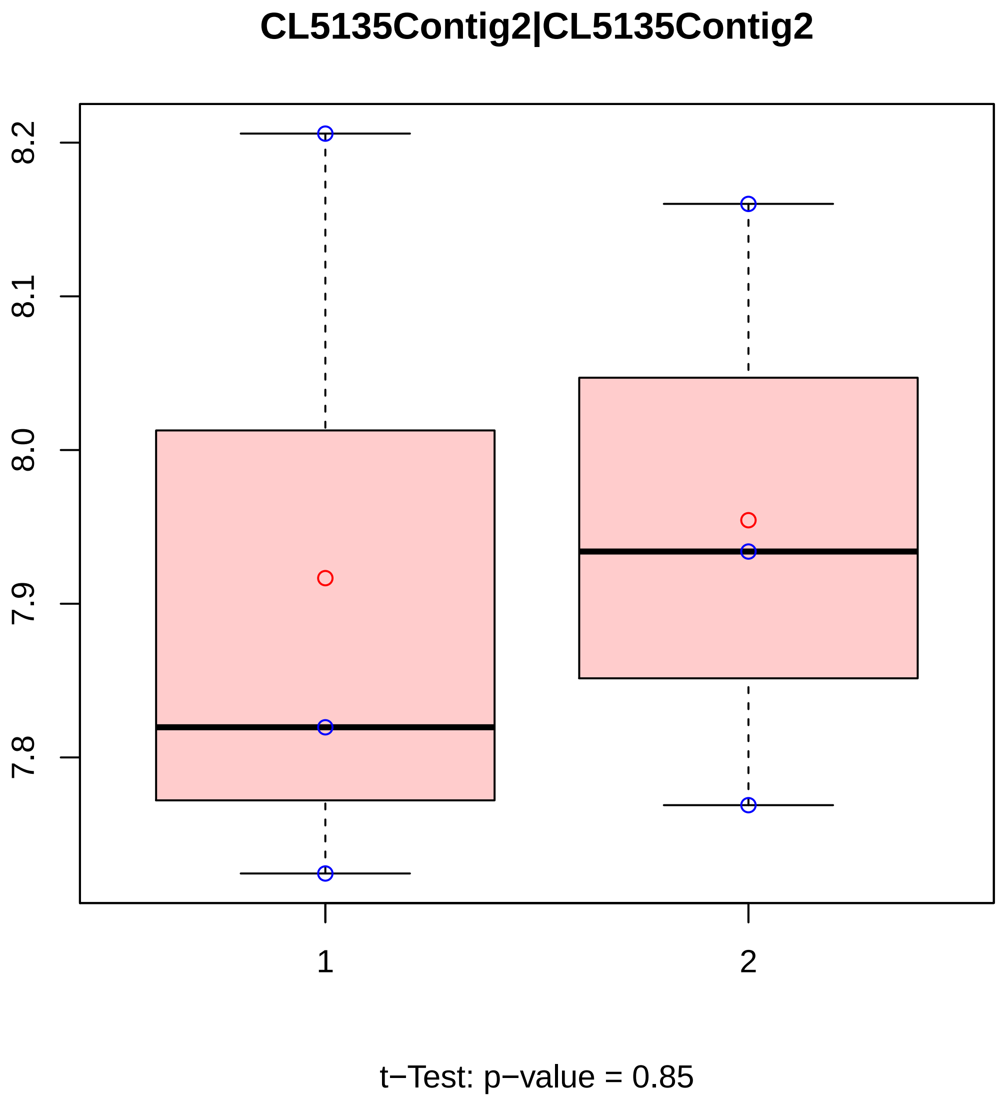
CL5135Contig2|CL5135Contig2
8.2
8.1
8.0
7.9
7.8
1
2
t−Test: p−value = 0.85

# CL513Contig10|CL513Contig10

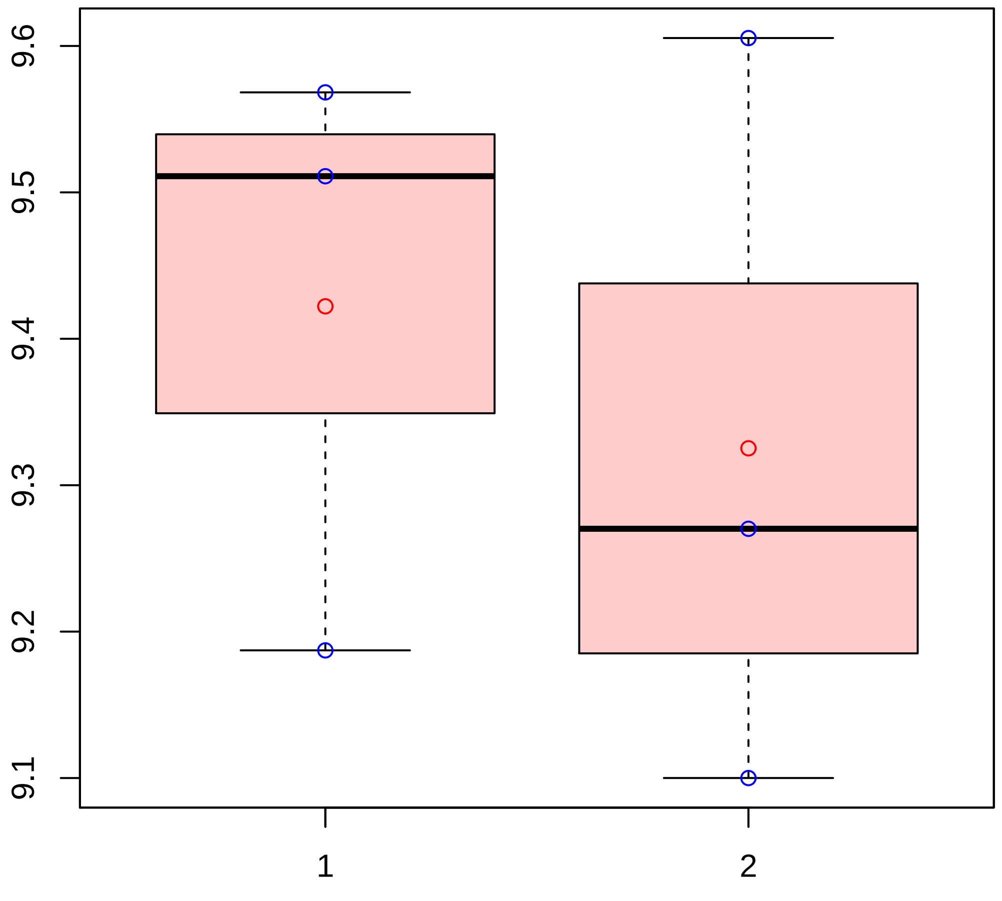


t−Test: p−value = 0.64

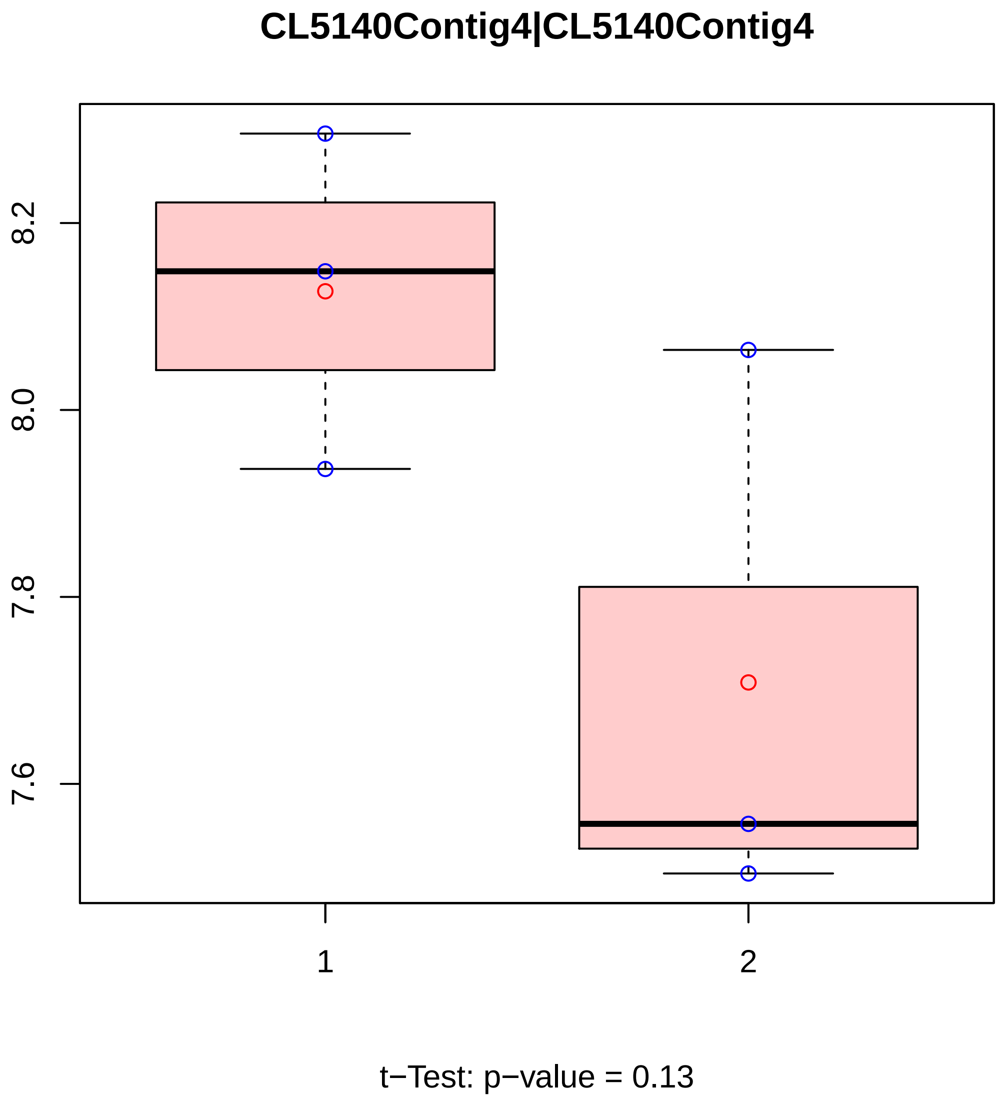
CL5140Contig4|CL5140Contig4
8.2
8.0
7.8
7.6
1
2
t−Test: p−value = 0.13

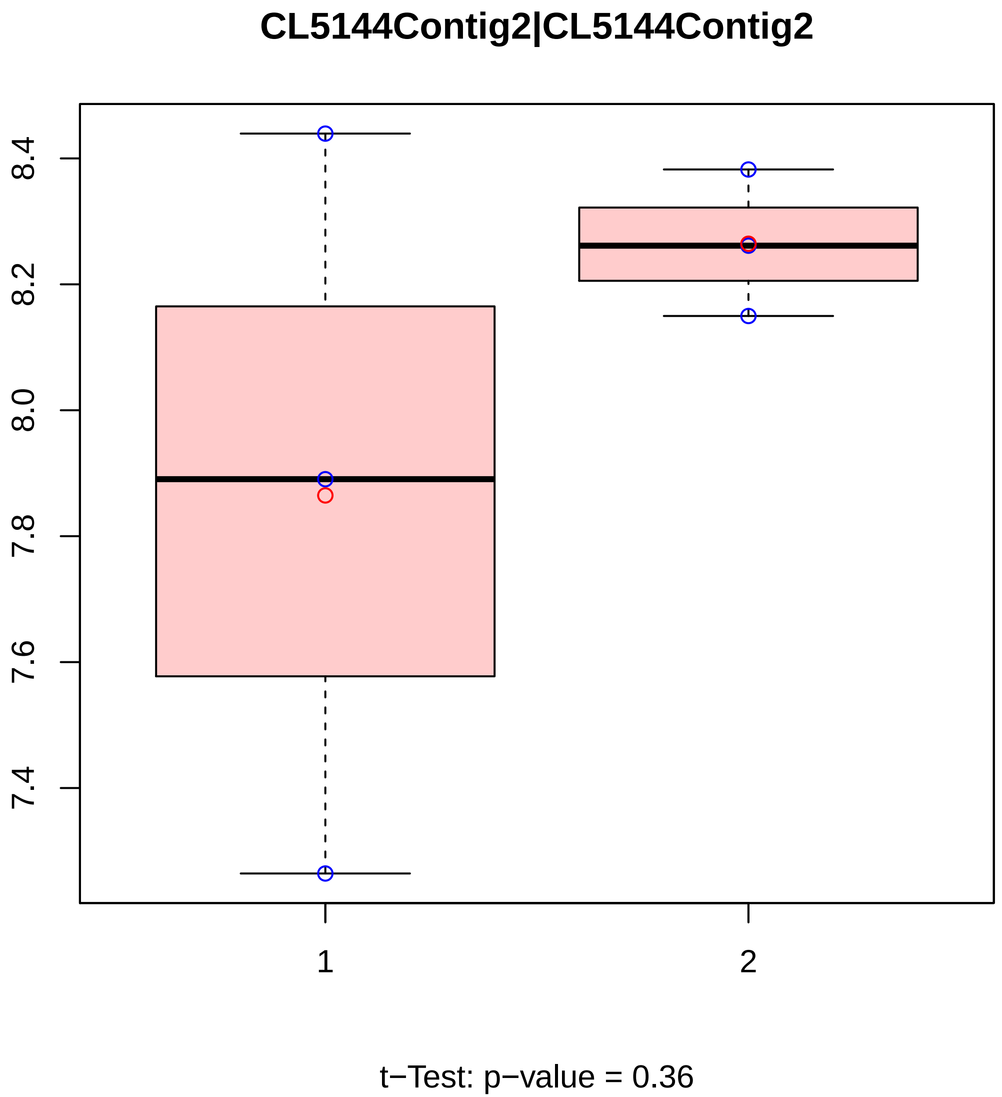

CL5144Contig2|CL5144Contig2
8.4
8.2
8.0
7.8
7.6
7.4
1
2
t−Test: p−value = 0.36

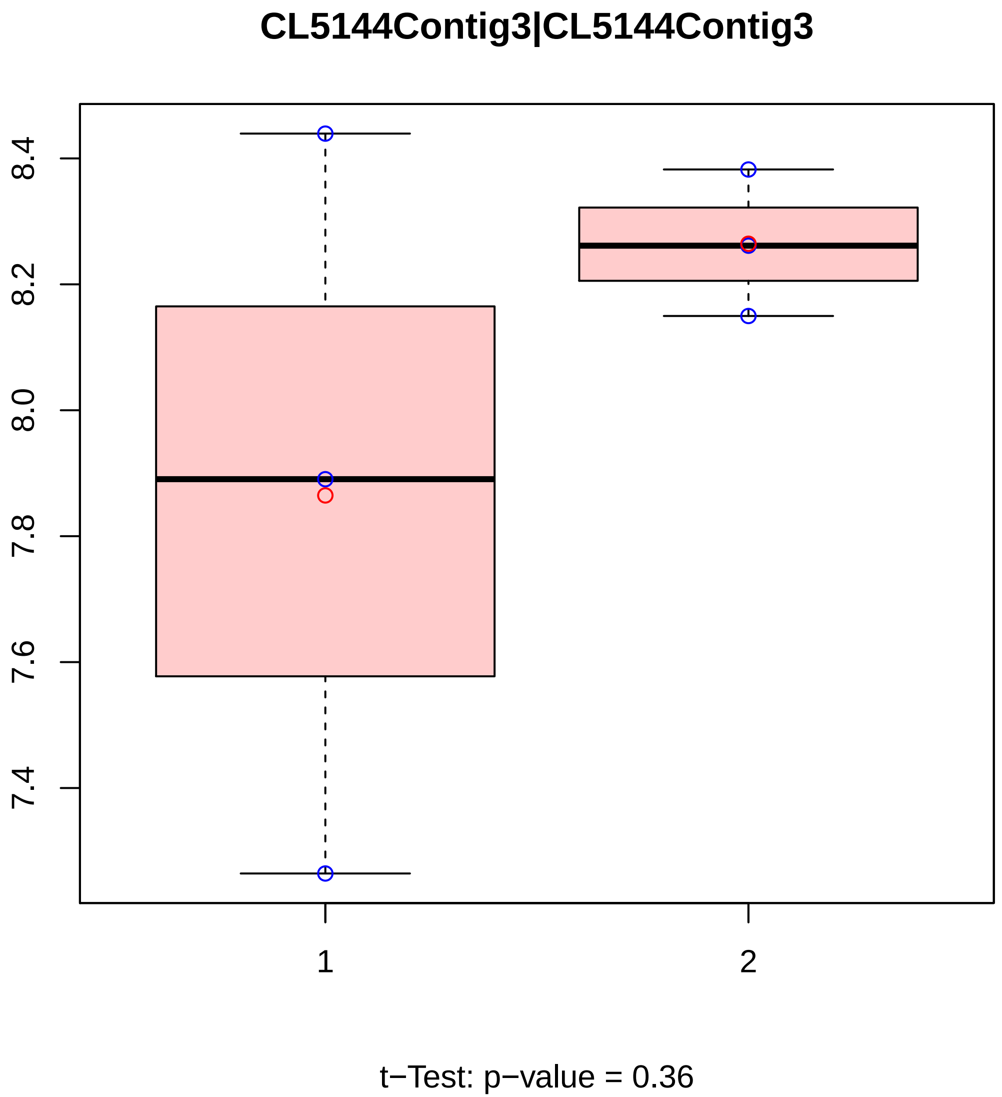
CL5144Contig3|CL5144Contig3
8.4
8.2
8.0
7.8
7.6
7.4
1
2
t−Test: p−value = 0.36

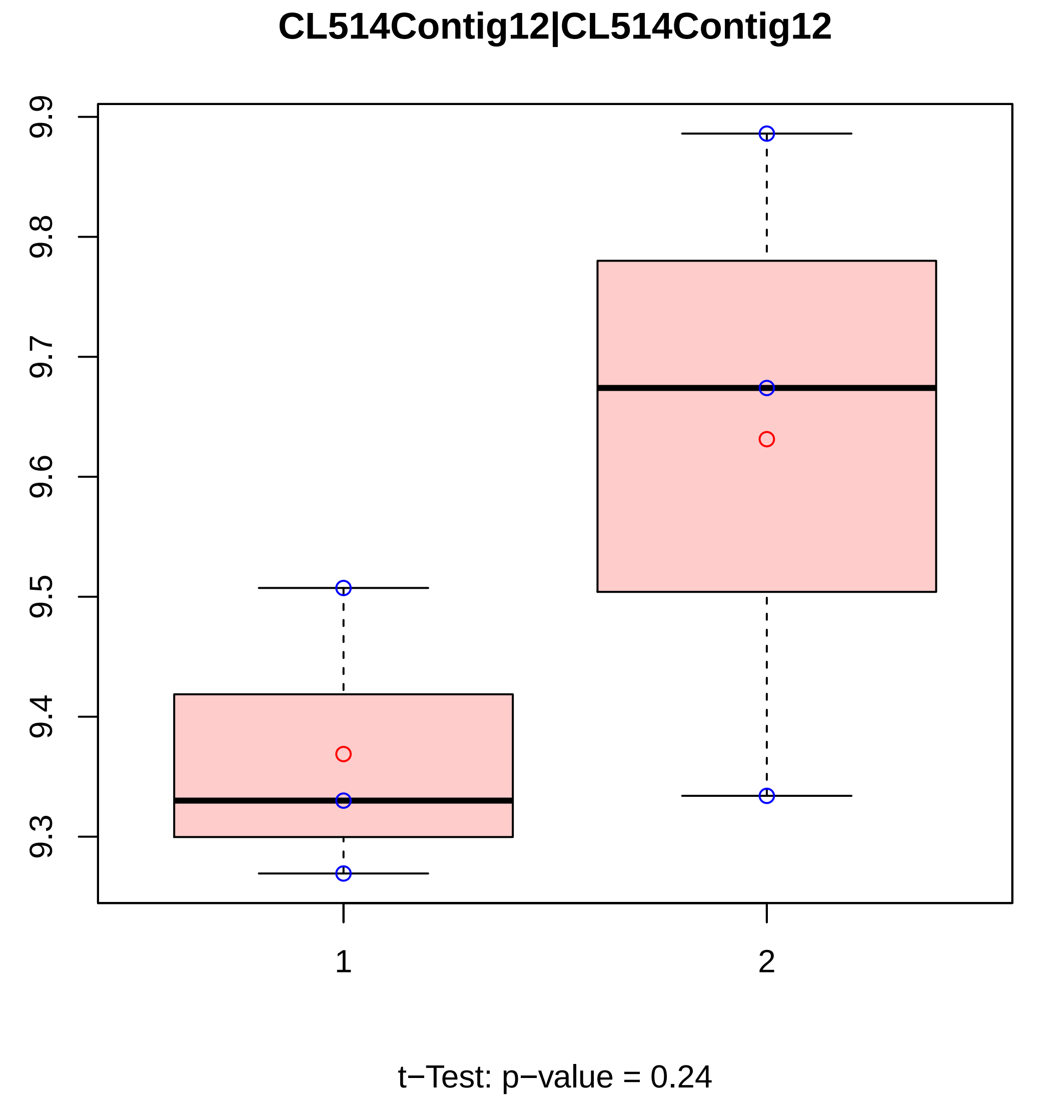
CL514Contig12|CL514Contig12
9.3
9.4
9.5
9.6
9.7
9.8
9.9
1
2
t−Test: p−value = 0.24

**CL514Contig1|CL514Contig1**

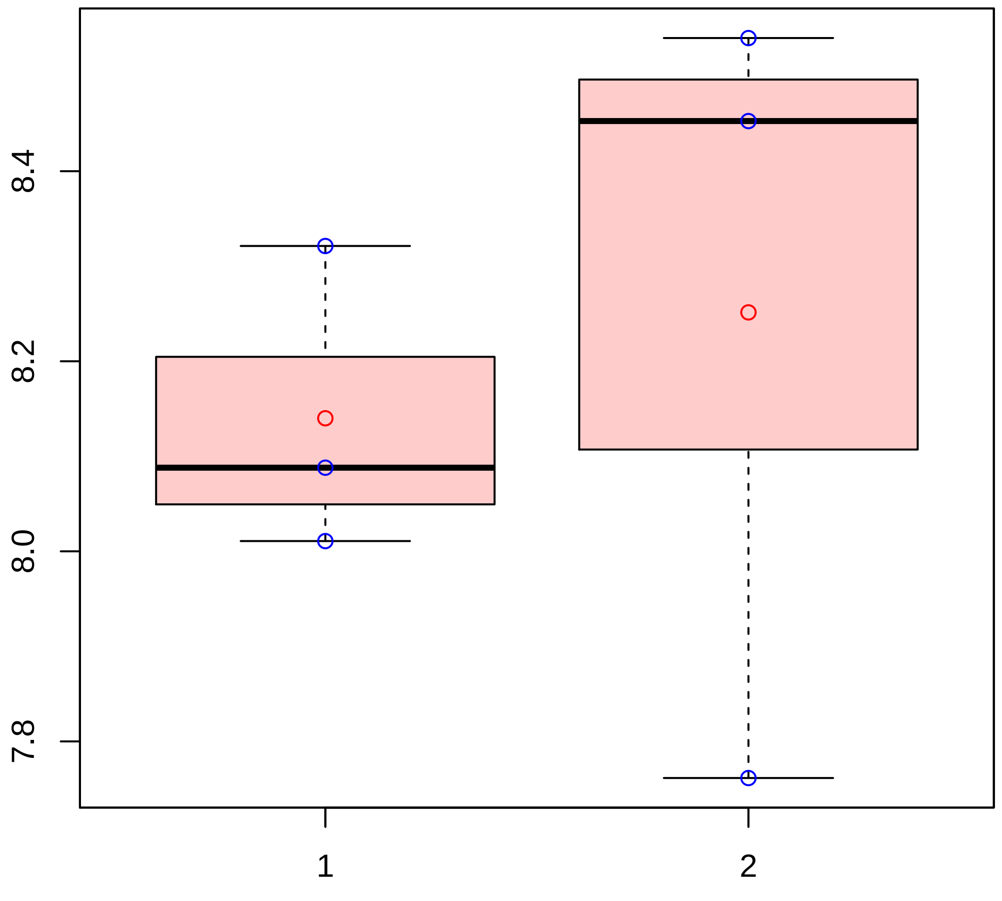


t−Test: p−value = 0.71

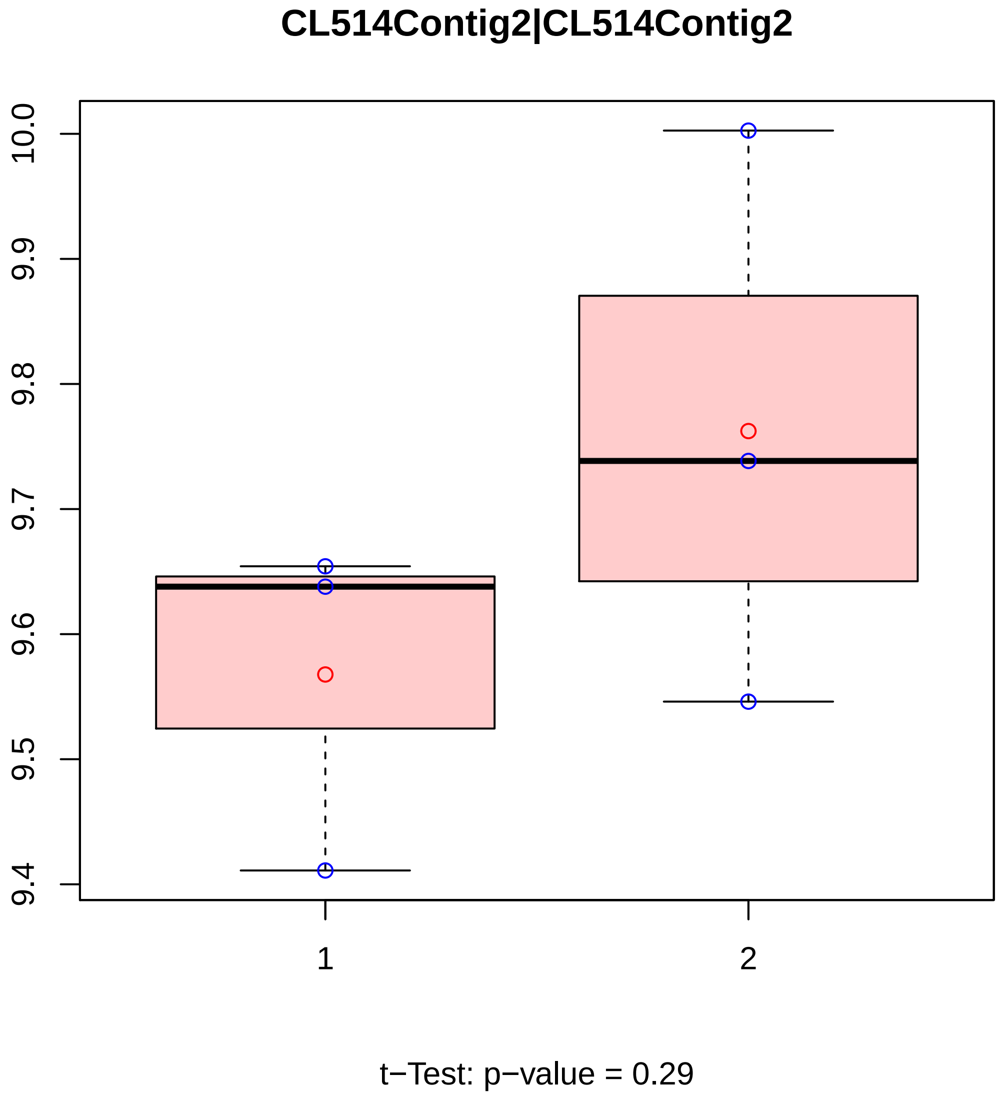
CL514Contig2|CL514Contig2
10.0
9.9
9.8
9.7
9.6
9.5
9.4
1
2
t−Test: p−value = 0.29

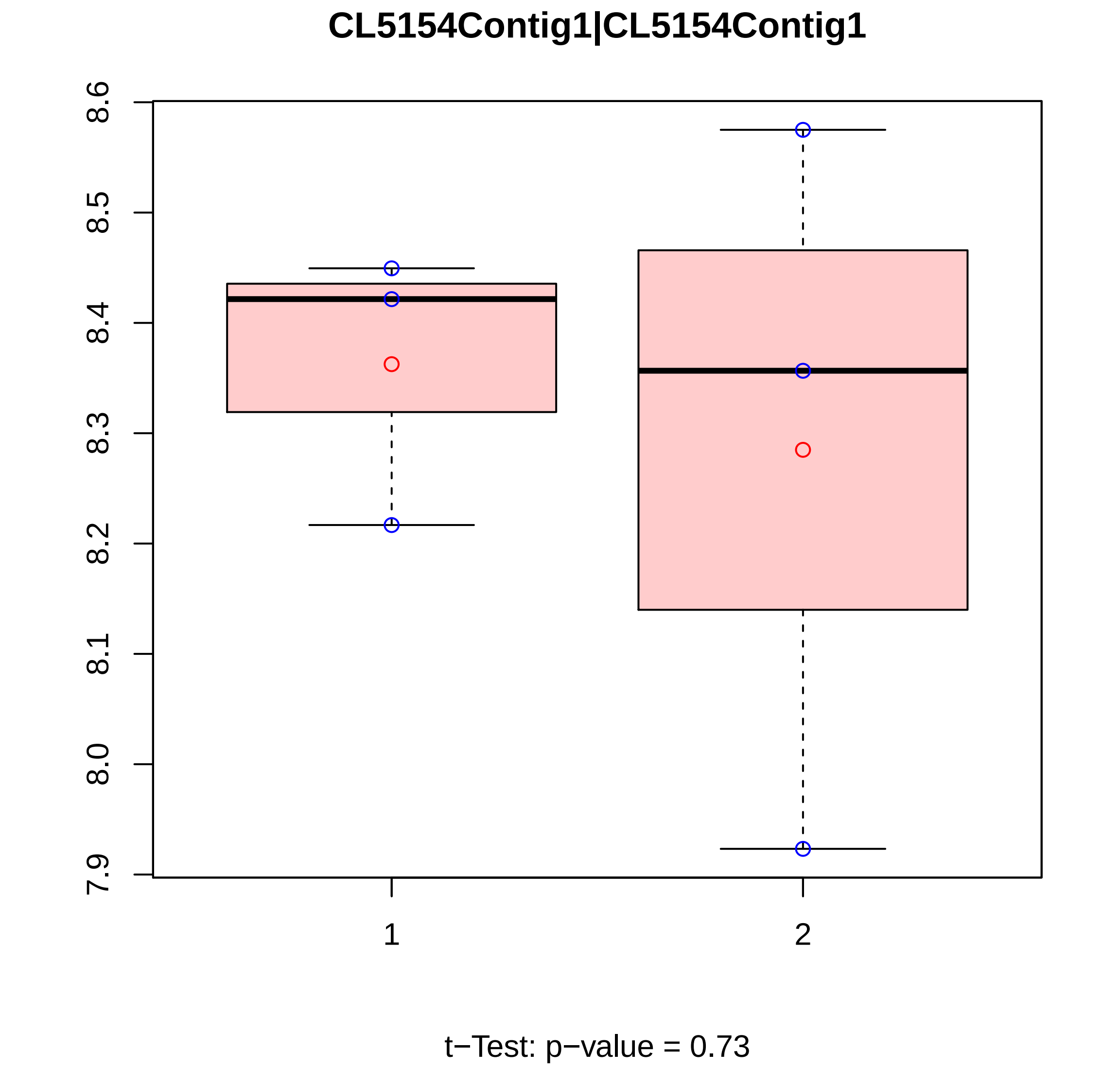
CL5154Contig1|CL5154Contig1
8.6
8.5
8.4
8.3
8.2
8.1
8.0
7.9
1
2
t−Test: p−value = 0.73

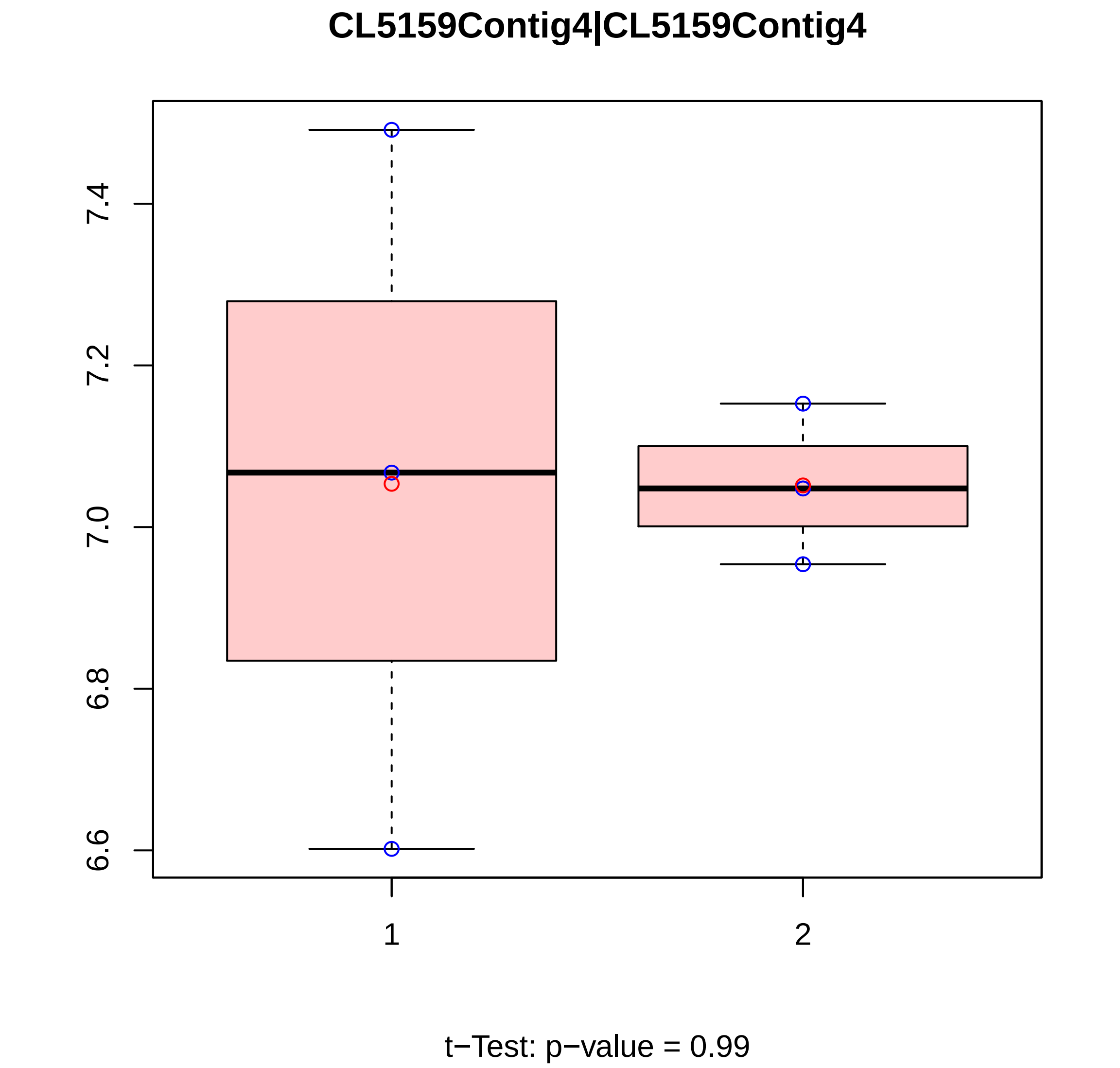
CL5159Contig4|CL5159Contig4
7.4
7.2
7.0
6.8
6.6
1
2
t−Test: p−value = 0.99

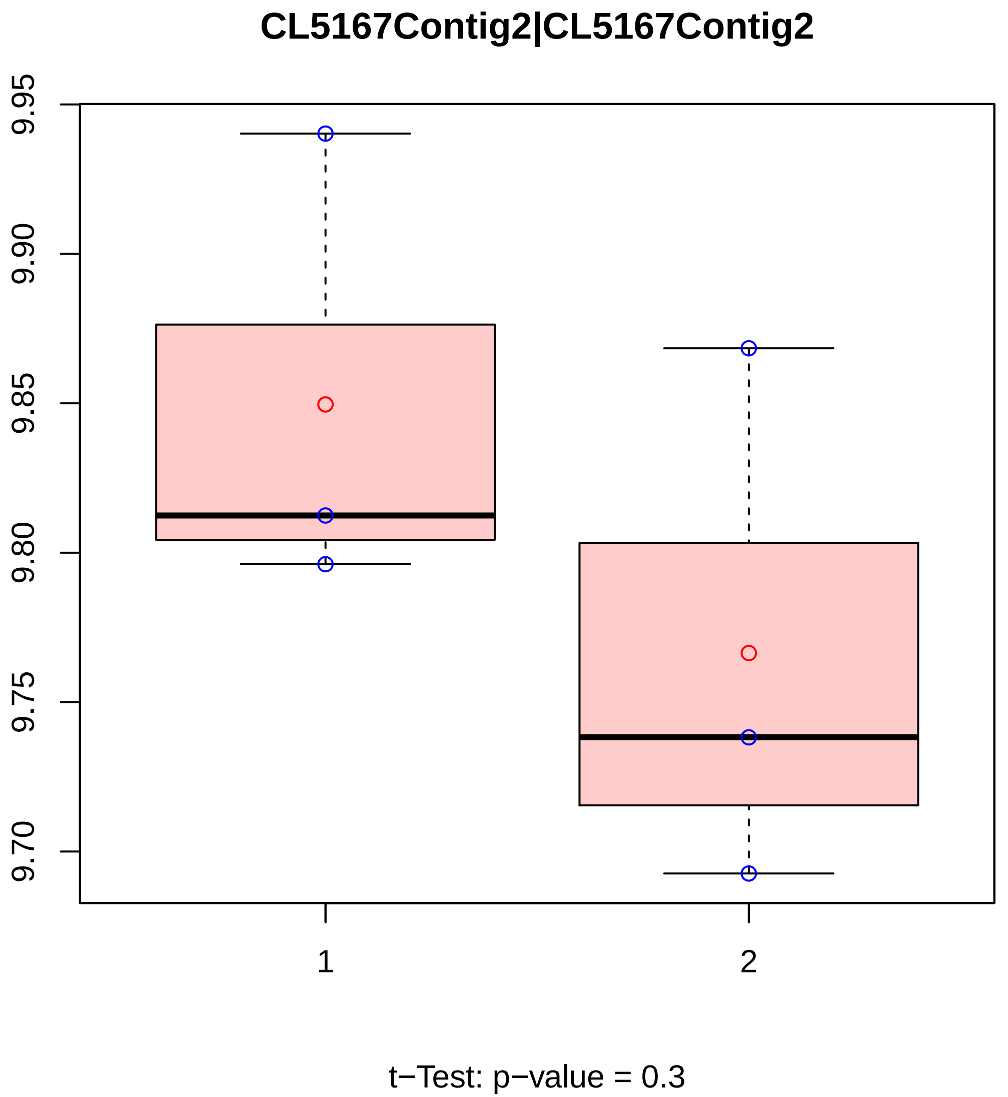
CL5167Contig2|CL5167Contig2
9.95
9.90
9.85
9.80
9.75
9.70
1
2
t−Test: p−value = 0.3

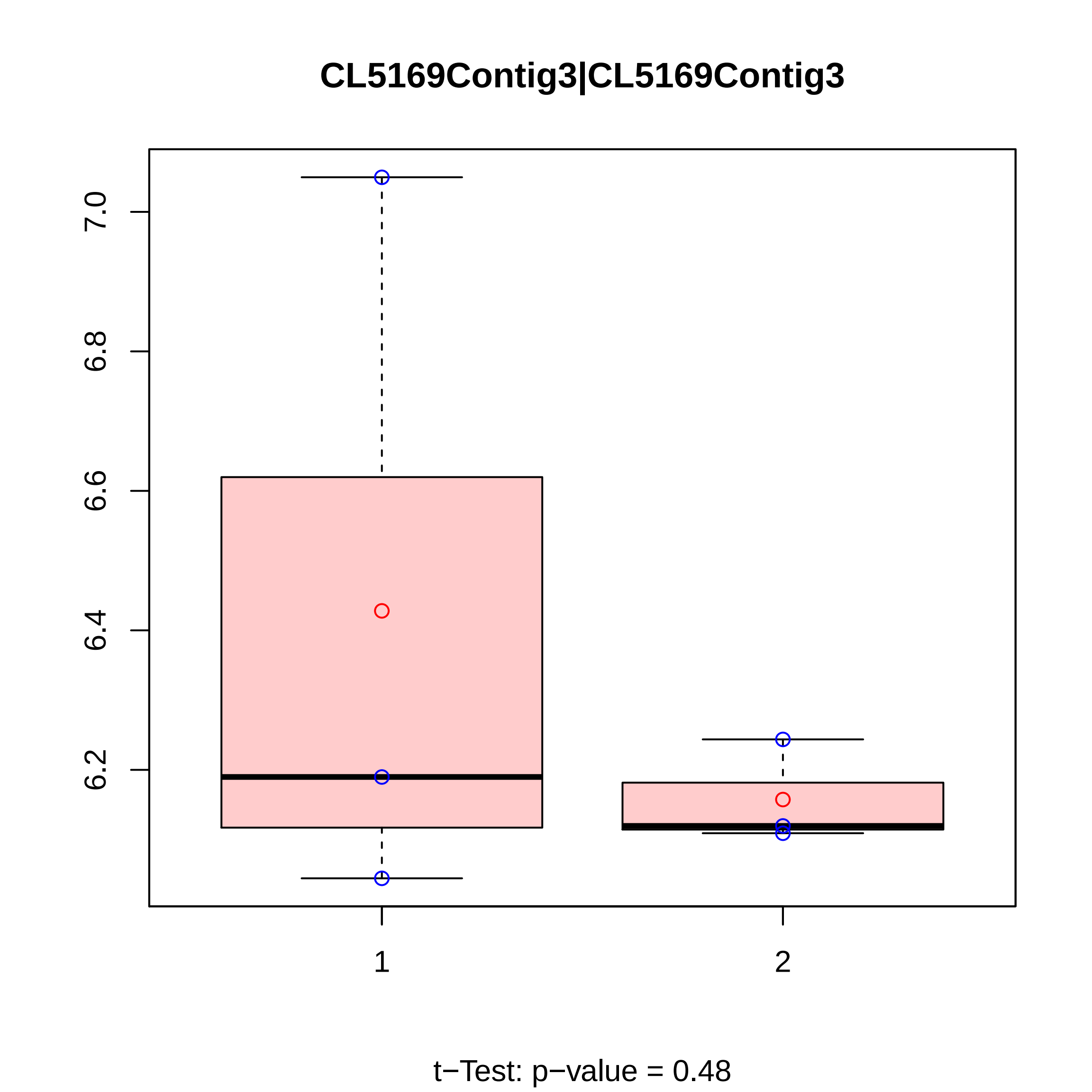
CL5169Contig3|CL5169Contig3
7.0
6.8
6.6
6.4
6.2
1
2
t−Test: p−value = 0.48

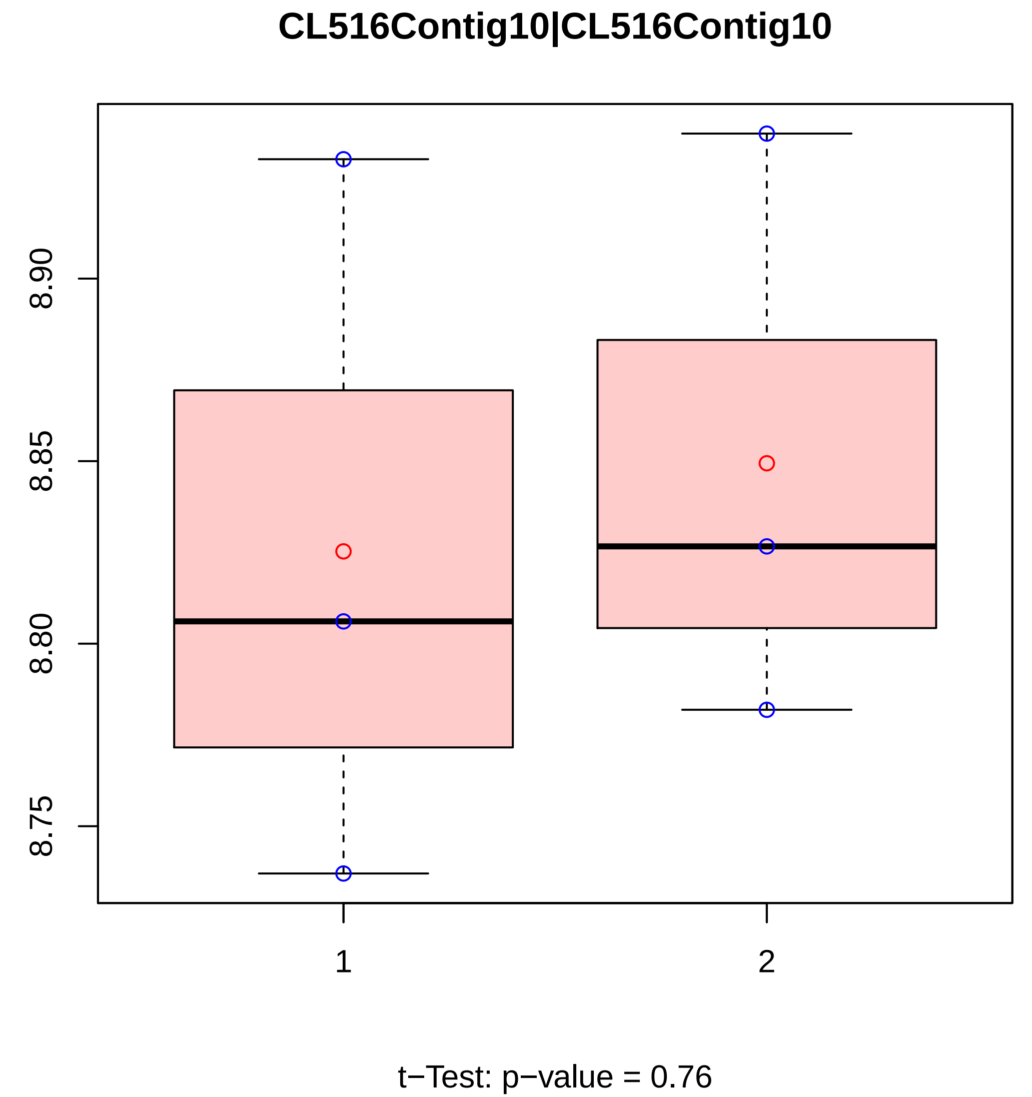

CL516Contig10|CL516Contig10
8.75
8.80
8.85
8.90
1
2
t−Test: p−value = 0.76

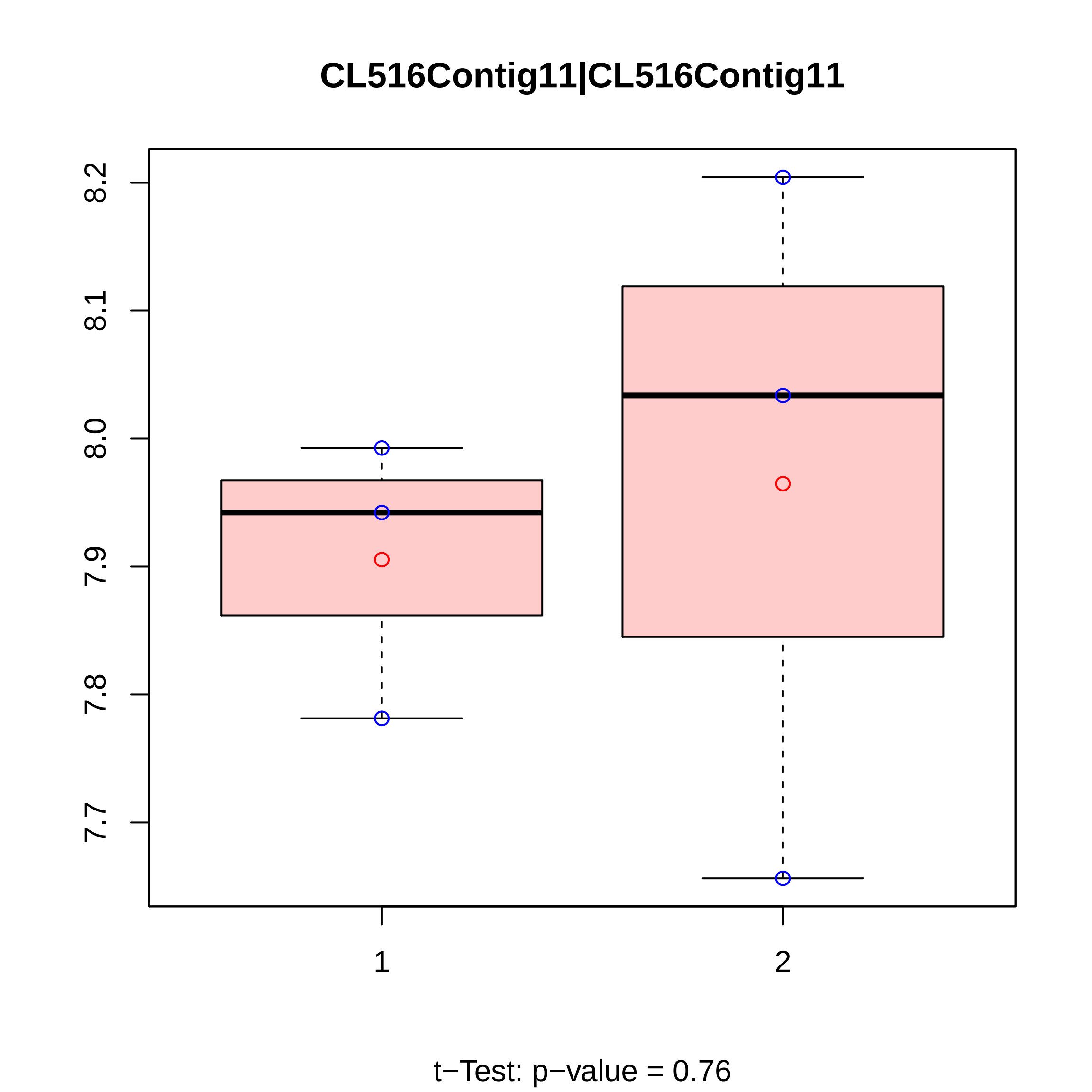

CL516Contig11|CL516Contig11
8.2
8.1
8.0
7.9
7.8
7.7
1
2
t−Test: p−value = 0.76

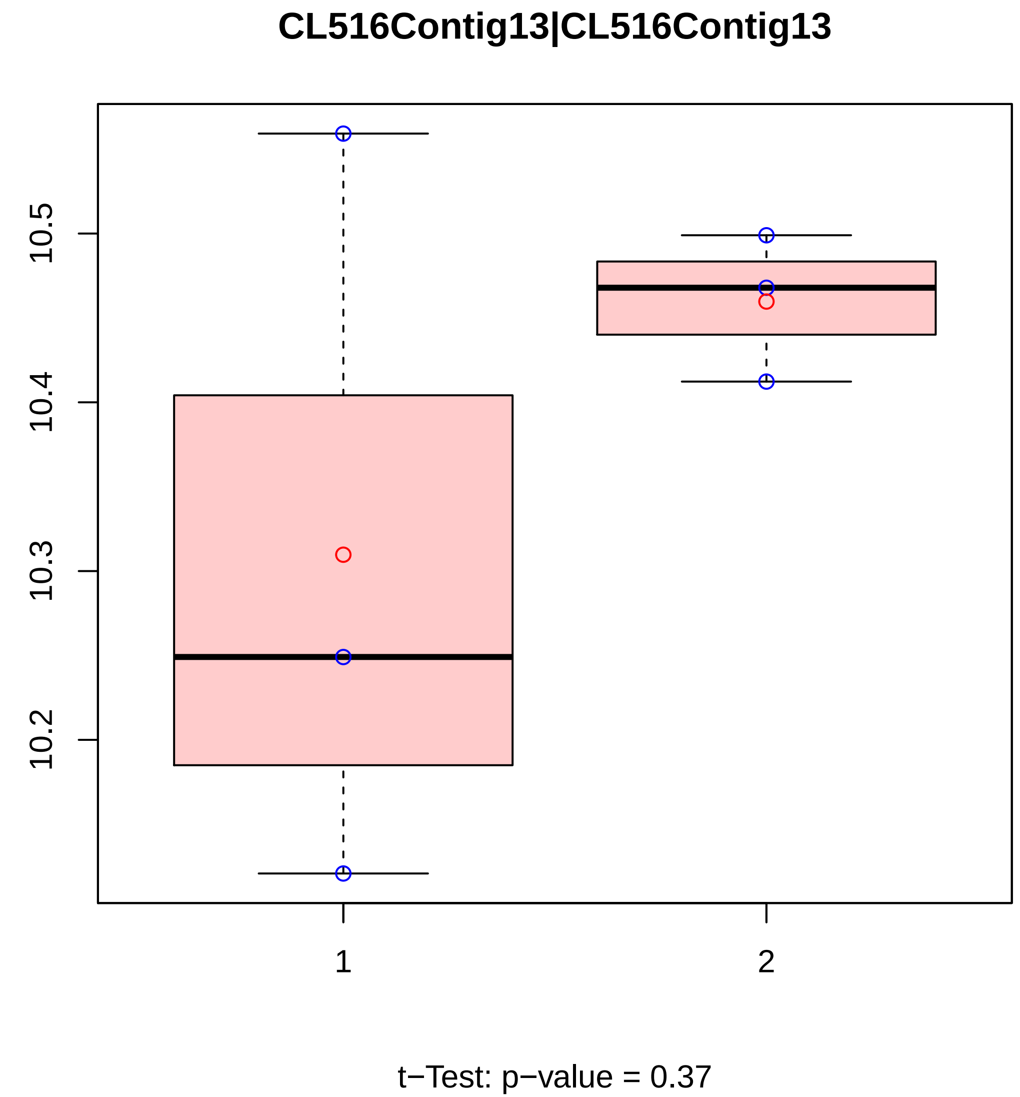
CL516Contig13|CL516Contig13
10.2
10.3
10.4
10.5
1
2
t−Test: p−value = 0.37

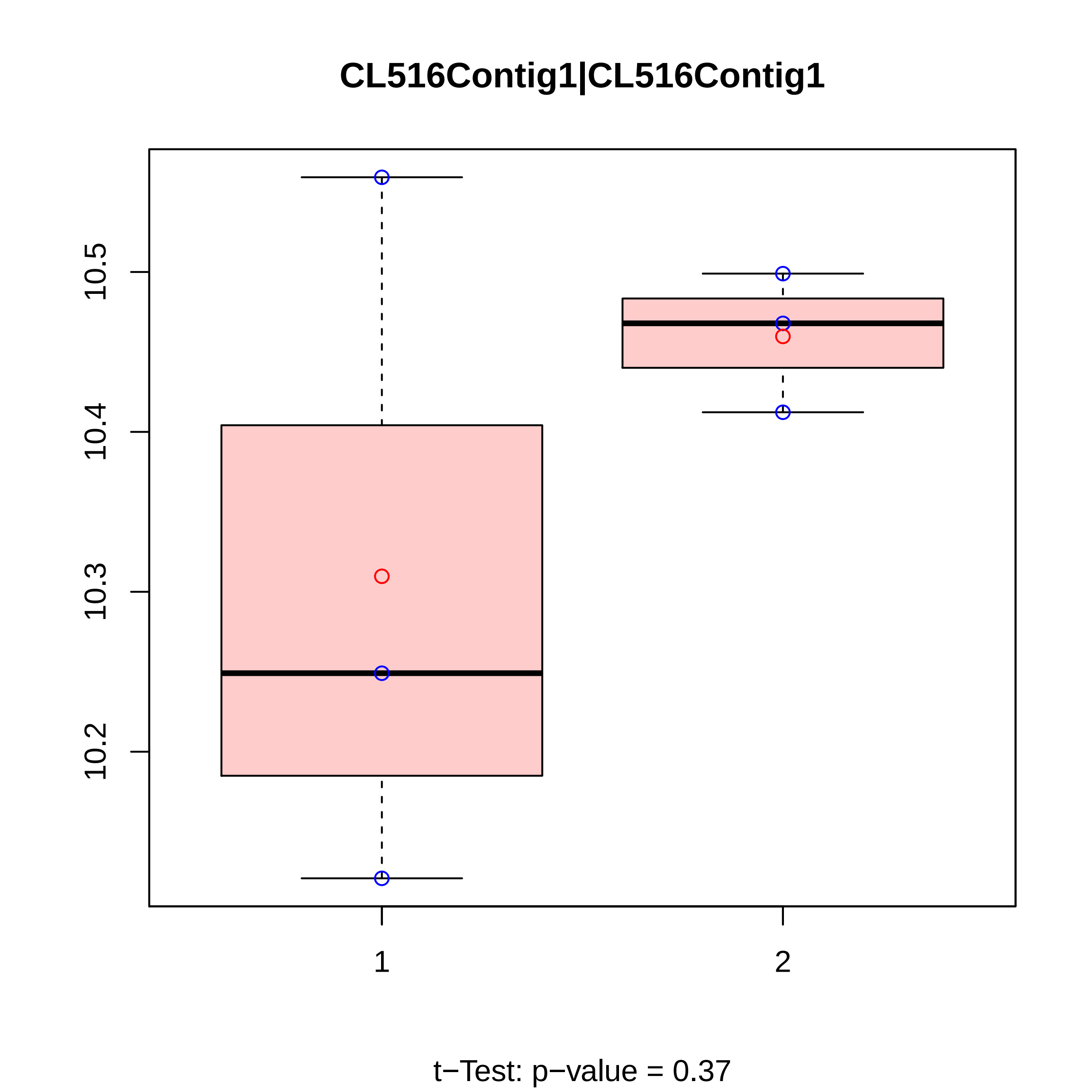

CL516Contig1|CL516Contig1
10.2
10.3
10.4
10.5
1
2
t−Test: p−value = 0.37

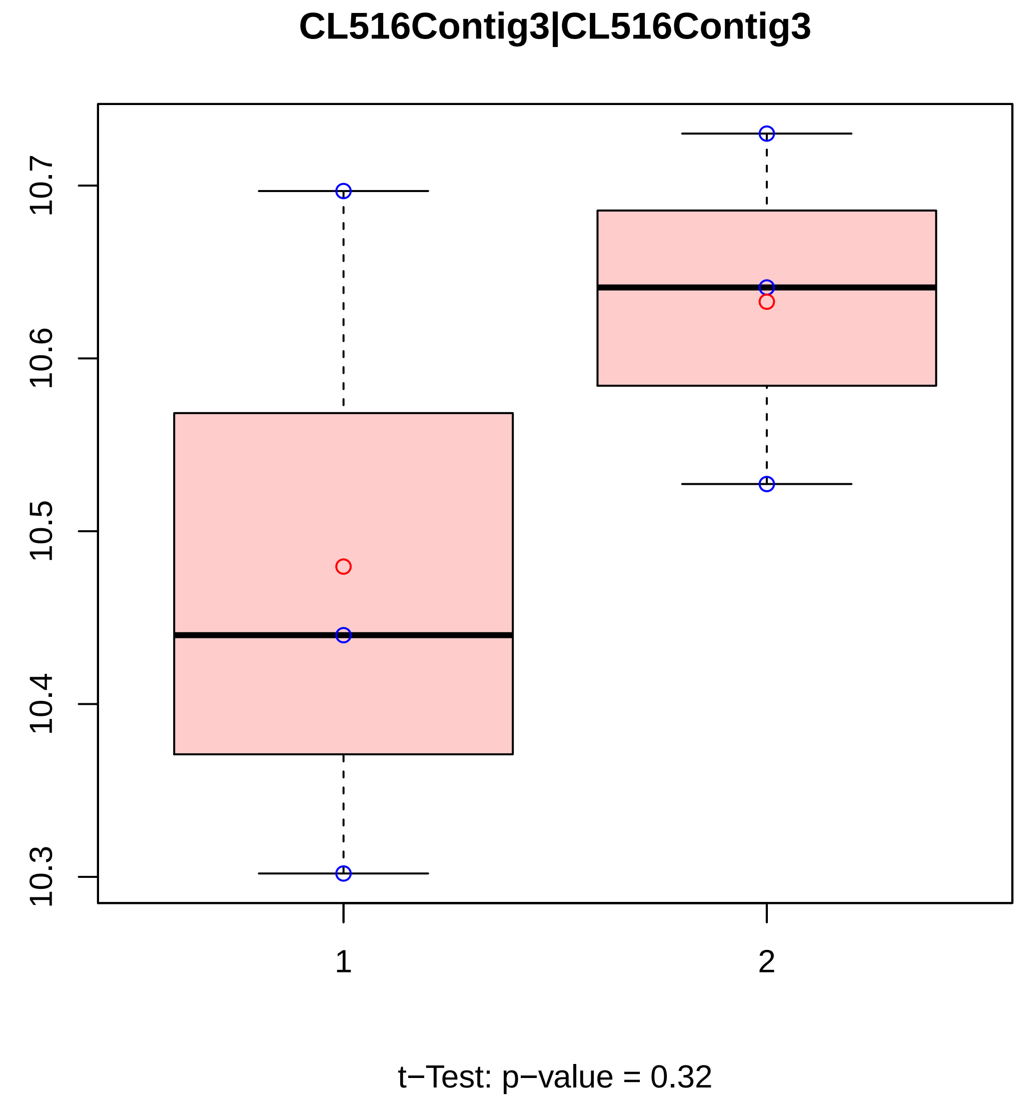
CL516Contig3|CL516Contig3
10.3
10.4
10.5
10.6
10.7
1
2
t−Test: p−value = 0.32

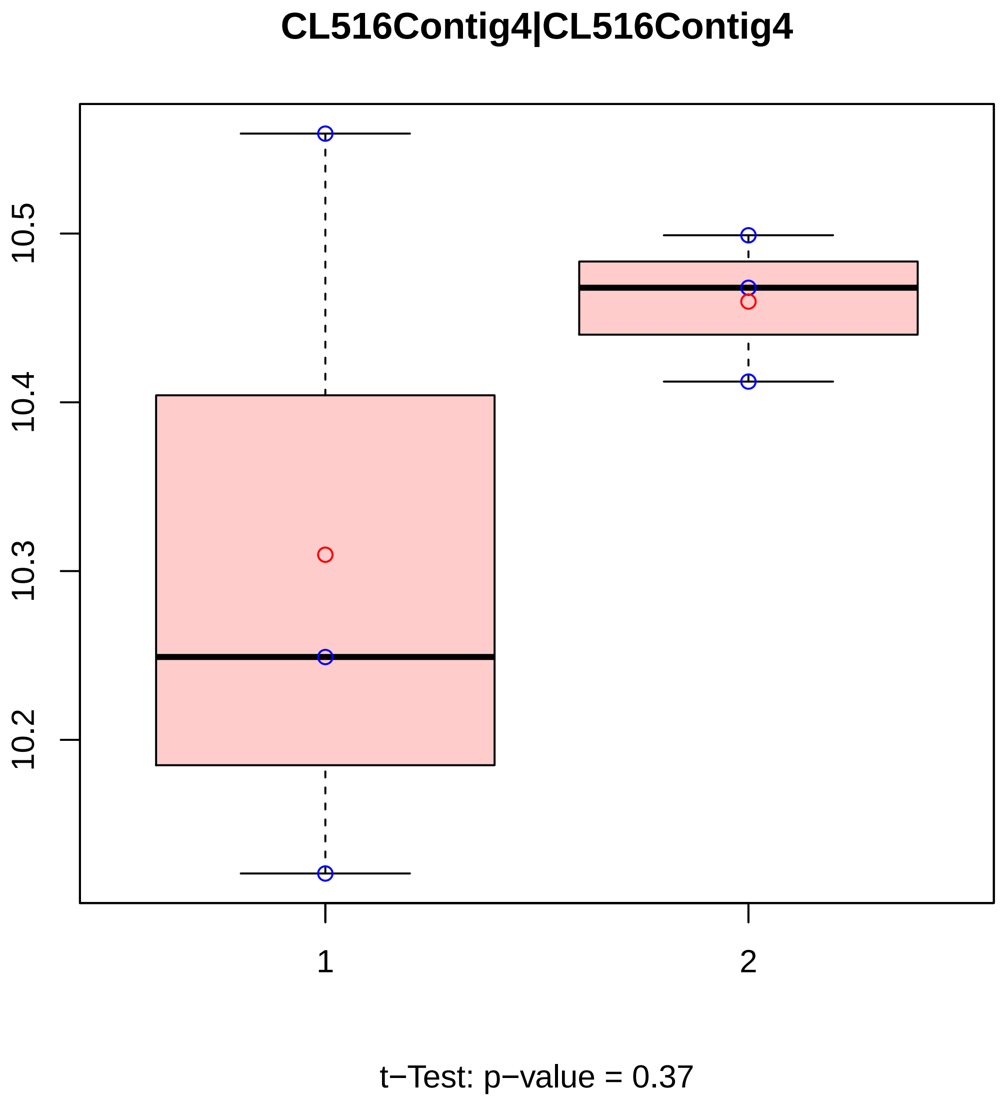

CL516Contig4|CL516Contig4
10.2
10.3
10.4
10.5
1
2
t−Test: p−value = 0.37

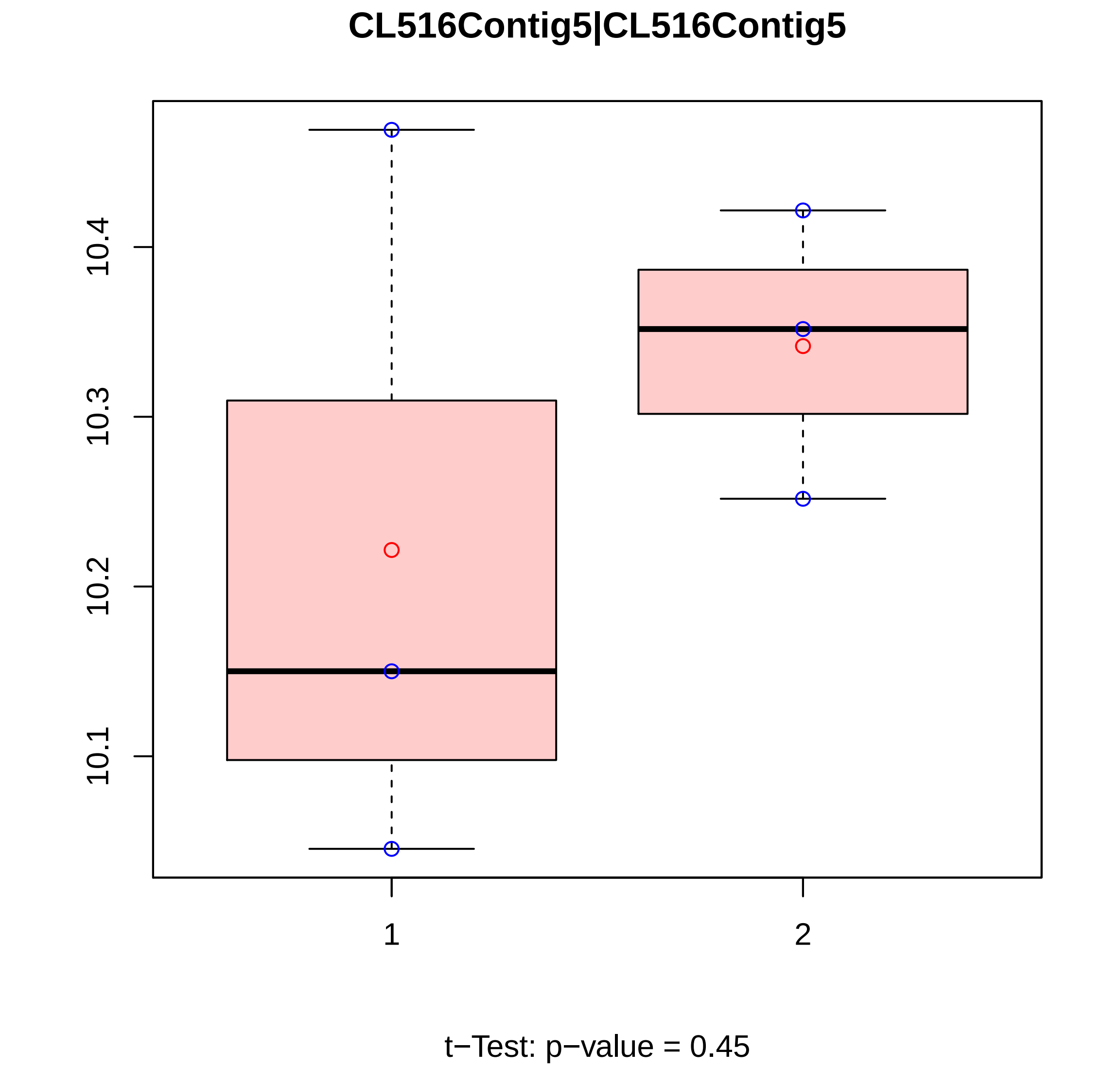
CL516Contig5|CL516Contig5
10.1
10.2
10.3
10.4
1
2
t−Test: p−value = 0.45

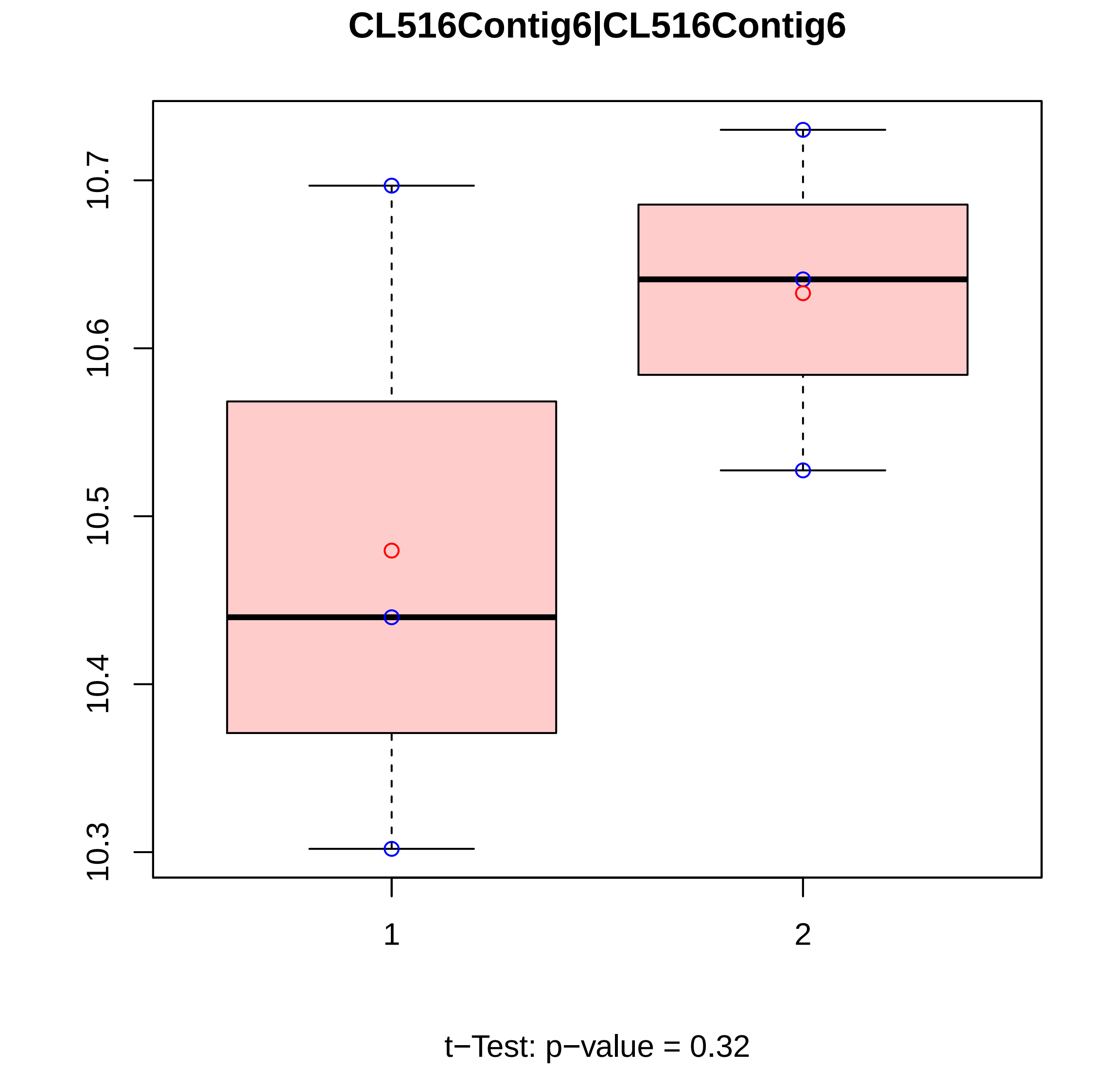
CL516Contig6|CL516Contig6
10.3
10.4
10.5
10.6
10.7
1
2
t−Test: p−value = 0.32

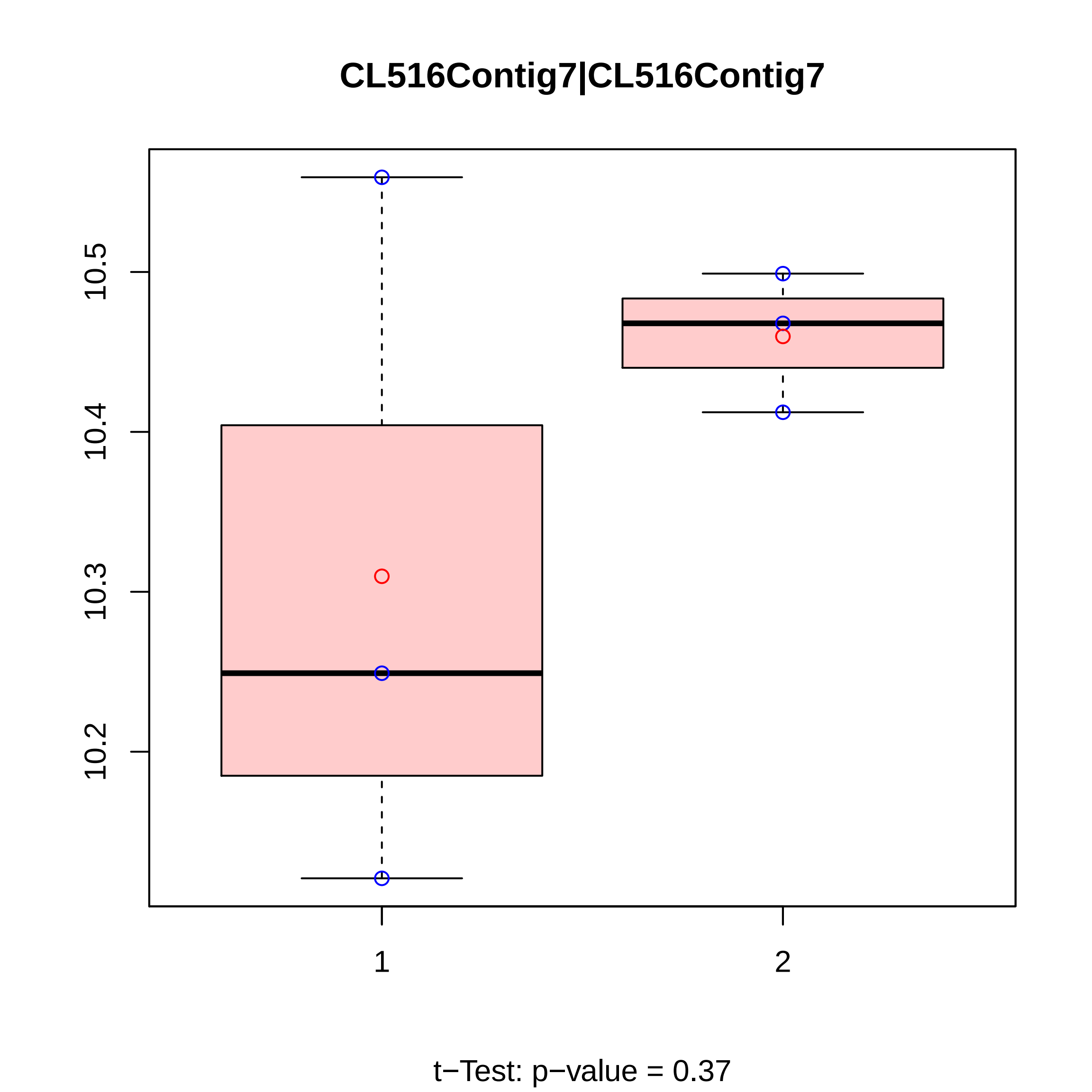
CL516Contig7|CL516Contig7
10.2
10.3
10.4
10.5
1
2
t−Test: p−value = 0.37

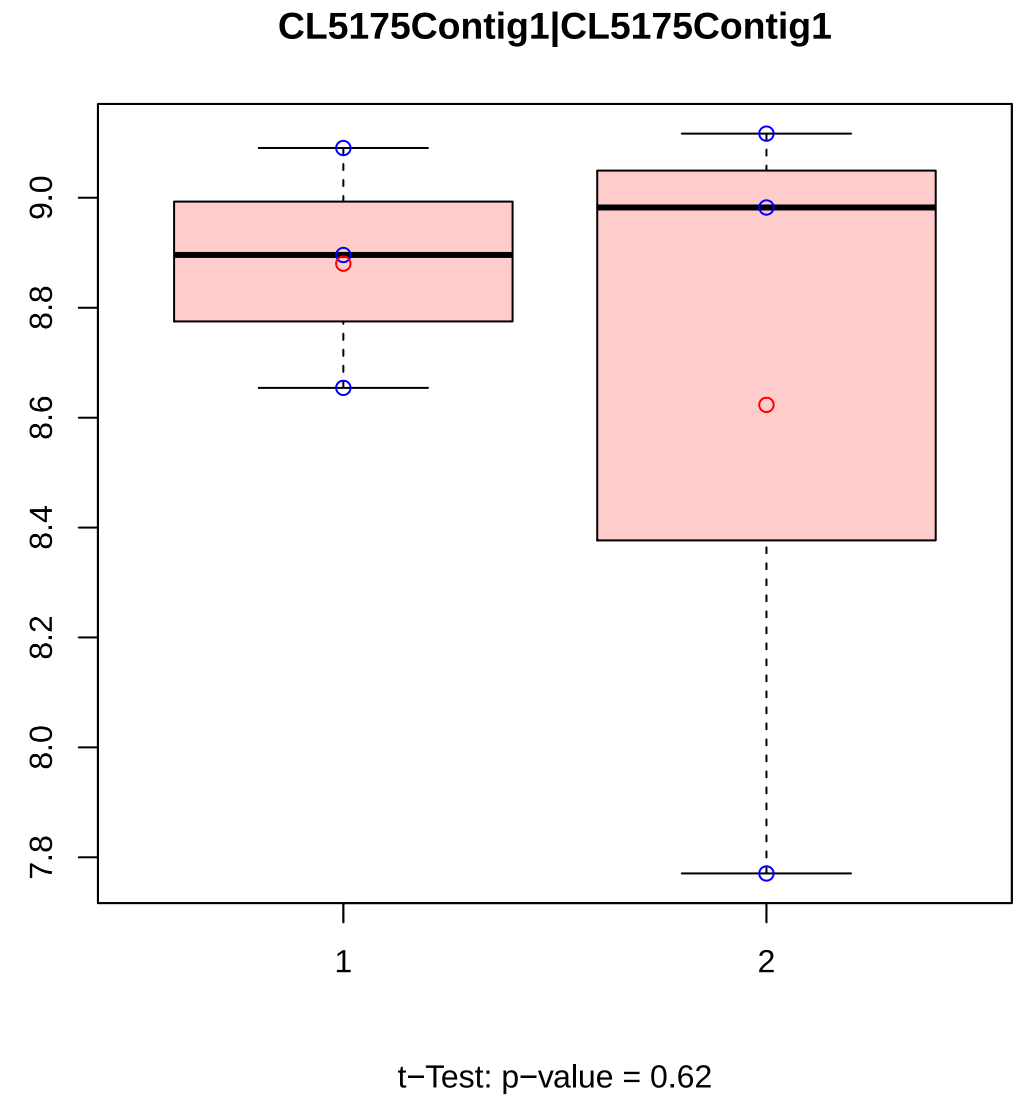

CL5175Contig1|CL5175Contig1
9.0
8.8
8.6
8.4
8.2
8.0
7.8
1
2
t−Test: p−value = 0.62

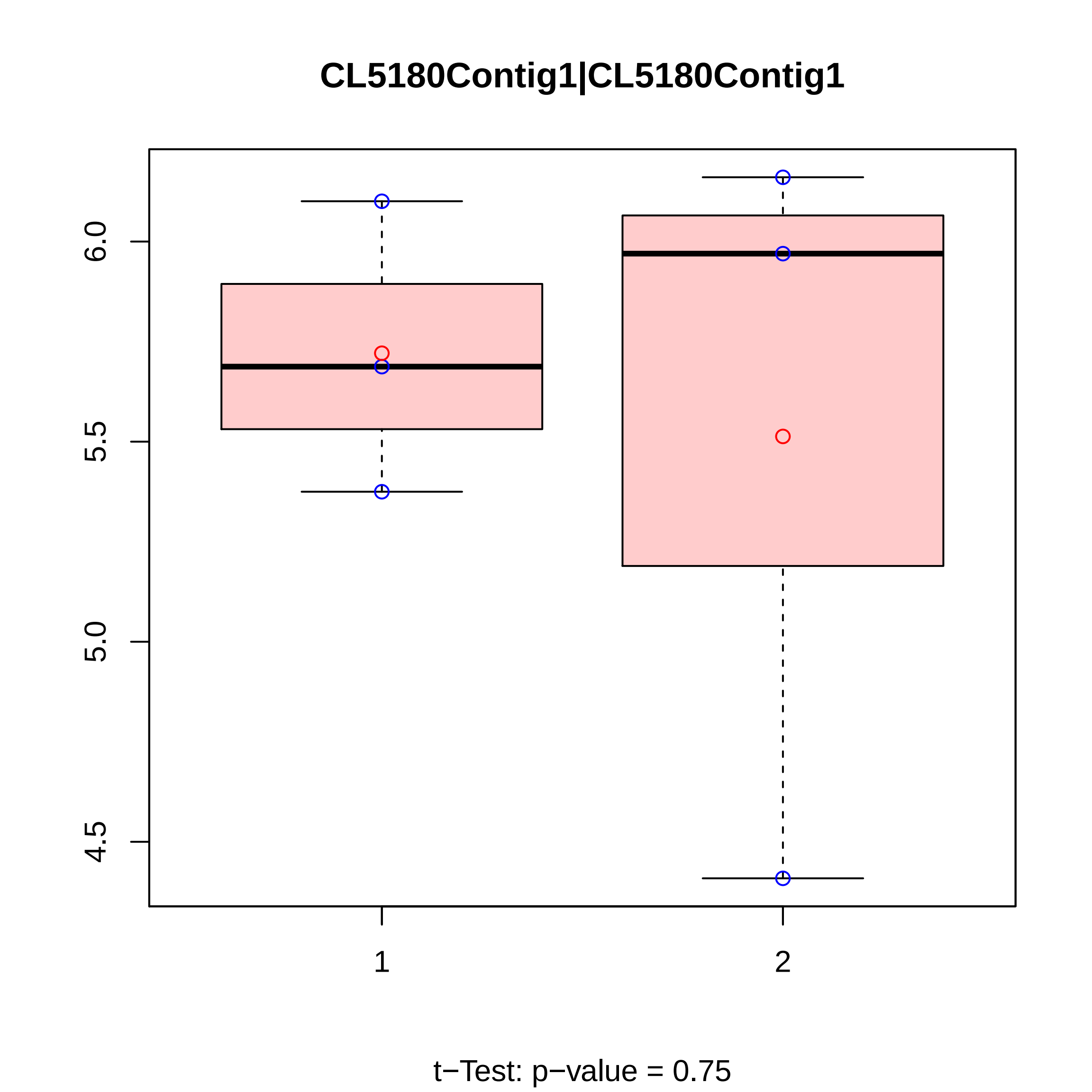

CL5180Contig1|CL5180Contig1
6.0
5.5
5.0
4.5
1
2
t−Test: p−value = 0.75

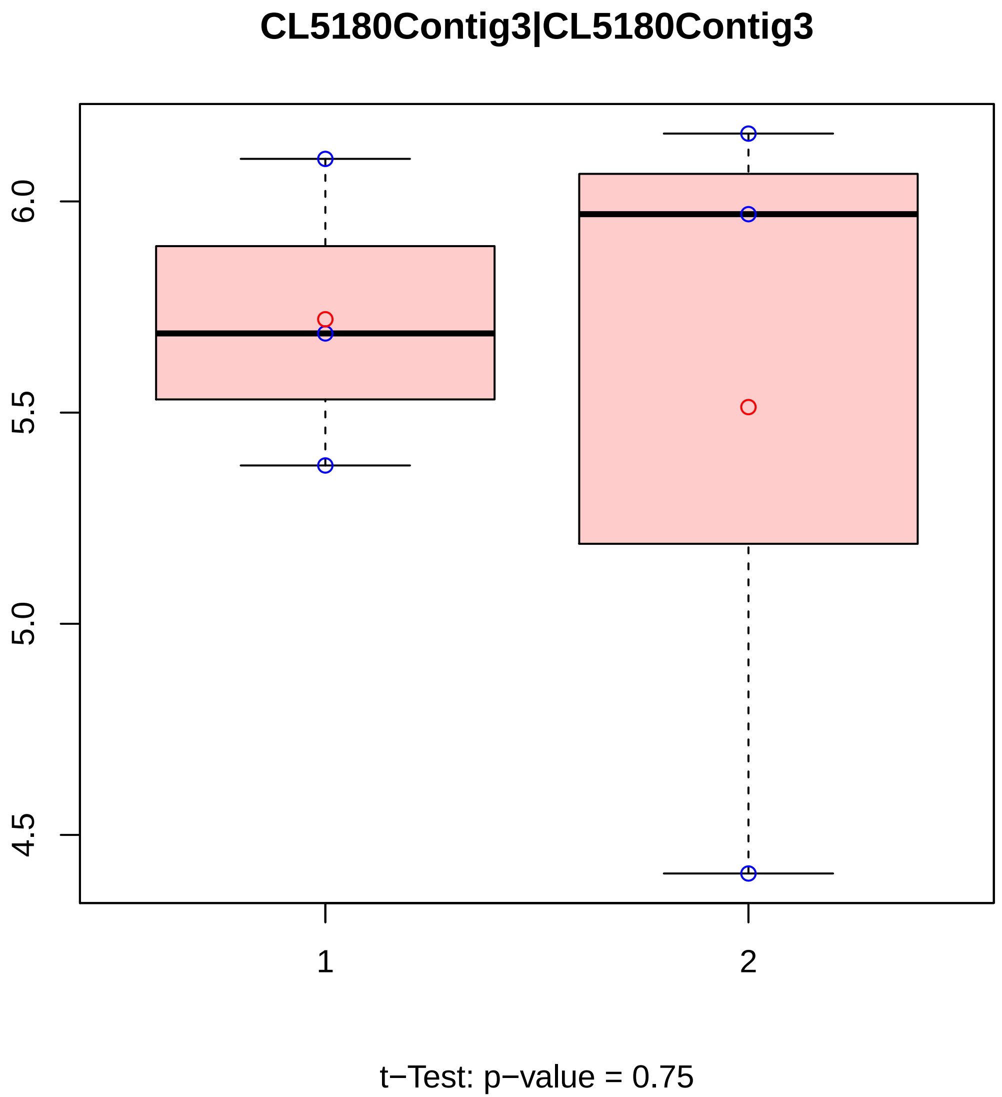

CL5180Contig3|CL5180Contig3
6.0
5.5
5.0
4.5
1
2
t−Test: p−value = 0.75

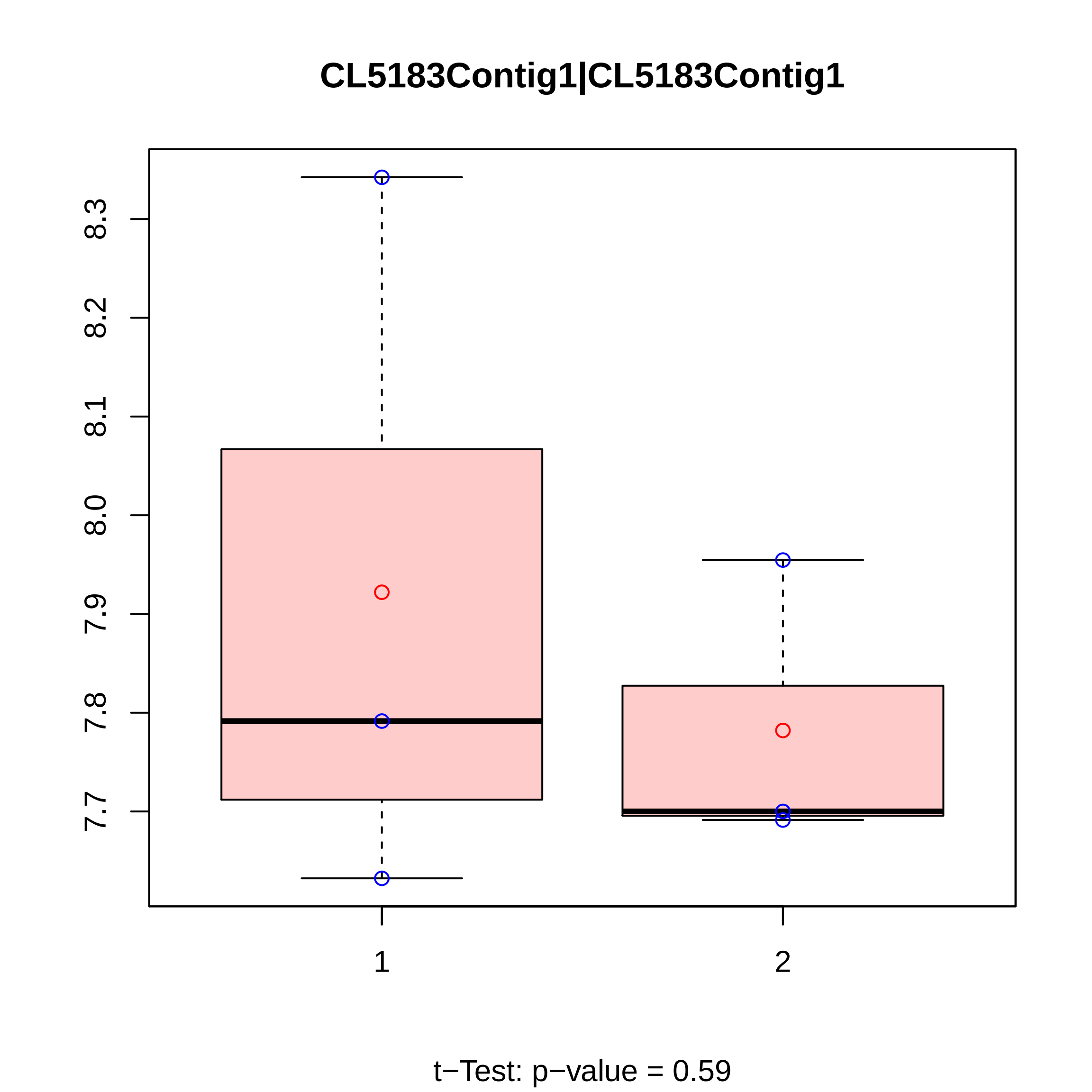

CL5183Contig1|CL5183Contig1
8.3
8.2
8.1
8.0
7.9
7.8
7.7
1
2
t−Test: p−value = 0.59

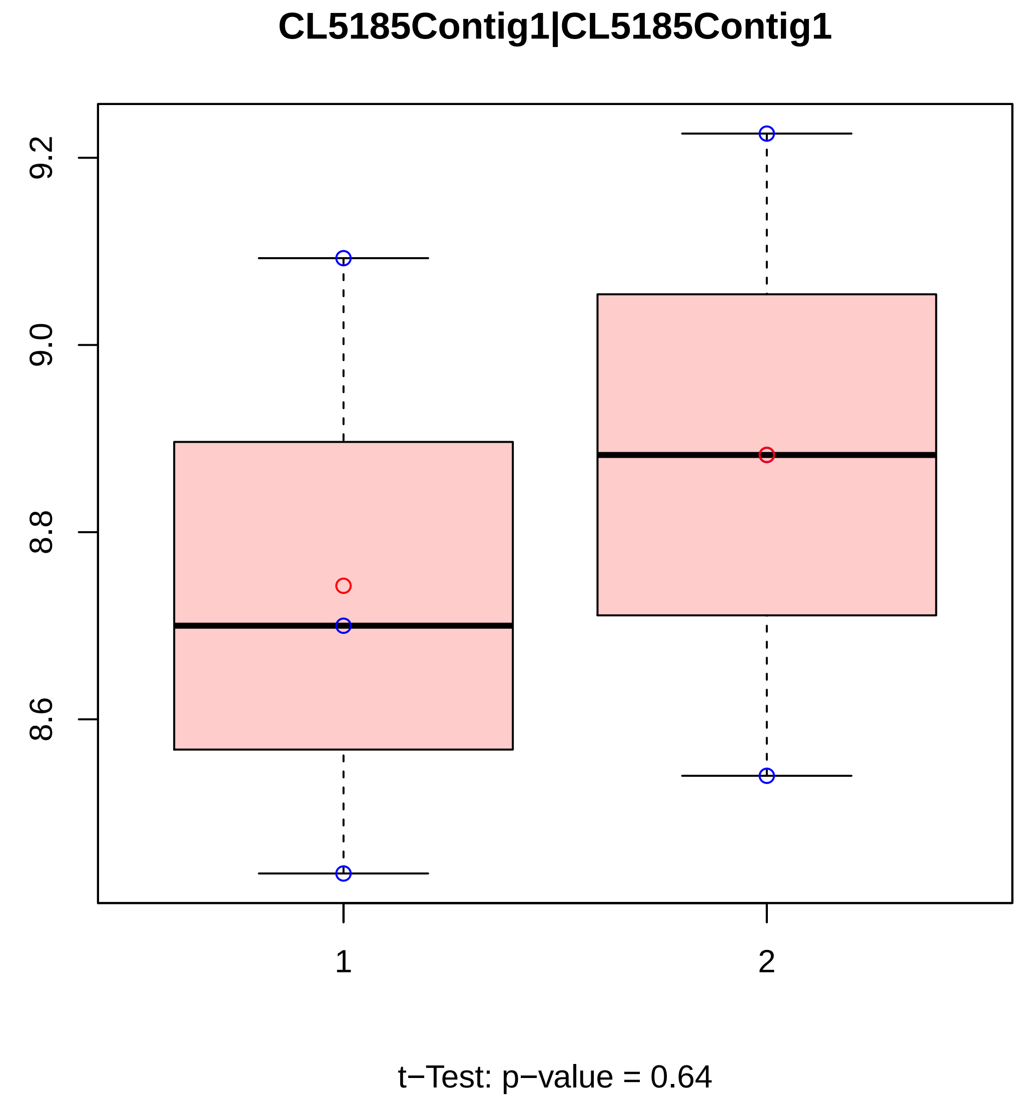

CL5185Contig1|CL5185Contig1
9.2
9.0
8.8
8.6
1
2
t−Test: p−value = 0.64

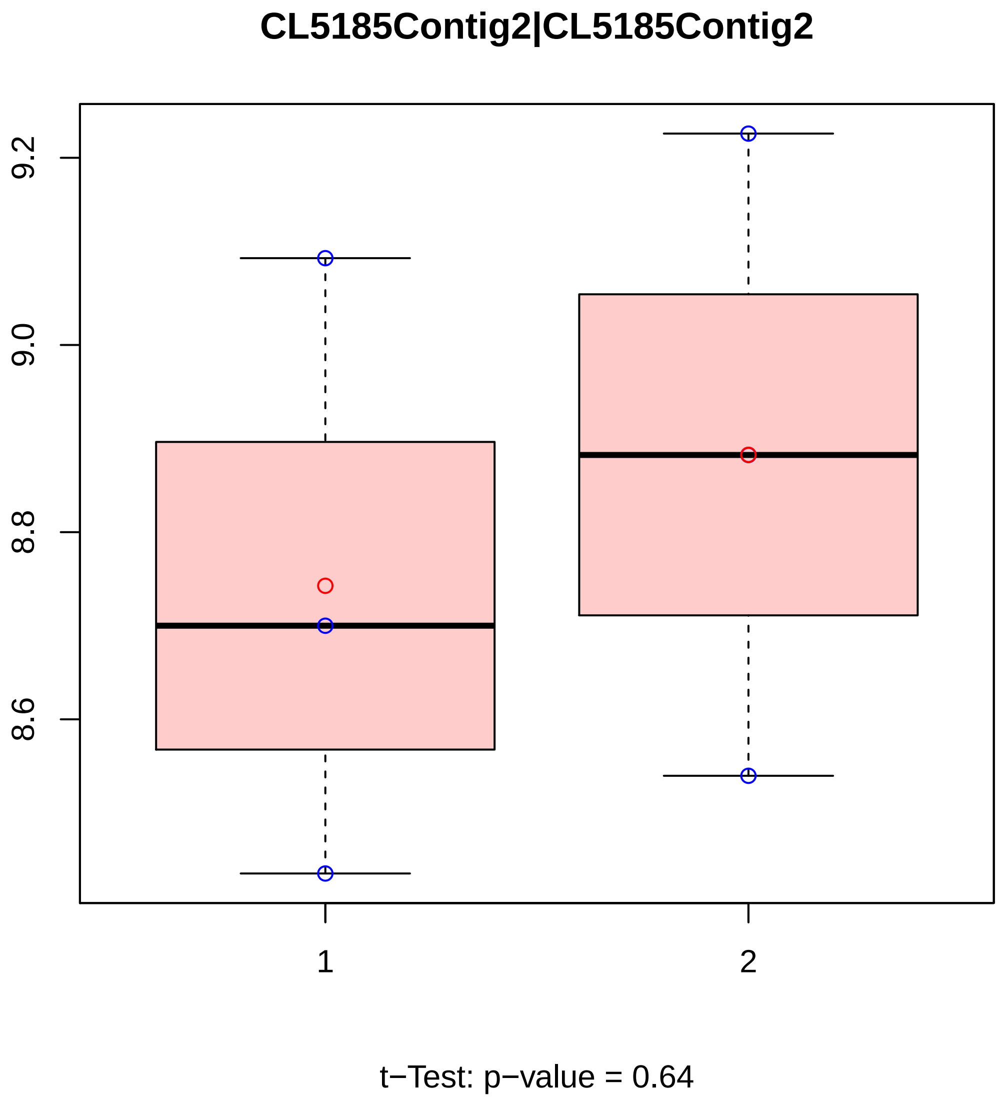
CL5185Contig2|CL5185Contig2
9.2
9.0
8.8
8.6
1
2
t−Test: p−value = 0.64

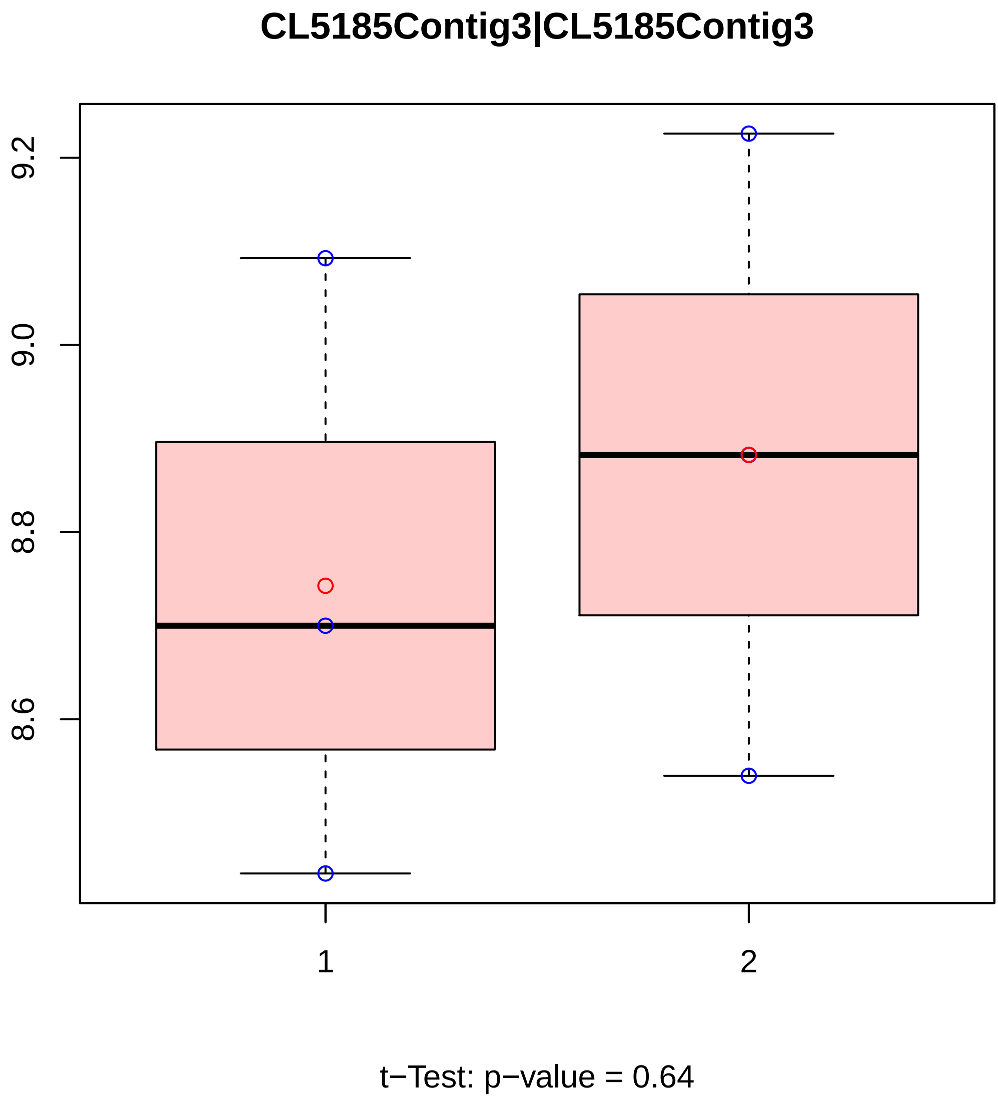

CL5185Contig3|CL5185Contig3
9.2
9.0
8.8
8.6
1
2
t−Test: p−value = 0.64

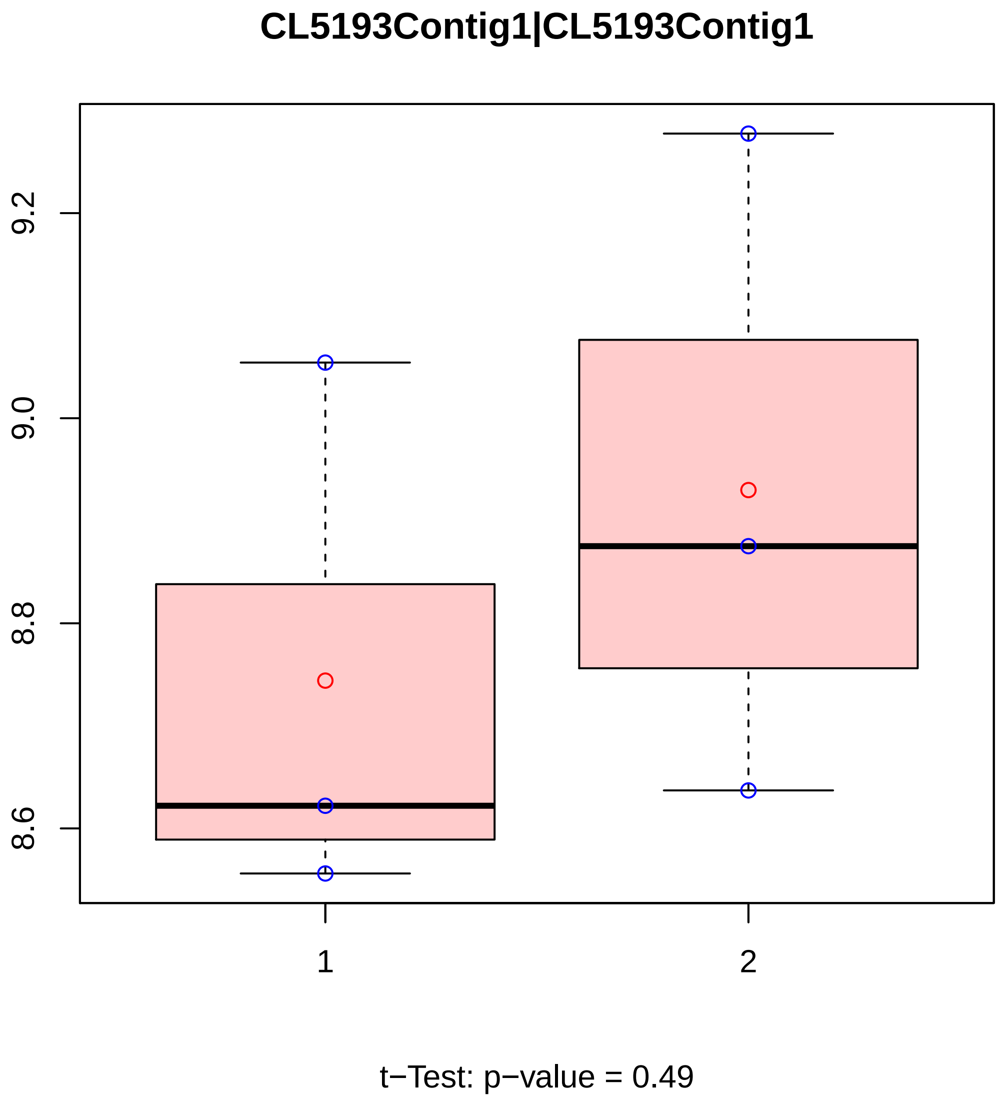
CL5193Contig1|CL5193Contig1
9.2
9.0
8.8
8.6
1
2
t−Test: p−value = 0.49

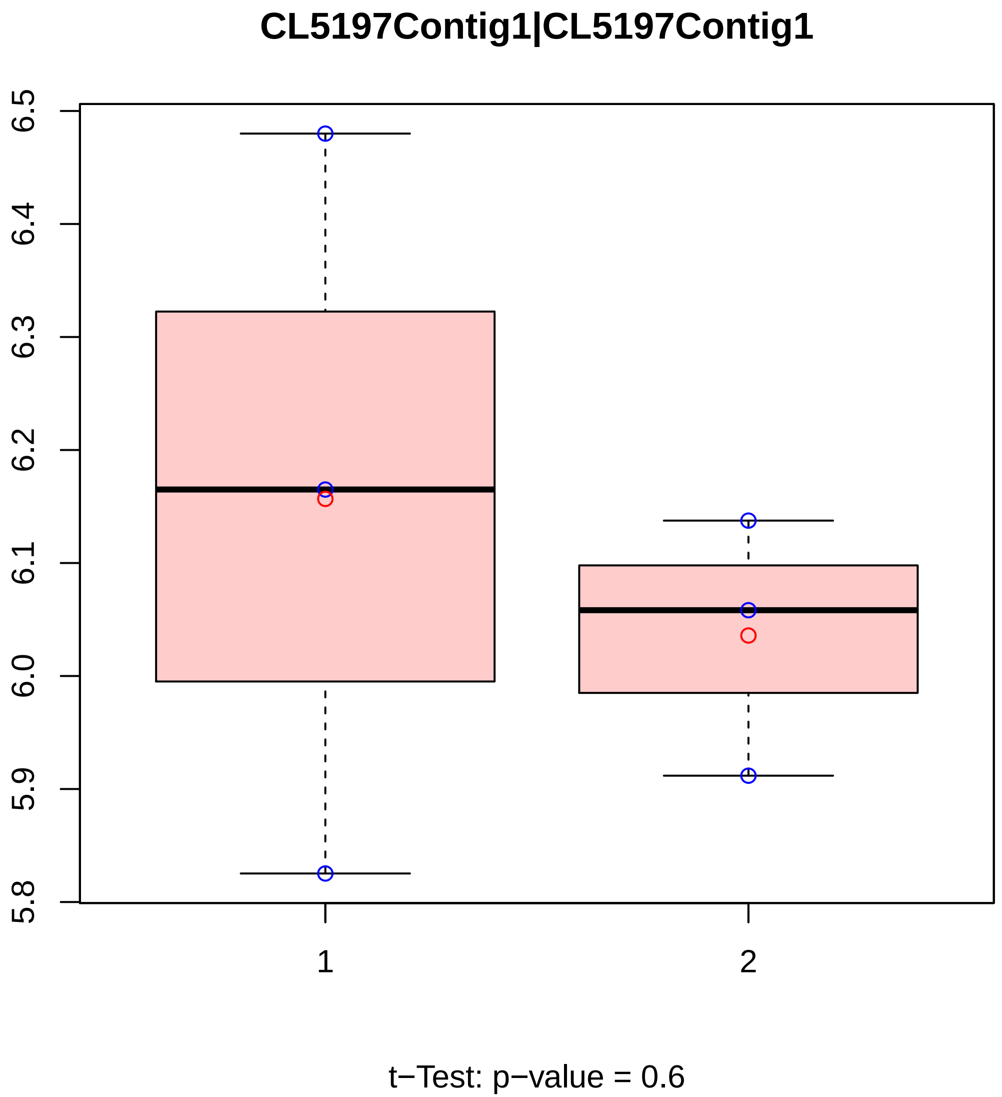

CL5197Contig1|CL5197Contig1
6.5
6.4
6.3
6.2
6.1
6.0
5.9
5.8
1
2
t−Test: p−value = 0.6

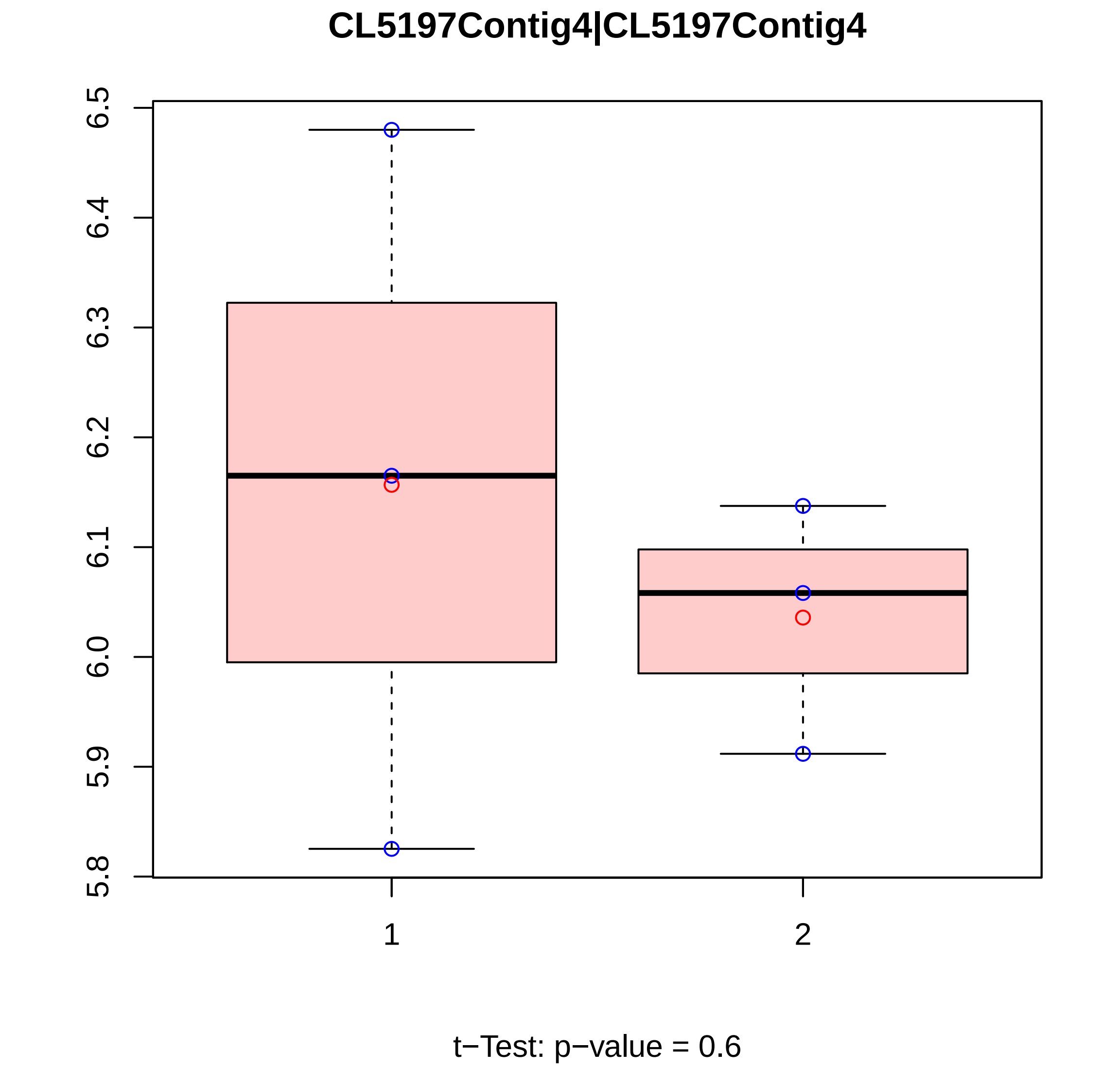

CL5197Contig4|CL5197Contig4
6.5
6.4
6.3
6.2
6.1
6.0
5.9
5.8
1
2
t−Test: p−value = 0.6

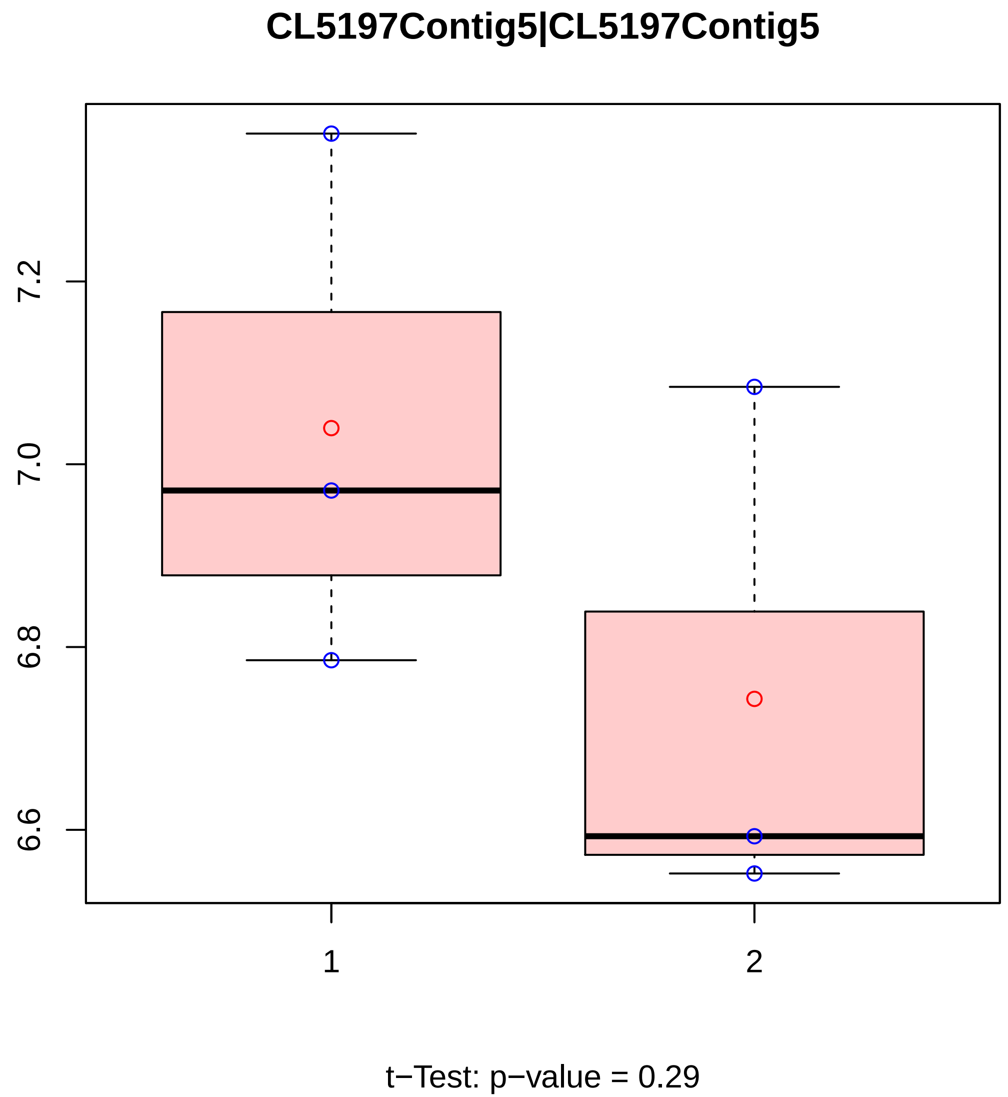

CL5197Contig5|CL5197Contig5
7.2
7.0
6.8
6.6
1
2
t−Test: p−value = 0.29

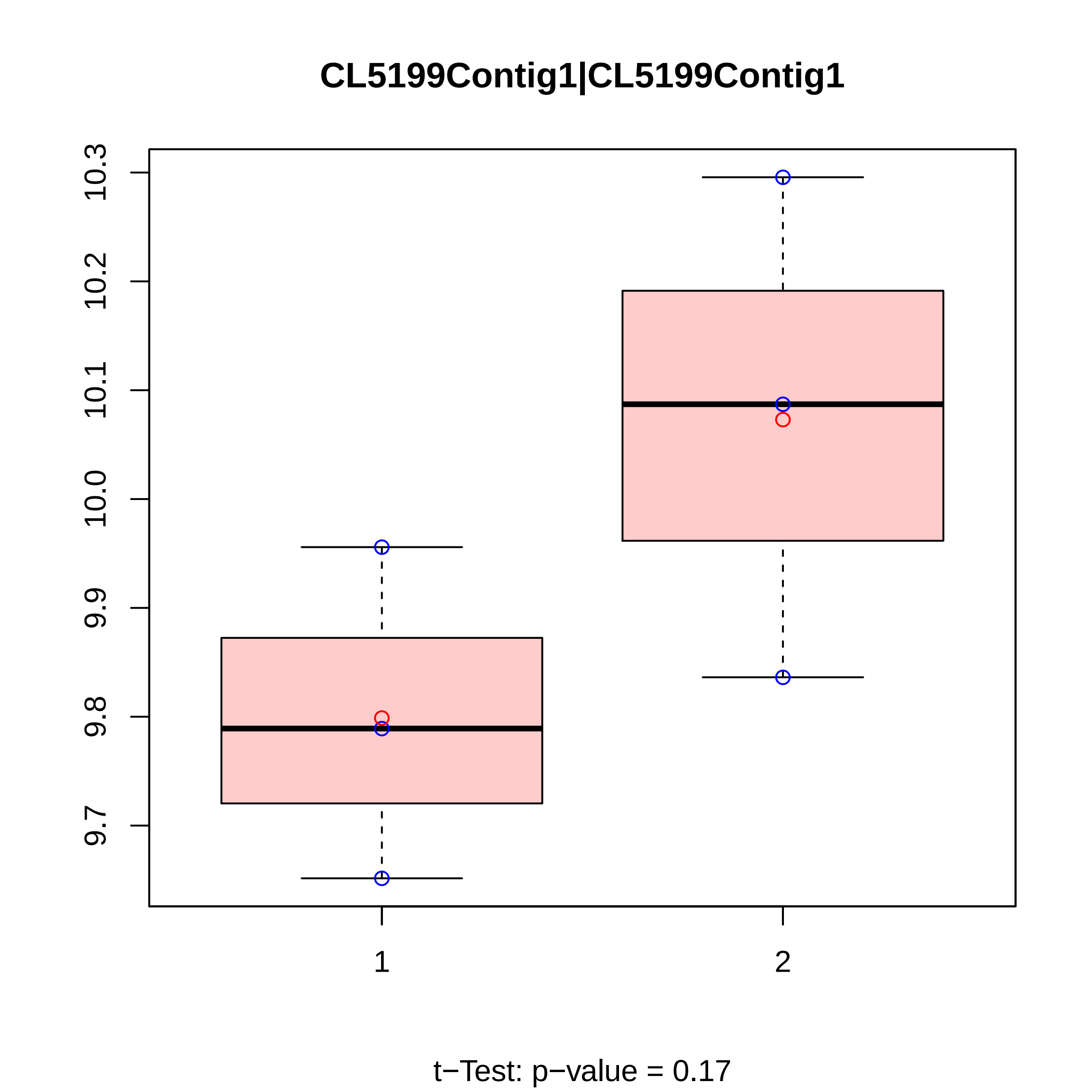
CL5199Contig1|CL5199Contig1
10.3
10.2
10.1
10.0
9.9
9.8
9.7
1
2
t−Test: p−value = 0.17

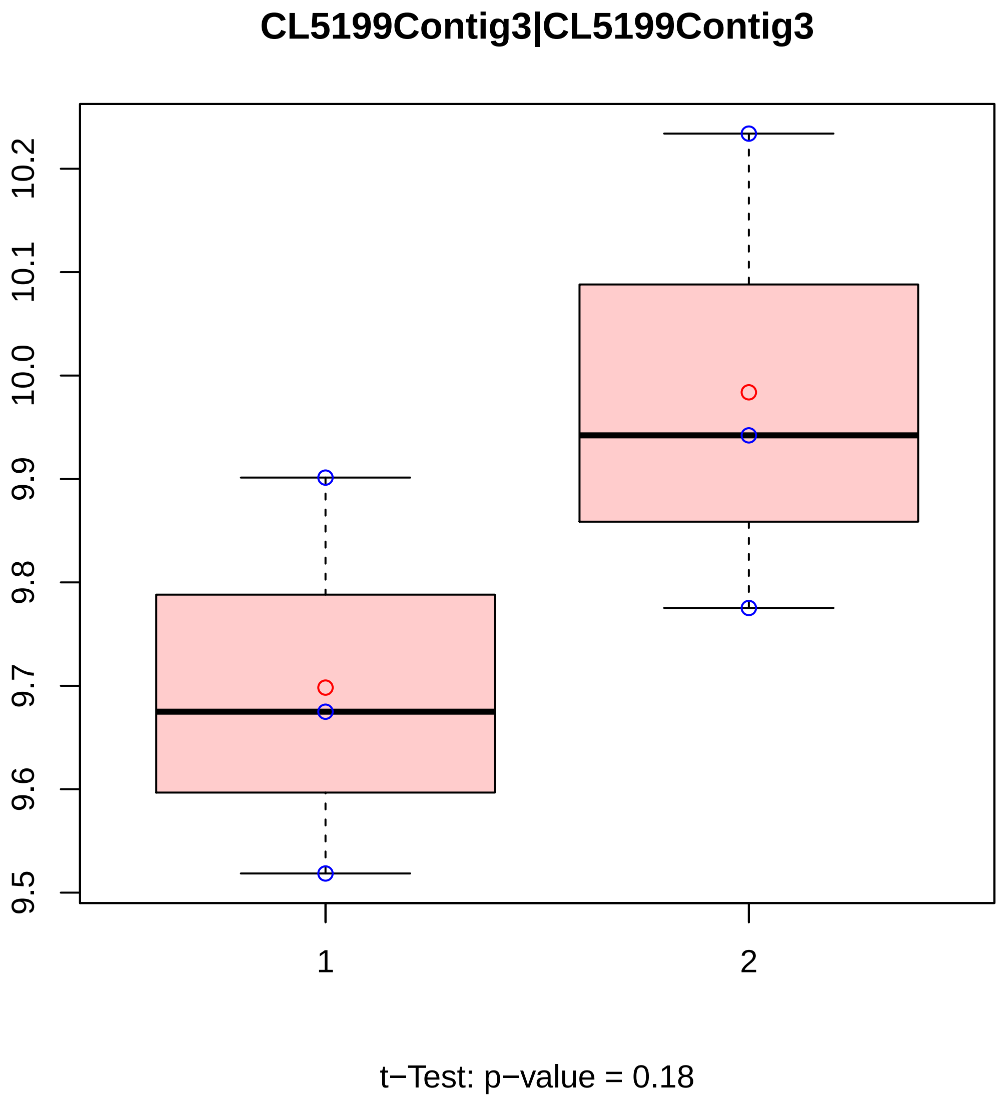
CL5199Contig3|CL5199Contig3
10.2
10.1
10.0
9.9
9.8
9.7
9.6
9.5
1
2
t−Test: p−value = 0.18

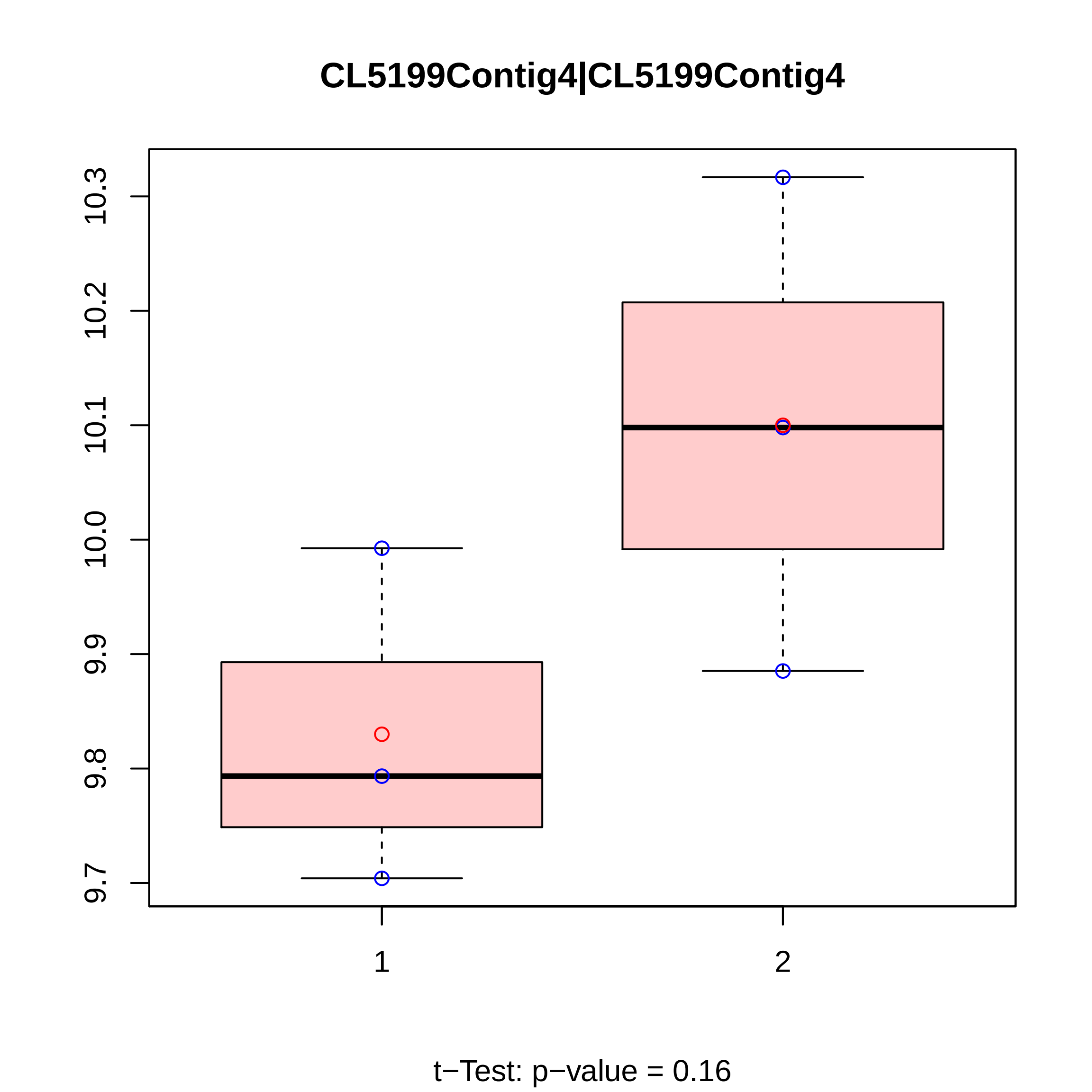
CL5199Contig4|CL5199Contig4
10.3
10.2
10.1
10.0
9.9
9.8
9.7
1
2
t−Test: p−value = 0.16

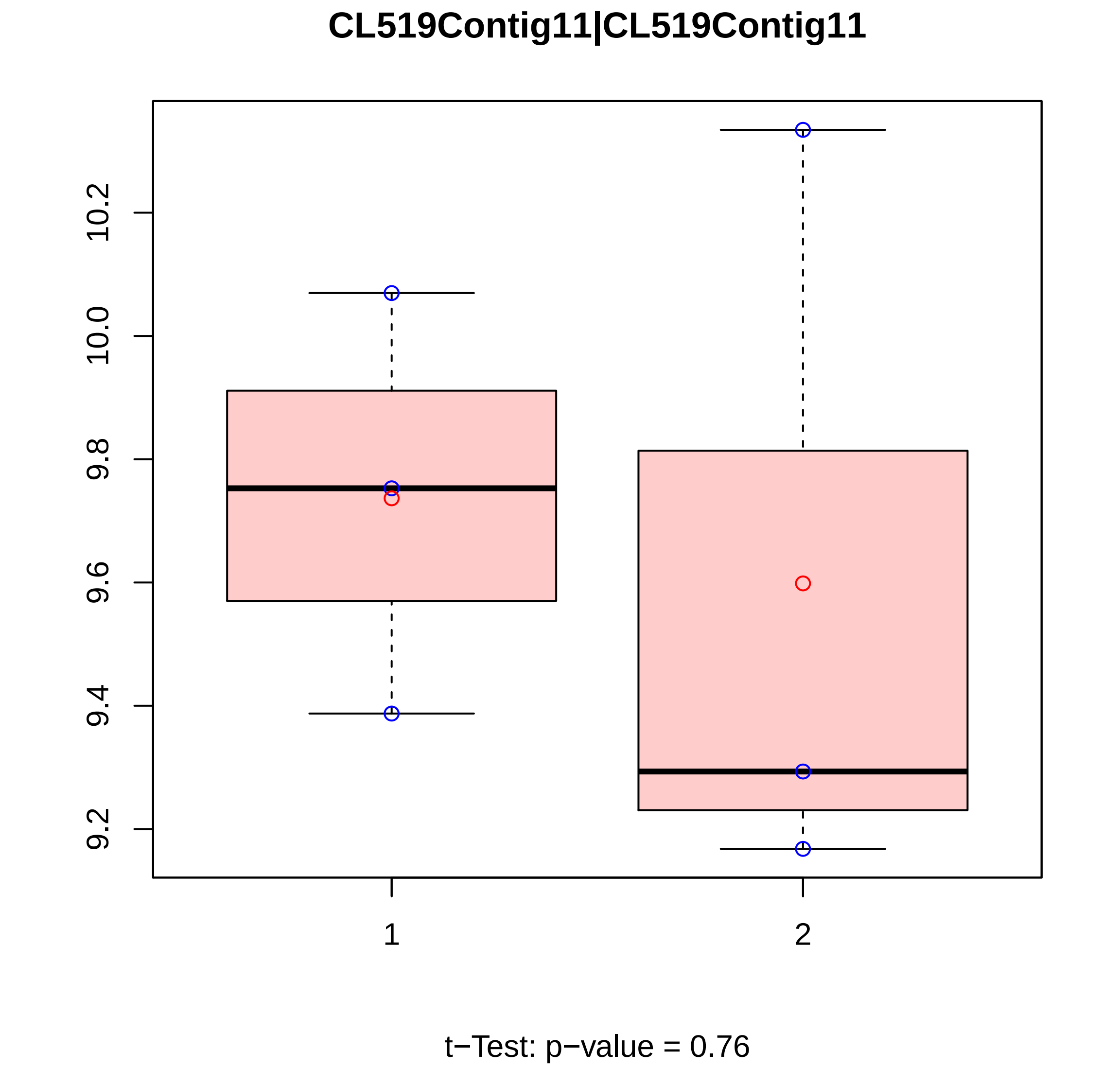
CL519Contig11|CL519Contig11
10.2
10.0
9.8
9.6
9.4
9.2
1
2
t−Test: p−value = 0.76

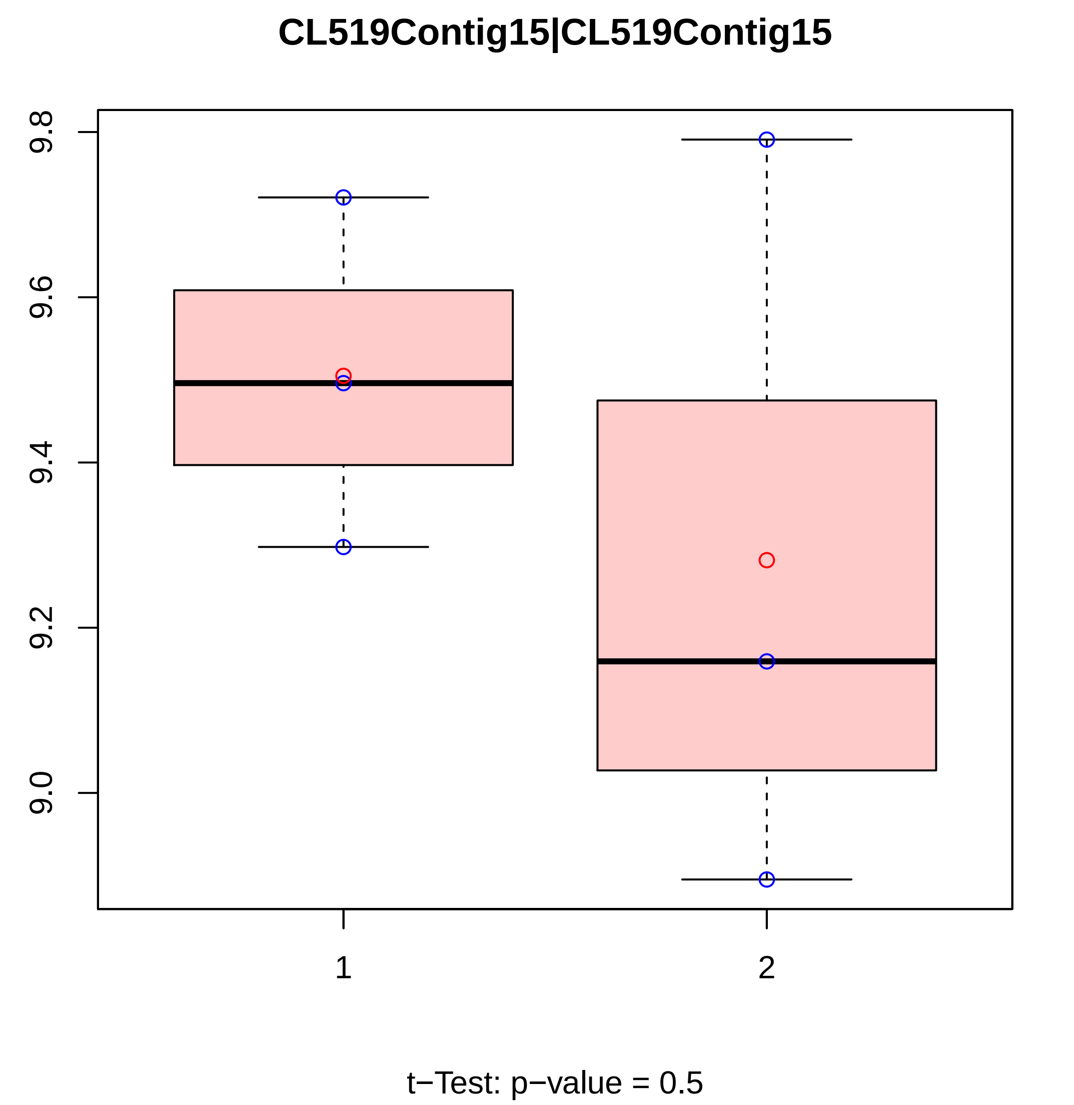
CL519Contig15|CL519Contig15
9.0
9.2
9.4
9.6
9.8
1
2
t−Test: p−value = 0.5

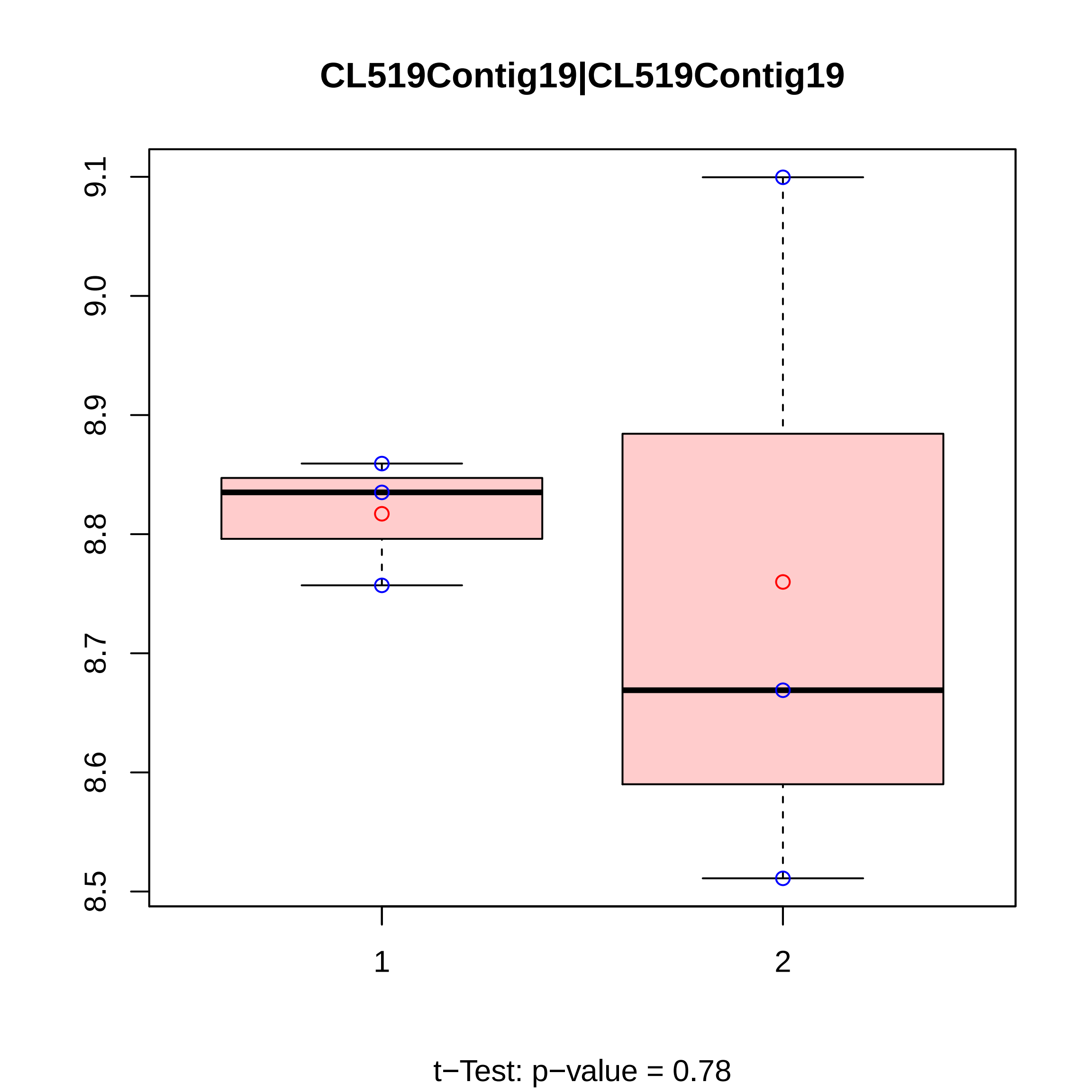
CL519Contig19|CL519Contig19
9.1
9.0
8.9
8.8
8.7
8.6
8.5
1
2
t−Test: p−value = 0.78

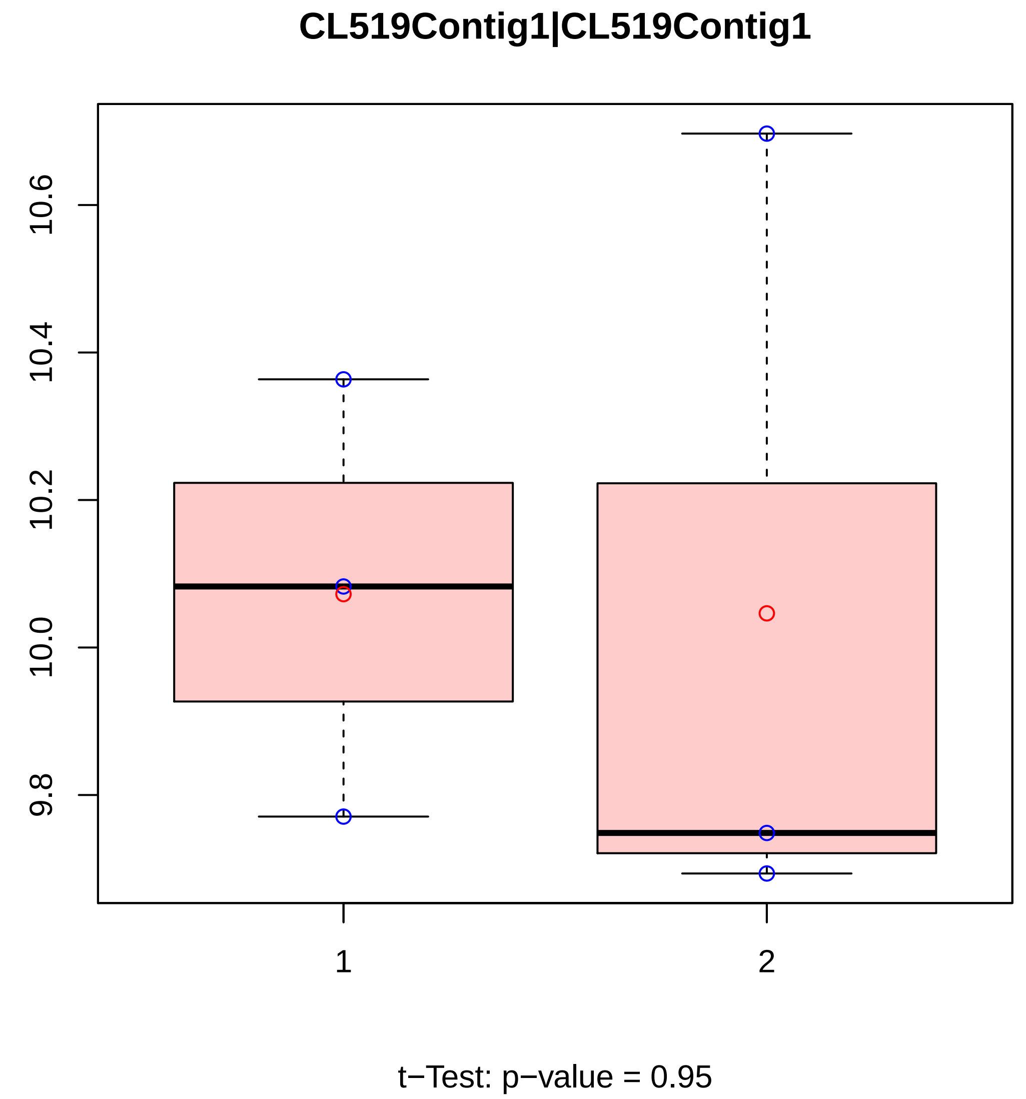

CL519Contig1|CL519Contig1
10.6
10.4
10.2
10.0
9.8
1
2
t−Test: p−value = 0.95

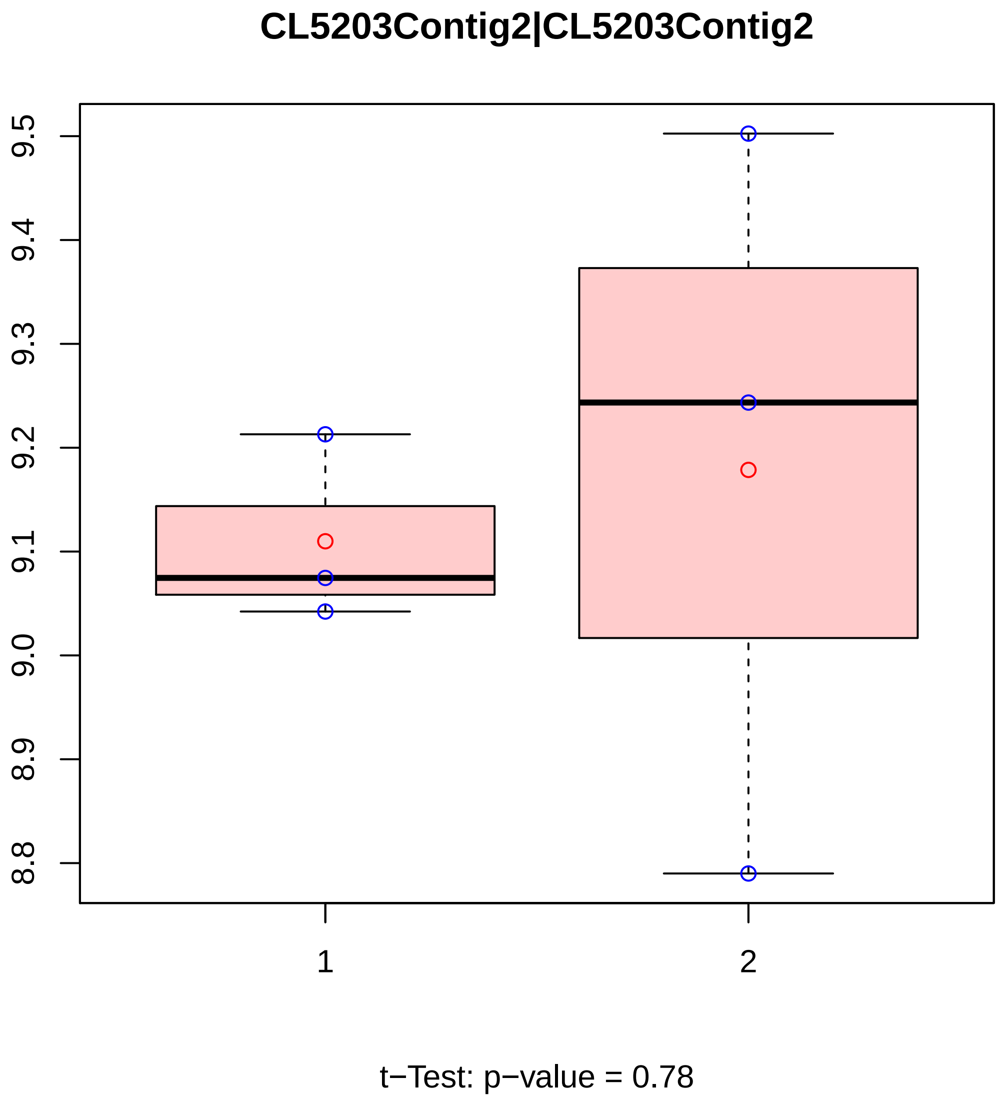

CL5203Contig2|CL5203Contig2
9.5
9.4
9.3
9.2
9.1
9.0
8.9
8.8
1
2
t−Test: p−value = 0.78

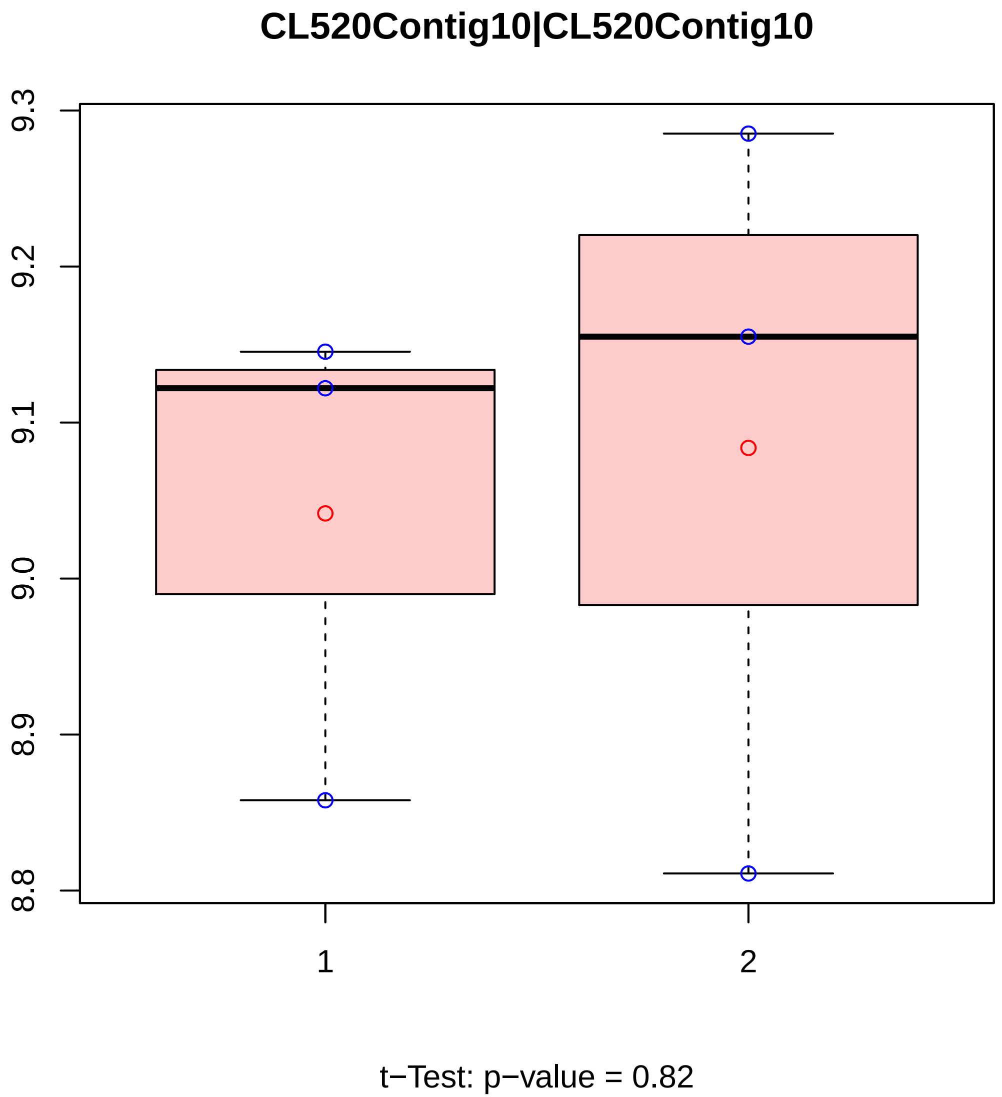

CL520Contig10|CL520Contig10
9.3
9.2
9.1
9.0
8.9
8.8
1
2
t−Test: p−value = 0.82

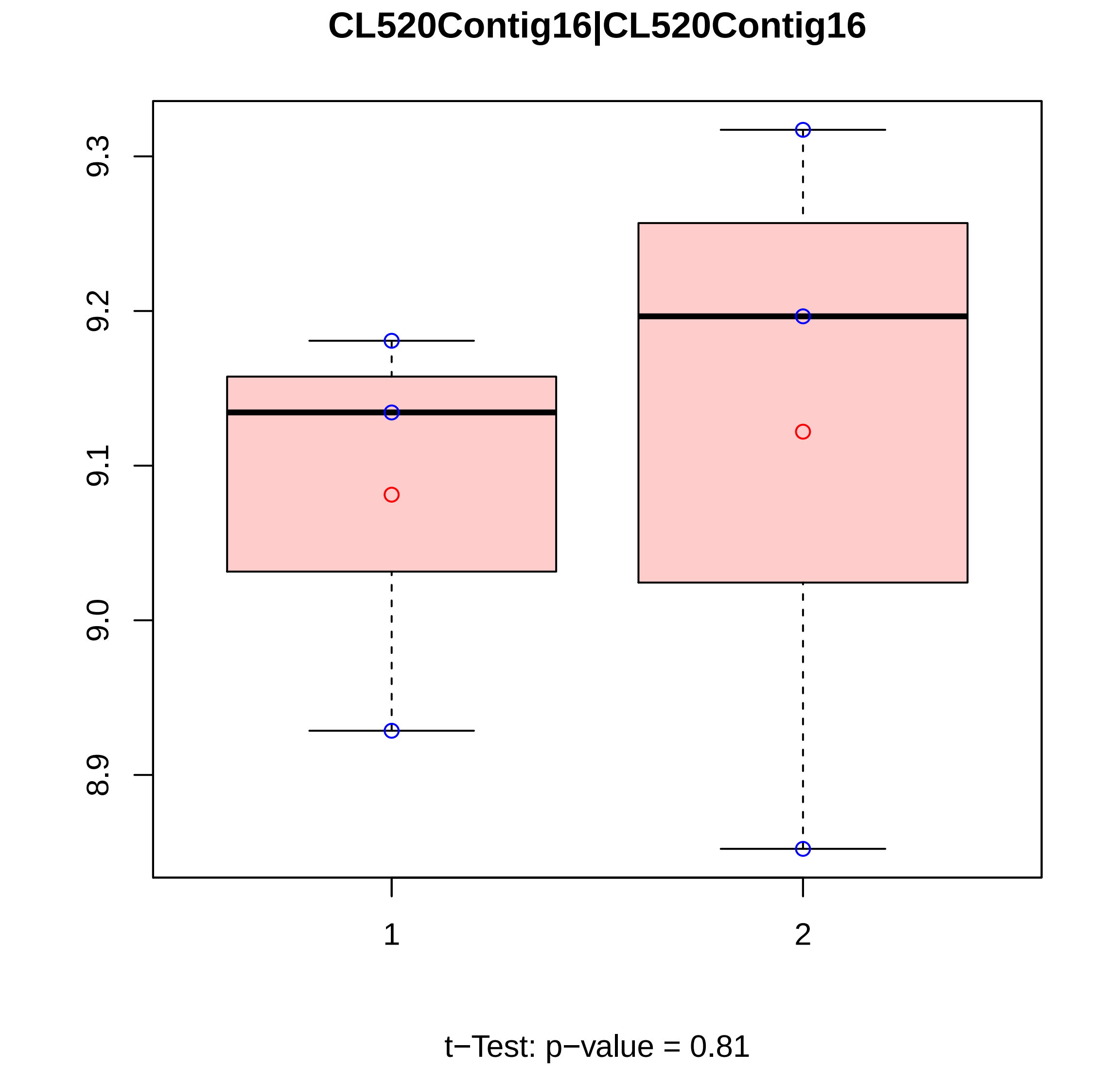
CL520Contig16|CL520Contig16
9.3
9.2
9.1
9.0
8.9
1
2
t−Test: p−value = 0.81

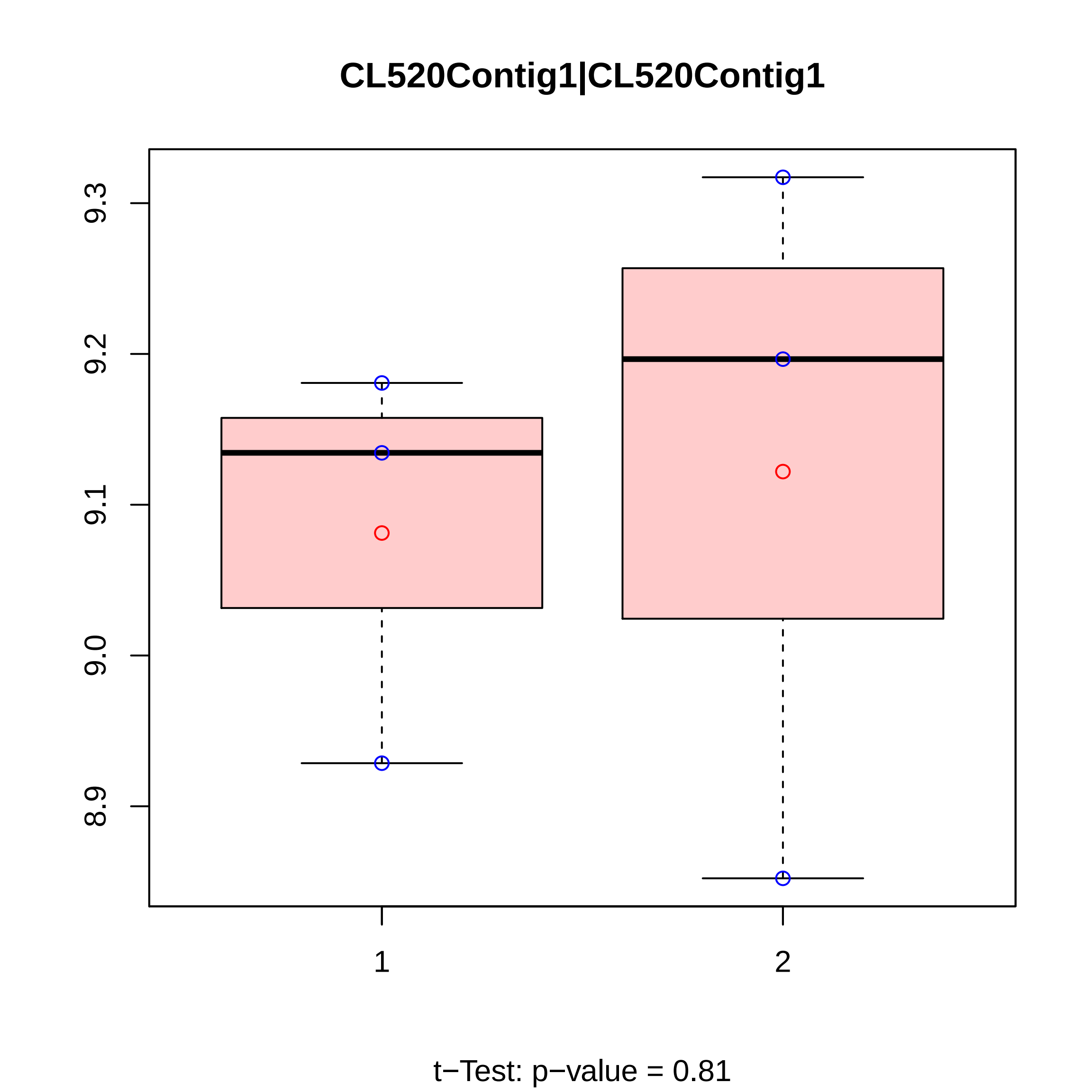
CL520Contig1|CL520Contig1
9.3
9.2
9.1
9.0
8.9
1
2
t−Test: p−value = 0.81

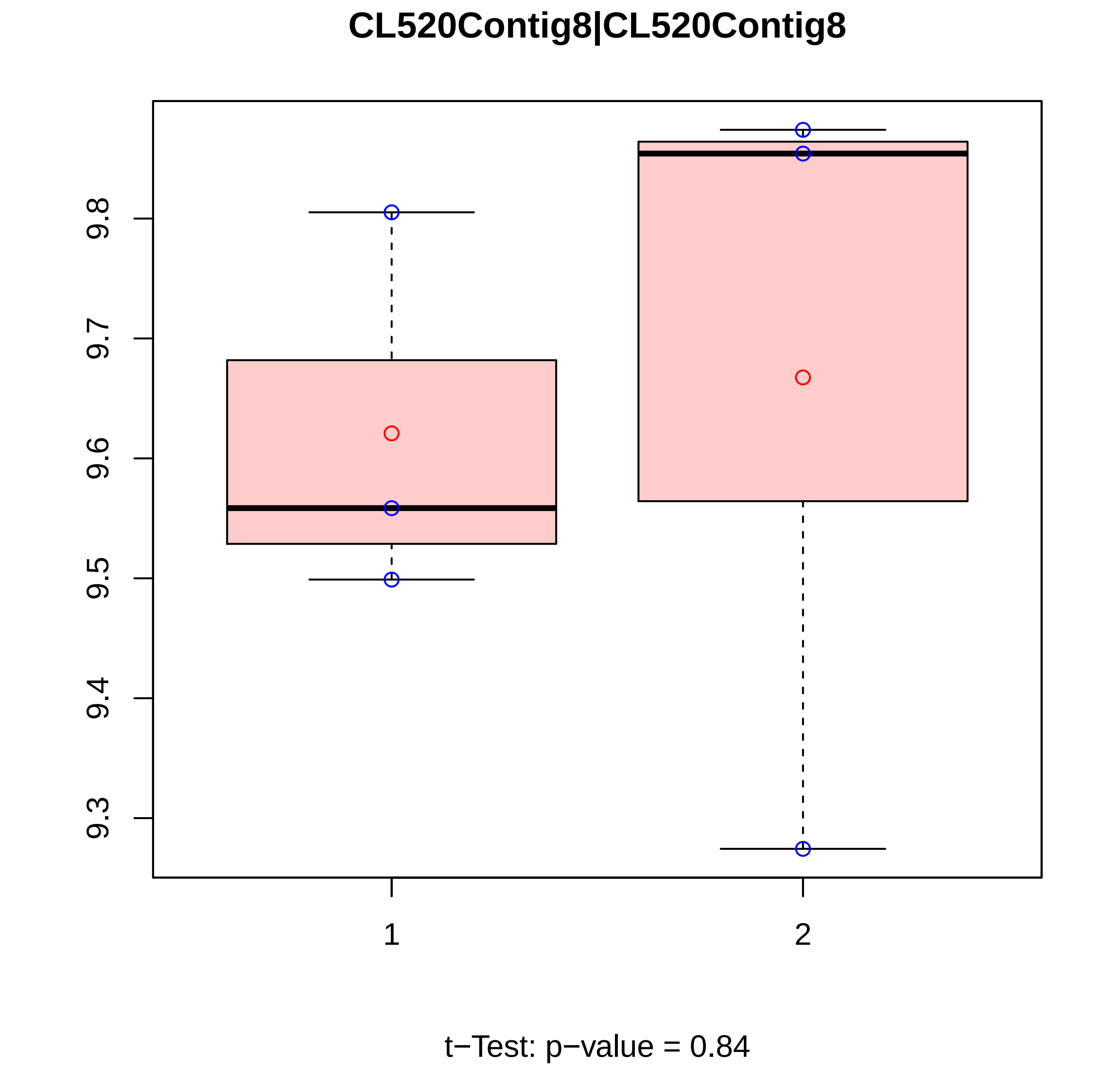

CL520Contig8|CL520Contig8
9.3
9.4
9.5
9.6
9.7
9.8
1
2
t−Test: p−value = 0.84

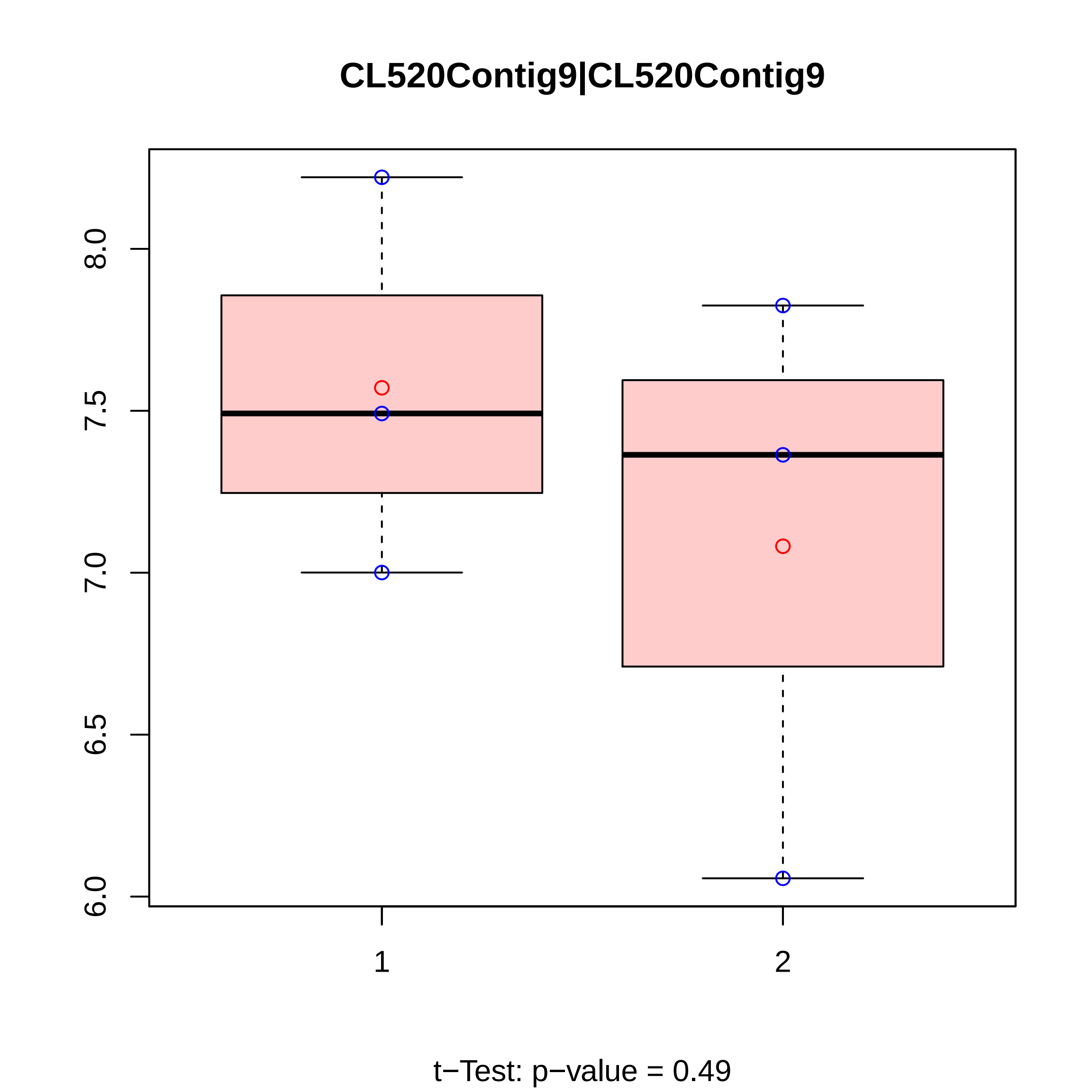

CL520Contig9|CL520Contig9
8.0
7.5
7.0
6.5
6.0
1
2
t−Test: p−value = 0.49

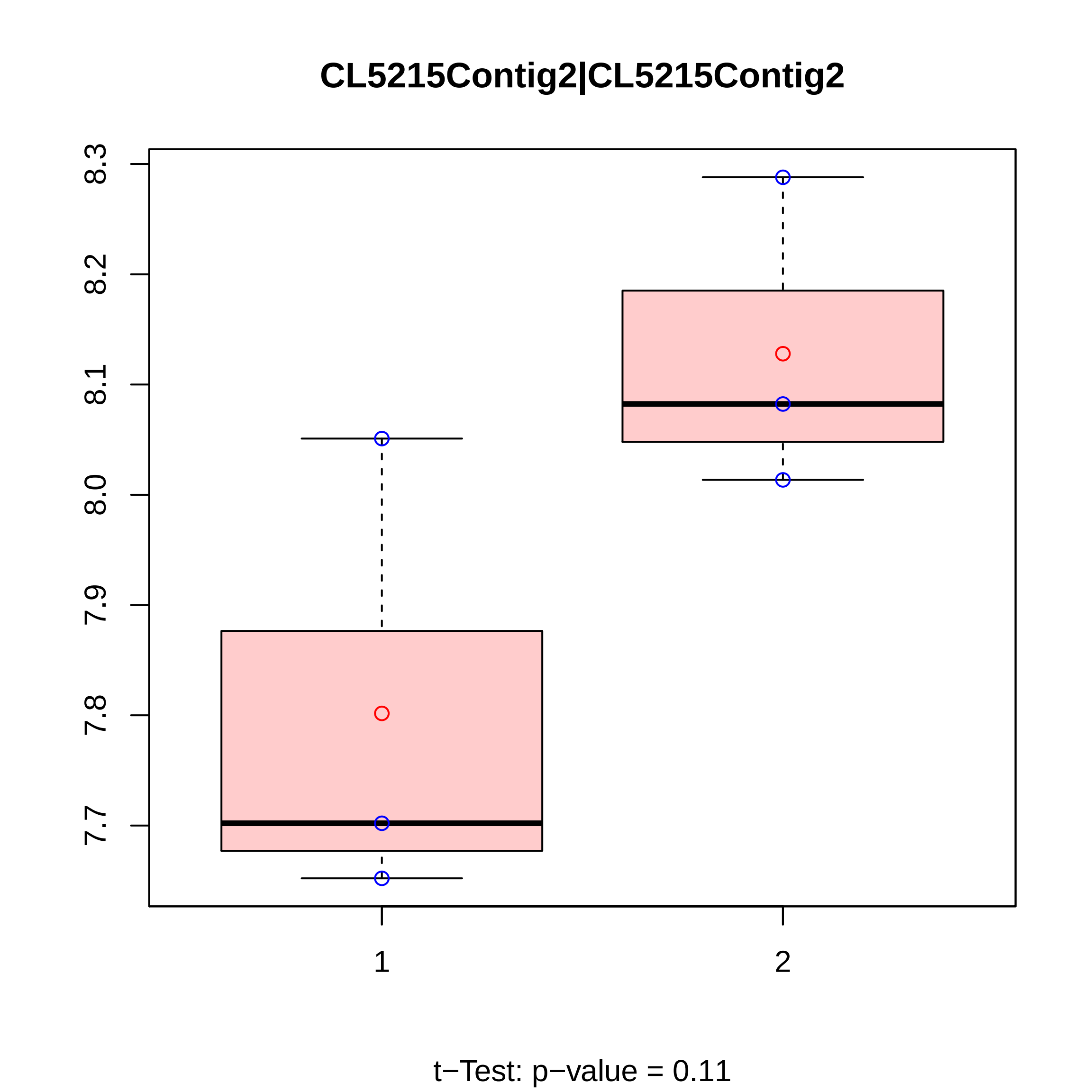
CL5215Contig2|CL5215Contig2
8.3
8.2
8.1
8.0
7.9
7.8
7.7
1
2
t−Test: p−value = 0.11

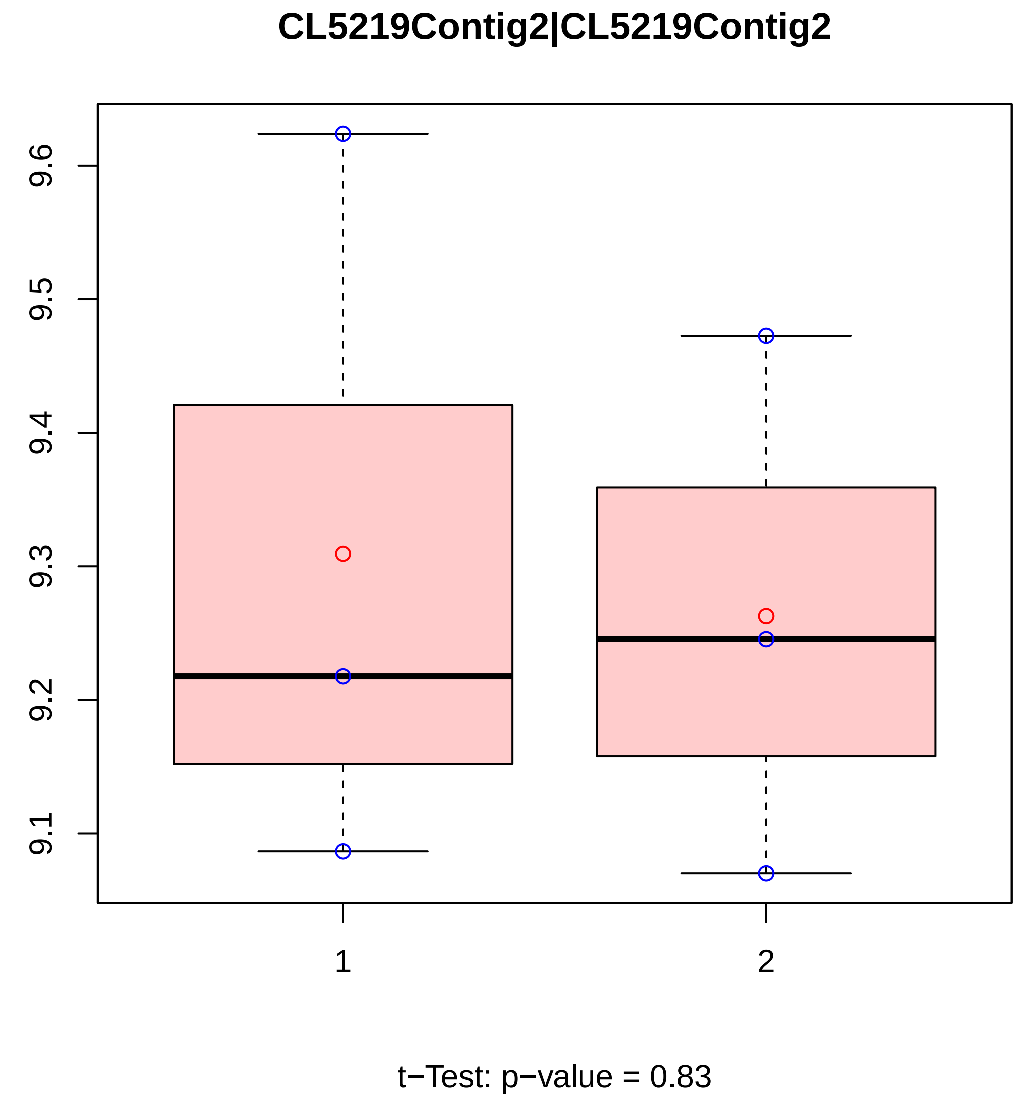
CL5219Contig2|CL5219Contig2
9.1
9.2
9.3
9.4
9.5
9.6
1
2
t−Test: p−value = 0.83

# CL5220Contig3|CL5220Contig3

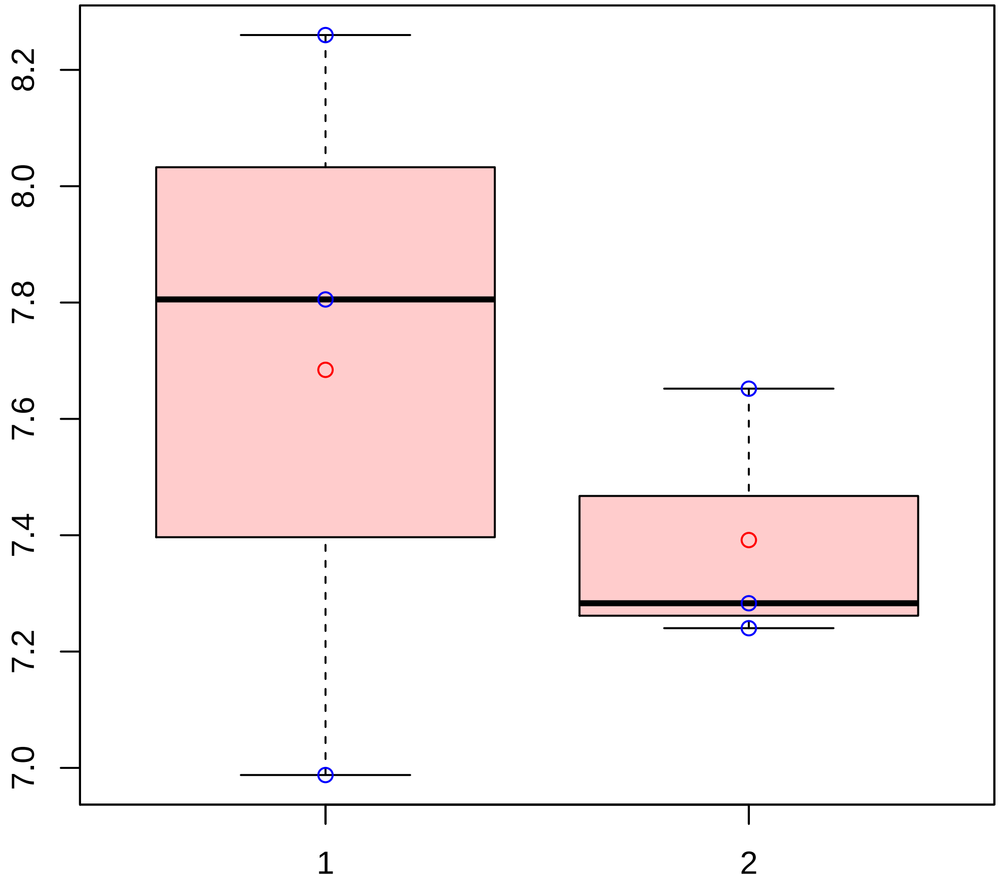


t−Test: p−value = 0.52

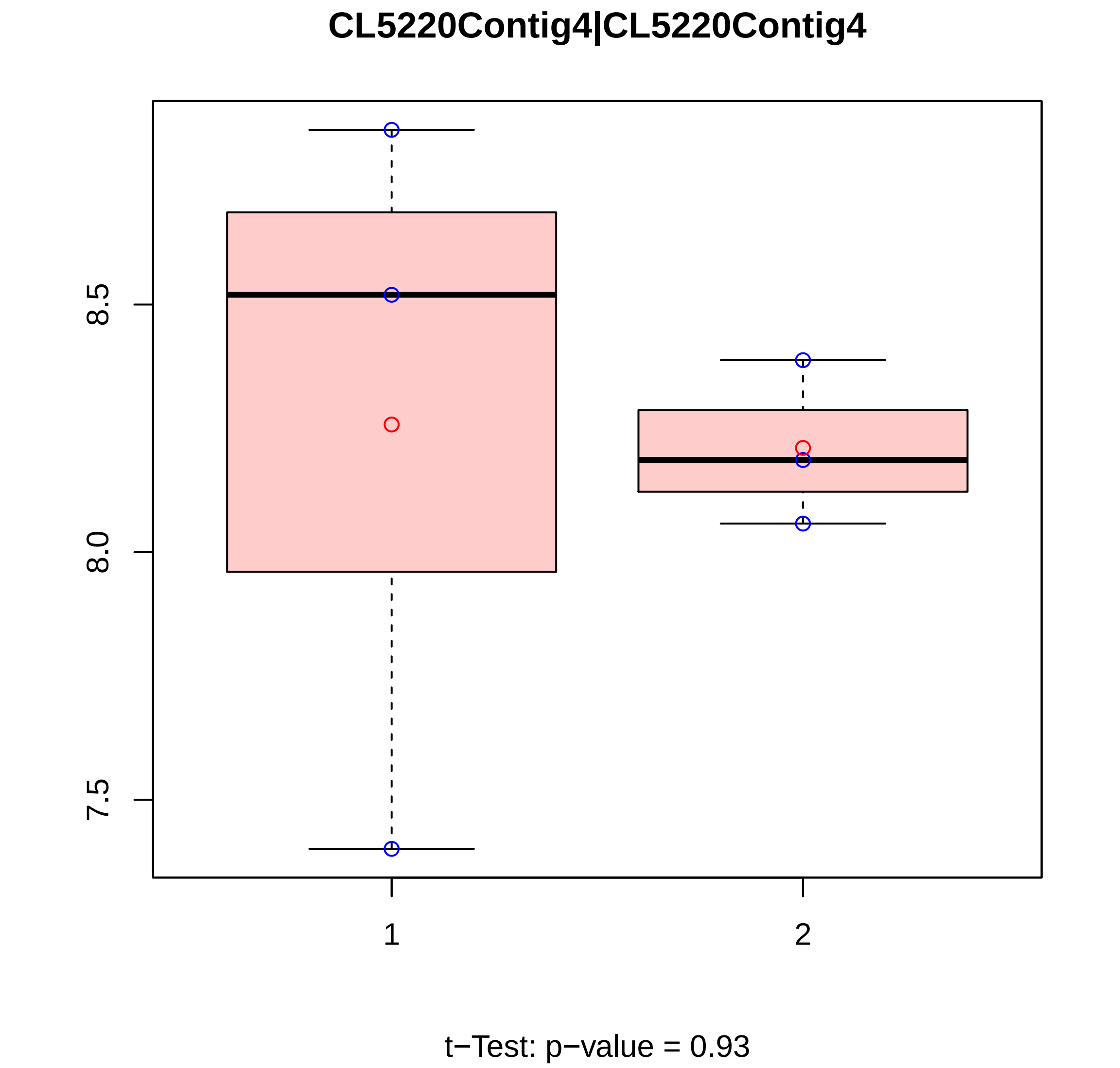

CL5220Contig4|CL5220Contig4
8.5
8.0
7.5
1
2
t−Test: p−value = 0.93

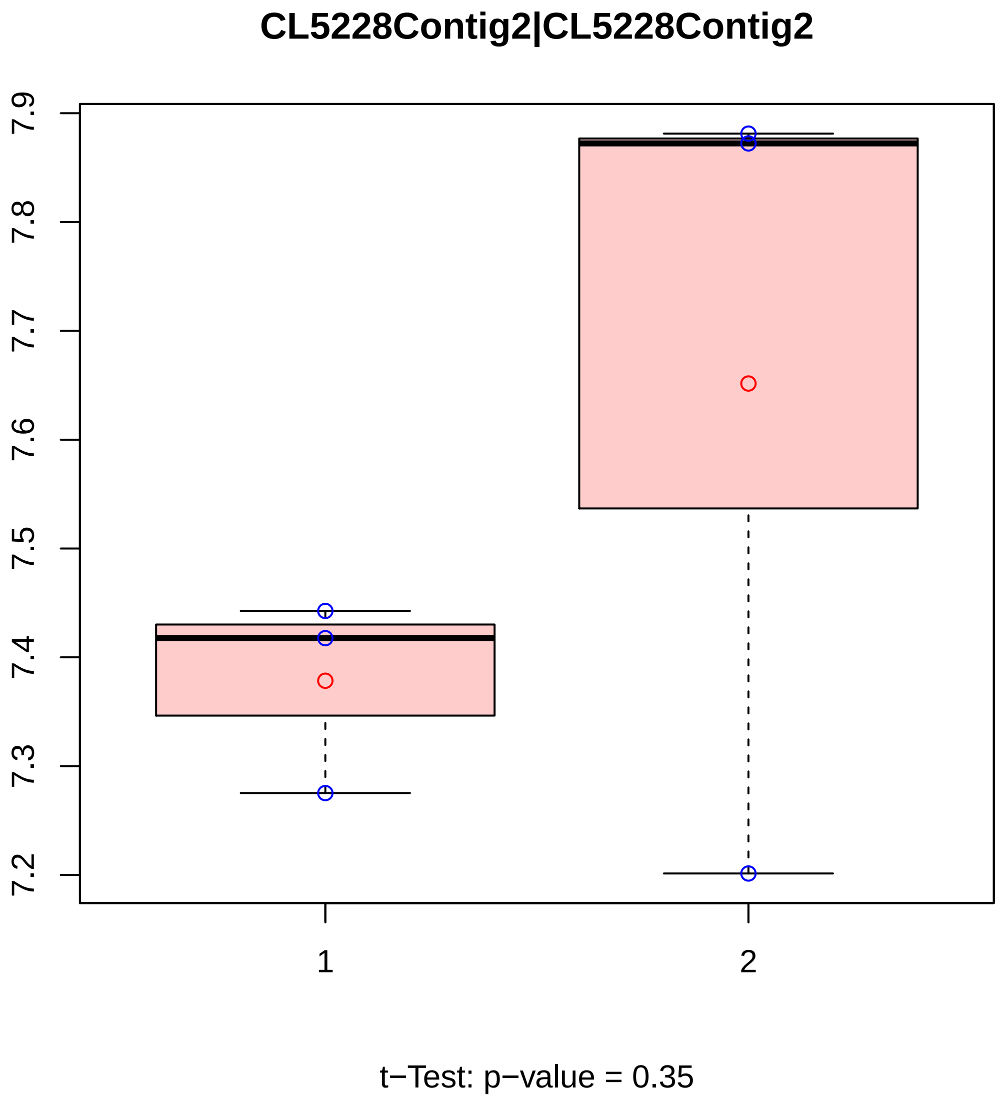
CL5228Contig2|CL5228Contig2
7.9
7.8
7.7
7.6
7.5
7.4
7.3
7.2
1
2
t−Test: p−value = 0.35

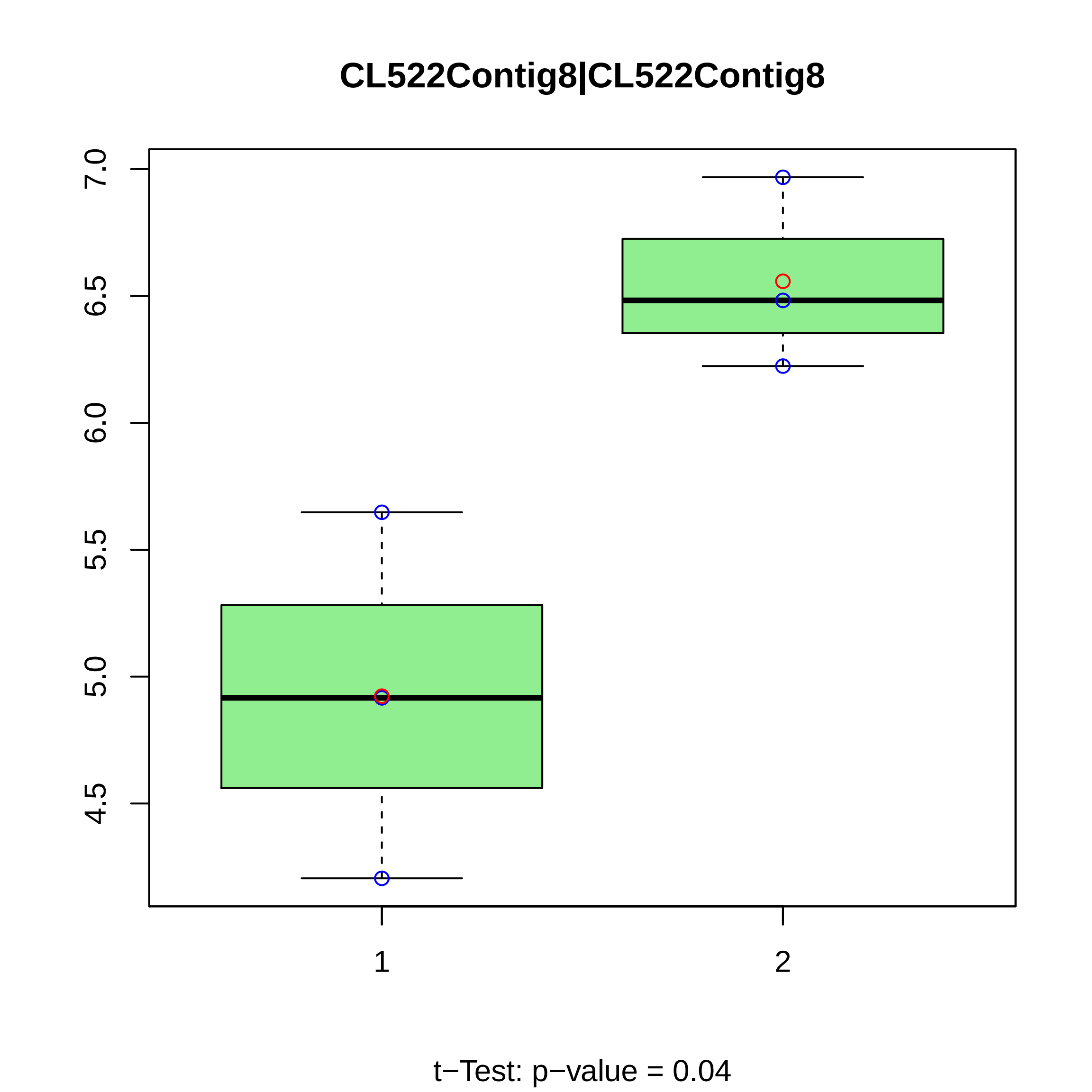
CL522Contig8|CL522Contig8
7.0
6.5
6.0
5.5
5.0
4.5
1
2
t−Test: p−value = 0.04

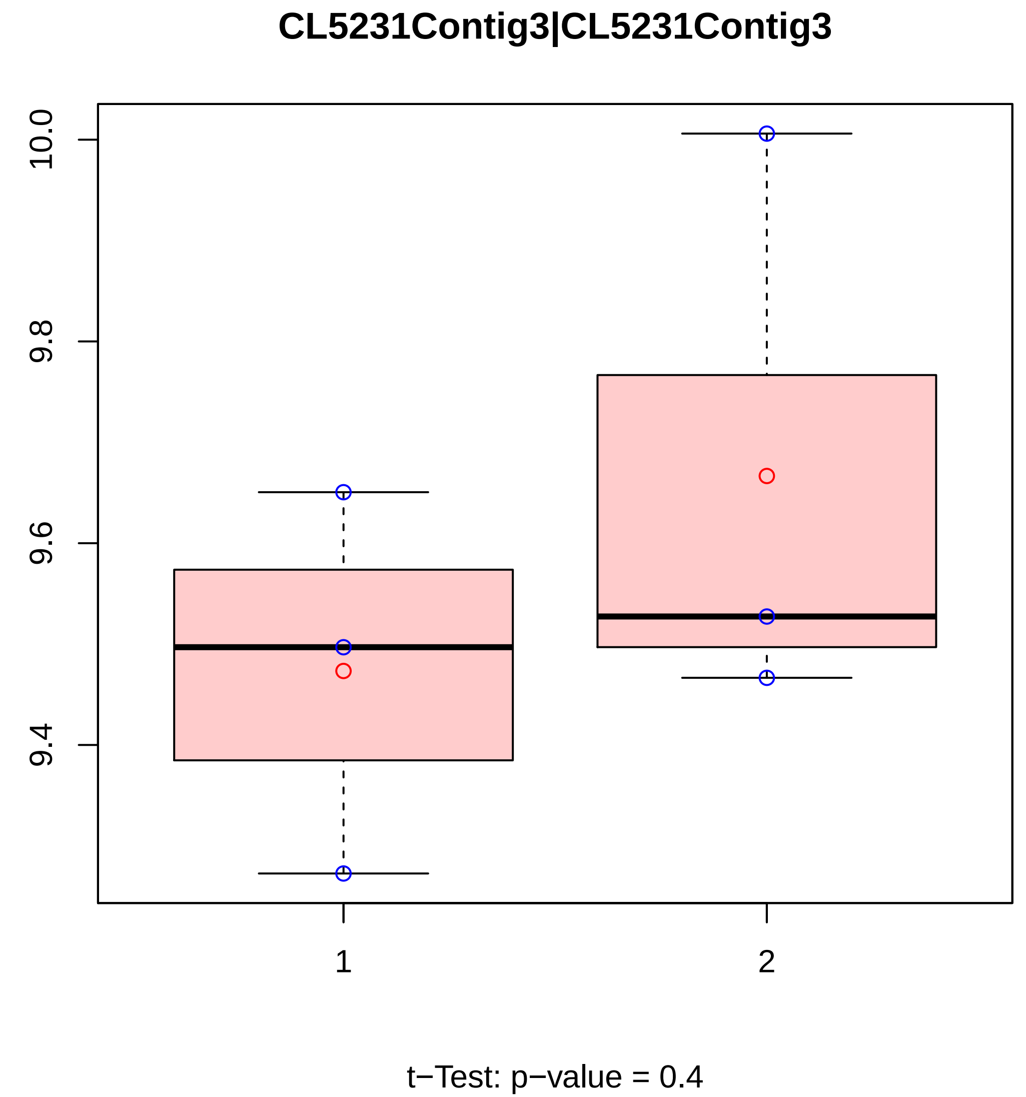
CL5231Contig3|CL5231Contig3
10.0
9.8
9.6
9.4
1
2
t−Test: p−value = 0.4

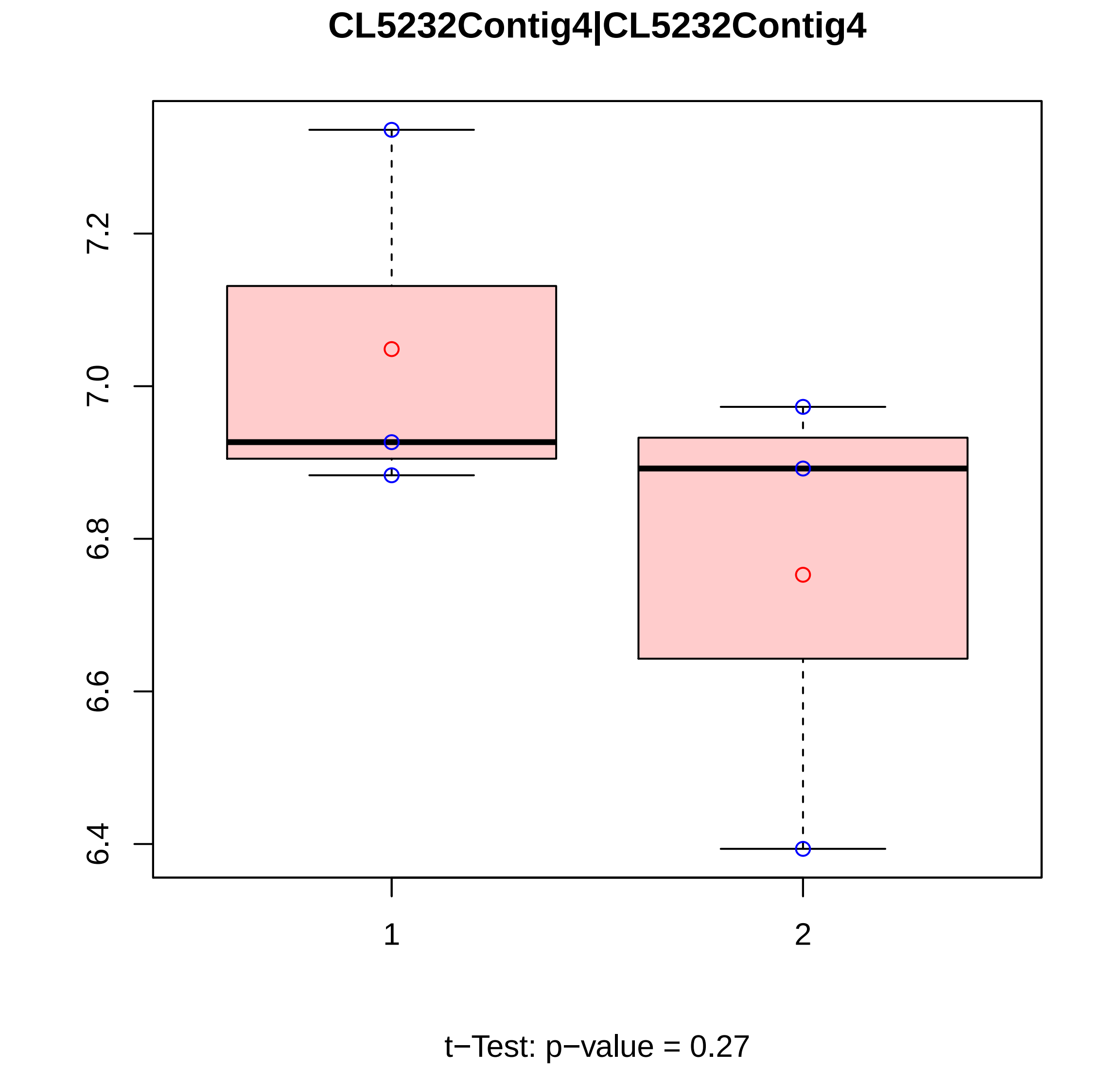
CL5232Contig4|CL5232Contig4
7.2
7.0
6.8
6.6
6.4
1
2
t−Test: p−value = 0.27

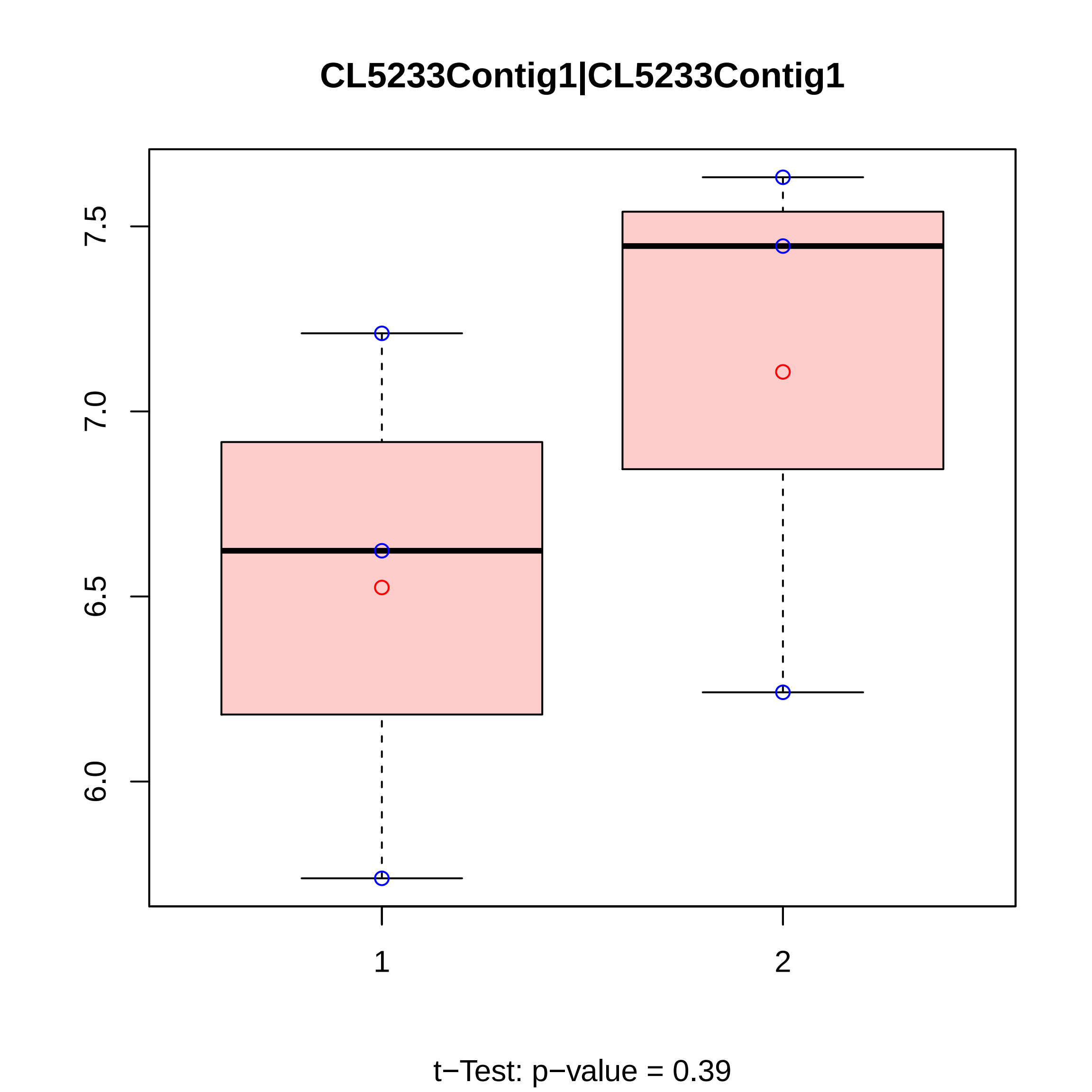

CL5233Contig1|CL5233Contig1
7.5
7.0
6.5
6.0
1
2
t−Test: p−value = 0.39

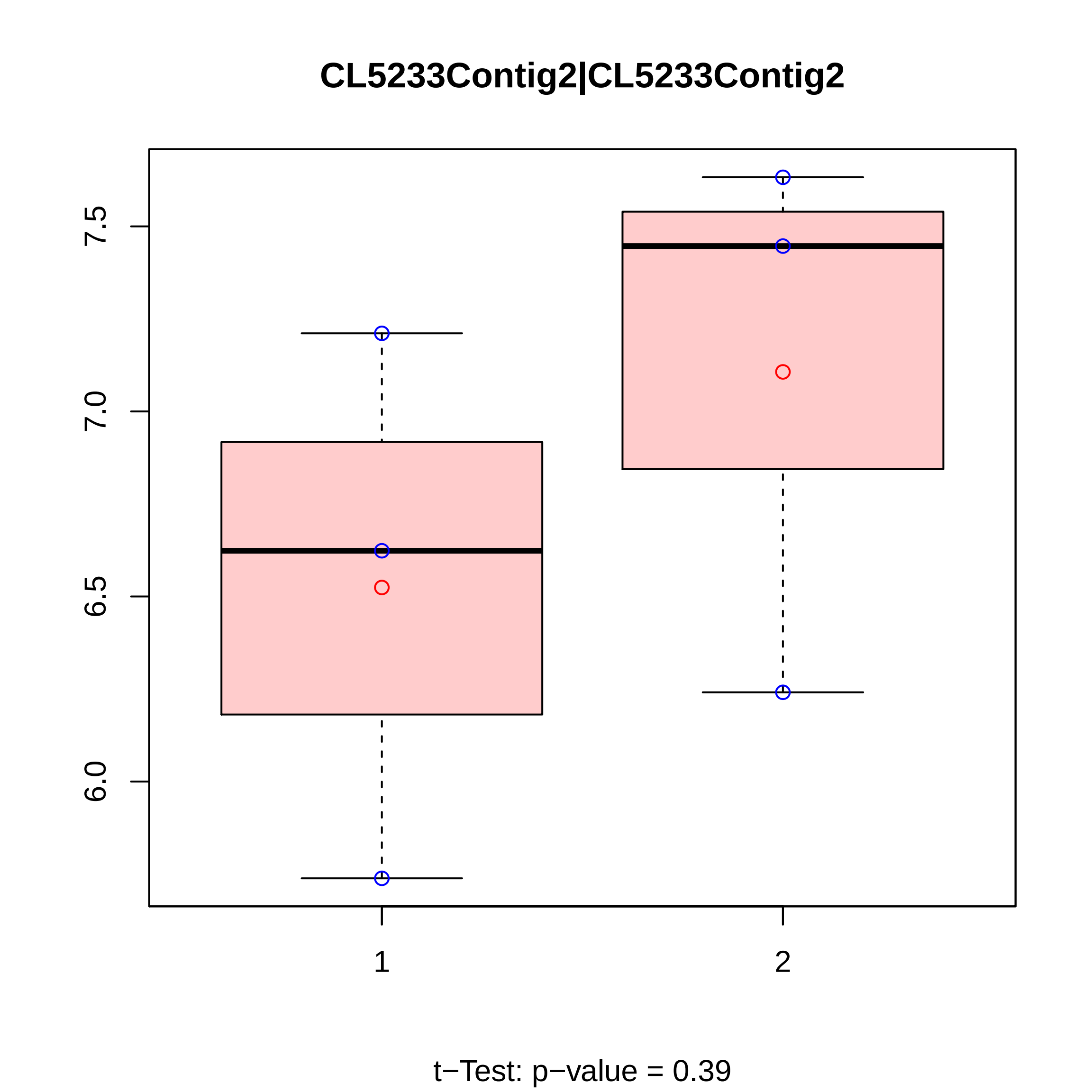

CL5233Contig2|CL5233Contig2
7.5
7.0
6.5
6.0
1
2
t−Test: p−value = 0.39

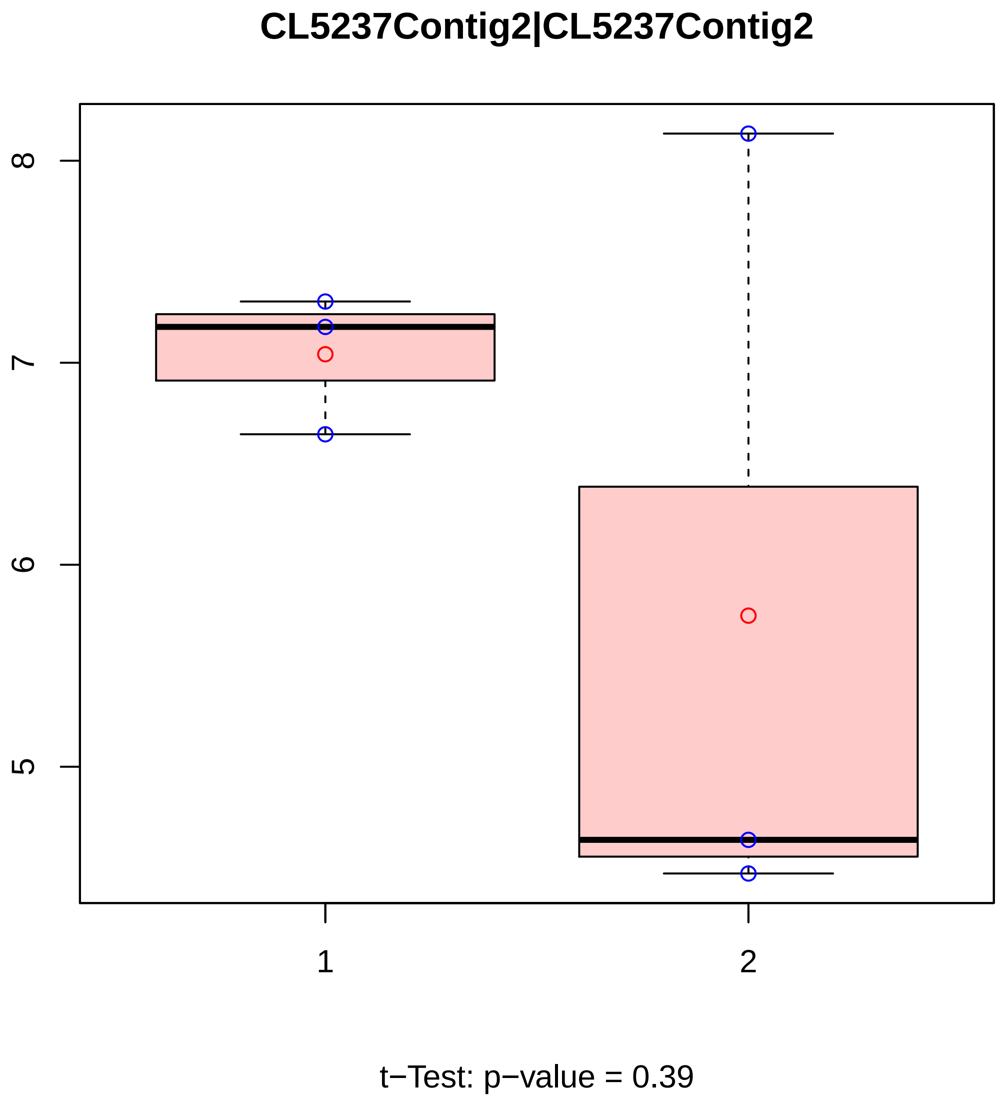

CL5237Contig2|CL5237Contig2
8
7
6
5
1
2
t−Test: p−value = 0.39

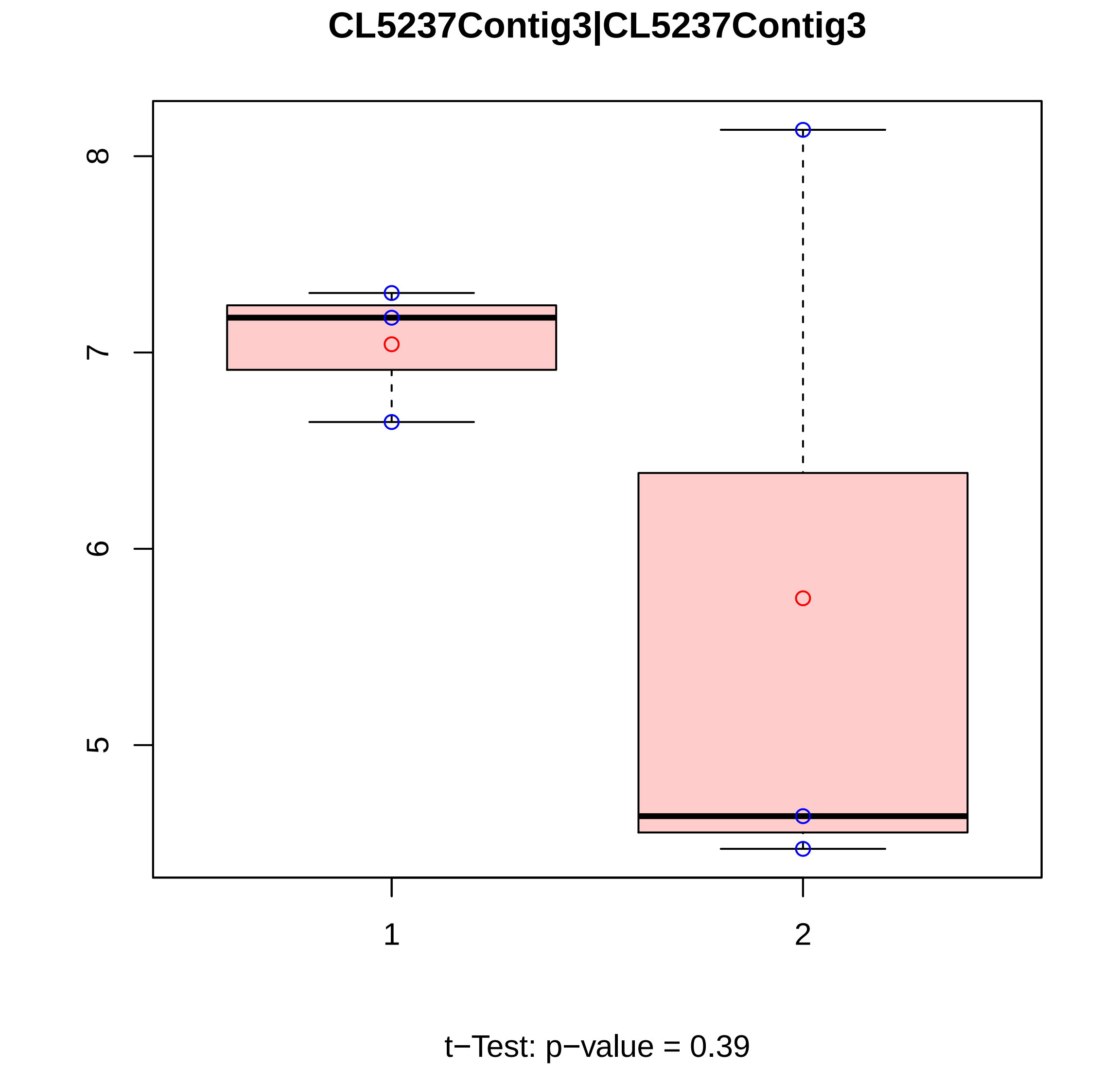
CL5237Contig3|CL5237Contig3
8
7
6
5
1
2
t−Test: p−value = 0.39

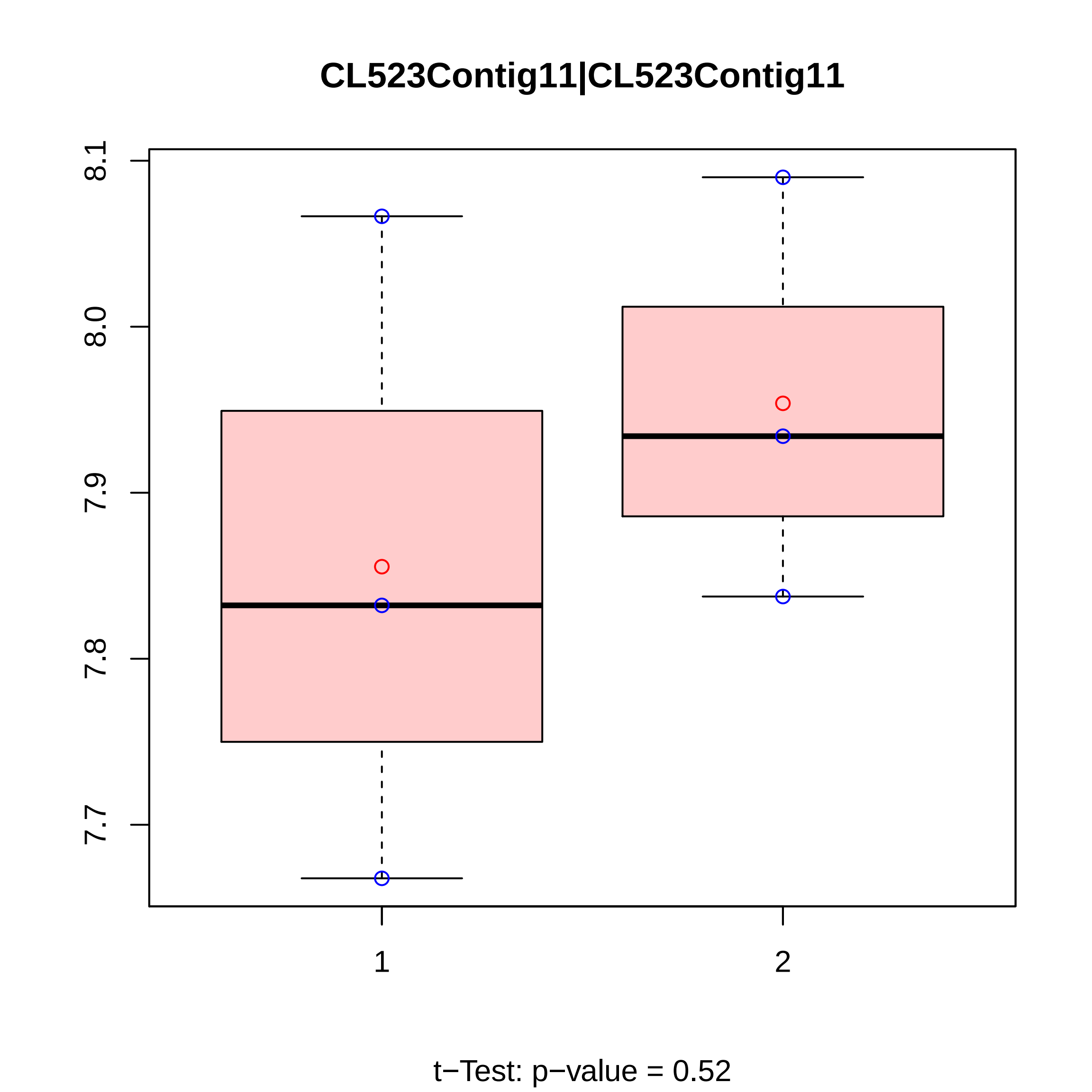

CL523Contig11|CL523Contig11
8.1
8.0
7.9
7.8
7.7
1
2
t−Test: p−value = 0.52

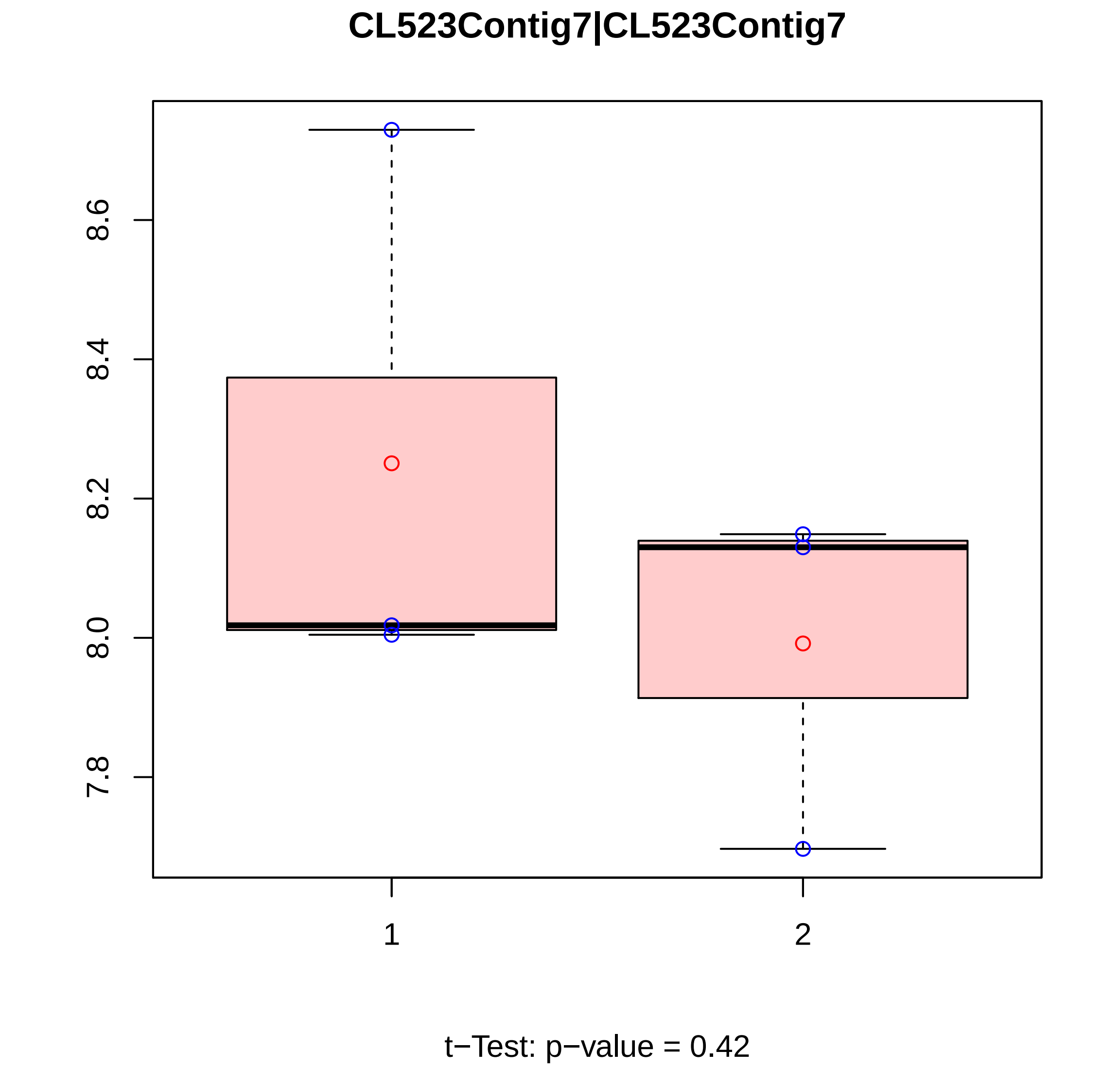
CL523Contig7|CL523Contig7
8.6
8.4
8.2
8.0
7.8
1
2
t−Test: p−value = 0.42

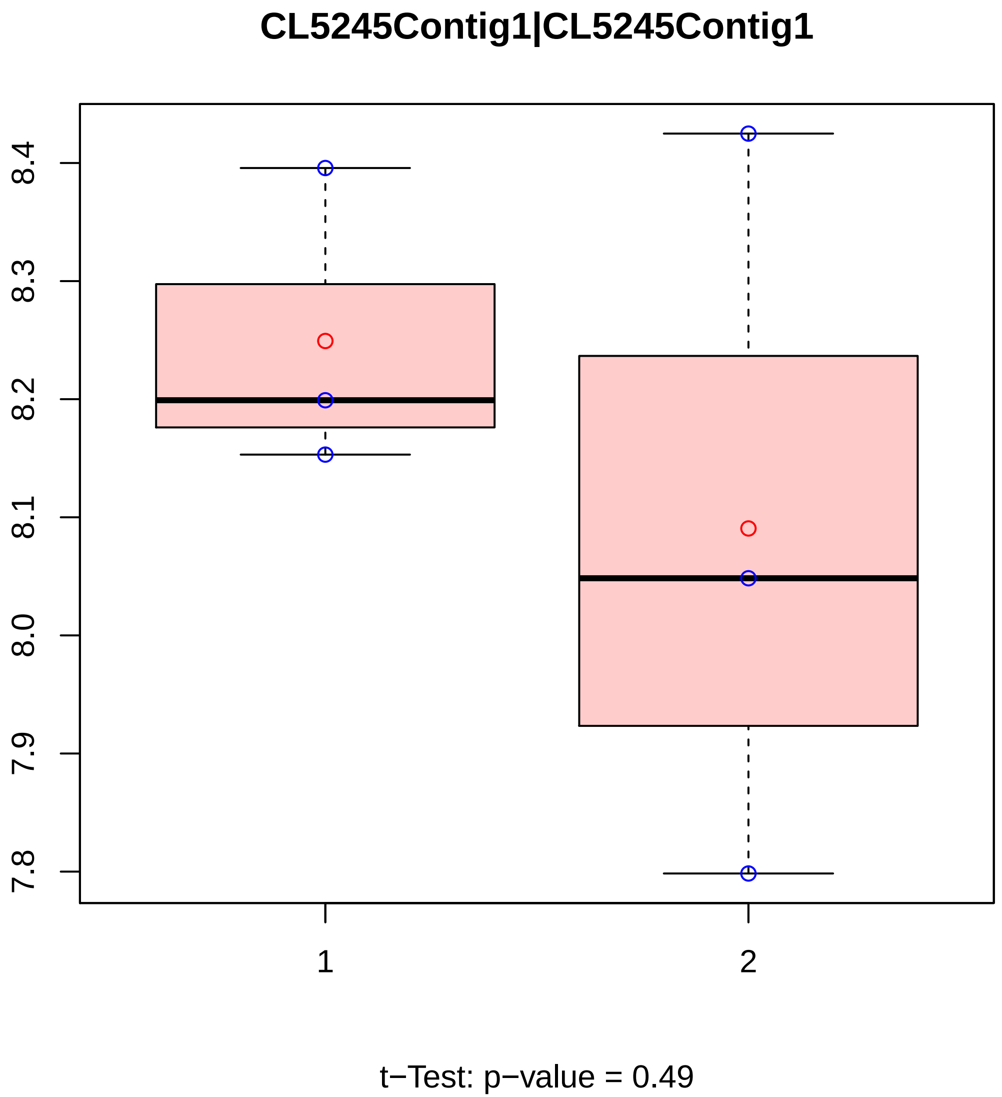
CL5245Contig1|CL5245Contig1
8.4
8.3
8.2
8.1
8.0
7.9
7.8
1
2
t−Test: p−value = 0.49

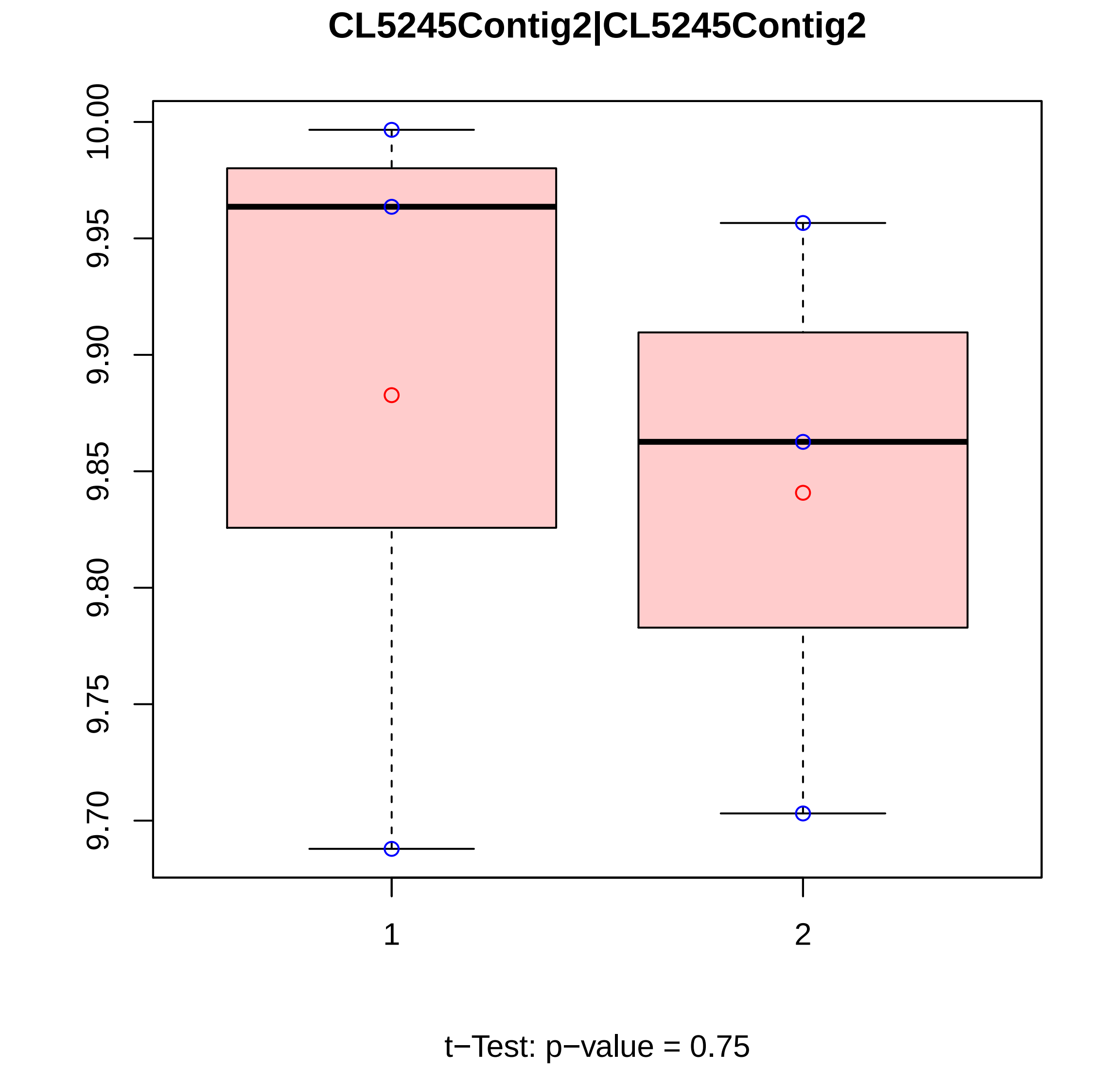

CL5245Contig2|CL5245Contig2
10.00
9.95
9.90
9.85
9.80
9.75
9.70
1
2
t−Test: p−value = 0.75

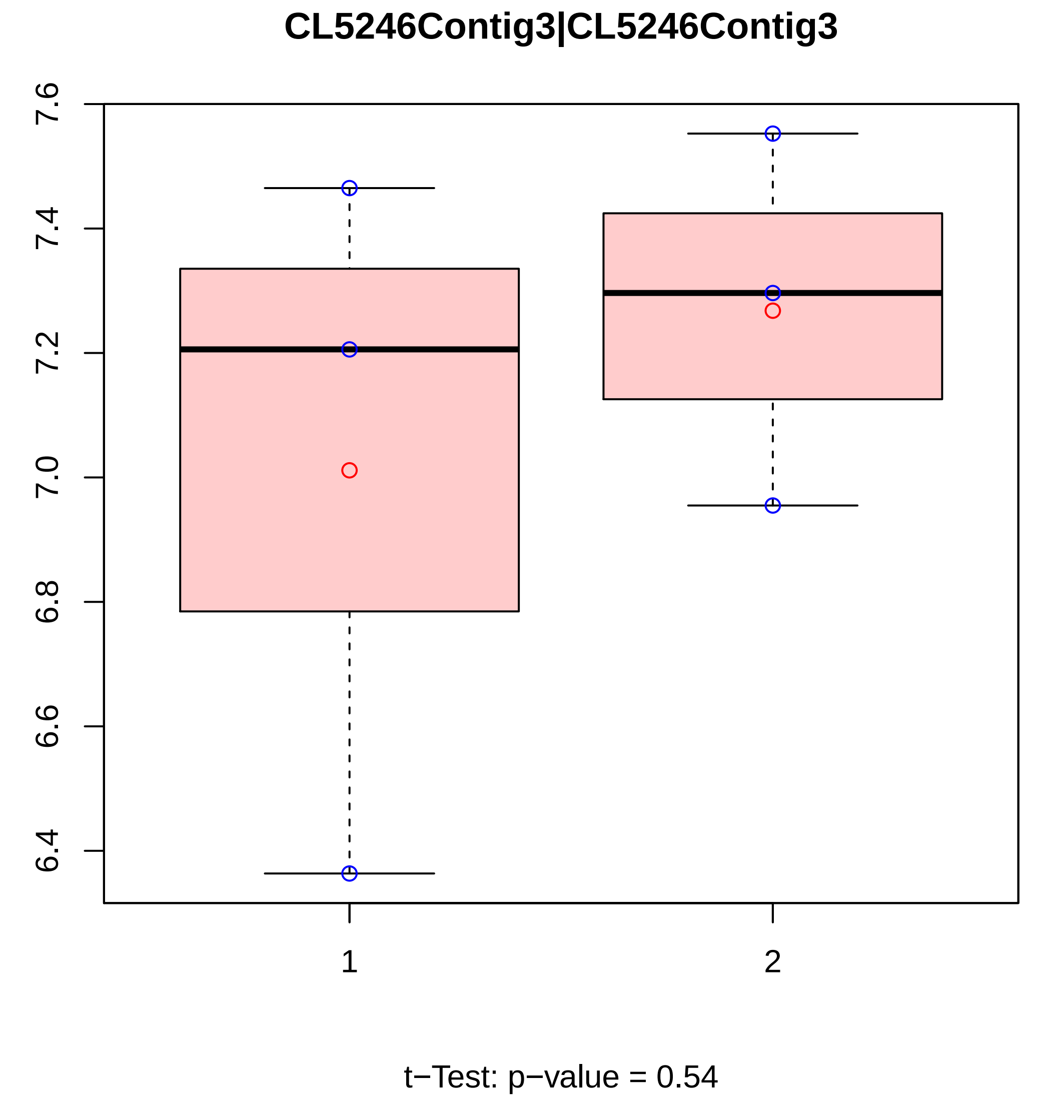
CL5246Contig3|CL5246Contig3
7.6
7.4
7.2
7.0
6.8
6.6
6.4
1
2
t−Test: p−value = 0.54

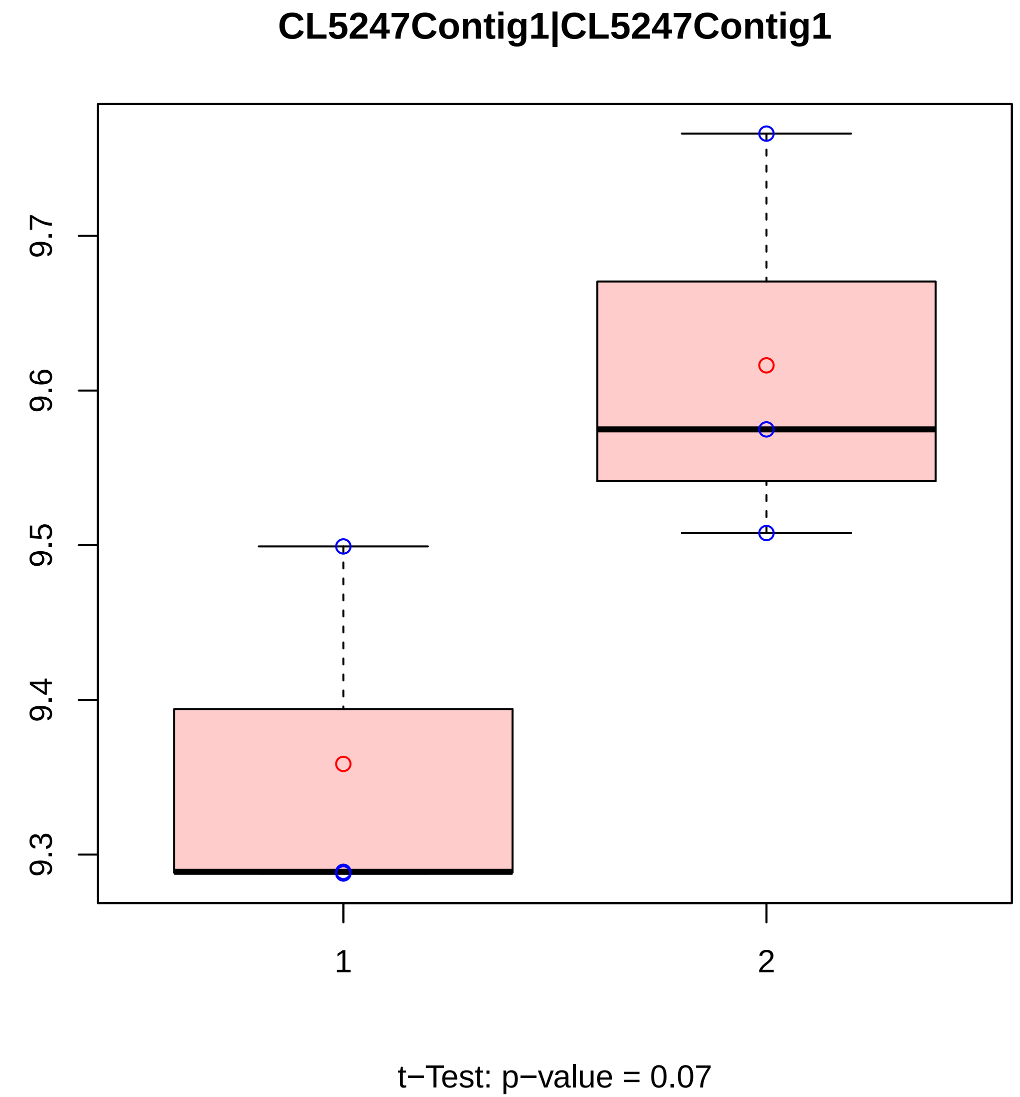
CL5247Contig1|CL5247Contig1
9.3
9.4
9.5
9.6
9.7
1
2
t−Test: p−value = 0.07

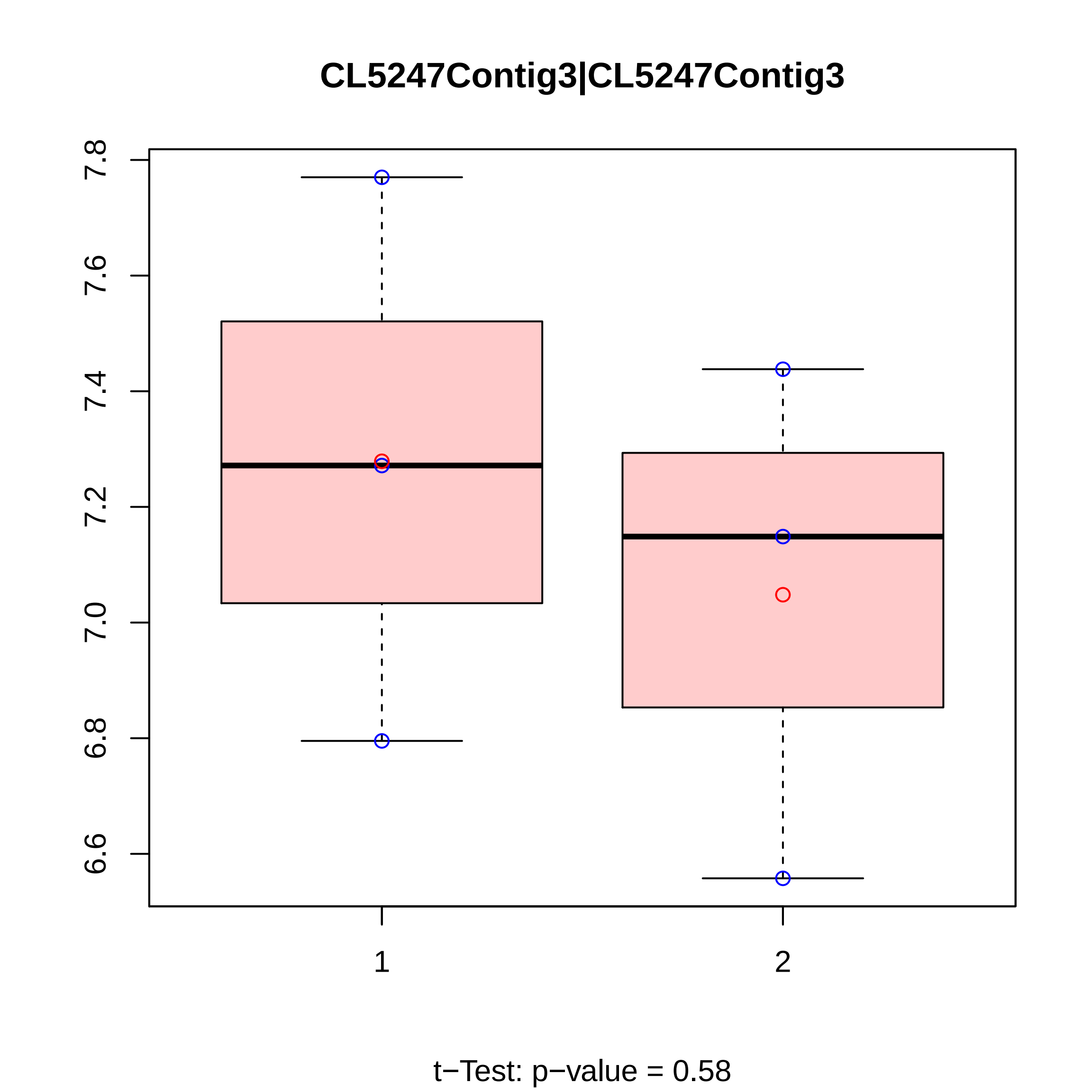

CL5247Contig3|CL5247Contig3
7.8
7.6
7.4
7.2
7.0
6.8
6.6
1
2
t−Test: p−value = 0.58

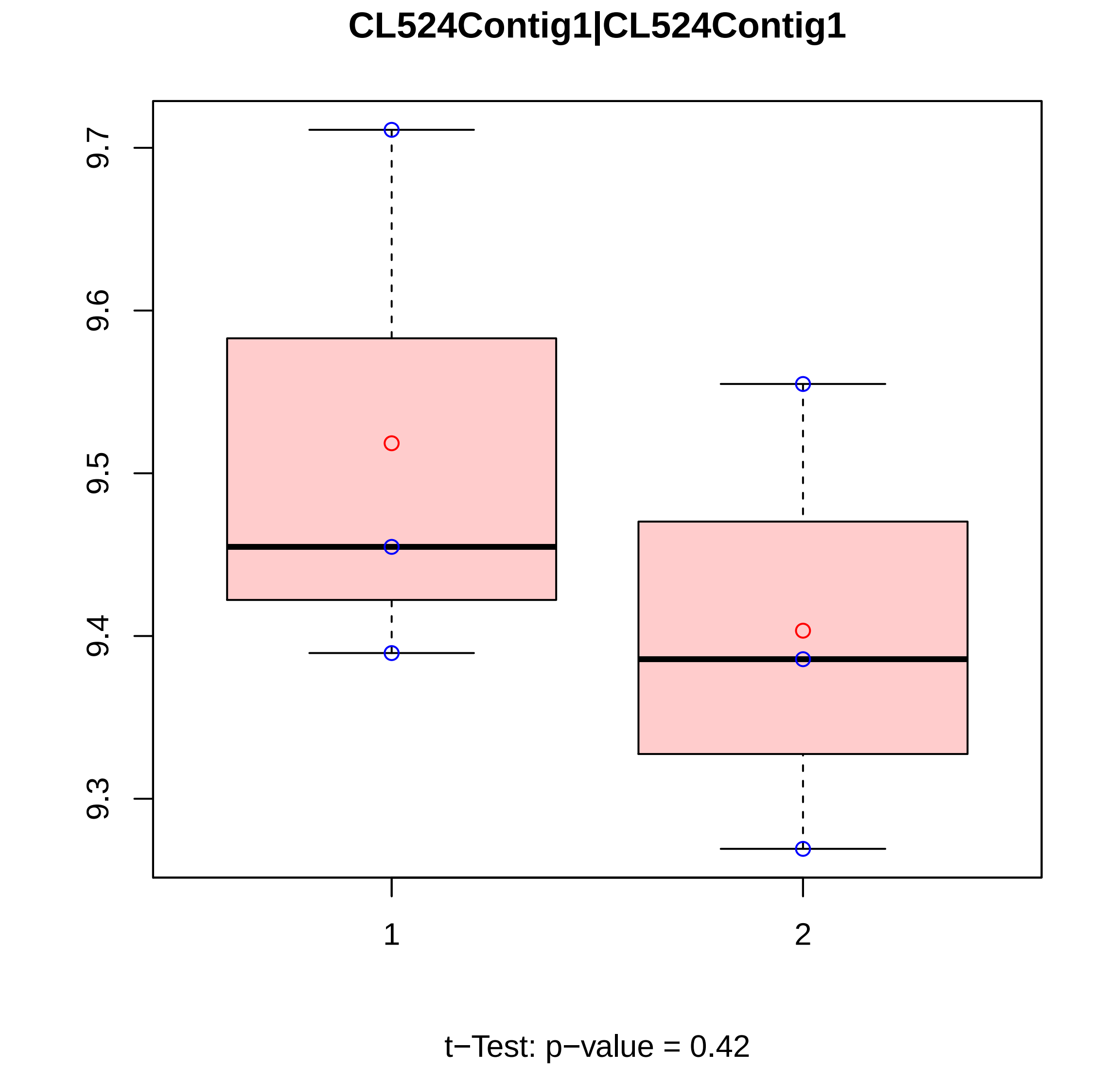

CL524Contig1|CL524Contig1
9.7
9.6
9.5
9.4
9.3
1
2
t−Test: p−value = 0.42

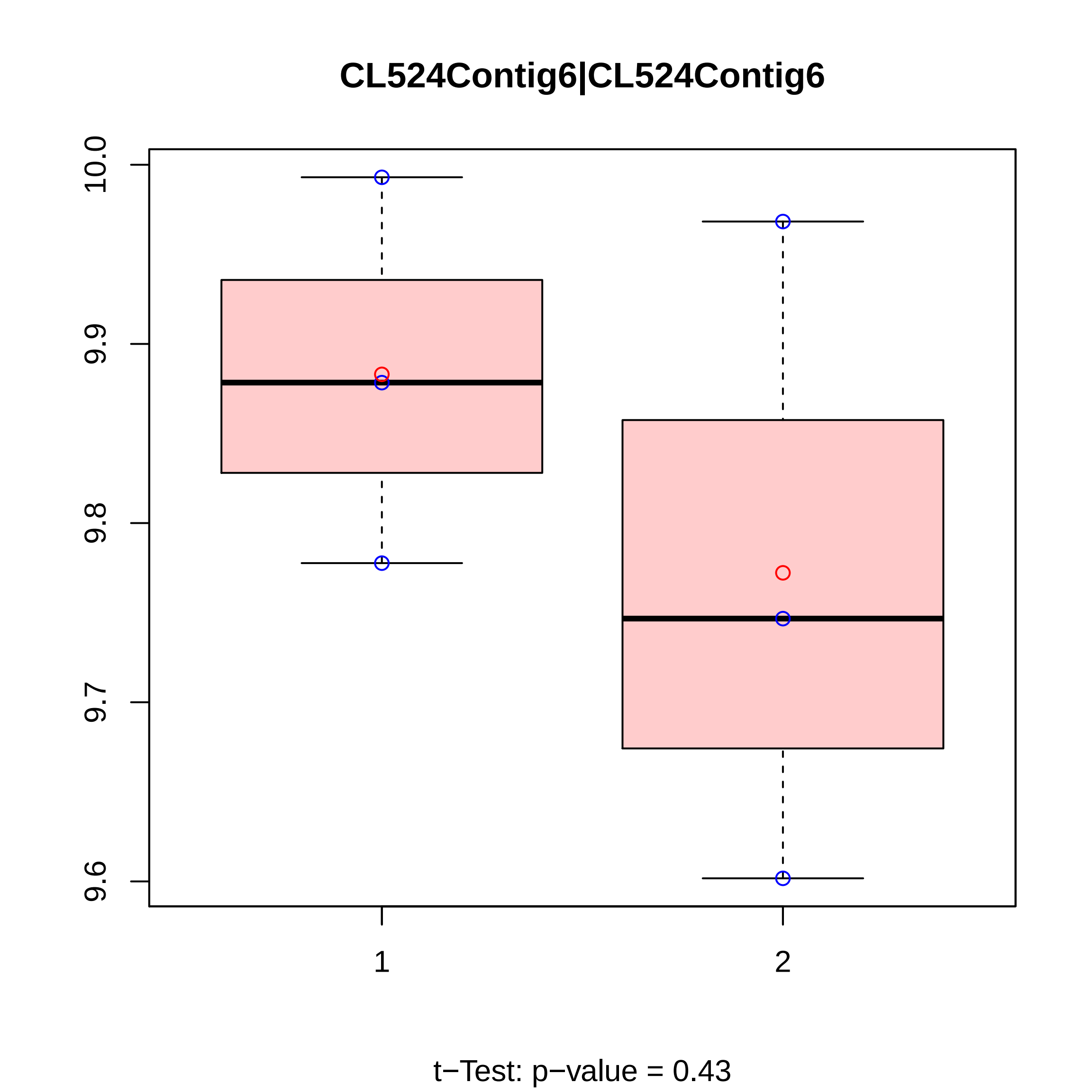
CL524Contig6|CL524Contig6
10.0
9.9
9.8
9.7
9.6
1
2
t−Test: p−value = 0.43

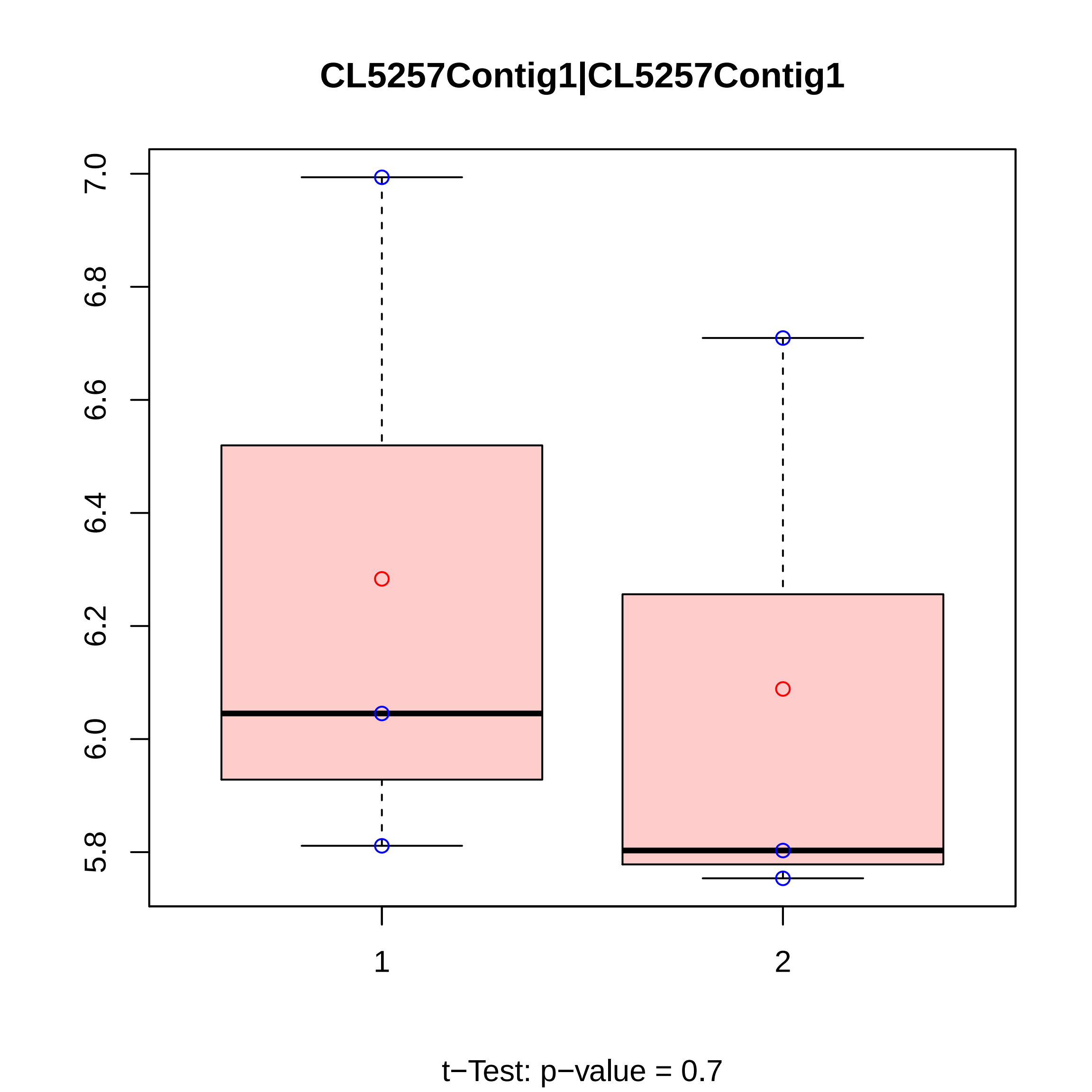
CL5257Contig1|CL5257Contig1
7.0
6.8
6.6
6.4
6.2
6.0
5.8
1
2
t−Test: p−value = 0.7

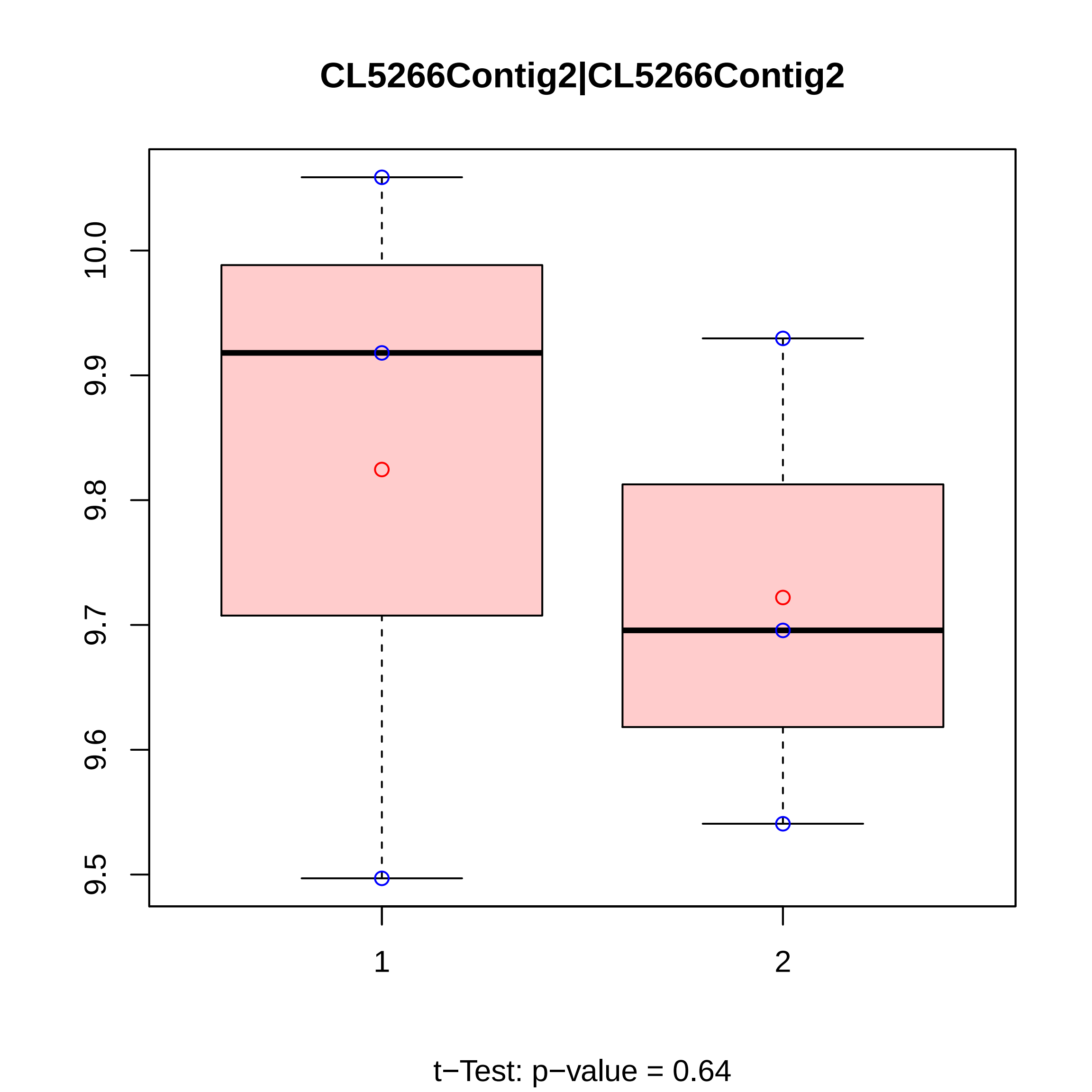

CL5266Contig2|CL5266Contig2
10.0
9.9
9.8
9.7
9.6
9.5
1
2
t−Test: p−value = 0.64

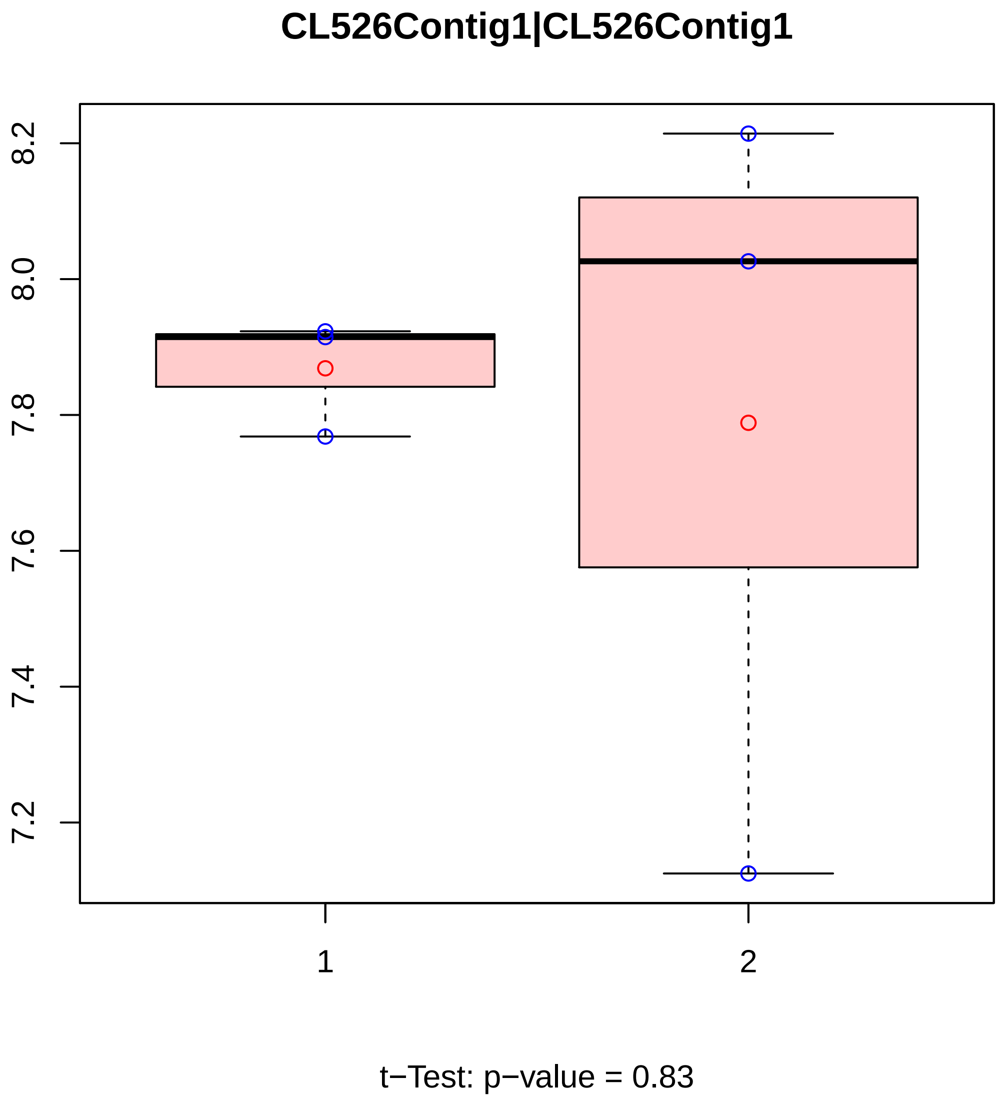

CL526Contig1|CL526Contig1
8.2
8.0
7.8
7.6
7.4
7.2
1
2
t−Test: p−value = 0.83

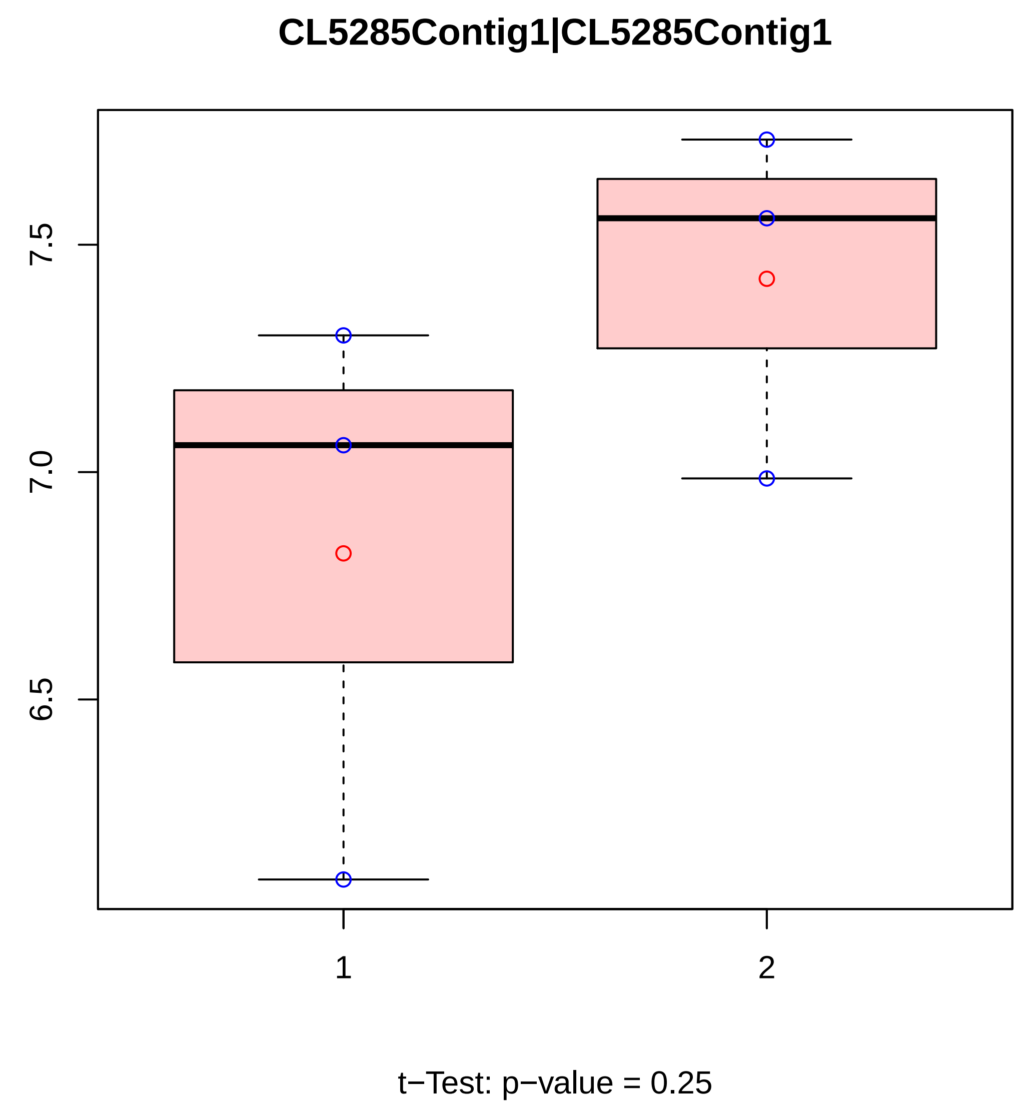
CL5285Contig1|CL5285Contig1
7.5
7.0
6.5
1
2
t−Test: p−value = 0.25

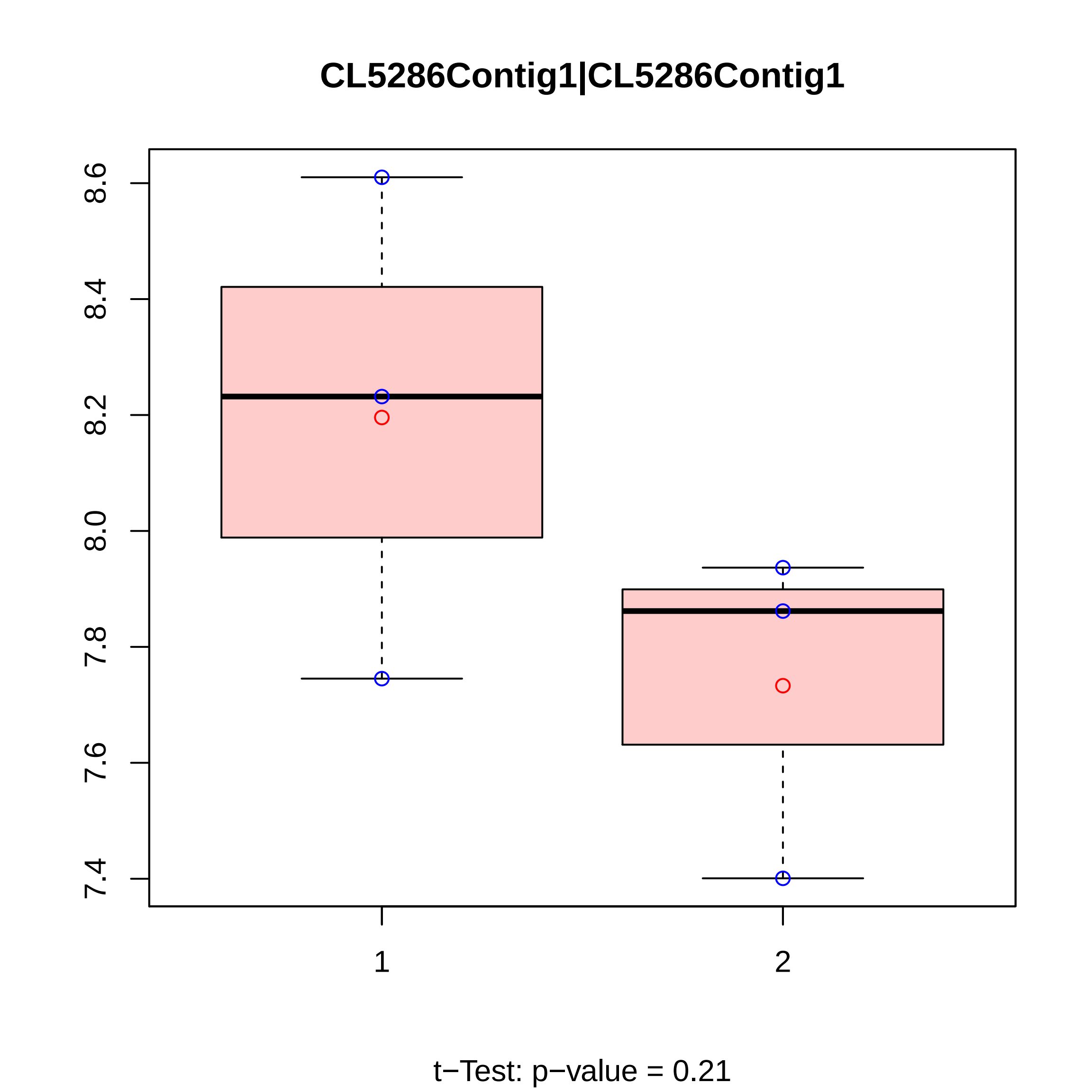

CL5286Contig1|CL5286Contig1
8.6
8.4
8.2
8.0
7.8
7.6
7.4
1
2
t−Test: p−value = 0.21

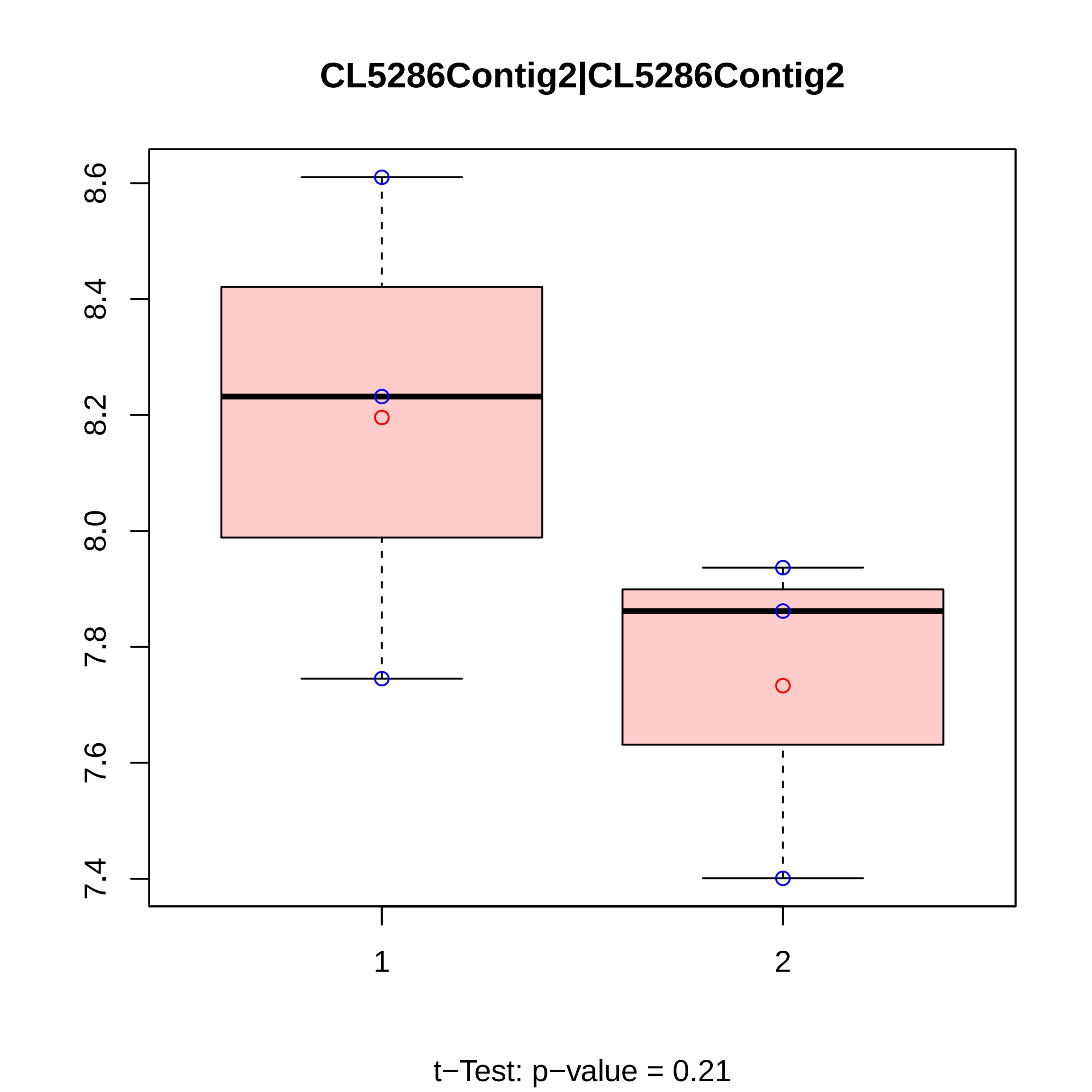

CL5286Contig2|CL5286Contig2
8.6
8.4
8.2
8.0
7.8
7.6
7.4
1
2
t−Test: p−value = 0.21

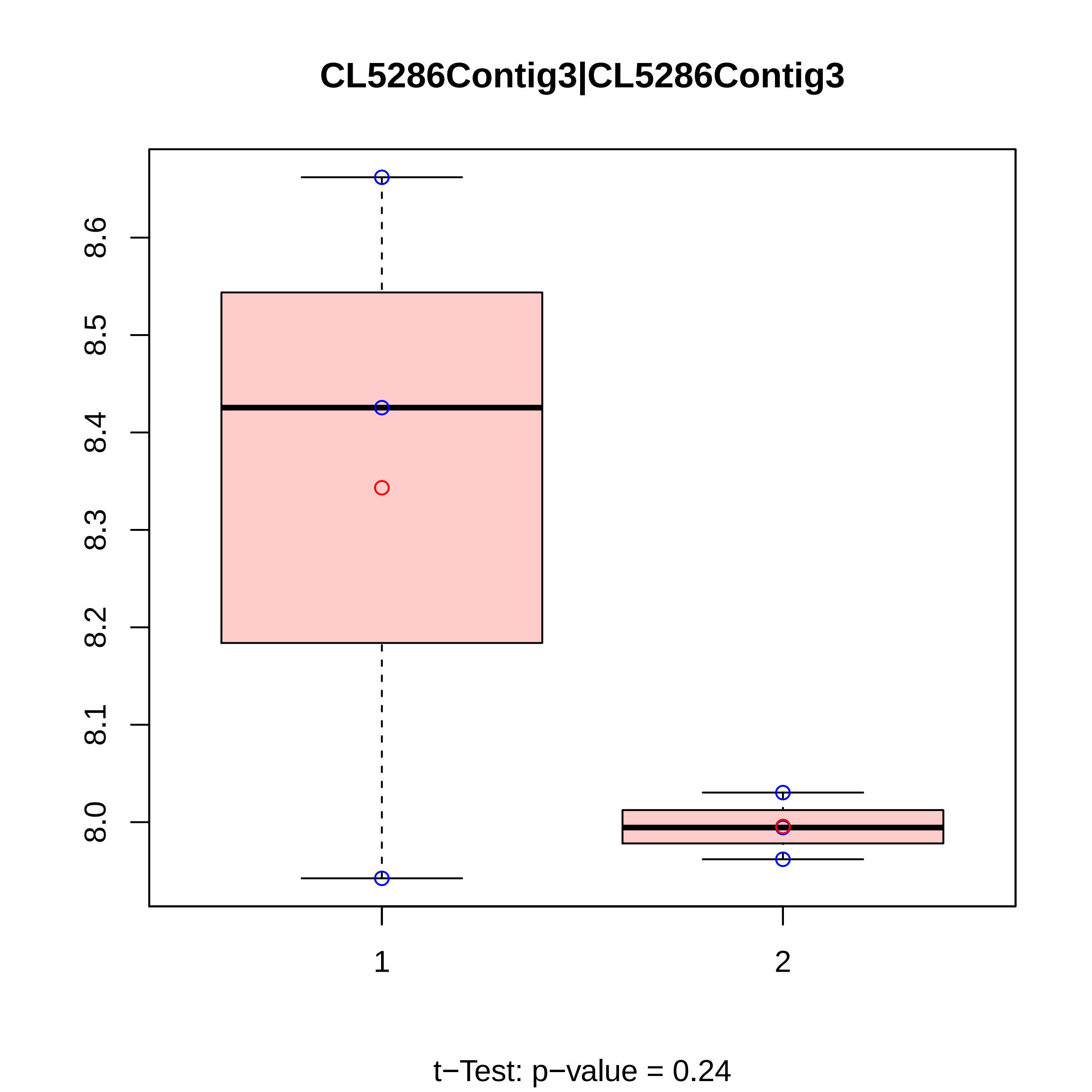
CL5286Contig3|CL5286Contig3
8.0
8.1
8.2
8.3
8.4
8.5
8.6
1
2
t−Test: p−value = 0.24

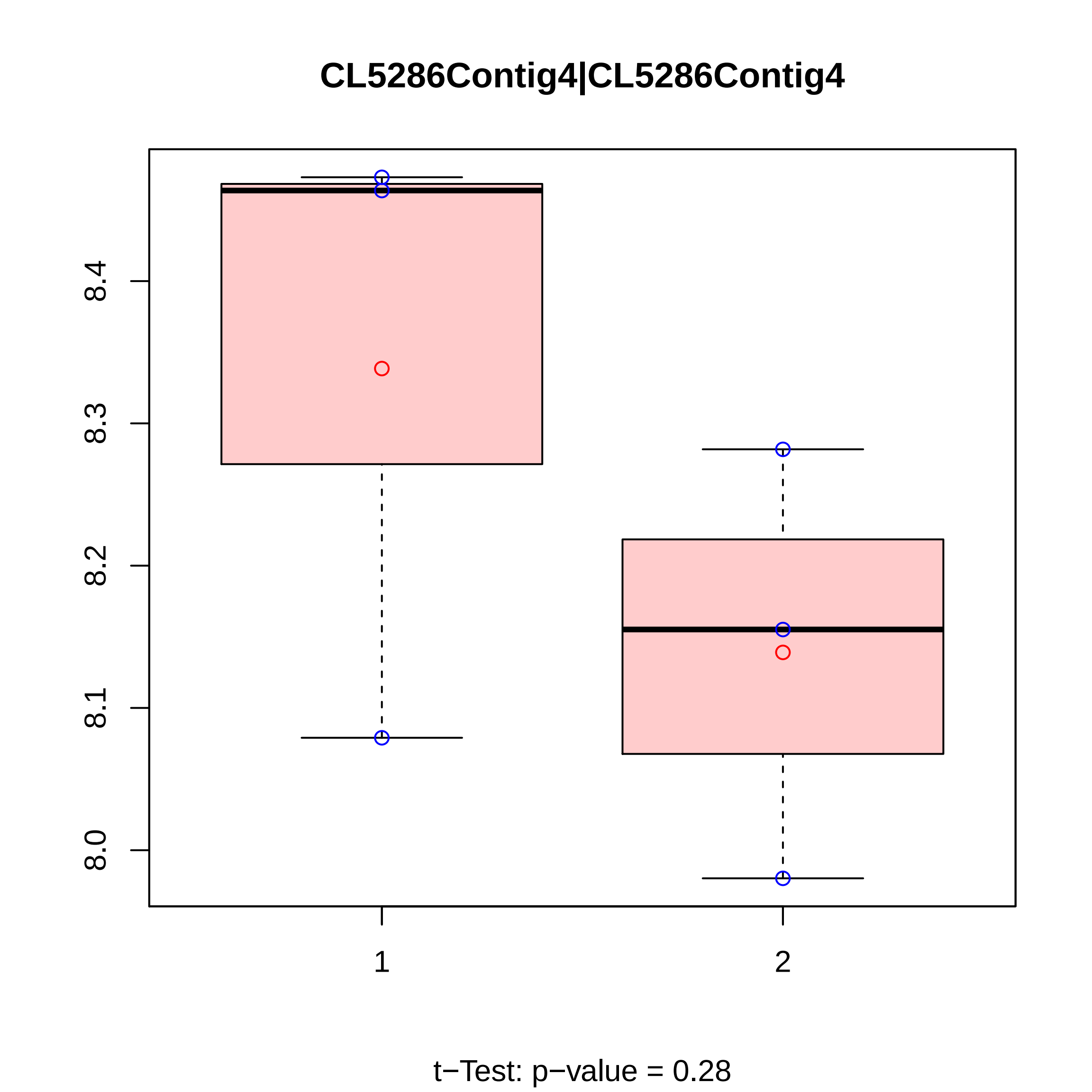
CL5286Contig4|CL5286Contig4
8.0
8.1
8.2
8.3
8.4
1
2
t−Test: p−value = 0.28

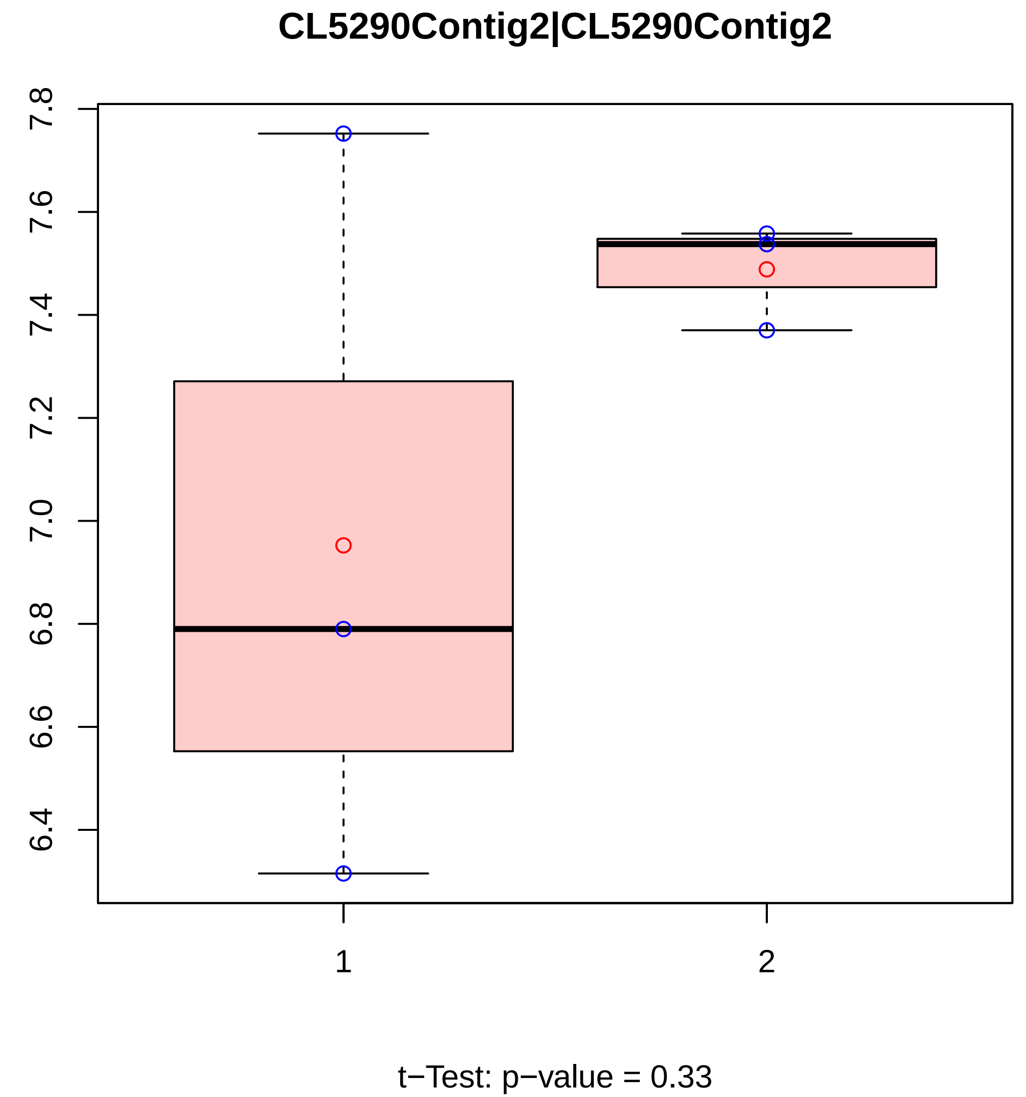
CL5290Contig2|CL5290Contig2
7.8
7.6
7.4
7.2
7.0
6.8
6.6
6.4
1
2
t−Test: p−value = 0.33

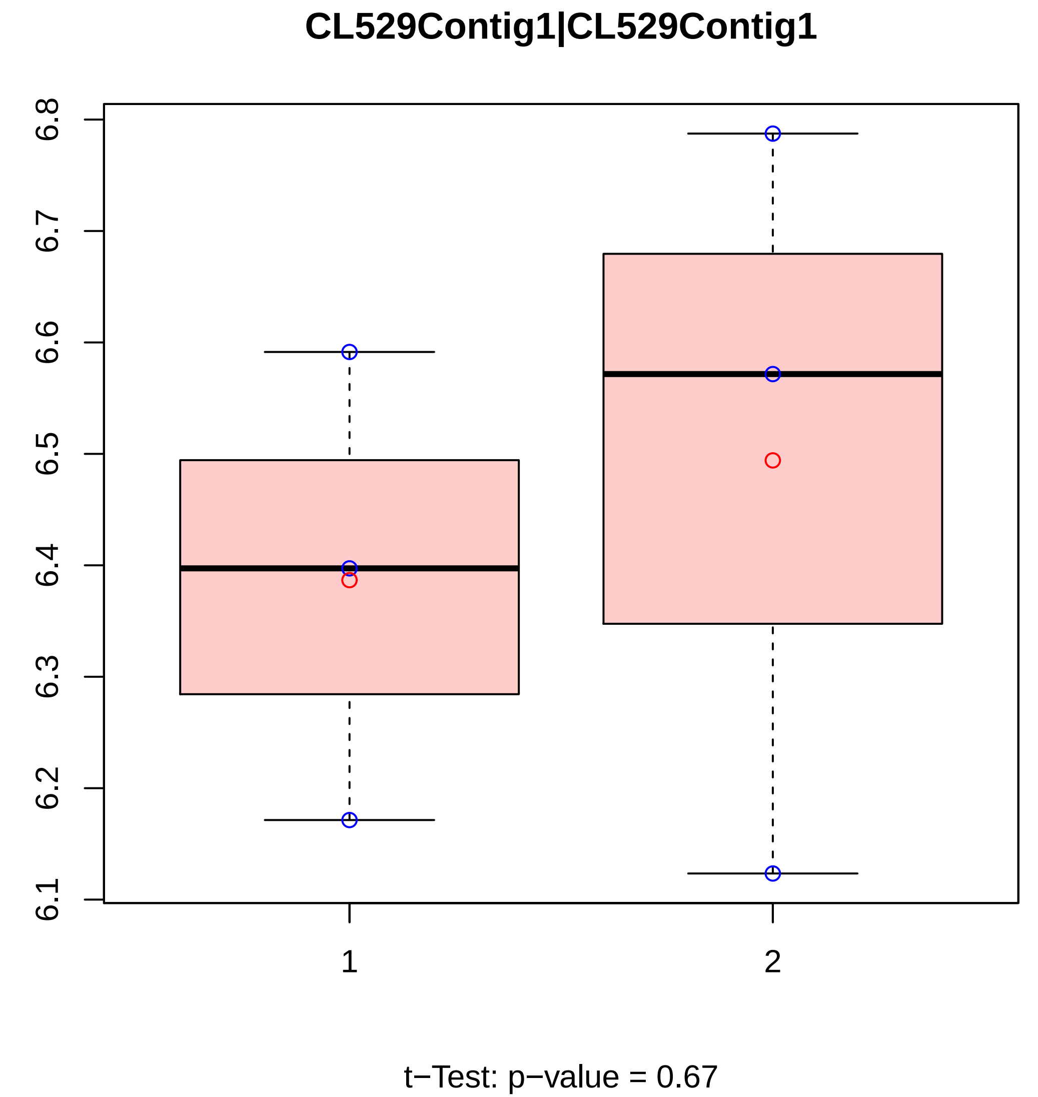
CL529Contig1|CL529Contig1
6.1
6.2
6.3
6.4
6.5
6.6
6.7
6.8
1
2
t−Test: p−value = 0.67

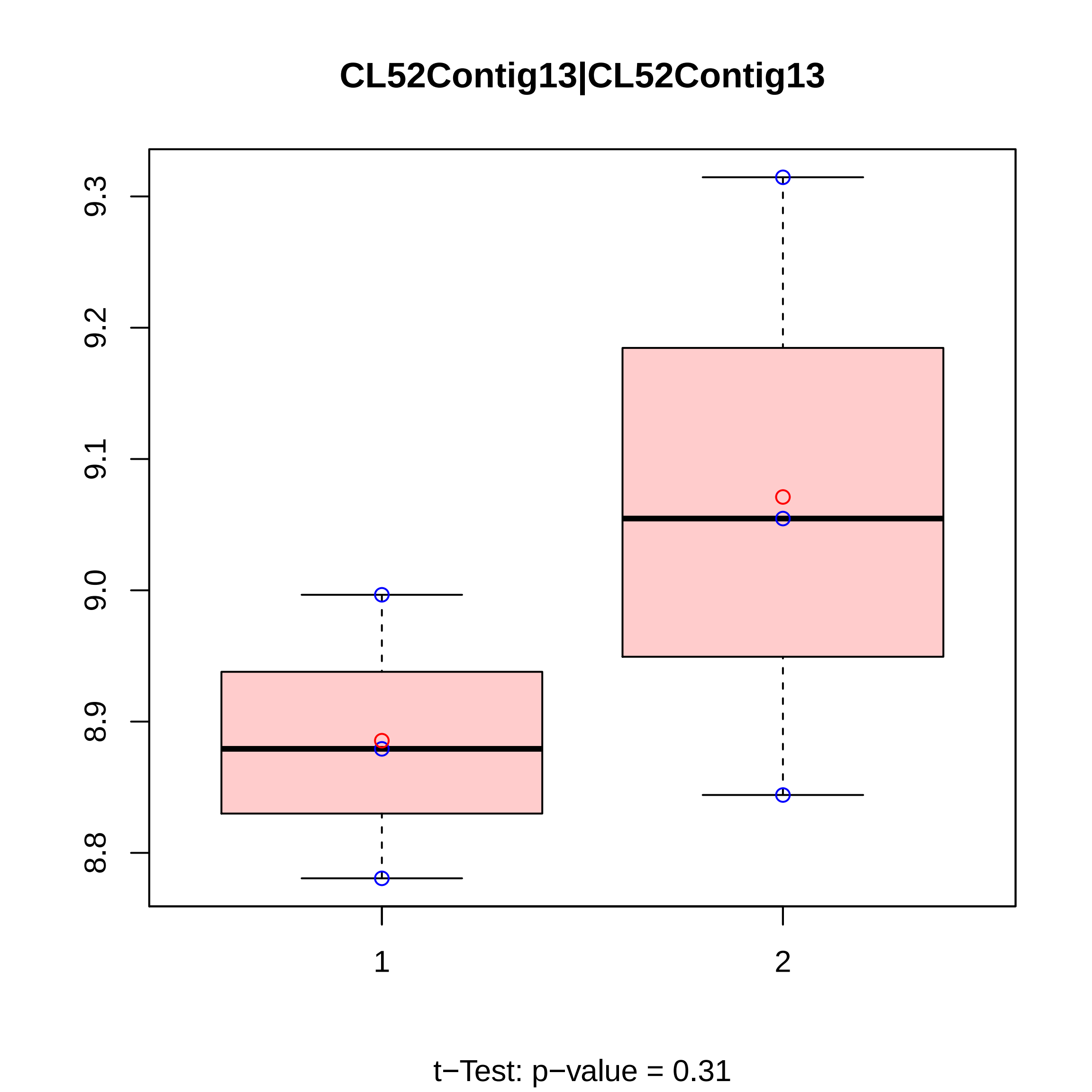
CL52Contig13|CL52Contig13
9.3
9.2
9.1
9.0
8.9
8.8
1
2
t−Test: p−value = 0.31

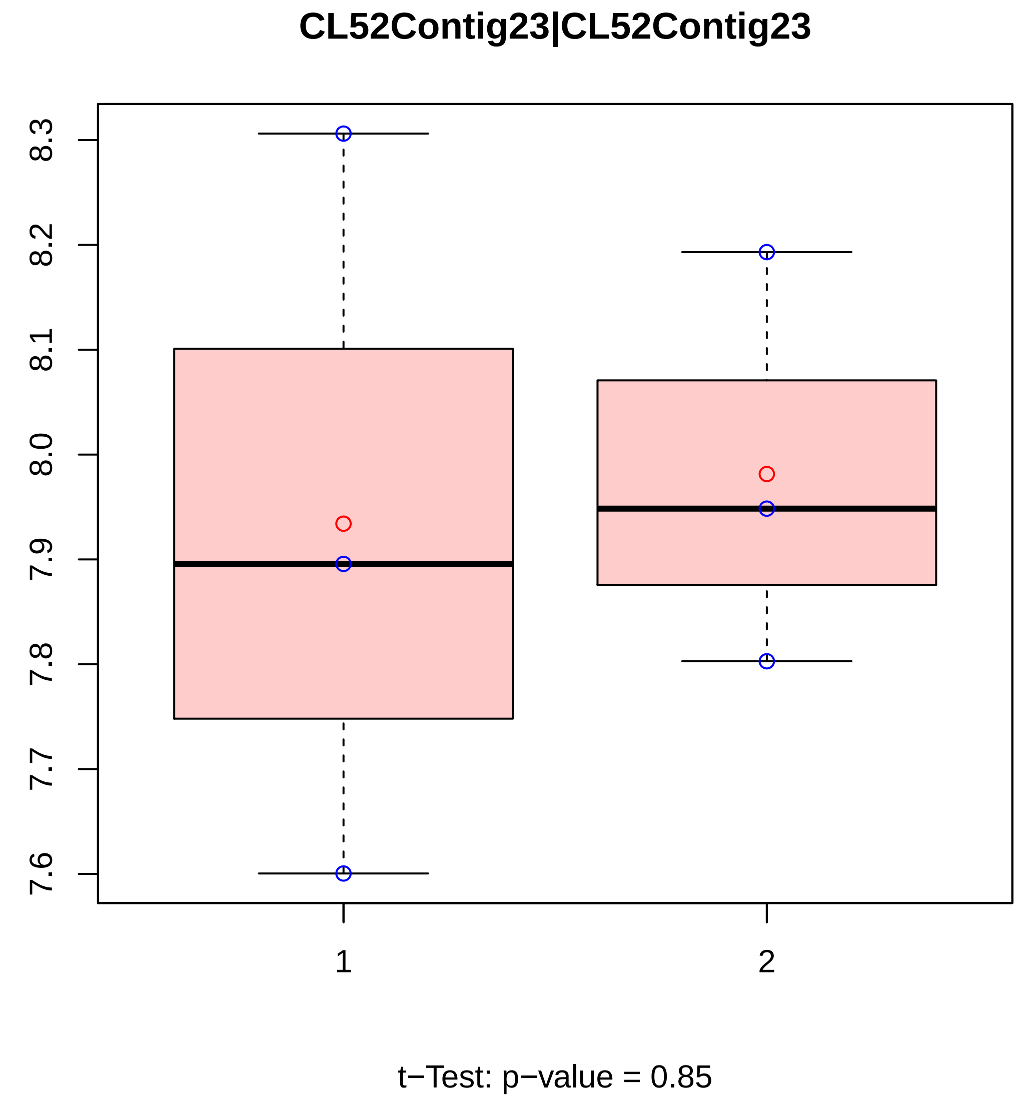
CL52Contig23|CL52Contig23
8.3
8.2
8.1
8.0
7.9
7.8
7.7
7.6
1
2
t−Test: p−value = 0.85

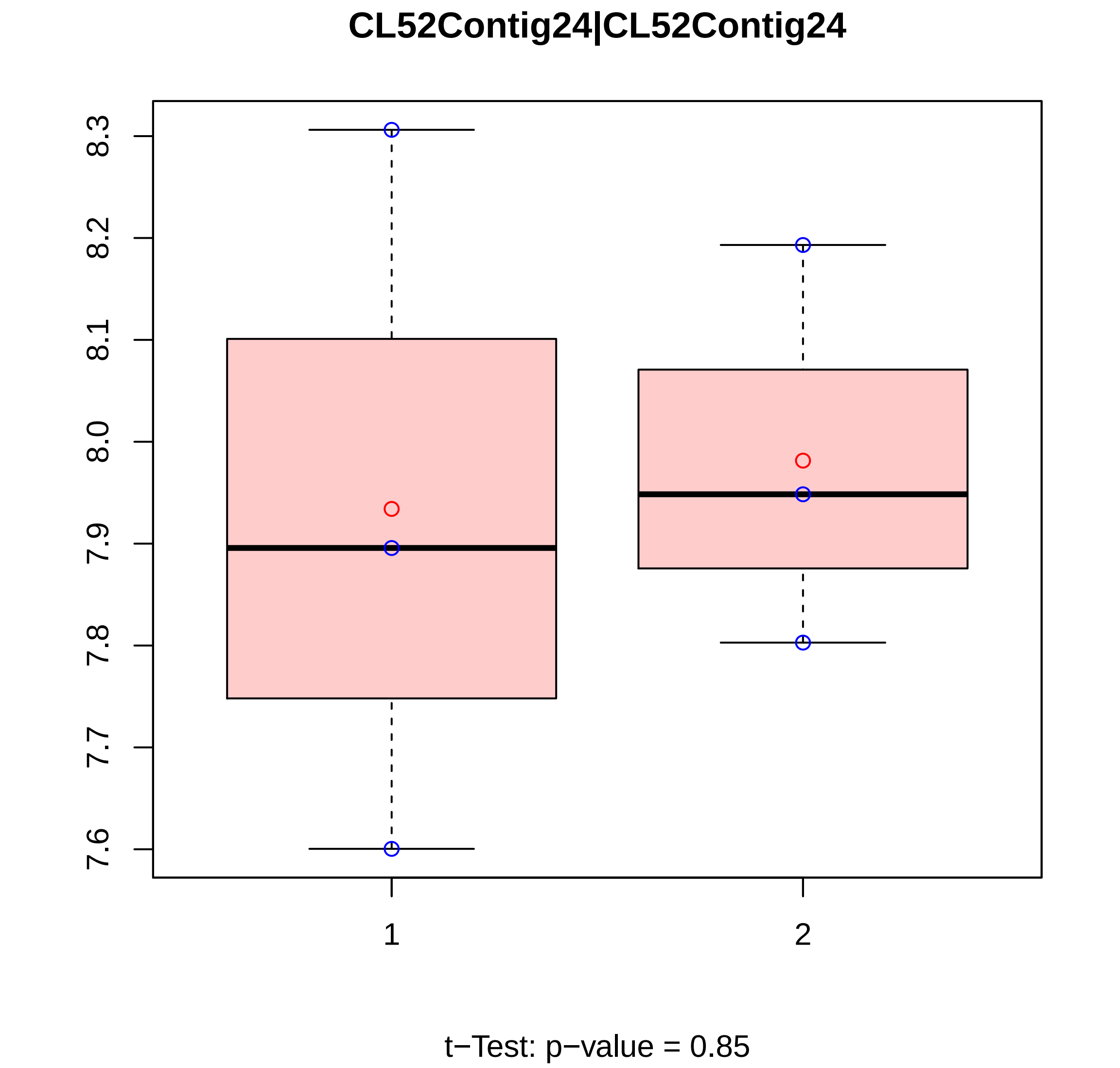

CL52Contig24|CL52Contig24
8.3
8.2
8.1
8.0
7.9
7.8
7.7
7.6
1
2
t−Test: p−value = 0.85

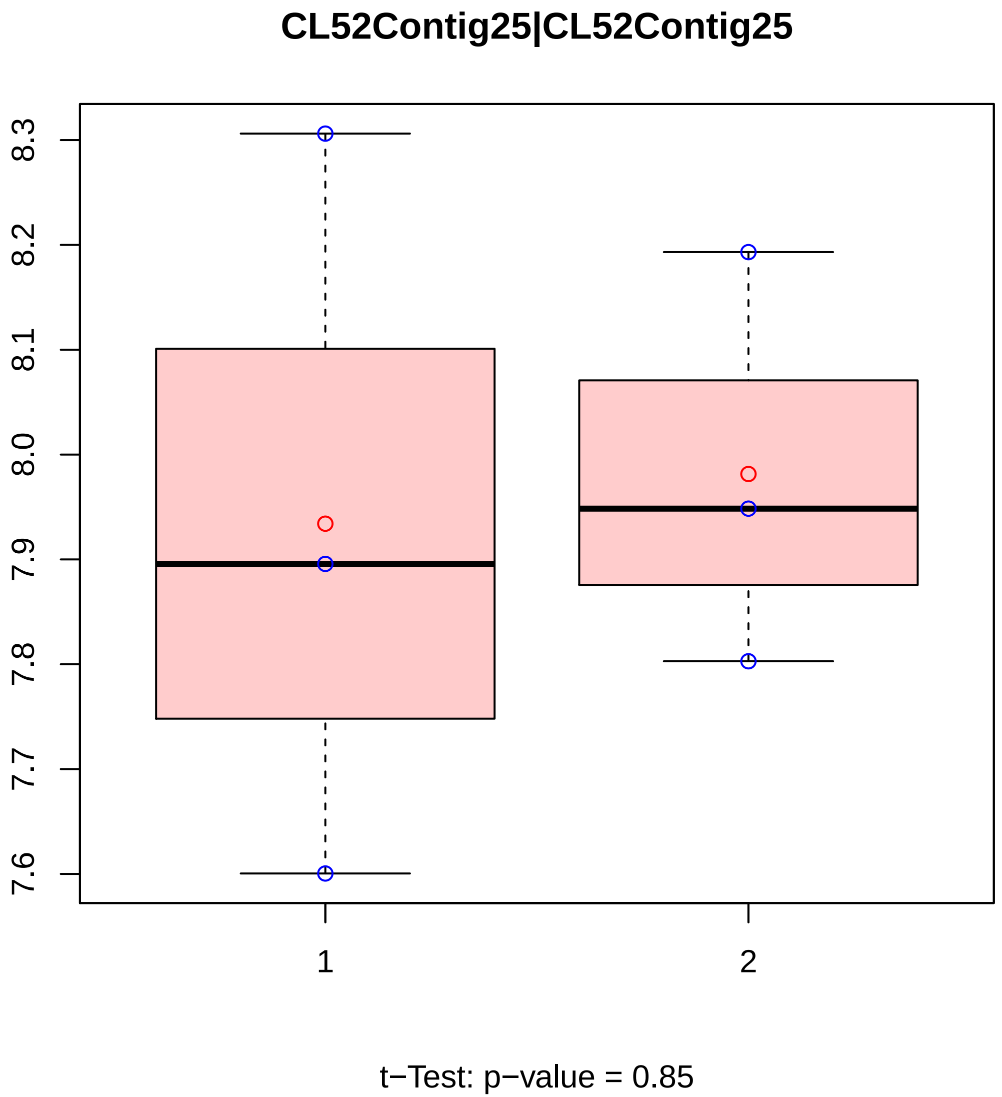
CL52Contig25|CL52Contig25
8.3
8.2
8.1
8.0
7.9
7.8
7.7
7.6
1
2
t−Test: p−value = 0.85

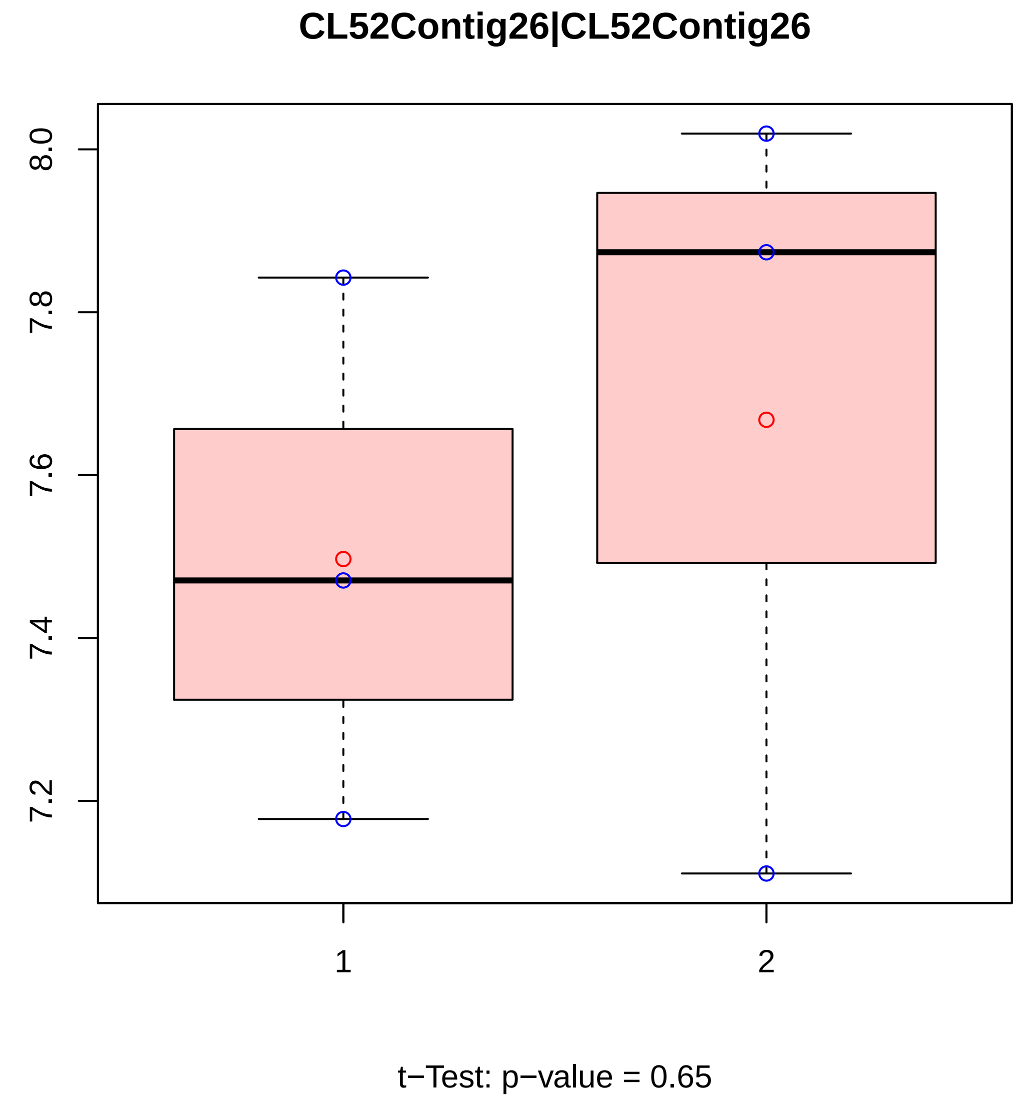
CL52Contig26|CL52Contig26
8.0
7.8
7.6
7.4
7.2
1
2
t−Test: p−value = 0.65

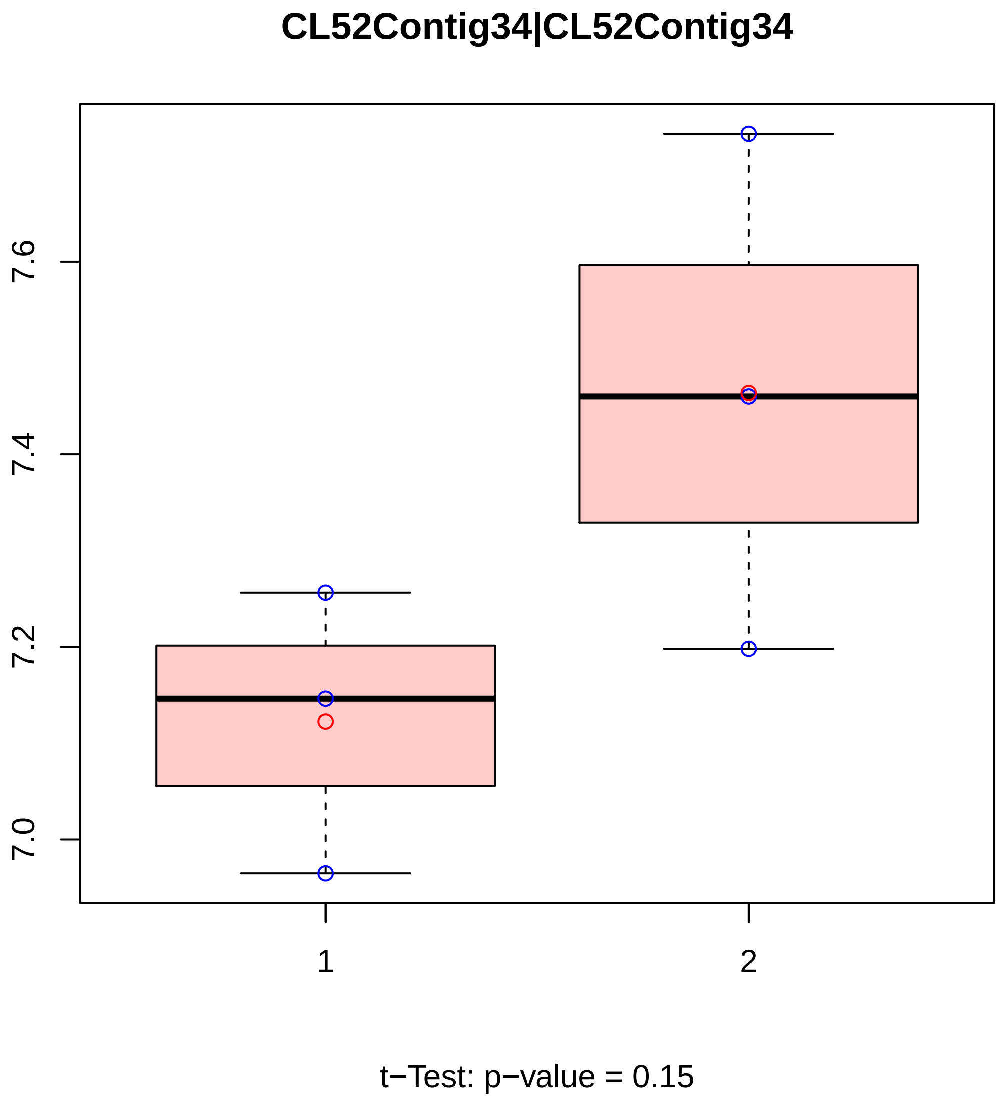

CL52Contig34|CL52Contig34
7.0
7.2
7.4
7.6
1
2
t−Test: p−value = 0.15

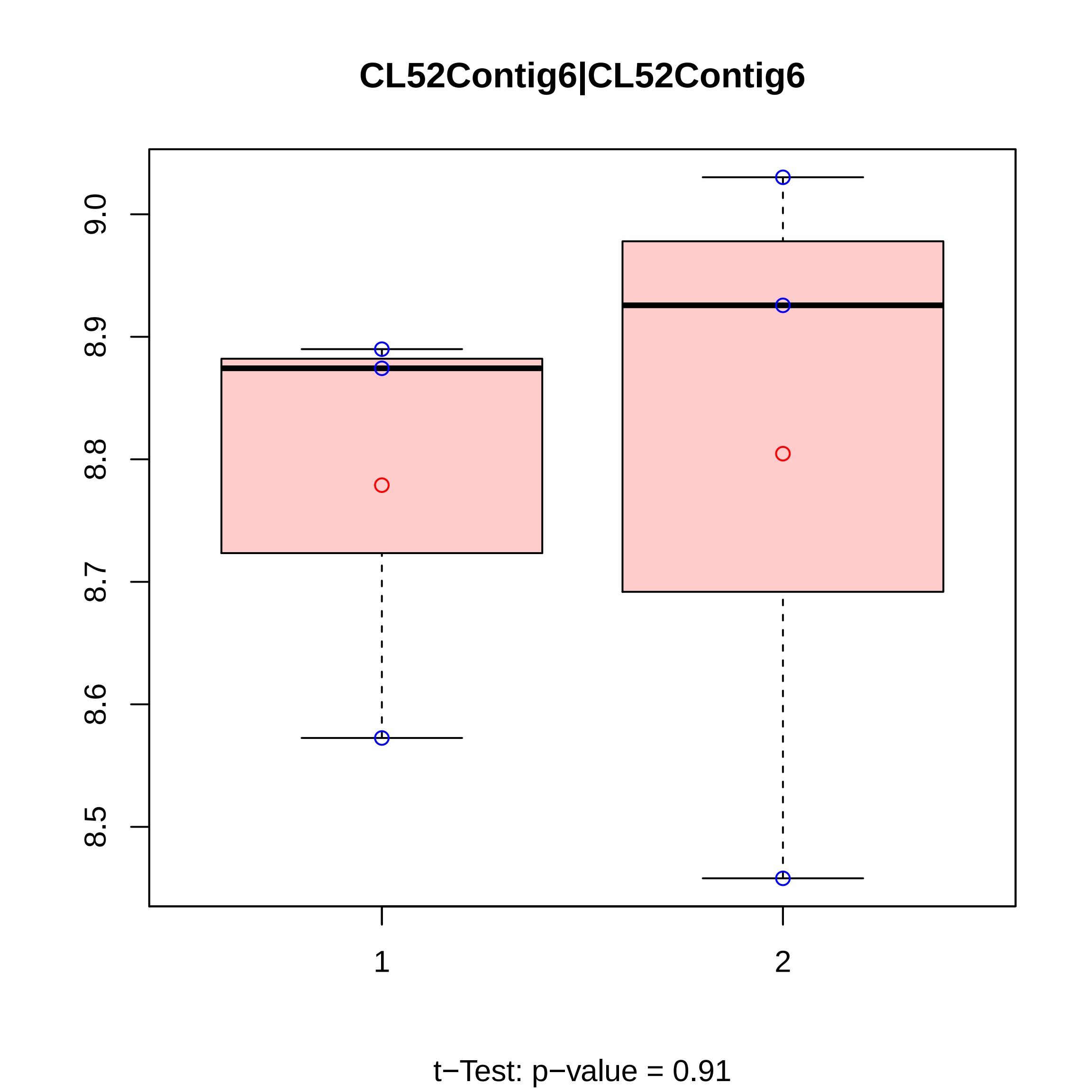

CL52Contig6|CL52Contig6
9.0
8.9
8.8
8.7
8.6
8.5
1
2
t−Test: p−value = 0.91

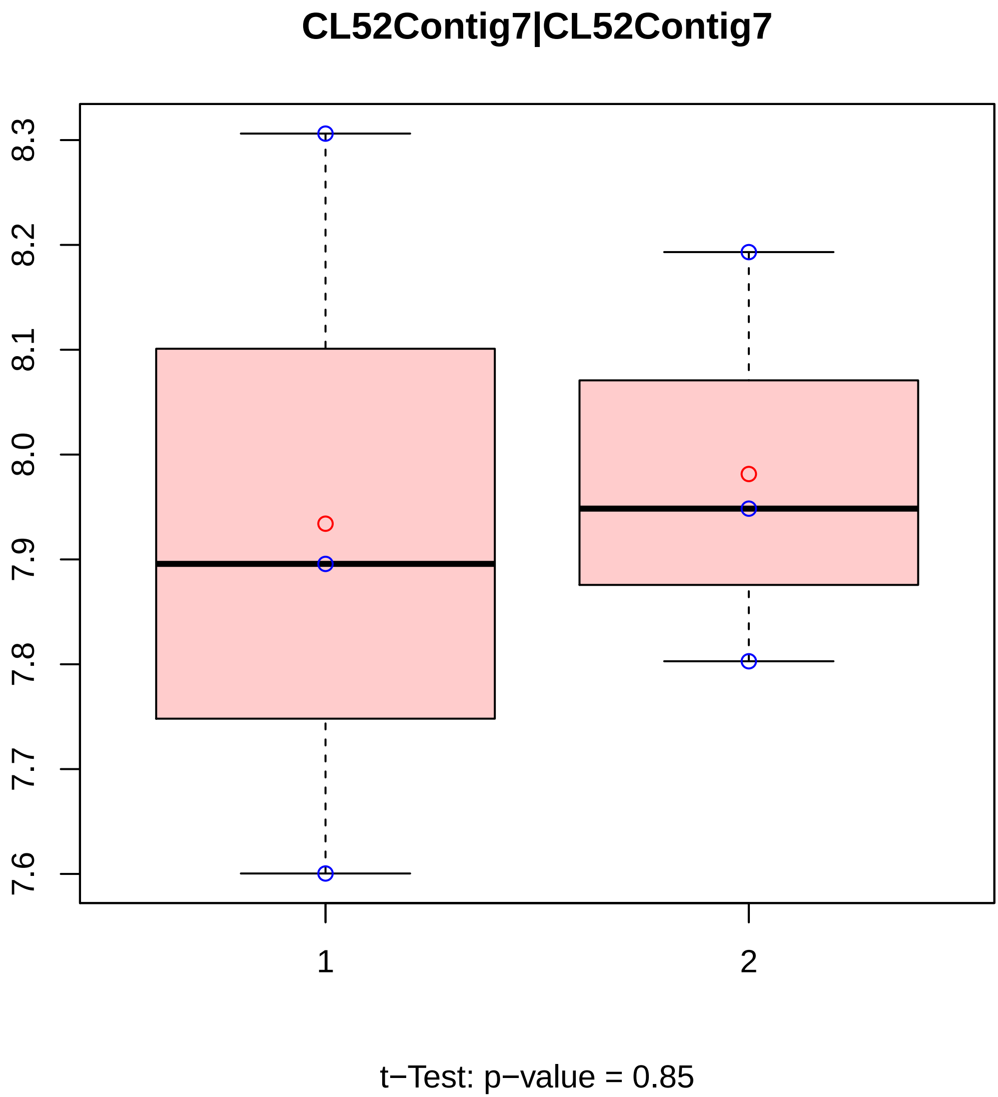

CL52Contig7|CL52Contig7
8.3
8.2
8.1
8.0
7.9
7.8
7.7
7.6
1
2
t−Test: p−value = 0.85

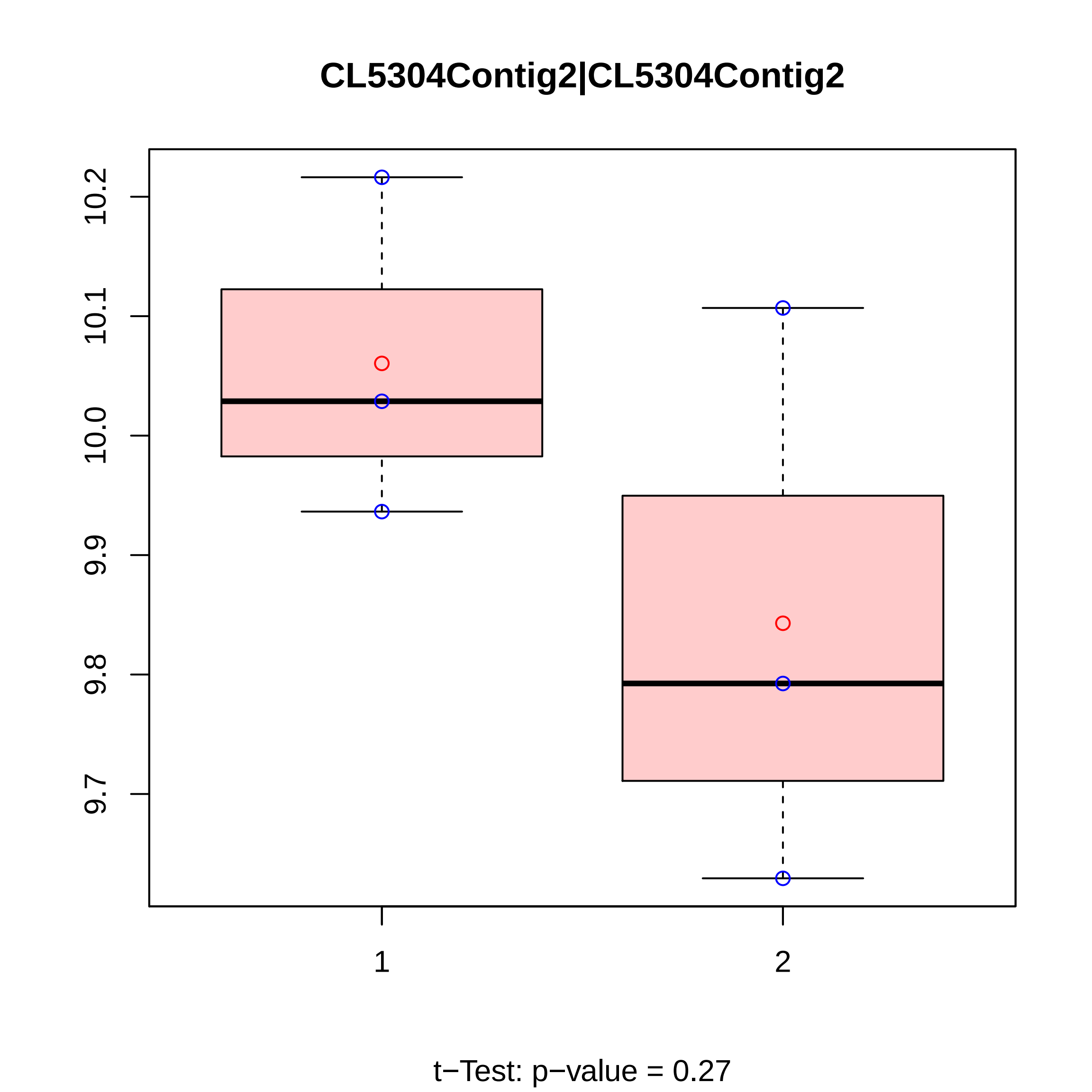

CL5304Contig2|CL5304Contig2
10.2
10.1
10.0
9.9
9.8
9.7
1
2
t−Test: p−value = 0.27

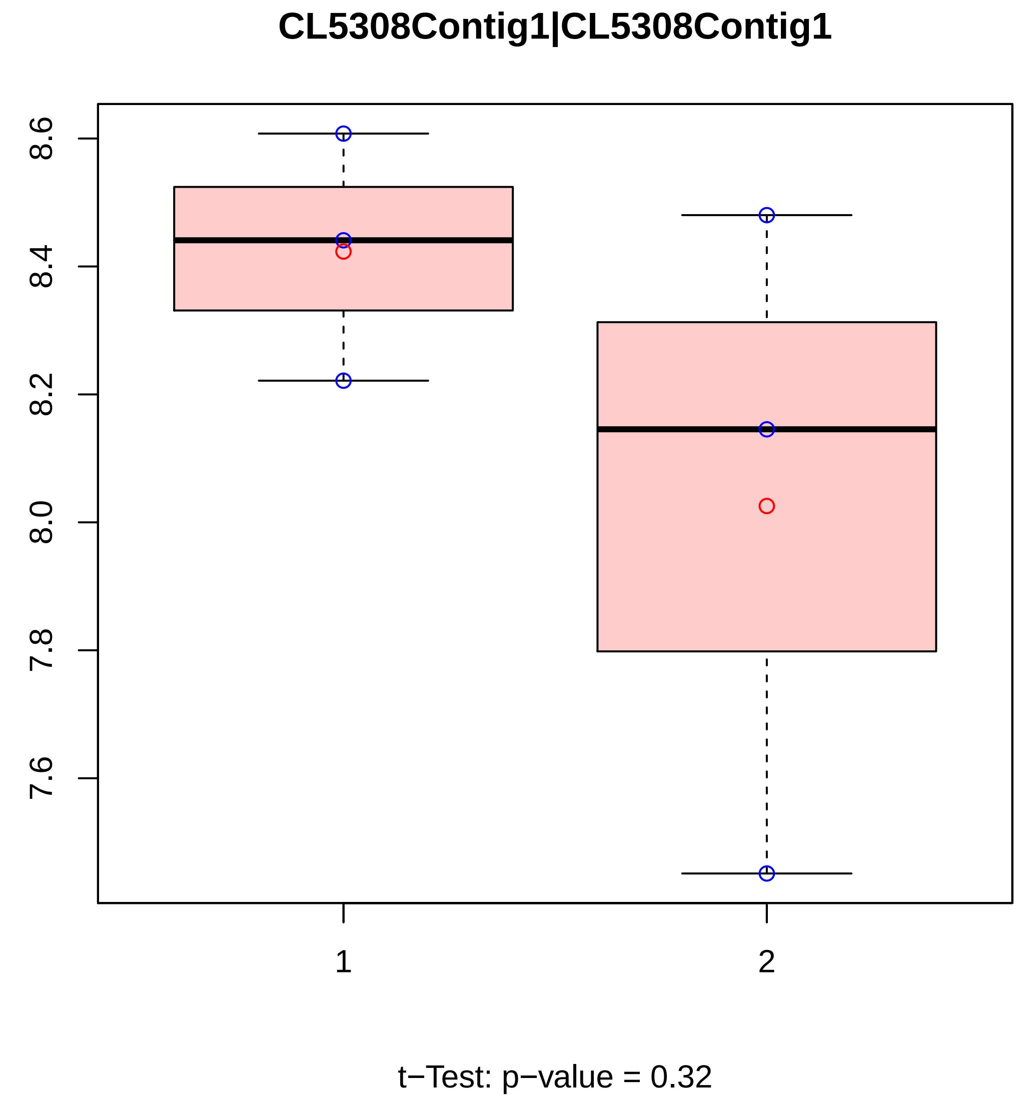

CL5308Contig1|CL5308Contig1
8.6
8.4
8.2
8.0
7.8
7.6
1
2
t−Test: p−value = 0.32

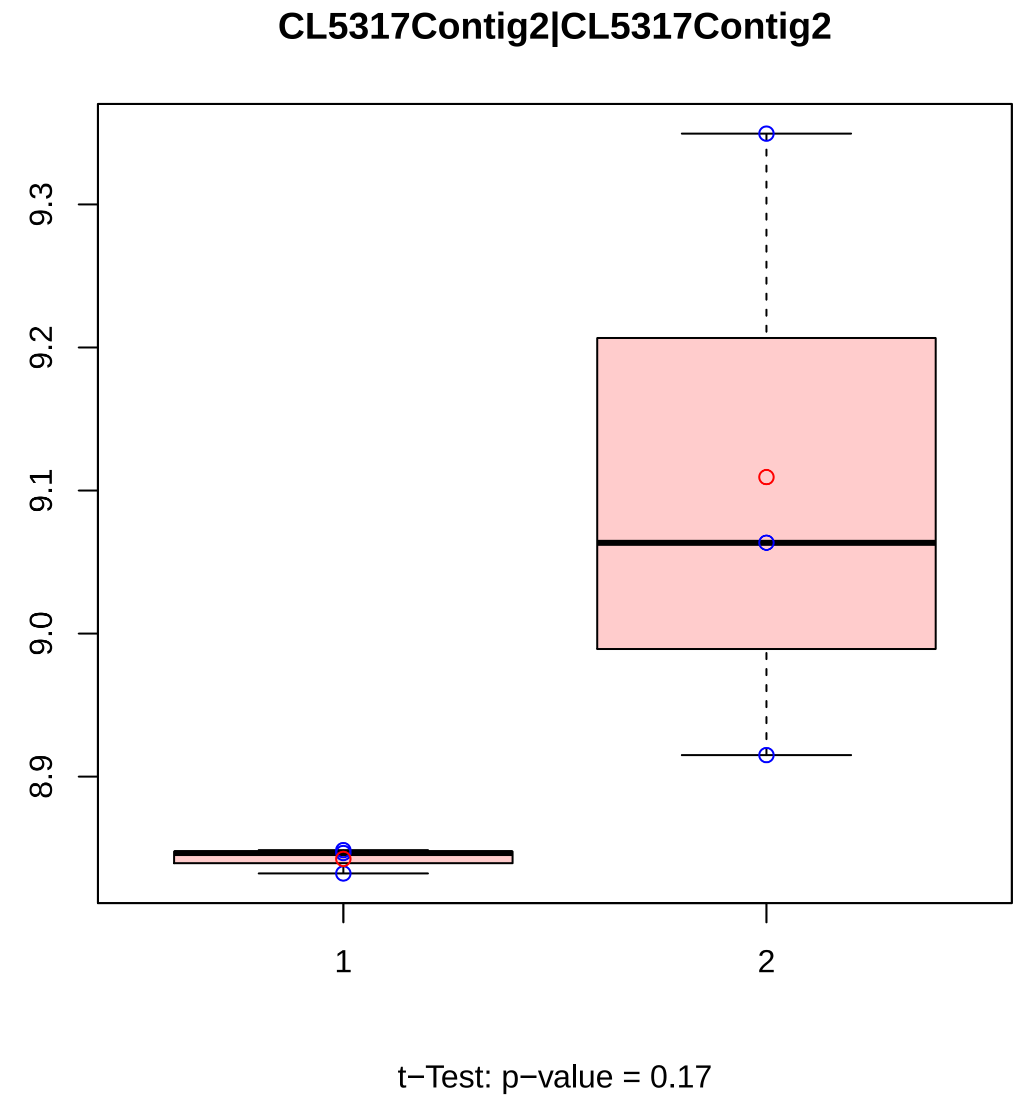

CL5317Contig2|CL5317Contig2
9.3
9.2
9.1
9.0
8.9
1
2
t−Test: p−value = 0.17

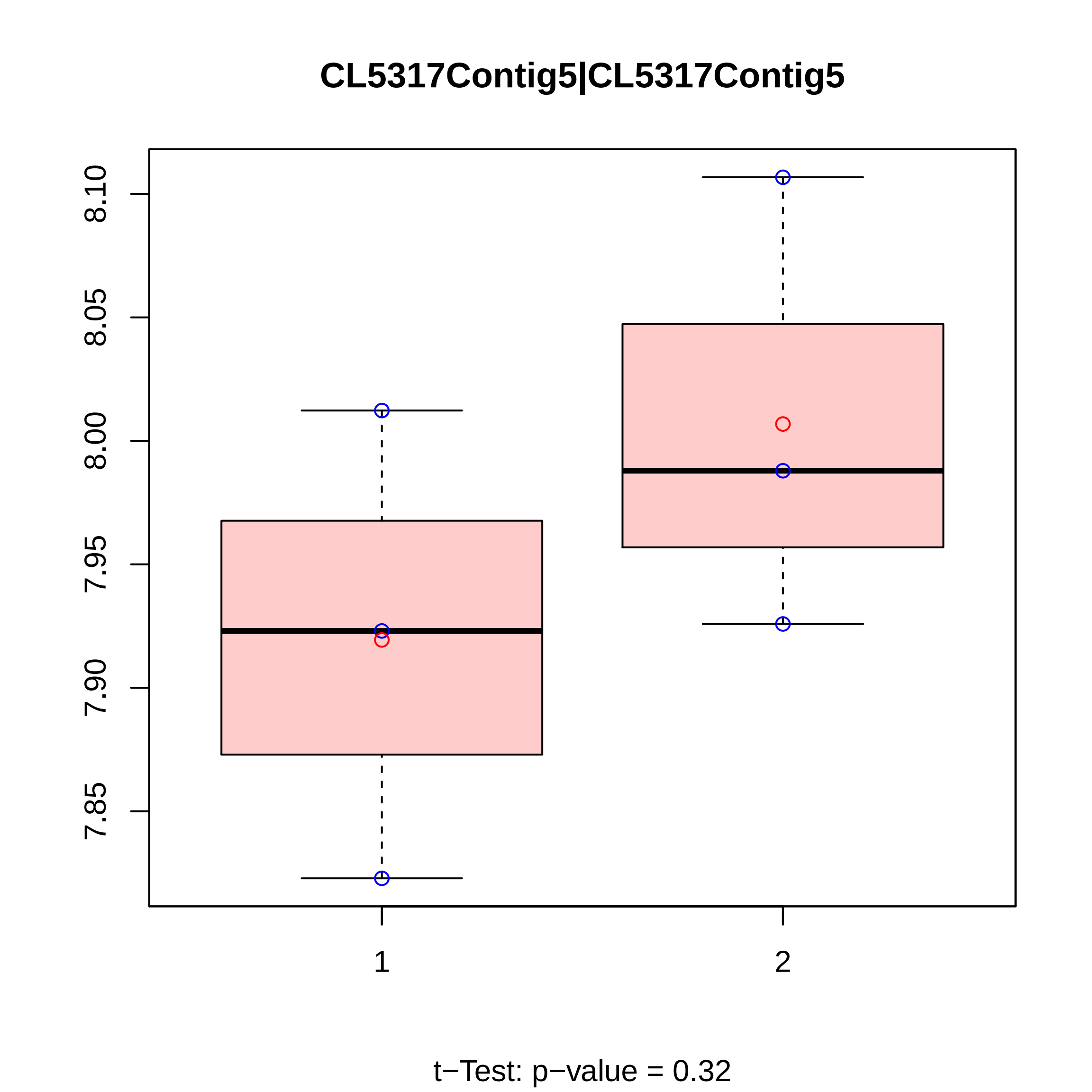
CL5317Contig5|CL5317Contig5
8.10
8.05
8.00
7.95
7.90
7.85
1
2
t−Test: p−value = 0.32

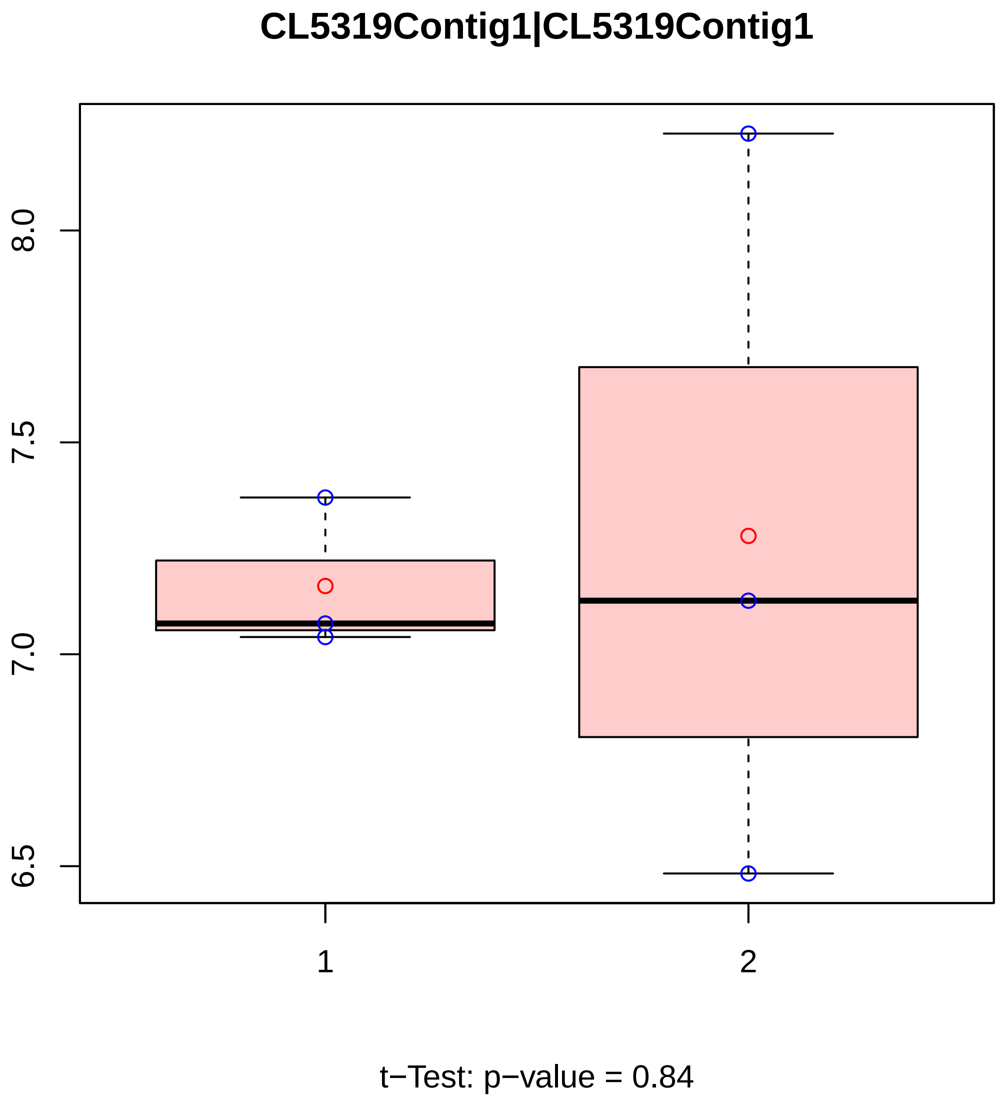
CL5319Contig1|CL5319Contig1
8.0
7.5
7.0
6.5
1
2
t−Test: p−value = 0.84

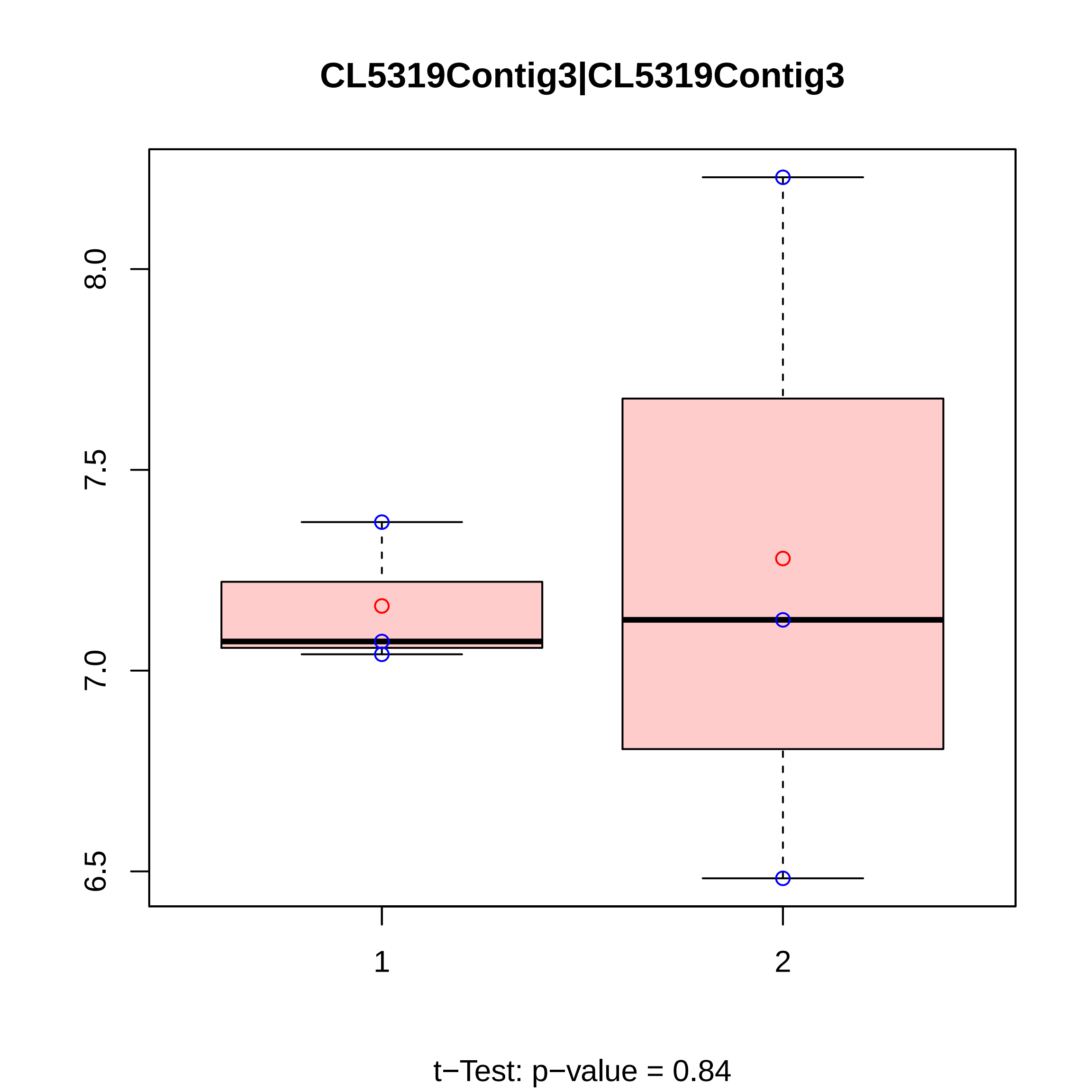

CL5319Contig3|CL5319Contig3
8.0
7.5
7.0
6.5
1
2
t−Test: p−value = 0.84

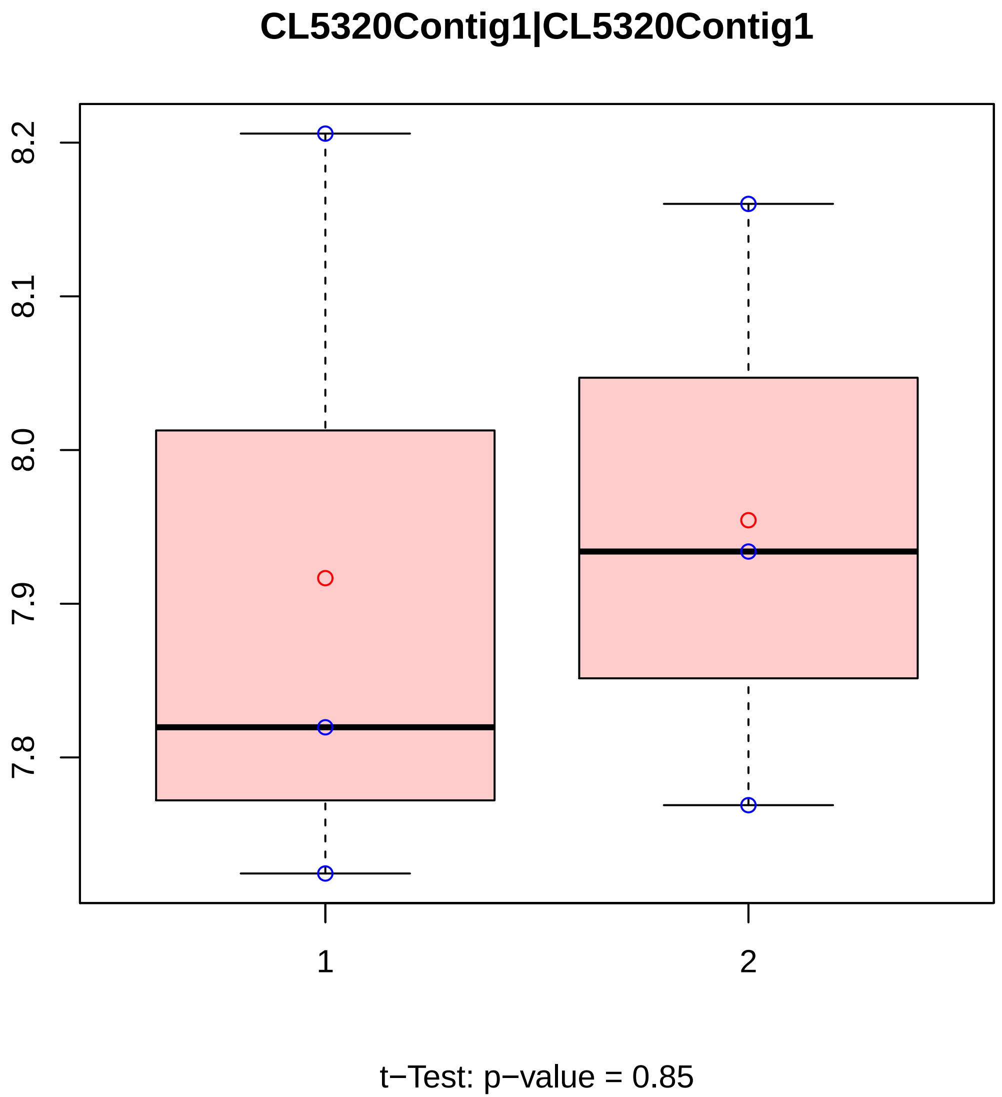
CL5320Contig1|CL5320Contig1
8.2
8.1
8.0
7.9
7.8
1
2
t−Test: p−value = 0.85

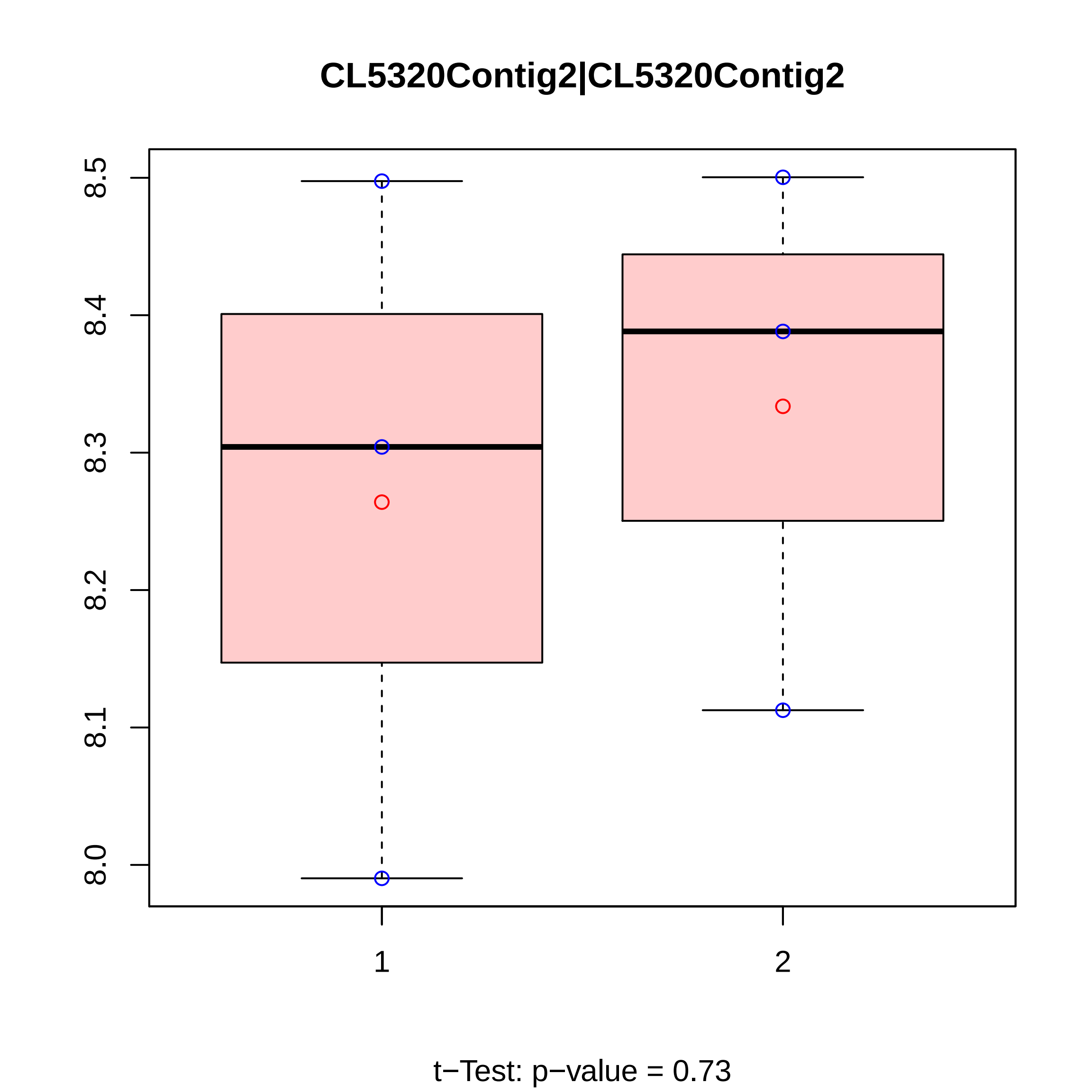
CL5320Contig2|CL5320Contig2
8.5
8.4
8.3
8.2
8.1
8.0
1
2
t−Test: p−value = 0.73

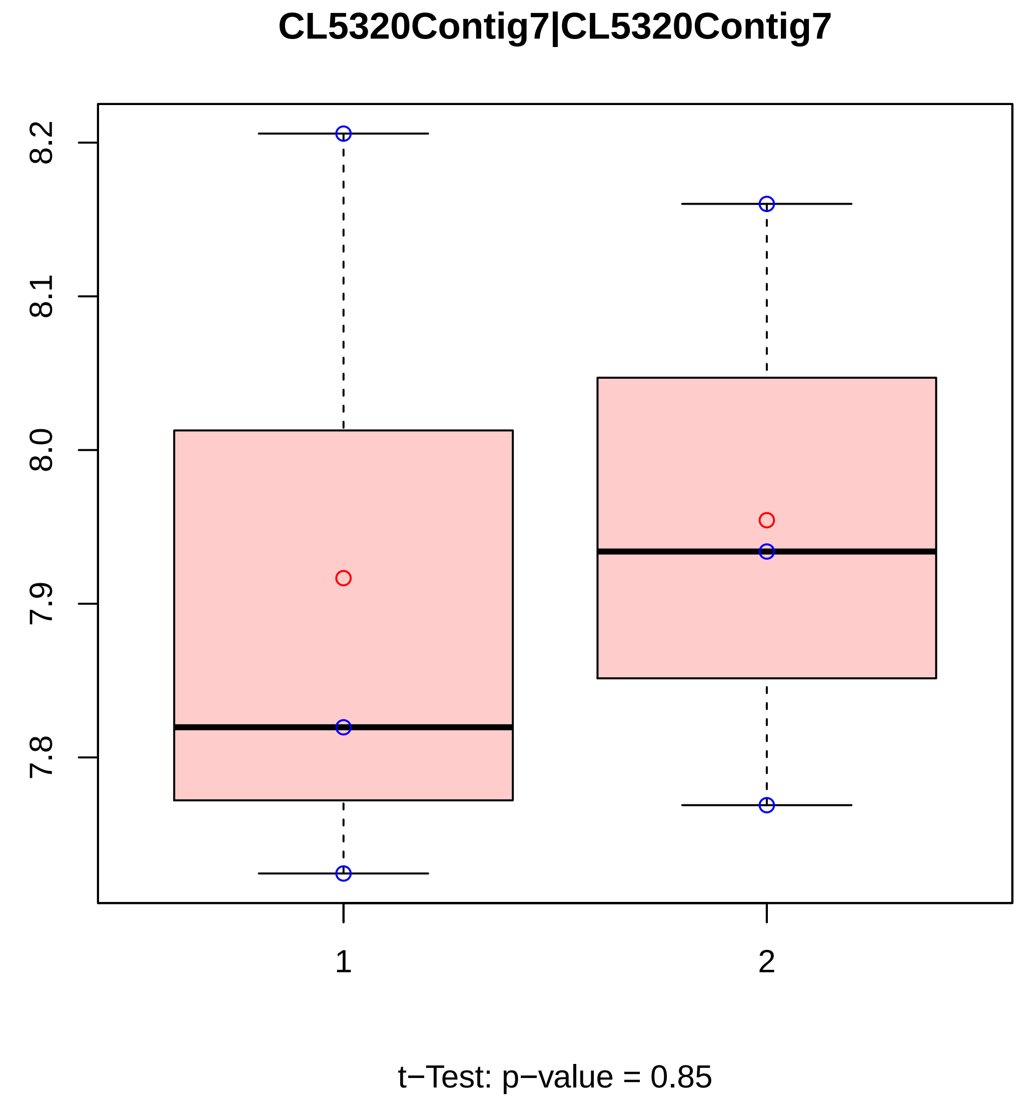
CL5320Contig7|CL5320Contig7
8.2
8.1
8.0
7.9
7.8
1
2
t−Test: p−value = 0.85

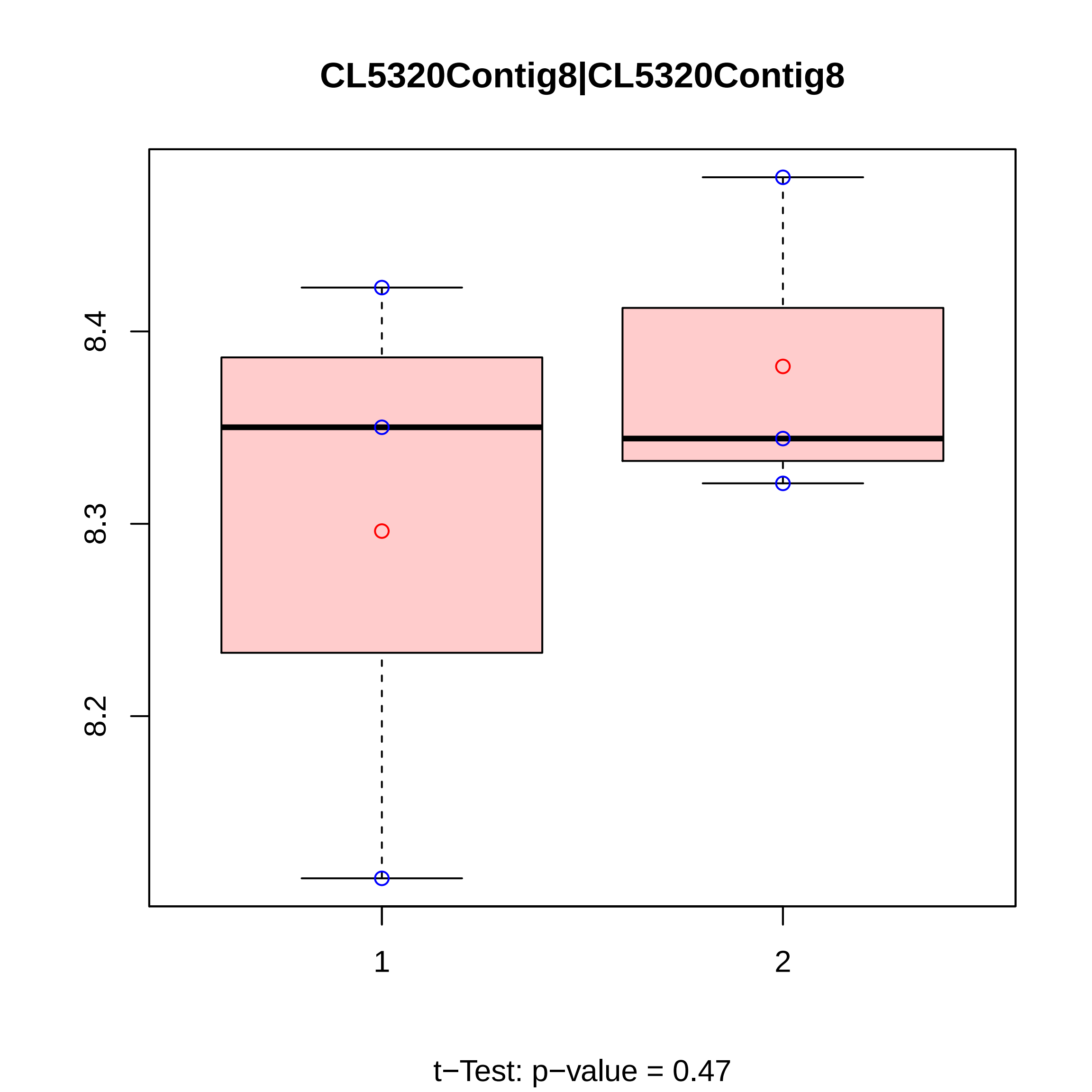
CL5320Contig8|CL5320Contig8
8.4
8.3
8.2
1
2
t−Test: p−value = 0.47

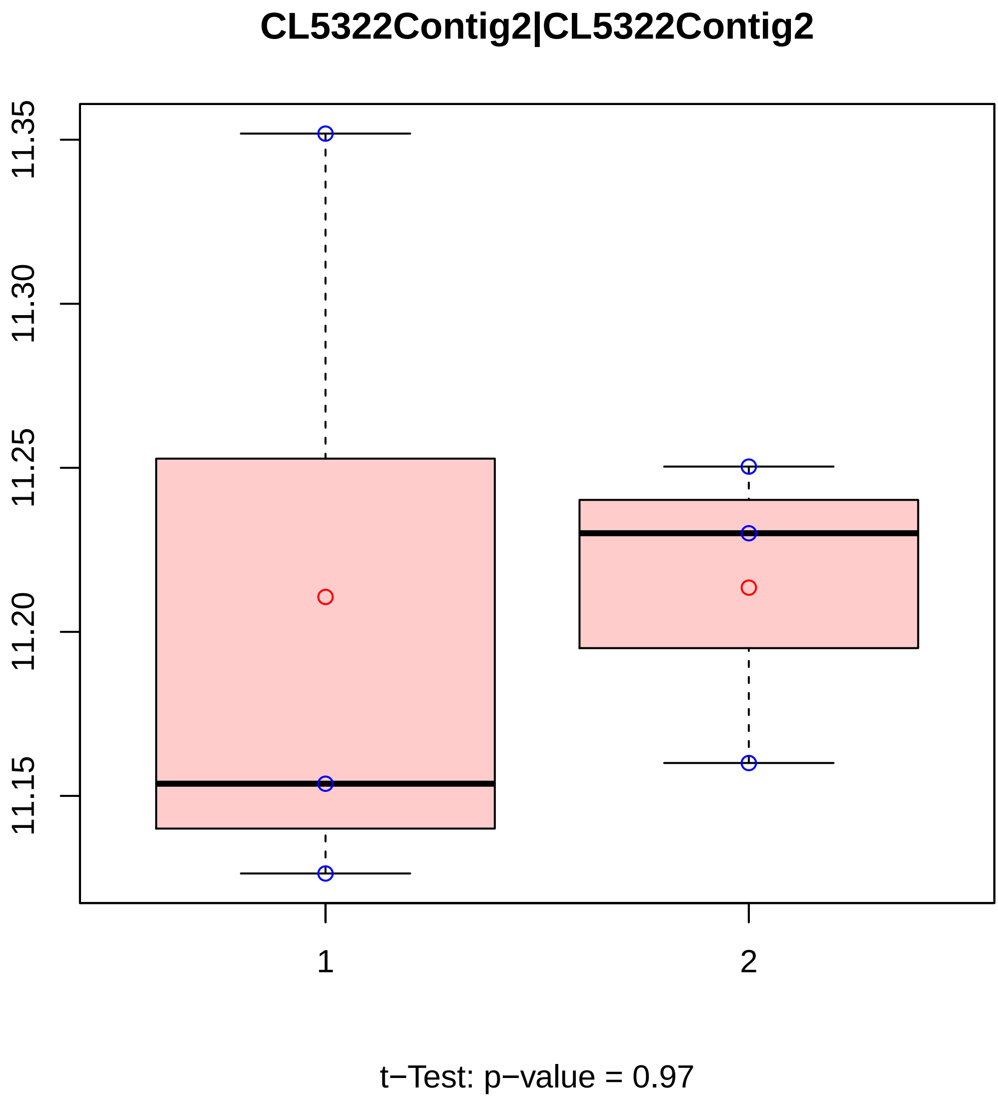
CL5322Contig2|CL5322Contig2
11.35
11.30
11.25
11.20
11.15
1
2
t−Test: p−value = 0.97

CL5322Contig3|CL5322Contig3

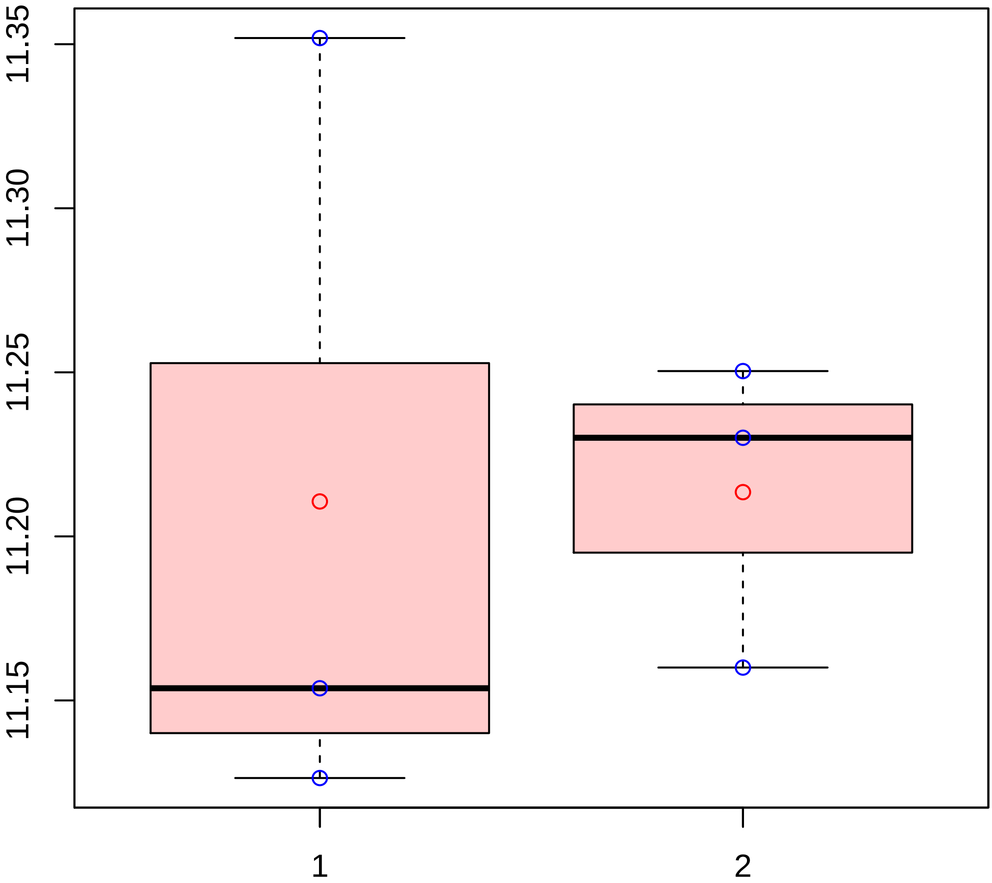


t−Test: p−value = 0.97

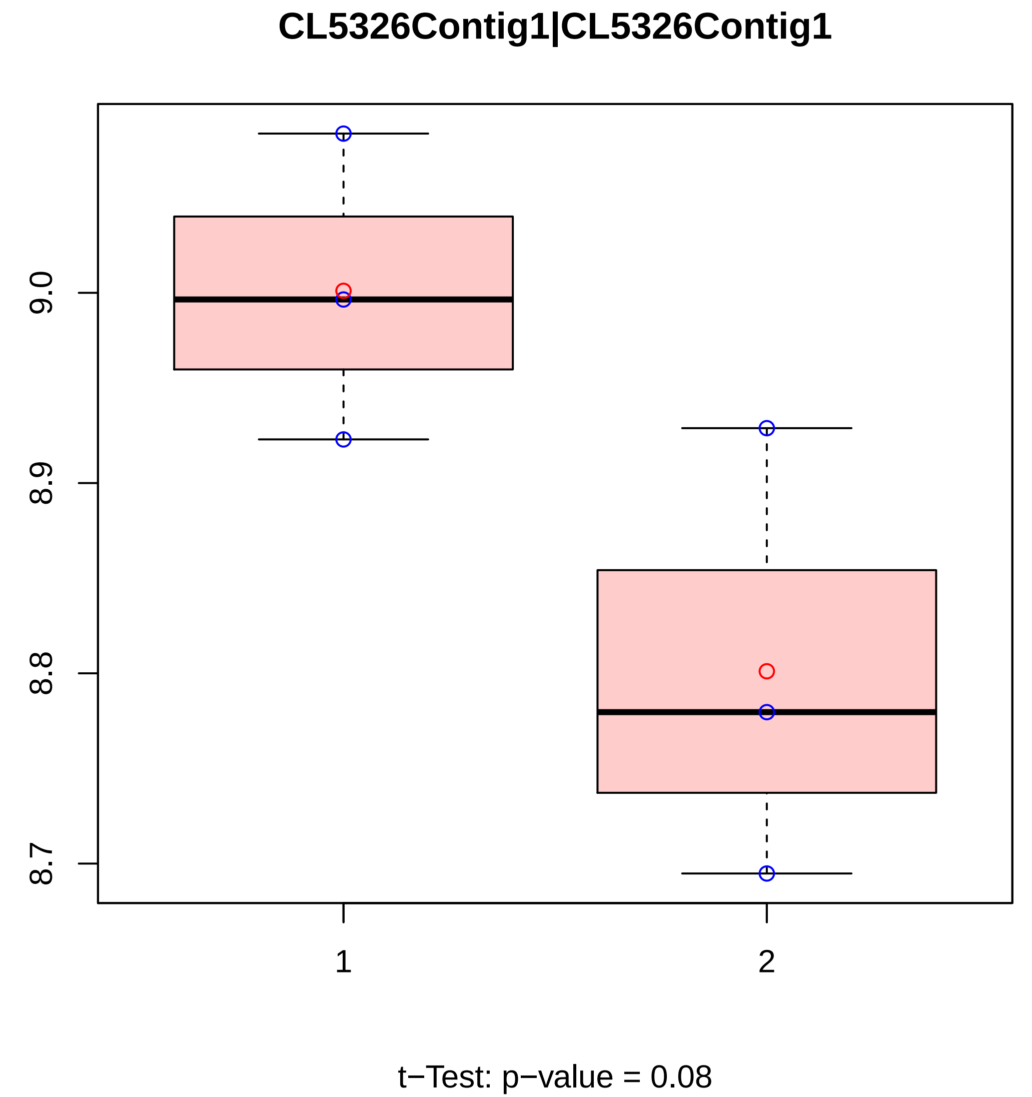
CL5326Contig1|CL5326Contig1
9.0
8.9
8.8
8.7
1
2
t−Test: p−value = 0.08

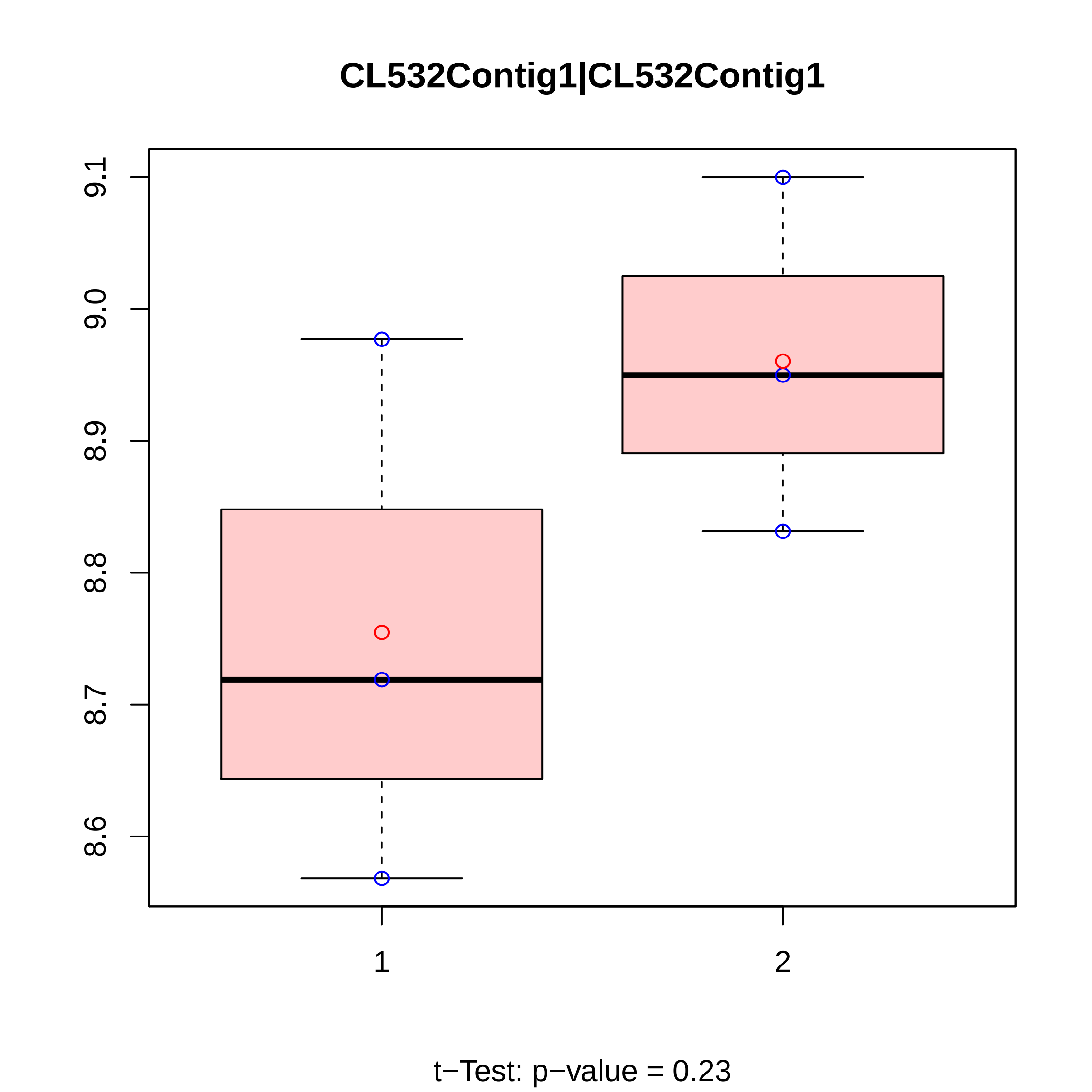
CL532Contig1|CL532Contig1
9.1
9.0
8.9
8.8
8.7
8.6
1
2
t−Test: p−value = 0.23

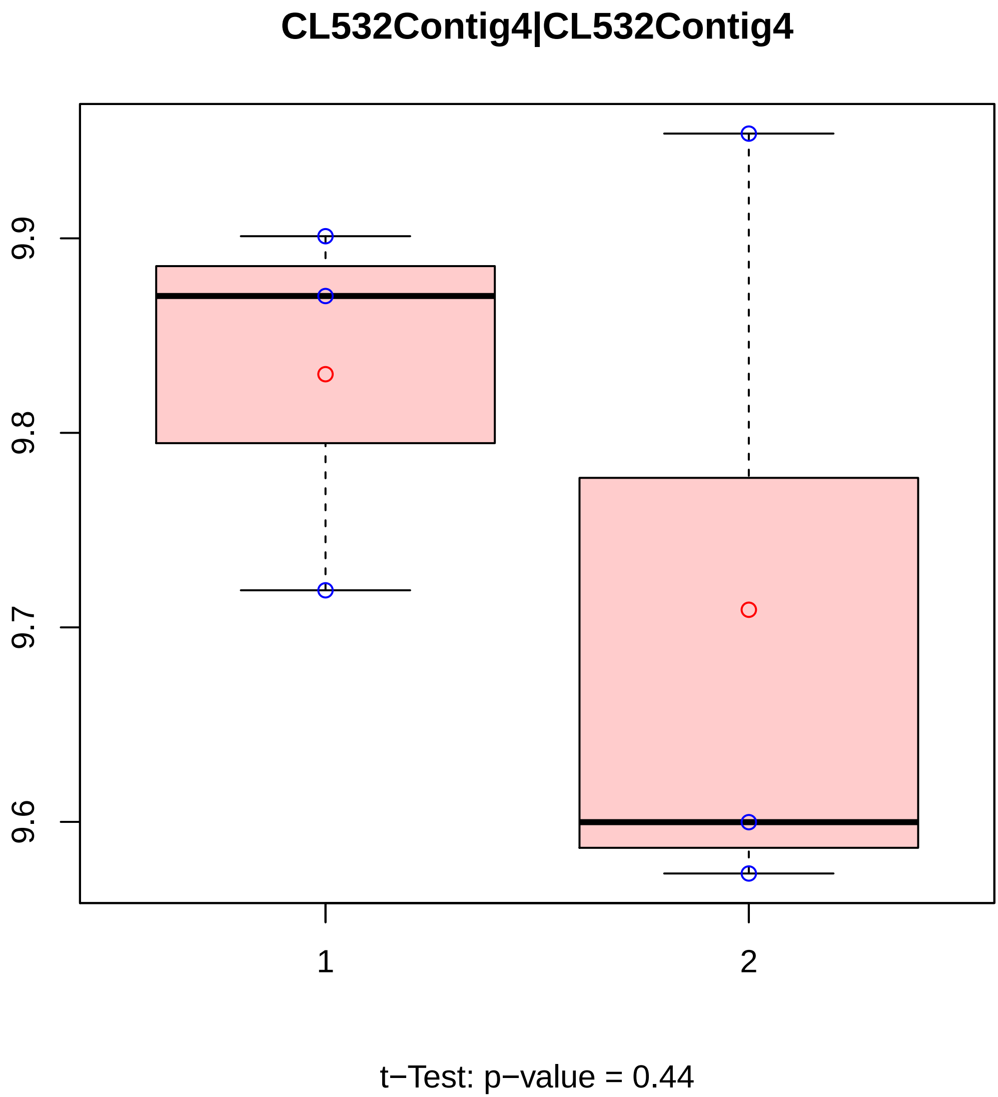

CL532Contig4|CL532Contig4
9.9
9.8
9.7
9.6
1
2
t−Test: p−value = 0.44

# CL5336Contig5|CL5336Contig5

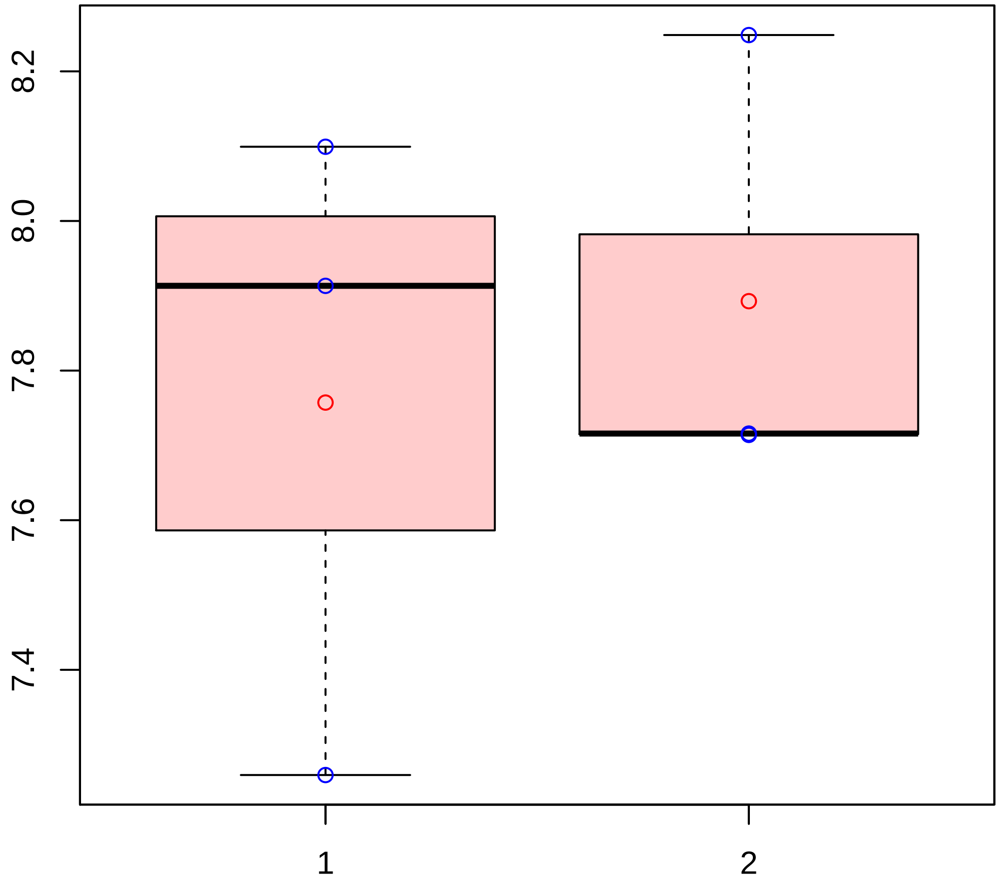


t−Test: p−value = 0.69

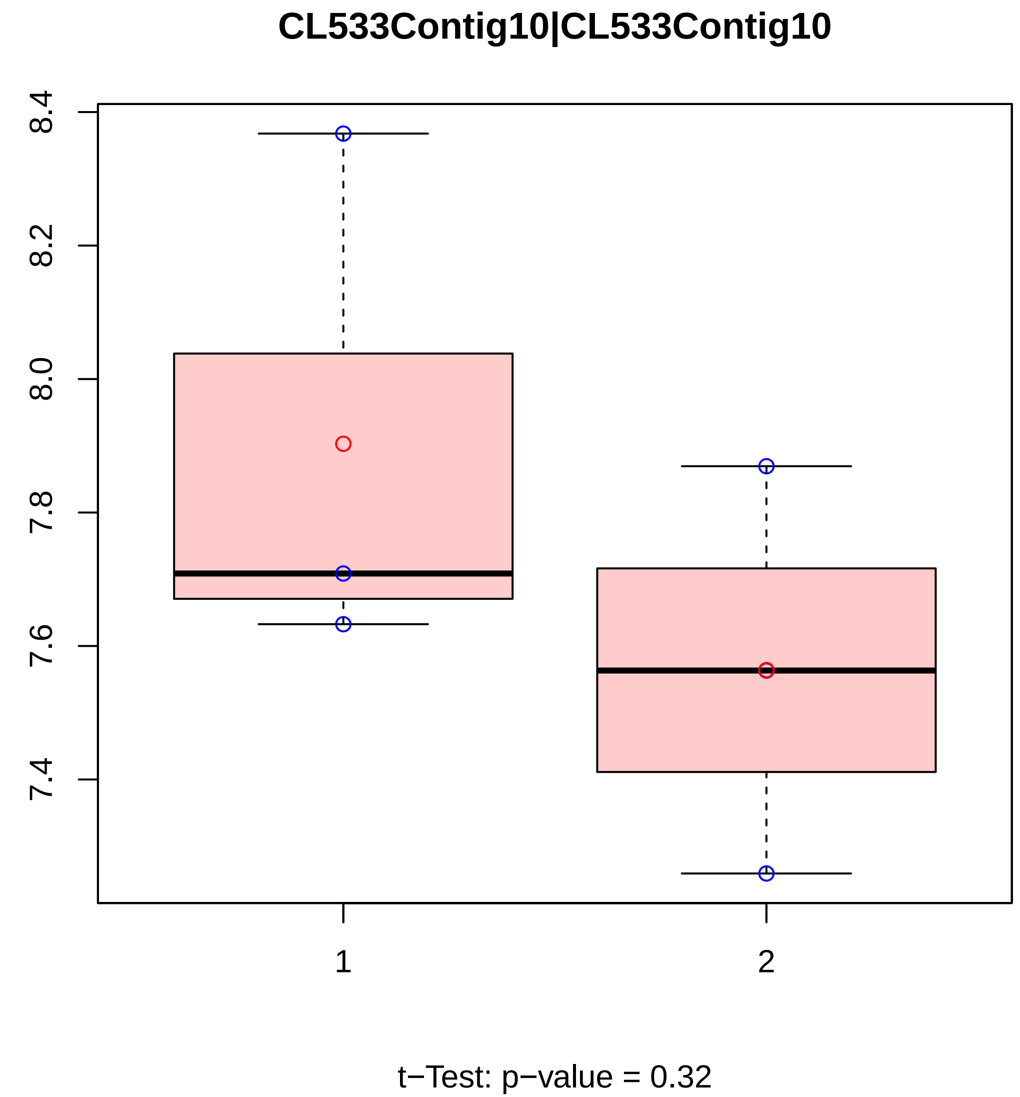
CL533Contig10|CL533Contig10
8.4
8.2
8.0
7.8
7.6
7.4
1
2
t−Test: p−value = 0.32

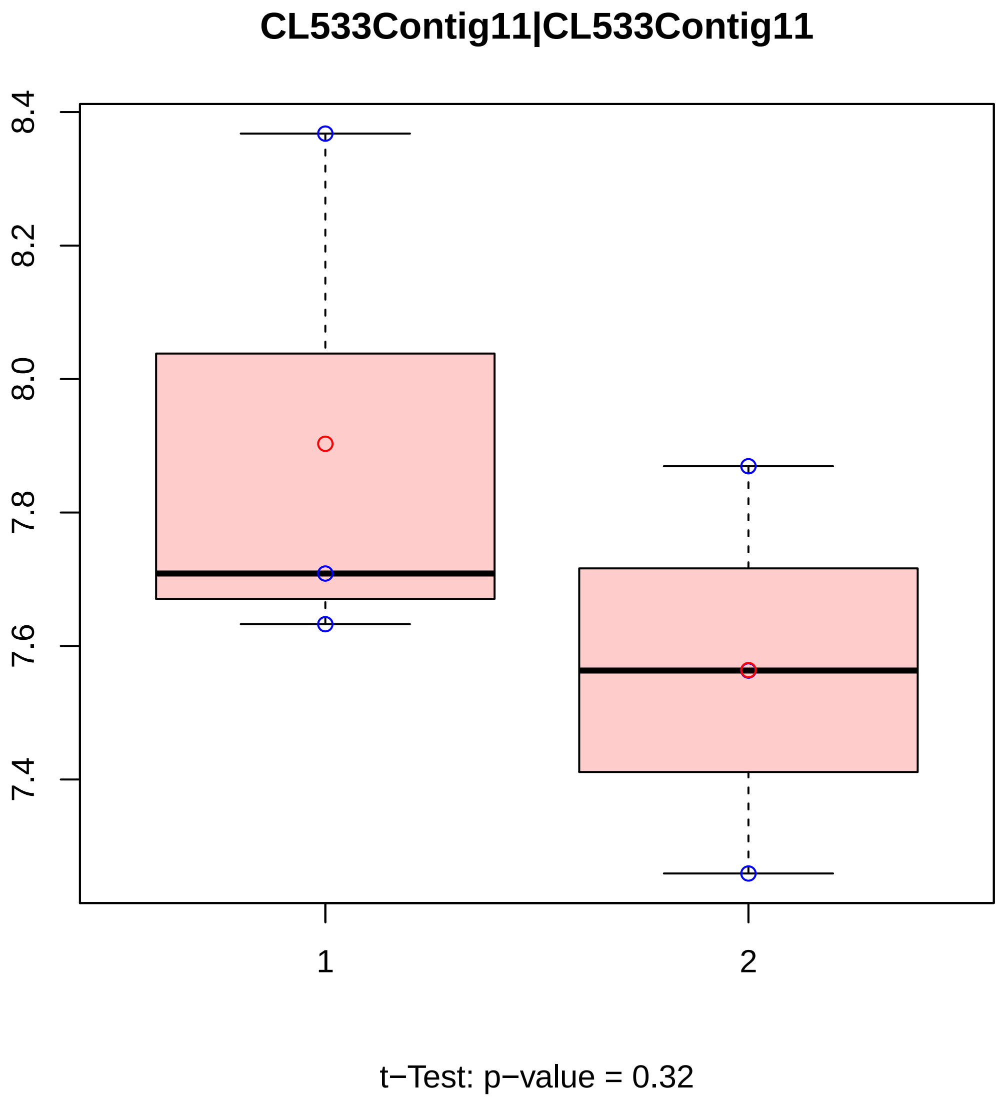

CL533Contig11|CL533Contig11
8.4
8.2
8.0
7.8
7.6
7.4
1
2
t−Test: p−value = 0.32

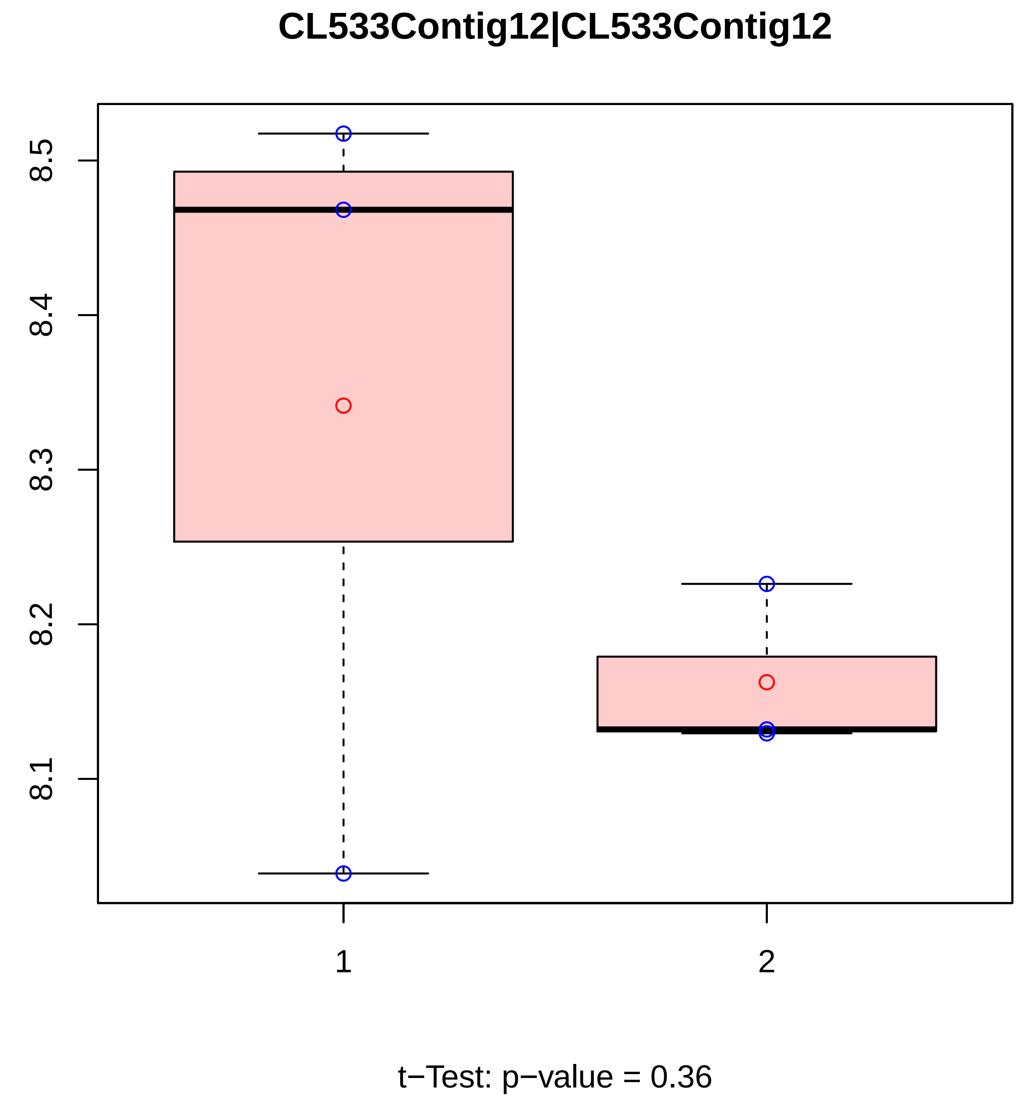
CL533Contig12|CL533Contig12
8.1
8.2
8.3
8.4
8.5
1
2
t−Test: p−value = 0.36

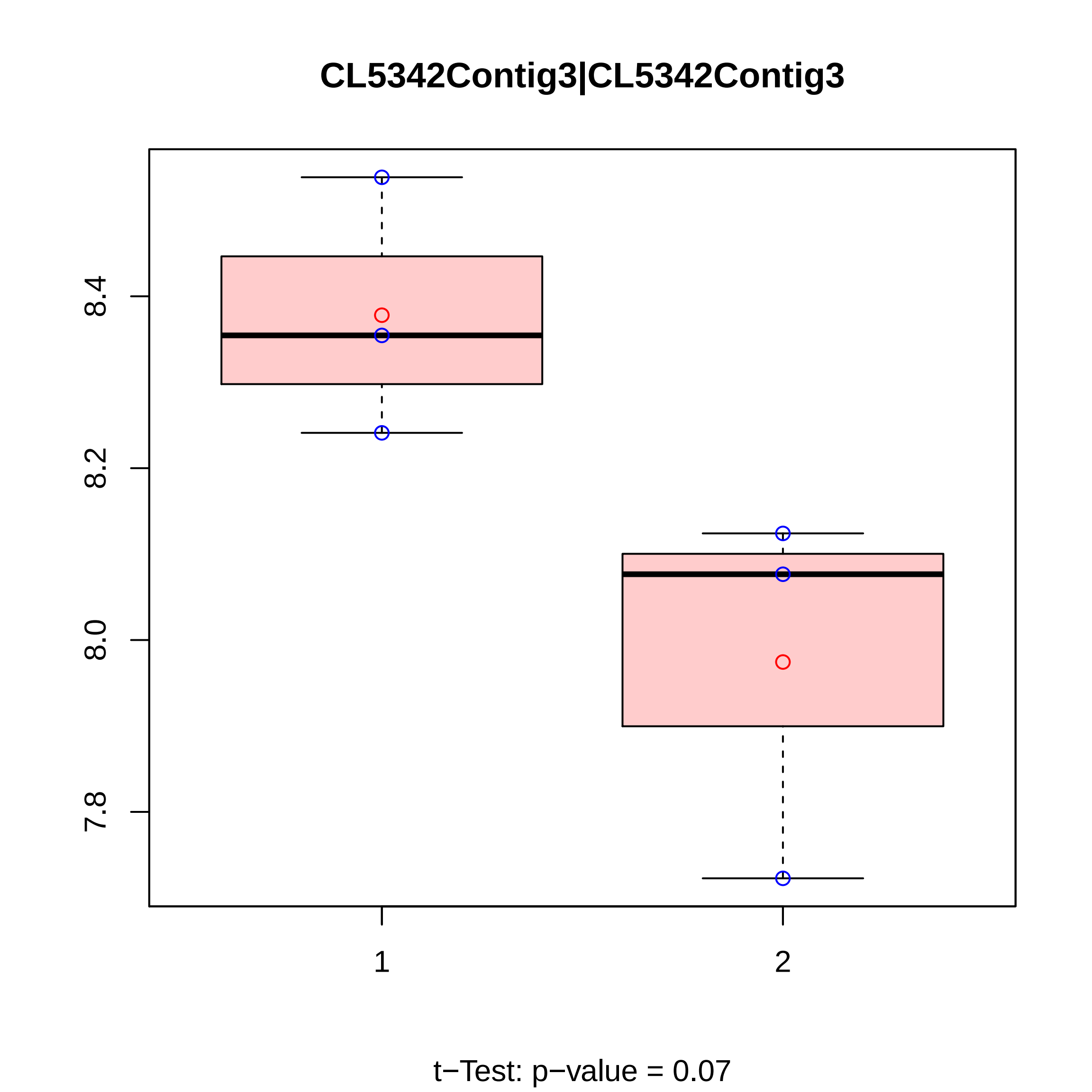
CL5342Contig3|CL5342Contig3
8.4
8.2
8.0
7.8
1
2
t−Test: p−value = 0.07

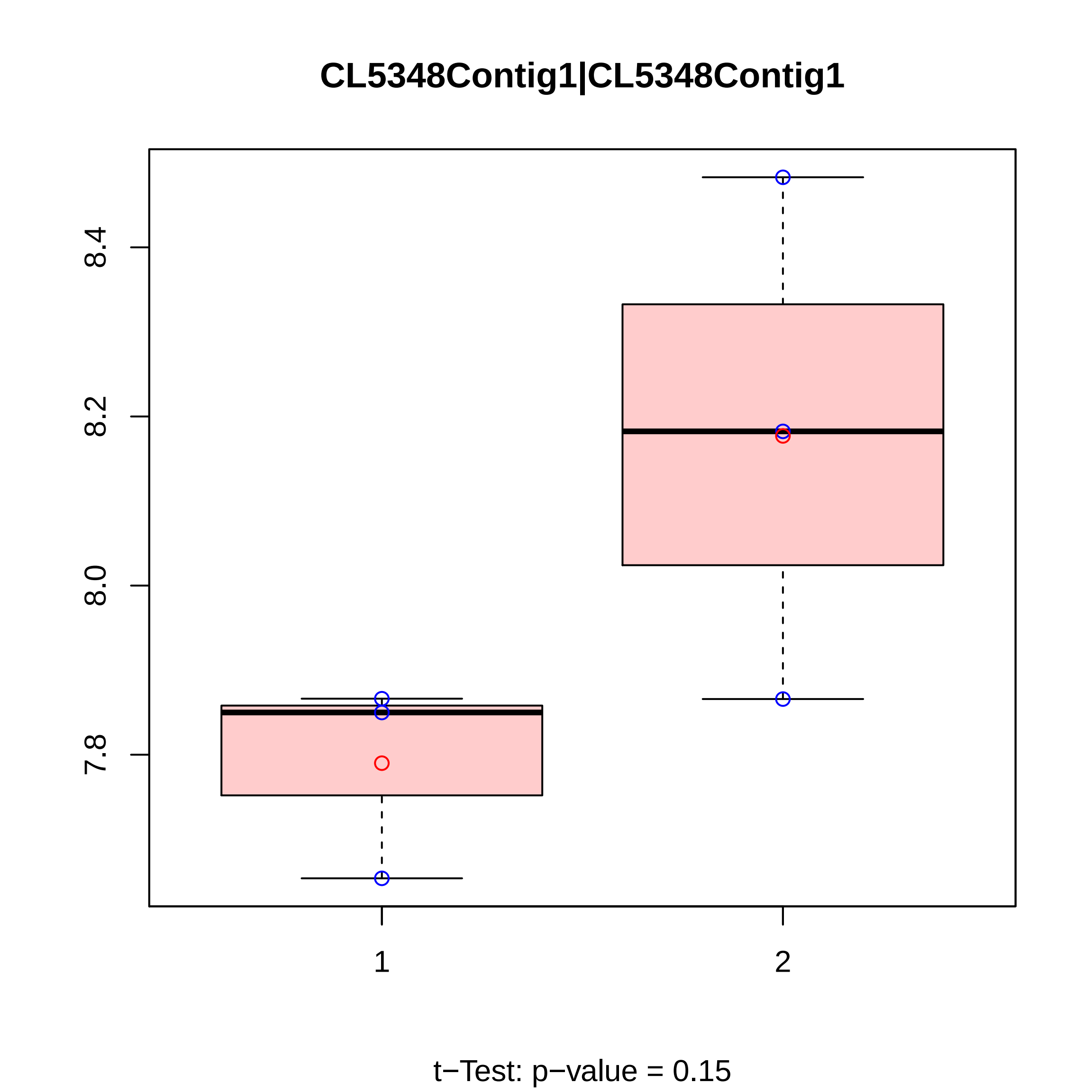

CL5348Contig1|CL5348Contig1
8.4
8.2
8.0
7.8
1
2
t−Test: p−value = 0.15

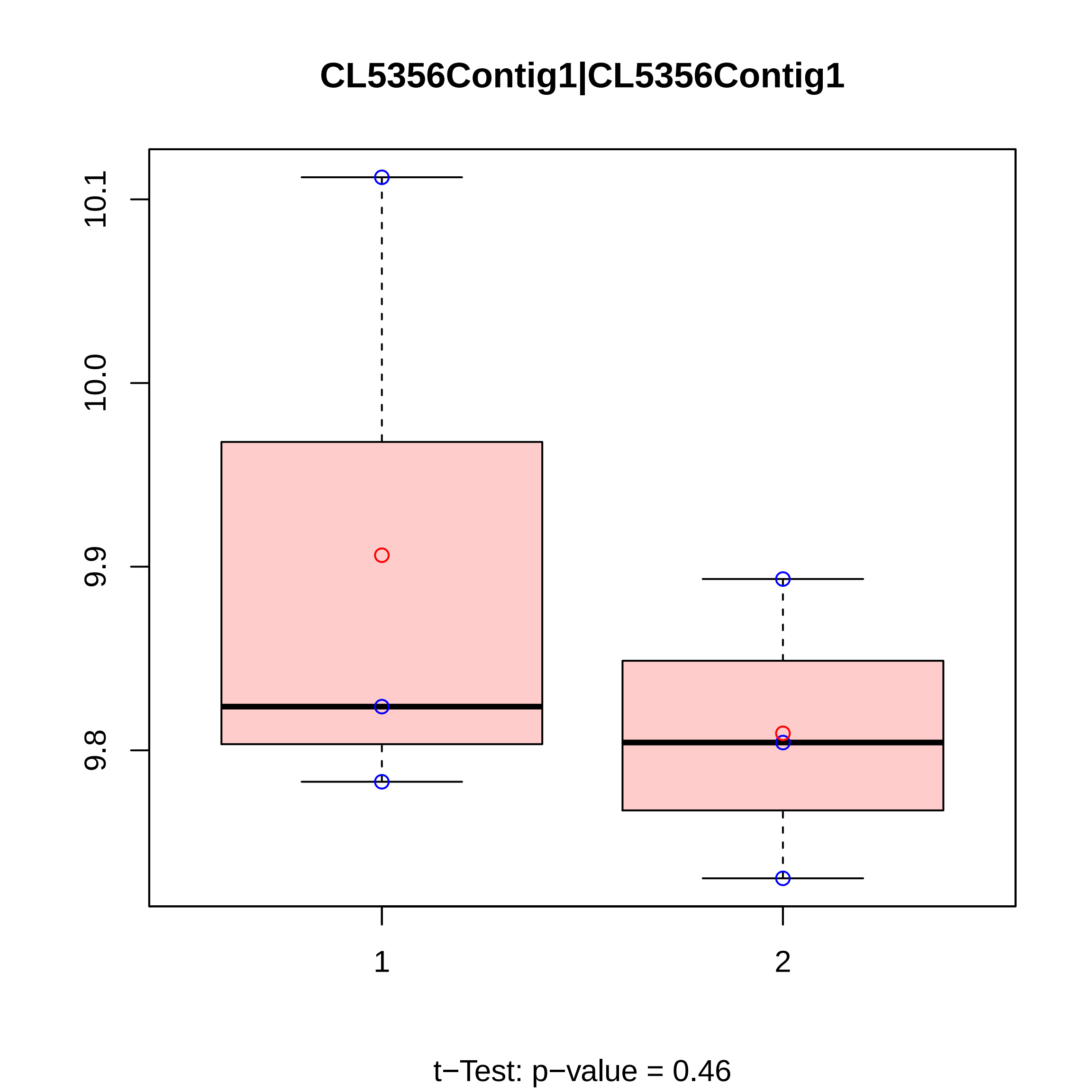

CL5356Contig1|CL5356Contig1
10.1
10.0
9.9
9.8
1
2
t−Test: p−value = 0.46

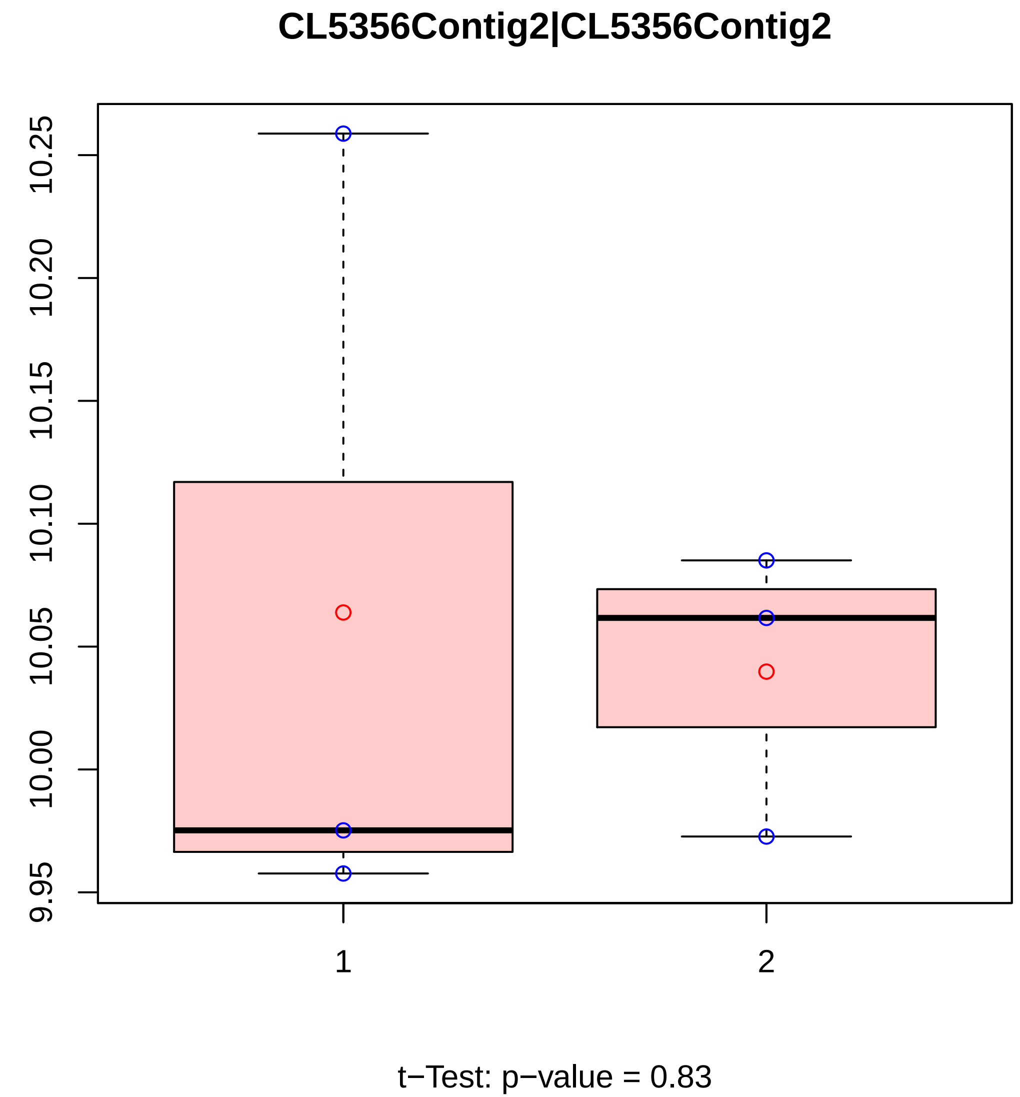
CL5356Contig2|CL5356Contig2
10.25
10.20
10.15
10.10
10.05
10.00
9.95
1
2
t−Test: p−value = 0.83

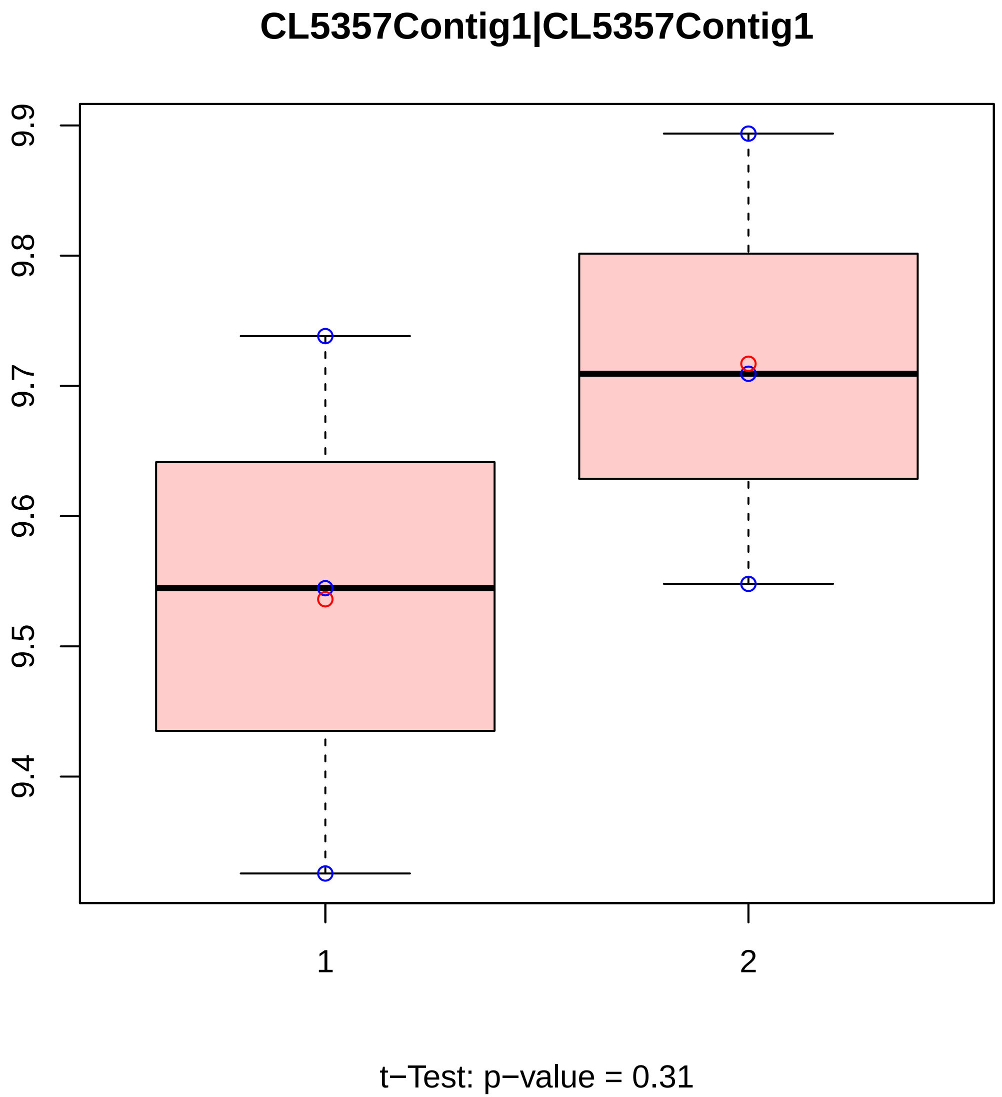
CL5357Contig1|CL5357Contig1
9.9
9.8
9.7
9.6
9.5
9.4
1
2
t−Test: p−value = 0.31

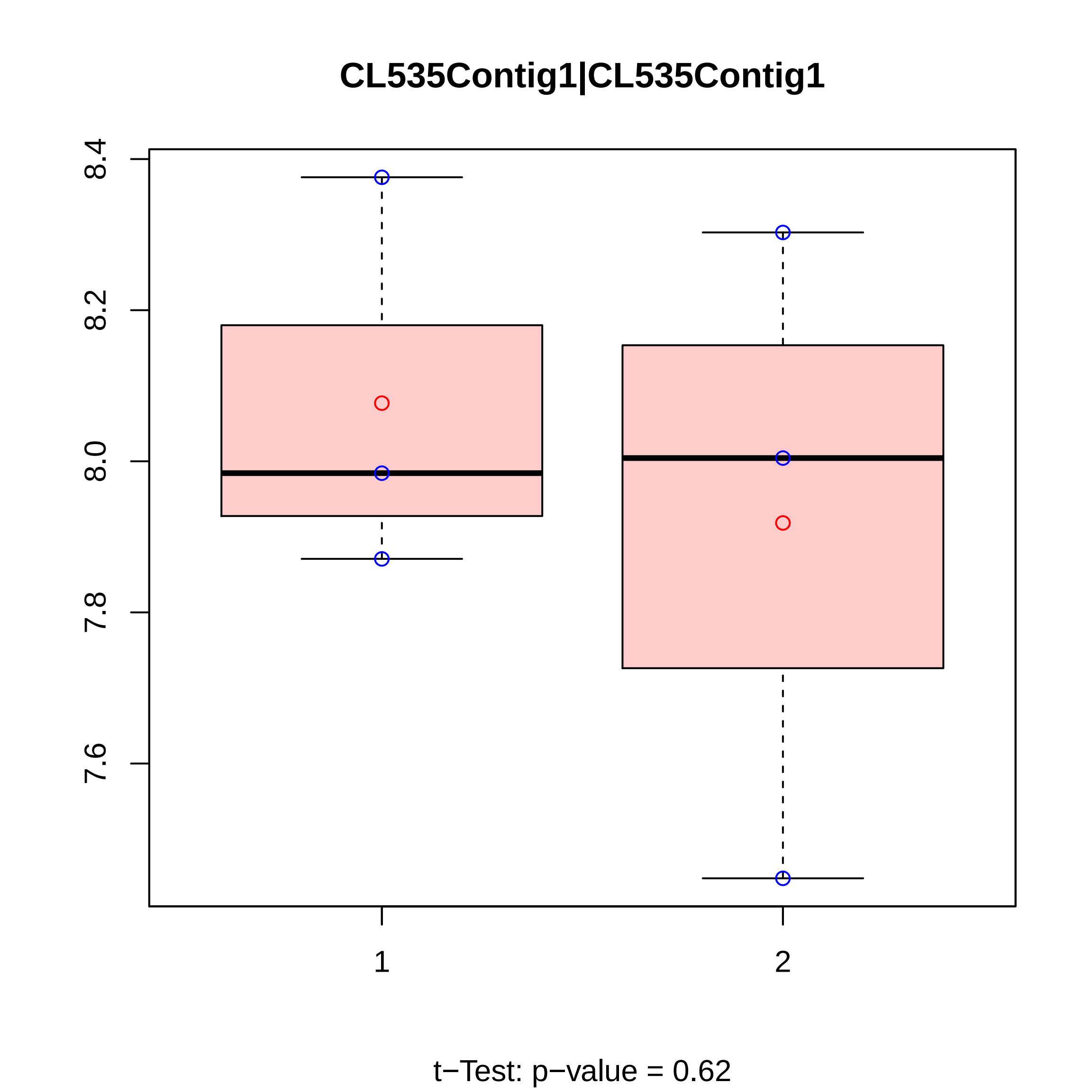

CL535Contig1|CL535Contig1
8.4
8.2
8.0
7.8
7.6
1
2
t−Test: p−value = 0.62

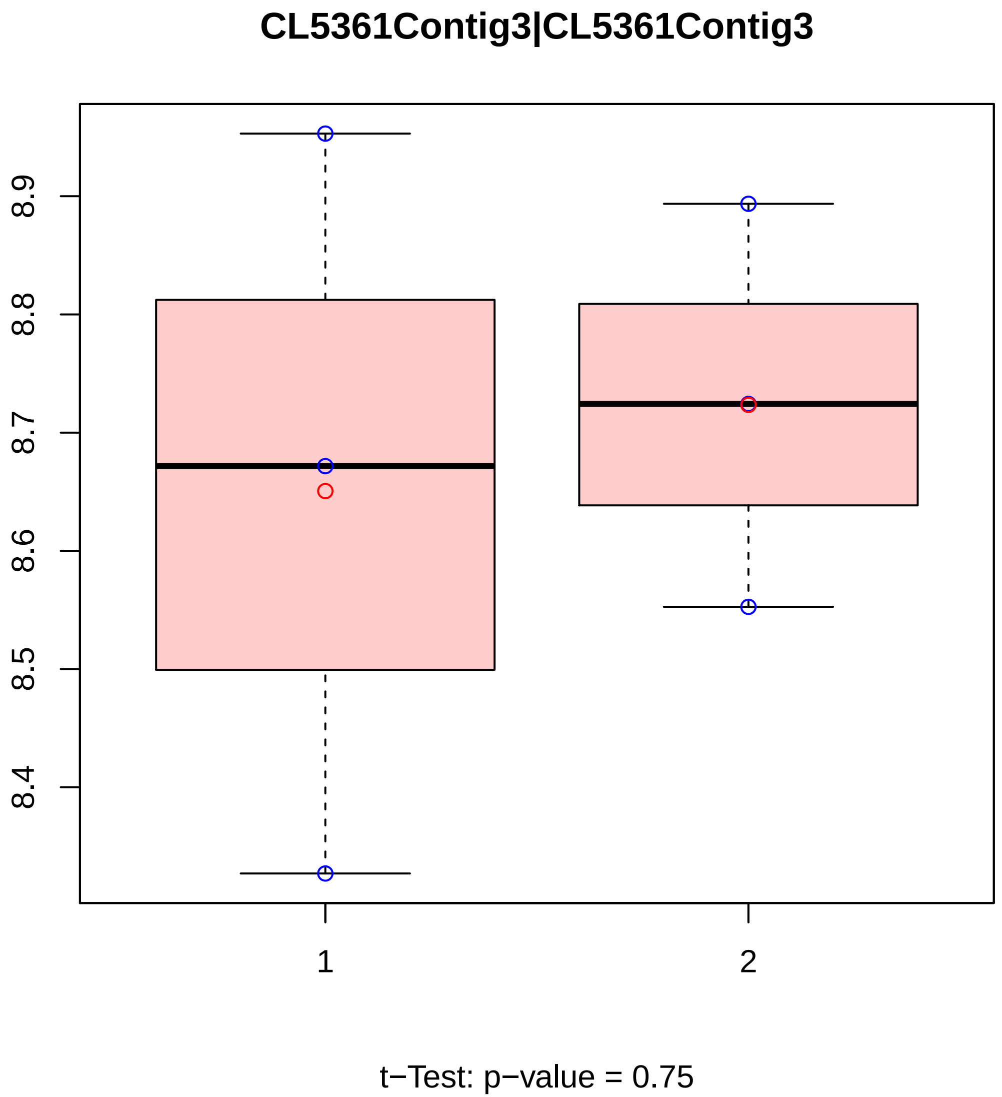
CL5361Contig3|CL5361Contig3
8.4
8.5
8.6
8.7
8.8
8.9
1
2
t−Test: p−value = 0.75

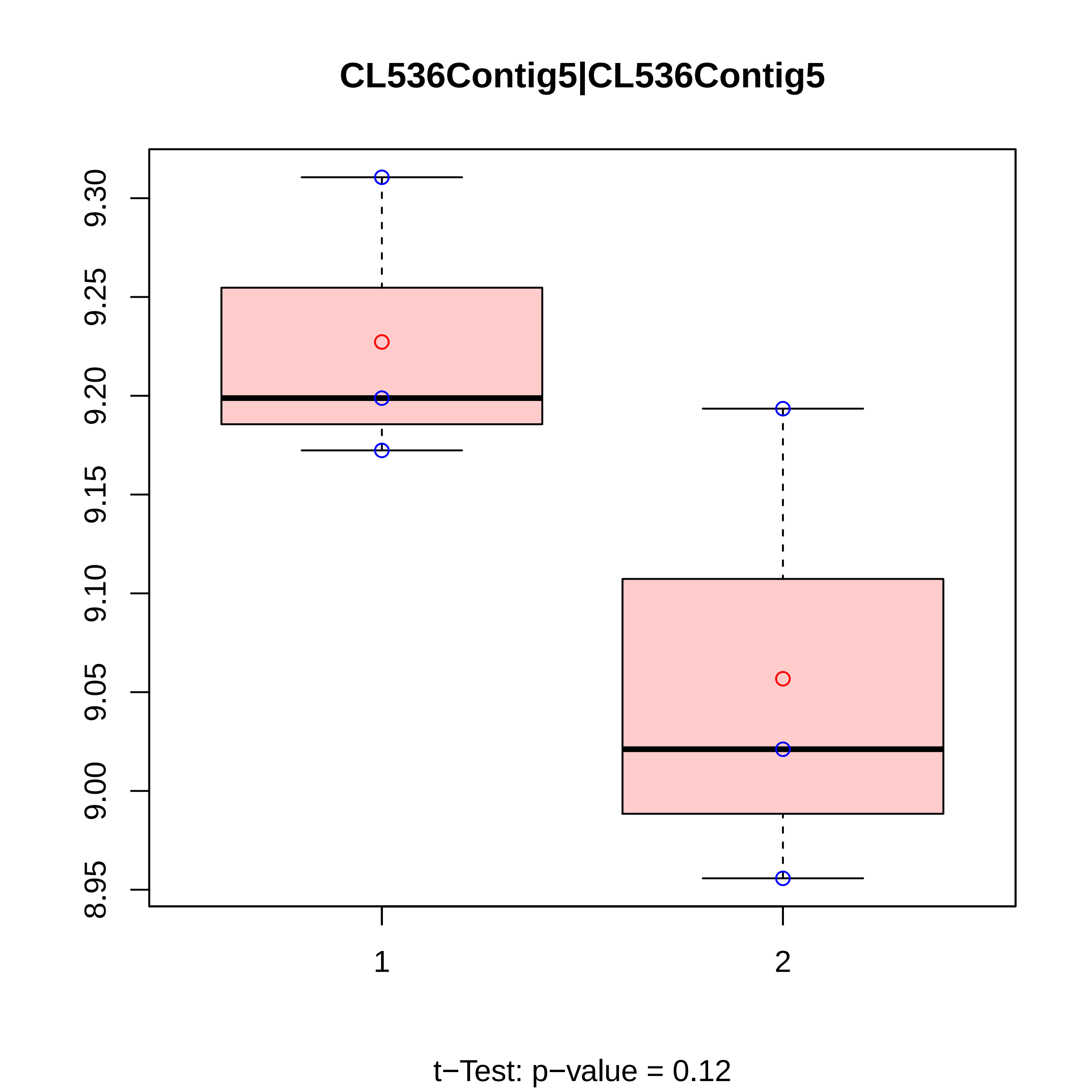
CL536Contig5|CL536Contig5
9.30
9.25
9.20
9.15
9.10
9.05
9.00
8.95
1
2
t−Test: p−value = 0.12

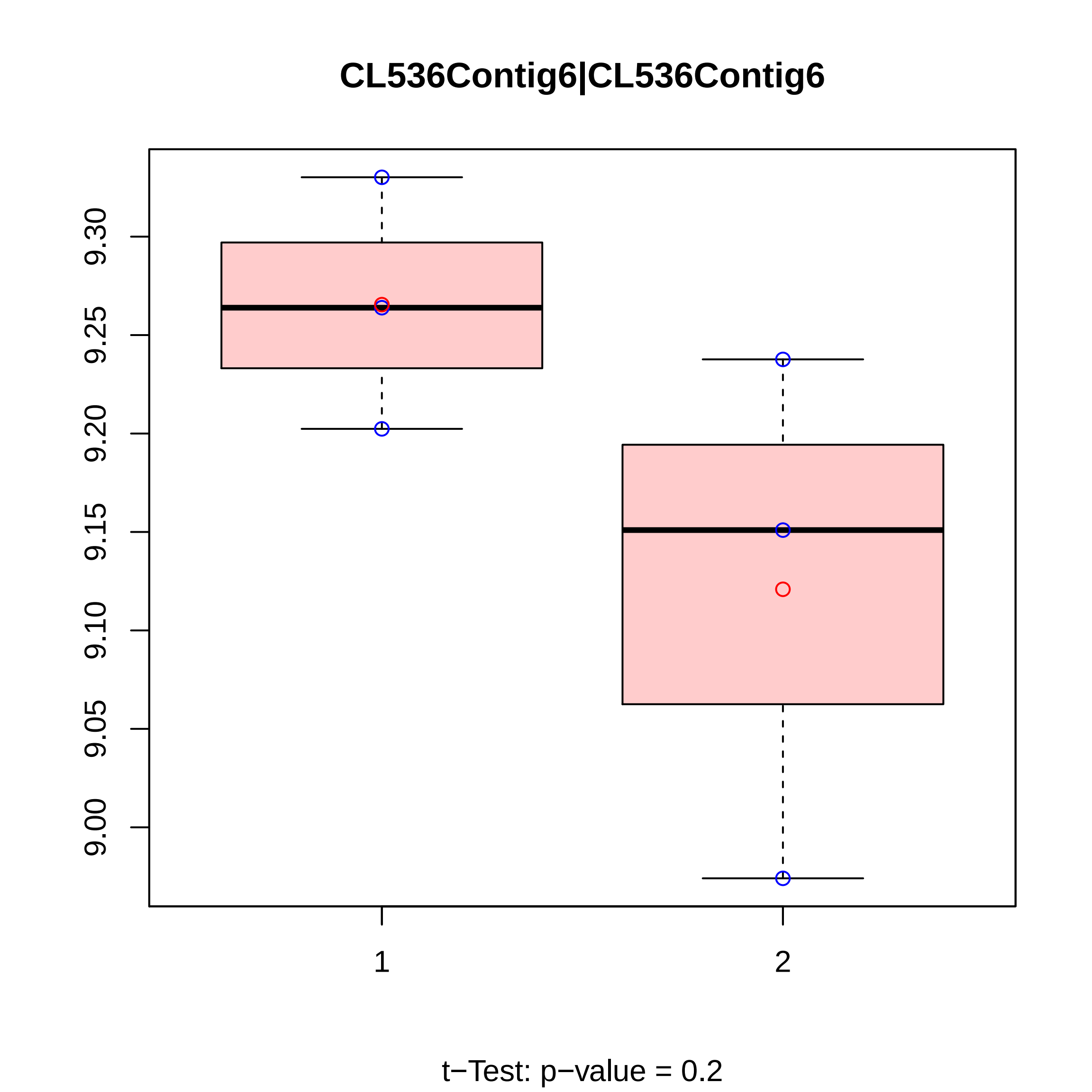

CL536Contig6|CL536Contig6
9.00
9.05
9.10
9.15
9.20
9.25
9.30
1
2
t−Test: p−value = 0.2

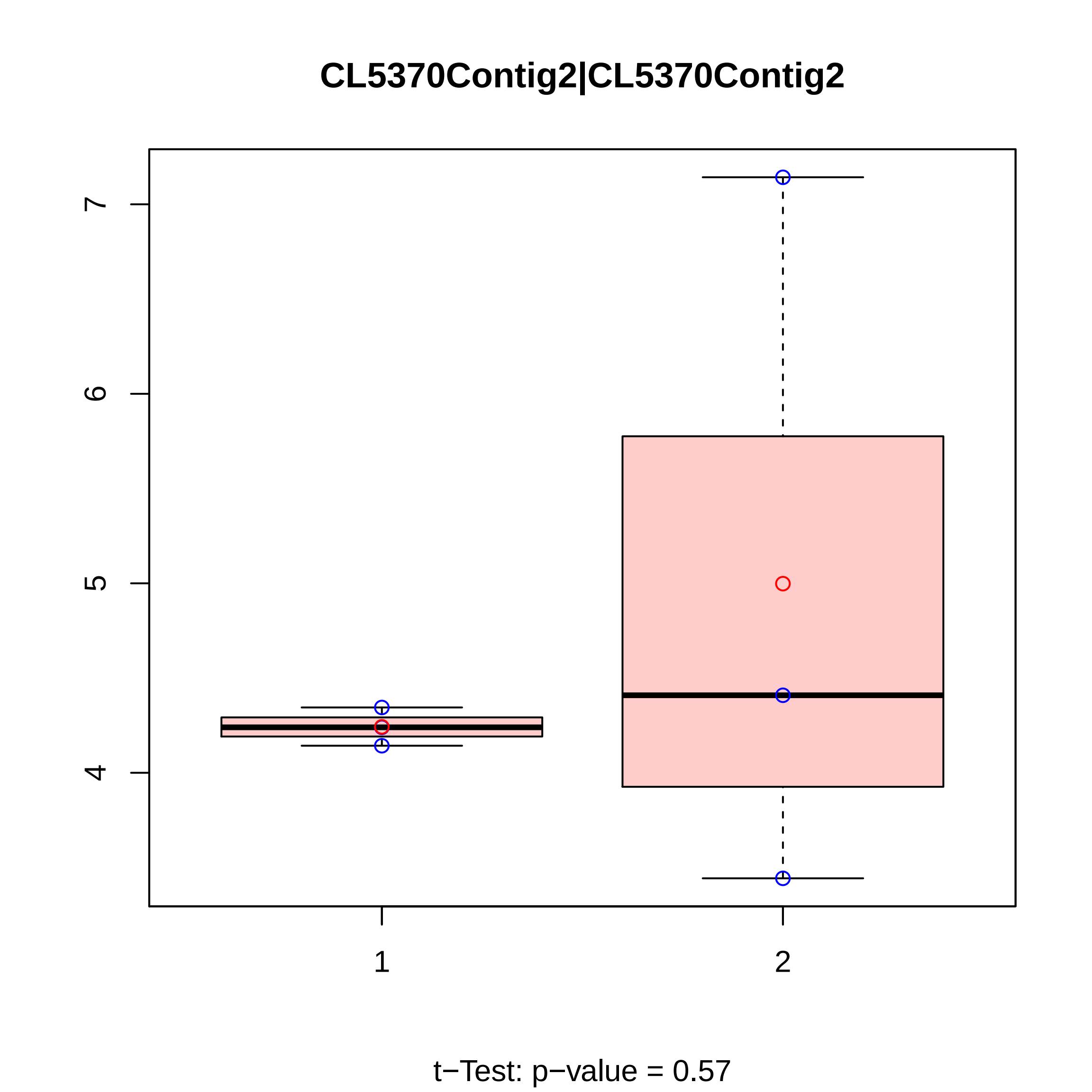

CL5370Contig2|CL5370Contig2
7
6
5
4
1
2
t−Test: p−value = 0.57

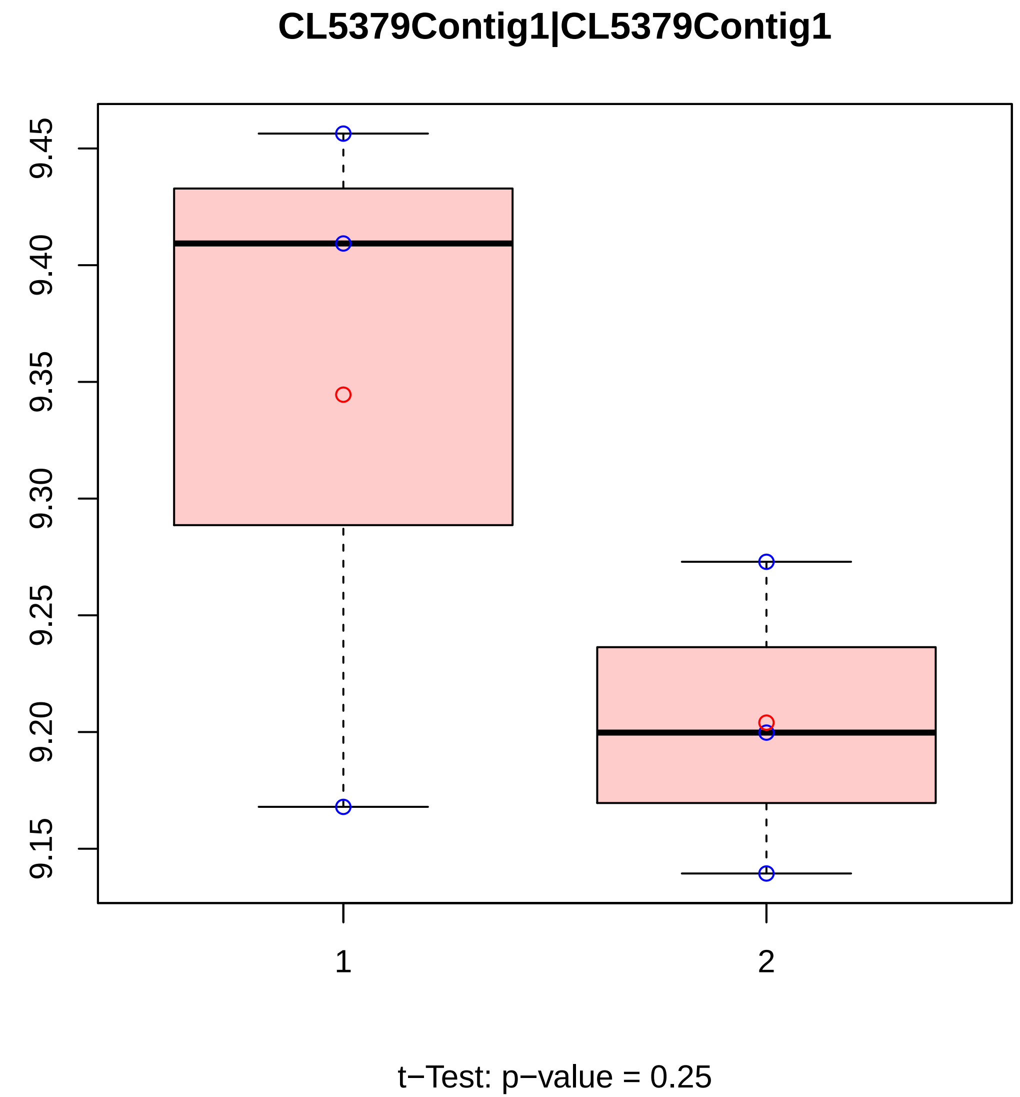
CL5379Contig1|CL5379Contig1
9.45
9.40
9.35
9.30
9.25
9.20
9.15
1
2
t−Test: p−value = 0.25

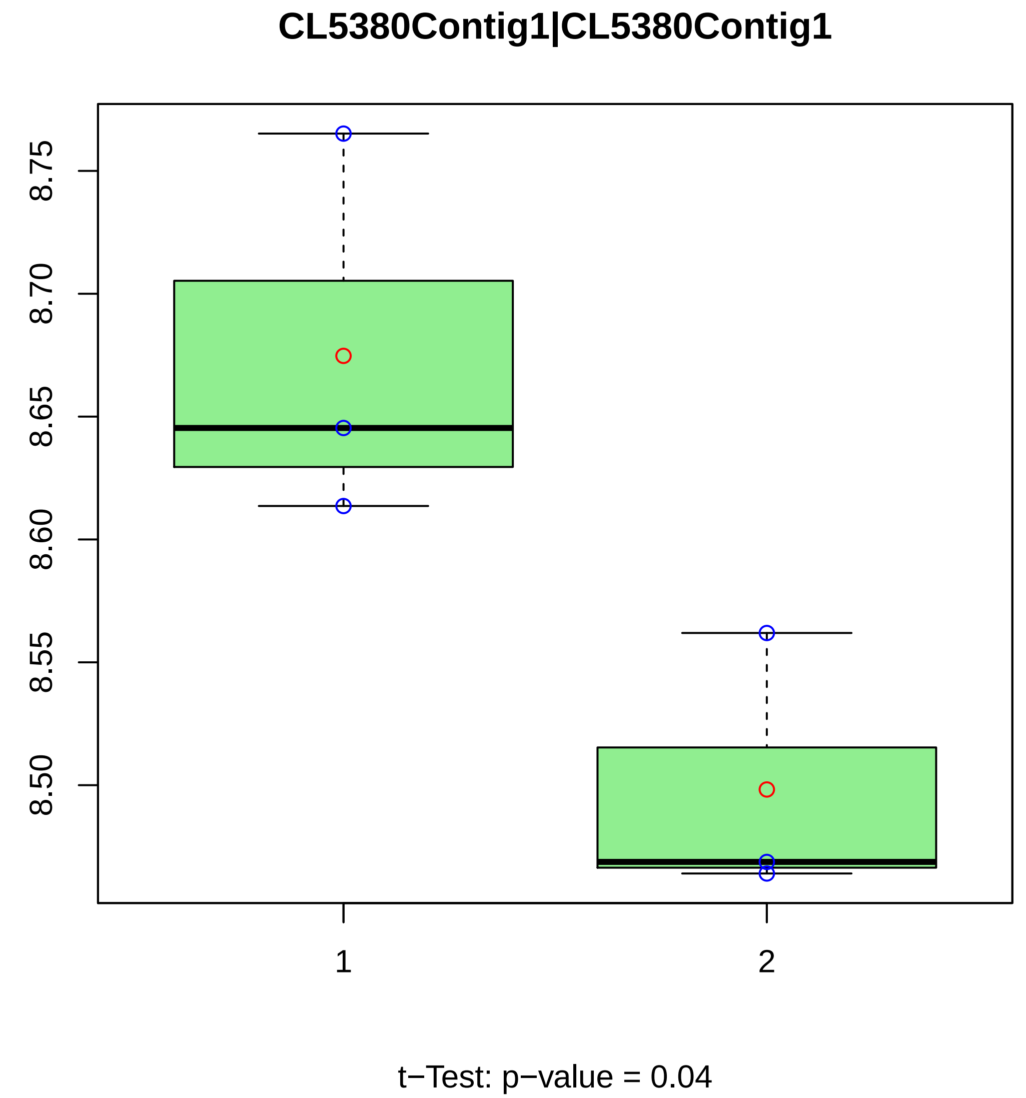
CL5380Contig1|CL5380Contig1
8.75
8.70
8.65
8.60
8.55
8.50
1
2
t−Test: p−value = 0.04

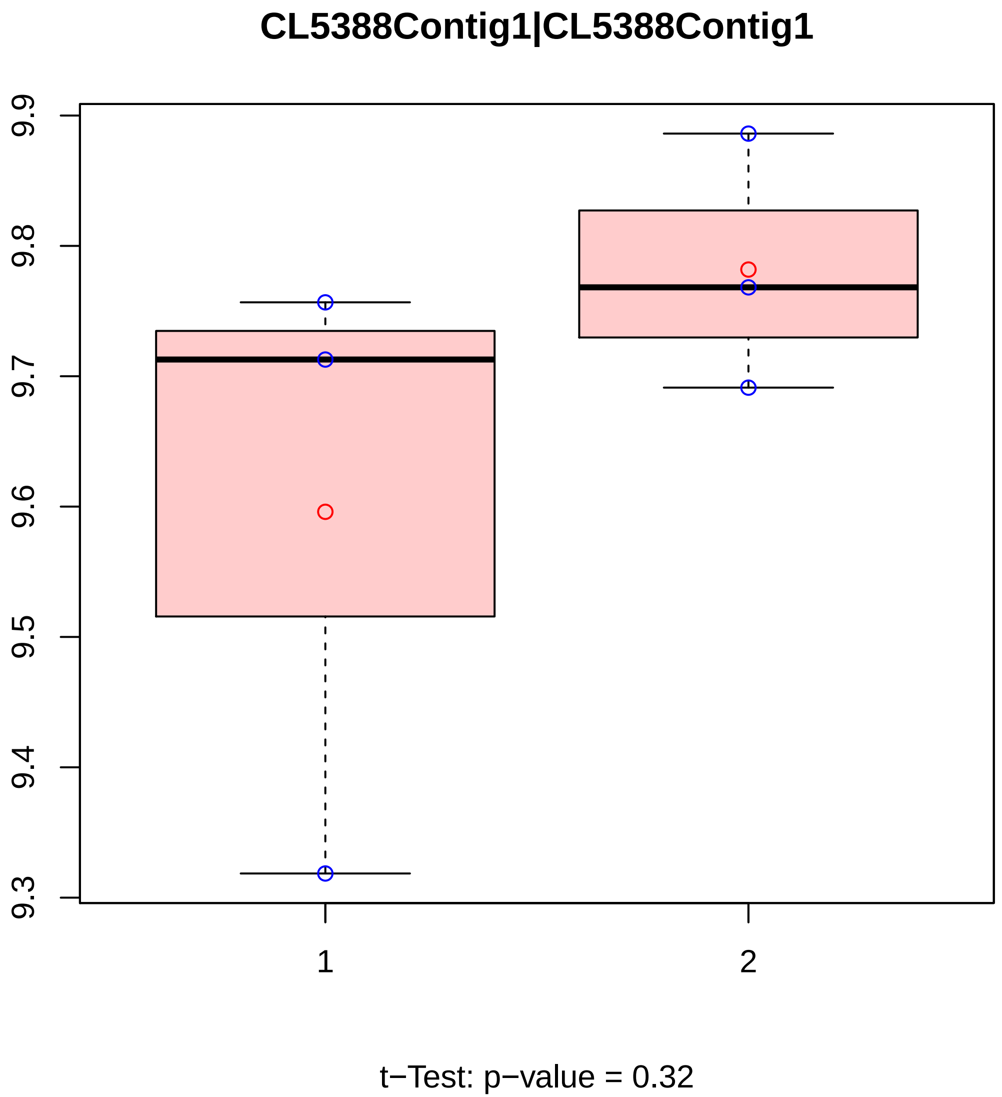

CL5388Contig1|CL5388Contig1
9.3
9.4
9.5
9.6
9.7
9.8
9.9
1
2
t−Test: p−value = 0.32

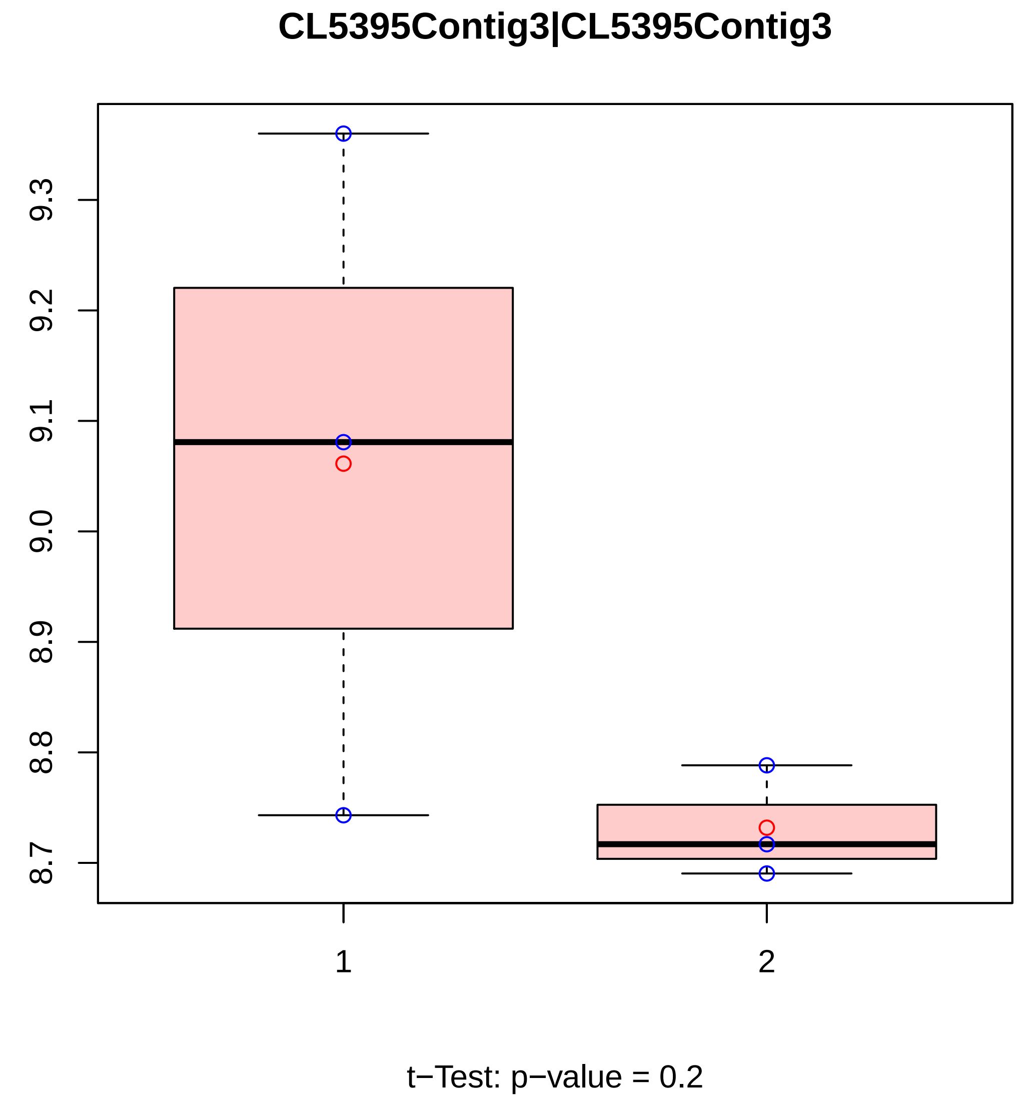
CL5395Contig3|CL5395Contig3
9.3
9.2
9.1
9.0
8.9
8.8
8.7
1
2
t−Test: p−value = 0.2

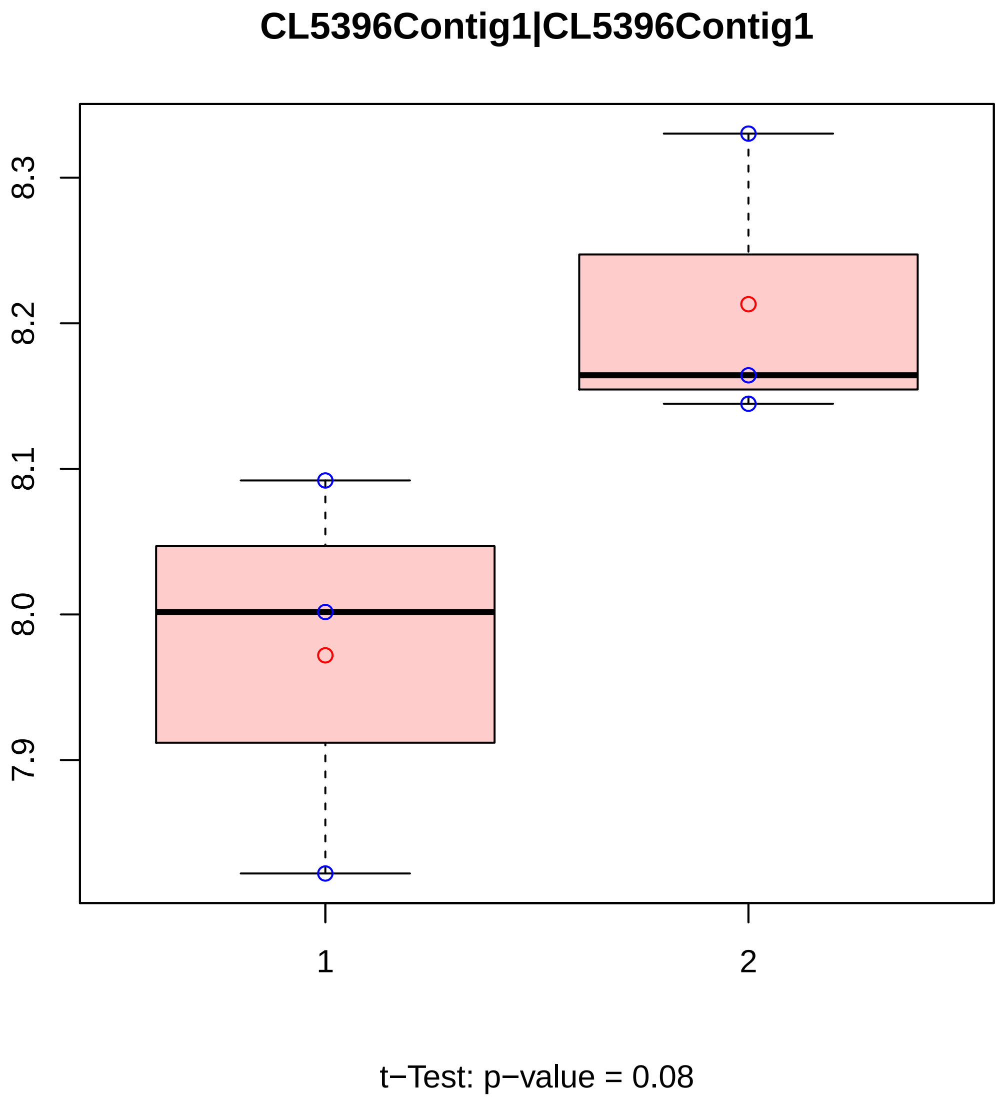
CL5396Contig1|CL5396Contig1
8.3
8.2
8.1
8.0
7.9
1
2
t−Test: p−value = 0.08

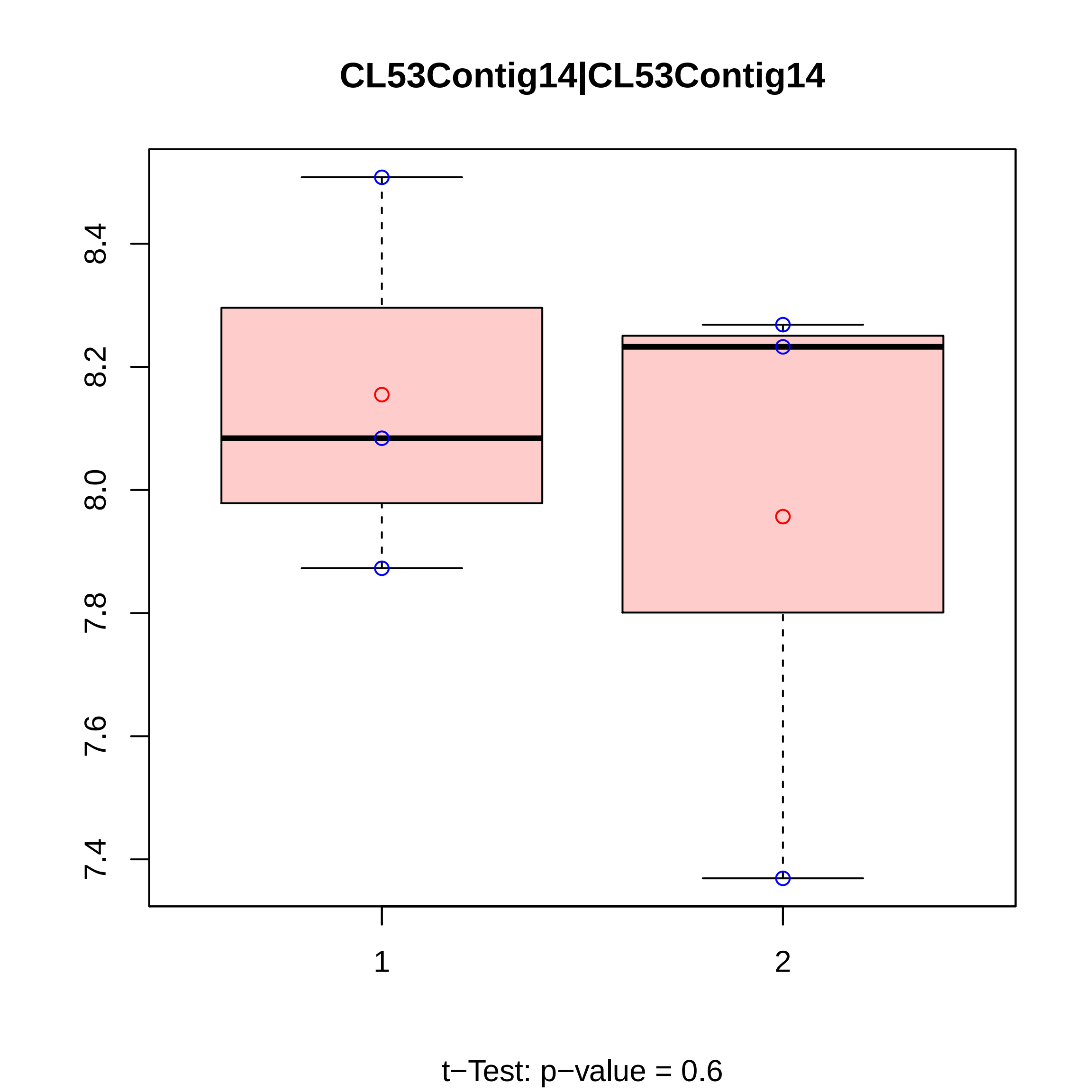

CL53Contig14|CL53Contig14
8.4
8.2
8.0
7.8
7.6
7.4
1
2
t−Test: p−value = 0.6

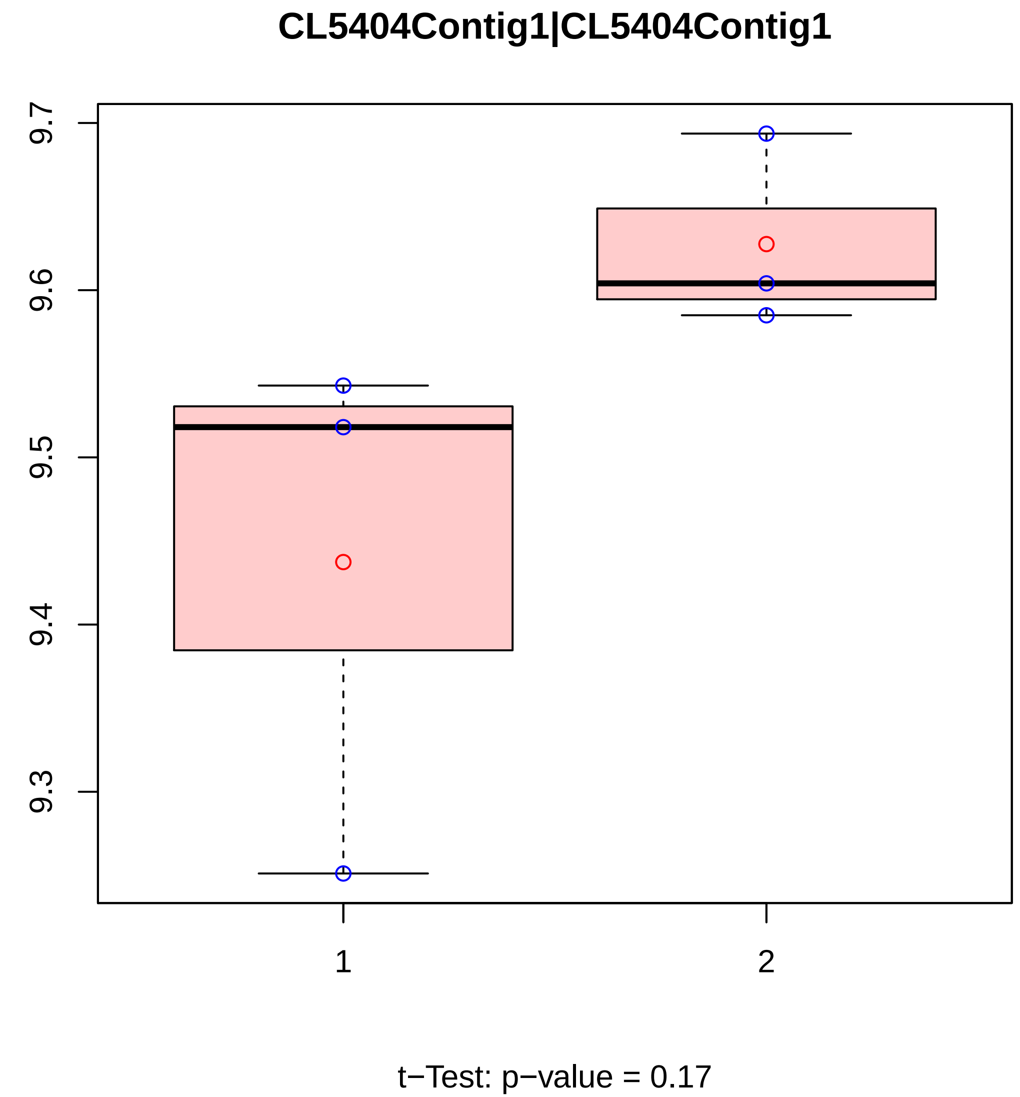
CL5404Contig1|CL5404Contig1
9.7
9.6
9.5
9.4
9.3
1
2
t−Test: p−value = 0.17

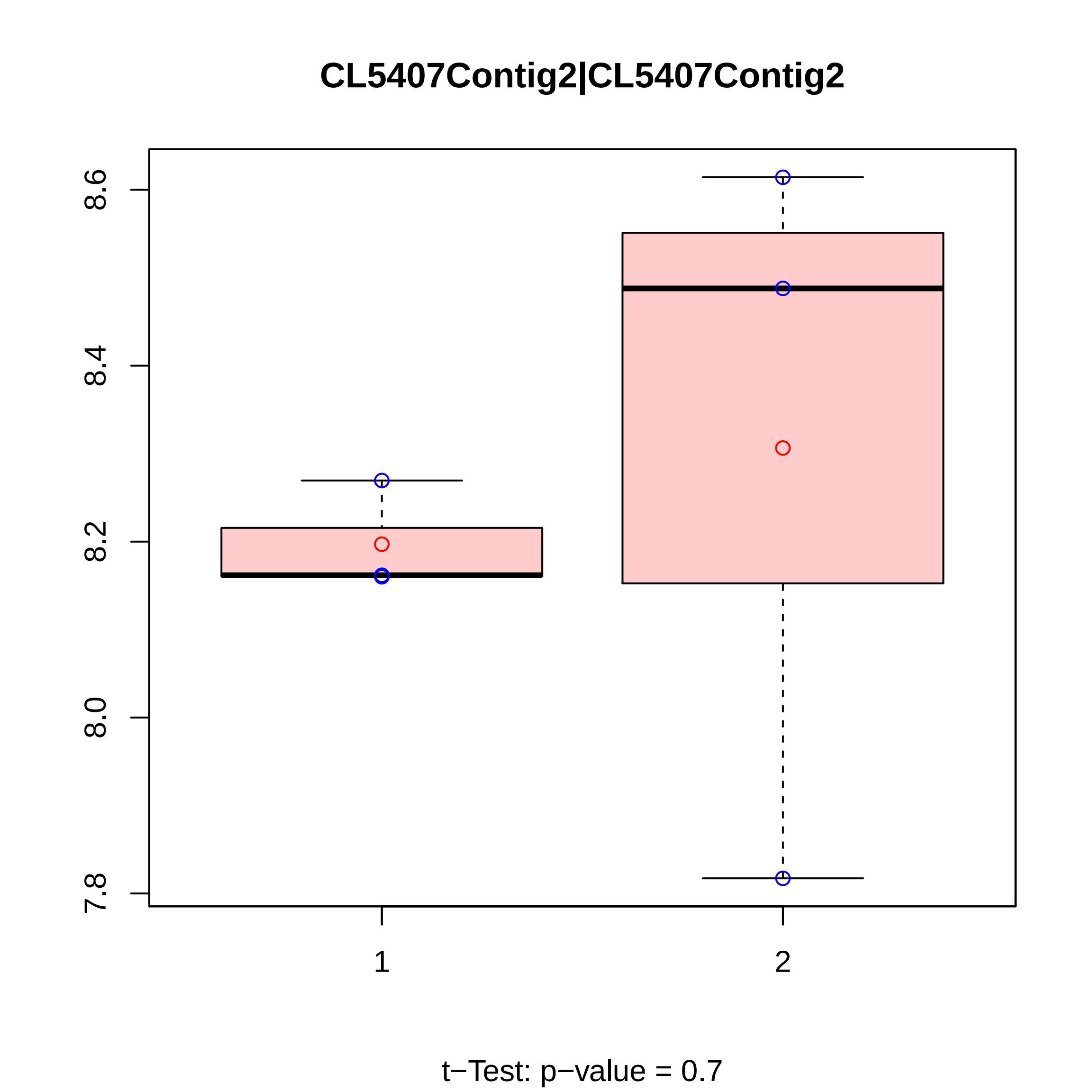
CL5407Contig2|CL5407Contig2
8.6
8.4
8.2
8.0
7.8
1
2
t−Test: p−value = 0.7

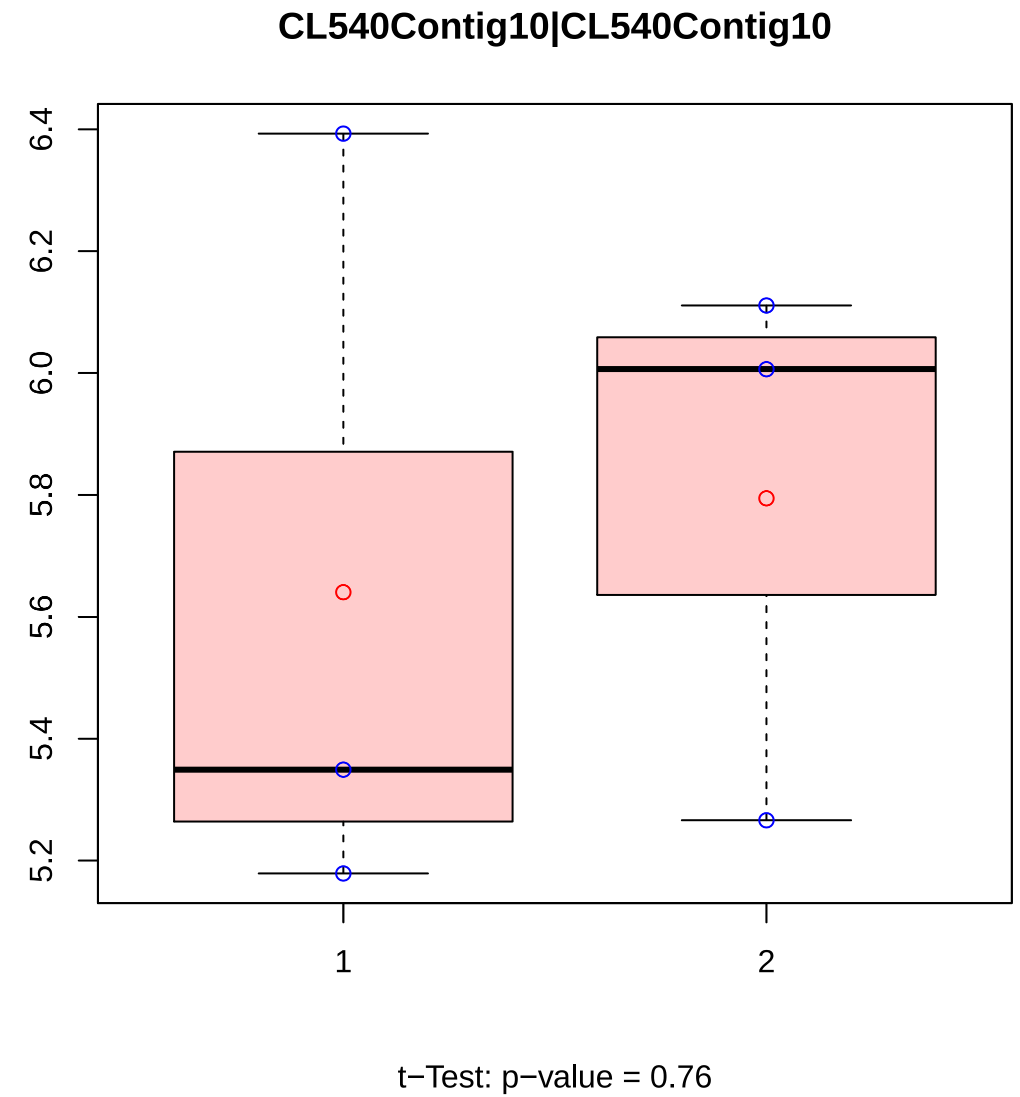

CL540Contig10|CL540Contig10
6.4
6.2
6.0
5.8
5.6
5.4
5.2
1
2
t−Test: p−value = 0.76

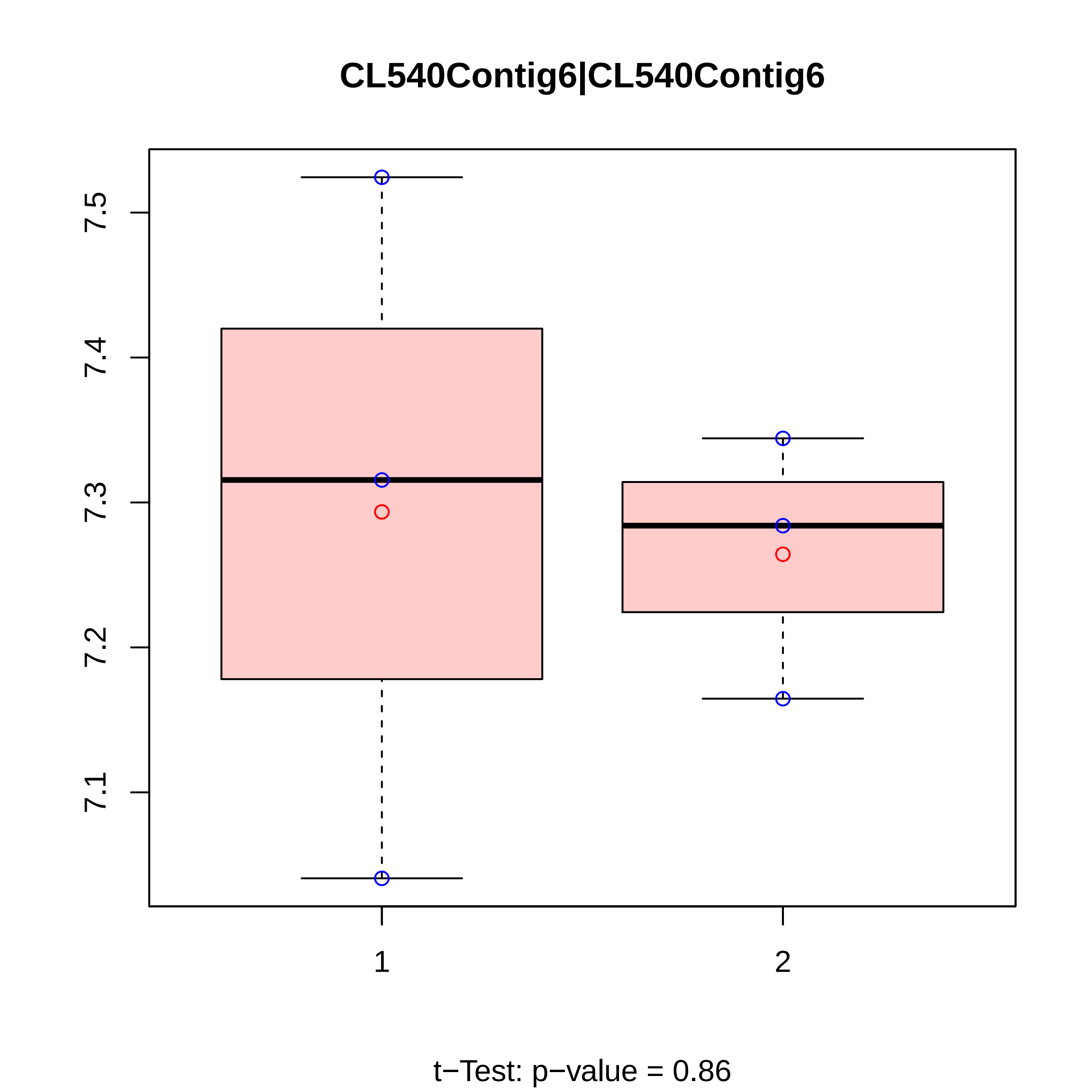
CL540Contig6|CL540Contig6
7.1
7.2
7.3
7.4
7.5
1
2
t−Test: p−value = 0.86

# CL5413Contig2|CL5413Contig2

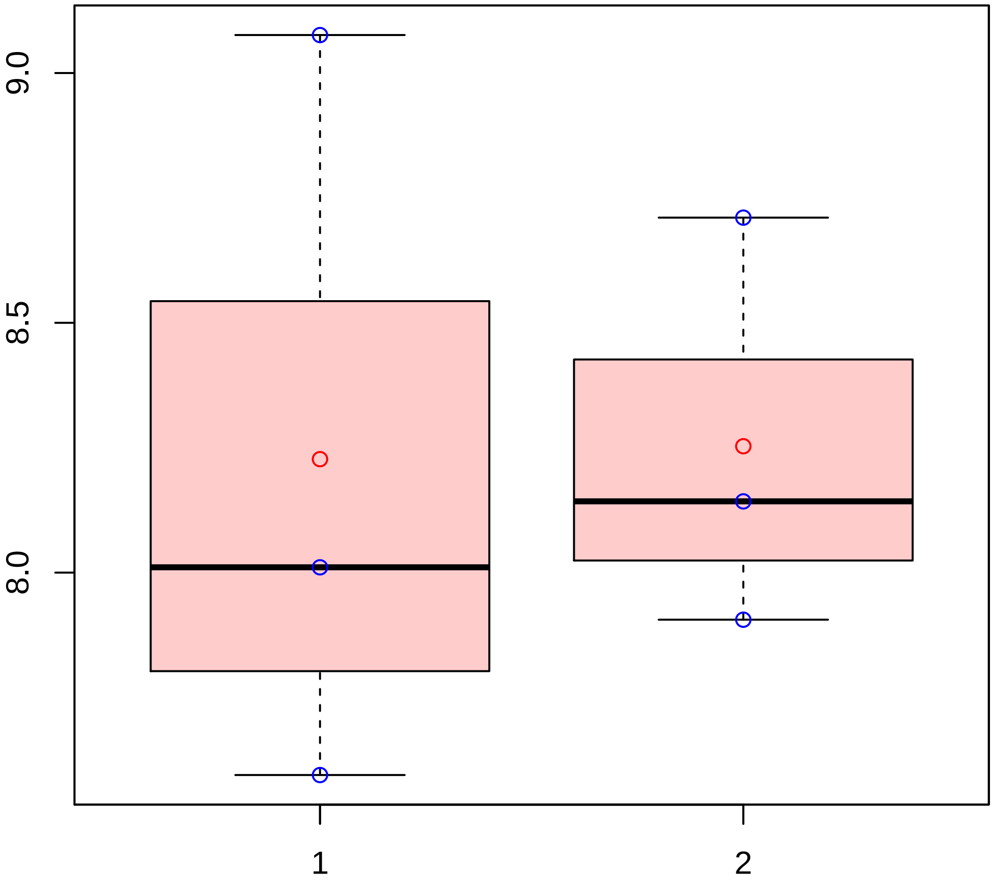


t−Test: p−value = 0.96

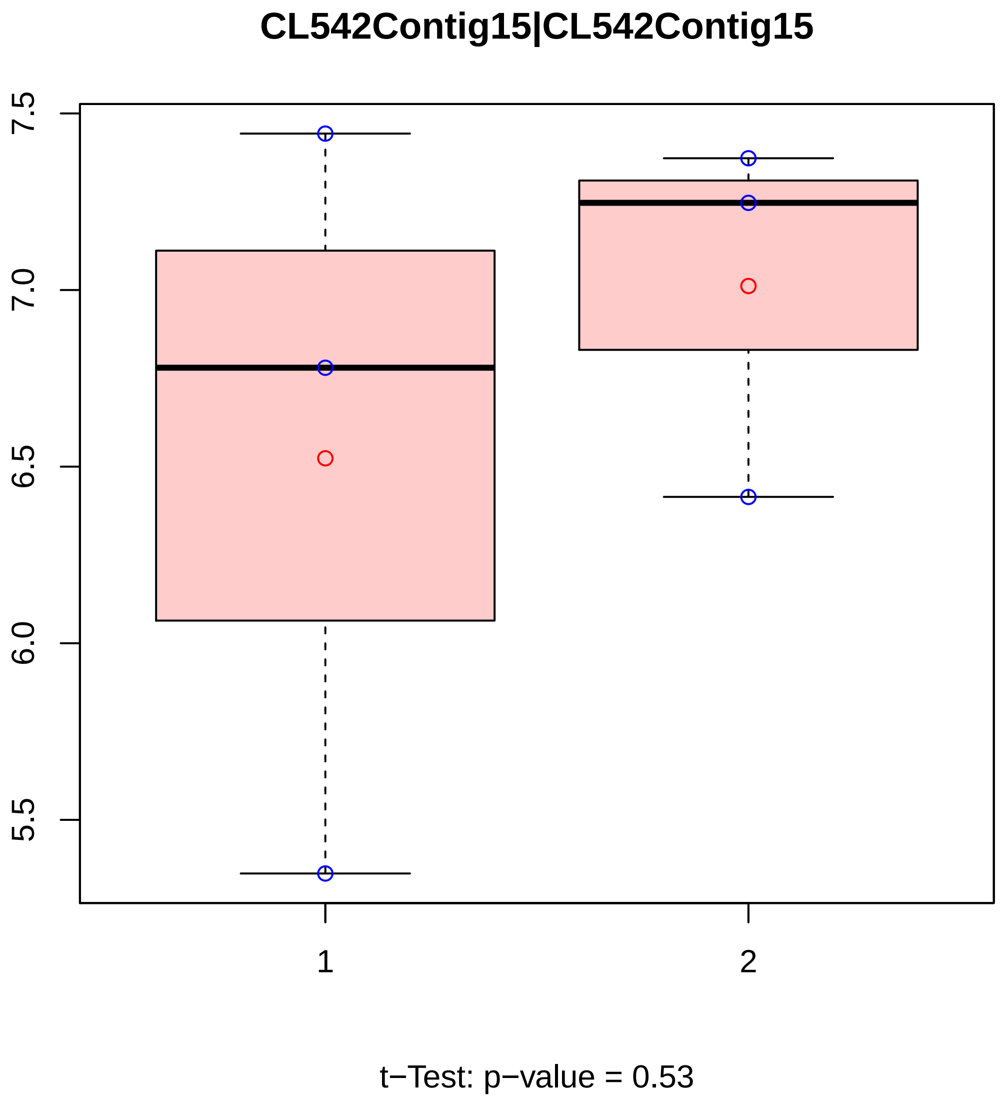
CL542Contig15|CL542Contig15
7.5
7.0
6.5
6.0
5.5
1
2
t−Test: p−value = 0.53

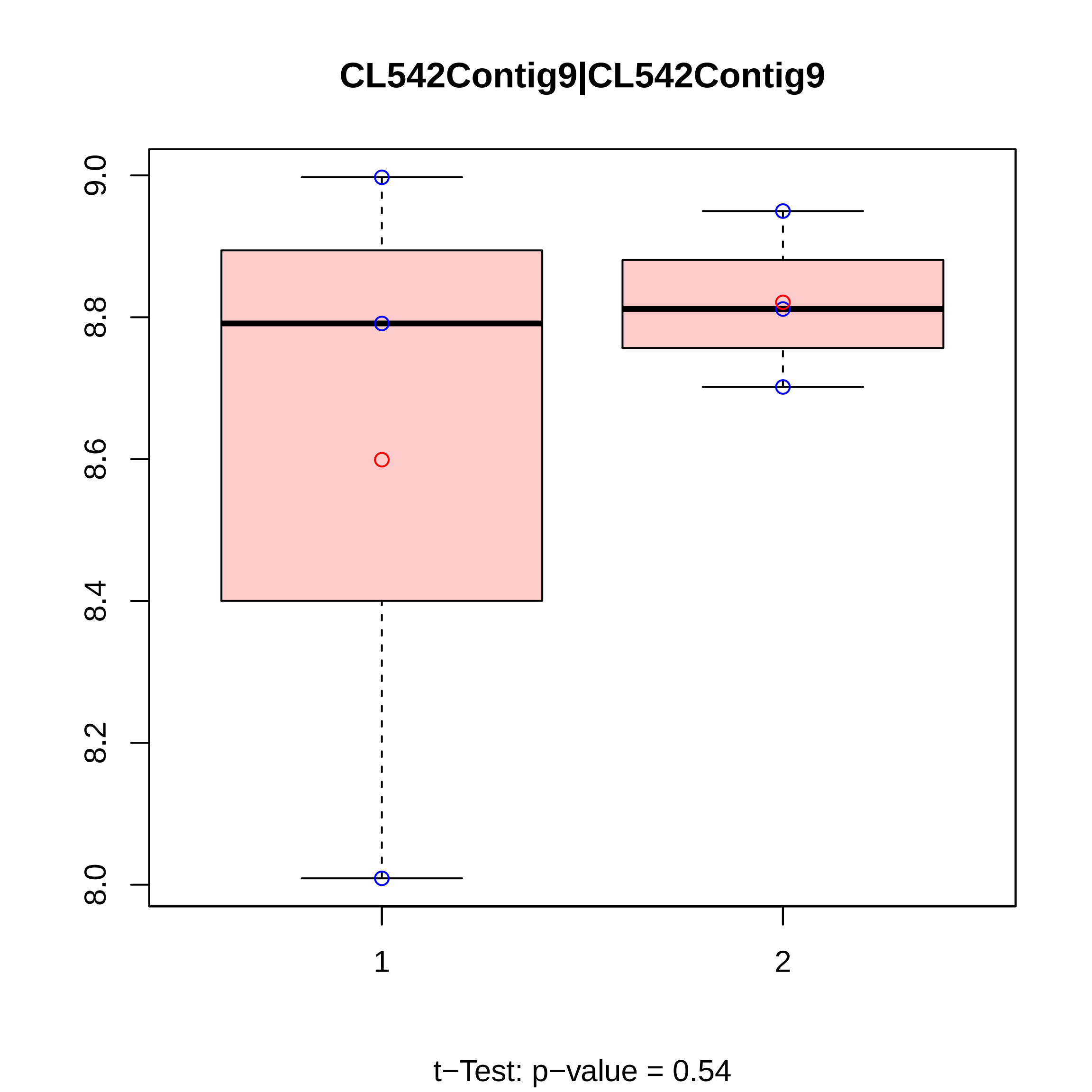

CL542Contig9|CL542Contig9
9.0
8.8
8.6
8.4
8.2
8.0
1
2
t−Test: p−value = 0.54

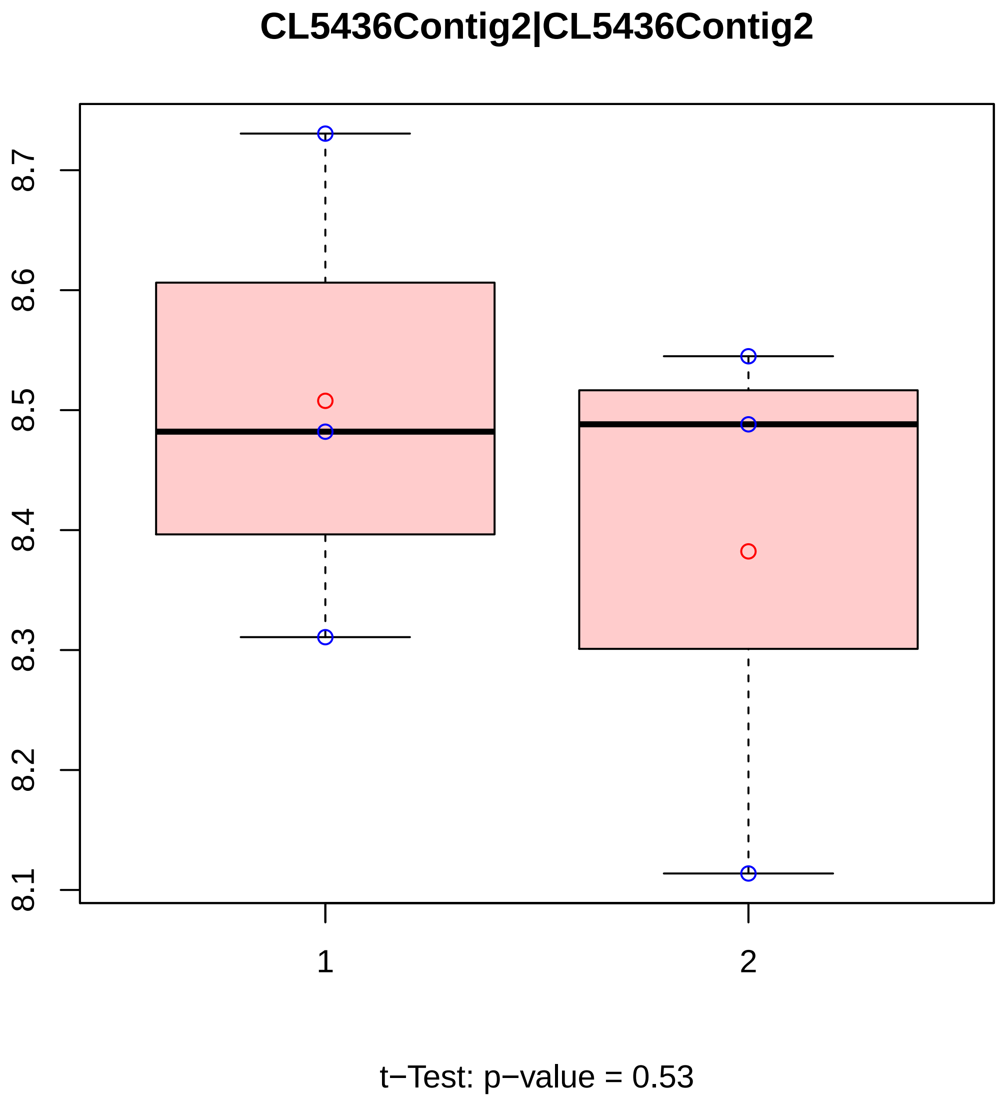

CL5436Contig2|CL5436Contig2
8.1
8.2
8.3
8.4
8.5
8.6
8.7
1
2
t−Test: p−value = 0.53

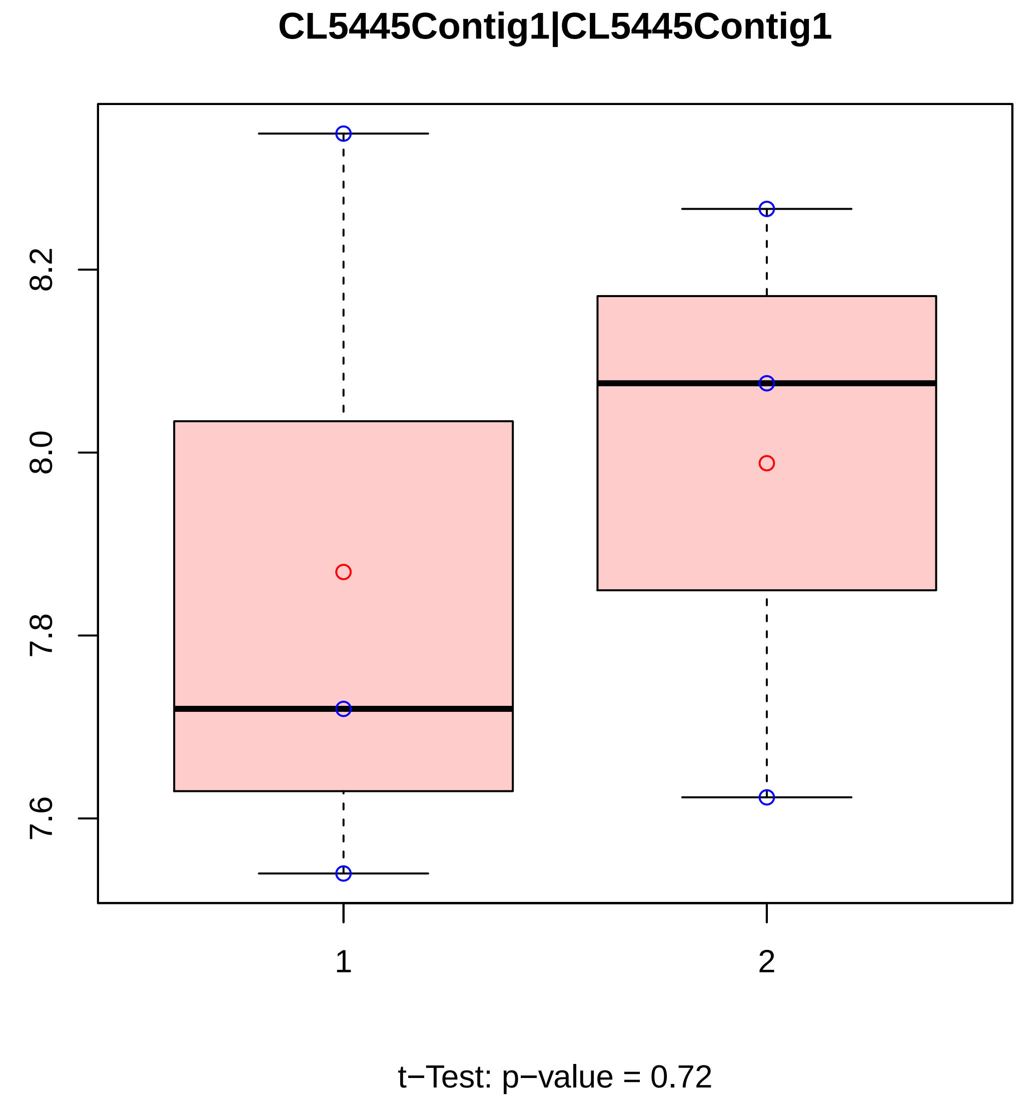

CL5445Contig1|CL5445Contig1
8.2
8.0
7.8
7.6
1
2
t−Test: p−value = 0.72

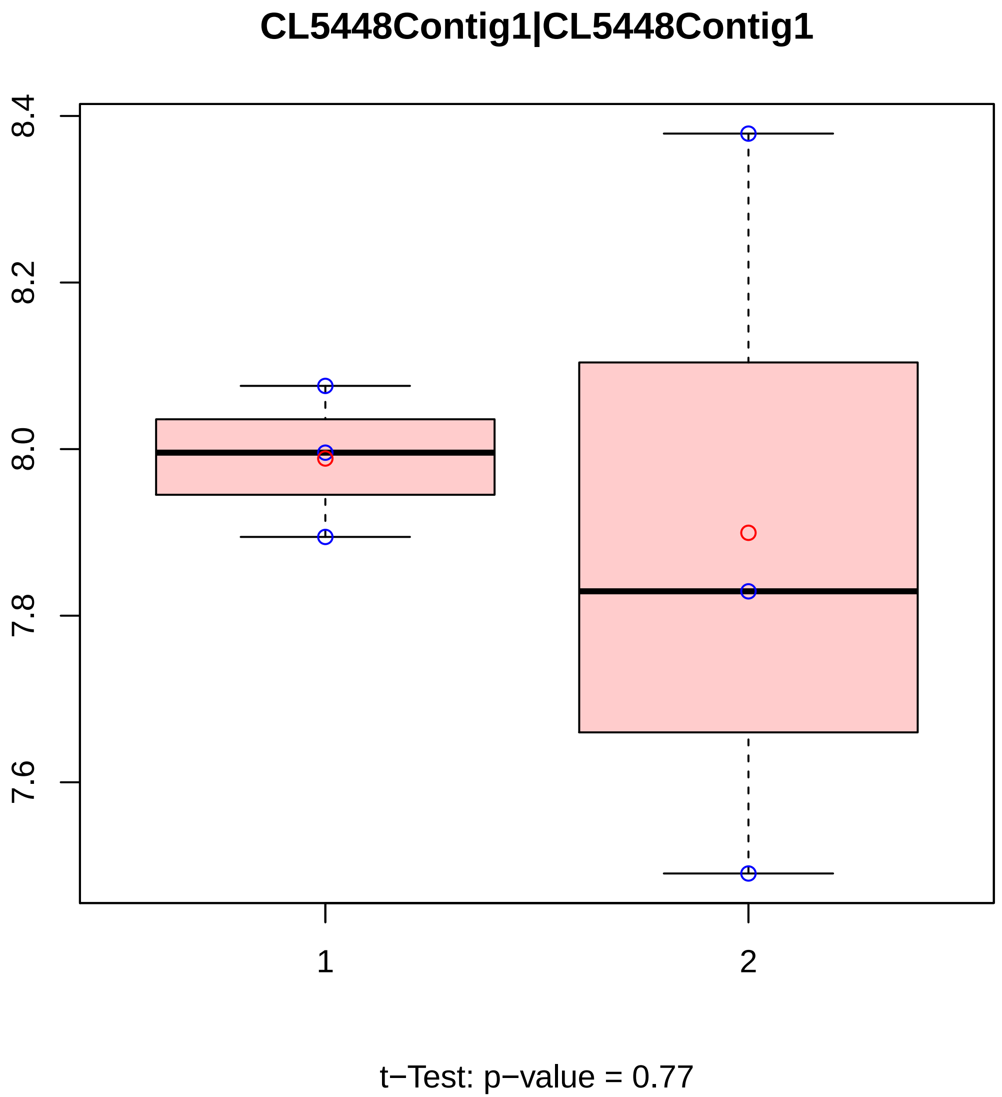
CL5448Contig1|CL5448Contig1
8.4
8.2
8.0
7.8
7.6
1
2
t−Test: p−value = 0.77

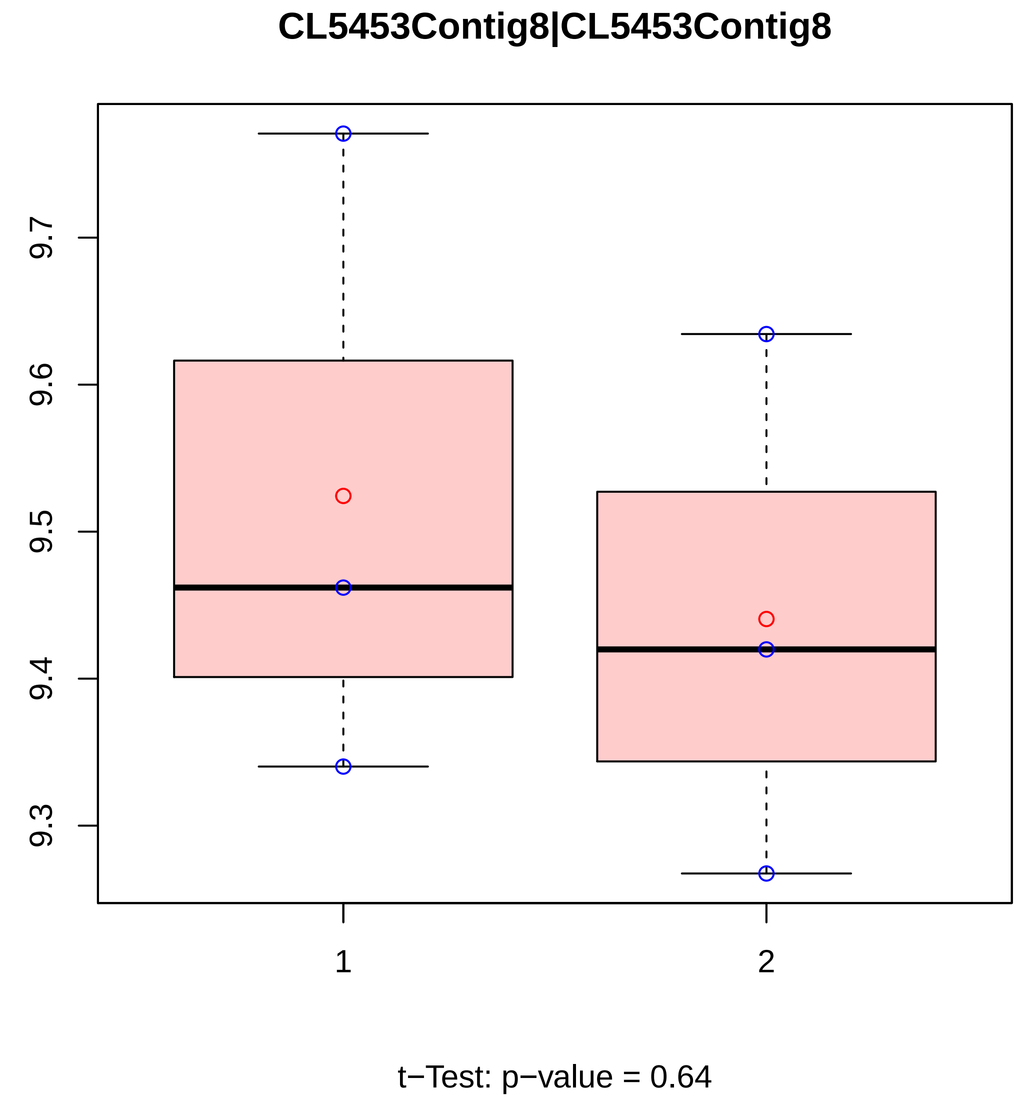
CL5453Contig8|CL5453Contig8
9.3
9.4
9.5
9.6
9.7
1
2
t−Test: p−value = 0.64

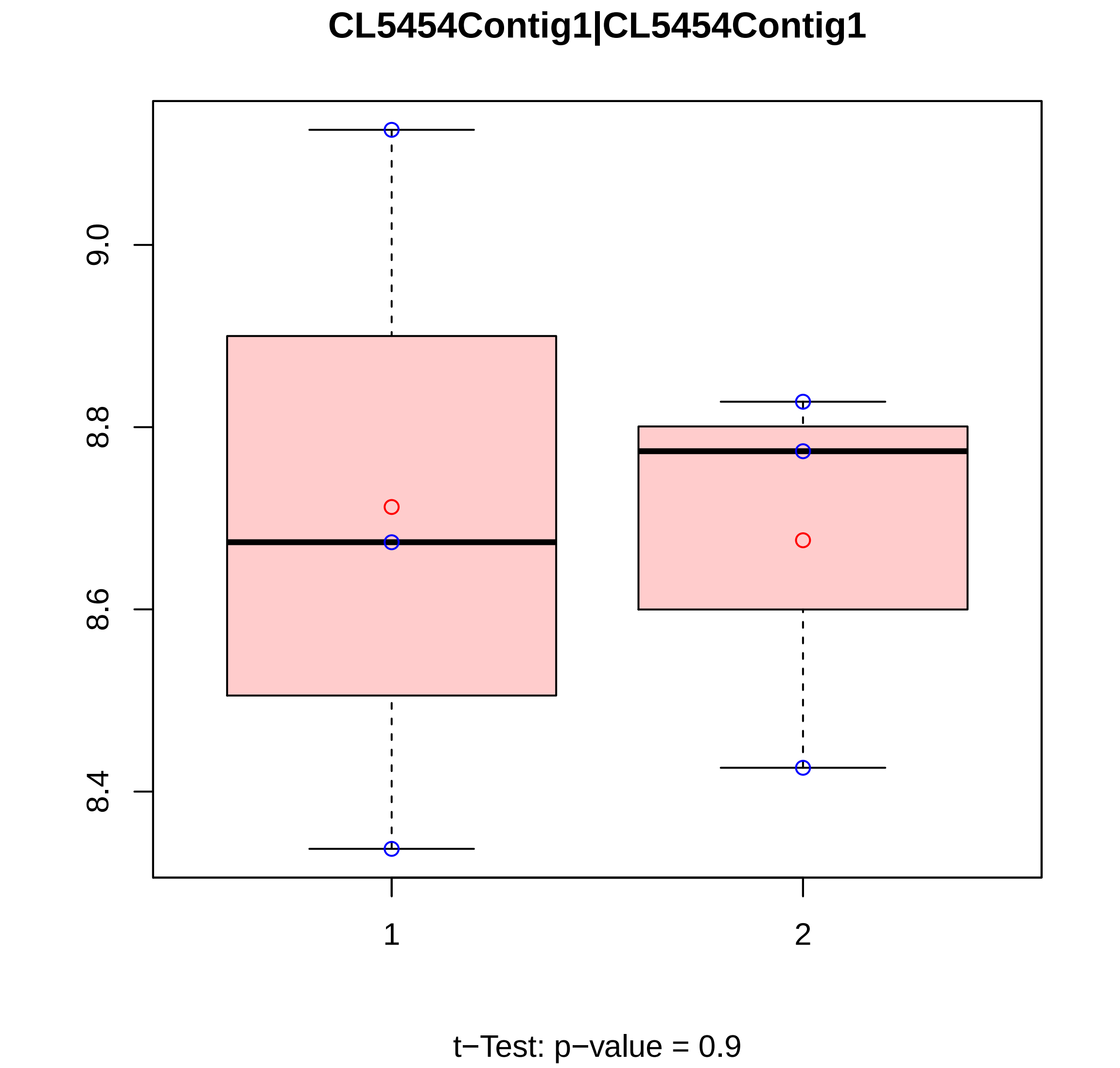
CL5454Contig1|CL5454Contig1
9.0
8.8
8.6
8.4
1
2
t−Test: p−value = 0.9

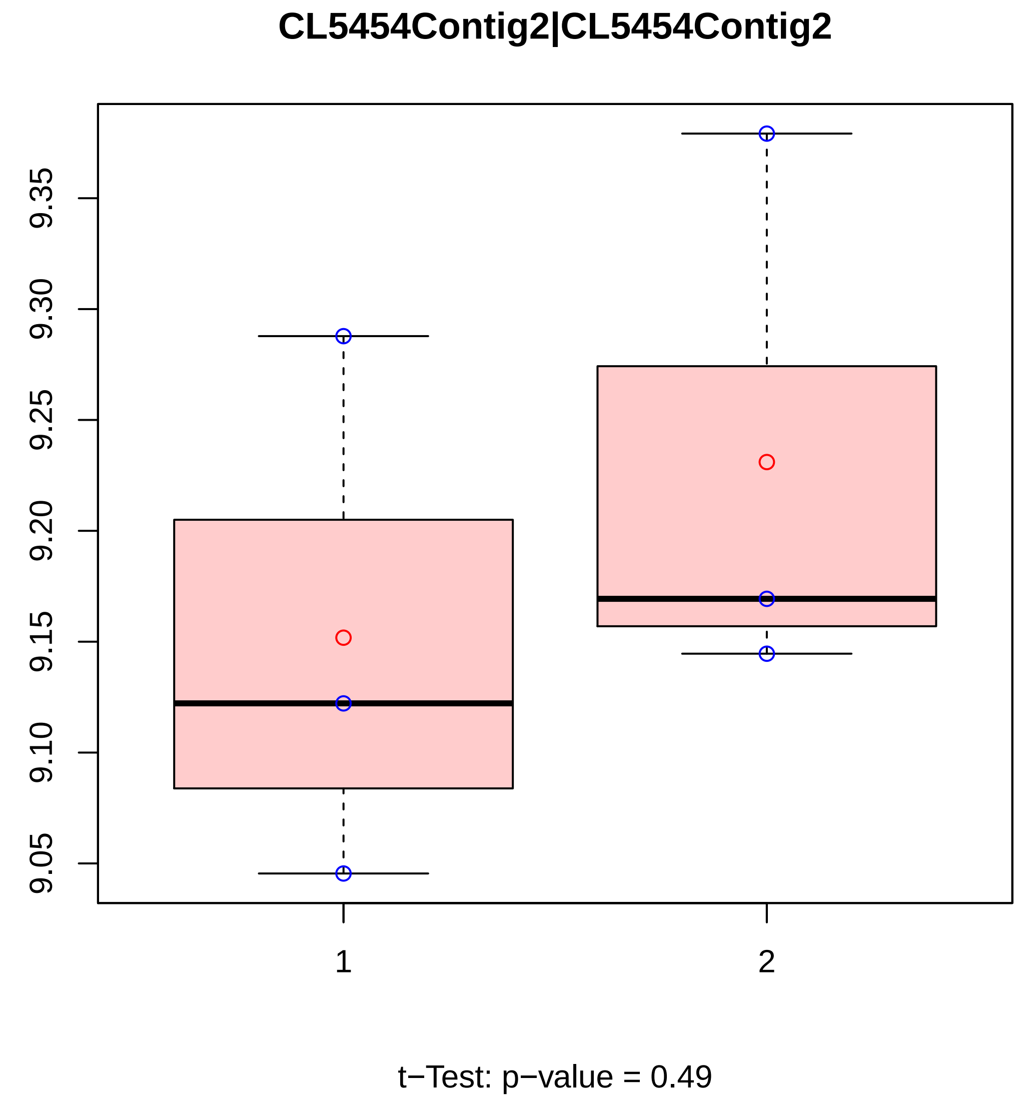
CL5454Contig2|CL5454Contig2
9.05
9.10
9.15
9.20
9.25
9.30
9.35
1
2
t−Test: p−value = 0.49

## CL5454Contig5|CL5454Contig5

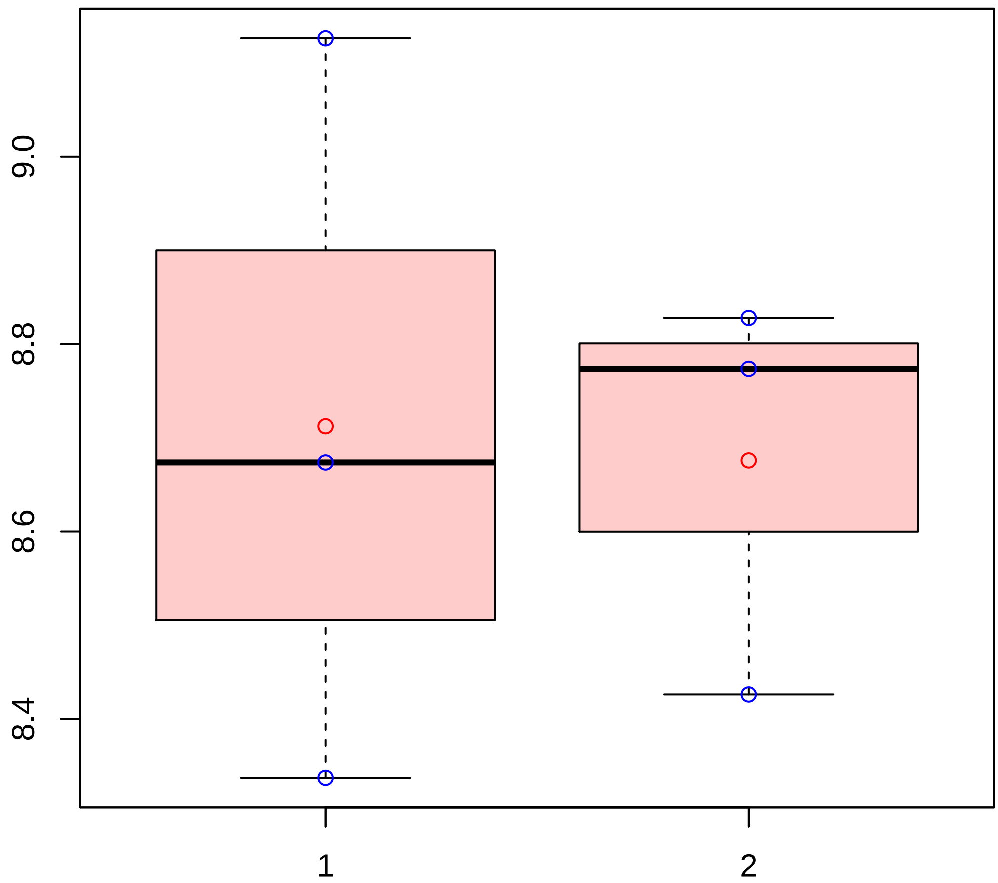


t−Test: p−value = 0.9

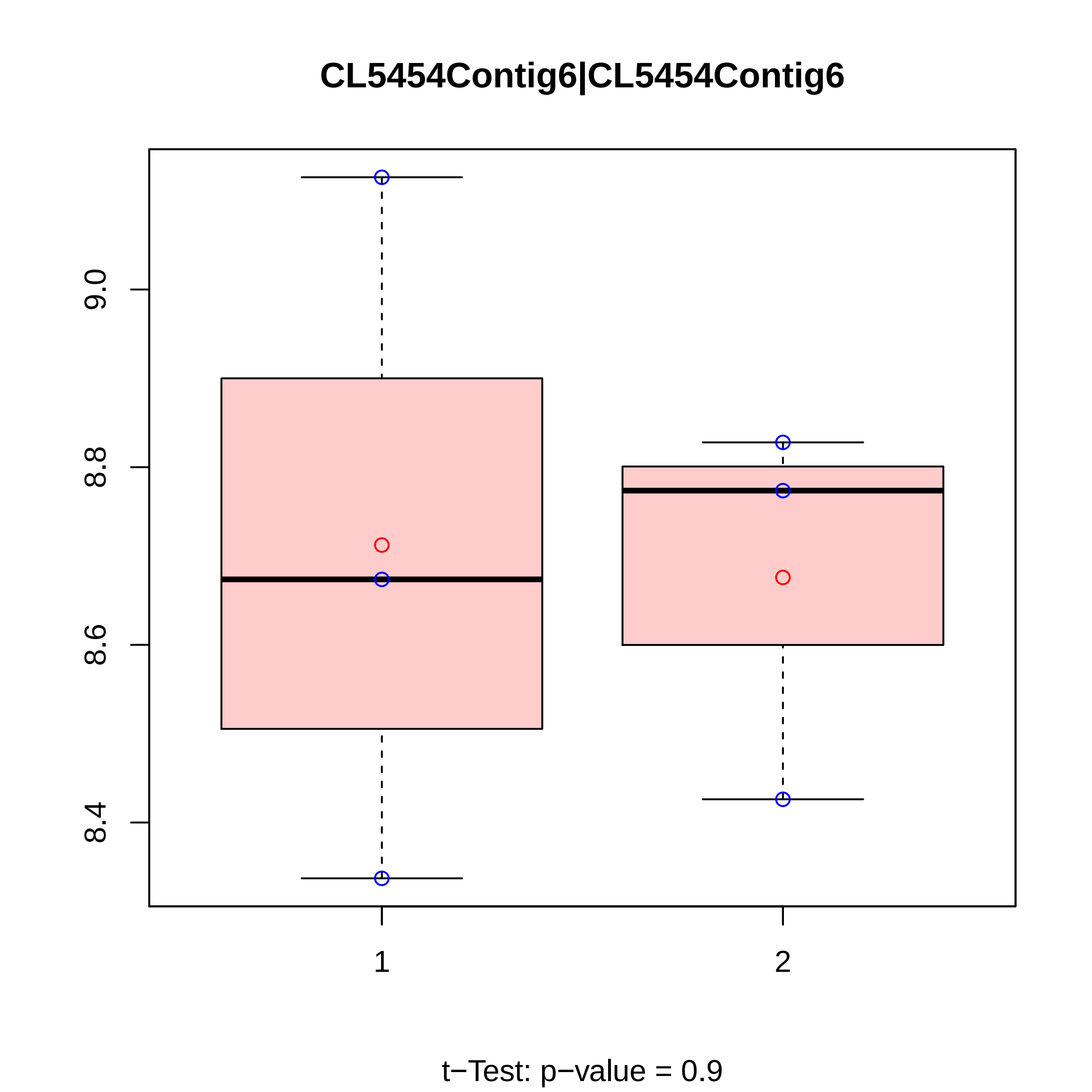
CL5454Contig6|CL5454Contig6
9.0
8.8
8.6
8.4
1
2
t−Test: p−value = 0.9

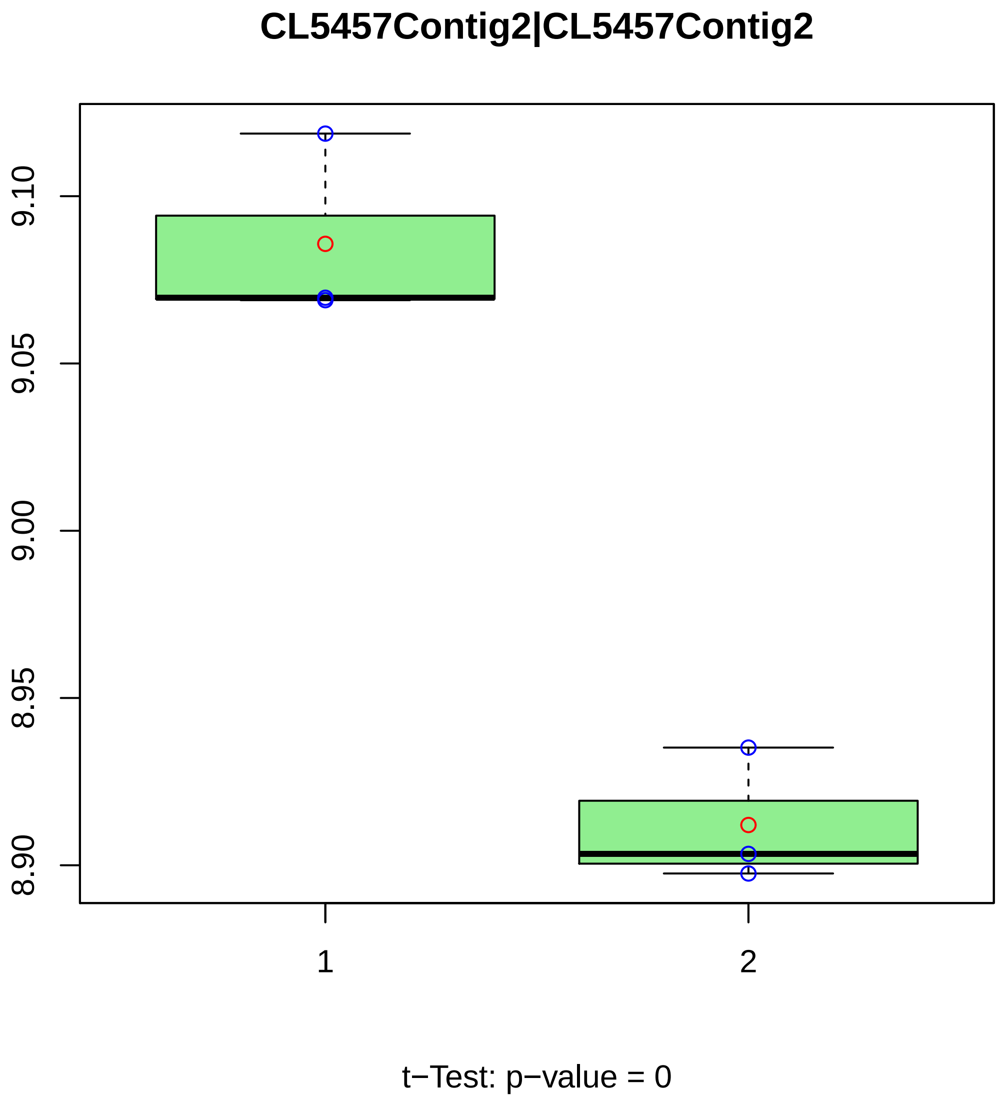

CL5457Contig2|CL5457Contig2
9.10
9.05
9.00
8.95
8.90
1
2
t−Test: p−value = 0

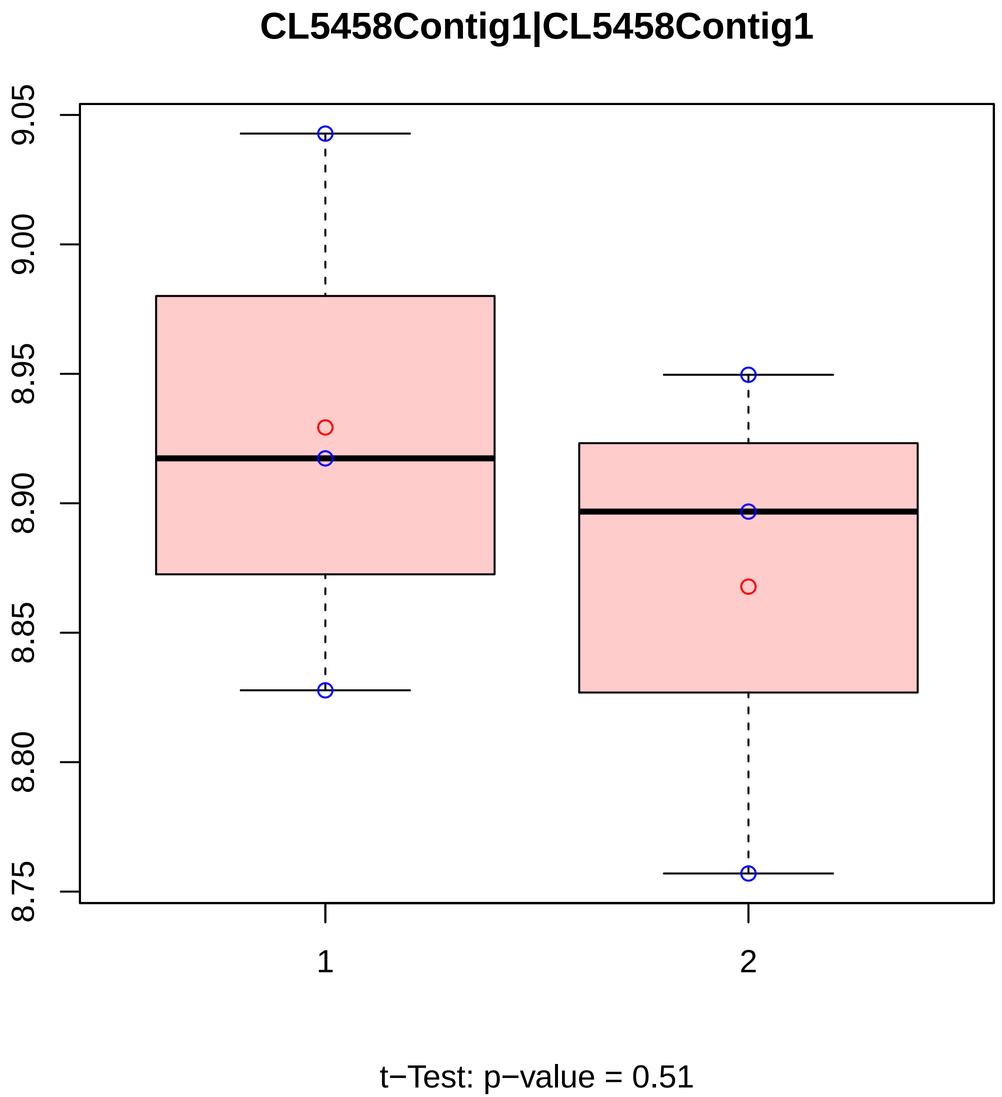
CL5458Contig1|CL5458Contig1
9.05
9.00
8.95
8.90
8.85
8.80
8.75
1
2
t−Test: p−value = 0.51

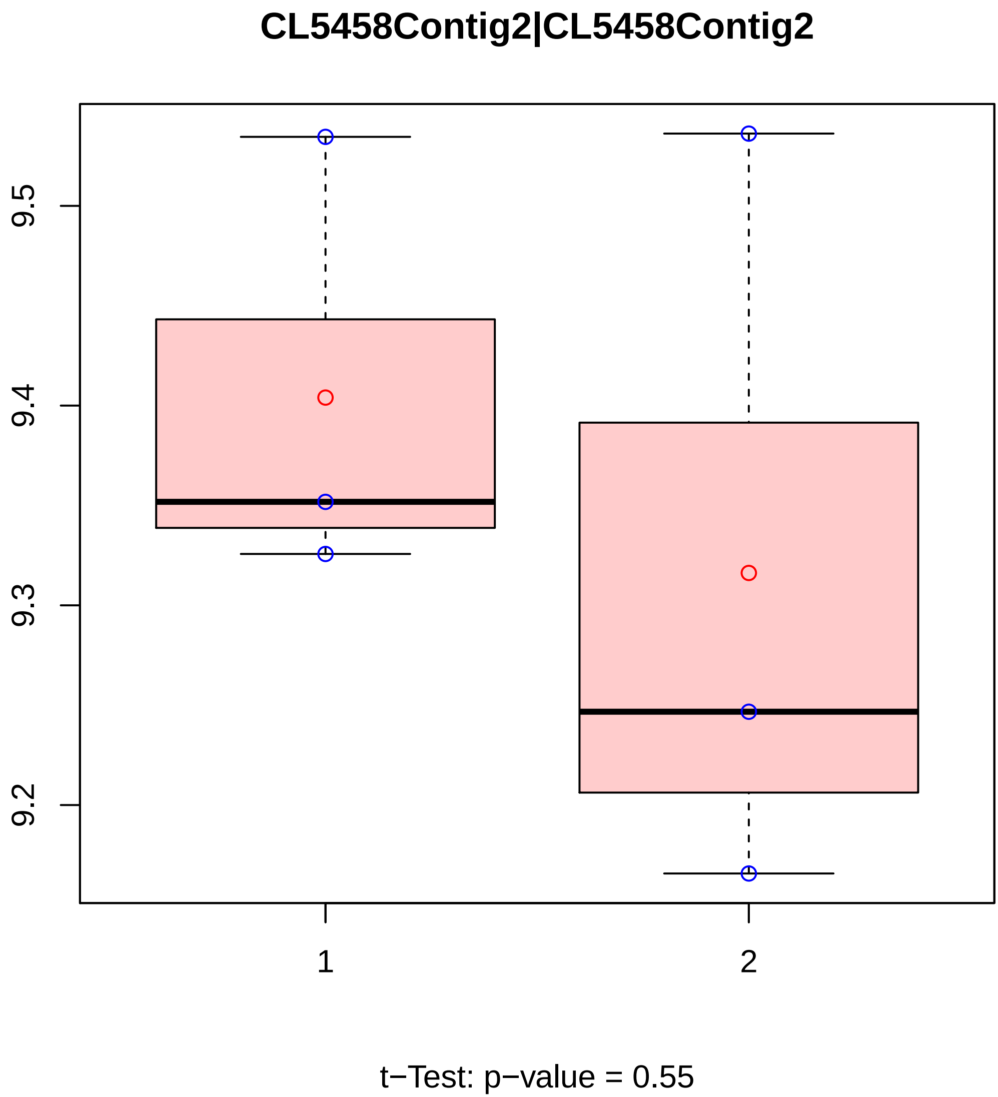
CL5458Contig2|CL5458Contig2
9.2
9.3
9.4
9.5
1
2
t−Test: p−value = 0.55

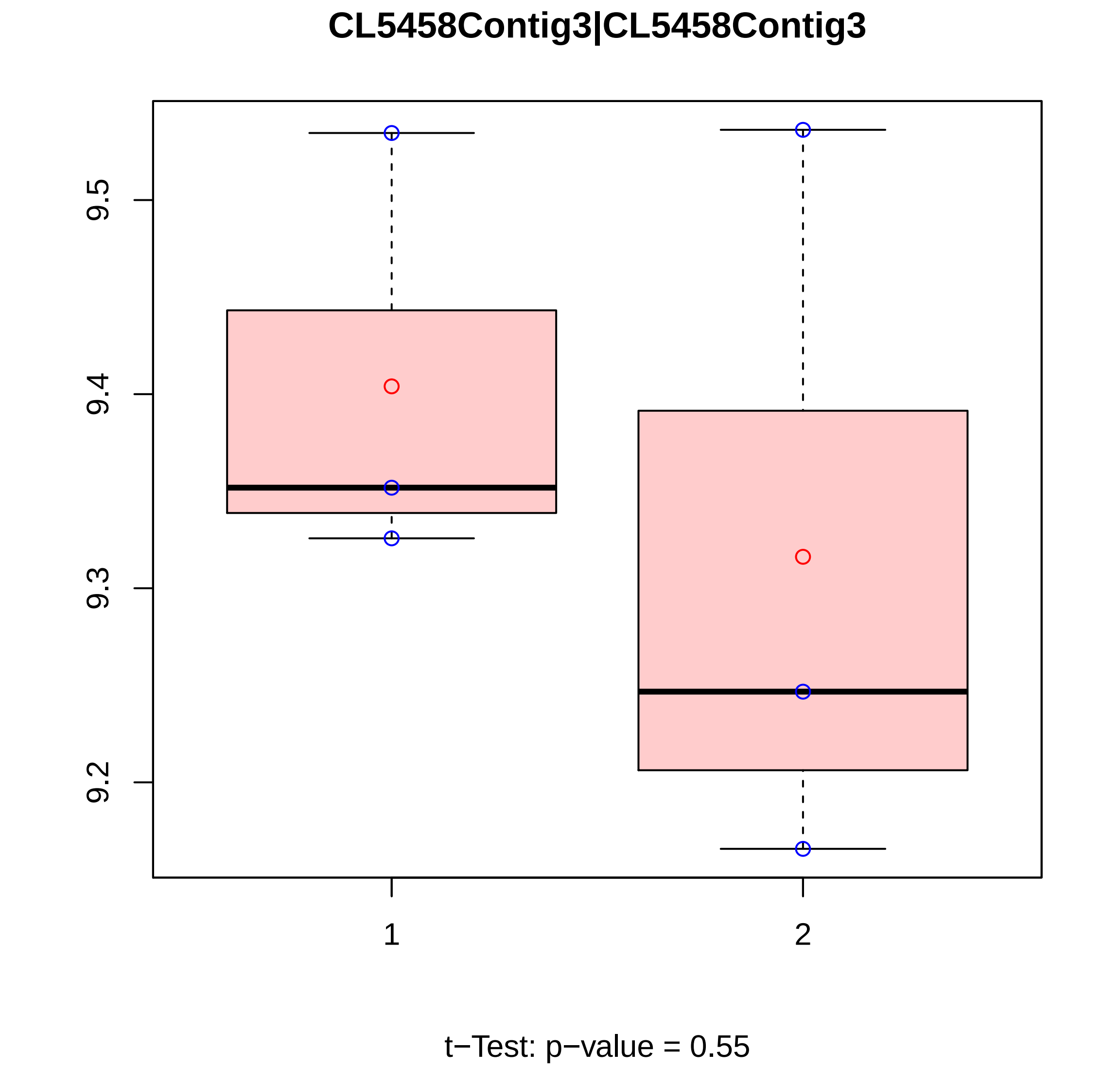

CL5458Contig3|CL5458Contig3
9.5
9.4
9.3
9.2
1
2
t−Test: p−value = 0.55

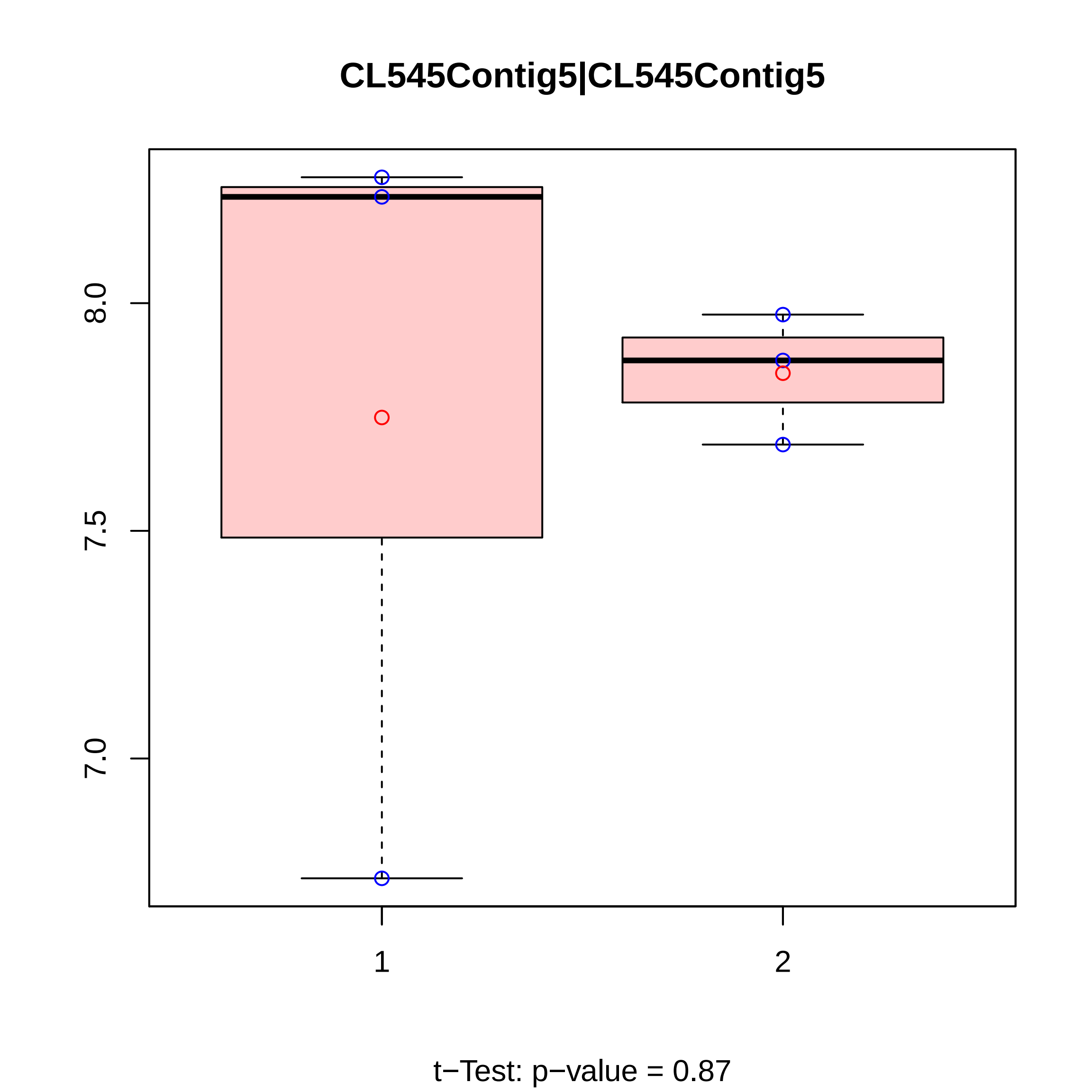

CL545Contig5|CL545Contig5
8.0
7.5
7.0
1
2
t−Test: p−value = 0.87

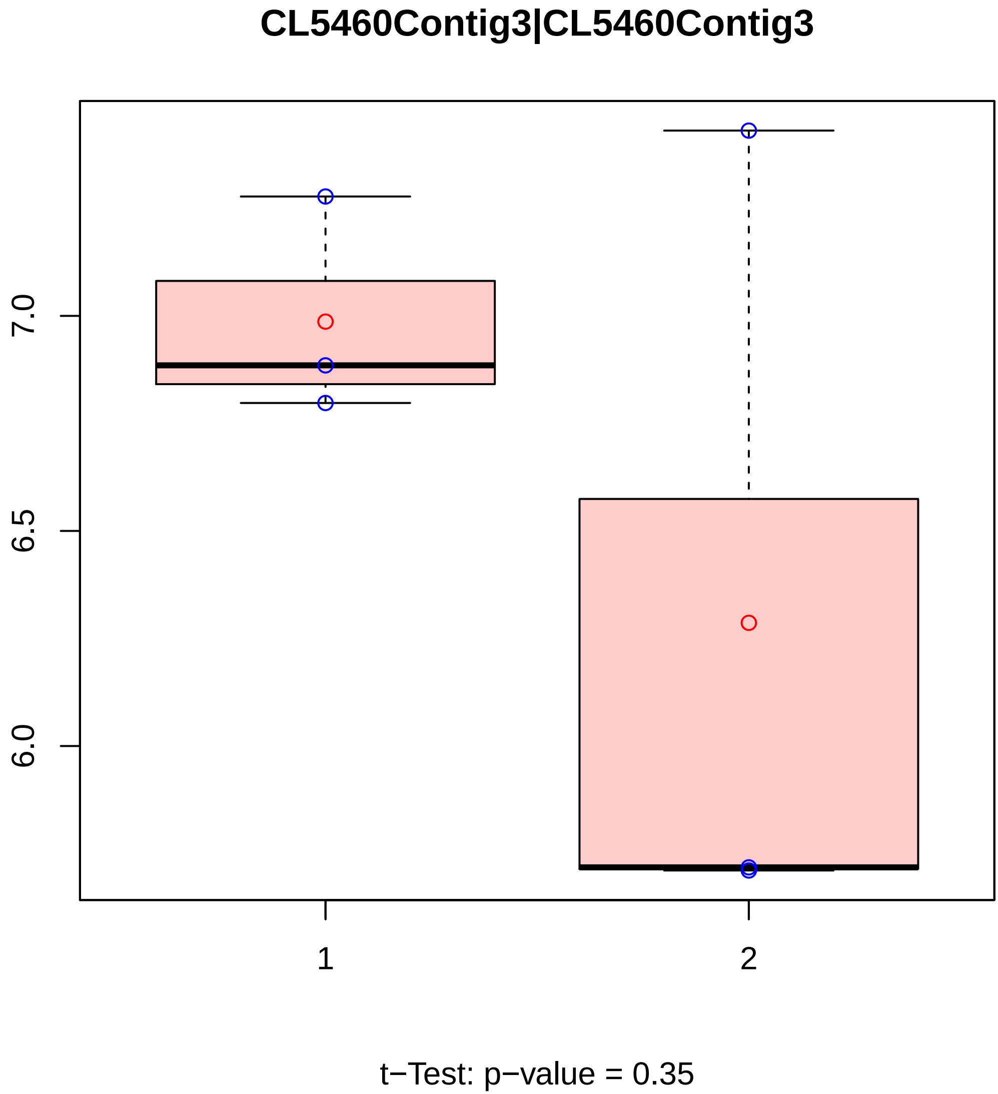
CL5460Contig3|CL5460Contig3
7.0
6.5
6.0
1
2
t−Test: p−value = 0.35

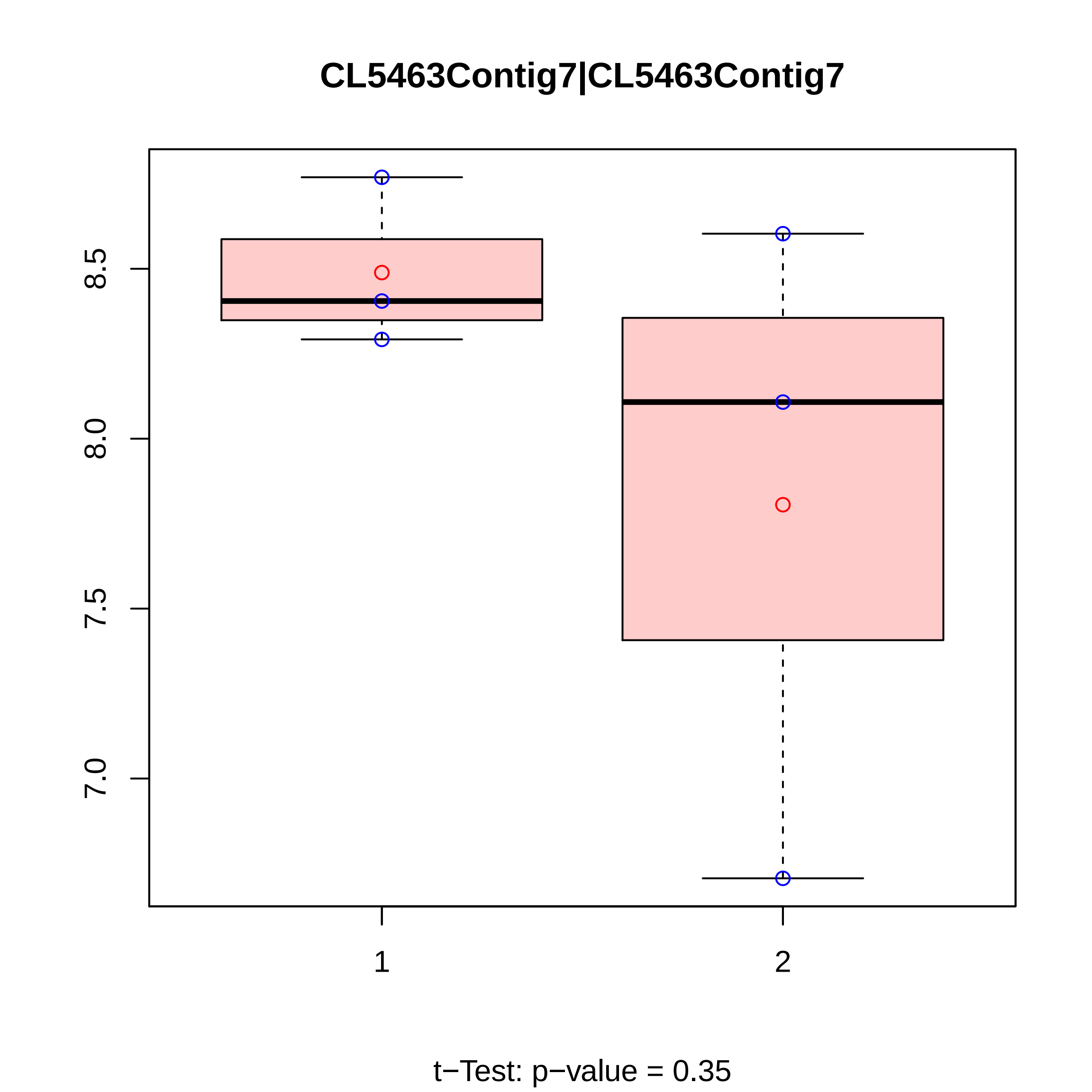

CL5463Contig7|CL5463Contig7
8.5
8.0
7.5
7.0
1
2
t−Test: p−value = 0.35

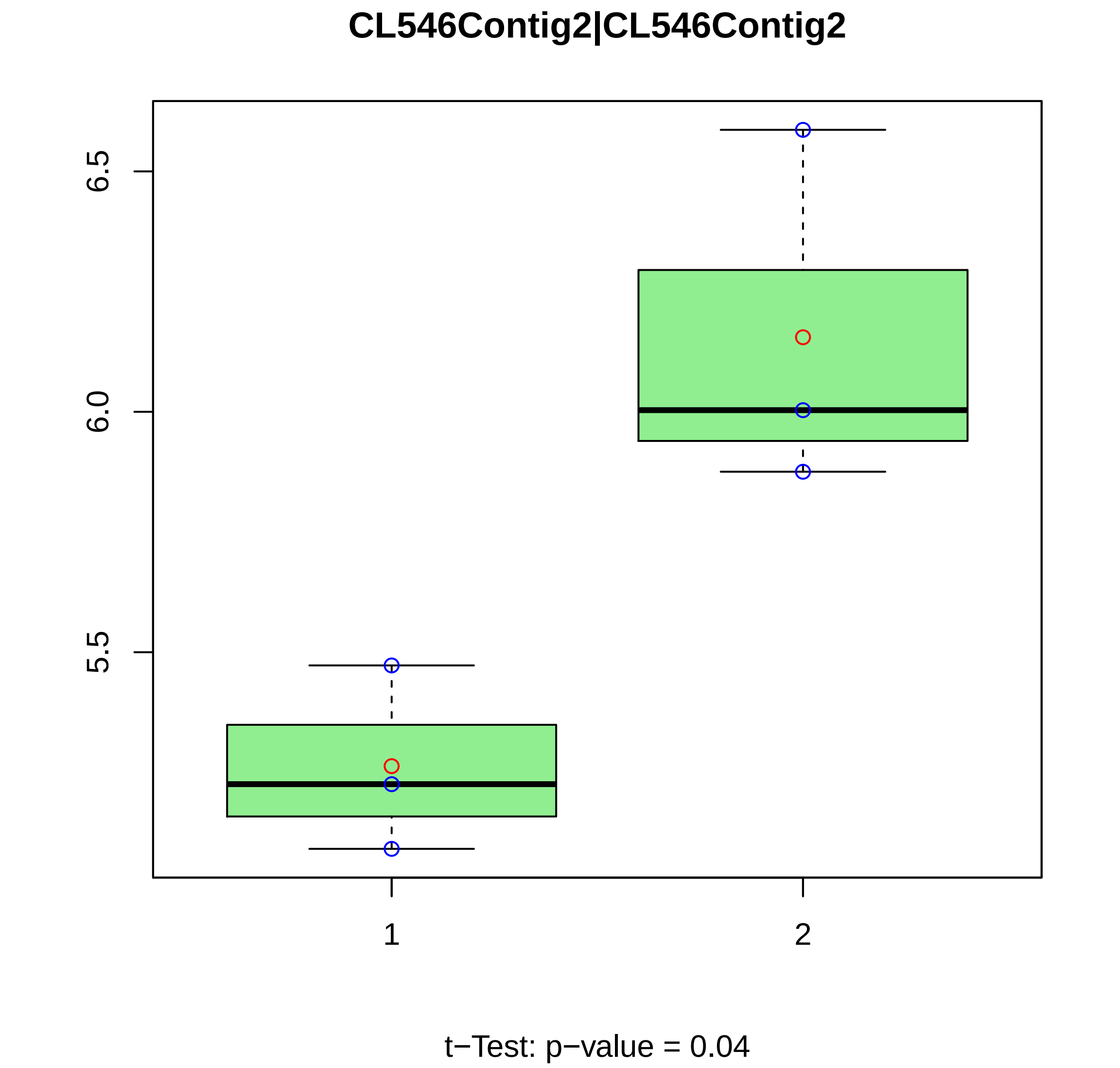
CL546Contig2|CL546Contig2
6.5
6.0
5.5
1
2
t−Test: p−value = 0.04

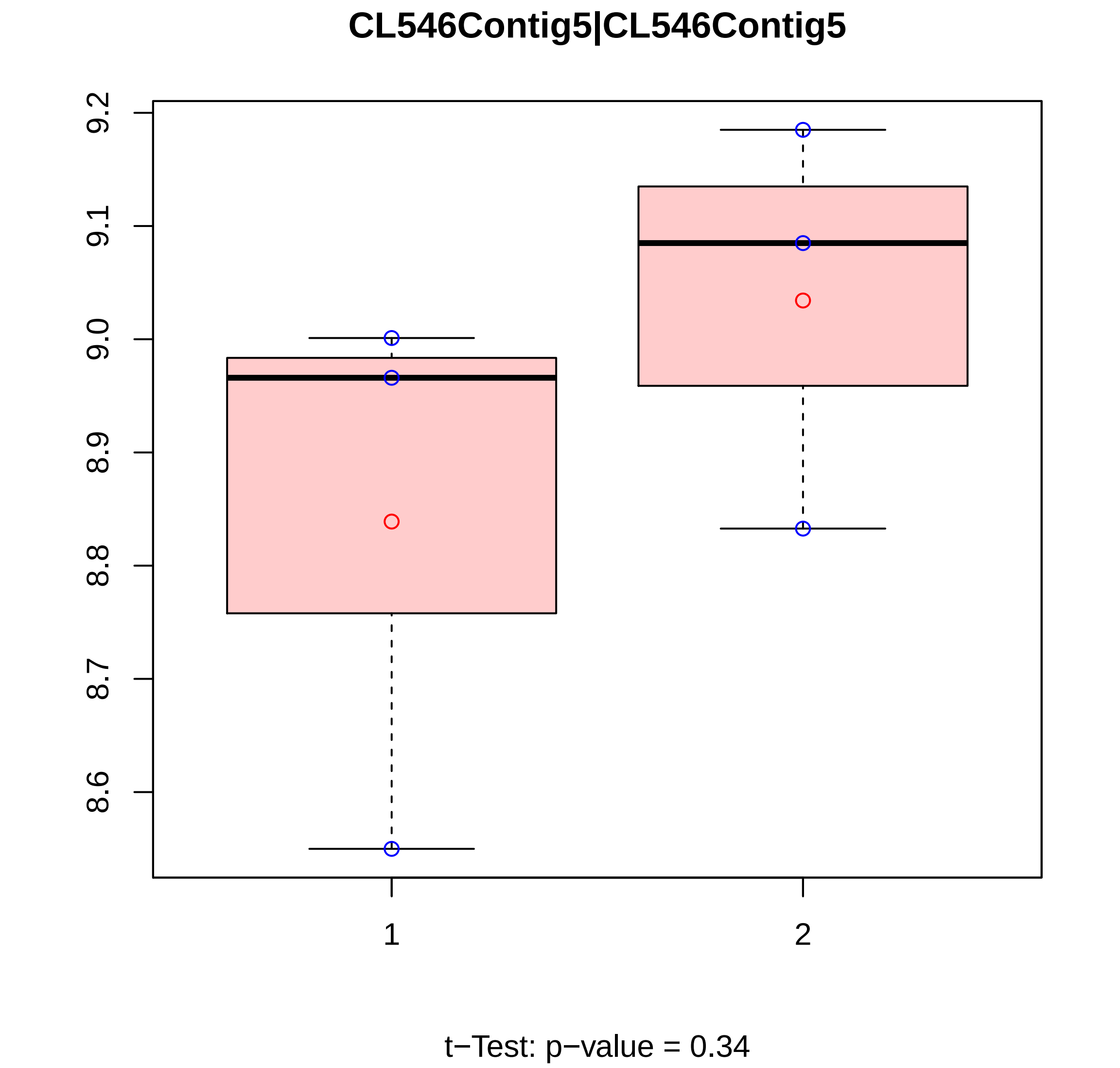
CL546Contig5|CL546Contig5
9.2
9.1
9.0
8.9
8.8
8.7
8.6
1
2
t−Test: p−value = 0.34

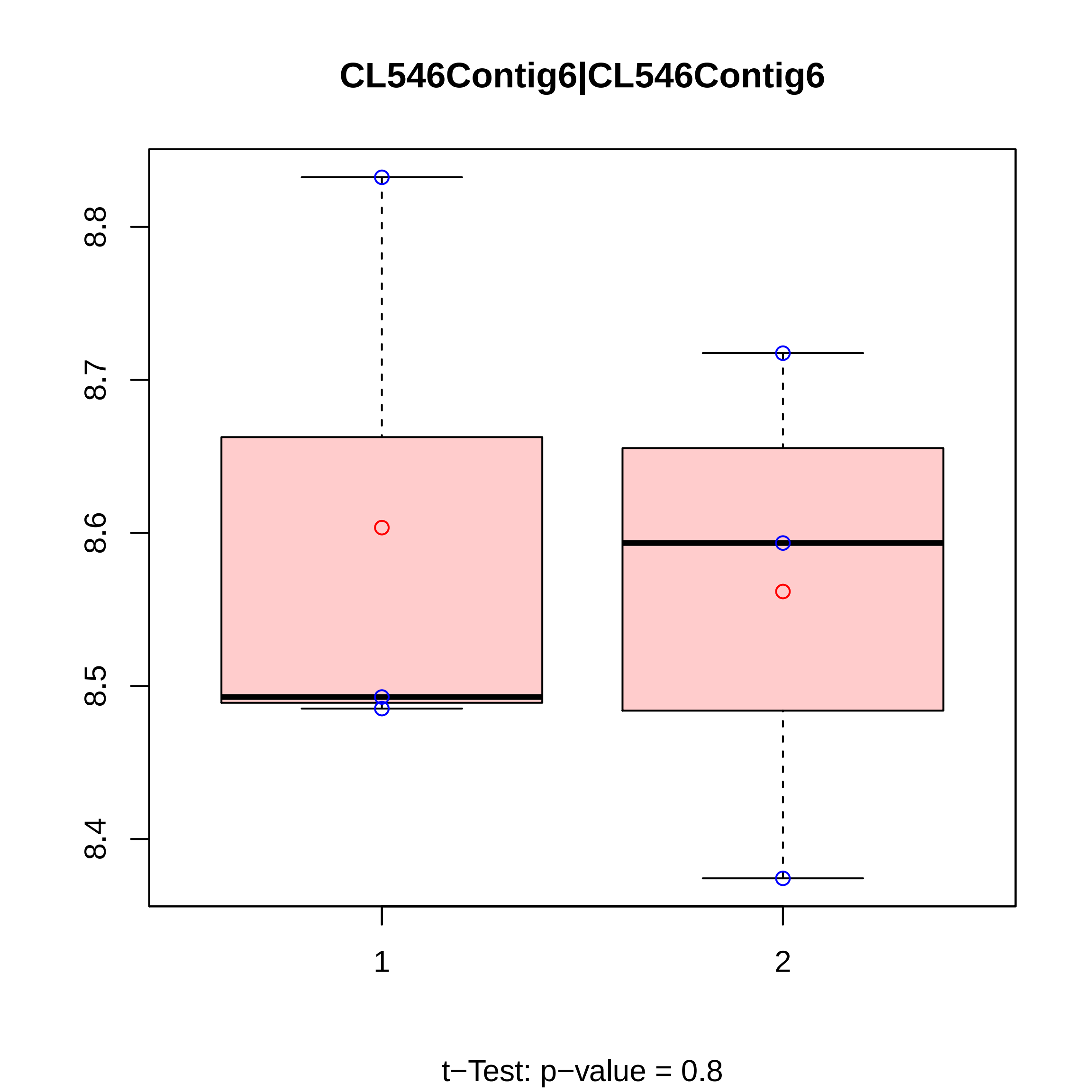

CL546Contig6|CL546Contig6
8.4
8.5
8.6
8.7
8.8
1
2
t−Test: p−value = 0.8

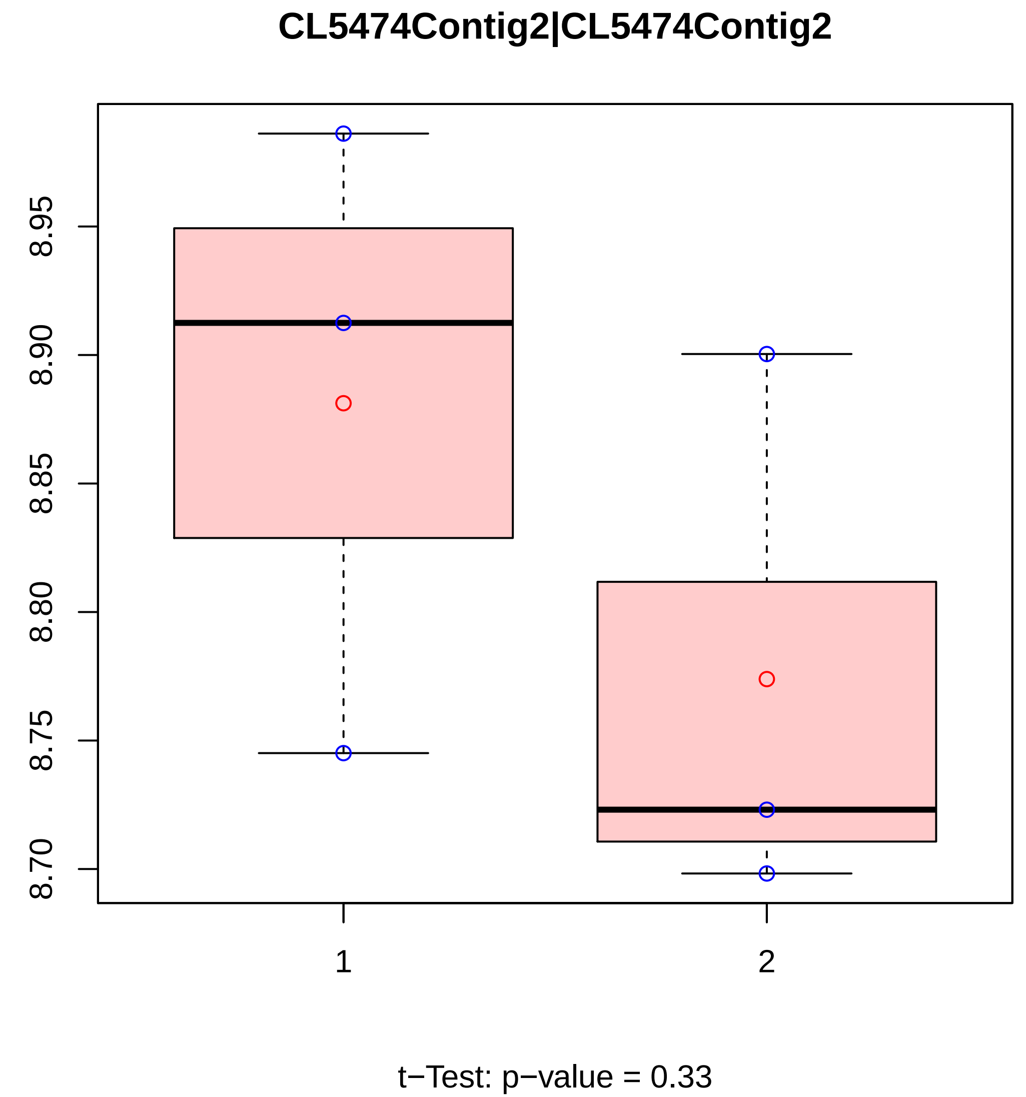

CL5474Contig2|CL5474Contig2
8.70
8.75
8.80
8.85
8.90
8.95
1
2
t−Test: p−value = 0.33

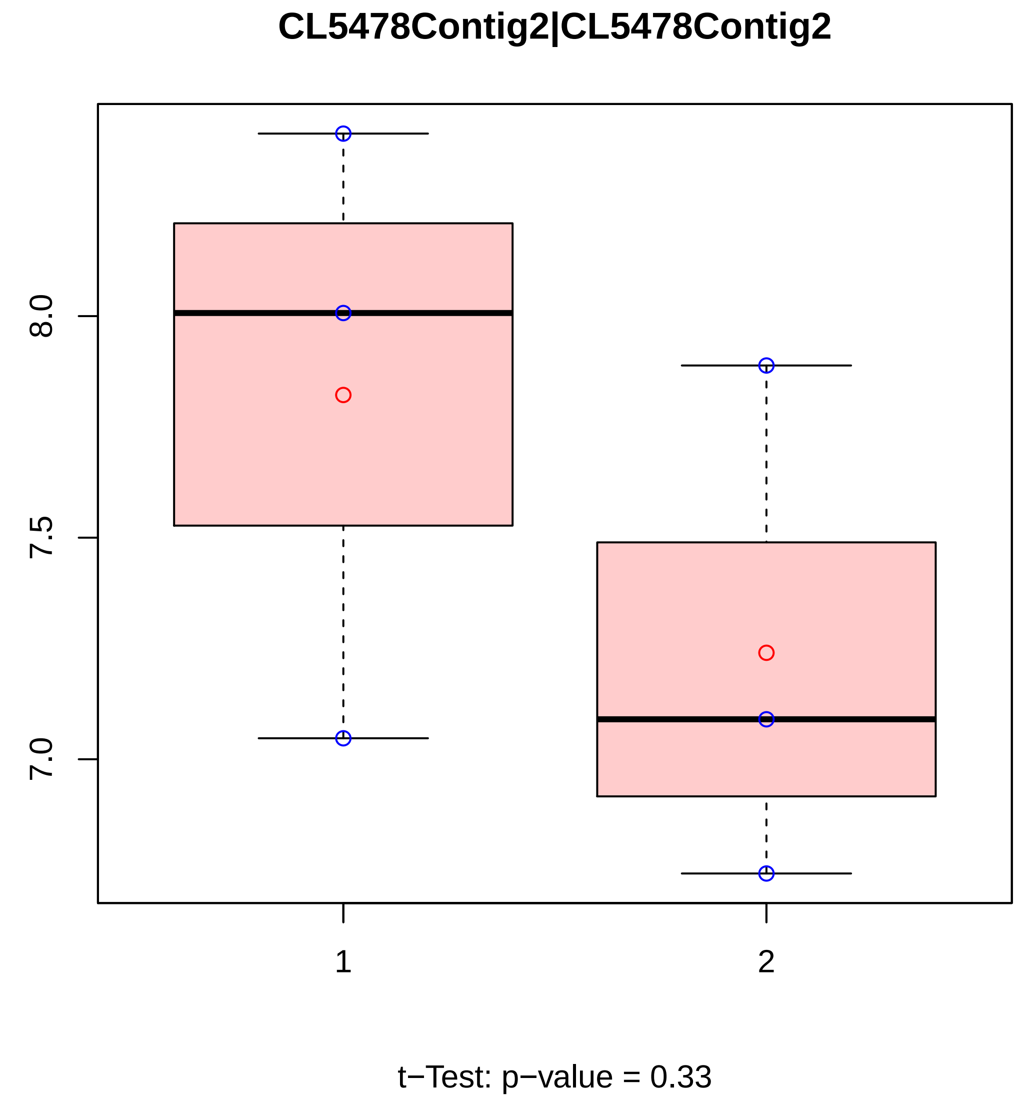
CL5478Contig2|CL5478Contig2
8.0
7.5
7.0
1
2
t−Test: p−value = 0.33

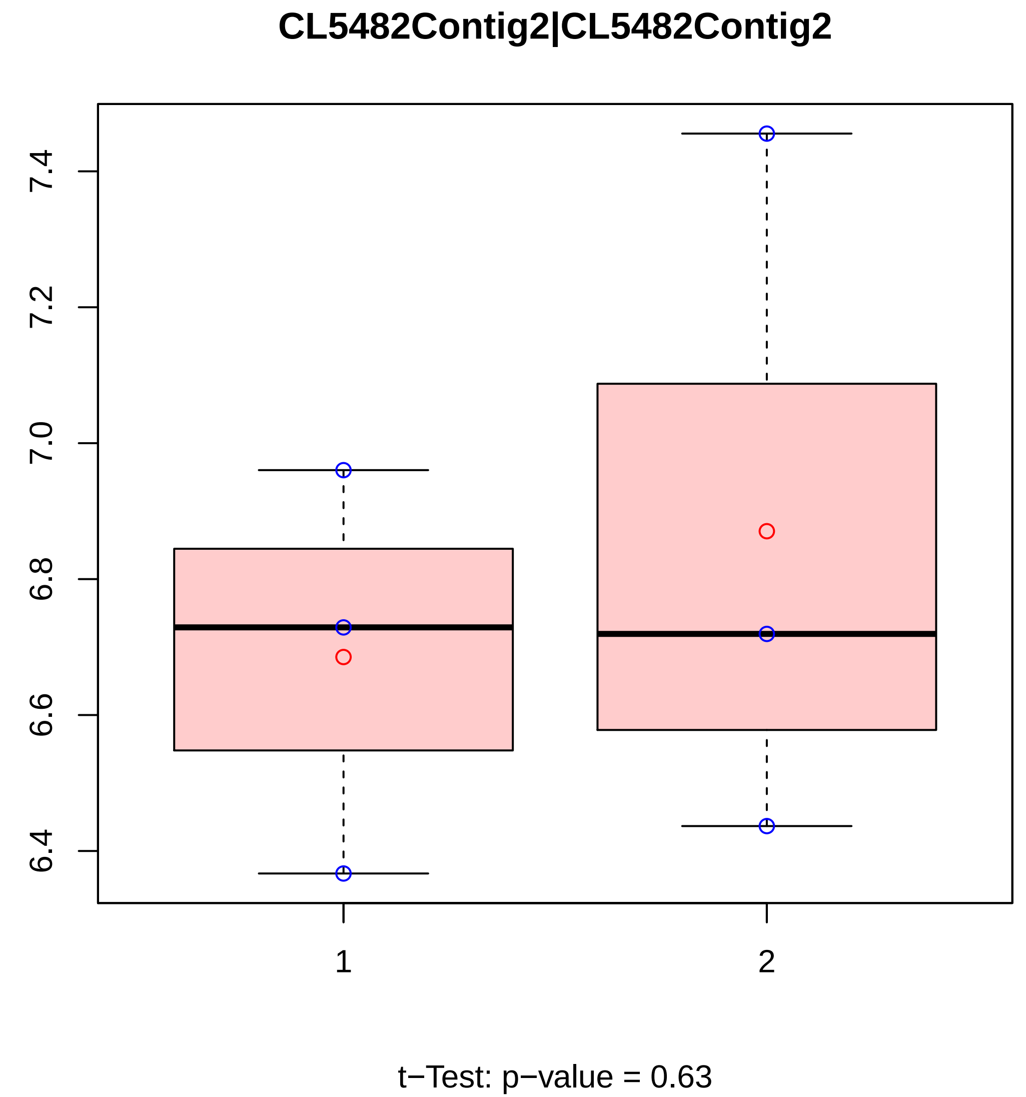

CL5482Contig2|CL5482Contig2
6.4
6.6
6.8
7.0
7.2
7.4
1
2
t−Test: p−value = 0.63

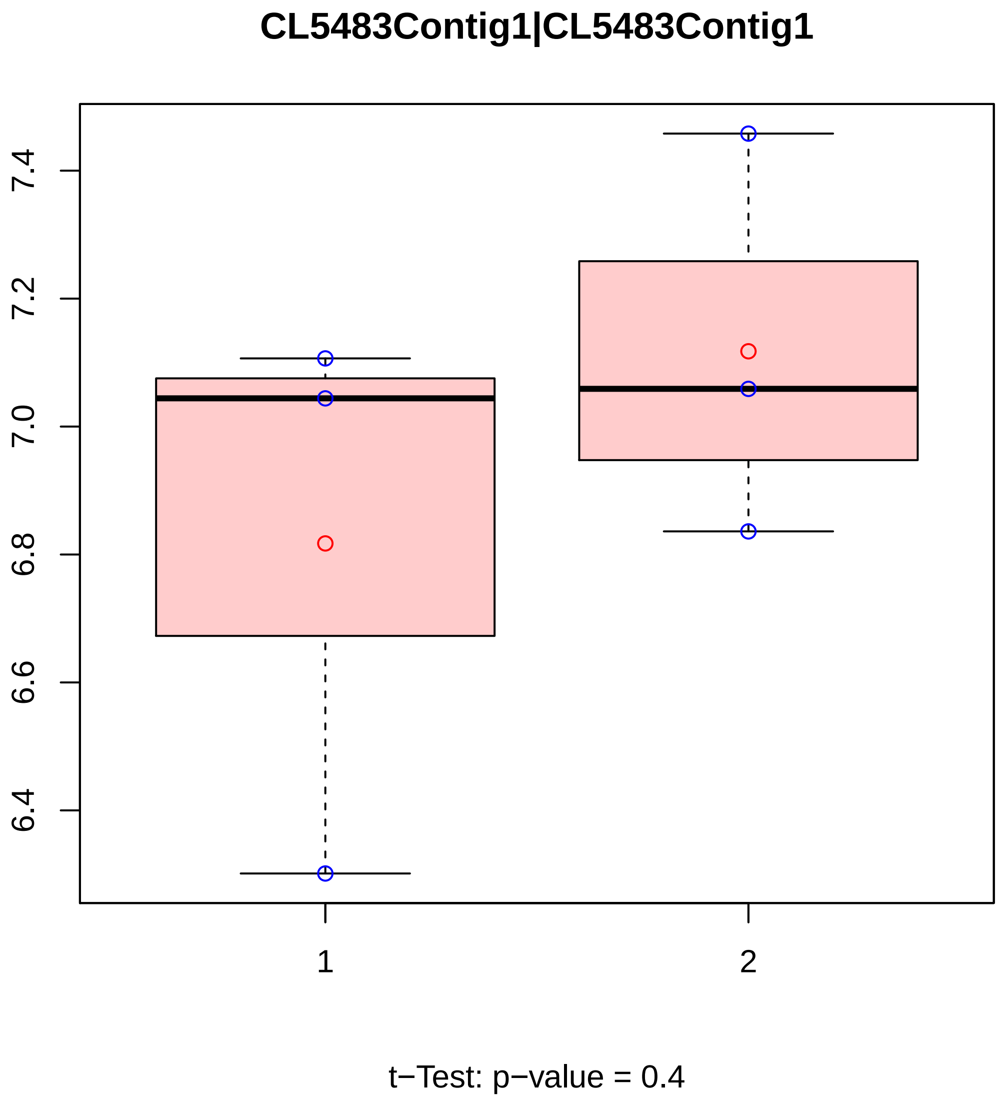

CL5483Contig1|CL5483Contig1
7.4
7.2
7.0
6.8
6.6
6.4
1
2
t−Test: p−value = 0.4

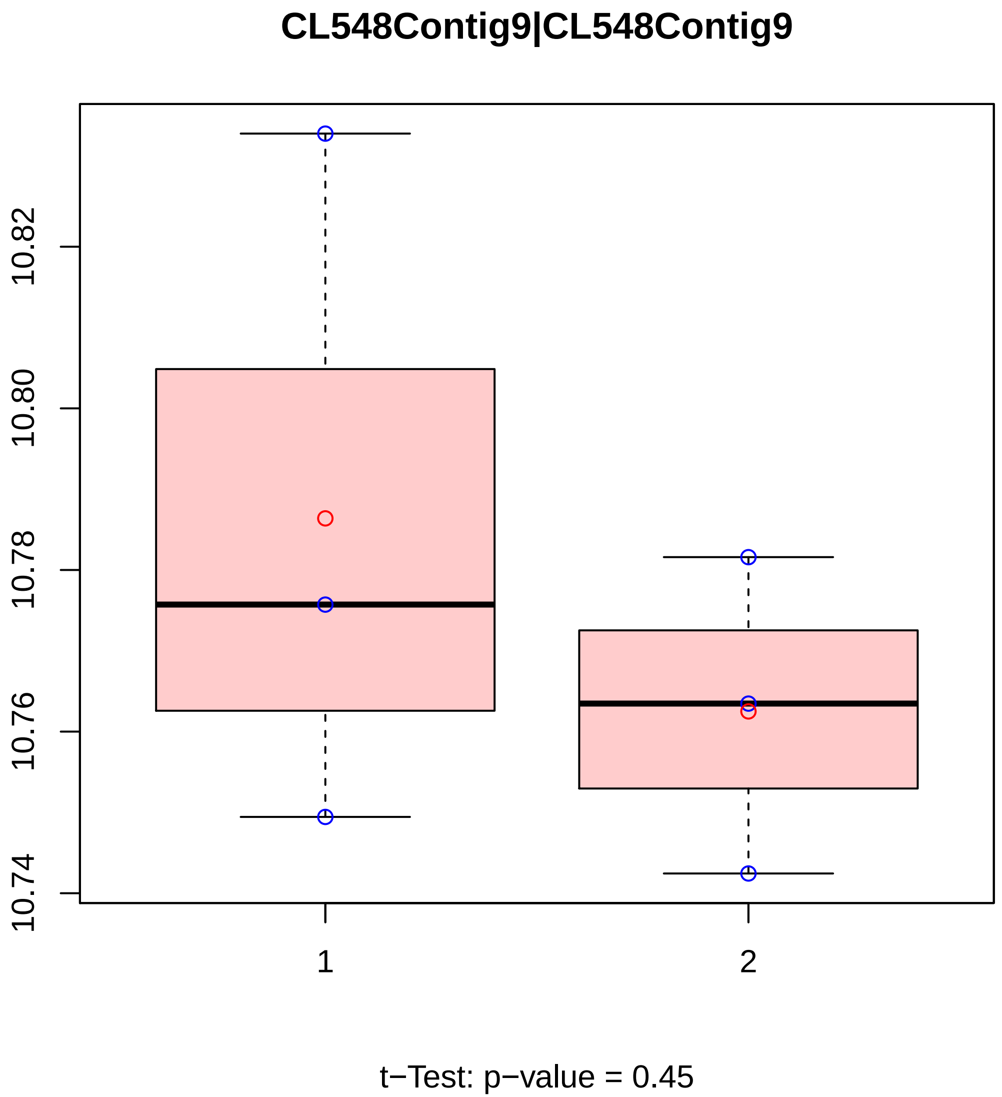
CL548Contig9|CL548Contig9
10.74
10.76
10.78
10.80
10.82
1
2
t−Test: p−value = 0.45

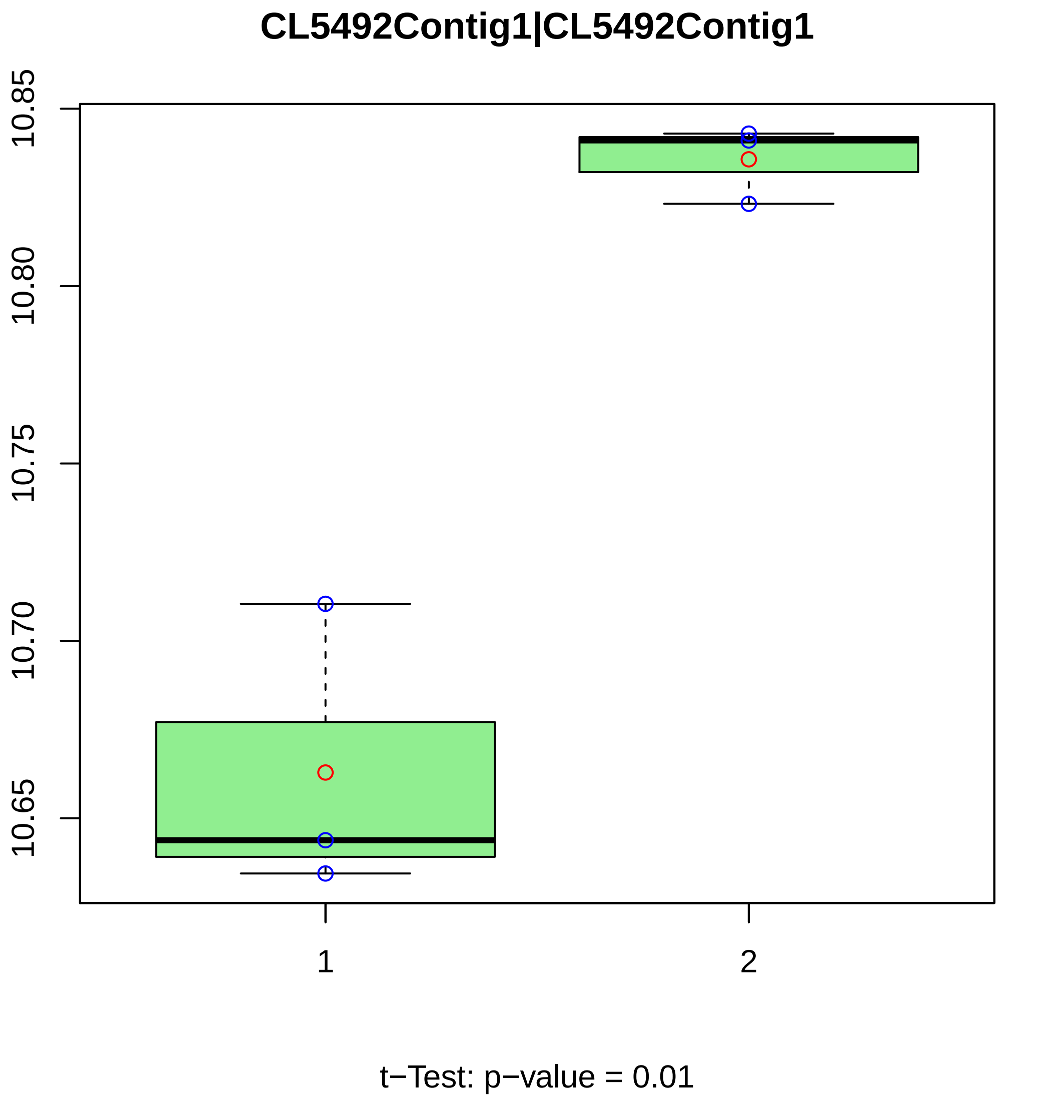

CL5492Contig1|CL5492Contig1
10.85
10.80
10.75
10.70
10.65
1
2
t−Test: p−value = 0.01

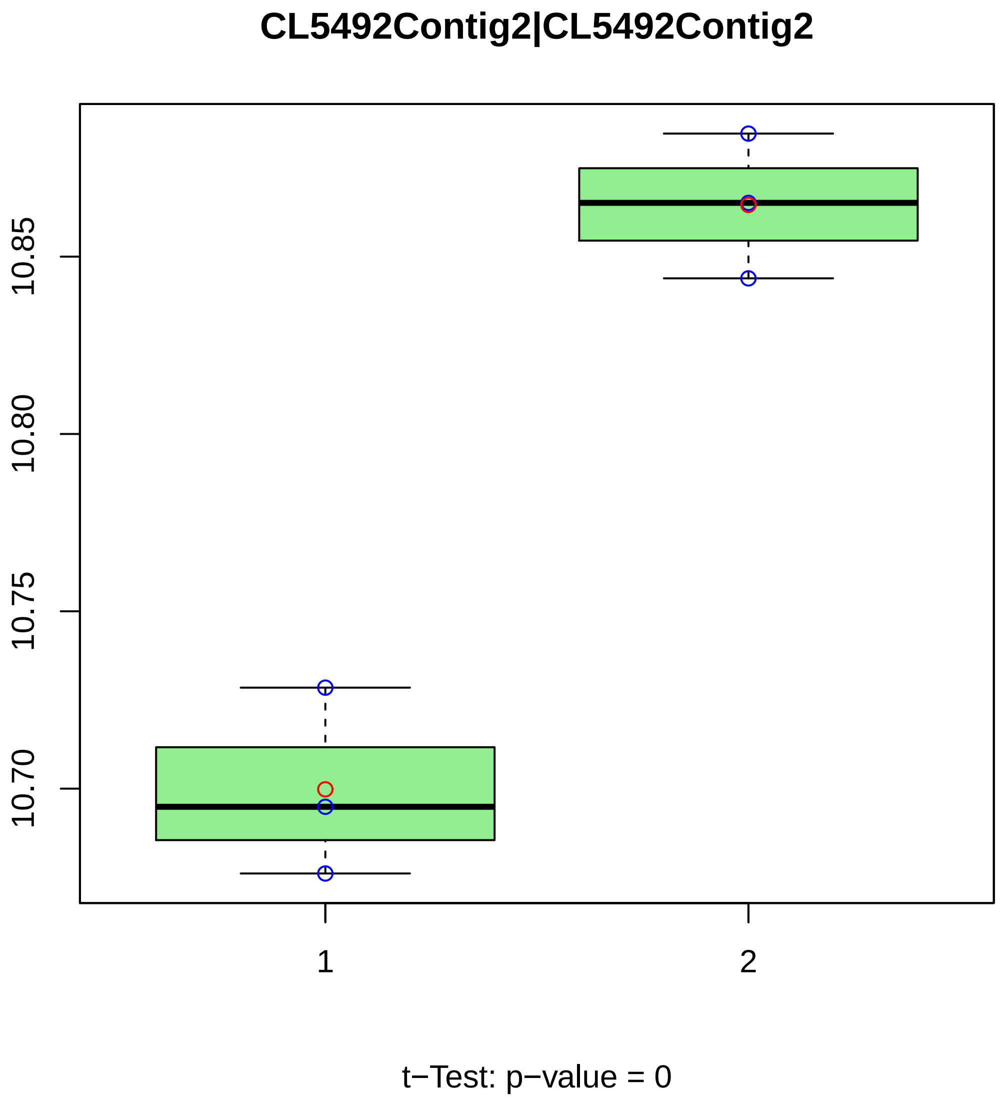
CL5492Contig2|CL5492Contig2
10.70
10.75
10.80
10.85
1
2
t−Test: p−value = 0

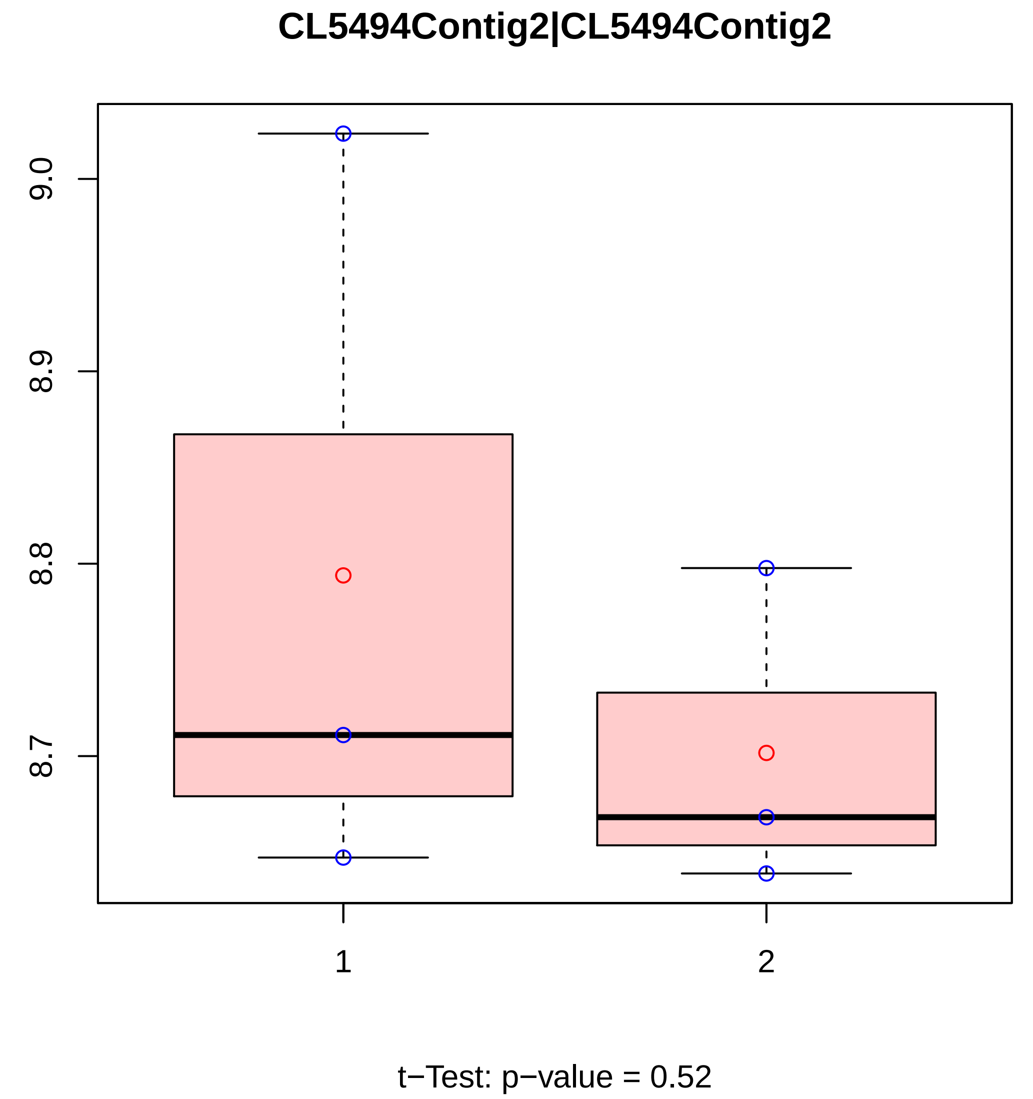
CL5494Contig2|CL5494Contig2
9.0
8.9
8.8
8.7
1
2
t−Test: p−value = 0.52

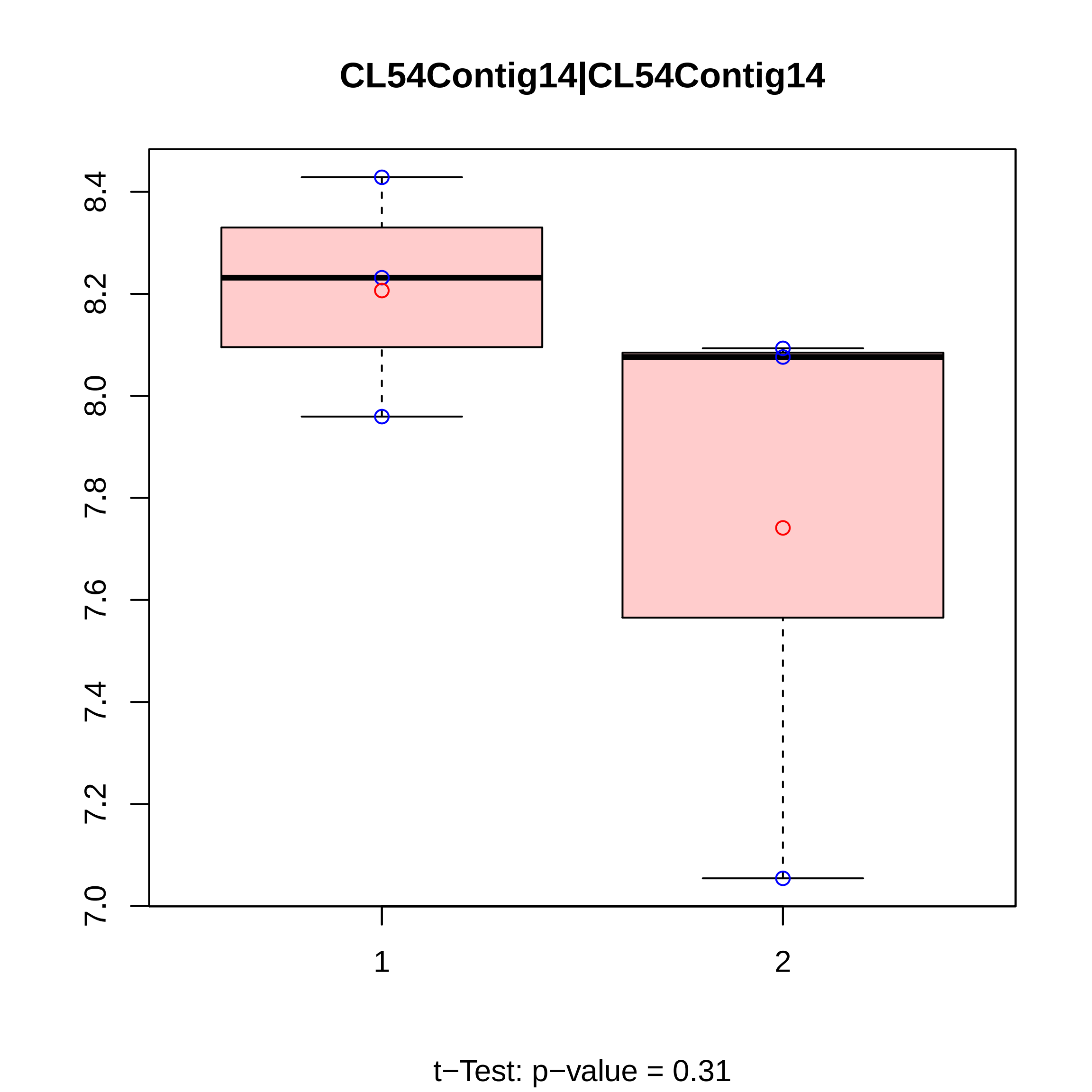

CL54Contig14|CL54Contig14
8.4
8.2
8.0
7.8
7.6
7.4
7.2
7.0
1
2
t−Test: p−value = 0.31

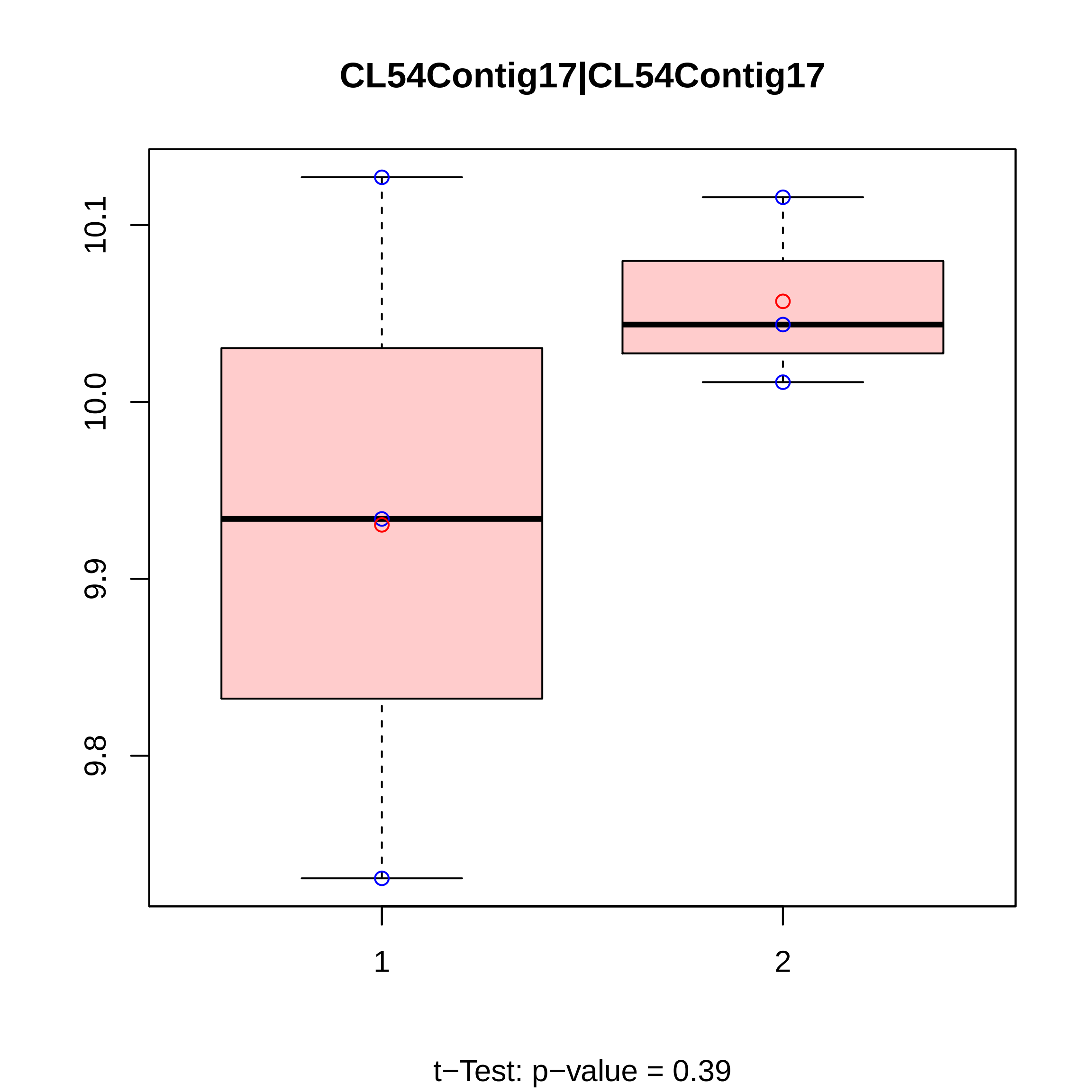

CL54Contig17|CL54Contig17
10.1
10.0
9.9
9.8
1
2
t−Test: p−value = 0.39

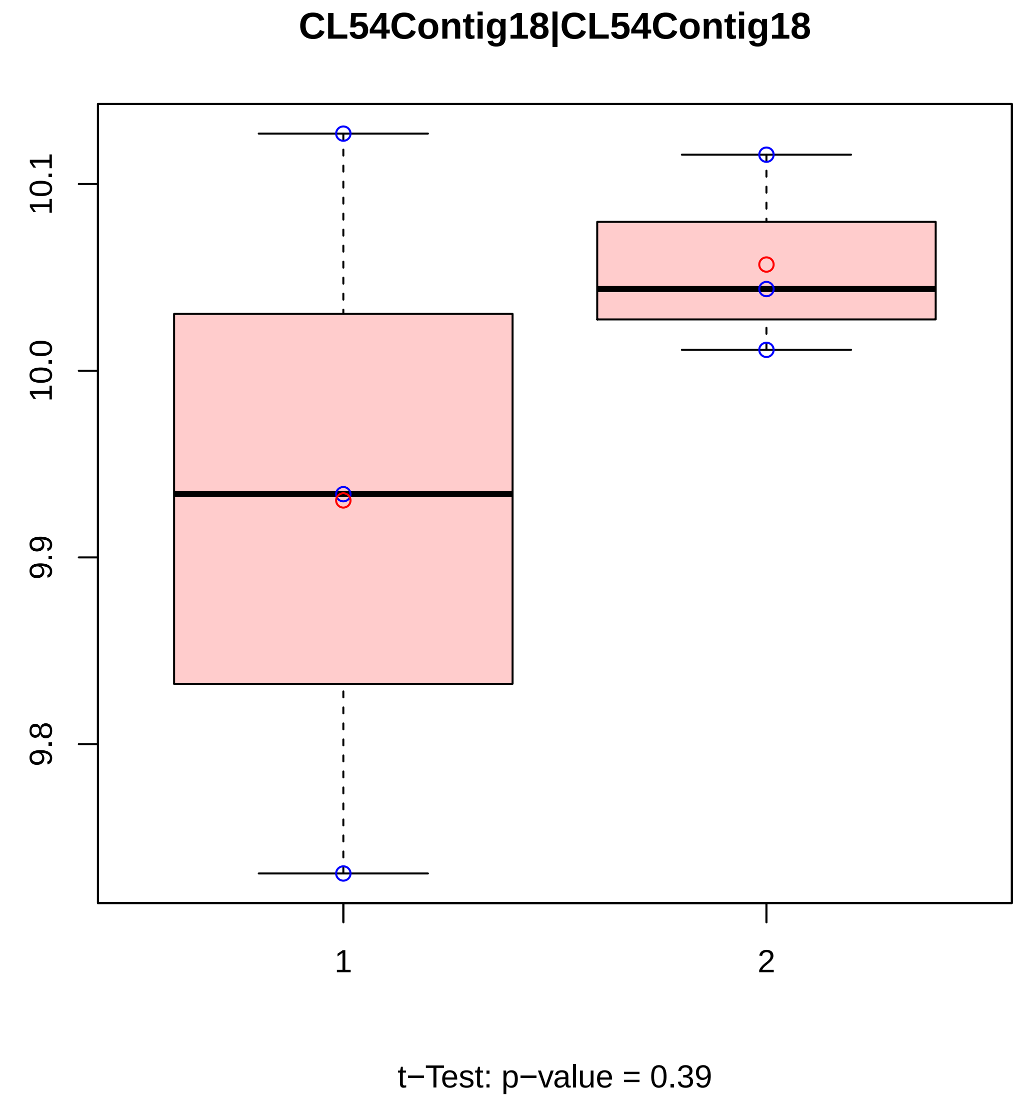
CL54Contig18|CL54Contig18
10.1
10.0
9.9
9.8
1
2
t−Test: p−value = 0.39

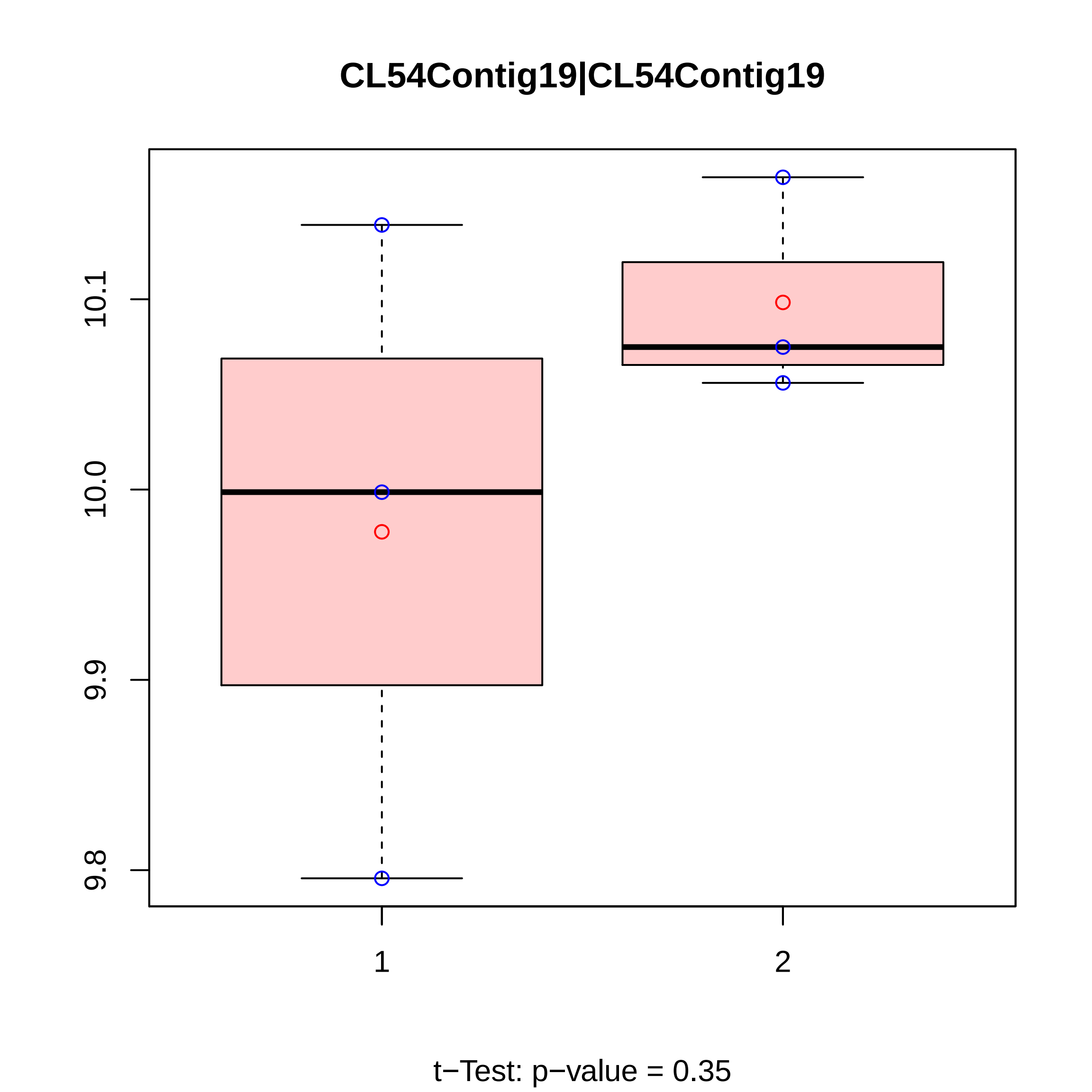

CL54Contig19|CL54Contig19
10.1
10.0
9.9
9.8
1
2
t−Test: p−value = 0.35

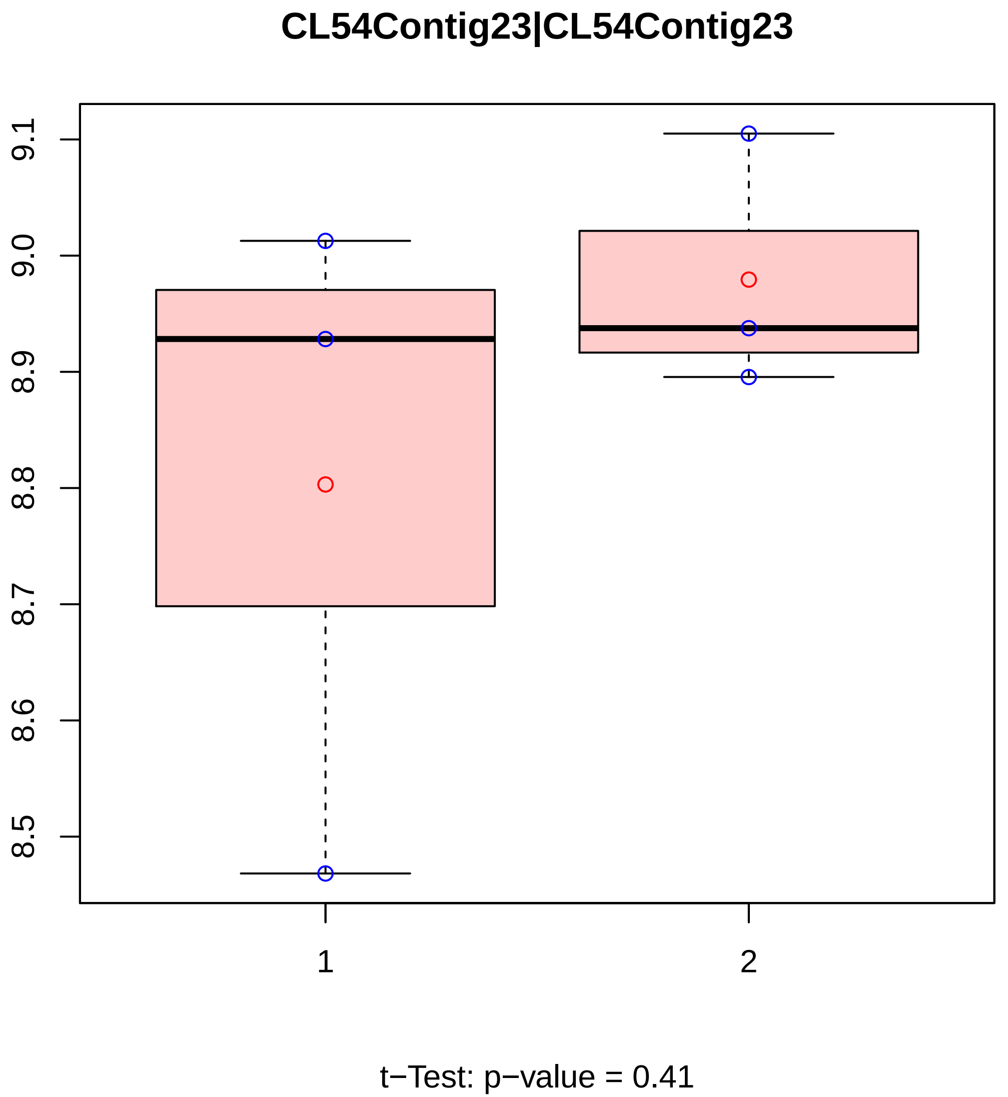
CL54Contig23|CL54Contig23
9.1
9.0
8.9
8.8
8.7
8.6
8.5
1
2
t−Test: p−value = 0.41

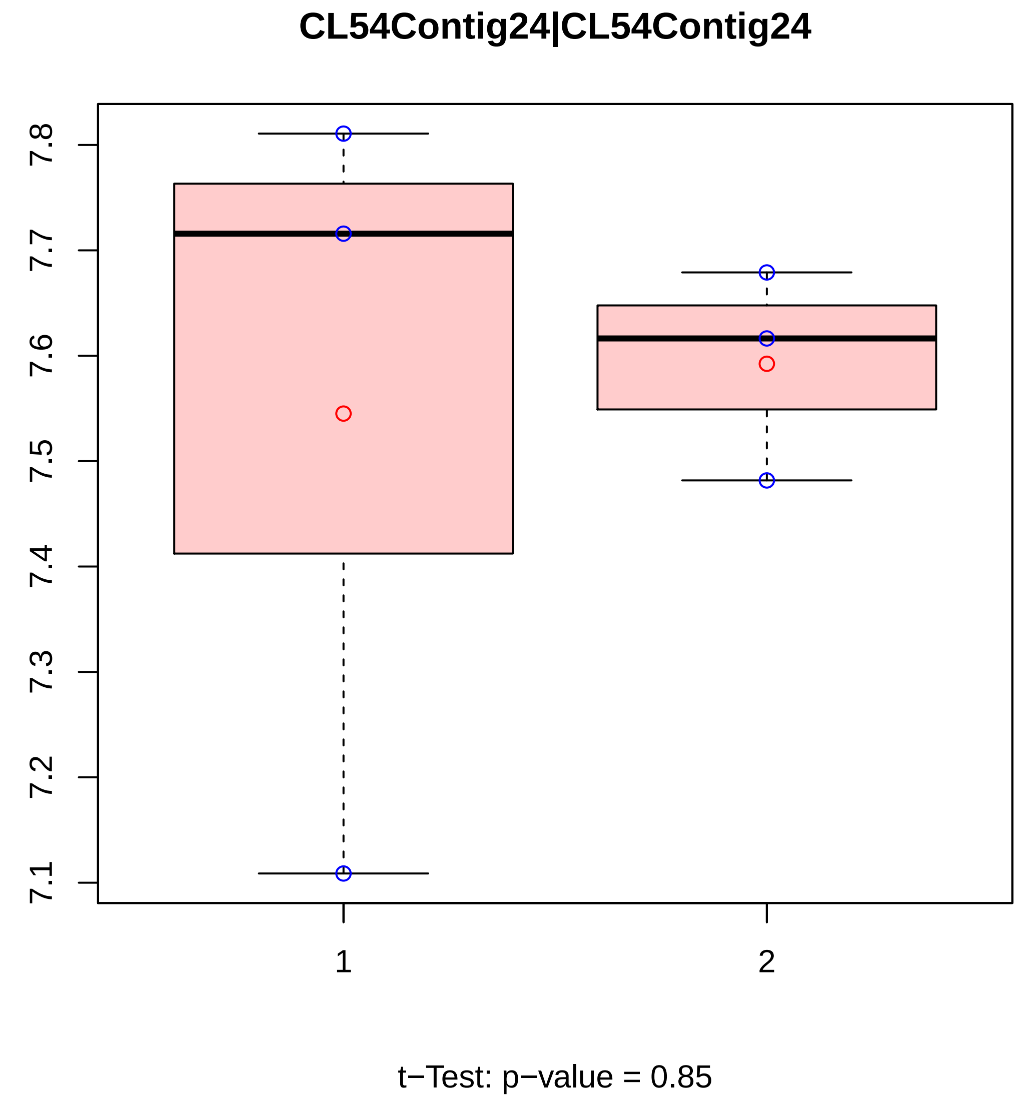

CL54Contig24|CL54Contig24
7.1
7.2
7.3
7.4
7.5
7.6
7.7
7.8
1
2
t−Test: p−value = 0.85

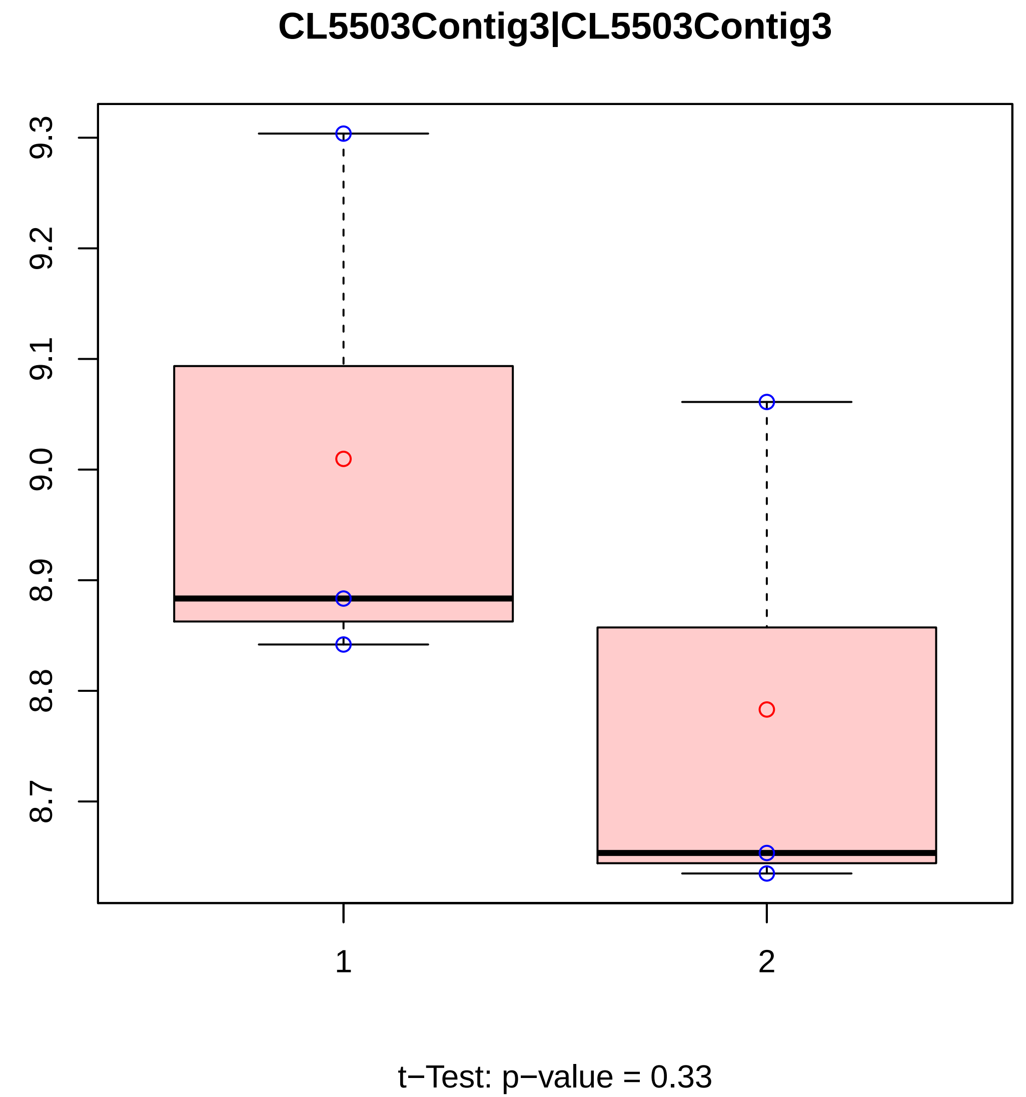
CL5503Contig3|CL5503Contig3
9.3
9.2
9.1
9.0
8.9
8.8
8.7
1
2
t−Test: p−value = 0.33

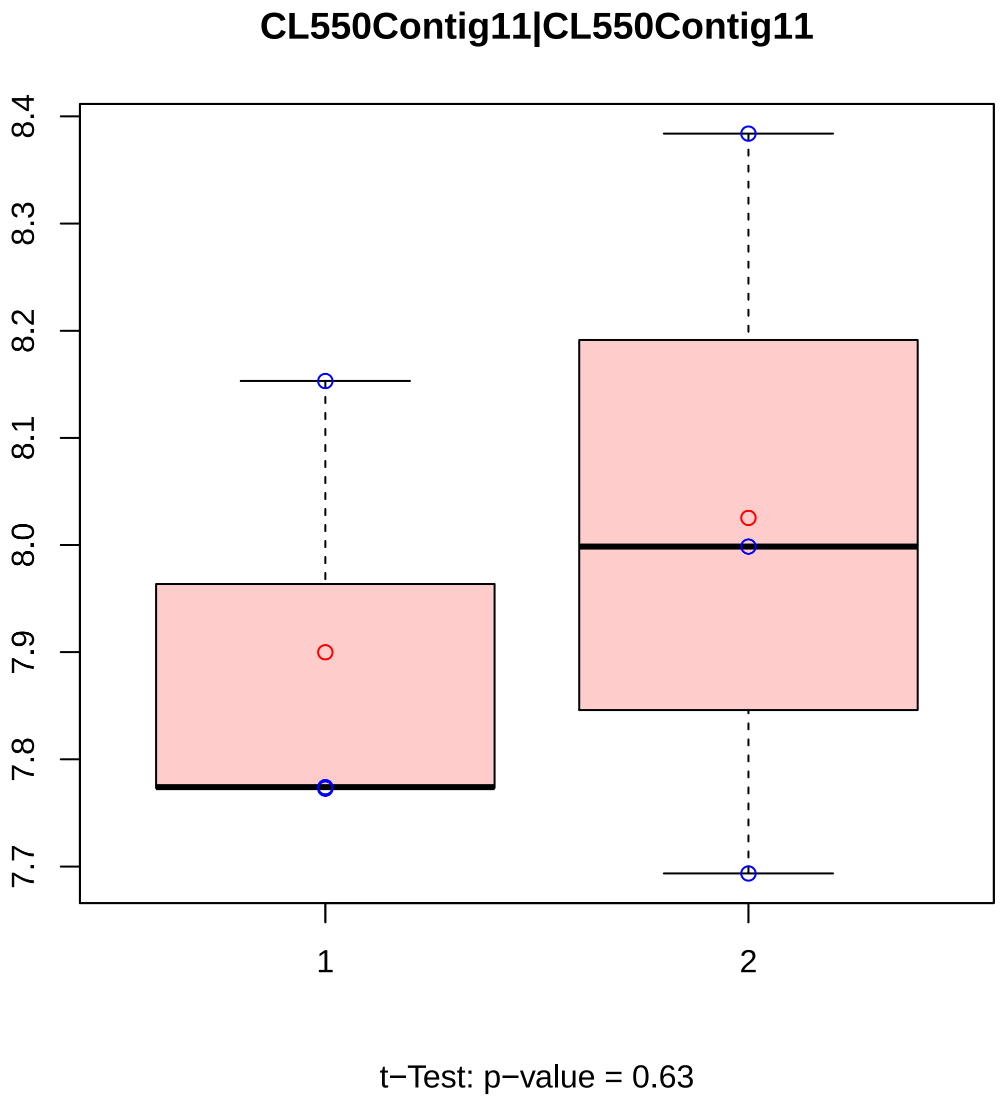

CL550Contig11|CL550Contig11
8.4
8.3
8.2
8.1
8.0
7.9
7.8
7.7
1
2
t−Test: p−value = 0.63

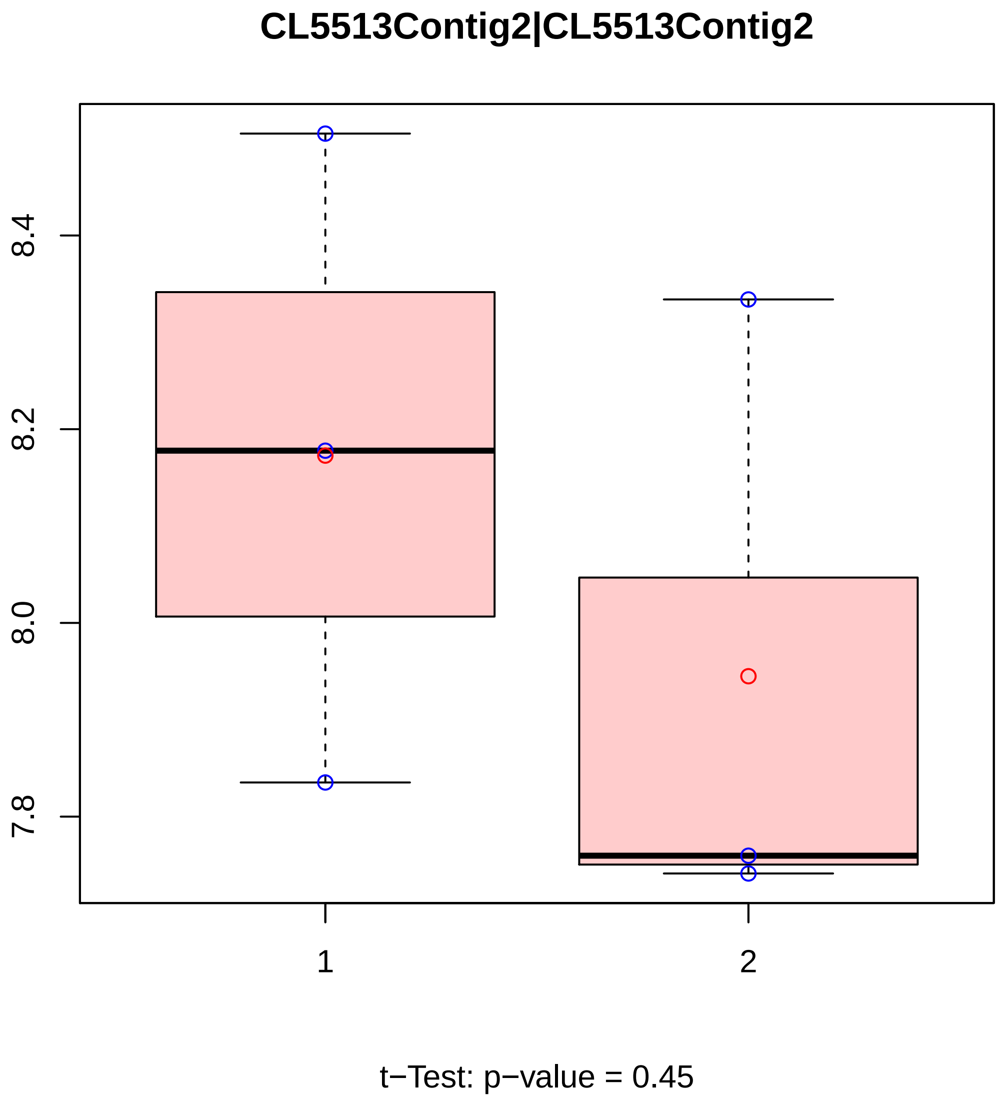
CL5513Contig2|CL5513Contig2
8.4
8.2
8.0
7.8
1
2
t−Test: p−value = 0.45

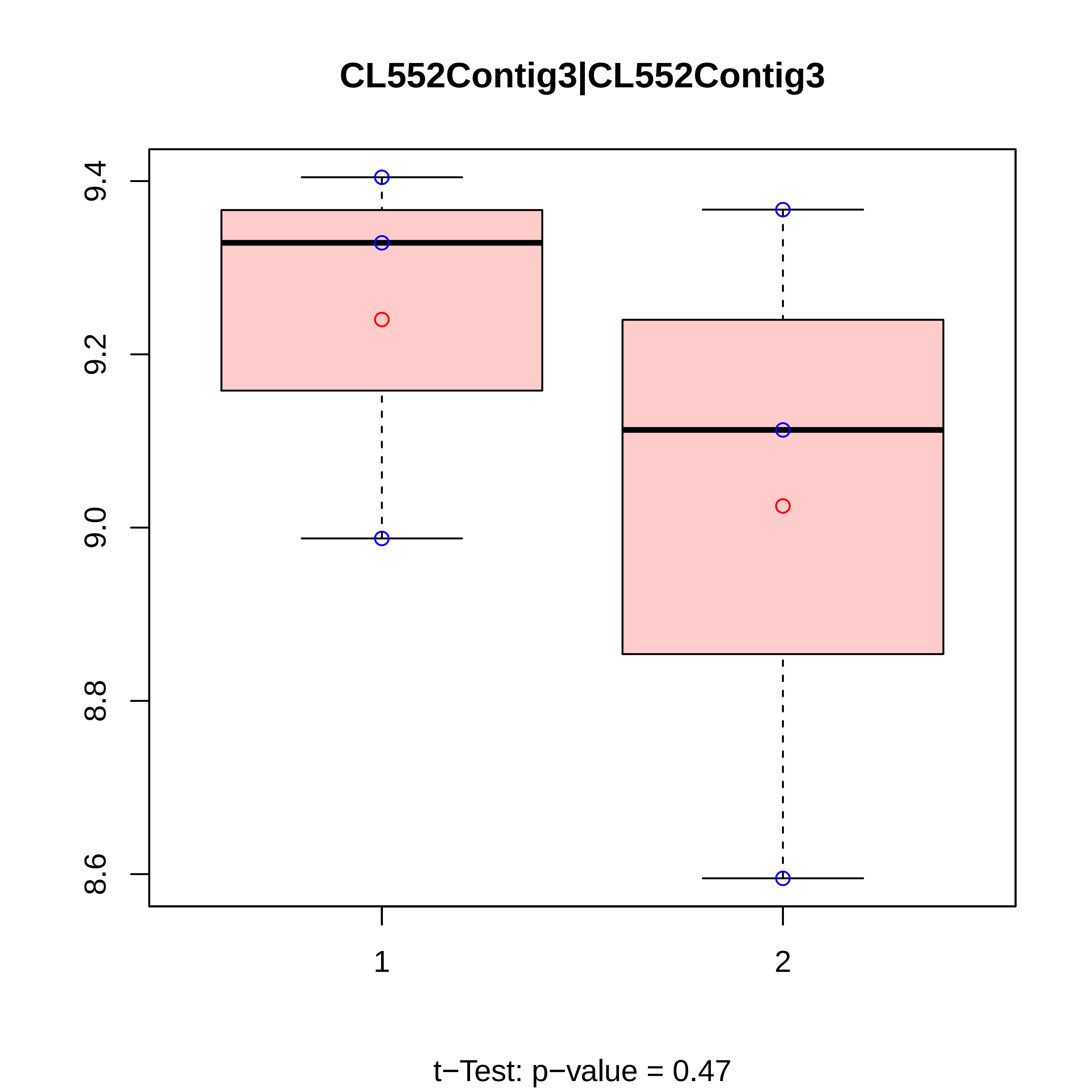
CL552Contig3|CL552Contig3
9.4
9.2
9.0
8.8
8.6
1
2
t−Test: p−value = 0.47

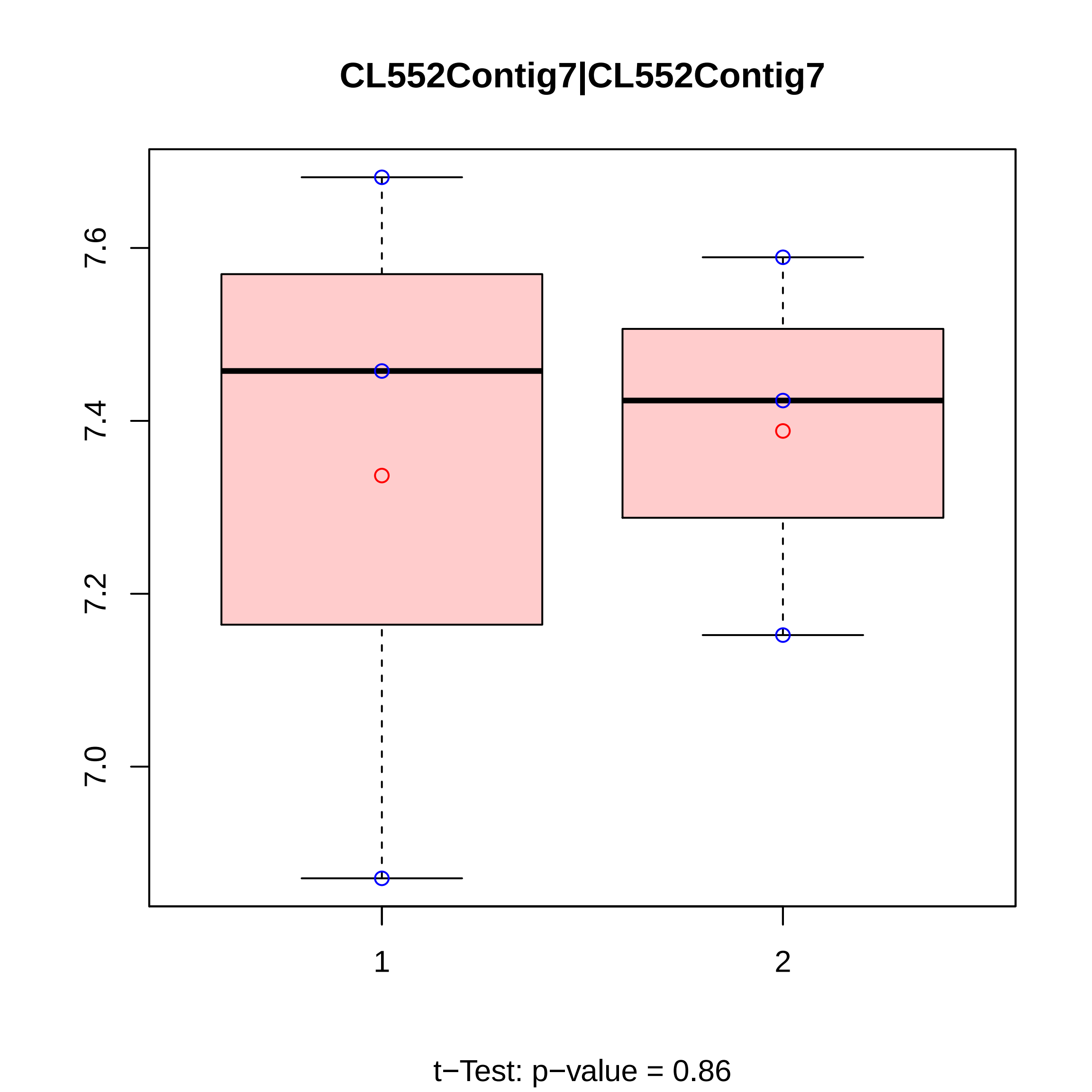
CL552Contig7|CL552Contig7
7.0
7.2
7.4
7.6
1
2
t−Test: p−value = 0.86

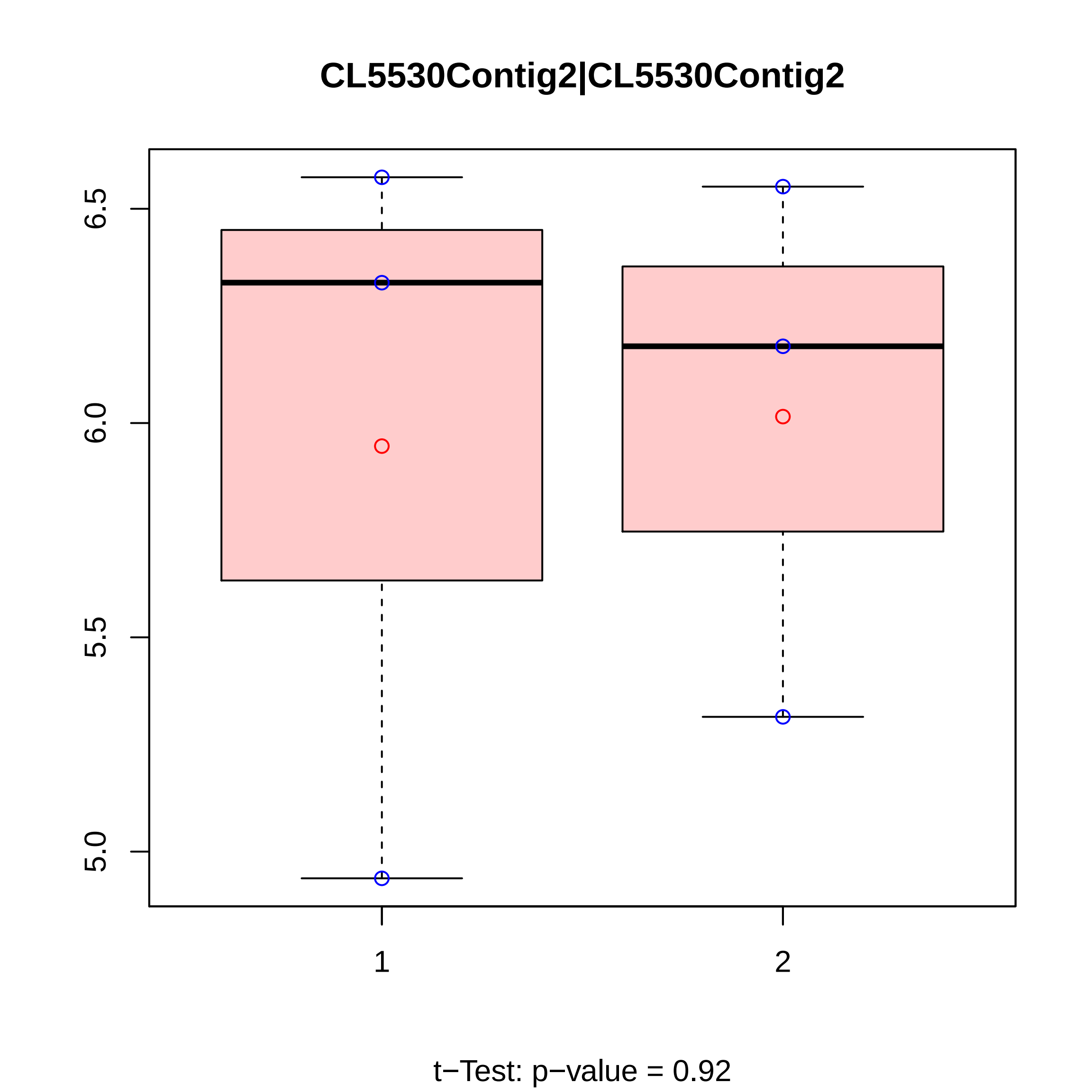

CL5530Contig2|CL5530Contig2
6.5
6.0
5.5
5.0
1
2
t−Test: p−value = 0.92

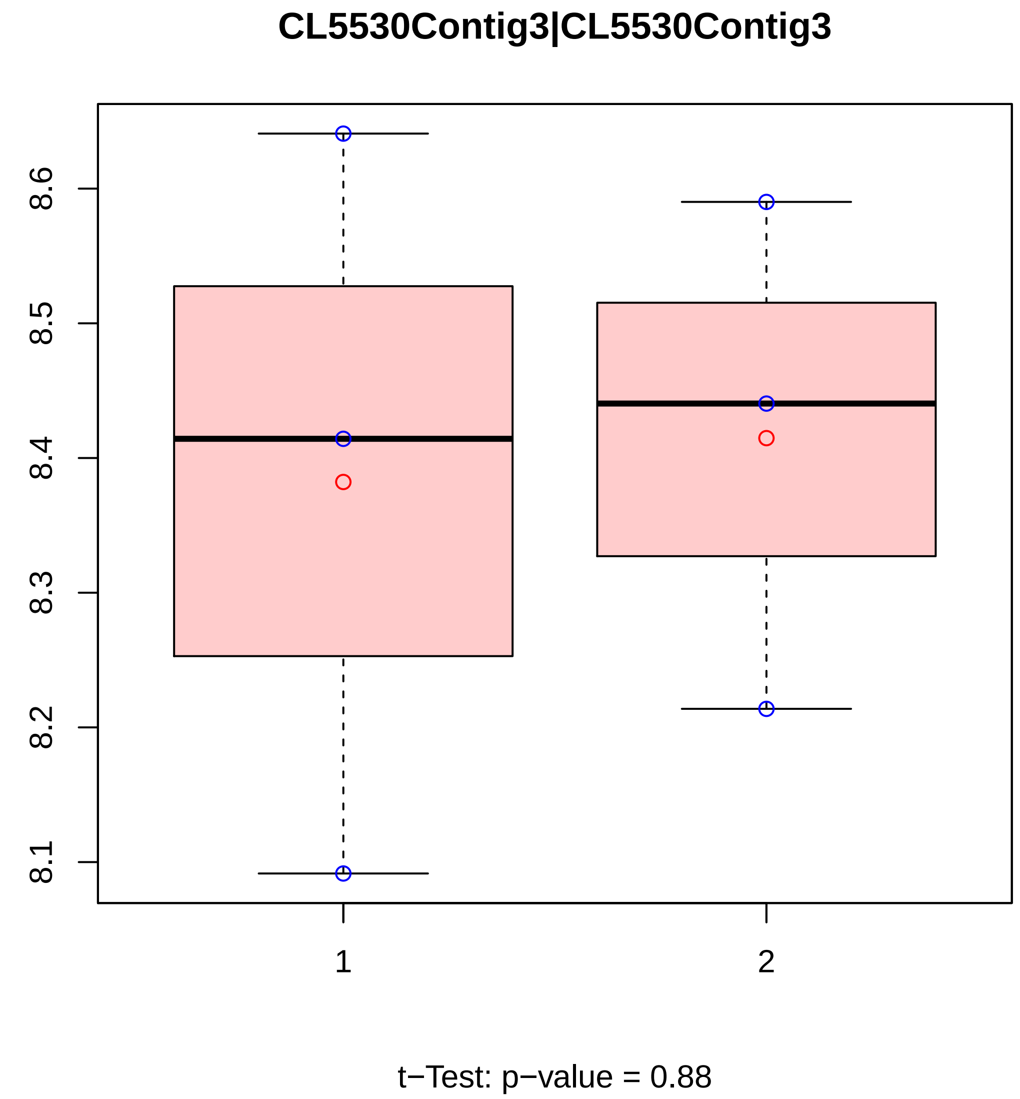
CL5530Contig3|CL5530Contig3
8.1
8.2
8.3
8.4
8.5
8.6
1
2
t−Test: p−value = 0.88

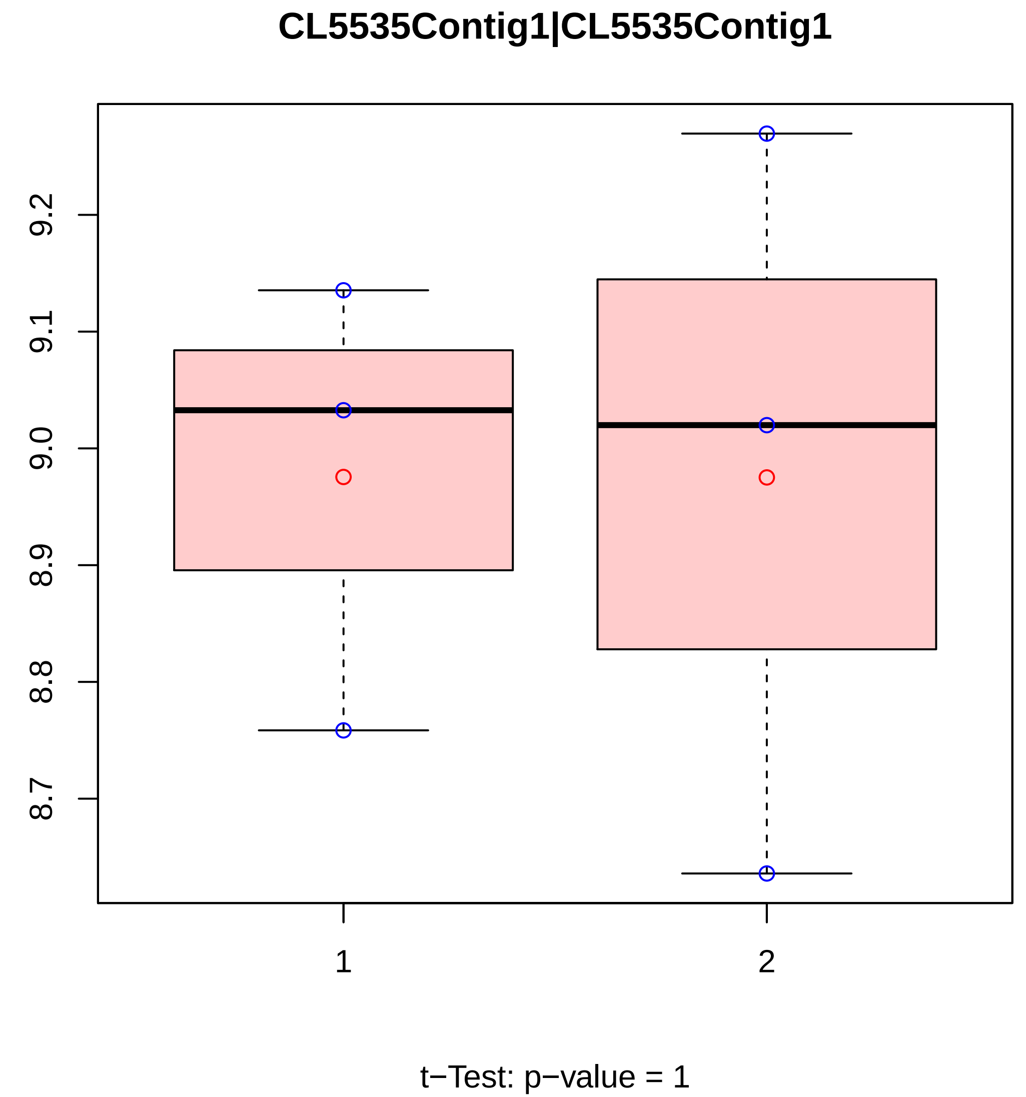
CL5535Contig1|CL5535Contig1
9.2
9.1
9.0
8.9
8.8
8.7
1
2
t−Test: p−value = 1

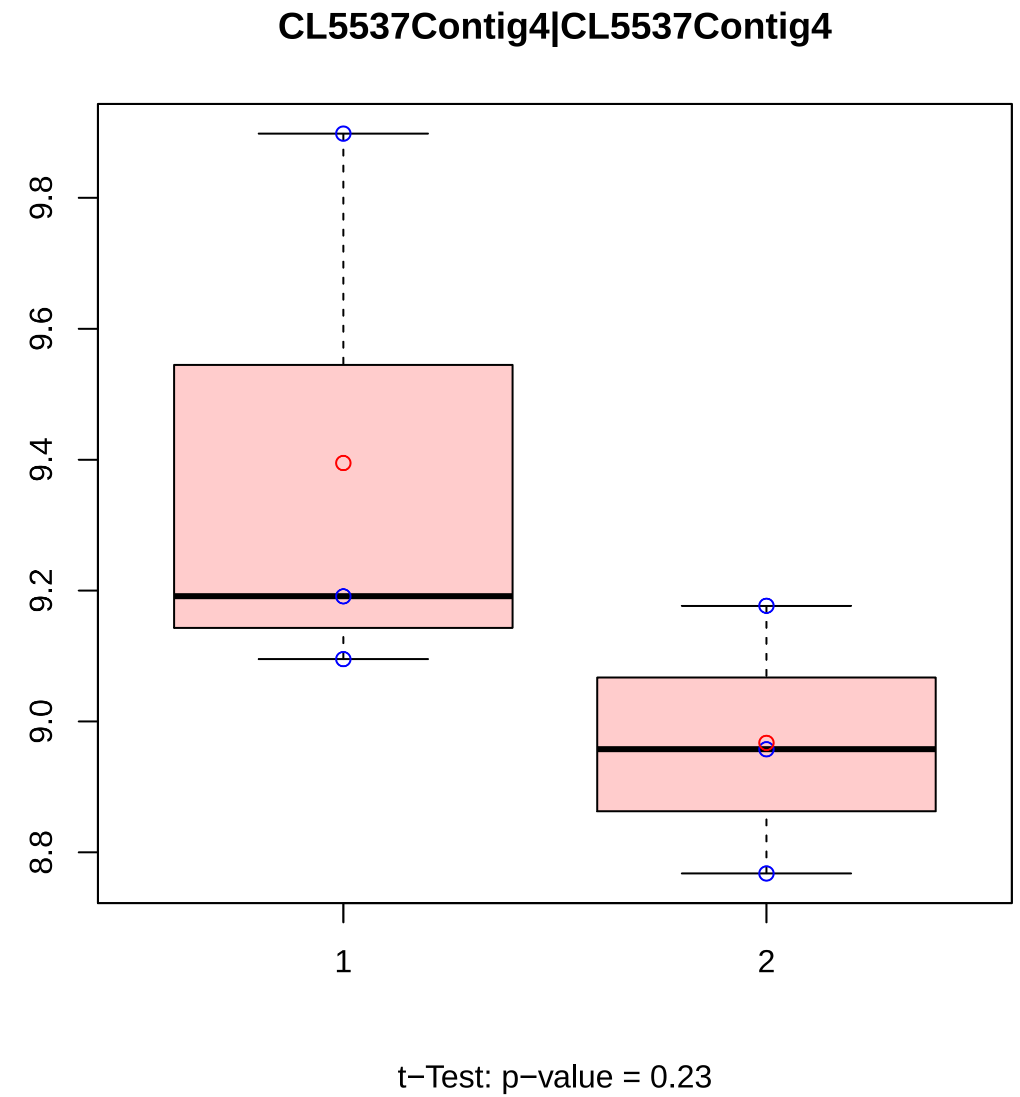

CL5537Contig4|CL5537Contig4
9.8
9.6
9.4
9.2
9.0
8.8
1
2
t−Test: p−value = 0.23

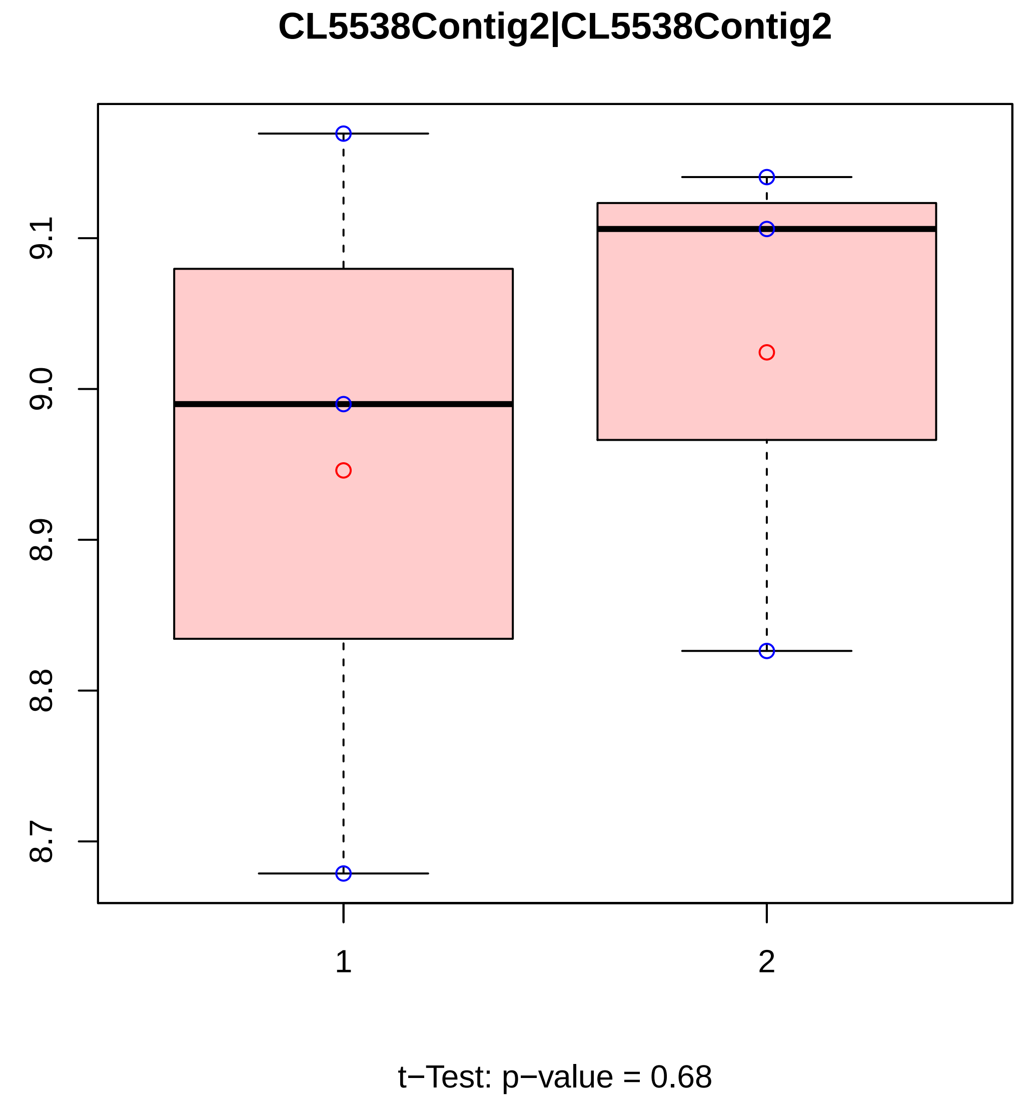

CL5538Contig2|CL5538Contig2
9.1
9.0
8.9
8.8
8.7
1
2
t−Test: p−value = 0.68

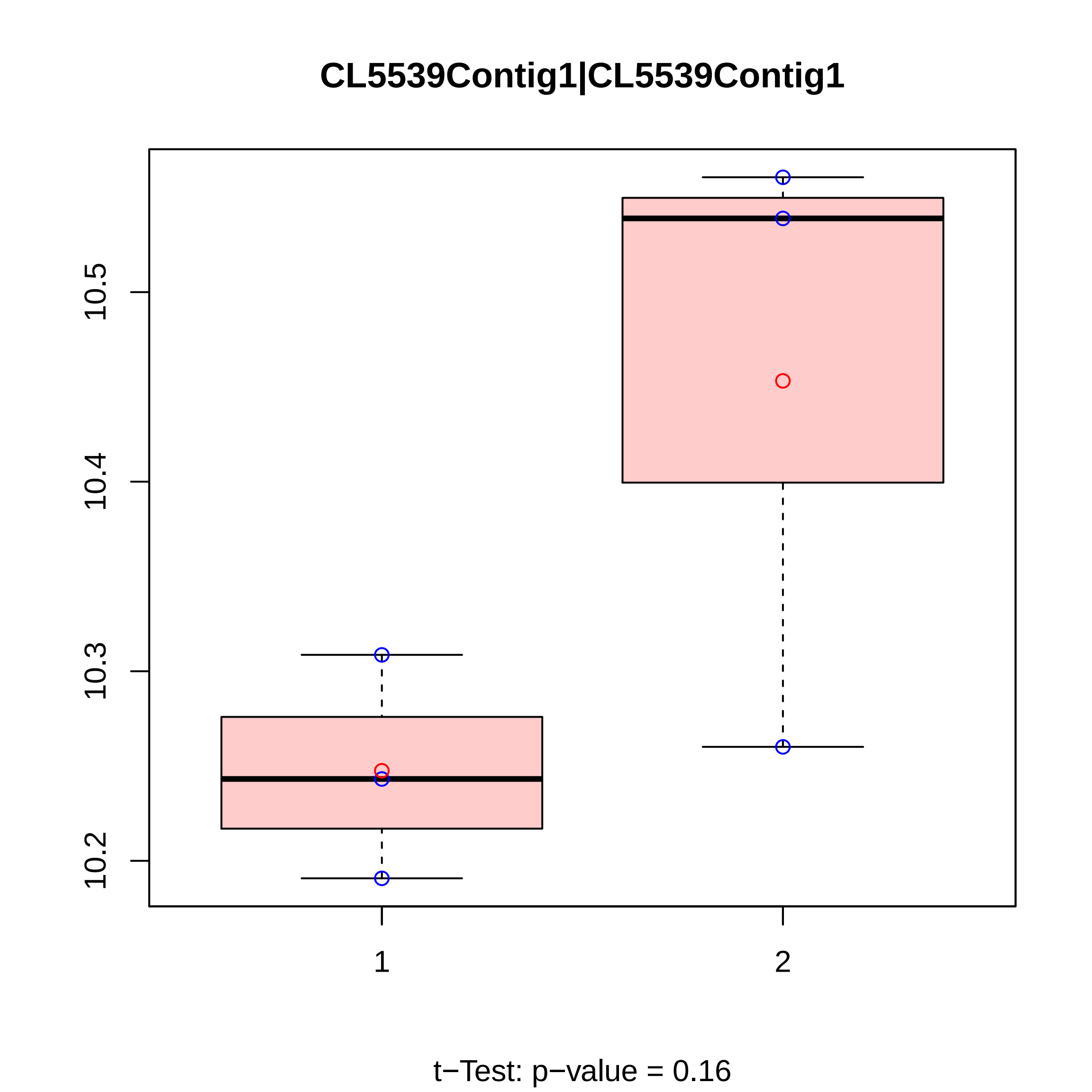
CL5539Contig1|CL5539Contig1
10.2
10.3
10.4
10.5
1
2
t−Test: p−value = 0.16

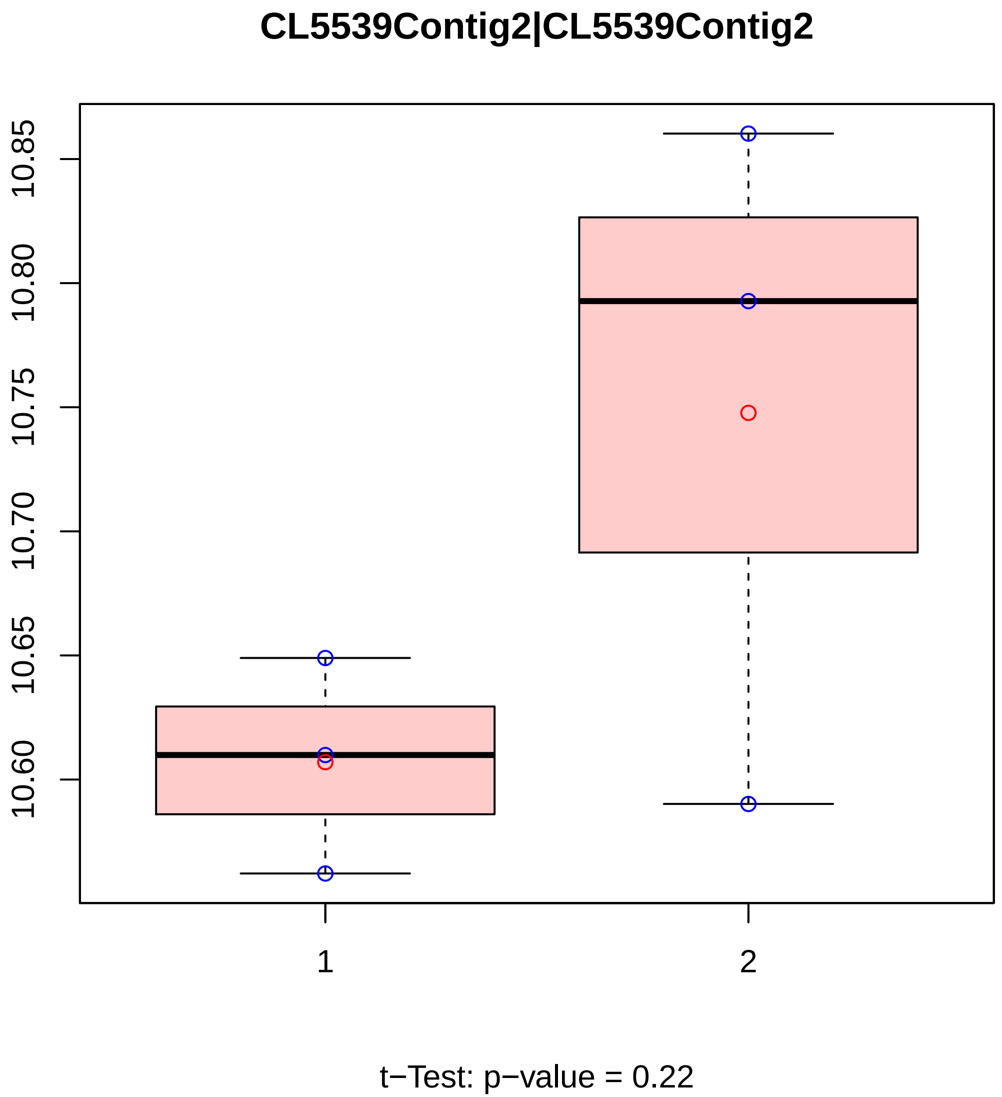
CL5539Contig2|CL5539Contig2
10.85
10.80
10.75
10.70
10.65
10.60
1
2
t−Test: p−value = 0.22

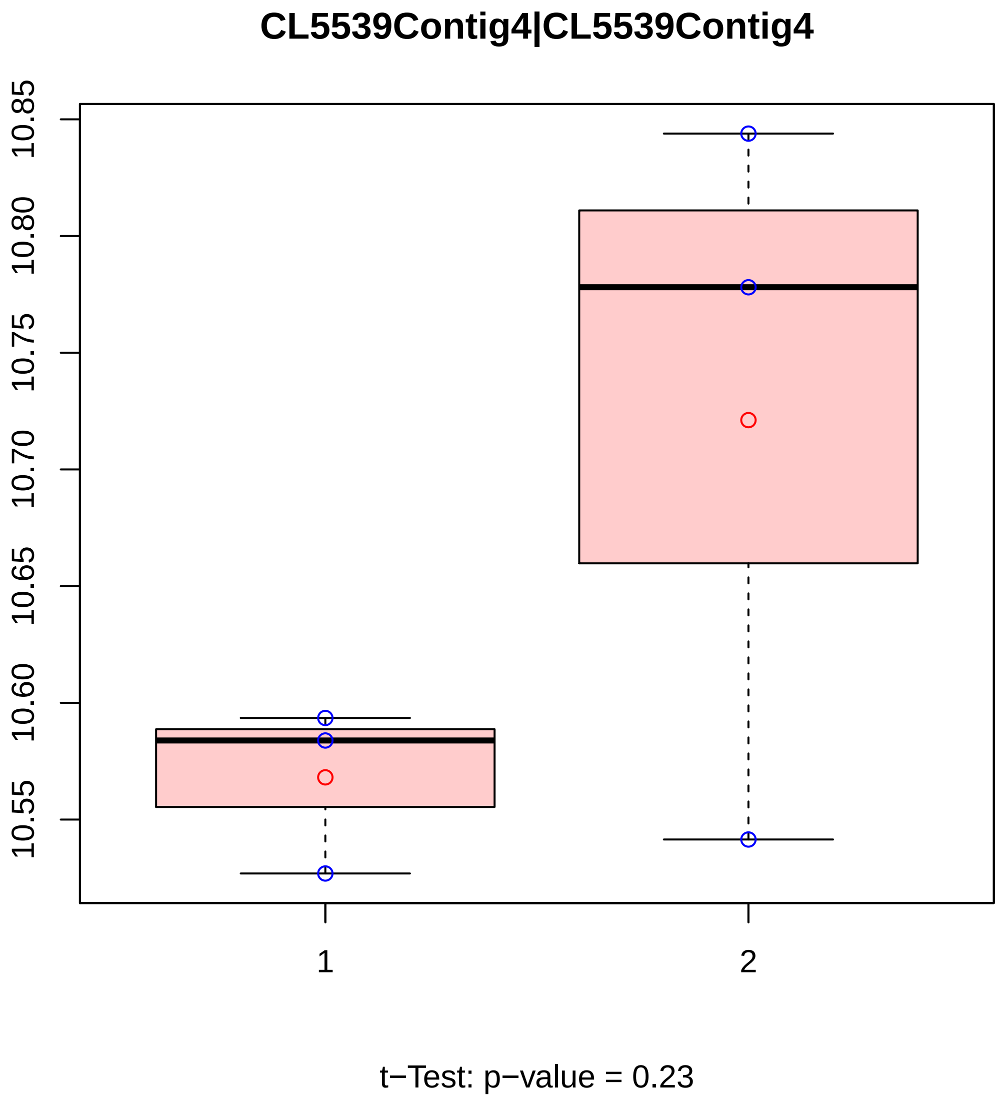

CL5539Contig4|CL5539Contig4
10.85
10.80
10.75
10.70
10.65
10.60
10.55
1
2
t−Test: p−value = 0.23

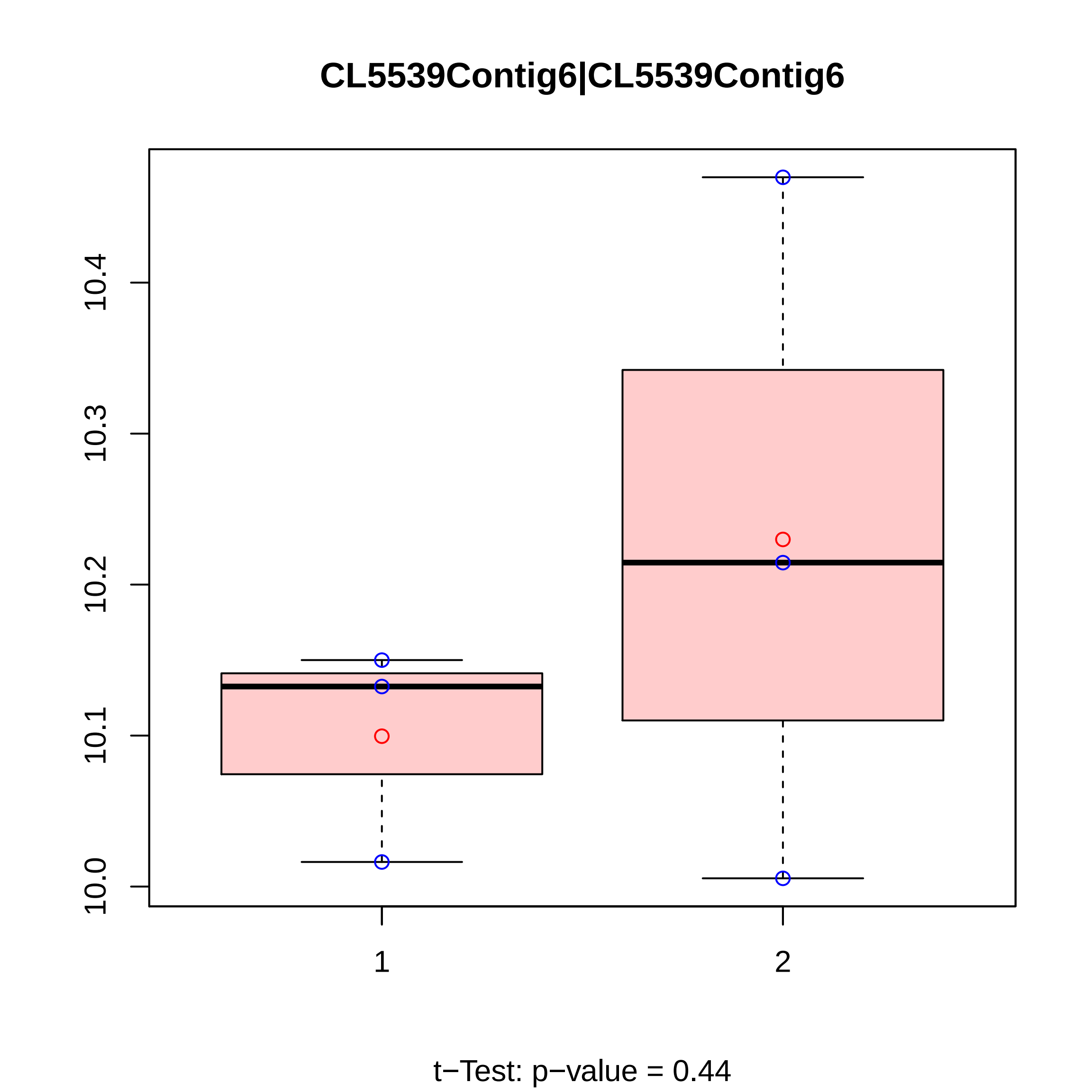

CL5539Contig6|CL5539Contig6
10.0
10.1
10.2
10.3
10.4
1
2
t−Test: p−value = 0.44

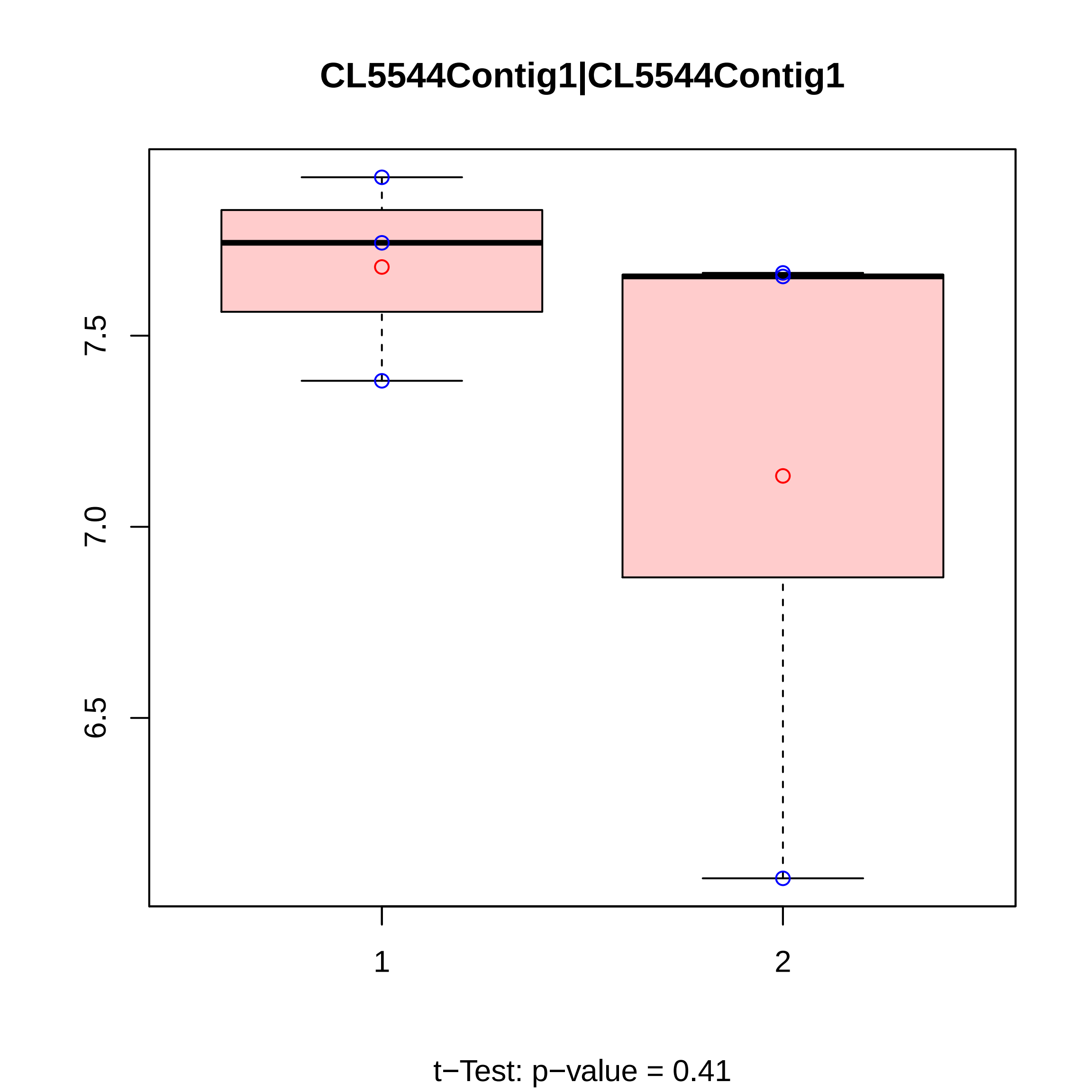
CL5544Contig1|CL5544Contig1
7.5
7.0
6.5
1
2
t−Test: p−value = 0.41

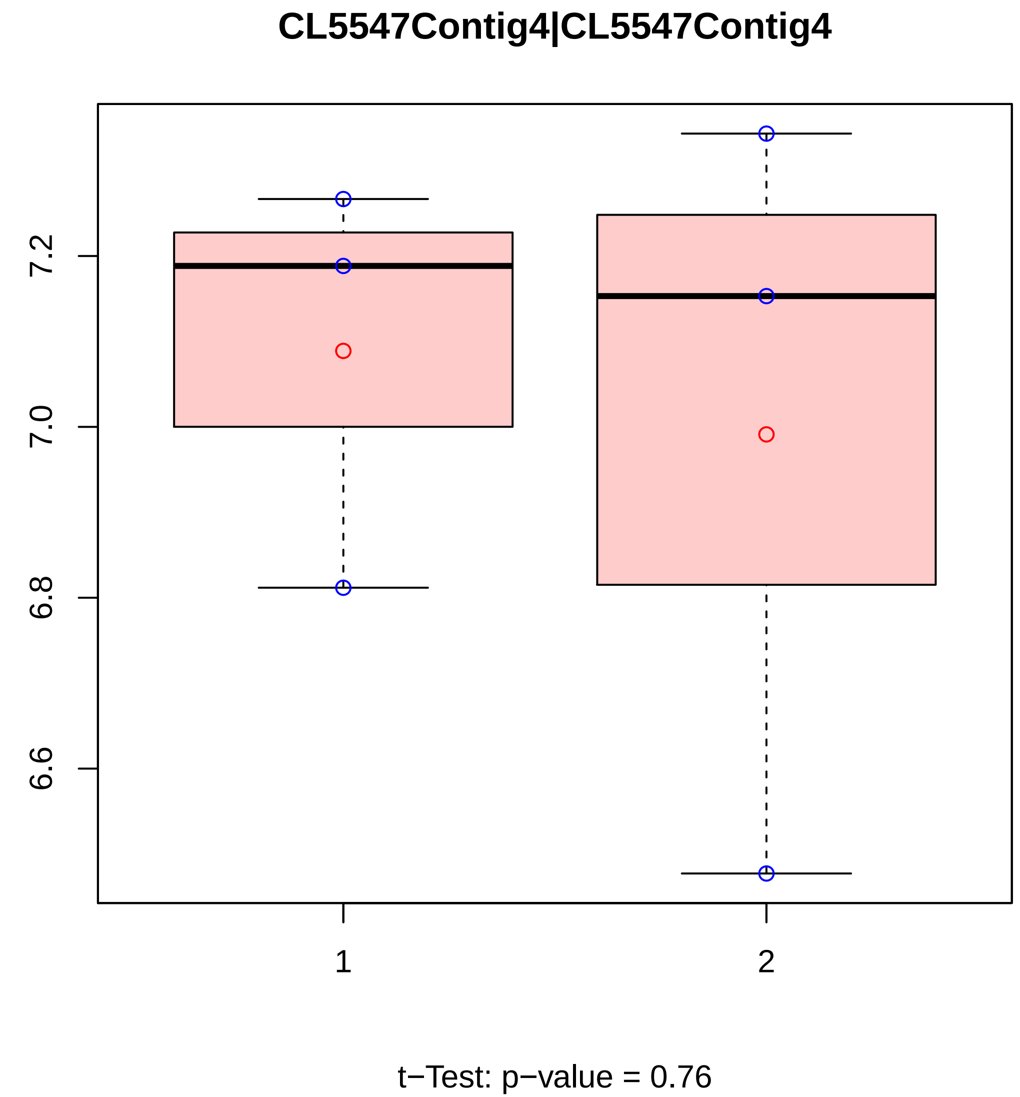
CL5547Contig4|CL5547Contig4
7.2
7.0
6.8
6.6
1
2
t−Test: p−value = 0.76

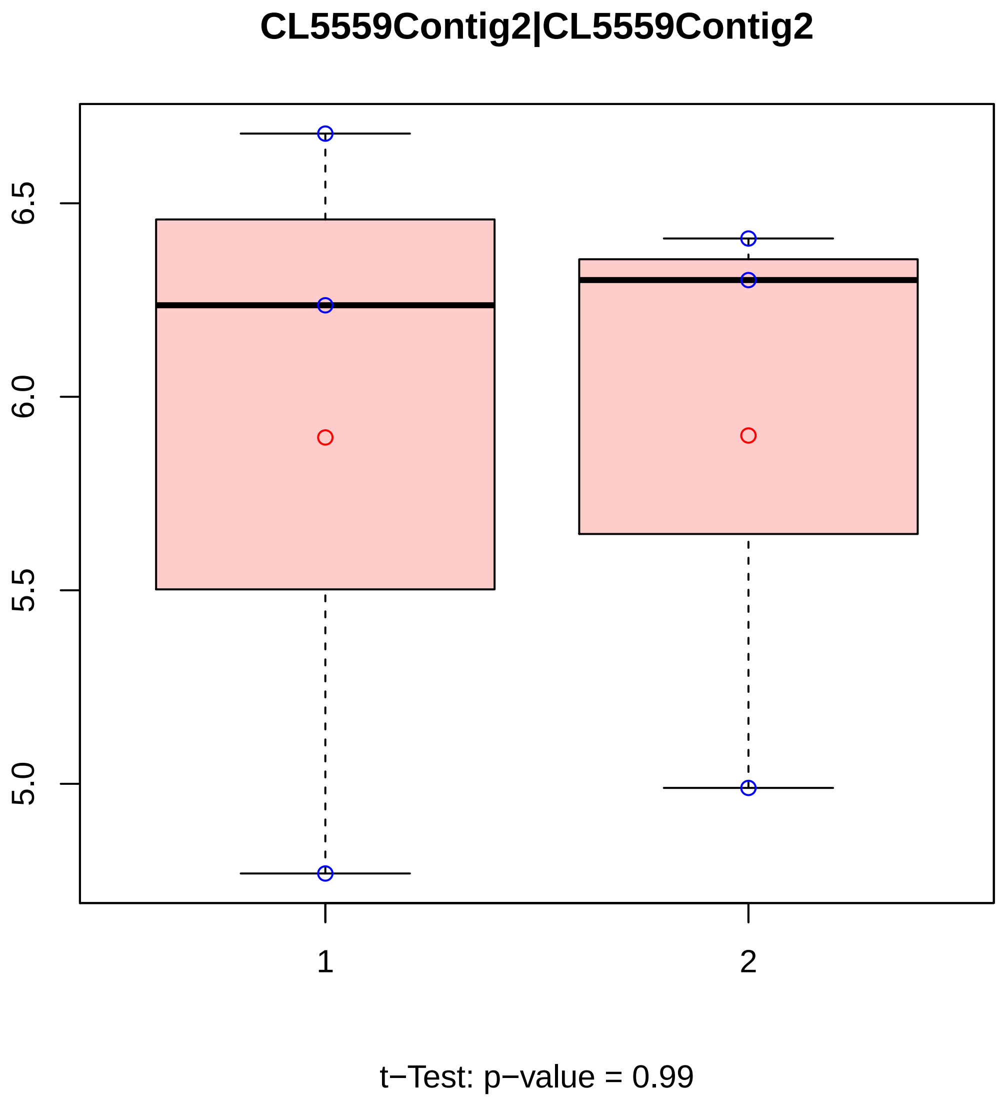

CL5559Contig2|CL5559Contig2
6.5
6.0
5.5
5.0
1
2
t−Test: p−value = 0.99

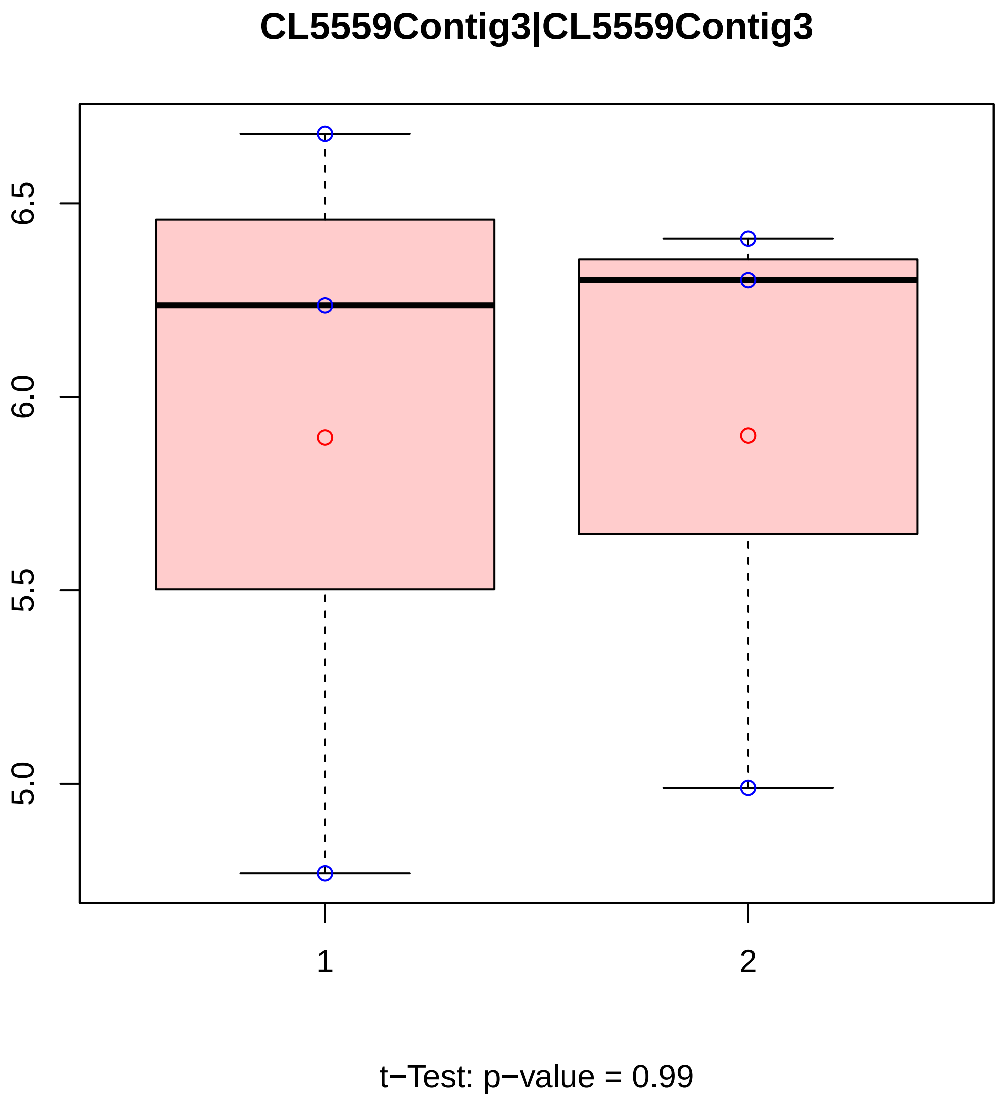
CL5559Contig3|CL5559Contig3
6.5
6.0
5.5
5.0
1
2
t−Test: p−value = 0.99

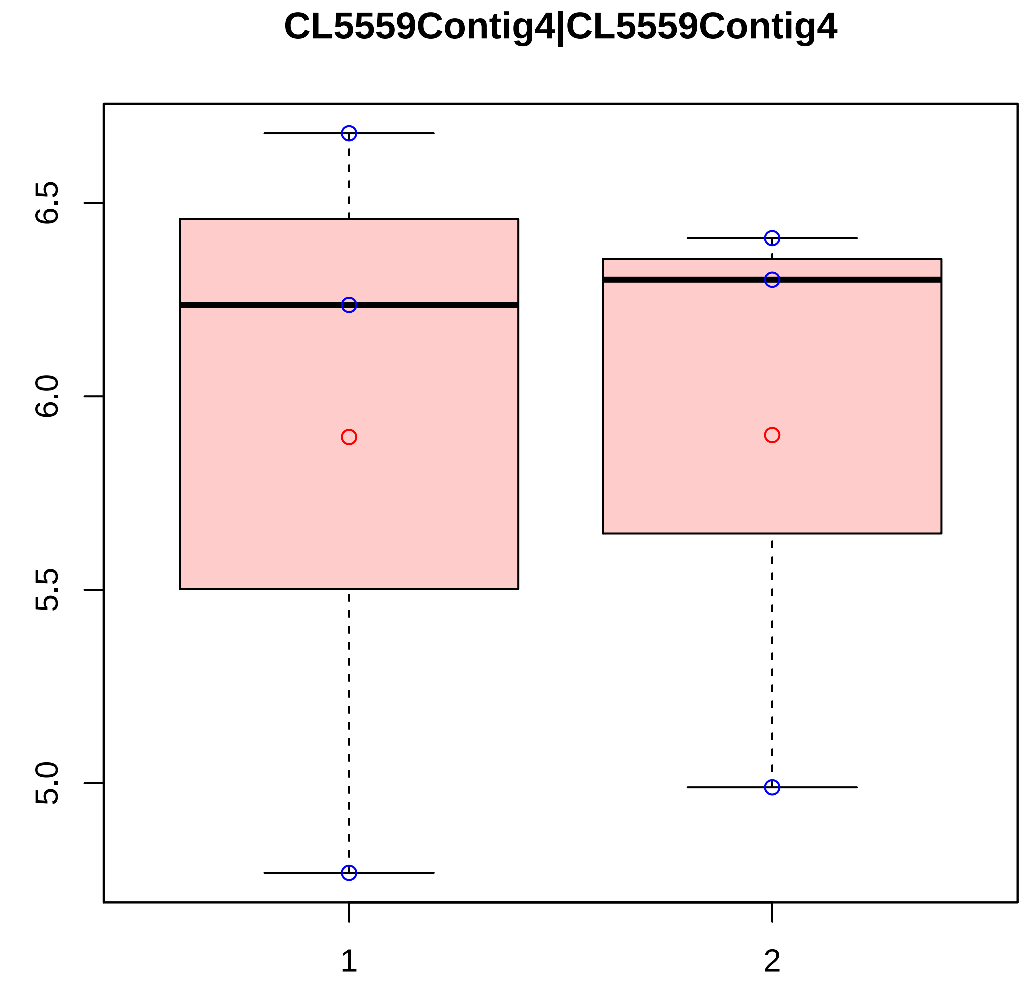


t−Test: p−value = 0.99

## CL555Contig11|CL555Contig11

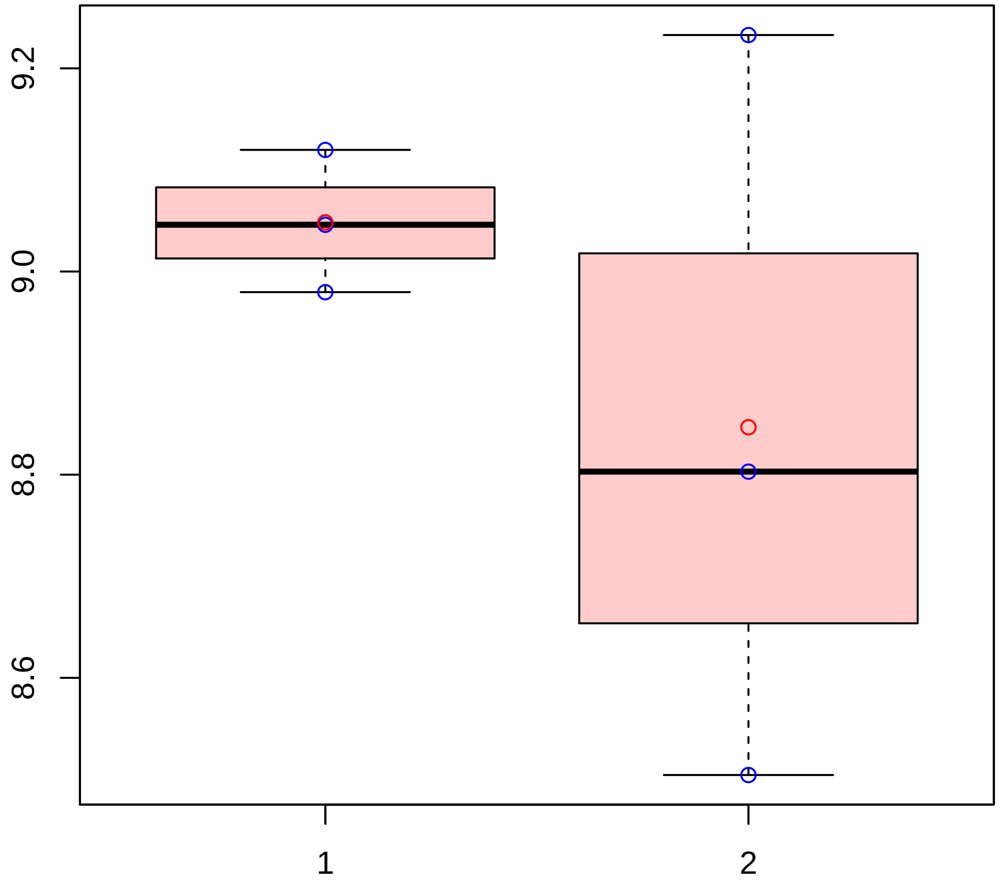


t−Test: p−value = 0.44

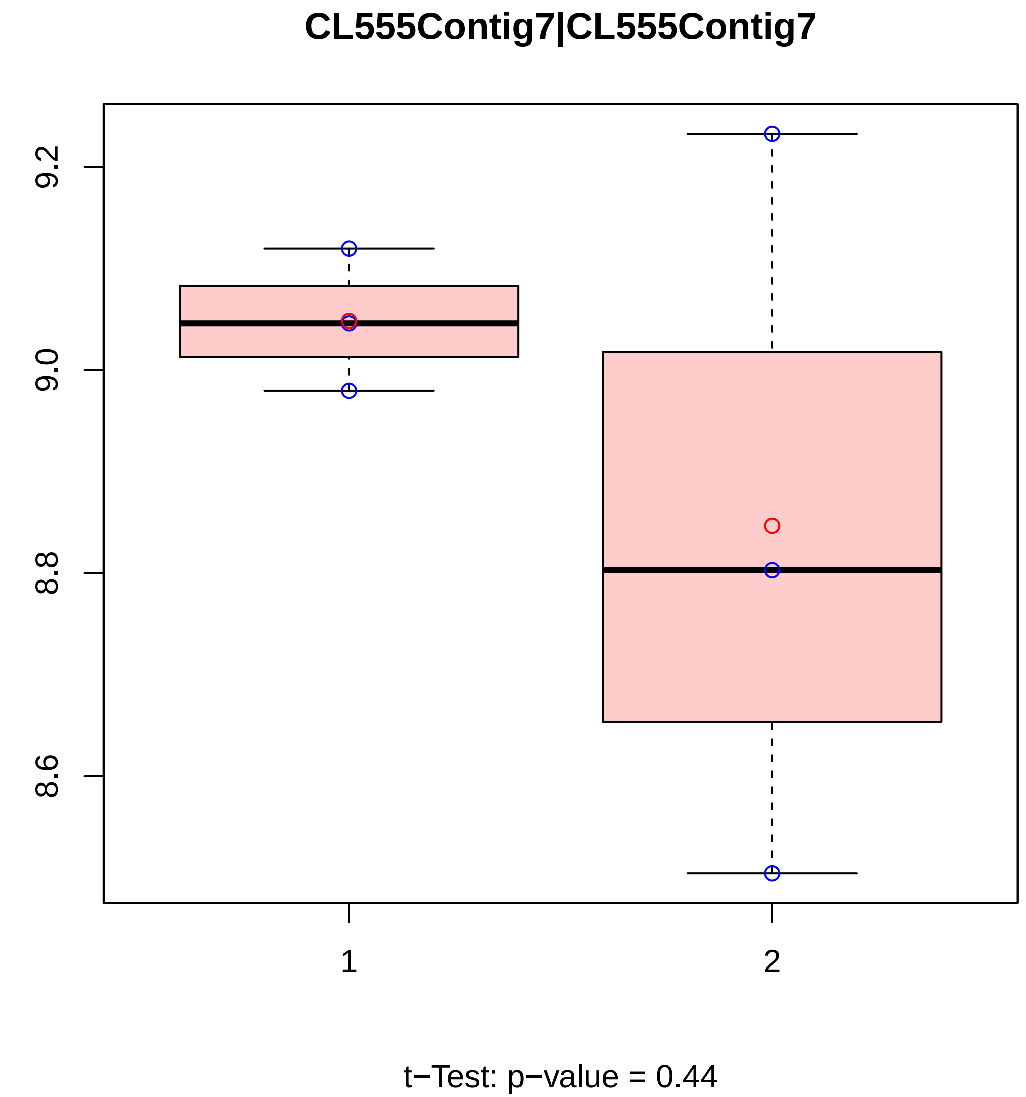
CL555Contig7|CL555Contig7
9.2
9.0
8.8
8.6
1
2
t−Test: p−value = 0.44

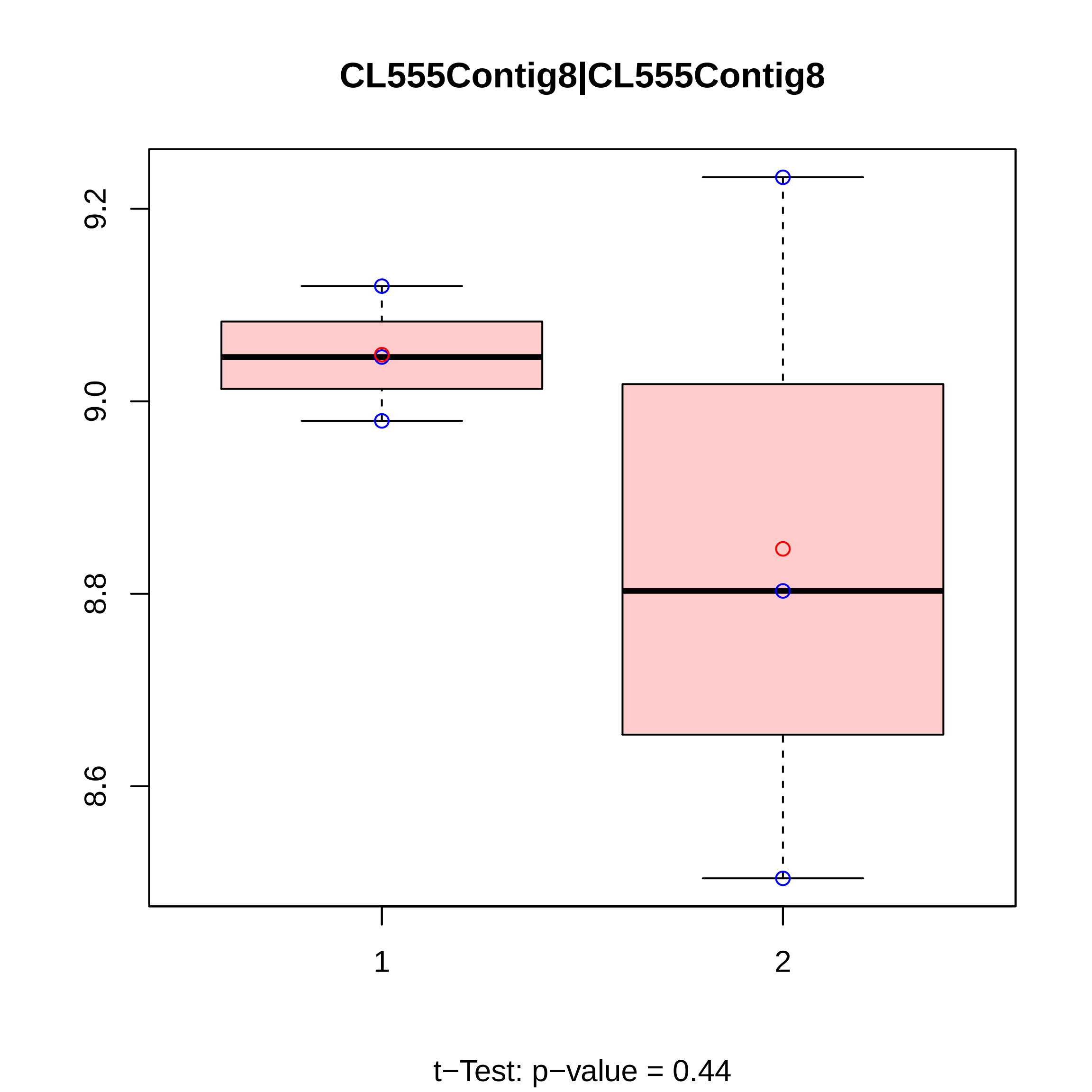

CL555Contig8|CL555Contig8
9.2
9.0
8.8
8.6
1
2
t−Test: p−value = 0.44

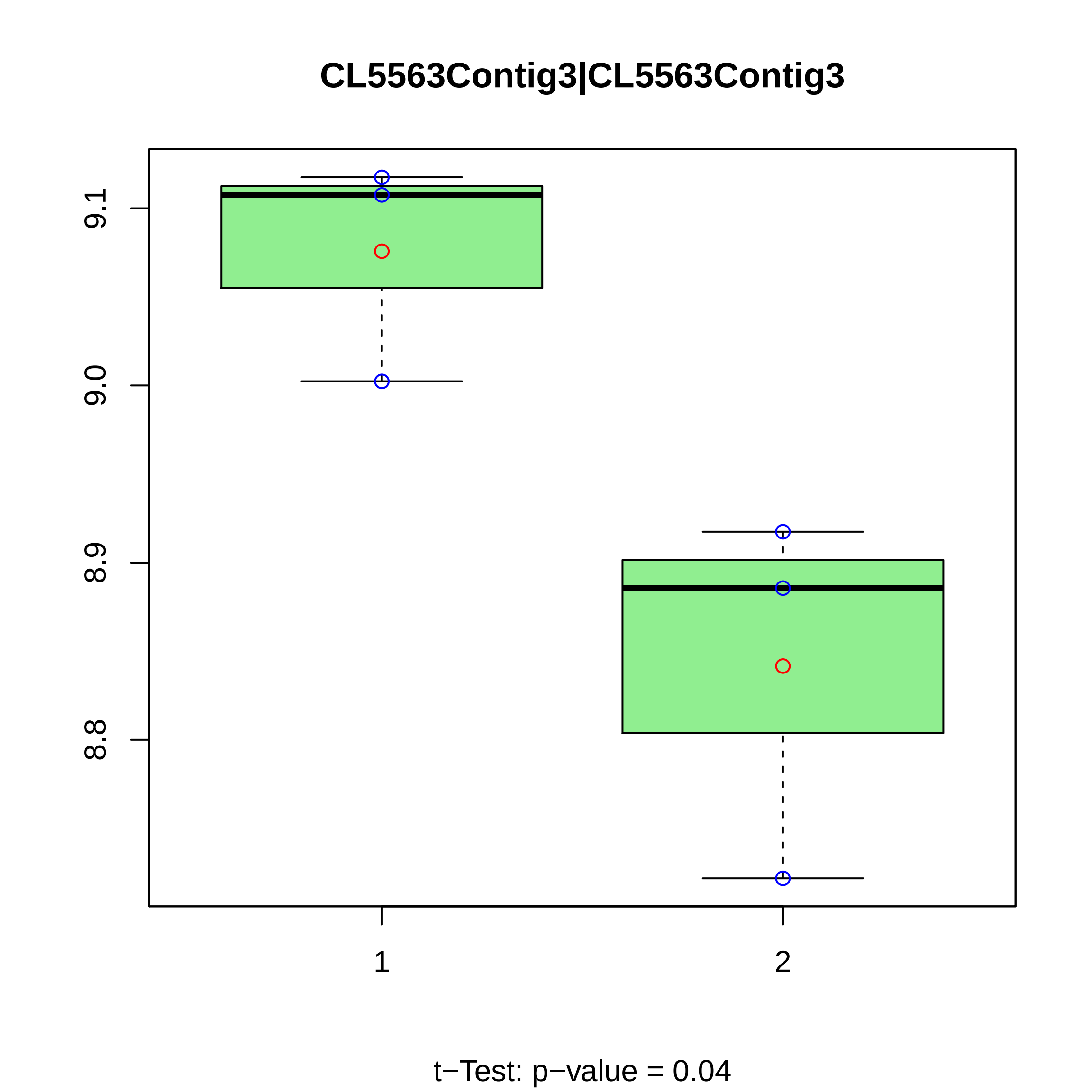

CL5563Contig3|CL5563Contig3
9.1
9.0
8.9
8.8
1
2
t−Test: p−value = 0.04

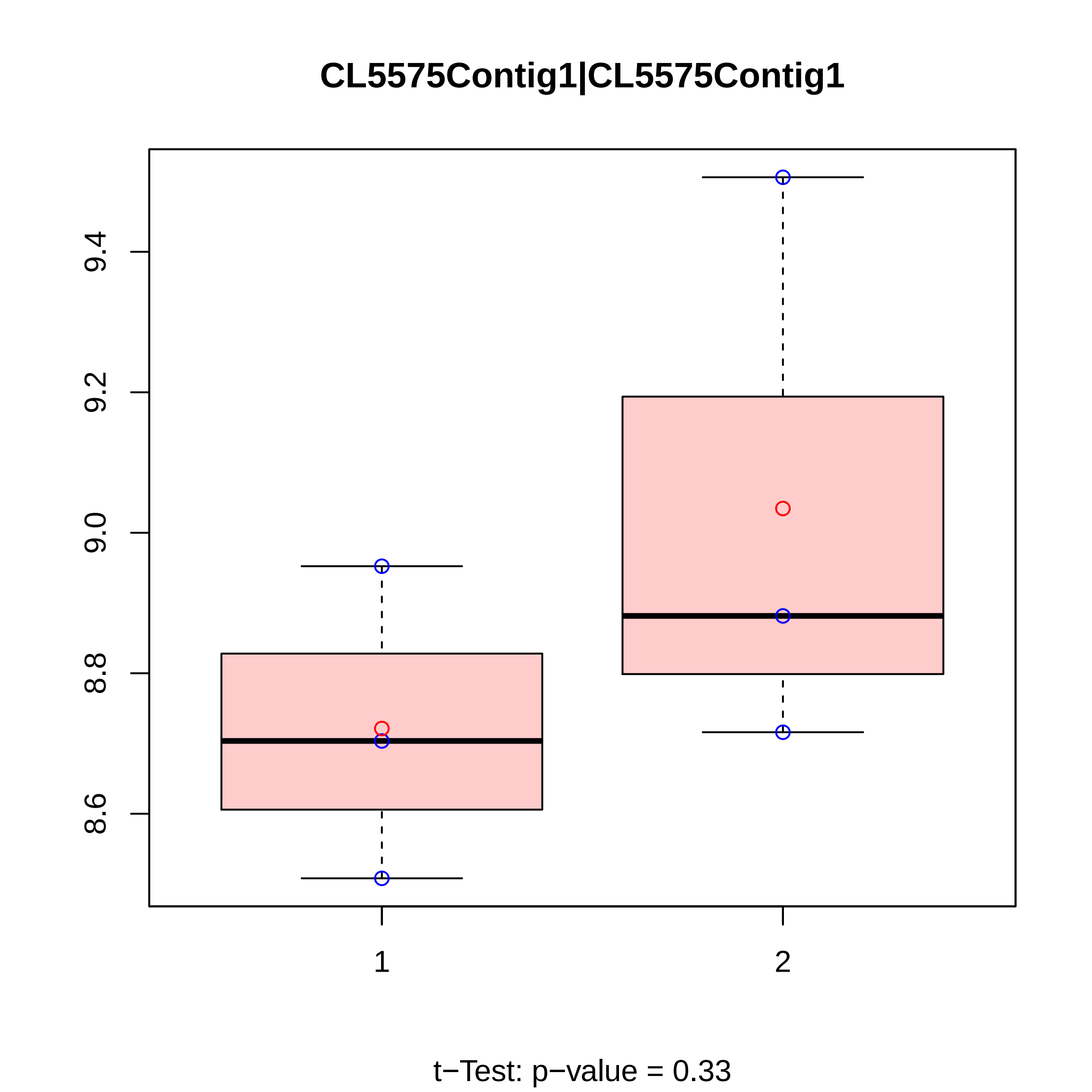

CL5575Contig1|CL5575Contig1
9.4
9.2
9.0
8.8
8.6
1
2
t−Test: p−value = 0.33

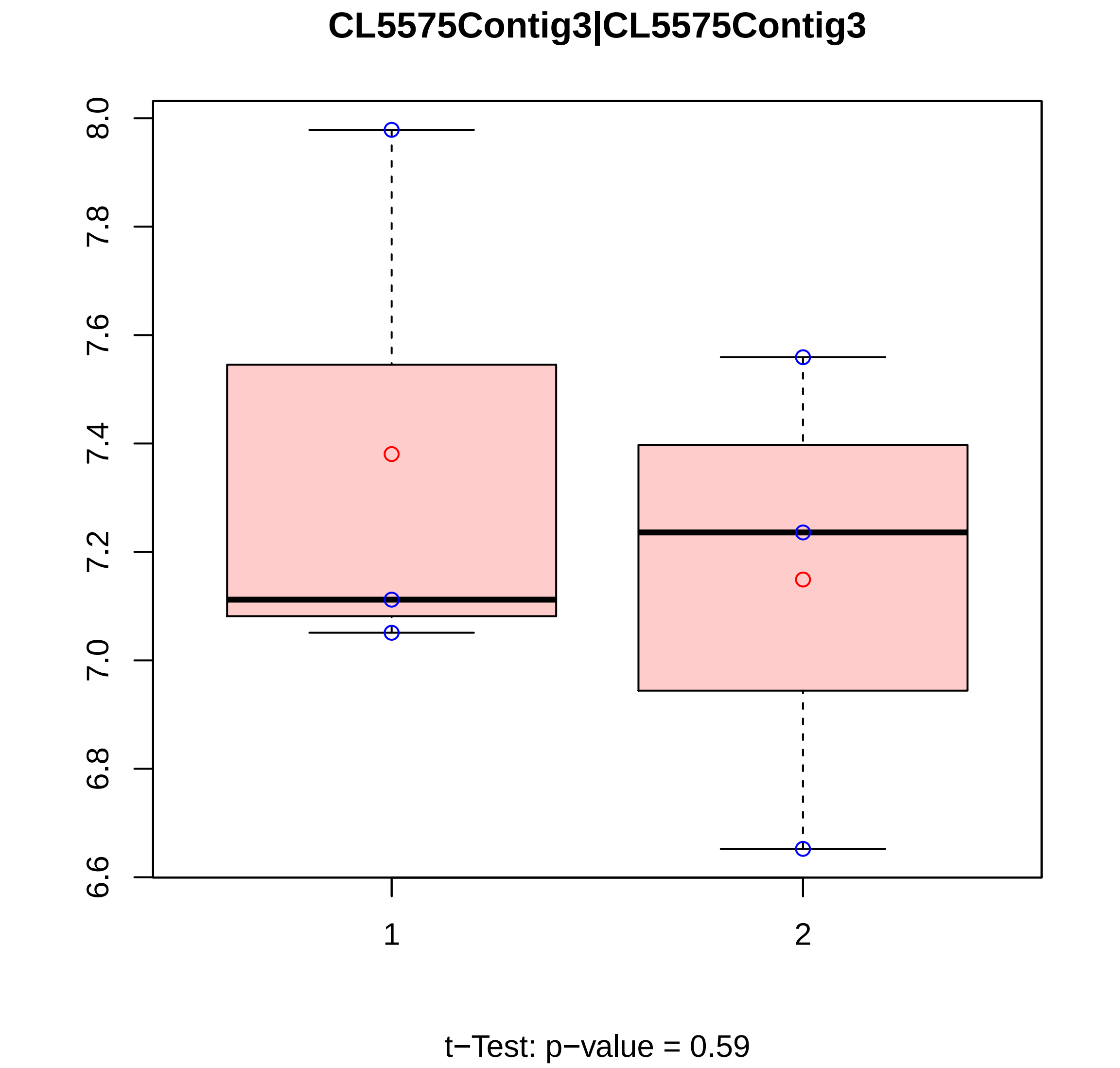

CL5575Contig3|CL5575Contig3
8.0
7.8
7.6
7.4
7.2
7.0
6.8
6.6
1
2
t−Test: p−value = 0.59

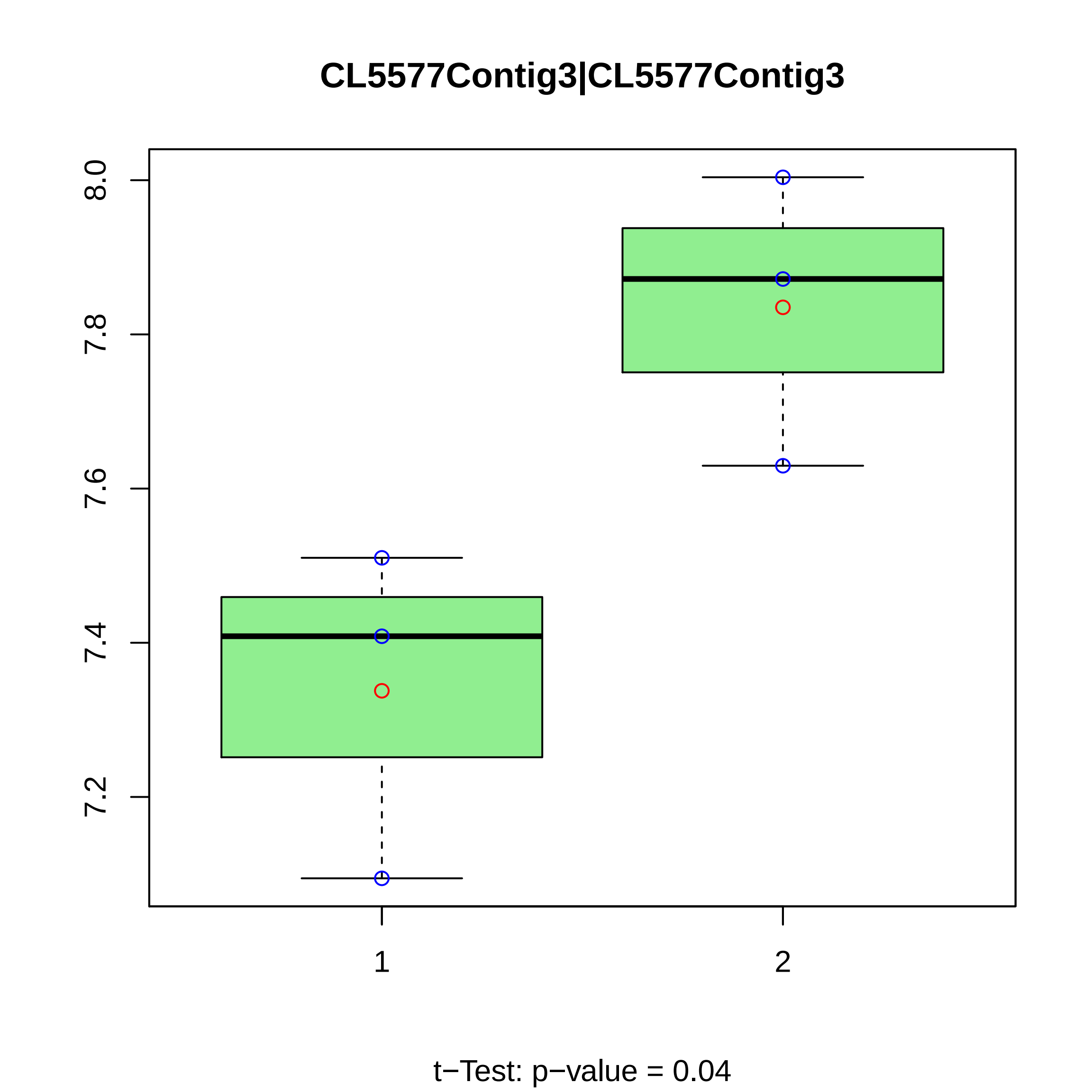

CL5577Contig3|CL5577Contig3
8.0
7.8
7.6
7.4
7.2
1
2
t−Test: p−value = 0.04

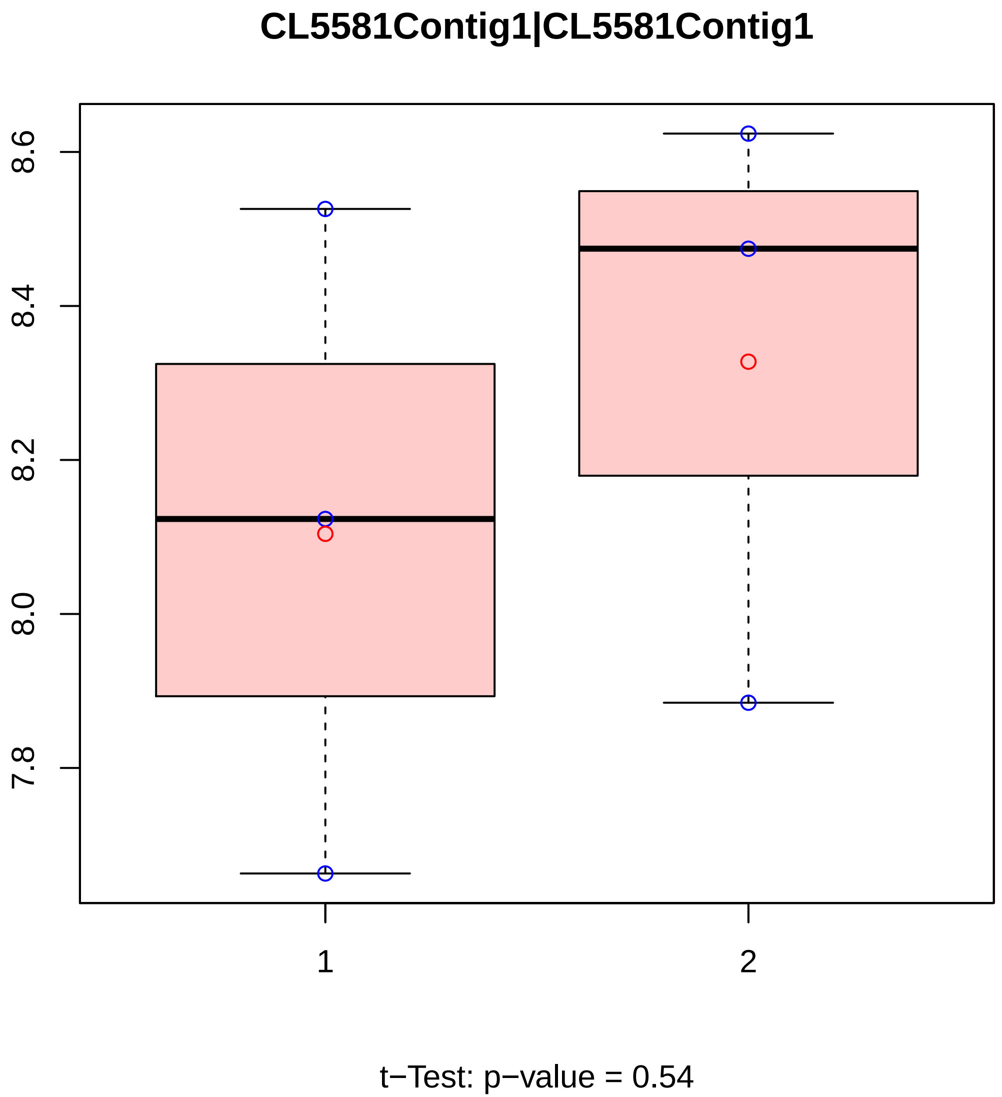
CL5581Contig1|CL5581Contig1
8.6
8.4
8.2
8.0
7.8
1
2
t−Test: p−value = 0.54

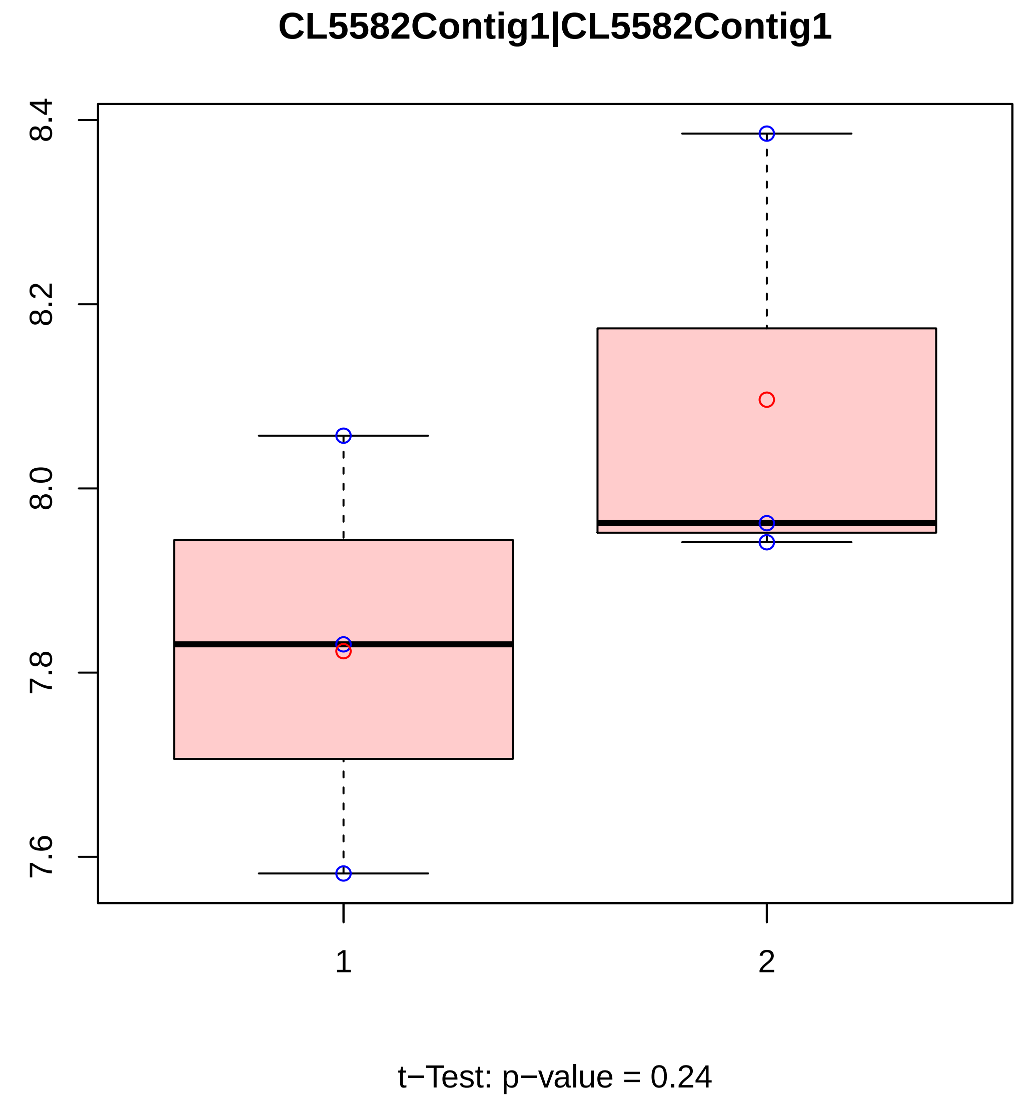
CL5582Contig1|CL5582Contig1
8.4
8.2
8.0
7.8
7.6
1
2
t−Test: p−value = 0.24

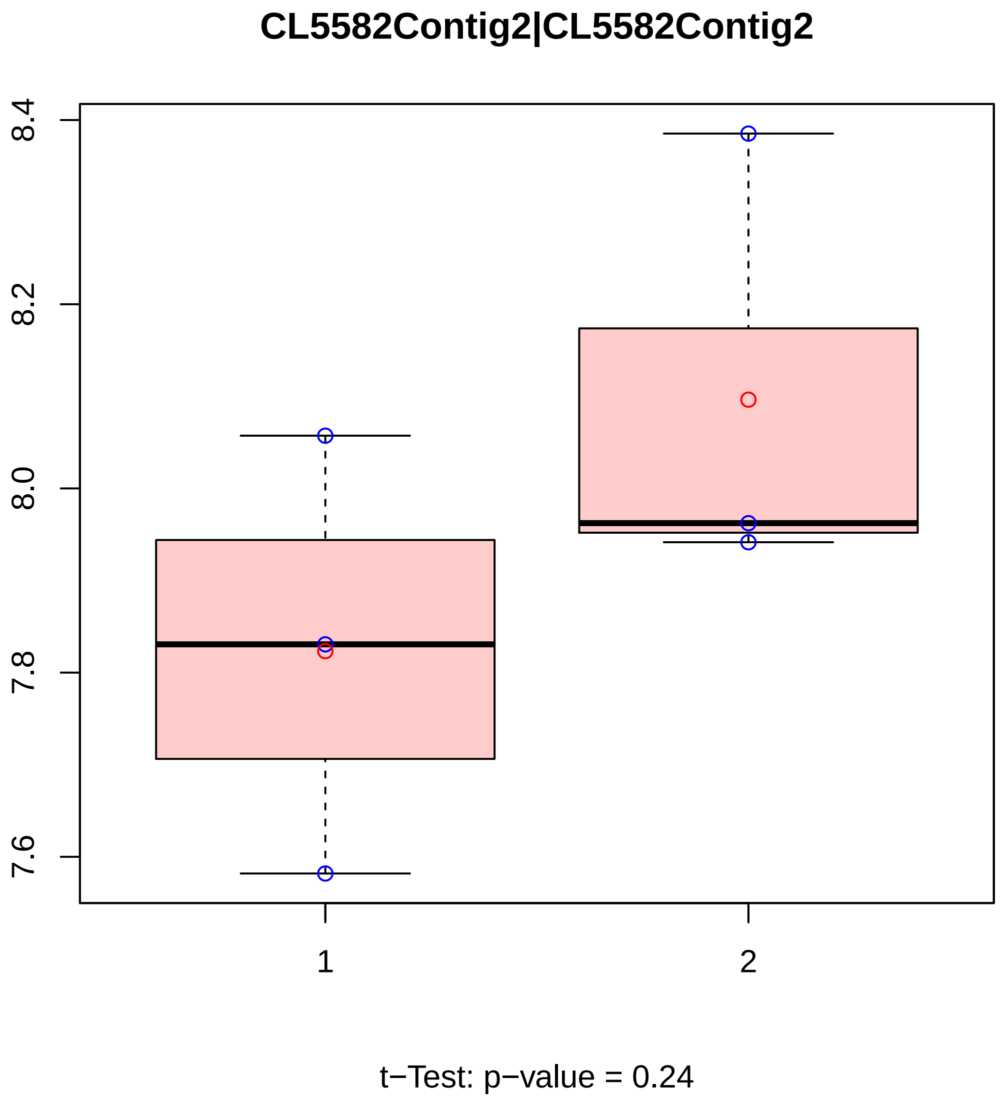

CL5582Contig2|CL5582Contig2
8.4
8.2
8.0
7.8
7.6
1
2
t−Test: p−value = 0.24

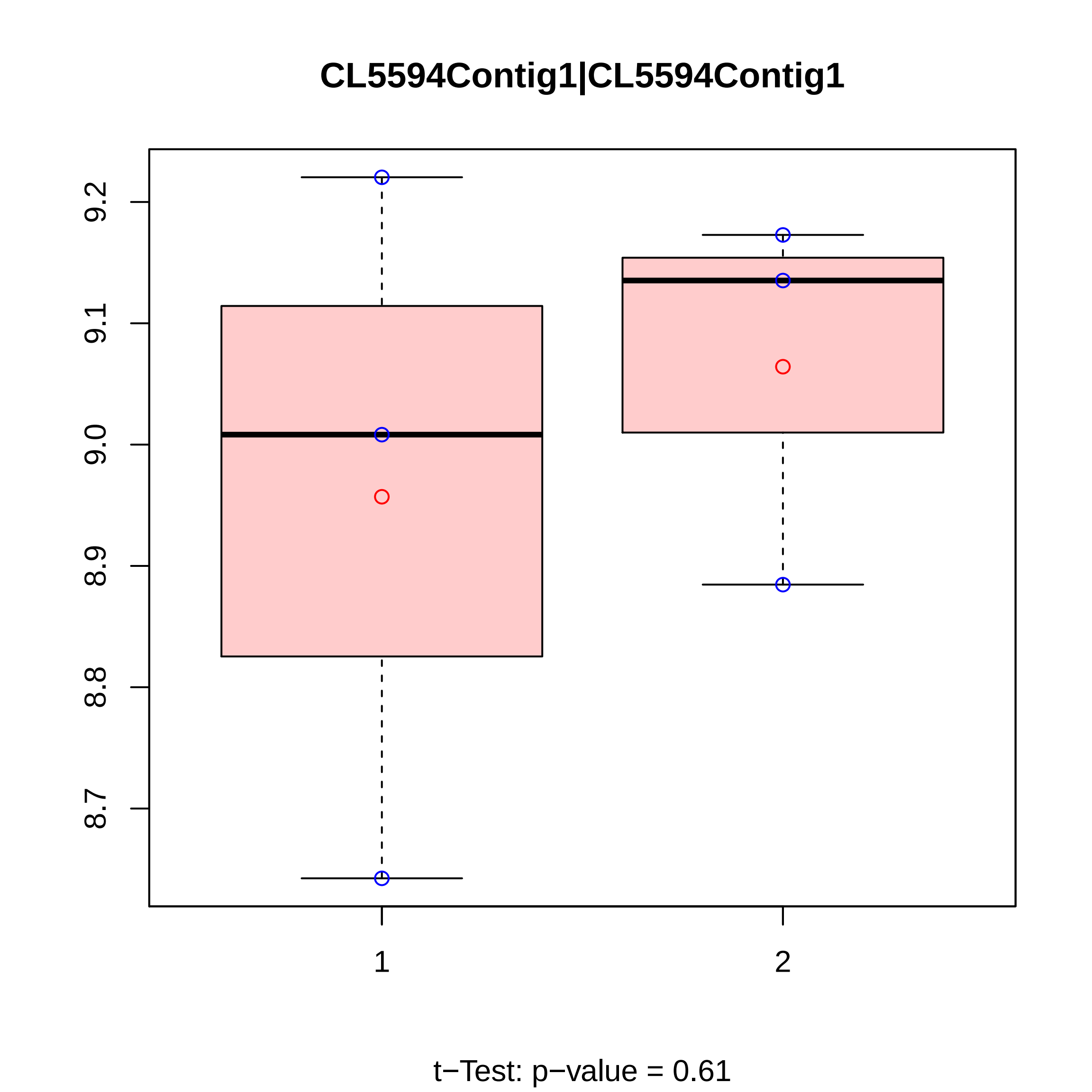

CL5594Contig1|CL5594Contig1
9.2
9.1
9.0
8.9
8.8
8.7
1
2
t−Test: p−value = 0.61

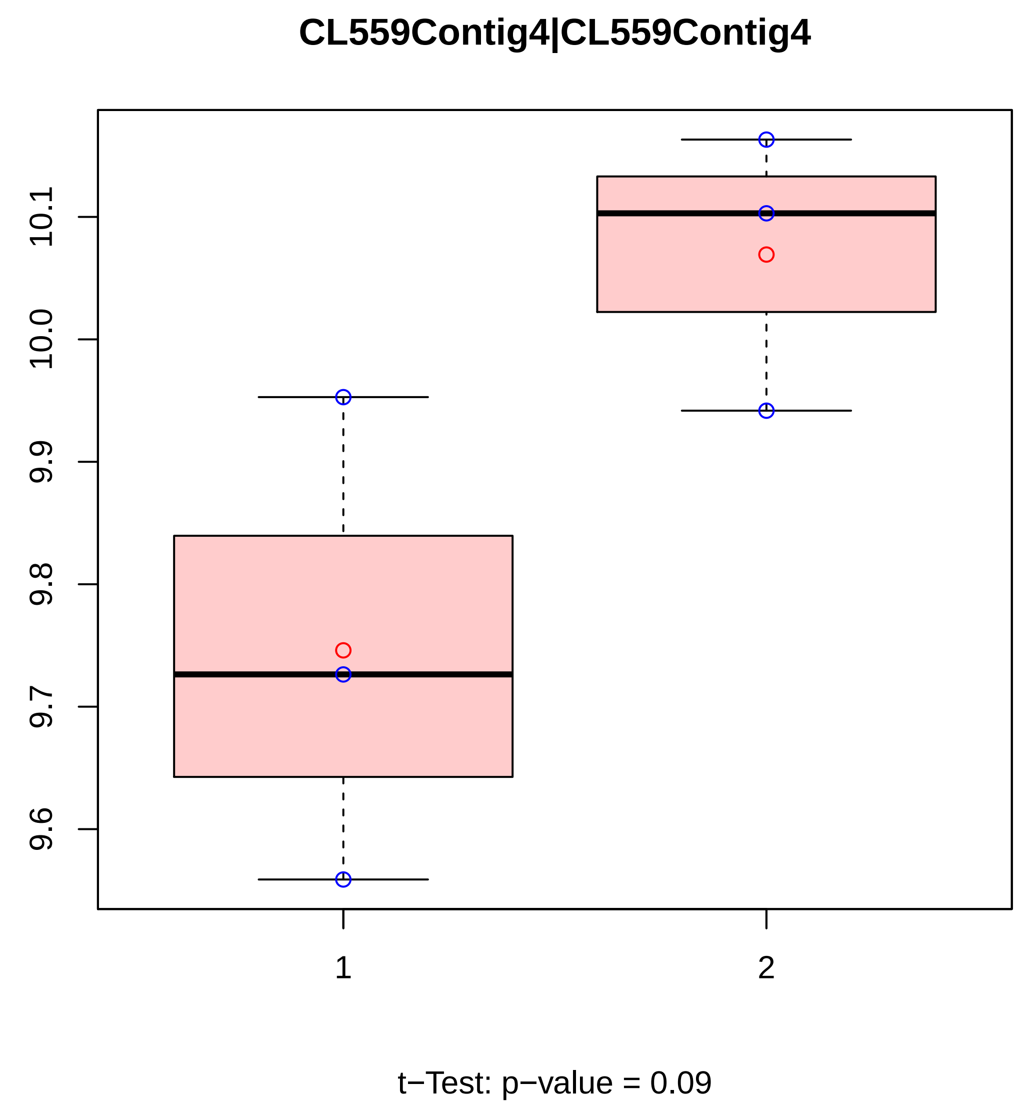
CL559Contig4|CL559Contig4
10.1
10.0
9.9
9.8
9.7
9.6
1
2
t−Test: p−value = 0.09

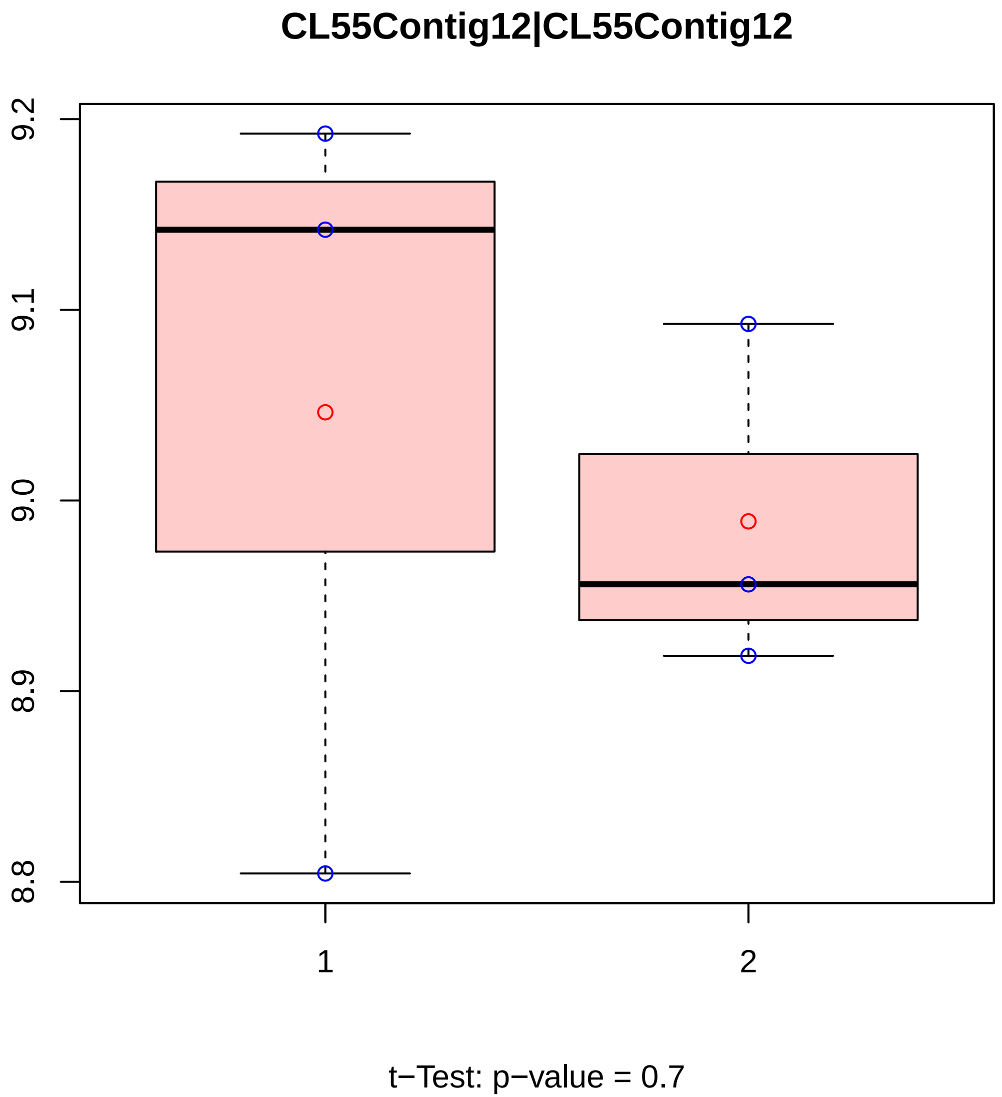
CL55Contig12|CL55Contig12
9.2
9.1
9.0
8.9
8.8
1
2
t−Test: p−value = 0.7

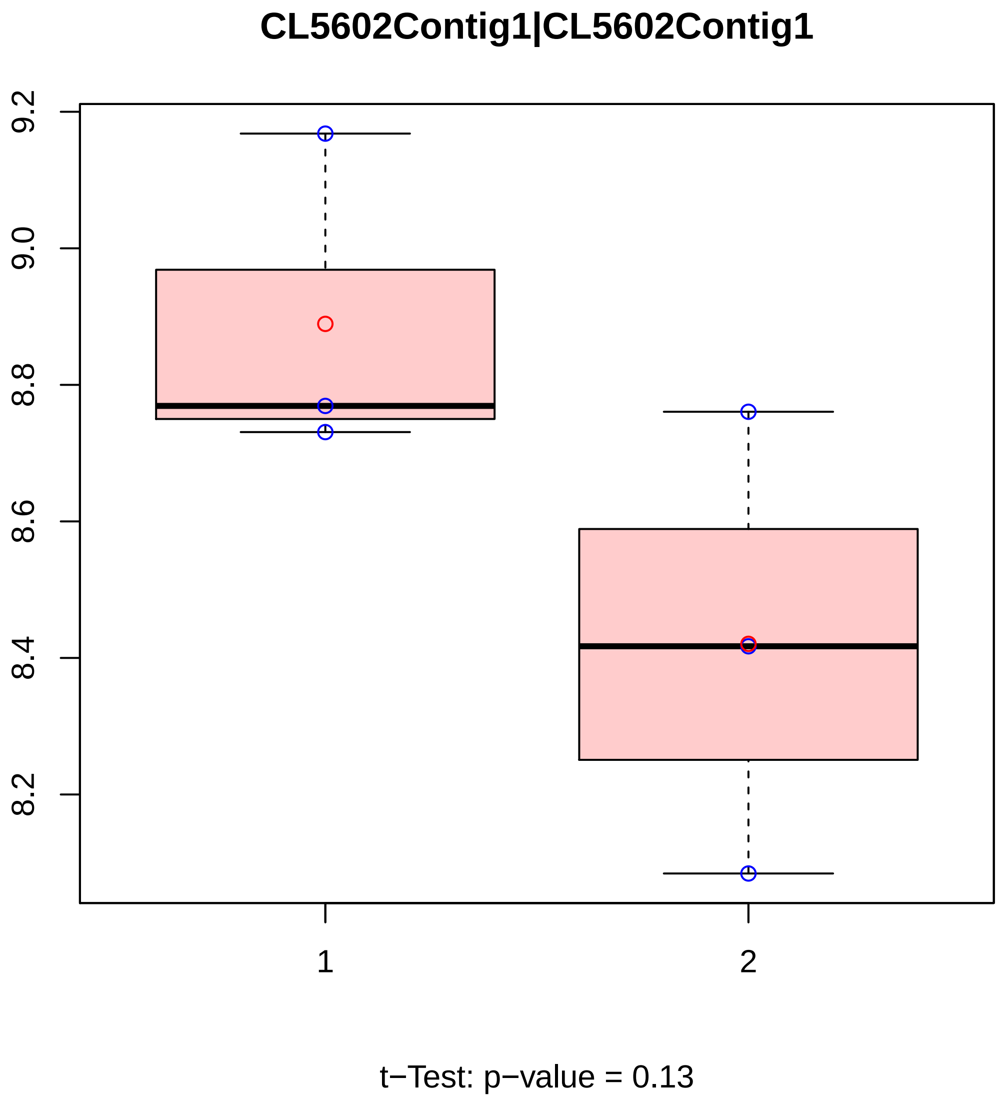
CL5602Contig1|CL5602Contig1
9.2
9.0
8.8
8.6
8.4
8.2
1
2
t−Test: p−value = 0.13

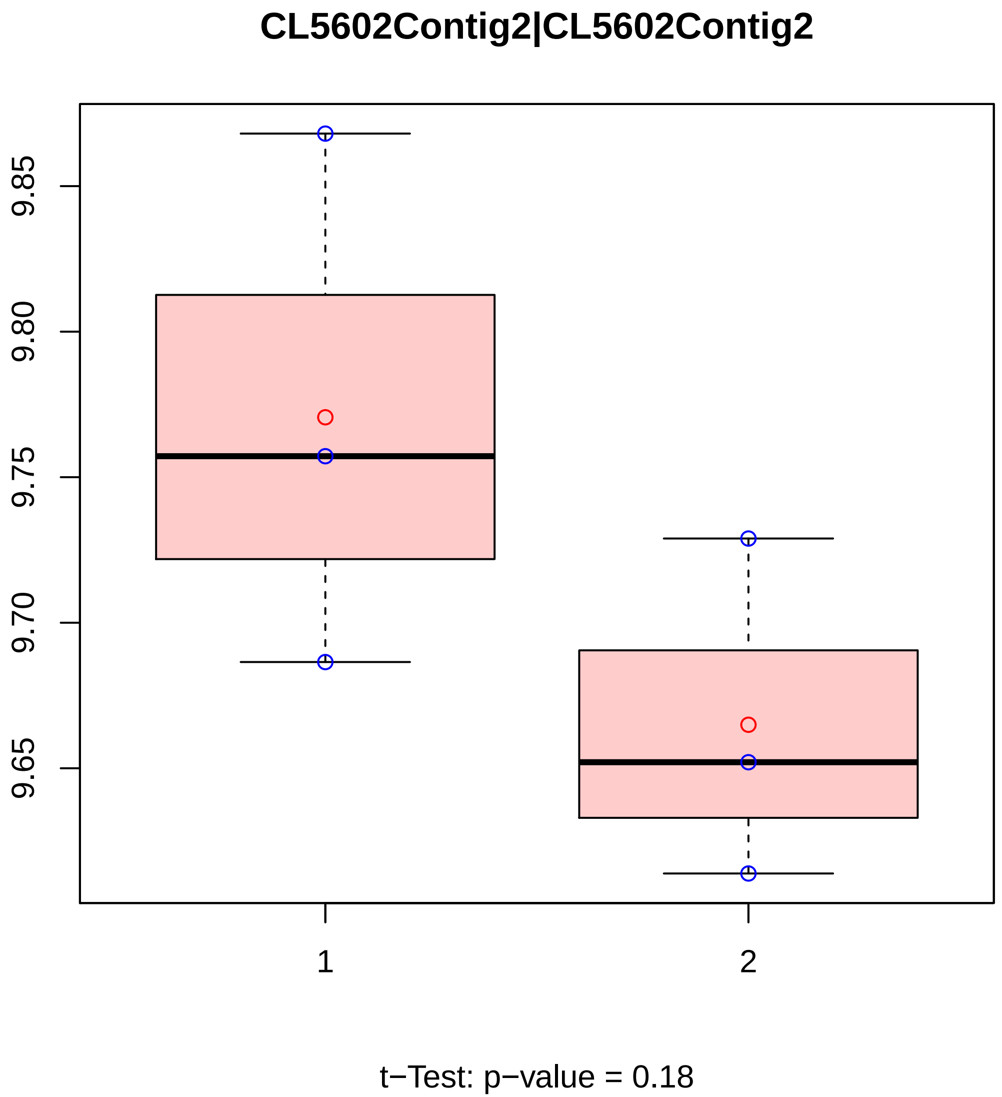

CL5602Contig2|CL5602Contig2
9.65
9.70
9.75
9.80
9.85
1
2
t−Test: p−value = 0.18

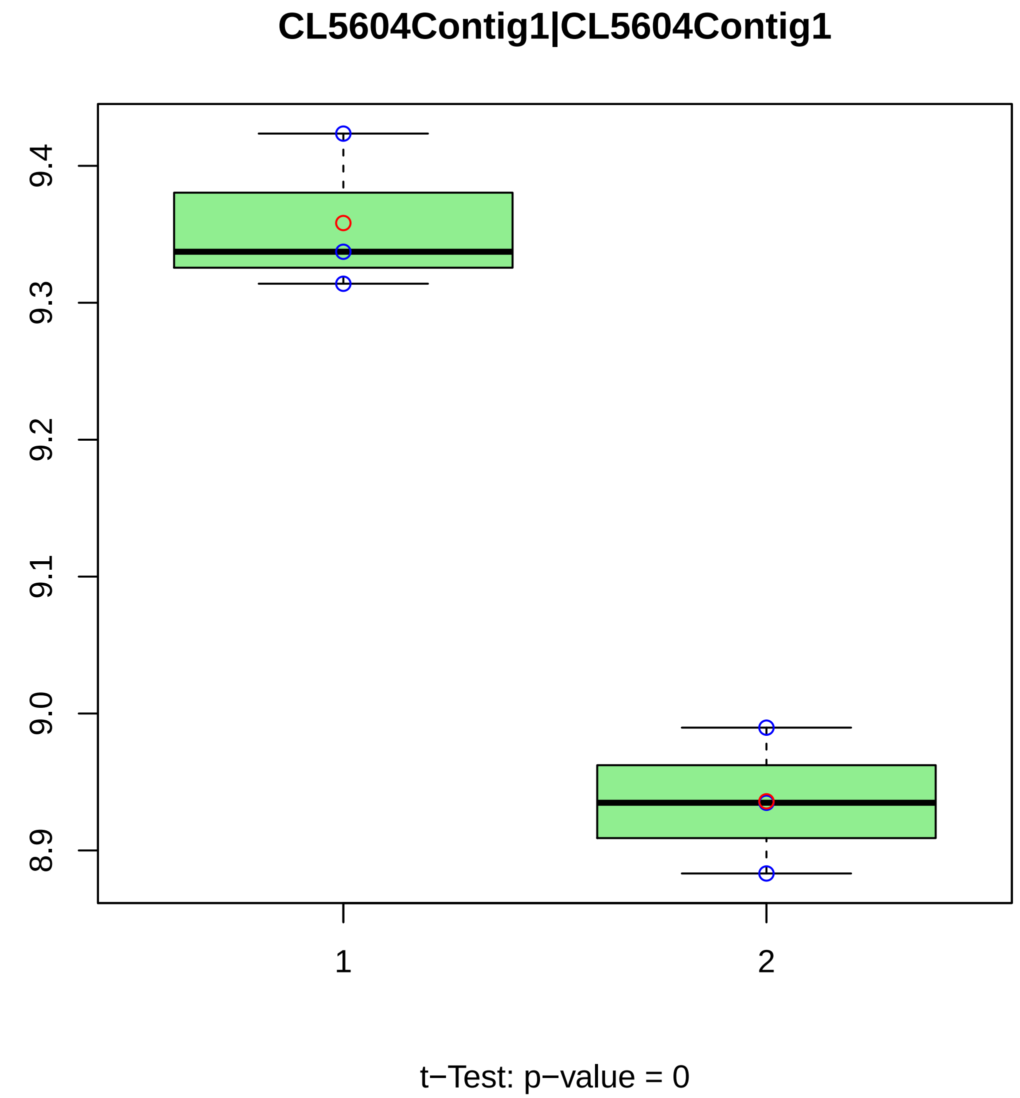
CL5604Contig1|CL5604Contig1
9.4
9.3
9.2
9.1
9.0
8.9
1
2
t−Test: p−value = 0

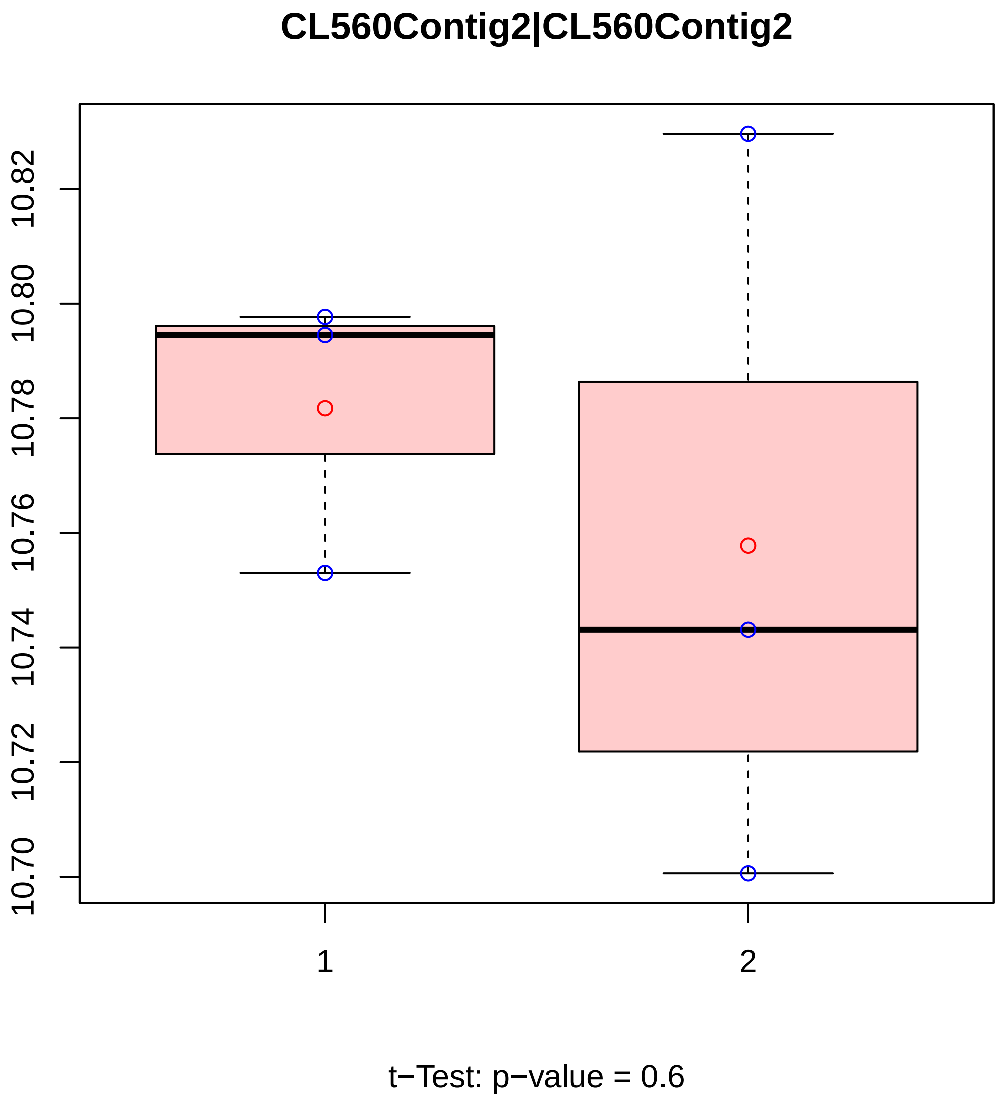
CL560Contig2|CL560Contig2
10.70
10.72
10.74
10.76
10.78
10.80
10.82
1
2
t−Test: p−value = 0.6

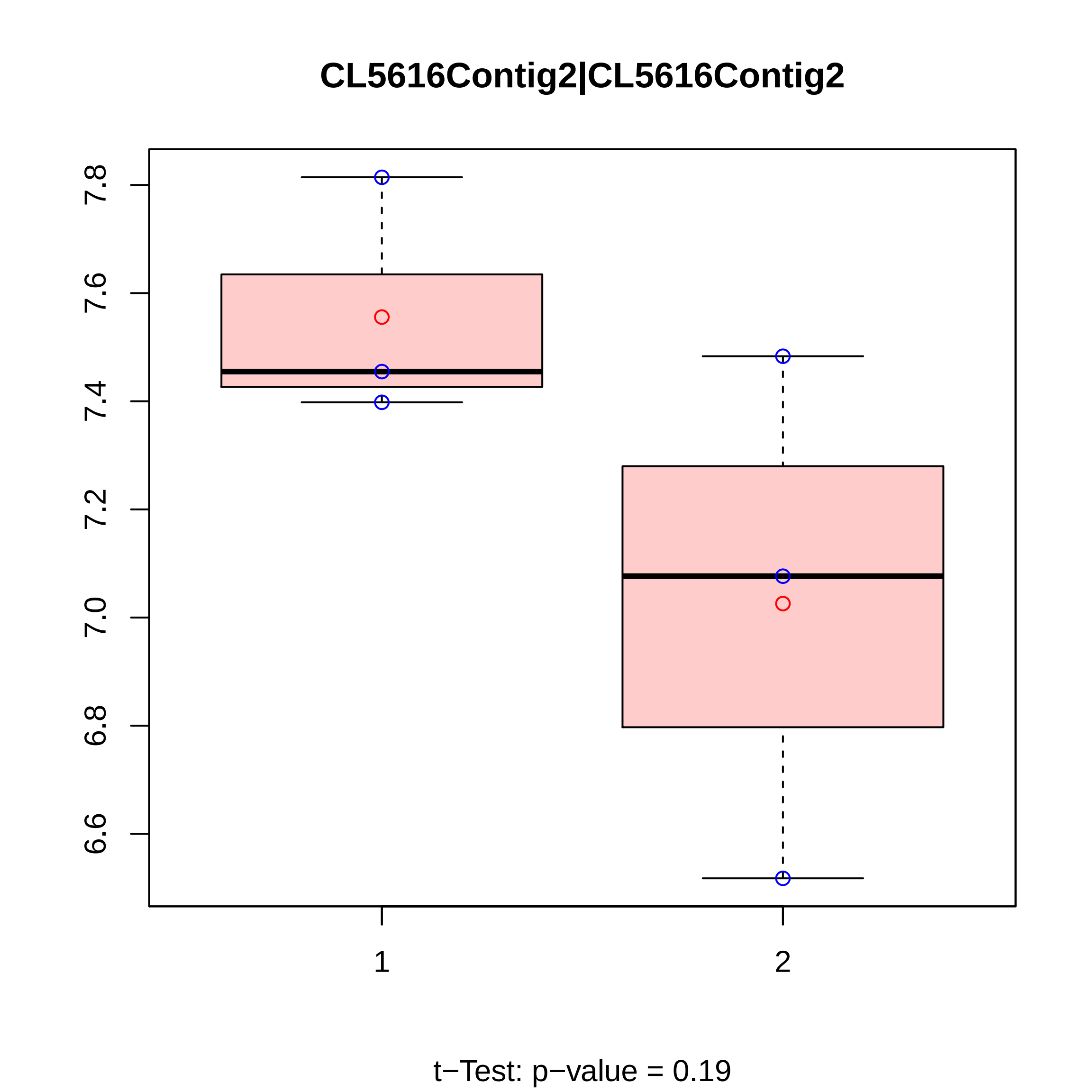

CL5616Contig2|CL5616Contig2
7.8
7.6
7.4
7.2
7.0
6.8
6.6
1
2
t−Test: p−value = 0.19

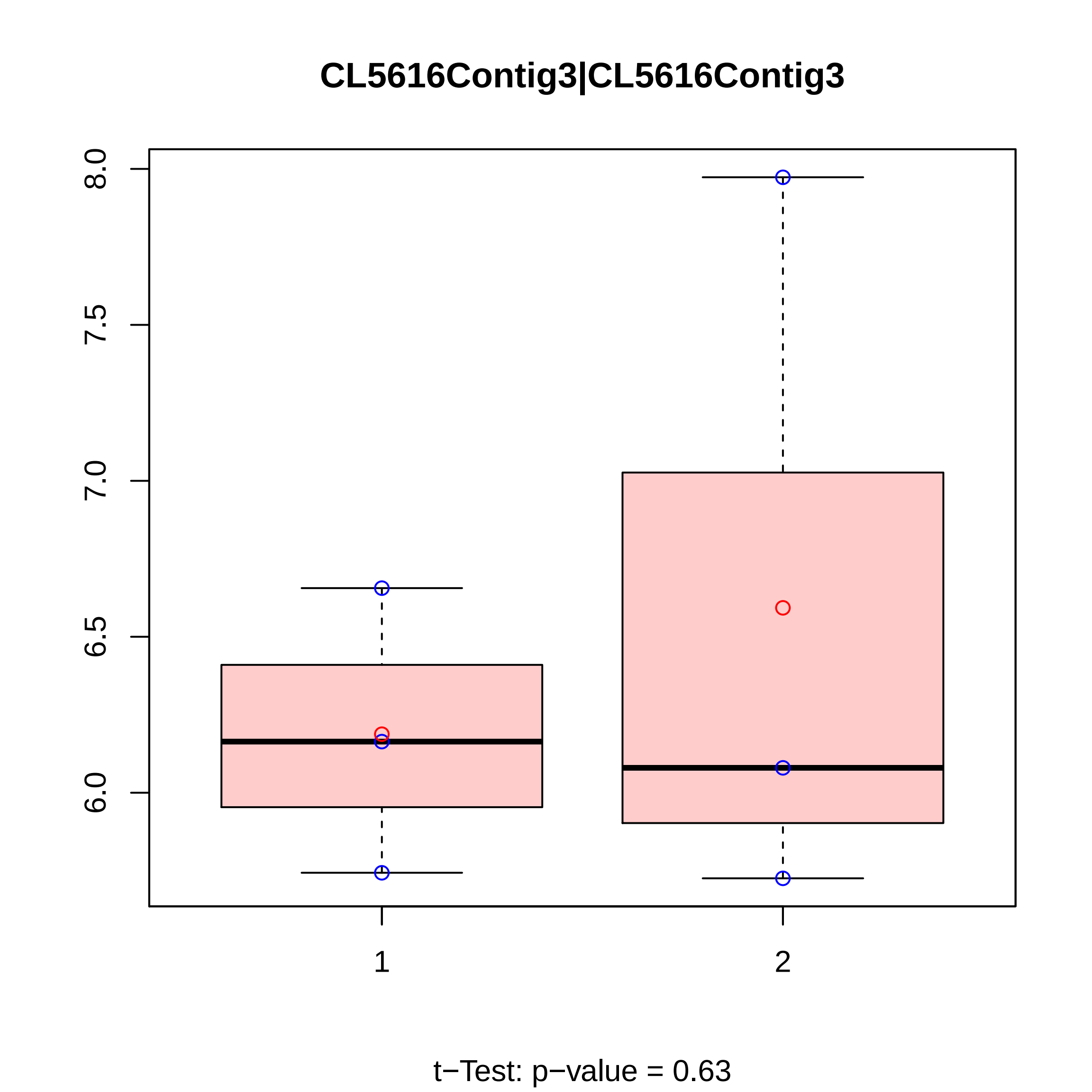

CL5616Contig3|CL5616Contig3
8.0
7.5
7.0
6.5
6.0
1
2
t−Test: p−value = 0.63

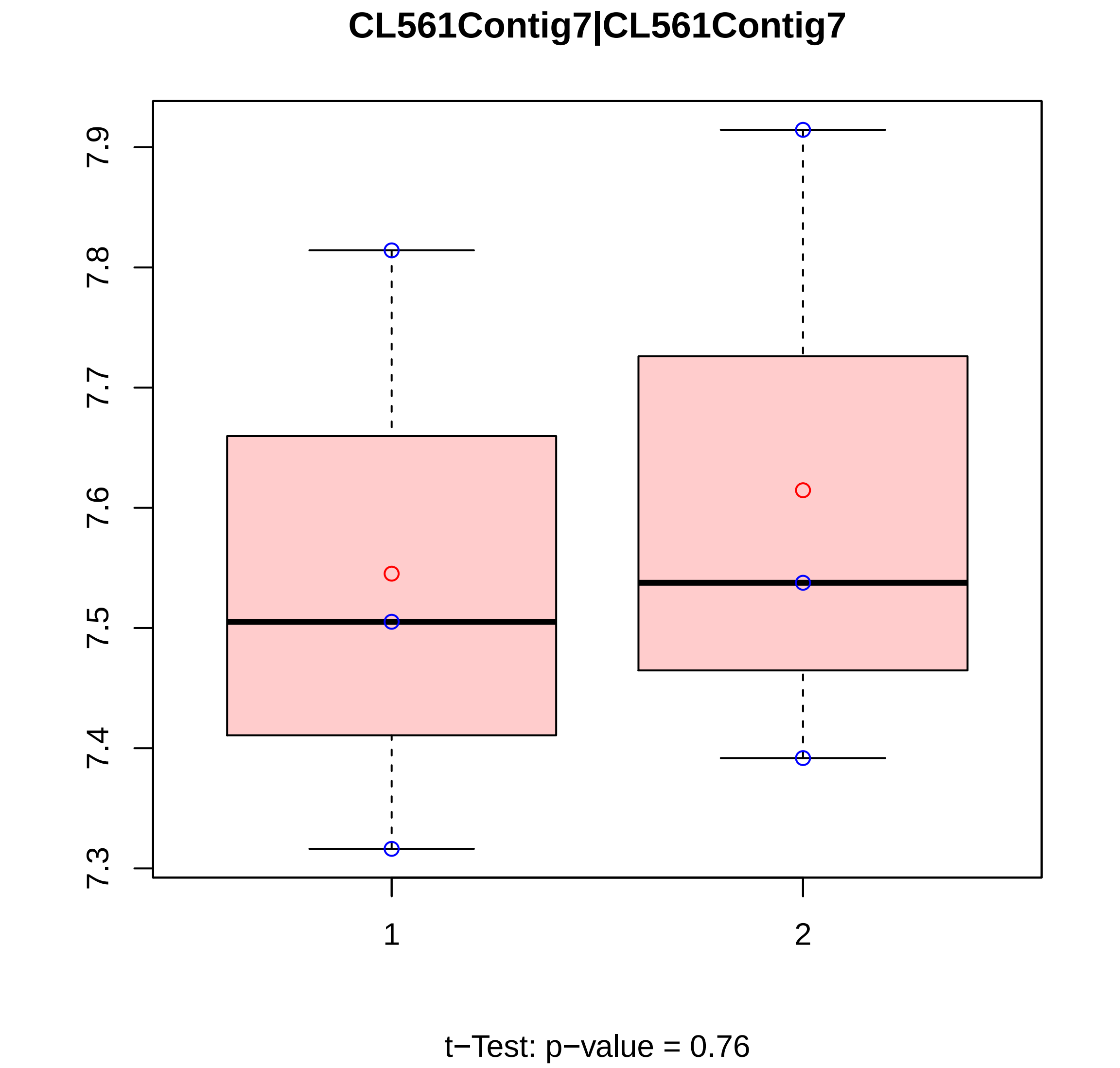
CL561Contig7|CL561Contig7
7.3
7.4
7.5
7.6
7.7
7.8
7.9
1
2
t−Test: p−value = 0.76

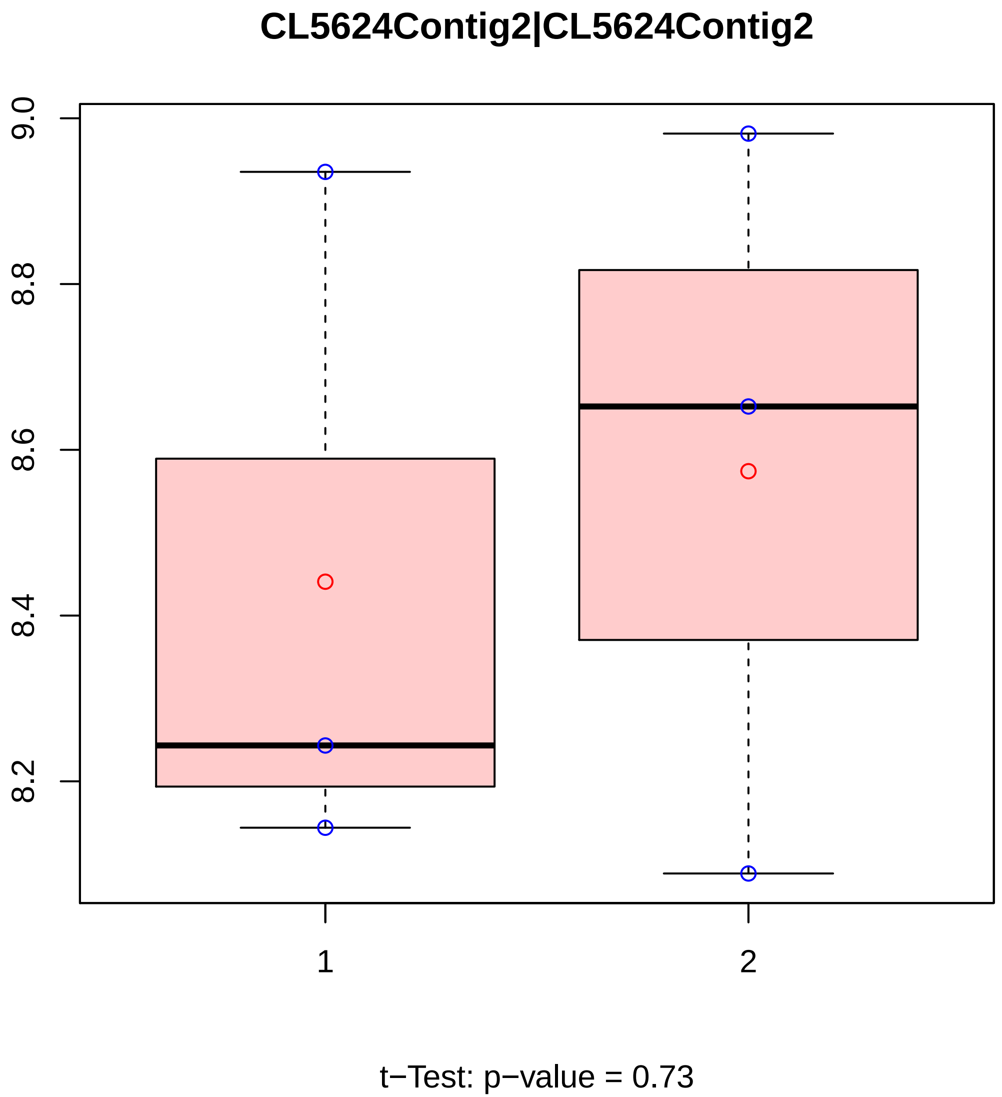
CL5624Contig2|CL5624Contig2
9.0
8.8
8.6
8.4
8.2
1
2
t−Test: p−value = 0.73

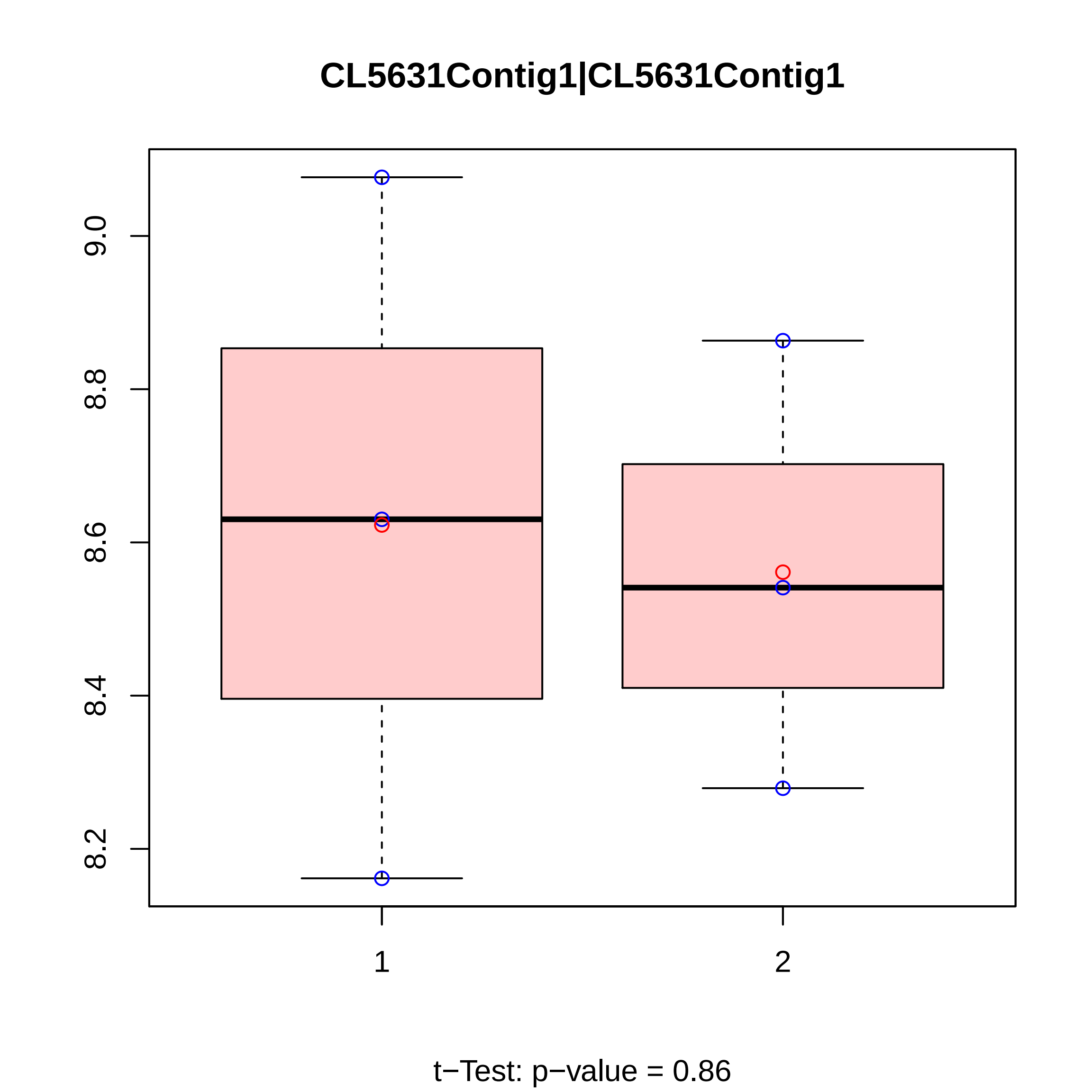

CL5631Contig1|CL5631Contig1
9.0
8.8
8.6
8.4
8.2
1
2
t−Test: p−value = 0.86

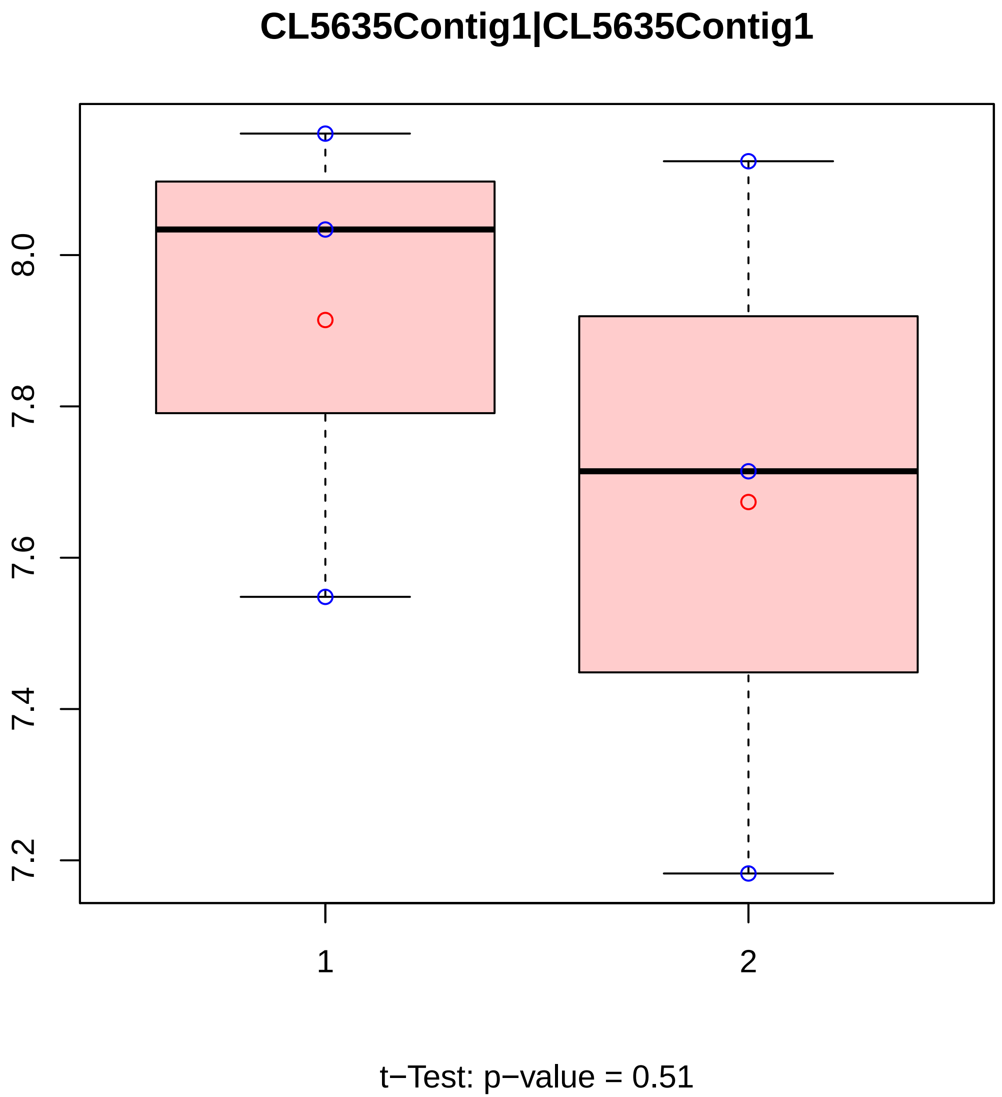

CL5635Contig1|CL5635Contig1
8.0
7.8
7.6
7.4
7.2
1
2
t−Test: p−value = 0.51

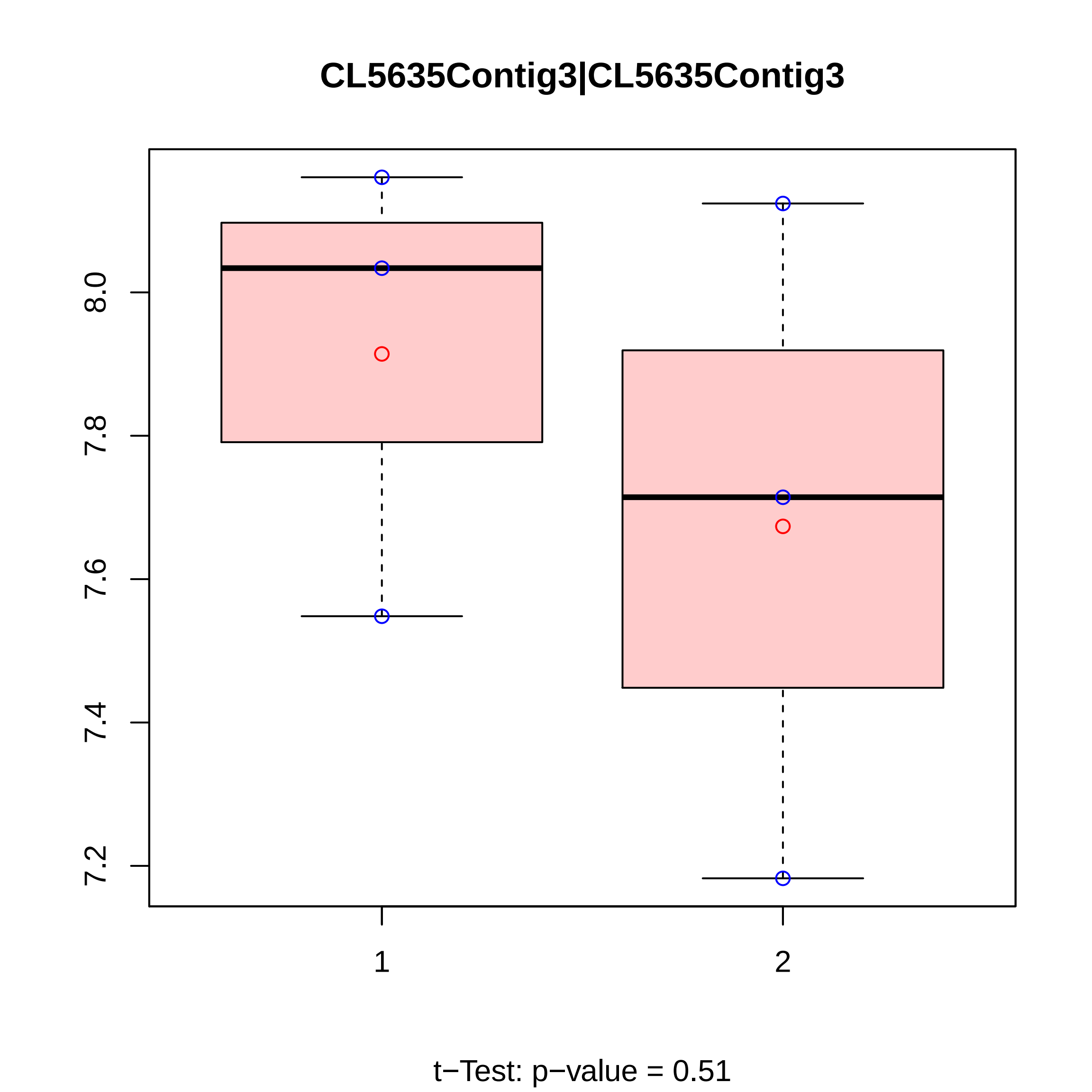

CL5635Contig3|CL5635Contig3
8.0
7.8
7.6
7.4
7.2
1
2
t−Test: p−value = 0.51

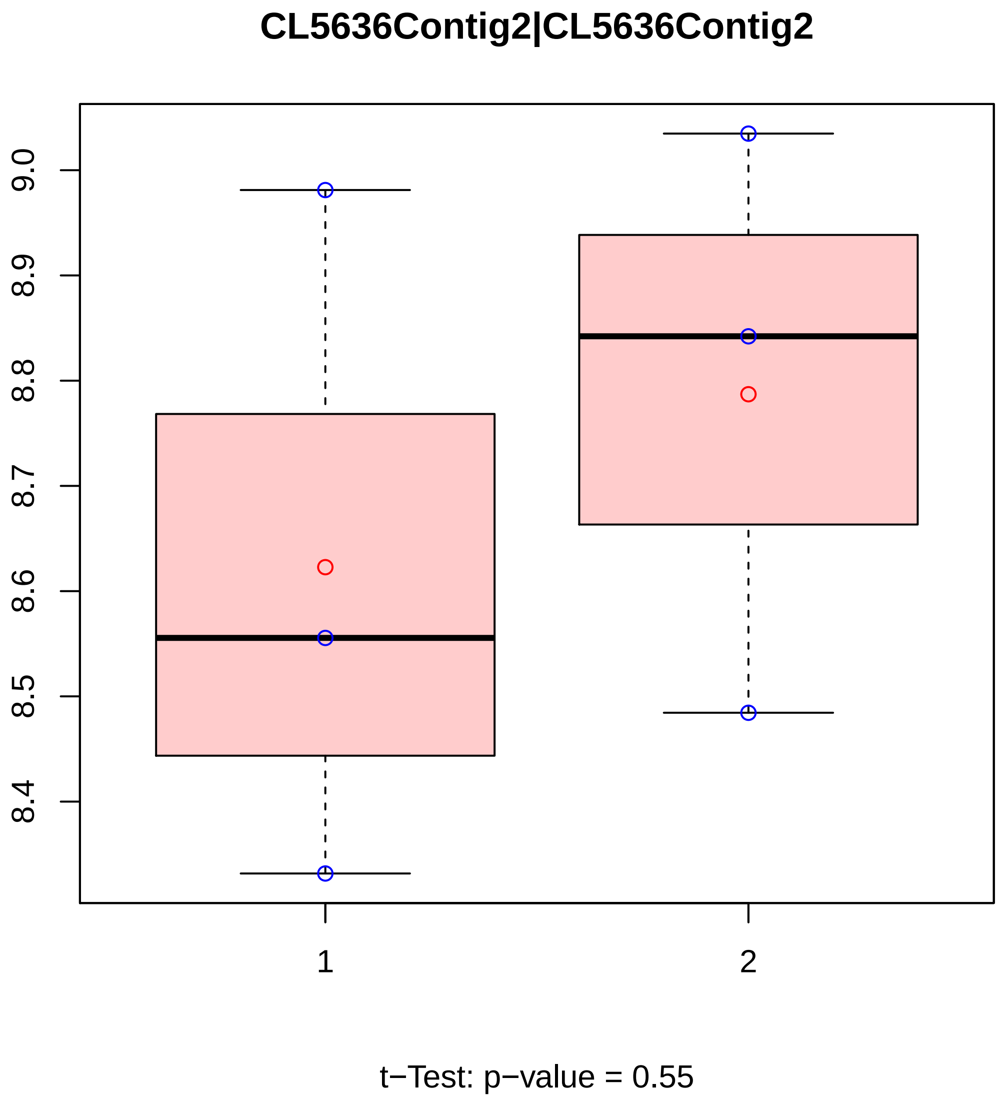
CL5636Contig2|CL5636Contig2
9.0
8.9
8.8
8.7
8.6
8.5
8.4
1
2
t−Test: p−value = 0.55

# CL5637Contig1|CL5637Contig1

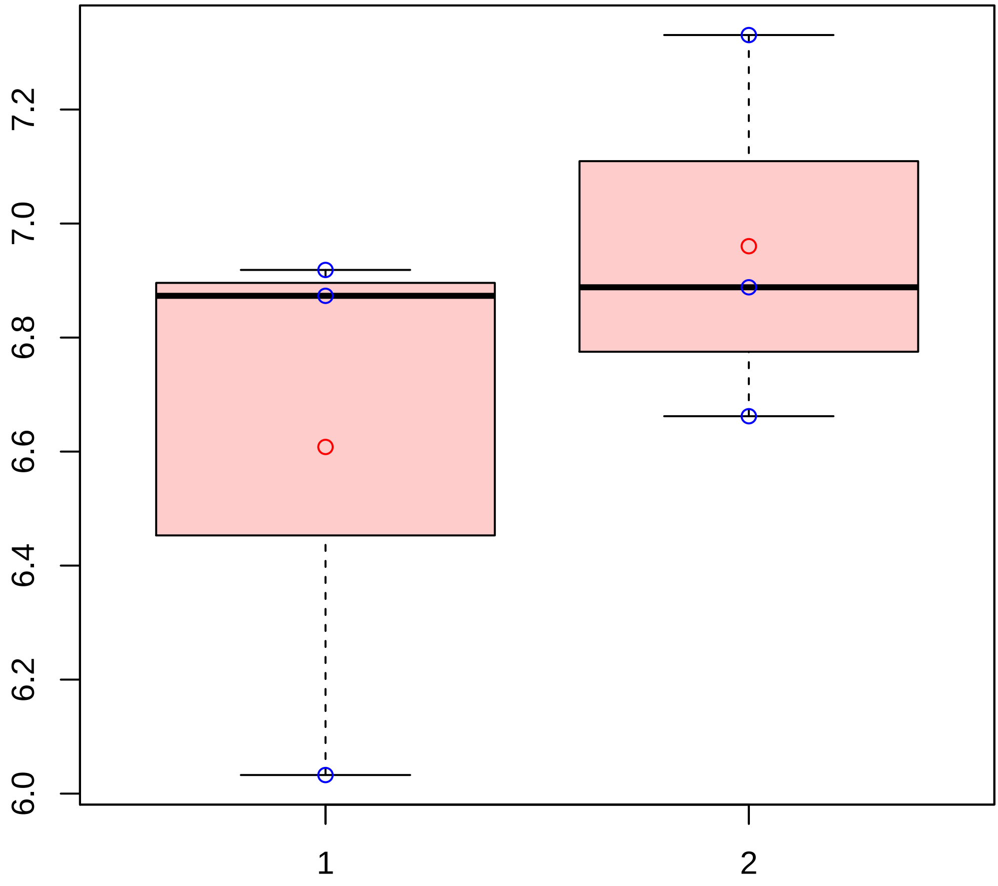


t−Test: p−value = 0.38

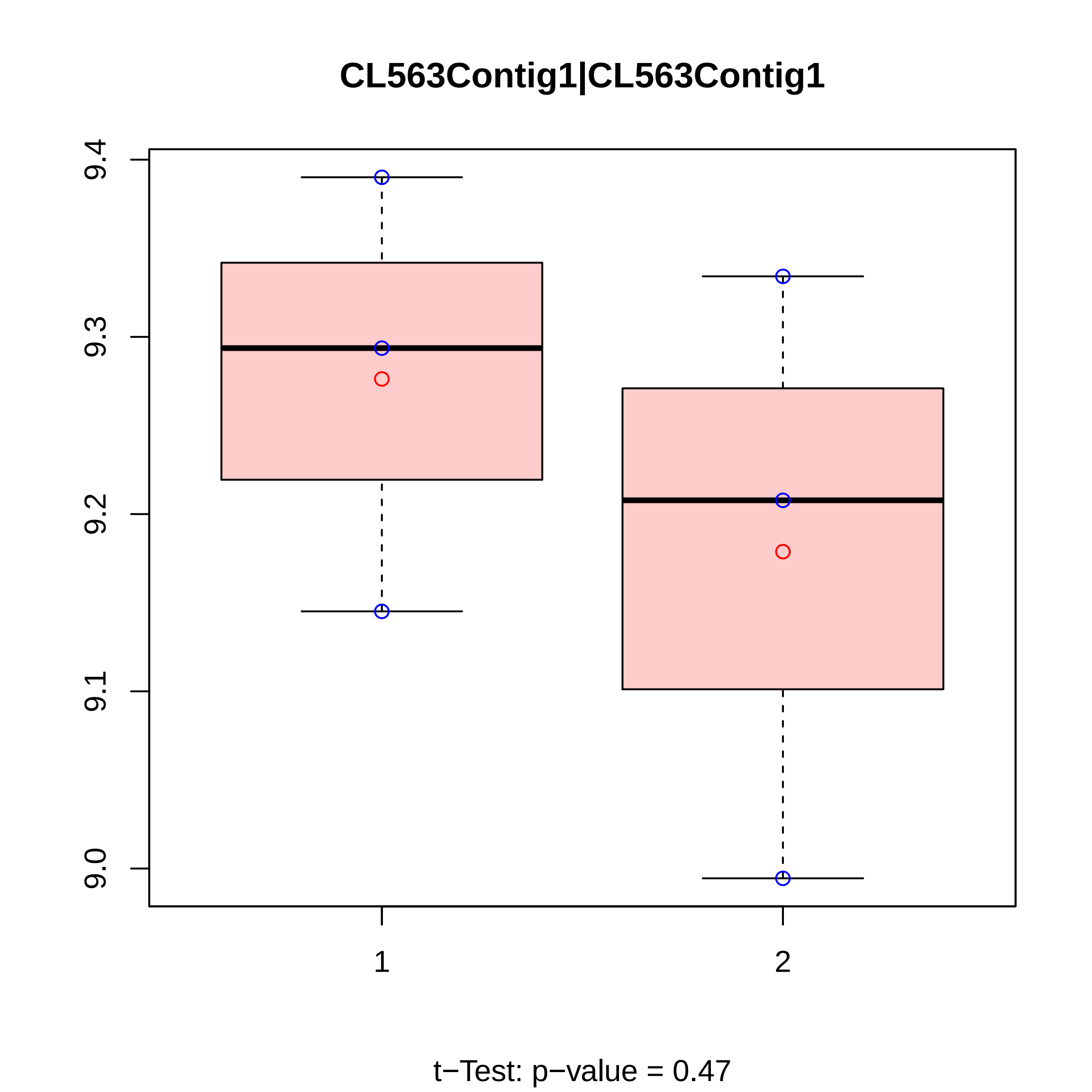

CL563Contig1|CL563Contig1
9.0
9.1
9.2
9.3
9.4
1
2
t−Test: p−value = 0.47

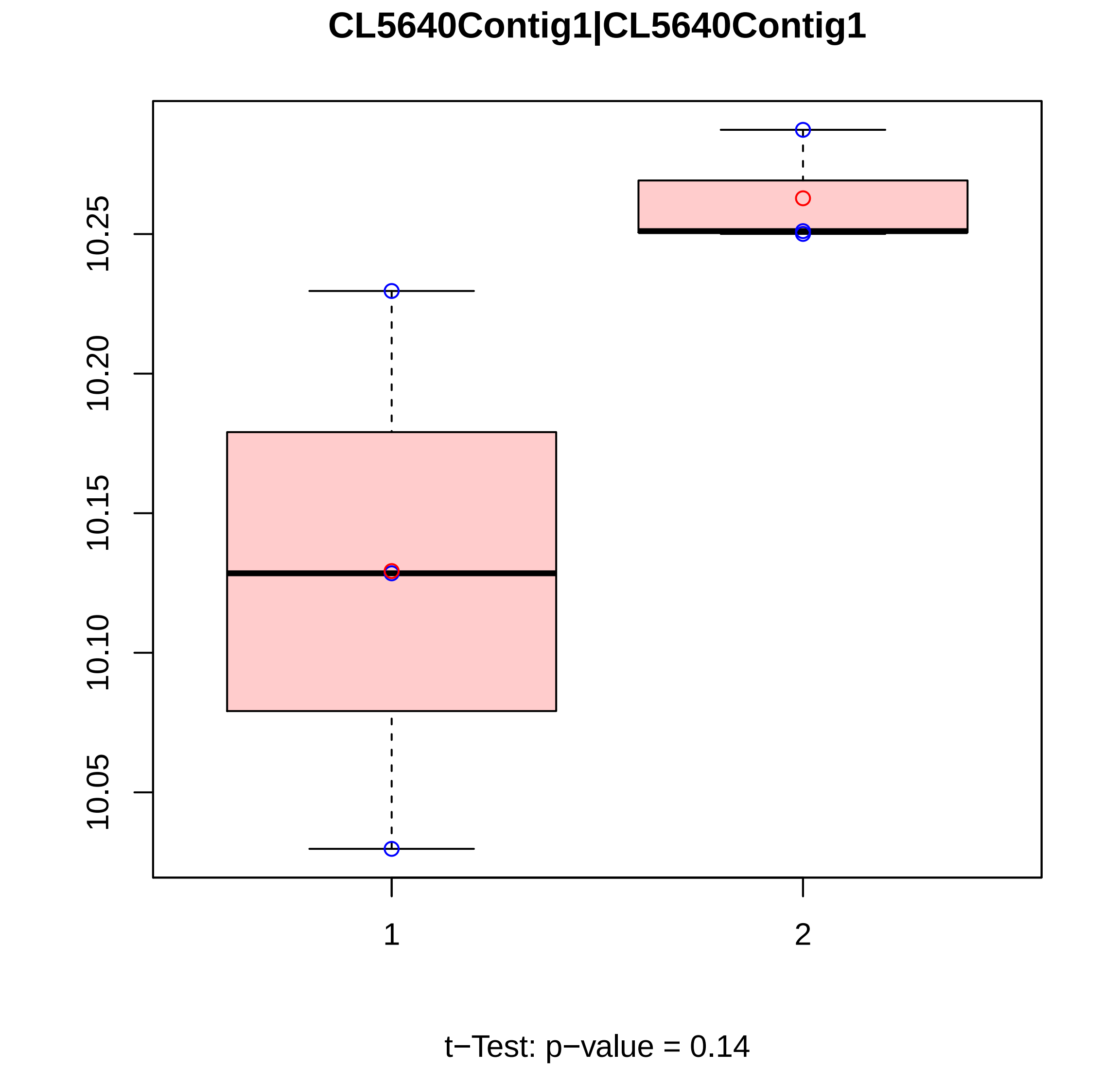

CL5640Contig1|CL5640Contig1
10.05
10.10
10.15
10.20
10.25
1
2
t−Test: p−value = 0.14

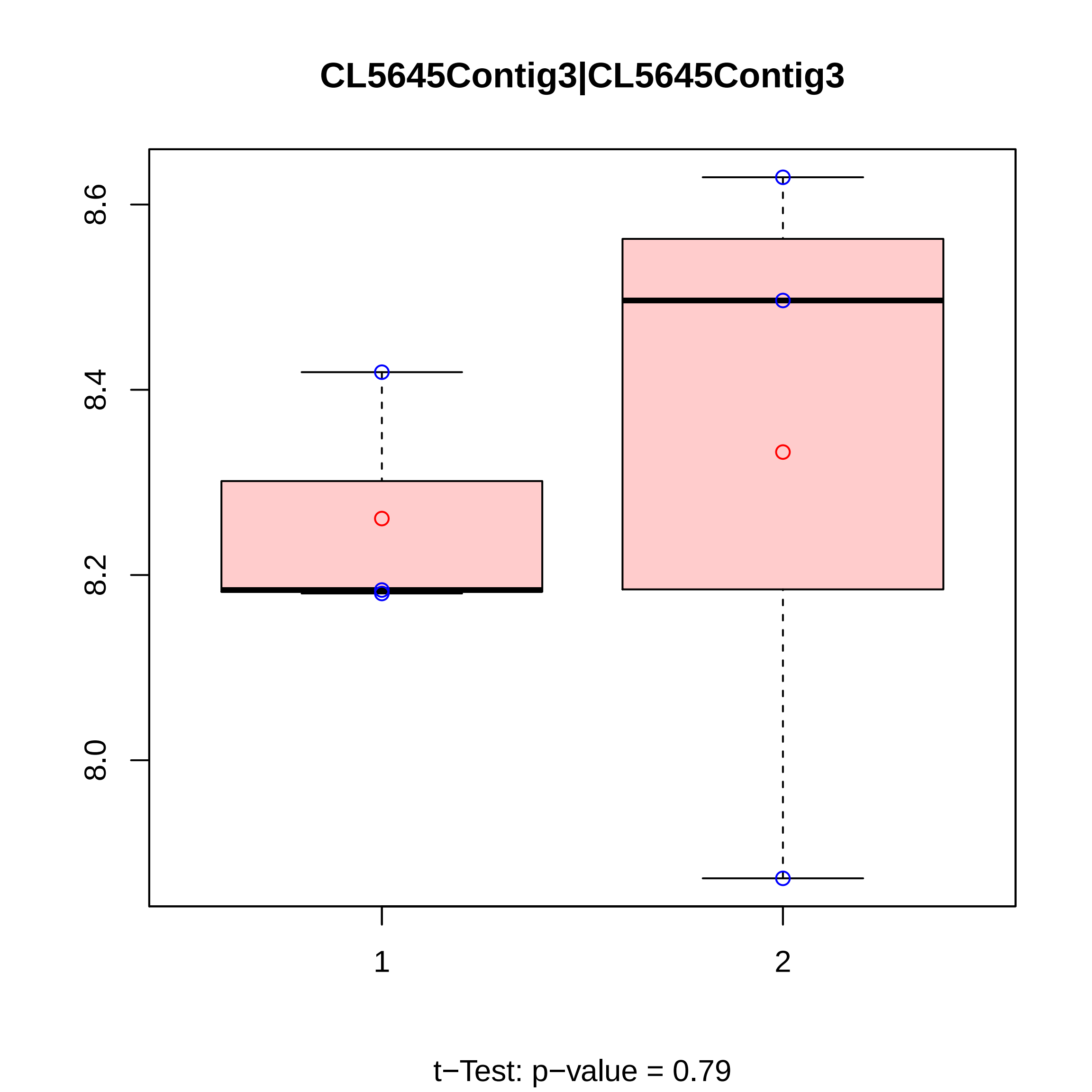

CL5645Contig3|CL5645Contig3
8.0
8.2
8.4
8.6
1
2
t−Test: p−value = 0.79

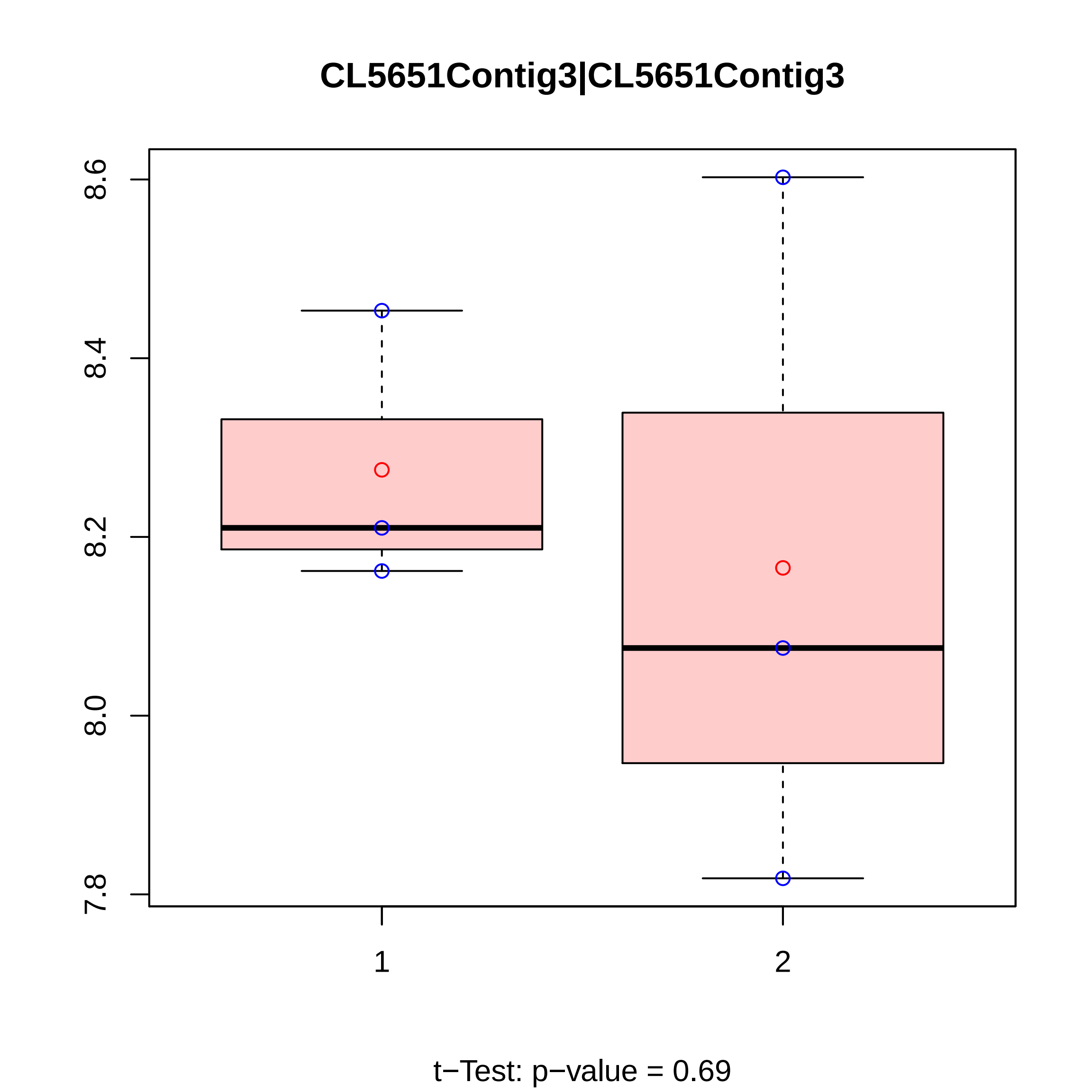
CL5651Contig3|CL5651Contig3
8.6
8.4
8.2
8.0
7.8
1
2
t−Test: p−value = 0.69

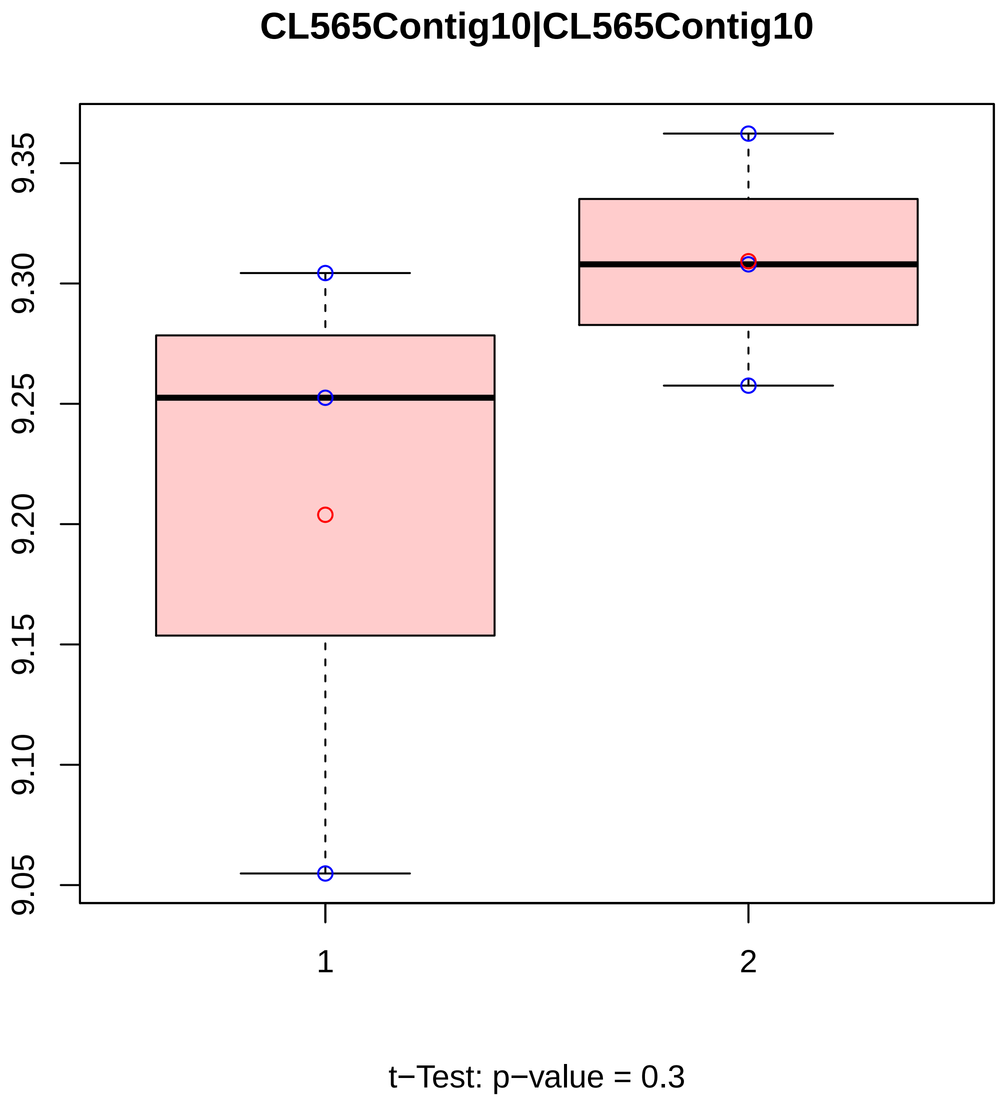
CL565Contig10|CL565Contig10
9.05
9.10
9.15
9.20
9.25
9.30
9.35
1
2
t−Test: p−value = 0.3

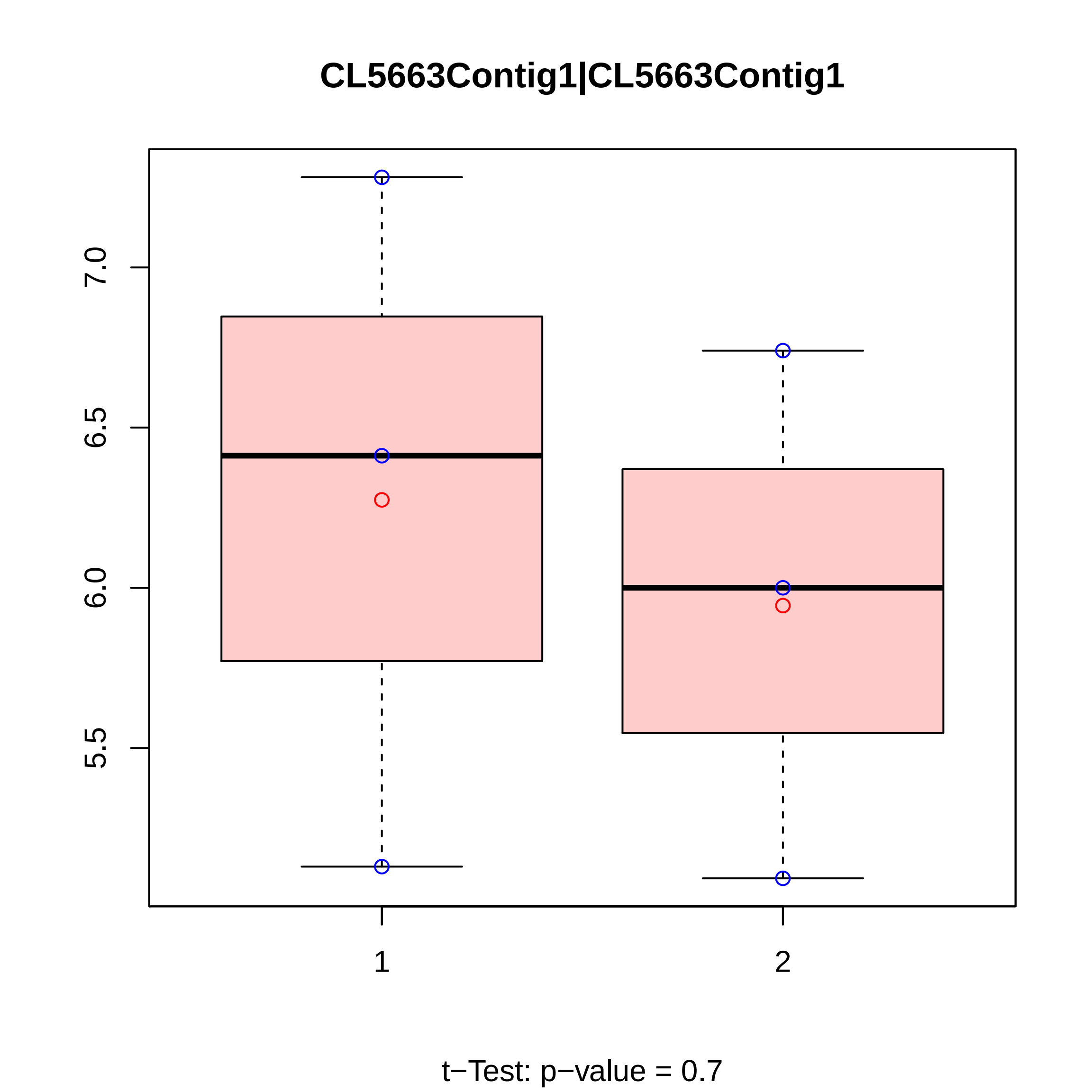
CL5663Contig1|CL5663Contig1
7.0
6.5
6.0
5.5
1
2
t−Test: p−value = 0.7

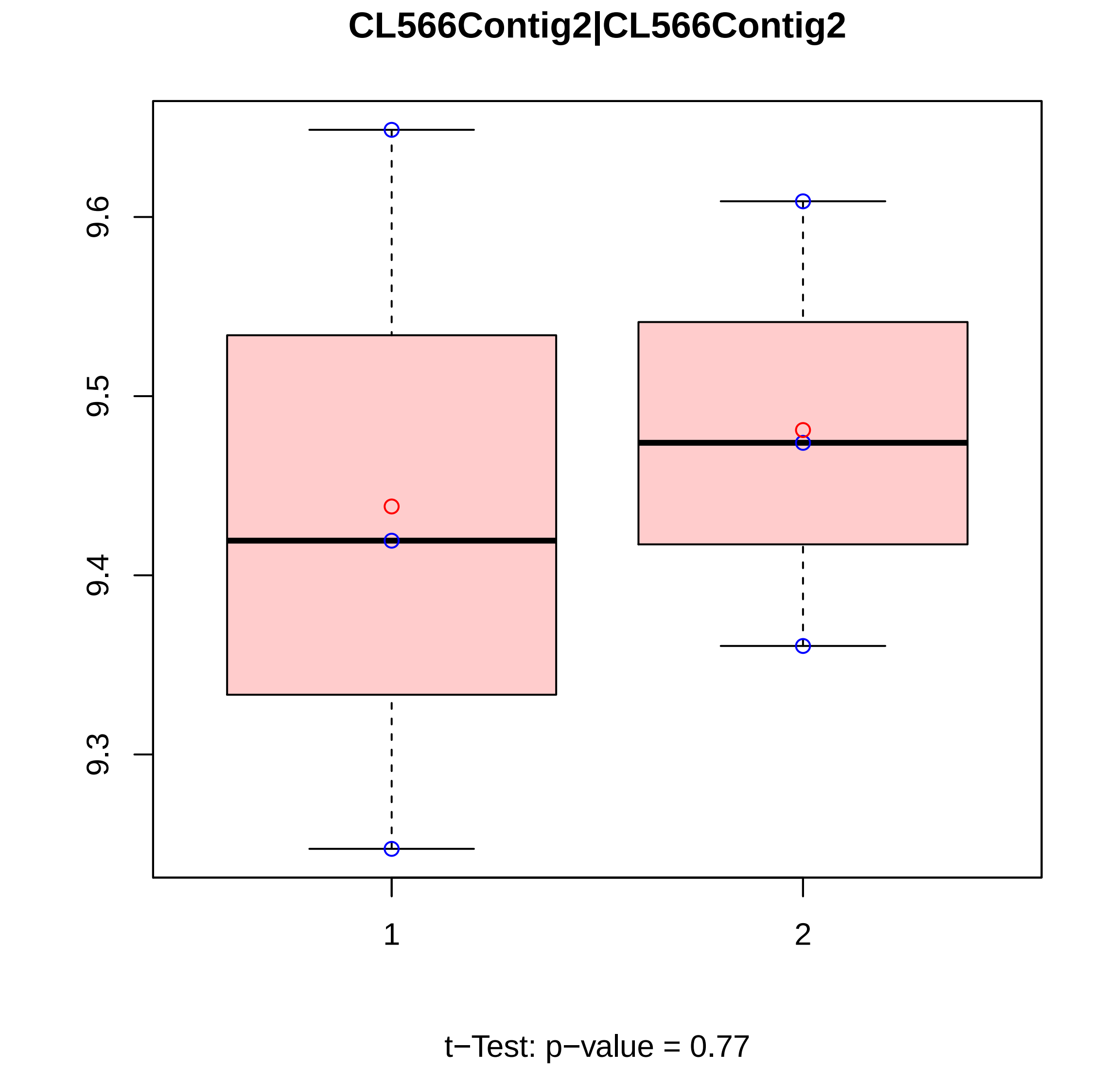
CL566Contig2|CL566Contig2
9.3
9.4
9.5
9.6
1
2
t−Test: p−value = 0.77

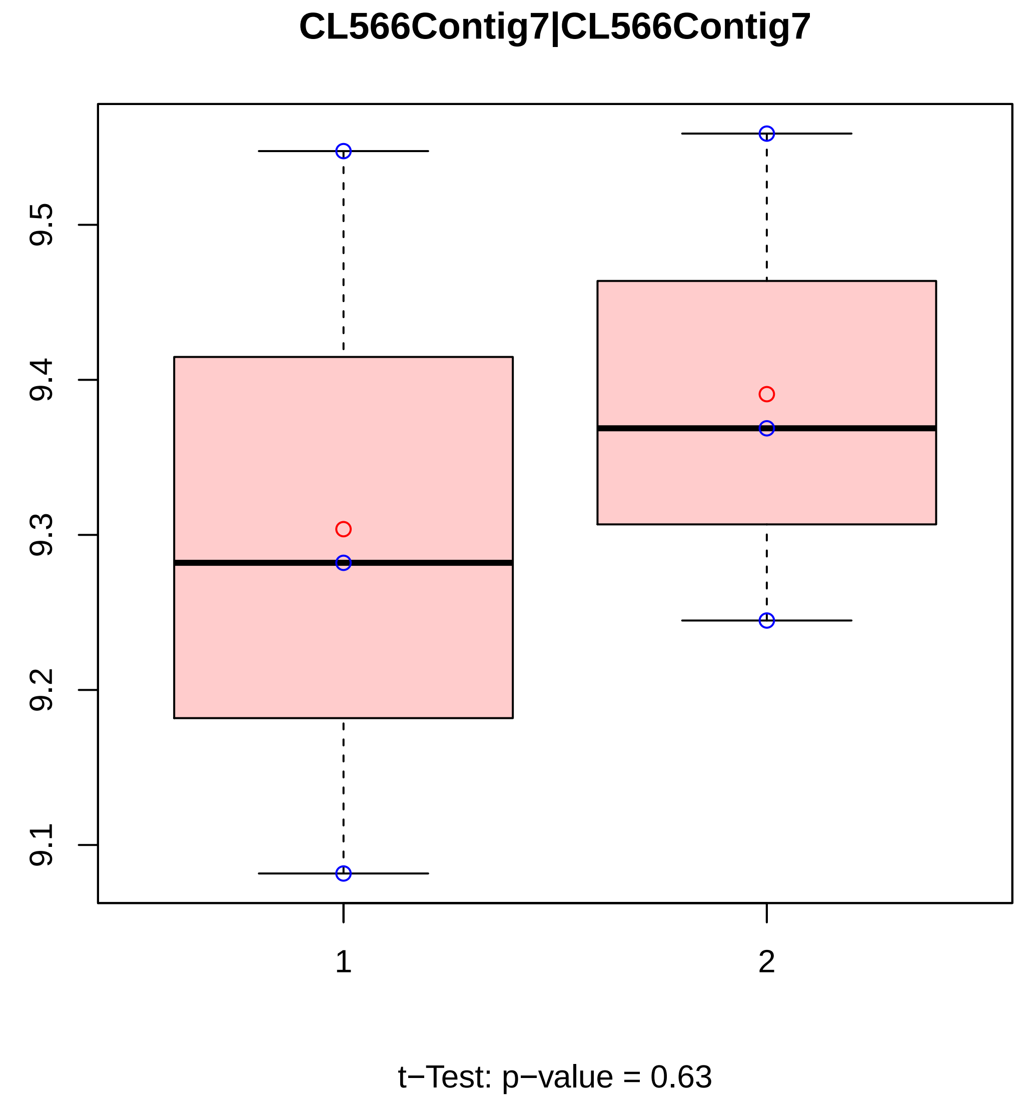

CL566Contig7|CL566Contig7
9.1
9.2
9.3
9.4
9.5
1
2
t−Test: p−value = 0.63

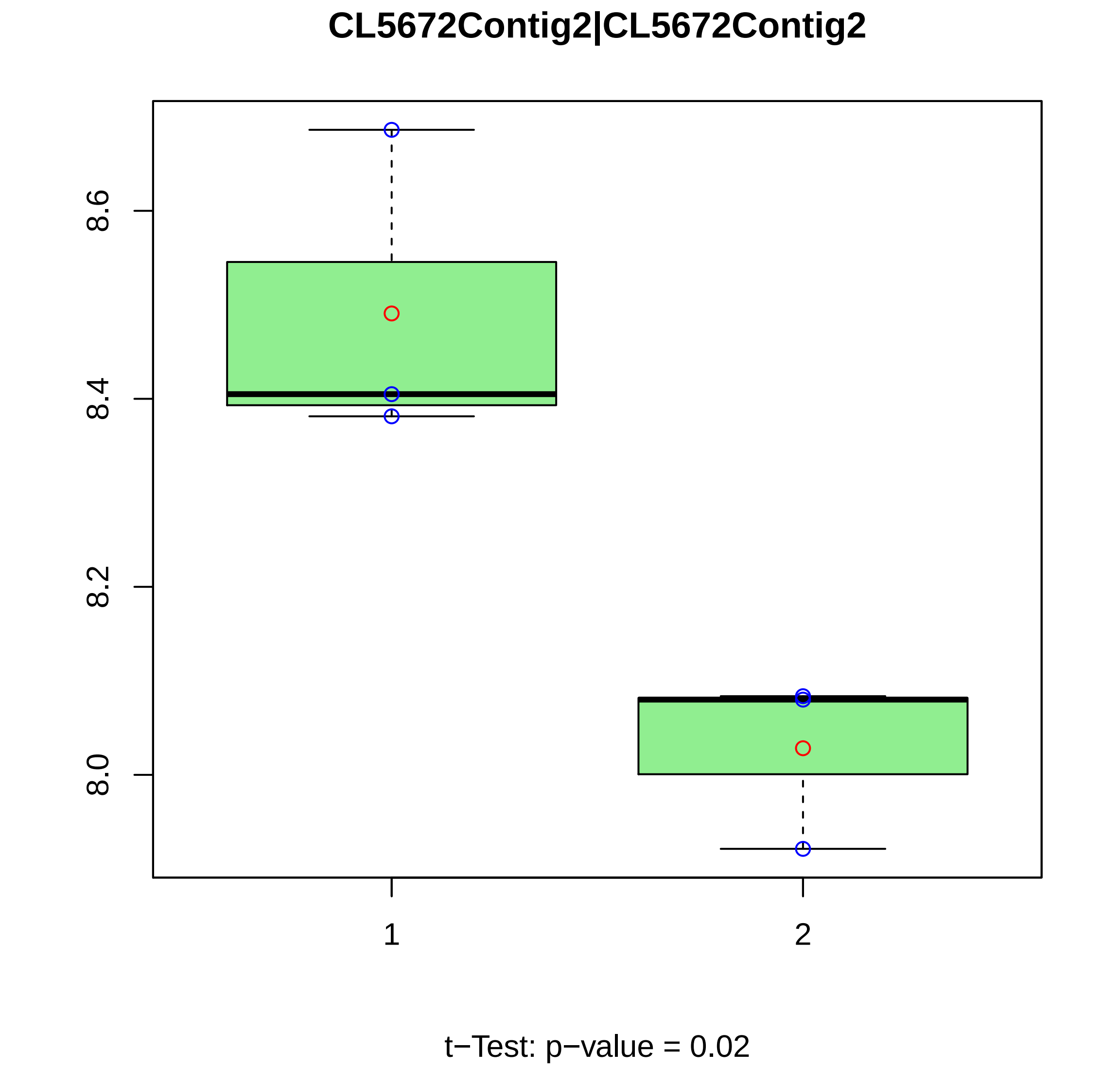
CL5672Contig2|CL5672Contig2
8.0
8.2
8.4
8.6
1
2
t−Test: p−value = 0.02

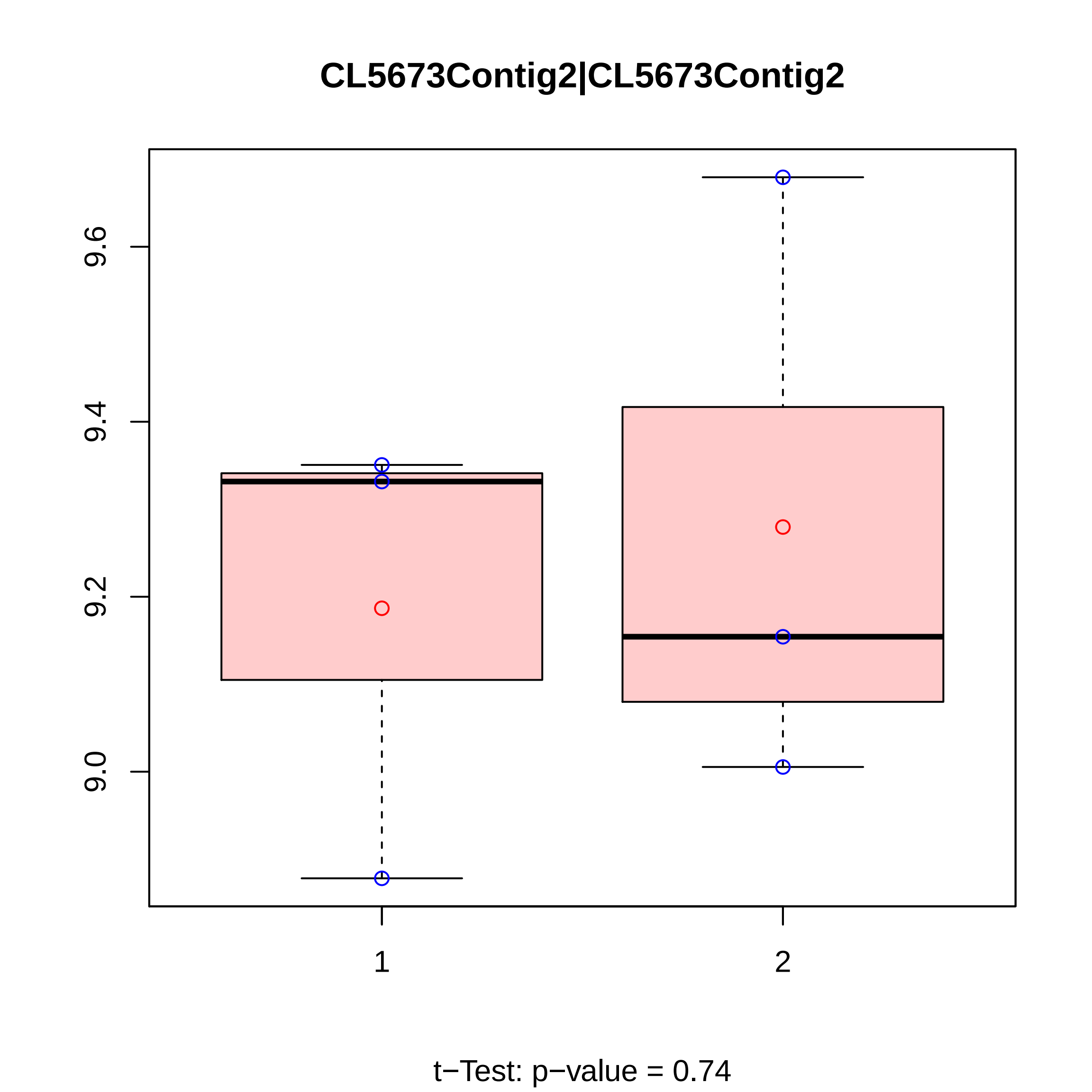
CL5673Contig2|CL5673Contig2
9.0
9.2
9.4
9.6
1
2
t−Test: p−value = 0.74

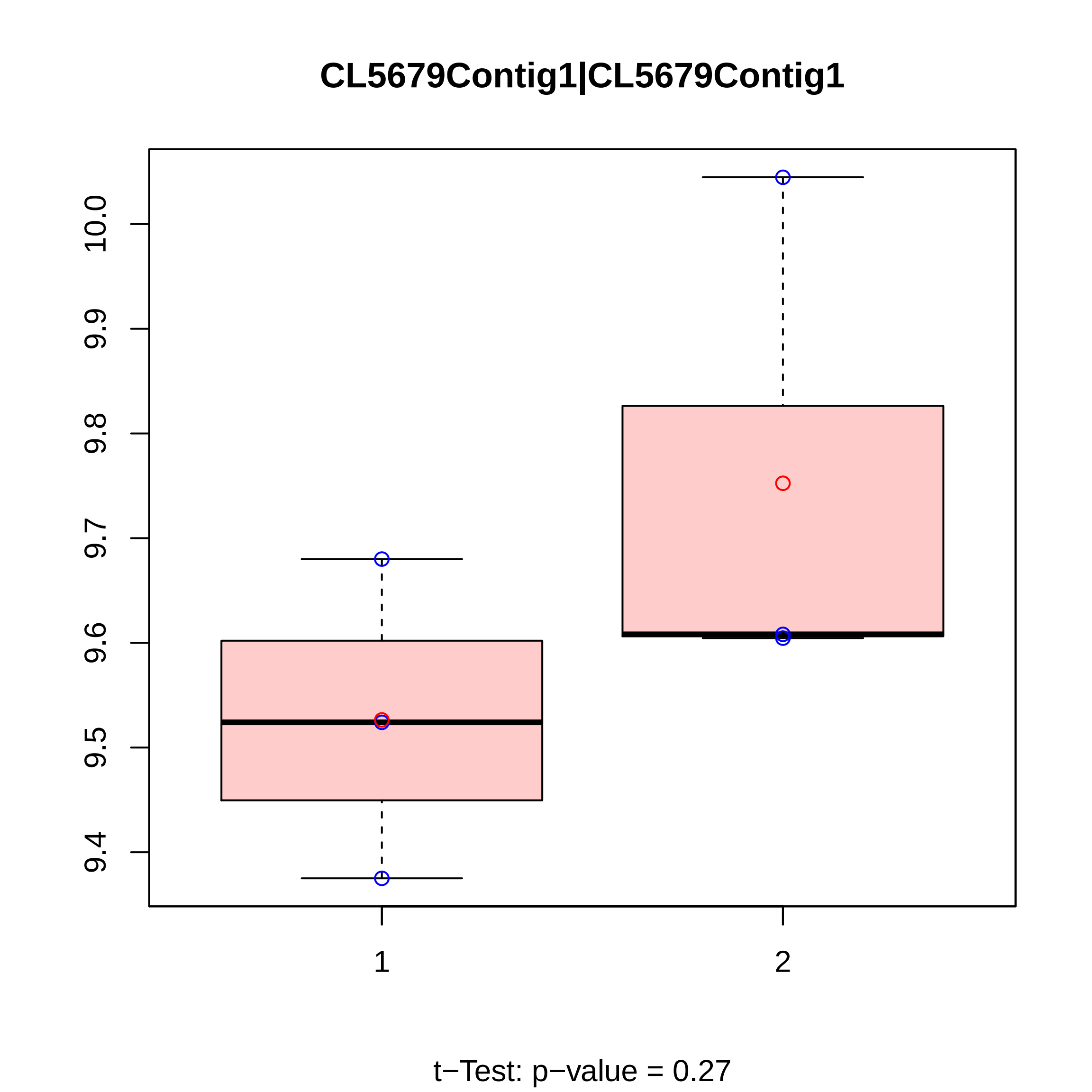

CL5679Contig1|CL5679Contig1
10.0
9.9
9.8
9.7
9.6
9.5
9.4
1
2
t−Test: p−value = 0.27

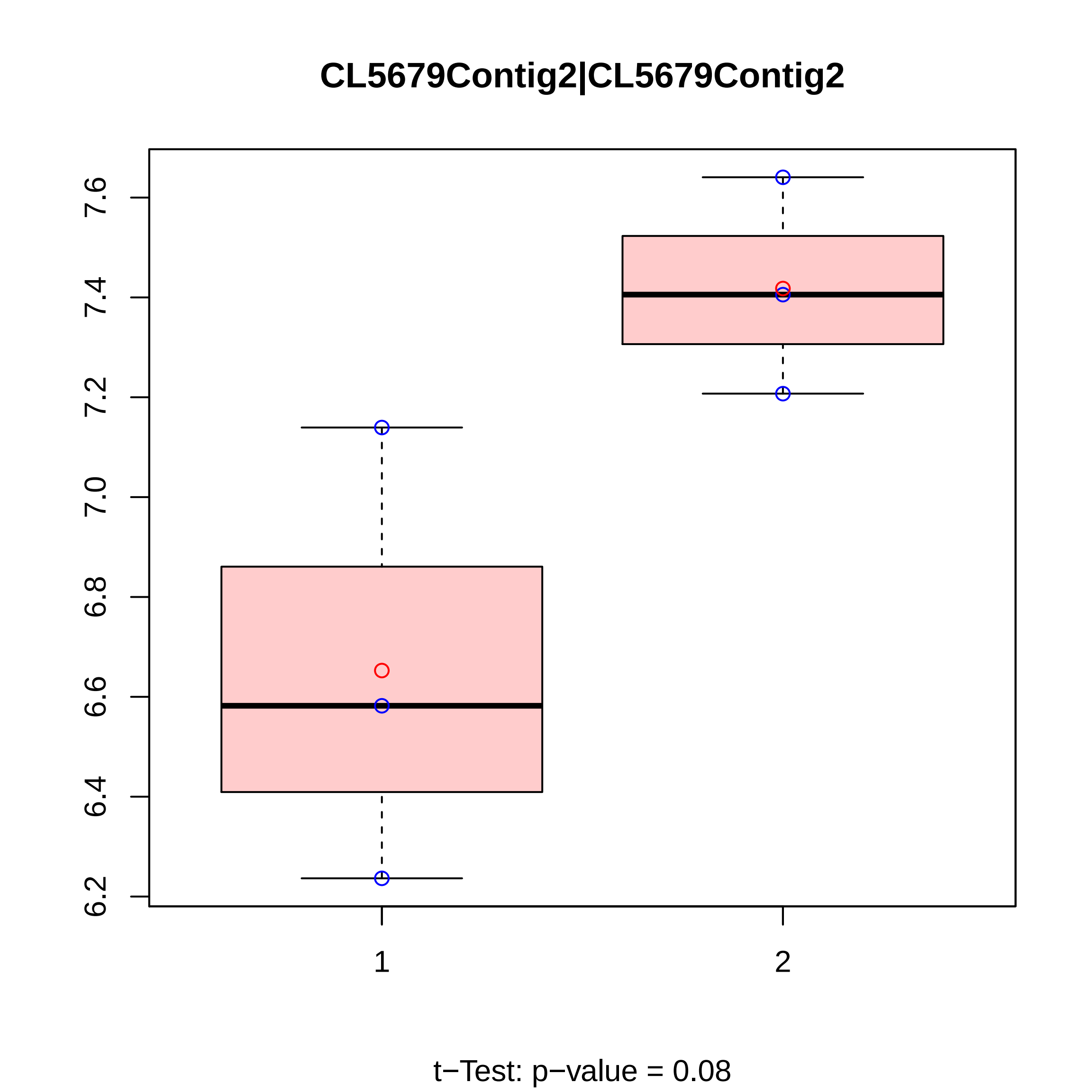
CL5679Contig2|CL5679Contig2
7.6
7.4
7.2
7.0
6.8
6.6
6.4
6.2
1
2
t−Test: p−value = 0.08

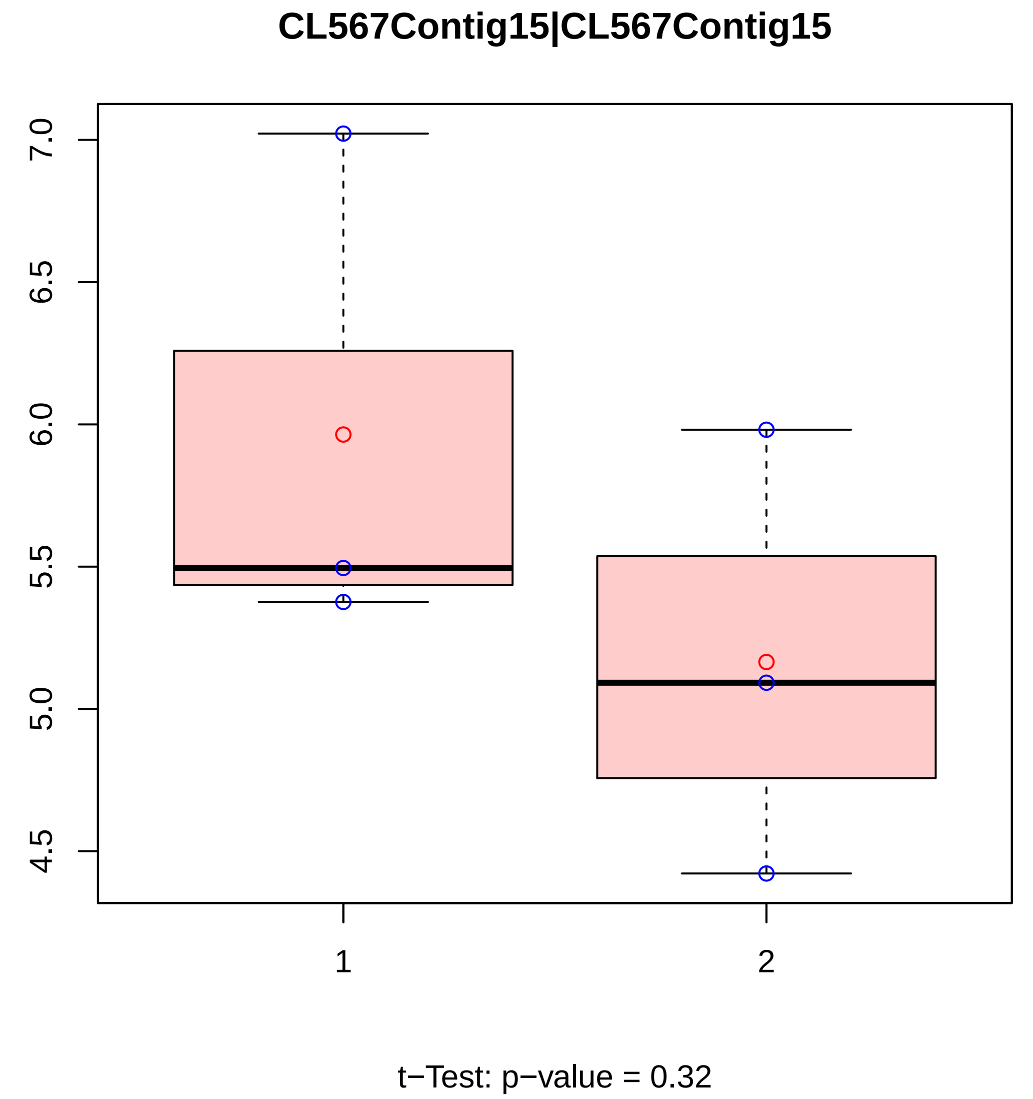
CL567Contig15|CL567Contig15
7.0
6.5
6.0
5.5
5.0
4.5
1
2
t−Test: p−value = 0.32

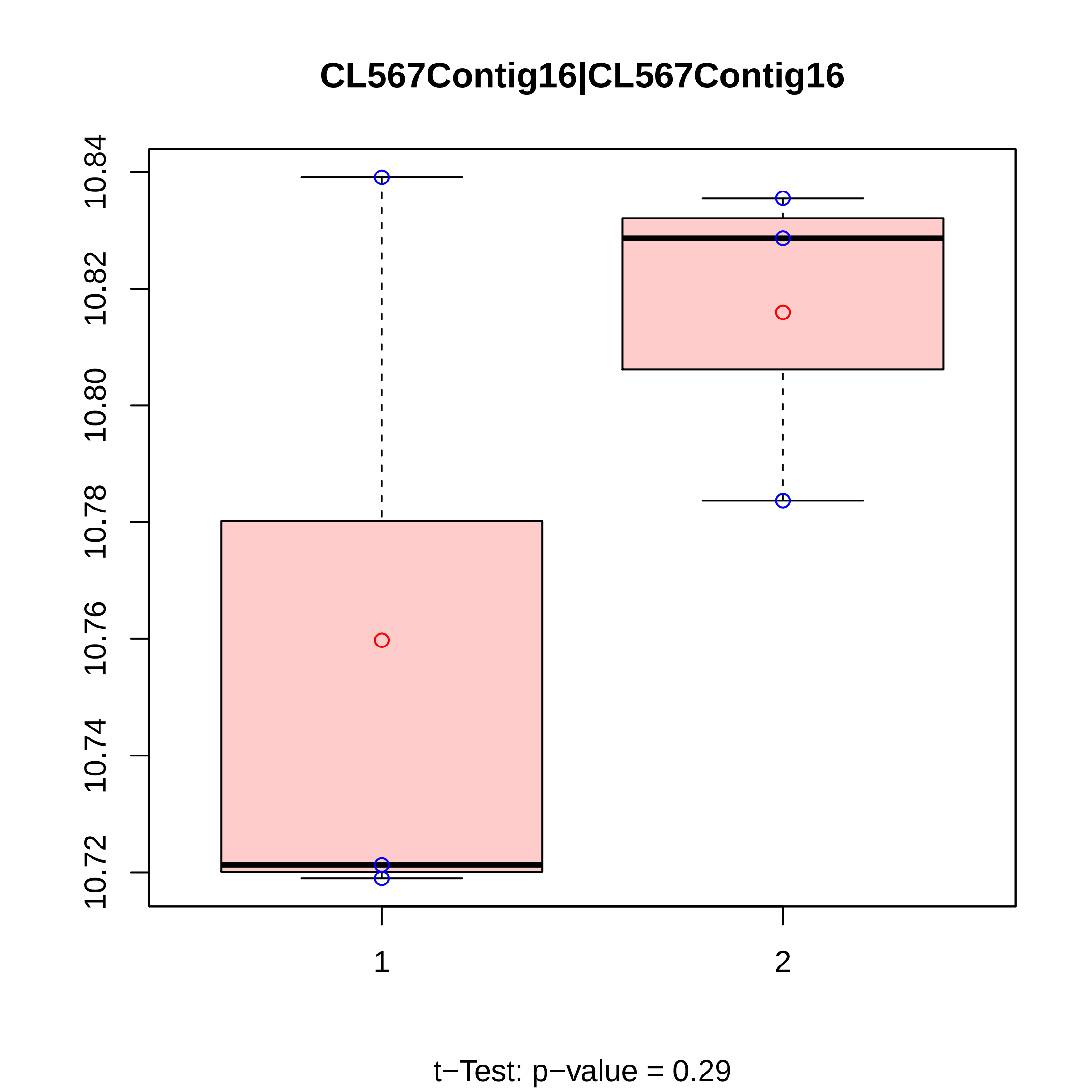

CL567Contig16|CL567Contig16
10.84
10.82
10.80
10.78
10.76
10.74
10.72
1
2
t−Test: p−value = 0.29

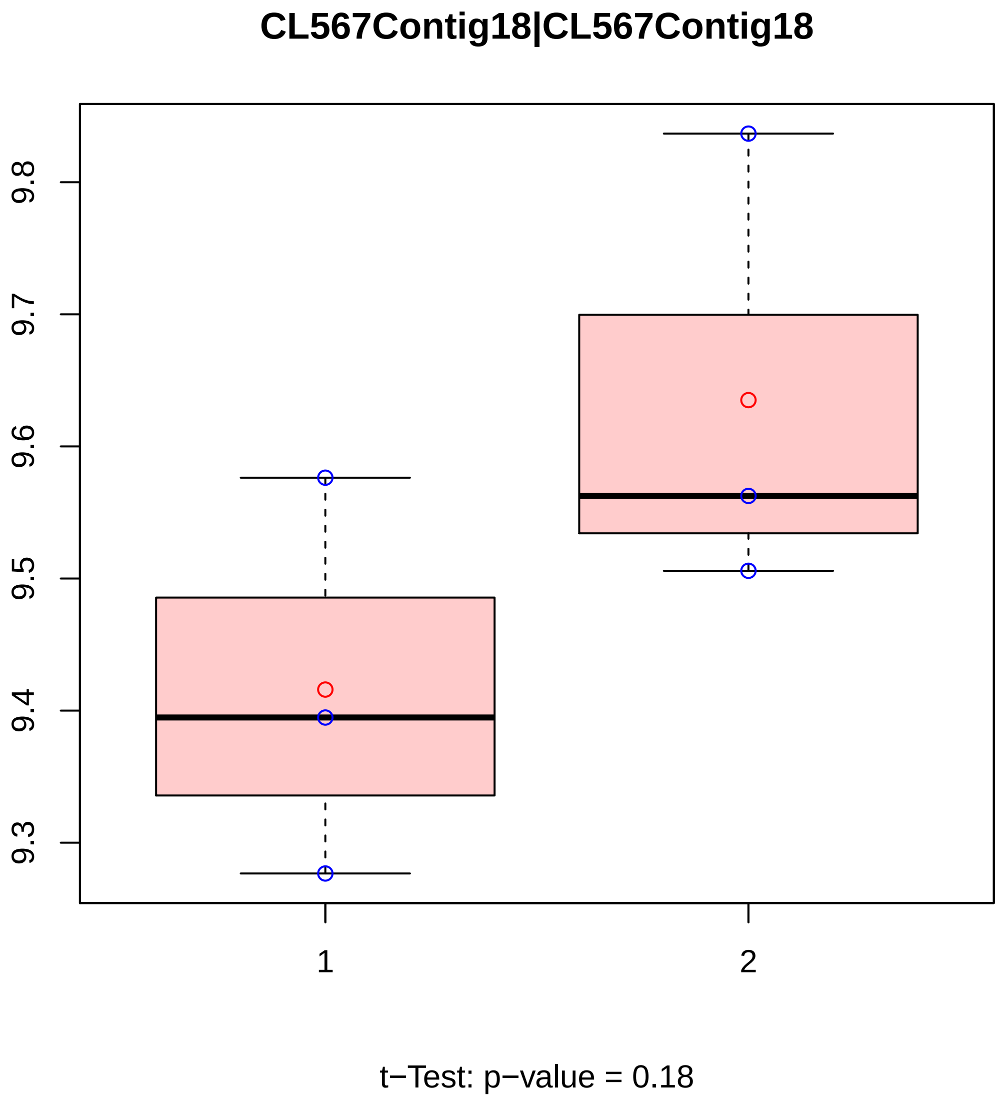
CL567Contig18|CL567Contig18
9.3
9.4
9.5
9.6
9.7
9.8
1
2
t−Test: p−value = 0.18

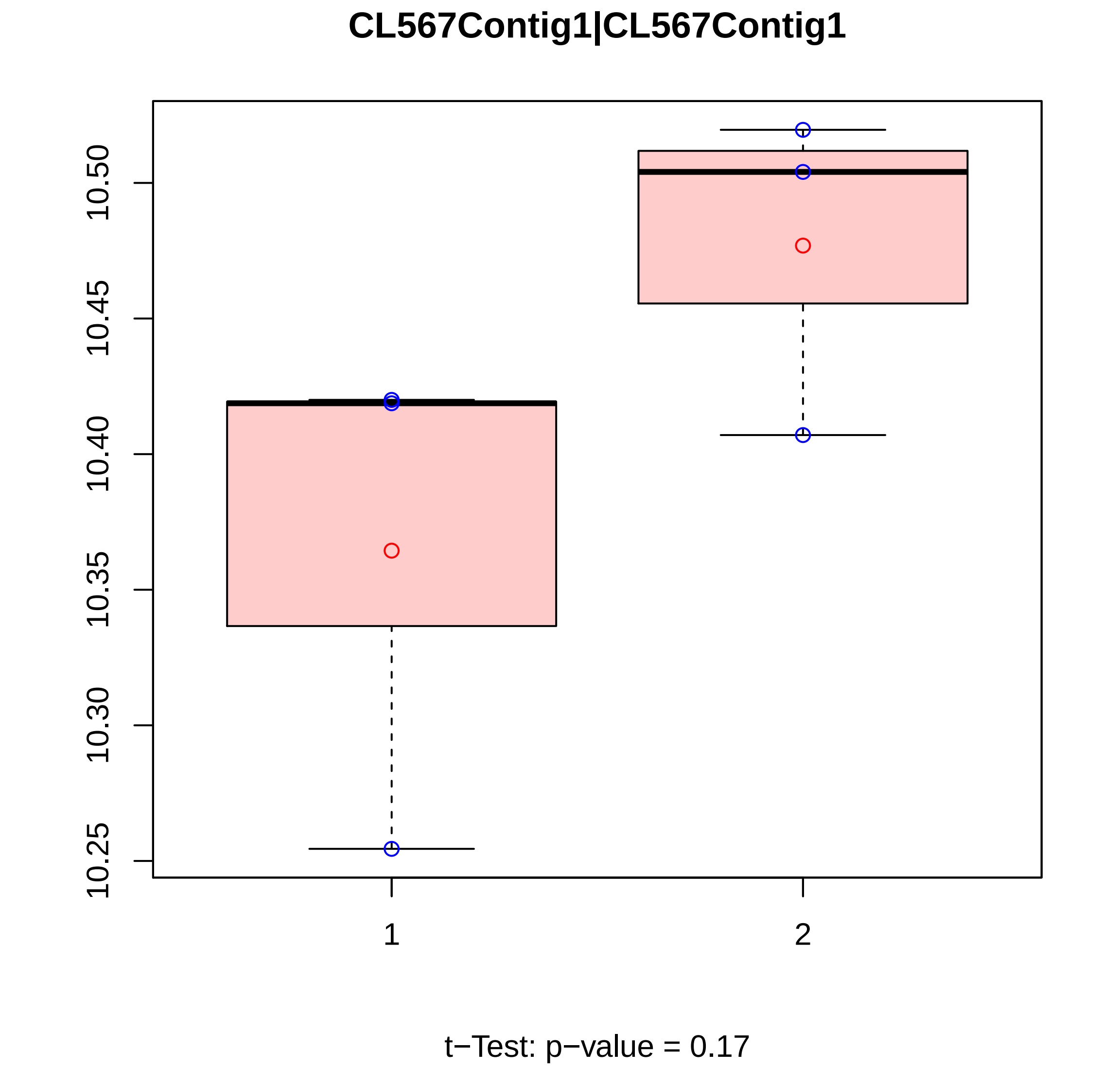

CL567Contig1|CL567Contig1
10.25
10.30
10.35
10.40
10.45
10.50
1
2
t−Test: p−value = 0.17

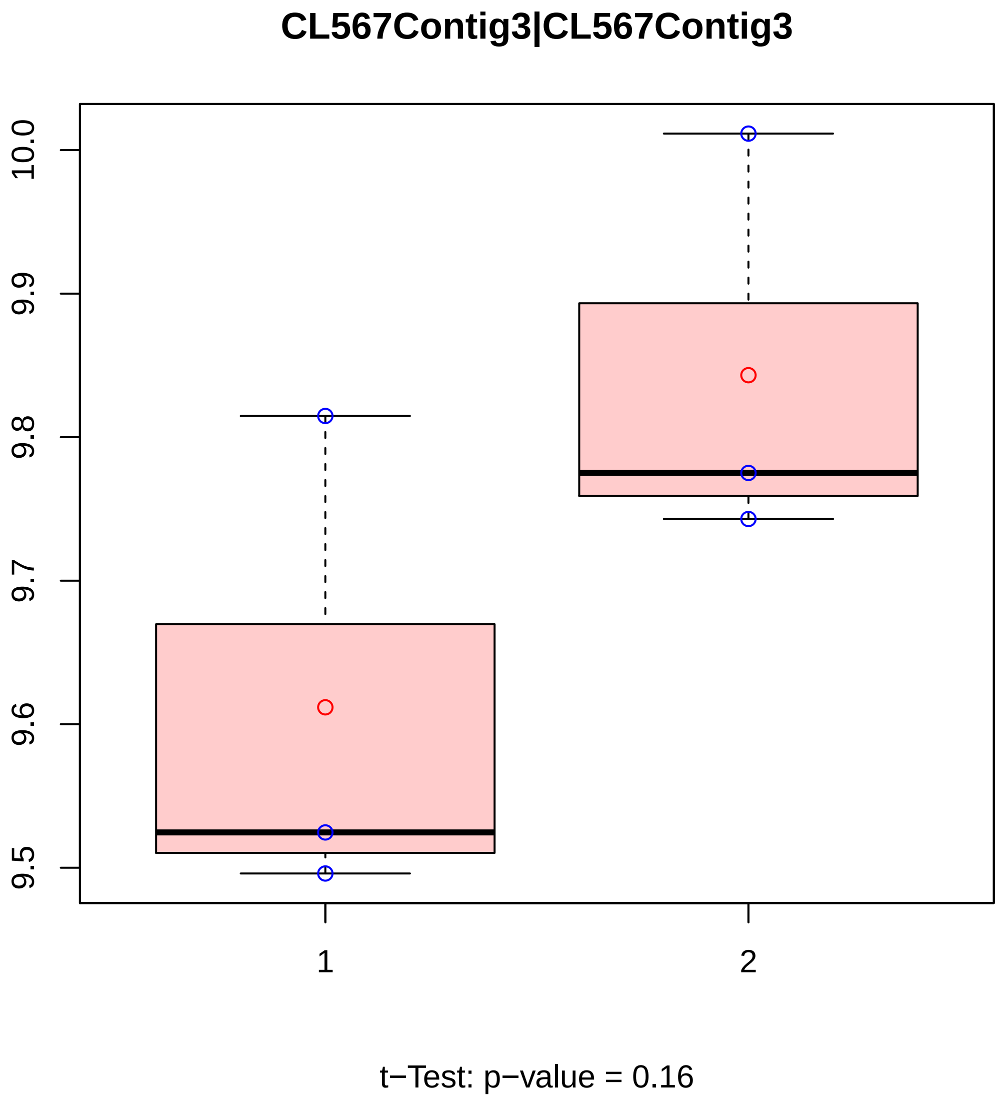

CL567Contig3|CL567Contig3
10.0
9.9
9.8
9.7
9.6
9.5
1
2
t−Test: p−value = 0.16

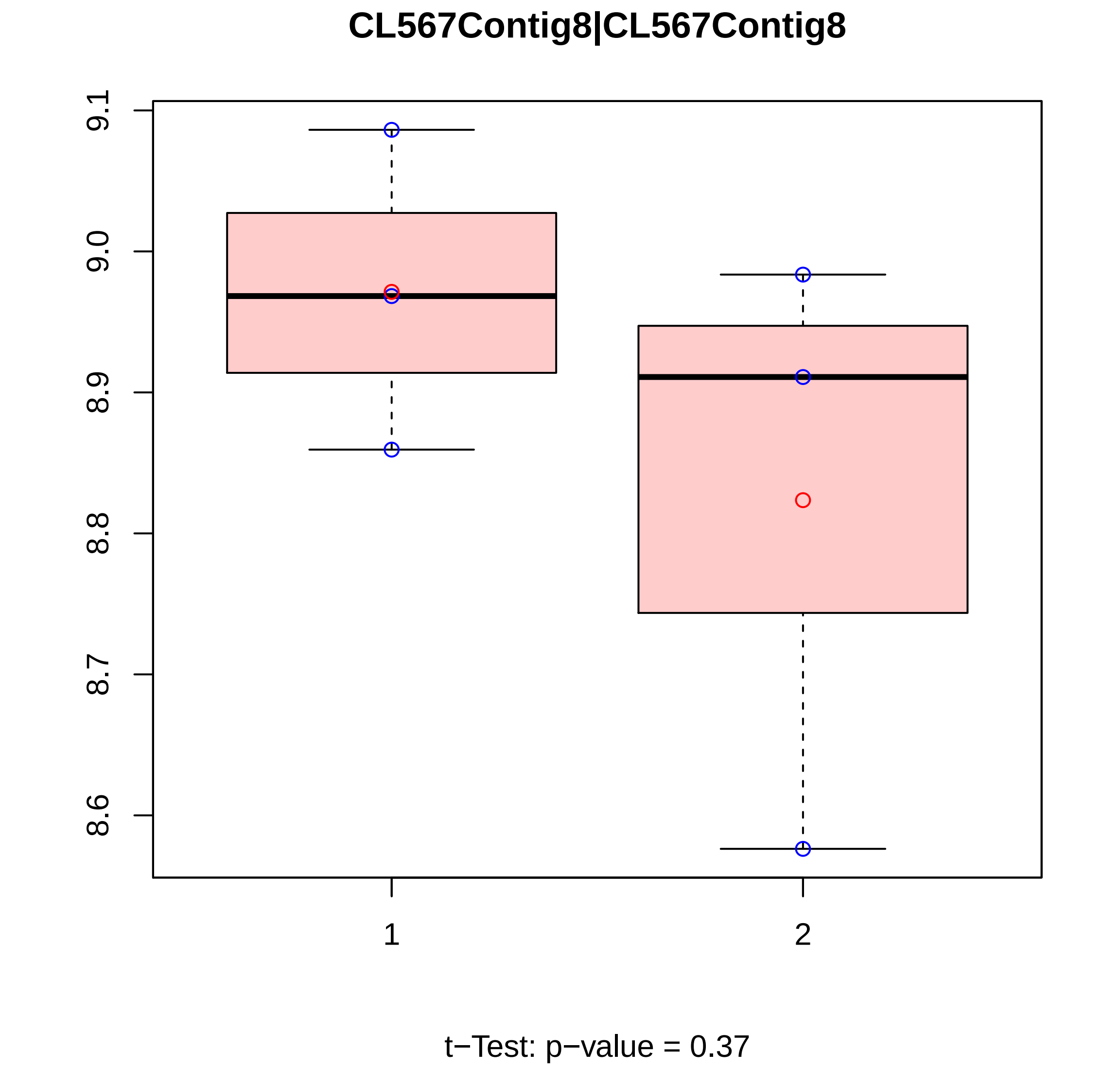
CL567Contig8|CL567Contig8
9.1
9.0
8.9
8.8
8.7
8.6
1
2
t−Test: p−value = 0.37

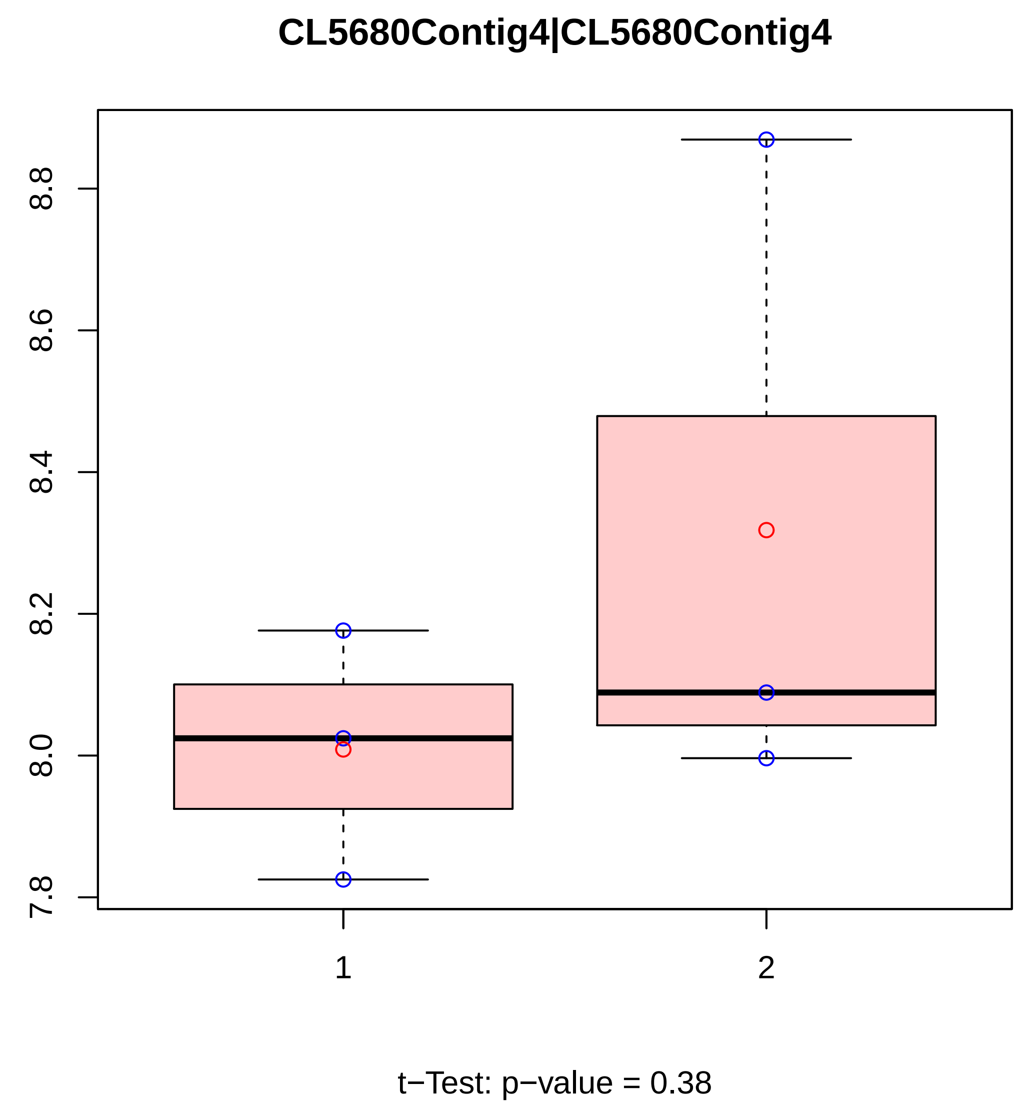

CL5680Contig4|CL5680Contig4
8.8
8.6
8.4
8.2
8.0
7.8
1
2
t−Test: p−value = 0.38

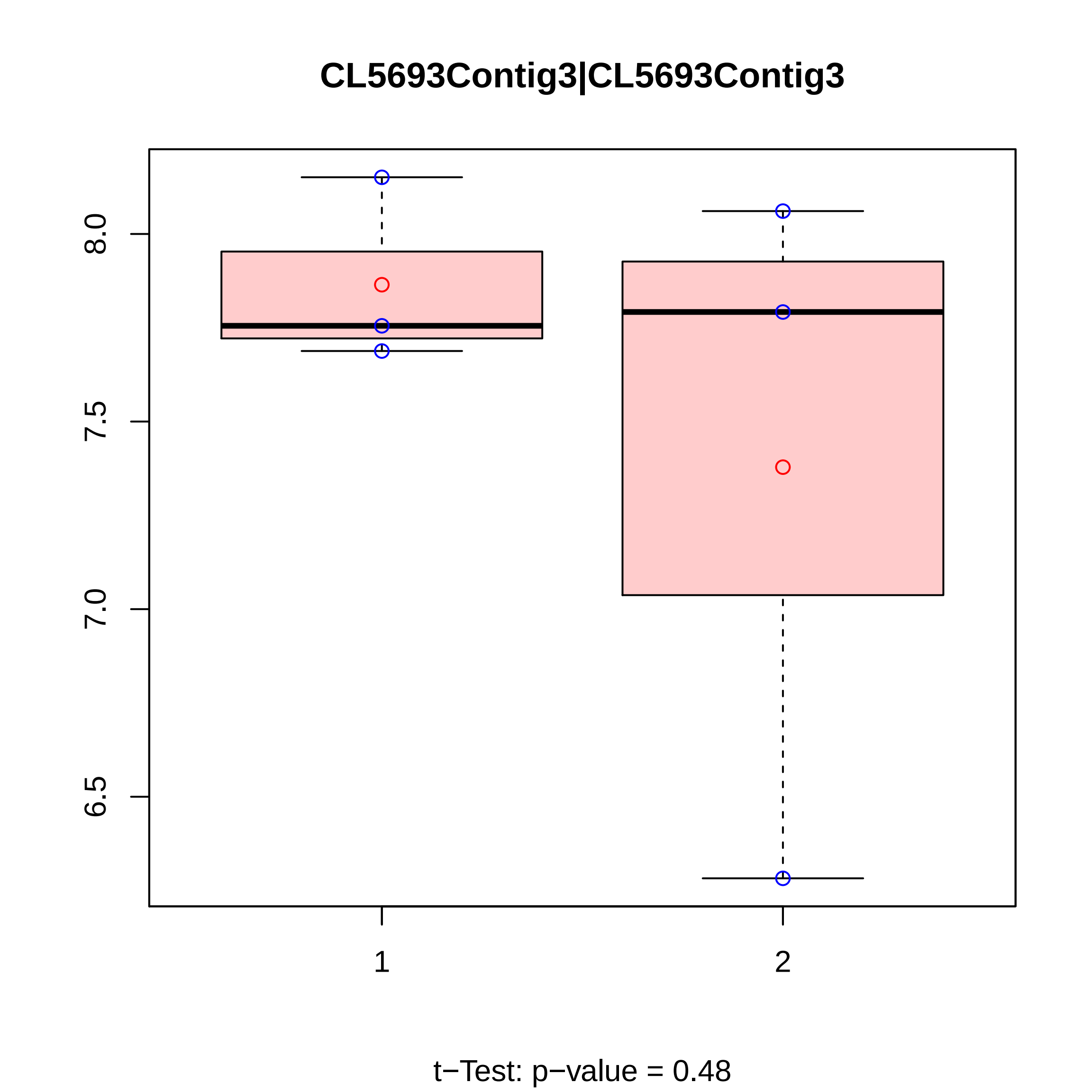
CL5693Contig3|CL5693Contig3
8.0
7.5
7.0
6.5
1
2
t−Test: p−value = 0.48

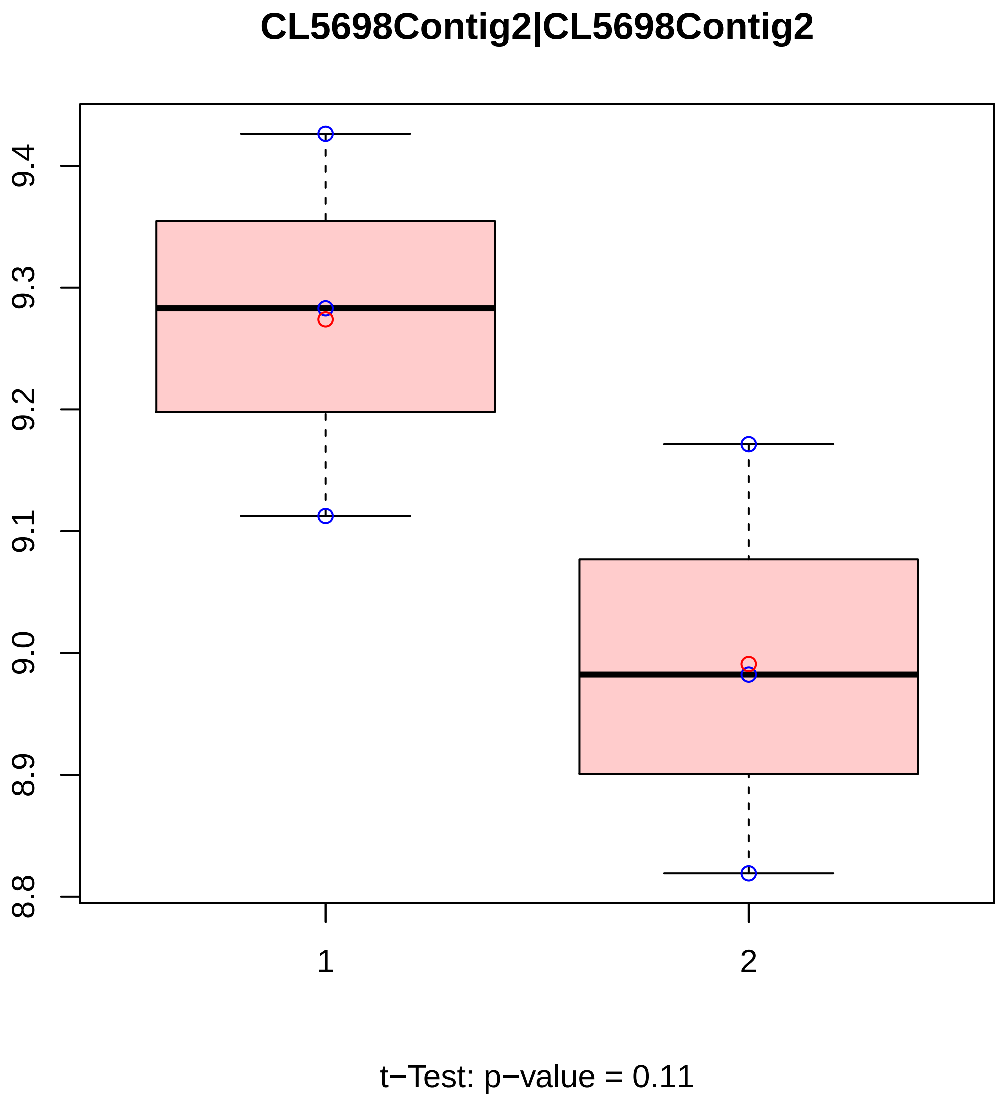
CL5698Contig2|CL5698Contig2
9.4
9.3
9.2
9.1
9.0
8.9
8.8
1
2
t−Test: p−value = 0.11

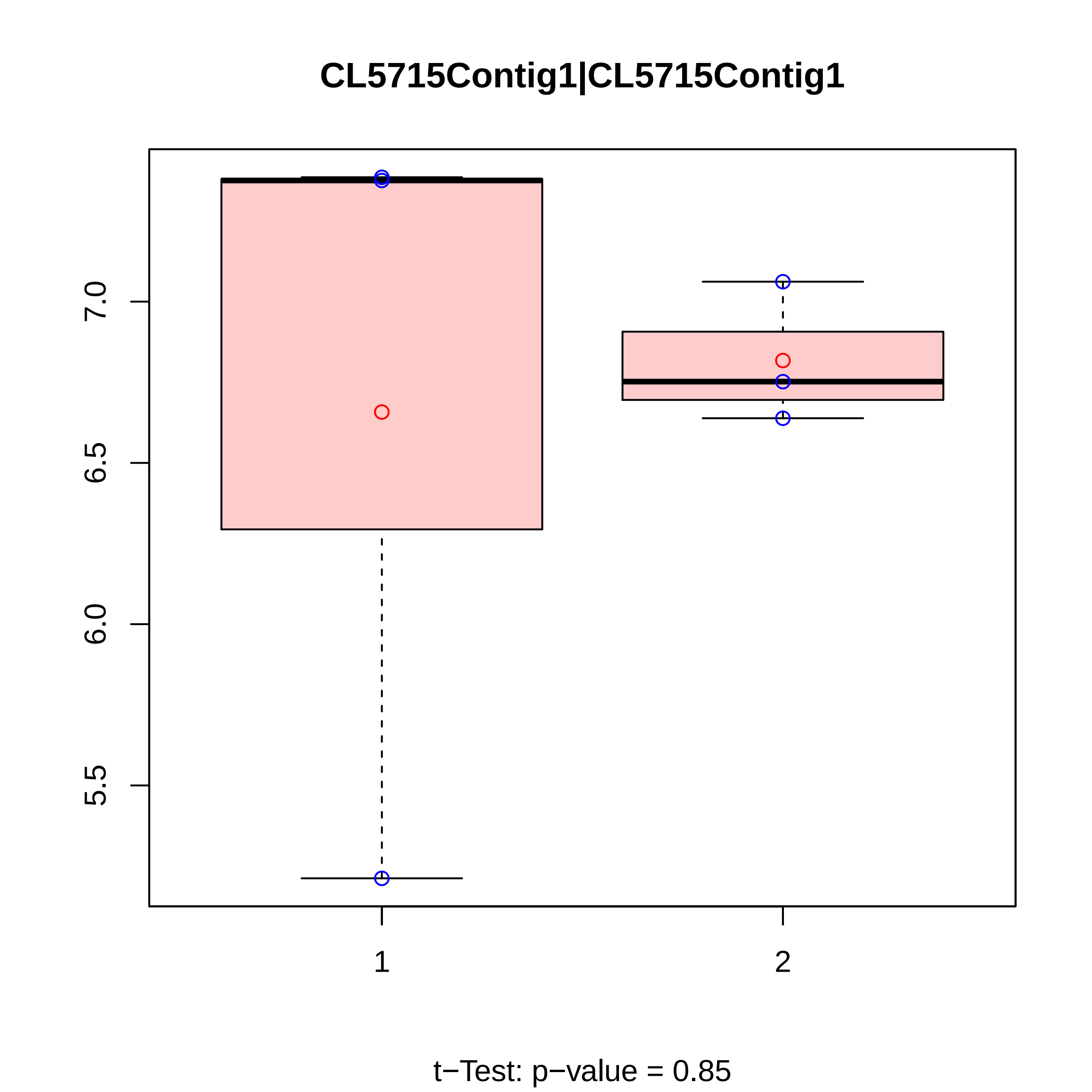
CL5715Contig1|CL5715Contig1
7.0
6.5
6.0
5.5
1
2
t−Test: p−value = 0.85

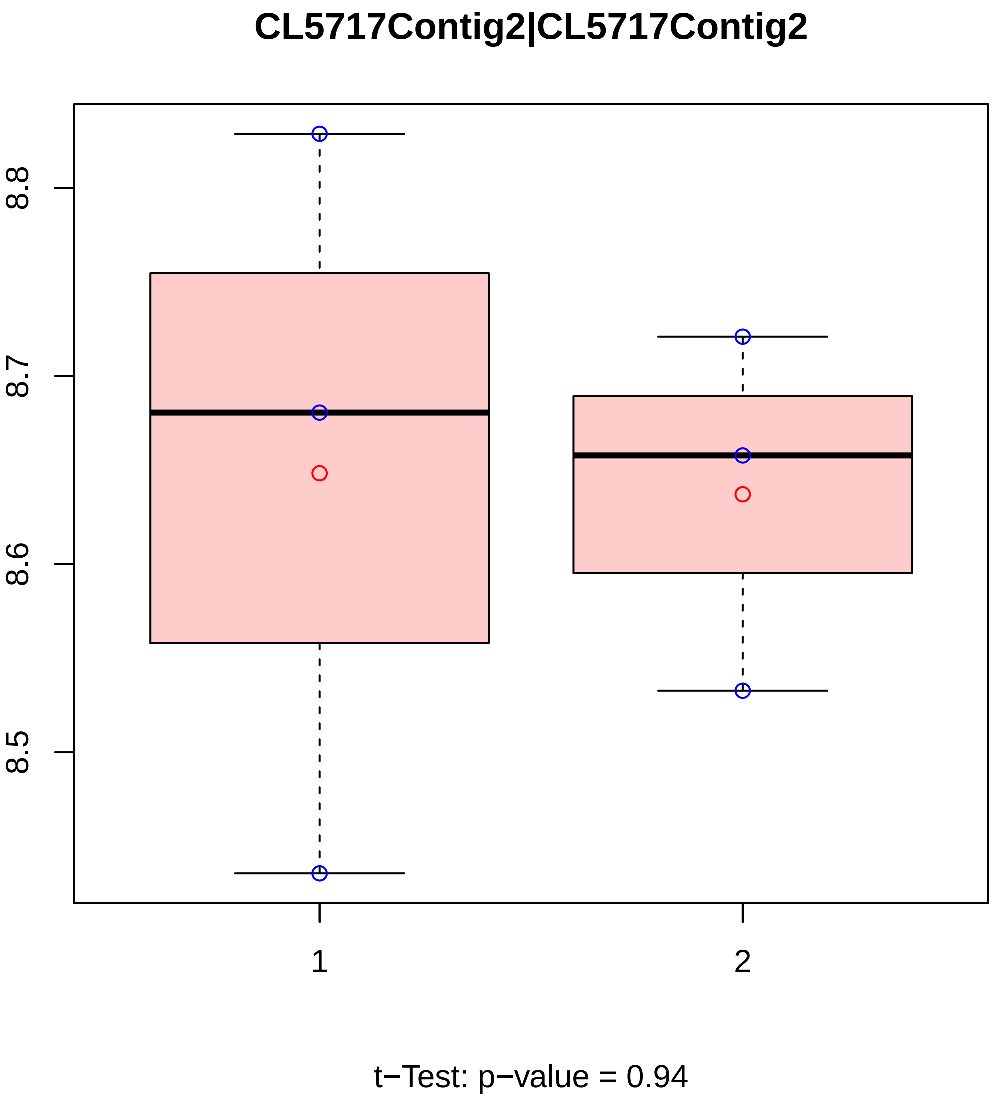

CL5717Contig2|CL5717Contig2
8.5
8.6
8.7
8.8
1
2
t−Test: p−value = 0.94

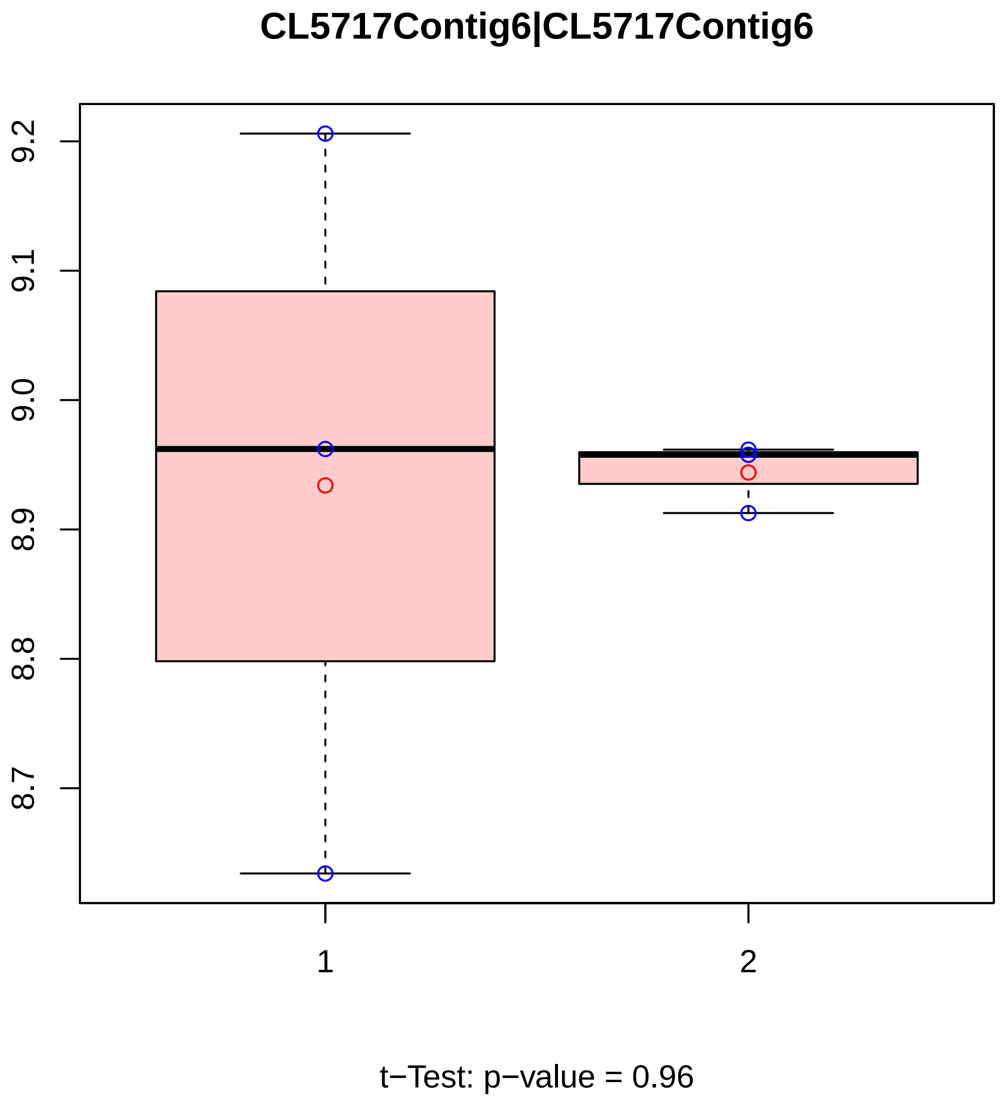
CL5717Contig6|CL5717Contig6
9.2
9.1
9.0
8.9
8.8
8.7
1
2
t−Test: p−value = 0.96

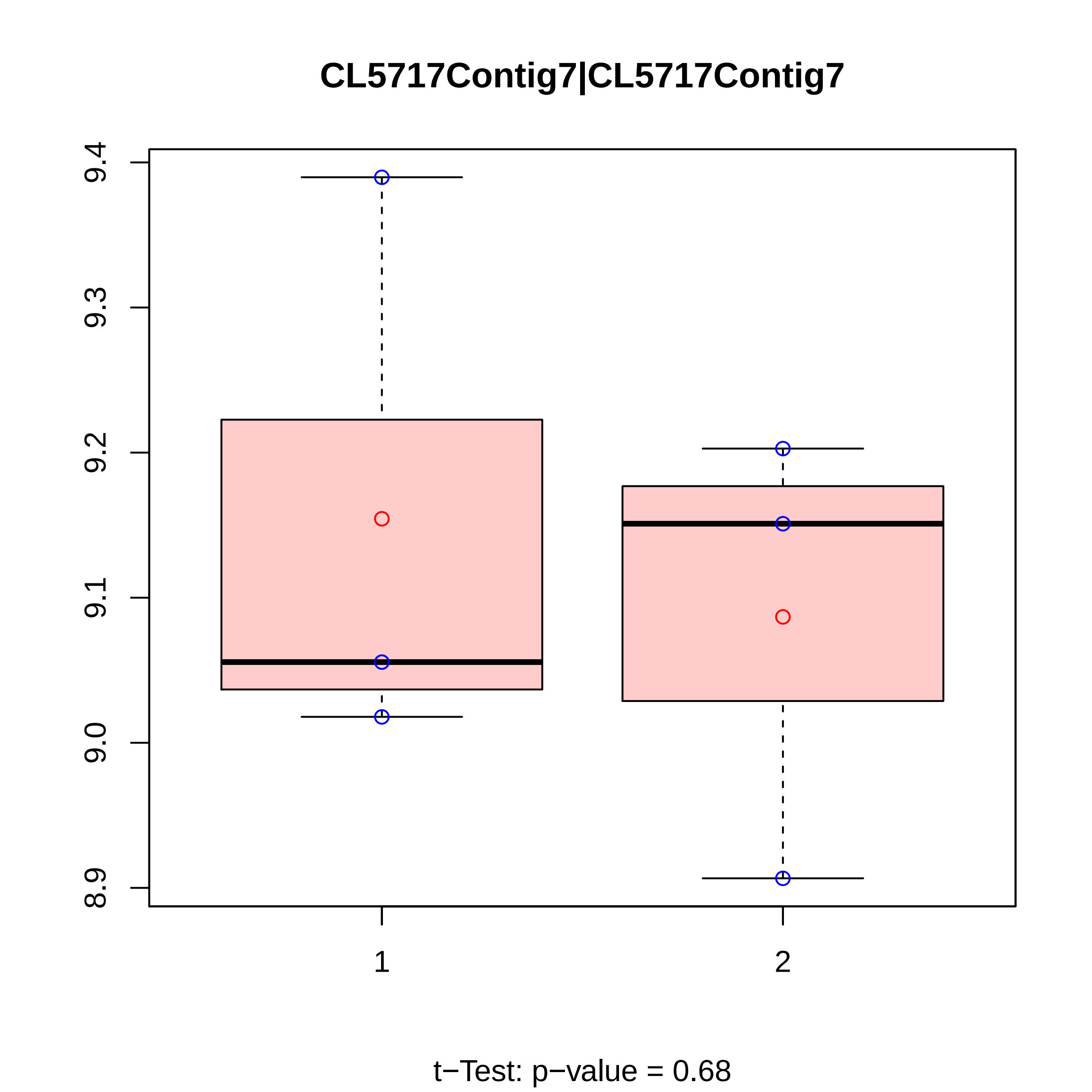
CL5717Contig7|CL5717Contig7
9.4
9.3
9.2
9.1
9.0
8.9
1
2
t−Test: p−value = 0.68

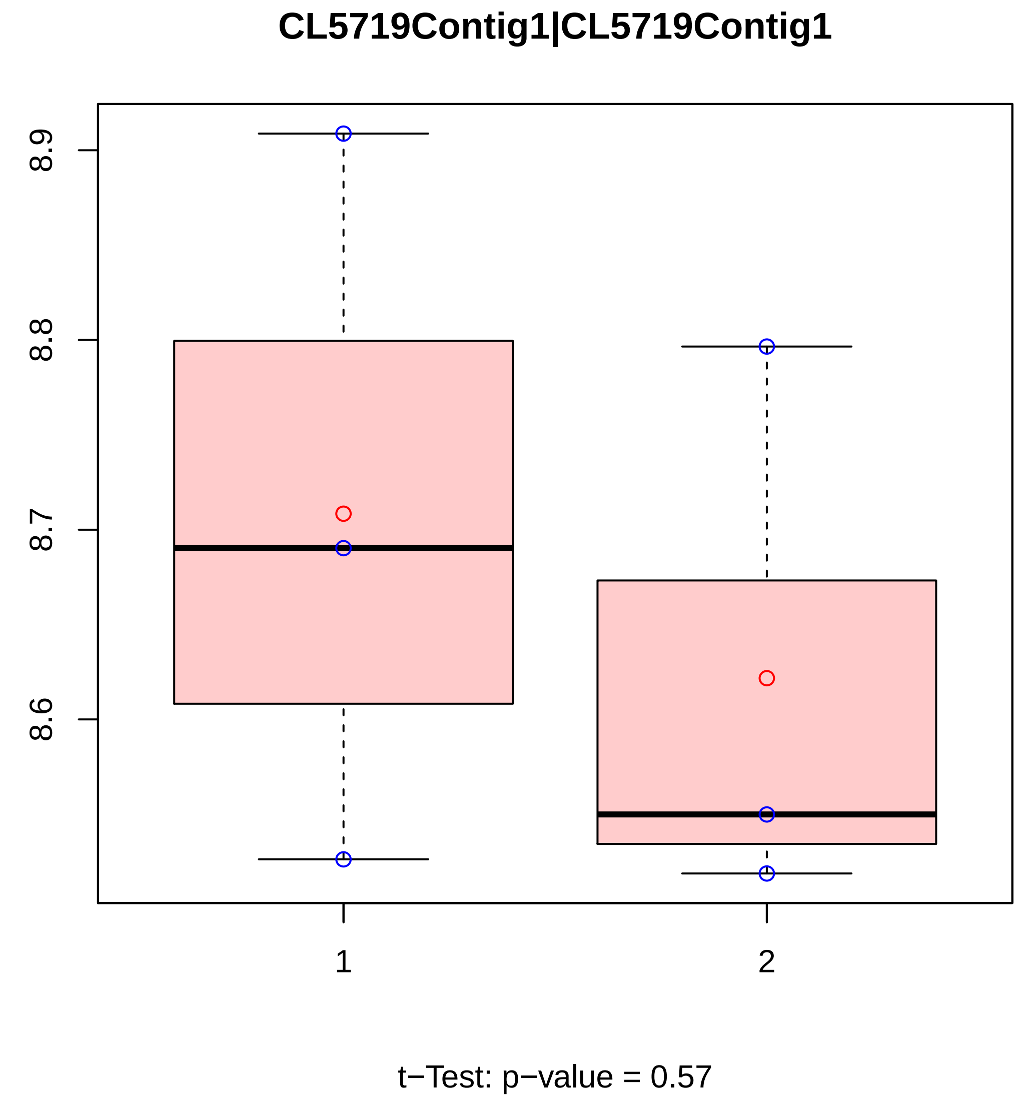
CL5719Contig1|CL5719Contig1
8.6
8.7
8.8
8.9
1
2
t−Test: p−value = 0.57

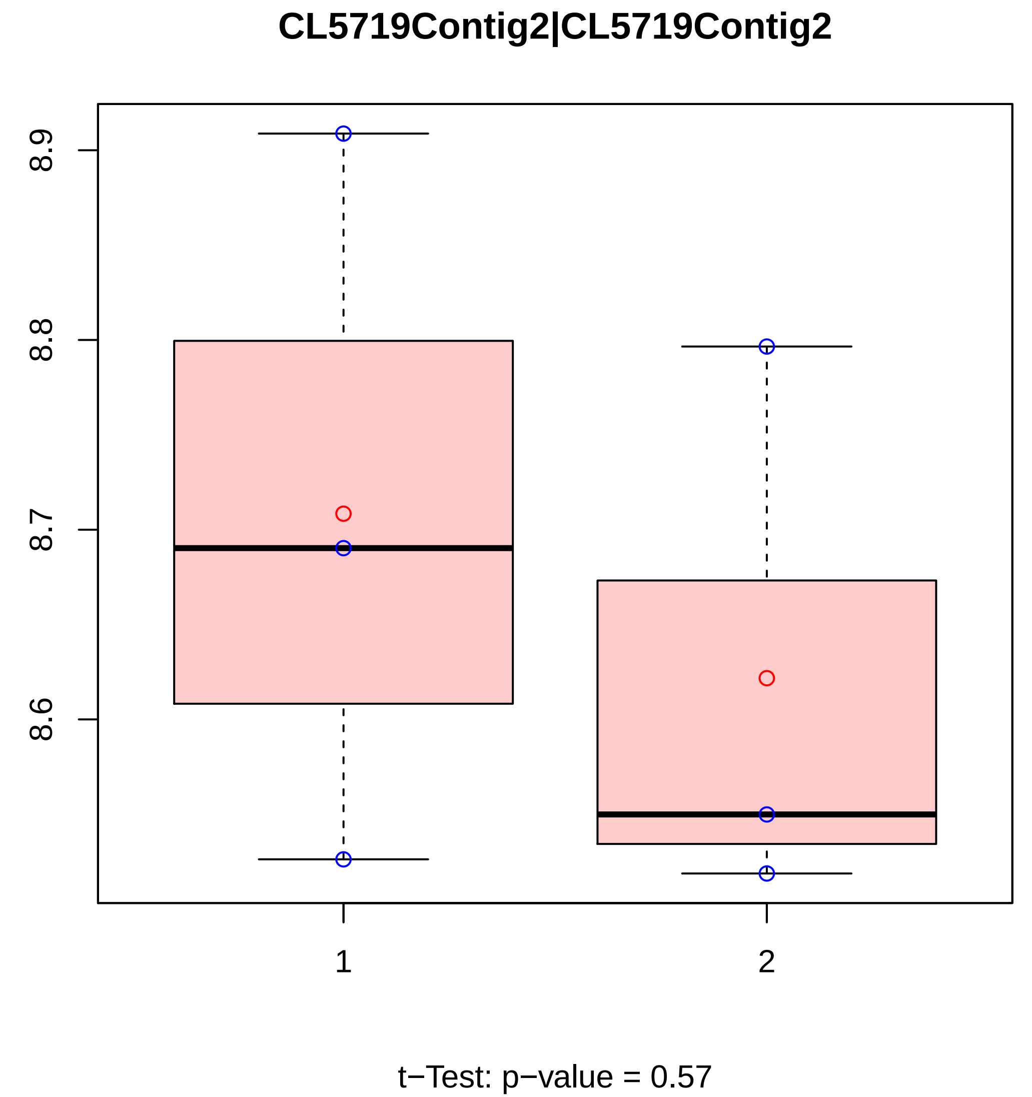
CL5719Contig2|CL5719Contig2
8.9
8.8
8.7
8.6
1
2
t−Test: p−value = 0.57

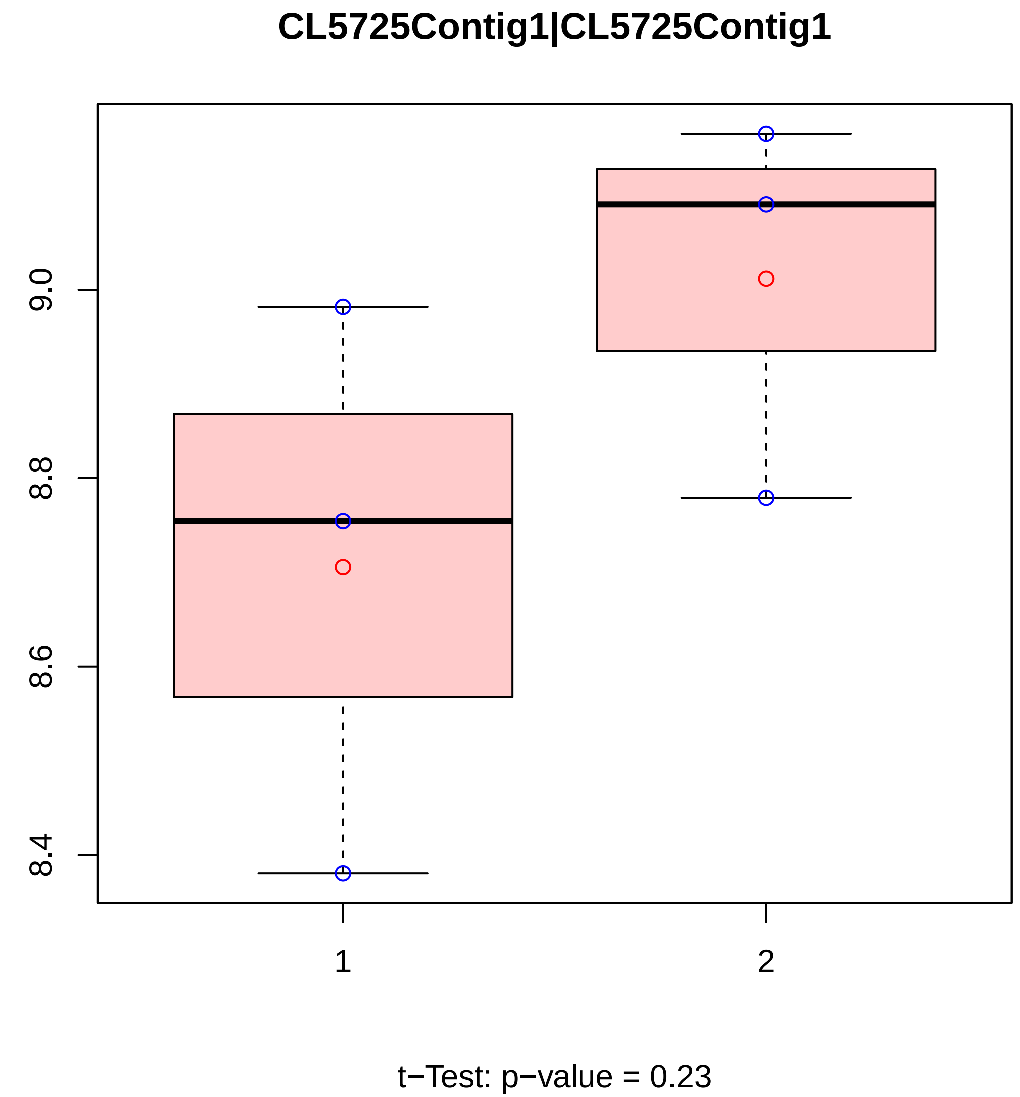
CL5725Contig1|CL5725Contig1
9.0
8.8
8.6
8.4
1
2
t−Test: p−value = 0.23

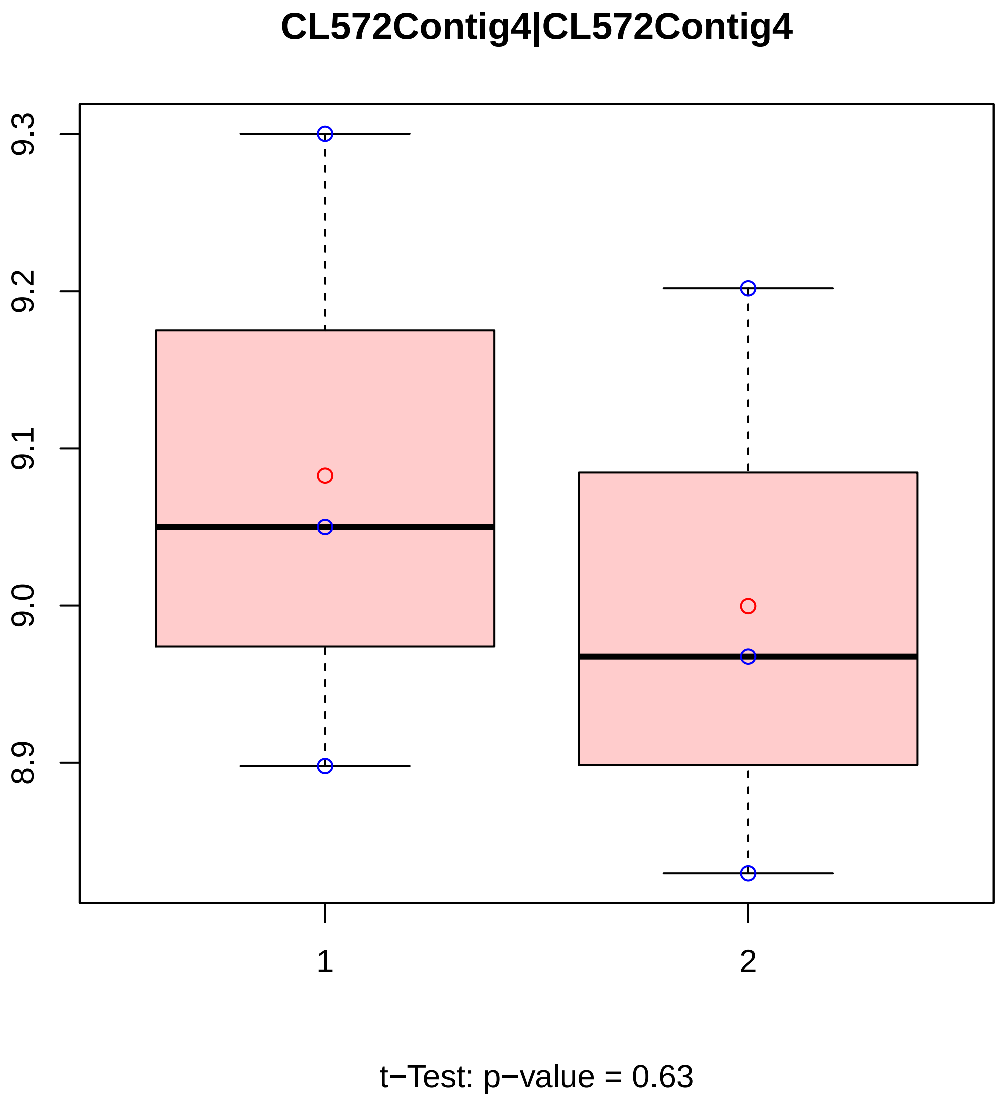

CL572Contig4|CL572Contig4
9.3
9.2
9.1
9.0
8.9
1
2
t−Test: p−value = 0.63

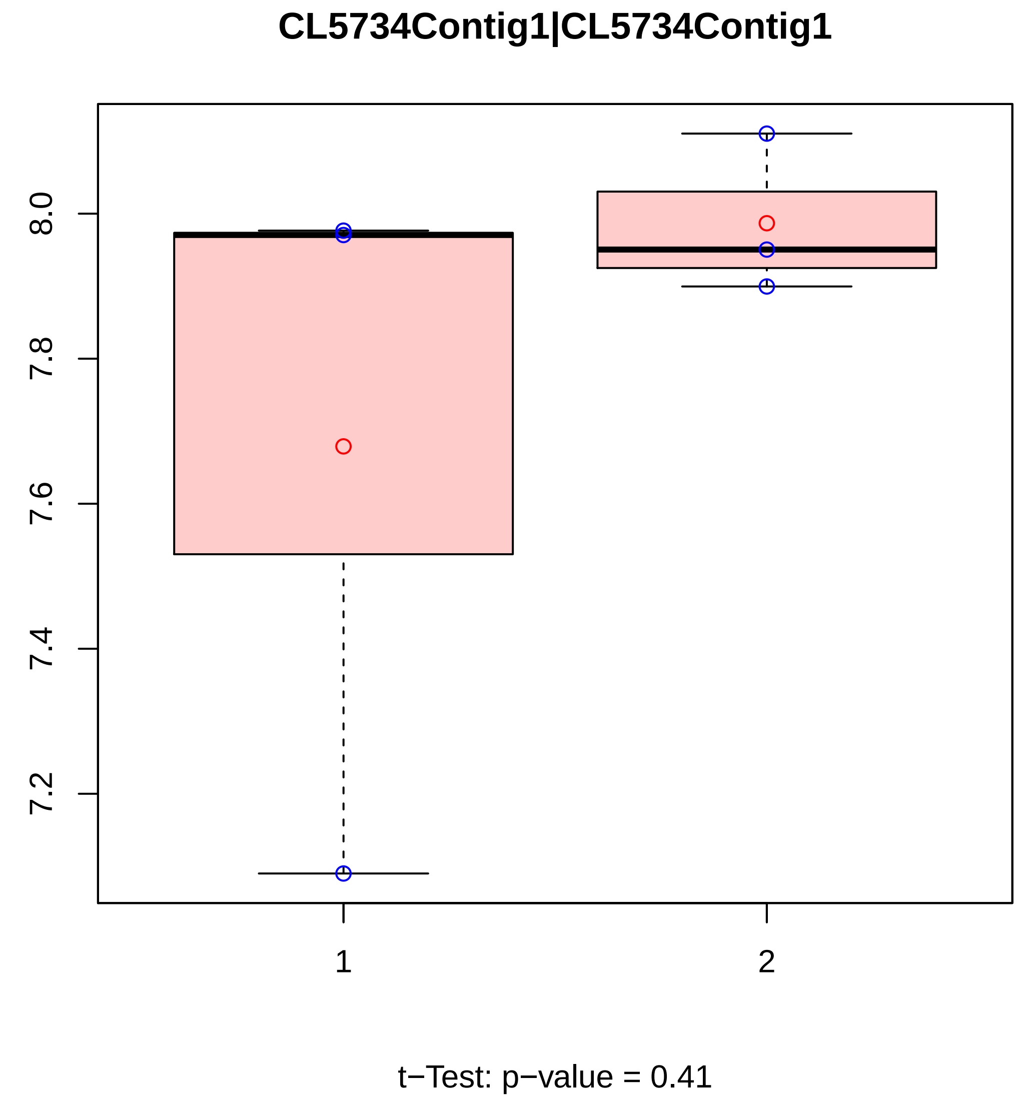
CL5734Contig1|CL5734Contig1
8.0
7.8
7.6
7.4
7.2
1
2
t−Test: p−value = 0.41

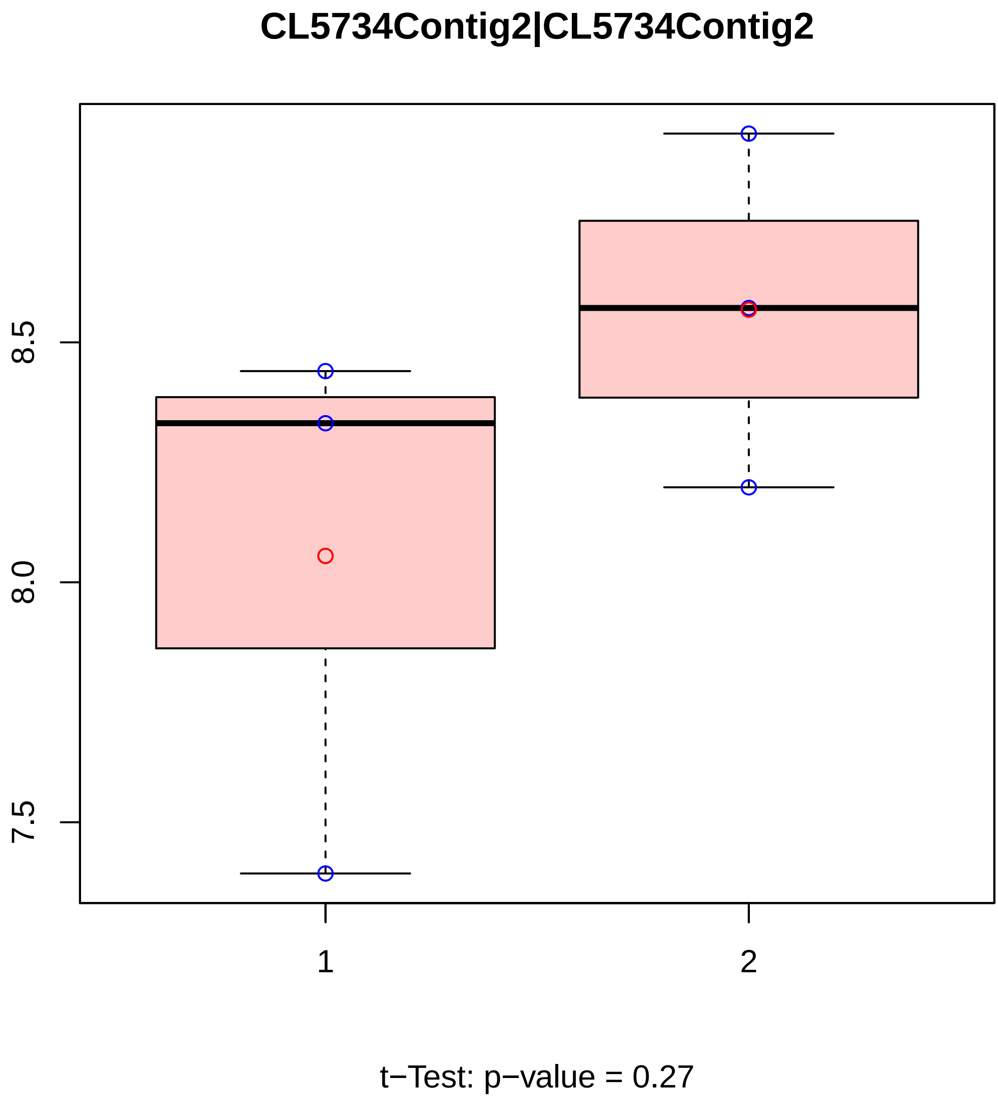
CL5734Contig2|CL5734Contig2
8.5
8.0
7.5
1
2
t−Test: p−value = 0.27

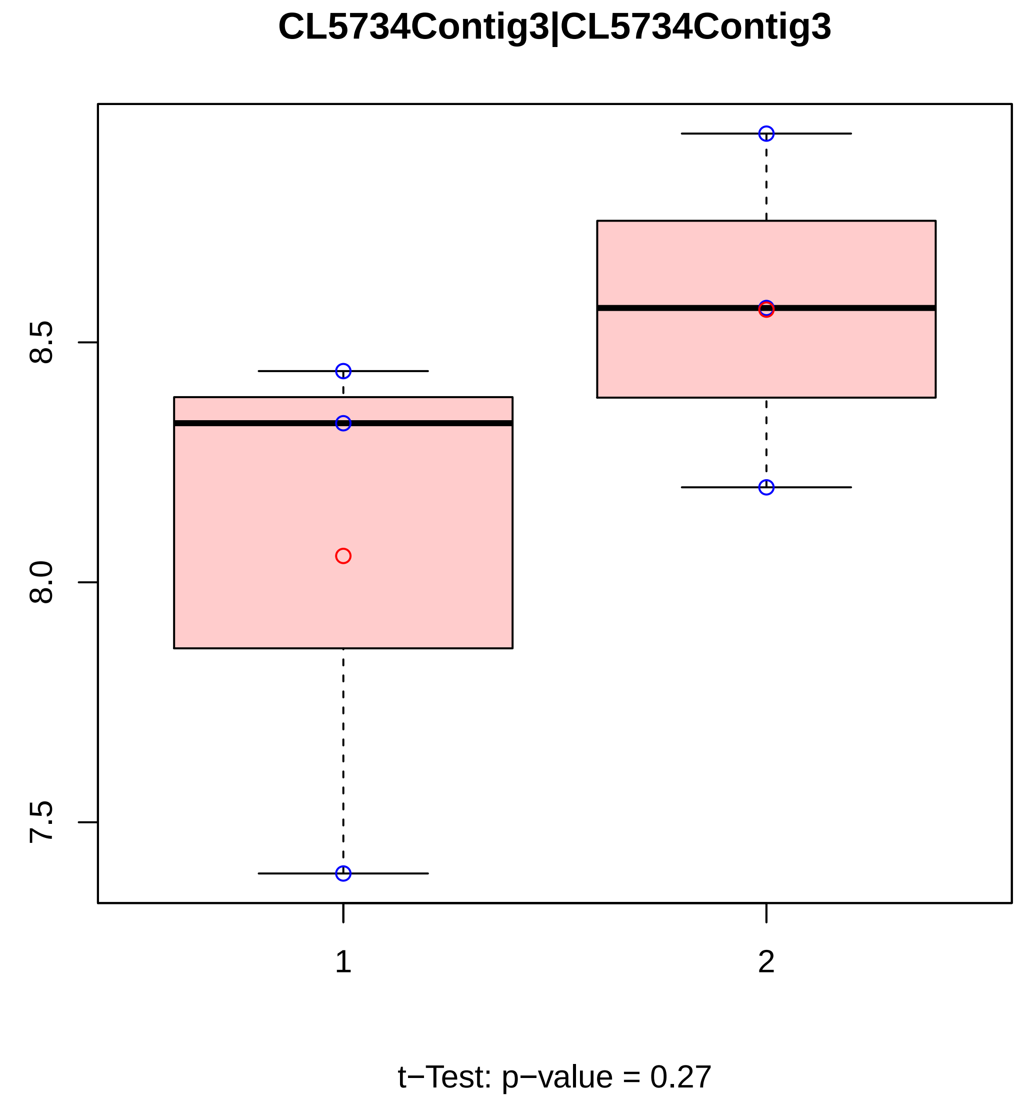
CL5734Contig3|CL5734Contig3
8.5
8.0
7.5
1
2
t−Test: p−value = 0.27

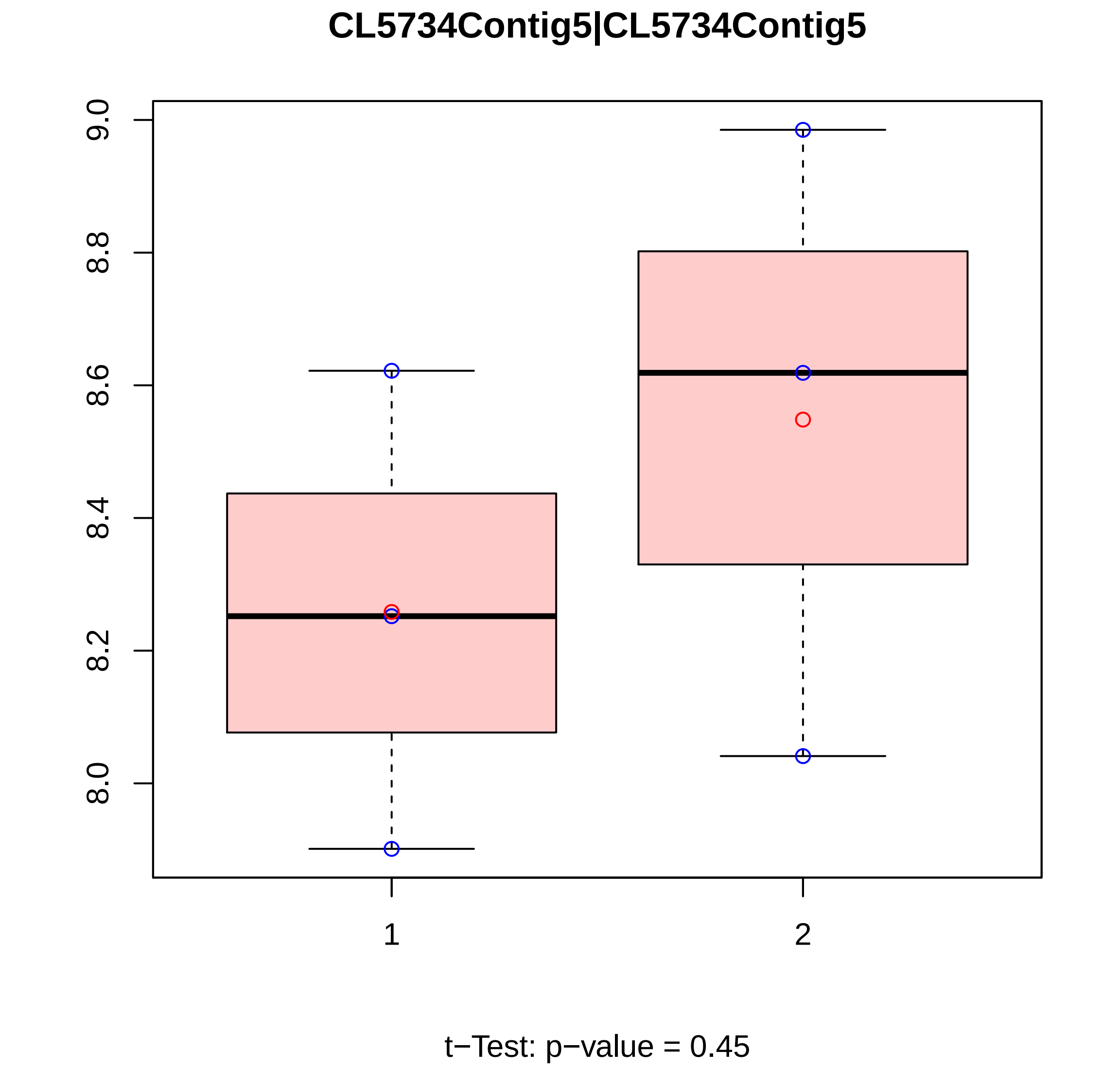
CL5734Contig5|CL5734Contig5
9.0
8.8
8.6
8.4
8.2
8.0
1
2
t−Test: p−value = 0.45

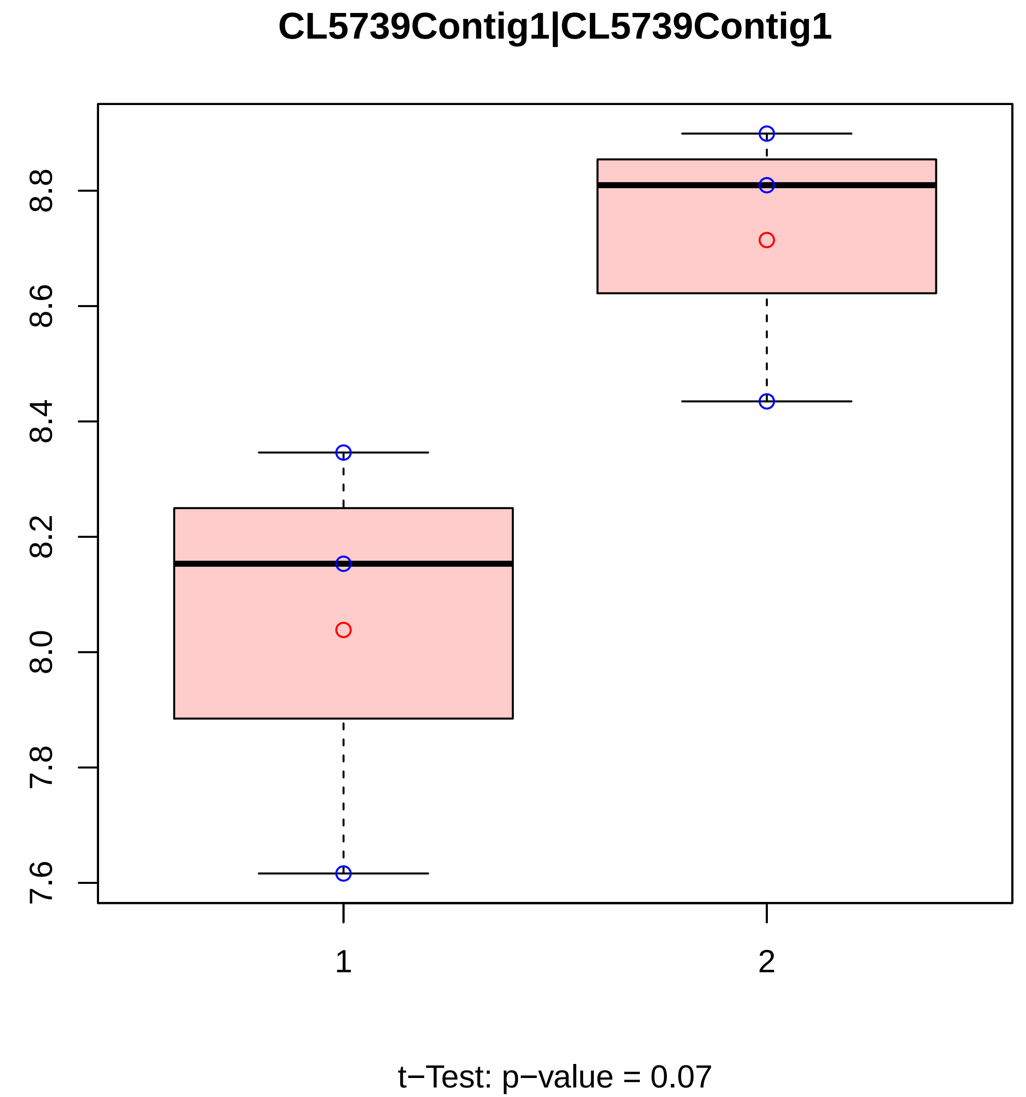

CL5739Contig1|CL5739Contig1
8.8
8.6
8.4
8.2
8.0
7.8
7.6
1
2
t−Test: p−value = 0.07

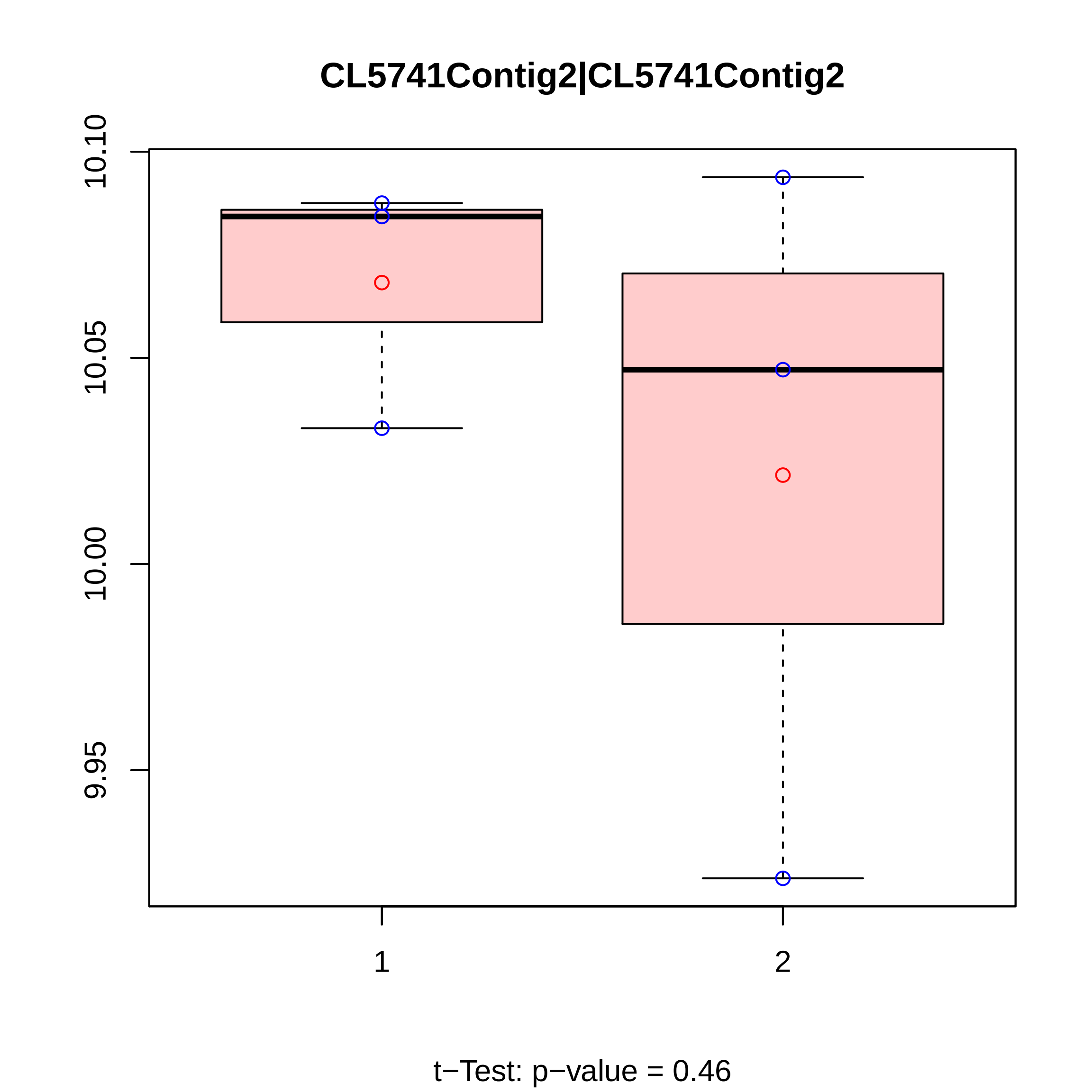
CL5741Contig2|CL5741Contig2
10.10
10.05
10.00
9.95
1
2
t−Test: p−value = 0.46

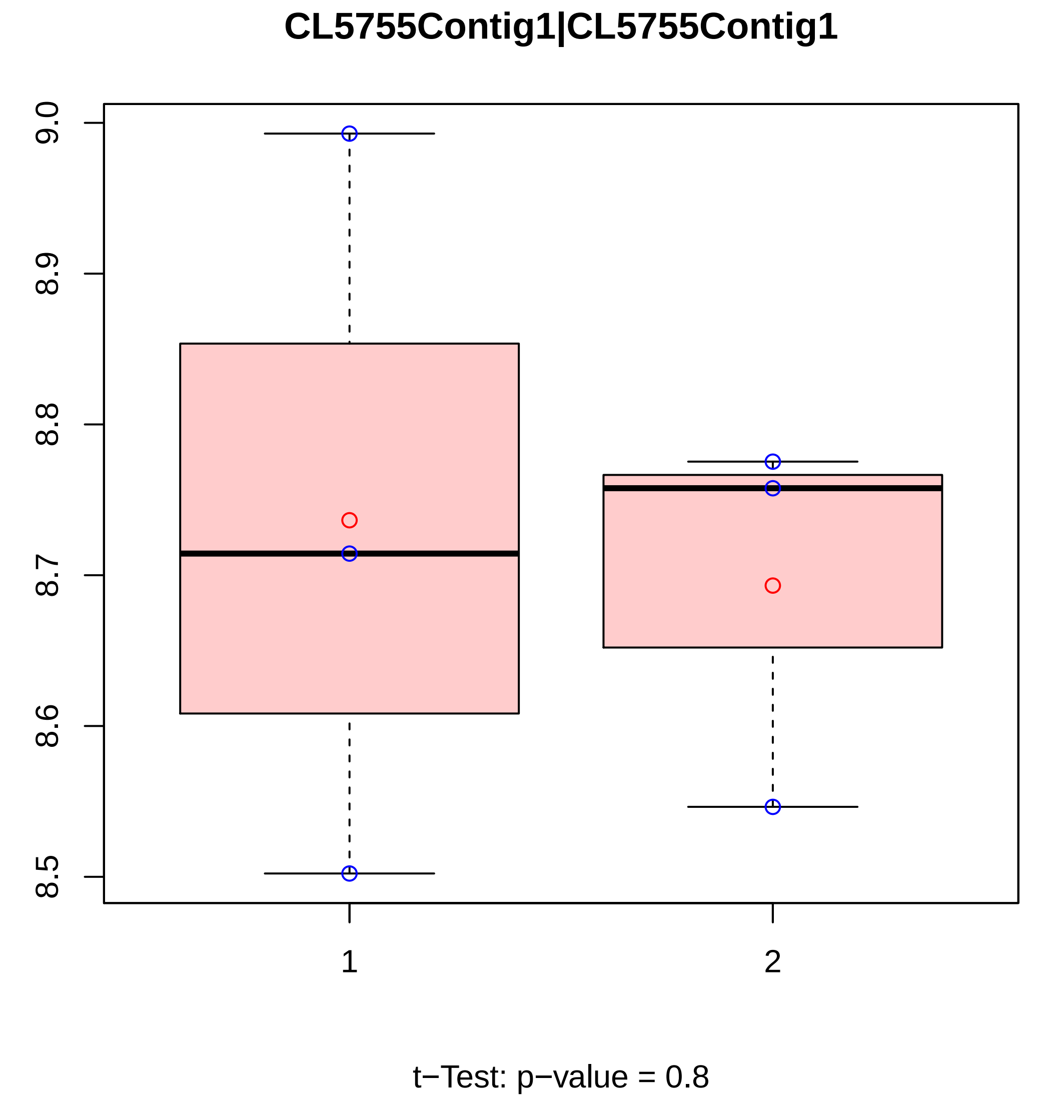

CL5755Contig1|CL5755Contig1
9.0
8.9
8.8
8.7
8.6
8.5
1
2
t−Test: p−value = 0.8

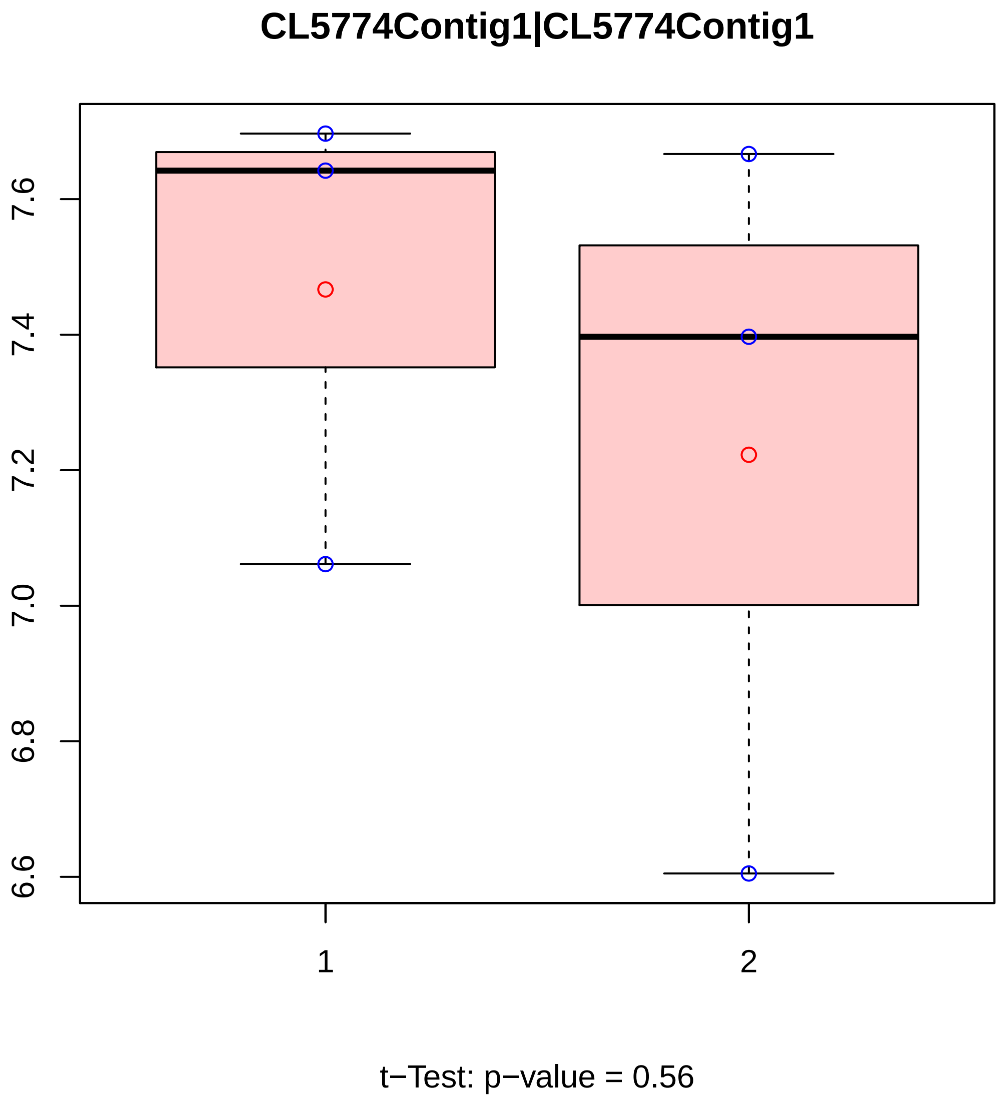

CL5774Contig1|CL5774Contig1
7.6
7.4
7.2
7.0
6.8
6.6
1
2
t−Test: p−value = 0.56

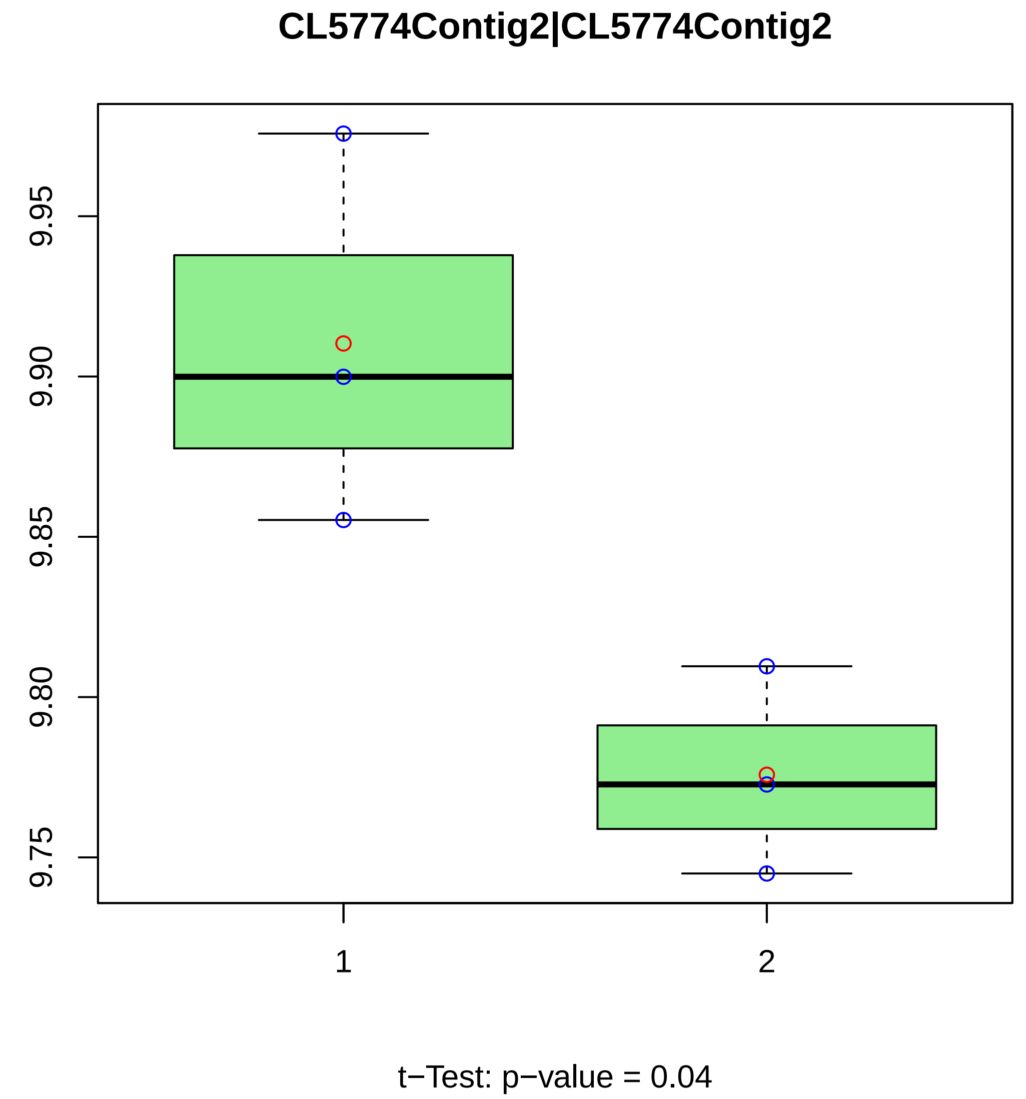

CL5774Contig2|CL5774Contig2
9.75
9.80
9.85
9.90
9.95
1
2
t−Test: p−value = 0.04

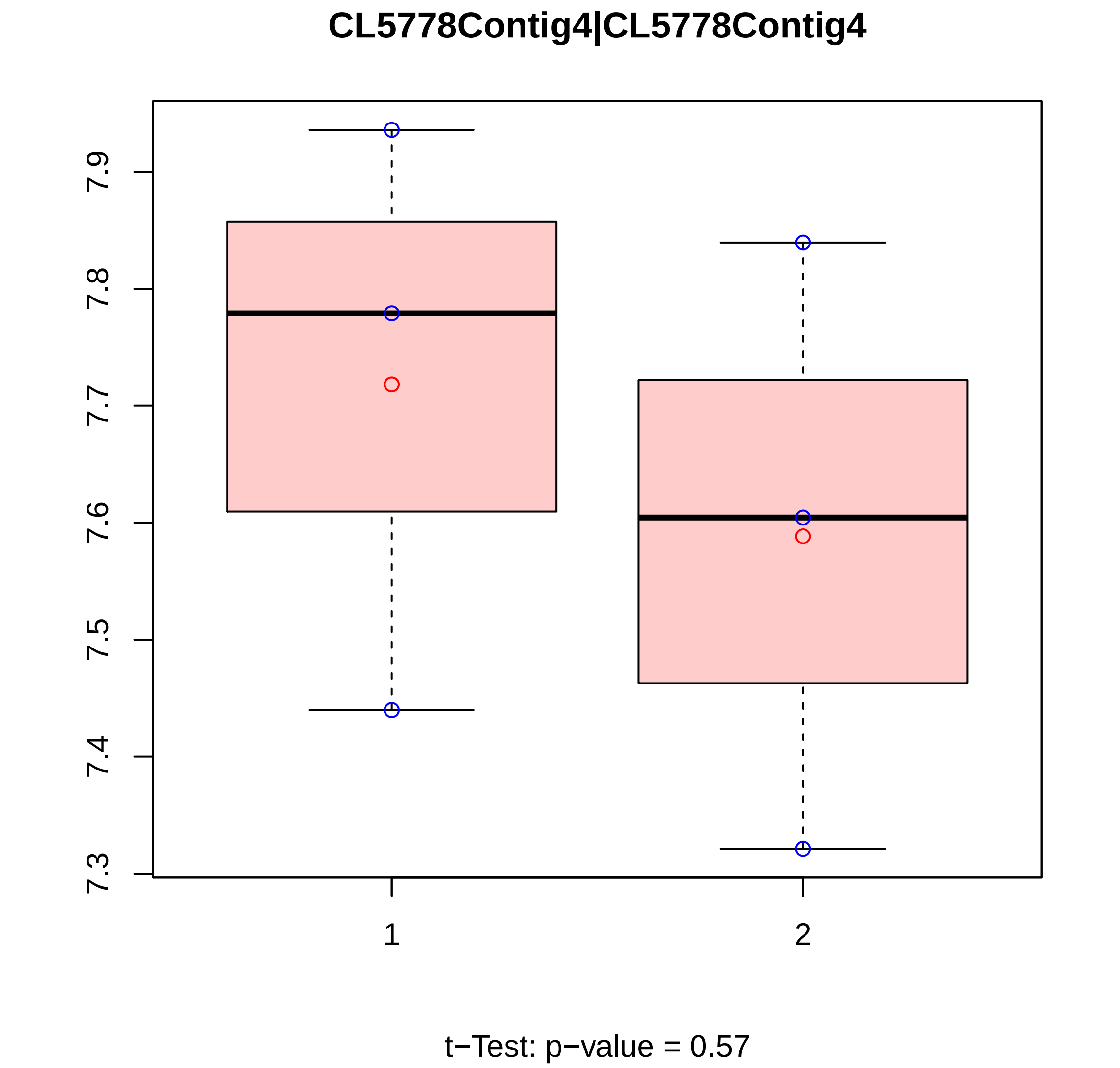

CL5778Contig4|CL5778Contig4
7.3
7.4
7.5
7.6
7.7
7.8
7.9
1
2
t−Test: p−value = 0.57

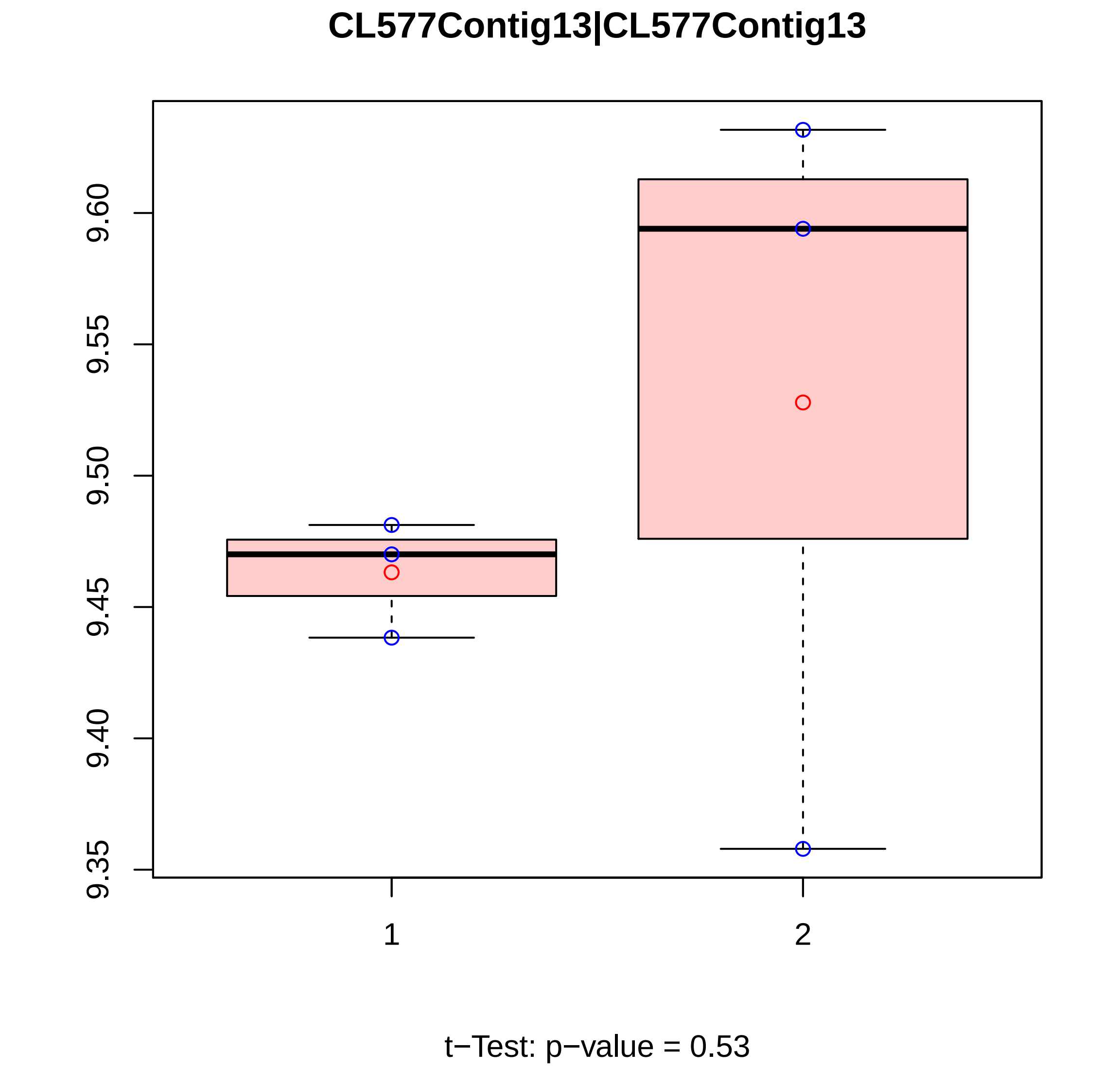

CL577Contig13|CL577Contig13
9.35
9.40
9.45
9.50
9.55
9.60
1
2
t−Test: p−value = 0.53

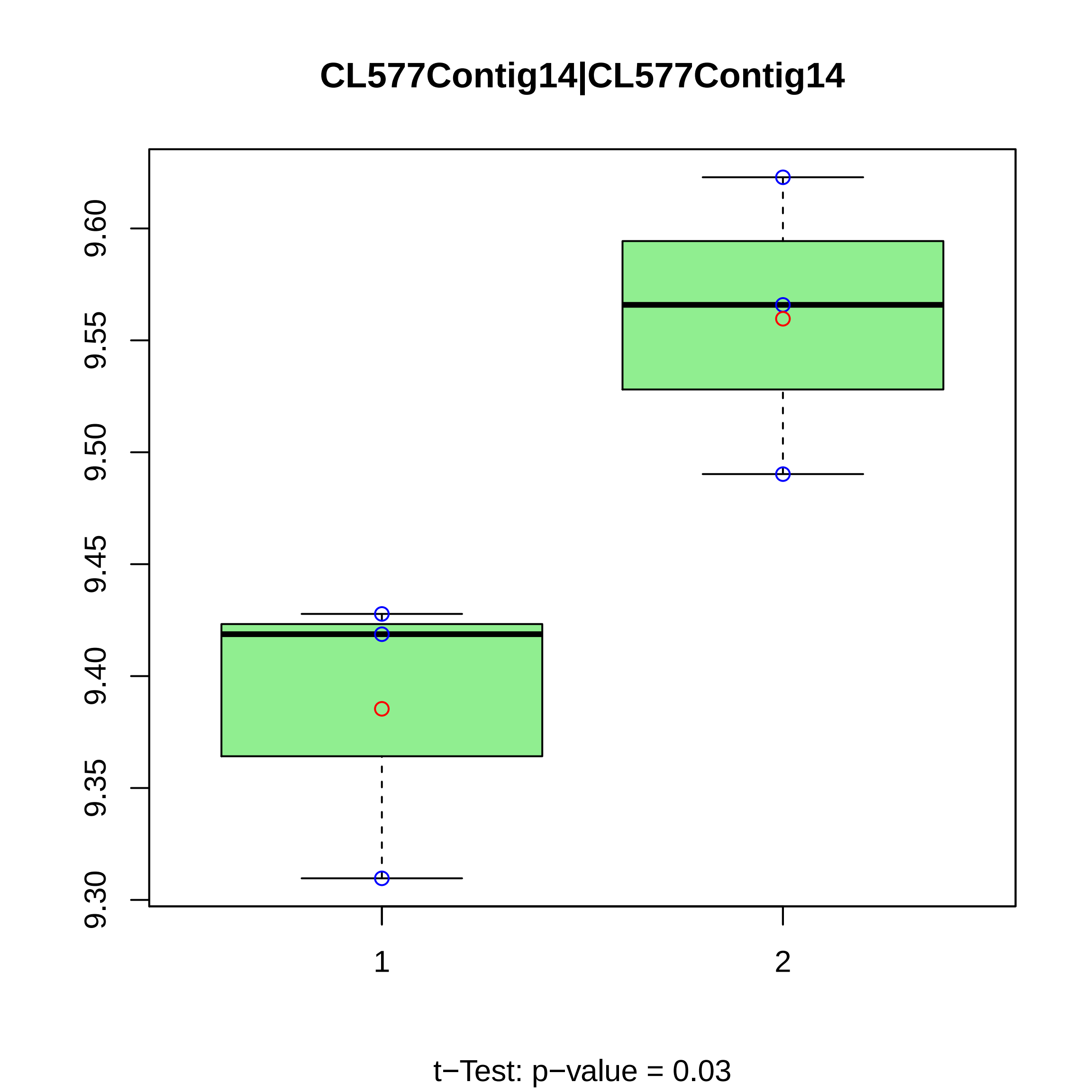
CL577Contig14|CL577Contig14
9.30
9.35
9.40
9.45
9.50
9.55
9.60
1
2
t−Test: p−value = 0.03

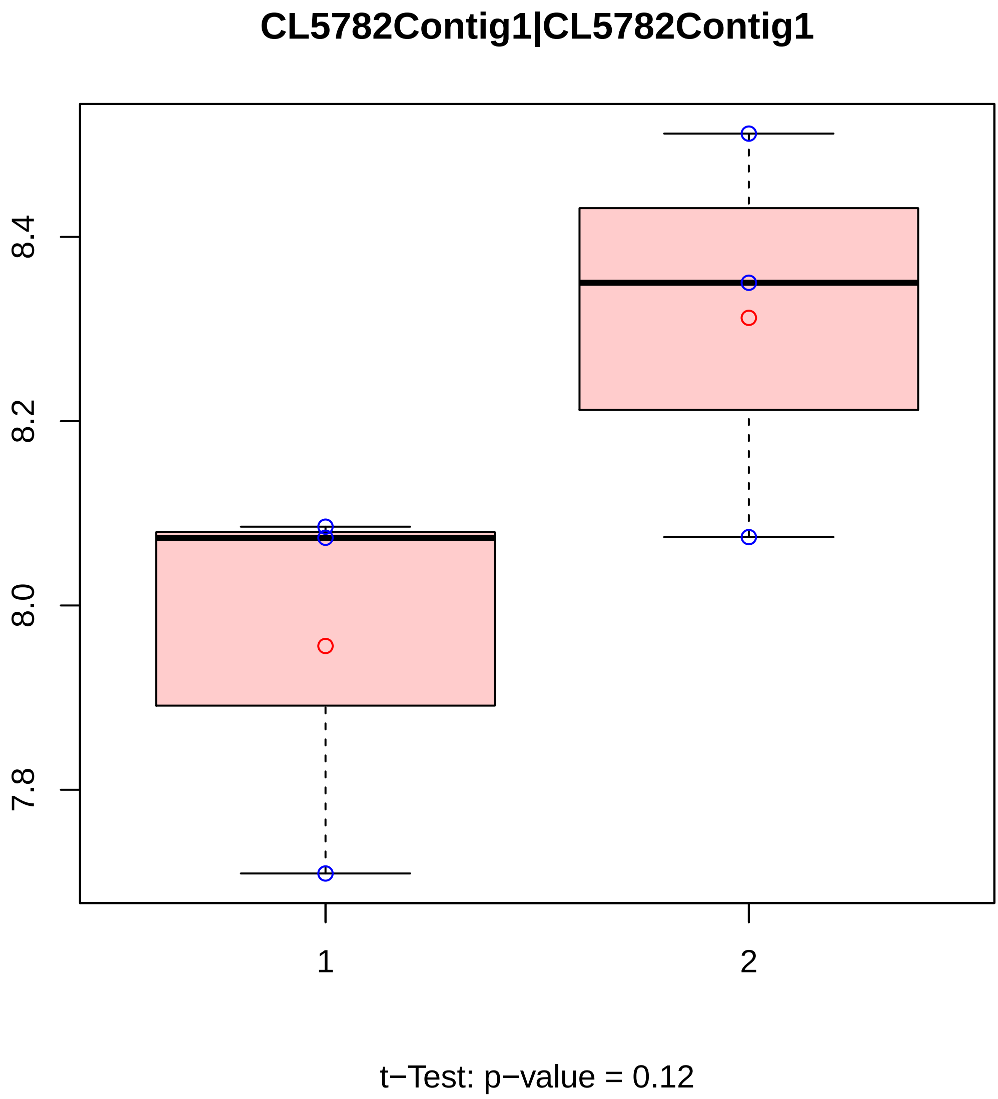

CL5782Contig1|CL5782Contig1
8.4
8.2
8.0
7.8
1
2
t−Test: p−value = 0.12

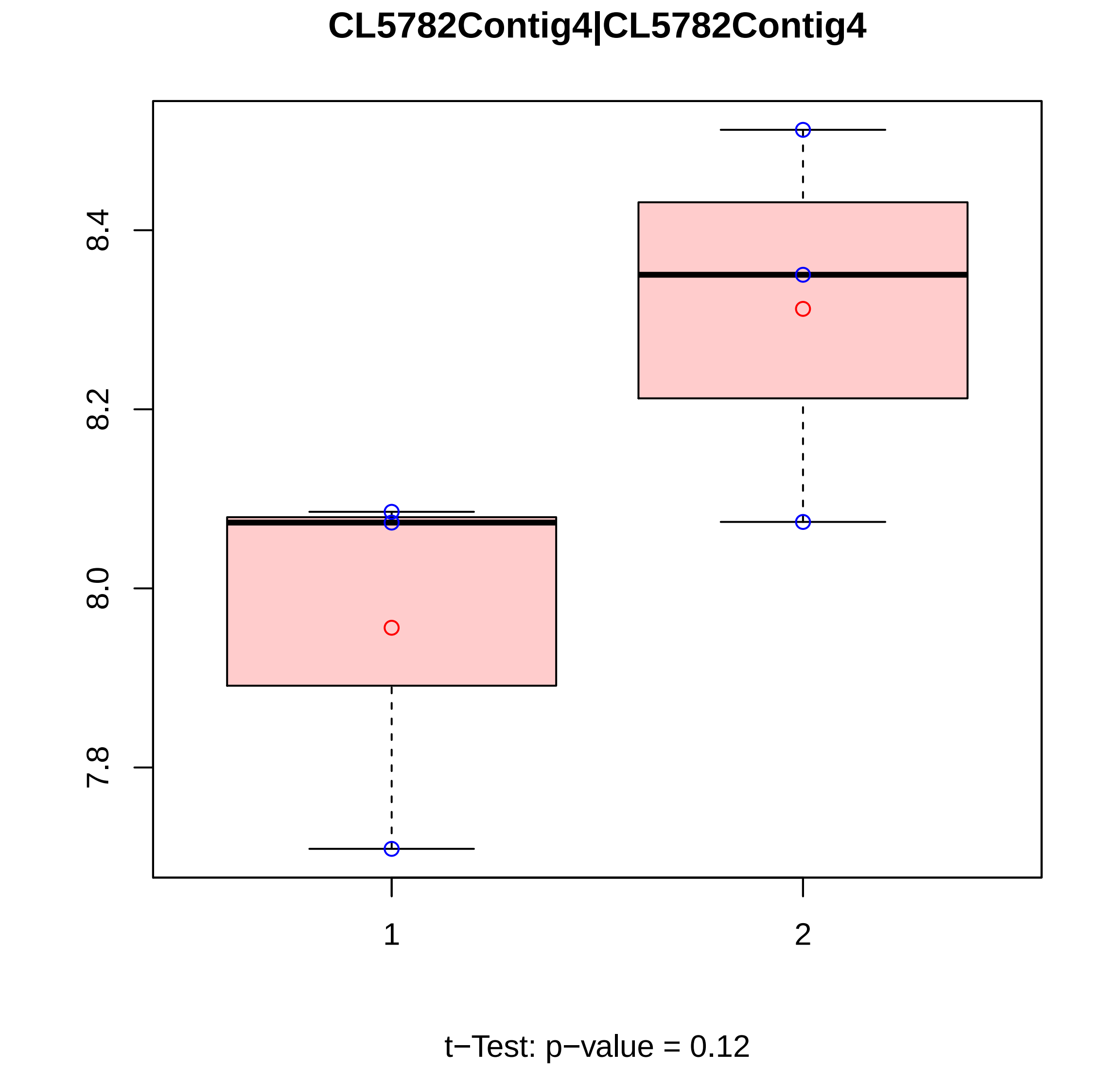
CL5782Contig4|CL5782Contig4
8.4
8.2
8.0
7.8
1
2
t−Test: p−value = 0.12

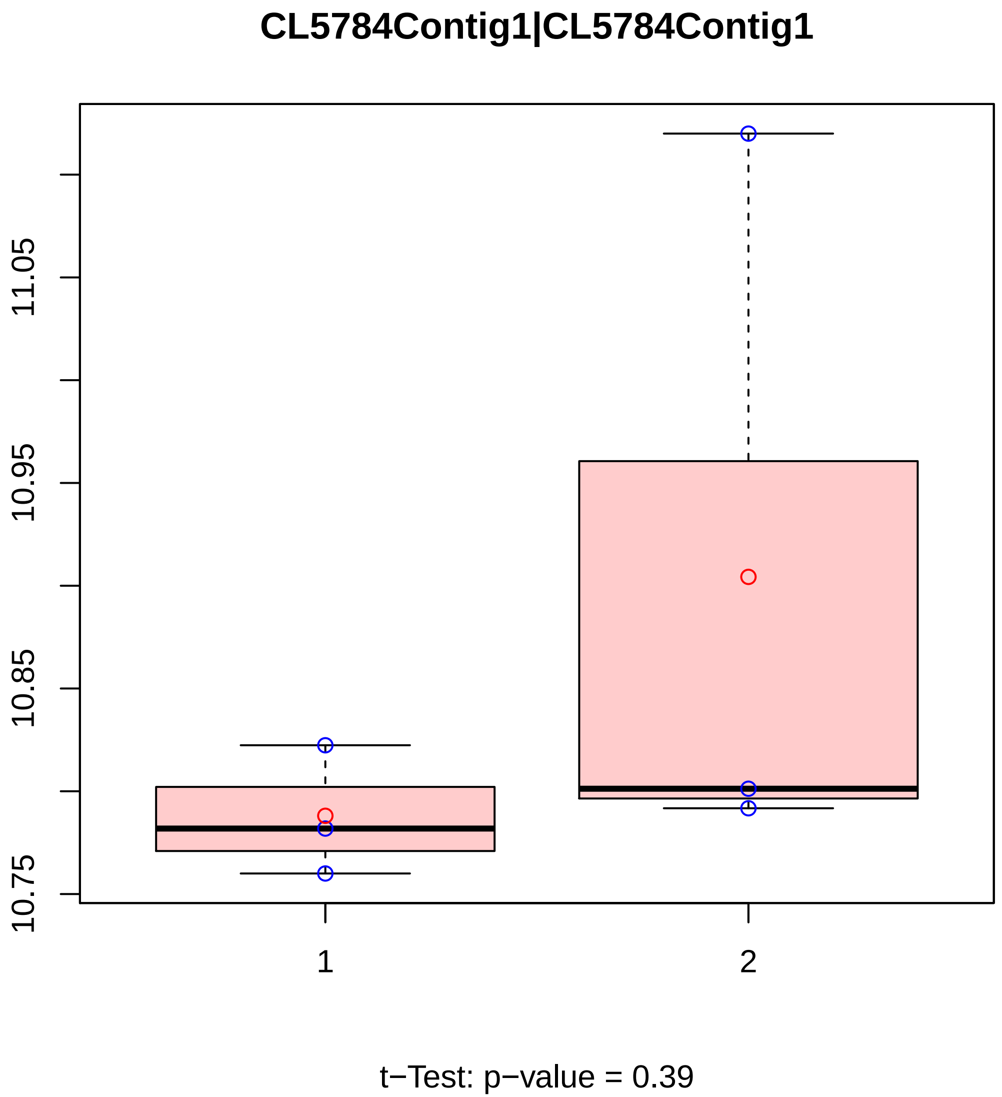

CL5784Contig1|CL5784Contig1
11.05
10.95
10.85
10.75
1
2
t−Test: p−value = 0.39

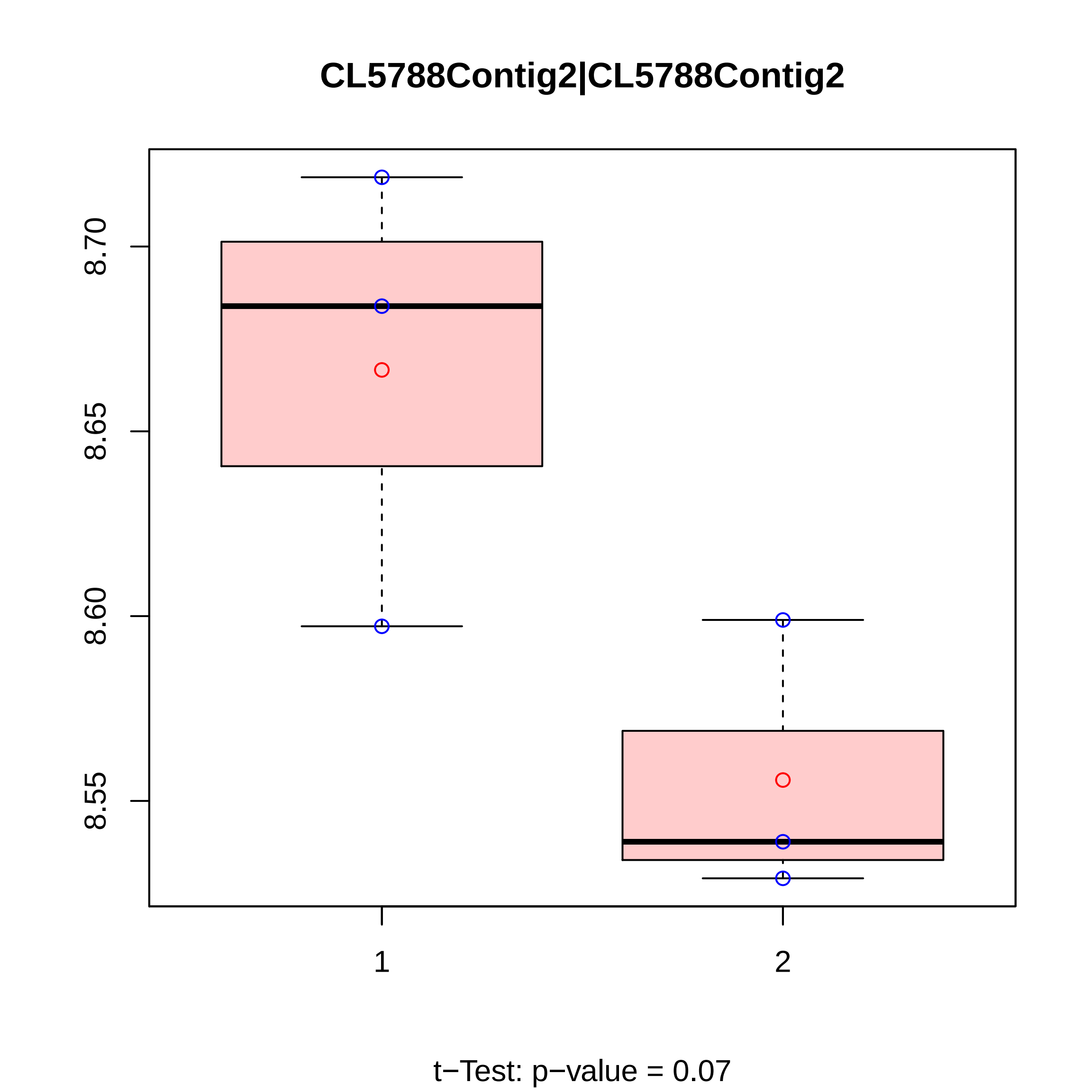

CL5788Contig2|CL5788Contig2
8.70
8.65
8.60
8.55
1
2
t−Test: p−value = 0.07

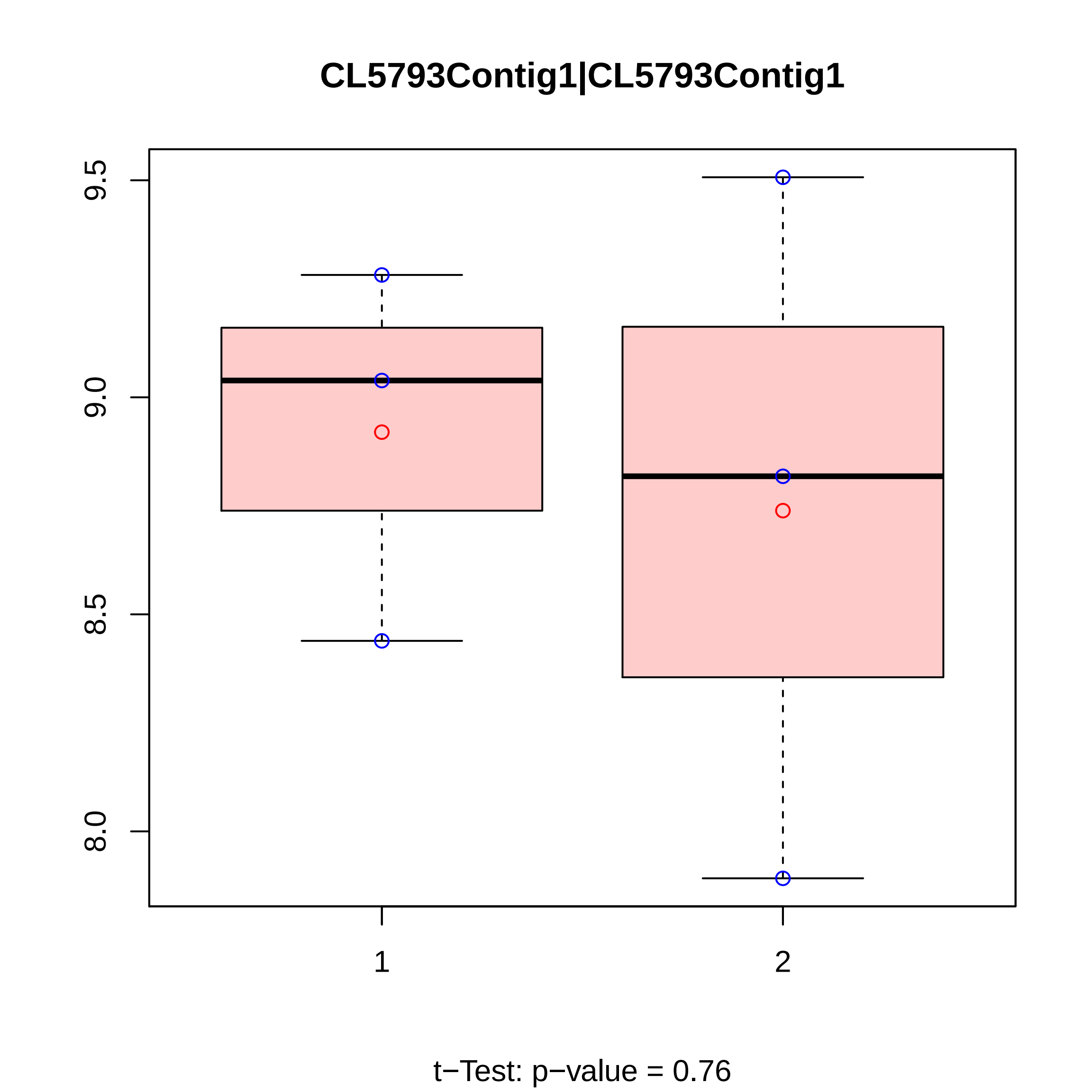
CL5793Contig1|CL5793Contig1
9.5
9.0
8.5
8.0
1
2
t−Test: p−value = 0.76

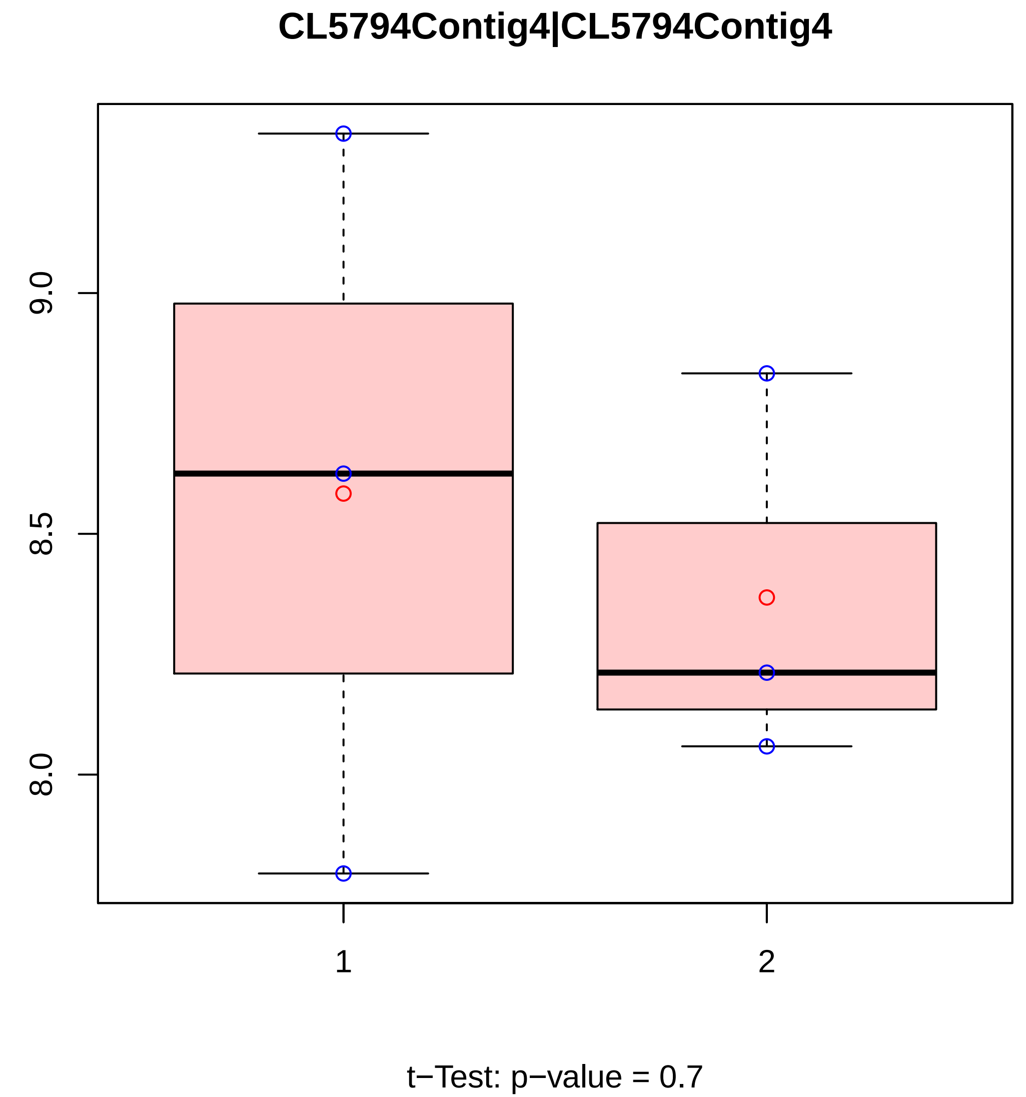
CL5794Contig4|CL5794Contig4
9.0
8.5
8.0
1
2
t−Test: p−value = 0.7

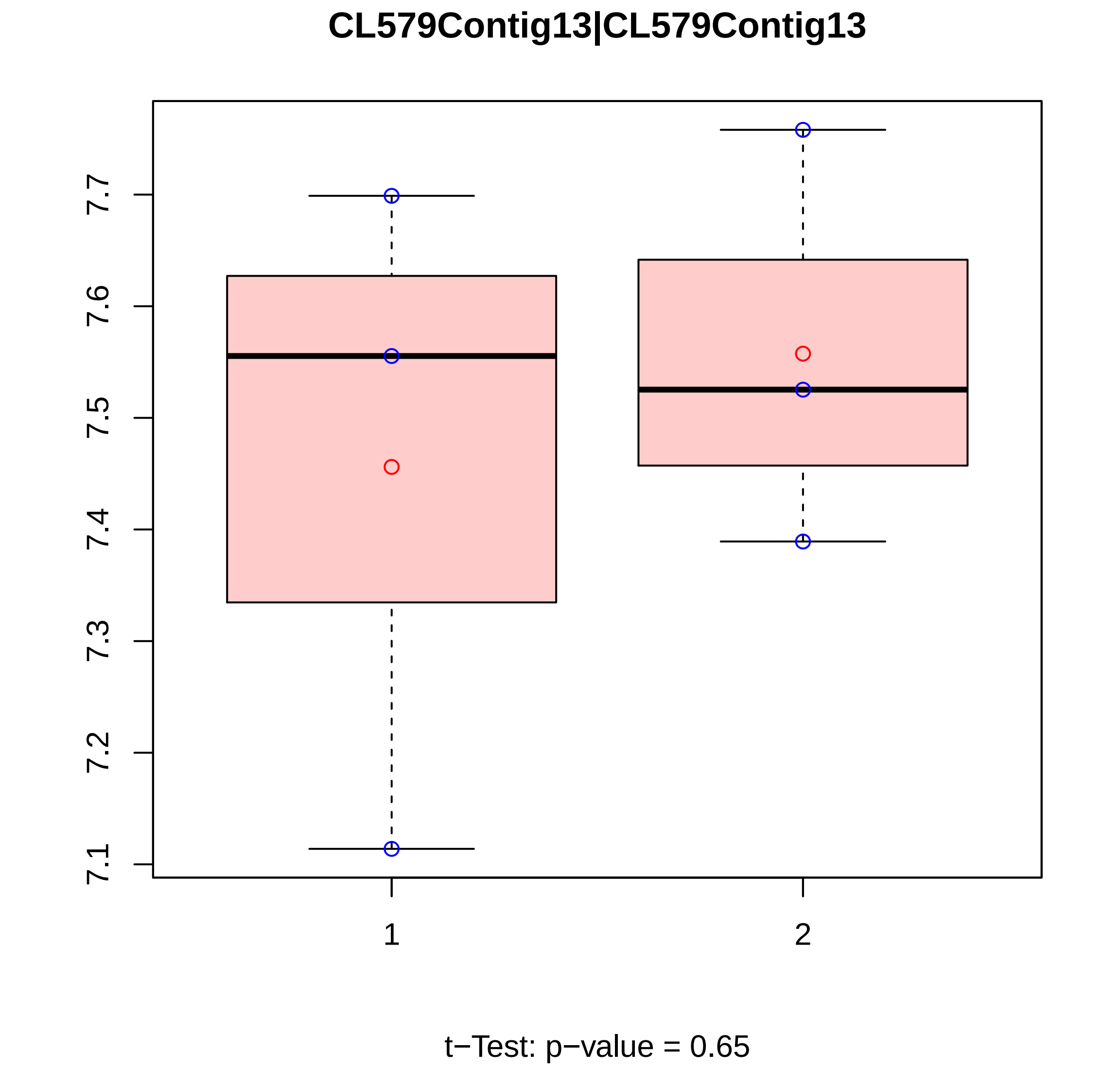
CL579Contig13|CL579Contig13
7.1
7.2
7.3
7.4
7.5
7.6
7.7
1
2
t−Test: p−value = 0.65

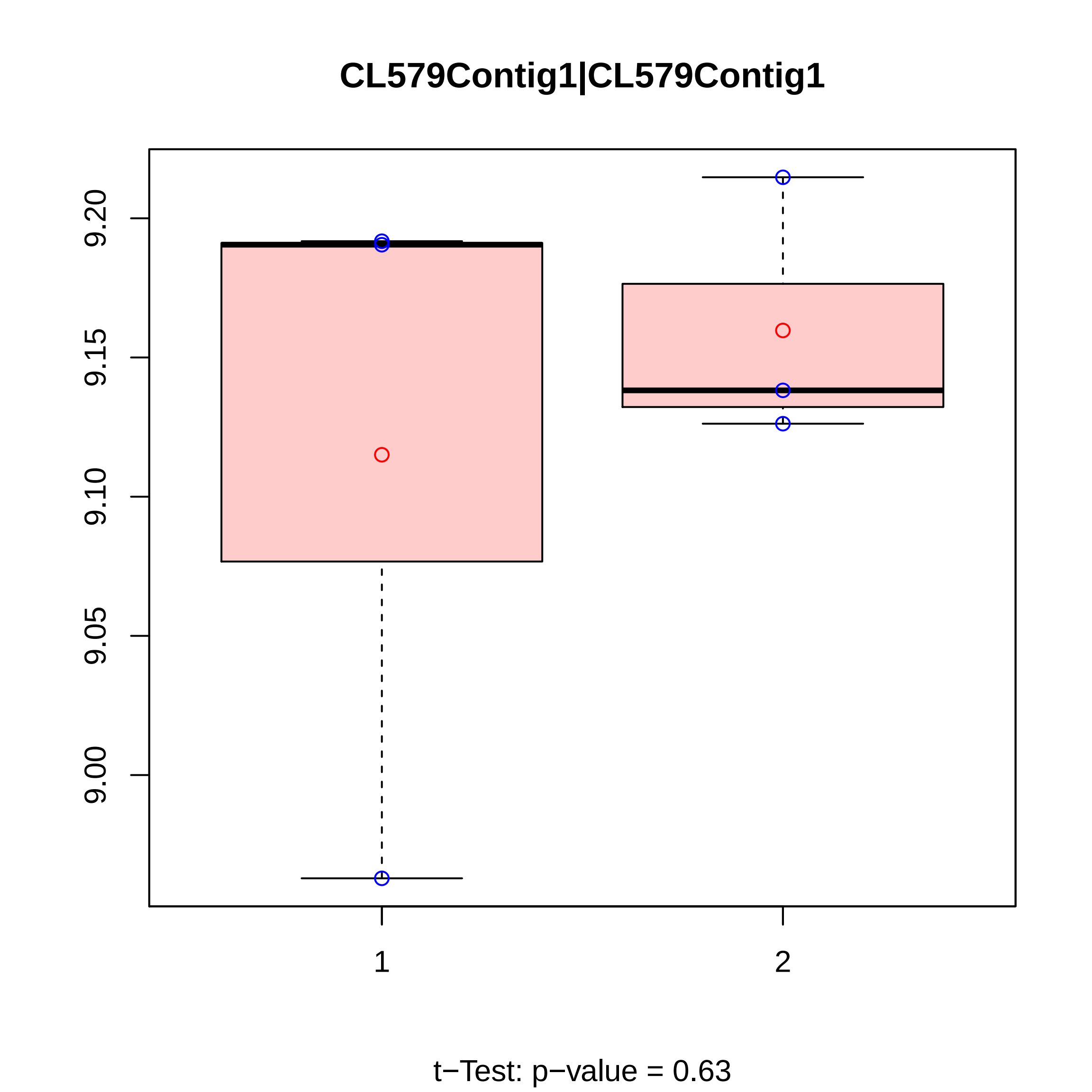

CL579Contig1|CL579Contig1
9.00
9.05
9.10
9.15
9.20
1
2
t−Test: p−value = 0.63

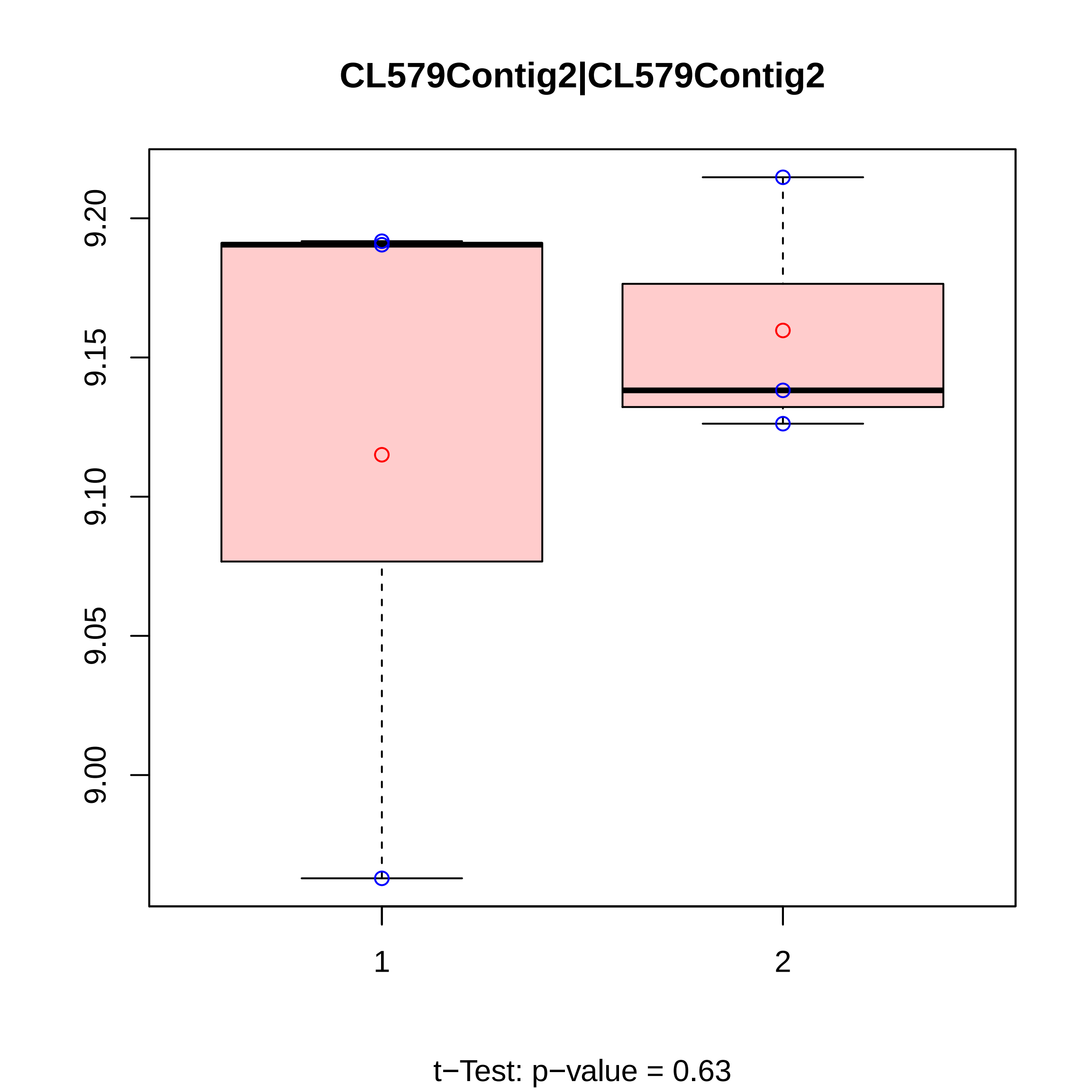

CL579Contig2|CL579Contig2
9.00
9.05
9.10
9.15
9.20
1
2
t−Test: p−value = 0.63

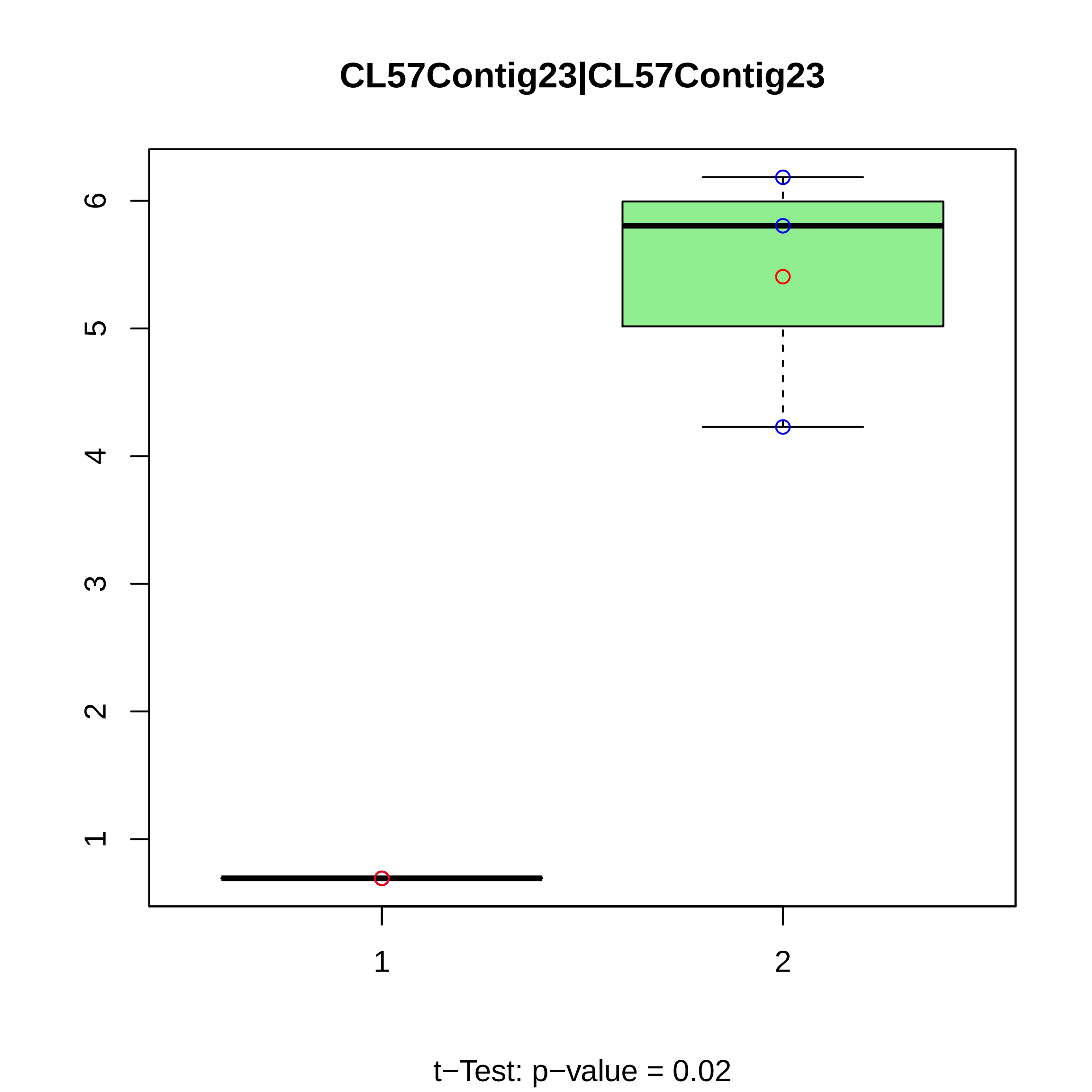
CL57Contig23|CL57Contig23
1
2
3
4
5
6
1
2
t−Test: p−value = 0.02

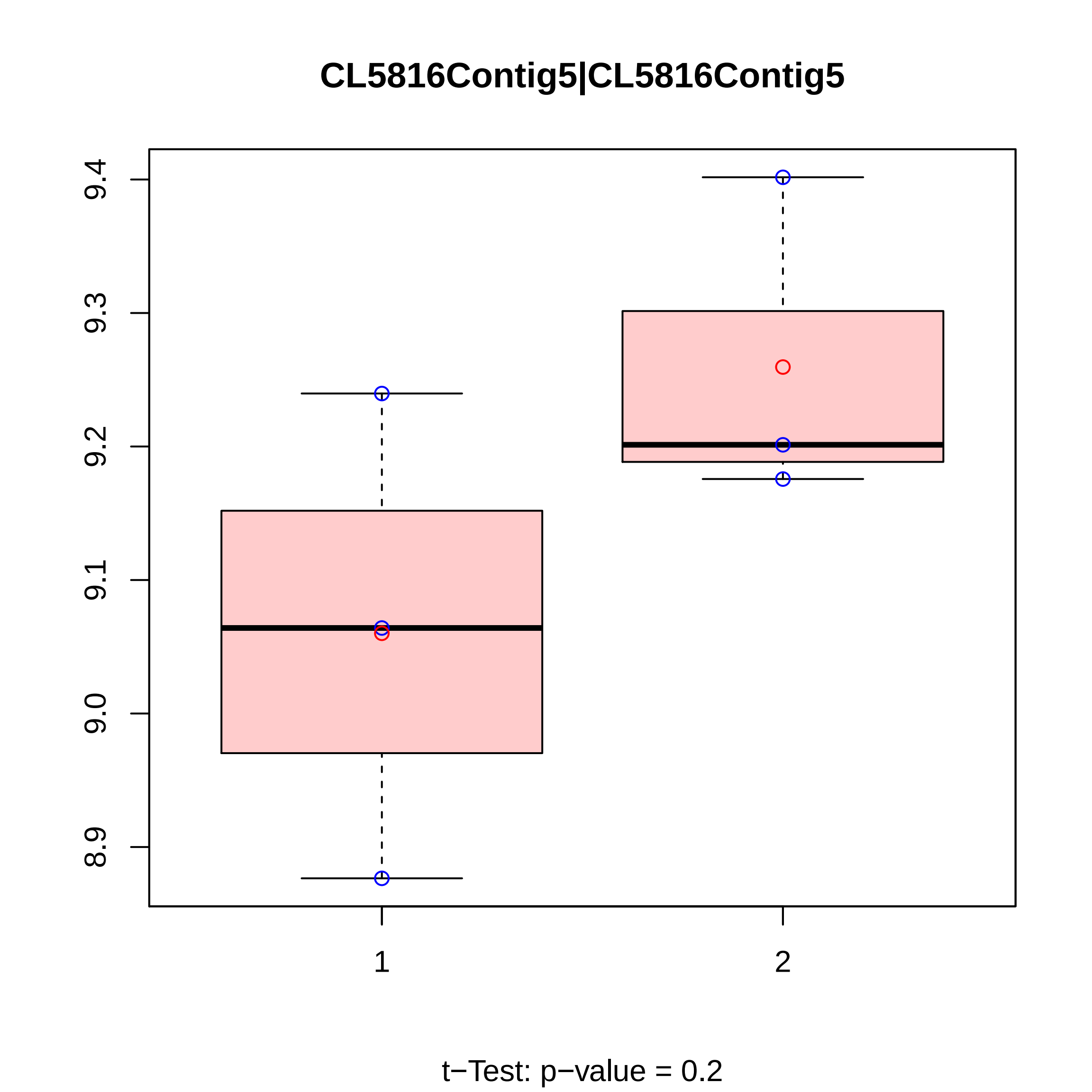
CL5816Contig5|CL5816Contig5
9.4
9.3
9.2
9.1
9.0
8.9
1
2
t−Test: p−value = 0.2

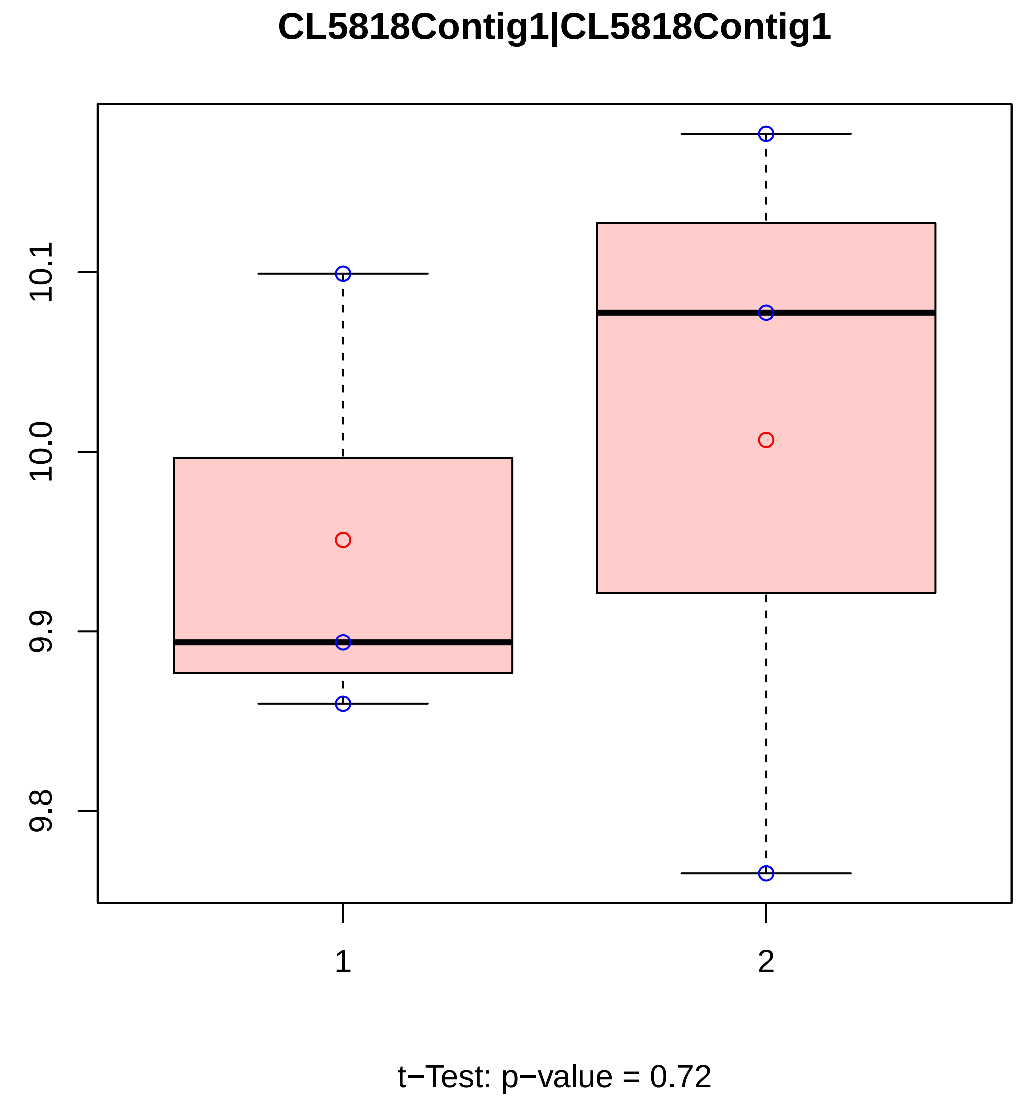
CL5818Contig1|CL5818Contig1
10.1
10.0
9.9
9.8
1
2
t−Test: p−value = 0.72

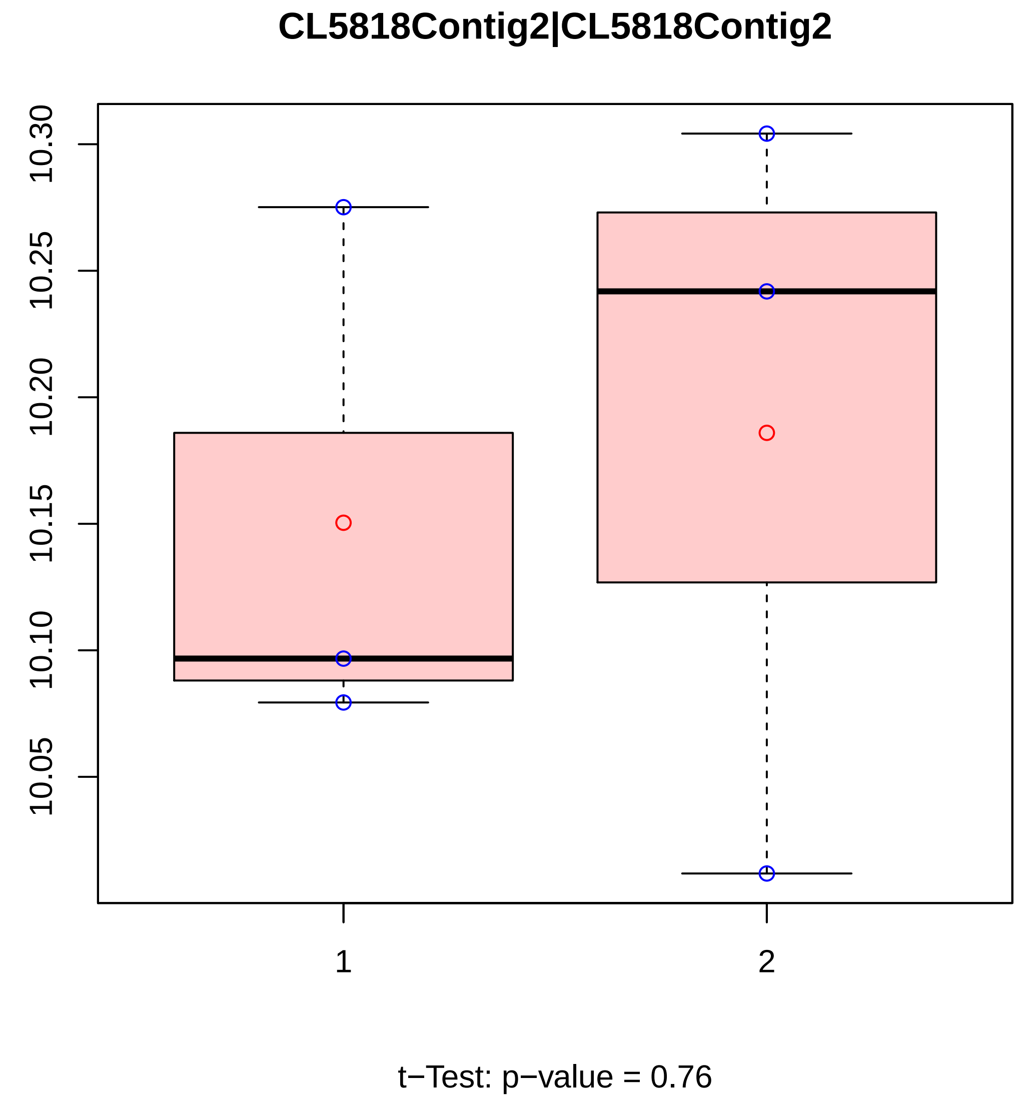

CL5818Contig2|CL5818Contig2
10.05
10.10
10.15
10.20
10.25
10.30
1
2
t−Test: p−value = 0.76

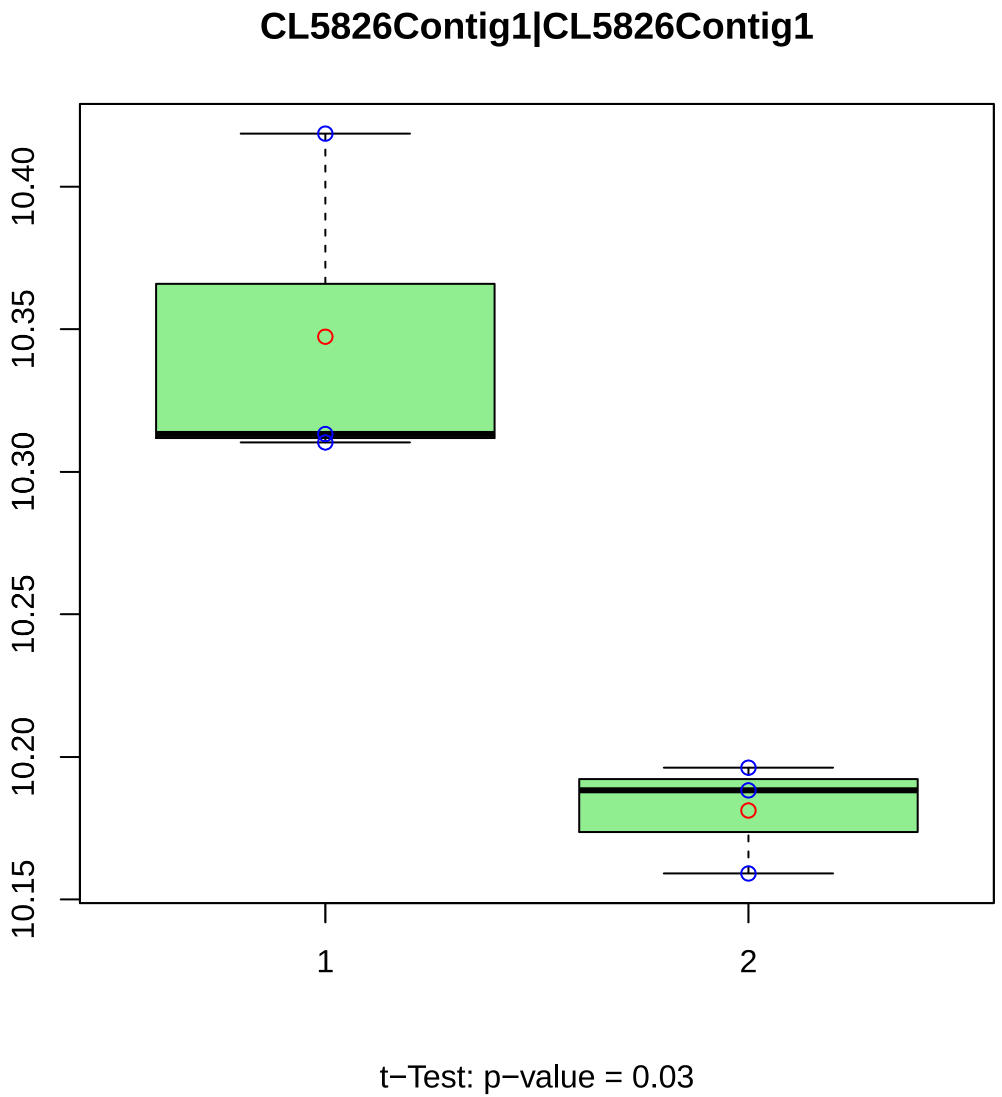
CL5826Contig1|CL5826Contig1
10.15
10.20
10.25
10.30
10.35
10.40
1
2
t−Test: p−value = 0.03

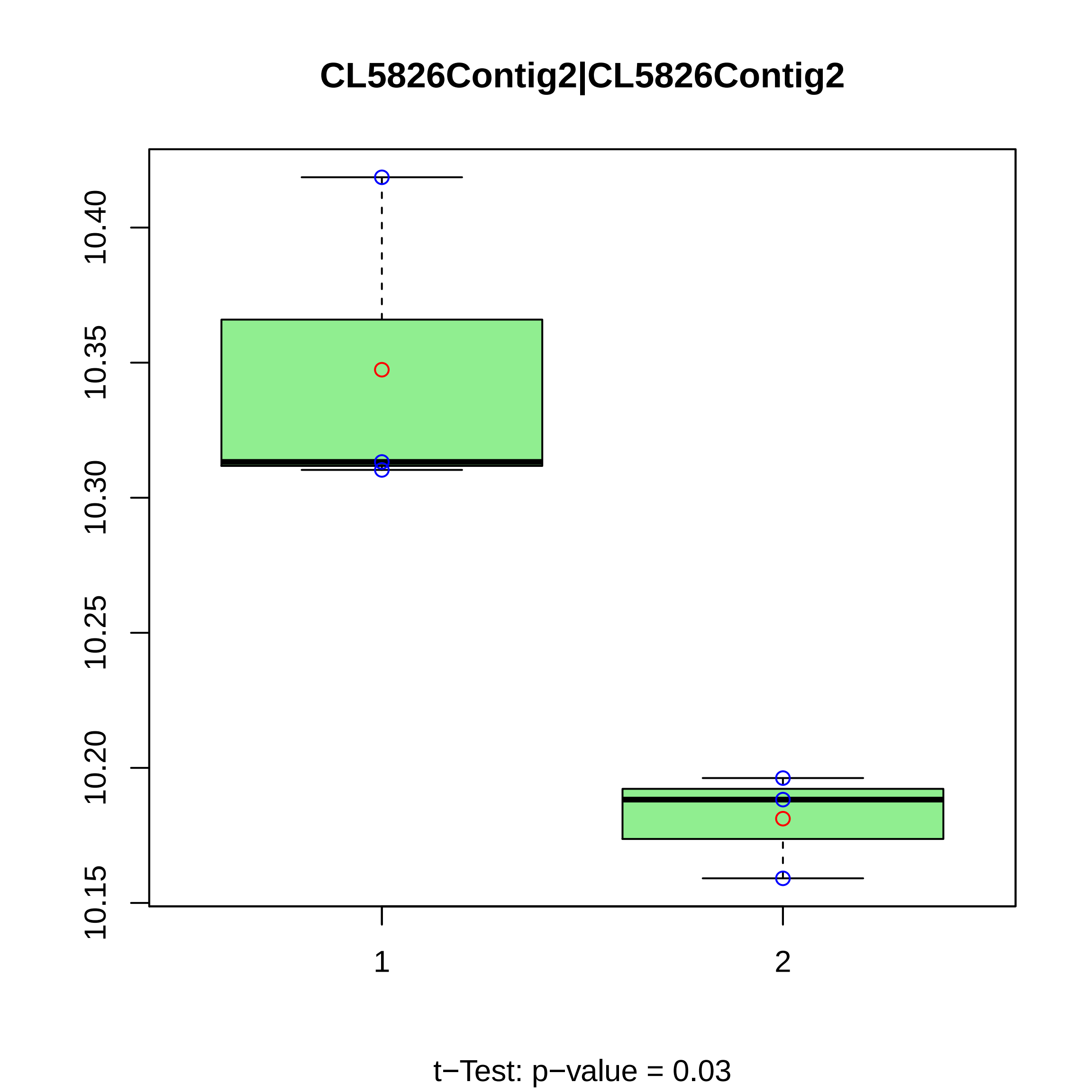
CL5826Contig2|CL5826Contig2
10.15
10.20
10.25
10.30
10.35
10.40
1
2
t−Test: p−value = 0.03

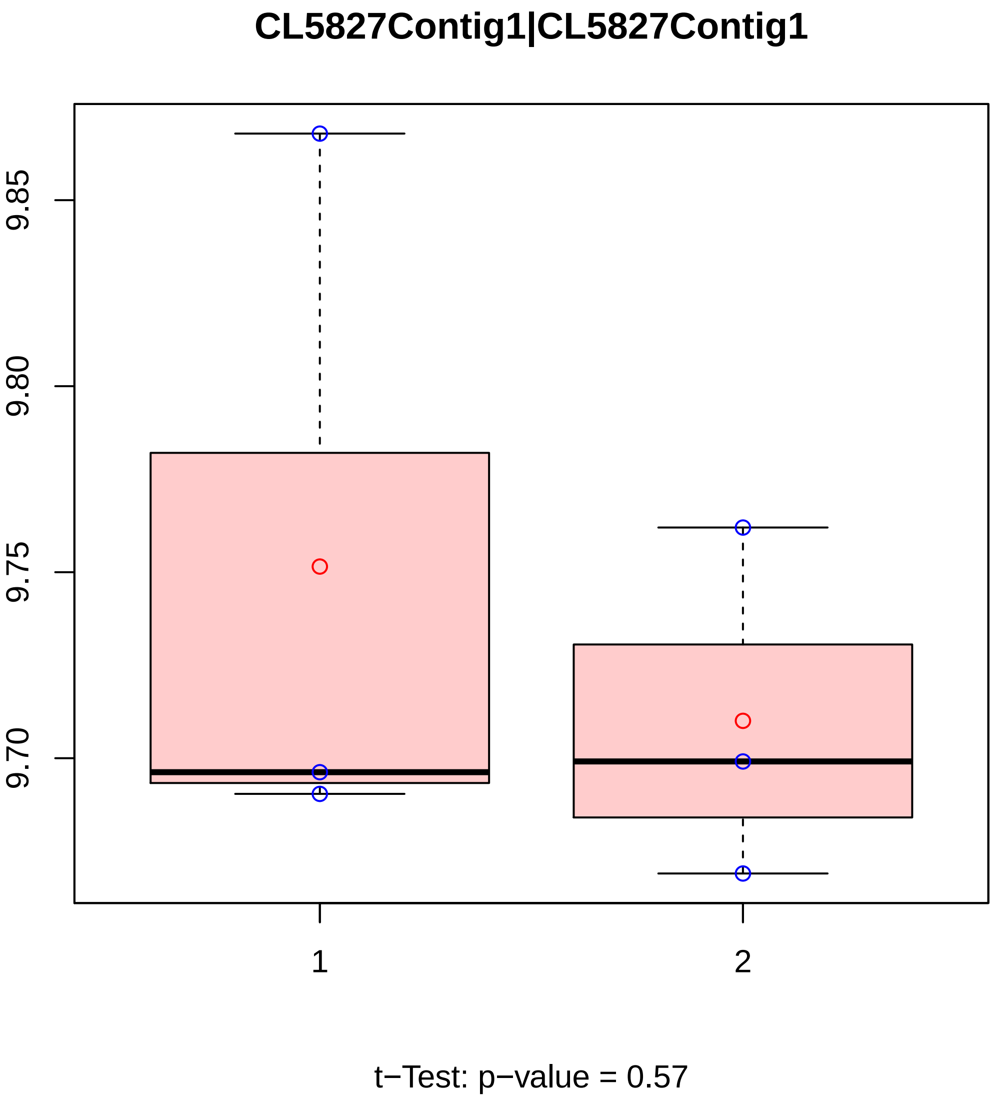

CL5827Contig1|CL5827Contig1
9.70
9.75
9.80
9.85
1
2
t−Test: p−value = 0.57

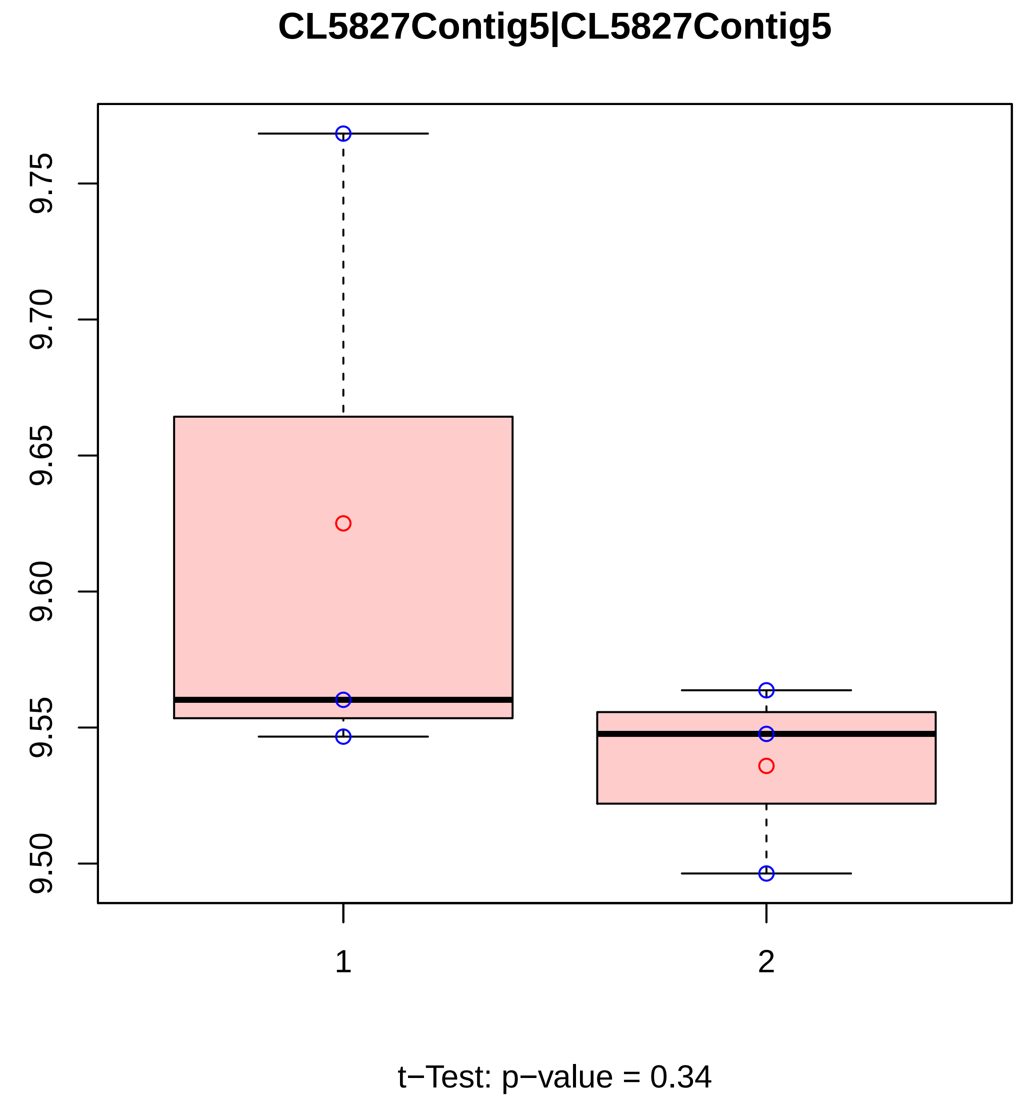

CL5827Contig5|CL5827Contig5
9.50
9.55
9.60
9.65
9.70
9.75
1
2
t−Test: p−value = 0.34

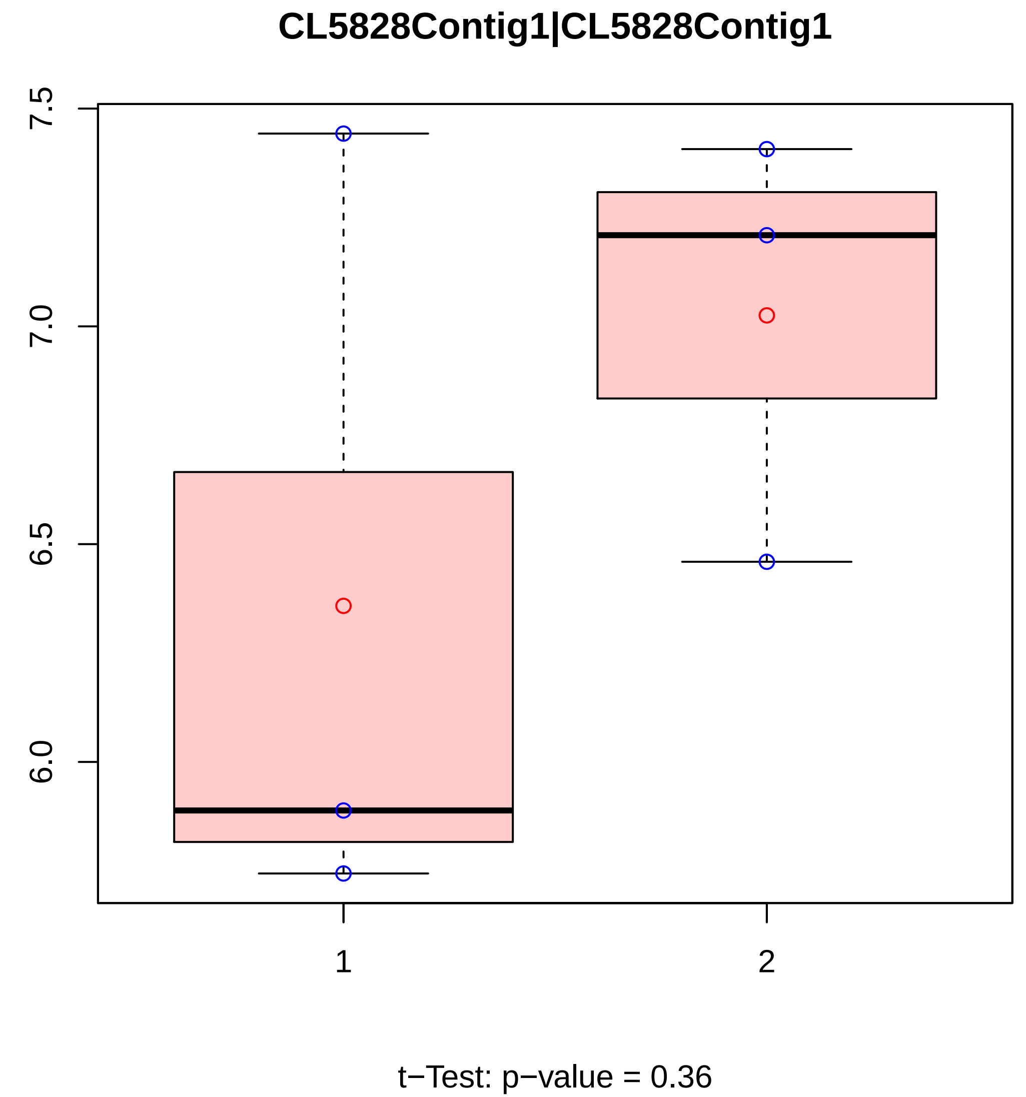
CL5828Contig1|CL5828Contig1
7.5
7.0
6.5
6.0
1
2
t−Test: p−value = 0.36

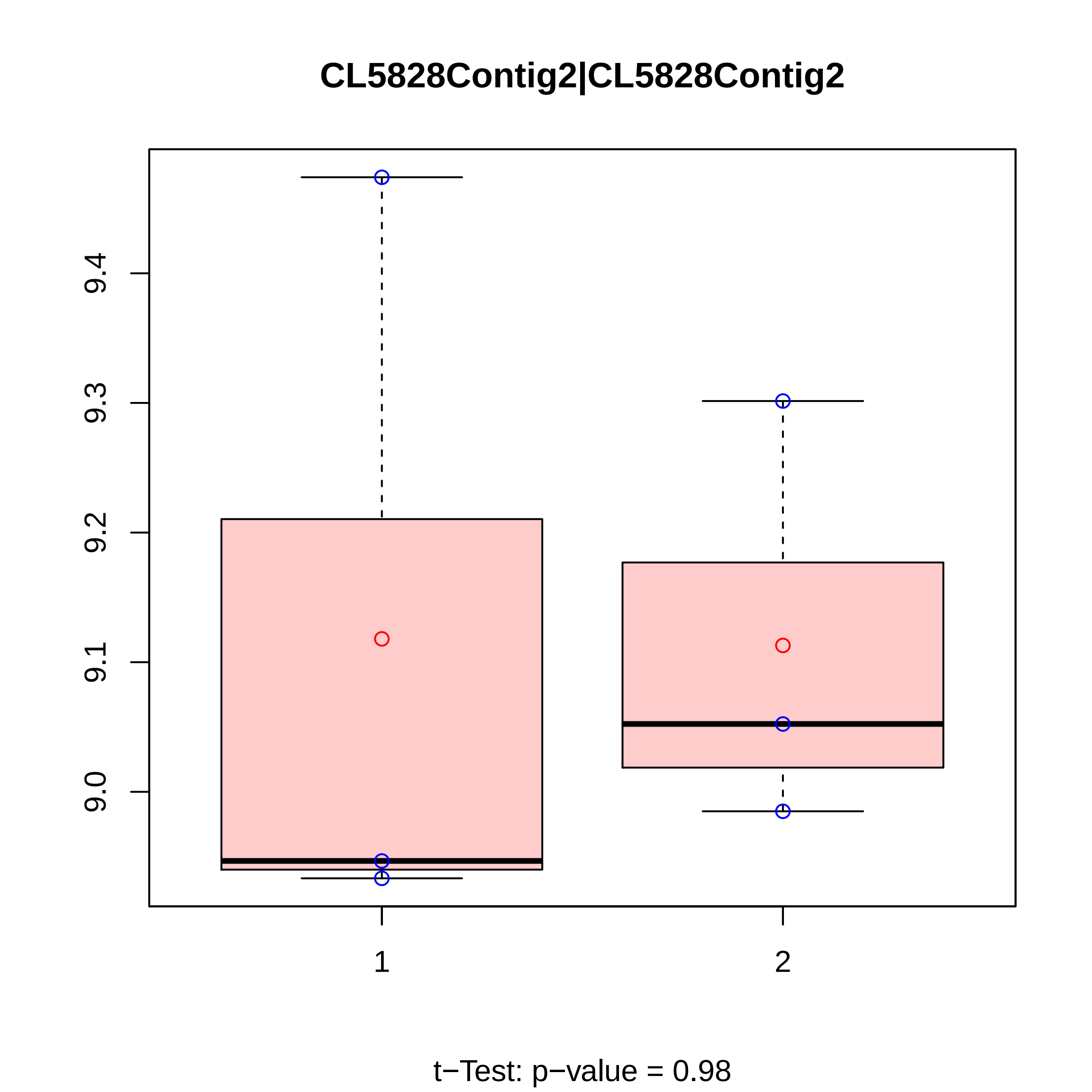
CL5828Contig2|CL5828Contig2
9.0
9.1
9.2
9.3
9.4
1
2
t−Test: p−value = 0.98

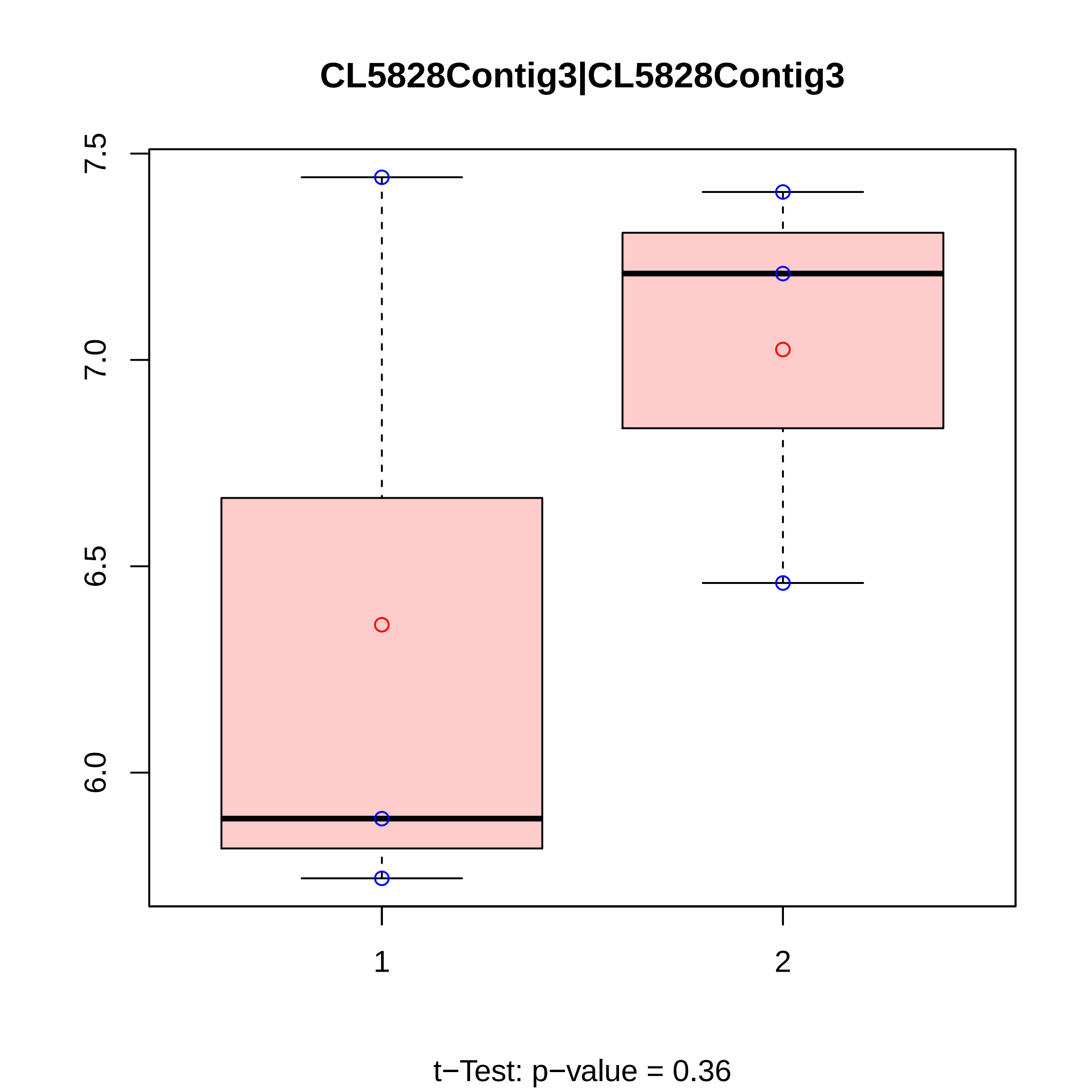

CL5828Contig3|CL5828Contig3
7.5
7.0
6.5
6.0
1
2
t−Test: p−value = 0.36

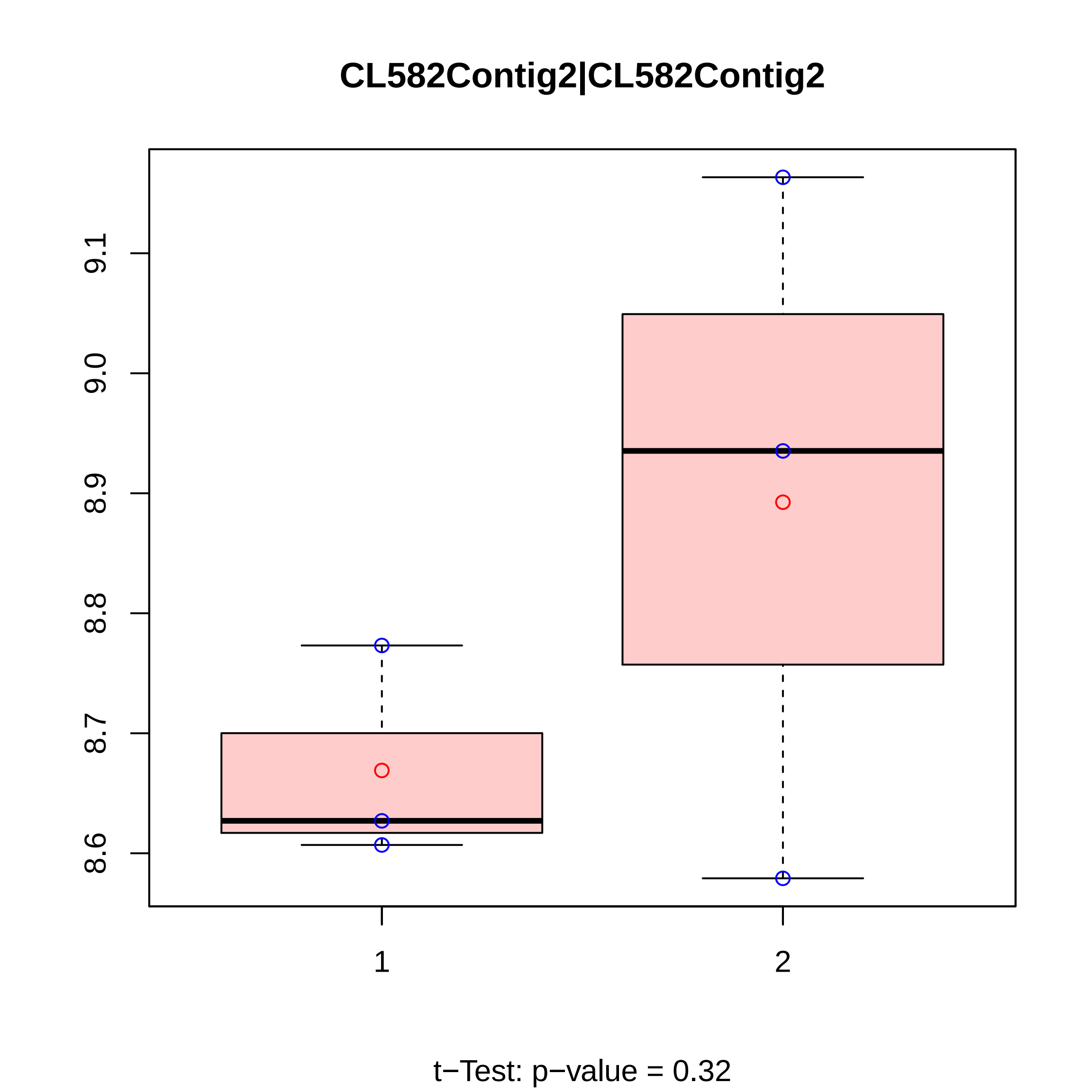
CL582Contig2|CL582Contig2
9.1
9.0
8.9
8.8
8.7
8.6
1
2
t−Test: p−value = 0.32

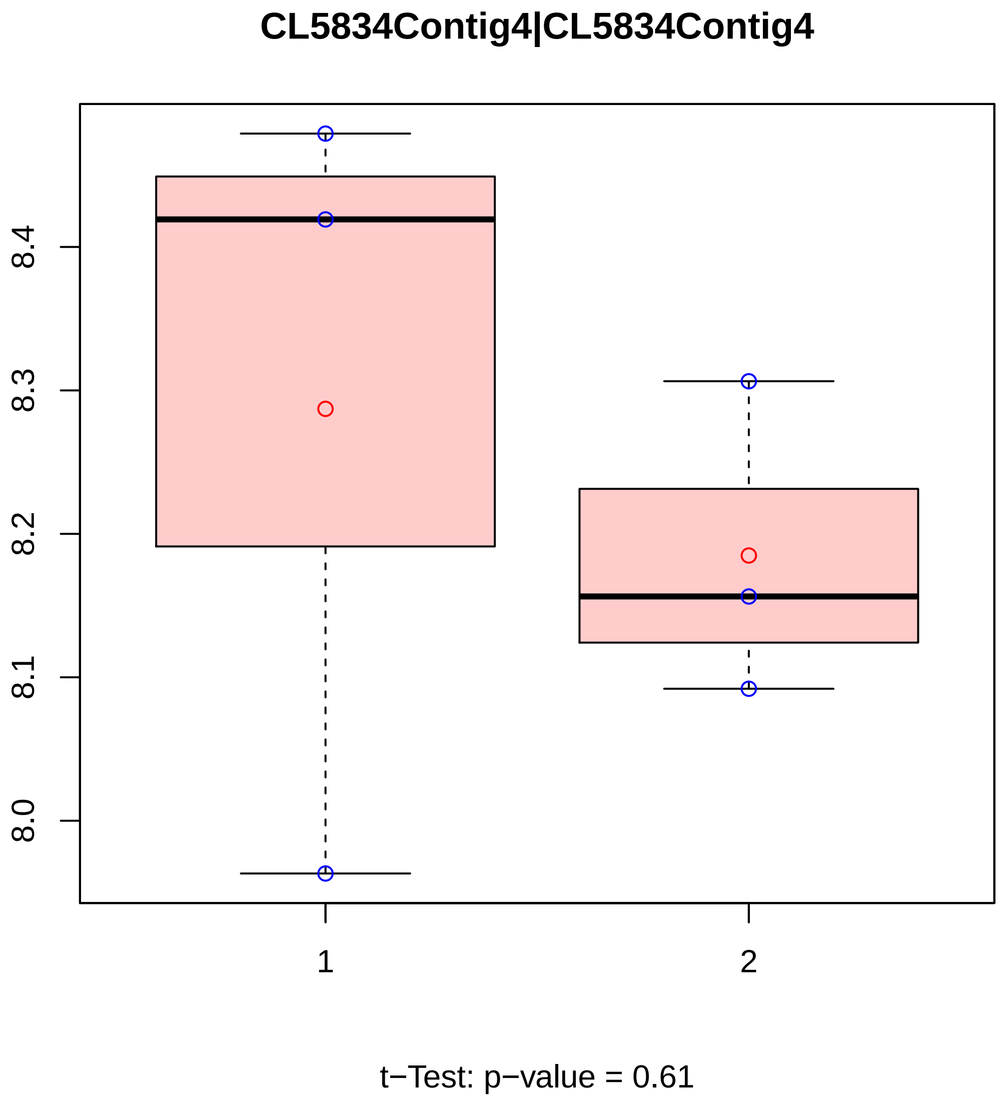

CL5834Contig4|CL5834Contig4
8.0
8.1
8.2
8.3
8.4
1
2
t−Test: p−value = 0.61

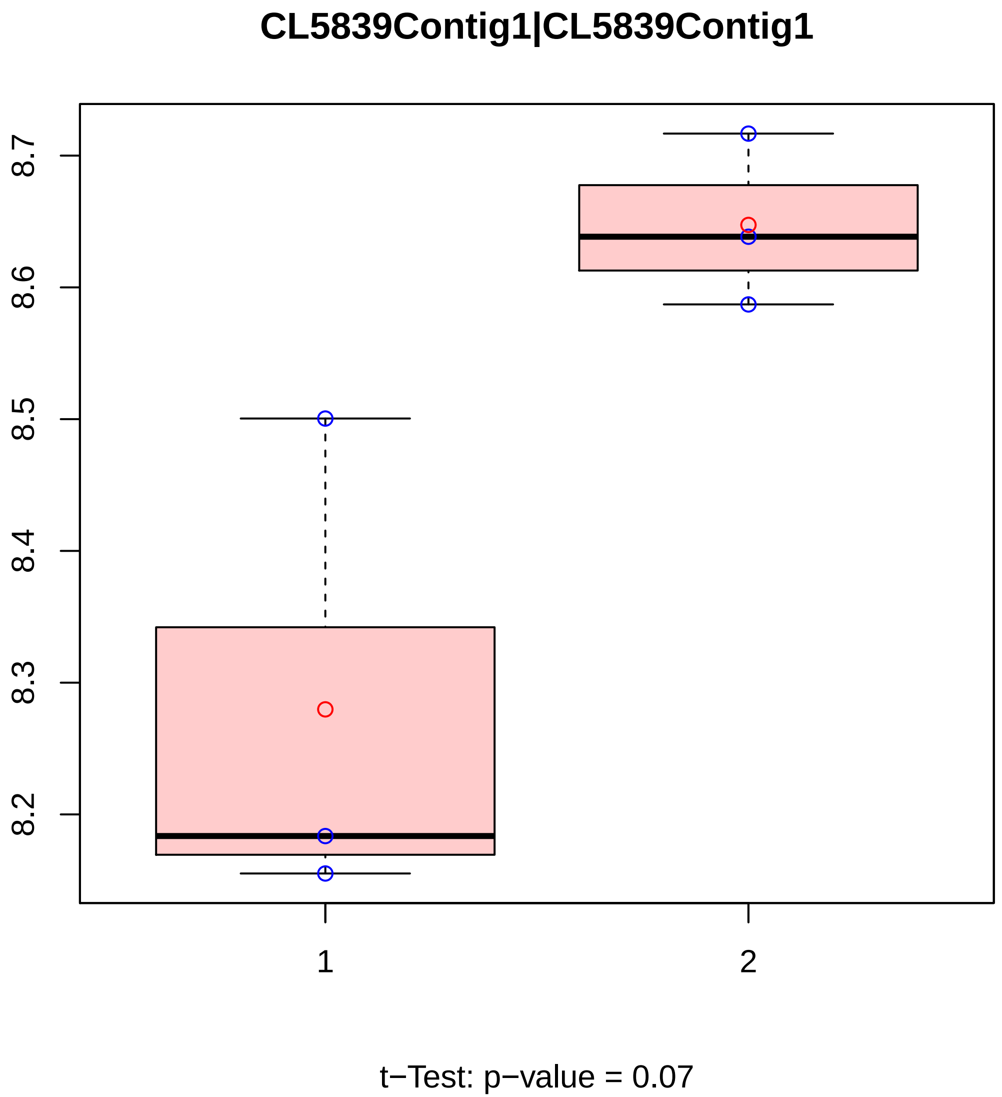
CL5839Contig1|CL5839Contig1
8.2
8.3
8.4
8.5
8.6
8.7
1
2
t−Test: p−value = 0.07

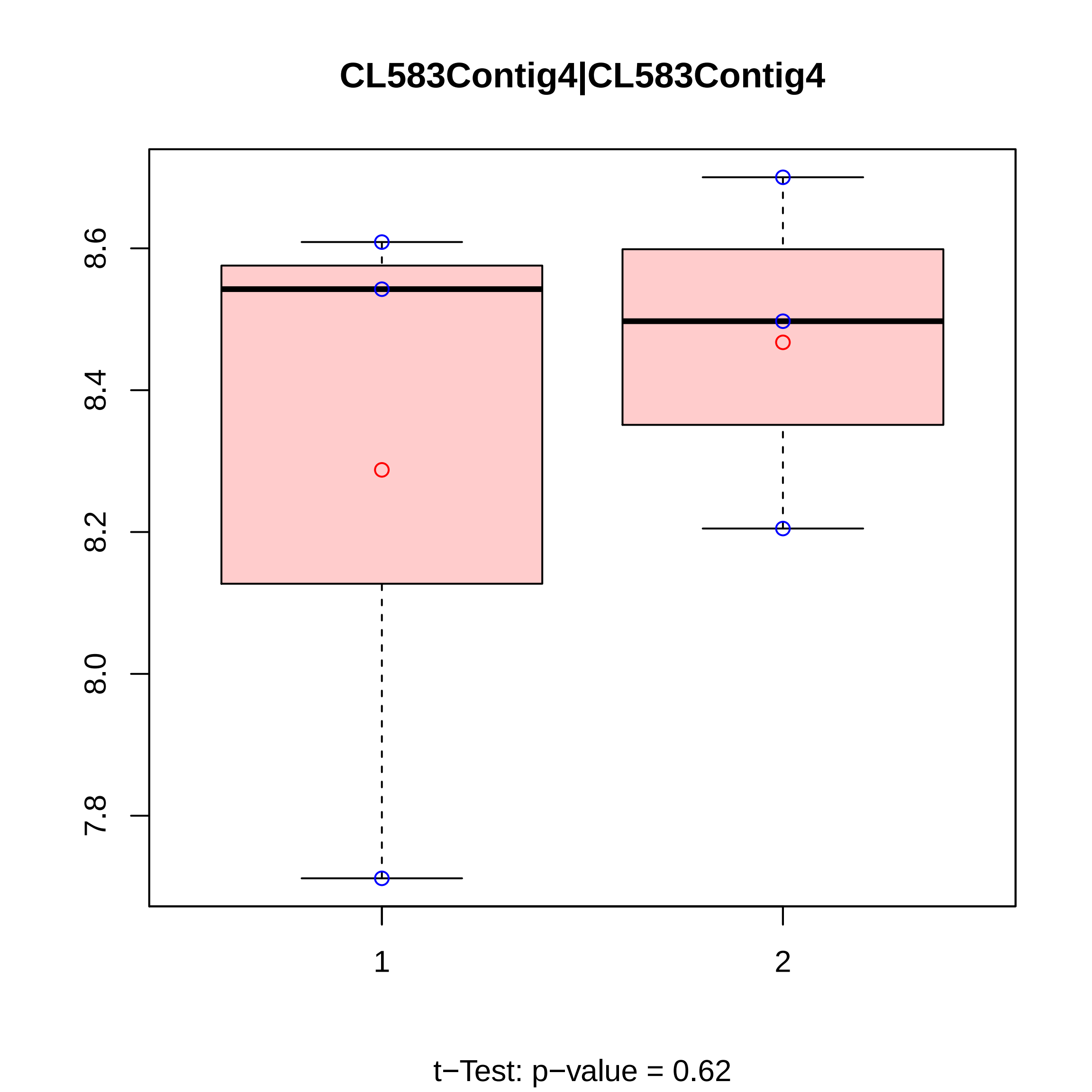
CL583Contig4|CL583Contig4
8.6
8.4
8.2
8.0
7.8
1
2
t−Test: p−value = 0.62

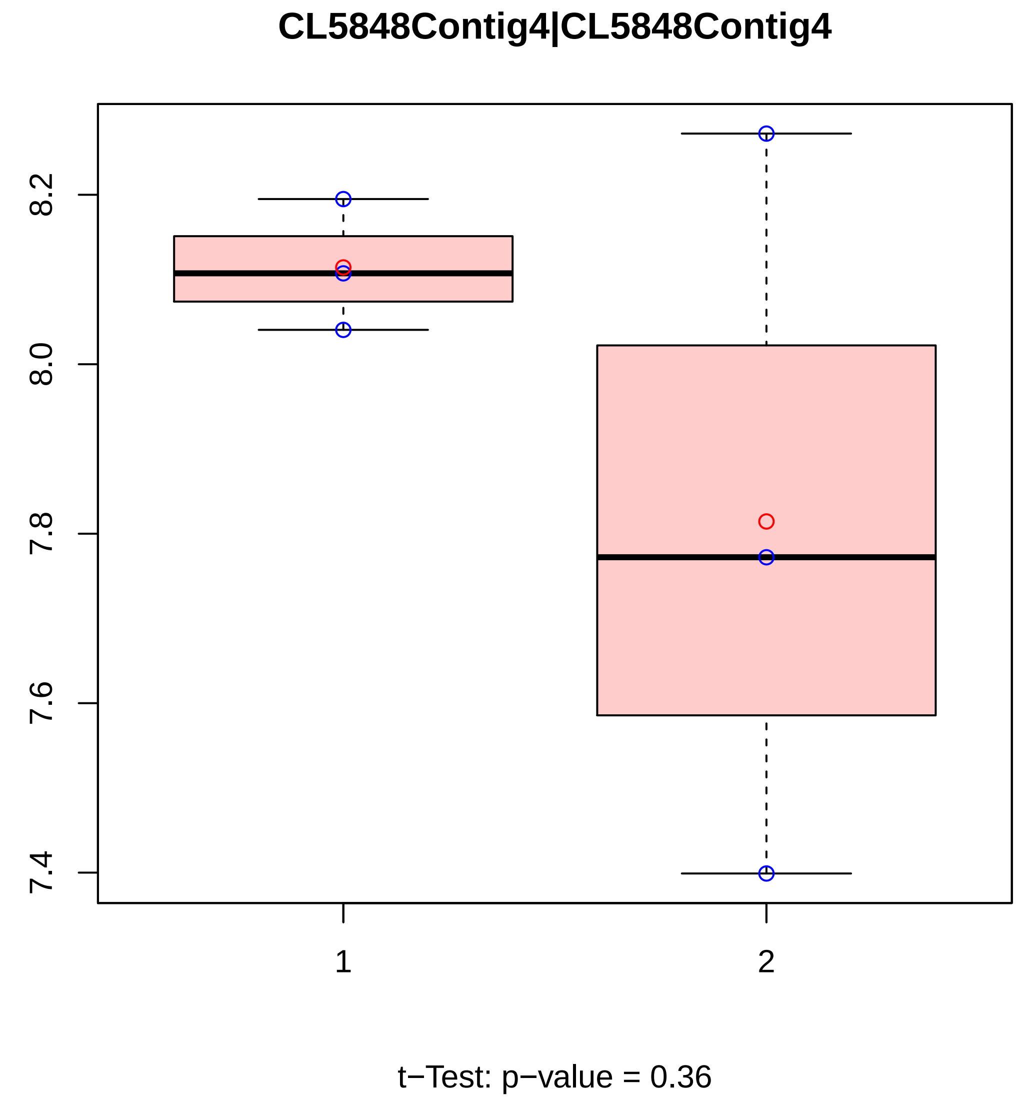

CL5848Contig4|CL5848Contig4
8.2
8.0
7.8
7.6
7.4
1
2
t−Test: p−value = 0.36

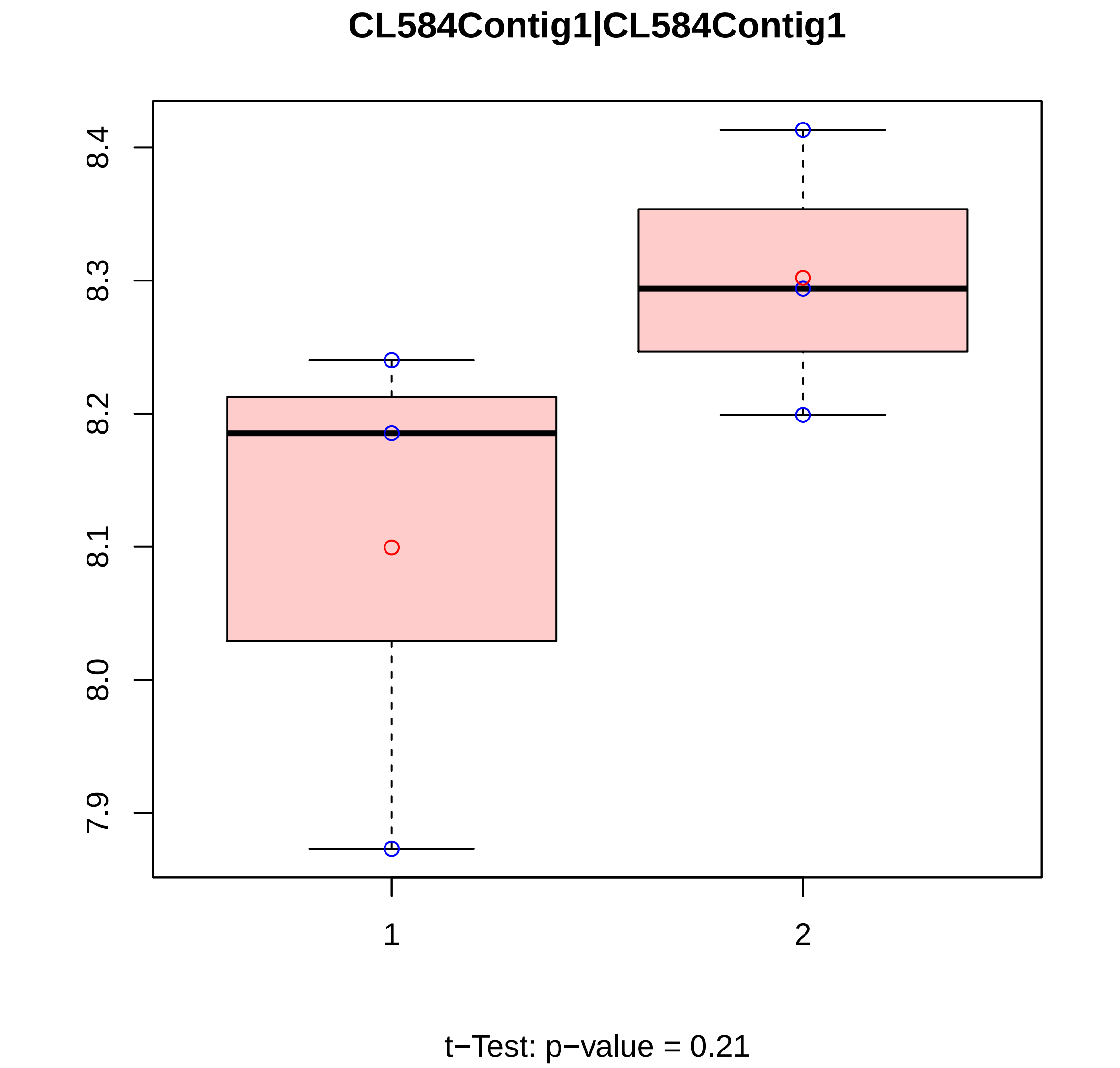
CL584Contig1|CL584Contig1
8.4
8.3
8.2
8.1
8.0
7.9
1
2
t−Test: p−value = 0.21

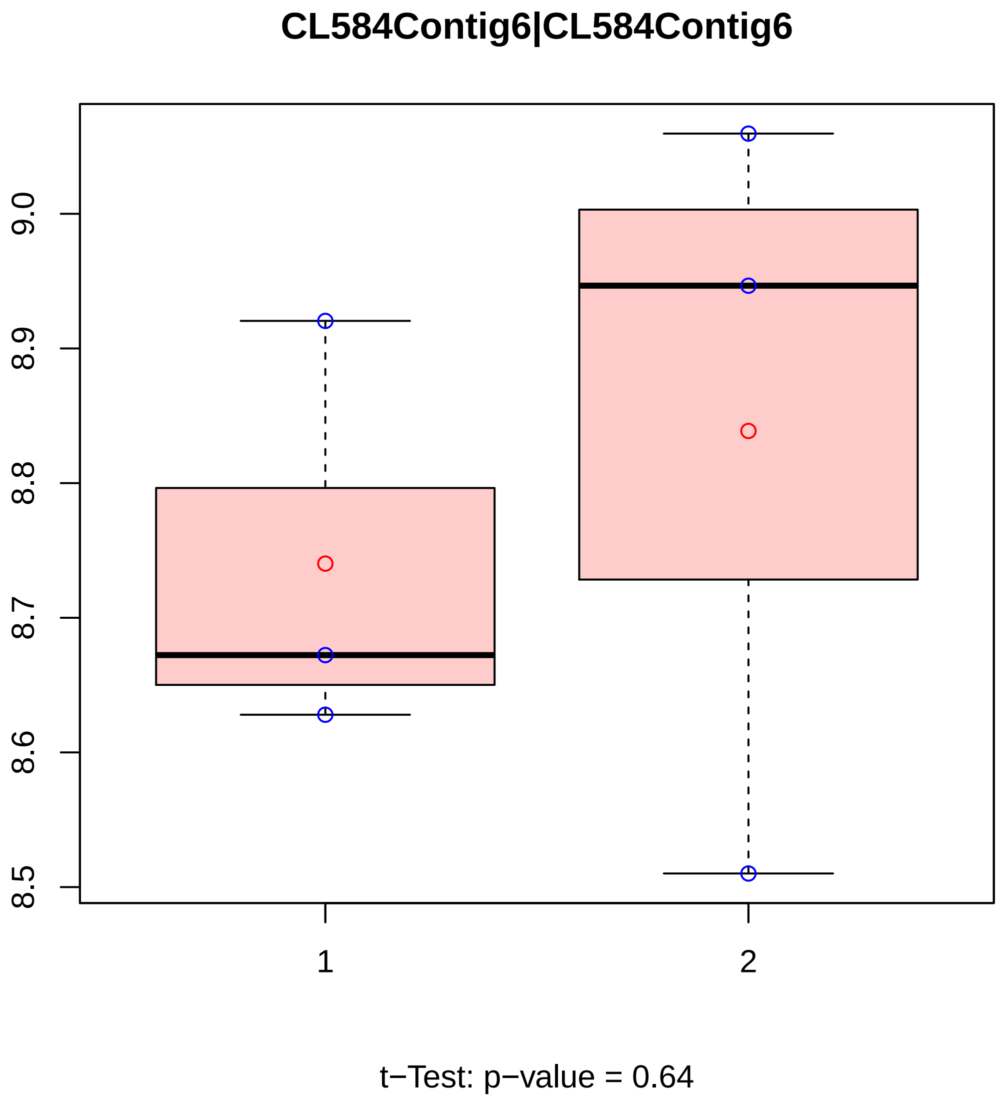
CL584Contig6|CL584Contig6
9.0
8.9
8.8
8.7
8.6
8.5
1
2
t−Test: p−value = 0.64

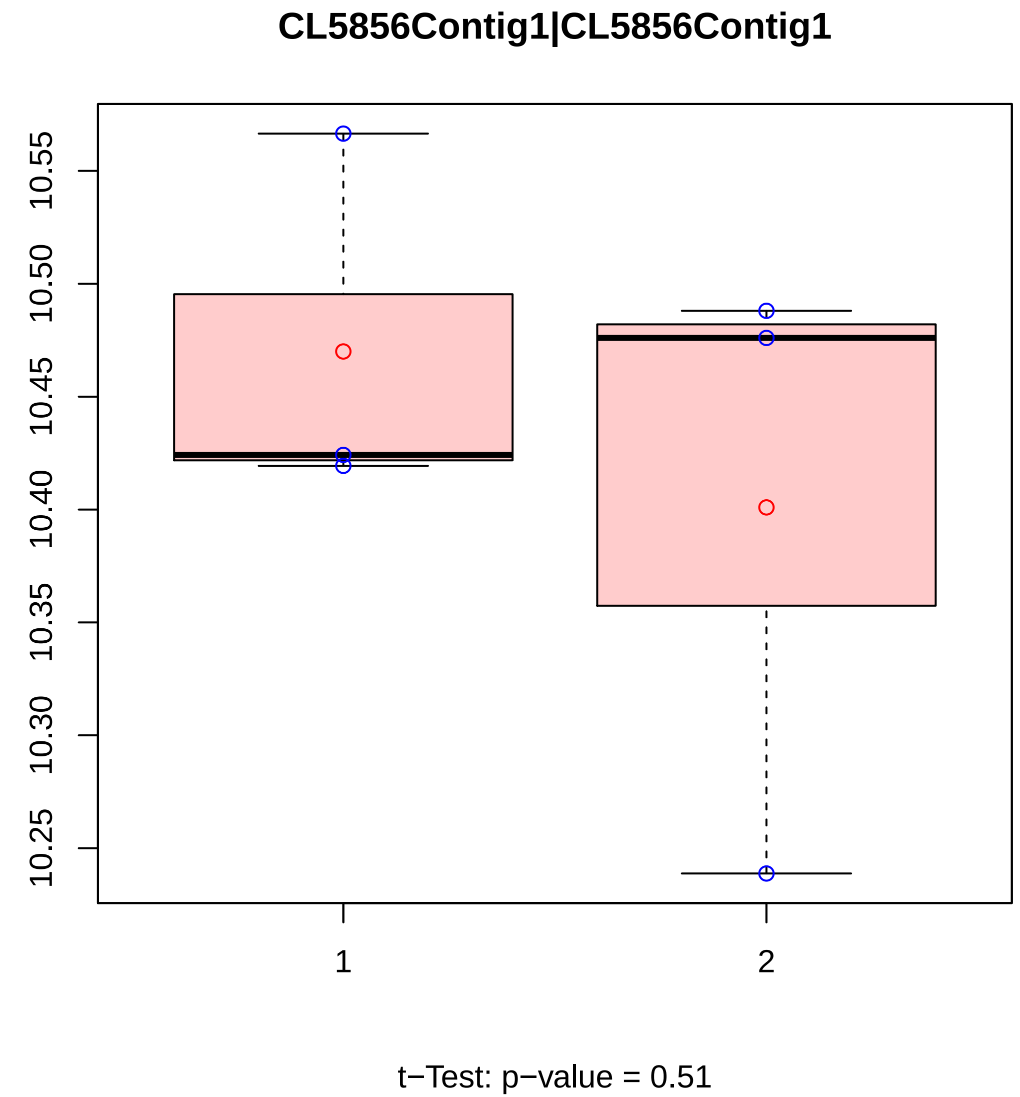
CL5856Contig1|CL5856Contig1
10.25
10.30
10.35
10.40
10.45
10.50
10.55
1
2
t−Test: p−value = 0.51

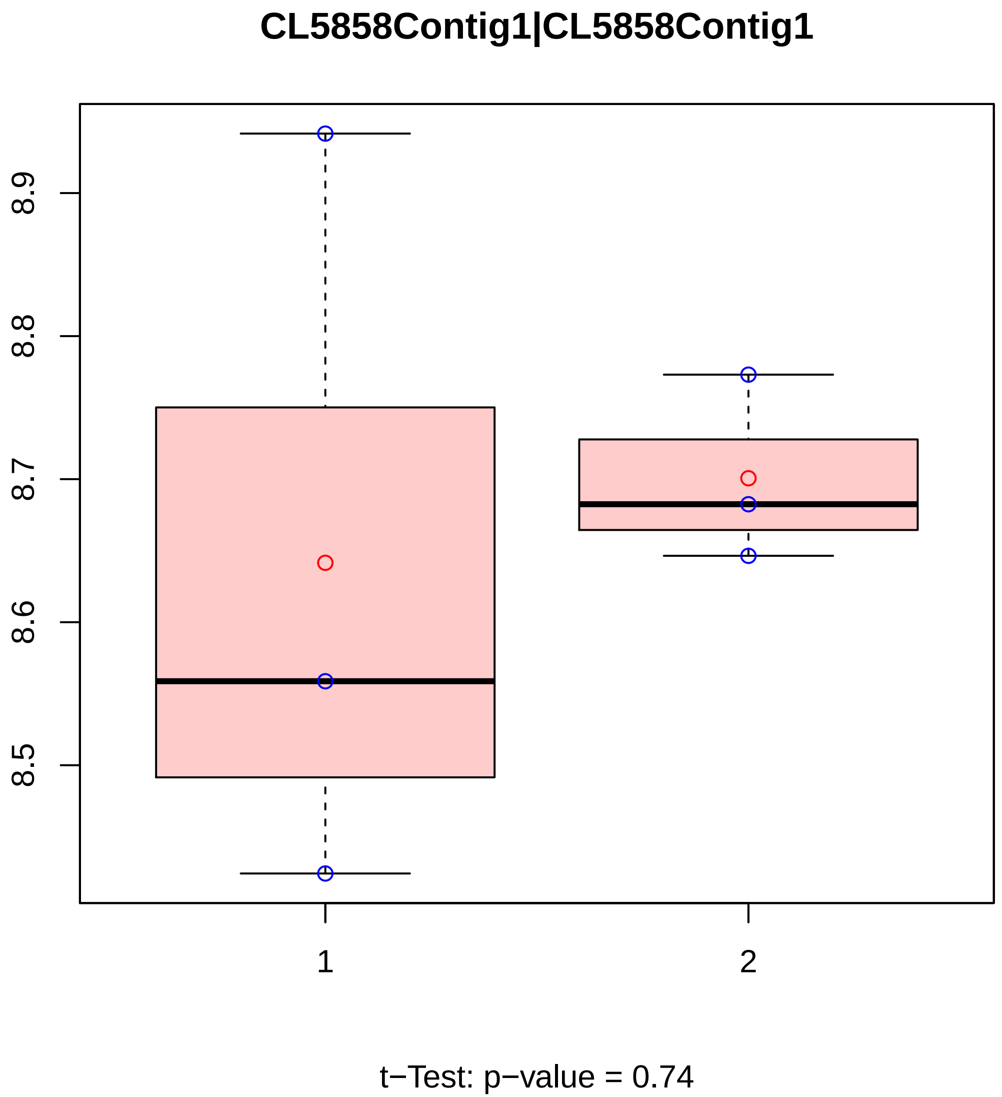
CL5858Contig1|CL5858Contig1
8.5
8.6
8.7
8.8
8.9
1
2
t−Test: p−value = 0.74

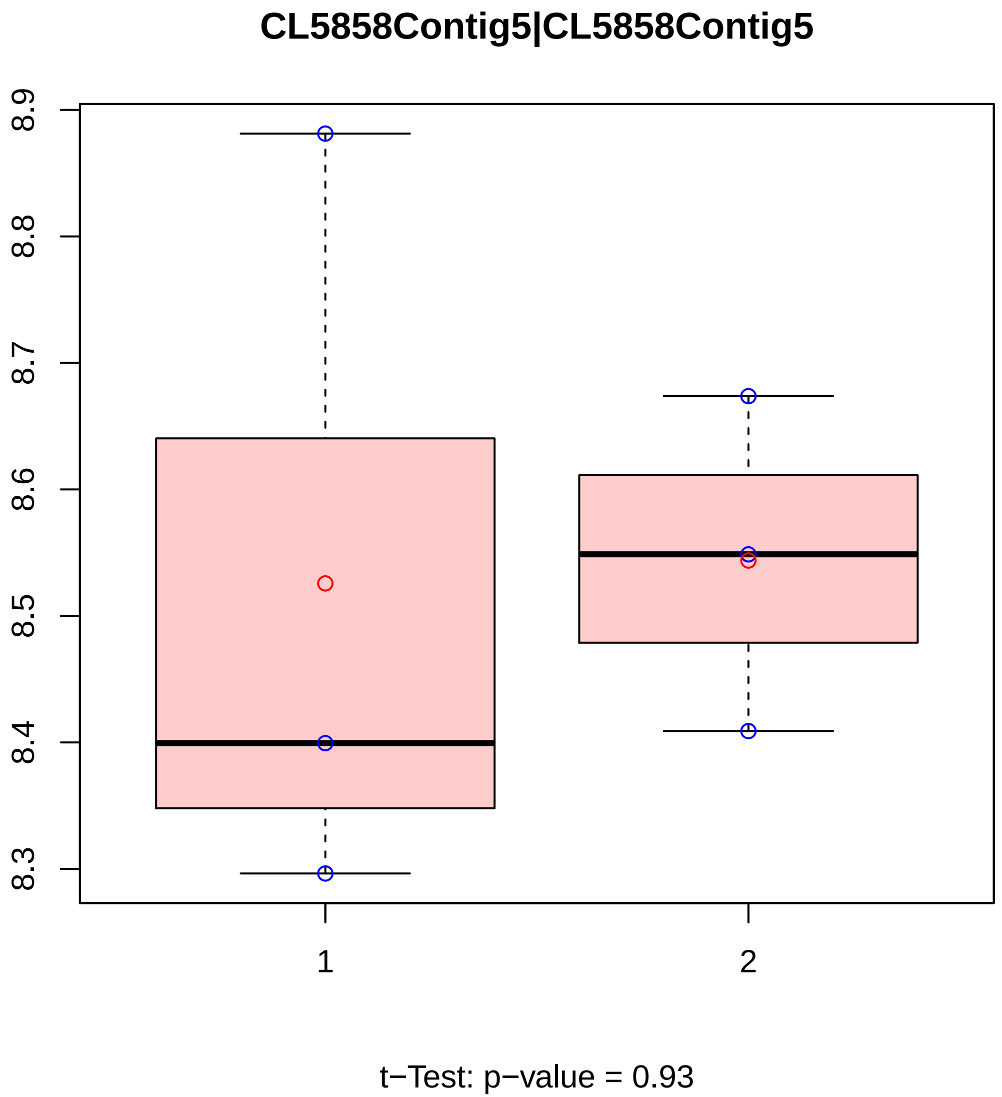
CL5858Contig5|CL5858Contig5
8.3
8.4
8.5
8.6
8.7
8.8
8.9
1
2
t−Test: p−value = 0.93

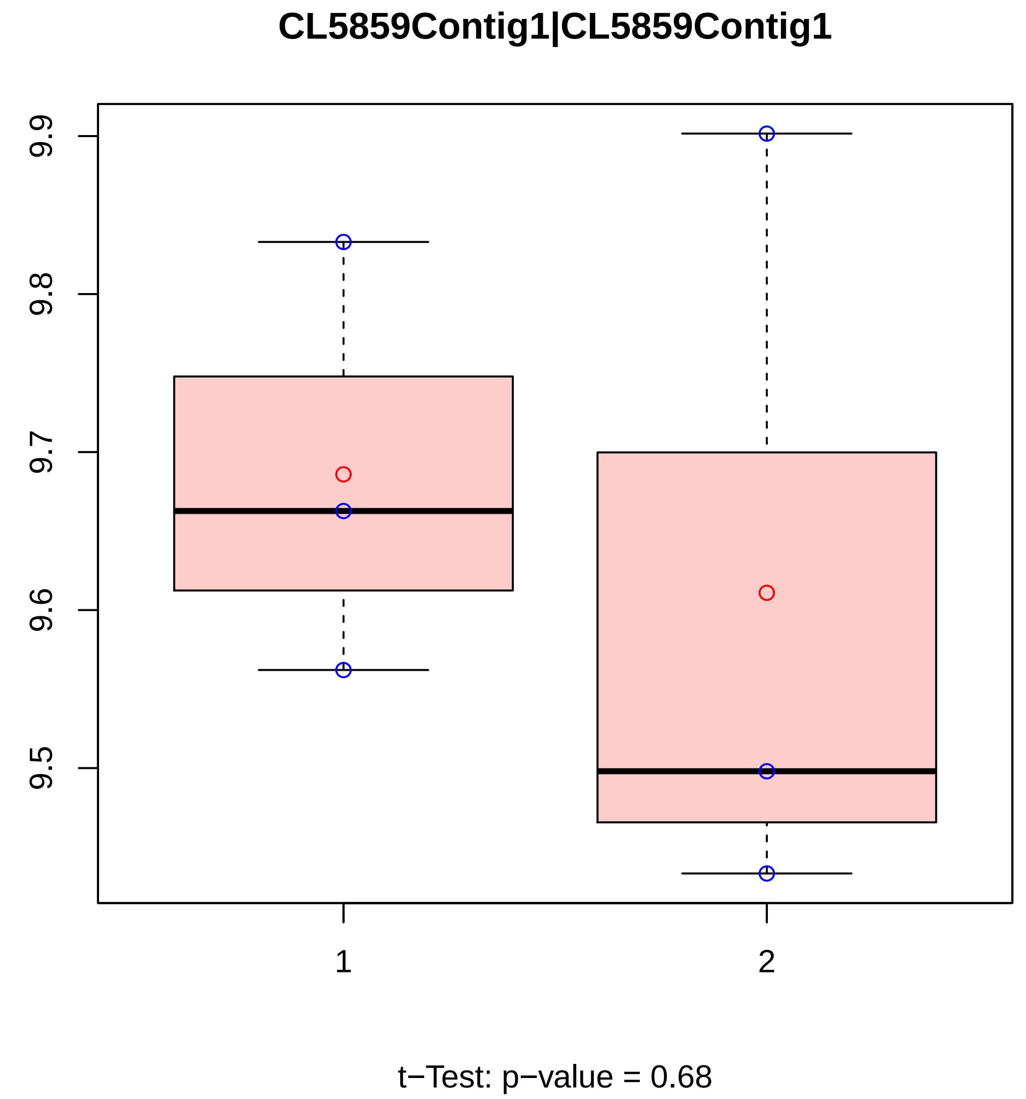
CL5859Contig1|CL5859Contig1
9.5
9.6
9.7
9.8
9.9
1
2
t−Test: p−value = 0.68

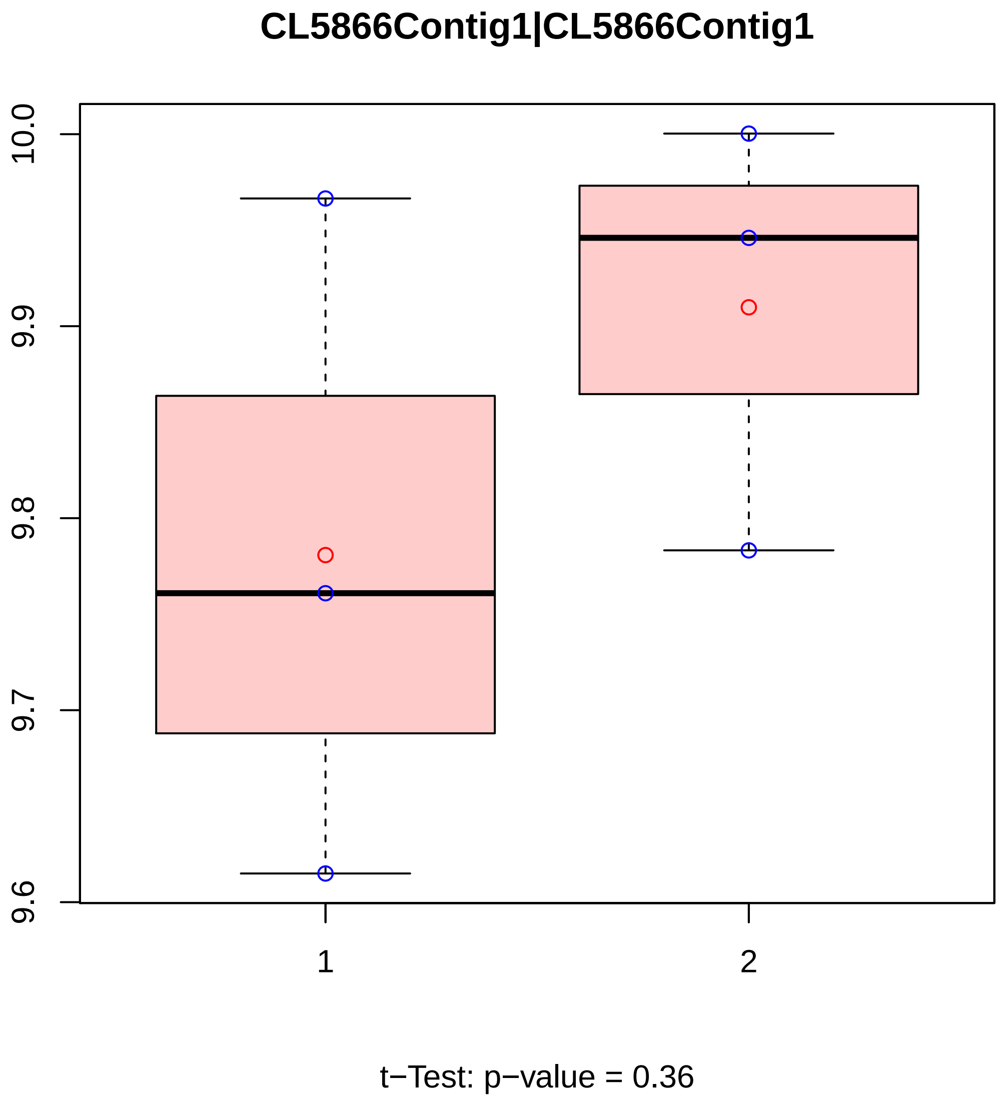
CL5866Contig1|CL5866Contig1
10.0
9.9
9.8
9.7
9.6
1
2
t−Test: p−value = 0.36

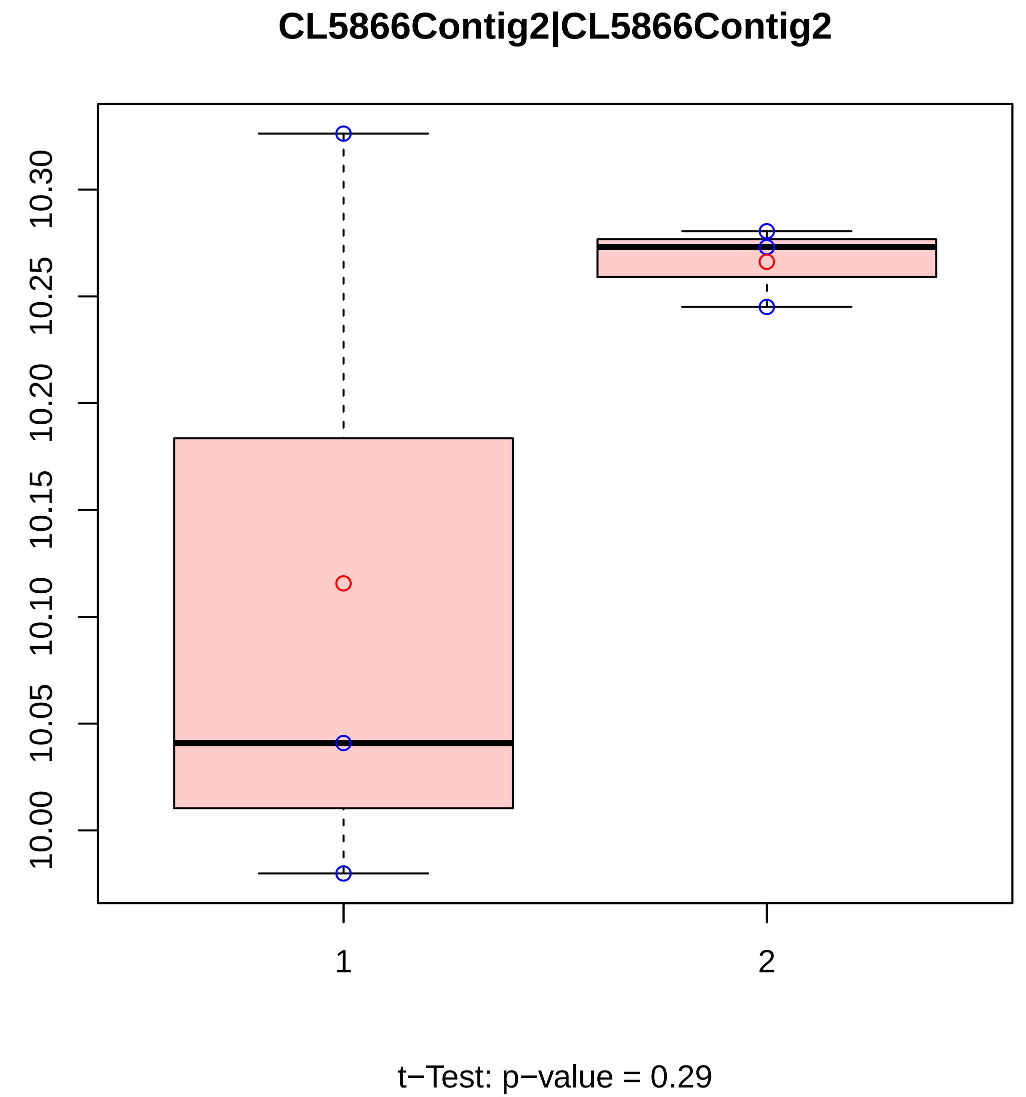
CL5866Contig2|CL5866Contig2
10.00
10.05
10.10
10.15
10.20
10.25
10.30
1
2
t−Test: p−value = 0.29

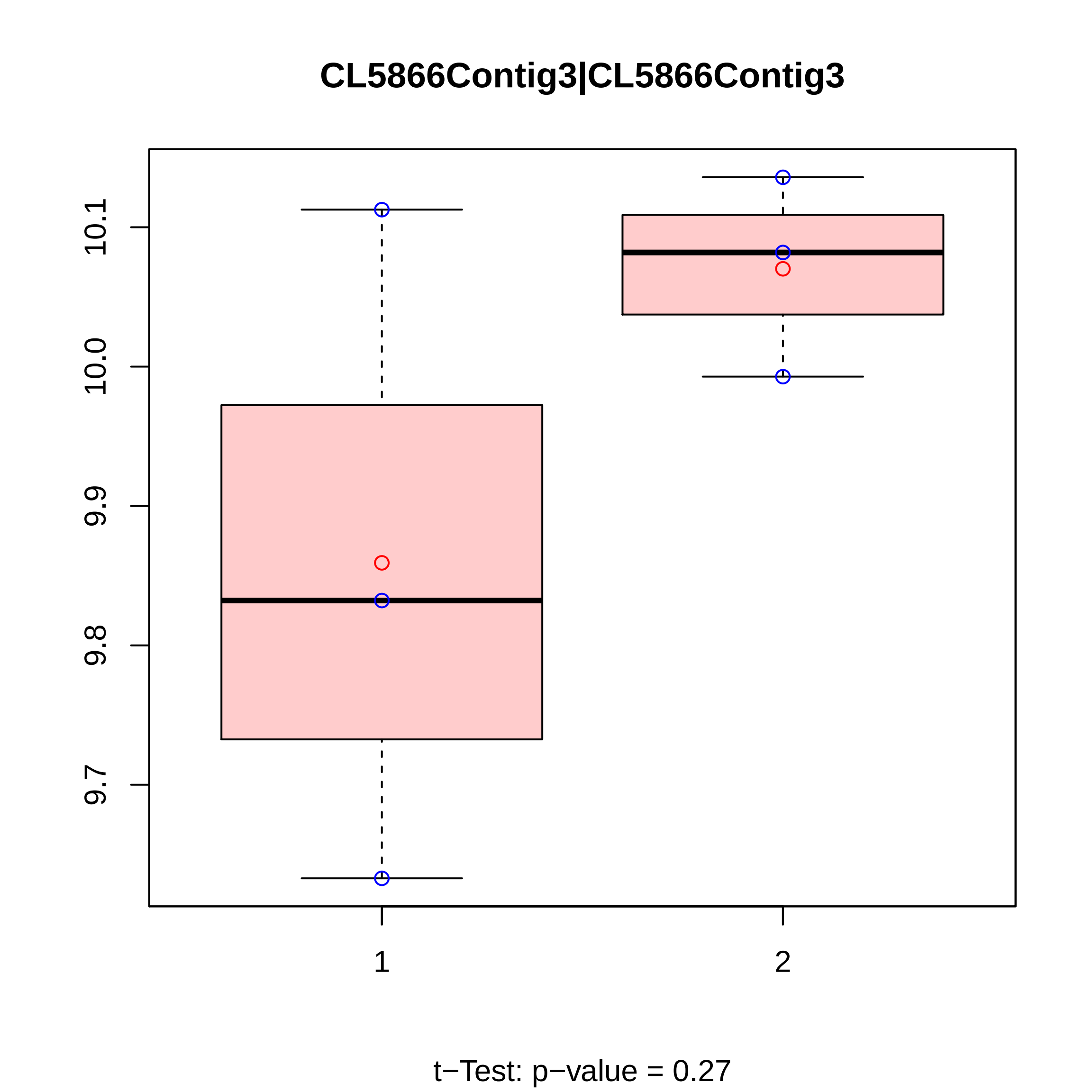
CL5866Contig3|CL5866Contig3
10.1
10.0
9.9
9.8
9.7
1
2
t−Test: p−value = 0.27

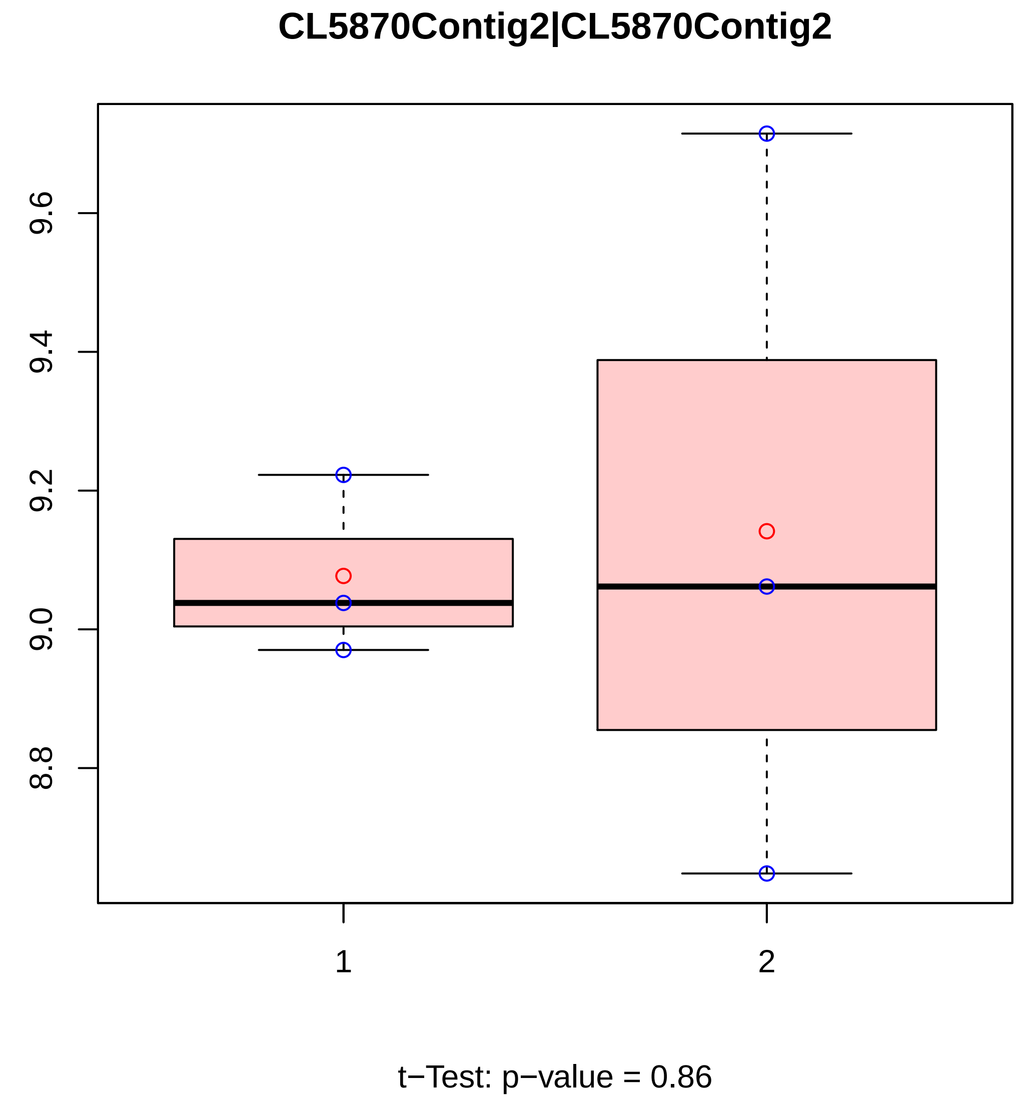
CL5870Contig2|CL5870Contig2
9.6
9.4
9.2
9.0
8.8
1
2
t−Test: p−value = 0.86

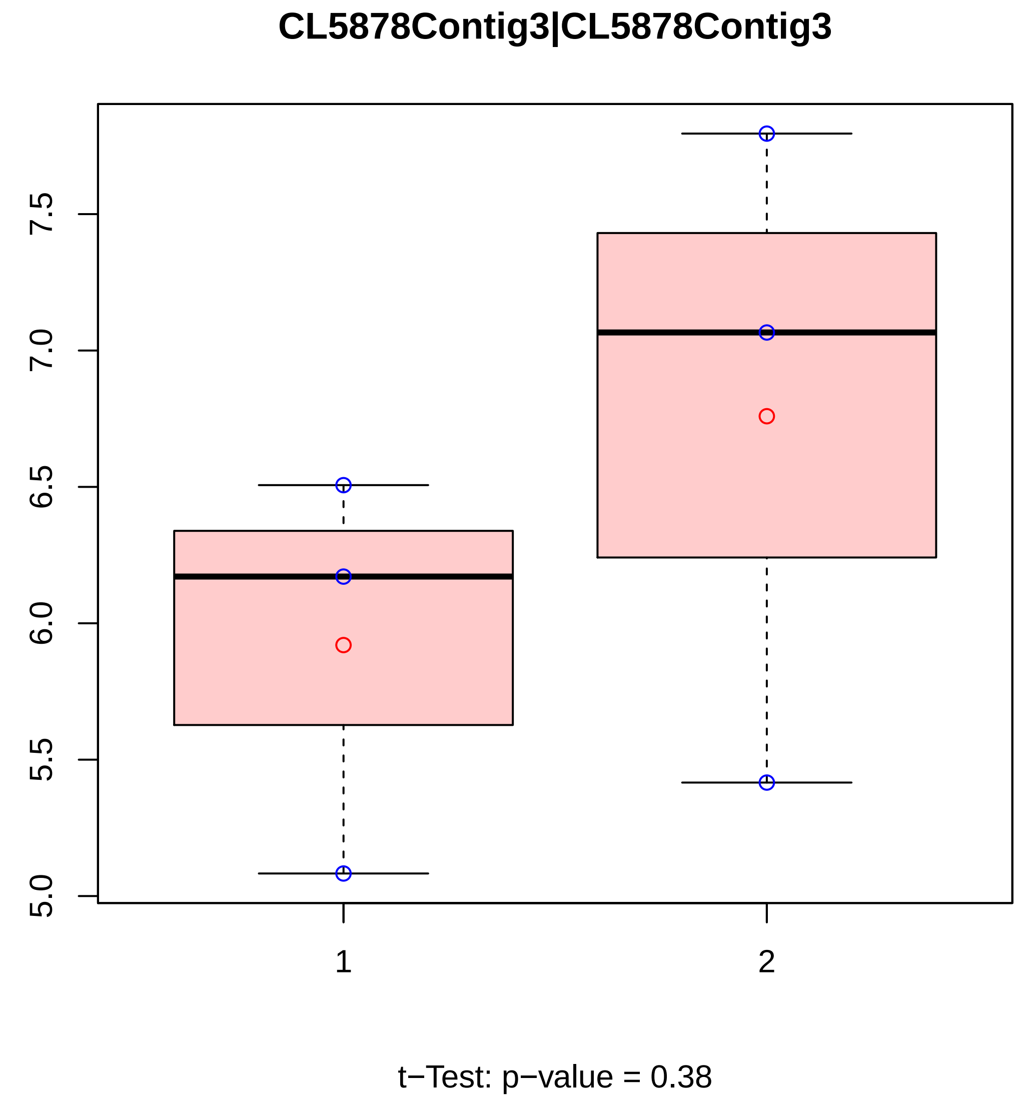
CL5878Contig3|CL5878Contig3
7.5
7.0
6.5
6.0
5.5
5.0
1
2
t−Test: p−value = 0.38

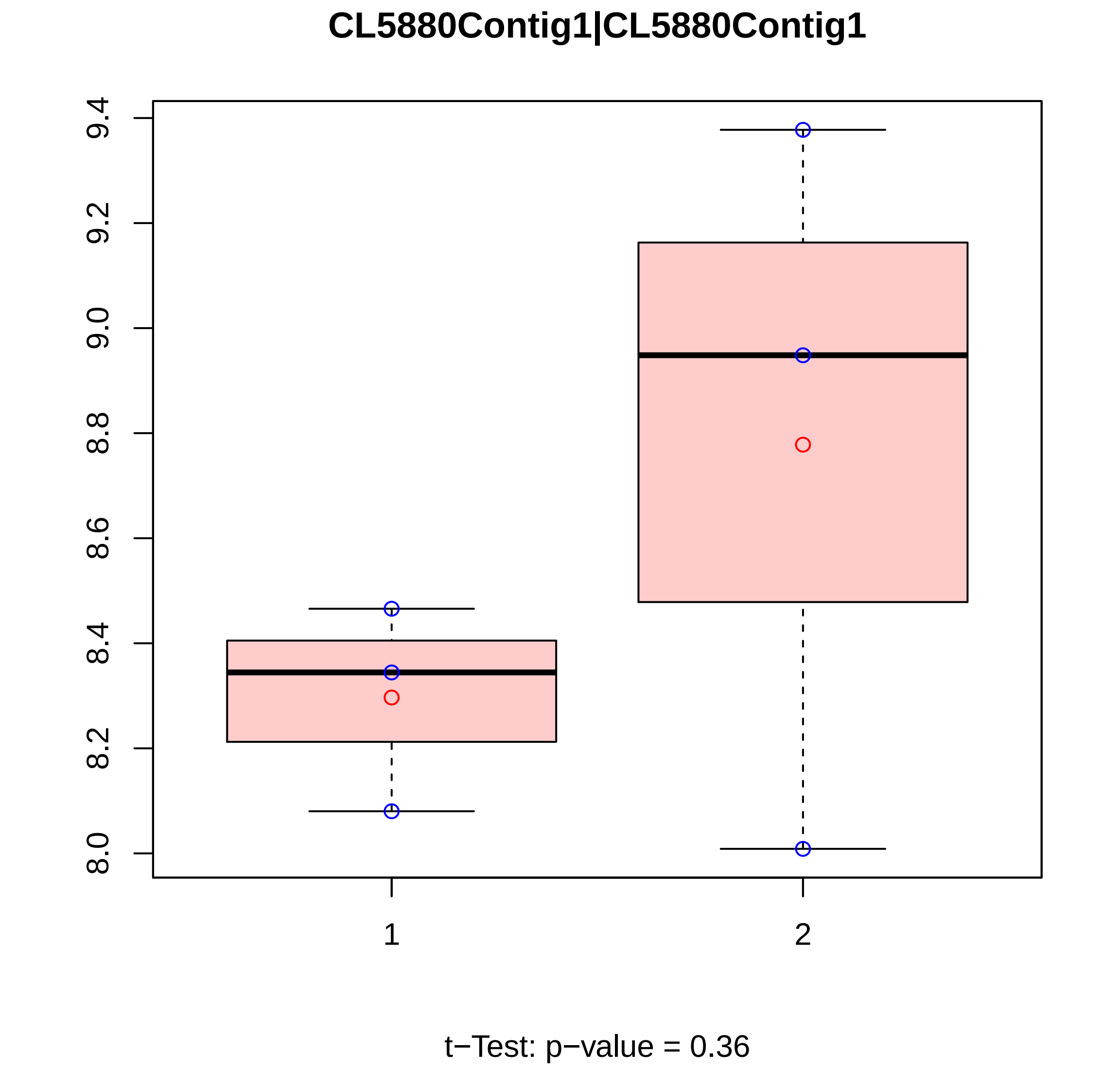
CL5880Contig1|CL5880Contig1
9.4
9.2
9.0
8.8
8.6
8.4
8.2
8.0
1
2
t−Test: p−value = 0.36

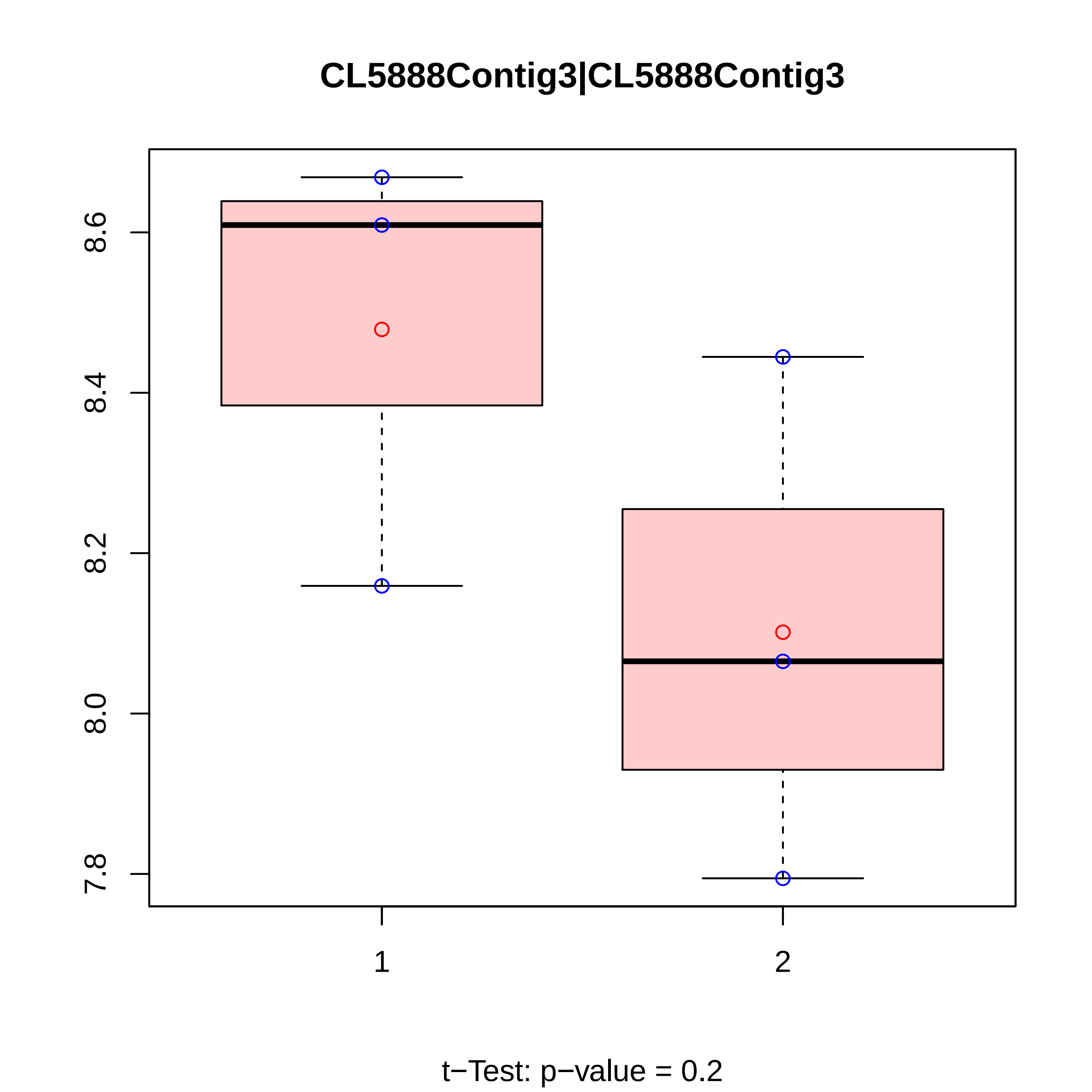

CL5888Contig3|CL5888Contig3
8.6
8.4
8.2
8.0
7.8
1
2
t−Test: p−value = 0.2

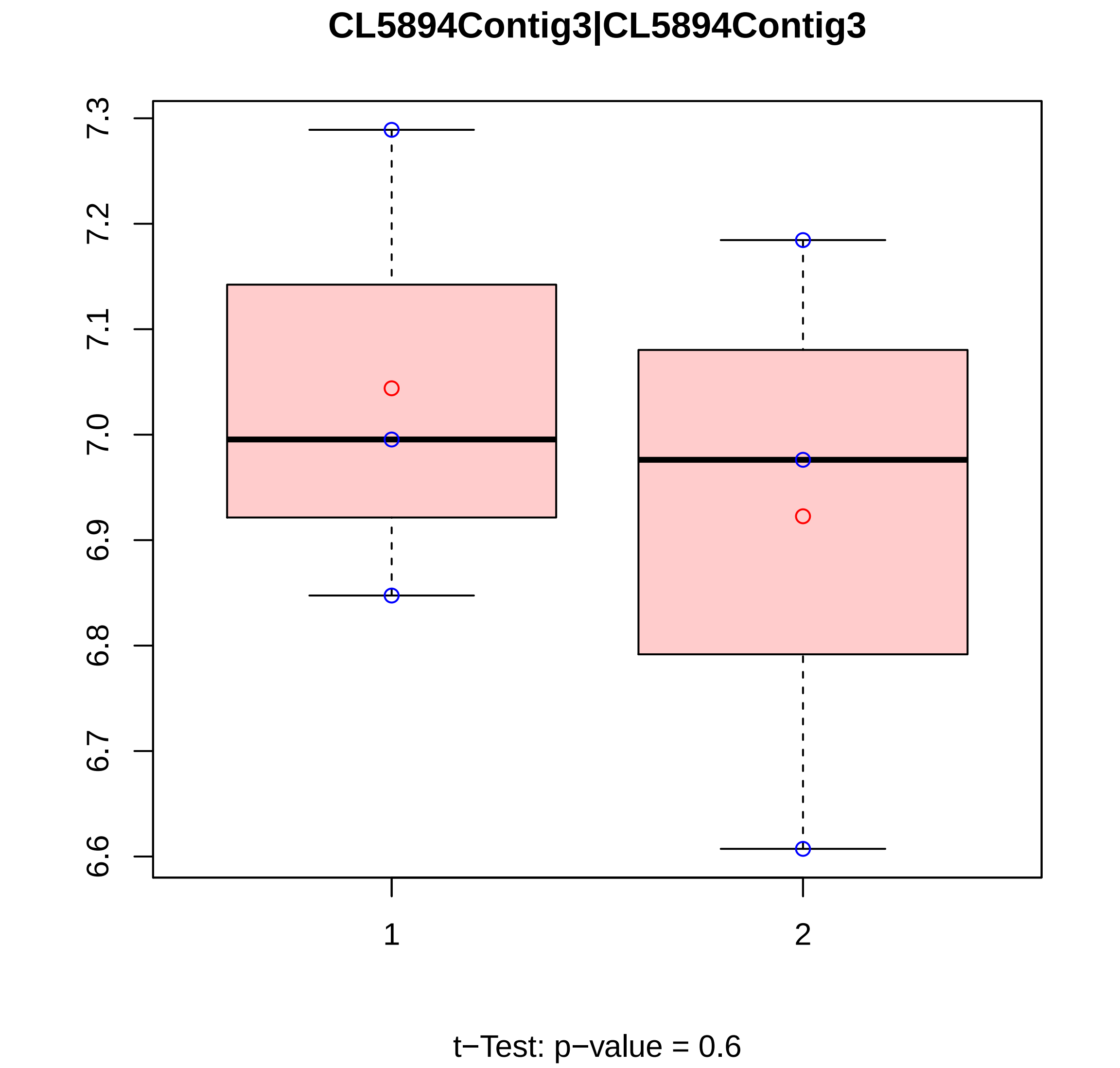

CL5894Contig3|CL5894Contig3
7.3
7.2
7.1
7.0
6.9
6.8
6.7
6.6
1
2
t−Test: p−value = 0.6

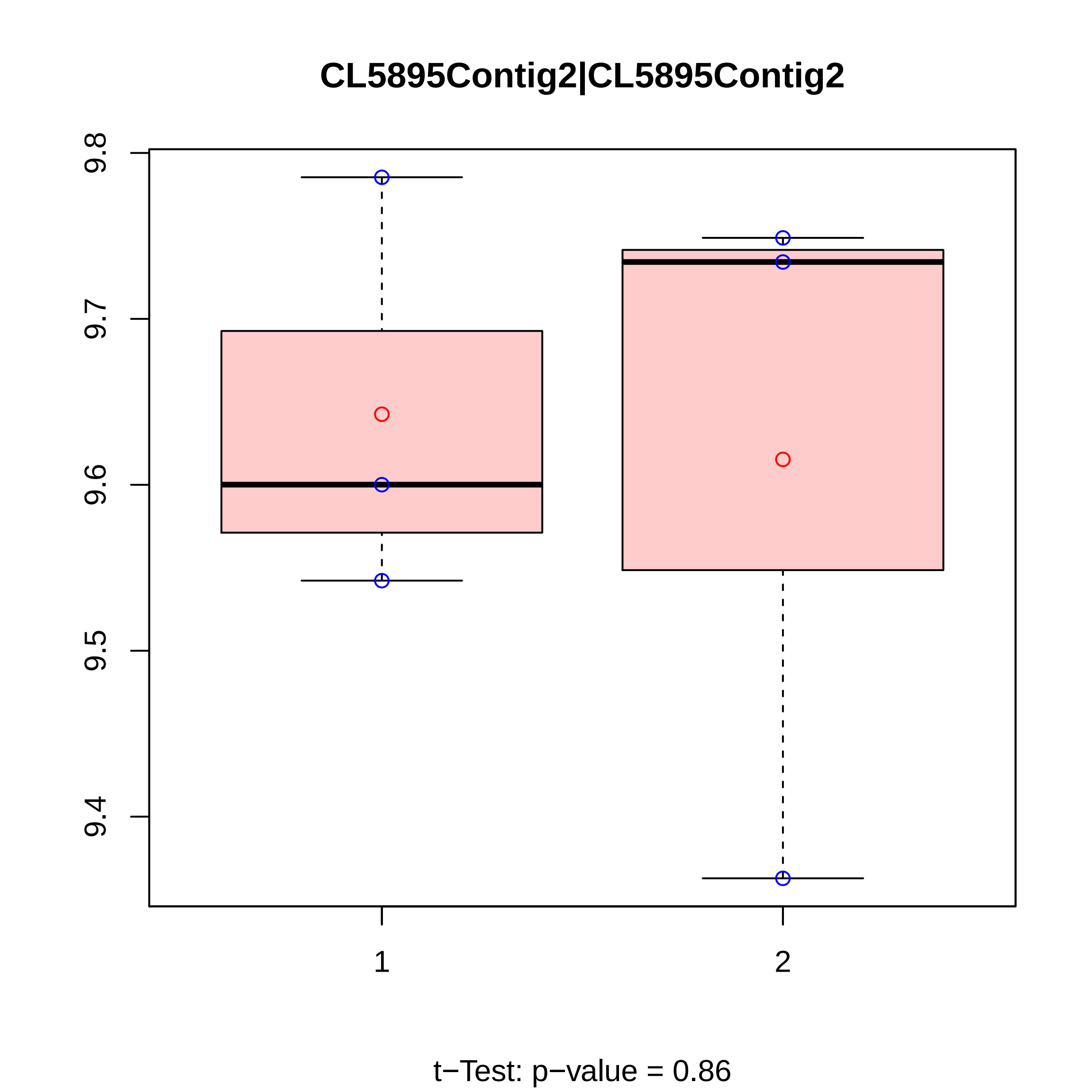
CL5895Contig2|CL5895Contig2
9.8
9.7
9.6
9.5
9.4
1
2
t−Test: p−value = 0.86

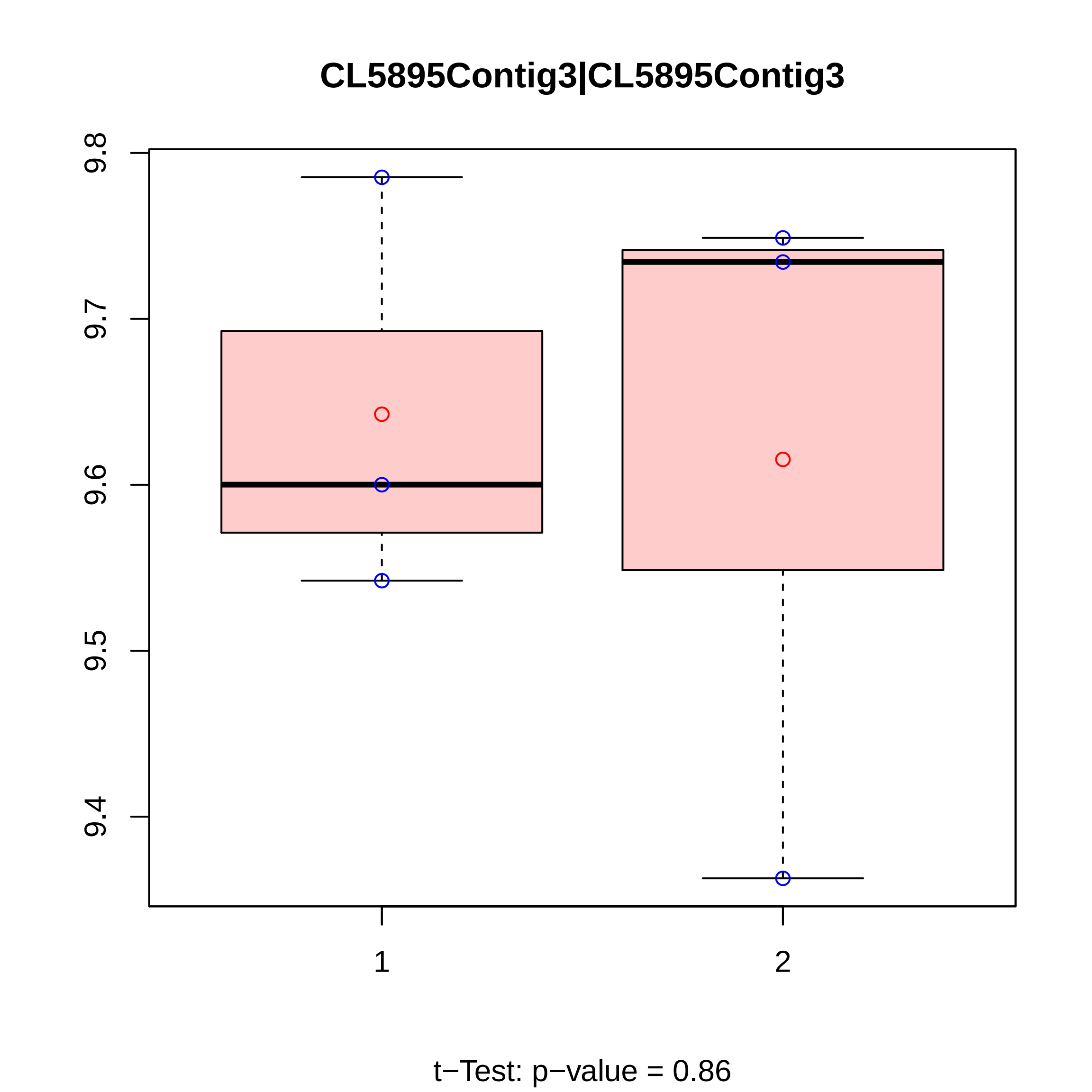
CL5895Contig3|CL5895Contig3
9.4
9.5
9.6
9.7
9.8
1
2
t−Test: p−value = 0.86

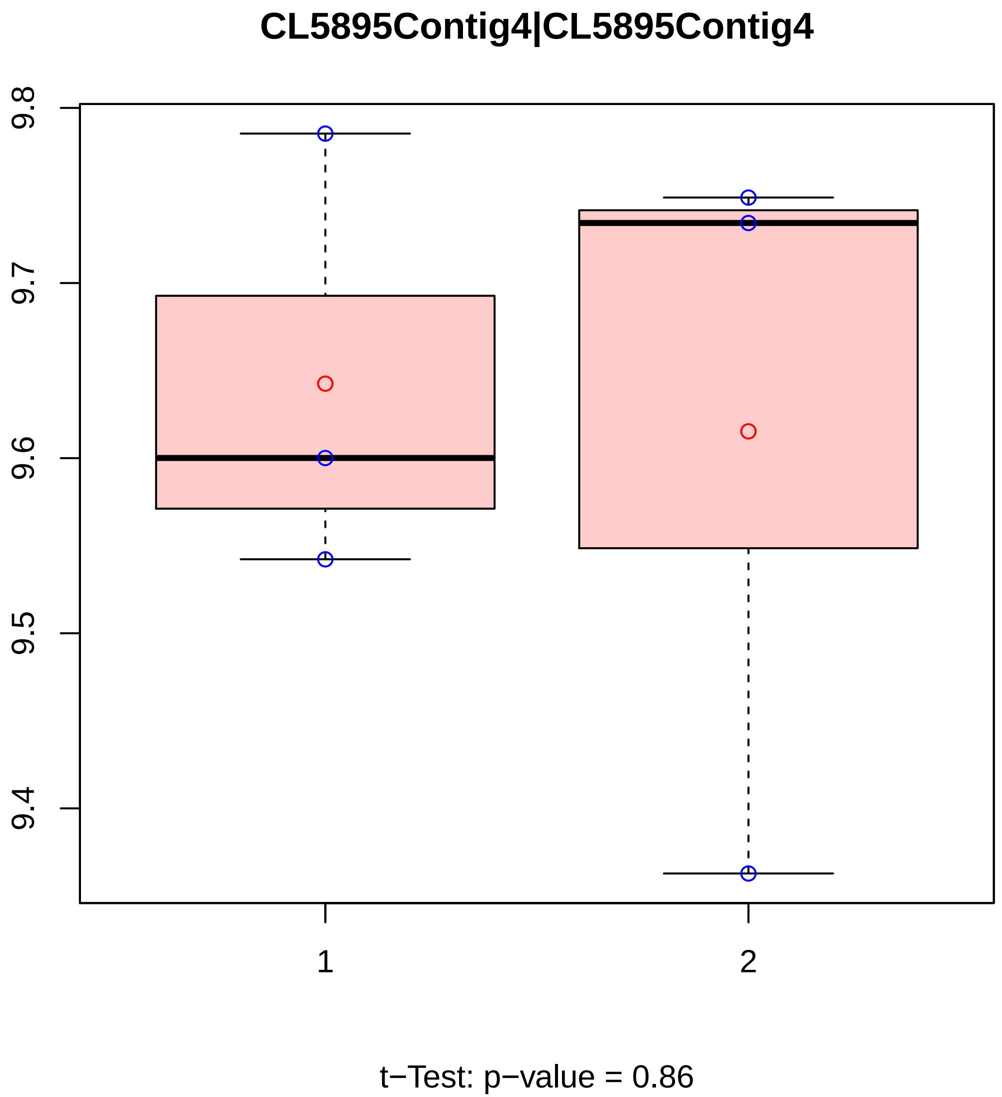
CL5895Contig4|CL5895Contig4
9.8
9.7
9.6
9.5
9.4
1
2
t−Test: p−value = 0.86

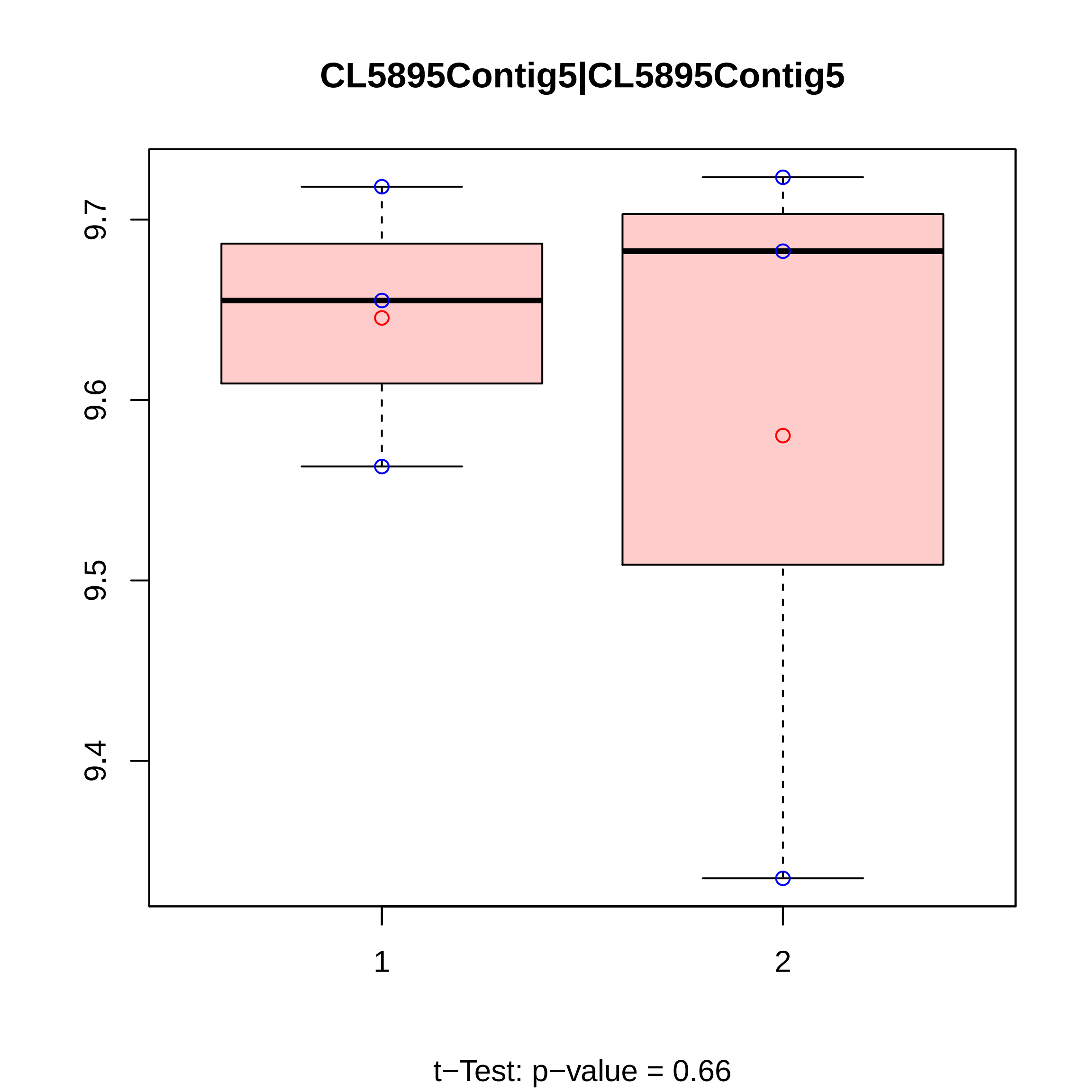

CL5895Contig5|CL5895Contig5
9.7
9.6
9.5
9.4
1
2
t−Test: p−value = 0.66

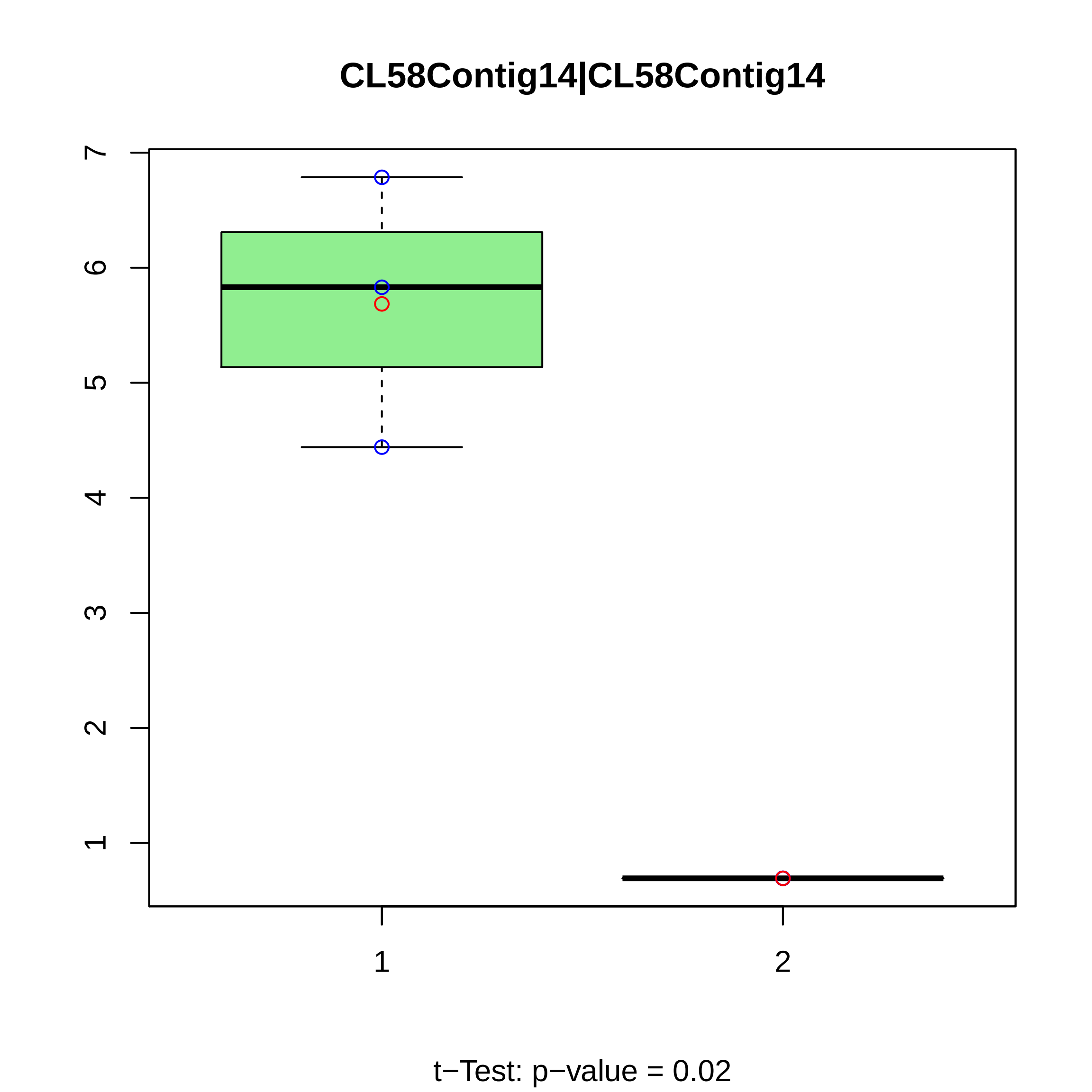
CL58Contig14|CL58Contig14
1
2
3
4
5
6
7
1
2
t−Test: p−value = 0.02

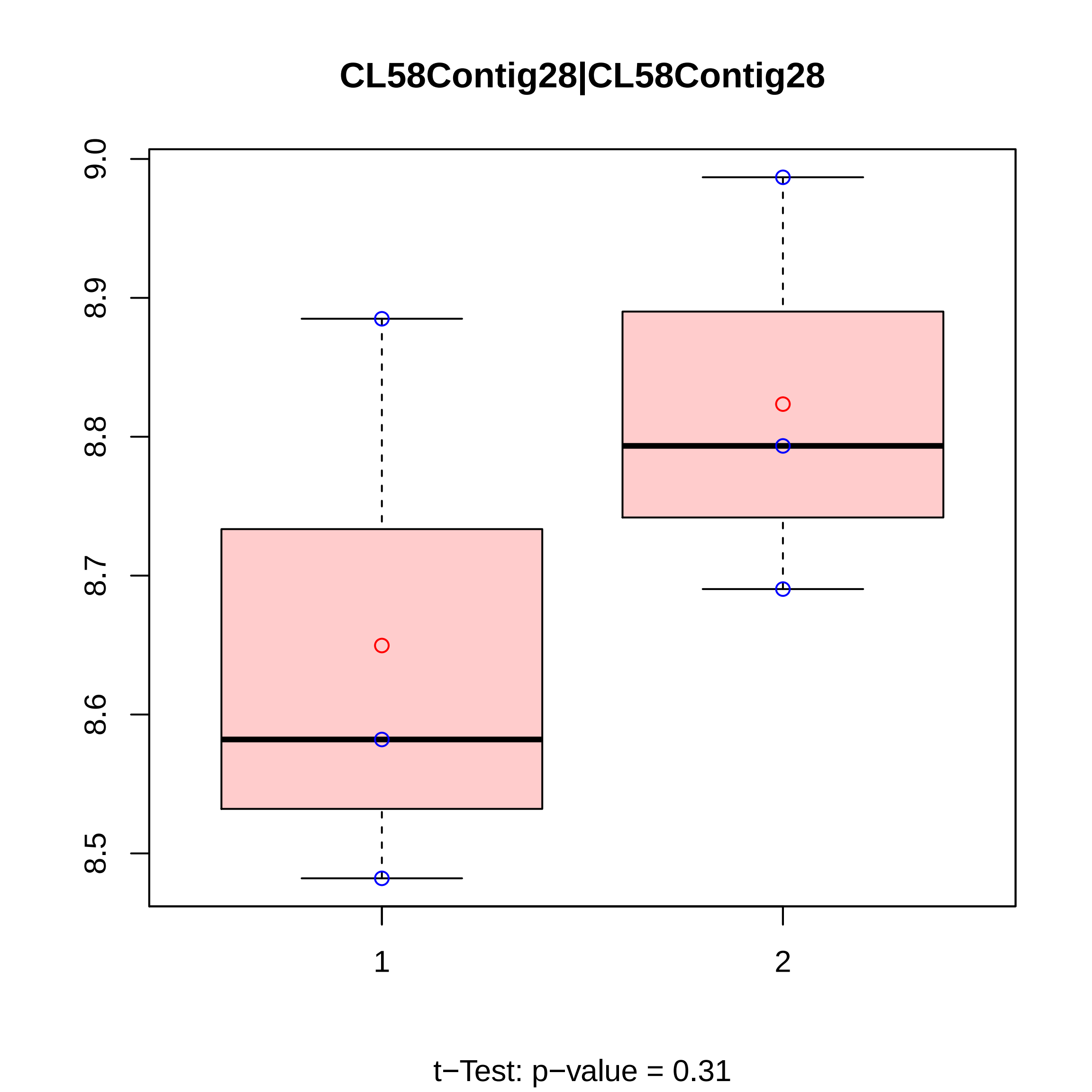
CL58Contig28|CL58Contig28
9.0
8.9
8.8
8.7
8.6
8.5
1
2
t−Test: p−value = 0.31

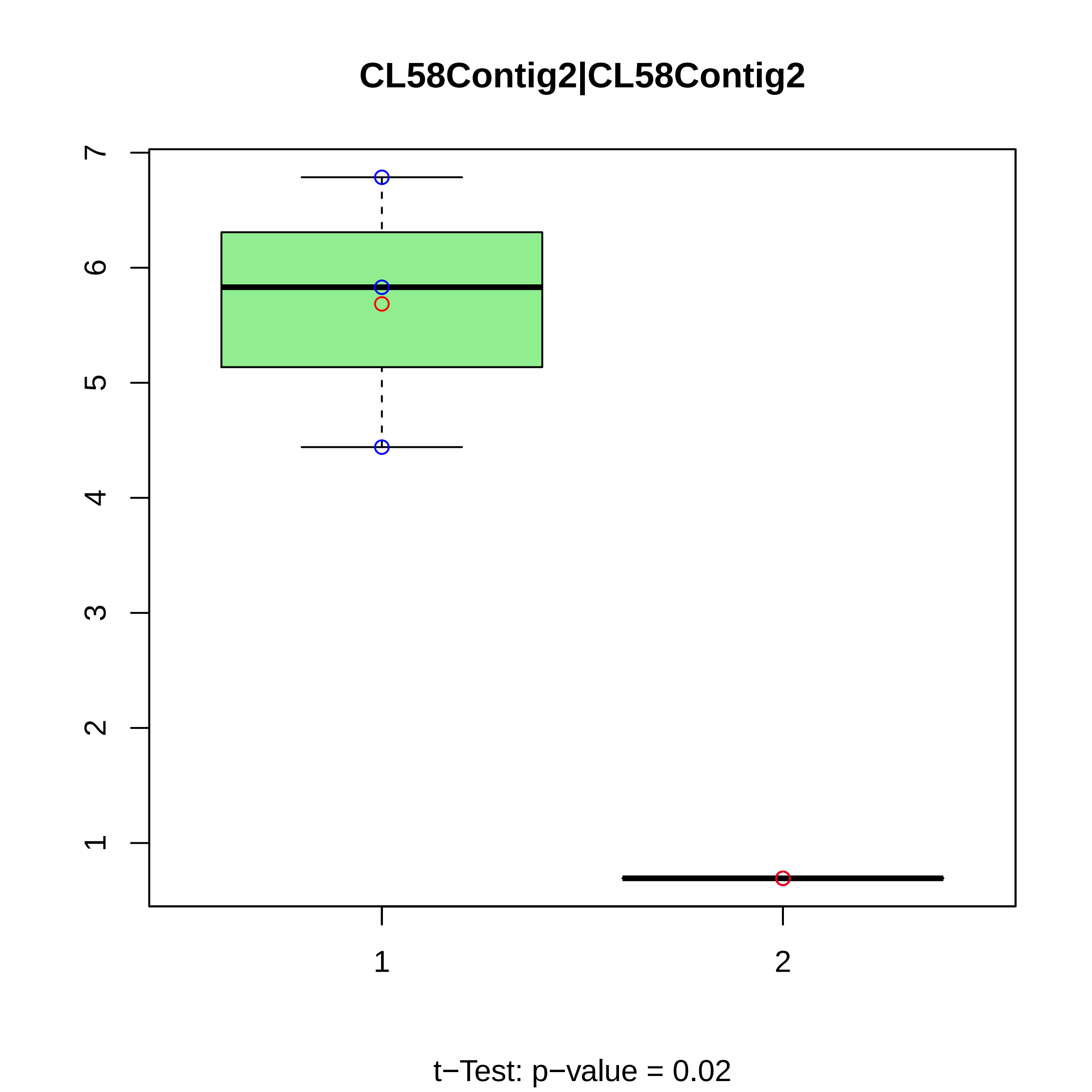

CL58Contig2|CL58Contig2
1
2
3
4
5
6
7
1
2
t−Test: p−value = 0.02

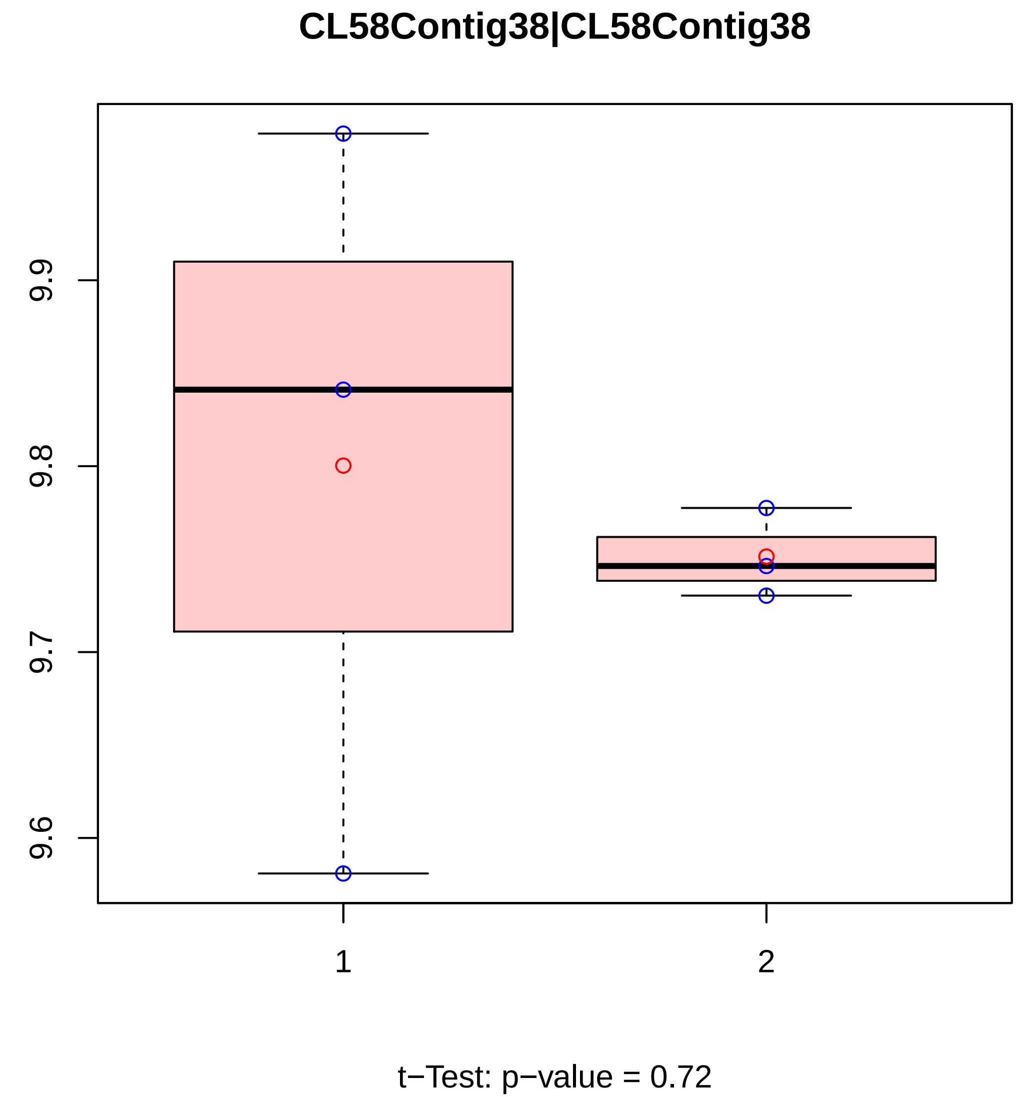

CL58Contig38|CL58Contig38
9.6
9.7
9.8
9.9
1
2
t−Test: p−value = 0.72

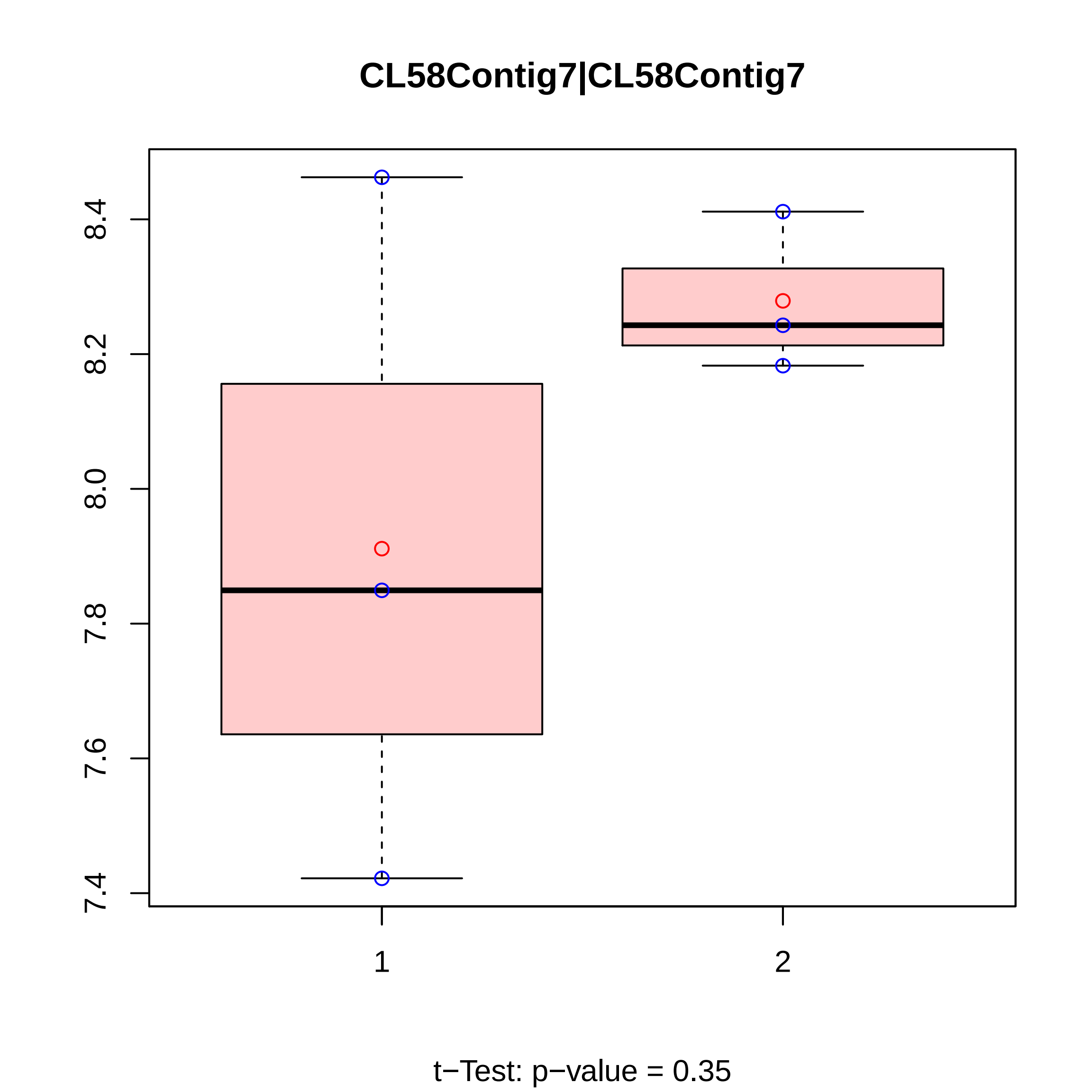
CL58Contig7|CL58Contig7
8.4
8.2
8.0
7.8
7.6
7.4
1
2
t−Test: p−value = 0.35

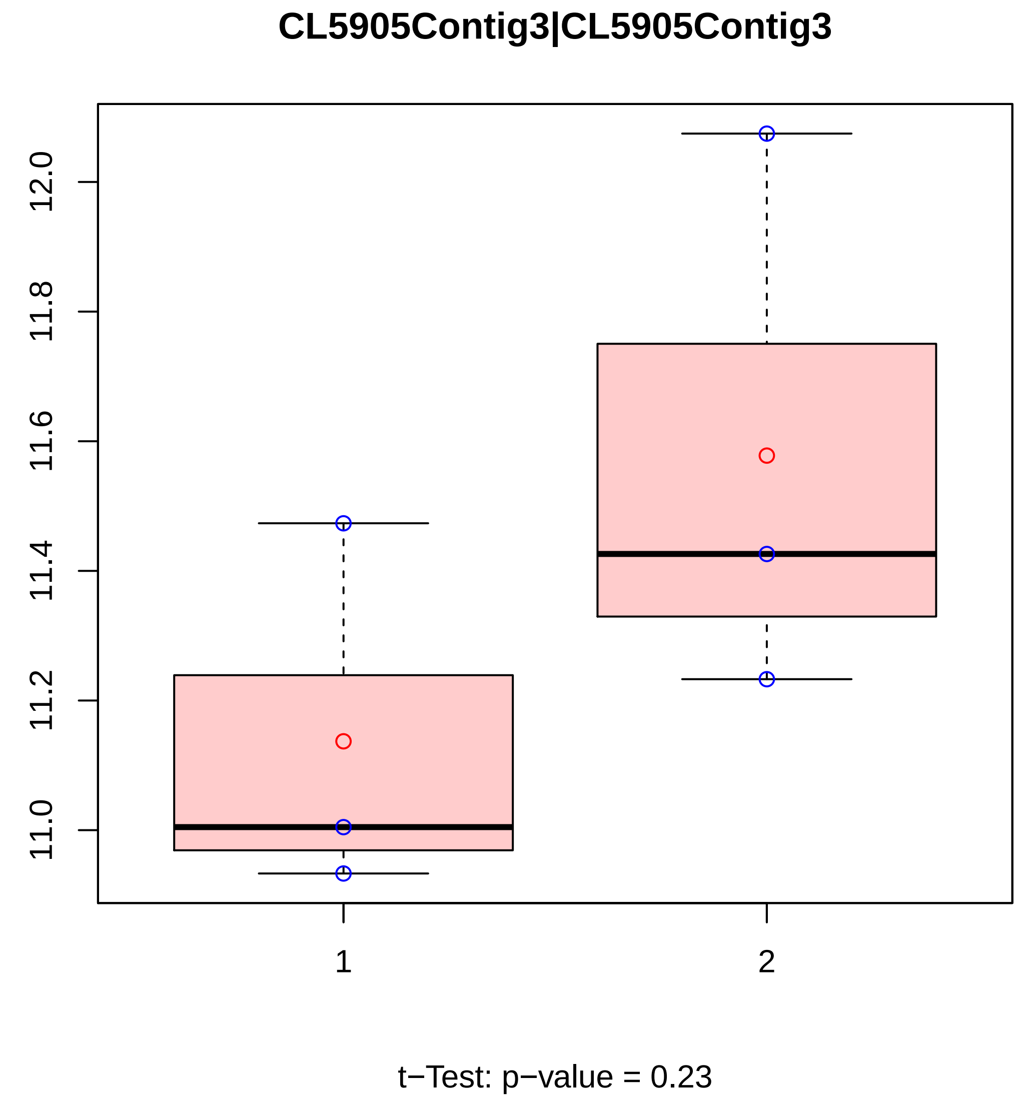

CL5905Contig3|CL5905Contig3
12.0
11.8
11.6
11.4
11.2
11.0
1
2
t−Test: p−value = 0.23

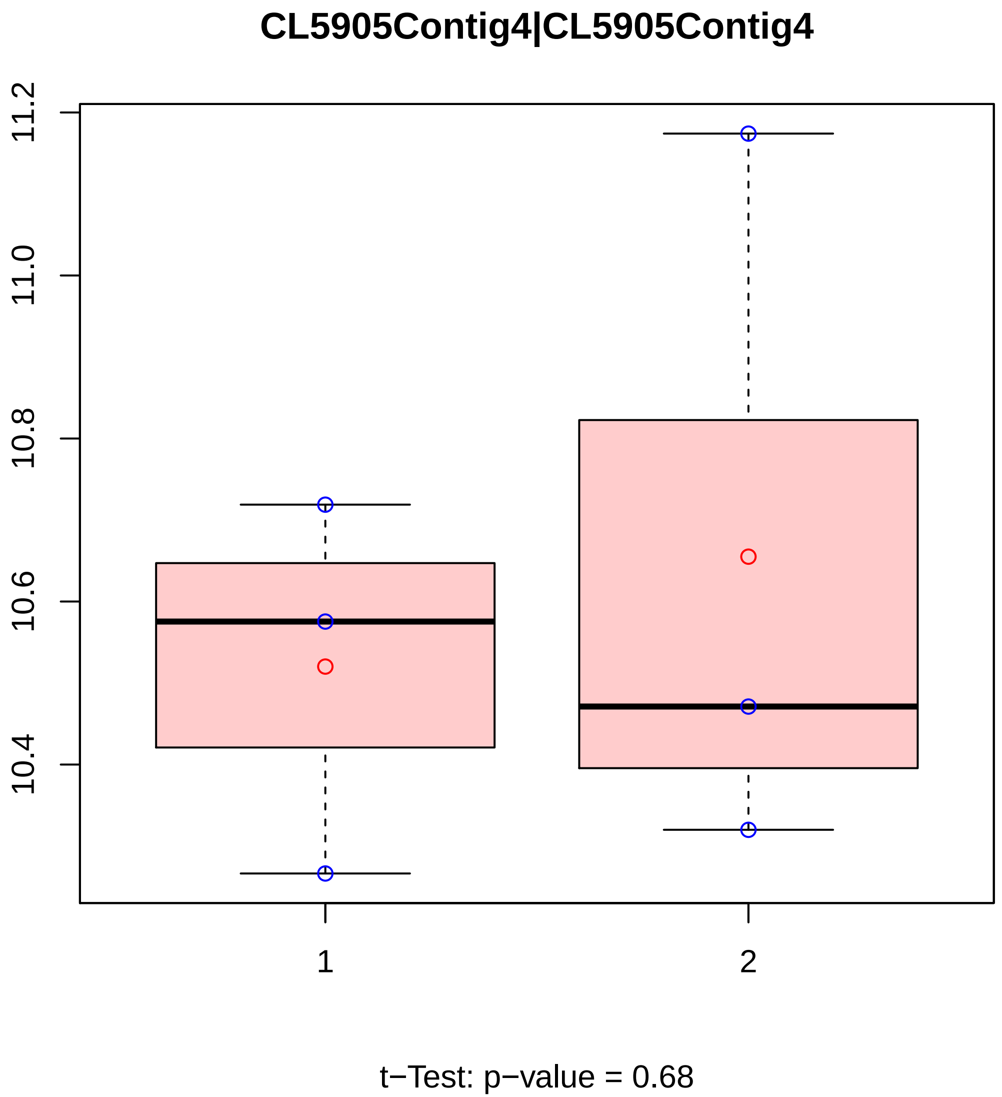

CL5905Contig4|CL5905Contig4
11.2
11.0
10.8
10.6
10.4
1
2
t−Test: p−value = 0.68

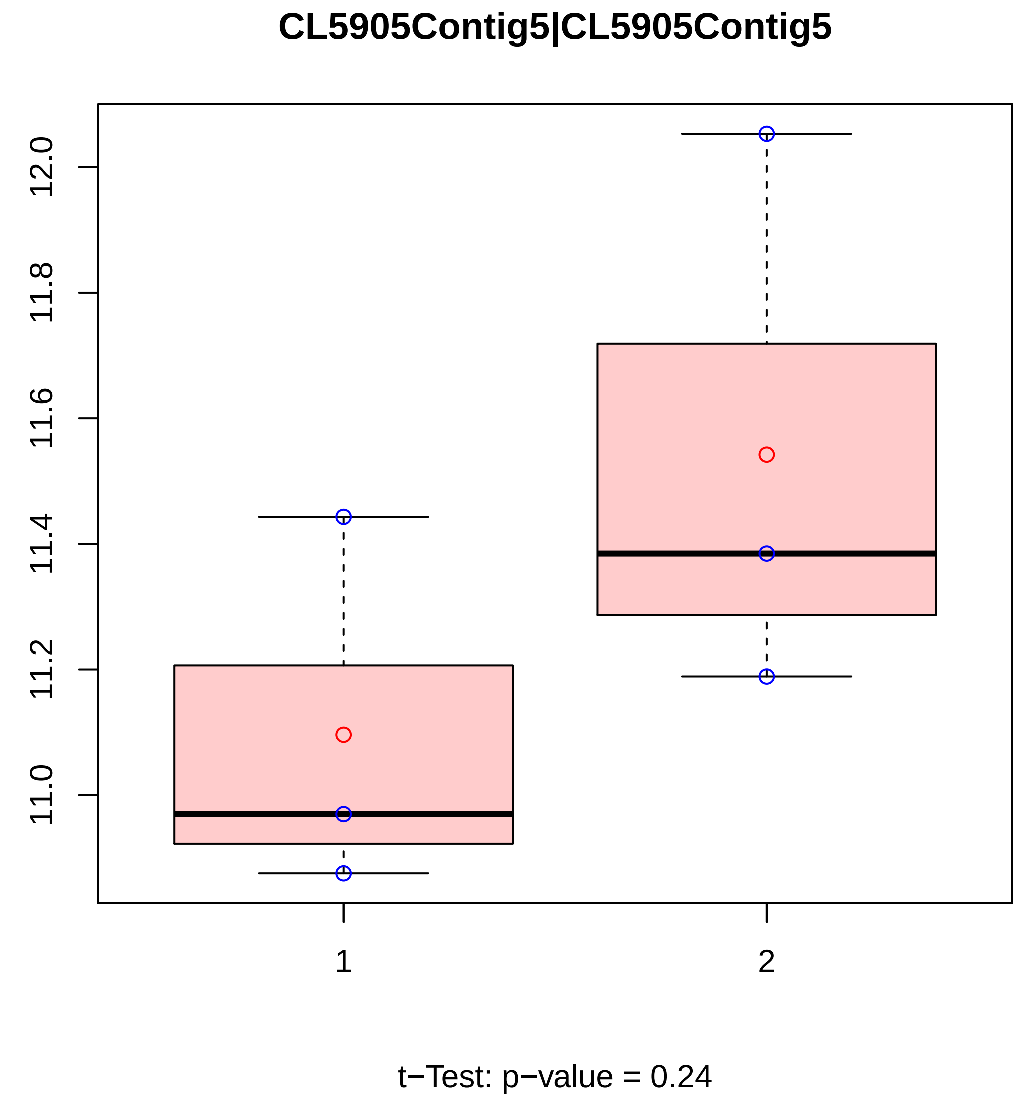
CL5905Contig5|CL5905Contig5
12.0
11.8
11.6
11.4
11.2
11.0
1
2
t−Test: p−value = 0.24

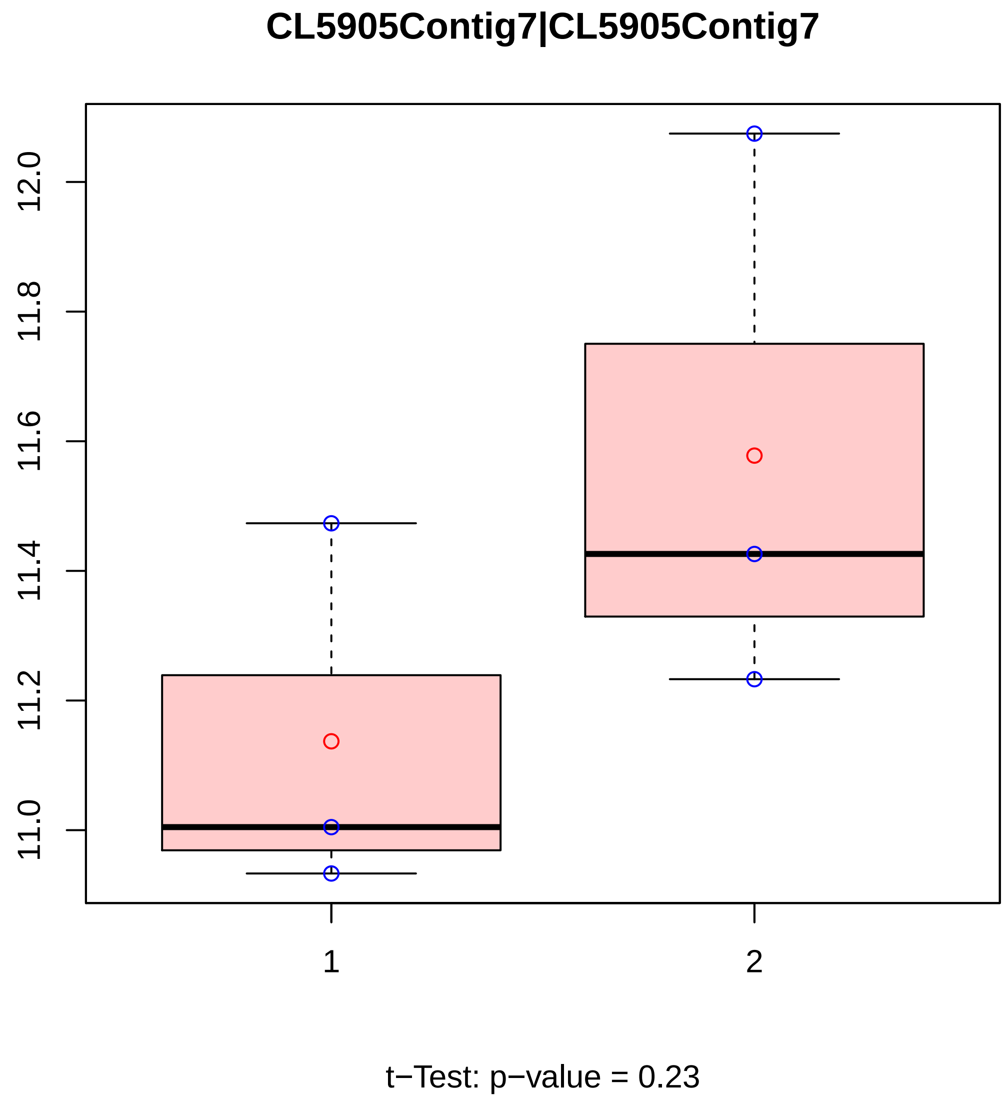
CL5905Contig7|CL5905Contig7
12.0
11.8
11.6
11.4
11.2
11.0
1
2
t−Test: p−value = 0.23

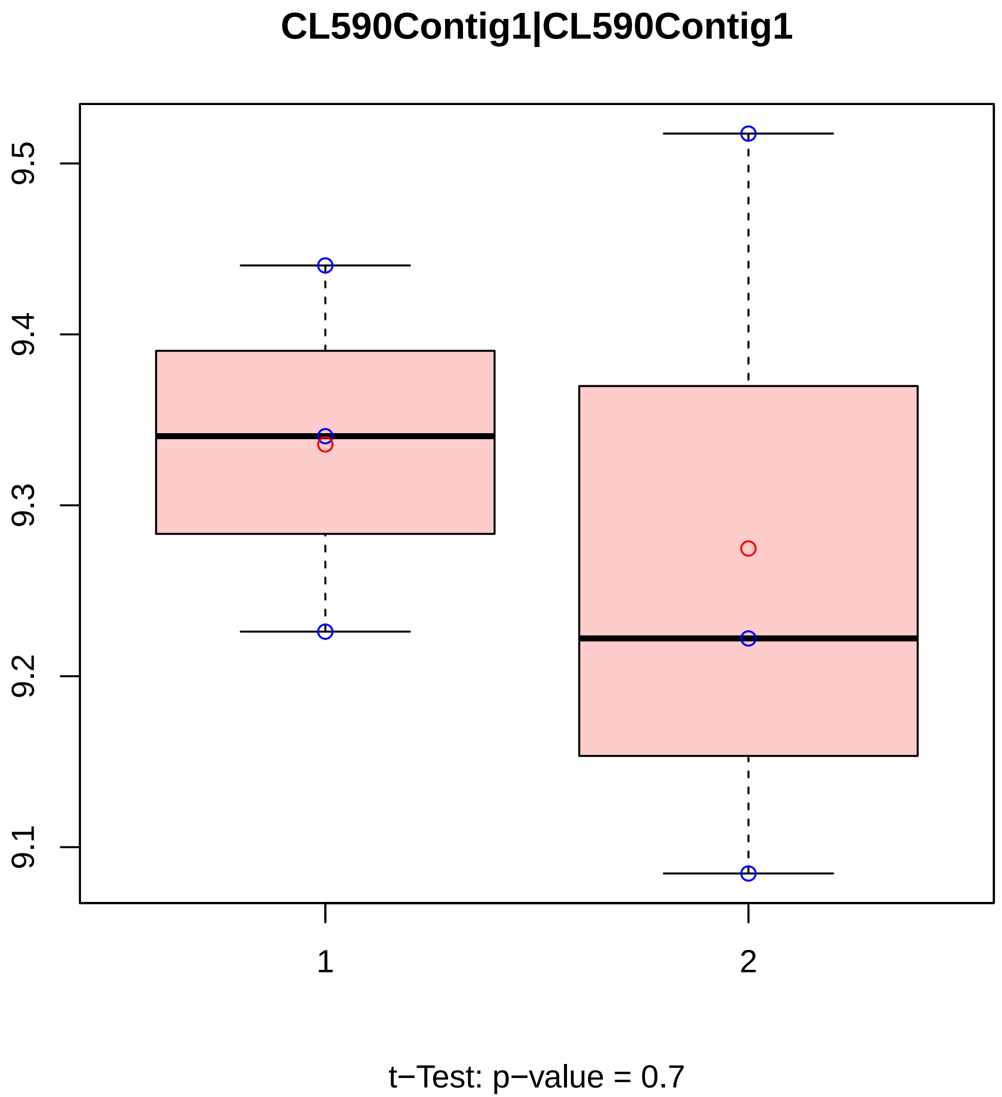
CL590Contig1|CL590Contig1
9.1
9.2
9.3
9.4
9.5
1
2
t−Test: p−value = 0.7

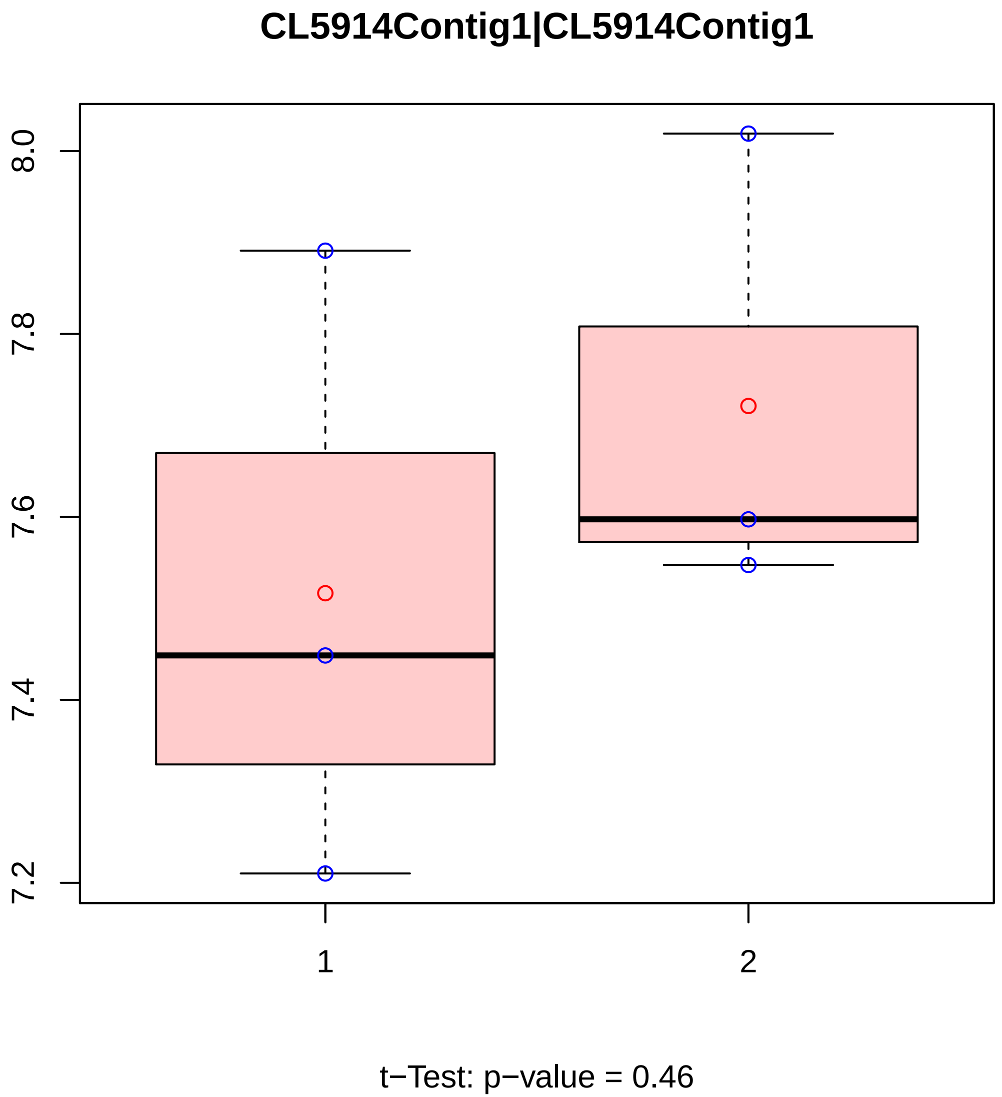

CL5914Contig1|CL5914Contig1
8.0
7.8
7.6
7.4
7.2
1
2
t−Test: p−value = 0.46

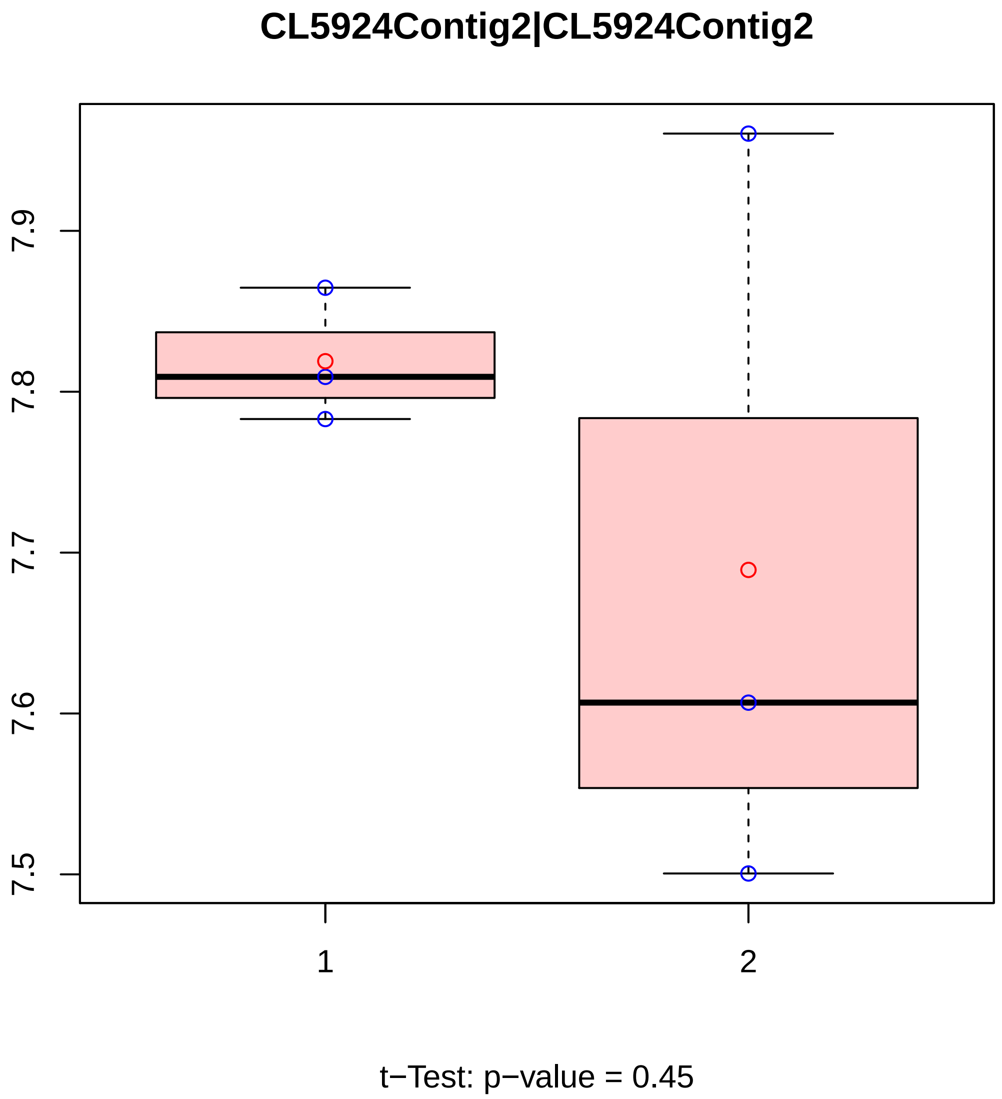

CL5924Contig2|CL5924Contig2
7.5
7.6
7.7
7.8
7.9
1
2
t−Test: p−value = 0.45

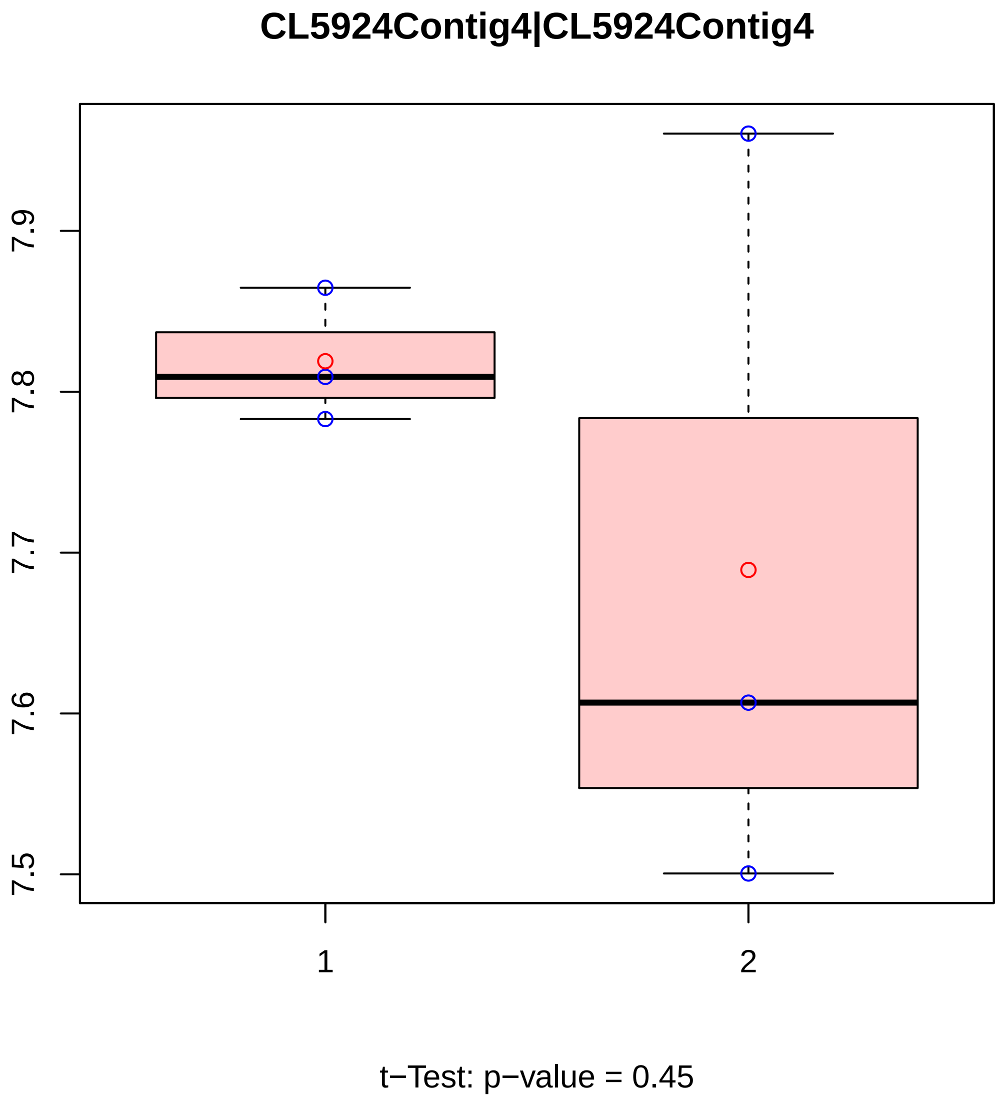
CL5924Contig4|CL5924Contig4
7.5
7.6
7.7
7.8
7.9
1
2
t−Test: p−value = 0.45

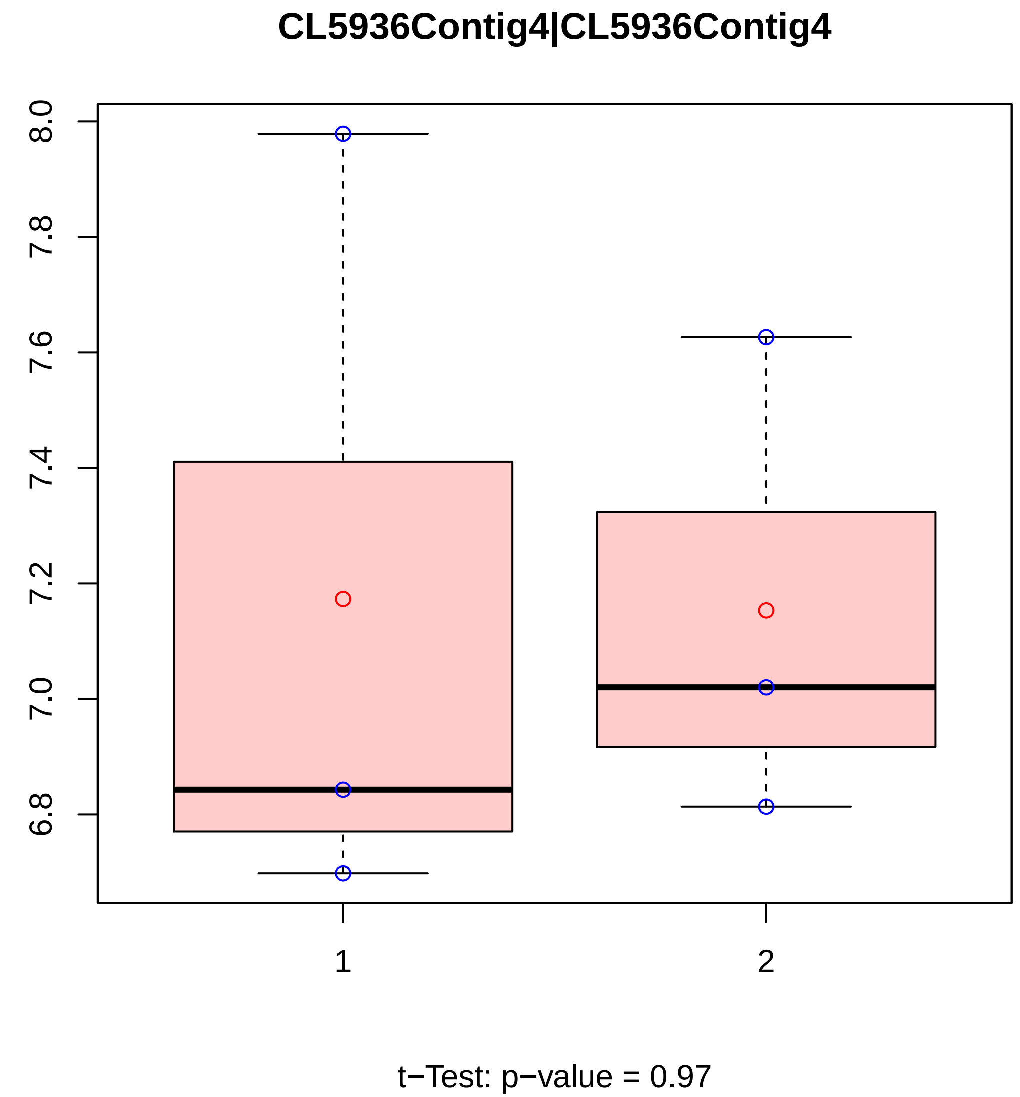

CL5936Contig4|CL5936Contig4
8.0
7.8
7.6
7.4
7.2
7.0
6.8
1
2
t−Test: p−value = 0.97

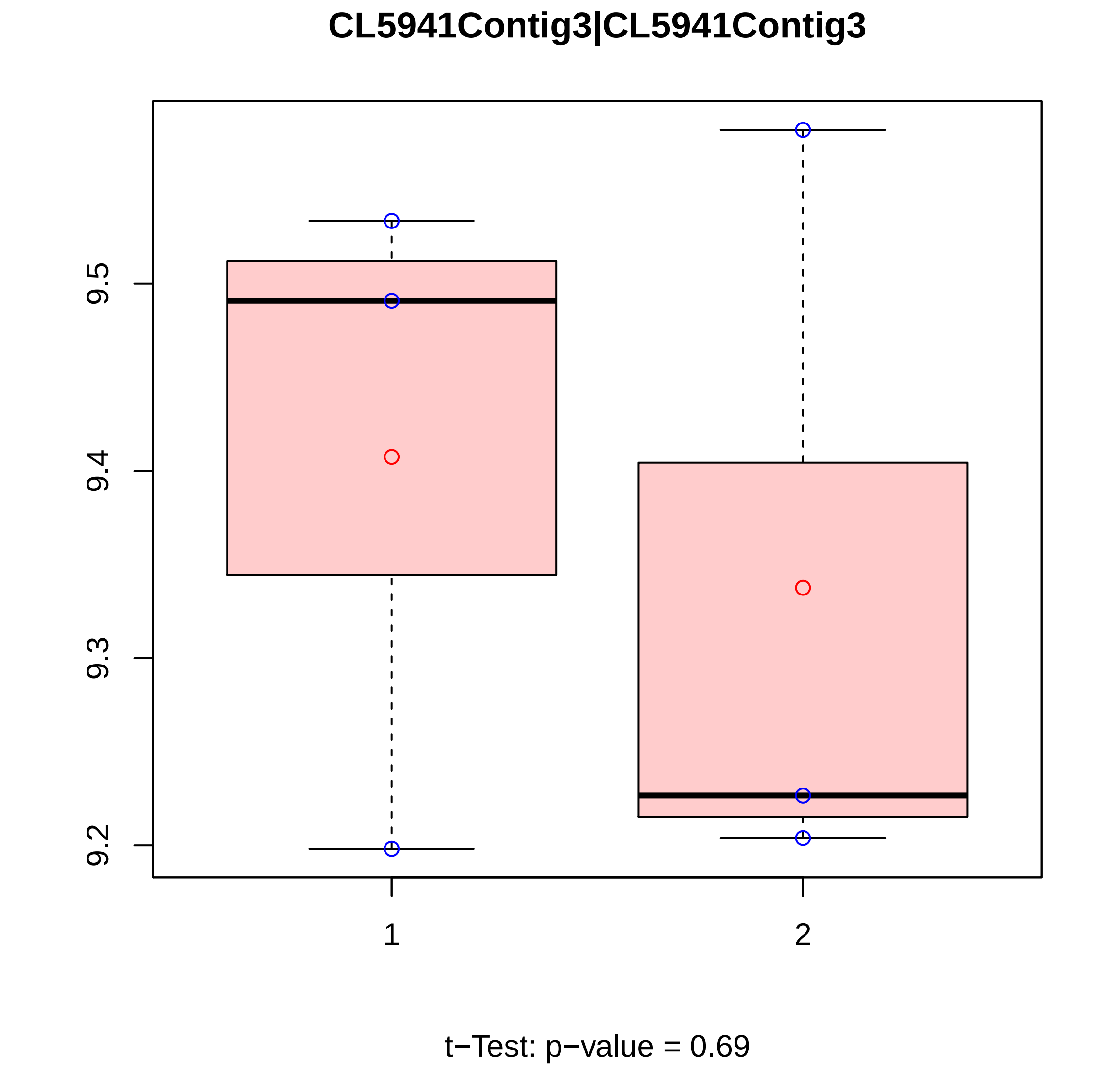

CL5941Contig3|CL5941Contig3
9.2
9.3
9.4
9.5
1
2
t−Test: p−value = 0.69

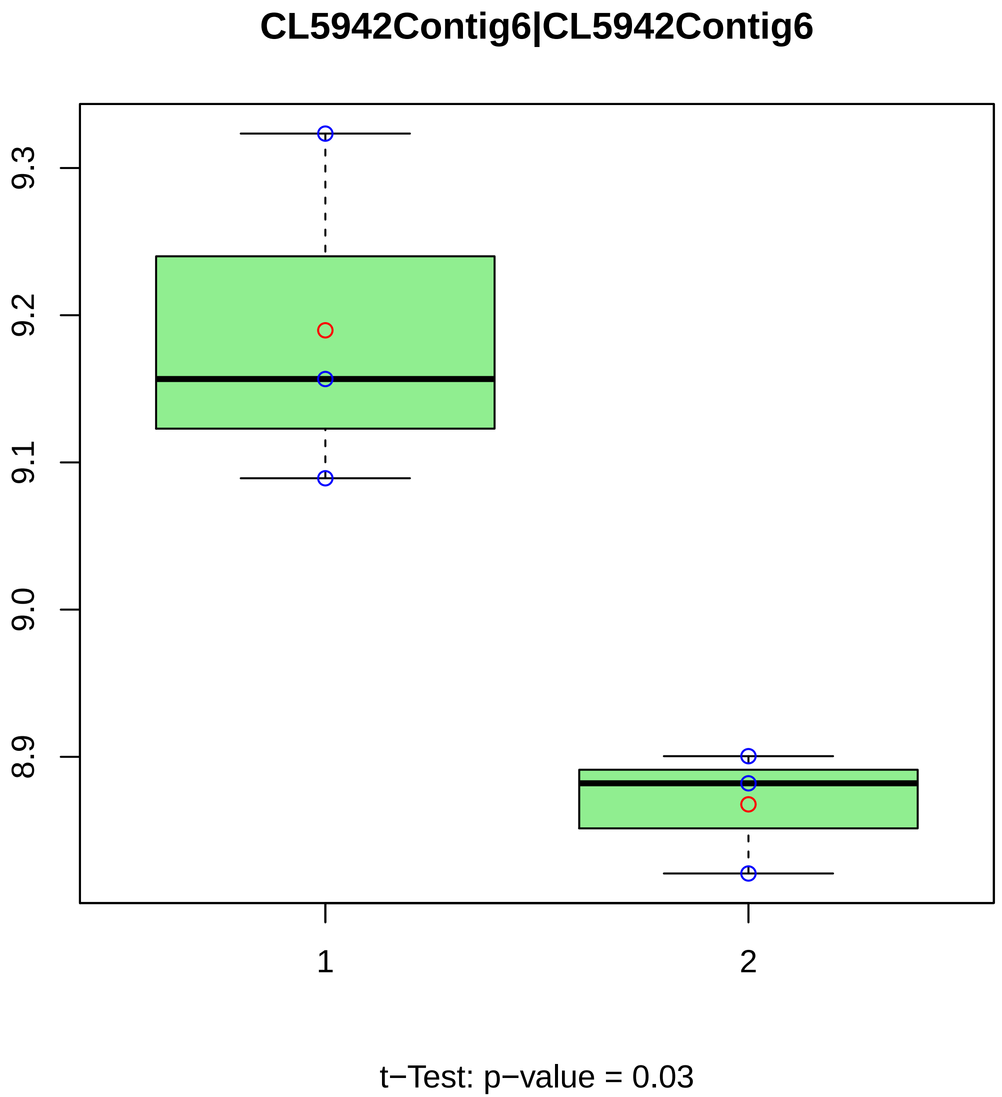
CL5942Contig6|CL5942Contig6
9.3
9.2
9.1
9.0
8.9
1
2
t−Test: p−value = 0.03

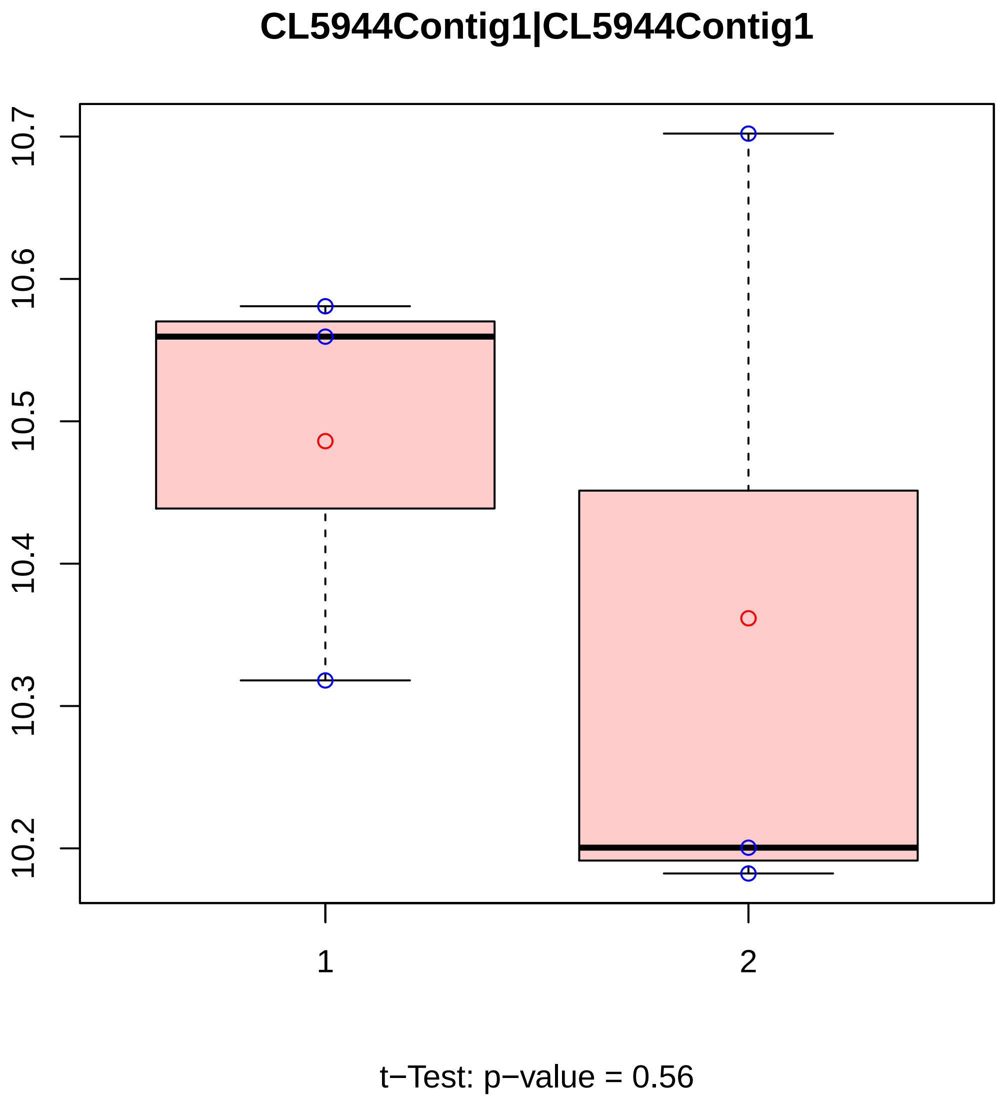

CL5944Contig1|CL5944Contig1
10.7
10.6
10.5
10.4
10.3
10.2
1
2
t−Test: p−value = 0.56

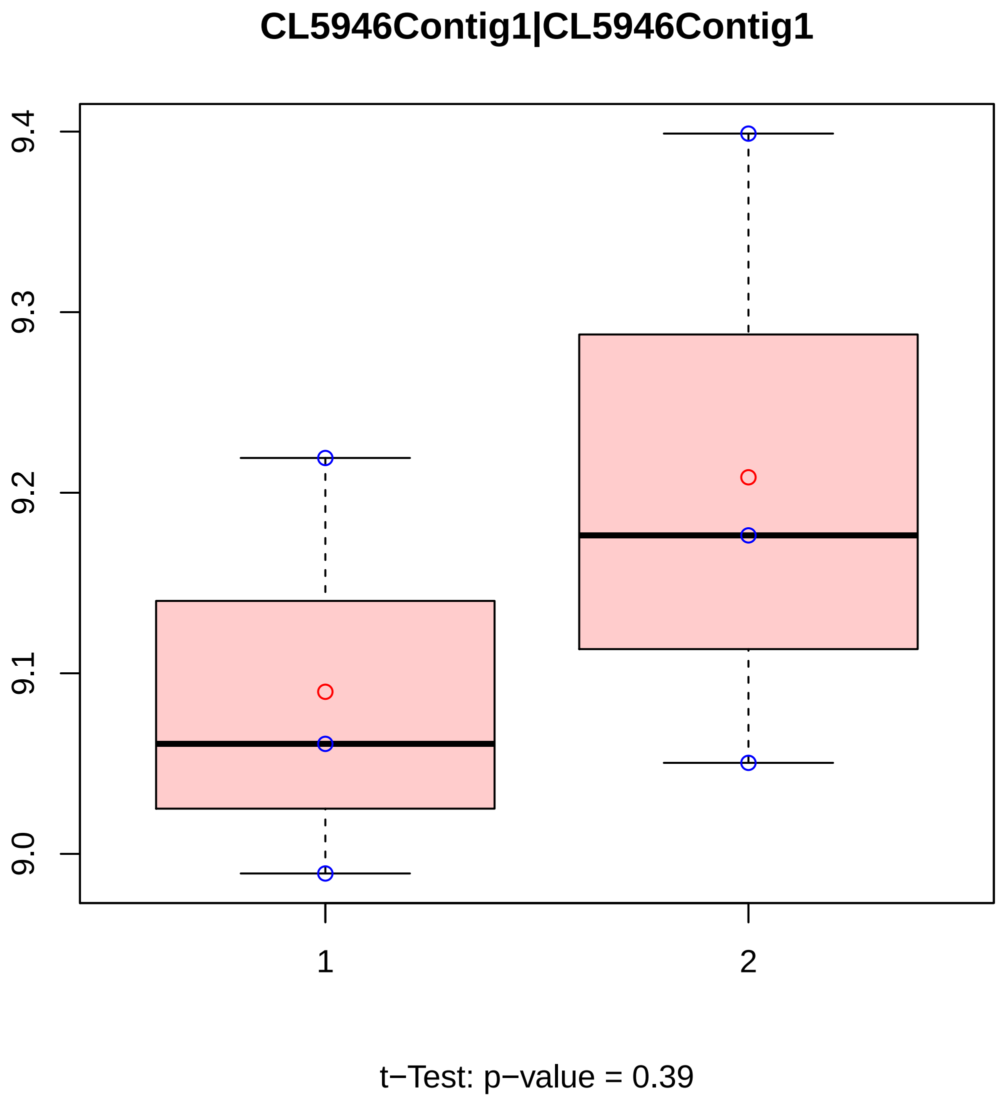
CL5946Contig1|CL5946Contig1
9.0
9.1
9.2
9.3
9.4
1
2
t−Test: p−value = 0.39

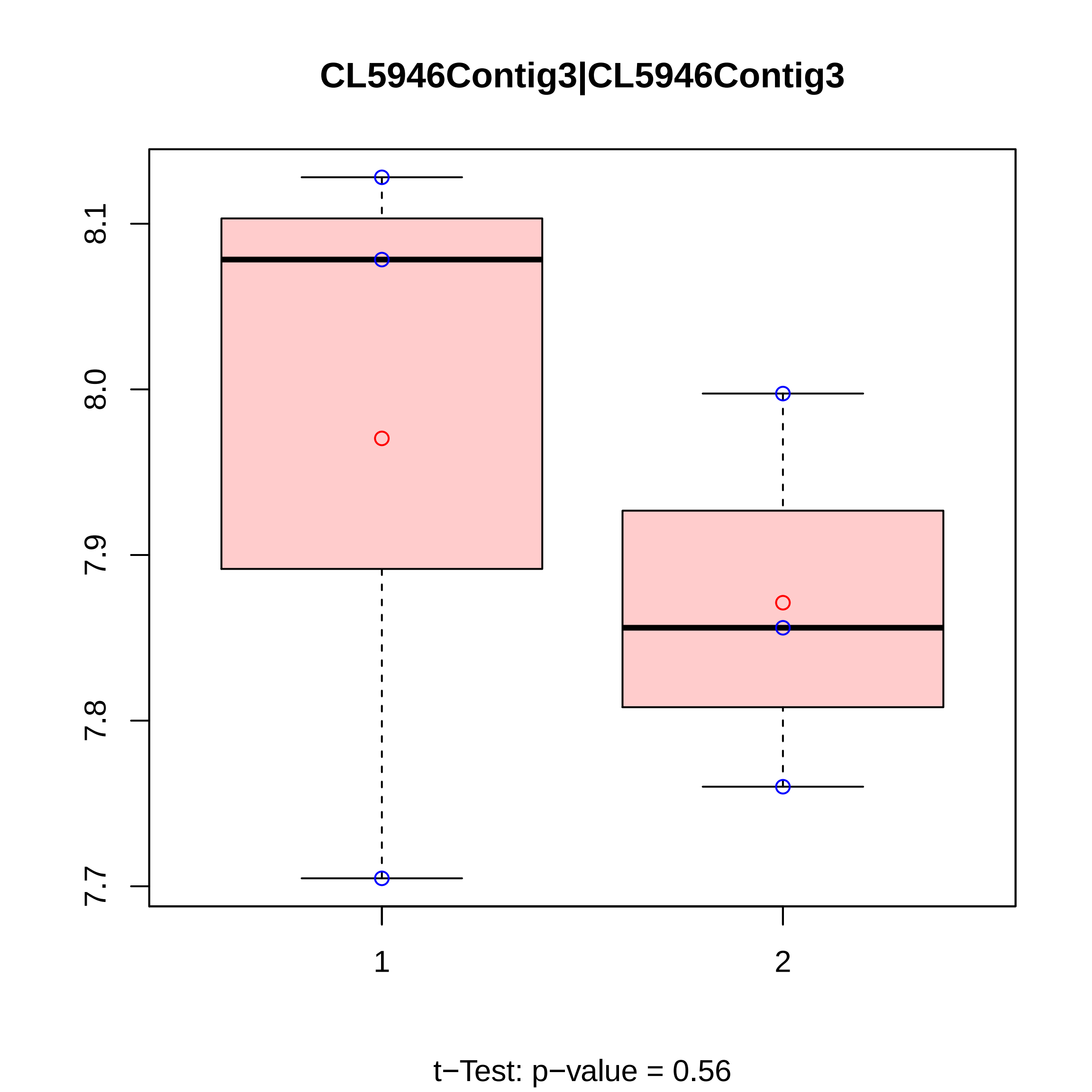

CL5946Contig3|CL5946Contig3
8.1
8.0
7.9
7.8
7.7
1
2
t−Test: p−value = 0.56

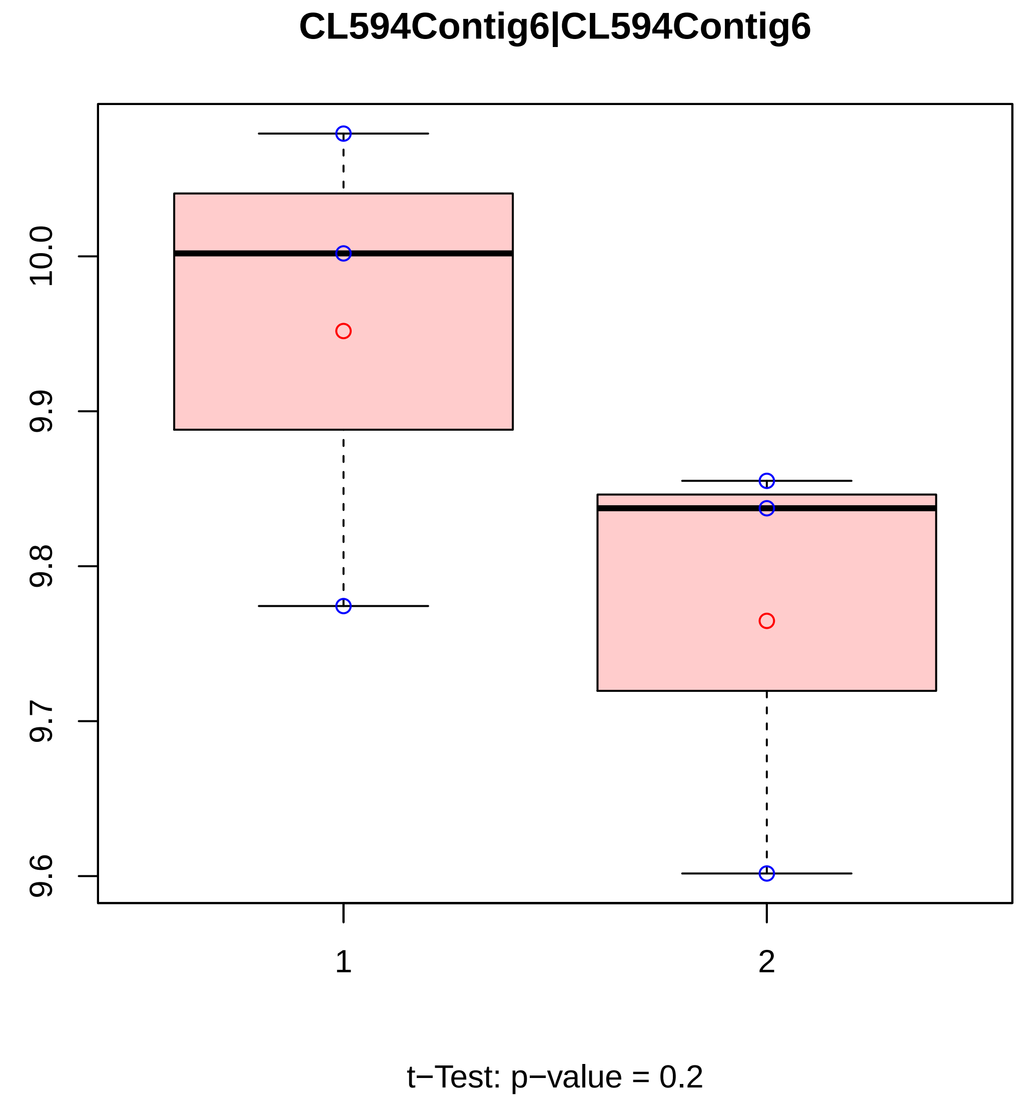
CL594Contig6|CL594Contig6
10.0
9.9
9.8
9.7
9.6
1
2
t−Test: p−value = 0.2

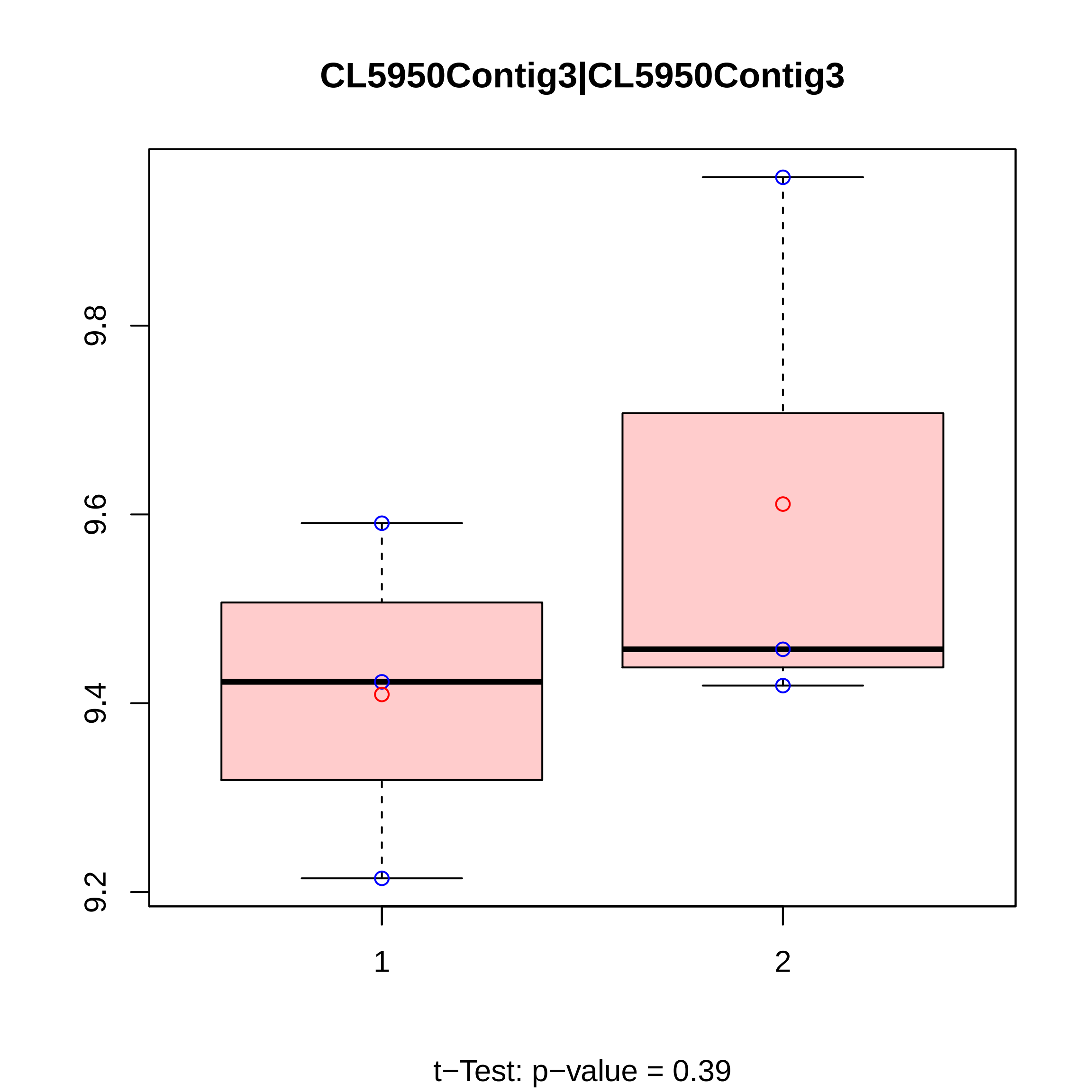

CL5950Contig3|CL5950Contig3
9.2
9.4
9.6
9.8
1
2
t−Test: p−value = 0.39

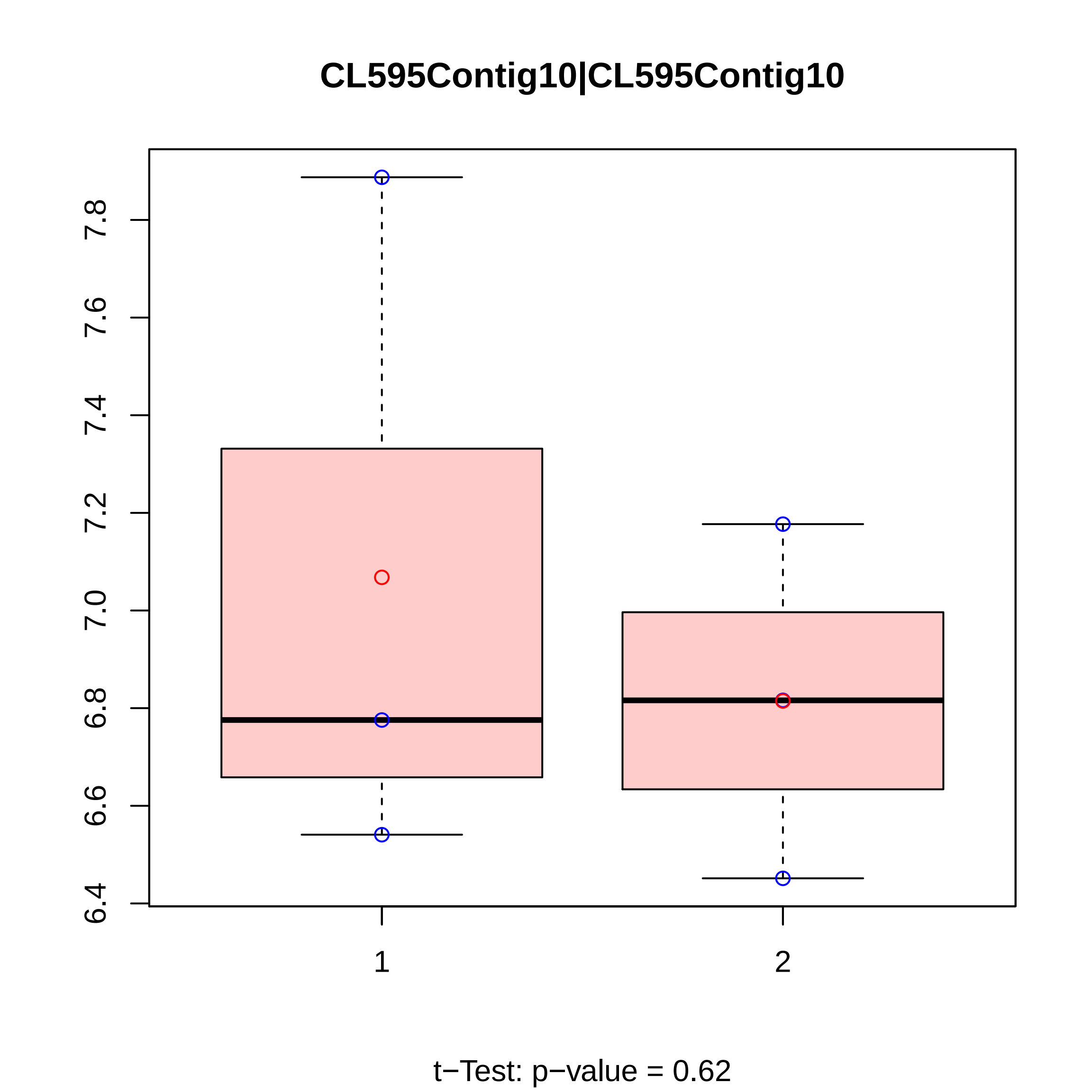
CL595Contig10|CL595Contig10
7.8
7.6
7.4
7.2
7.0
6.8
6.6
6.4
1
2
t−Test: p−value = 0.62

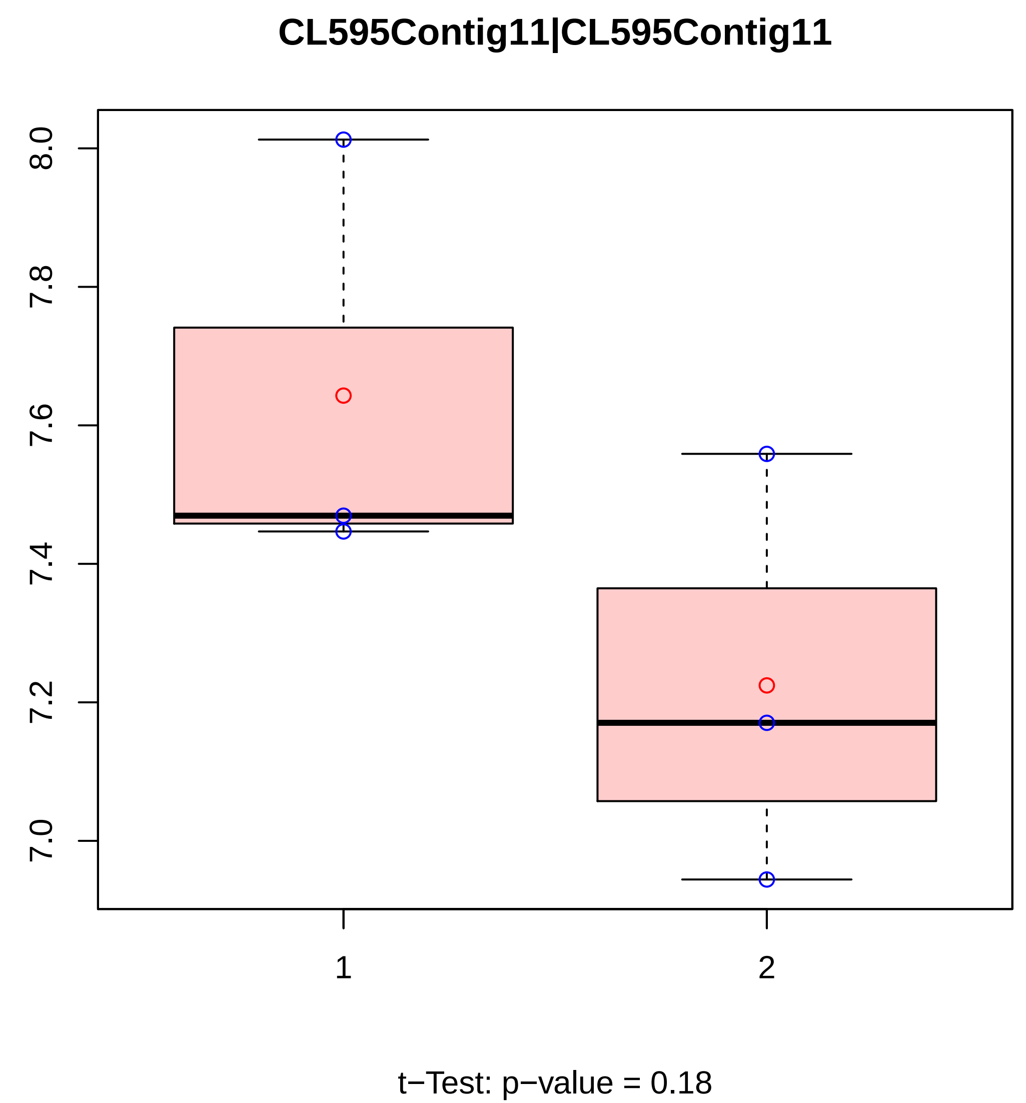

CL595Contig11|CL595Contig11
8.0
7.8
7.6
7.4
7.2
7.0
1
2
t−Test: p−value = 0.18

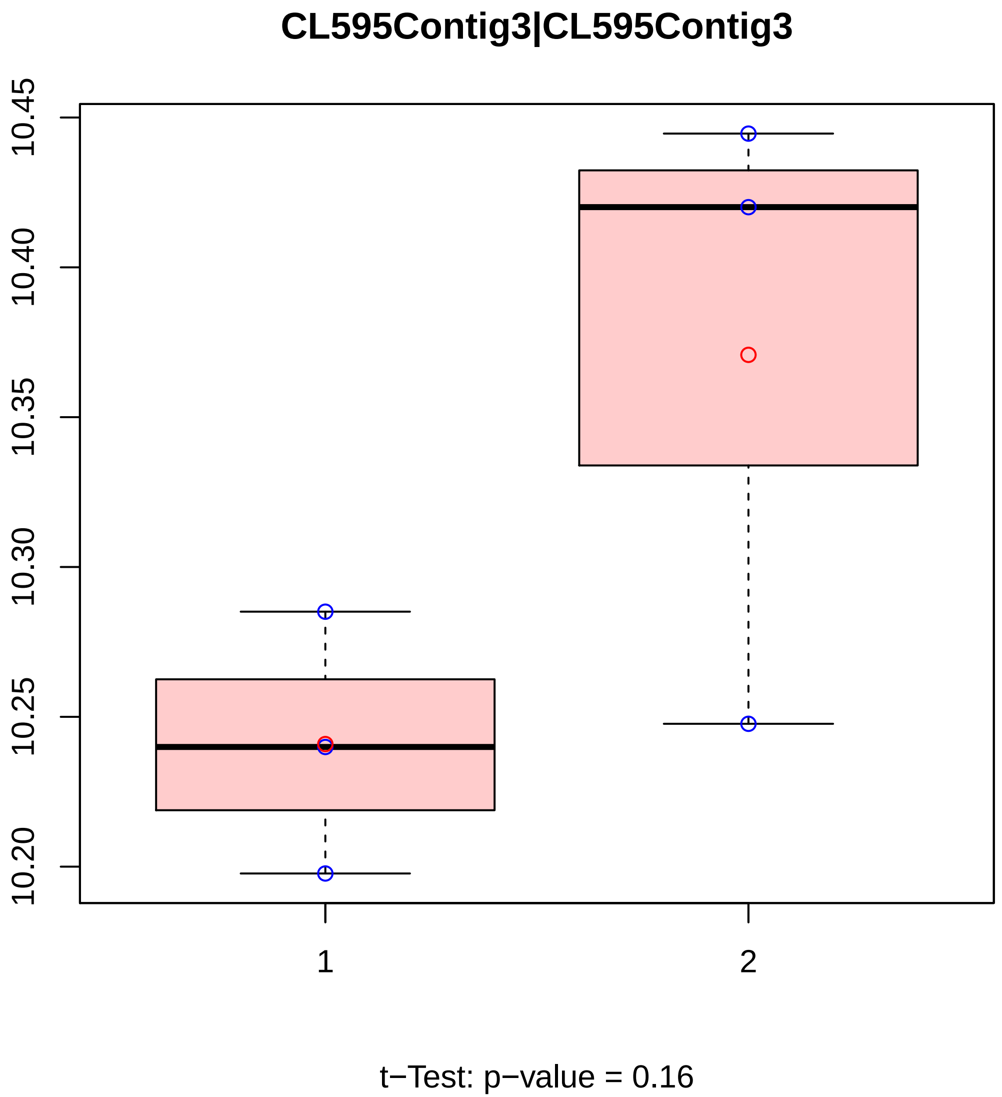
CL595Contig3|CL595Contig3
10.45
10.40
10.35
10.30
10.25
10.20
1
2
t−Test: p−value = 0.16

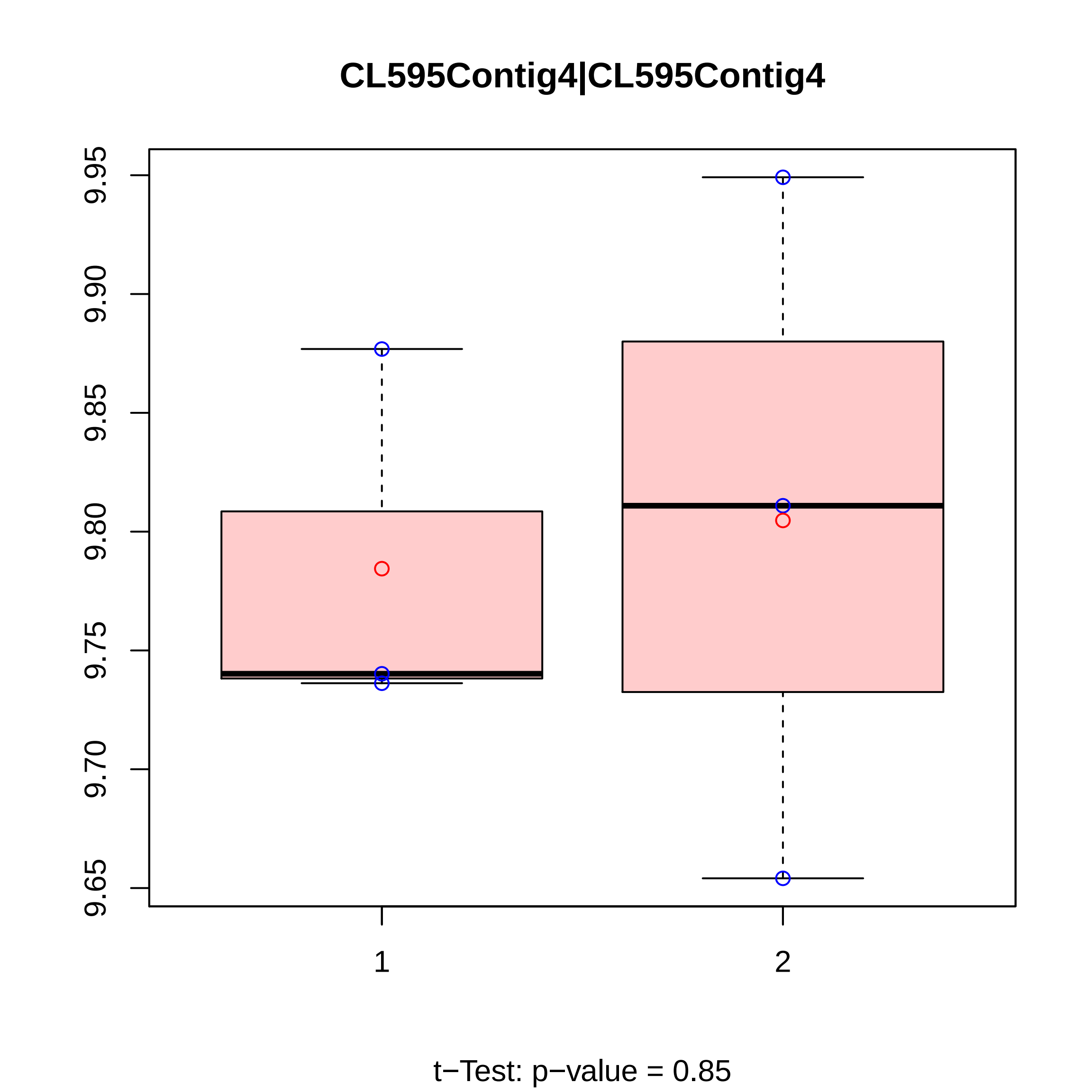
CL595Contig4|CL595Contig4
9.65
9.70
9.75
9.80
9.85
9.90
9.95
1
2
t−Test: p−value = 0.85

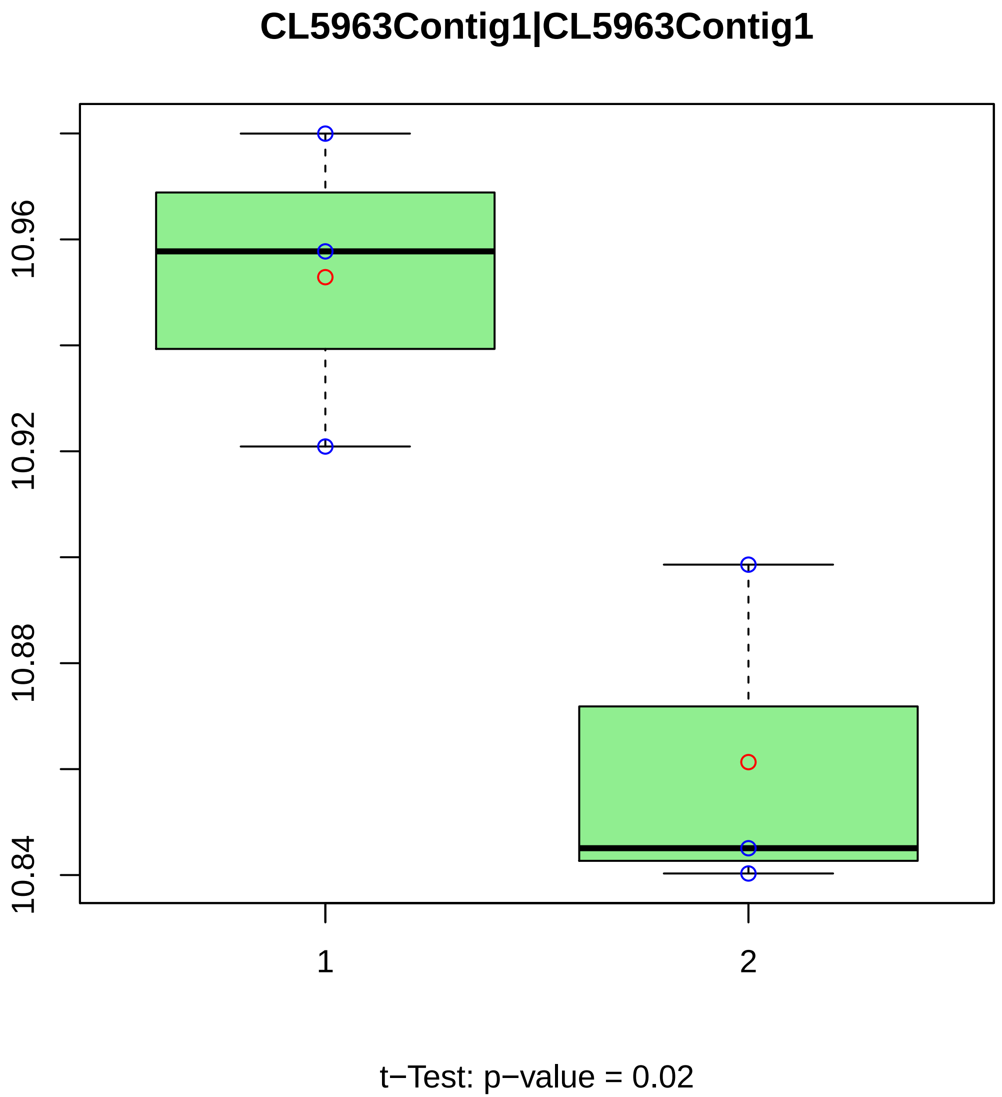

CL5963Contig1|CL5963Contig1
10.84
10.88
10.92
10.96
1
2
t−Test: p−value = 0.02

CL5968Contig1|CL5968Contig1

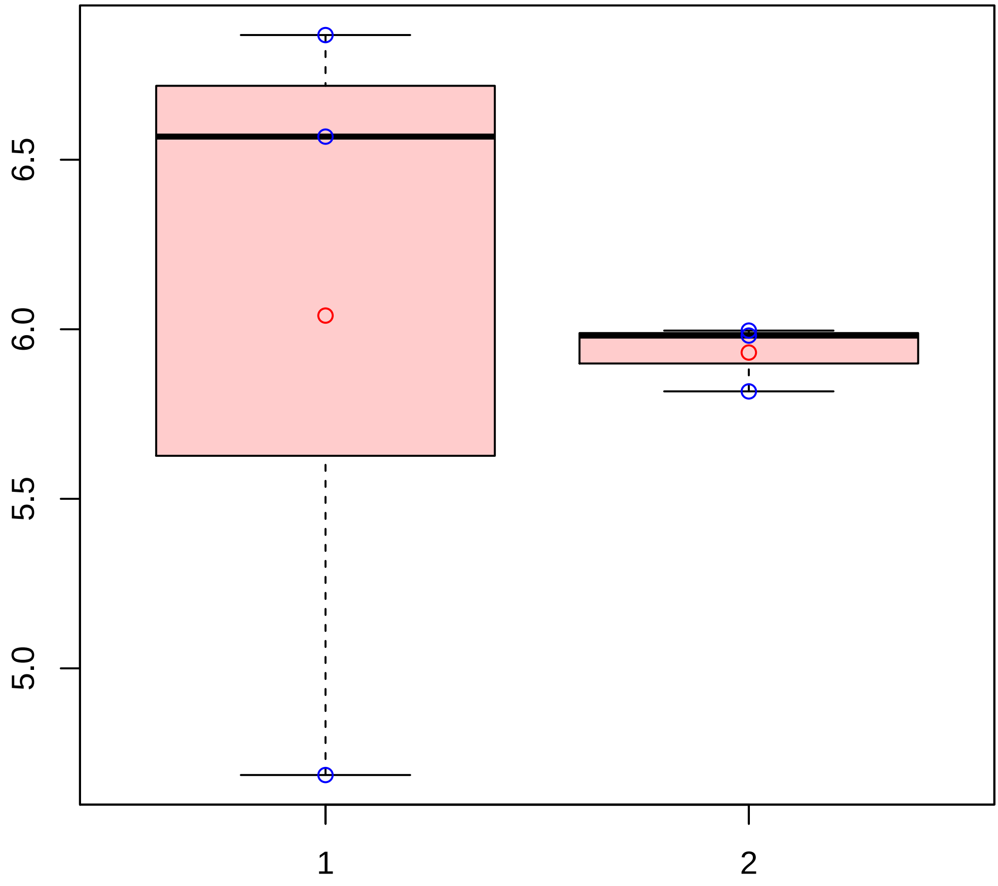


t−Test: p−value = 0.89

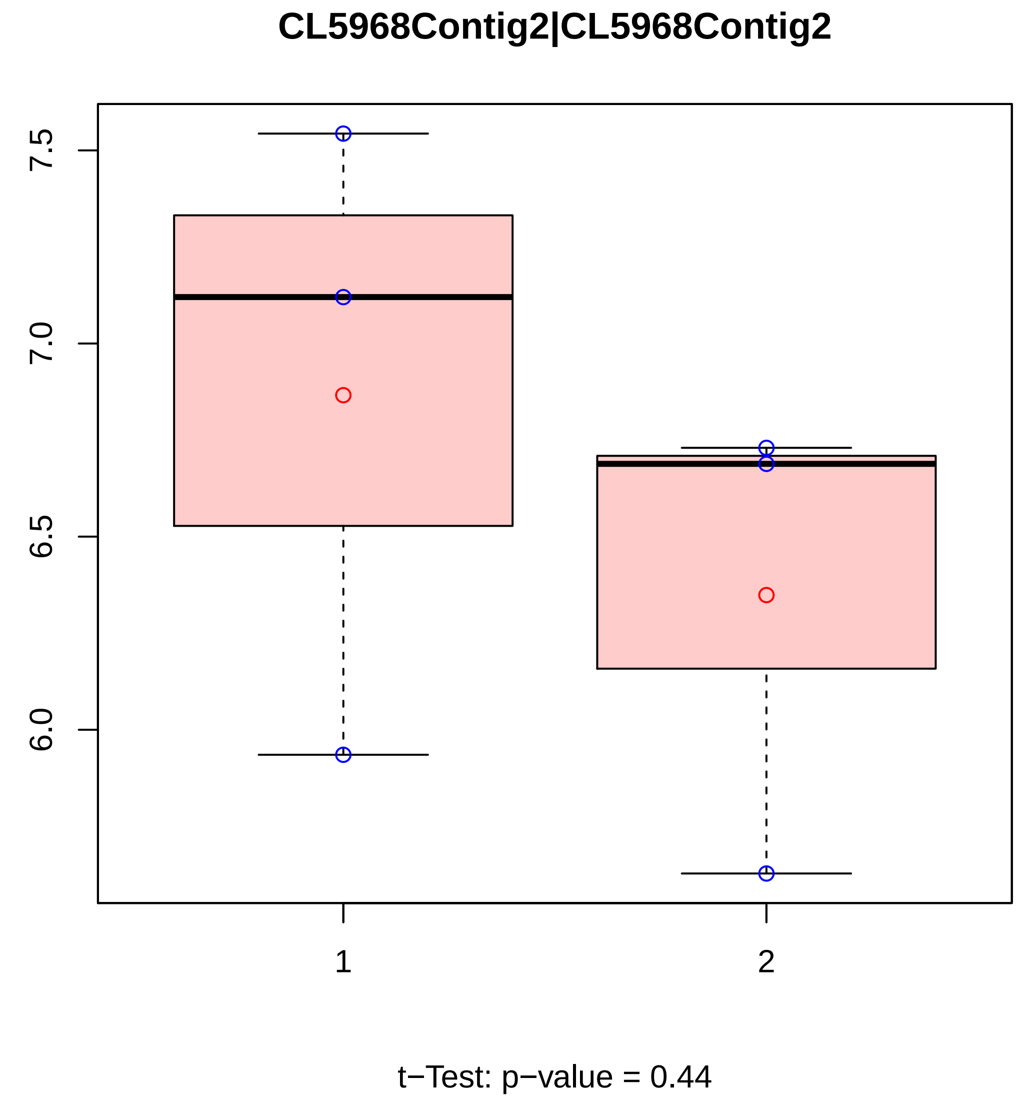

CL5968Contig2|CL5968Contig2
7.5
7.0
6.5
6.0
1
2
t−Test: p−value = 0.44

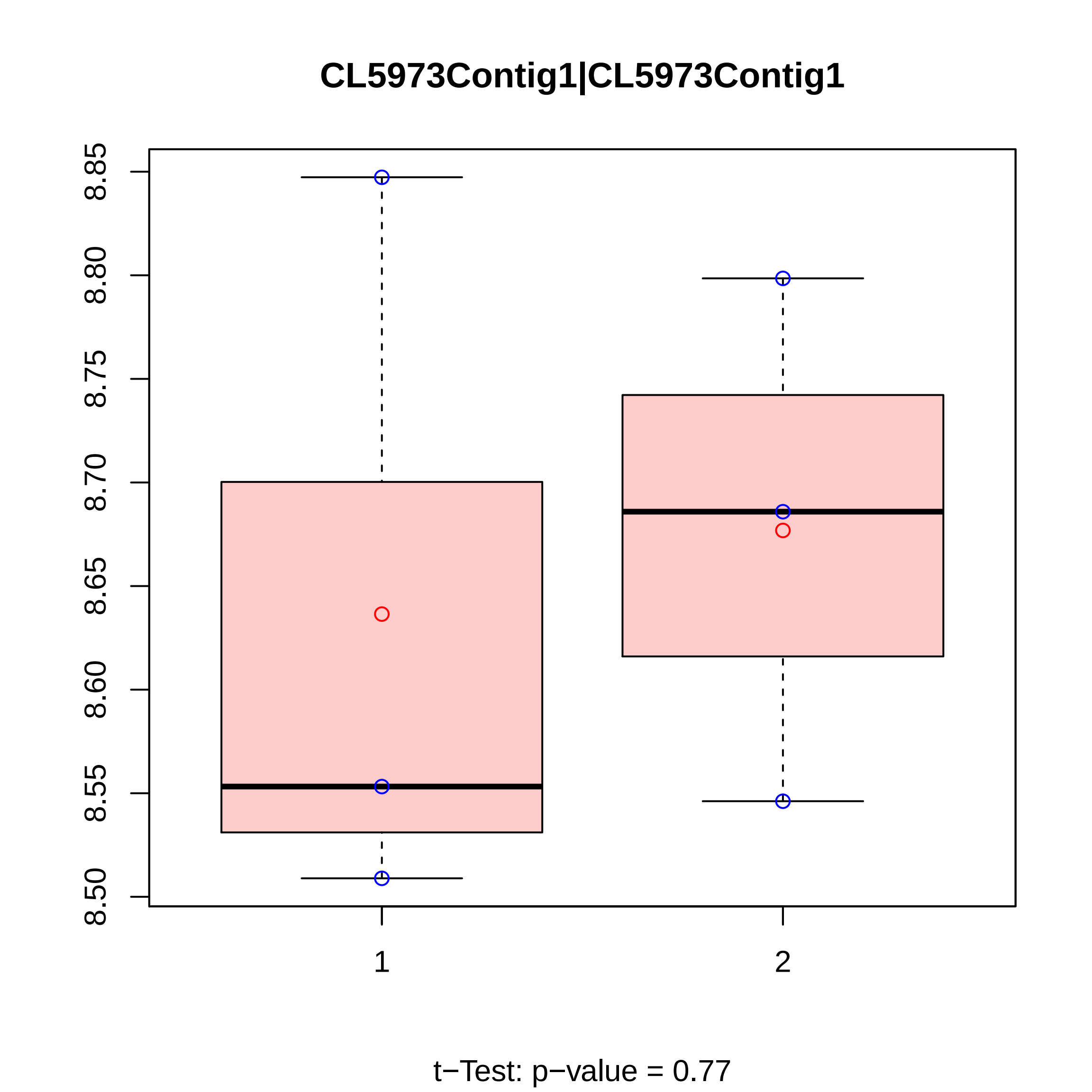

CL5973Contig1|CL5973Contig1
8.50
8.55
8.60
8.65
8.70
8.75
8.80
8.85
1
2
t−Test: p−value = 0.77

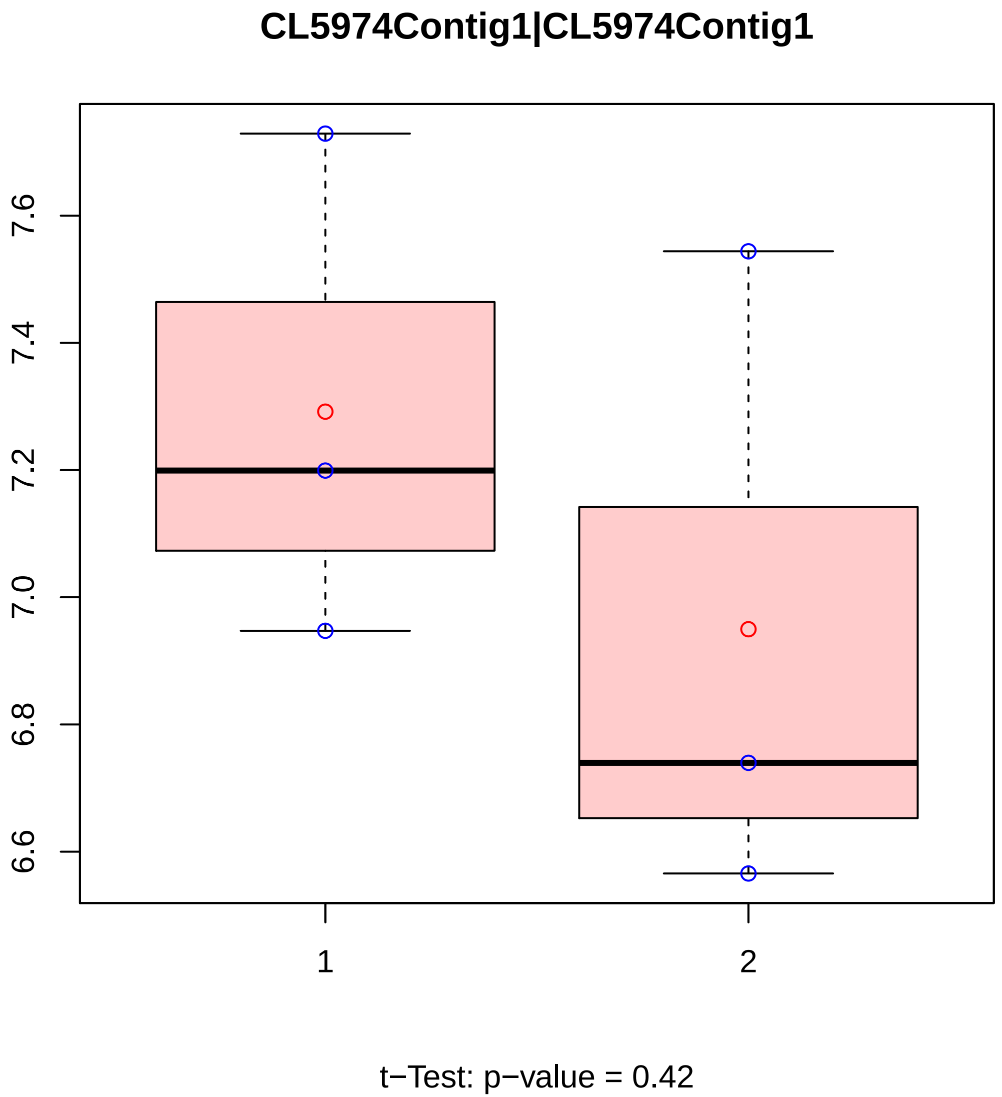
CL5974Contig1|CL5974Contig1
7.6
7.4
7.2
7.0
6.8
6.6
1
2
t−Test: p−value = 0.42

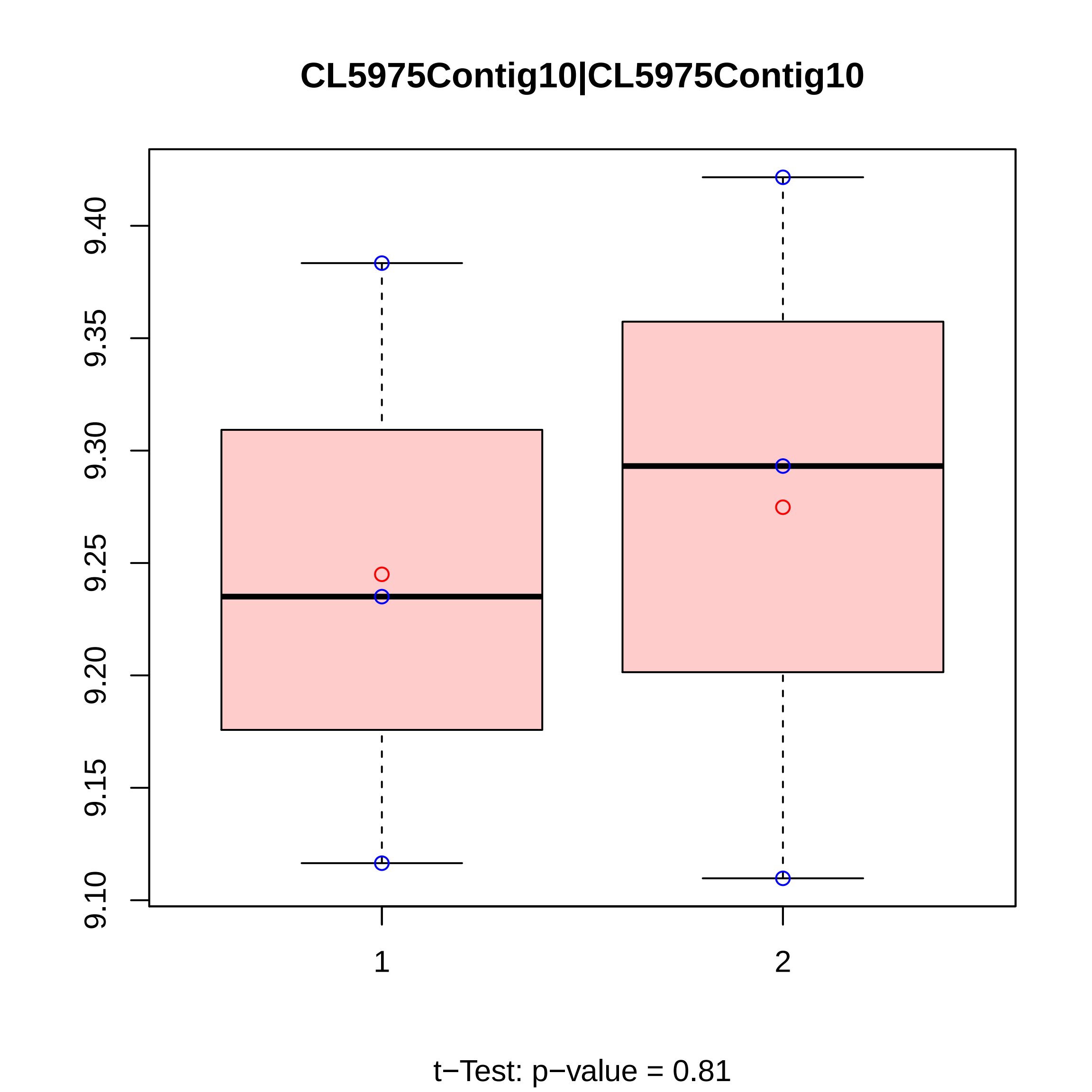
CL5975Contig10|CL5975Contig10
9.10
9.15
9.20
9.25
9.30
9.35
9.40
1
2
t−Test: p−value = 0.81

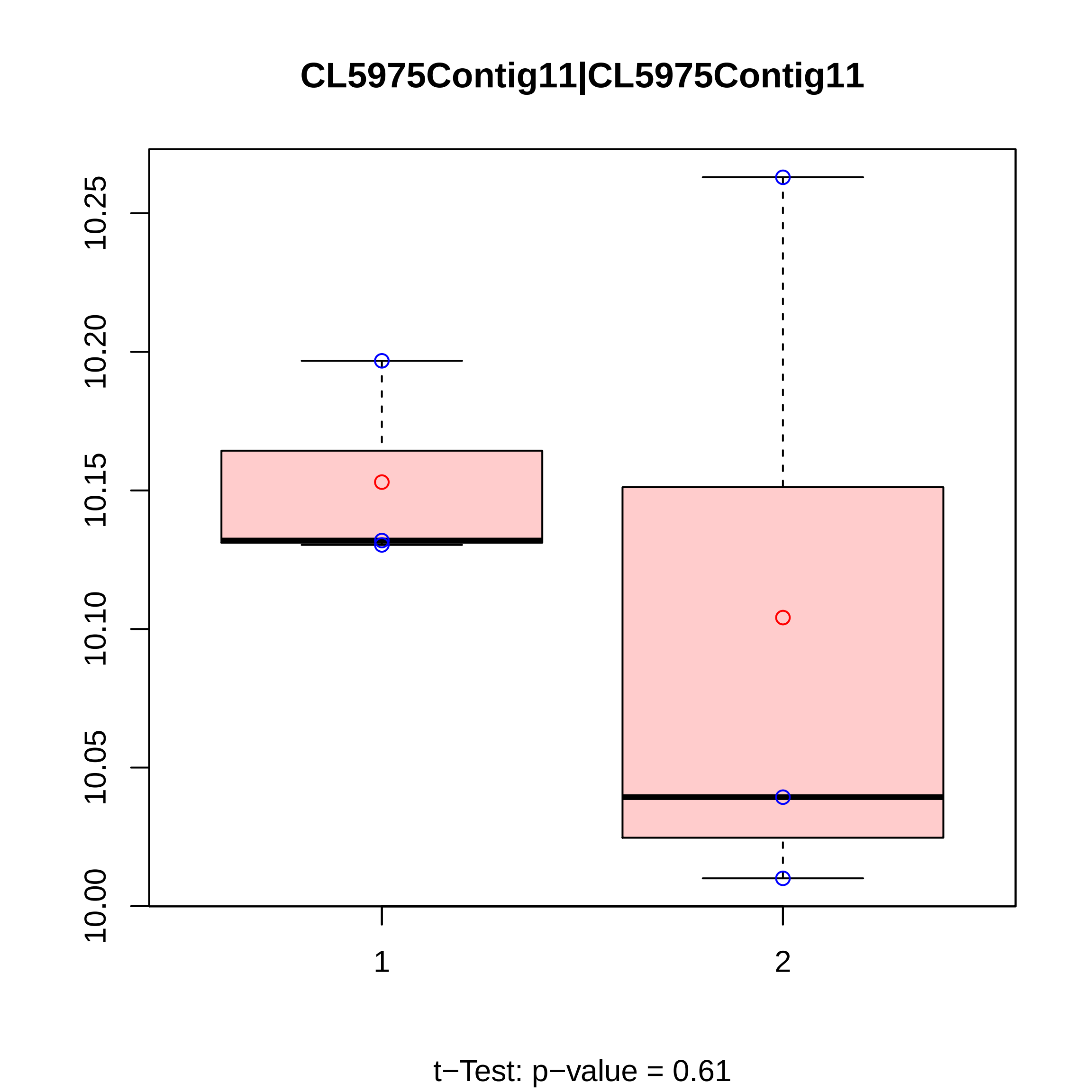

CL5975Contig11|CL5975Contig11
10.00
10.05
10.10
10.15
10.20
10.25
1
2
t−Test: p−value = 0.61

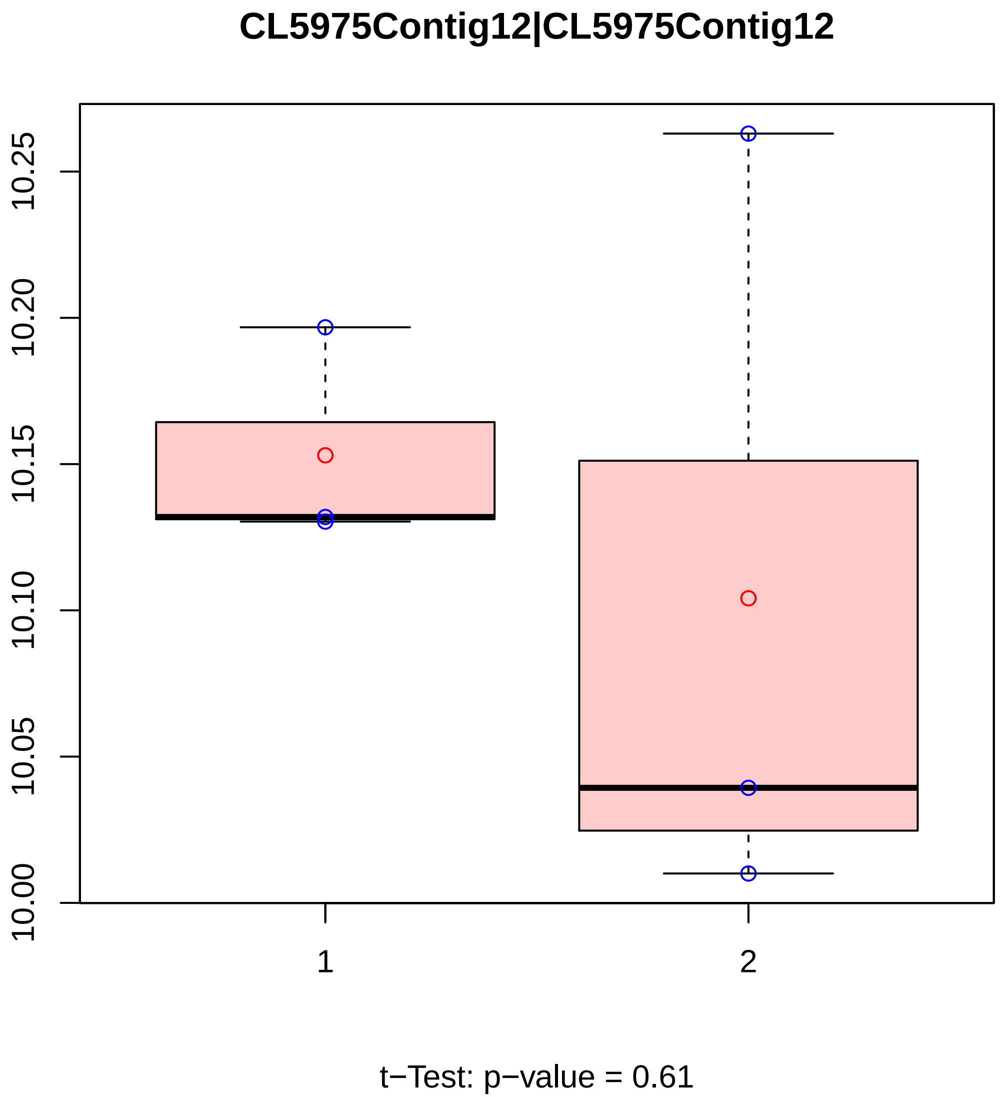

CL5975Contig12|CL5975Contig12
10.00
10.05
10.10
10.15
10.20
10.25
1
2
t−Test: p−value = 0.61

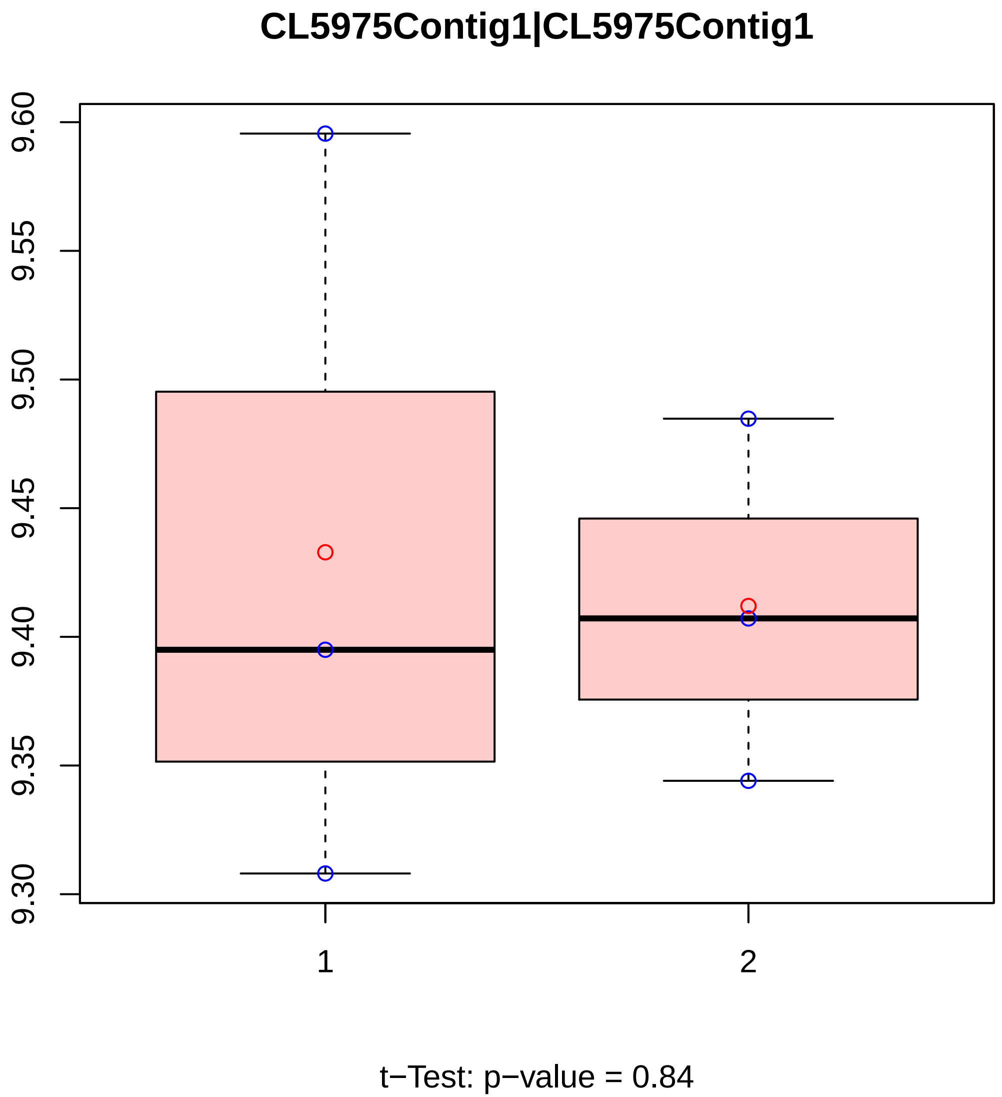
CL5975Contig1|CL5975Contig1
9.30
9.35
9.40
9.45
9.50
9.55
9.60
1
2
t−Test: p−value = 0.84

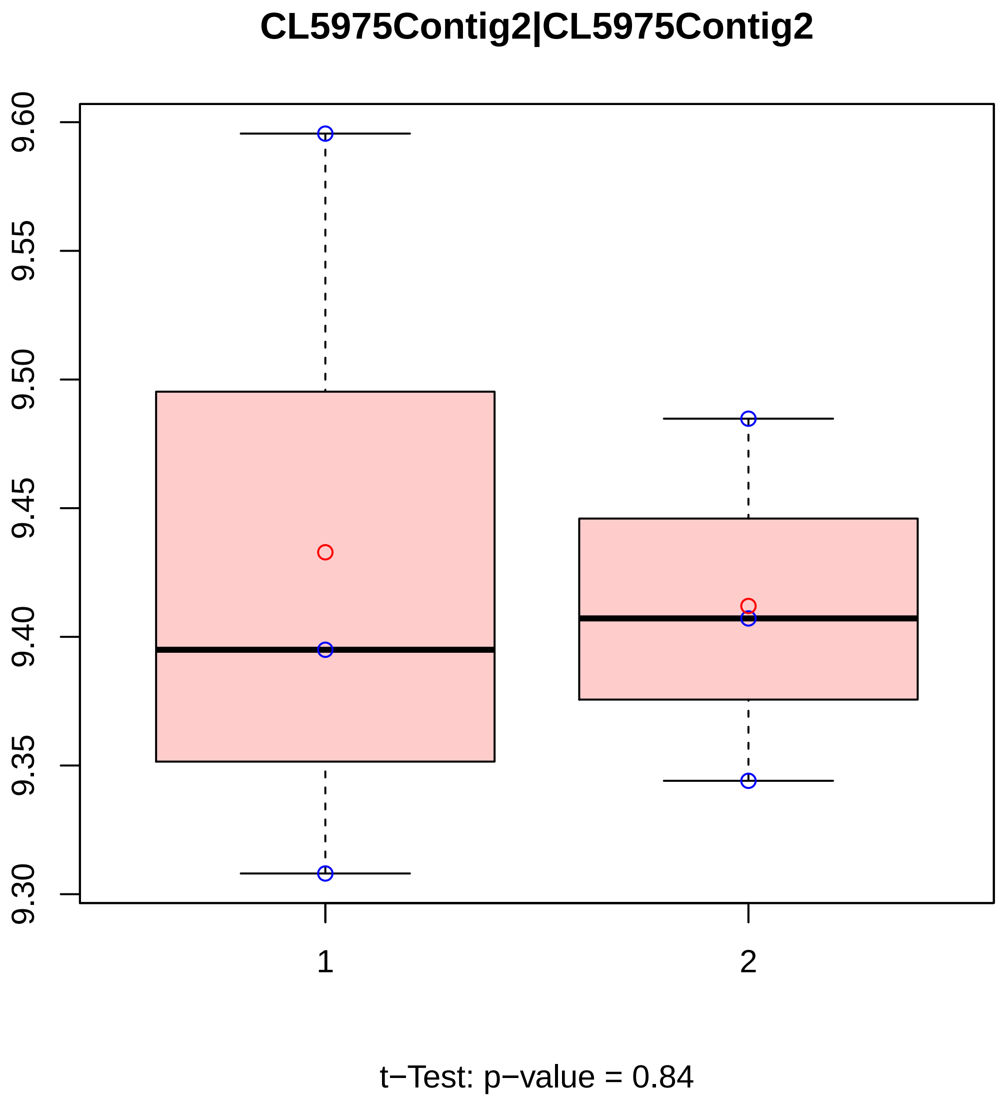

CL5975Contig2|CL5975Contig2
9.60
9.55
9.50
9.45
9.40
9.35
9.30
1
2
t−Test: p−value = 0.84

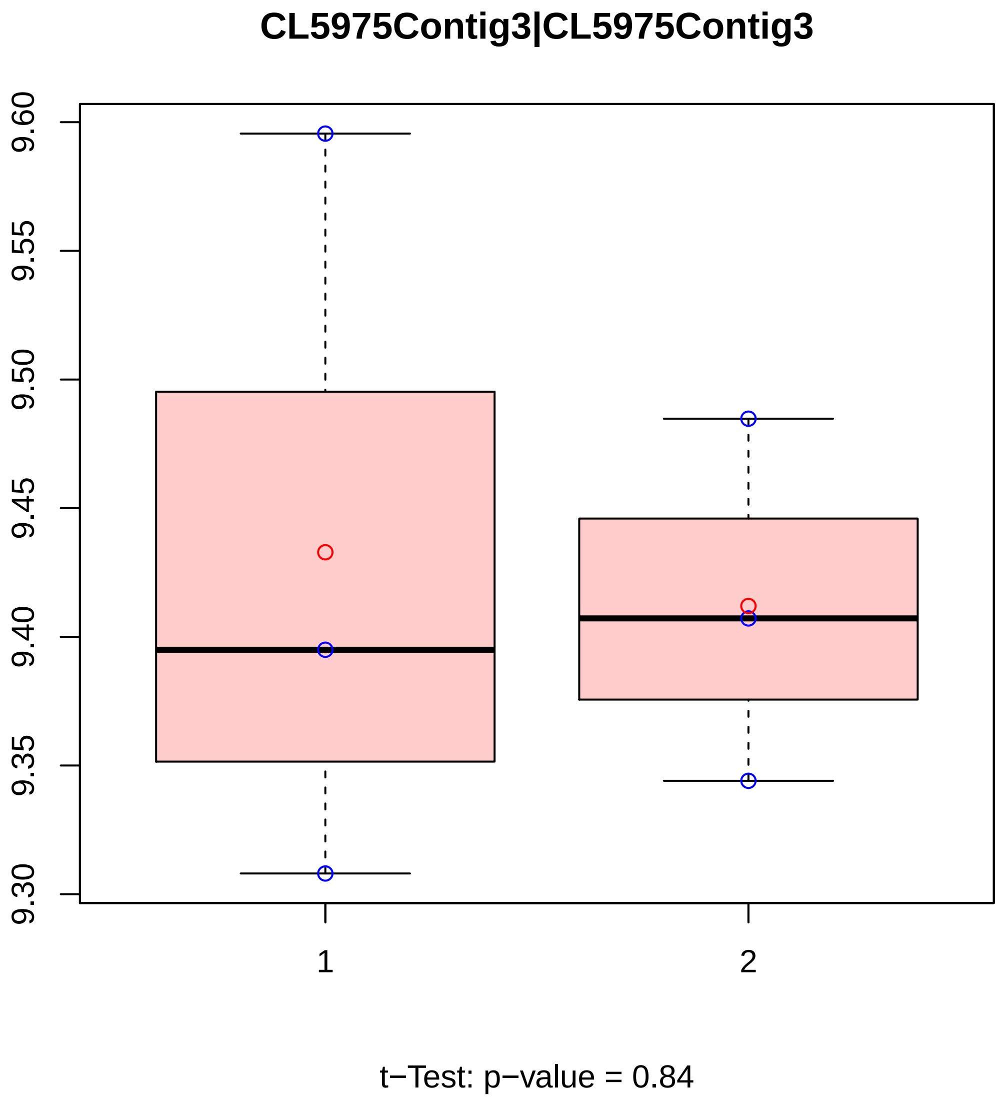

CL5975Contig3|CL5975Contig3
9.30
9.35
9.40
9.45
9.50
9.55
9.60
1
2
t−Test: p−value = 0.84

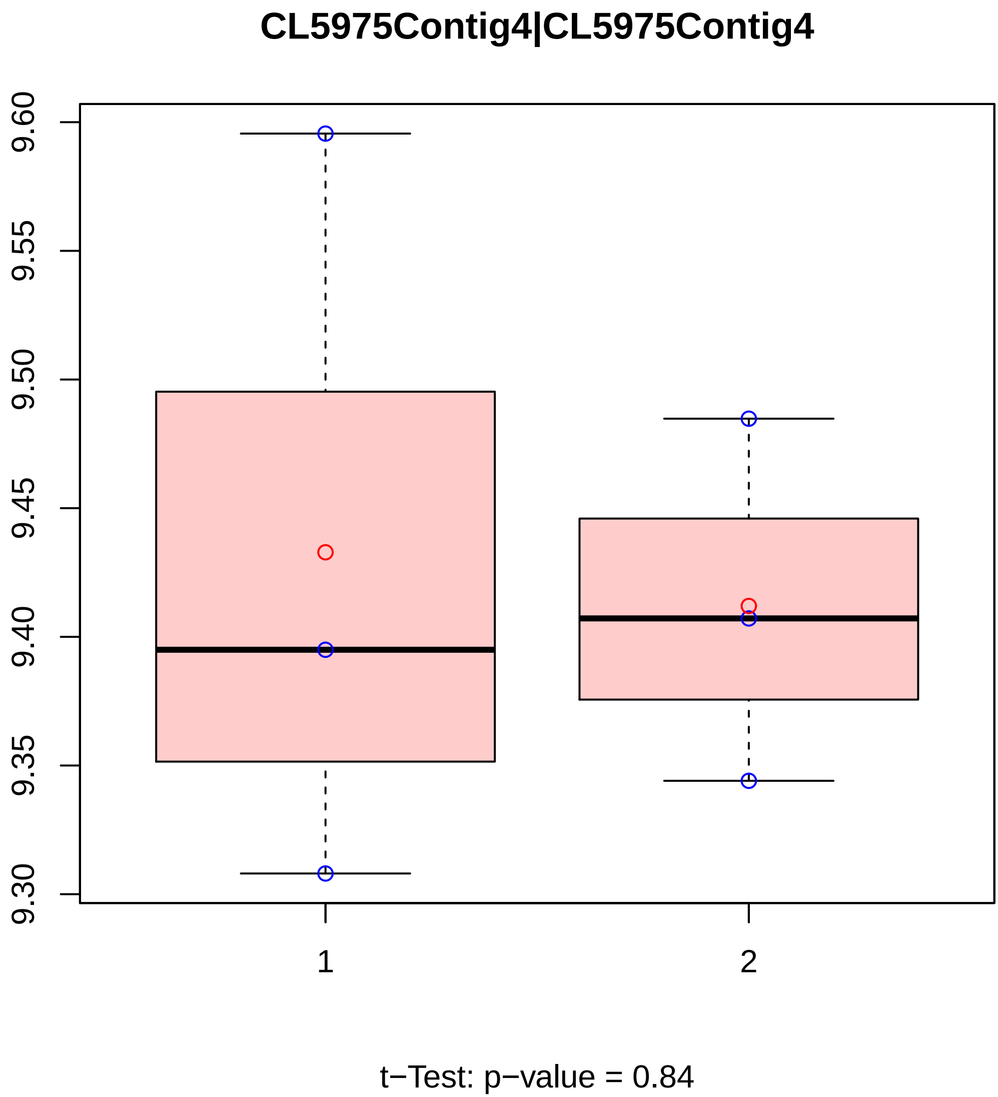
CL5975Contig4|CL5975Contig4
9.30
9.35
9.40
9.45
9.50
9.55
9.60
1
2
t−Test: p−value = 0.84

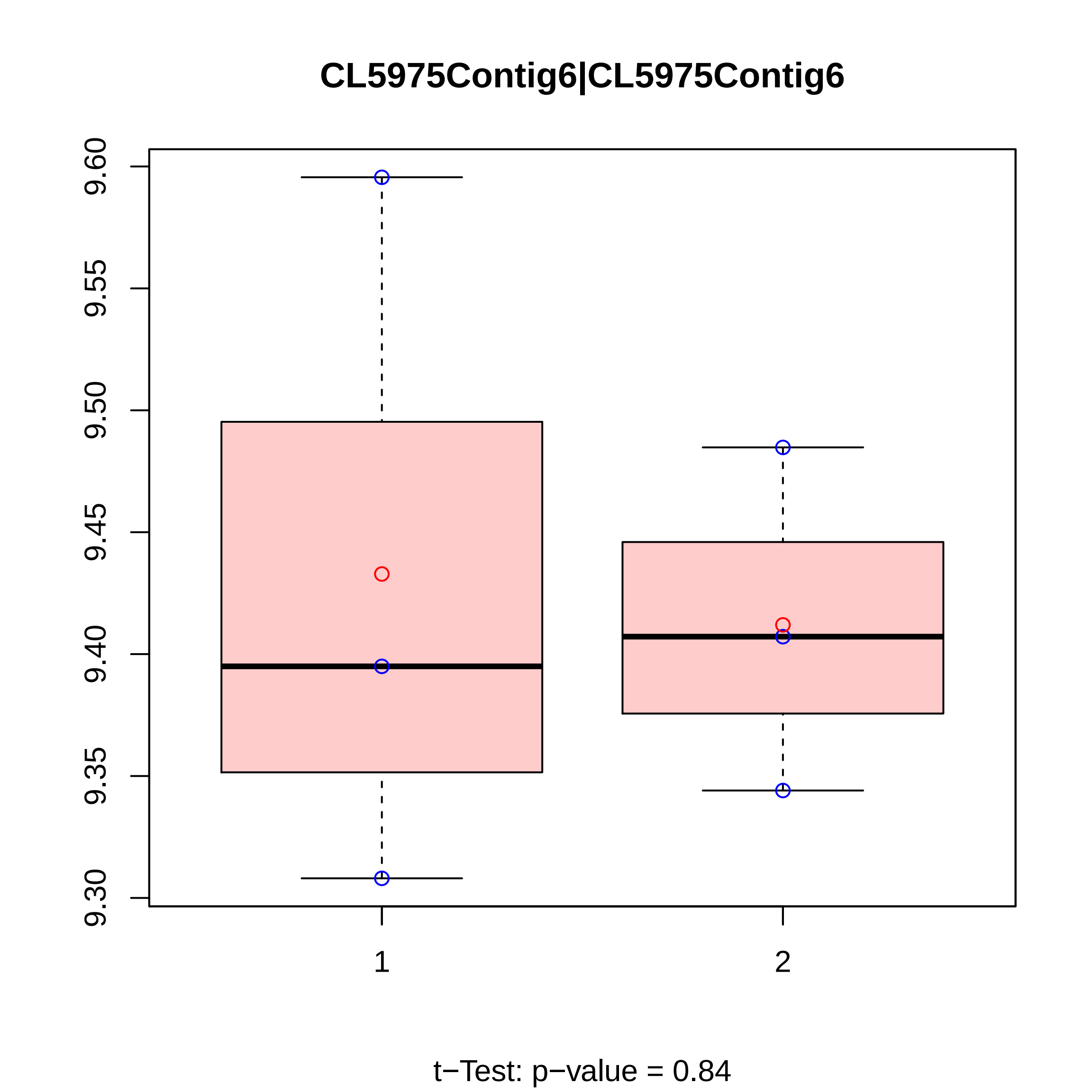
CL5975Contig6|CL5975Contig6
9.30
9.35
9.40
9.45
9.50
9.55
9.60
1
2
t−Test: p−value = 0.84

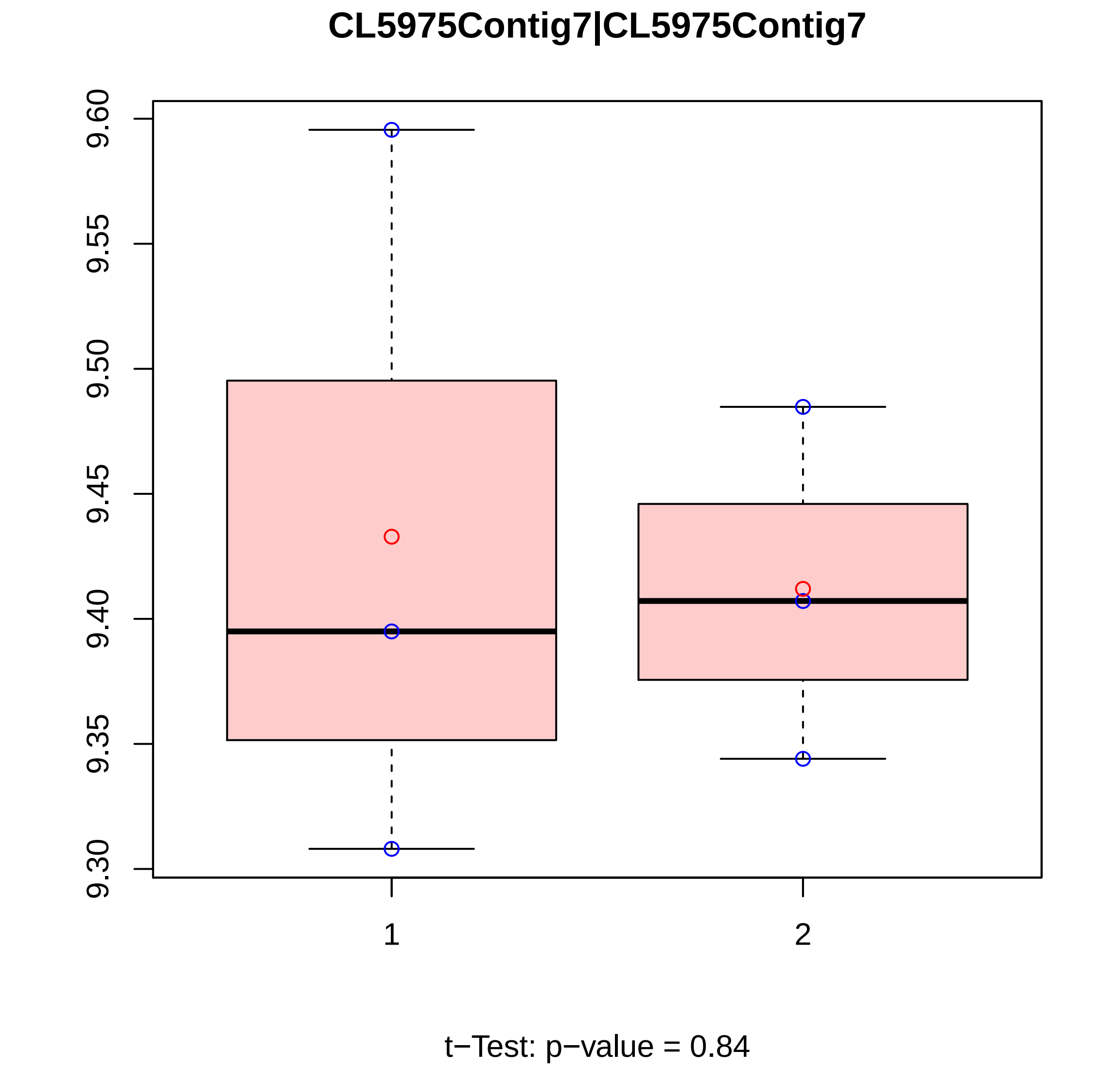

CL5975Contig7|CL5975Contig7
9.30
9.35
9.40
9.45
9.50
9.55
9.60
1
2
t−Test: p−value = 0.84

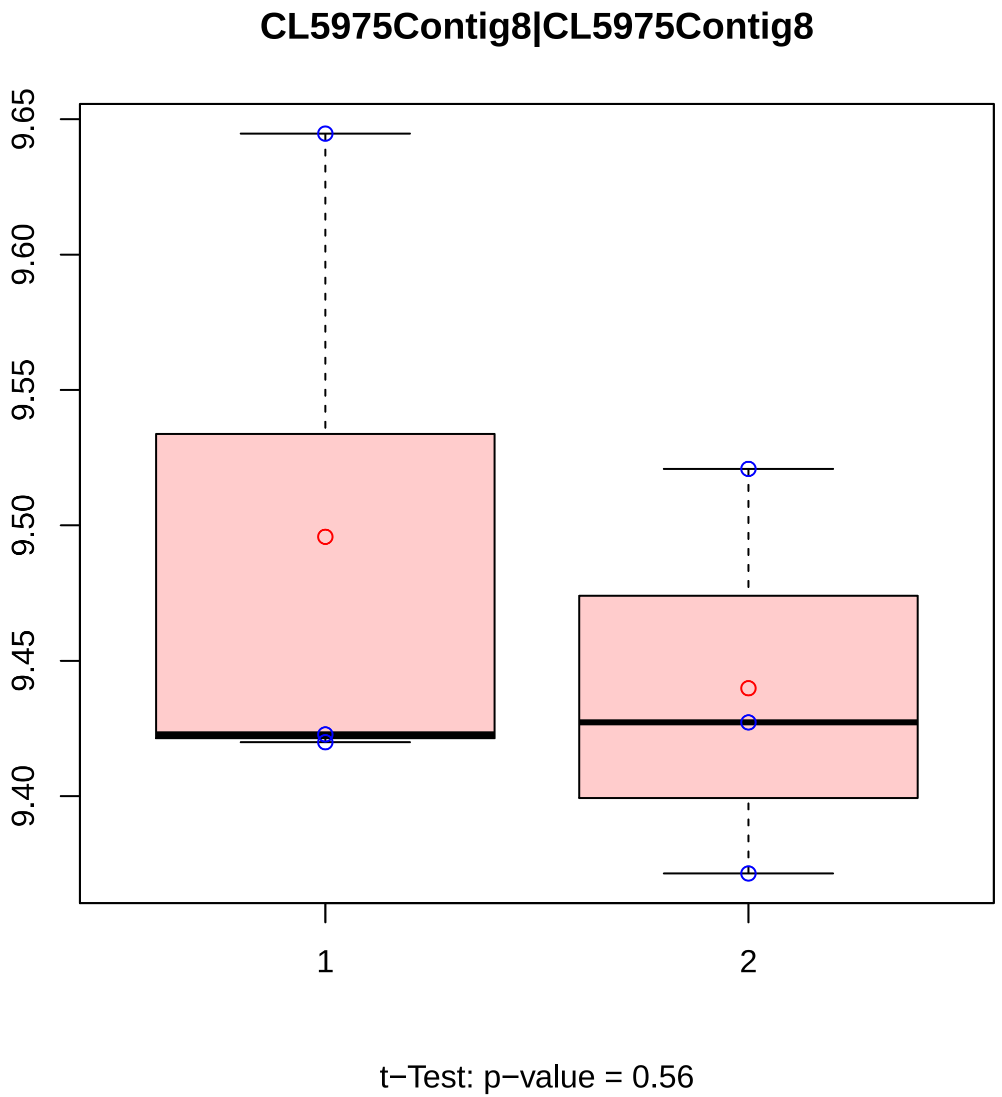

CL5975Contig8|CL5975Contig8
9.65
9.60
9.55
9.50
9.45
9.40
1
2
t−Test: p−value = 0.56

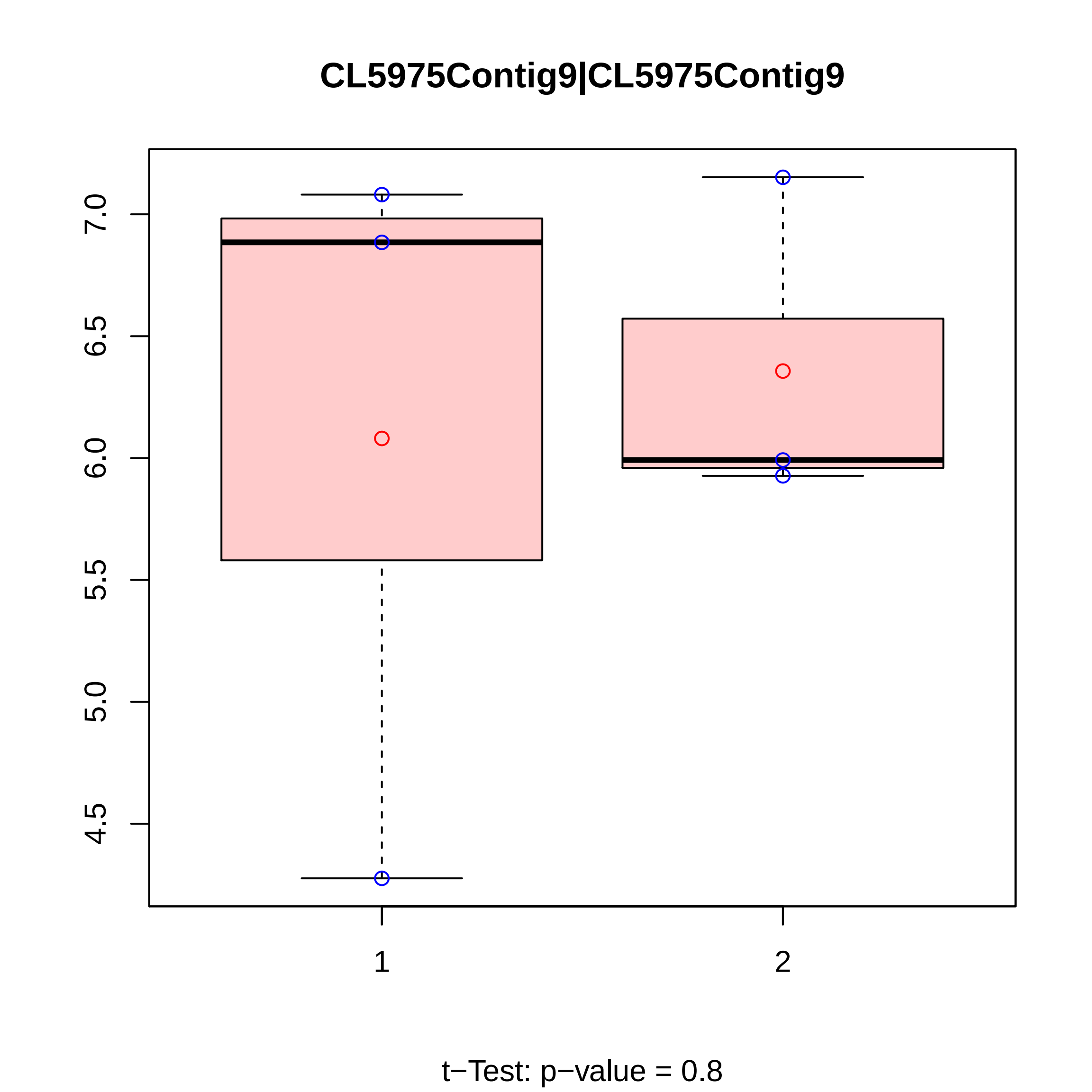
CL5975Contig9|CL5975Contig9
7.0
6.5
6.0
5.5
5.0
4.5
1
2
t−Test: p−value = 0.8

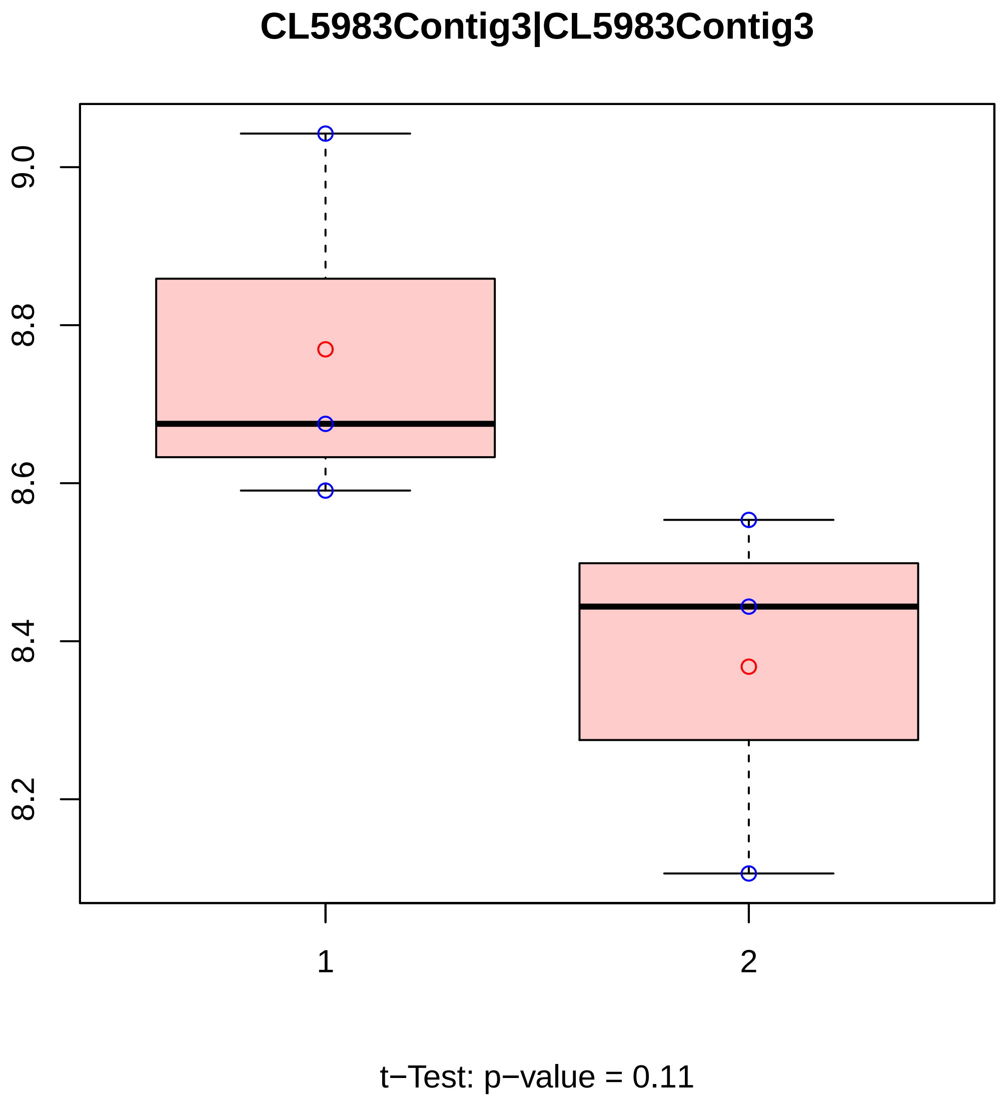

CL5983Contig3|CL5983Contig3
9.0
8.8
8.6
8.4
8.2
1
2
t−Test: p−value = 0.11

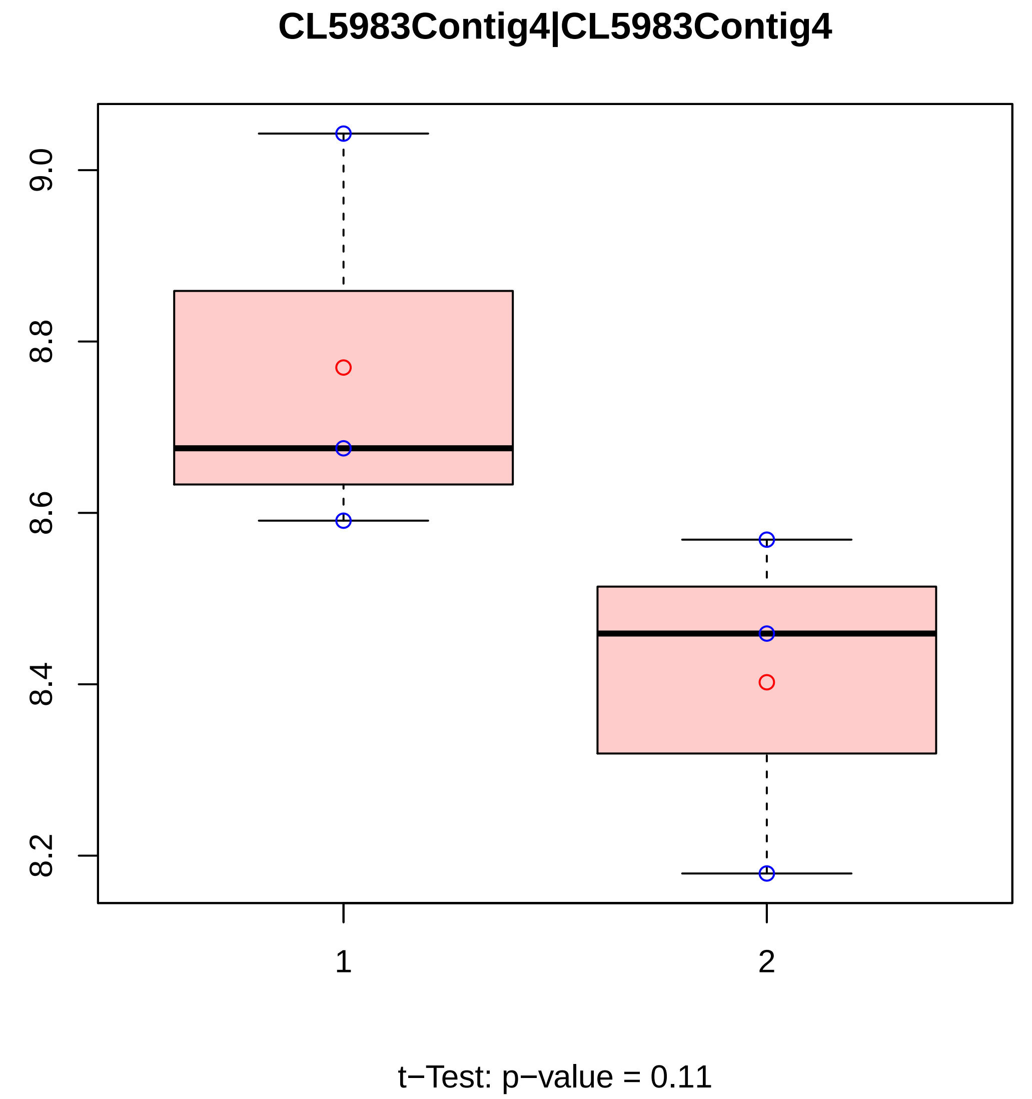

CL5983Contig4|CL5983Contig4
9.0
8.8
8.6
8.4
8.2
1
2
t−Test: p−value = 0.11

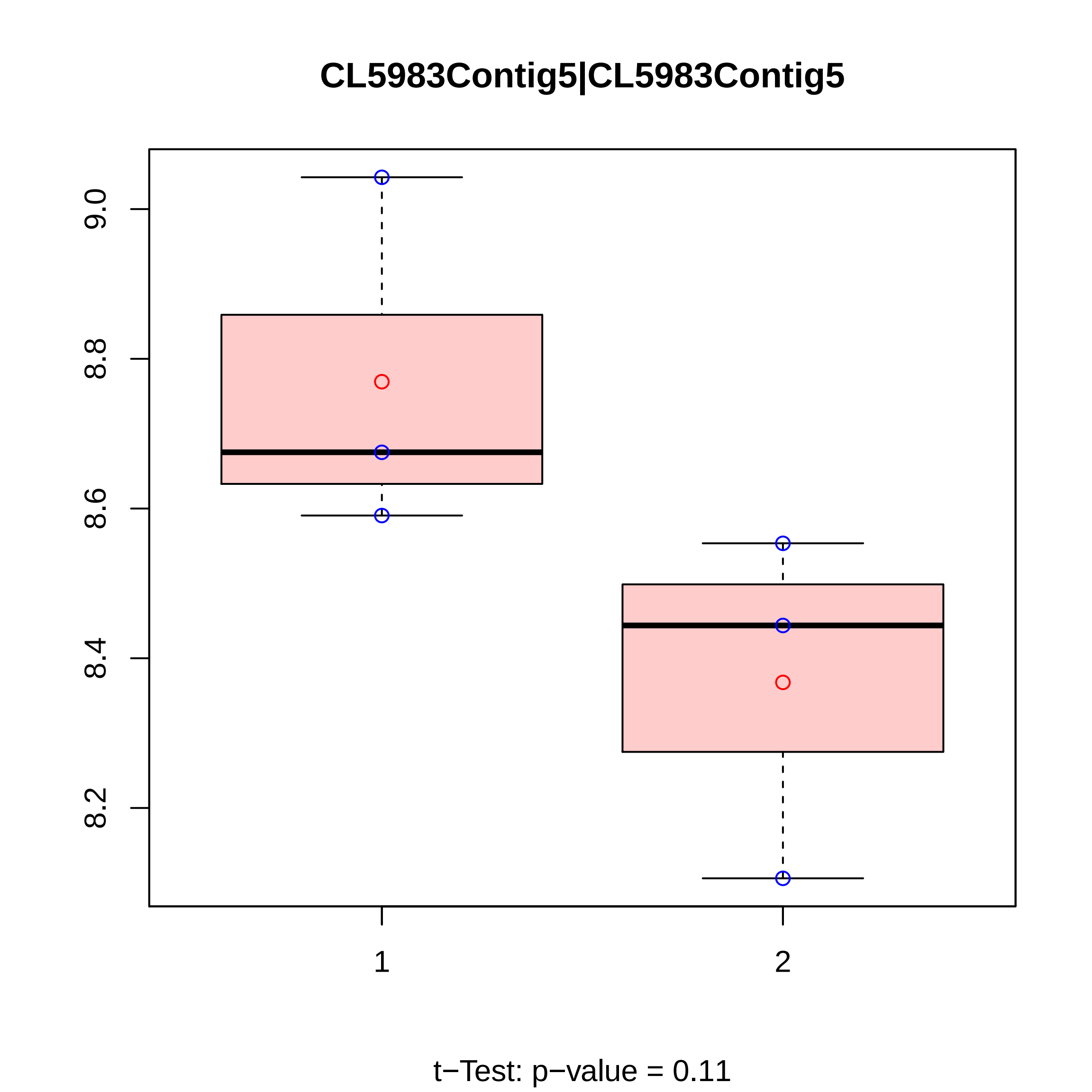
CL5983Contig5|CL5983Contig5
9.0
8.8
8.6
8.4
8.2
1
2
t−Test: p−value = 0.11

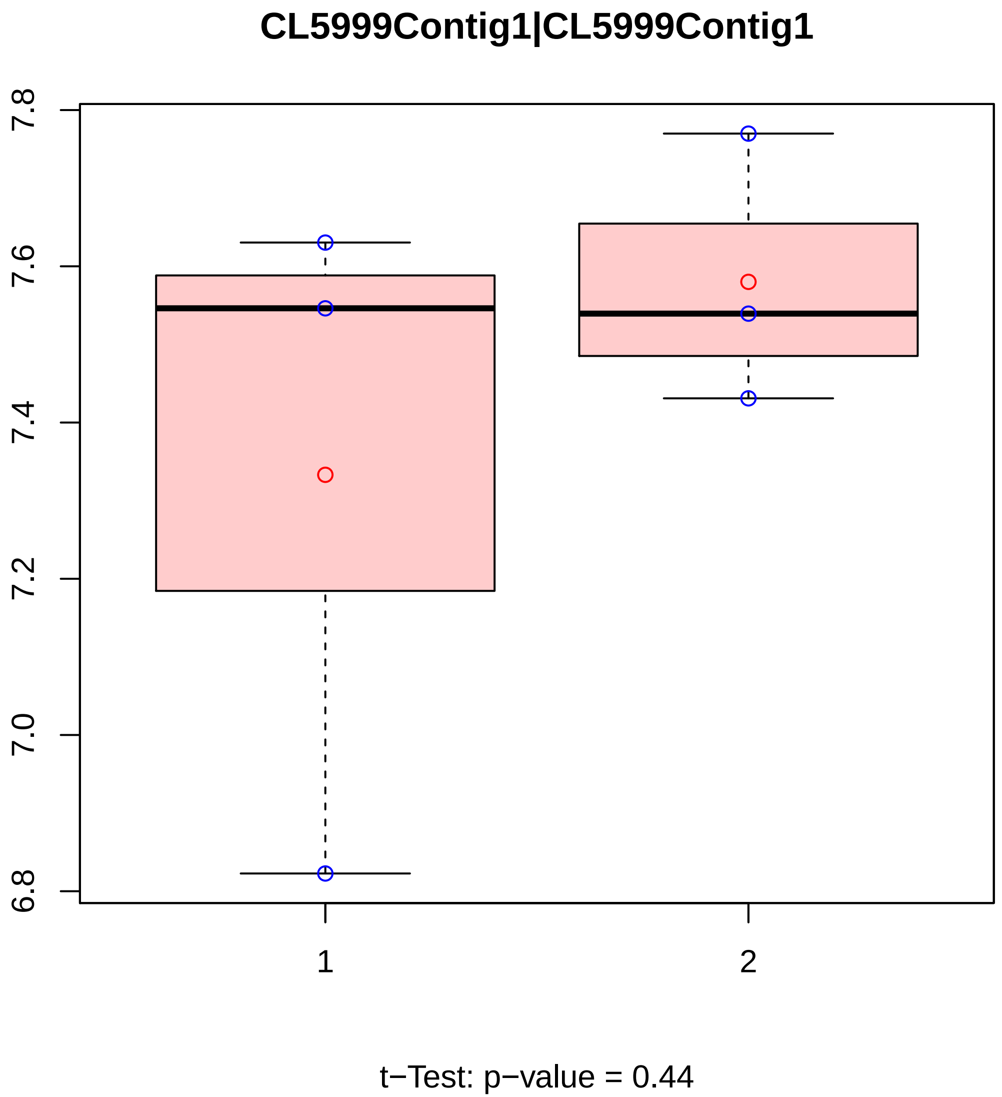
CL5999Contig1|CL5999Contig1
7.8
7.6
7.4
7.2
7.0
6.8
1
2
t−Test: p−value = 0.44

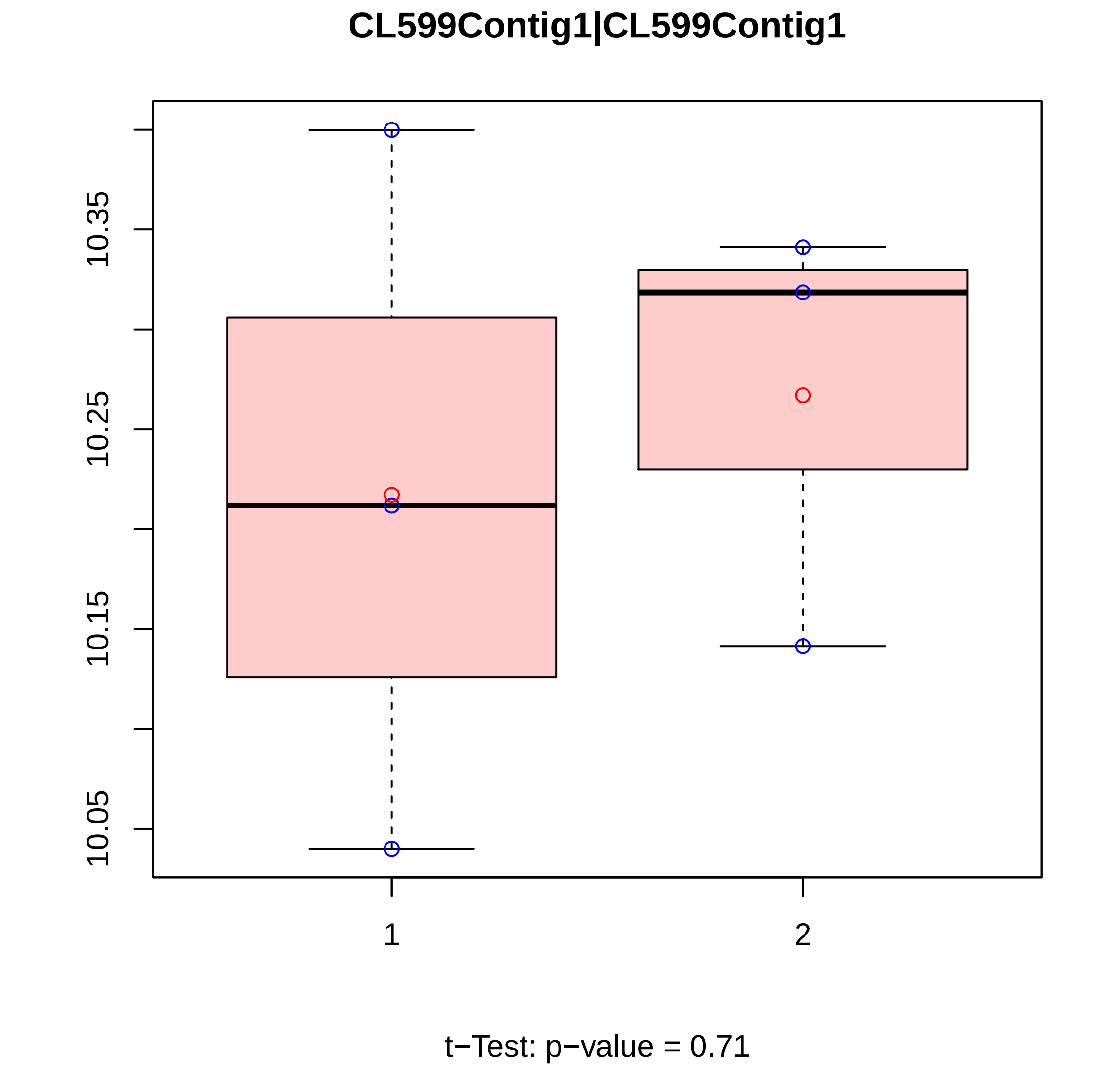
CL599Contig1|CL599Contig1
10.05
10.15
10.25
10.35
1
2
t−Test: p−value = 0.71

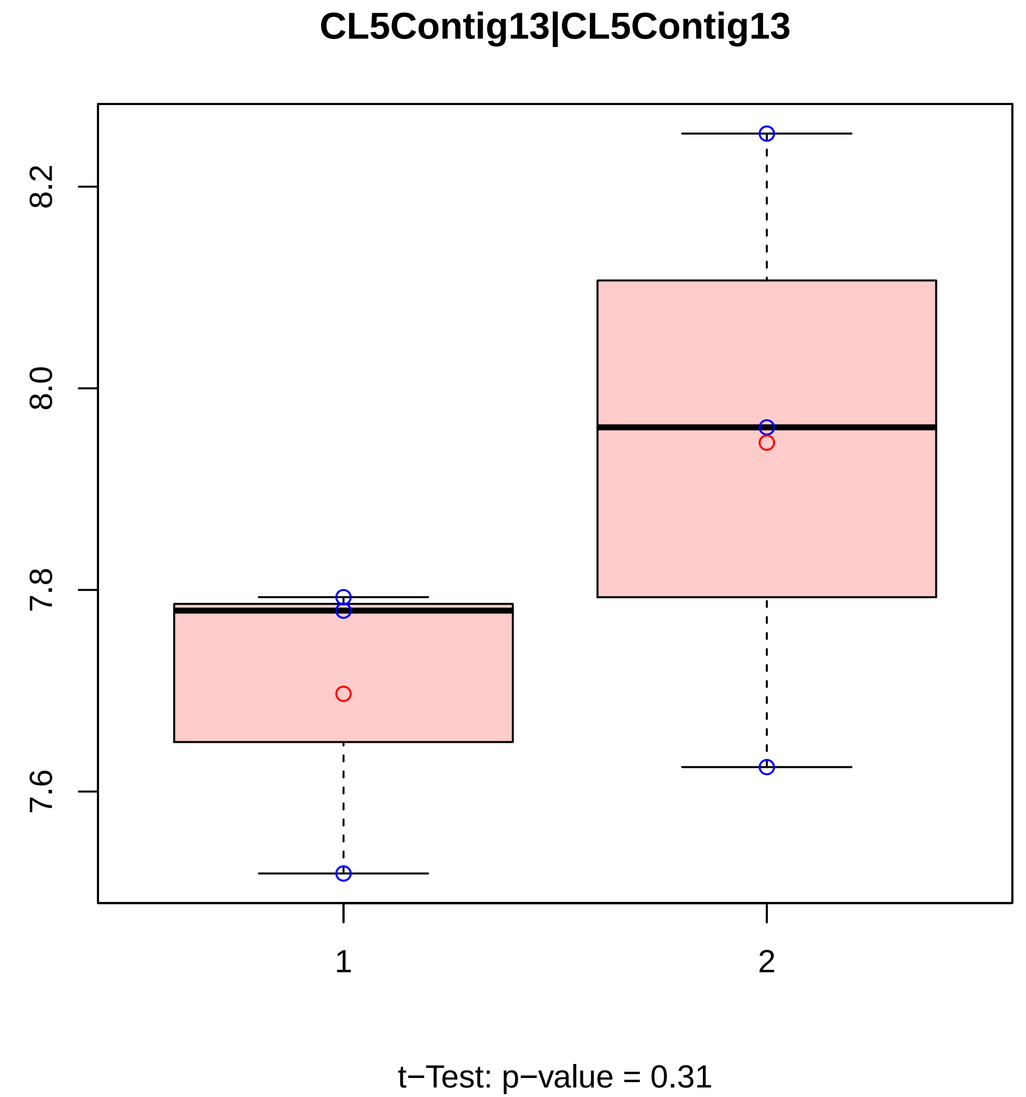
CL5Contig13|CL5Contig13
8.2
8.0
7.8
7.6
1
2
t−Test: p−value = 0.31

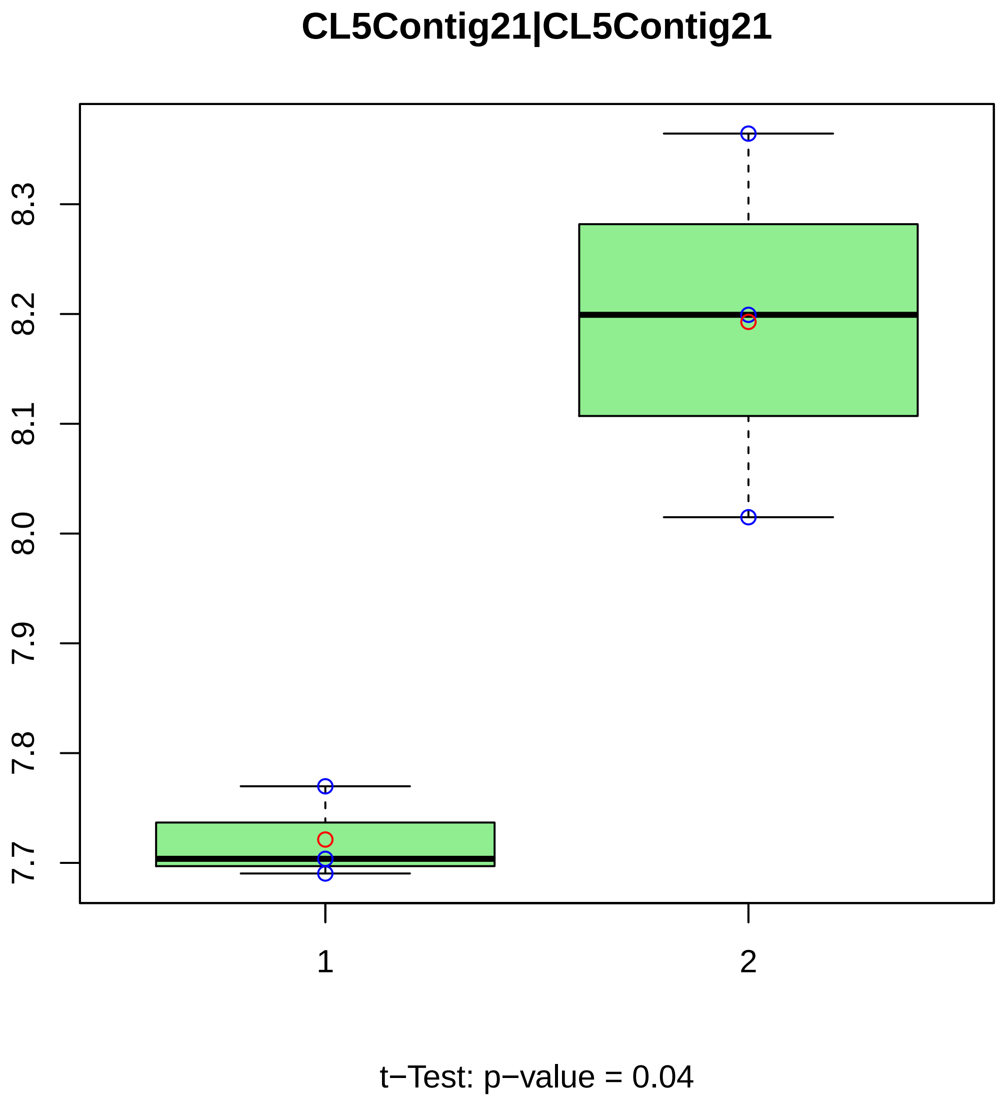

CL5Contig21|CL5Contig21
8.3
8.2
8.1
8.0
7.9
7.8
7.7
1
2
t−Test: p−value = 0.04

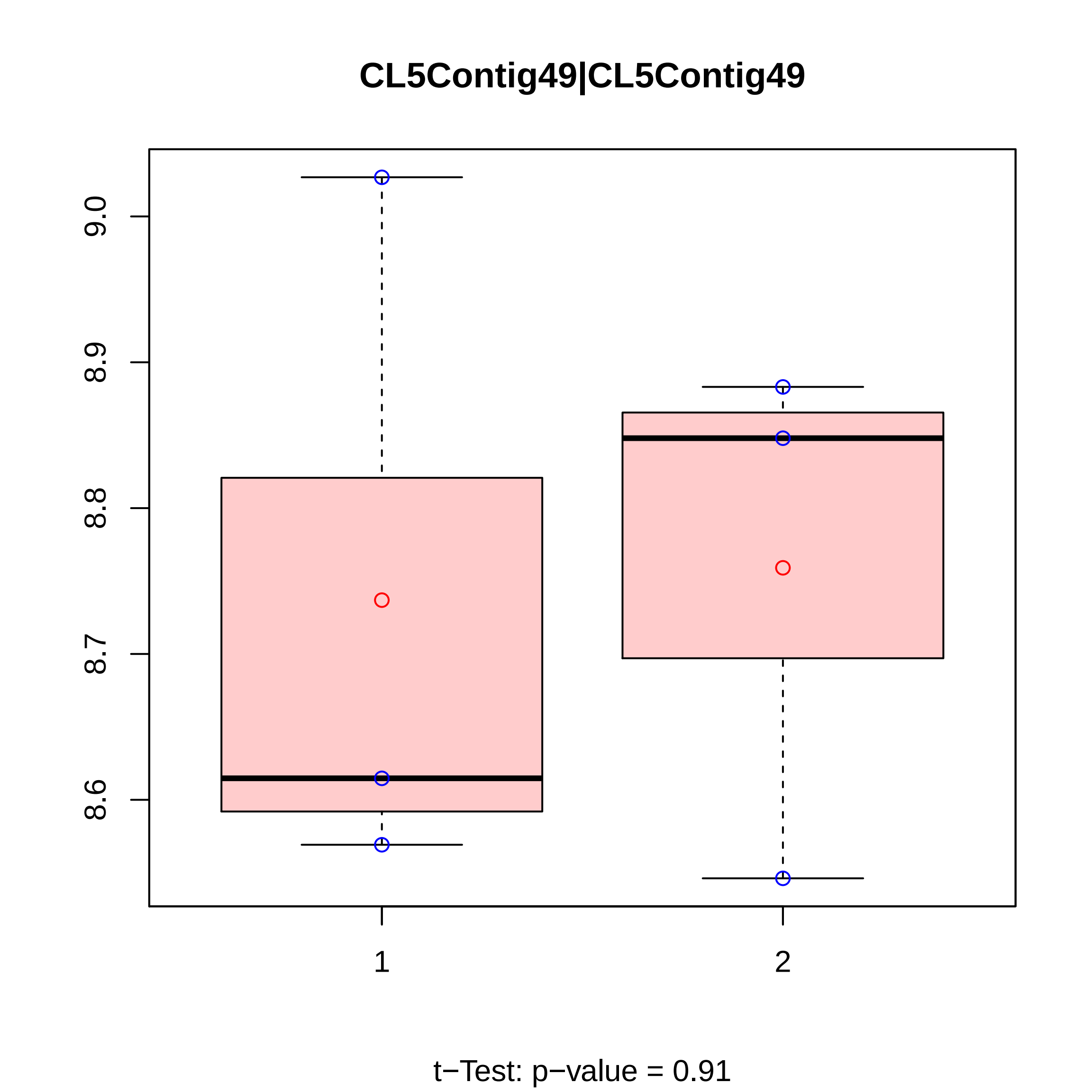

CL5Contig49|CL5Contig49
9.0
8.9
8.8
8.7
8.6
1
2
t−Test: p−value = 0.91

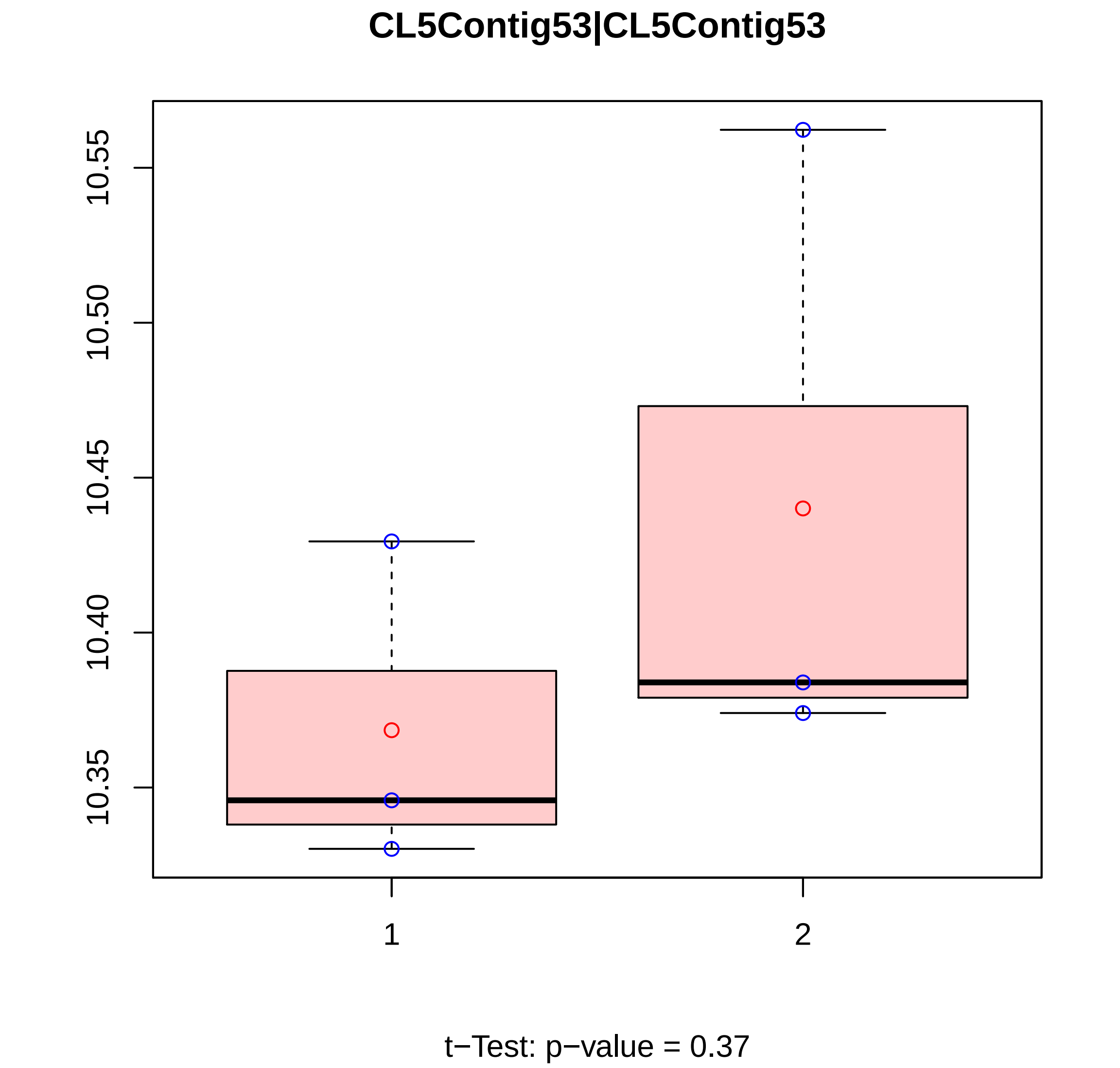

CL5Contig53|CL5Contig53
10.35
10.40
10.45
10.50
10.55
1
2
t−Test: p−value = 0.37

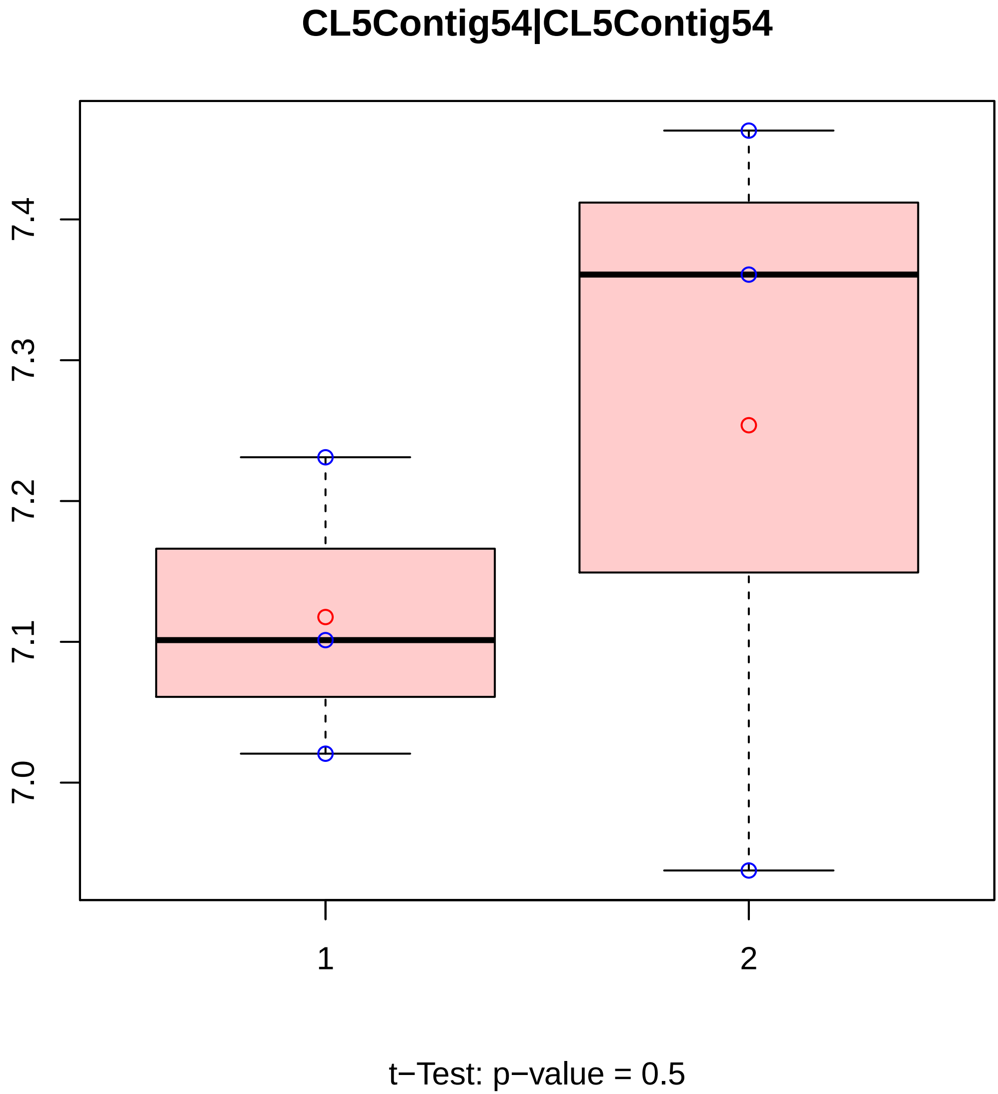
CL5Contig54|CL5Contig54
7.0
7.1
7.2
7.3
7.4
1
2
t−Test: p−value = 0.5

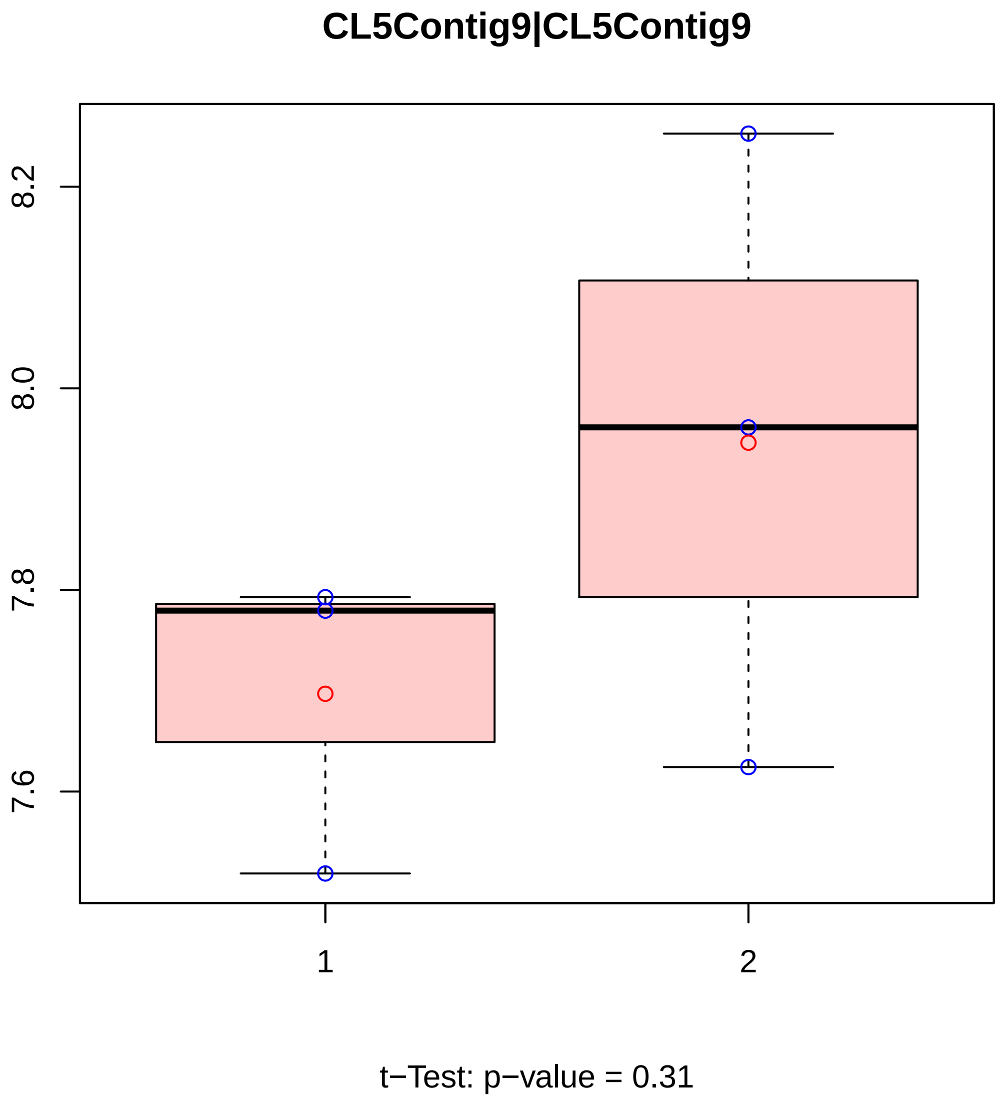
CL5Contig9|CL5Contig9
8.2
8.0
7.8
7.6
1
2
t−Test: p−value = 0.31

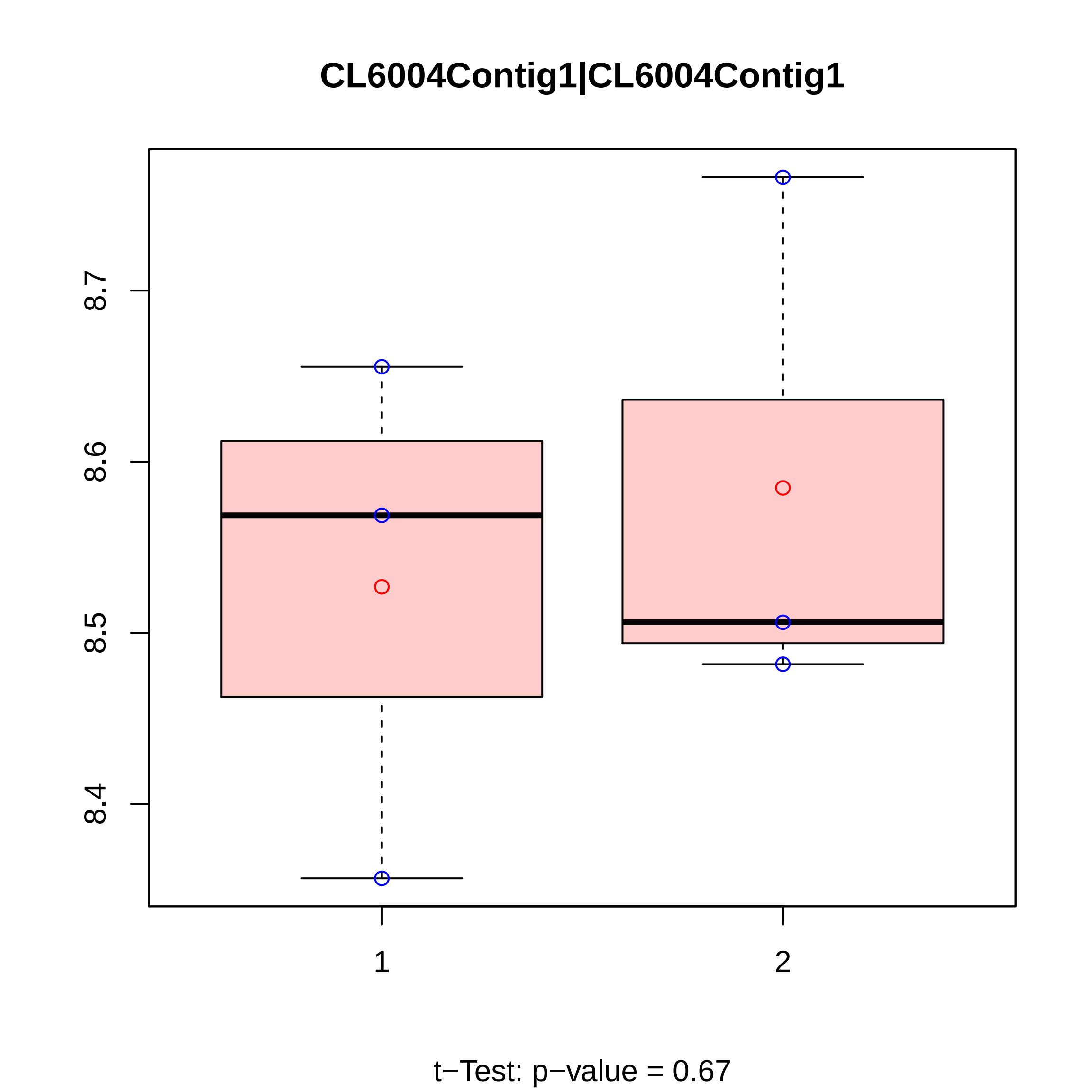

CL6004Contig1|CL6004Contig1
8.4
8.5
8.6
8.7
1
2
t−Test: p−value = 0.67

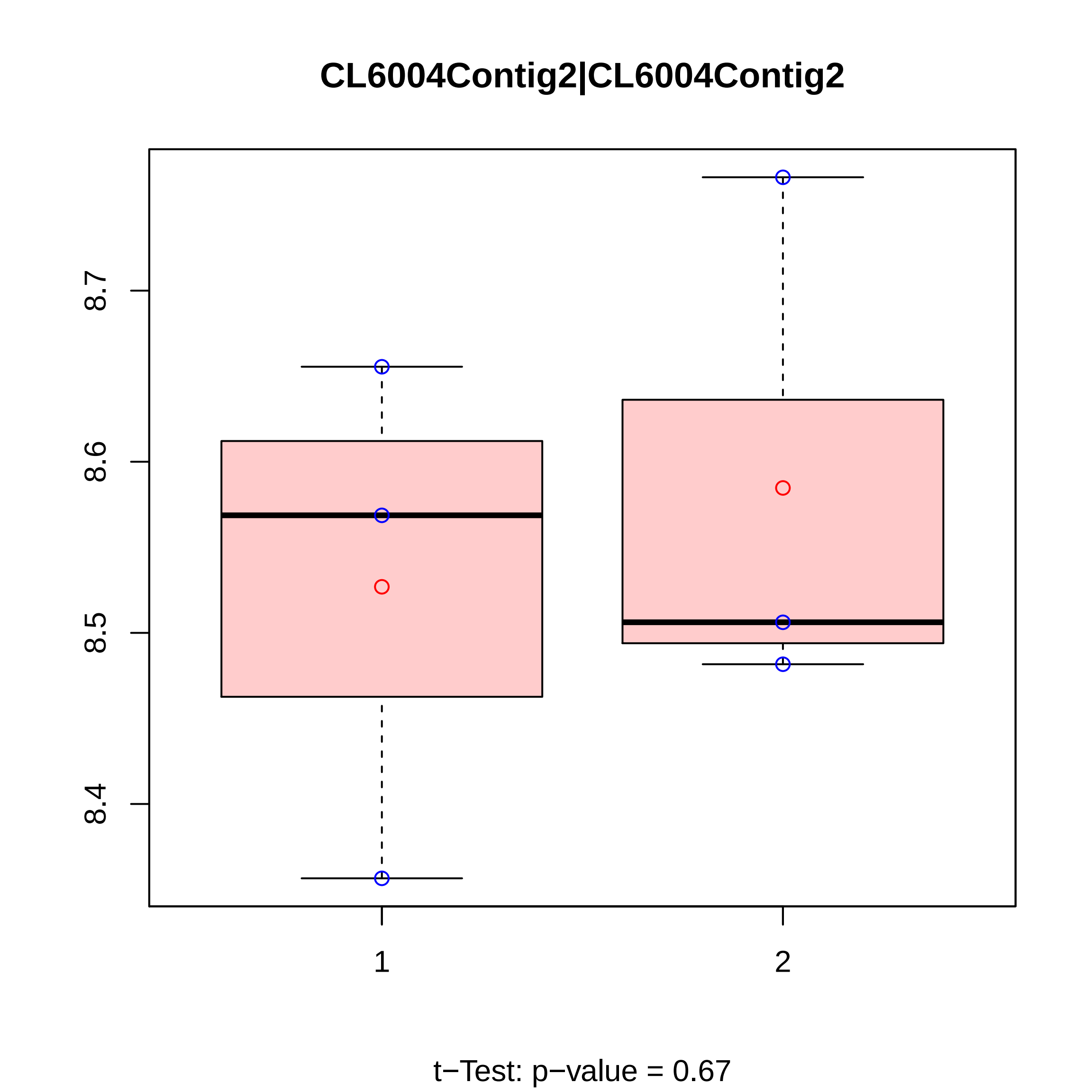
CL6004Contig2|CL6004Contig2
8.4
8.5
8.6
8.7
1
2
t−Test: p−value = 0.67

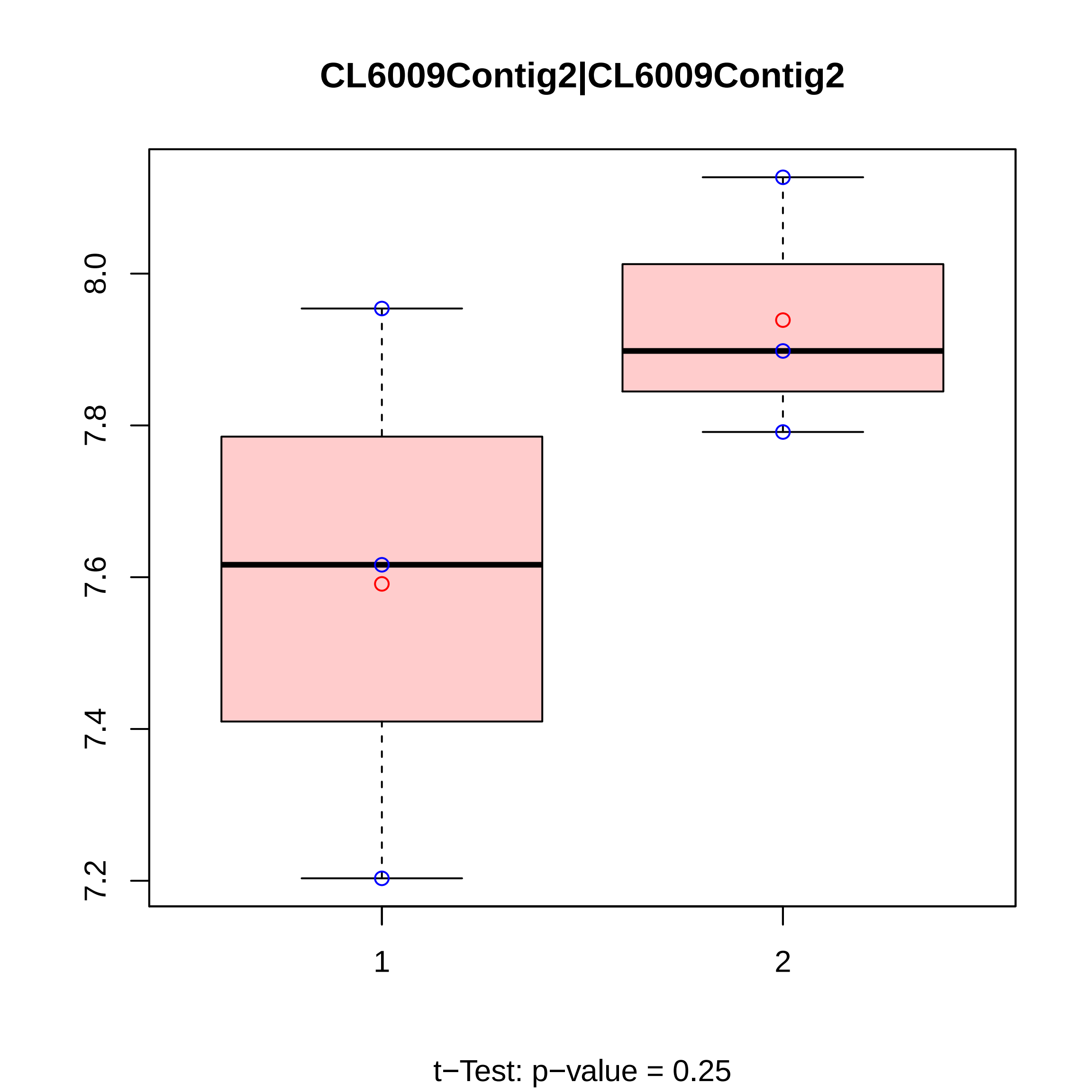

CL6009Contig2|CL6009Contig2
8.0
7.8
7.6
7.4
7.2
1
2
t−Test: p−value = 0.25

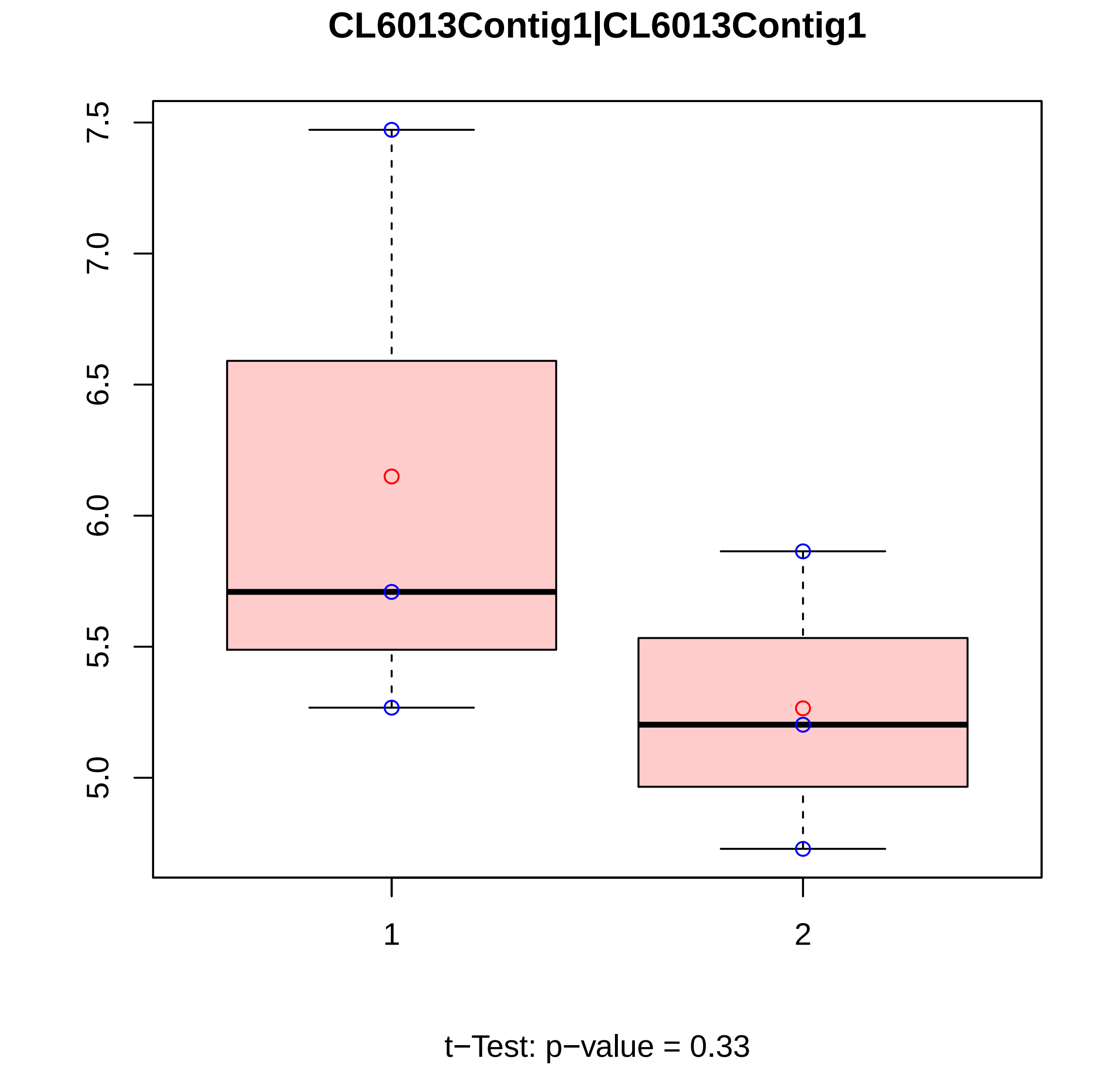

CL6013Contig1|CL6013Contig1
7.5
7.0
6.5
6.0
5.5
5.0
1
2
t−Test: p−value = 0.33

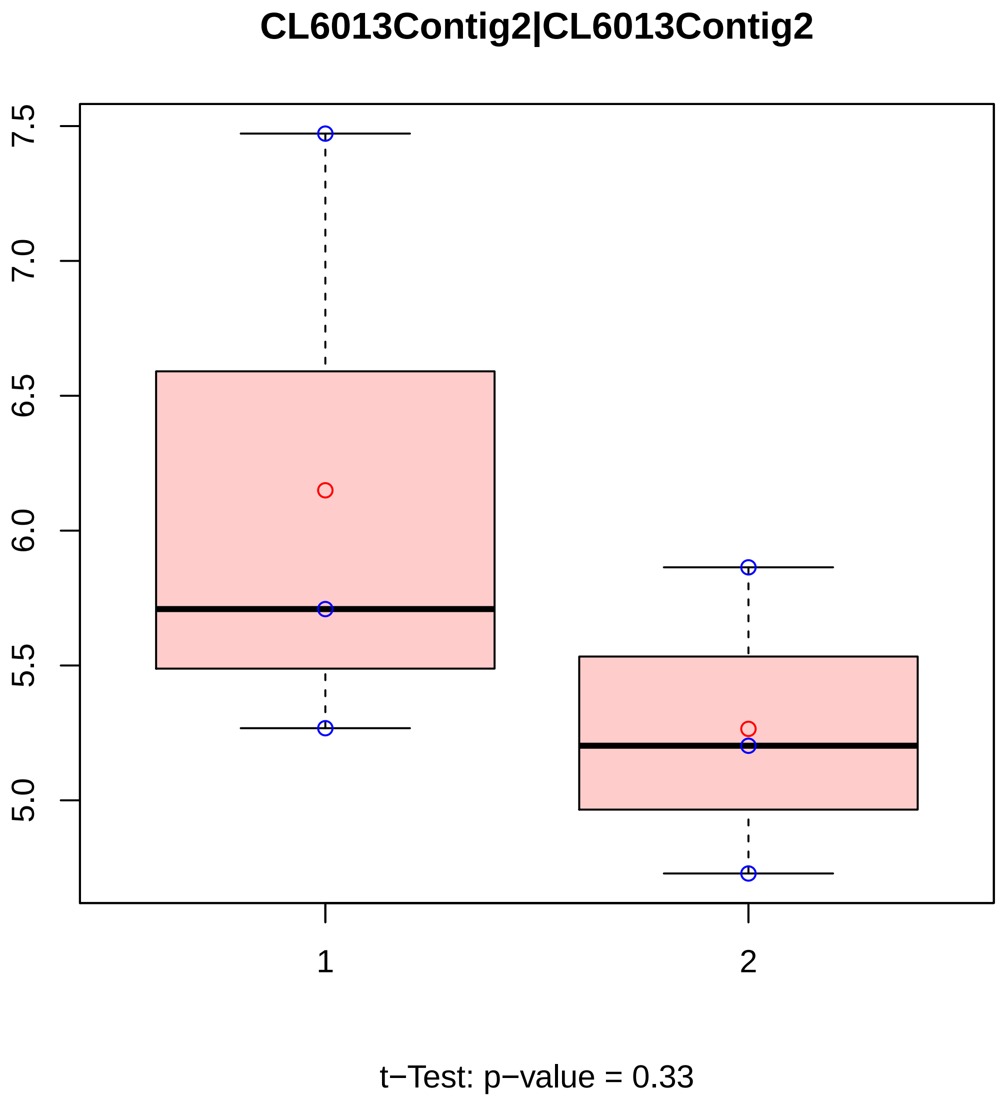
CL6013Contig2|CL6013Contig2
7.5
7.0
6.5
6.0
5.5
5.0
1
2
t−Test: p−value = 0.33

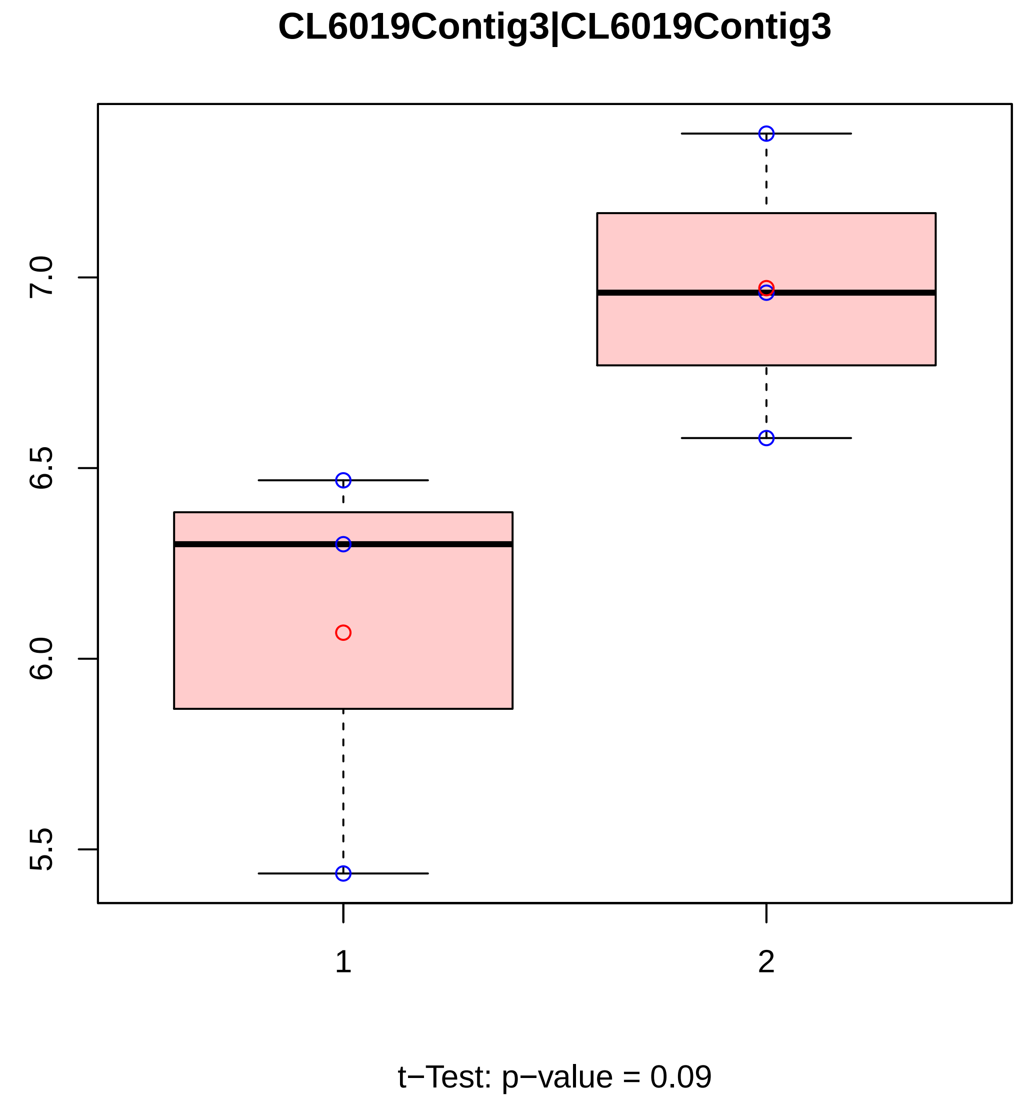
CL6019Contig3|CL6019Contig3
7.0
6.5
6.0
5.5
1
2
t−Test: p−value = 0.09

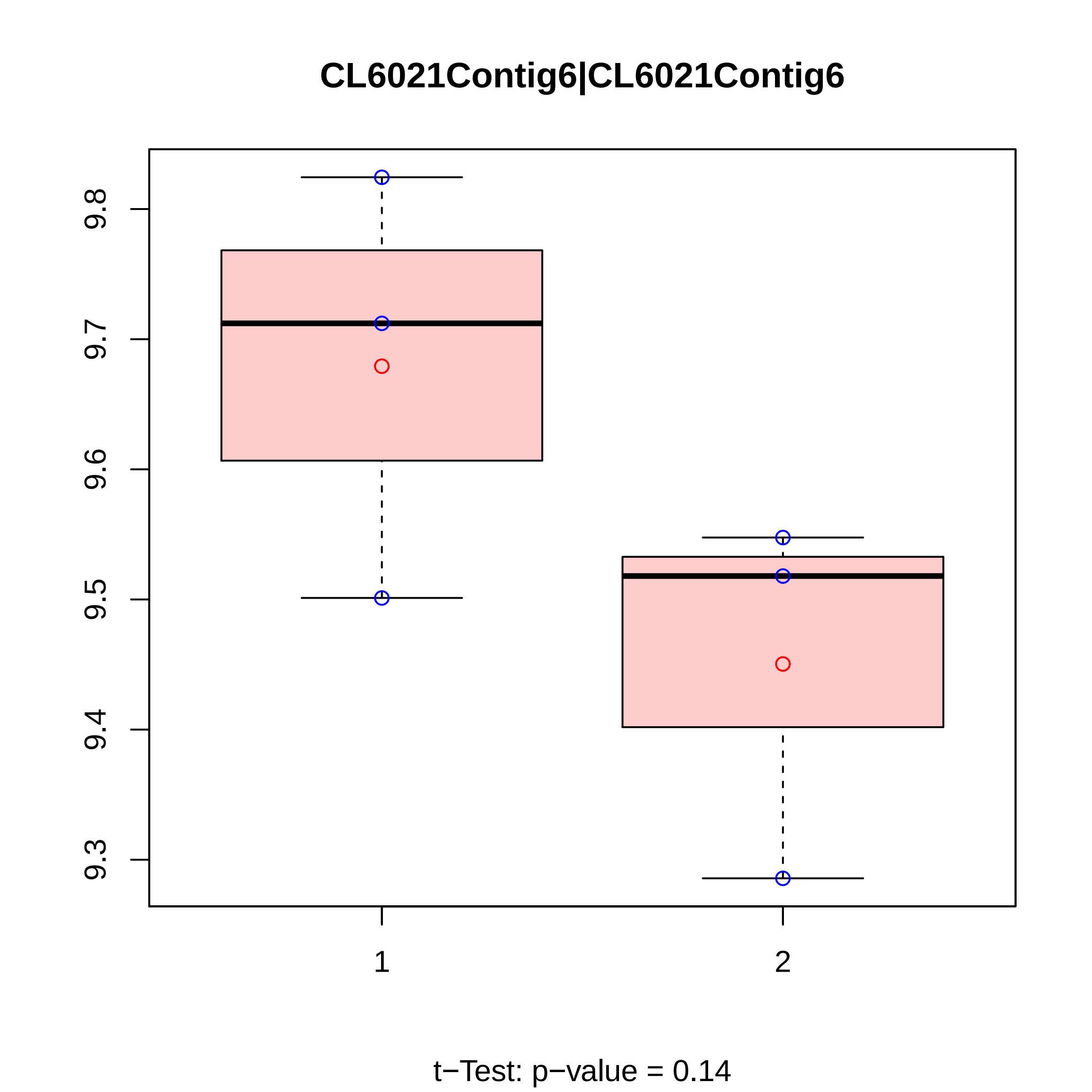
CL6021Contig6|CL6021Contig6
9.3
9.4
9.5
9.6
9.7
9.8
1
2
t−Test: p−value = 0.14

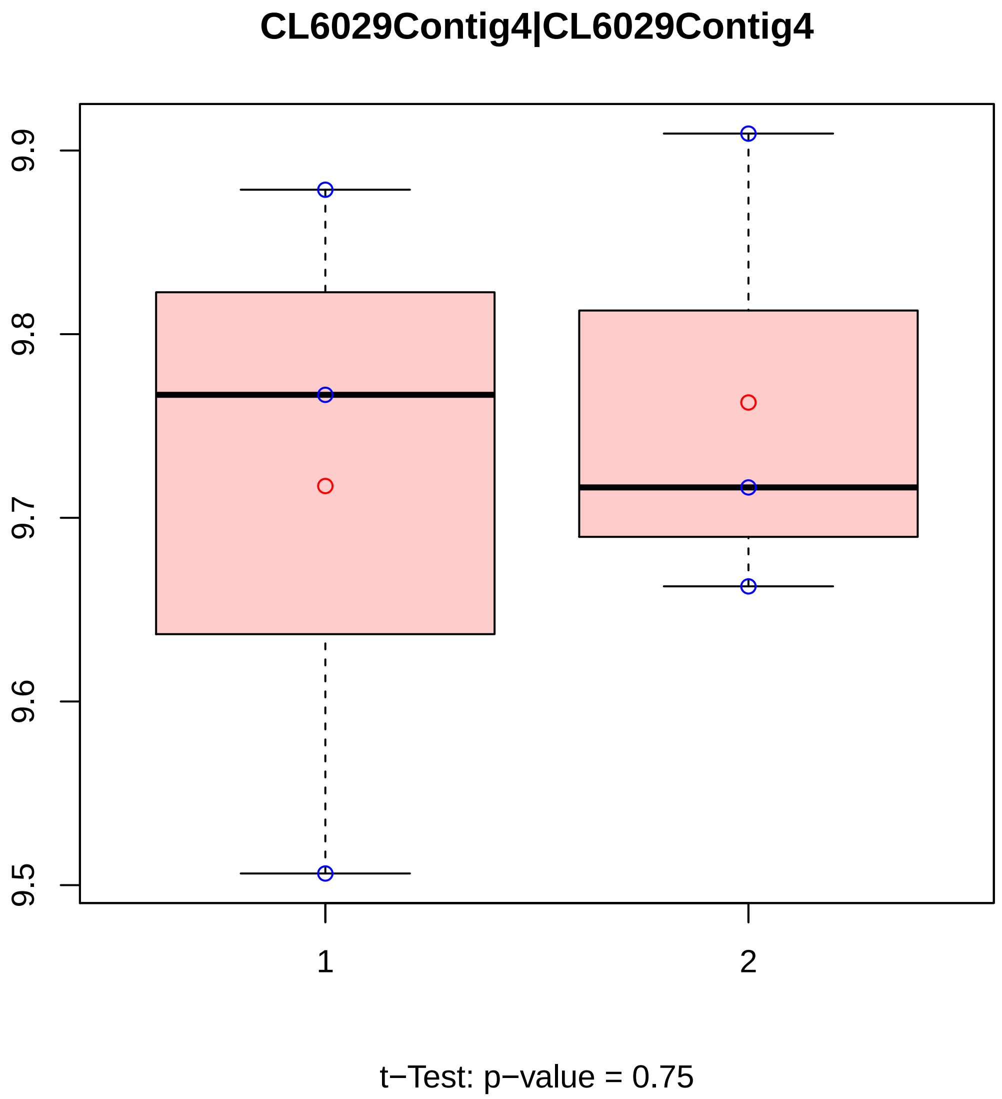

CL6029Contig4|CL6029Contig4
9.5
9.6
9.7
9.8
9.9
1
2
t−Test: p−value = 0.75

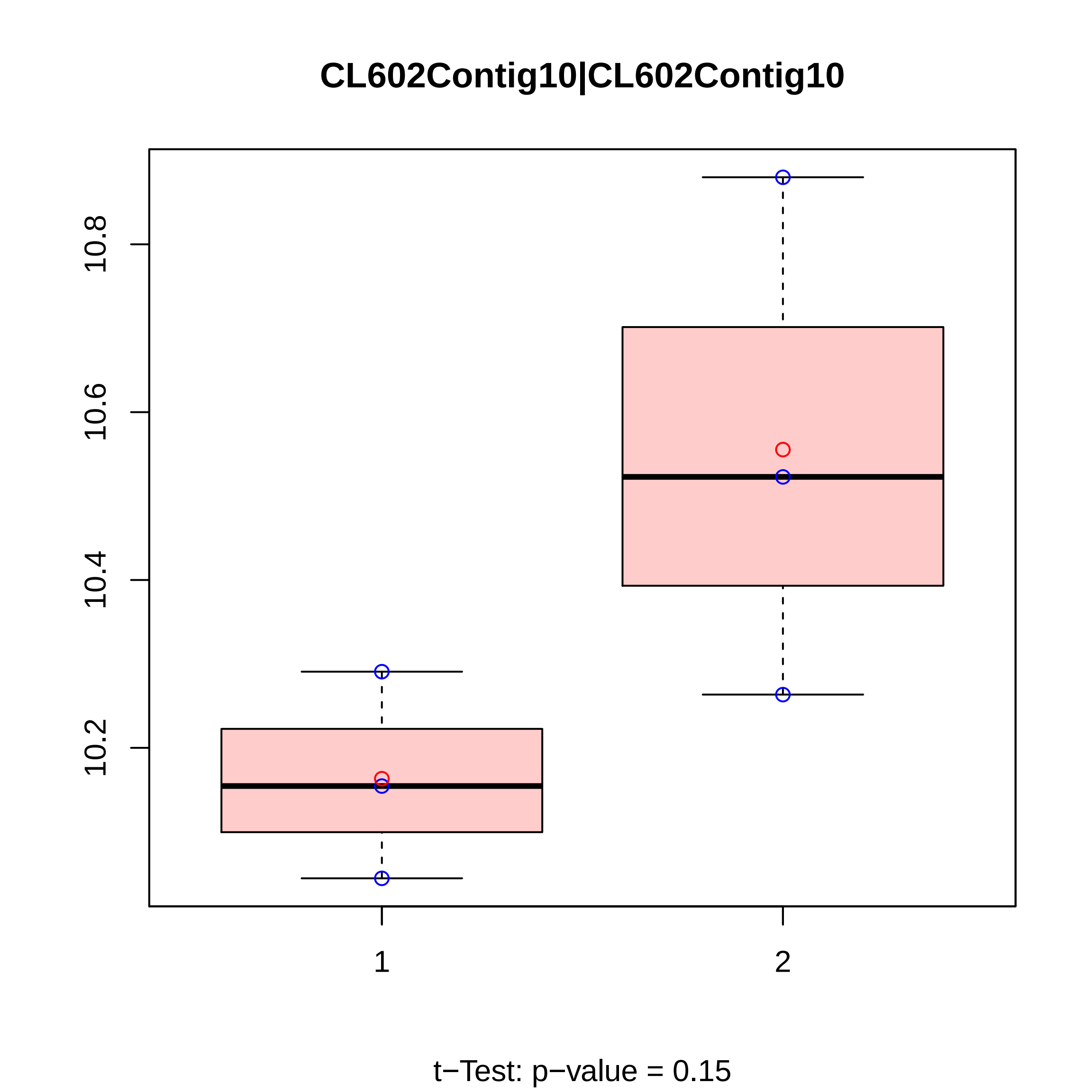
CL602Contig10|CL602Contig10
10.2
10.4
10.6
10.8
1
2
t−Test: p−value = 0.15

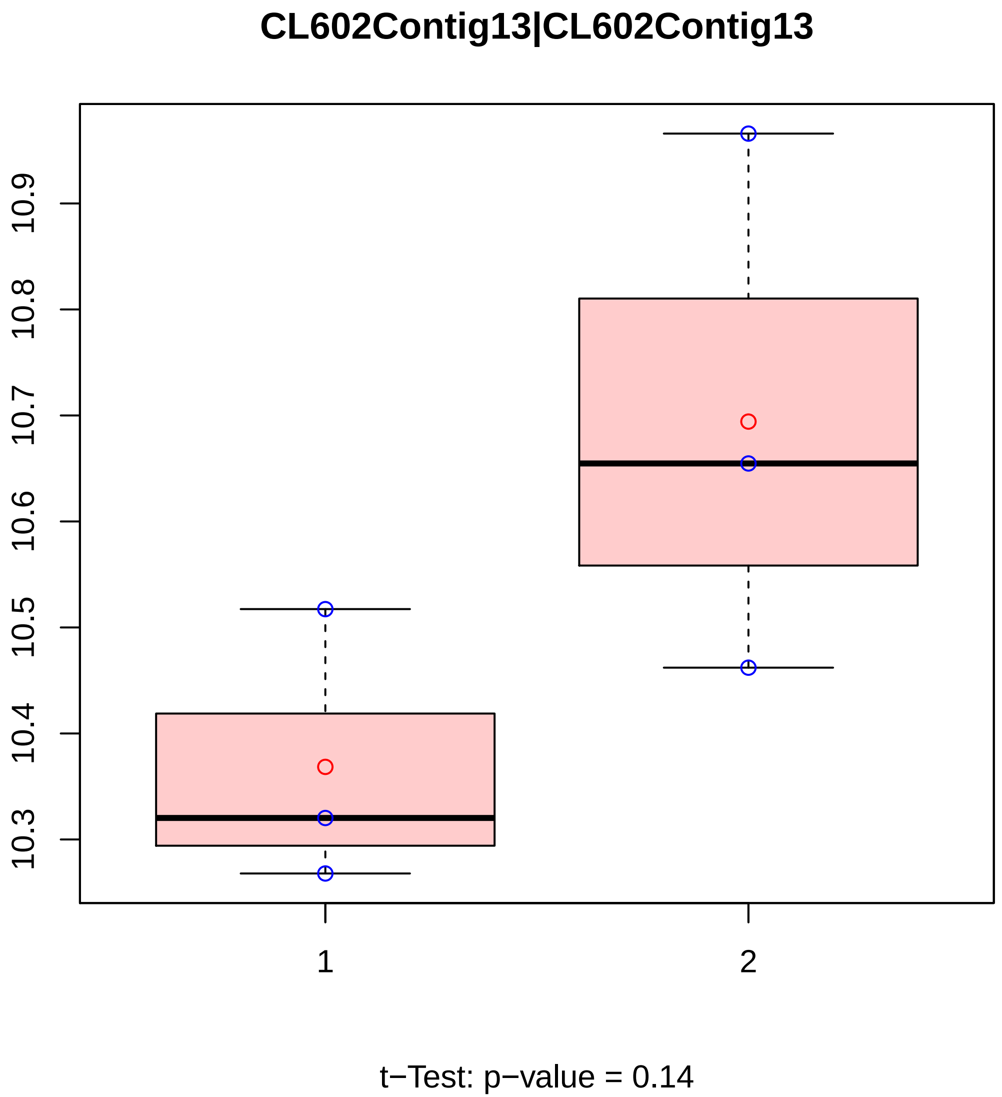
CL602Contig13|CL602Contig13
10.3
10.4
10.5
10.6
10.7
10.8
10.9
1
2
t−Test: p−value = 0.14

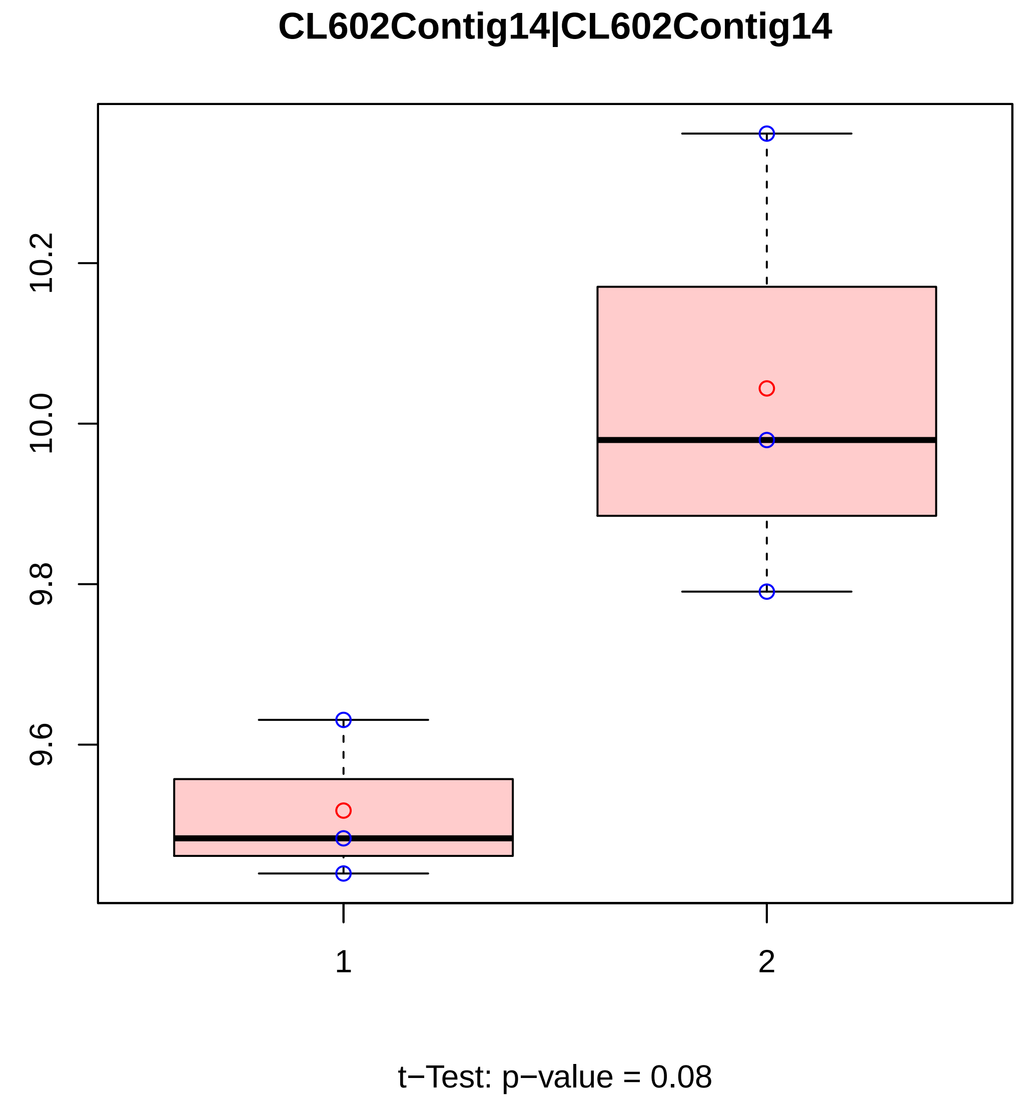
CL602Contig14|CL602Contig14
10.2
10.0
9.8
9.6
1
2
t−Test: p−value = 0.08

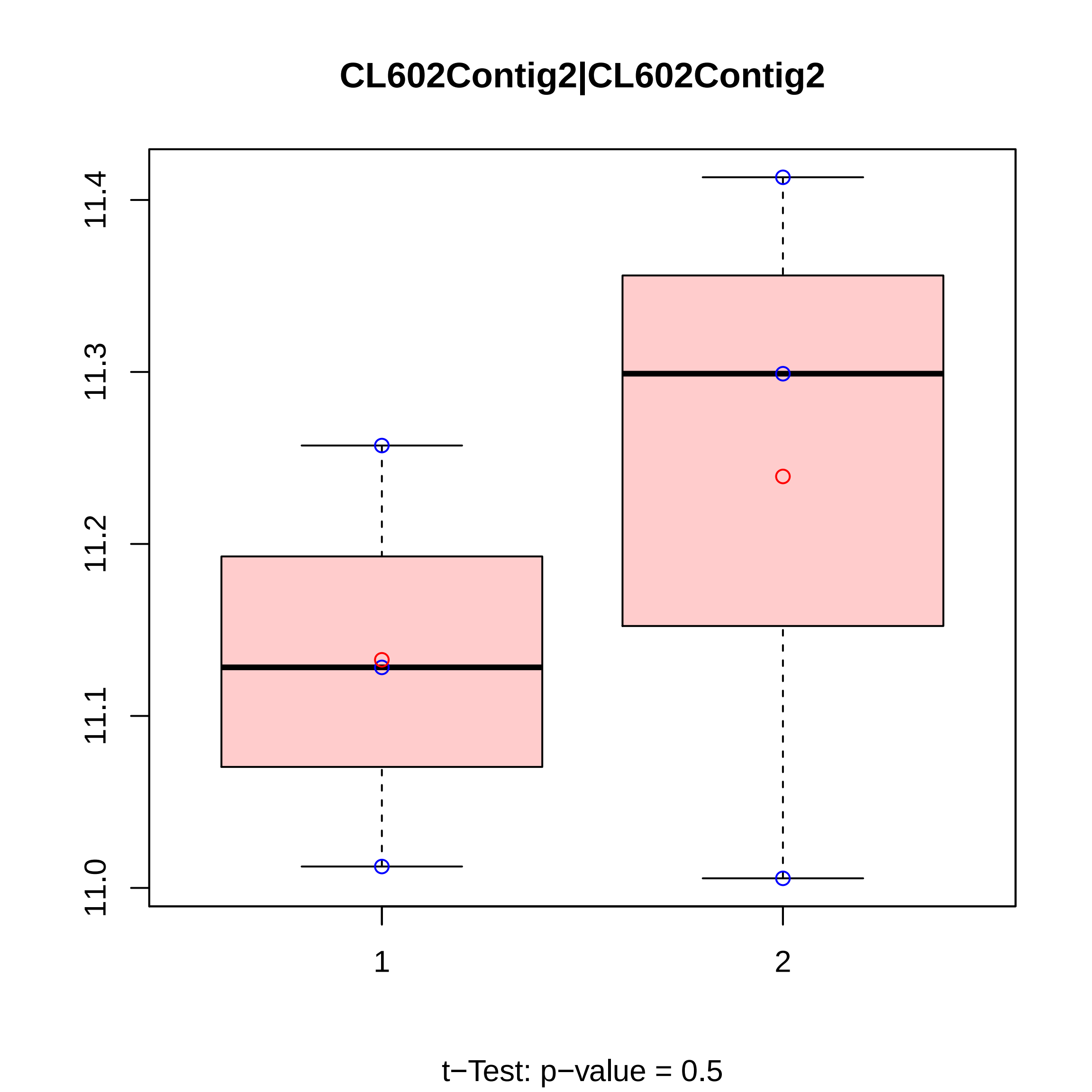
CL602Contig2|CL602Contig2
11.0
11.1
11.2
11.3
11.4
1
2
t−Test: p−value = 0.5

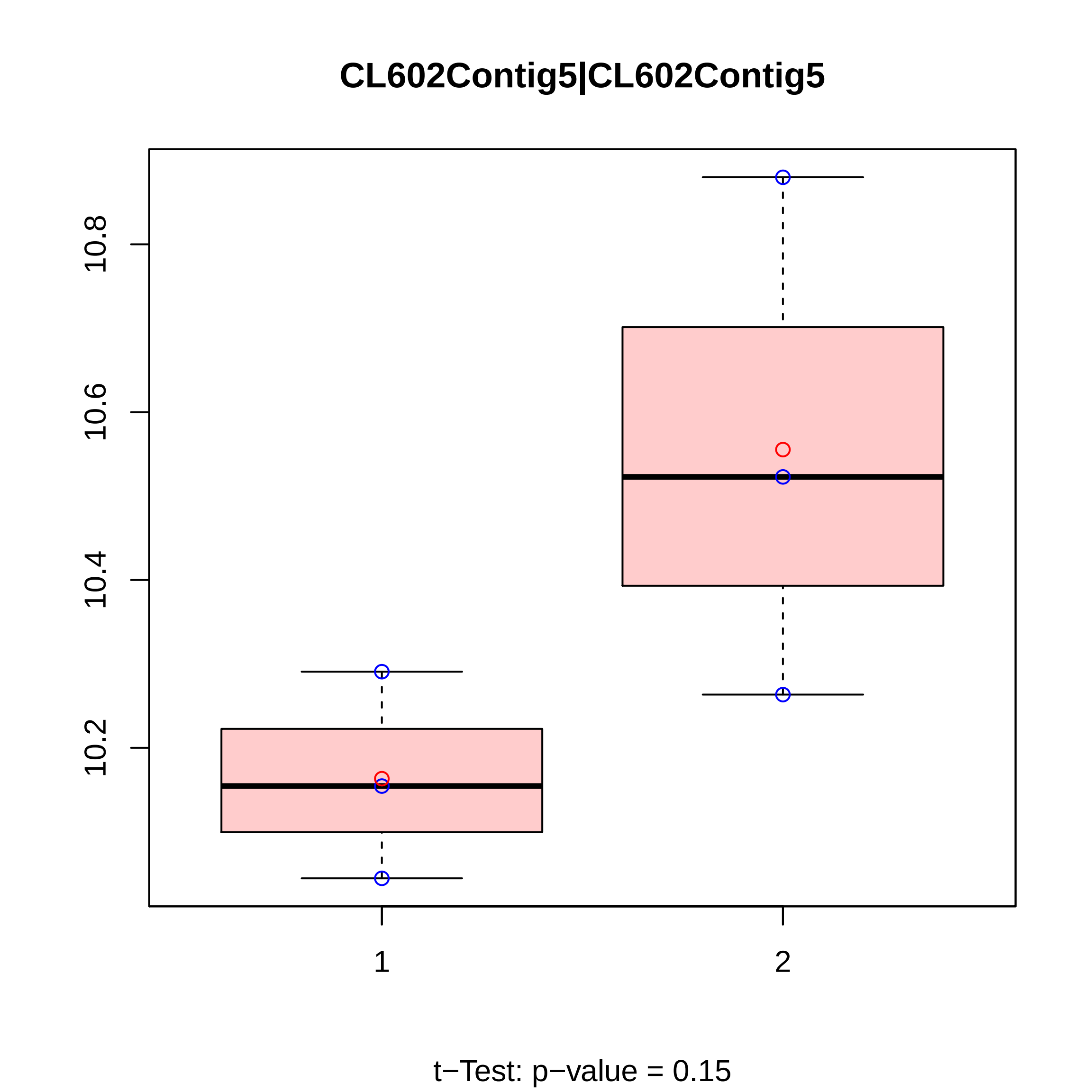

CL602Contig5|CL602Contig5
10.2
10.4
10.6
10.8
1
2
t−Test: p−value = 0.15

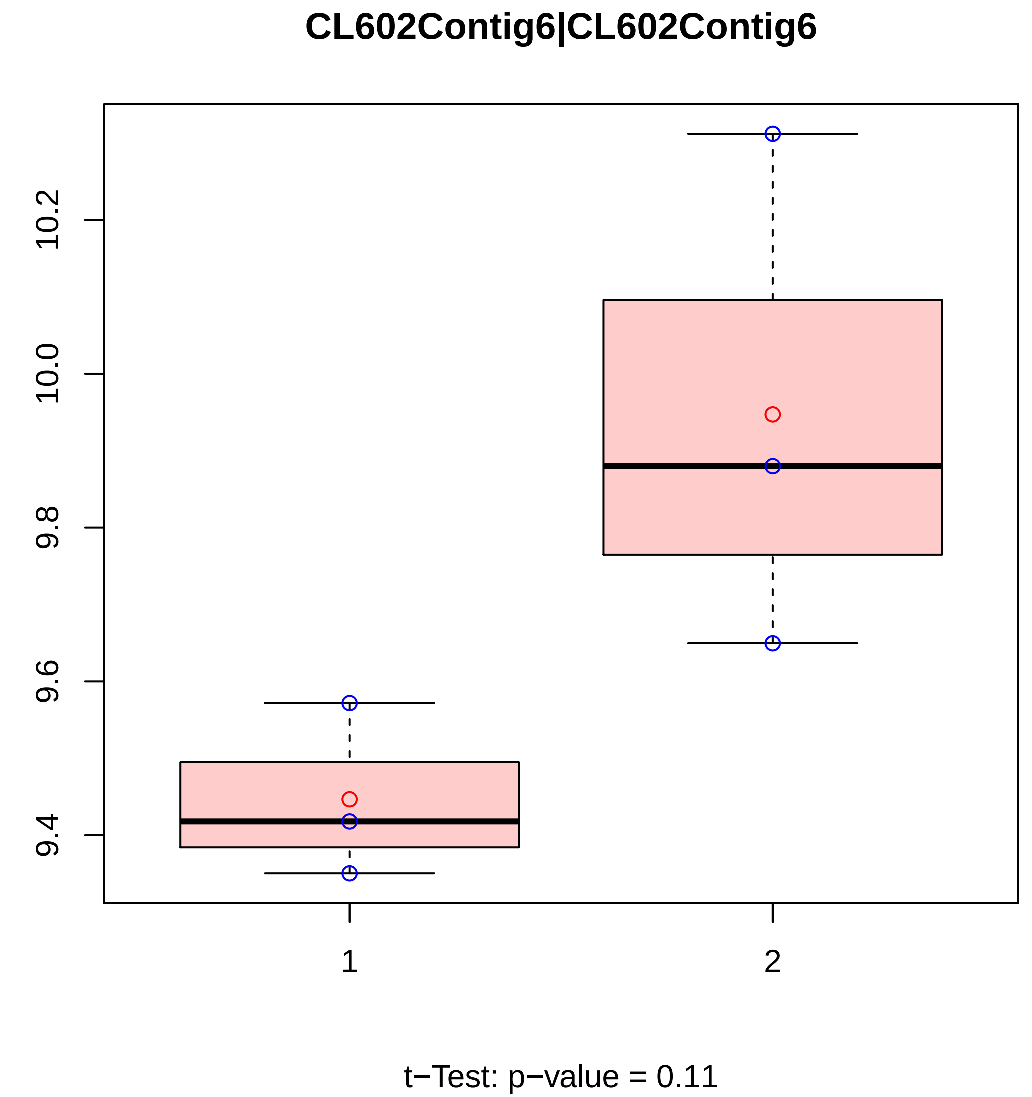
CL602Contig6|CL602Contig6
10.2
10.0
9.8
9.6
9.4
1
2
t−Test: p−value = 0.11

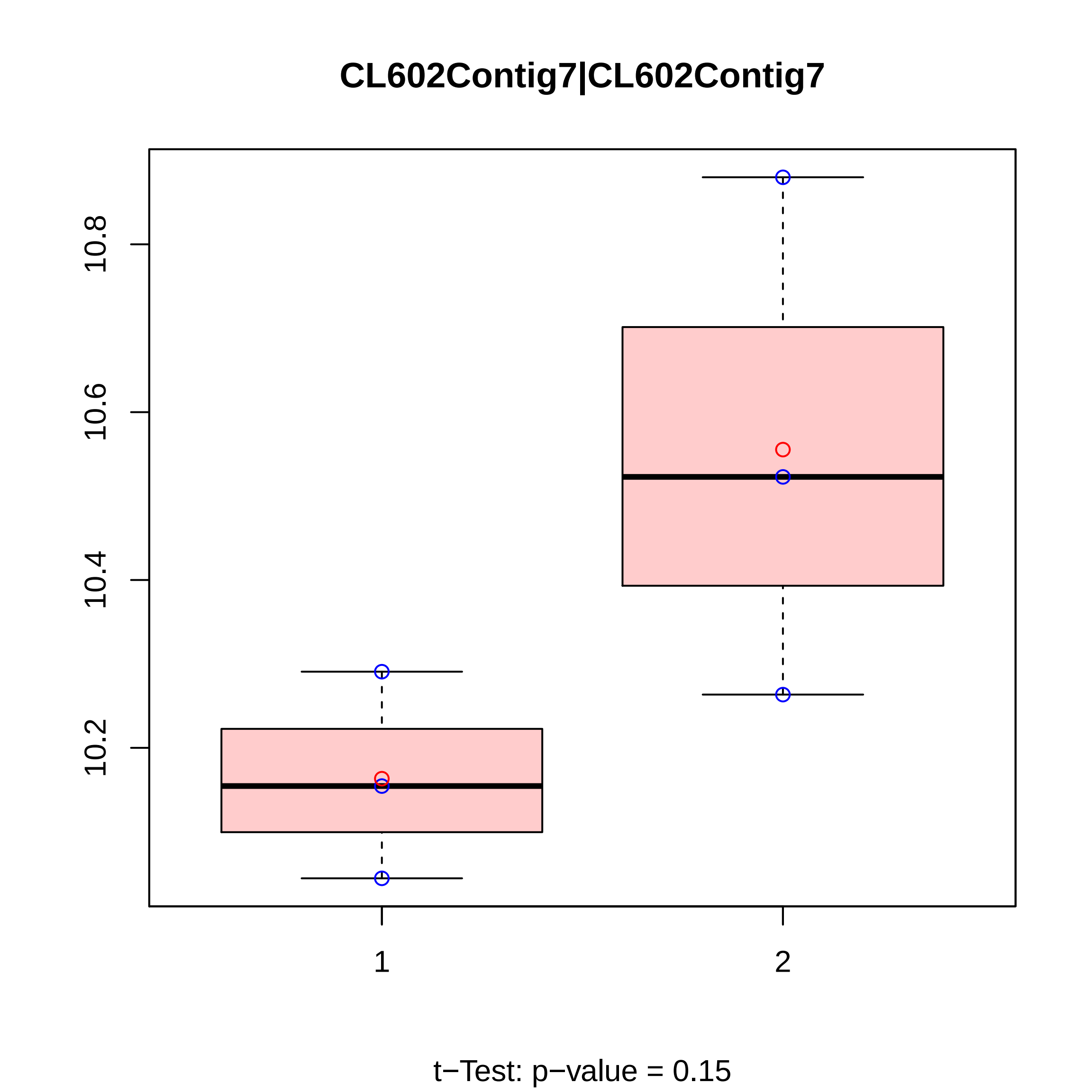
CL602Contig7|CL602Contig7
10.2
10.4
10.6
10.8
1
2
t−Test: p−value = 0.15

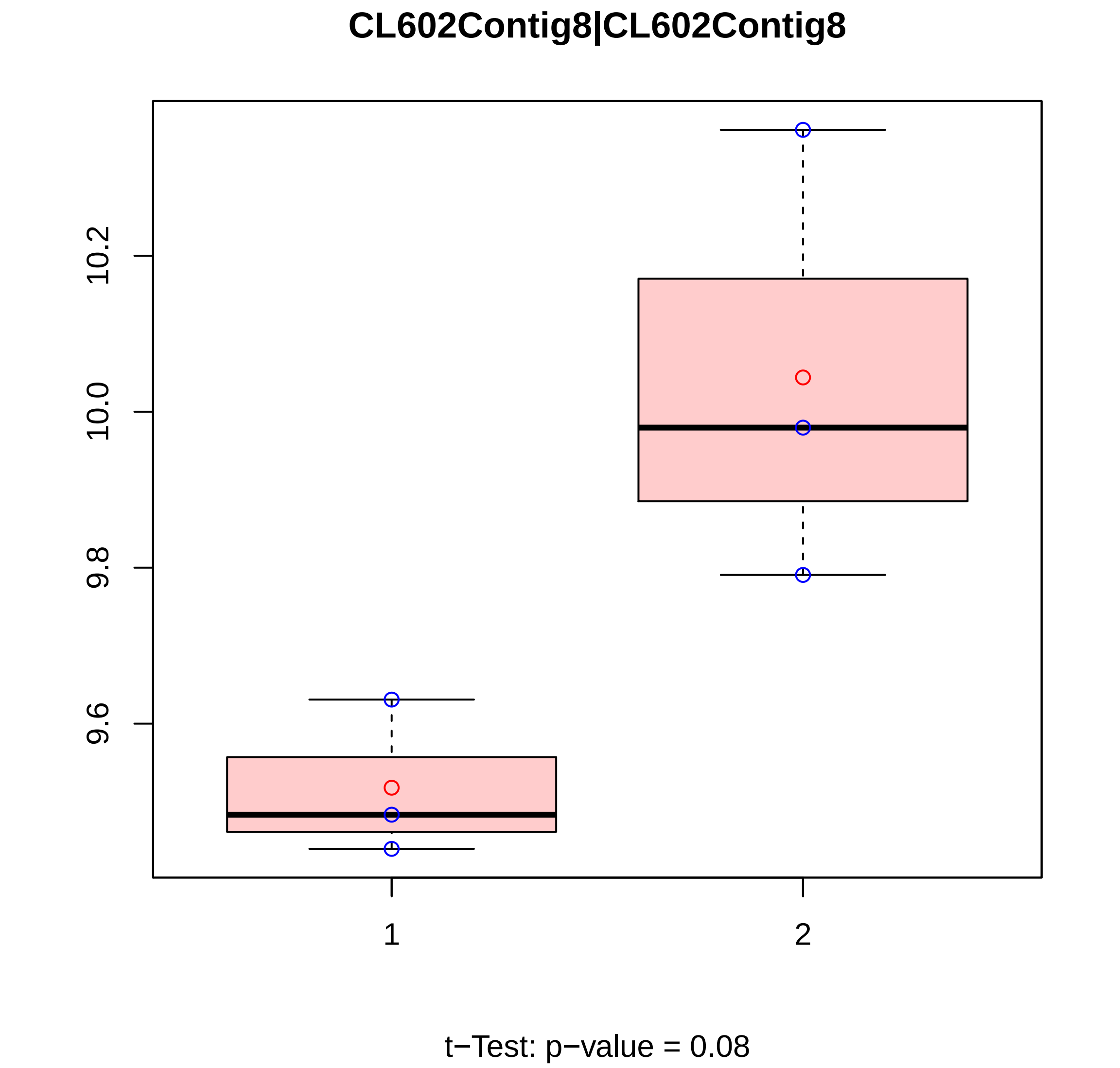
CL602Contig8|CL602Contig8
10.2
10.0
9.8
9.6
1
2
t−Test: p−value = 0.08

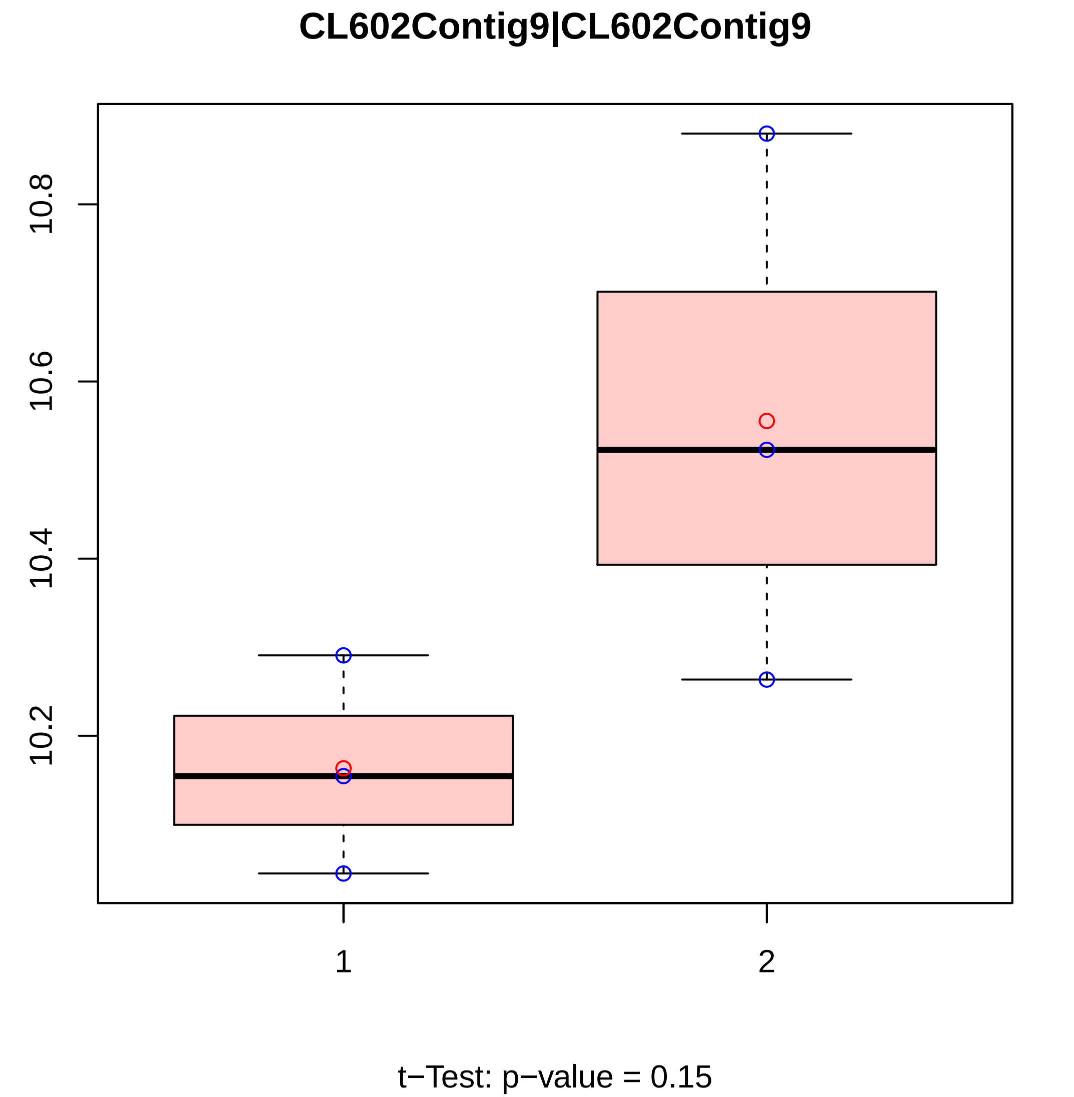
CL602Contig9|CL602Contig9
10.2
10.4
10.6
10.8
1
2
t−Test: p−value = 0.15

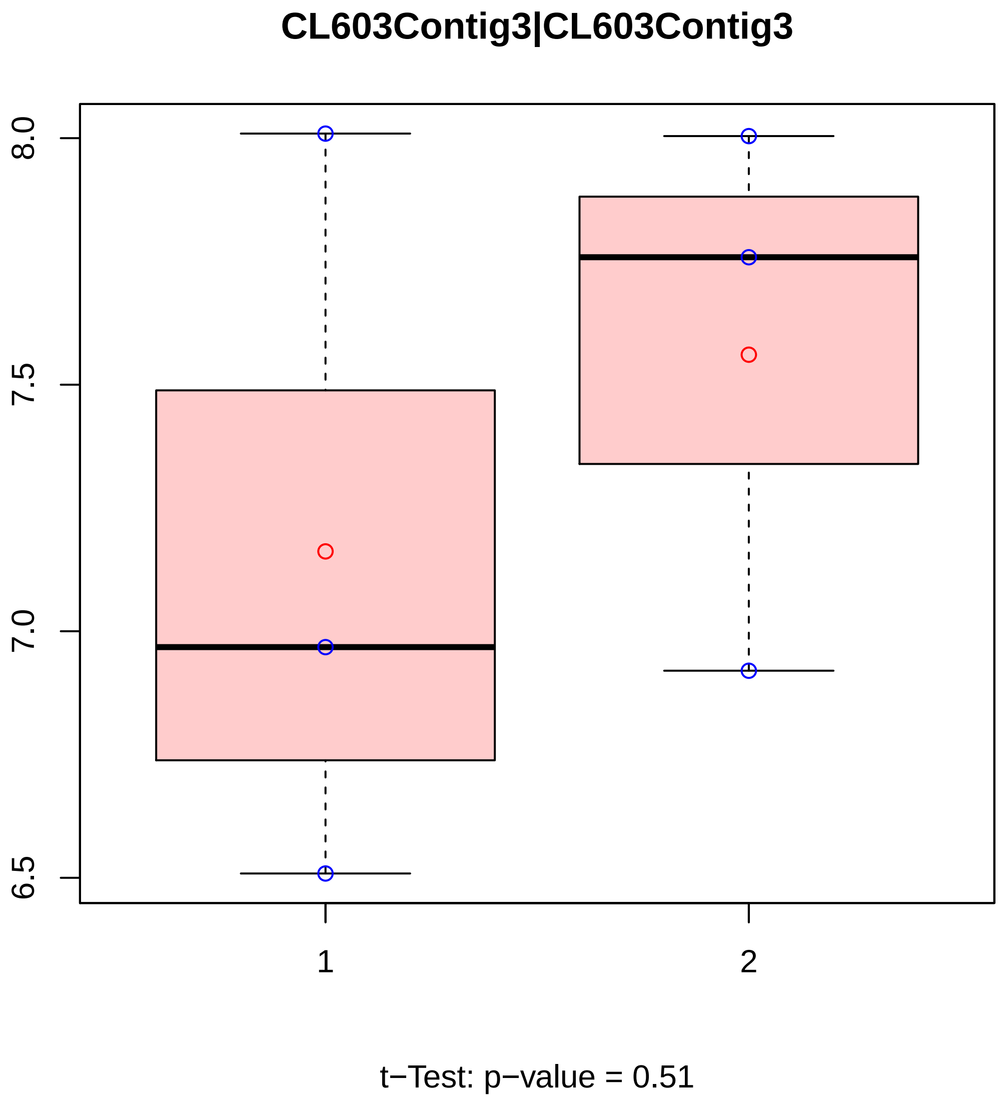
CL603Contig3|CL603Contig3
8.0
7.5
7.0
6.5
1
2
t−Test: p−value = 0.51

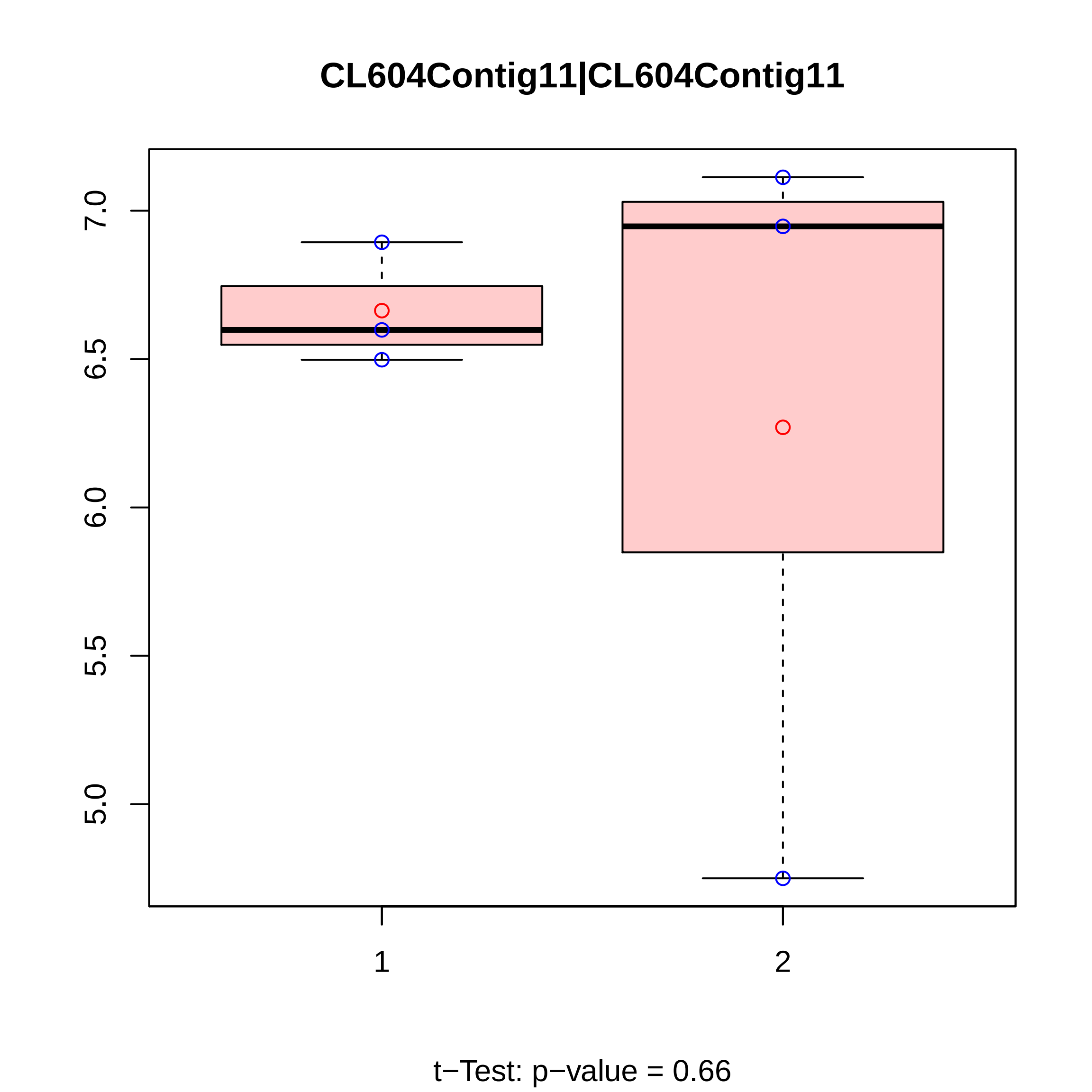
CL604Contig11|CL604Contig11
7.0
6.5
6.0
5.5
5.0
1
2
t−Test: p−value = 0.66

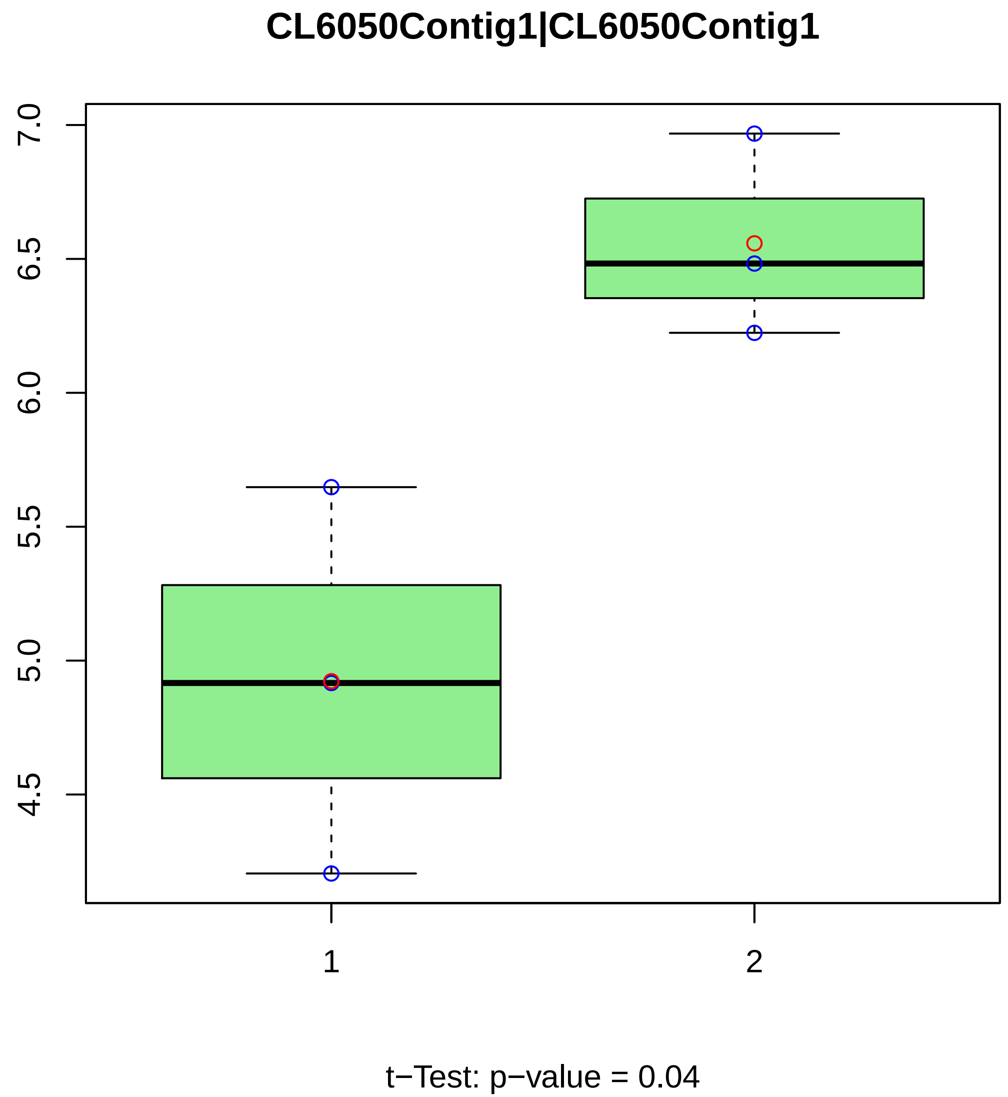
CL6050Contig1|CL6050Contig1
7.0
6.5
6.0
5.5
5.0
4.5
1
2
t−Test: p−value = 0.04

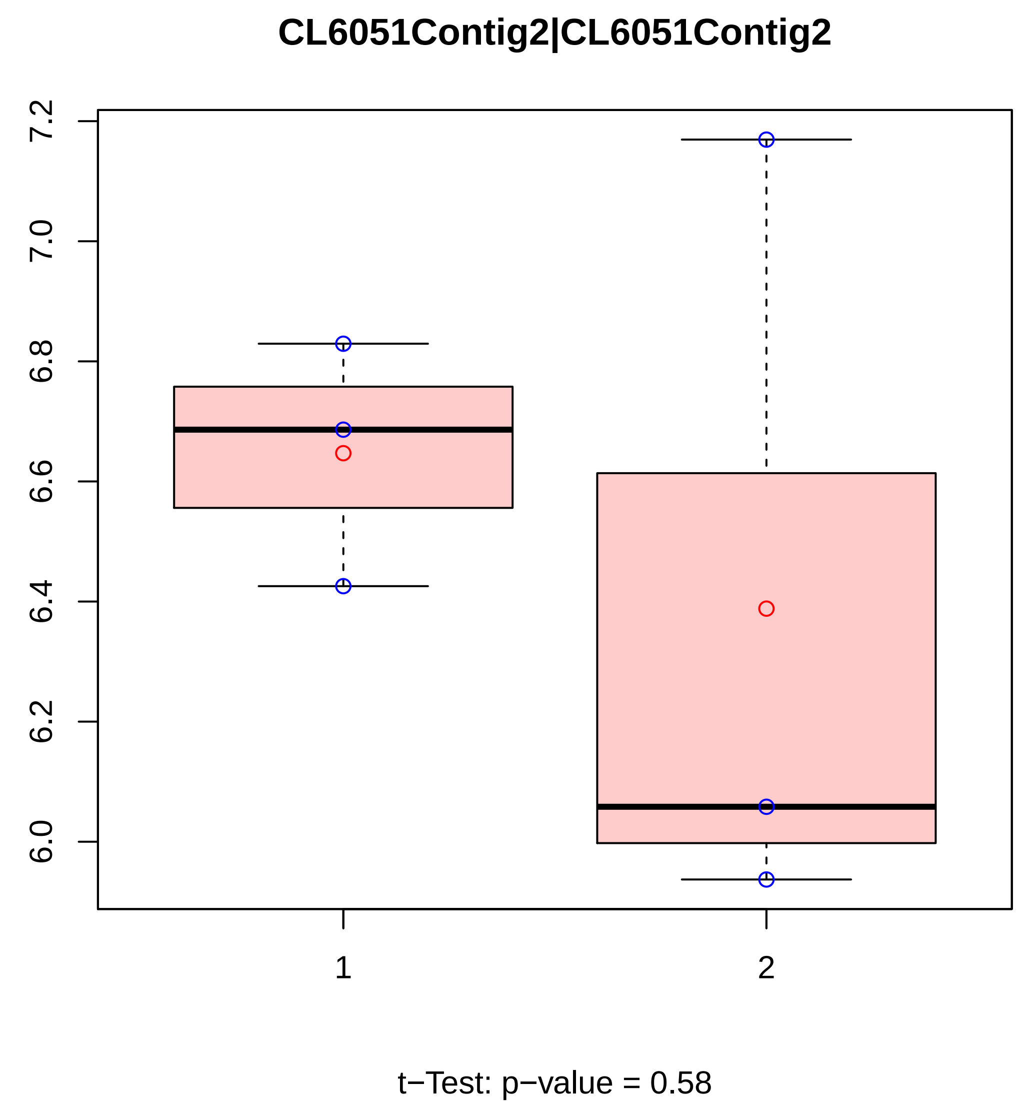
CL6051Contig2|CL6051Contig2
7.2
7.0
6.8
6.6
6.4
6.2
6.0
1
2
t−Test: p−value = 0.58

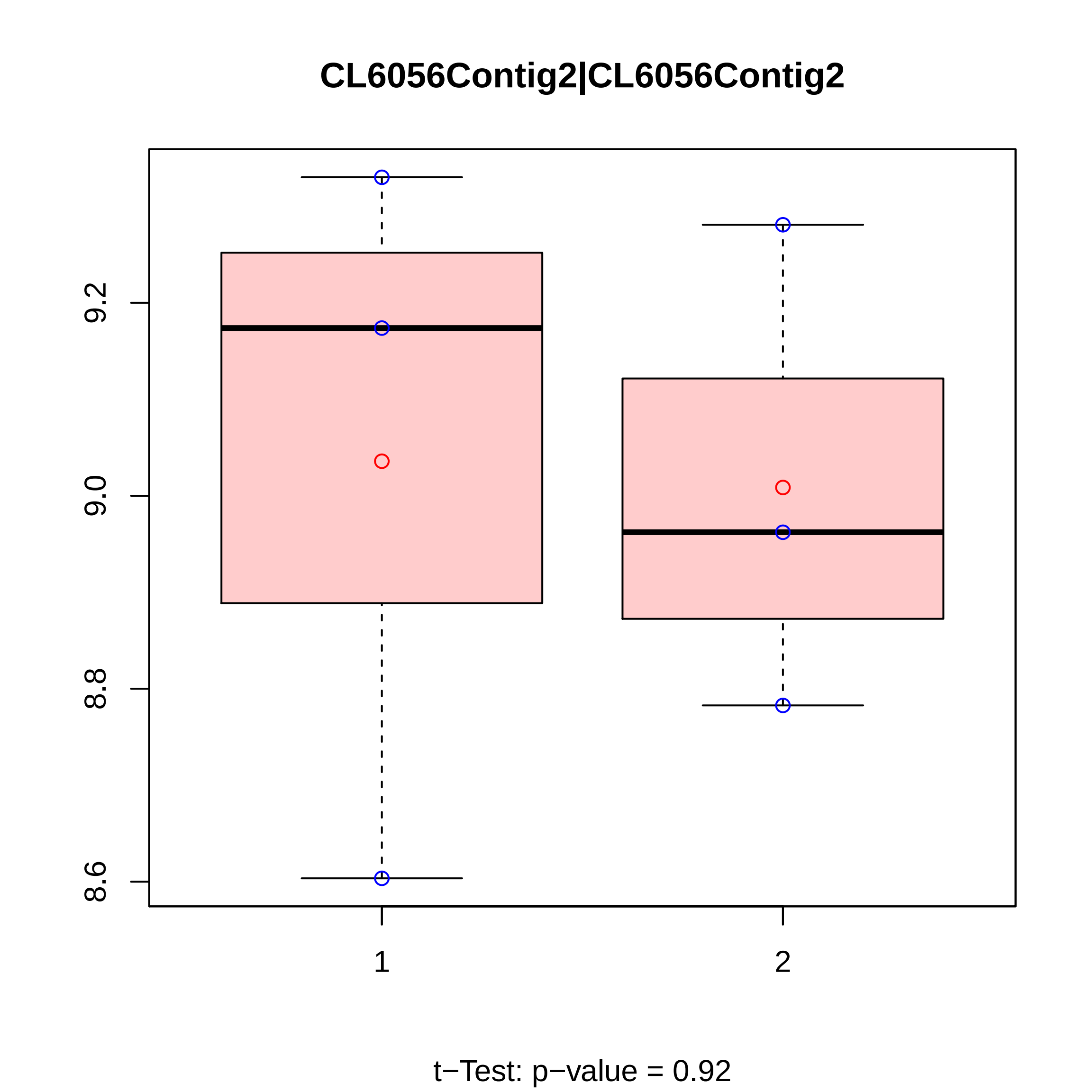
CL6056Contig2|CL6056Contig2
9.2
9.0
8.8
8.6
1
2
t−Test: p−value = 0.92

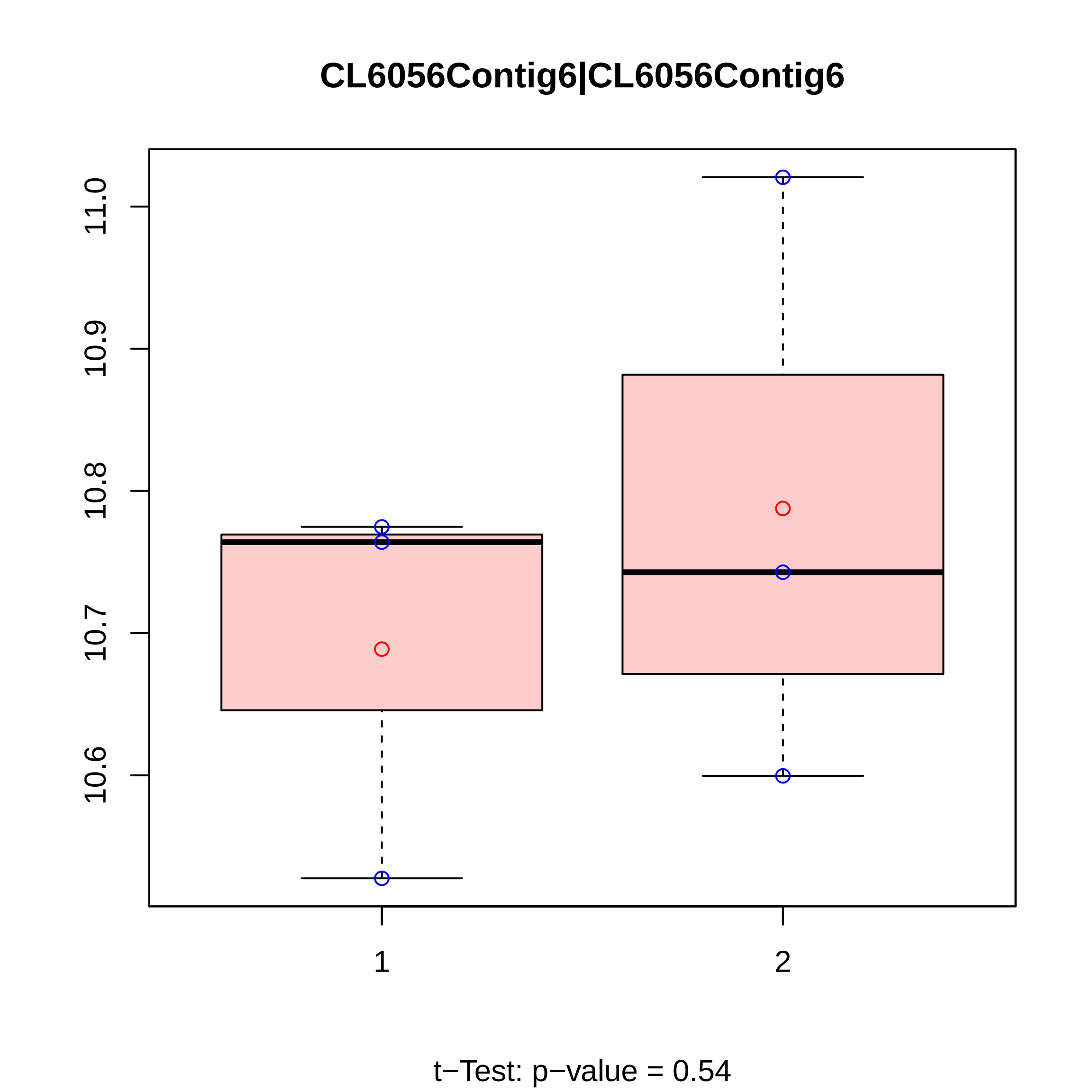
CL6056Contig6|CL6056Contig6
11.0
10.9
10.8
10.7
10.6
1
2
t−Test: p−value = 0.54

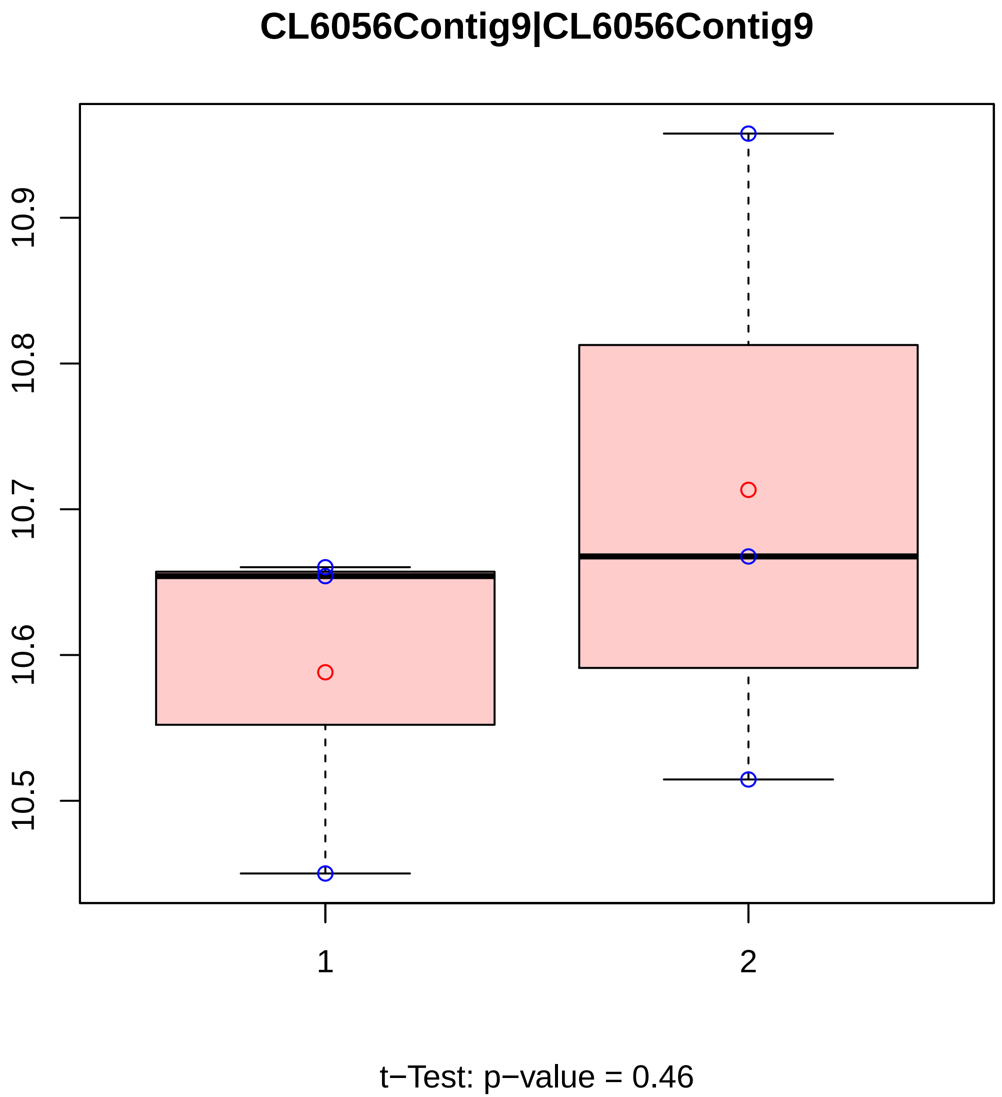

CL6056Contig9|CL6056Contig9
10.5
10.6
10.7
10.8
10.9
1
2
t−Test: p−value = 0.46

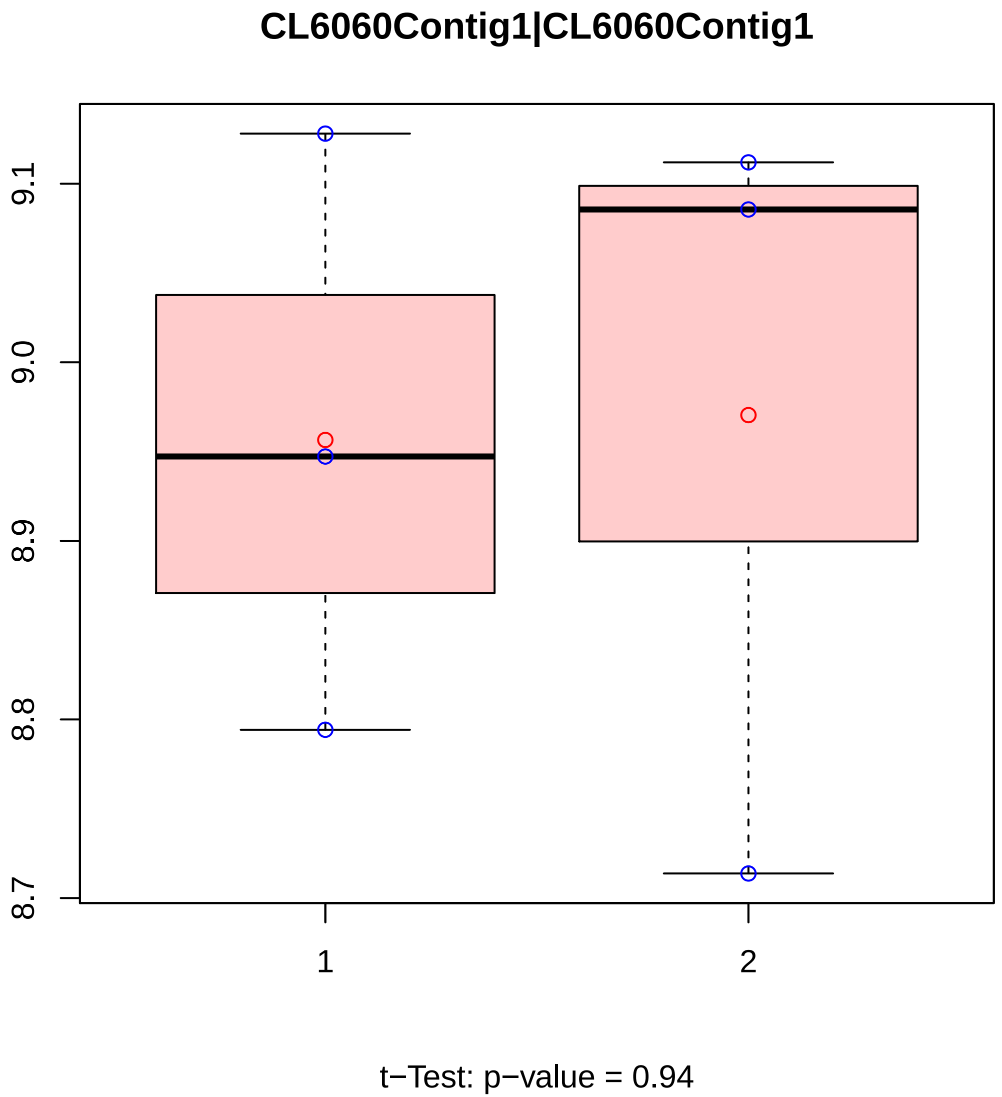
CL6060Contig1|CL6060Contig1
9.1
9.0
8.9
8.8
8.7
1
2
t−Test: p−value = 0.94

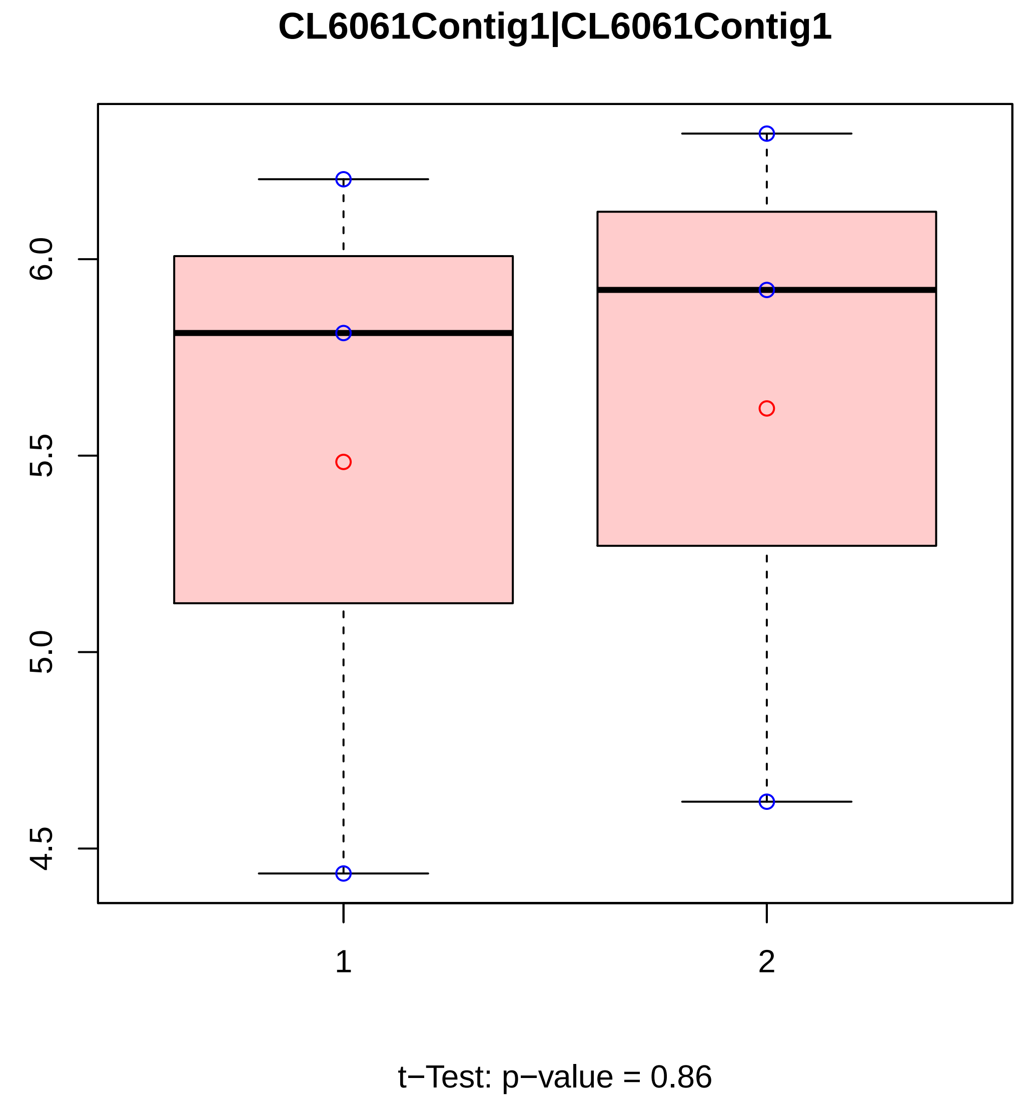

CL6061Contig1|CL6061Contig1
6.0
5.5
5.0
4.5
1
2
t−Test: p−value = 0.86

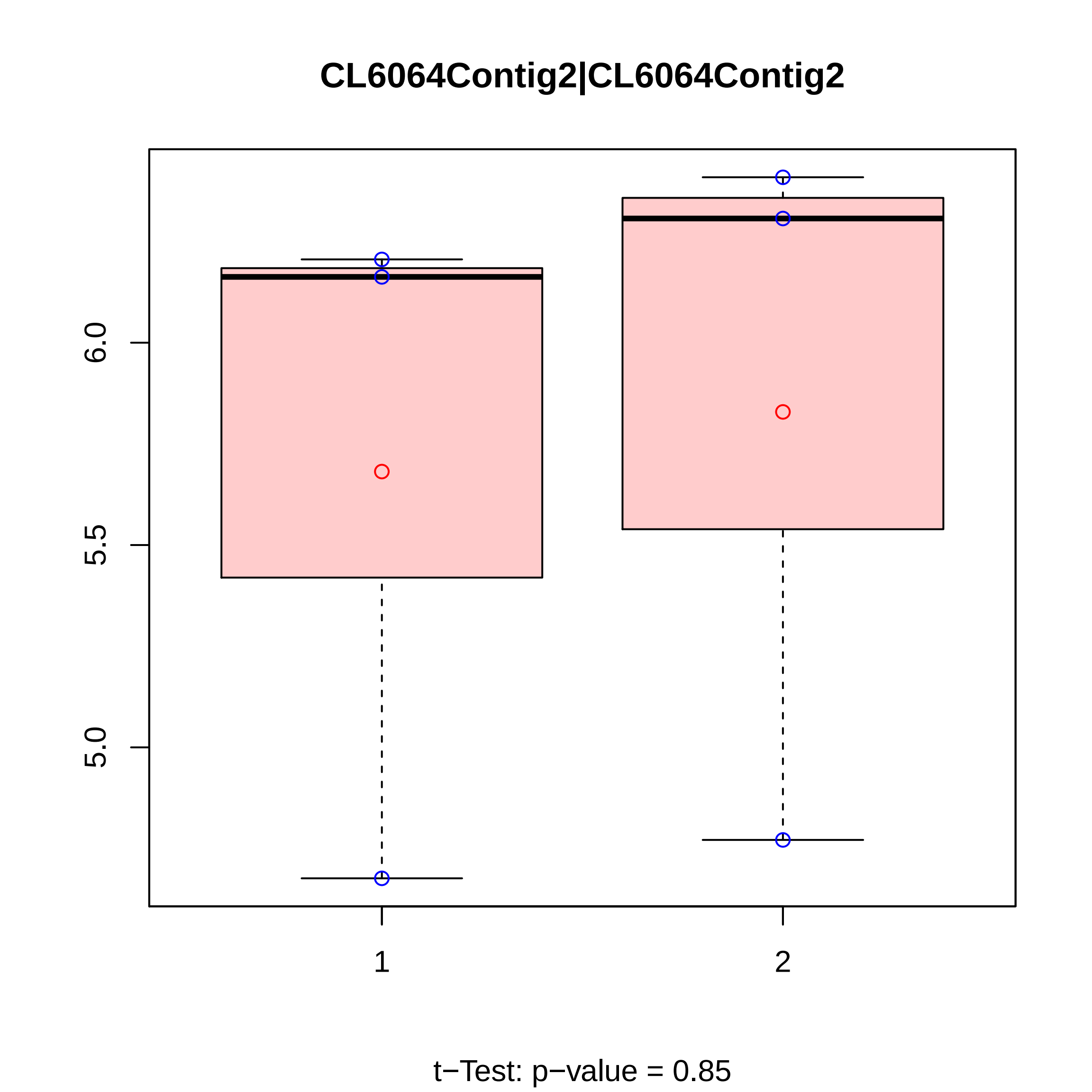

CL6064Contig2|CL6064Contig2
6.0
5.5
5.0
1
2
t−Test: p−value = 0.85

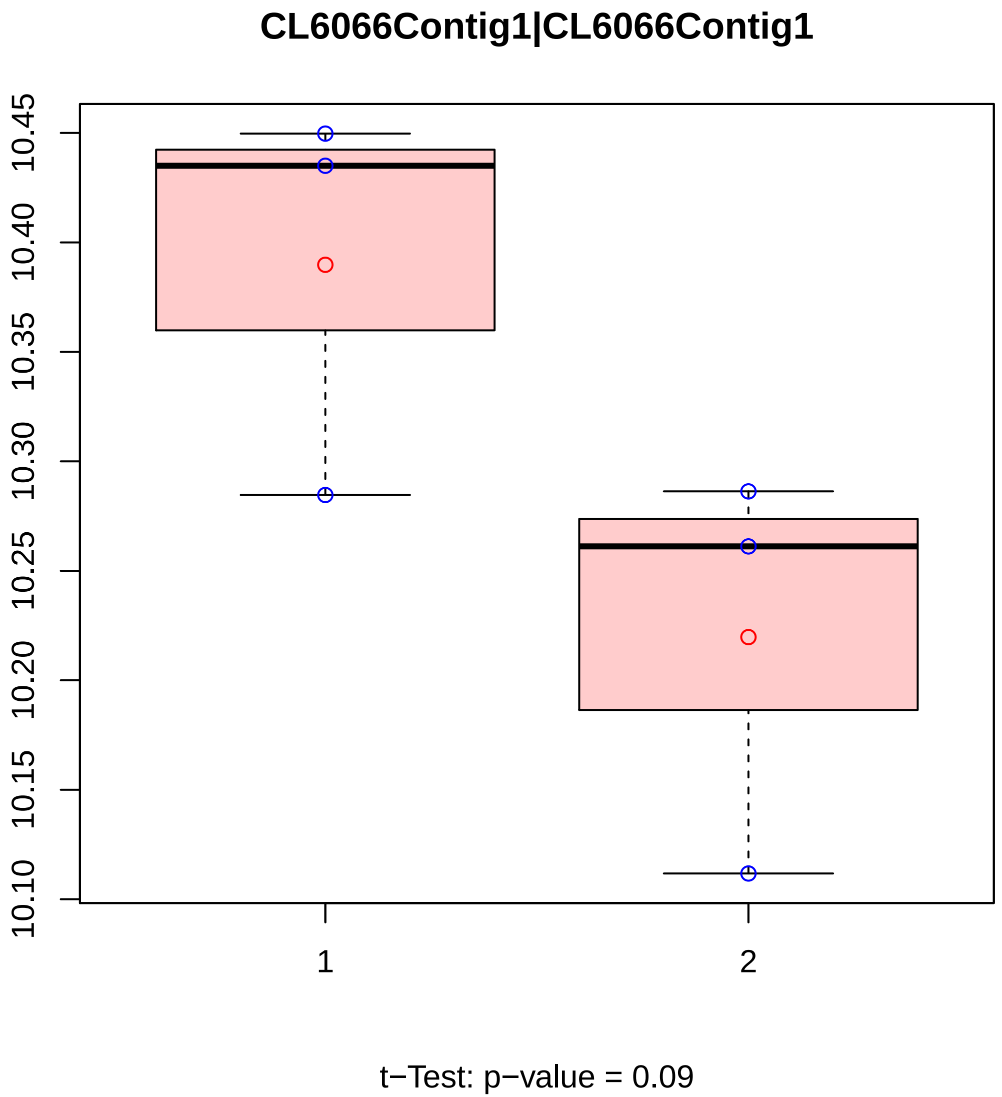
CL6066Contig1|CL6066Contig1
10.10
10.15
10.20
10.25
10.30
10.35
10.40
10.45
1
2
t−Test: p−value = 0.09

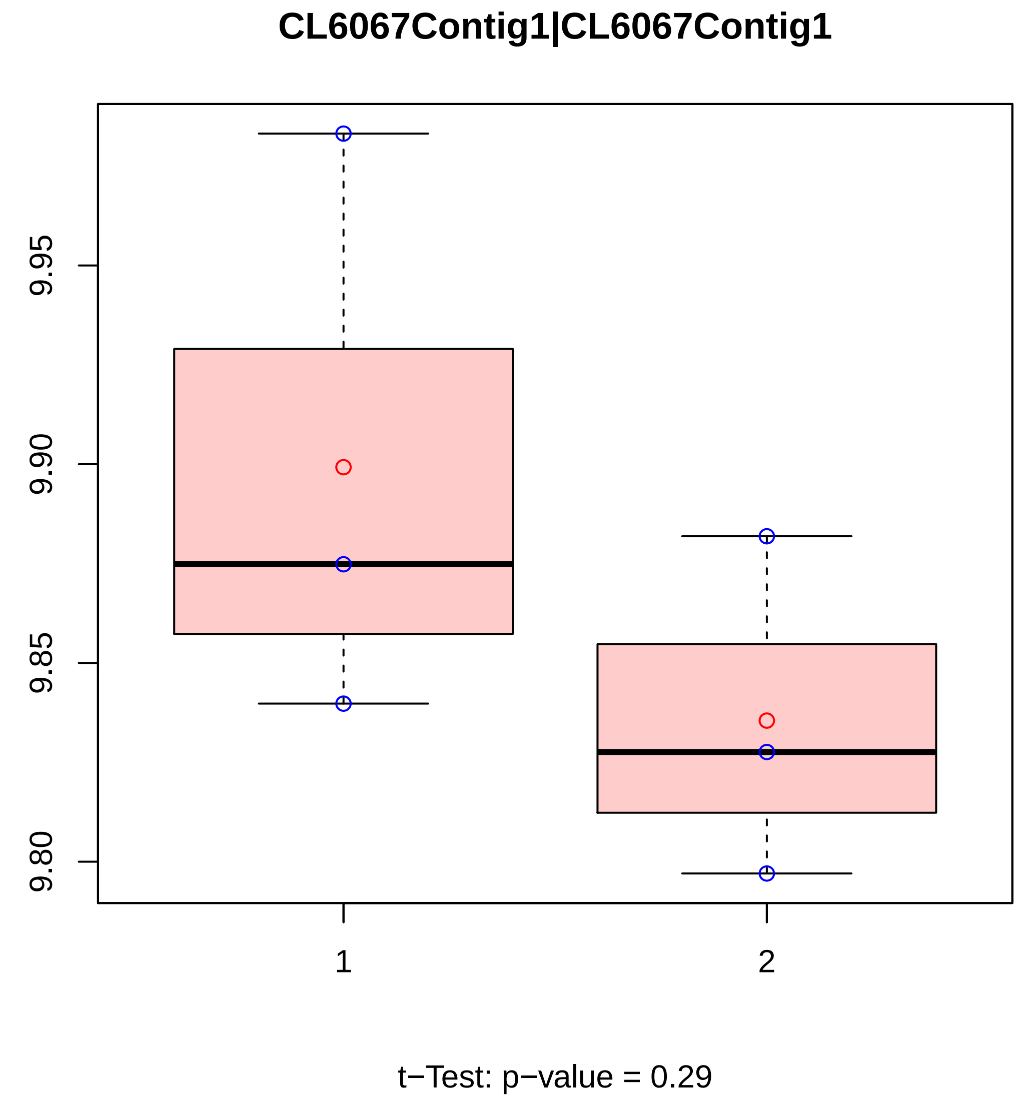

CL6067Contig1|CL6067Contig1
9.80
9.85
9.90
9.95
1
2
t−Test: p−value = 0.29

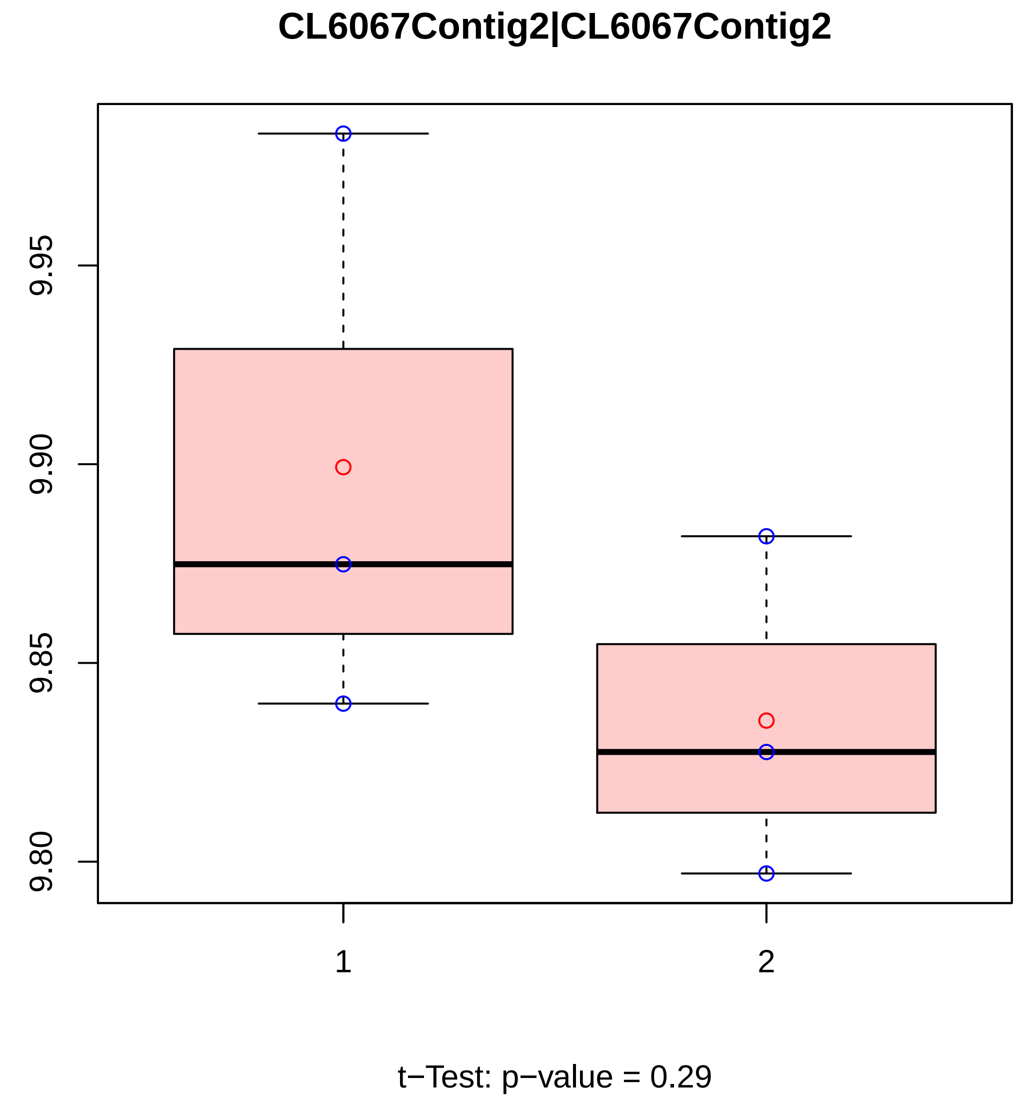
CL6067Contig2|CL6067Contig2
9.80
9.85
9.90
9.95
1
2
t−Test: p−value = 0.29

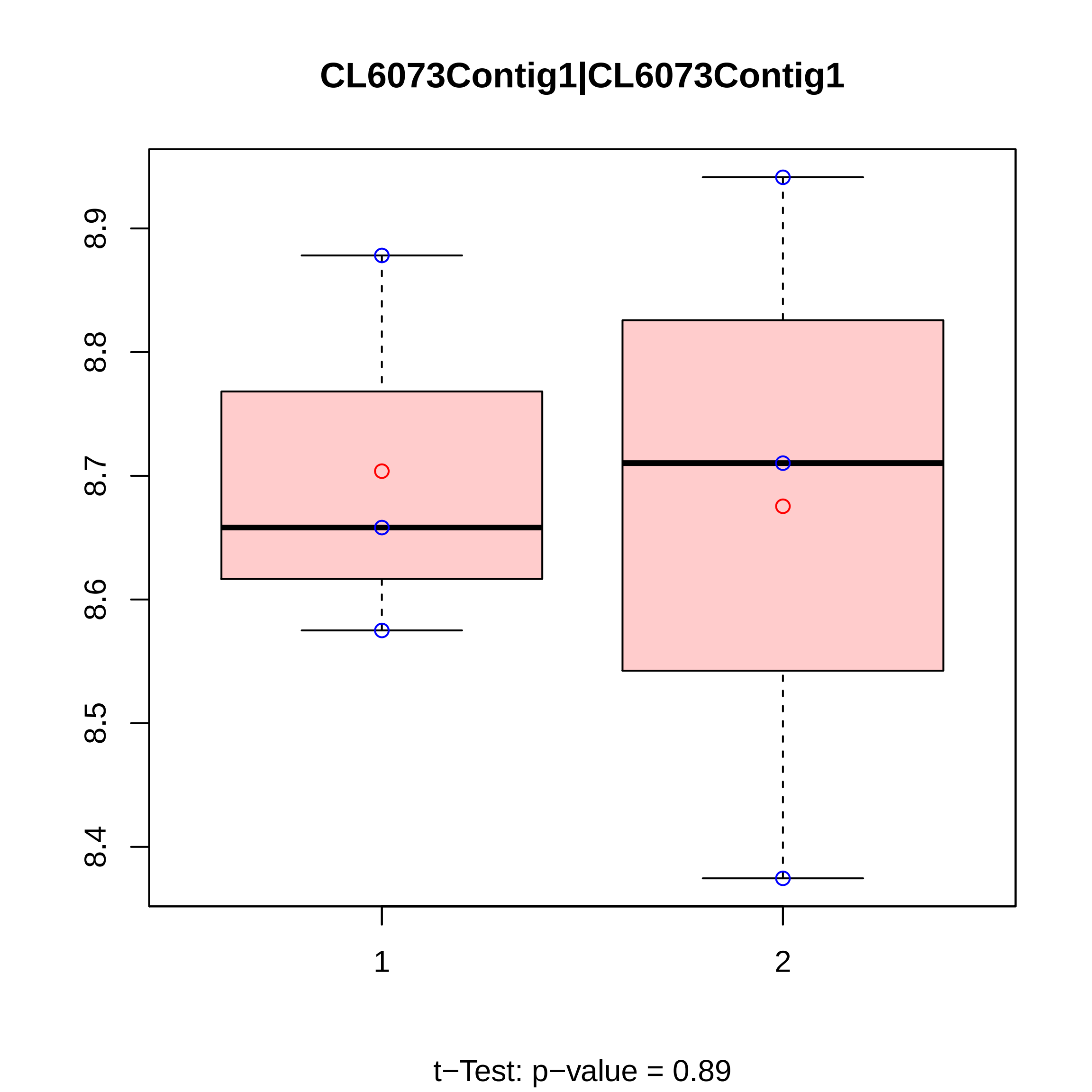
CL6073Contig1|CL6073Contig1
8.4
8.5
8.6
8.7
8.8
8.9
1
2
t−Test: p−value = 0.89

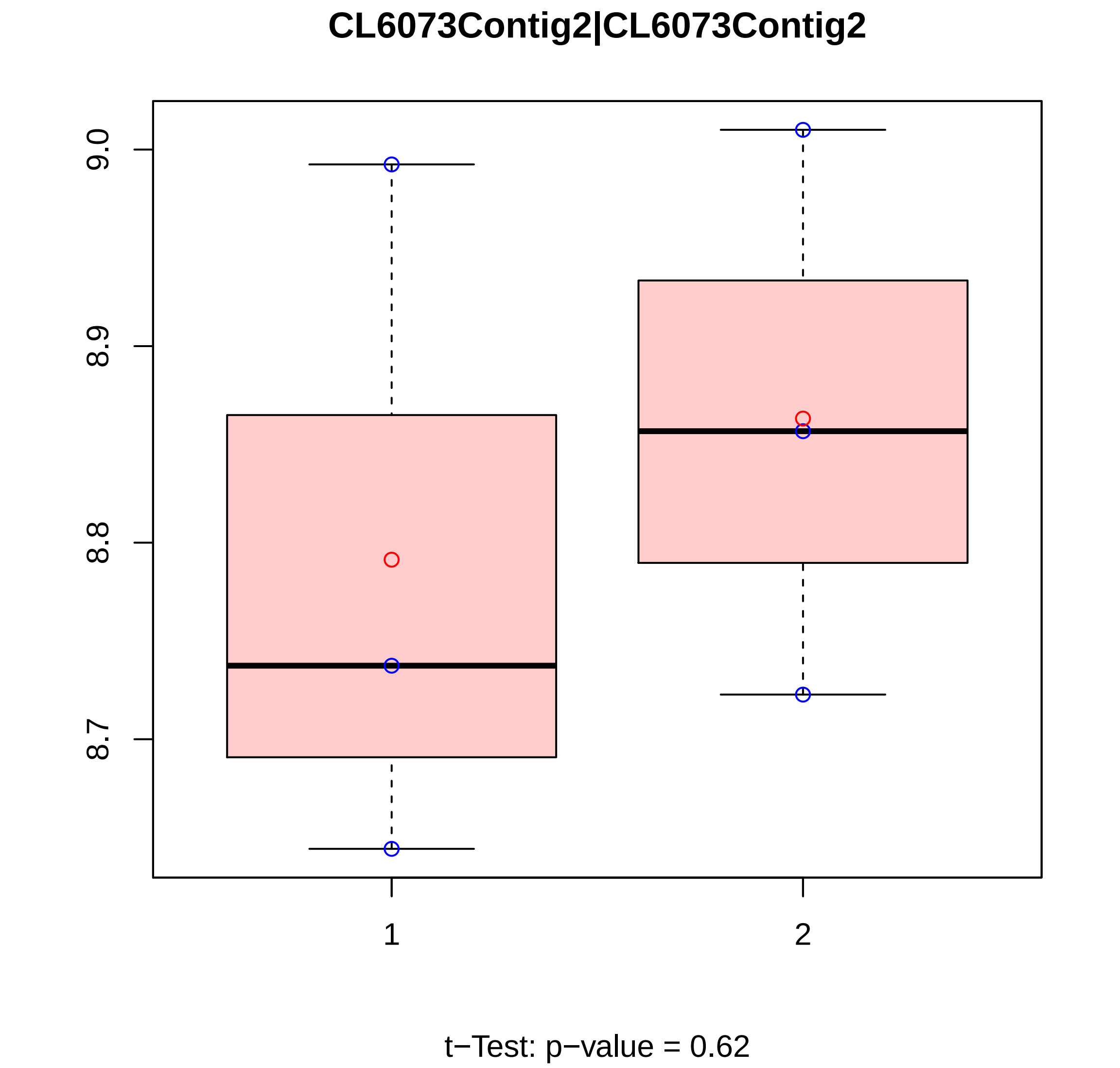
CL6073Contig2|CL6073Contig2
9.0
8.9
8.8
8.7
1
2
t−Test: p−value = 0.62

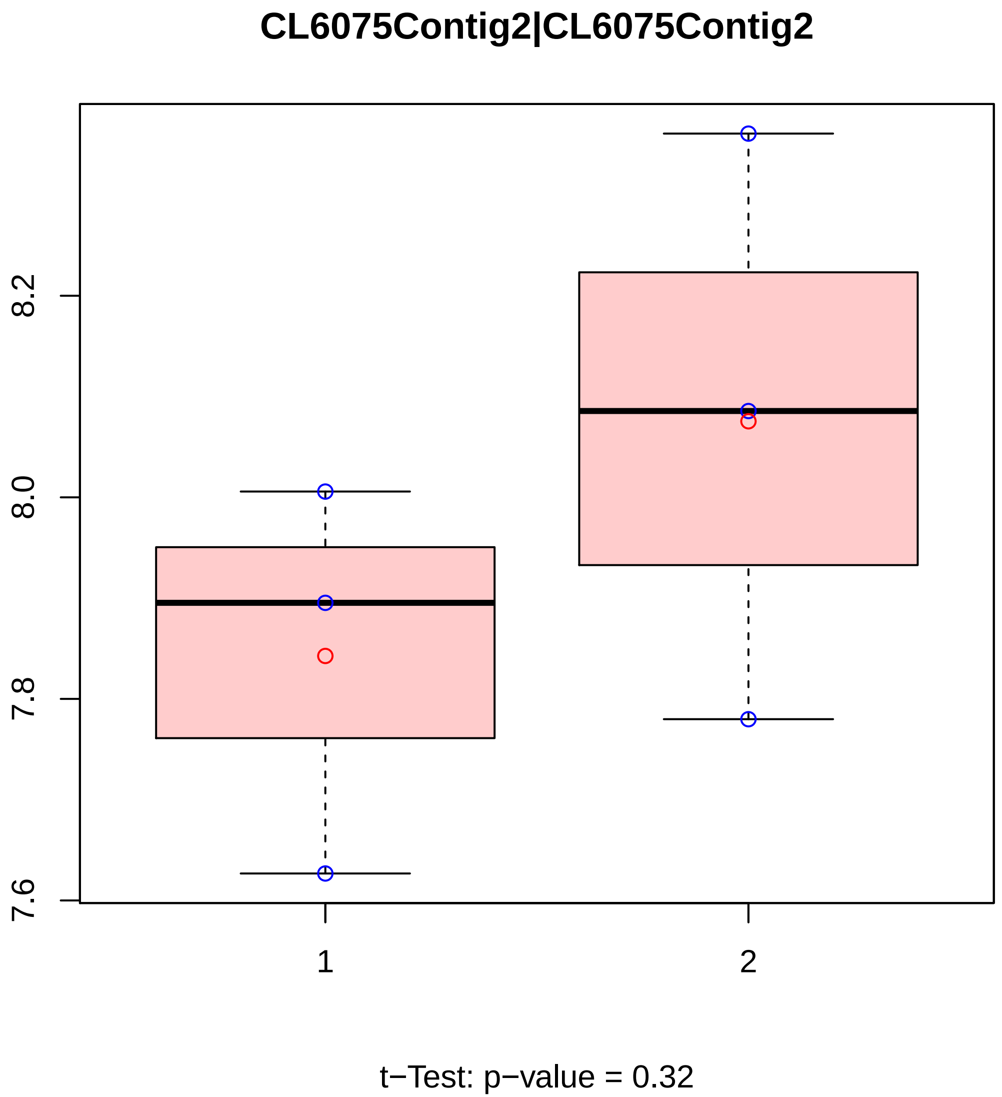

CL6075Contig2|CL6075Contig2
7.6
7.8
8.0
8.2
1
2
t−Test: p−value = 0.32

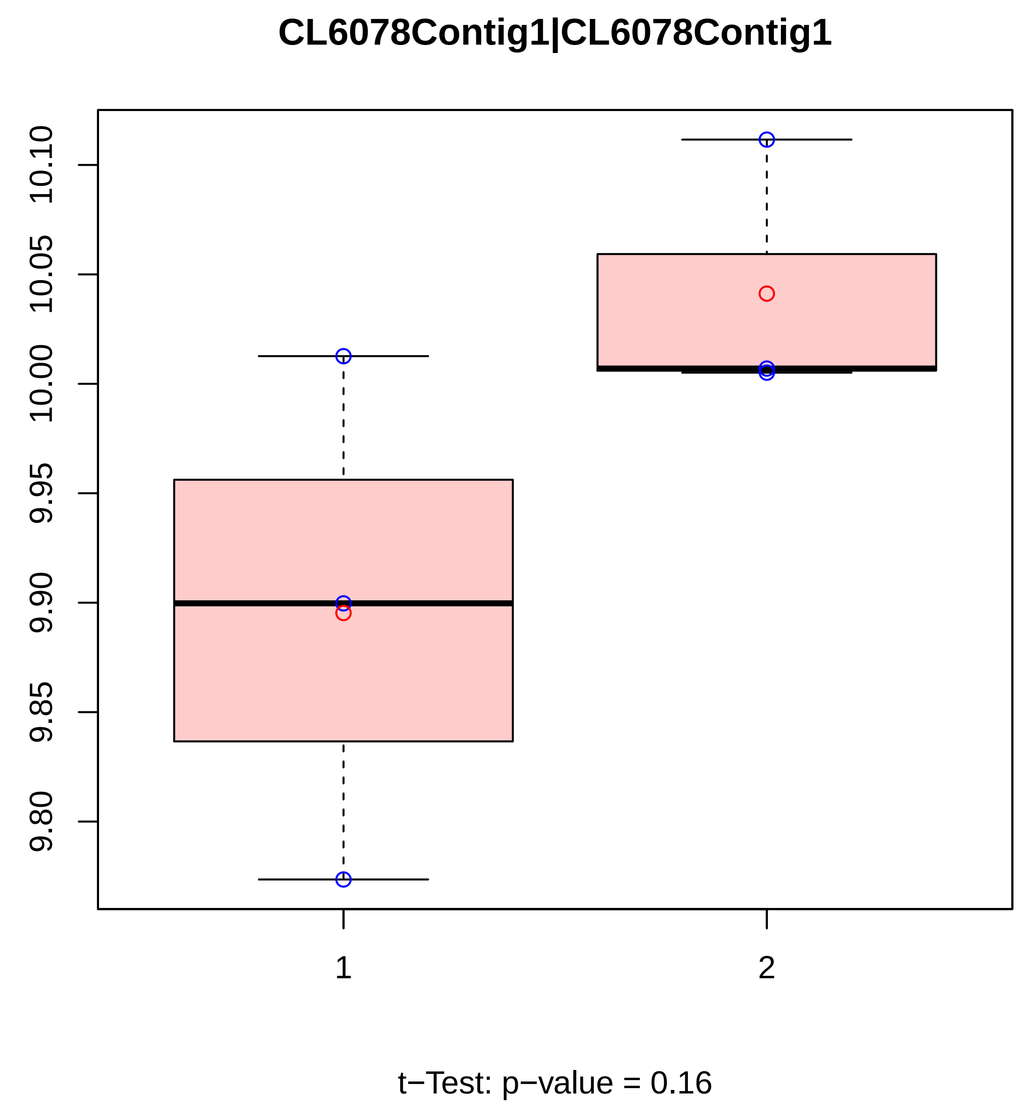

CL6078Contig1|CL6078Contig1
10.10
10.05
10.00
9.95
9.90
9.85
9.80
1
2
t−Test: p−value = 0.16

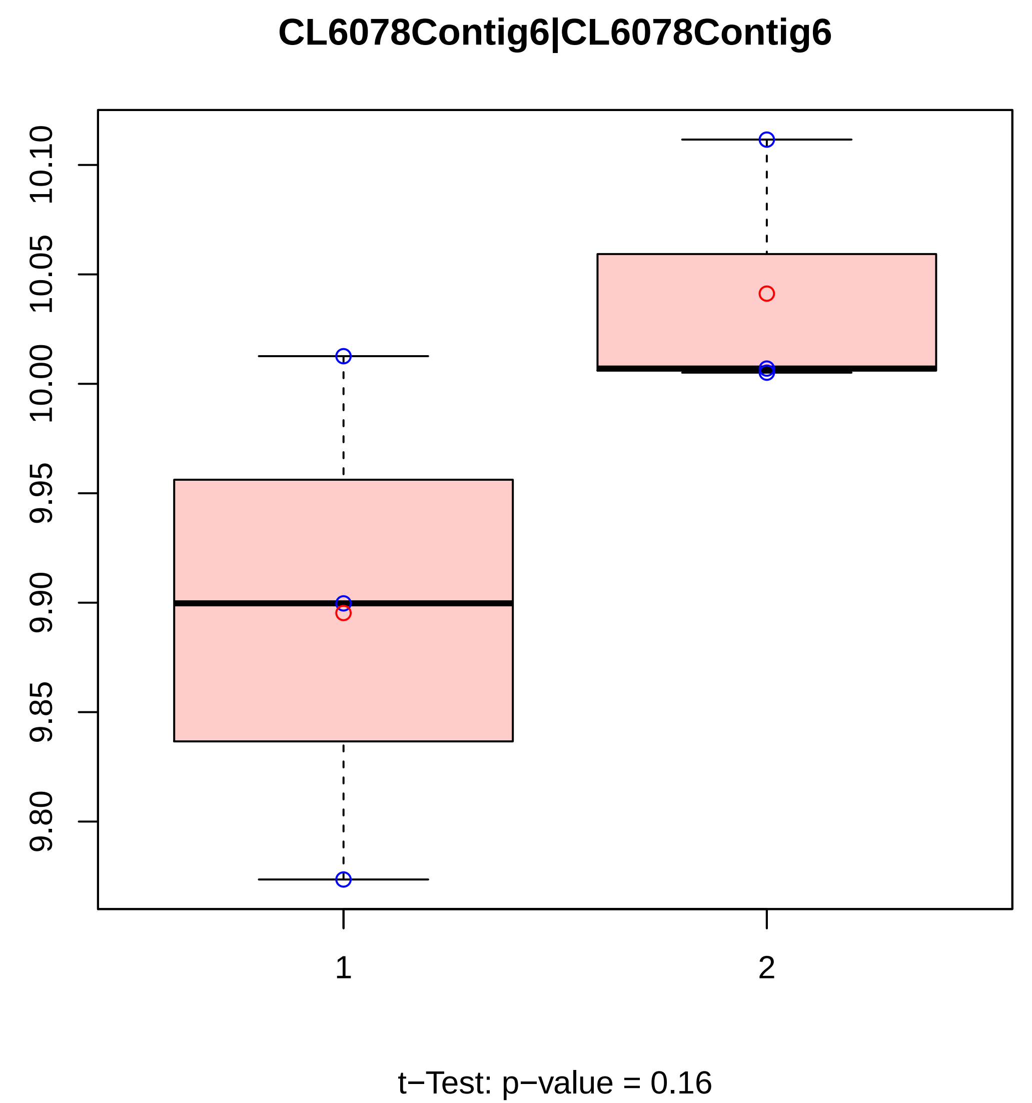
CL6078Contig6|CL6078Contig6
10.10
10.05
10.00
9.95
9.90
9.85
9.80
1
2
t−Test: p−value = 0.16

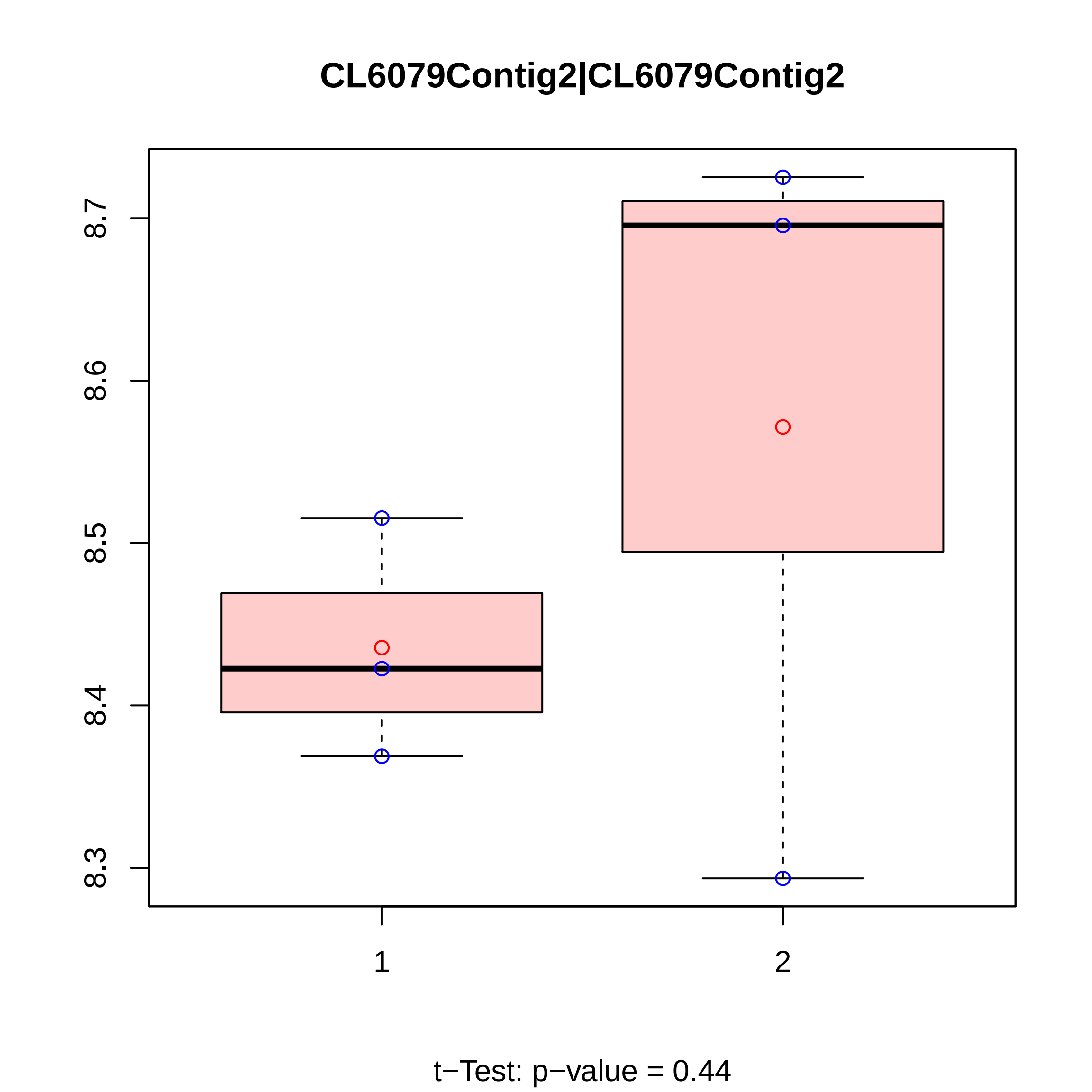
CL6079Contig2|CL6079Contig2
8.7
8.6
8.5
8.4
8.3
1
2
t−Test: p−value = 0.44

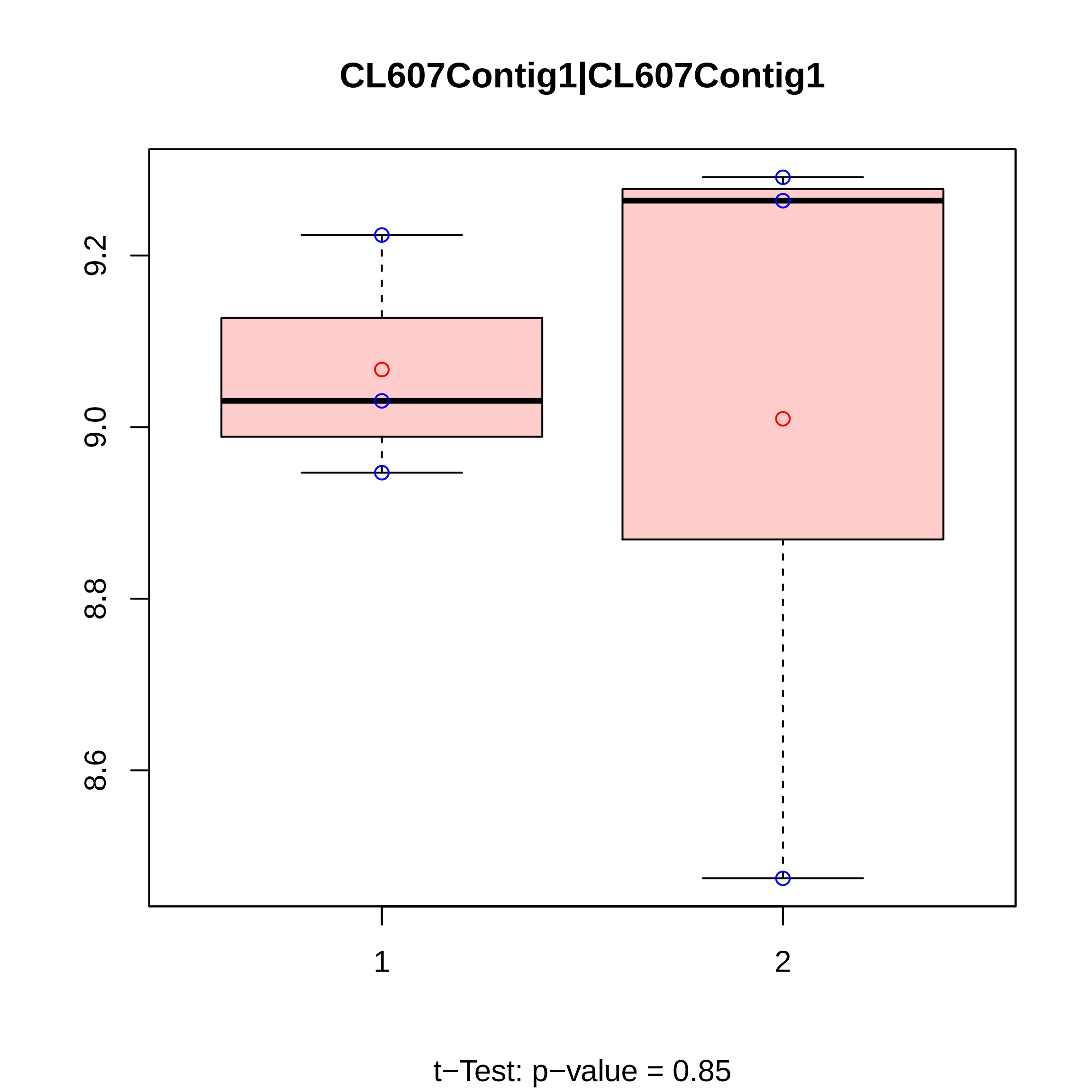

CL607Contig1|CL607Contig1
9.2
9.0
8.8
8.6
1
2
t−Test: p−value = 0.85

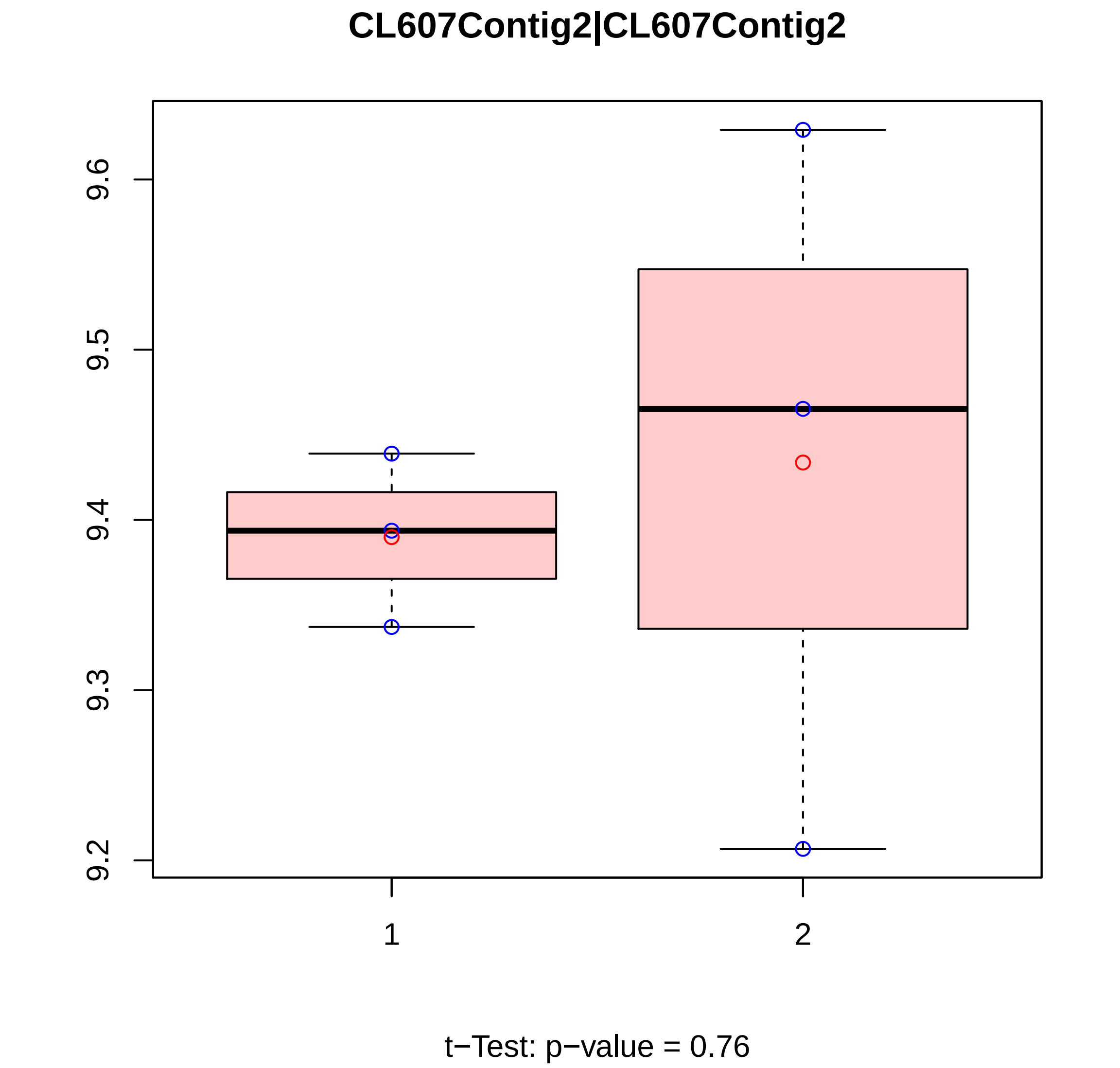
CL607Contig2|CL607Contig2
9.2
9.3
9.4
9.5
9.6
1
2
t−Test: p−value = 0.76

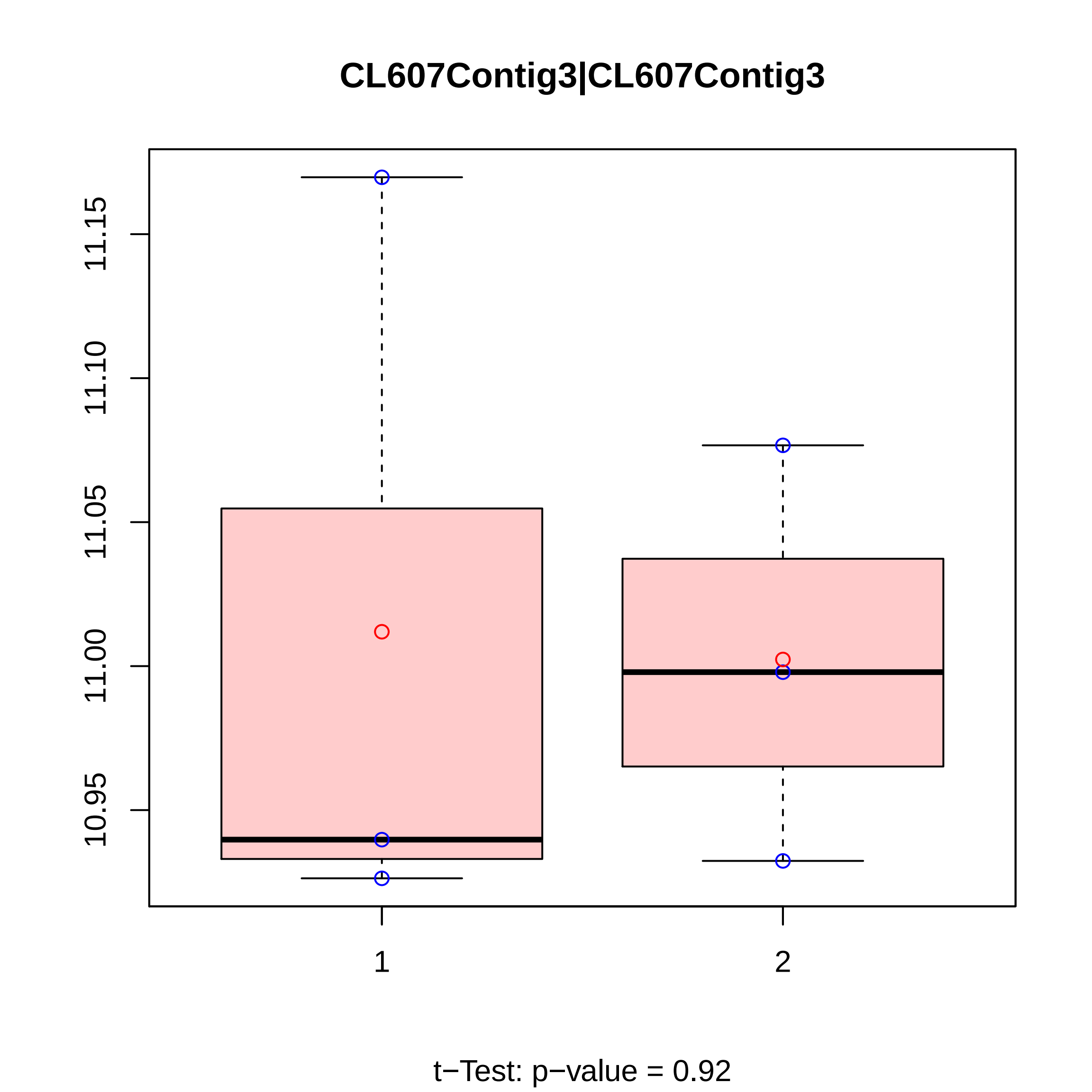
CL607Contig3|CL607Contig3
11.15
11.10
11.05
11.00
10.95
1
2
t−Test: p−value = 0.92

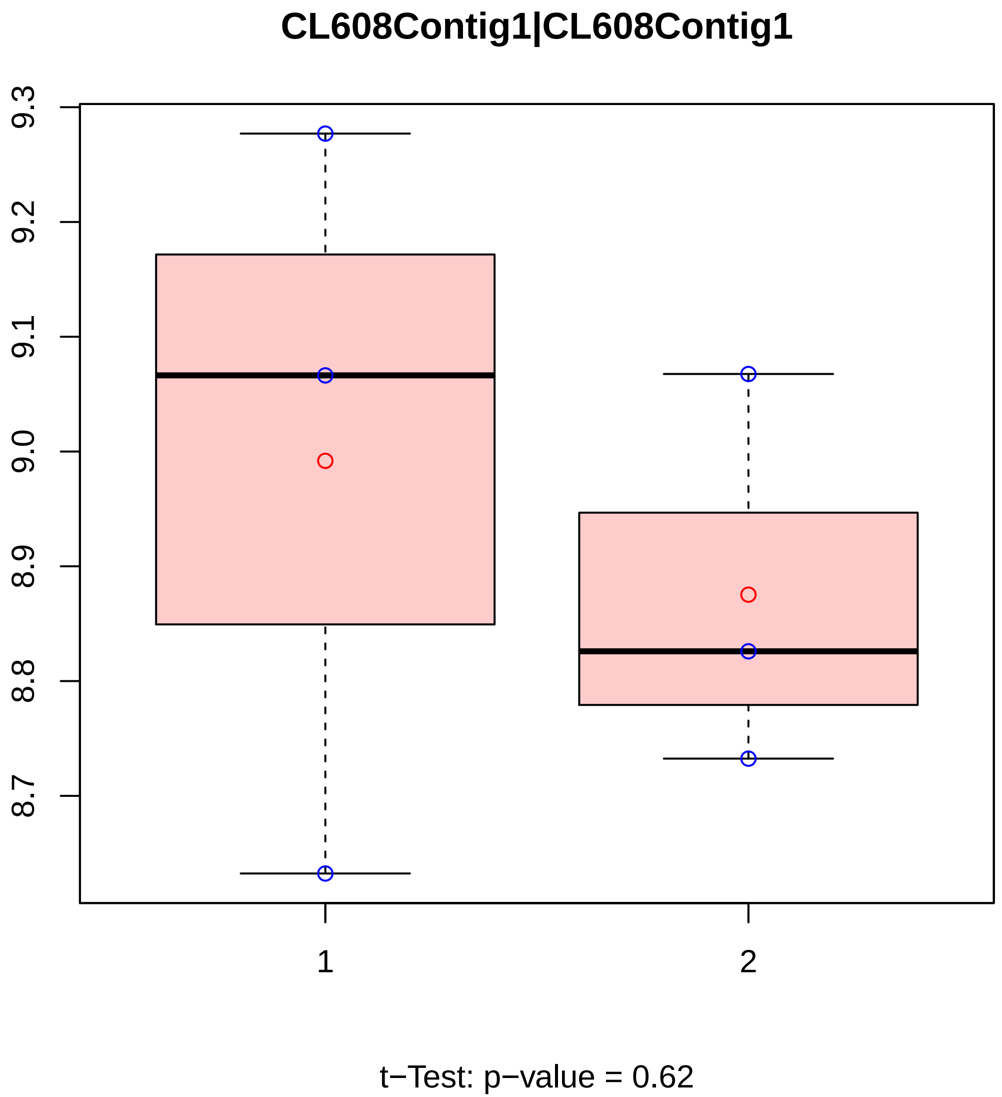
CL608Contig1|CL608Contig1
9.3
9.2
9.1
9.0
8.9
8.8
8.7
1
2
t−Test: p−value = 0.62

CL60Contig22|CL60Contig22

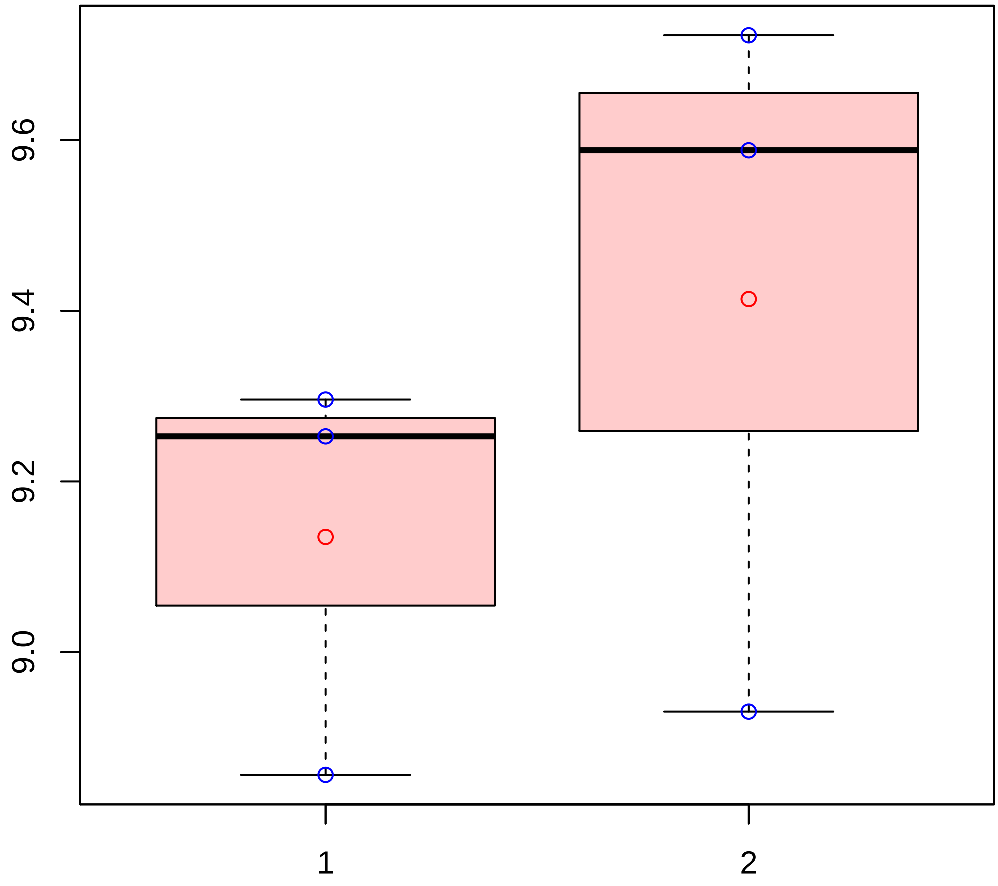


t−Test: p−value = 0.39

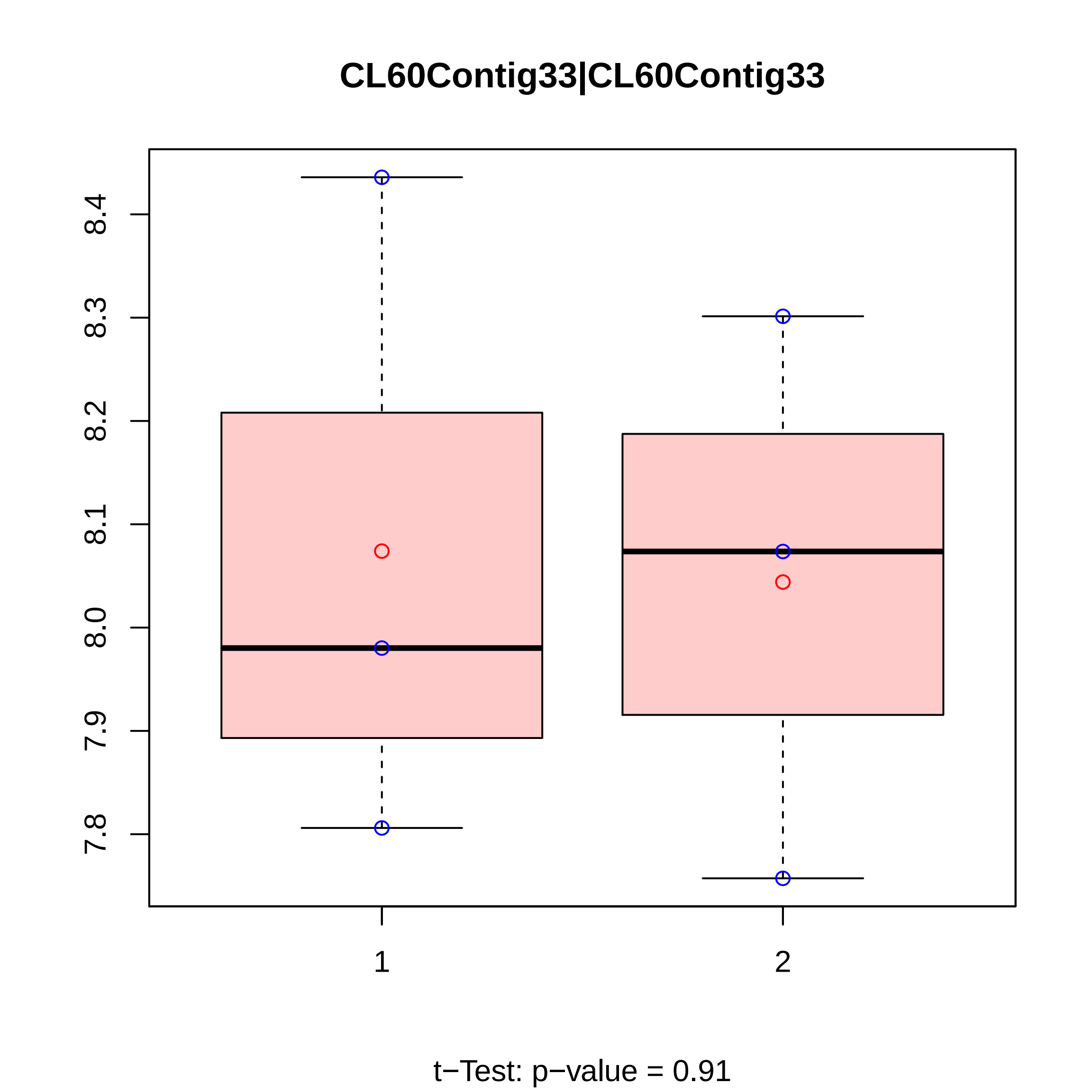
CL60Contig33|CL60Contig33
8.4
8.3
8.2
8.1
8.0
7.9
7.8
1
2
t−Test: p−value = 0.91

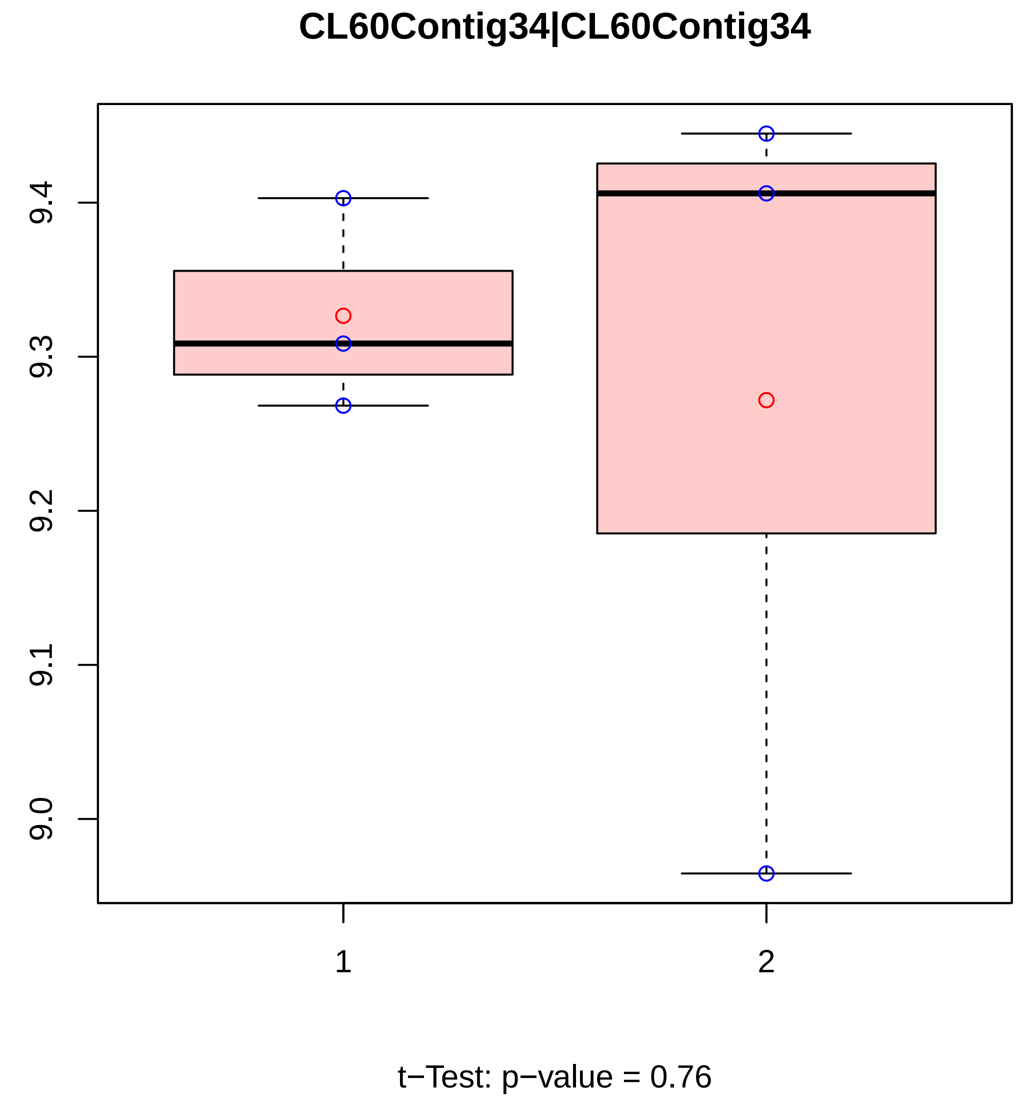
CL60Contig34|CL60Contig34
9.0
9.1
9.2
9.3
9.4
1
2
t−Test: p−value = 0.76

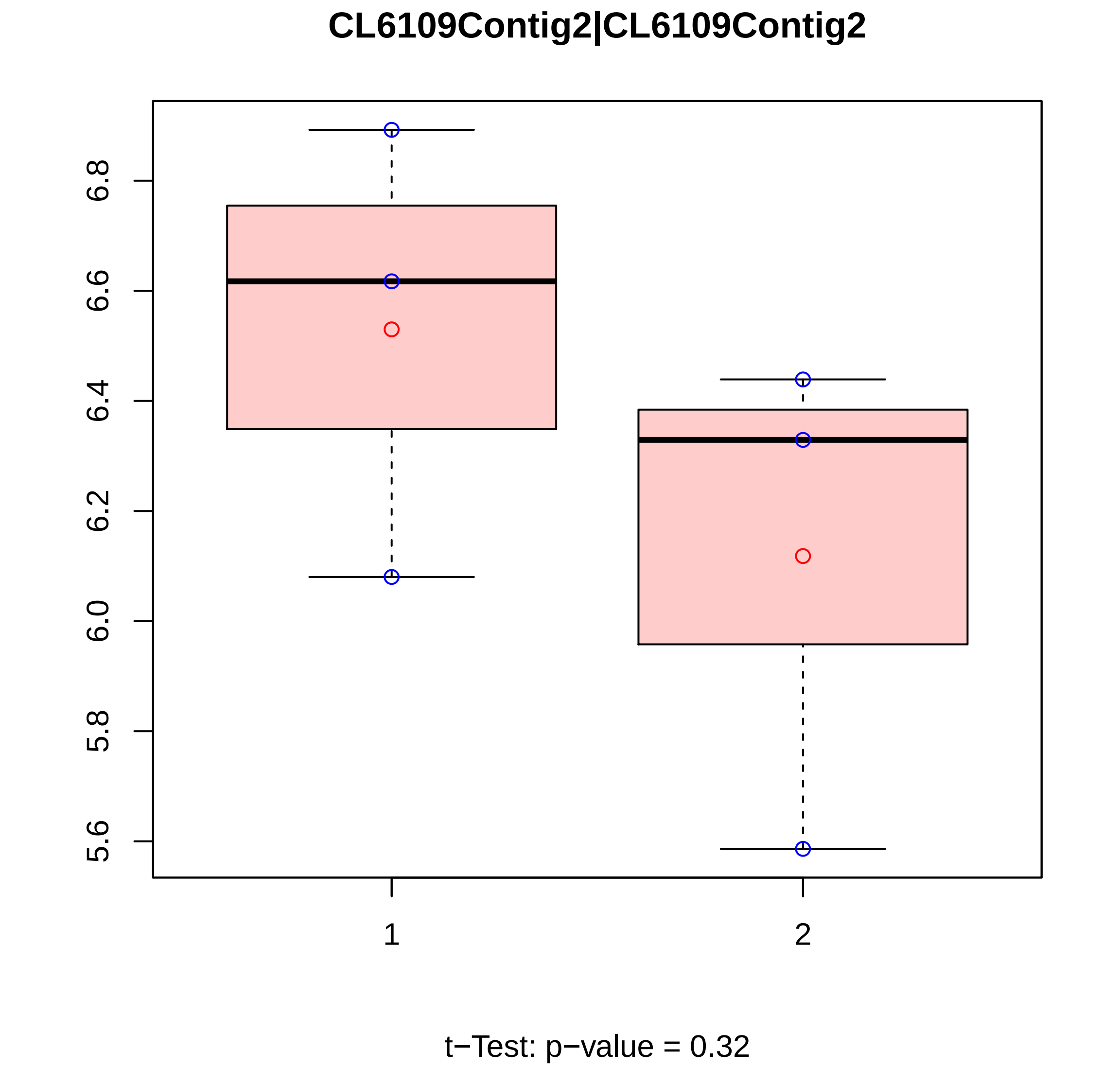
CL6109Contig2|CL6109Contig2
6.8
6.6
6.4
6.2
6.0
5.8
5.6
1
2
t−Test: p−value = 0.32

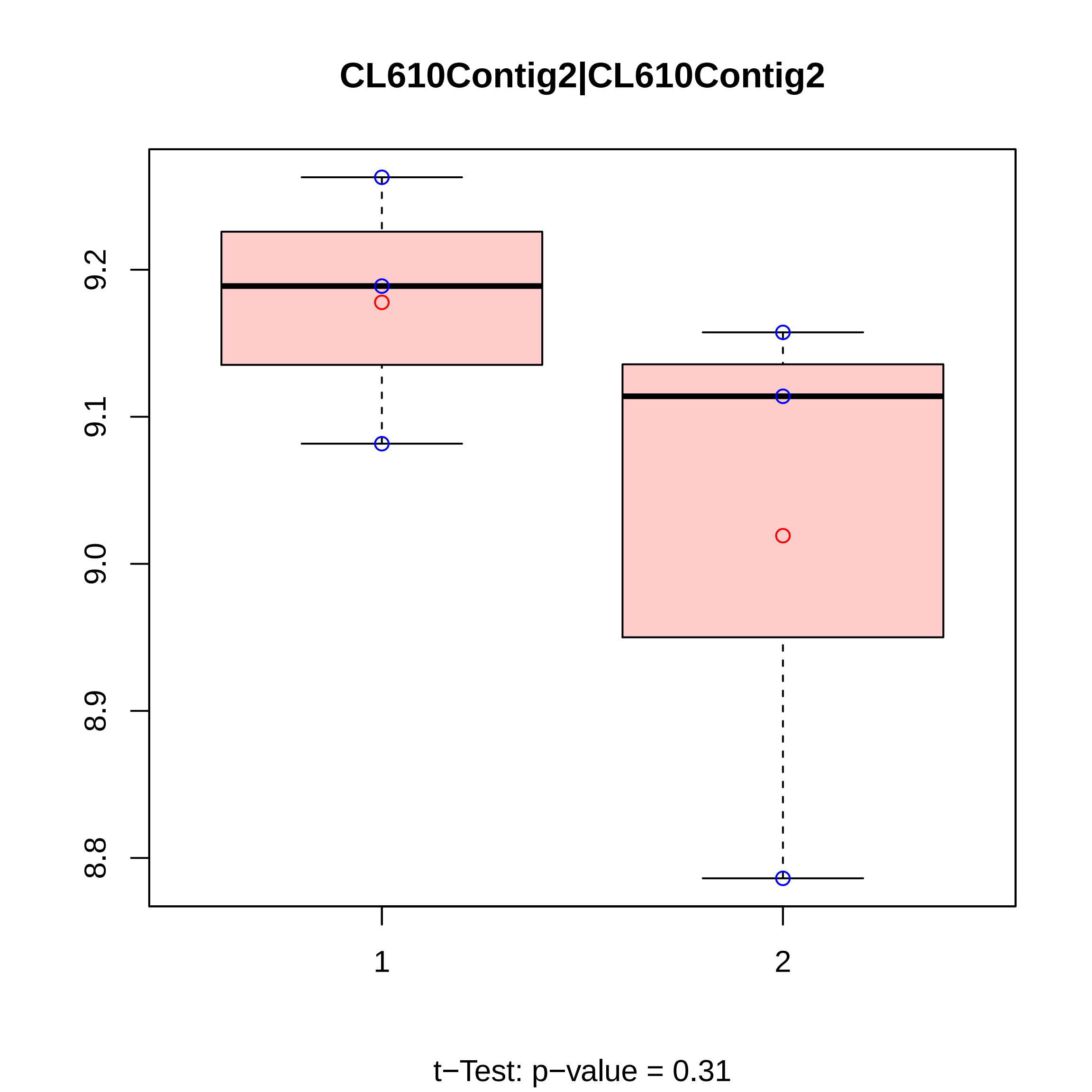

CL610Contig2|CL610Contig2
9.2
9.1
9.0
8.9
8.8
1
2
t−Test: p−value = 0.31

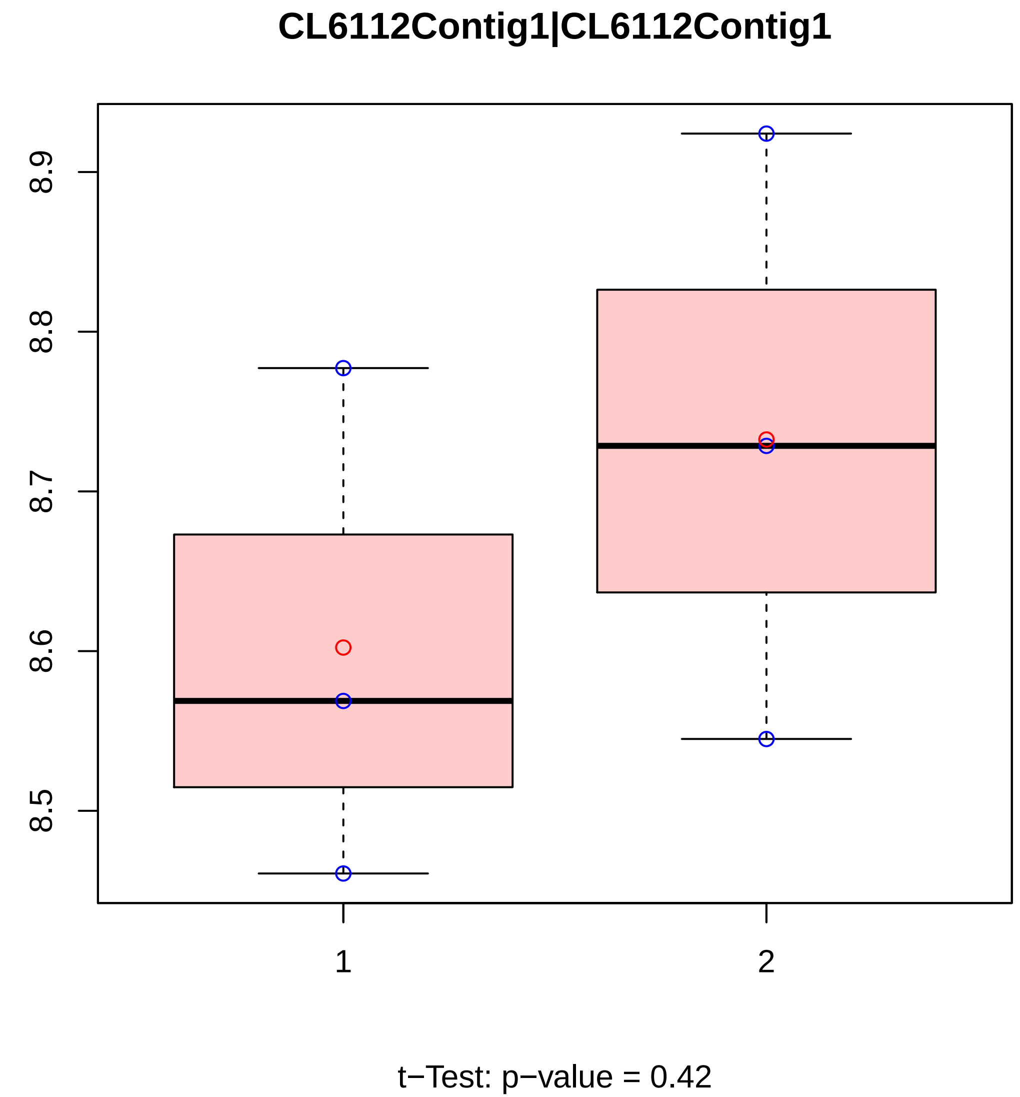
CL6112Contig1|CL6112Contig1
8.5
8.6
8.7
8.8
8.9
1
2
t−Test: p−value = 0.42

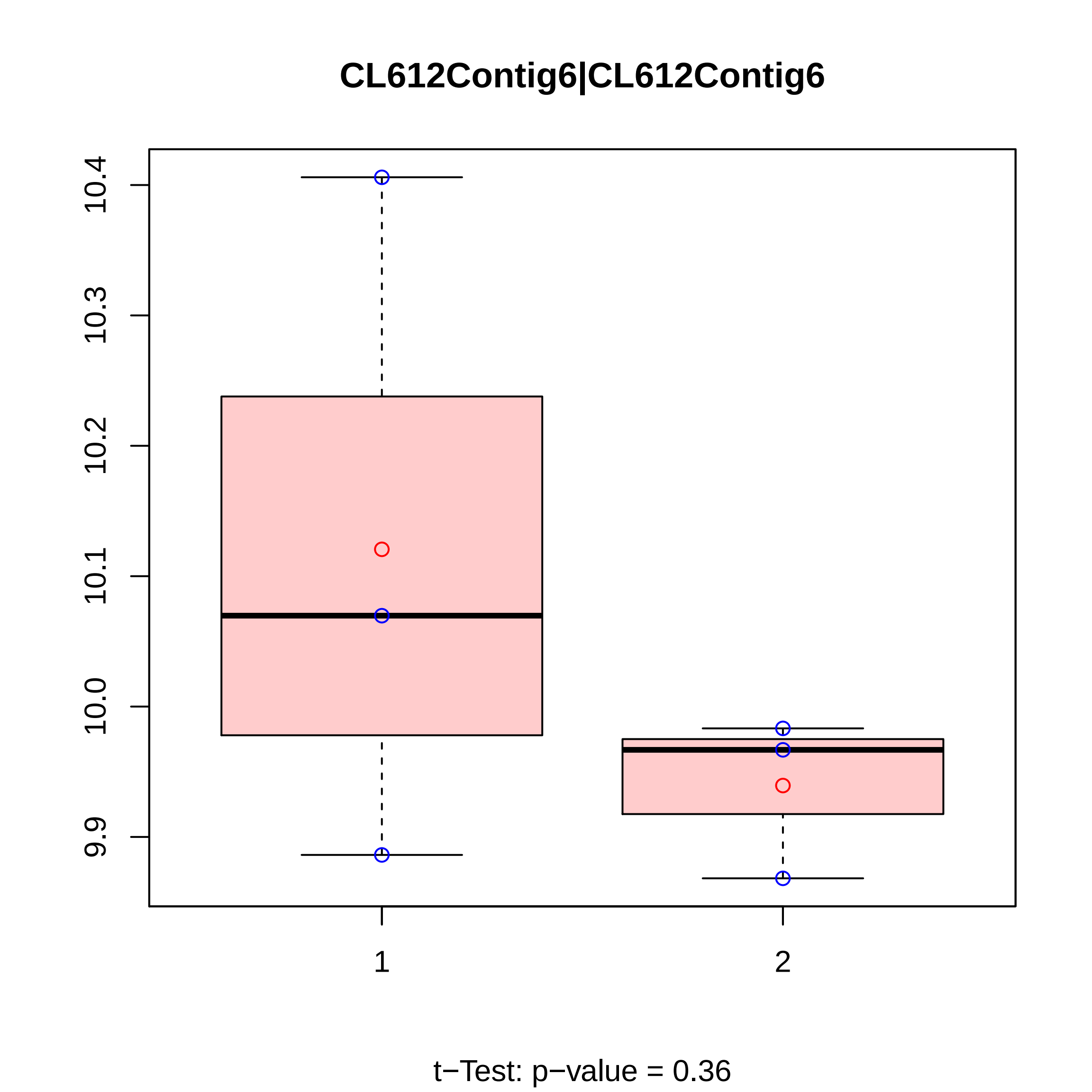
CL612Contig6|CL612Contig6
10.4
10.3
10.2
10.1
10.0
9.9
1
2
t−Test: p−value = 0.36

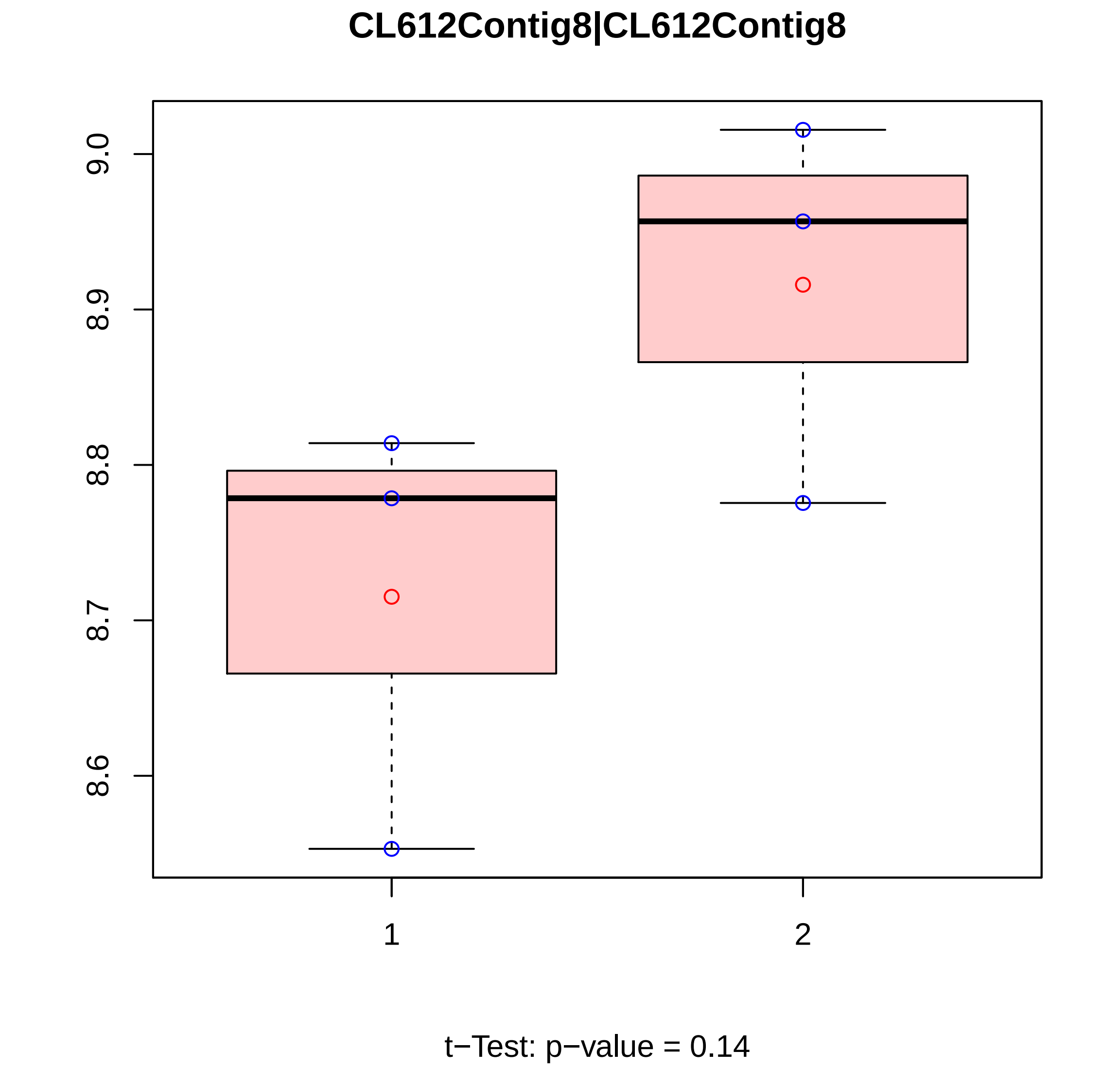
CL612Contig8|CL612Contig8
9.0
8.9
8.8
8.7
8.6
1
2
t−Test: p−value = 0.14

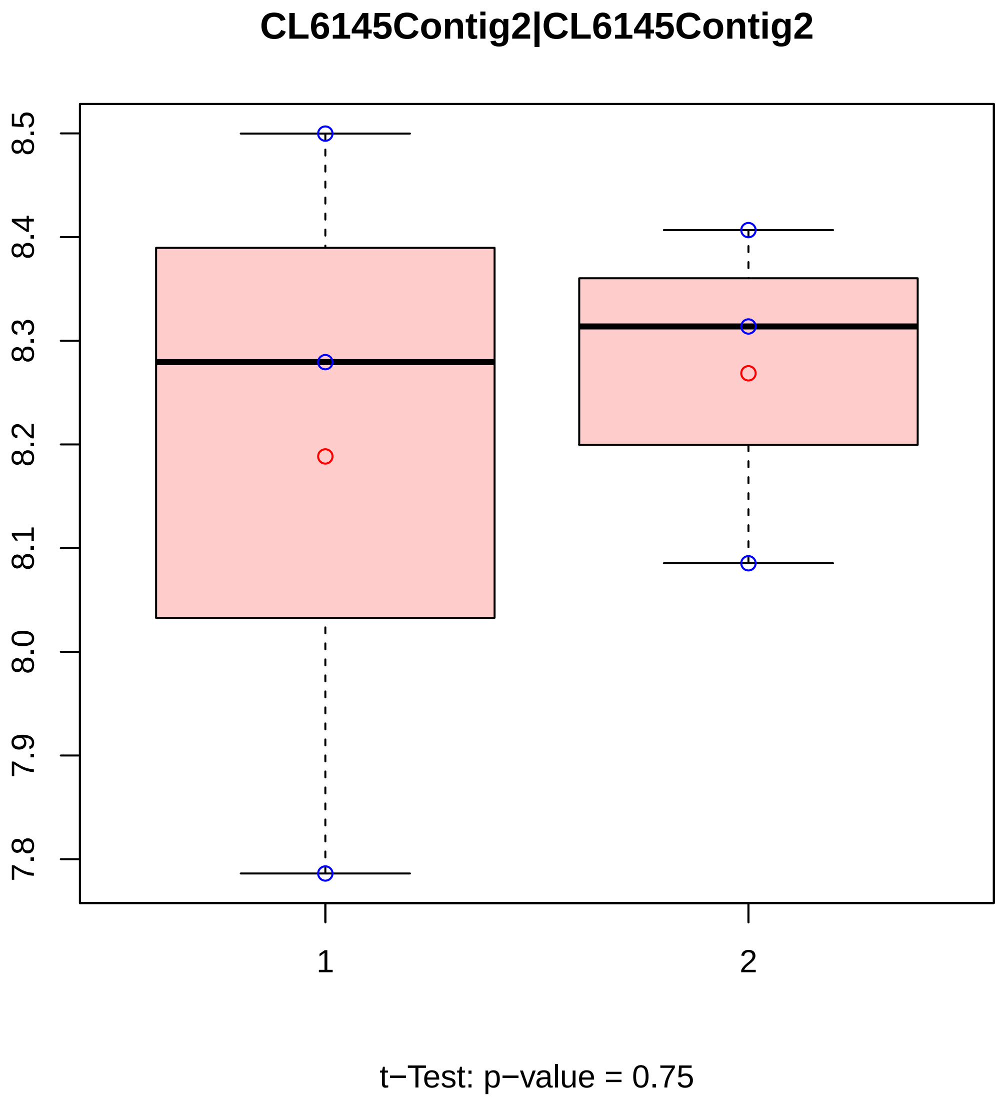

CL6145Contig2|CL6145Contig2
8.5
8.4
8.3
8.2
8.1
8.0
7.9
7.8
1
2
t−Test: p−value = 0.75

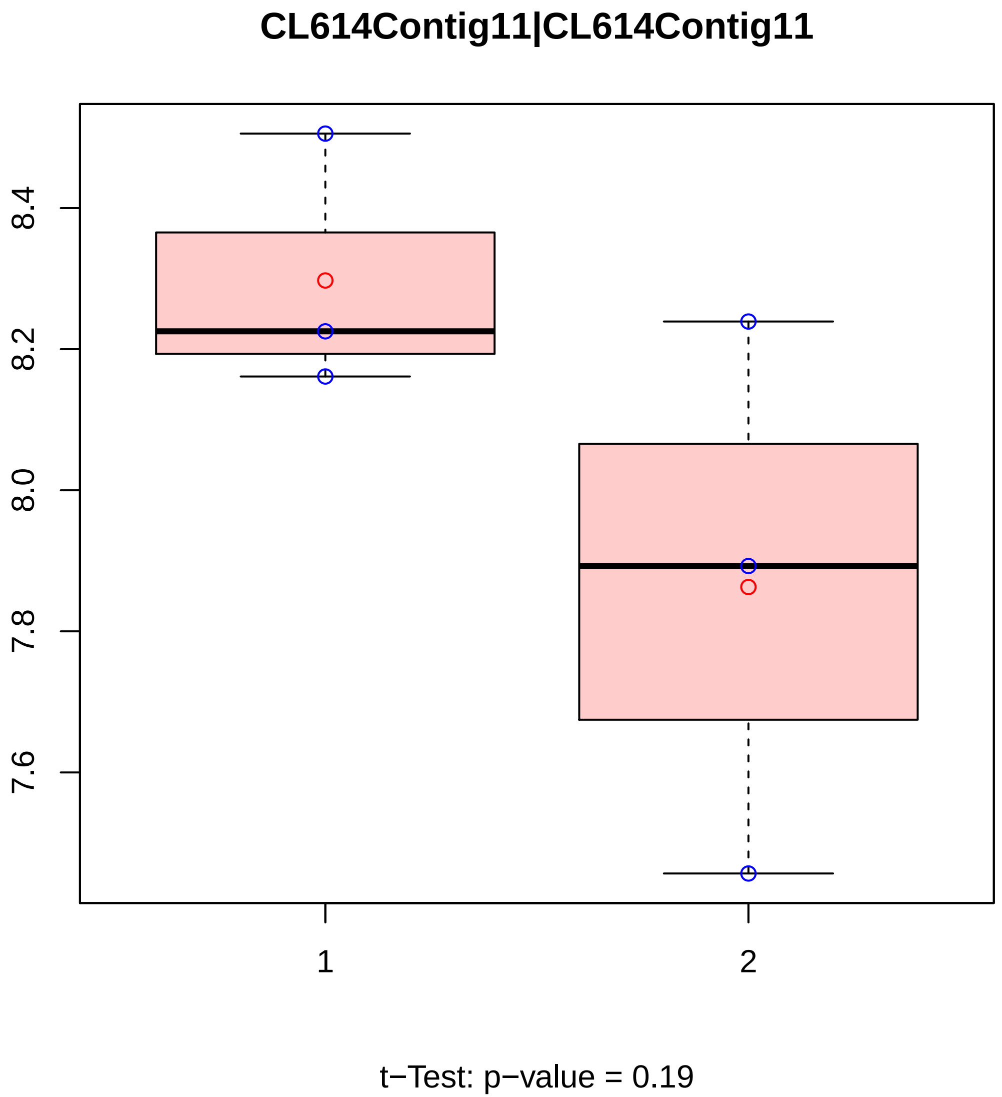

CL614Contig11|CL614Contig11
8.4
8.2
8.0
7.8
7.6
1
2
t−Test: p−value = 0.19

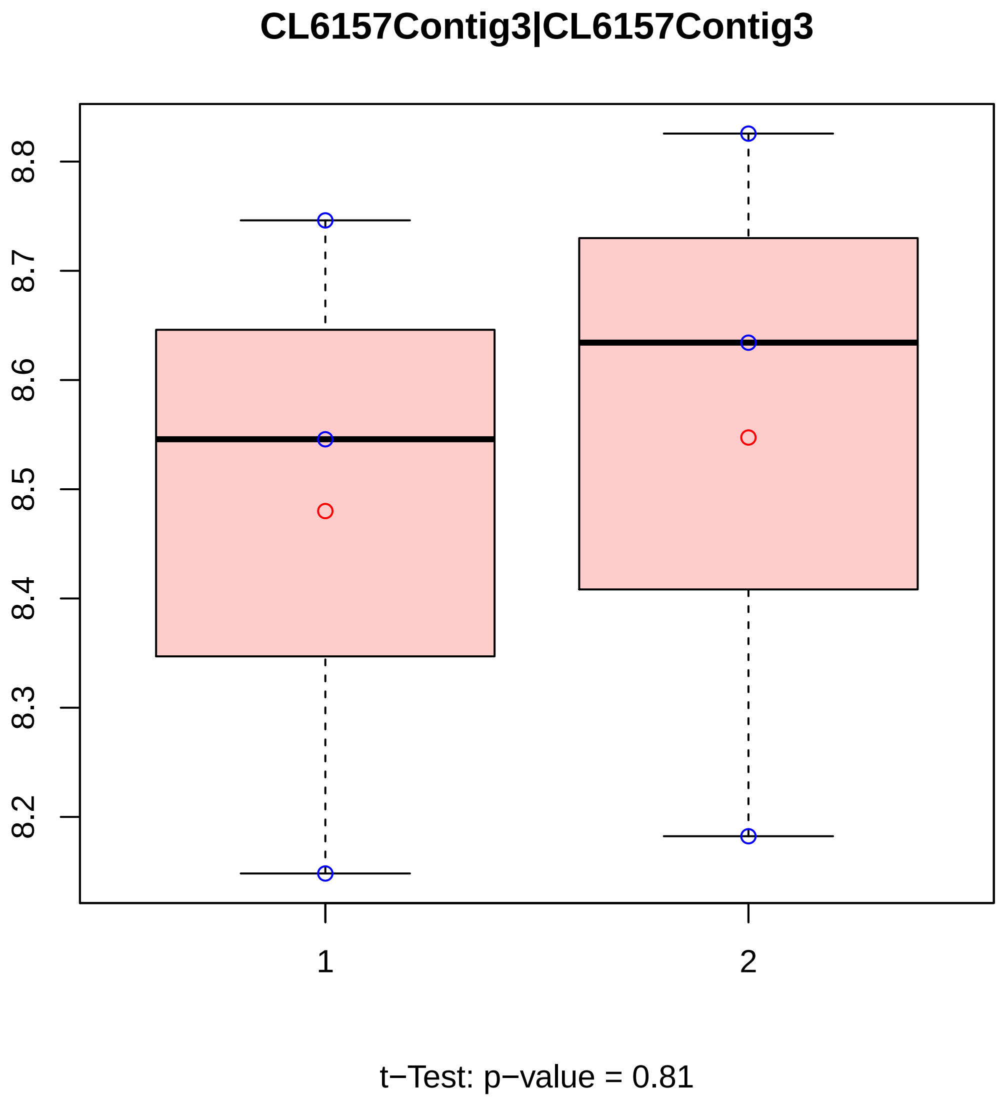

CL6157Contig3|CL6157Contig3
8.2
8.3
8.4
8.5
8.6
8.7
8.8
1
2
t−Test: p−value = 0.81

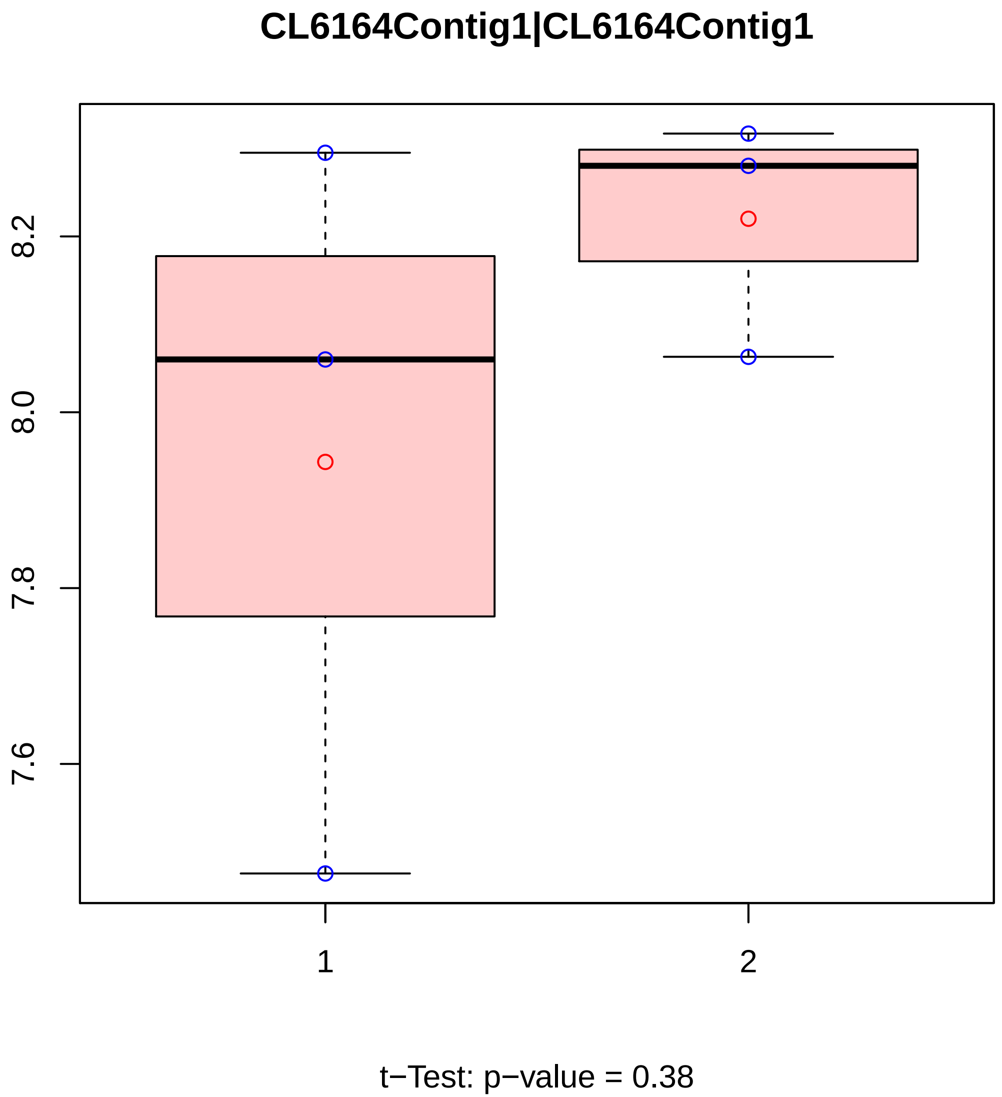

CL6164Contig1|CL6164Contig1
8.2
8.0
7.8
7.6
1
2
t−Test: p−value = 0.38

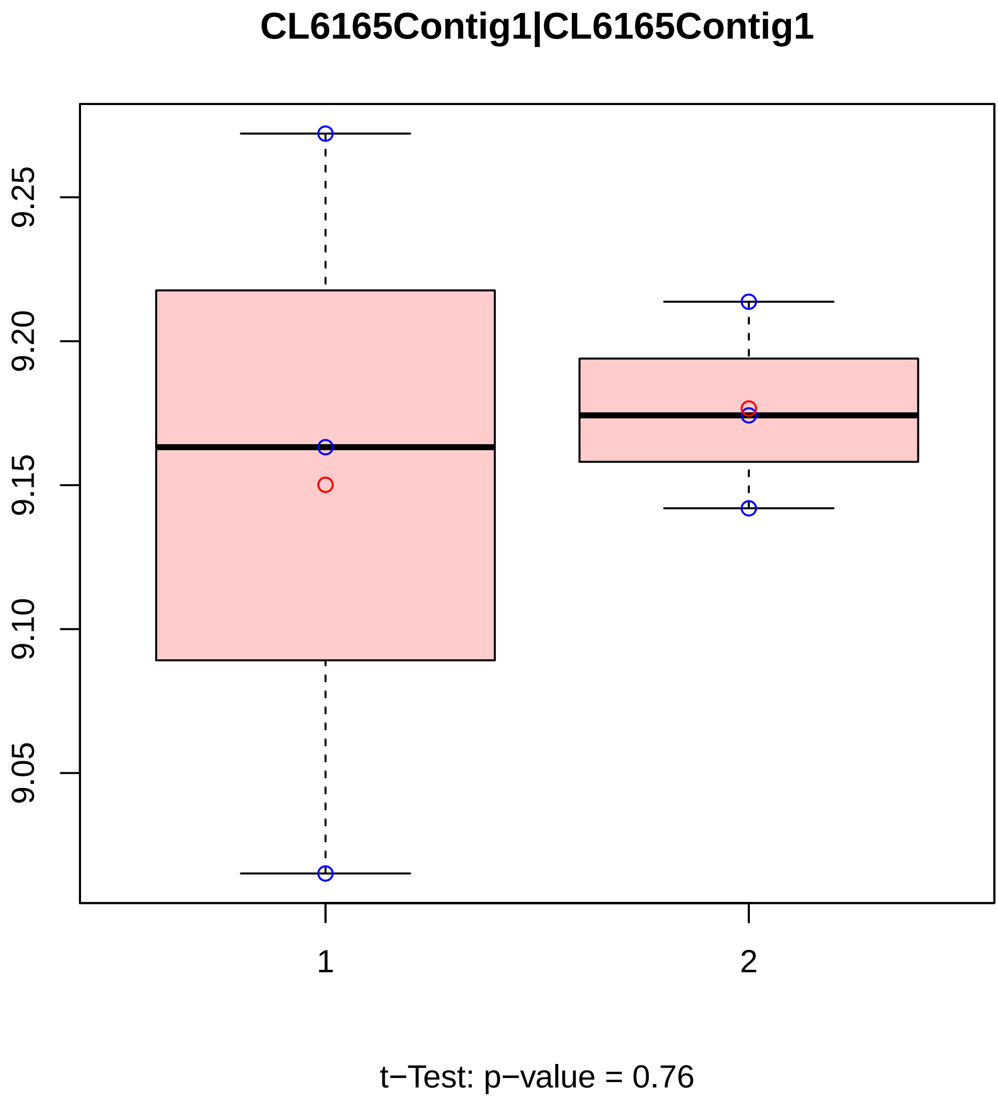

CL6165Contig1|CL6165Contig1
9.05
9.10
9.15
9.20
9.25
1
2
t−Test: p−value = 0.76

# CL616Contig5|CL616Contig5

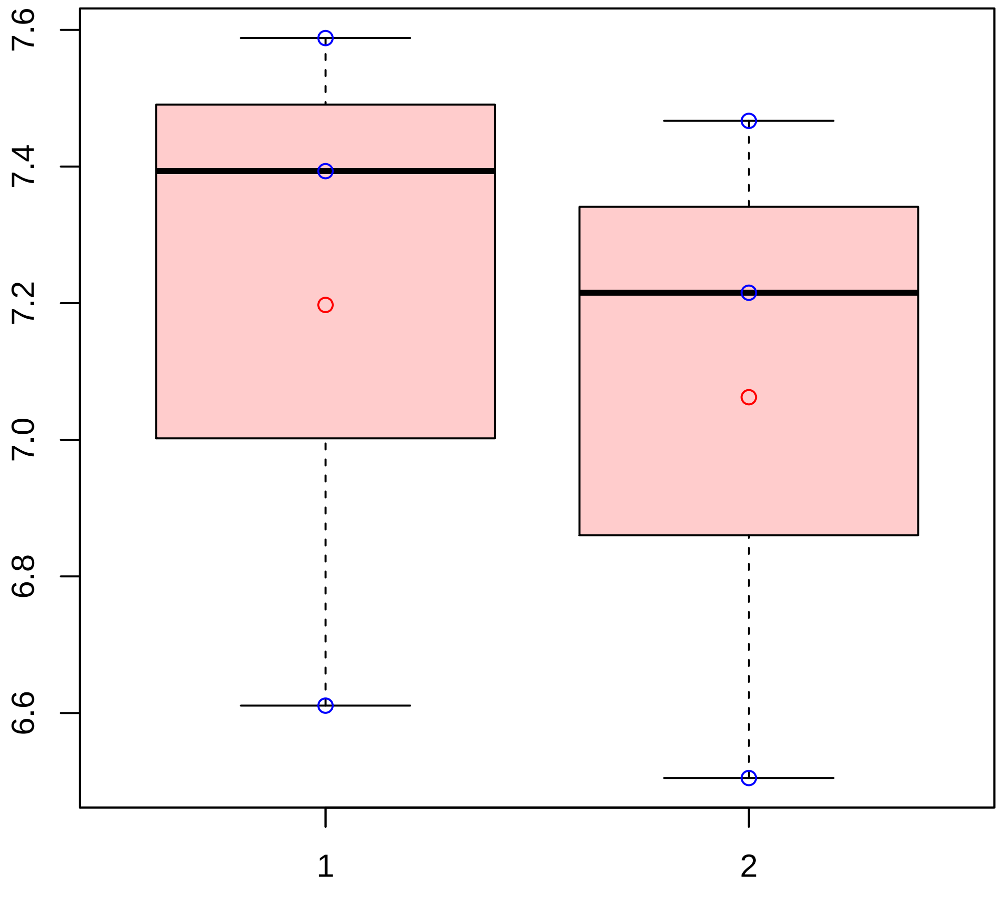


t−Test: p−value = 0.76

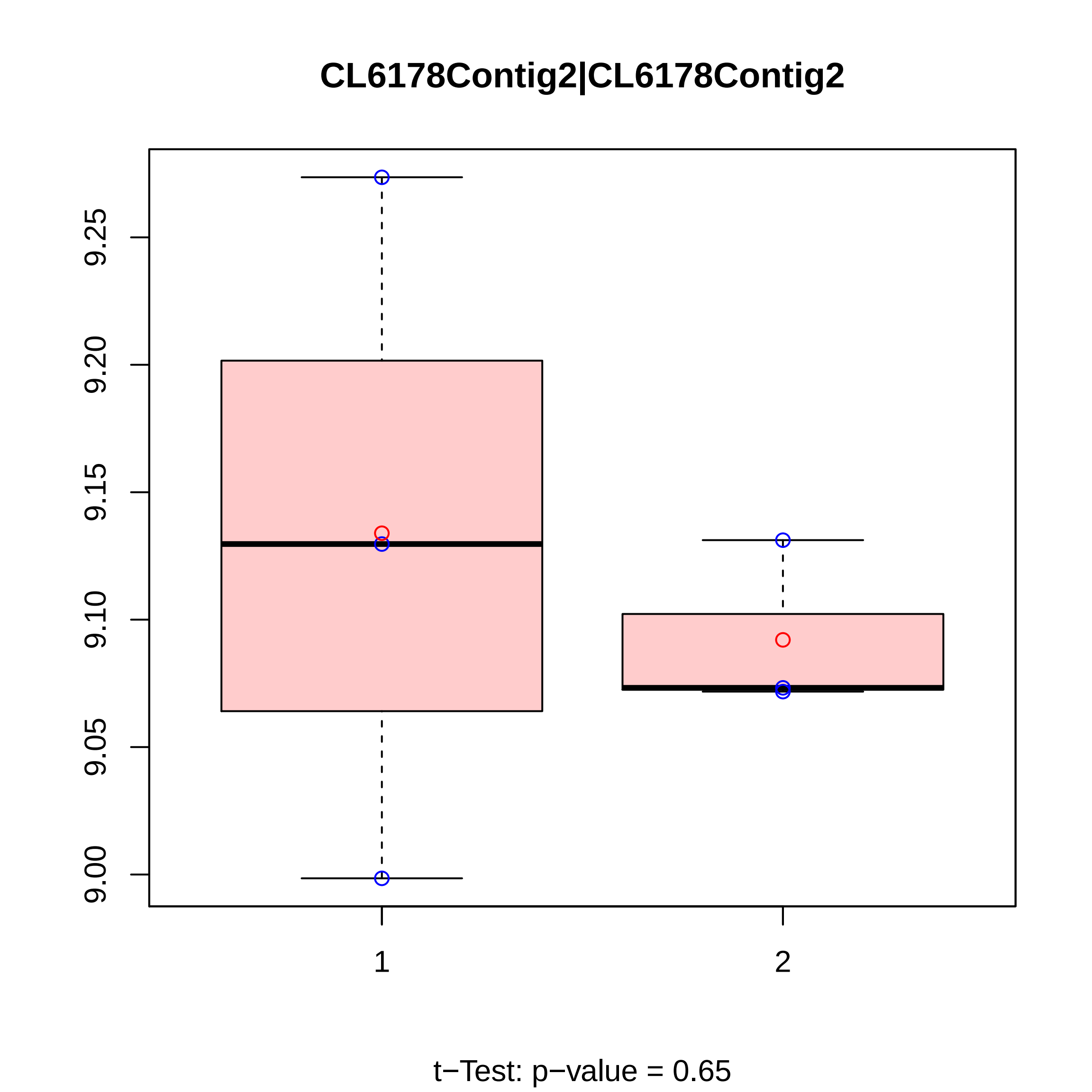
CL6178Contig2|CL6178Contig2
9.00
9.05
9.10
9.15
9.20
9.25
1
2
t−Test: p−value = 0.65

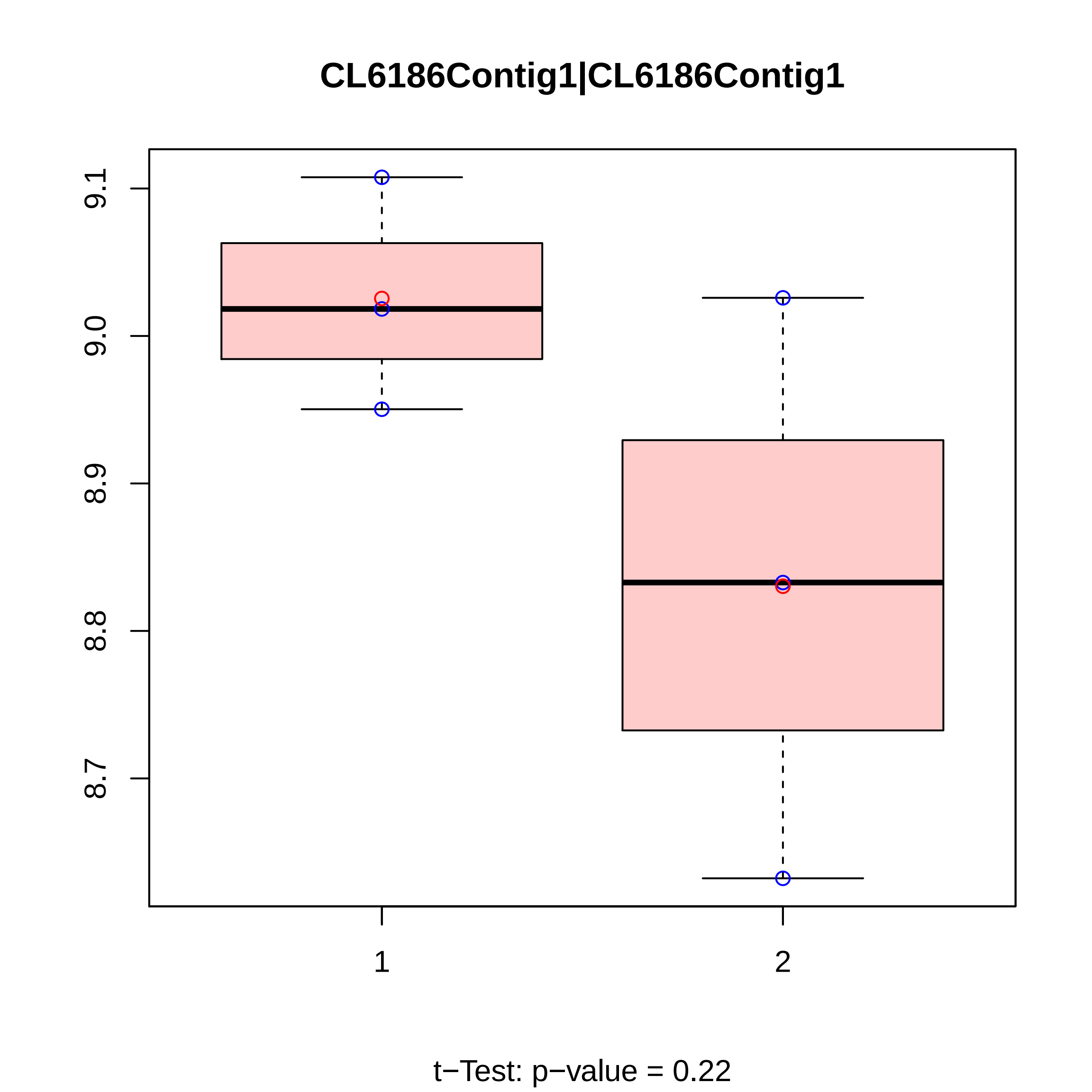
CL6186Contig1|CL6186Contig1
9.1
9.0
8.9
8.8
8.7
1
2
t−Test: p−value = 0.22

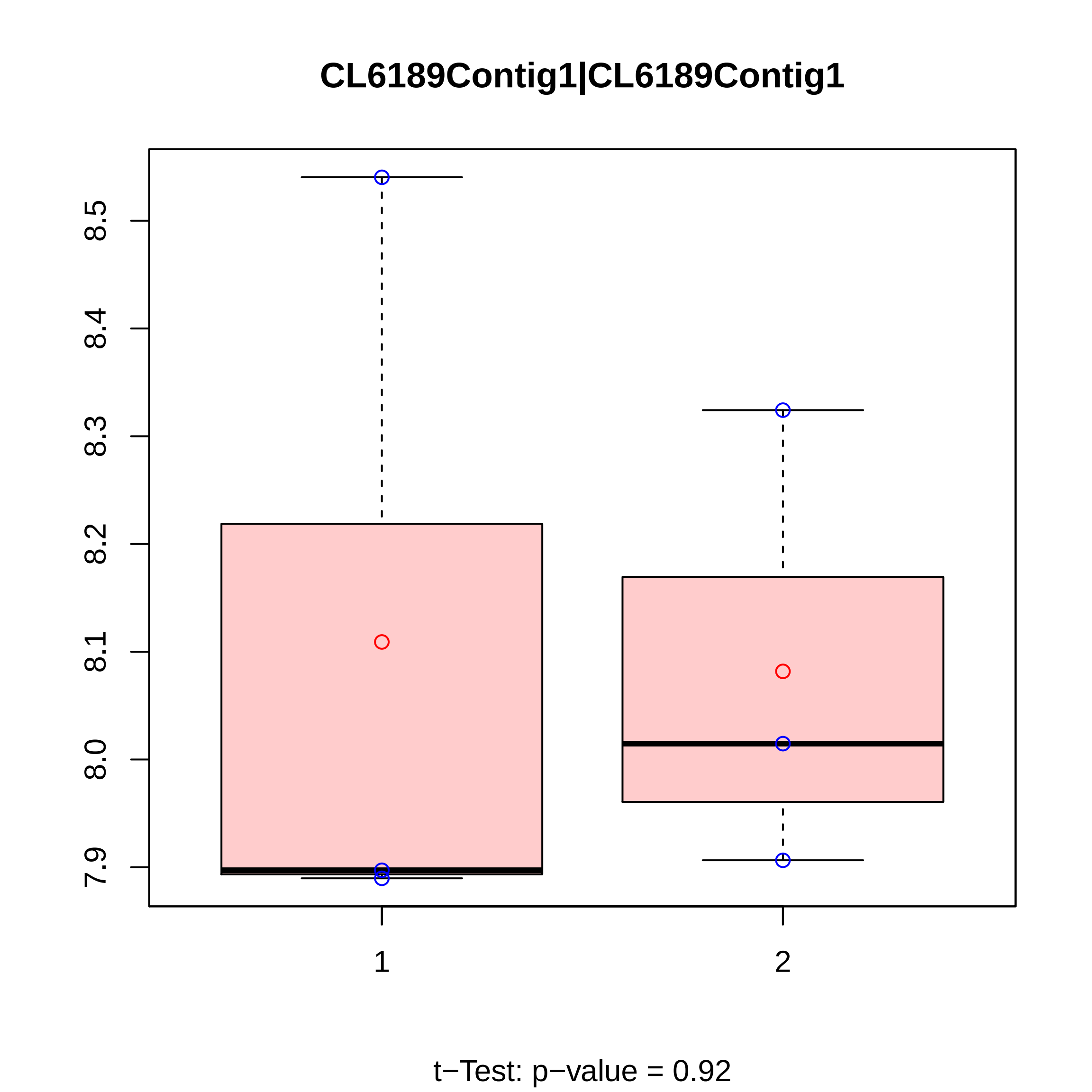
CL6189Contig1|CL6189Contig1
8.5
8.4
8.3
8.2
8.1
8.0
7.9
1
2
t−Test: p−value = 0.92

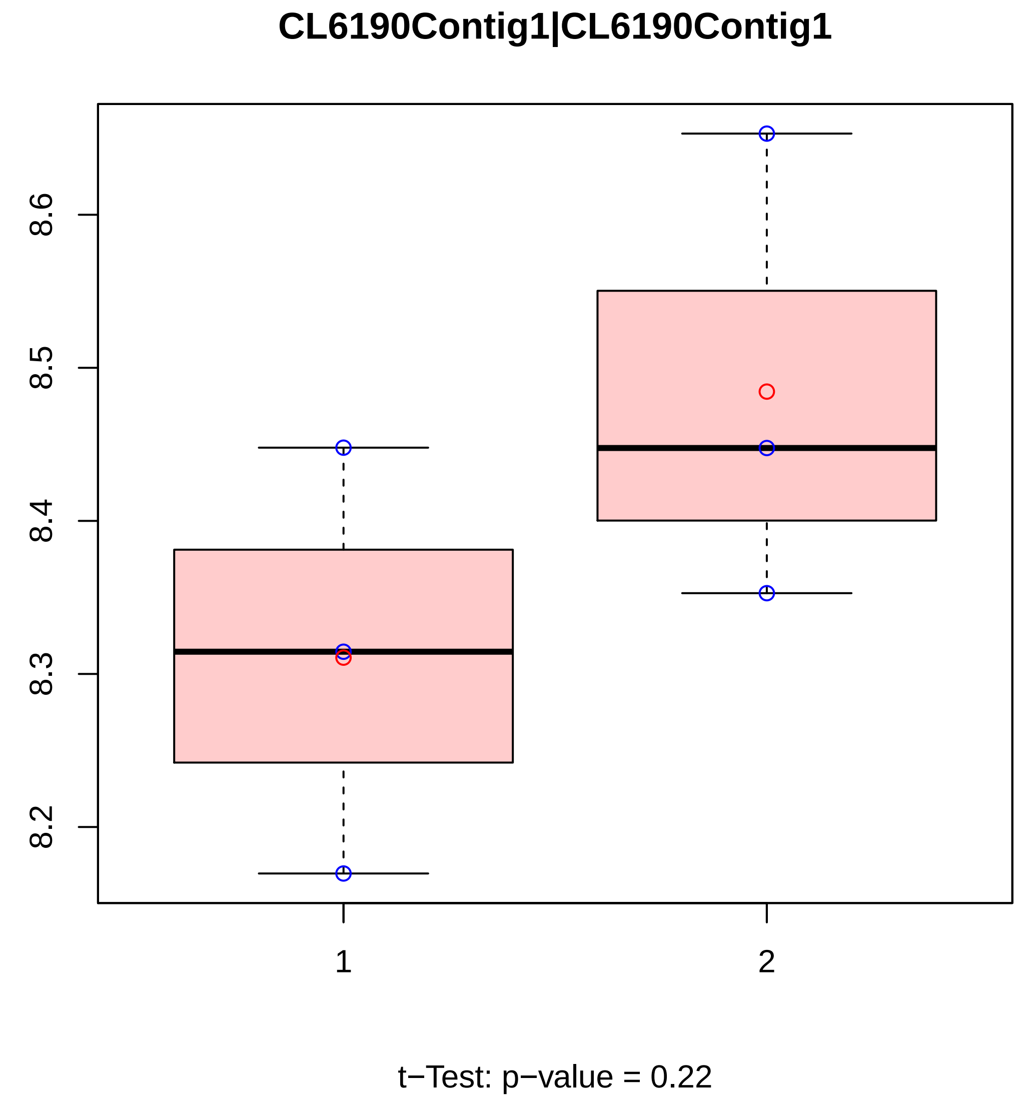

CL6190Contig1|CL6190Contig1
8.2
8.3
8.4
8.5
8.6
1
2
t−Test: p−value = 0.22

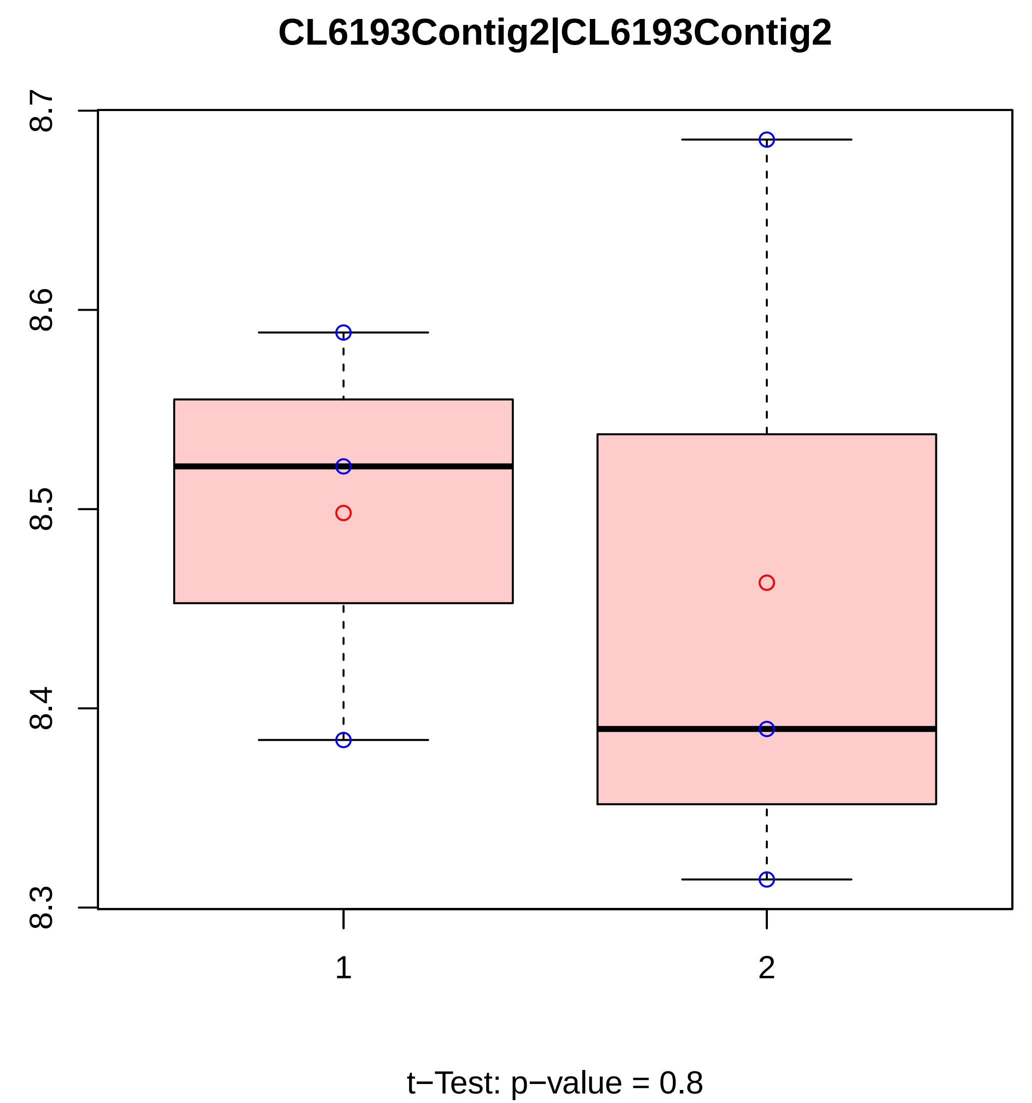
CL6193Contig2|CL6193Contig2
8.7
8.6
8.5
8.4
8.3
1
2
t−Test: p−value = 0.8

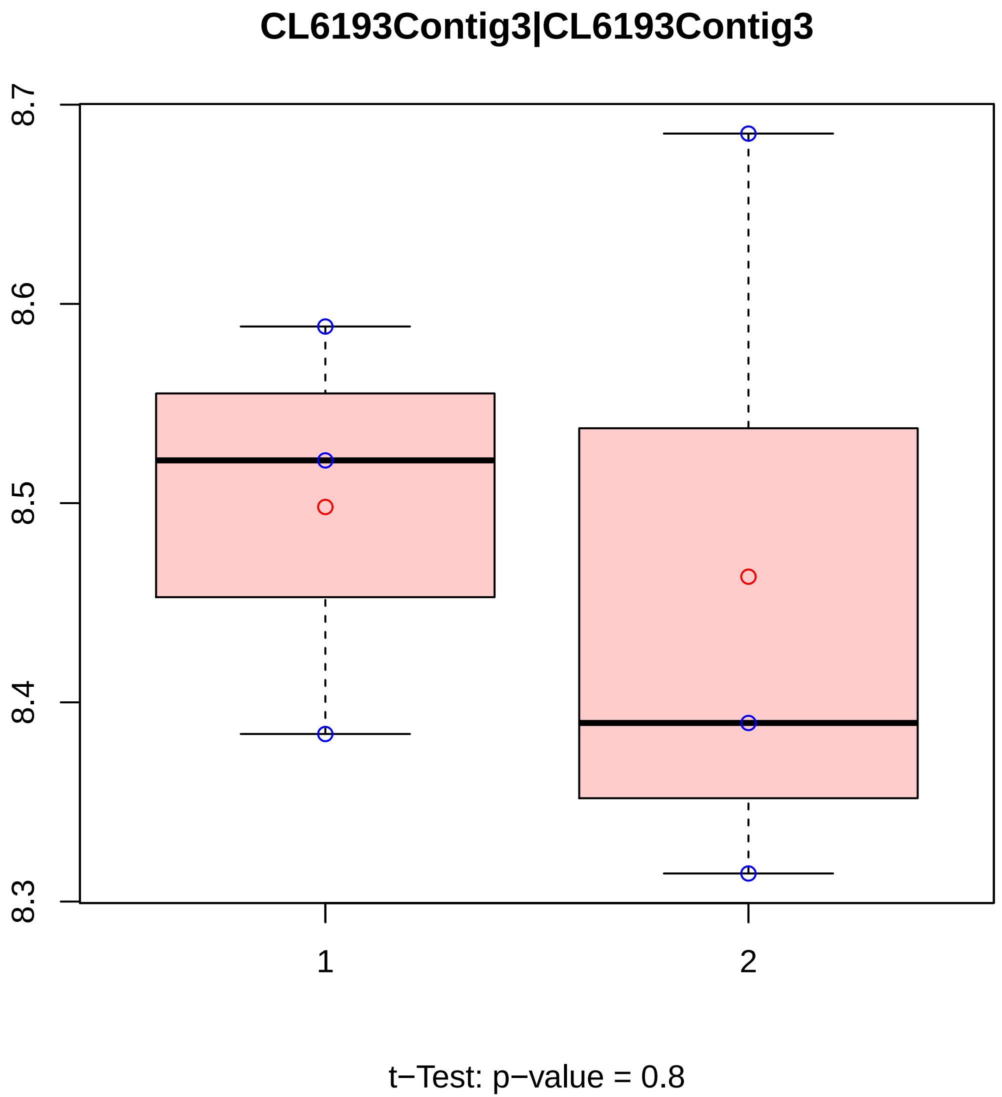
CL6193Contig3|CL6193Contig3
8.3
8.4
8.5
8.6
8.7
1
2
t−Test: p−value = 0.8

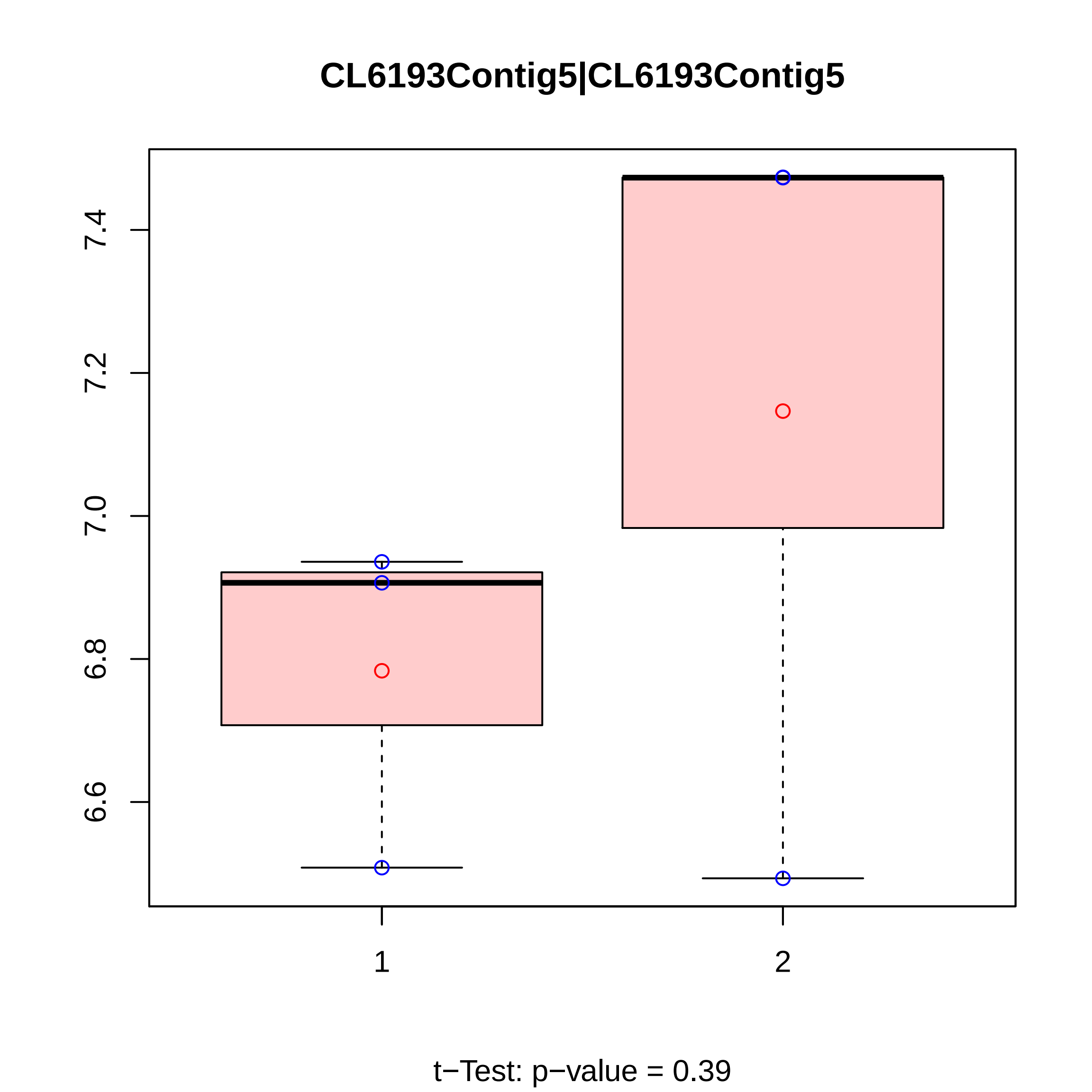
CL6193Contig5|CL6193Contig5
7.4
7.2
7.0
6.8
6.6
1
2
t−Test: p−value = 0.39

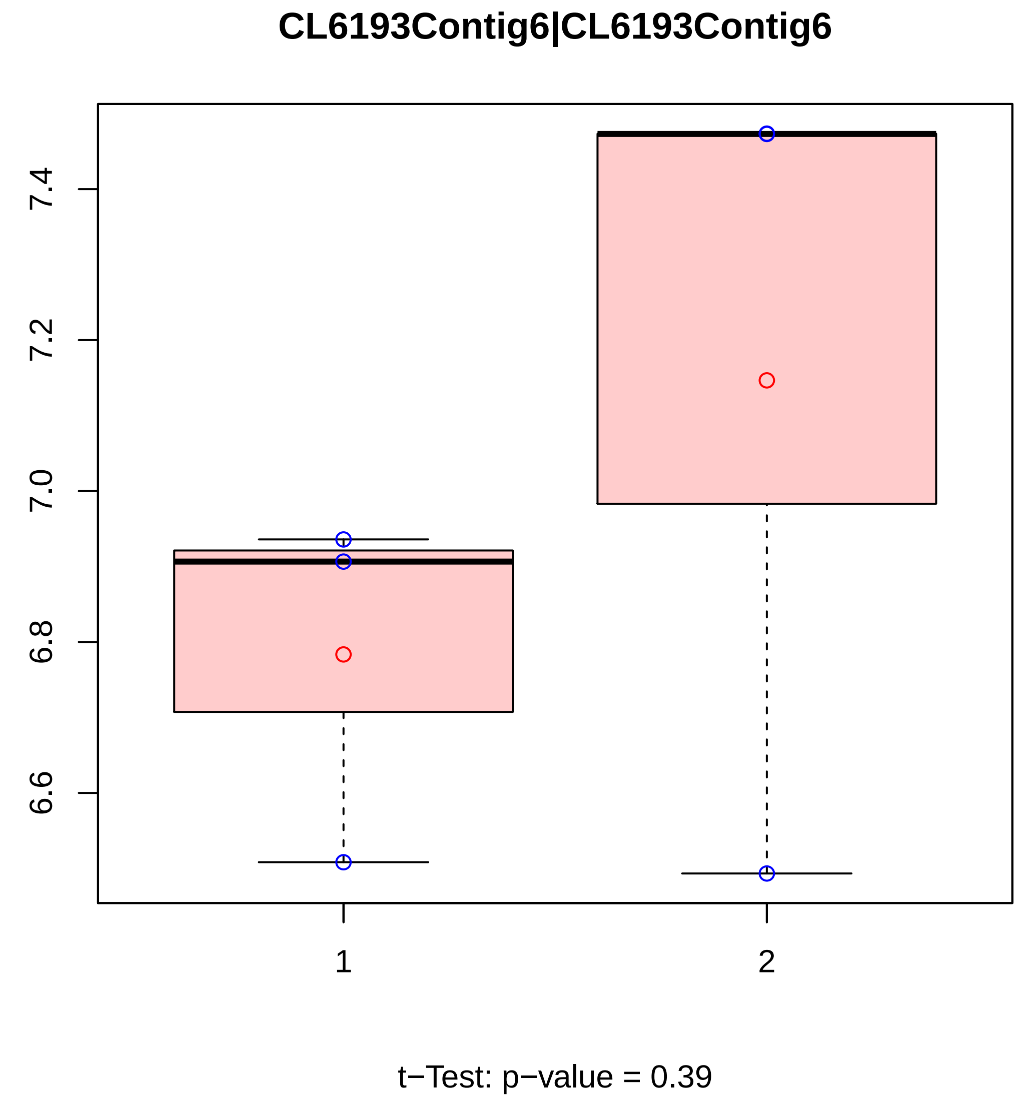
CL6193Contig6|CL6193Contig6
7.4
7.2
7.0
6.8
6.6
1
2
t−Test: p−value = 0.39

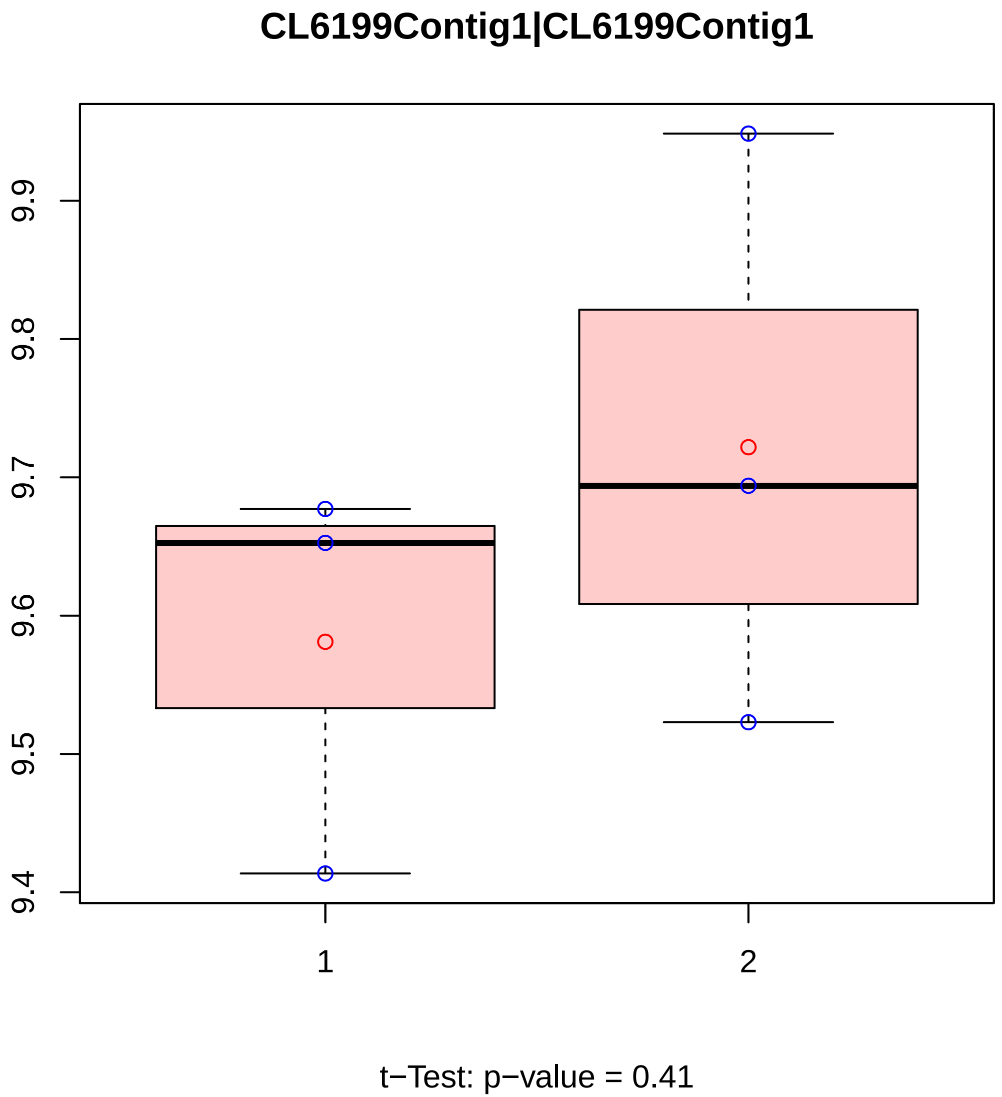

CL6199Contig1|CL6199Contig1
9.4
9.5
9.6
9.7
9.8
9.9
1
2
t−Test: p−value = 0.41

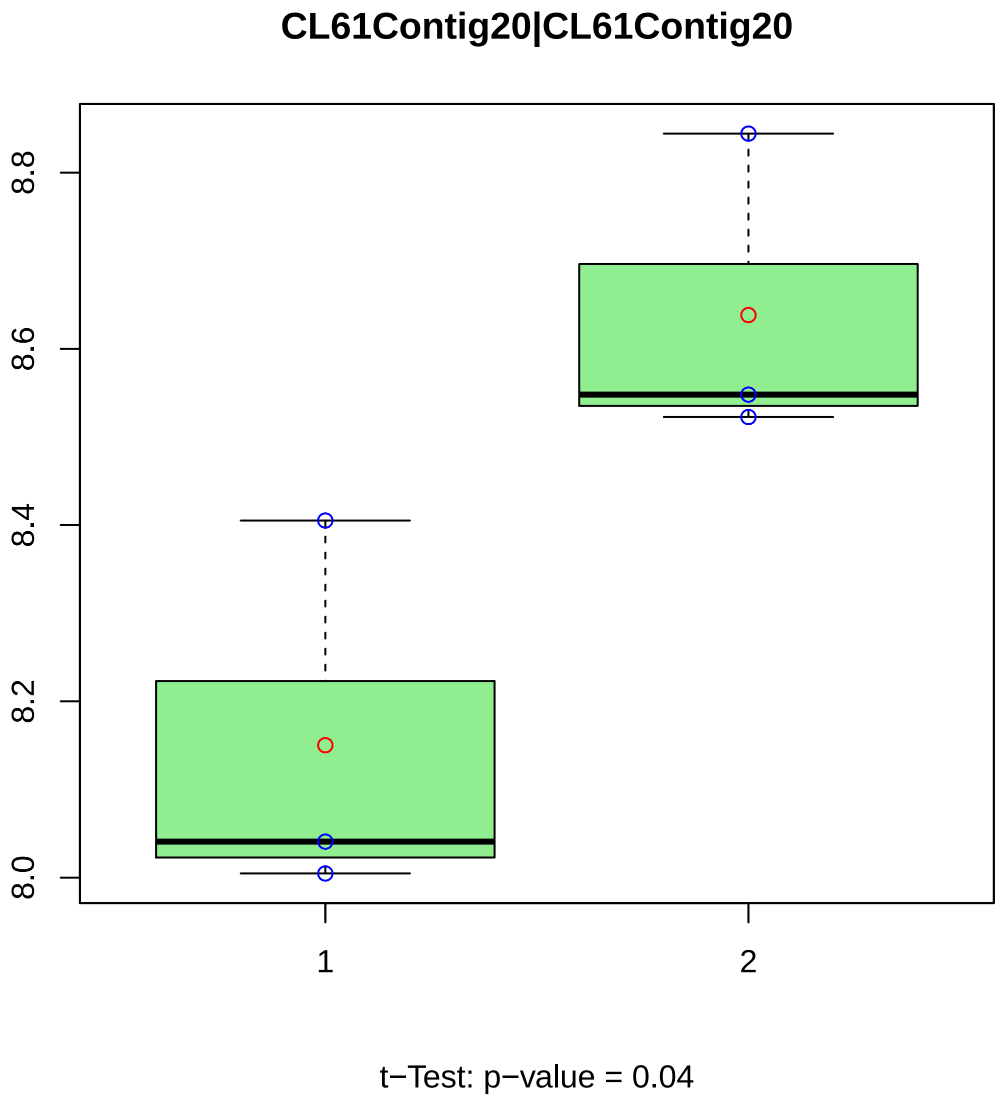
CL61Contig20|CL61Contig20
8.0
8.2
8.4
8.6
8.8
1
2
t−Test: p−value = 0.04

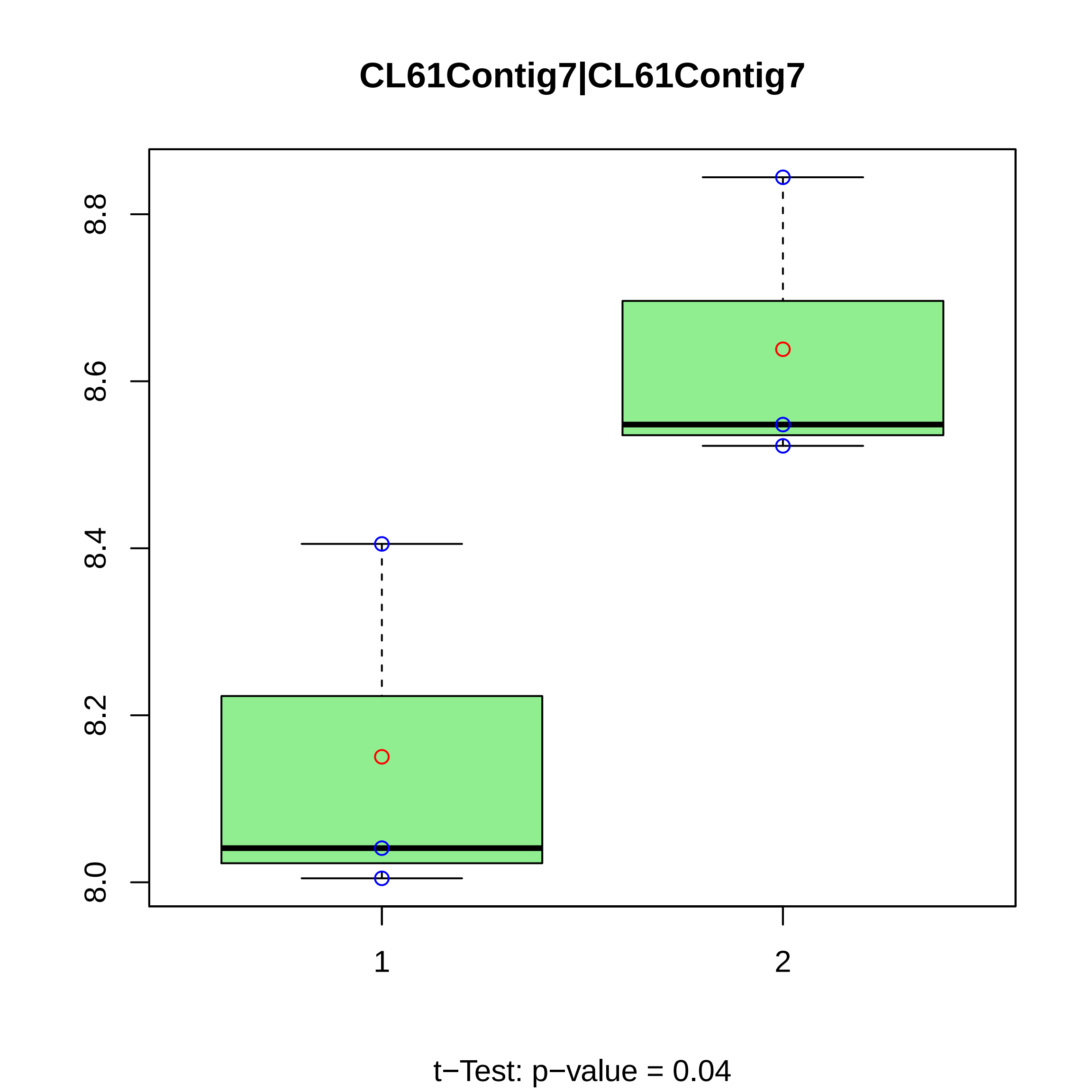
CL61Contig7|CL61Contig7
8.0
8.2
8.4
8.6
8.8
1
2
t−Test: p−value = 0.04

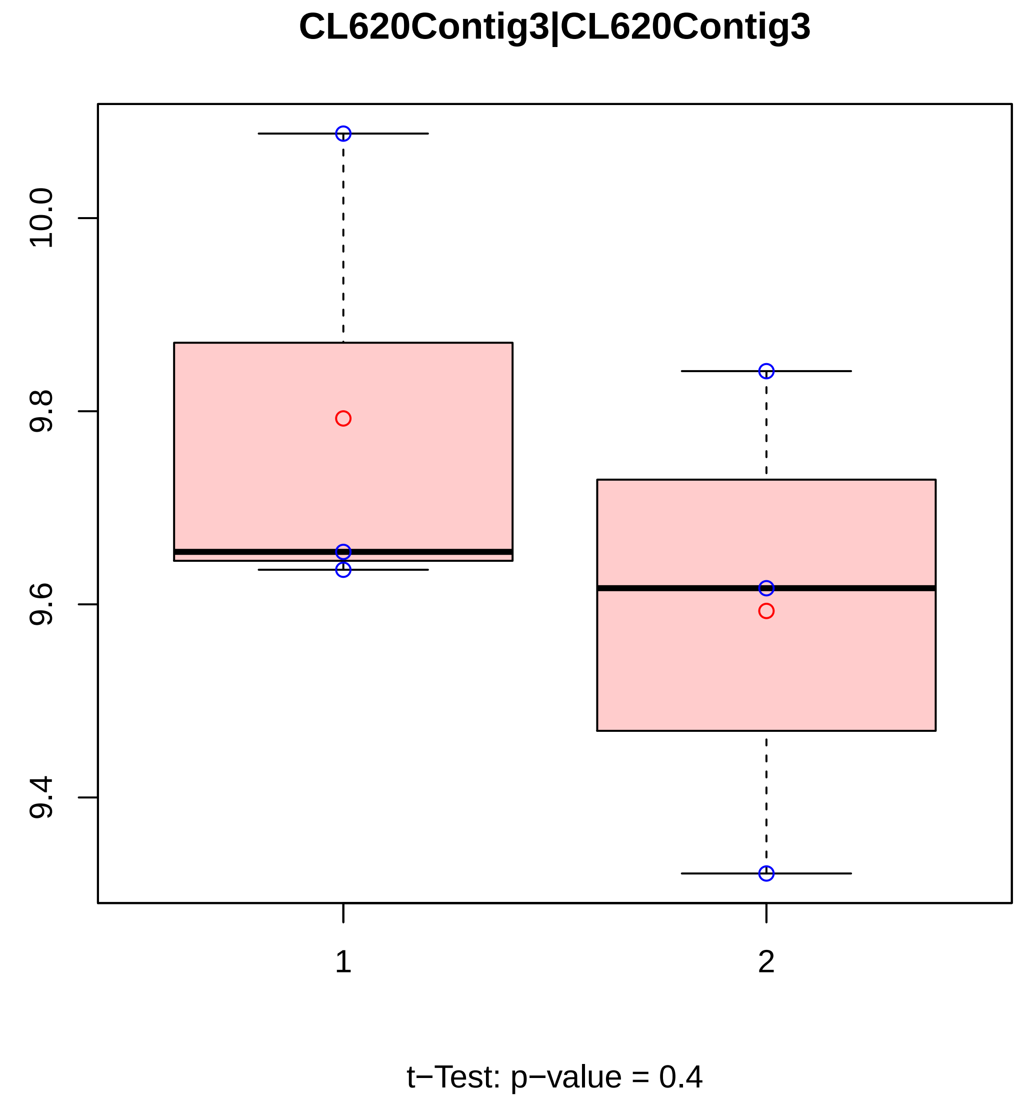
CL620Contig3|CL620Contig3
10.0
9.8
9.6
9.4
1
2
t−Test: p−value = 0.4

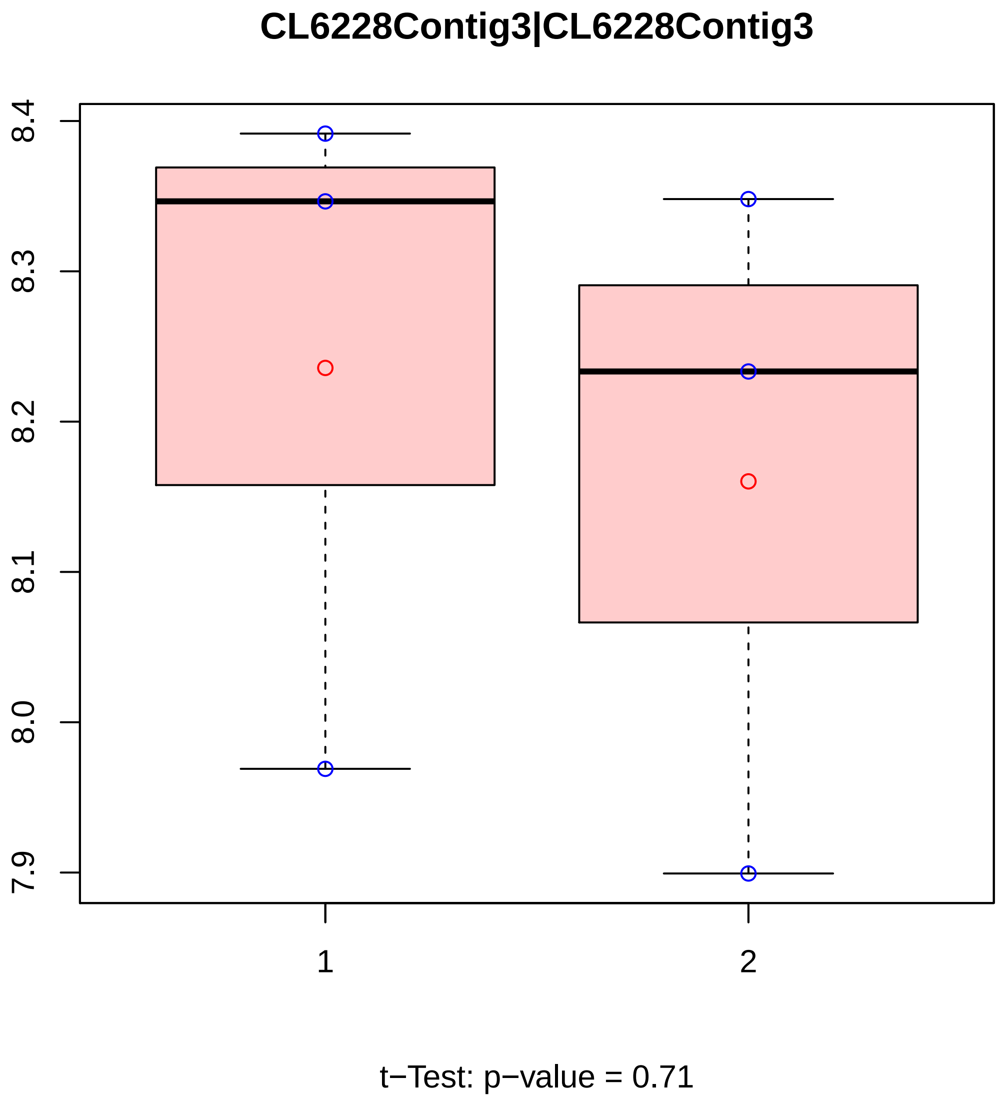

CL6228Contig3|CL6228Contig3
8.4
8.3
8.2
8.1
8.0
7.9
1
2
t−Test: p−value = 0.71

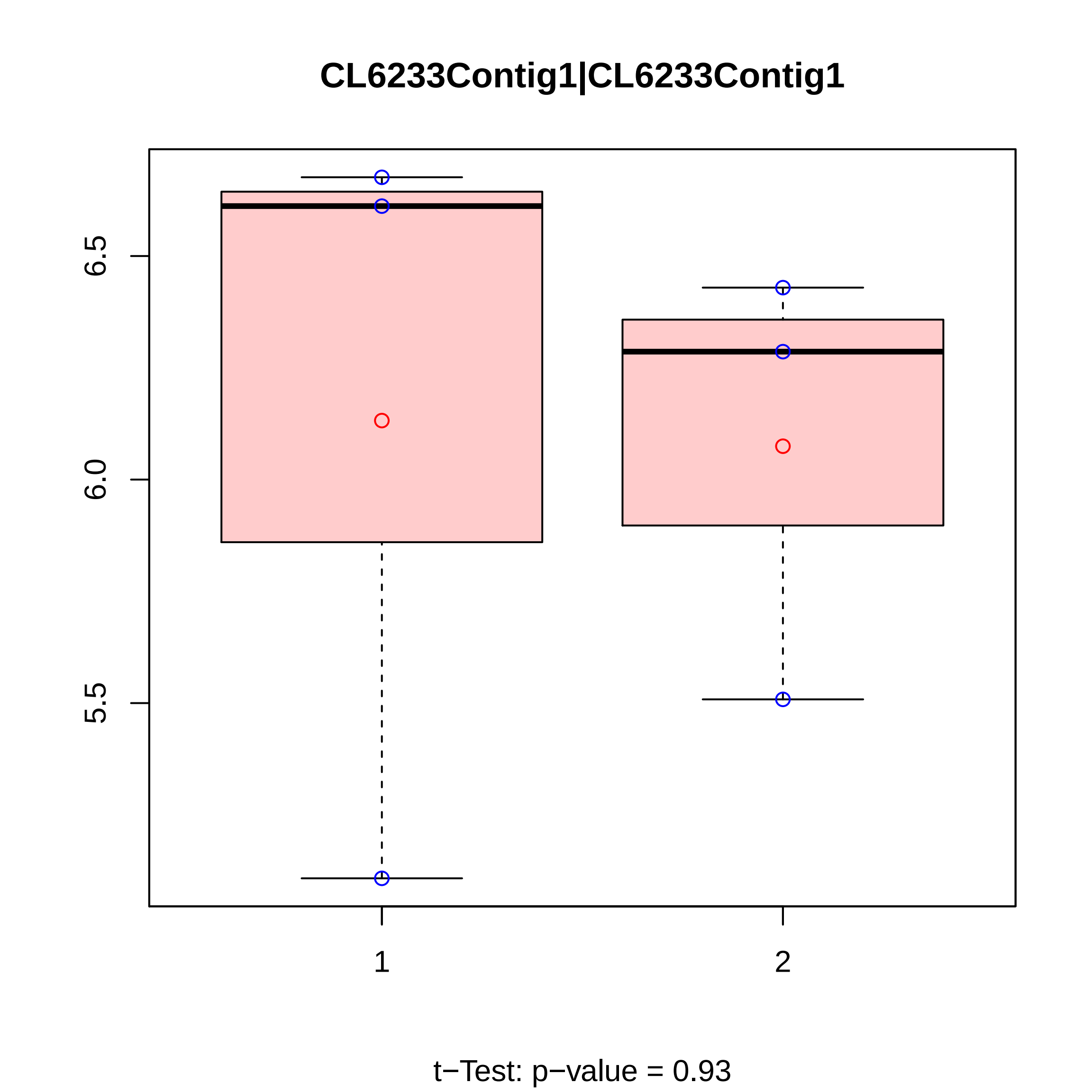
CL6233Contig1|CL6233Contig1
6.5
6.0
5.5
1
2
t−Test: p−value = 0.93

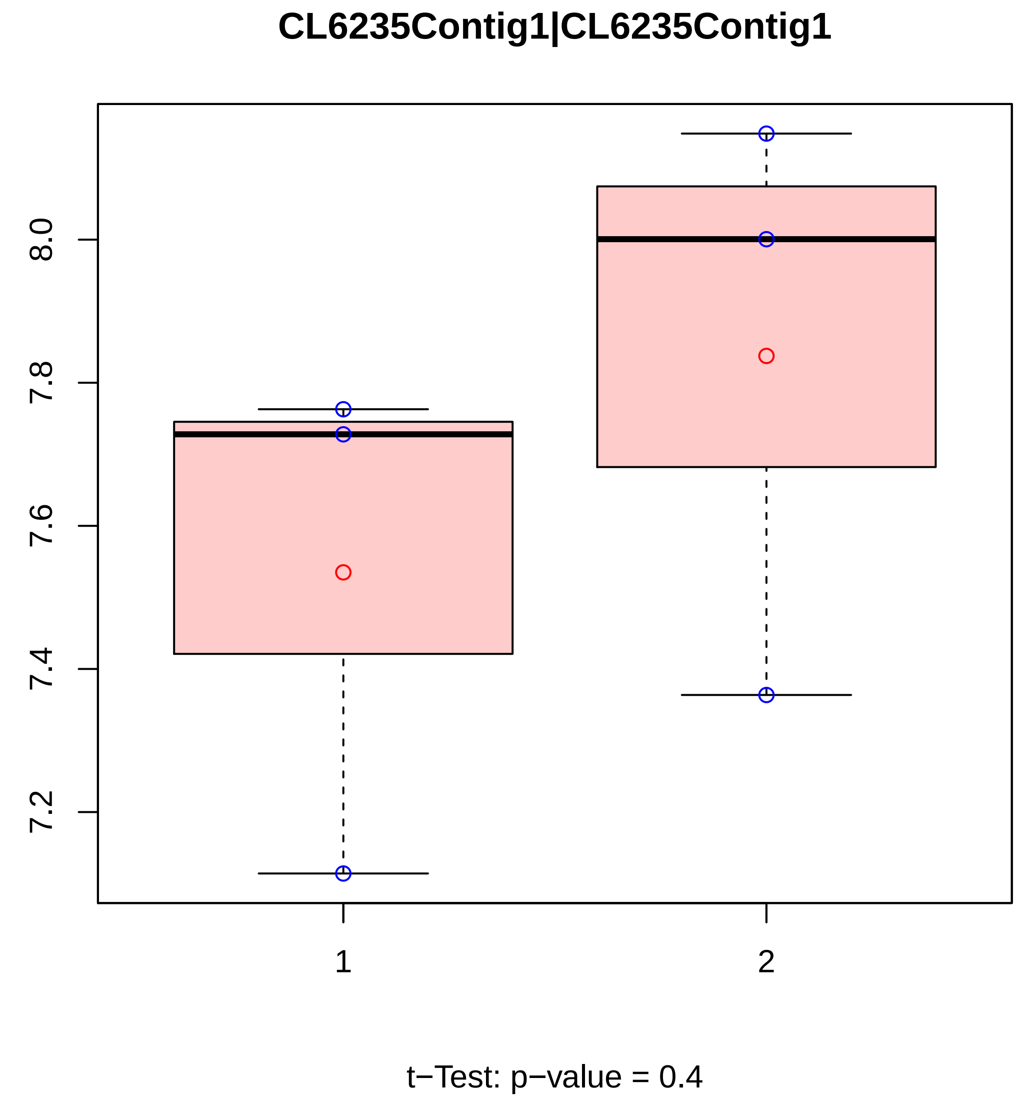
CL6235Contig1|CL6235Contig1
8.0
7.8
7.6
7.4
7.2
1
2
t−Test: p−value = 0.4

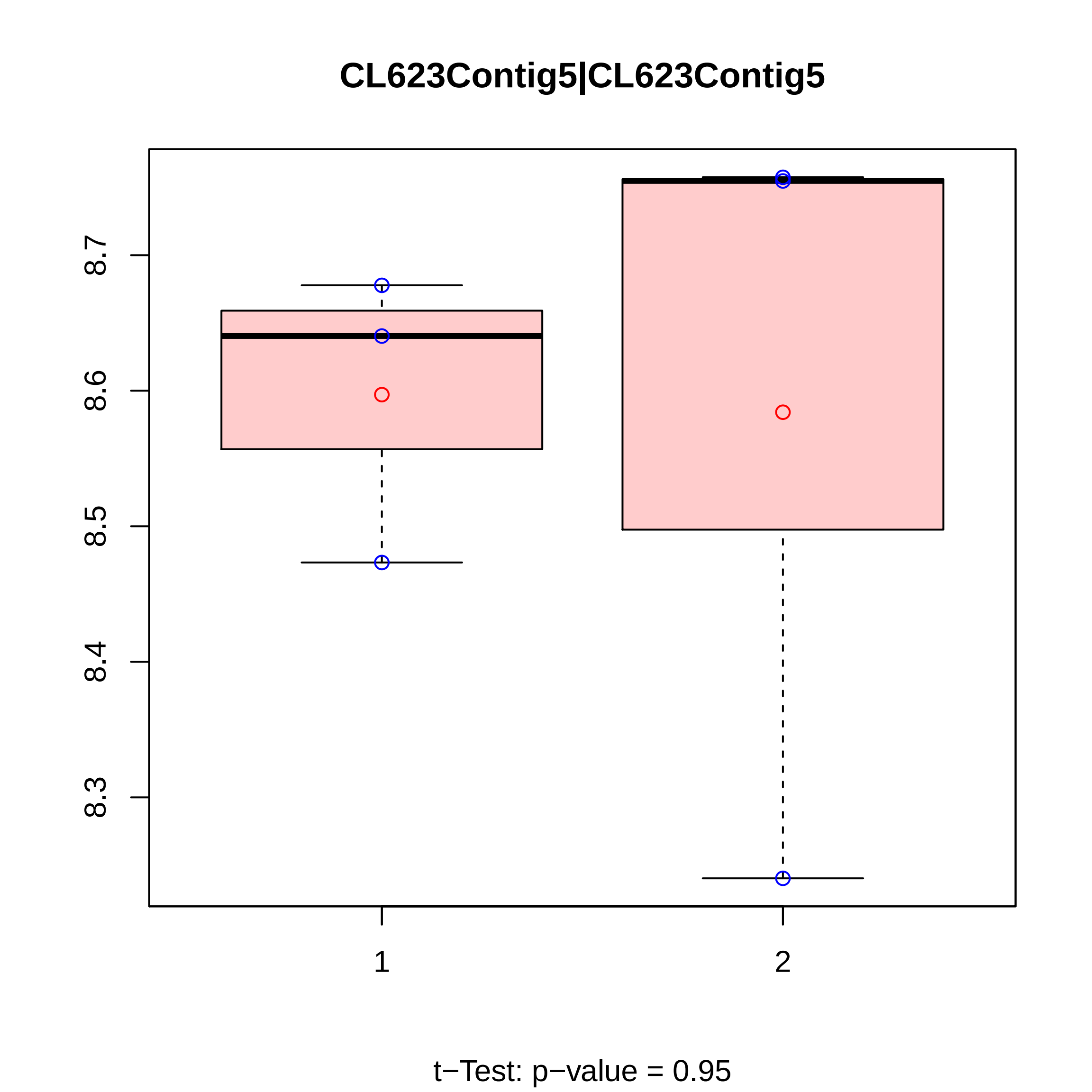
CL623Contig5|CL623Contig5
8.3
8.4
8.5
8.6
8.7
1
2
t−Test: p−value = 0.95

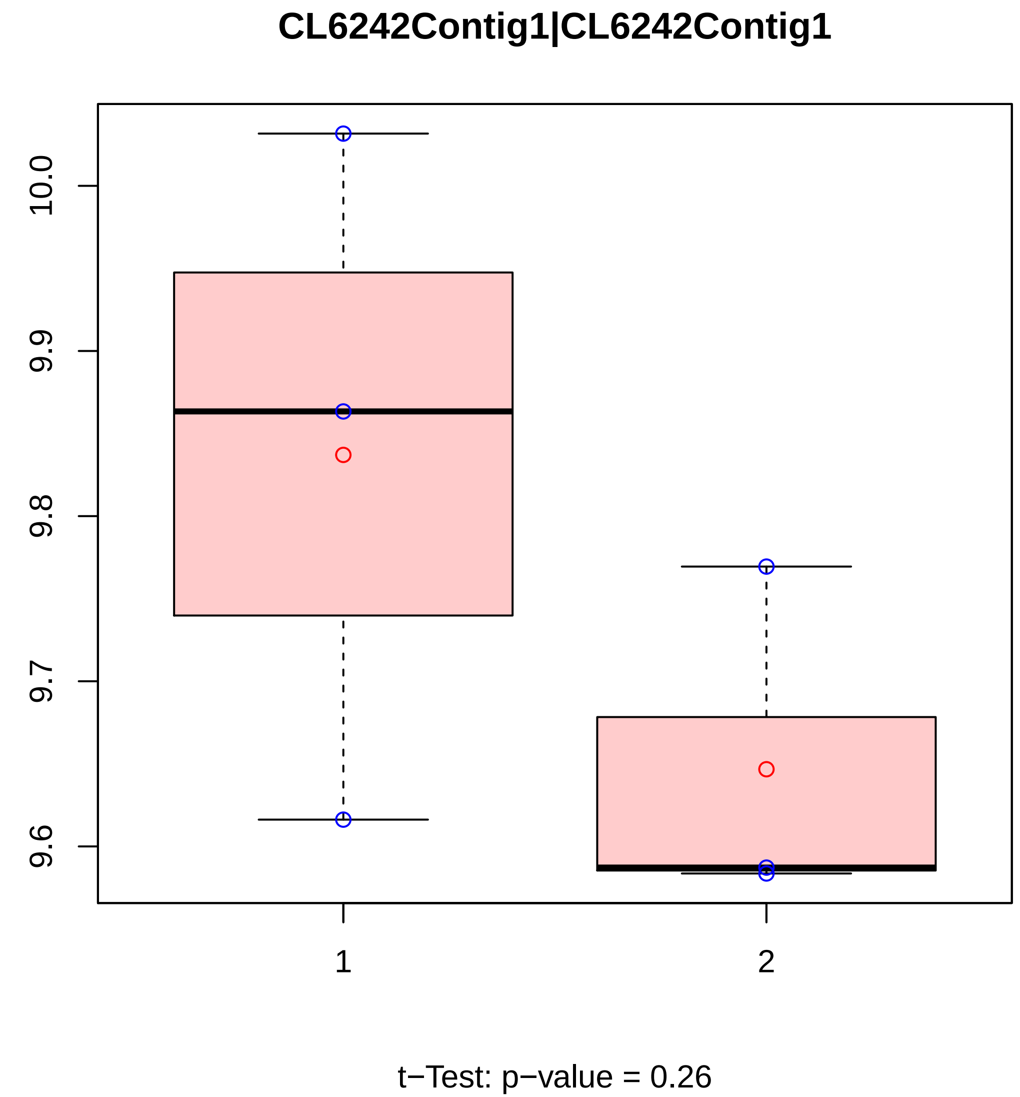

CL6242Contig1|CL6242Contig1
10.0
9.9
9.8
9.7
9.6
1
2
t−Test: p−value = 0.26

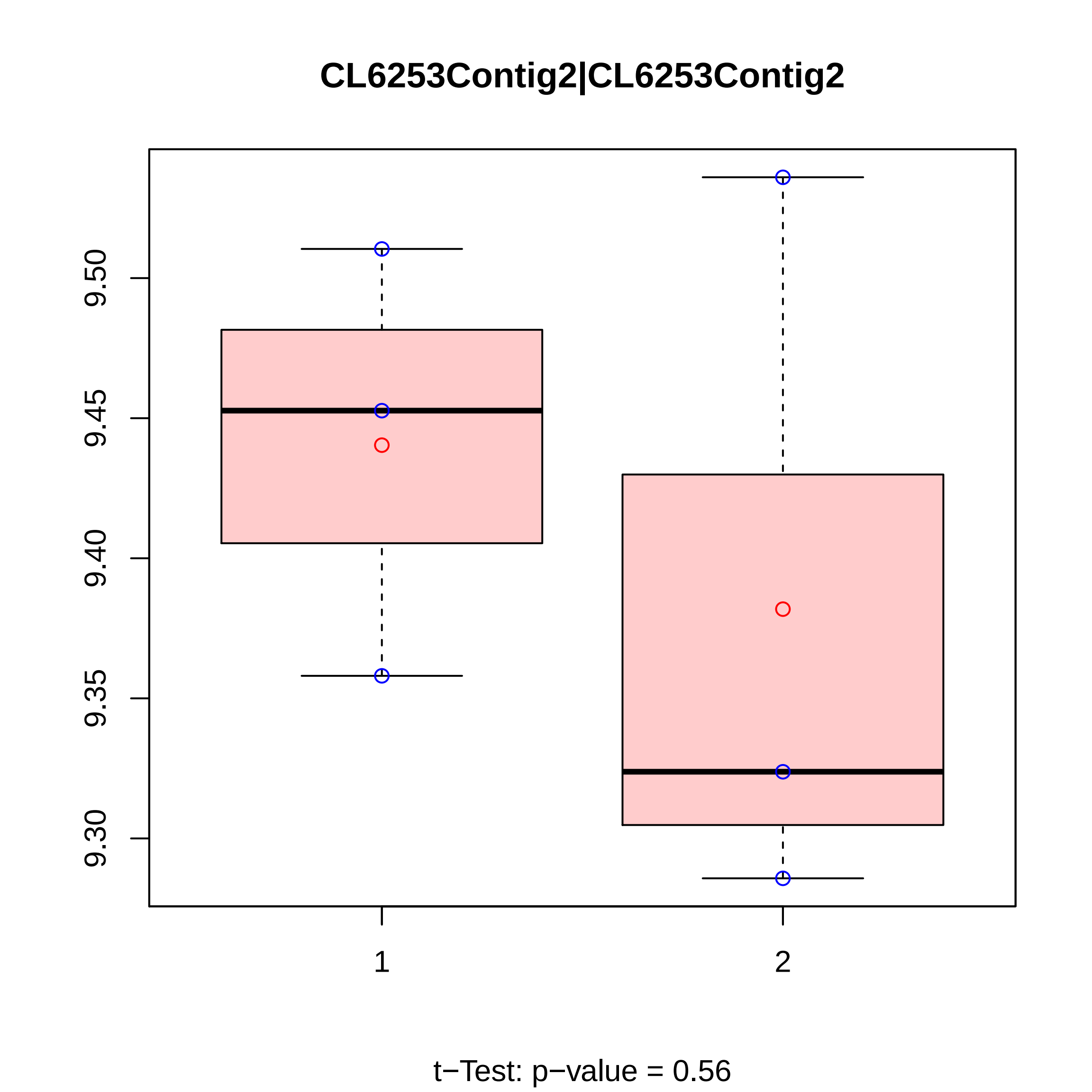
CL6253Contig2|CL6253Contig2
9.30
9.35
9.40
9.45
9.50
1
2
t−Test: p−value = 0.56

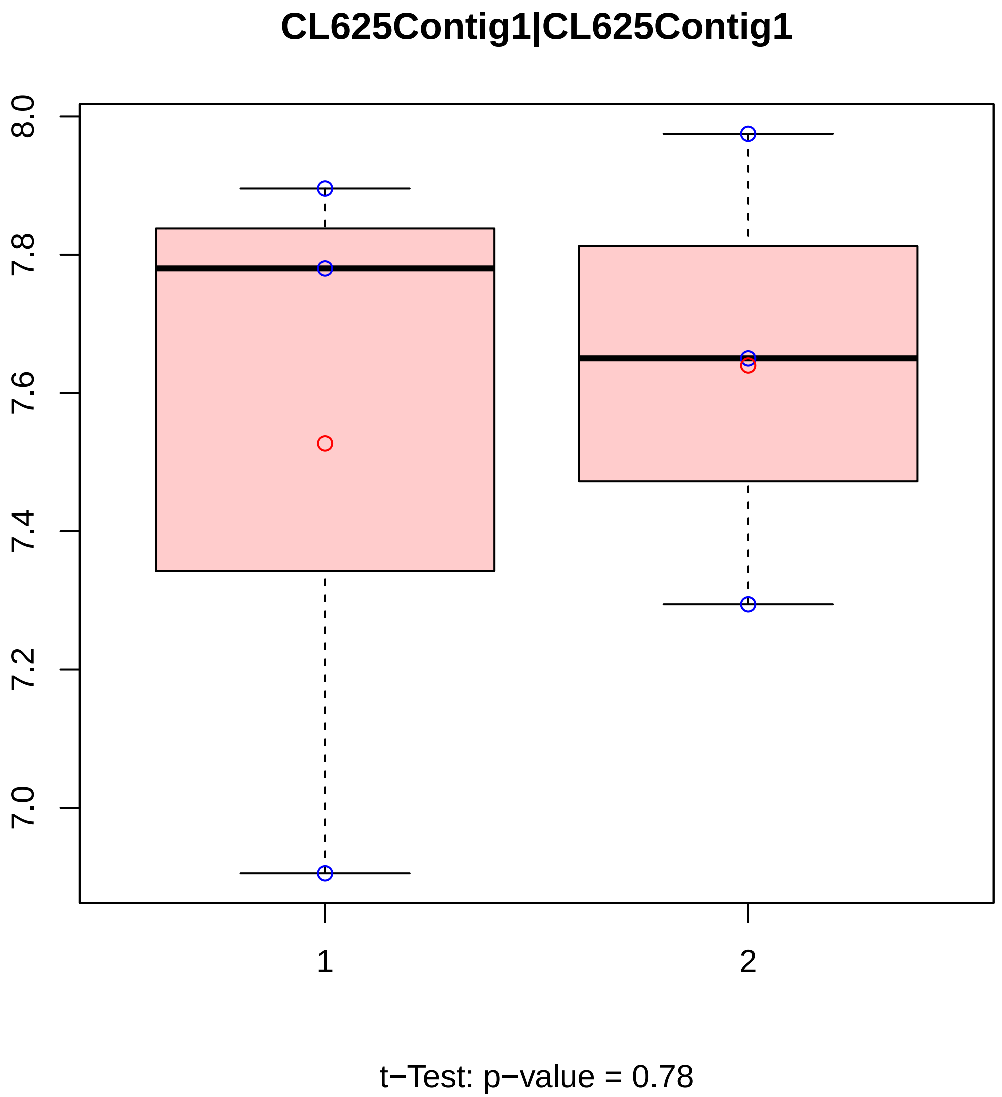
CL625Contig1|CL625Contig1
8.0
7.8
7.6
7.4
7.2
7.0
1
2
t−Test: p−value = 0.78

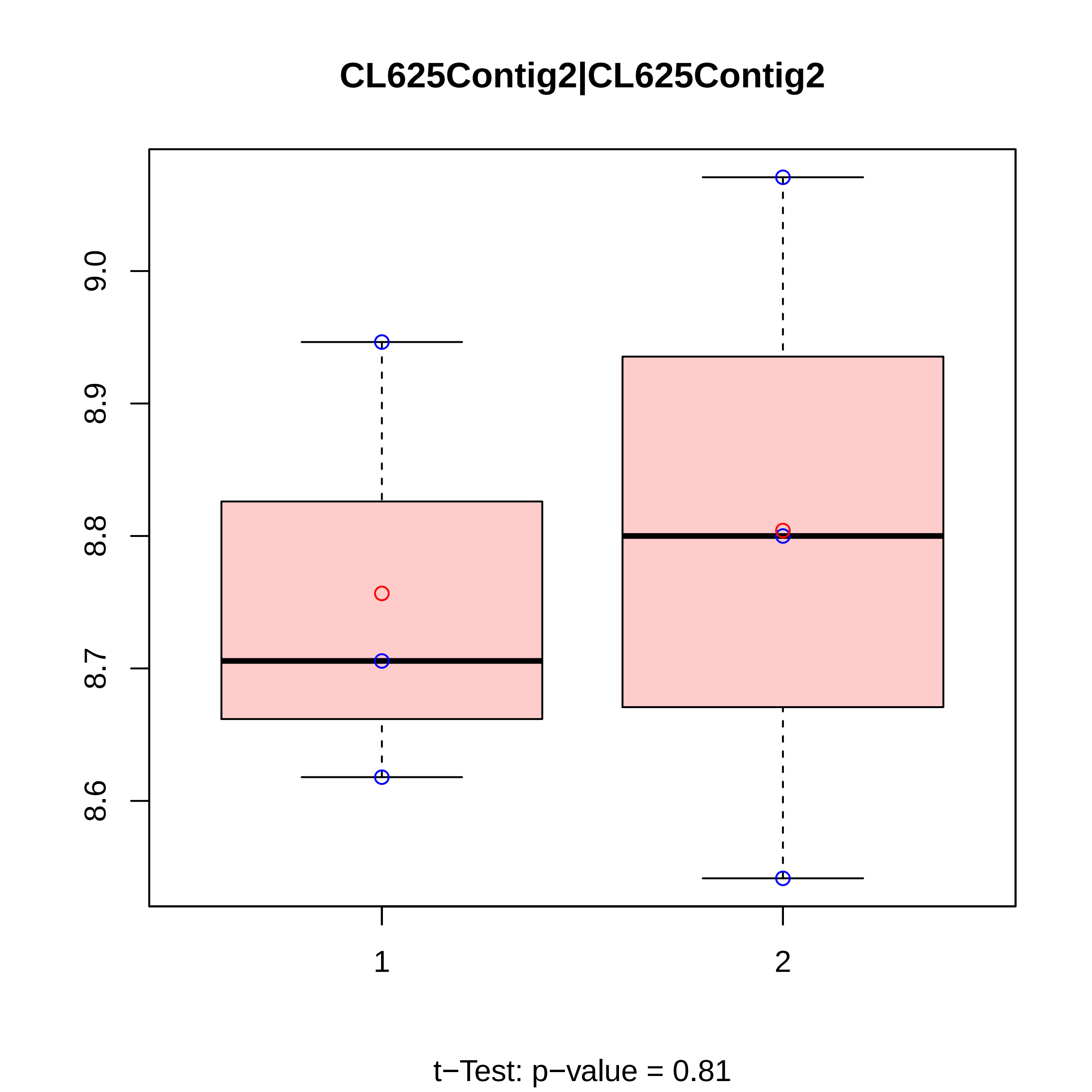

CL625Contig2|CL625Contig2
9.0
8.9
8.8
8.7
8.6
1
2
t−Test: p−value = 0.81

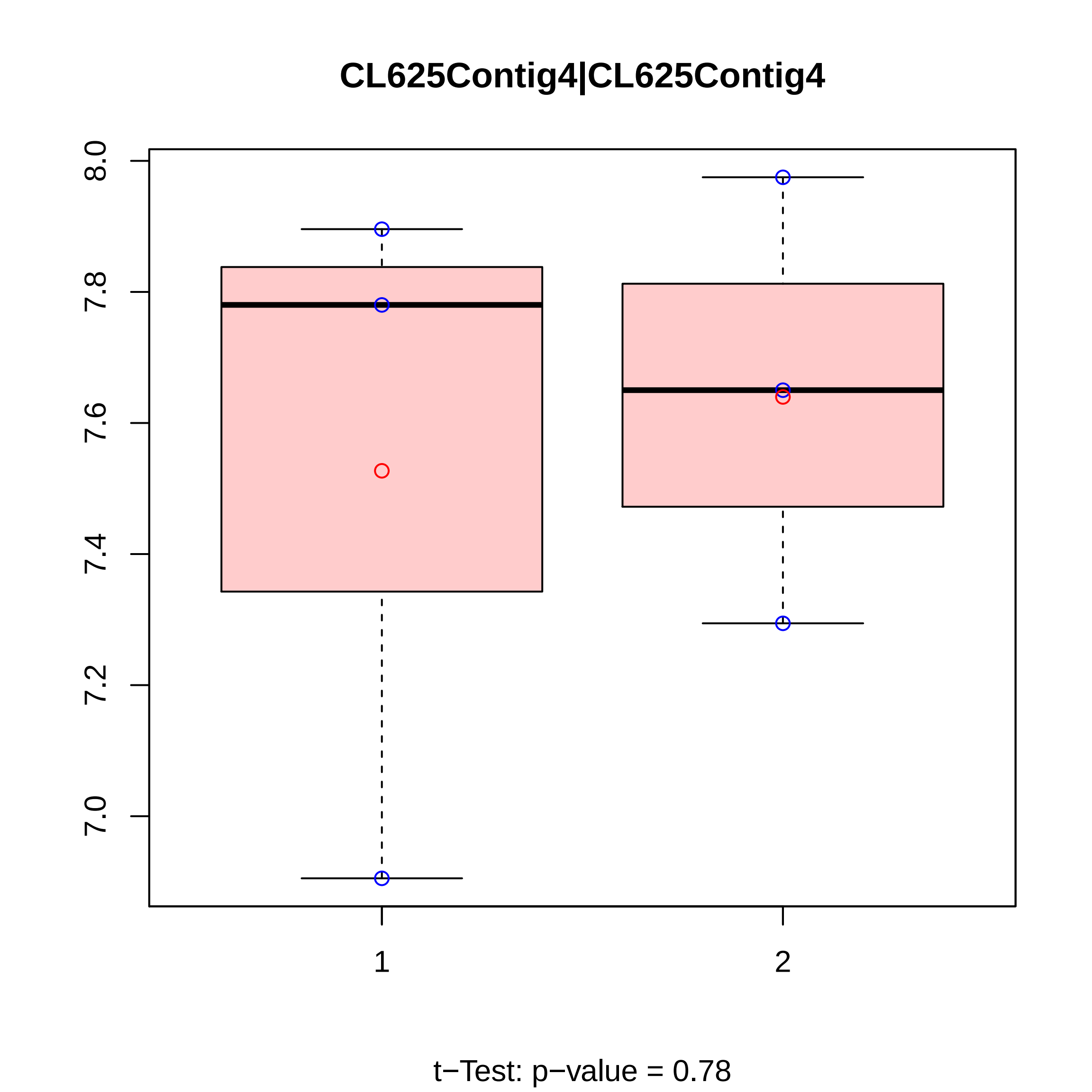
CL625Contig4|CL625Contig4
8.0
7.8
7.6
7.4
7.2
7.0
1
2
t−Test: p−value = 0.78

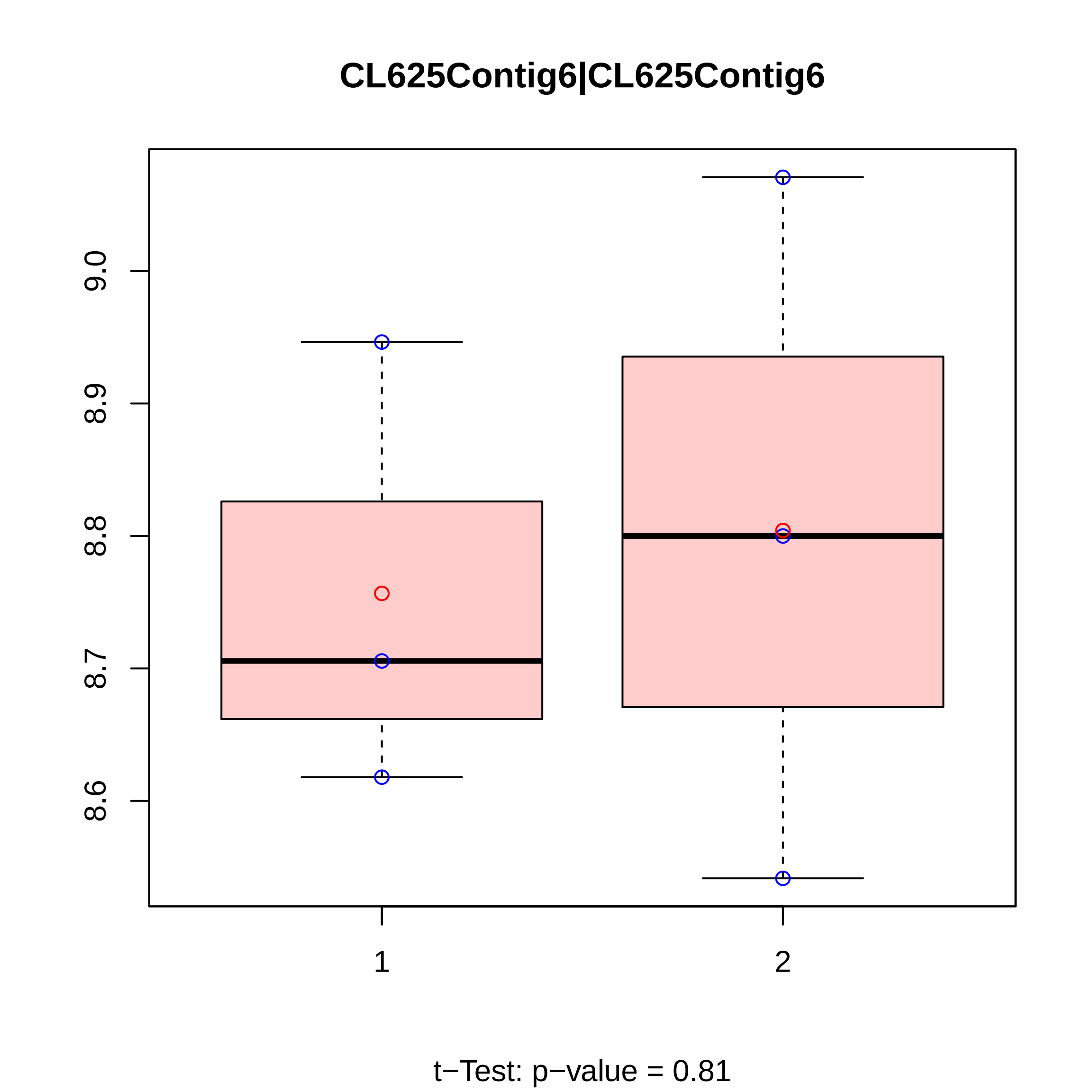
CL625Contig6|CL625Contig6
9.0
8.9
8.8
8.7
8.6
1
2
t−Test: p−value = 0.81

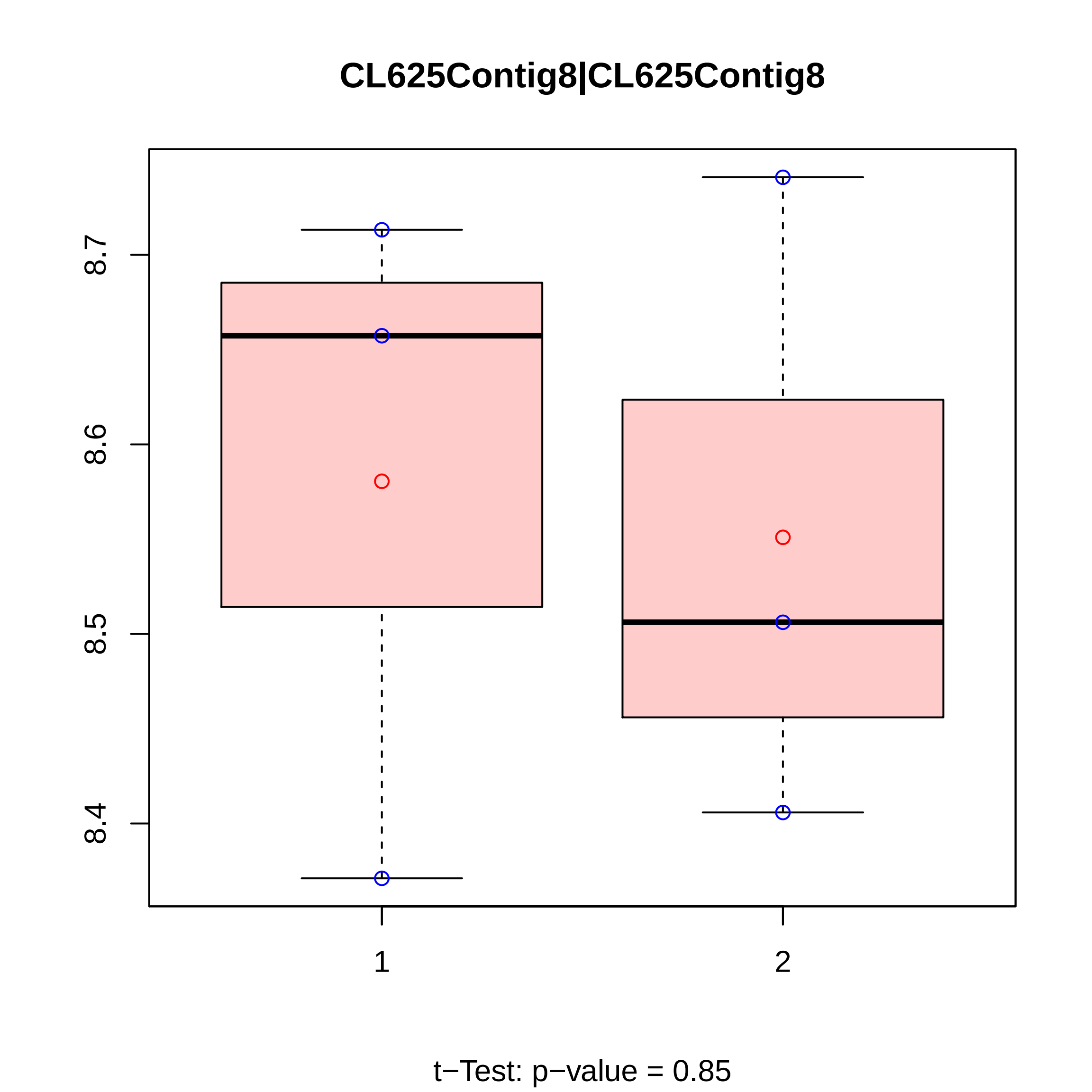

CL625Contig8|CL625Contig8
8.4
8.5
8.6
8.7
1
2
t−Test: p−value = 0.85

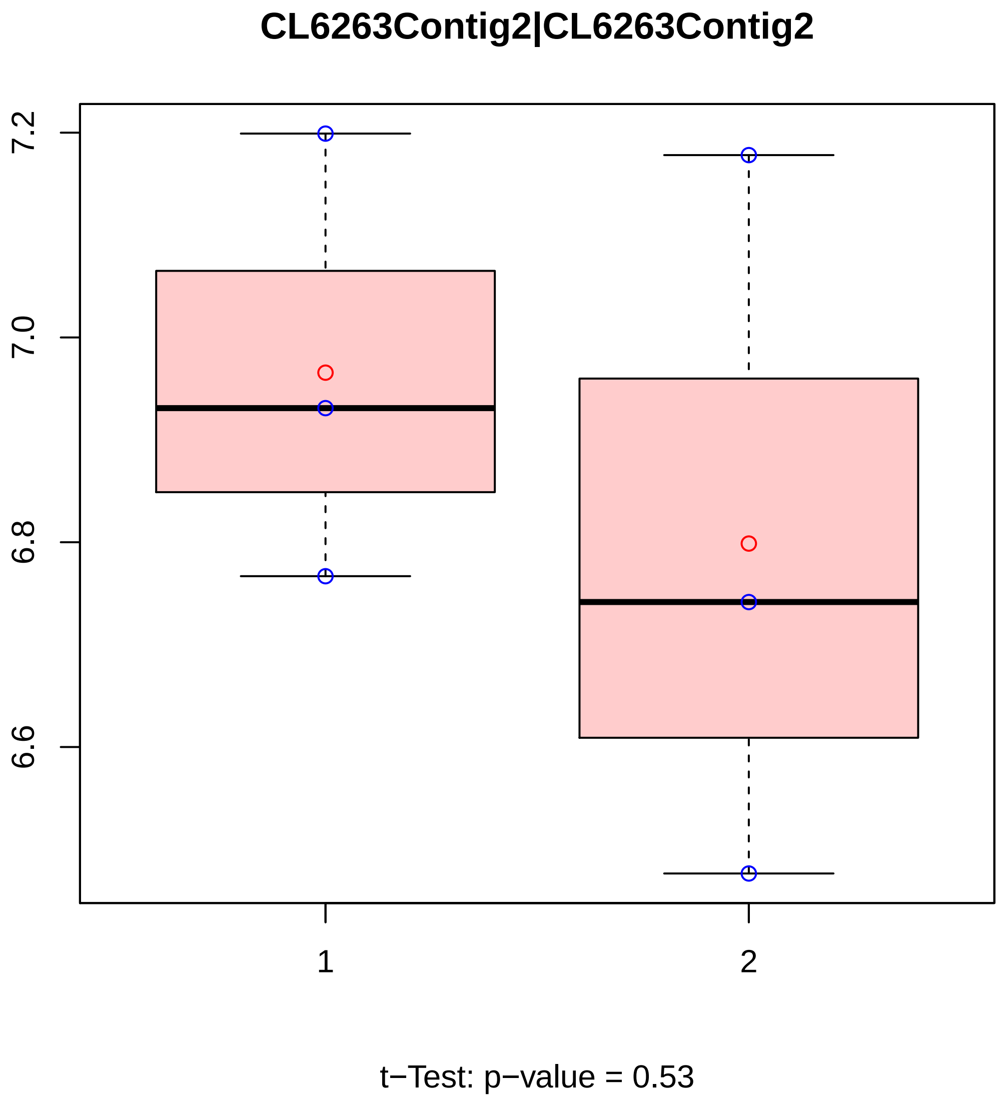
CL6263Contig2|CL6263Contig2
7.2
7.0
6.8
6.6
1
2
t−Test: p−value = 0.53

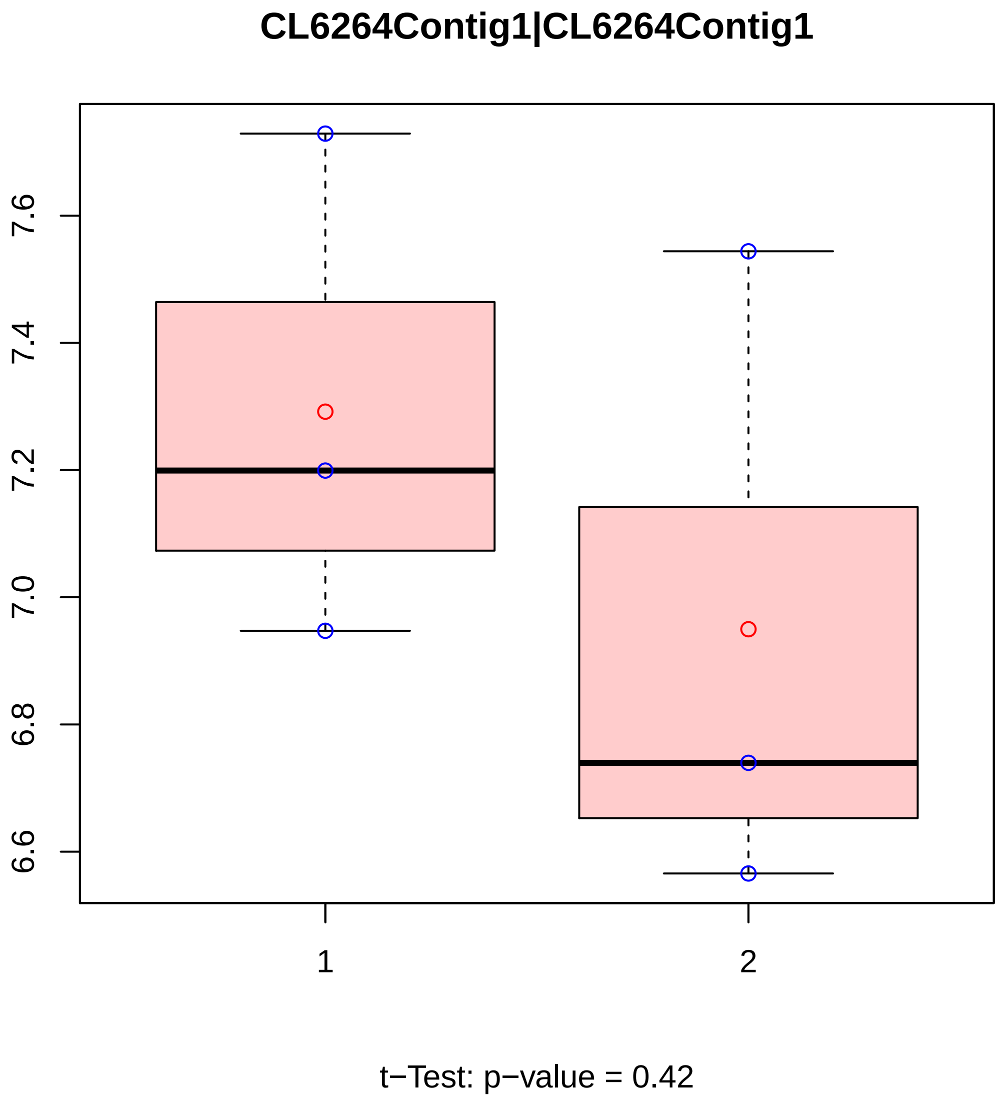
CL6264Contig1|CL6264Contig1
7.6
7.4
7.2
7.0
6.8
6.6
1
2
t−Test: p−value = 0.42

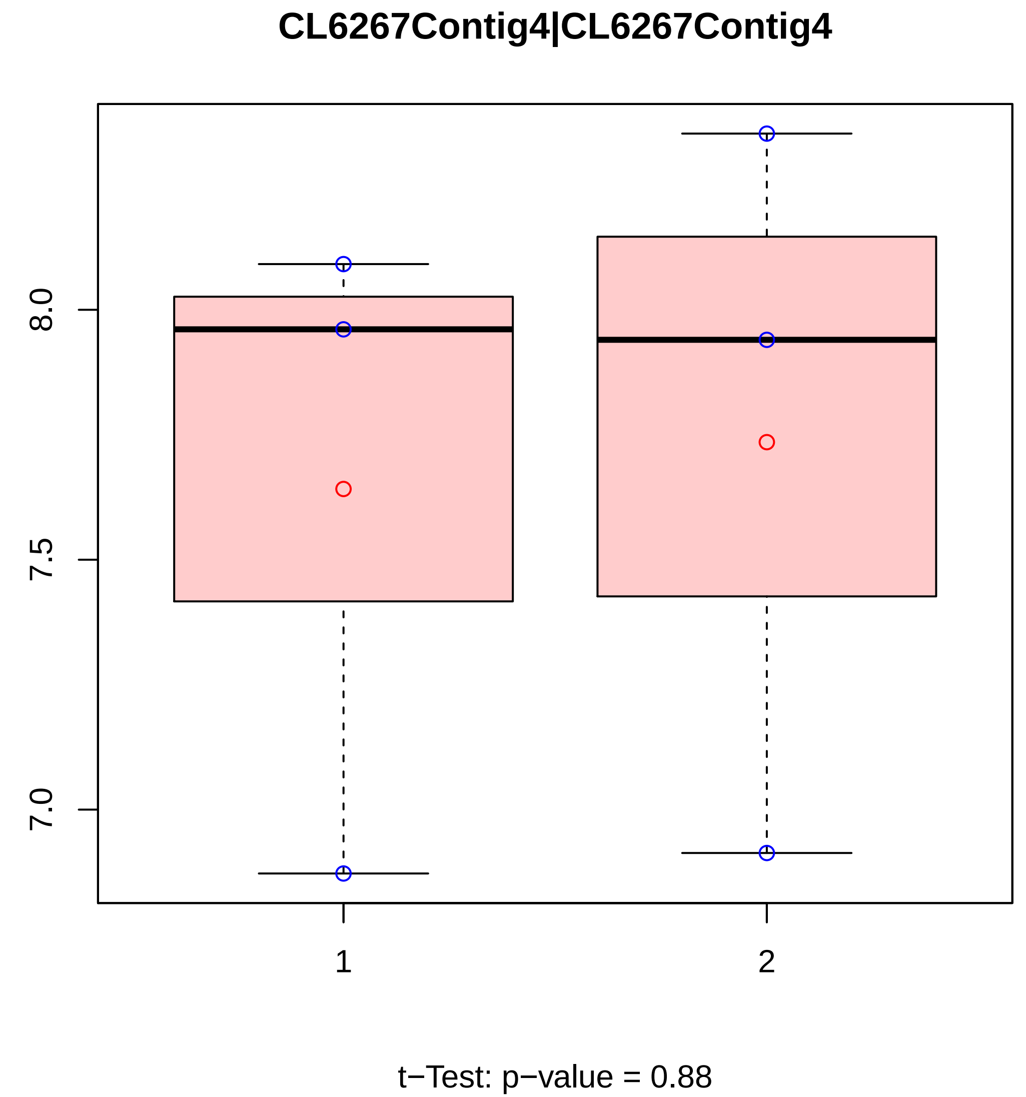
CL6267Contig4|CL6267Contig4
8.0
7.5
7.0
1
2
t−Test: p−value = 0.88

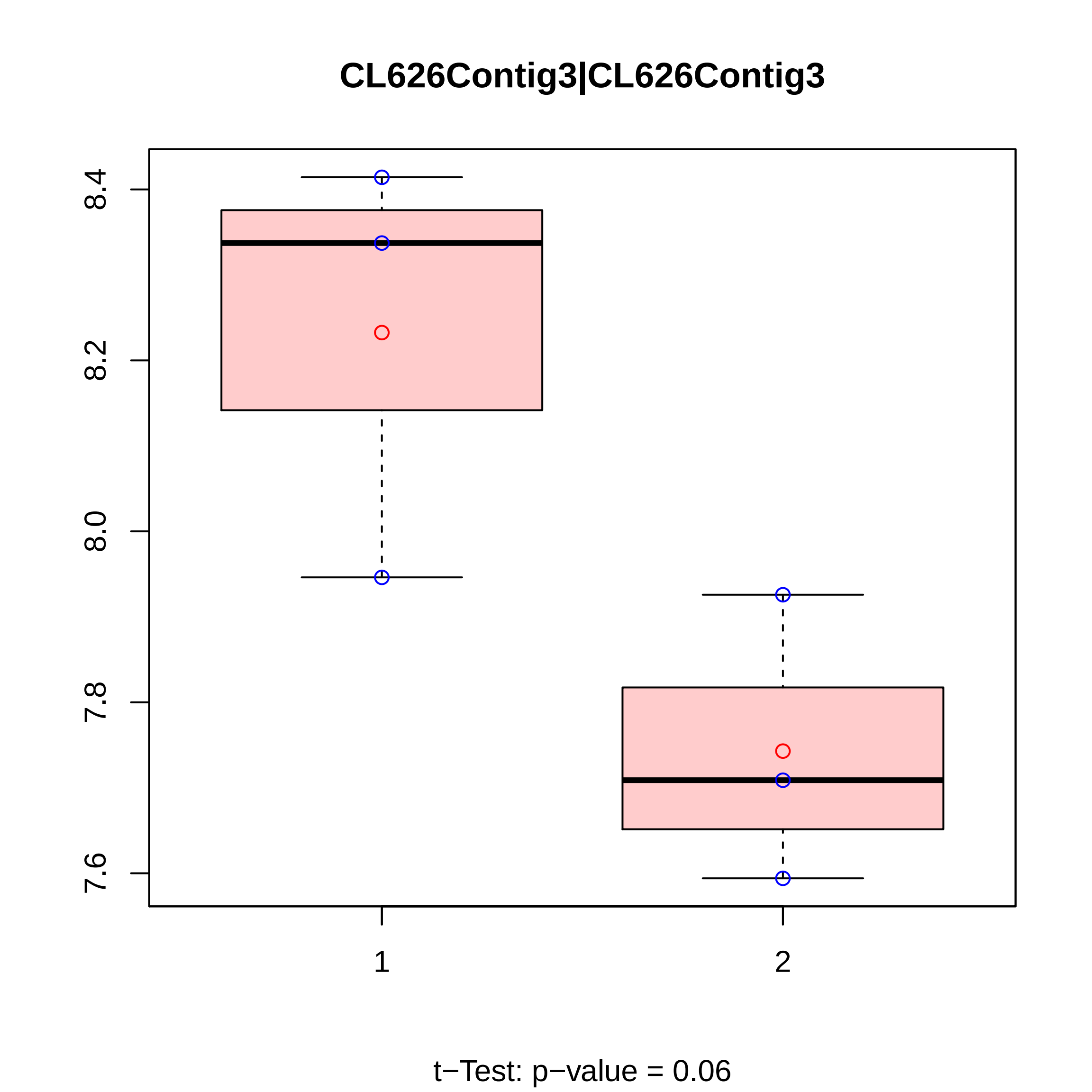
CL626Contig3|CL626Contig3
8.4
8.2
8.0
7.8
7.6
1
2
t−Test: p−value = 0.06

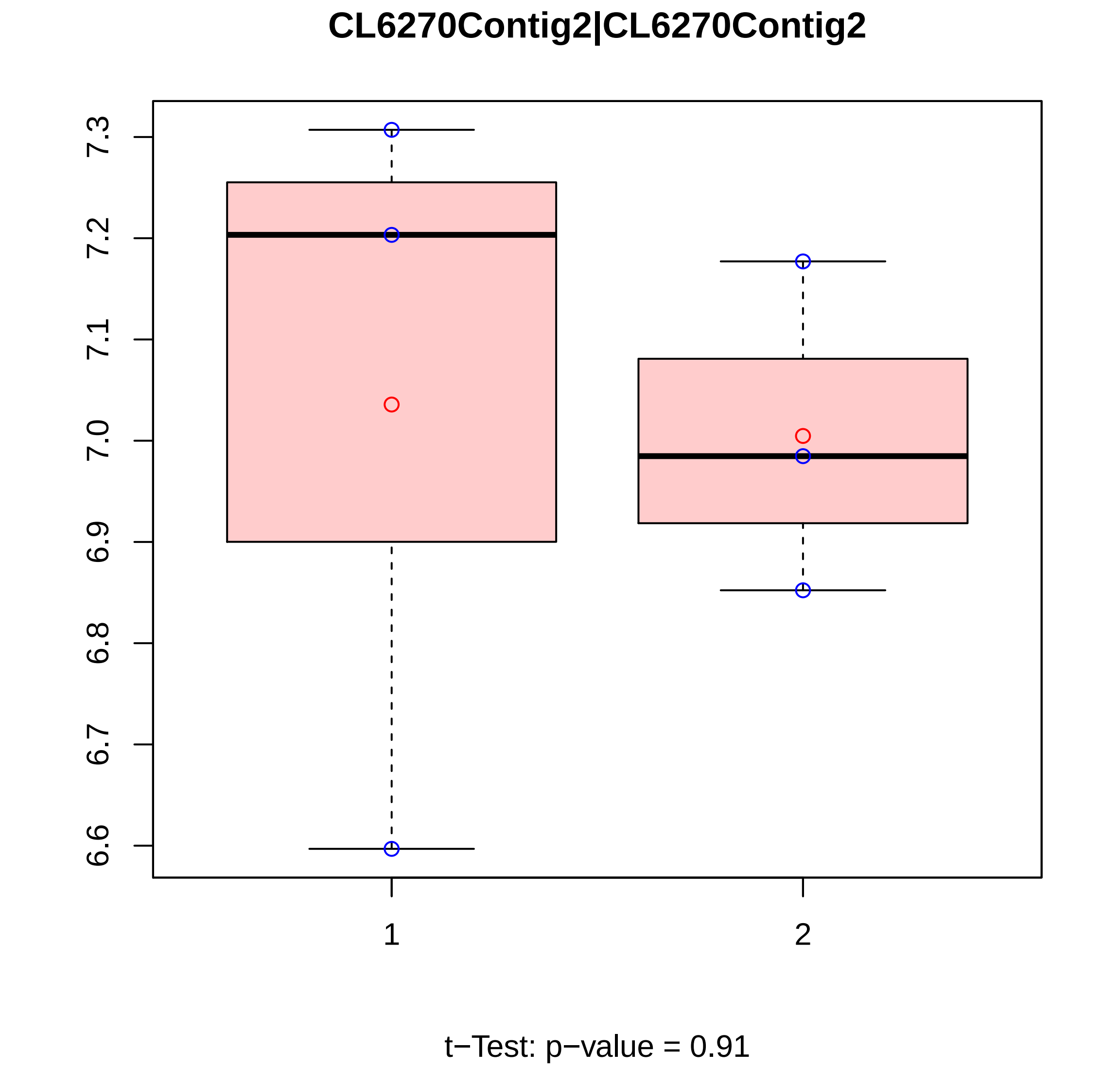
CL6270Contig2|CL6270Contig2
7.3
7.2
7.1
7.0
6.9
6.8
6.7
6.6
1
2
t−Test: p−value = 0.91

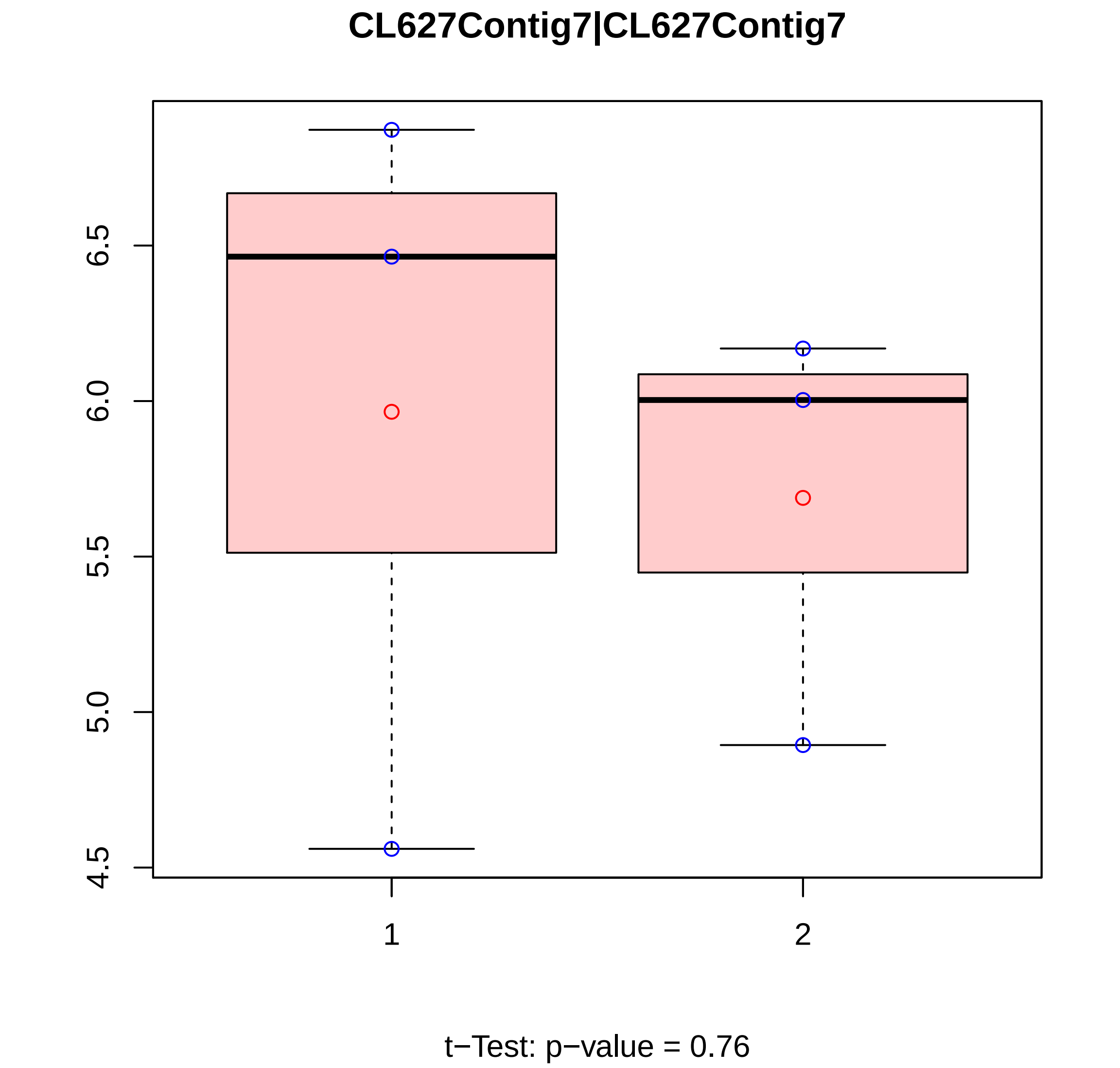
CL627Contig7|CL627Contig7
6.5
6.0
5.5
5.0
4.5
1
2
t−Test: p−value = 0.76

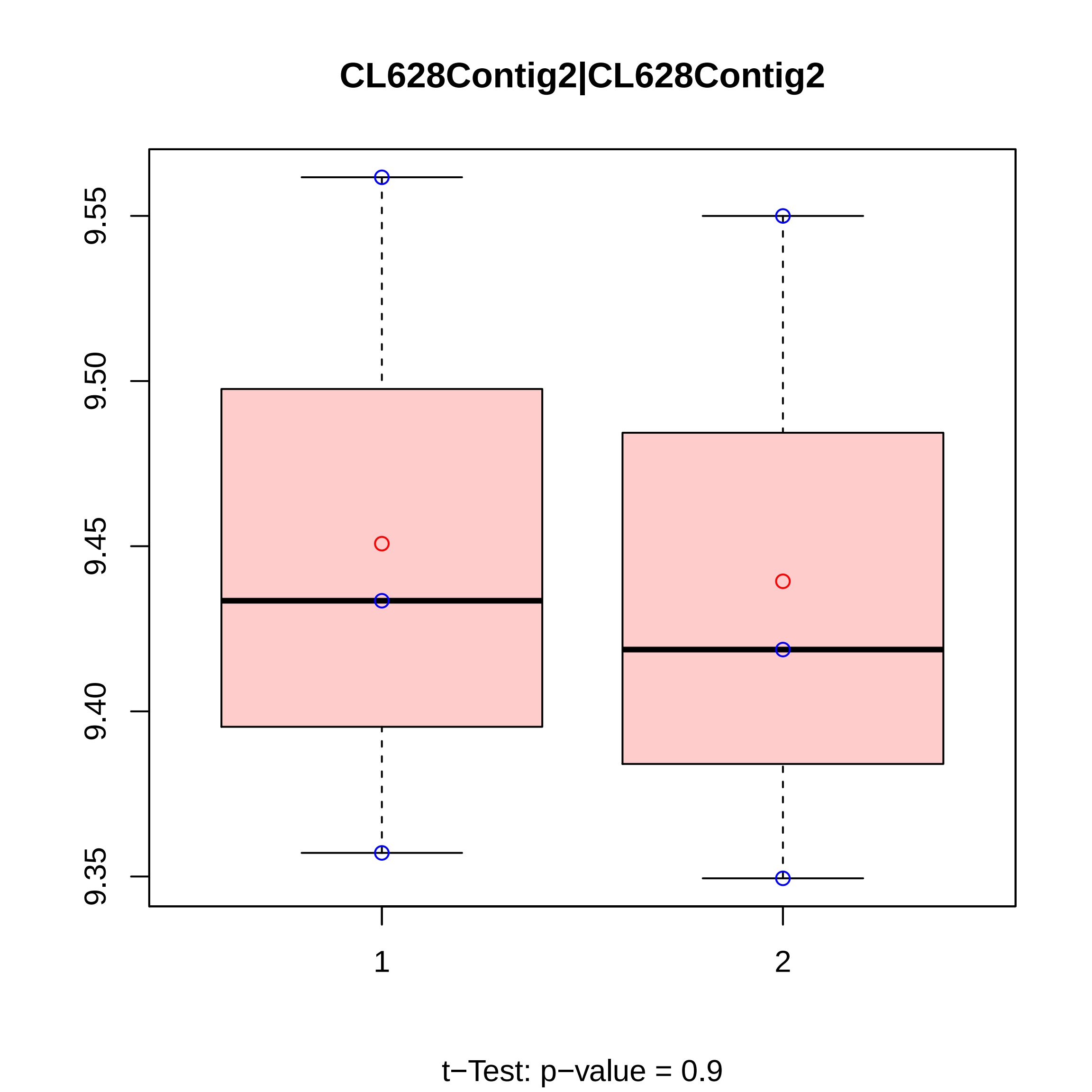
CL628Contig2|CL628Contig2
9.35
9.40
9.45
9.50
9.55
1
2
t−Test: p−value = 0.9

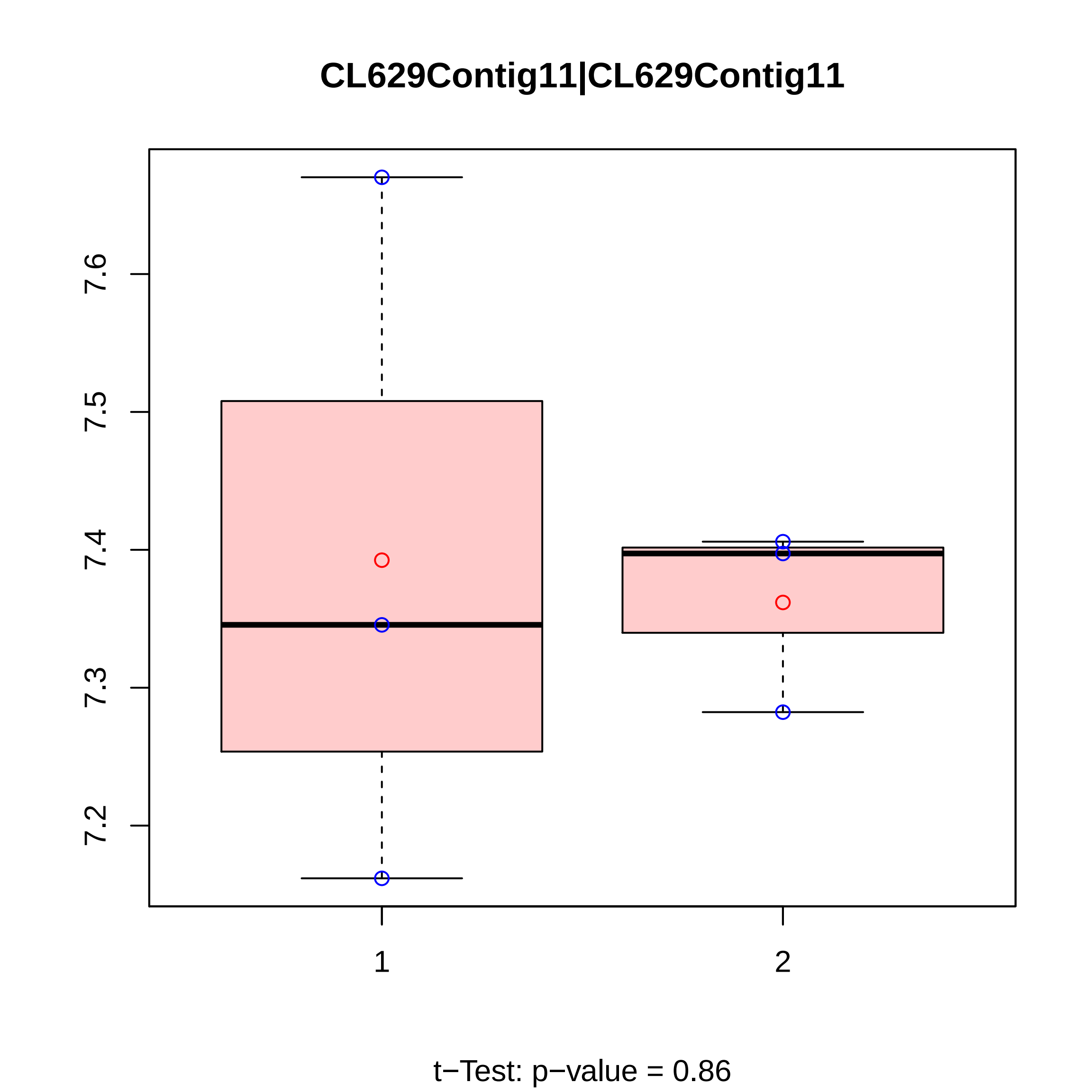
CL629Contig11|CL629Contig11
7.2
7.3
7.4
7.5
7.6
1
2
t−Test: p−value = 0.86

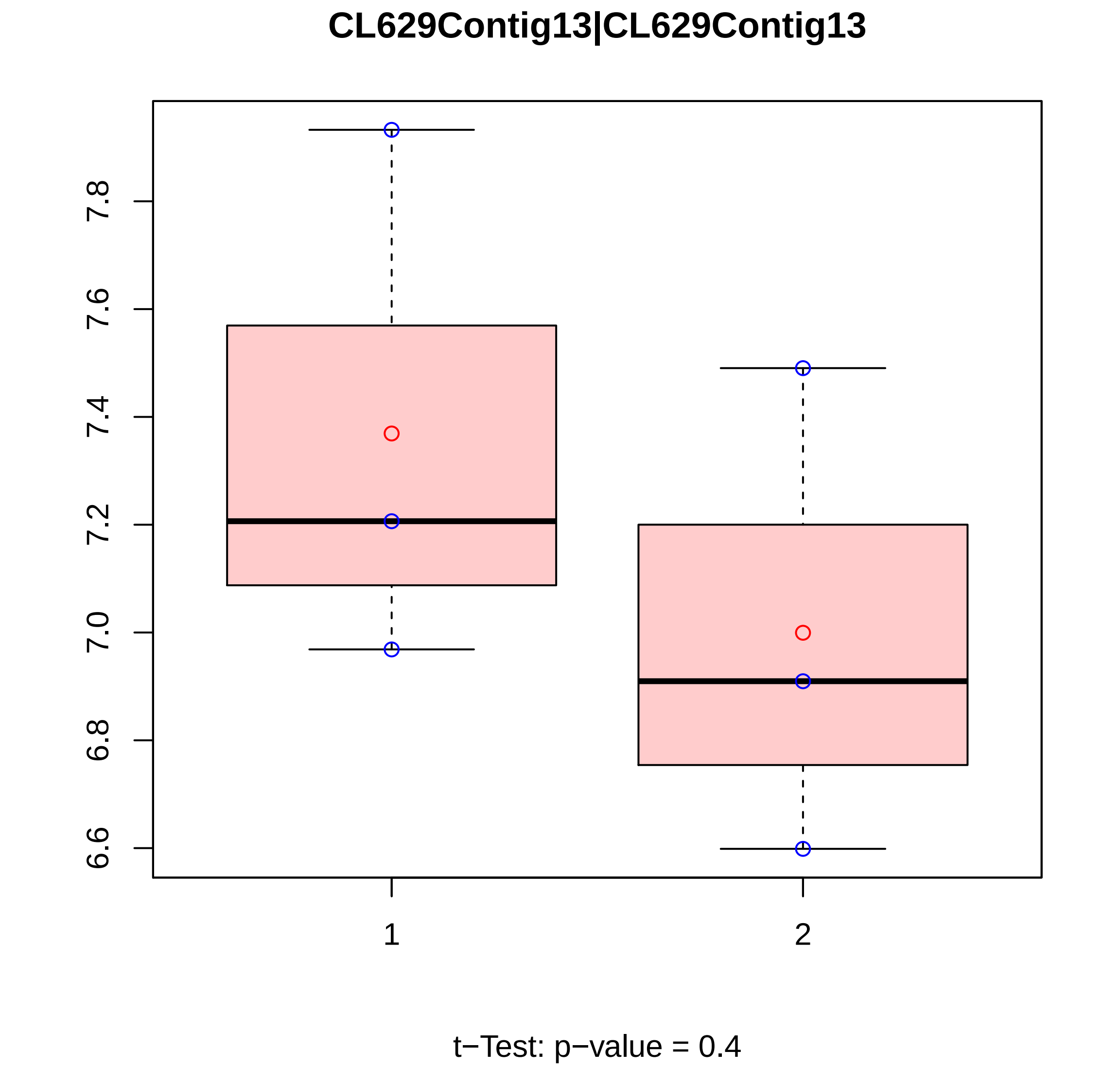
CL629Contig13|CL629Contig13
7.8
7.6
7.4
7.2
7.0
6.8
6.6
1
2
t−Test: p−value = 0.4

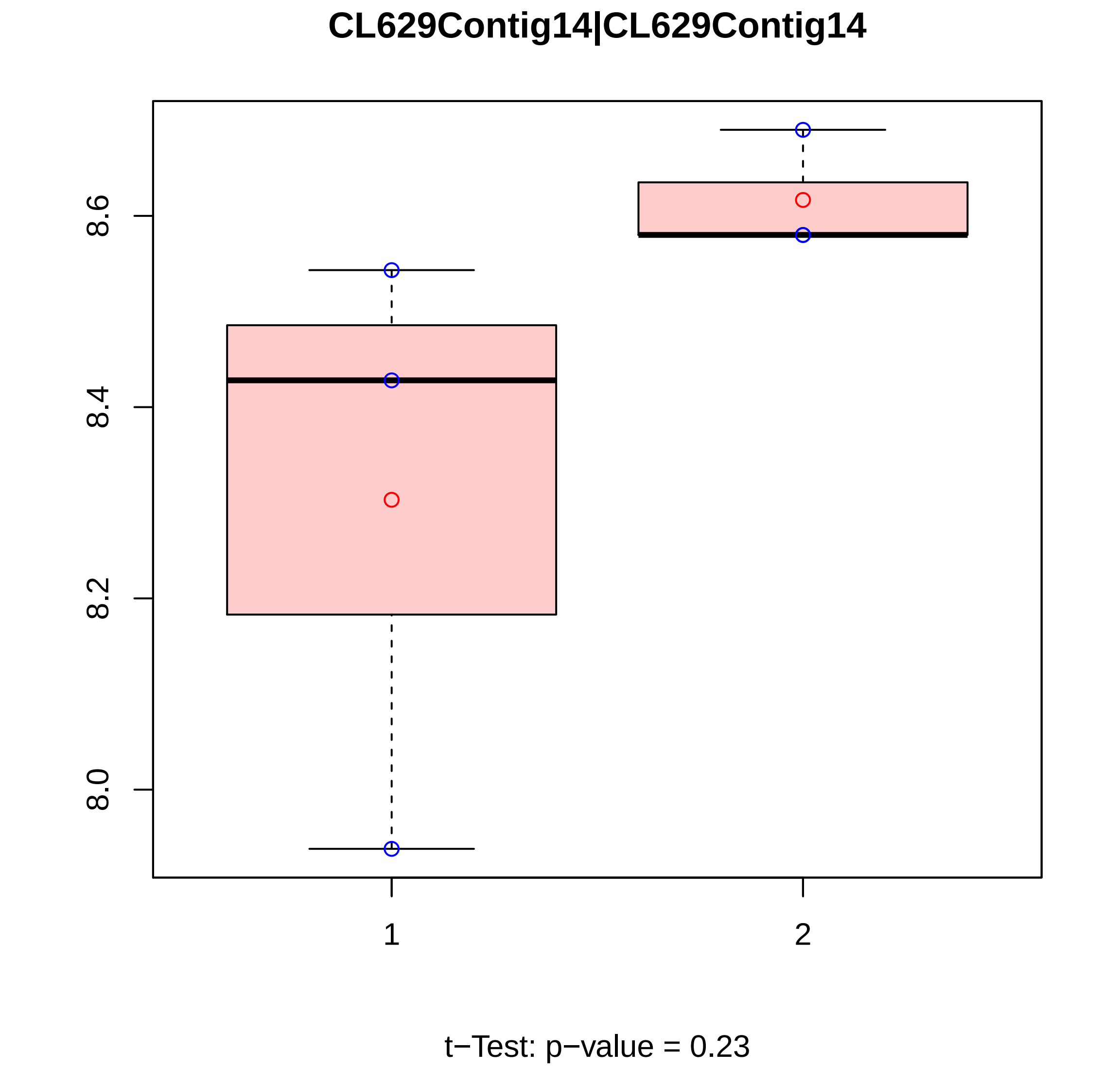

CL629Contig14|CL629Contig14
8.0
8.2
8.4
8.6
1
2
t−Test: p−value = 0.23

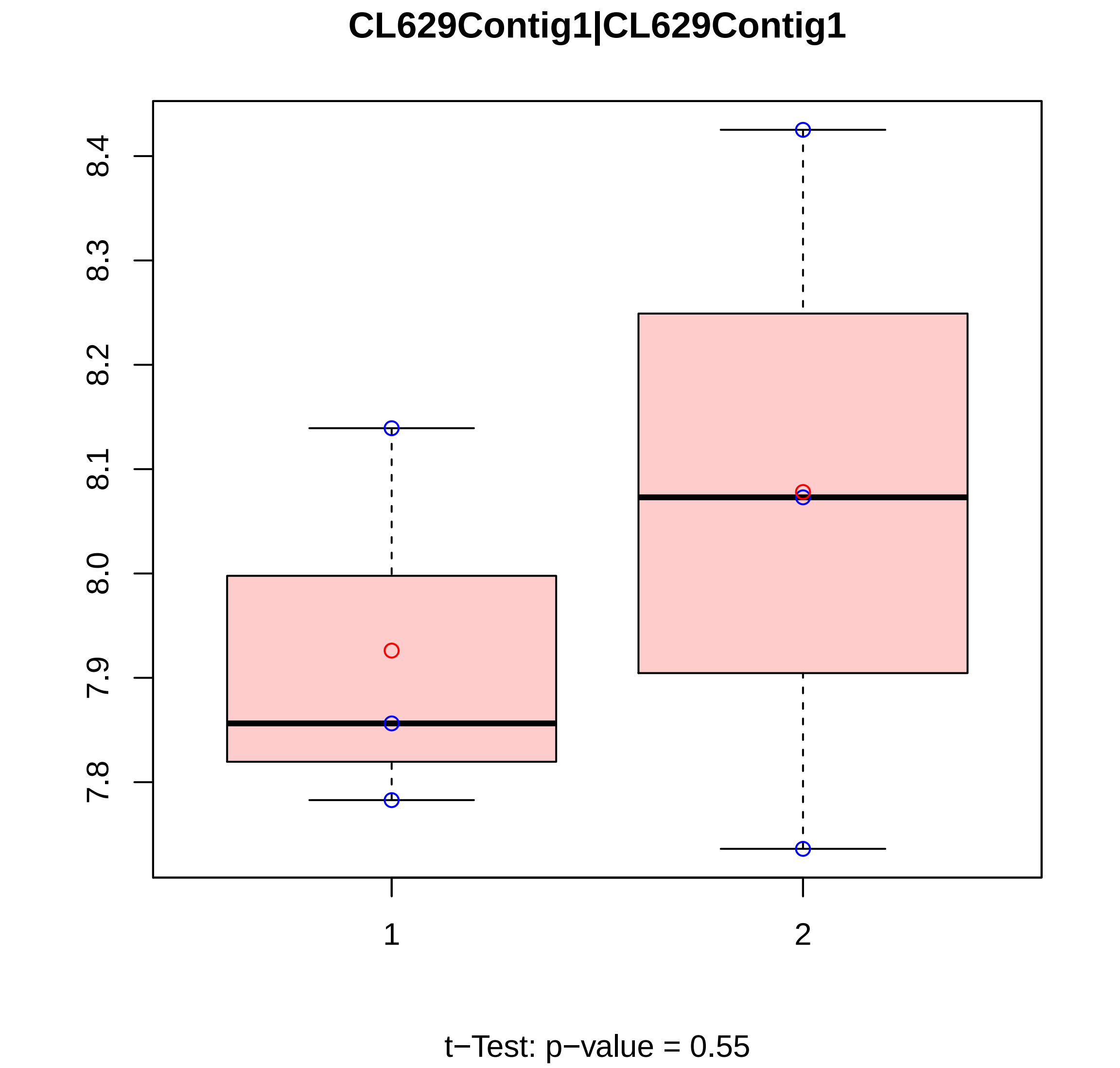
CL629Contig1|CL629Contig1
8.4
8.3
8.2
8.1
8.0
7.9
7.8
1
2
t−Test: p−value = 0.55

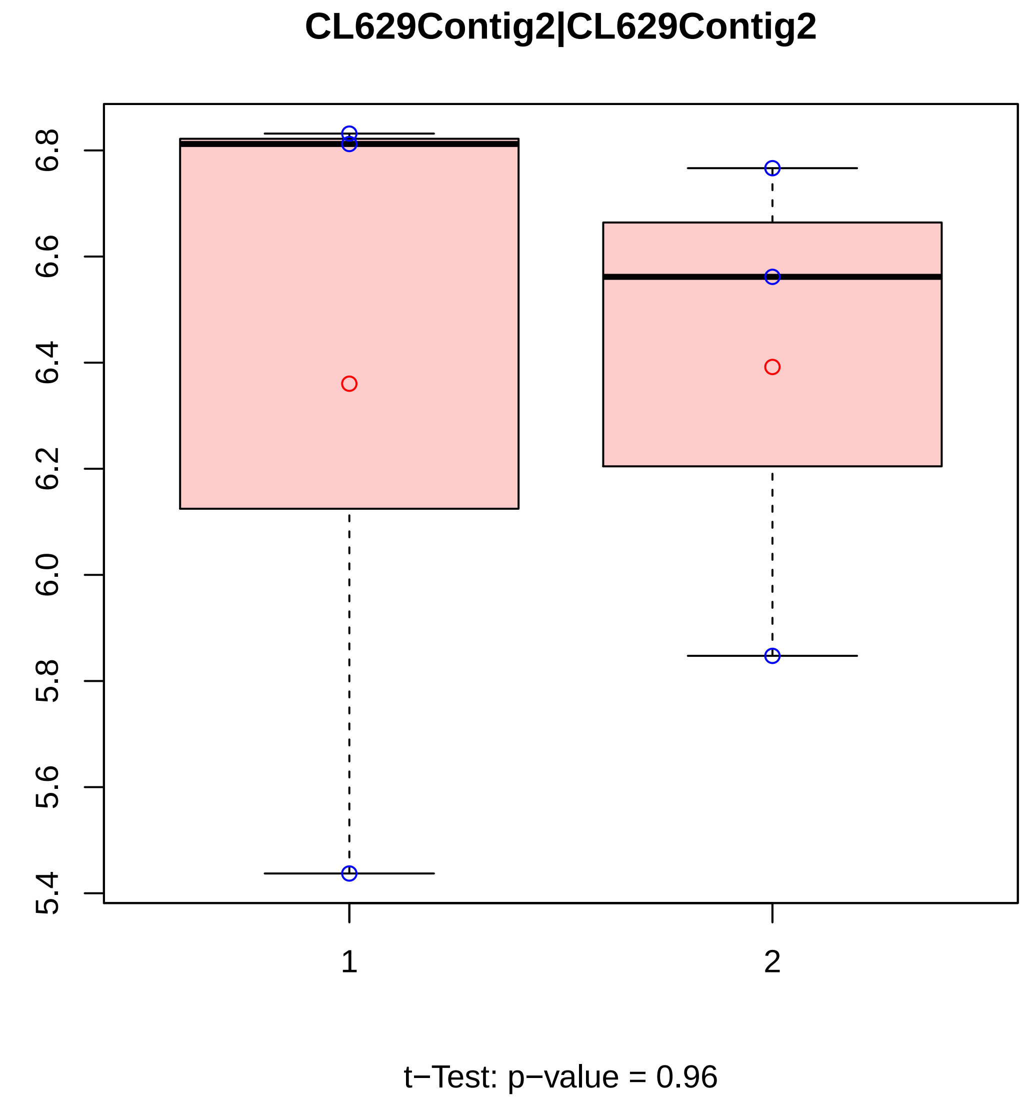
CL629Contig2|CL629Contig2
6.8
6.6
6.4
6.2
6.0
5.8
5.6
5.4
1
2
t−Test: p−value = 0.96

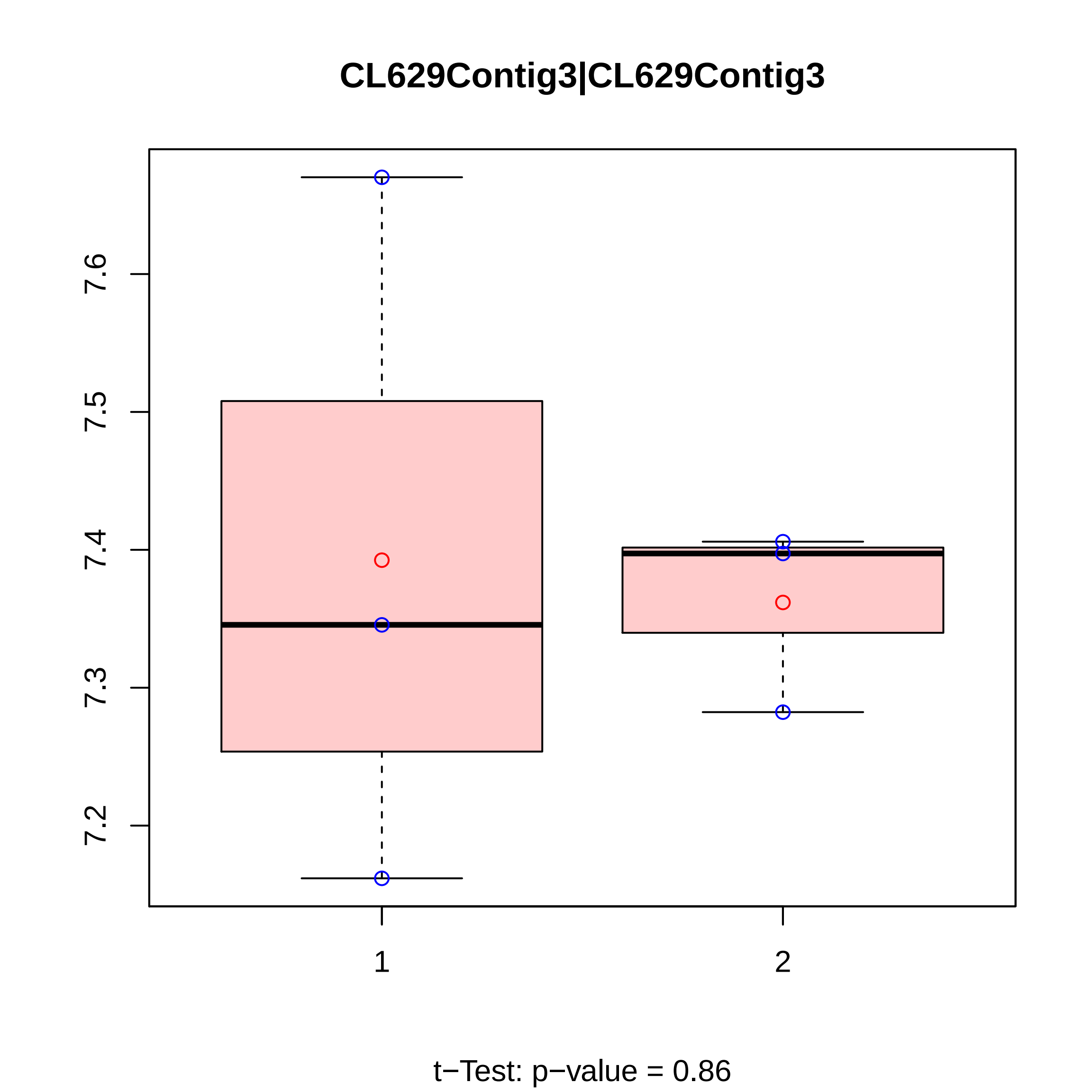
CL629Contig3|CL629Contig3
7.2
7.3
7.4
7.5
7.6
1
2
t−Test: p−value = 0.86

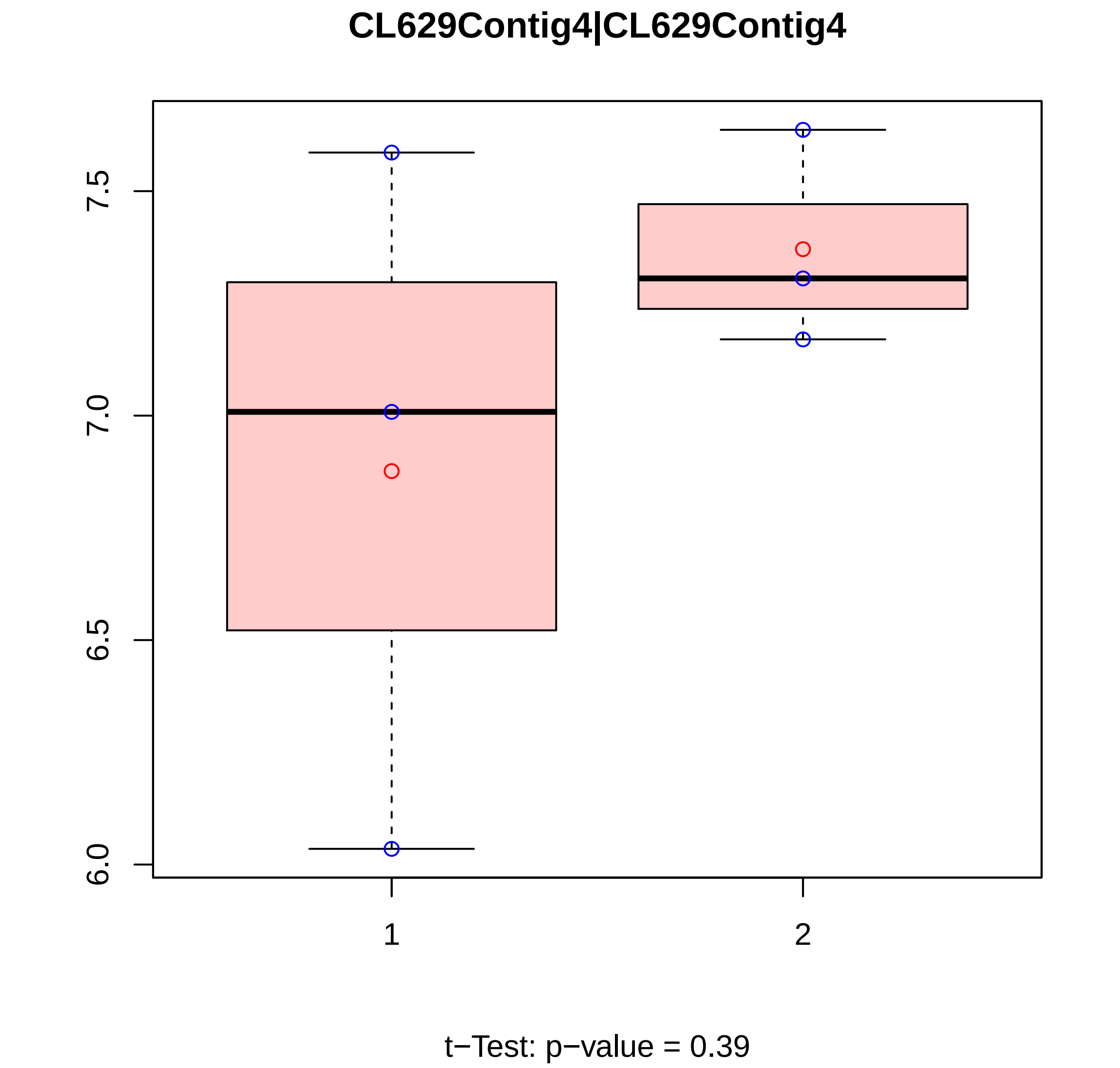

CL629Contig4|CL629Contig4
7.5
7.0
6.5
6.0
1
2
t−Test: p−value = 0.39

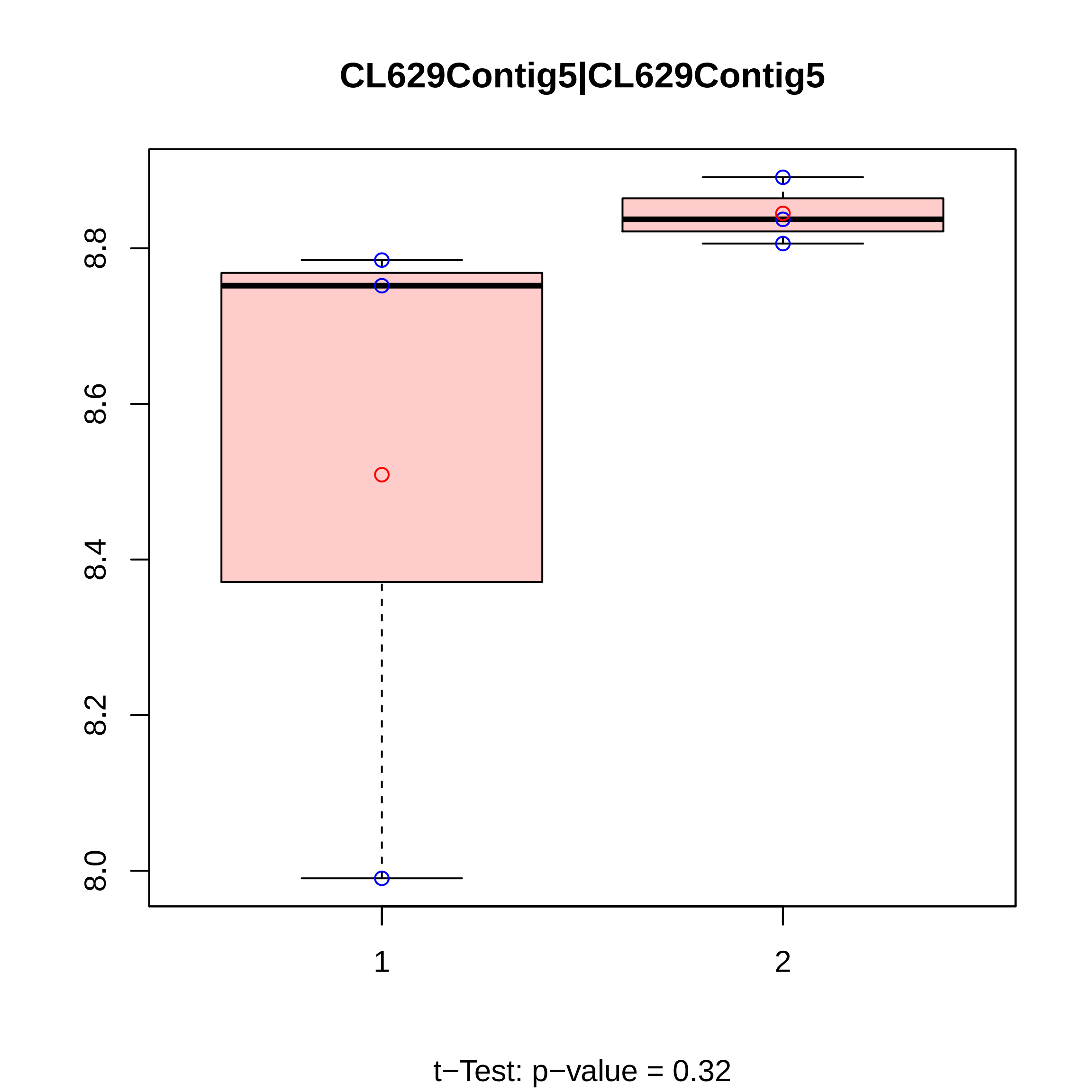

CL629Contig5|CL629Contig5
8.0
8.2
8.4
8.6
8.8
1
2
t−Test: p−value = 0.32

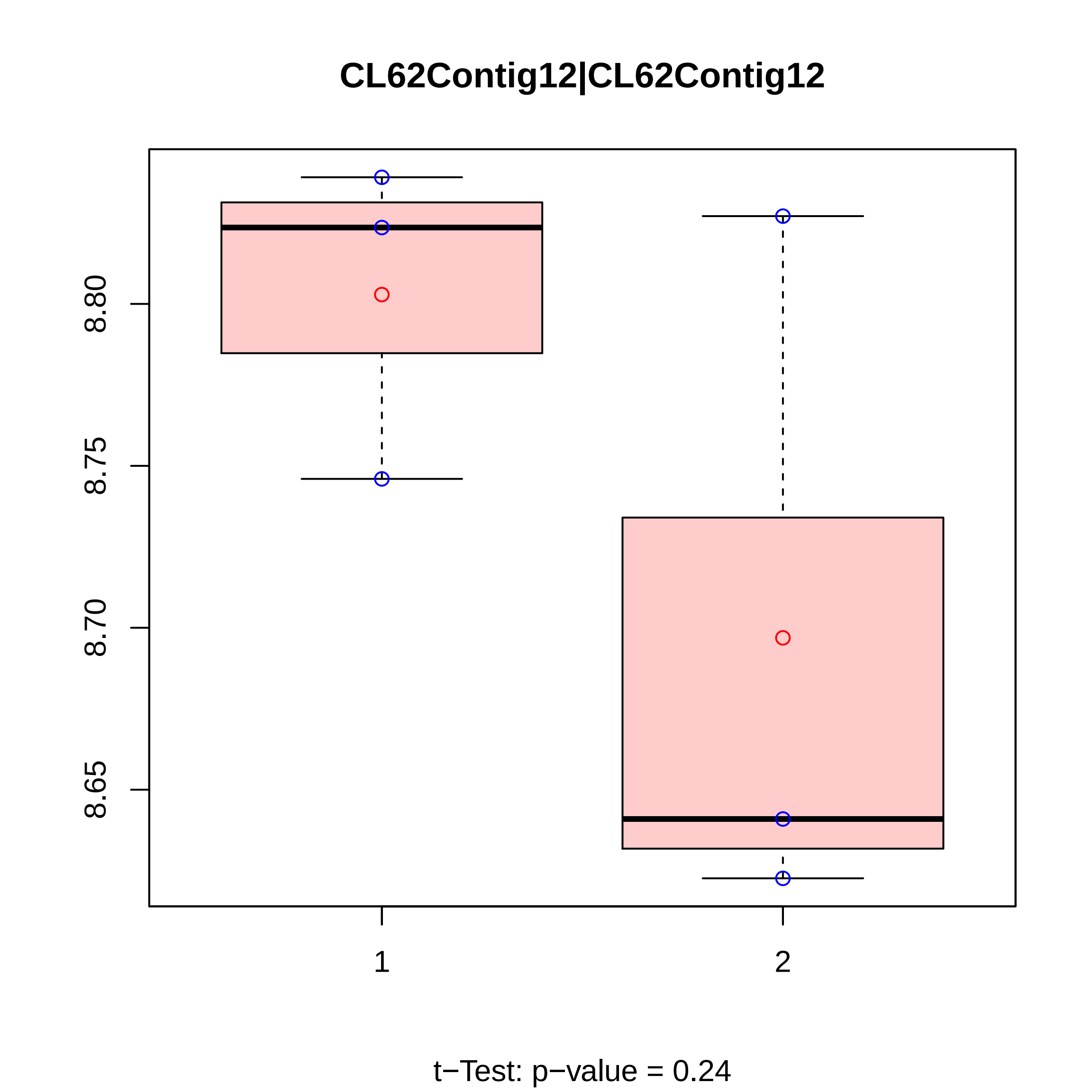
CL62Contig12|CL62Contig12
8.65
8.70
8.75
8.80
1
2
t−Test: p−value = 0.24

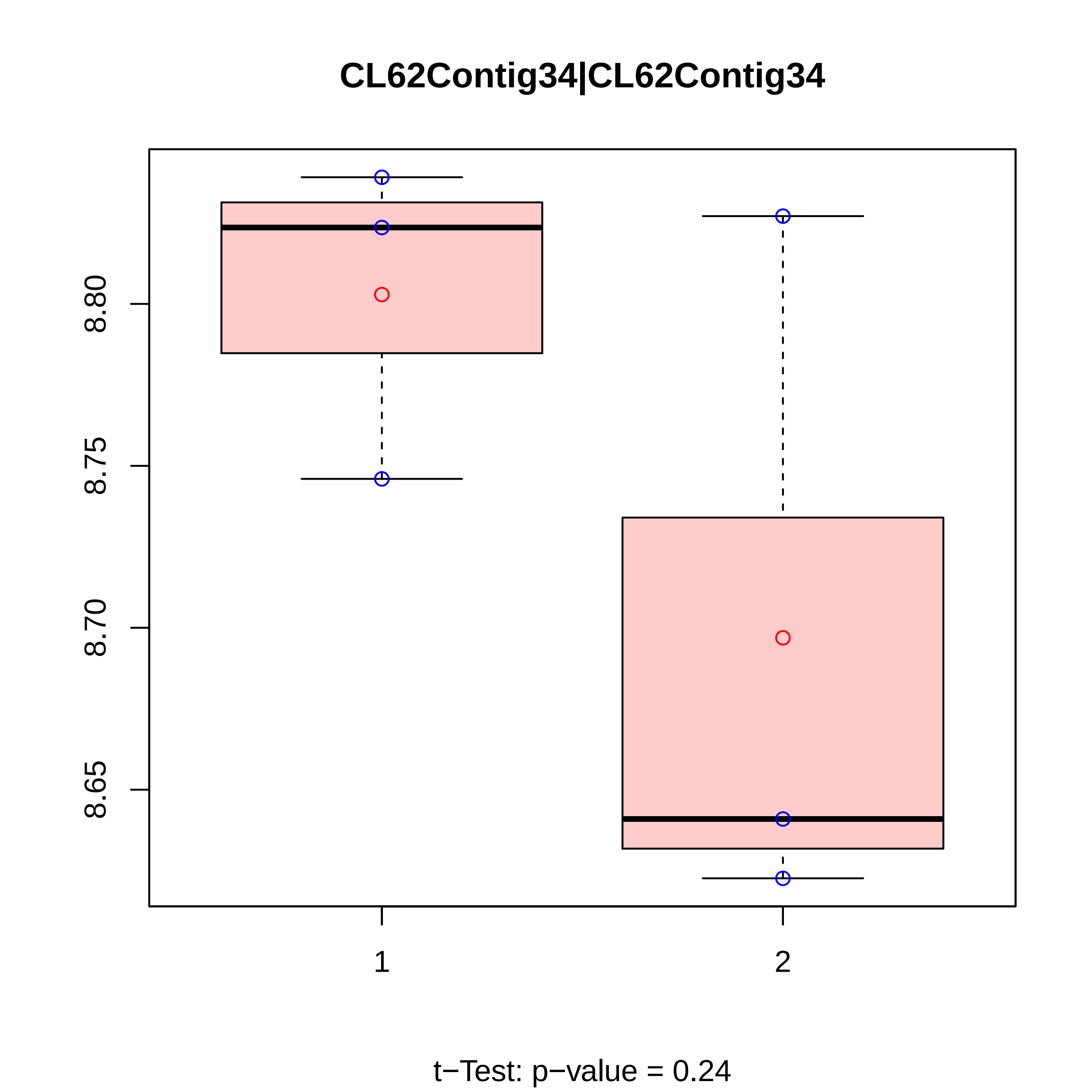
CL62Contig34|CL62Contig34
8.65
8.70
8.75
8.80
1
2
t−Test: p−value = 0.24

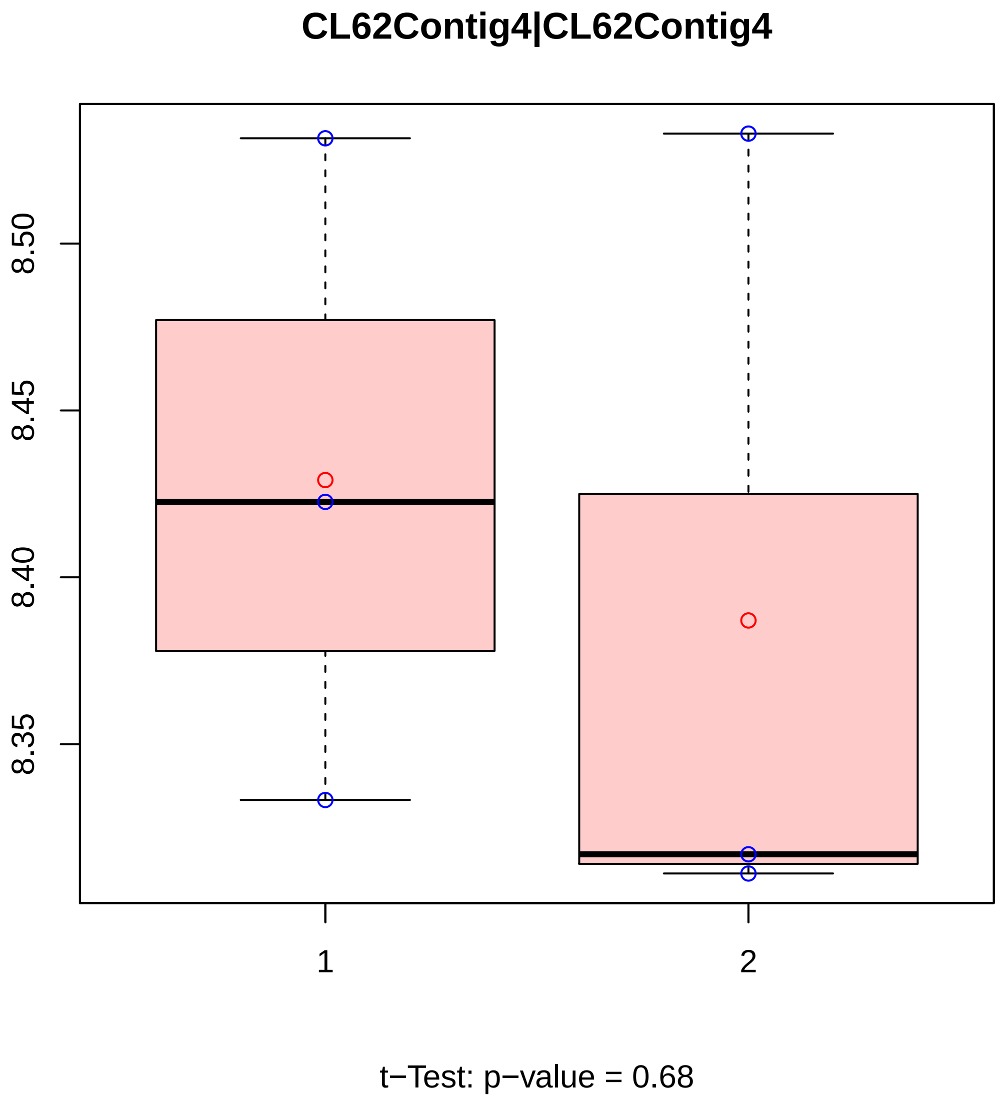
CL62Contig4|CL62Contig4
8.35
8.40
8.45
8.50
1
2
t−Test: p−value = 0.68

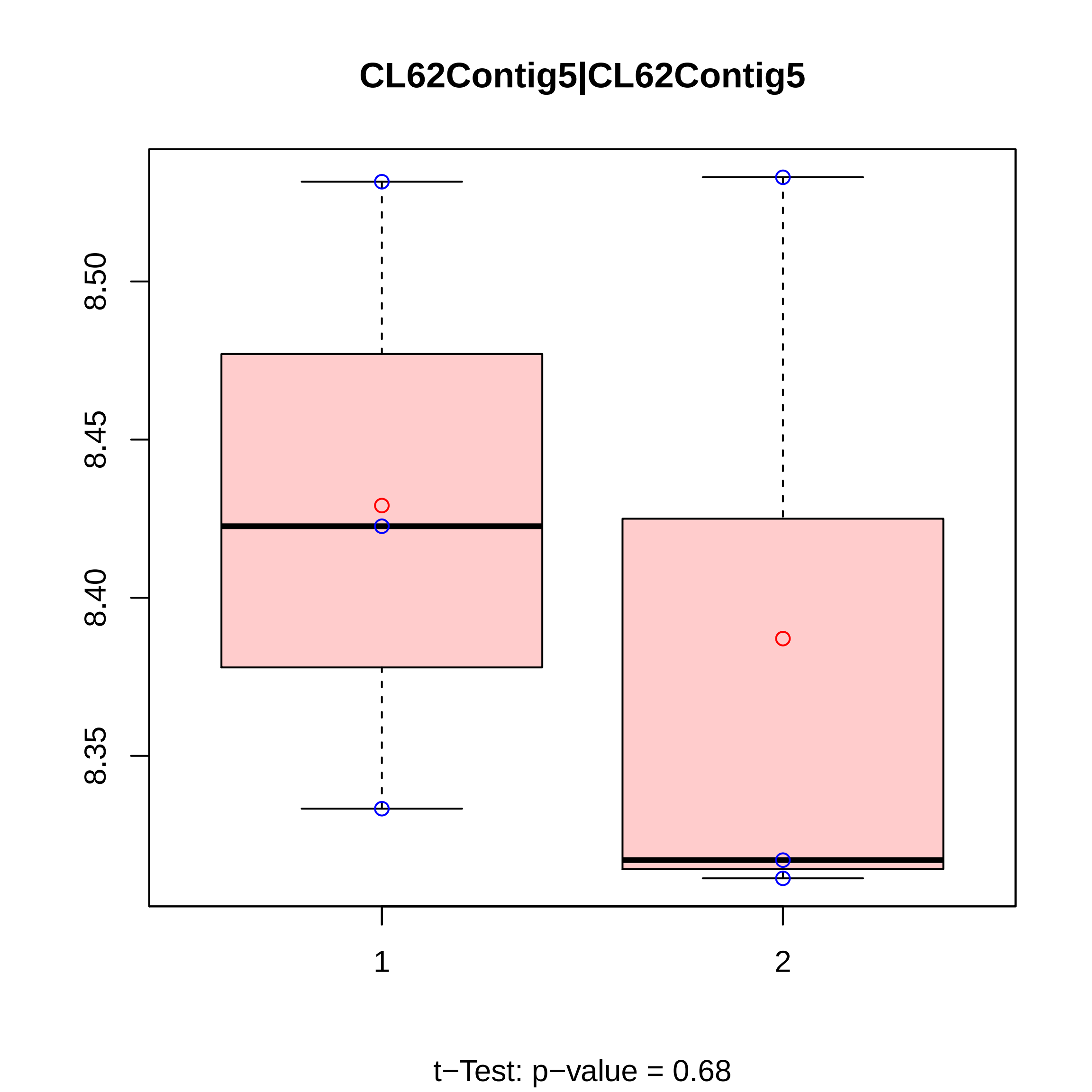
CL62Contig5|CL62Contig5
8.35
8.40
8.45
8.50
1
2
t−Test: p−value = 0.68

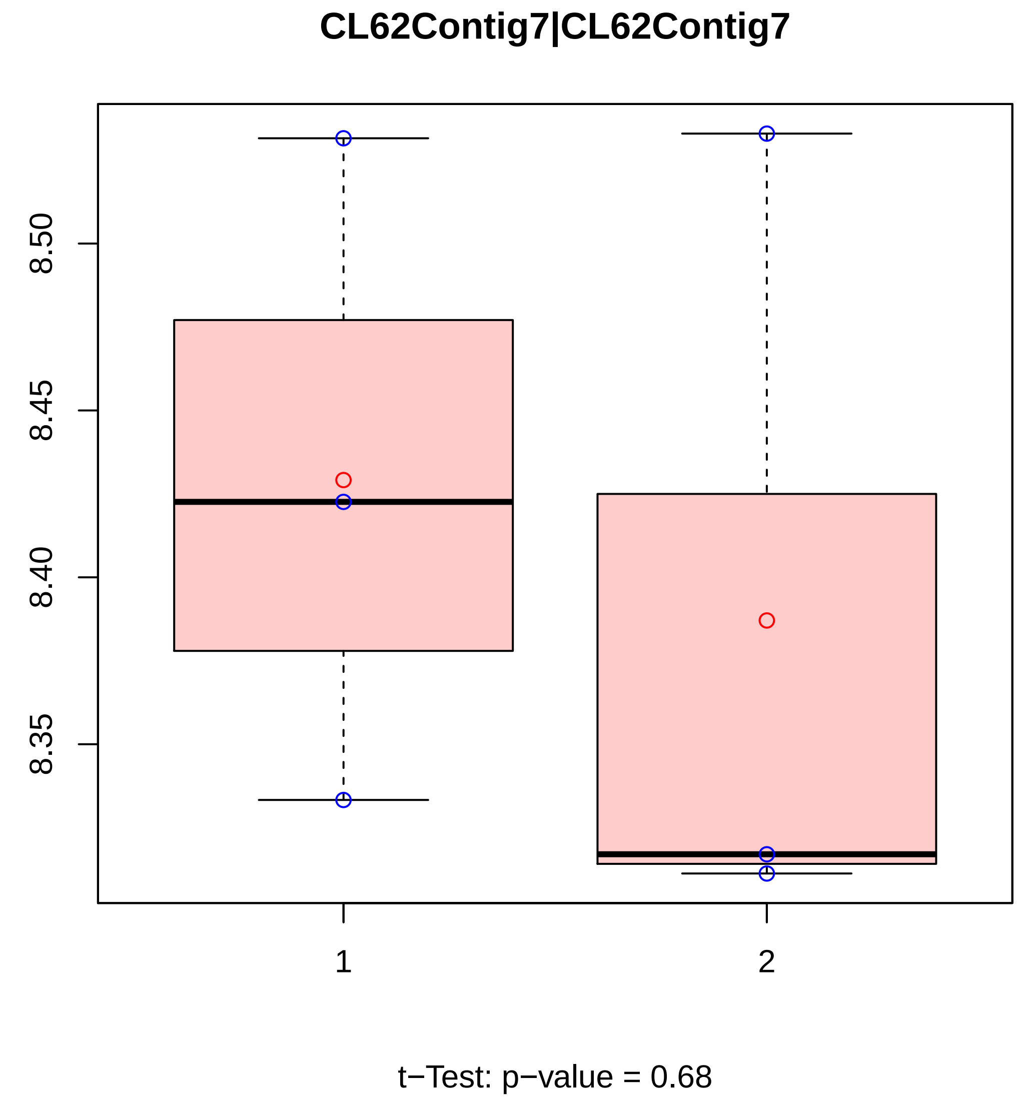
CL62Contig7|CL62Contig7
8.35
8.40
8.45
8.50
1
2
t−Test: p−value = 0.68

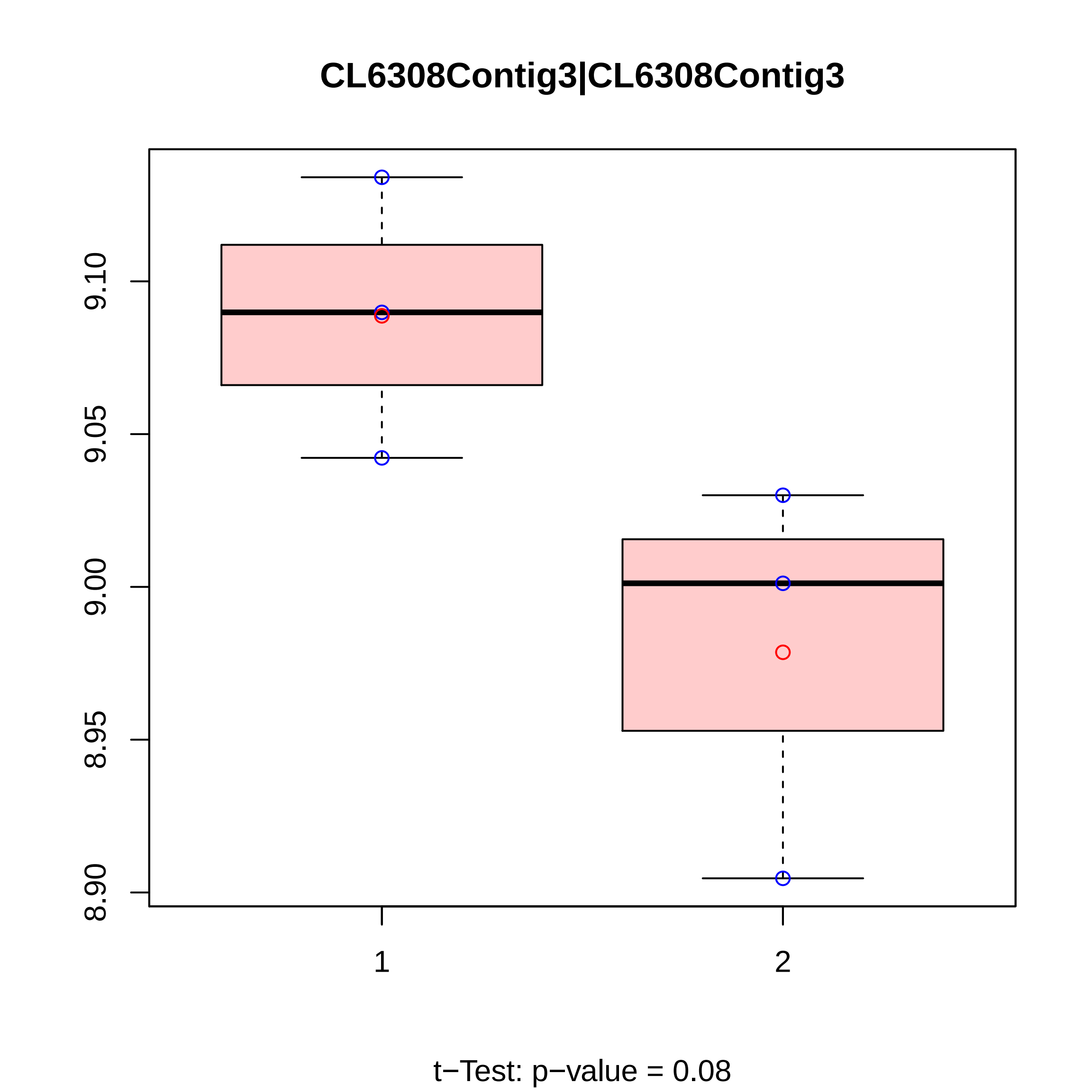

CL6308Contig3|CL6308Contig3
9.10
9.05
9.00
8.95
8.90
1
2
t−Test: p−value = 0.08

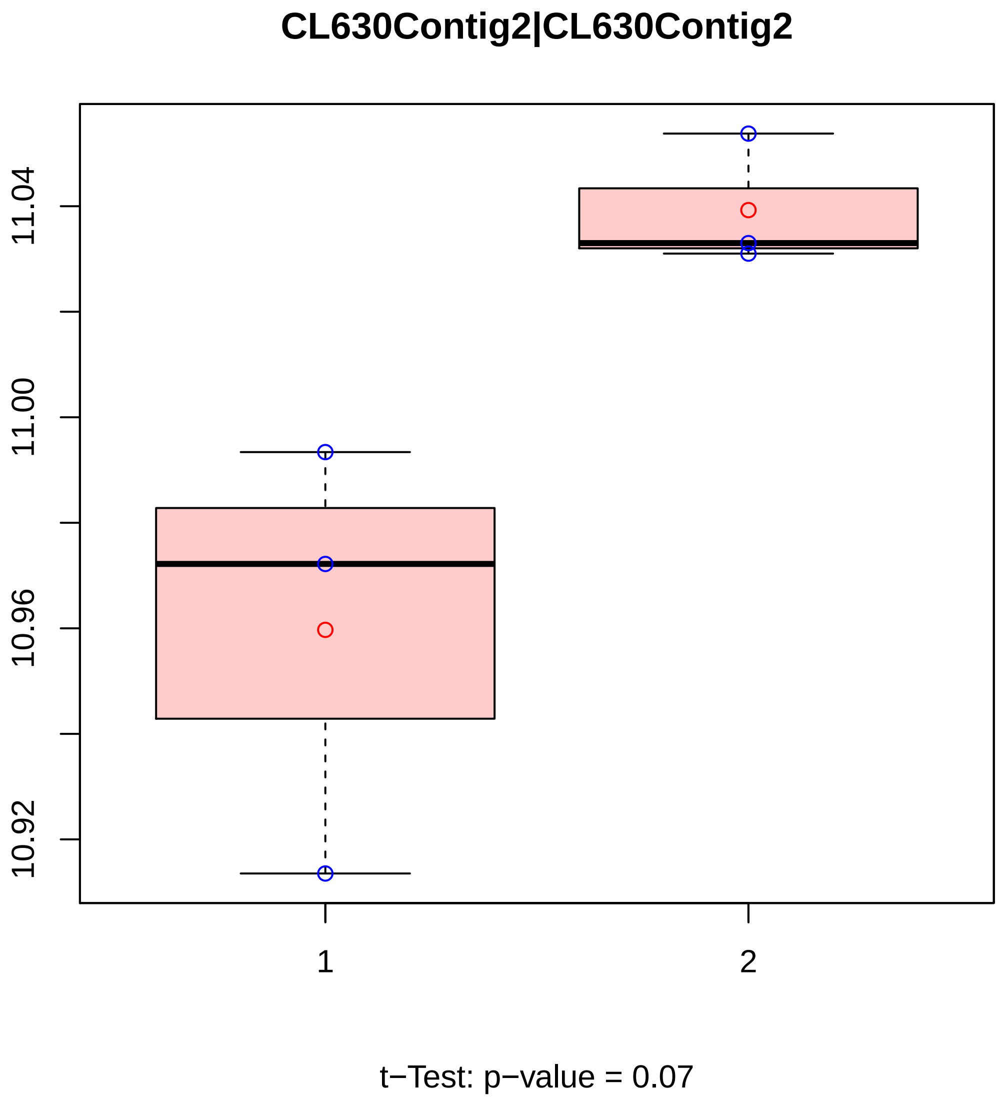
CL630Contig2|CL630Contig2
11.04
11.00
10.96
10.92
1
2
t−Test: p−value = 0.07

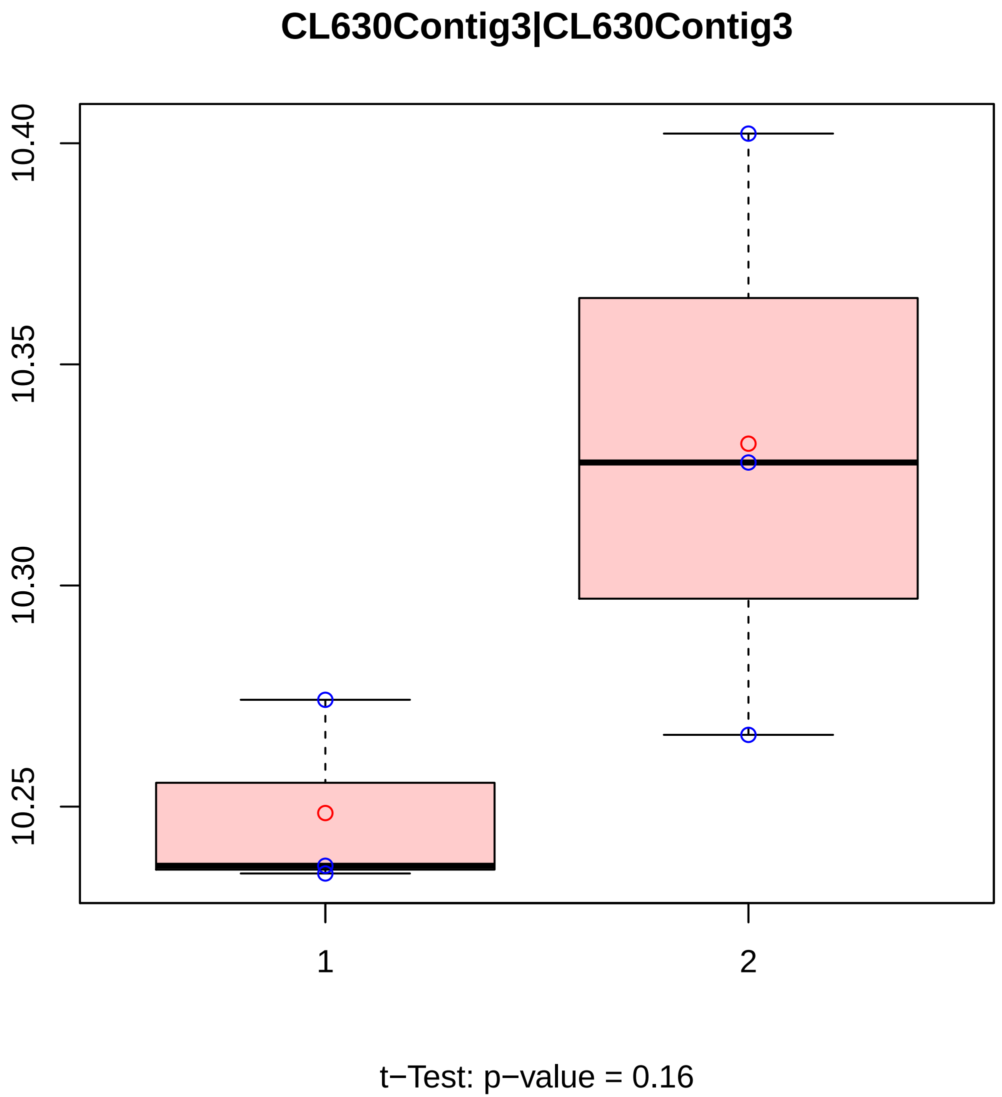

CL630Contig3|CL630Contig3
10.40
10.35
10.30
10.25
1
2
t−Test: p−value = 0.16

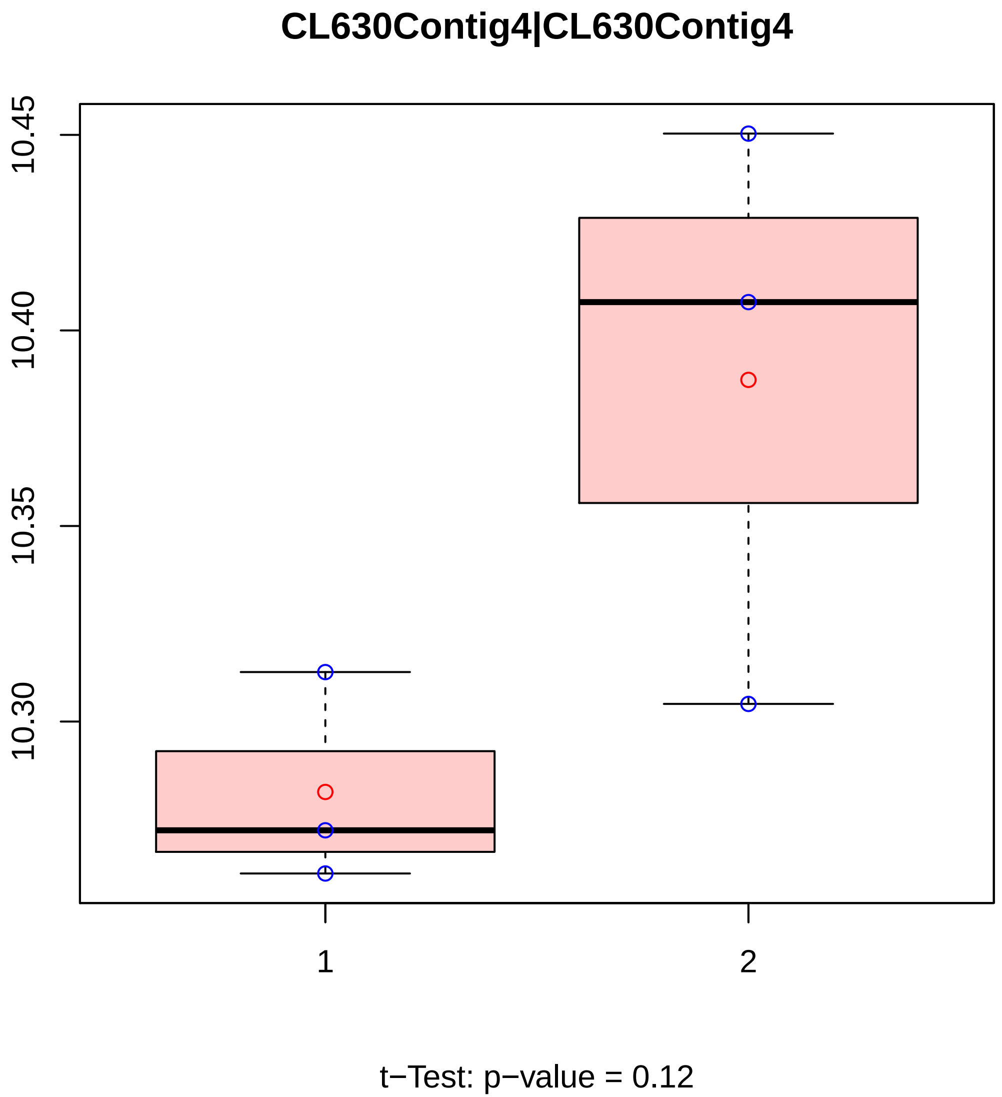

CL630Contig4|CL630Contig4
10.45
10.40
10.35
10.30
1
2
t−Test: p−value = 0.12

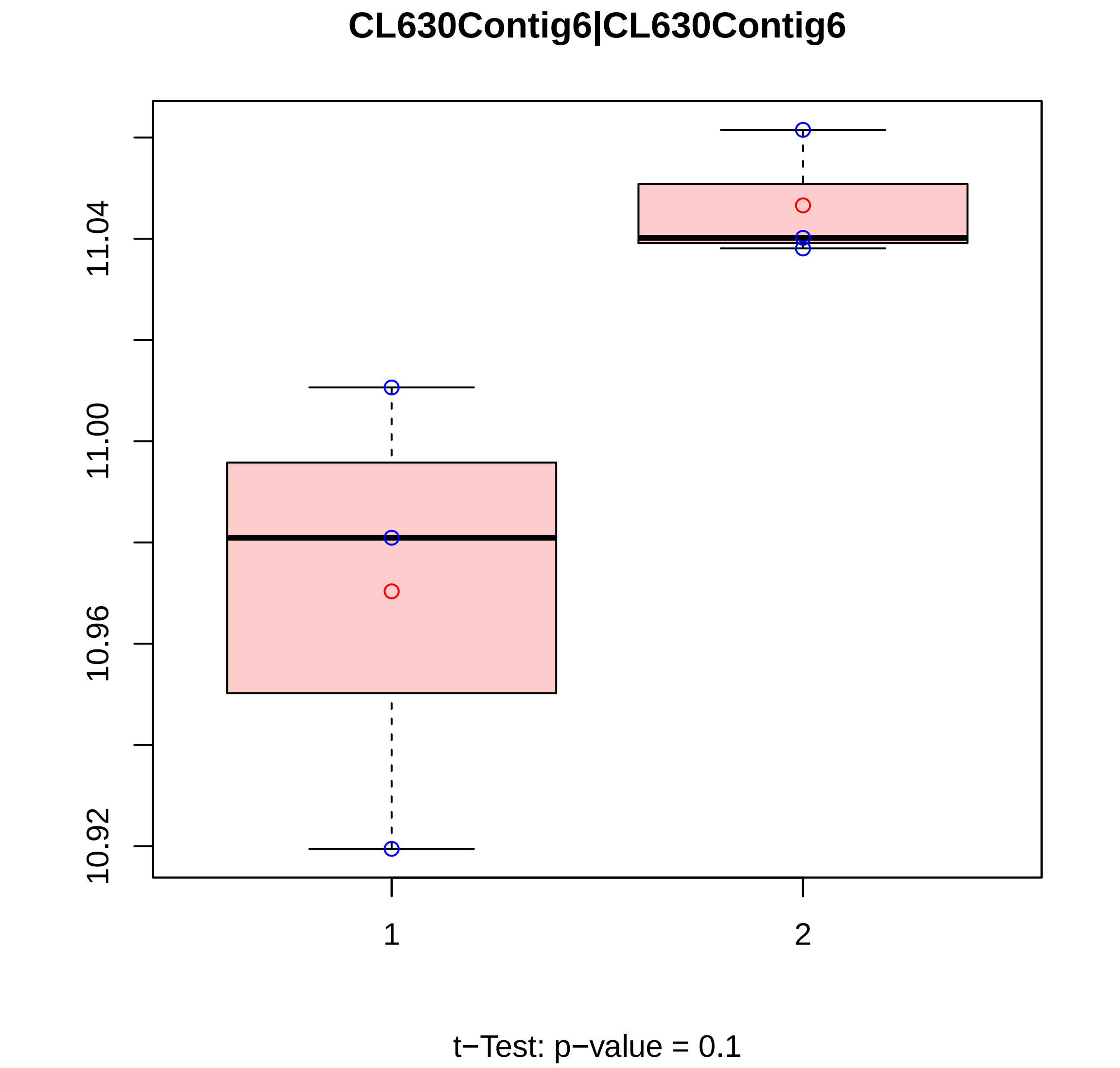

CL630Contig6|CL630Contig6
11.04
11.00
10.96
10.92
1
2
t−Test: p−value = 0.1

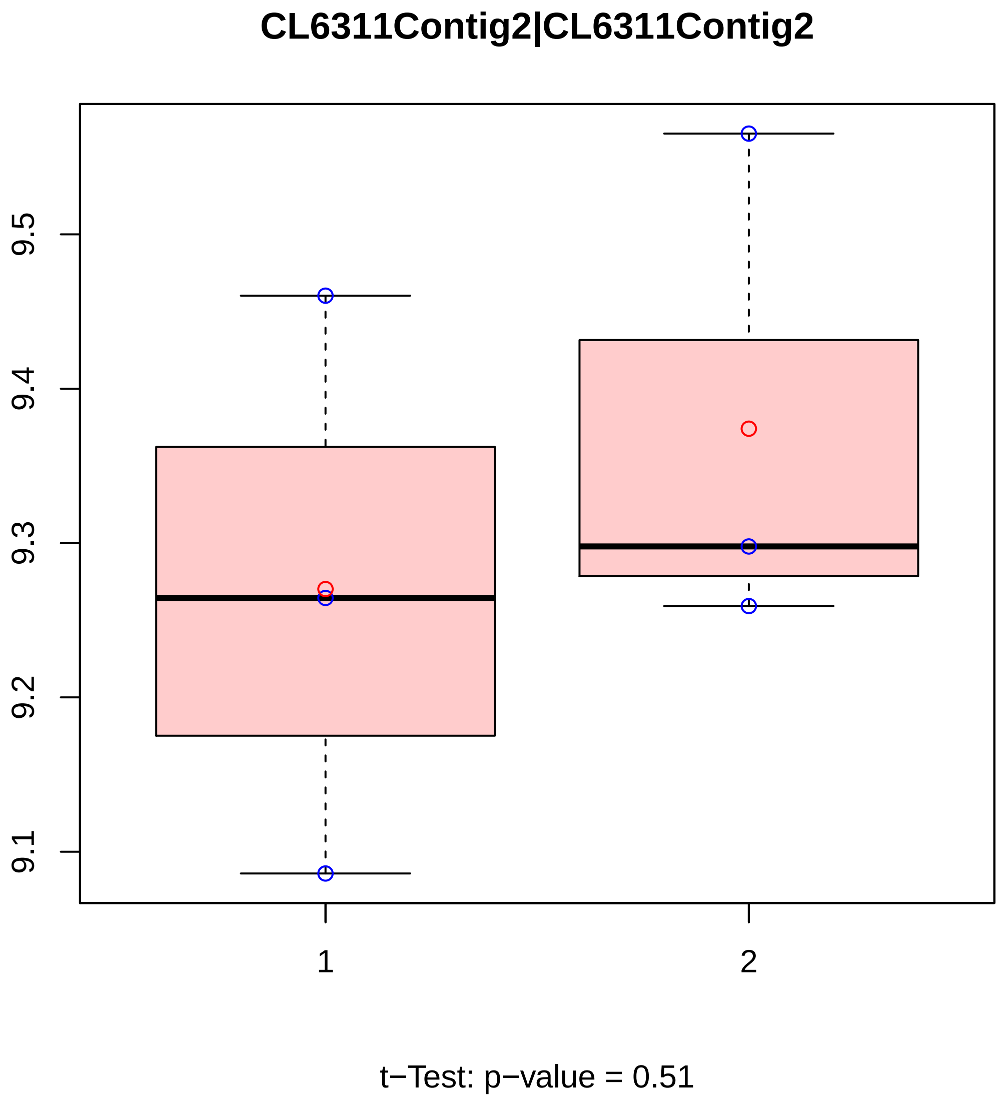
CL6311Contig2|CL6311Contig2
9.1
9.2
9.3
9.4
9.5
1
2
t−Test: p−value = 0.51

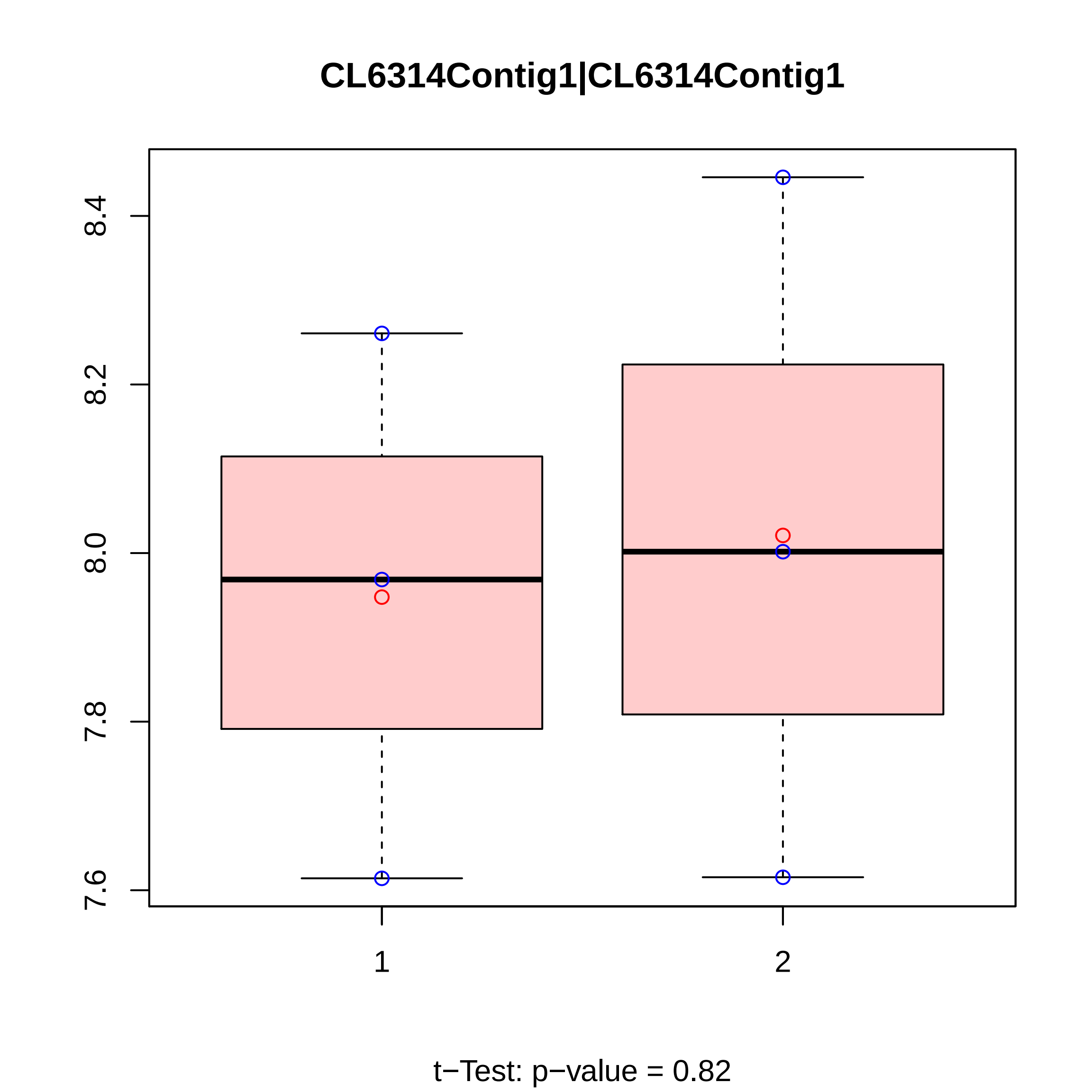
CL6314Contig1|CL6314Contig1
8.4
8.2
8.0
7.8
7.6
1
2
t−Test: p−value = 0.82

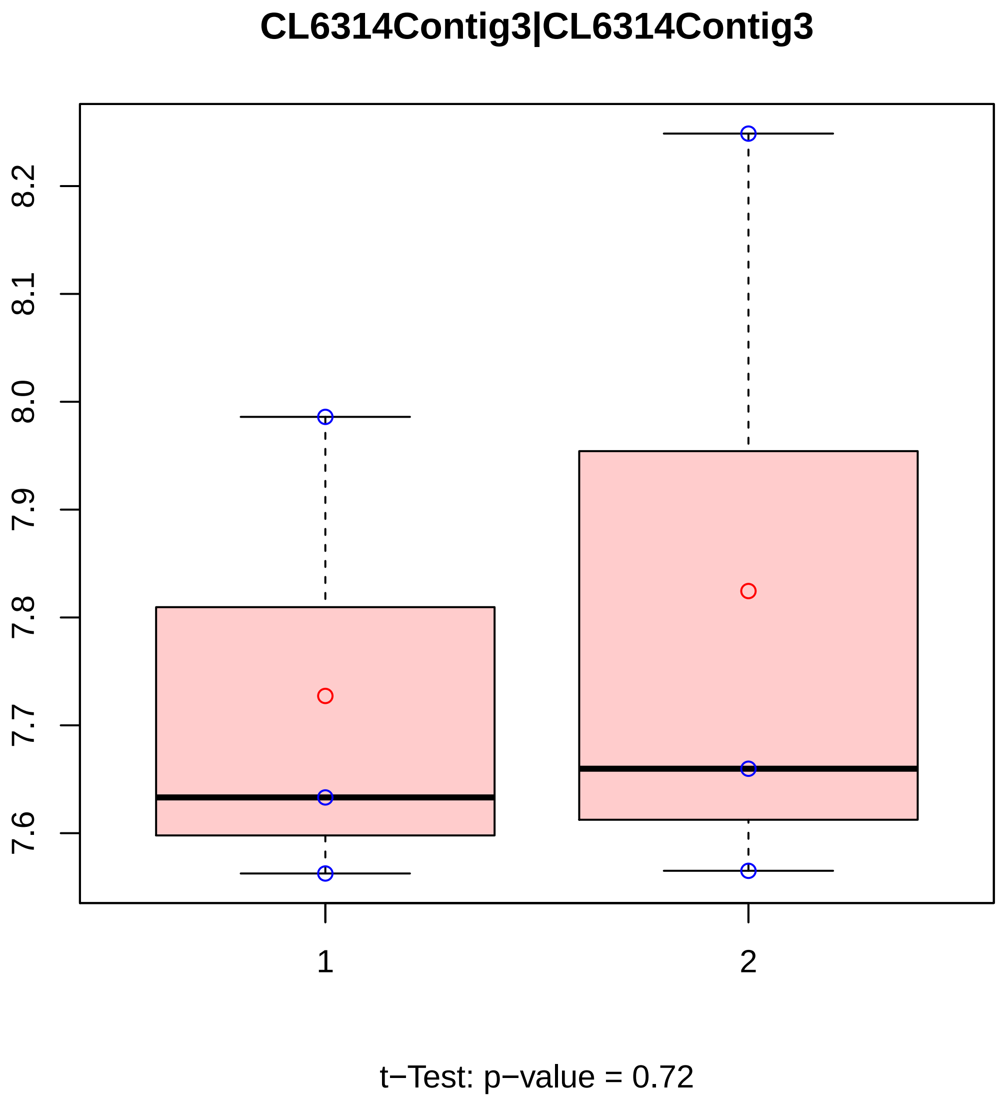

CL6314Contig3|CL6314Contig3
8.2
8.1
8.0
7.9
7.8
7.7
7.6
1
2
t−Test: p−value = 0.72

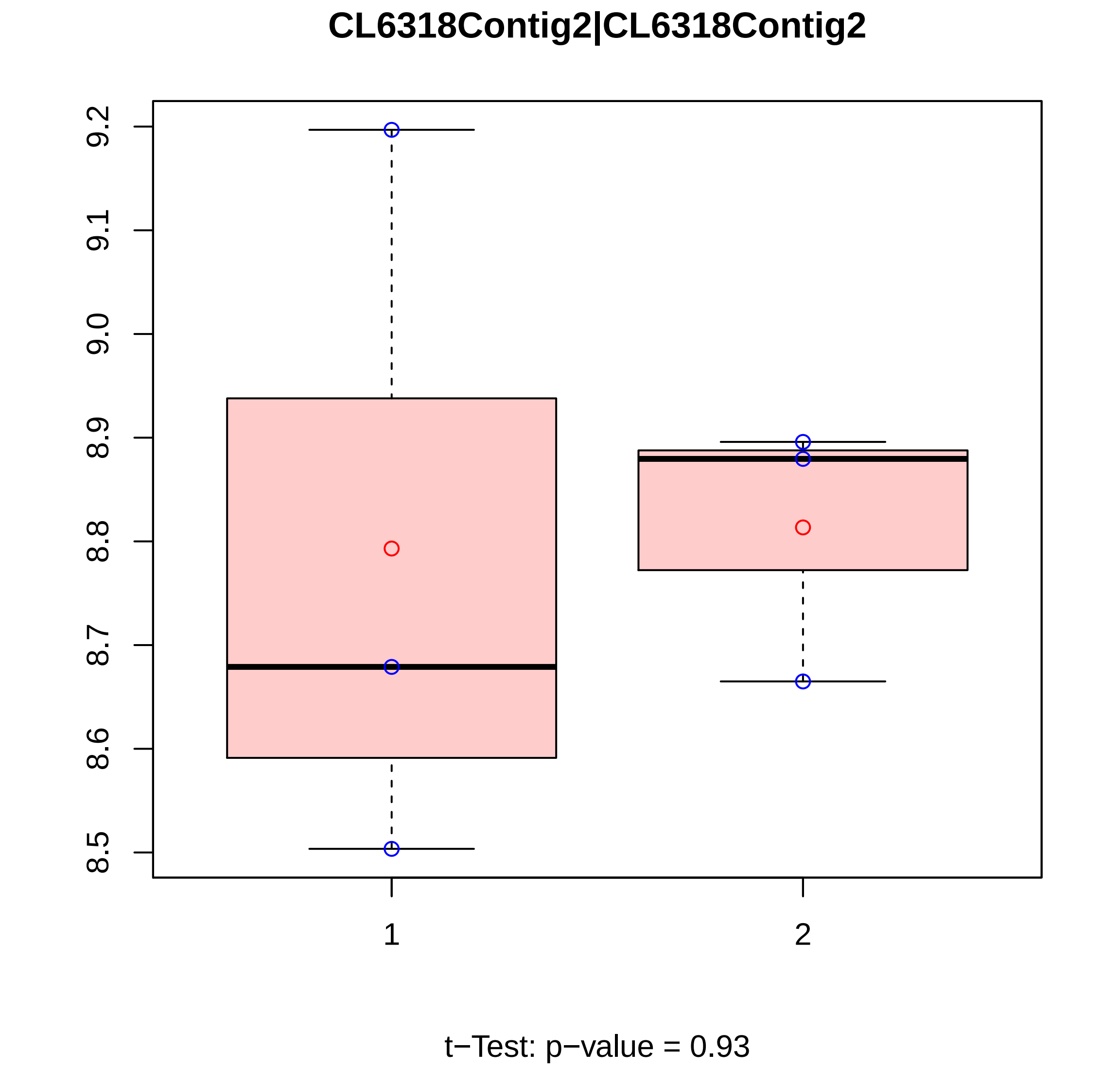
CL6318Contig2|CL6318Contig2
9.2
9.1
9.0
8.9
8.8
8.7
8.6
8.5
1
2
t−Test: p−value = 0.93

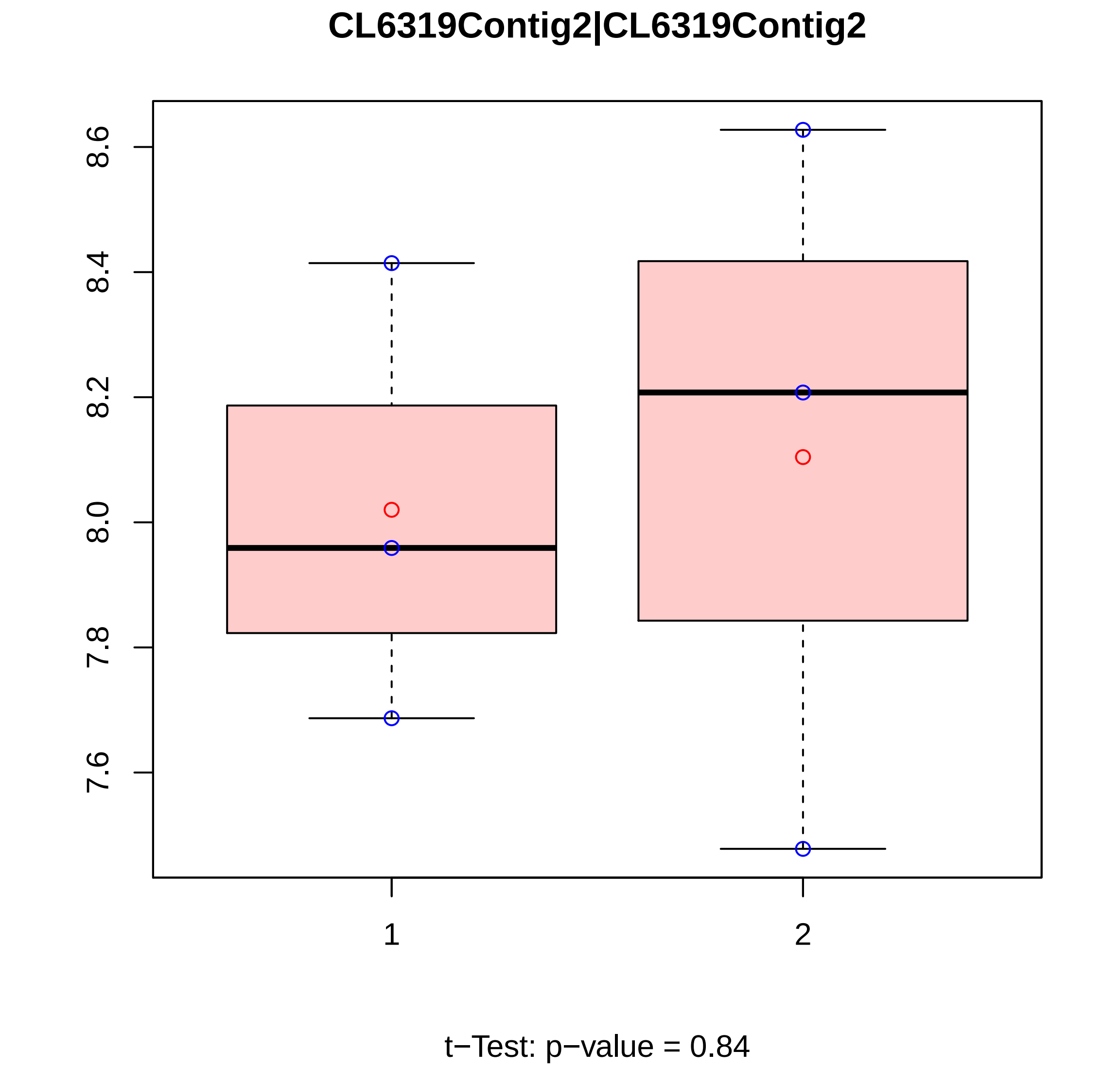

CL6319Contig2|CL6319Contig2
8.6
8.4
8.2
8.0
7.8
7.6
1
2
t−Test: p−value = 0.84

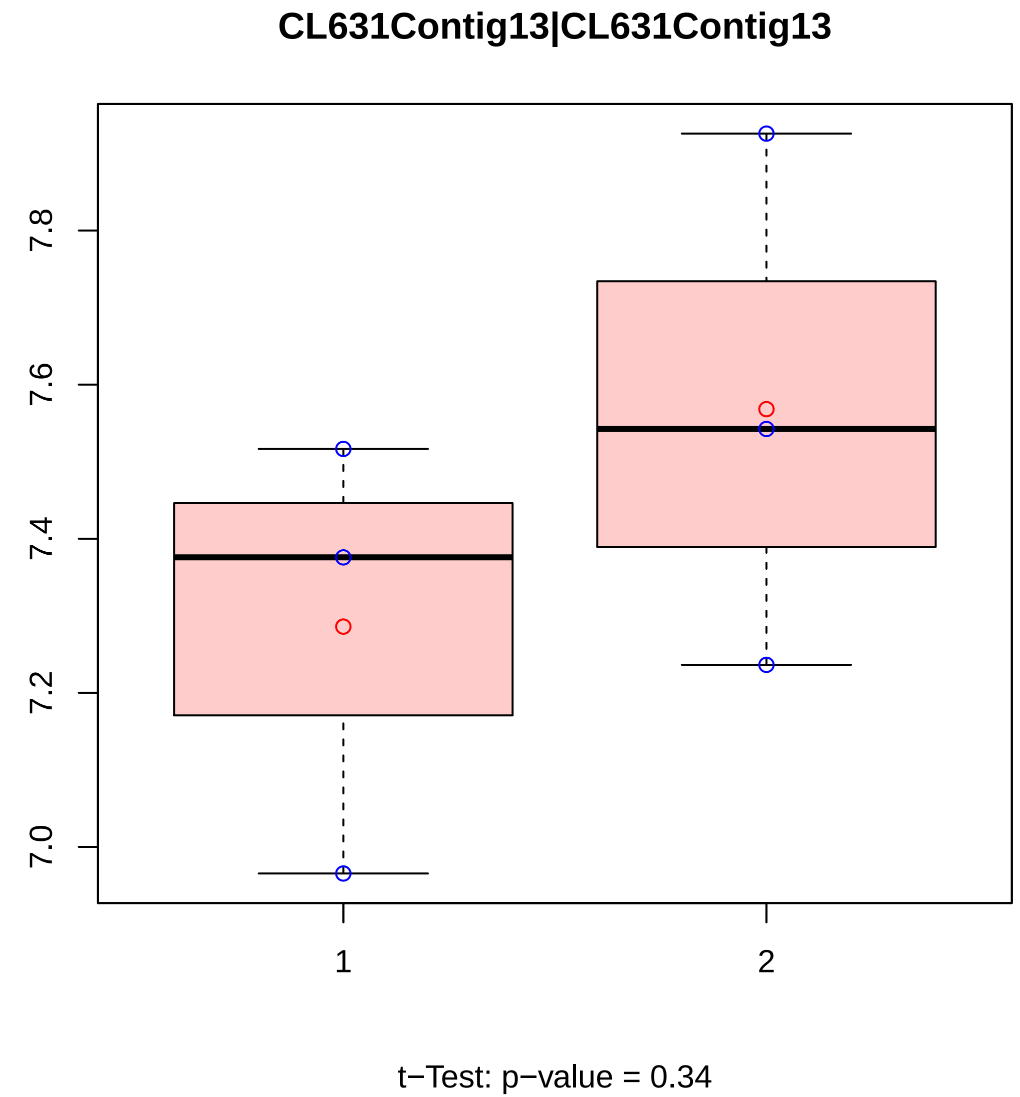
CL631Contig13|CL631Contig13
7.0
7.2
7.4
7.6
7.8
1
2
t−Test: p−value = 0.34

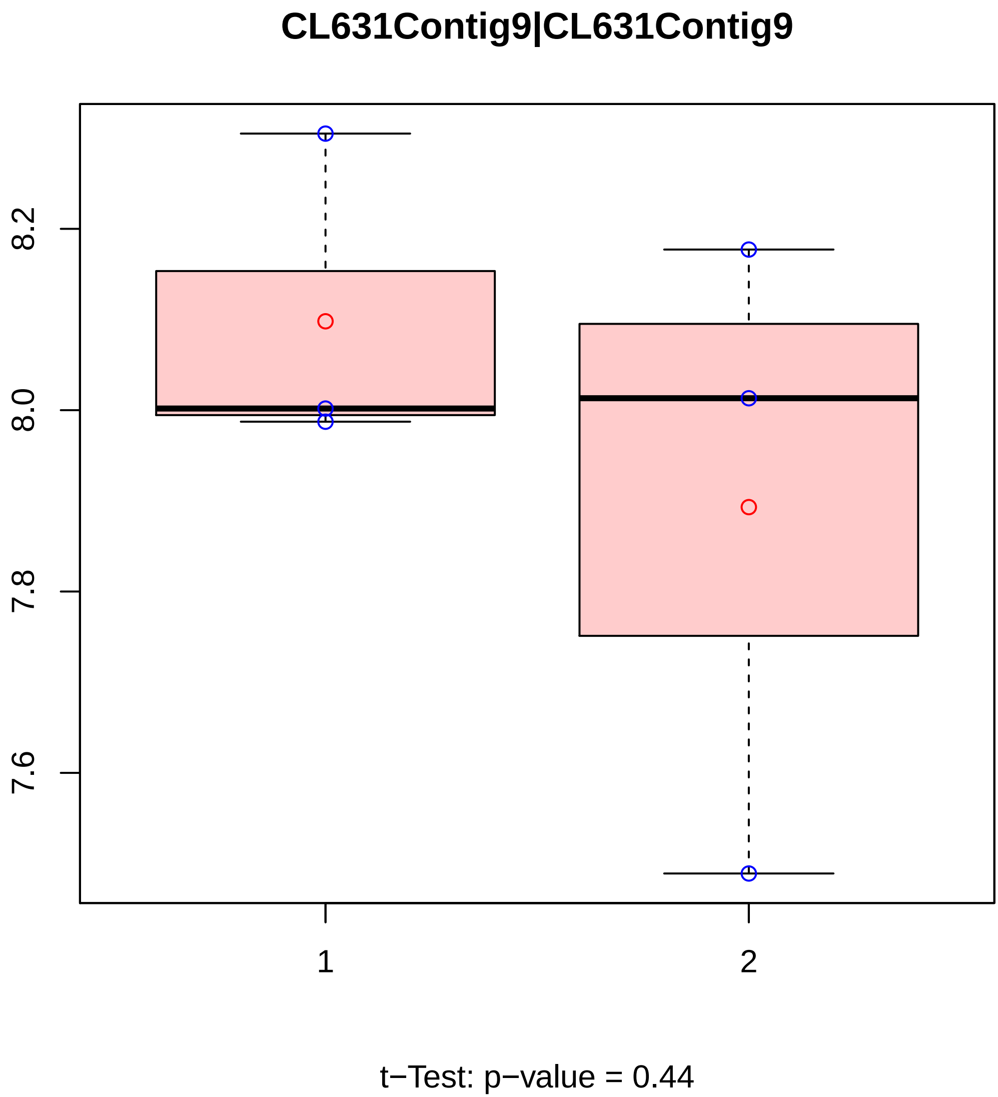
CL631Contig9|CL631Contig9
8.2
8.0
7.8
7.6
1
2
t−Test: p−value = 0.44

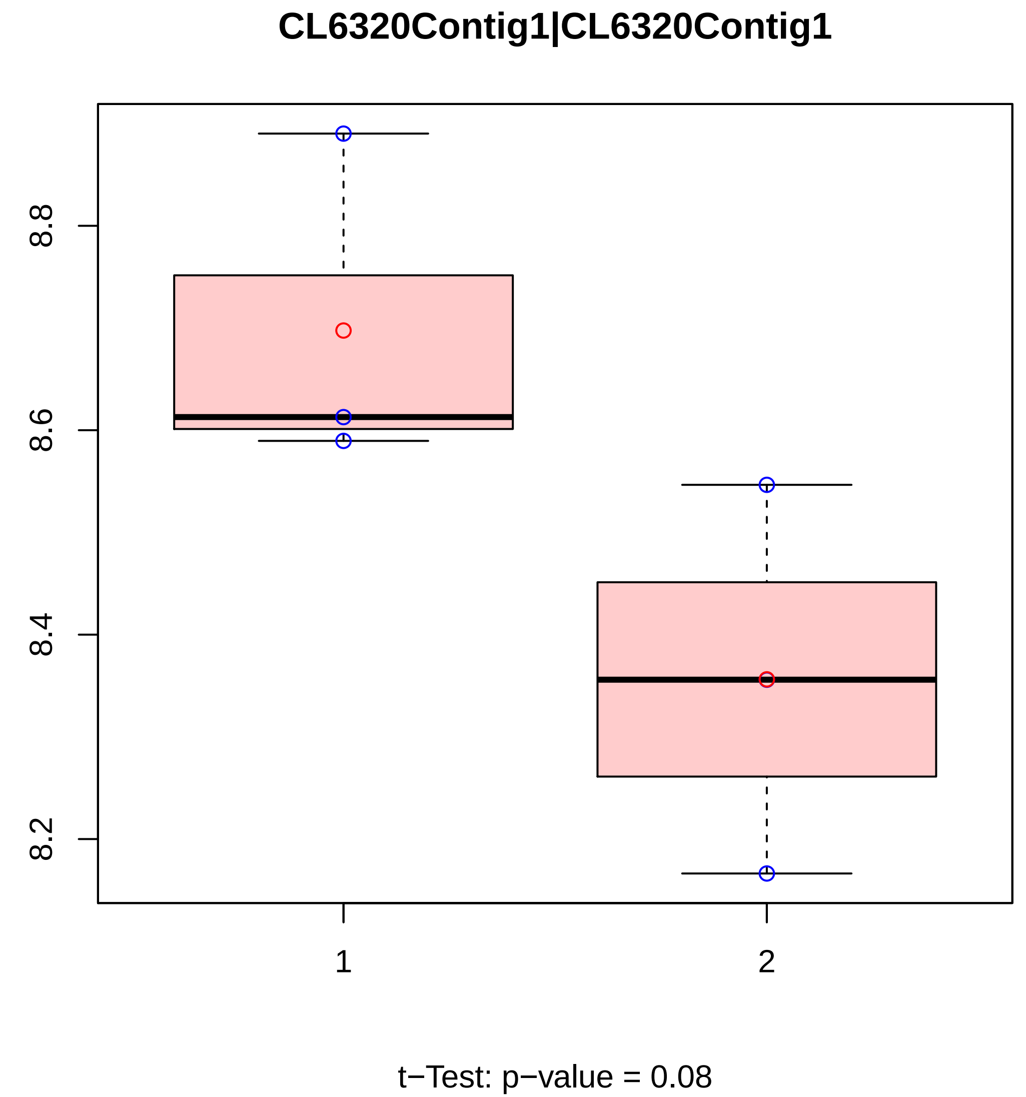
CL6320Contig1|CL6320Contig1
8.8
8.6
8.4
8.2
1
2
t−Test: p−value = 0.08

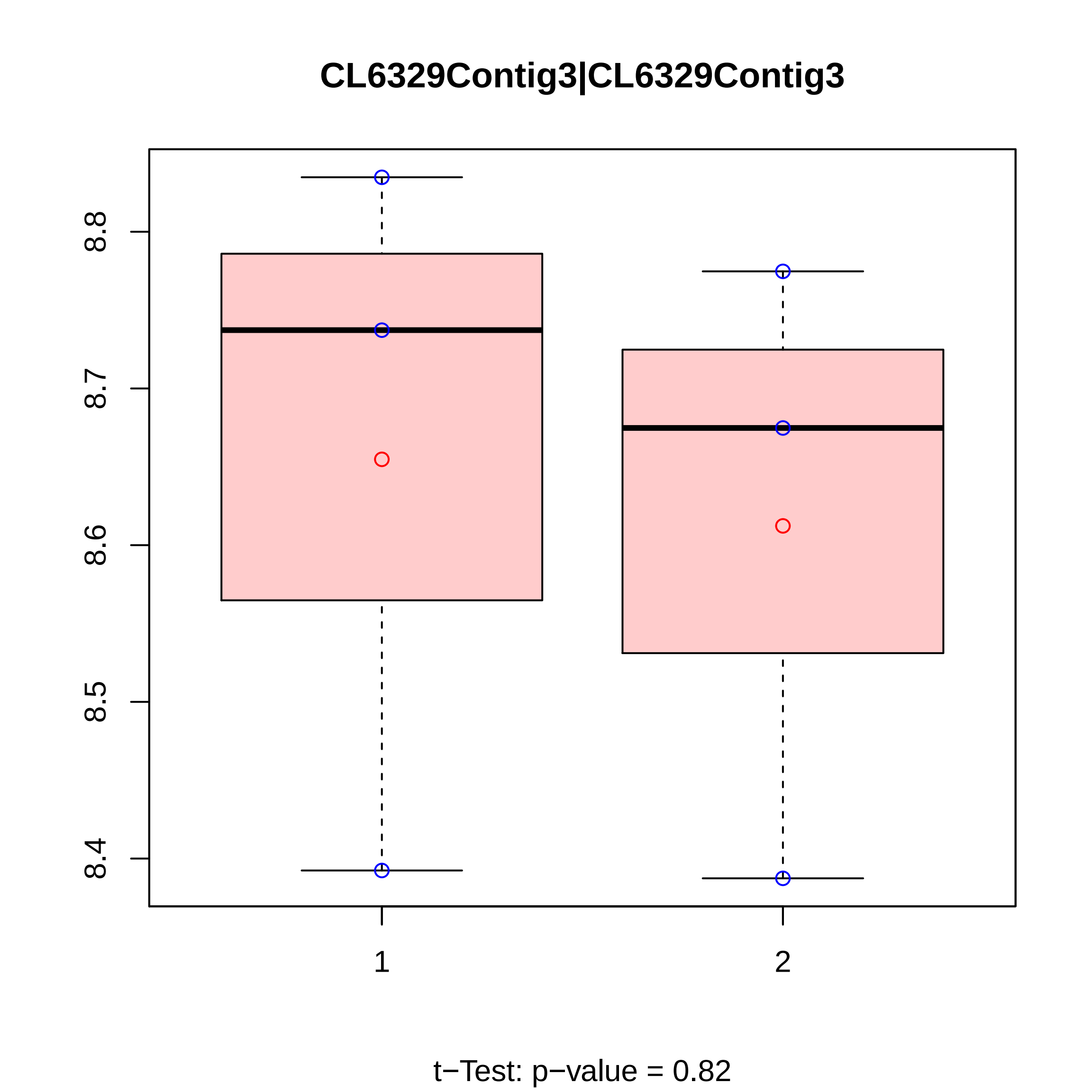

CL6329Contig3|CL6329Contig3
8.4
8.5
8.6
8.7
8.8
1
2
t−Test: p−value = 0.82

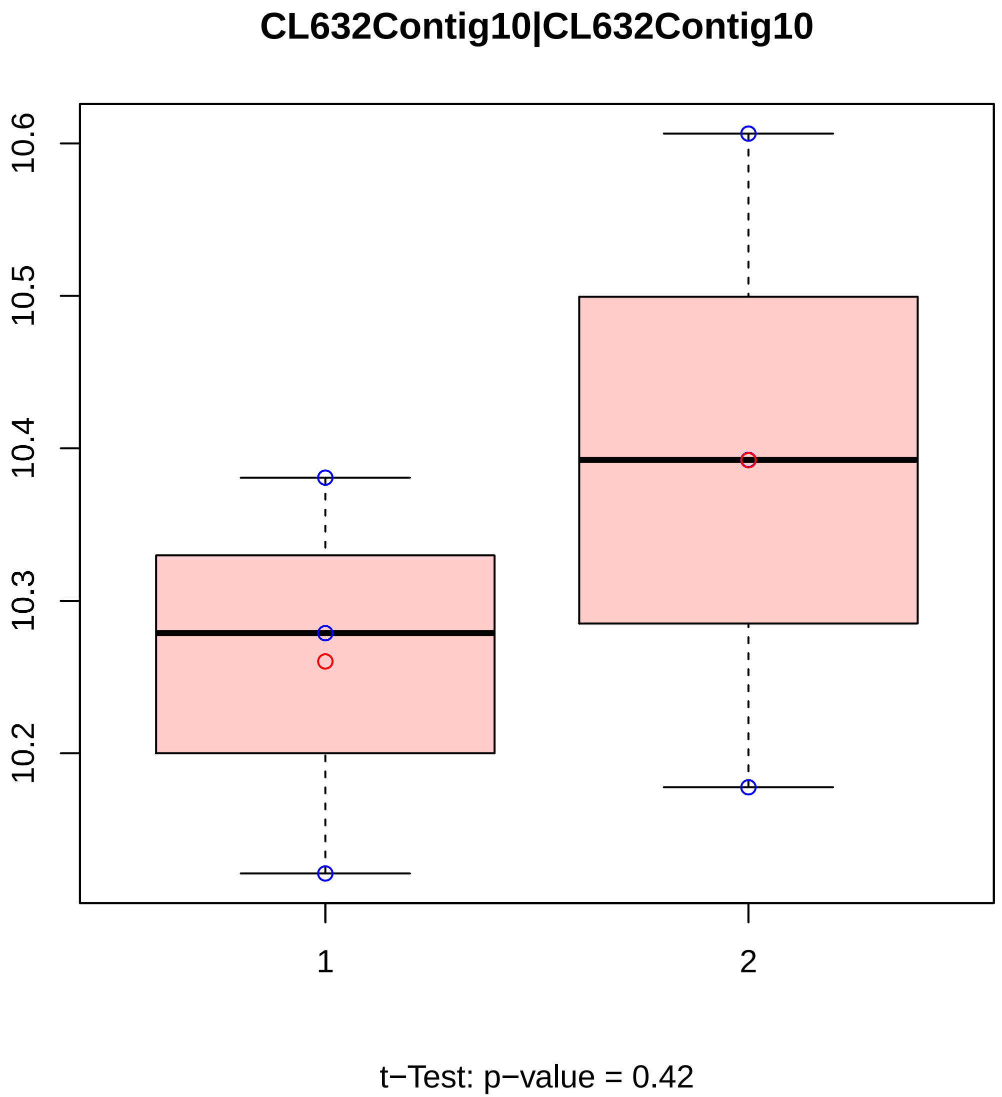
CL632Contig10|CL632Contig10
10.2
10.3
10.4
10.5
10.6
1
2
t−Test: p−value = 0.42

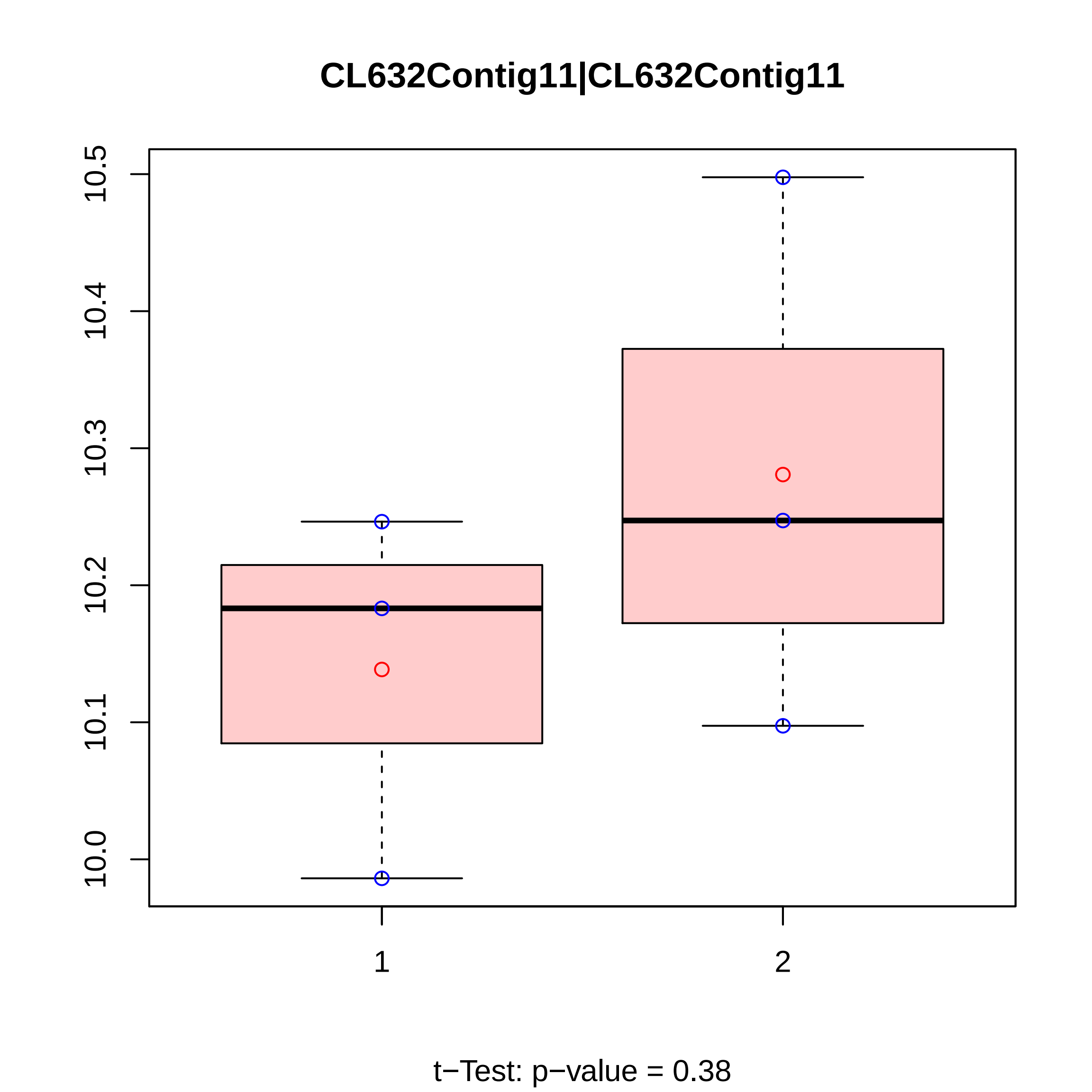
CL632Contig11|CL632Contig11
10.0
10.1
10.2
10.3
10.4
10.5
1
2
t−Test: p−value = 0.38

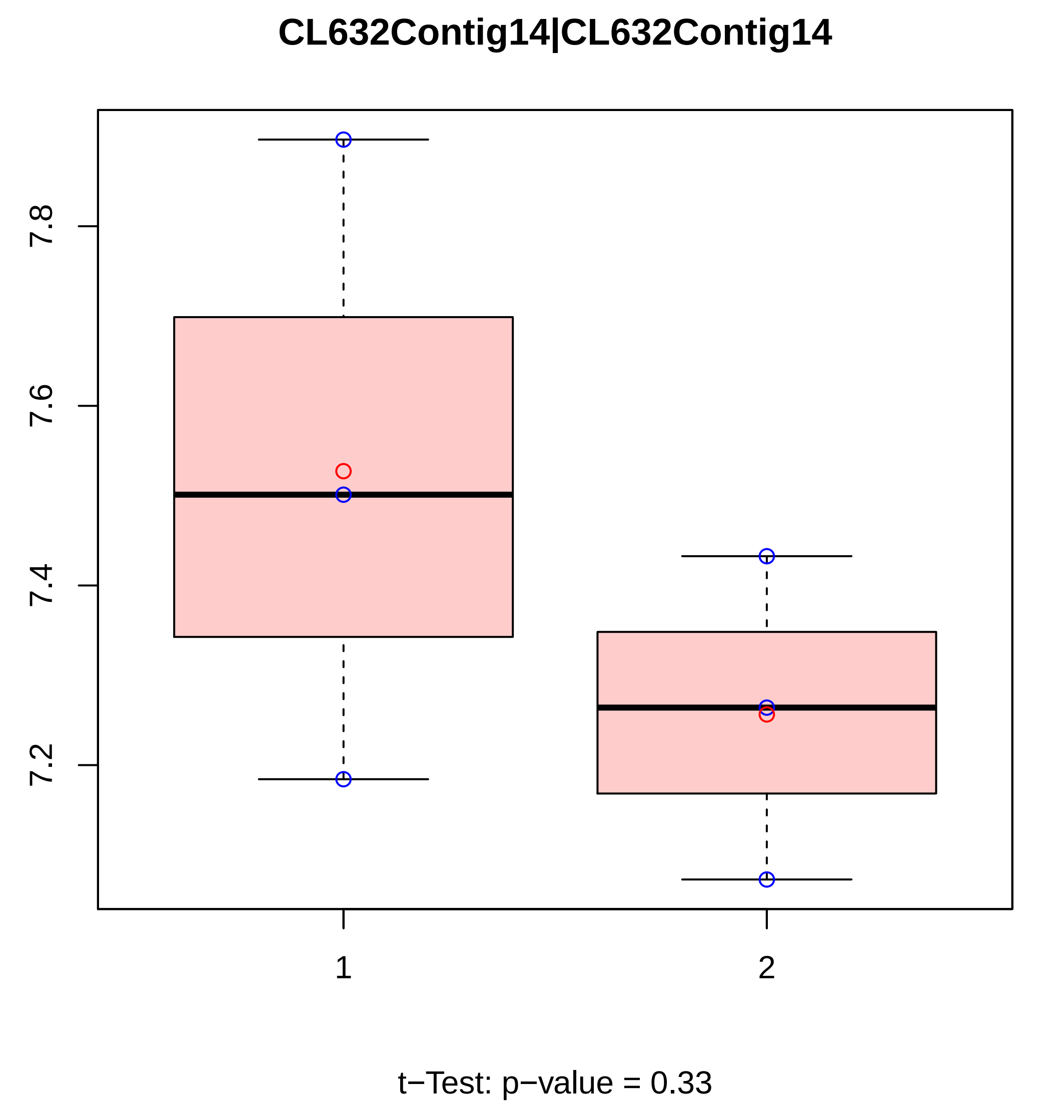
CL632Contig14|CL632Contig14
7.2
7.4
7.6
7.8
1
2
t−Test: p−value = 0.33

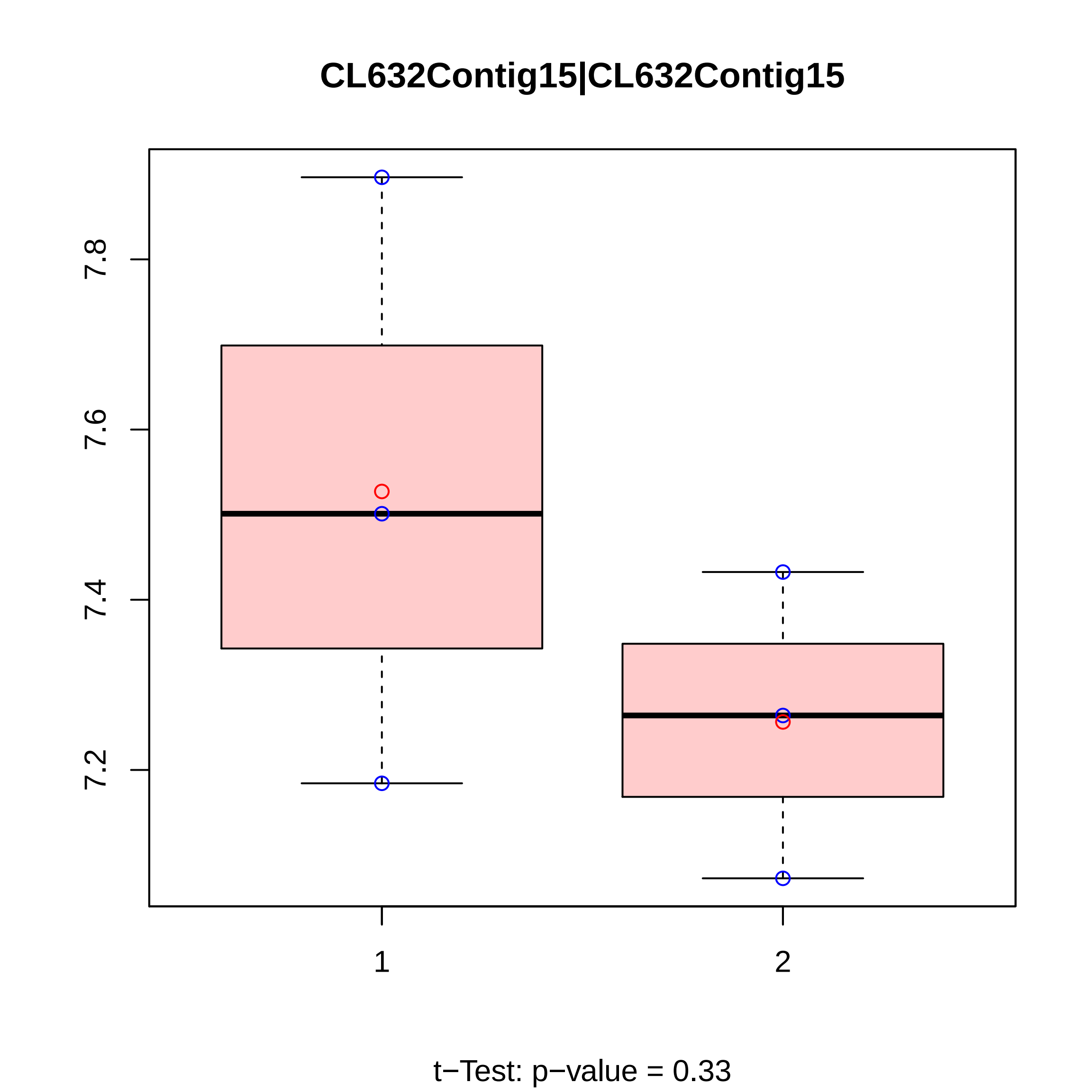

CL632Contig15|CL632Contig15
7.2
7.4
7.6
7.8
1
2
t−Test: p−value = 0.33

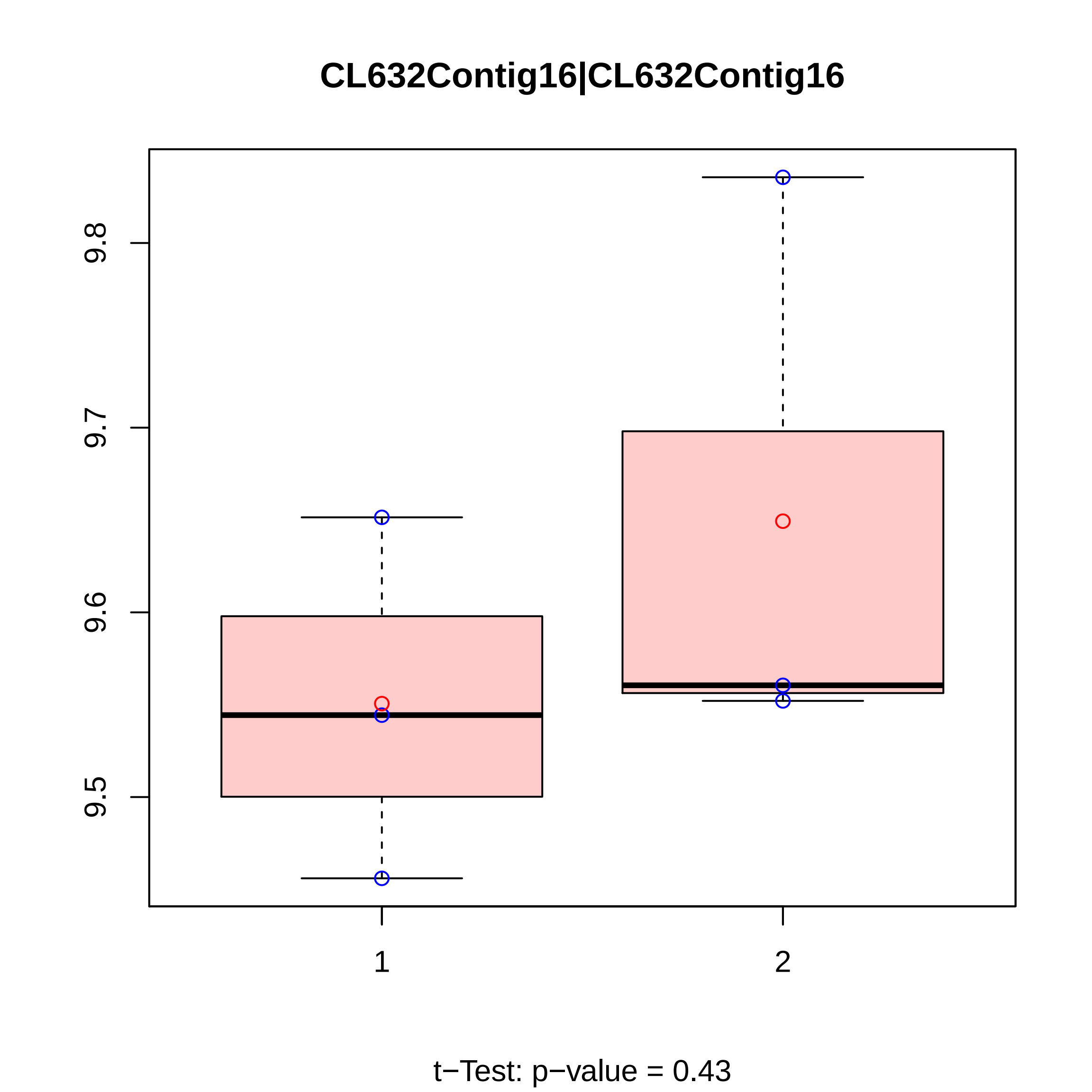

CL632Contig16|CL632Contig16
9.5
9.6
9.7
9.8
1
2
t−Test: p−value = 0.43

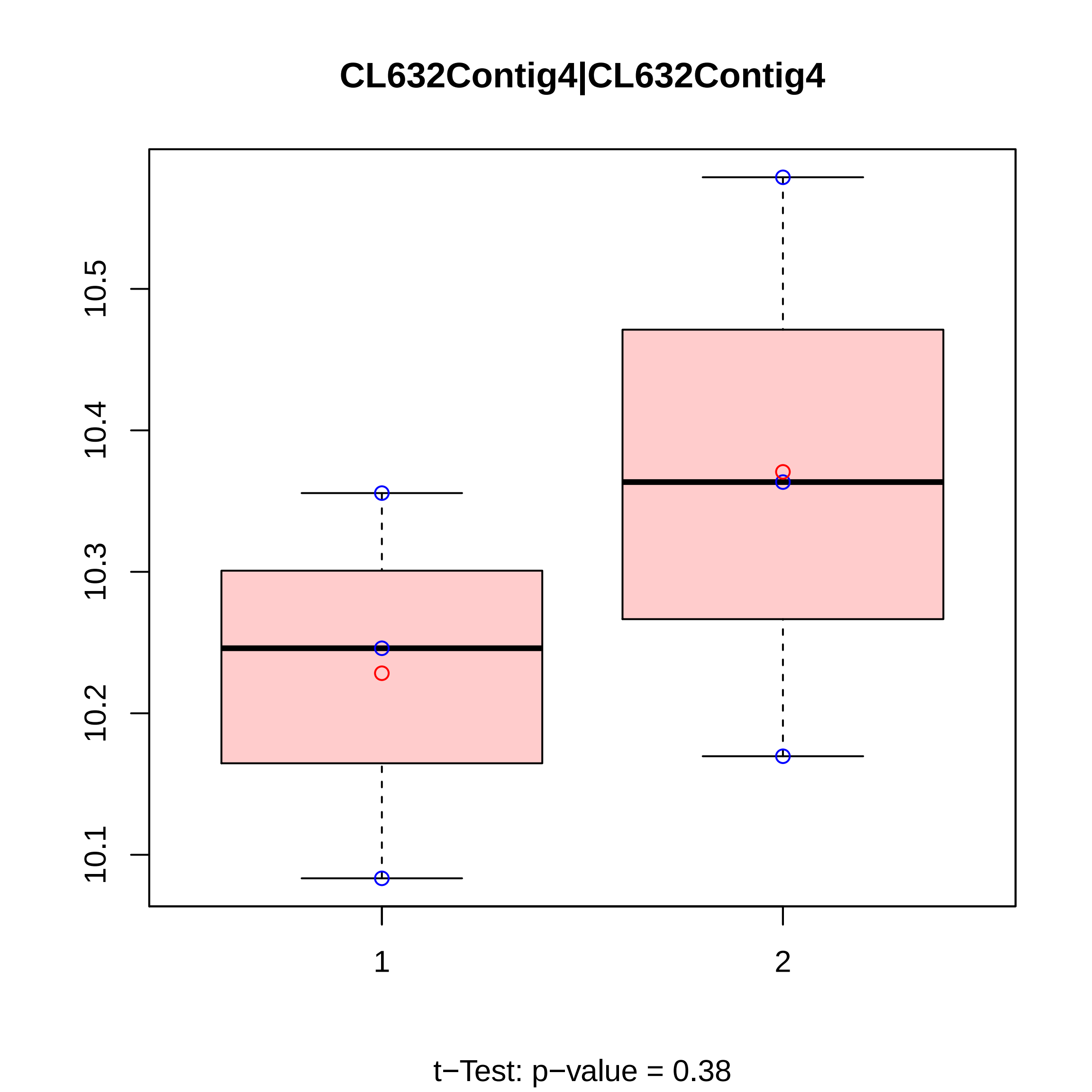
CL632Contig4|CL632Contig4
10.1
10.2
10.3
10.4
10.5
1
2
t−Test: p−value = 0.38

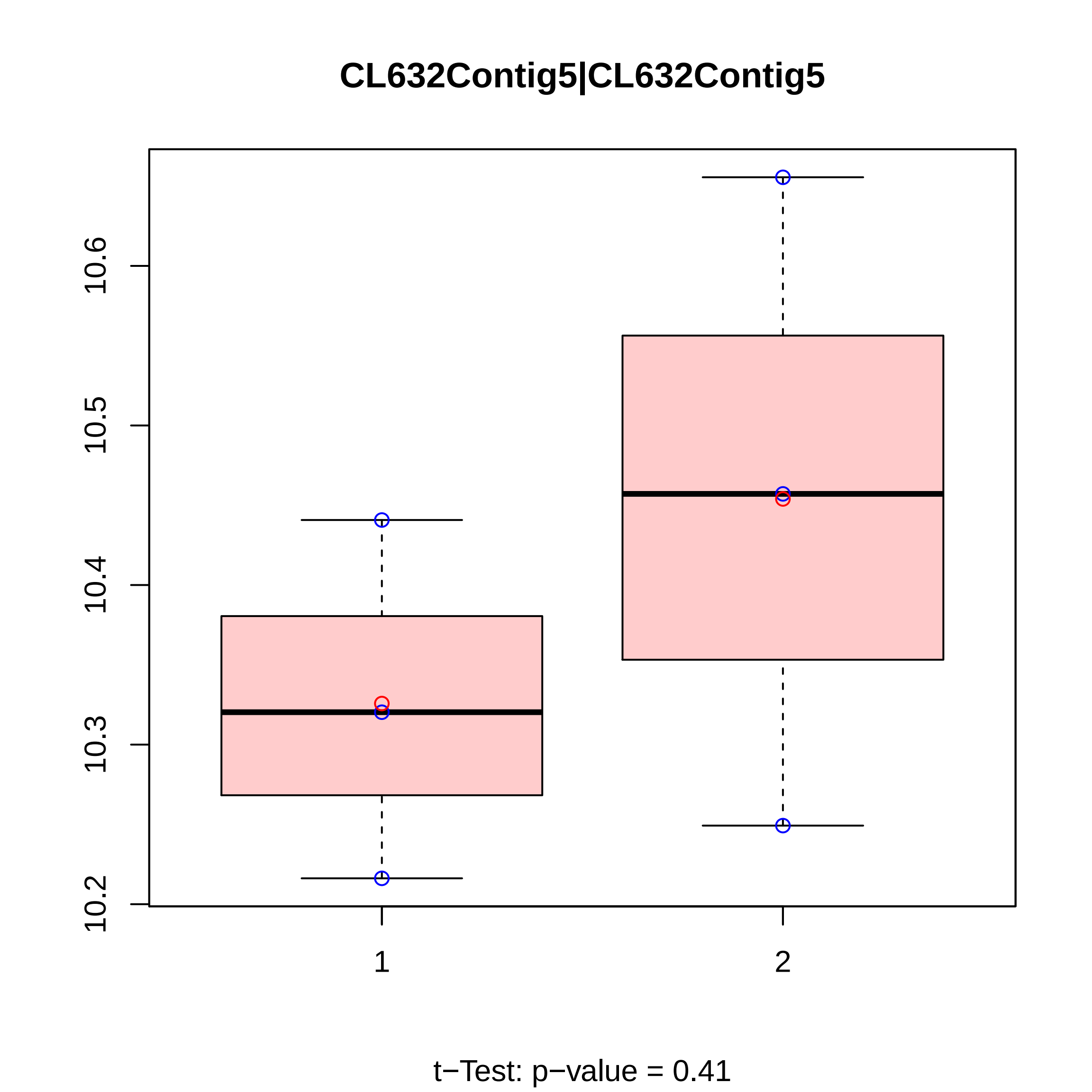
CL632Contig5|CL632Contig5
10.2
10.3
10.4
10.5
10.6
1
2
t−Test: p−value = 0.41

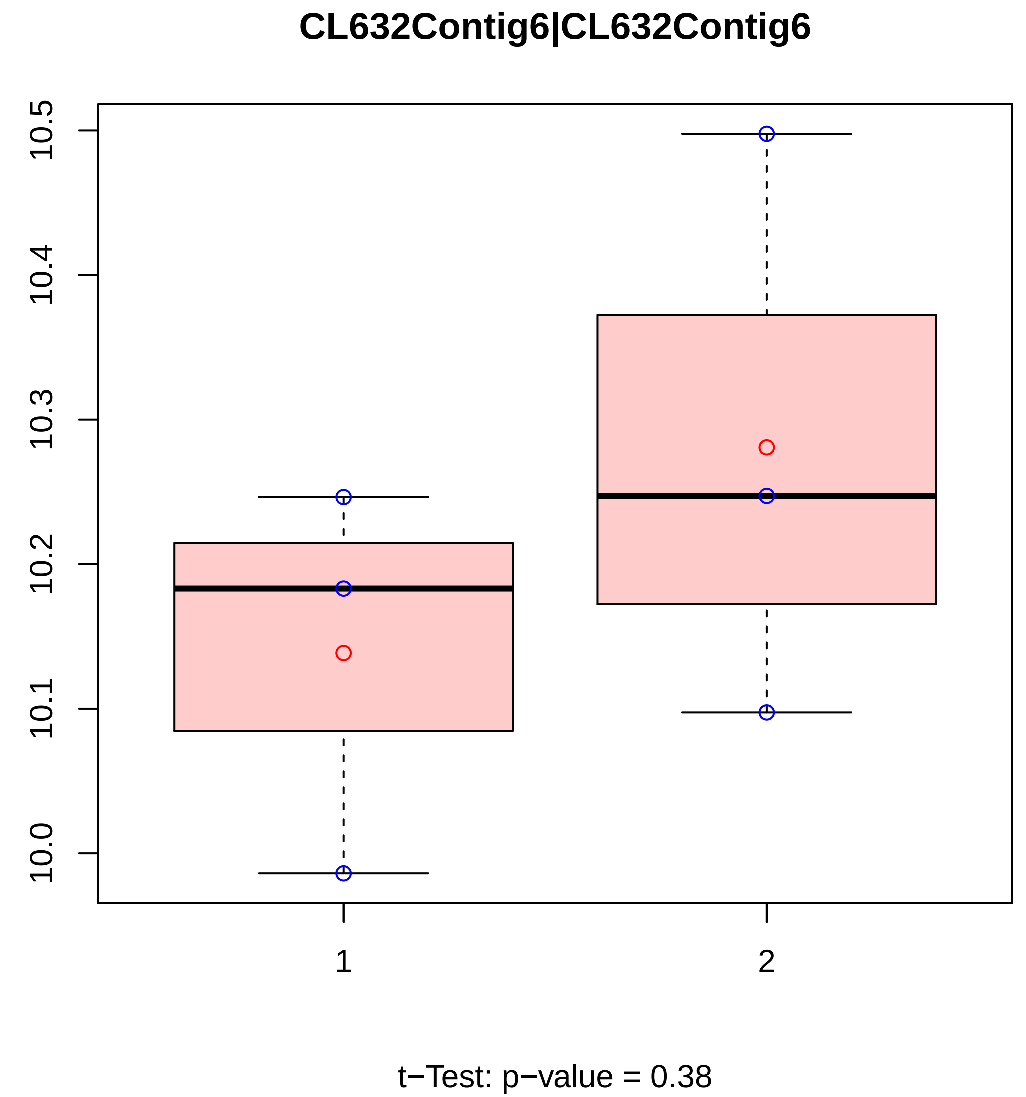

CL632Contig6|CL632Contig6
10.0
10.1
10.2
10.3
10.4
10.5
1
2
t−Test: p−value = 0.38

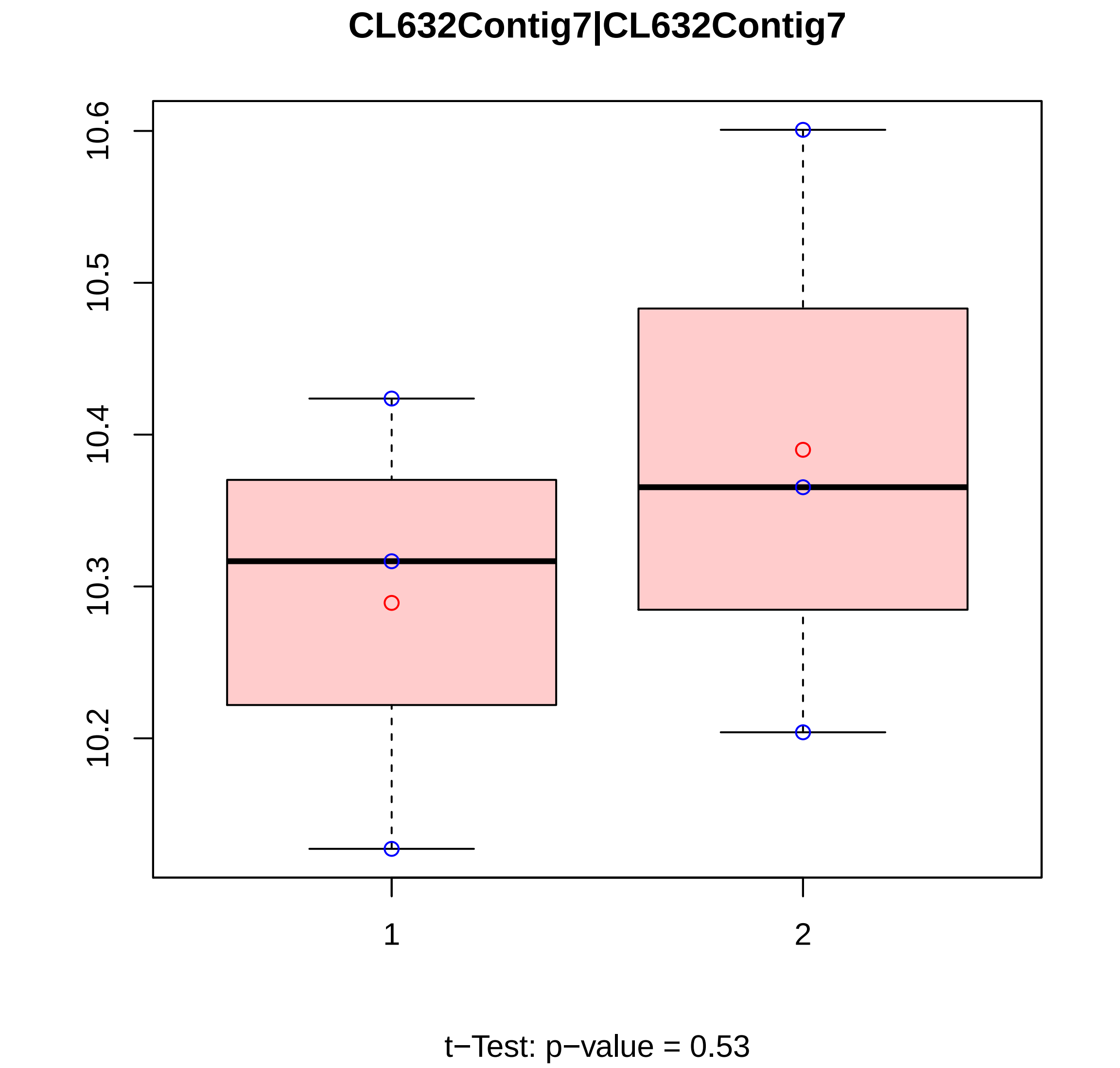
CL632Contig7|CL632Contig7
10.2
10.3
10.4
10.5
10.6
1
2
t−Test: p−value = 0.53

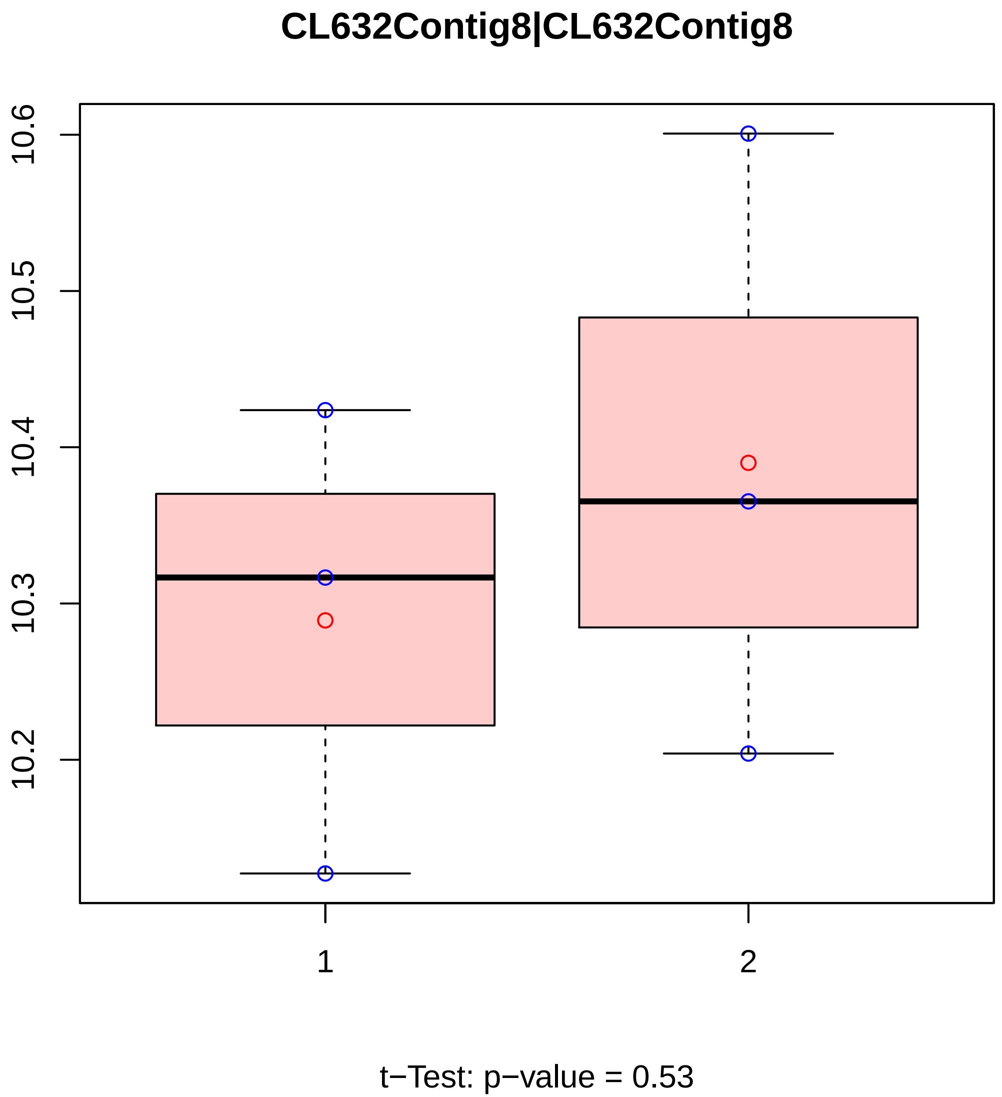
CL632Contig8|CL632Contig8
10.2
10.3
10.4
10.5
10.6
1
2
t−Test: p−value = 0.53

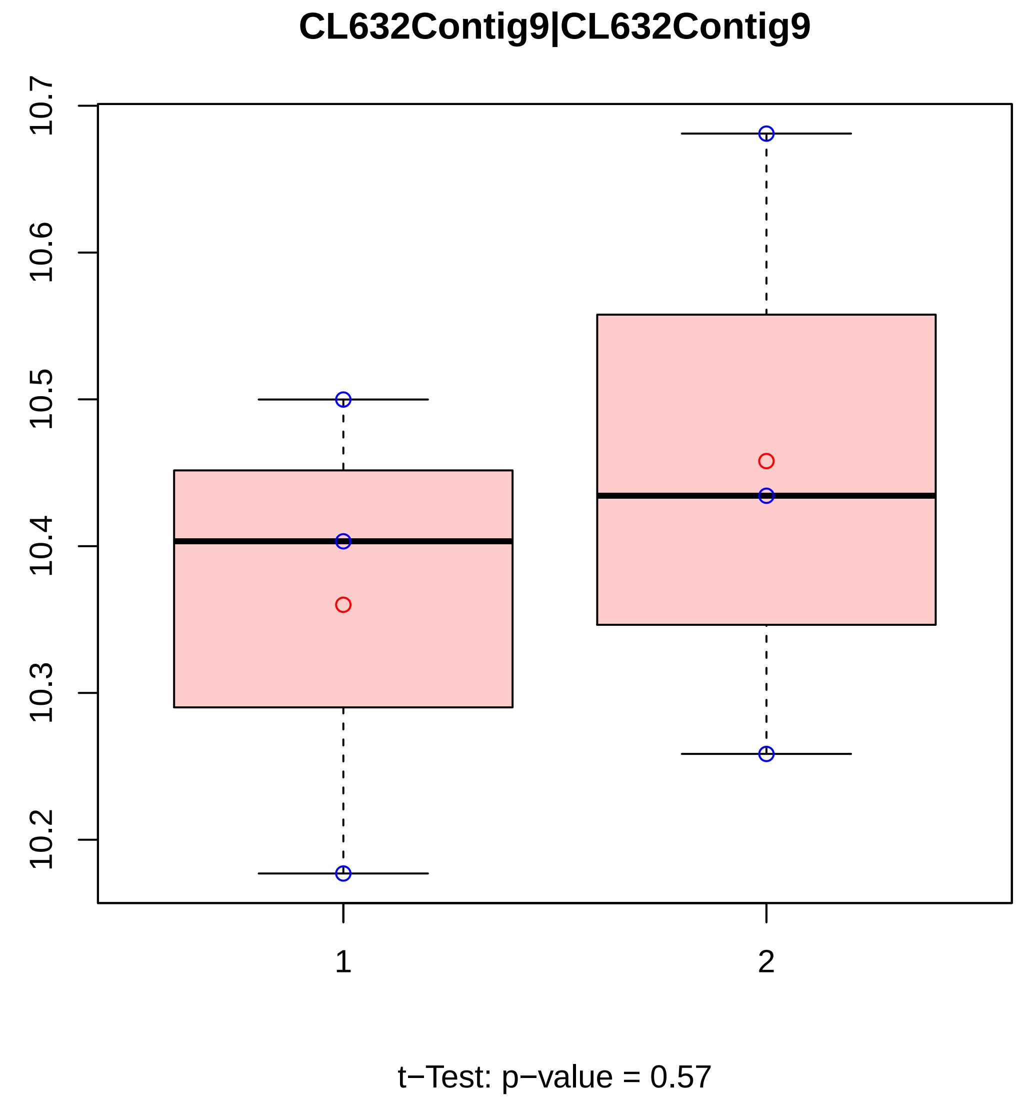
CL632Contig9|CL632Contig9
10.2
10.3
10.4
10.5
10.6
10.7
1
2
t−Test: p−value = 0.57

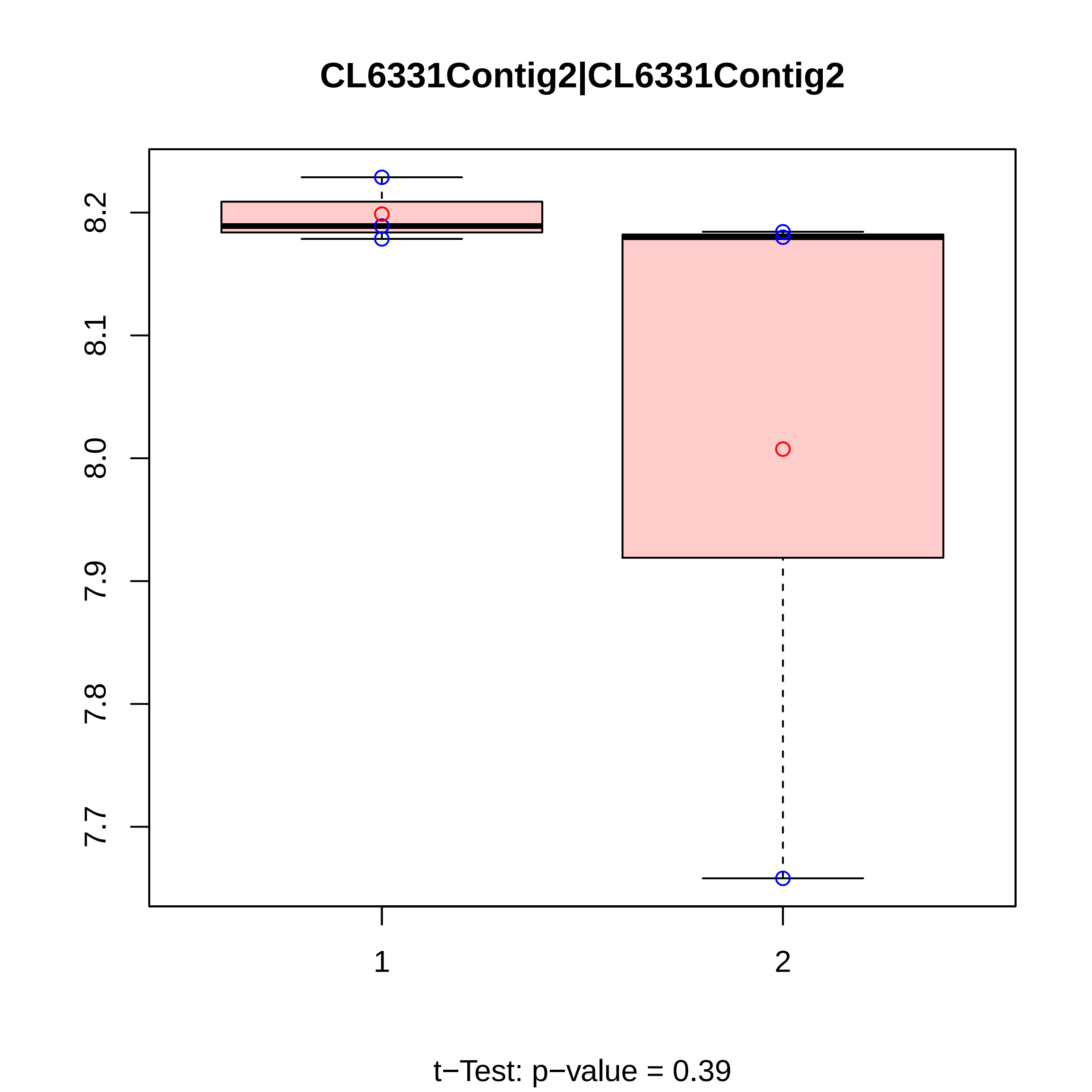
CL6331Contig2|CL6331Contig2
8.2
8.1
8.0
7.9
7.8
7.7
1
2
t−Test: p−value = 0.39

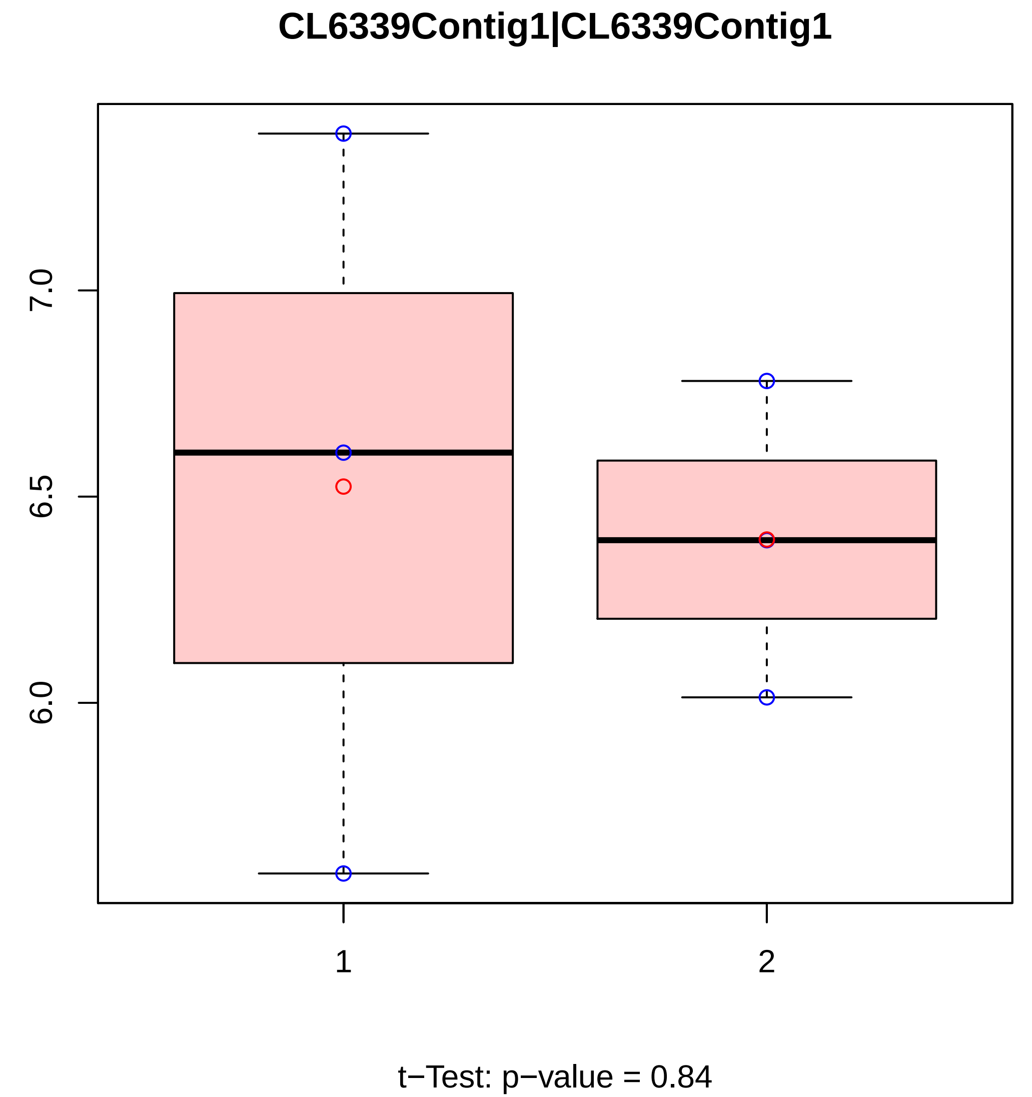

CL6339Contig1|CL6339Contig1
7.0
6.5
6.0
1
2
t−Test: p−value = 0.84

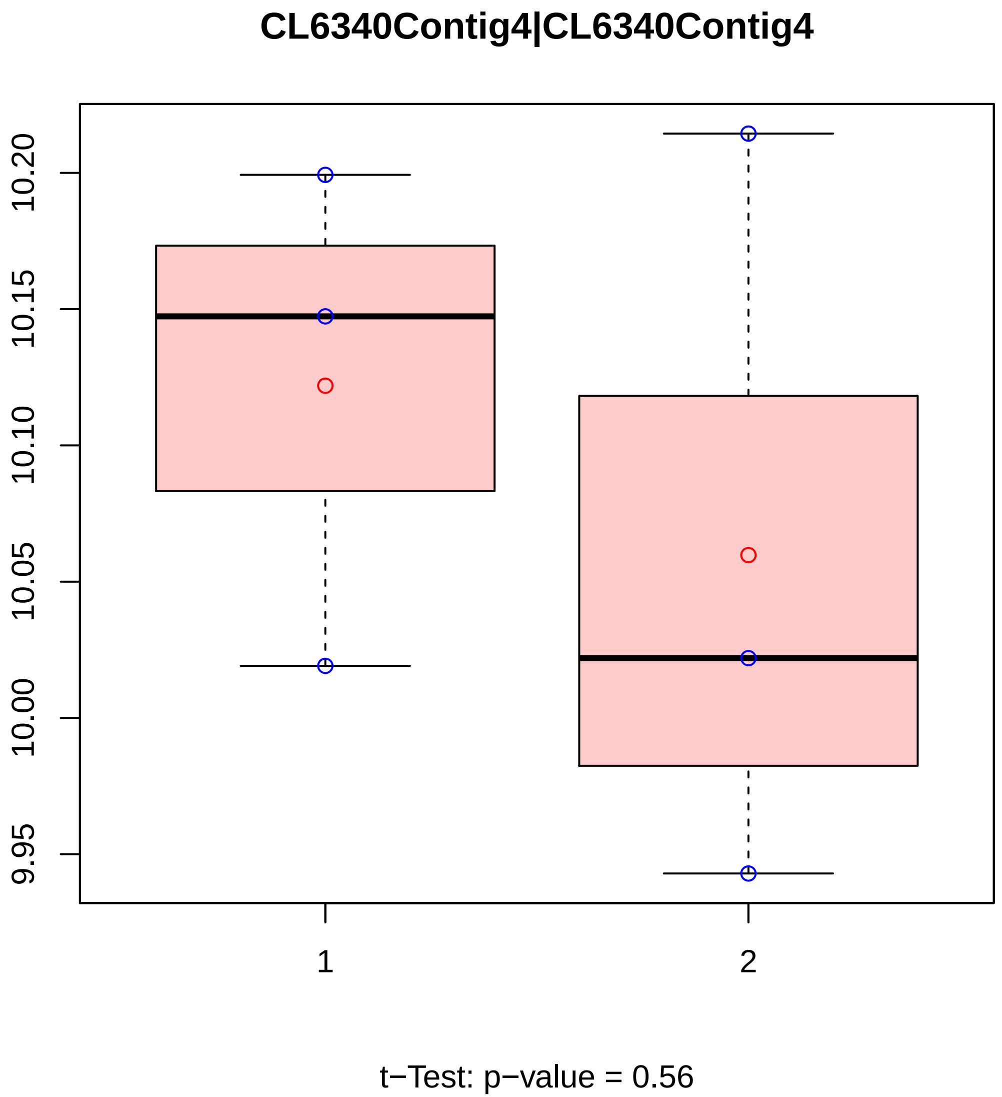
CL6340Contig4|CL6340Contig4
10.20
10.15
10.10
10.05
10.00
9.95
1
2
t−Test: p−value = 0.56

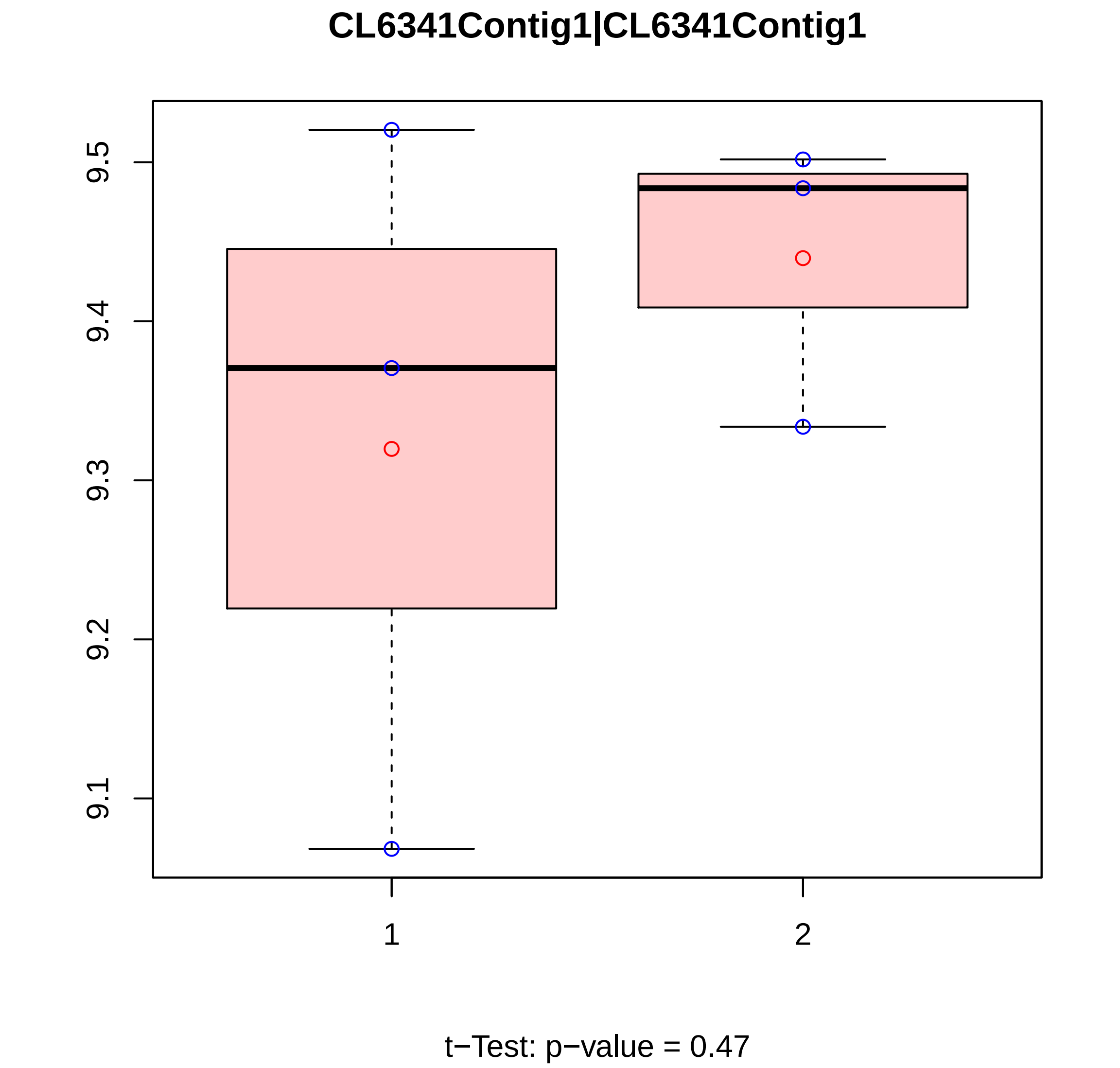

CL6341Contig1|CL6341Contig1
9.1
9.2
9.3
9.4
9.5
1
2
t−Test: p−value = 0.47

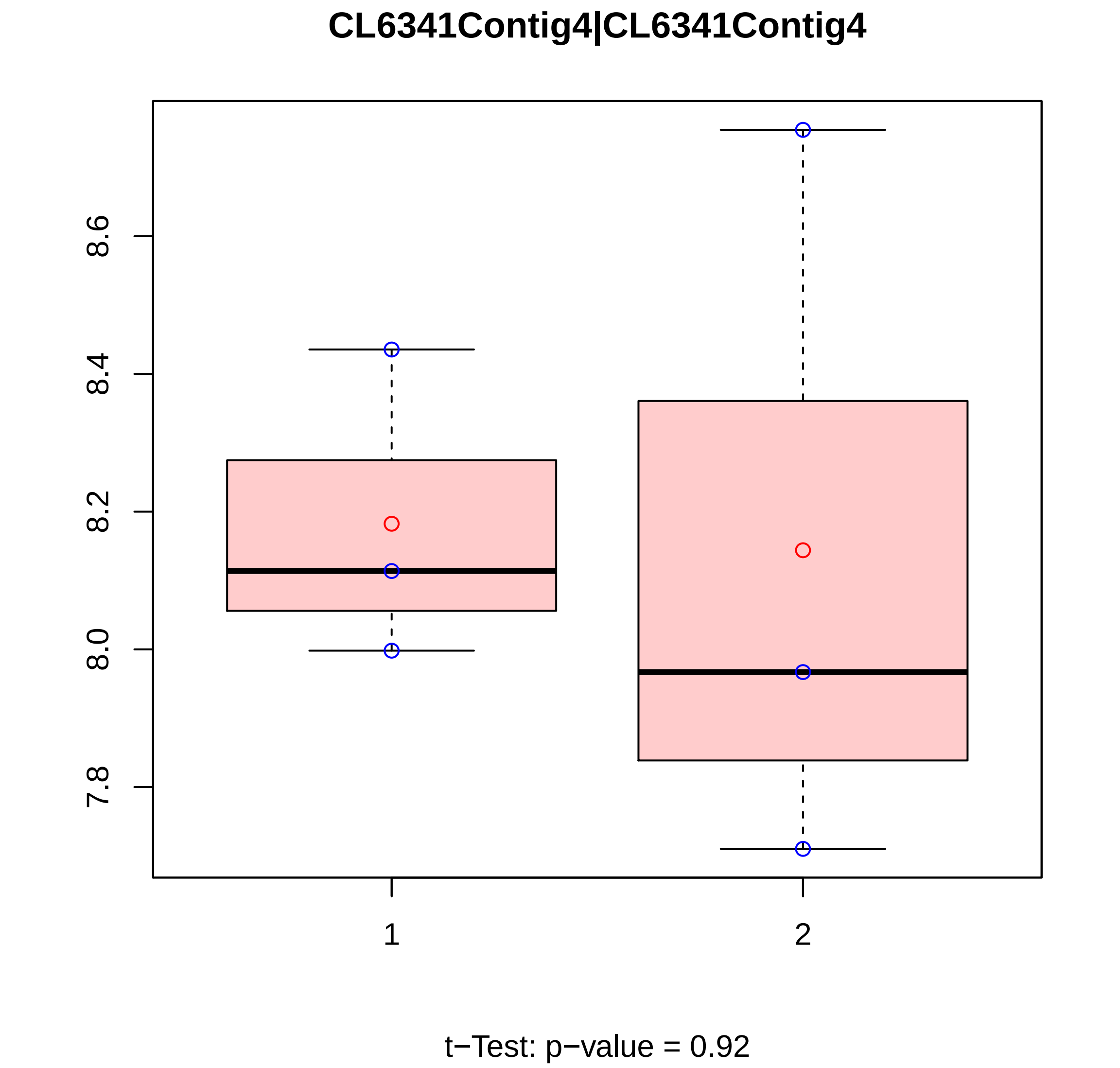
CL6341Contig4|CL6341Contig4
8.6
8.4
8.2
8.0
7.8
1
2
t−Test: p−value = 0.92

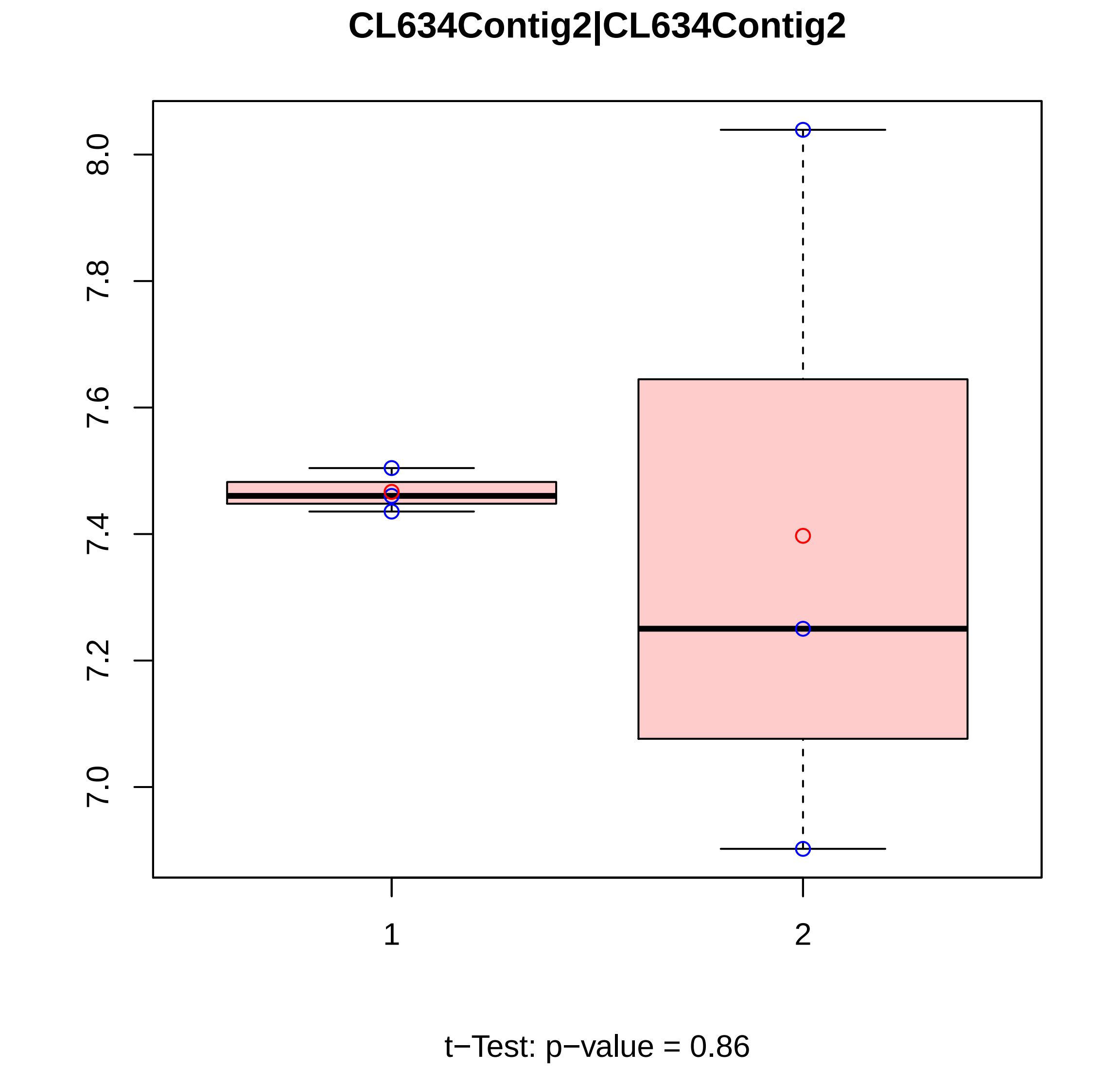

CL634Contig2|CL634Contig2
8.0
7.8
7.6
7.4
7.2
7.0
1
2
t−Test: p−value = 0.86

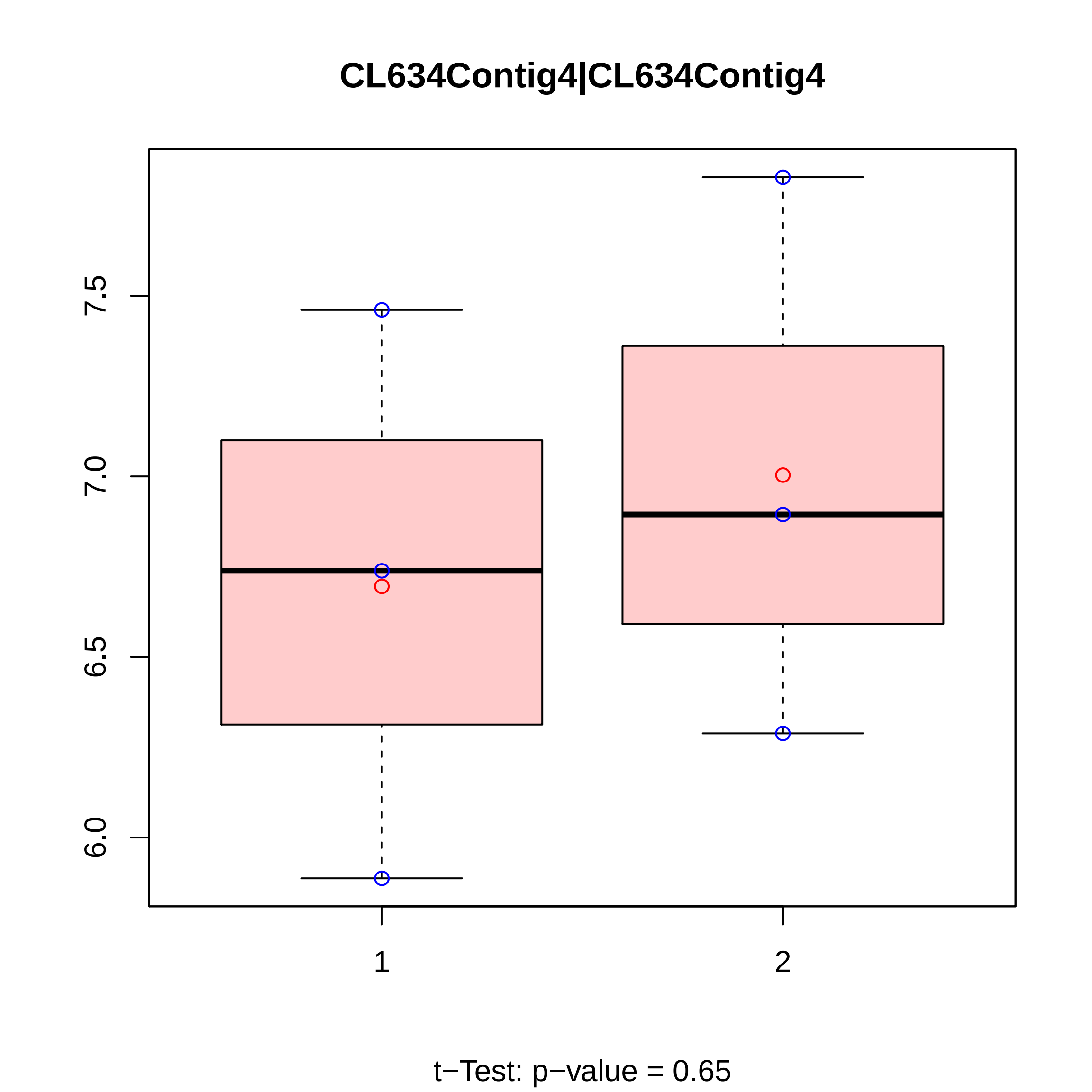

CL634Contig4|CL634Contig4
7.5
7.0
6.5
6.0
1
2
t−Test: p−value = 0.65

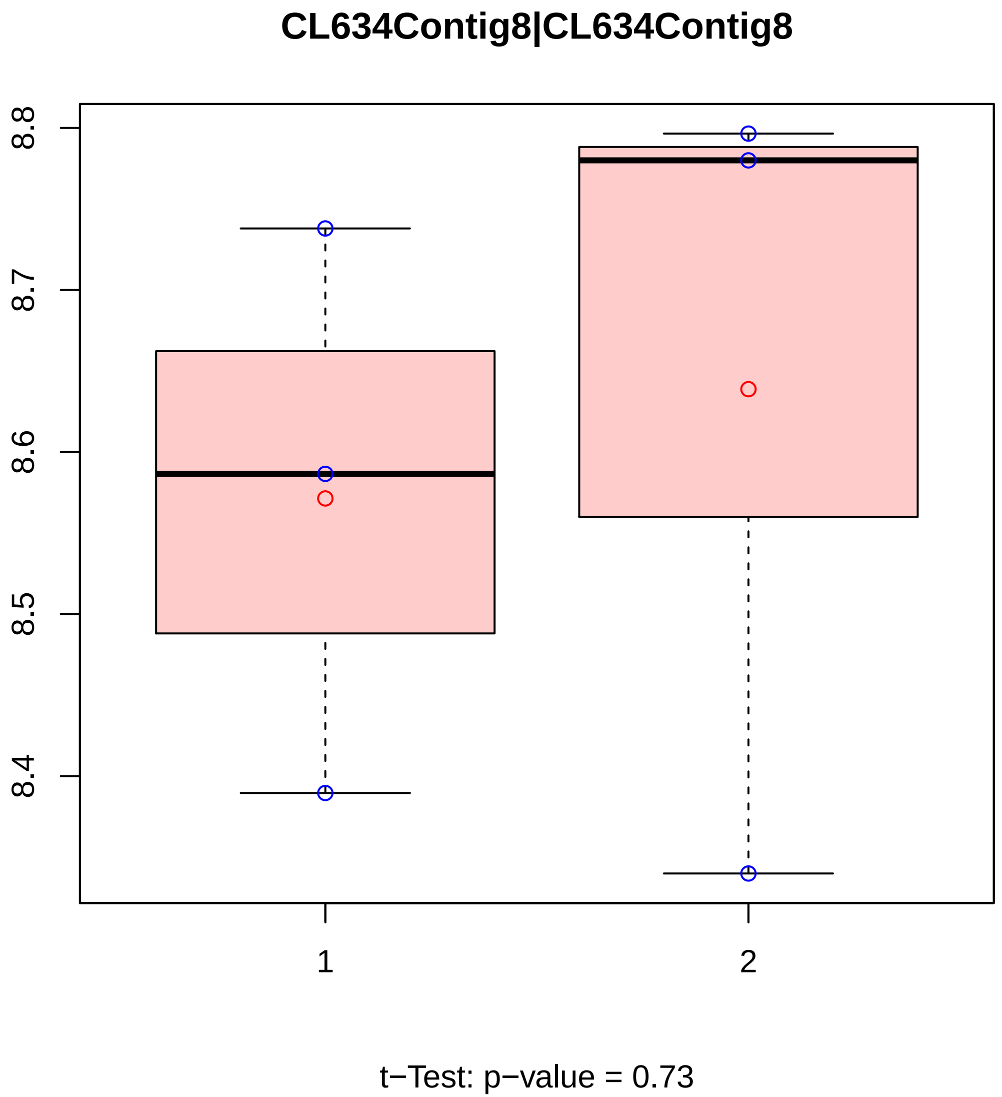

CL634Contig8|CL634Contig8
8.8
8.7
8.6
8.5
8.4
1
2
t−Test: p−value = 0.73

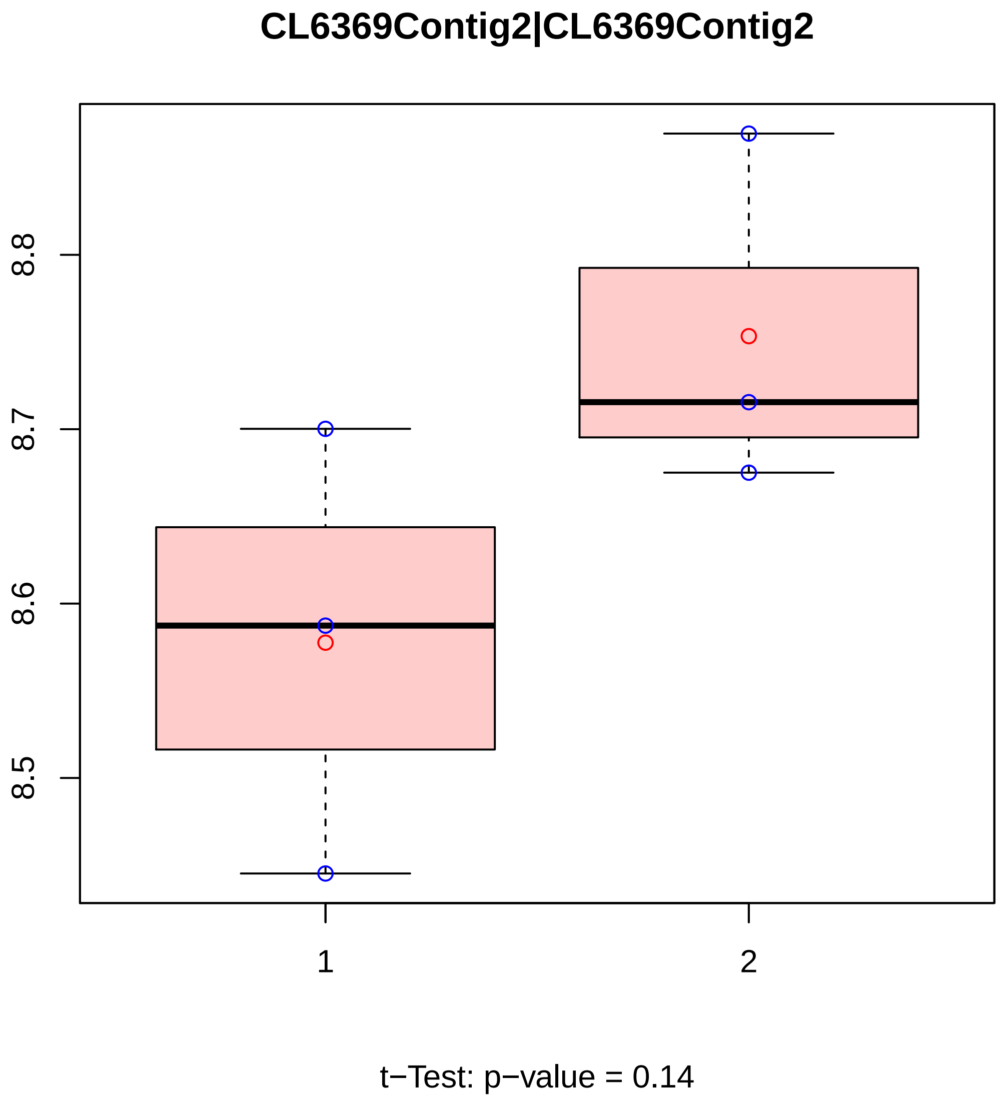
CL6369Contig2|CL6369Contig2
8.5
8.6
8.7
8.8
1
2
t−Test: p−value = 0.14

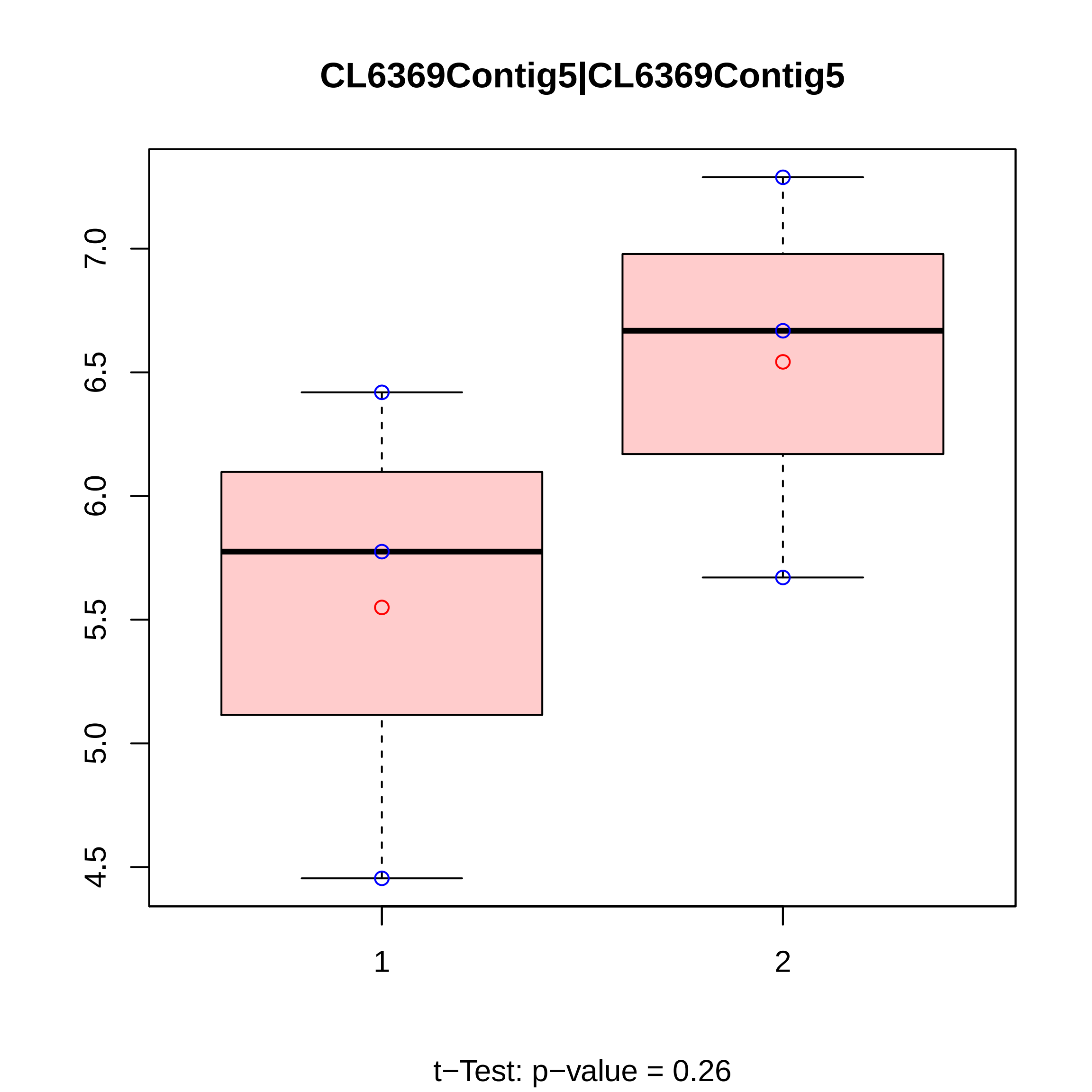
CL6369Contig5|CL6369Contig5
7.0
6.5
6.0
5.5
5.0
4.5
1
2
t−Test: p−value = 0.26

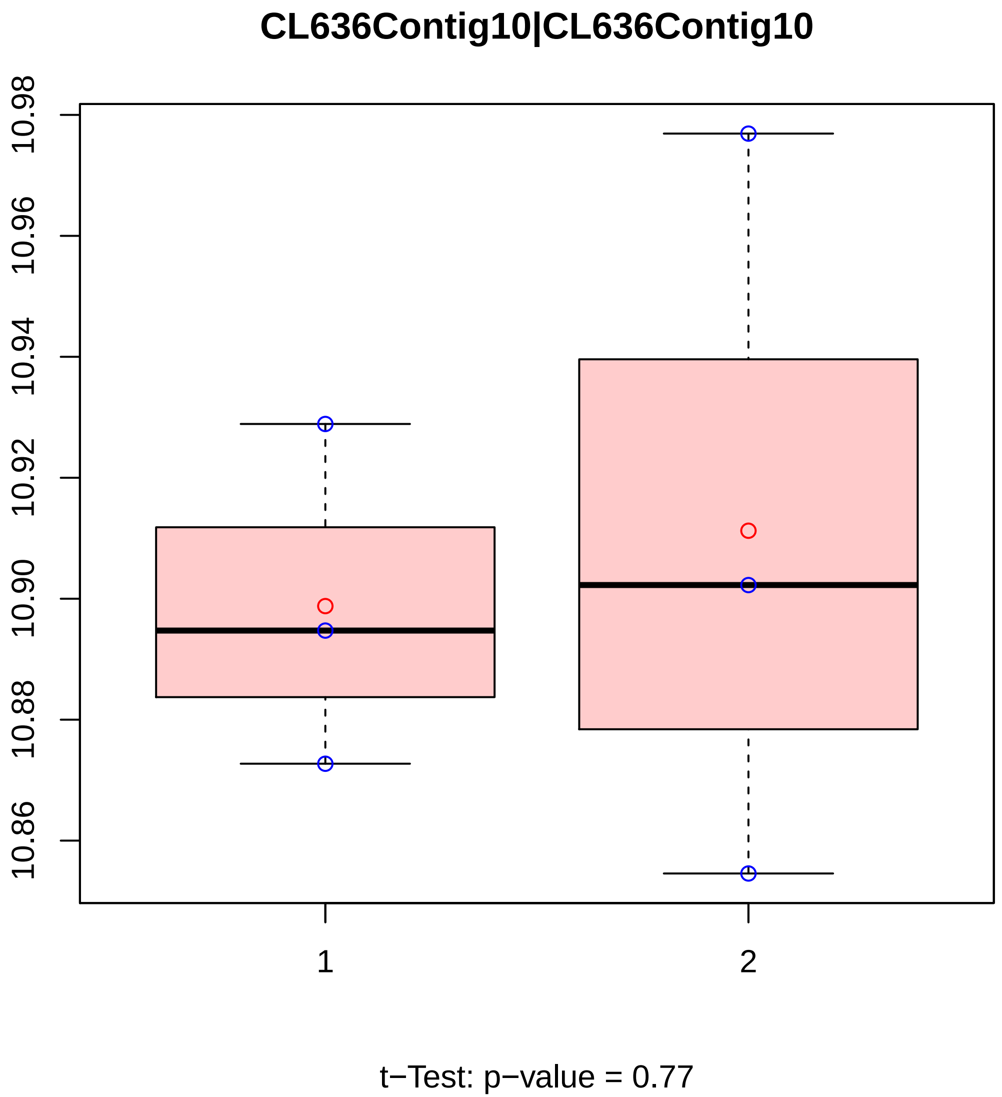

CL636Contig10|CL636Contig10
10.98
10.96
10.94
10.92
10.90
10.88
10.86
1
2
t−Test: p−value = 0.77

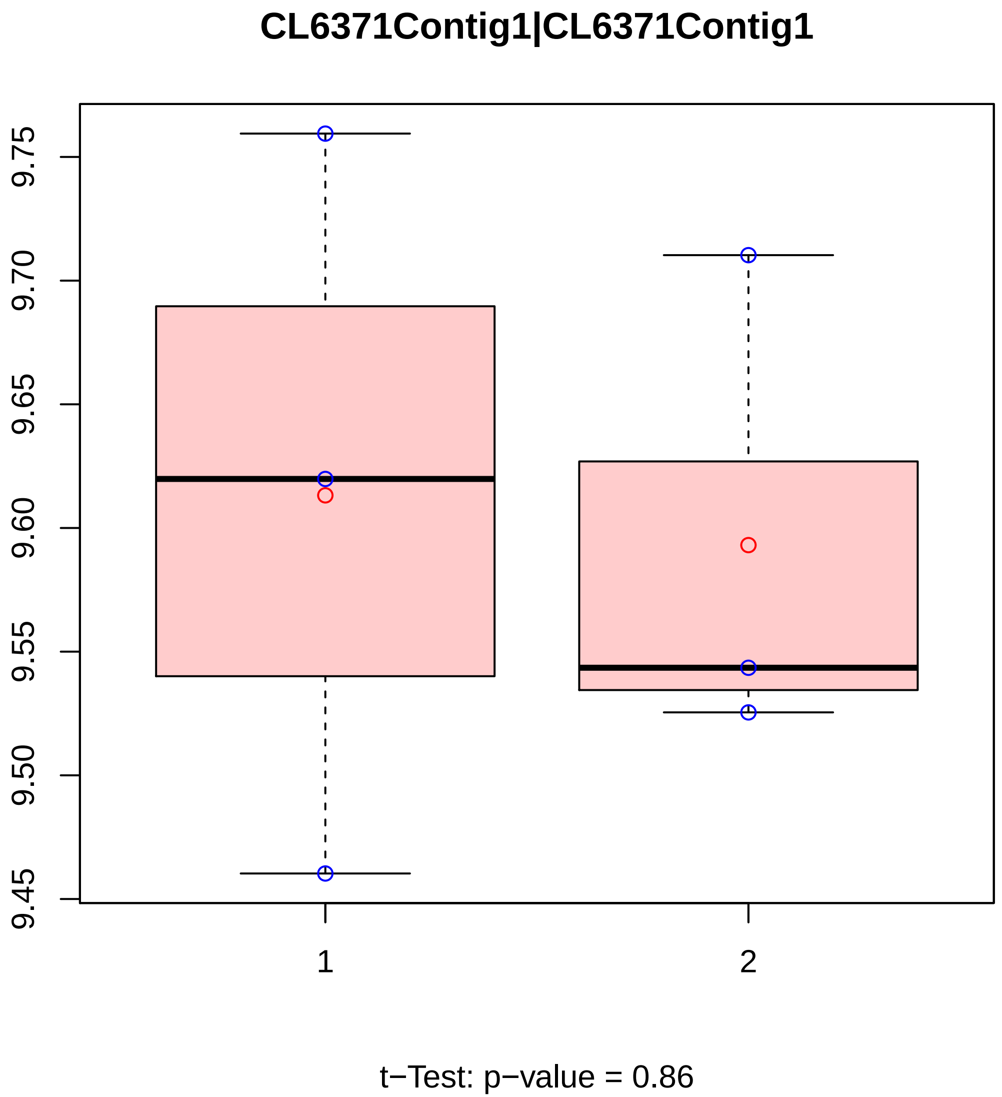

CL6371Contig1|CL6371Contig1
9.45
9.50
9.55
9.60
9.65
9.70
9.75
1
2
t−Test: p−value = 0.86

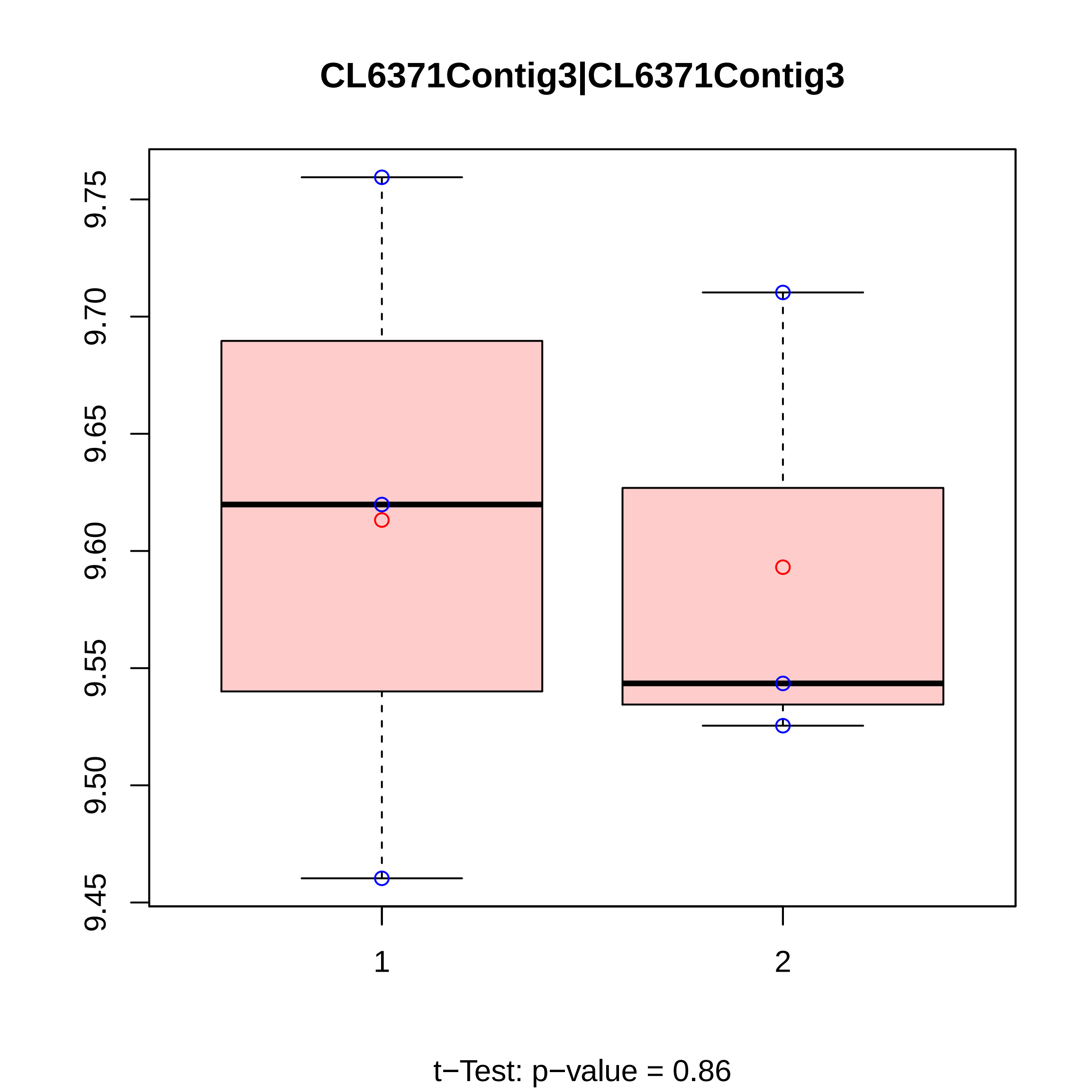
CL6371Contig3|CL6371Contig3
9.45
9.50
9.55
9.60
9.65
9.70
9.75
1
2
t−Test: p−value = 0.86

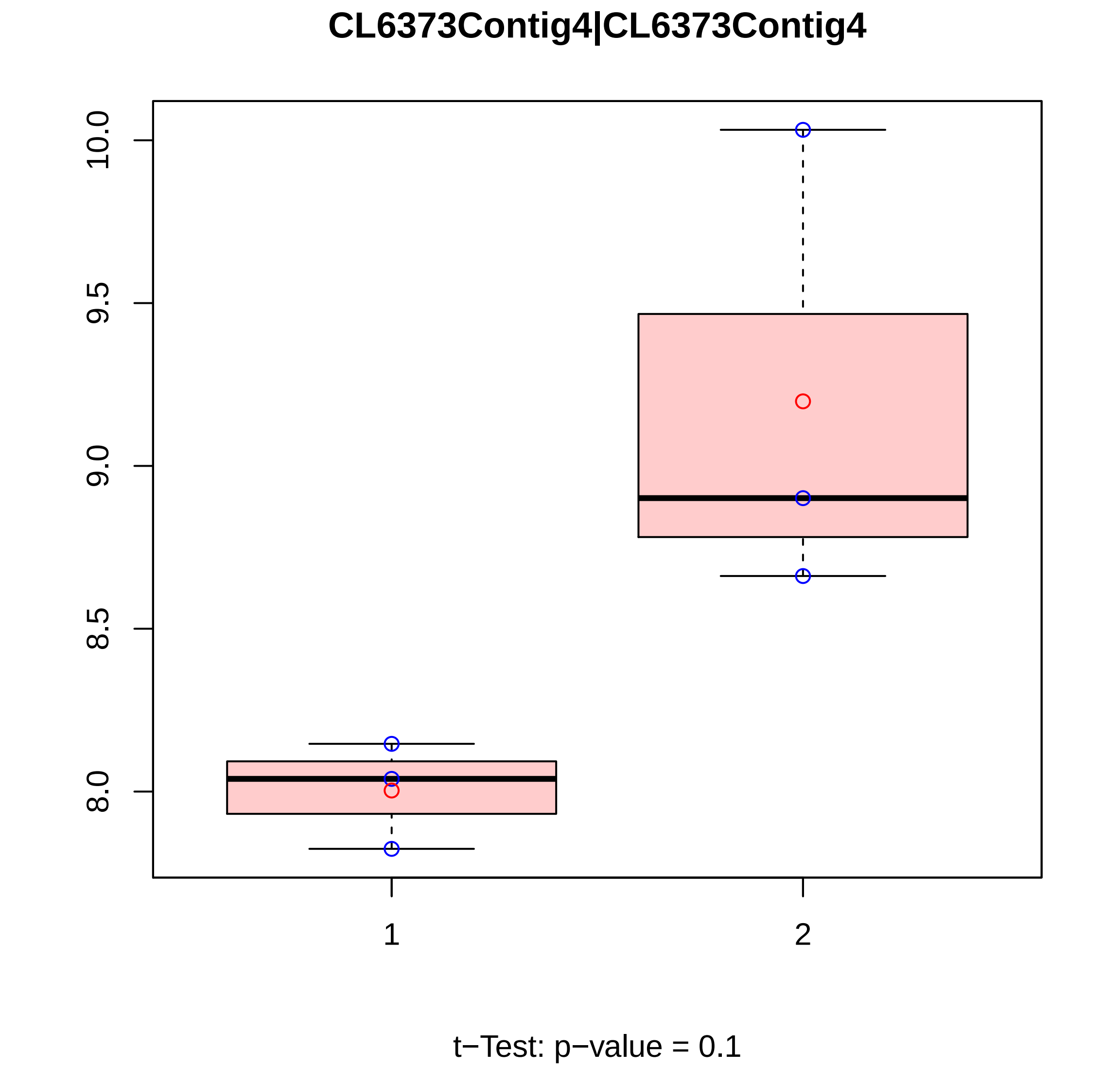

CL6373Contig4|CL6373Contig4
10.0
9.5
9.0
8.5
8.0
1
2
t−Test: p−value = 0.1

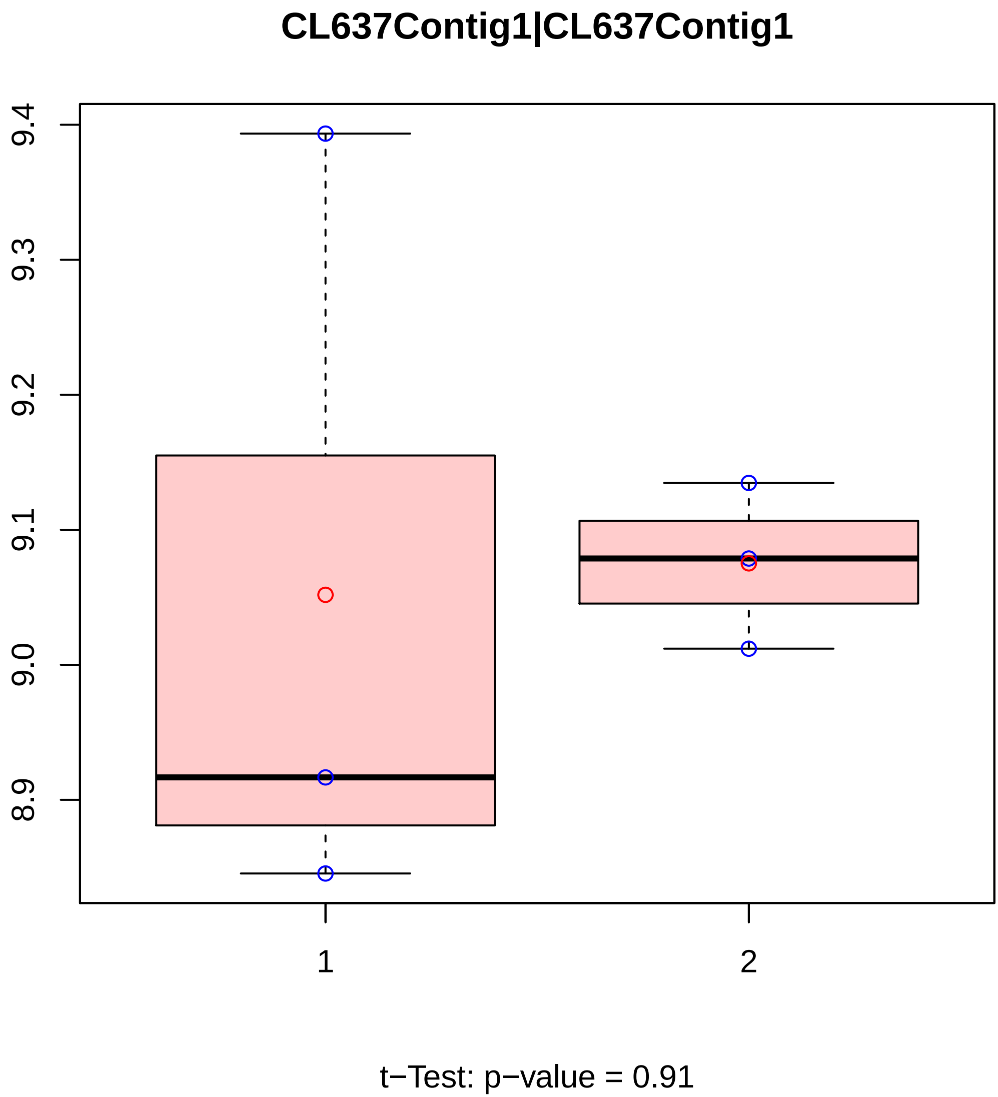
CL637Contig1|CL637Contig1
9.4
9.3
9.2
9.1
9.0
8.9
1
2
t−Test: p−value = 0.91

# CL6385Contig2|CL6385Contig2

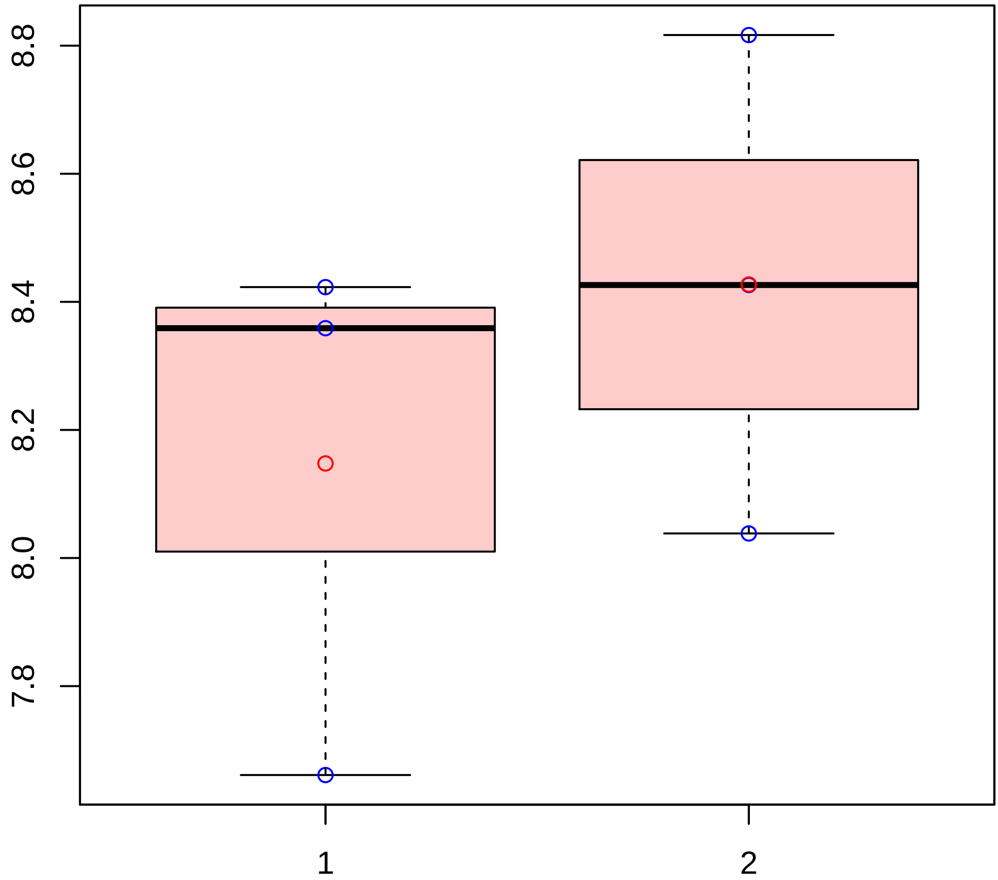


t−Test: p−value = 0.45

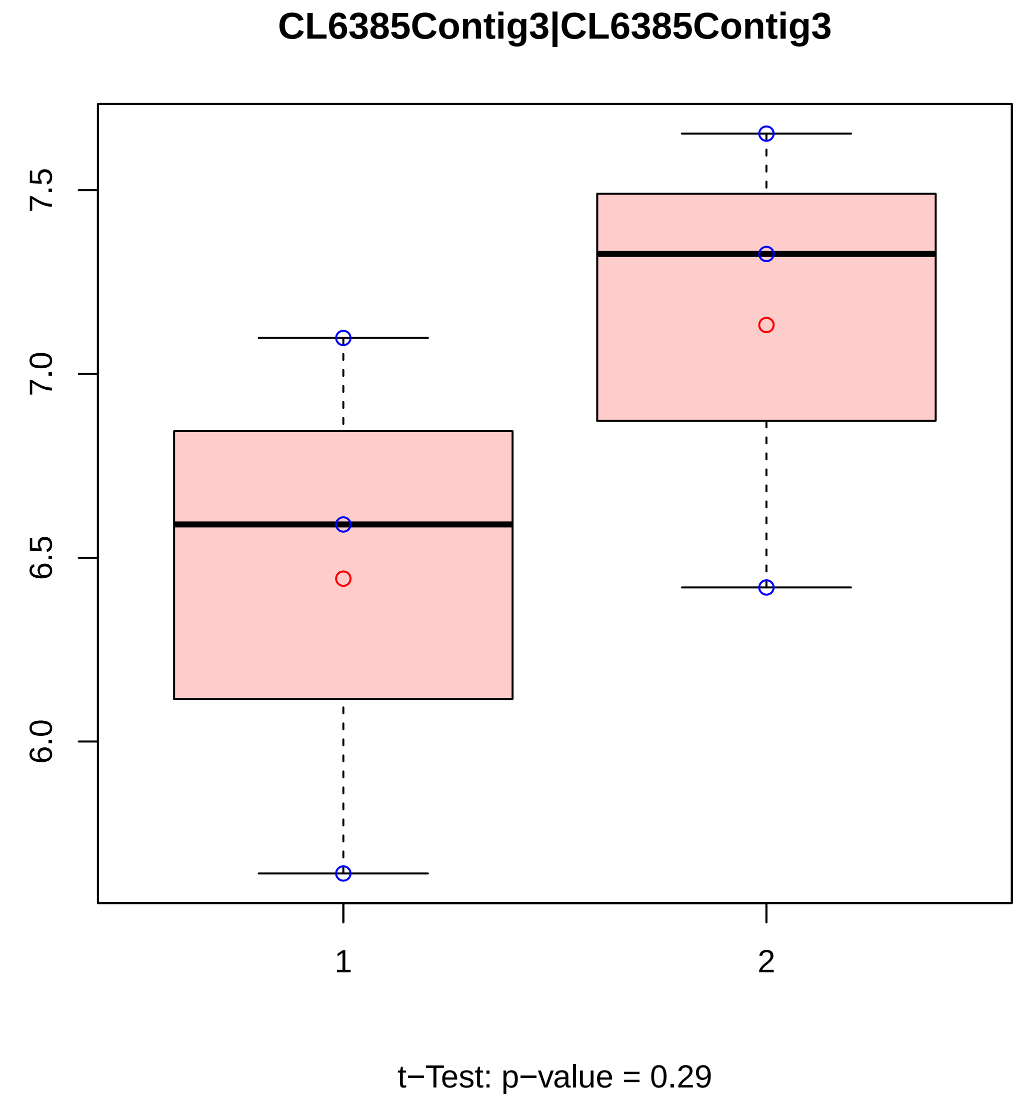
CL6385Contig3|CL6385Contig3
7.5
7.0
6.5
6.0
1
2
t−Test: p−value = 0.29

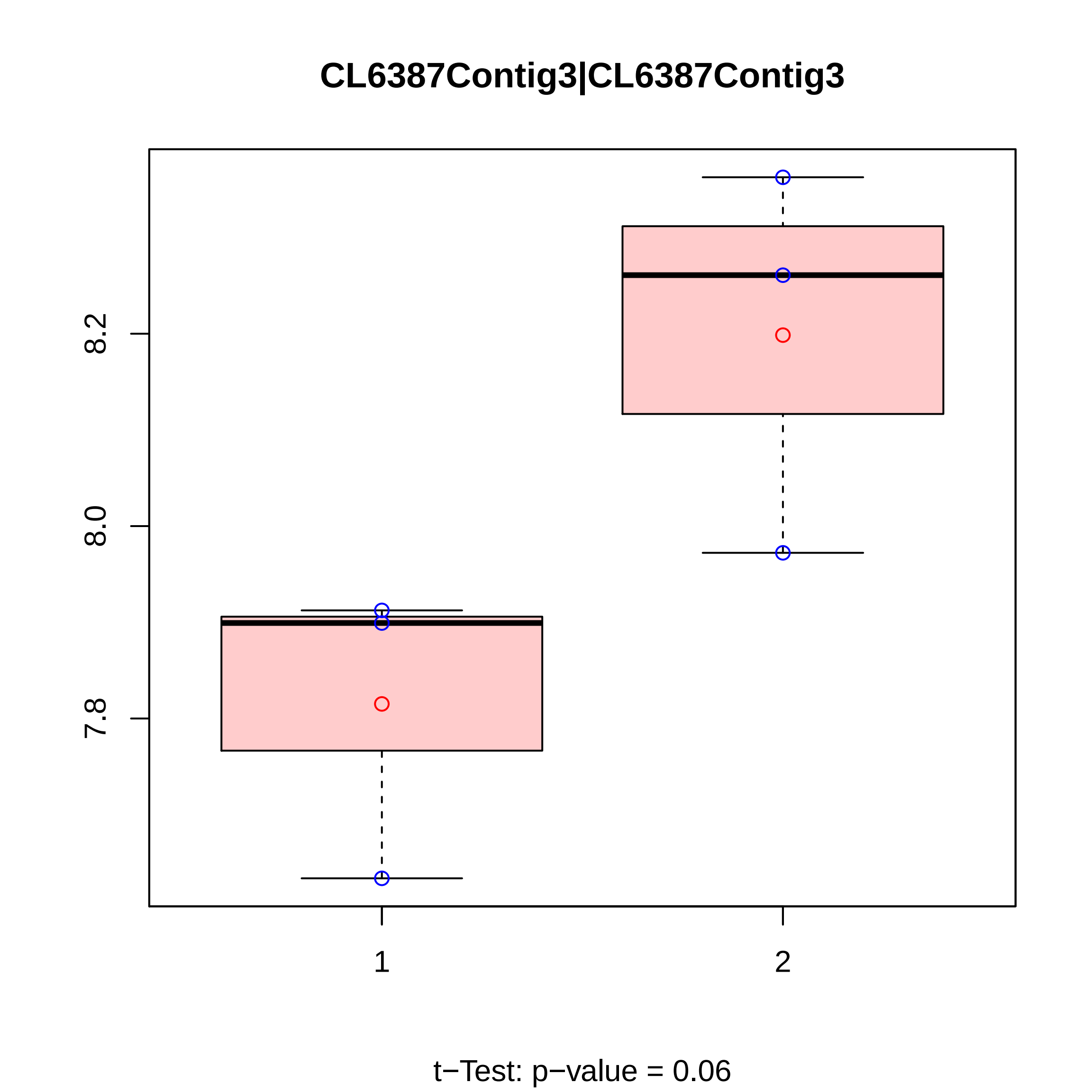

CL6387Contig3|CL6387Contig3
8.2
8.0
7.8
1
2
t−Test: p−value = 0.06

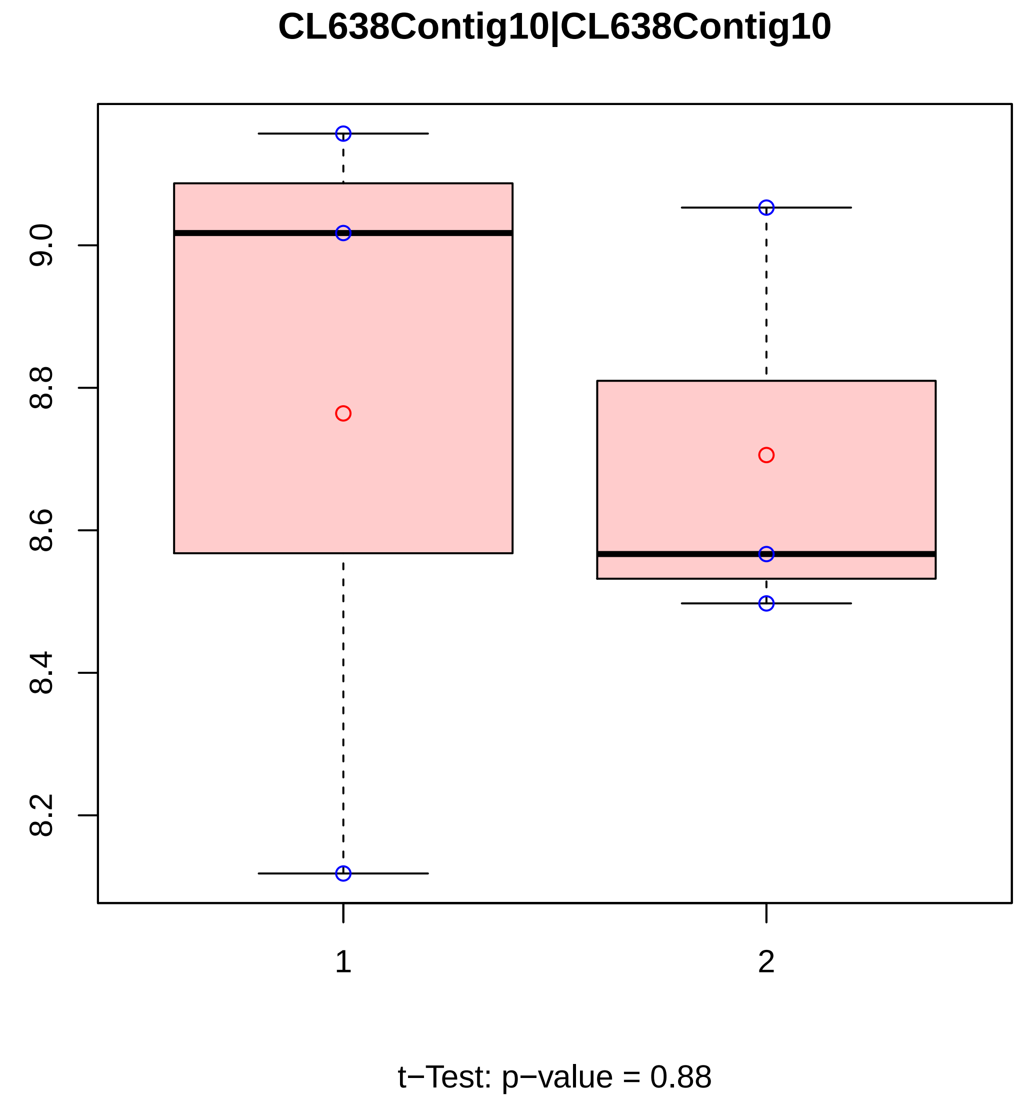
CL638Contig10|CL638Contig10
9.0
8.8
8.6
8.4
8.2
1
2
t−Test: p−value = 0.88

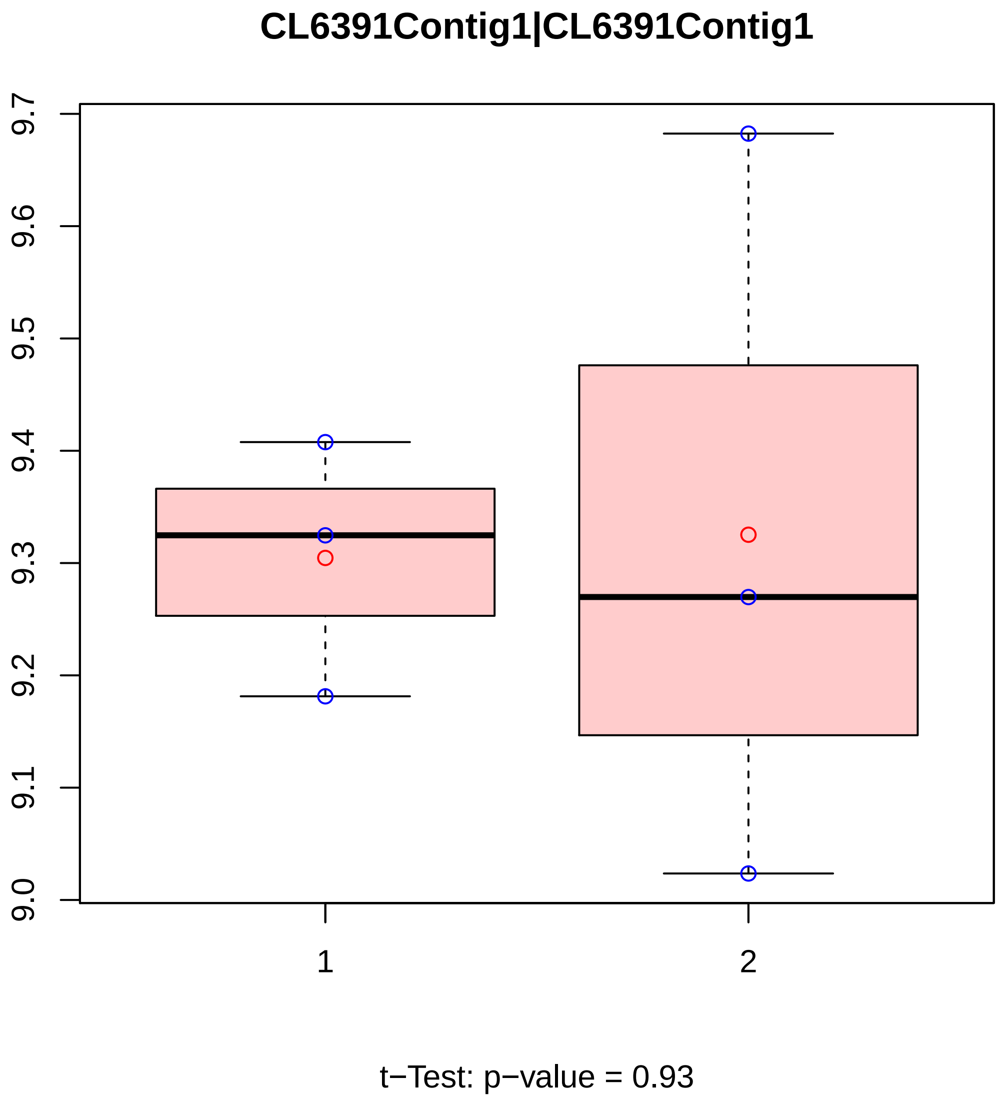
CL6391Contig1|CL6391Contig1
9.0
9.1
9.2
9.3
9.4
9.5
9.6
9.7
1
2
t−Test: p−value = 0.93

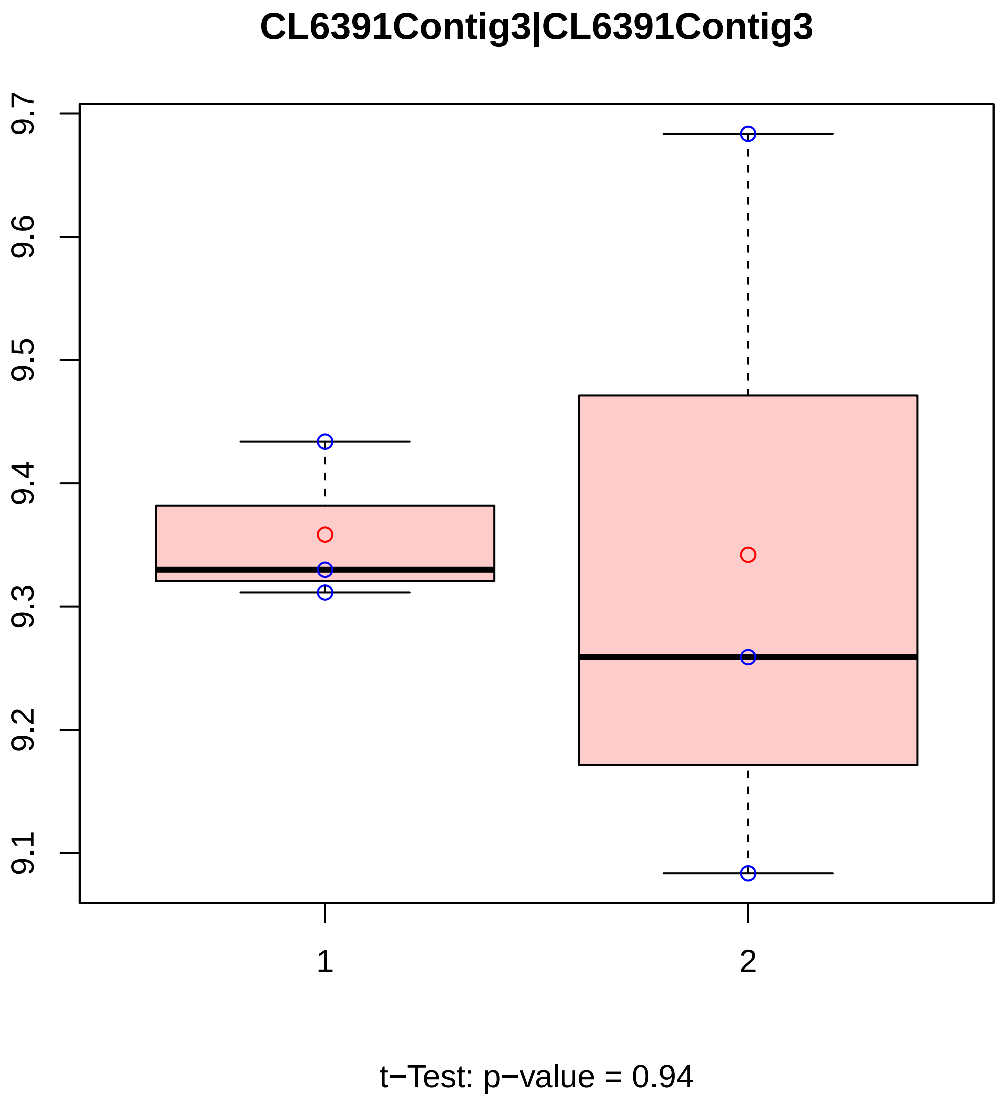
CL6391Contig3|CL6391Contig3
9.7
9.6
9.5
9.4
9.3
9.2
9.1
1
2
t−Test: p−value = 0.94

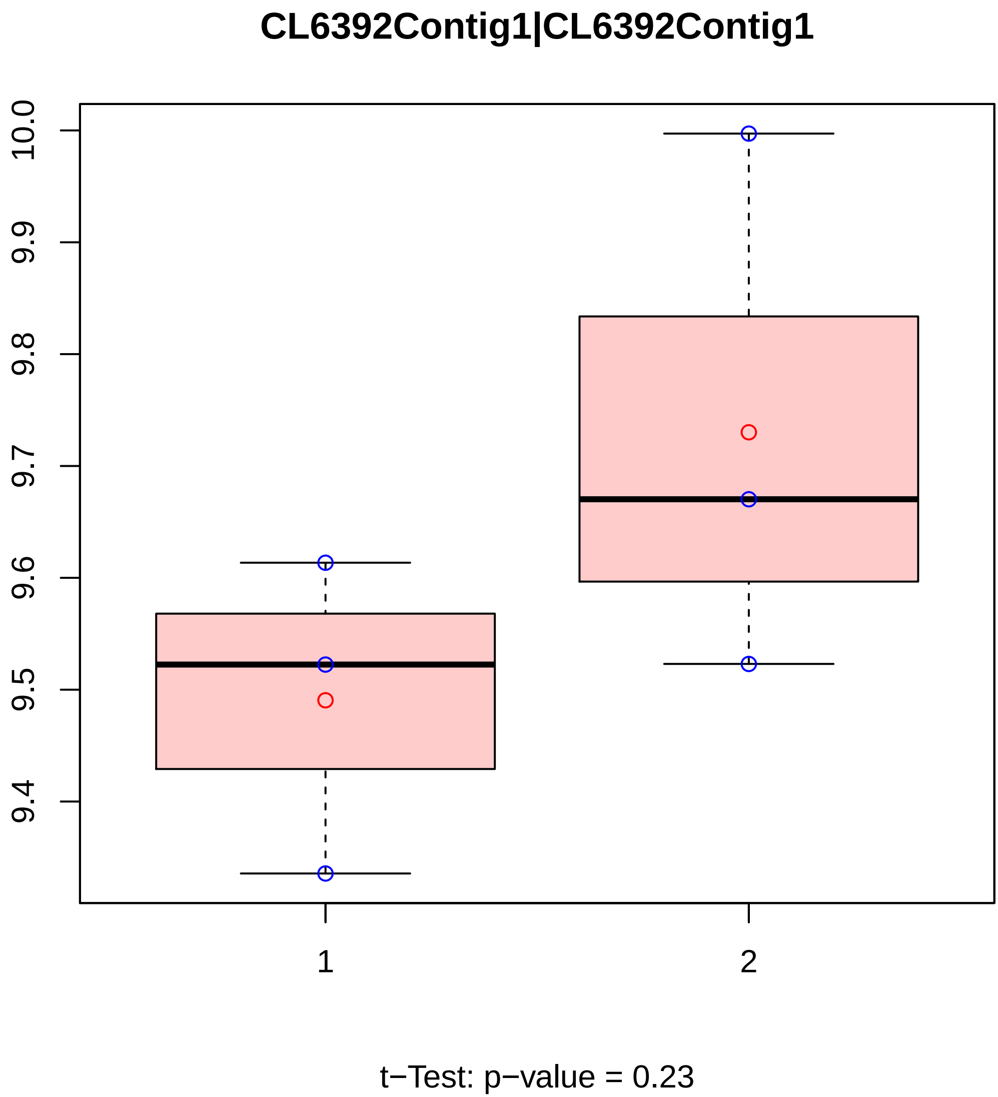

CL6392Contig1|CL6392Contig1
10.0
9.9
9.8
9.7
9.6
9.5
9.4
1
2
t−Test: p−value = 0.23

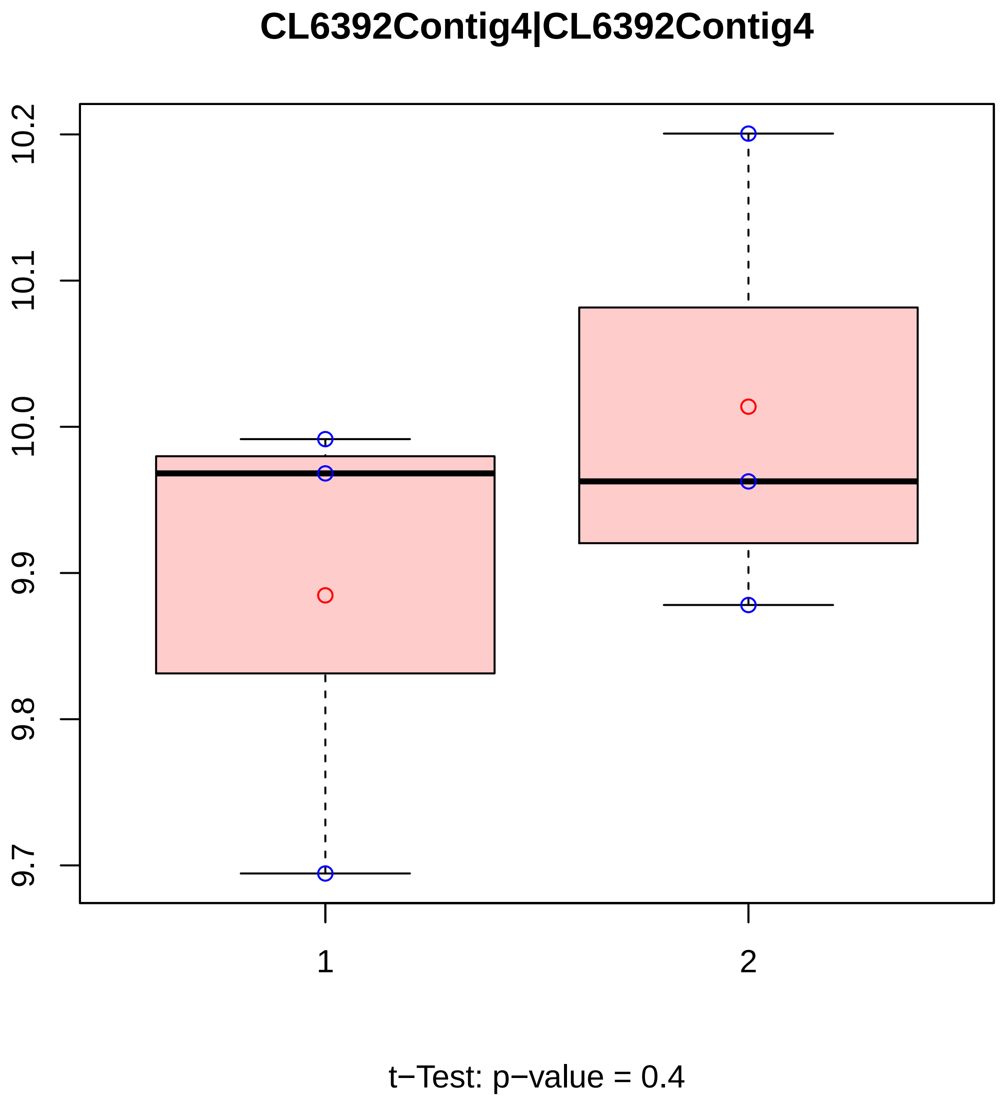
CL6392Contig4|CL6392Contig4
10.2
10.1
10.0
9.9
9.8
9.7
1
2
t−Test: p−value = 0.4

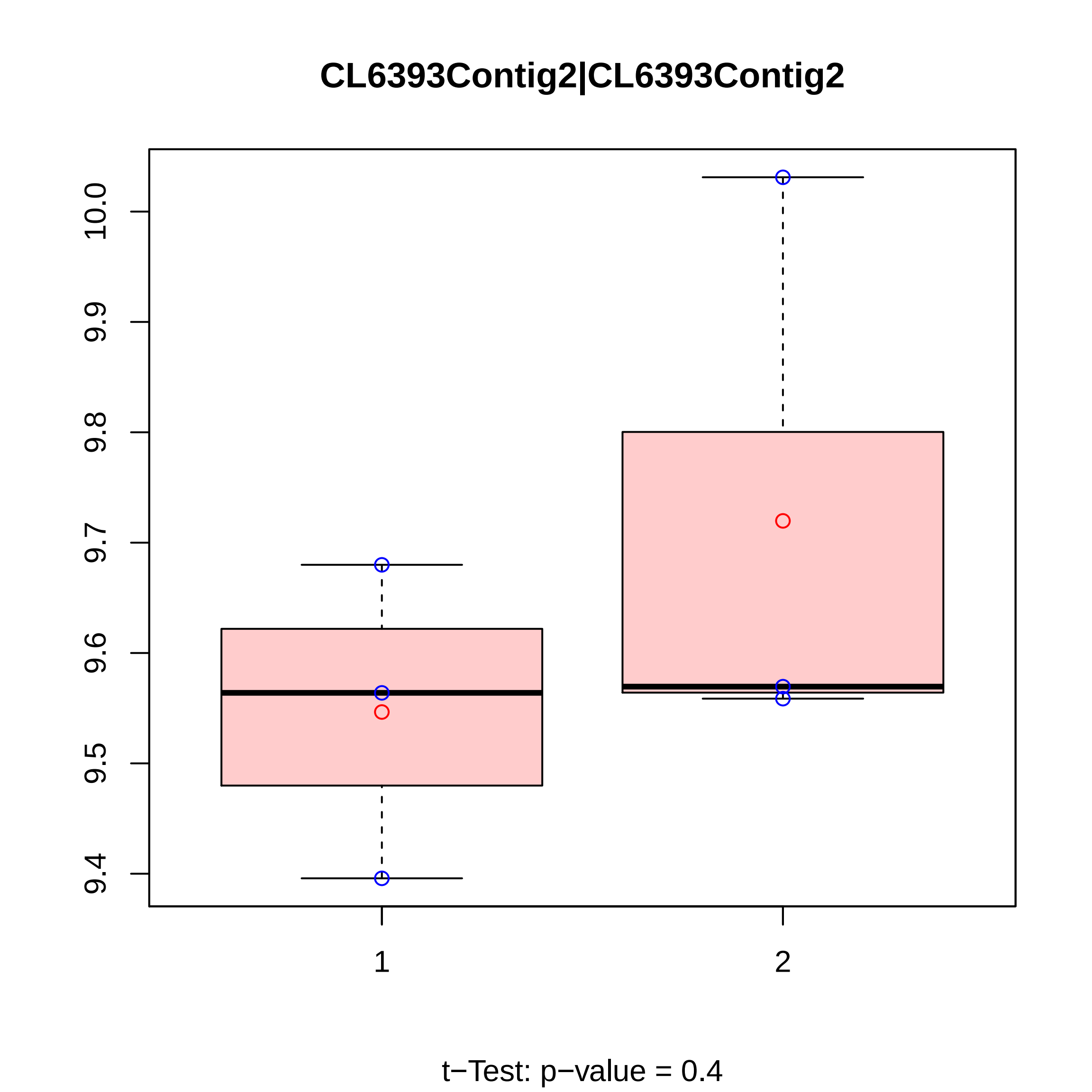
CL6393Contig2|CL6393Contig2
10.0
9.9
9.8
9.7
9.6
9.5
9.4
1
2
t−Test: p−value = 0.4

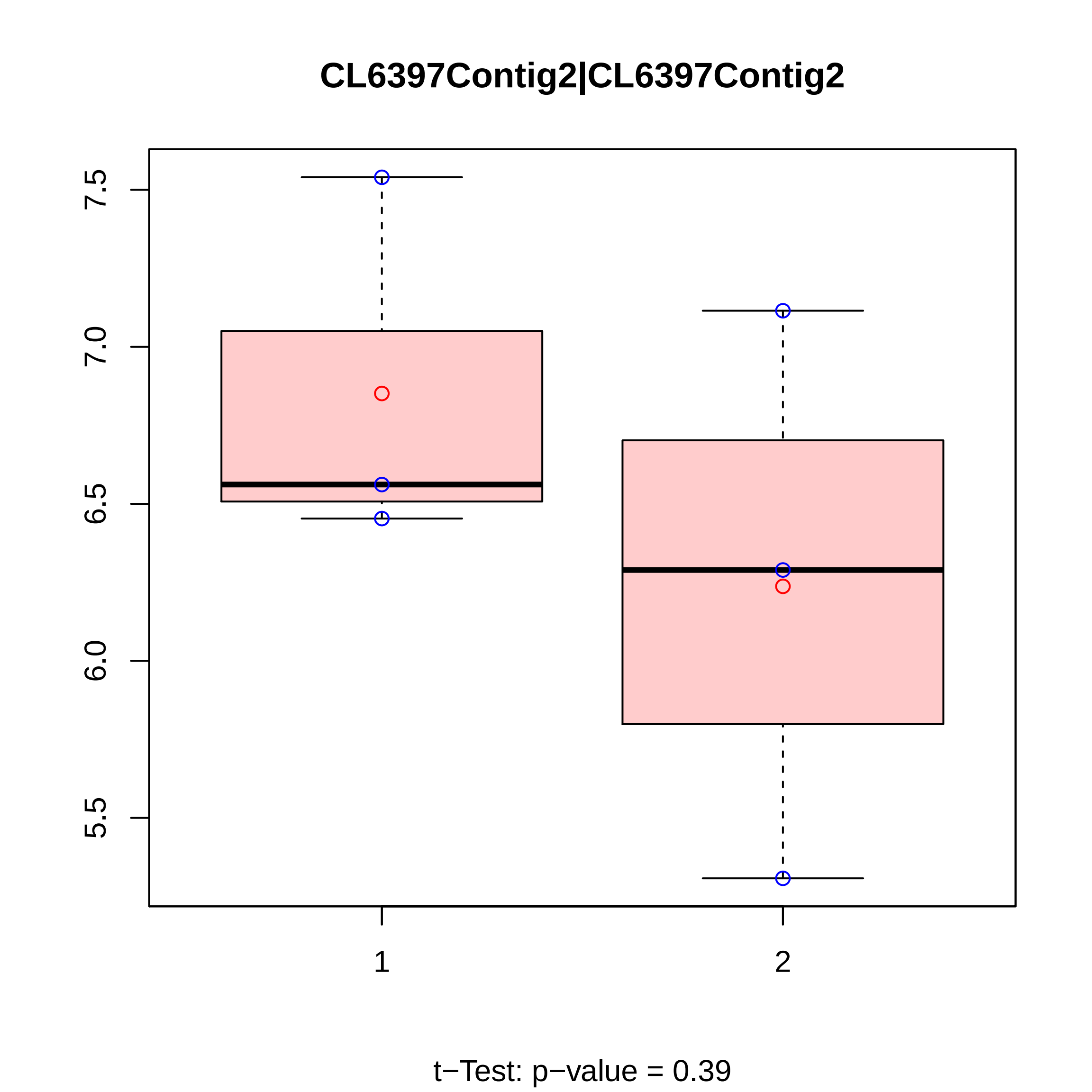

CL6397Contig2|CL6397Contig2
7.5
7.0
6.5
6.0
5.5
1
2
t−Test: p−value = 0.39

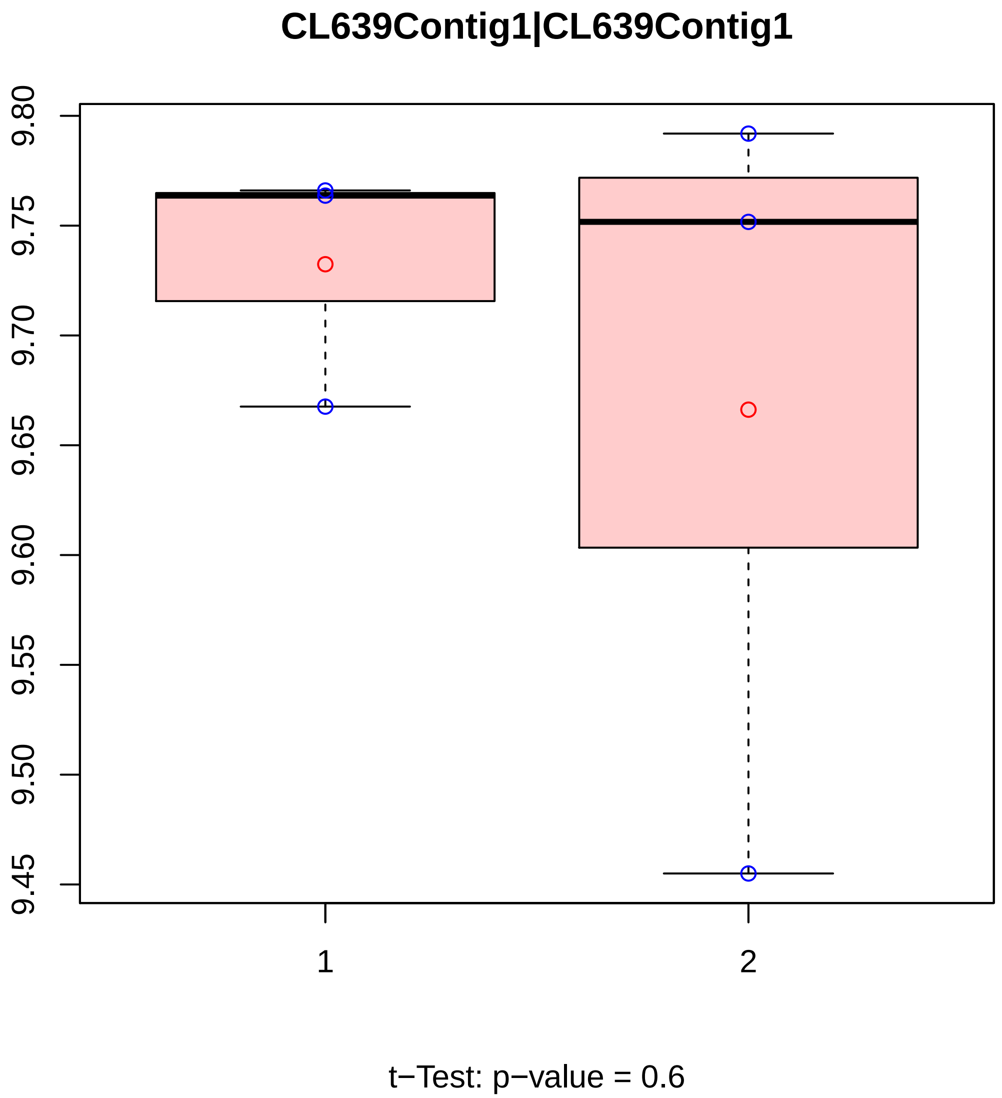
CL639Contig1|CL639Contig1
9.80
9.75
9.70
9.65
9.60
9.55
9.50
9.45
1
2
t−Test: p−value = 0.6

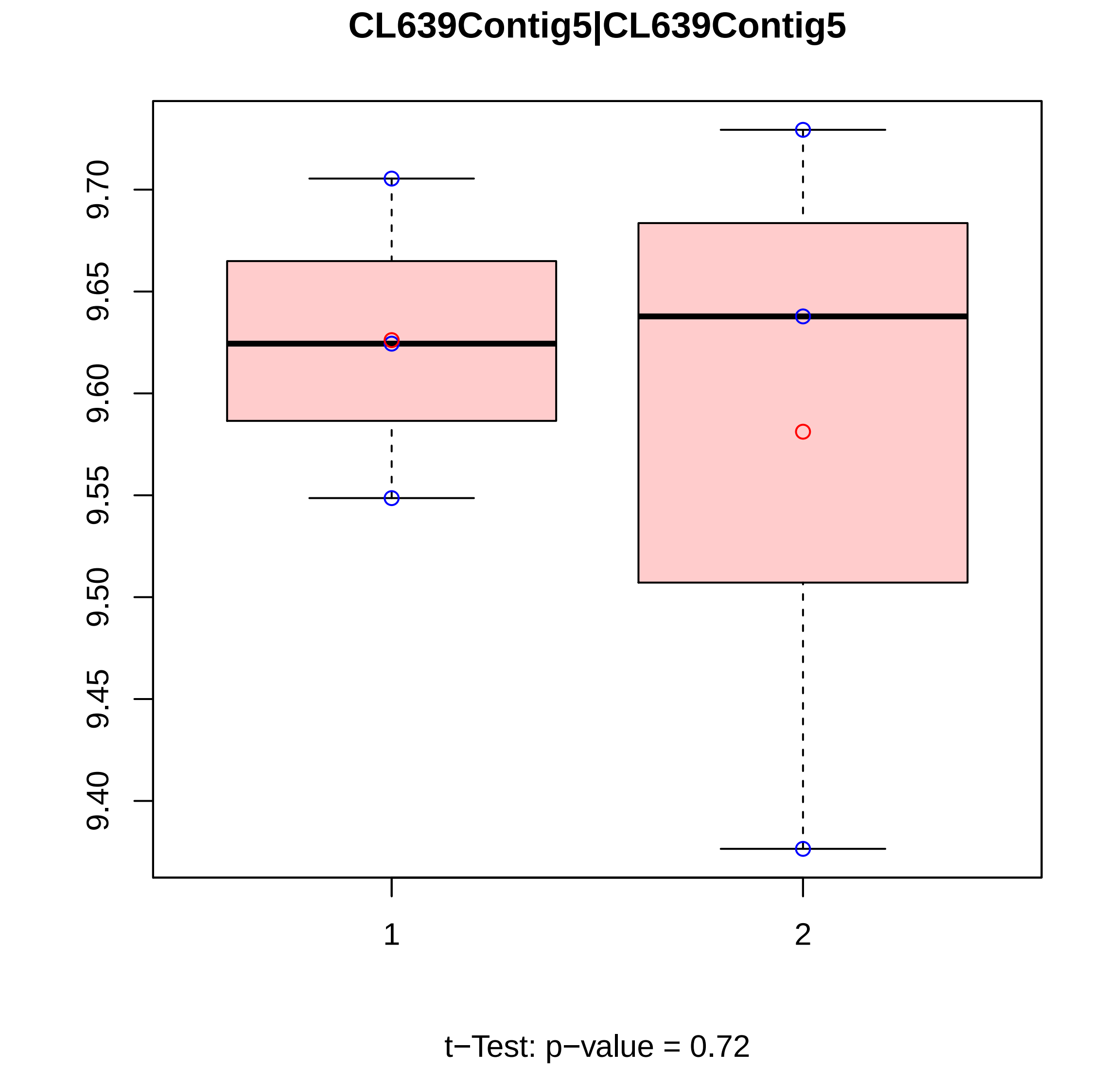
CL639Contig5|CL639Contig5
9.40
9.45
9.50
9.55
9.60
9.65
9.70
1
2
t−Test: p−value = 0.72

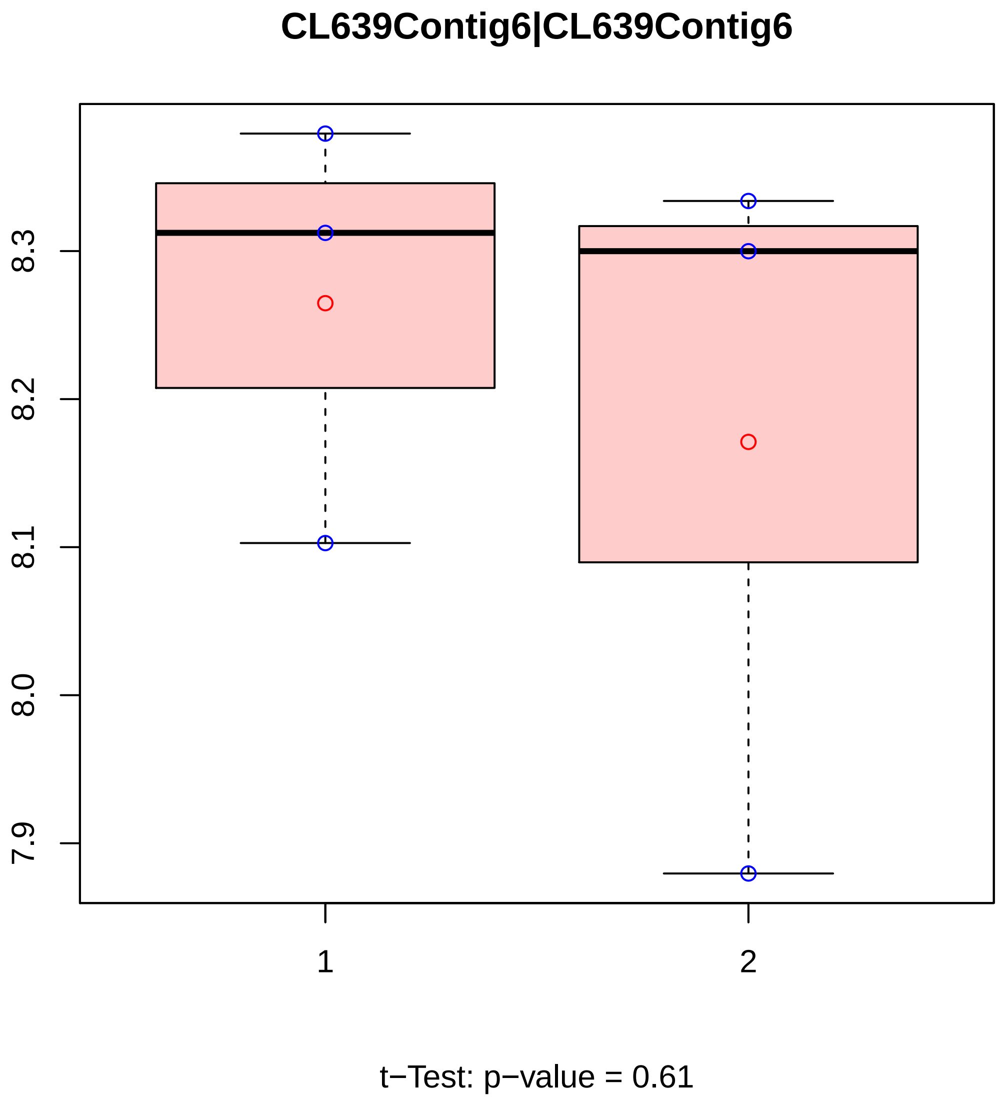
CL639Contig6|CL639Contig6
8.3
8.2
8.1
8.0
7.9
1
2
t−Test: p−value = 0.61

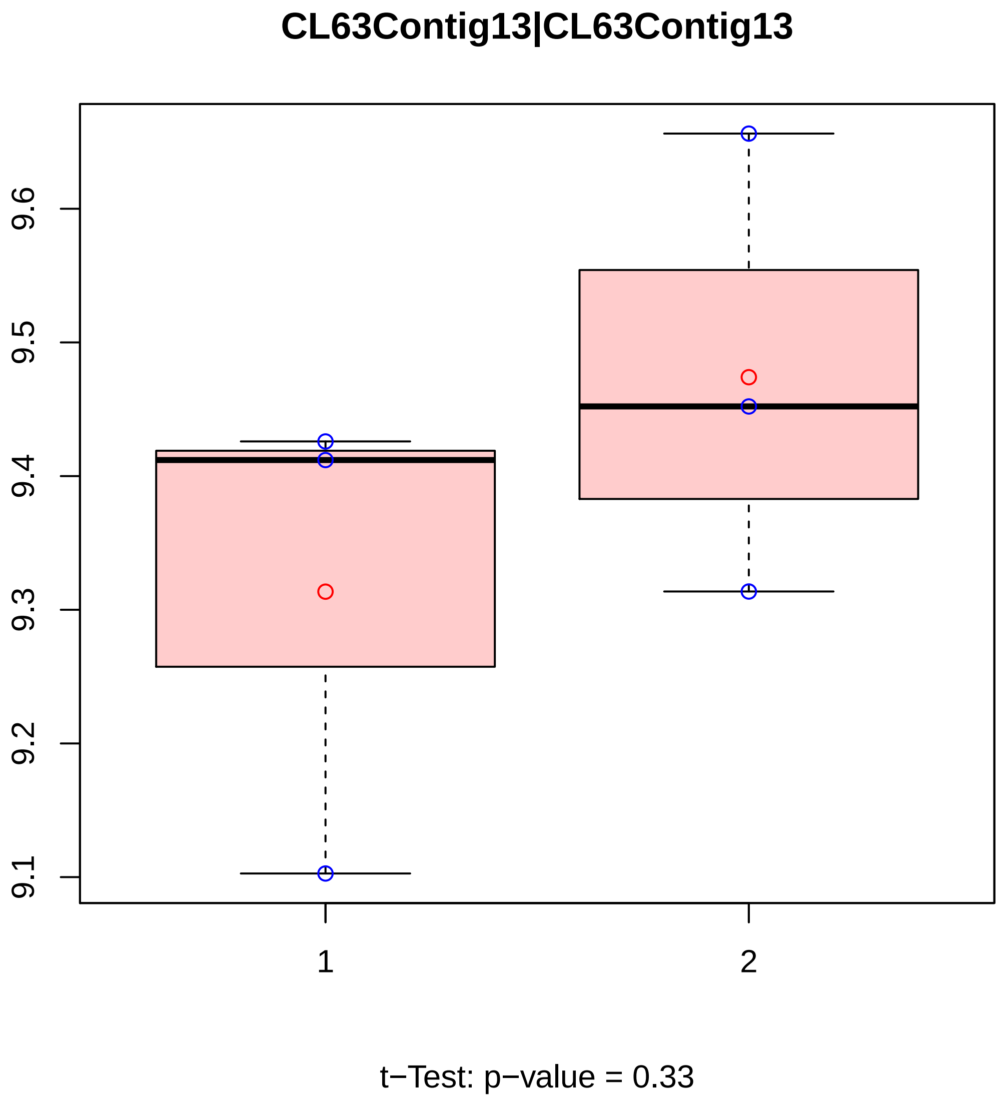
CL63Contig13|CL63Contig13
9.1
9.2
9.3
9.4
9.5
9.6
1
2
t−Test: p−value = 0.33

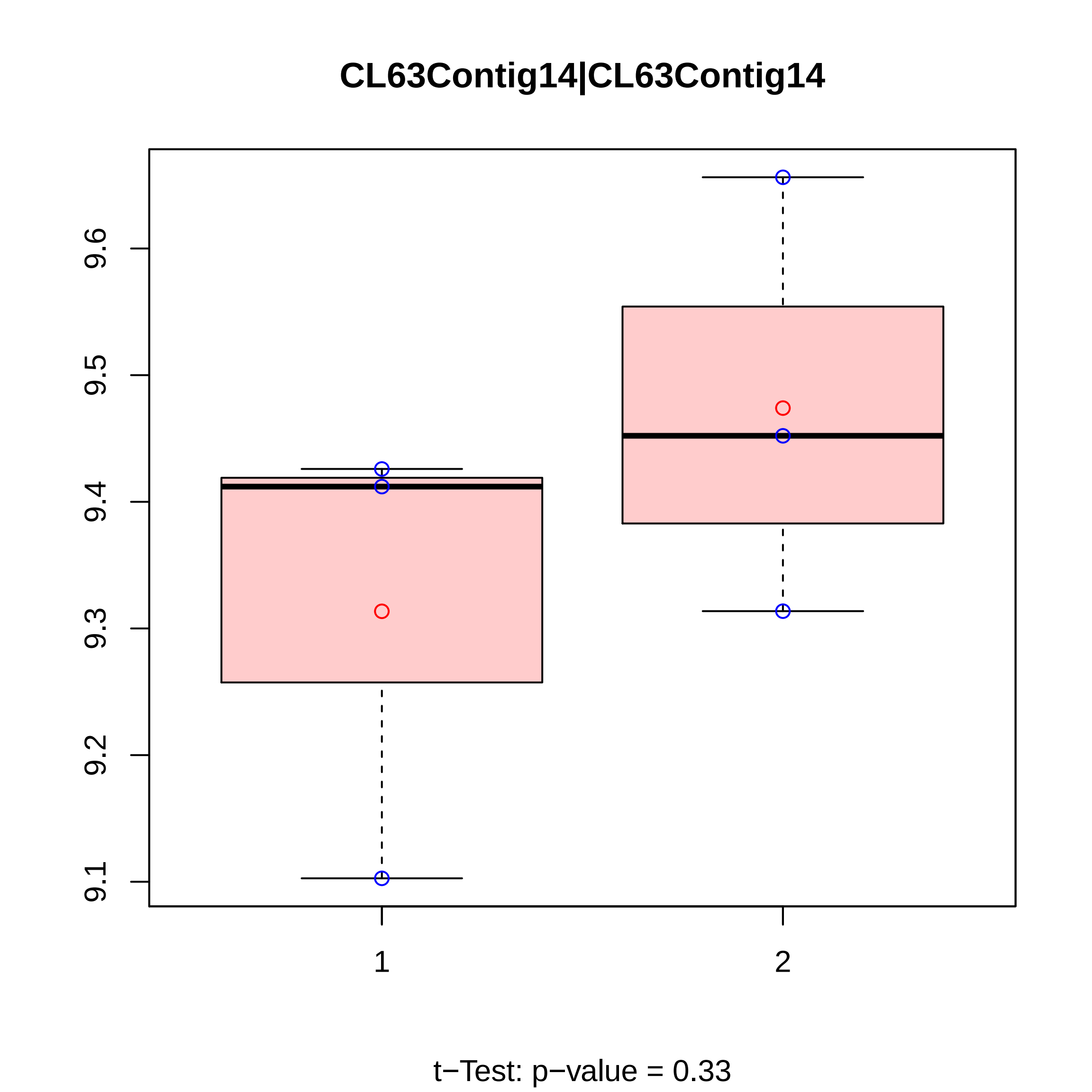
CL63Contig14|CL63Contig14
9.1
9.2
9.3
9.4
9.5
9.6
1
2
t−Test: p−value = 0.33

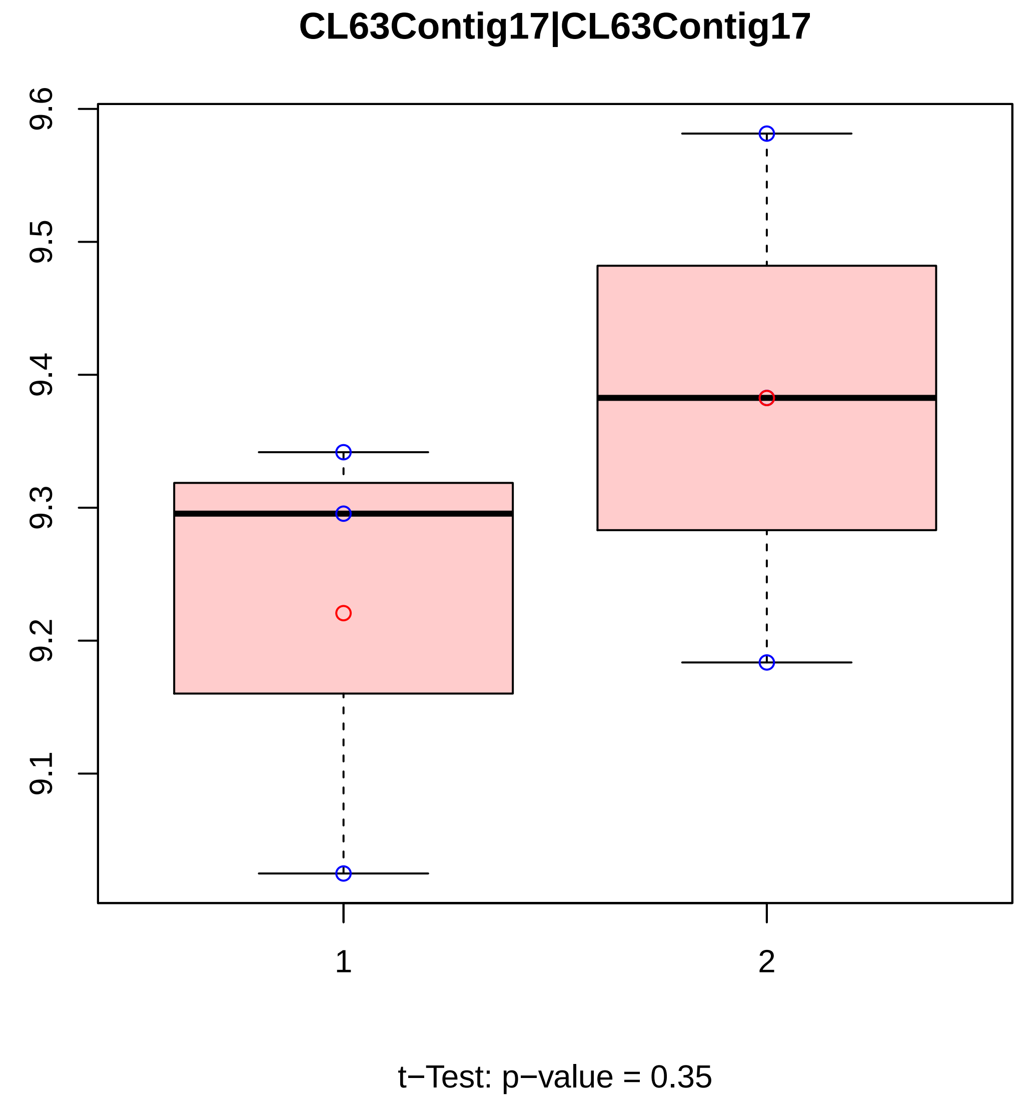
CL63Contig17|CL63Contig17
9.6
9.5
9.4
9.3
9.2
9.1
1
2
t−Test: p−value = 0.35

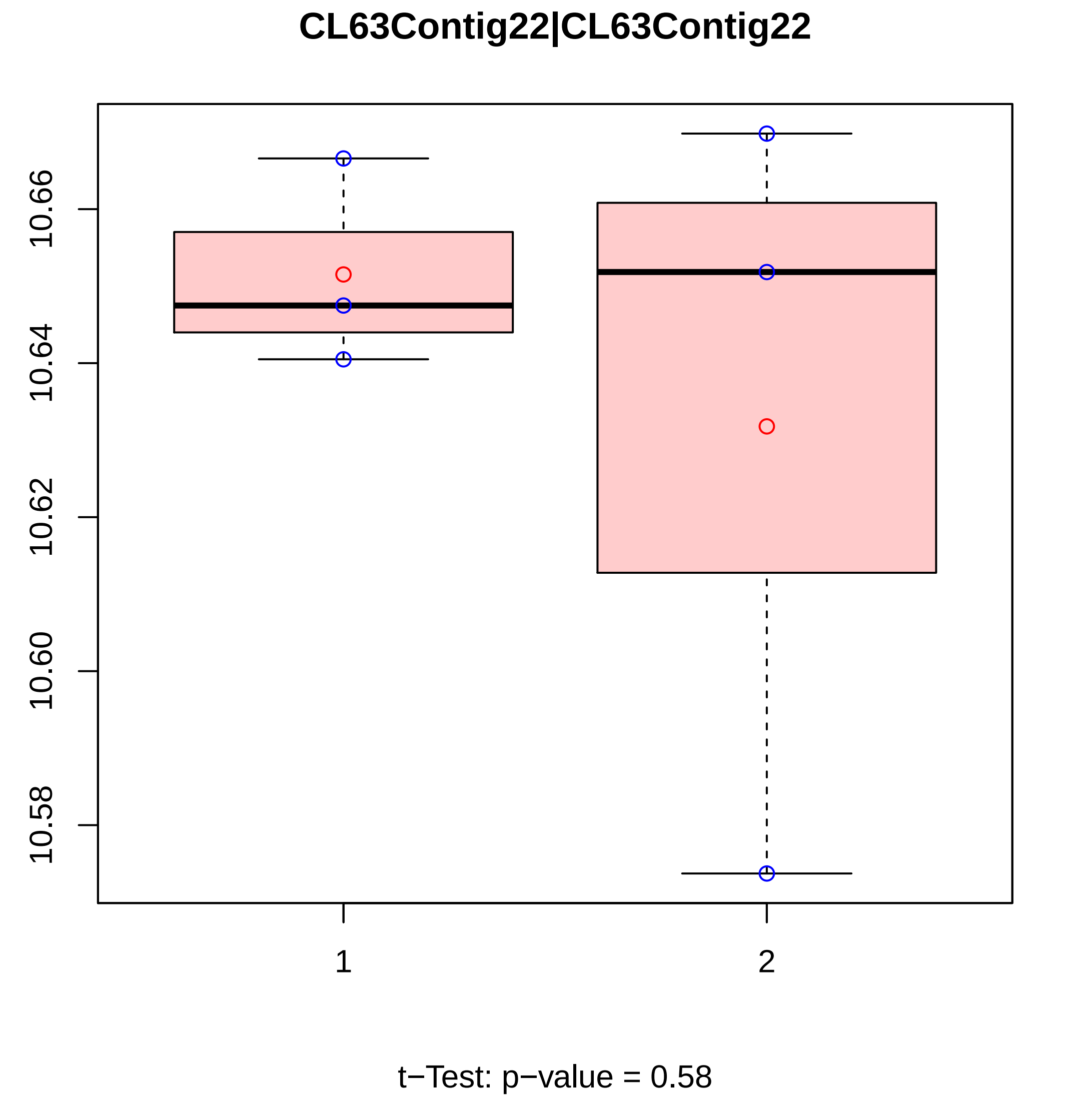

CL63Contig22|CL63Contig22
10.58
10.60
10.62
10.64
10.66
1
2
t−Test: p−value = 0.58

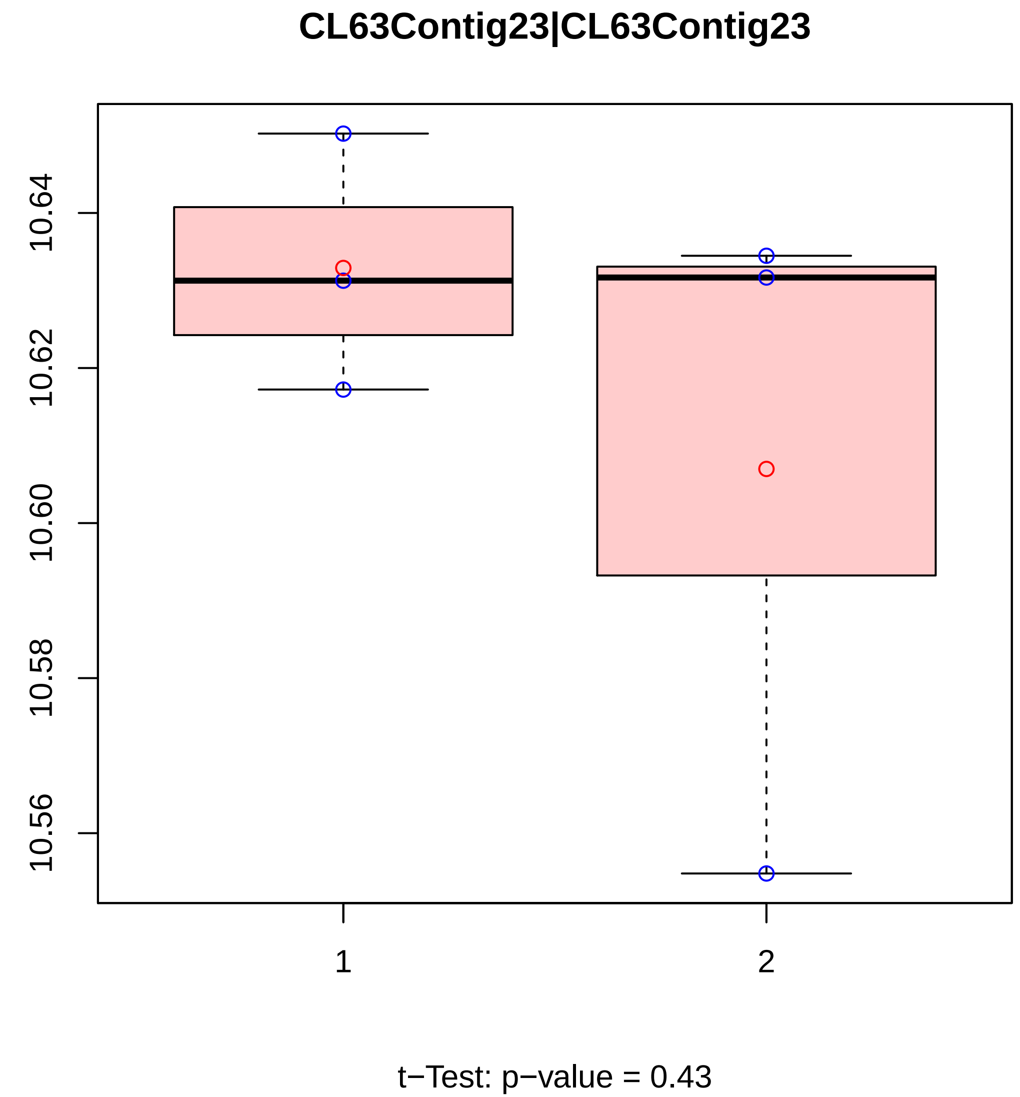
CL63Contig23|CL63Contig23
10.56
10.58
10.60
10.62
10.64
1
2
t−Test: p−value = 0.43

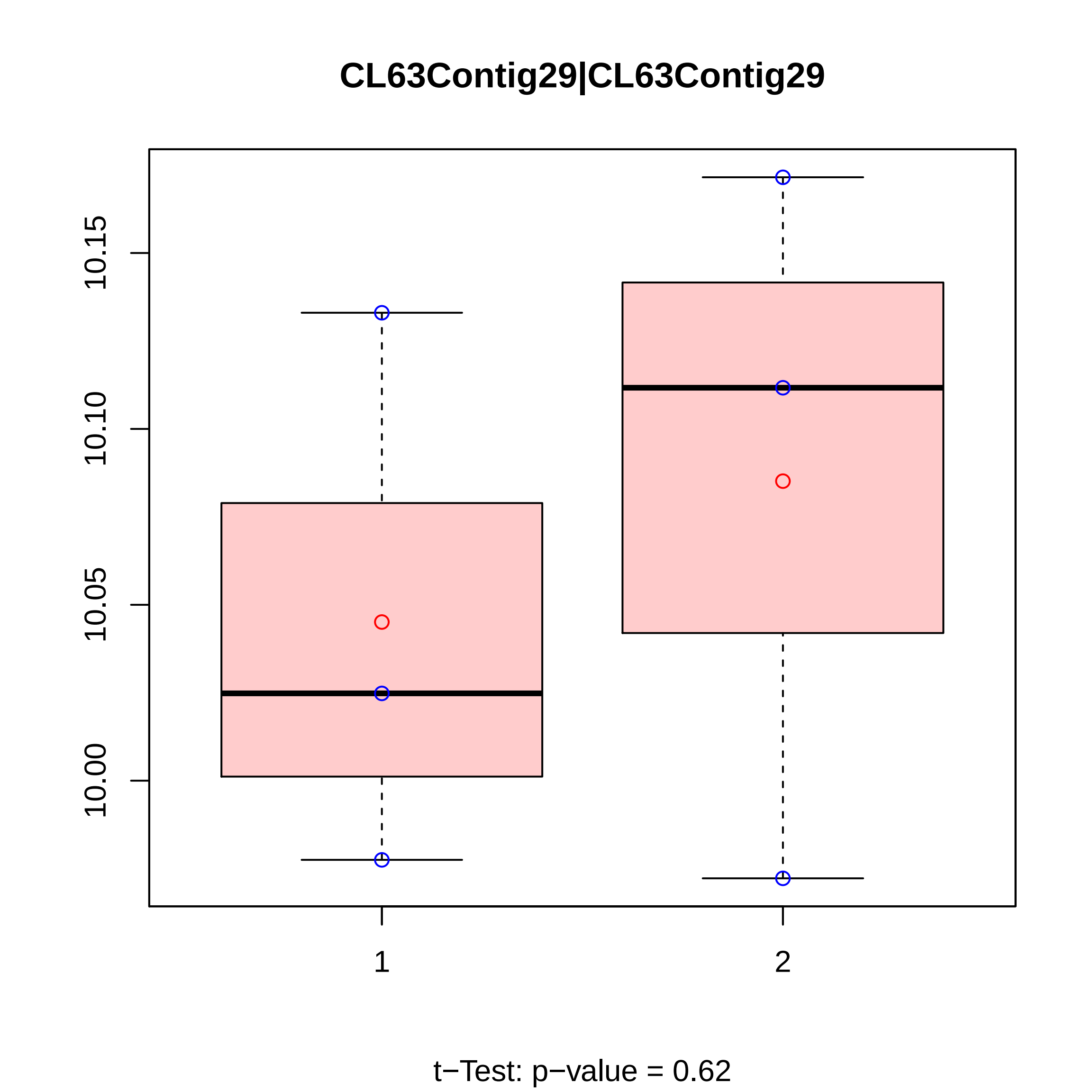

CL63Contig29|CL63Contig29
10.00
10.05
10.10
10.15
1
2
t−Test: p−value = 0.62

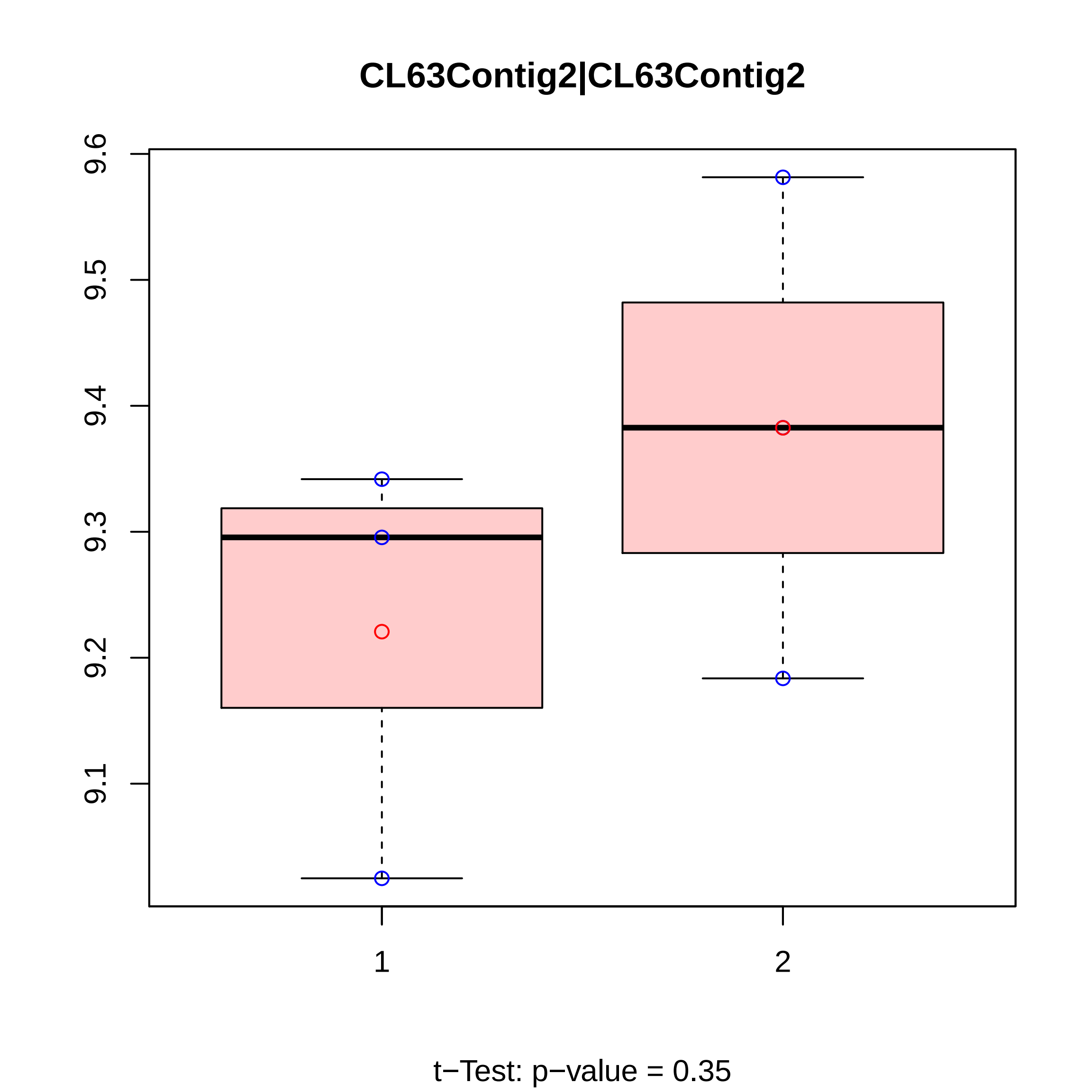

CL63Contig2|CL63Contig2
9.6
9.5
9.4
9.3
9.2
9.1
1
2
t−Test: p−value = 0.35

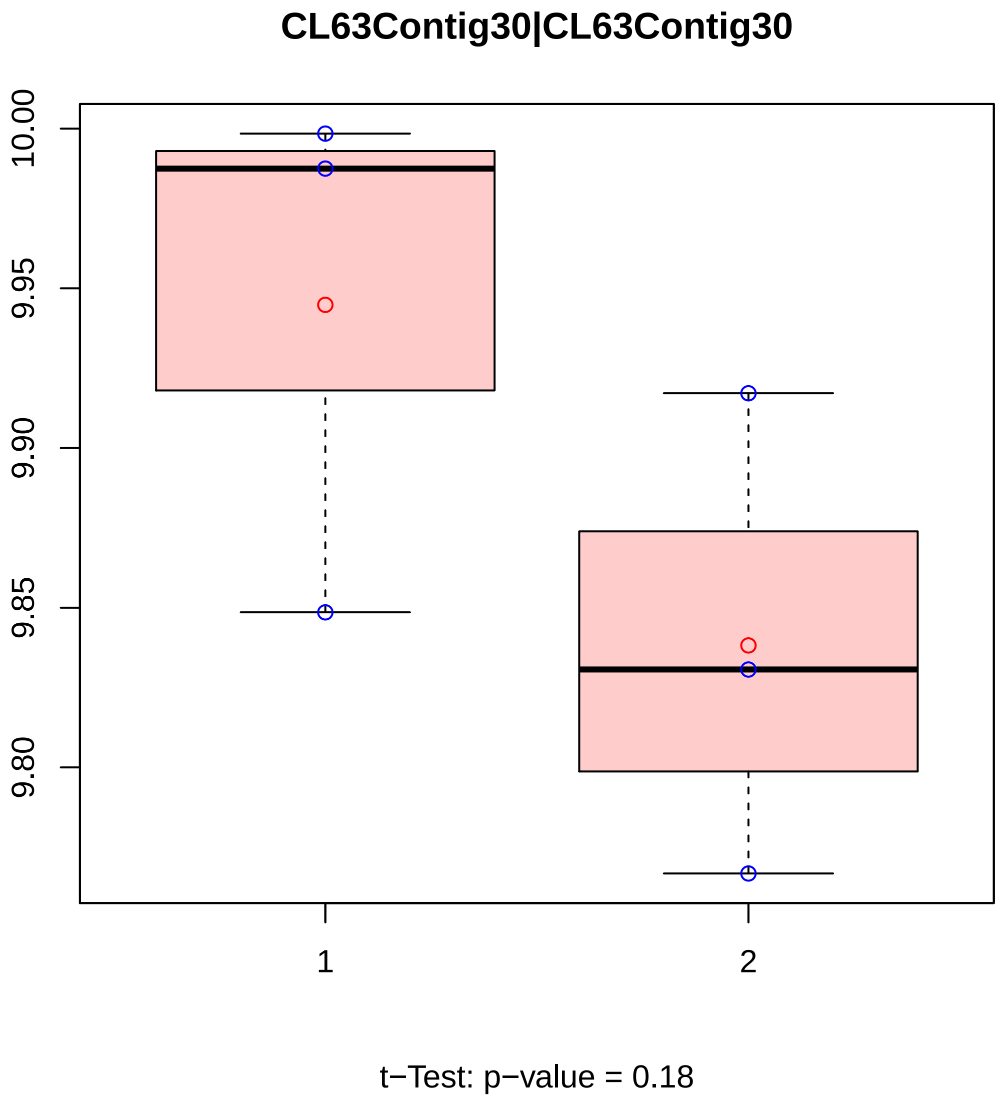
CL63Contig30|CL63Contig30
10.00
9.95
9.90
9.85
9.80
1
2
t−Test: p−value = 0.18

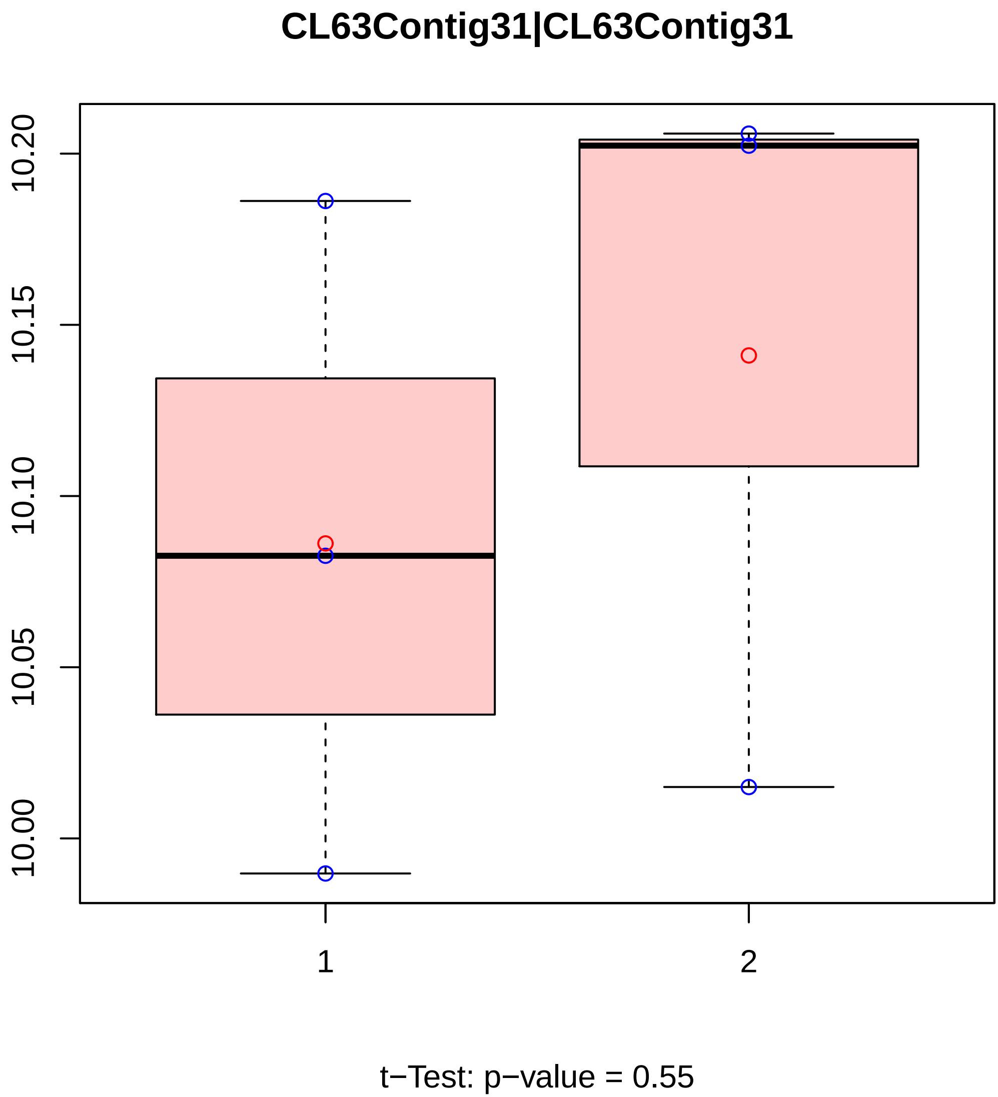

CL63Contig31|CL63Contig31
10.20
10.15
10.10
10.05
10.00
1
2
t−Test: p−value = 0.55

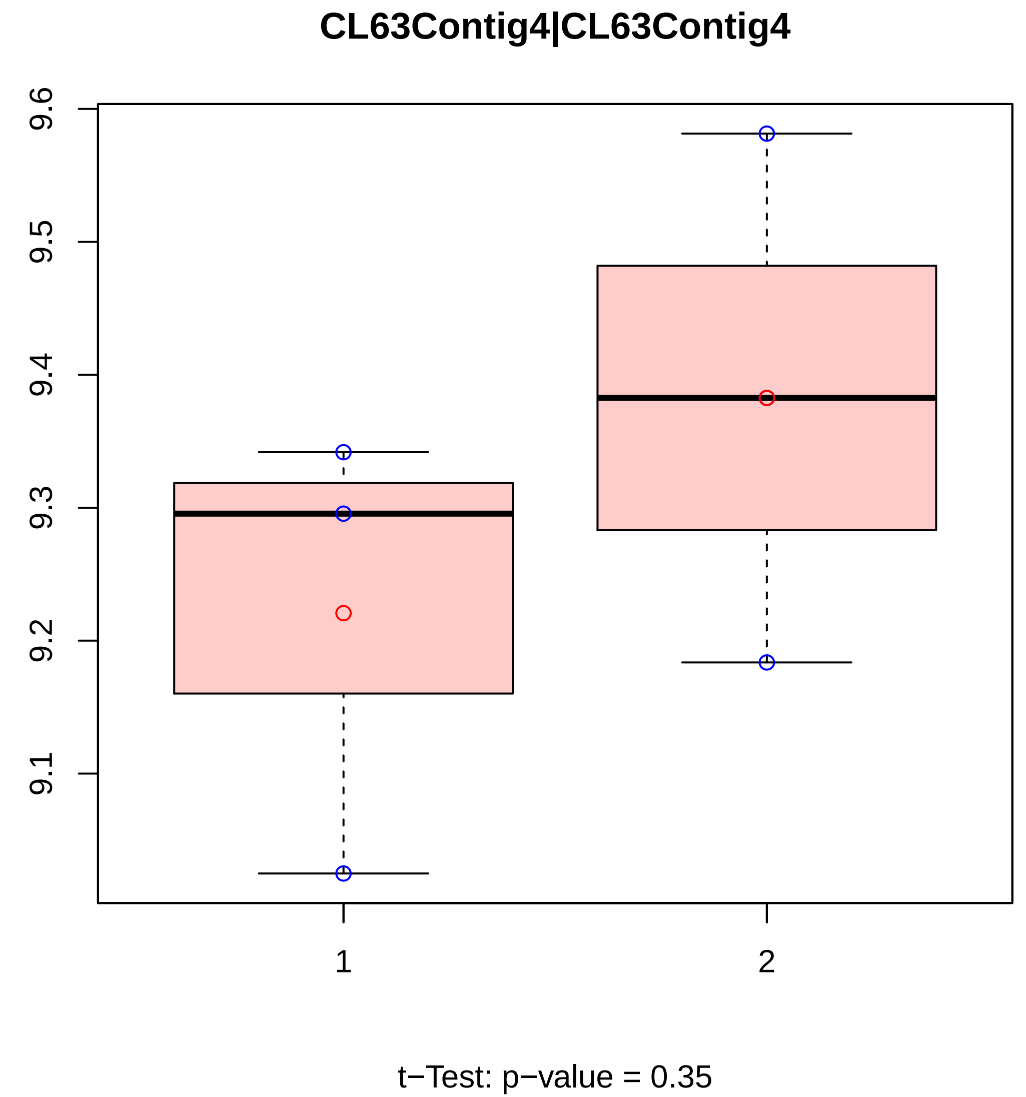
CL63Contig4|CL63Contig4
9.1
9.2
9.3
9.4
9.5
9.6
1
2
t−Test: p−value = 0.35

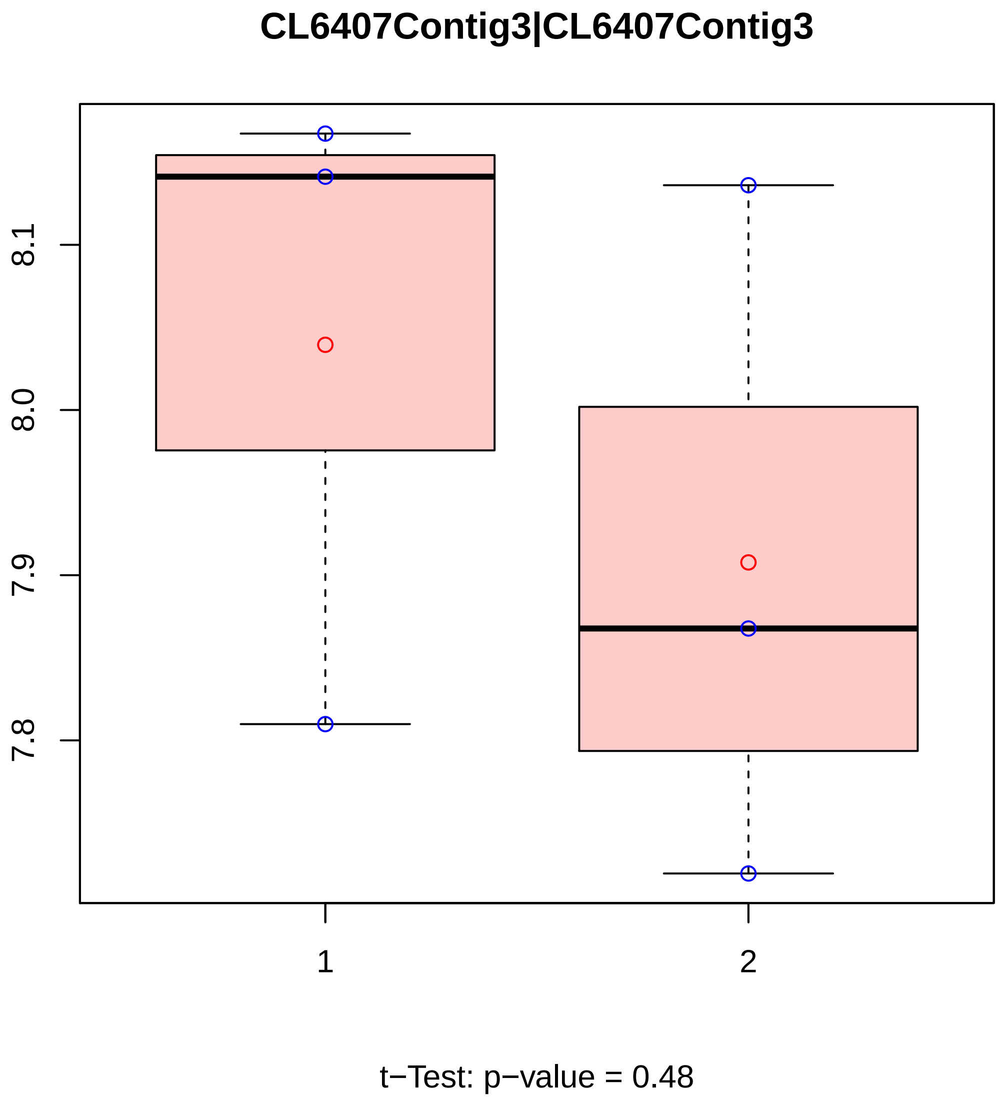

CL6407Contig3|CL6407Contig3
8.1
8.0
7.9
7.8
1
2
t−Test: p−value = 0.48

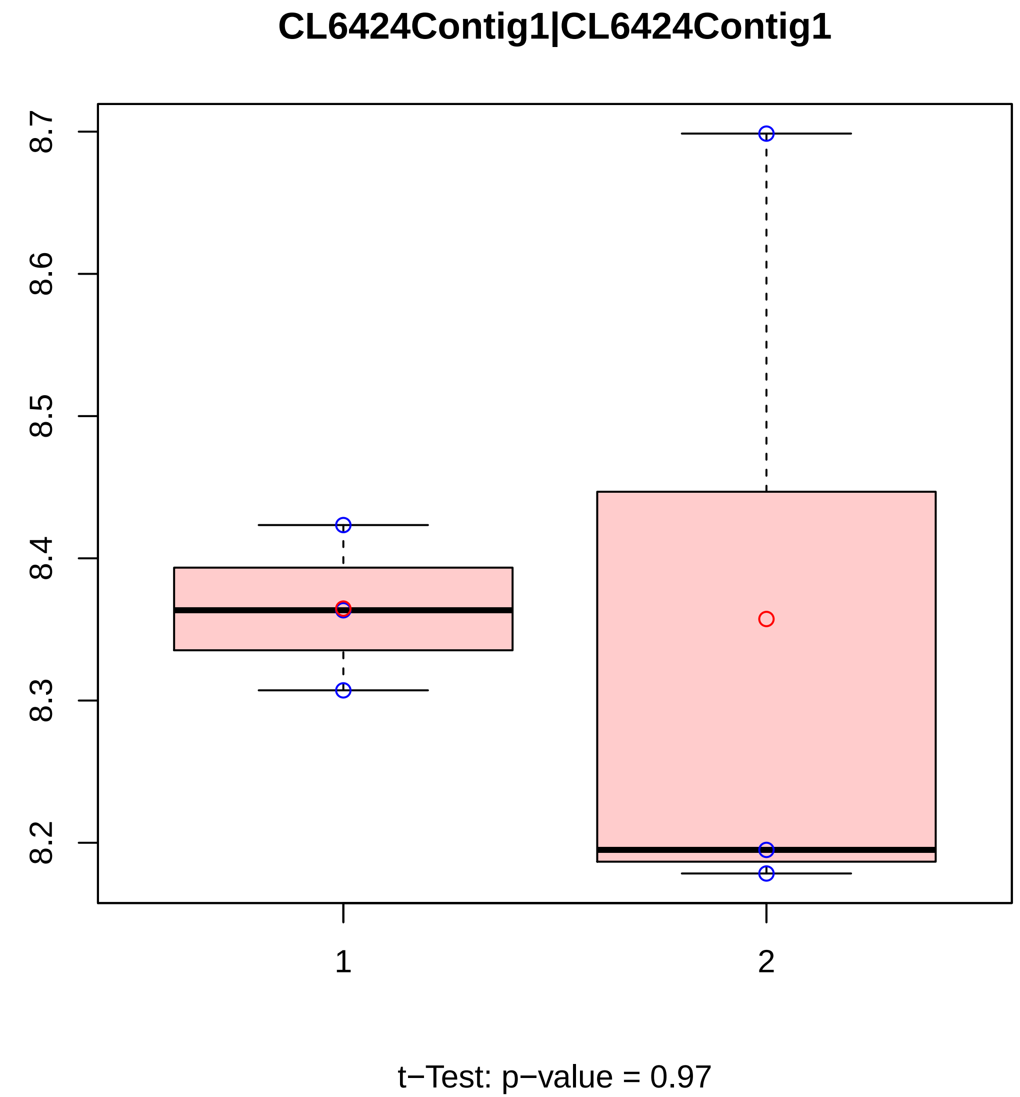
CL6424Contig1|CL6424Contig1
8.2
8.3
8.4
8.5
8.6
8.7
1
2
t−Test: p−value = 0.97

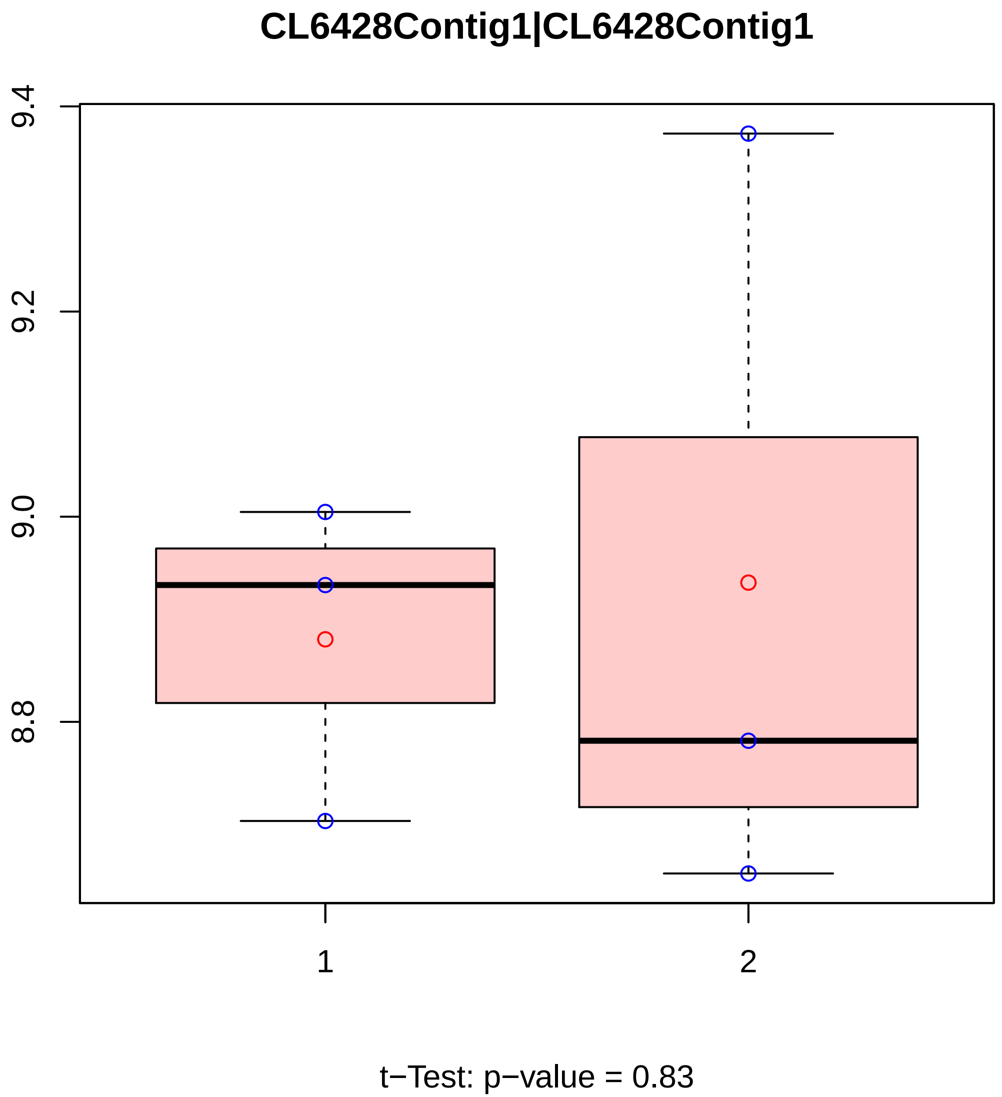

CL6428Contig1|CL6428Contig1
9.4
9.2
9.0
8.8
1
2
t−Test: p−value = 0.83

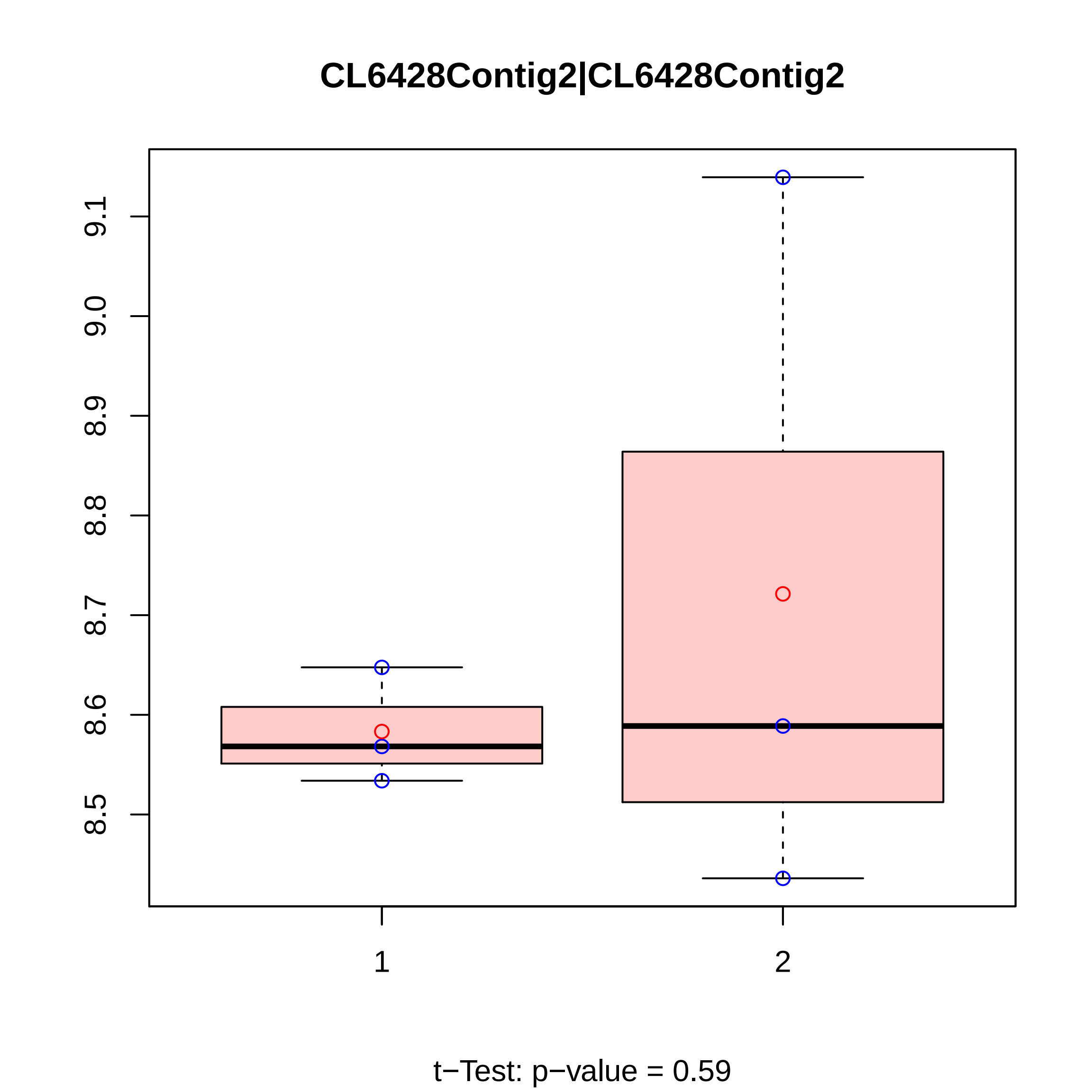
CL6428Contig2|CL6428Contig2
9.1
9.0
8.9
8.8
8.7
8.6
8.5
1
2
t−Test: p−value = 0.59

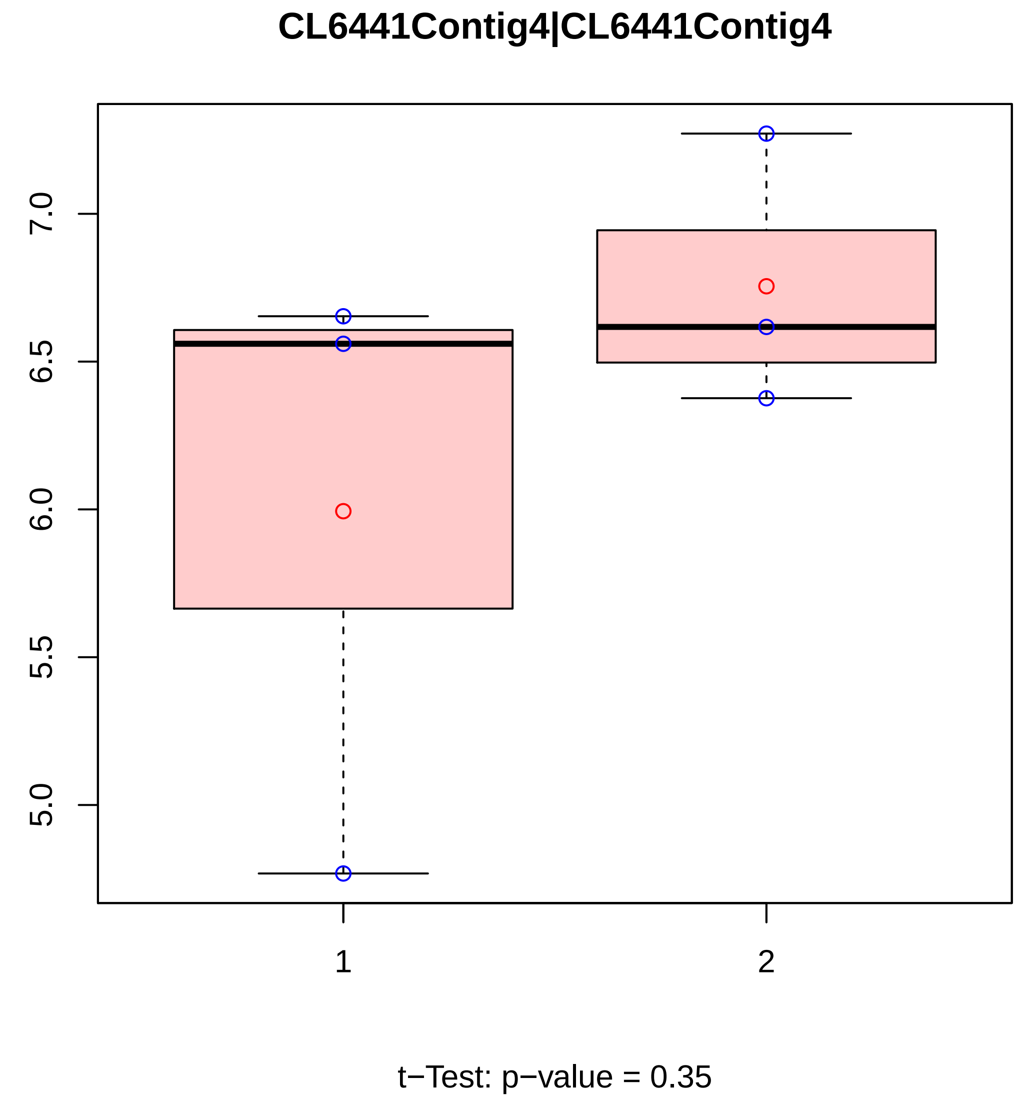

CL6441Contig4|CL6441Contig4
7.0
6.5
6.0
5.5
5.0
1
2
t−Test: p−value = 0.35

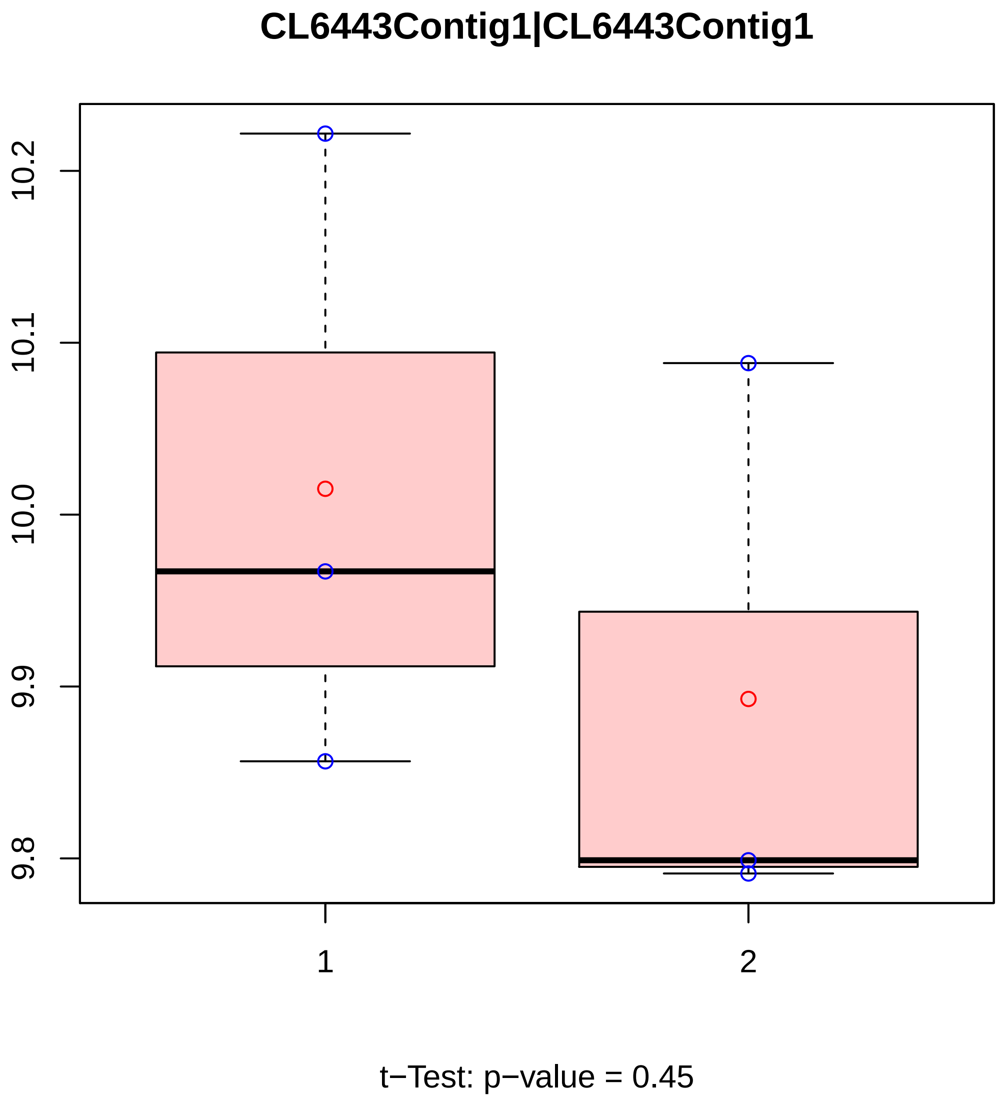
CL6443Contig1|CL6443Contig1
10.2
10.1
10.0
9.9
9.8
1
2
t−Test: p−value = 0.45

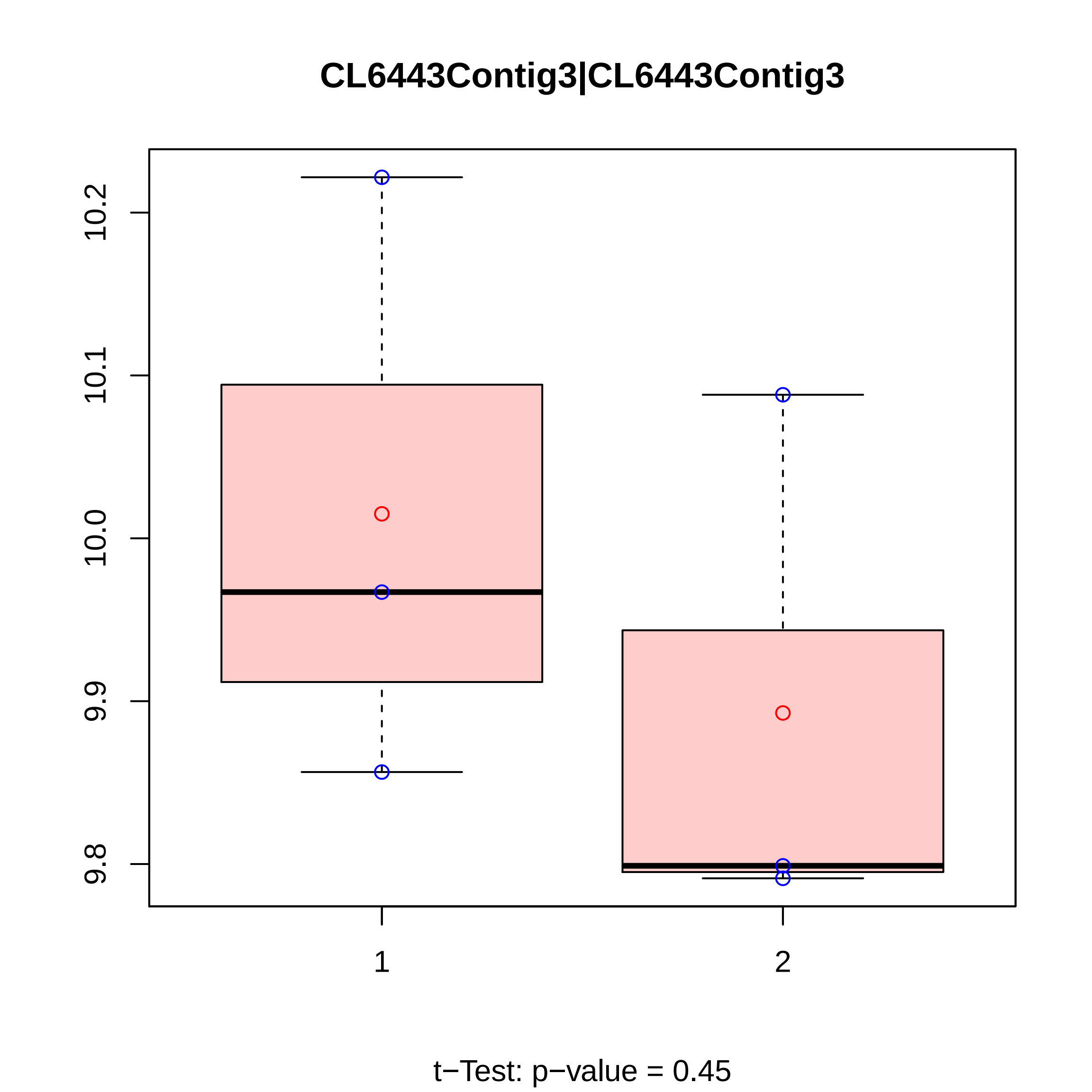

CL6443Contig3|CL6443Contig3
10.2
10.1
10.0
9.9
9.8
1
2
t−Test: p−value = 0.45

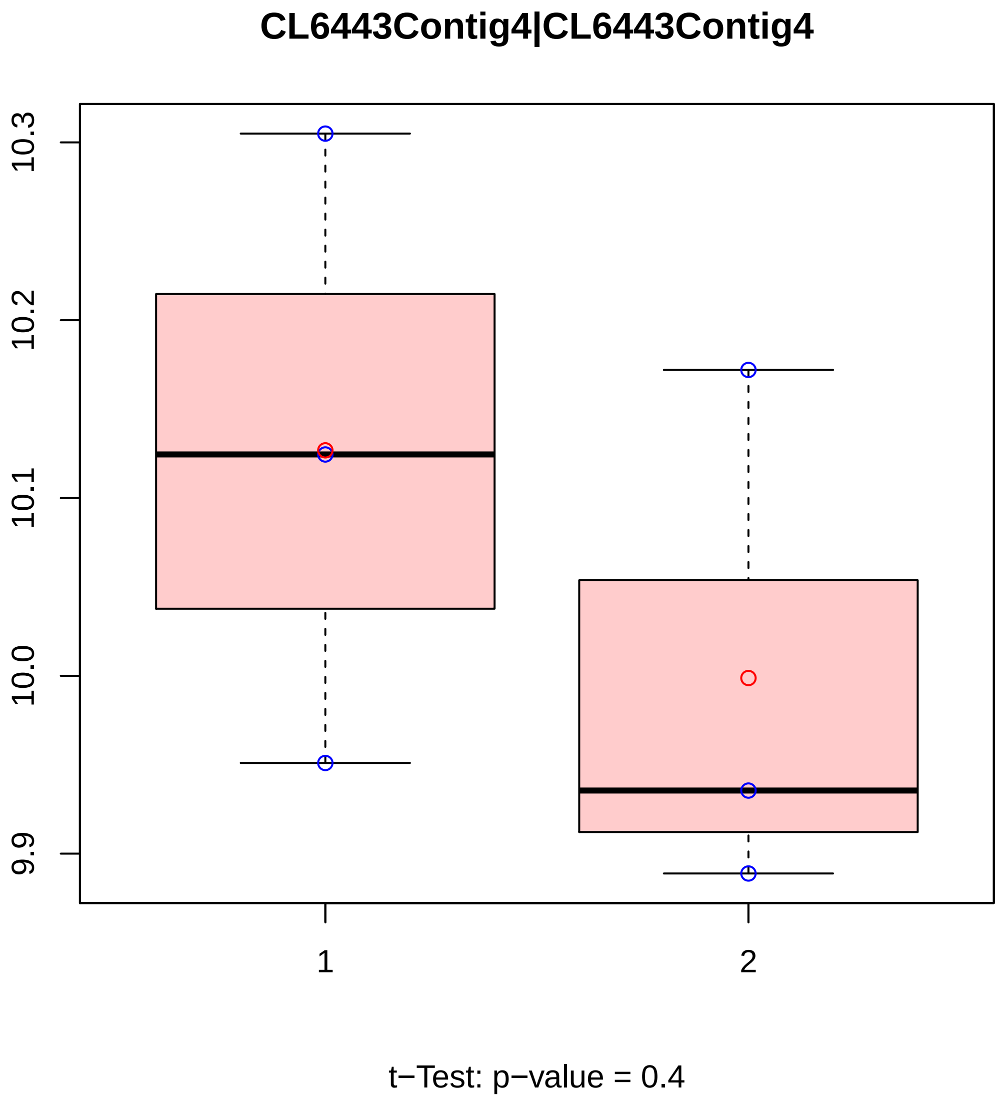

CL6443Contig4|CL6443Contig4
10.3
10.2
10.1
10.0
9.9
1
2
t−Test: p−value = 0.4

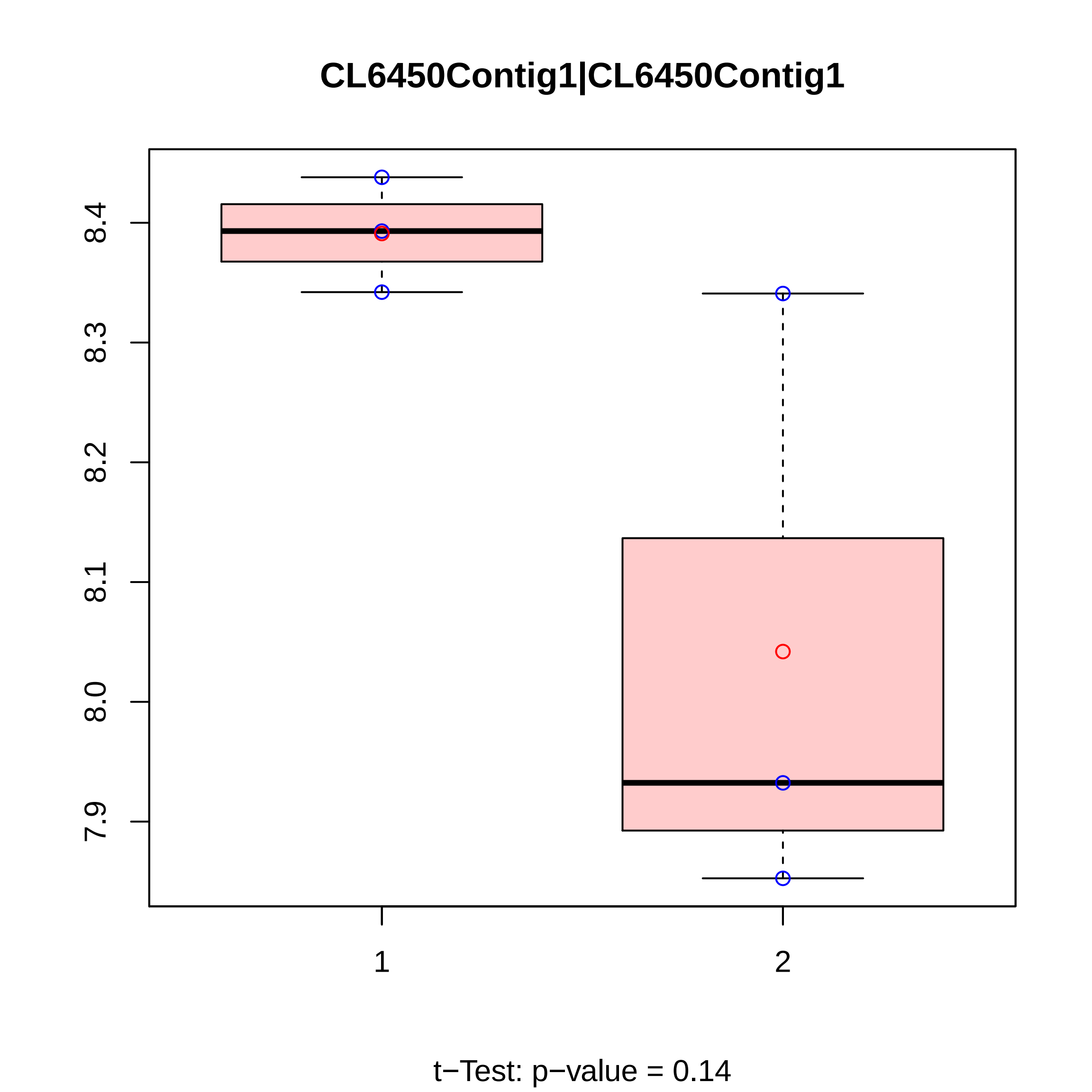

CL6450Contig1|CL6450Contig1
8.4
8.3
8.2
8.1
8.0
7.9
1
2
t−Test: p−value = 0.14

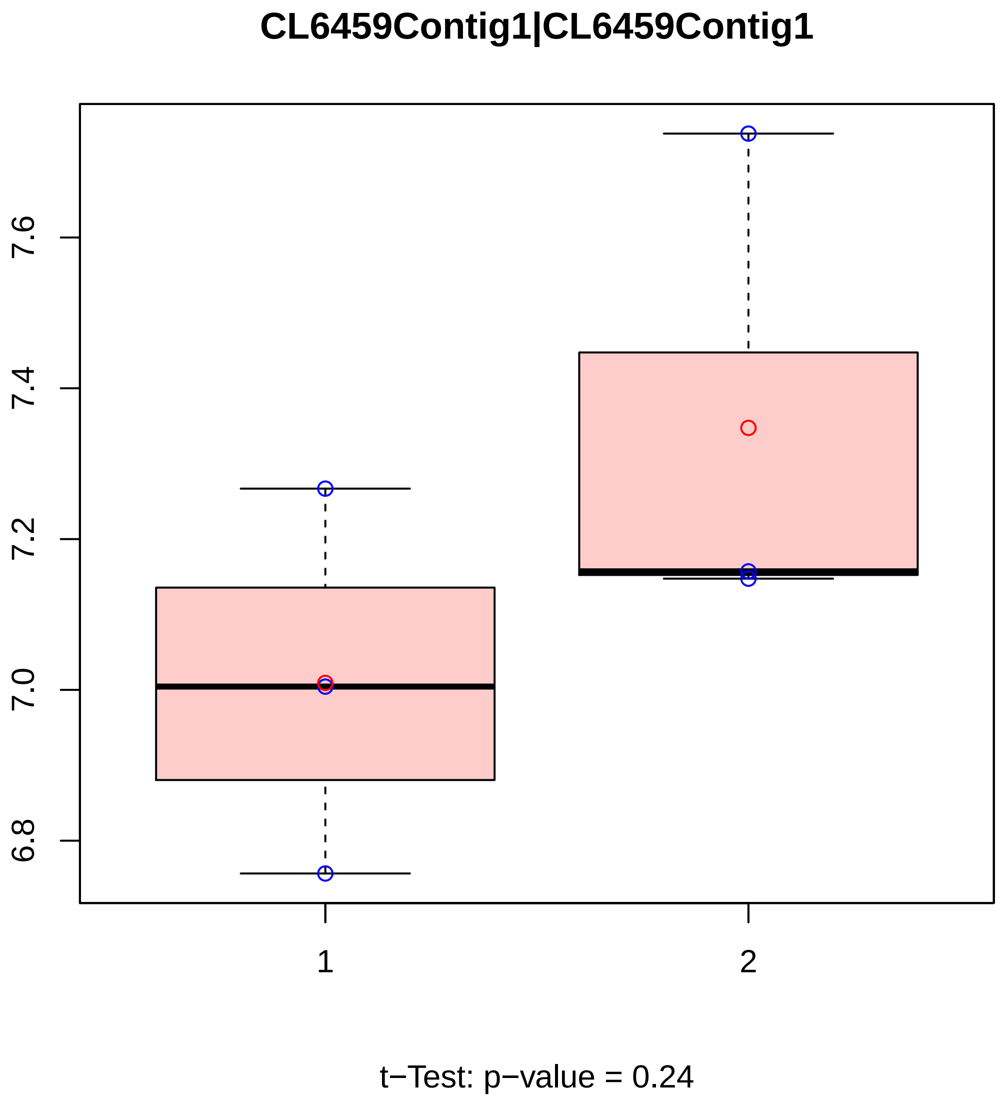
CL6459Contig1|CL6459Contig1
6.8
7.0
7.2
7.4
7.6
1
2
t−Test: p−value = 0.24

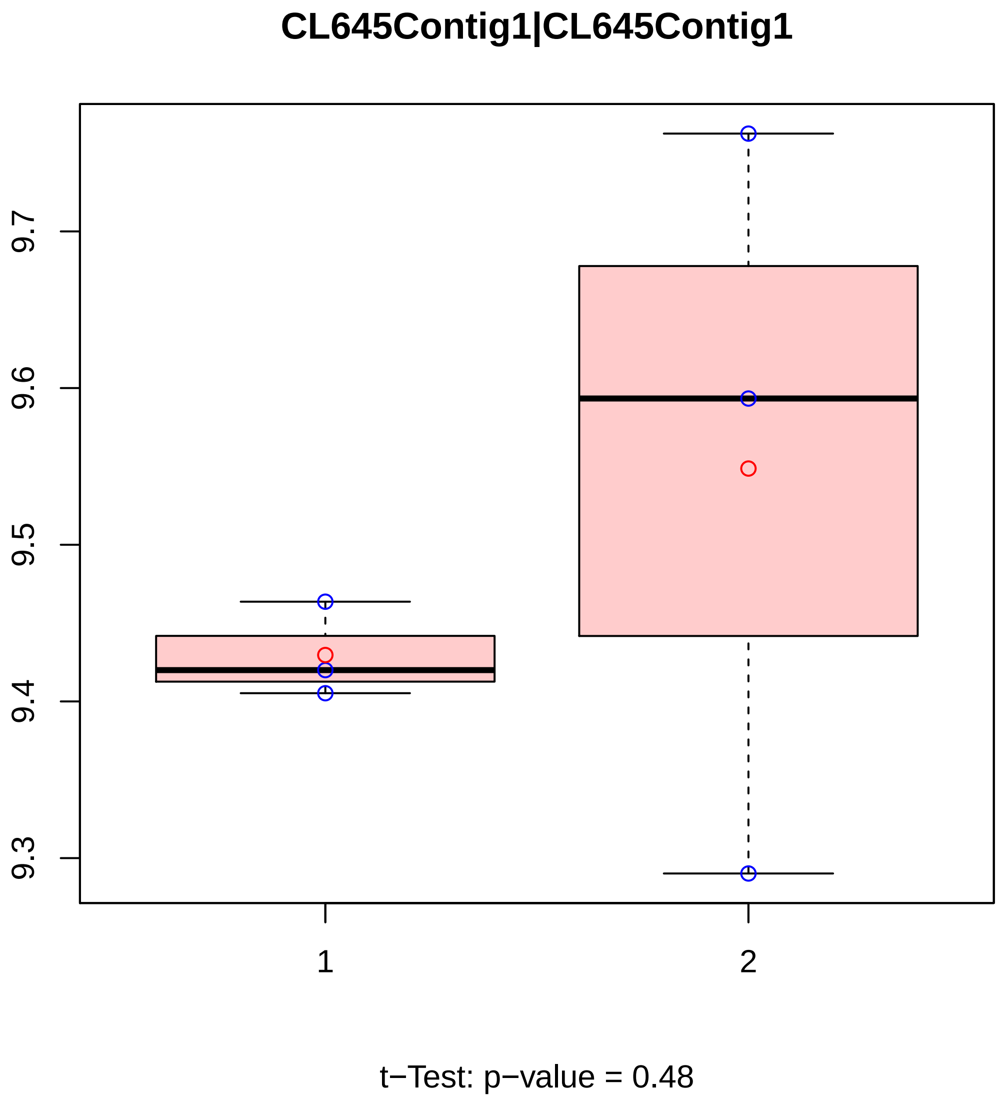
CL645Contig1|CL645Contig1
9.3
9.4
9.5
9.6
9.7
1
2
t−Test: p−value = 0.48

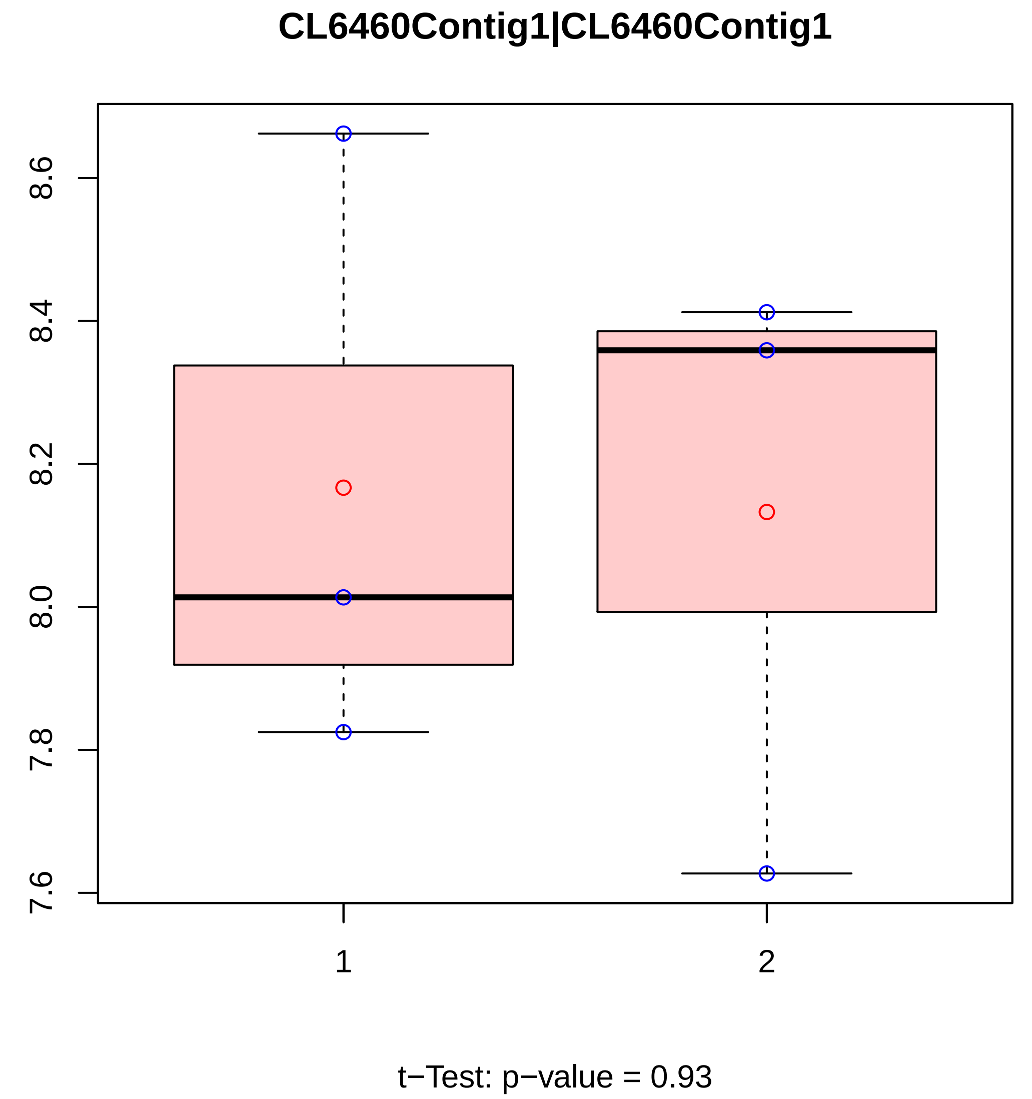

CL6460Contig1|CL6460Contig1
8.6
8.4
8.2
8.0
7.8
7.6
1
2
t−Test: p−value = 0.93

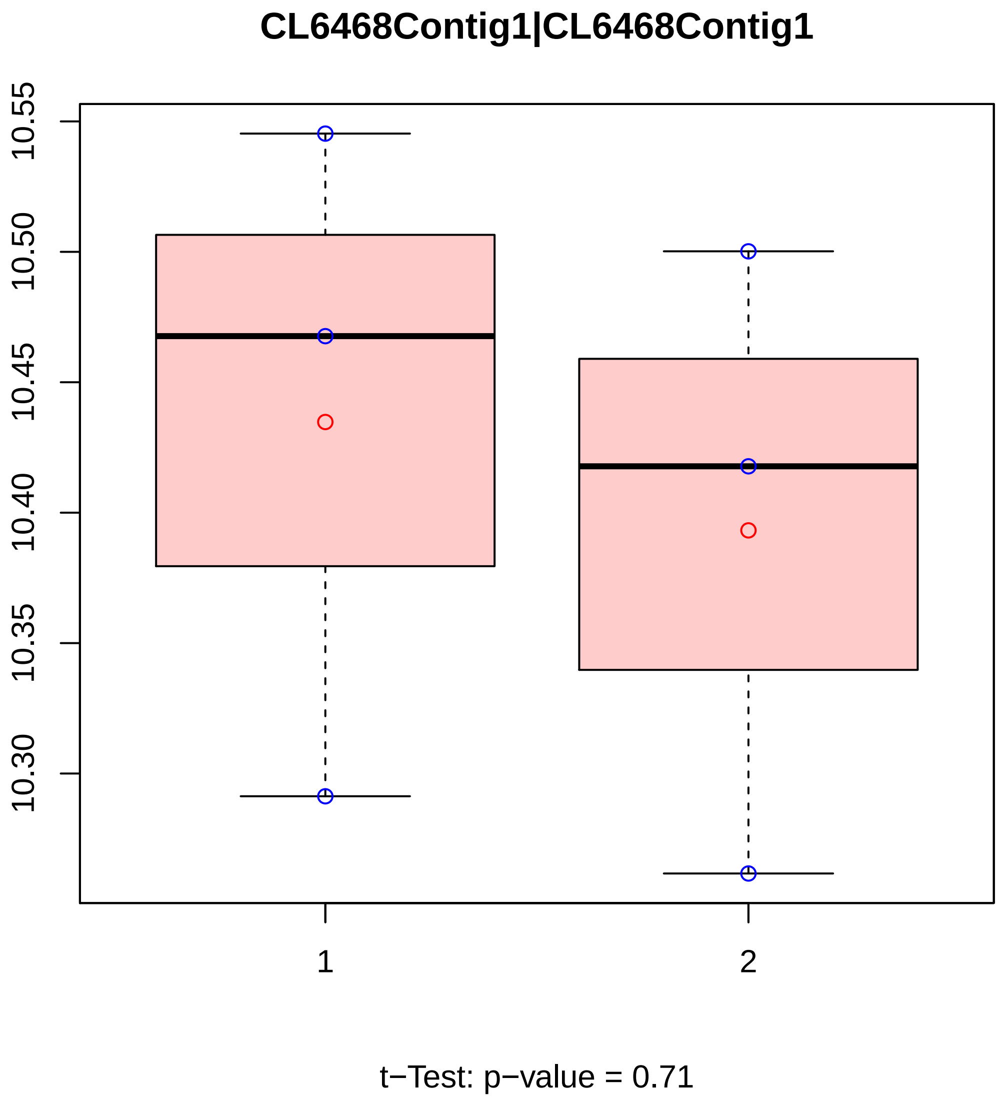
CL6468Contig1|CL6468Contig1
10.30
10.35
10.40
10.45
10.50
10.55
1
2
t−Test: p−value = 0.71

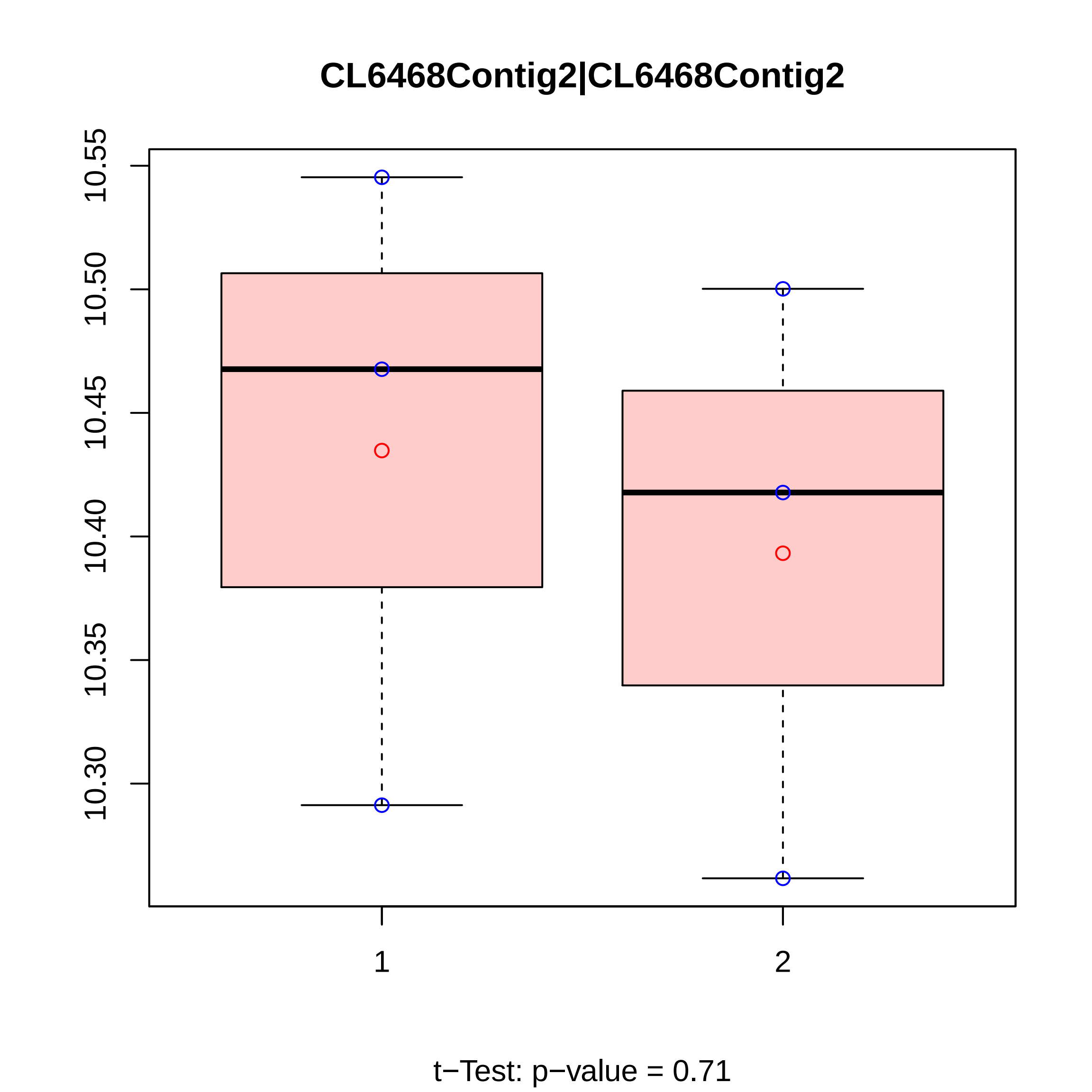

CL6468Contig2|CL6468Contig2
10.55
10.50
10.45
10.40
10.35
10.30
1
2
t−Test: p−value = 0.71

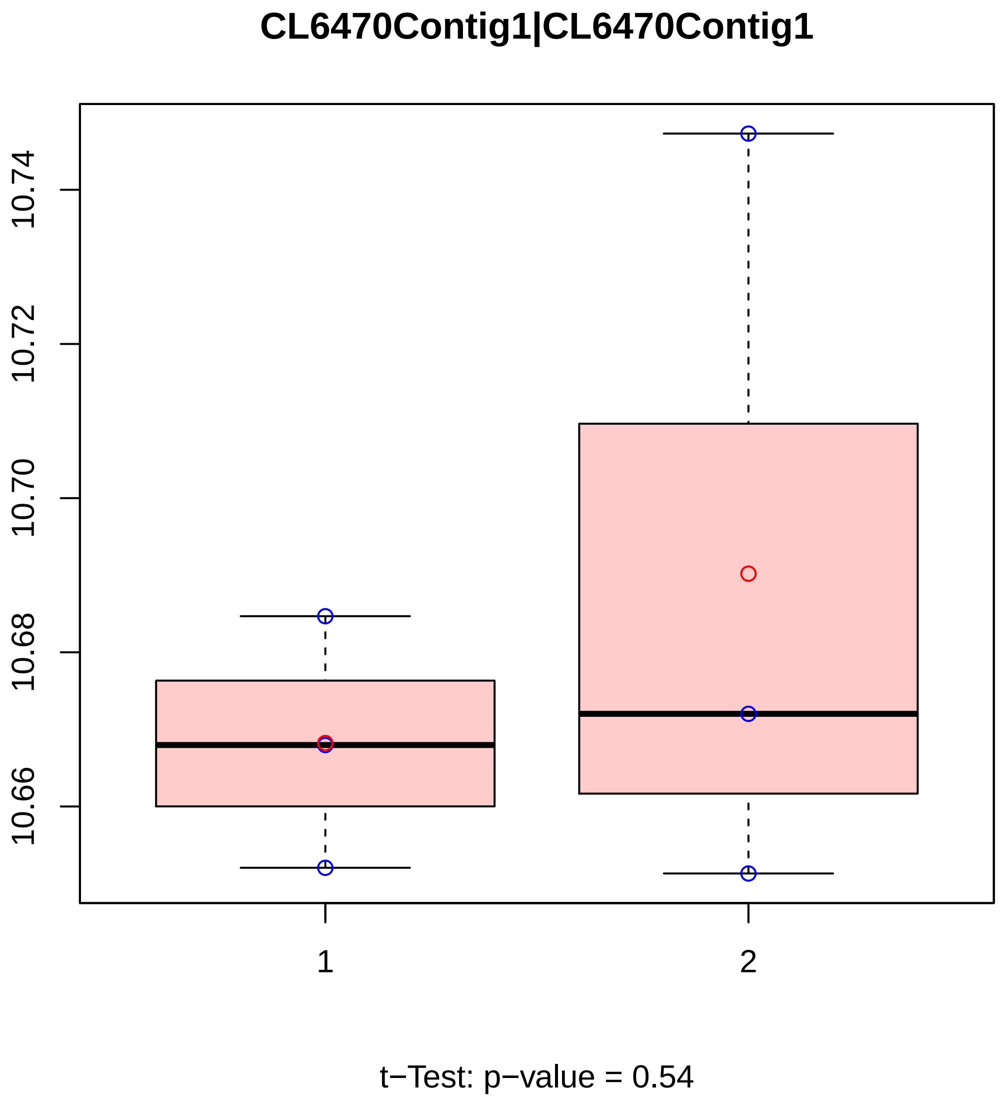
CL6470Contig1|CL6470Contig1
10.66
10.68
10.70
10.72
10.74
1
2
t−Test: p−value = 0.54

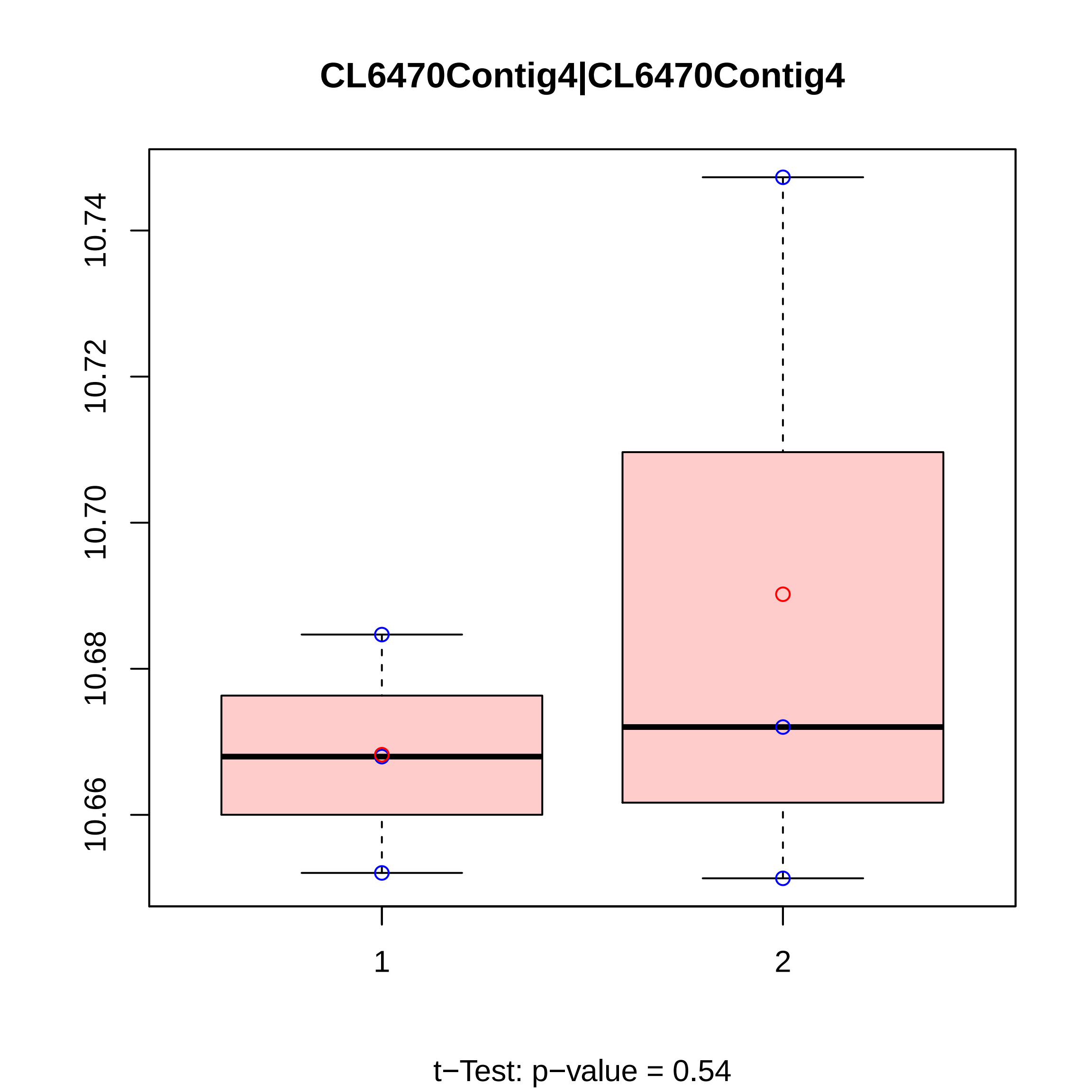
CL6470Contig4|CL6470Contig4
10.66
10.68
10.70
10.72
10.74
1
2
t−Test: p−value = 0.54

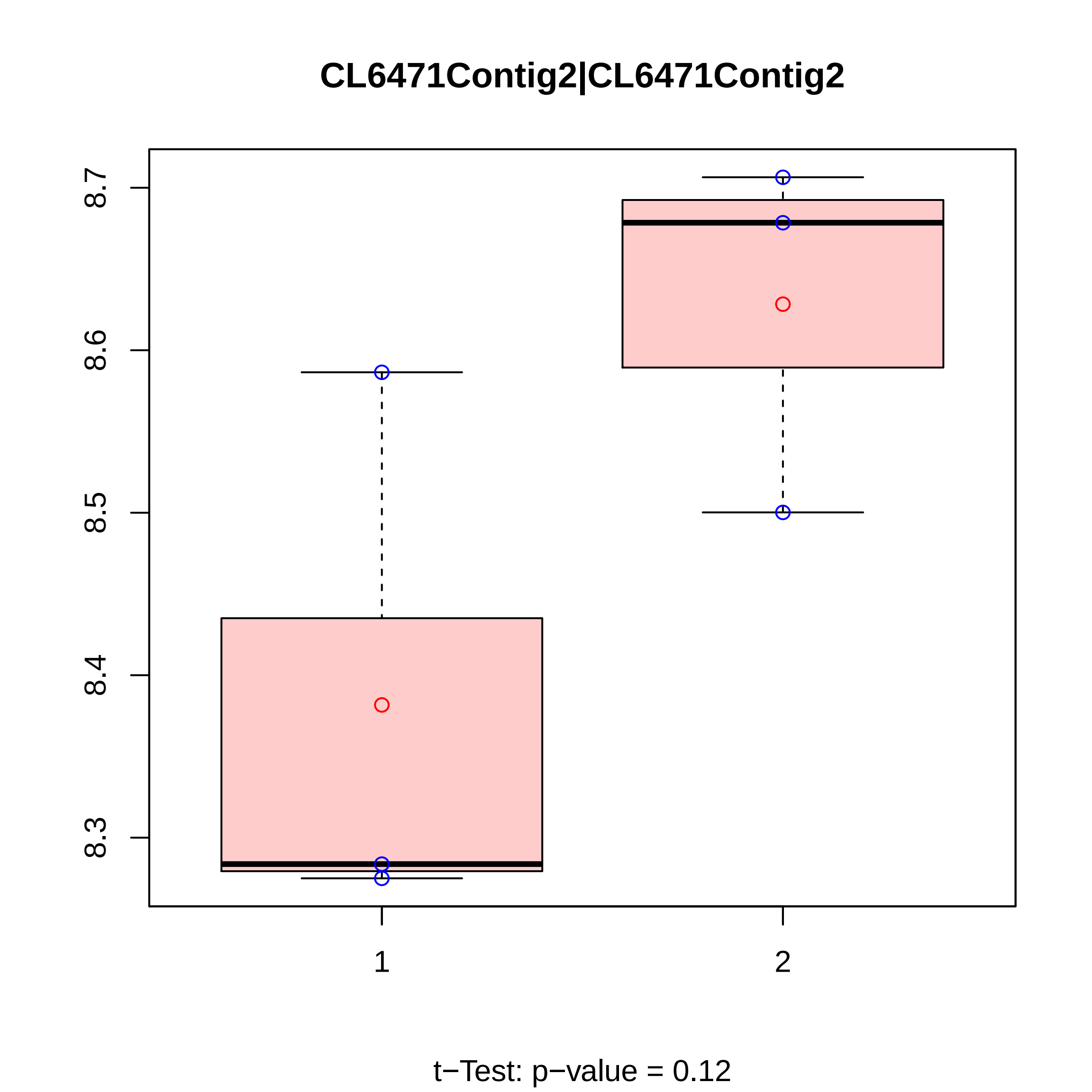
CL6471Contig2|CL6471Contig2
8.3
8.4
8.5
8.6
8.7
1
2
t−Test: p−value = 0.12

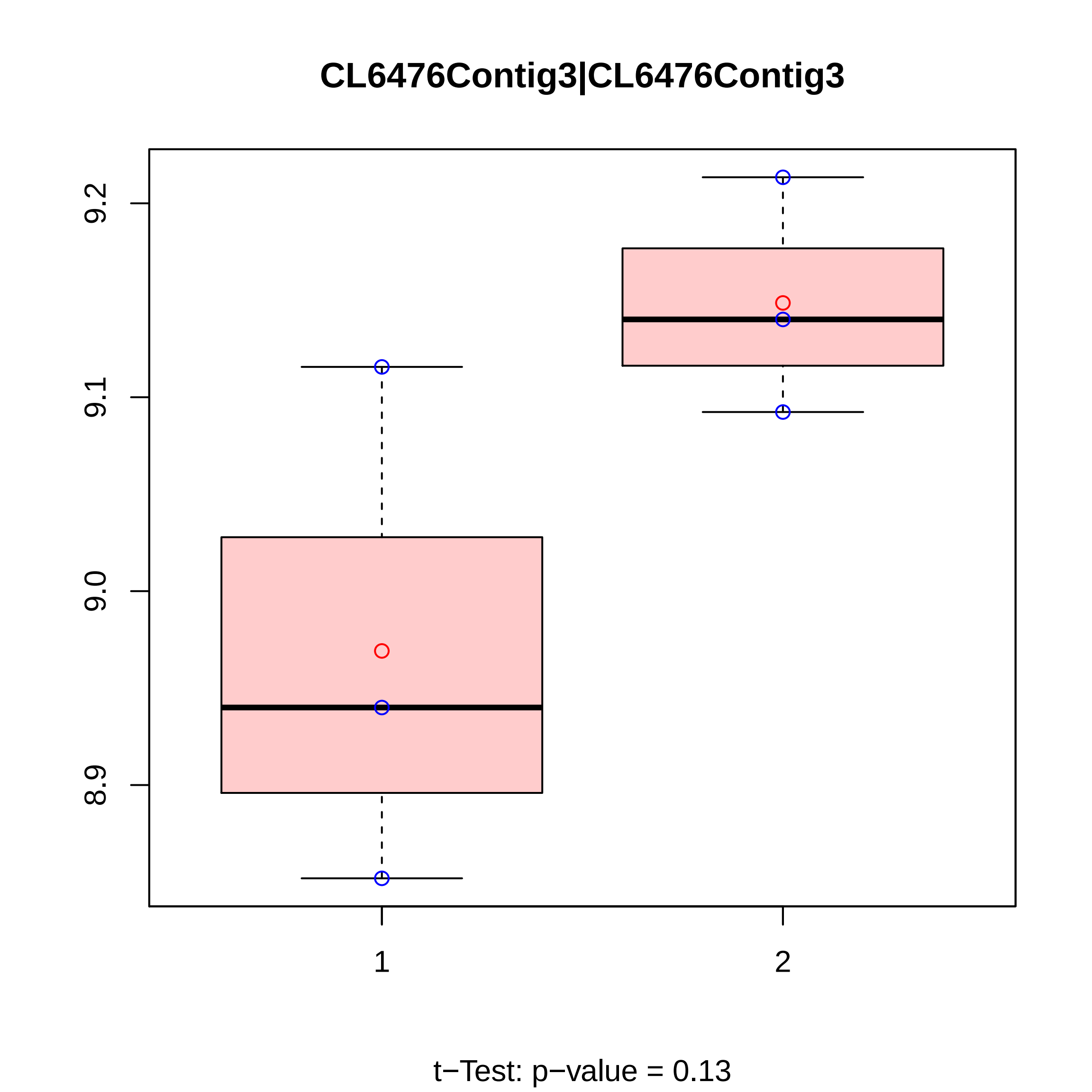
CL6476Contig3|CL6476Contig3
9.2
9.1
9.0
8.9
1
2
t−Test: p−value = 0.13

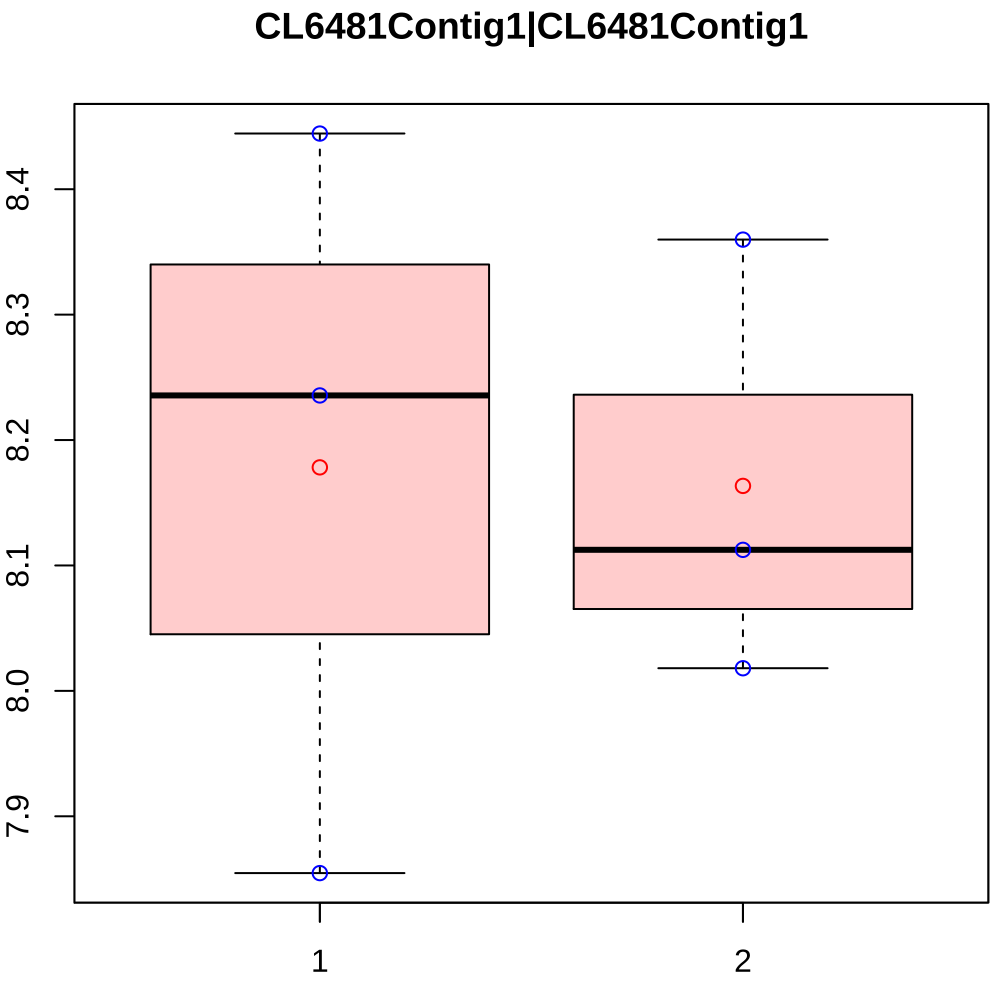


t−Test: p−value = 0.95

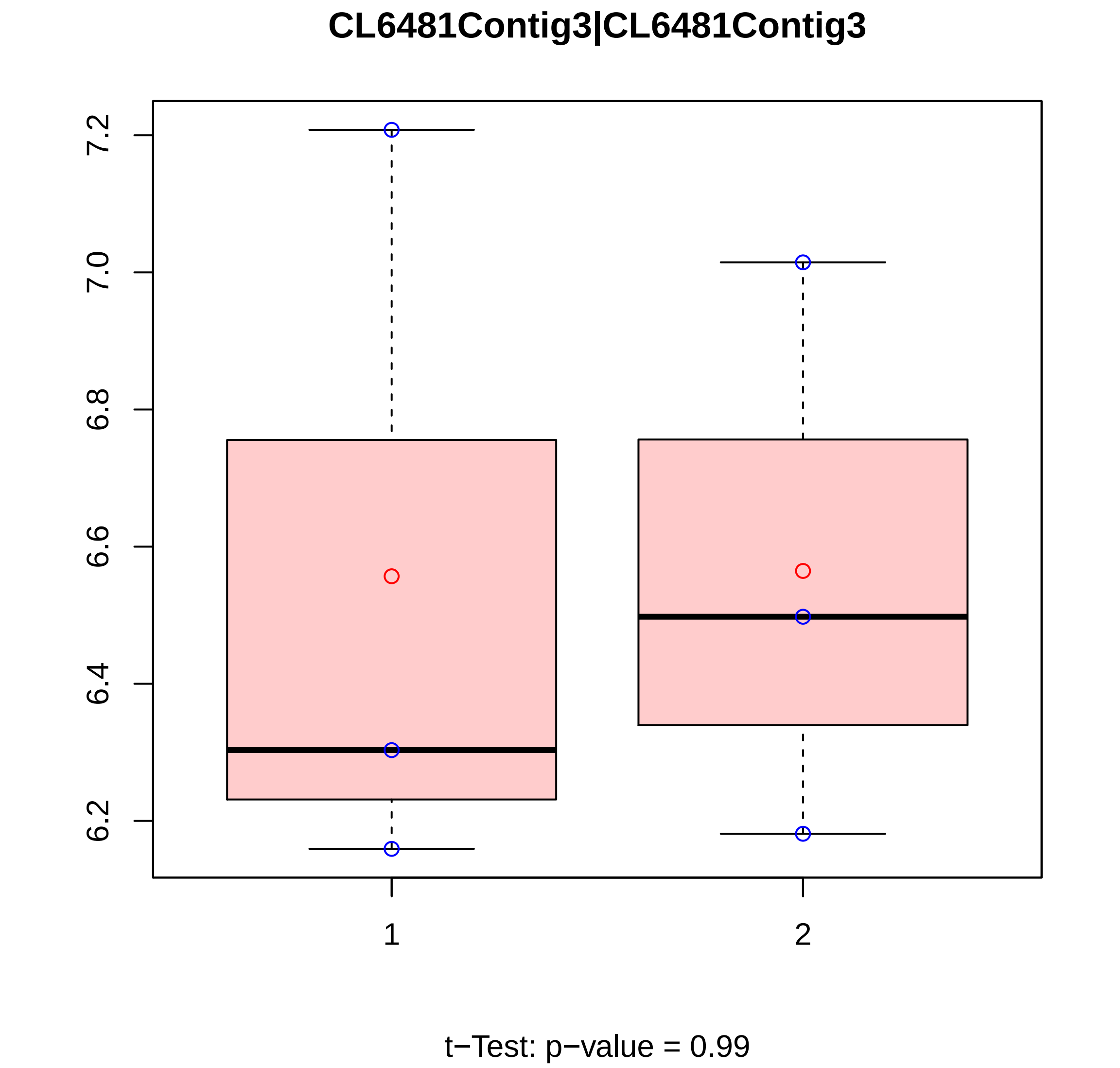
CL6481Contig3|CL6481Contig3
7.2
7.0
6.8
6.6
6.4
6.2
1
2
t−Test: p−value = 0.99

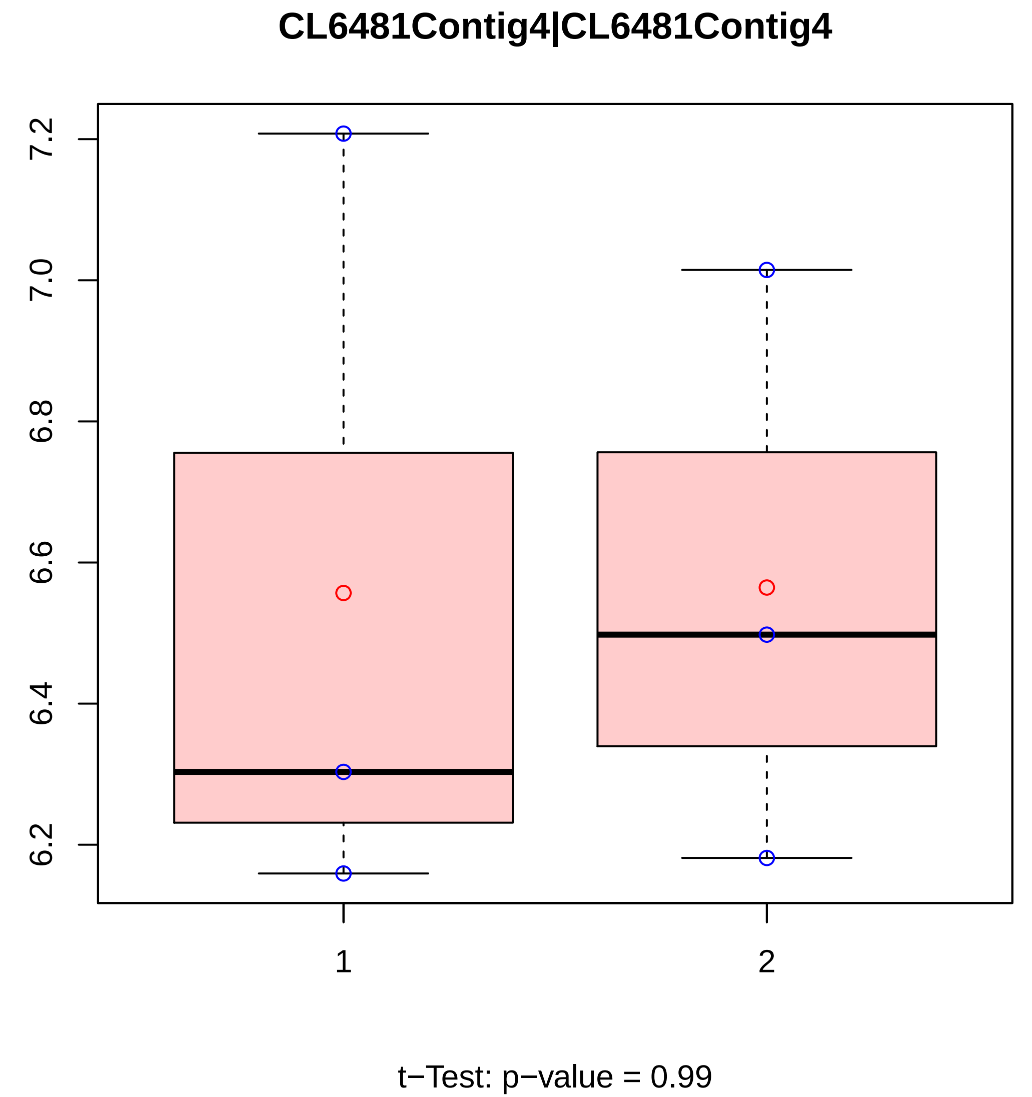

CL6481Contig4|CL6481Contig4
7.2
7.0
6.8
6.6
6.4
6.2
1
2
t−Test: p−value = 0.99

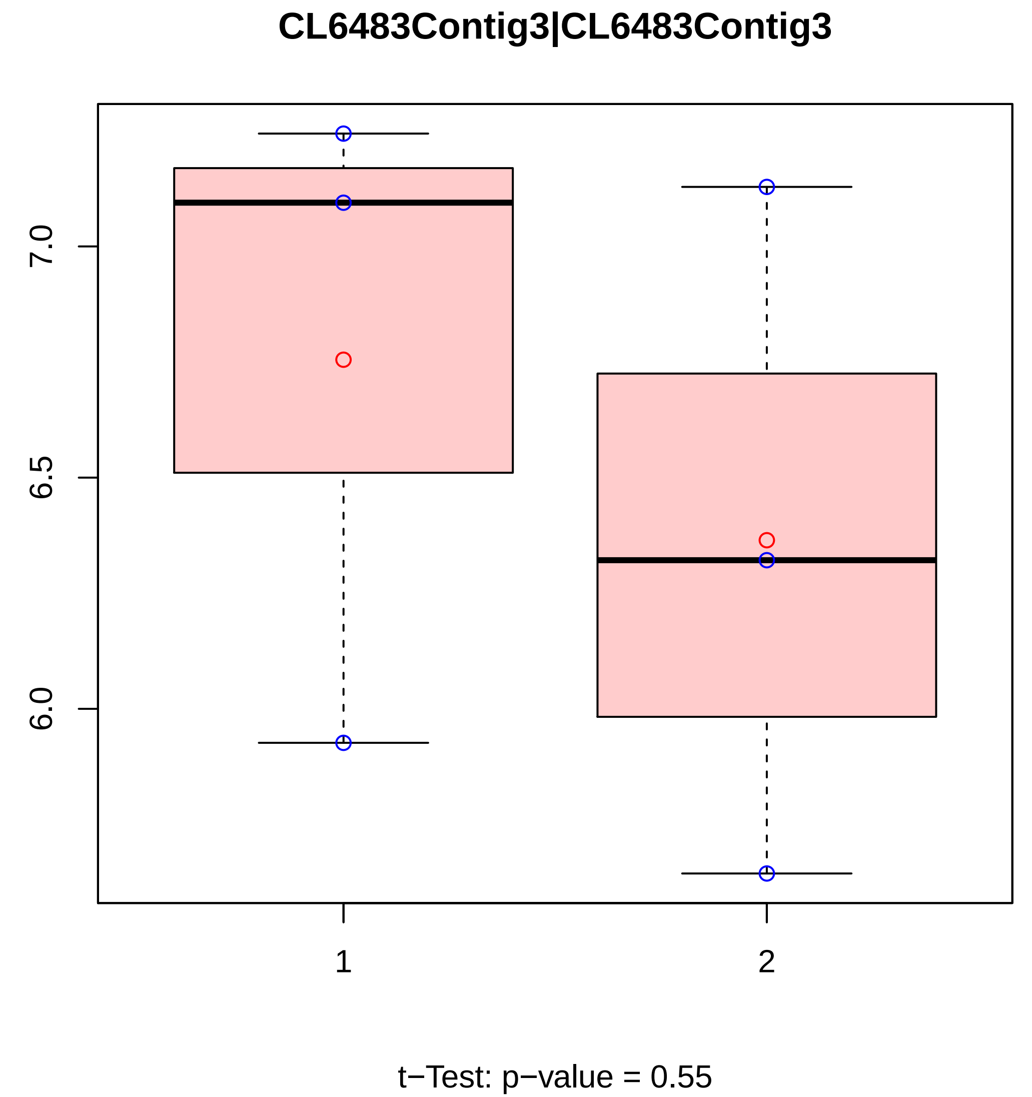
CL6483Contig3|CL6483Contig3
7.0
6.5
6.0
1
2
t−Test: p−value = 0.55

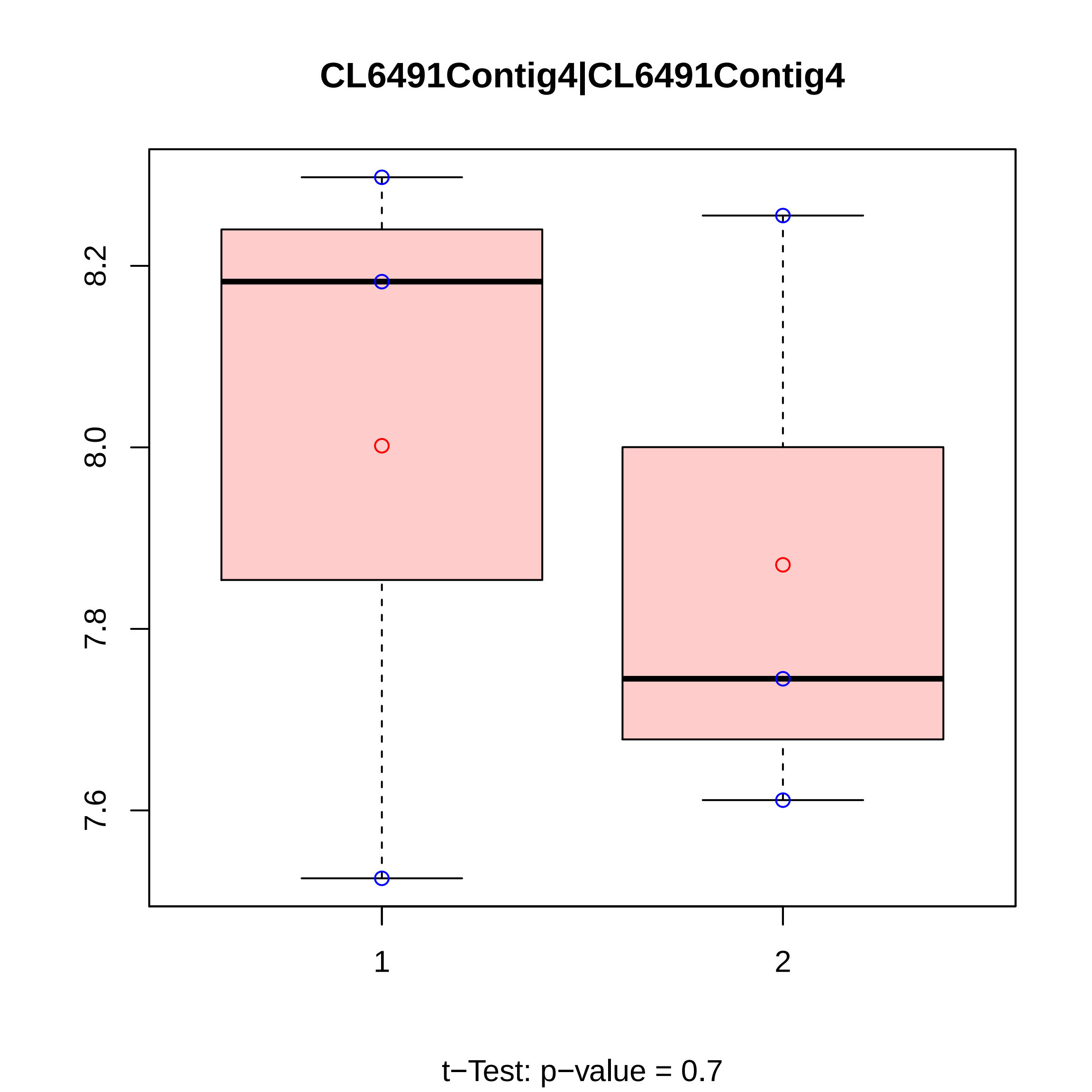
CL6491Contig4|CL6491Contig4
8.2
8.0
7.8
7.6
1
2
t−Test: p−value = 0.7

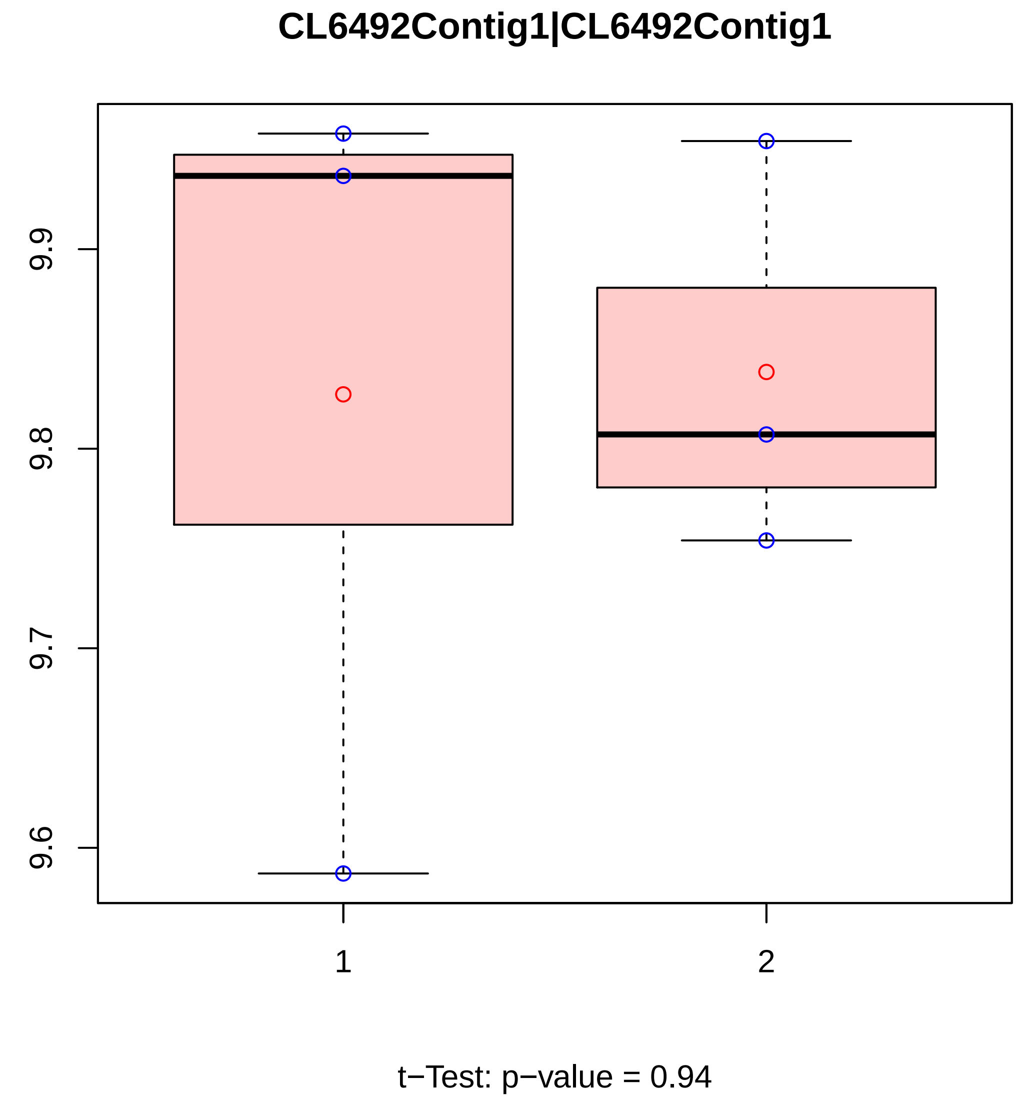
CL6492Contig1|CL6492Contig1
9.6
9.7
9.8
9.9
1
2
t−Test: p−value = 0.94

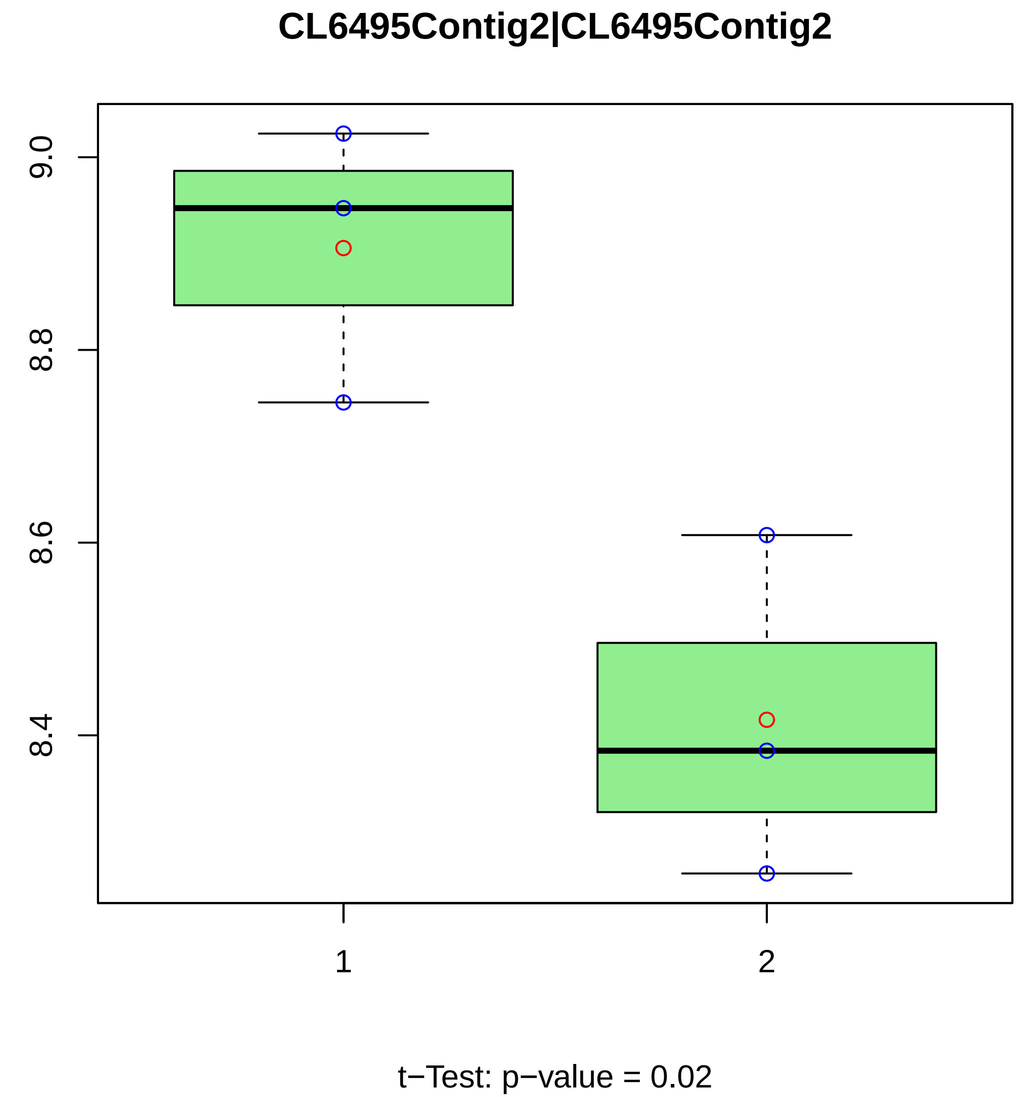
CL6495Contig2|CL6495Contig2
9.0
8.8
8.6
8.4
1
2
t−Test: p−value = 0.02

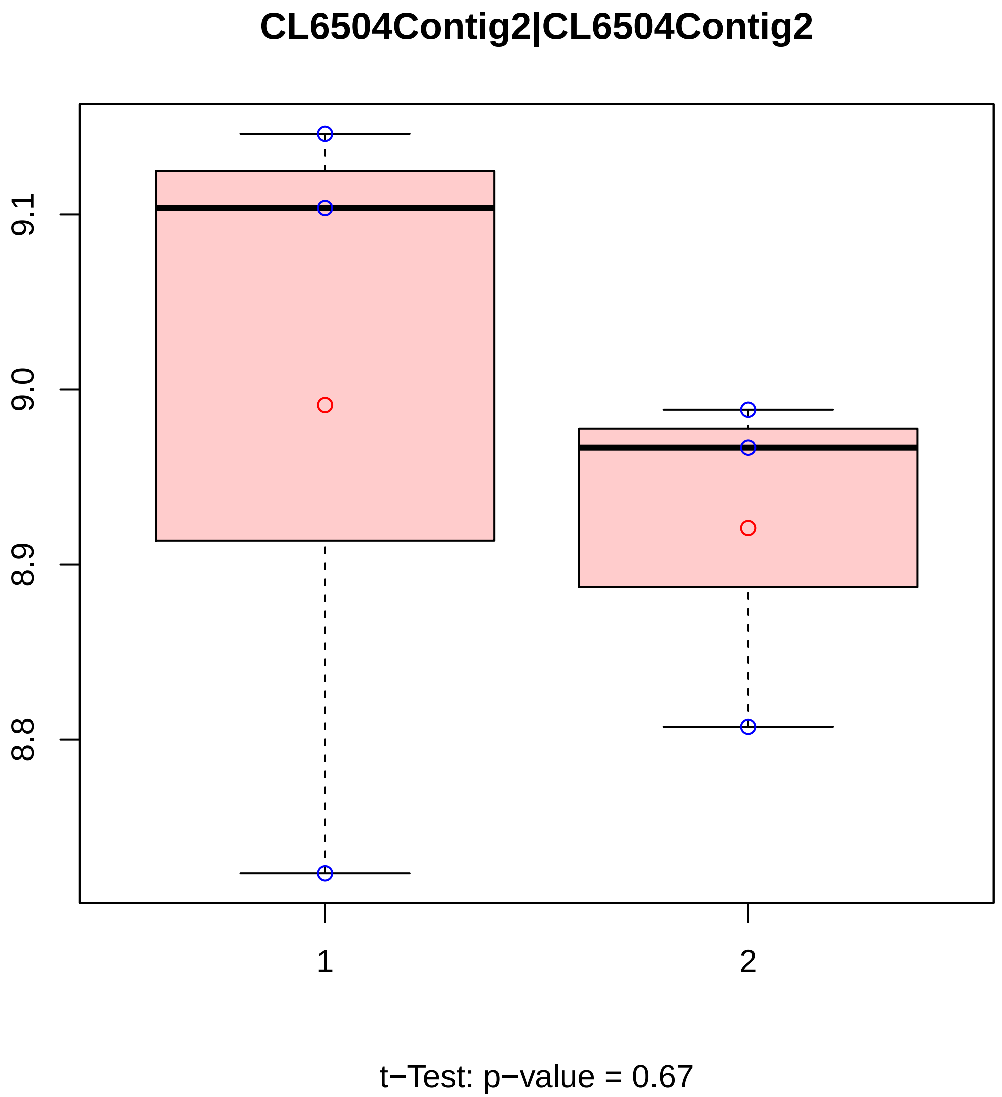

CL6504Contig2|CL6504Contig2
9.1
9.0
8.9
8.8
1
2
t−Test: p−value = 0.67

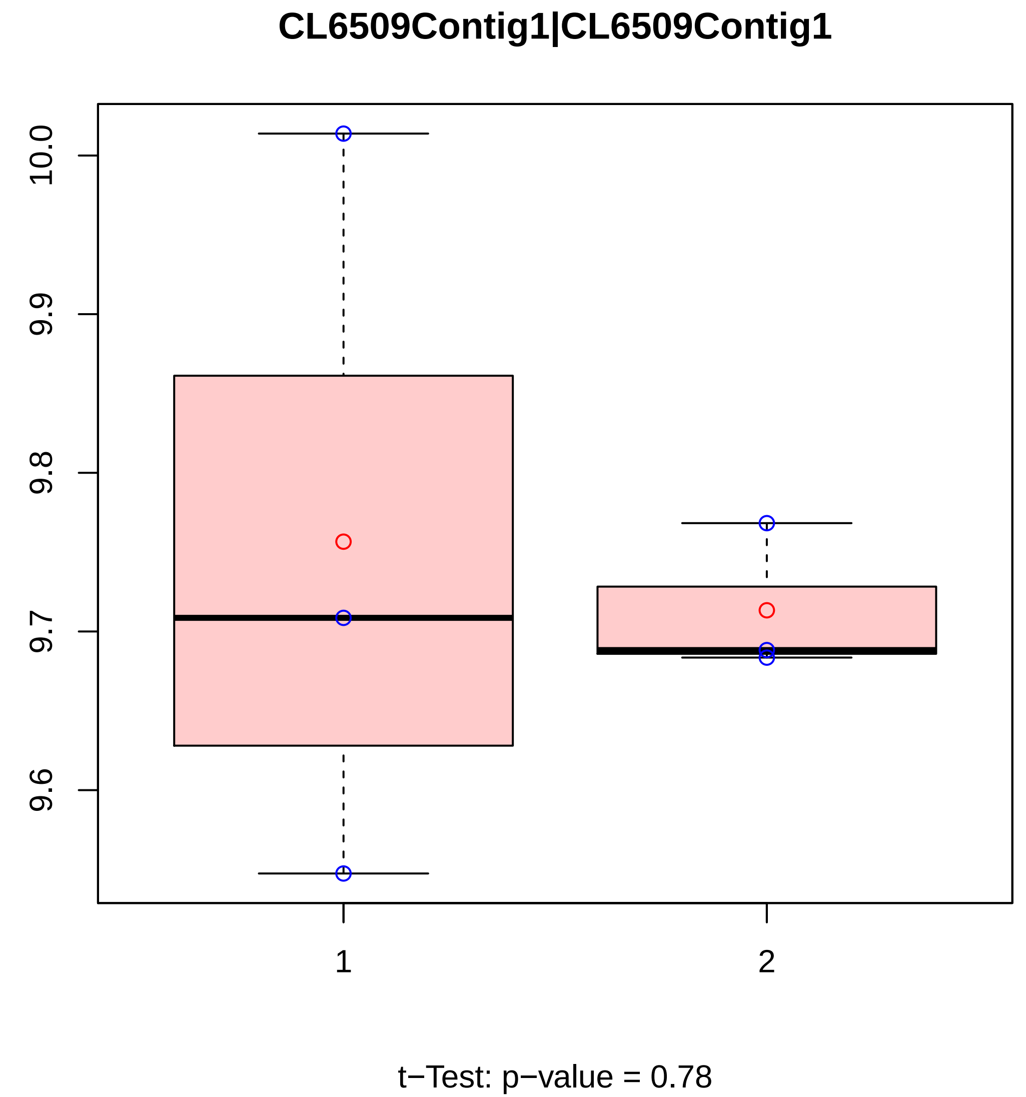

CL6509Contig1|CL6509Contig1
10.0
9.9
9.8
9.7
9.6
1
2
t−Test: p−value = 0.78

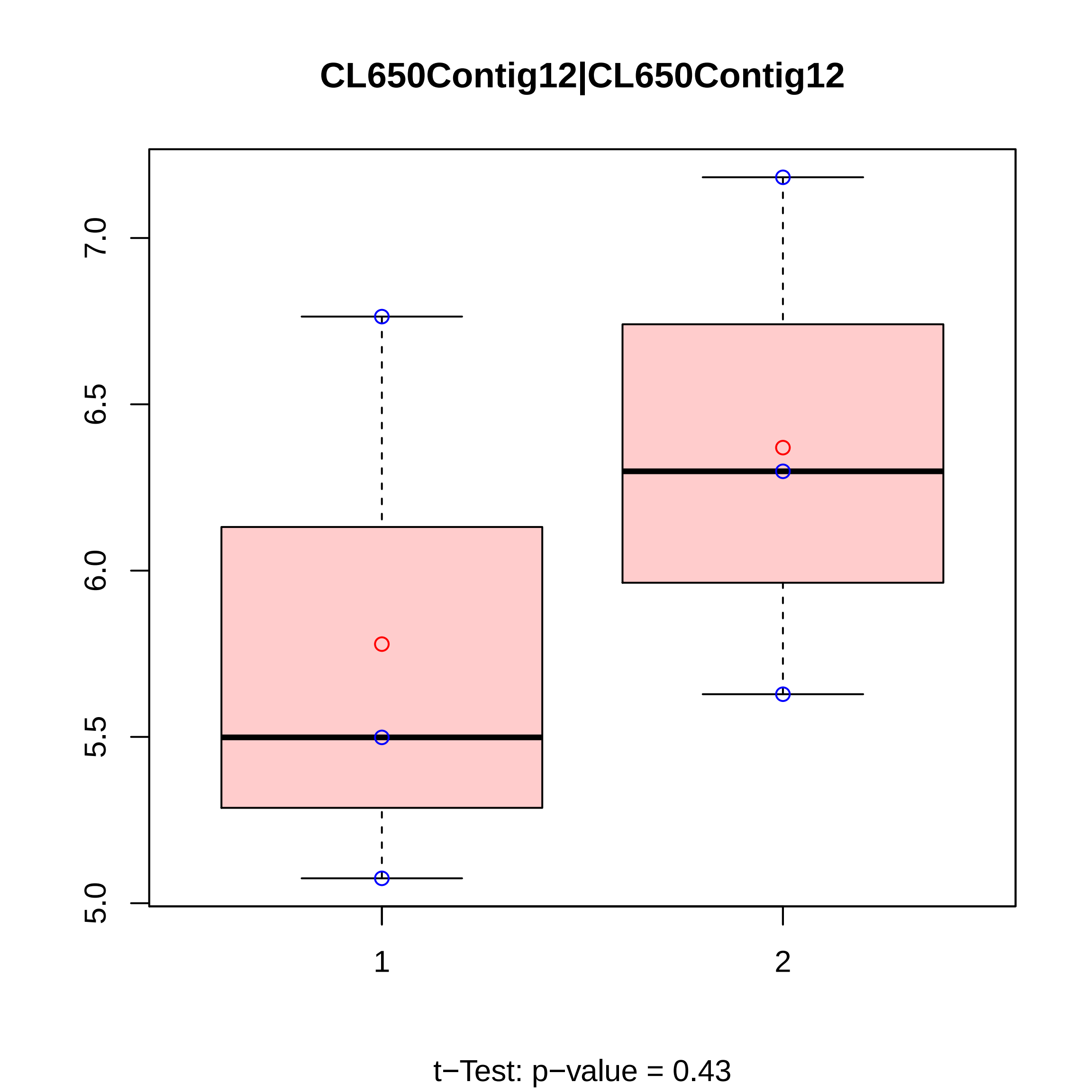
CL650Contig12|CL650Contig12
7.0
6.5
6.0
5.5
5.0
1
2
t−Test: p−value = 0.43

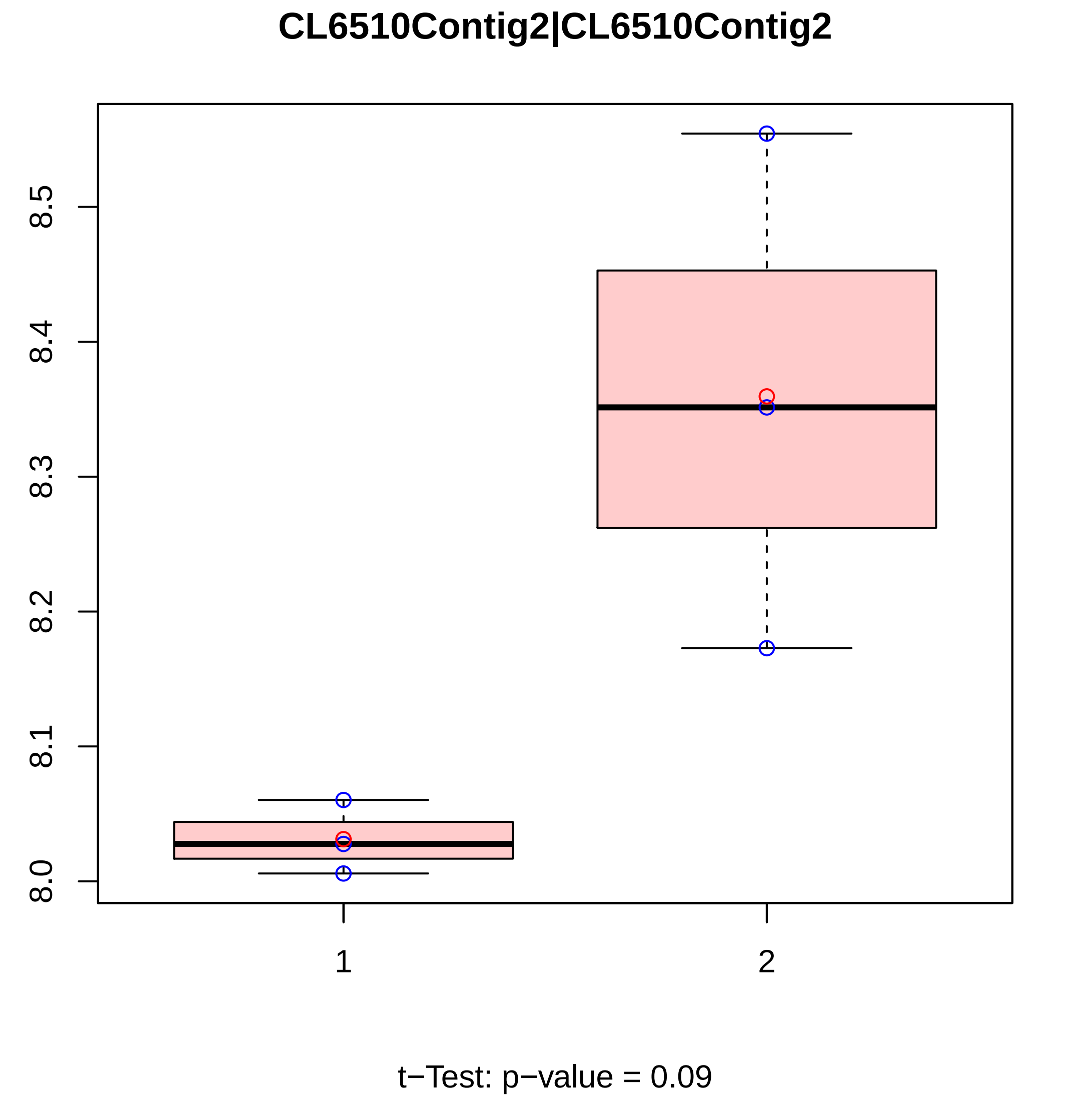

CL6510Contig2|CL6510Contig2
8.0
8.1
8.2
8.3
8.4
8.5
1
2
t−Test: p−value = 0.09

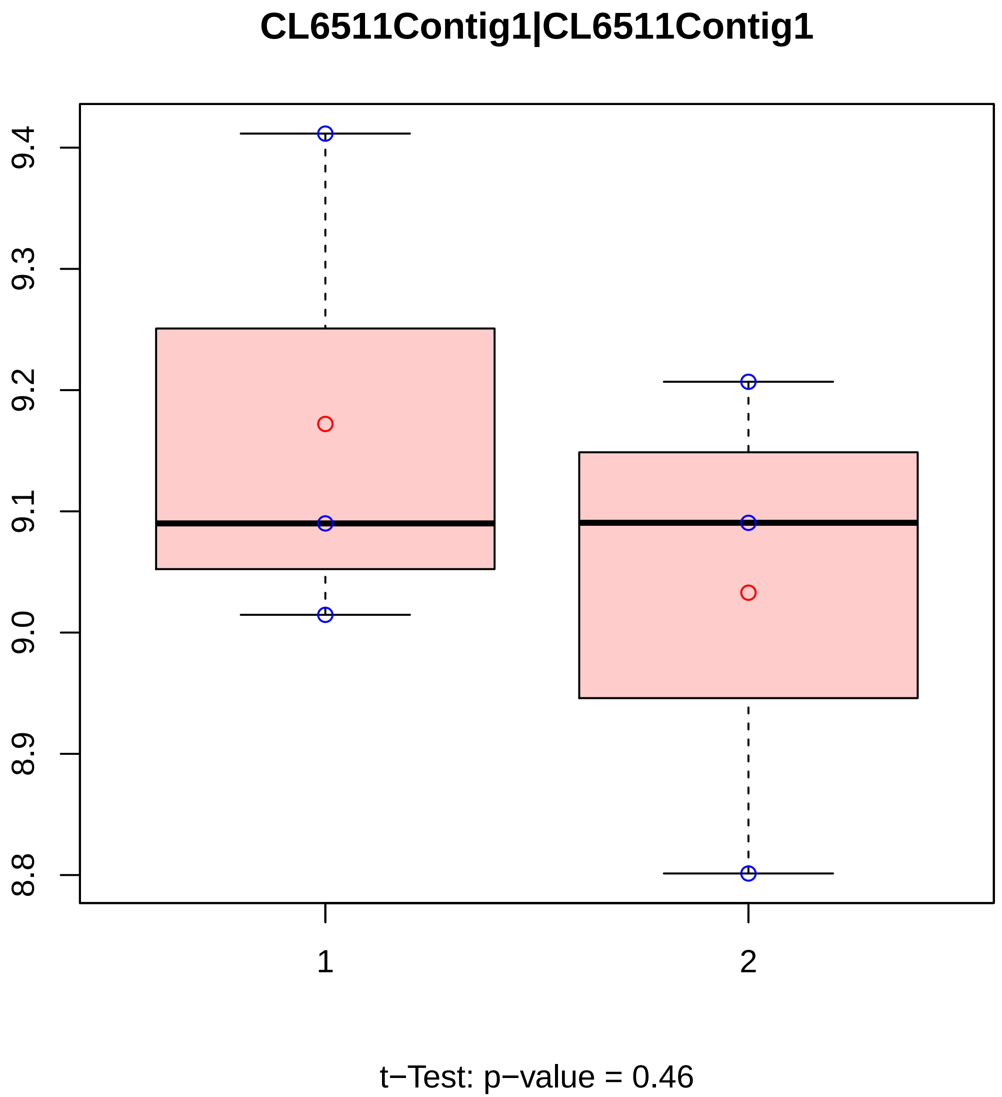

CL6511Contig1|CL6511Contig1
9.4
9.3
9.2
9.1
9.0
8.9
8.8
1
2
t−Test: p−value = 0.46

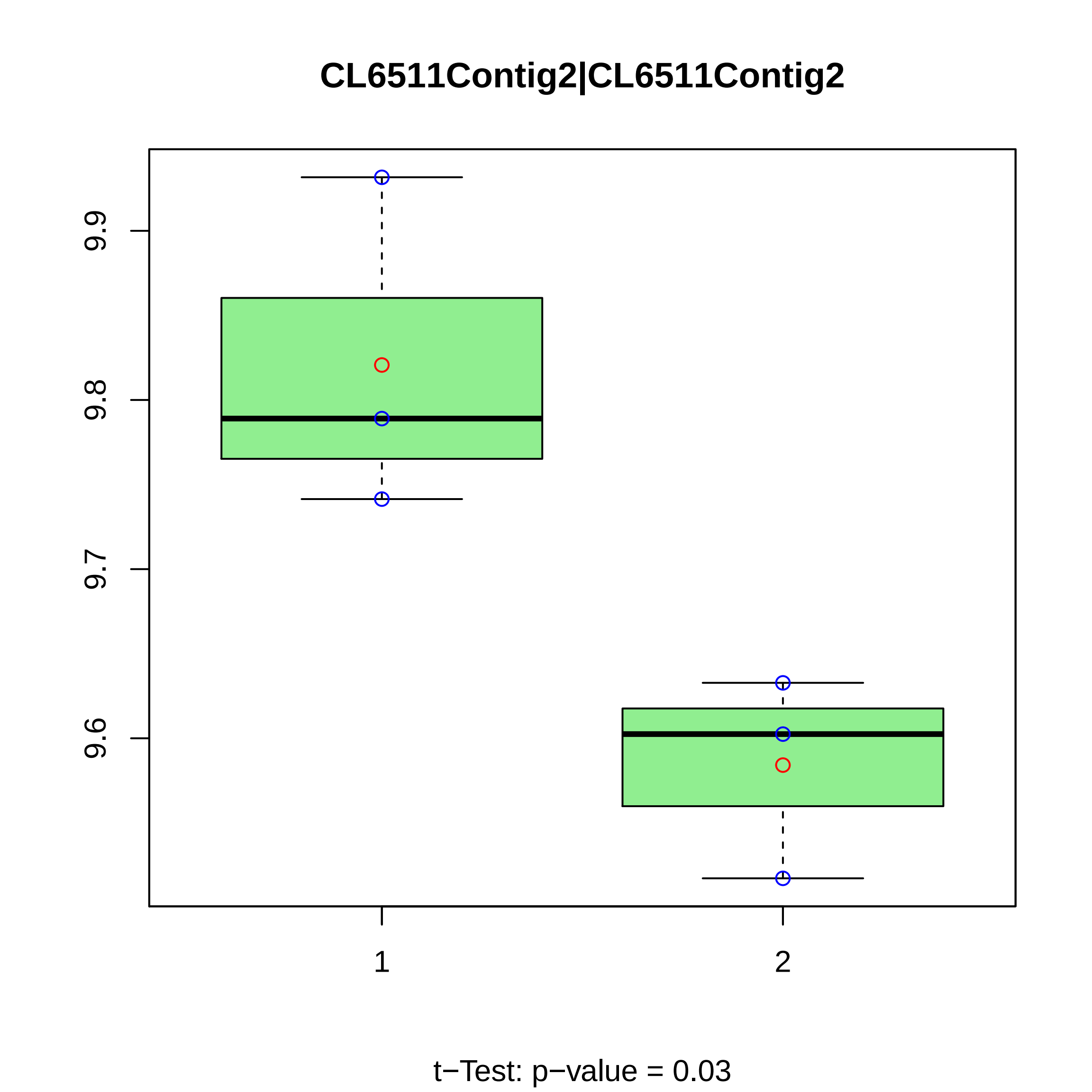

CL6511Contig2|CL6511Contig2
9.9
9.8
9.7
9.6
1
2
t−Test: p−value = 0.03

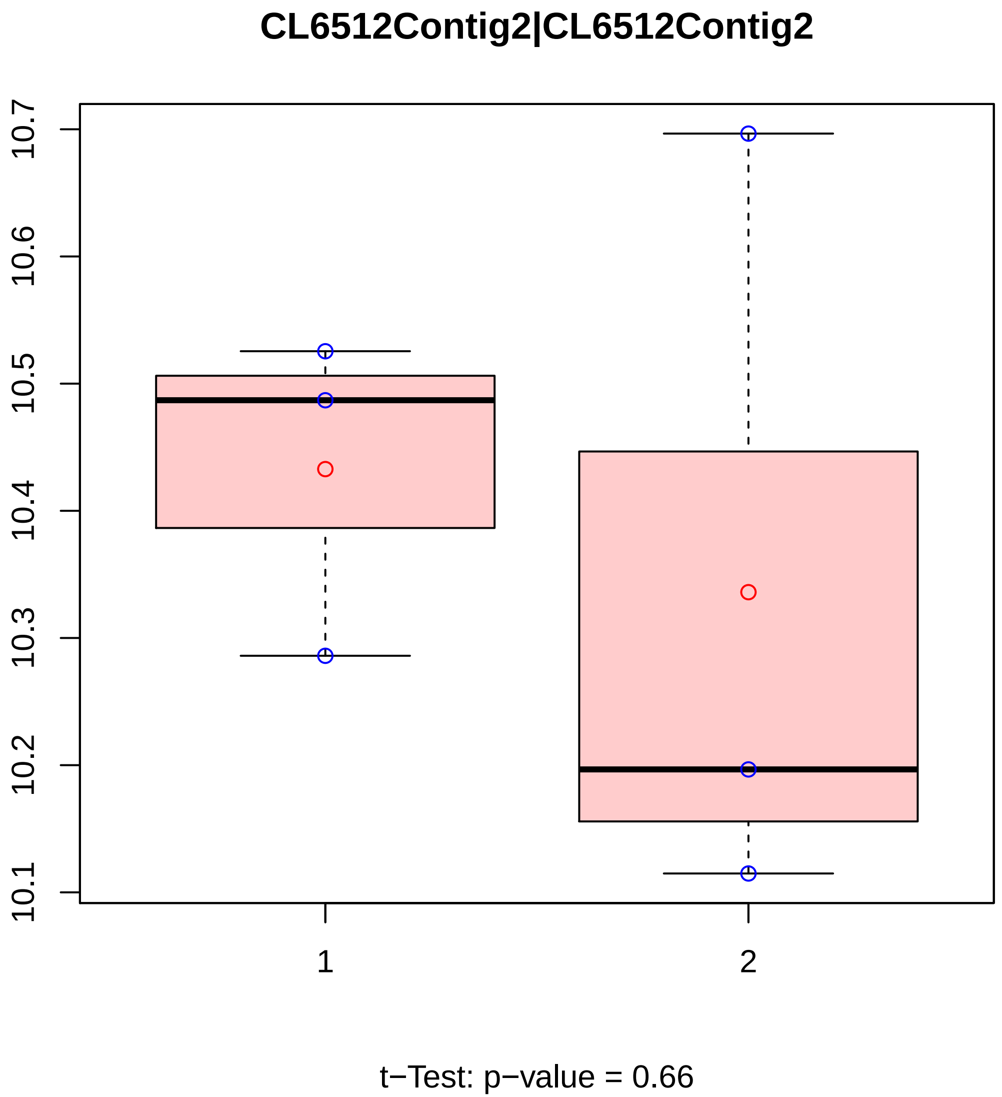

CL6512Contig2|CL6512Contig2
10.1
10.2
10.3
10.4
10.5
10.6
10.7
1
2
t−Test: p−value = 0.66

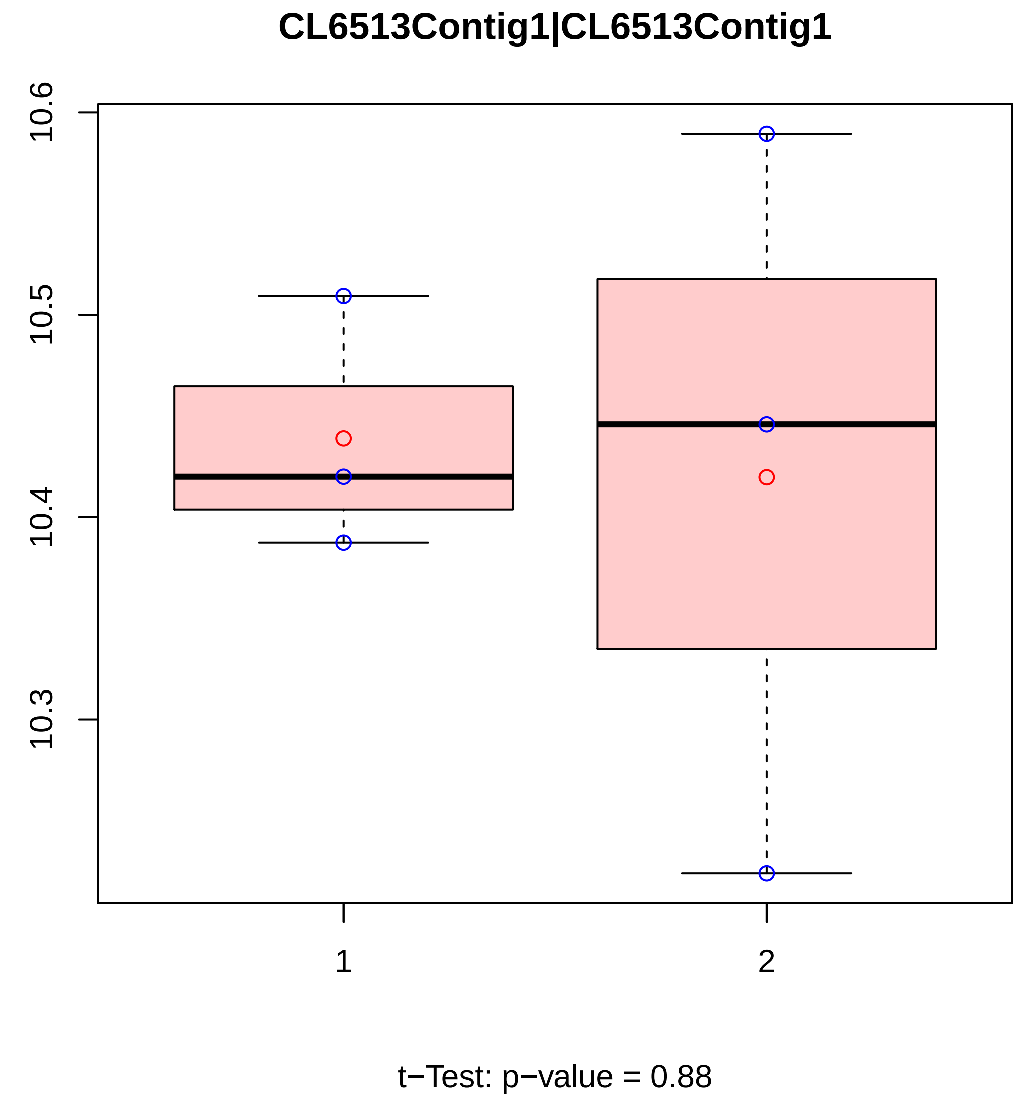
CL6513Contig1|CL6513Contig1
10.6
10.5
10.4
10.3
1
2
t−Test: p−value = 0.88

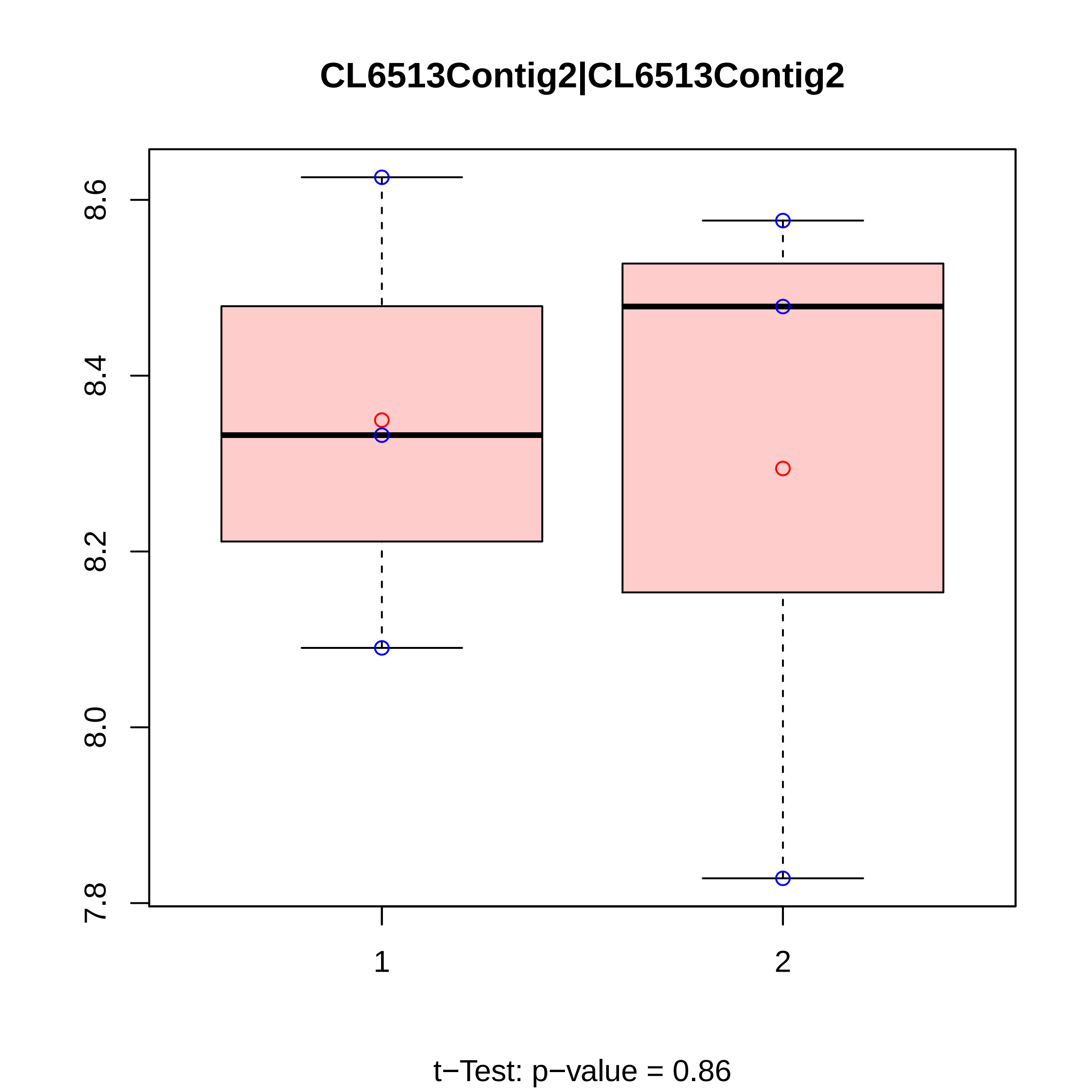
CL6513Contig2|CL6513Contig2
8.6
8.4
8.2
8.0
7.8
1
2
t−Test: p−value = 0.86

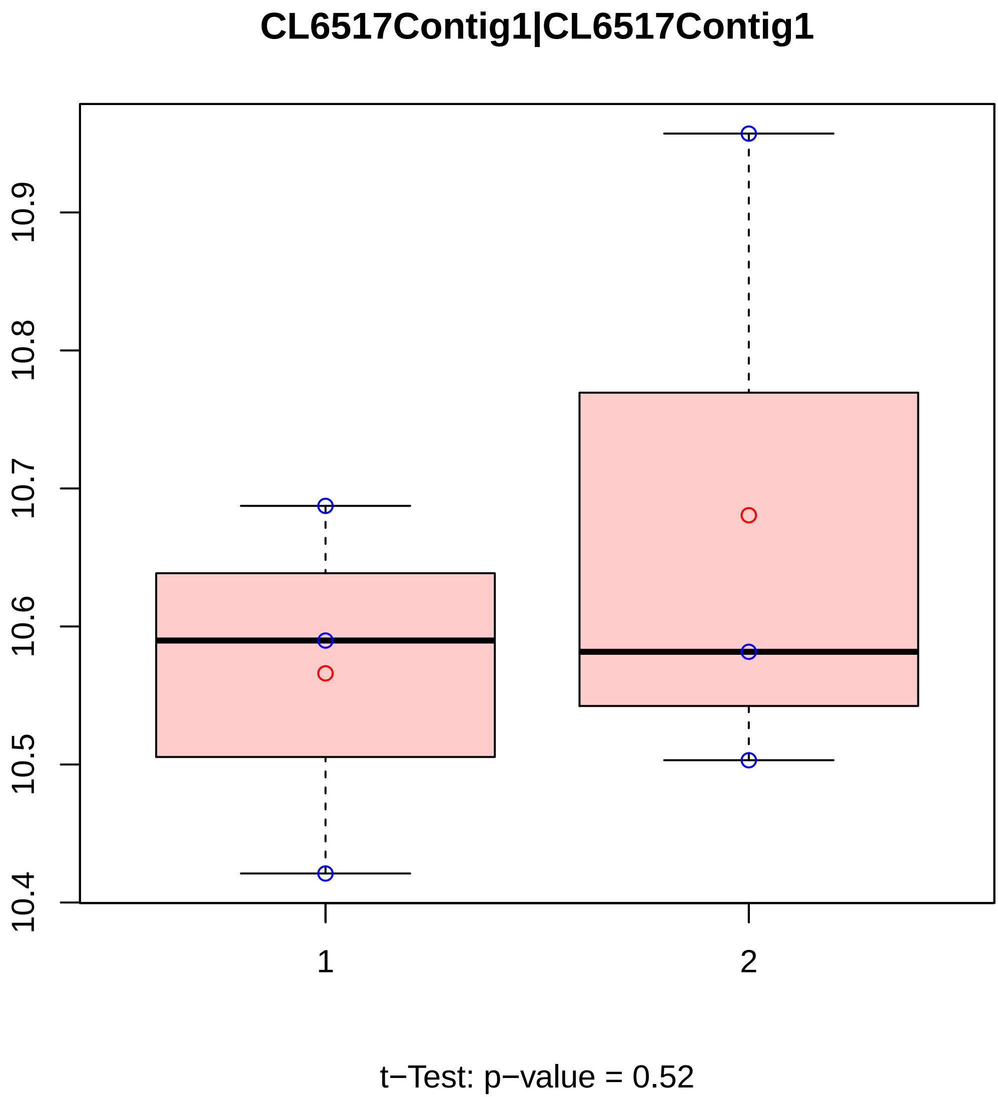

CL6517Contig1|CL6517Contig1
10.4
10.5
10.6
10.7
10.8
10.9
1
2
t−Test: p−value = 0.52

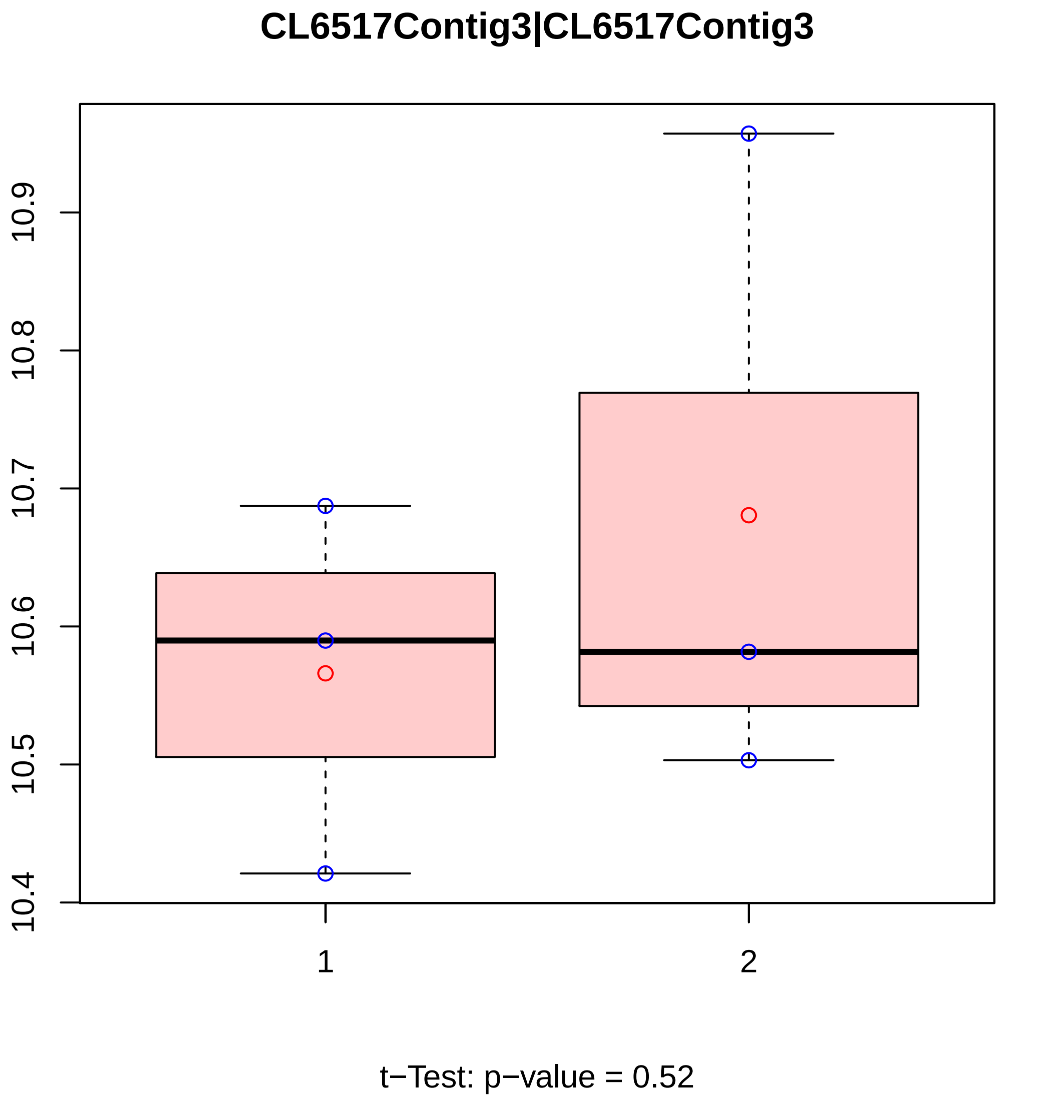

CL6517Contig3|CL6517Contig3
10.4
10.5
10.6
10.7
10.8
10.9
1
2
t−Test: p−value = 0.52

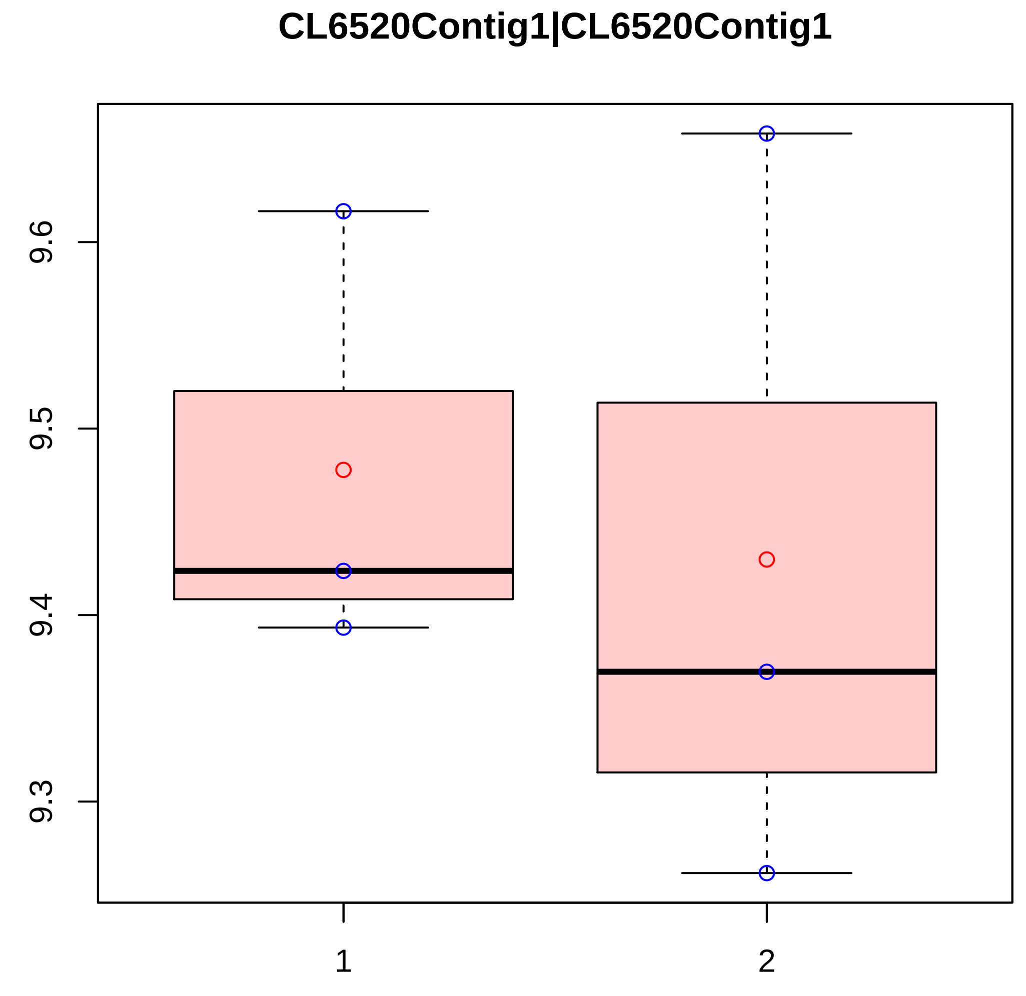

t−Test: p−value = 0.75

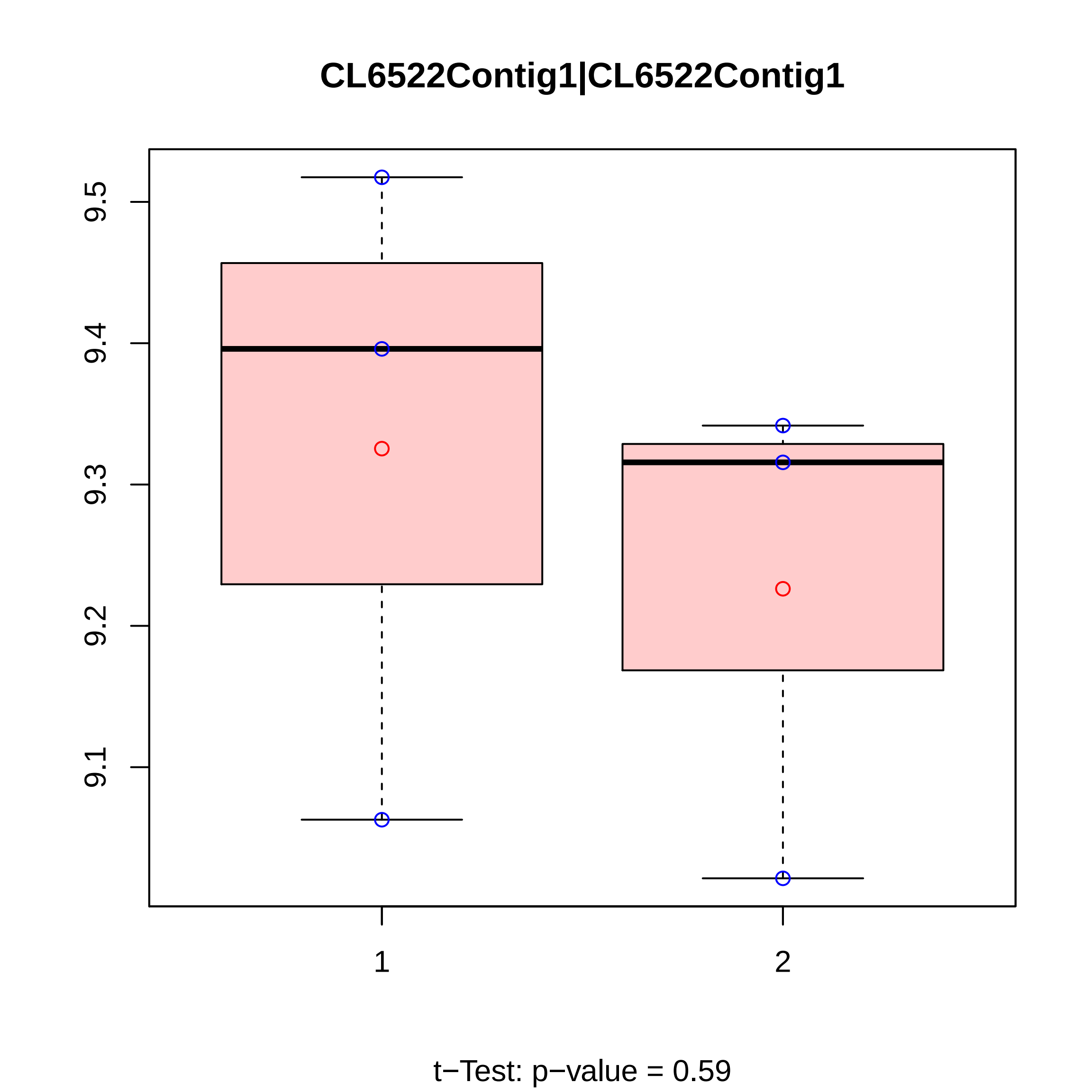

CL6522Contig1|CL6522Contig1
9.1
9.2
9.3
9.4
9.5
1
2
t−Test: p−value = 0.59

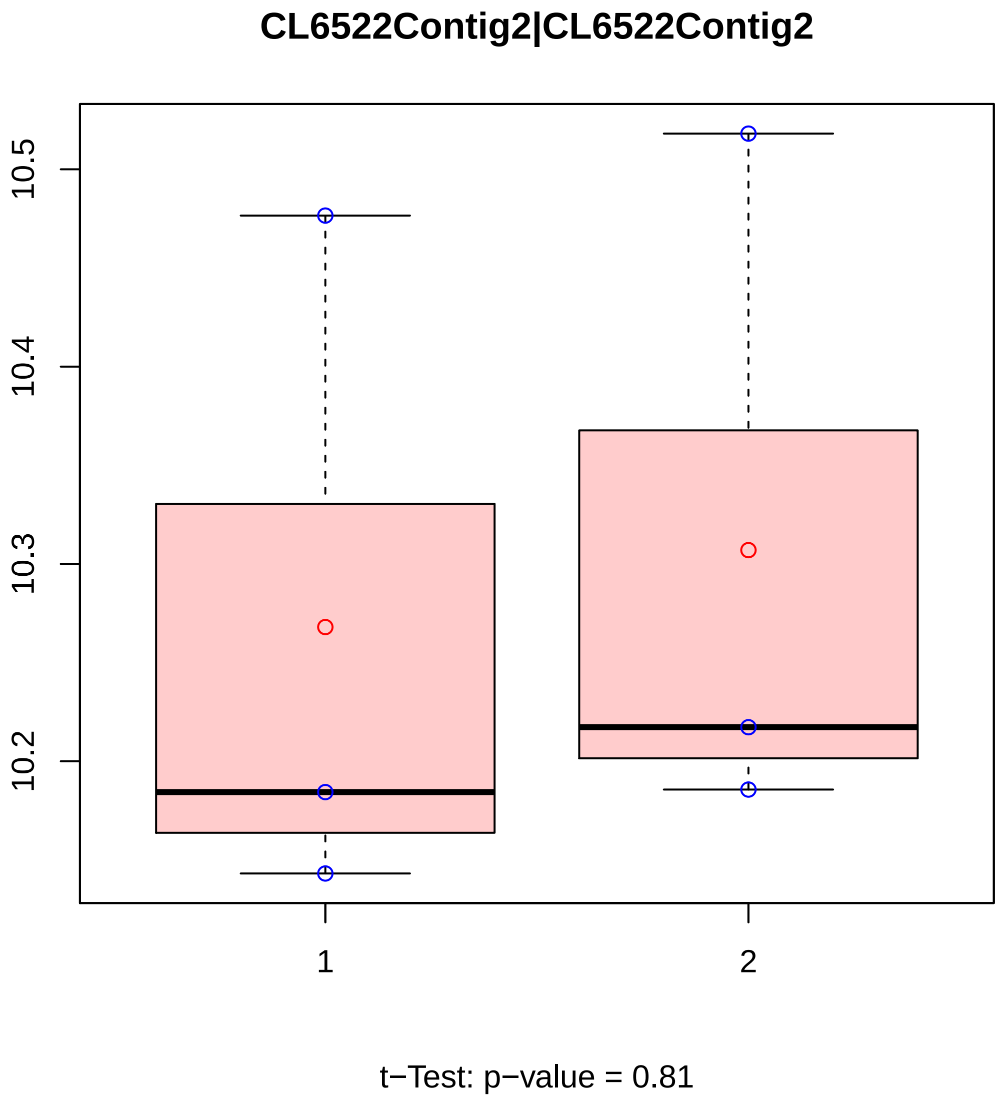

CL6522Contig2|CL6522Contig2
10.2
10.3
10.4
10.5
1
2
t−Test: p−value = 0.81

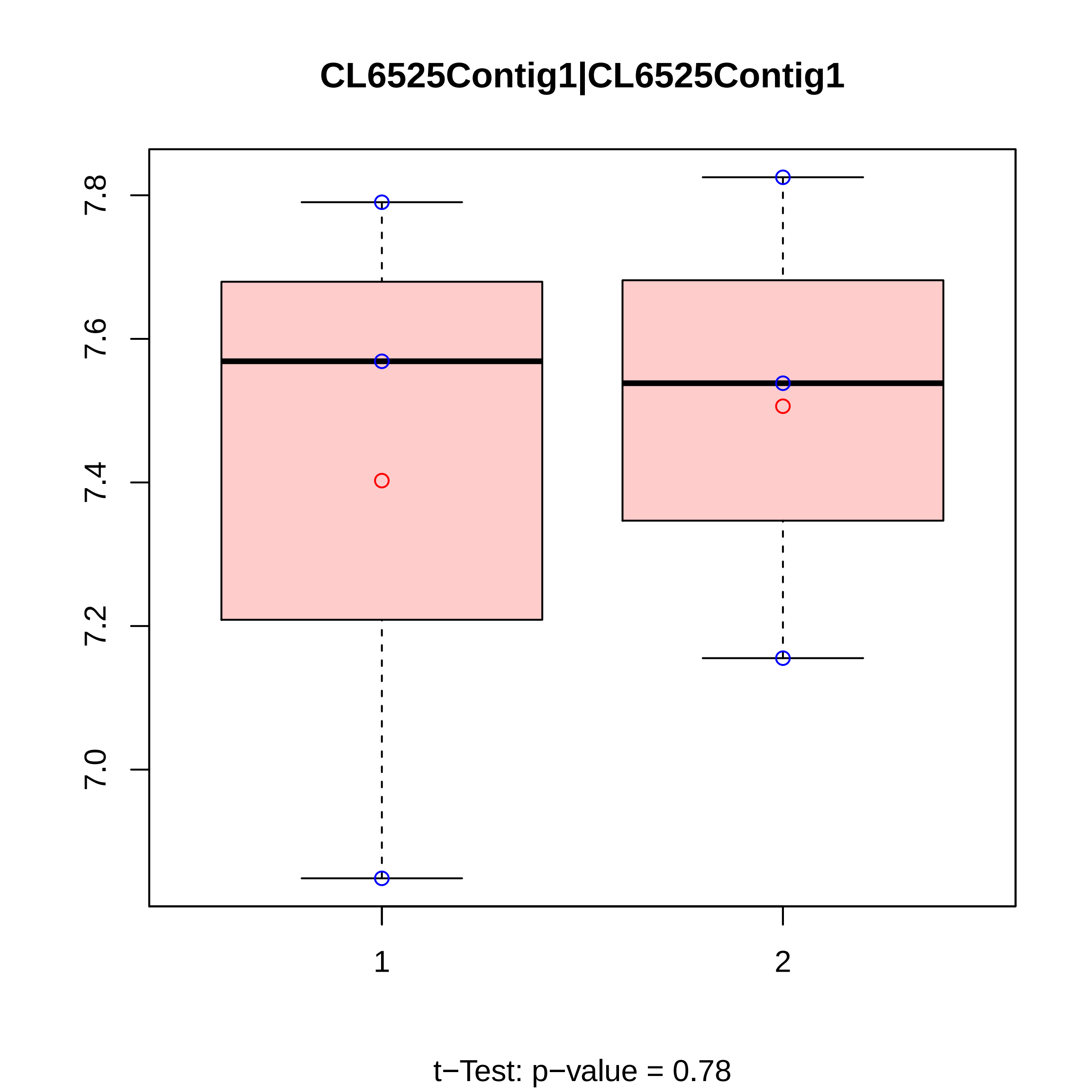
CL6525Contig1|CL6525Contig1
7.0
7.2
7.4
7.6
7.8
1
2
t−Test: p−value = 0.78

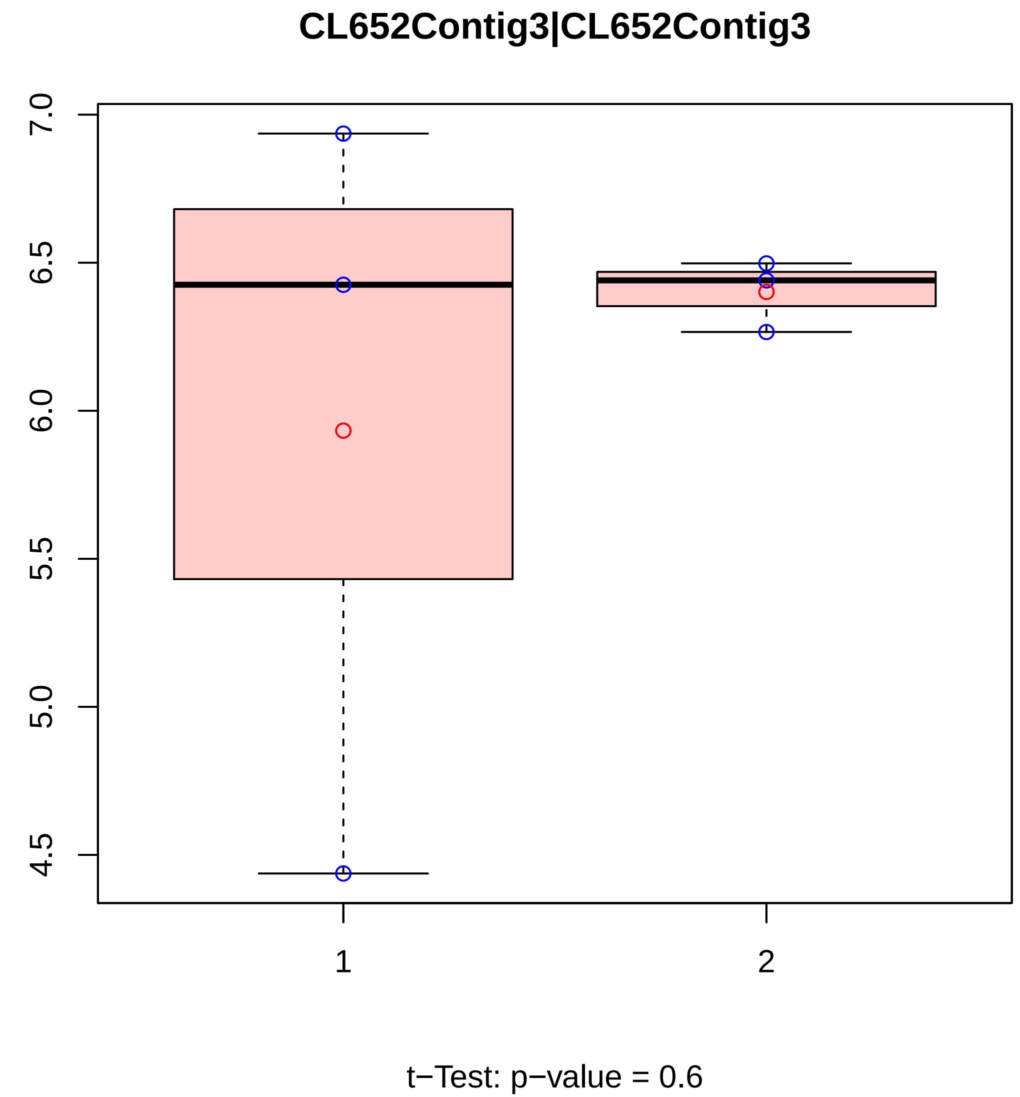
CL652Contig3|CL652Contig3
7.0
6.5
6.0
5.5
5.0
4.5
1
2
t−Test: p−value = 0.6

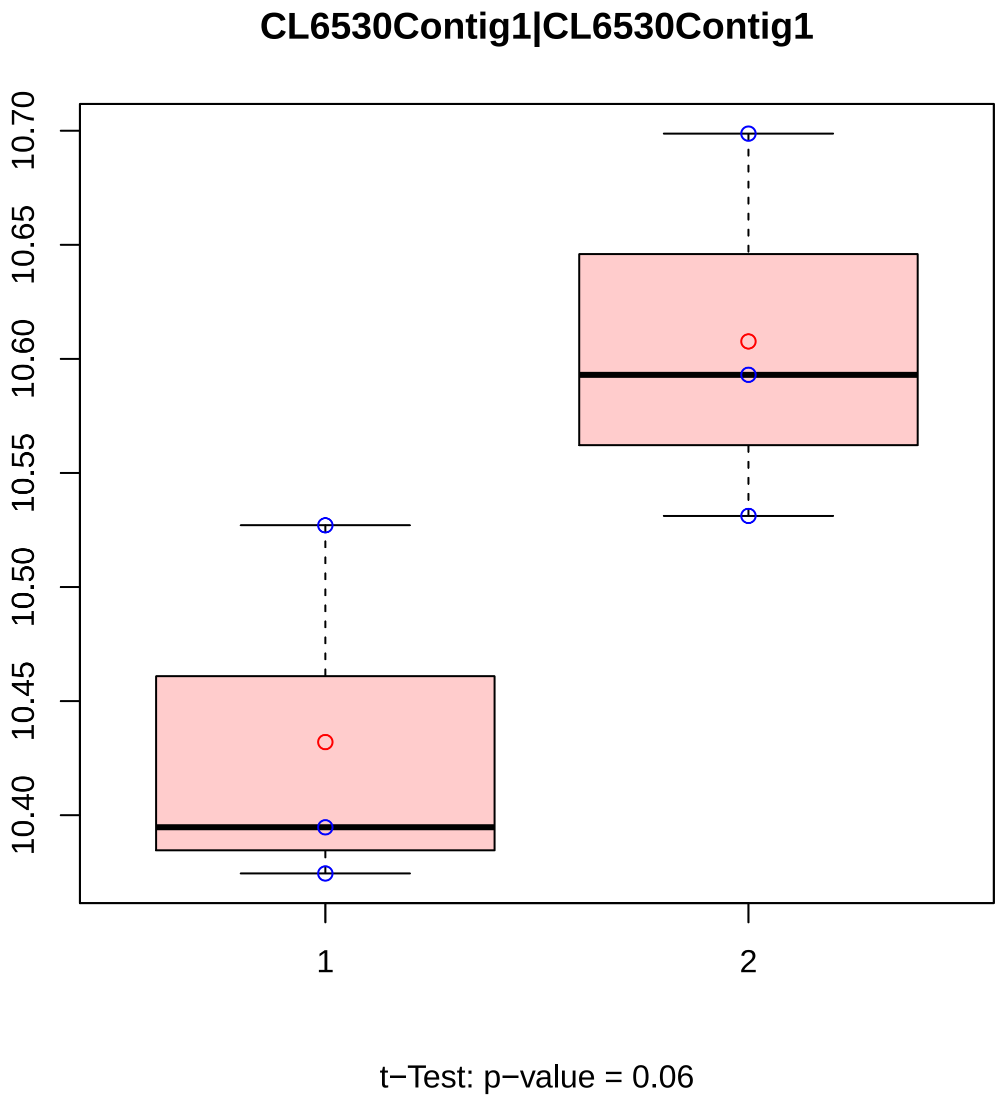
CL6530Contig1|CL6530Contig1
10.40
10.45
10.50
10.55
10.60
10.65
10.70
1
2
t−Test: p−value = 0.06

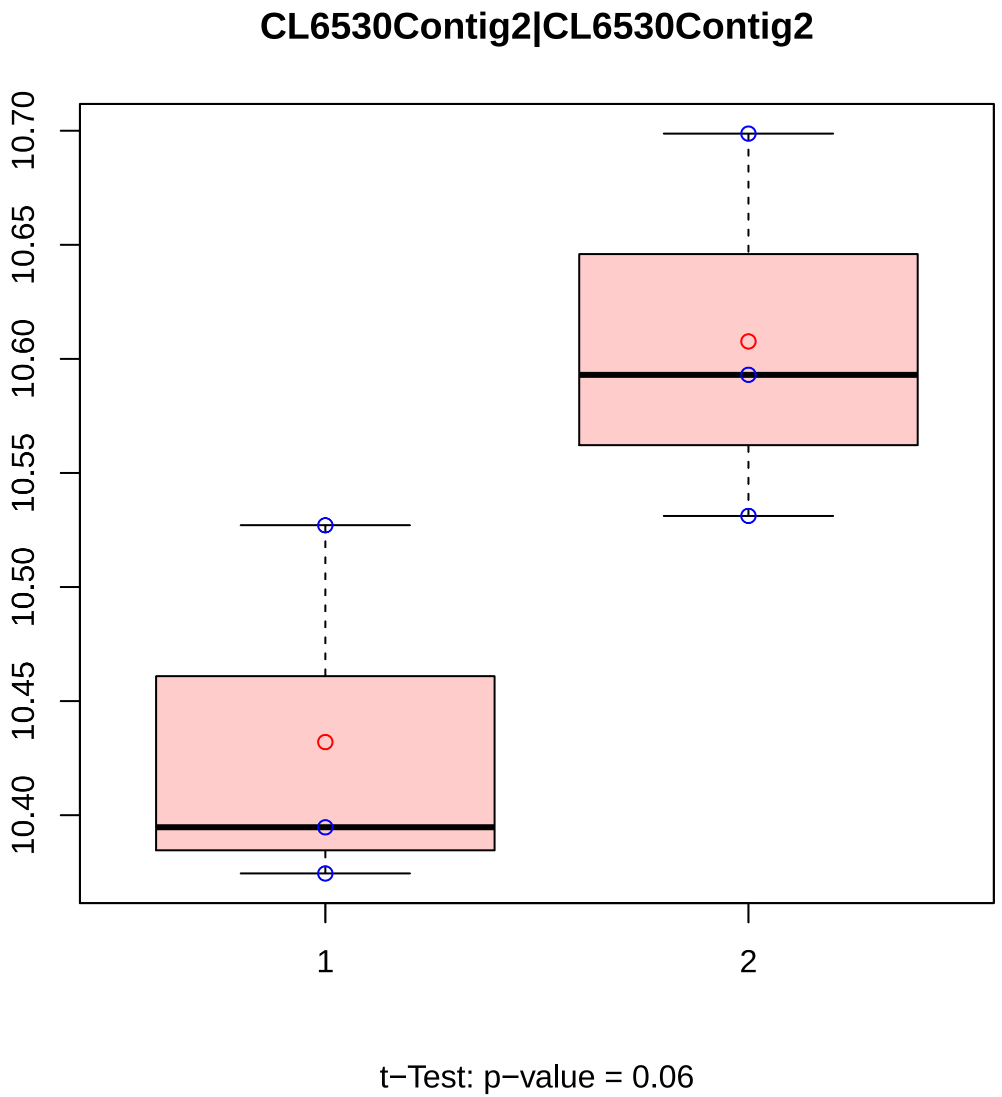
CL6530Contig2|CL6530Contig2
10.70
10.65
10.60
10.55
10.50
10.45
10.40
1
2
t−Test: p−value = 0.06

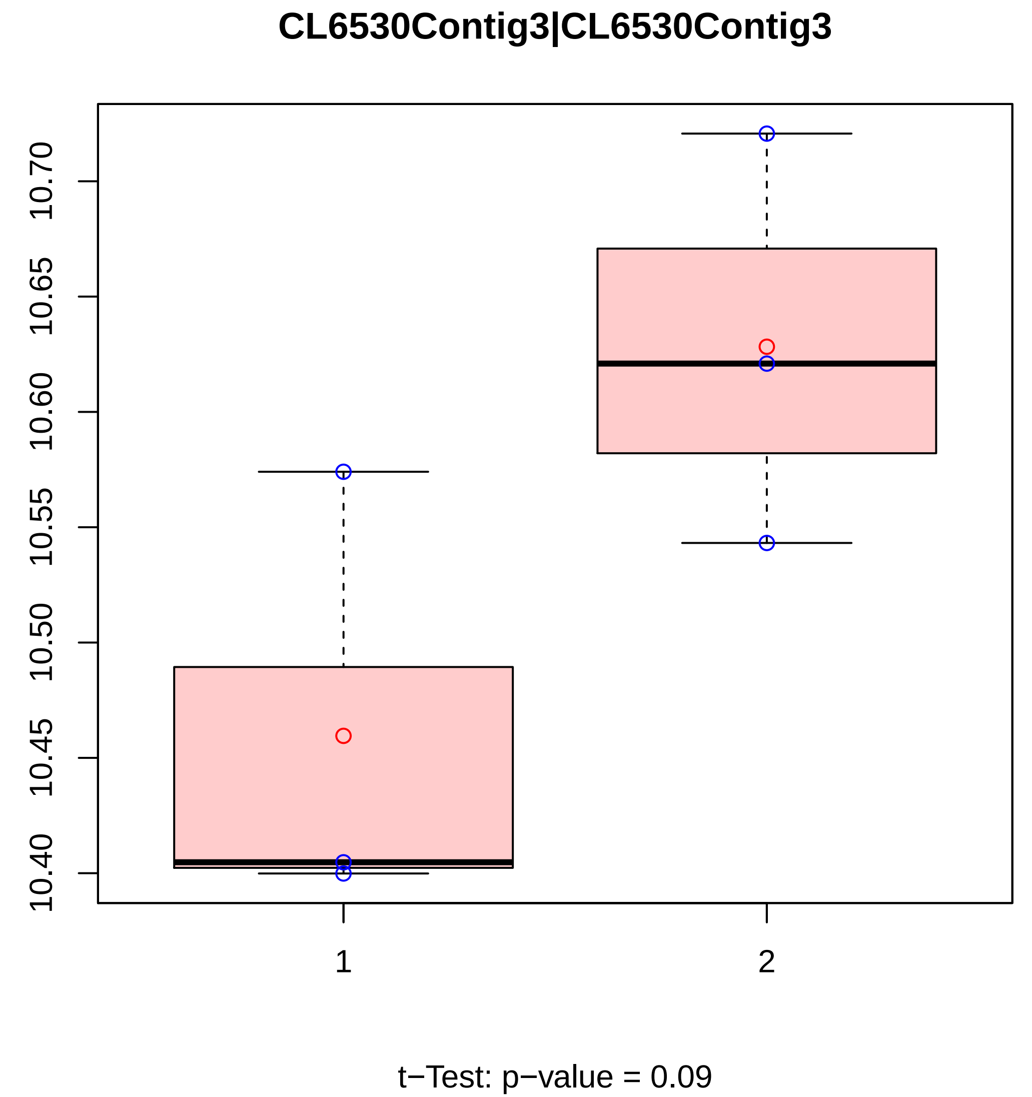
CL6530Contig3|CL6530Contig3
10.40
10.45
10.50
10.55
10.60
10.65
10.70
1
2
t−Test: p−value = 0.09

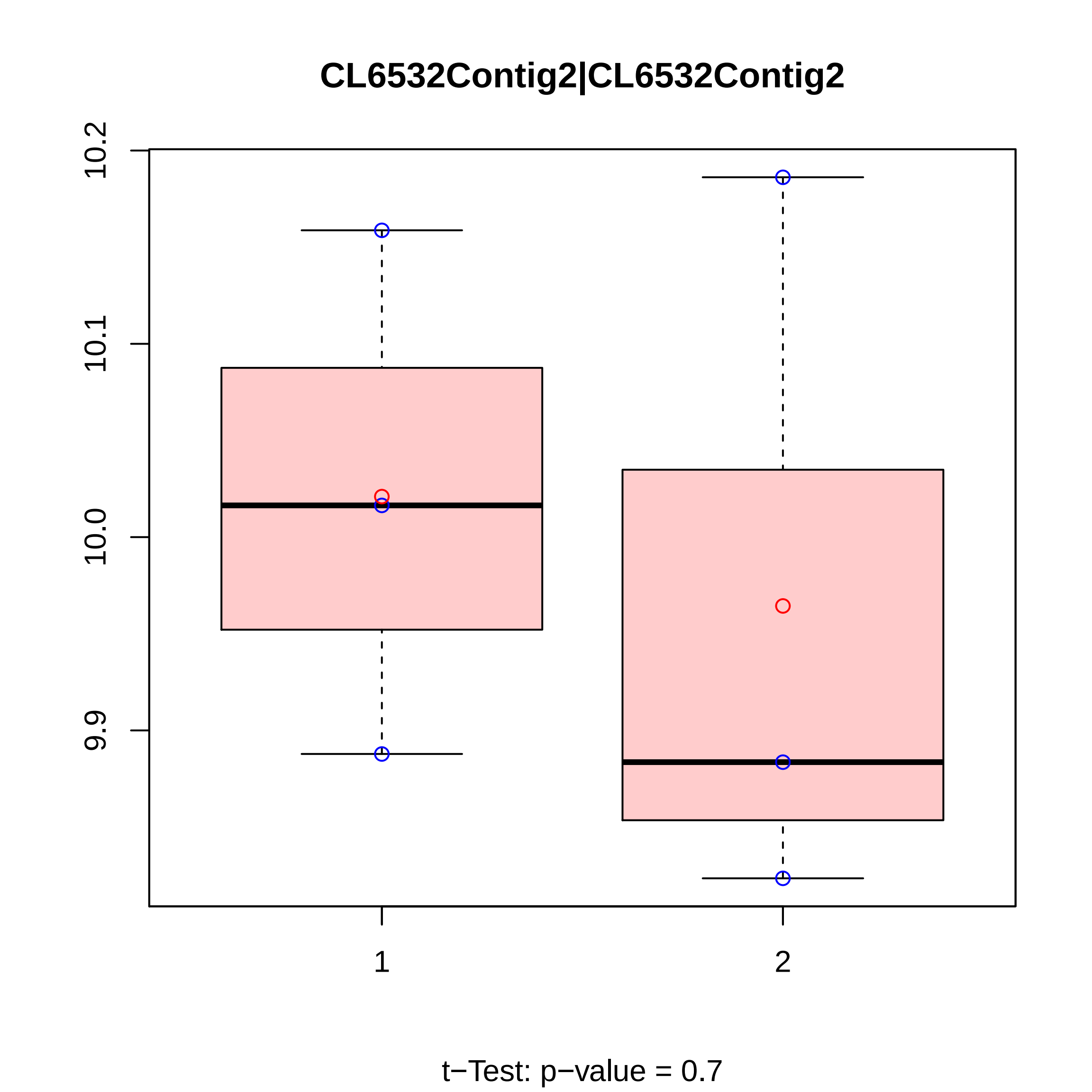
CL6532Contig2|CL6532Contig2
10.2
10.1
10.0
9.9
1
2
t−Test: p−value = 0.7

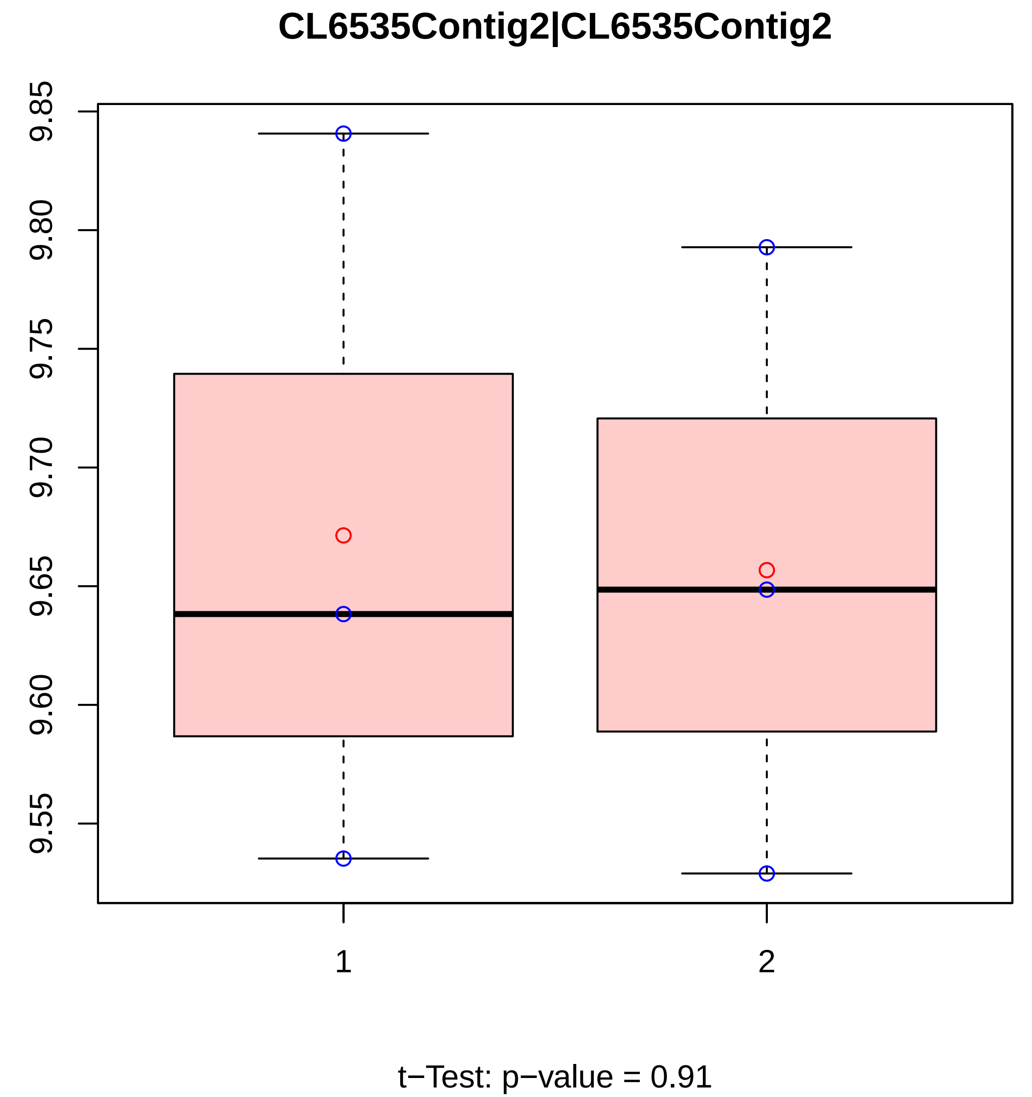
CL6535Contig2|CL6535Contig2
9.85
9.80
9.75
9.70
9.65
9.60
9.55
1
2
t−Test: p−value = 0.91

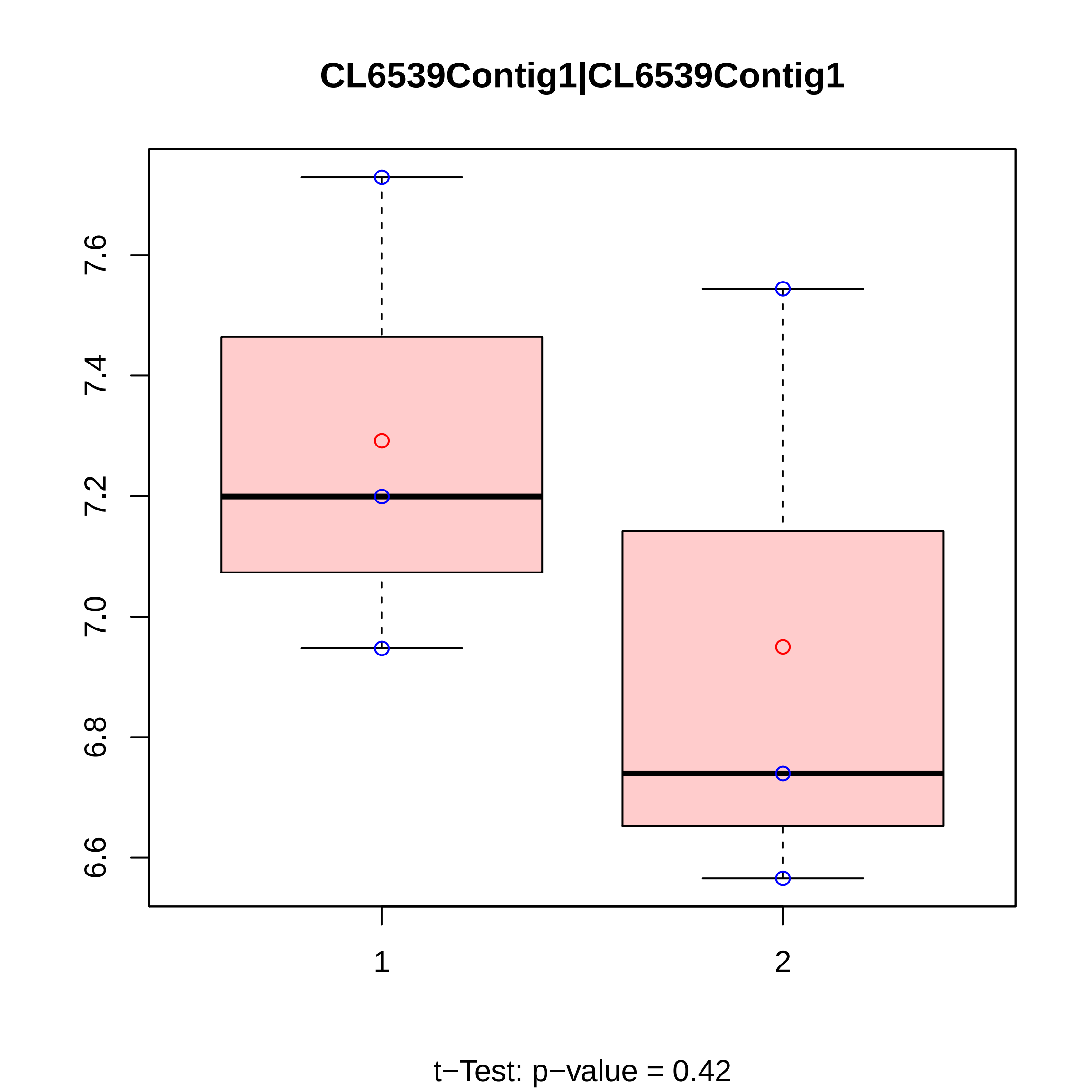

CL6539Contig1|CL6539Contig1
7.6
7.4
7.2
7.0
6.8
6.6
1
2
t−Test: p−value = 0.42

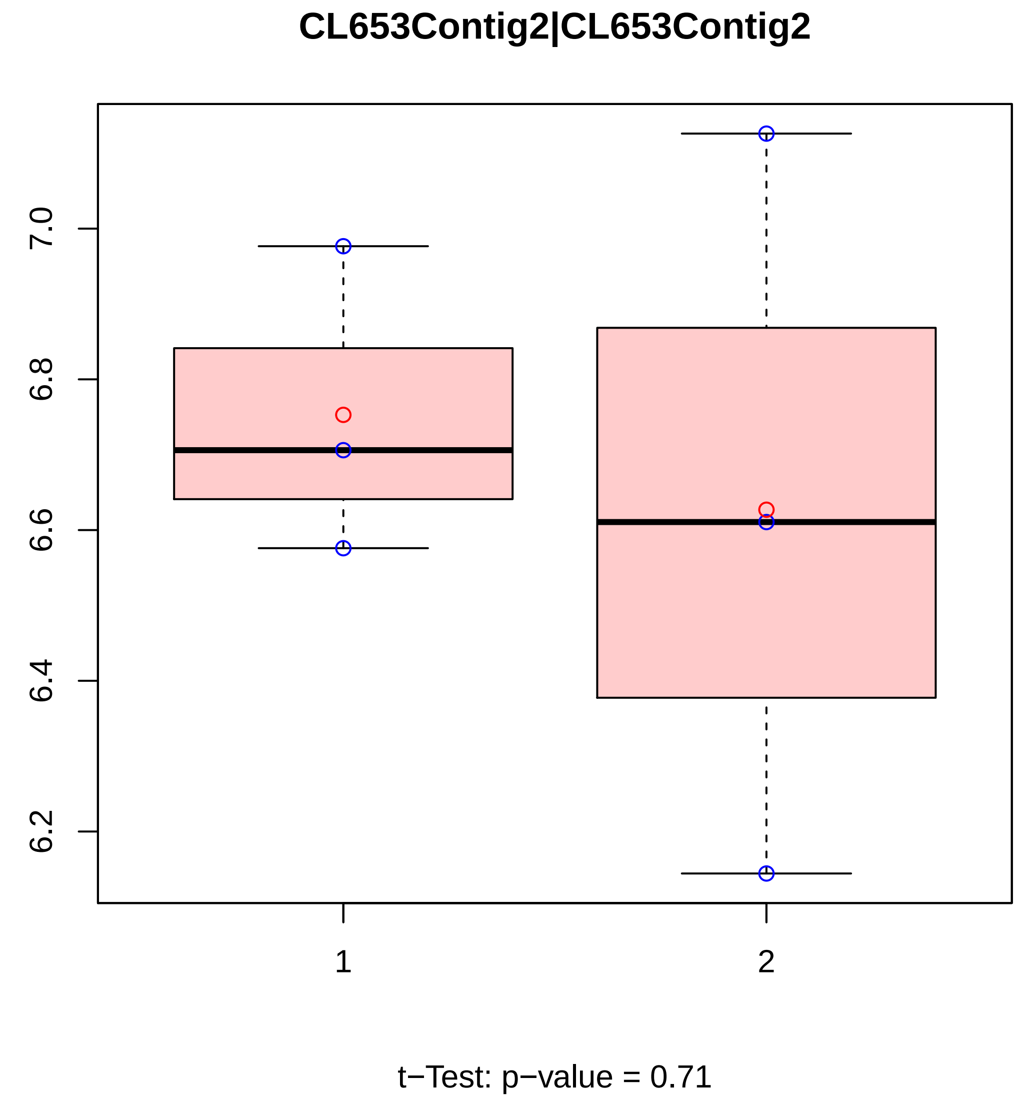
CL653Contig2|CL653Contig2
7.0
6.8
6.6
6.4
6.2
1
2
t−Test: p−value = 0.71

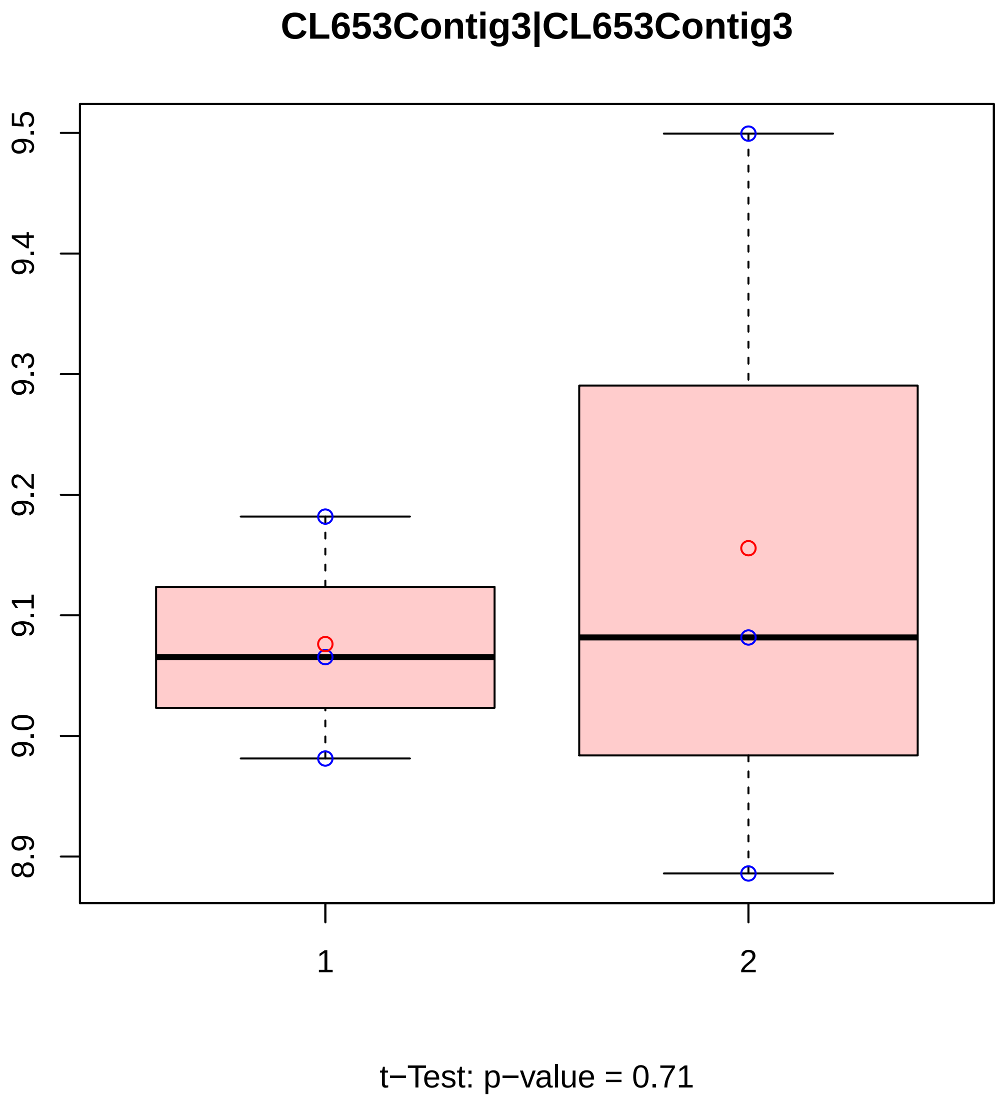
CL653Contig3|CL653Contig3
9.5
9.4
9.3
9.2
9.1
9.0
8.9
1
2
t−Test: p−value = 0.71

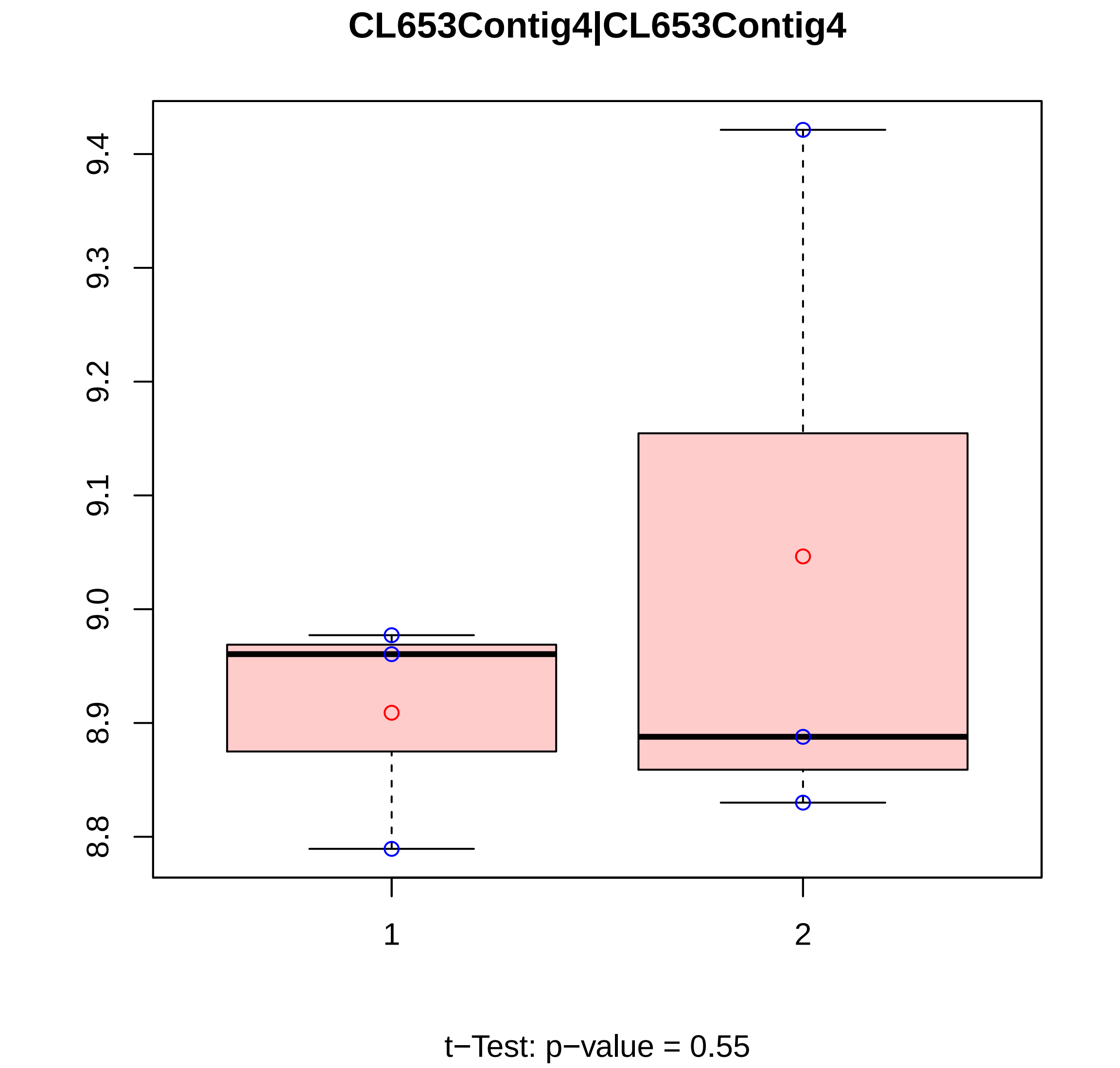
CL653Contig4|CL653Contig4
9.4
9.3
9.2
9.1
9.0
8.9
8.8
1
2
t−Test: p−value = 0.55

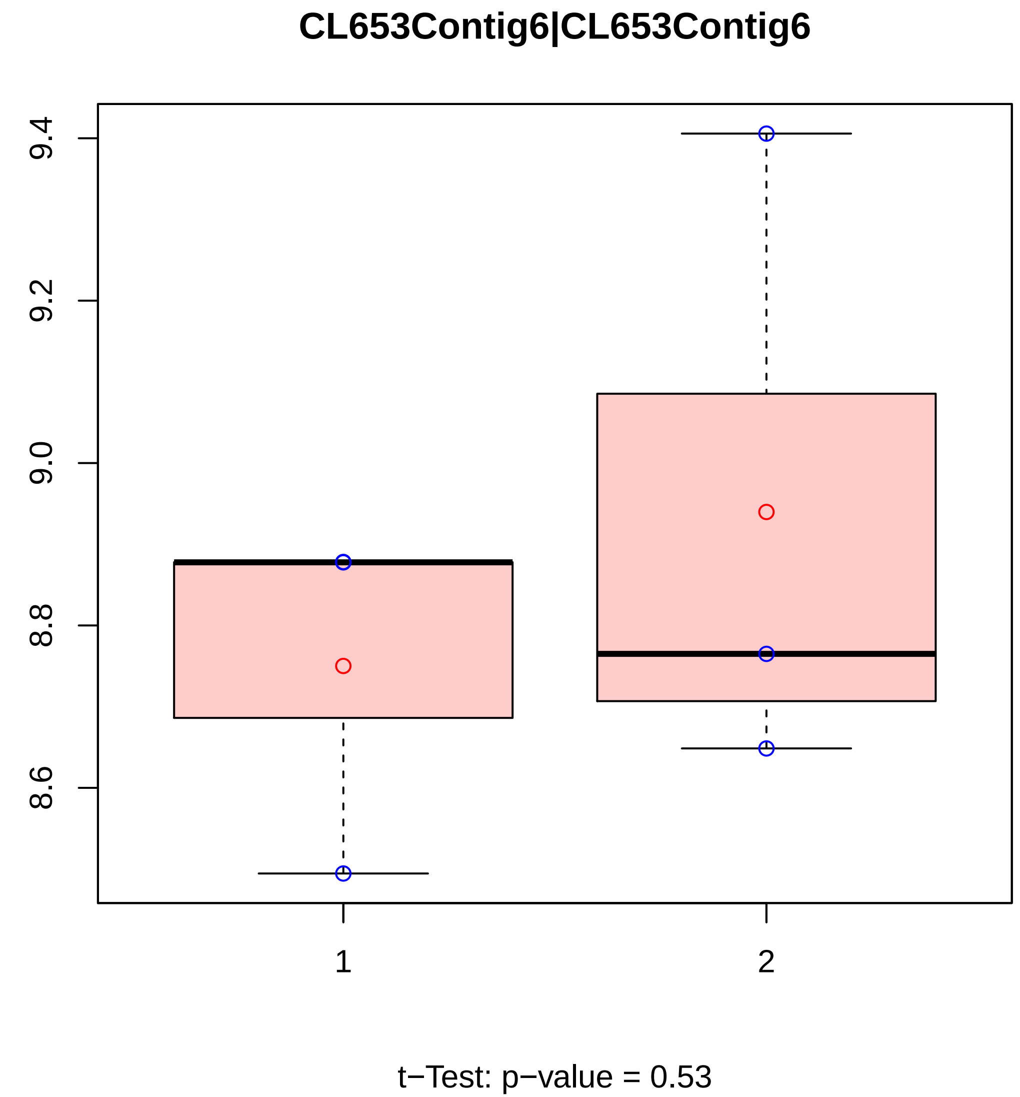

CL653Contig6|CL653Contig6
9.4
9.2
9.0
8.8
8.6
1
2
t−Test: p−value = 0.53

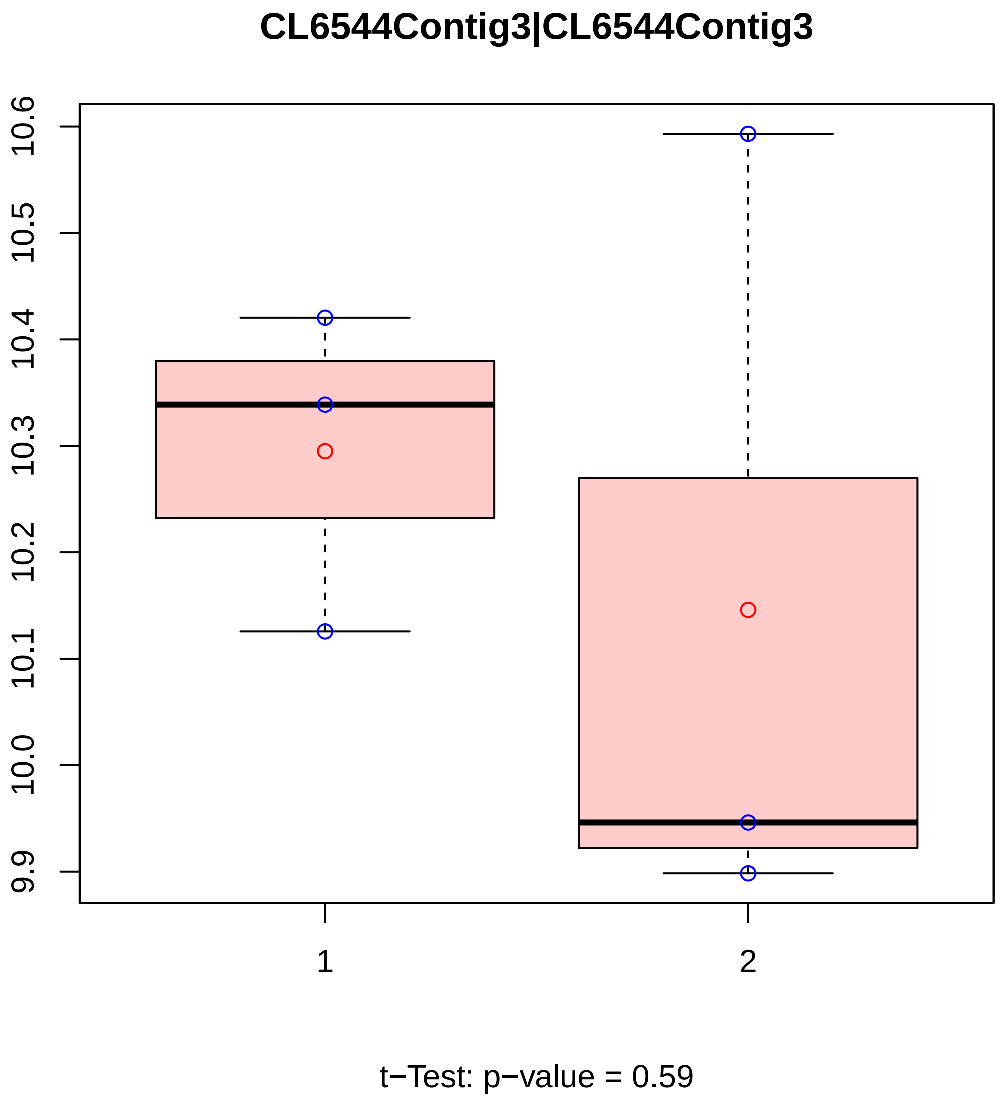
CL6544Contig3|CL6544Contig3
10.6
10.5
10.4
10.3
10.2
10.1
10.0
9.9
1
2
t−Test: p−value = 0.59

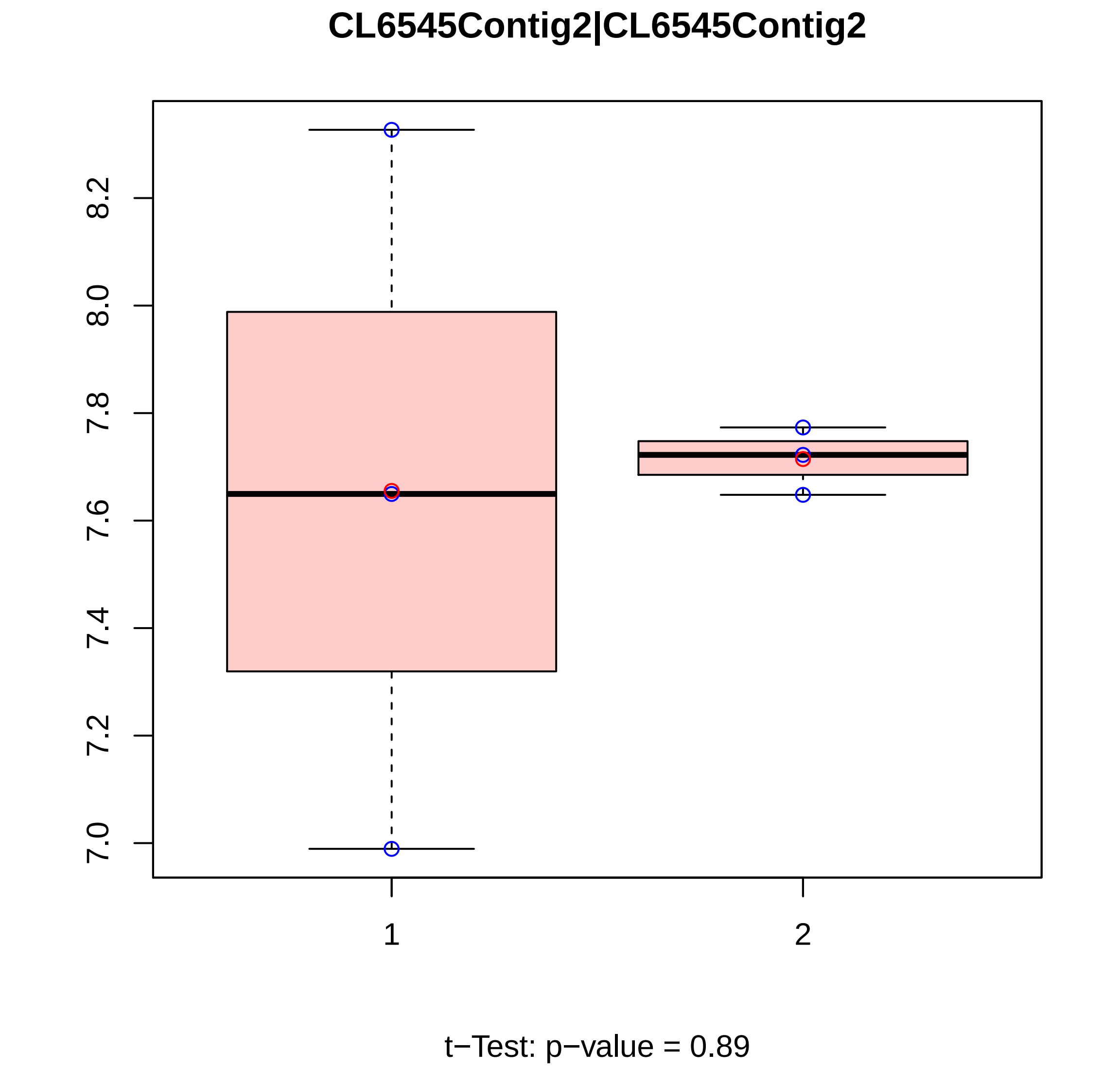
CL6545Contig2|CL6545Contig2
7.0
7.2
7.4
7.6
7.8
8.0
8.2
1
2
t−Test: p−value = 0.89

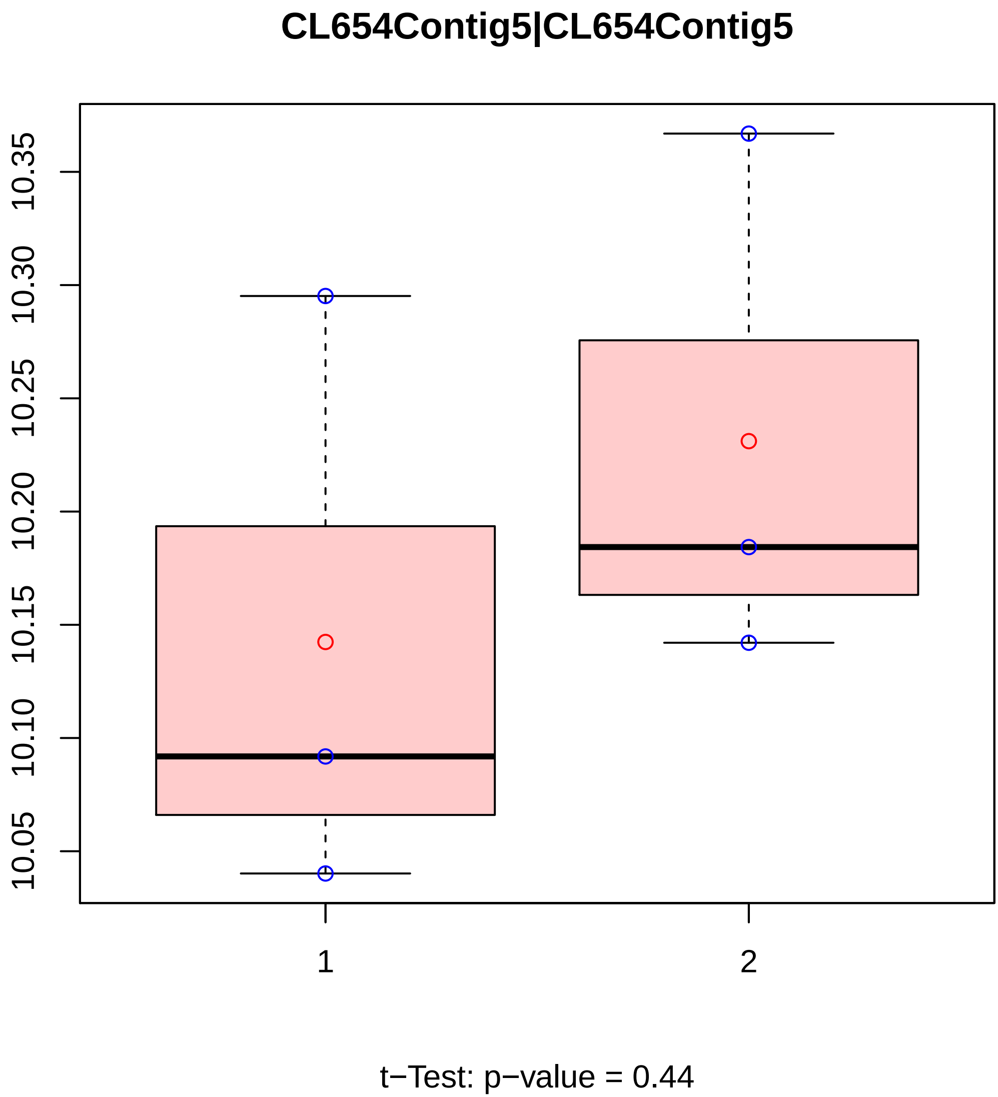

CL654Contig5|CL654Contig5
10.05
10.10
10.15
10.20
10.25
10.30
10.35
1
2
t−Test: p−value = 0.44

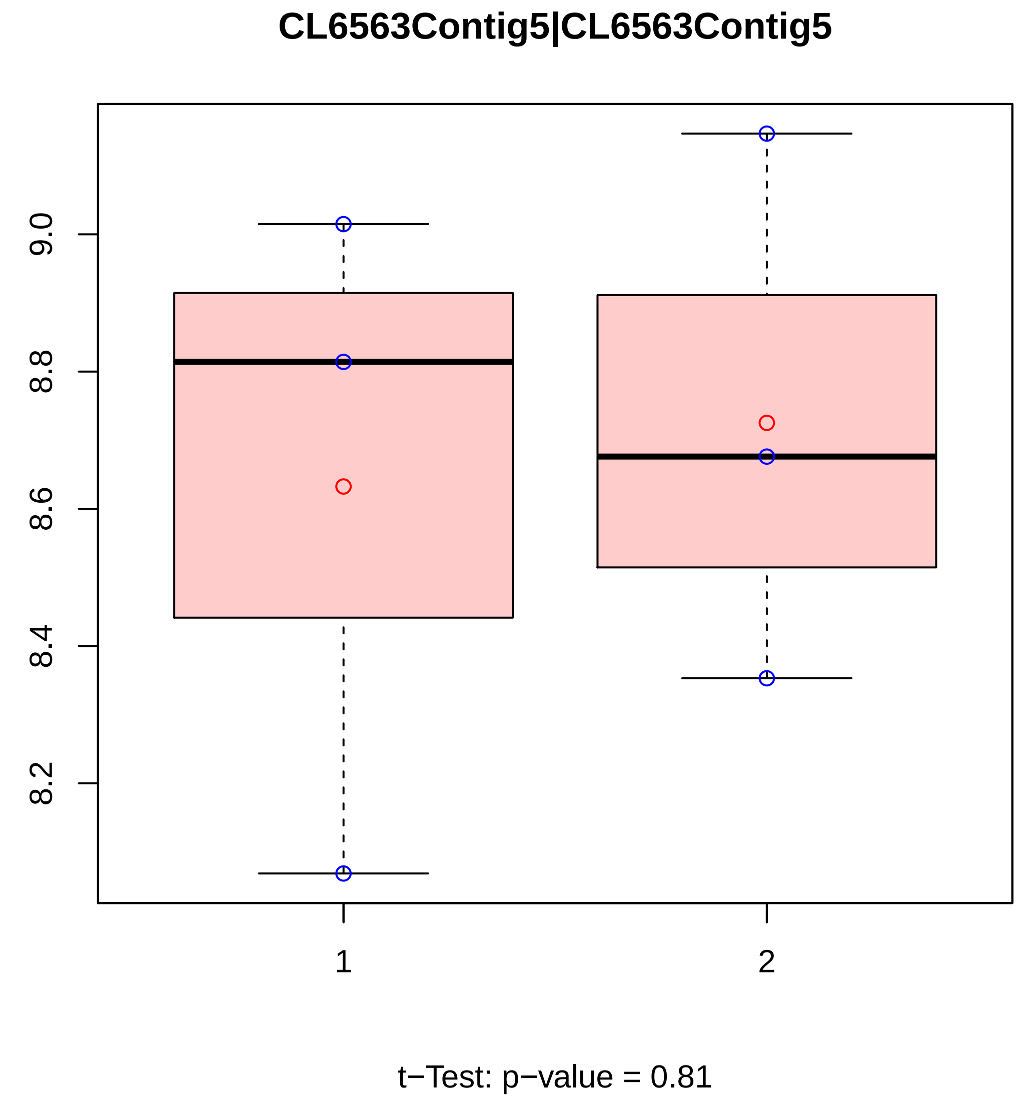

CL6563Contig5|CL6563Contig5
9.0
8.8
8.6
8.4
8.2
1
2
t−Test: p−value = 0.81

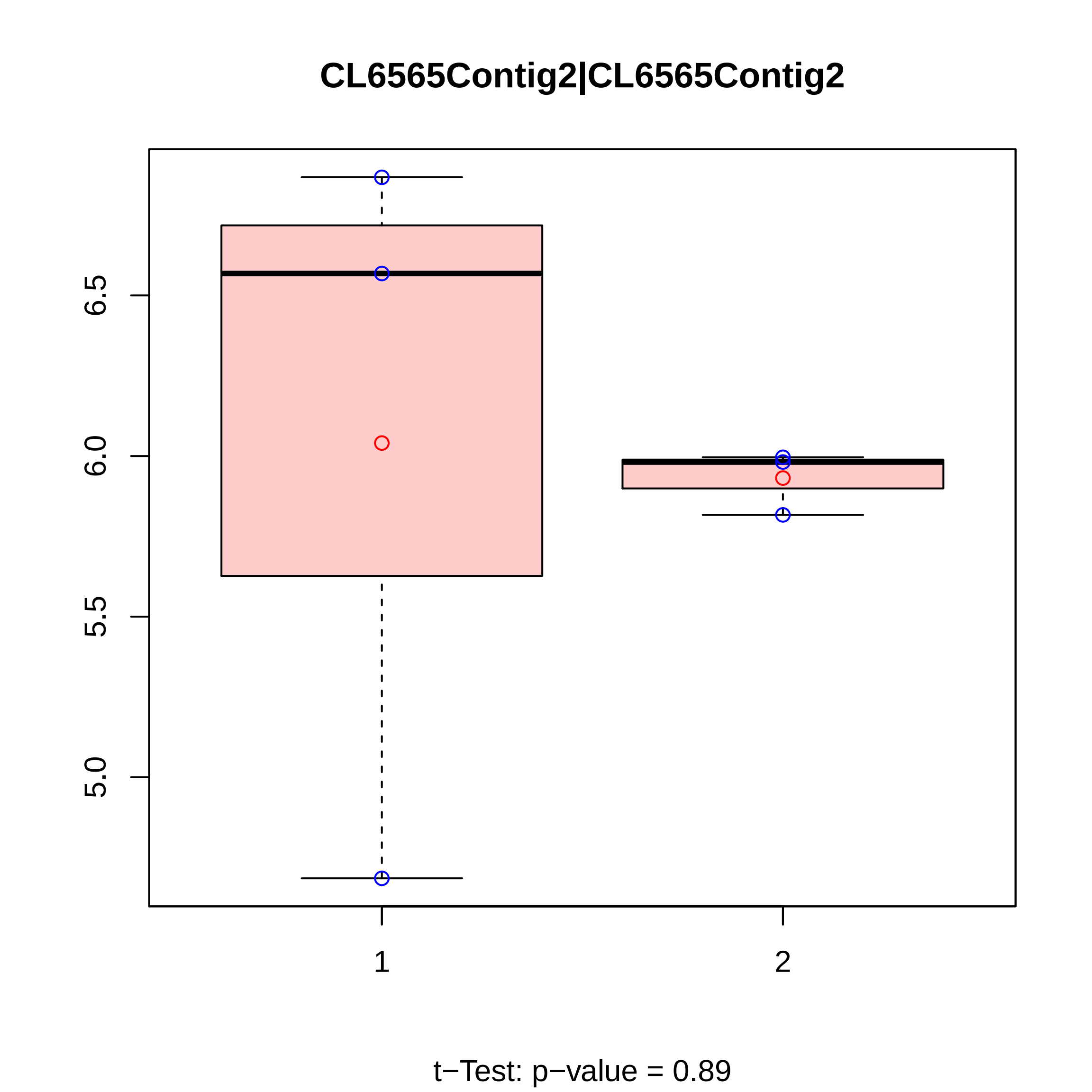
CL6565Contig2|CL6565Contig2
6.5
6.0
5.5
5.0
1
2
t−Test: p−value = 0.89

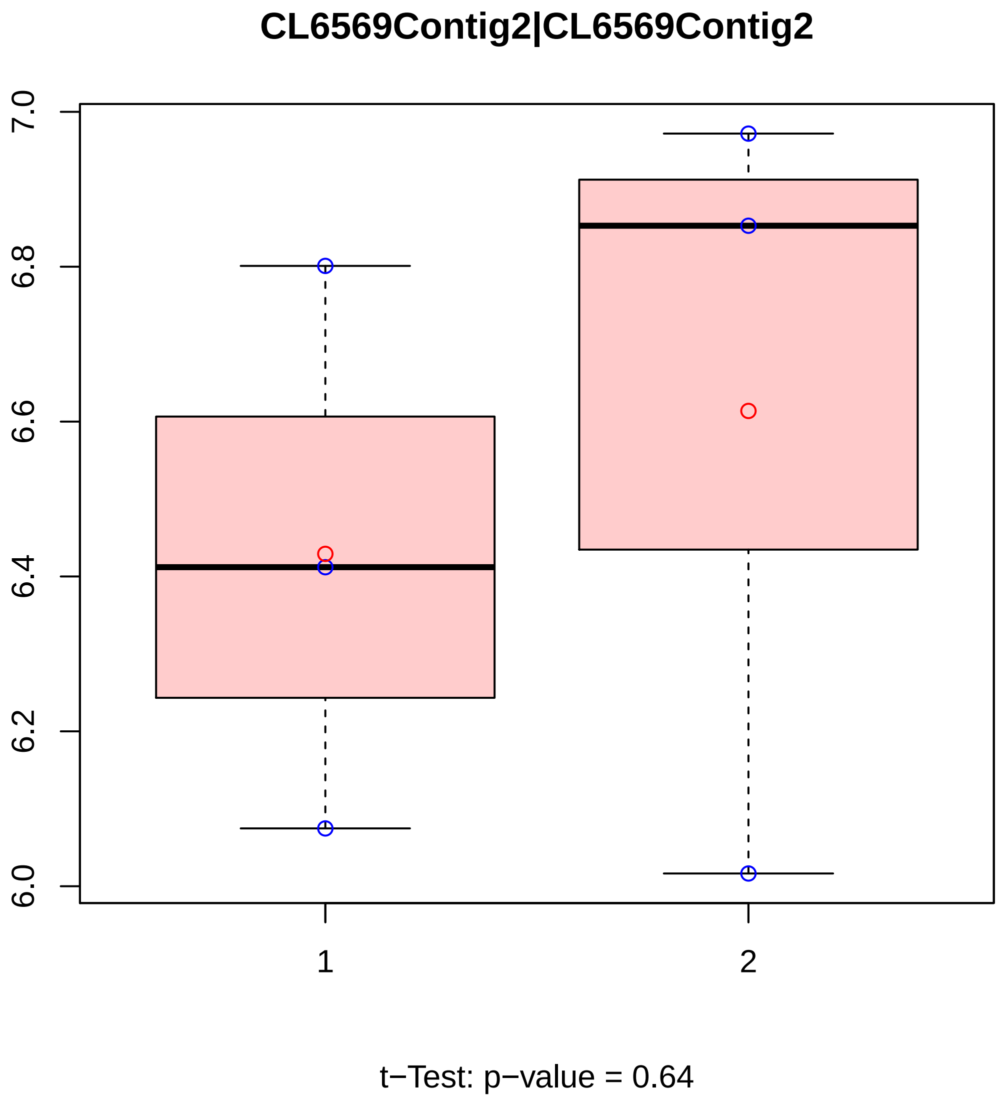
CL6569Contig2|CL6569Contig2
7.0
6.8
6.6
6.4
6.2
6.0
1
2
t−Test: p−value = 0.64

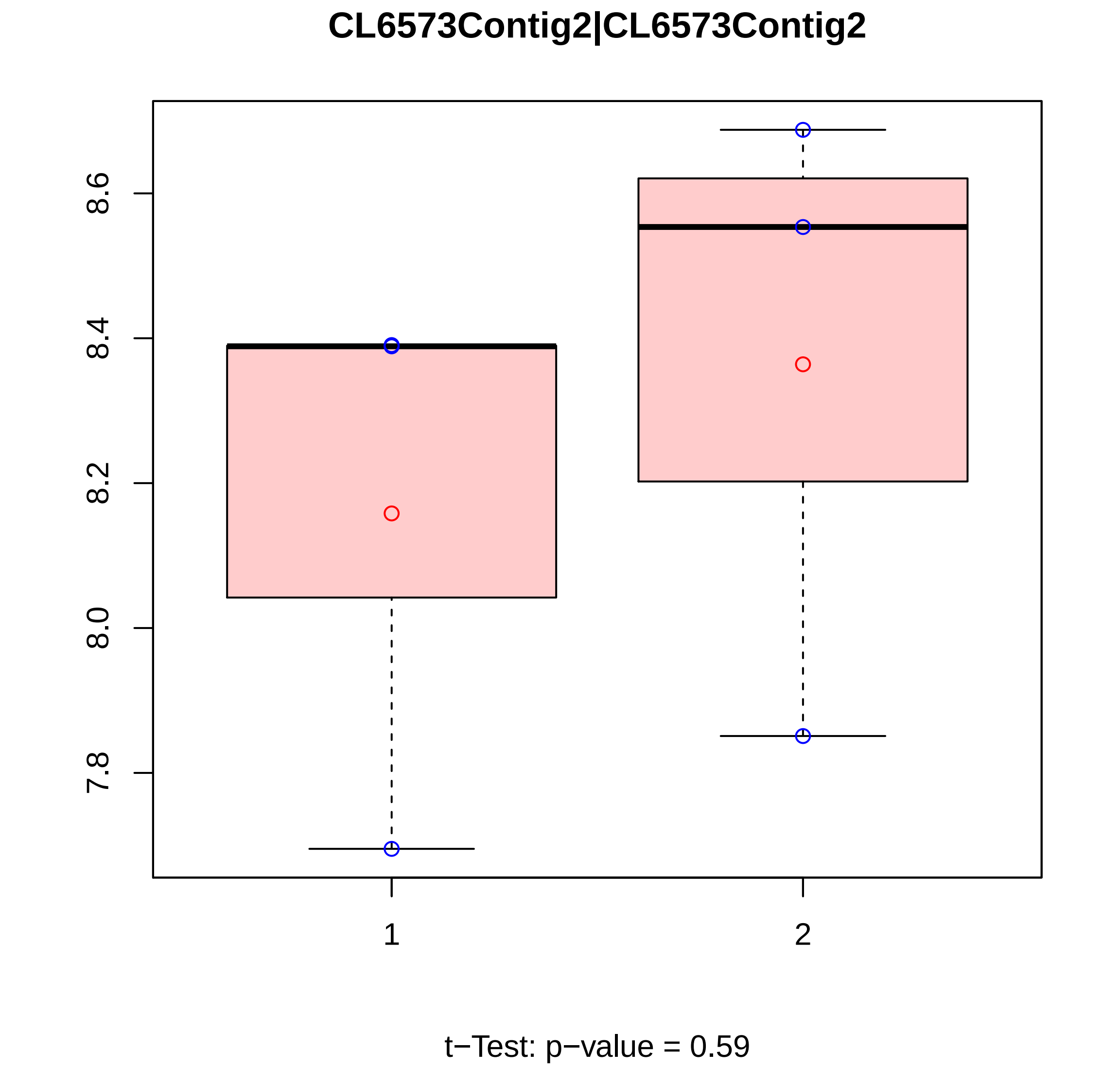

CL6573Contig2|CL6573Contig2
8.6
8.4
8.2
8.0
7.8
1
2
t−Test: p−value = 0.59

# CL65Contig6|CL65Contig6

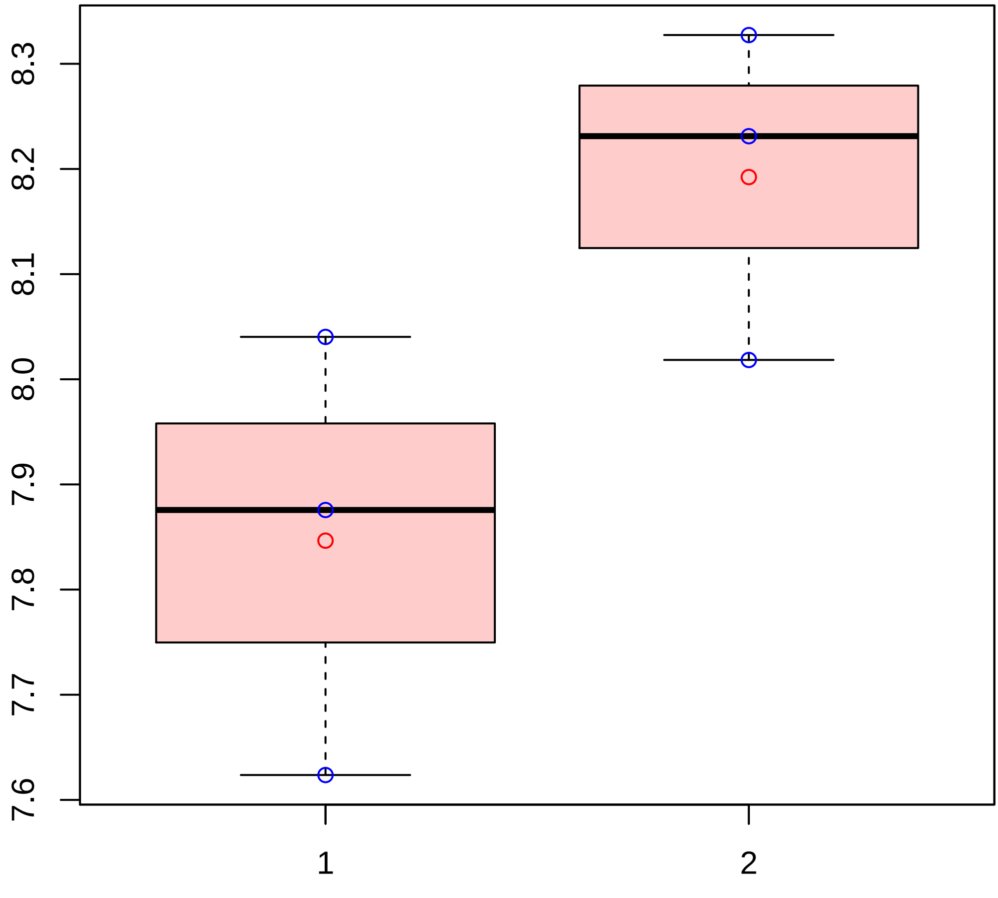


t−Test: p−value = 0.09

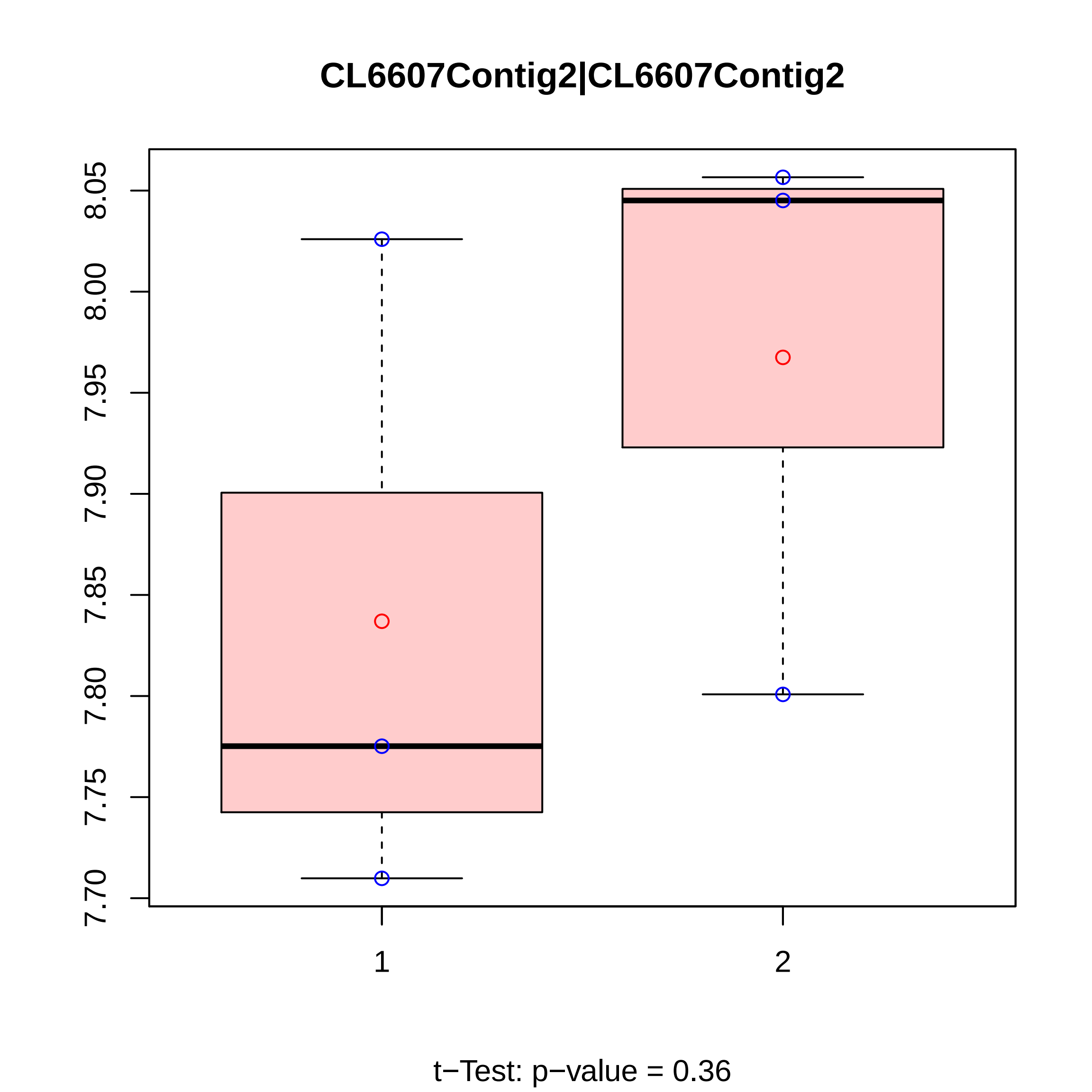

CL6607Contig2|CL6607Contig2
8.05
8.00
7.95
7.90
7.85
7.80
7.75
7.70
1
2
t−Test: p−value = 0.36

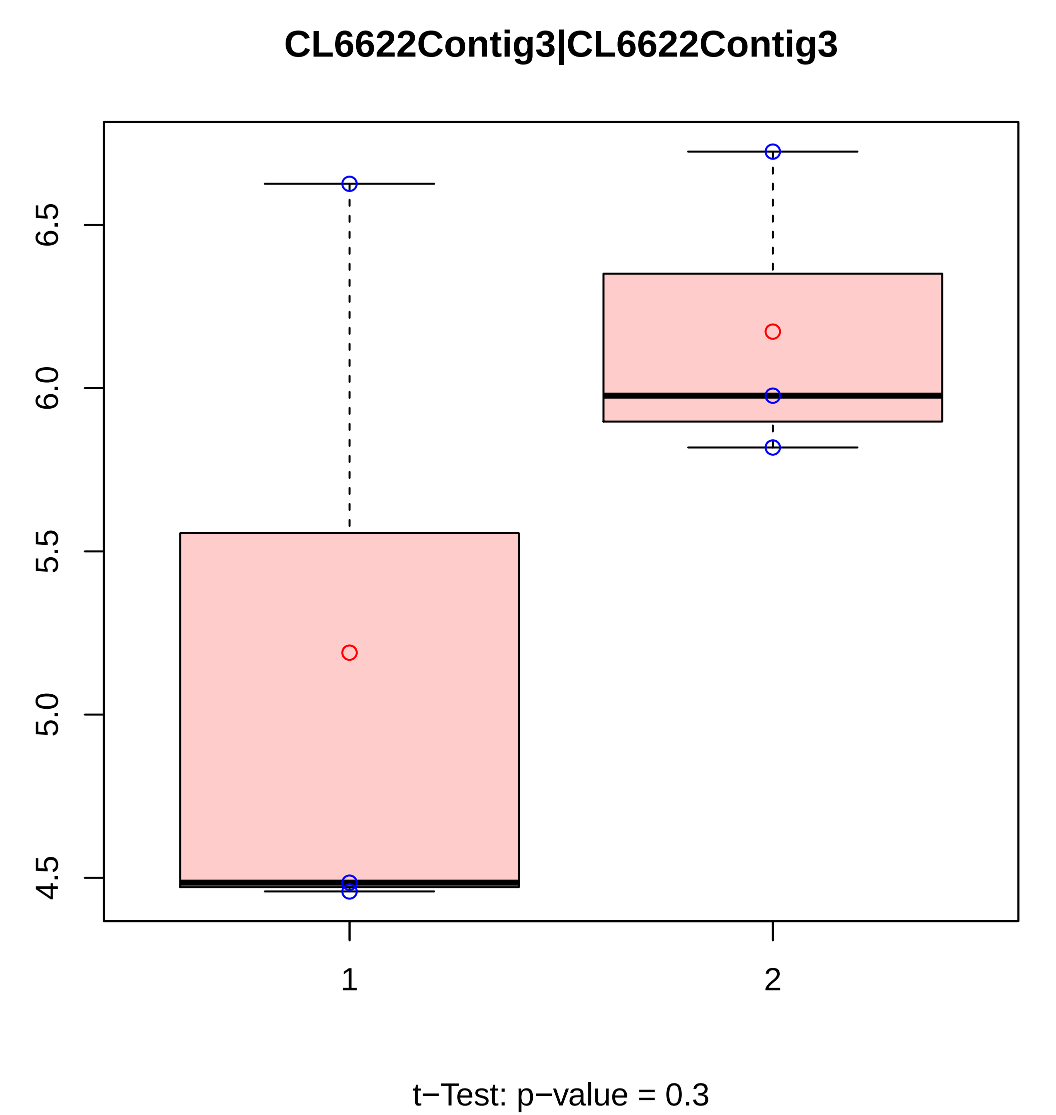
CL6622Contig3|CL6622Contig3
6.5
6.0
5.5
5.0
4.5
1
2
t−Test: p−value = 0.3

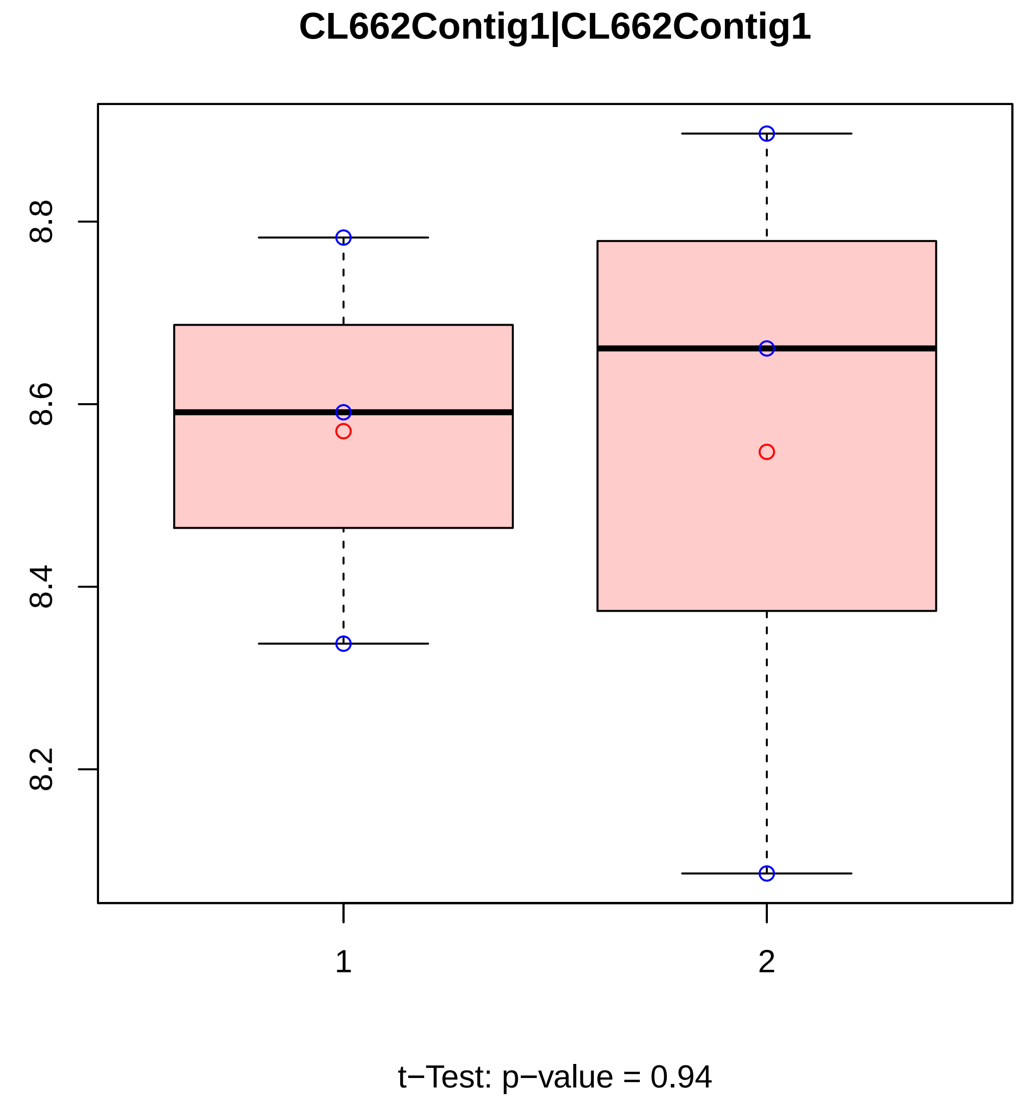
CL662Contig1|CL662Contig1
8.2
8.4
8.6
8.8
1
2
t−Test: p−value = 0.94

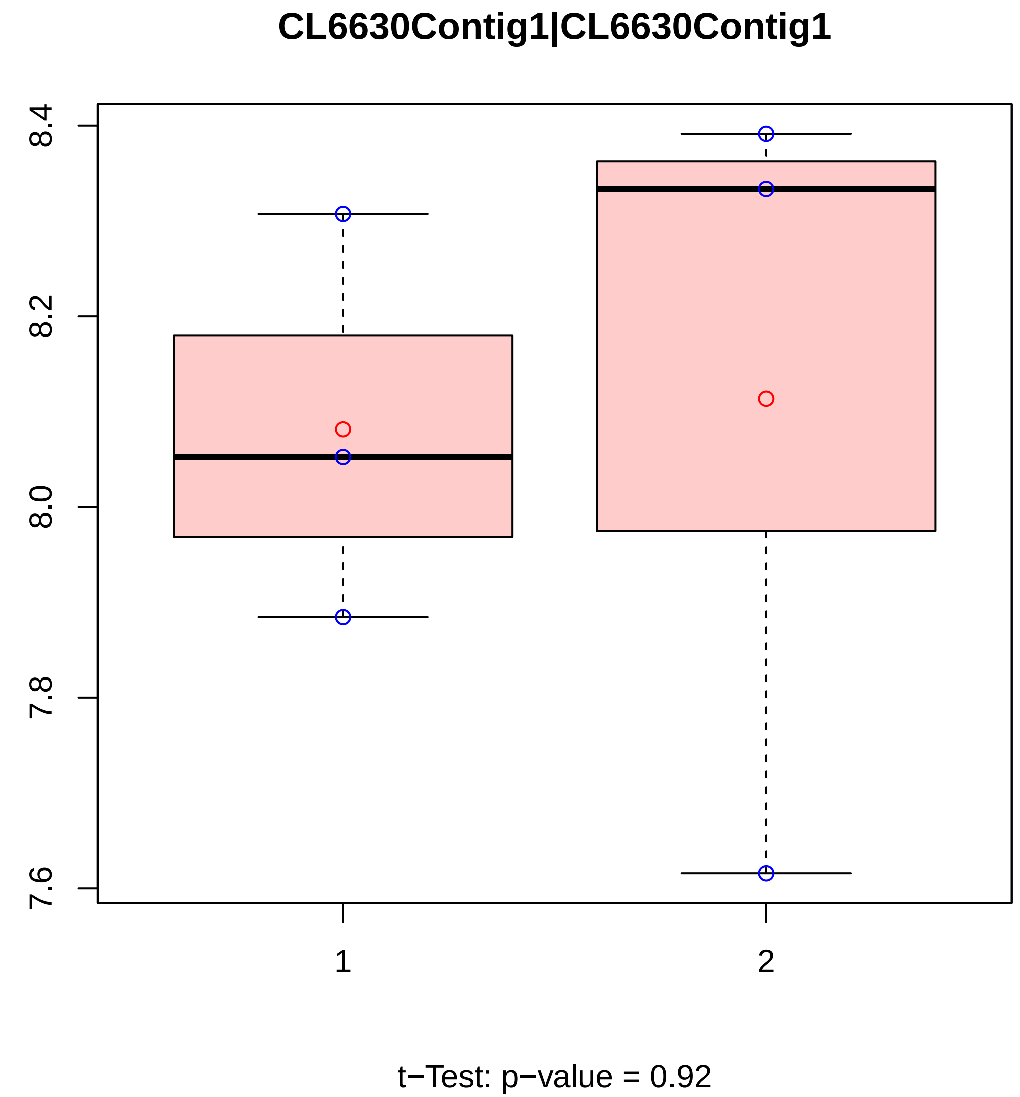

CL6630Contig1|CL6630Contig1
8.4
8.2
8.0
7.8
7.6
1
2
t−Test: p−value = 0.92

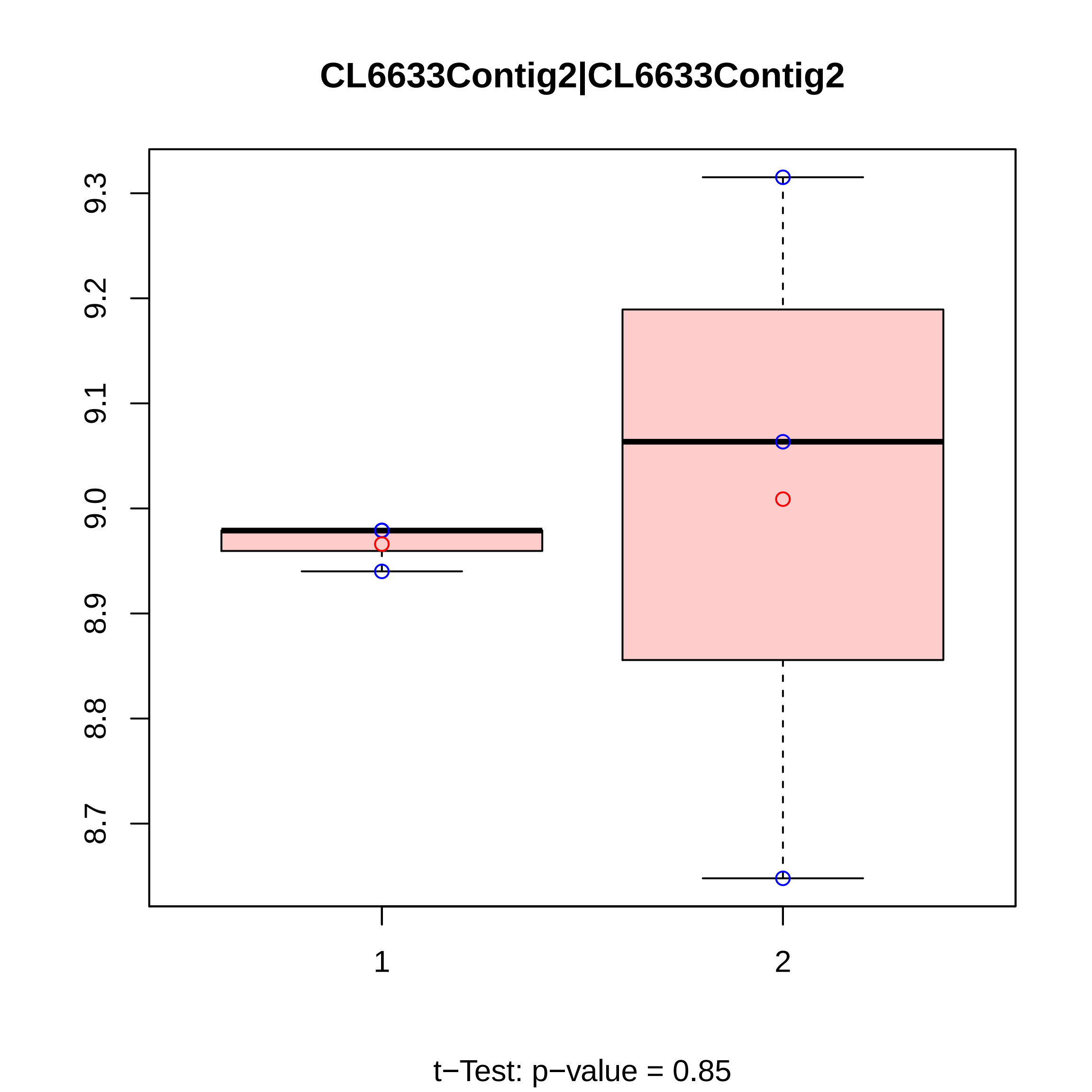
CL6633Contig2|CL6633Contig2
9.3
9.2
9.1
9.0
8.9
8.8
8.7
1
2
t−Test: p−value = 0.85

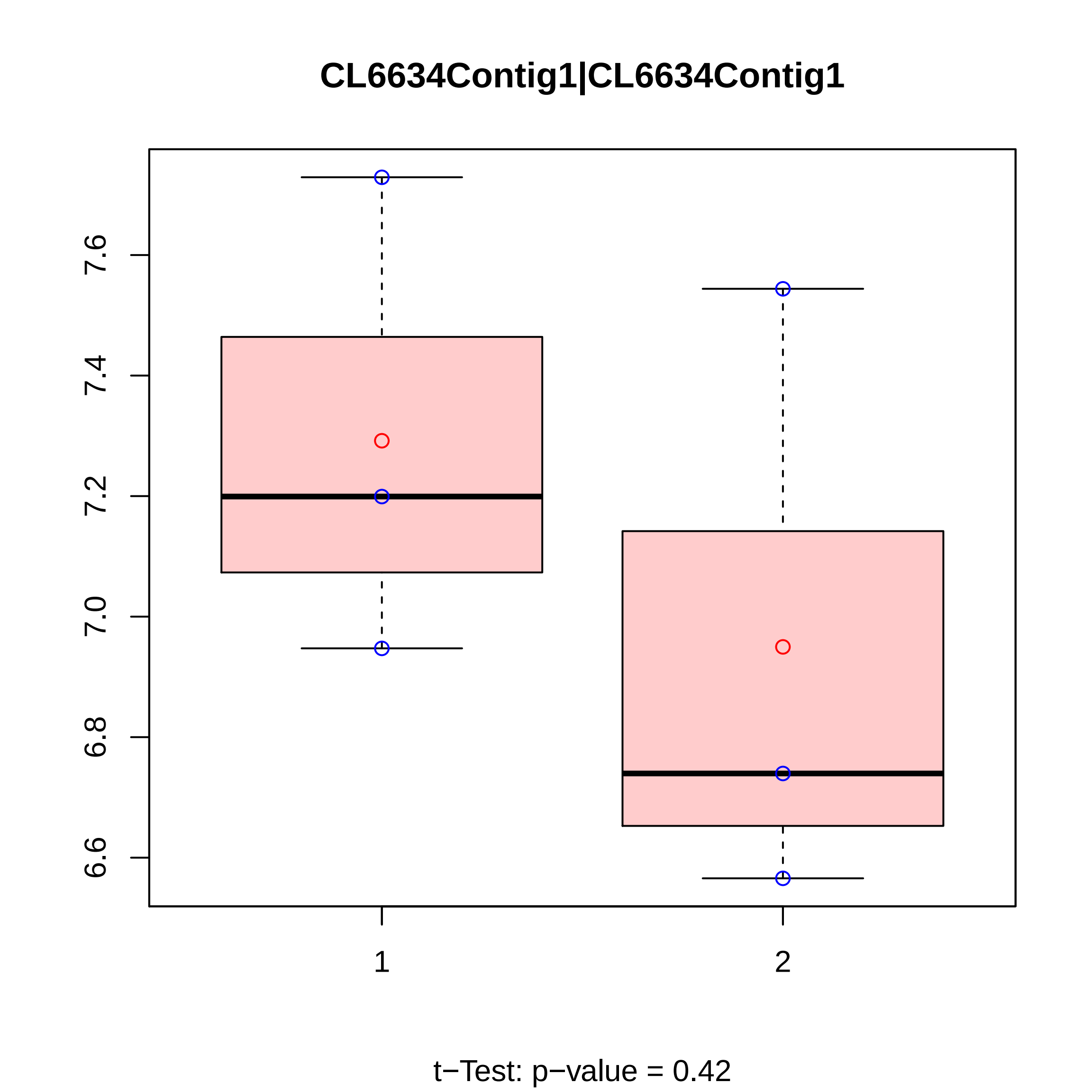
CL6634Contig1|CL6634Contig1
7.6
7.4
7.2
7.0
6.8
6.6
1
2
t−Test: p−value = 0.42

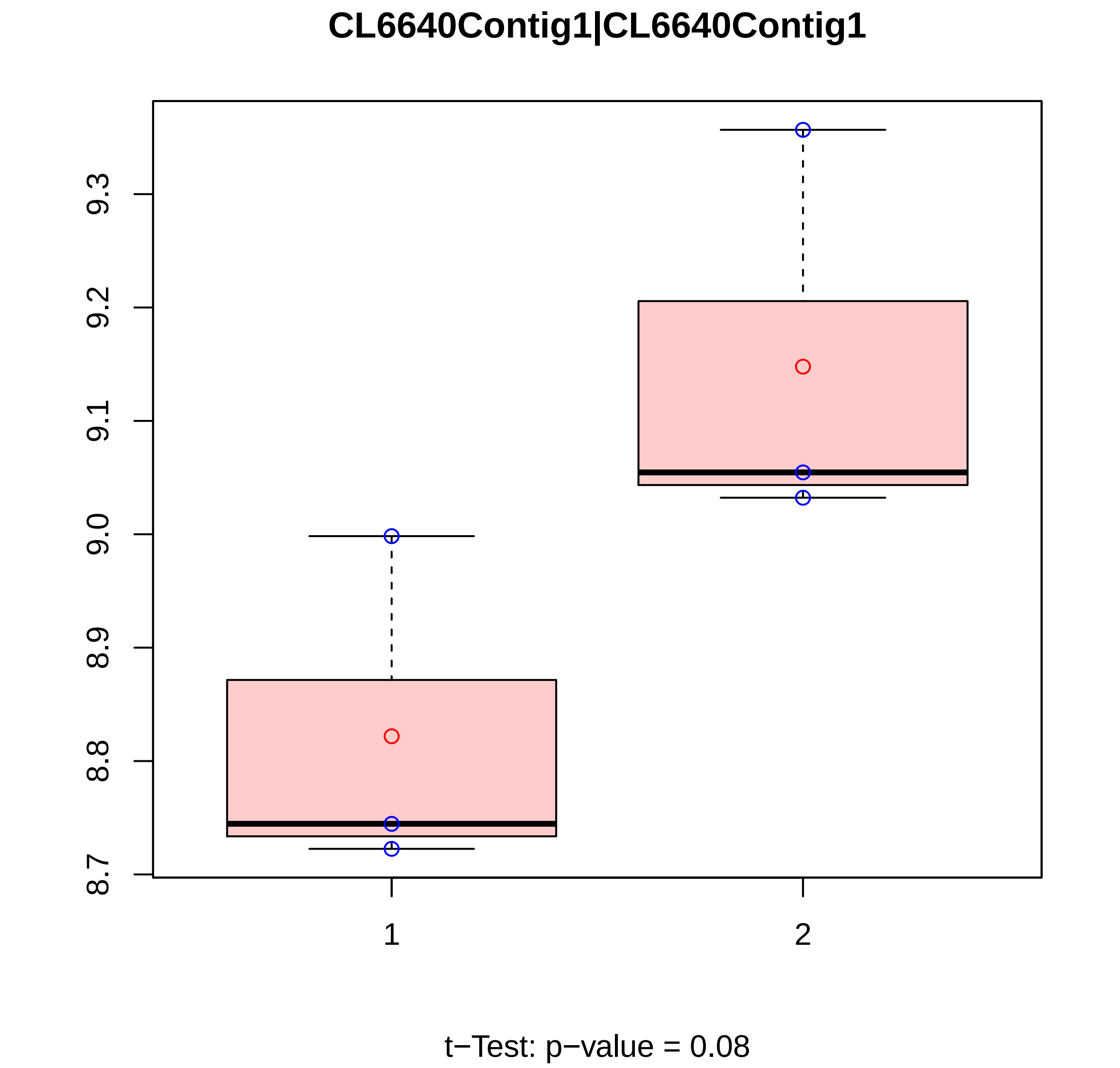

CL6640Contig1|CL6640Contig1
9.3
9.2
9.1
9.0
8.9
8.8
8.7
1
2
t−Test: p−value = 0.08

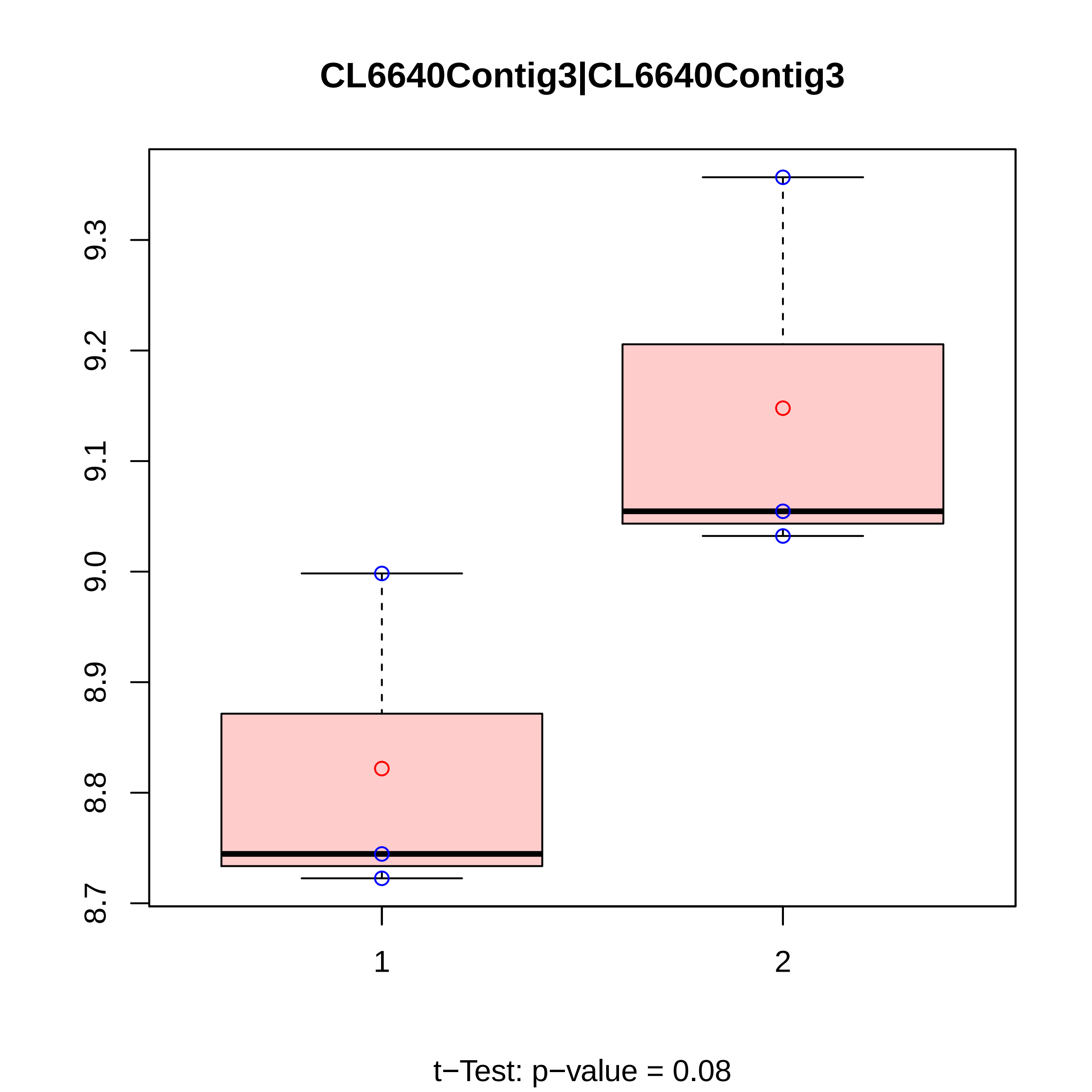

CL6640Contig3|CL6640Contig3
9.3
9.2
9.1
9.0
8.9
8.8
8.7
1
2
t−Test: p−value = 0.08

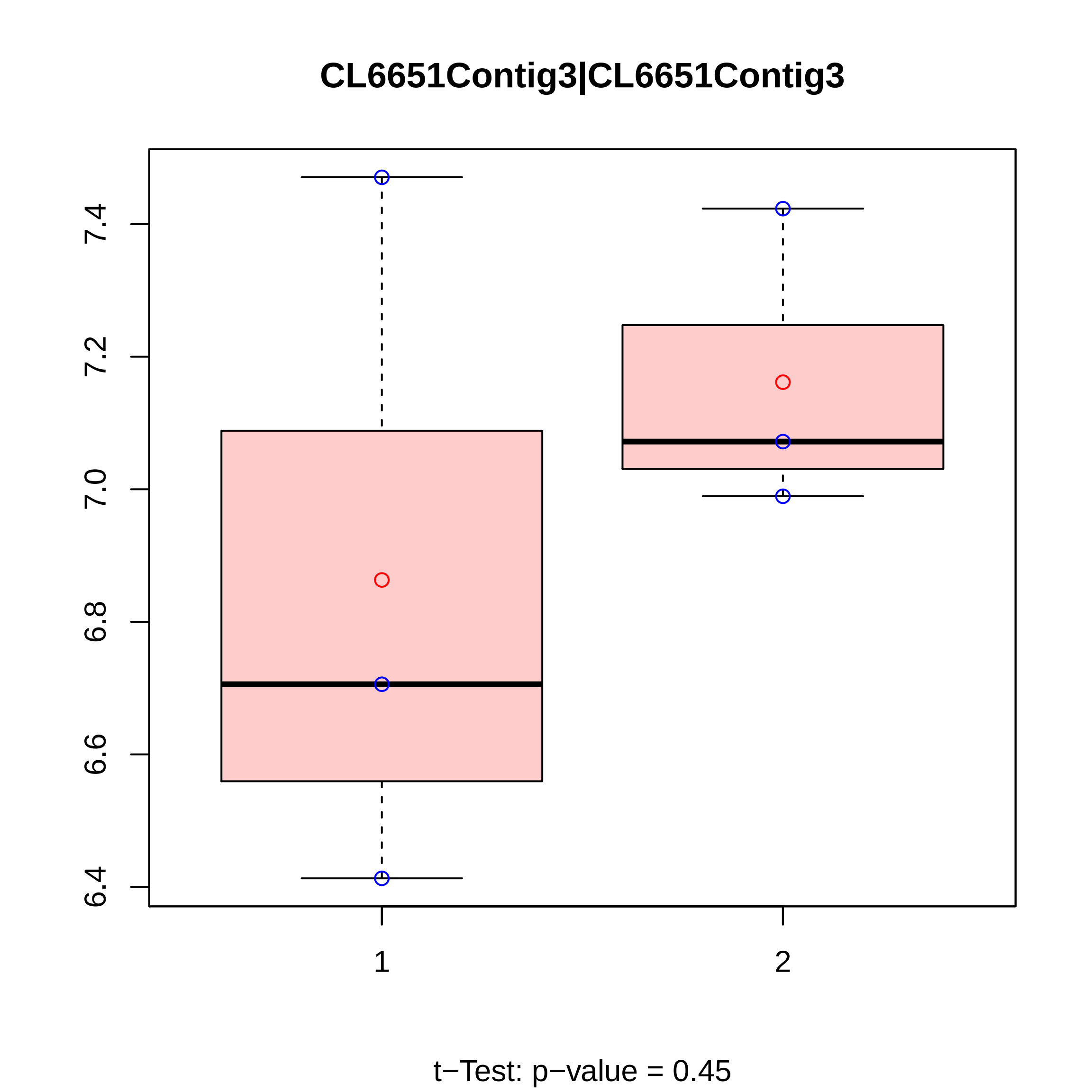
CL6651Contig3|CL6651Contig3
7.4
7.2
7.0
6.8
6.6
6.4
1
2
t−Test: p−value = 0.45

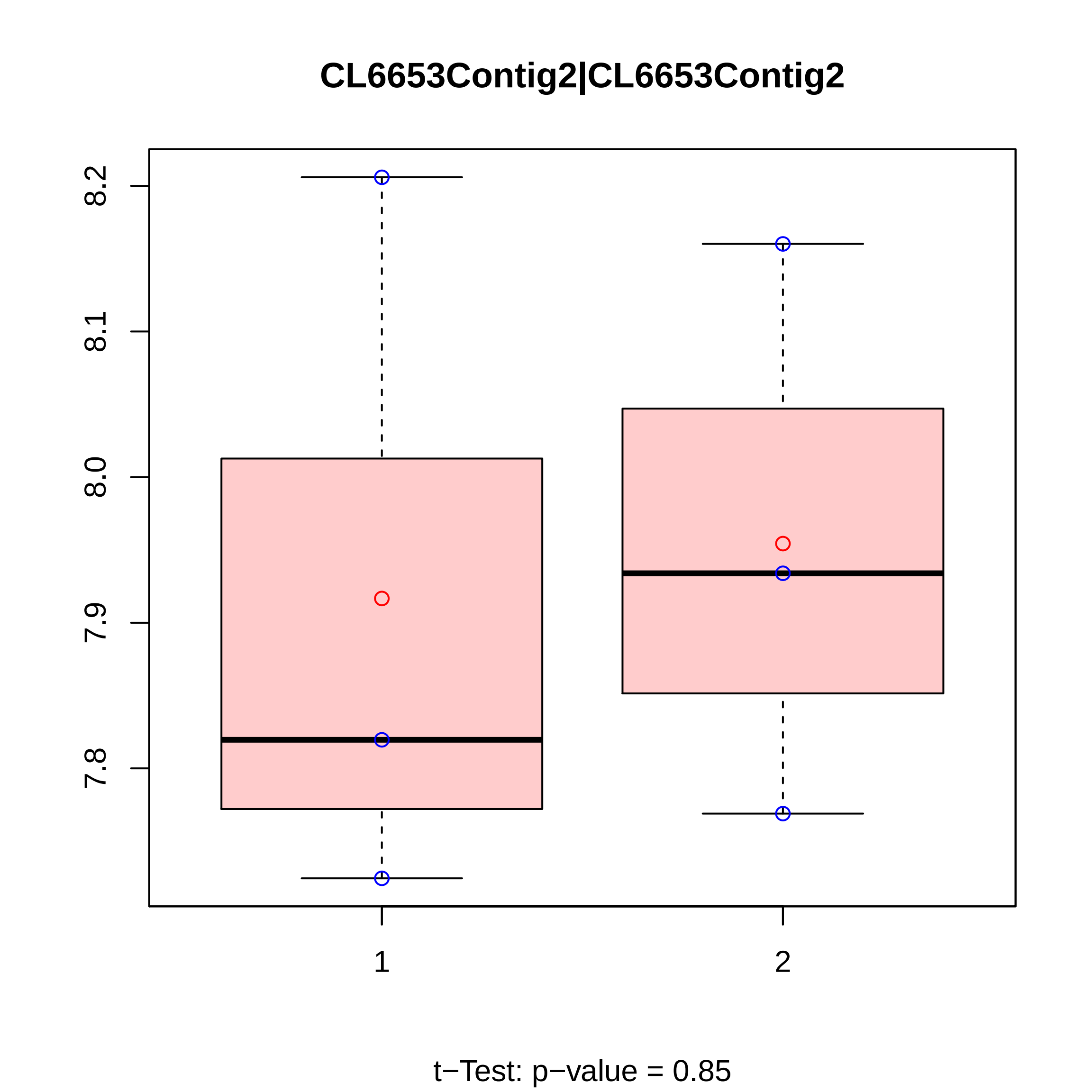
CL6653Contig2|CL6653Contig2
8.2
8.1
8.0
7.9
7.8
1
2
t−Test: p−value = 0.85

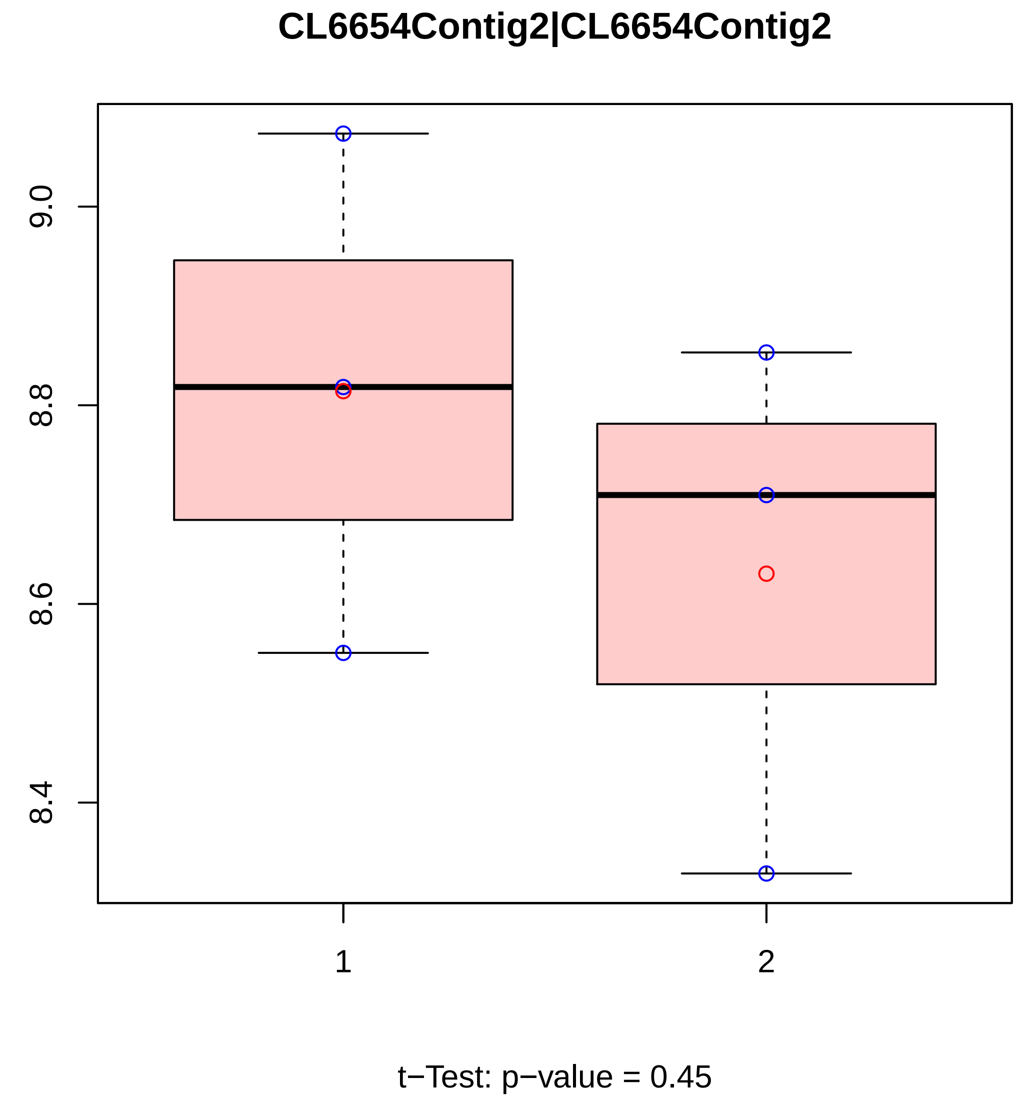
CL6654Contig2|CL6654Contig2
9.0
8.8
8.6
8.4
1
2
t−Test: p−value = 0.45

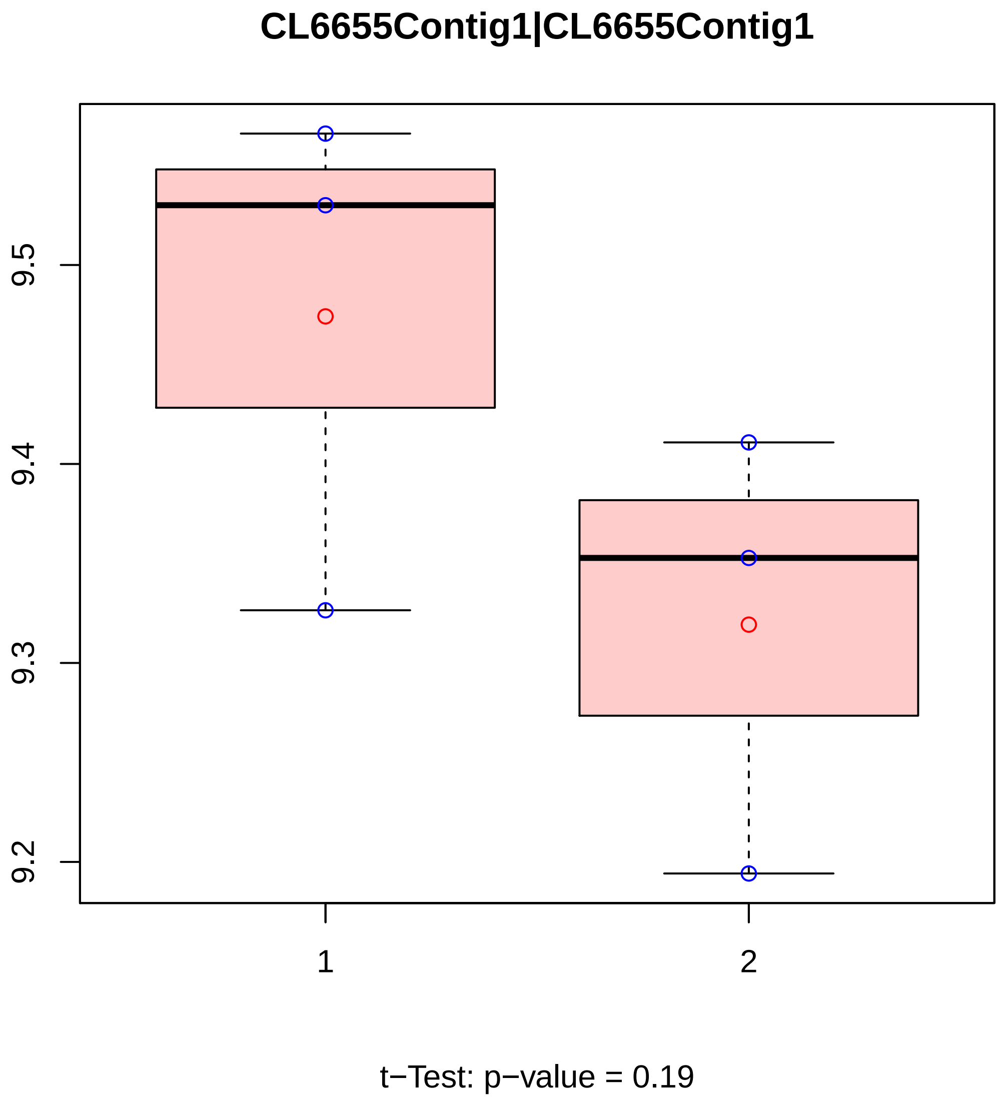

CL6655Contig1|CL6655Contig1
9.2
9.3
9.4
9.5
1
2
t−Test: p−value = 0.19

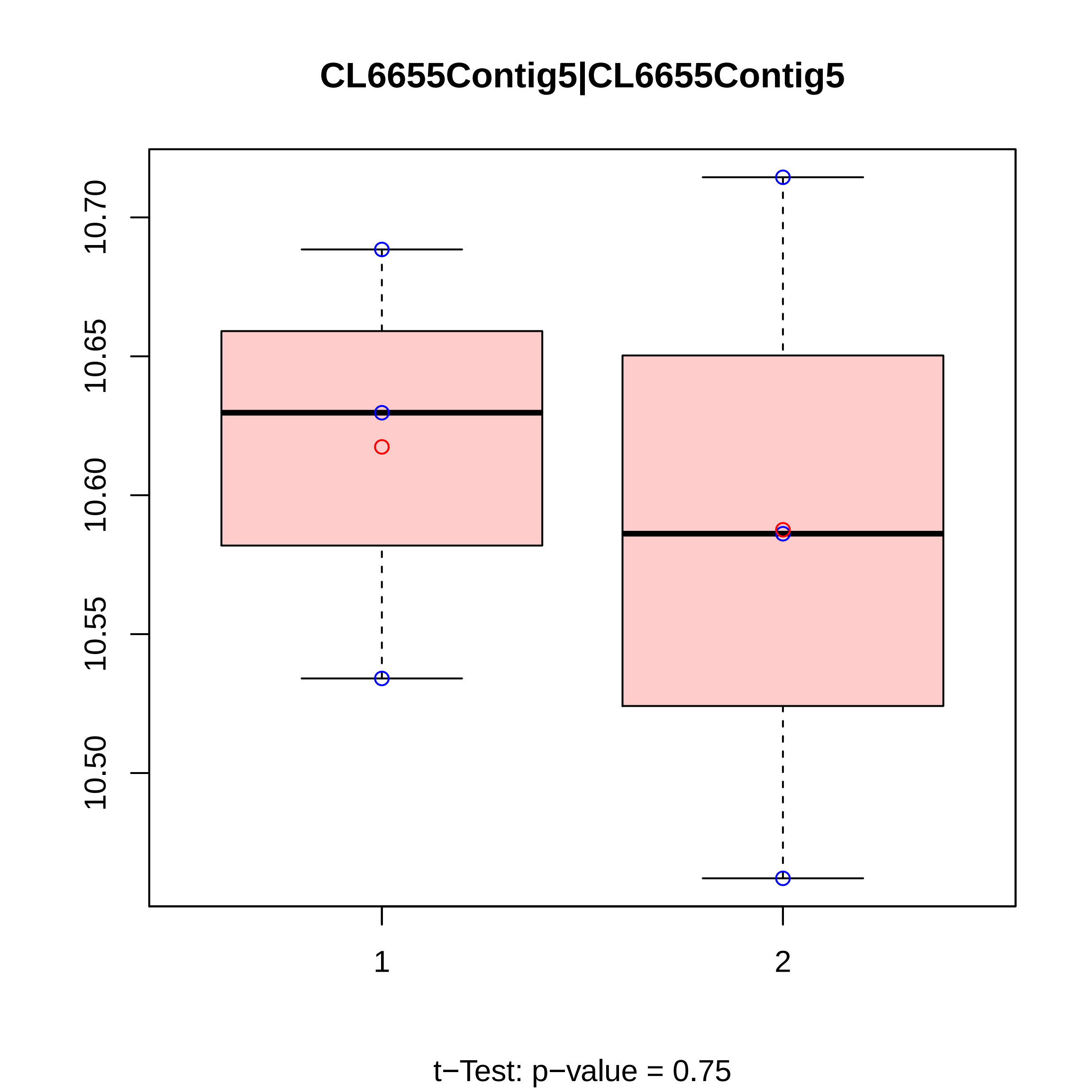

CL6655Contig5|CL6655Contig5
10.50
10.55
10.60
10.65
10.70
1
2
t−Test: p−value = 0.75

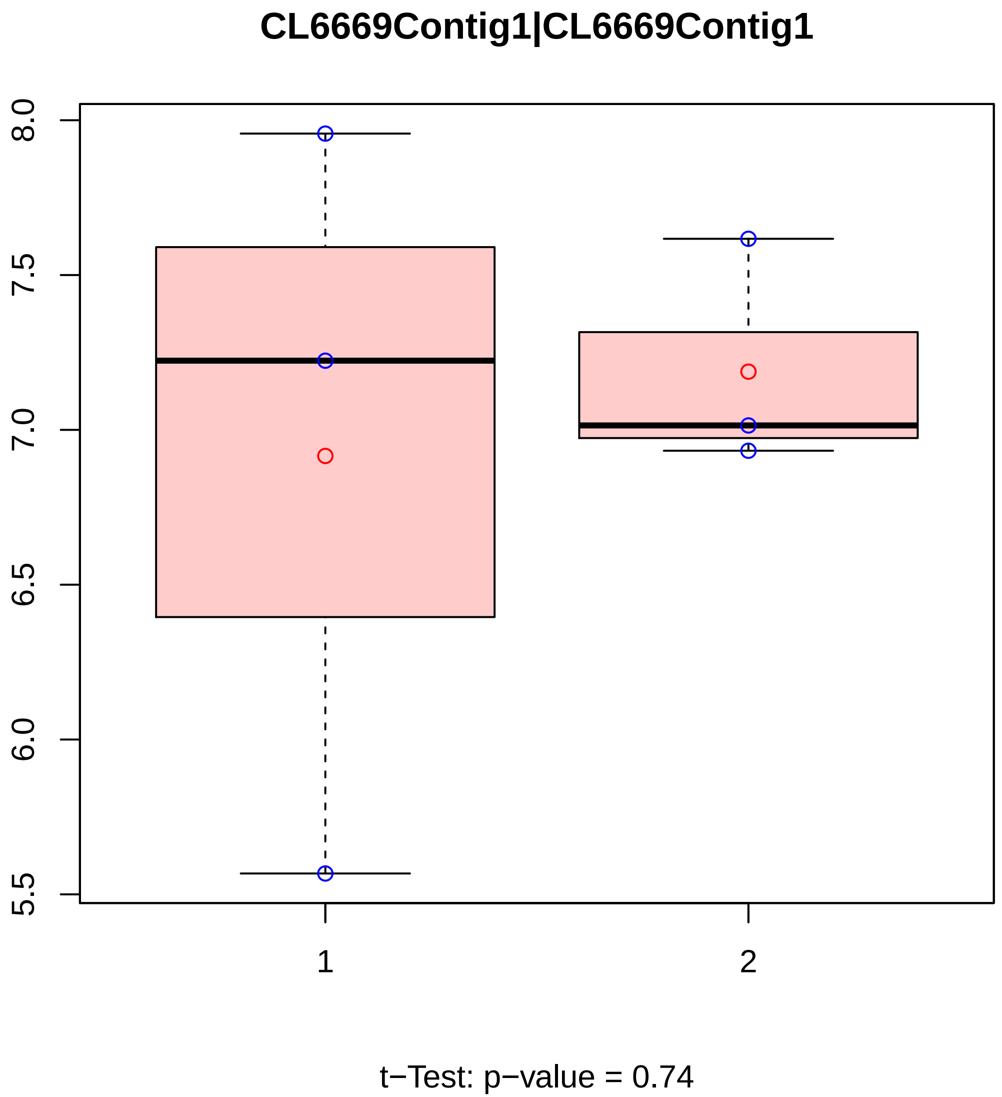

CL6669Contig1|CL6669Contig1
8.0
7.5
7.0
6.5
6.0
5.5
1
2
t−Test: p−value = 0.74

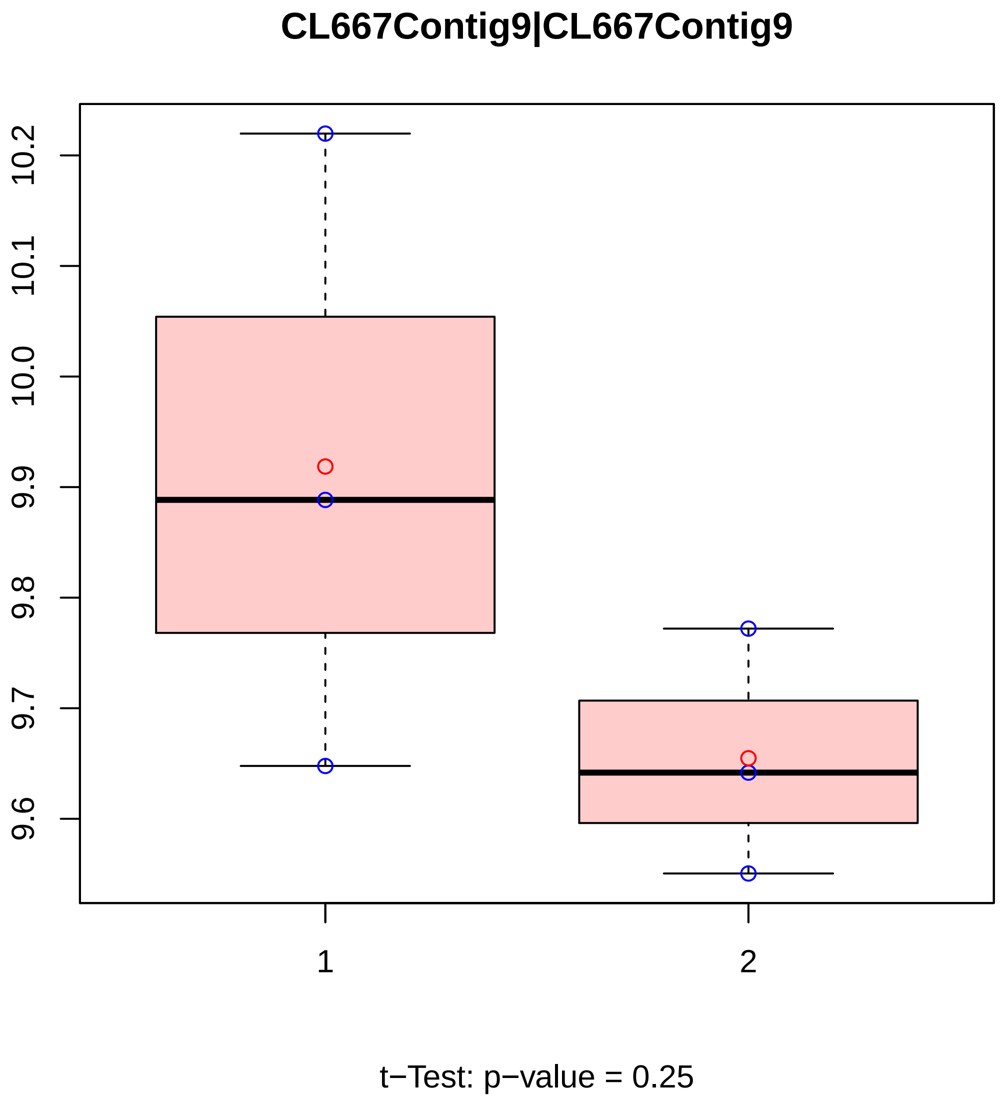
CL667Contig9|CL667Contig9
10.2
10.1
10.0
9.9
9.8
9.7
9.6
1
2
t−Test: p−value = 0.25

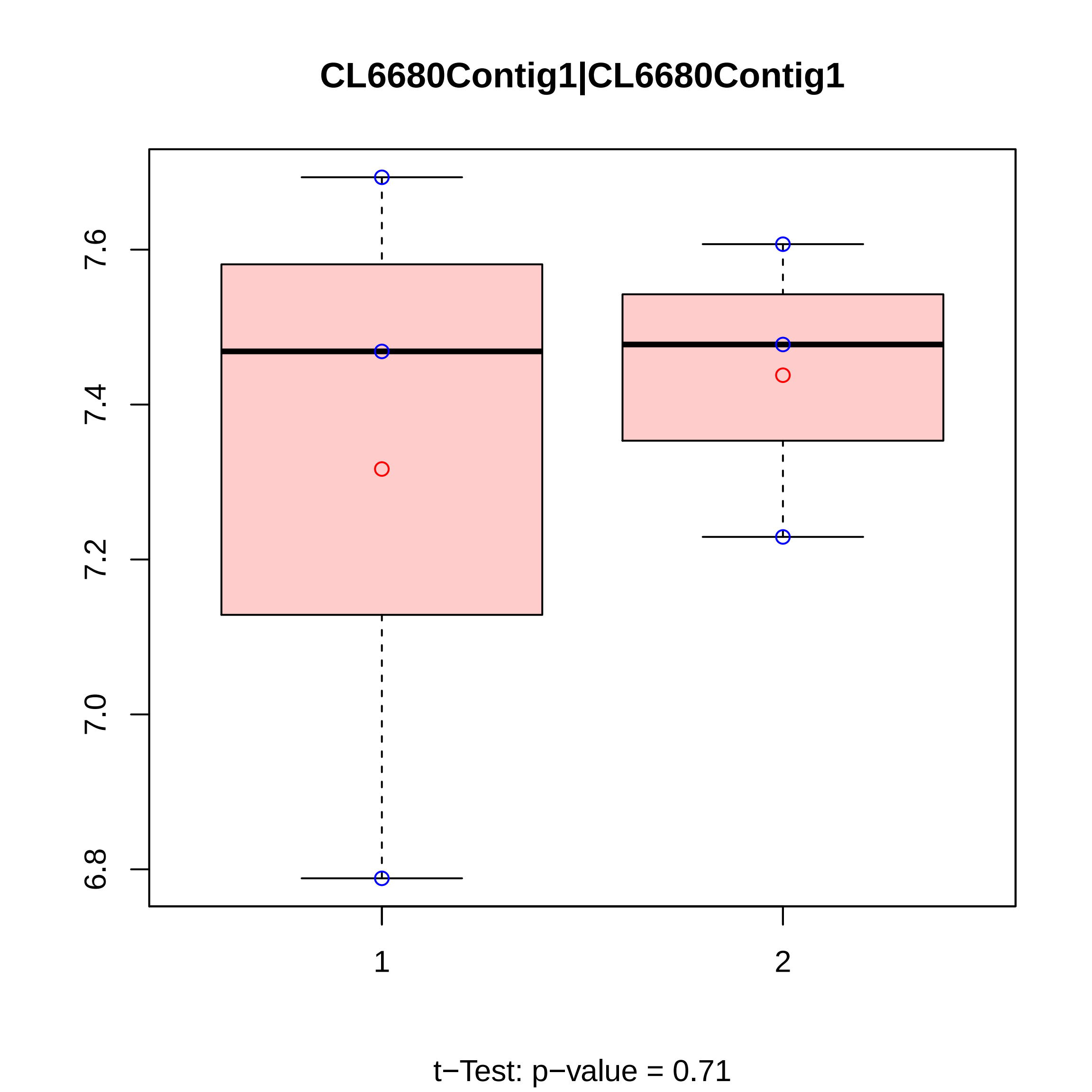
CL6680Contig1|CL6680Contig1
7.6
7.4
7.2
7.0
6.8
1
2
t−Test: p−value = 0.71

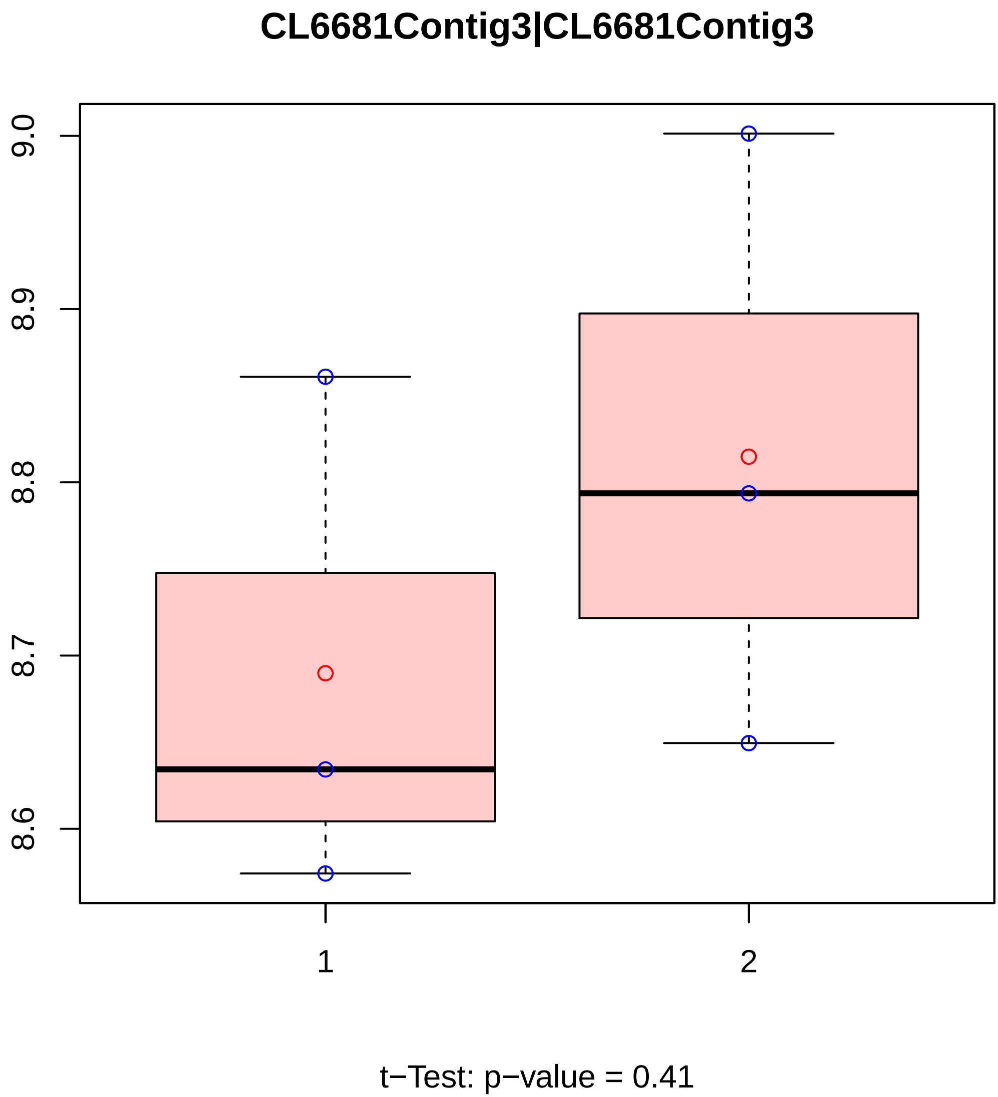
CL6681Contig3|CL6681Contig3
9.0
8.9
8.8
8.7
8.6
1
2
t−Test: p−value = 0.41

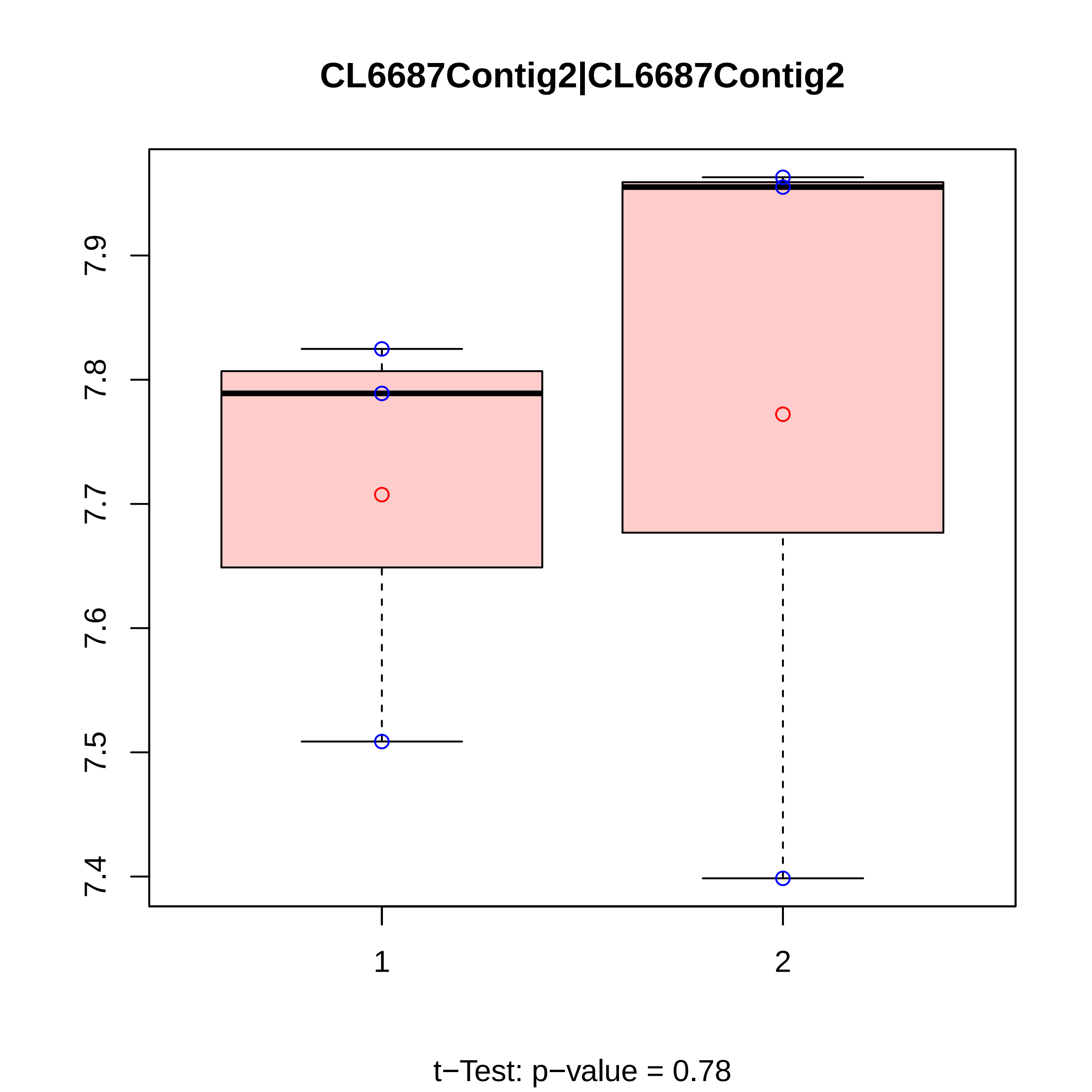
CL6687Contig2|CL6687Contig2
7.4
7.5
7.6
7.7
7.8
7.9
1
2
t−Test: p−value = 0.78

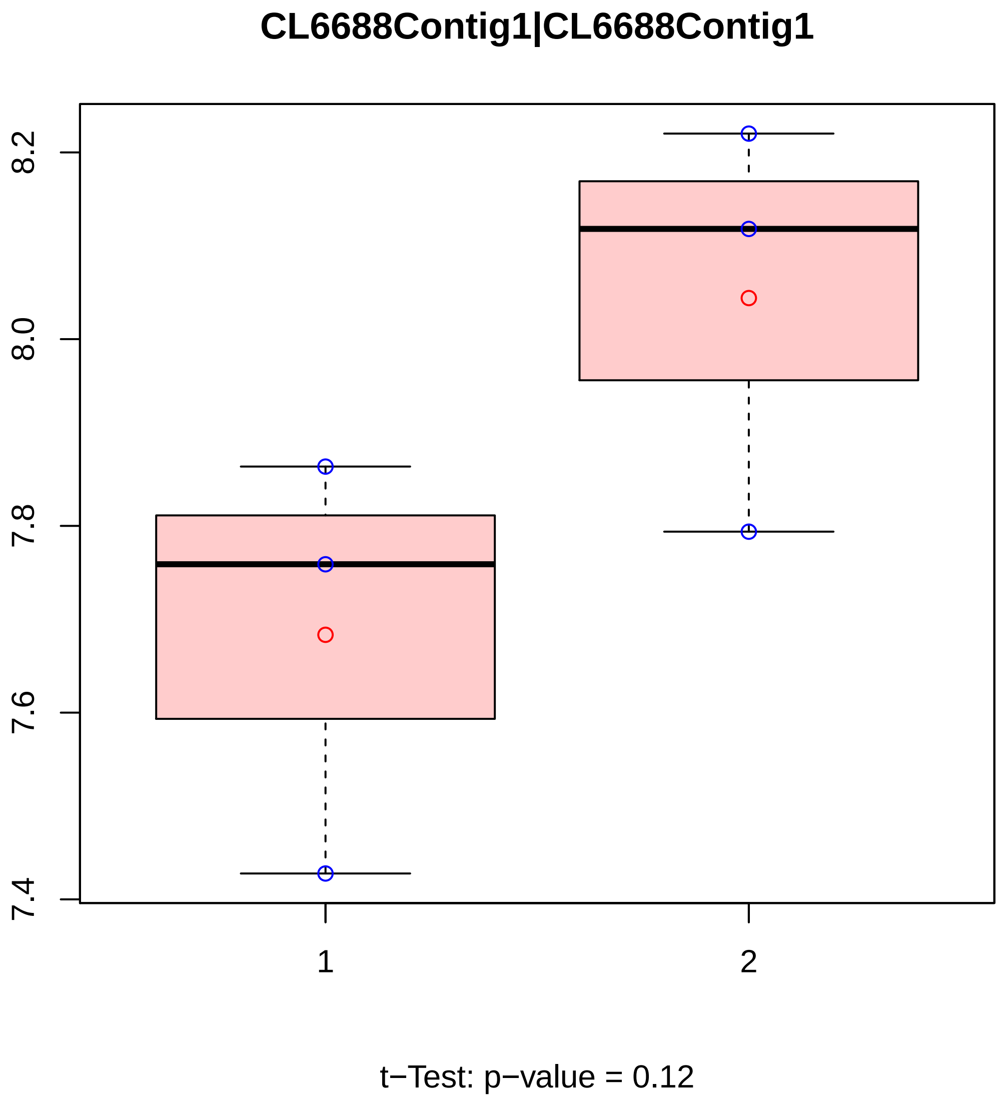

CL6688Contig1|CL6688Contig1
8.2
8.0
7.8
7.6
7.4
1
2
t−Test: p−value = 0.12

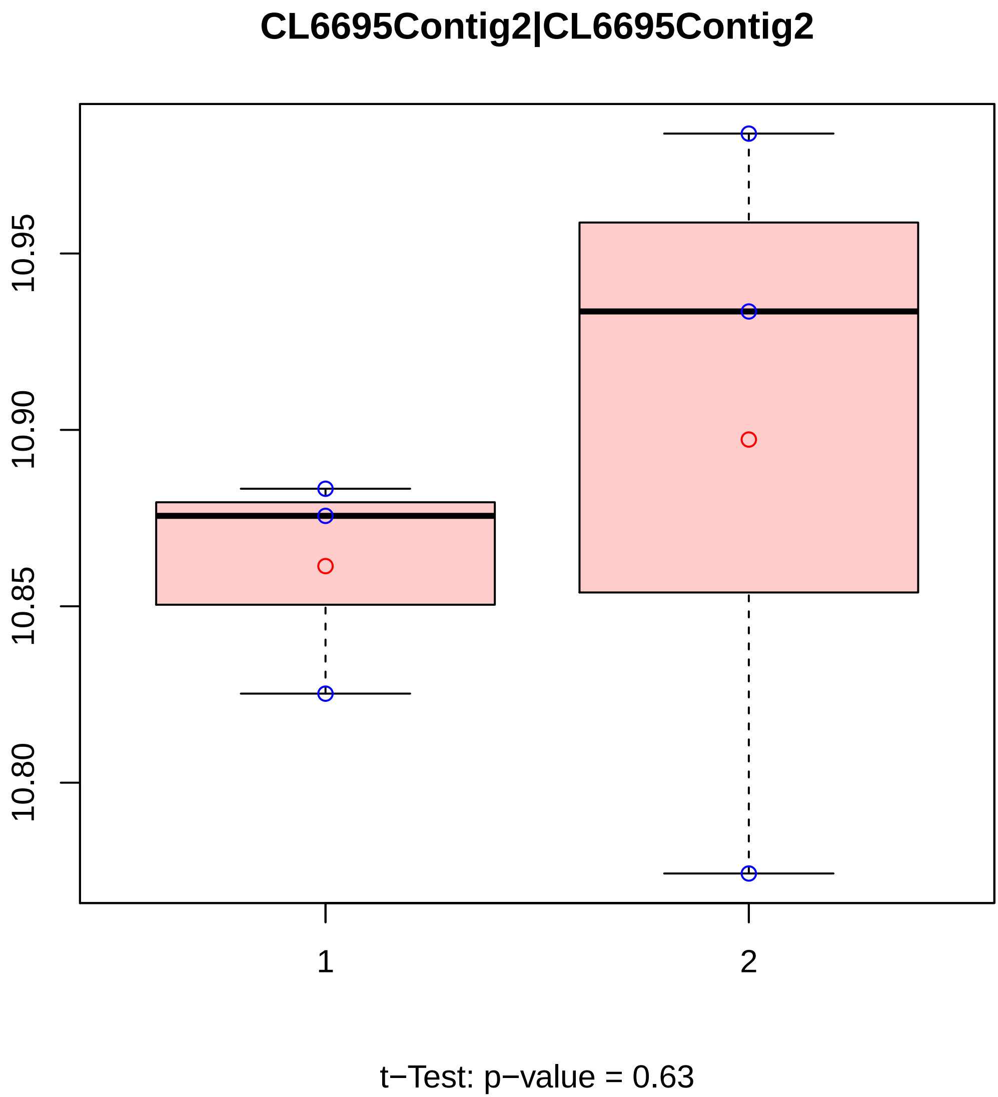

CL6695Contig2|CL6695Contig2
10.80
10.85
10.90
10.95
1
2
t−Test: p−value = 0.63

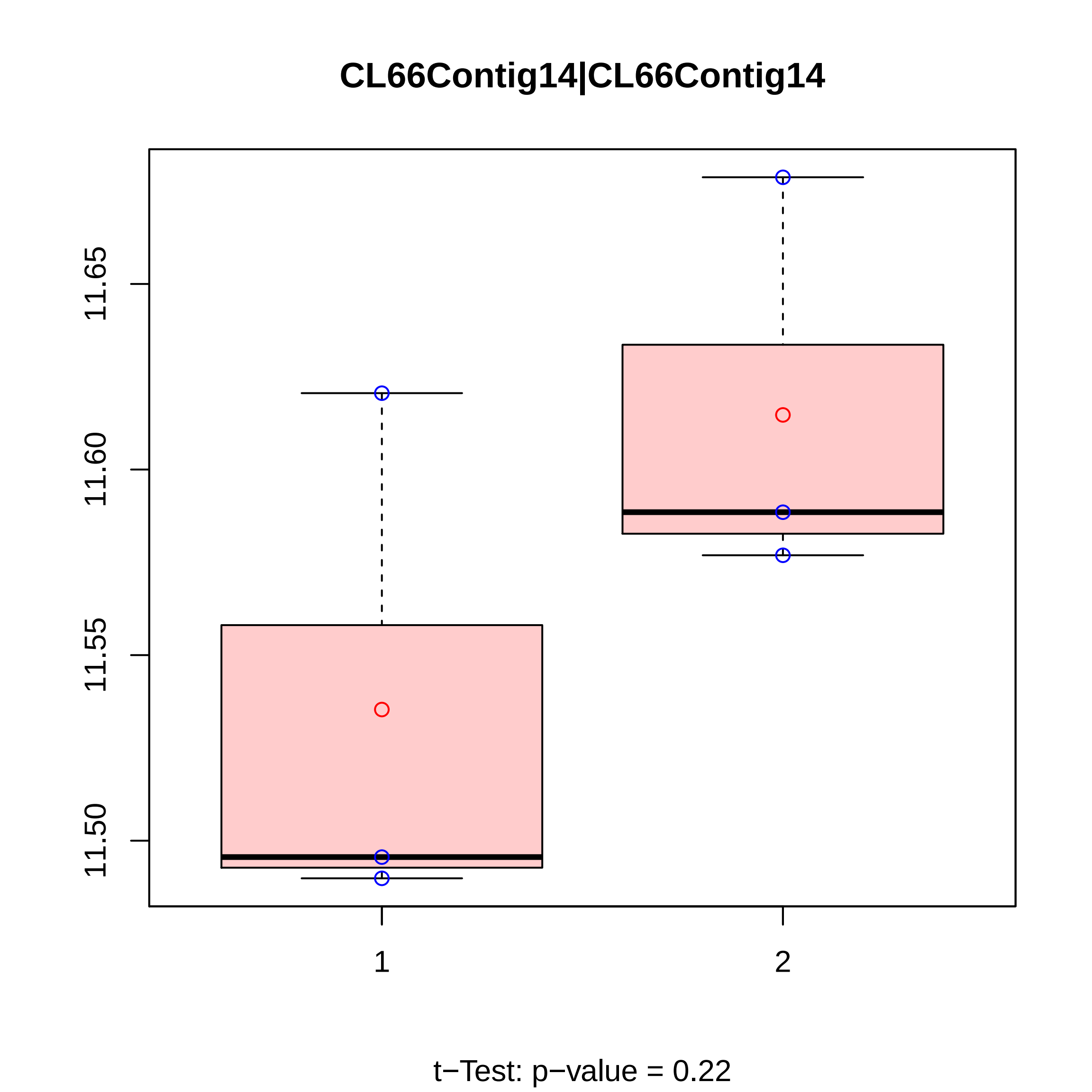
CL66Contig14|CL66Contig14
11.50
11.55
11.60
11.65
1
2
t−Test: p−value = 0.22

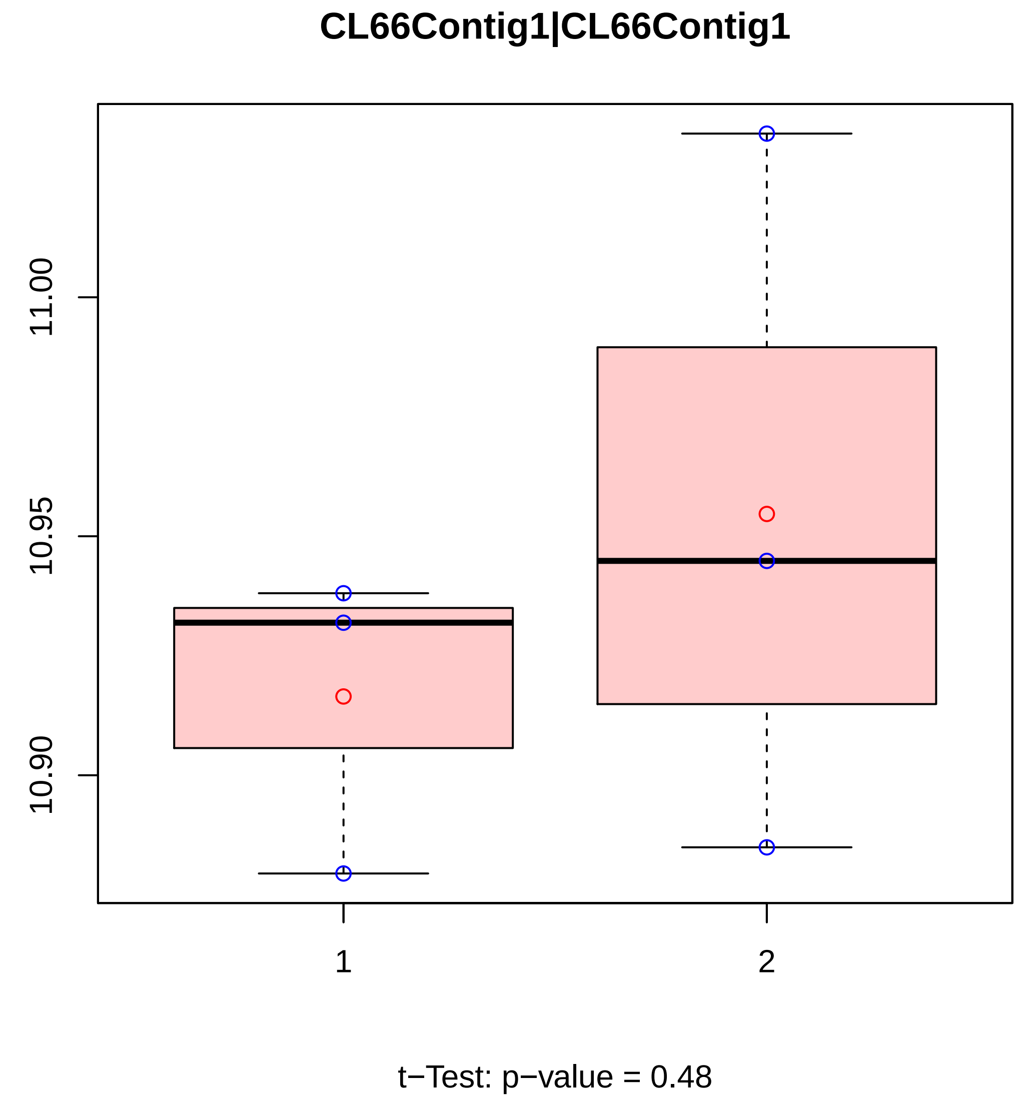
CL66Contig1|CL66Contig1
11.00
10.95
10.90
1
2
t−Test: p−value = 0.48

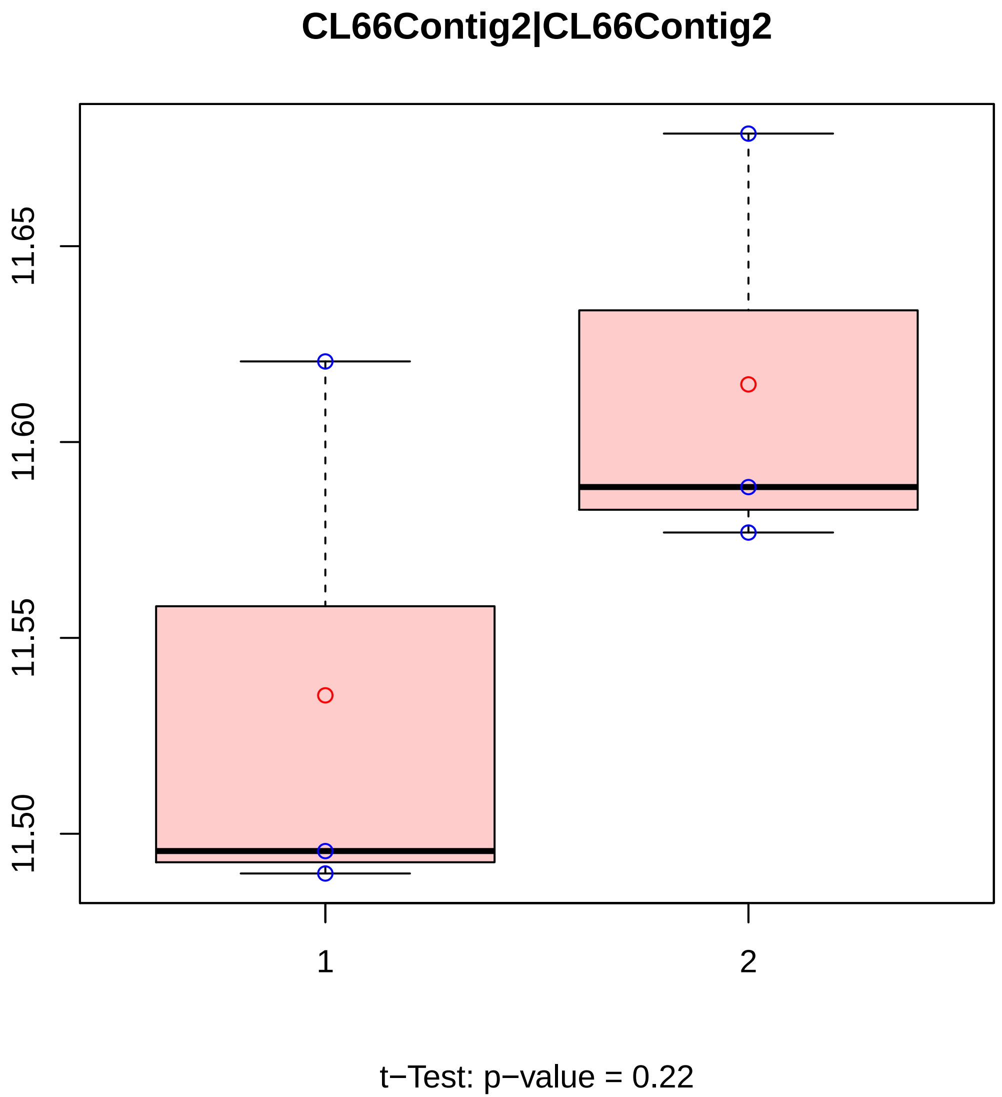
CL66Contig2|CL66Contig2
11.50
11.55
11.60
11.65
1
2
t−Test: p−value = 0.22

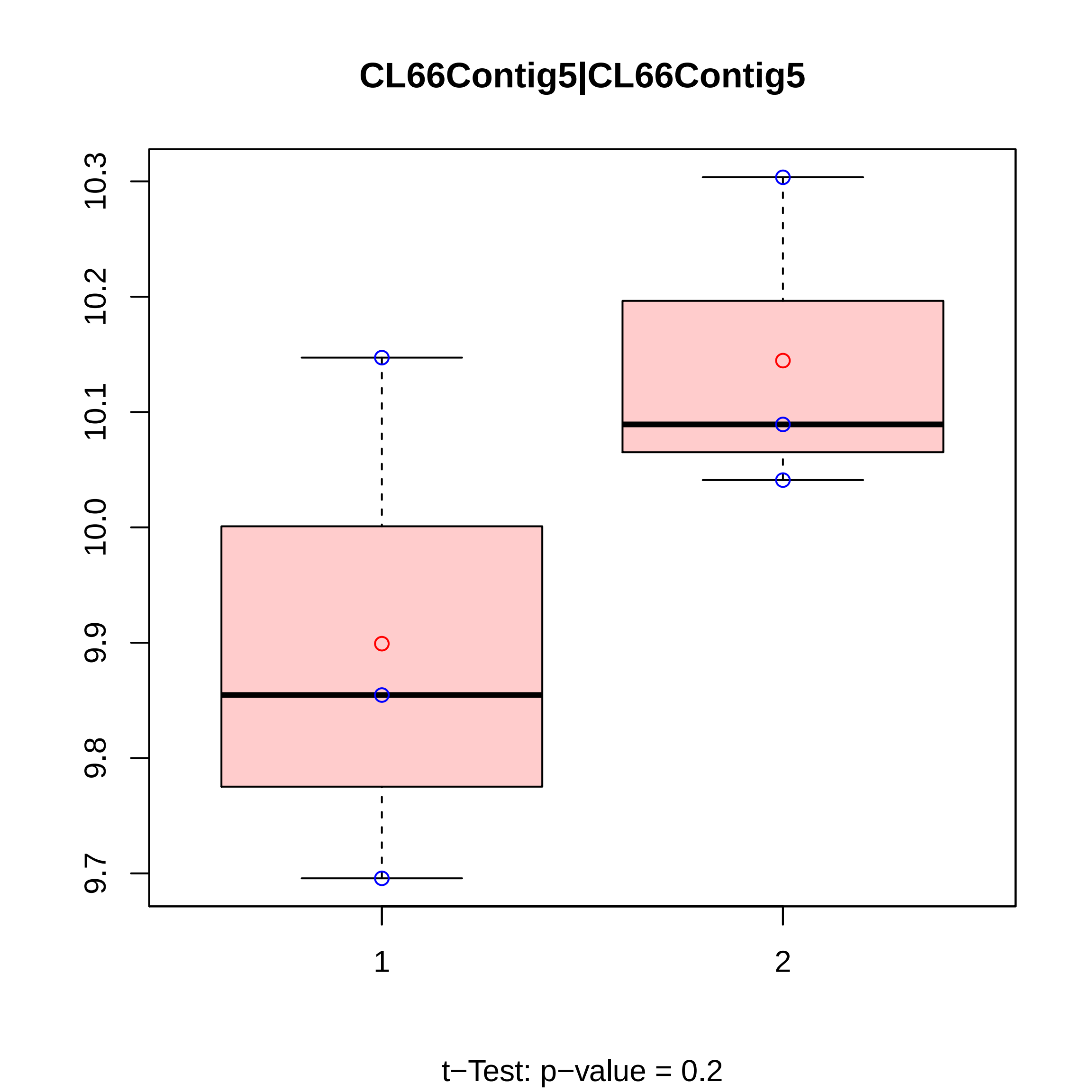
CL66Contig5|CL66Contig5
10.3
10.2
10.1
10.0
9.9
9.8
9.7
1
2
t−Test: p−value = 0.2

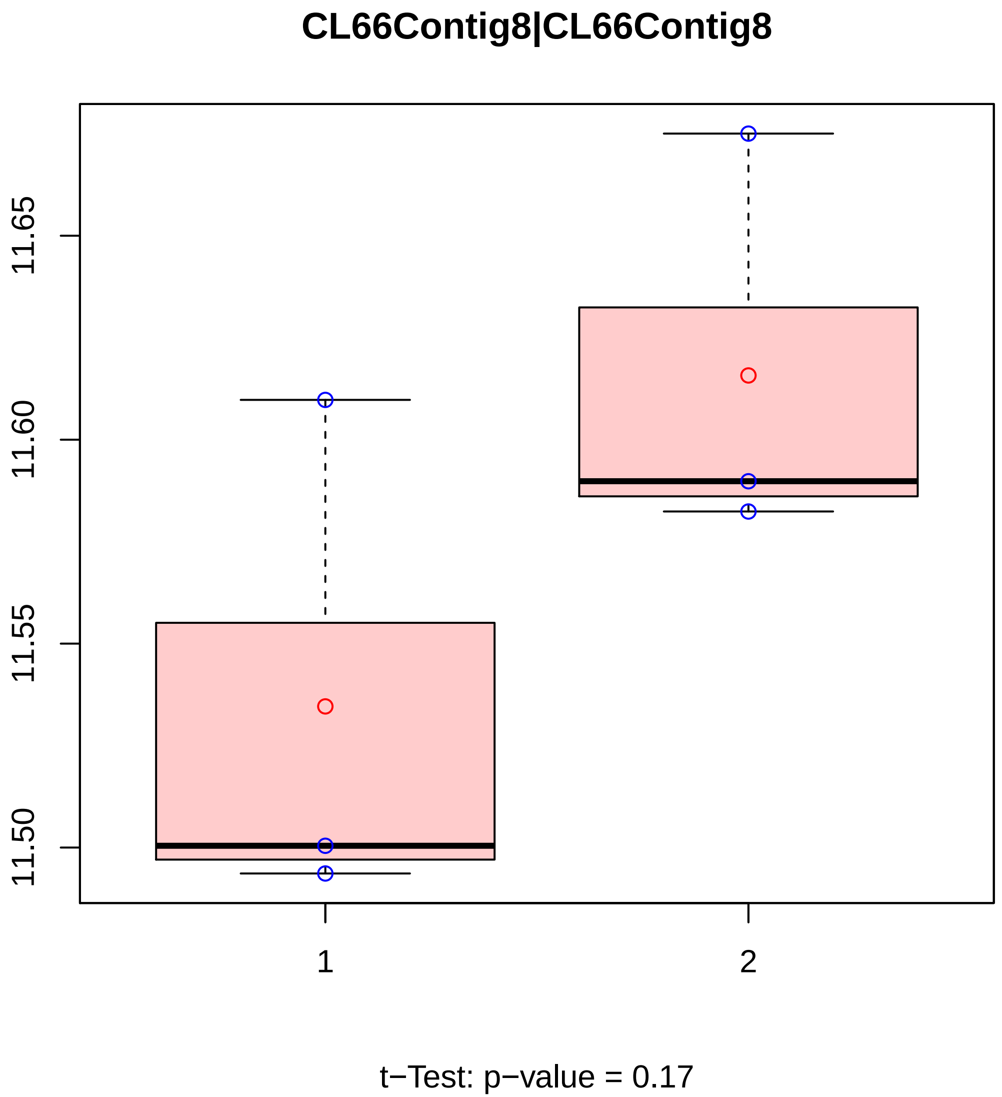
CL66Contig8|CL66Contig8
11.50
11.55
11.60
11.65
1
2
t−Test: p−value = 0.17

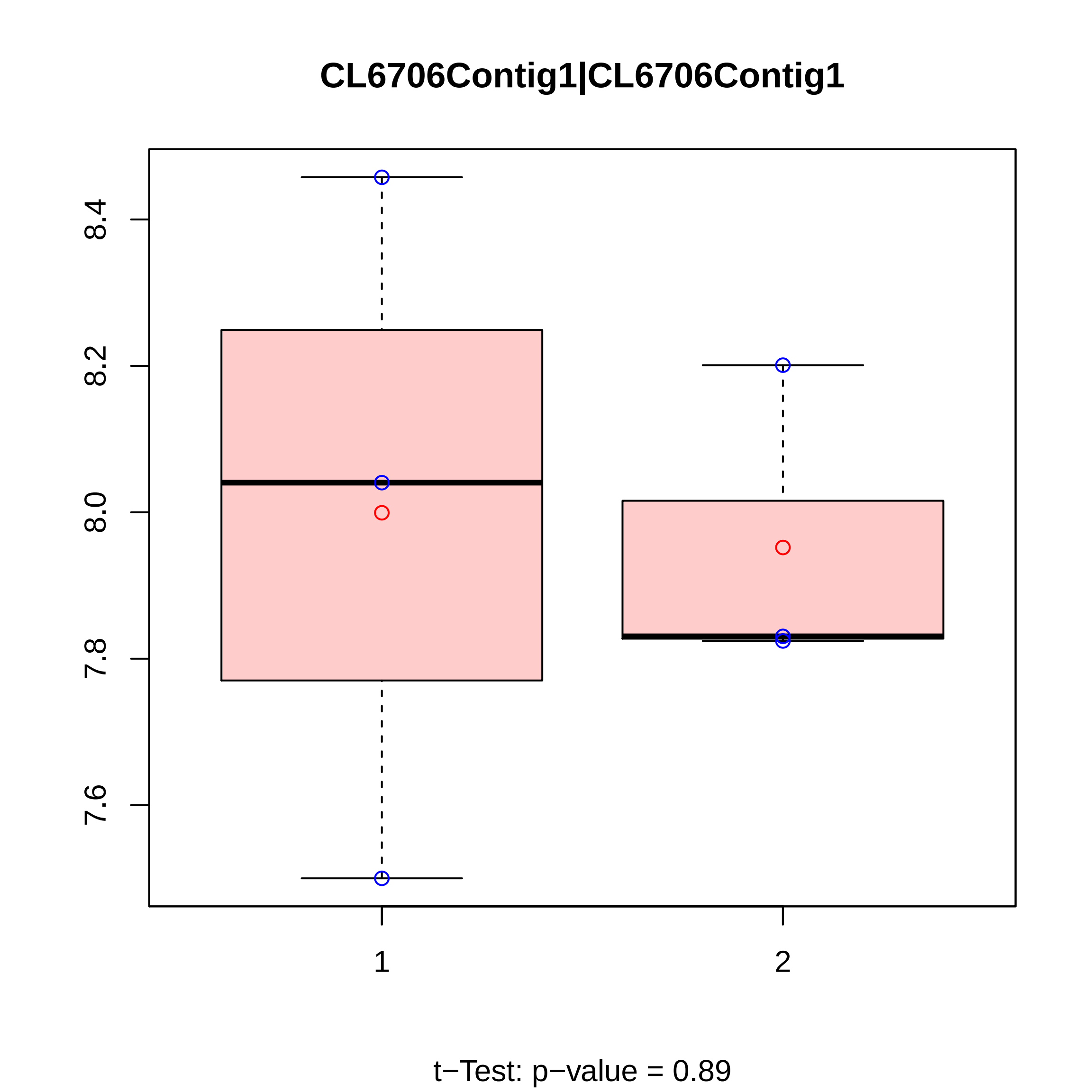
CL6706Contig1|CL6706Contig1
8.4
8.2
8.0
7.8
7.6
1
2
t−Test: p−value = 0.89

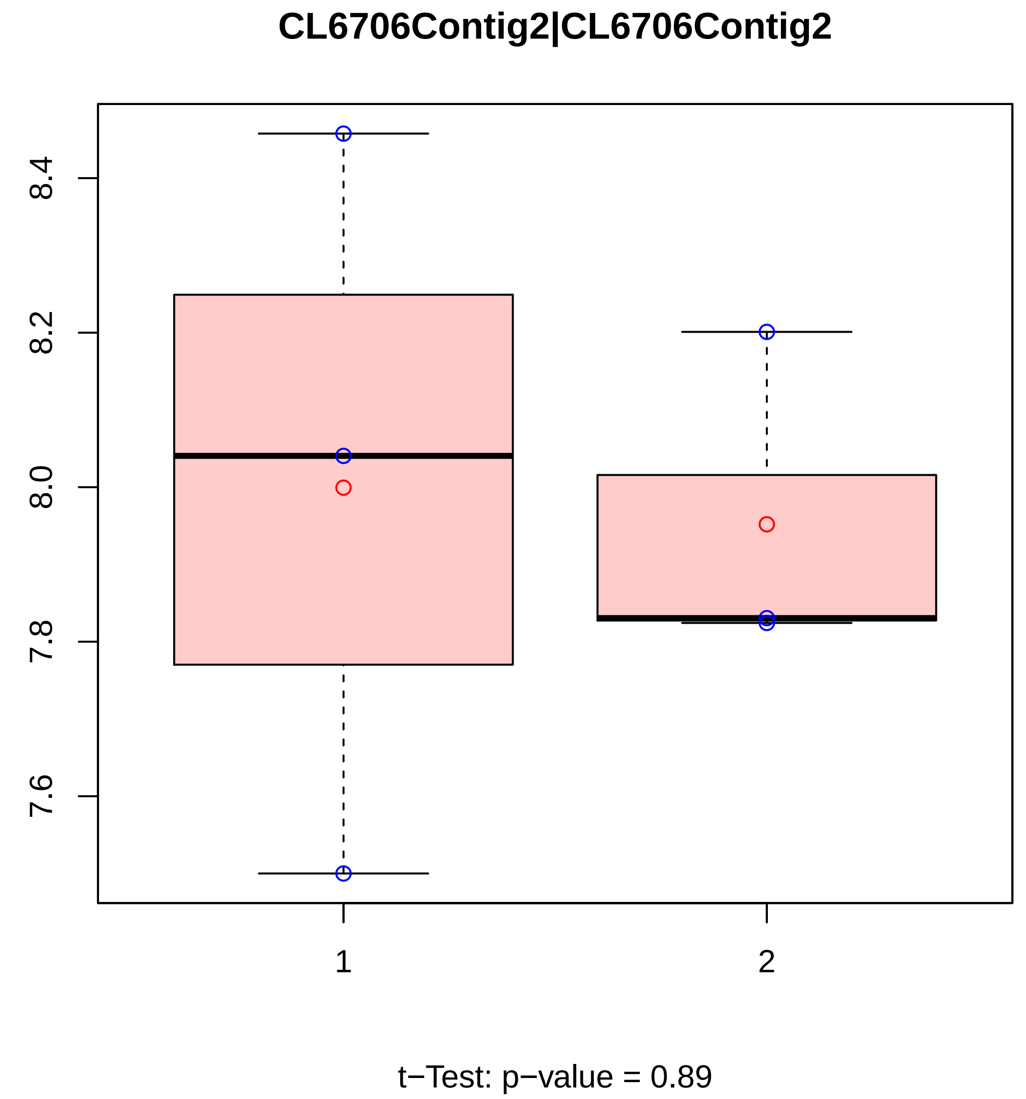
CL6706Contig2|CL6706Contig2
8.4
8.2
8.0
7.8
7.6
1
2
t−Test: p−value = 0.89

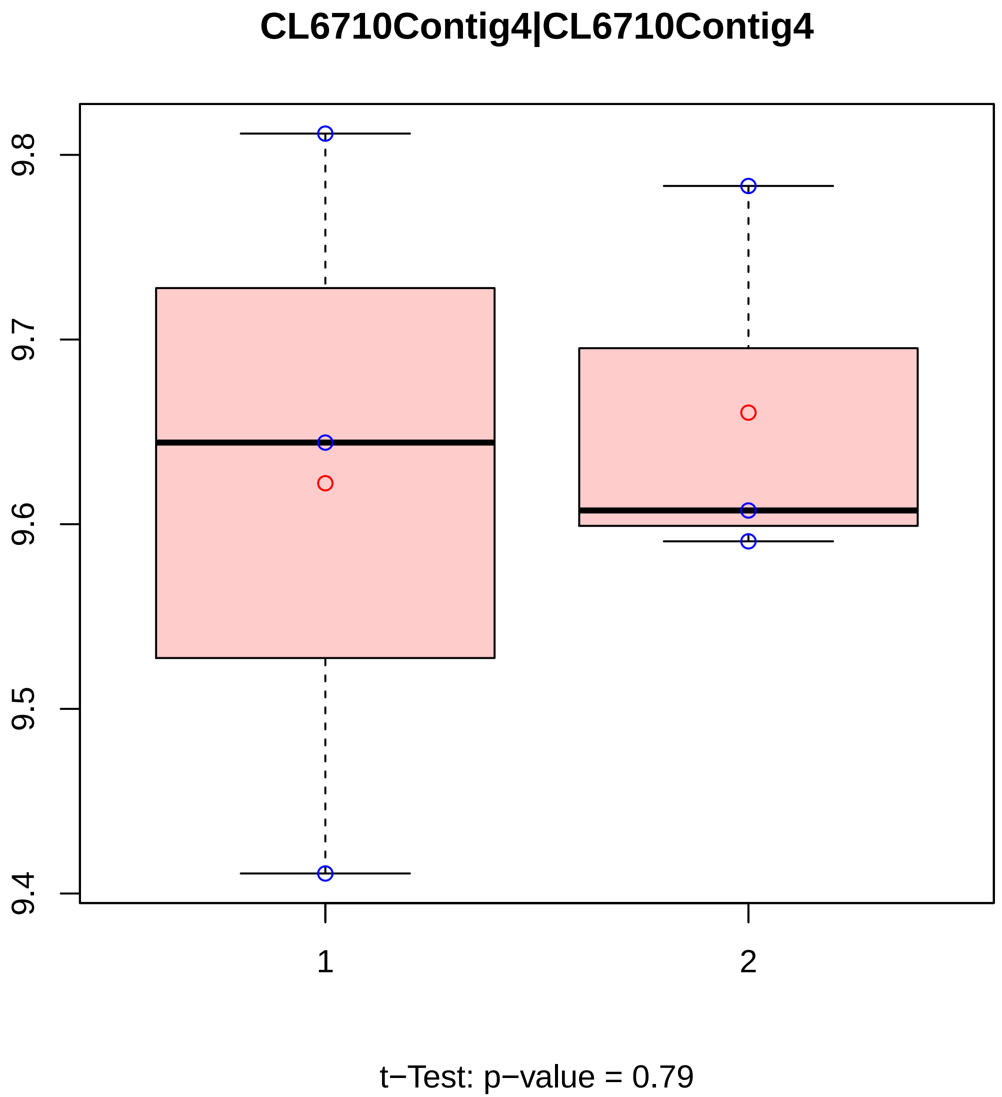
CL6710Contig4|CL6710Contig4
9.4
9.5
9.6
9.7
9.8
1
2
t−Test: p−value = 0.79

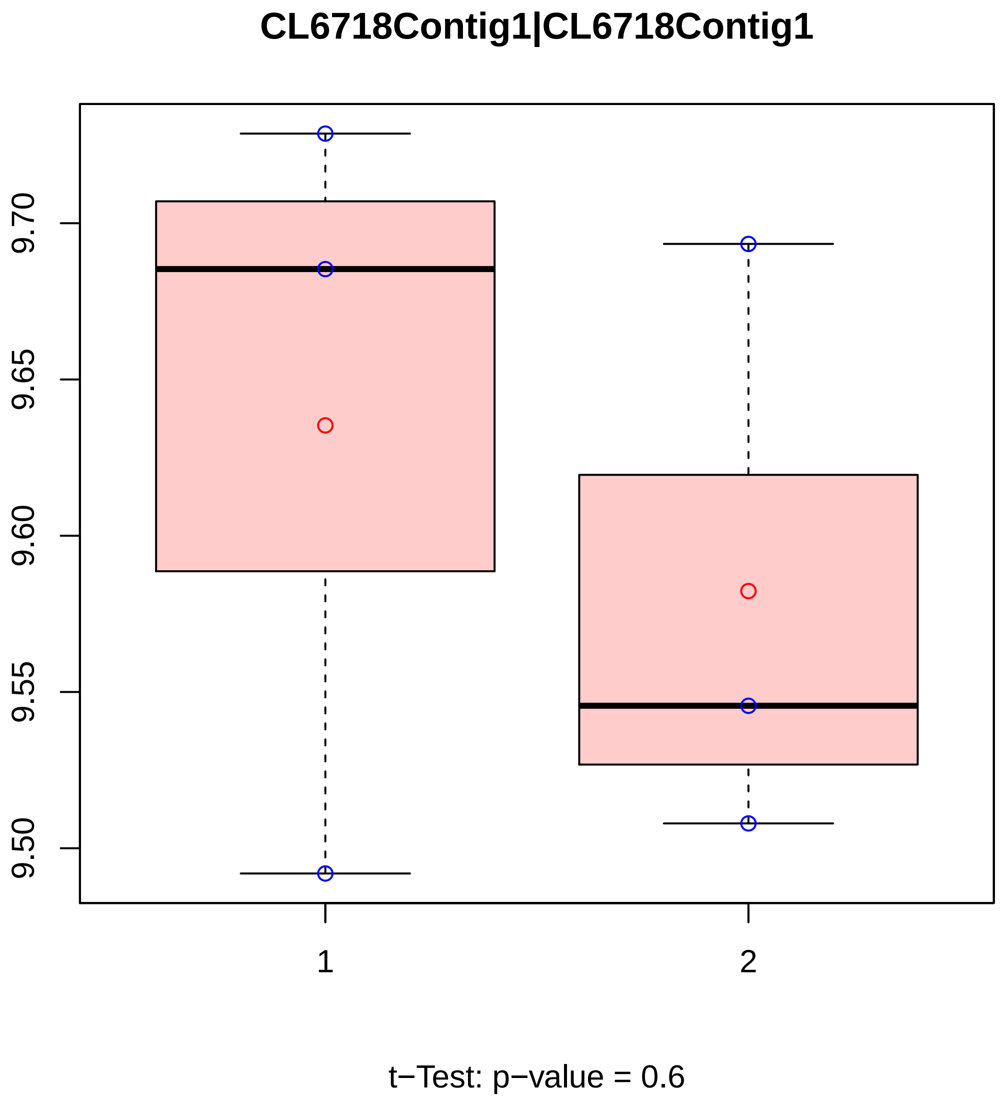
CL6718Contig1|CL6718Contig1
9.50
9.55
9.60
9.65
9.70
1
2
t−Test: p−value = 0.6

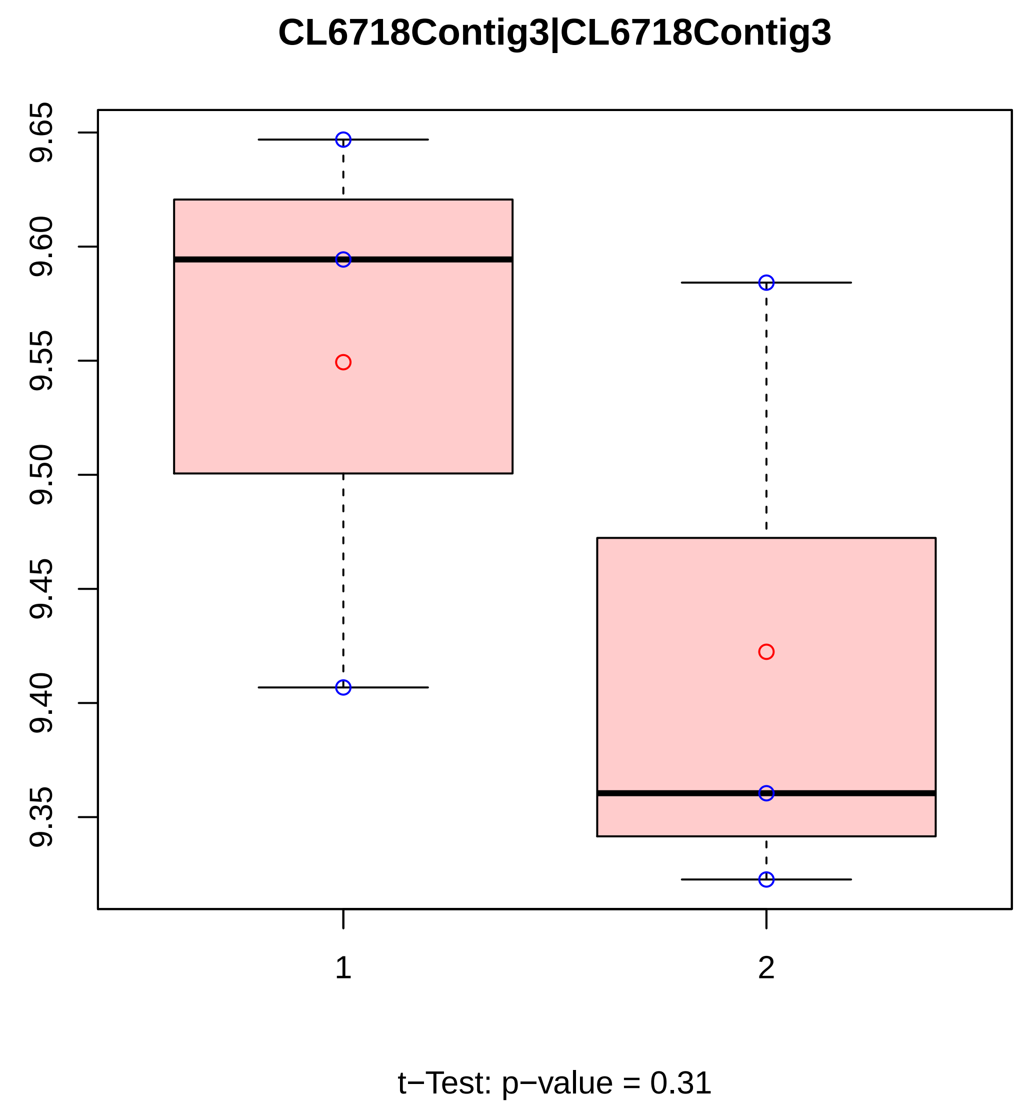
CL6718Contig3|CL6718Contig3
9.65
9.60
9.55
9.50
9.45
9.40
9.35
1
2
t−Test: p−value = 0.31

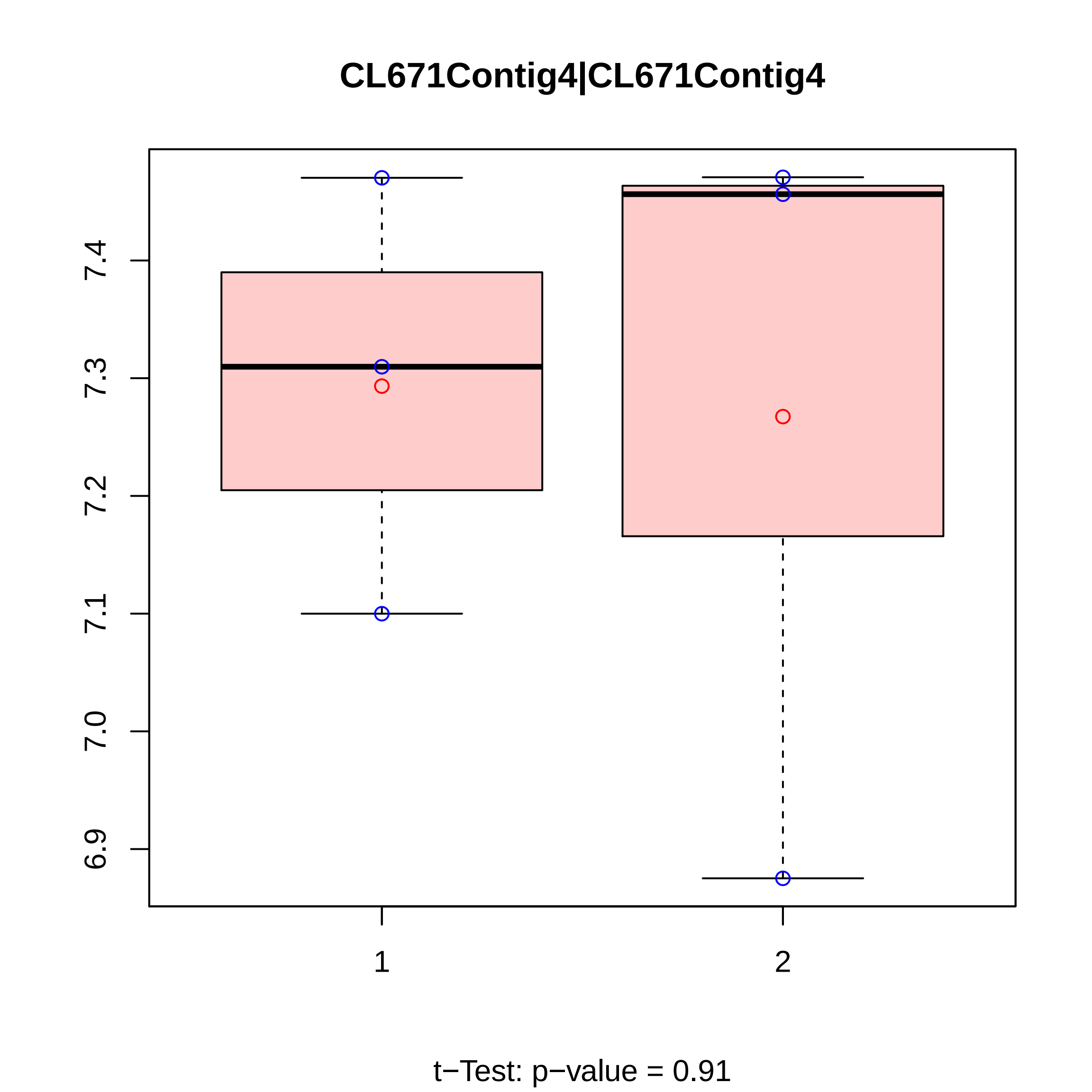
CL671Contig4|CL671Contig4
7.4
7.3
7.2
7.1
7.0
6.9
1
2
t−Test: p−value = 0.91

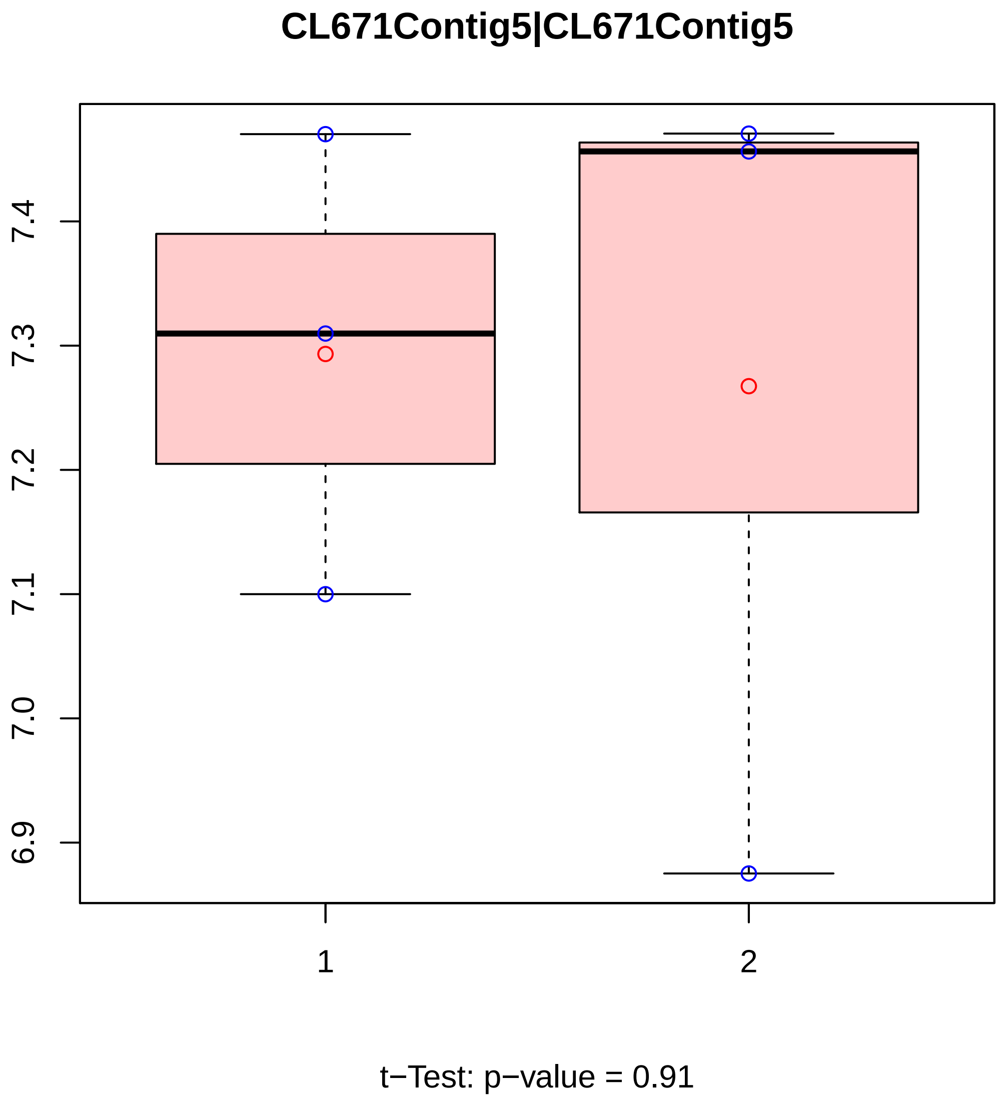
CL671Contig5|CL671Contig5
7.4
7.3
7.2
7.1
7.0
6.9
1
2
t−Test: p−value = 0.91

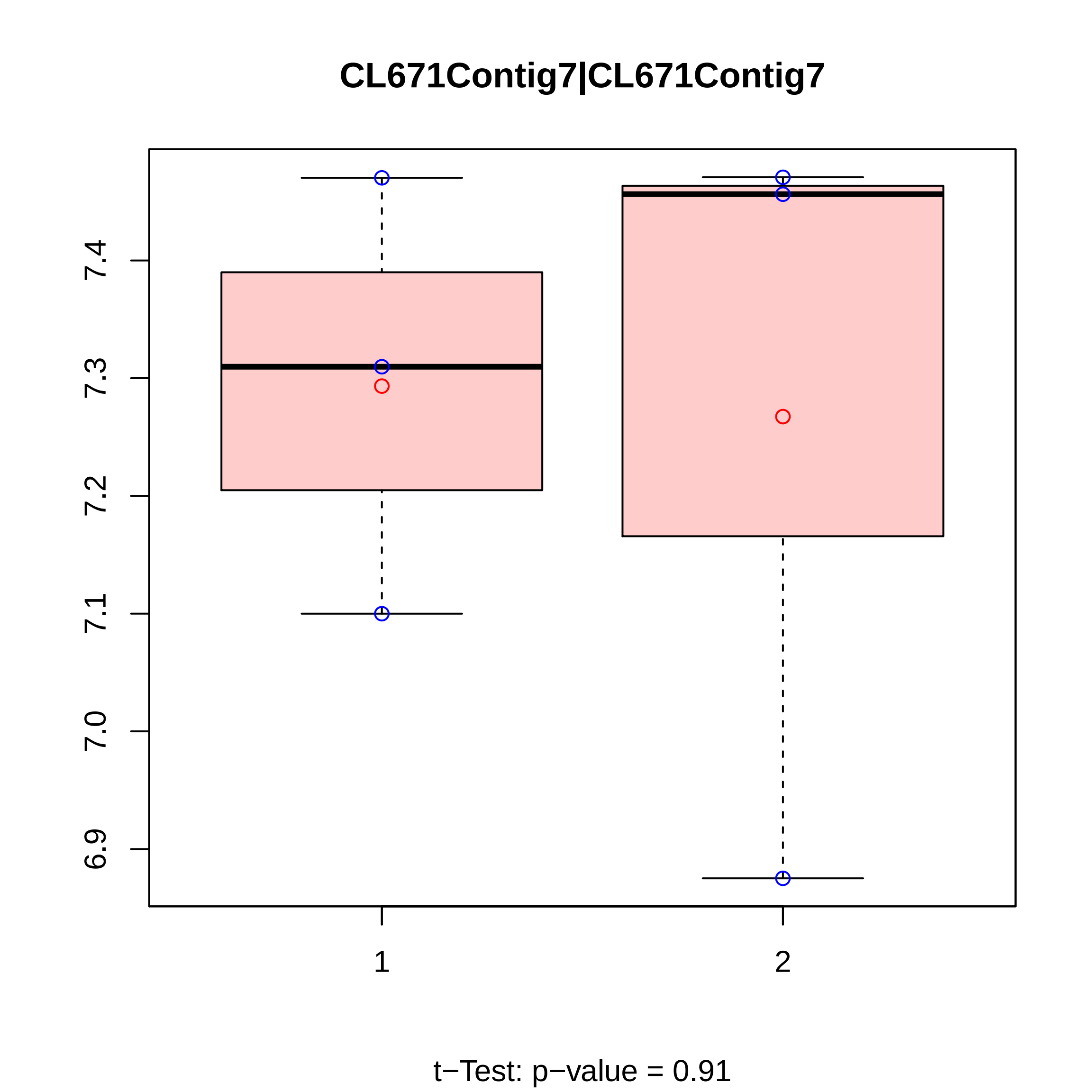
CL671Contig7|CL671Contig7
7.4
7.3
7.2
7.1
7.0
6.9
1
2
t−Test: p−value = 0.91

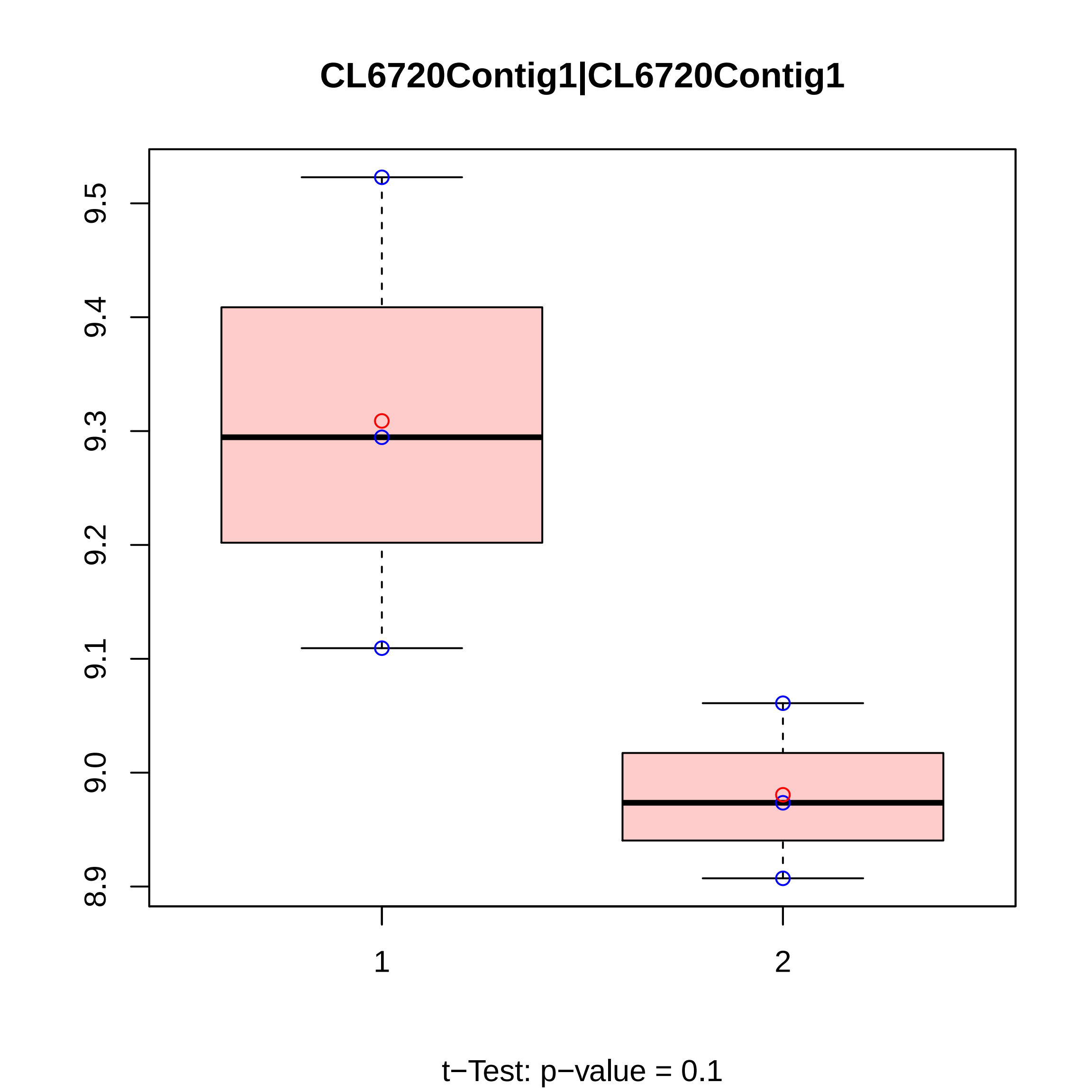
CL6720Contig1|CL6720Contig1
9.5
9.4
9.3
9.2
9.1
9.0
8.9
1
2
t−Test: p−value = 0.1

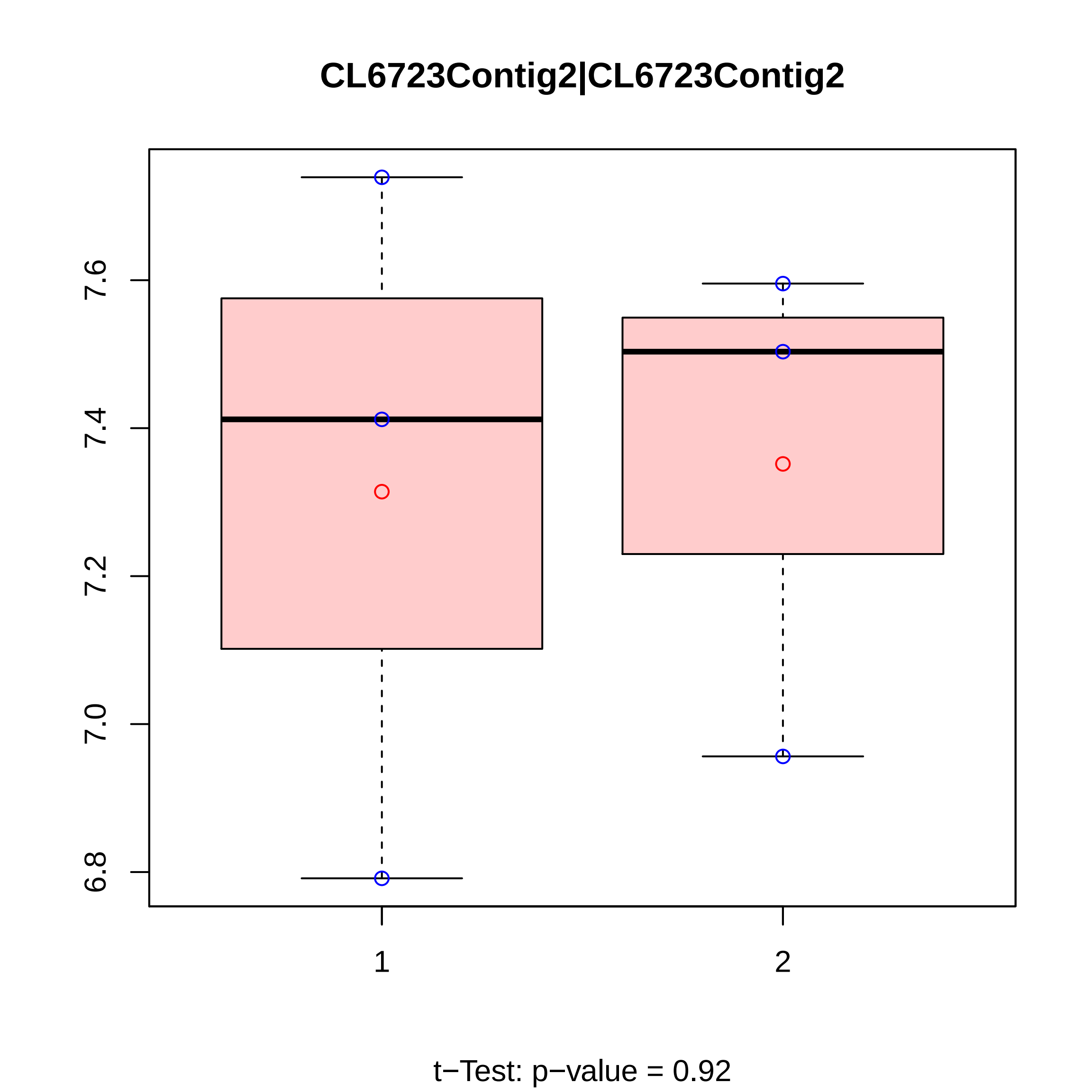
CL6723Contig2|CL6723Contig2
7.6
7.4
7.2
7.0
6.8
1
2
t−Test: p−value = 0.92

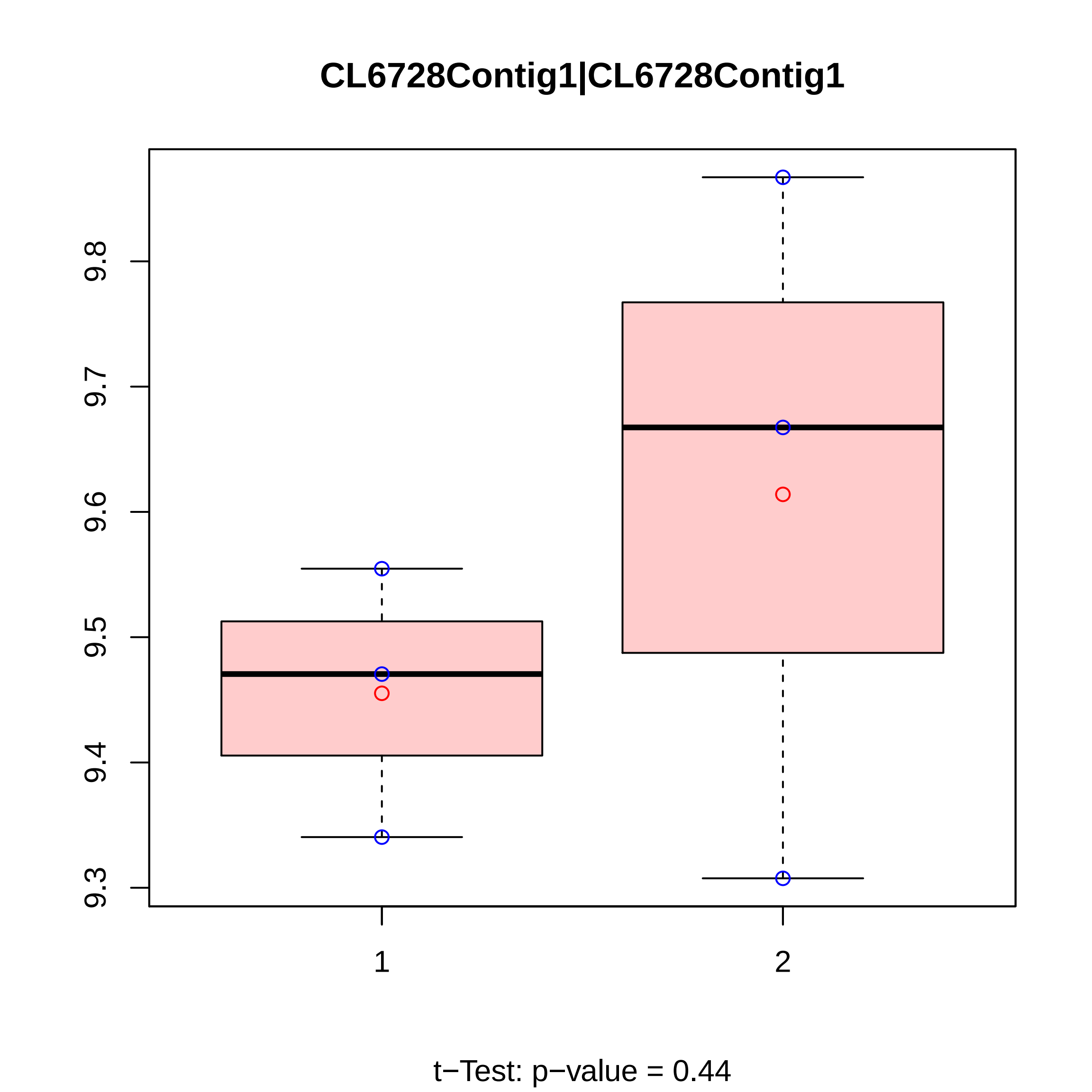

CL6728Contig1|CL6728Contig1
9.3
9.4
9.5
9.6
9.7
9.8
1
2
t−Test: p−value = 0.44

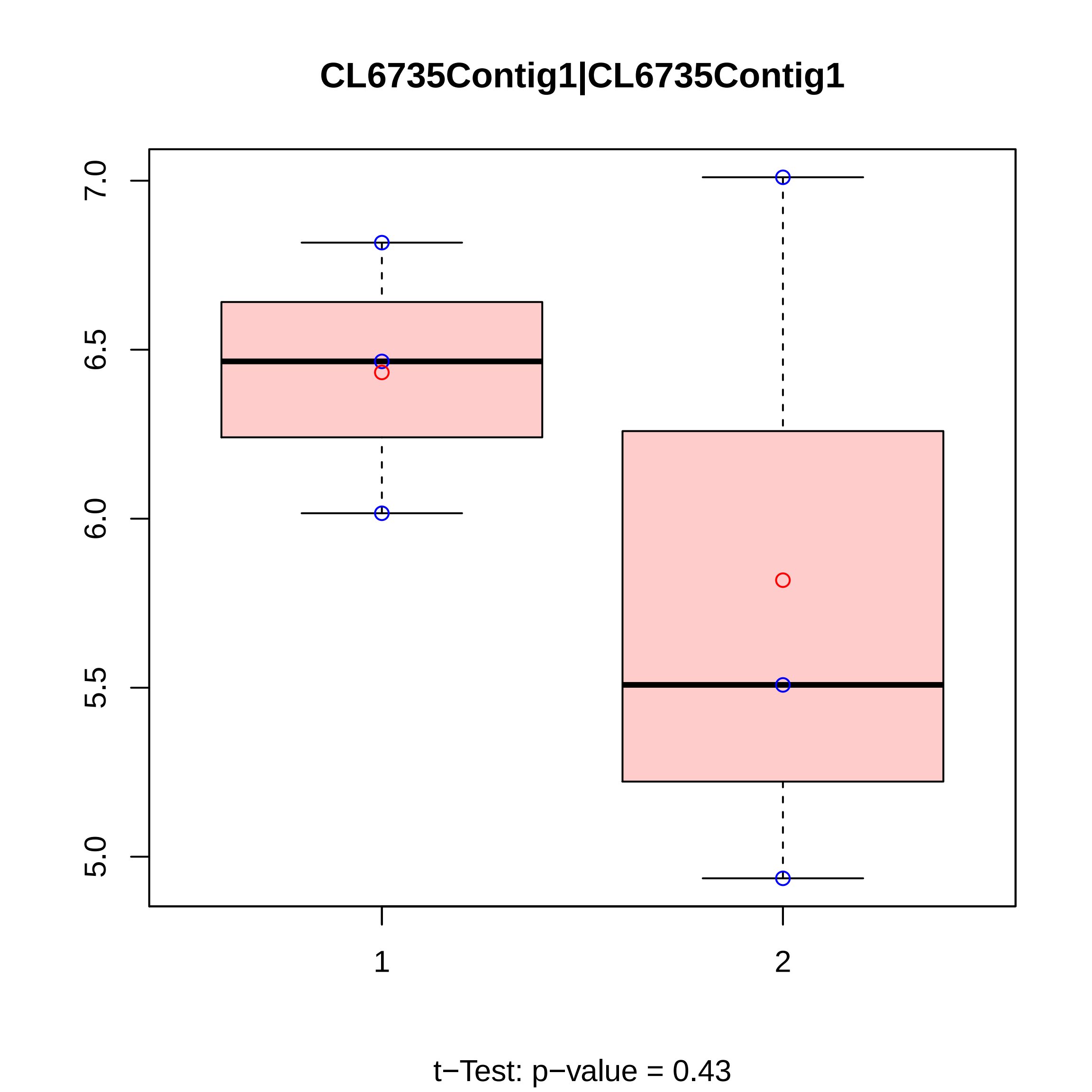

CL6735Contig1|CL6735Contig1
7.0
6.5
6.0
5.5
5.0
1
2
t−Test: p−value = 0.43

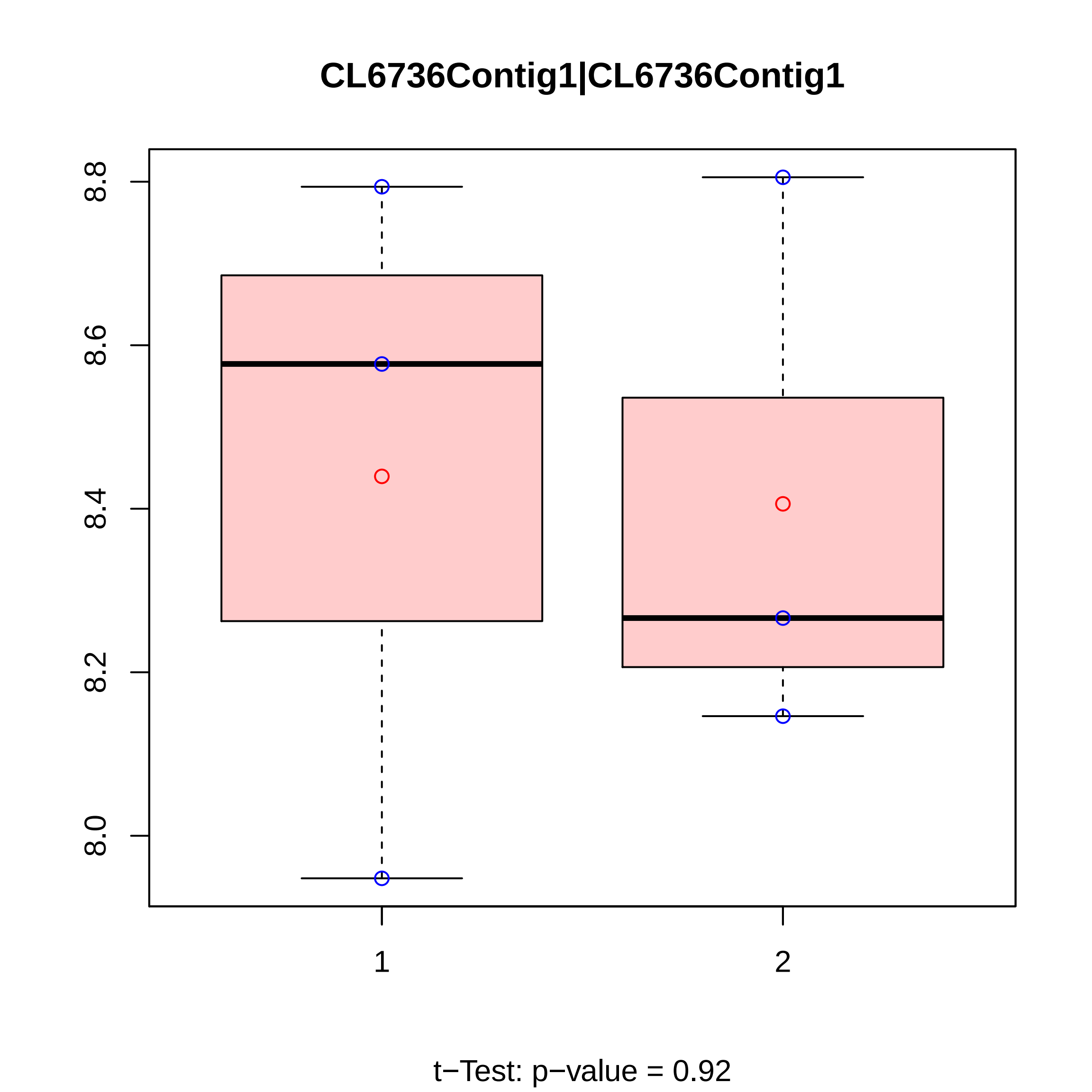
CL6736Contig1|CL6736Contig1
8.0
8.2
8.4
8.6
8.8
1
2
t−Test: p−value = 0.92

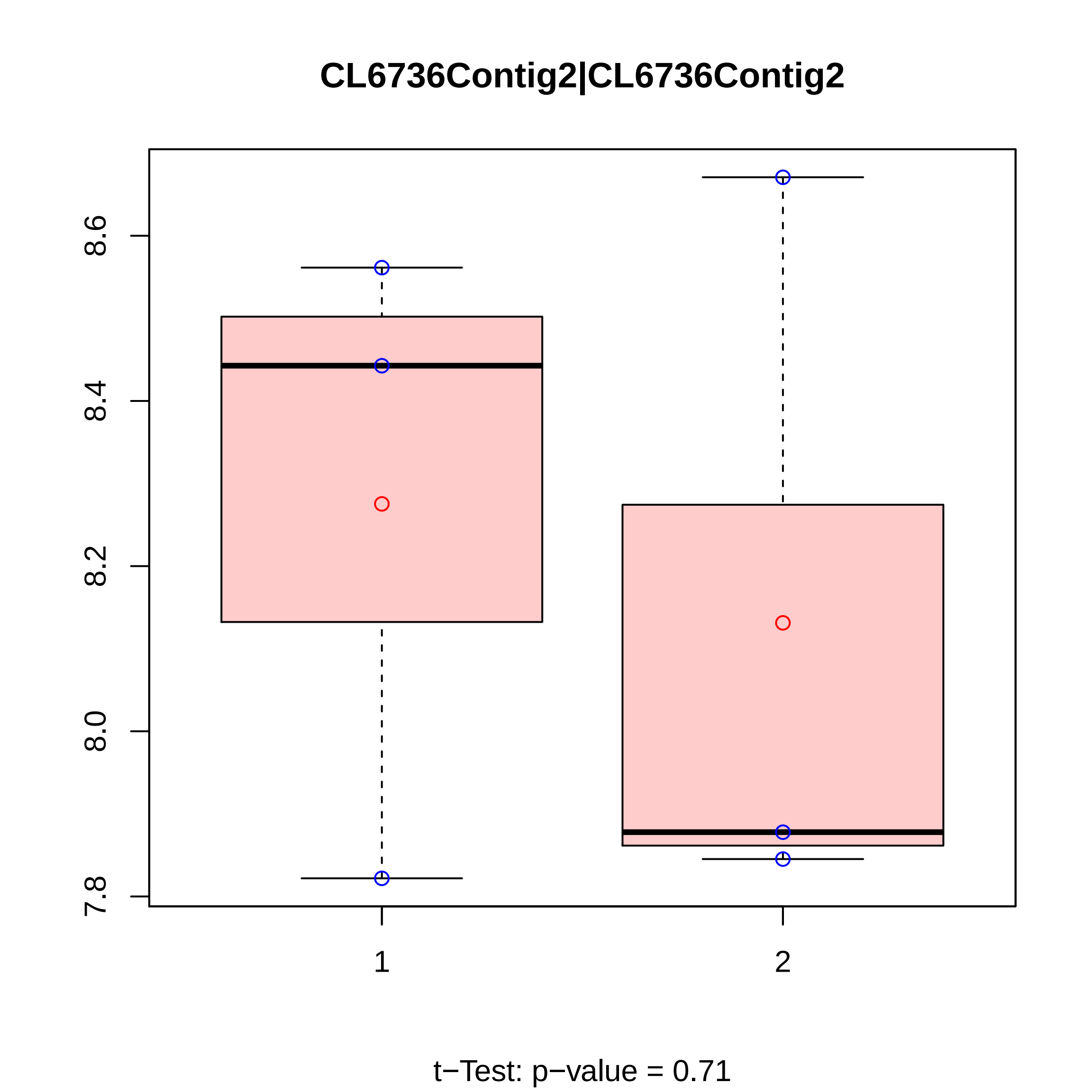
CL6736Contig2|CL6736Contig2
8.6
8.4
8.2
8.0
7.8
1
2
t−Test: p−value = 0.71

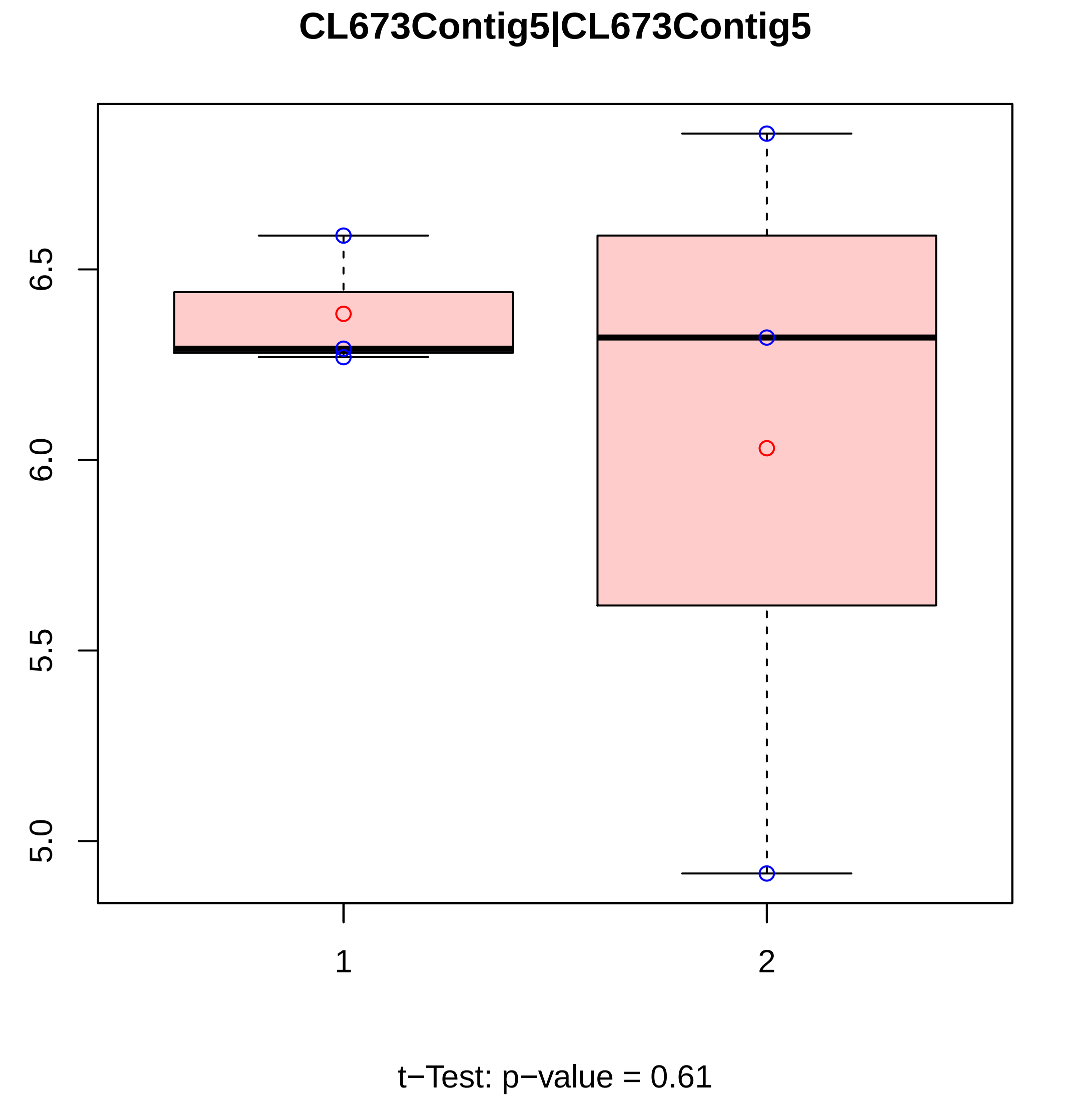
CL673Contig5|CL673Contig5
6.5
6.0
5.5
5.0
1
2
t−Test: p−value = 0.61

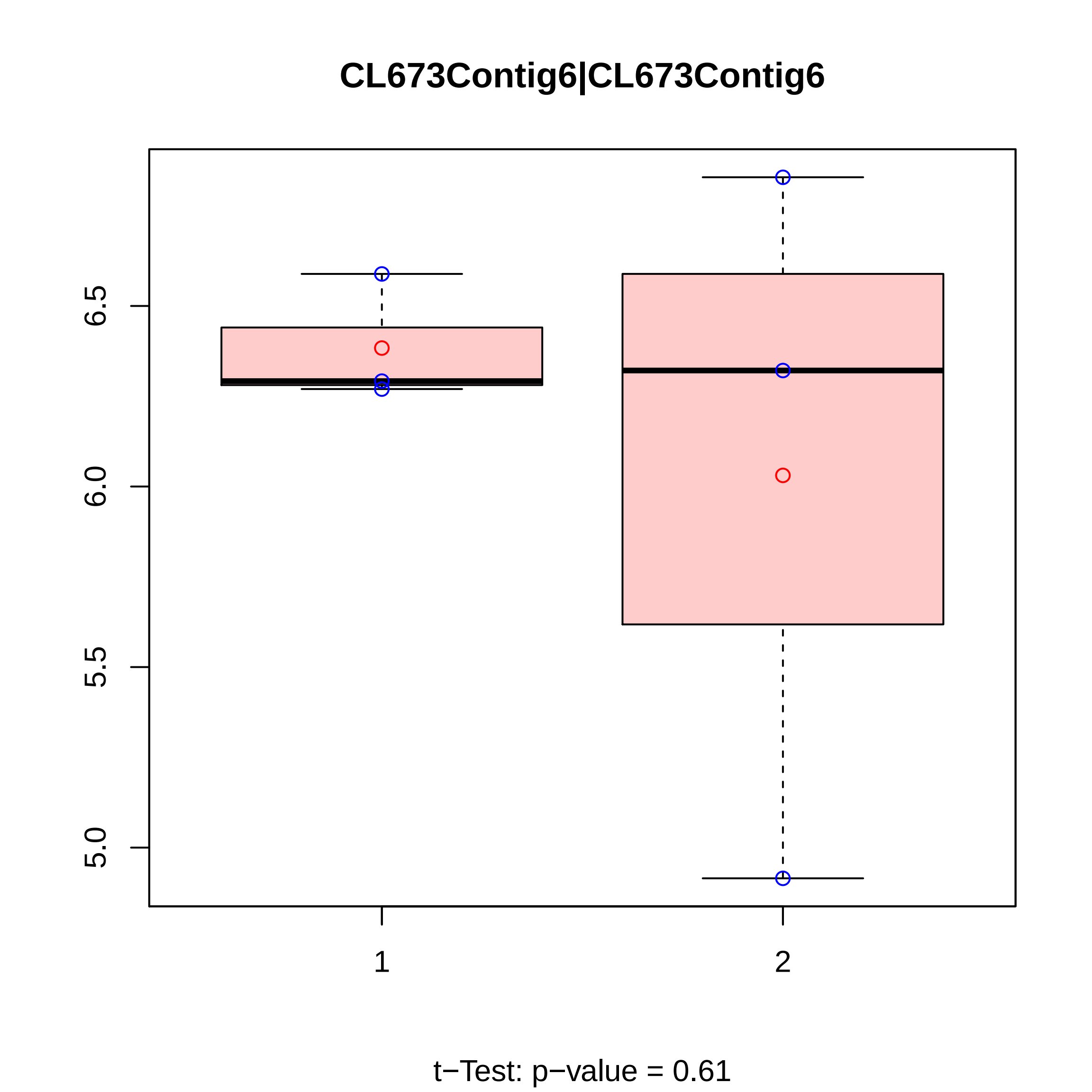

CL673Contig6|CL673Contig6
6.5
6.0
5.5
5.0
1
2
t−Test: p−value = 0.61

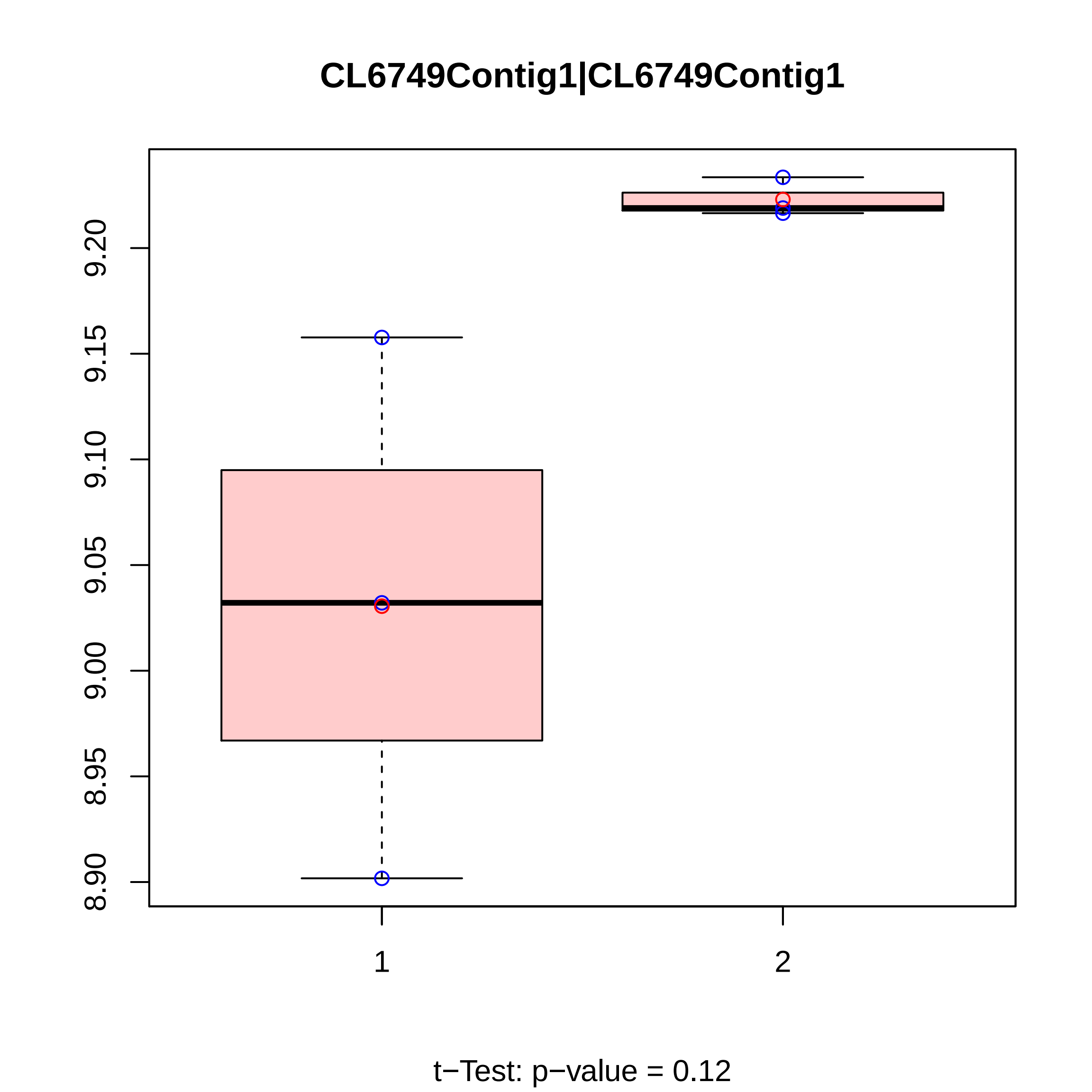
CL6749Contig1|CL6749Contig1
9.20
9.15
9.10
9.05
9.00
8.95
8.90
1
2
t−Test: p−value = 0.12

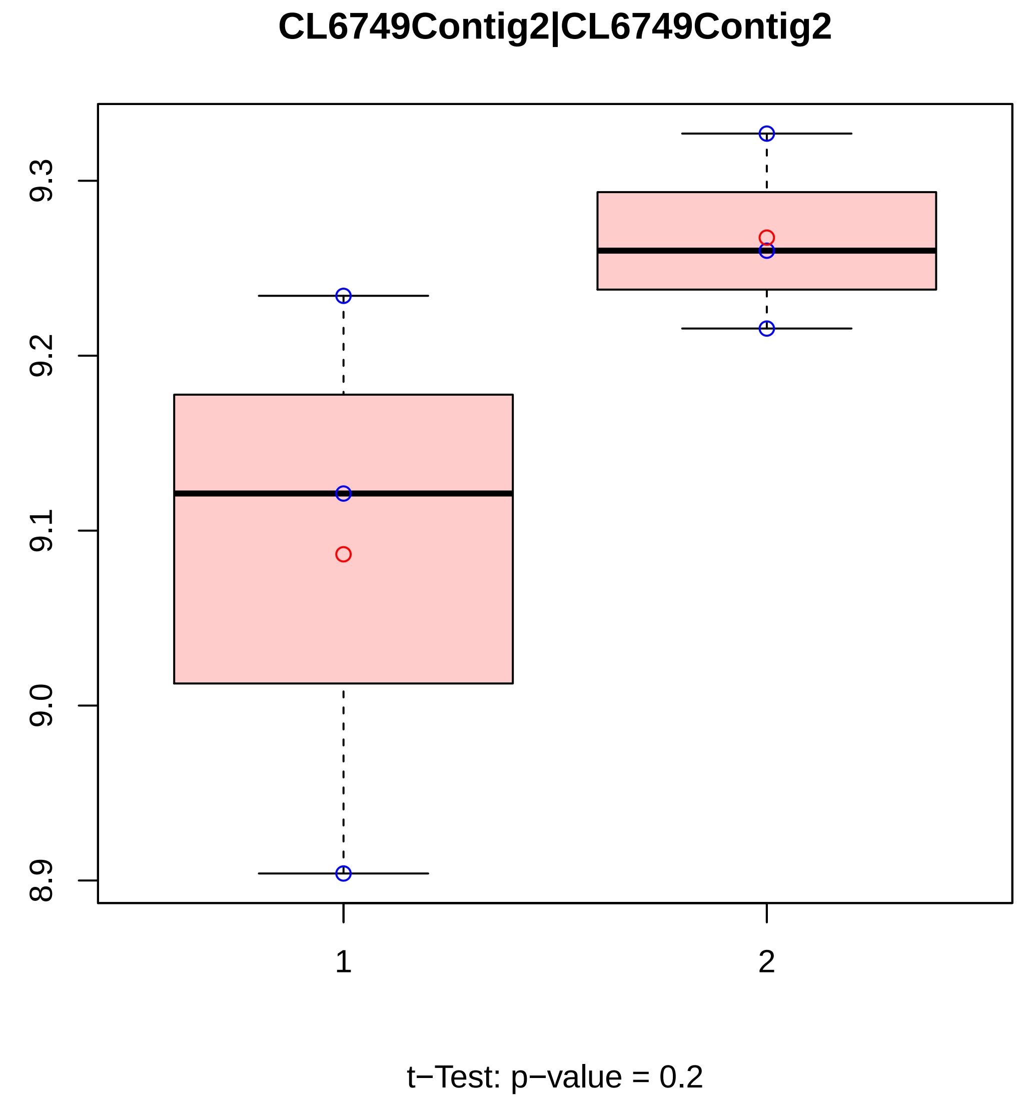

CL6749Contig2|CL6749Contig2
9.3
9.2
9.1
9.0
8.9
1
2
t−Test: p−value = 0.2

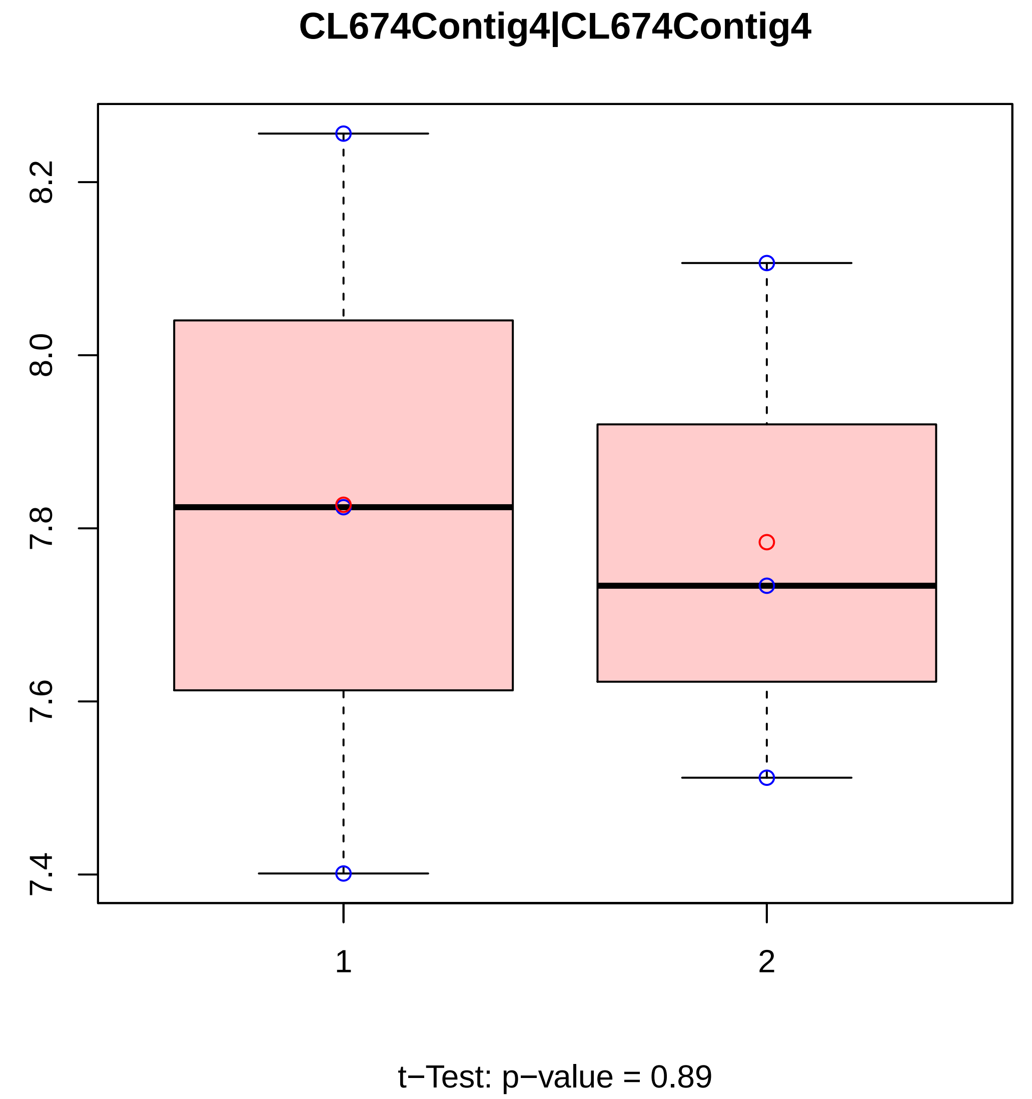
CL674Contig4|CL674Contig4
8.2
8.0
7.8
7.6
7.4
1
2
t−Test: p−value = 0.89

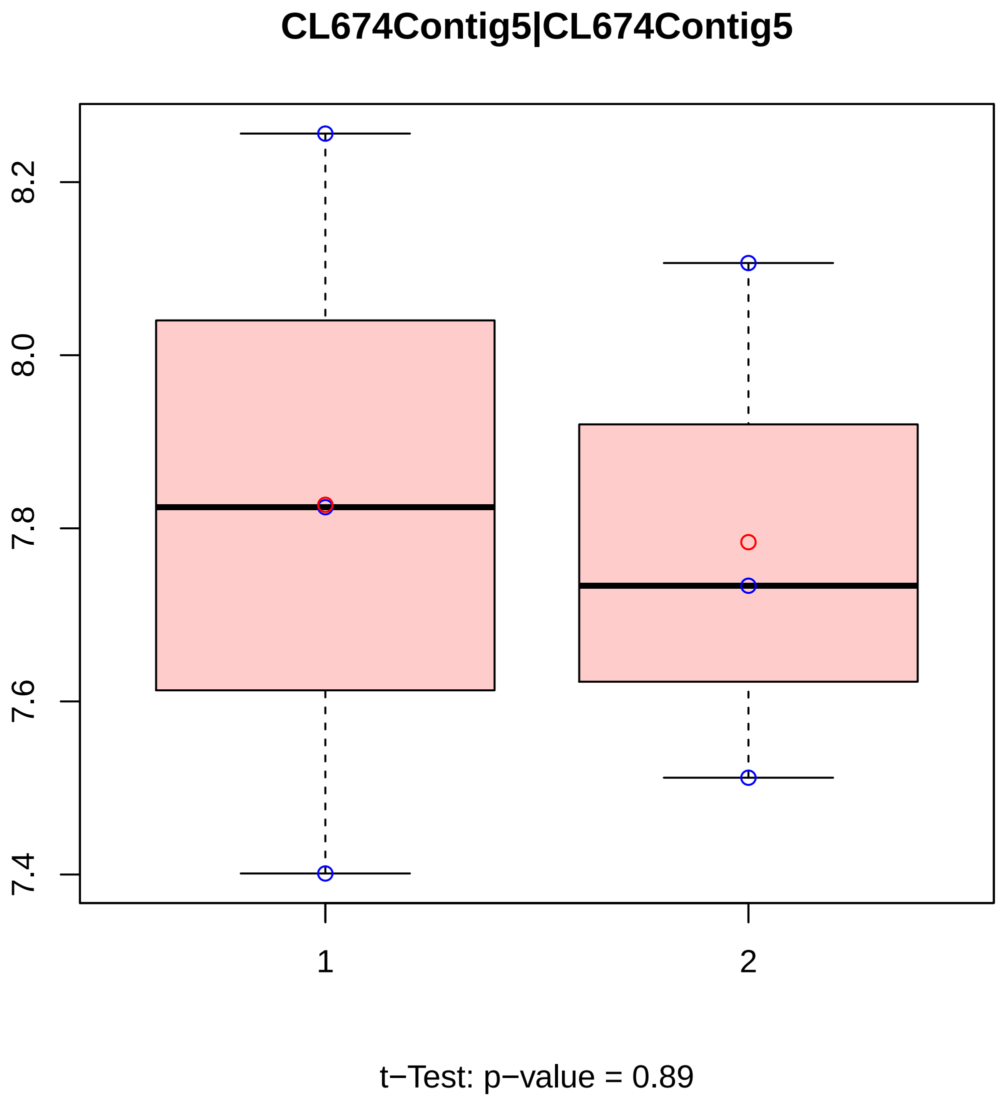
CL674Contig5|CL674Contig5
8.2
8.0
7.8
7.6
7.4
1
2
t−Test: p−value = 0.89

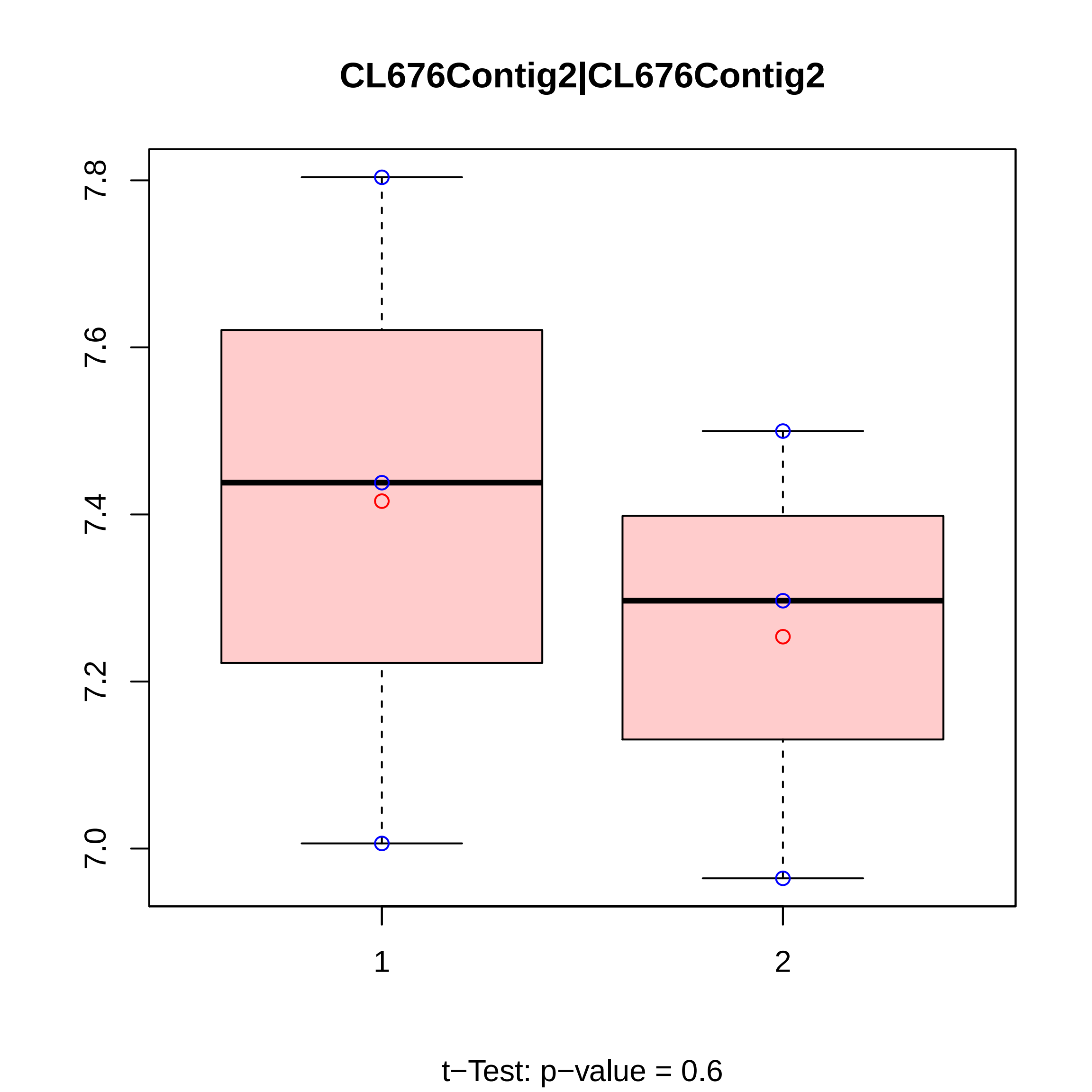
CL676Contig2|CL676Contig2
7.8
7.6
7.4
7.2
7.0
1
2
t−Test: p−value = 0.6

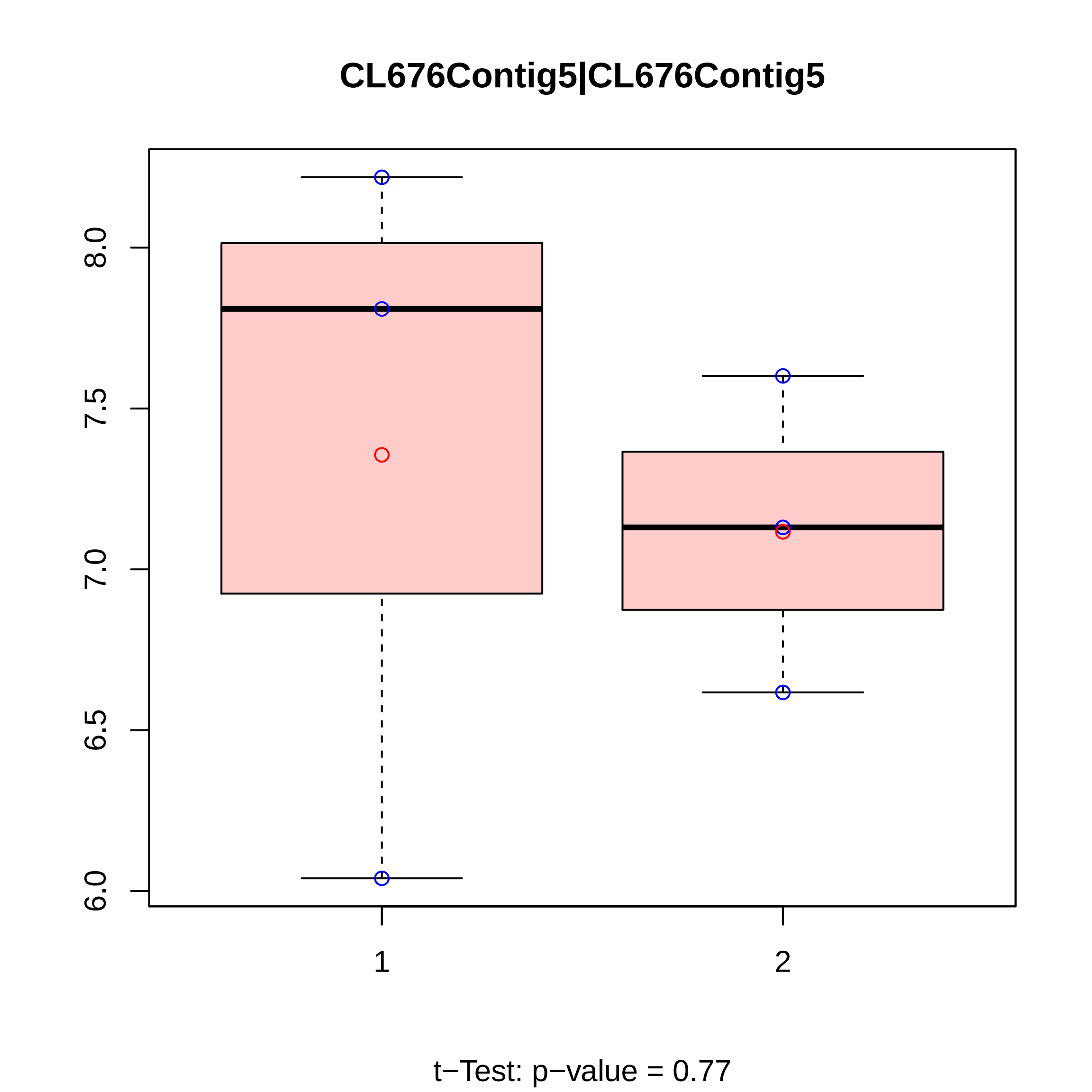

CL676Contig5|CL676Contig5
8.0
7.5
7.0
6.5
6.0
1
2
t−Test: p−value = 0.77

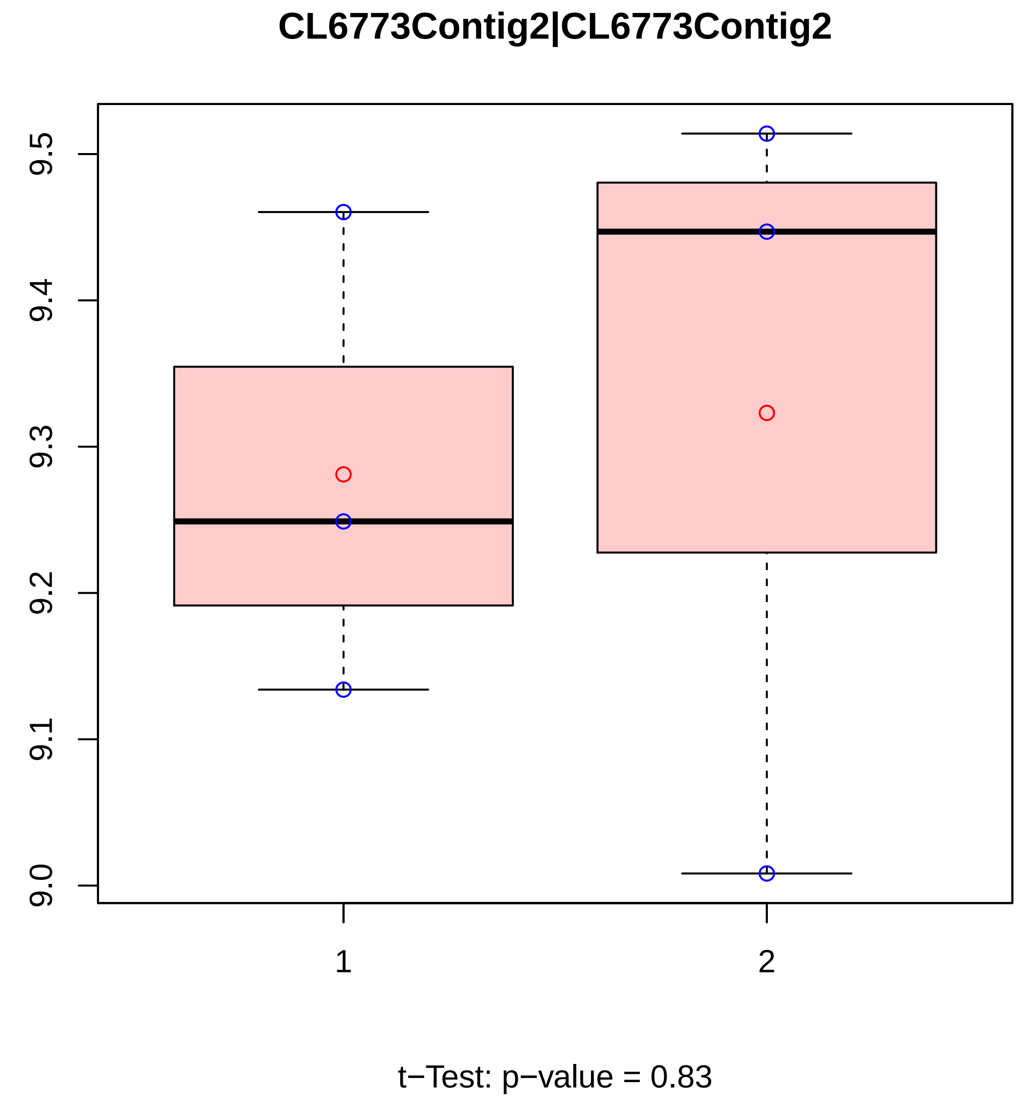
CL6773Contig2|CL6773Contig2
9.0
9.1
9.2
9.3
9.4
9.5
1
2
t−Test: p−value = 0.83

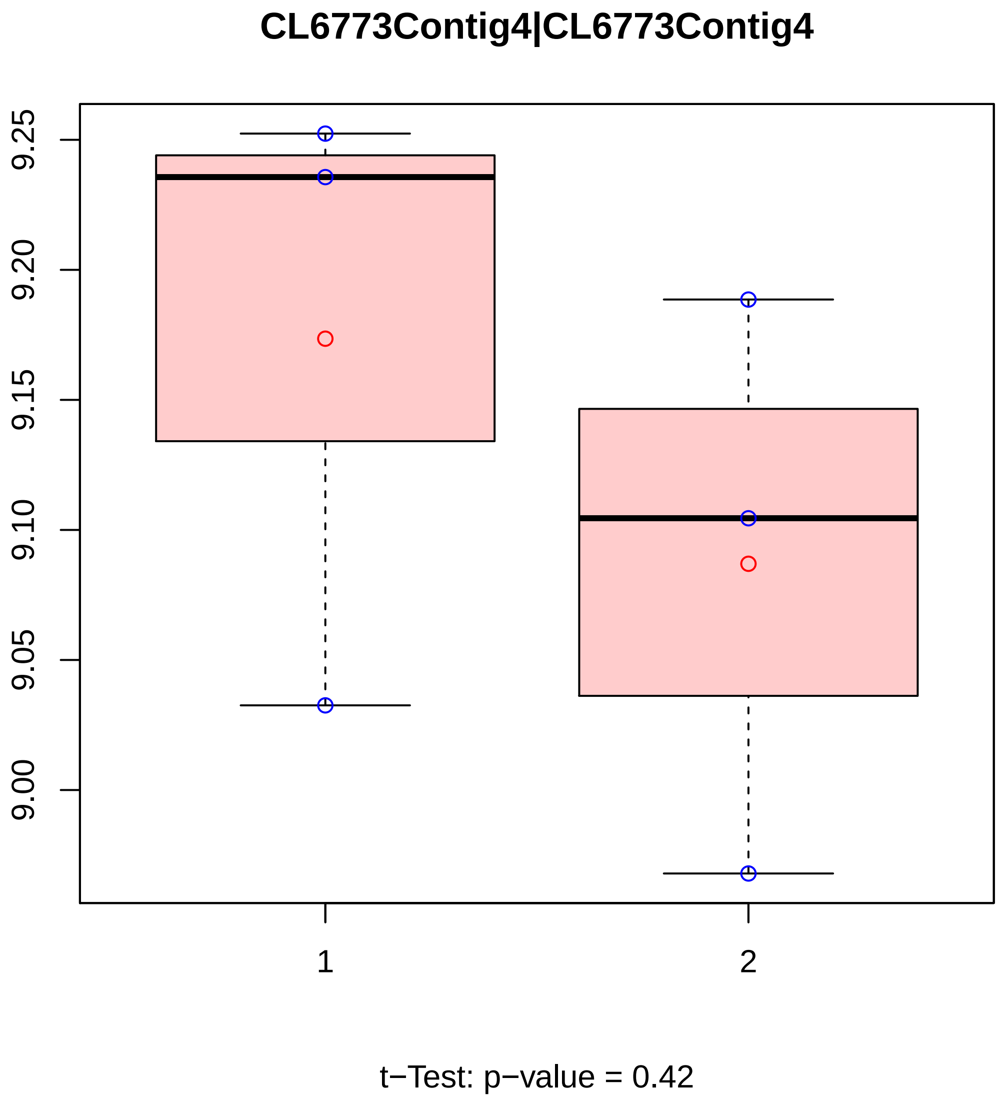
CL6773Contig4|CL6773Contig4
9.00
9.05
9.10
9.15
9.20
9.25
1
2
t−Test: p−value = 0.42

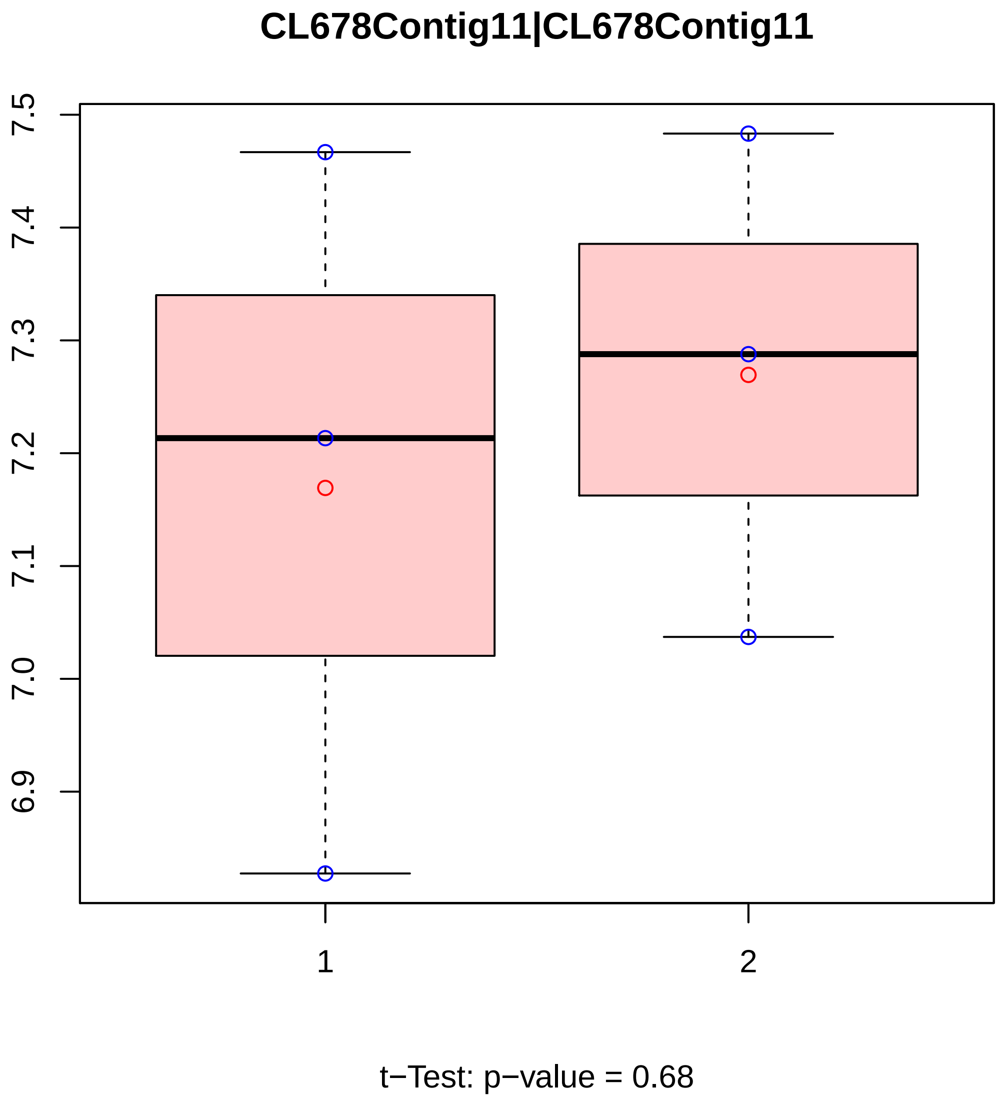

CL678Contig11|CL678Contig11
7.5
7.4
7.3
7.2
7.1
7.0
6.9
1
2
t−Test: p−value = 0.68

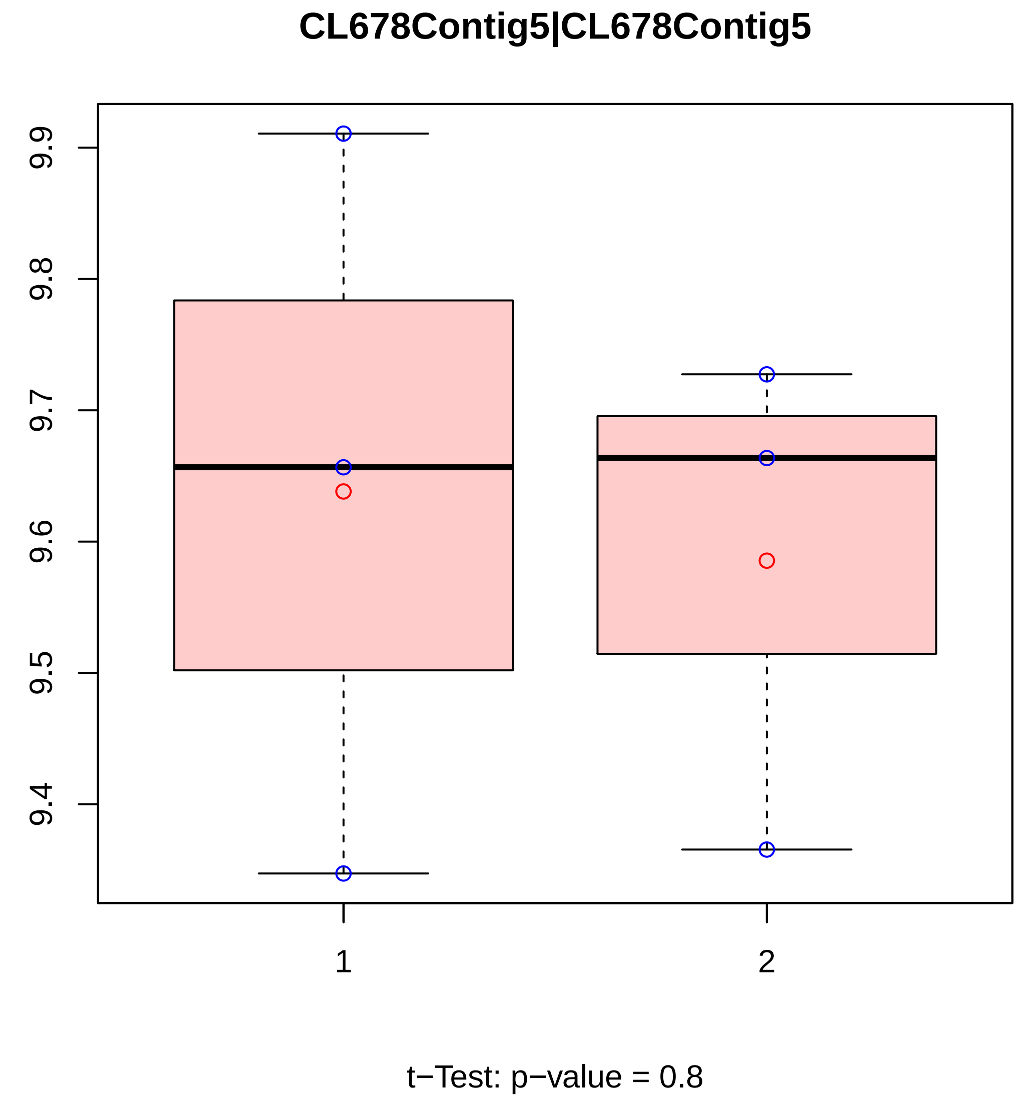

CL678Contig5|CL678Contig5
9.4
9.5
9.6
9.7
9.8
9.9
1
2
t−Test: p−value = 0.8

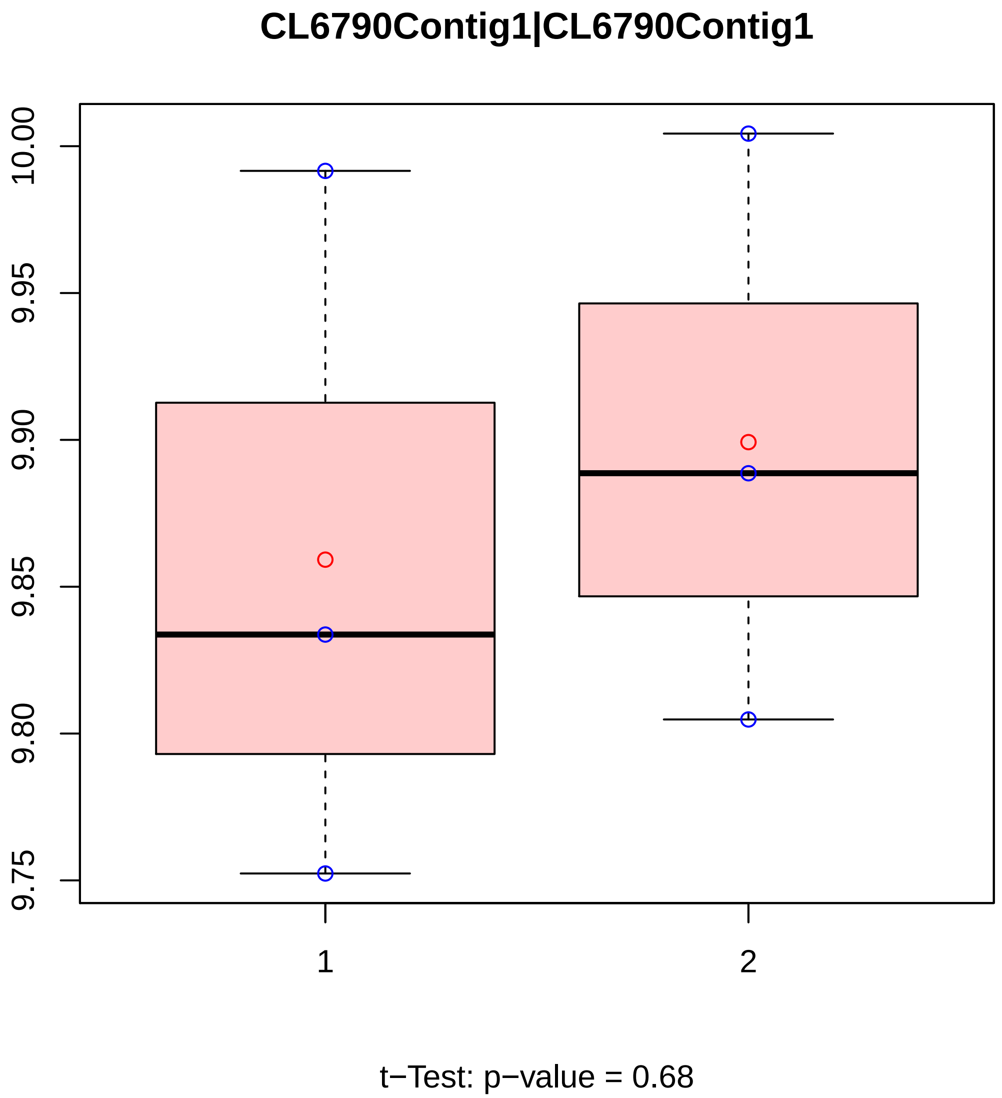
CL6790Contig1|CL6790Contig1
10.00
9.95
9.90
9.85
9.80
9.75
1
2
t−Test: p−value = 0.68

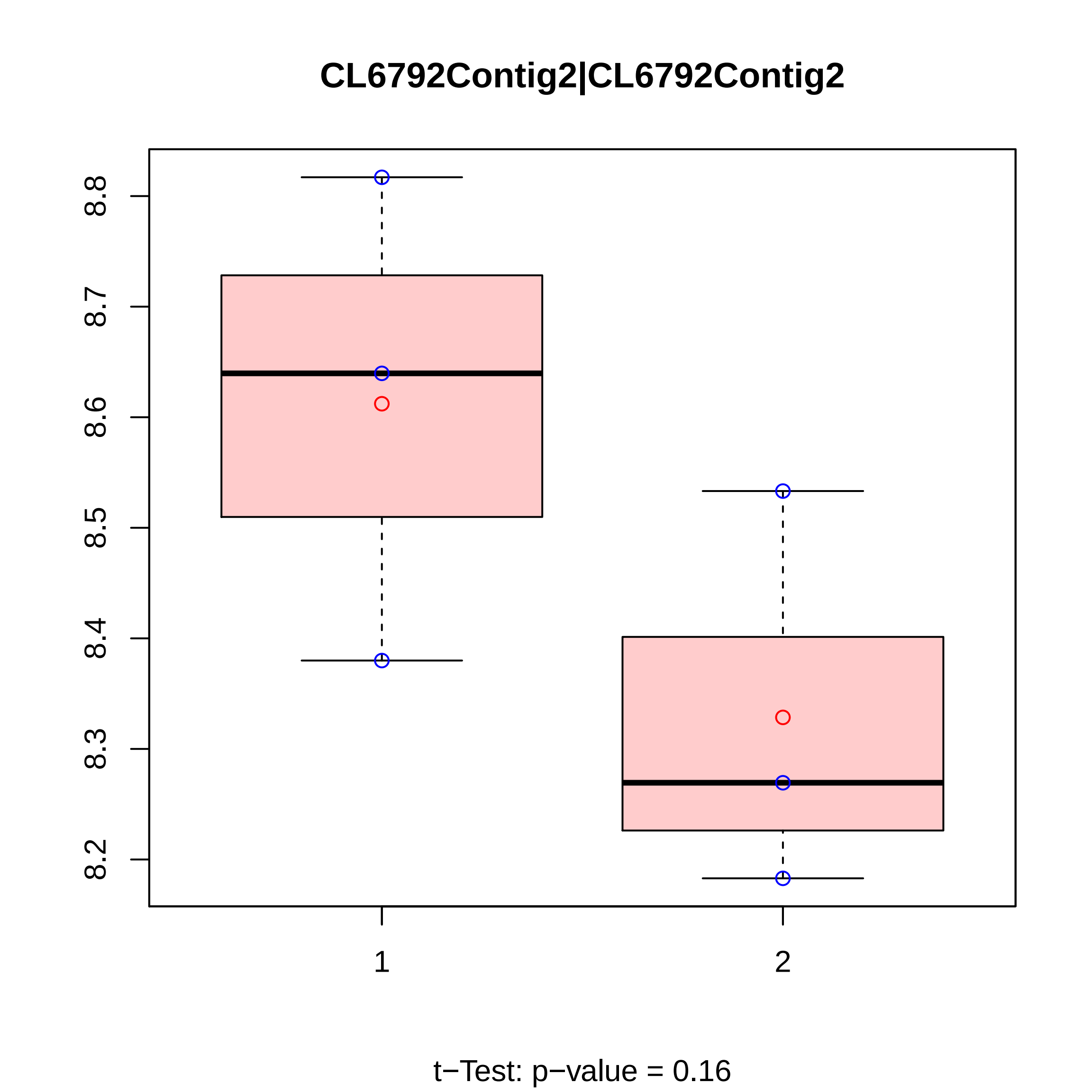

CL6792Contig2|CL6792Contig2
8.2
8.3
8.4
8.5
8.6
8.7
8.8
1
2
t−Test: p−value = 0.16

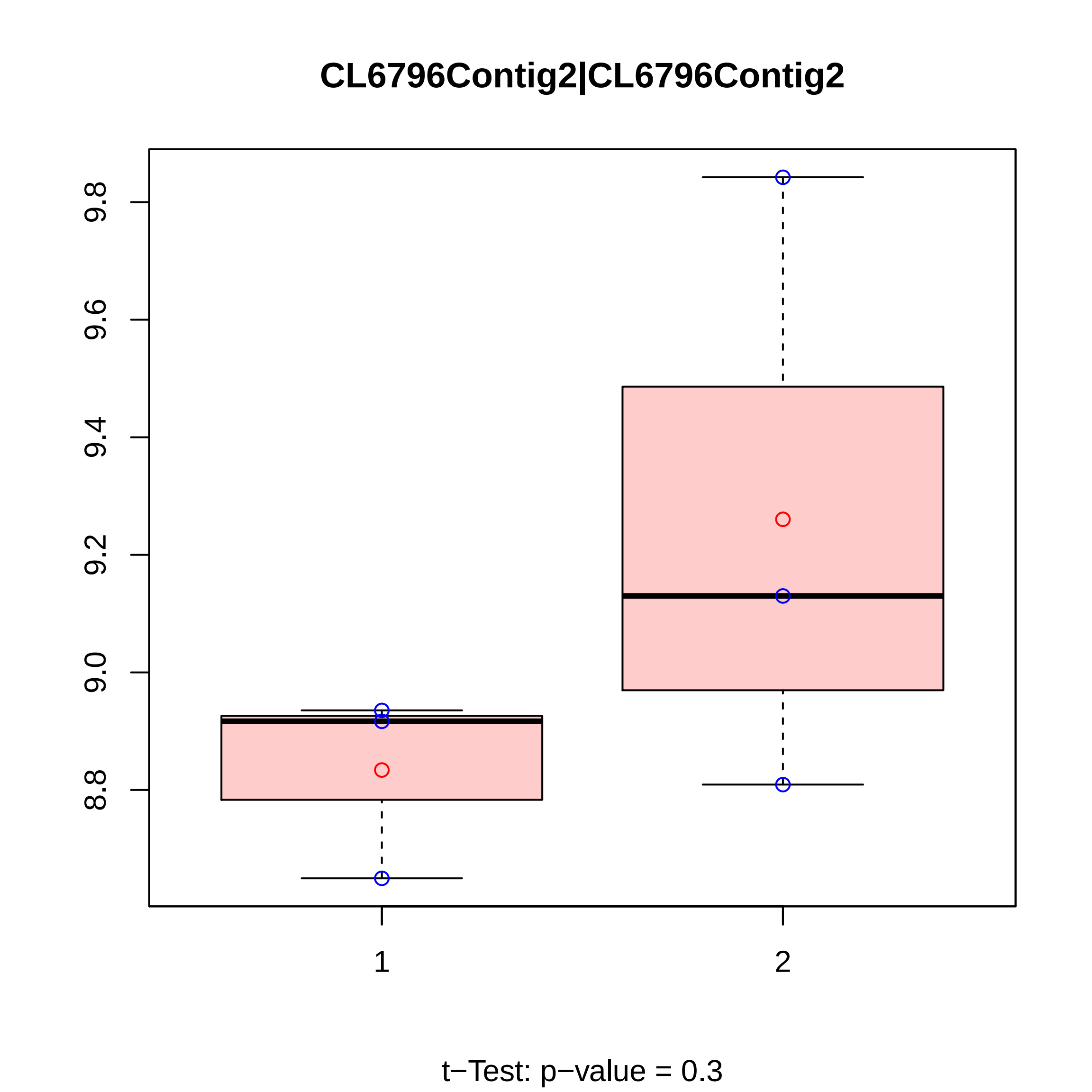
CL6796Contig2|CL6796Contig2
9.8
9.6
9.4
9.2
9.0
8.8
1
2
t−Test: p−value = 0.3

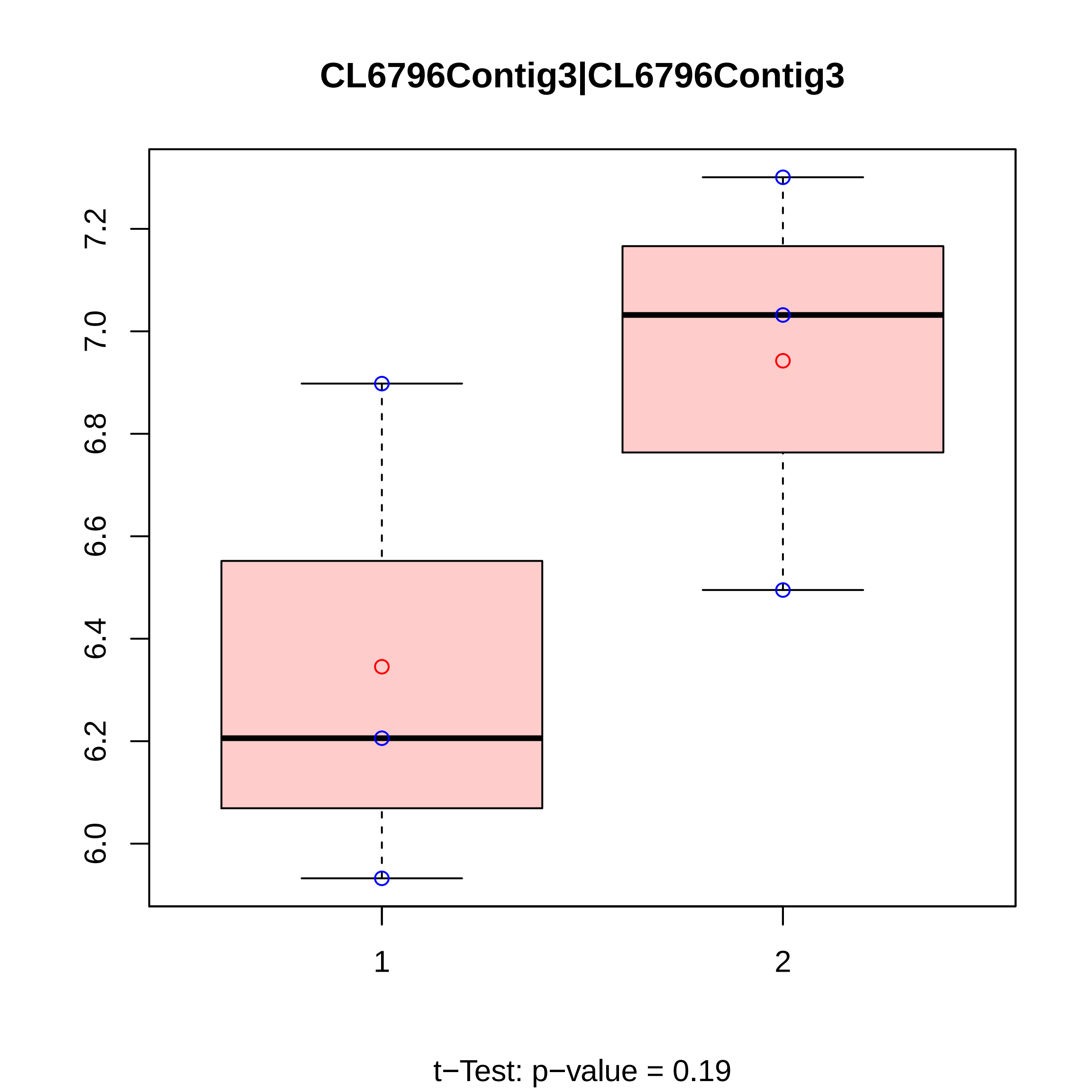
CL6796Contig3|CL6796Contig3
7.2
7.0
6.8
6.6
6.4
6.2
6.0
1
2
t−Test: p−value = 0.19

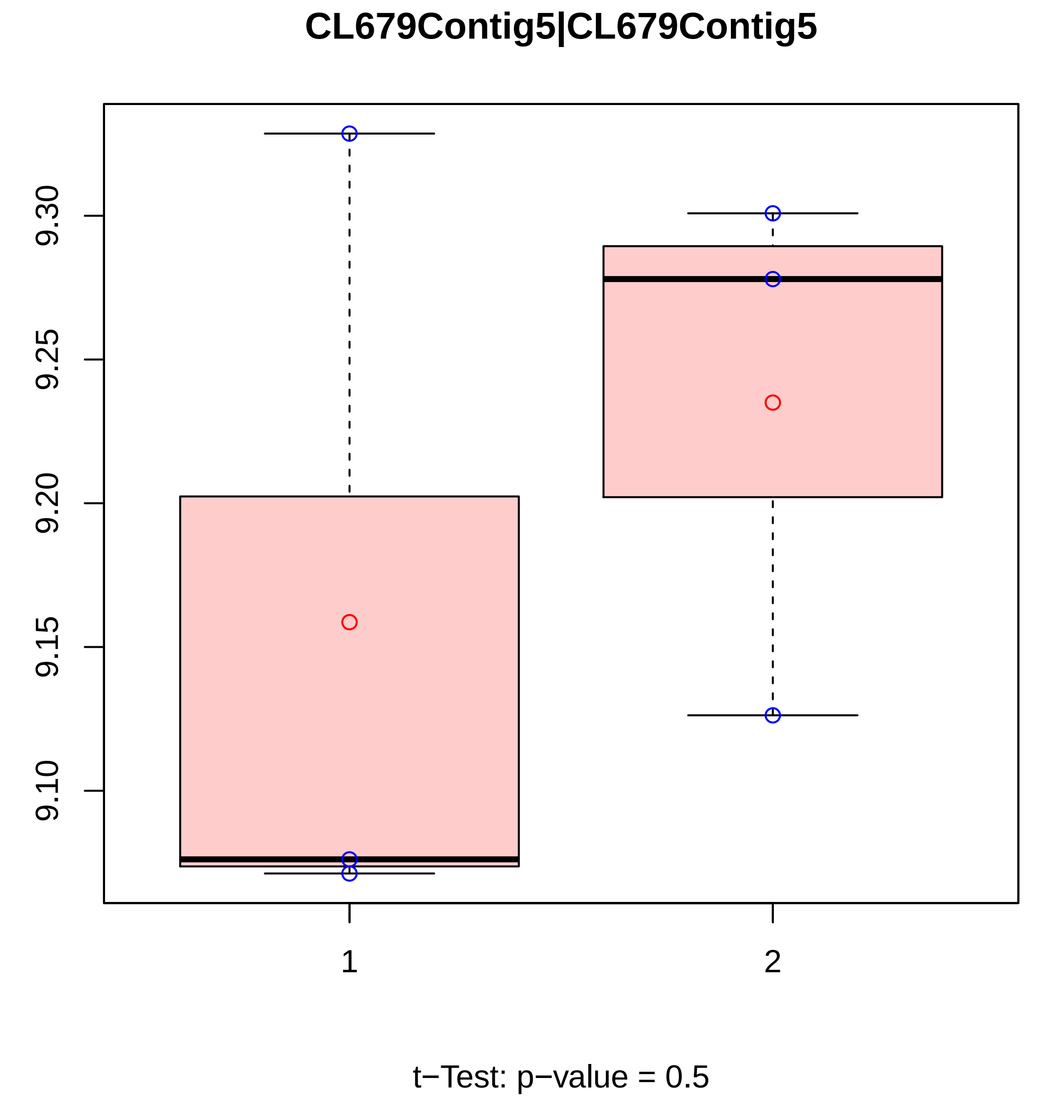

CL679Contig5|CL679Contig5
9.10
9.15
9.20
9.25
9.30
1
2
t−Test: p−value = 0.5

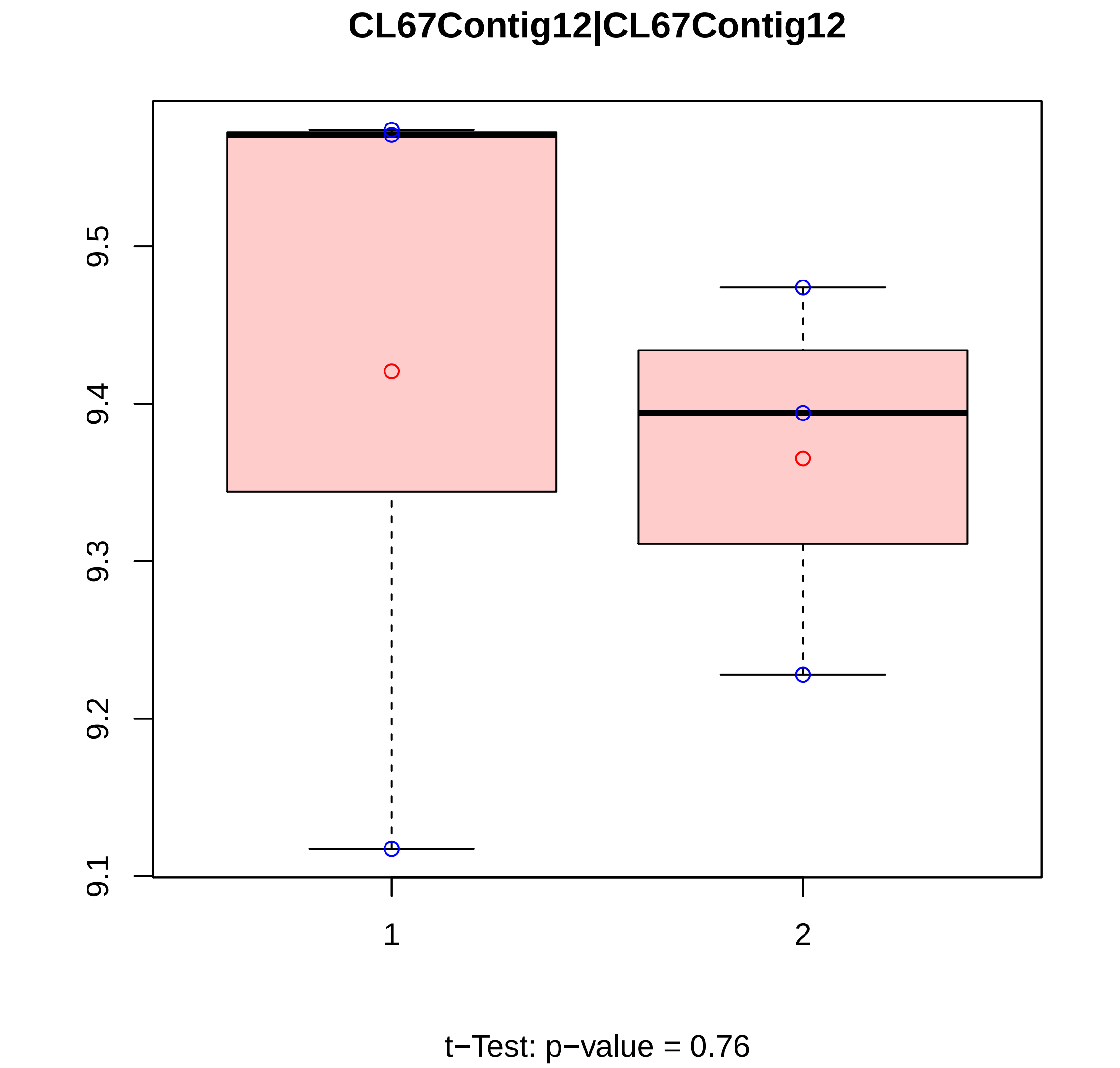
CL67Contig12|CL67Contig12
9.1
9.2
9.3
9.4
9.5
1
2
t−Test: p−value = 0.76

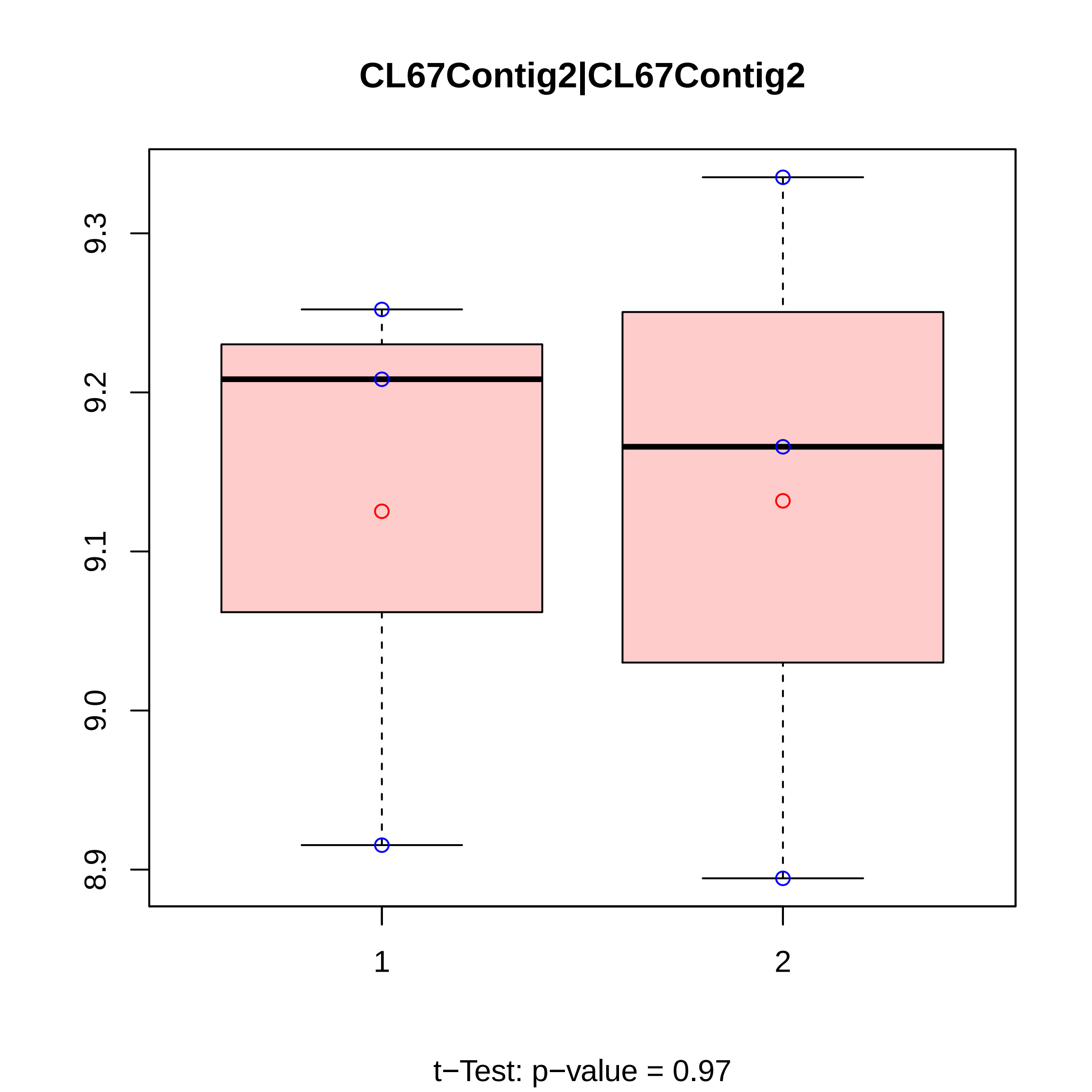

CL67Contig2|CL67Contig2
9.3
9.2
9.1
9.0
8.9
1
2
t−Test: p−value = 0.97

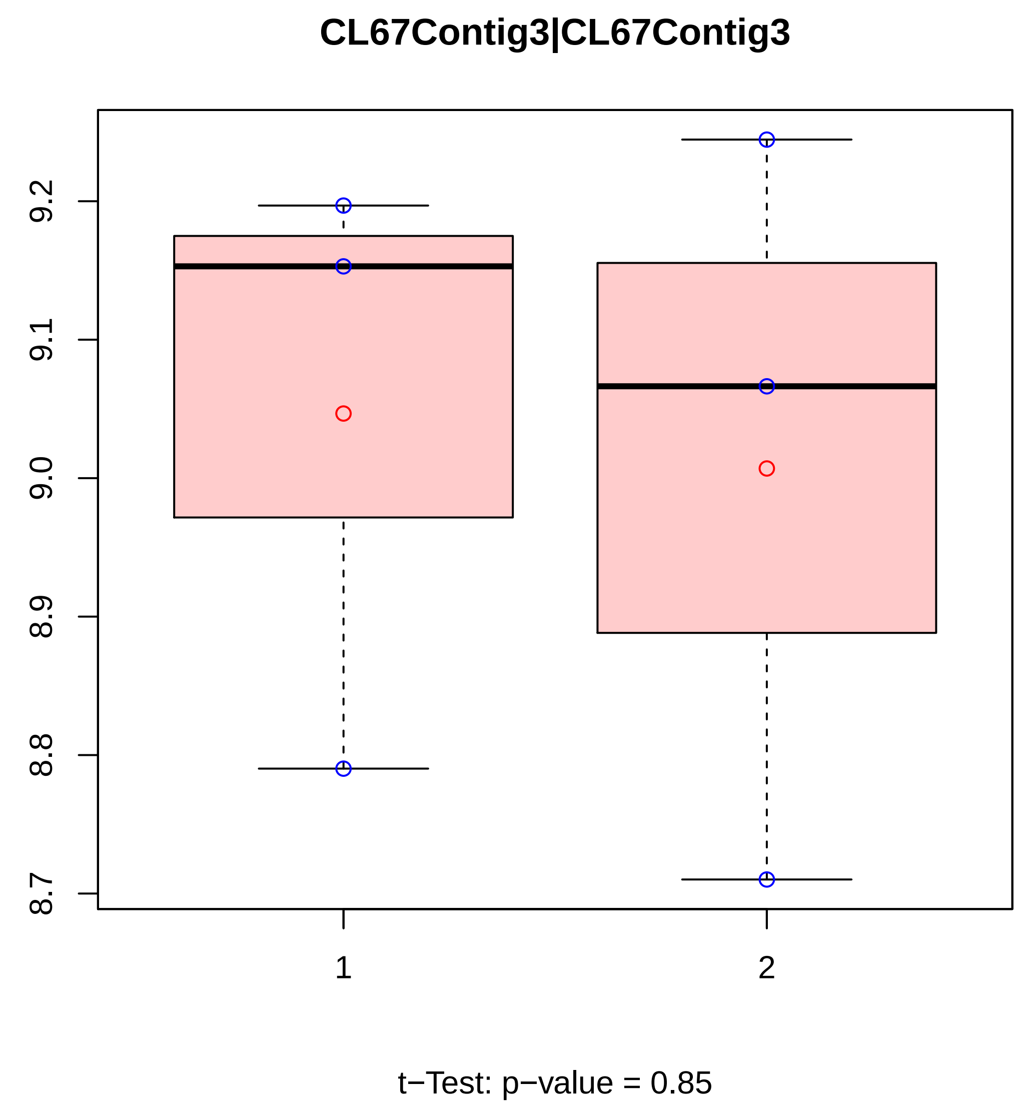

CL67Contig3|CL67Contig3
9.2
9.1
9.0
8.9
8.8
8.7
1
2
t−Test: p−value = 0.85

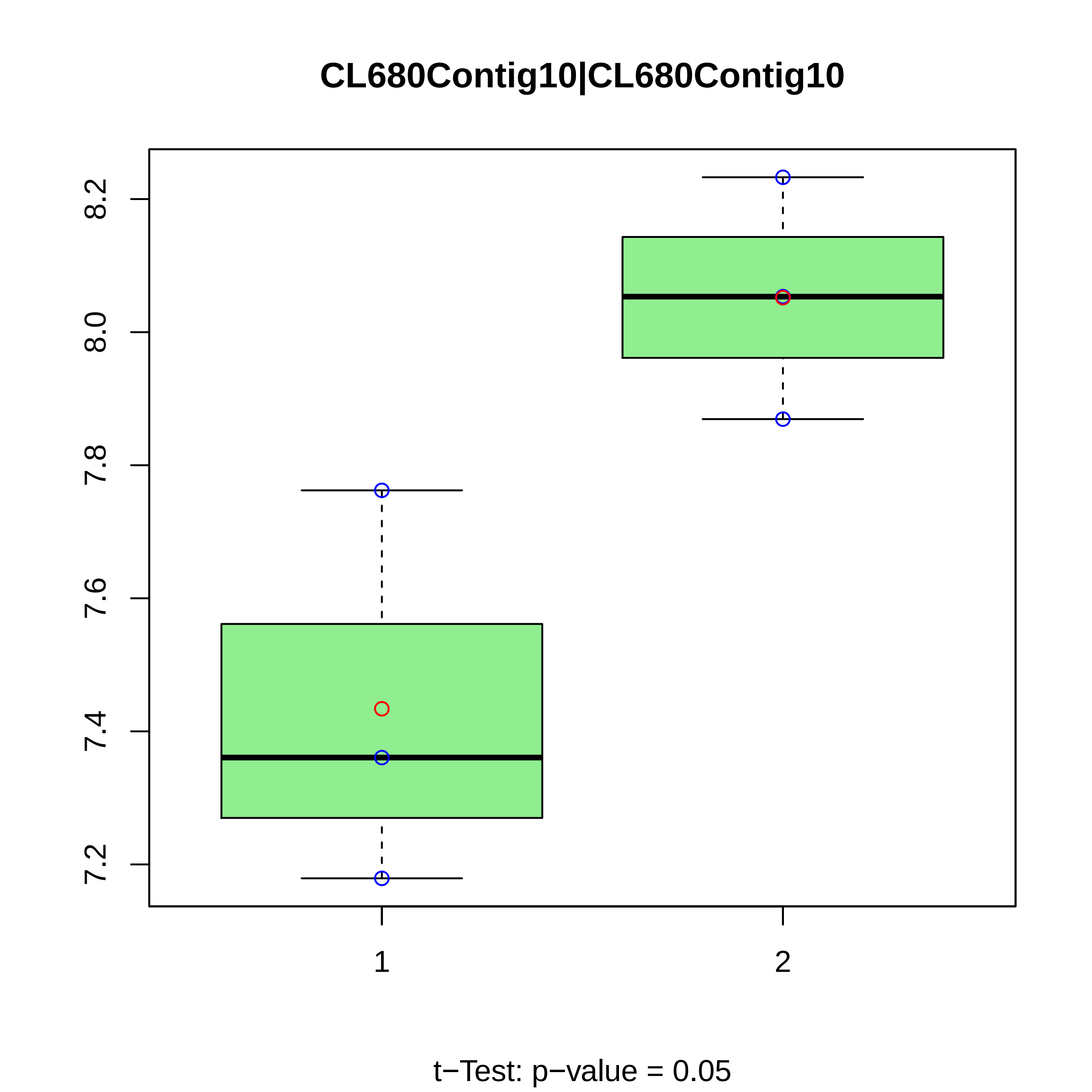

CL680Contig10|CL680Contig10
8.2
8.0
7.8
7.6
7.4
7.2
1
2
t−Test: p−value = 0.05

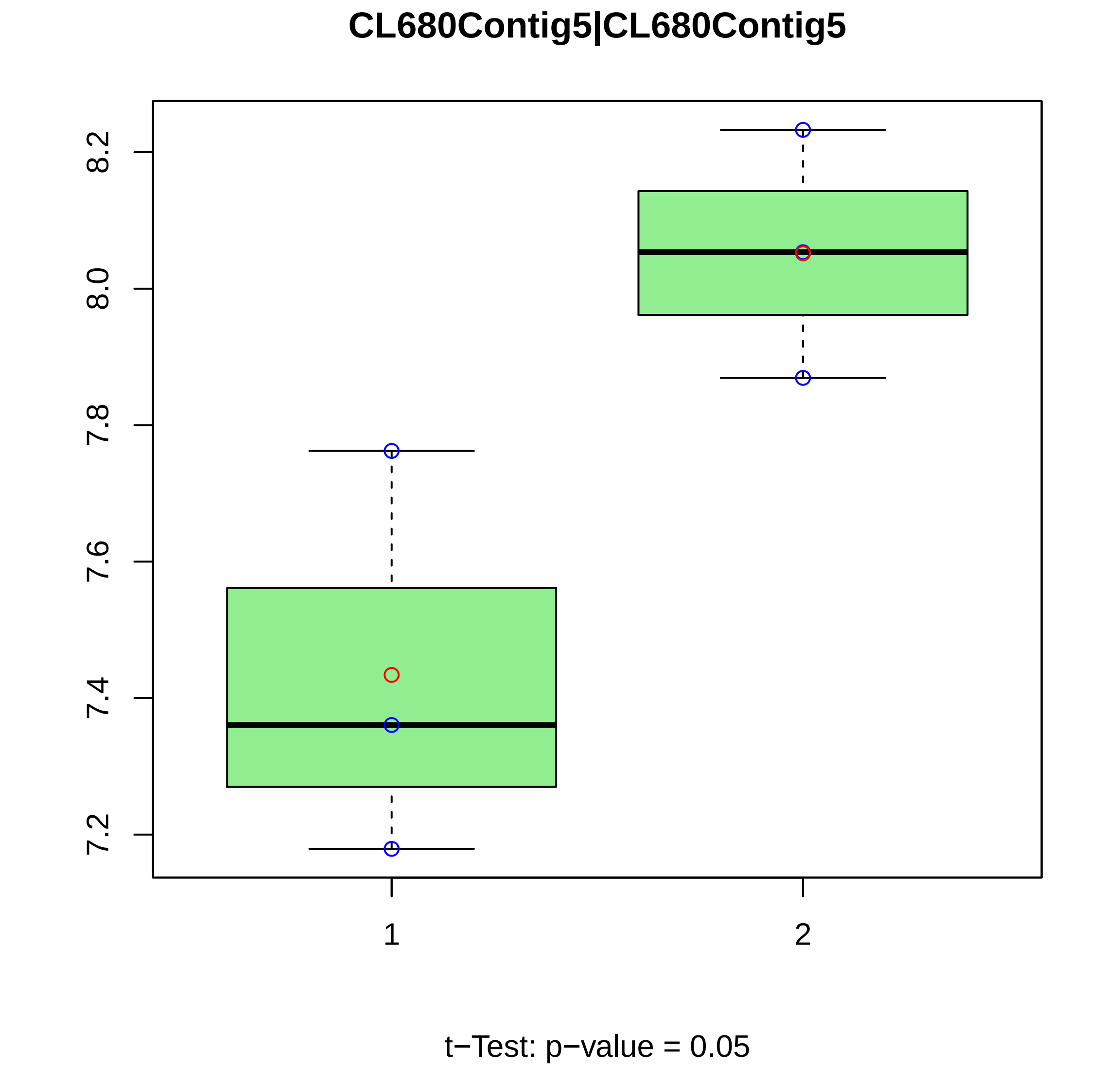
CL680Contig5|CL680Contig5
8.2
8.0
7.8
7.6
7.4
7.2
1
2
t−Test: p−value = 0.05

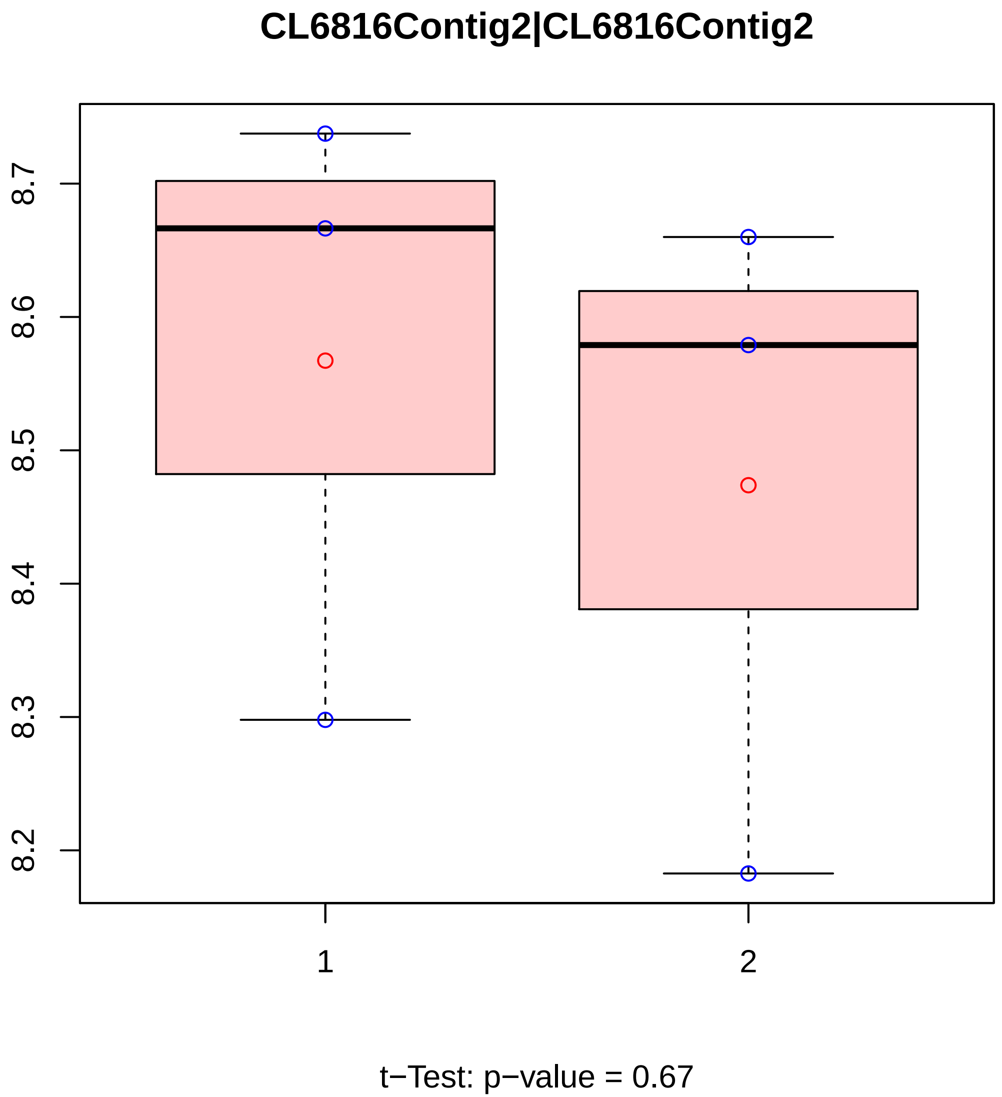
CL6816Contig2|CL6816Contig2
8.2
8.3
8.4
8.5
8.6
8.7
1
2
t−Test: p−value = 0.67

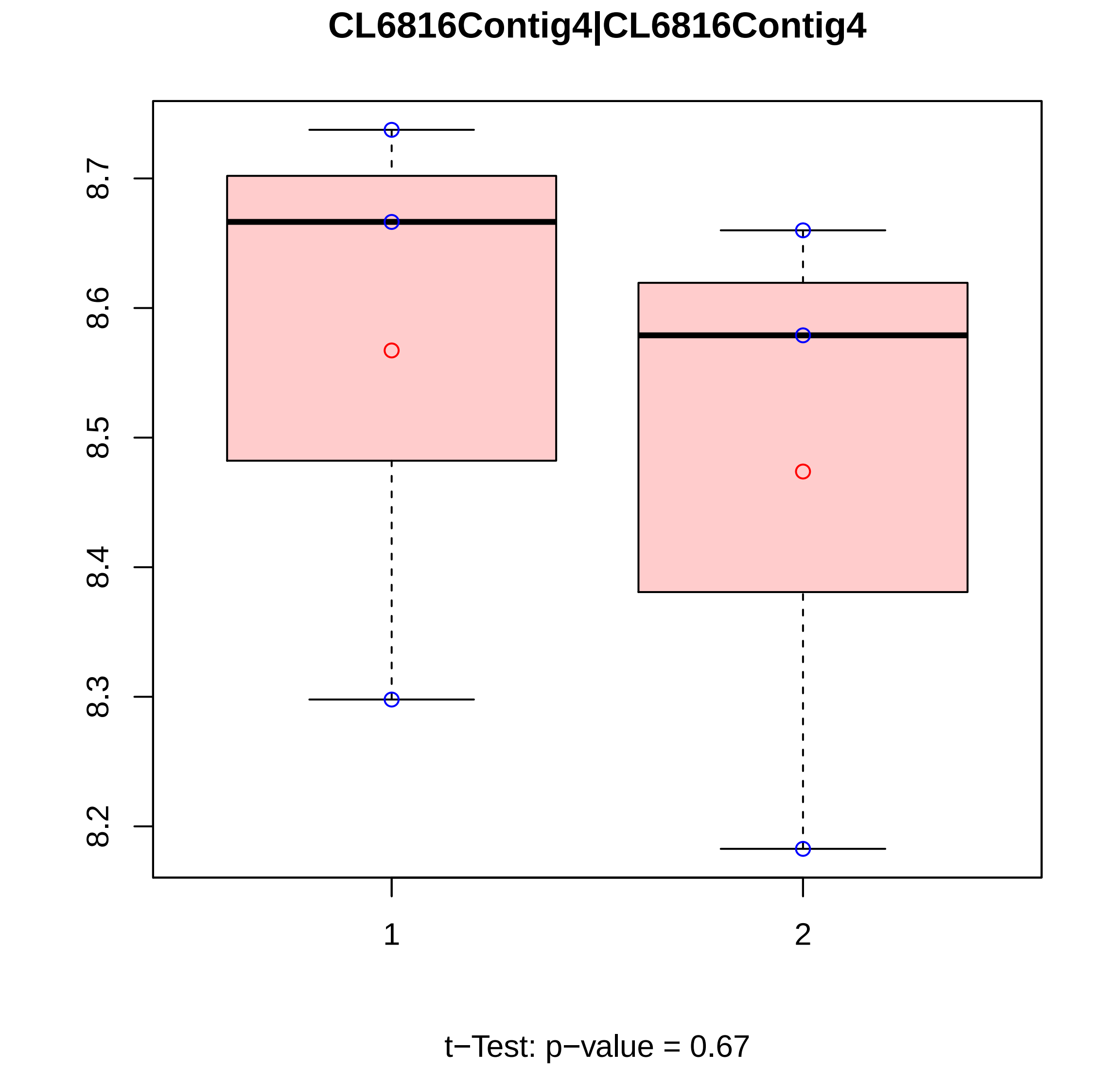
CL6816Contig4|CL6816Contig4
8.2
8.3
8.4
8.5
8.6
8.7
1
2
t−Test: p−value = 0.67

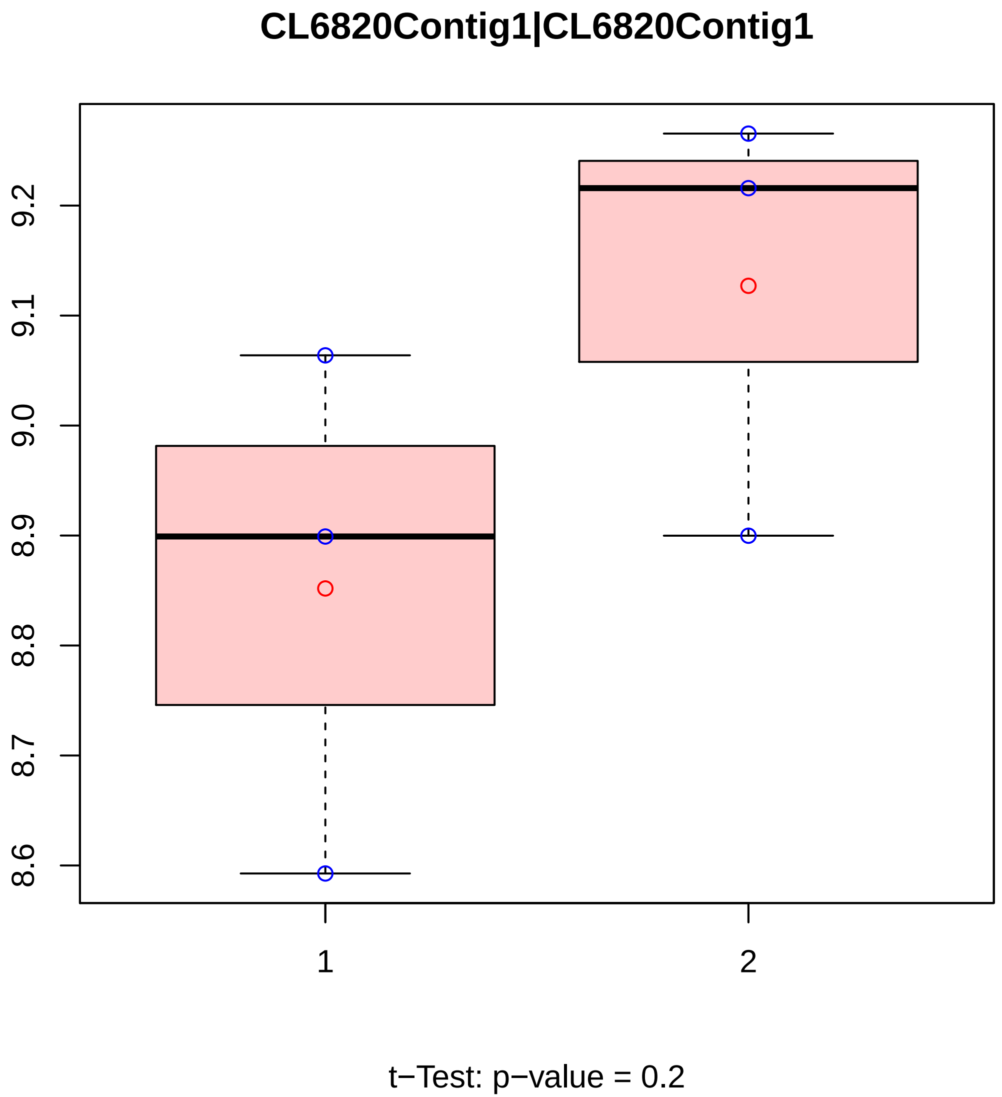

CL6820Contig1|CL6820Contig1
9.2
9.1
9.0
8.9
8.8
8.7
8.6
1
2
t−Test: p−value = 0.2

# CL6822Contig1|CL6822Contig1

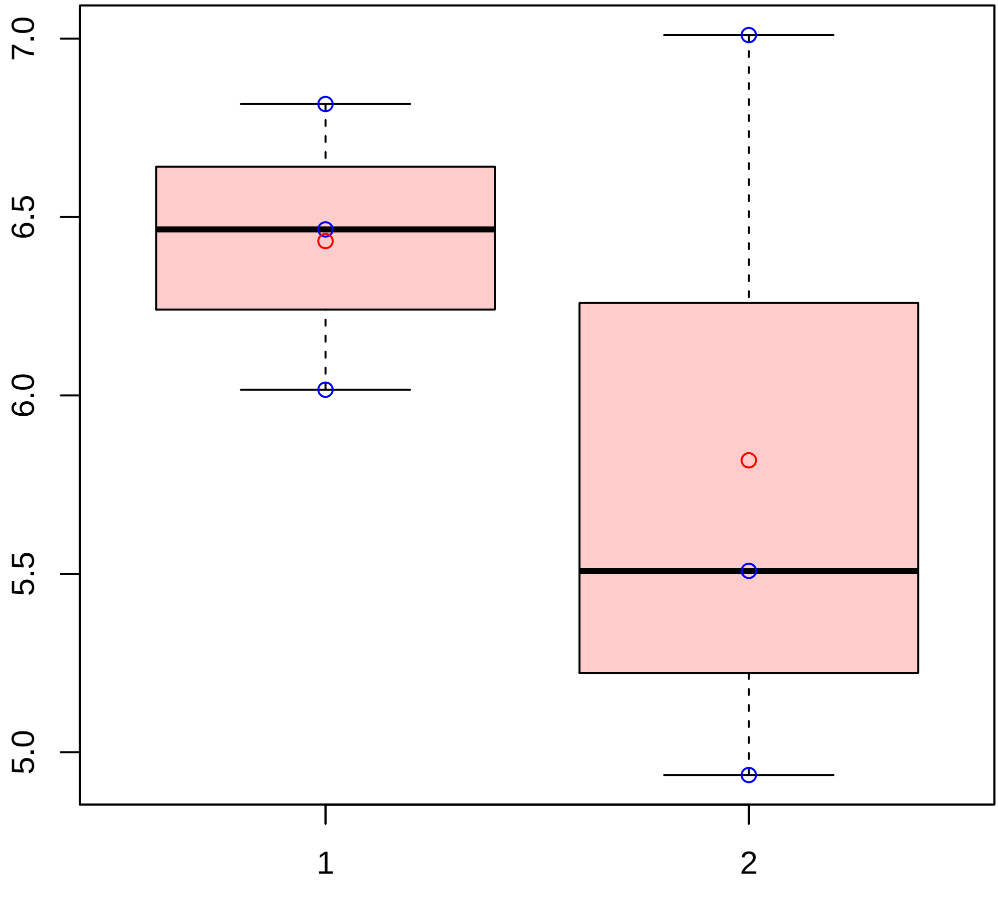


t−Test: p−value = 0.43

## CL682Contig1|CL682Contig1

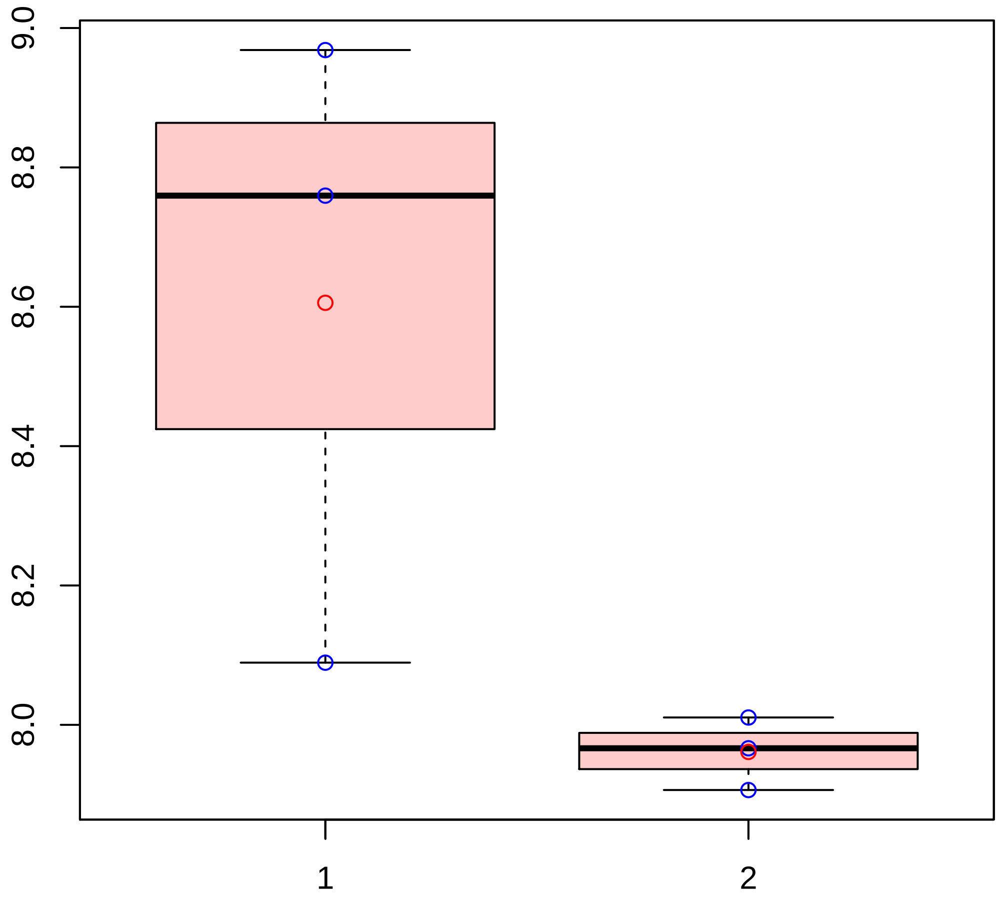


t−Test: p−value = 0.13

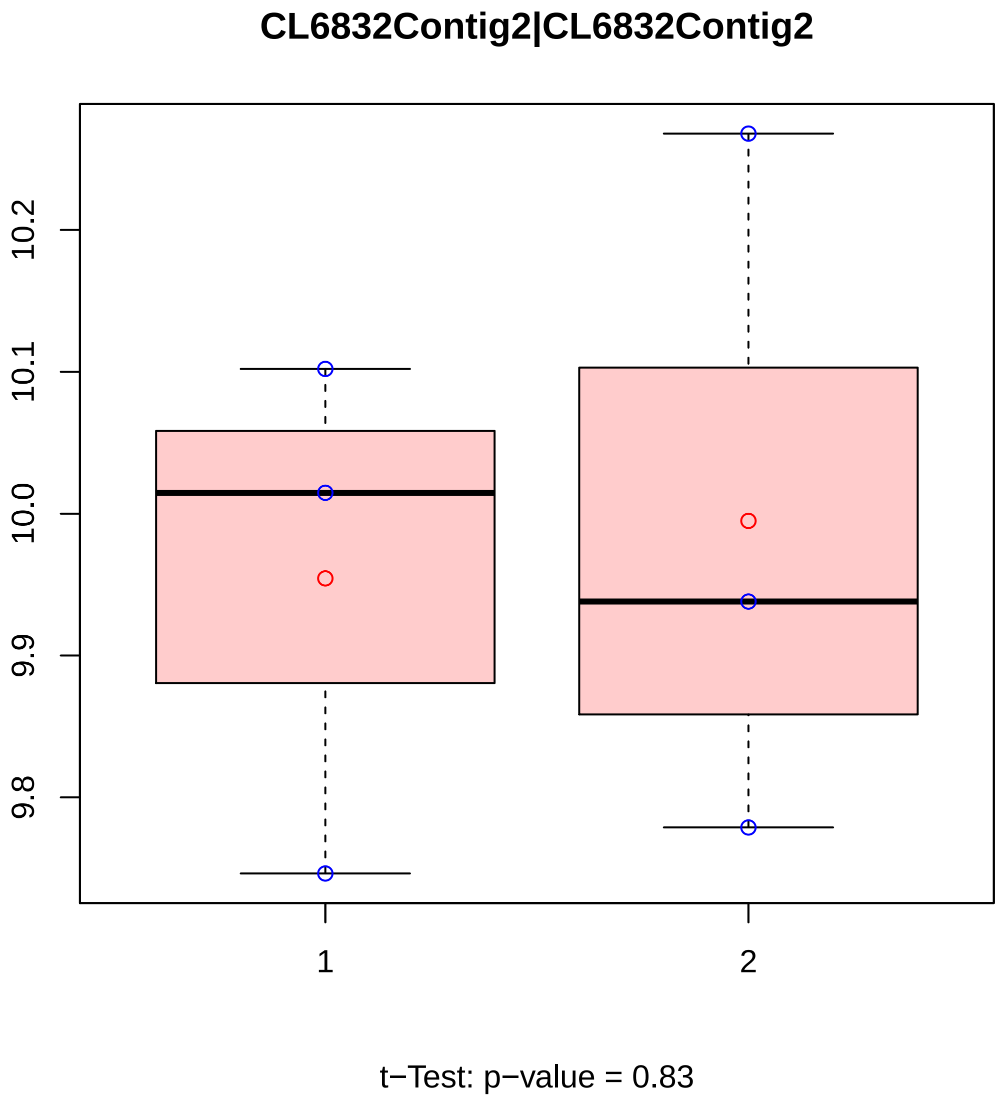

CL6832Contig2|CL6832Contig2
10.2
10.1
10.0
9.9
9.8
1
2
t−Test: p−value = 0.83

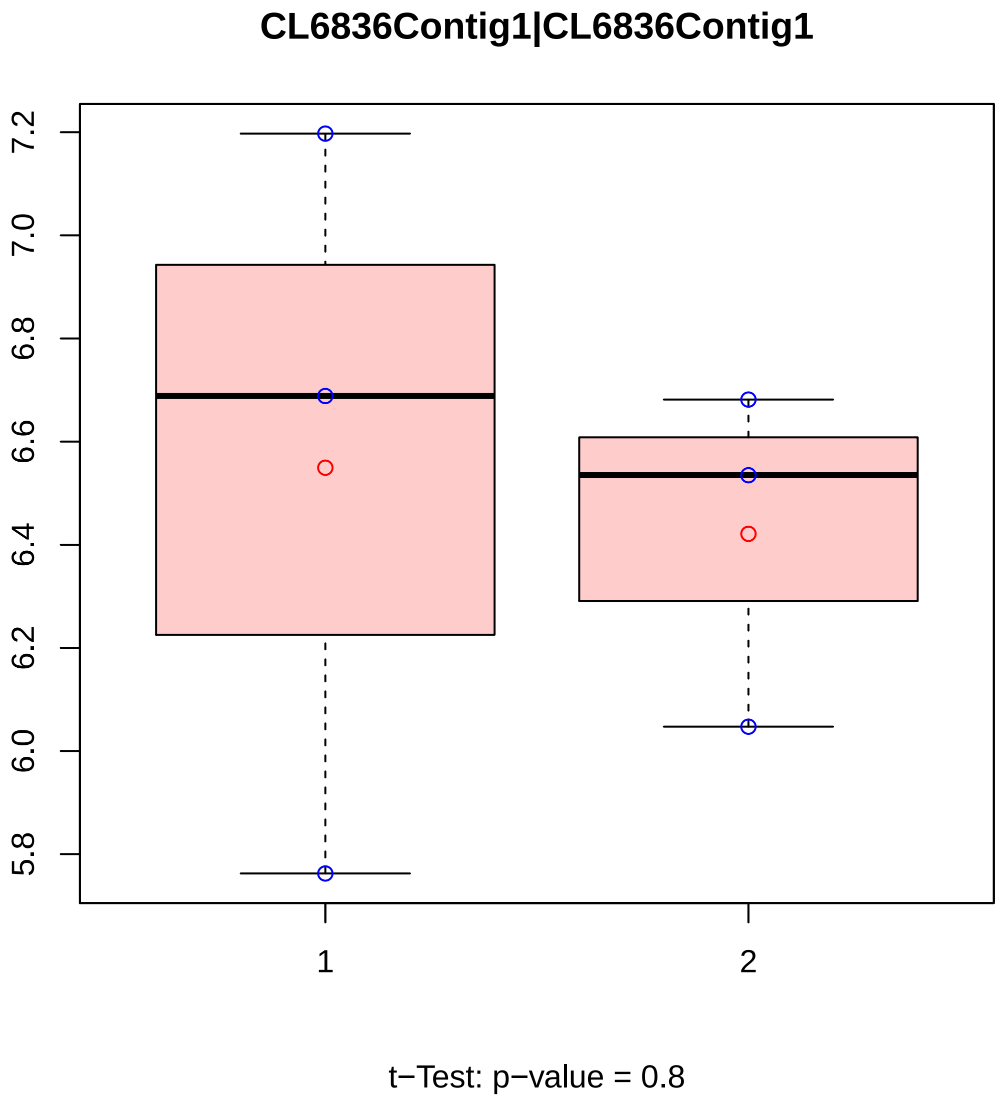
CL6836Contig1|CL6836Contig1
7.2
7.0
6.8
6.6
6.4
6.2
6.0
5.8
1
2
t−Test: p−value = 0.8

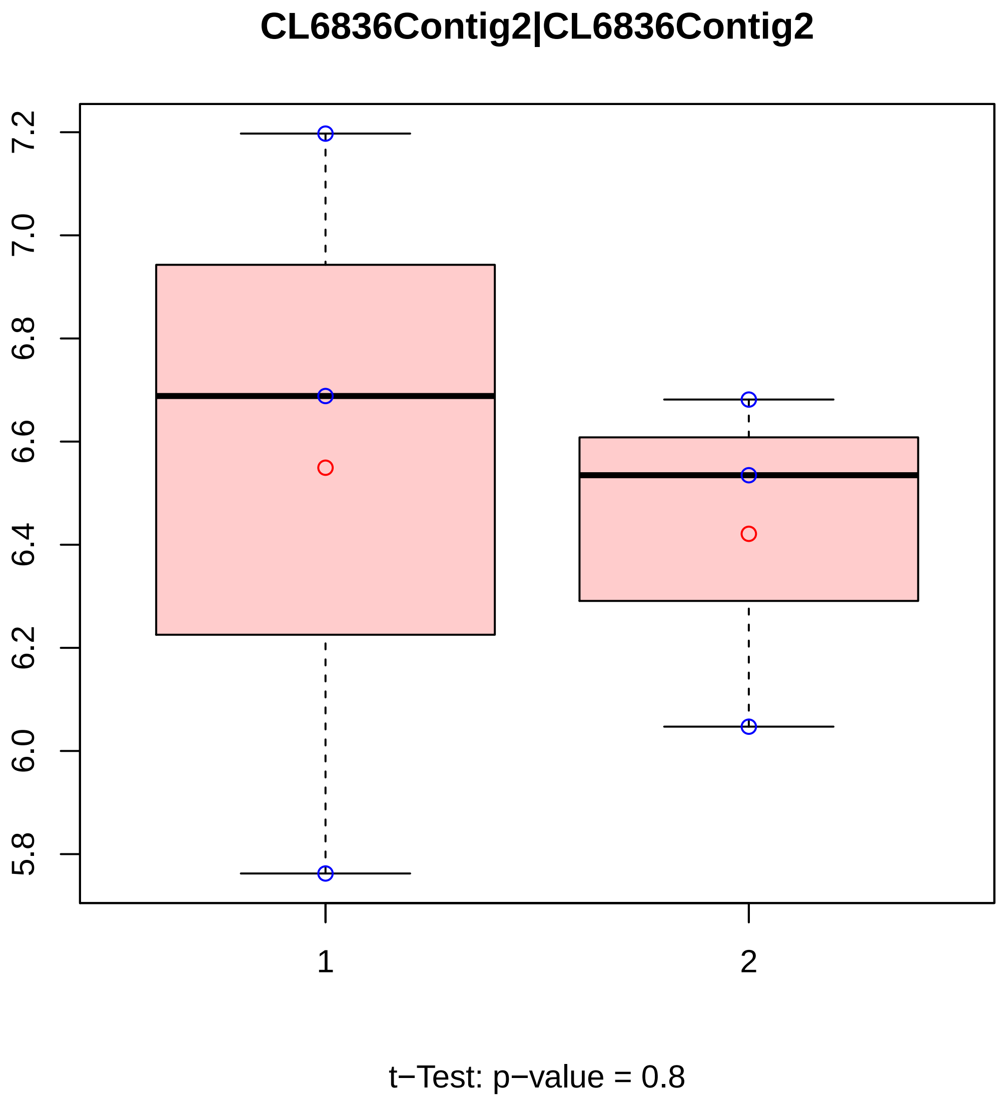

CL6836Contig2|CL6836Contig2
7.2
7.0
6.8
6.6
6.4
6.2
6.0
5.8
1
2
t−Test: p−value = 0.8

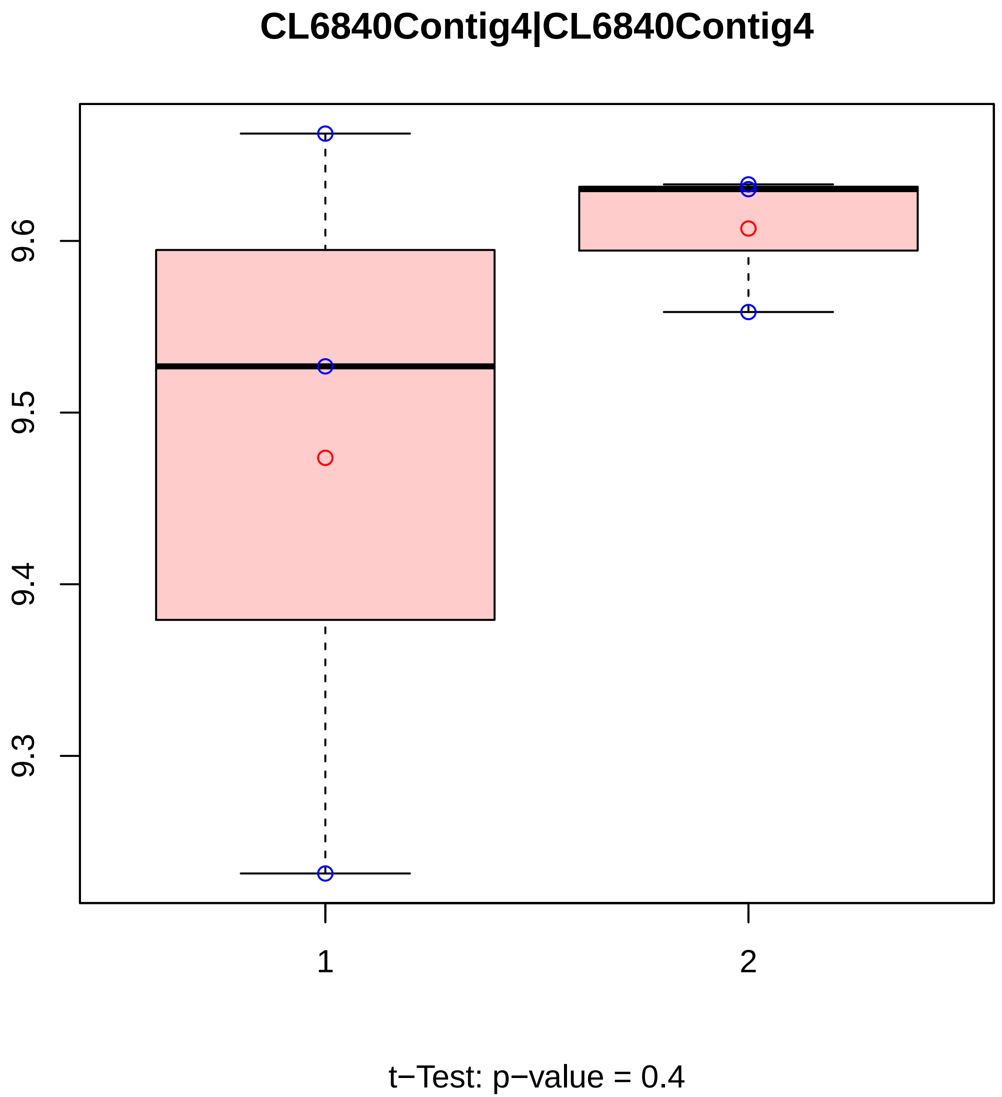

CL6840Contig4|CL6840Contig4
9.3
9.4
9.5
9.6
1
2
t−Test: p−value = 0.4

# CL6841Contig1|CL6841Contig1

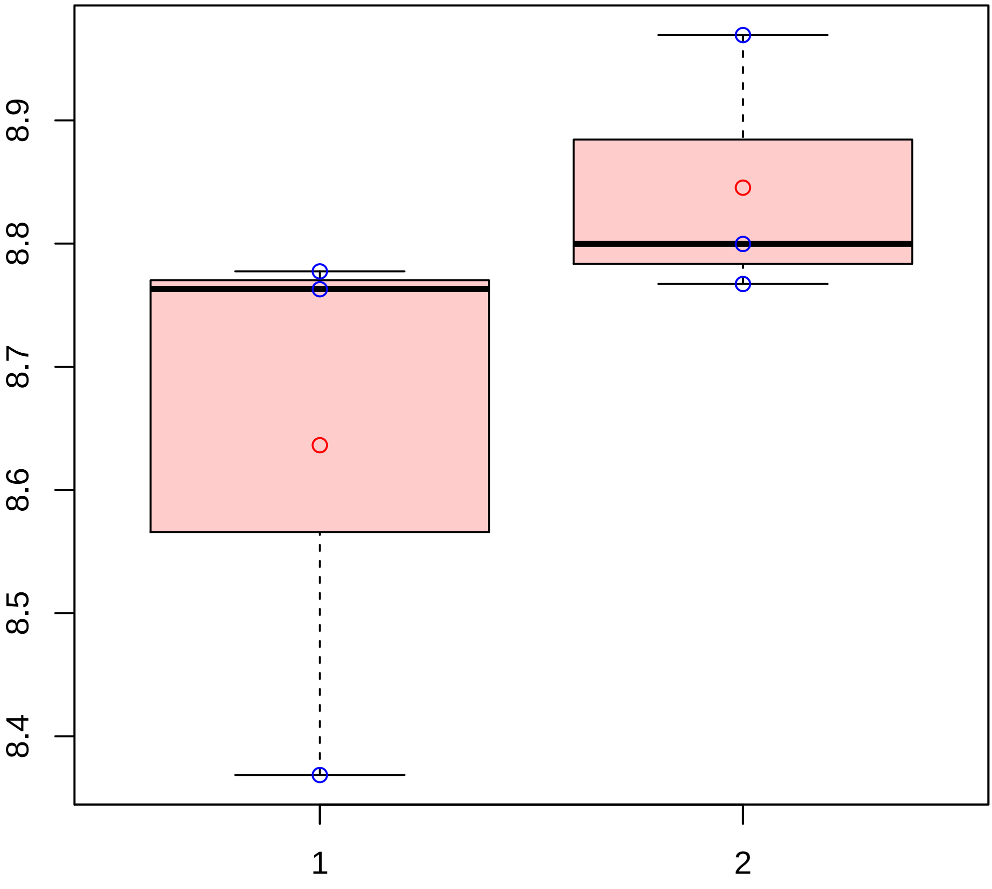


t−Test: p−value = 0.26

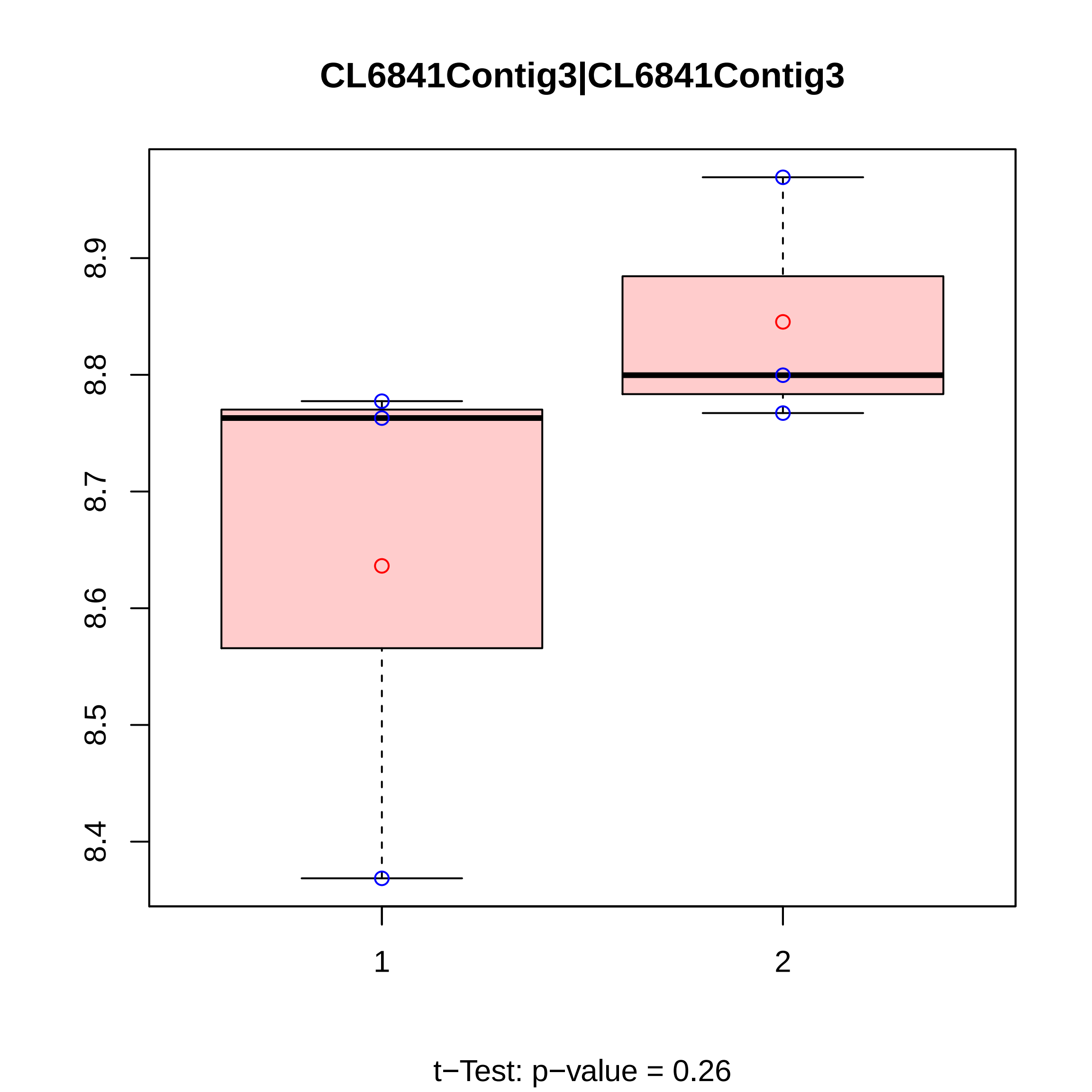
CL6841Contig3|CL6841Contig3
8.4
8.5
8.6
8.7
8.8
8.9
1
2
t−Test: p−value = 0.26

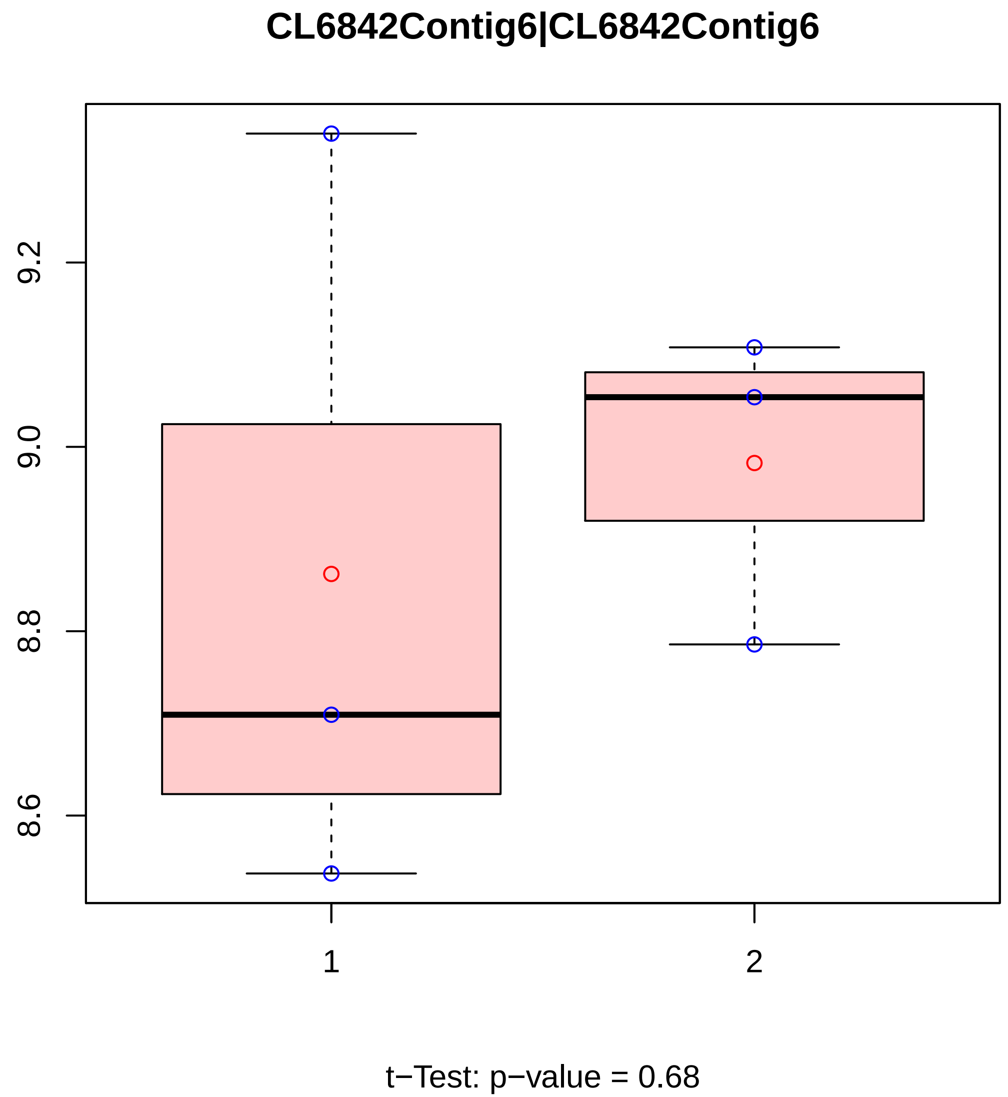

CL6842Contig6|CL6842Contig6
9.2
9.0
8.8
8.6
1
2
t−Test: p−value = 0.68

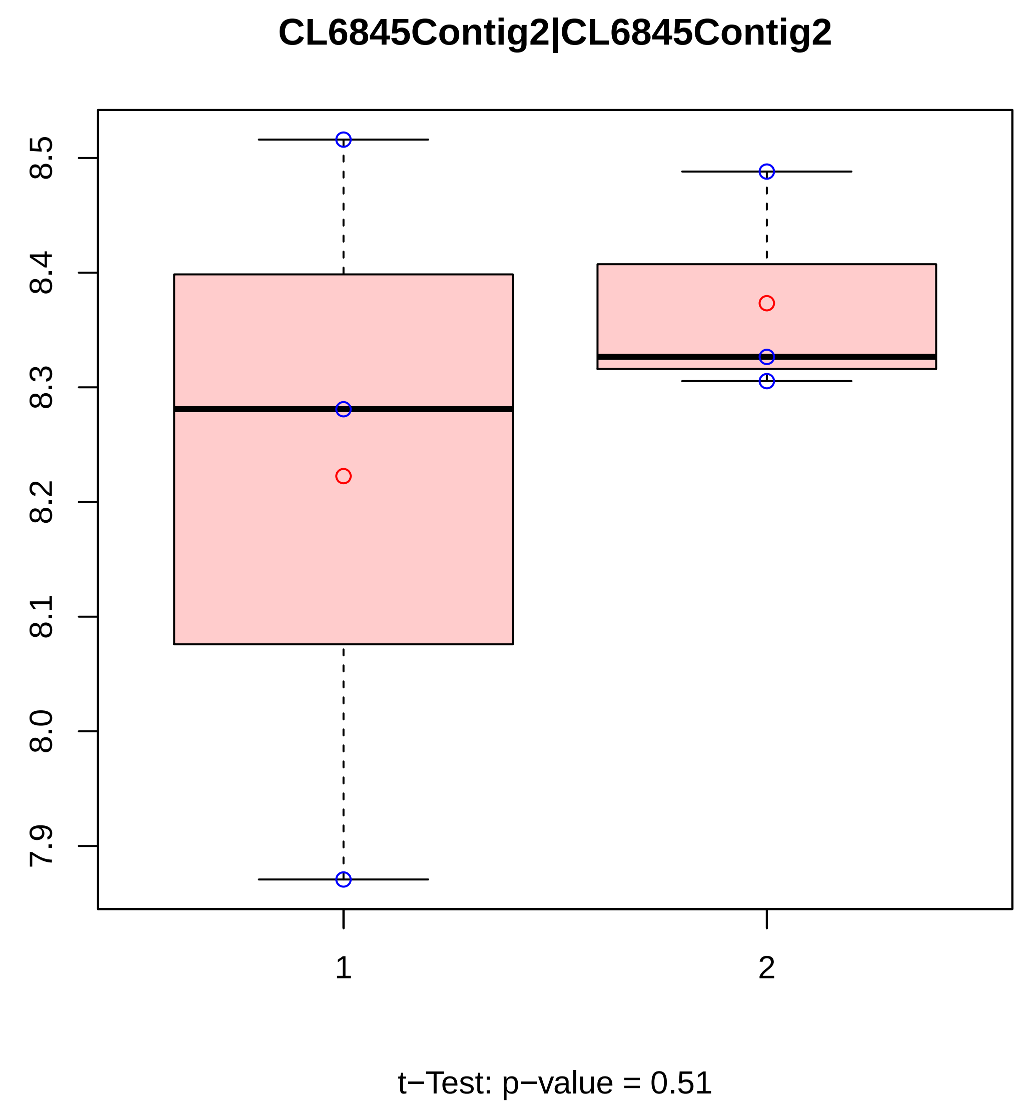
CL6845Contig2|CL6845Contig2
8.5
8.4
8.3
8.2
8.1
8.0
7.9
1
2
t−Test: p−value = 0.51

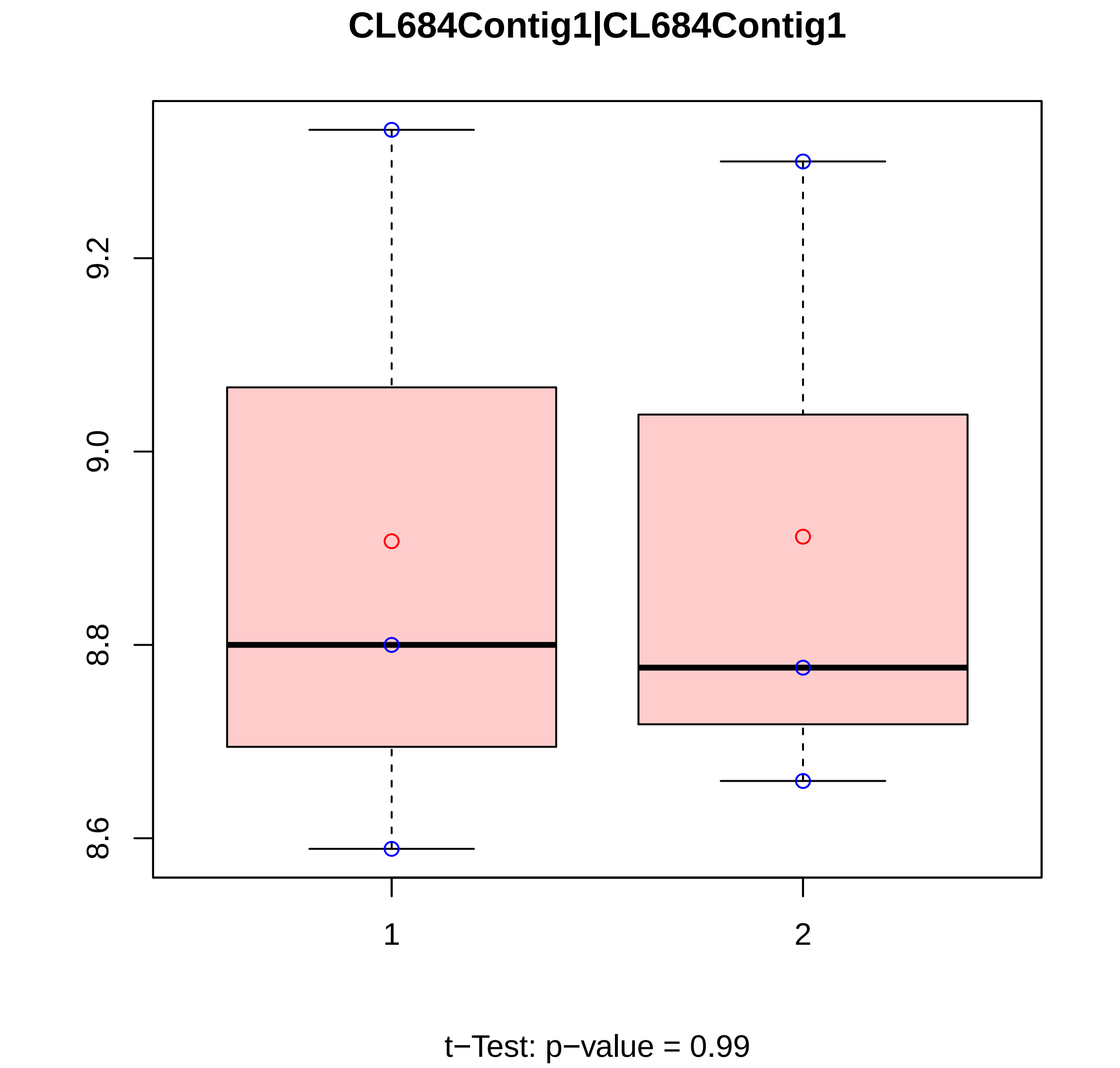
CL684Contig1|CL684Contig1
9.2
9.0
8.8
8.6
1
2
t−Test: p−value = 0.99

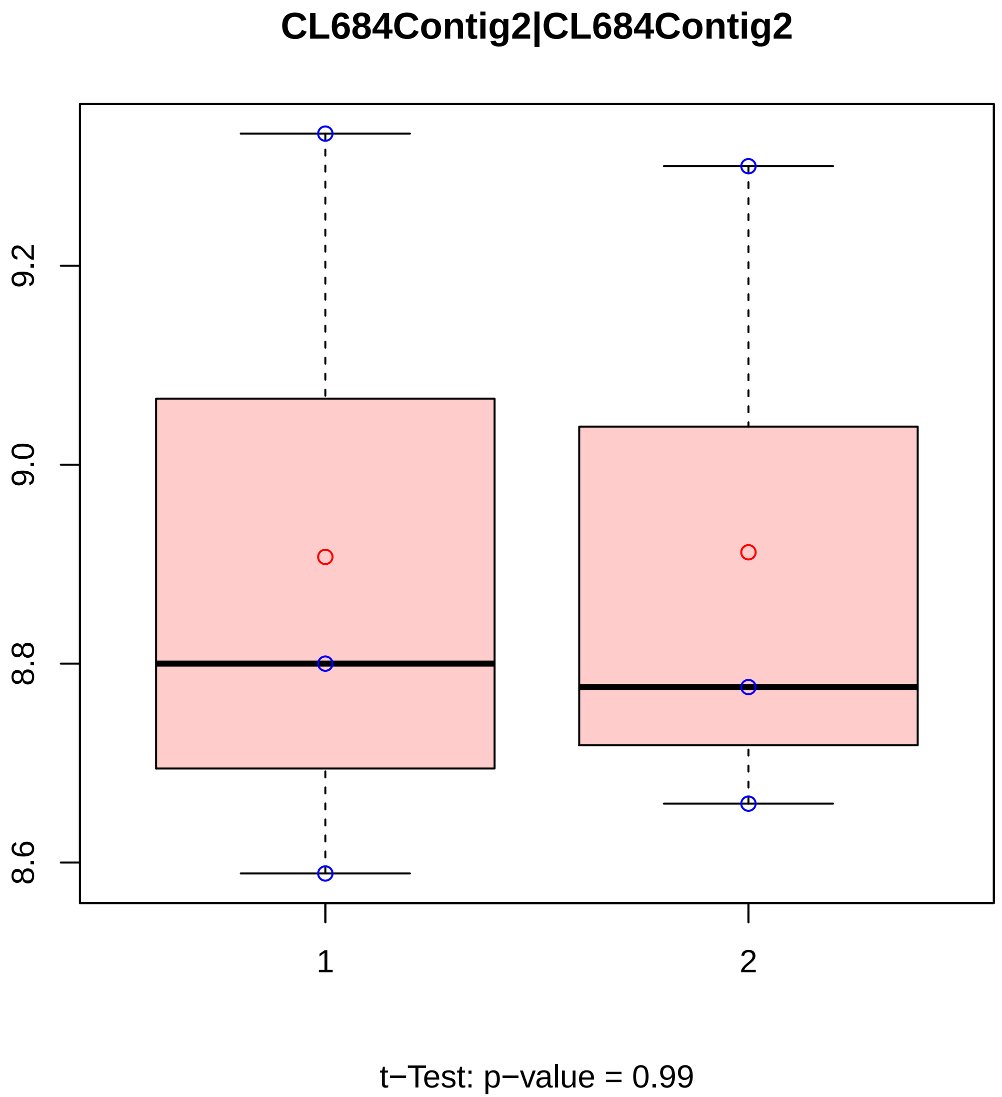
CL684Contig2|CL684Contig2
9.2
9.0
8.8
8.6
1
2
t−Test: p−value = 0.99

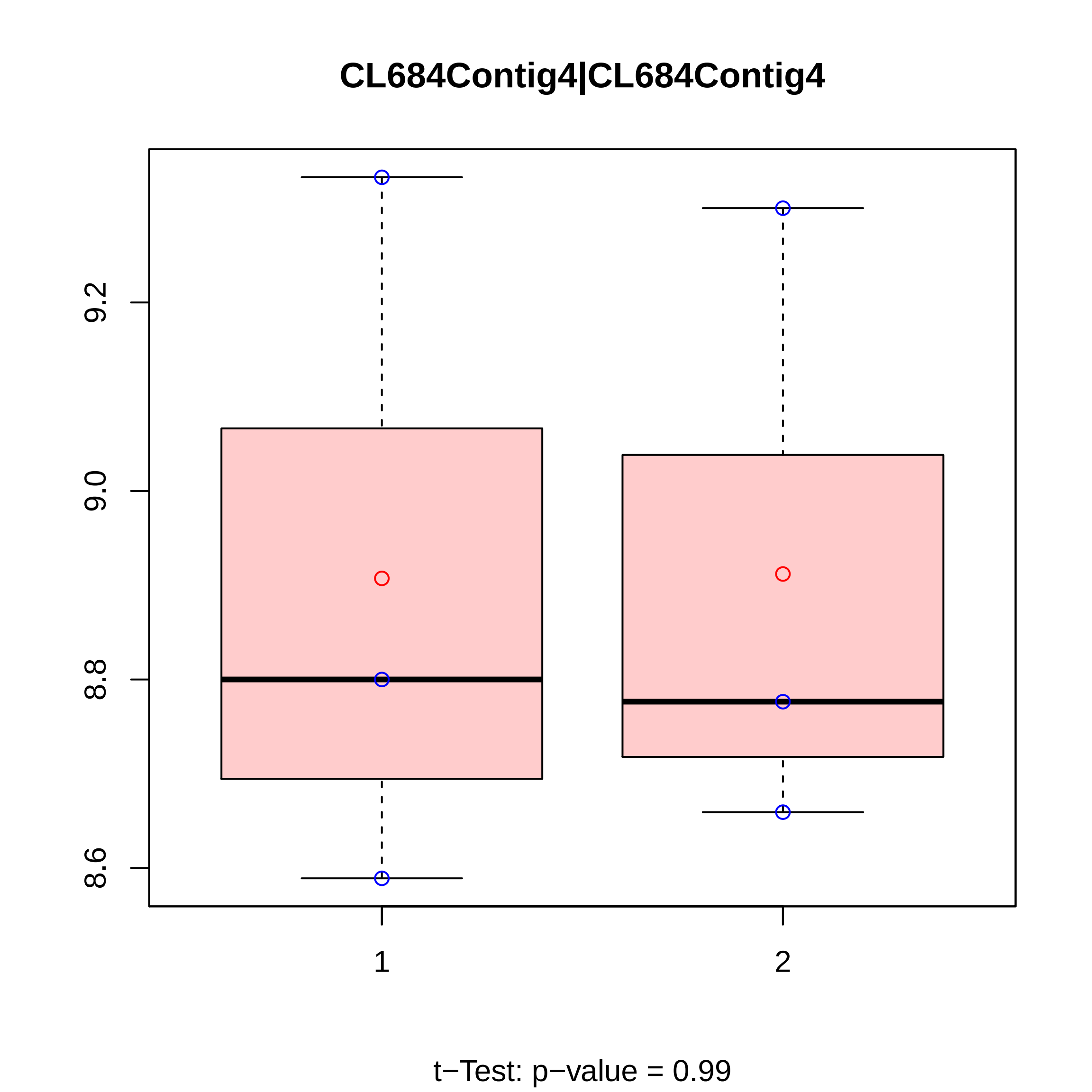

CL684Contig4|CL684Contig4
9.2
9.0
8.8
8.6
1
2
t−Test: p−value = 0.99

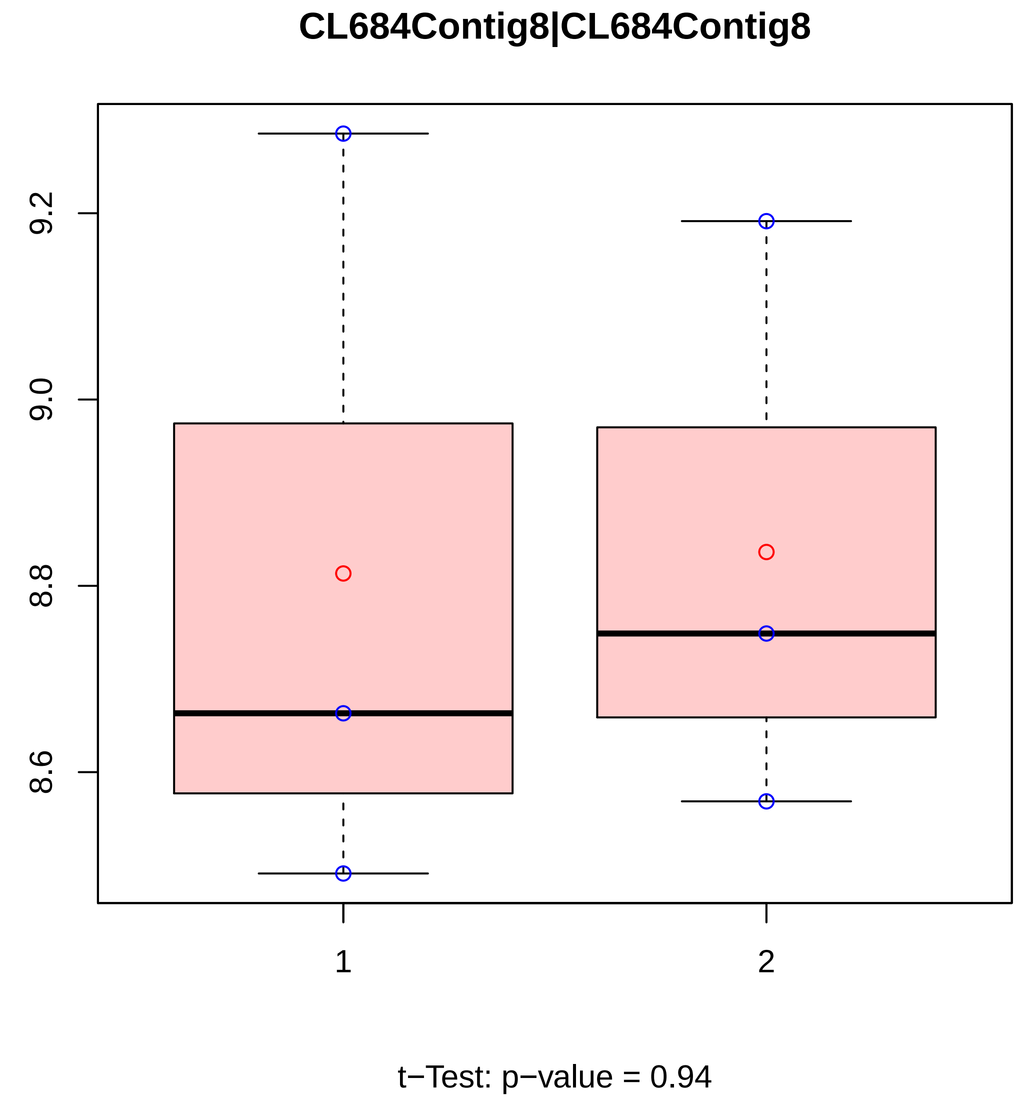
CL684Contig8|CL684Contig8
9.2
9.0
8.8
8.6
1
2
t−Test: p−value = 0.94

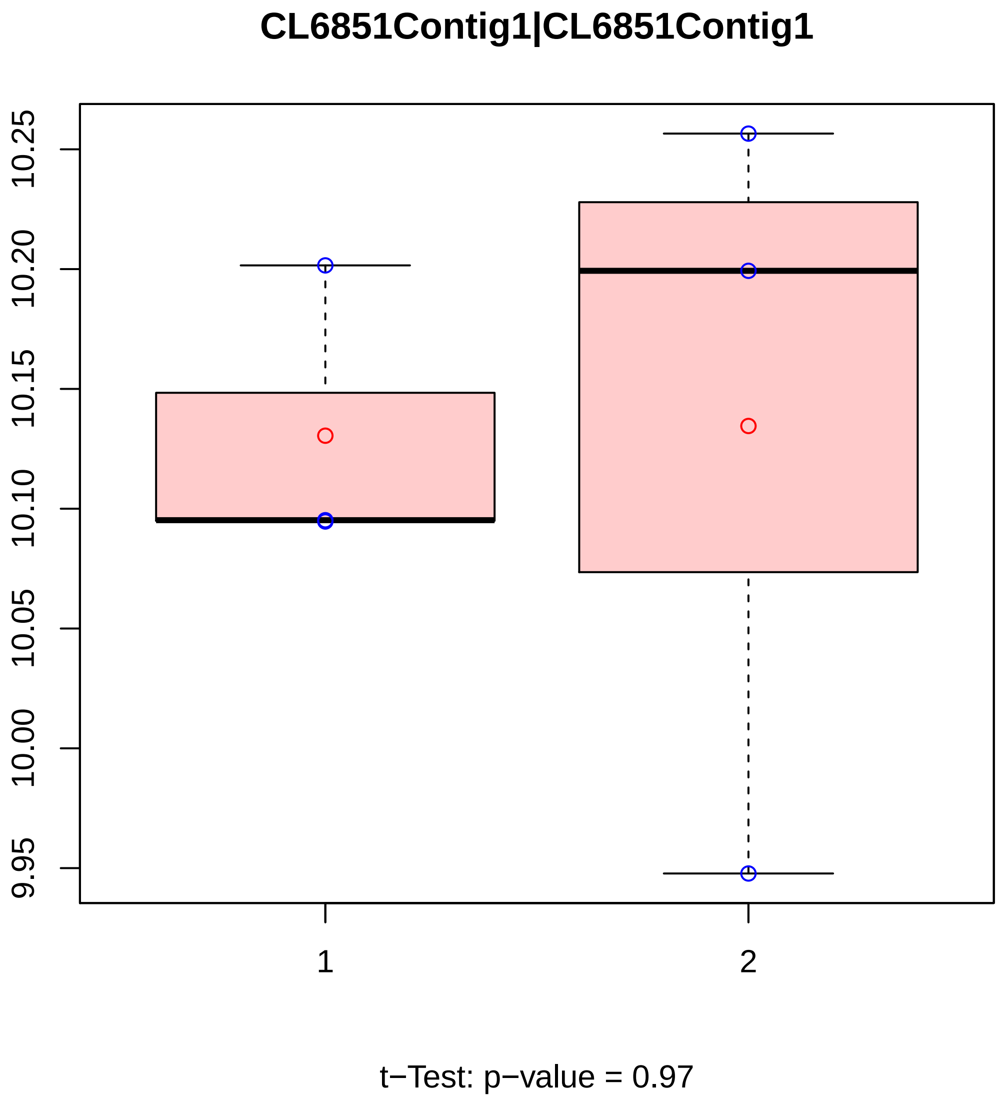
CL6851Contig1|CL6851Contig1
10.25
10.20
10.15
10.10
10.05
10.00
9.95
1
2
t−Test: p−value = 0.97

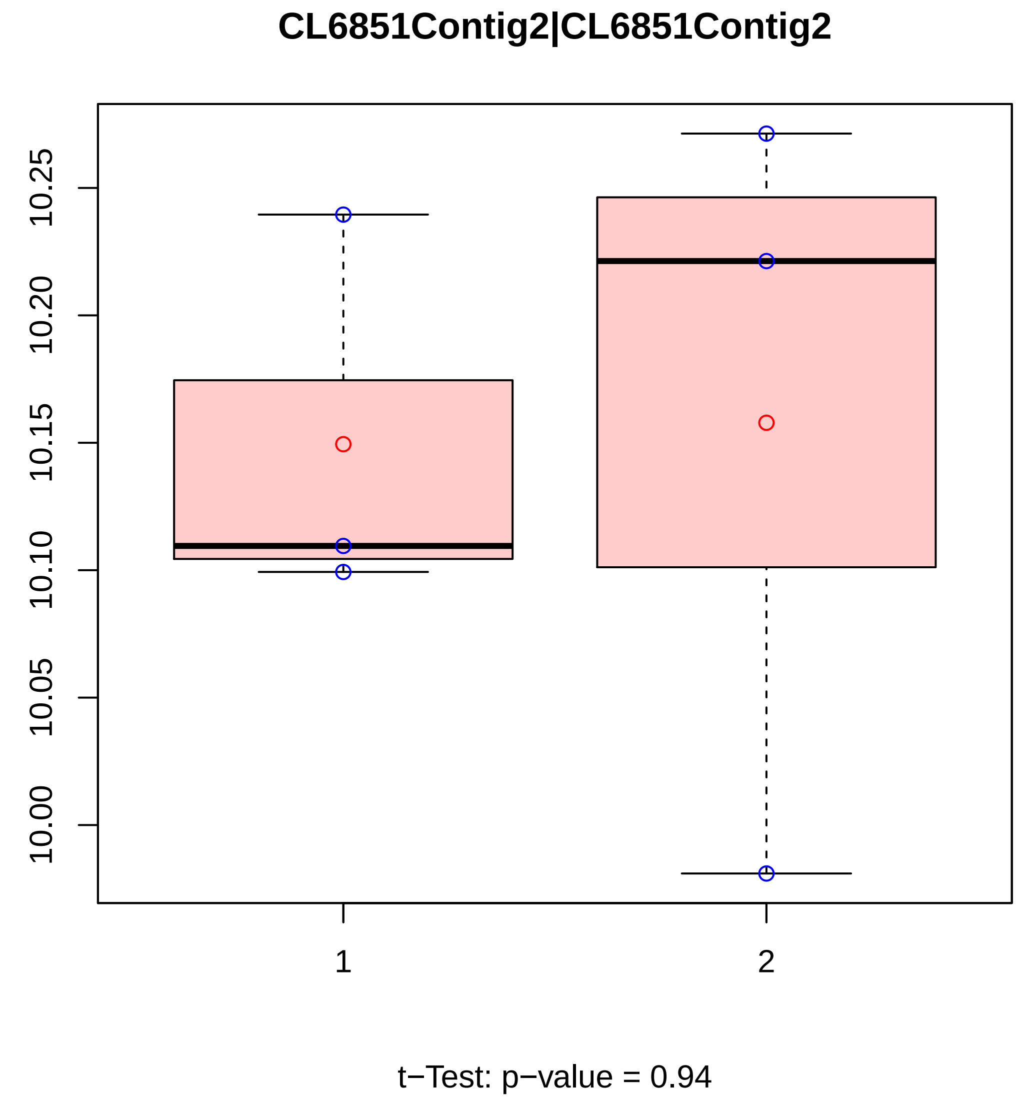

CL6851Contig2|CL6851Contig2
10.00
10.05
10.10
10.15
10.20
10.25
1
2
t−Test: p−value = 0.94

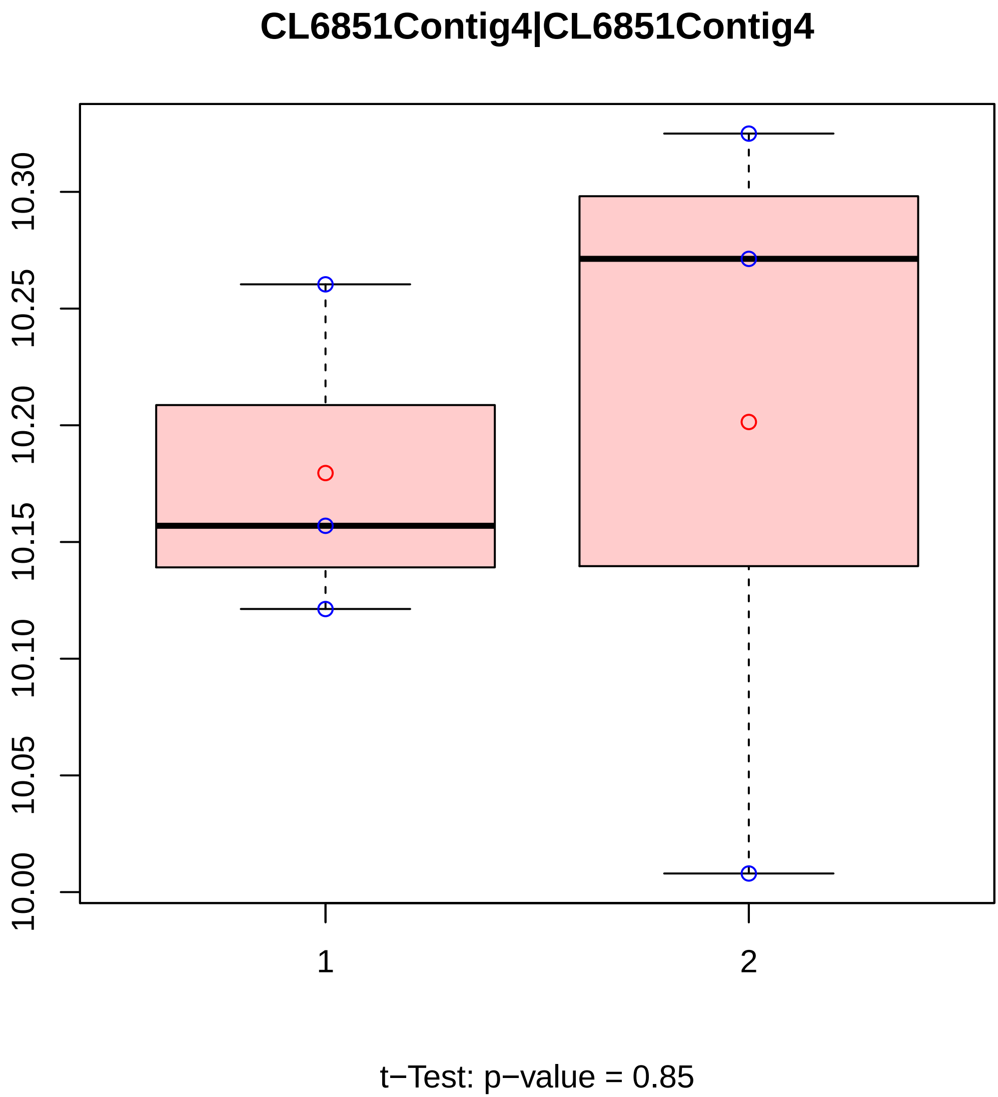

CL6851Contig4|CL6851Contig4
10.00
10.05
10.10
10.15
10.20
10.25
10.30
1
2
t−Test: p−value = 0.85

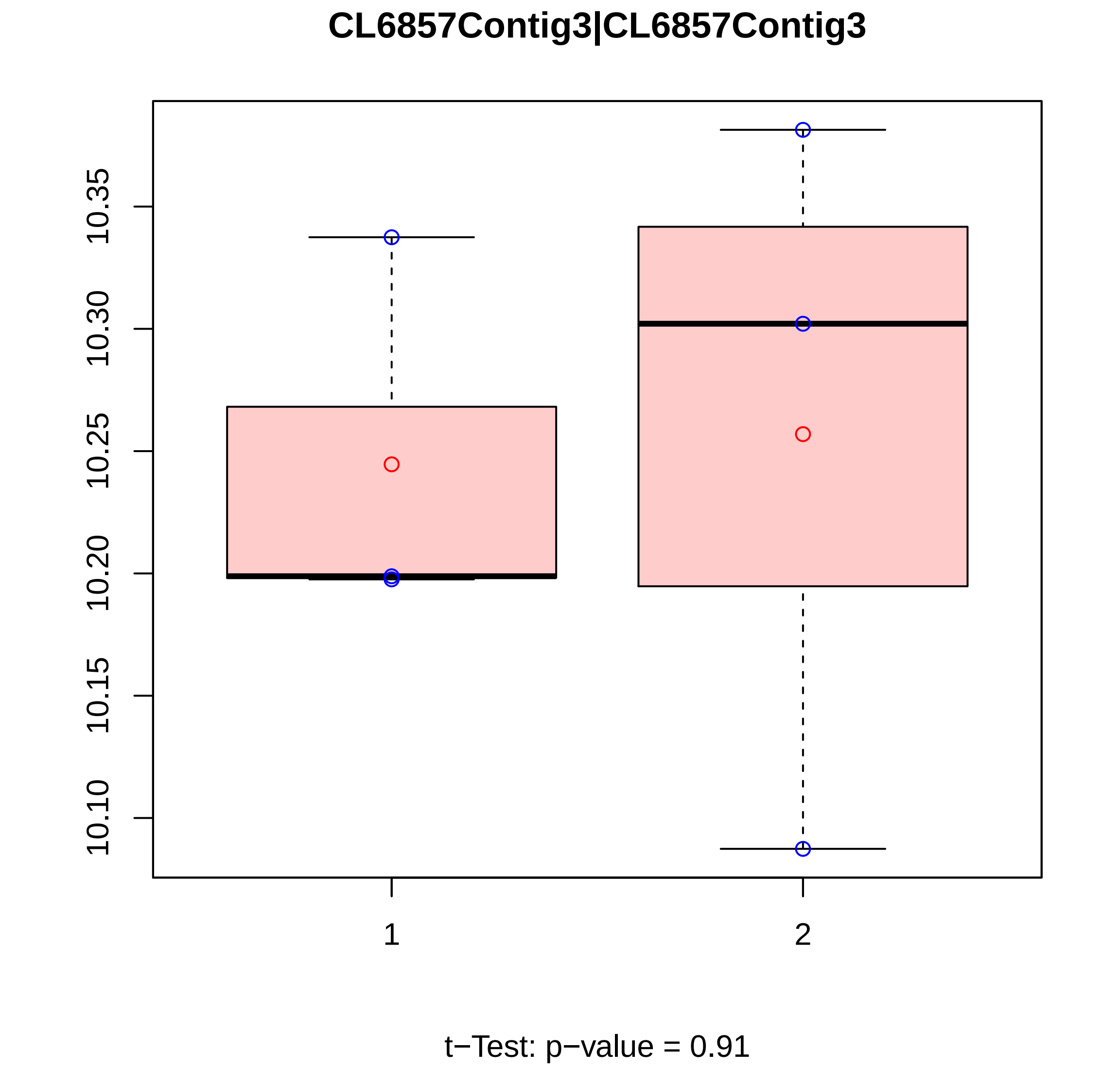

CL6857Contig3|CL6857Contig3
10.10
10.15
10.20
10.25
10.30
10.35
1
2
t−Test: p−value = 0.91

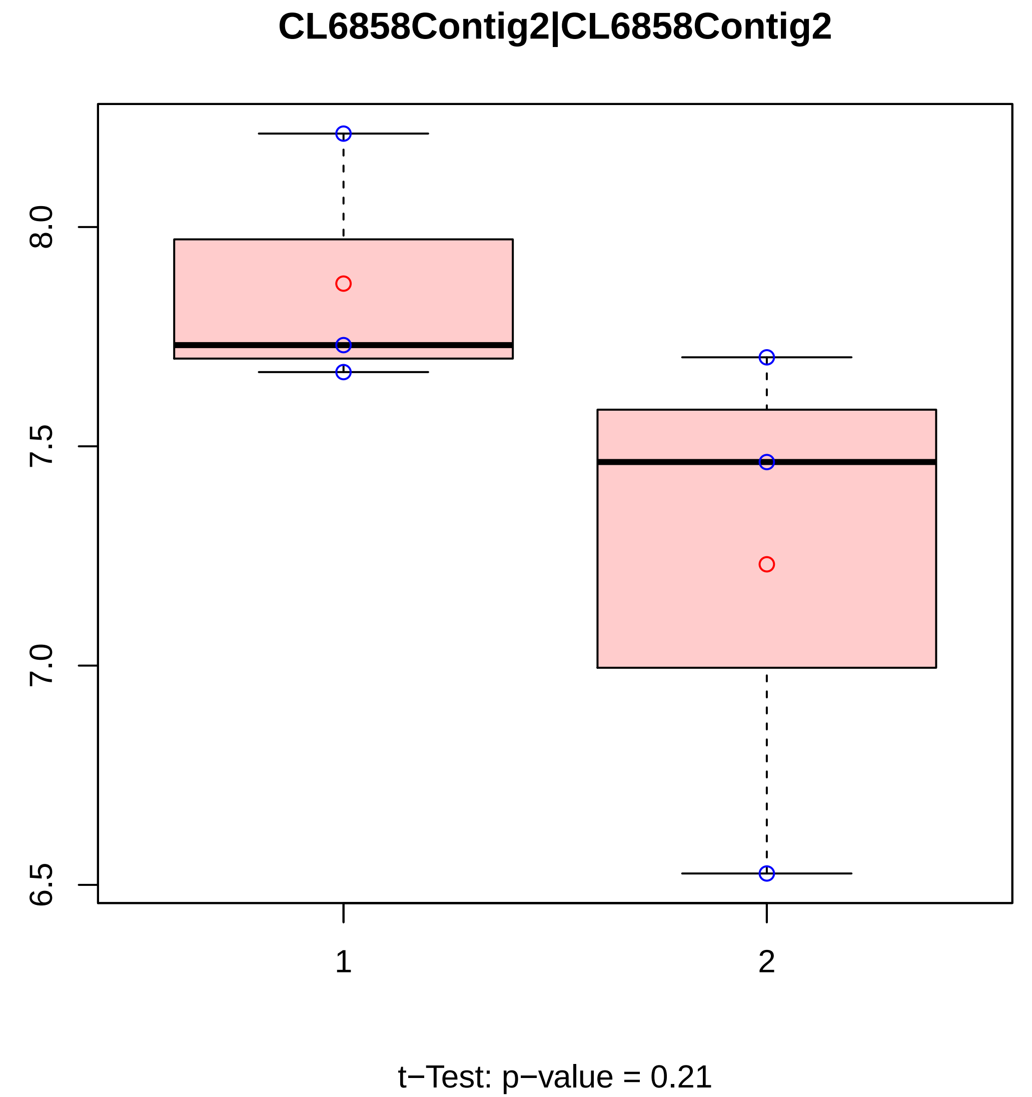
CL6858Contig2|CL6858Contig2
8.0
7.5
7.0
6.5
1
2
t−Test: p−value = 0.21

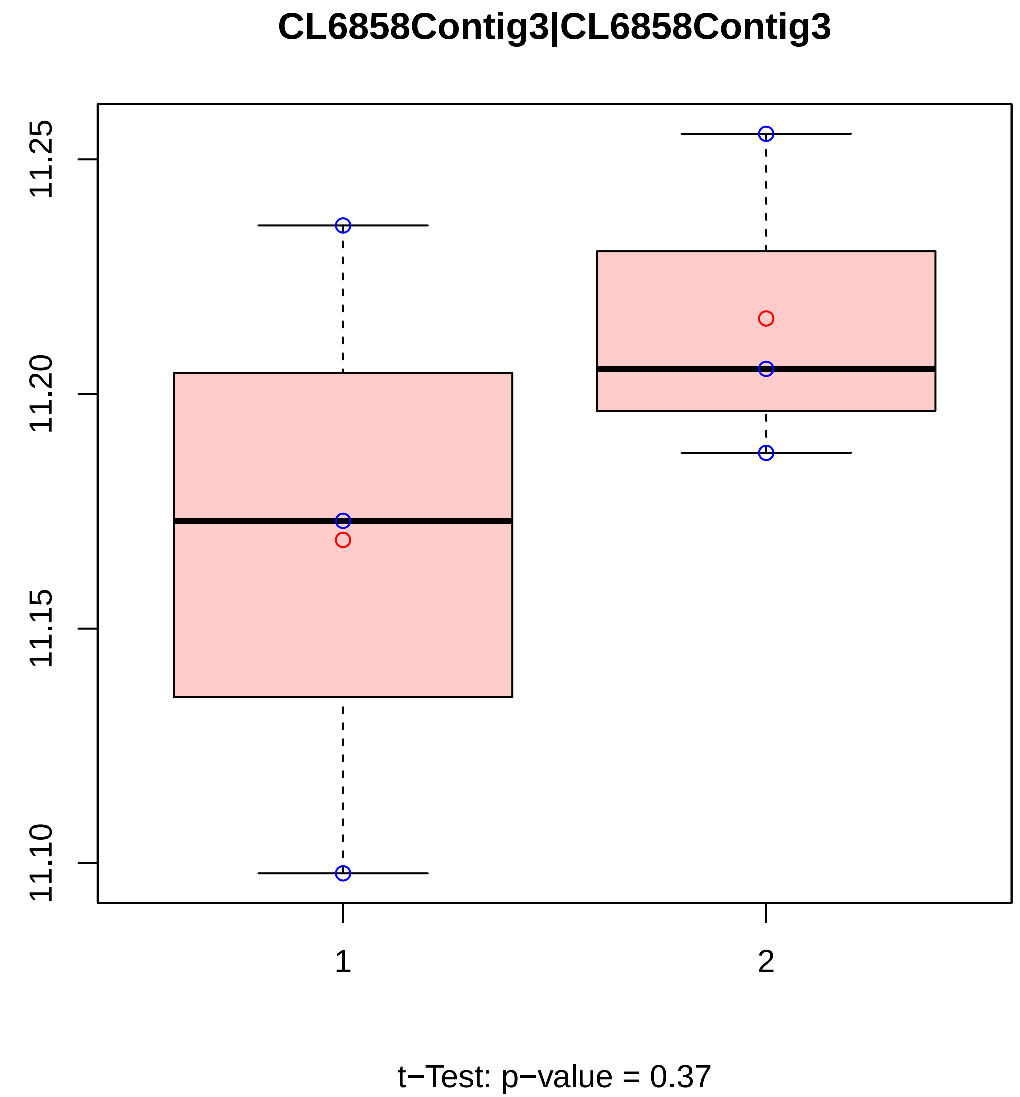

CL6858Contig3|CL6858Contig3
11.25
11.20
11.15
11.10
1
2
t−Test: p−value = 0.37

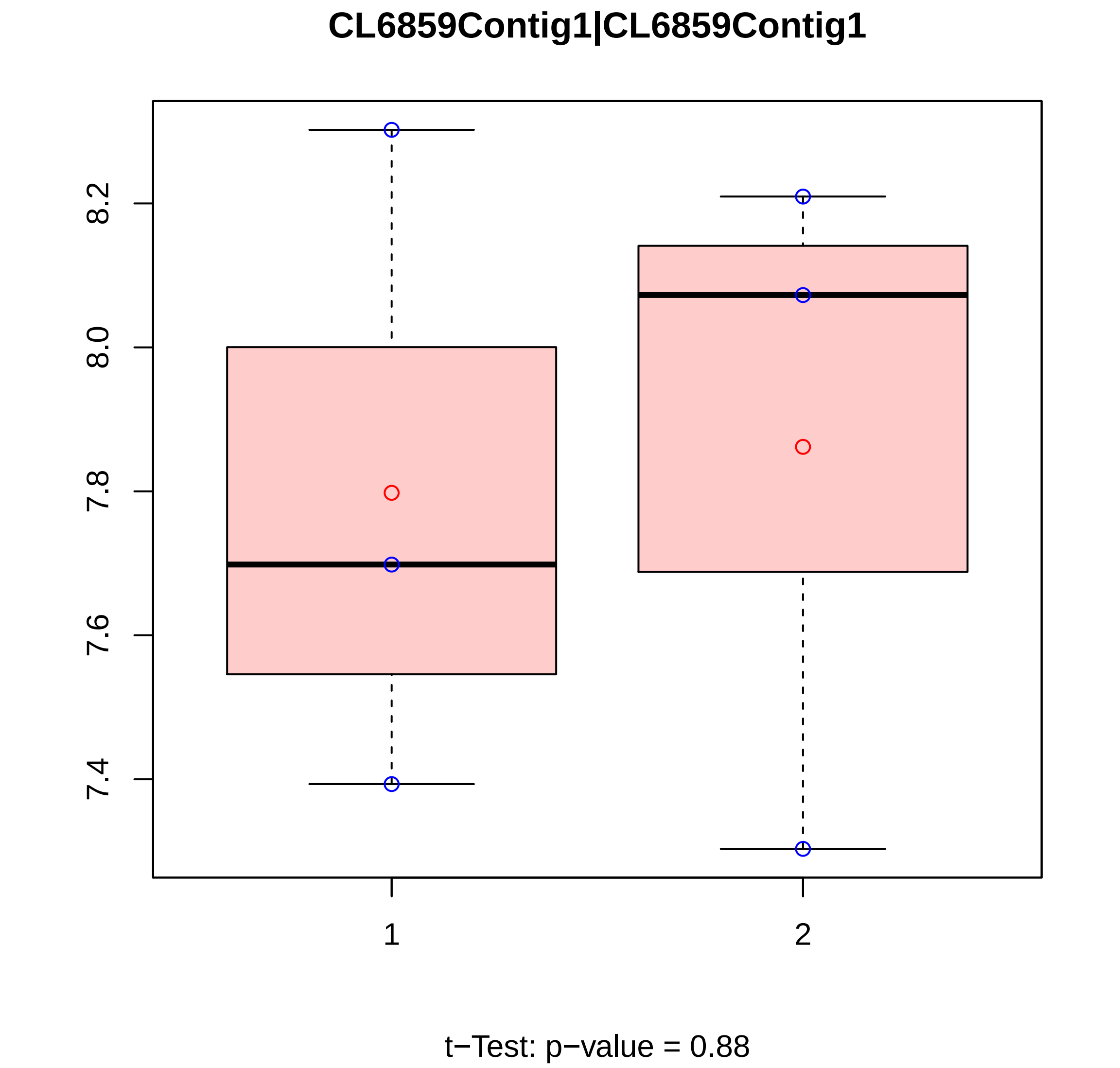
CL6859Contig1|CL6859Contig1
8.2
8.0
7.8
7.6
7.4
1
2
t−Test: p−value = 0.88

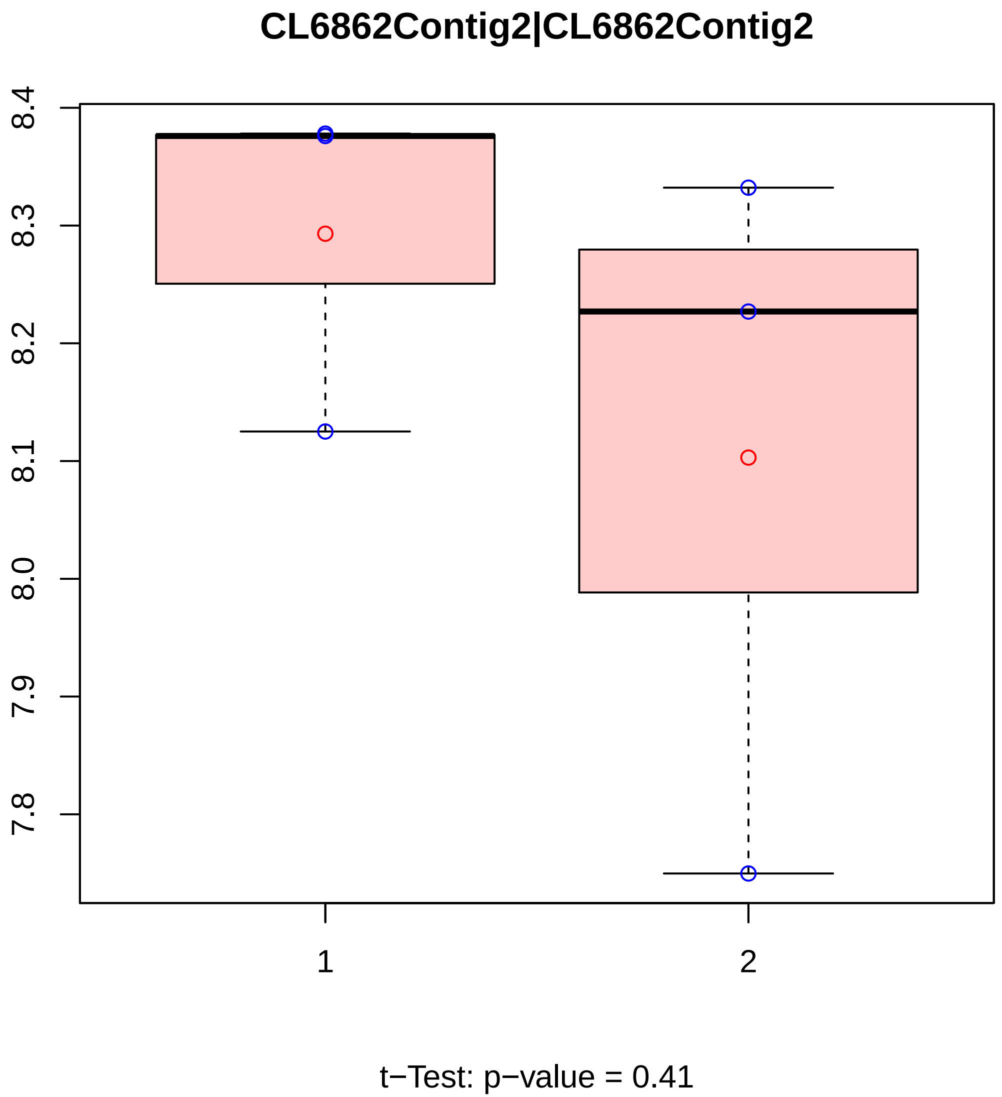
CL6862Contig2|CL6862Contig2
8.4
8.3
8.2
8.1
8.0
7.9
7.8
1
2
t−Test: p−value = 0.41

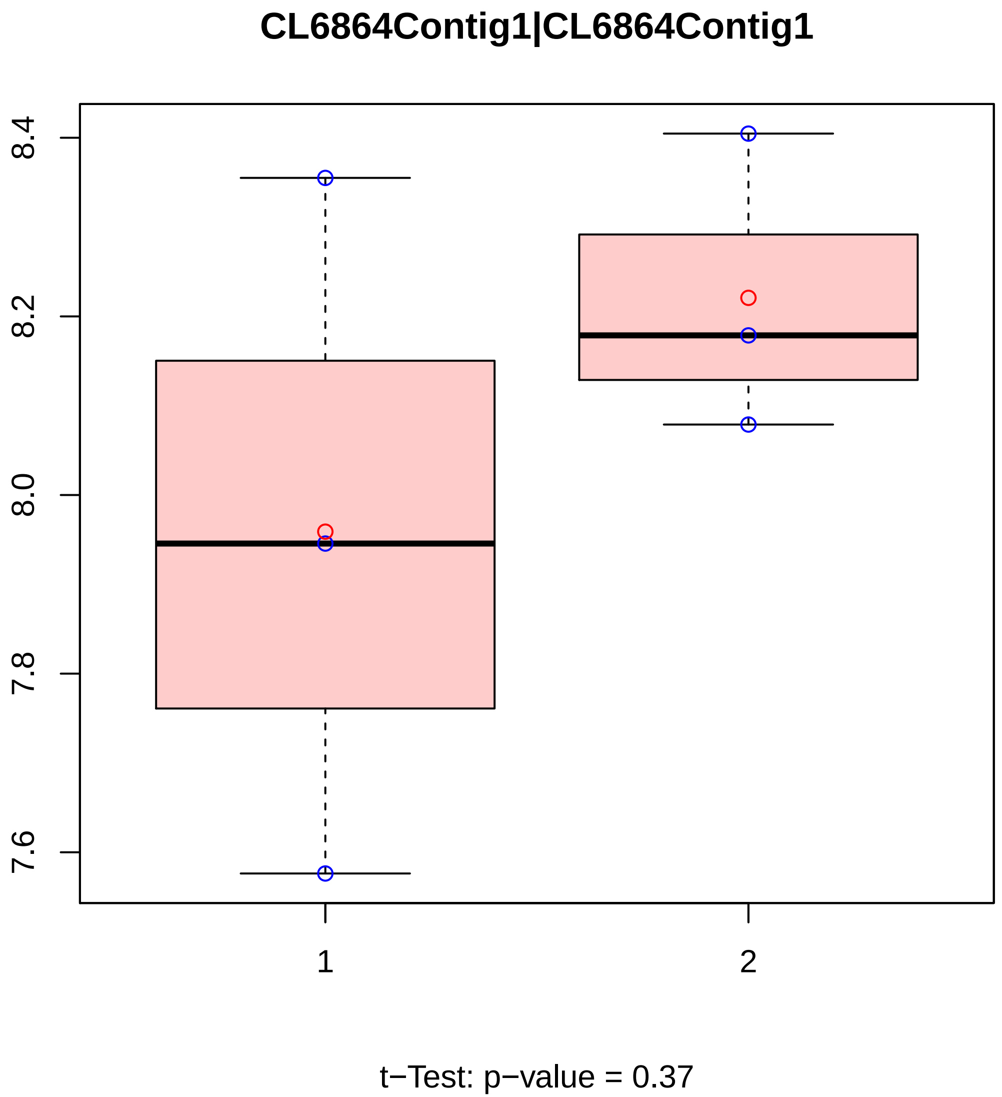
CL6864Contig1|CL6864Contig1
8.4
8.2
8.0
7.8
7.6
1
2
t−Test: p−value = 0.37

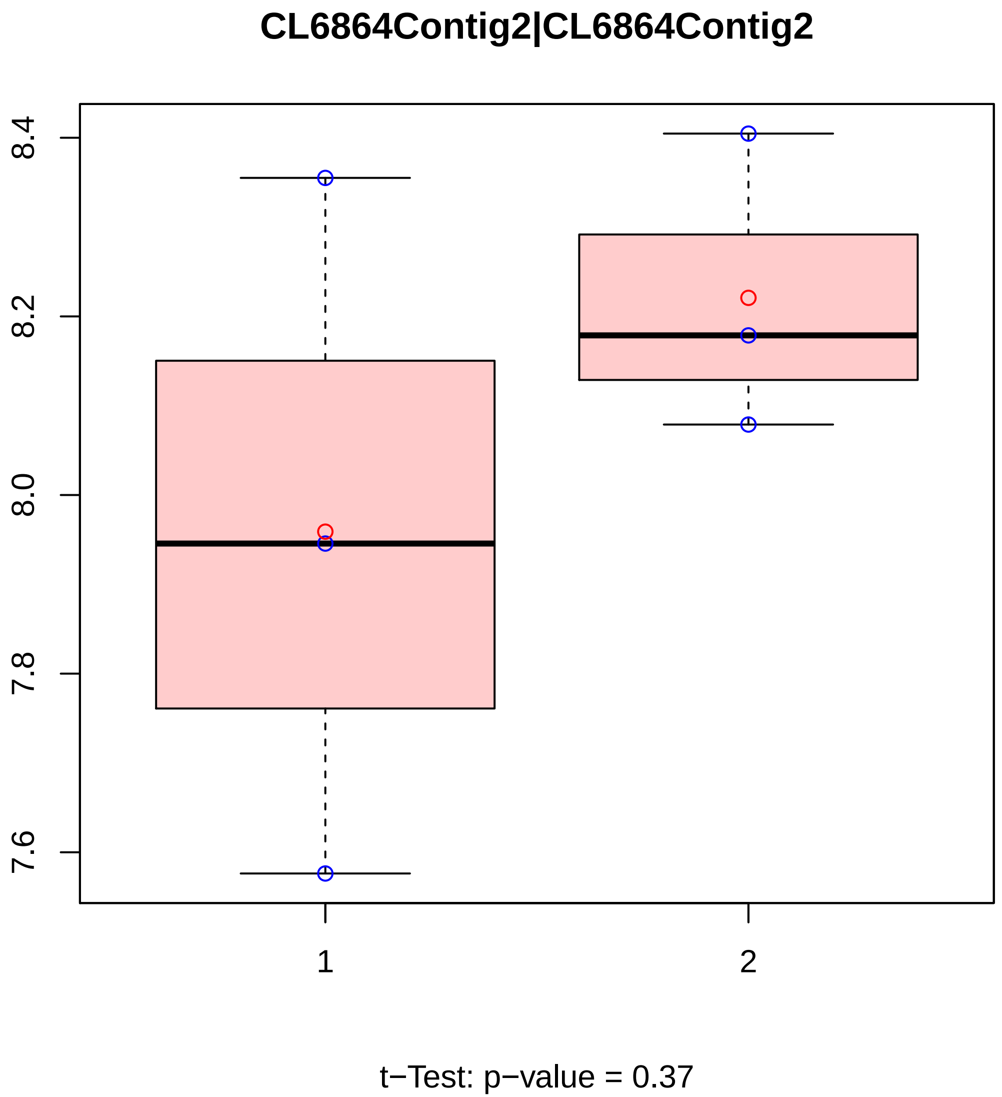

CL6864Contig2|CL6864Contig2
8.4
8.2
8.0
7.8
7.6
1
2
t−Test: p−value = 0.37

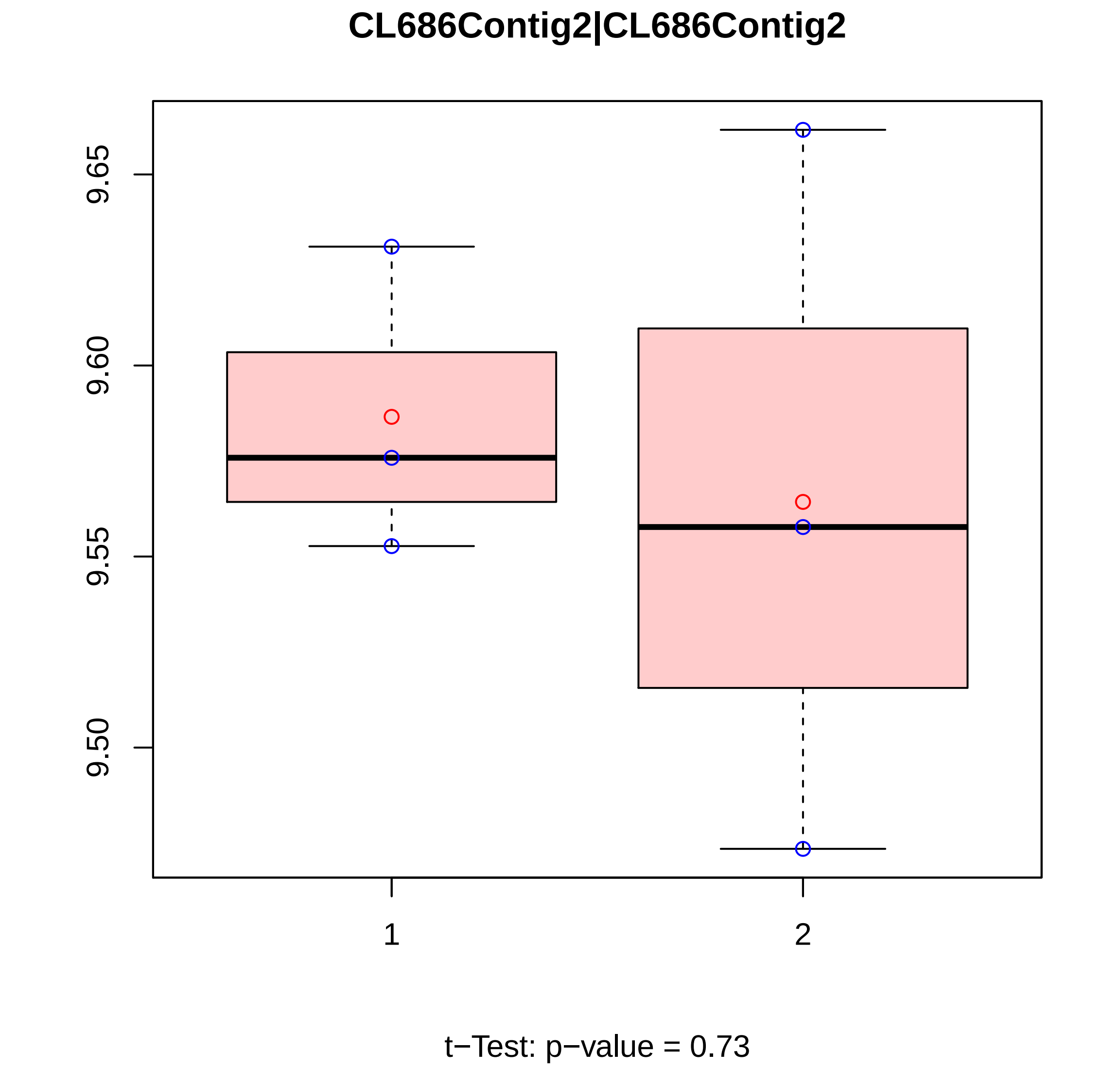
CL686Contig2|CL686Contig2
9.50
9.55
9.60
9.65
1
2
t−Test: p−value = 0.73

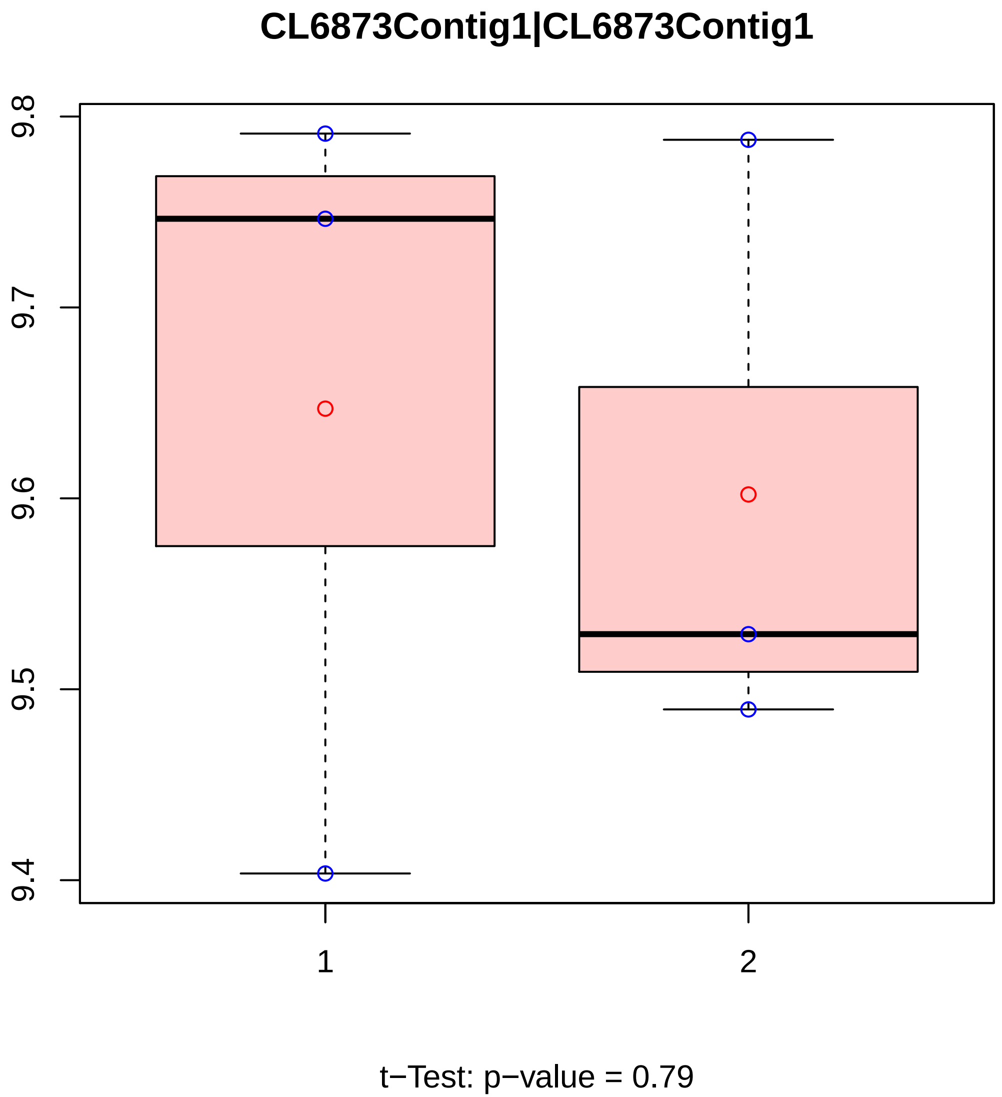
CL6873Contig1|CL6873Contig1
9.4
9.5
9.6
9.7
9.8
1
2
t−Test: p−value = 0.79

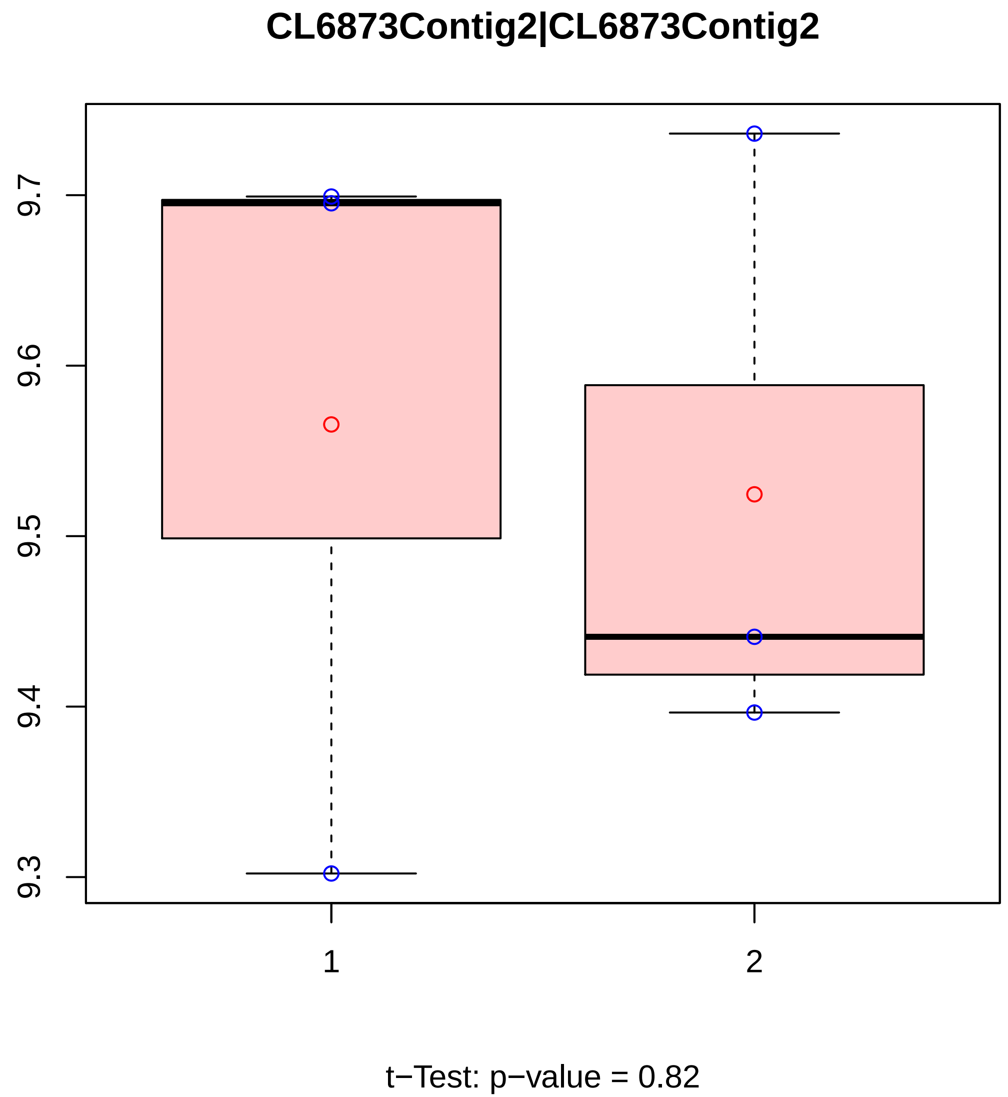
CL6873Contig2|CL6873Contig2
9.3
9.4
9.5
9.6
9.7
1
2
t−Test: p−value = 0.82

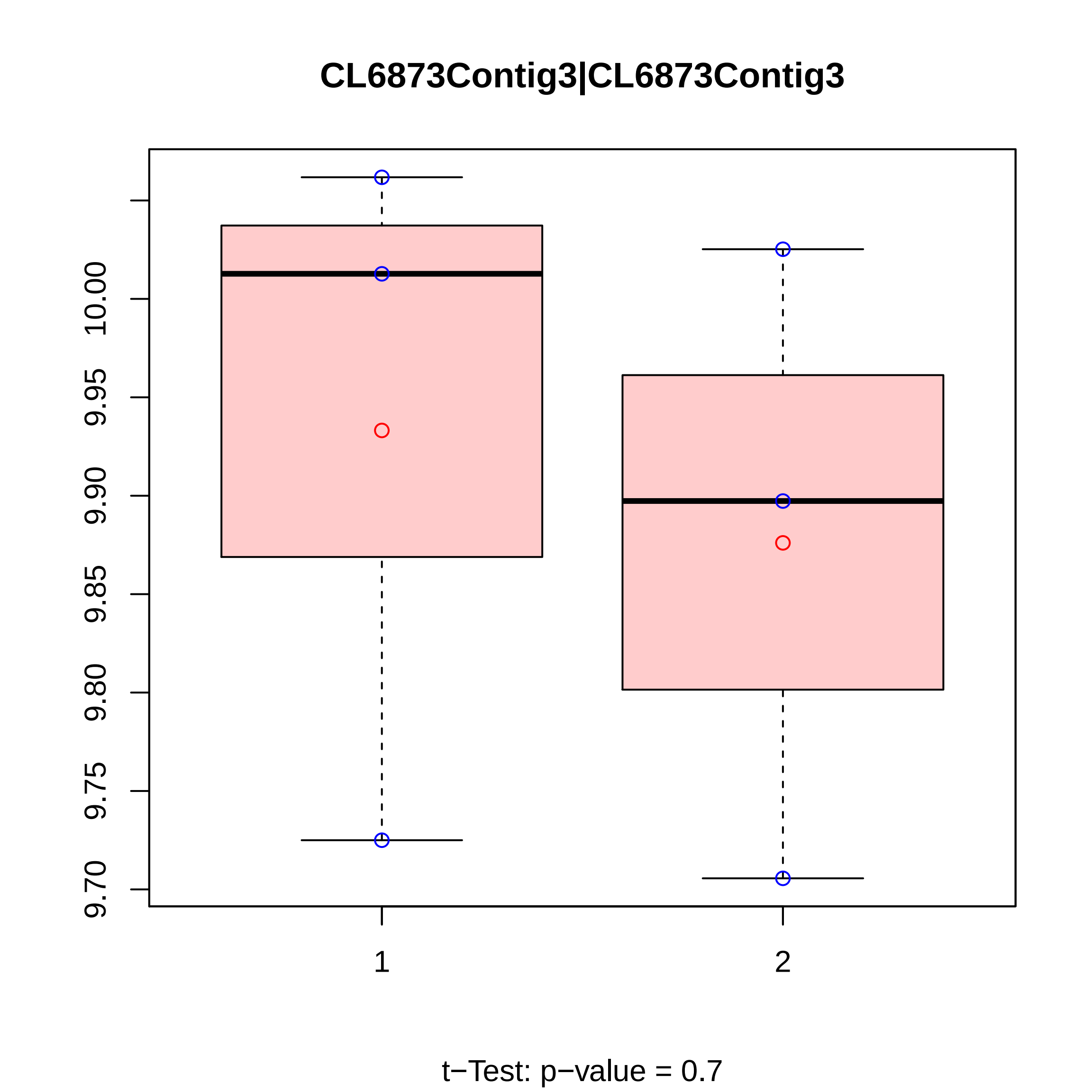
CL6873Contig3|CL6873Contig3
10.00
9.95
9.90
9.85
9.80
9.75
9.70
1
2
t−Test: p−value = 0.7

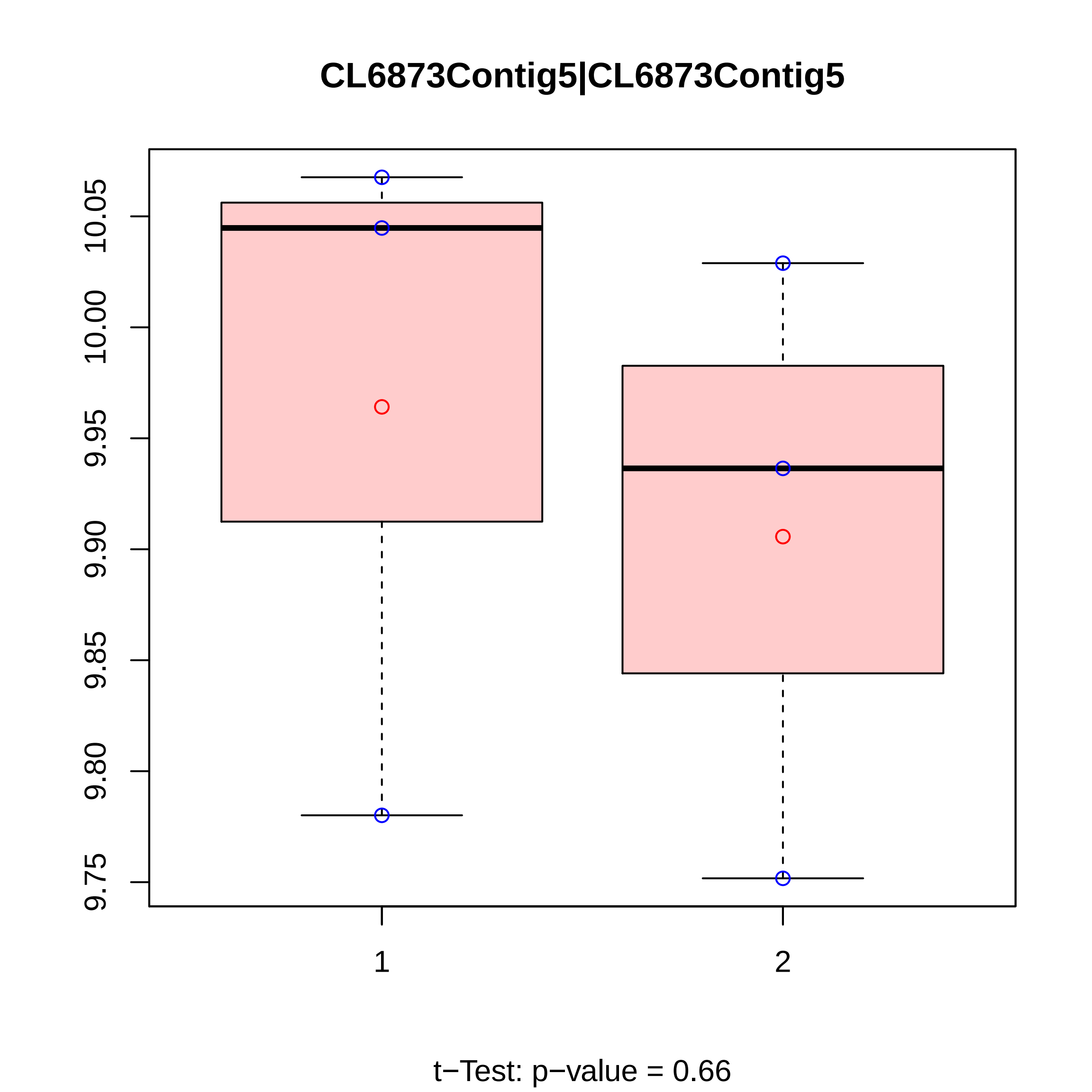
CL6873Contig5|CL6873Contig5
10.05
10.00
9.95
9.90
9.85
9.80
9.75
1
2
t−Test: p−value = 0.66

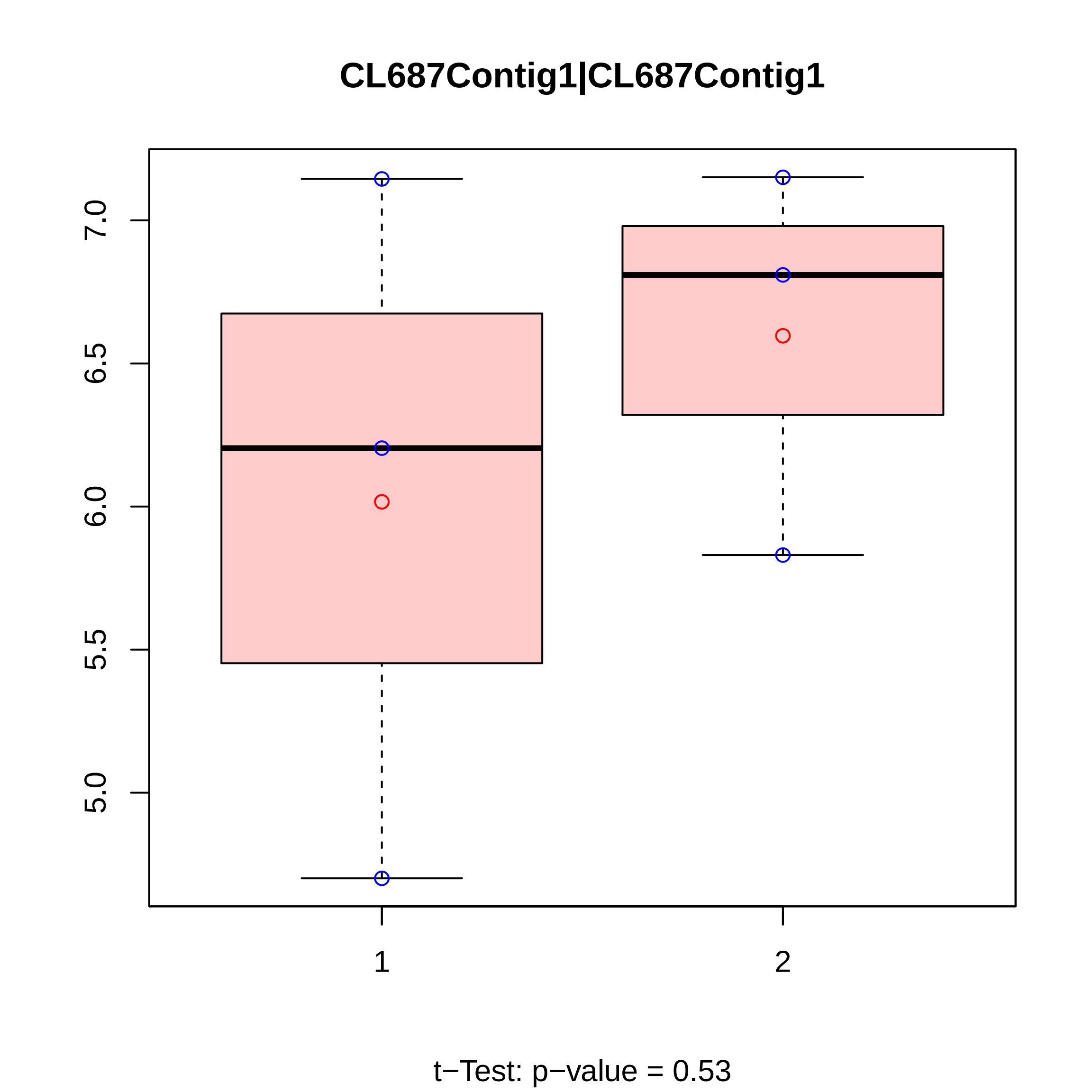
CL687Contig1|CL687Contig1
7.0
6.5
6.0
5.5
5.0
1
2
t−Test: p−value = 0.53

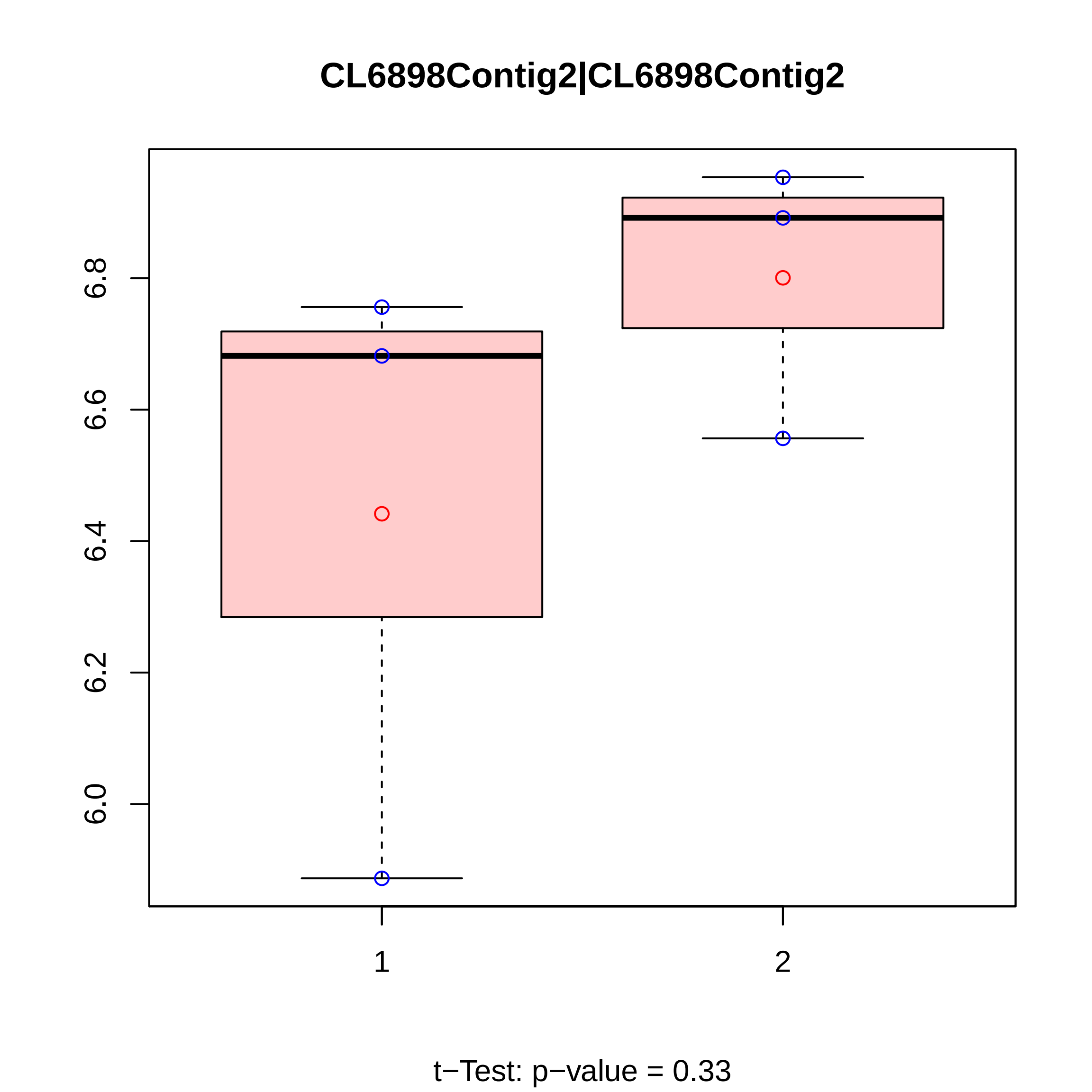
CL6898Contig2|CL6898Contig2
6.0
6.2
6.4
6.6
6.8
1
2
t−Test: p−value = 0.33

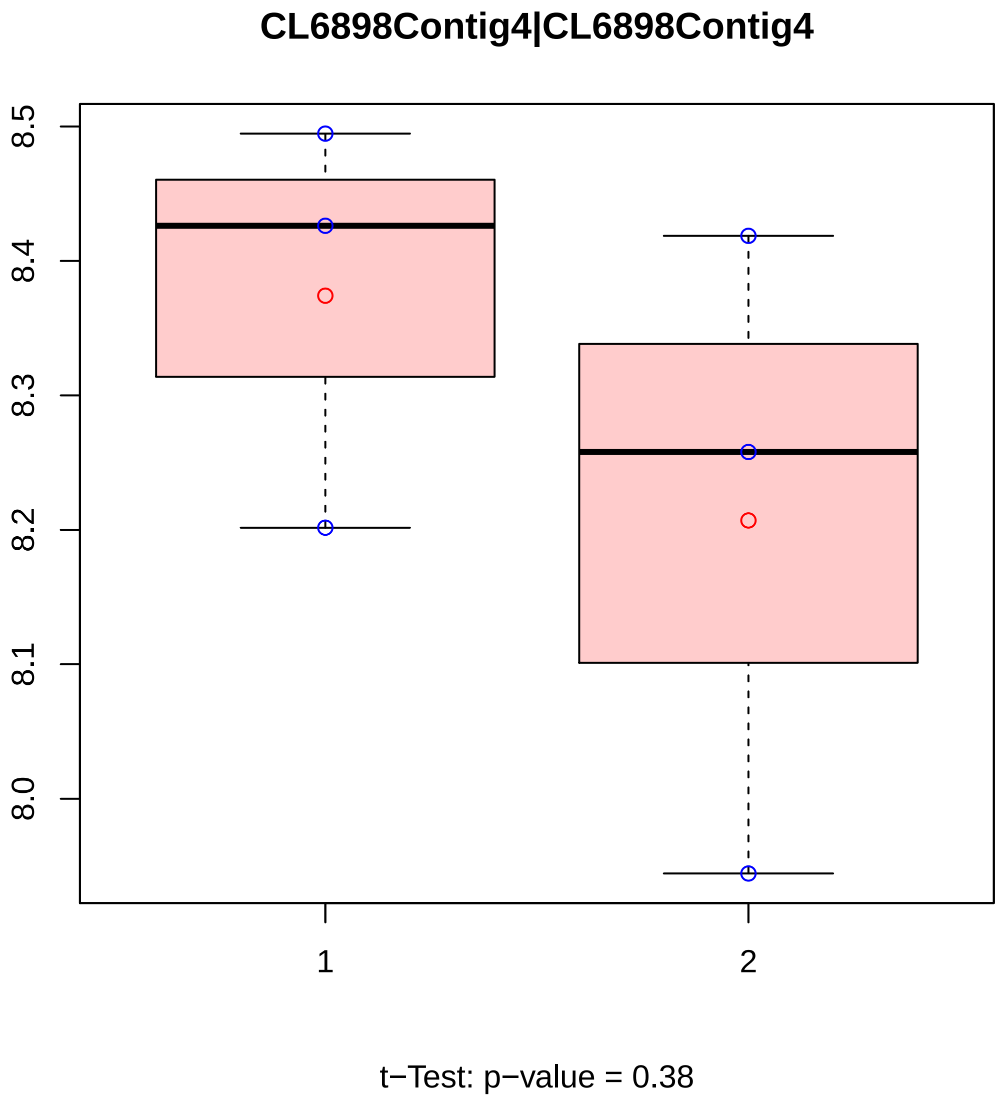
CL6898Contig4|CL6898Contig4
8.0
8.1
8.2
8.3
8.4
8.5
1
2
t−Test: p−value = 0.38

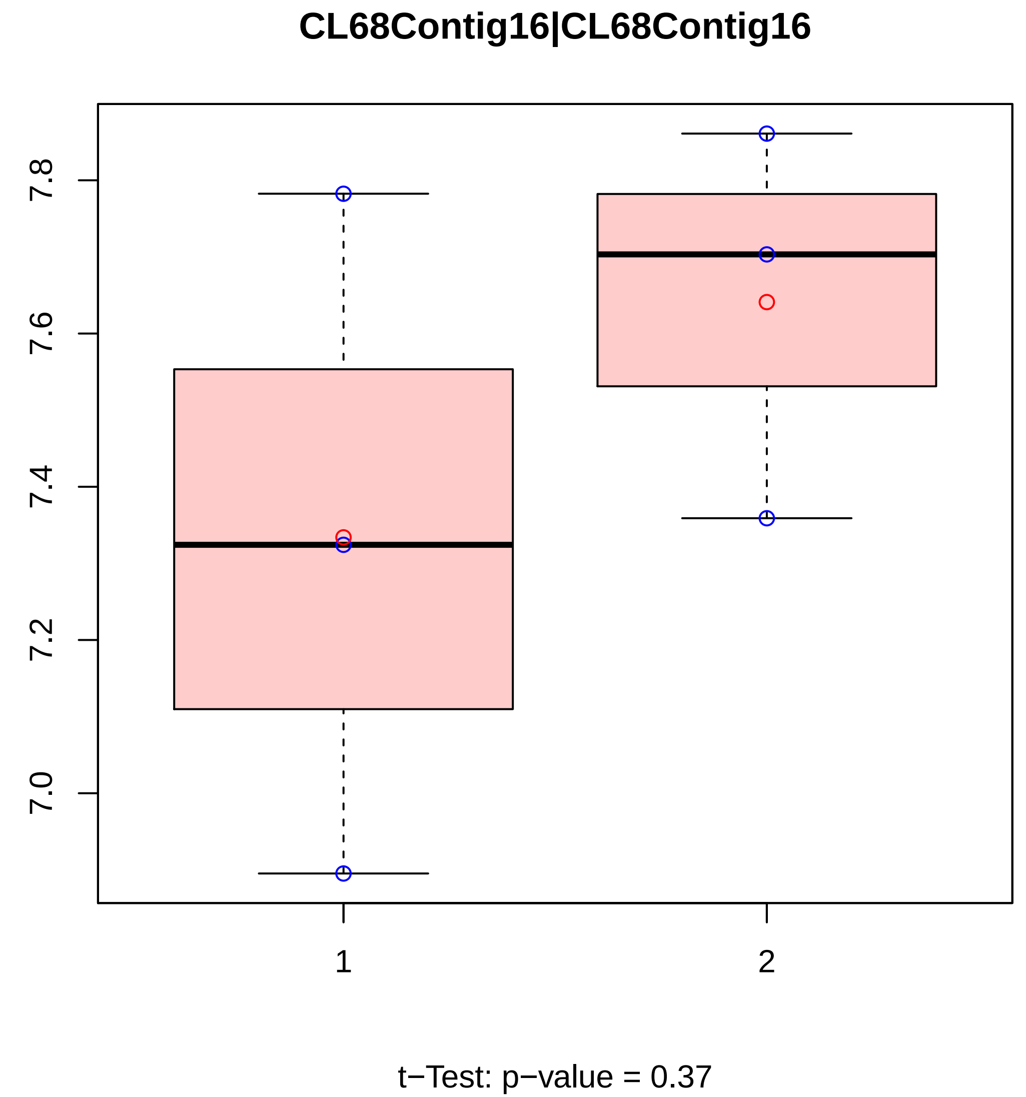

CL68Contig16|CL68Contig16
7.0
7.2
7.4
7.6
7.8
1
2
t−Test: p−value = 0.37

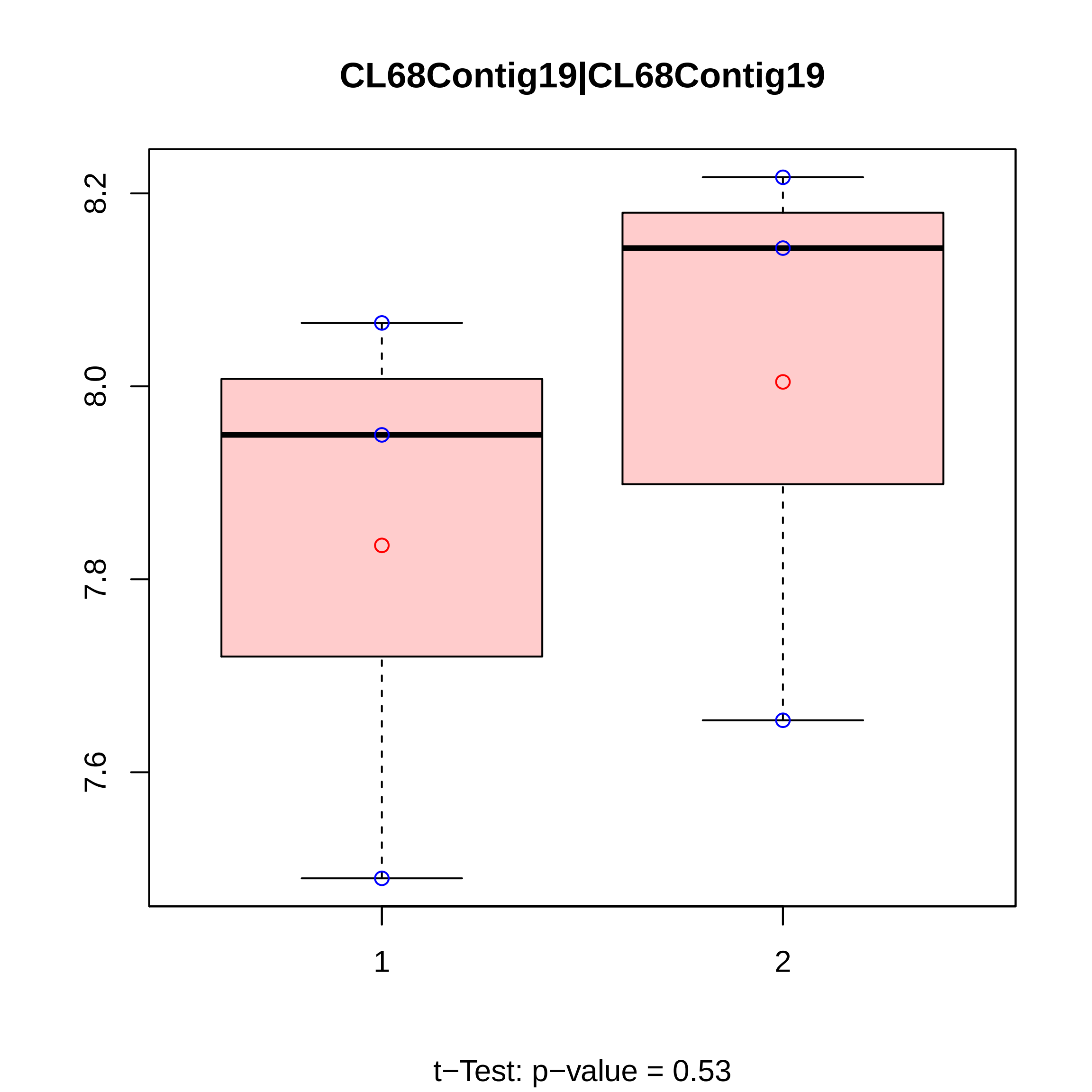
CL68Contig19|CL68Contig19
8.2
8.0
7.8
7.6
1
2
t−Test: p−value = 0.53

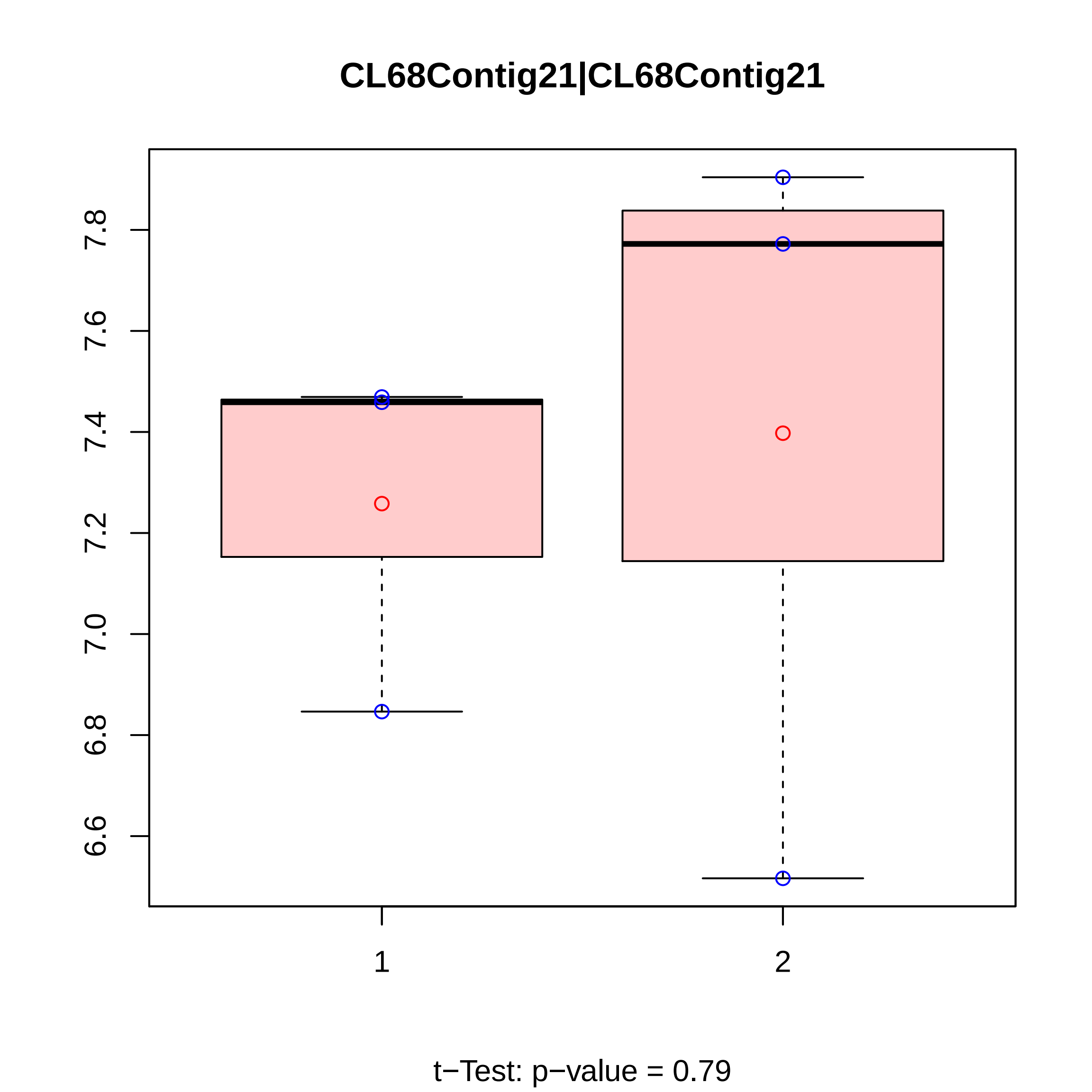
CL68Contig21|CL68Contig21
7.8
7.6
7.4
7.2
7.0
6.8
6.6
1
2
t−Test: p−value = 0.79

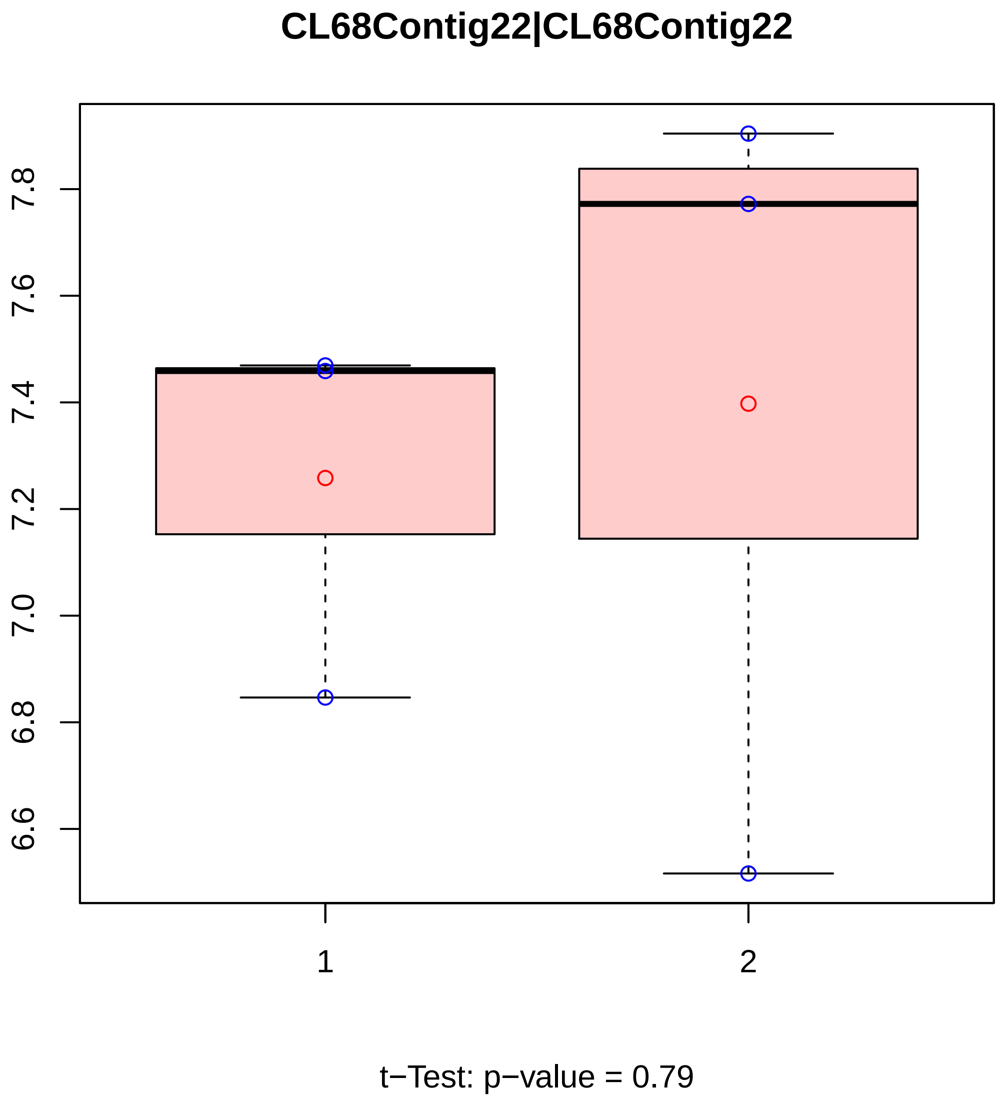
CL68Contig22|CL68Contig22
7.8
7.6
7.4
7.2
7.0
6.8
6.6
1
2
t−Test: p−value = 0.79

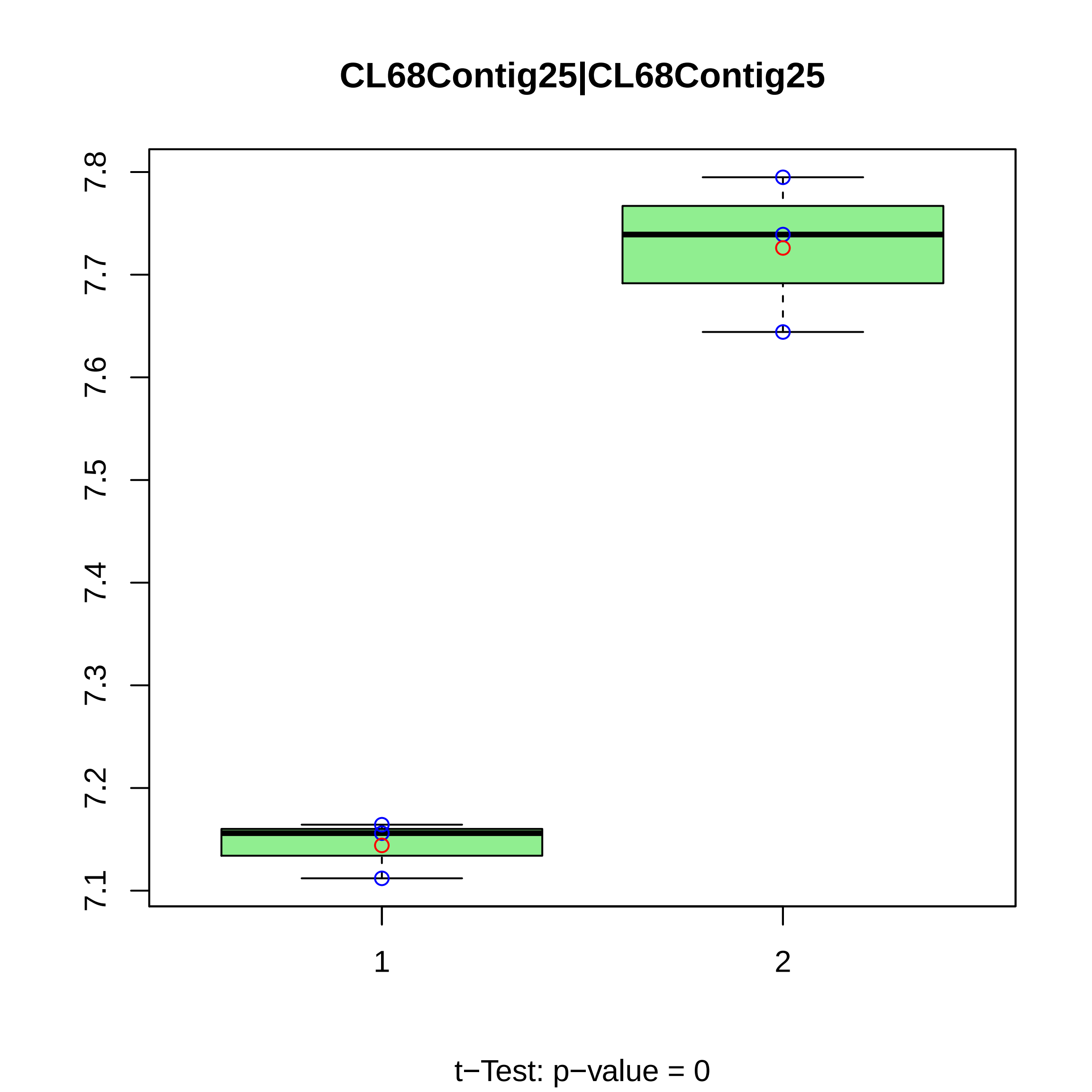
CL68Contig25|CL68Contig25
7.1
7.2
7.3
7.4
7.5
7.6
7.7
7.8
1
2
t−Test: p−value = 0

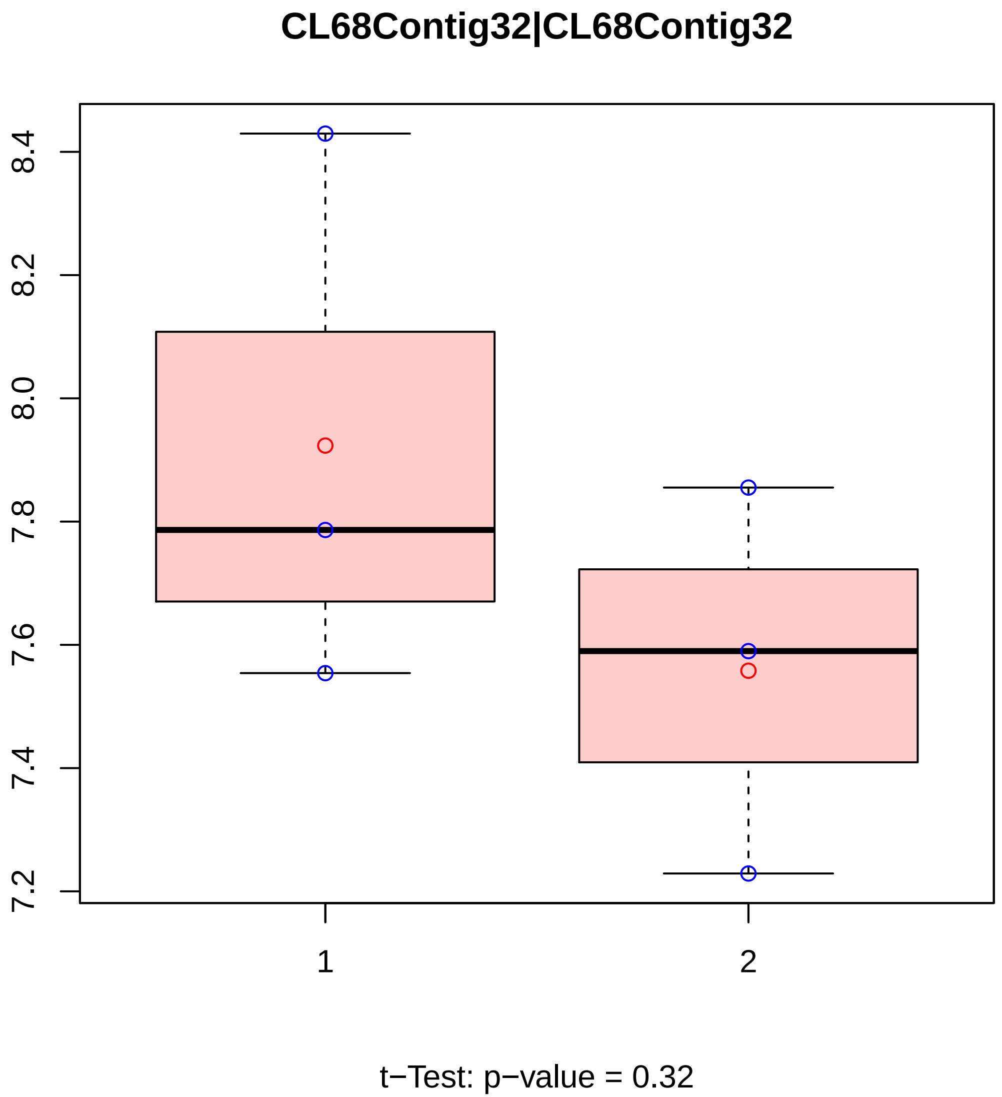
CL68Contig32|CL68Contig32
8.4
8.2
8.0
7.8
7.6
7.4
7.2
1
2
t−Test: p−value = 0.32

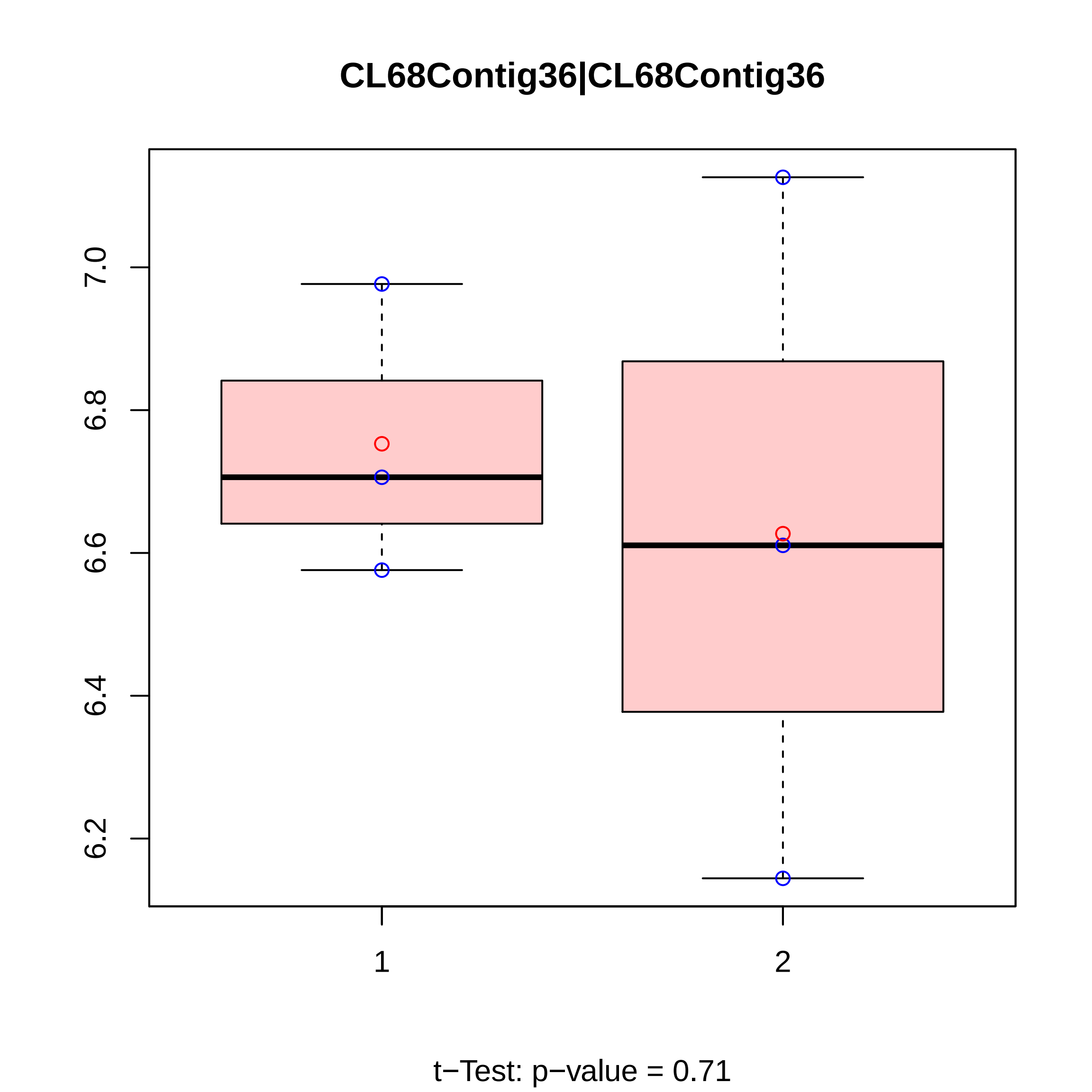
CL68Contig36|CL68Contig36
7.0
6.8
6.6
6.4
6.2
1
2
t−Test: p−value = 0.71

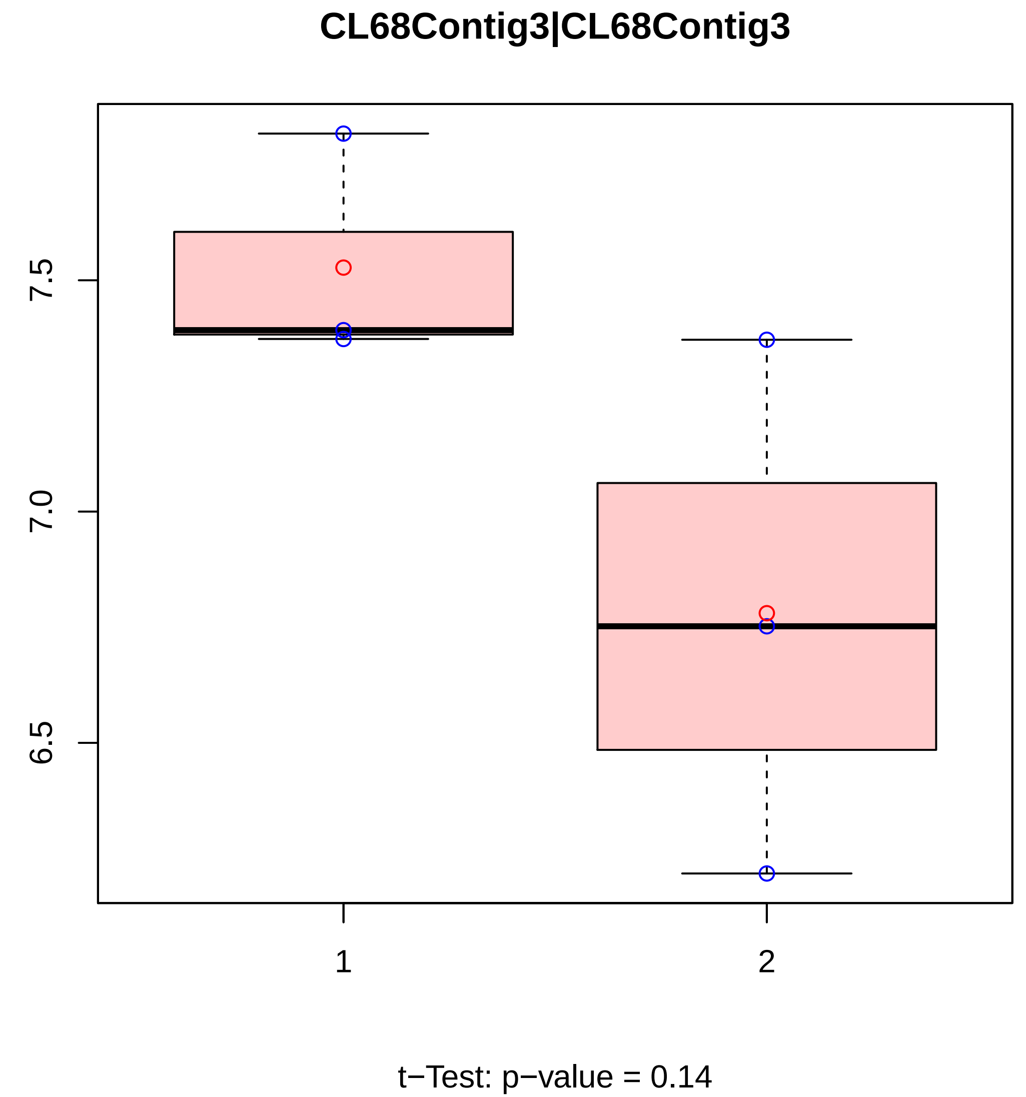
CL68Contig3|CL68Contig3
7.5
7.0
6.5
1
2
t−Test: p−value = 0.14

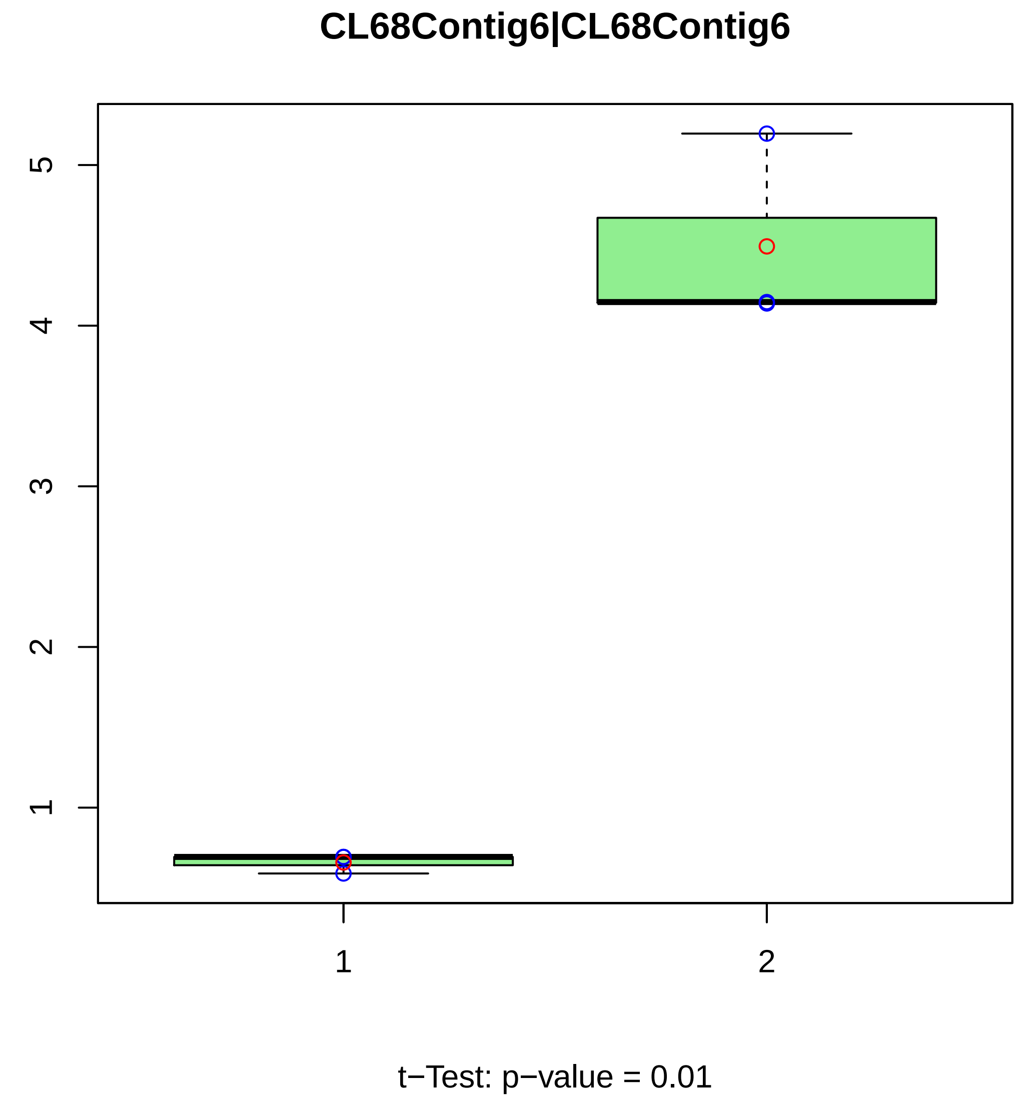
CL68Contig6|CL68Contig6
1
2
3
4
5
1
2
t−Test: p−value = 0.01

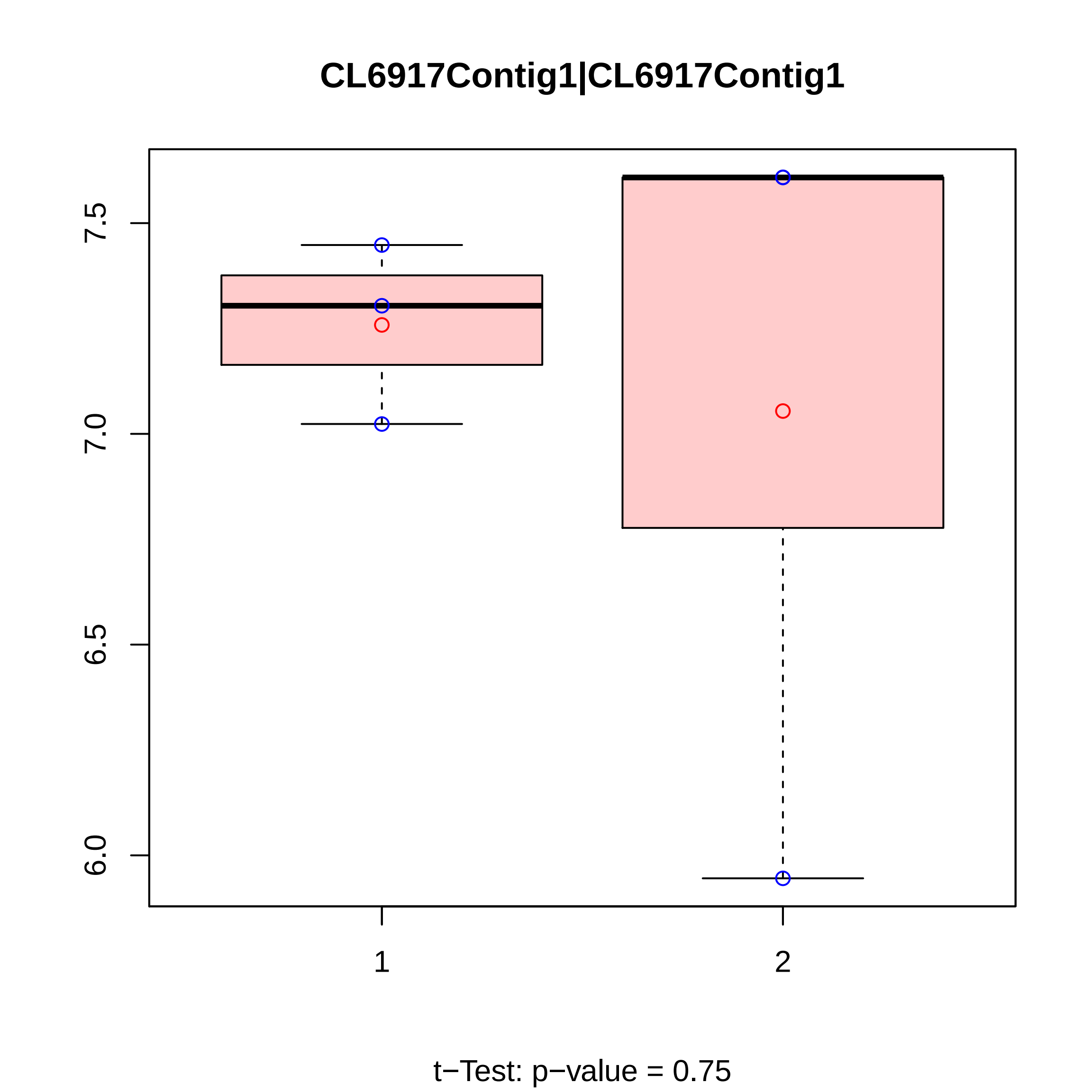

CL6917Contig1|CL6917Contig1
7.5
7.0
6.5
6.0
1
2
t−Test: p−value = 0.75

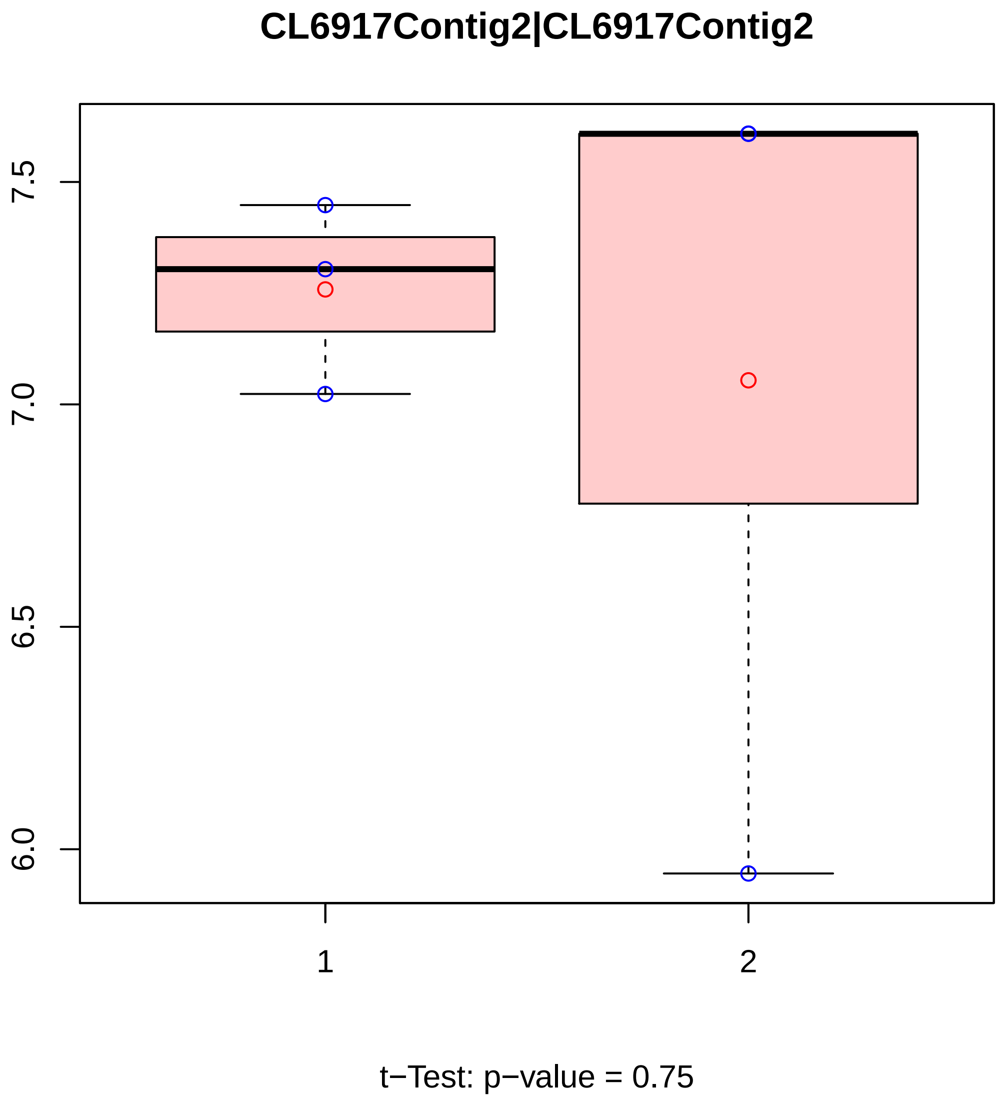

CL6917Contig2|CL6917Contig2
7.5
7.0
6.5
6.0
1
2
t−Test: p−value = 0.75

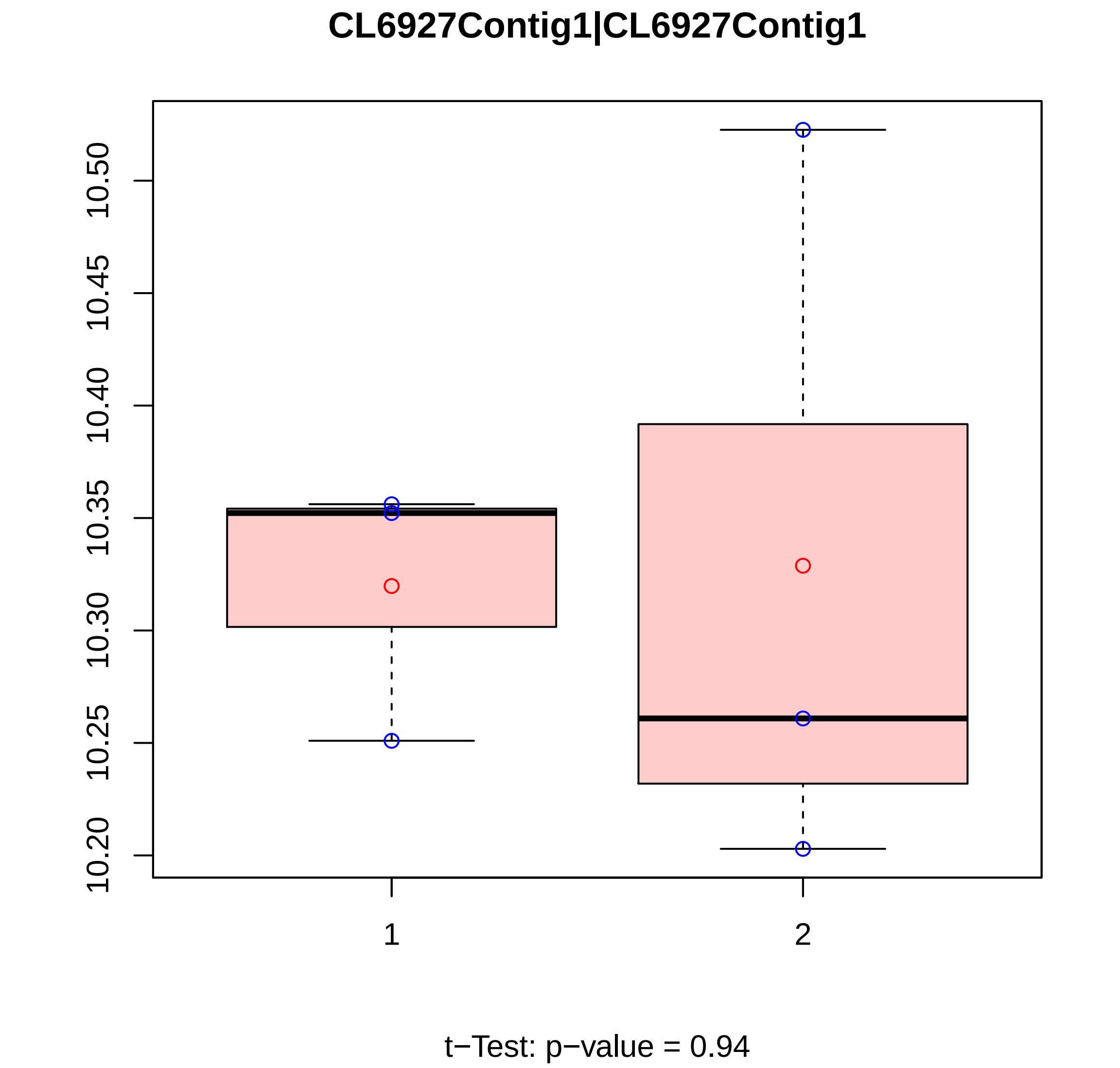

CL6927Contig1|CL6927Contig1
10.20
10.25
10.30
10.35
10.40
10.45
10.50
1
2
t−Test: p−value = 0.94

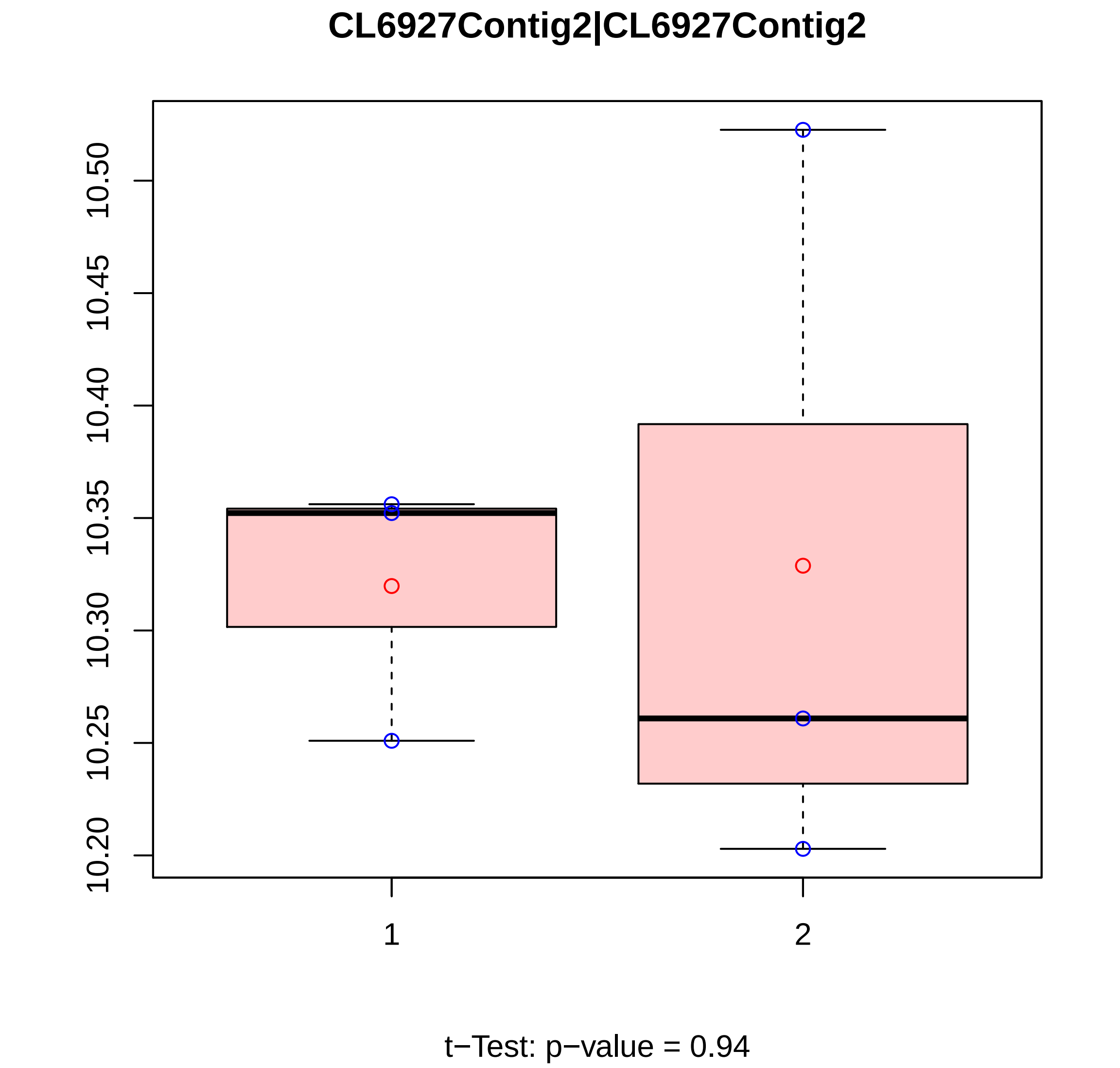

CL6927Contig2|CL6927Contig2
10.20
10.25
10.30
10.35
10.40
10.45
10.50
1
2
t−Test: p−value = 0.94

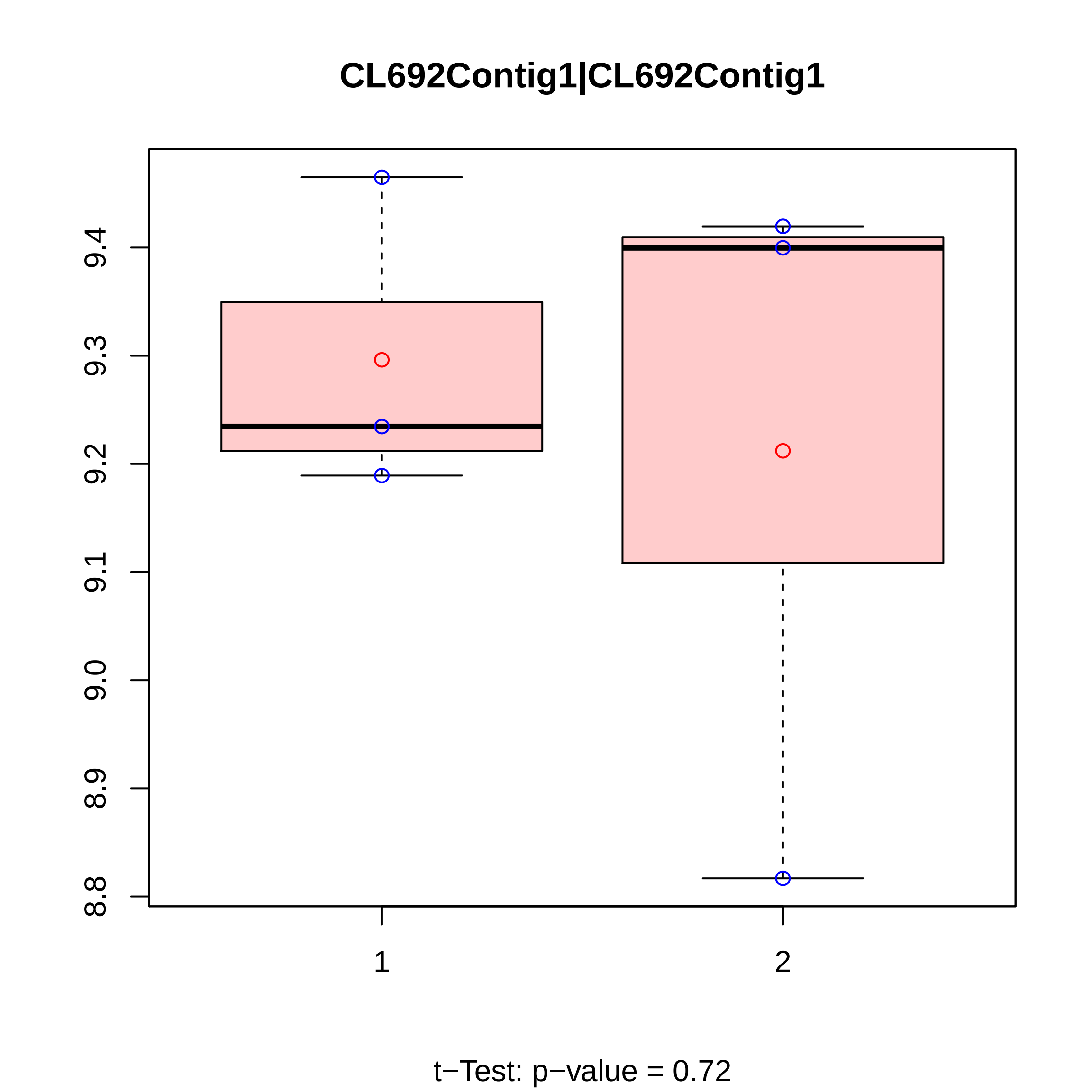
CL692Contig1|CL692Contig1
9.4
9.3
9.2
9.1
9.0
8.9
8.8
1
2
t−Test: p−value = 0.72

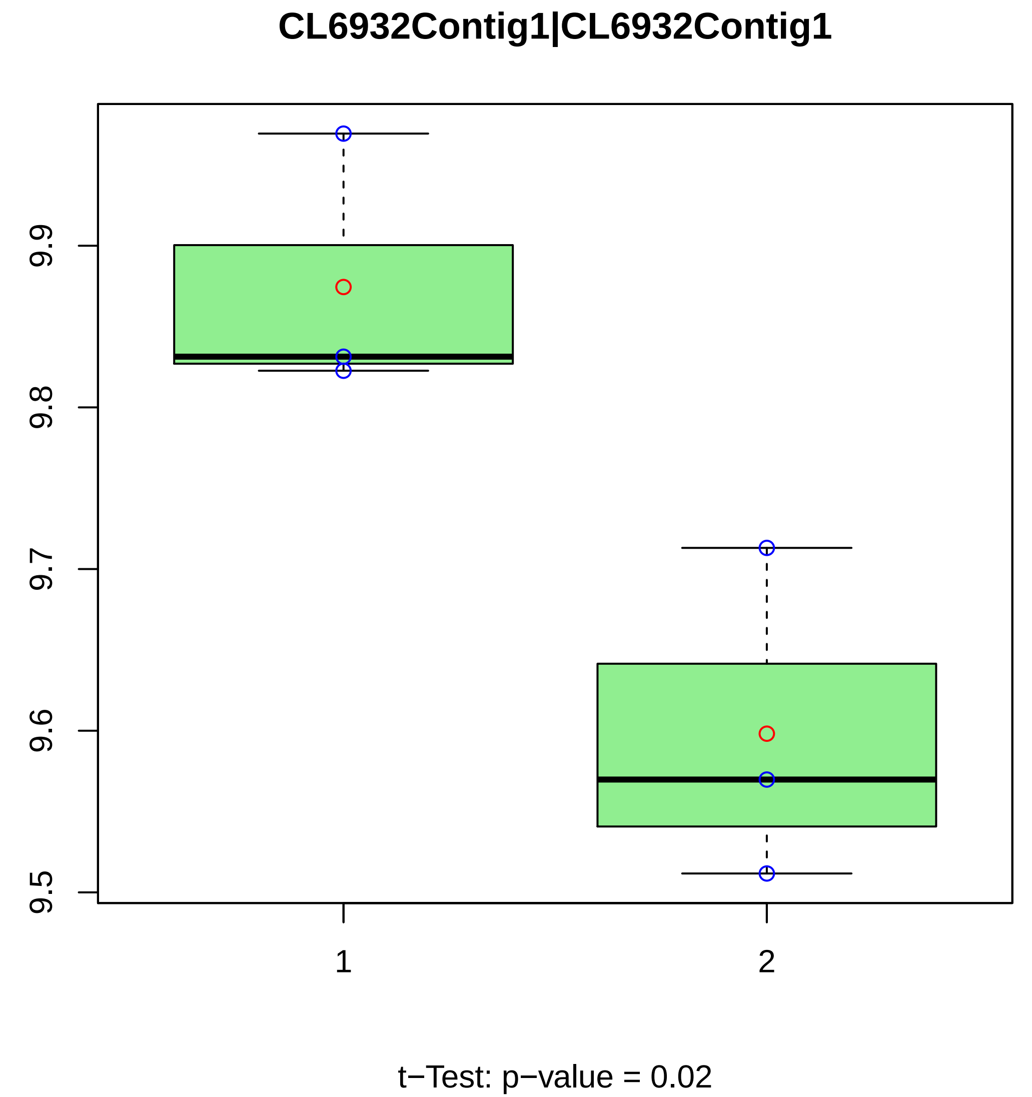
CL6932Contig1|CL6932Contig1
9.5
9.6
9.7
9.8
9.9
1
2
t−Test: p−value = 0.02

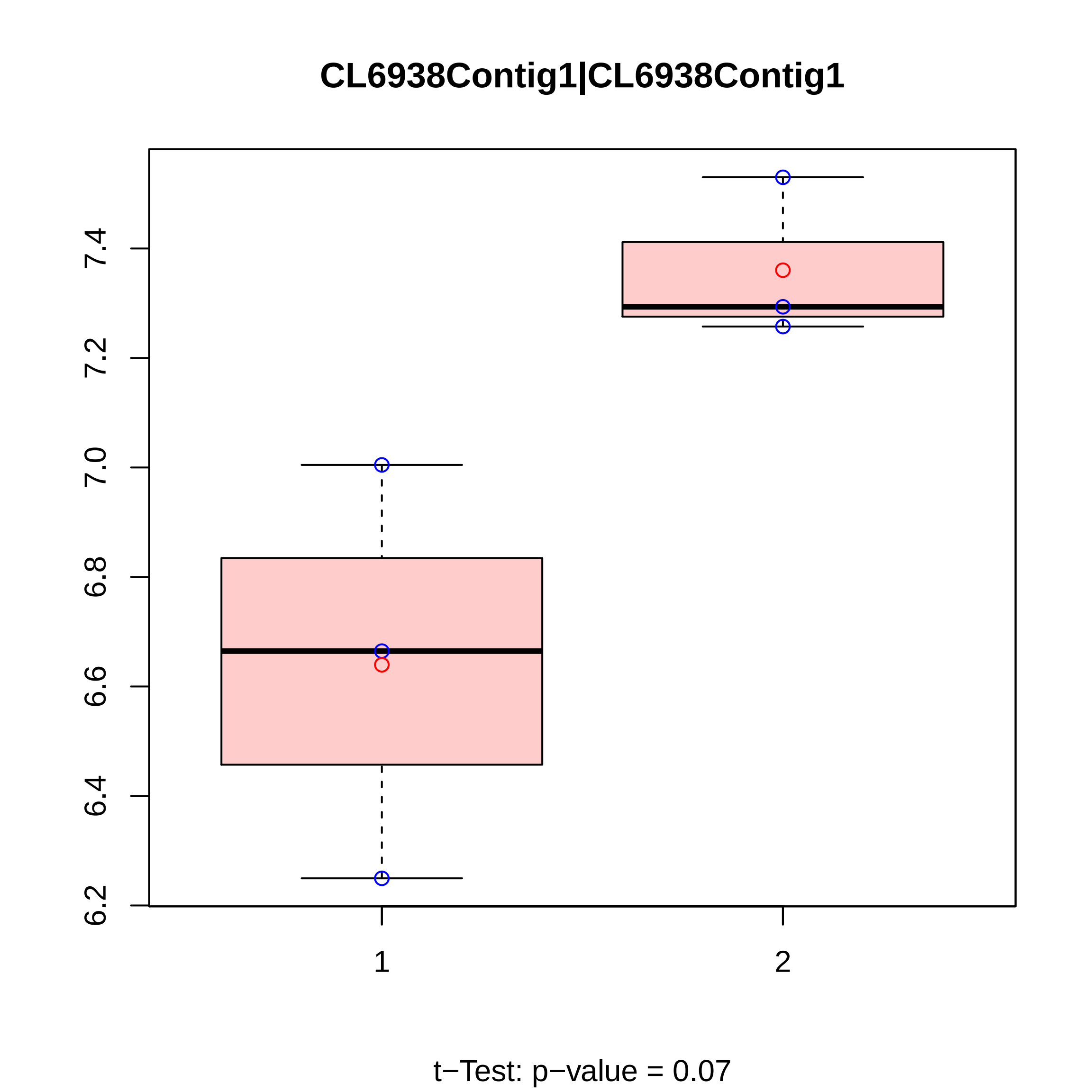

CL6938Contig1|CL6938Contig1
6.2
6.4
6.6
6.8
7.0
7.2
7.4
1
2
t−Test: p−value = 0.07

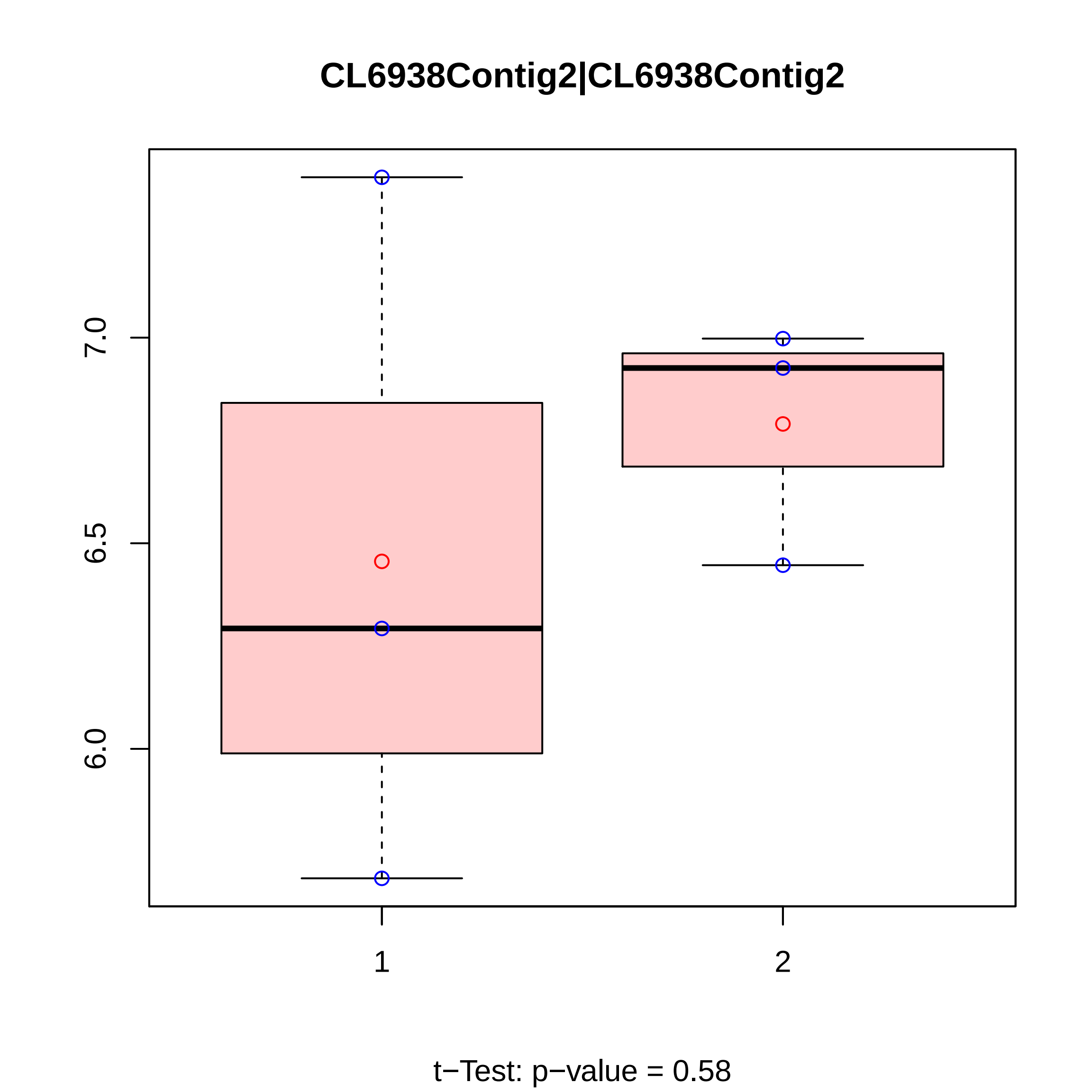
CL6938Contig2|CL6938Contig2
7.0
6.5
6.0
1
2
t−Test: p−value = 0.58

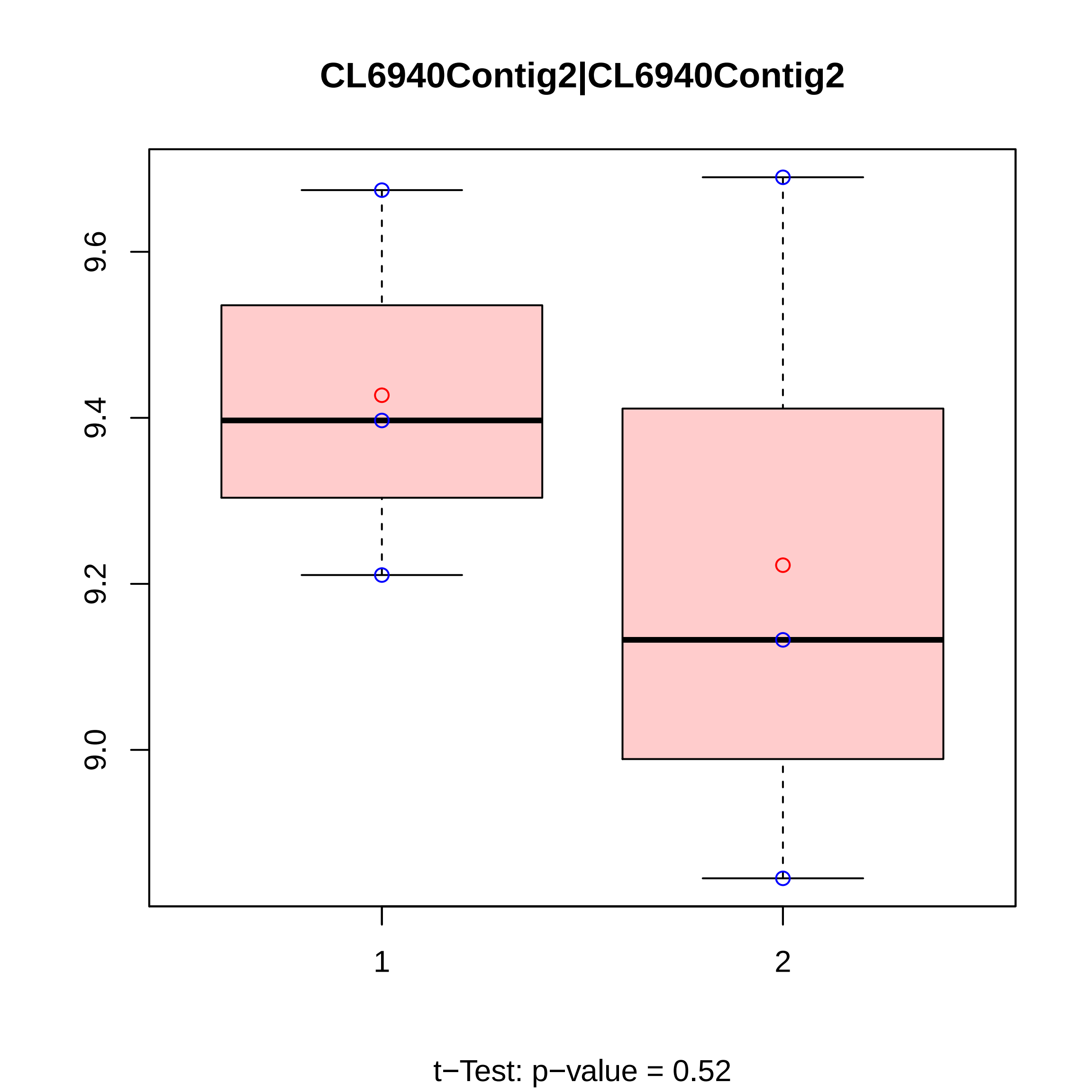
CL6940Contig2|CL6940Contig2
9.0
9.2
9.4
9.6
1
2
t−Test: p−value = 0.52

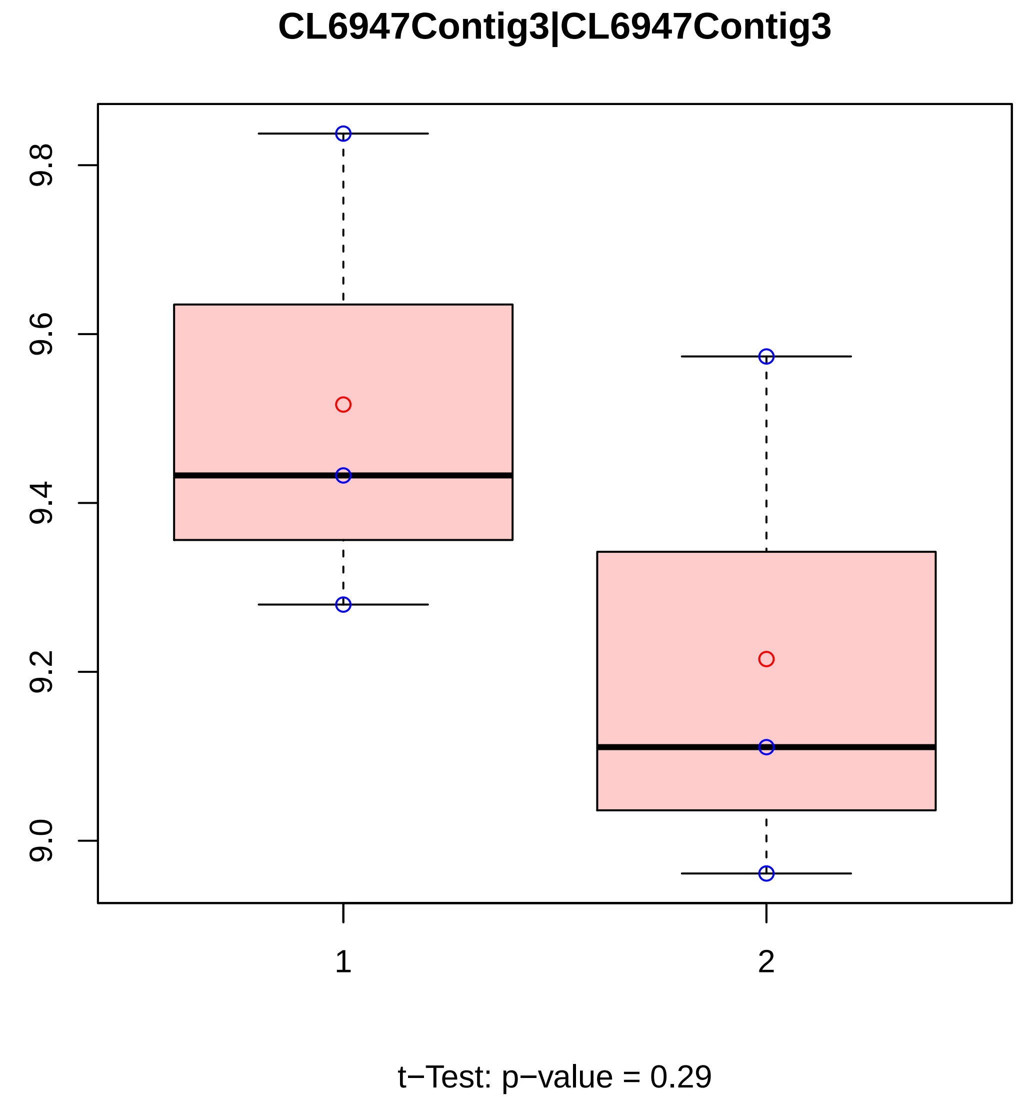
CL6947Contig3|CL6947Contig3
9.0
9.2
9.4
9.6
9.8
1
2
t−Test: p−value = 0.29

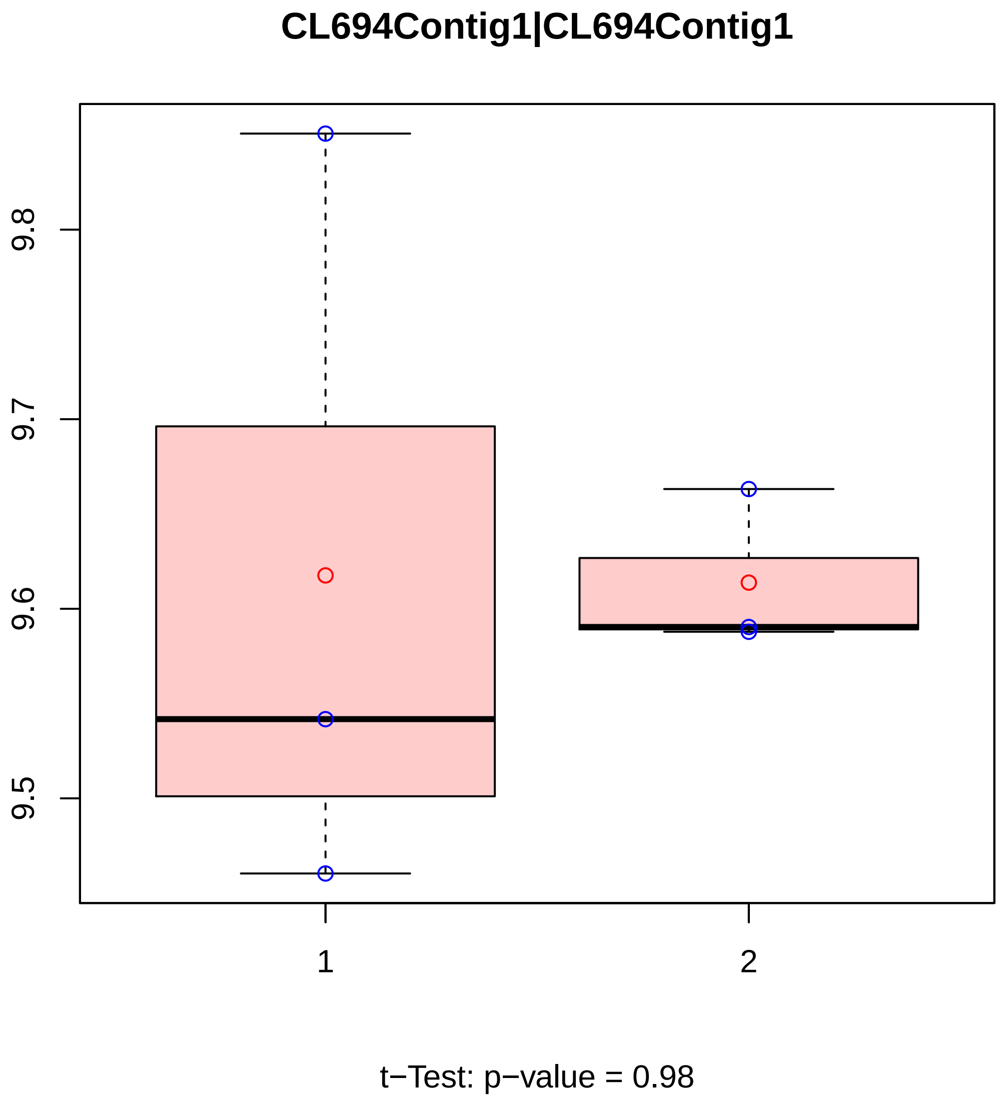

CL694Contig1|CL694Contig1
9.5
9.6
9.7
9.8
1
2
t−Test: p−value = 0.98

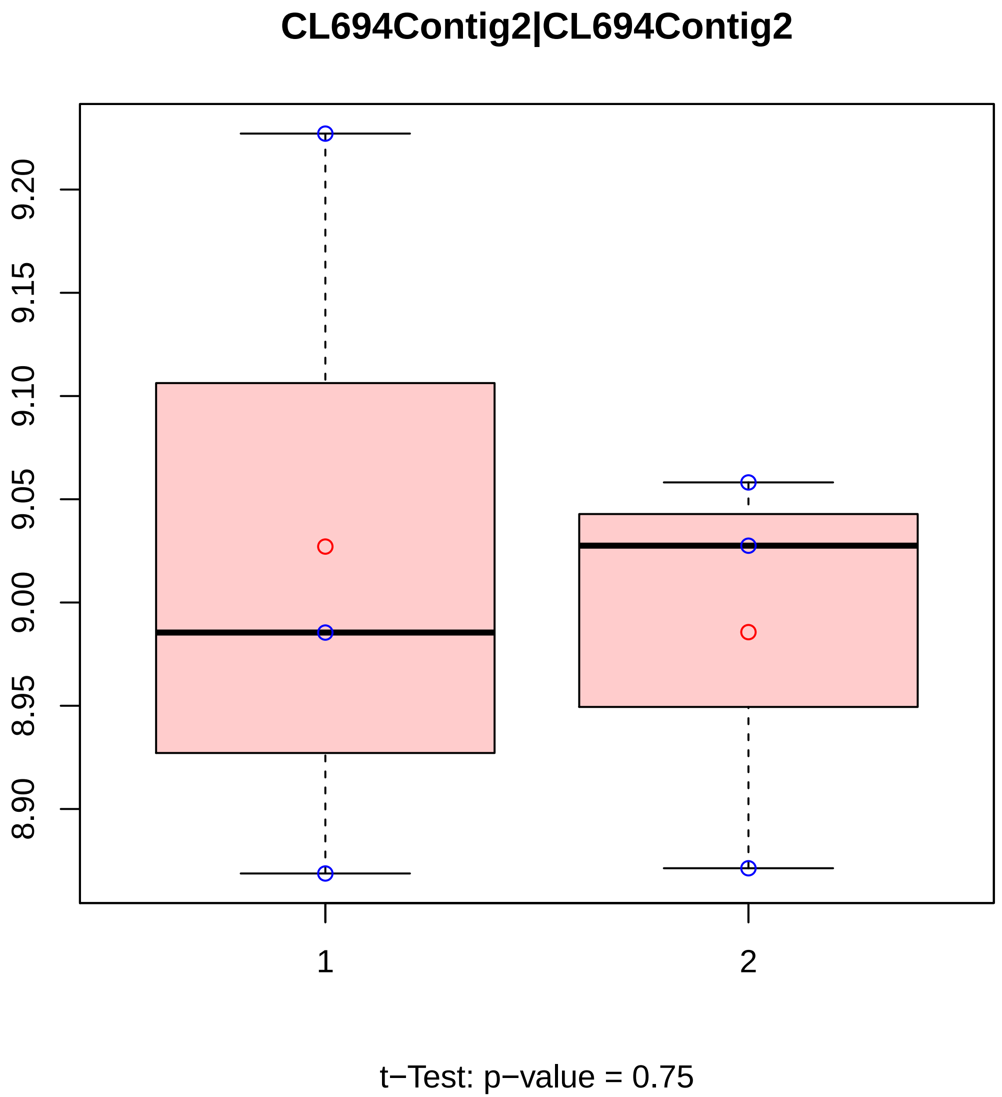
CL694Contig2|CL694Contig2
9.20
9.15
9.10
9.05
9.00
8.95
8.90
1
2
t−Test: p−value = 0.75

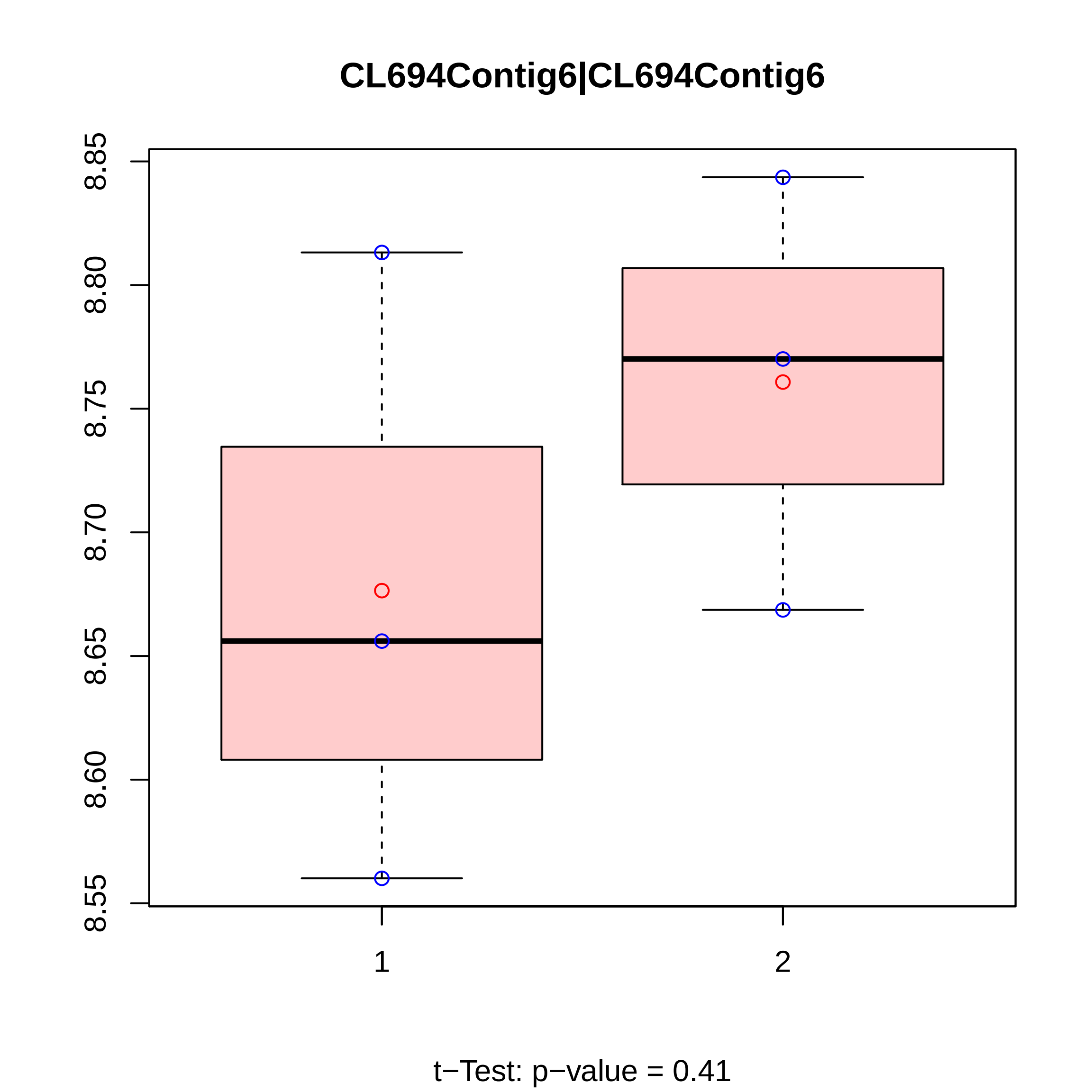

CL694Contig6|CL694Contig6
8.55
8.60
8.65
8.70
8.75
8.80
8.85
1
2
t−Test: p−value = 0.41

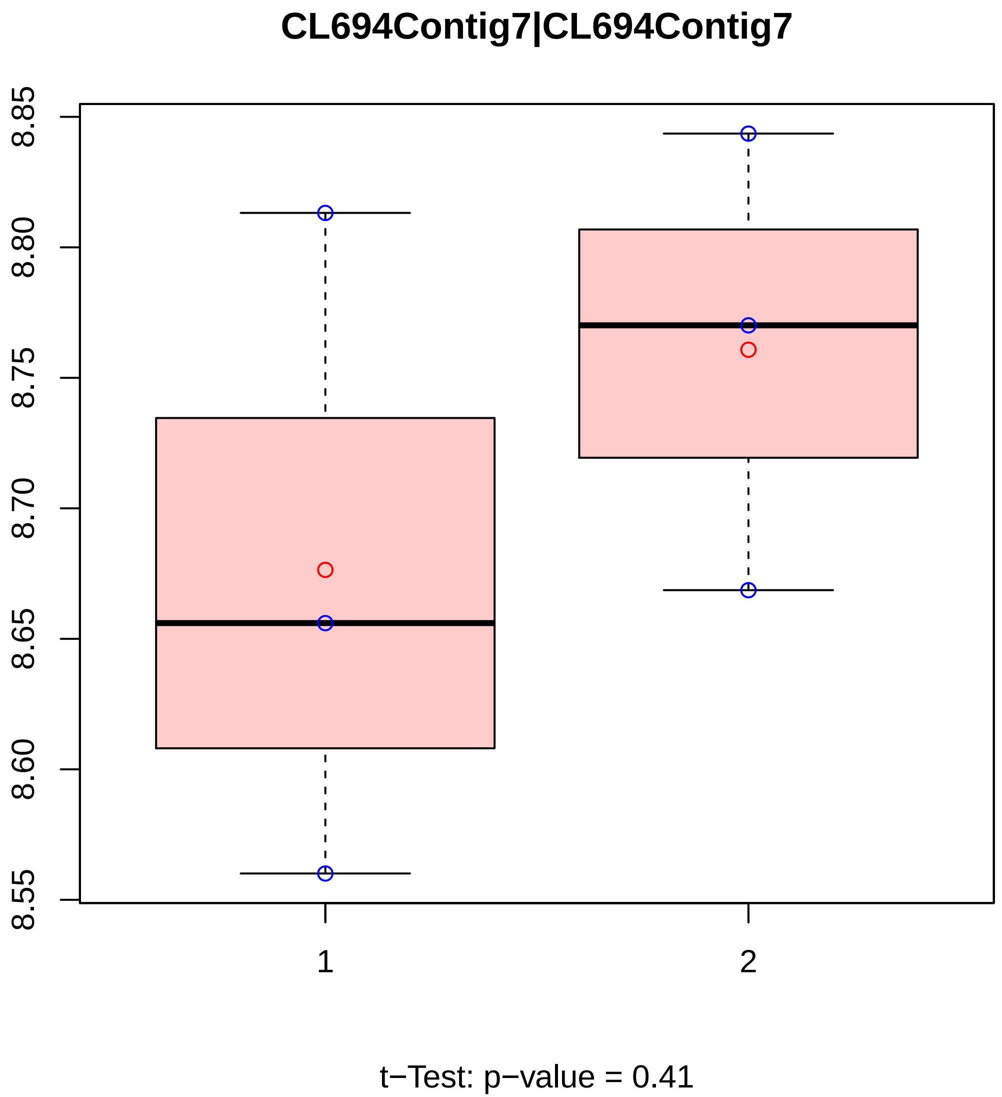
CL694Contig7|CL694Contig7
8.55
8.60
8.65
8.70
8.75
8.80
8.85
1
2
t−Test: p−value = 0.41

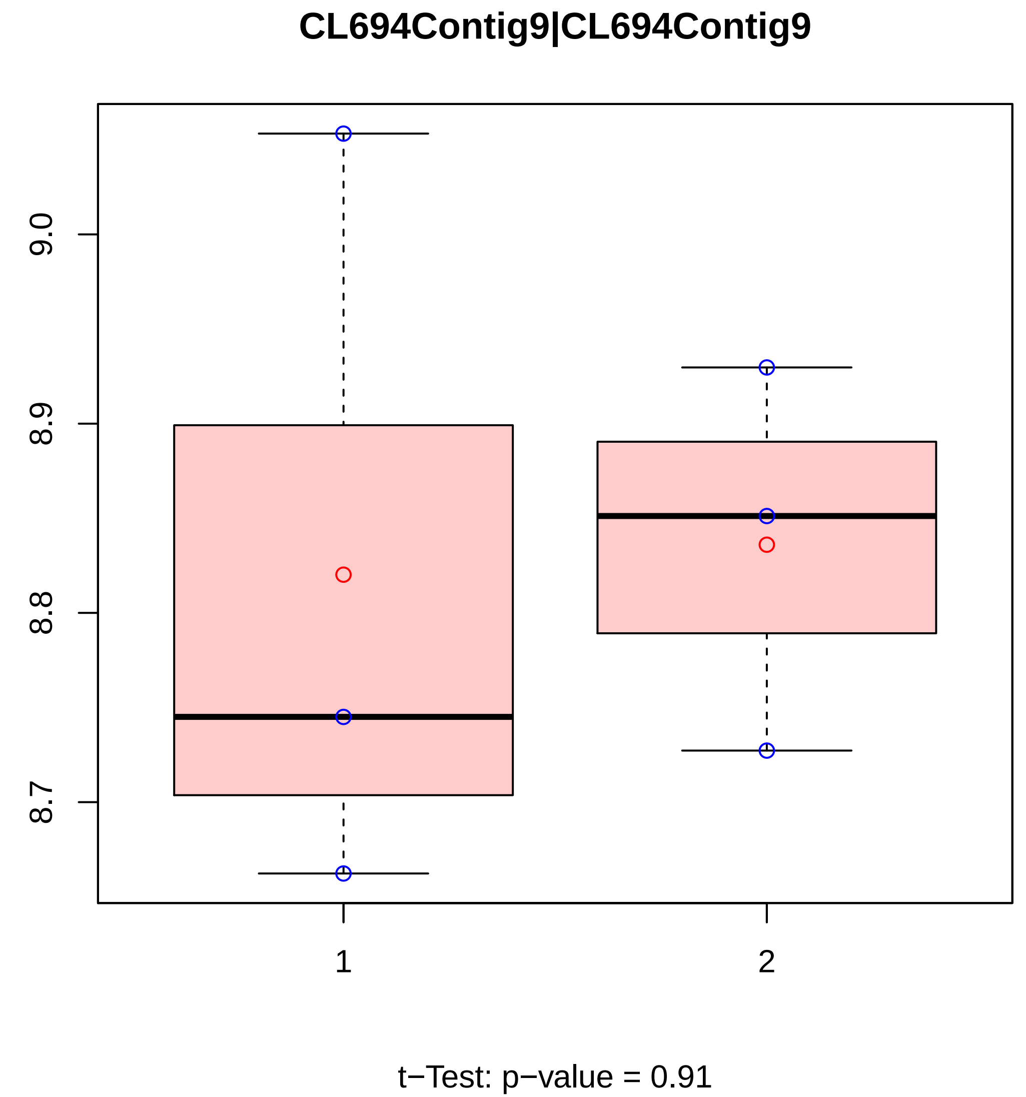

CL694Contig9|CL694Contig9
9.0
8.9
8.8
8.7
1
2
t−Test: p−value = 0.91

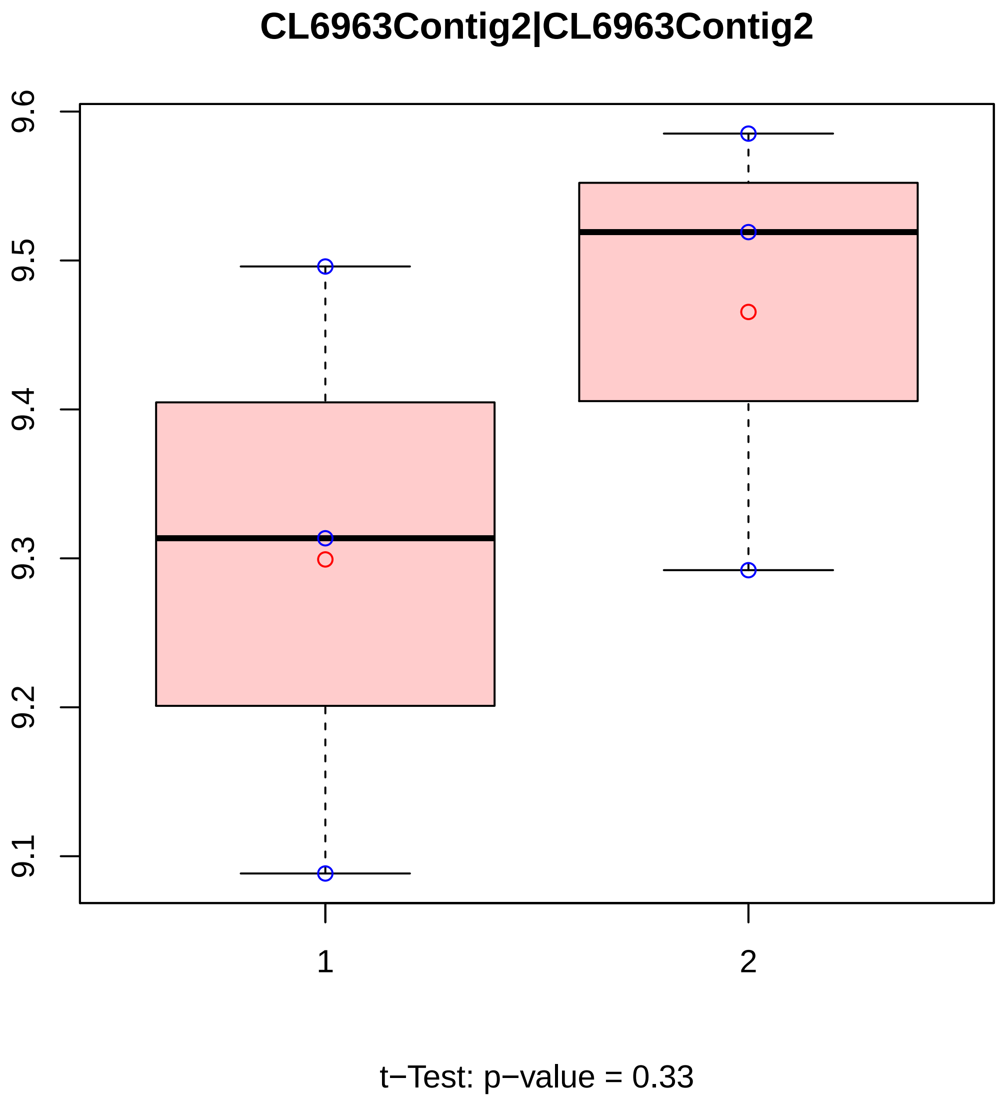
CL6963Contig2|CL6963Contig2
9.1
9.2
9.3
9.4
9.5
9.6
1
2
t−Test: p−value = 0.33

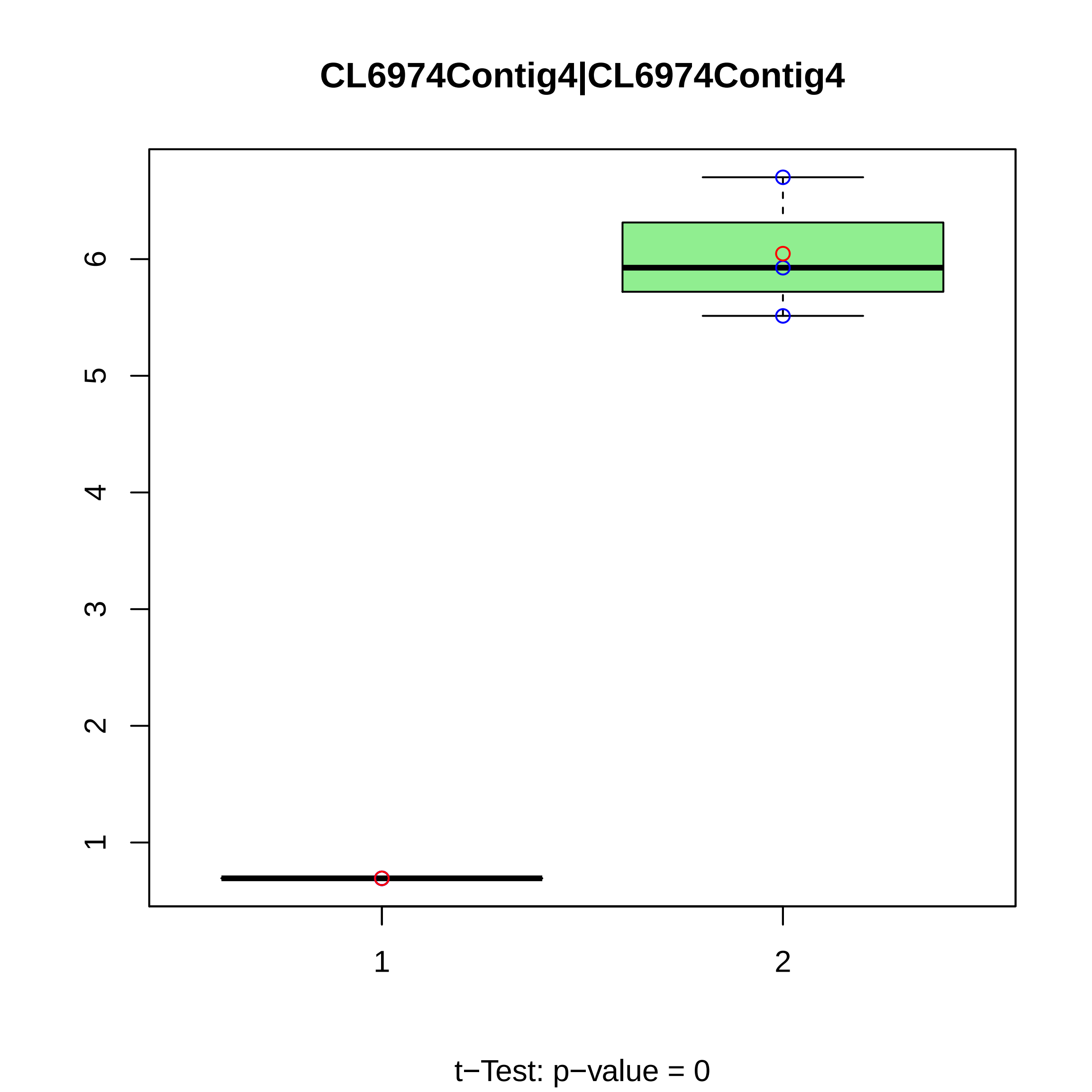
CL6974Contig4|CL6974Contig4
1
2
3
4
5
6
1
2
t−Test: p−value = 0

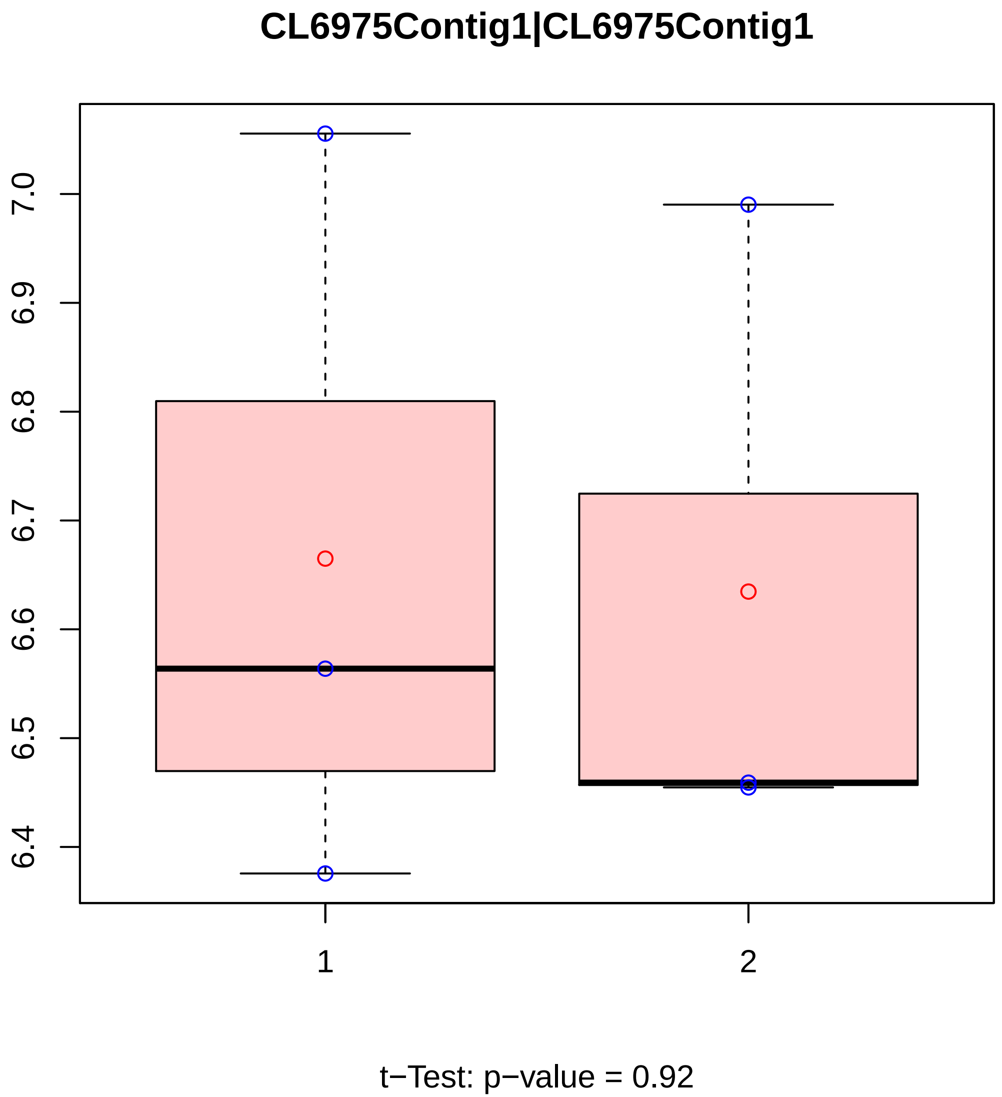
CL6975Contig1|CL6975Contig1
7.0
6.9
6.8
6.7
6.6
6.5
6.4
1
2
t−Test: p−value = 0.92

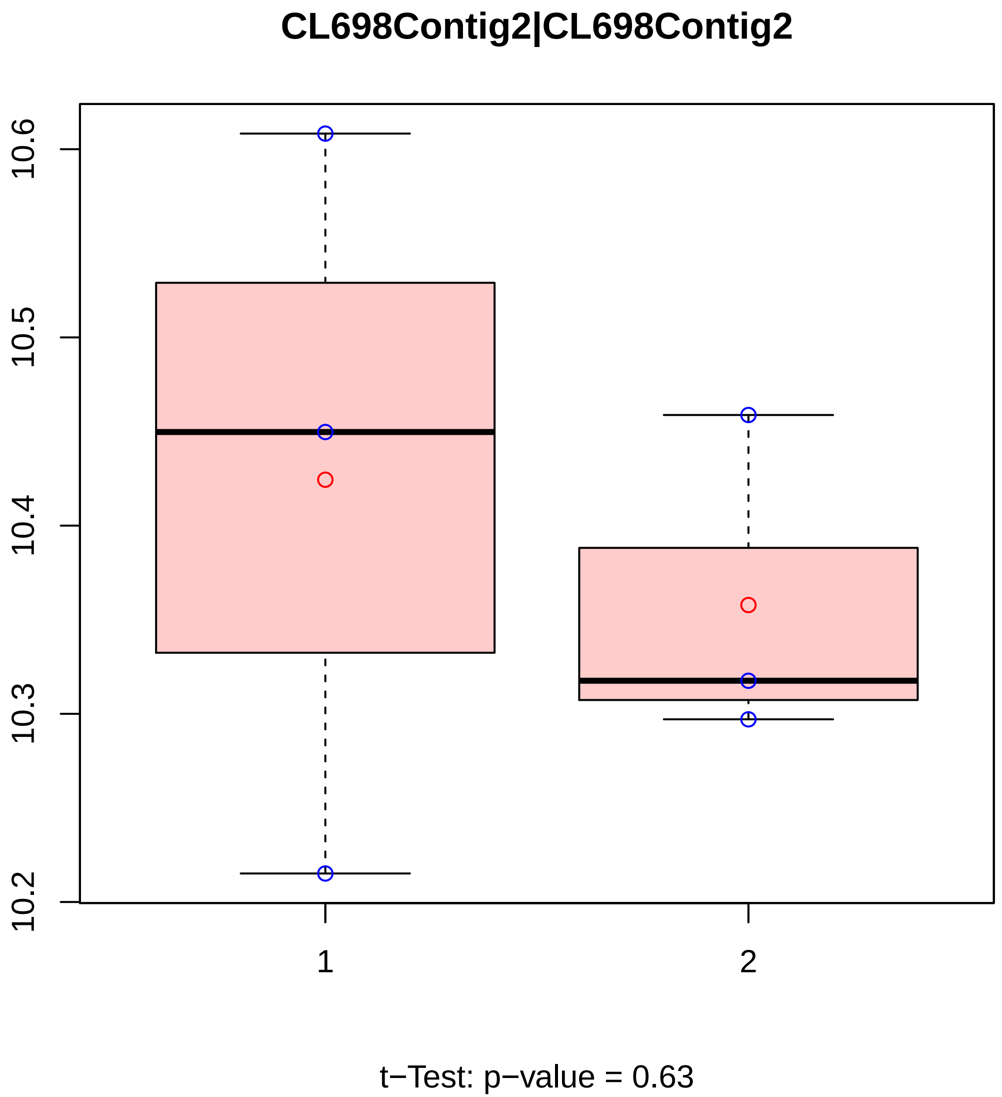
CL698Contig2|CL698Contig2
10.2
10.3
10.4
10.5
10.6
1
2
t−Test: p−value = 0.63

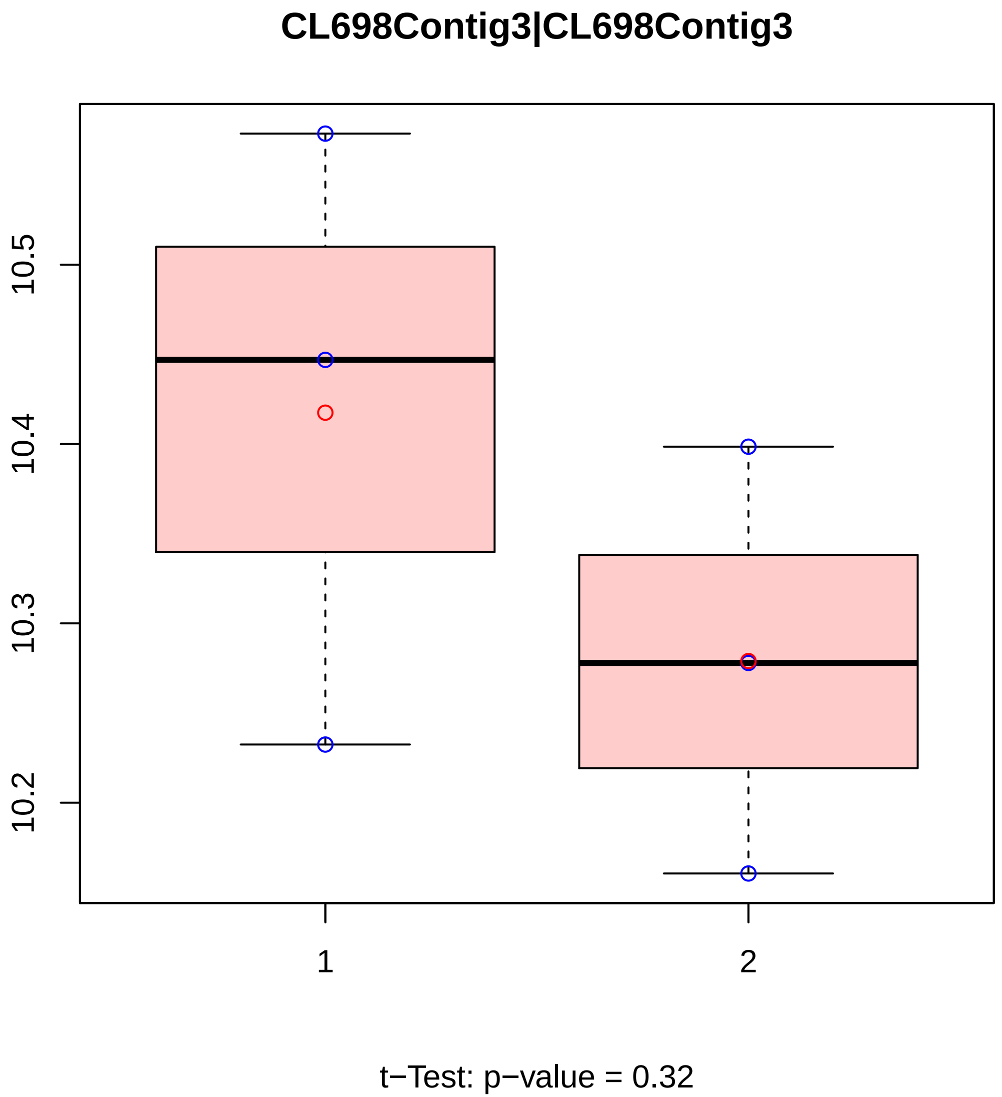

CL698Contig3|CL698Contig3
10.2
10.3
10.4
10.5
1
2
t−Test: p−value = 0.32

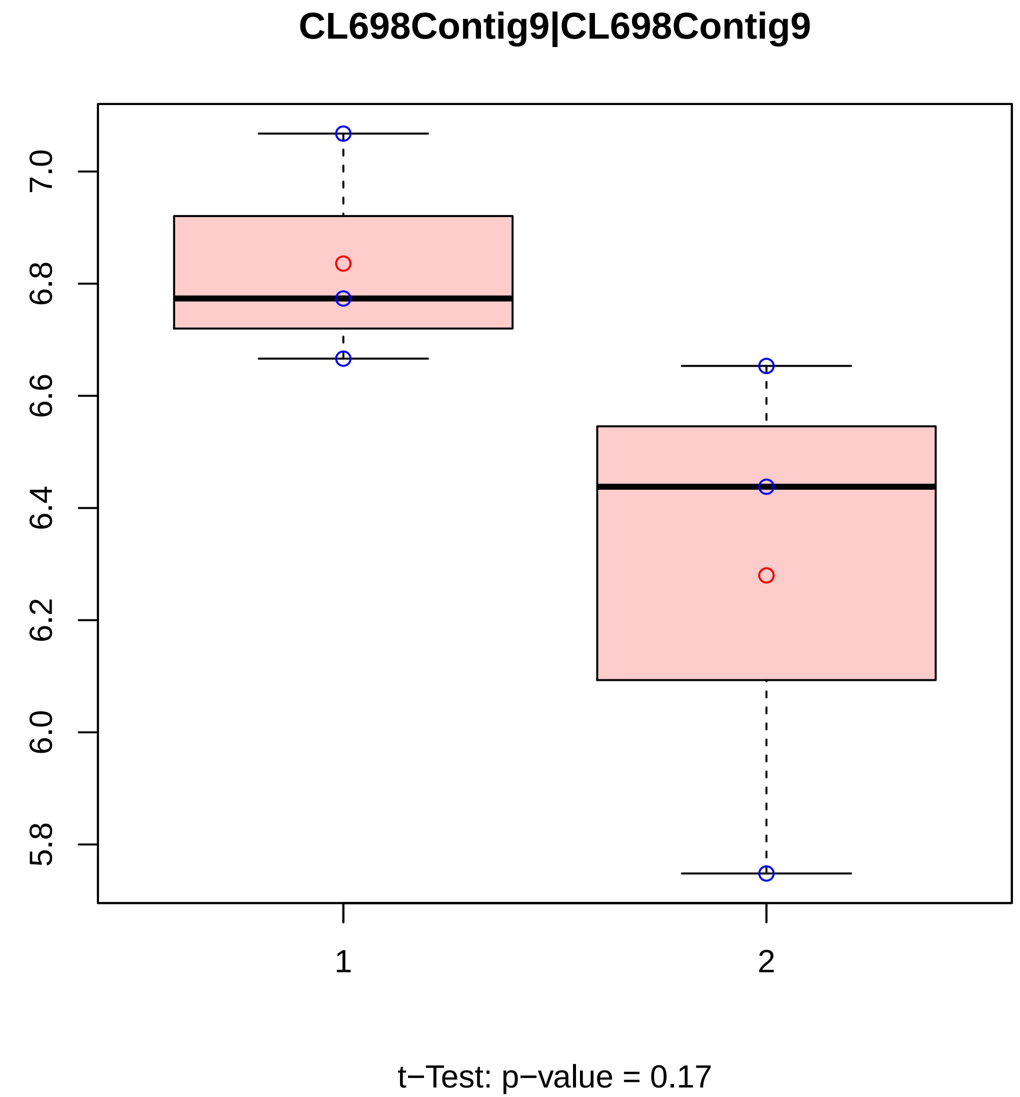
CL698Contig9|CL698Contig9
7.0
6.8
6.6
6.4
6.2
6.0
5.8
1
2
t−Test: p−value = 0.17

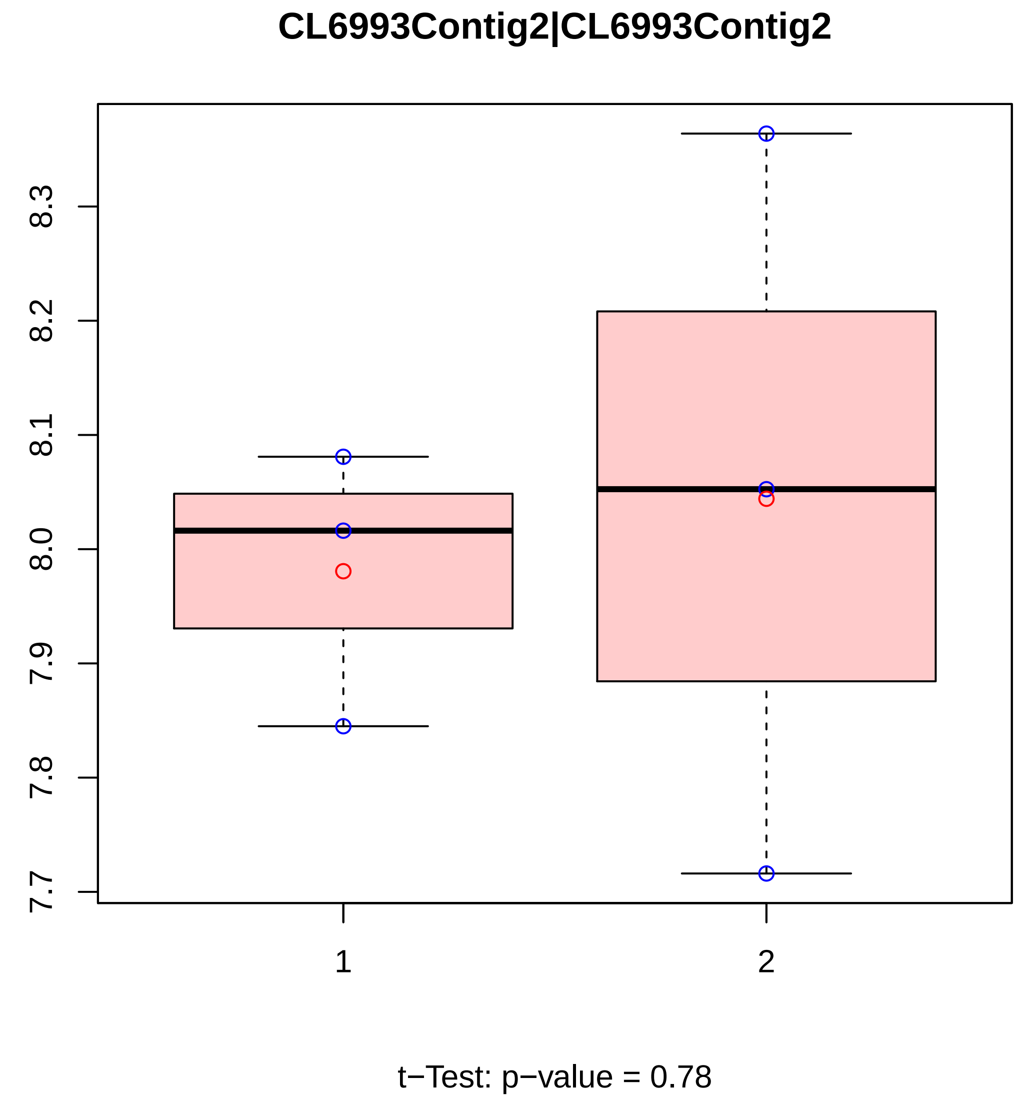
CL6993Contig2|CL6993Contig2
8.3
8.2
8.1
8.0
7.9
7.8
7.7
1
2
t−Test: p−value = 0.78

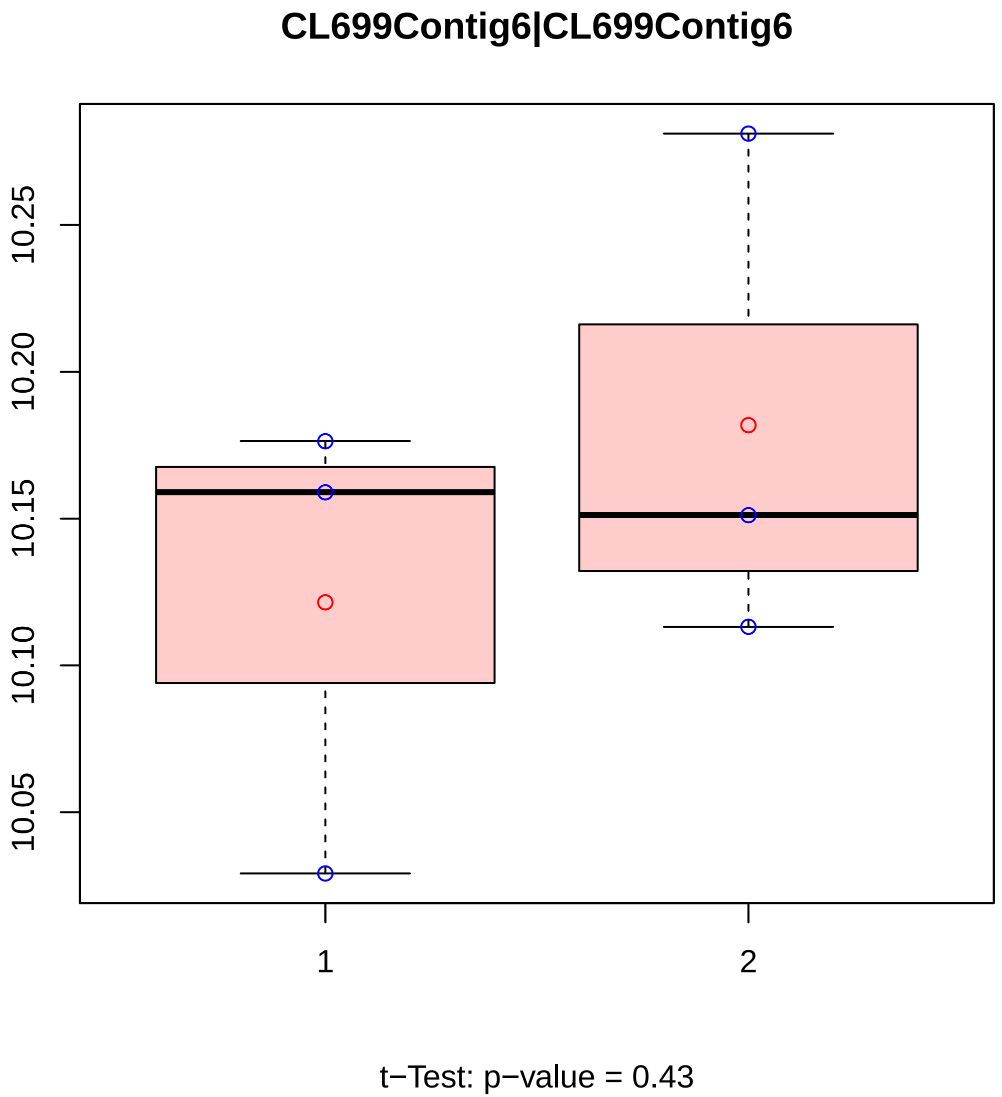

CL699Contig6|CL699Contig6
10.05
10.10
10.15
10.20
10.25
1
2
t−Test: p−value = 0.43

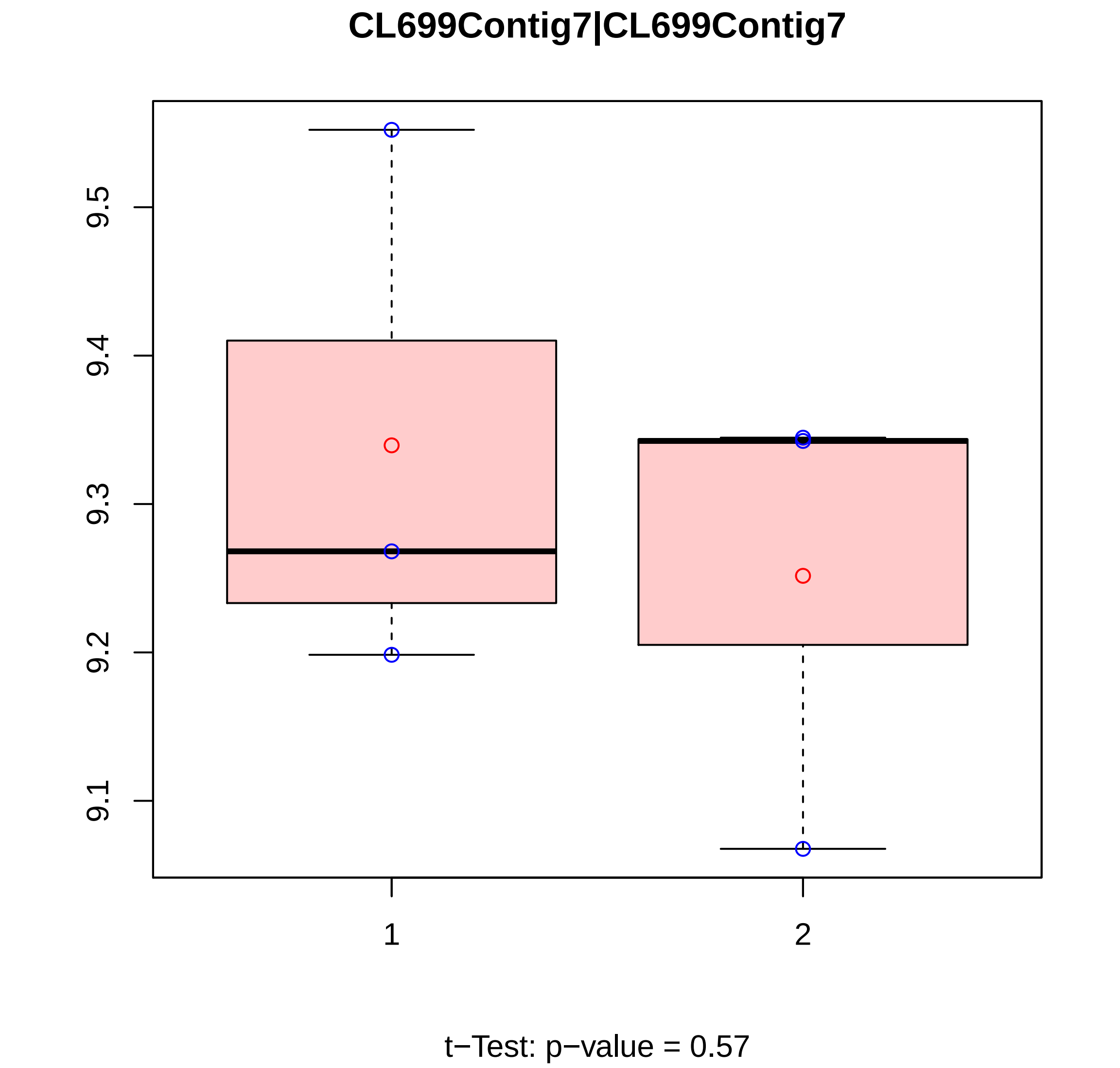
CL699Contig7|CL699Contig7
9.1
9.2
9.3
9.4
9.5
1
2
t−Test: p−value = 0.57

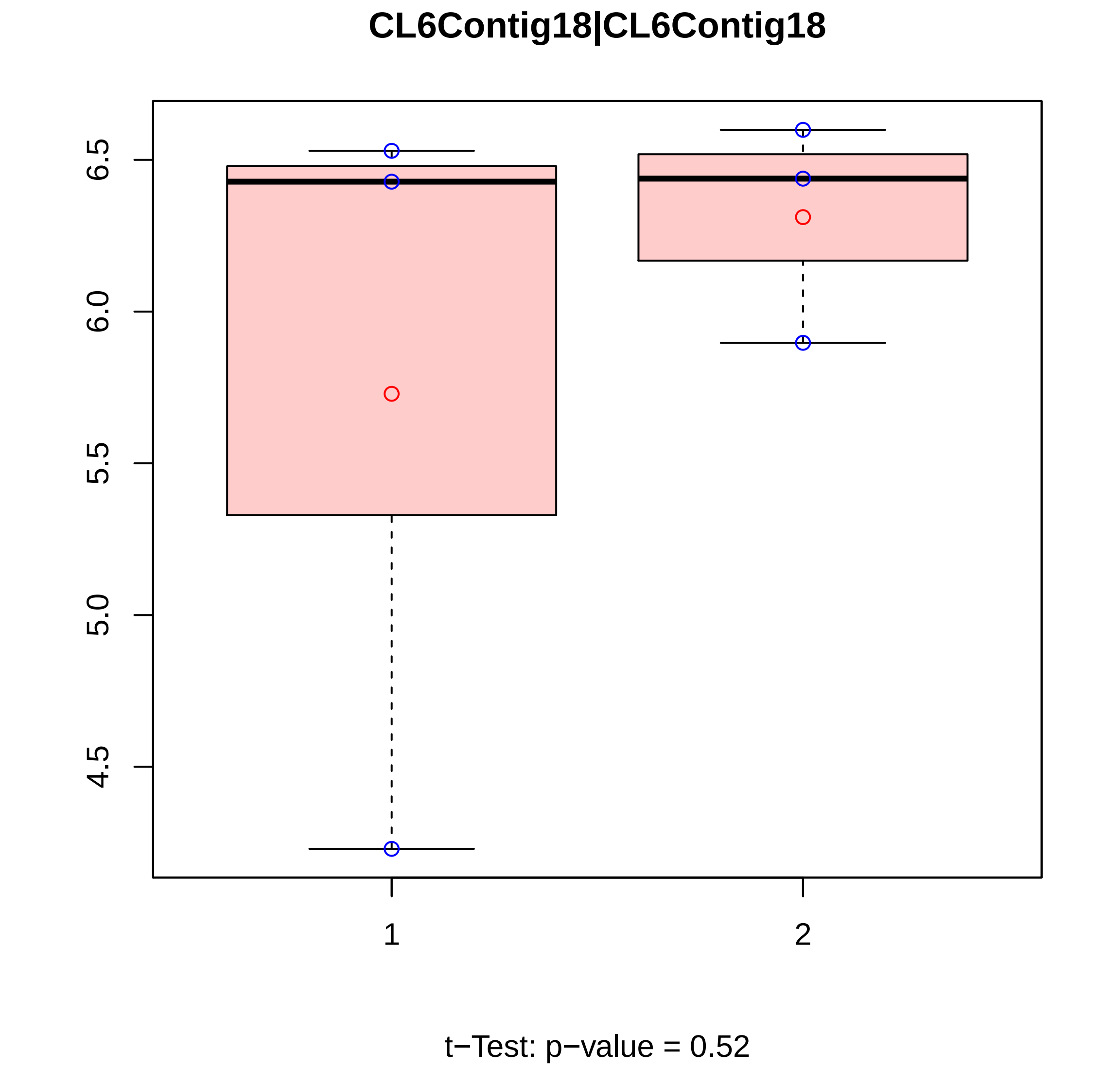
CL6Contig18|CL6Contig18
6.5
6.0
5.5
5.0
4.5
1
2
t−Test: p−value = 0.52

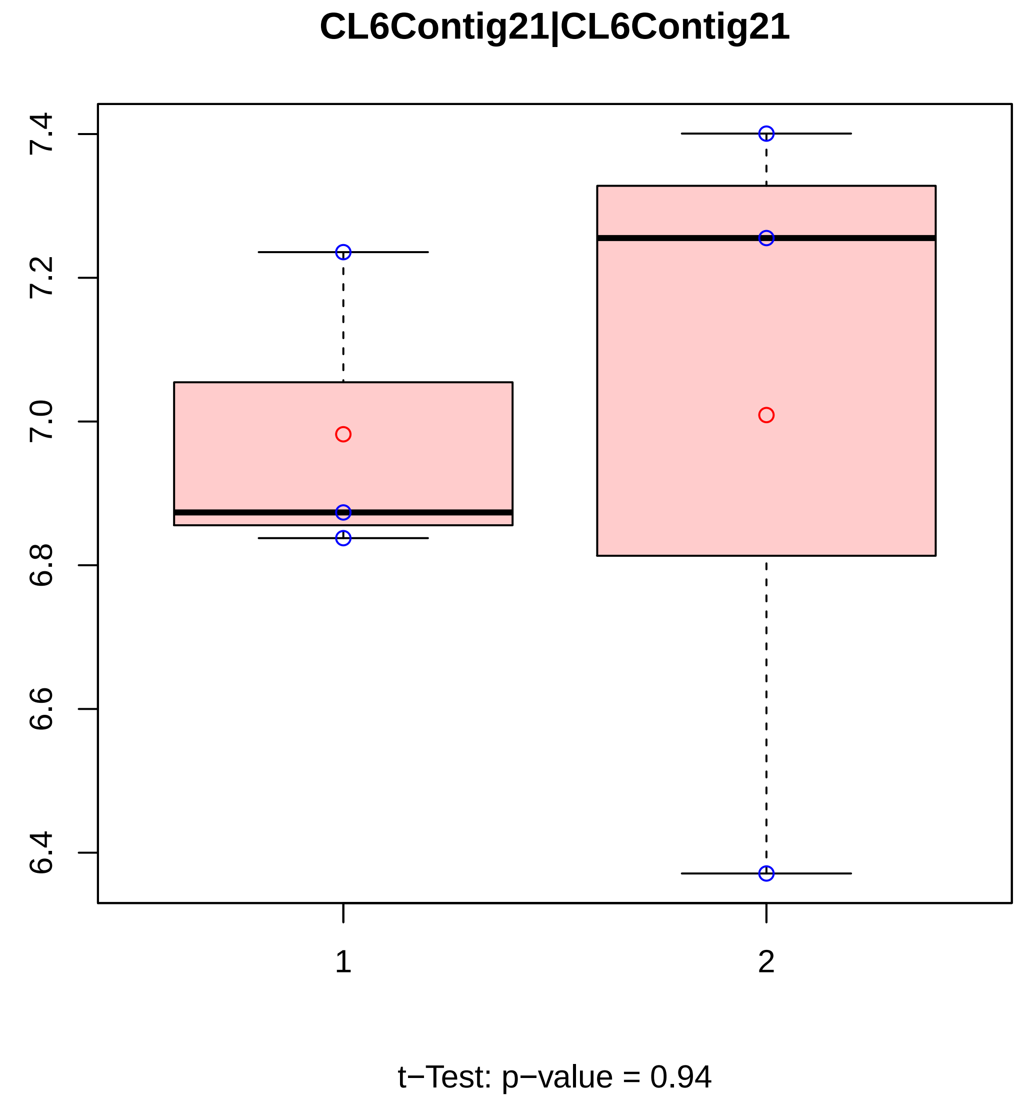

CL6Contig21|CL6Contig21
7.4
7.2
7.0
6.8
6.6
6.4
1
2
t−Test: p−value = 0.94

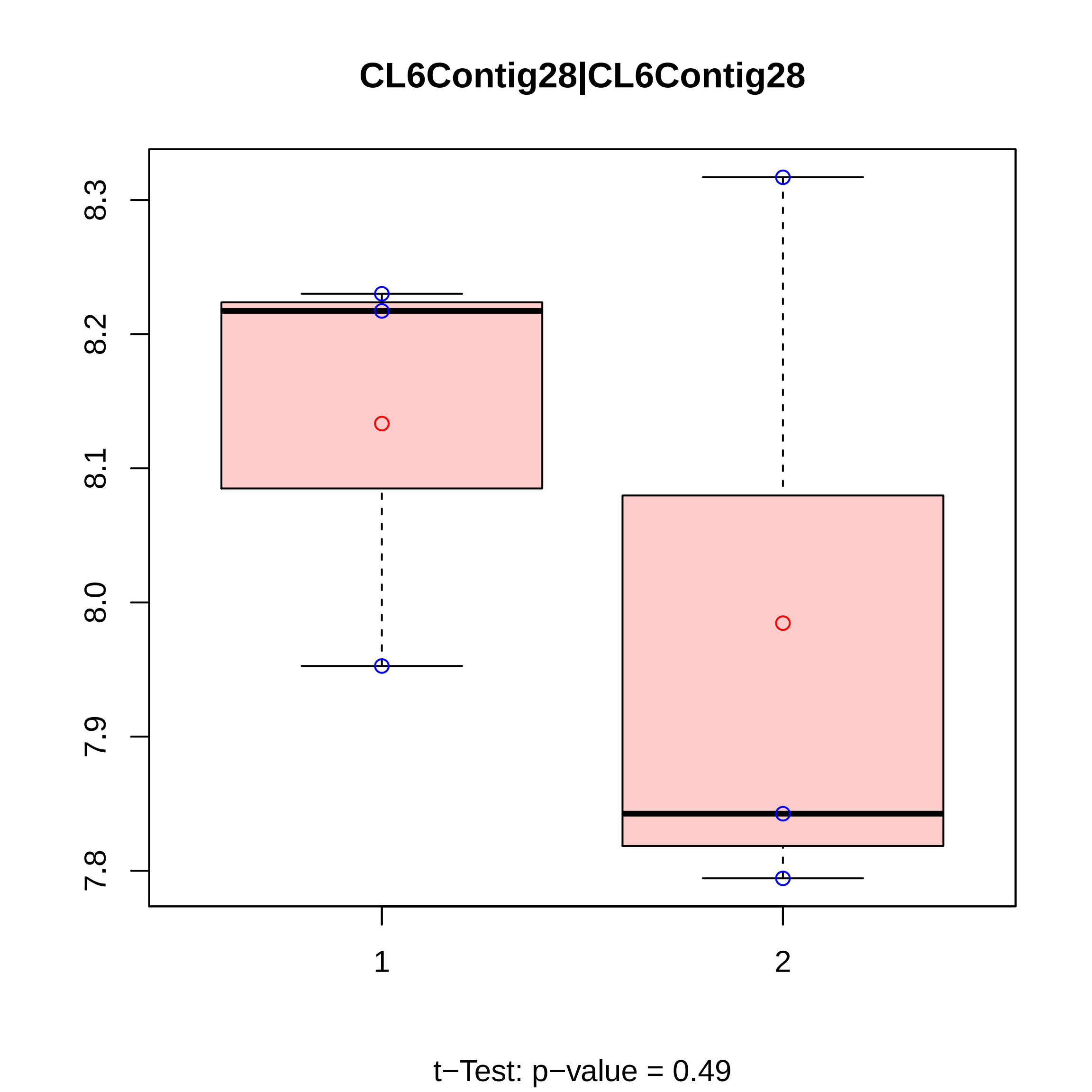
CL6Contig28|CL6Contig28
8.3
8.2
8.1
8.0
7.9
7.8
1
2
t−Test: p−value = 0.49

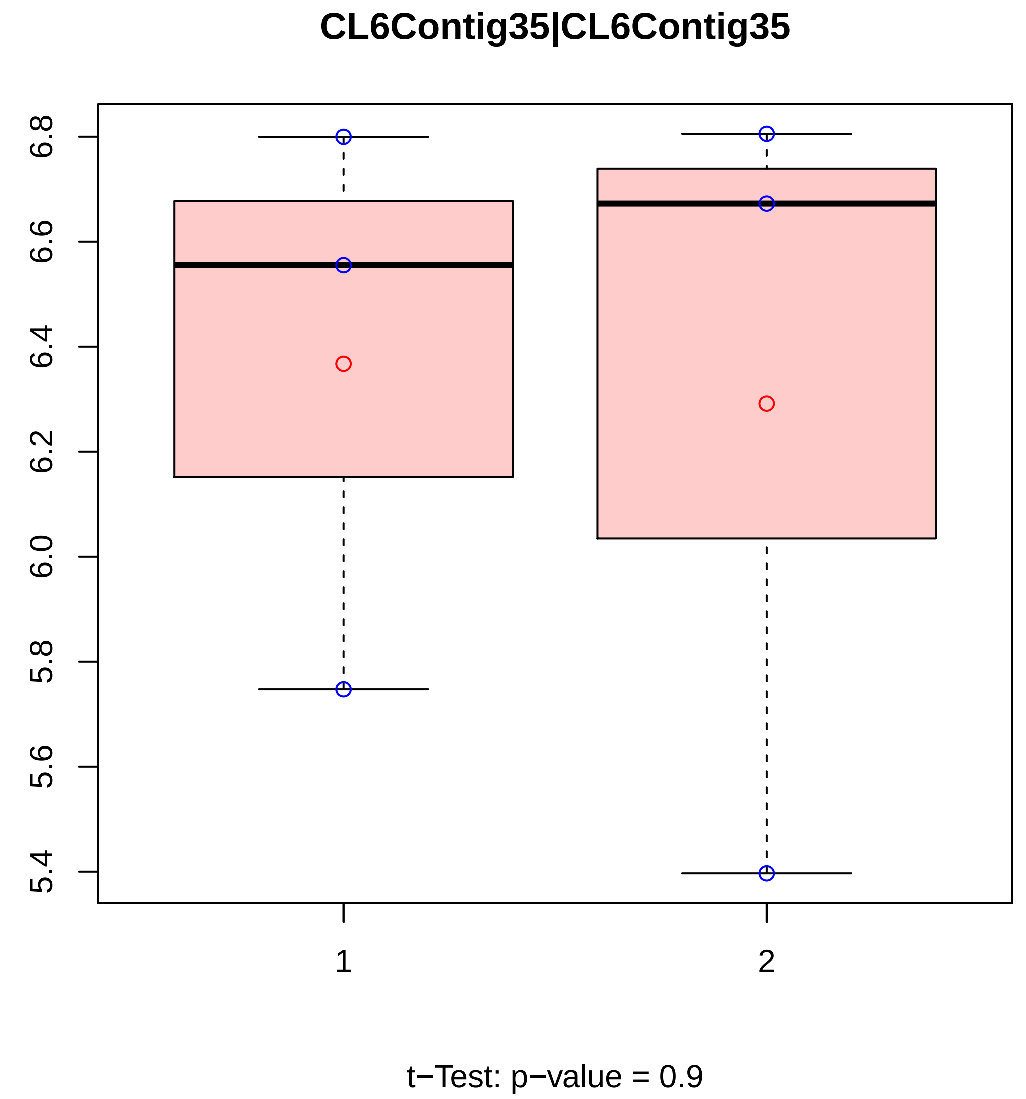
CL6Contig35|CL6Contig35
6.8
6.6
6.4
6.2
6.0
5.8
5.6
5.4
1
2
t−Test: p−value = 0.9

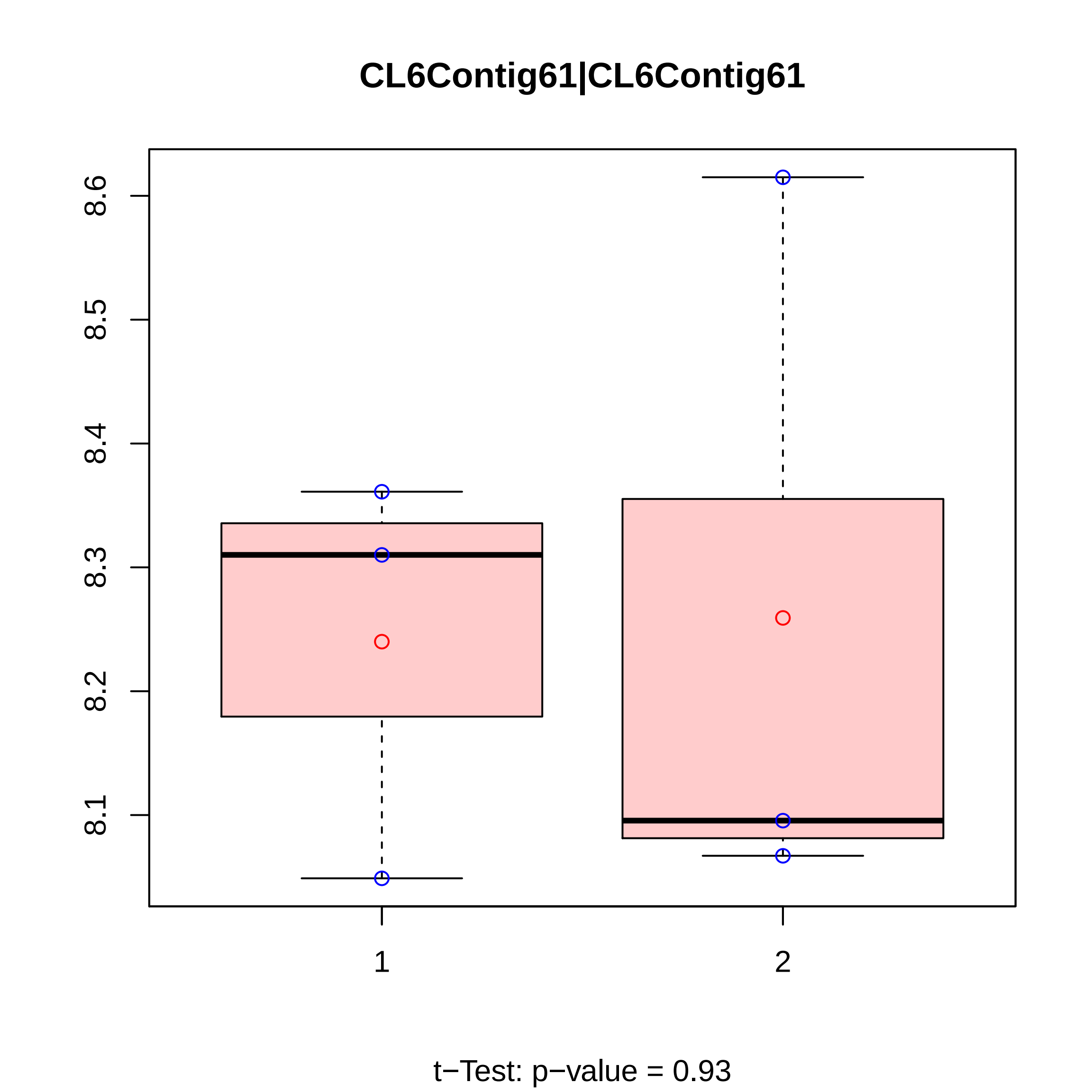
CL6Contig61|CL6Contig61
8.1
8.2
8.3
8.4
8.5
8.6
1
2
t−Test: p−value = 0.93

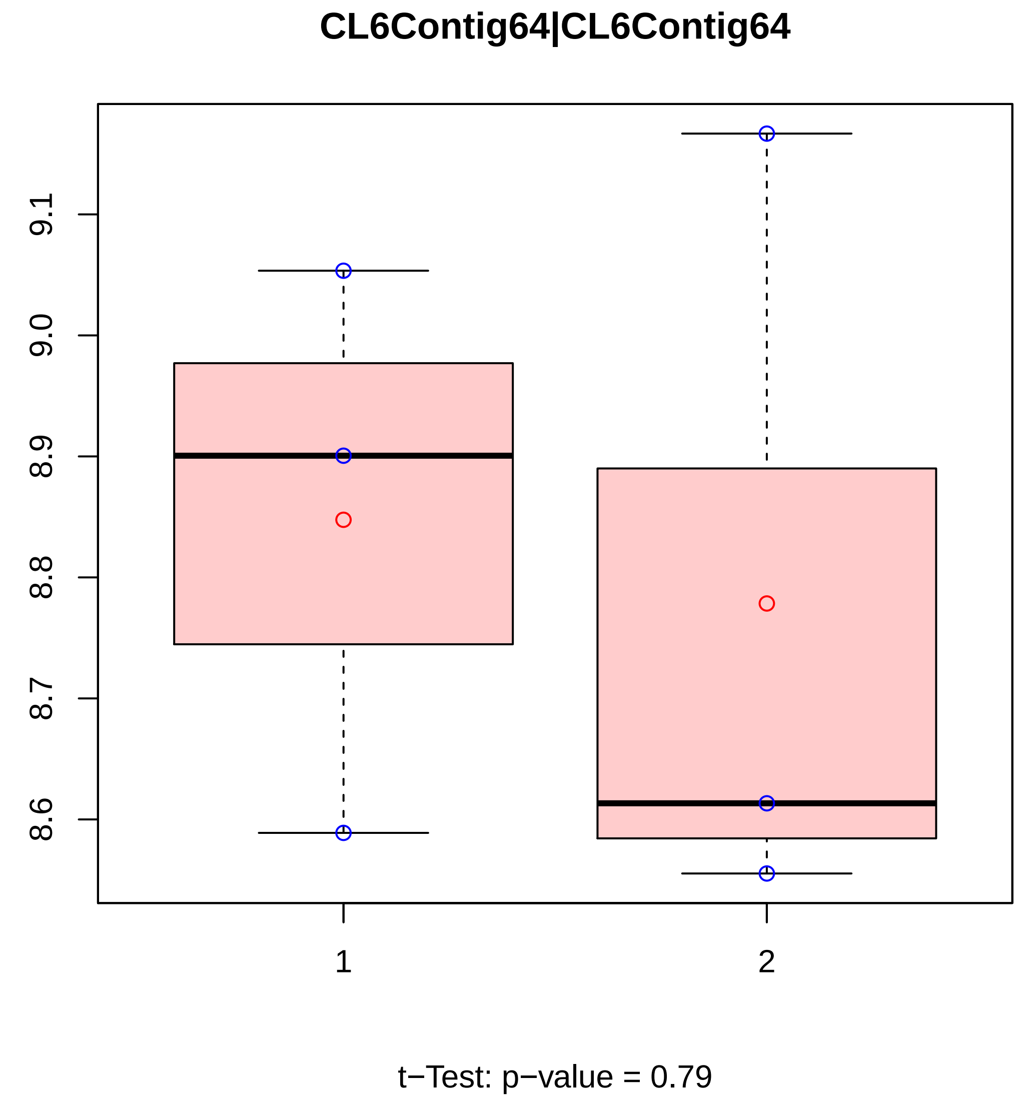
CL6Contig64|CL6Contig64
9.1
9.0
8.9
8.8
8.7
8.6
1
2
t−Test: p−value = 0.79

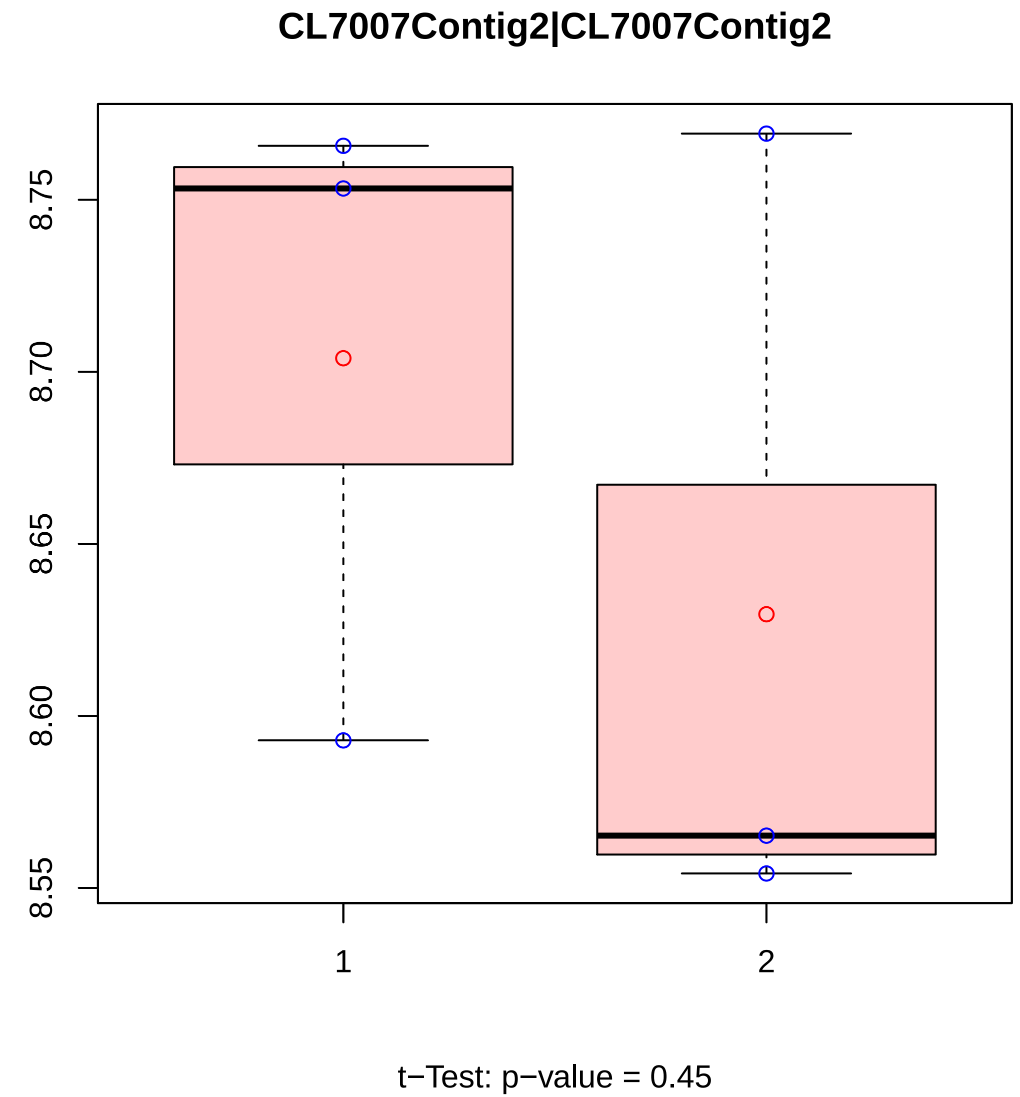
CL7007Contig2|CL7007Contig2
8.55
8.60
8.65
8.70
8.75
1
2
t−Test: p−value = 0.45

# CL7007Contig3|CL7007Contig3

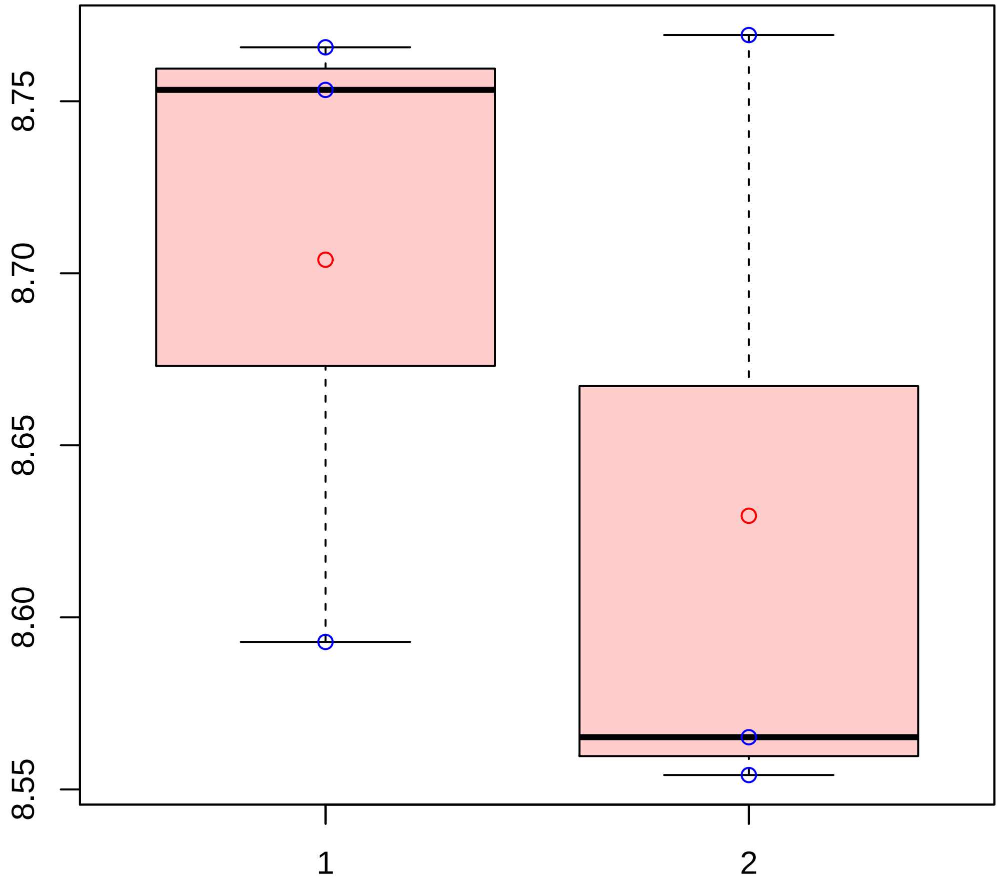


t−Test: p−value = 0.45

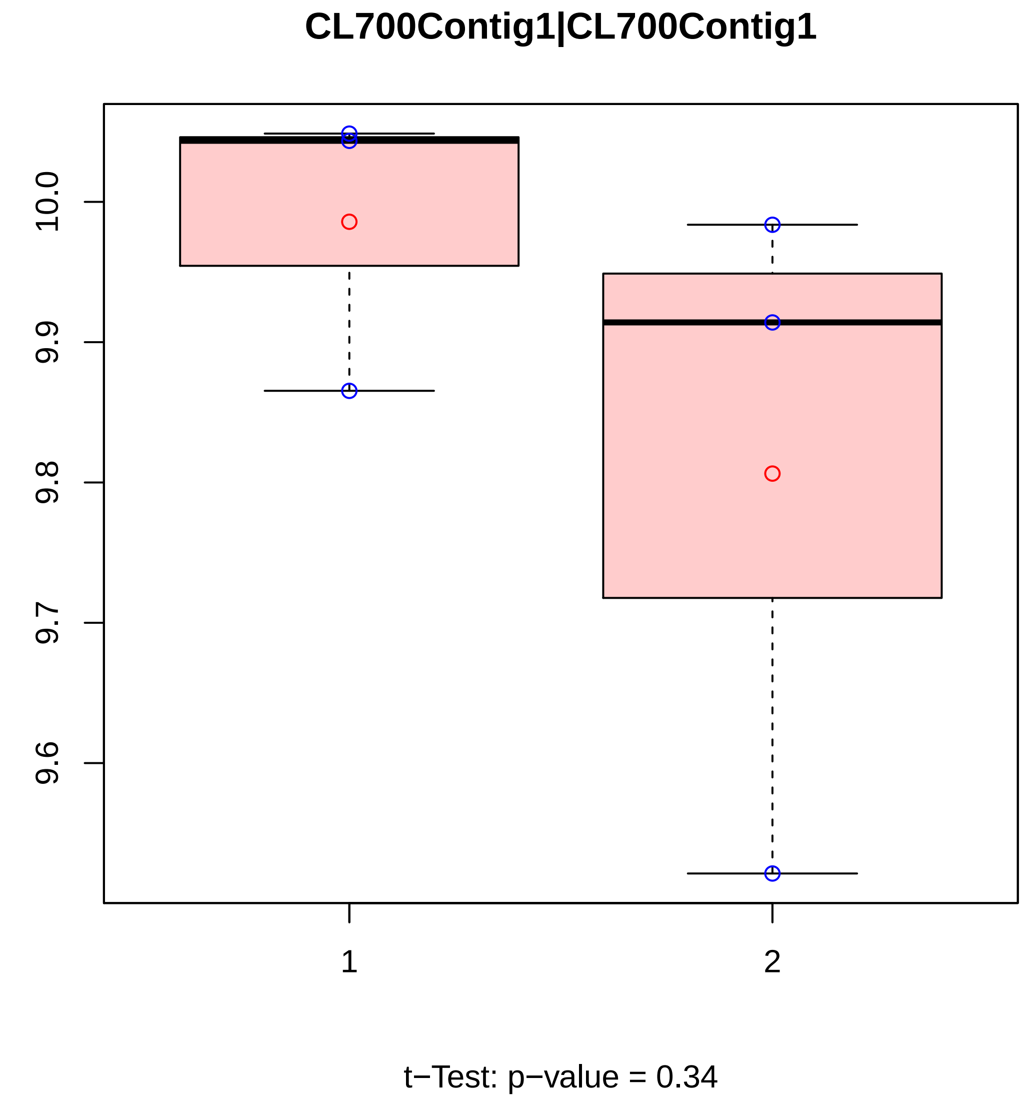
CL700Contig1|CL700Contig1
10.0
9.9
9.8
9.7
9.6
1
2
t−Test: p−value = 0.34

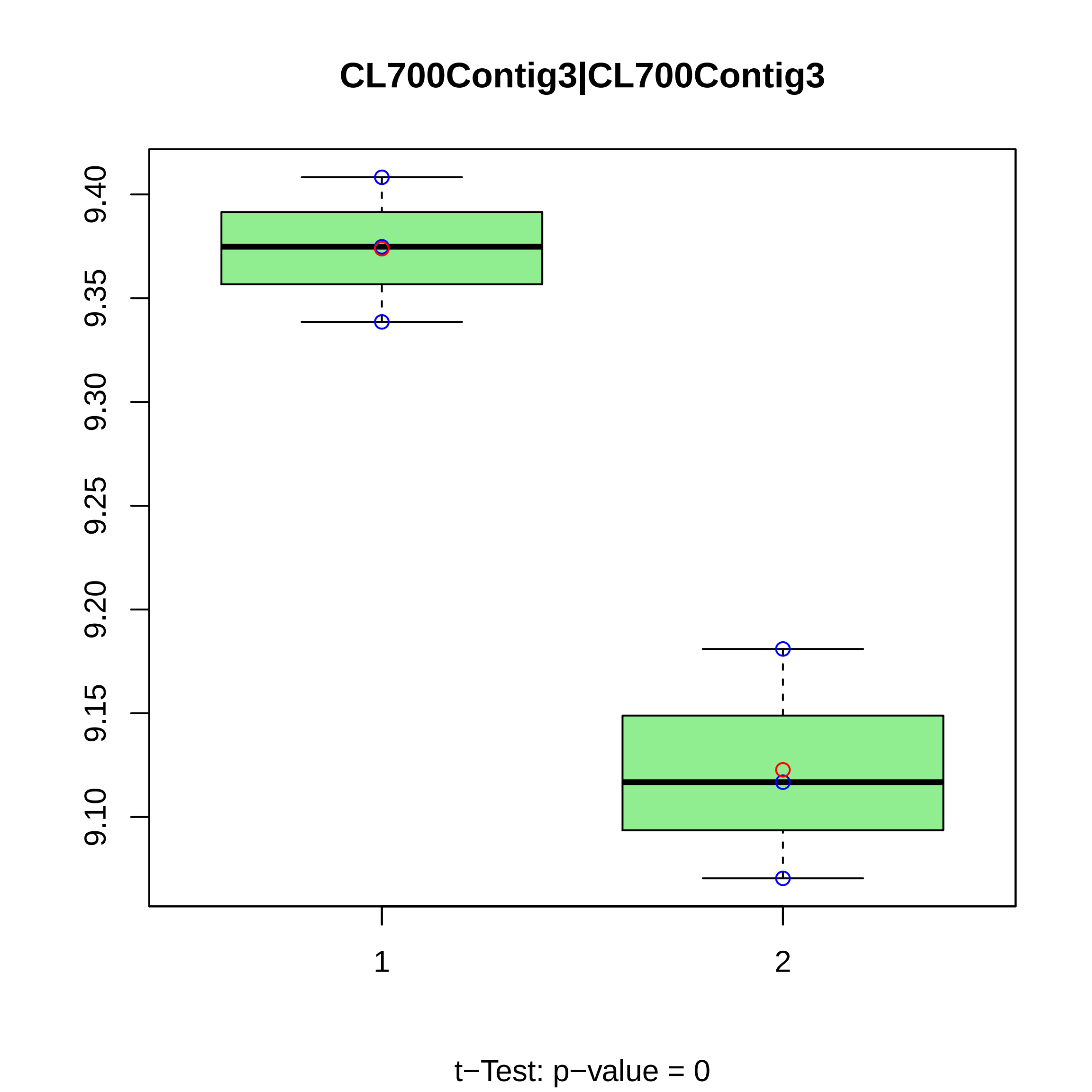

CL700Contig3|CL700Contig3
9.40
9.35
9.30
9.25
9.20
9.15
9.10
1
2
t−Test: p−value = 0

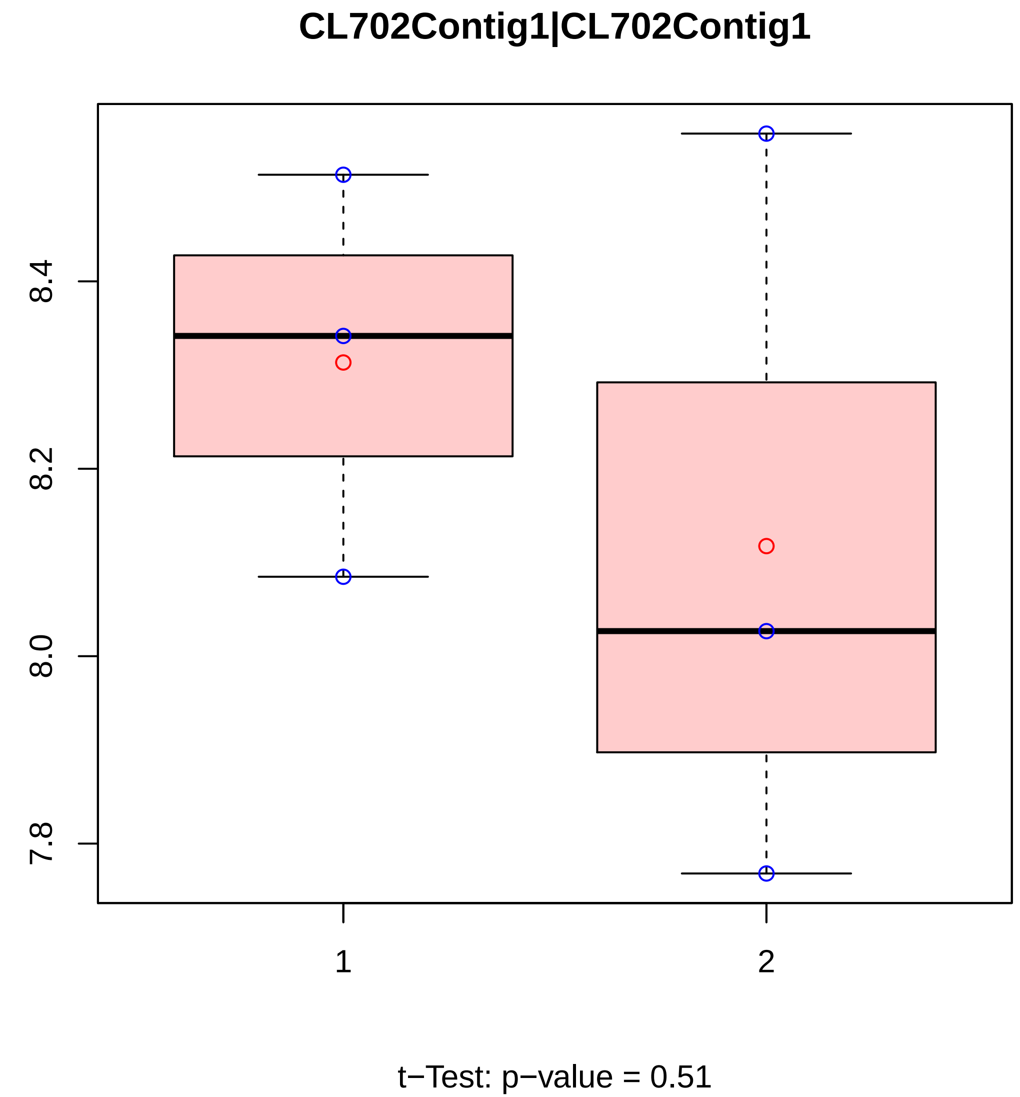

CL702Contig1|CL702Contig1
8.4
8.2
8.0
7.8
1
2
t−Test: p−value = 0.51

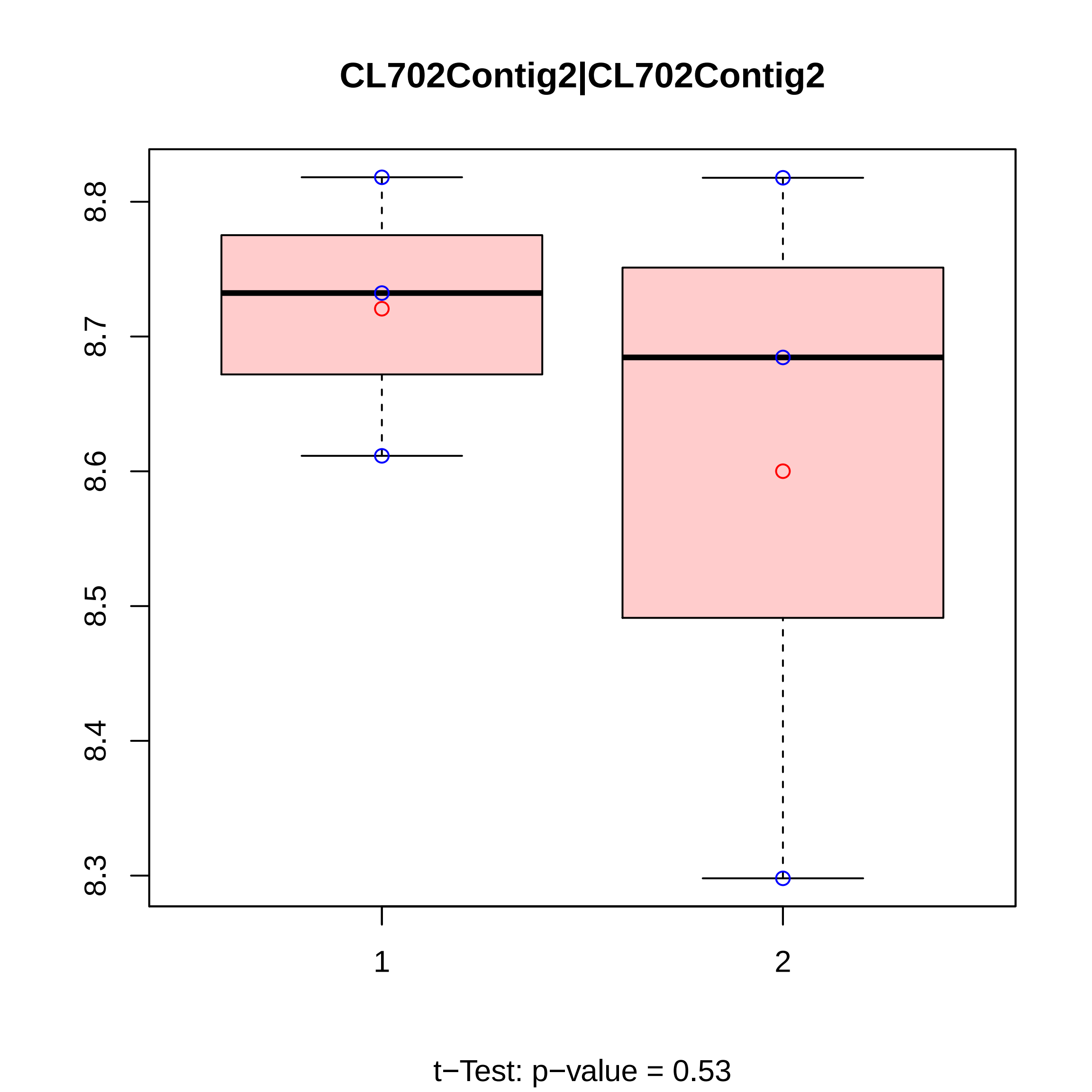
CL702Contig2|CL702Contig2
8.3
8.4
8.5
8.6
8.7
8.8
1
2
t−Test: p−value = 0.53

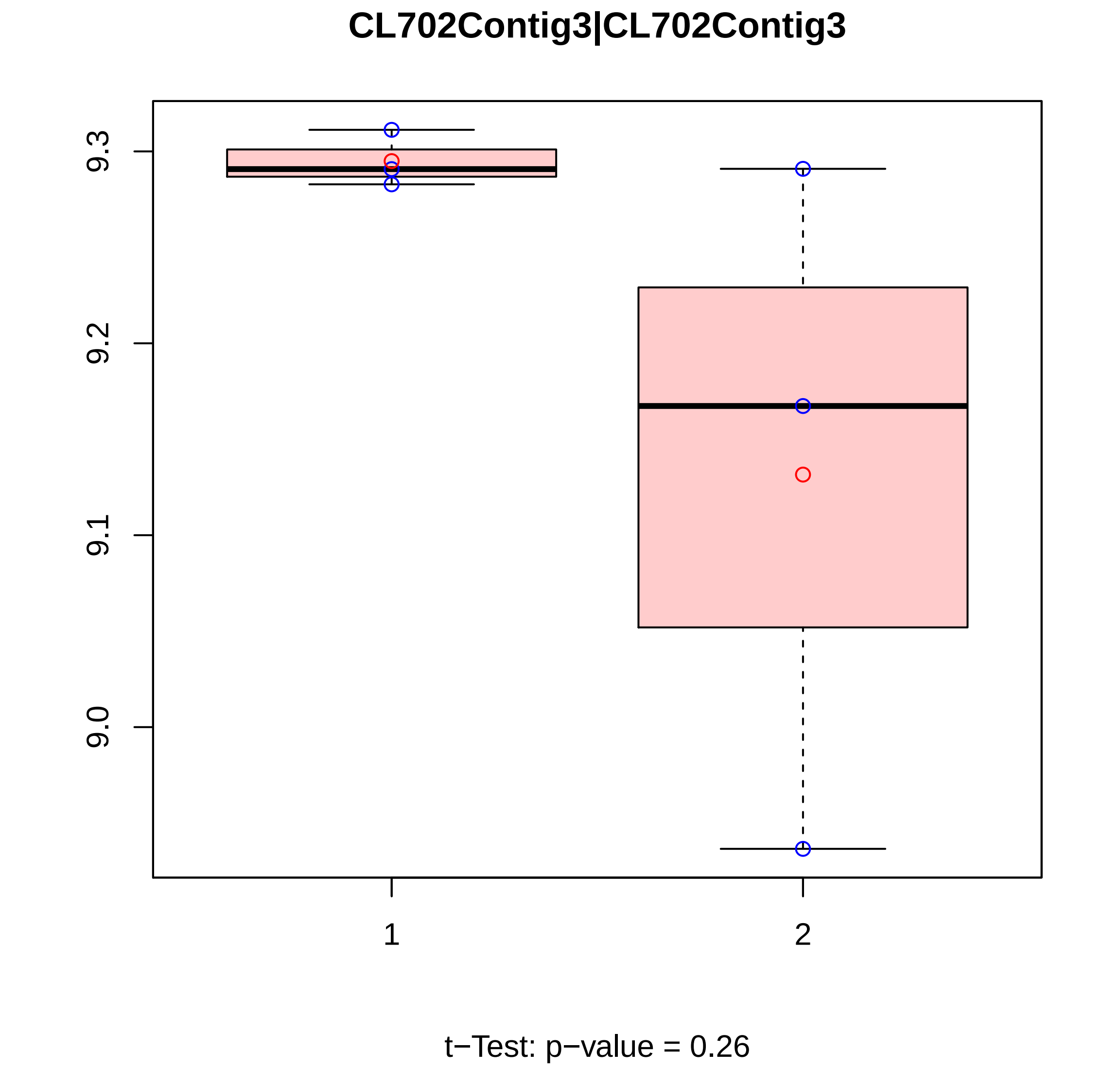

CL702Contig3|CL702Contig3
9.0
9.1
9.2
9.3
1
2
t−Test: p−value = 0.26

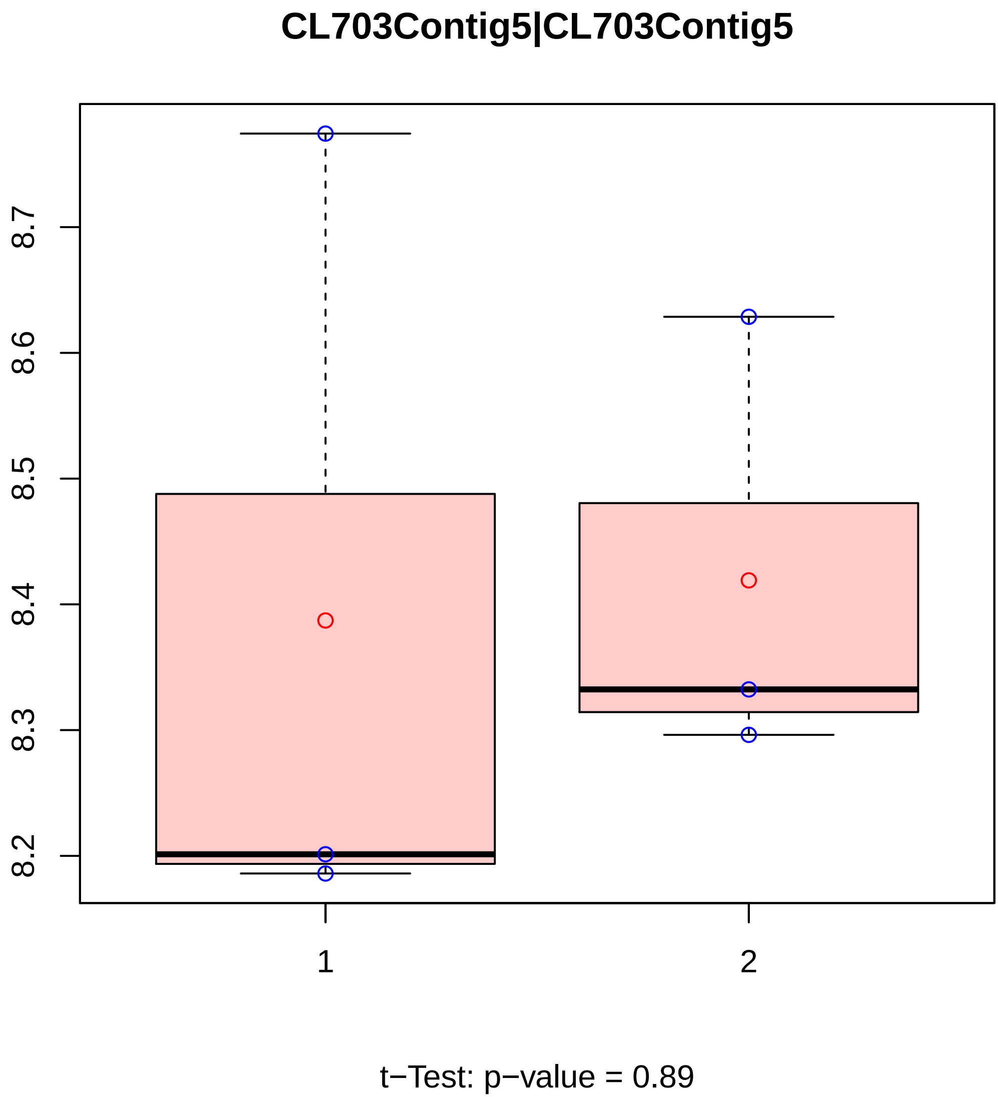
CL703Contig5|CL703Contig5
8.2
8.3
8.4
8.5
8.6
8.7
1
2
t−Test: p−value = 0.89

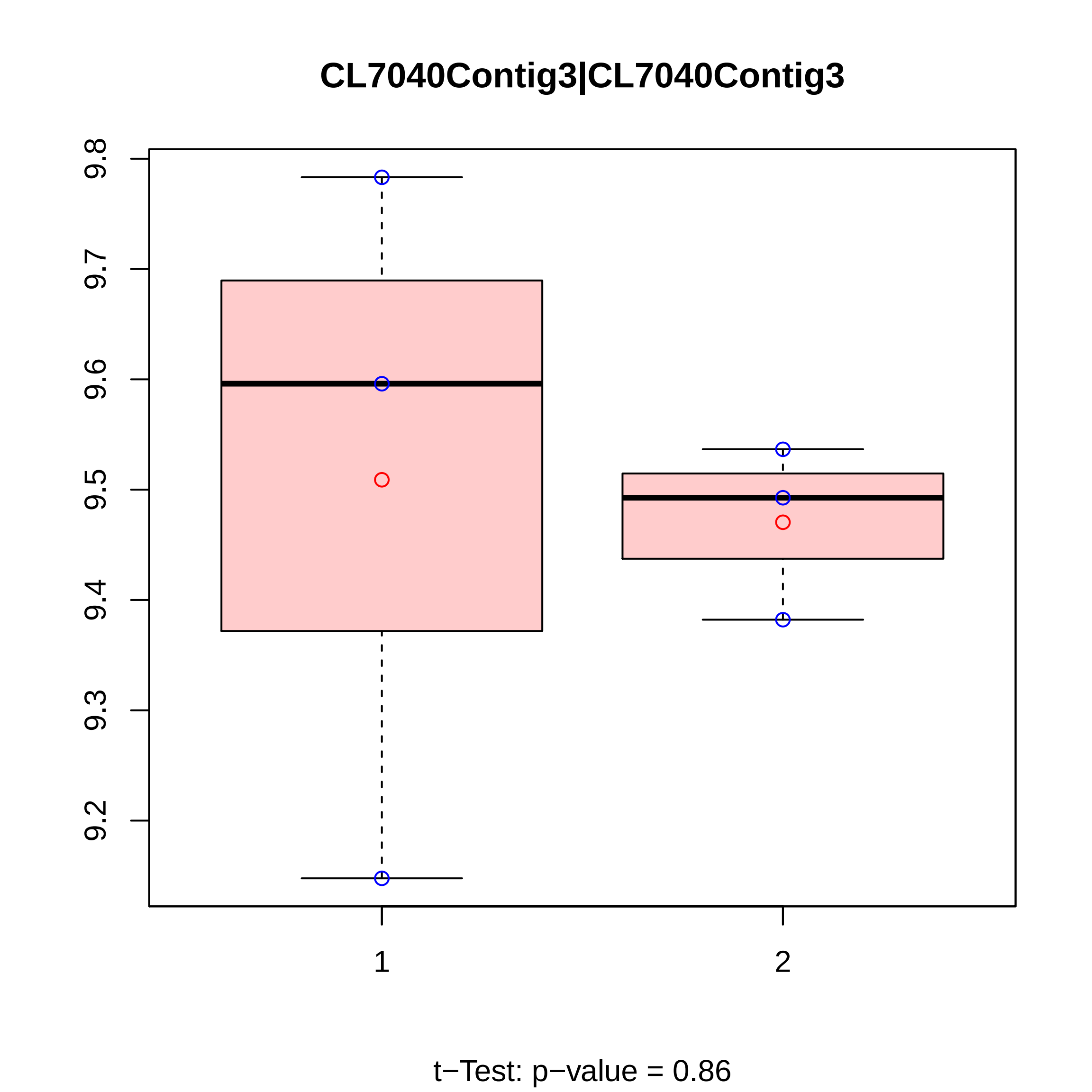
CL7040Contig3|CL7040Contig3
9.2
9.3
9.4
9.5
9.6
9.7
9.8
1
2
t−Test: p−value = 0.86

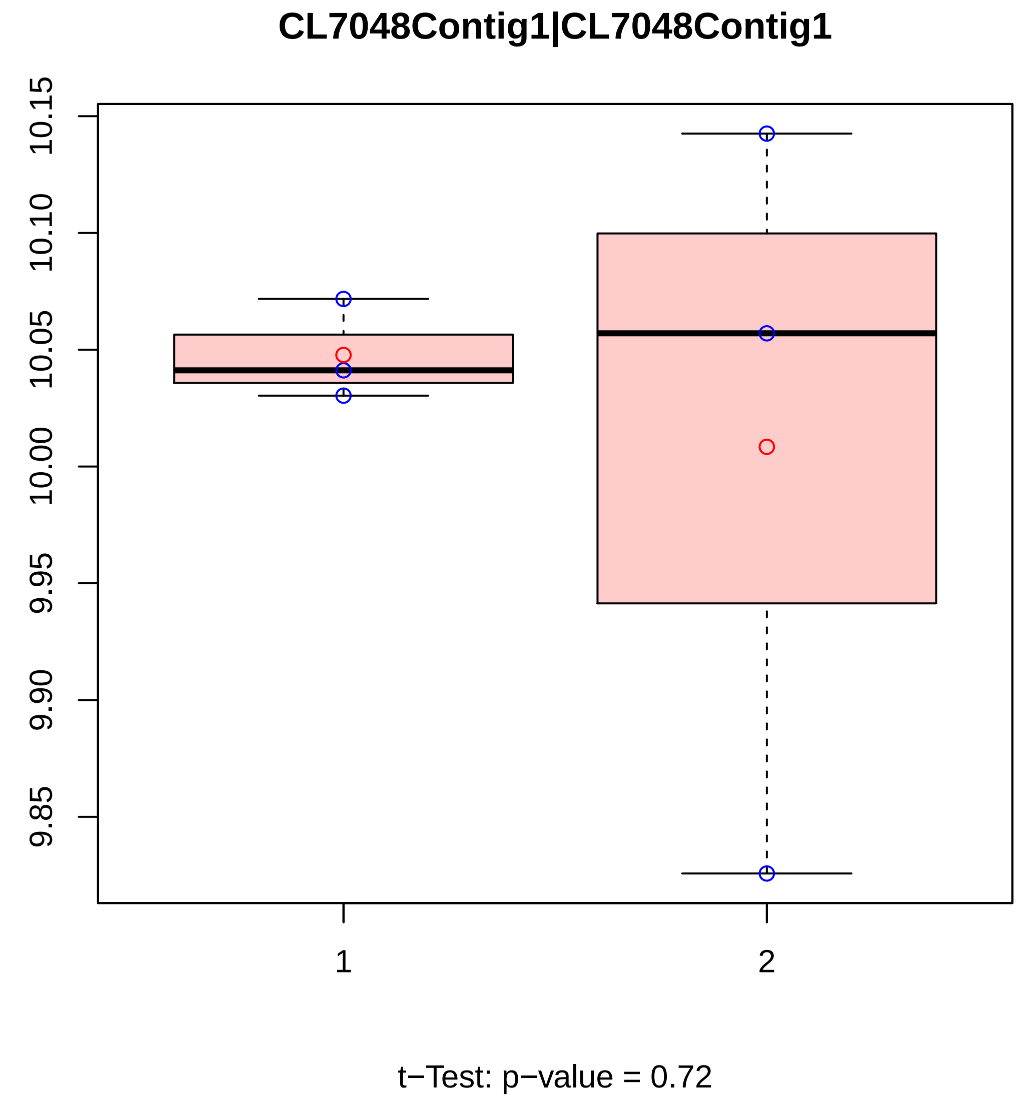

CL7048Contig1|CL7048Contig1
10.15
10.10
10.05
10.00
9.95
9.90
9.85
1
2
t−Test: p−value = 0.72

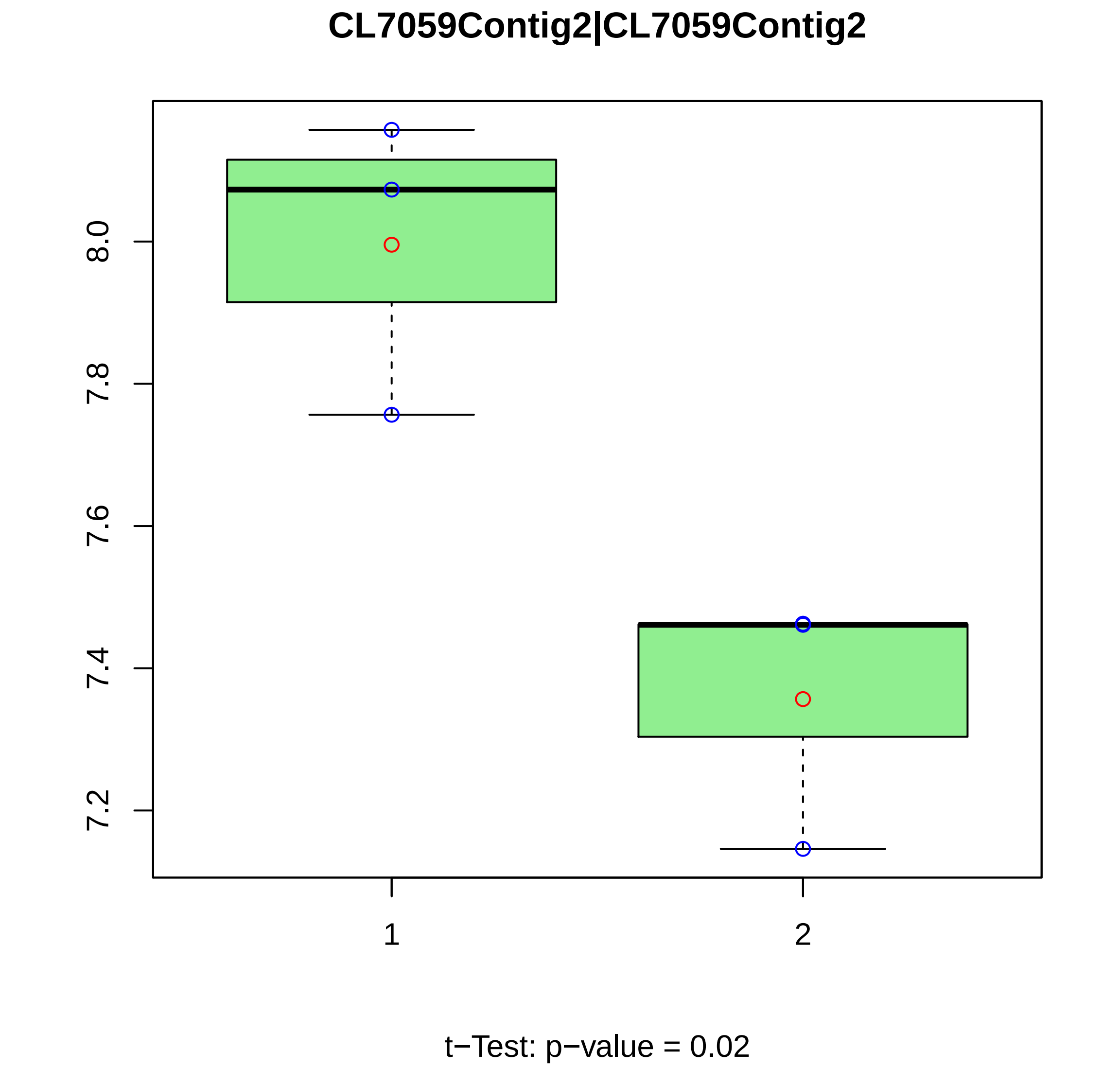

CL7059Contig2|CL7059Contig2
8.0
7.8
7.6
7.4
7.2
1
2
t−Test: p−value = 0.02

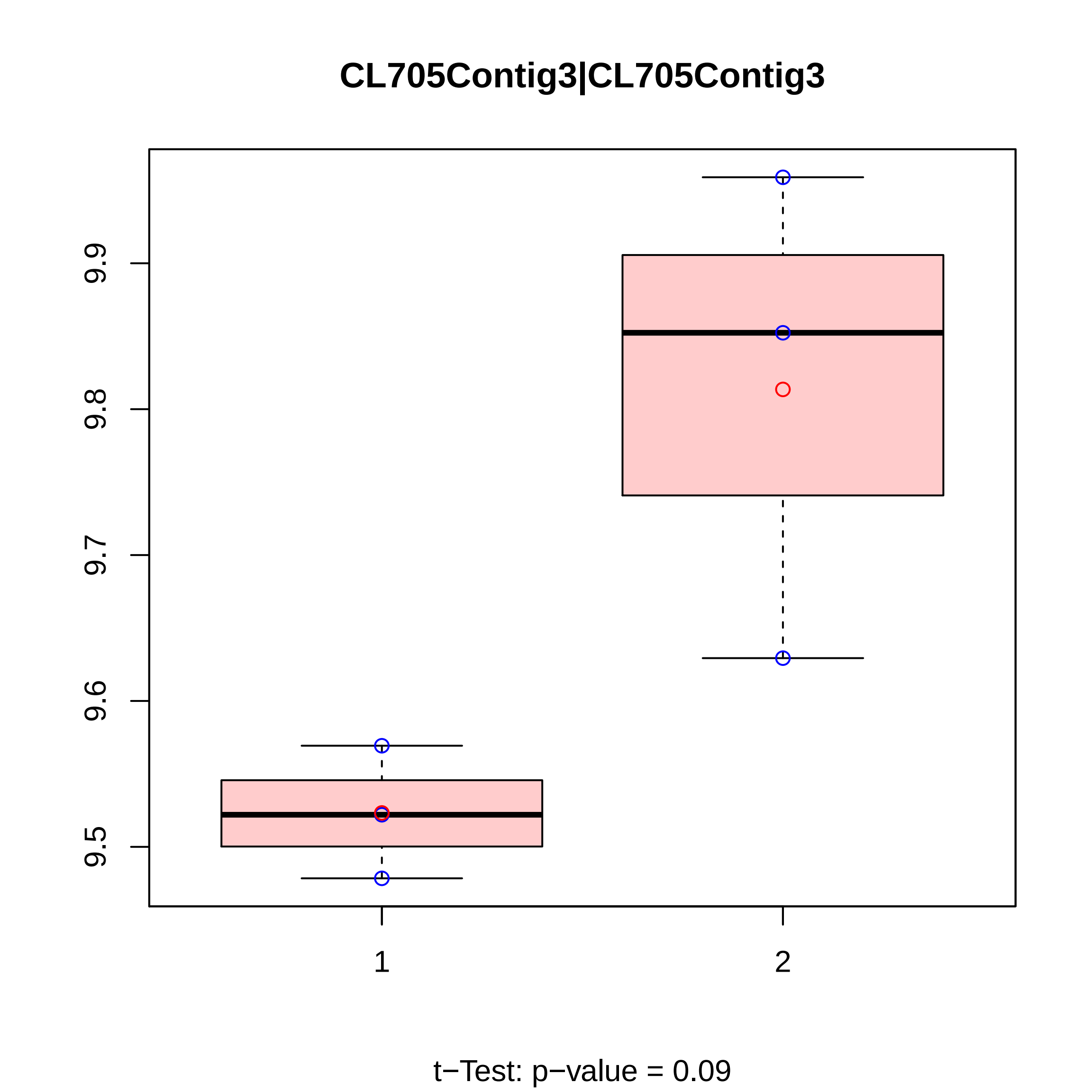
CL705Contig3|CL705Contig3
9.5
9.6
9.7
9.8
9.9
1
2
t−Test: p−value = 0.09

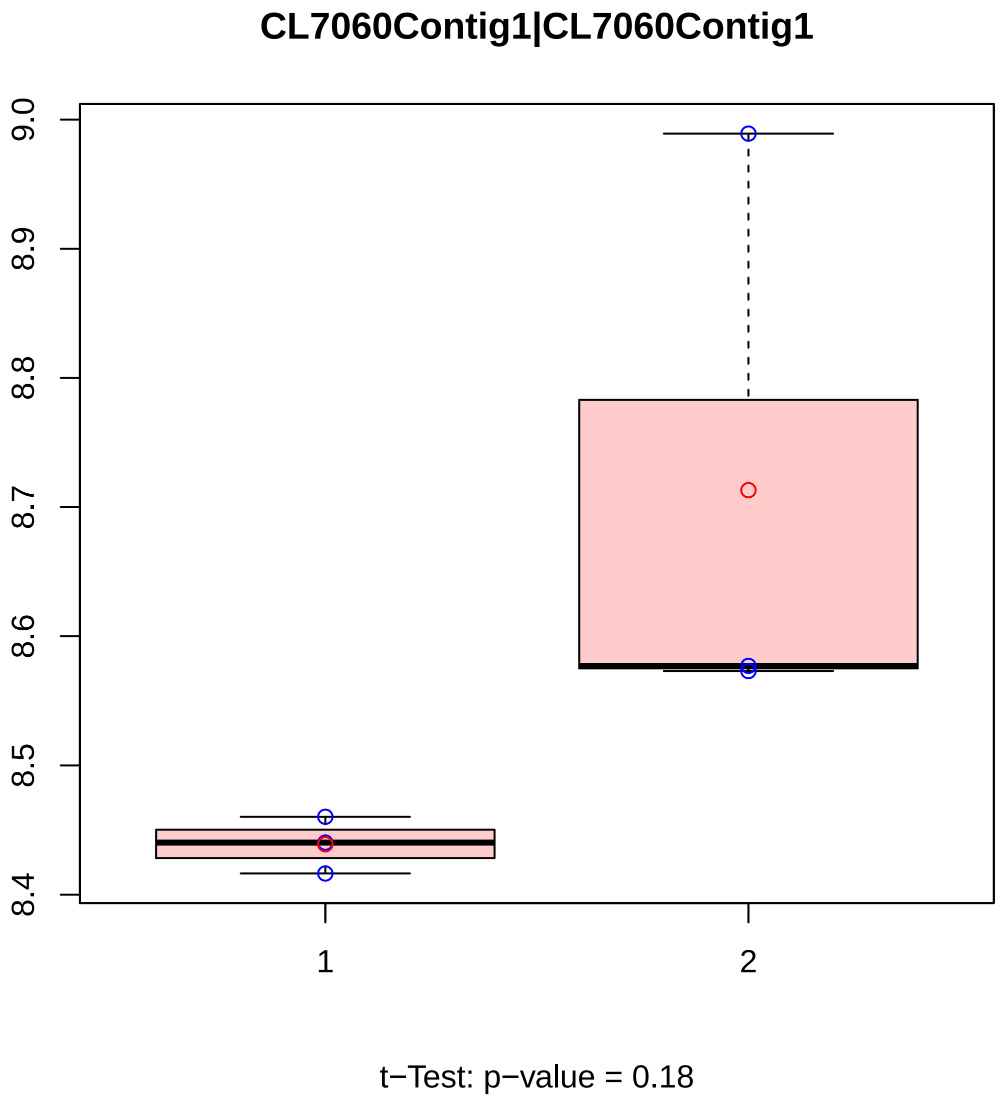

CL7060Contig1|CL7060Contig1
9.0
8.9
8.8
8.7
8.6
8.5
8.4
1
2
t−Test: p−value = 0.18

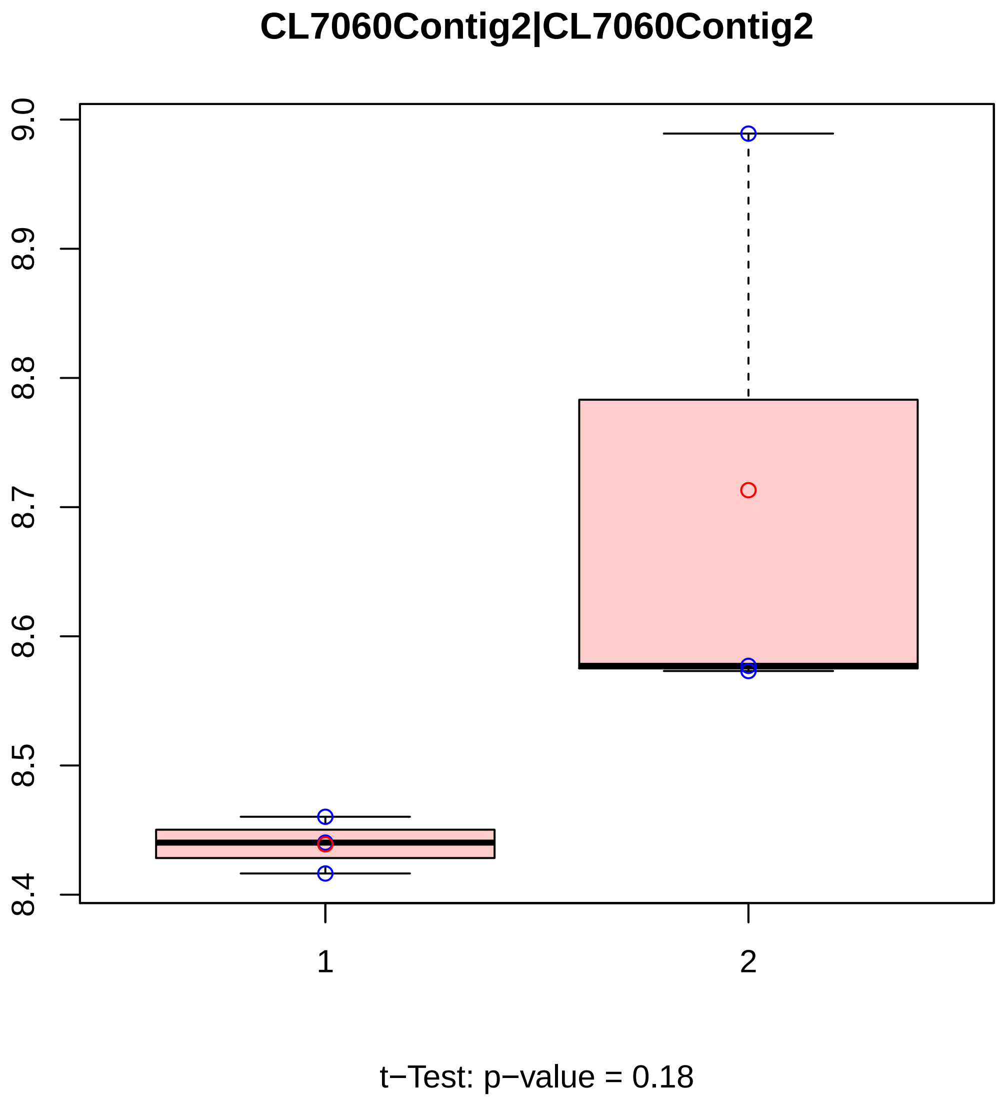

CL7060Contig2|CL7060Contig2
9.0
8.9
8.8
8.7
8.6
8.5
8.4
1
2
t−Test: p−value = 0.18

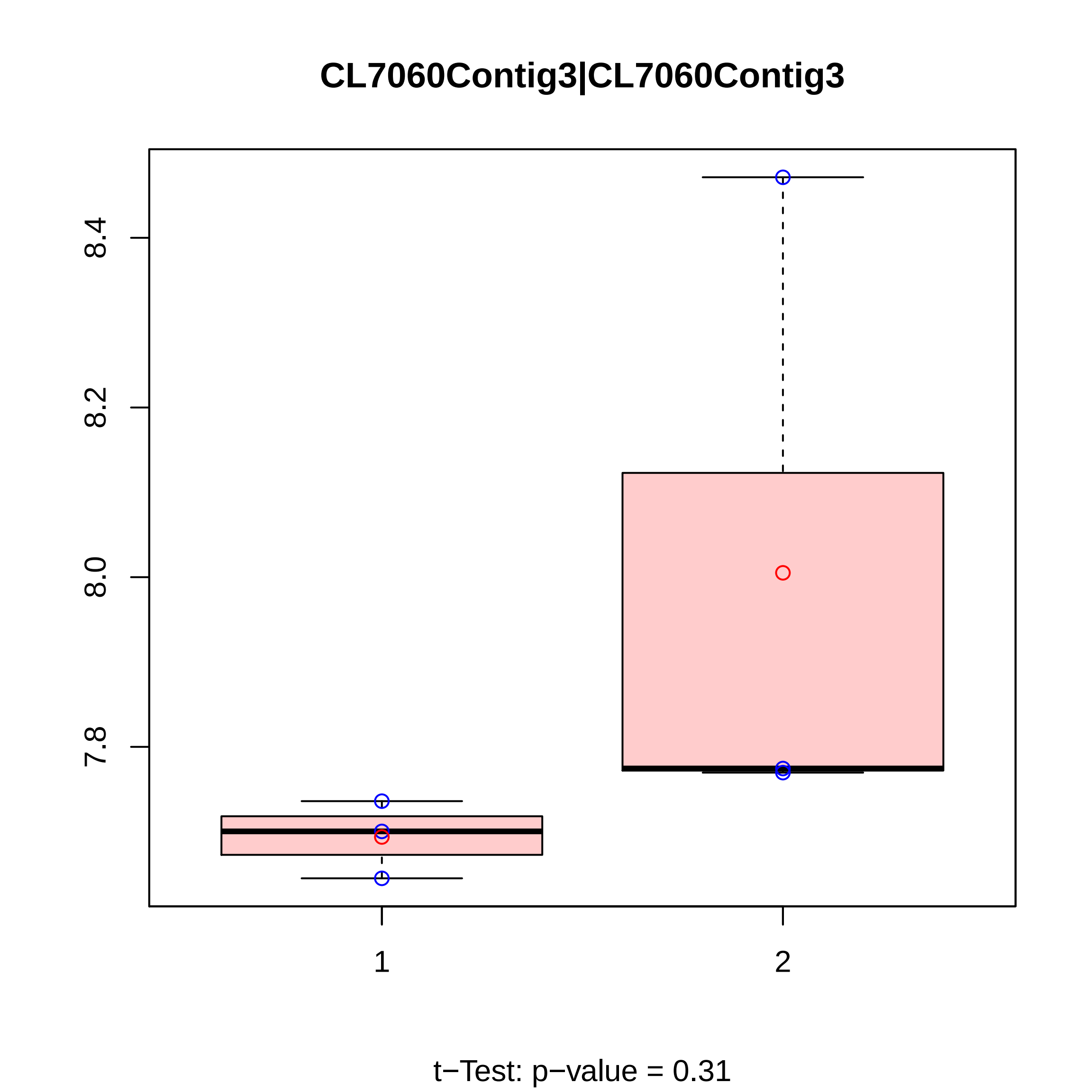
CL7060Contig3|CL7060Contig3
8.4
8.2
8.0
7.8
1
2
t−Test: p−value = 0.31

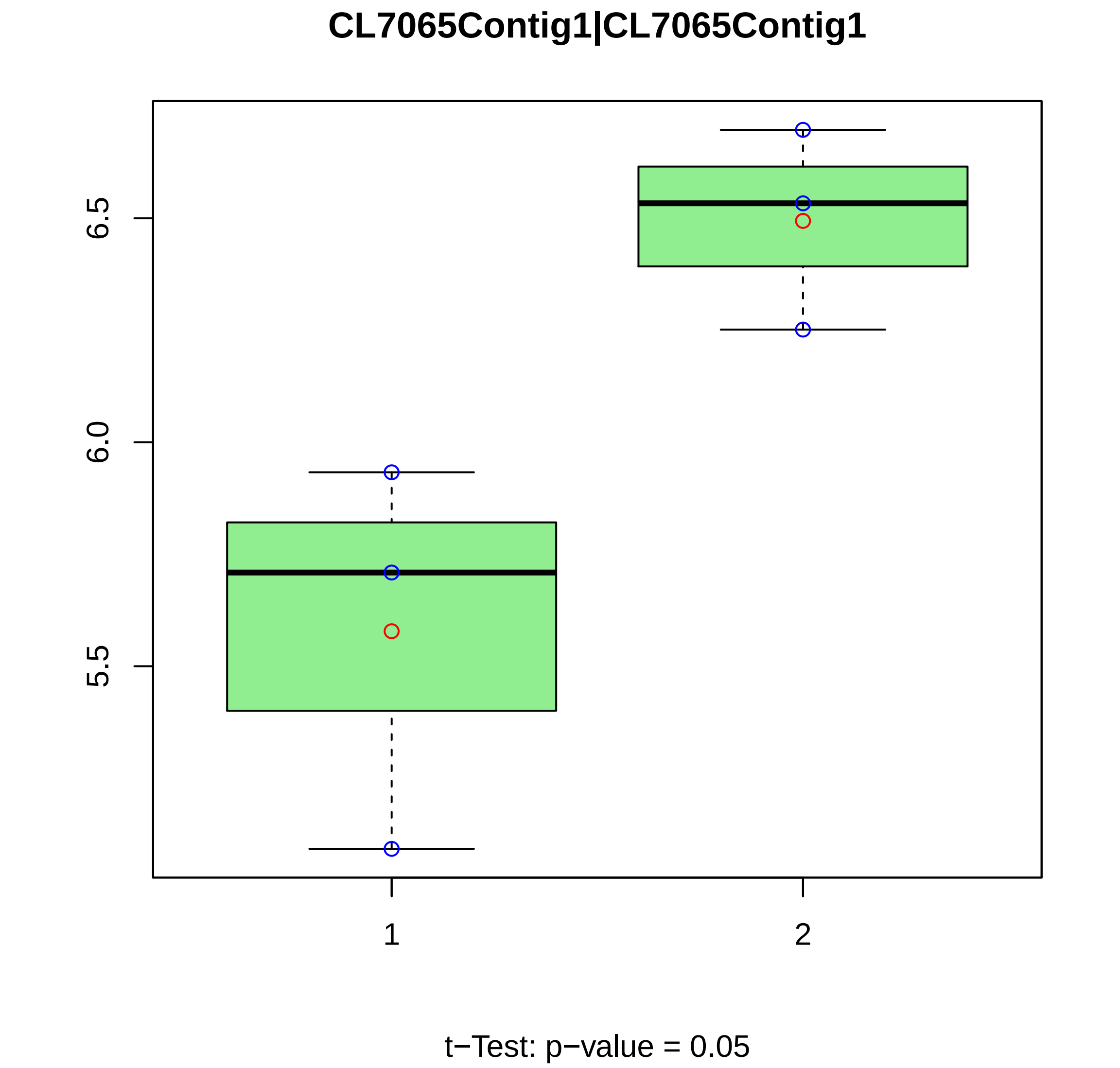
CL7065Contig1|CL7065Contig1
6.5
6.0
5.5
1
2
t−Test: p−value = 0.05

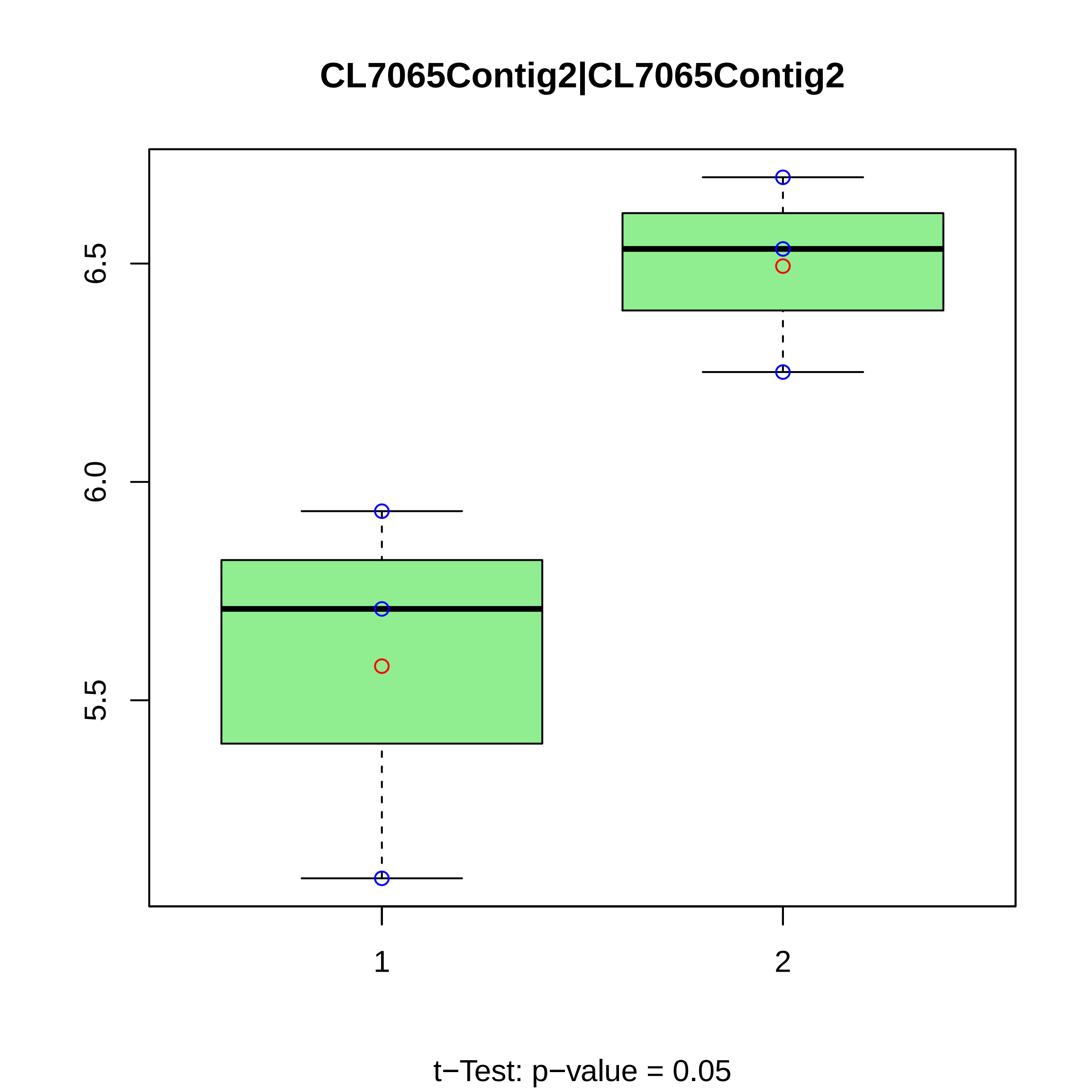

CL7065Contig2|CL7065Contig2
6.5
6.0
5.5
1
2
t−Test: p−value = 0.05

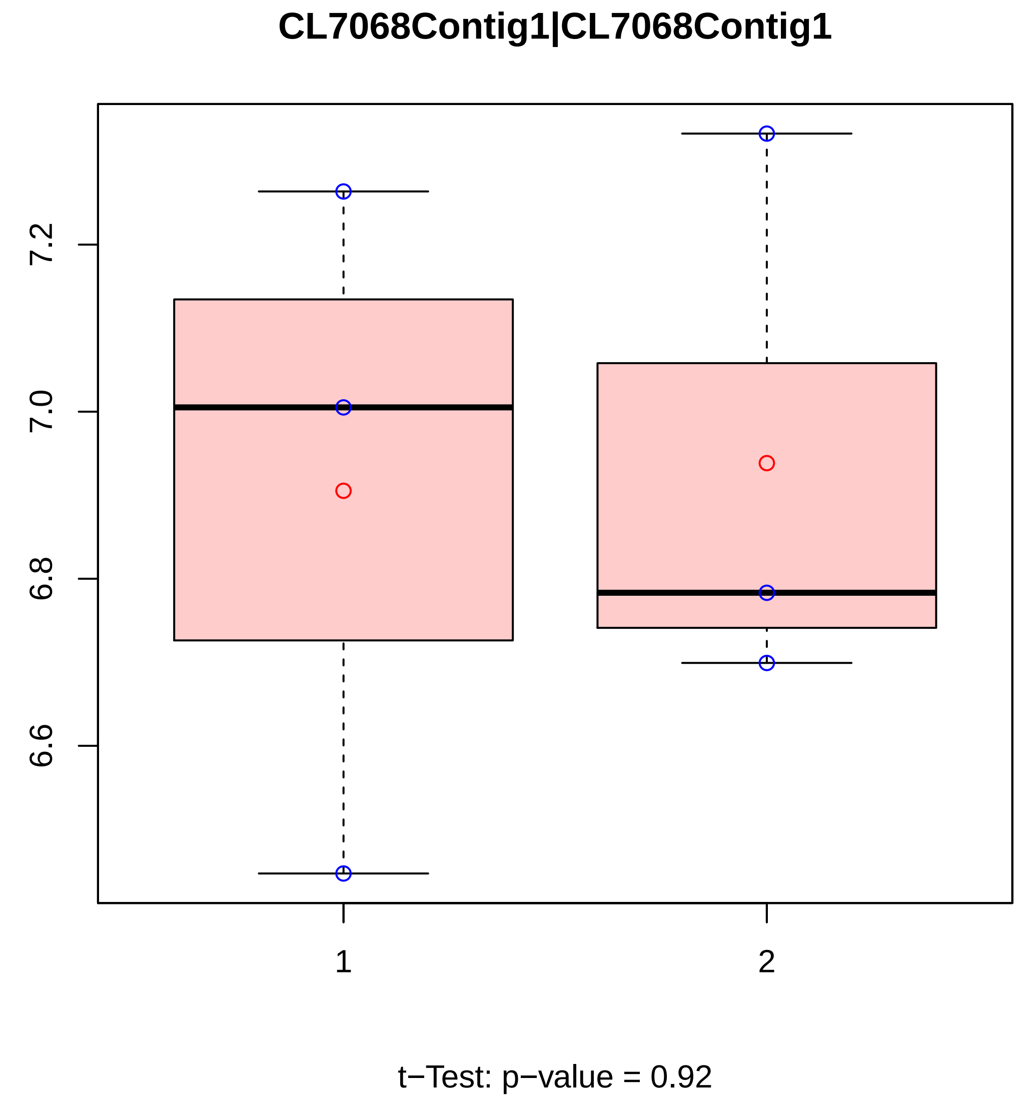

CL7068Contig1|CL7068Contig1
7.2
7.0
6.8
6.6
1
2
t−Test: p−value = 0.92

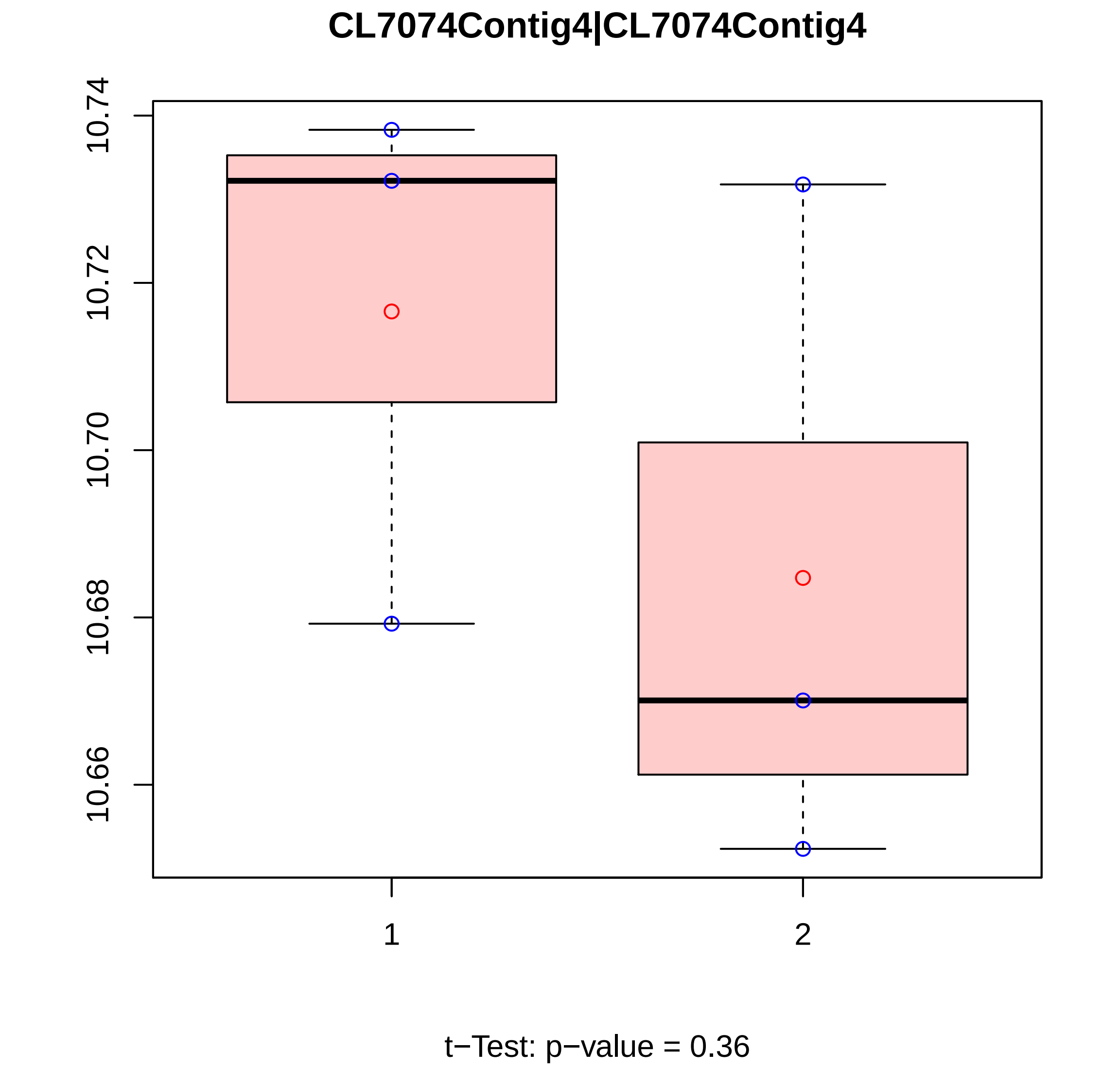

CL7074Contig4|CL7074Contig4
10.74
10.72
10.70
10.68
10.66
1
2
t−Test: p−value = 0.36

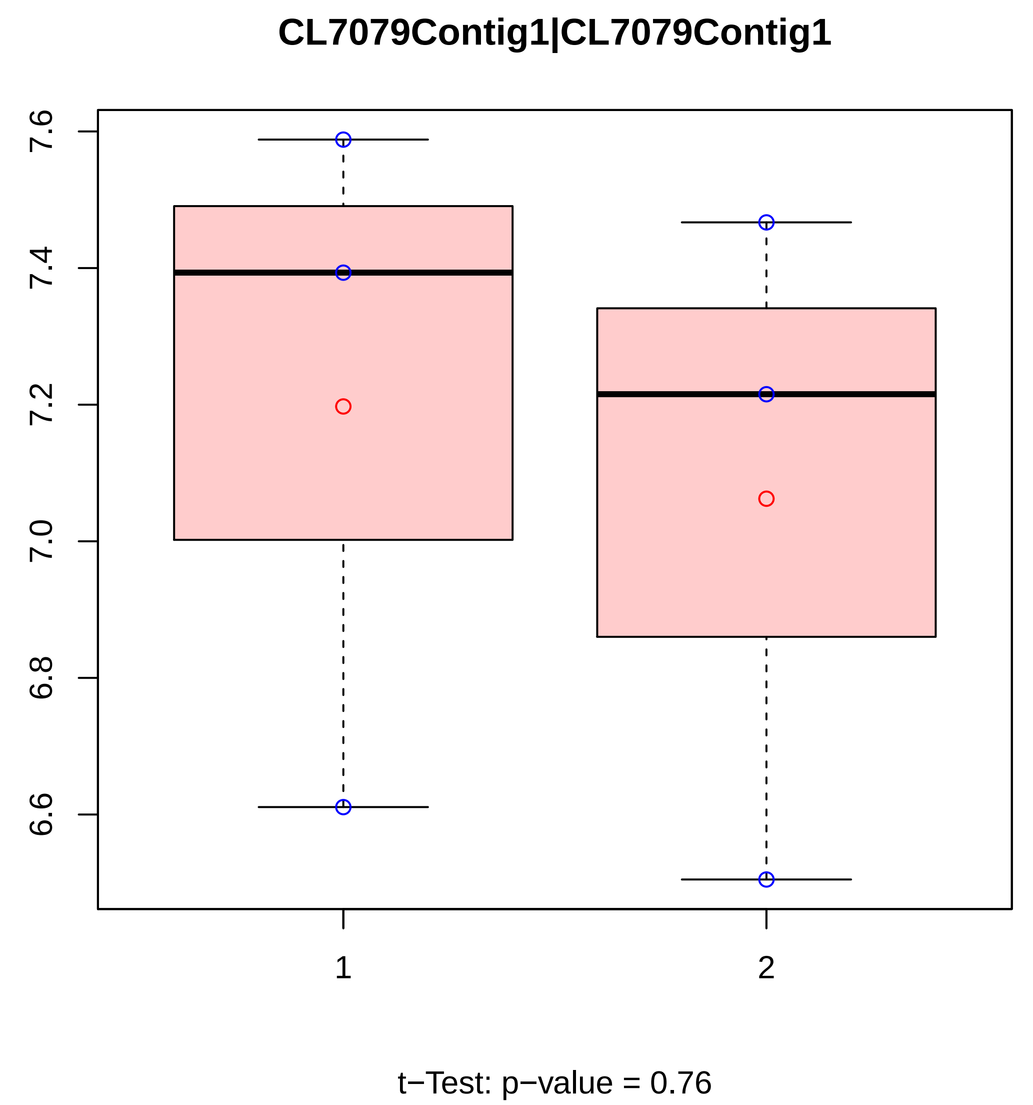
CL7079Contig1|CL7079Contig1
7.6
7.4
7.2
7.0
6.8
6.6
1
2
t−Test: p−value = 0.76

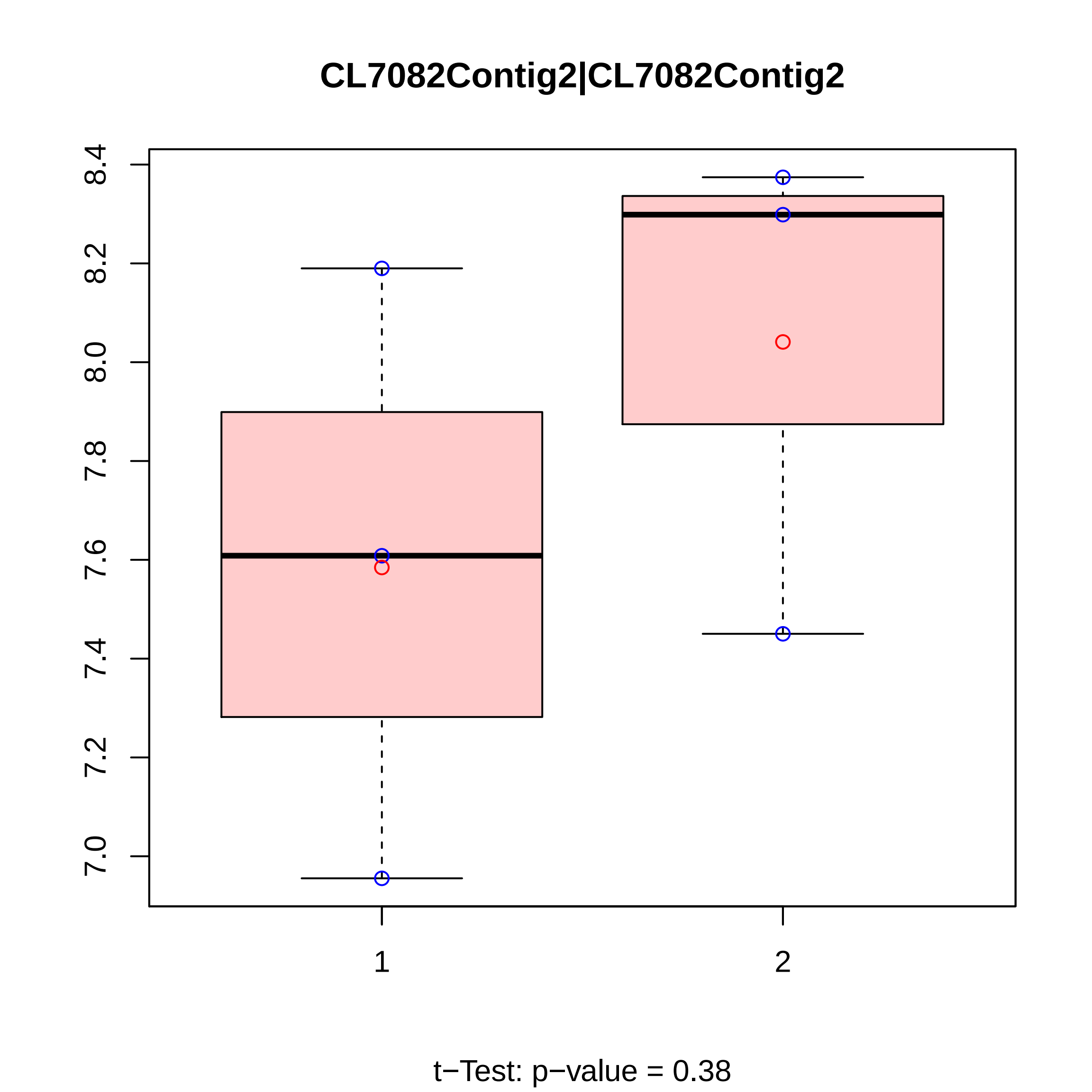

CL7082Contig2|CL7082Contig2
8.4
8.2
8.0
7.8
7.6
7.4
7.2
7.0
1
2
t−Test: p−value = 0.38

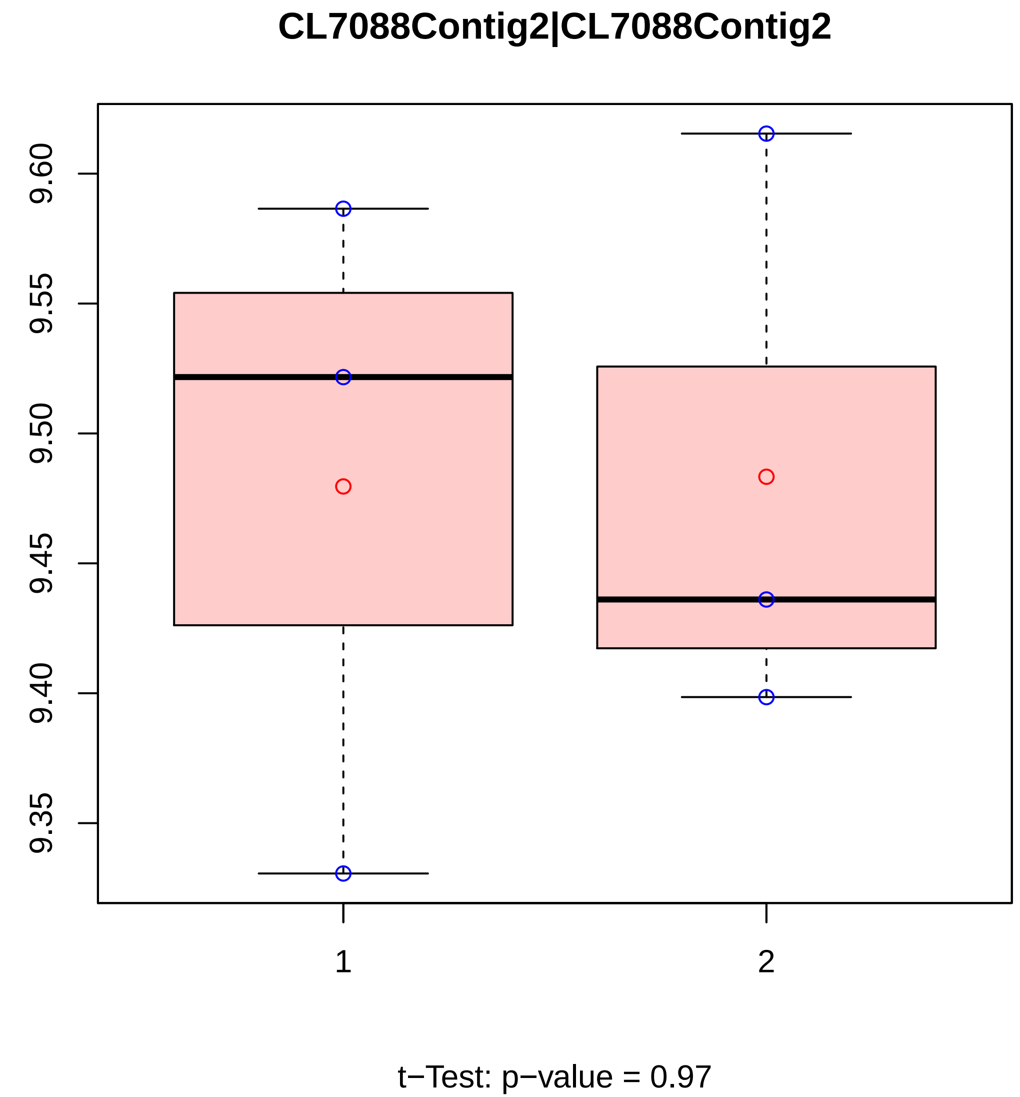
CL7088Contig2|CL7088Contig2
9.35
9.40
9.45
9.50
9.55
9.60
1
2
t−Test: p−value = 0.97

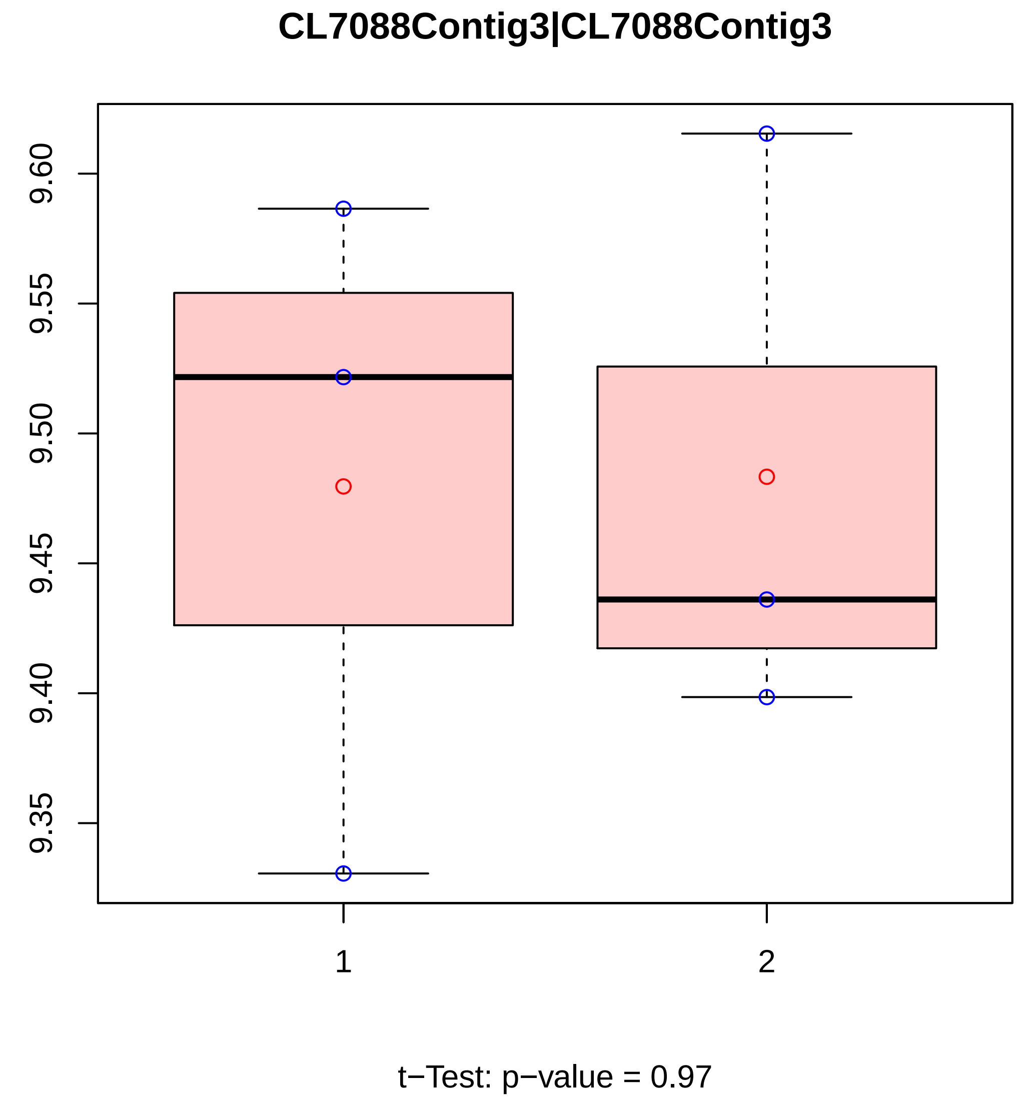
CL7088Contig3|CL7088Contig3
9.35
9.40
9.45
9.50
9.55
9.60
1
2
t−Test: p−value = 0.97

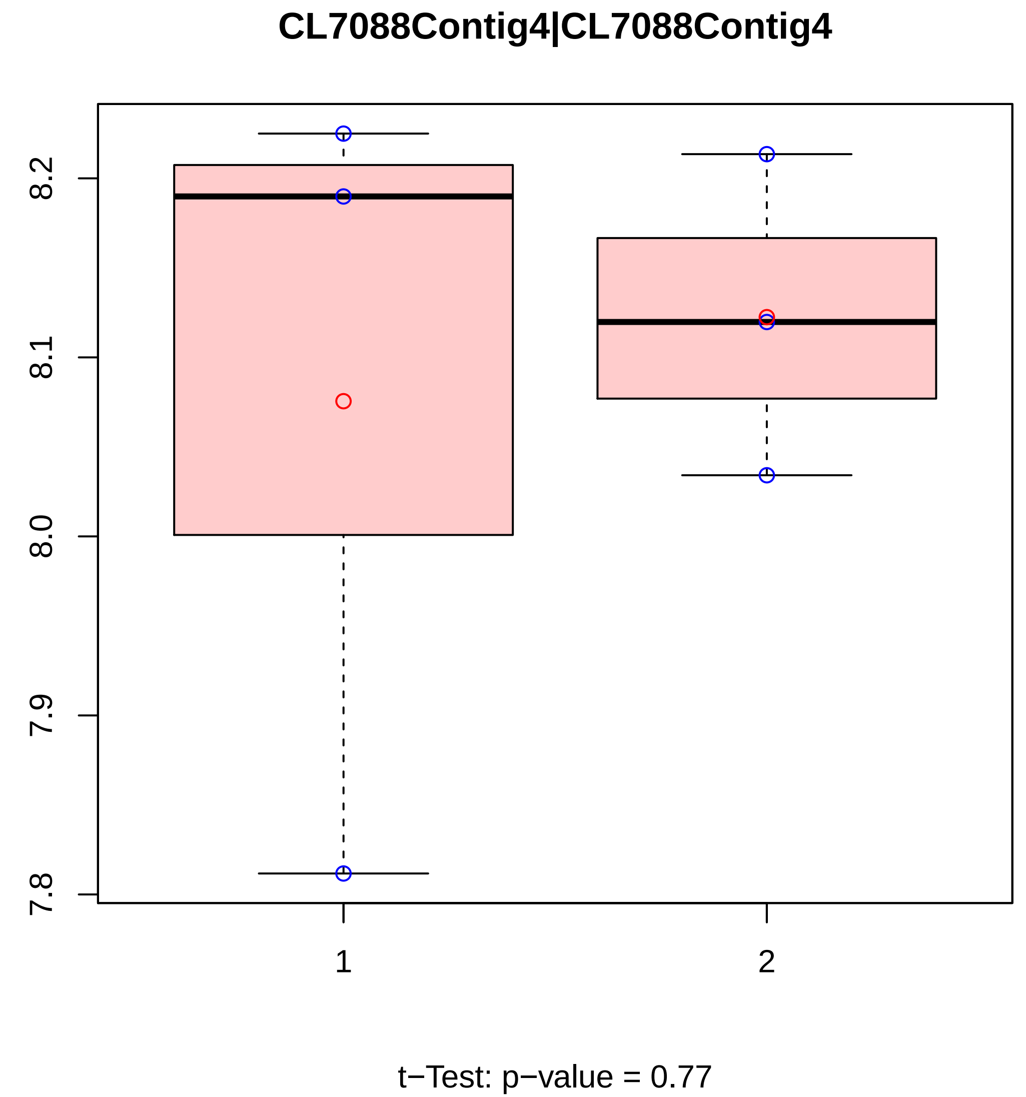
CL7088Contig4|CL7088Contig4
8.2
8.1
8.0
7.9
7.8
1
2
t−Test: p−value = 0.77

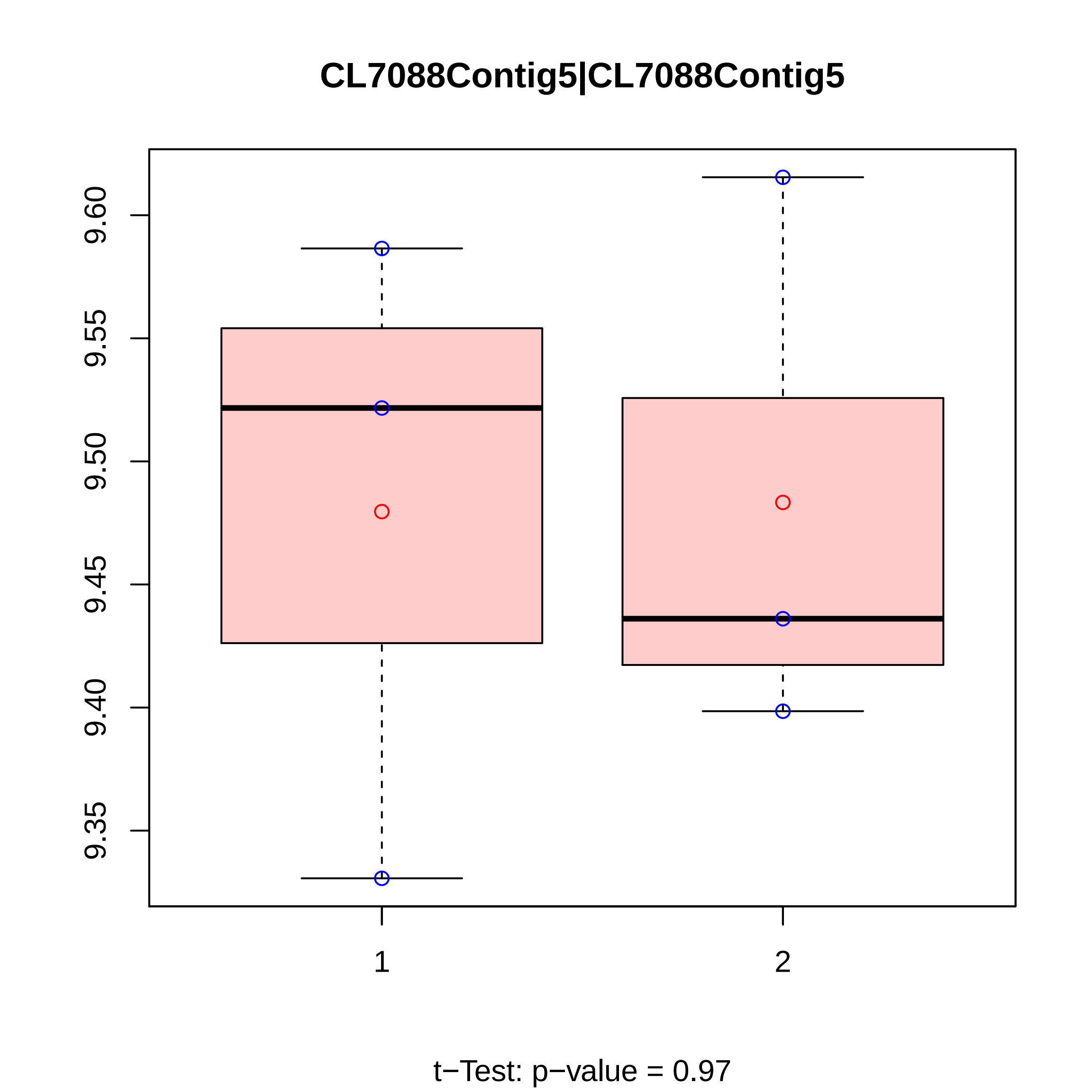

CL7088Contig5|CL7088Contig5
9.35
9.40
9.45
9.50
9.55
9.60
1
2
t−Test: p−value = 0.97

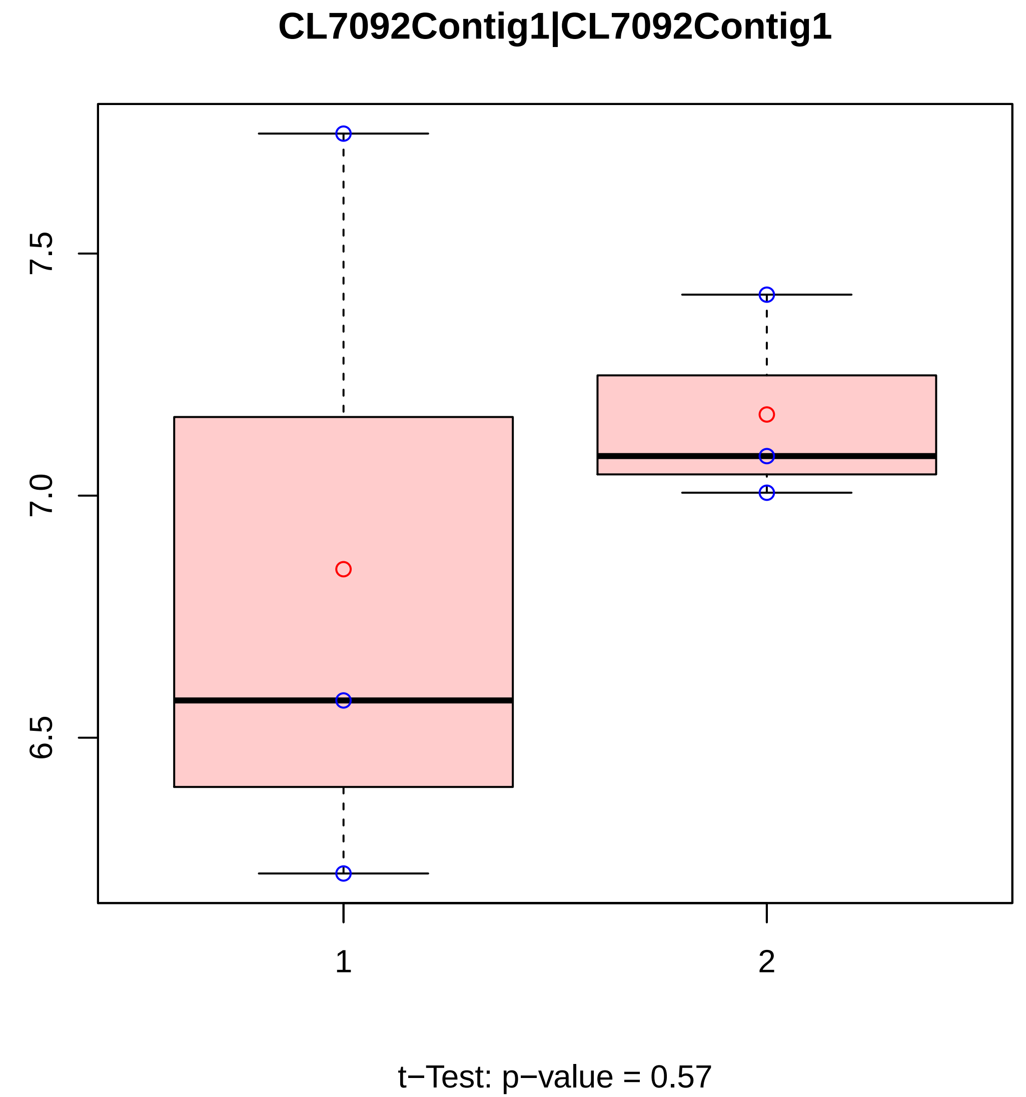
CL7092Contig1|CL7092Contig1
7.5
7.0
6.5
1
2
t−Test: p−value = 0.57

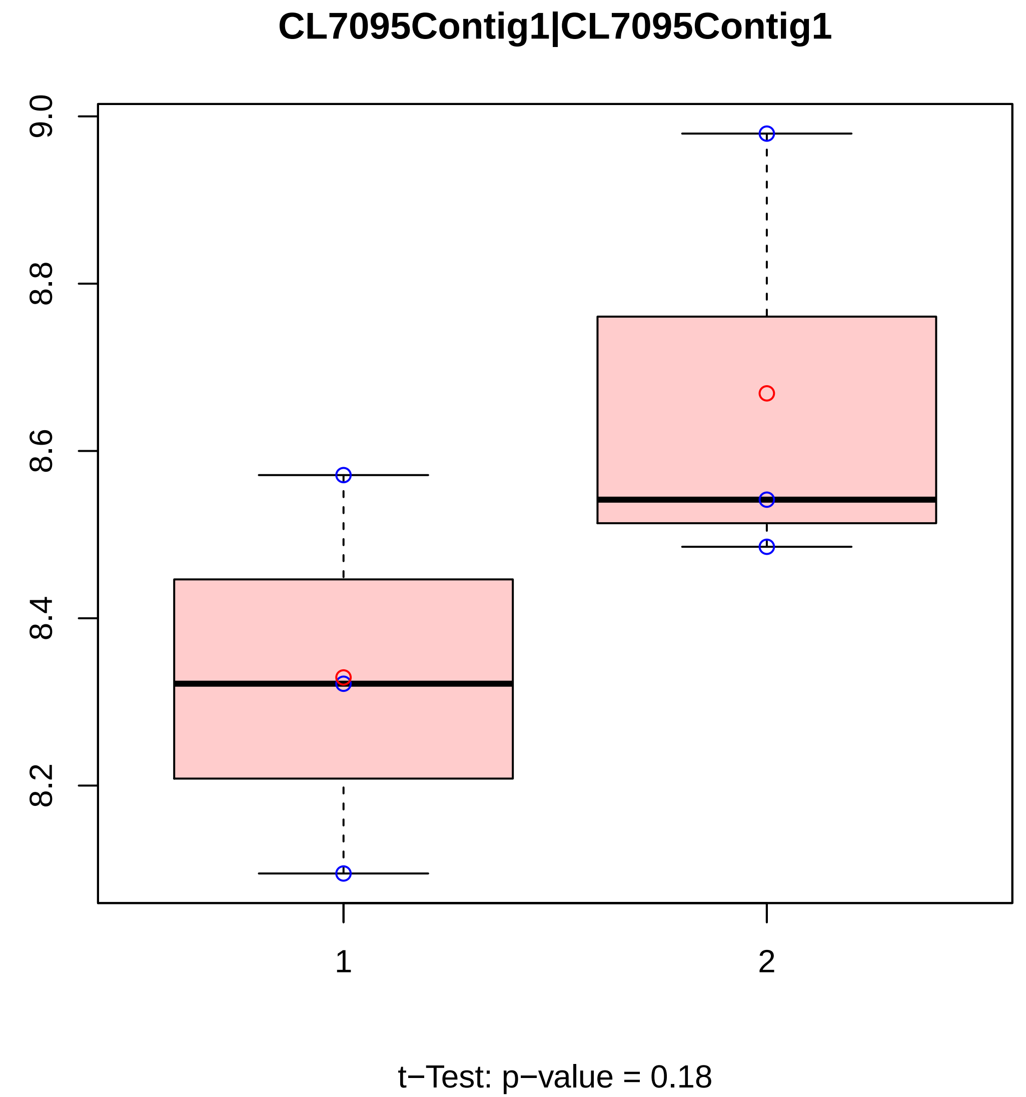

CL7095Contig1|CL7095Contig1
9.0
8.8
8.6
8.4
8.2
1
2
t−Test: p−value = 0.18

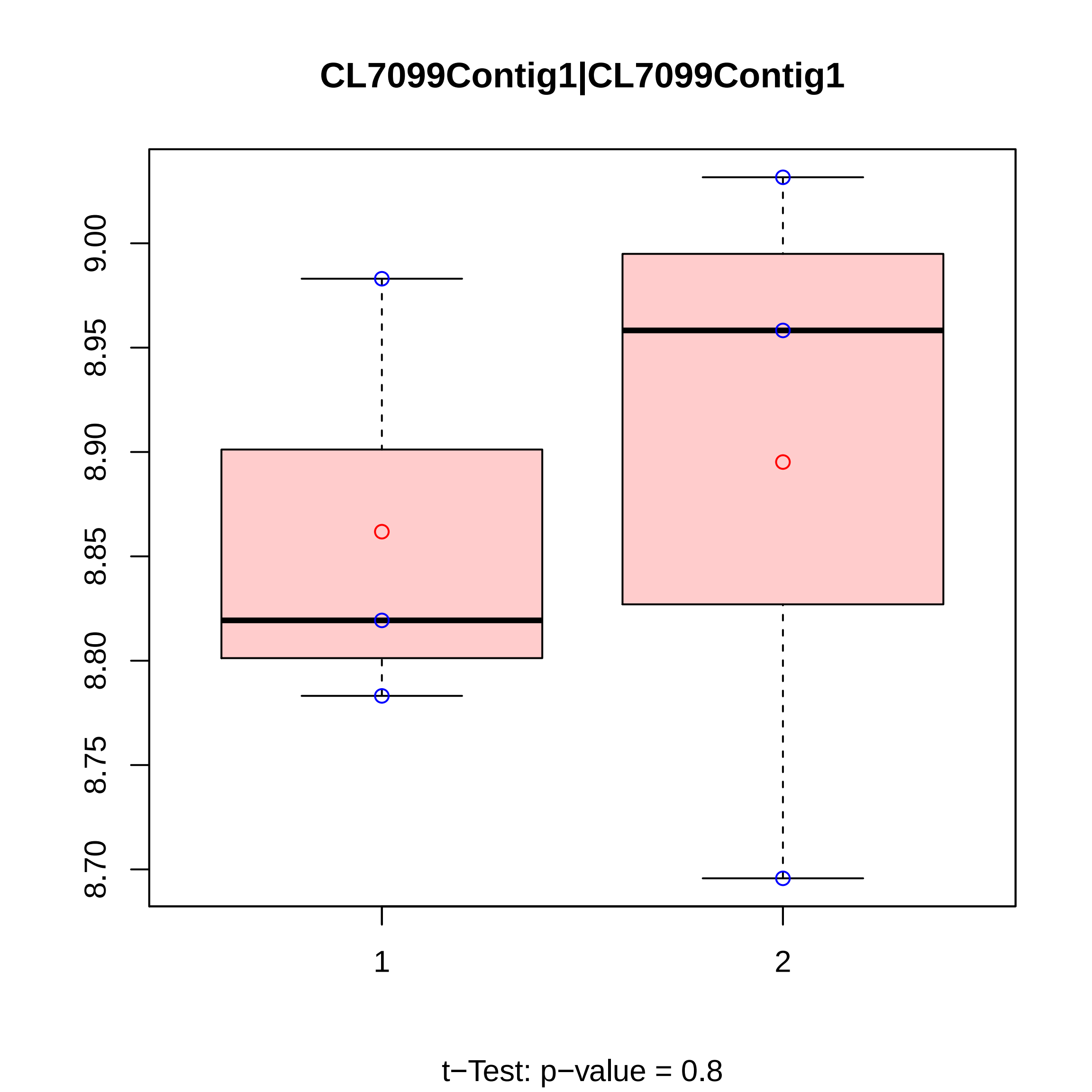
CL7099Contig1|CL7099Contig1
9.00
8.95
8.90
8.85
8.80
8.75
8.70
1
2
t−Test: p−value = 0.8

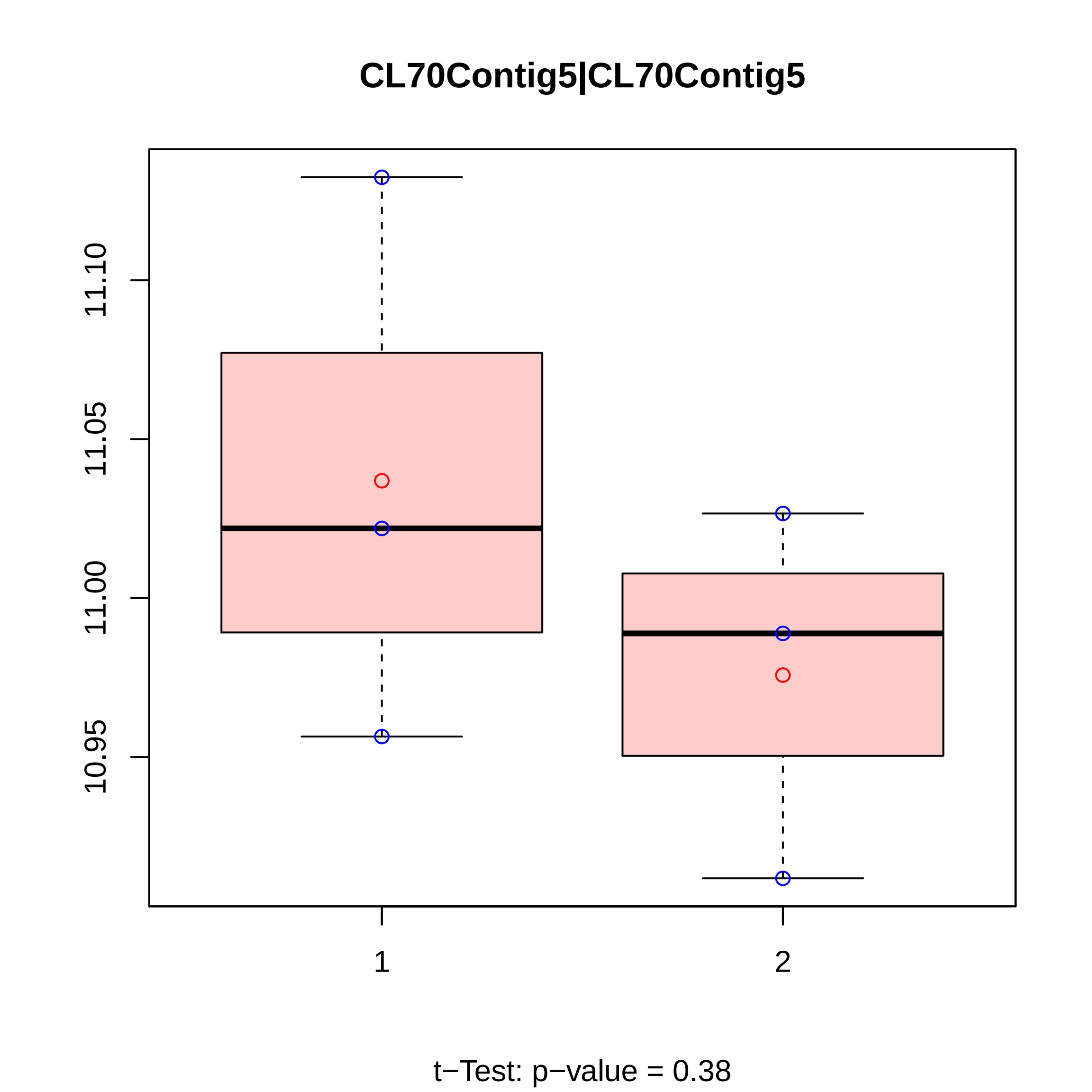

CL70Contig5|CL70Contig5
11.10
11.05
11.00
10.95
1
2
t−Test: p−value = 0.38

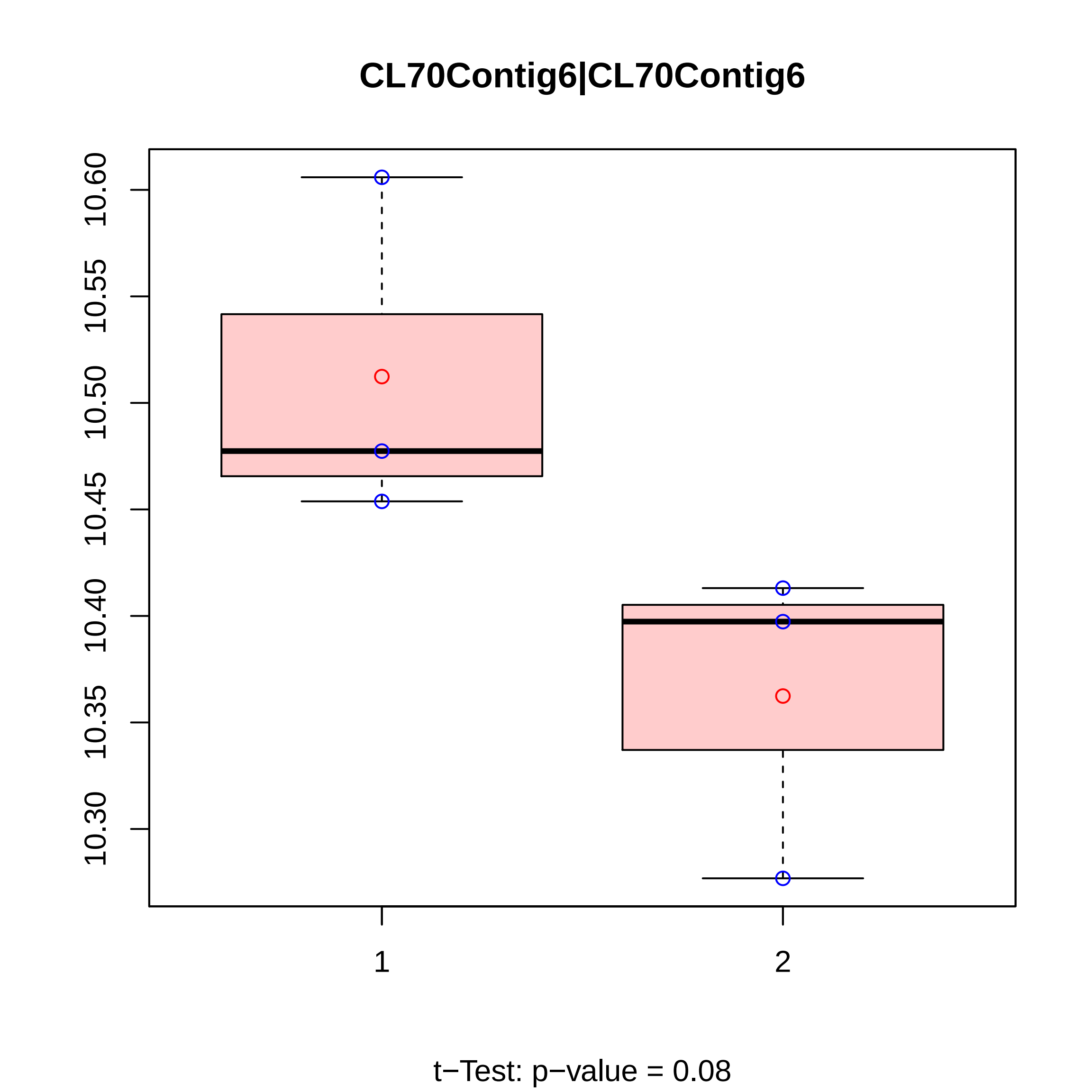
CL70Contig6|CL70Contig6
10.30
10.35
10.40
10.45
10.50
10.55
10.60
1
2
t−Test: p−value = 0.08

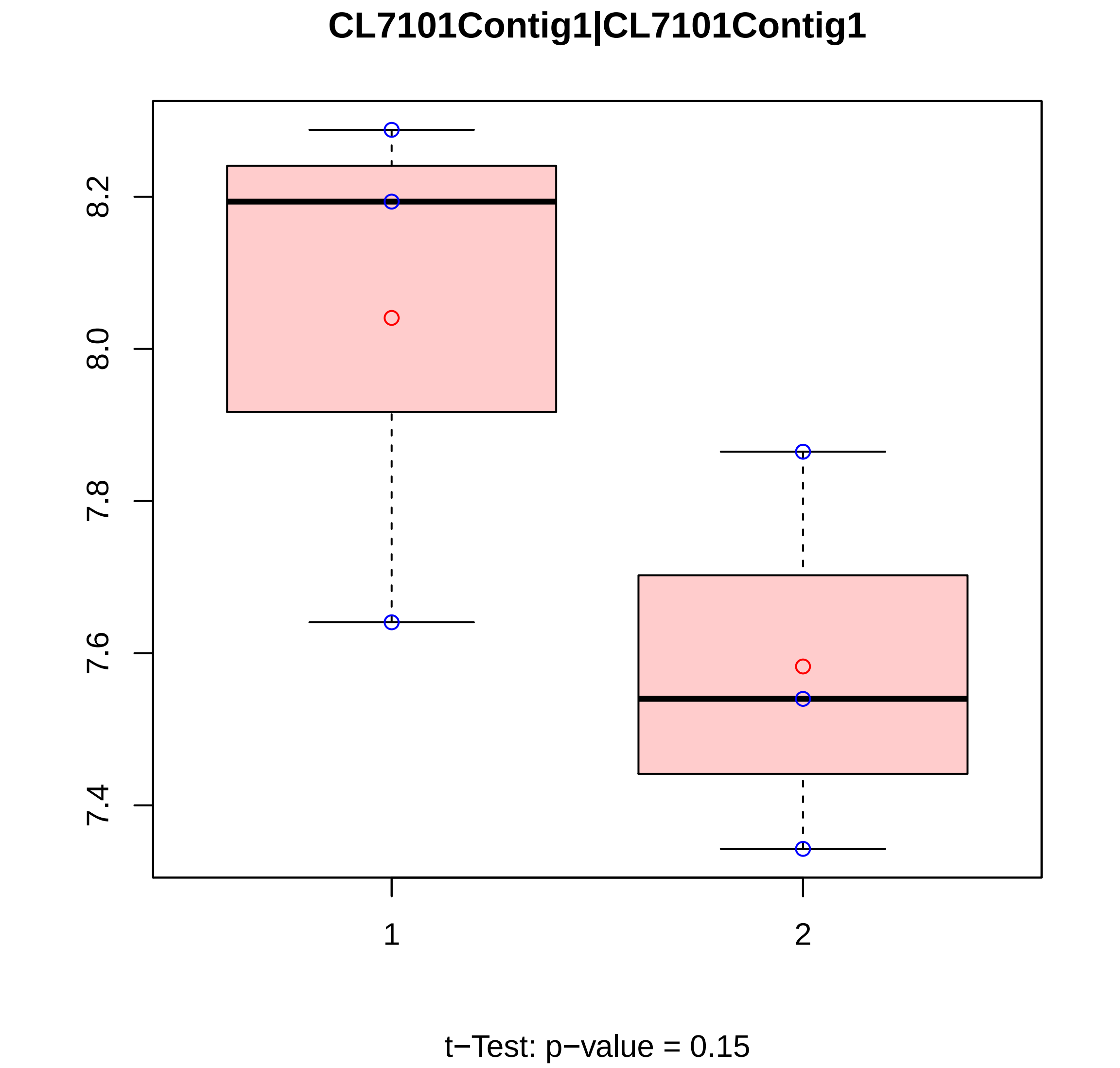

CL7101Contig1|CL7101Contig1
8.2
8.0
7.8
7.6
7.4
1
2
t−Test: p−value = 0.15

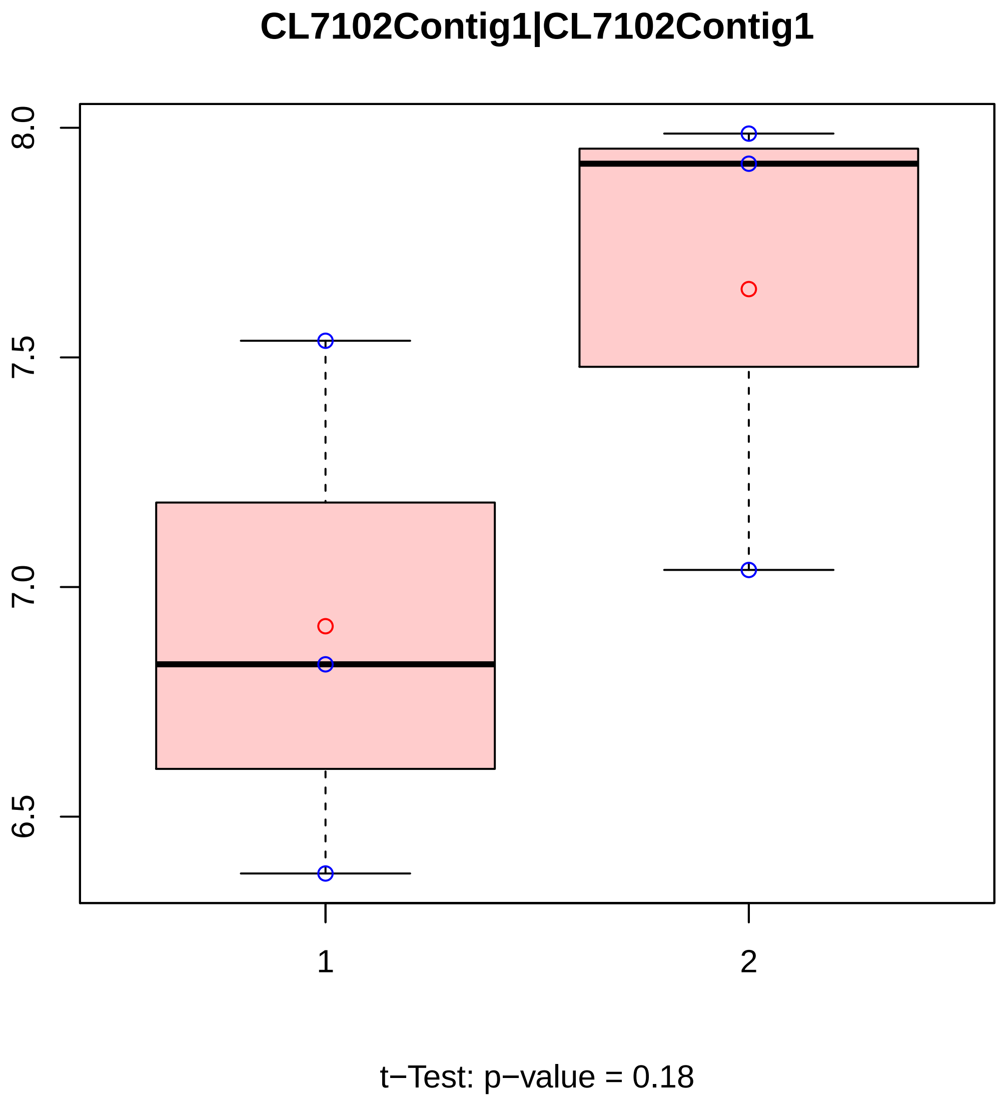
CL7102Contig1|CL7102Contig1
8.0
7.5
7.0
6.5
1
2
t−Test: p−value = 0.18

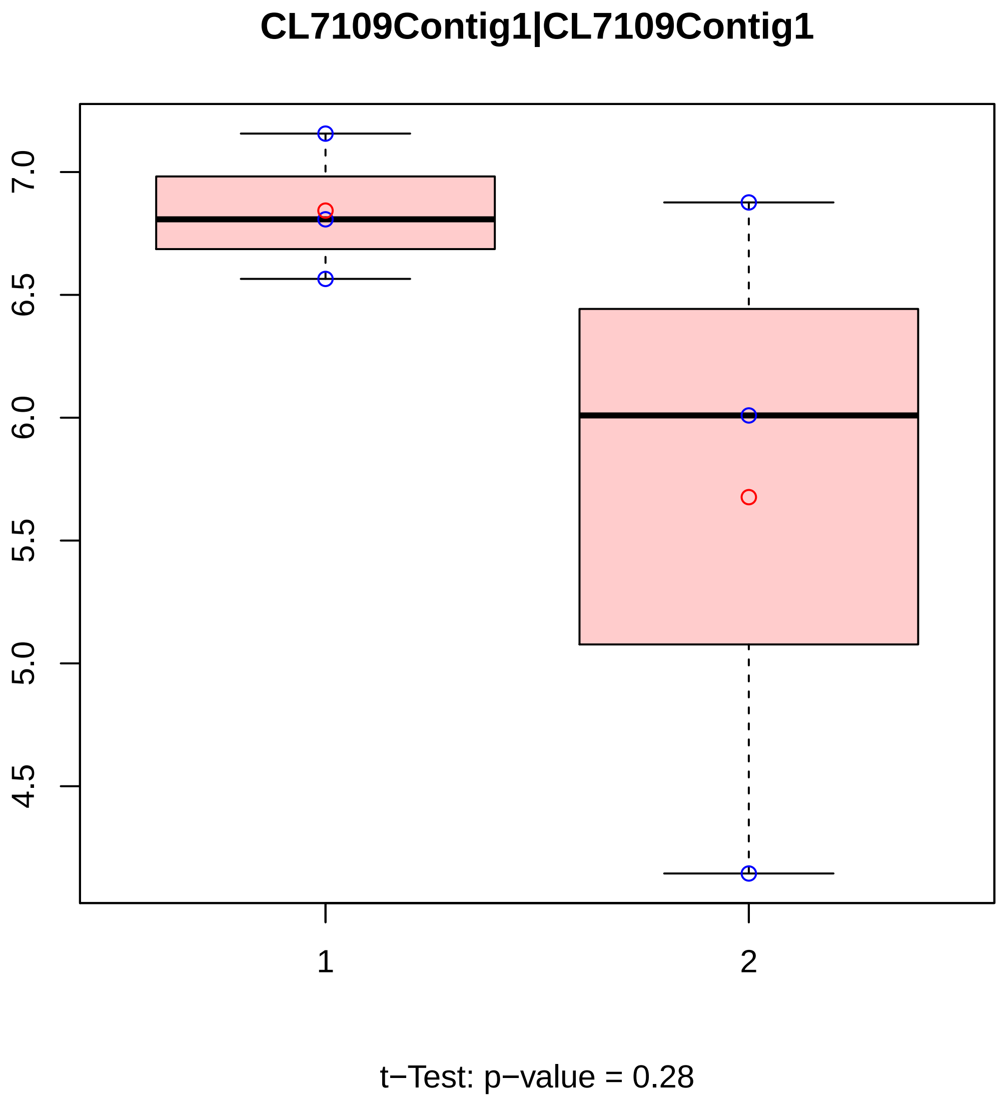

CL7109Contig1|CL7109Contig1
7.0
6.5
6.0
5.5
5.0
4.5
1
2
t−Test: p−value = 0.28

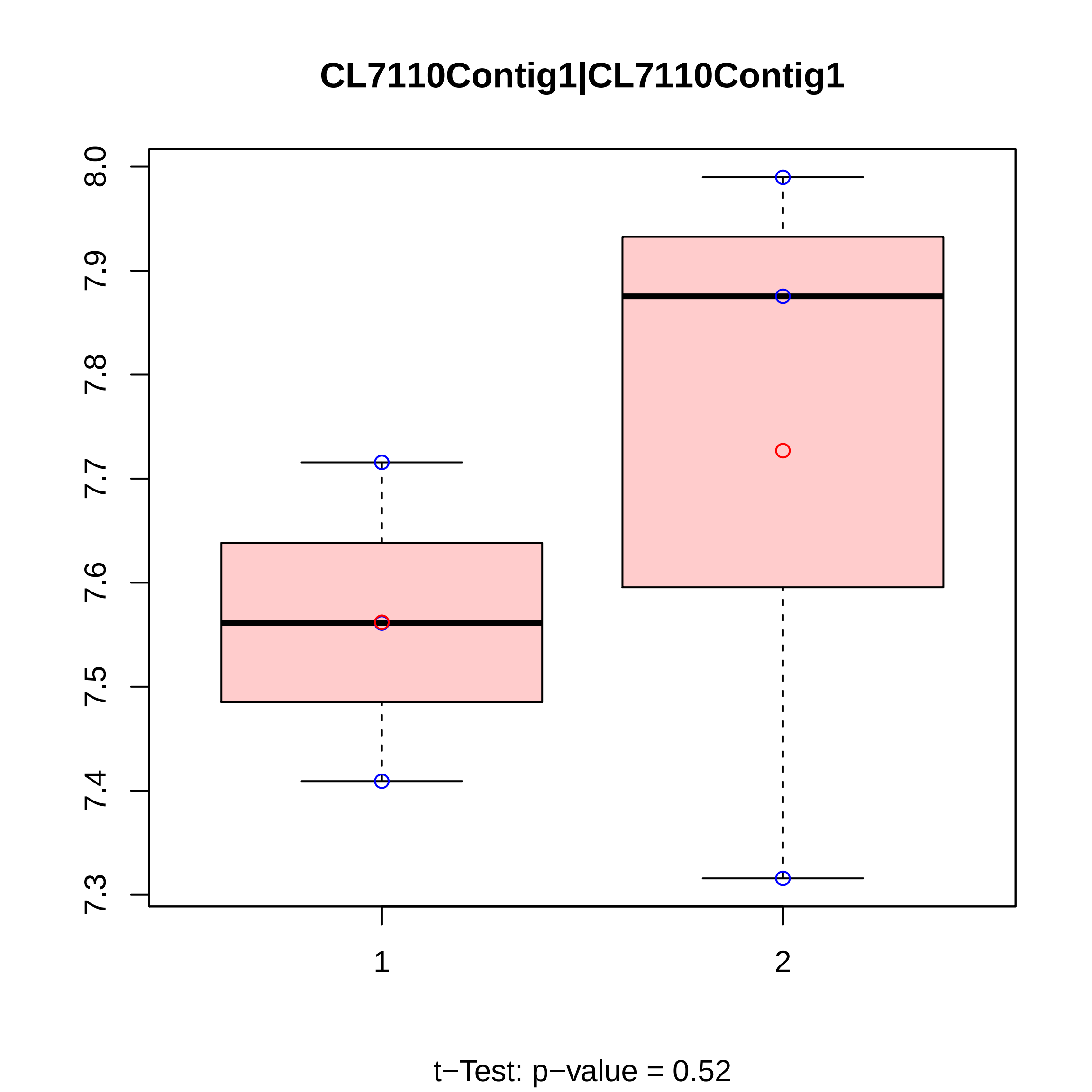
CL7110Contig1|CL7110Contig1
8.0
7.9
7.8
7.7
7.6
7.5
7.4
7.3
1
2
t−Test: p−value = 0.52

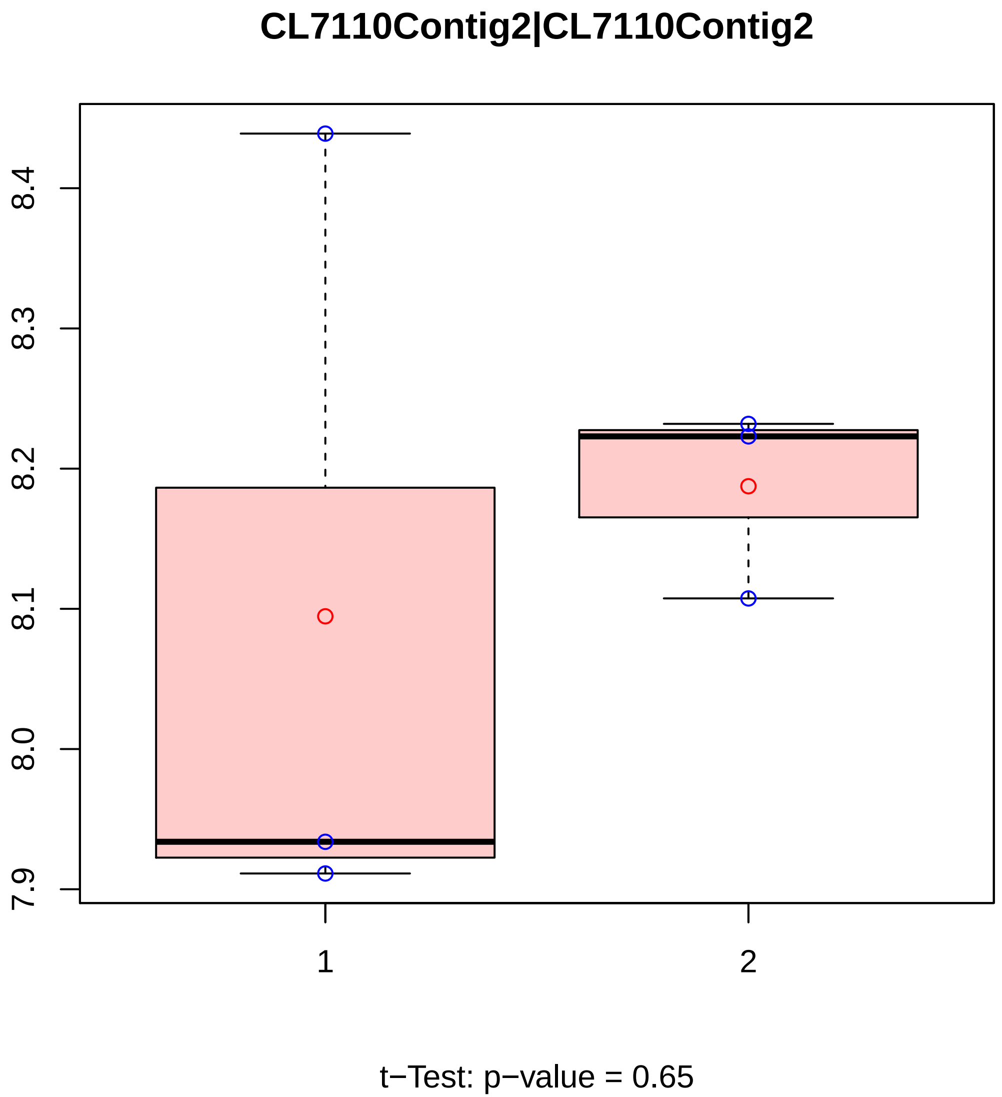

CL7110Contig2|CL7110Contig2
8.4
8.3
8.2
8.1
8.0
7.9
1
2
t−Test: p−value = 0.65

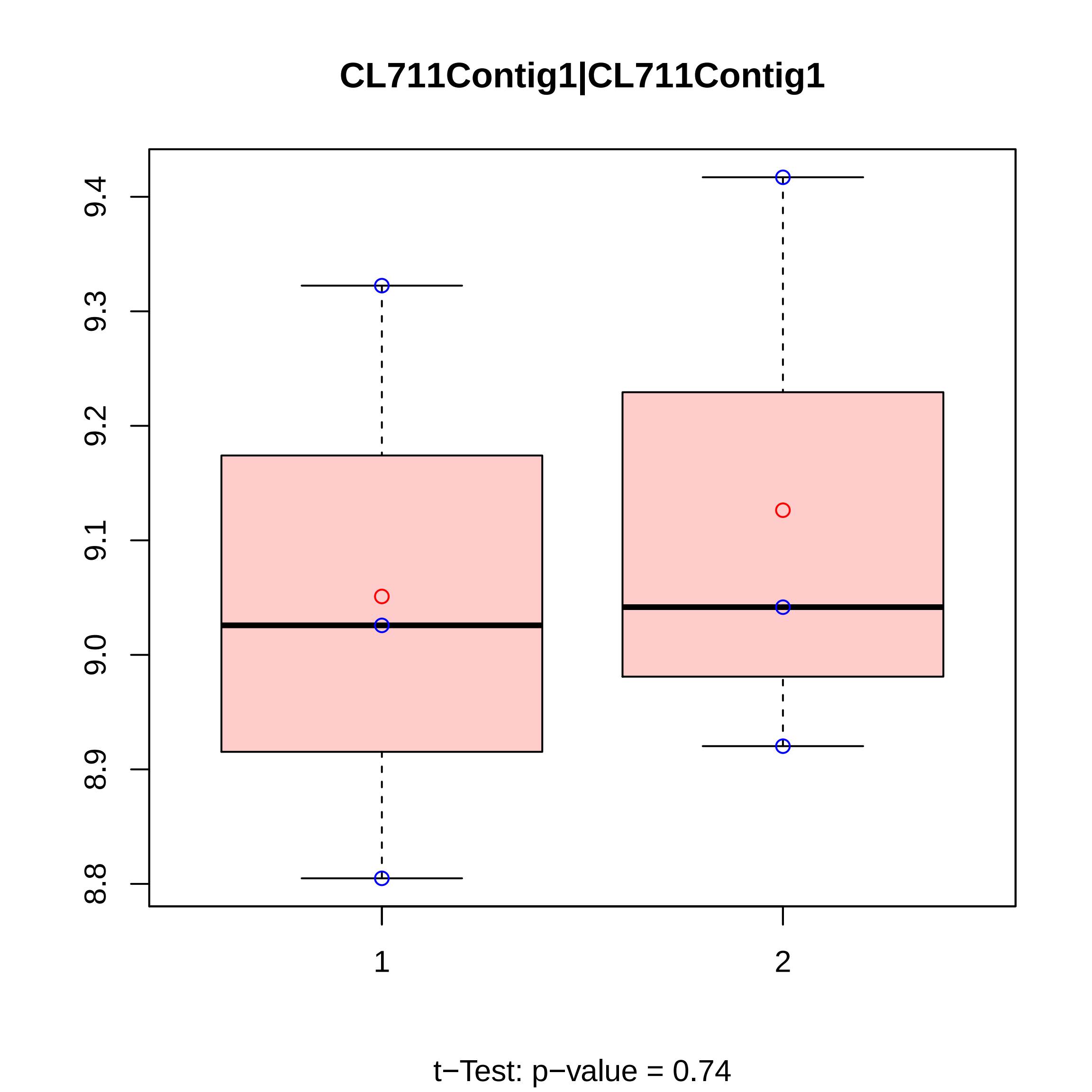

CL711Contig1|CL711Contig1
9.4
9.3
9.2
9.1
9.0
8.9
8.8
1
2
t−Test: p−value = 0.74

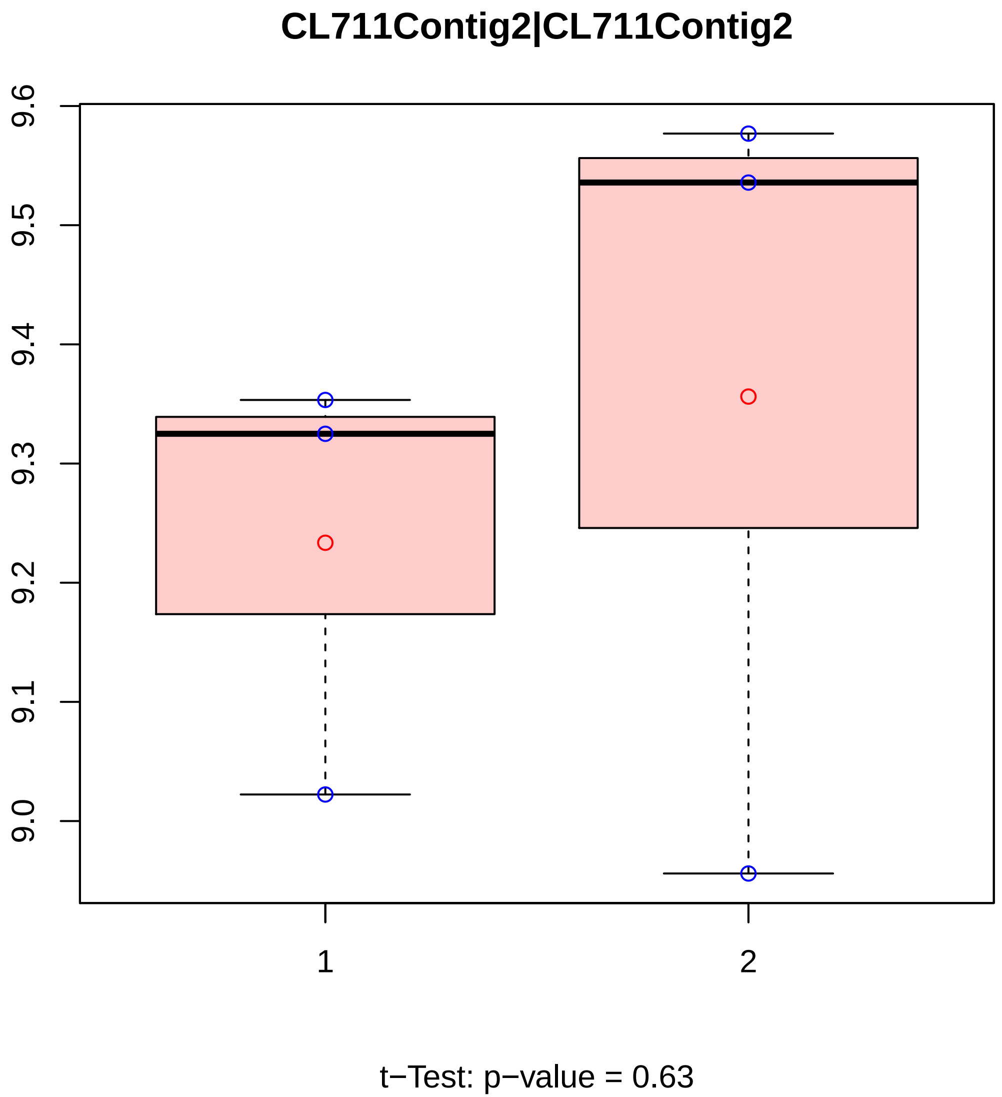
CL711Contig2|CL711Contig2
9.0
9.1
9.2
9.3
9.4
9.5
9.6
1
2
t−Test: p−value = 0.63

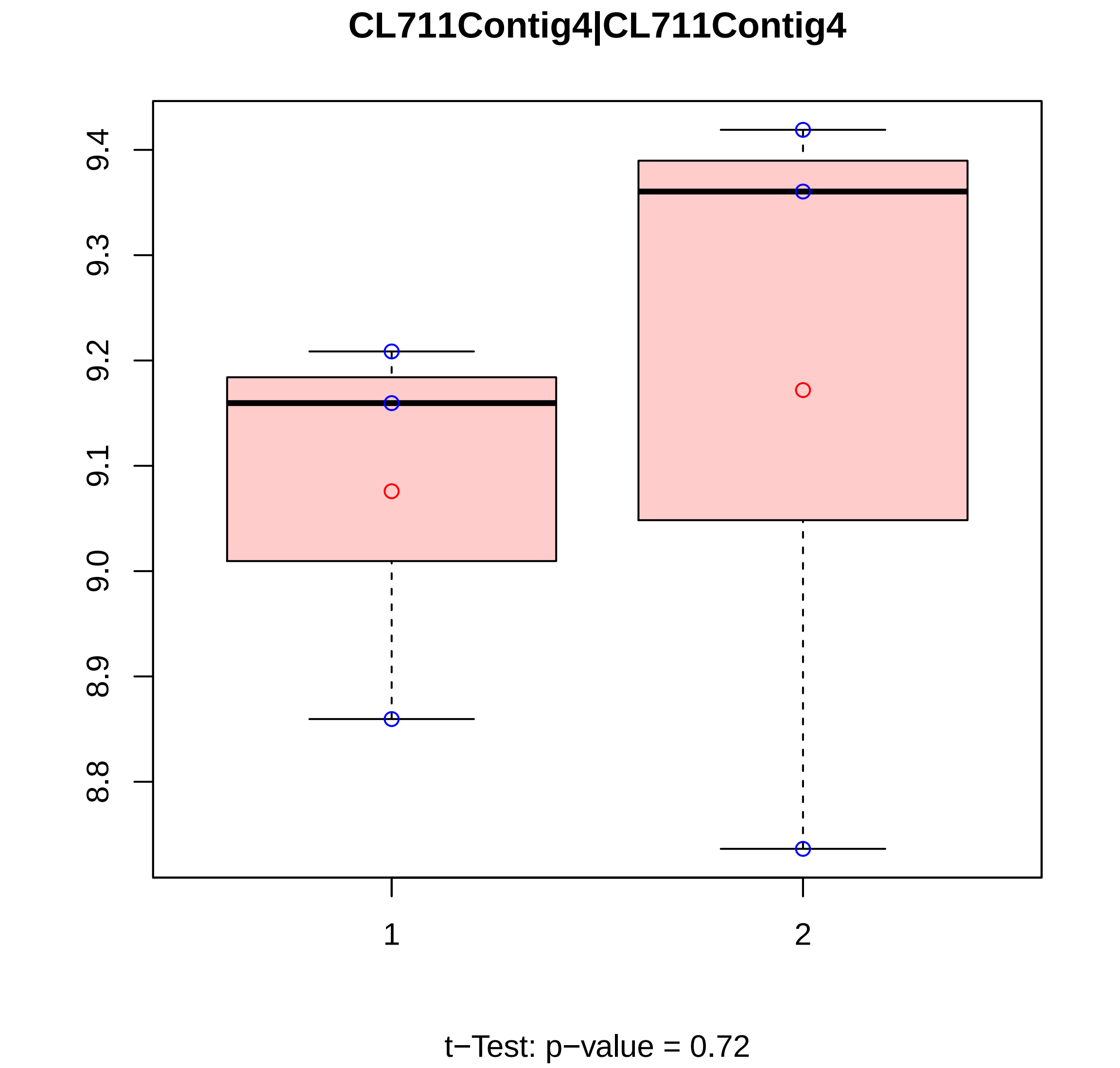

CL711Contig4|CL711Contig4
9.4
9.3
9.2
9.1
9.0
8.9
8.8
1
2
t−Test: p−value = 0.72

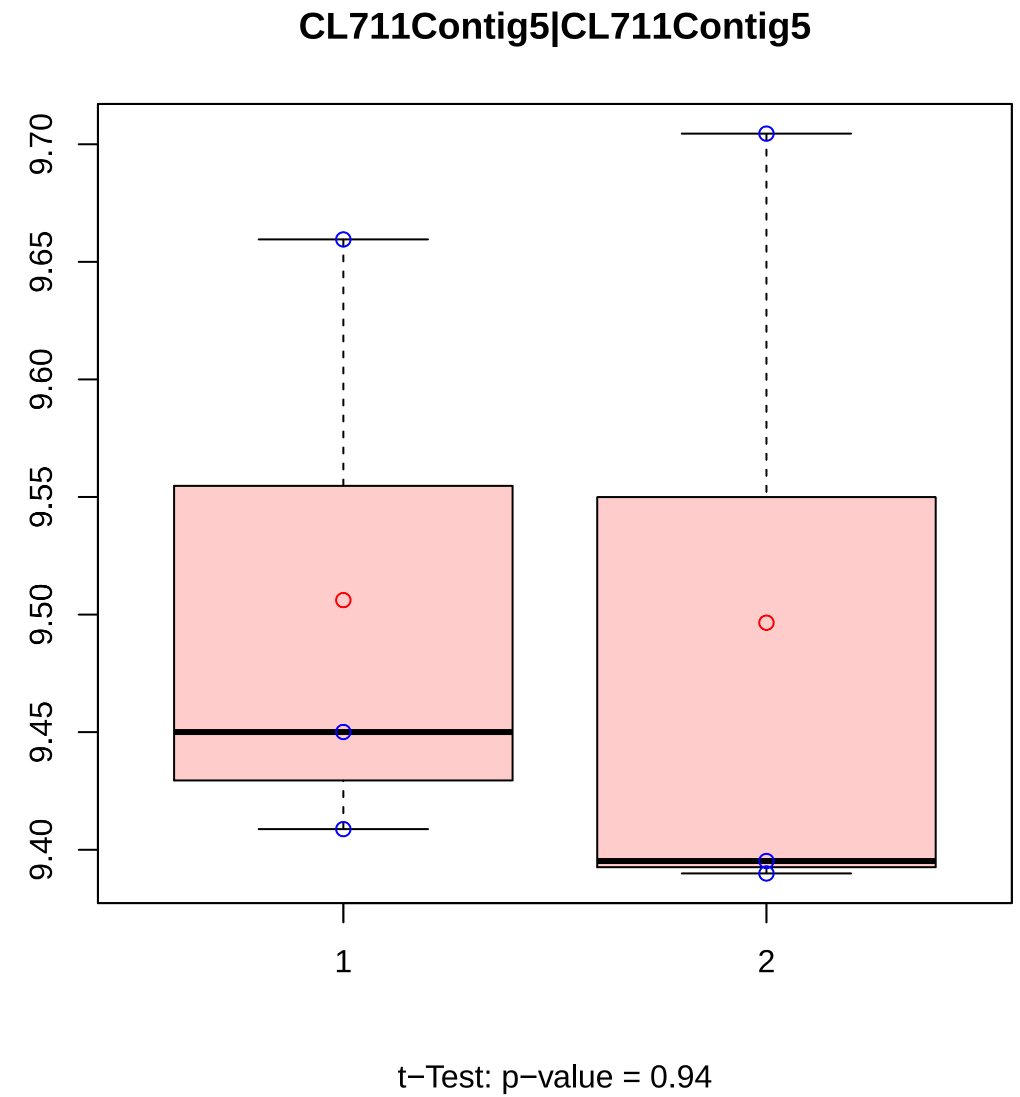
CL711Contig5|CL711Contig5
9.40
9.45
9.50
9.55
9.60
9.65
9.70
1
2
t−Test: p−value = 0.94

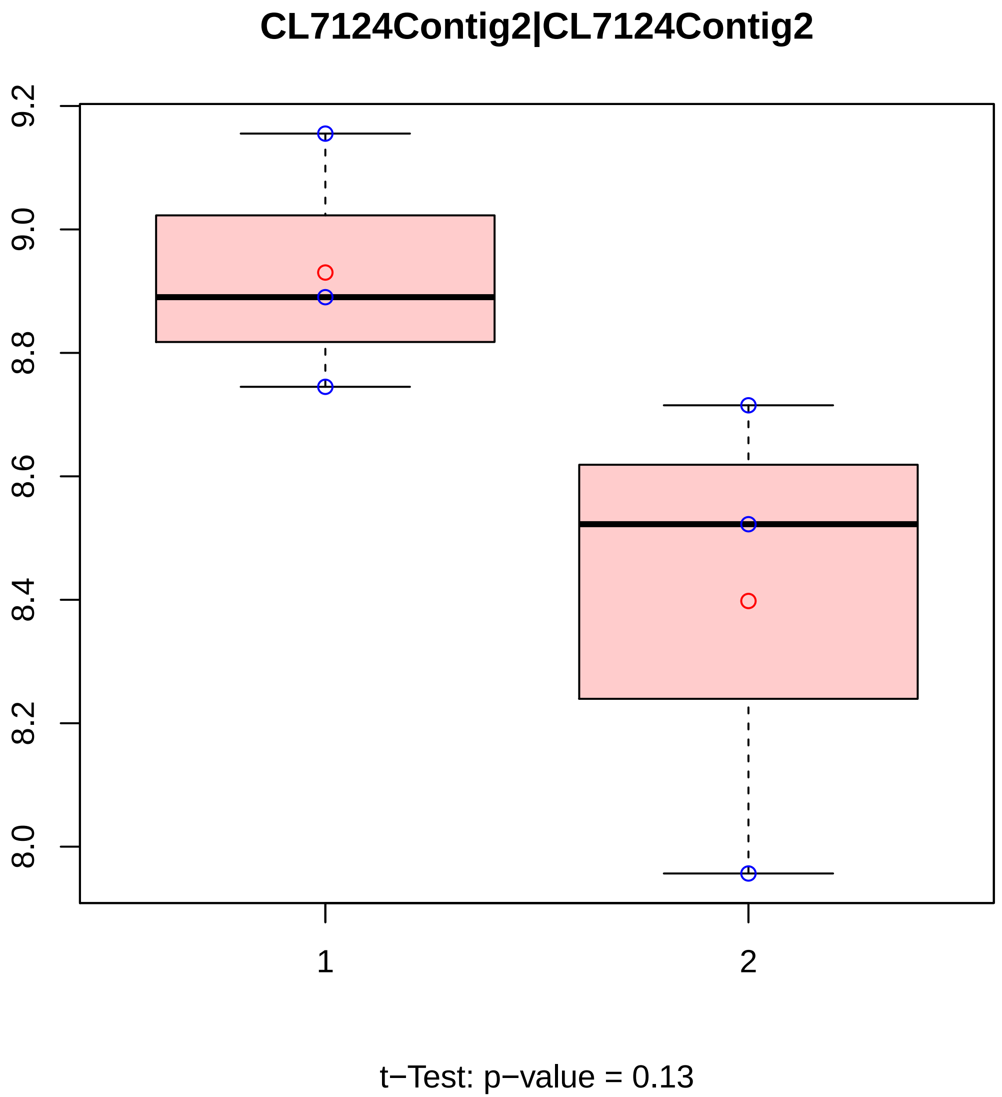

CL7124Contig2|CL7124Contig2
9.2
9.0
8.8
8.6
8.4
8.2
8.0
1
2
t−Test: p−value = 0.13

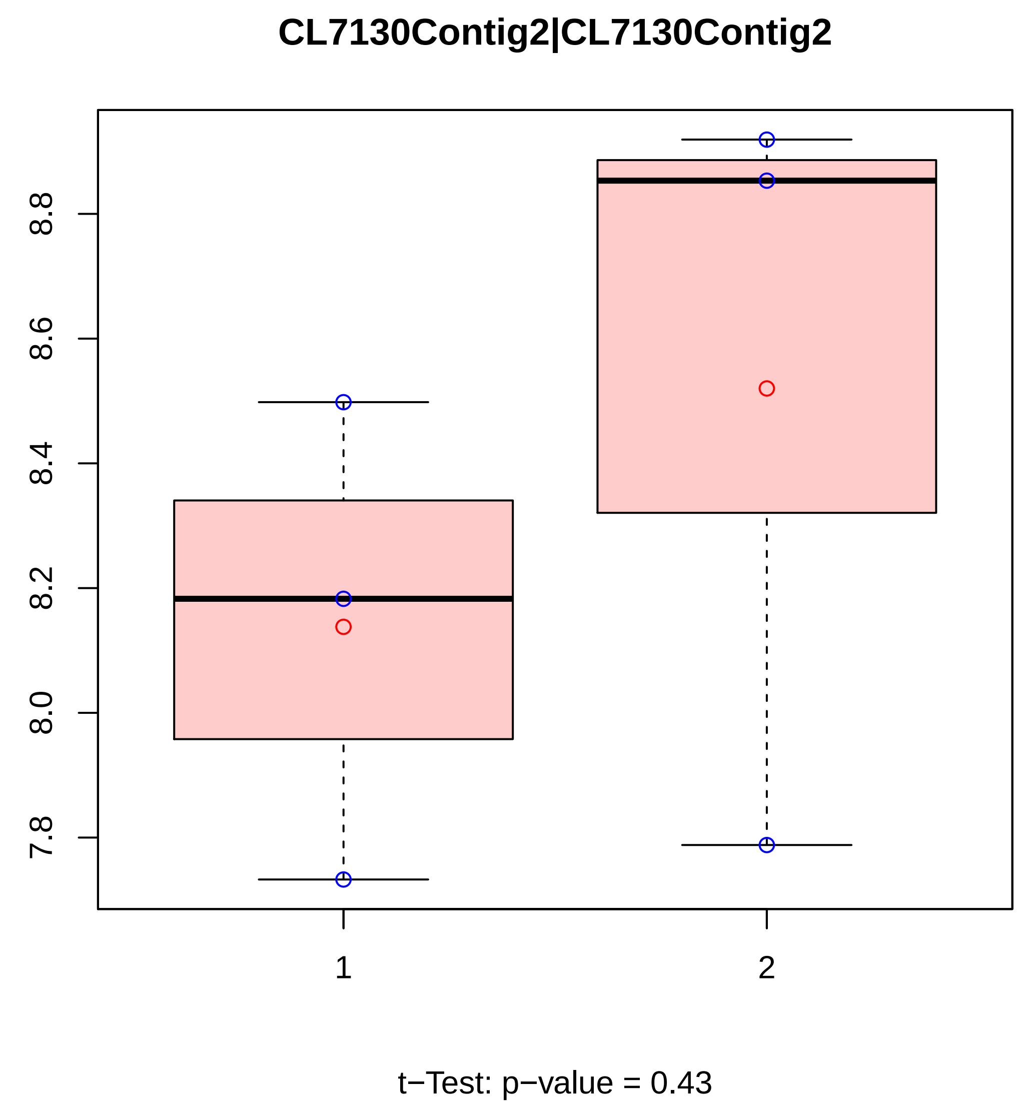
CL7130Contig2|CL7130Contig2
8.8
8.6
8.4
8.2
8.0
7.8
1
2
t−Test: p−value = 0.43

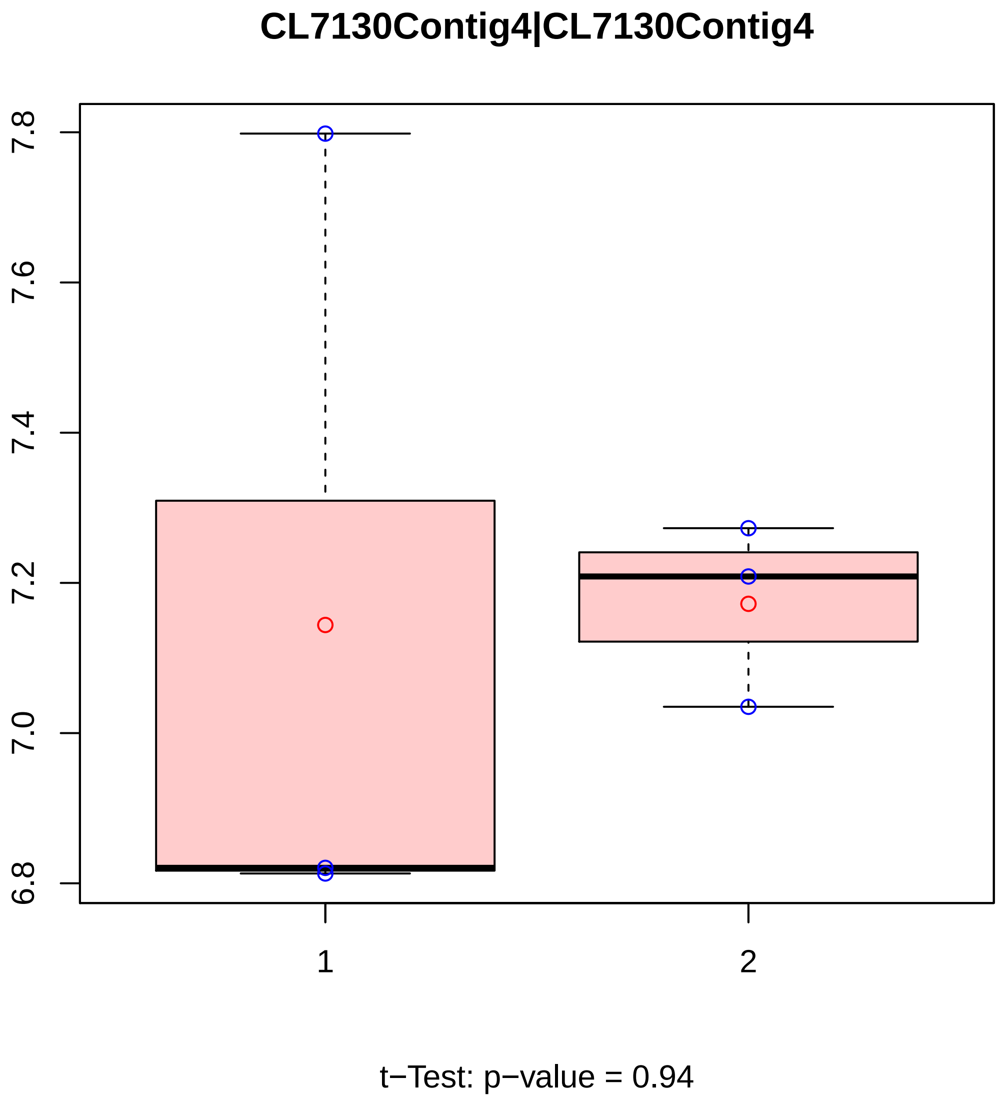
CL7130Contig4|CL7130Contig4
7.8
7.6
7.4
7.2
7.0
6.8
1
2
t−Test: p−value = 0.94

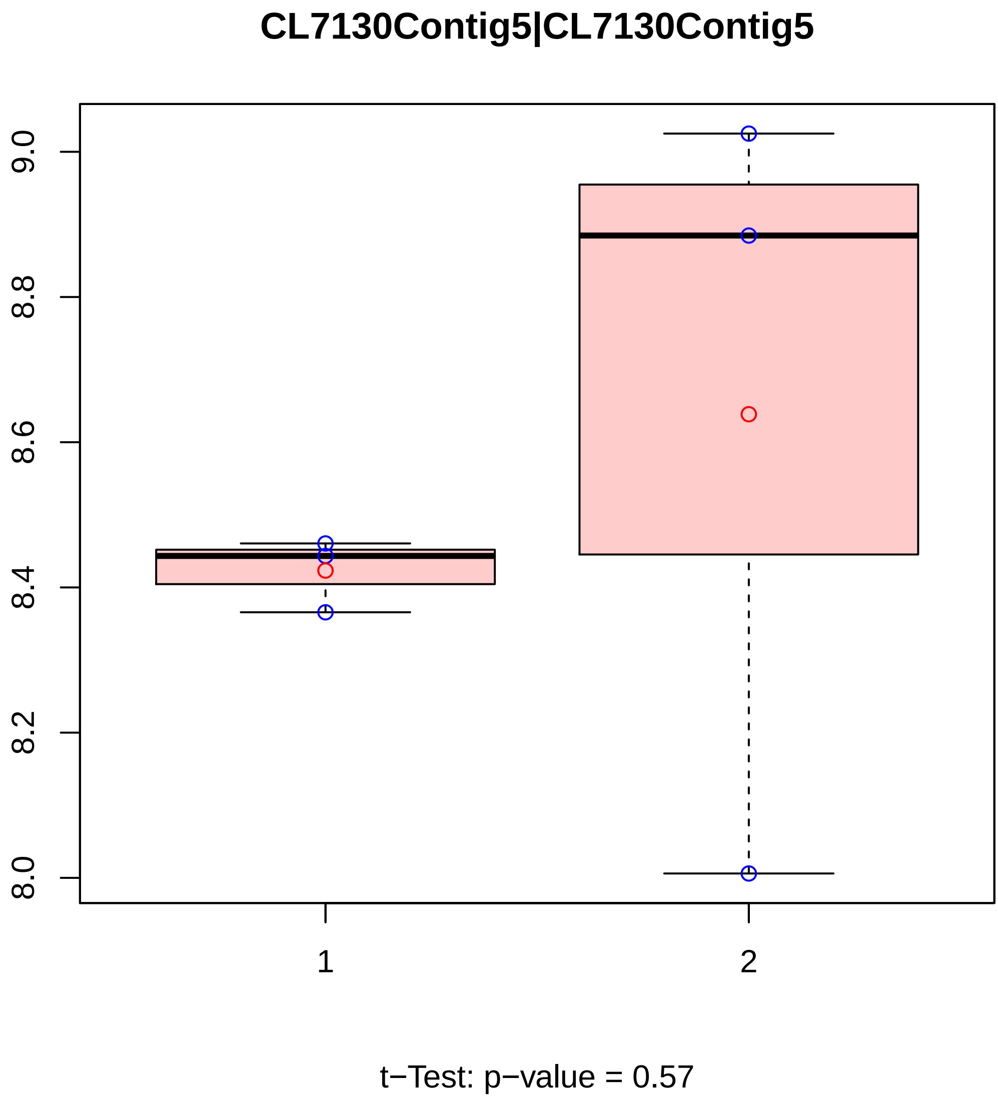
CL7130Contig5|CL7130Contig5
9.0
8.8
8.6
8.4
8.2
8.0
1
2
t−Test: p−value = 0.57

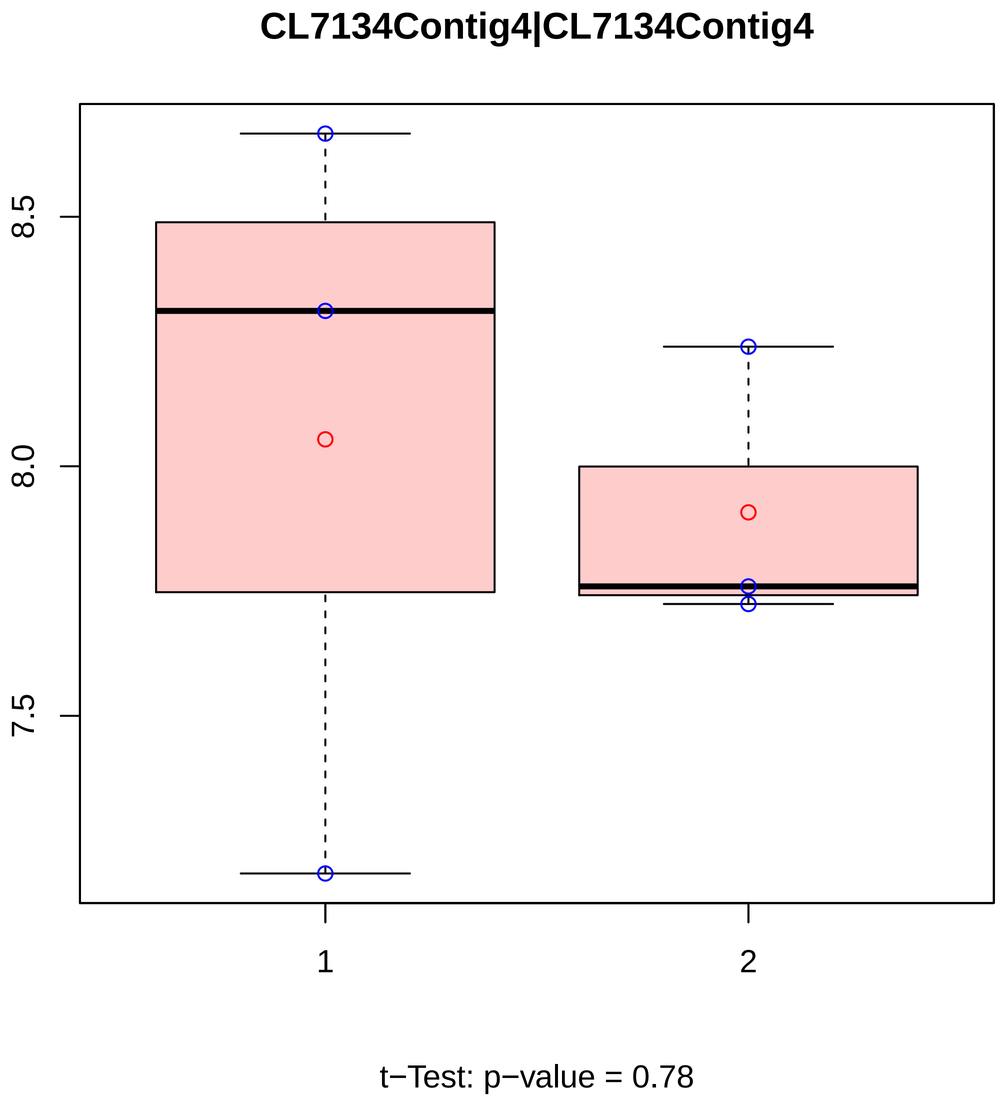

CL7134Contig4|CL7134Contig4
8.5
8.0
7.5
1
2
t−Test: p−value = 0.78

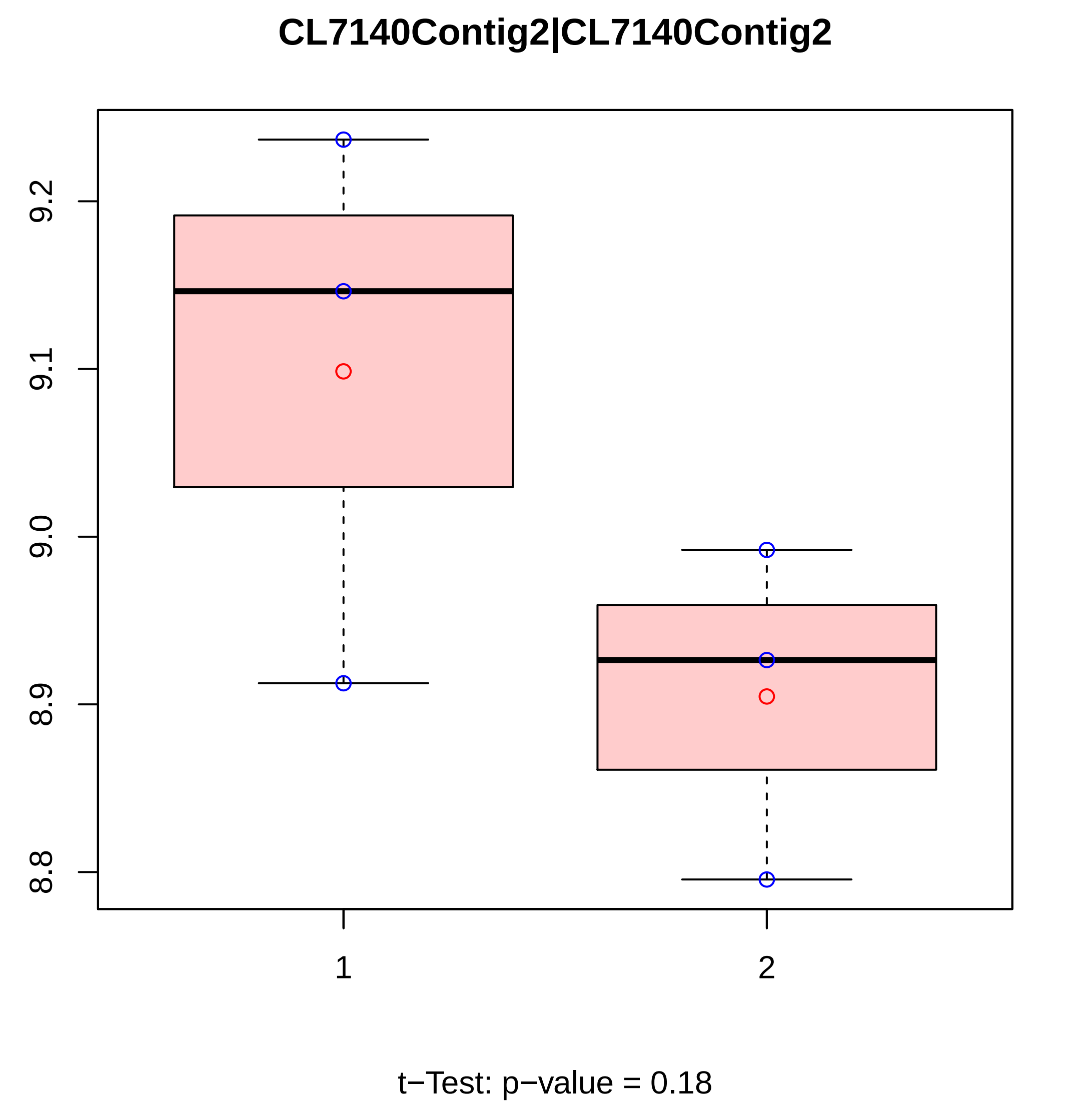
CL7140Contig2|CL7140Contig2
9.2
9.1
9.0
8.9
8.8
1
2
t−Test: p−value = 0.18

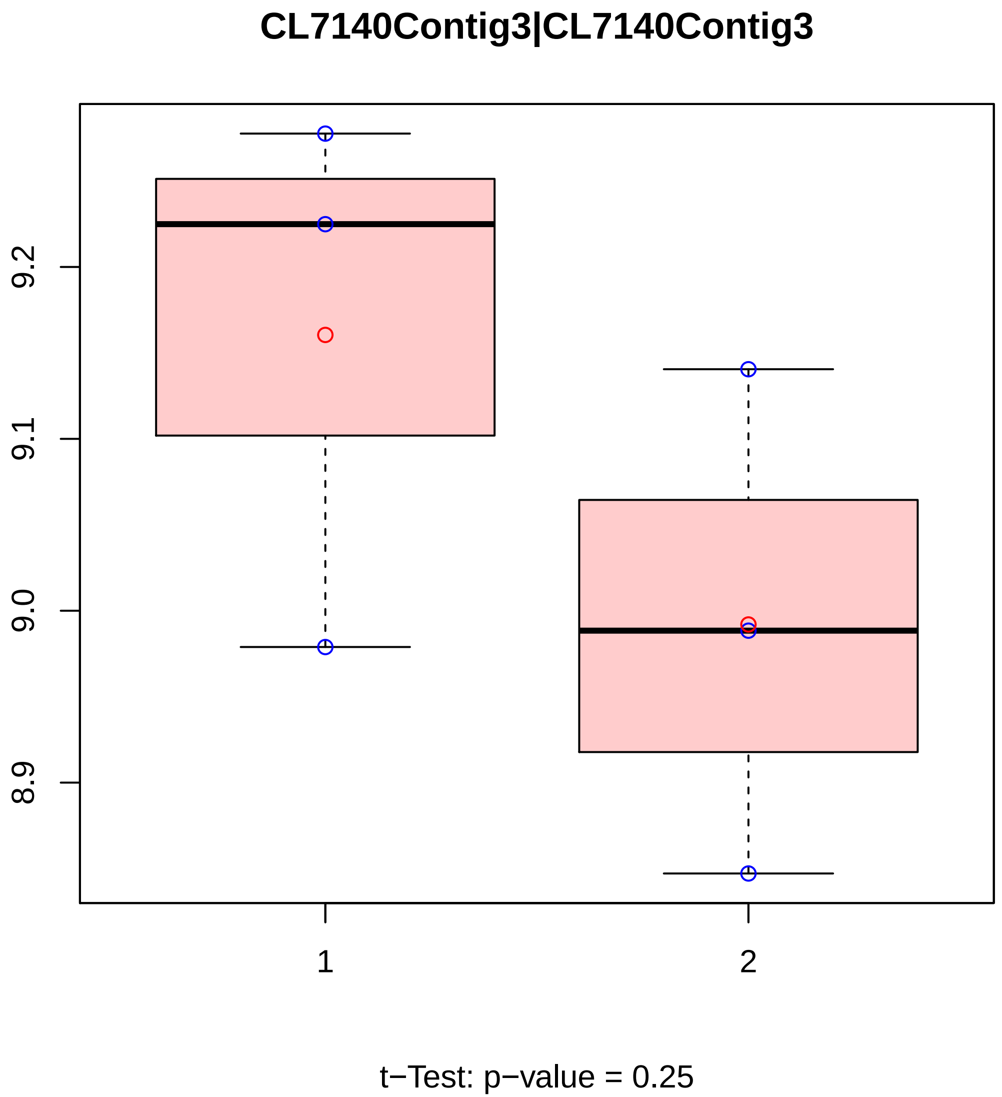

CL7140Contig3|CL7140Contig3
9.2
9.1
9.0
8.9
1
2
t−Test: p−value = 0.25

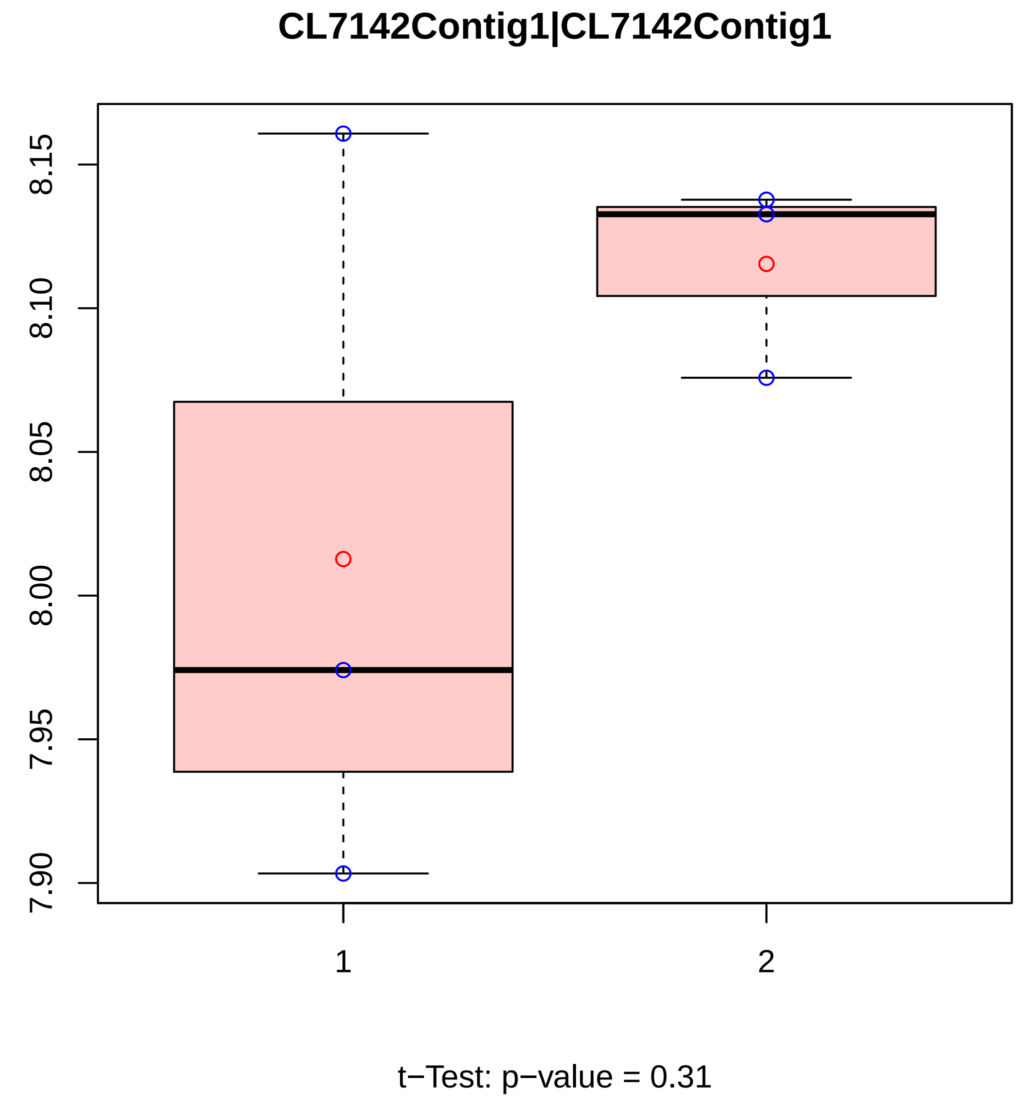
CL7142Contig1|CL7142Contig1
8.15
8.10
8.05
8.00
7.95
7.90
1
2
t−Test: p−value = 0.31

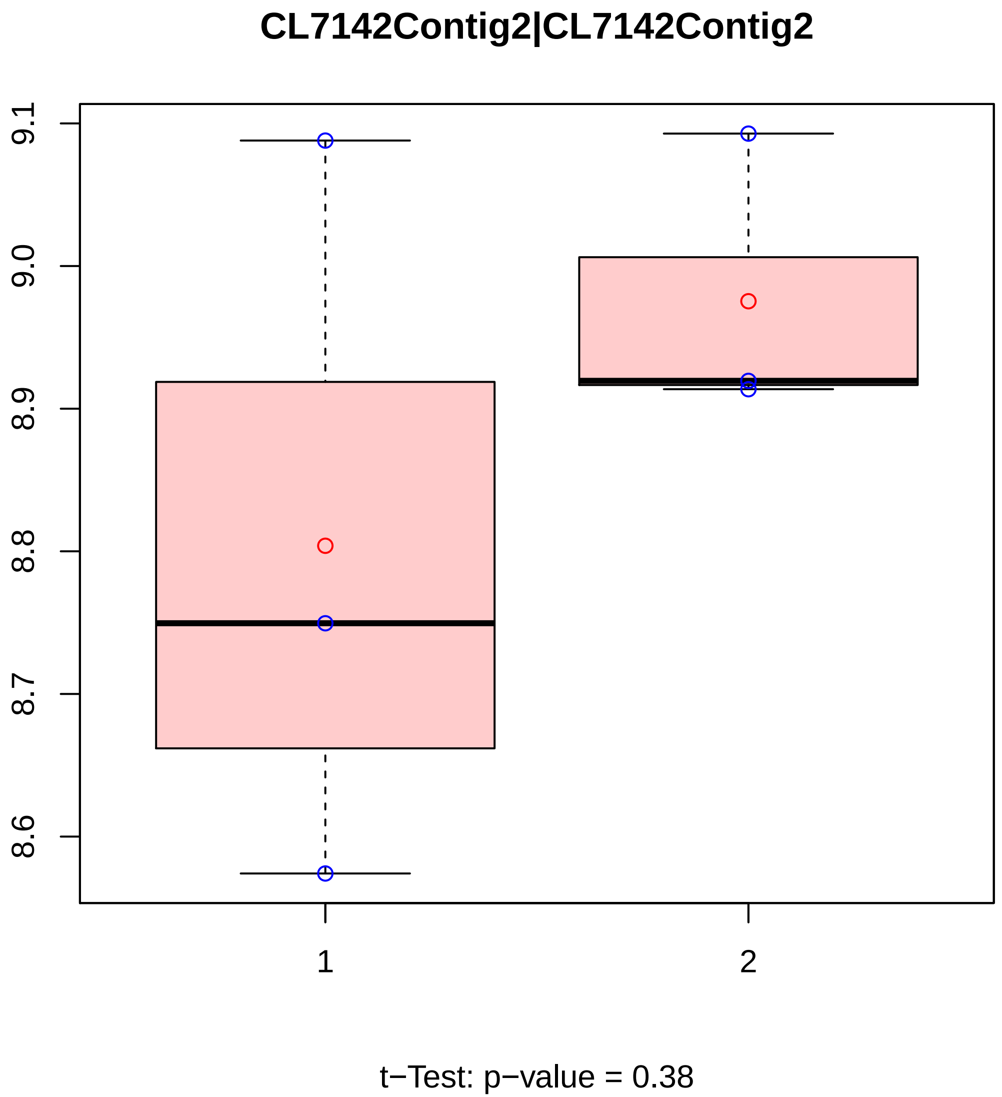
CL7142Contig2|CL7142Contig2
9.1
9.0
8.9
8.8
8.7
8.6
1
2
t−Test: p−value = 0.38

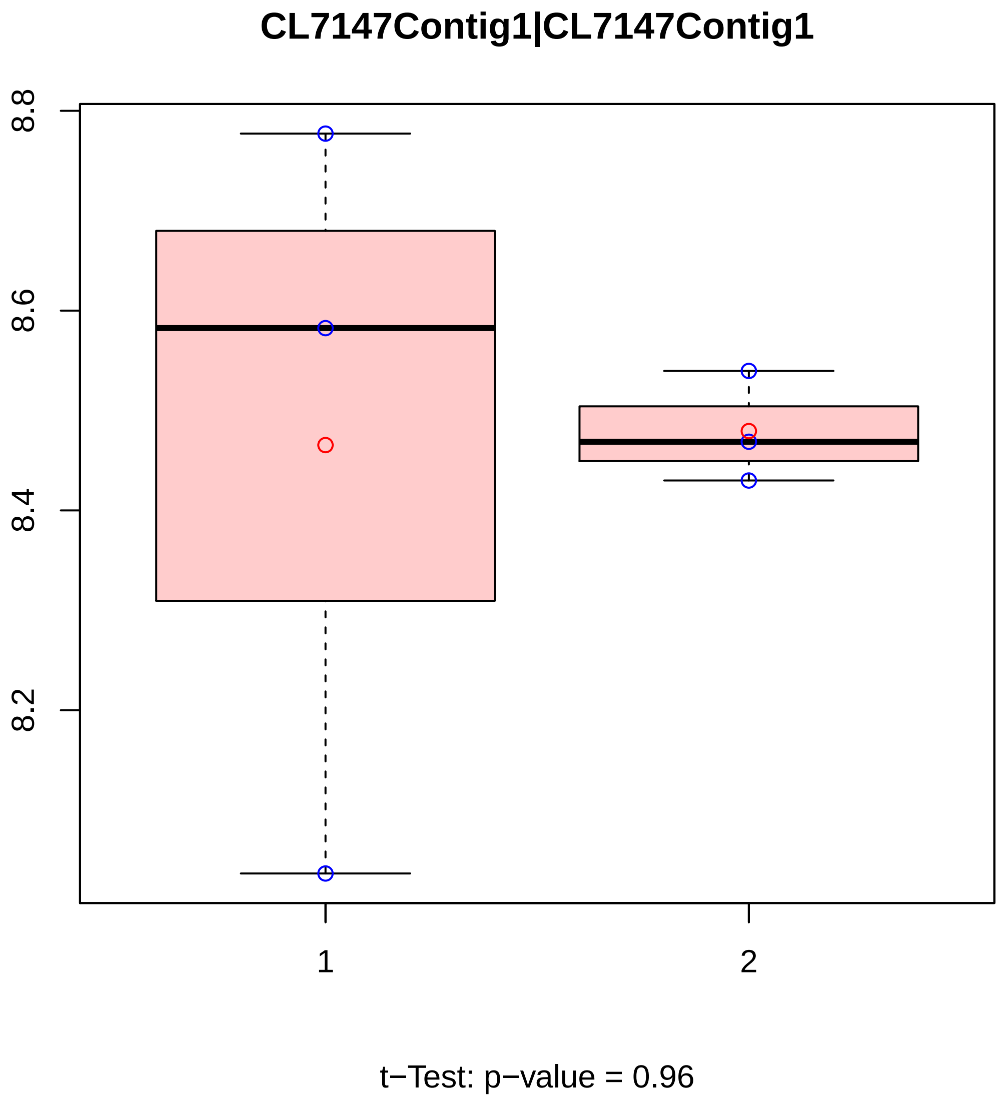
CL7147Contig1|CL7147Contig1
8.8
8.6
8.4
8.2
1
2
t−Test: p−value = 0.96

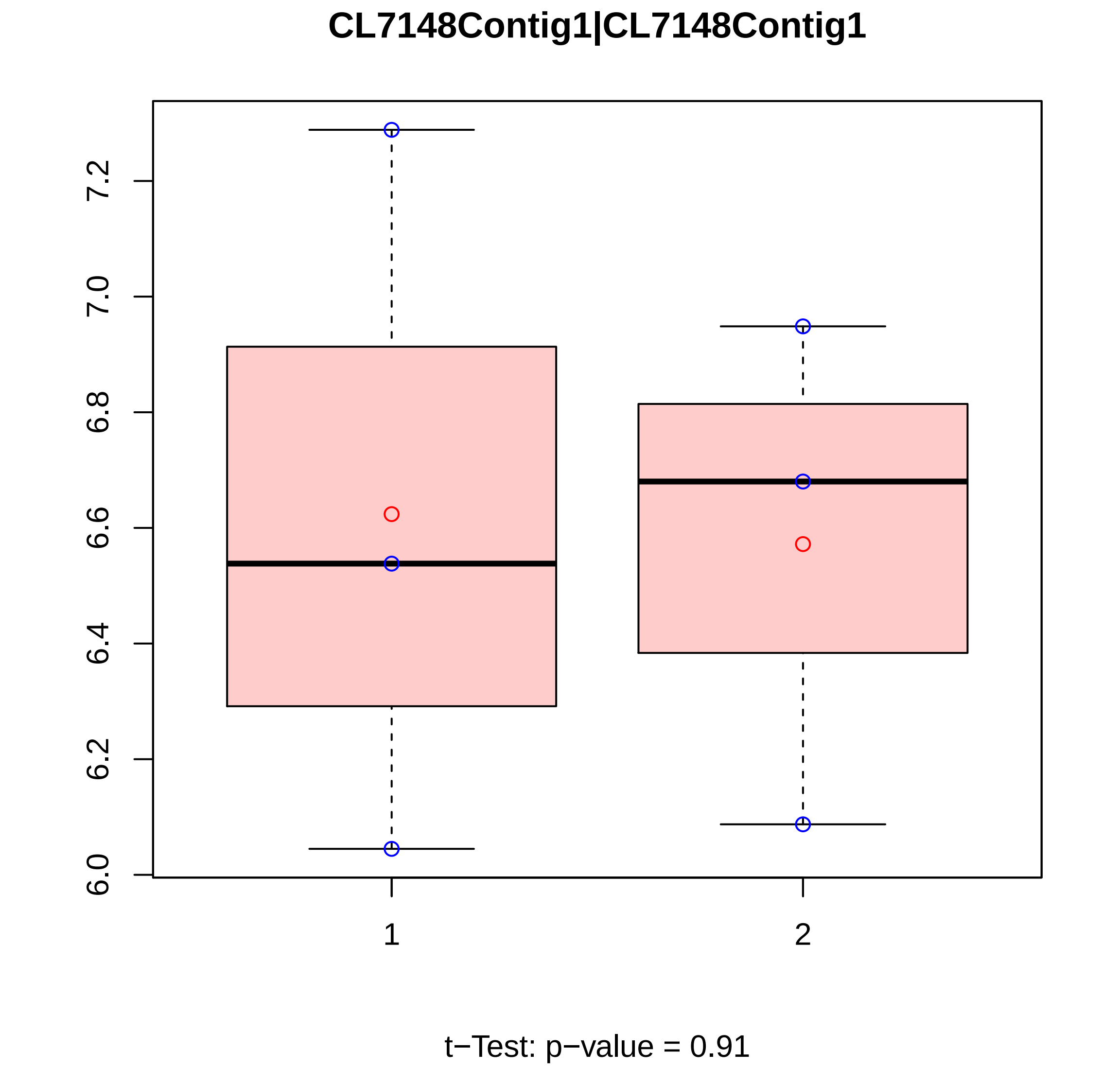
CL7148Contig1|CL7148Contig1
7.2
7.0
6.8
6.6
6.4
6.2
6.0
1
2
t−Test: p−value = 0.91

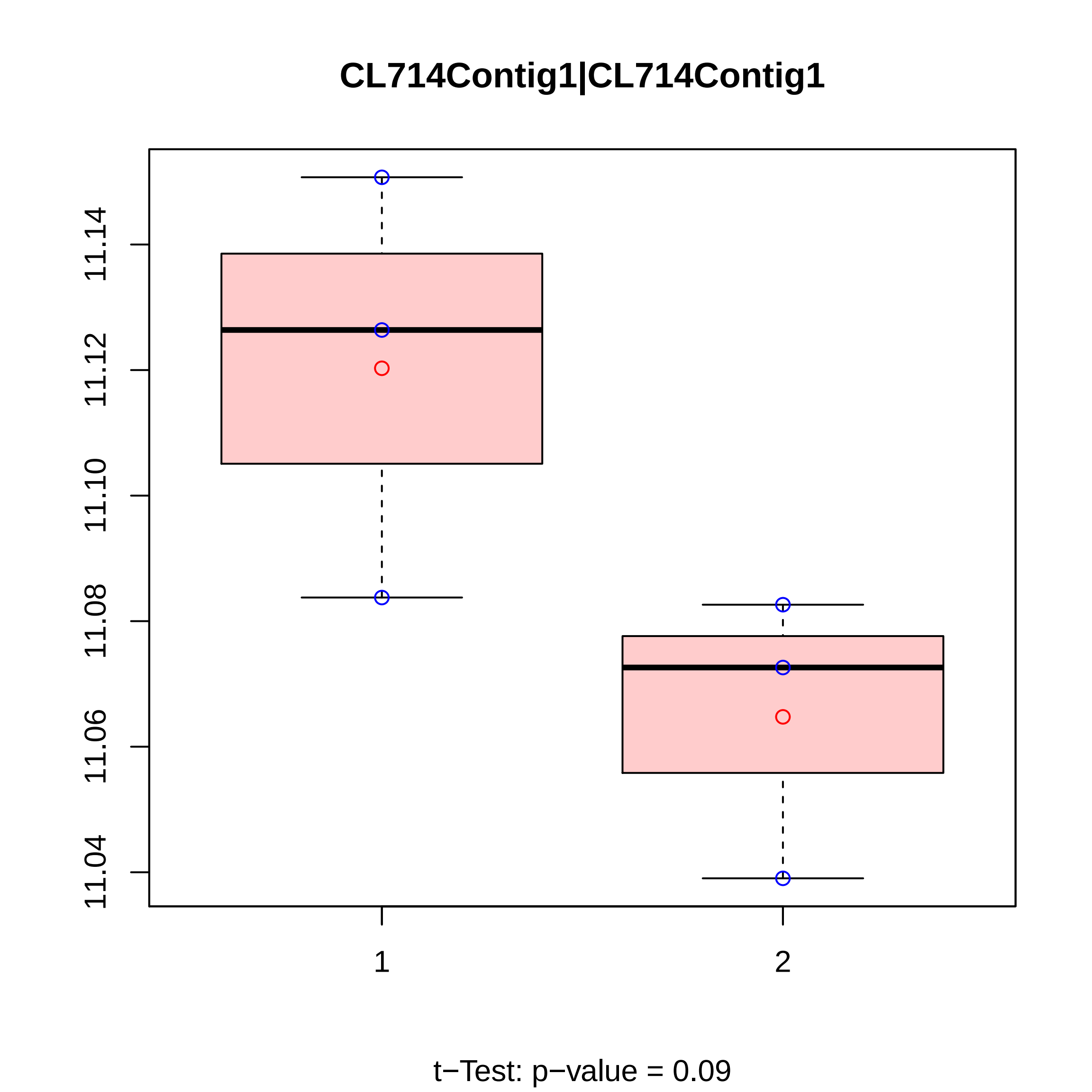
CL714Contig1|CL714Contig1
11.04
11.06
11.08
11.10
11.12
11.14
1
2
t−Test: p−value = 0.09

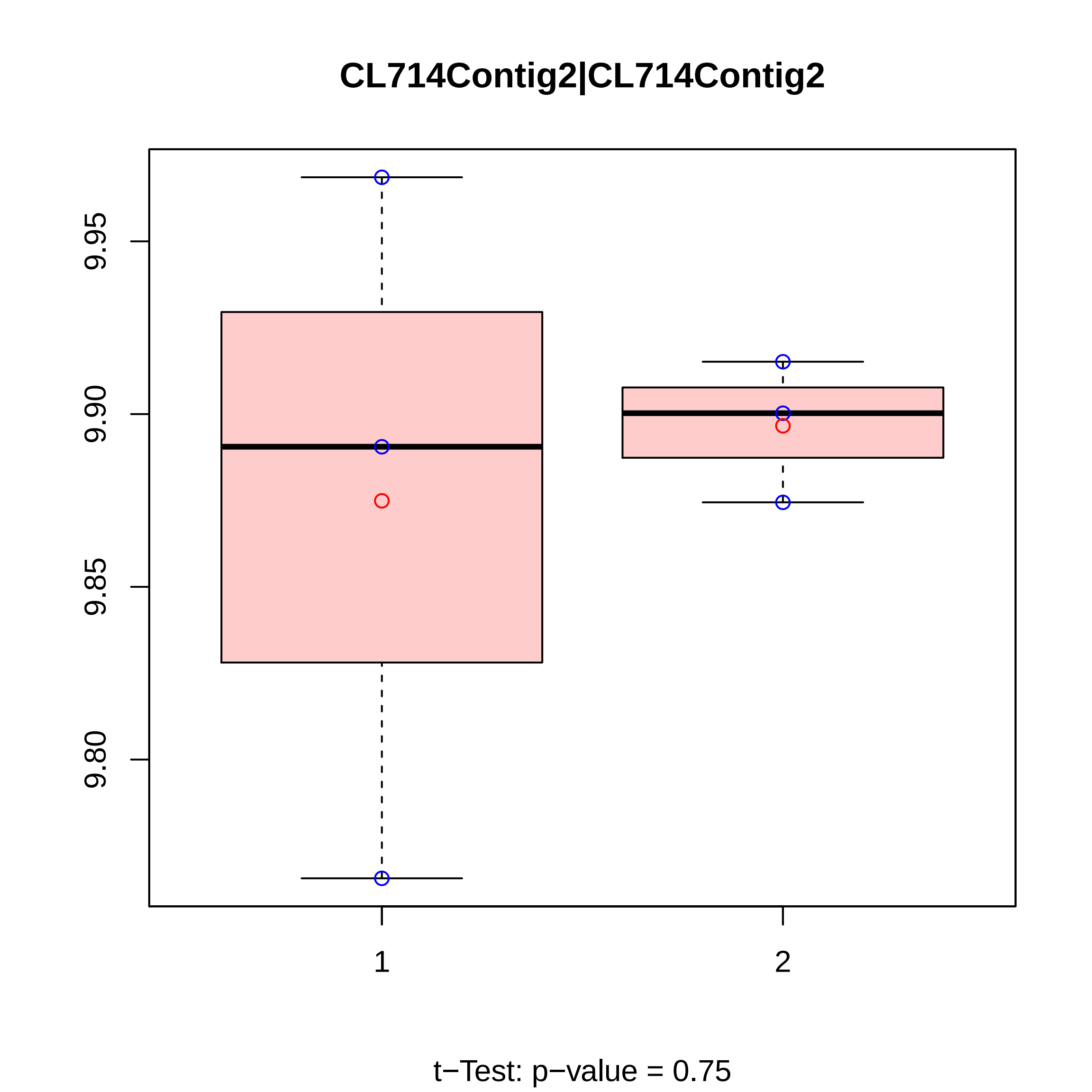

CL714Contig2|CL714Contig2
9.95
9.90
9.85
9.80
1
2
t−Test: p−value = 0.75

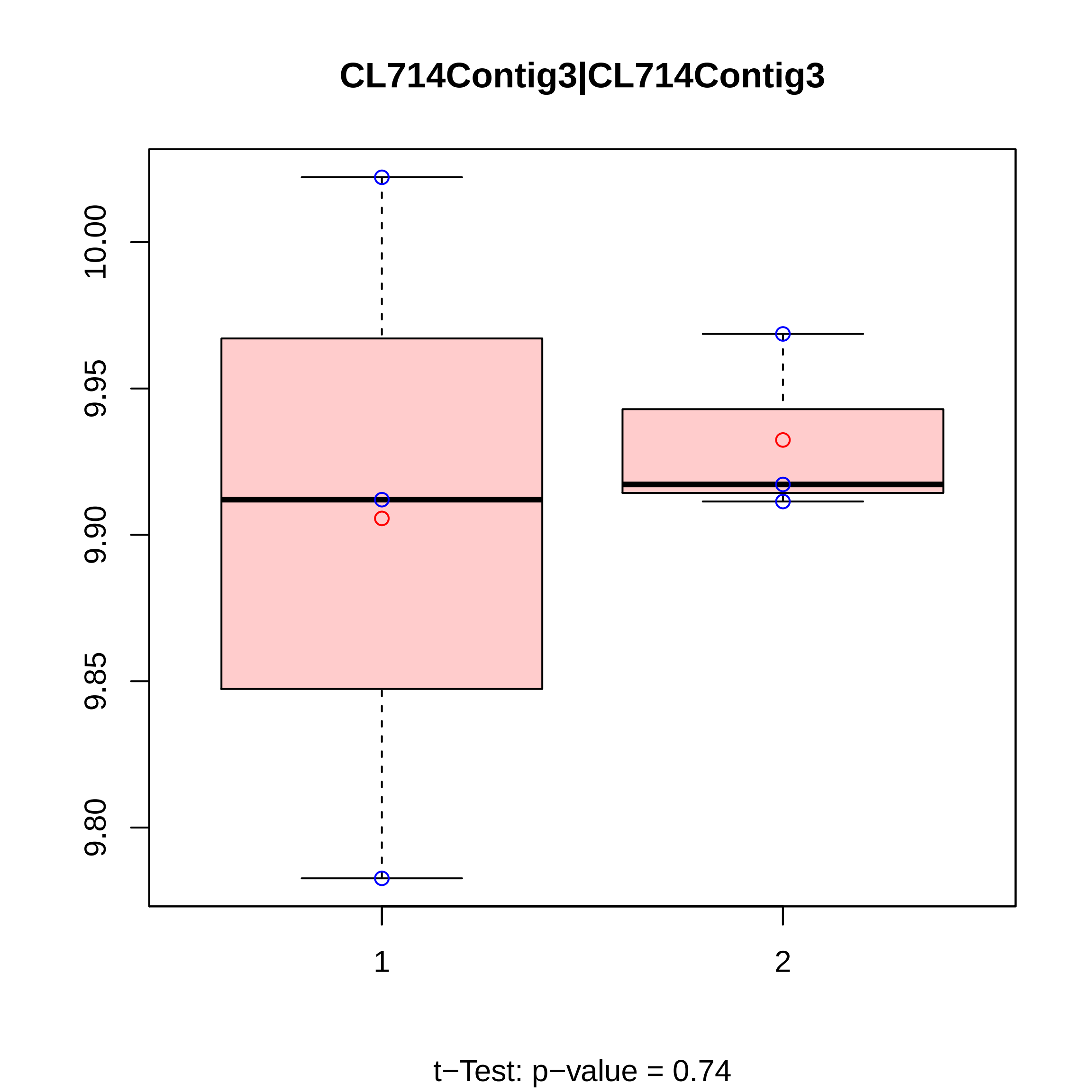

CL714Contig3|CL714Contig3
10.00
9.95
9.90
9.85
9.80
1
2
t−Test: p−value = 0.74

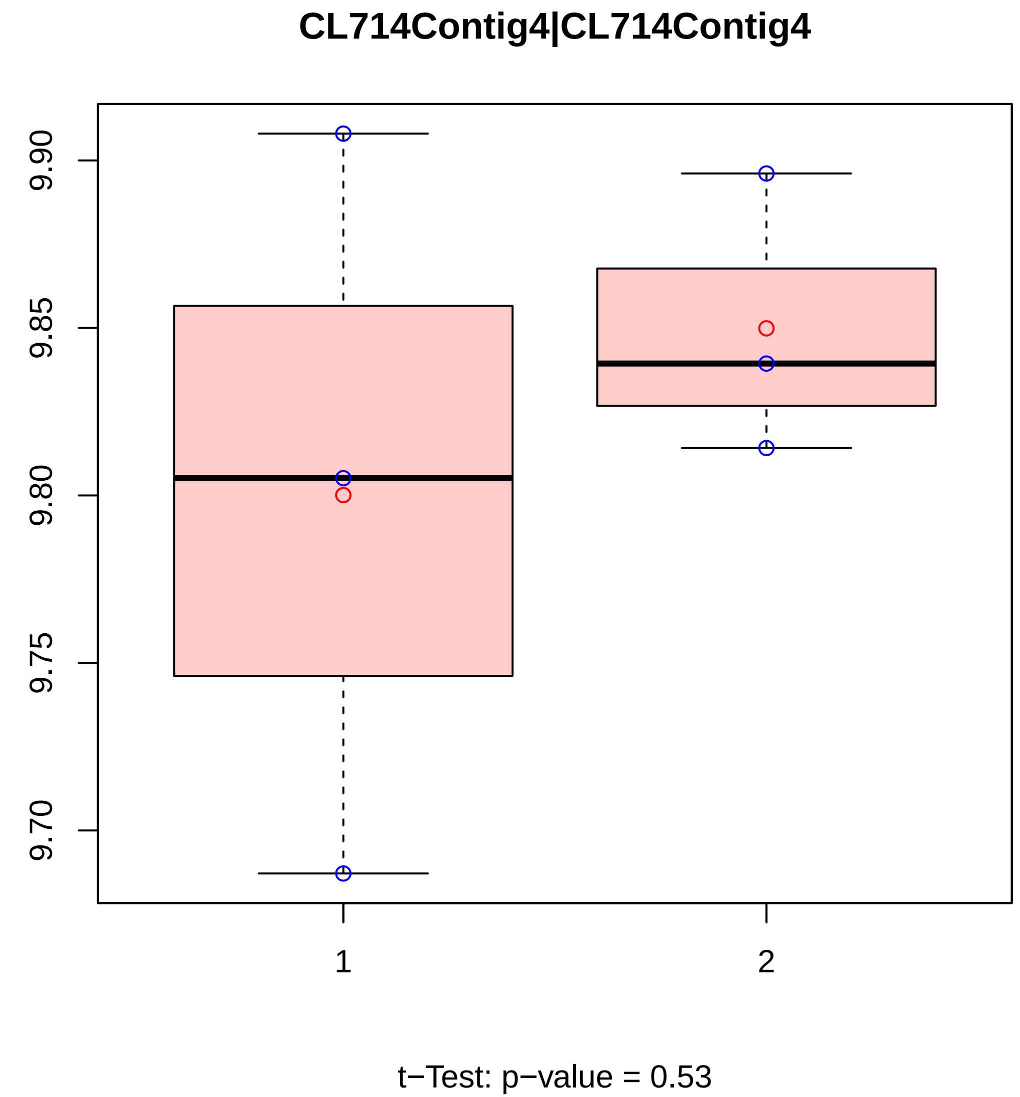
CL714Contig4|CL714Contig4
9.90
9.85
9.80
9.75
9.70
1
2
t−Test: p−value = 0.53

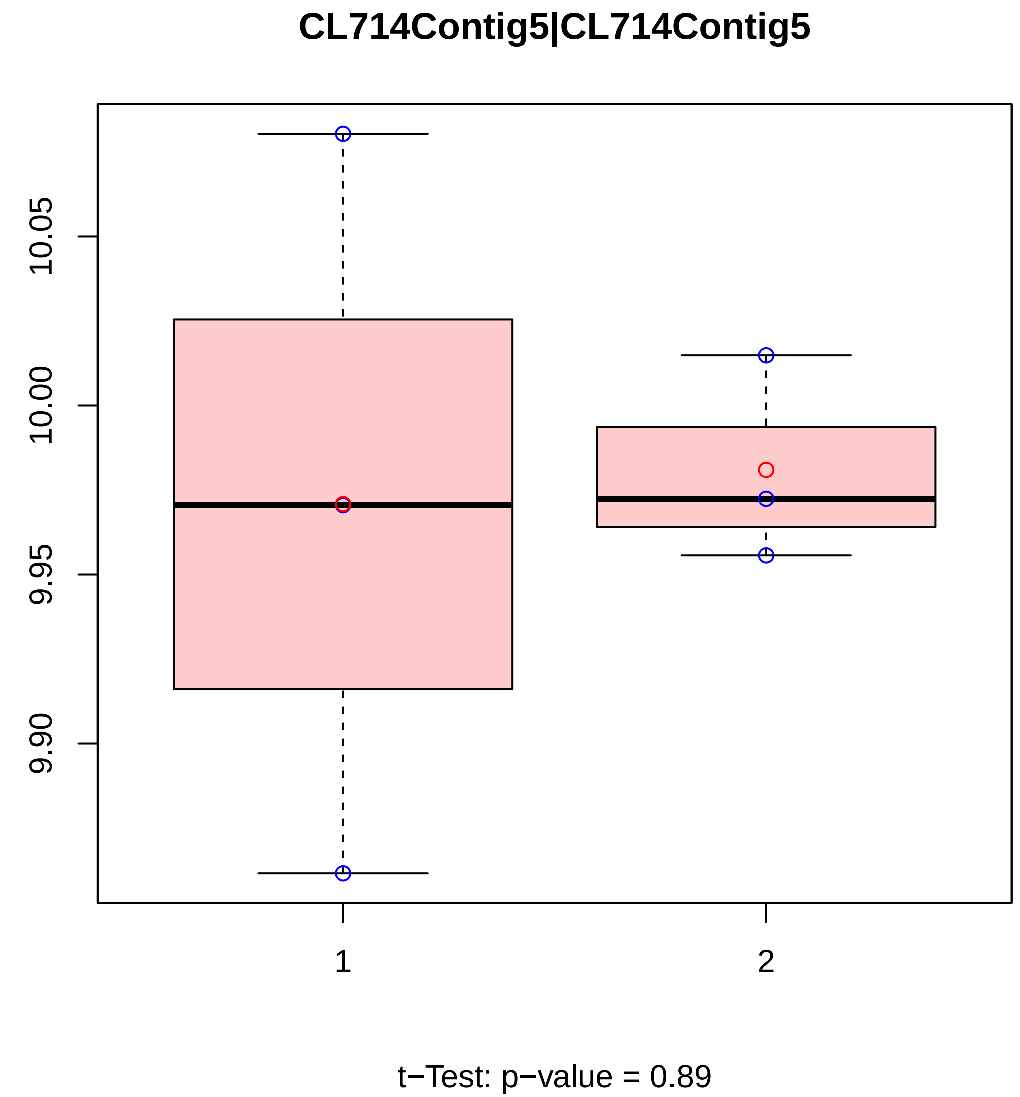
CL714Contig5|CL714Contig5
10.05
10.00
9.95
9.90
1
2
t−Test: p−value = 0.89

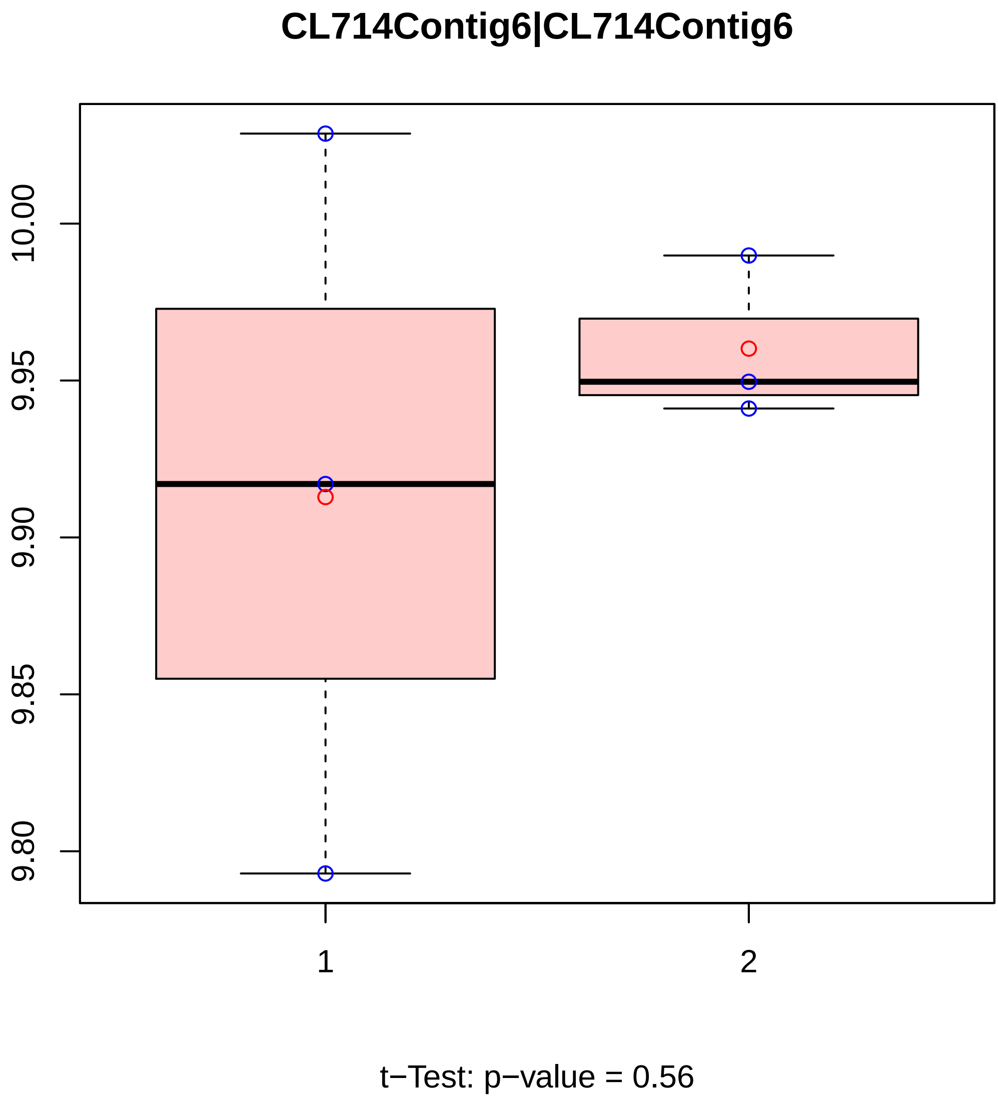

CL714Contig6|CL714Contig6
10.00
9.95
9.90
9.85
9.80
1
2
t−Test: p−value = 0.56

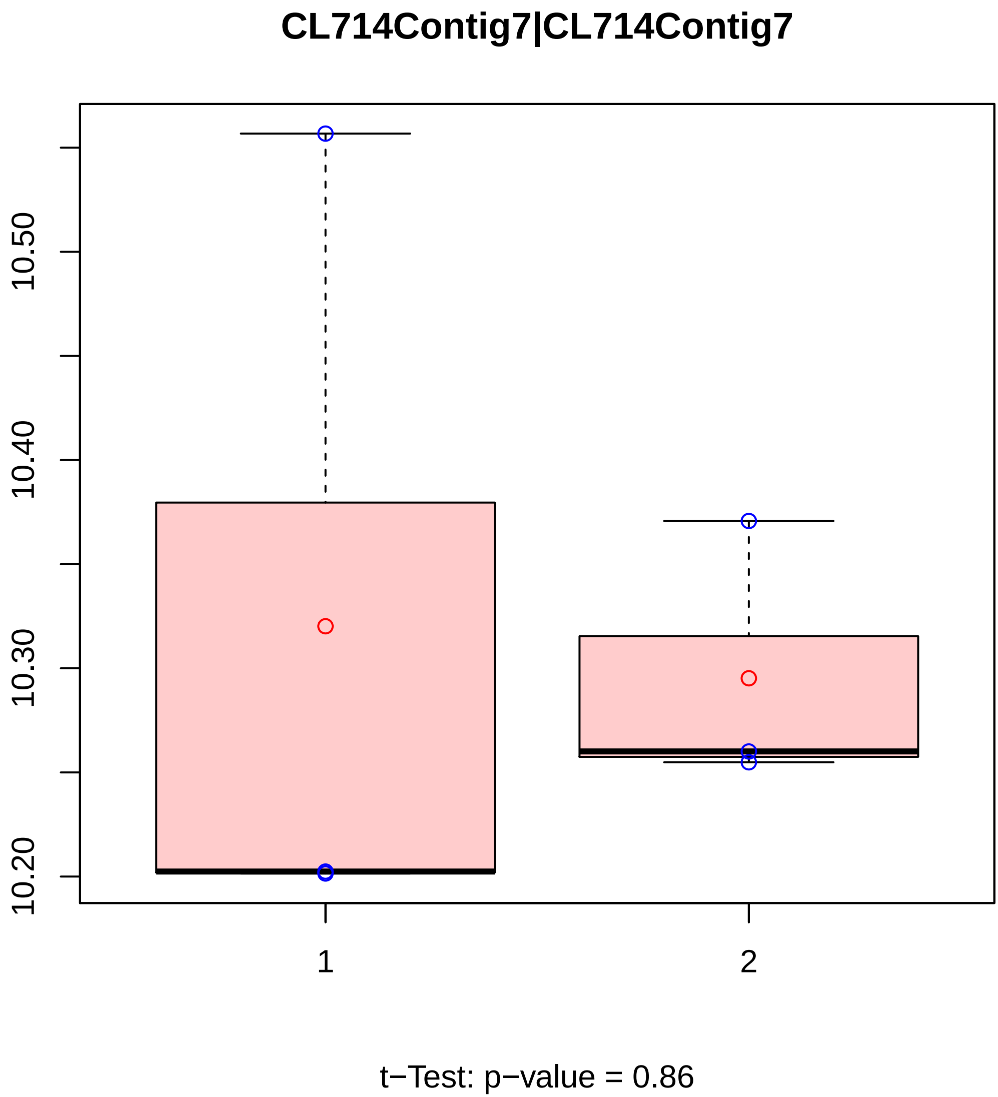
CL714Contig7|CL714Contig7
10.20
10.30
10.40
10.50
1
2
t−Test: p−value = 0.86

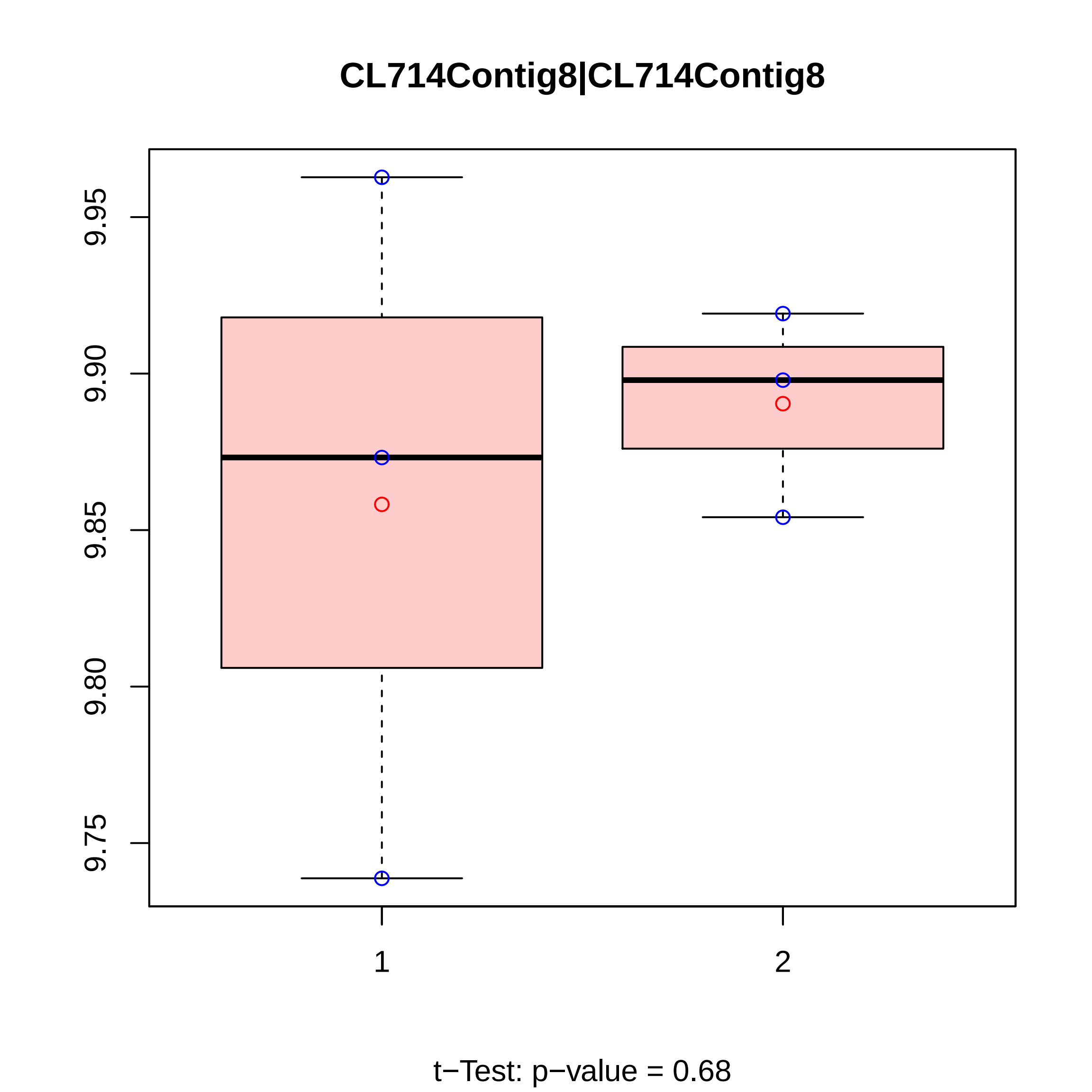
CL714Contig8|CL714Contig8
9.75
9.80
9.85
9.90
9.95
1
2
t−Test: p−value = 0.68

# CL7151Contig1|CL7151Contig1

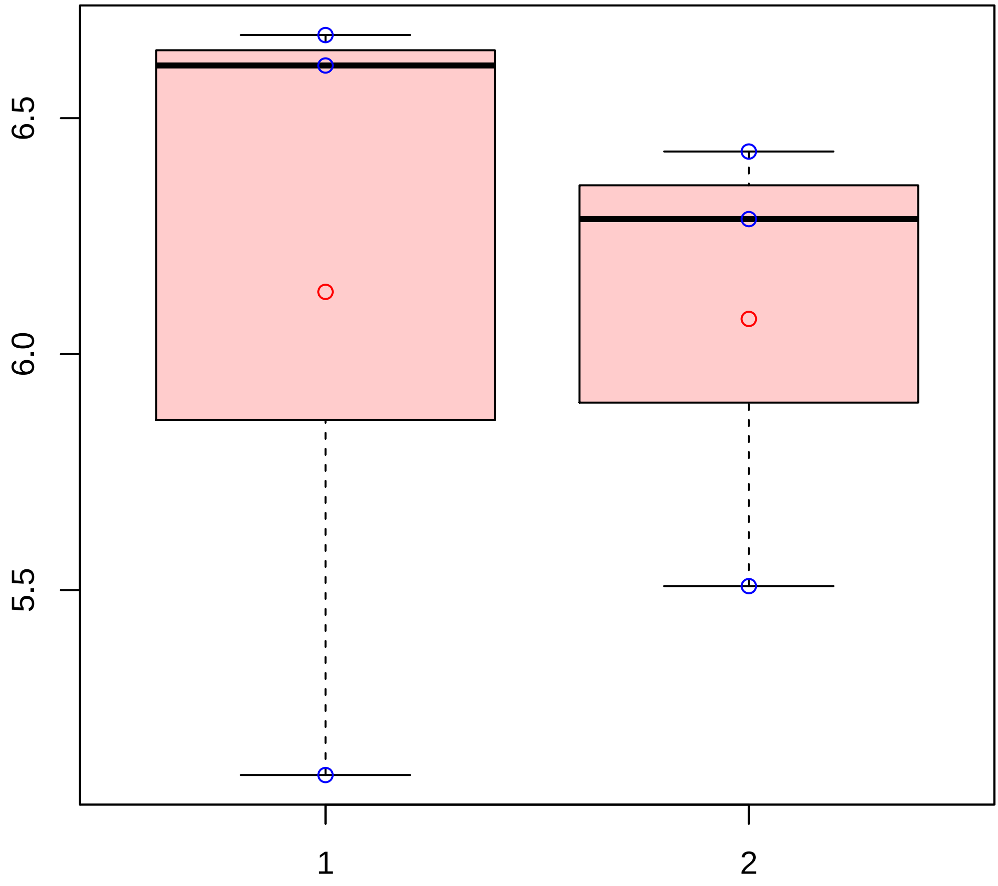


t−Test: p−value = 0.93

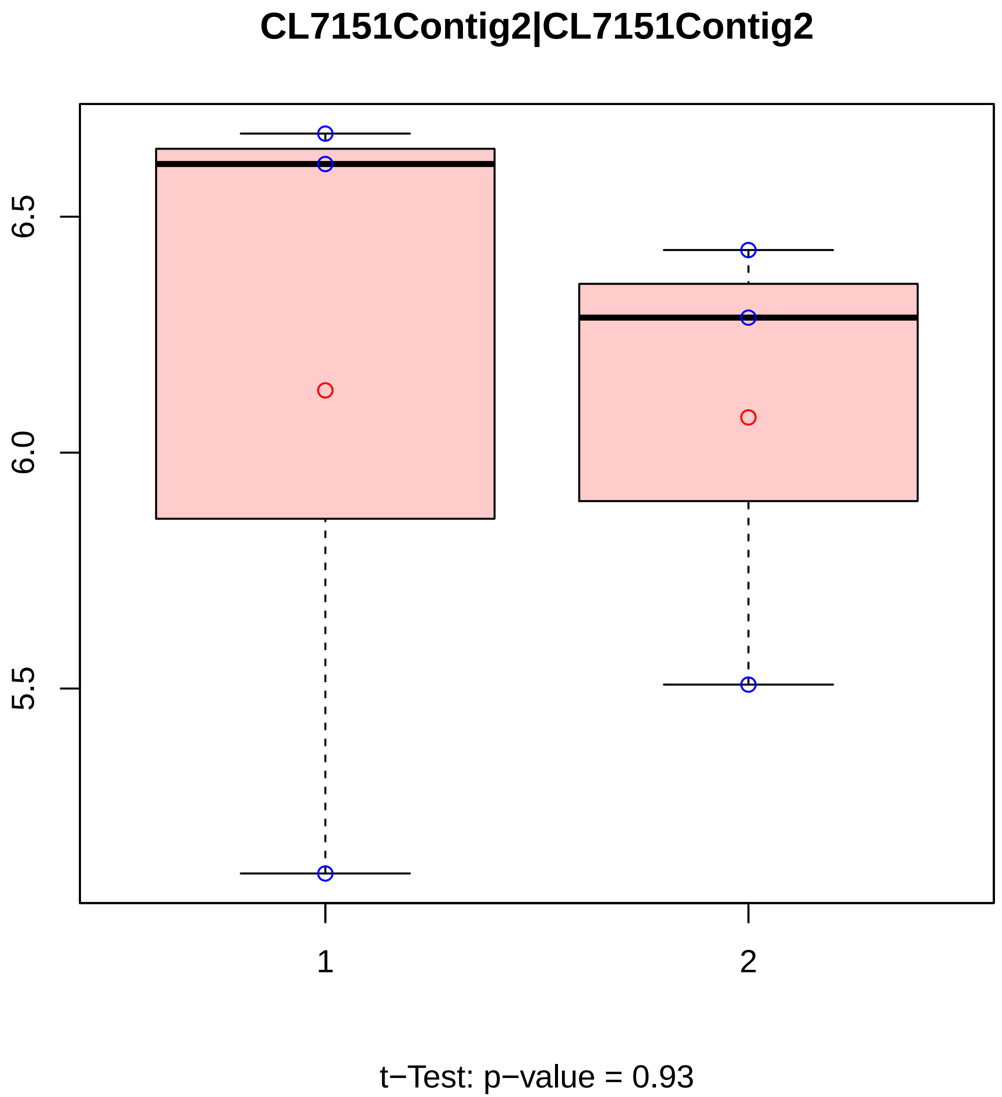

CL7151Contig2|CL7151Contig2
6.5
6.0
5.5
1
2
t−Test: p−value = 0.93

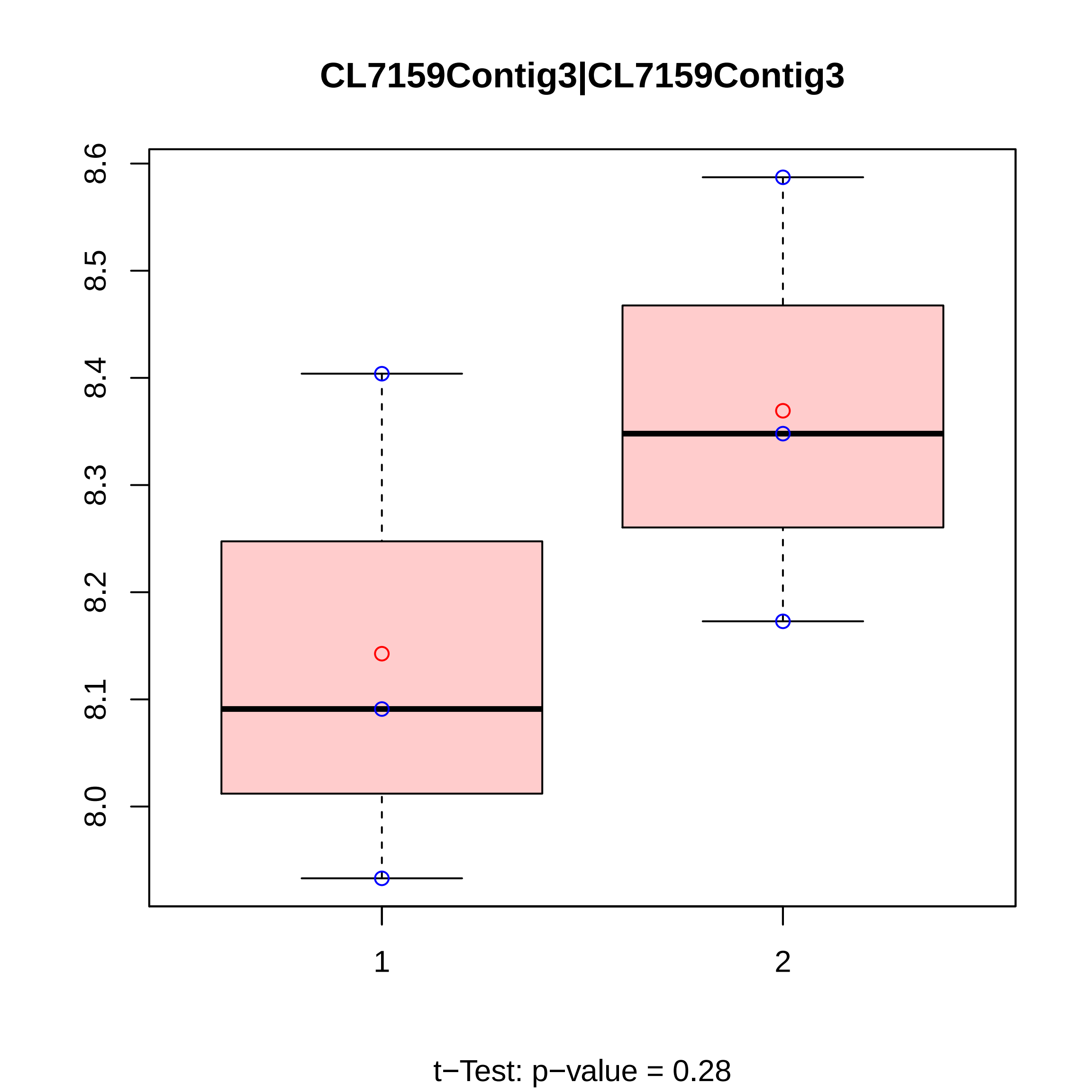
CL7159Contig3|CL7159Contig3
8.6
8.5
8.4
8.3
8.2
8.1
8.0
1
2
t−Test: p−value = 0.28

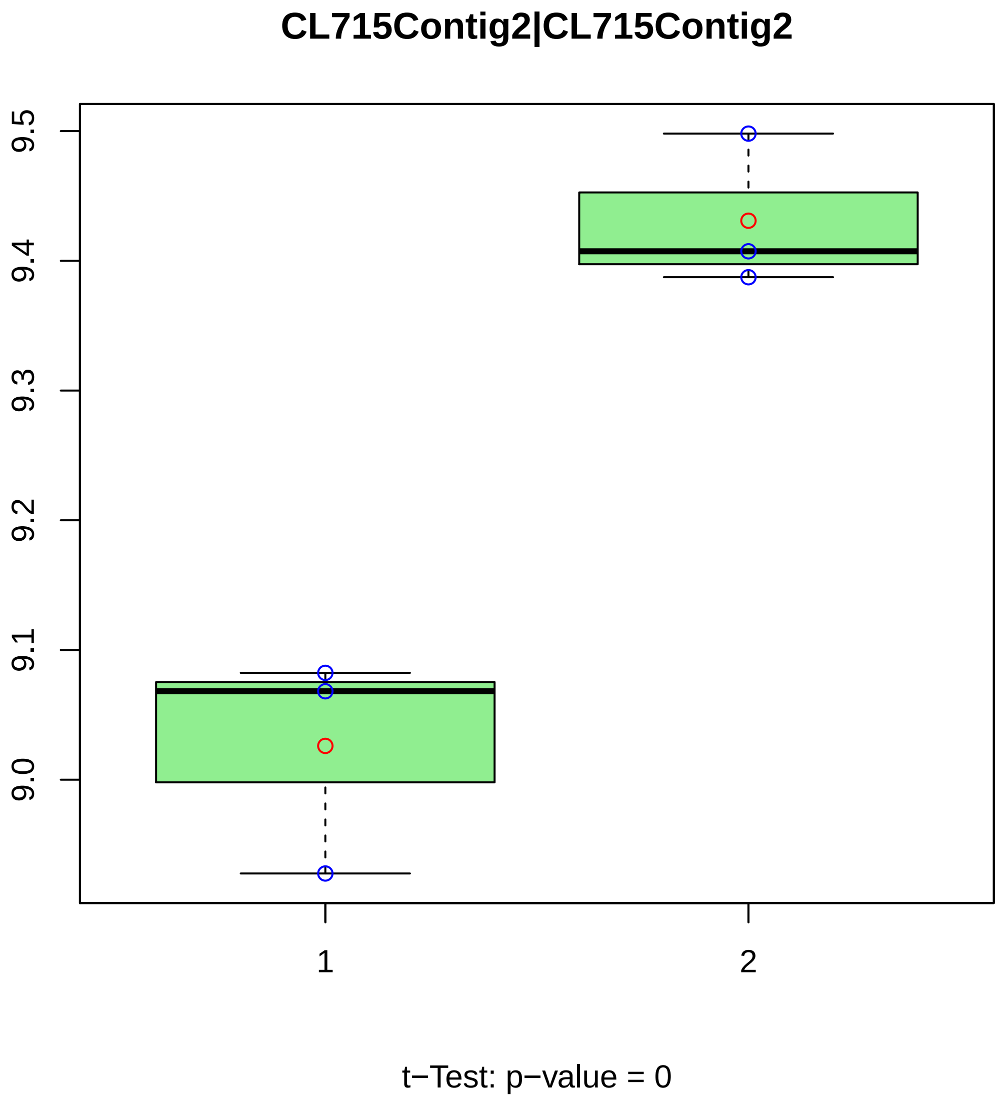
CL715Contig2|CL715Contig2
9.0
9.1
9.2
9.3
9.4
9.5
1
2
t−Test: p−value = 0

# CL7169Contig2|CL7169Contig2

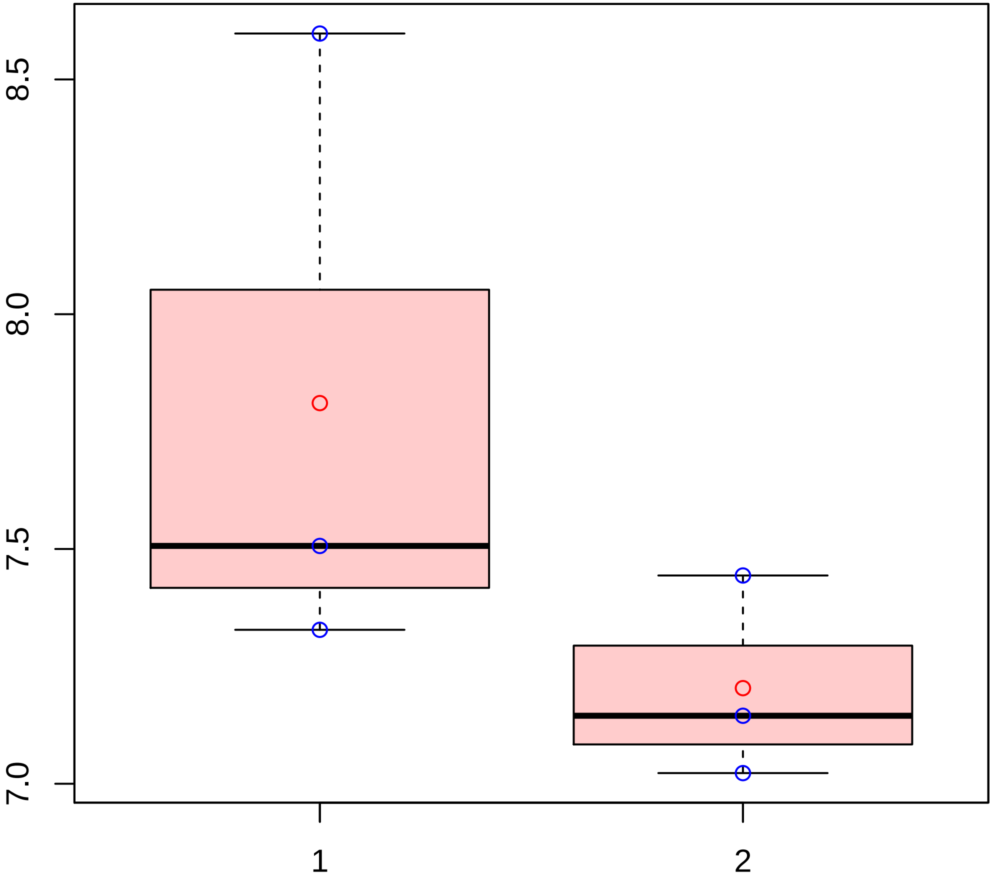


t−Test: p−value = 0.26

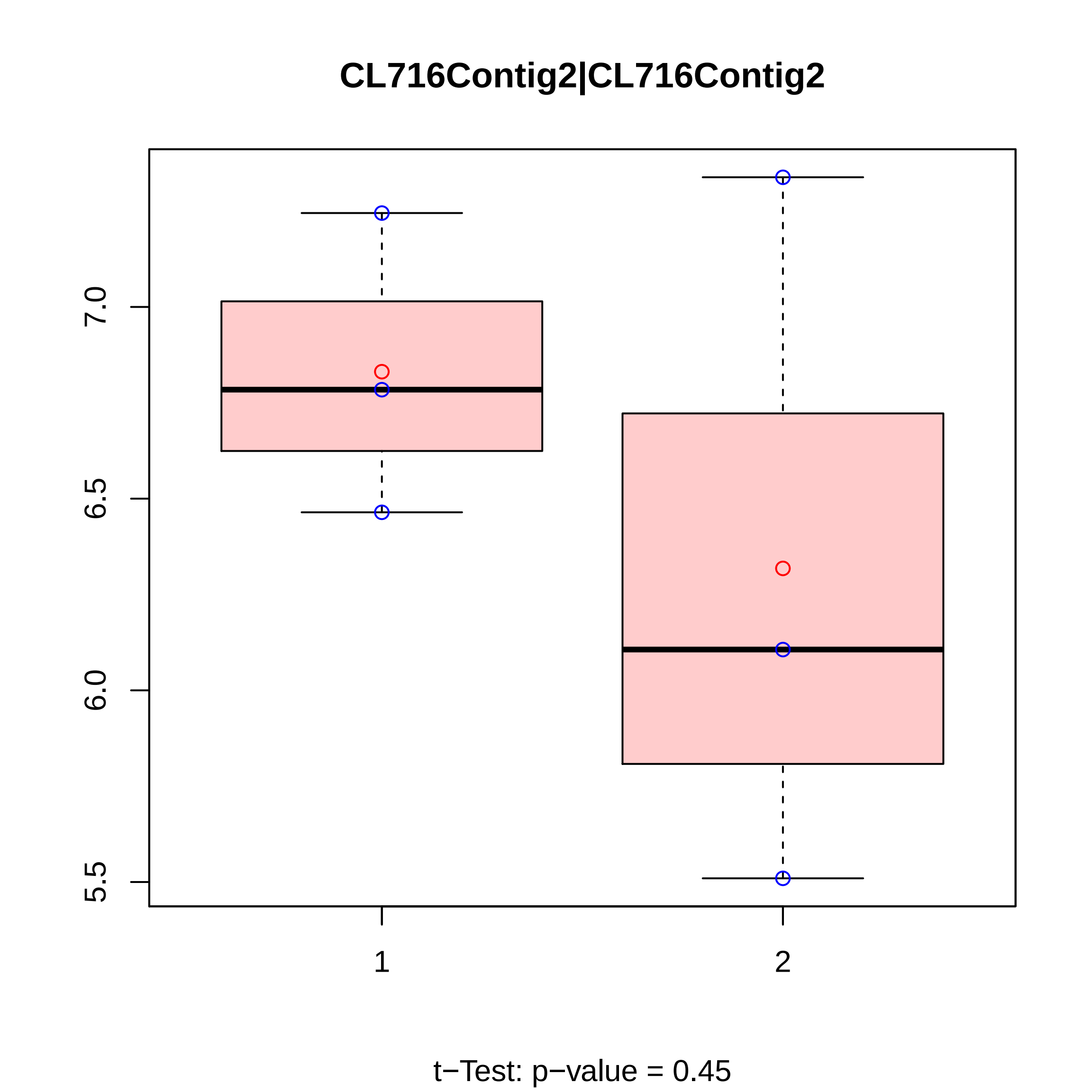
CL716Contig2|CL716Contig2
7.0
6.5
6.0
5.5
1
2
t−Test: p−value = 0.45

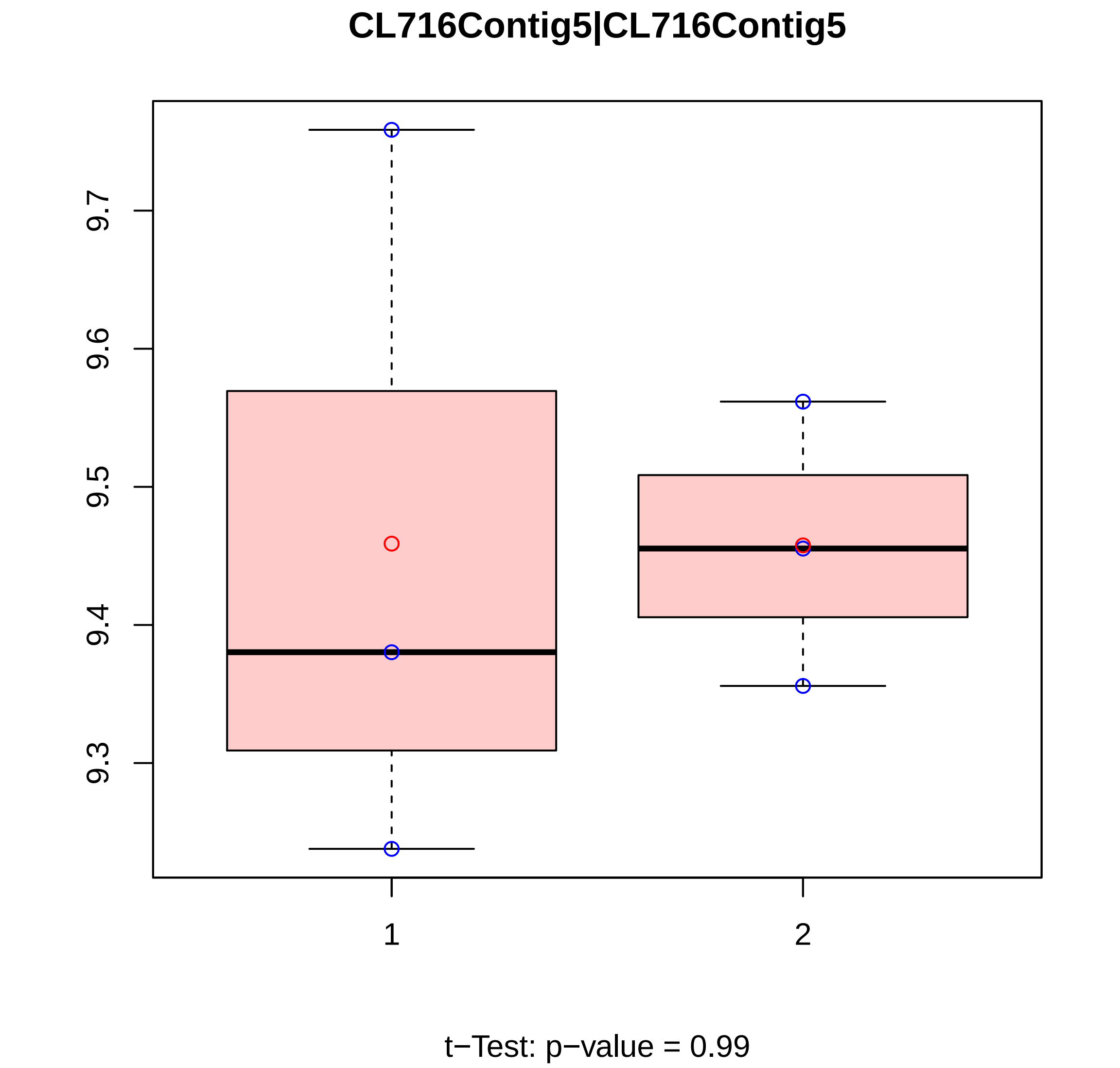
CL716Contig5|CL716Contig5
9.3
9.4
9.5
9.6
9.7
1
2
t−Test: p−value = 0.99

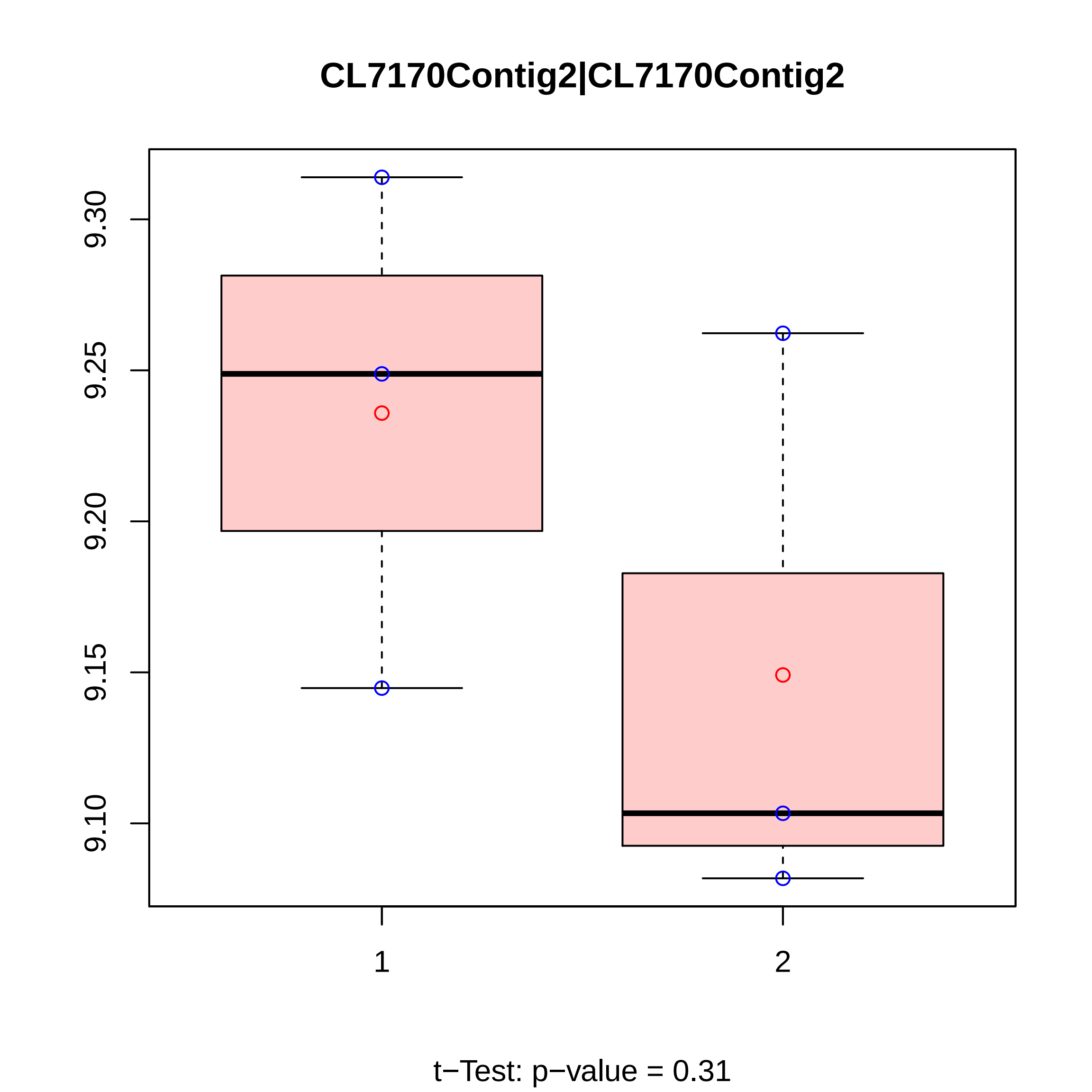
CL7170Contig2|CL7170Contig2
9.10
9.15
9.20
9.25
9.30
1
2
t−Test: p−value = 0.31

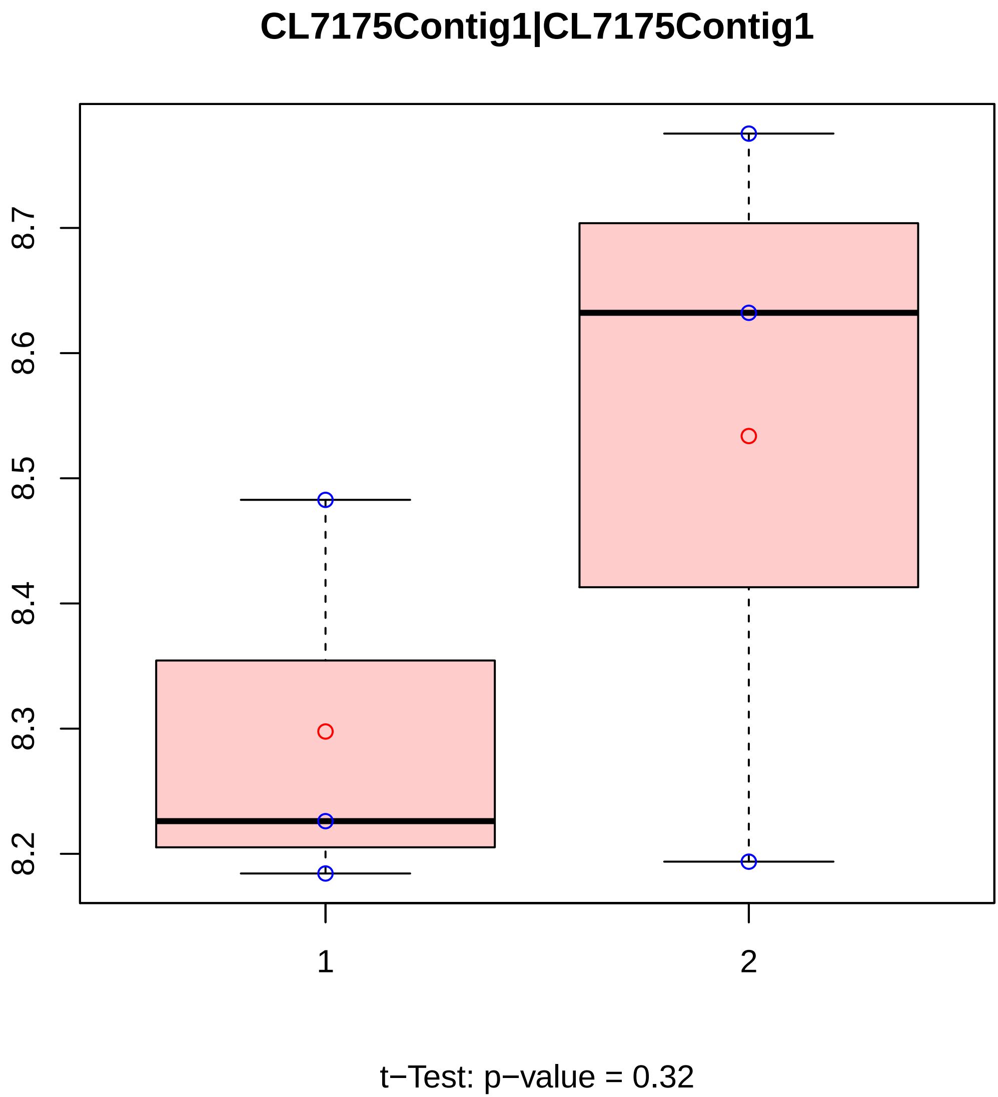
CL7175Contig1|CL7175Contig1
8.2
8.3
8.4
8.5
8.6
8.7
1
2
t−Test: p−value = 0.32

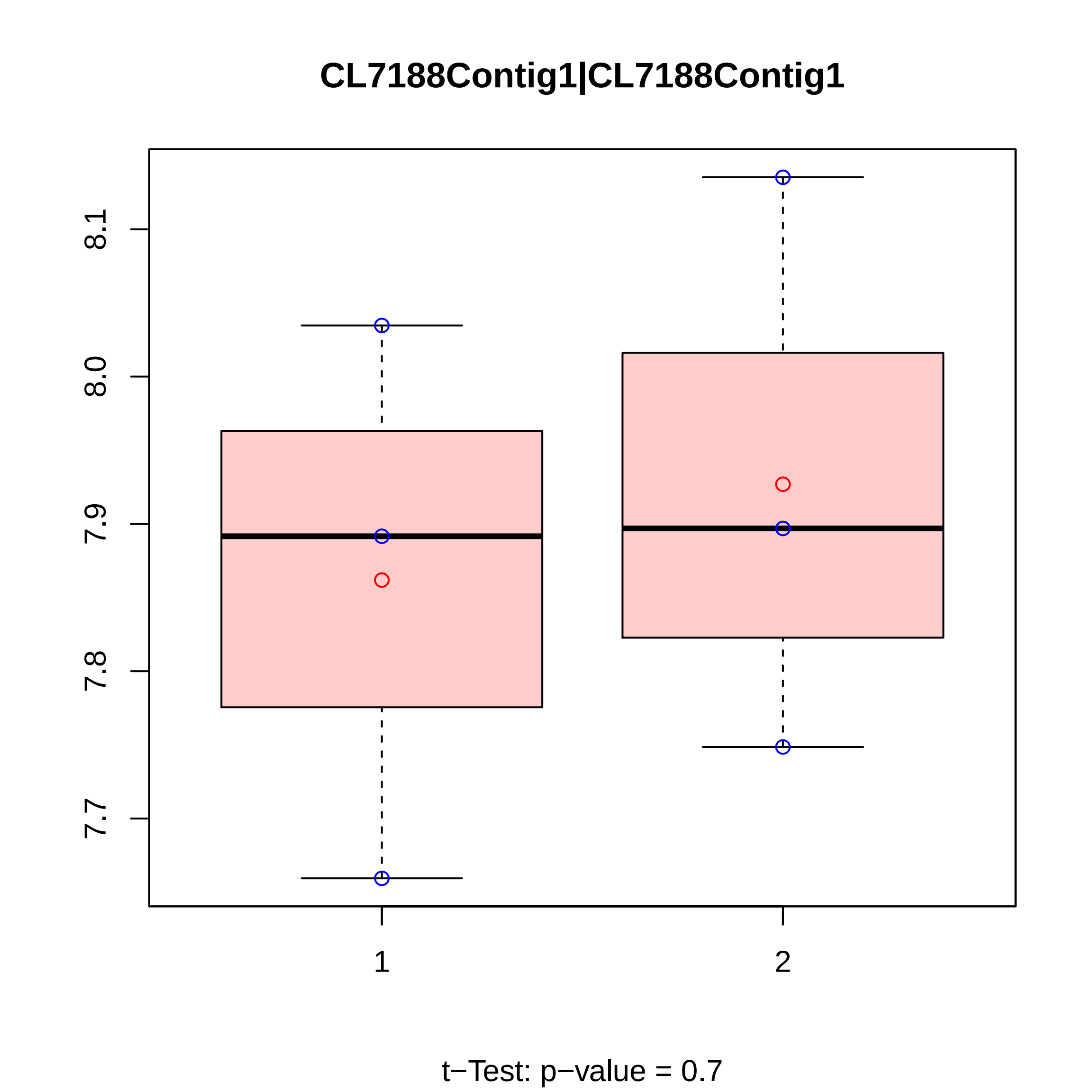
CL7188Contig1|CL7188Contig1
8.1
8.0
7.9
7.8
7.7
1
2
t−Test: p−value = 0.7

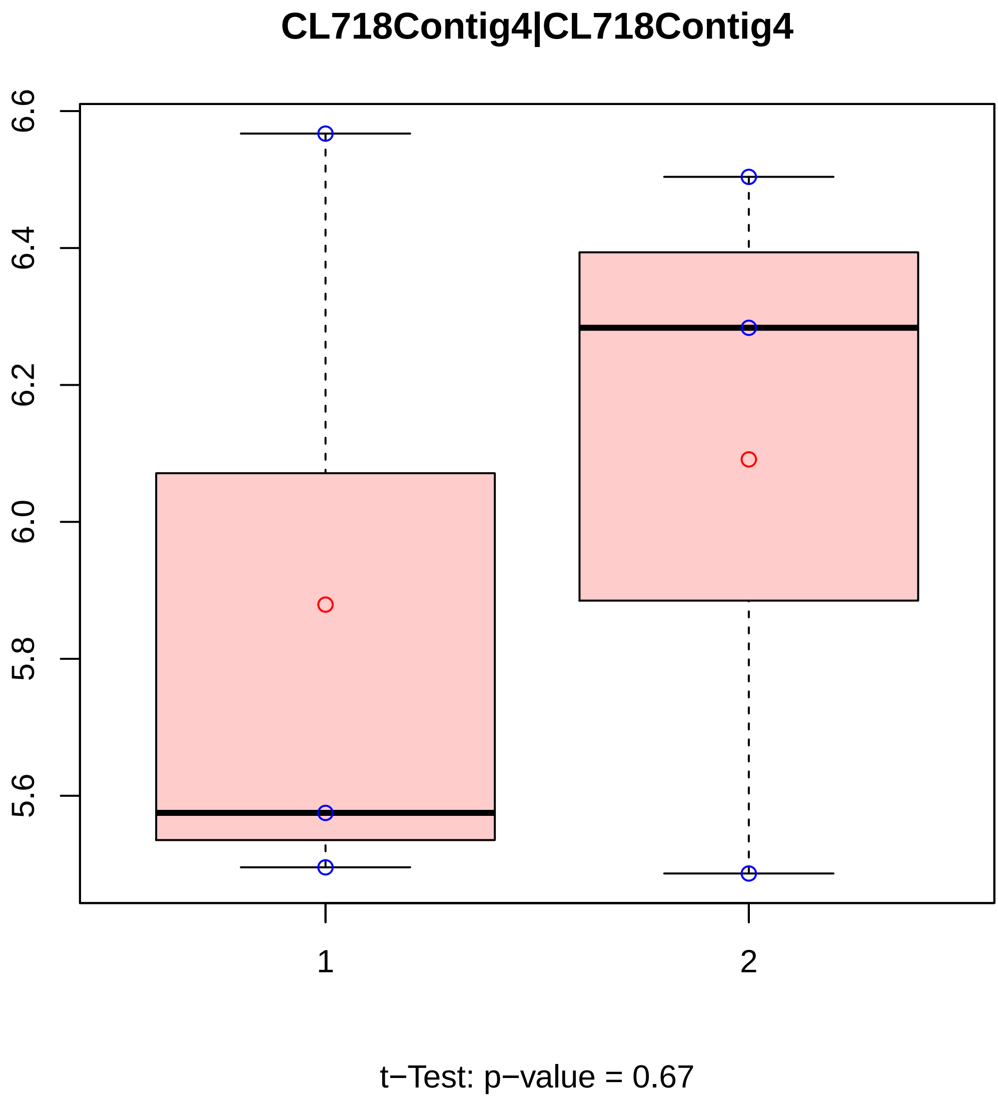
CL718Contig4|CL718Contig4
6.6
6.4
6.2
6.0
5.8
5.6
1
2
t−Test: p−value = 0.67

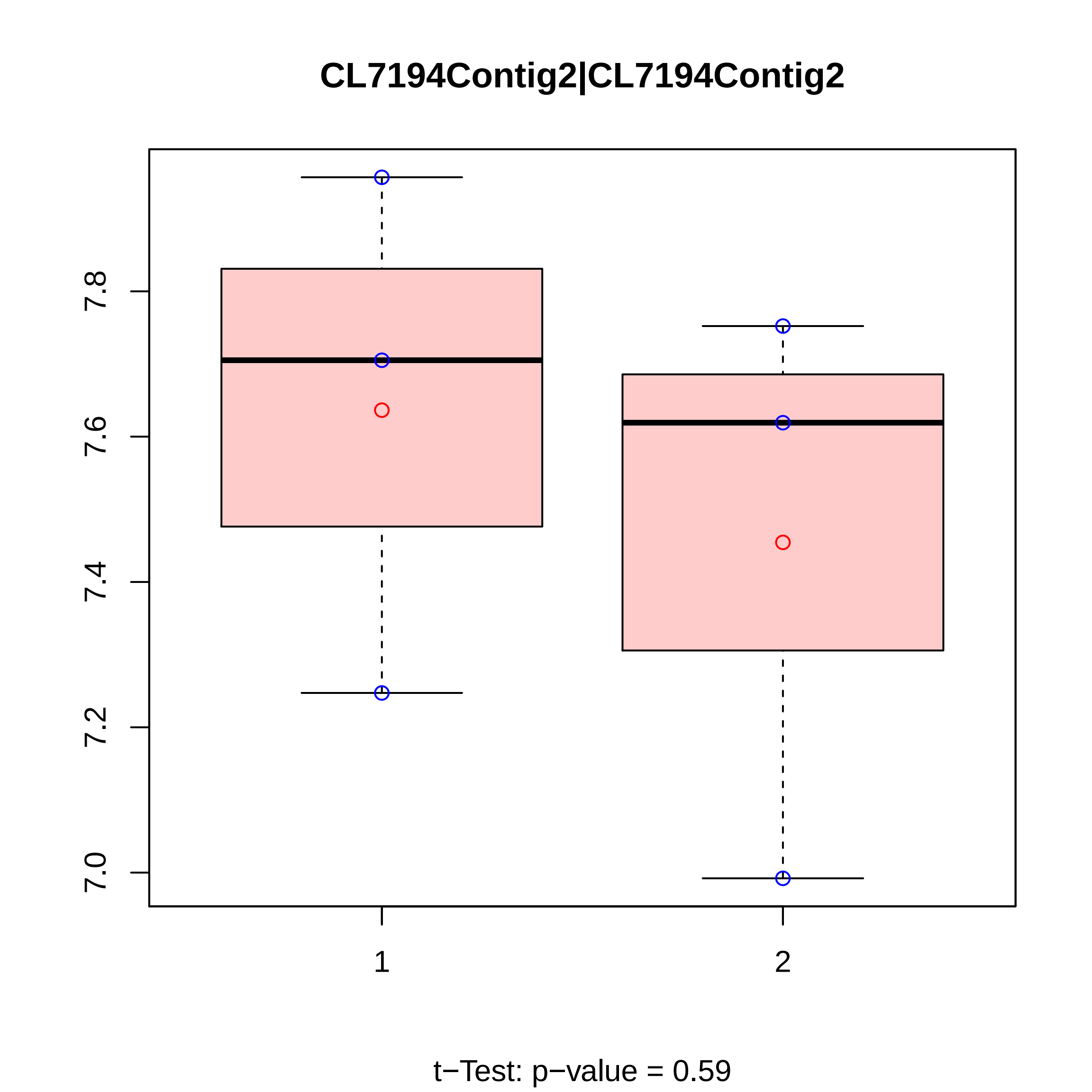

CL7194Contig2|CL7194Contig2
7.0
7.2
7.4
7.6
7.8
1
2
t−Test: p−value = 0.59

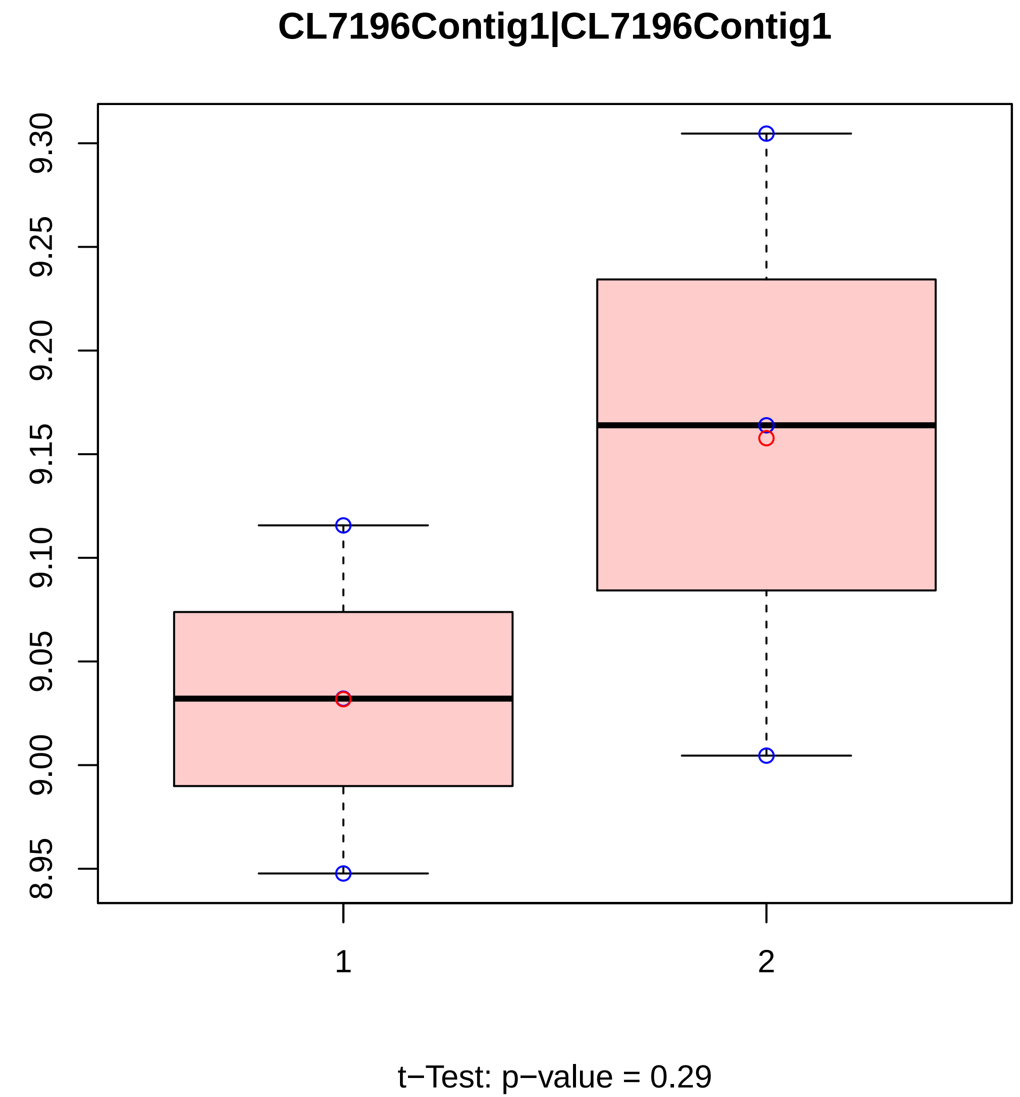
CL7196Contig1|CL7196Contig1
9.30
9.25
9.20
9.15
9.10
9.05
9.00
8.95
1
2
t−Test: p−value = 0.29

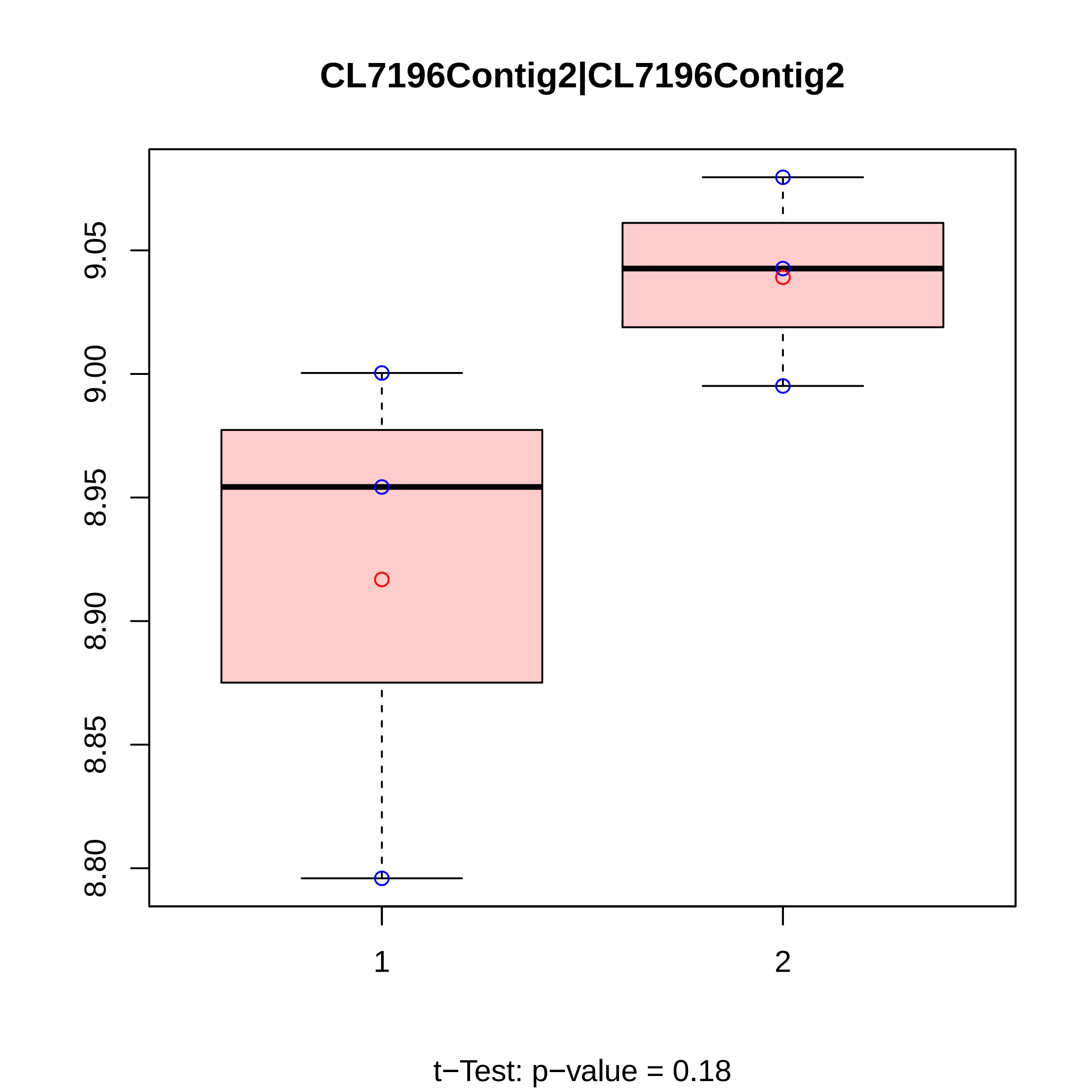
CL7196Contig2|CL7196Contig2
9.05
9.00
8.95
8.90
8.85
8.80
1
2
t−Test: p−value = 0.18

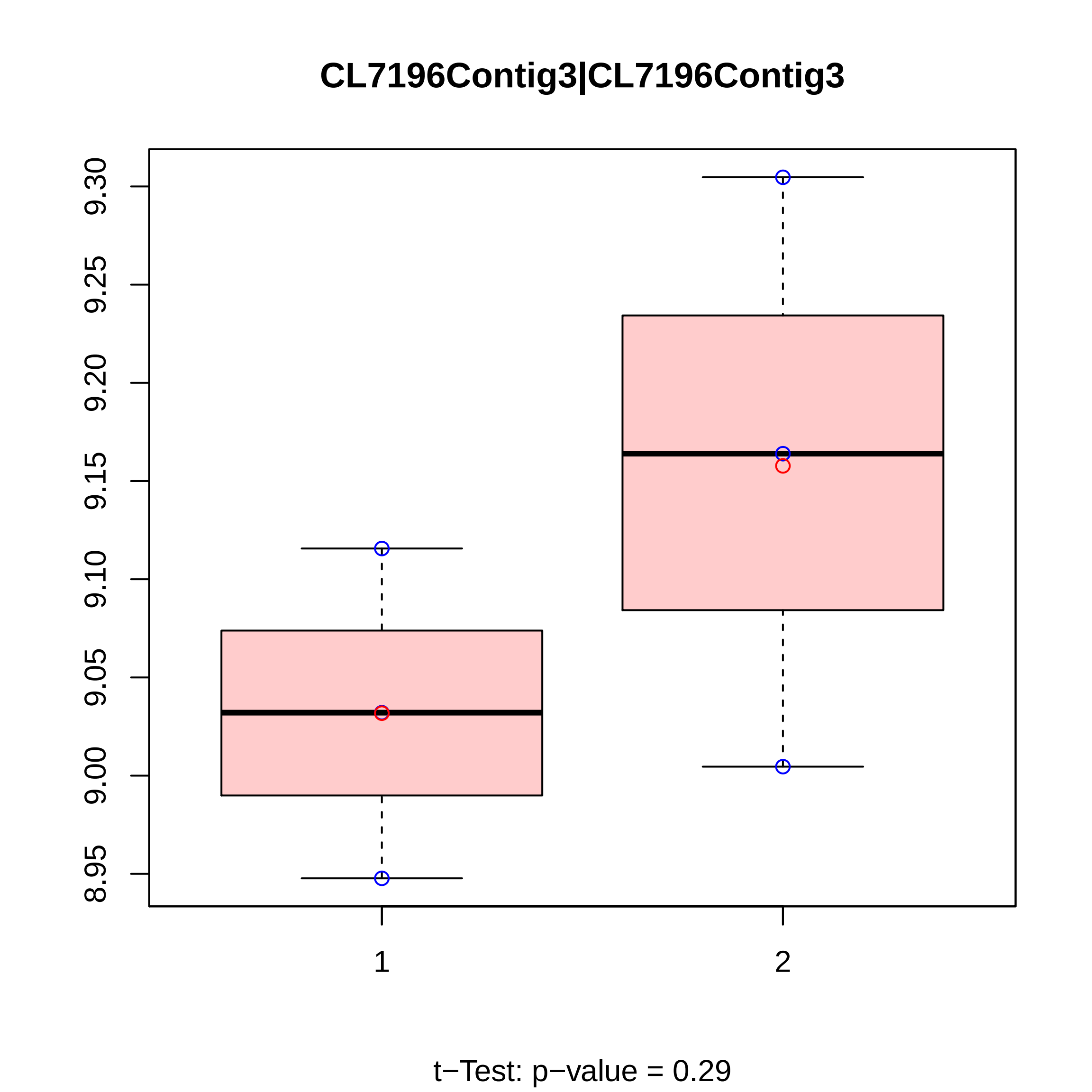

CL7196Contig3|CL7196Contig3
8.95
9.00
9.05
9.10
9.15
9.20
9.25
9.30
1
2
t−Test: p−value = 0.29

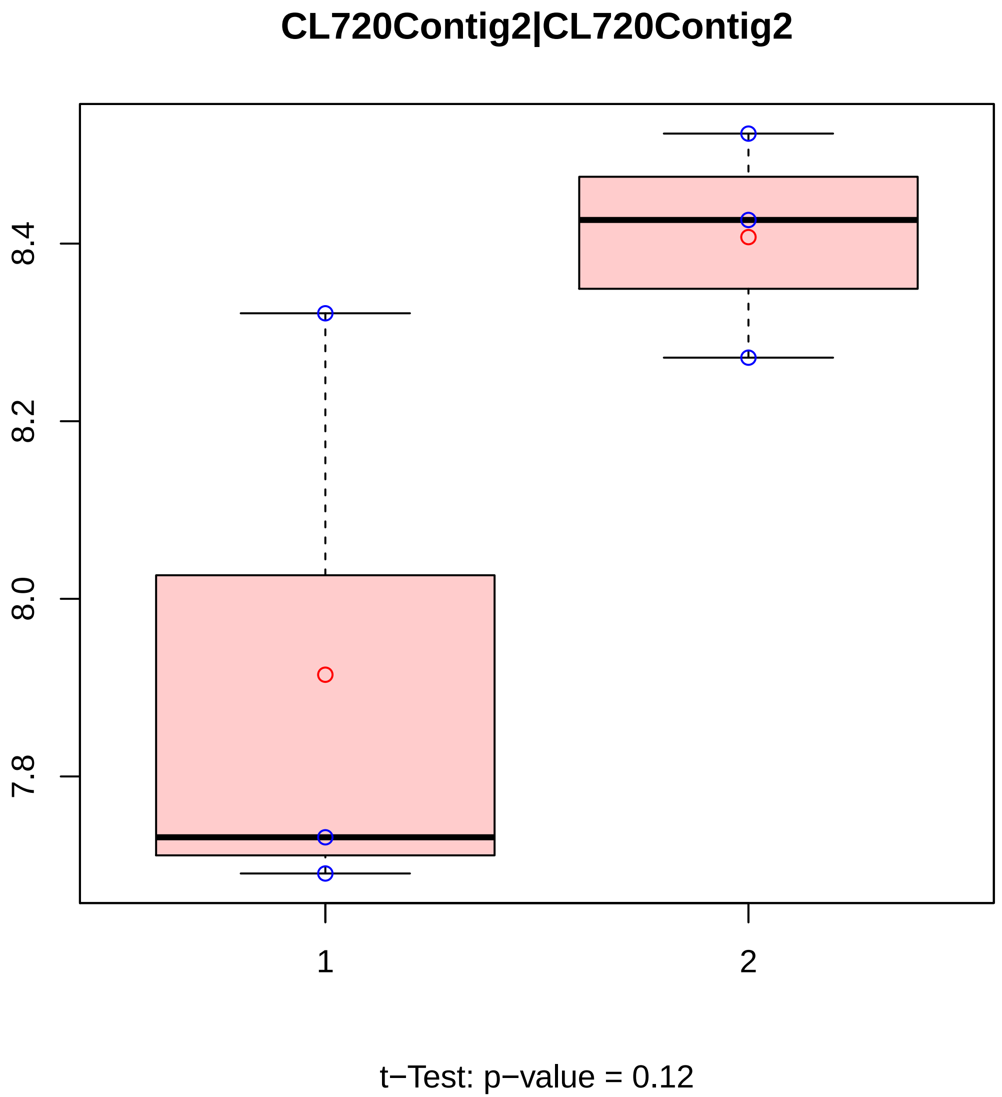
CL720Contig2|CL720Contig2
8.4
8.2
8.0
7.8
1
2
t−Test: p−value = 0.12

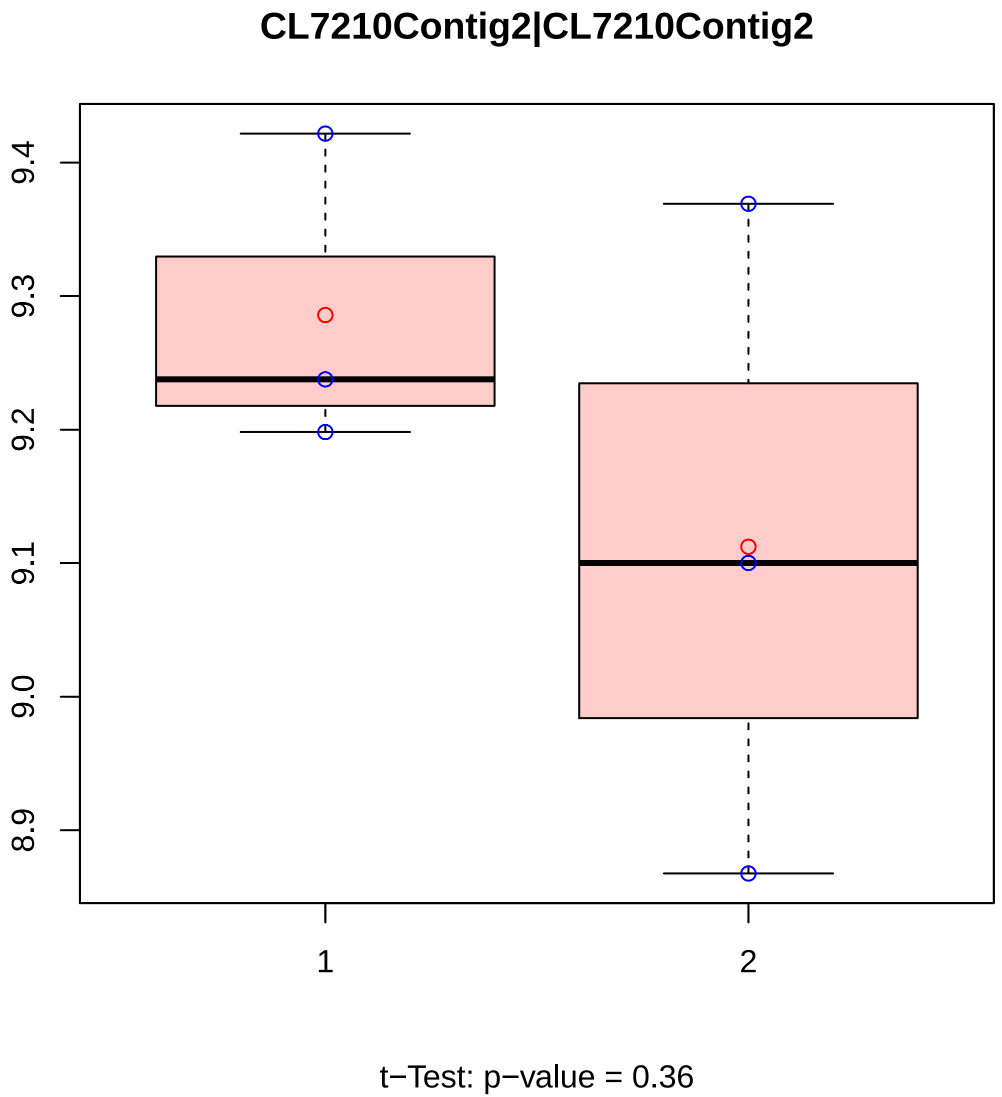

CL7210Contig2|CL7210Contig2
9.4
9.3
9.2
9.1
9.0
8.9
1
2
t−Test: p−value = 0.36

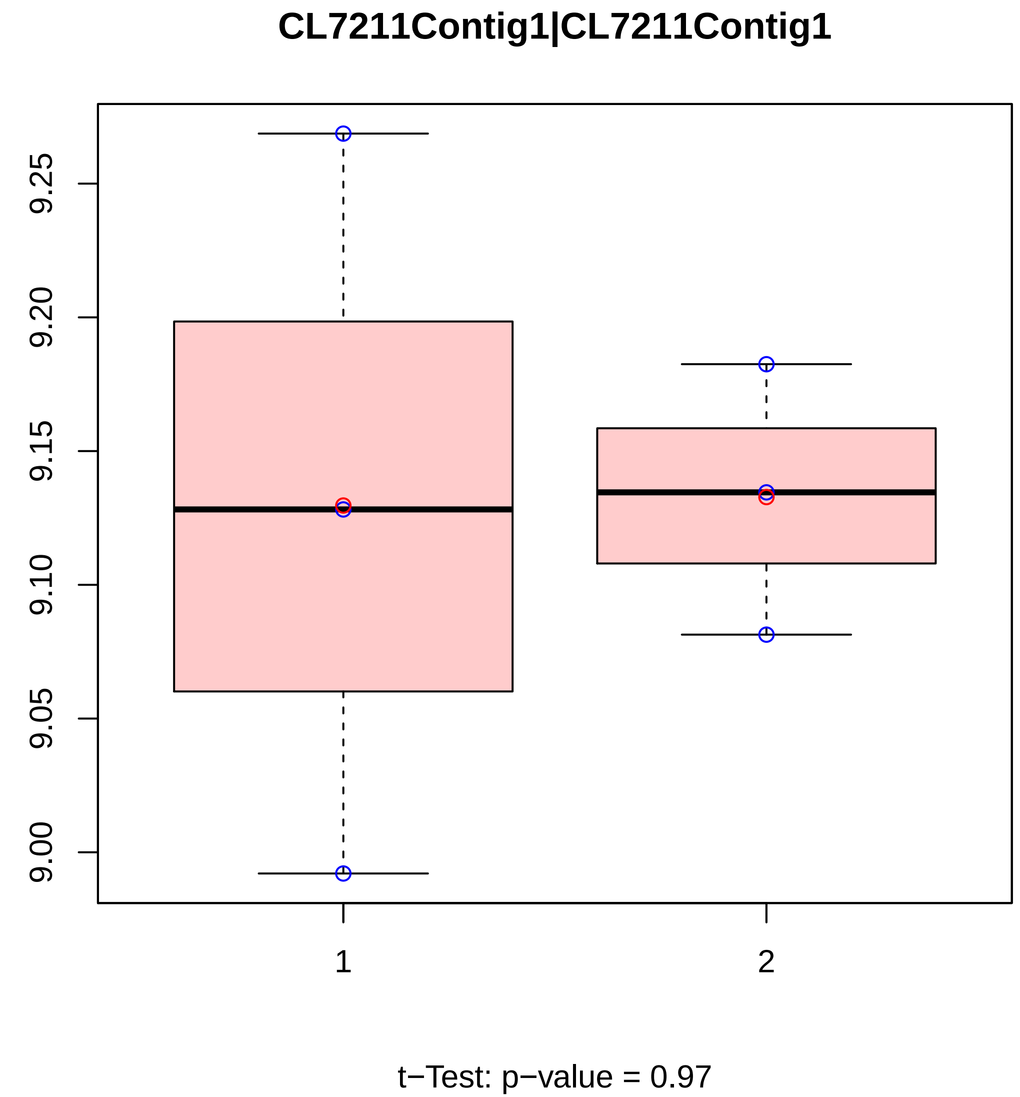
CL7211Contig1|CL7211Contig1
9.00
9.05
9.10
9.15
9.20
9.25
1
2
t−Test: p−value = 0.97

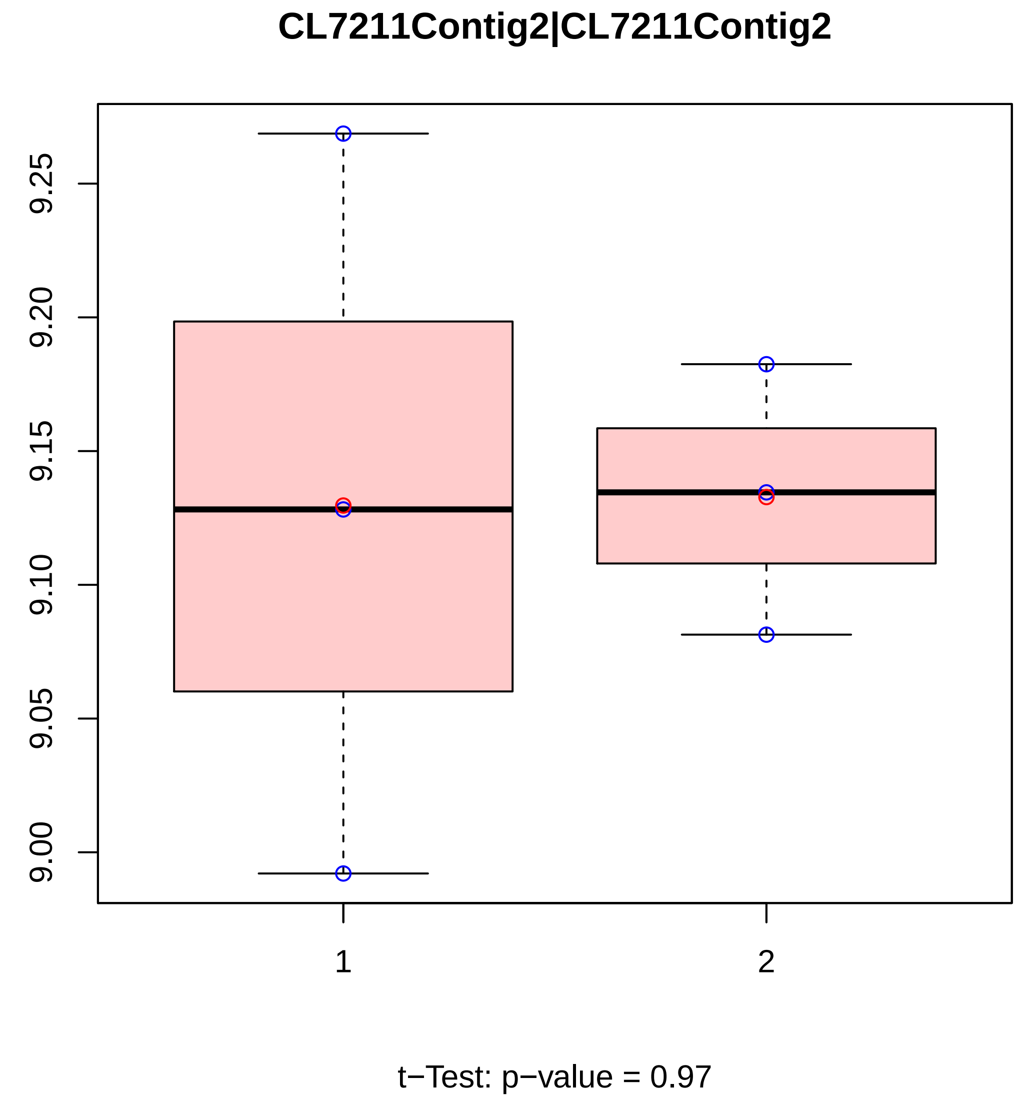

CL7211Contig2|CL7211Contig2
9.00
9.05
9.10
9.15
9.20
9.25
1
2
t−Test: p−value = 0.97

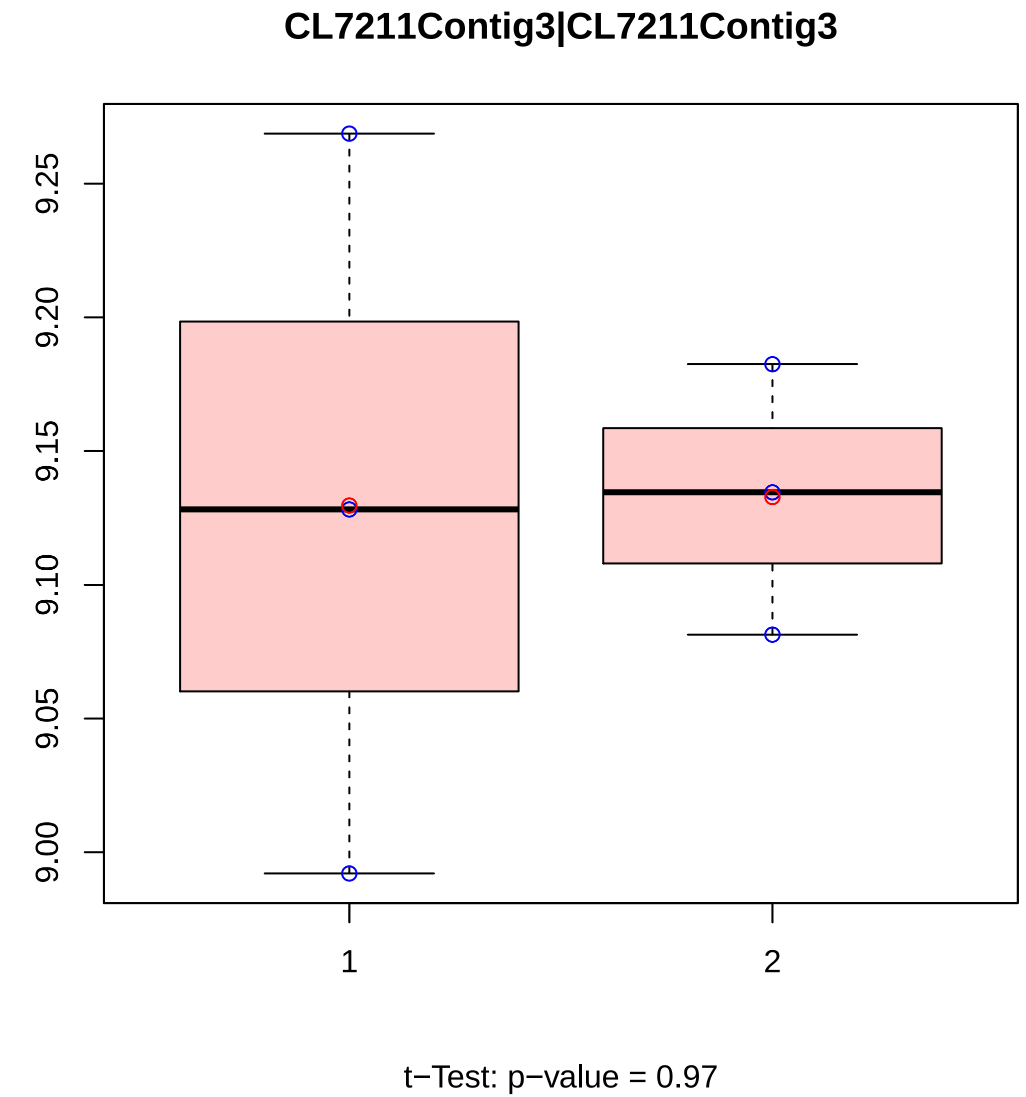
CL7211Contig3|CL7211Contig3
9.00
9.05
9.10
9.15
9.20
9.25
1
2
t−Test: p−value = 0.97

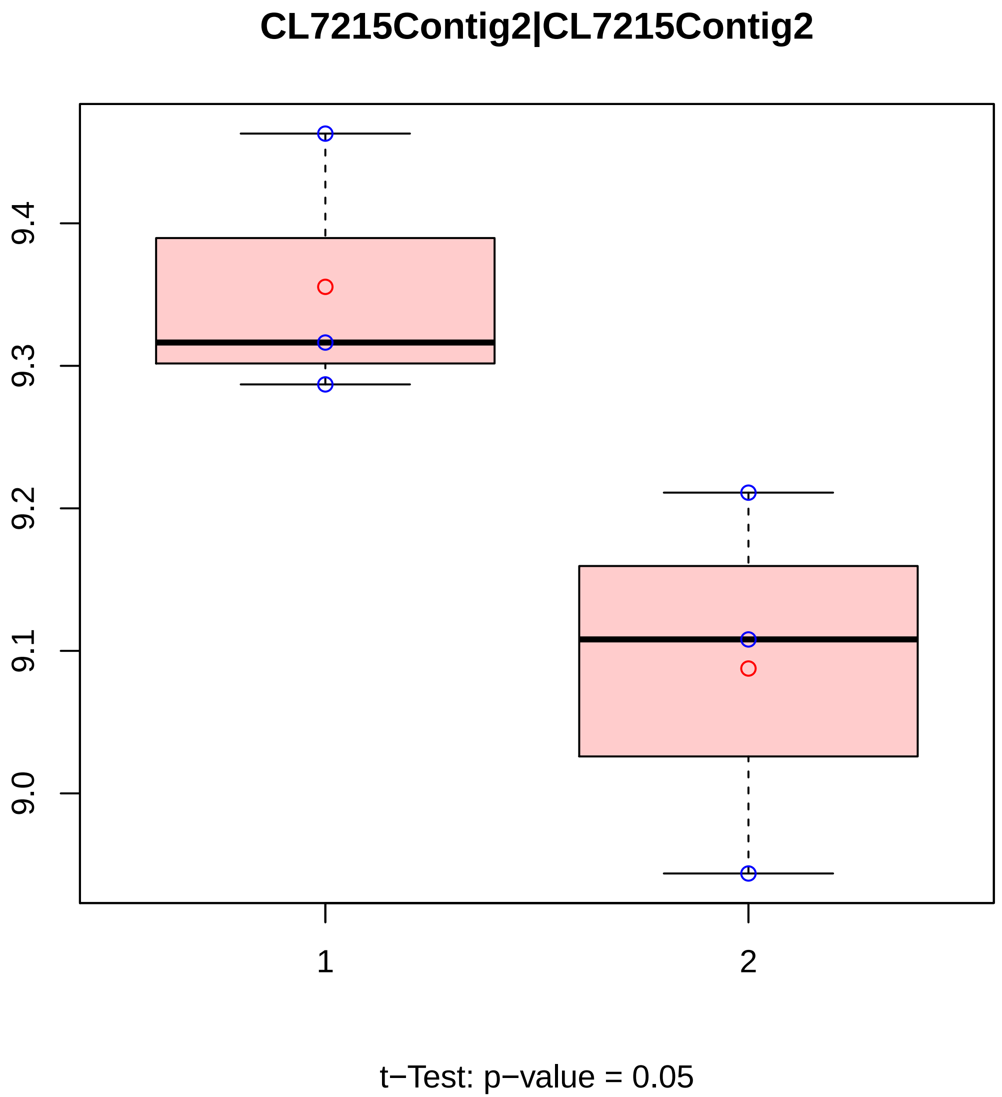
CL7215Contig2|CL7215Contig2
9.0
9.1
9.2
9.3
9.4
1
2
t−Test: p−value = 0.05

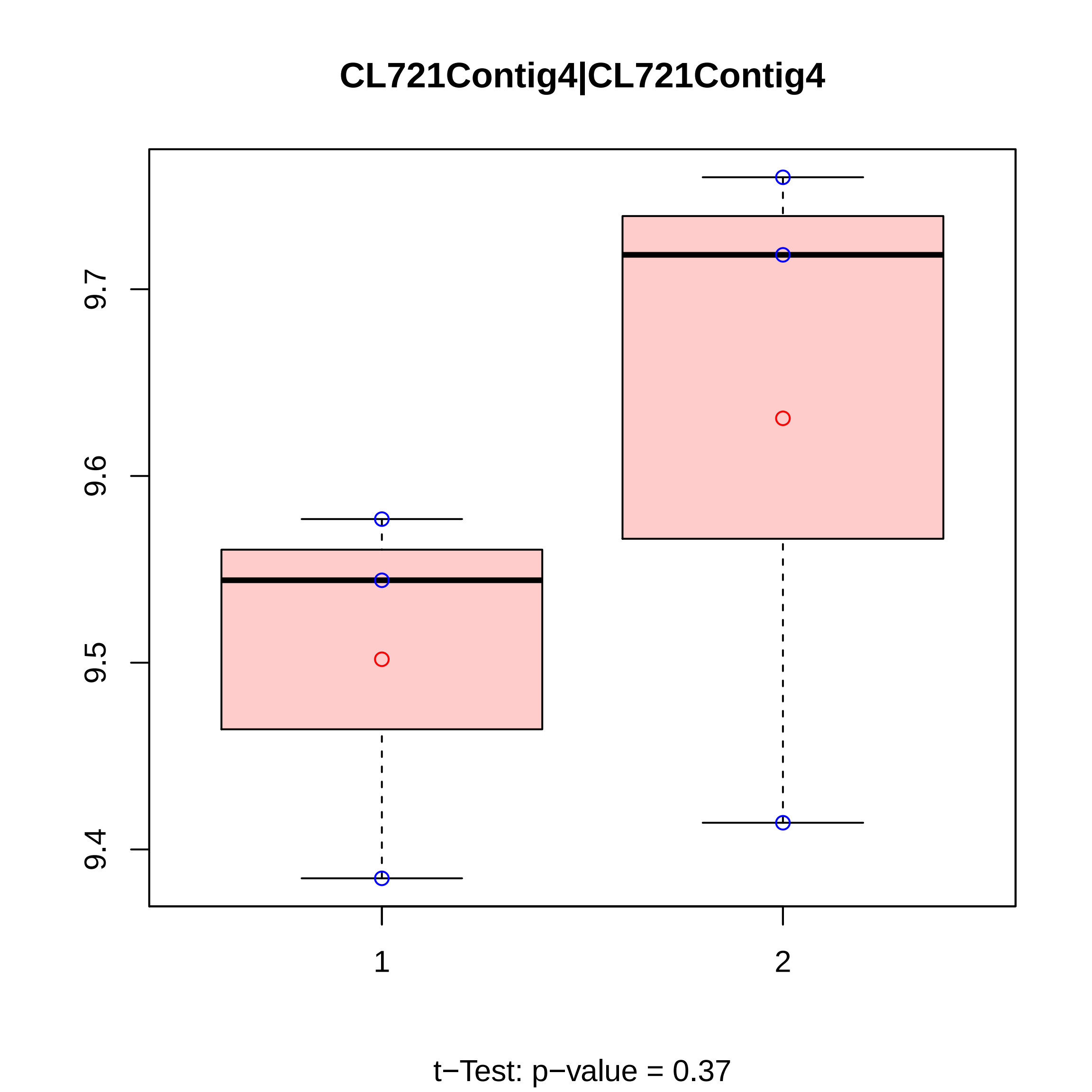
CL721Contig4|CL721Contig4
9.4
9.5
9.6
9.7
1
2
t−Test: p−value = 0.37

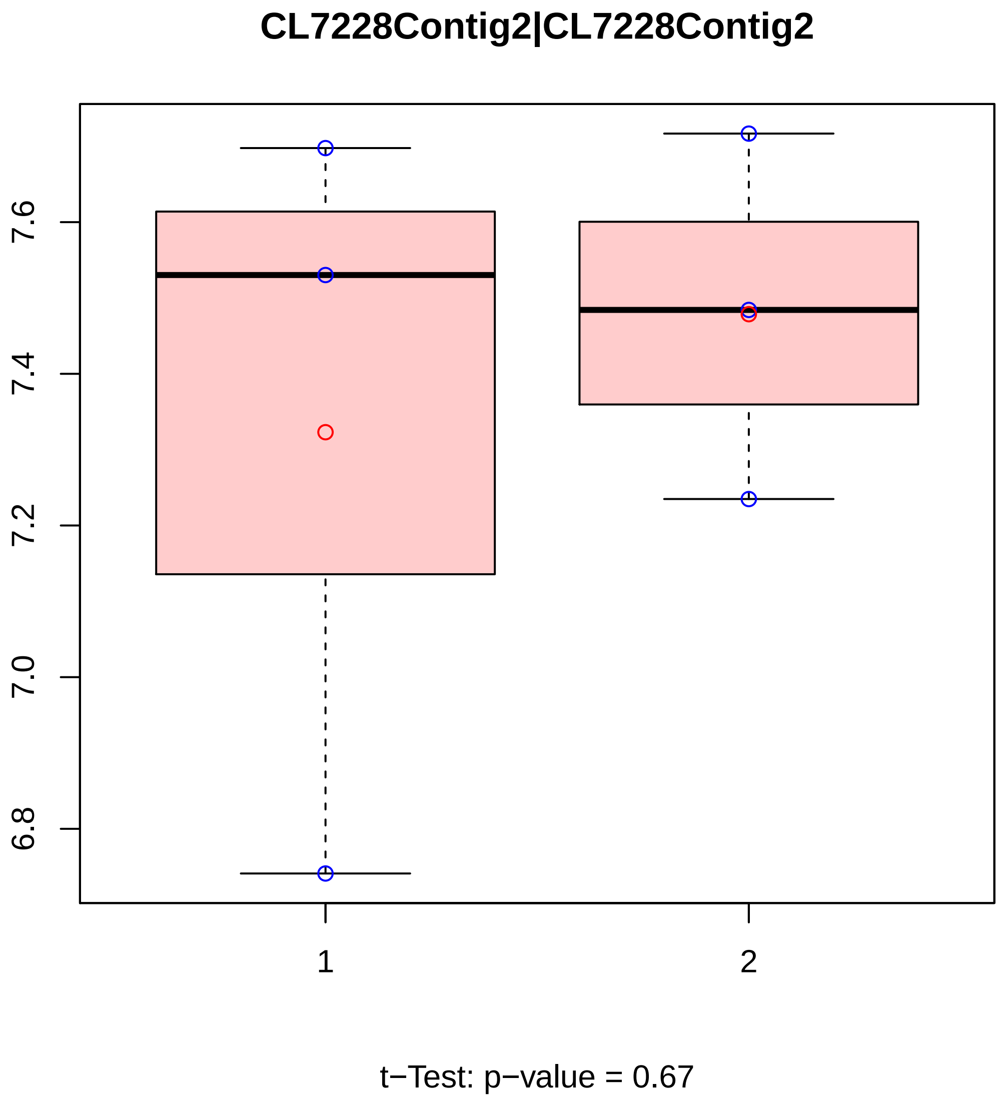
CL7228Contig2|CL7228Contig2
7.6
7.4
7.2
7.0
6.8
1
2
t−Test: p−value = 0.67

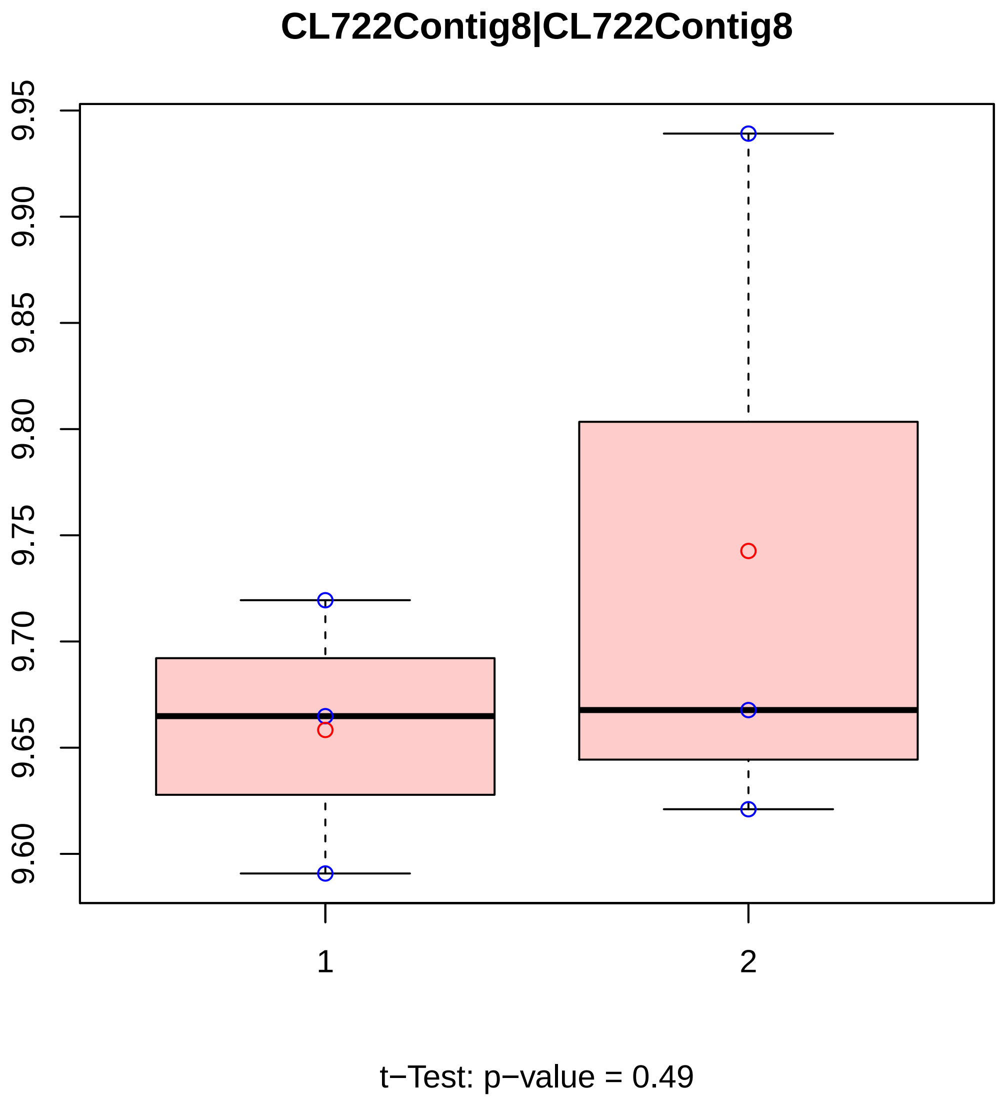
CL722Contig8|CL722Contig8
9.60
9.65
9.70
9.75
9.80
9.85
9.90
9.95
1
2
t−Test: p−value = 0.49

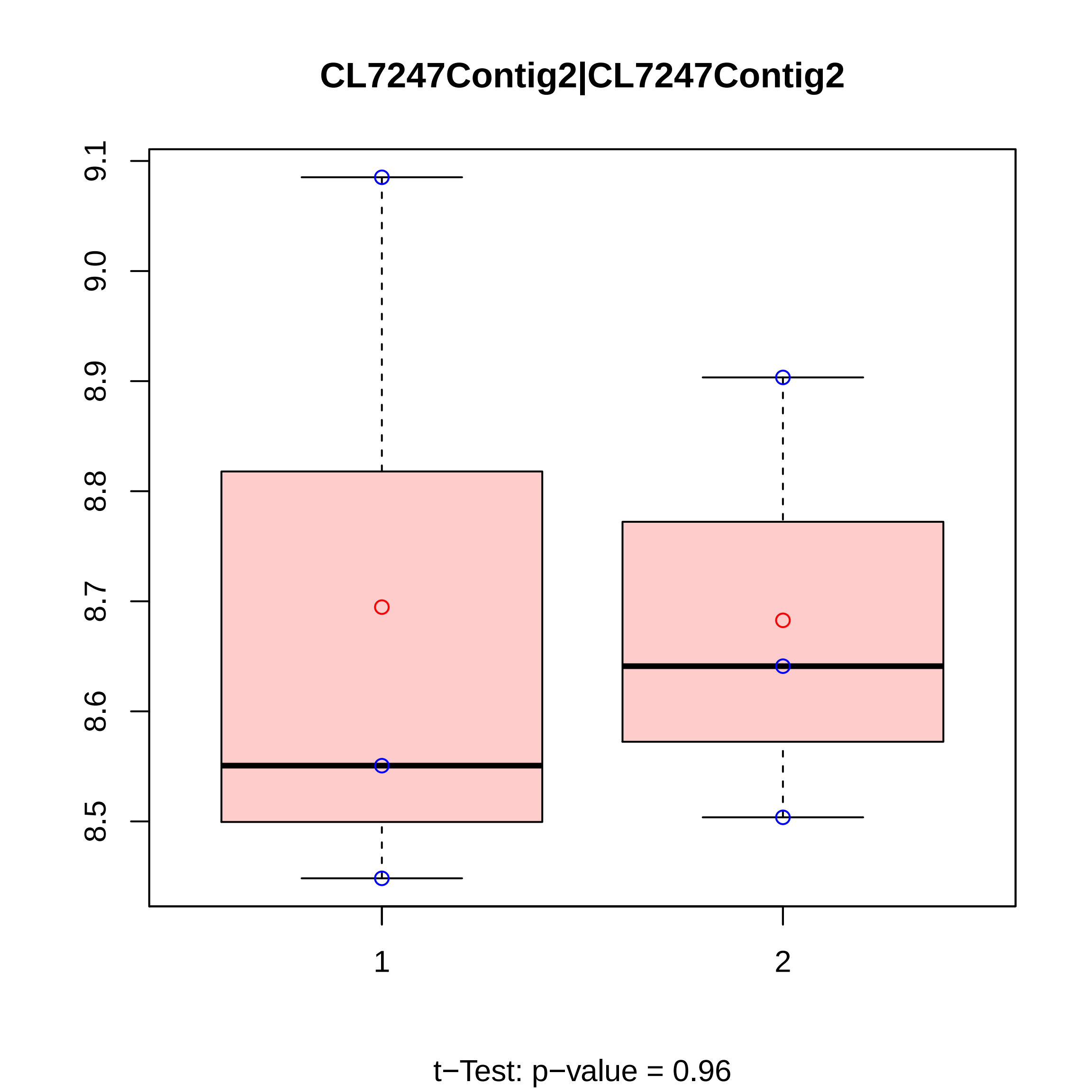

CL7247Contig2|CL7247Contig2
9.1
9.0
8.9
8.8
8.7
8.6
8.5
1
2
t−Test: p−value = 0.96

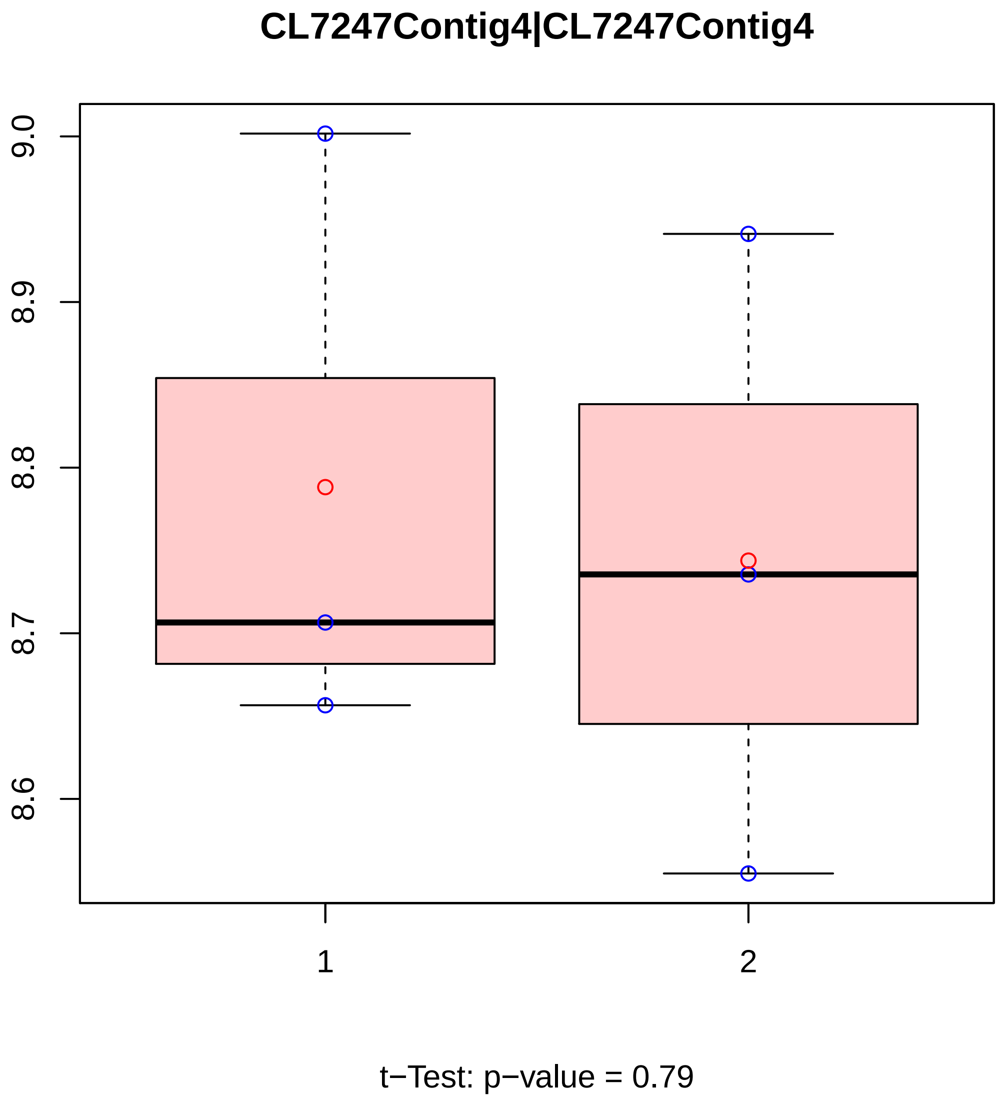

CL7247Contig4|CL7247Contig4
9.0
8.9
8.8
8.7
8.6
1
2
t−Test: p−value = 0.79

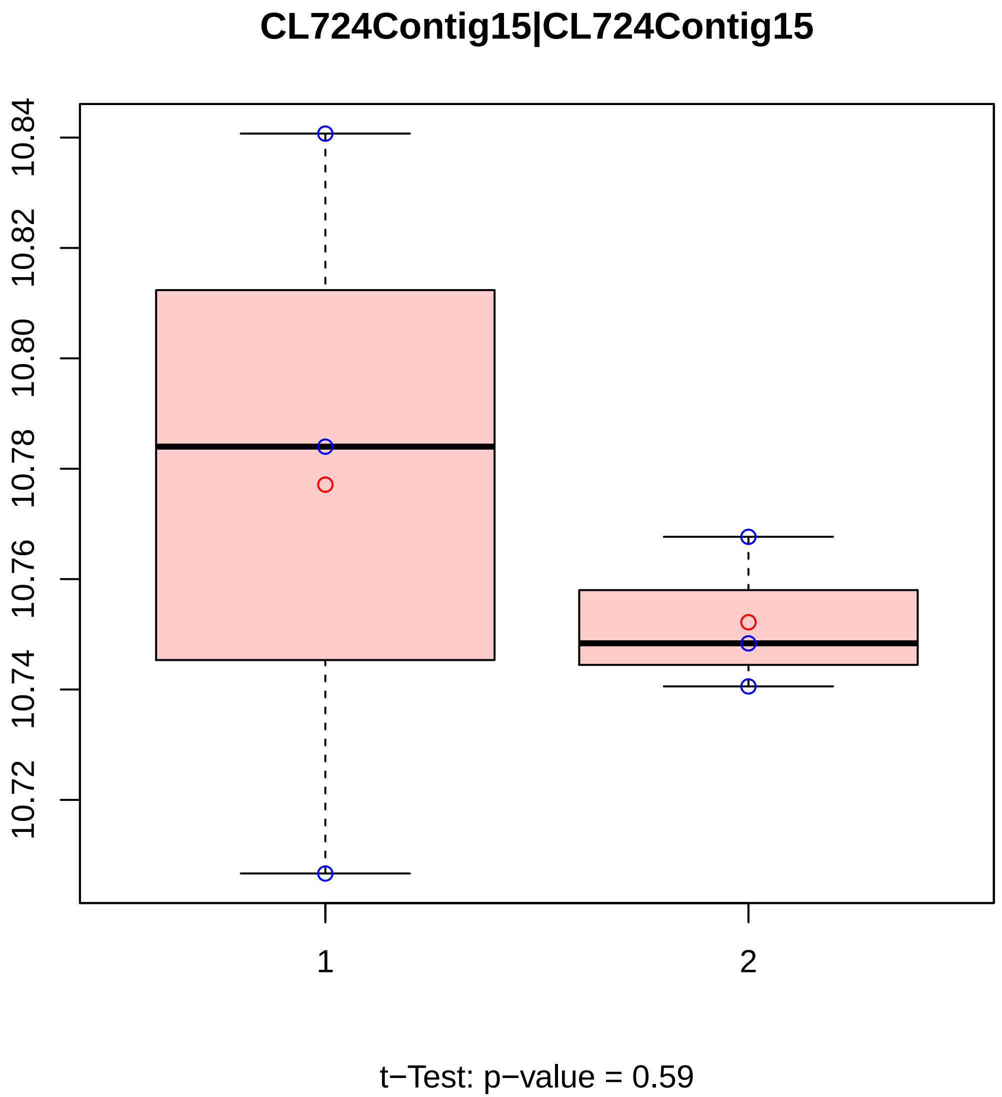

CL724Contig15|CL724Contig15
10.72
10.74
10.76
10.78
10.80
10.82
10.84
1
2
t−Test: p−value = 0.59

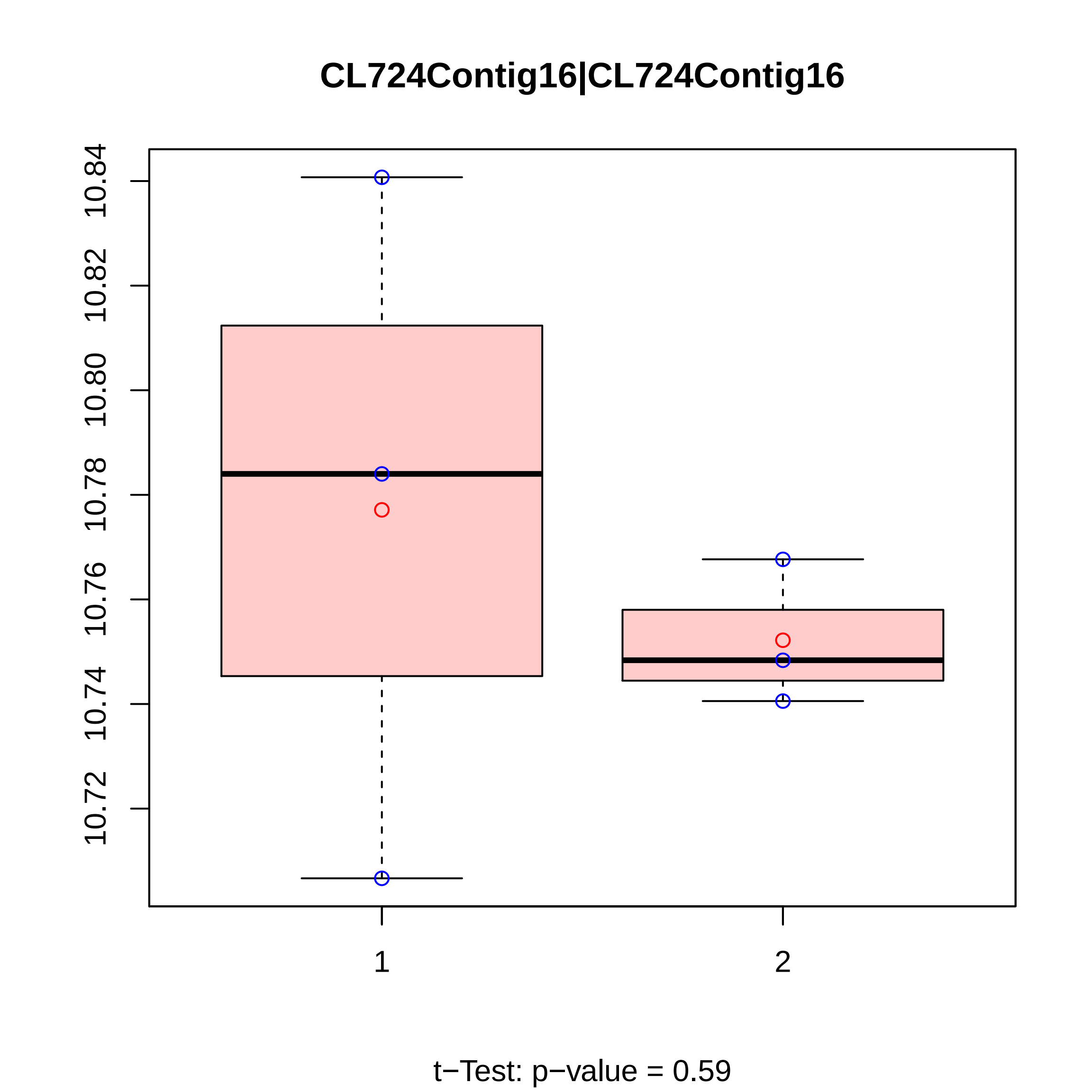

CL724Contig16|CL724Contig16
10.72
10.74
10.76
10.78
10.80
10.82
10.84
1
2
t−Test: p−value = 0.59

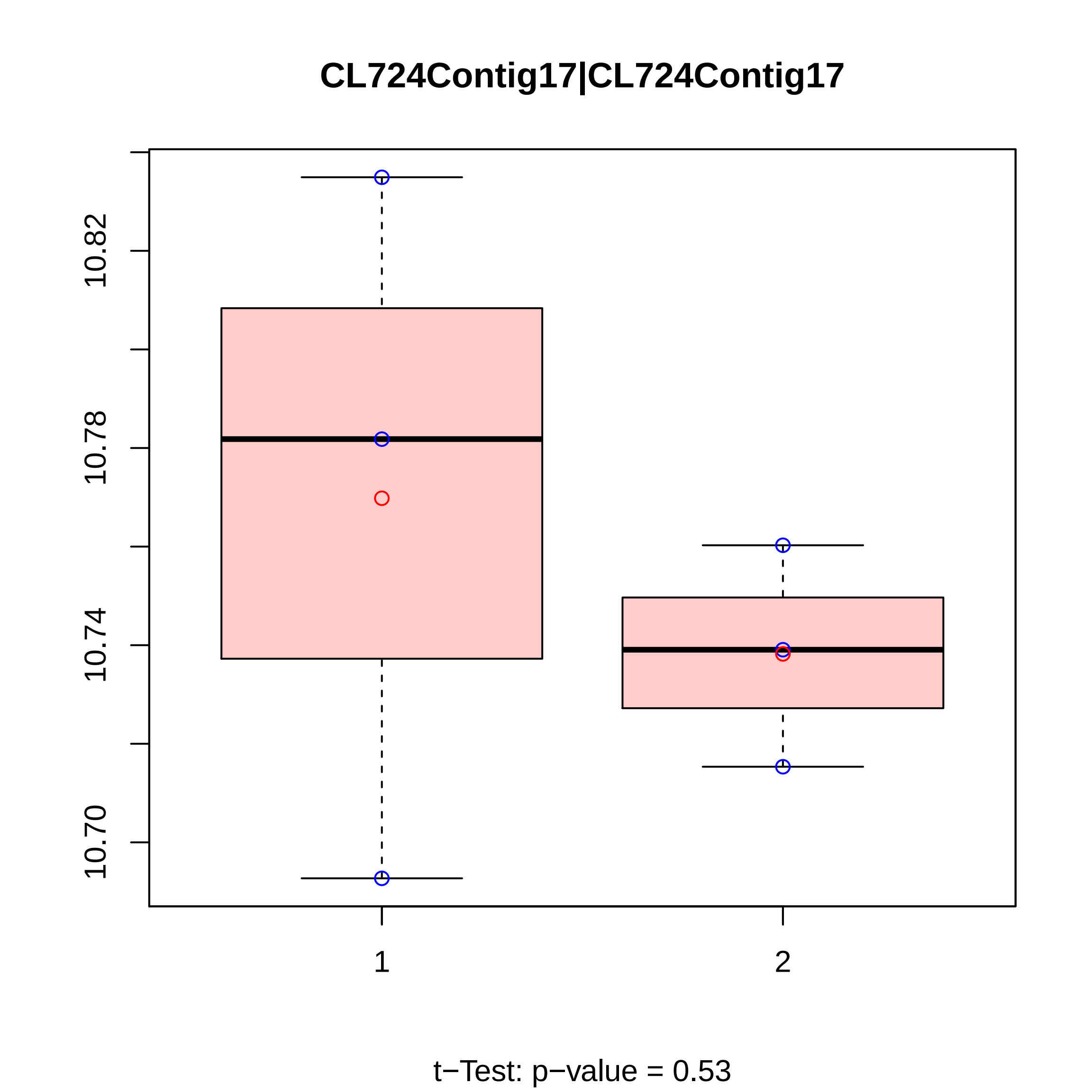
CL724Contig17|CL724Contig17
10.70
10.74
10.78
10.82
1
2
t−Test: p−value = 0.53

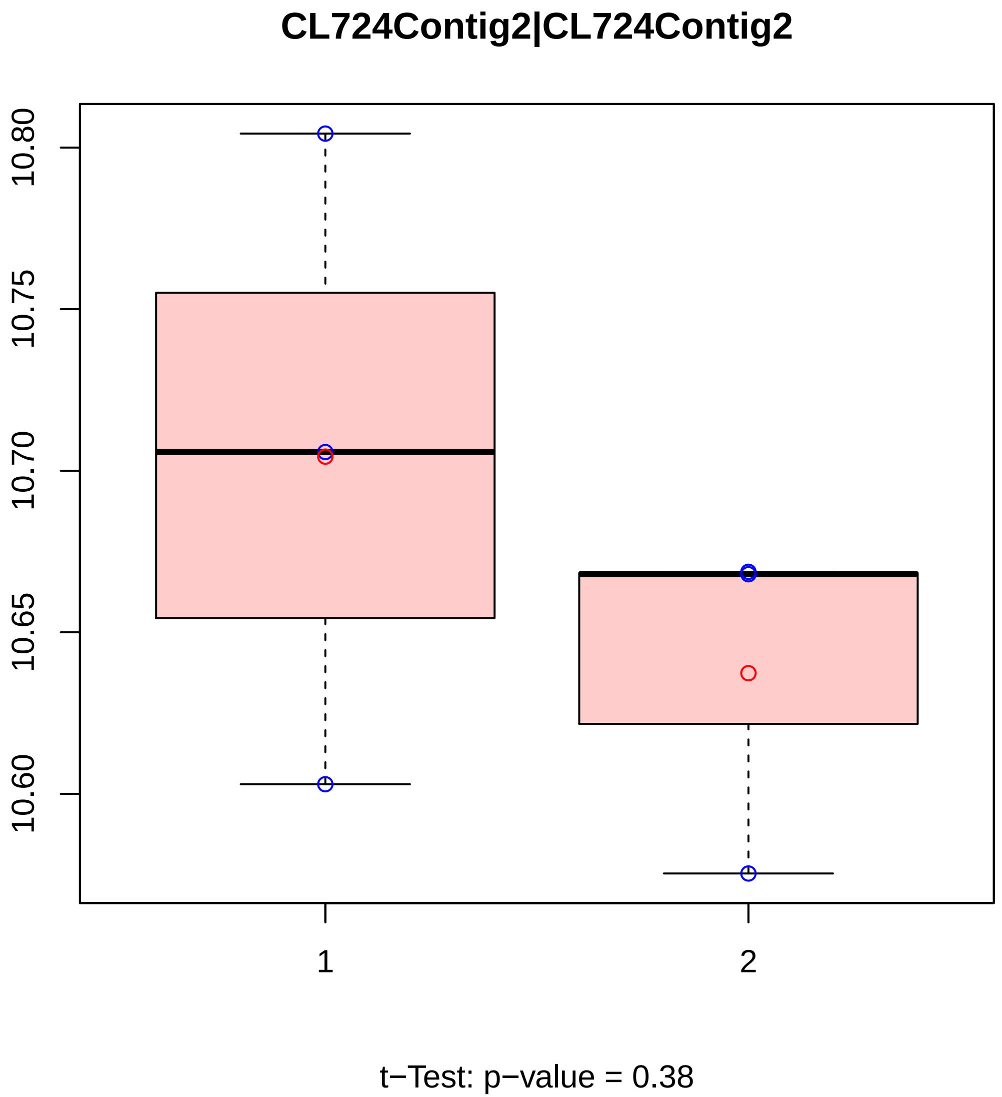
CL724Contig2|CL724Contig2
10.80
10.75
10.70
10.65
10.60
1
2
t−Test: p−value = 0.38

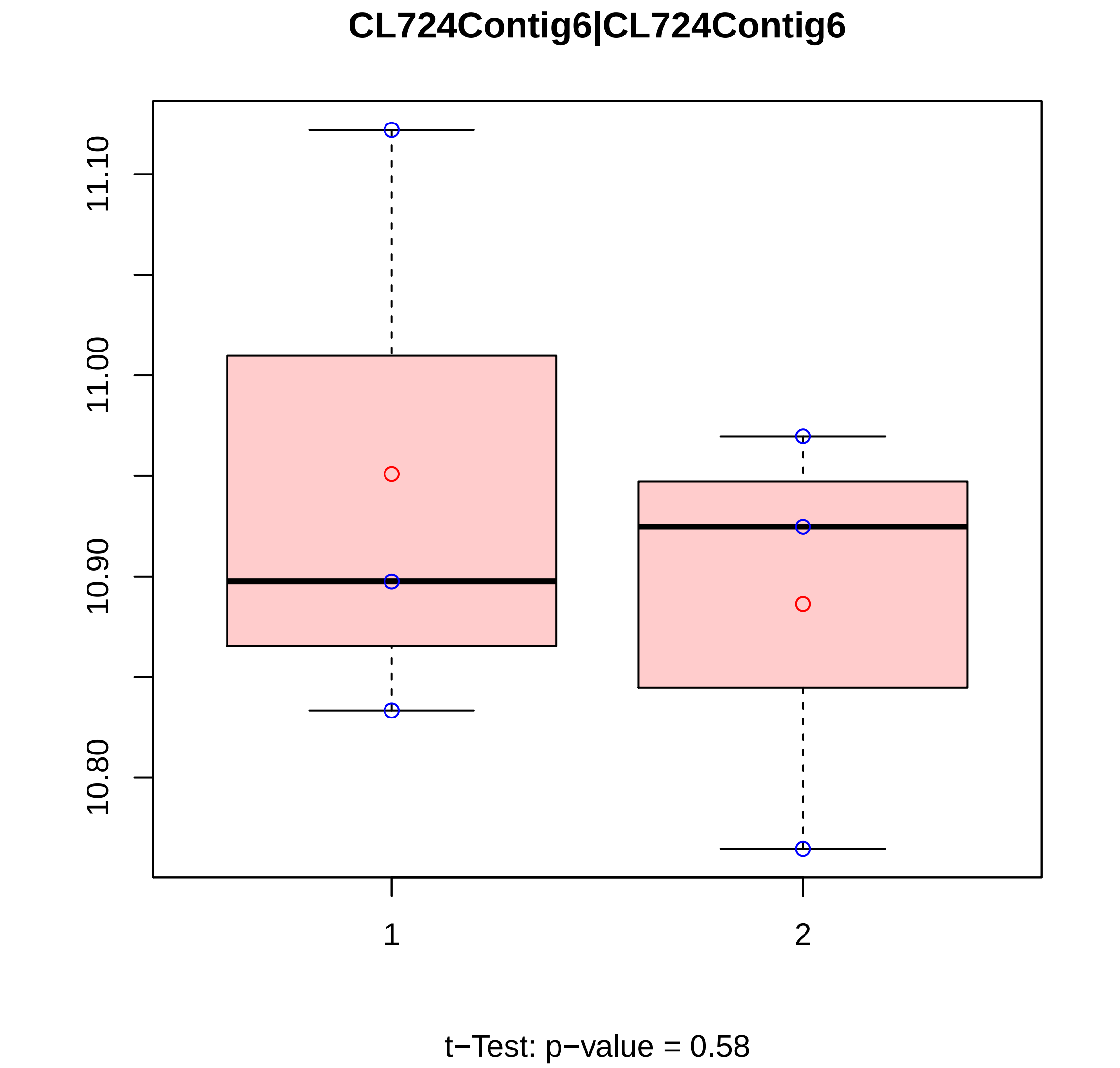
CL724Contig6|CL724Contig6
11.10
11.00
10.90
10.80
1
2
t−Test: p−value = 0.58

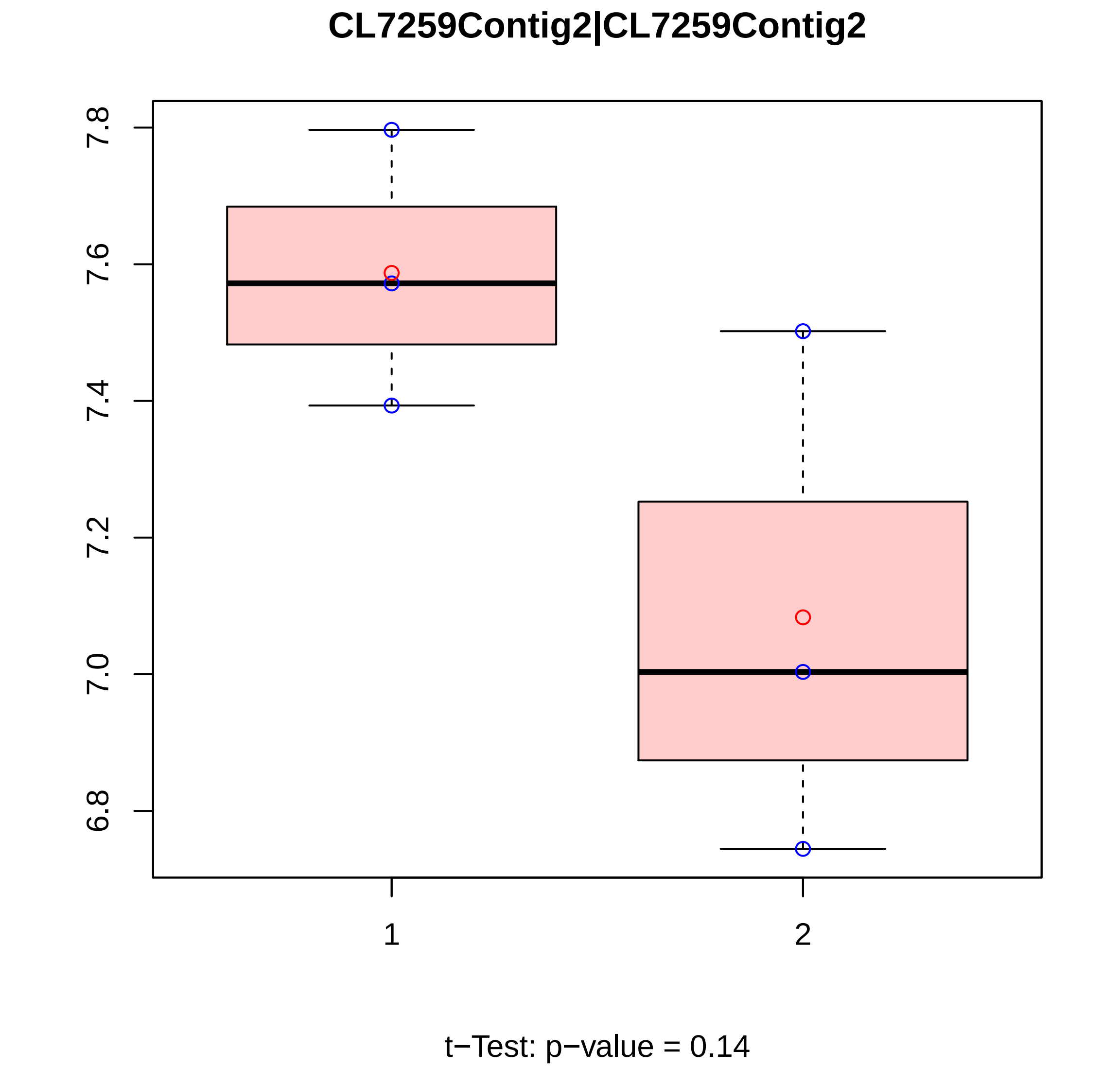
CL7259Contig2|CL7259Contig2
7.8
7.6
7.4
7.2
7.0
6.8
1
2
t−Test: p−value = 0.14

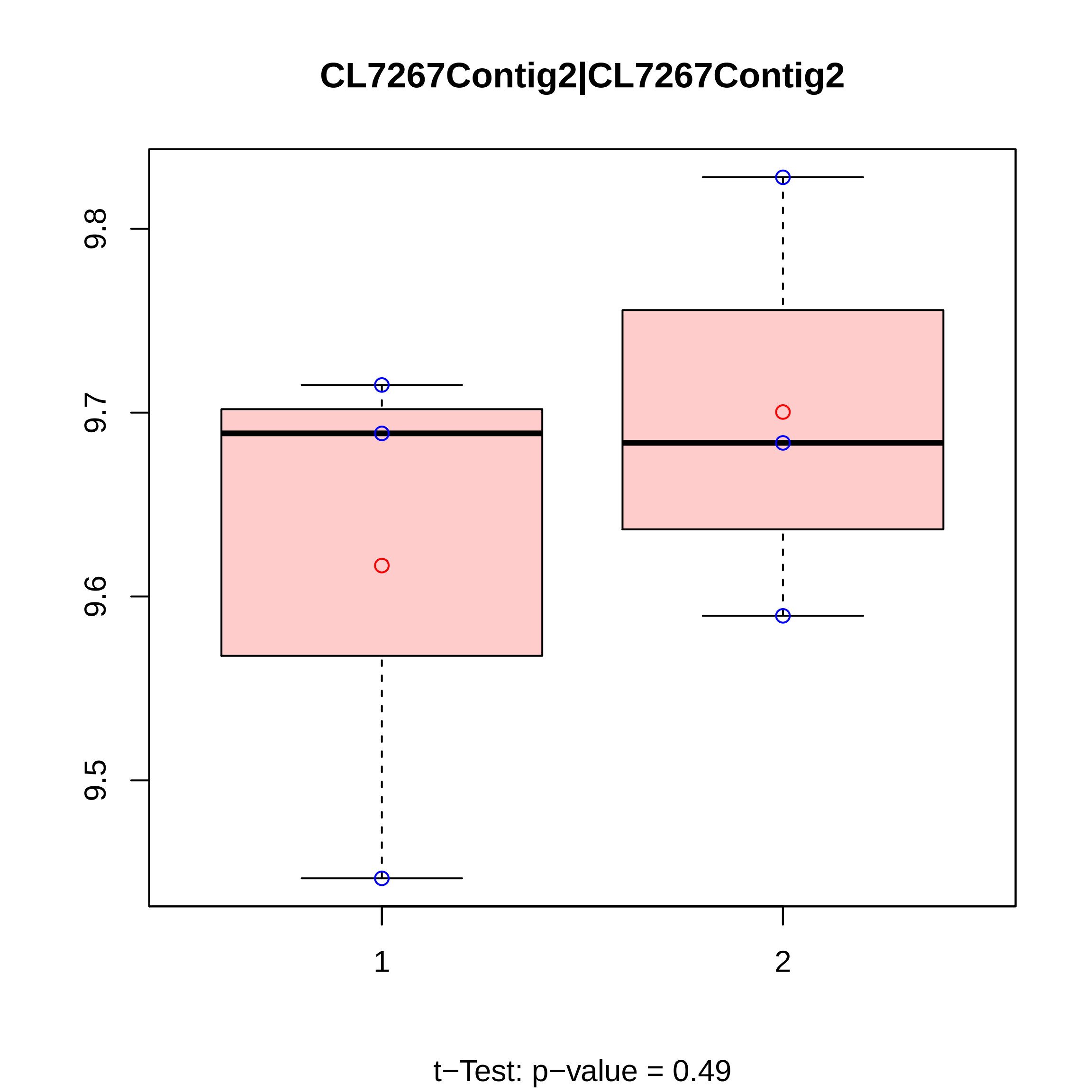

CL7267Contig2|CL7267Contig2
9.8
9.7
9.6
9.5
1
2
t−Test: p−value = 0.49

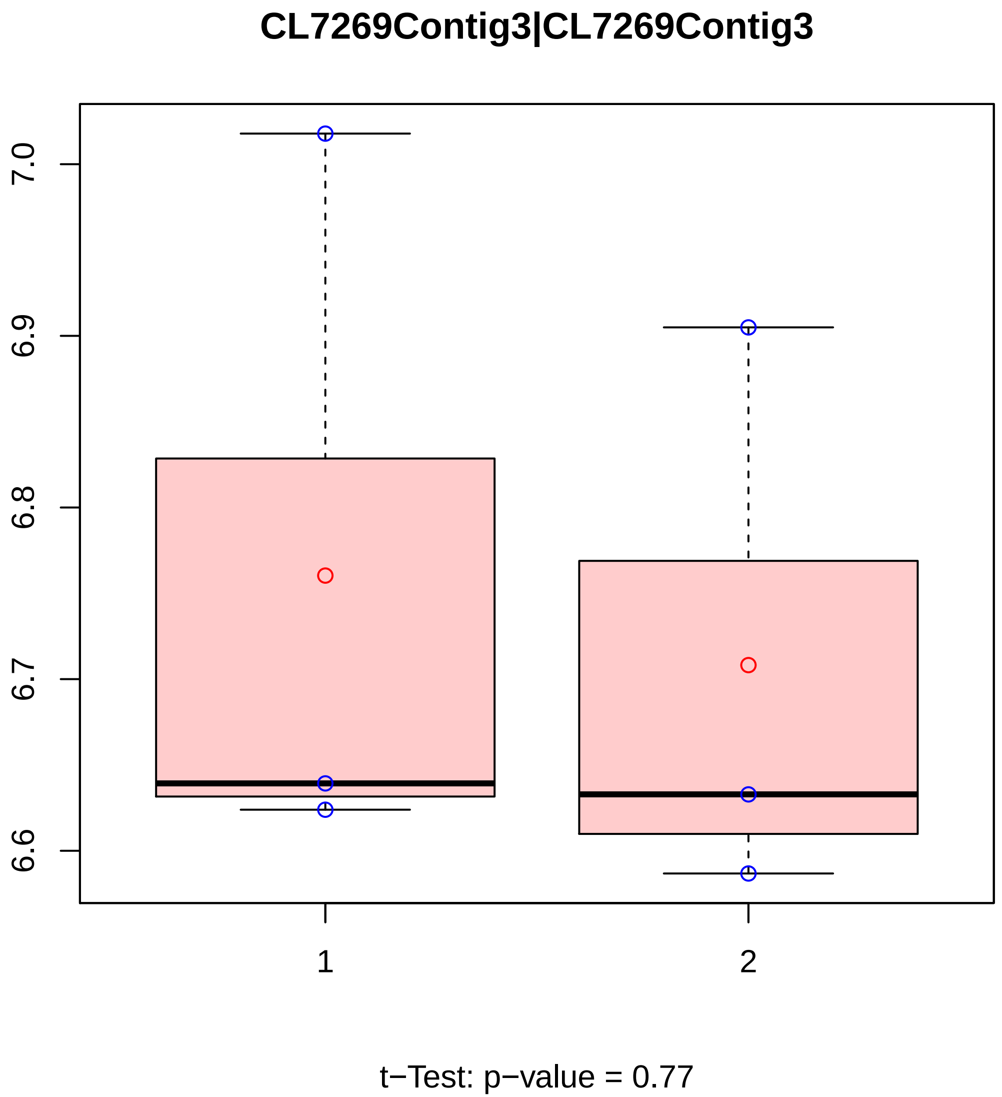
CL7269Contig3|CL7269Contig3
7.0
6.9
6.8
6.7
6.6
1
2
t−Test: p−value = 0.77

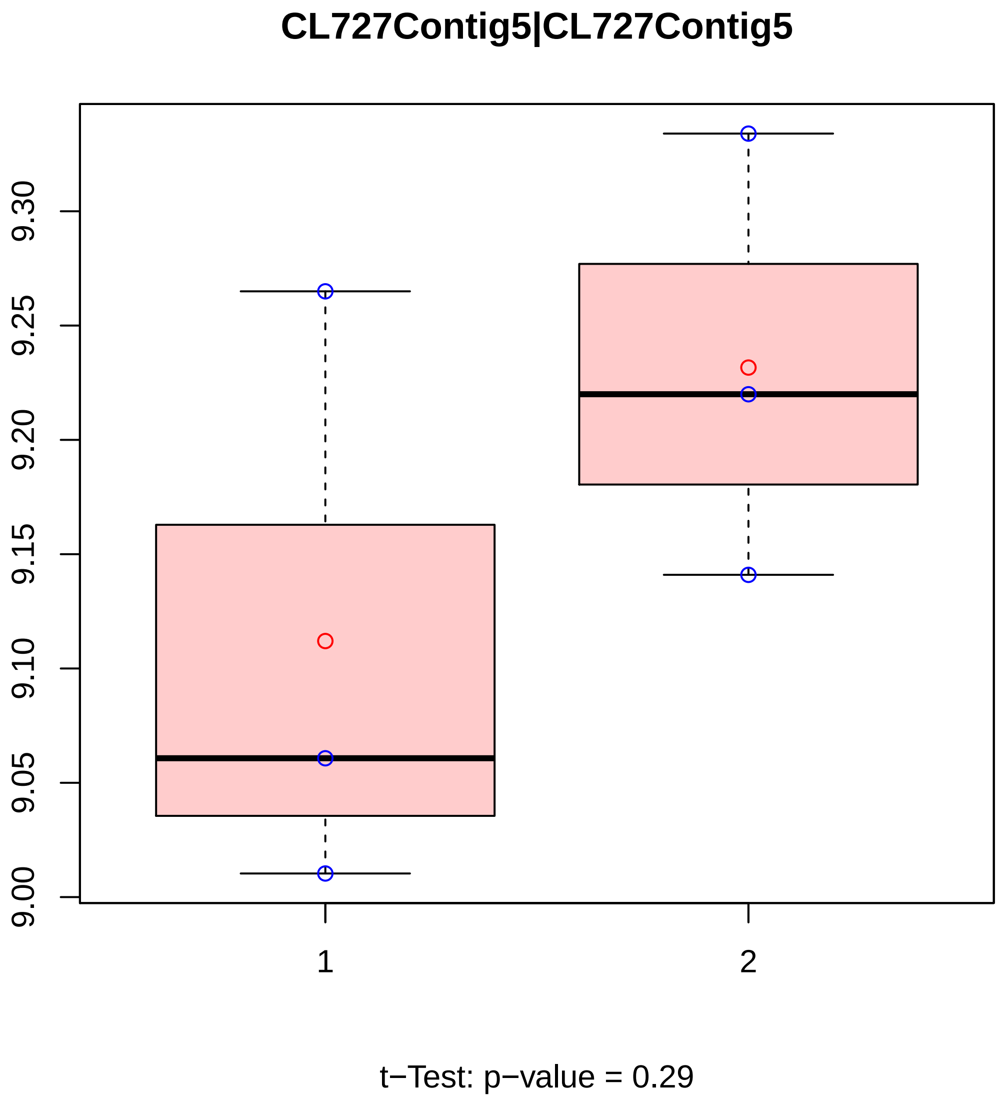
CL727Contig5|CL727Contig5
9.00
9.05
9.10
9.15
9.20
9.25
9.30
1
2
t−Test: p−value = 0.29

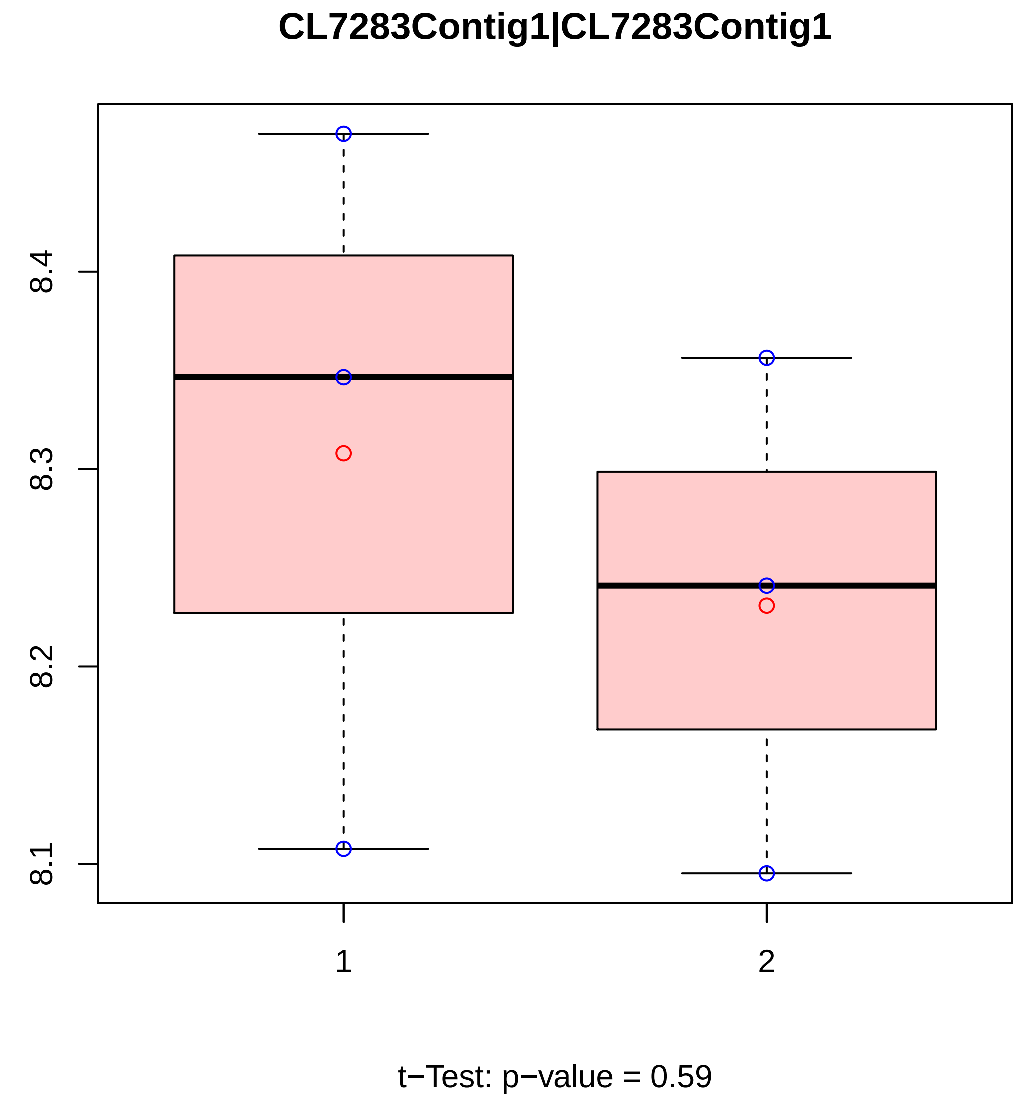
CL7283Contig1|CL7283Contig1
8.1
8.2
8.3
8.4
1
2
t−Test: p−value = 0.59

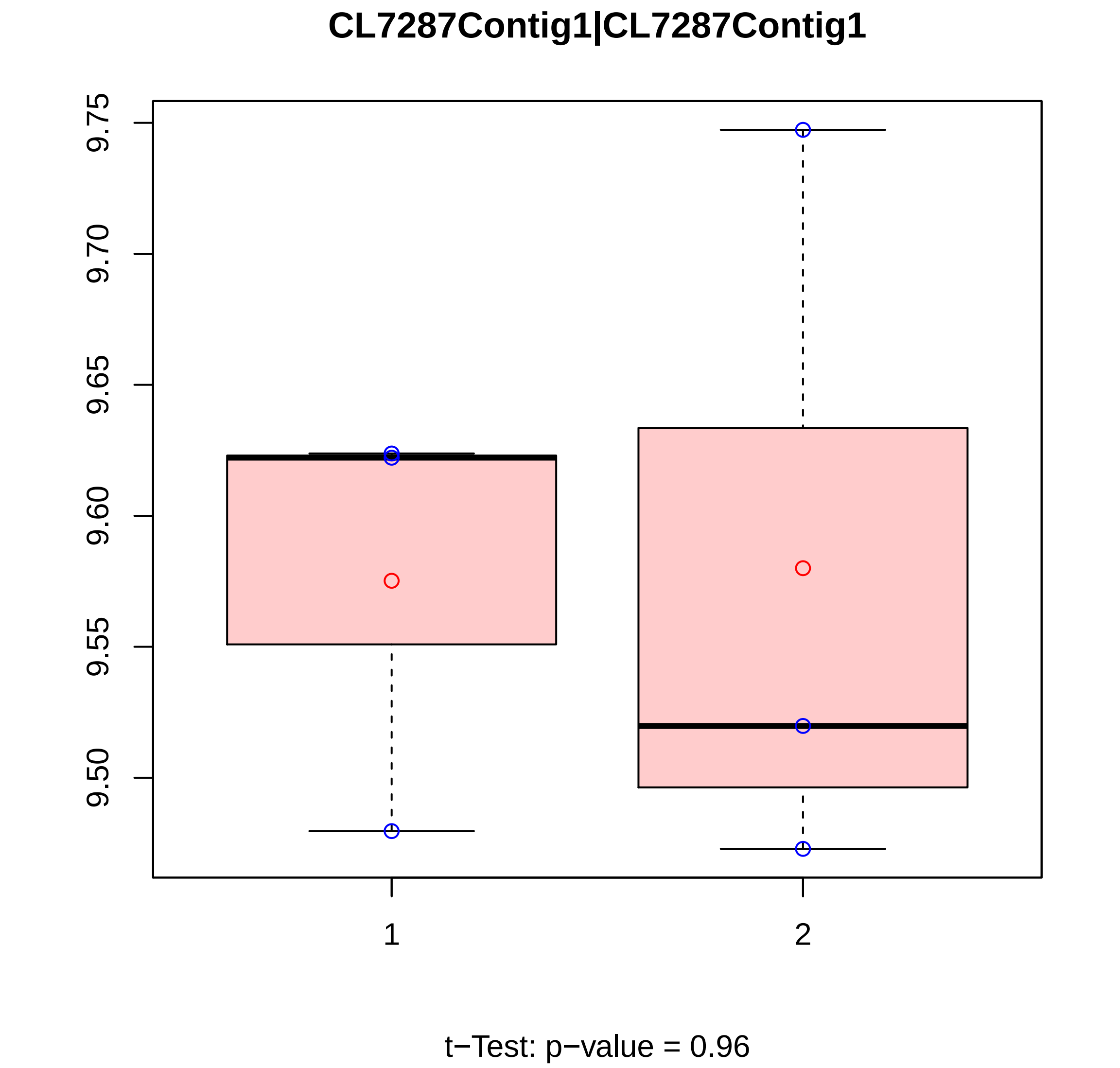
CL7287Contig1|CL7287Contig1
9.50
9.55
9.60
9.65
9.70
9.75
1
2
t−Test: p−value = 0.96

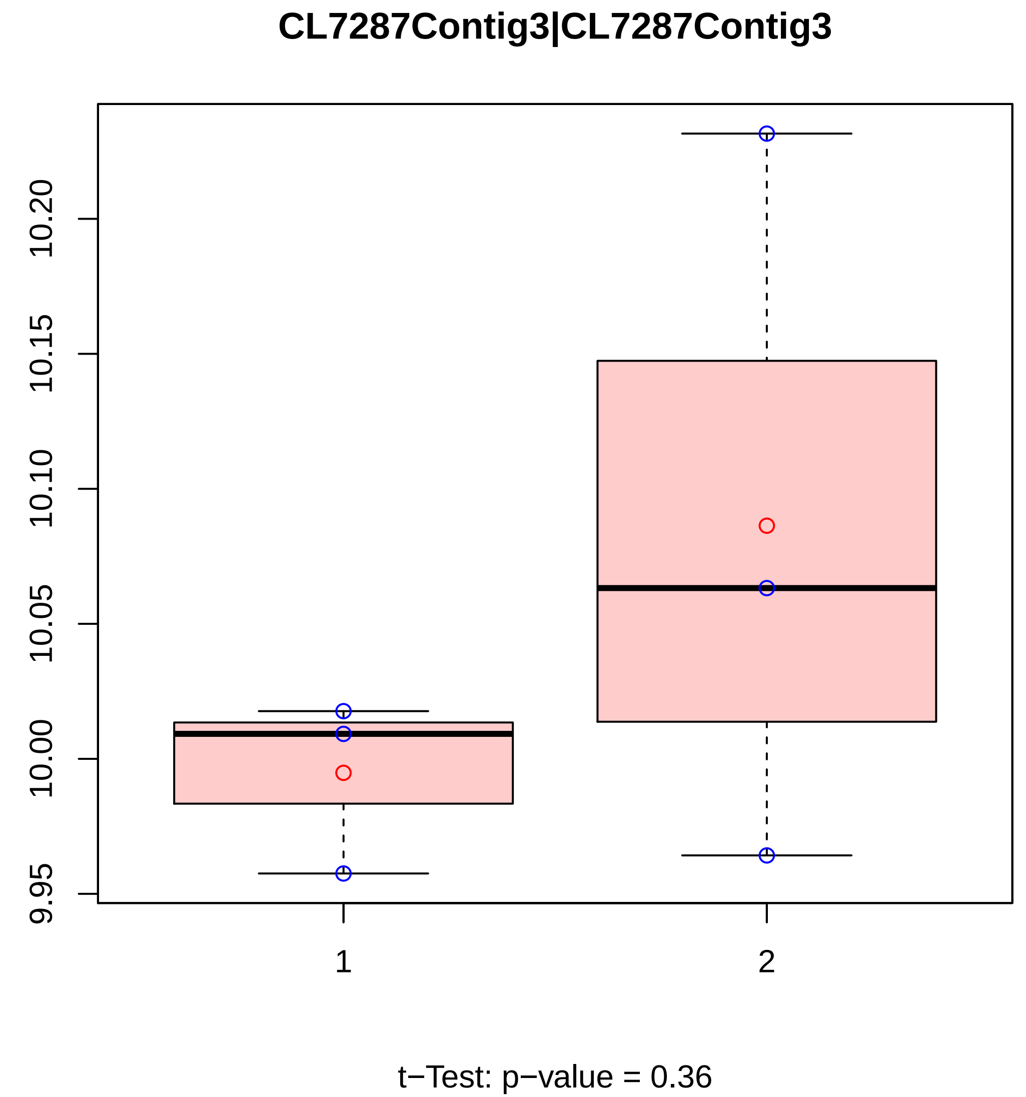
CL7287Contig3|CL7287Contig3
10.20
10.15
10.10
10.05
10.00
9.95
1
2
t−Test: p−value = 0.36

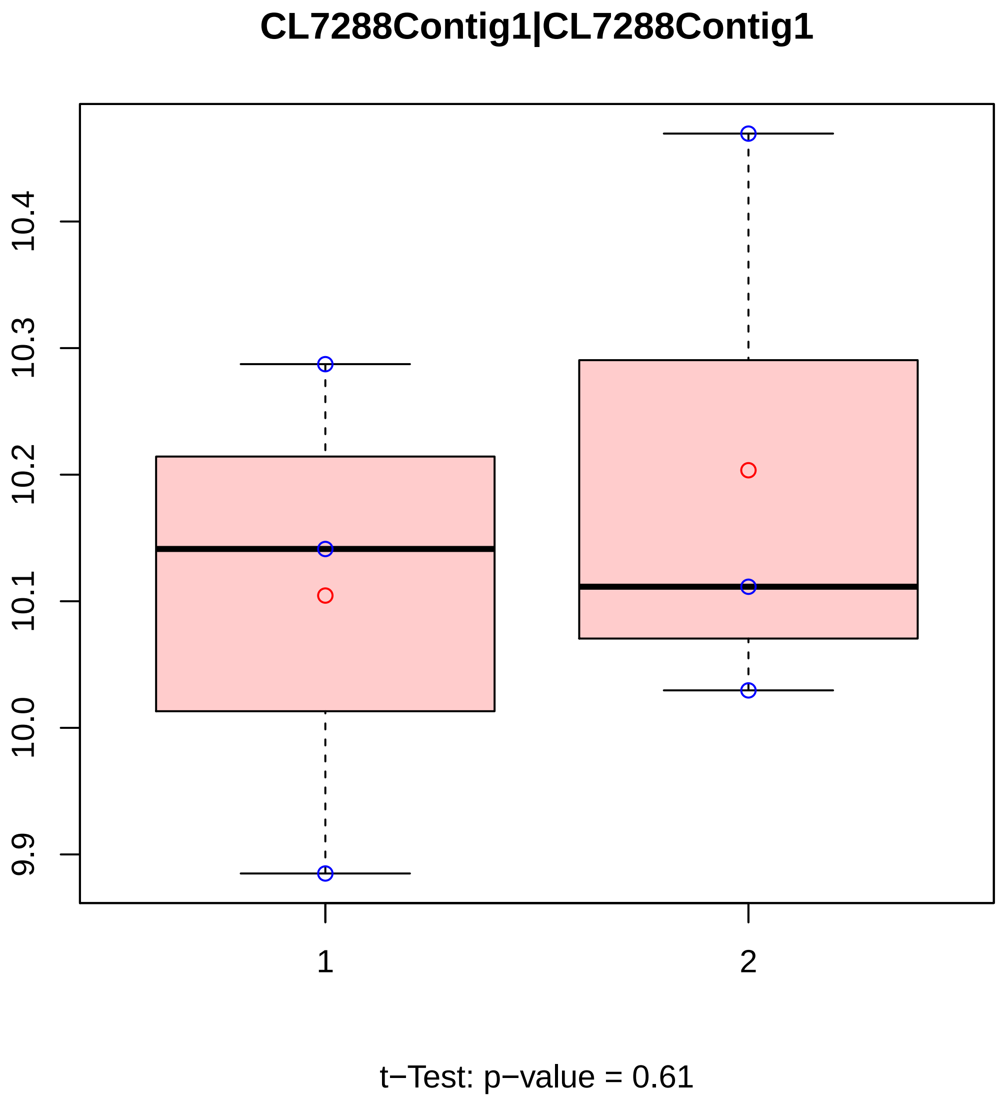

CL7288Contig1|CL7288Contig1
9.9
10.0
10.1
10.2
10.3
10.4
1
2
t−Test: p−value = 0.61

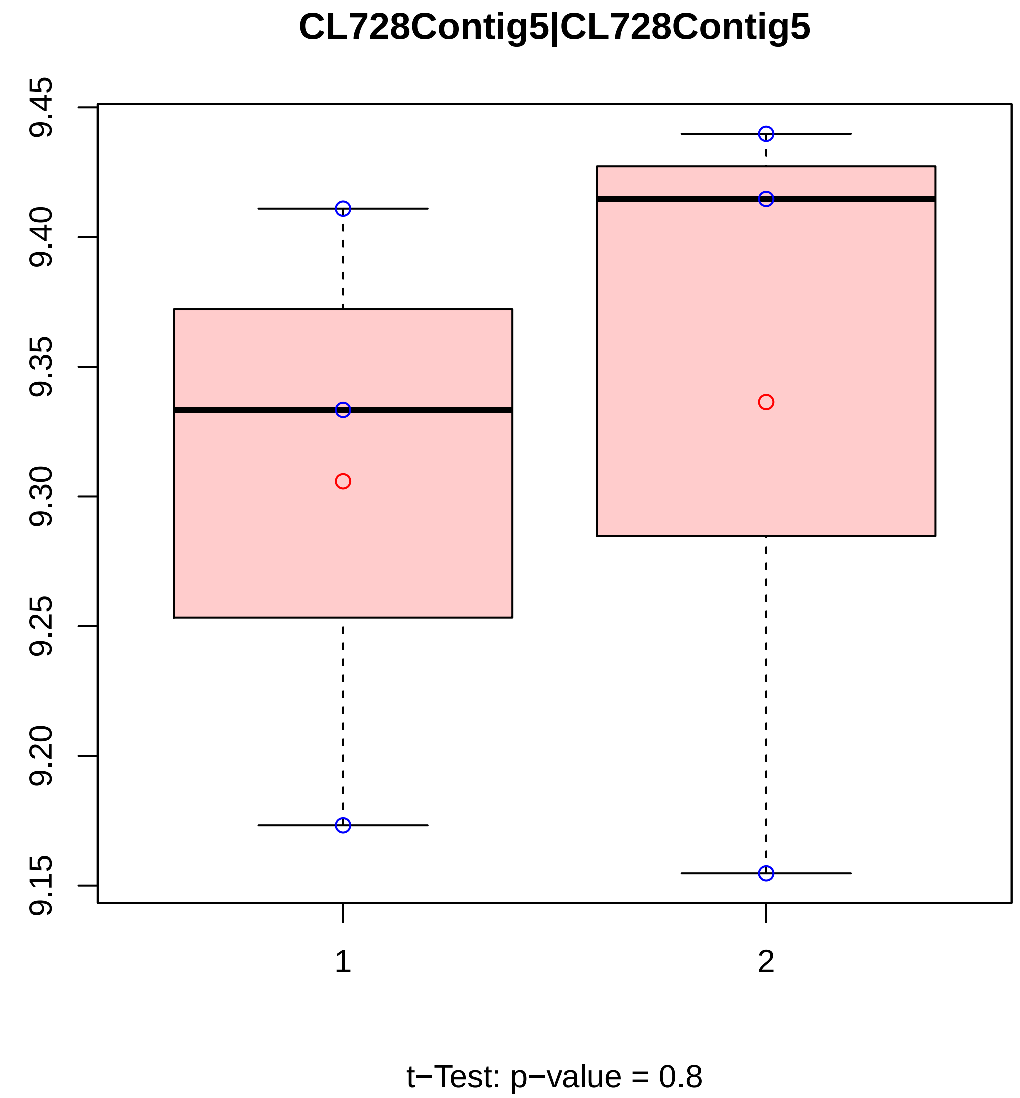

CL728Contig5|CL728Contig5
9.15
9.20
9.25
9.30
9.35
9.40
9.45
1
2
t−Test: p−value = 0.8

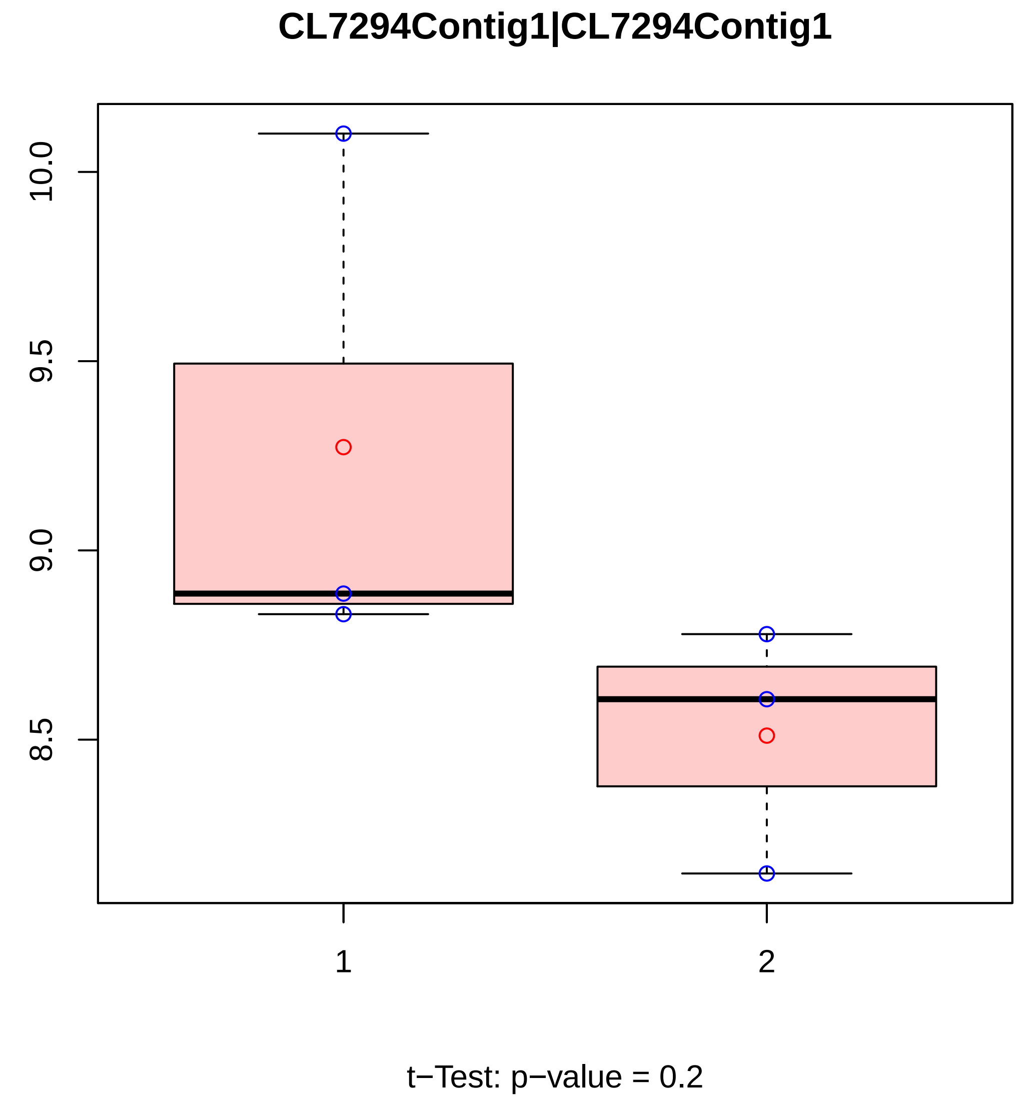

CL7294Contig1|CL7294Contig1
10.0
9.5
9.0
8.5
1
2
t−Test: p−value = 0.2

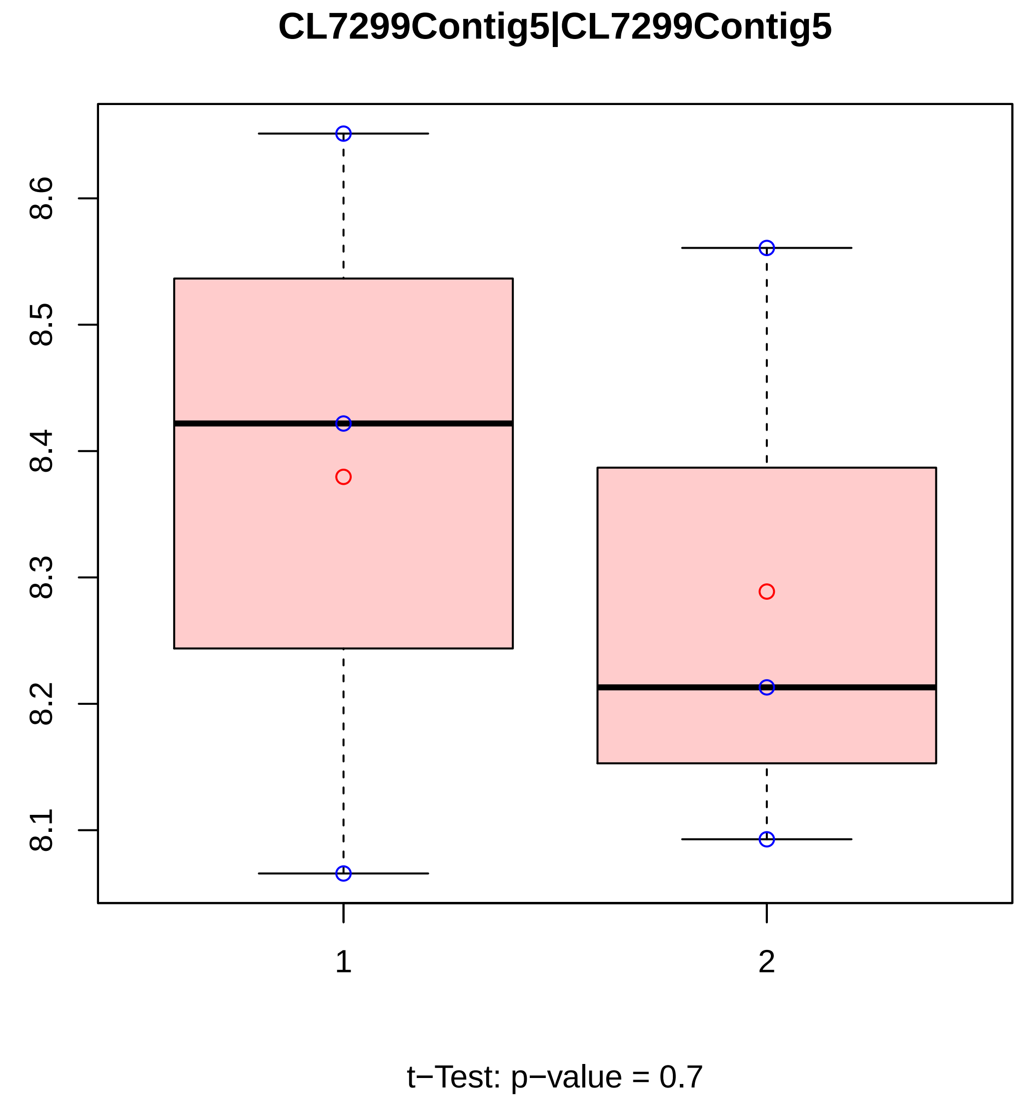
CL7299Contig5|CL7299Contig5
8.1
8.2
8.3
8.4
8.5
8.6
1
2
t−Test: p−value = 0.7

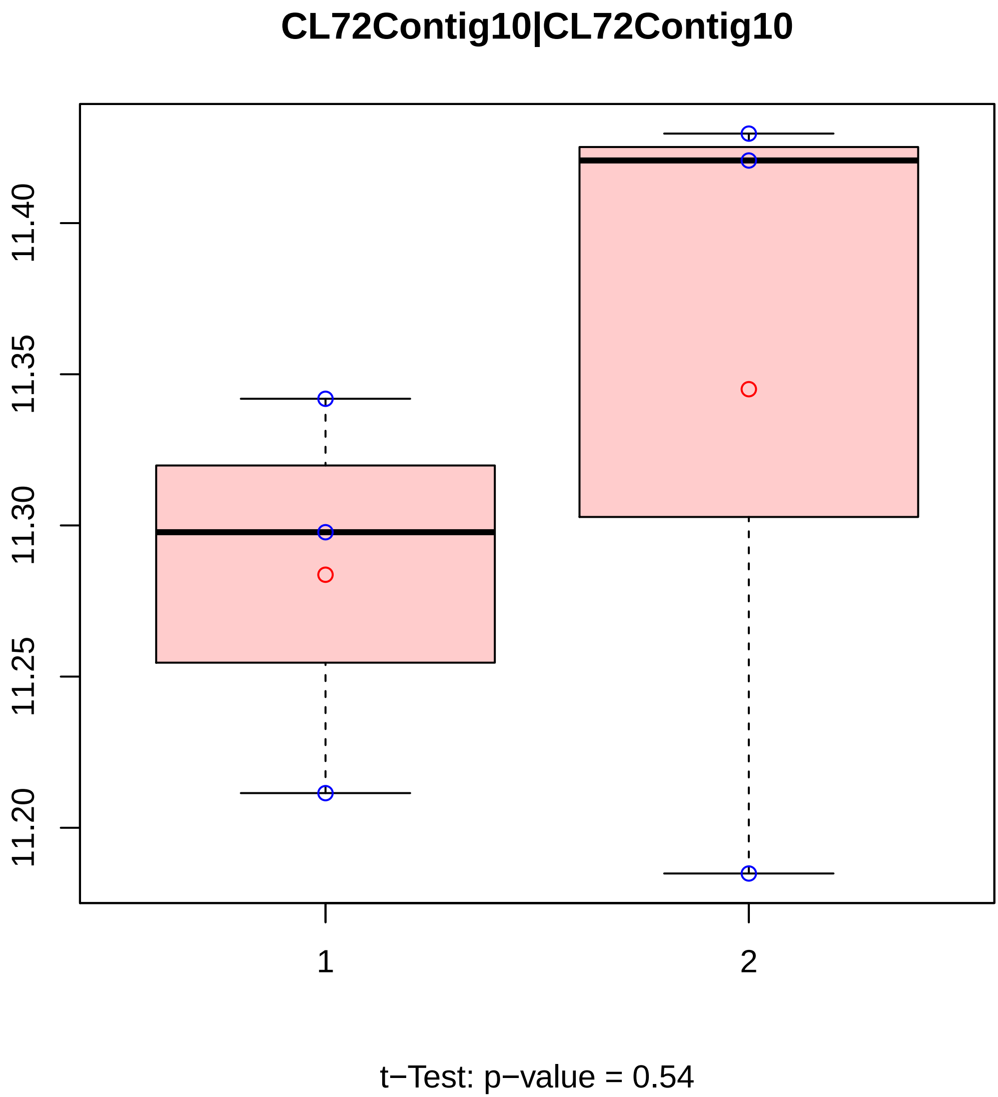
CL72Contig10|CL72Contig10
11.20
11.25
11.30
11.35
11.40
1
2
t−Test: p−value = 0.54

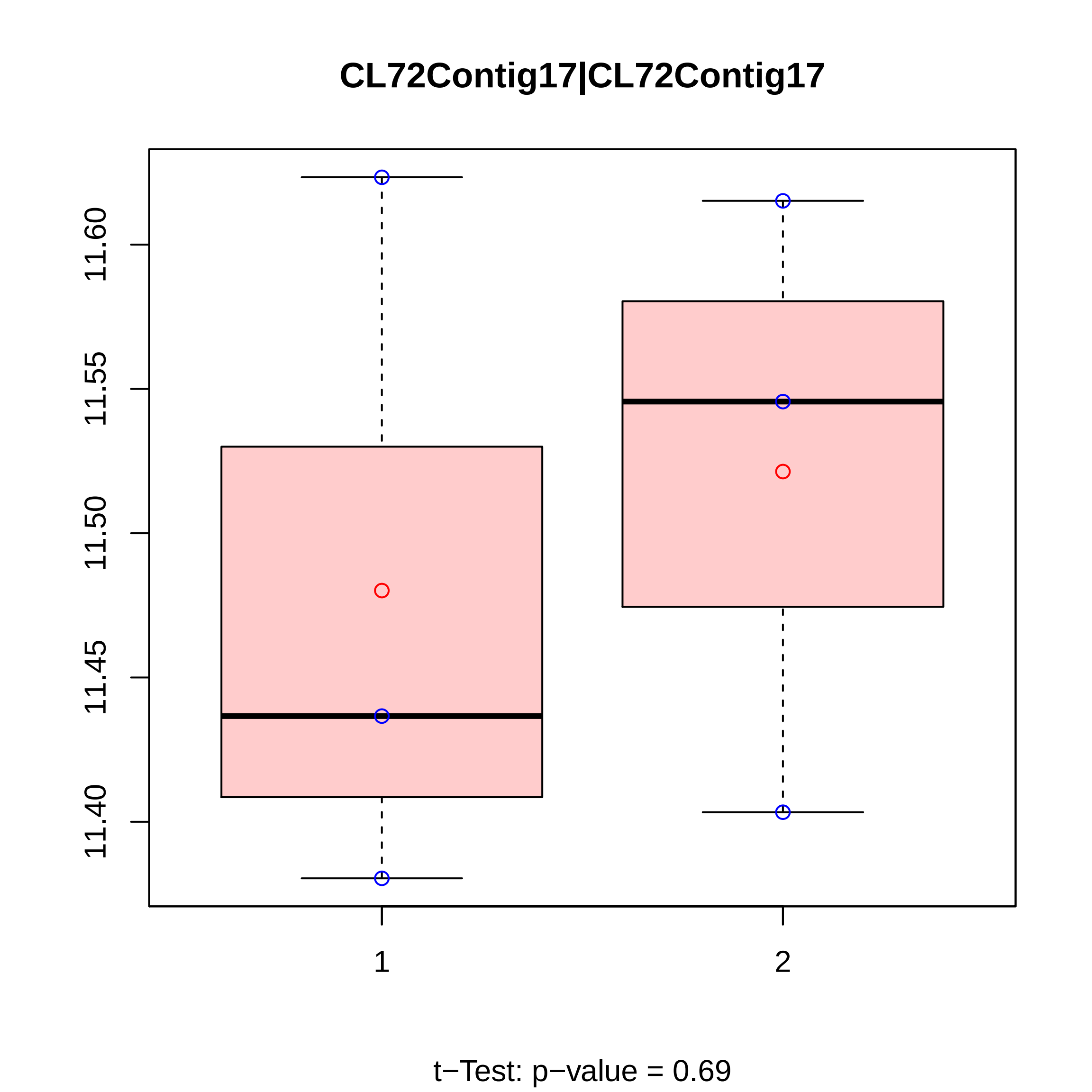
CL72Contig17|CL72Contig17
11.40
11.45
11.50
11.55
11.60
1
2
t−Test: p−value = 0.69

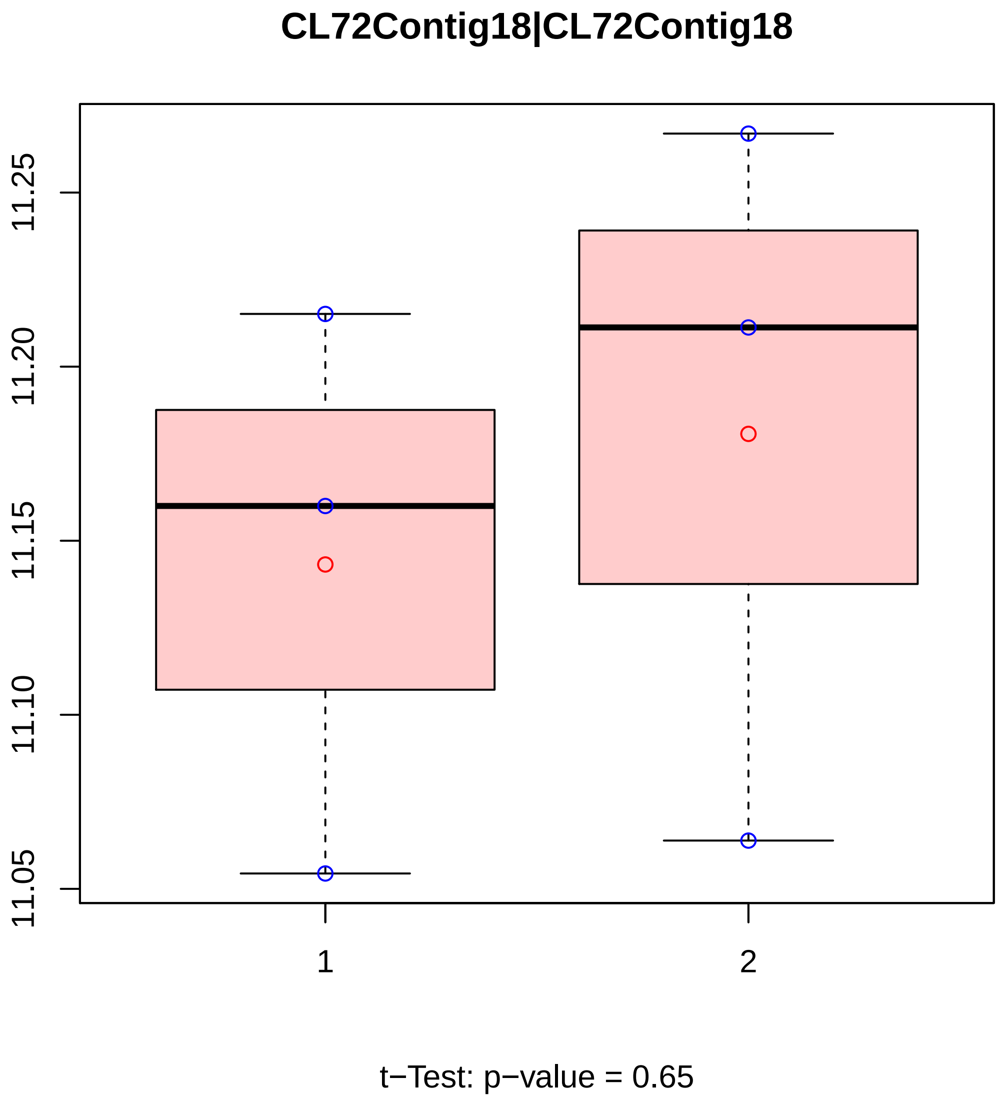

CL72Contig18|CL72Contig18
11.05
11.10
11.15
11.20
11.25
1
2
t−Test: p−value = 0.65

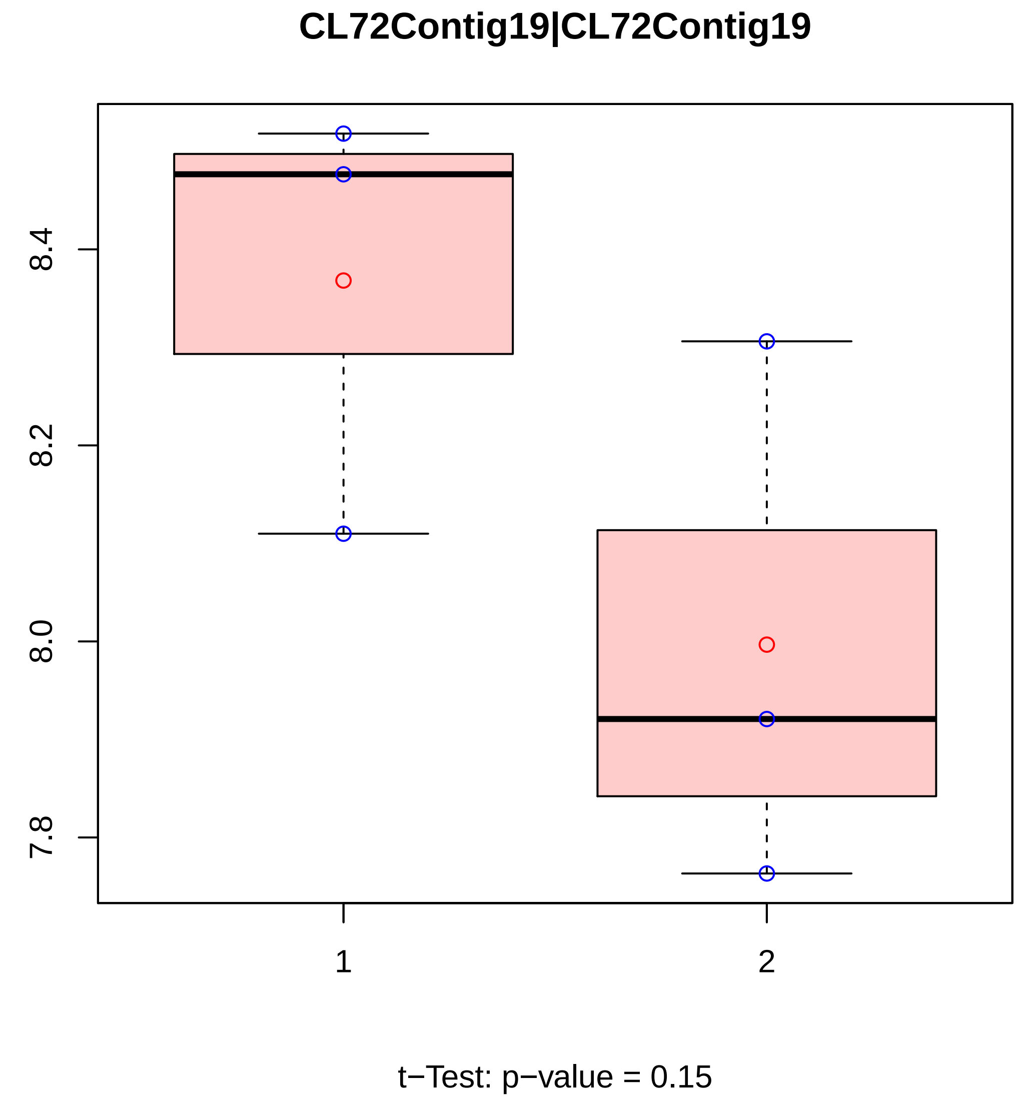
CL72Contig19|CL72Contig19
8.4
8.2
8.0
7.8
1
2
t−Test: p−value = 0.15

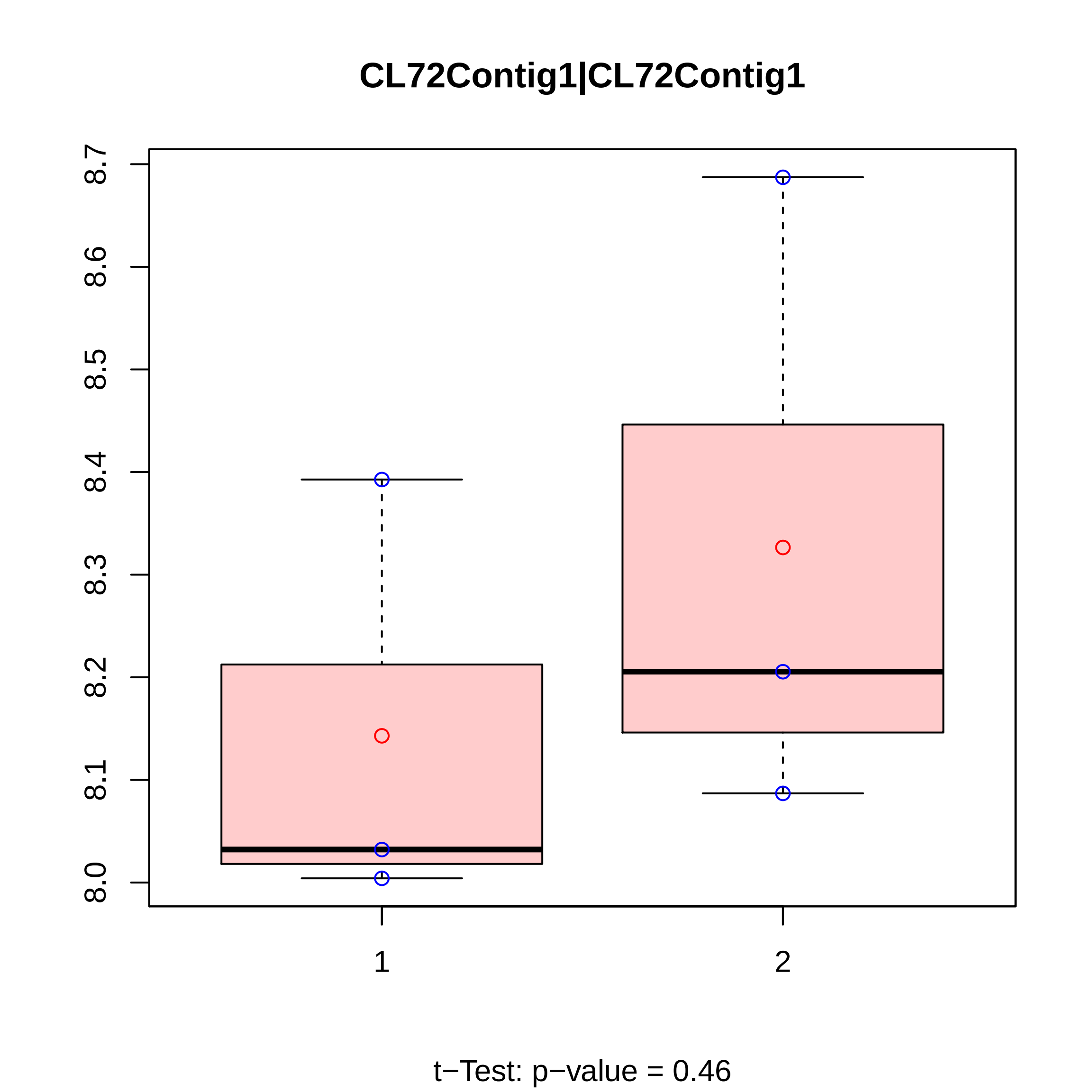

CL72Contig1|CL72Contig1
8.0
8.1
8.2
8.3
8.4
8.5
8.6
8.7
1
2
t−Test: p−value = 0.46

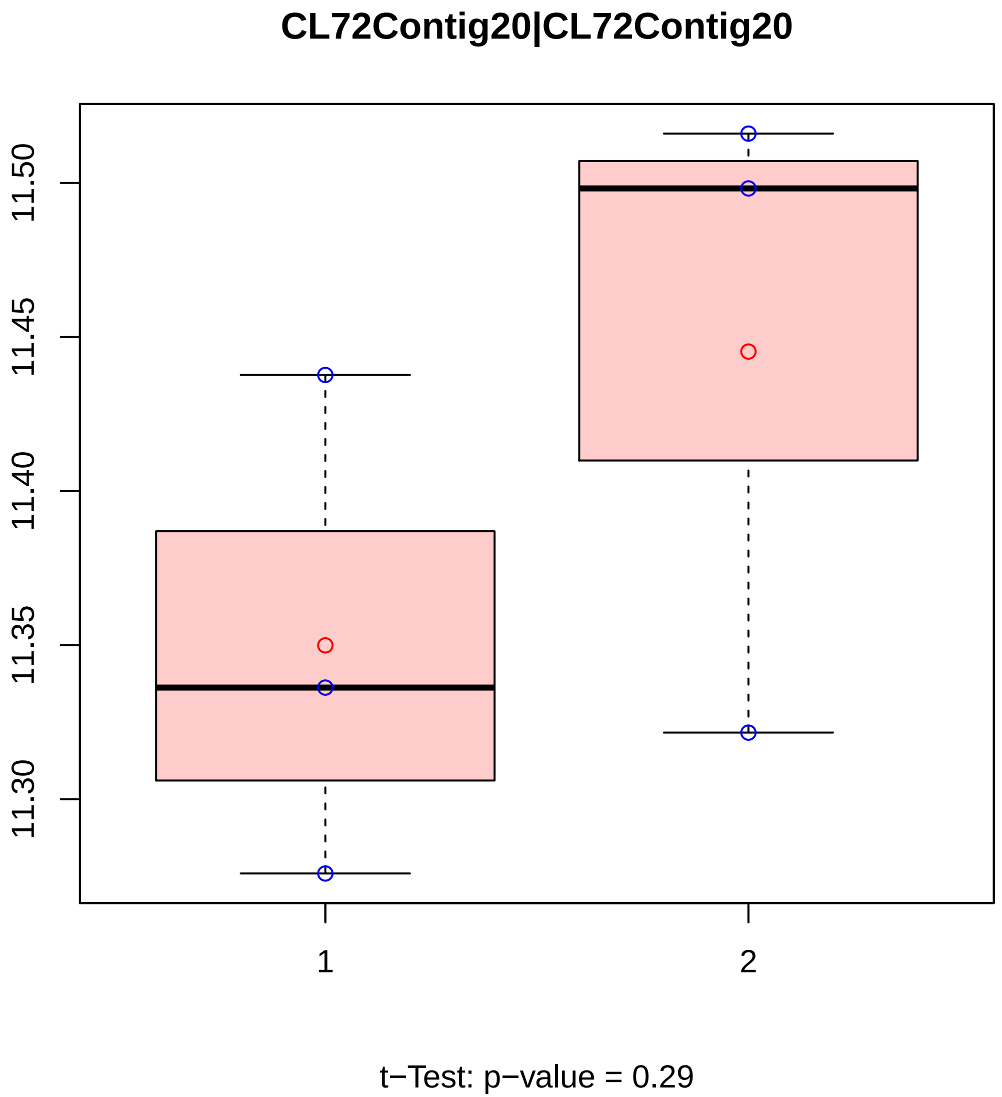
CL72Contig20|CL72Contig20
11.30
11.35
11.40
11.45
11.50
1
2
t−Test: p−value = 0.29

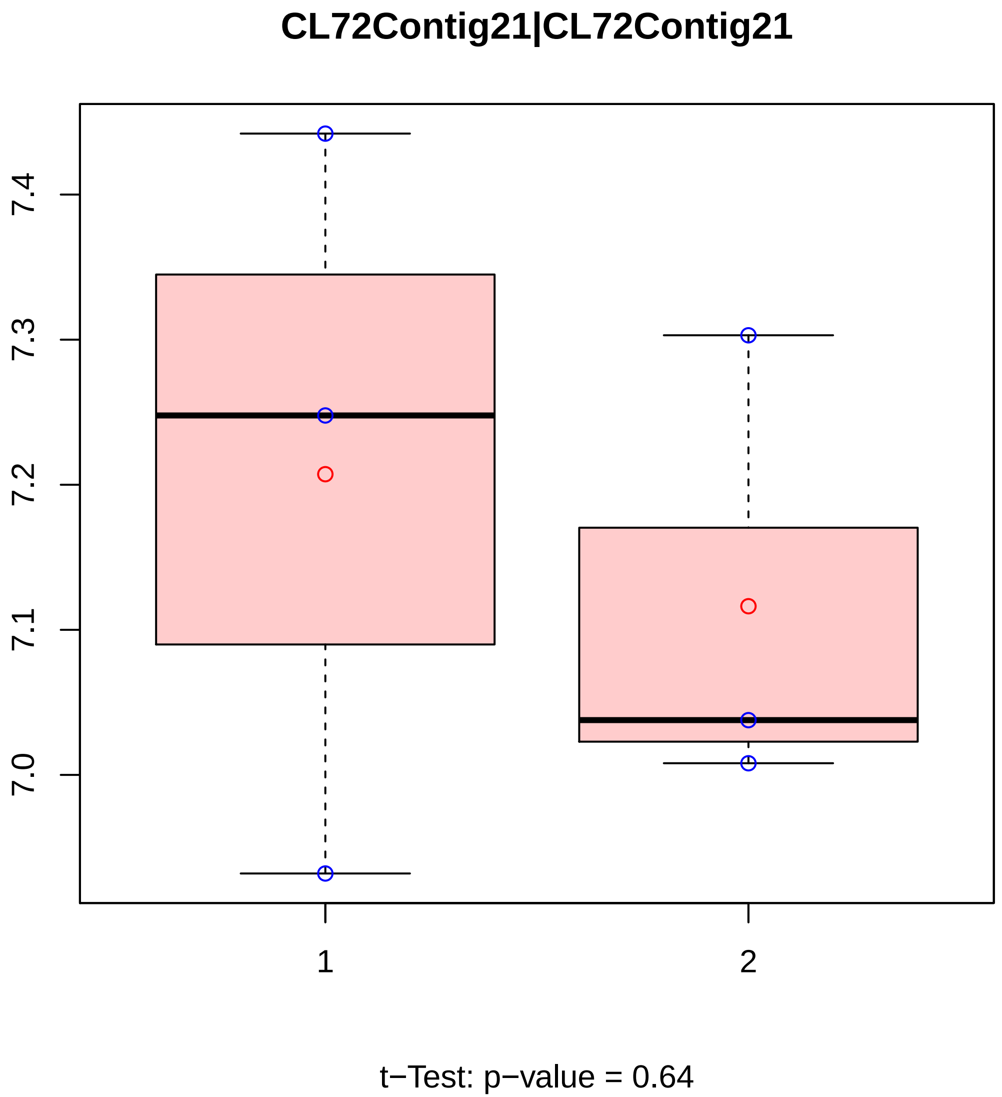
CL72Contig21|CL72Contig21
7.0
7.1
7.2
7.3
7.4
1
2
t−Test: p−value = 0.64

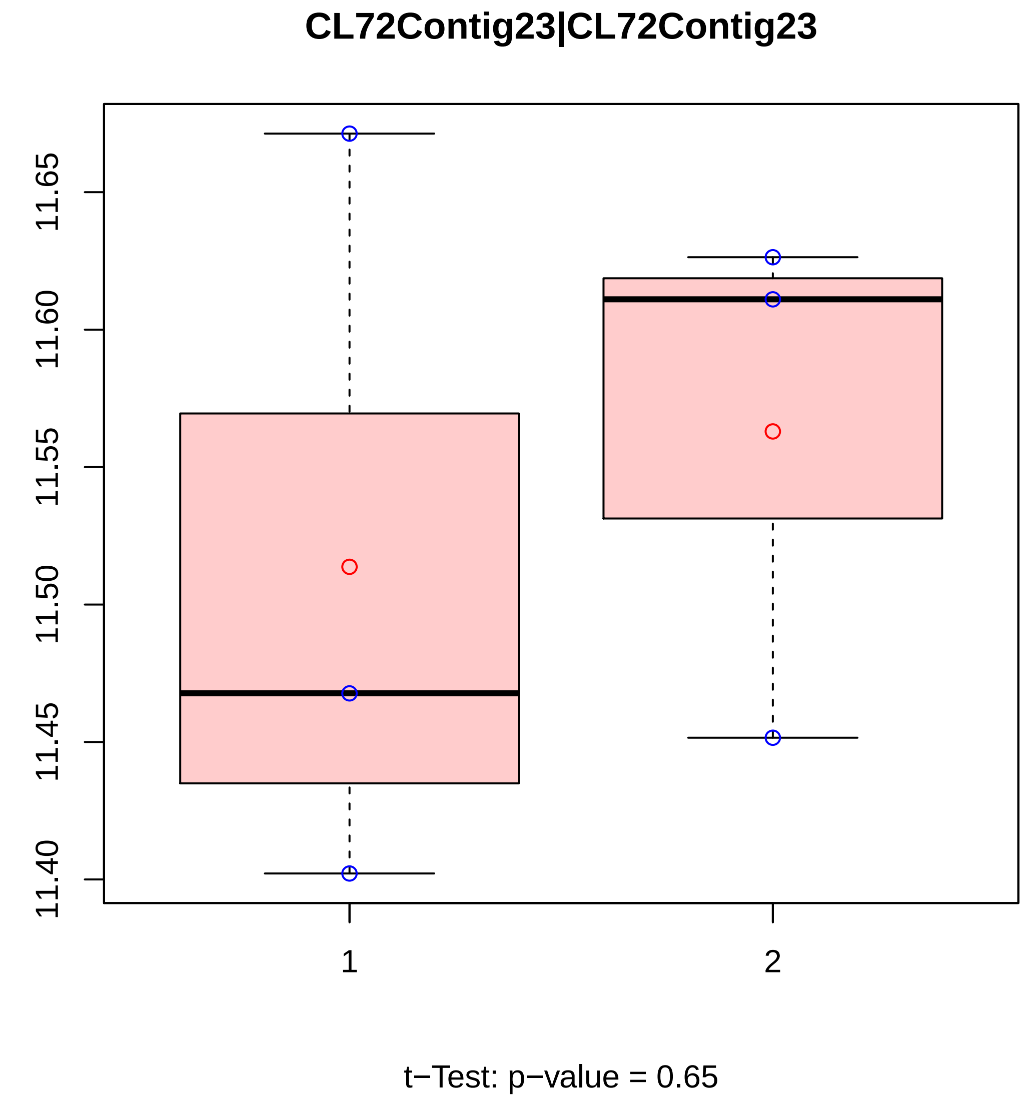
CL72Contig23|CL72Contig23
11.40
11.45
11.50
11.55
11.60
11.65
1
2
t−Test: p−value = 0.65

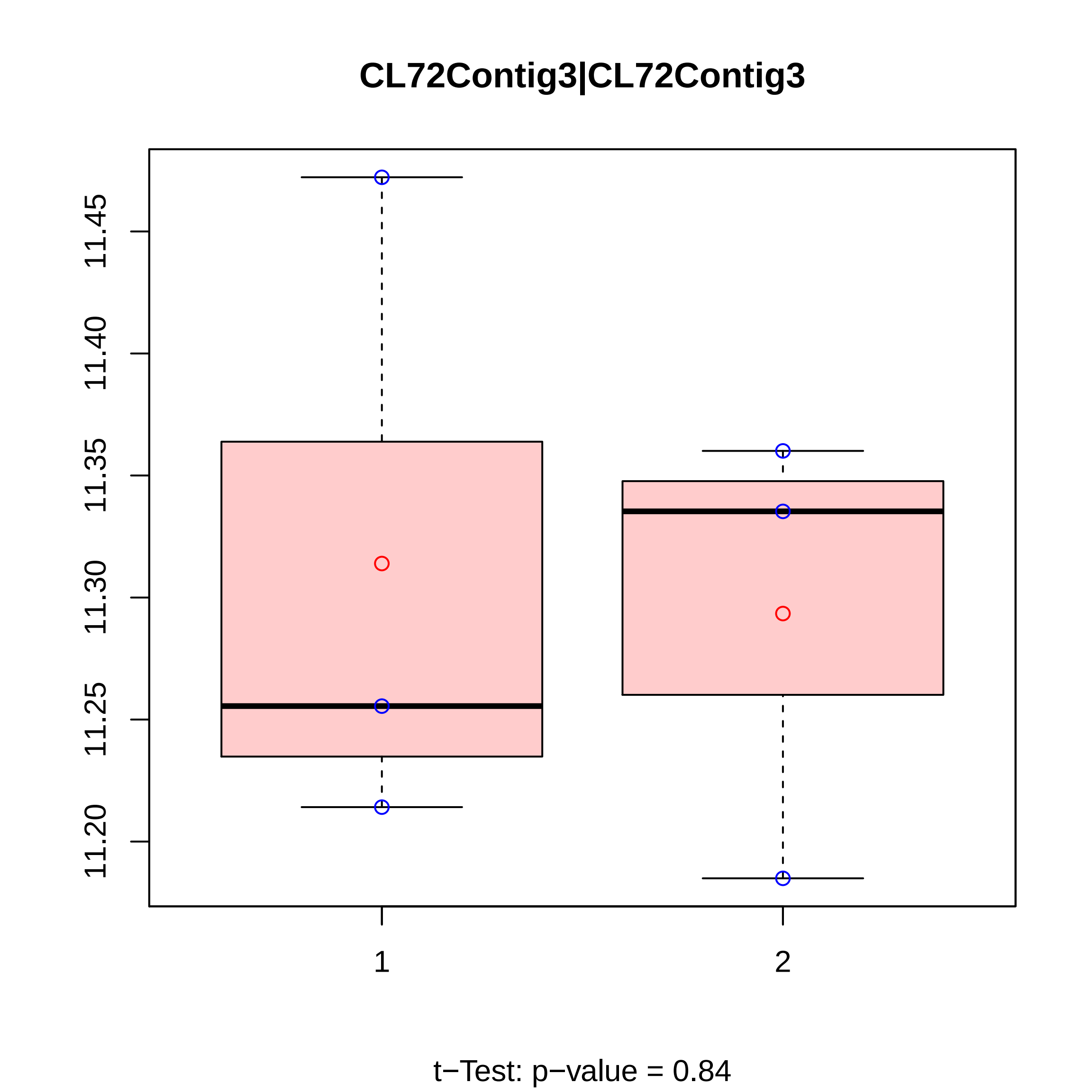
CL72Contig3|CL72Contig3
11.20
11.25
11.30
11.35
11.40
11.45
1
2
t−Test: p−value = 0.84

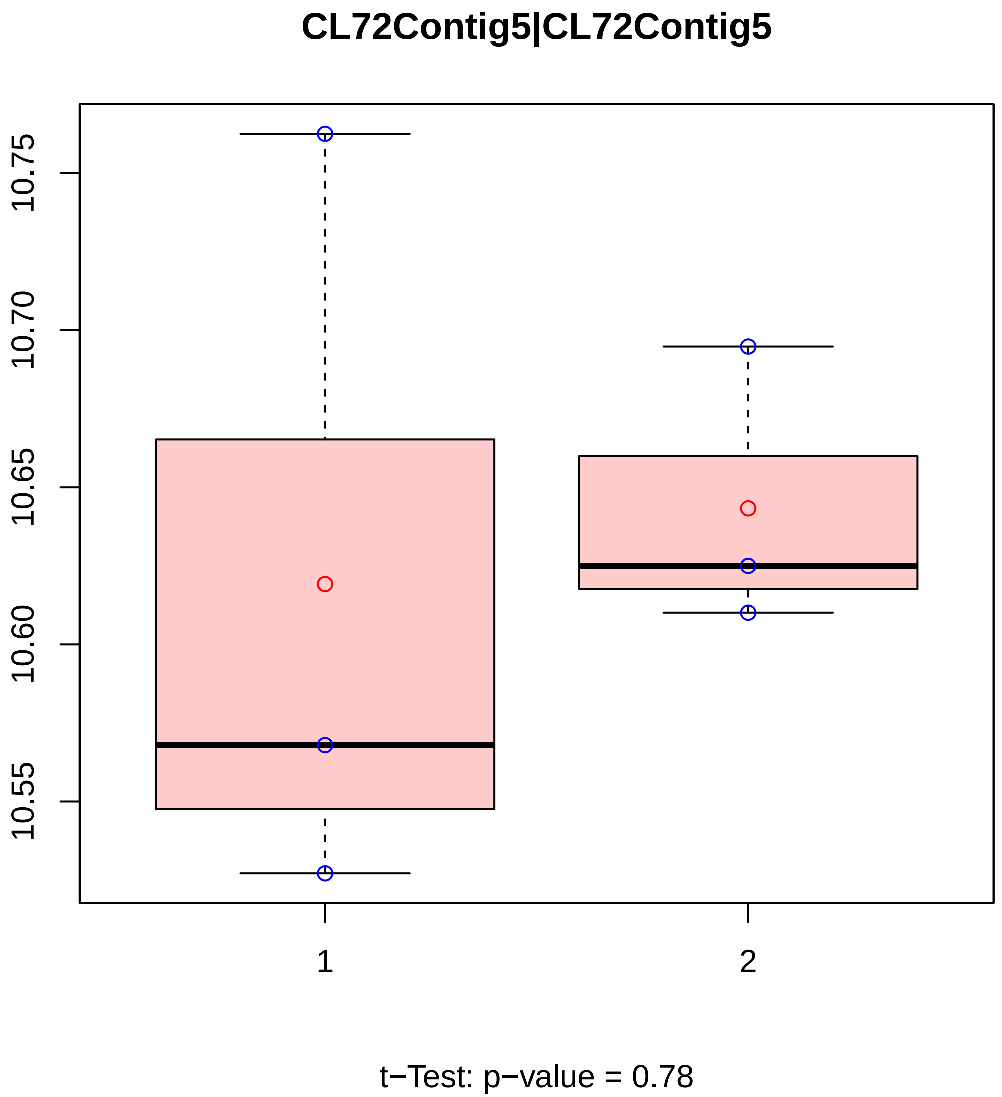

CL72Contig5|CL72Contig5
10.55
10.60
10.65
10.70
10.75
1
2
t−Test: p−value = 0.78

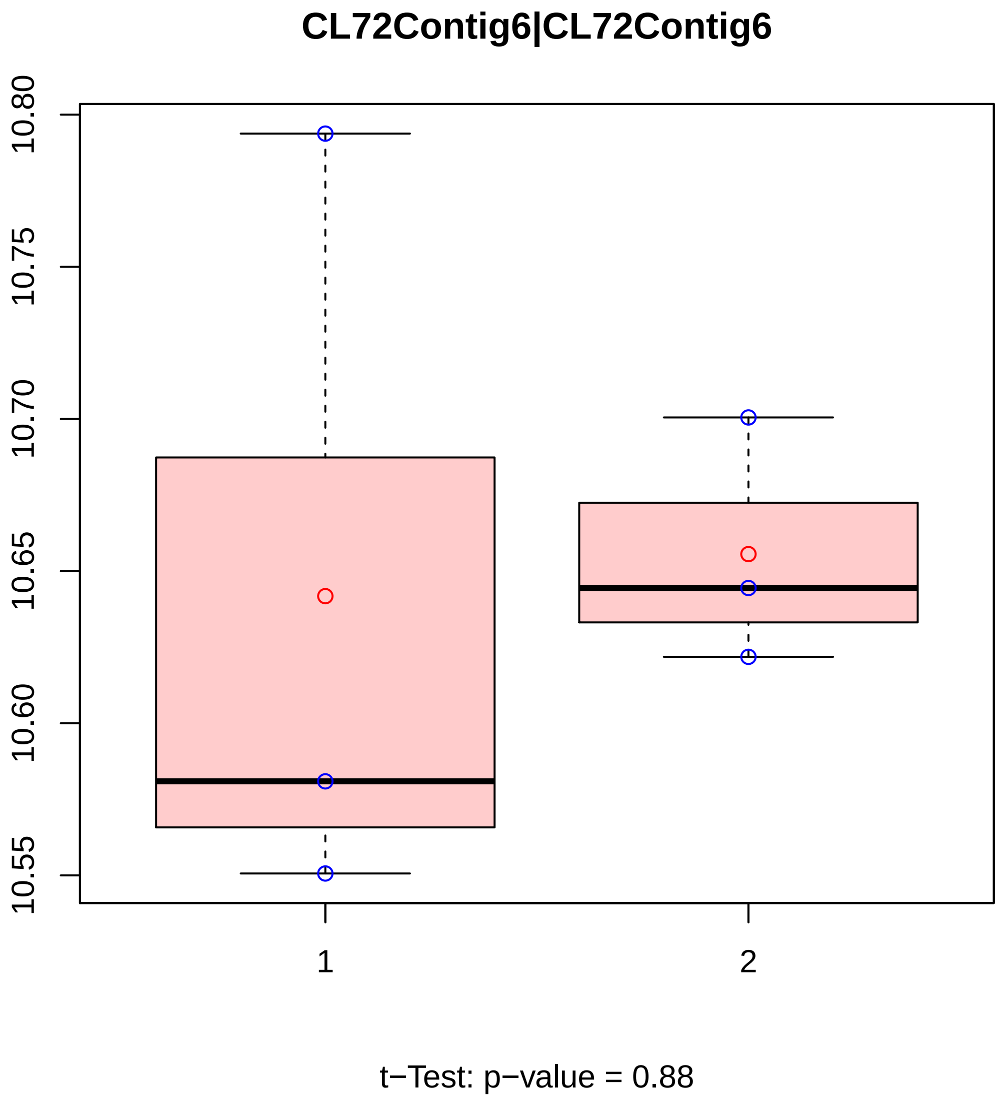

CL72Contig6|CL72Contig6
10.80
10.75
10.70
10.65
10.60
10.55
1
2
t−Test: p−value = 0.88

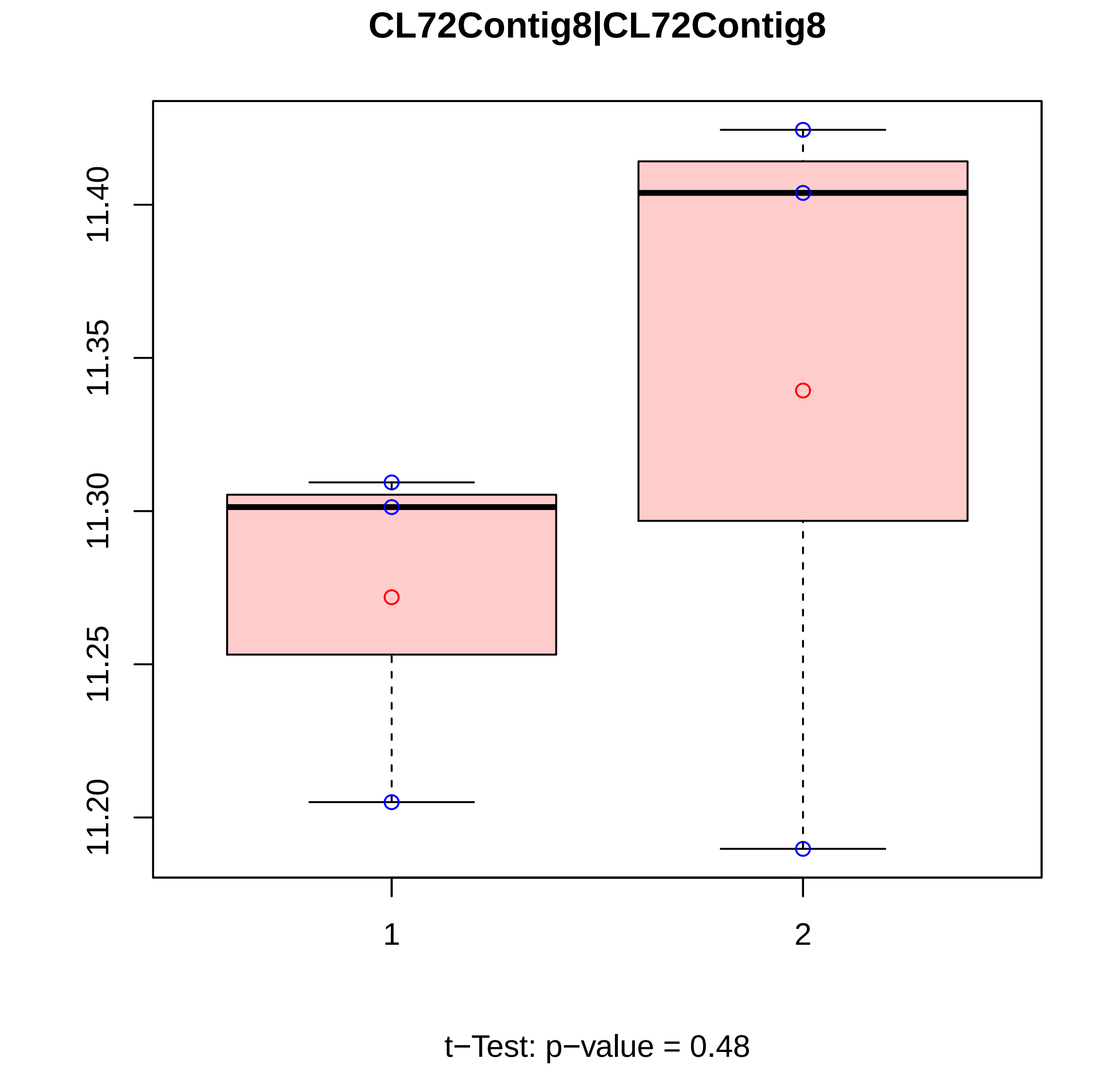
CL72Contig8|CL72Contig8
11.20
11.25
11.30
11.35
11.40
1
2
t−Test: p−value = 0.48

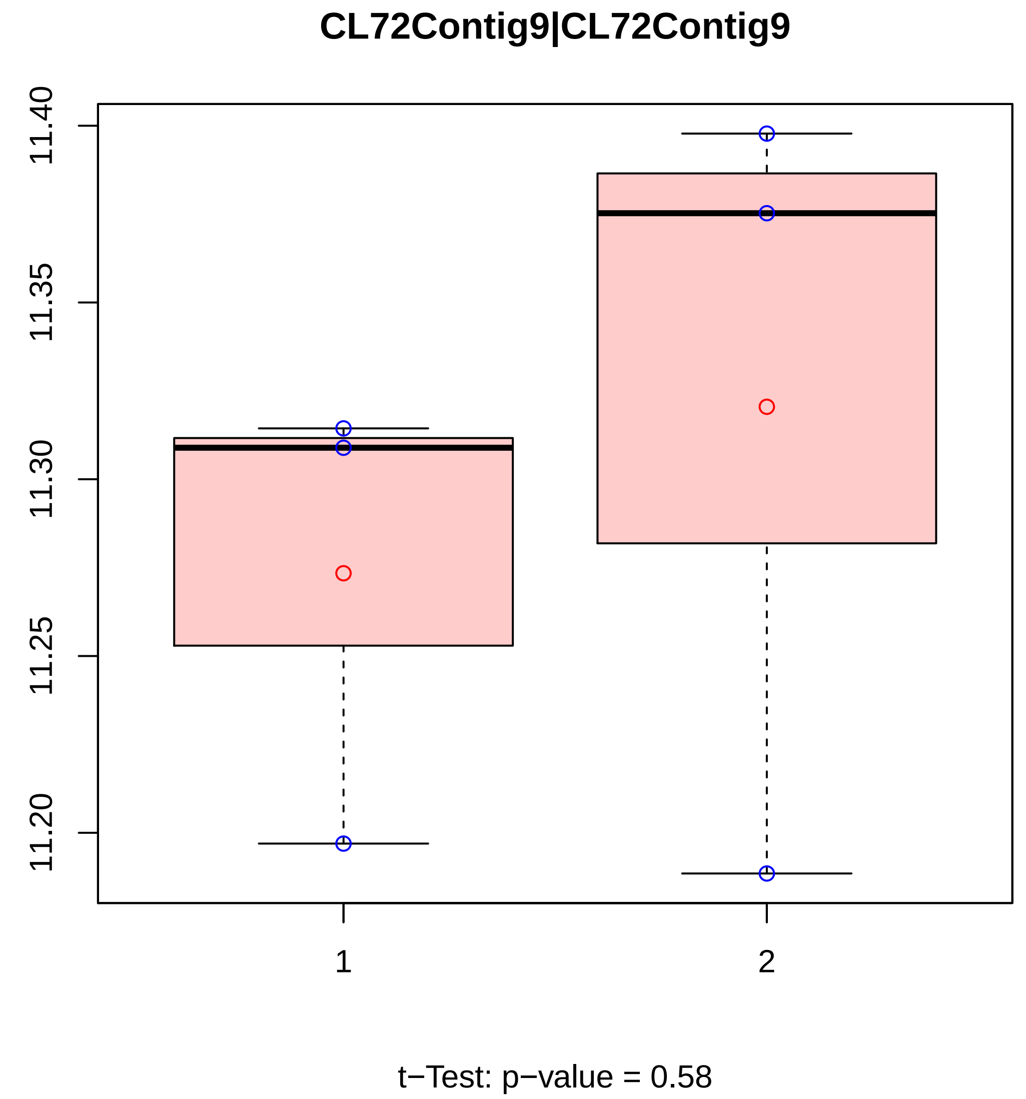

CL72Contig9|CL72Contig9
11.40
11.35
11.30
11.25
11.20
1
2
t−Test: p−value = 0.58

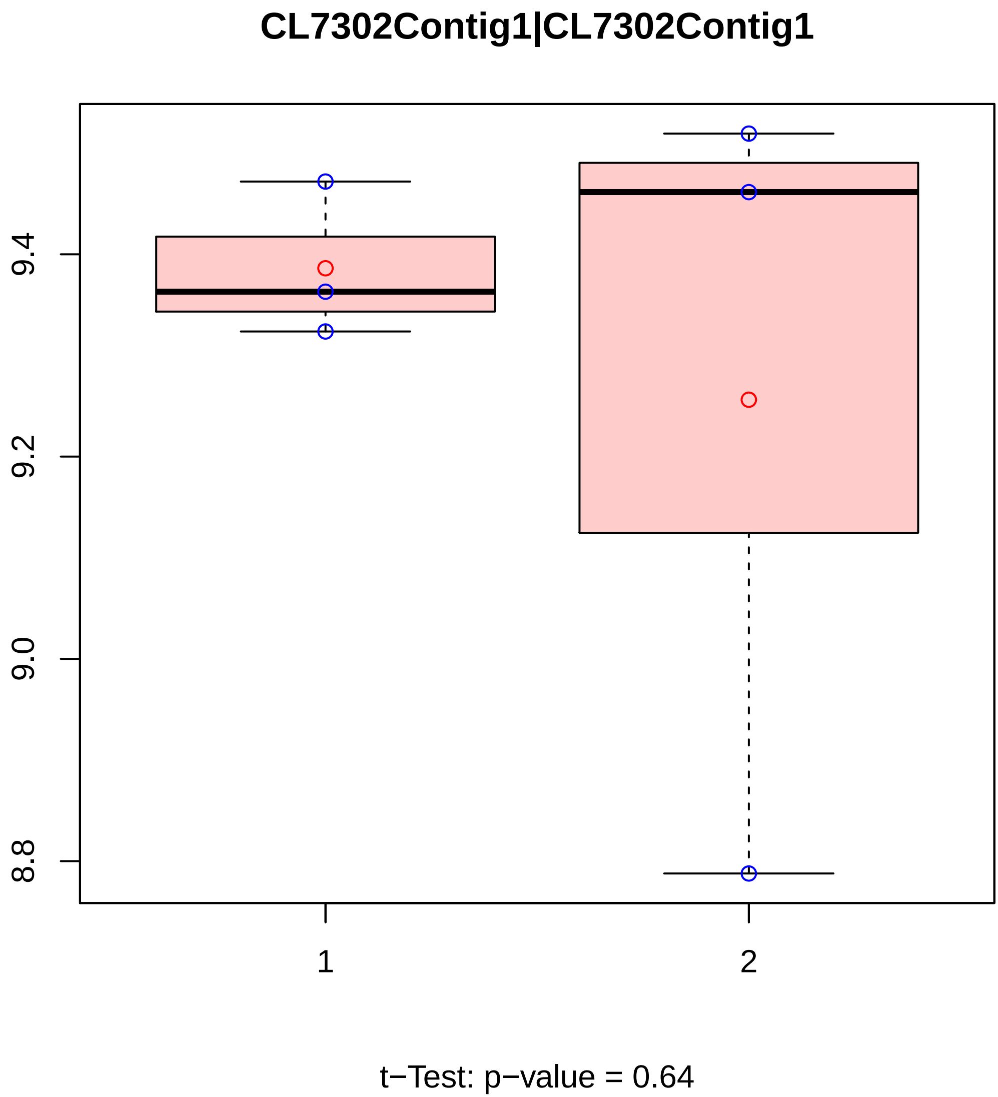
CL7302Contig1|CL7302Contig1
9.4
9.2
9.0
8.8
1
2
t−Test: p−value = 0.64

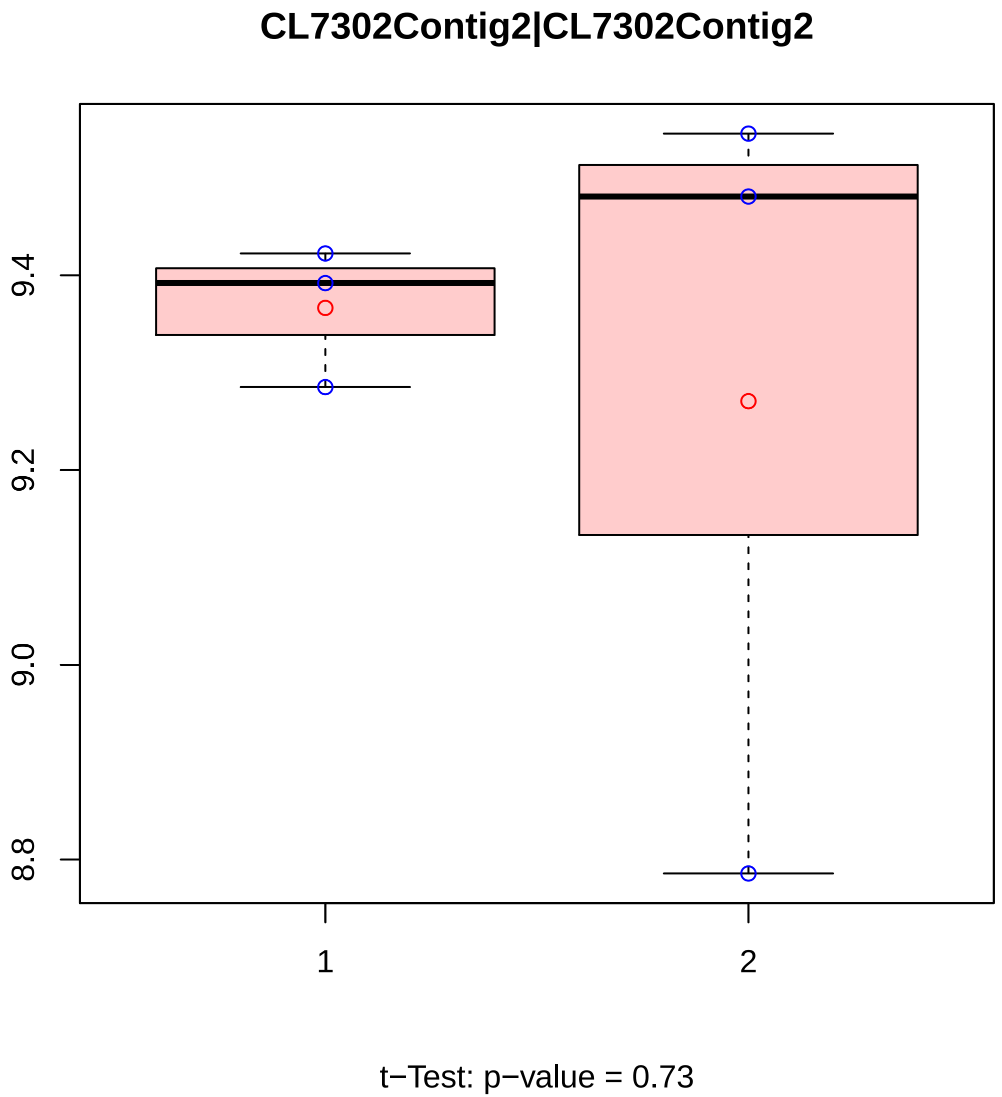

CL7302Contig2|CL7302Contig2
9.4
9.2
9.0
8.8
1
2
t−Test: p−value = 0.73

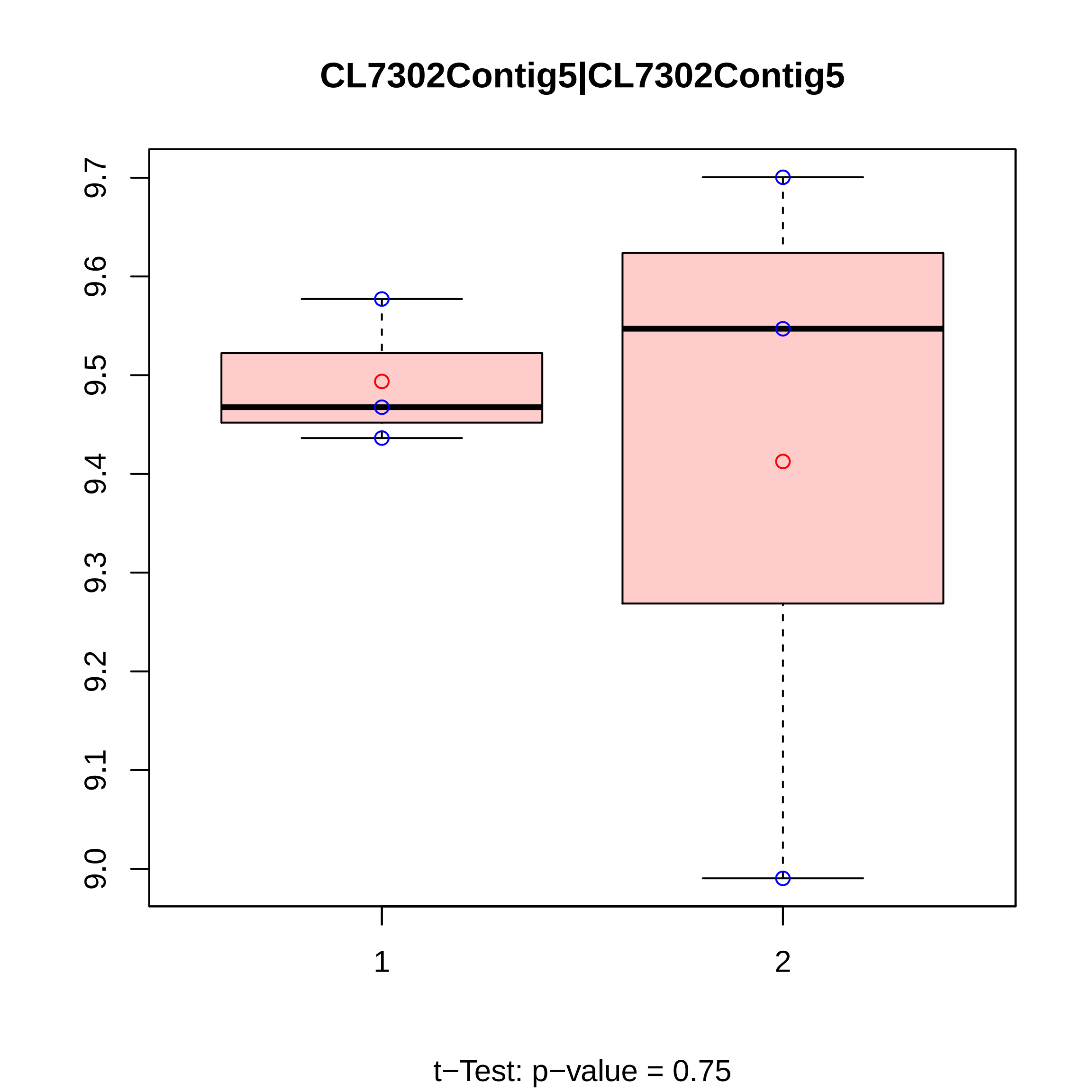
CL7302Contig5|CL7302Contig5
9.0
9.1
9.2
9.3
9.4
9.5
9.6
9.7
1
2
t−Test: p−value = 0.75

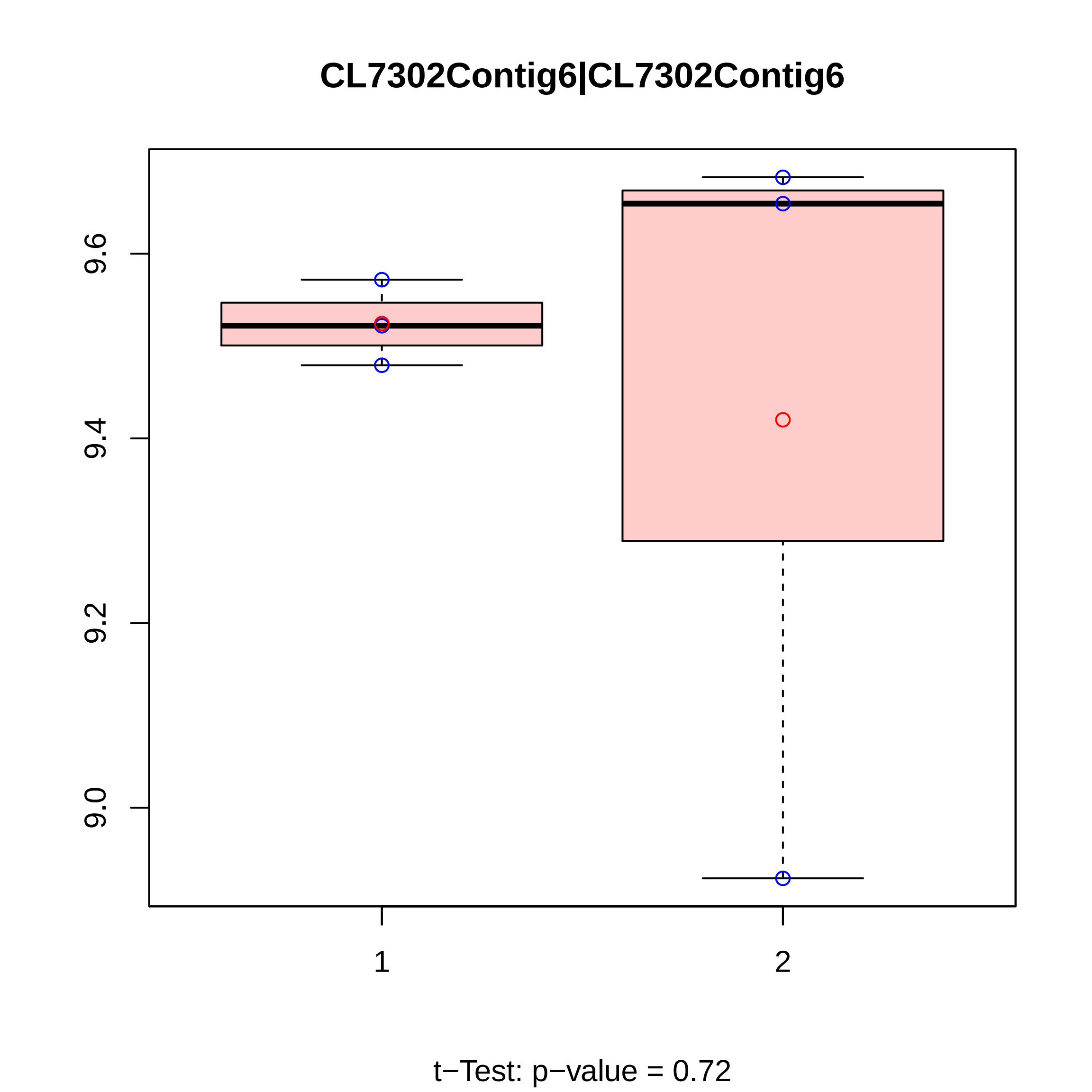

CL7302Contig6|CL7302Contig6
9.0
9.2
9.4
9.6
1
2
t−Test: p−value = 0.72

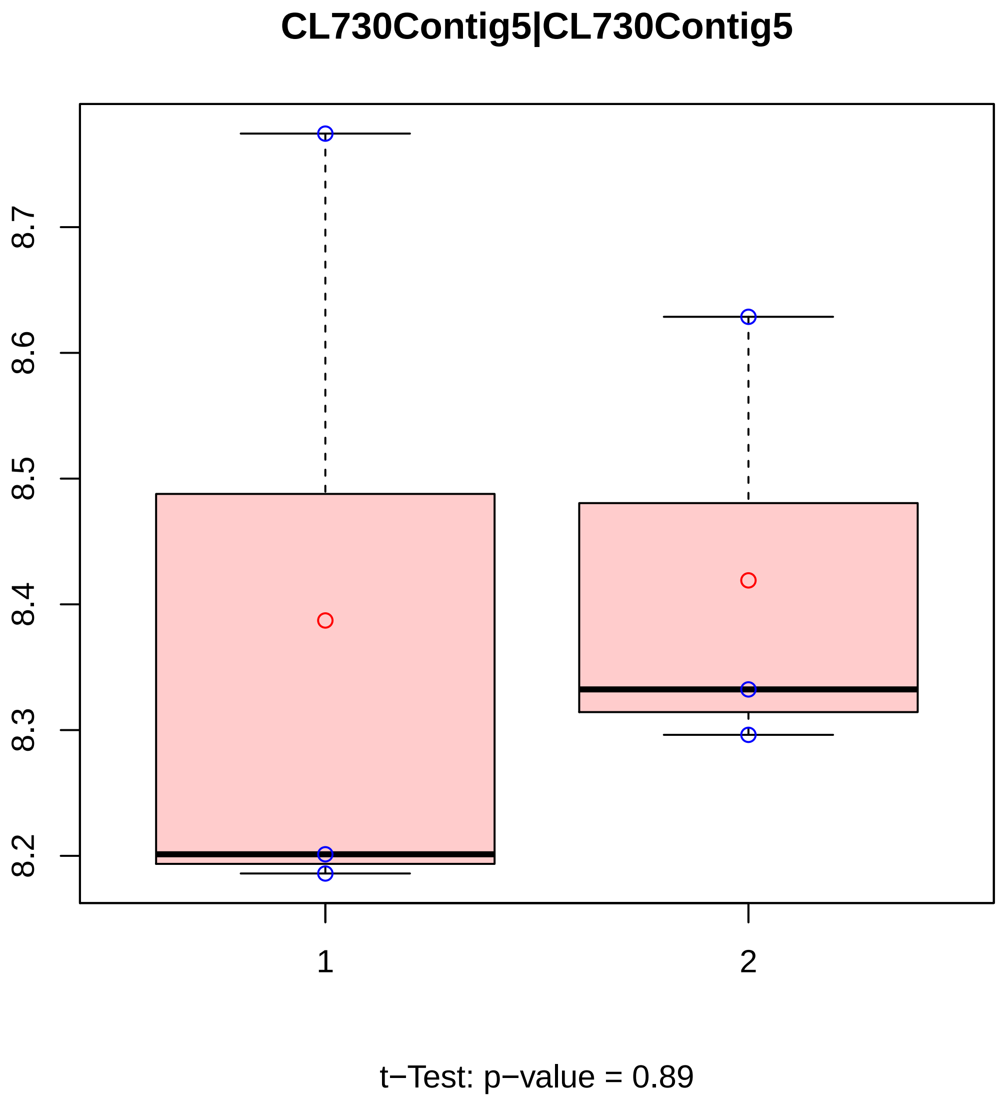

CL730Contig5|CL730Contig5
8.2
8.3
8.4
8.5
8.6
8.7
1
2
t−Test: p−value = 0.89

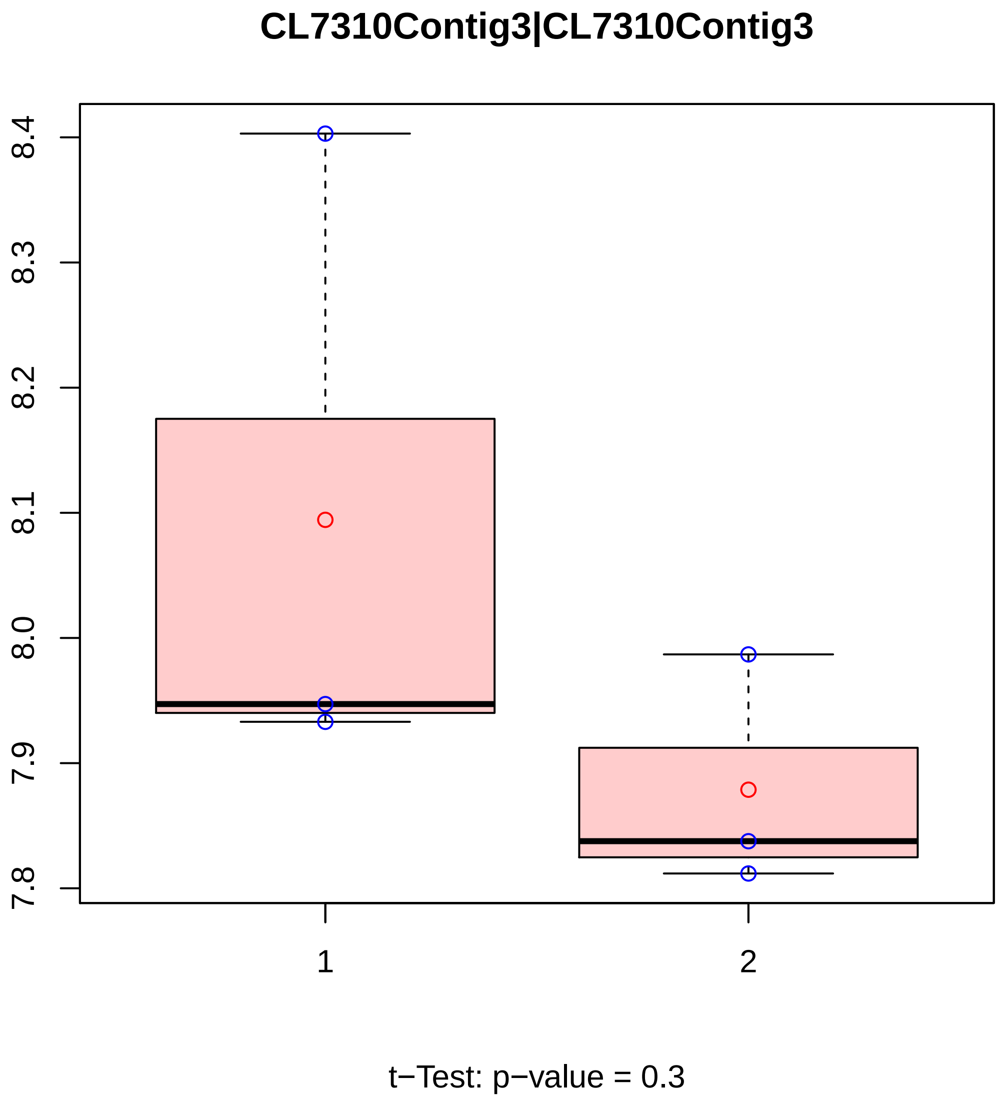

CL7310Contig3|CL7310Contig3
8.4
8.3
8.2
8.1
8.0
7.9
7.8
1
2
t−Test: p−value = 0.3

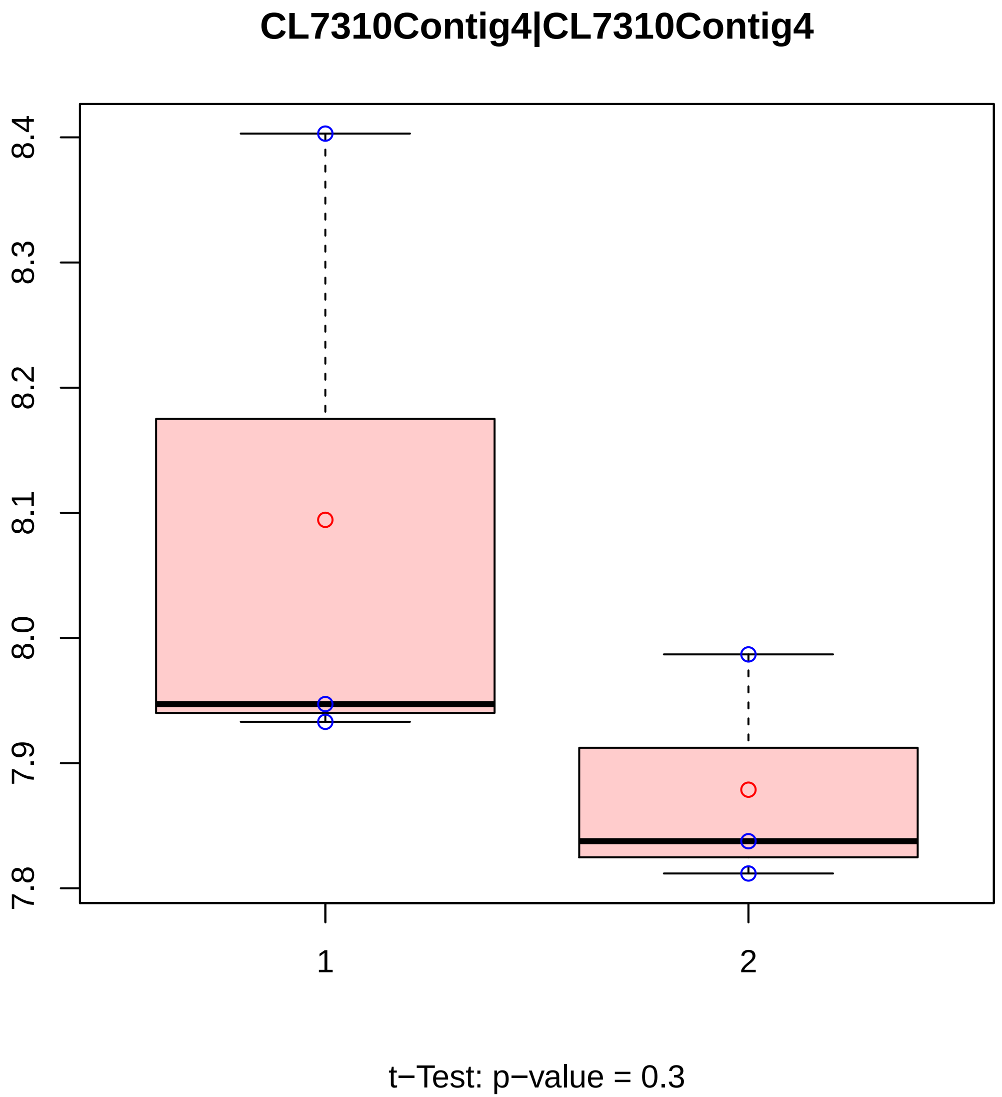
CL7310Contig4|CL7310Contig4
8.4
8.3
8.2
8.1
8.0
7.9
7.8
1
2
t−Test: p−value = 0.3

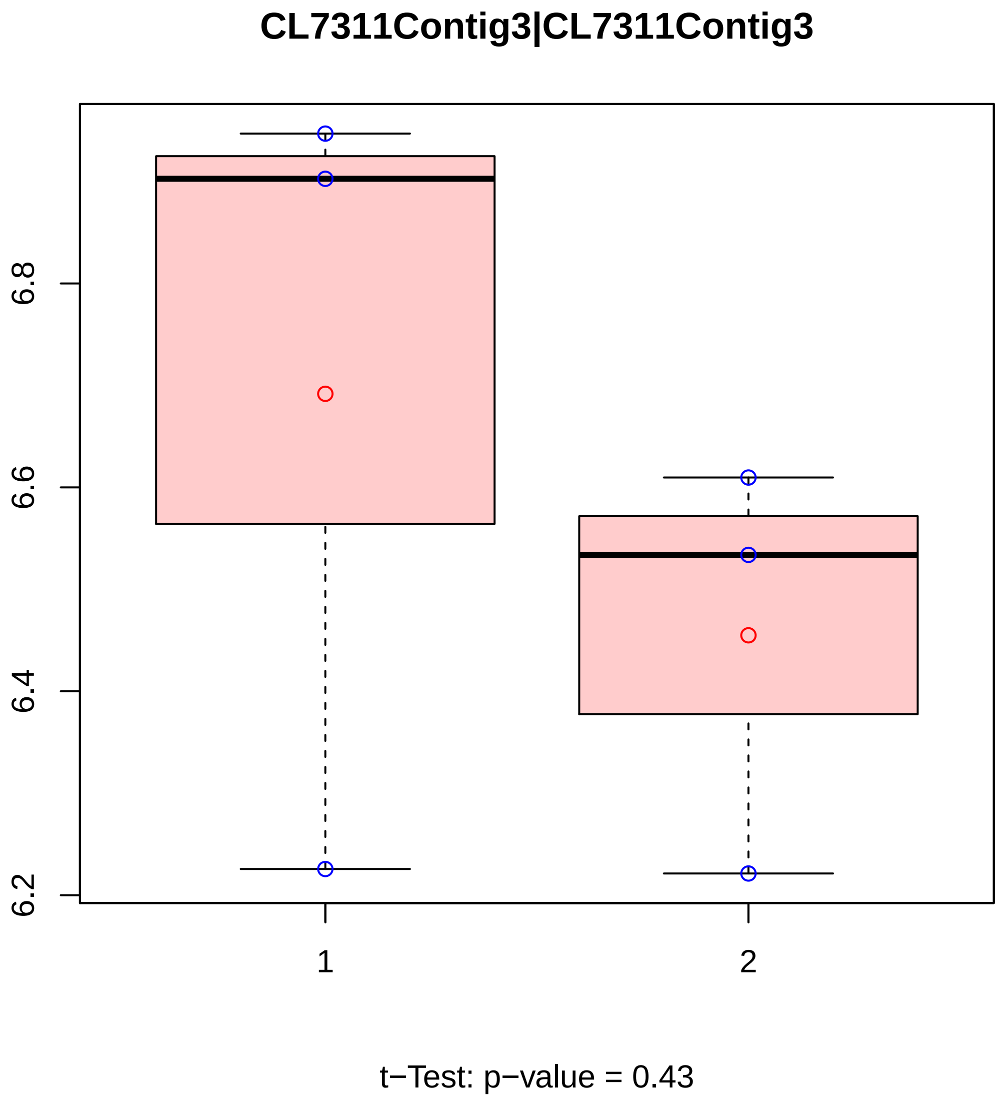
CL7311Contig3|CL7311Contig3
6.2
6.4
6.6
6.8
1
2
t−Test: p−value = 0.43

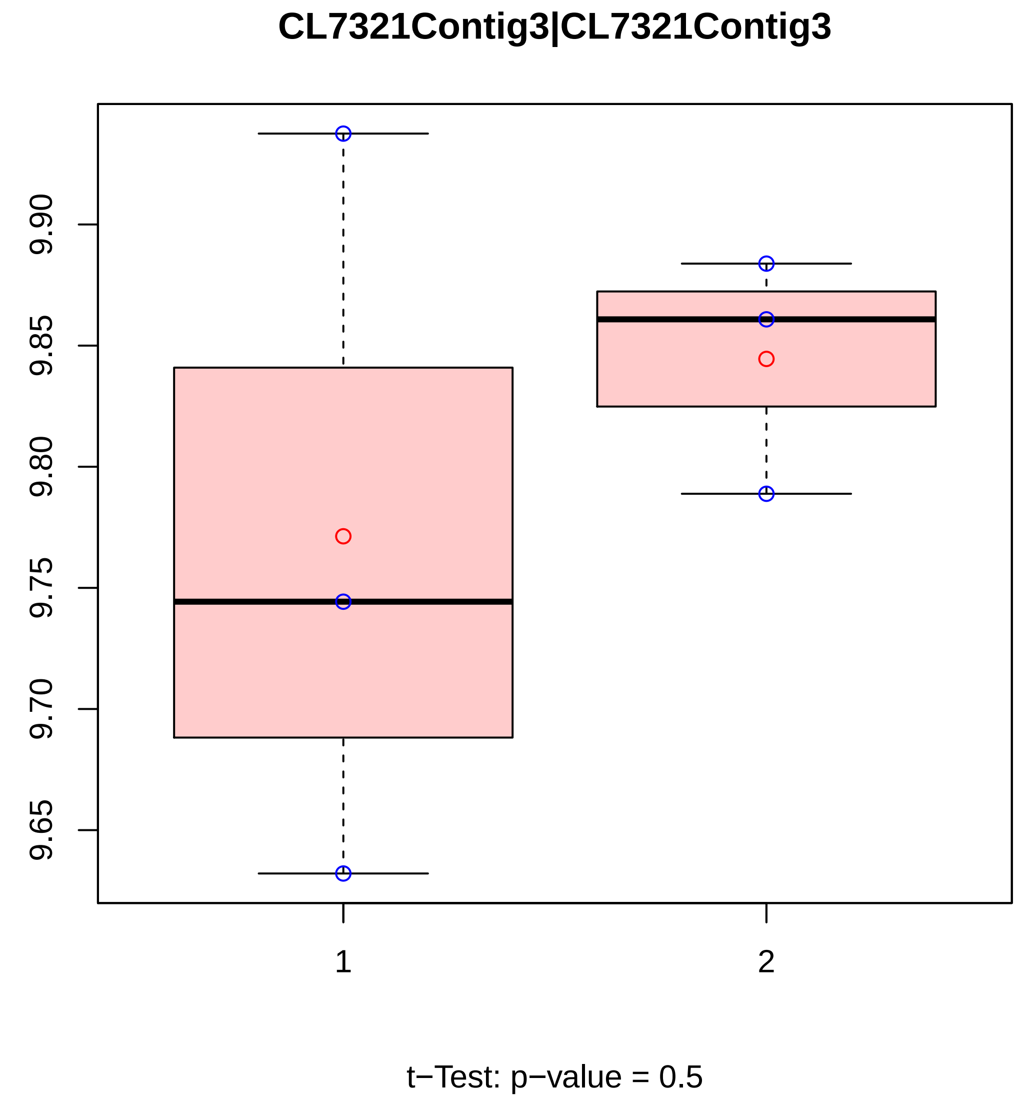
CL7321Contig3|CL7321Contig3
9.65
9.70
9.75
9.80
9.85
9.90
1
2
t−Test: p−value = 0.5

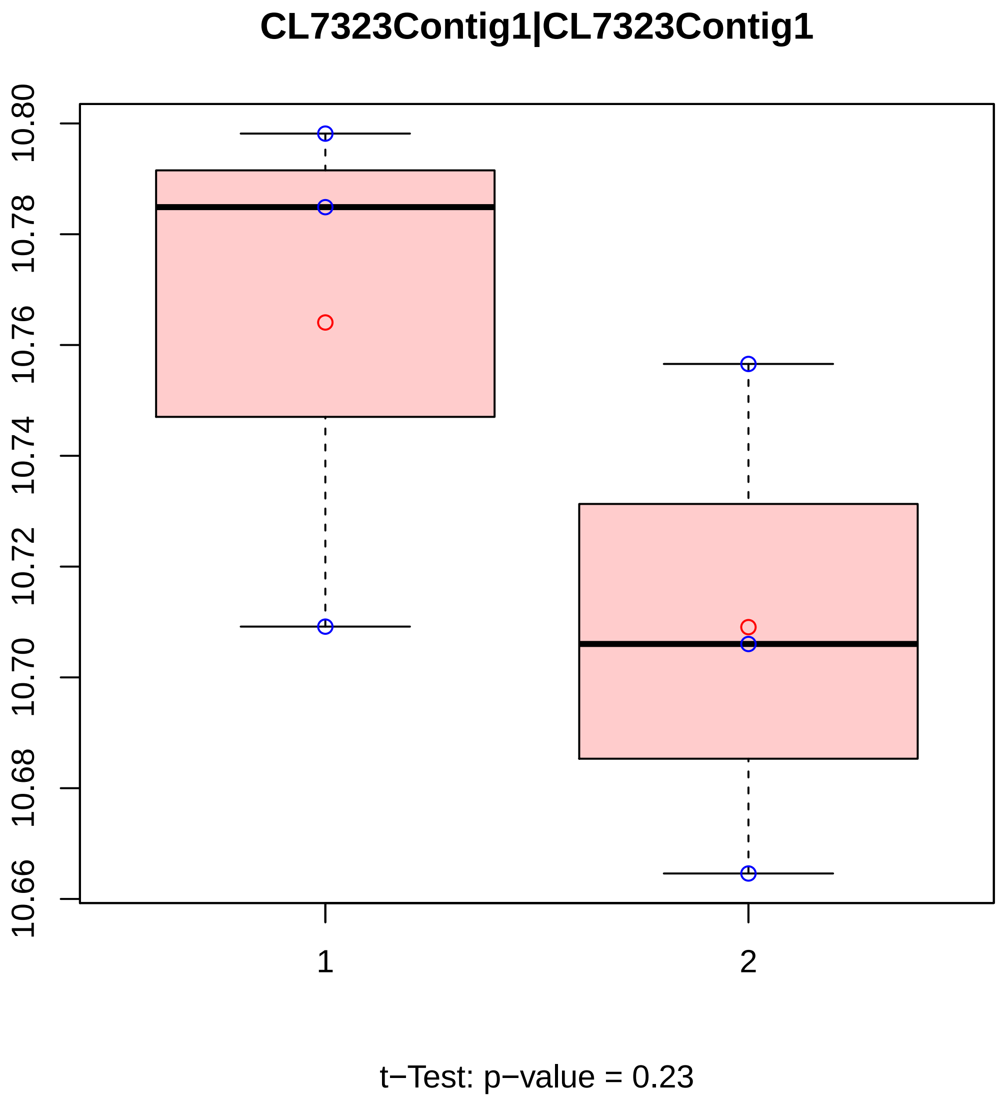

CL7323Contig1|CL7323Contig1
10.66
10.68
10.70
10.72
10.74
10.76
10.78
10.80
1
2
t−Test: p−value = 0.23

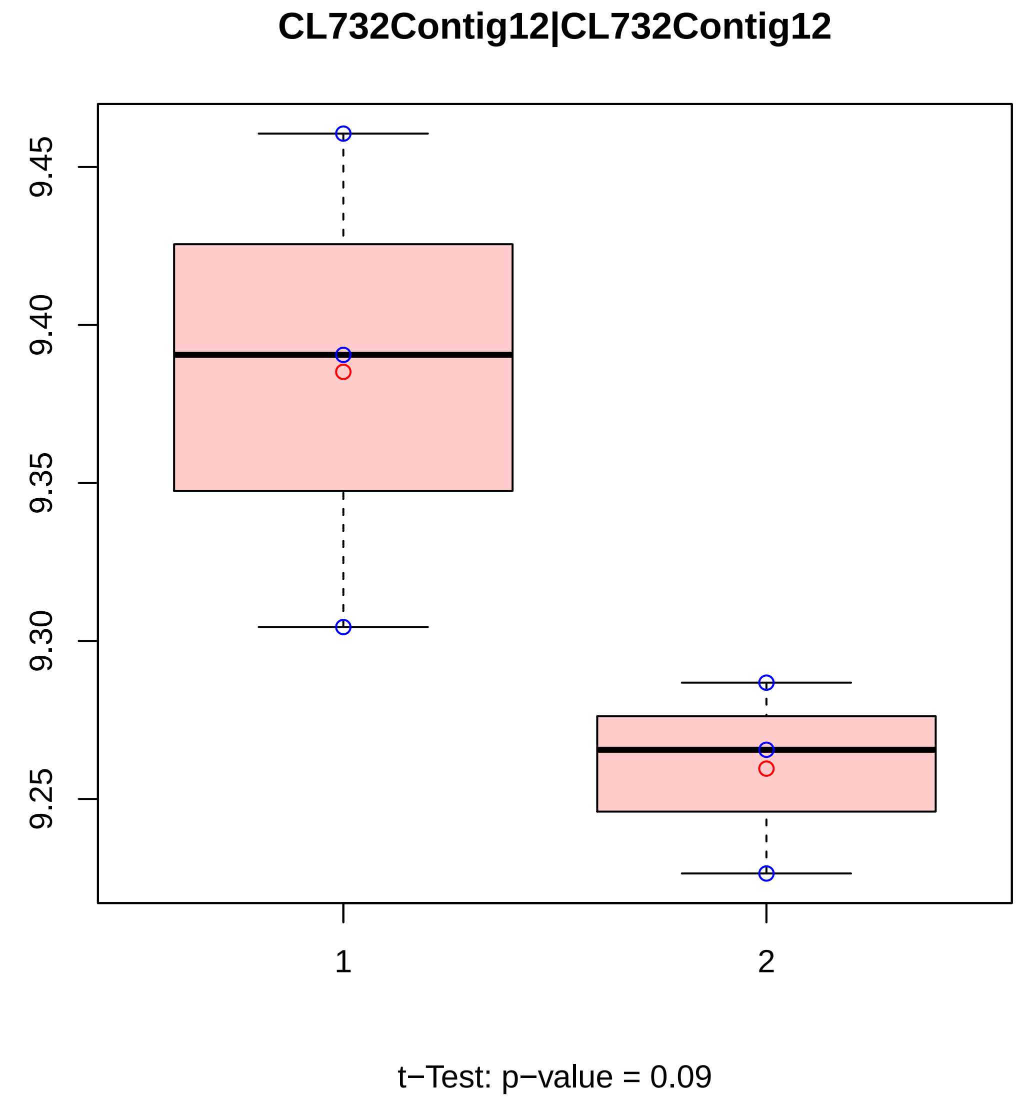
CL732Contig12|CL732Contig12
9.45
9.40
9.35
9.30
9.25
1
2
t−Test: p−value = 0.09

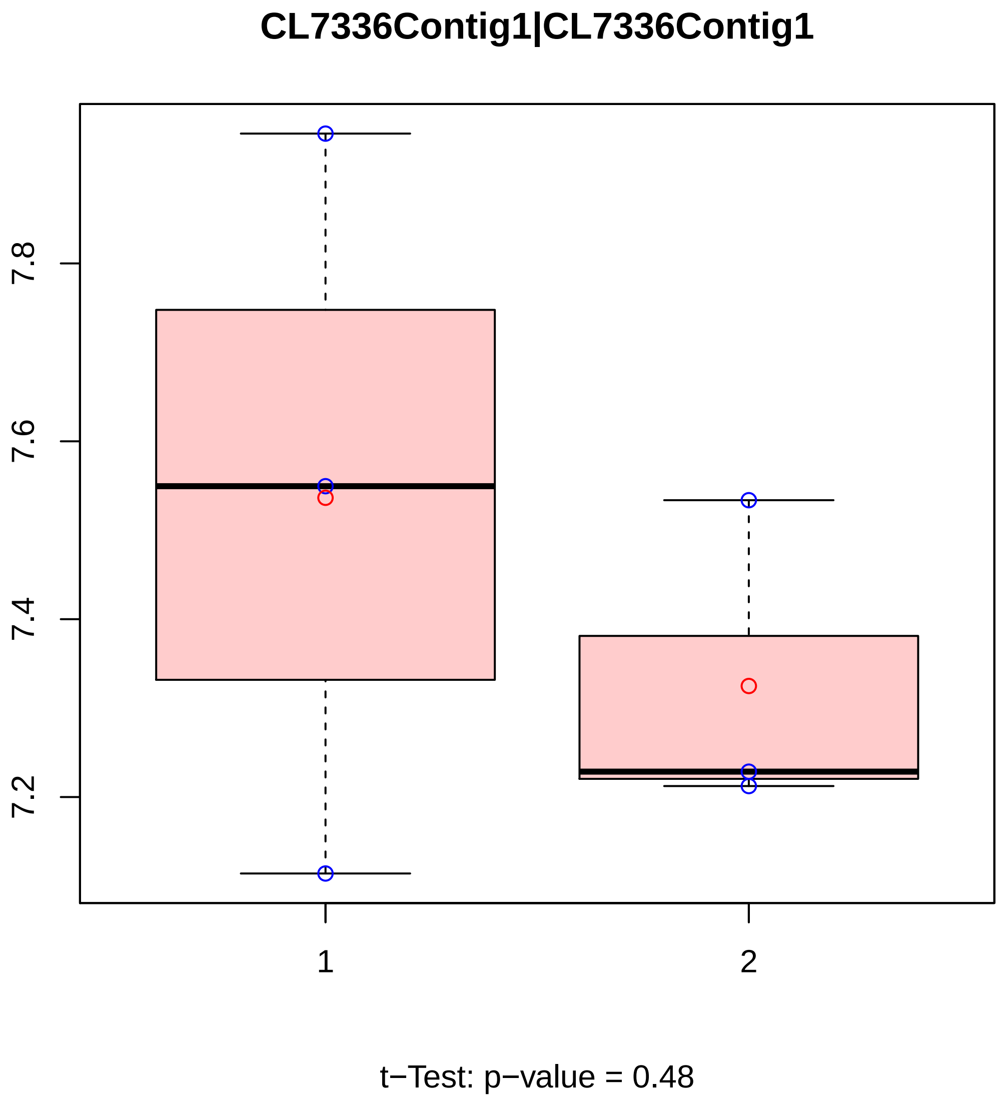
CL7336Contig1|CL7336Contig1
7.2
7.4
7.6
7.8
1
2
t−Test: p−value = 0.48

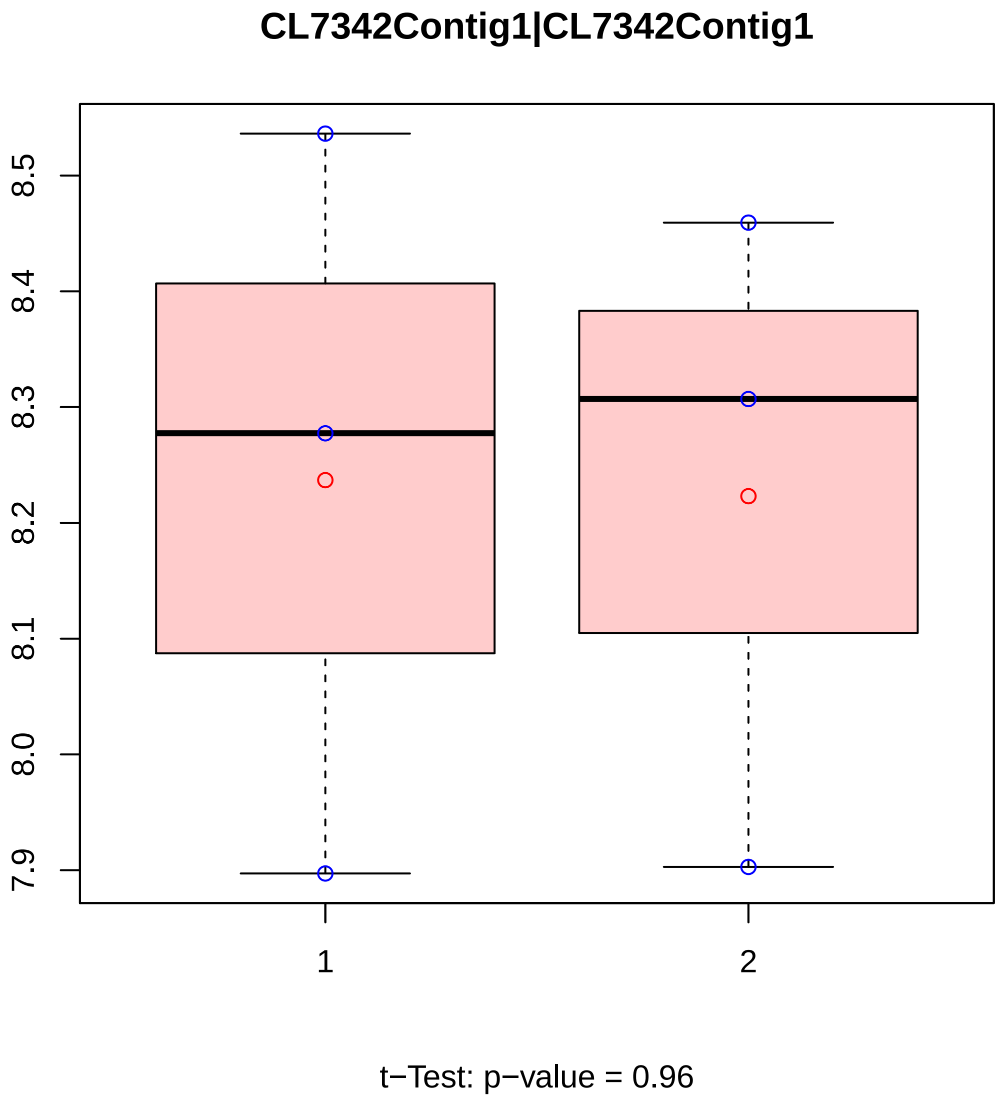
CL7342Contig1|CL7342Contig1
8.5
8.4
8.3
8.2
8.1
8.0
7.9
1
2
t−Test: p−value = 0.96

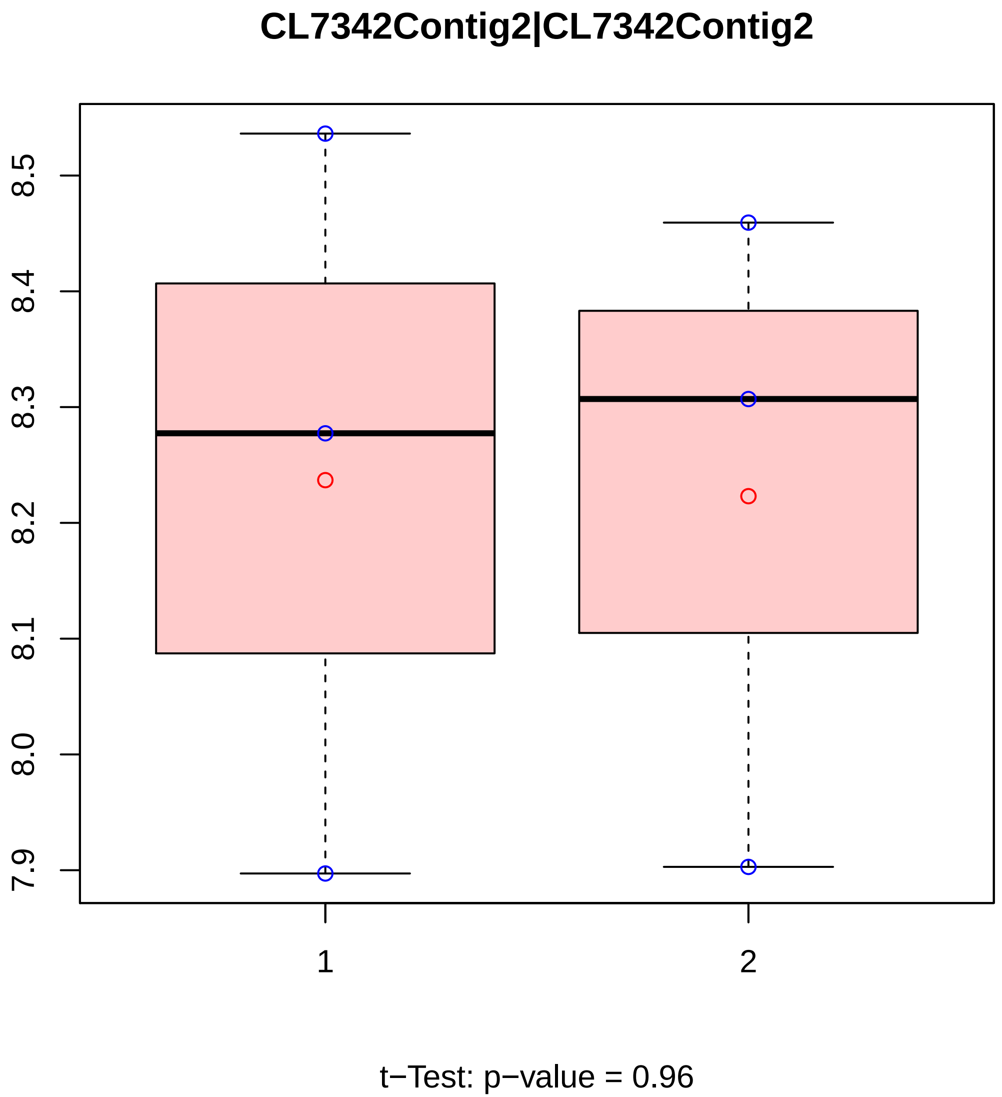
CL7342Contig2|CL7342Contig2
8.5
8.4
8.3
8.2
8.1
8.0
7.9
1
2
t−Test: p−value = 0.96

CL7347Contig2|CL7347Contig2

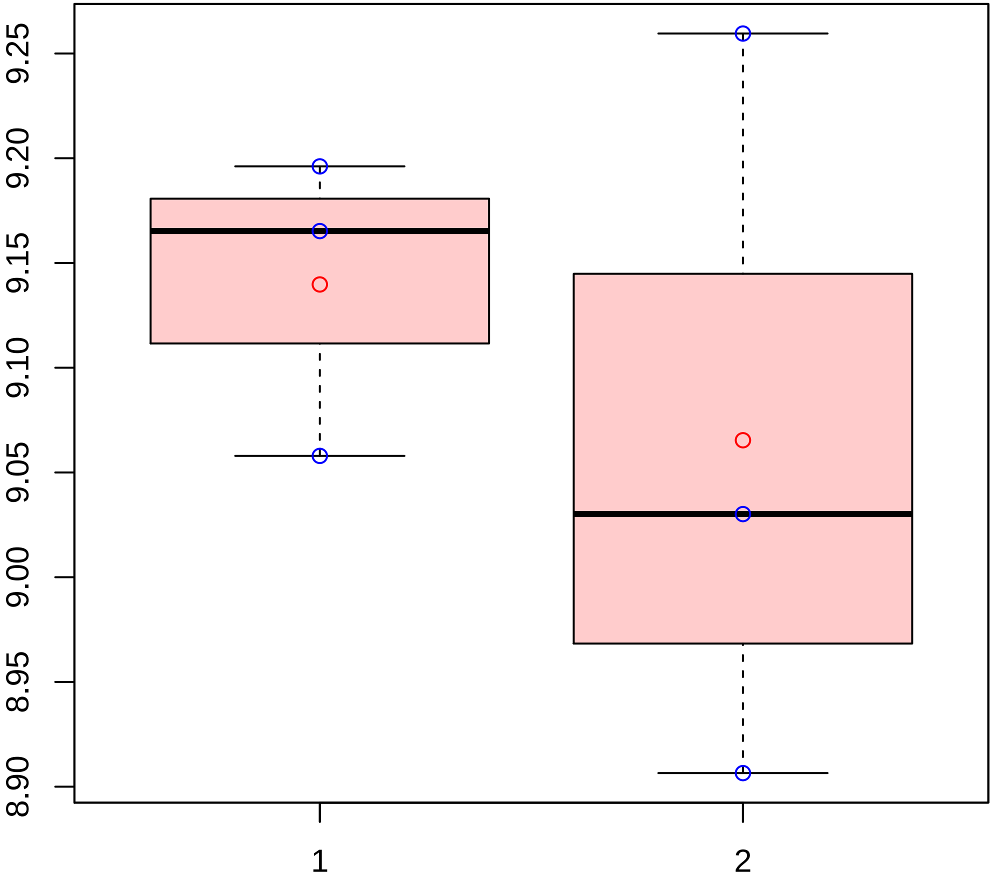


t−Test: p−value = 0.56

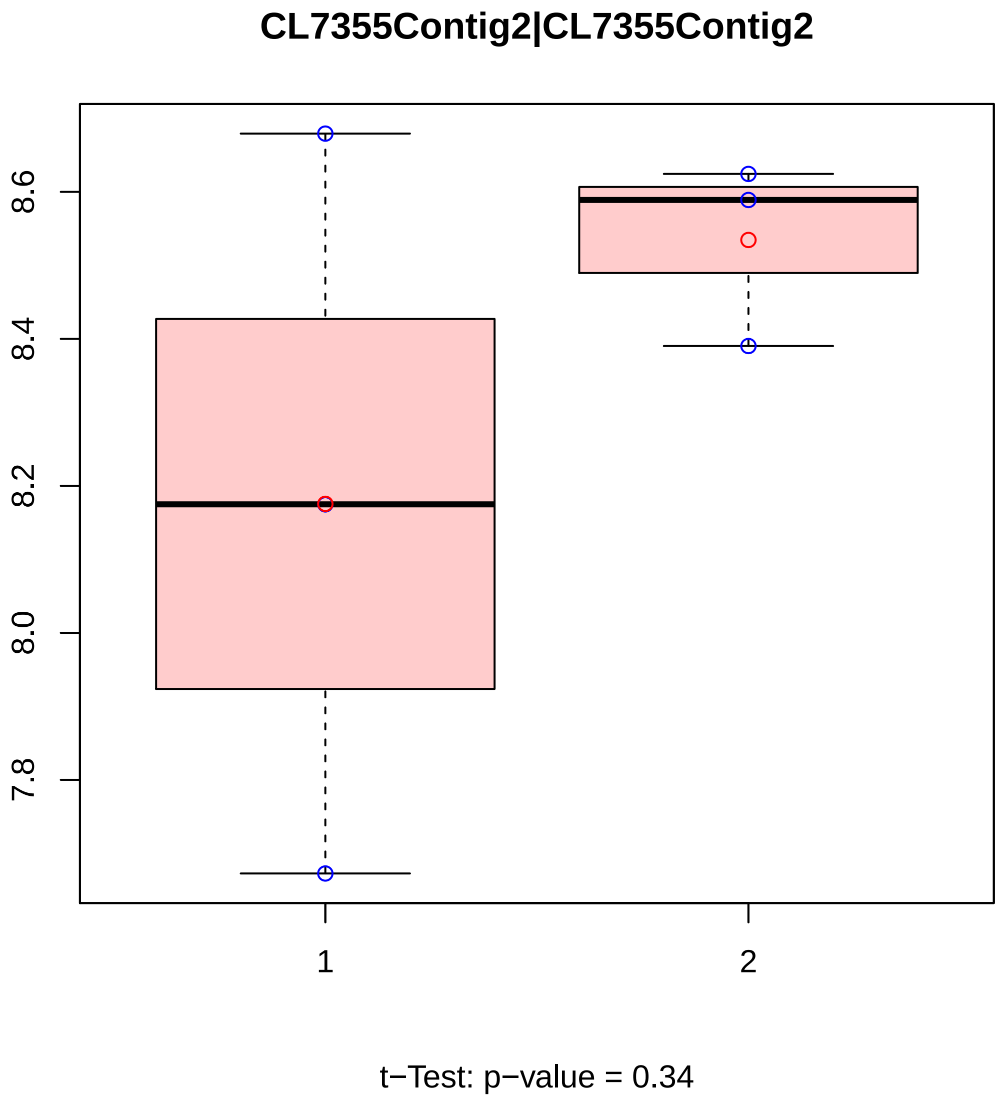

CL7355Contig2|CL7355Contig2
8.6
8.4
8.2
8.0
7.8
1
2
t−Test: p−value = 0.34

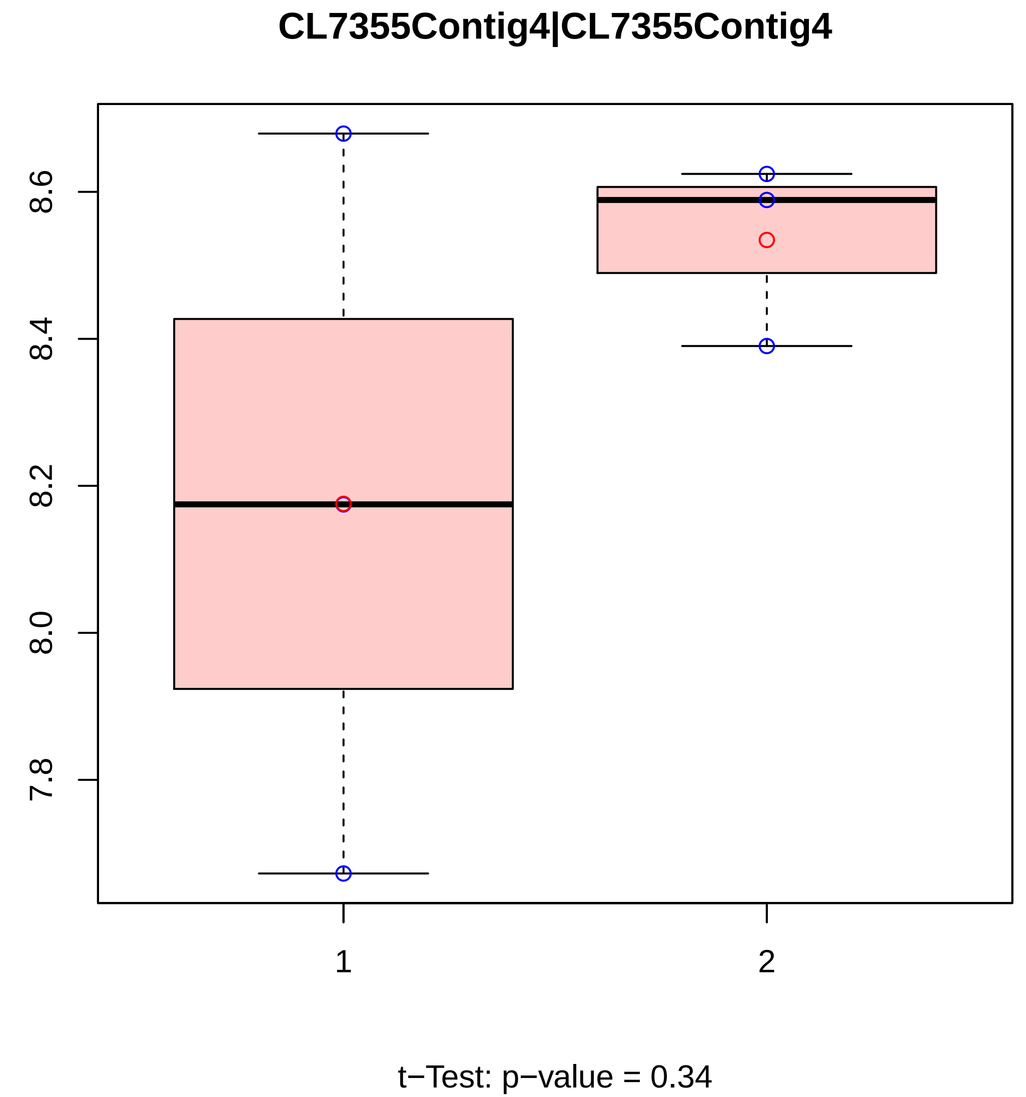

CL7355Contig4|CL7355Contig4
8.6
8.4
8.2
8.0
7.8
1
2
t−Test: p−value = 0.34

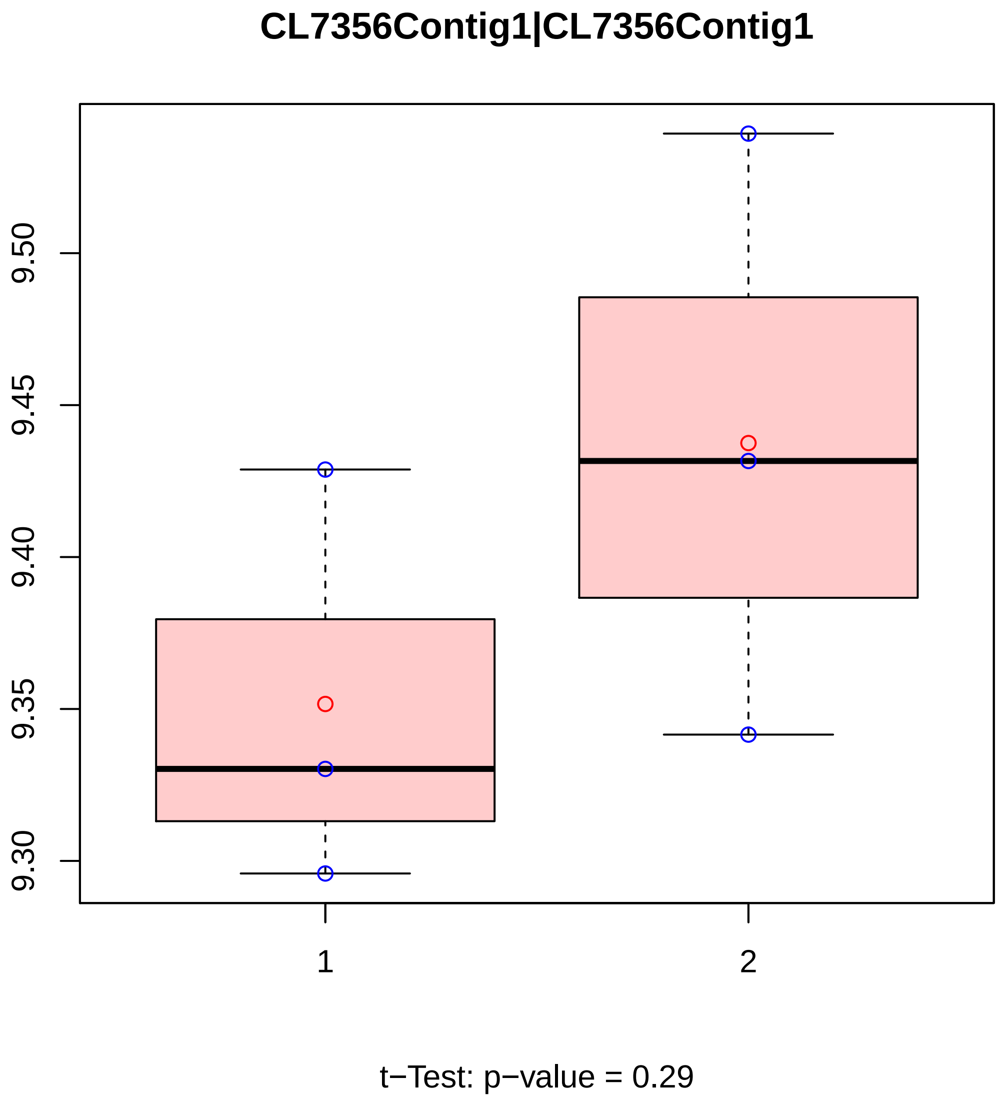

CL7356Contig1|CL7356Contig1
9.30
9.35
9.40
9.45
9.50
1
2
t−Test: p−value = 0.29

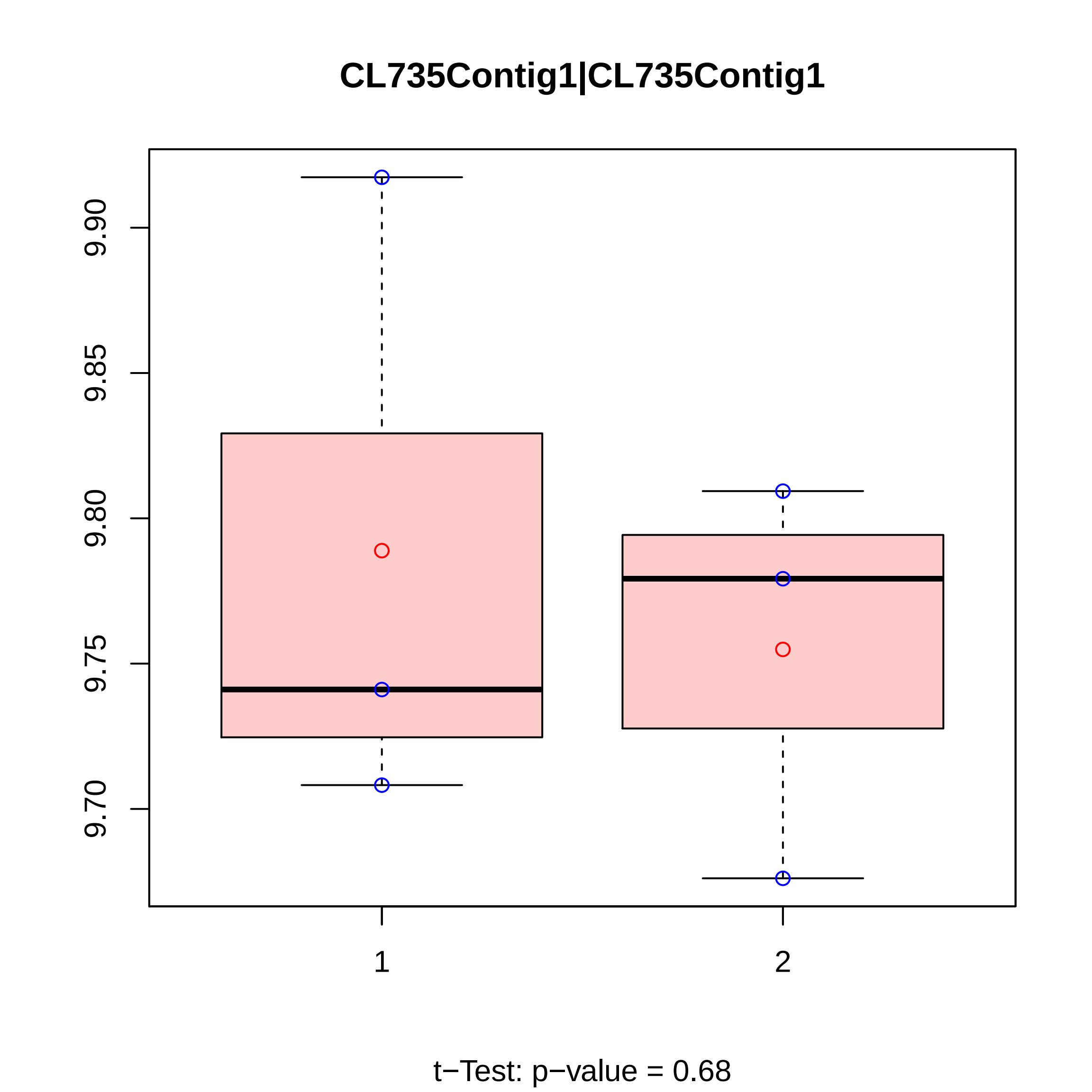

CL735Contig1|CL735Contig1
9.70
9.75
9.80
9.85
9.90
1
2
t−Test: p−value = 0.68

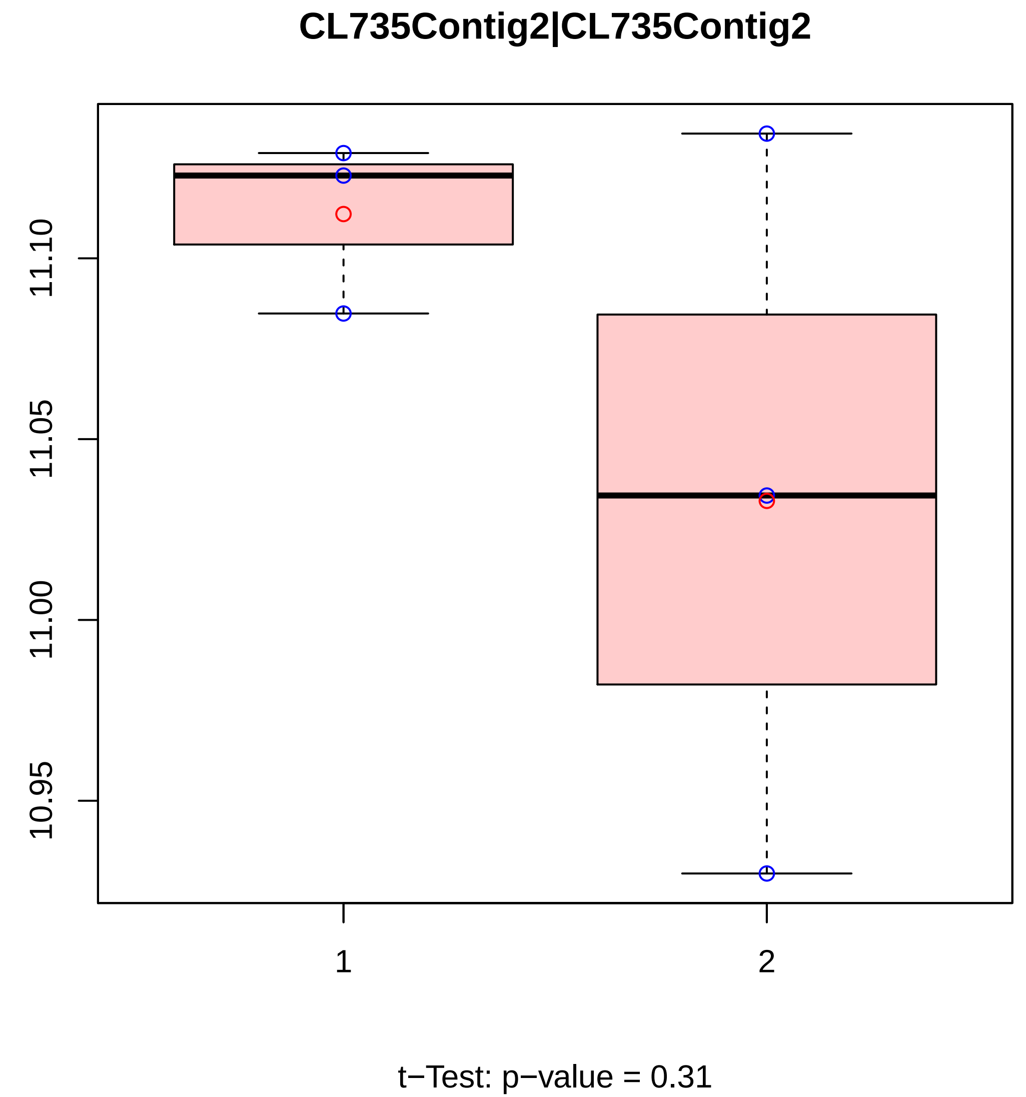
CL735Contig2|CL735Contig2
11.10
11.05
11.00
10.95
1
2
t−Test: p−value = 0.31

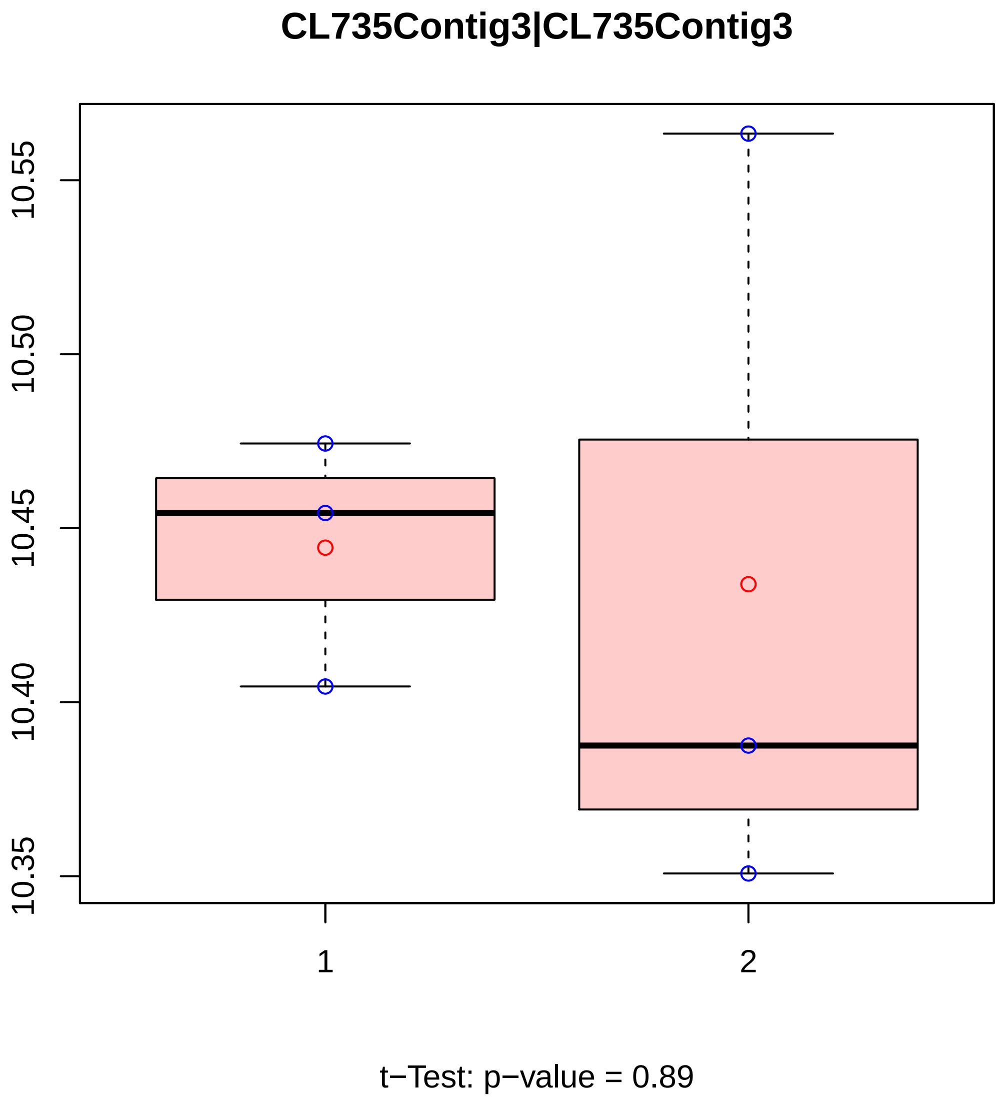

CL735Contig3|CL735Contig3
10.35
10.40
10.45
10.50
10.55
1
2
t−Test: p−value = 0.89

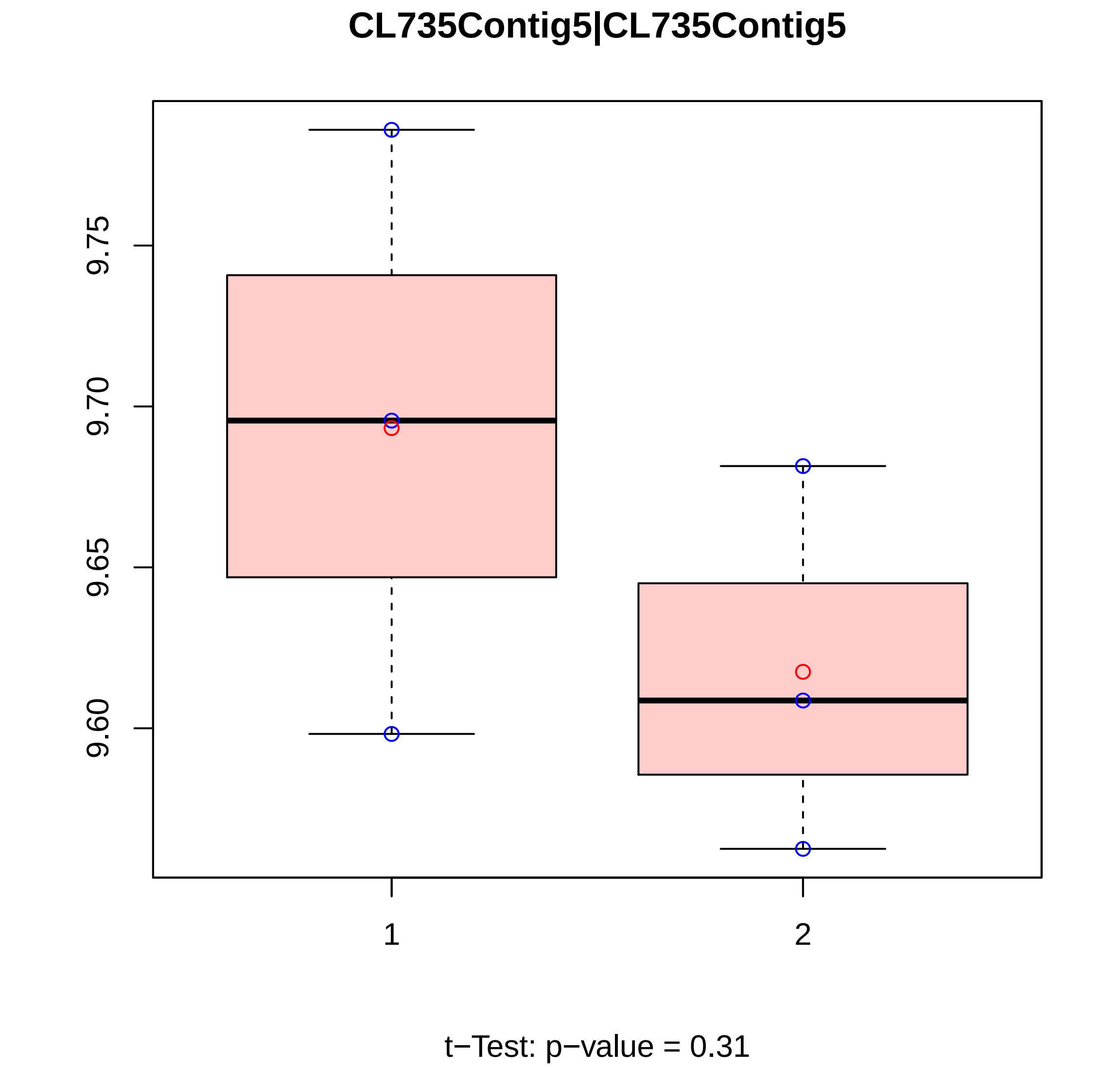
CL735Contig5|CL735Contig5
9.60
9.65
9.70
9.75
1
2
t−Test: p−value = 0.31

## CL7361Contig1|CL7361Contig1

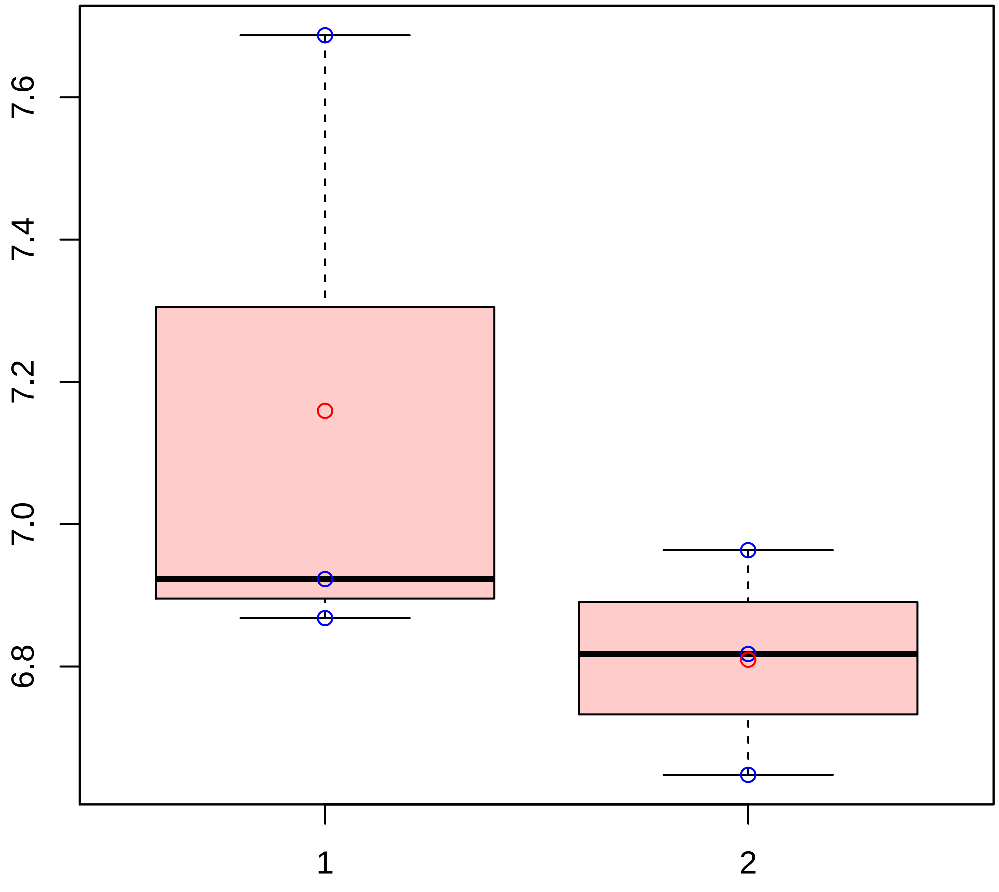


t−Test: p−value = 0.32

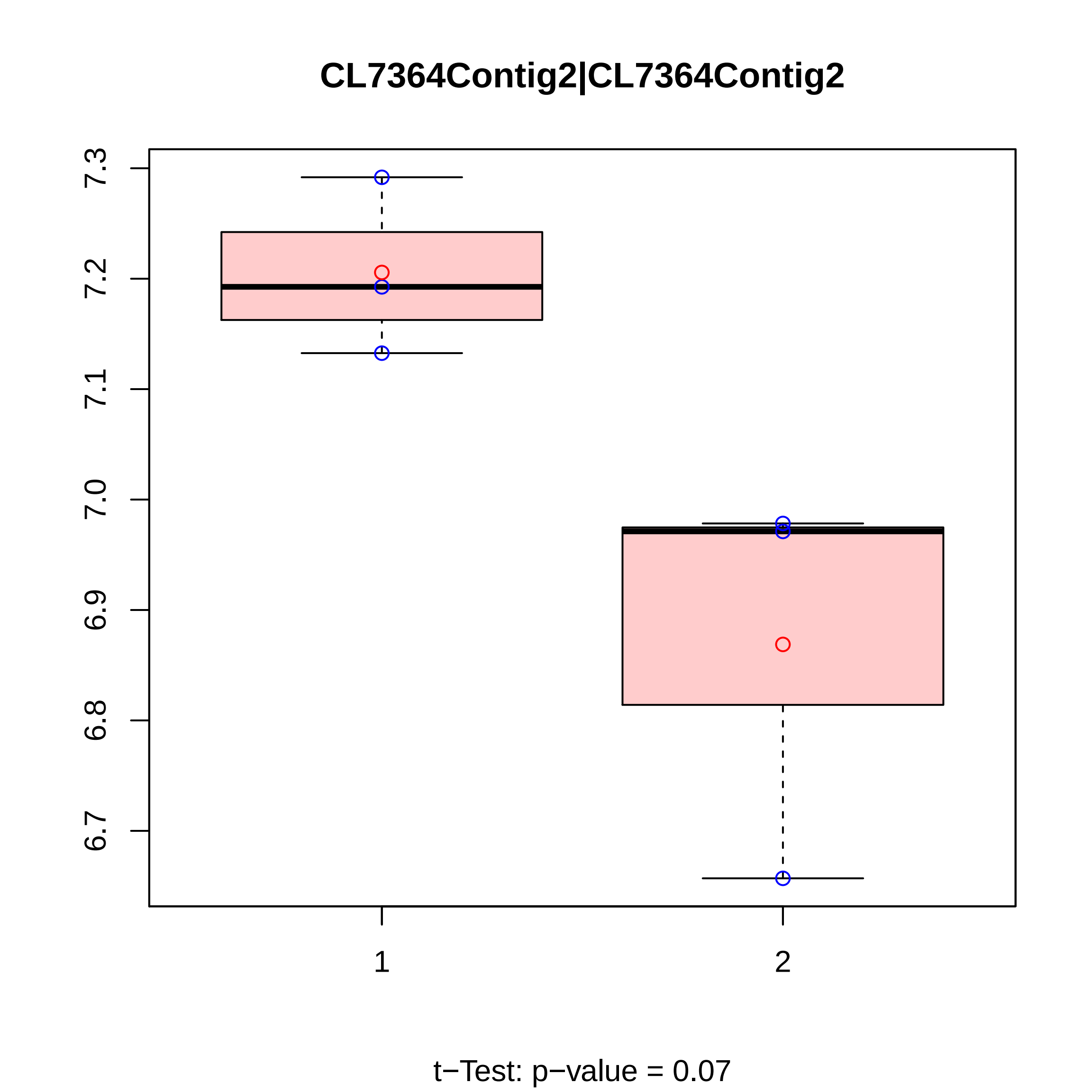
CL7364Contig2|CL7364Contig2
7.3
7.2
7.1
7.0
6.9
6.8
6.7
1
2
t−Test: p−value = 0.07

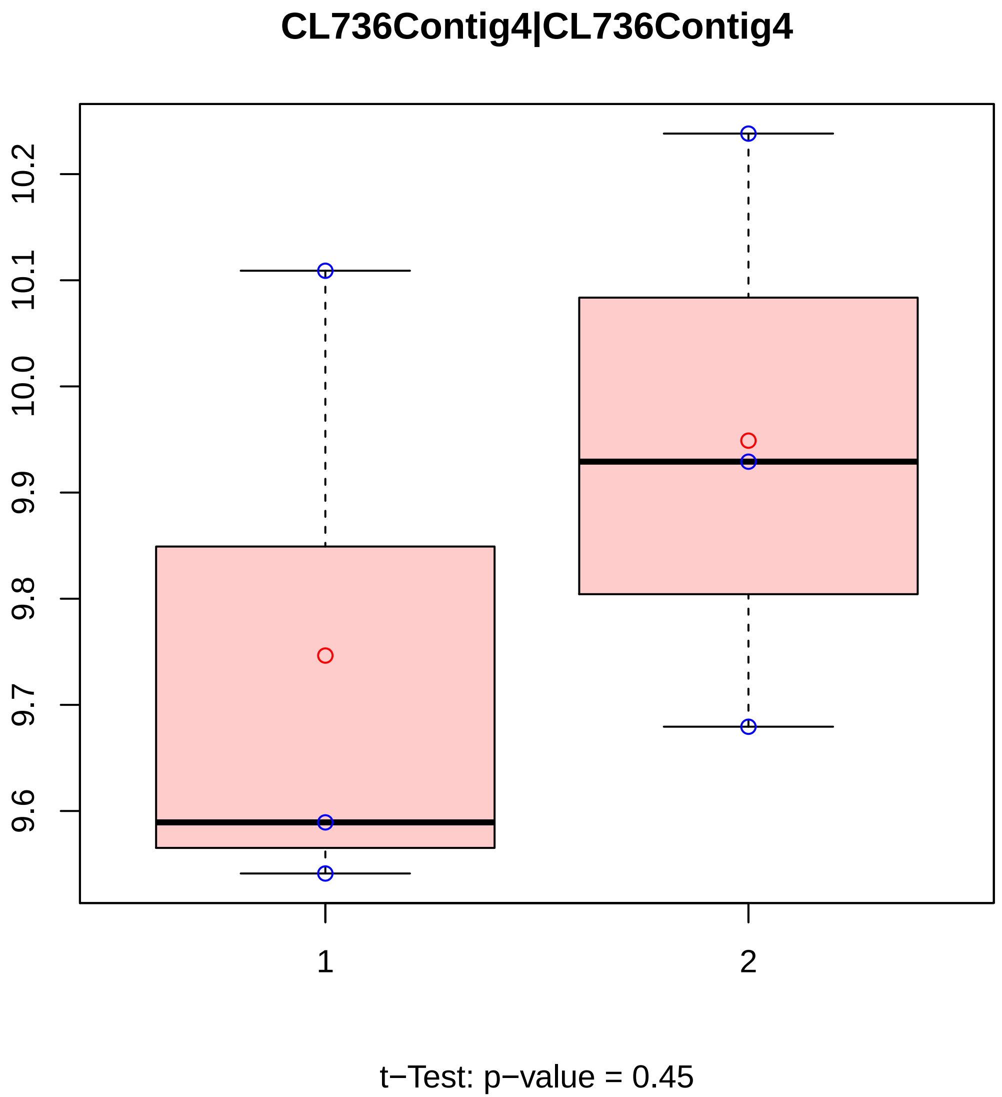

CL736Contig4|CL736Contig4
10.2
10.1
10.0
9.9
9.8
9.7
9.6
1
2
t−Test: p−value = 0.45

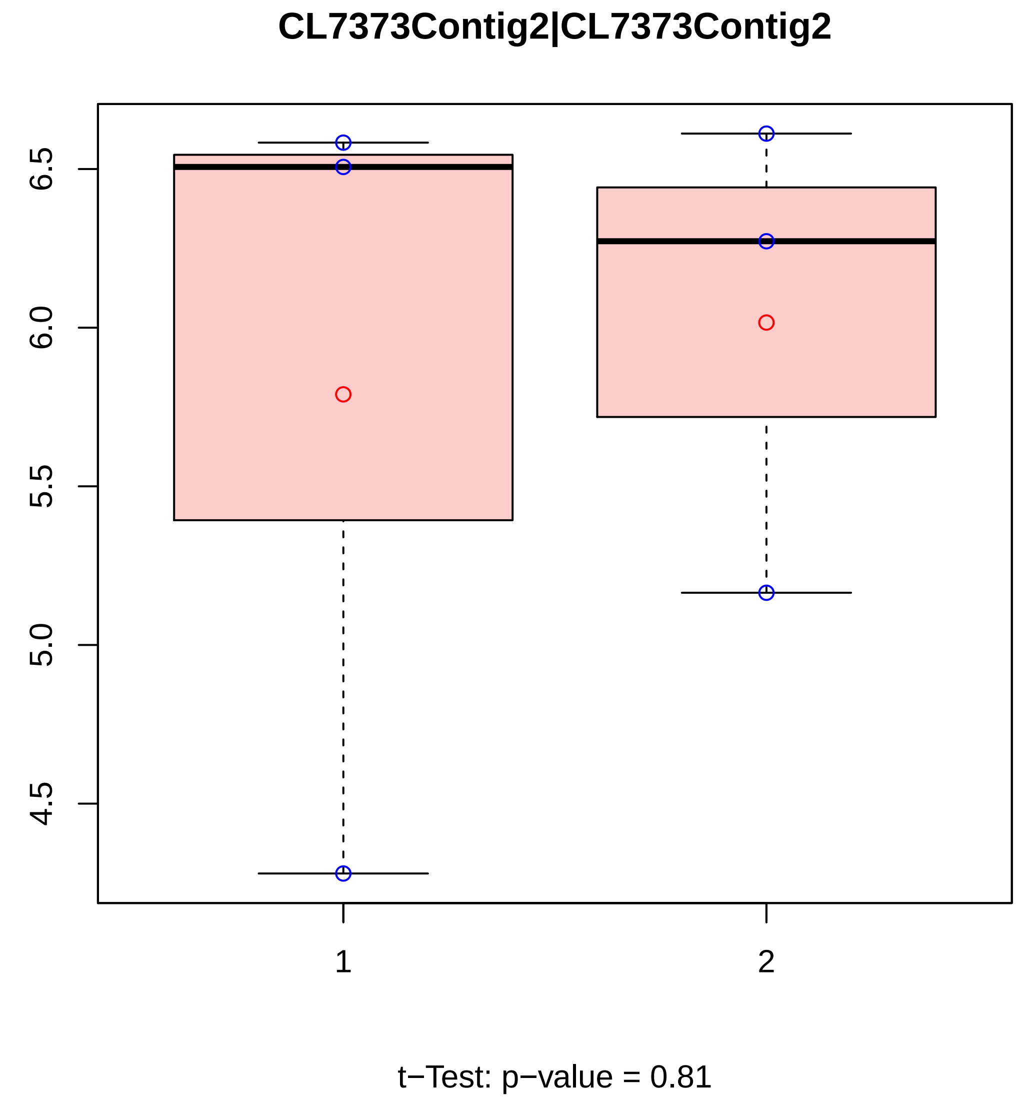
CL7373Contig2|CL7373Contig2
6.5
6.0
5.5
5.0
4.5
1
2
t−Test: p−value = 0.81

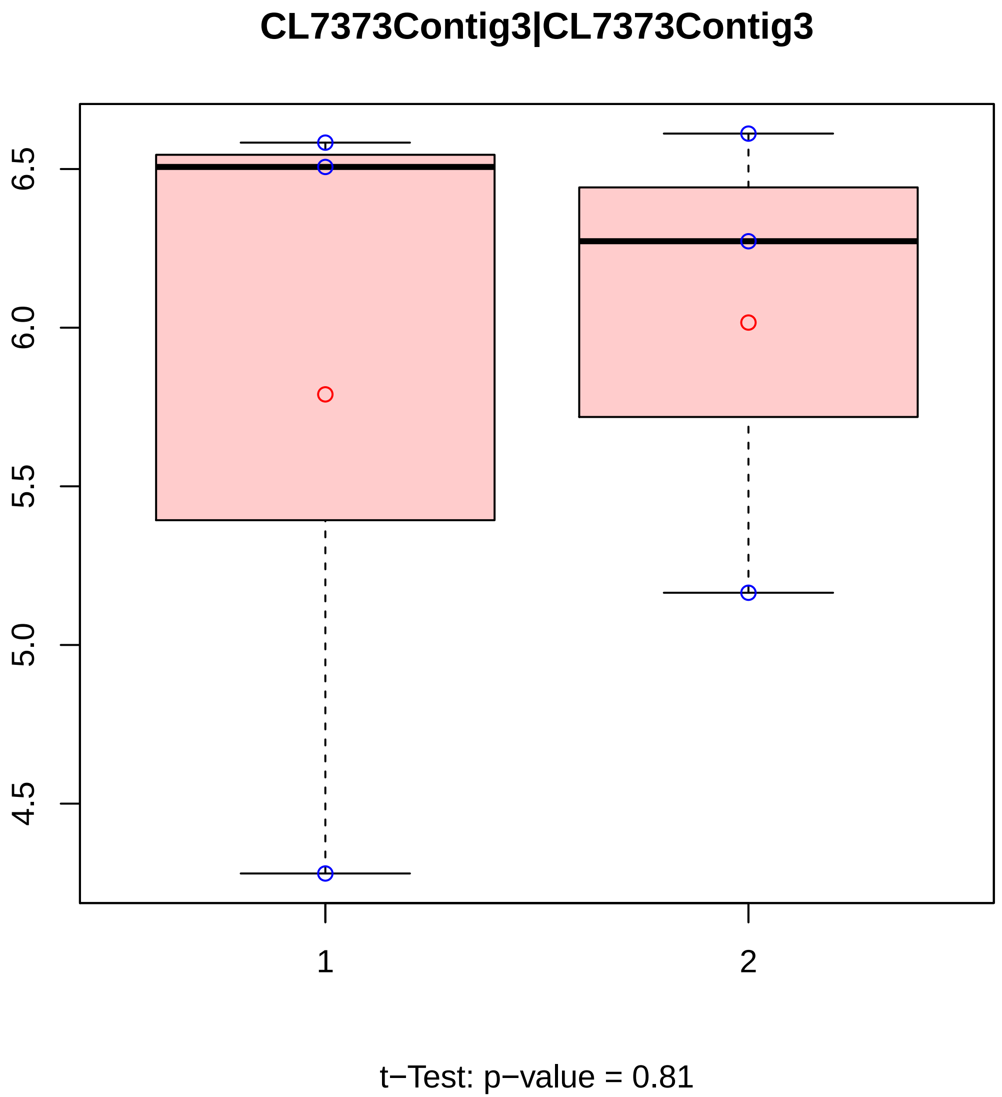
CL7373Contig3|CL7373Contig3
6.5
6.0
5.5
5.0
4.5
1
2
t−Test: p−value = 0.81

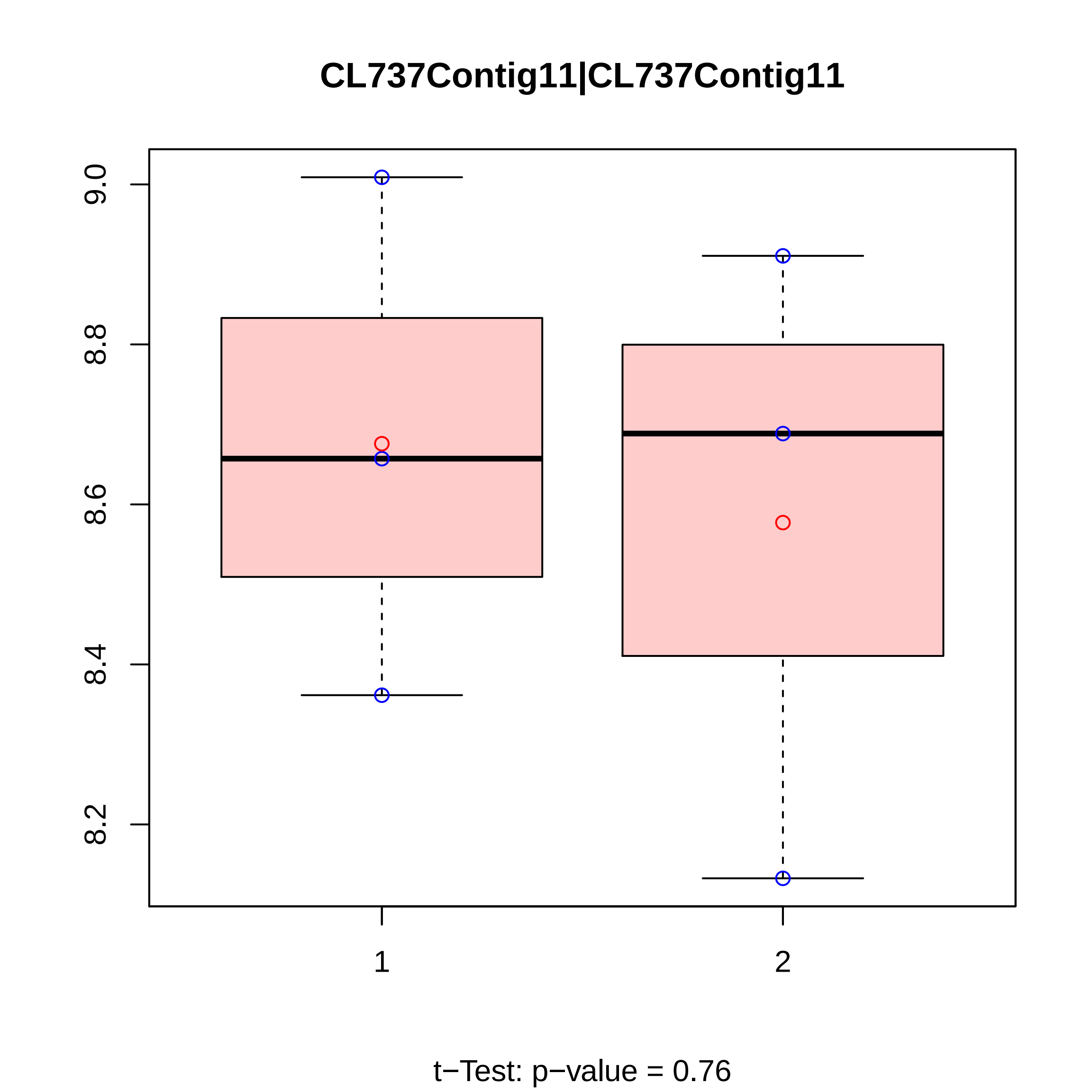
CL737Contig11|CL737Contig11
9.0
8.8
8.6
8.4
8.2
1
2
t−Test: p−value = 0.76

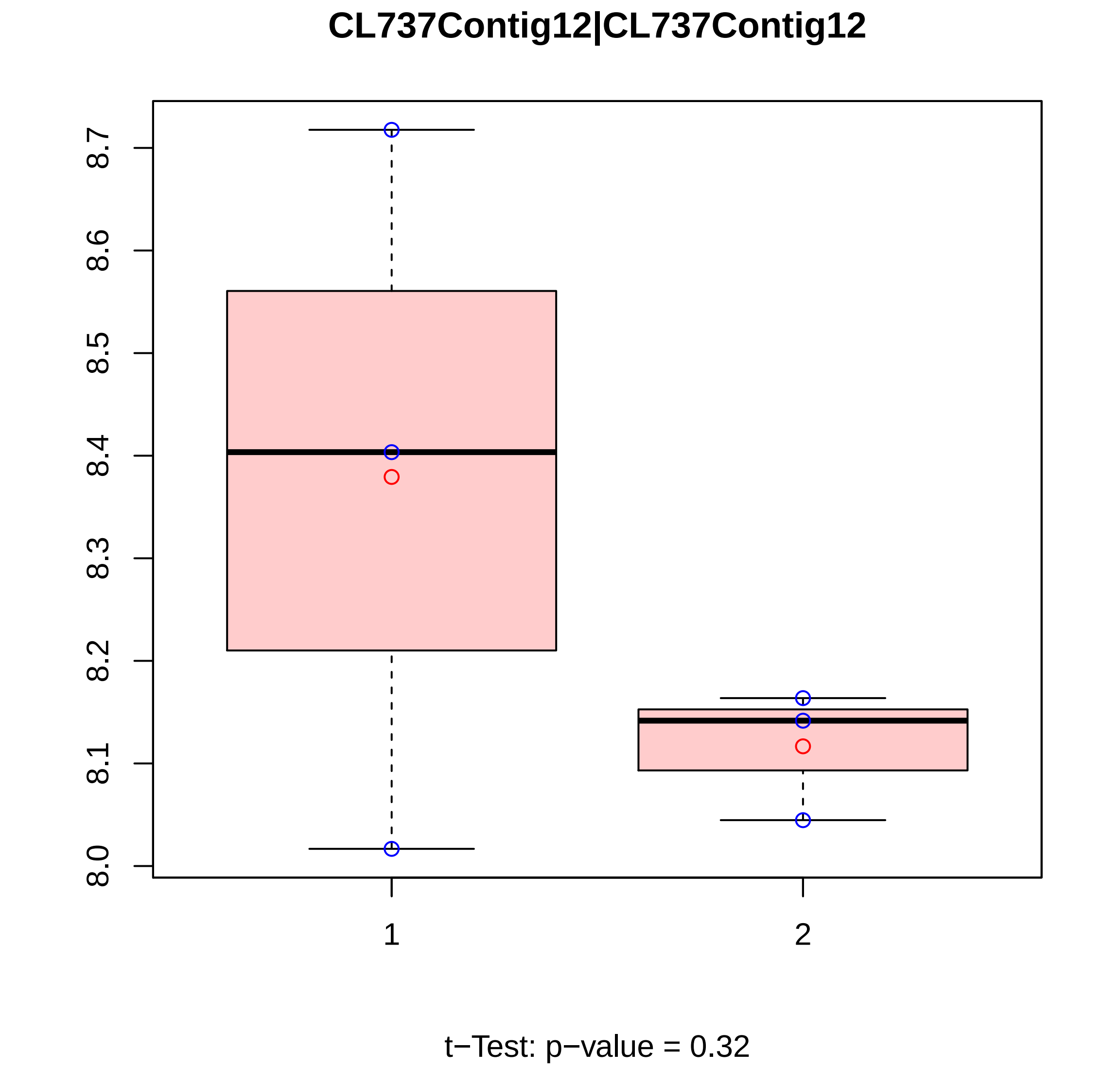

CL737Contig12|CL737Contig12
8.0
8.1
8.2
8.3
8.4
8.5
8.6
8.7
1
2
t−Test: p−value = 0.32

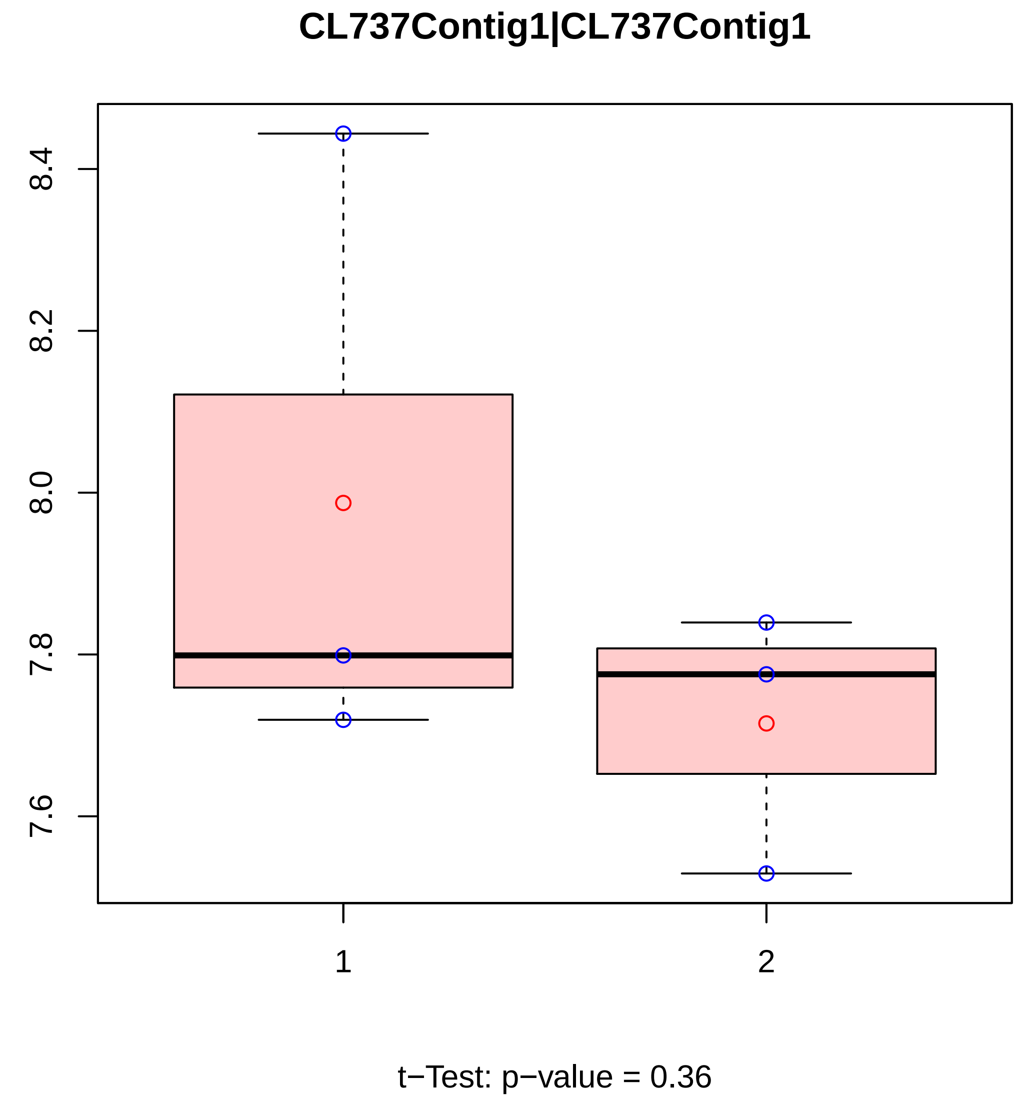
CL737Contig1|CL737Contig1
8.4
8.2
8.0
7.8
7.6
1
2
t−Test: p−value = 0.36

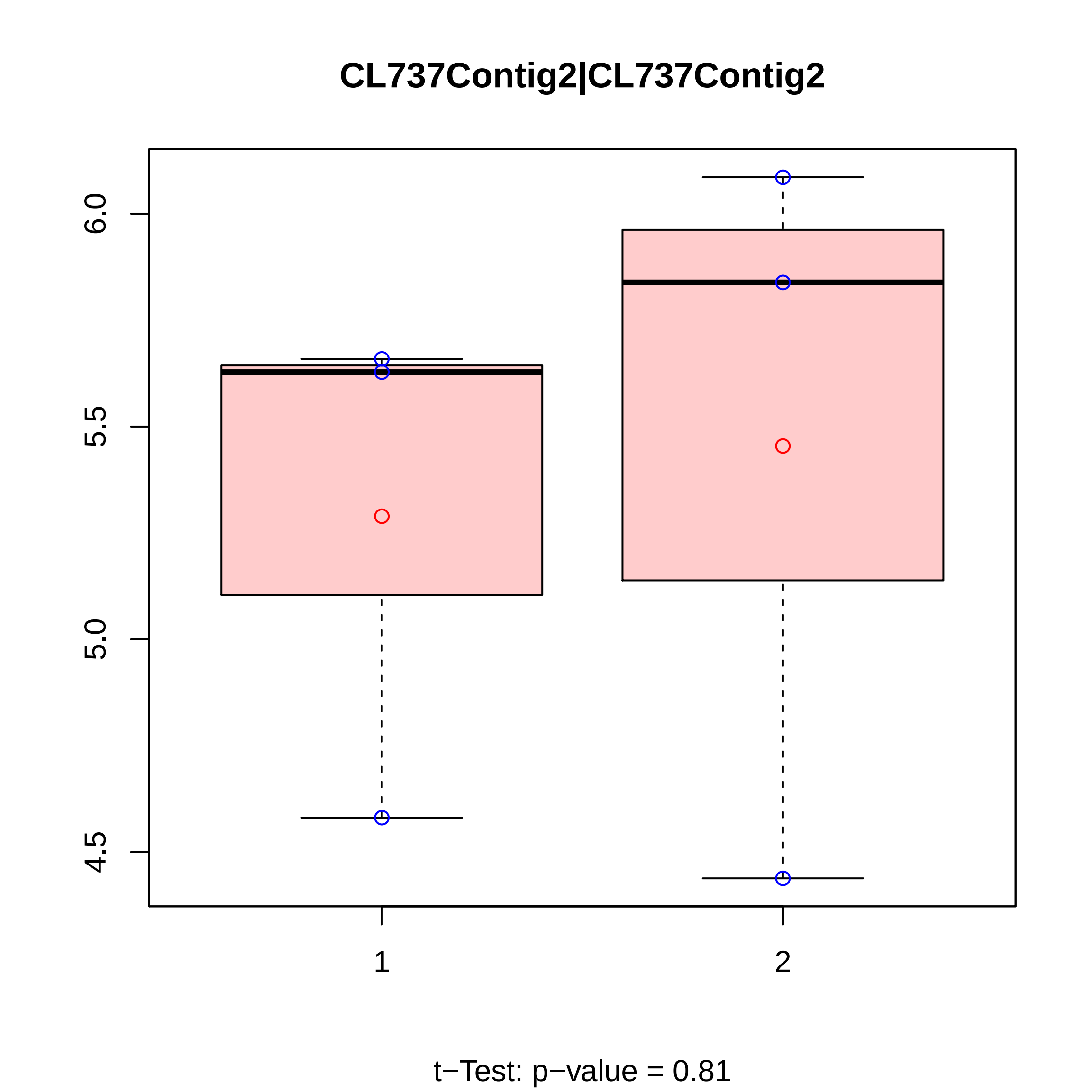

CL737Contig2|CL737Contig2
6.0
5.5
5.0
4.5
1
2
t−Test: p−value = 0.81

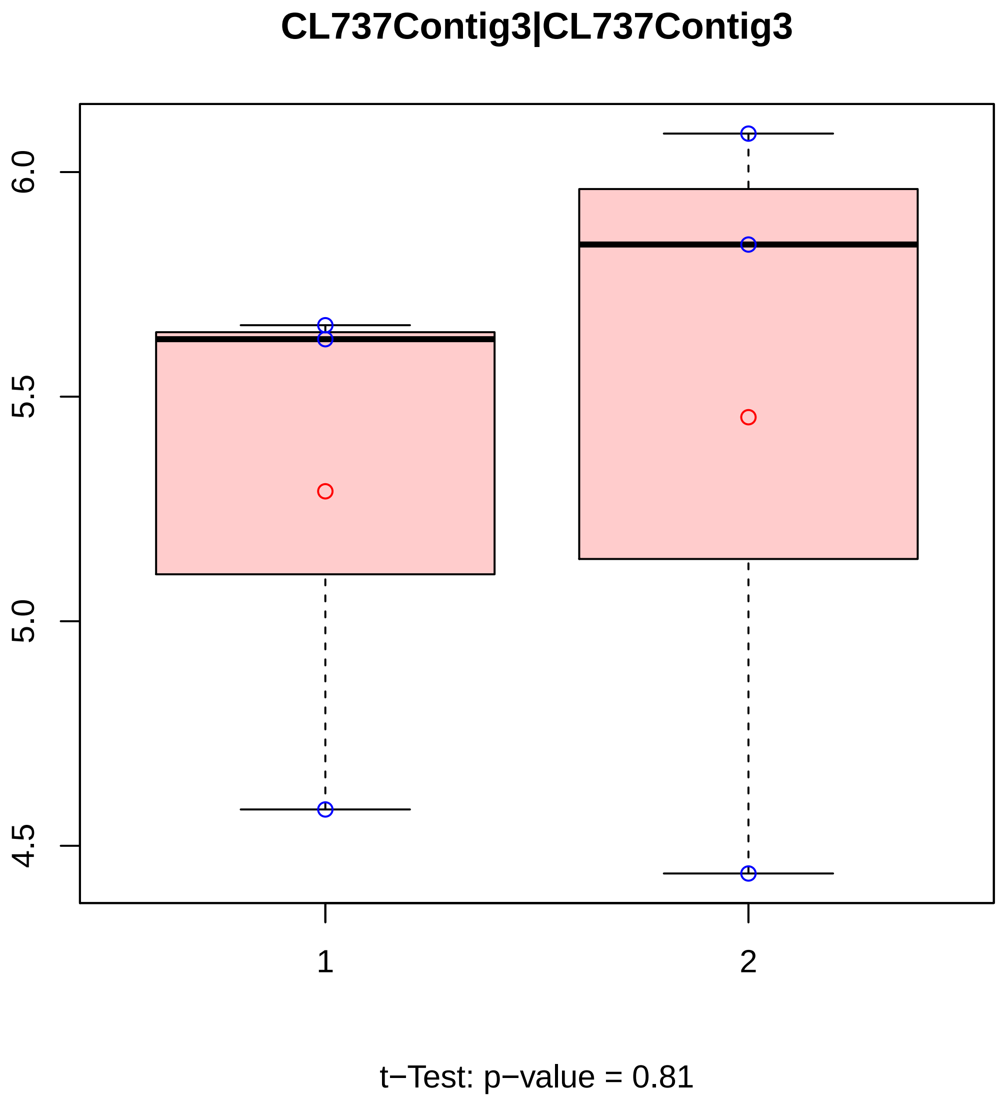

CL737Contig3|CL737Contig3
6.0
5.5
5.0
4.5
1
2
t−Test: p−value = 0.81

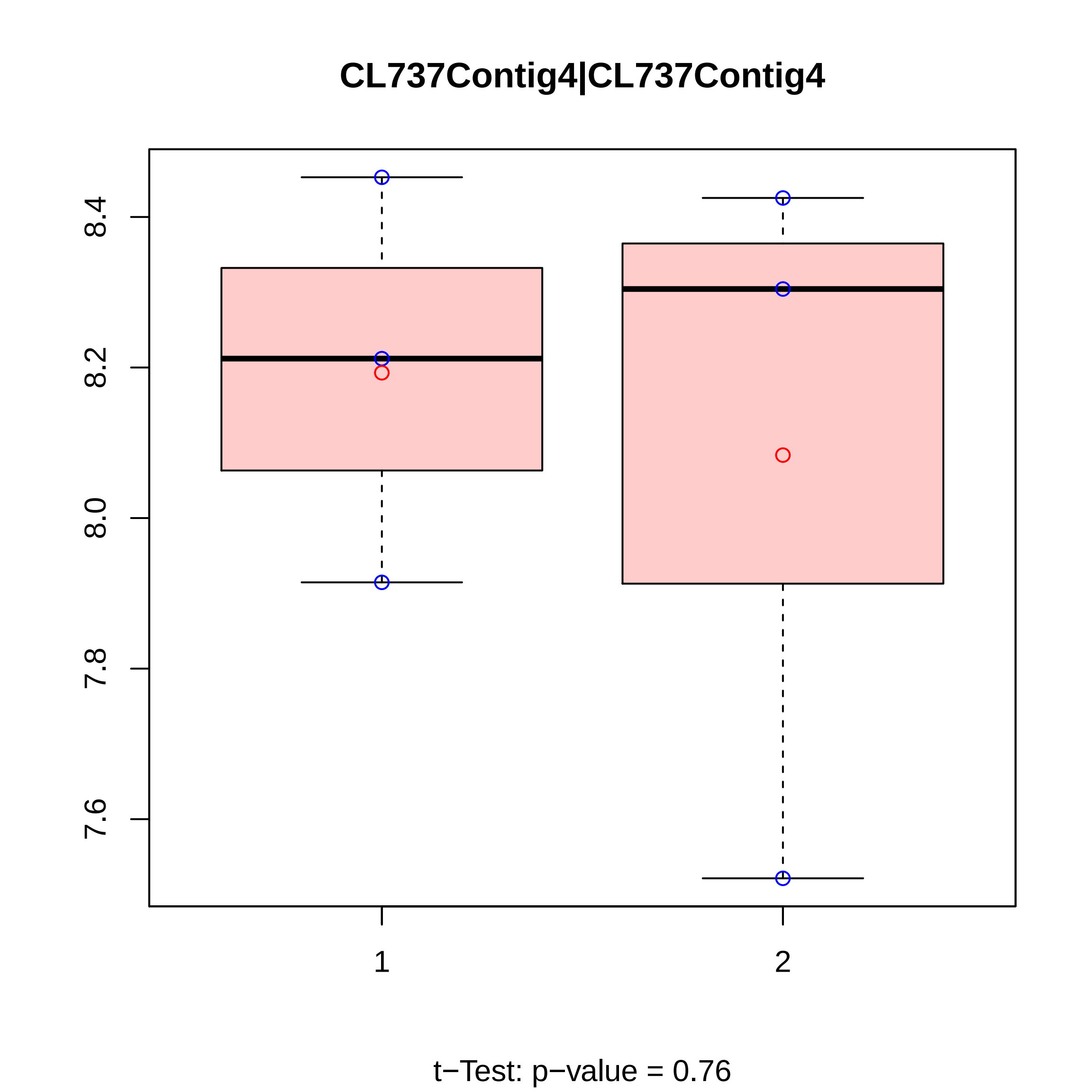

CL737Contig4|CL737Contig4
8.4
8.2
8.0
7.8
7.6
1
2
t−Test: p−value = 0.76

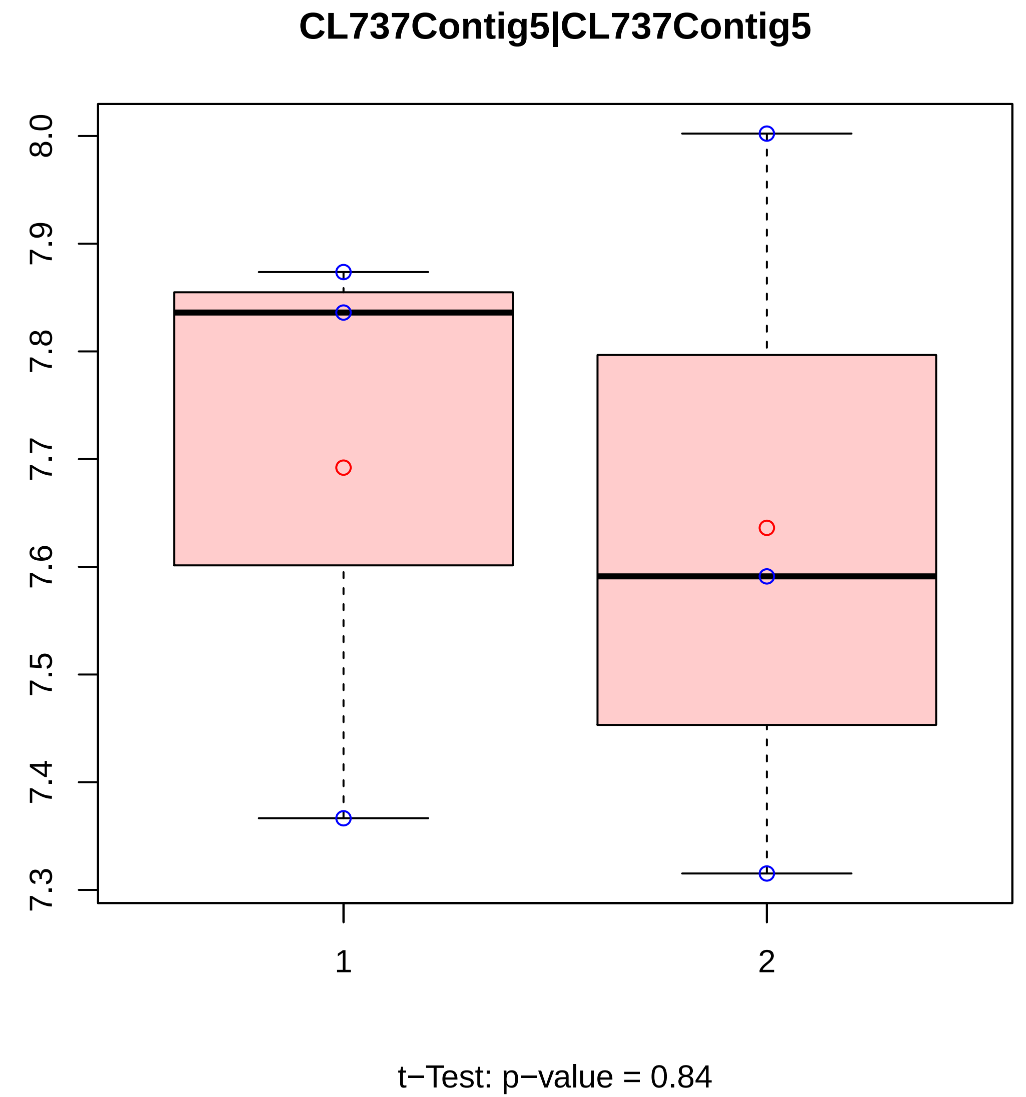
CL737Contig5|CL737Contig5
8.0
7.9
7.8
7.7
7.6
7.5
7.4
7.3
1
2
t−Test: p−value = 0.84

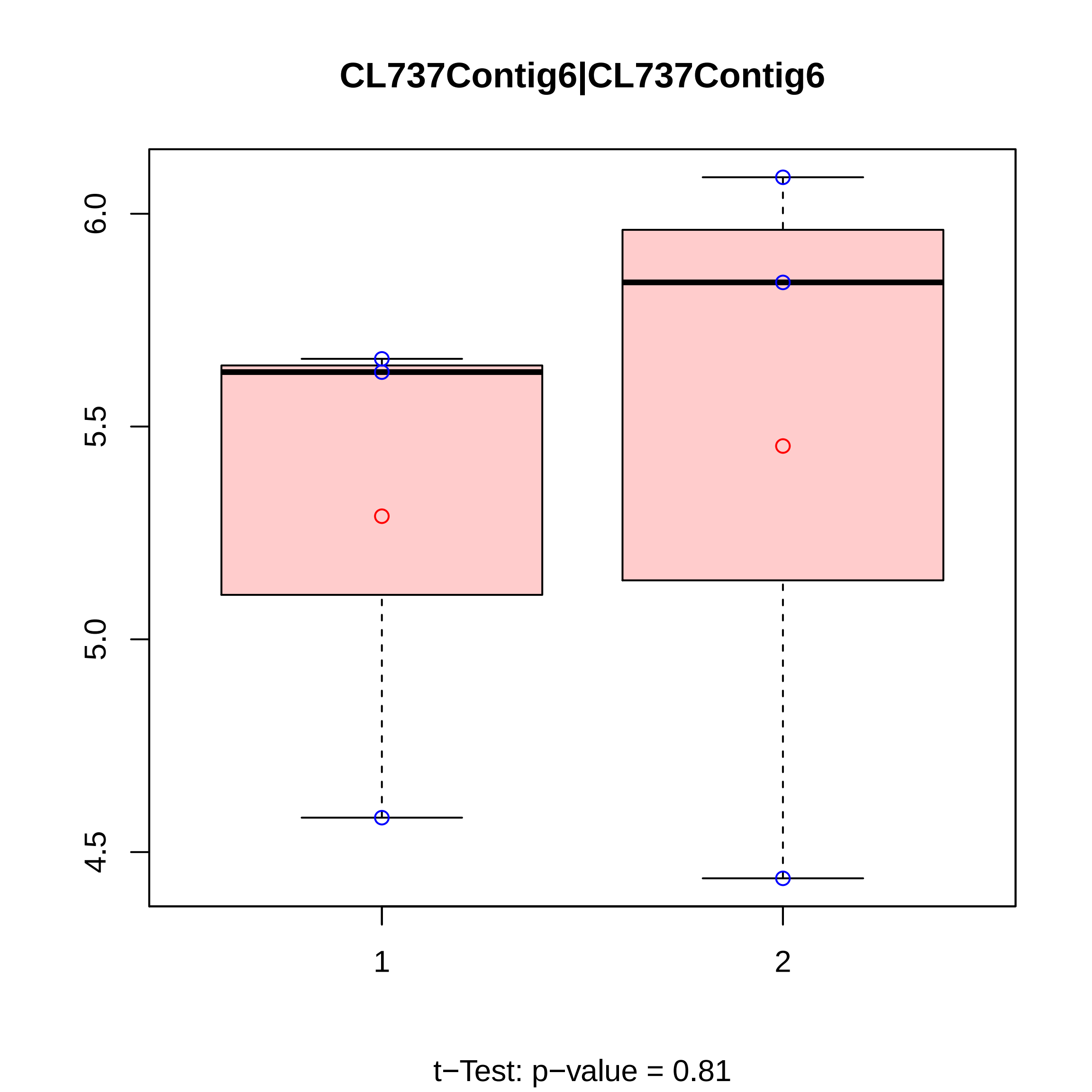
CL737Contig6|CL737Contig6
6.0
5.5
5.0
4.5
1
2
t−Test: p−value = 0.81

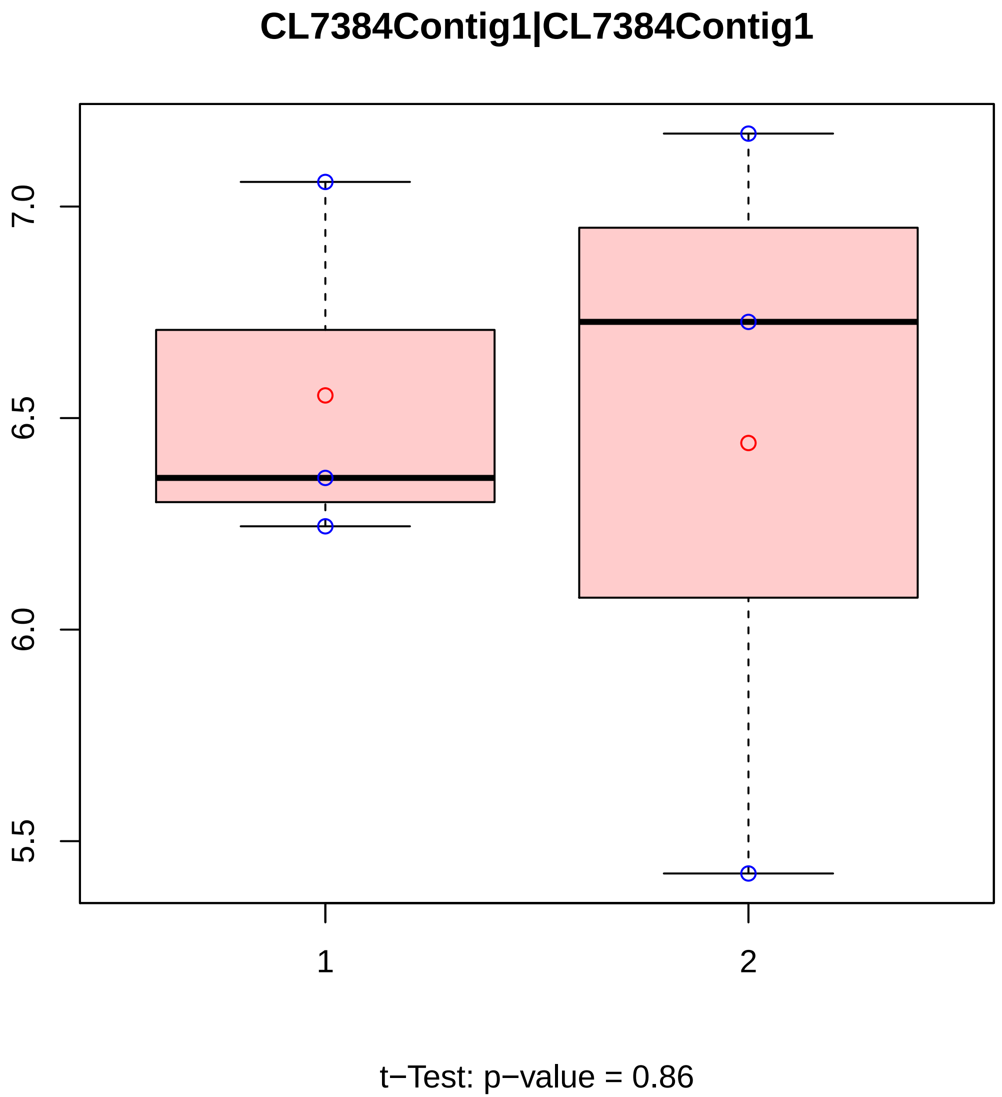
CL7384Contig1|CL7384Contig1
7.0
6.5
6.0
5.5
1
2
t−Test: p−value = 0.86

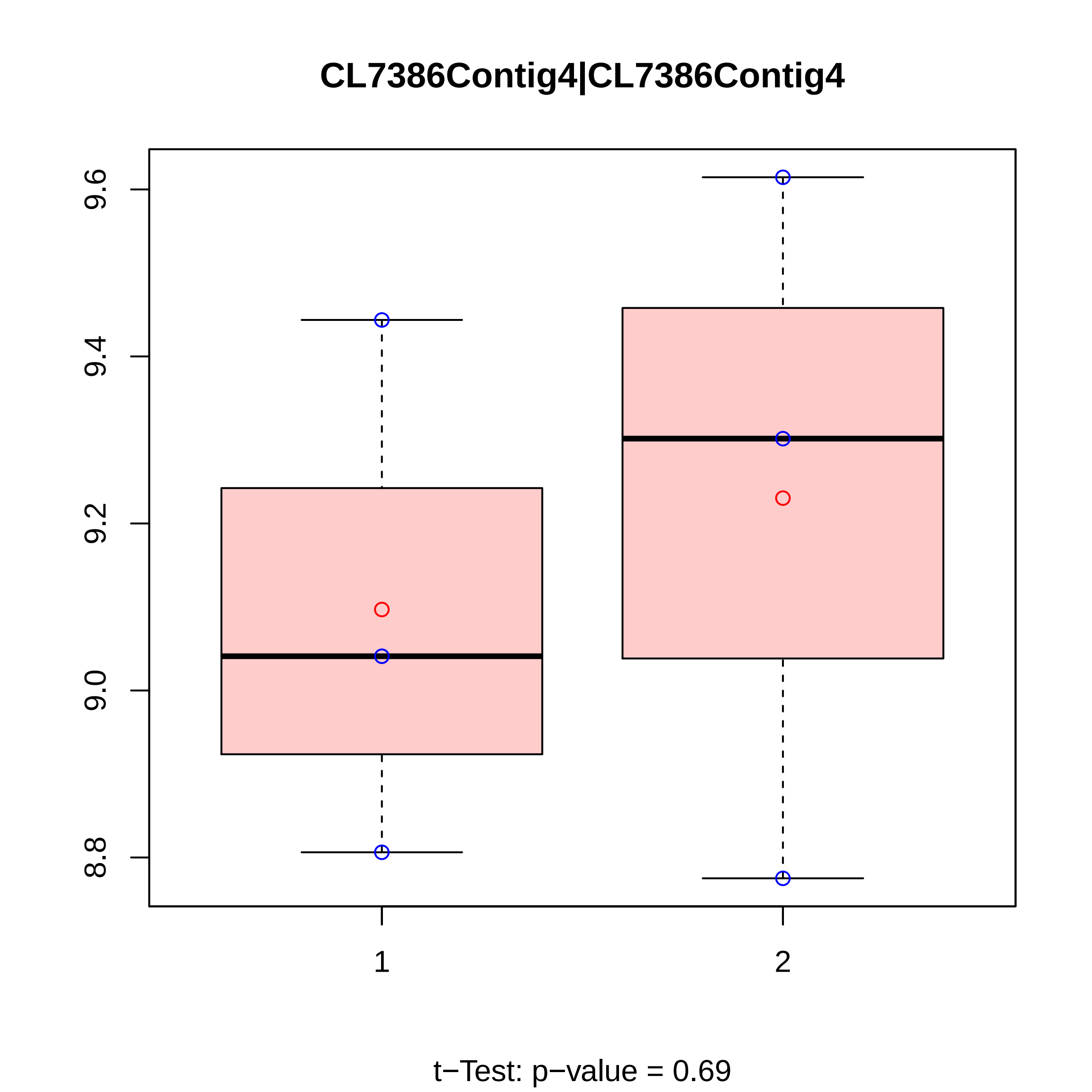

CL7386Contig4|CL7386Contig4
9.6
9.4
9.2
9.0
8.8
1
2
t−Test: p−value = 0.69

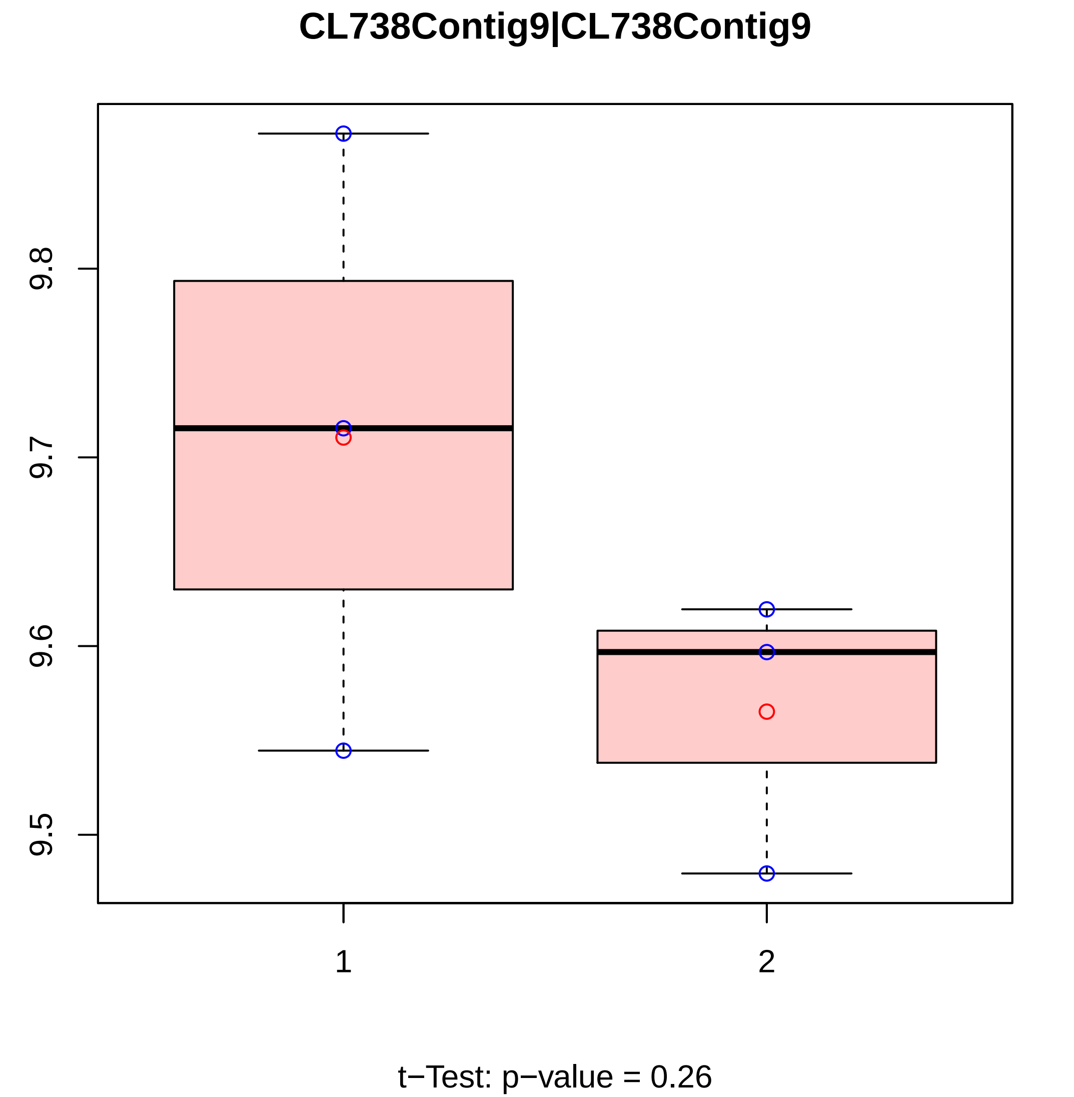

CL738Contig9|CL738Contig9
9.5
9.6
9.7
9.8
1
2
t−Test: p−value = 0.26

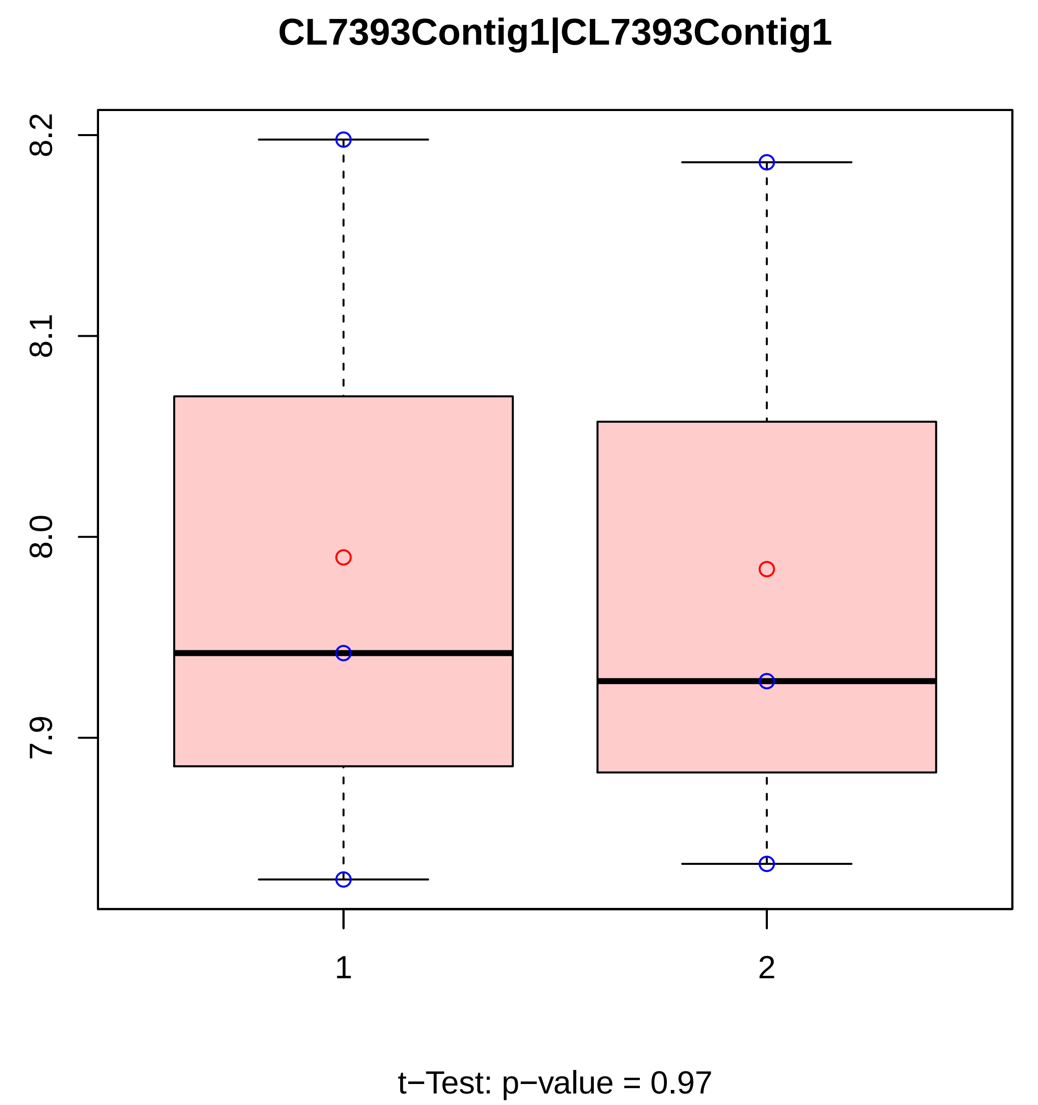
CL7393Contig1|CL7393Contig1
8.2
8.1
8.0
7.9
1
2
t−Test: p−value = 0.97

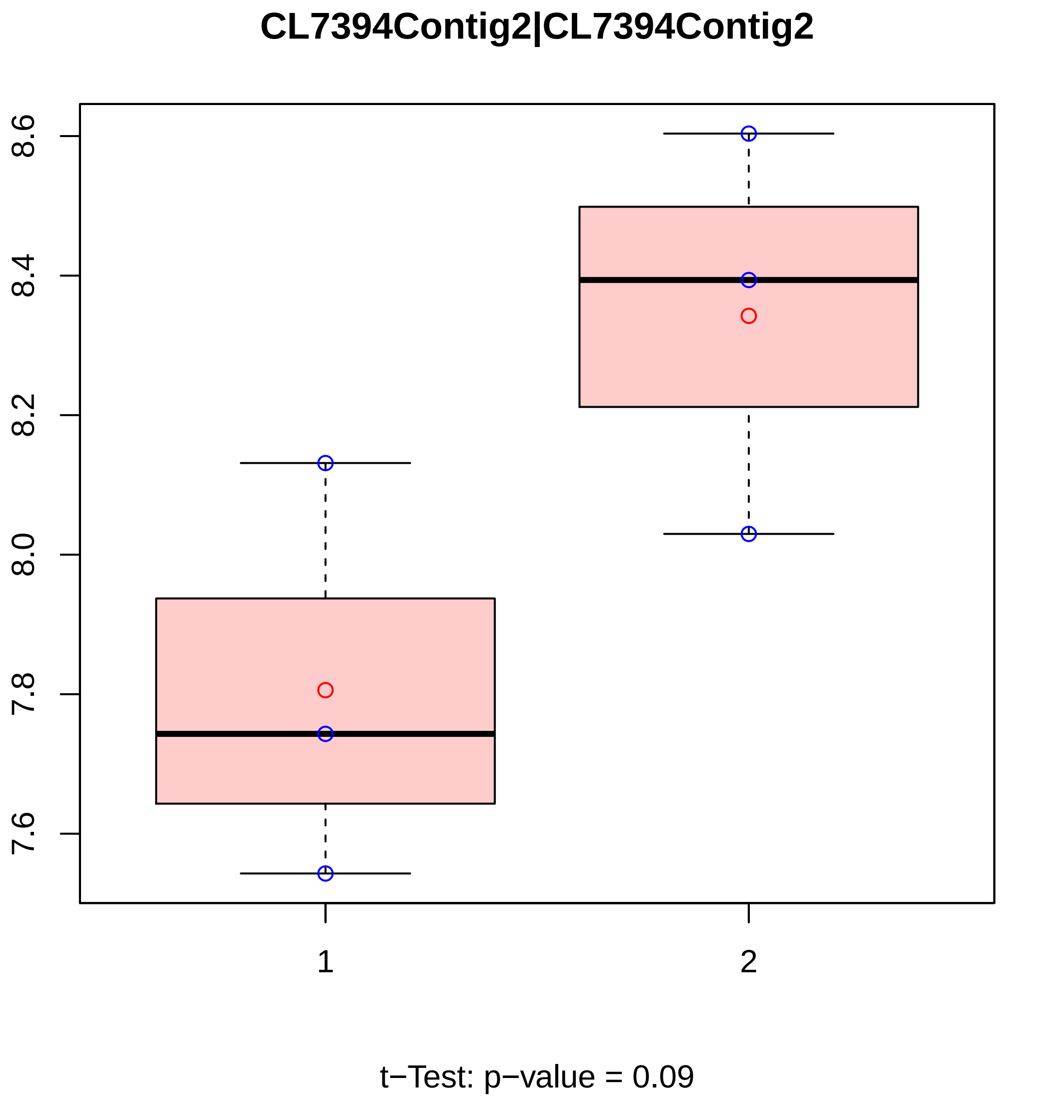
CL7394Contig2|CL7394Contig2
8.6
8.4
8.2
8.0
7.8
7.6
1
2
t−Test: p−value = 0.09

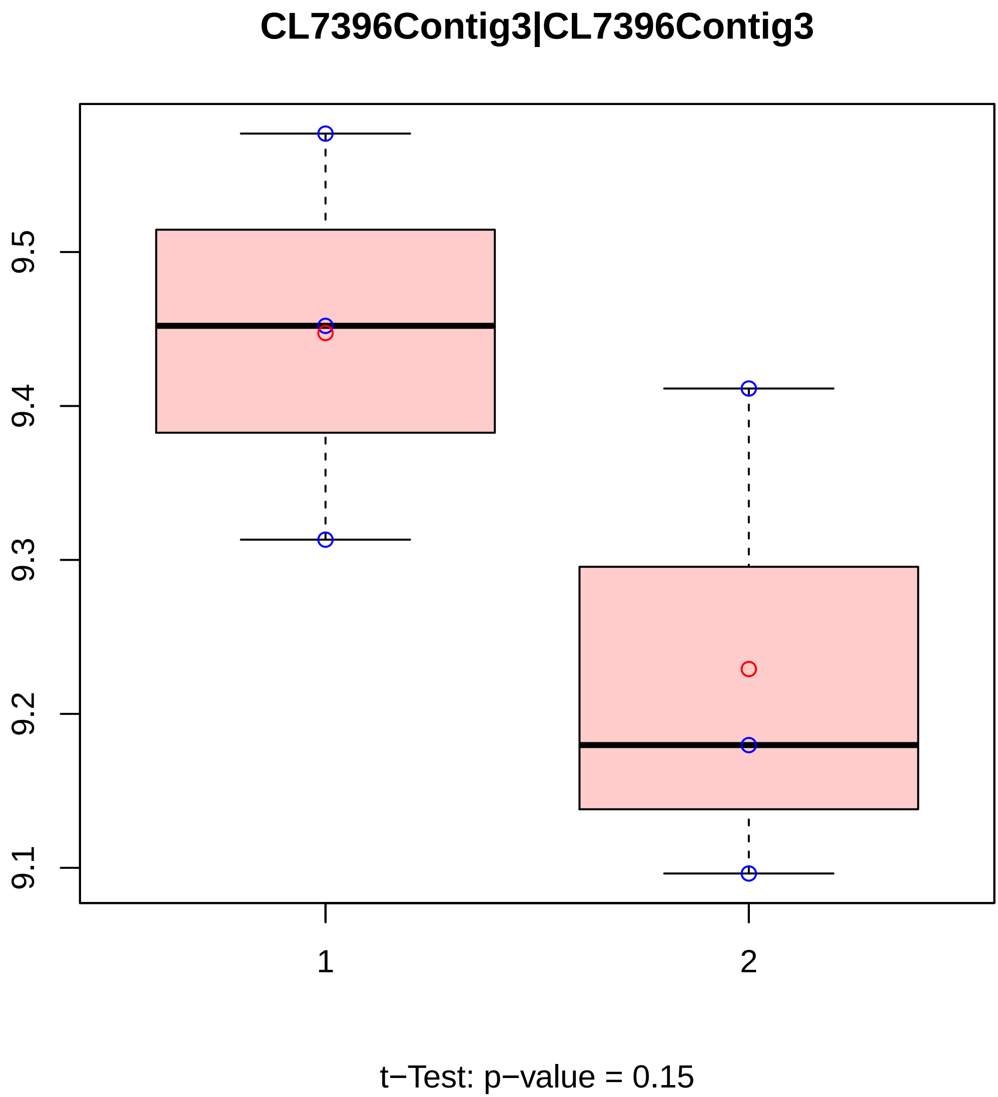

CL7396Contig3|CL7396Contig3
9.1
9.2
9.3
9.4
9.5
1
2
t−Test: p−value = 0.15

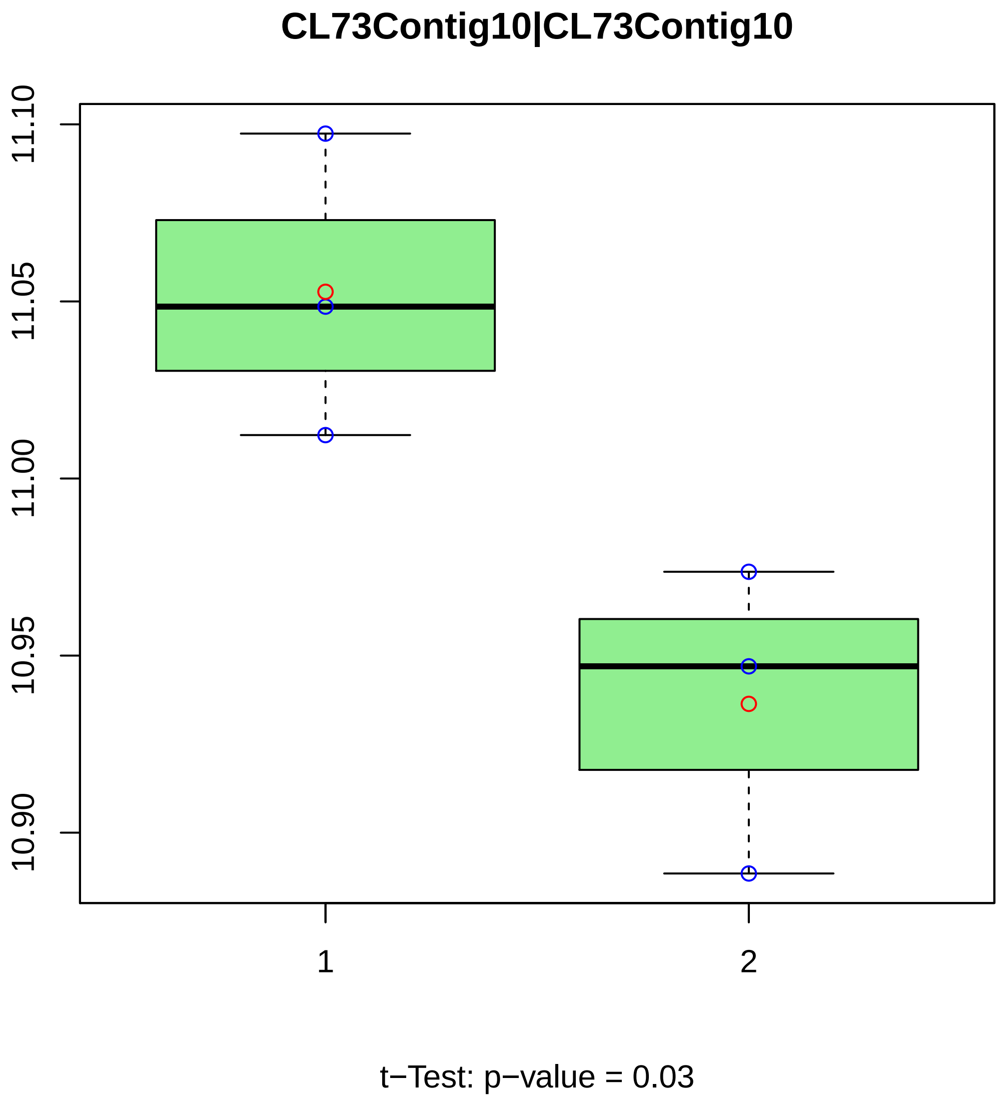

CL73Contig10|CL73Contig10
11.10
11.05
11.00
10.95
10.90
1
2
t−Test: p−value = 0.03

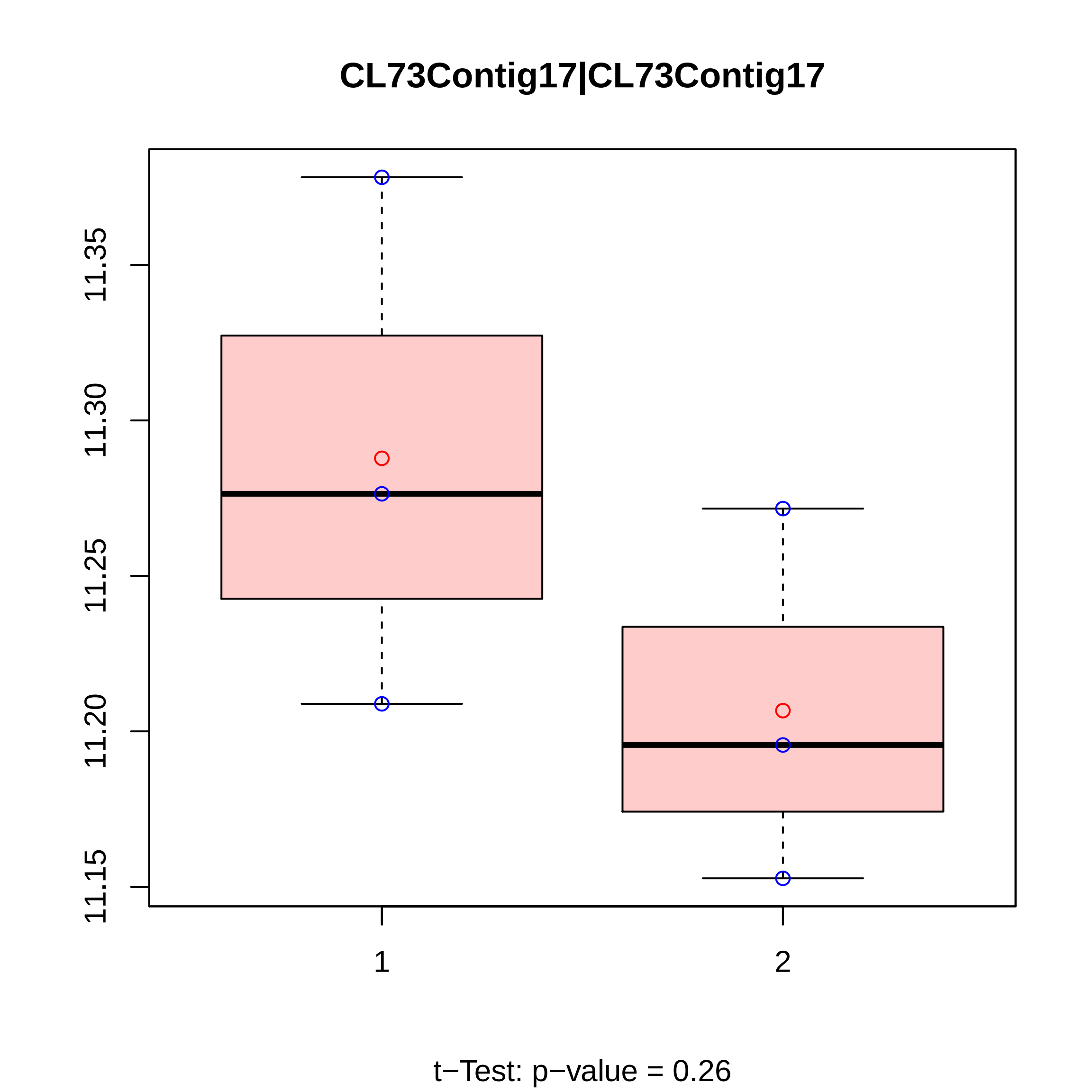

CL73Contig17|CL73Contig17
11.15
11.20
11.25
11.30
11.35
1
2
t−Test: p−value = 0.26

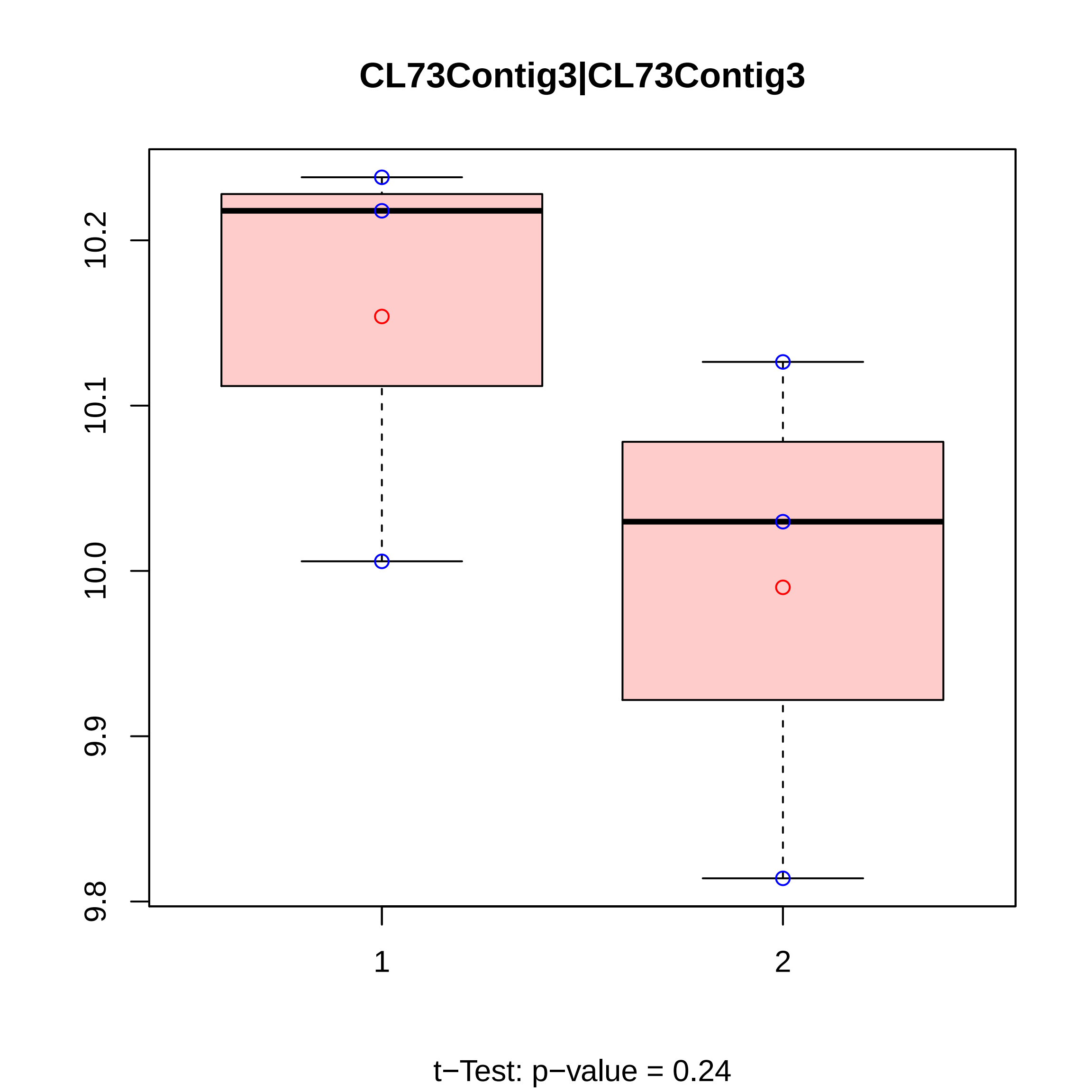
CL73Contig3|CL73Contig3
10.2
10.1
10.0
9.9
9.8
1
2
t−Test: p−value = 0.24

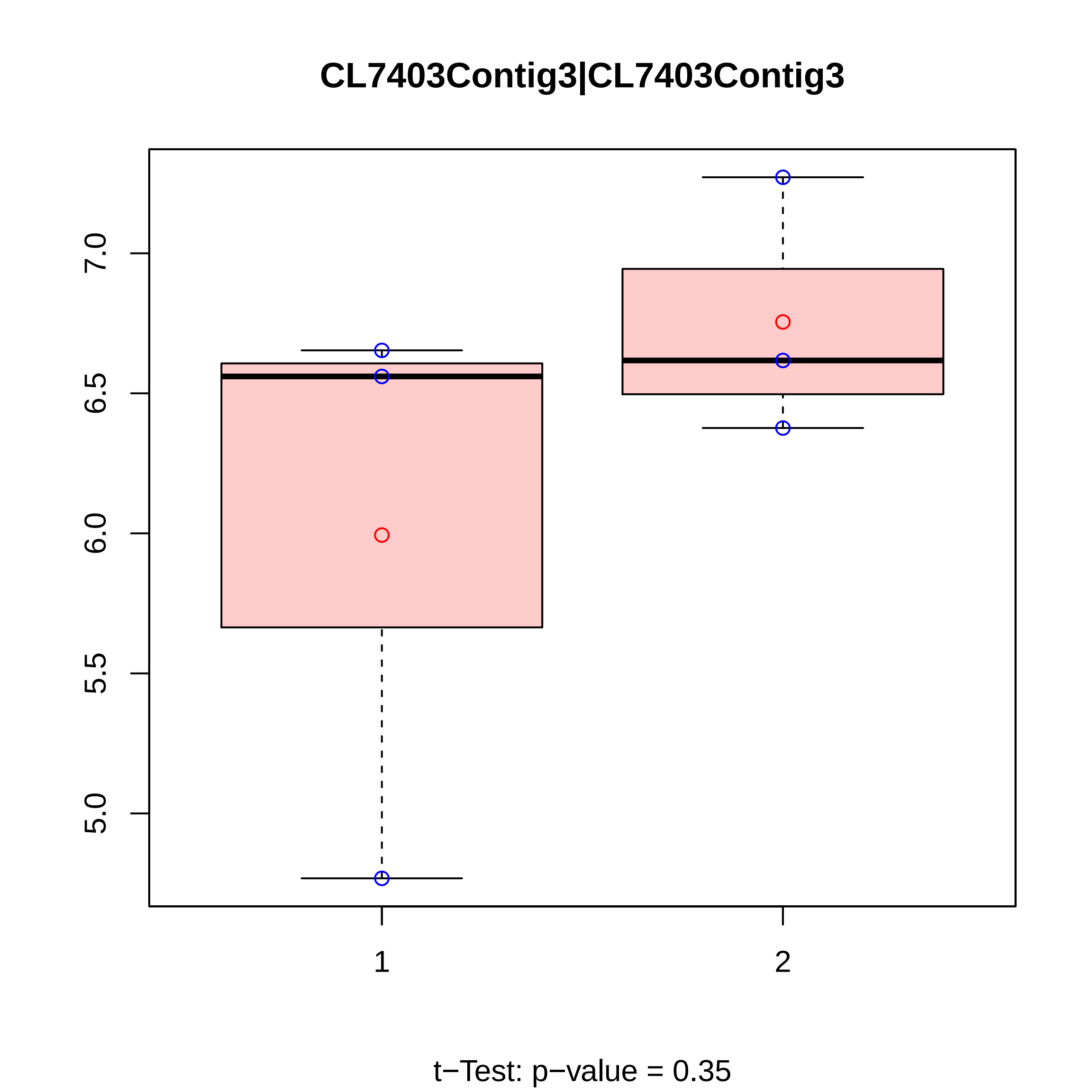

CL7403Contig3|CL7403Contig3
7.0
6.5
6.0
5.5
5.0
1
2
t−Test: p−value = 0.35

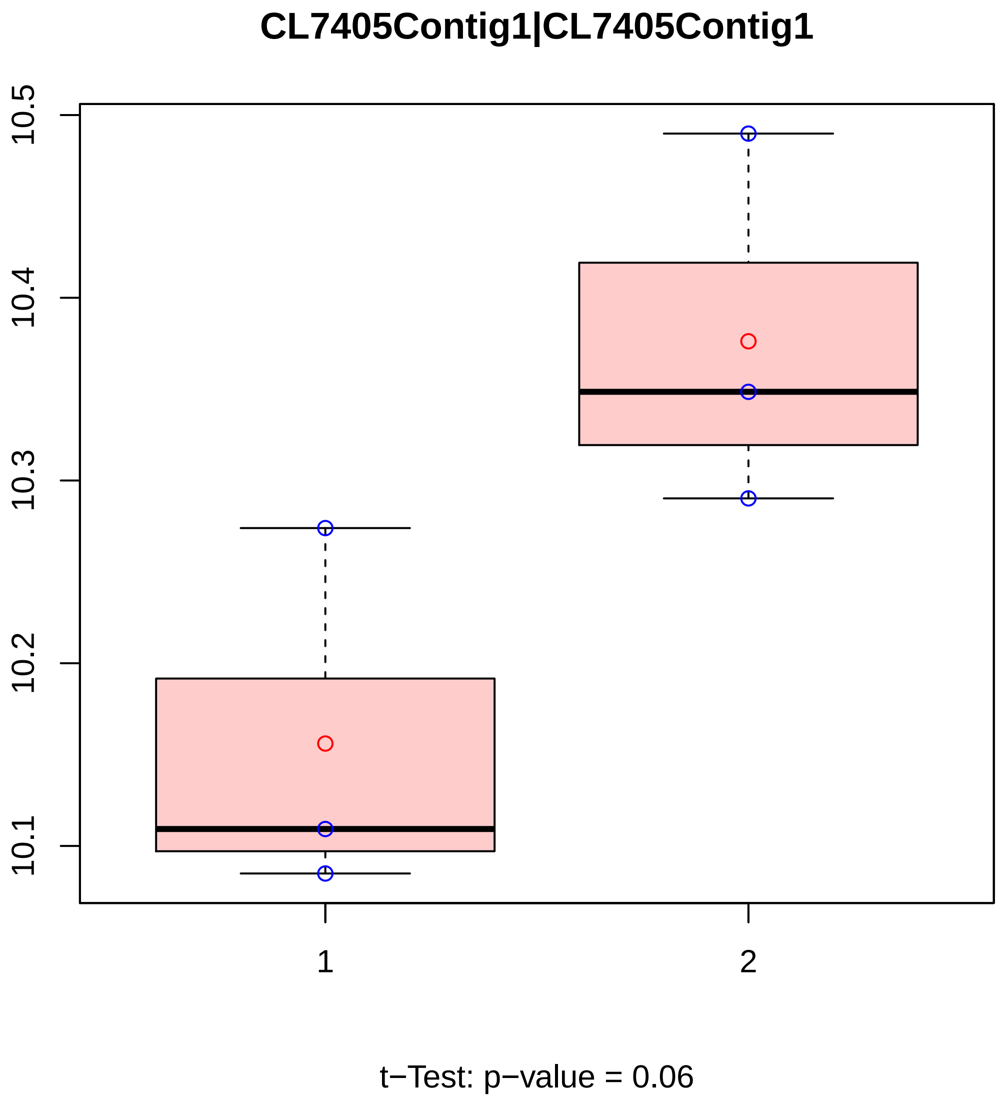
CL7405Contig1|CL7405Contig1
10.1
10.2
10.3
10.4
10.5
1
2
t−Test: p−value = 0.06

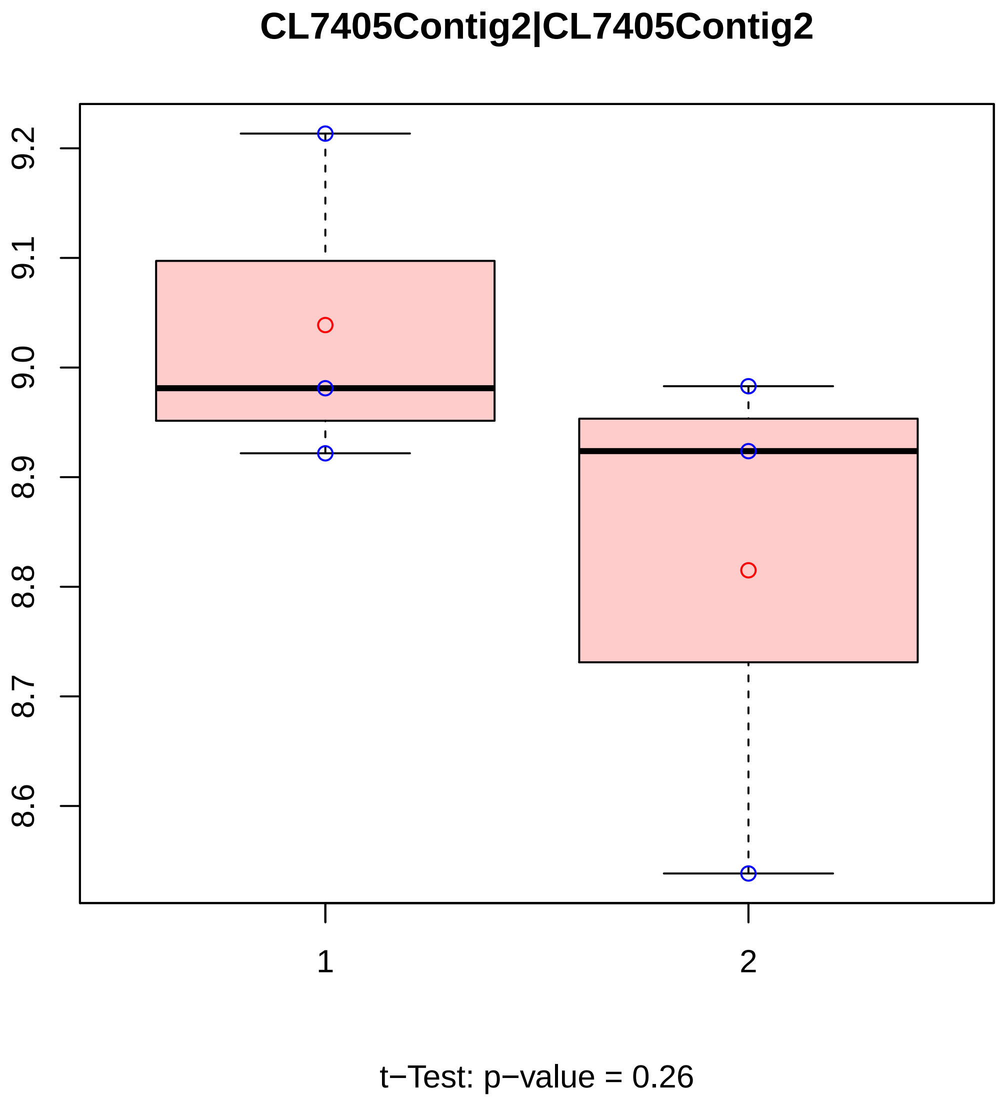

CL7405Contig2|CL7405Contig2
9.2
9.1
9.0
8.9
8.8
8.7
8.6
1
2
t−Test: p−value = 0.26

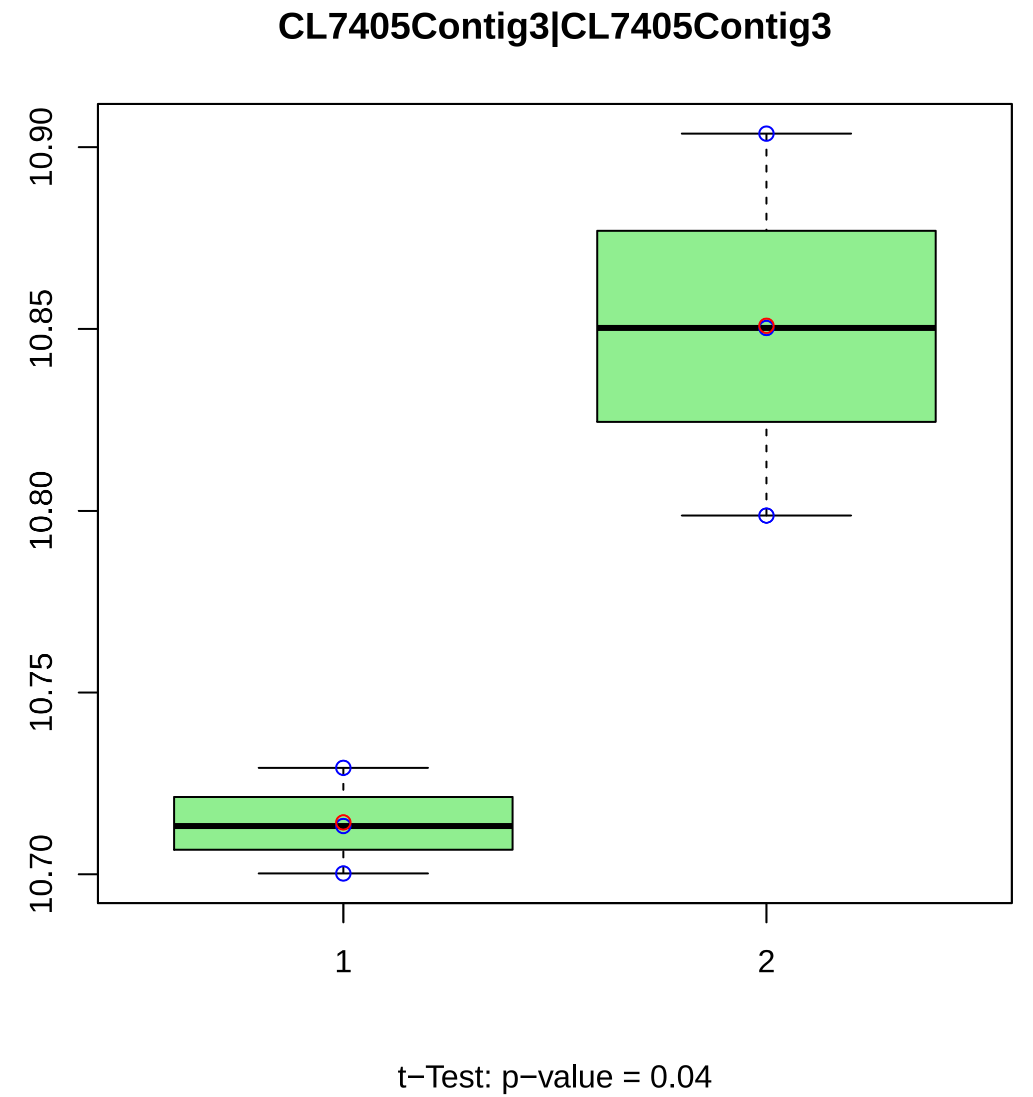

CL7405Contig3|CL7405Contig3
10.70
10.75
10.80
10.85
10.90
1
2
t−Test: p−value = 0.04

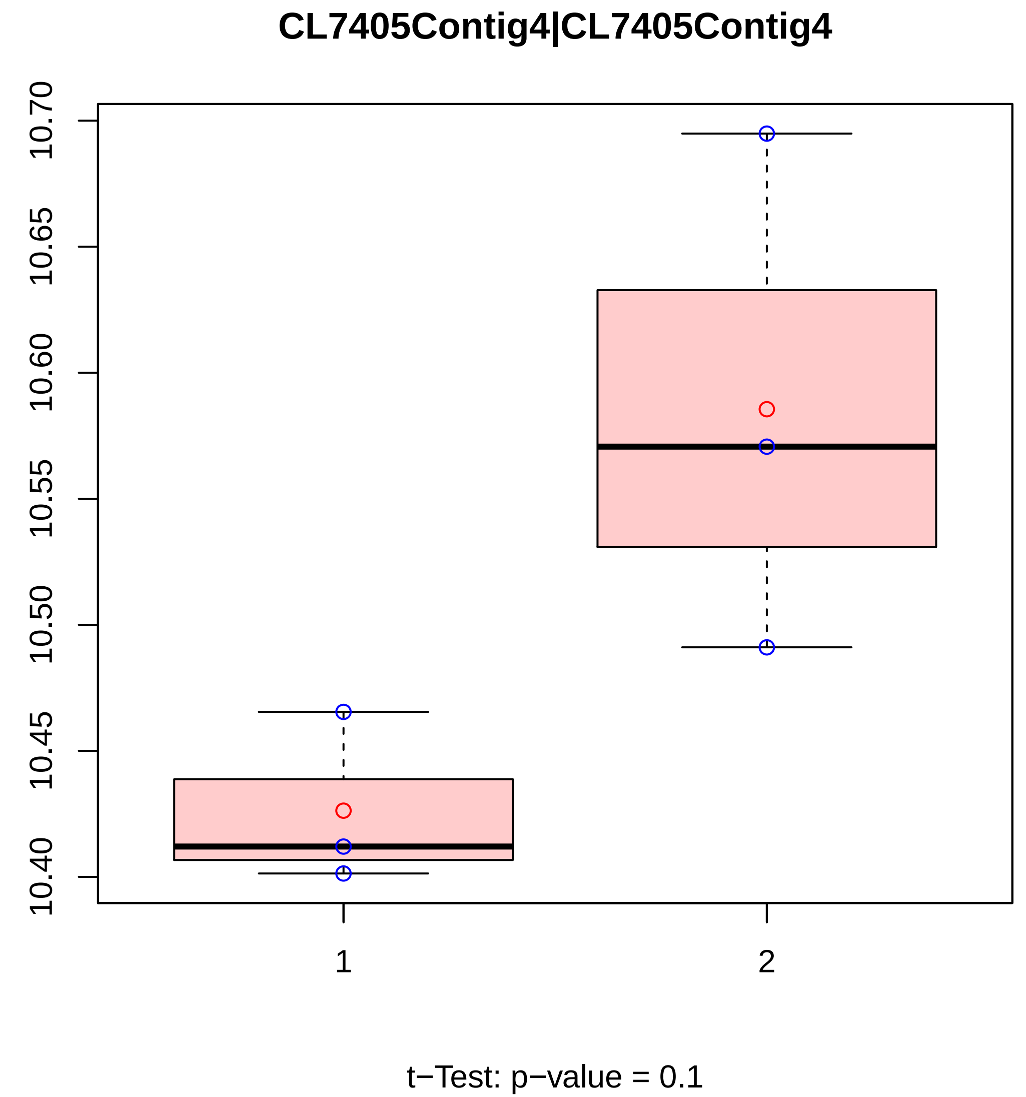

CL7405Contig4|CL7405Contig4
10.40
10.45
10.50
10.55
10.60
10.65
10.70
1
2
t−Test: p−value = 0.1

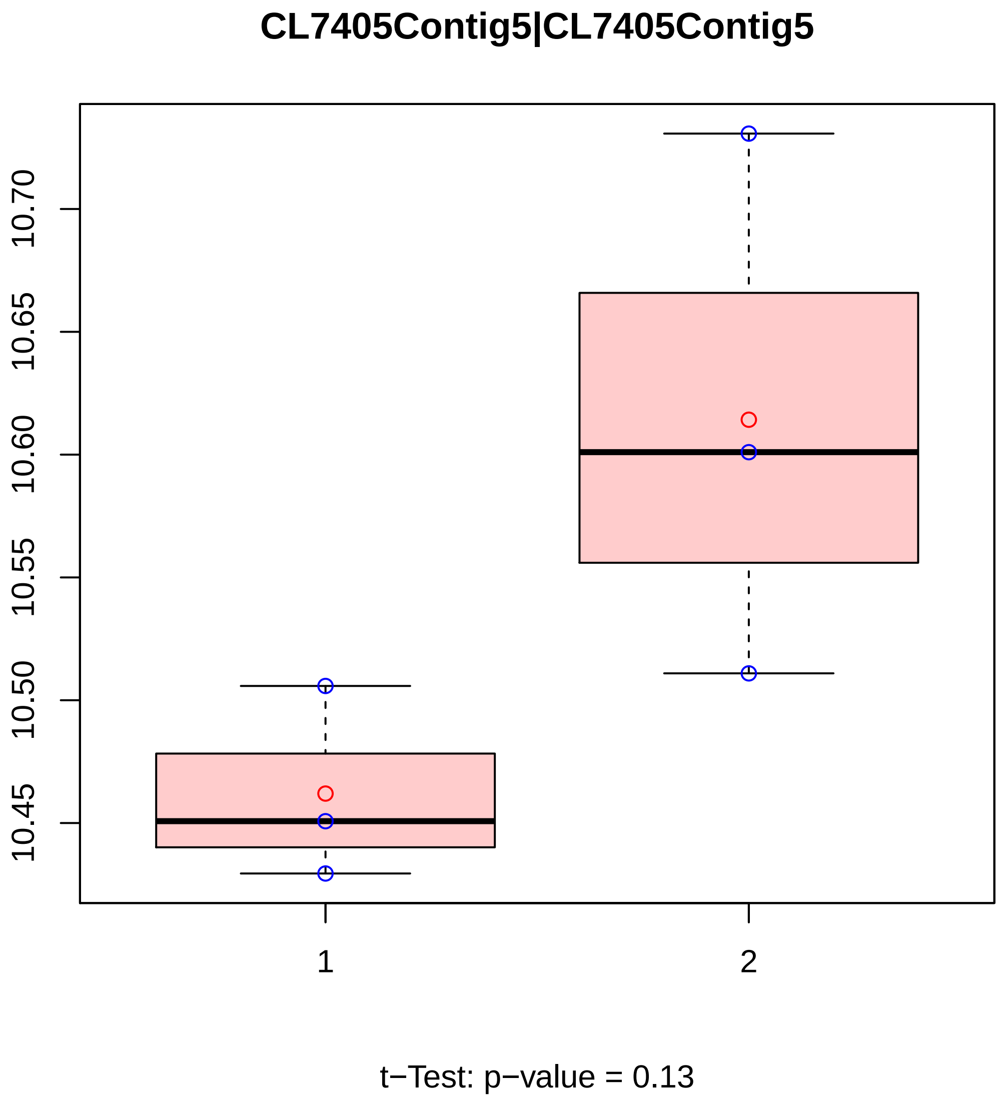
CL7405Contig5|CL7405Contig5
10.45
10.50
10.55
10.60
10.65
10.70
1
2
t−Test: p−value = 0.13

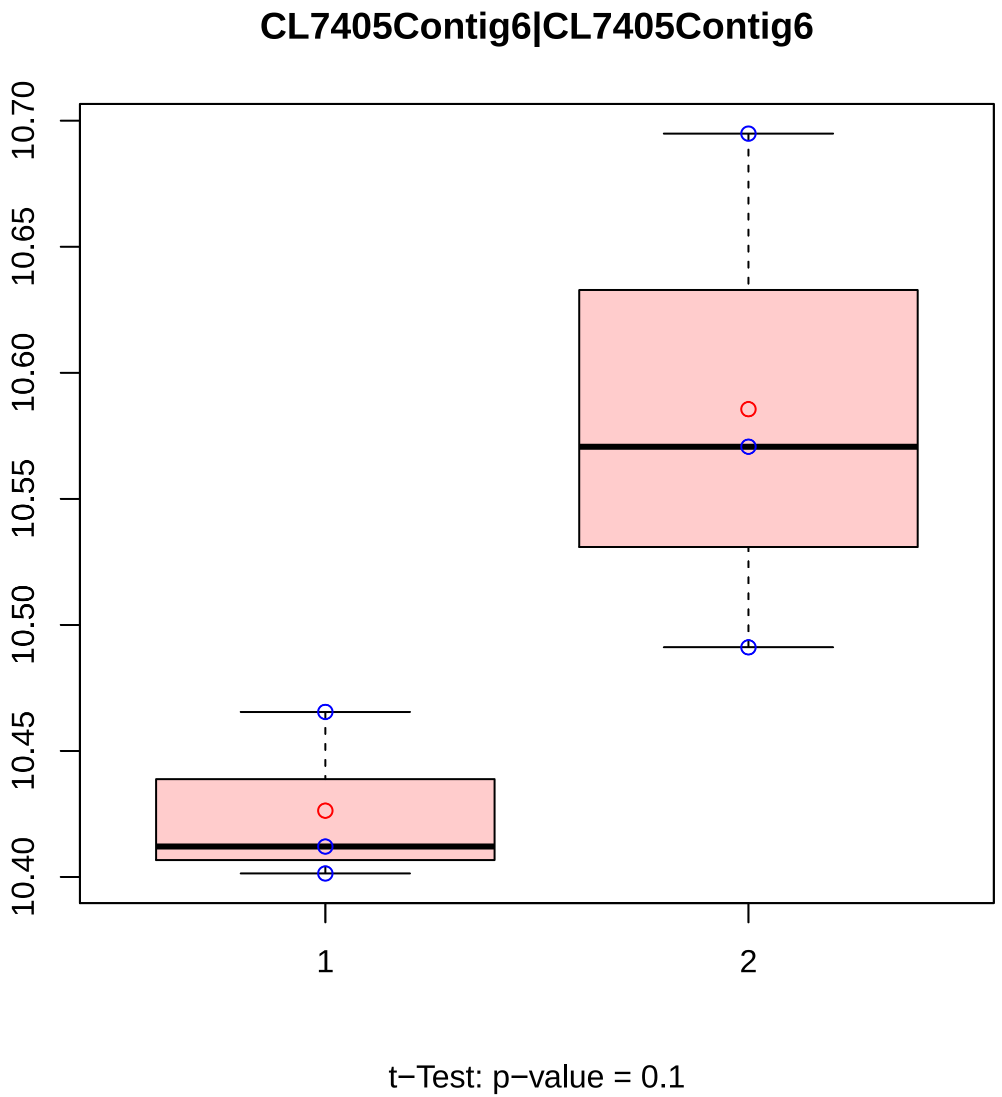

CL7405Contig6|CL7405Contig6
10.40
10.45
10.50
10.55
10.60
10.65
10.70
1
2
t−Test: p−value = 0.1

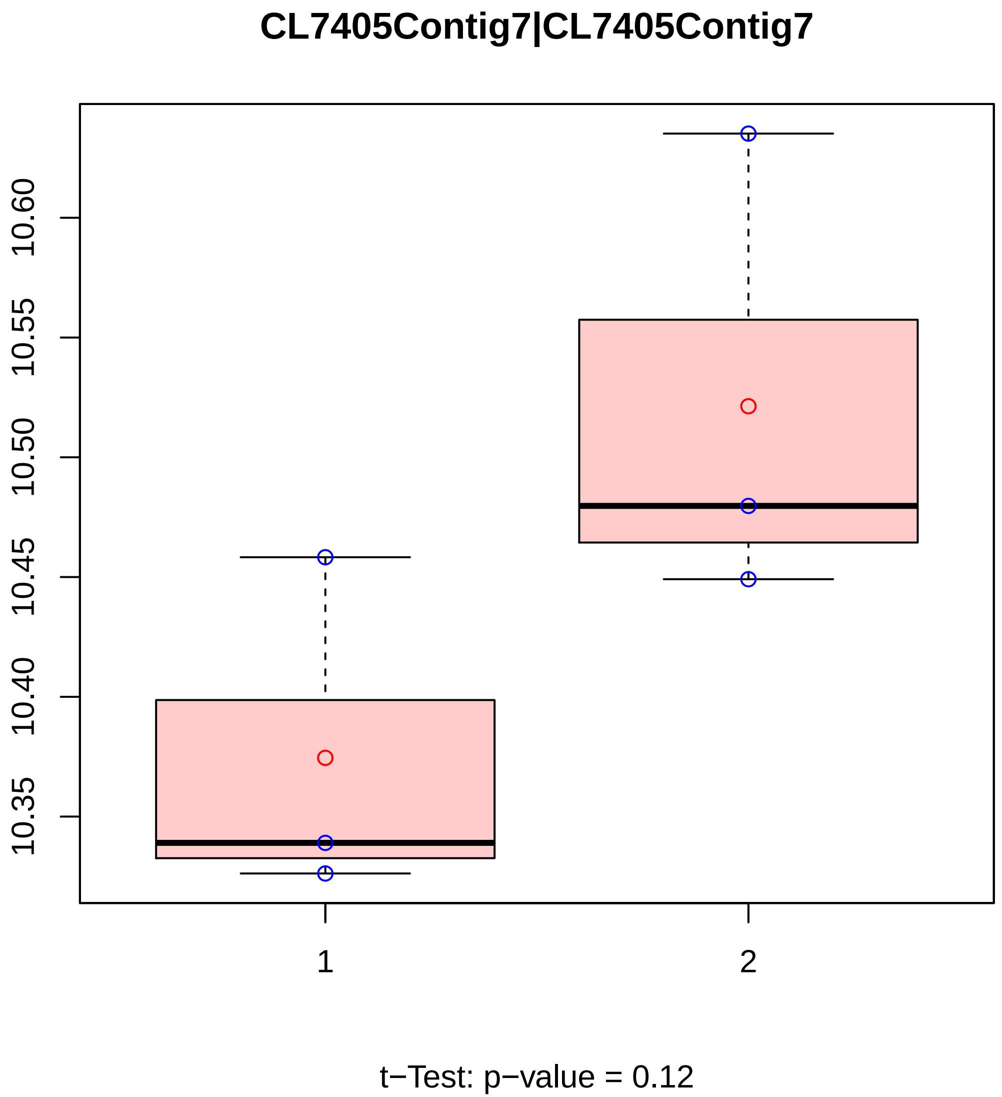

CL7405Contig7|CL7405Contig7
10.35
10.40
10.45
10.50
10.55
10.60
1
2
t−Test: p−value = 0.12

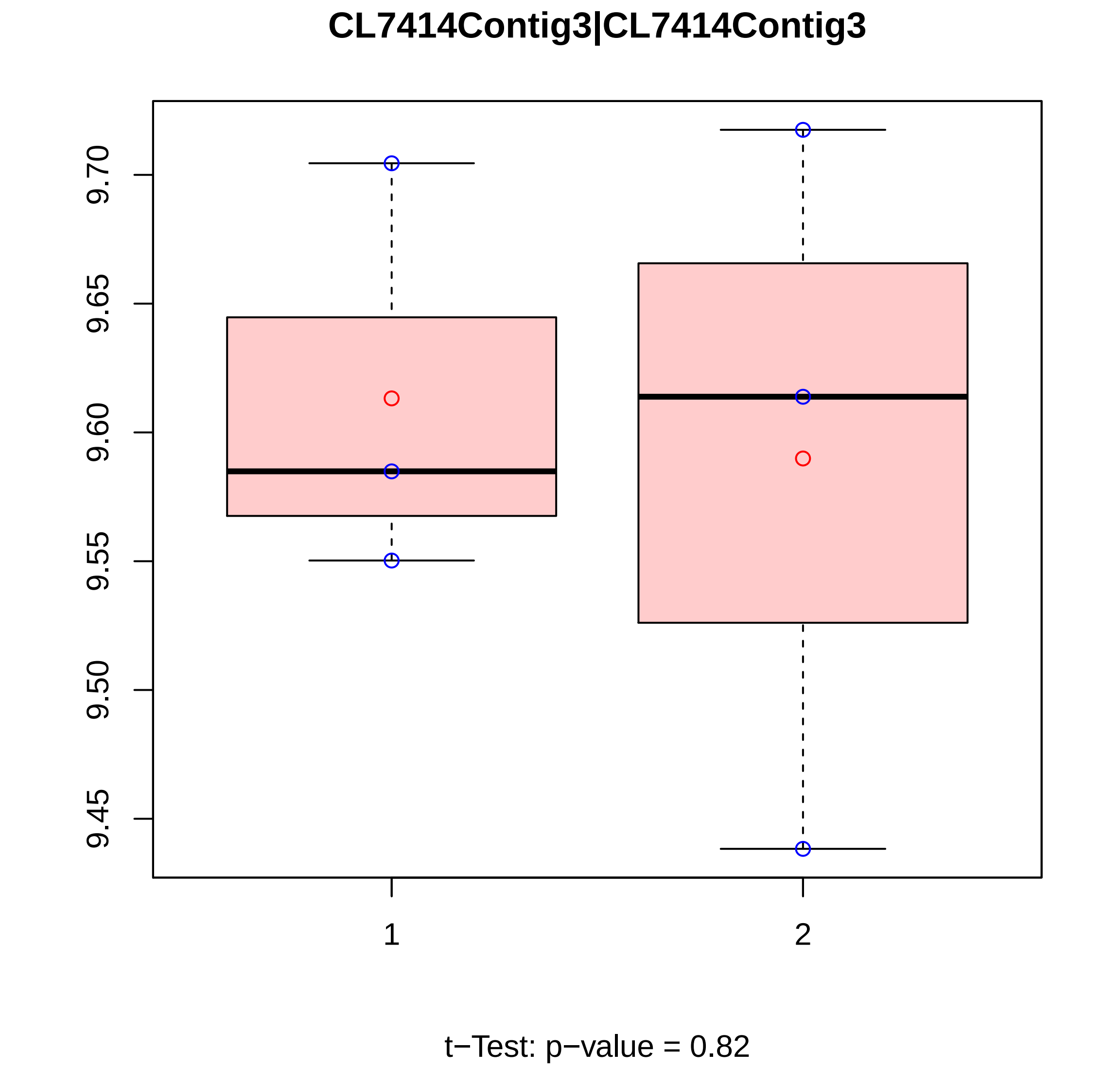

CL7414Contig3|CL7414Contig3
9.45
9.50
9.55
9.60
9.65
9.70
1
2
t−Test: p−value = 0.82

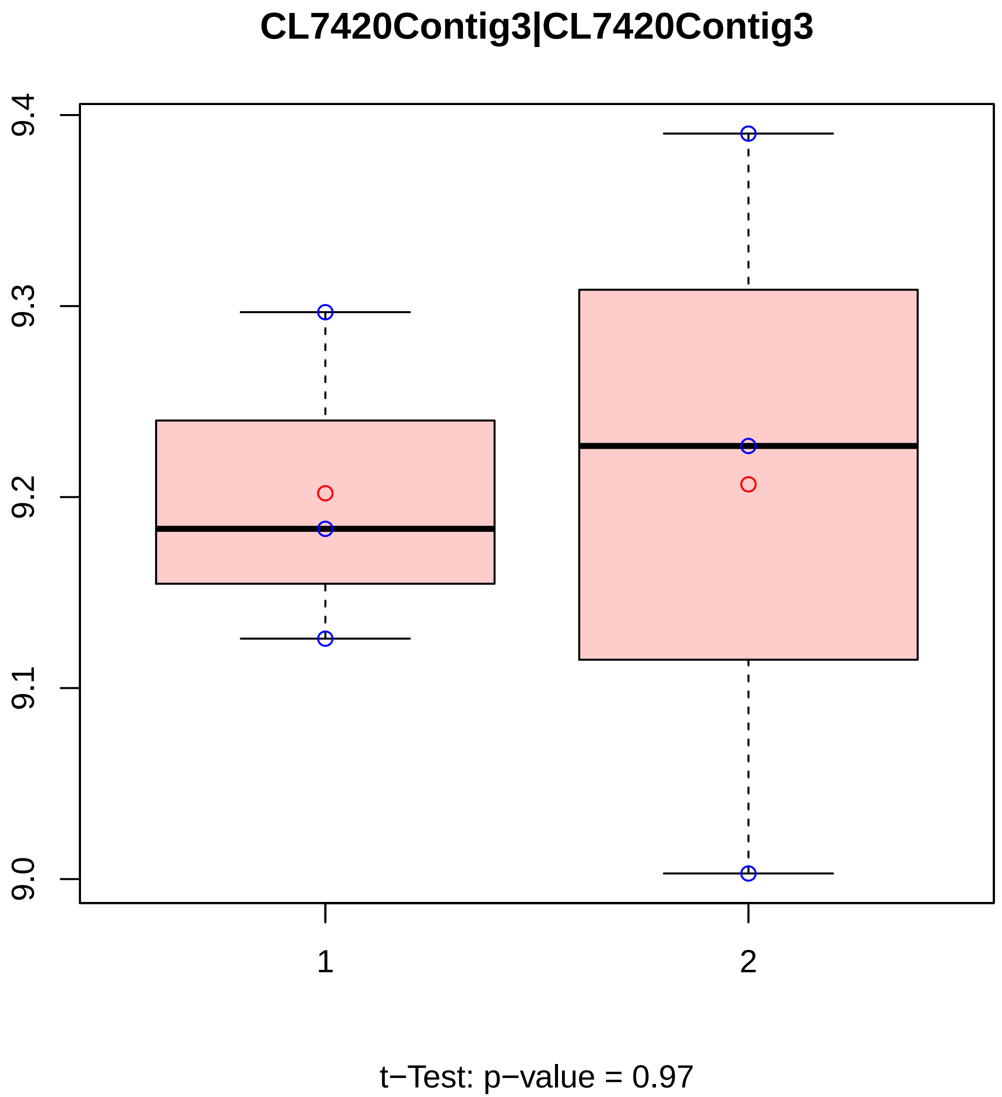
CL7420Contig3|CL7420Contig3
9.0
9.1
9.2
9.3
9.4
1
2
t−Test: p−value = 0.97

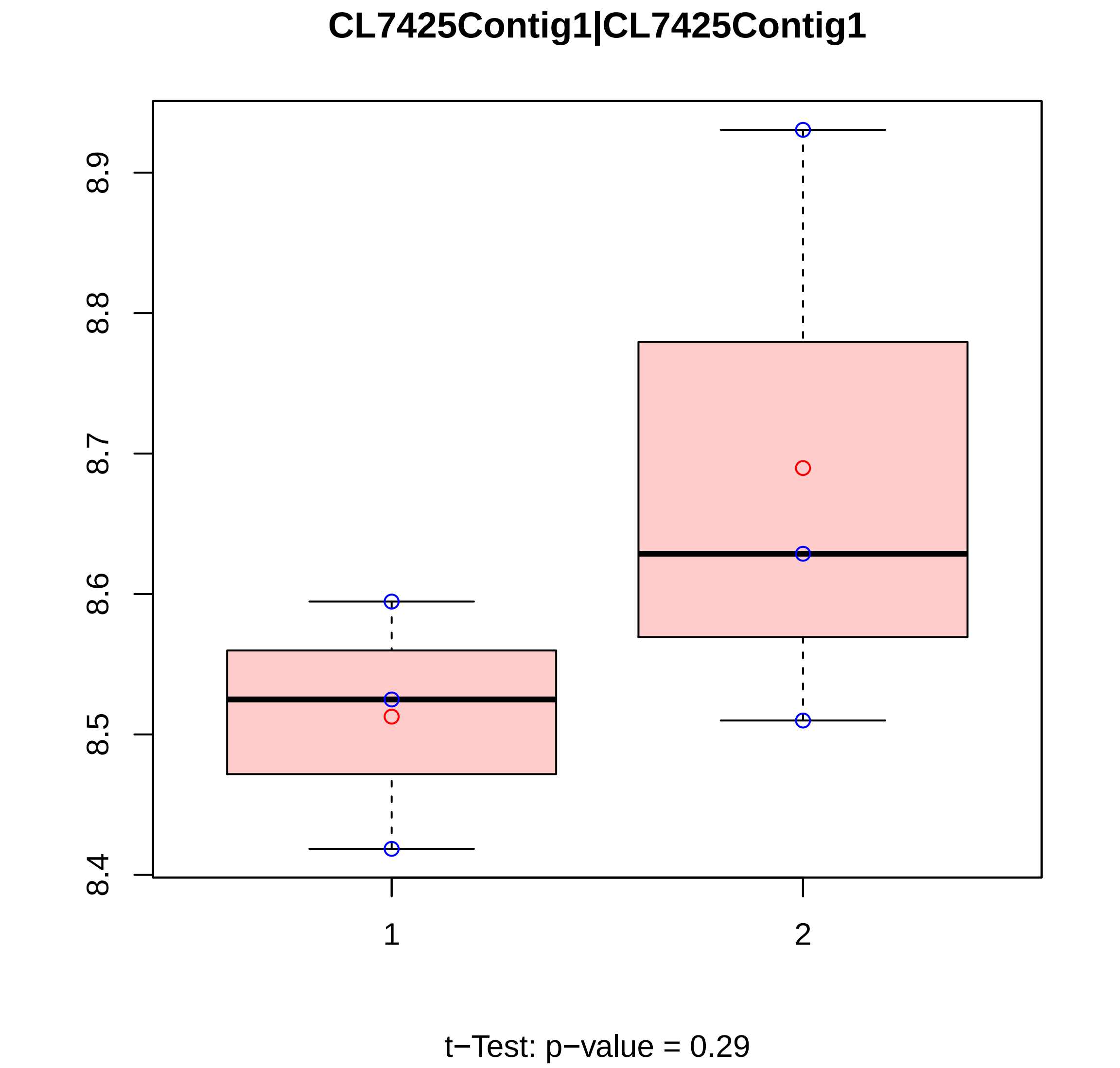
CL7425Contig1|CL7425Contig1
8.4
8.5
8.6
8.7
8.8
8.9
1
2
t−Test: p−value = 0.29

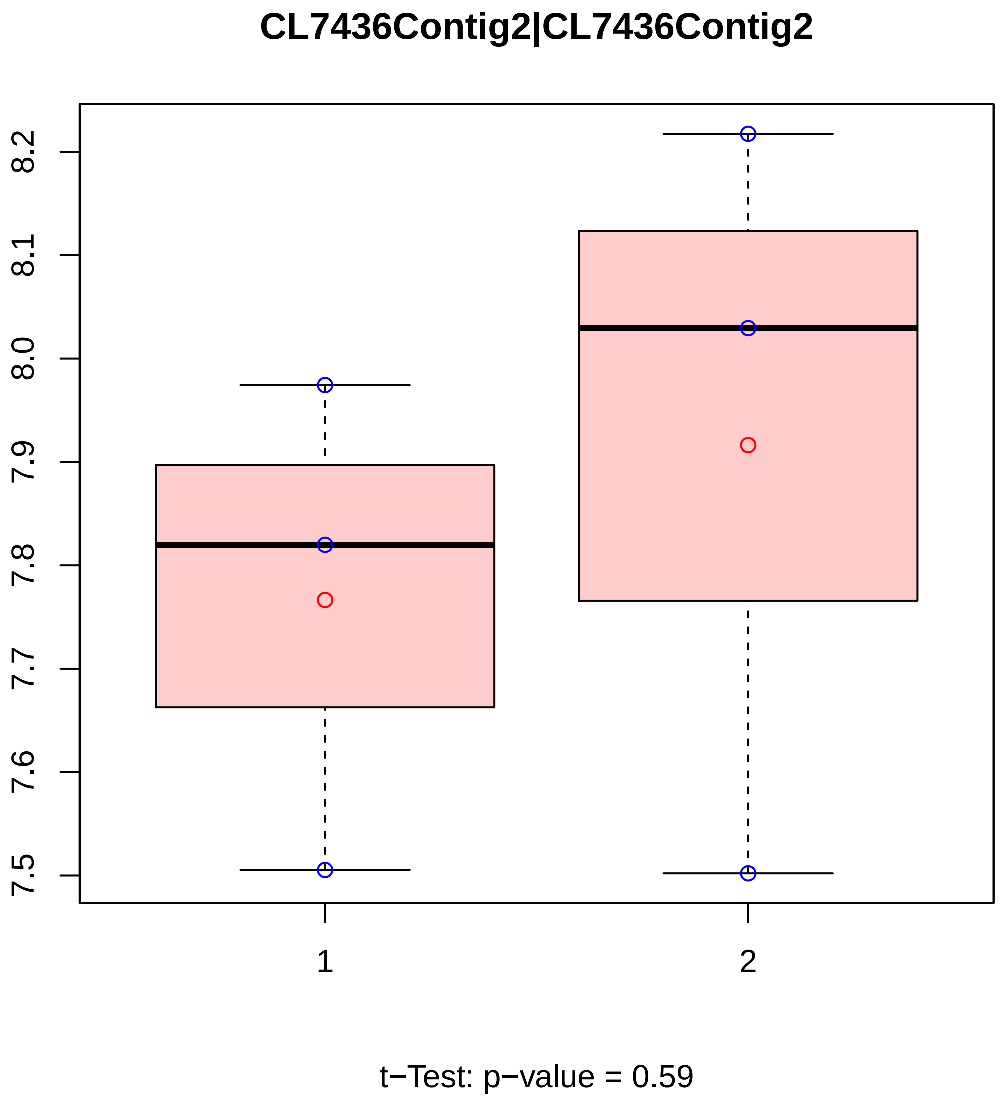
CL7436Contig2|CL7436Contig2
8.2
8.1
8.0
7.9
7.8
7.7
7.6
7.5
1
2
t−Test: p−value = 0.59

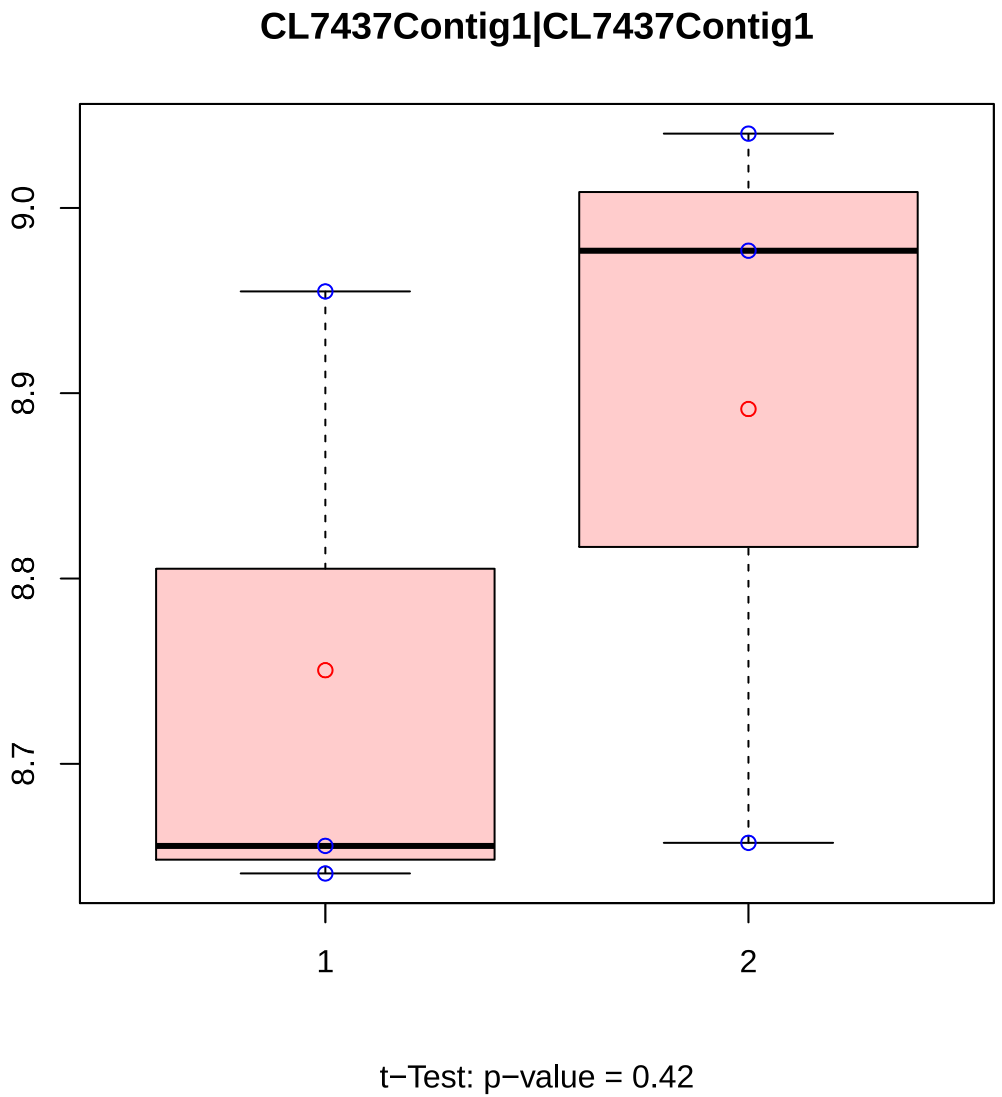

CL7437Contig1|CL7437Contig1
9.0
8.9
8.8
8.7
1
2
t−Test: p−value = 0.42

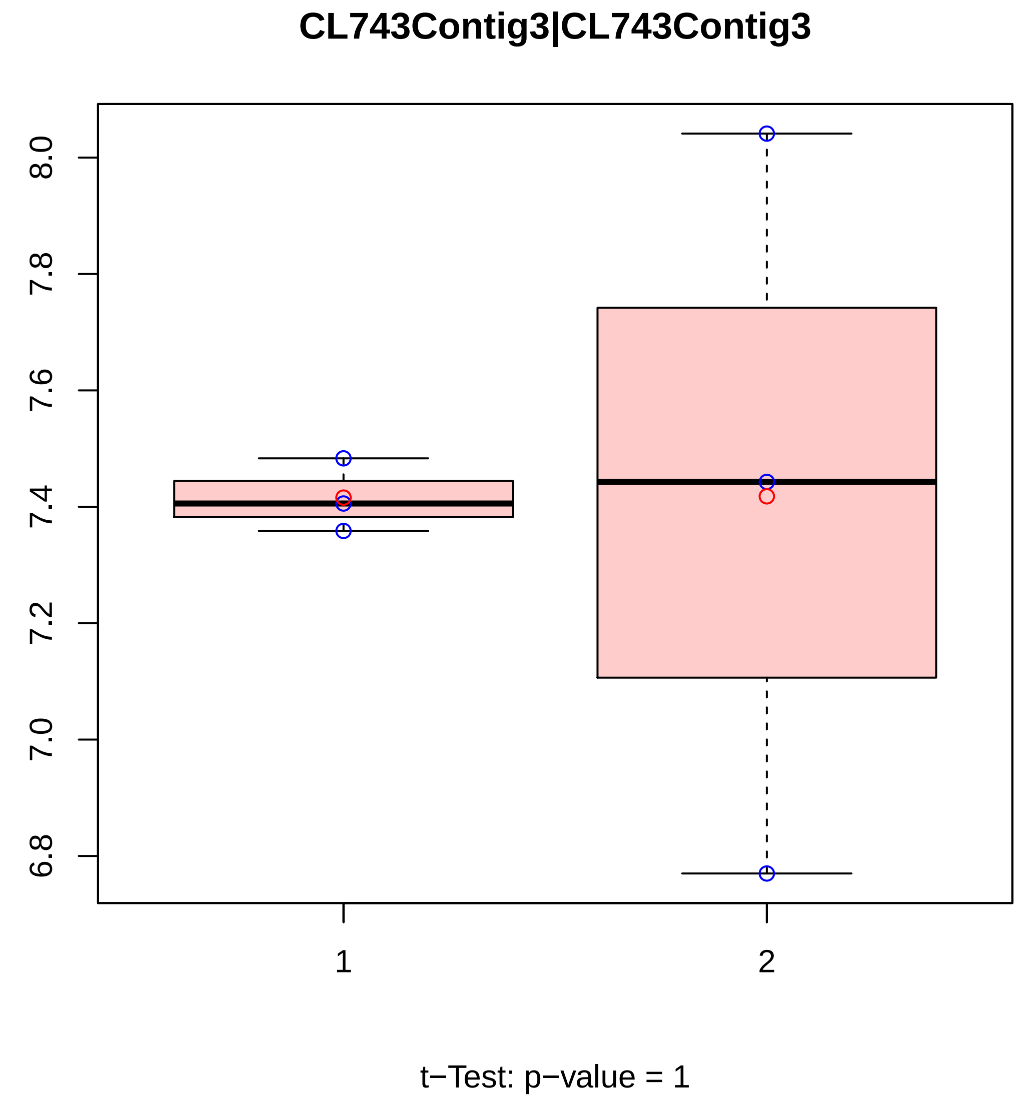

CL743Contig3|CL743Contig3
8.0
7.8
7.6
7.4
7.2
7.0
6.8
1
2
t−Test: p−value = 1

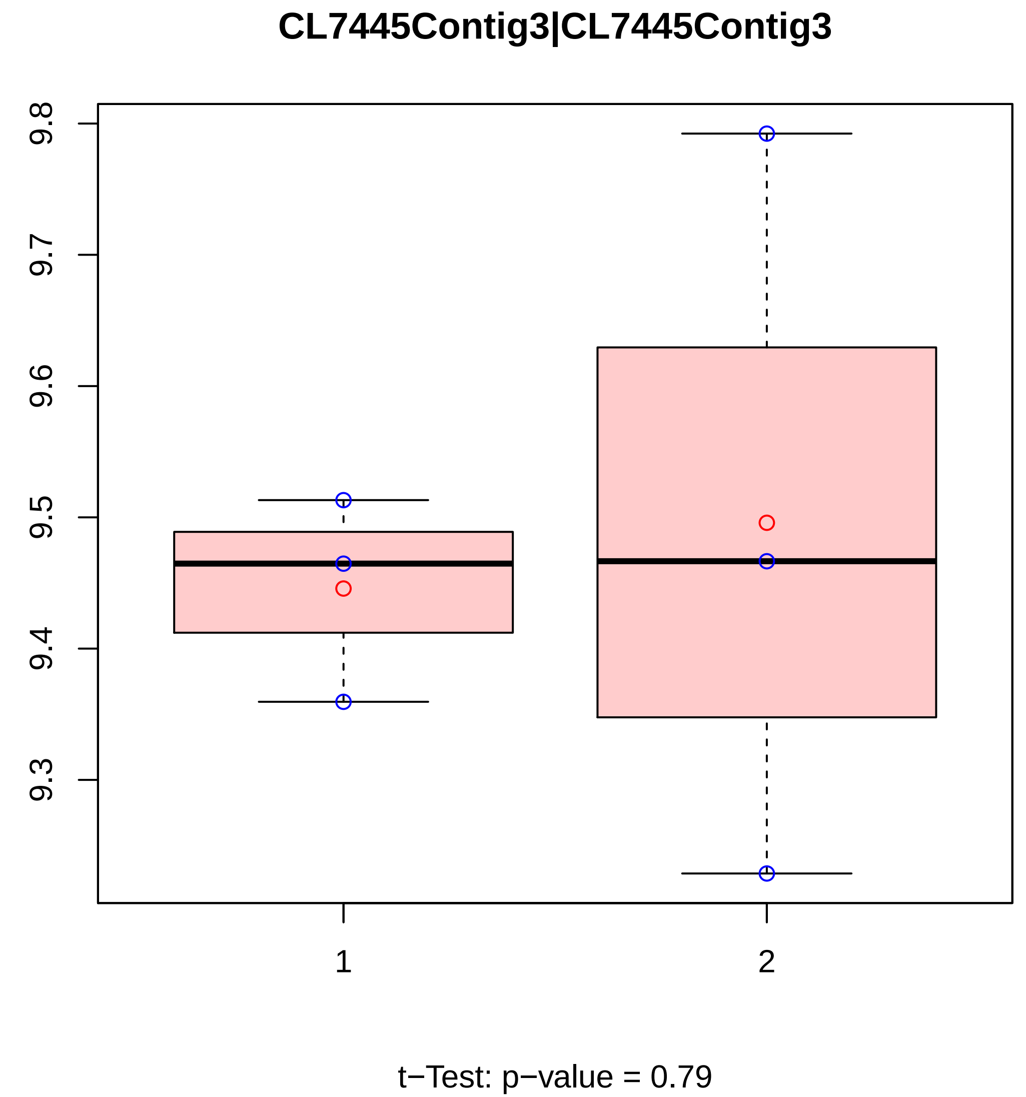
CL7445Contig3|CL7445Contig3
9.3
9.4
9.5
9.6
9.7
9.8
1
2
t−Test: p−value = 0.79

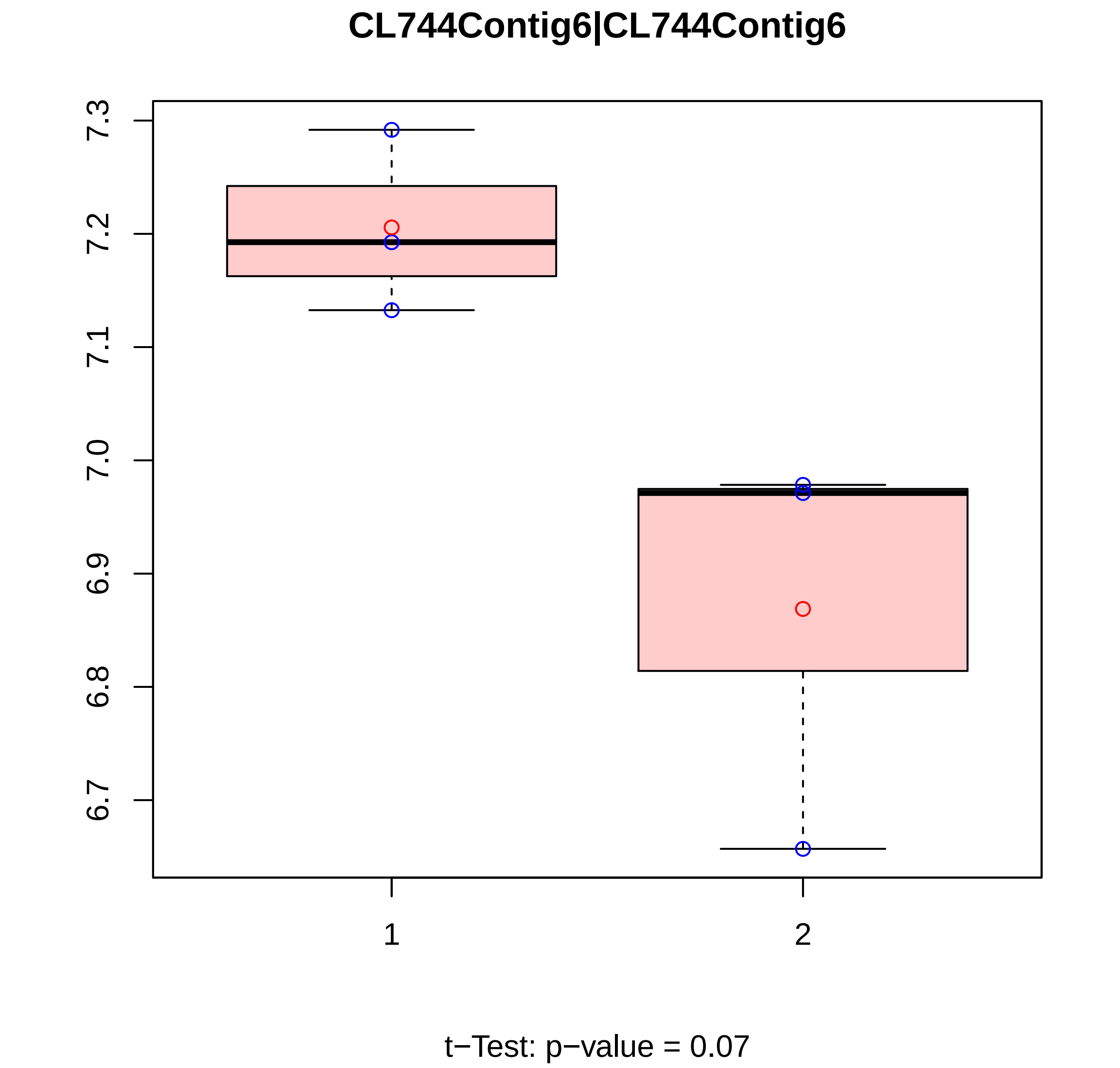

CL744Contig6|CL744Contig6
7.3
7.2
7.1
7.0
6.9
6.8
6.7
1
2
t−Test: p−value = 0.07

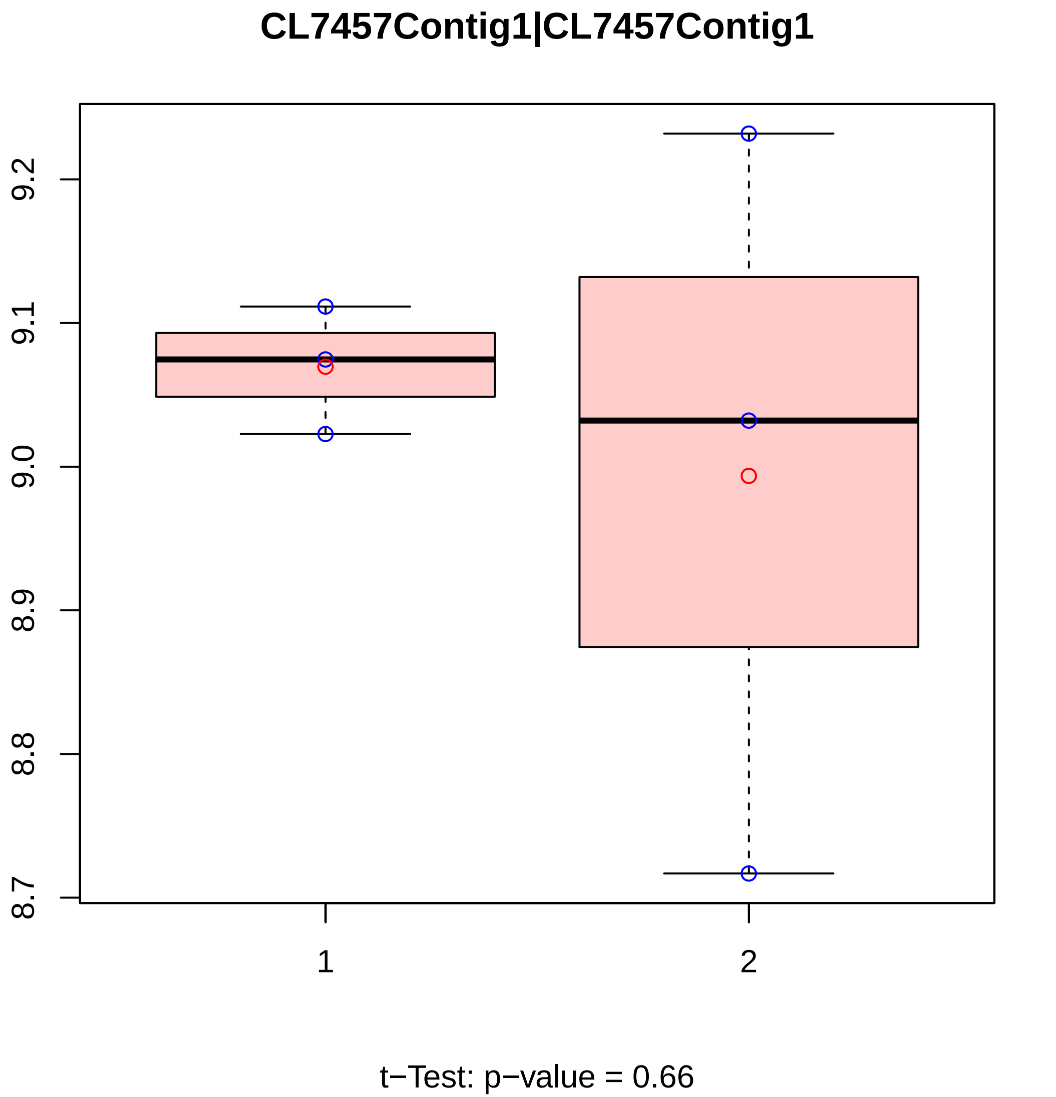
CL7457Contig1|CL7457Contig1
9.2
9.1
9.0
8.9
8.8
8.7
1
2
t−Test: p−value = 0.66

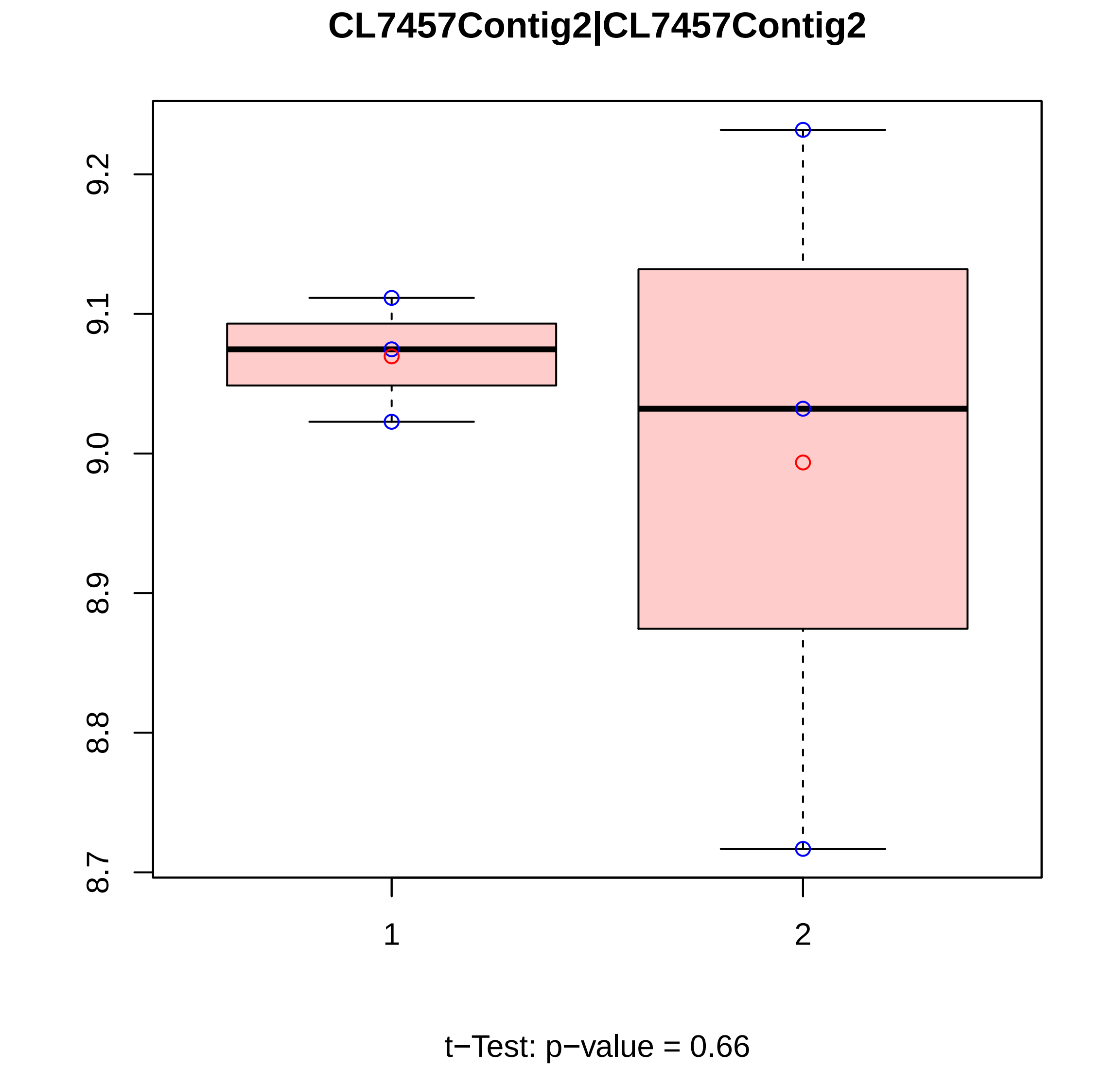
CL7457Contig2|CL7457Contig2
9.2
9.1
9.0
8.9
8.8
8.7
1
2
t−Test: p−value = 0.66

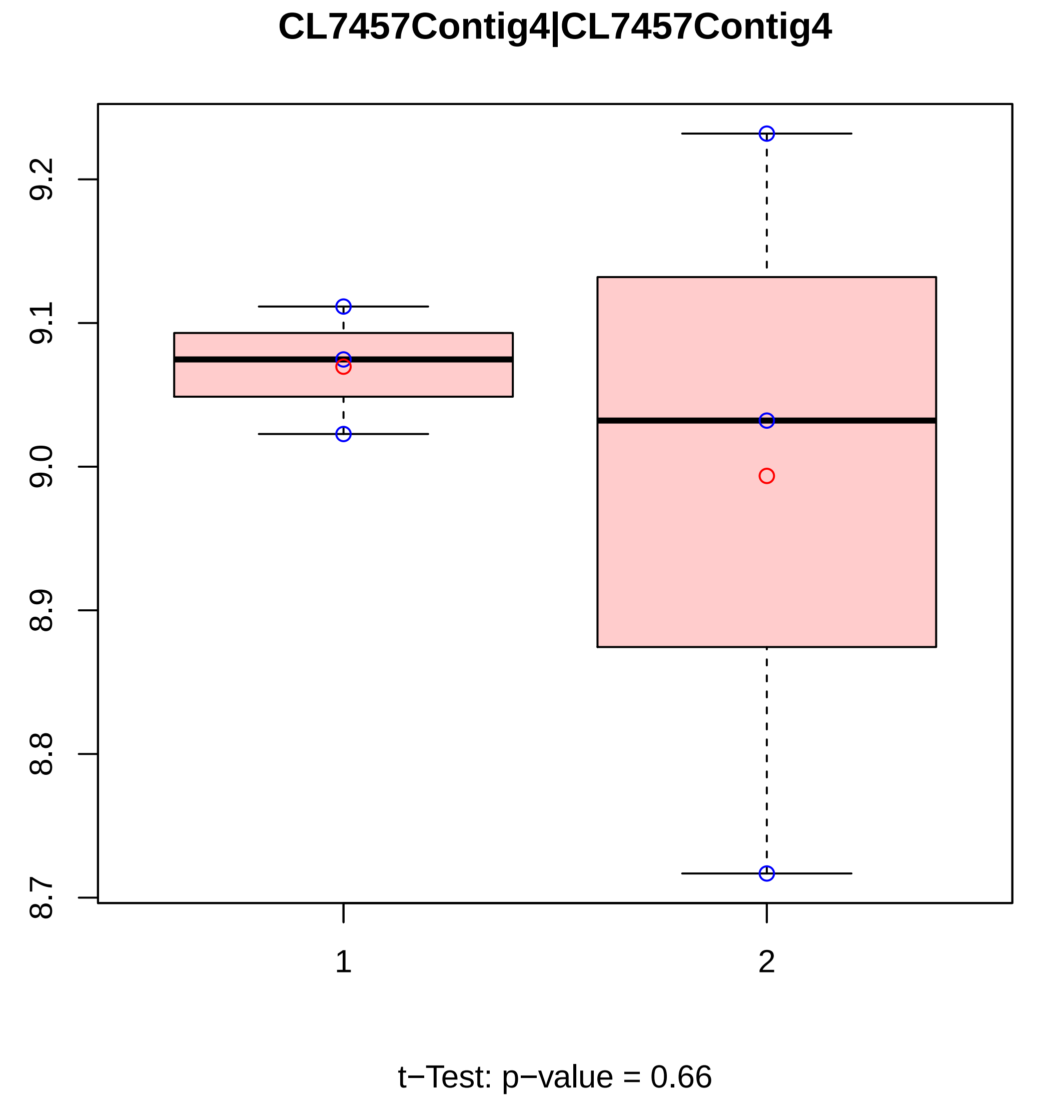
CL7457Contig4|CL7457Contig4
9.2
9.1
9.0
8.9
8.8
8.7
1
2
t−Test: p−value = 0.66

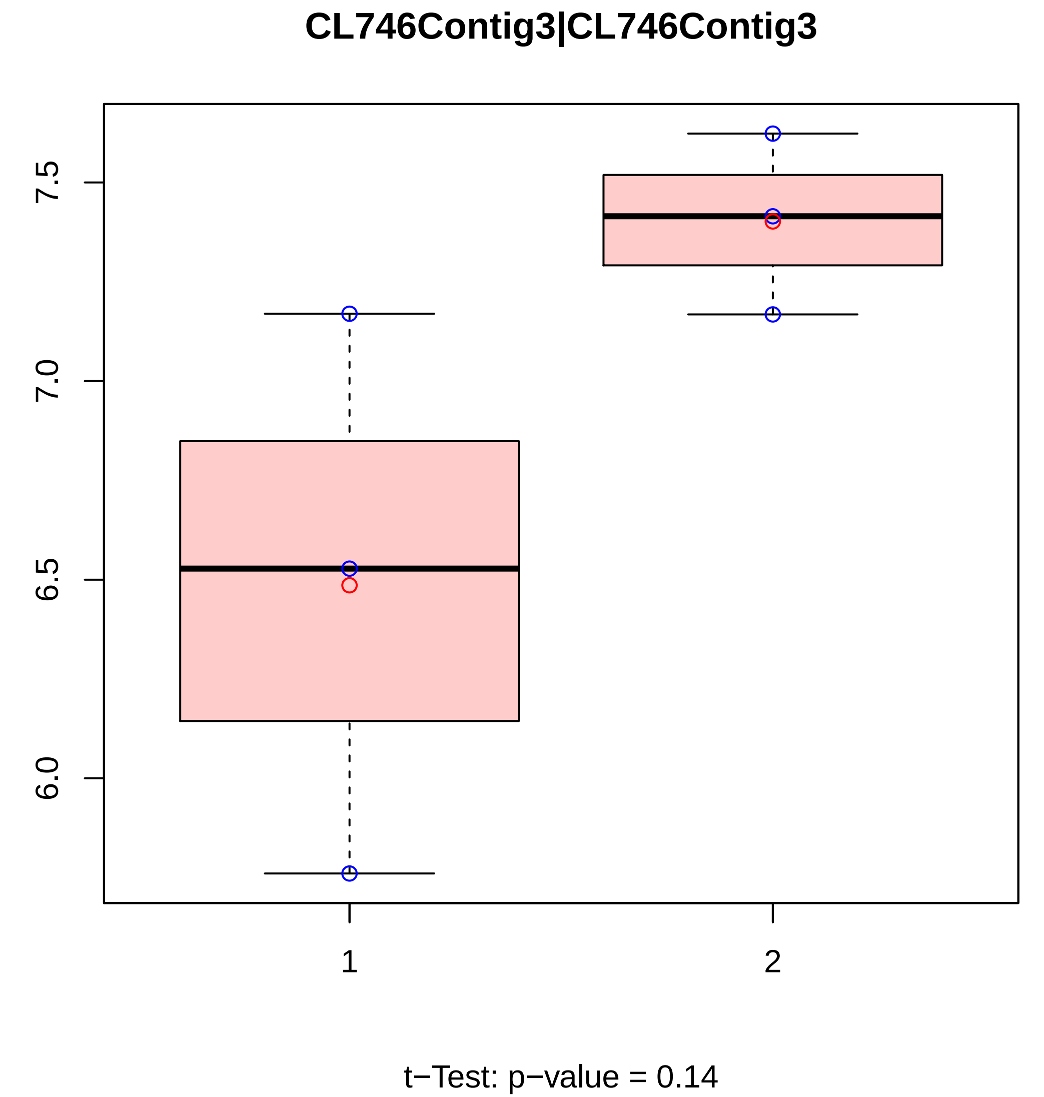
CL746Contig3|CL746Contig3
7.5
7.0
6.5
6.0
1
2
t−Test: p−value = 0.14

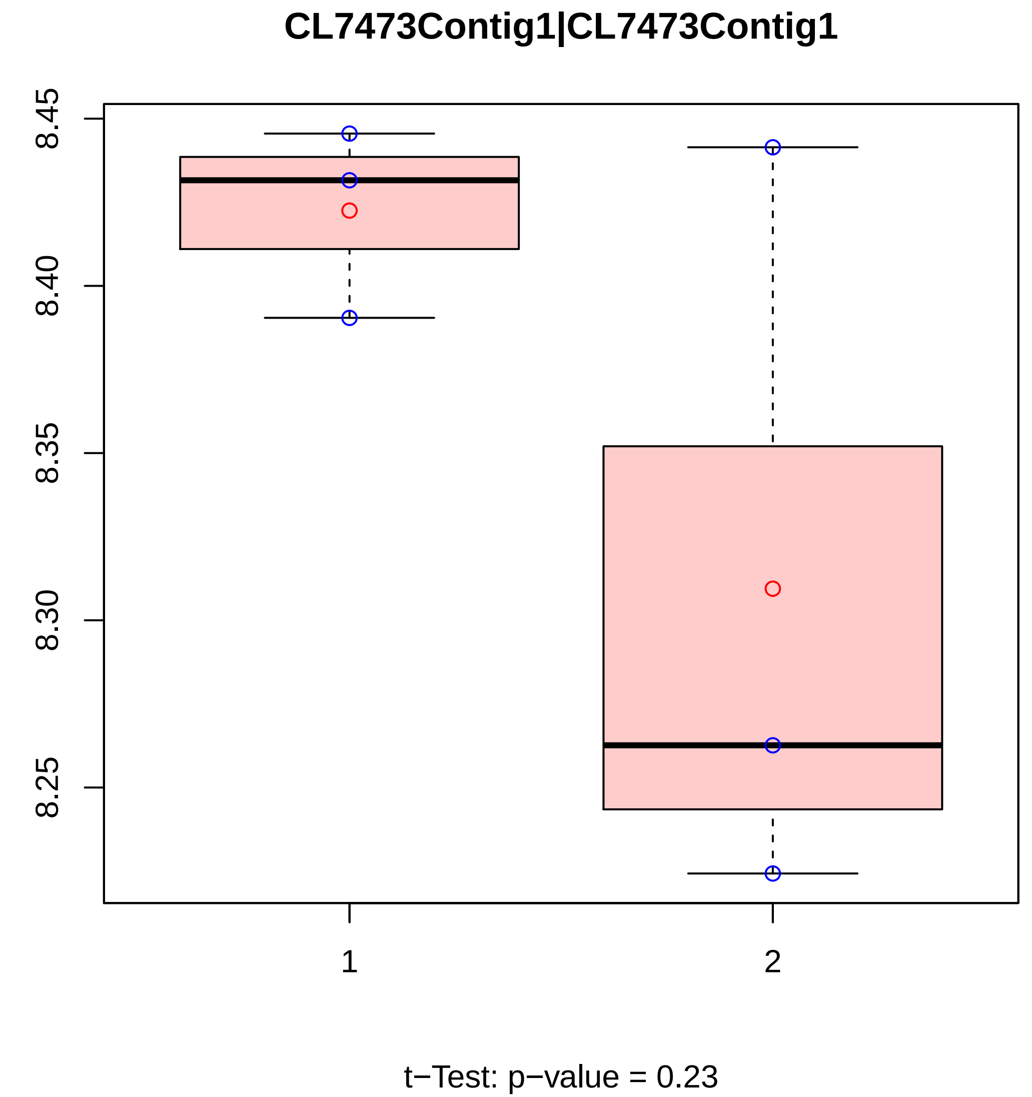
CL7473Contig1|CL7473Contig1
8.45
8.40
8.35
8.30
8.25
1
2
t−Test: p−value = 0.23

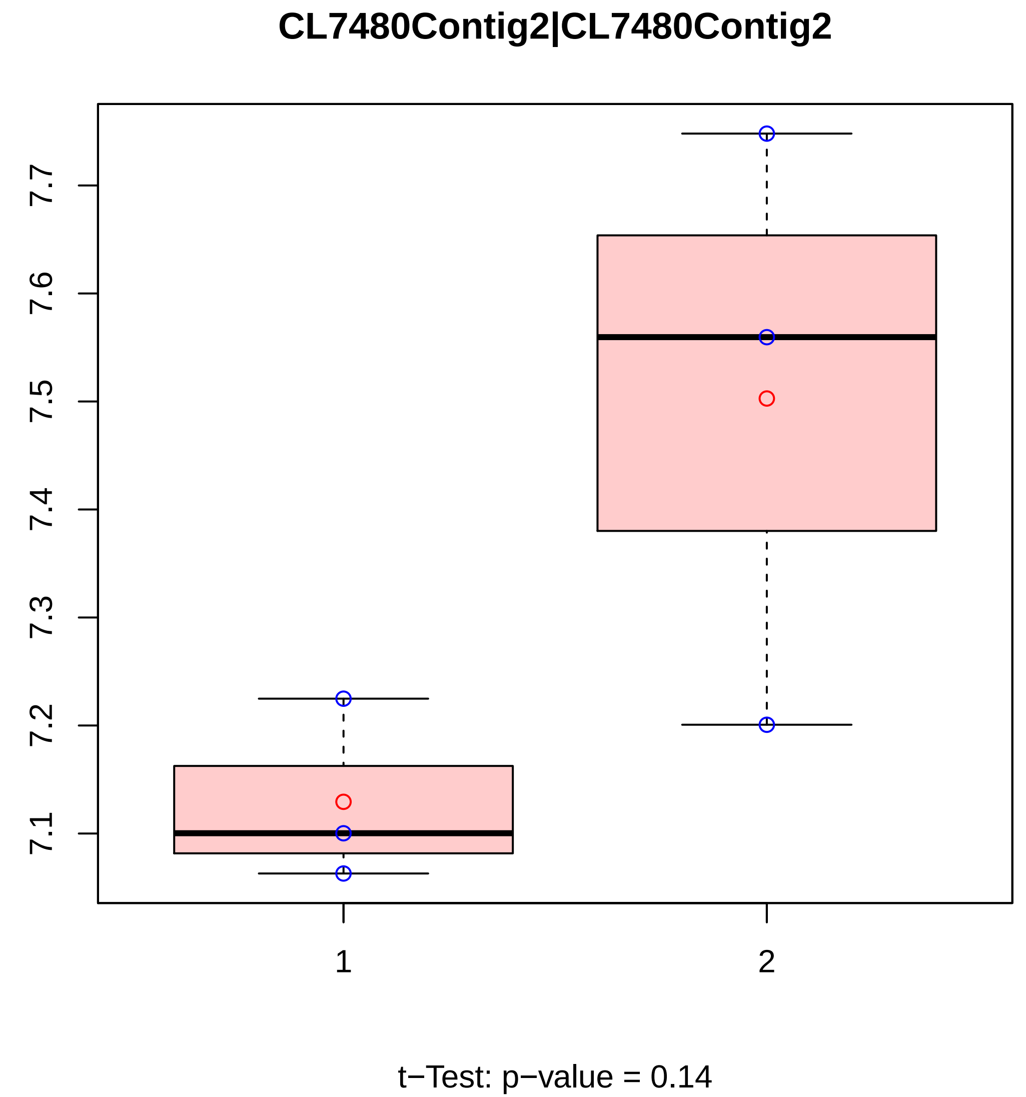
CL7480Contig2|CL7480Contig2
7.1
7.2
7.3
7.4
7.5
7.6
7.7
1
2
t−Test: p−value = 0.14

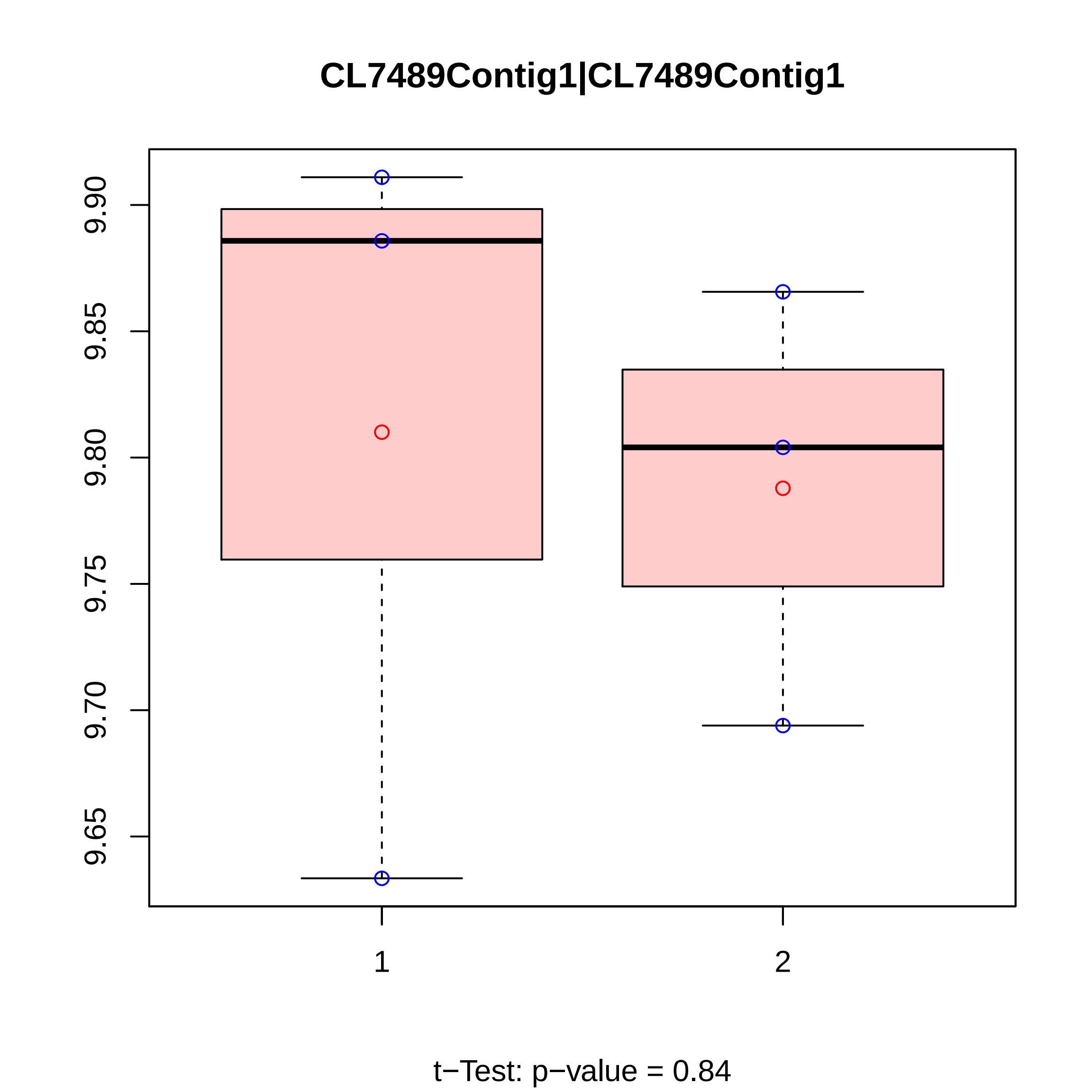

CL7489Contig1|CL7489Contig1
9.65
9.70
9.75
9.80
9.85
9.90
1
2
t−Test: p−value = 0.84

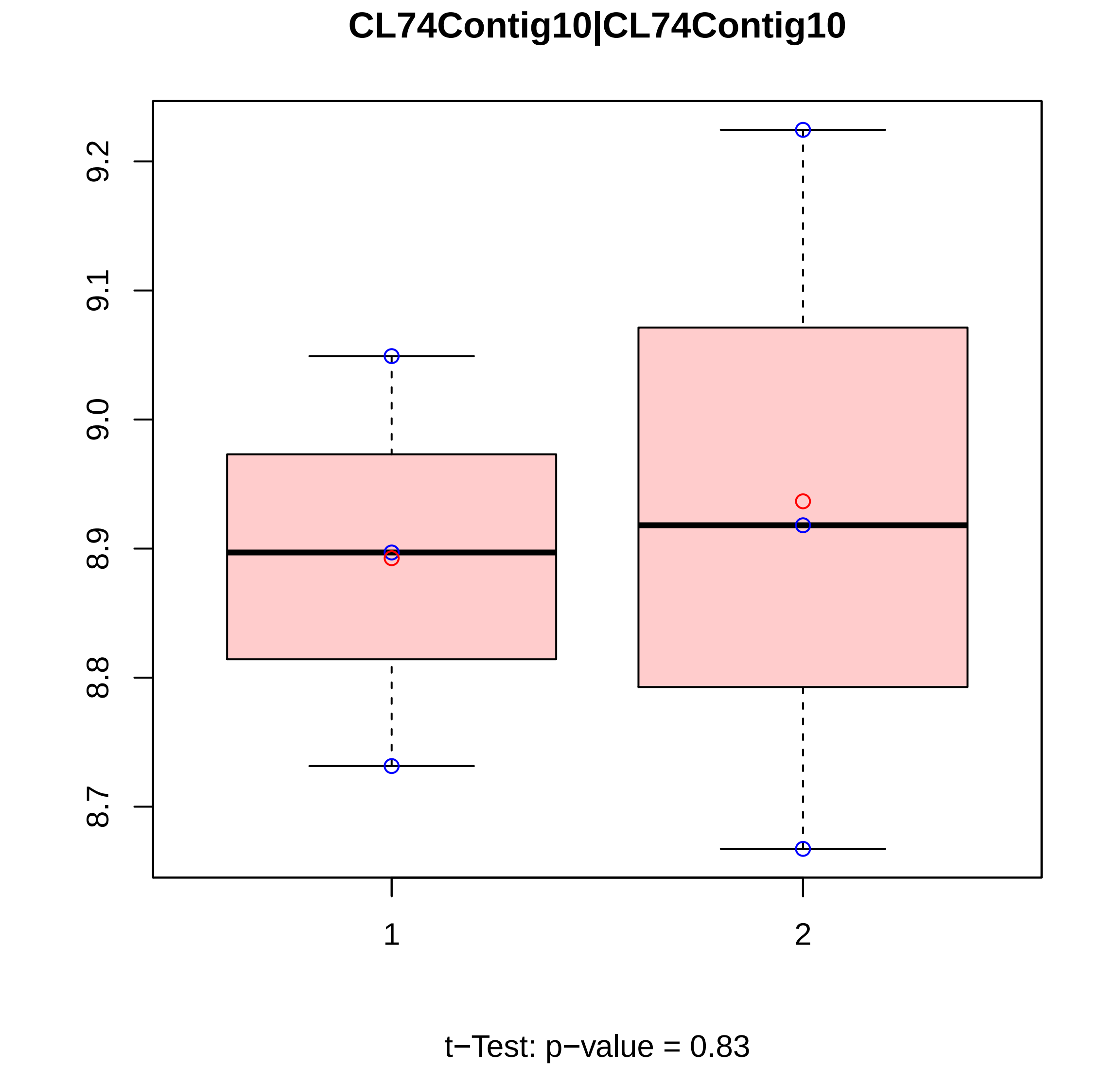
CL74Contig10|CL74Contig10
9.2
9.1
9.0
8.9
8.8
8.7
1
2
t−Test: p−value = 0.83

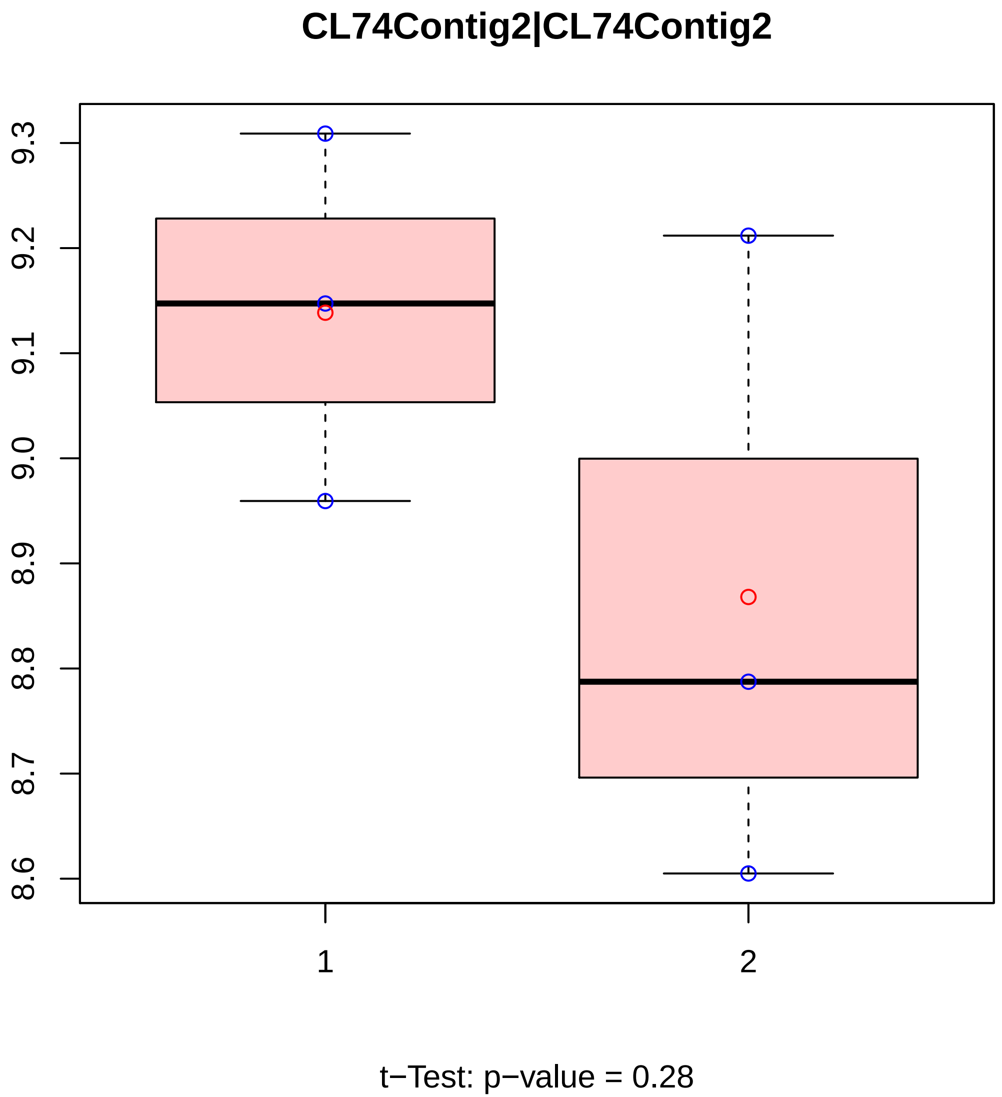
CL74Contig2|CL74Contig2
9.3
9.2
9.1
9.0
8.9
8.8
8.7
8.6
1
2
t−Test: p−value = 0.28

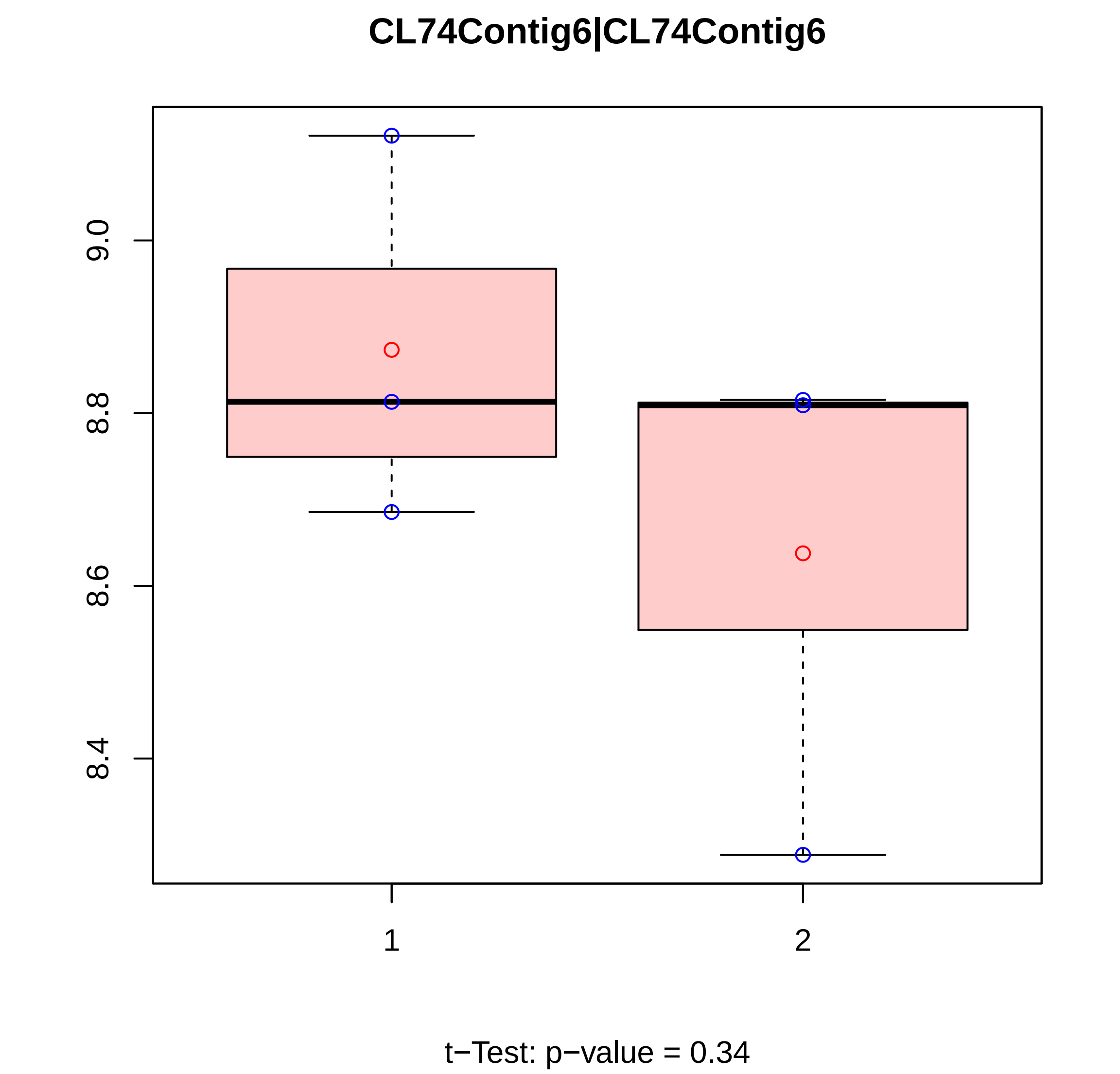

CL74Contig6|CL74Contig6
9.0
8.8
8.6
8.4
1
2
t−Test: p−value = 0.34

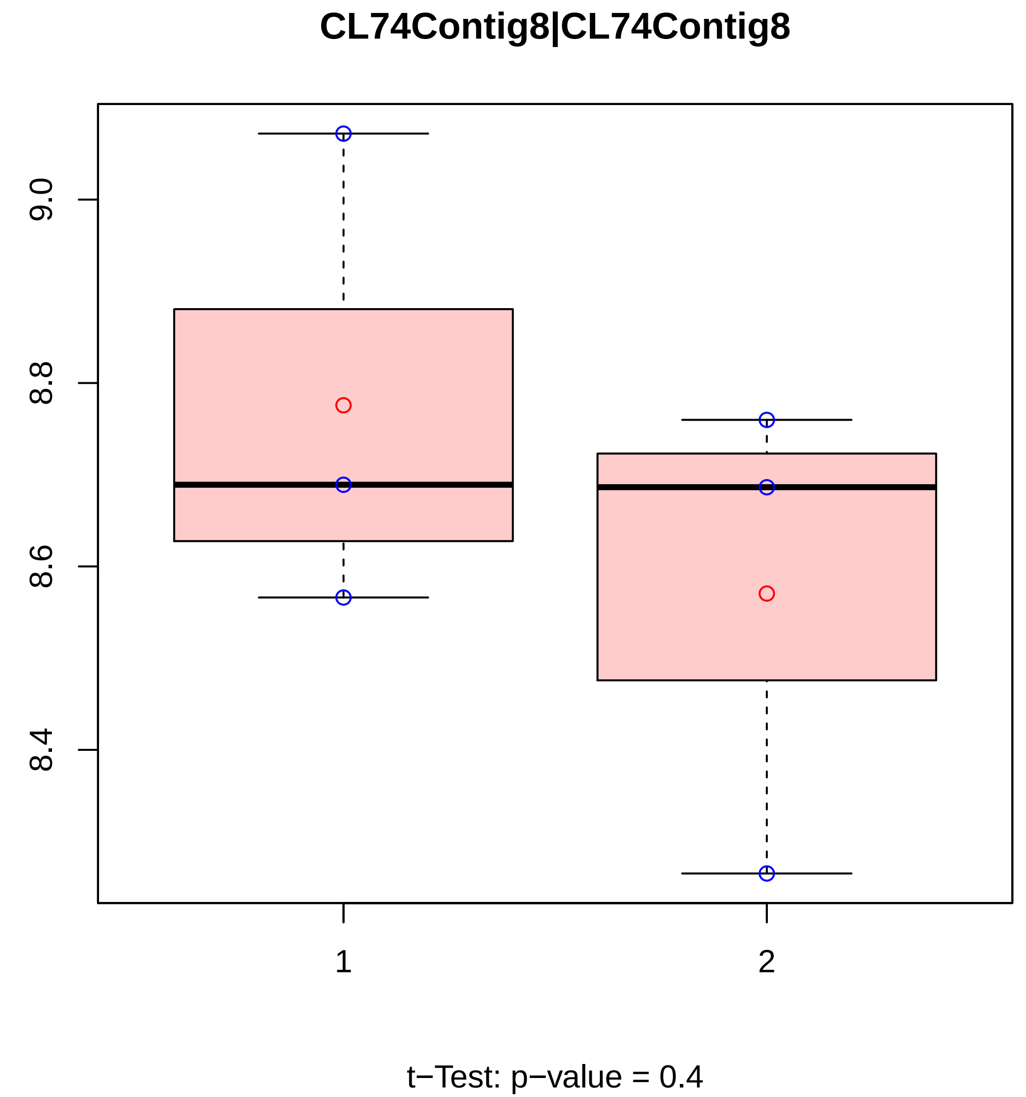
CL74Contig8|CL74Contig8
9.0
8.8
8.6
8.4
1
2
t−Test: p−value = 0.4

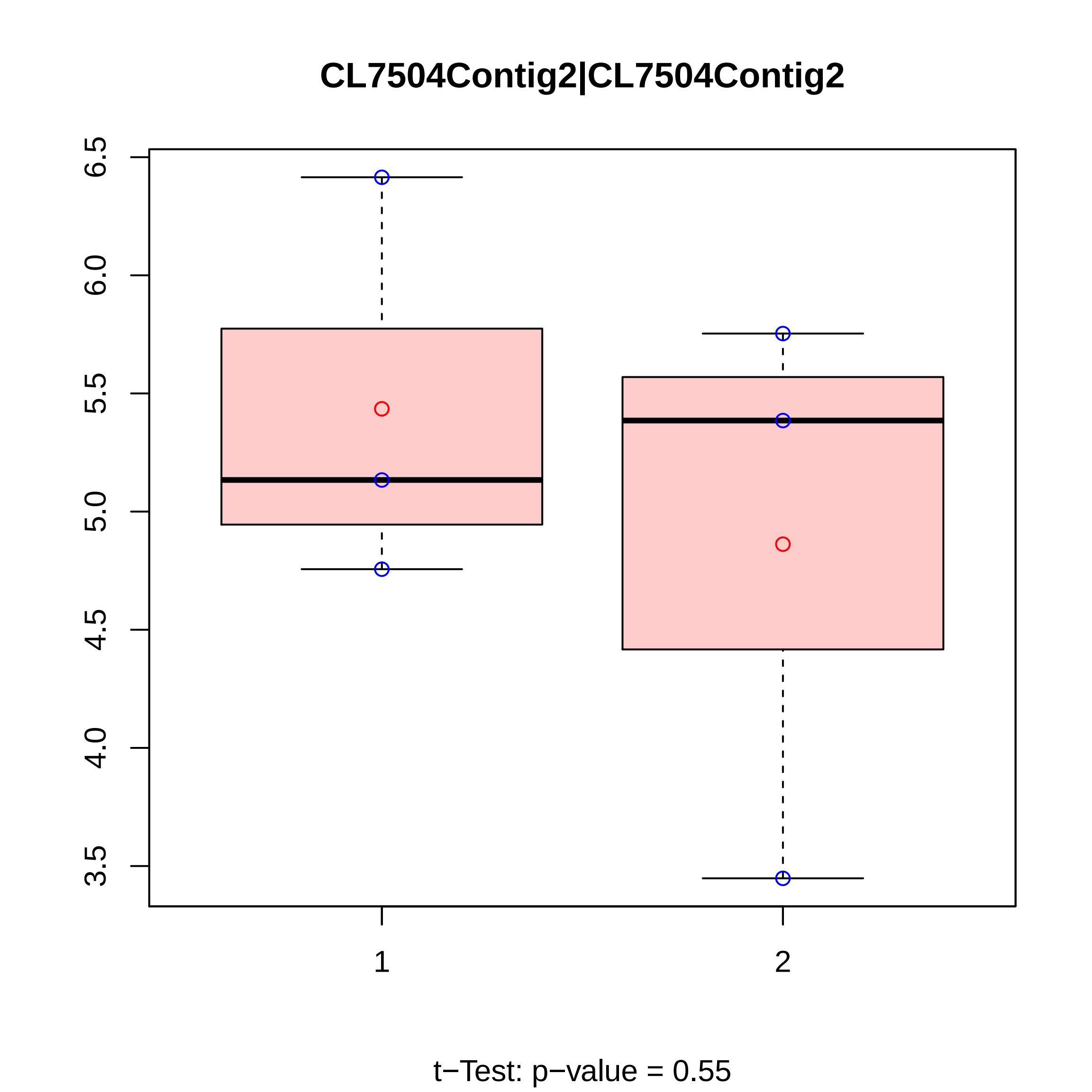

CL7504Contig2|CL7504Contig2
6.5
6.0
5.5
5.0
4.5
4.0
3.5
1
2
t−Test: p−value = 0.55

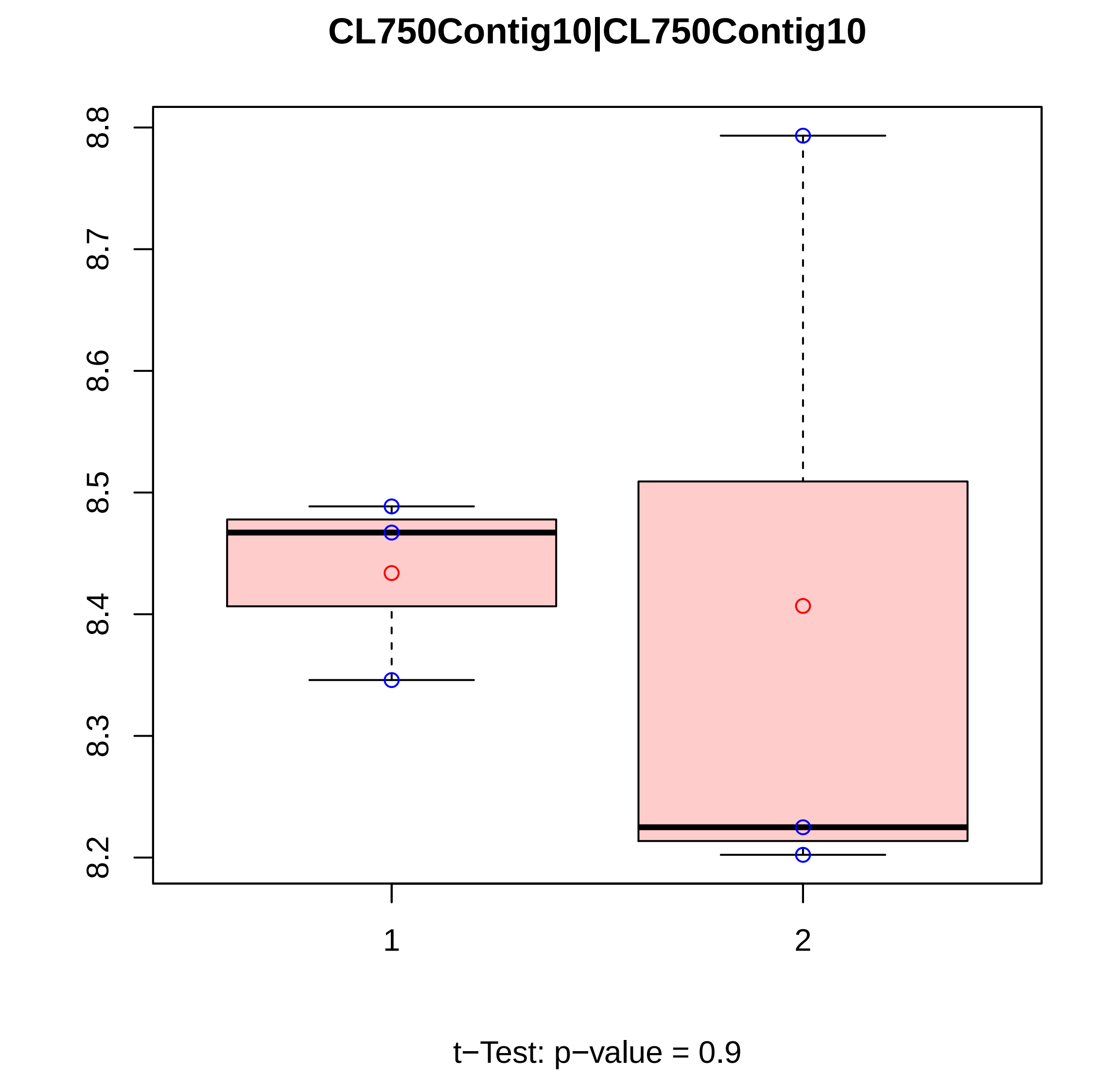

CL750Contig10|CL750Contig10
8.2
8.3
8.4
8.5
8.6
8.7
8.8
1
2
t−Test: p−value = 0.9

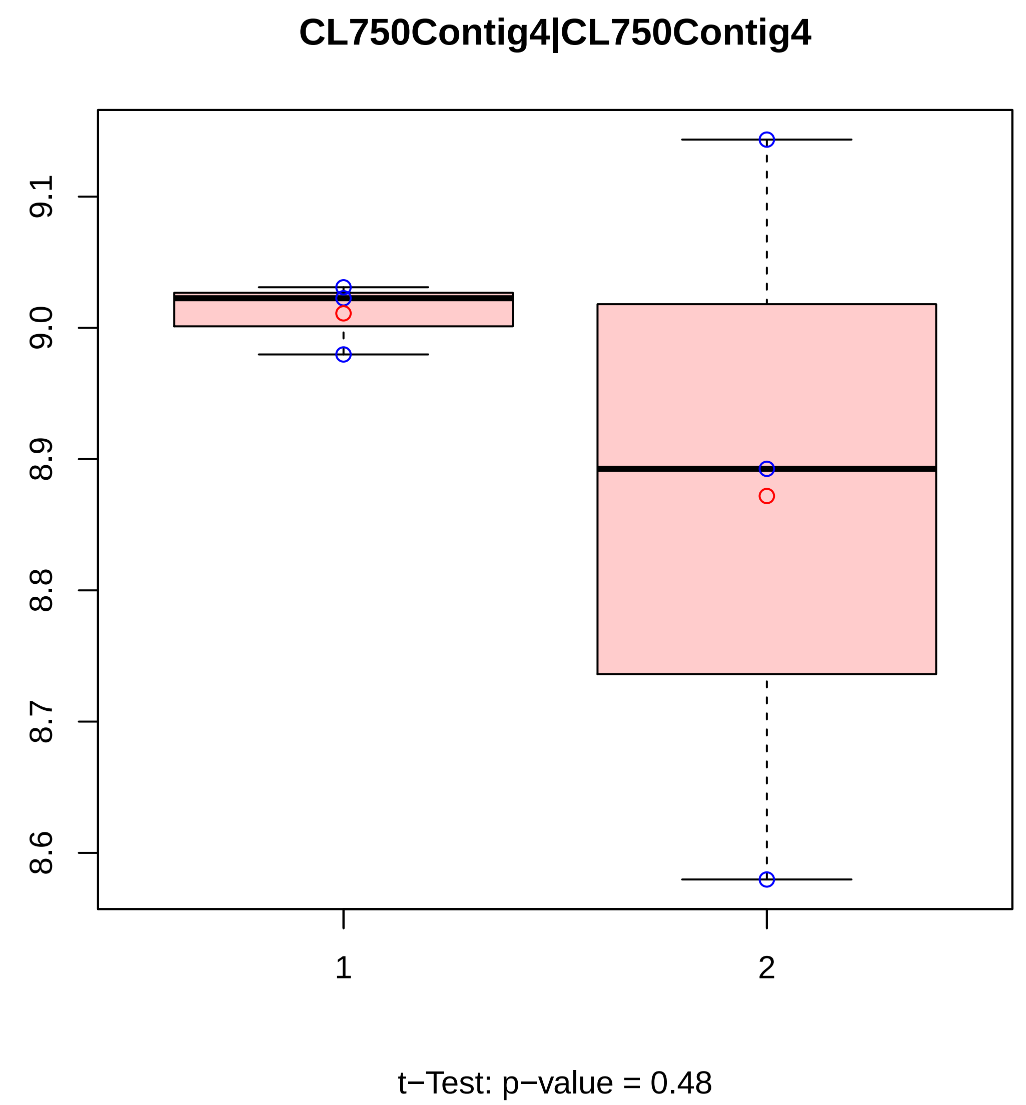

CL750Contig4|CL750Contig4
9.1
9.0
8.9
8.8
8.7
8.6
1
2
t−Test: p−value = 0.48

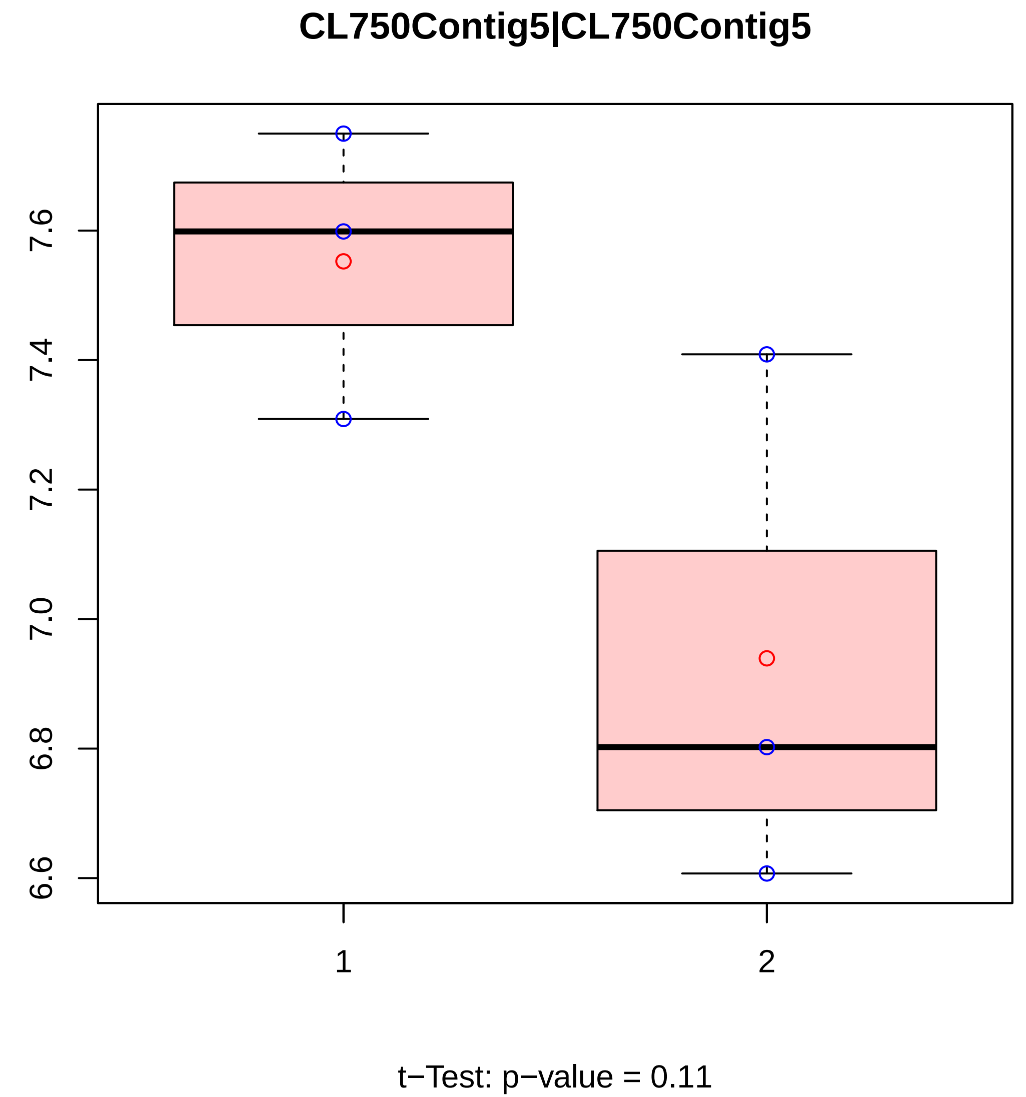

CL750Contig5|CL750Contig5
7.6
7.4
7.2
7.0
6.8
6.6
1
2
t−Test: p−value = 0.11

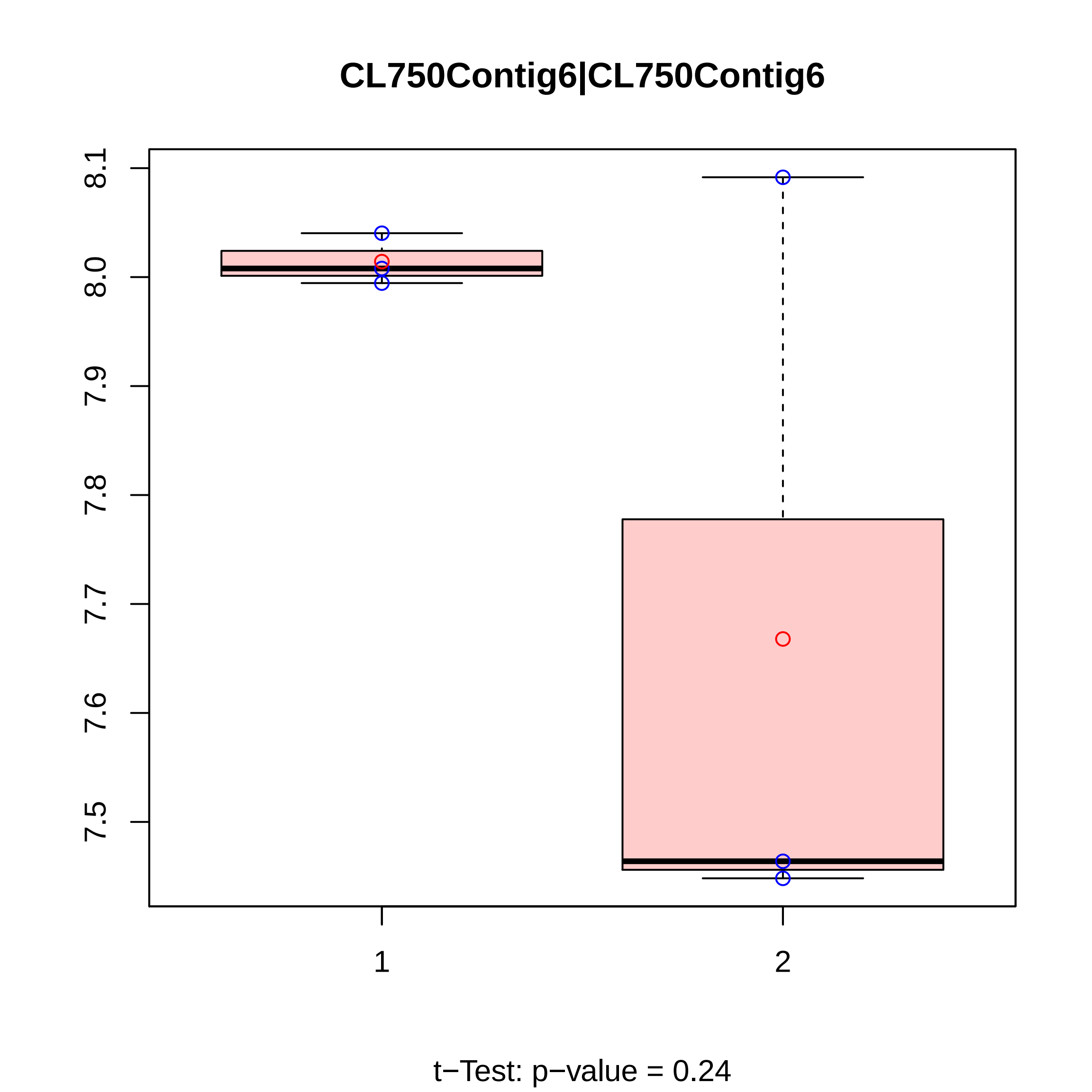

CL750Contig6|CL750Contig6
8.1
8.0
7.9
7.8
7.7
7.6
7.5
1
2
t−Test: p−value = 0.24

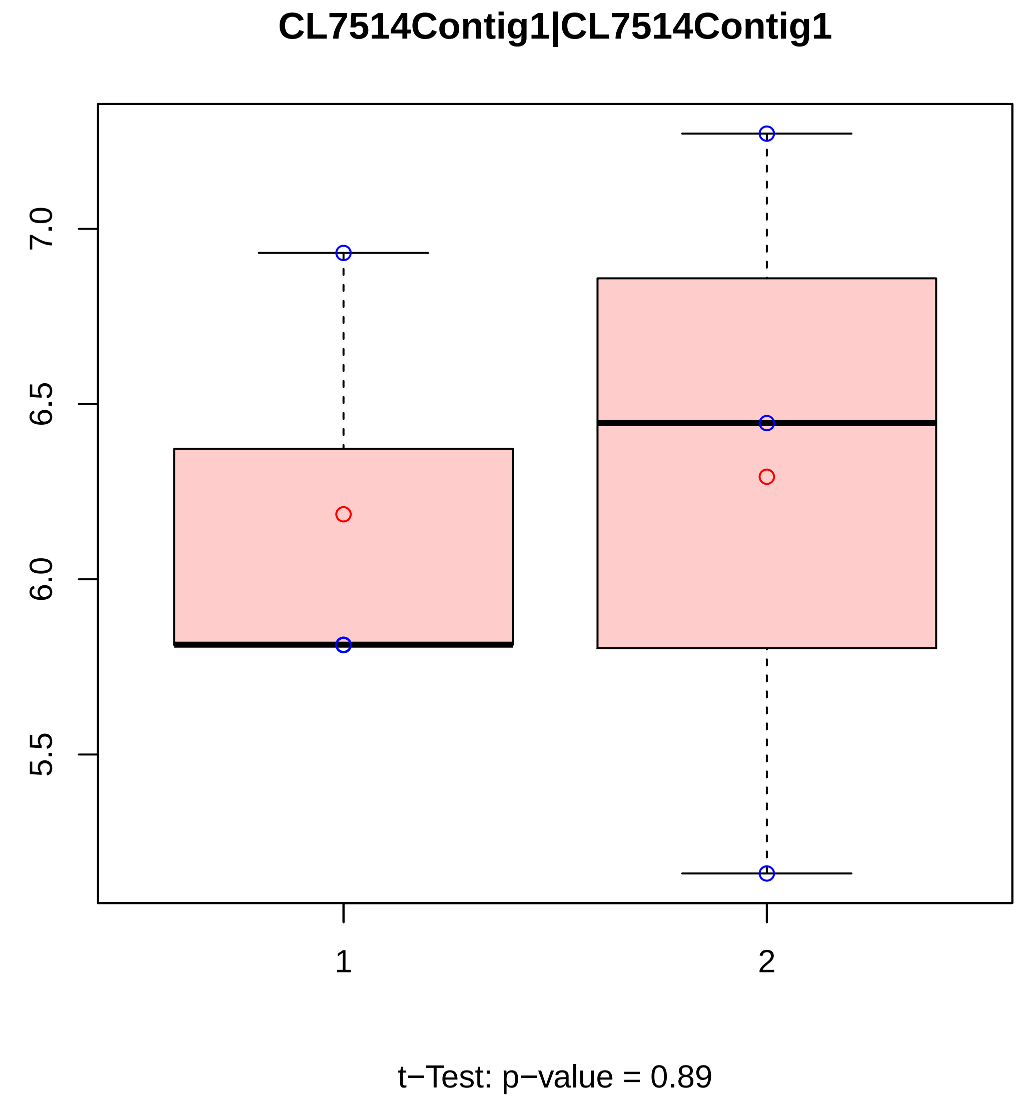

CL7514Contig1|CL7514Contig1
7.0
6.5
6.0
5.5
1
2
t−Test: p−value = 0.89

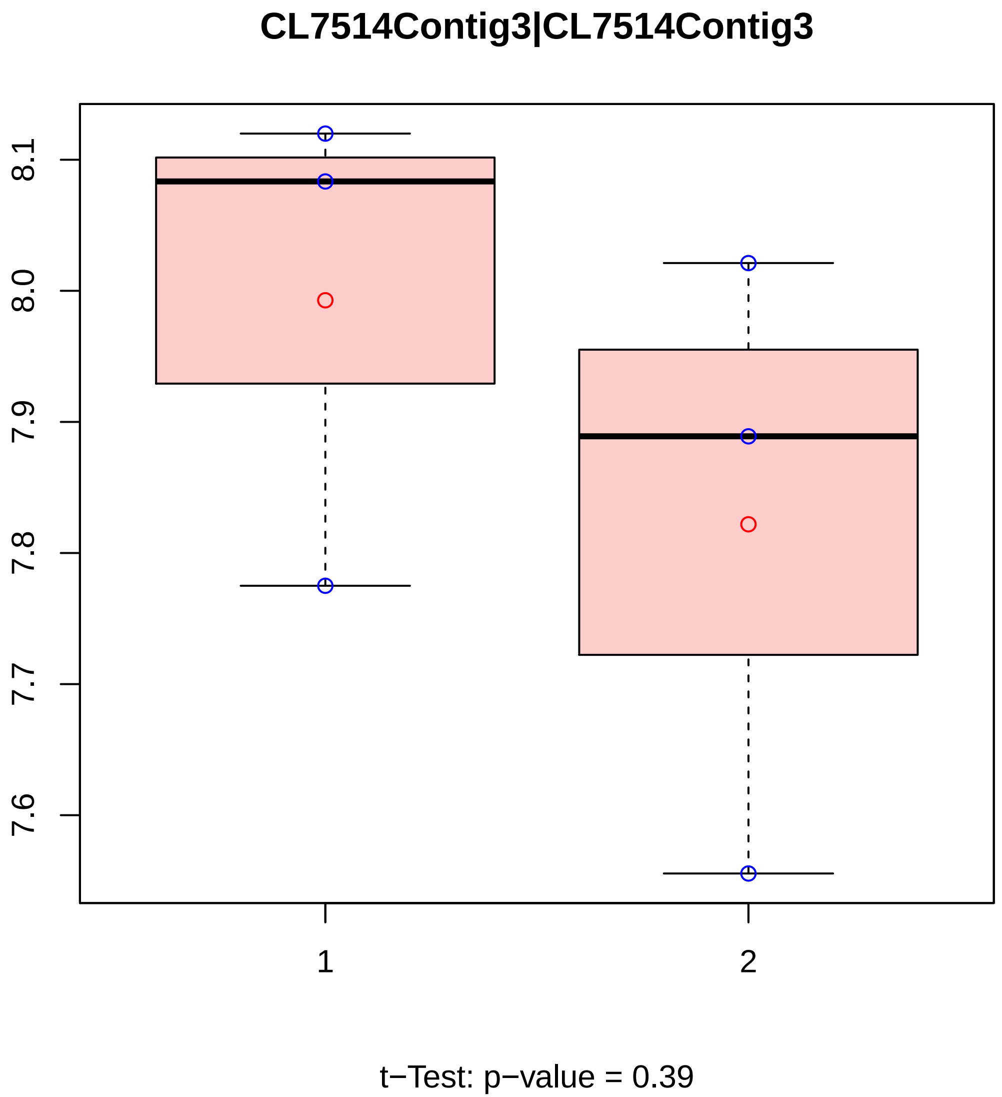

CL7514Contig3|CL7514Contig3
8.1
8.0
7.9
7.8
7.7
7.6
1
2
t−Test: p−value = 0.39

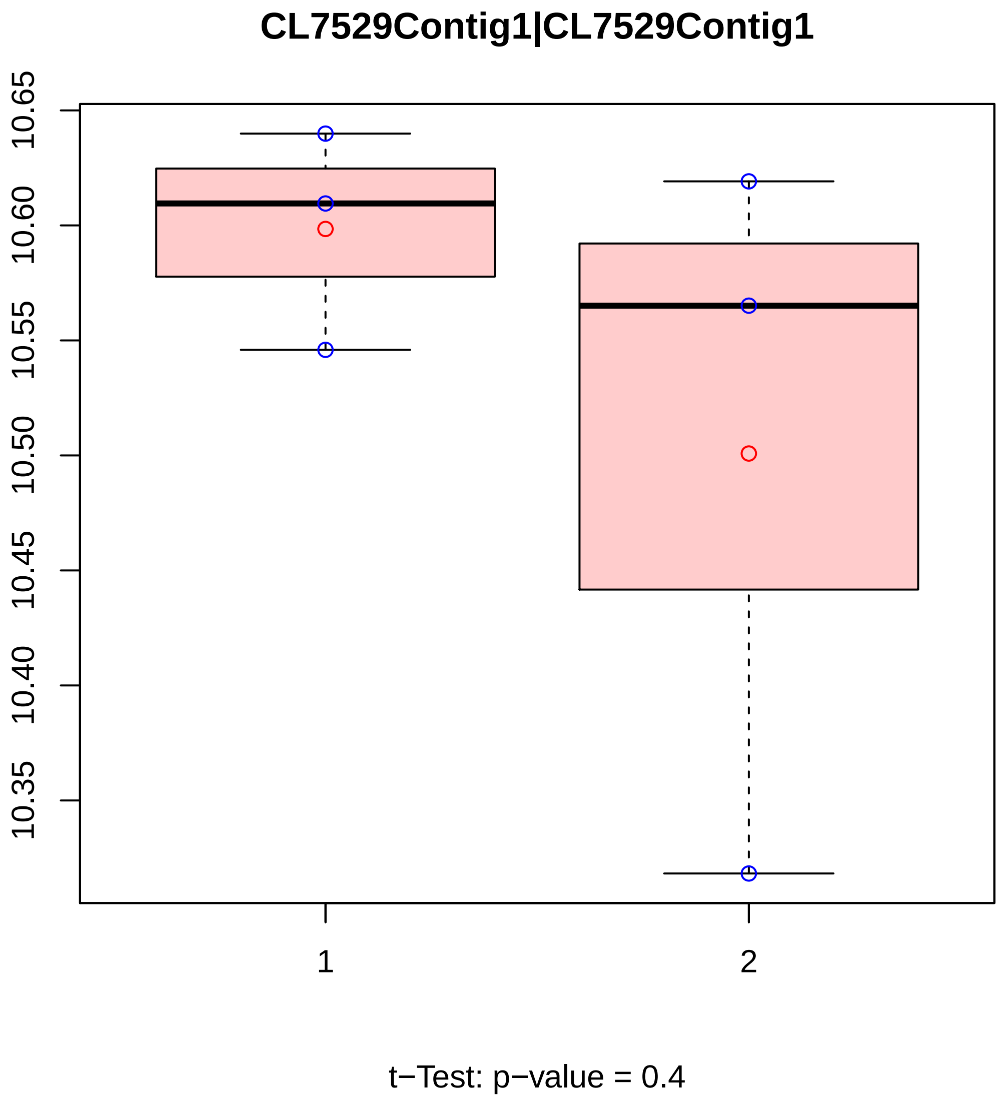
CL7529Contig1|CL7529Contig1
10.35
10.40
10.45
10.50
10.55
10.60
10.65
1
2
t−Test: p−value = 0.4

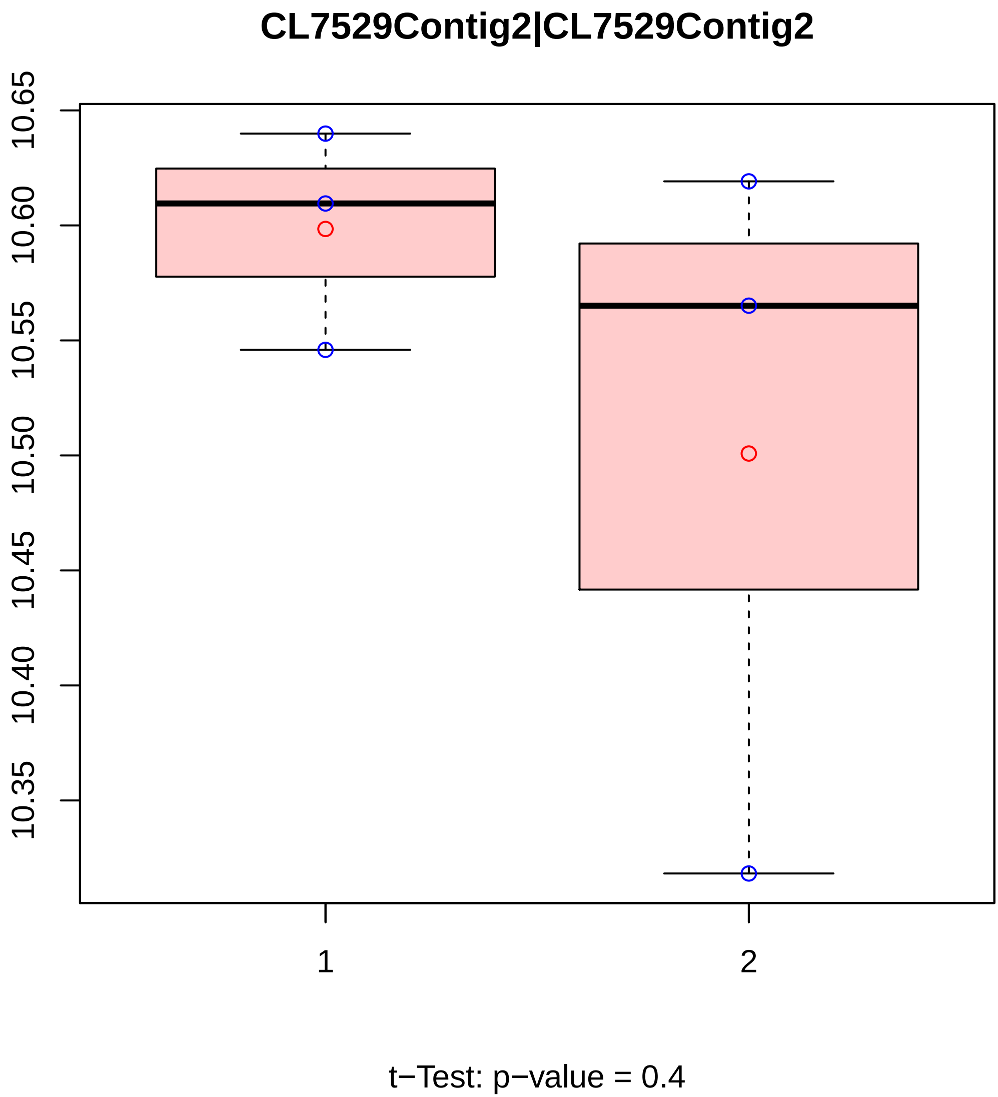

CL7529Contig2|CL7529Contig2
10.65
10.60
10.55
10.50
10.45
10.40
10.35
1
2
t−Test: p−value = 0.4

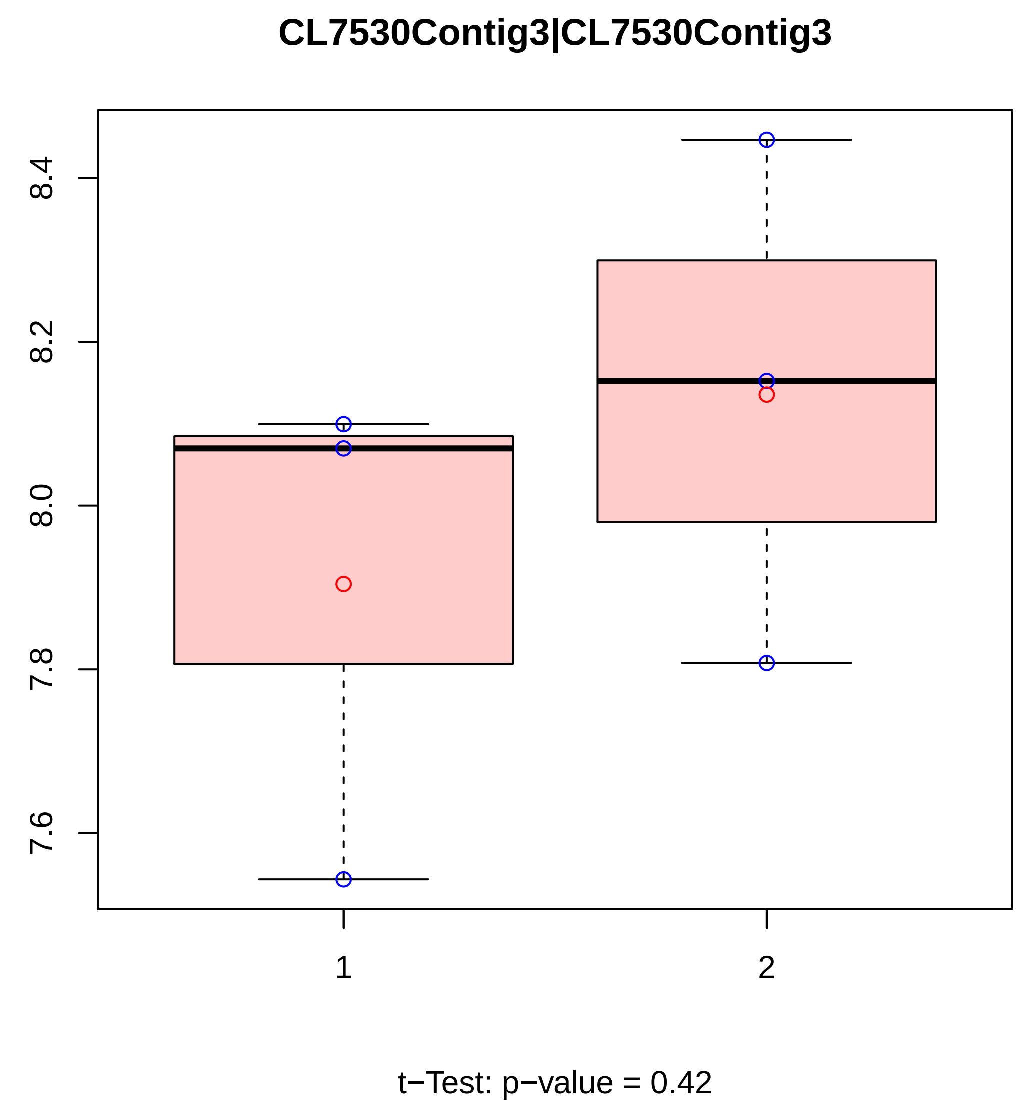

CL7530Contig3|CL7530Contig3
8.4
8.2
8.0
7.8
7.6
1
2
t−Test: p−value = 0.42

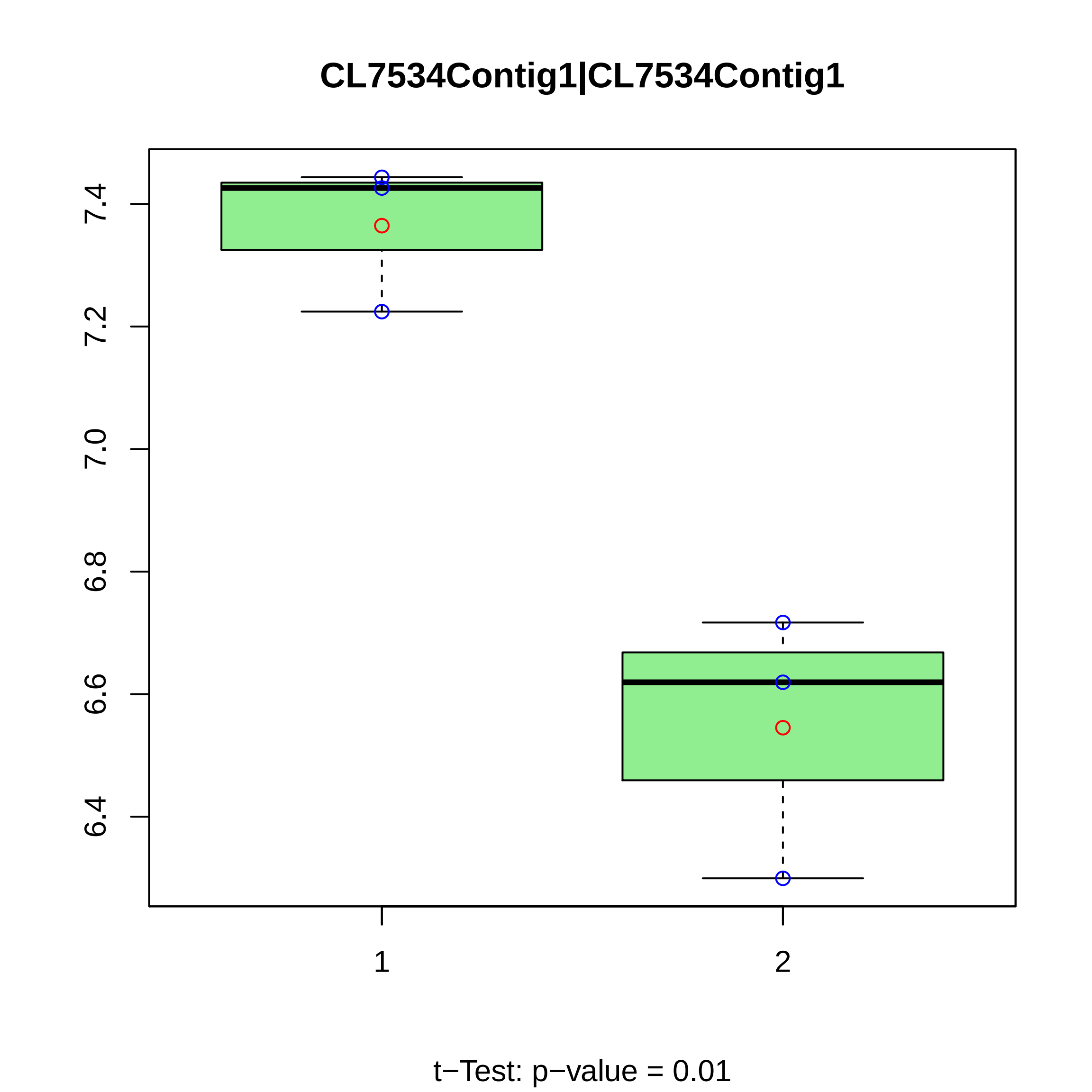
CL7534Contig1|CL7534Contig1
7.4
7.2
7.0
6.8
6.6
6.4
1
2
t−Test: p−value = 0.01

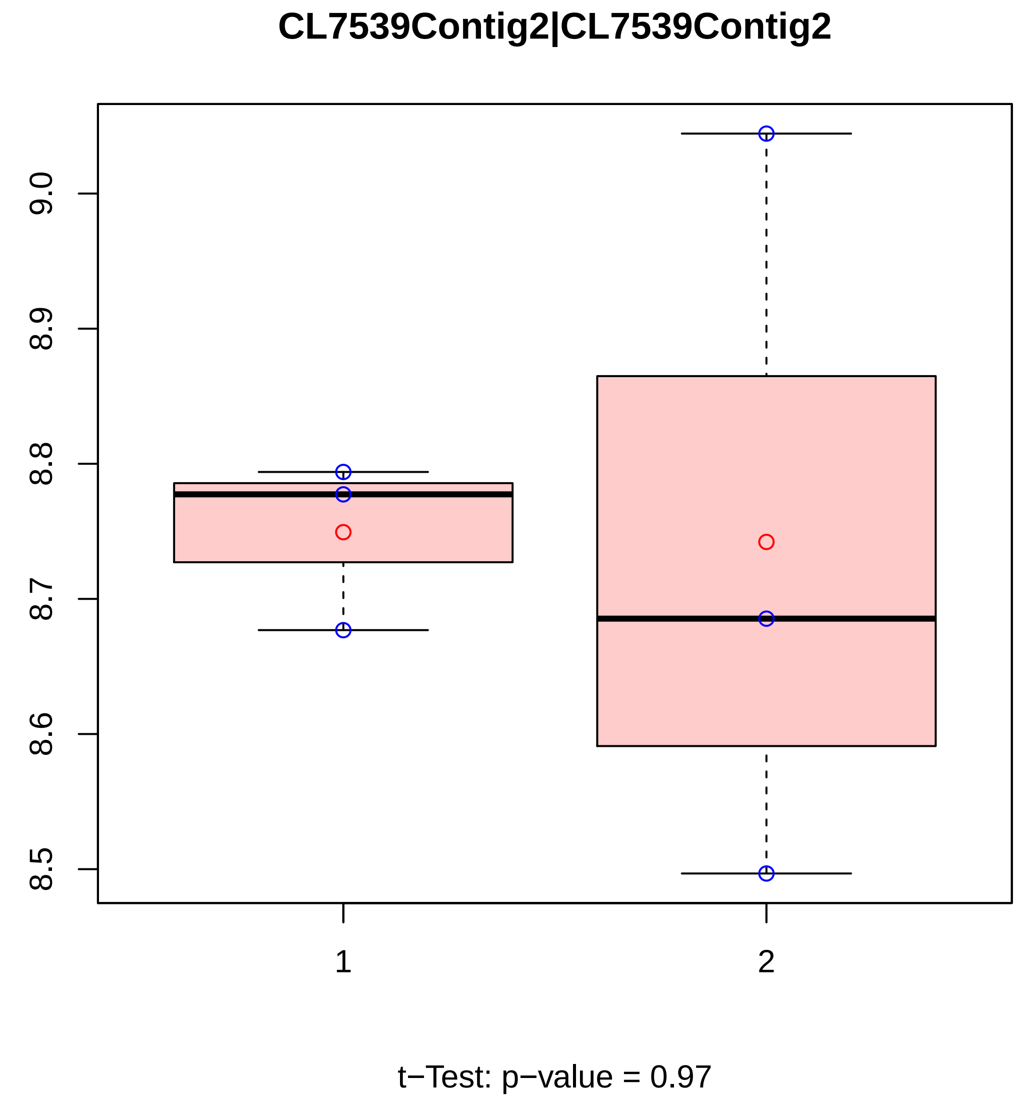

CL7539Contig2|CL7539Contig2
9.0
8.9
8.8
8.7
8.6
8.5
1
2
t−Test: p−value = 0.97

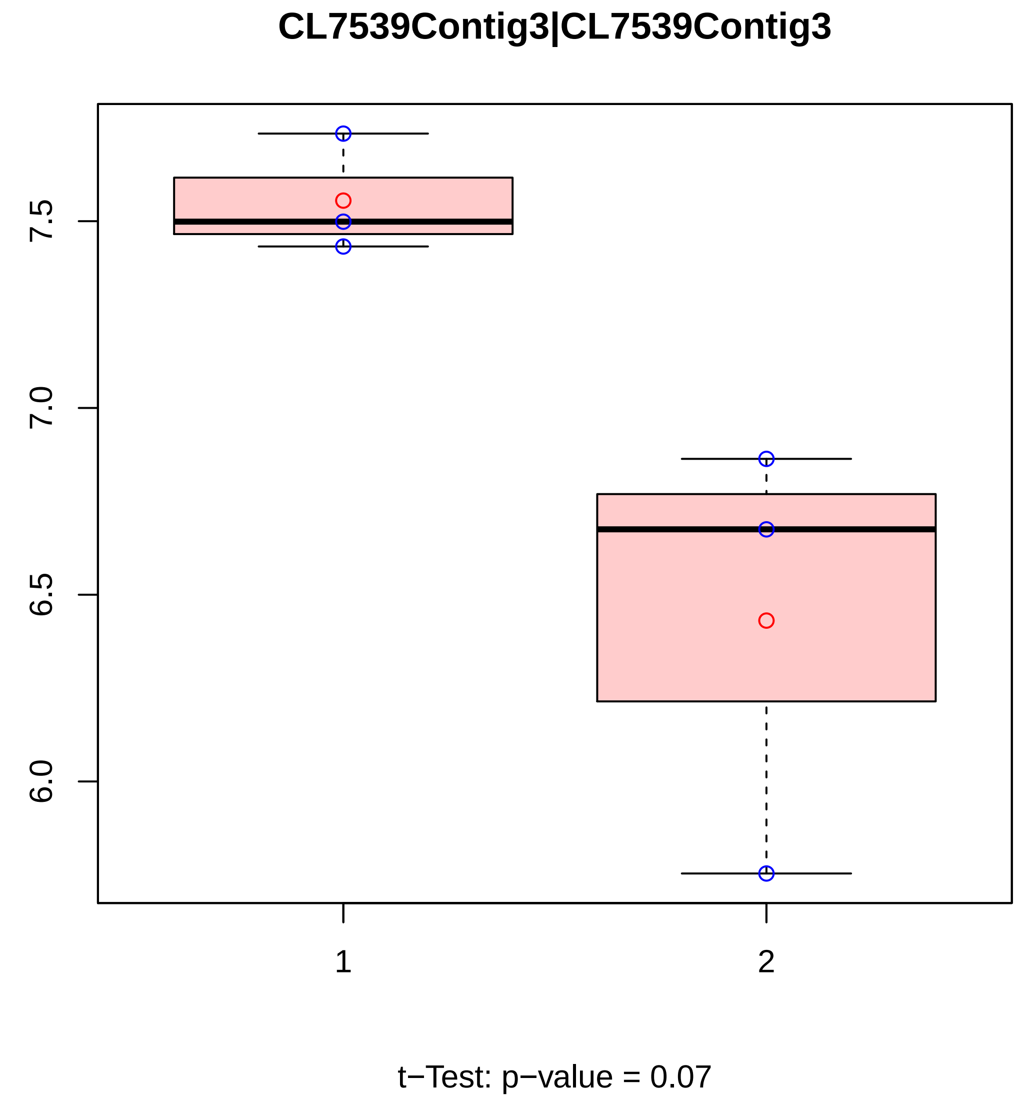
CL7539Contig3|CL7539Contig3
7.5
7.0
6.5
6.0
1
2
t−Test: p−value = 0.07

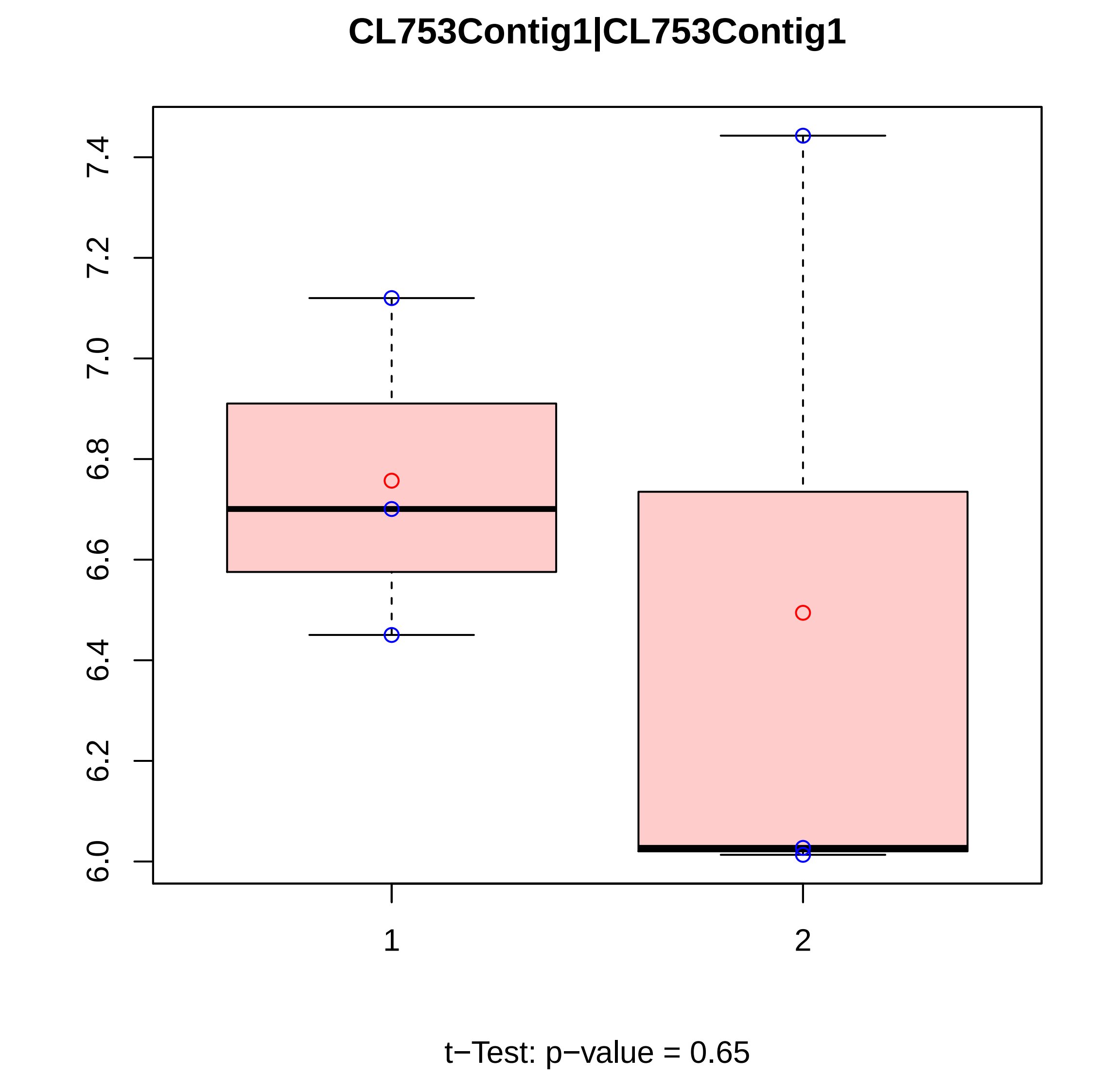

CL753Contig1|CL753Contig1
7.4
7.2
7.0
6.8
6.6
6.4
6.2
6.0
1
2
t−Test: p−value = 0.65

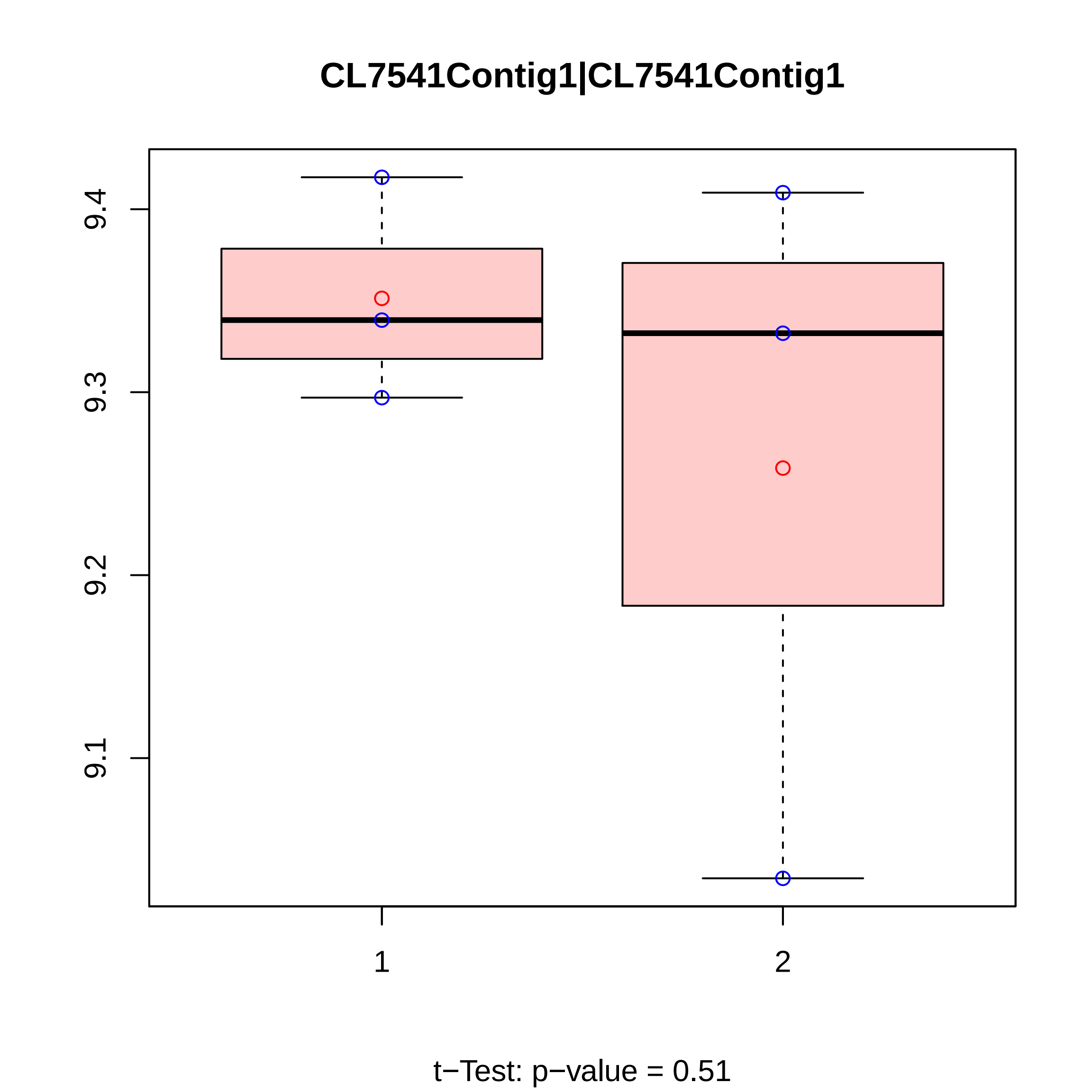
CL7541Contig1|CL7541Contig1
9.4
9.3
9.2
9.1
1
2
t−Test: p−value = 0.51

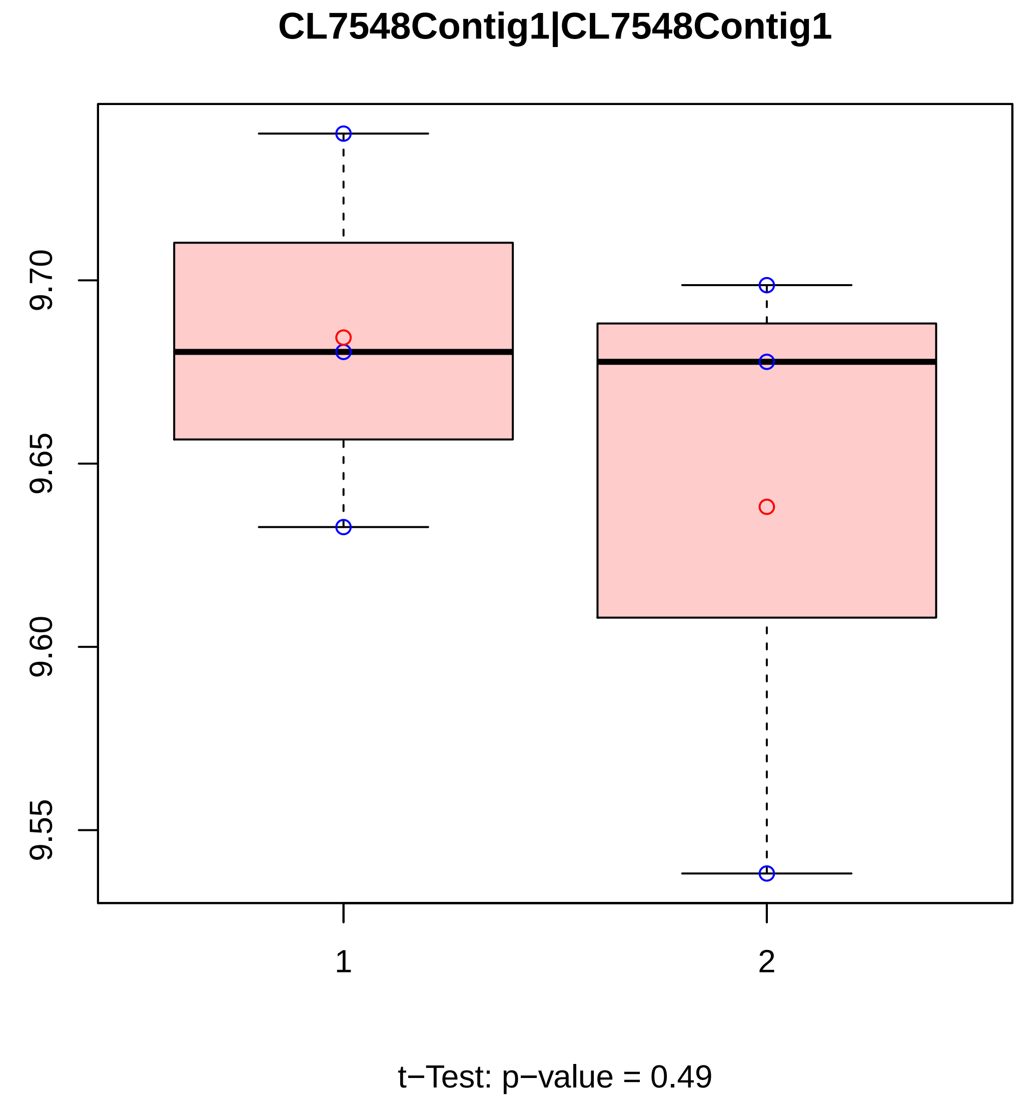
CL7548Contig1|CL7548Contig1
9.55
9.60
9.65
9.70
1
2
t−Test: p−value = 0.49

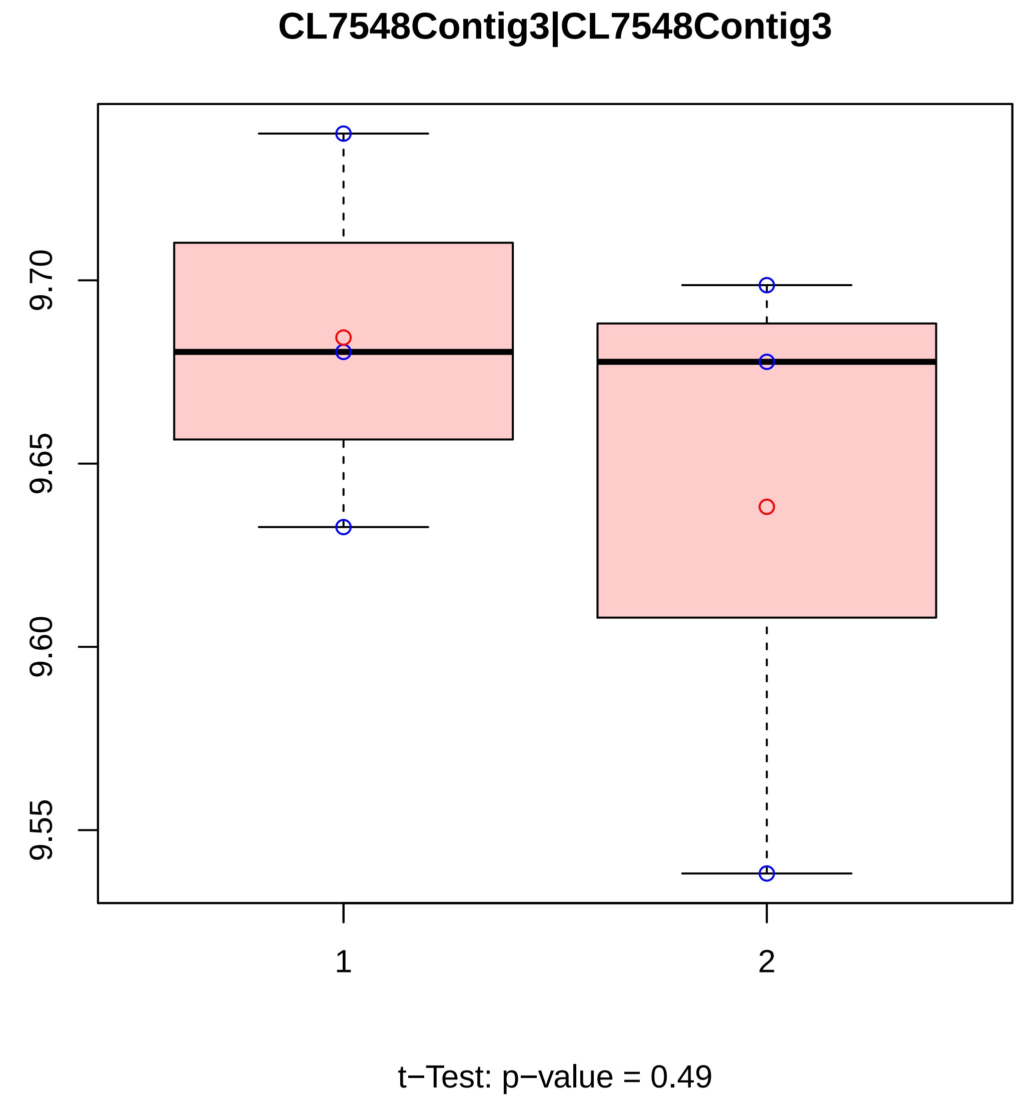
CL7548Contig3|CL7548Contig3
9.70
9.65
9.60
9.55
1
2
t−Test: p−value = 0.49

# CL7549Contig1|CL7549Contig1

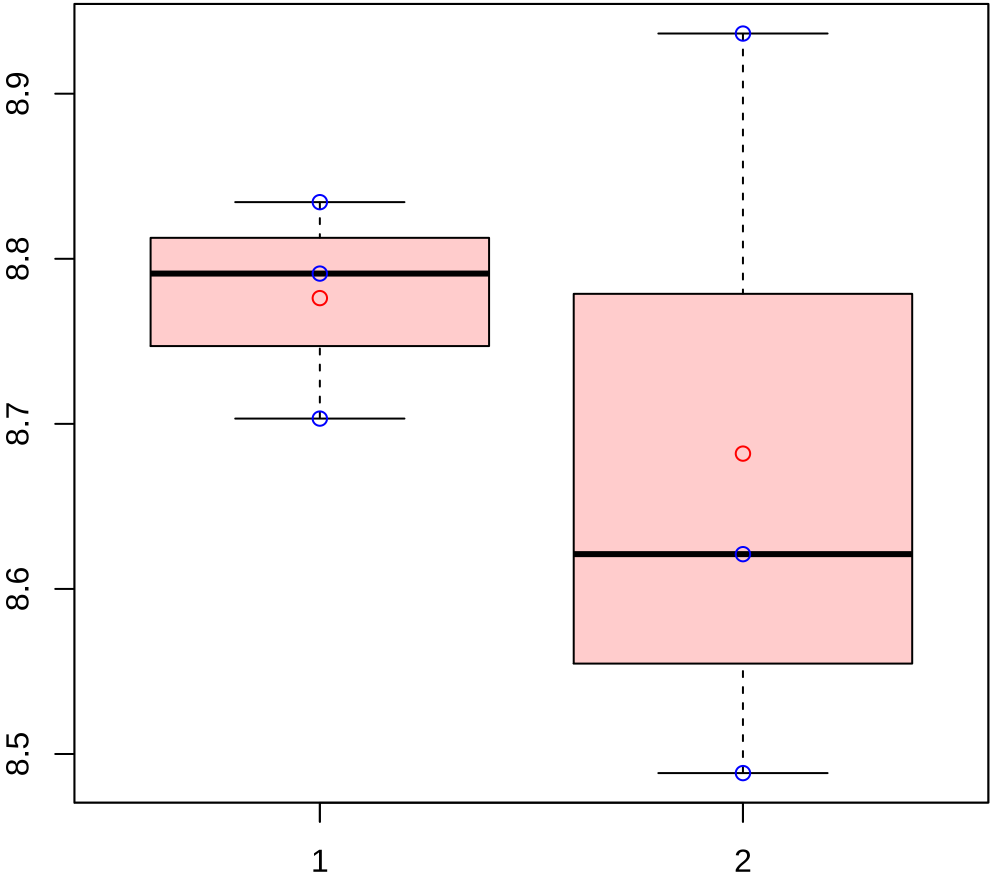


t−Test: p−value = 0.56

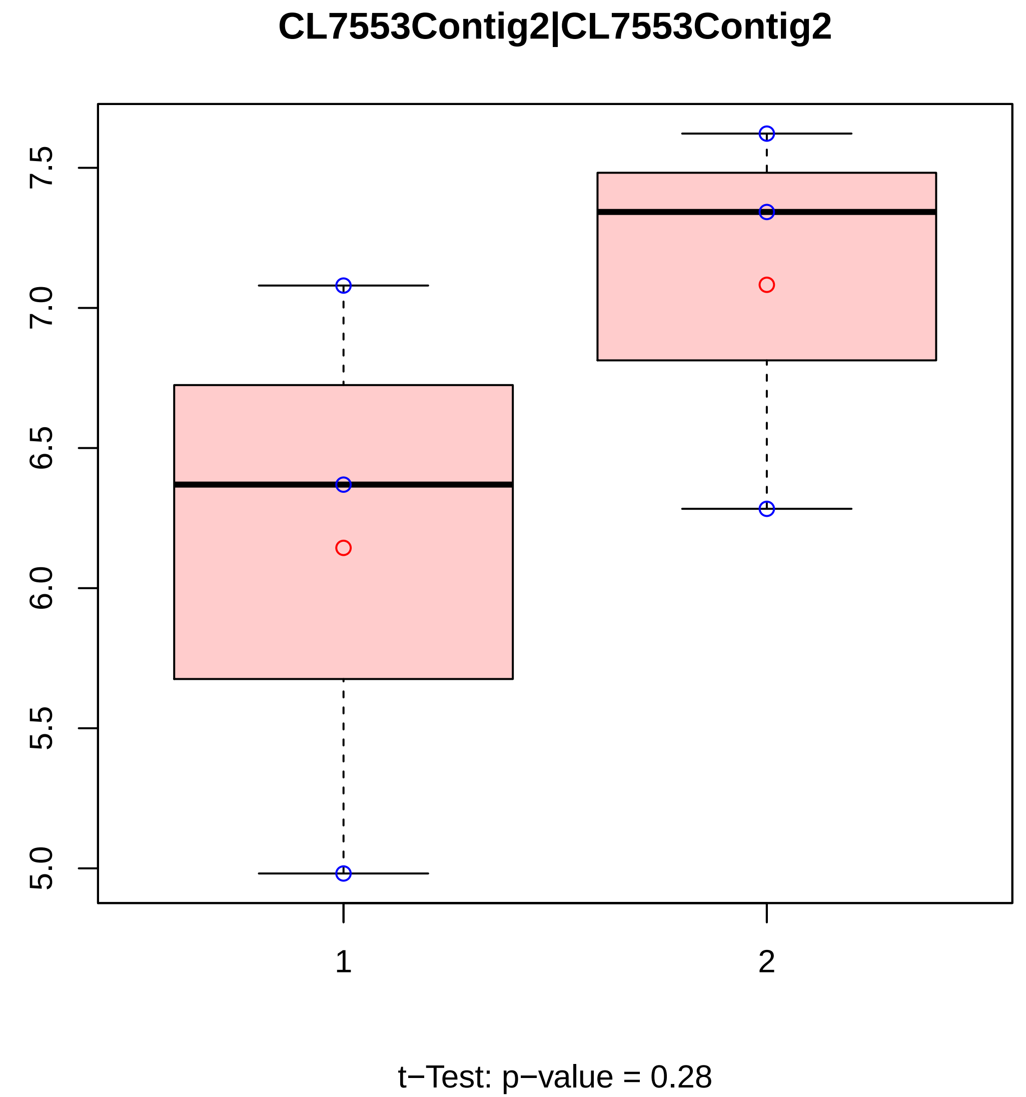
CL7553Contig2|CL7553Contig2
7.5
7.0
6.5
6.0
5.5
5.0
1
2
t−Test: p−value = 0.28

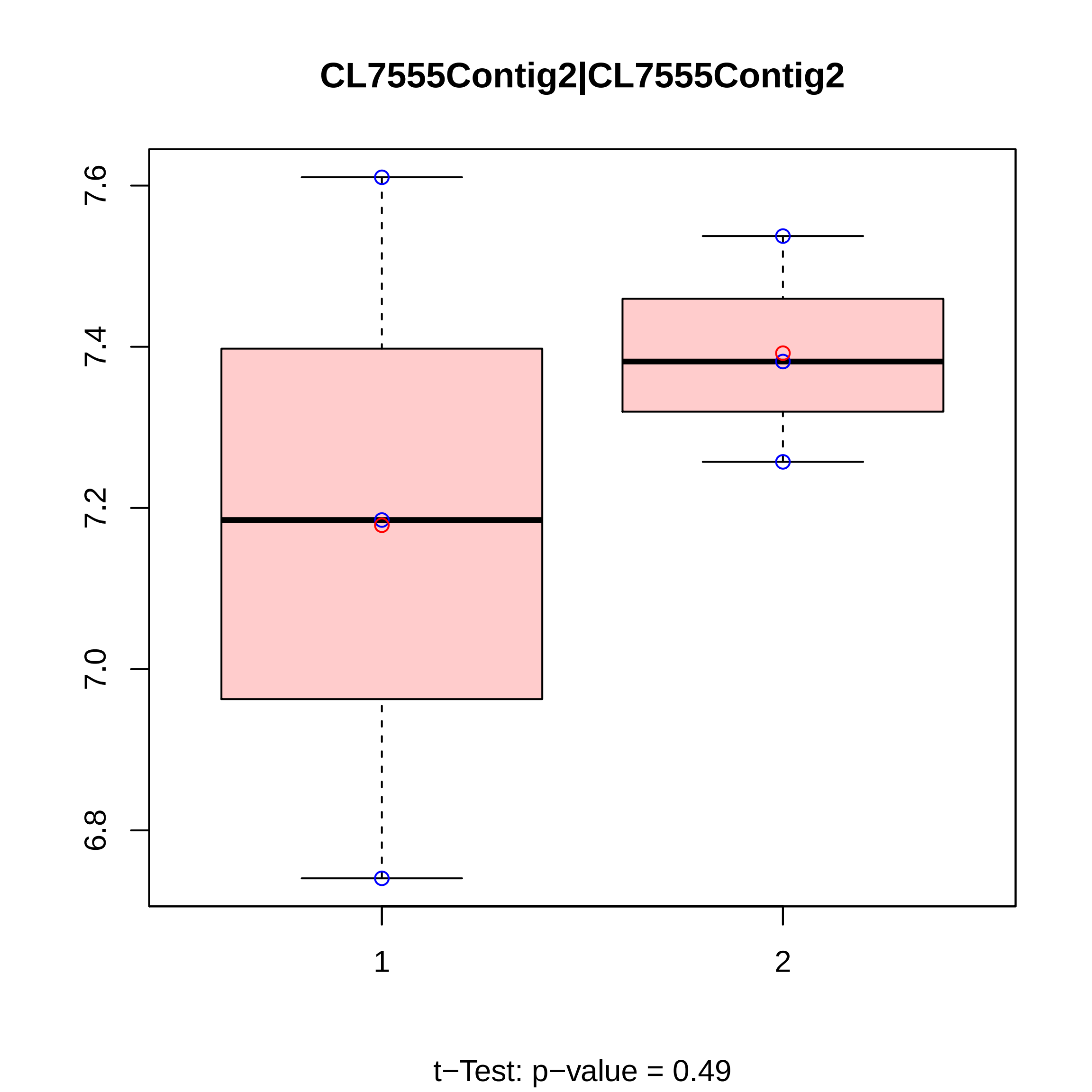

CL7555Contig2|CL7555Contig2
7.6
7.4
7.2
7.0
6.8
1
2
t−Test: p−value = 0.49

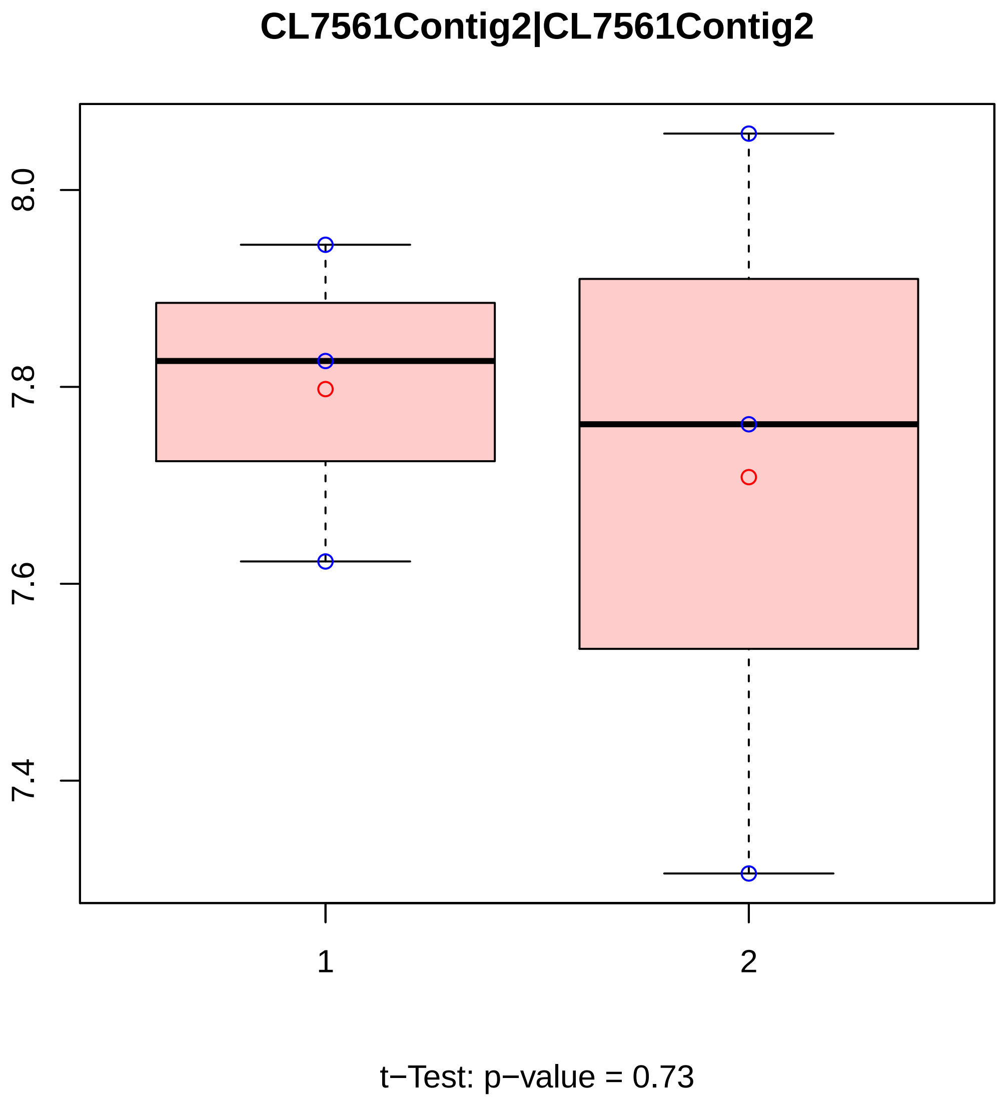
CL7561Contig2|CL7561Contig2
8.0
7.8
7.6
7.4
1
2
t−Test: p−value = 0.73

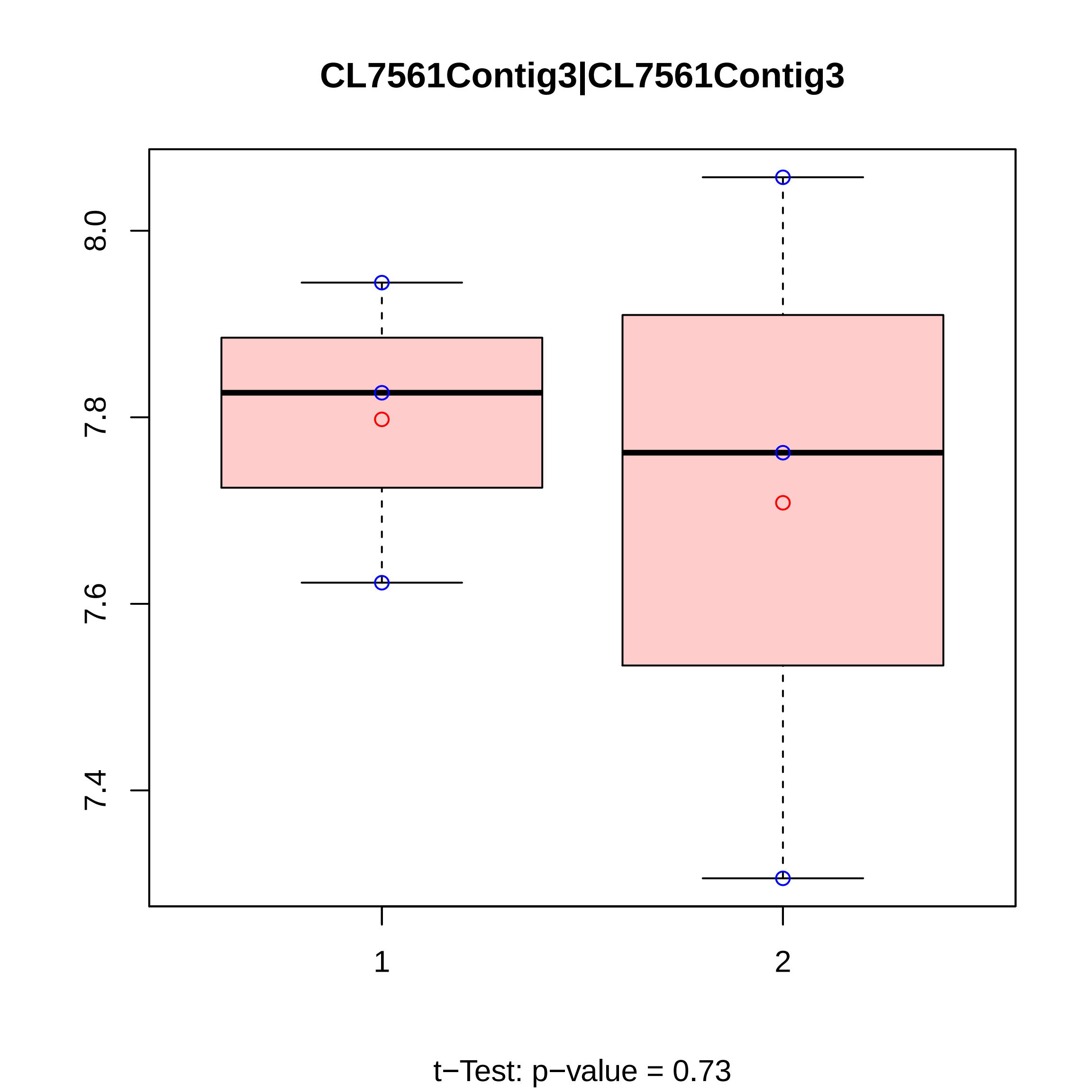

CL7561Contig3|CL7561Contig3
8.0
7.8
7.6
7.4
1
2
t−Test: p−value = 0.73

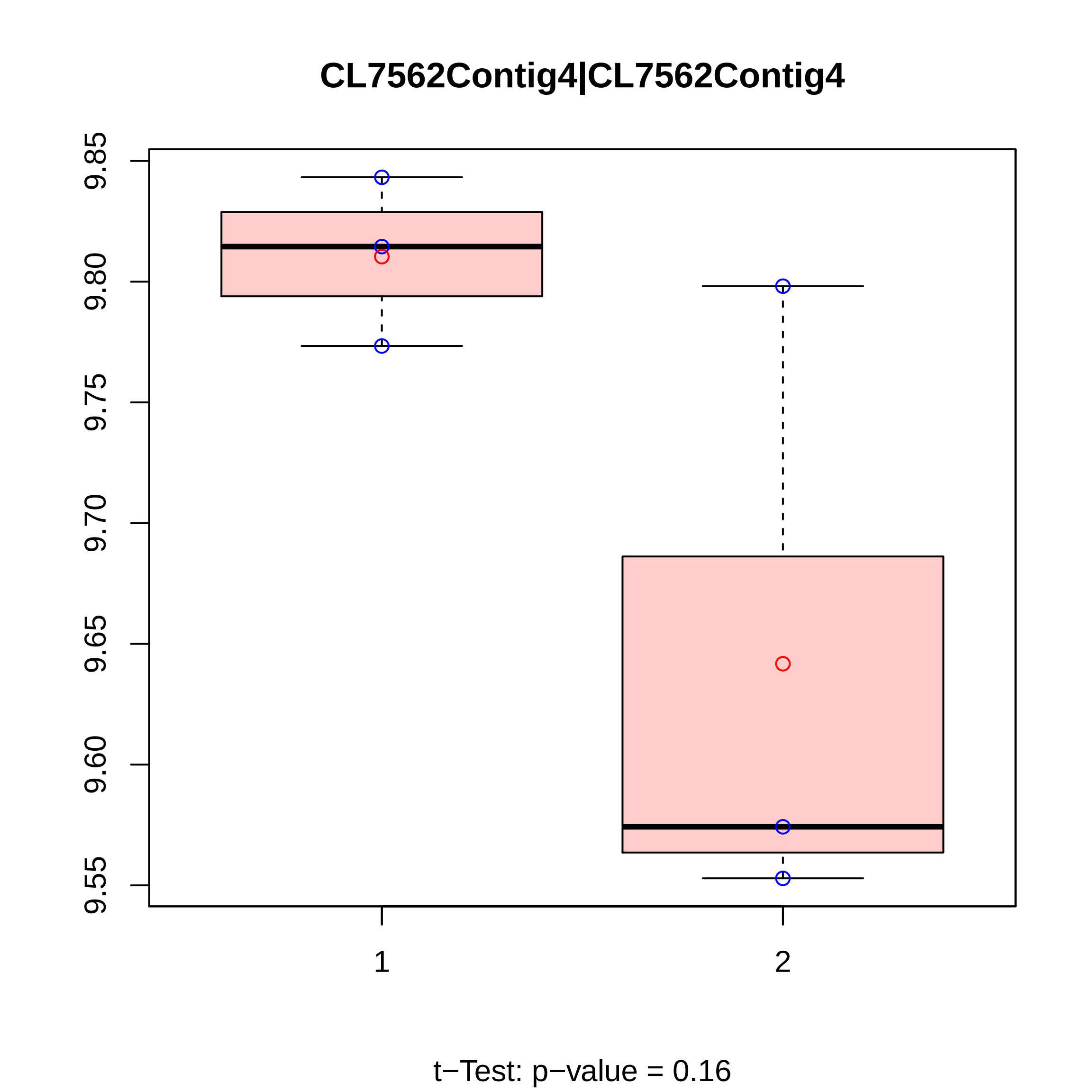

CL7562Contig4|CL7562Contig4
9.55
9.60
9.65
9.70
9.75
9.80
9.85
1
2
t−Test: p−value = 0.16

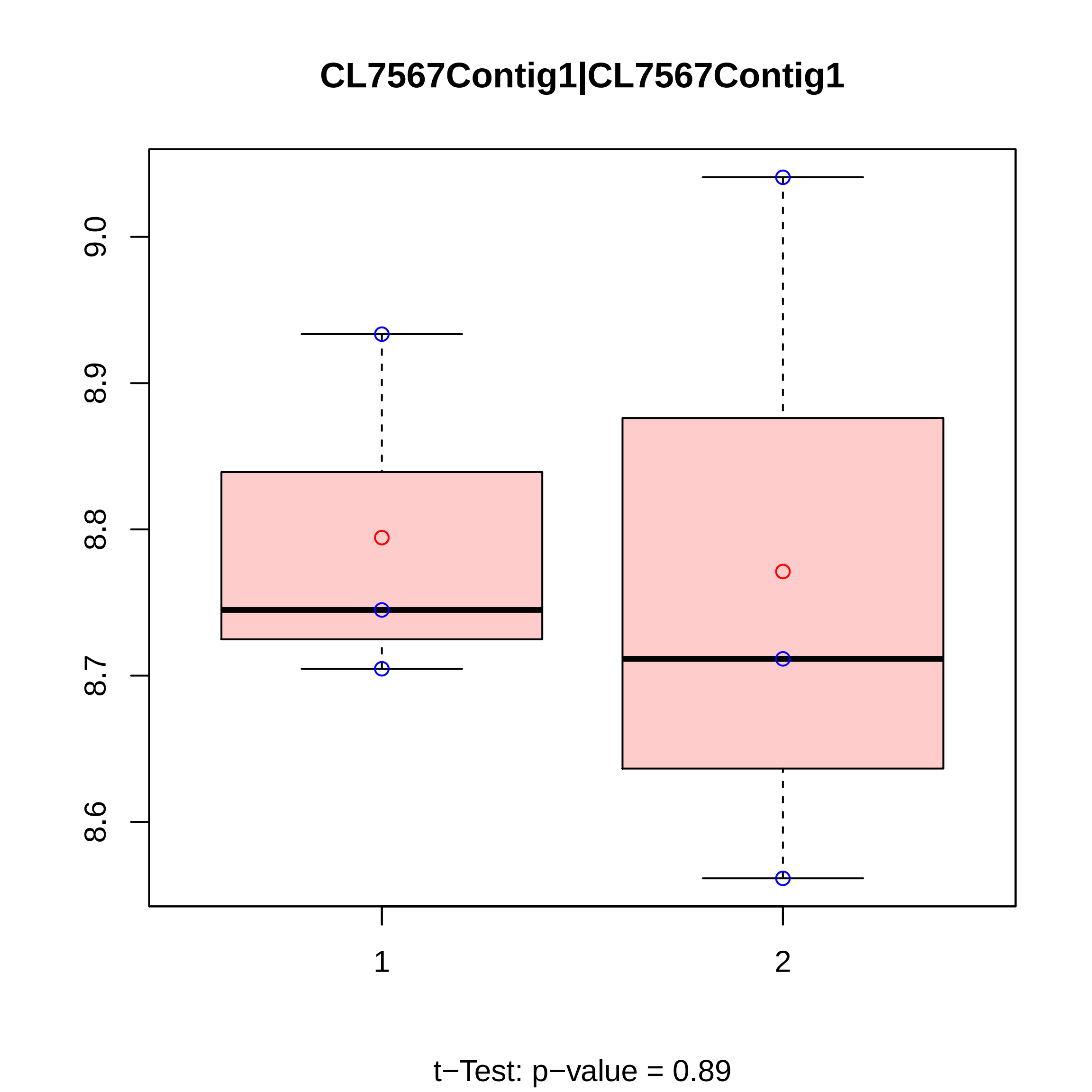

CL7567Contig1|CL7567Contig1
9.0
8.9
8.8
8.7
8.6
1
2
t−Test: p−value = 0.89

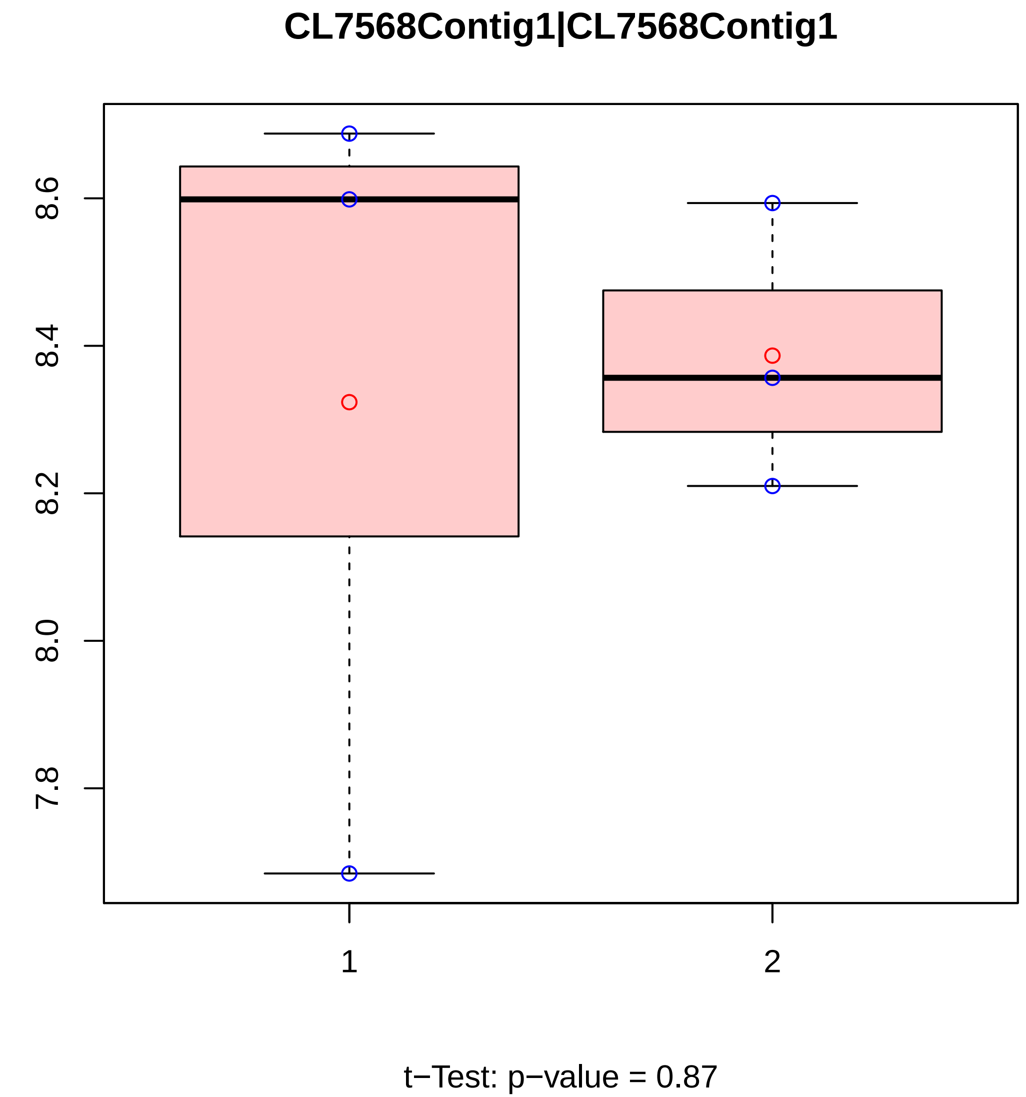
CL7568Contig1|CL7568Contig1
8.6
8.4
8.2
8.0
7.8
1
2
t−Test: p−value = 0.87

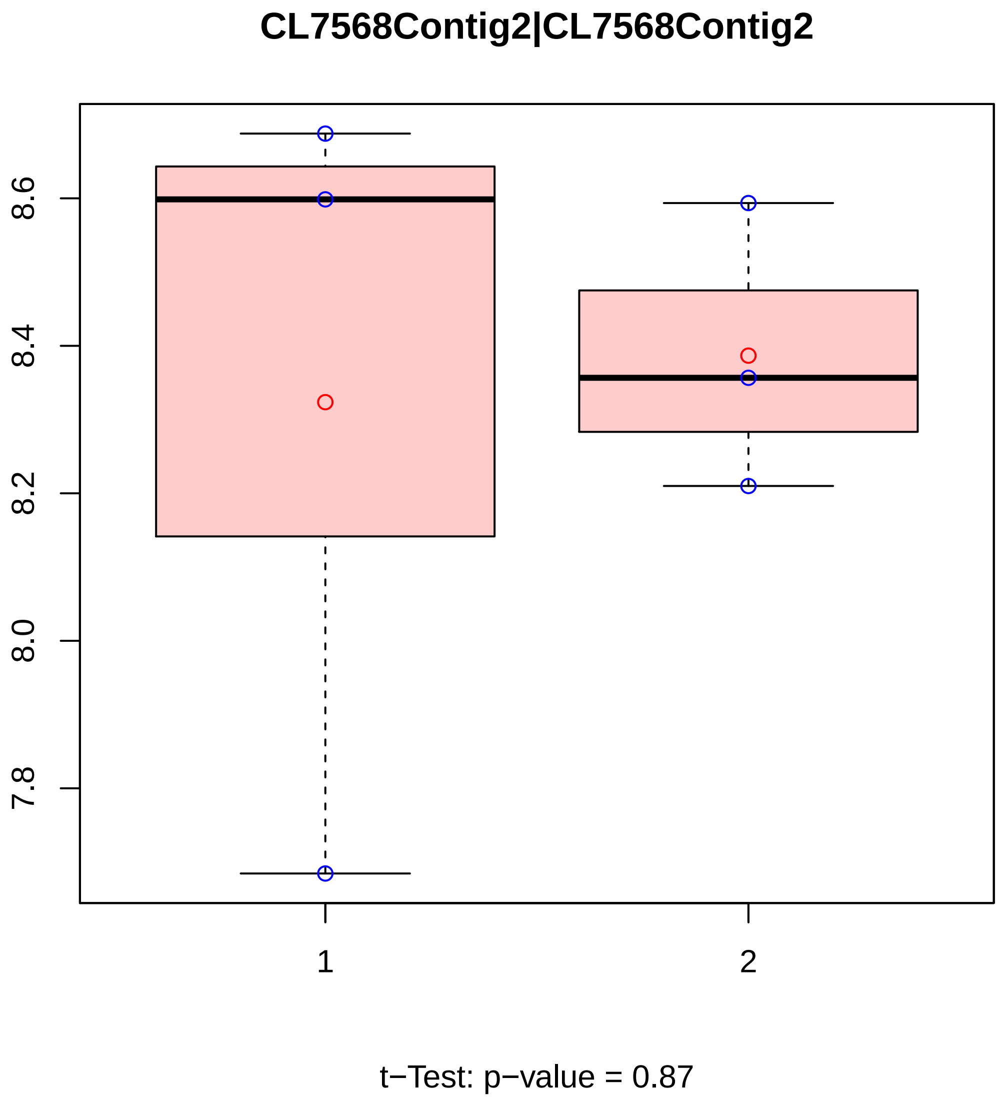
CL7568Contig2|CL7568Contig2
8.6
8.4
8.2
8.0
7.8
1
2
t−Test: p−value = 0.87

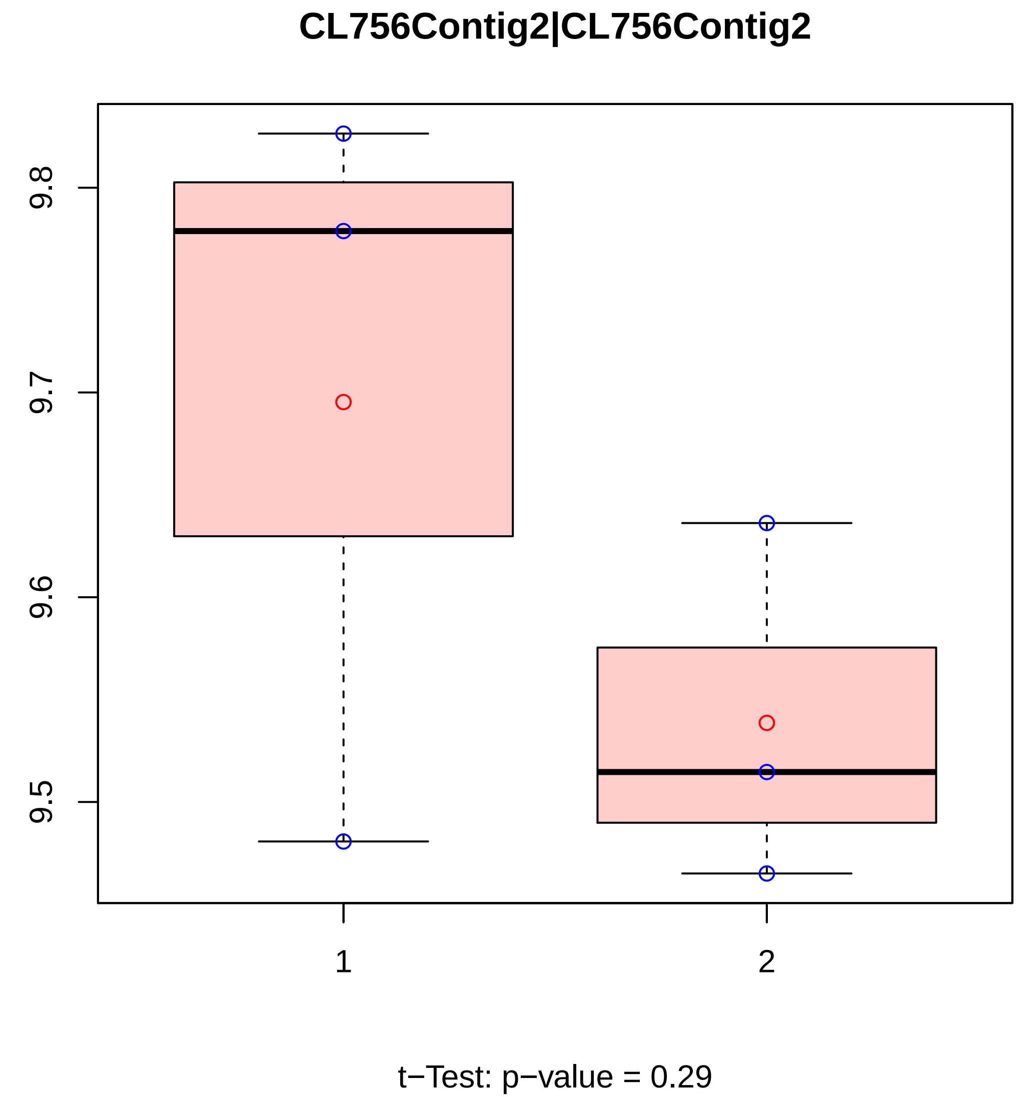
CL756Contig2|CL756Contig2
9.5
9.6
9.7
9.8
1
2
t−Test: p−value = 0.29

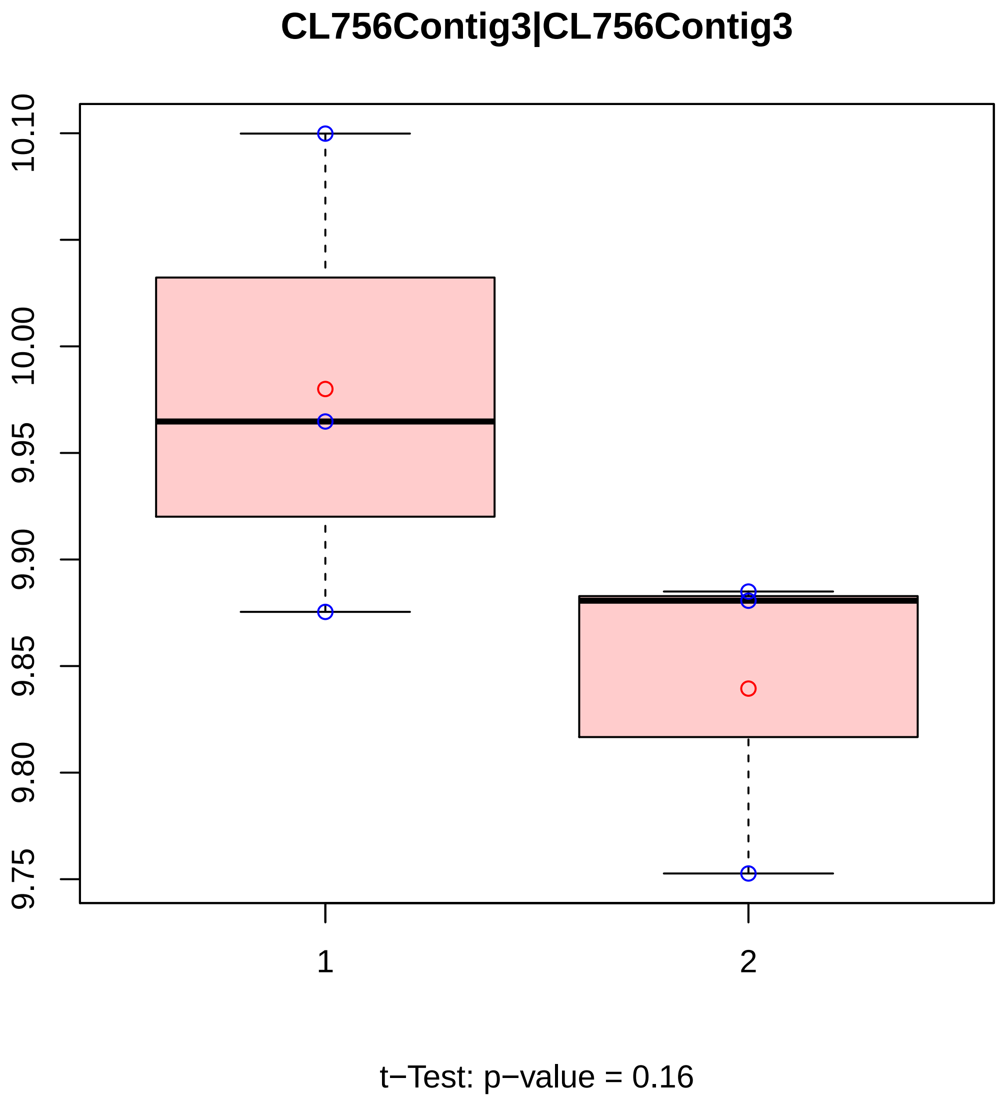
CL756Contig3|CL756Contig3
10.10
10.00
9.95
9.90
9.85
9.80
9.75
1
2
t−Test: p−value = 0.16

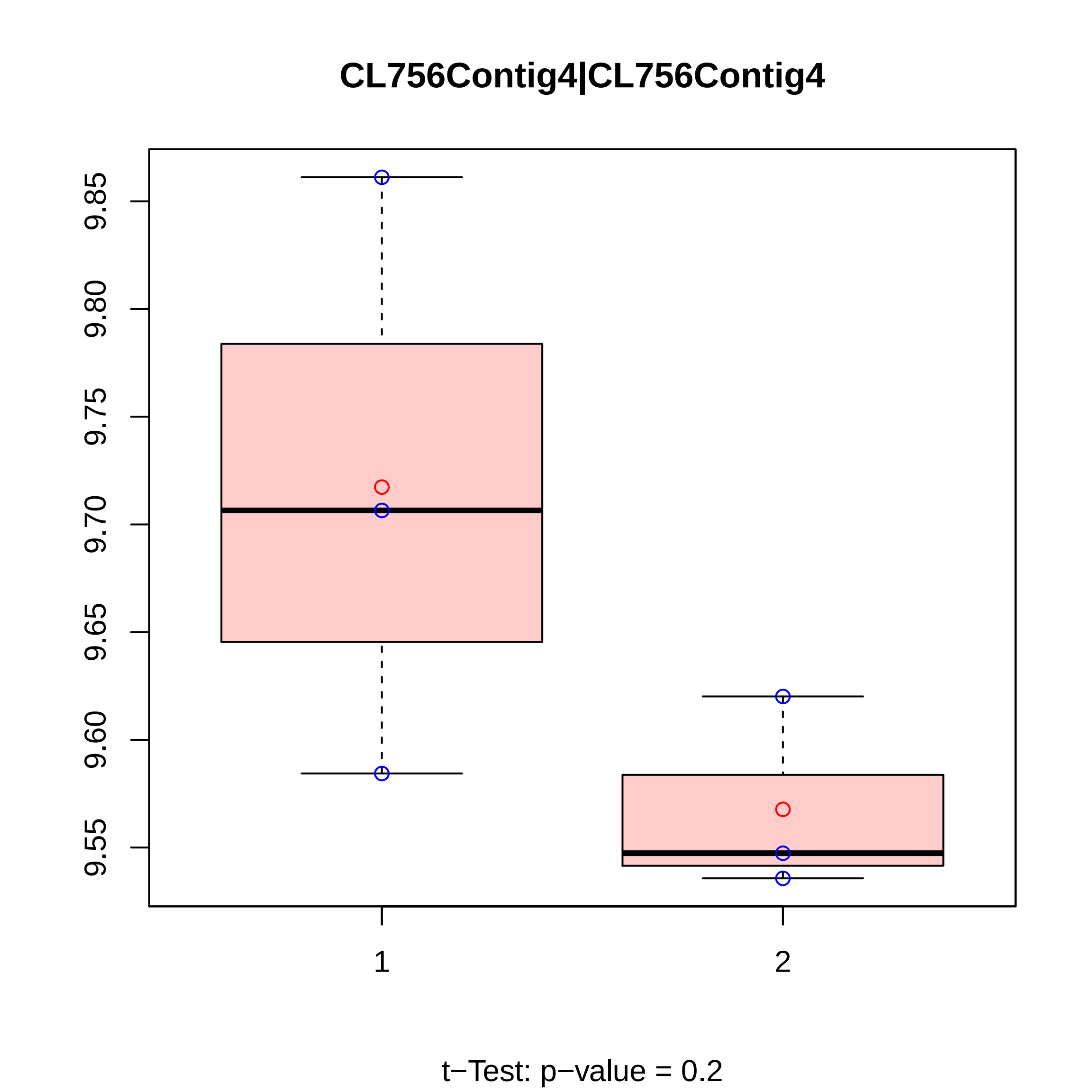
CL756Contig4|CL756Contig4
9.85
9.80
9.75
9.70
9.65
9.60
9.55
1
2
t−Test: p−value = 0.2

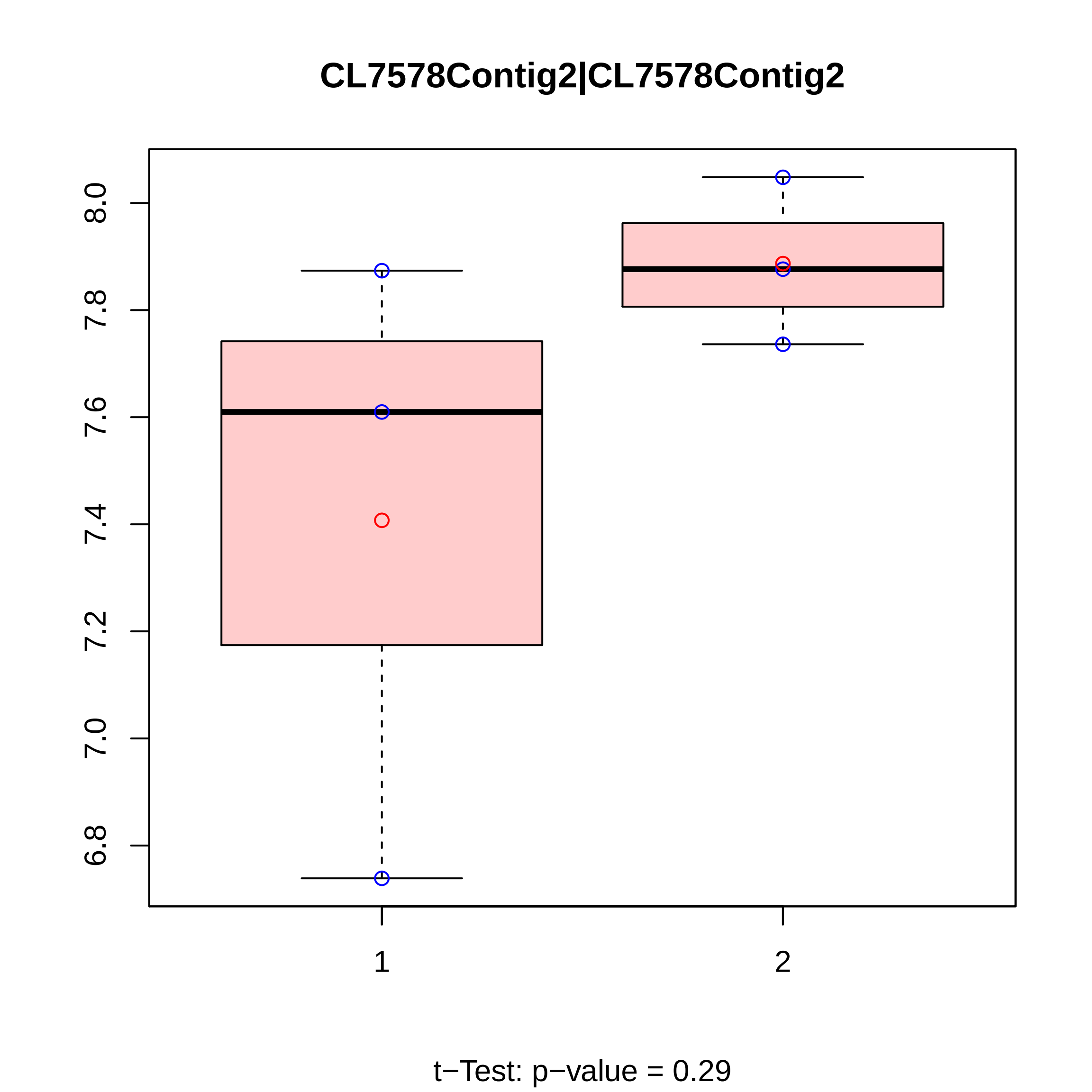
CL7578Contig2|CL7578Contig2
8.0
7.8
7.6
7.4
7.2
7.0
6.8
1
2
t−Test: p−value = 0.29

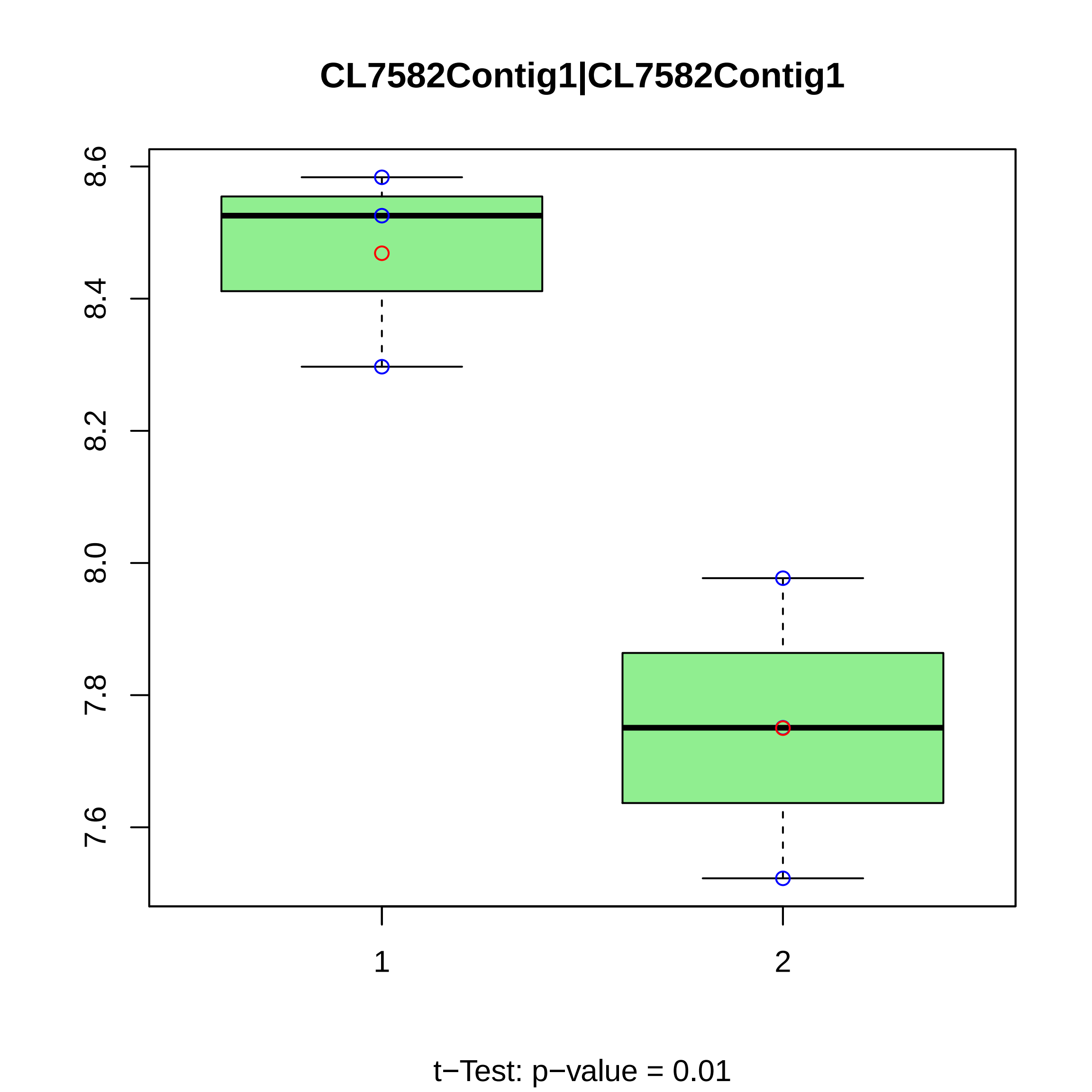

CL7582Contig1|CL7582Contig1
8.6
8.4
8.2
8.0
7.8
7.6
1
2
t−Test: p−value = 0.01

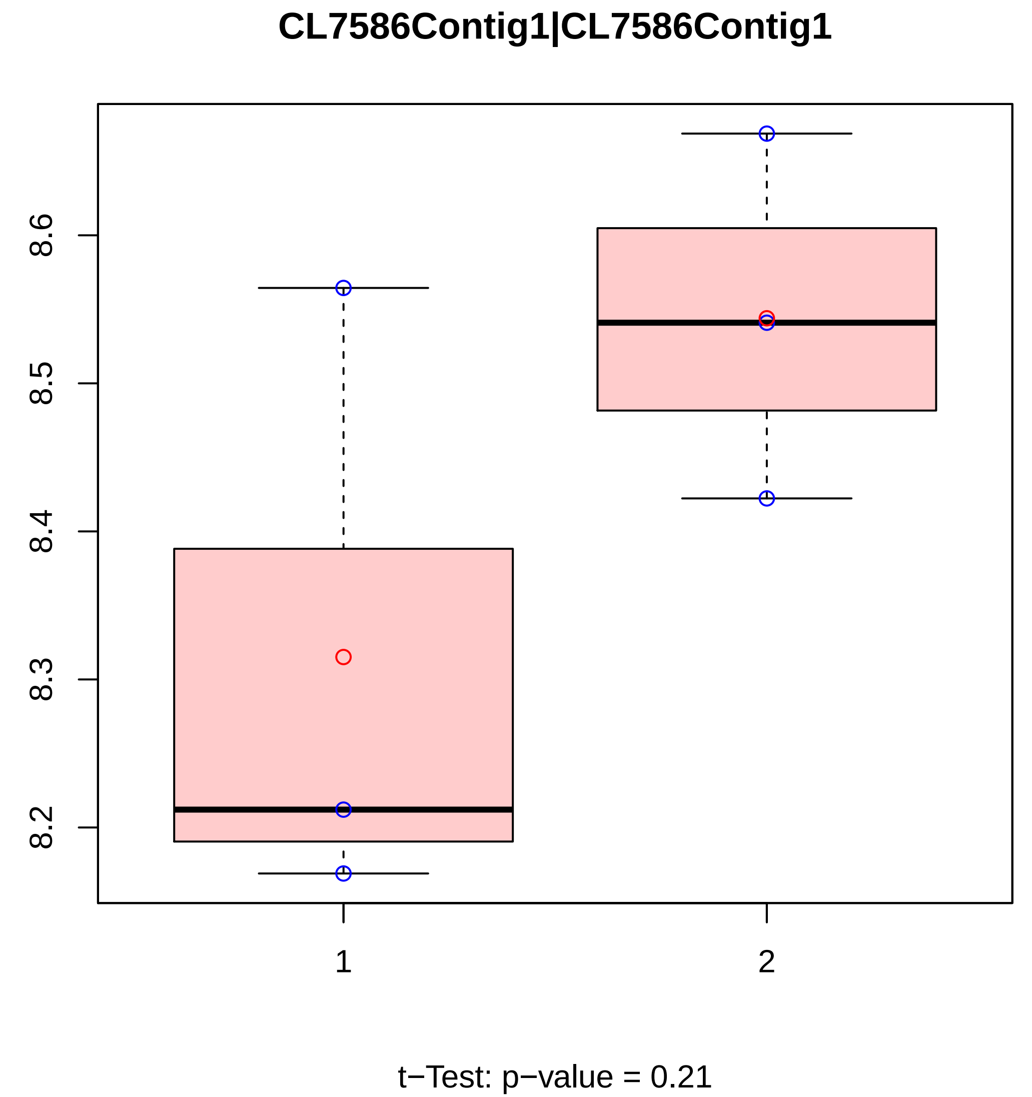
CL7586Contig1|CL7586Contig1
8.2
8.3
8.4
8.5
8.6
1
2
t−Test: p−value = 0.21

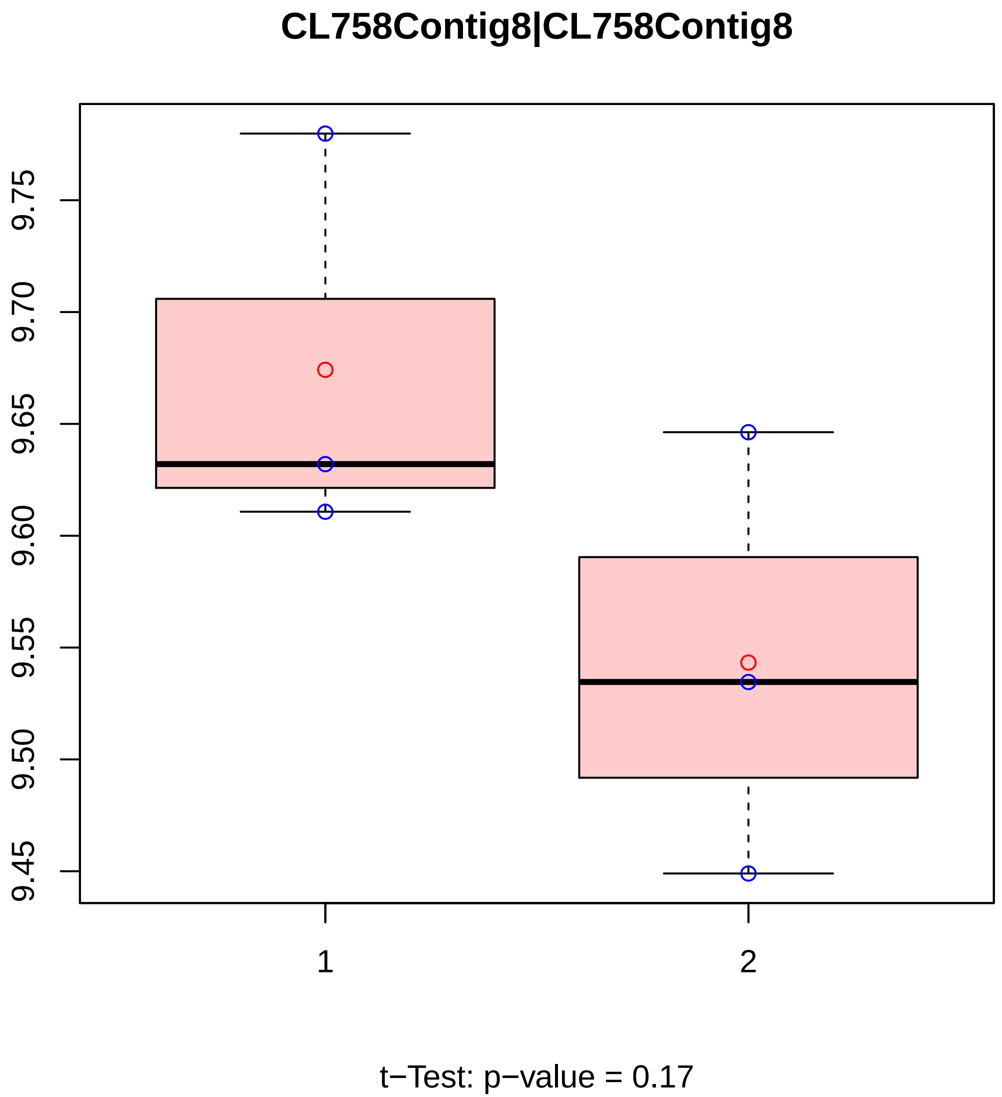

CL758Contig8|CL758Contig8
9.45
9.50
9.55
9.60
9.65
9.70
9.75
1
2
t−Test: p−value = 0.17

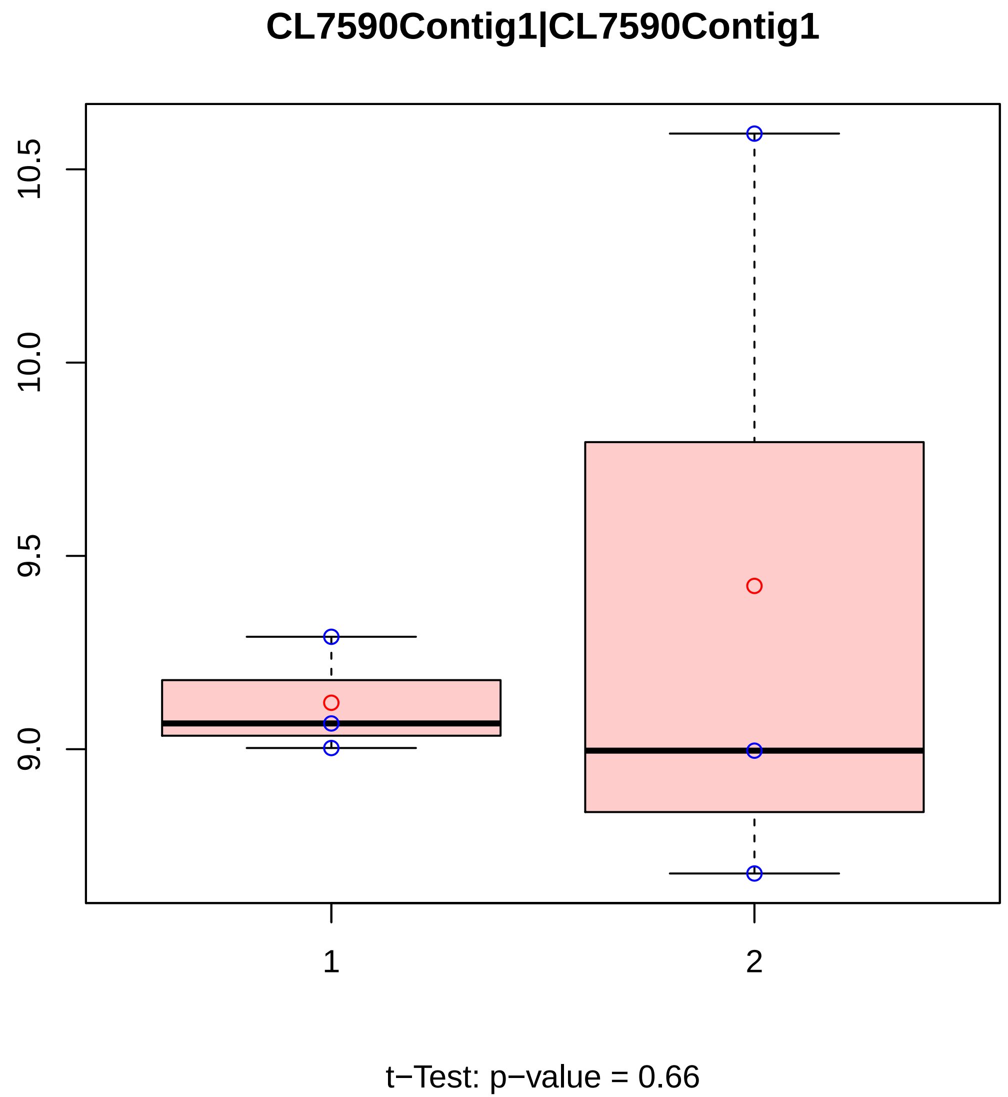

CL7590Contig1|CL7590Contig1
10.5
10.0
9.5
9.0
1
2
t−Test: p−value = 0.66

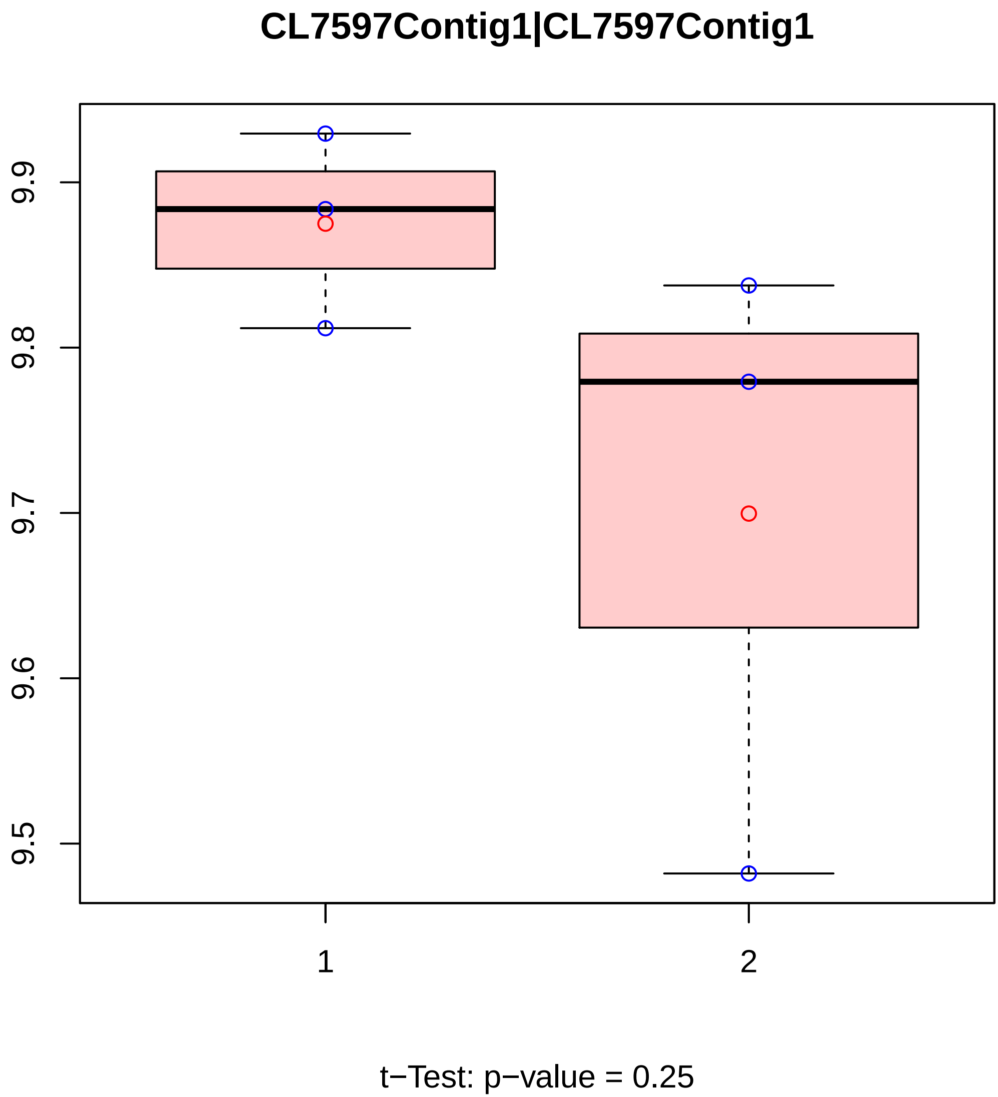
CL7597Contig1|CL7597Contig1
9.9
9.8
9.7
9.6
9.5
1
2
t−Test: p−value = 0.25

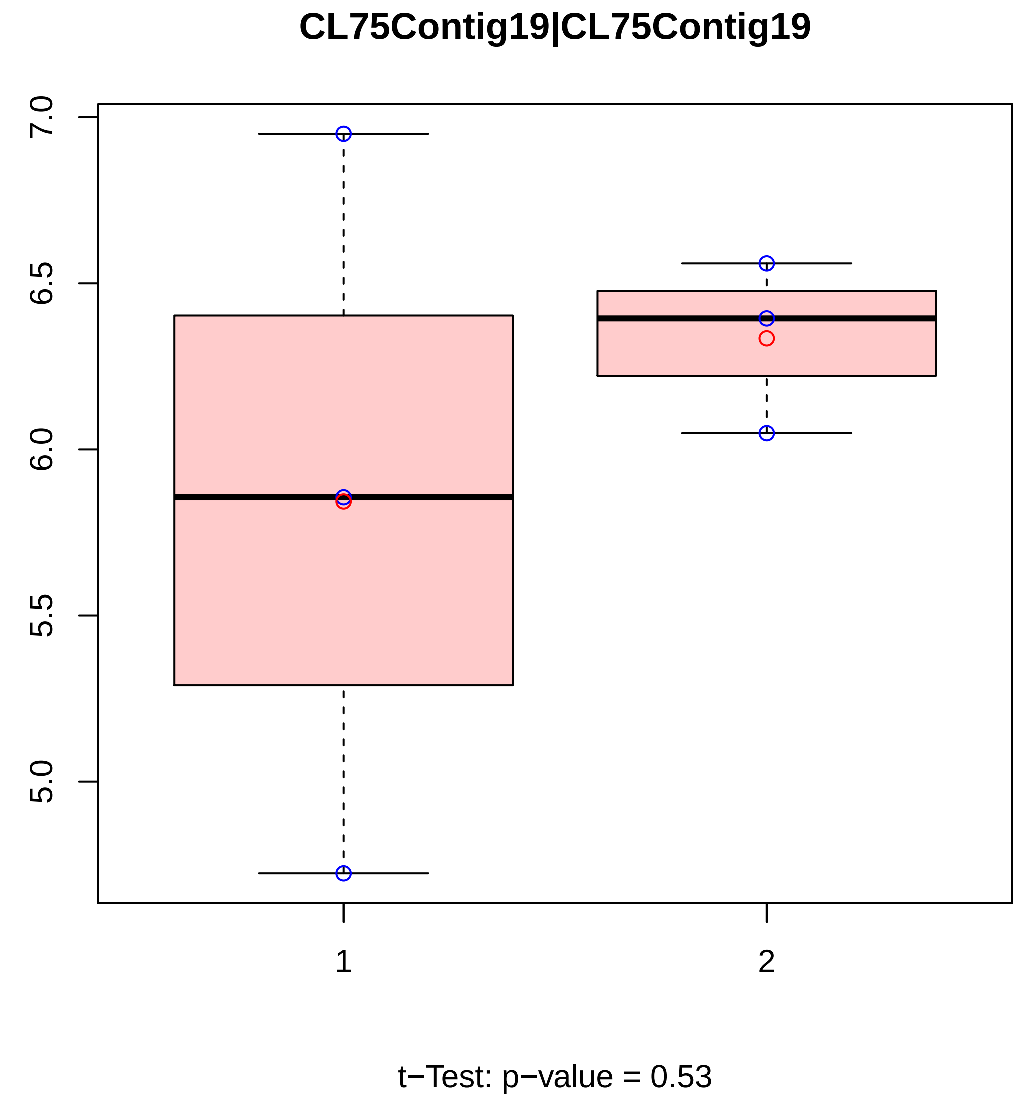
CL75Contig19|CL75Contig19
7.0
6.5
6.0
5.5
5.0
1
2
t−Test: p−value = 0.53

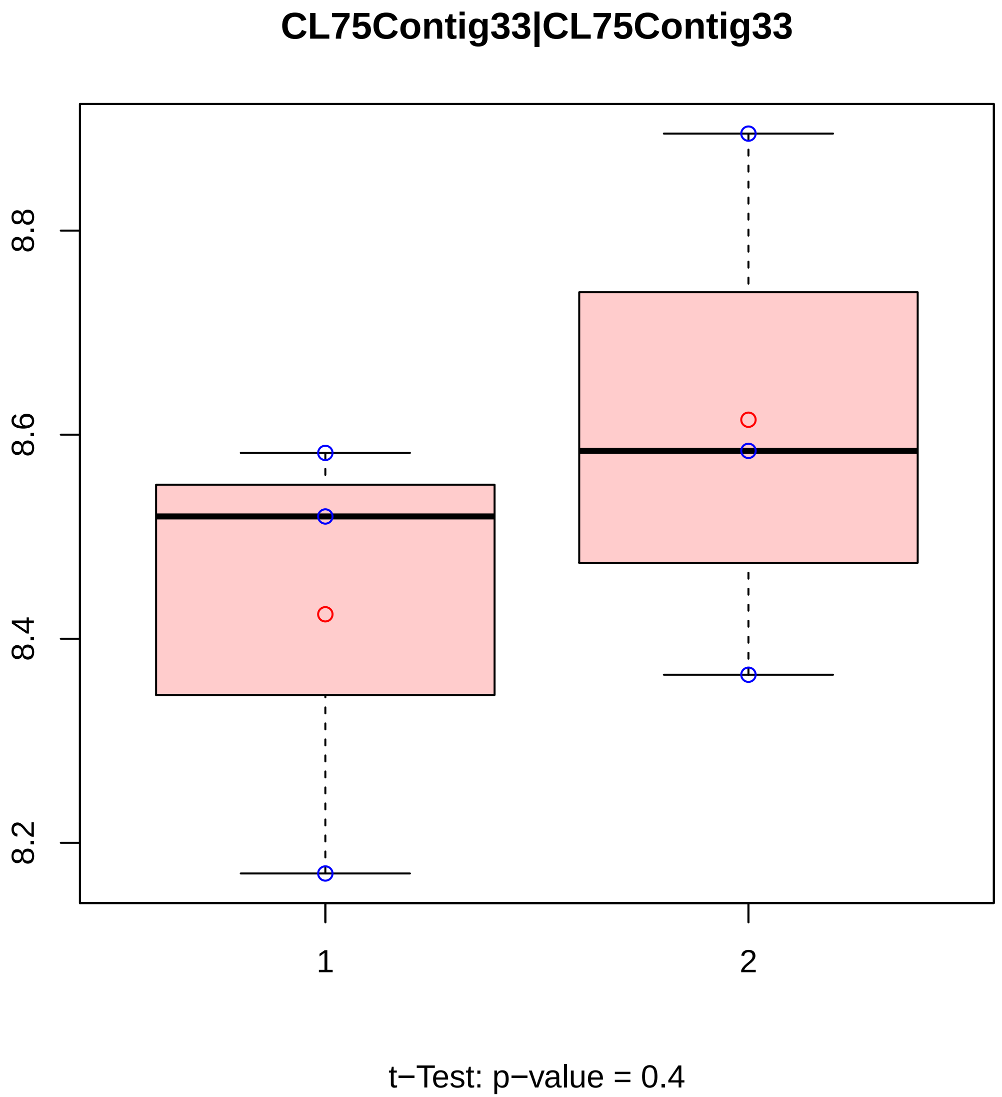

CL75Contig33|CL75Contig33
8.2
8.4
8.6
8.8
1
2
t−Test: p−value = 0.4

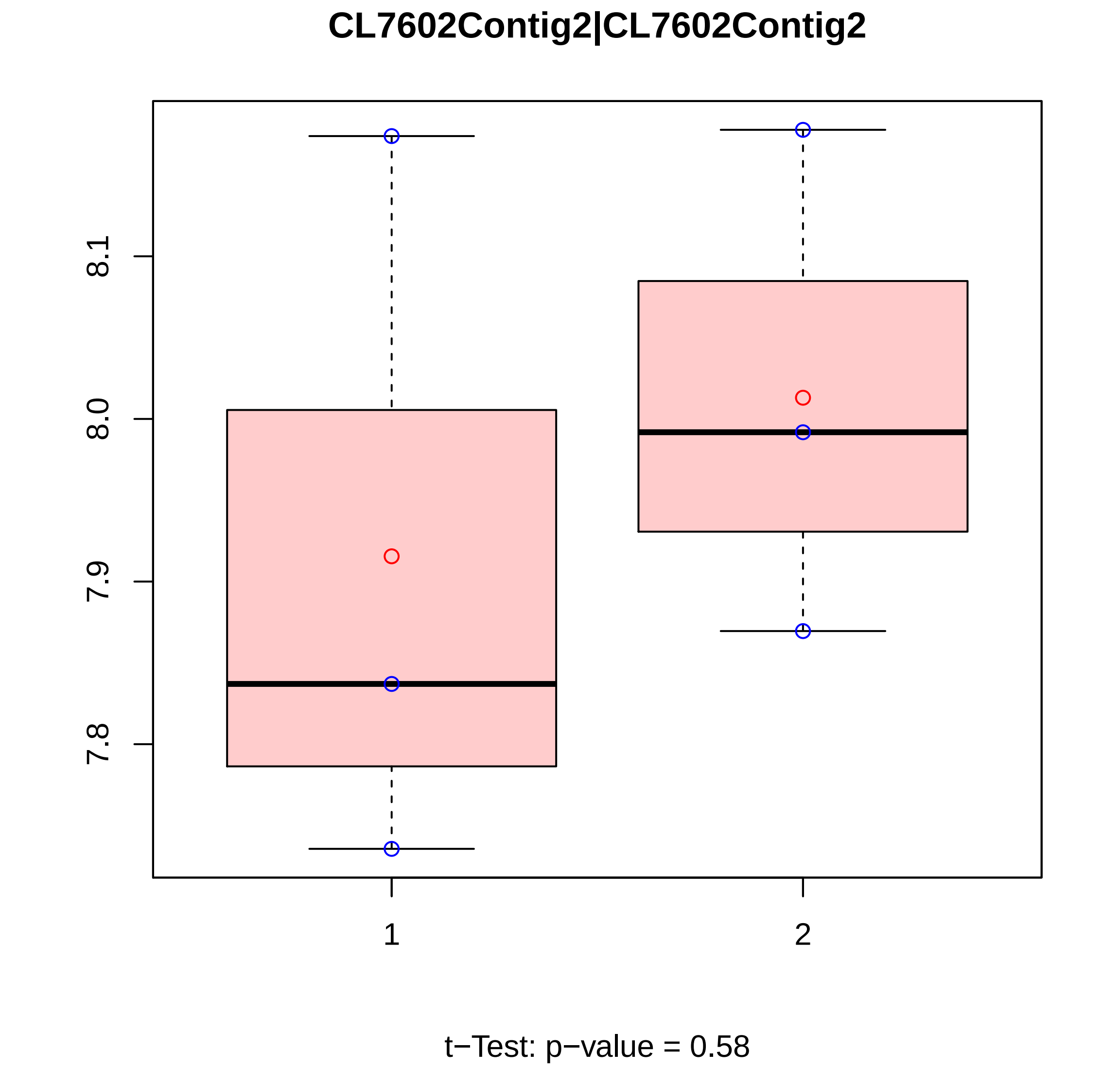
CL7602Contig2|CL7602Contig2
8.1
8.0
7.9
7.8
1
2
t−Test: p−value = 0.58

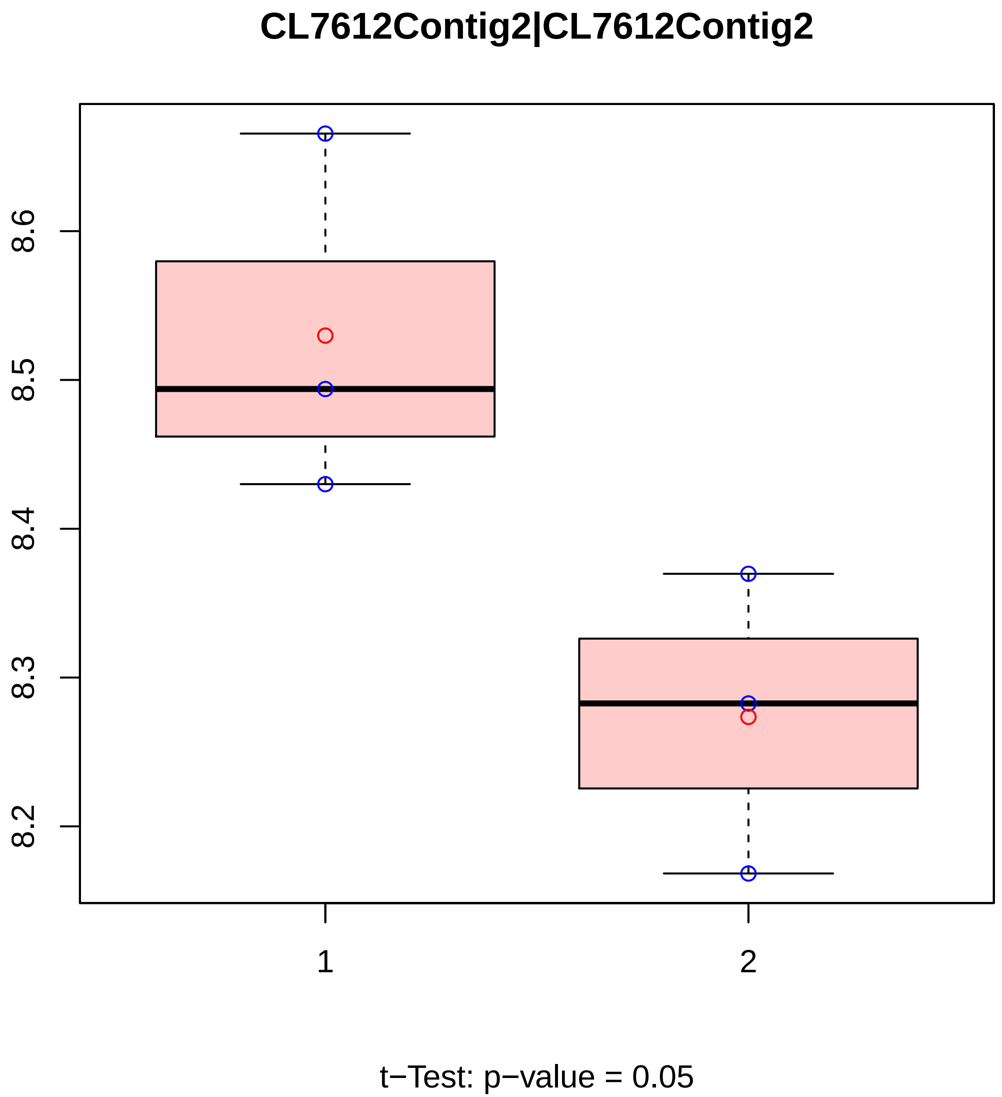
CL7612Contig2|CL7612Contig2
8.2
8.3
8.4
8.5
8.6
1
2
t−Test: p−value = 0.05

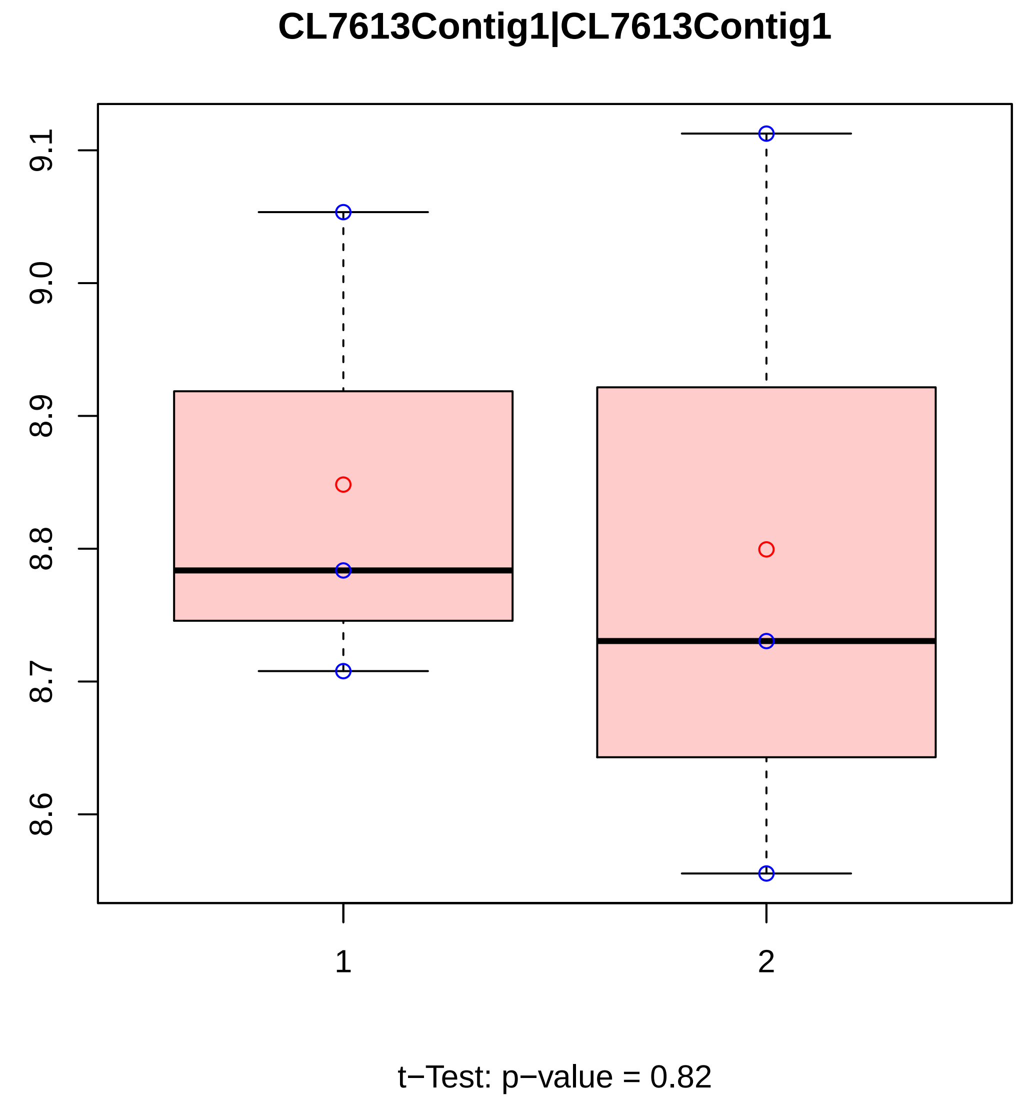

CL7613Contig1|CL7613Contig1
9.1
9.0
8.9
8.8
8.7
8.6
1
2
t−Test: p−value = 0.82

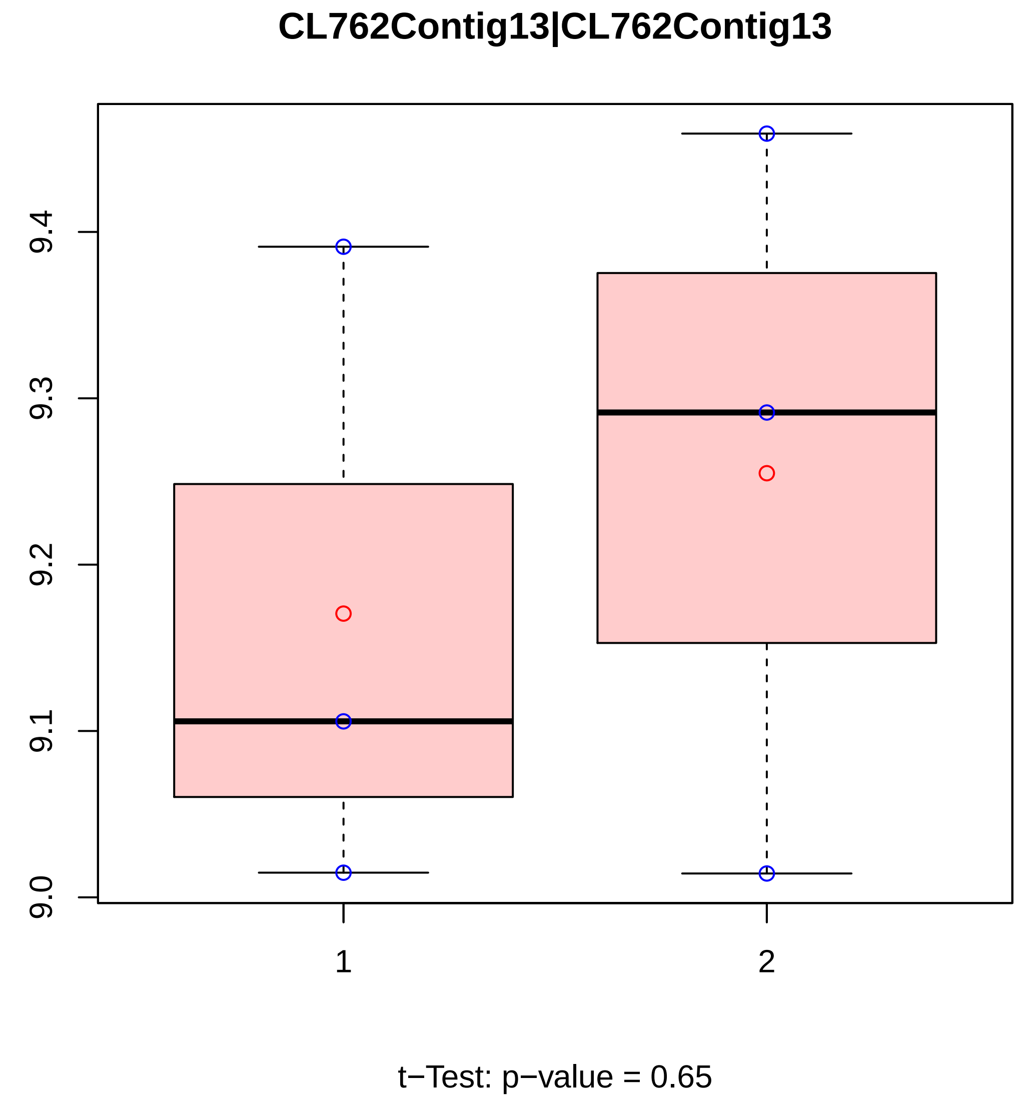

CL762Contig13|CL762Contig13
9.0
9.1
9.2
9.3
9.4
1
2
t−Test: p−value = 0.65

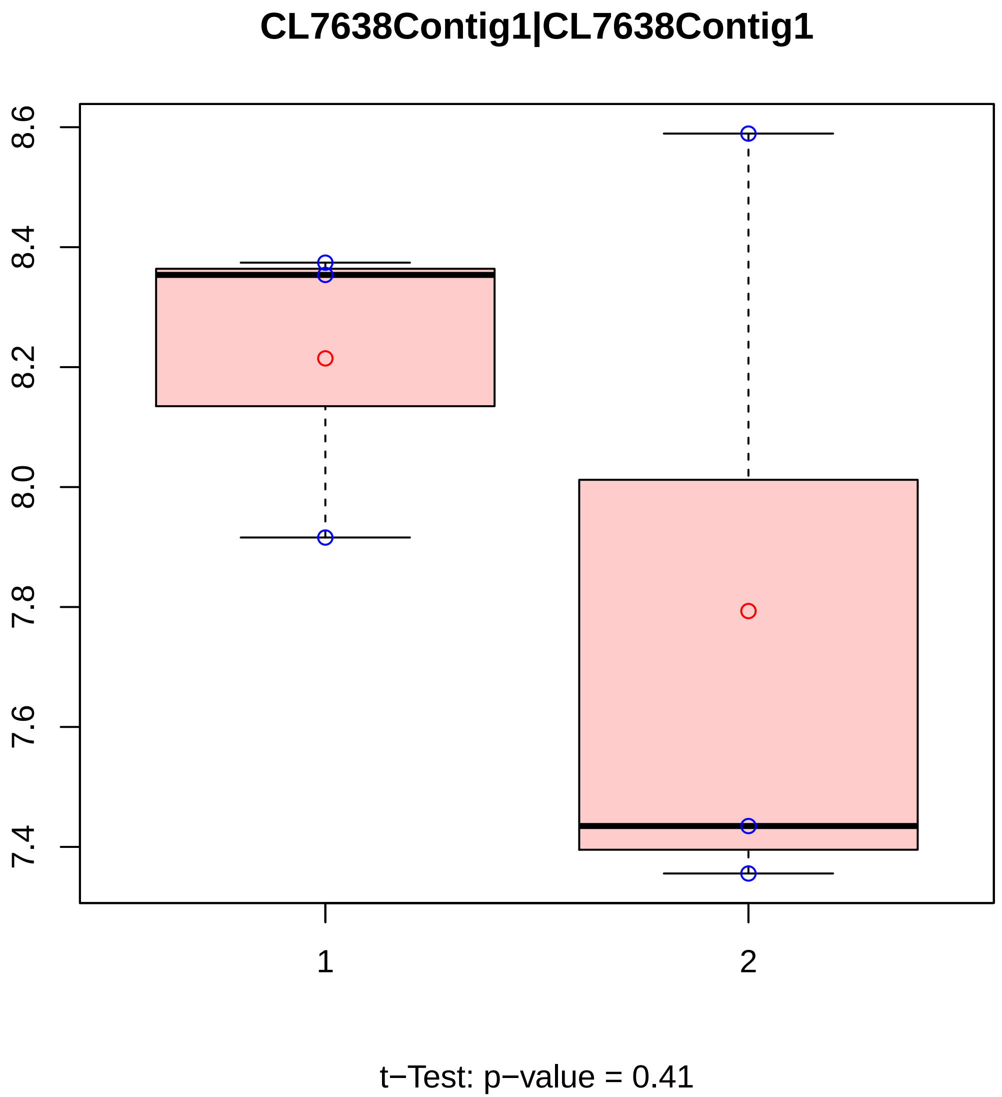

CL7638Contig1|CL7638Contig1
8.6
8.4
8.2
8.0
7.8
7.6
7.4
1
2
t−Test: p−value = 0.41

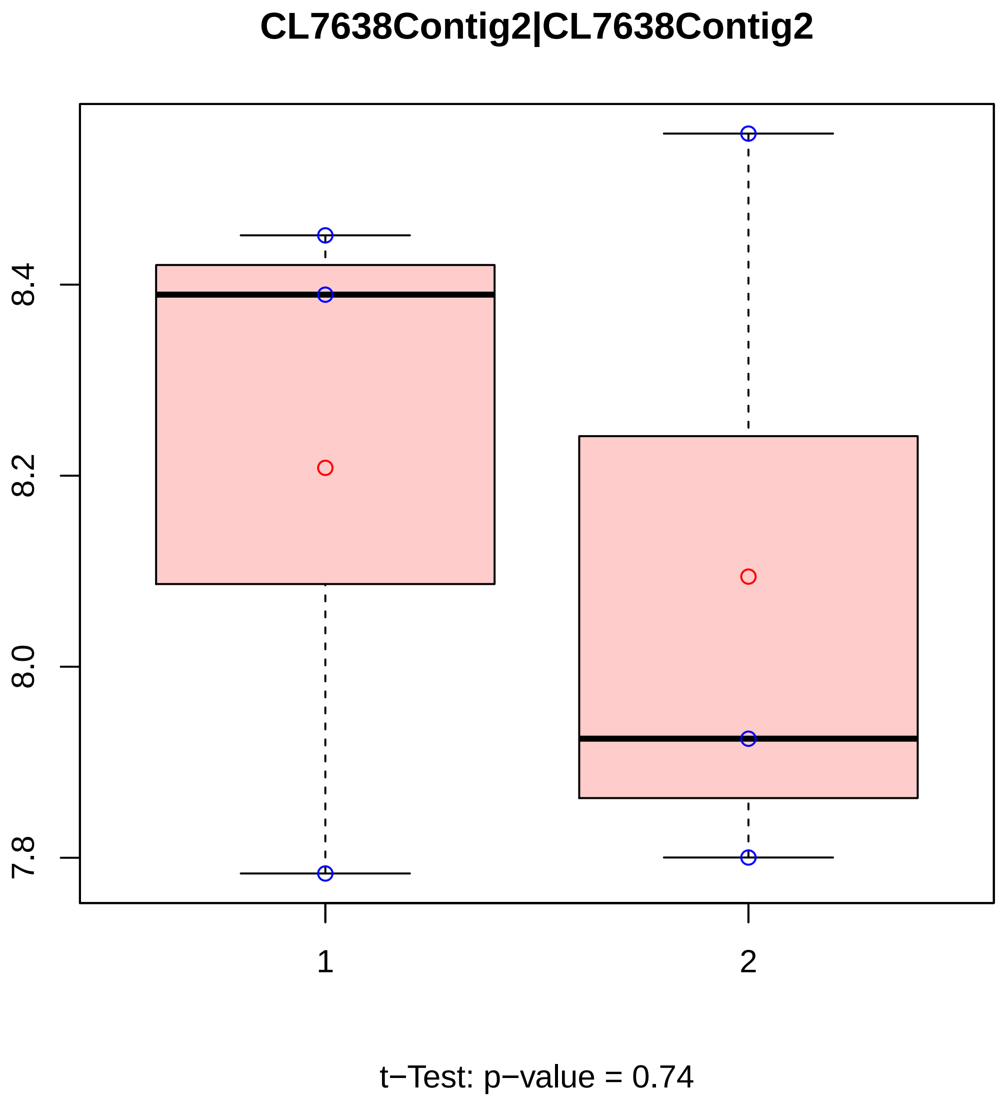
CL7638Contig2|CL7638Contig2
8.4
8.2
8.0
7.8
1
2
t−Test: p−value = 0.74

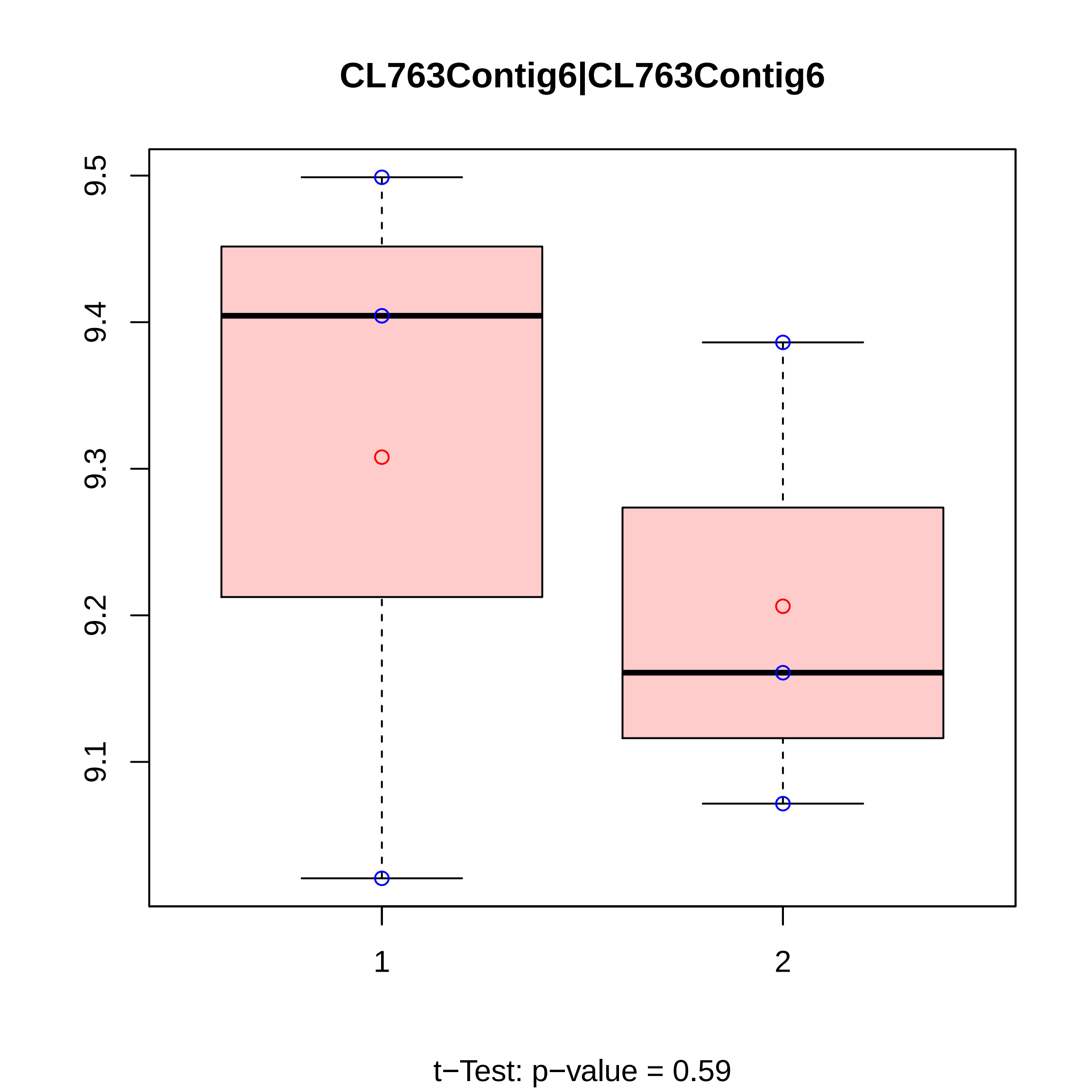
CL763Contig6|CL763Contig6
9.5
9.4
9.3
9.2
9.1
1
2
t−Test: p−value = 0.59

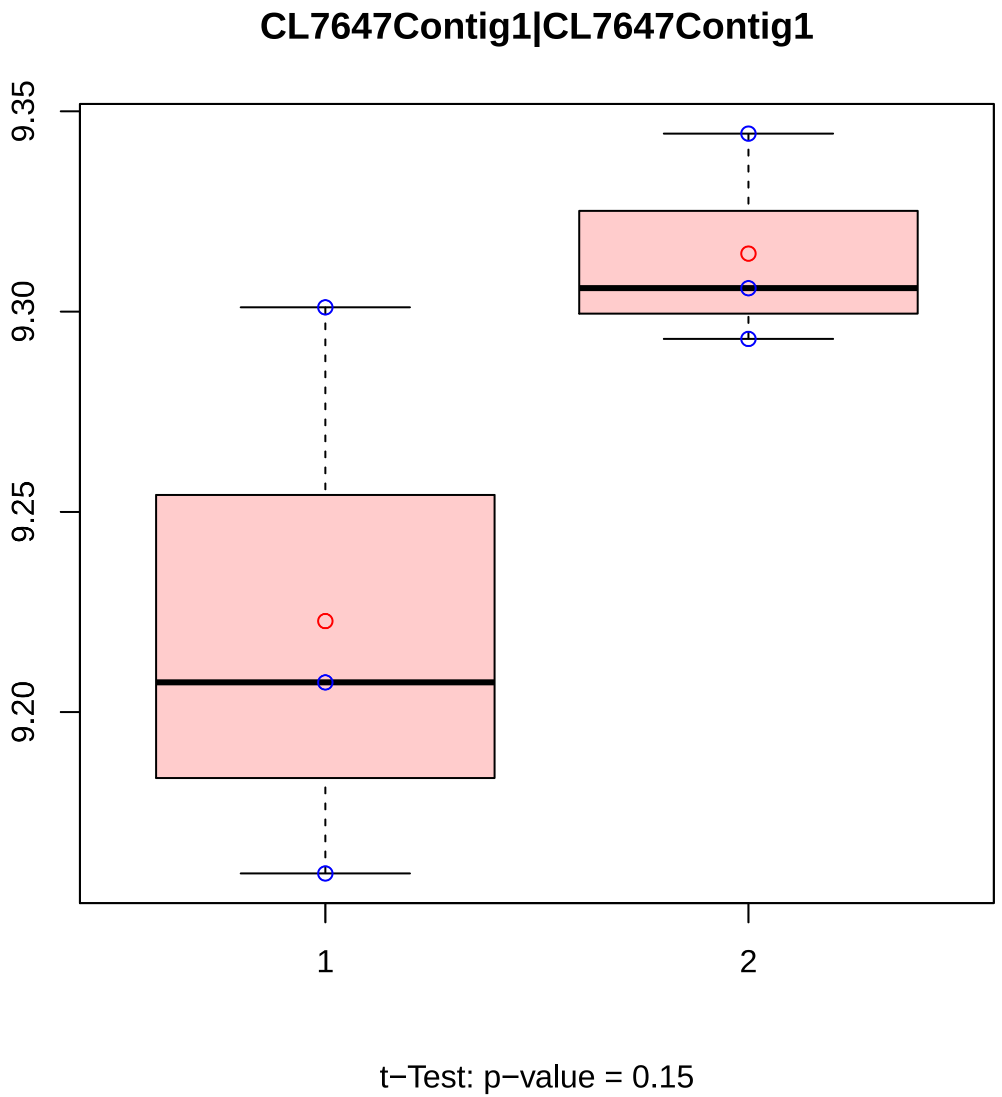
CL7647Contig1|CL7647Contig1
9.35
9.30
9.25
9.20
1
2
t−Test: p−value = 0.15

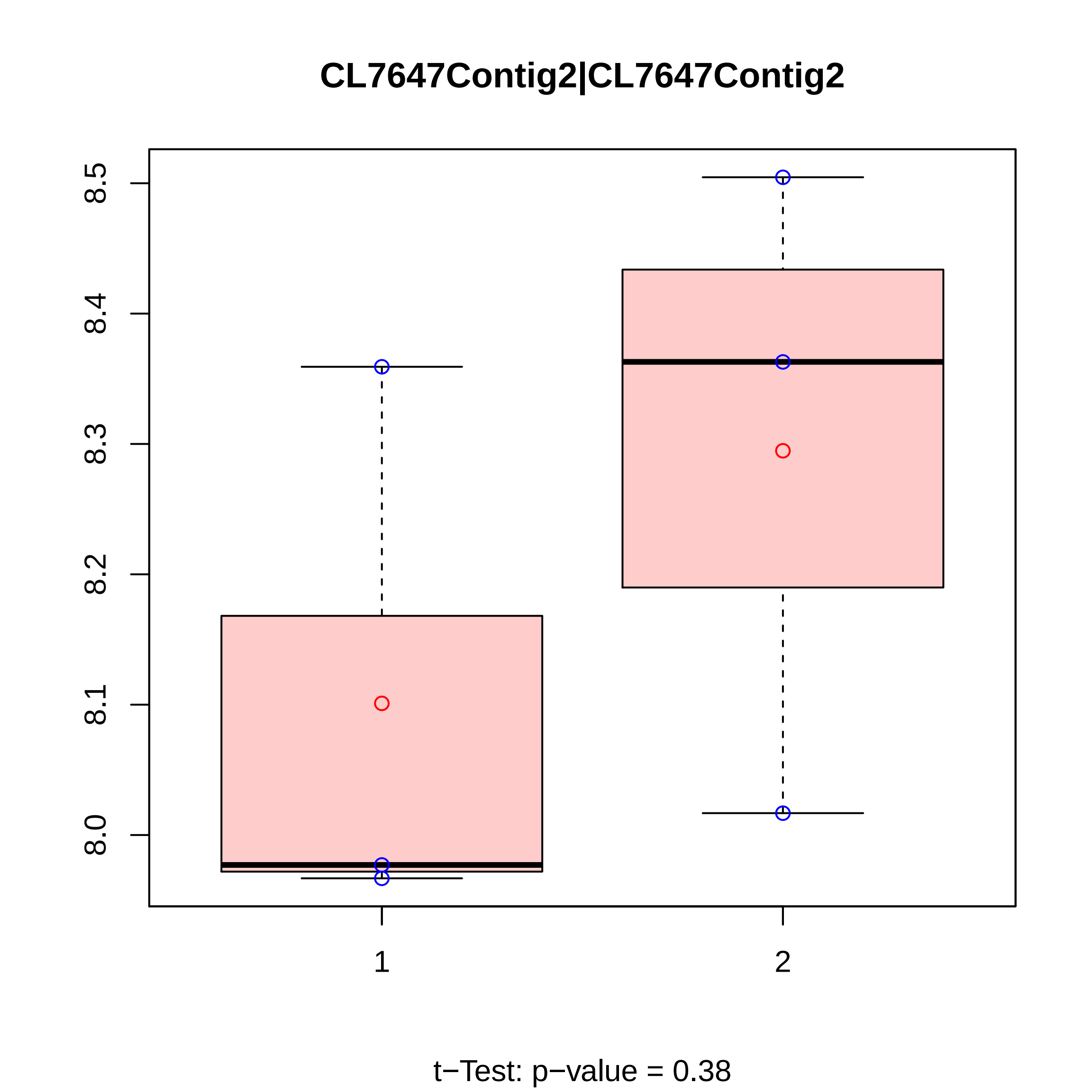

CL7647Contig2|CL7647Contig2
8.0
8.1
8.2
8.3
8.4
8.5
1
2
t−Test: p−value = 0.38

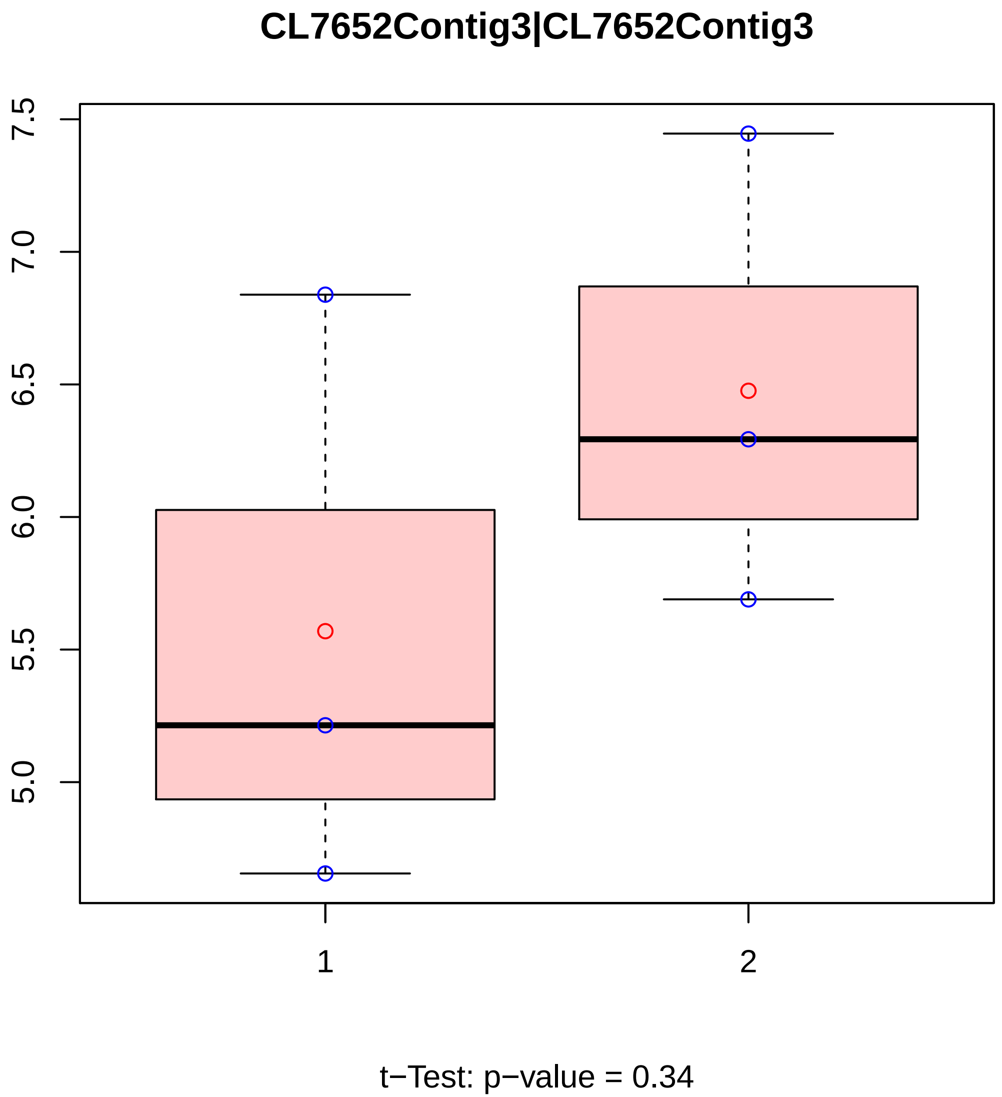

CL7652Contig3|CL7652Contig3
7.5
7.0
6.5
6.0
5.5
5.0
1
2
t−Test: p−value = 0.34

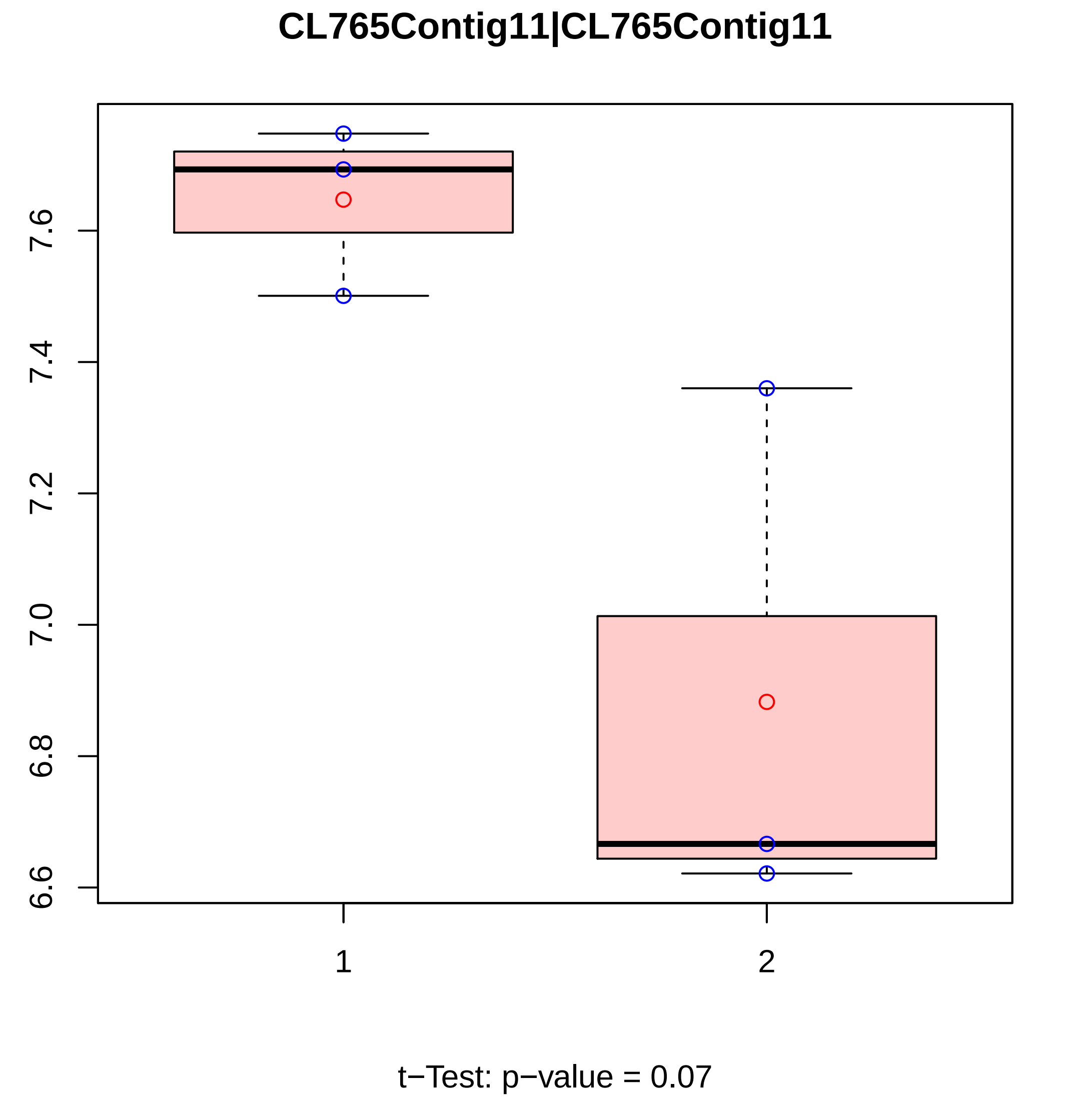

CL765Contig11|CL765Contig11
7.6
7.4
7.2
7.0
6.8
6.6
1
2
t−Test: p−value = 0.07

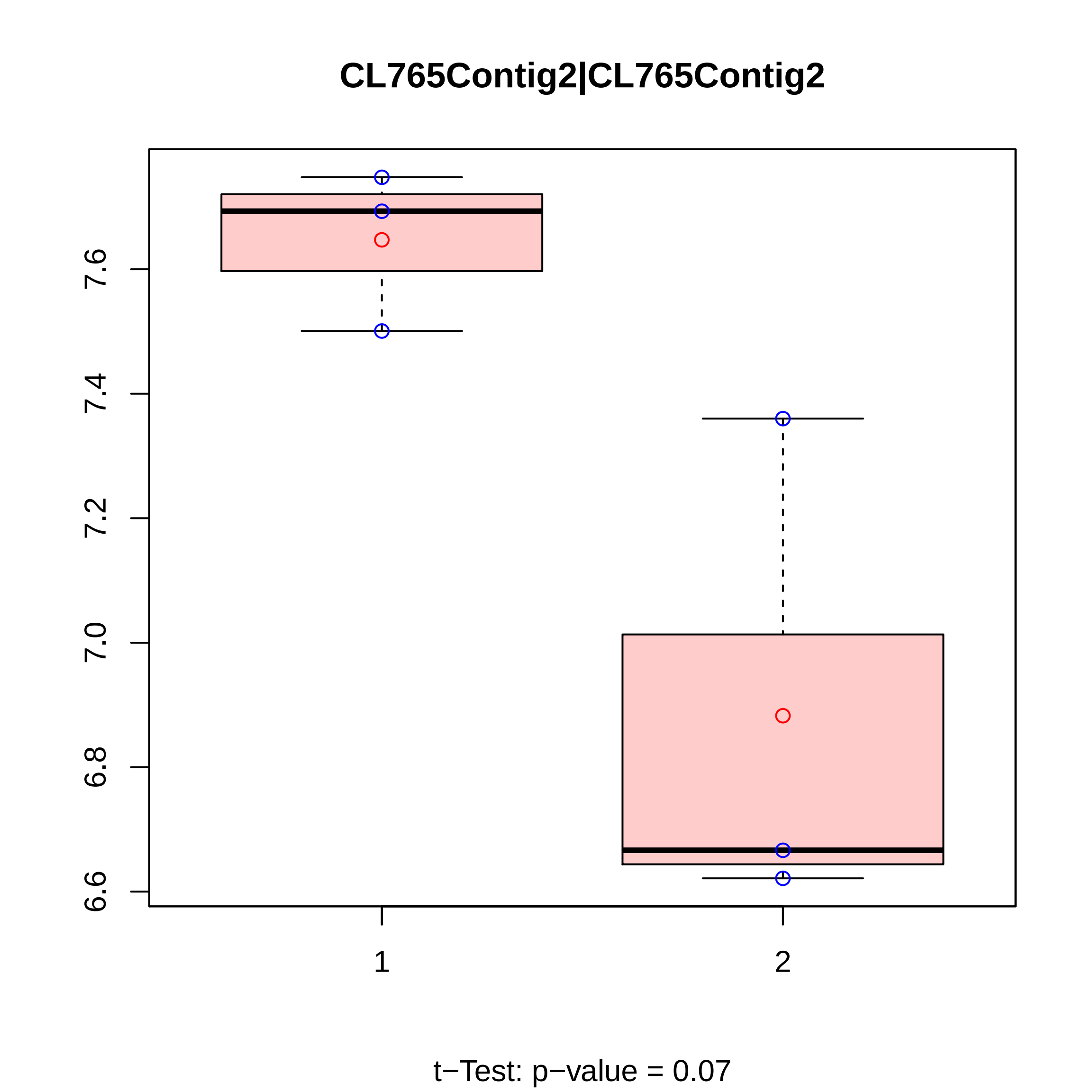
CL765Contig2|CL765Contig2
7.6
7.4
7.2
7.0
6.8
6.6
1
2
t−Test: p−value = 0.07

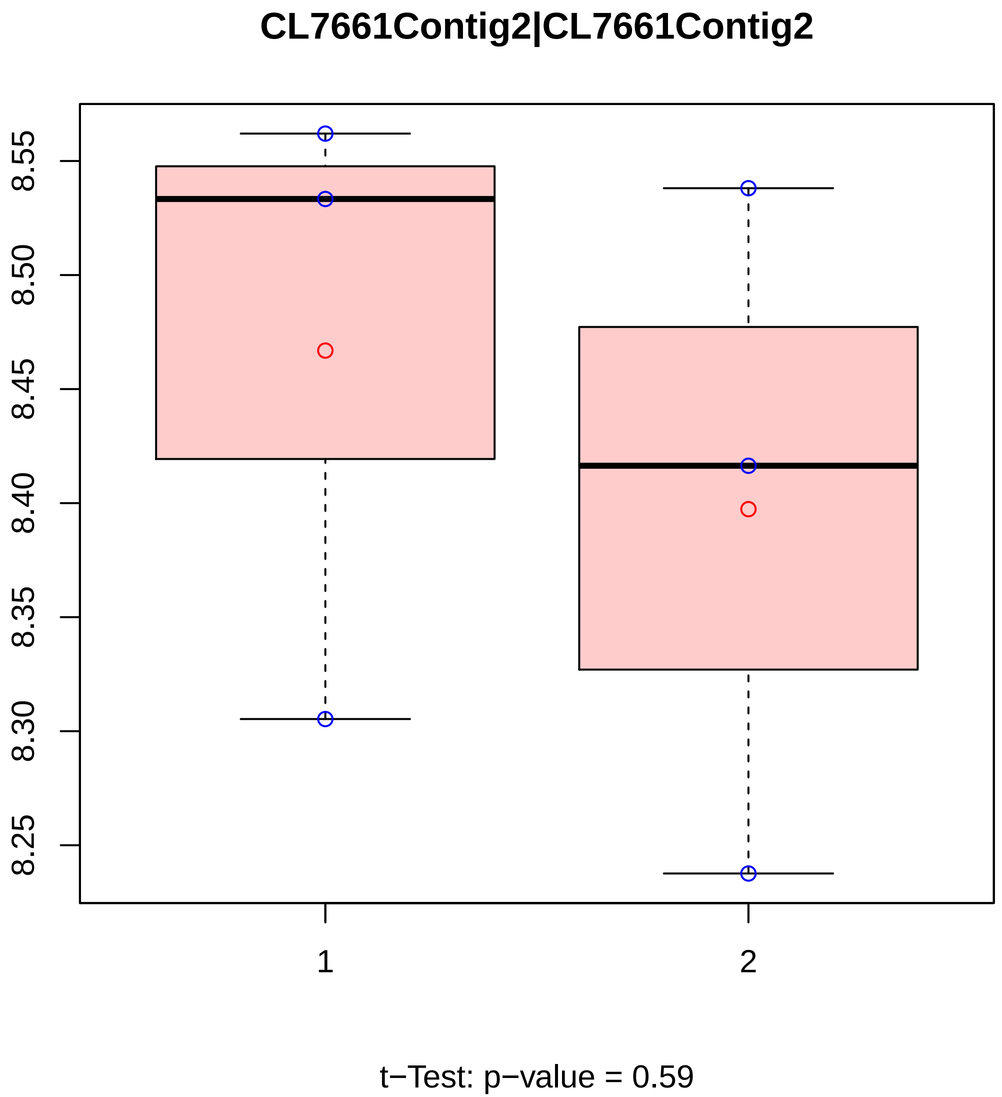

CL7661Contig2|CL7661Contig2
8.25
8.30
8.35
8.40
8.45
8.50
8.55
1
2
t−Test: p−value = 0.59

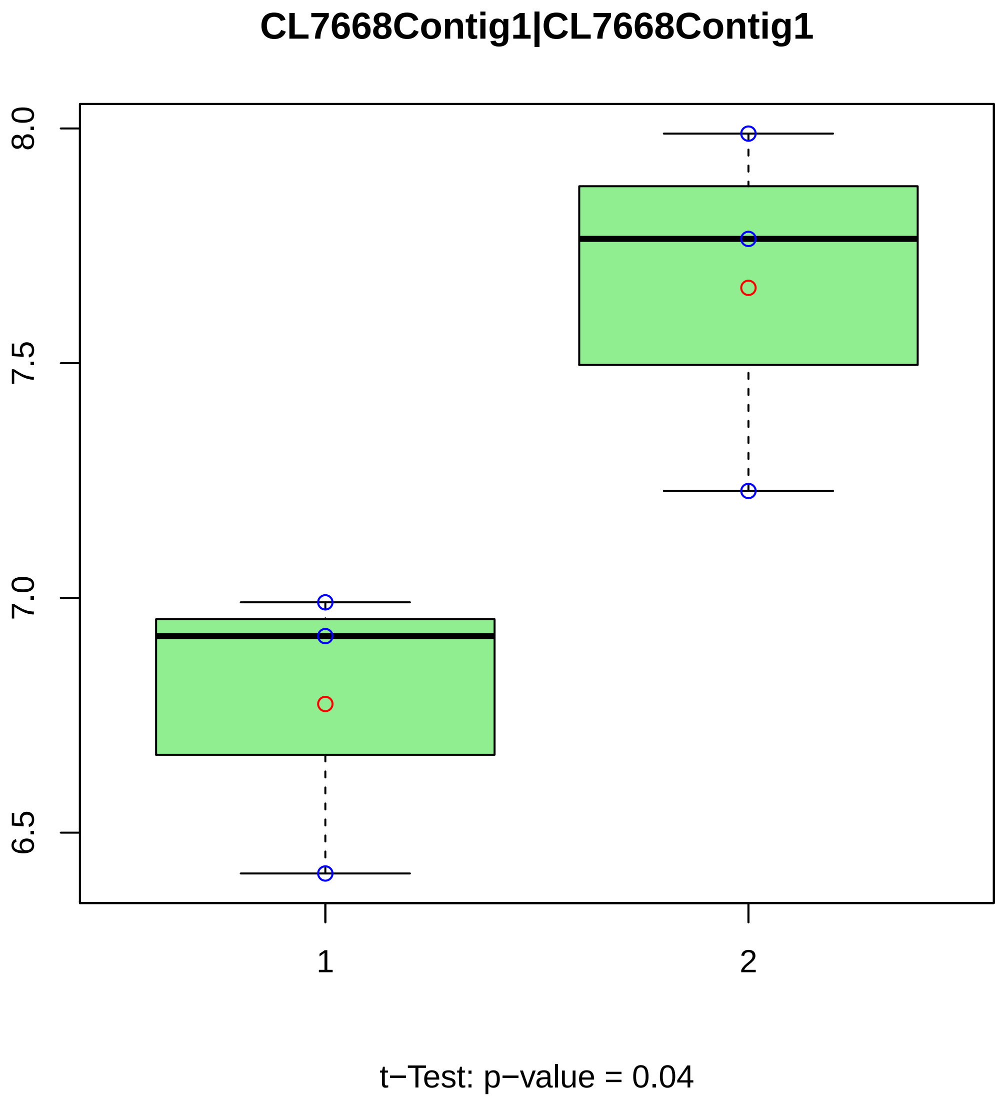

CL7668Contig1|CL7668Contig1
8.0
7.5
7.0
6.5
1
2
t−Test: p−value = 0.04

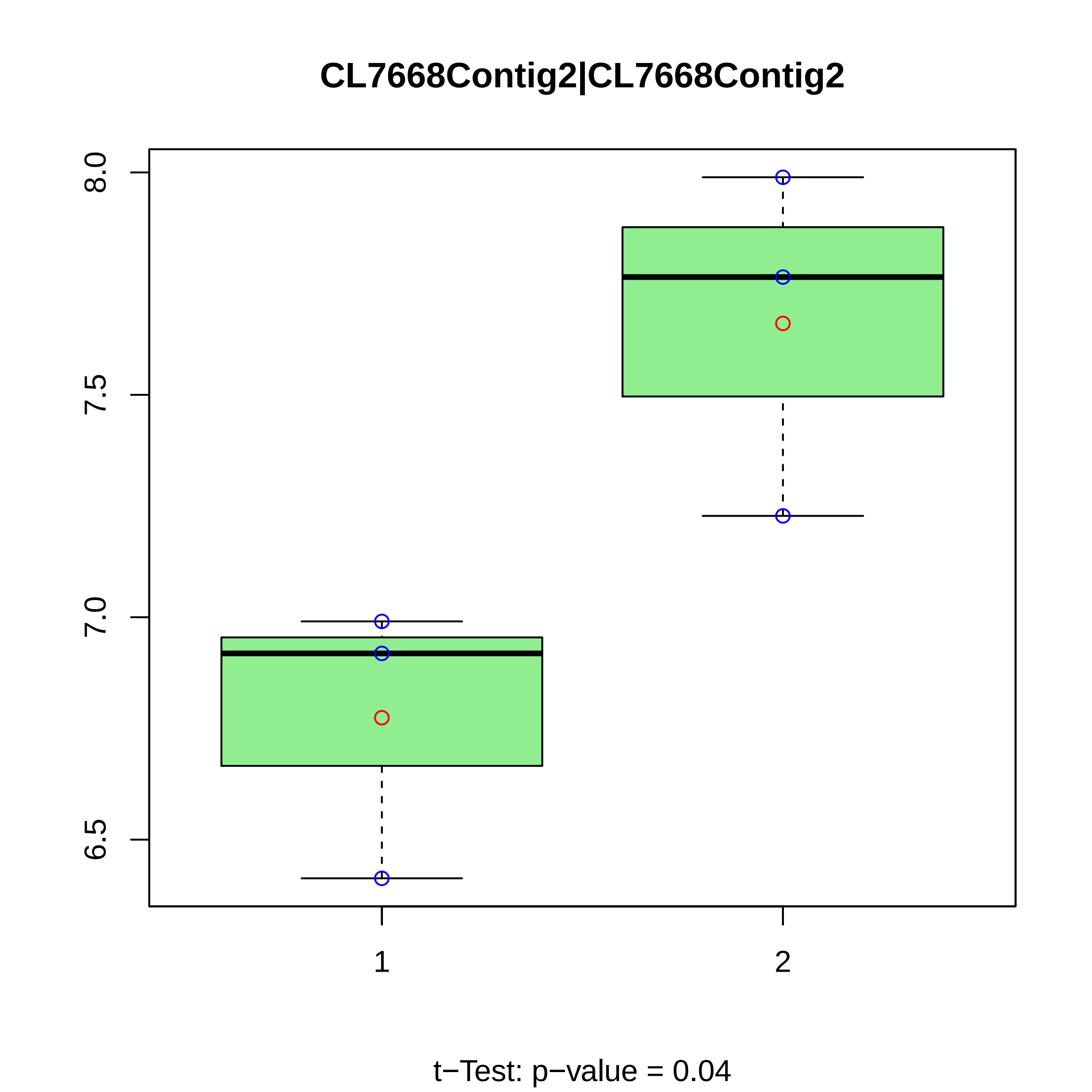
CL7668Contig2|CL7668Contig2
8.0
7.5
7.0
6.5
1
2
t−Test: p−value = 0.04

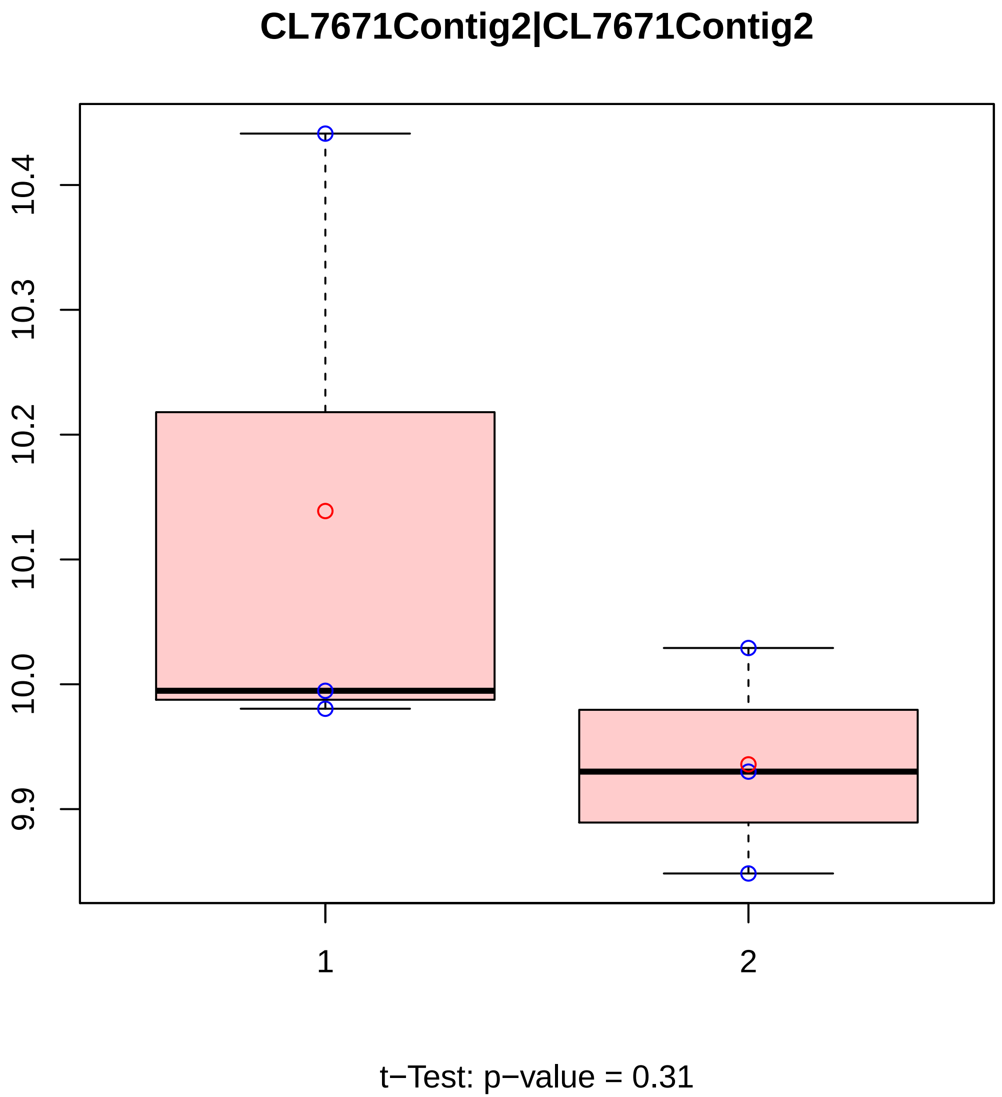

CL7671Contig2|CL7671Contig2
10.4
10.3
10.2
10.1
10.0
9.9
1
2
t−Test: p−value = 0.31

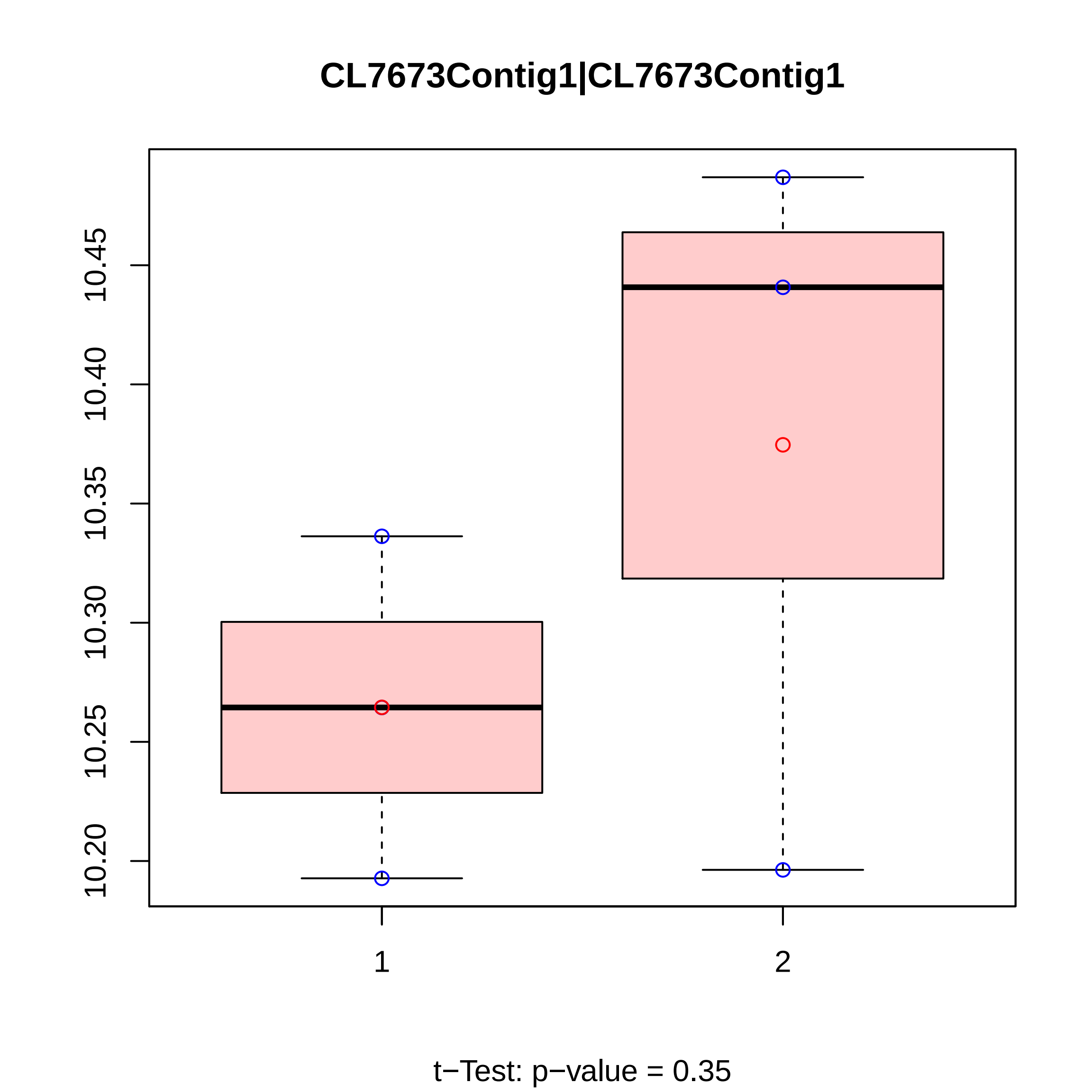
CL7673Contig1|CL7673Contig1
10.20
10.25
10.30
10.35
10.40
10.45
1
2
t−Test: p−value = 0.35

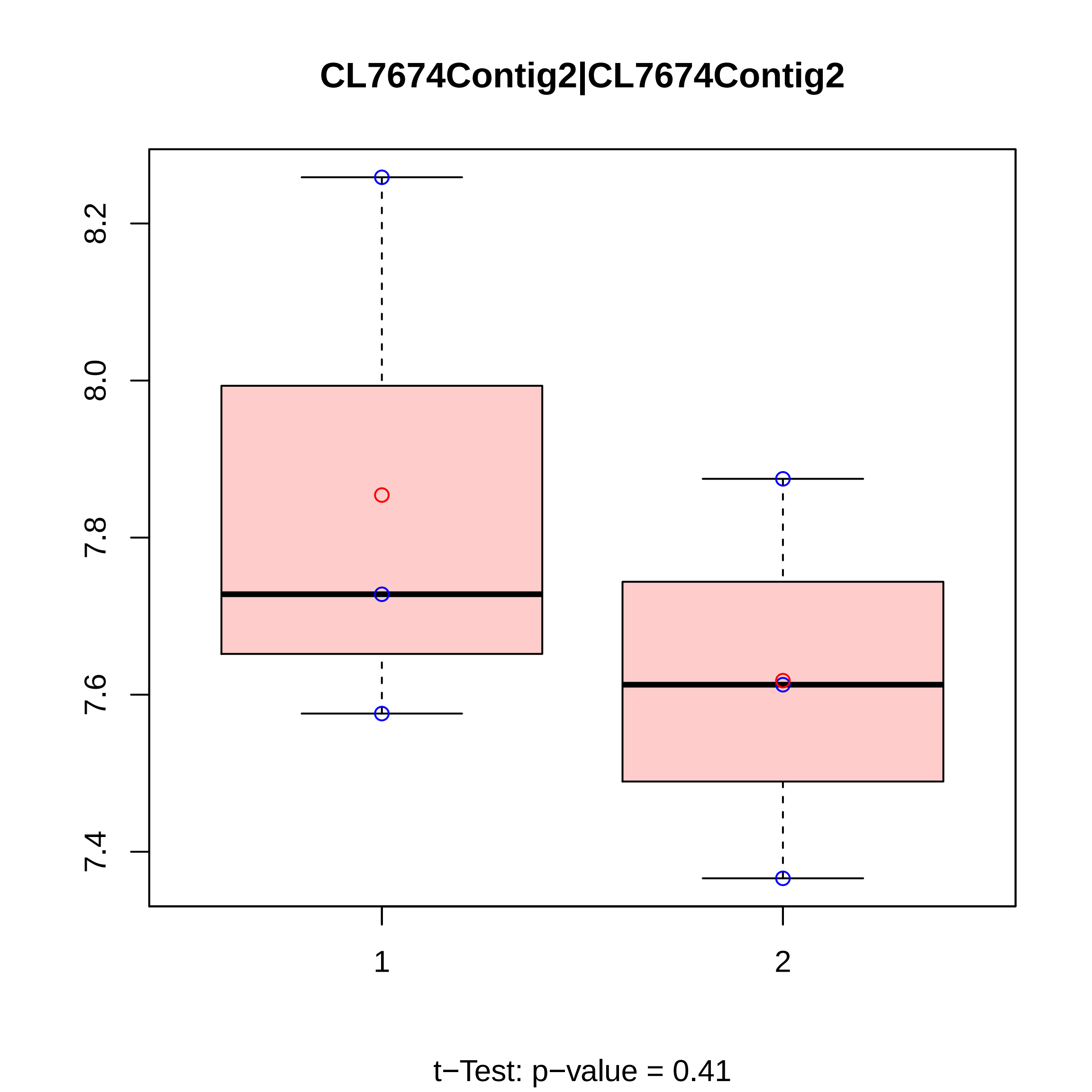
CL7674Contig2|CL7674Contig2
8.2
8.0
7.8
7.6
7.4
1
2
t−Test: p−value = 0.41

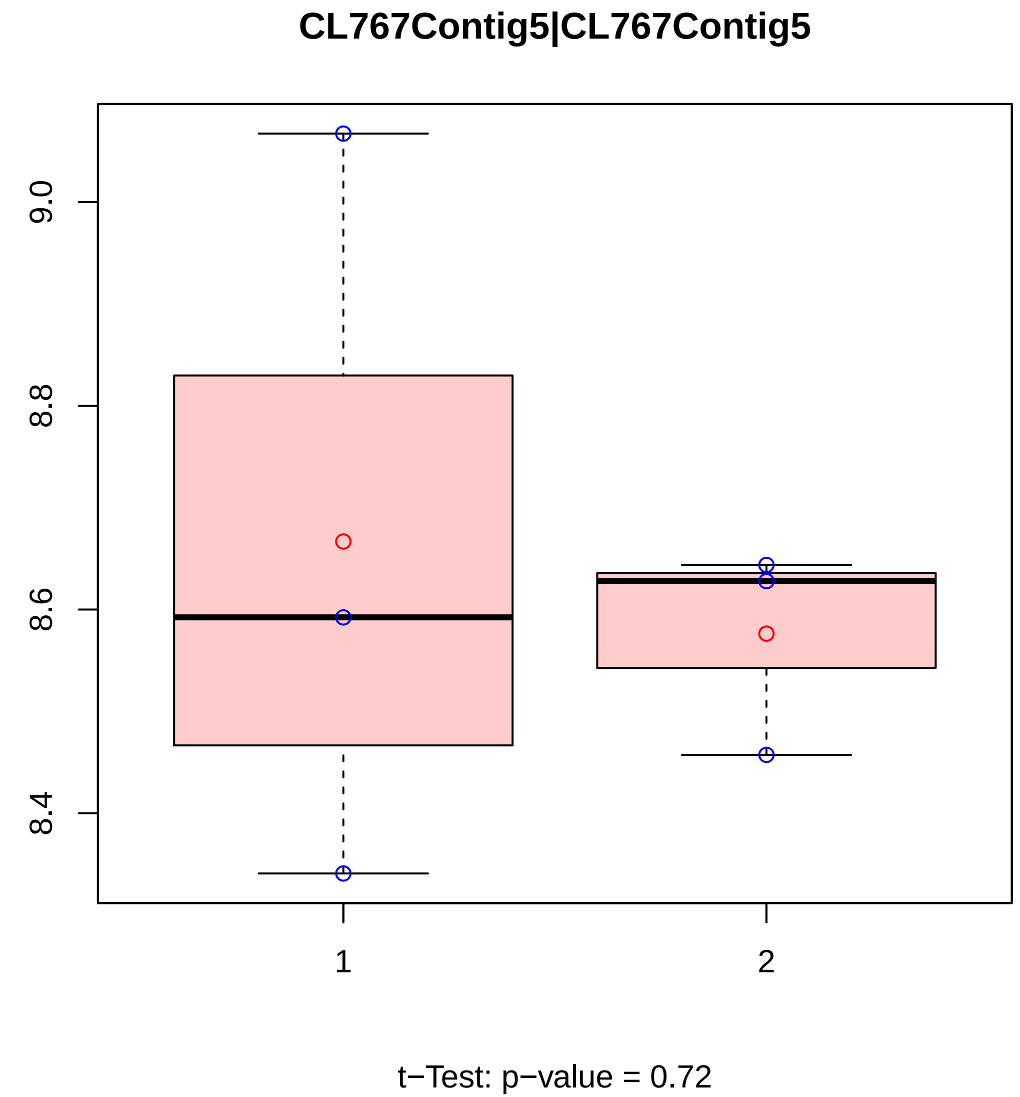
CL767Contig5|CL767Contig5
9.0
8.8
8.6
8.4
1
2
t−Test: p−value = 0.72

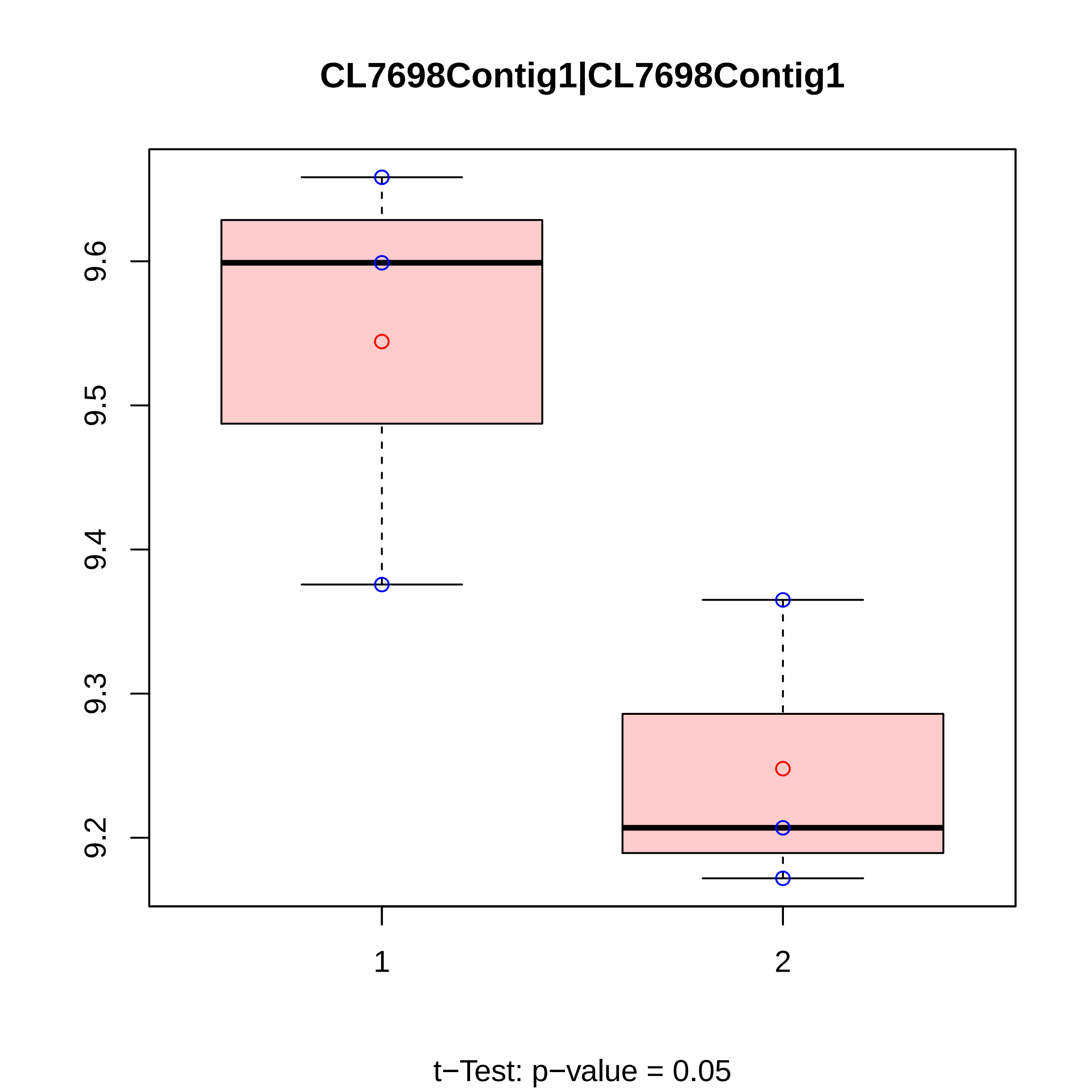

CL7698Contig1|CL7698Contig1
9.2
9.3
9.4
9.5
9.6
1
2
t−Test: p−value = 0.05

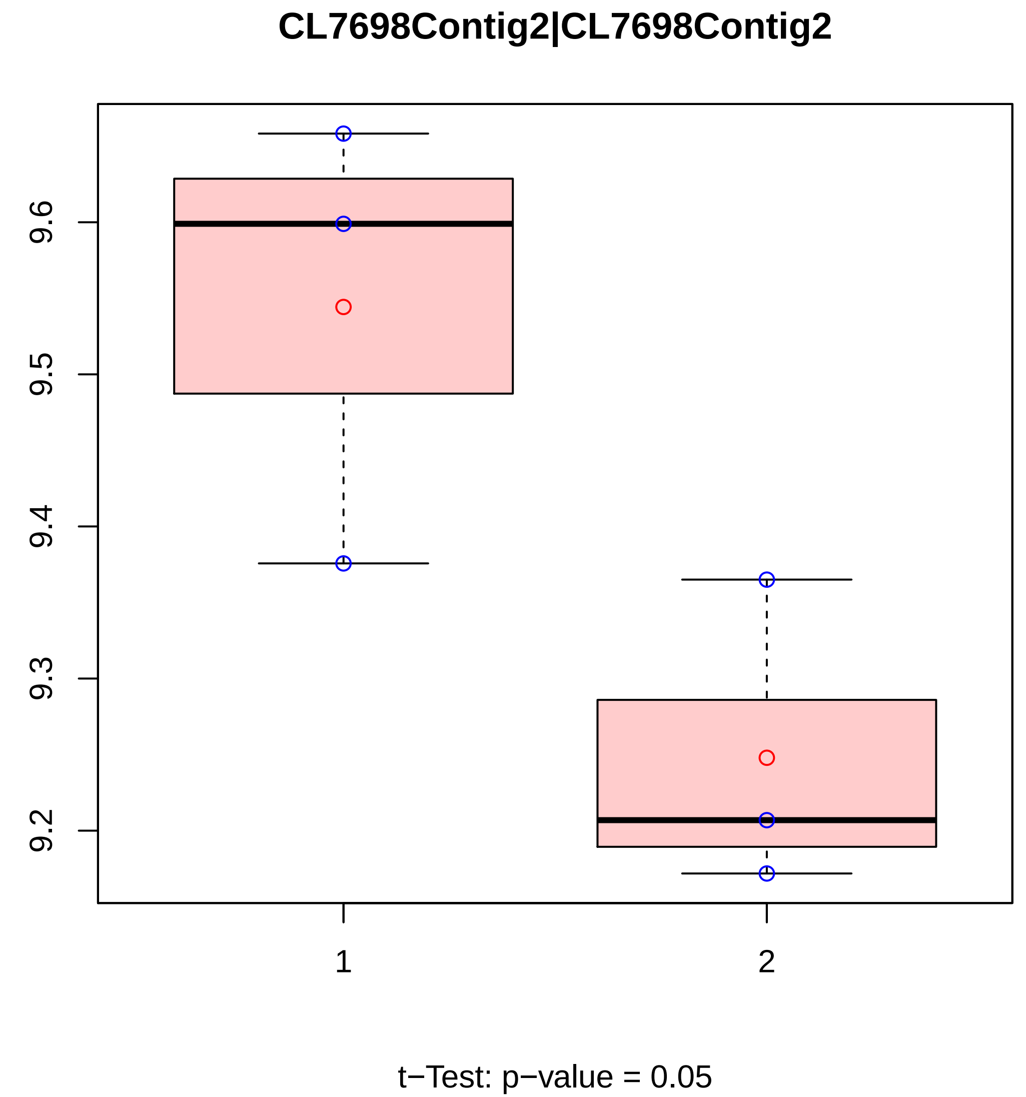

CL7698Contig2|CL7698Contig2
9.2
9.3
9.4
9.5
9.6
1
2
t−Test: p−value = 0.05

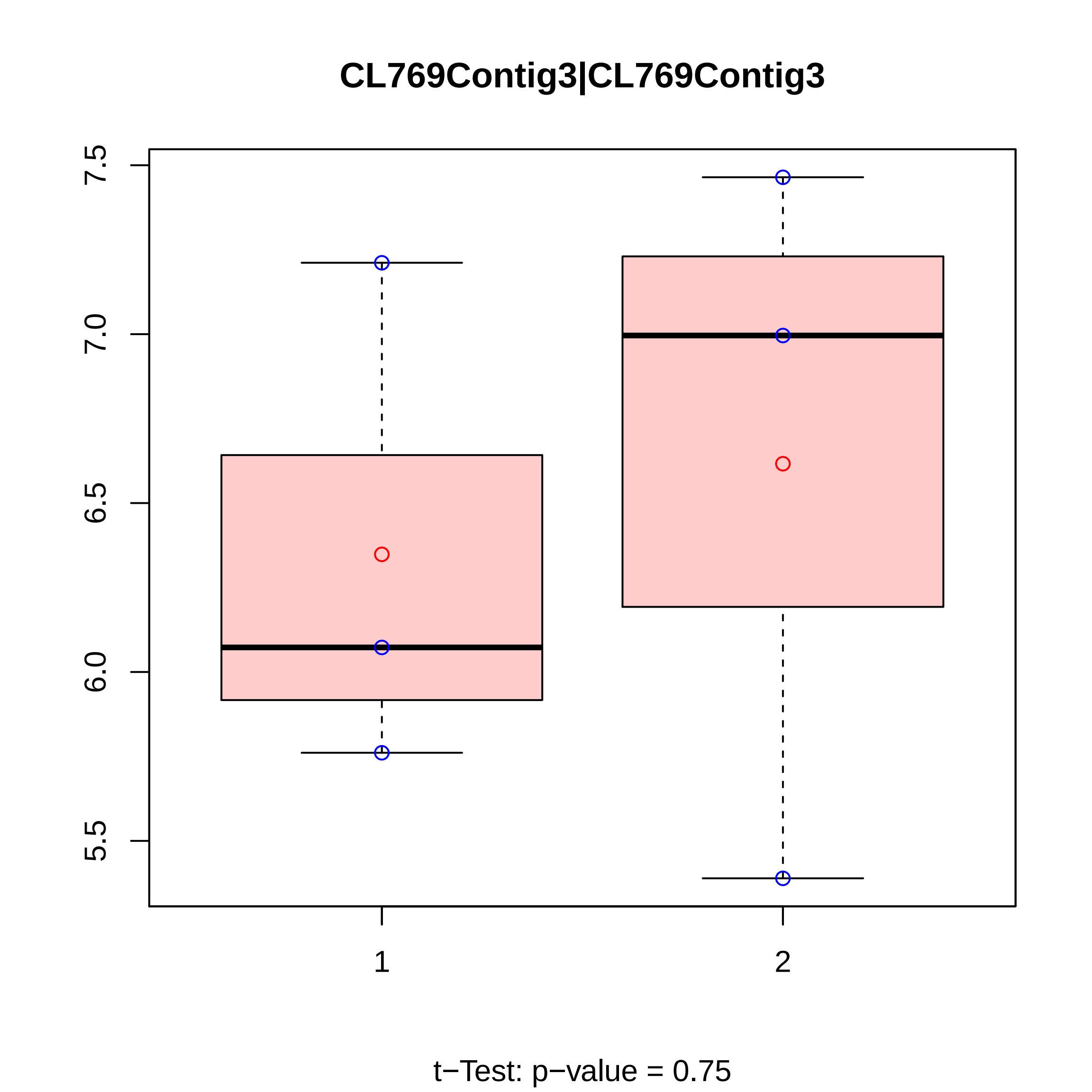
CL769Contig3|CL769Contig3
7.5
7.0
6.5
6.0
5.5
1
2
t−Test: p−value = 0.75

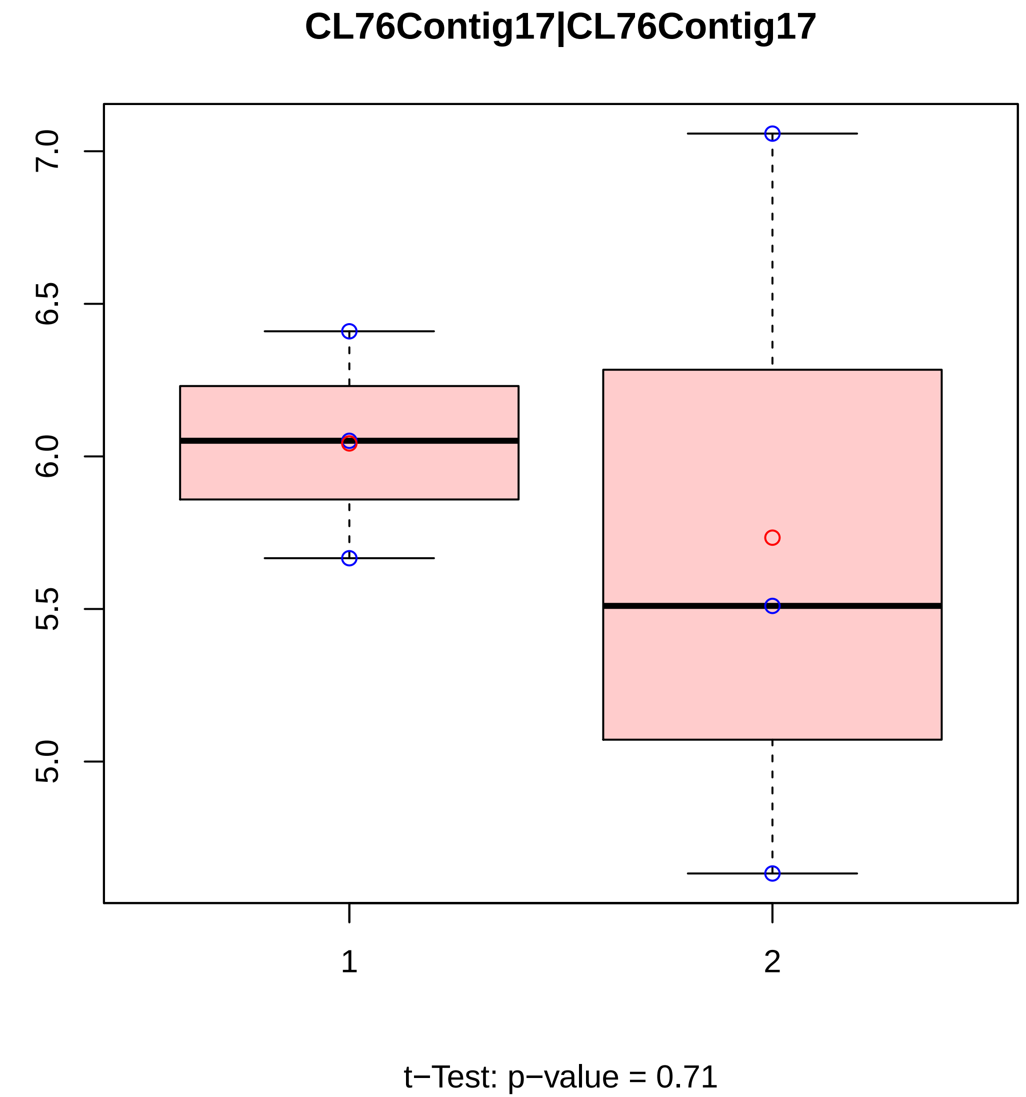
CL76Contig17|CL76Contig17
7.0
6.5
6.0
5.5
5.0
1
2
t−Test: p−value = 0.71

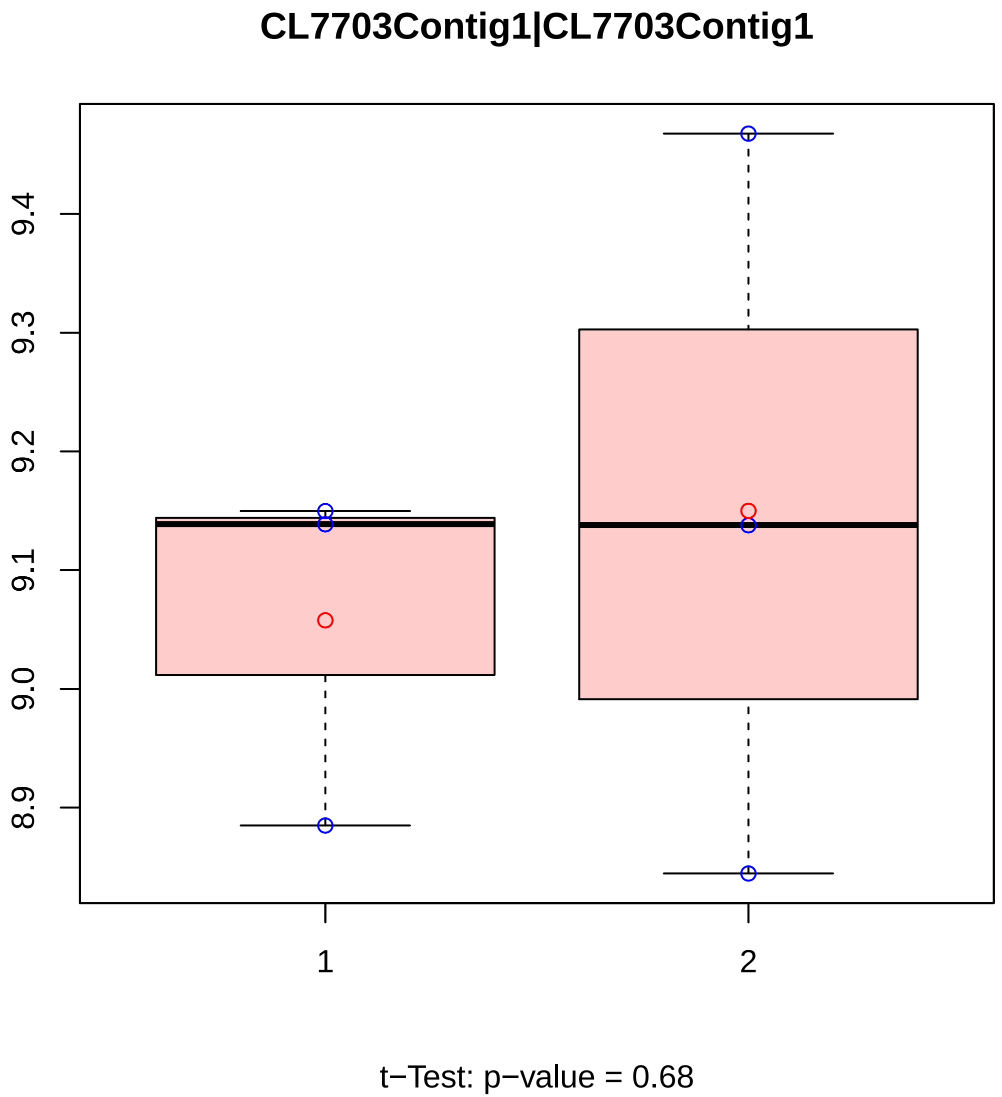

CL7703Contig1|CL7703Contig1
9.4
9.3
9.2
9.1
9.0
8.9
1
2
t−Test: p−value = 0.68

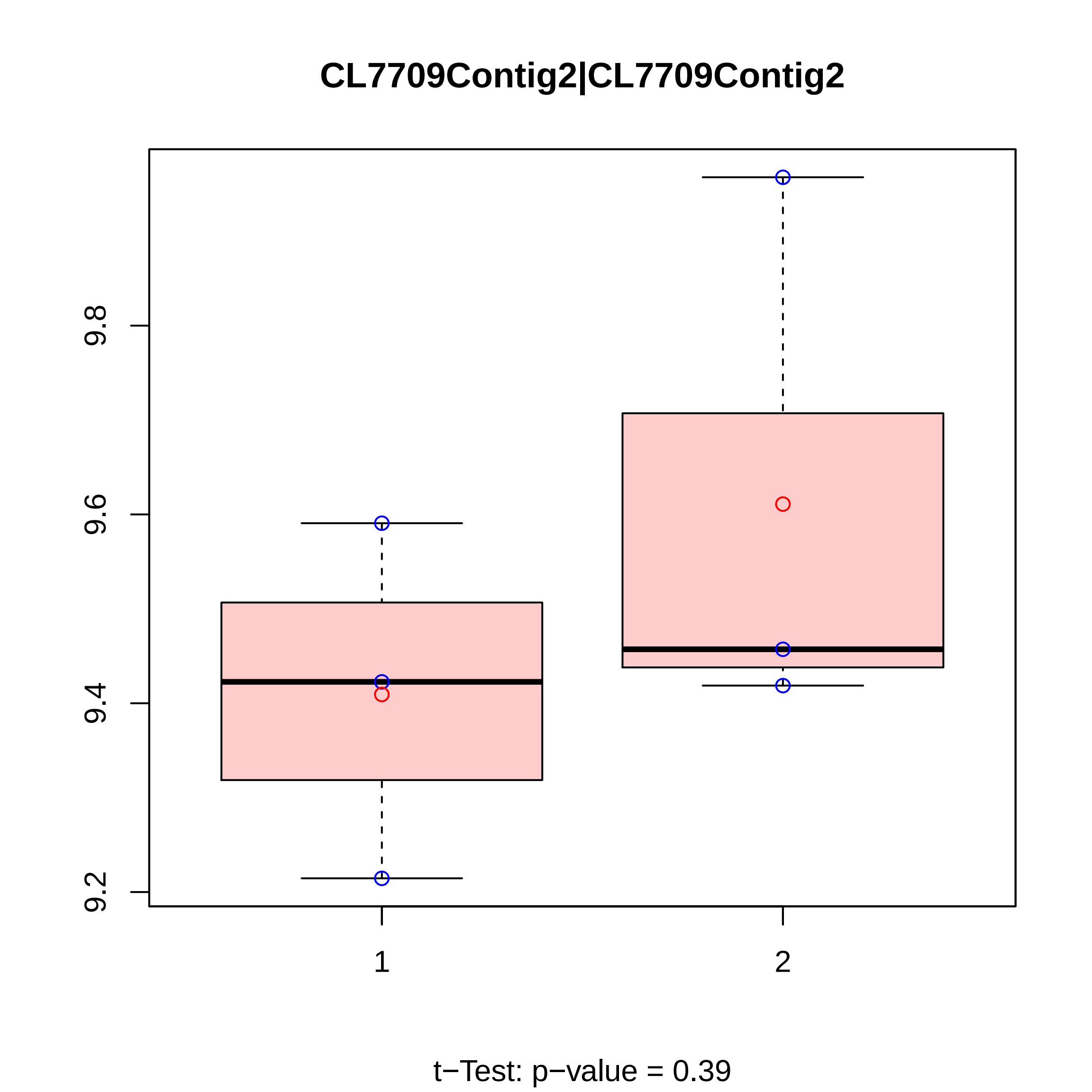
CL7709Contig2|CL7709Contig2
9.2
9.4
9.6
9.8
1
2
t−Test: p−value = 0.39

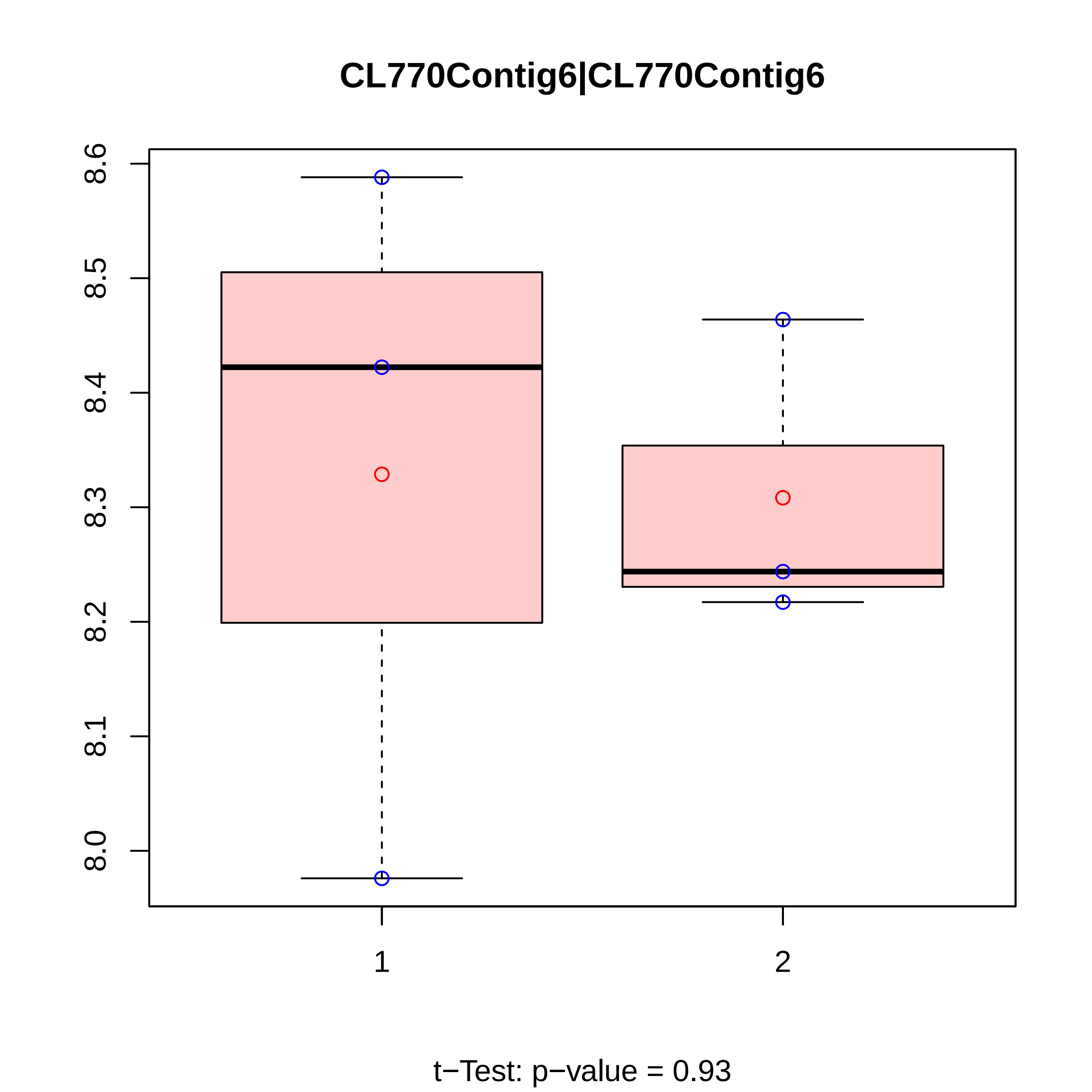
CL770Contig6|CL770Contig6
8.0
8.1
8.2
8.3
8.4
8.5
8.6
1
2
t−Test: p−value = 0.93

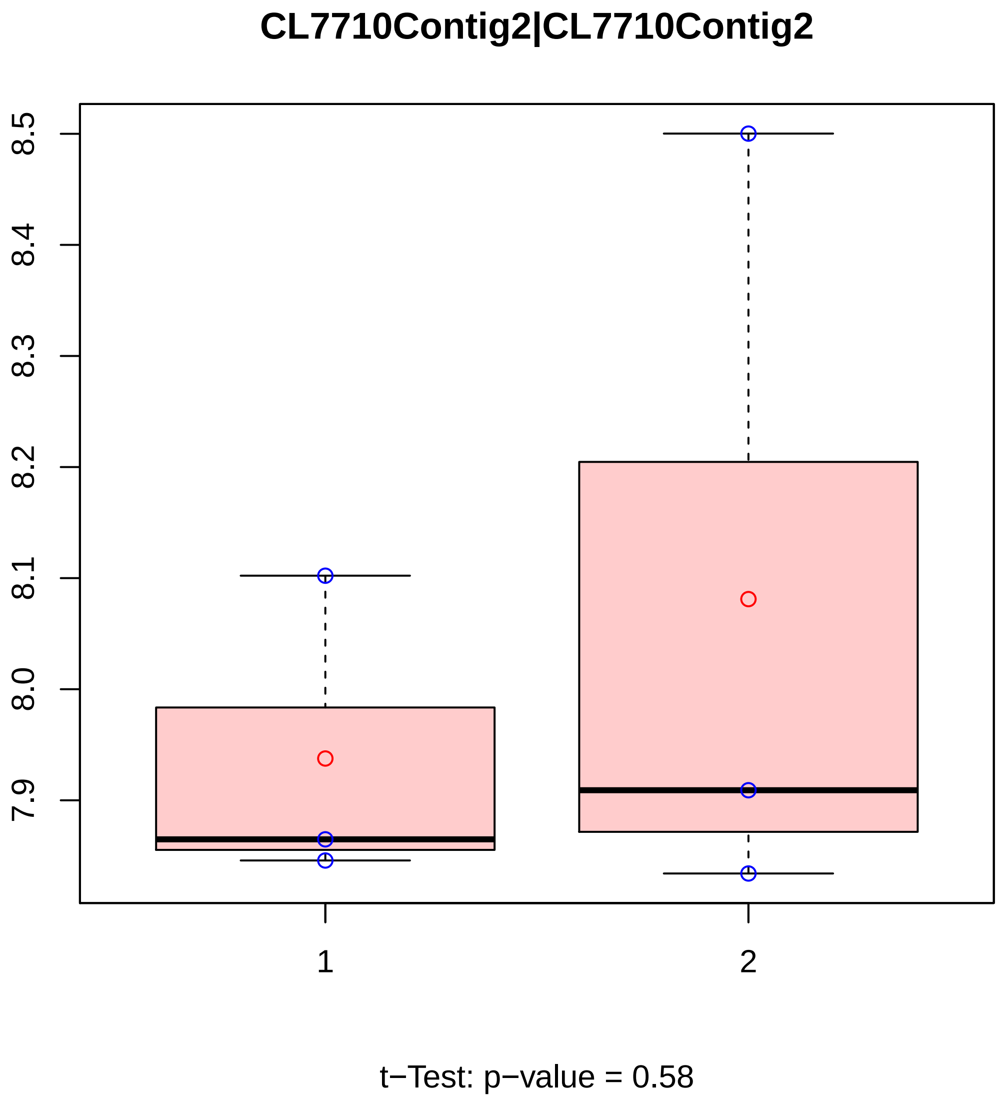
CL7710Contig2|CL7710Contig2
8.5
8.4
8.3
8.2
8.1
8.0
7.9
1
2
t−Test: p−value = 0.58

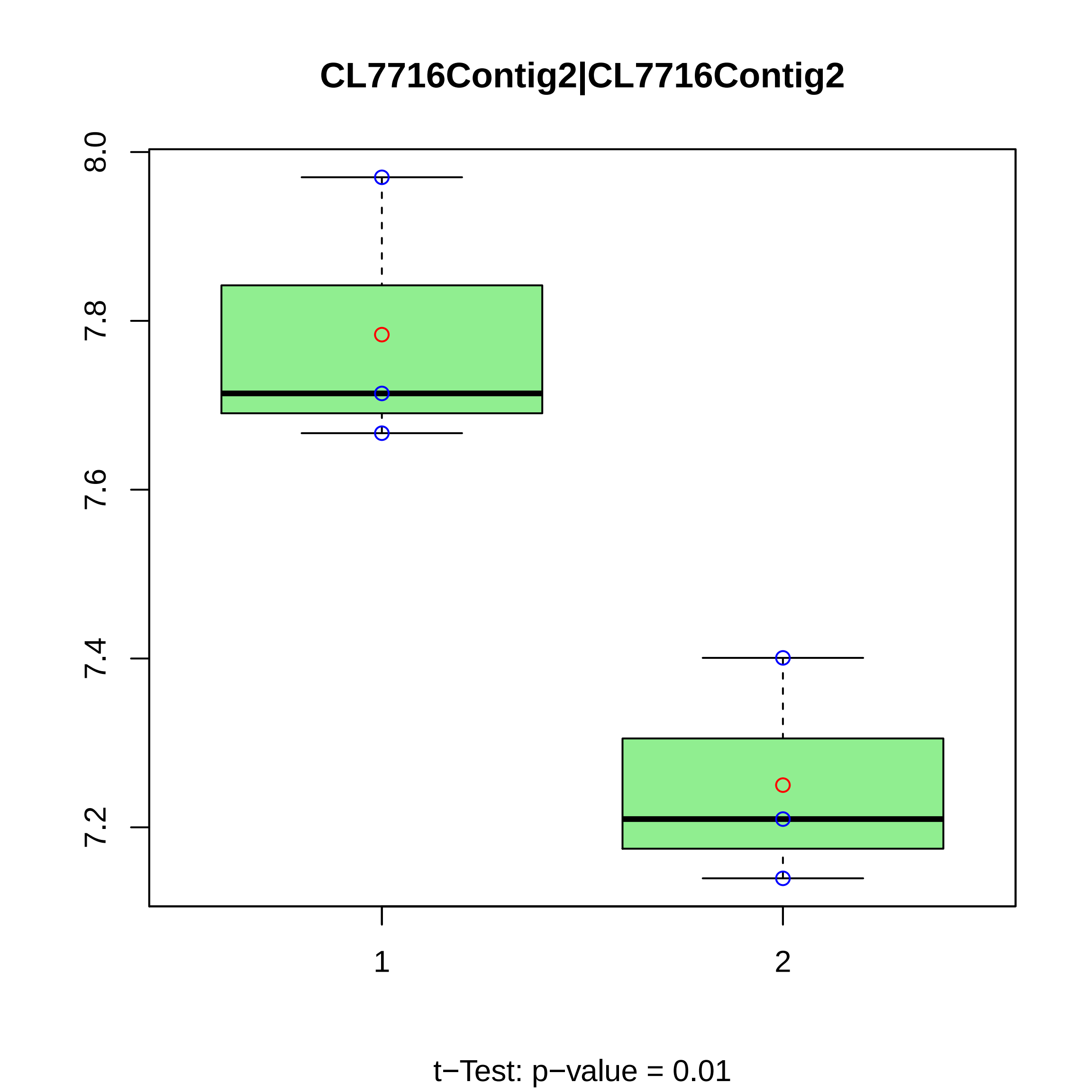
CL7716Contig2|CL7716Contig2
8.0
7.8
7.6
7.4
7.2
1
2
t−Test: p−value = 0.01

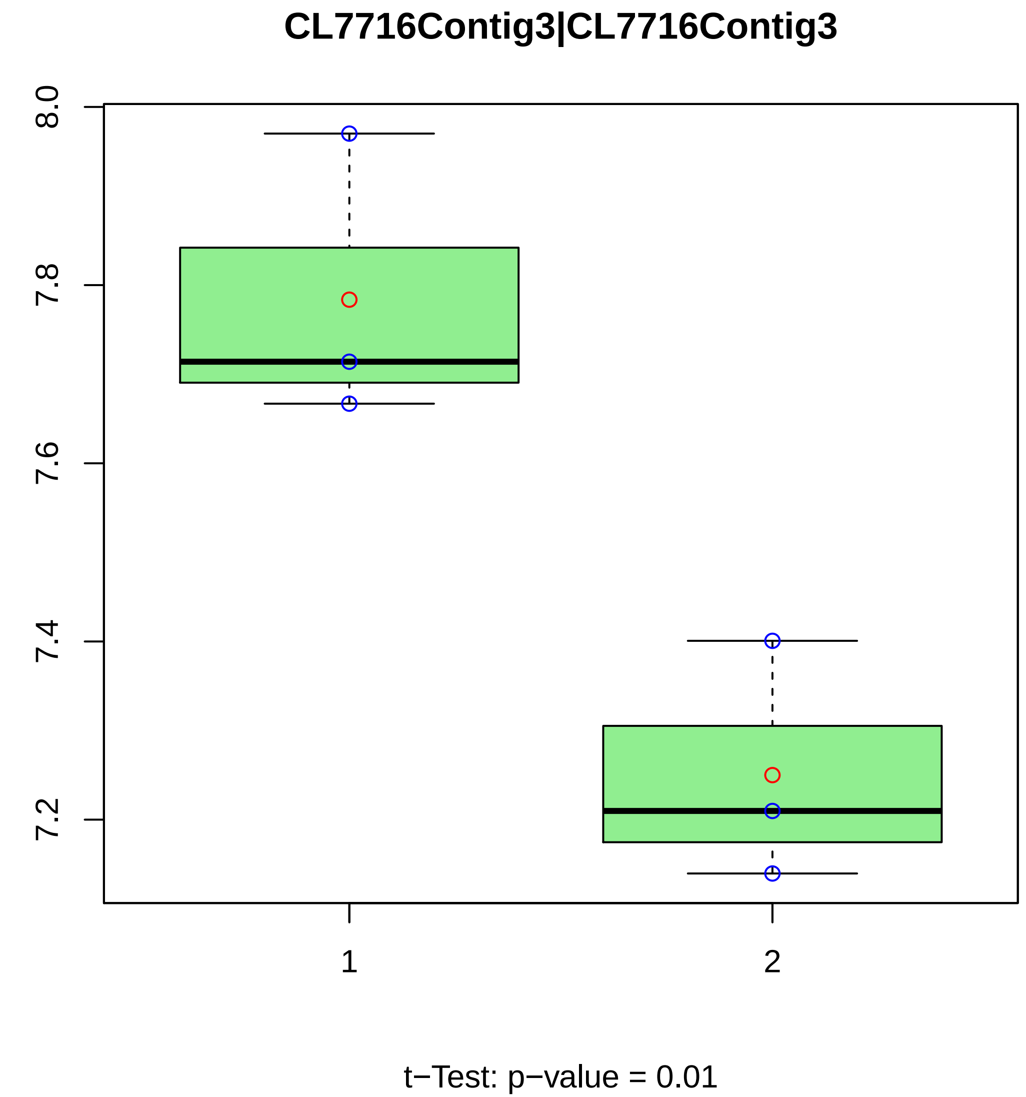
CL7716Contig3|CL7716Contig3
8.0
7.8
7.6
7.4
7.2
1
2
t−Test: p−value = 0.01

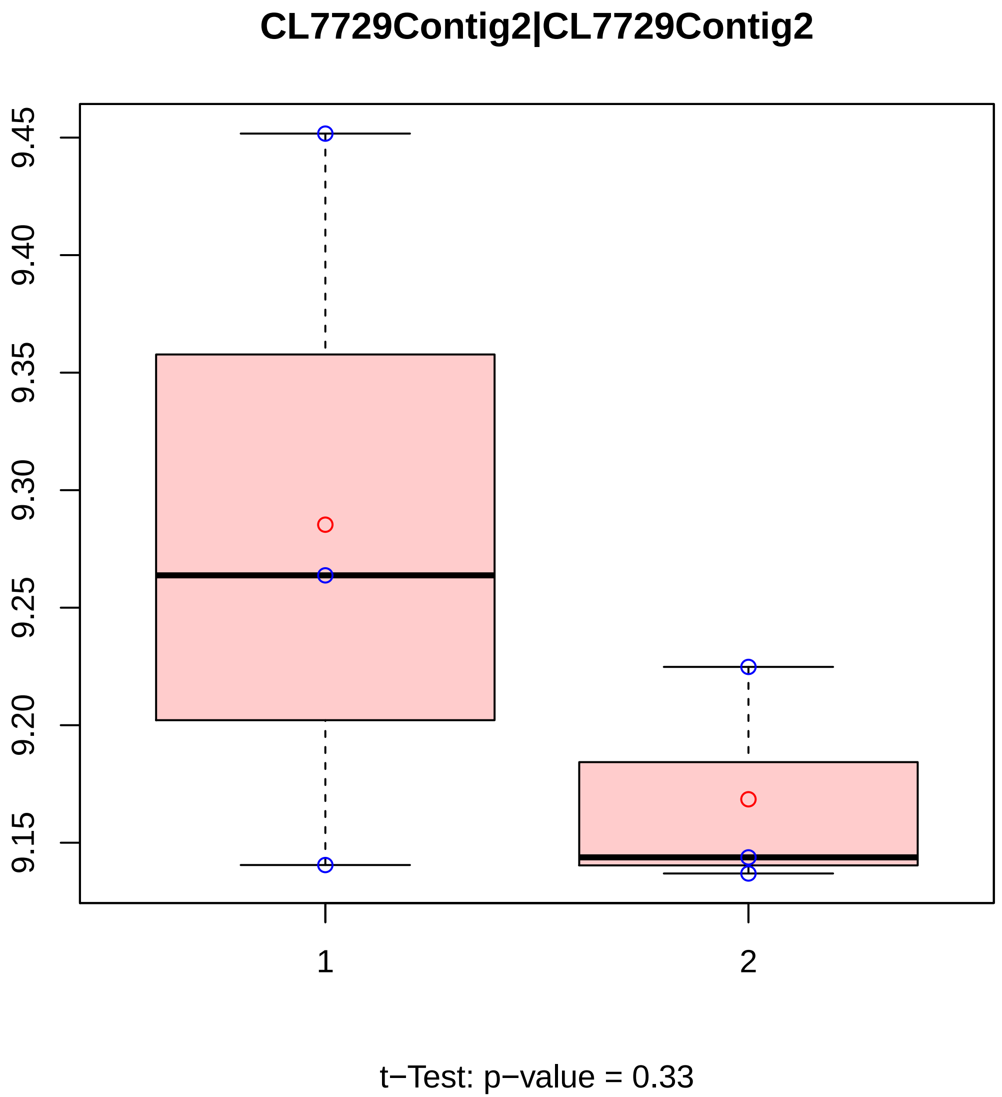
CL7729Contig2|CL7729Contig2
9.15
9.20
9.25
9.30
9.35
9.40
9.45
1
2
t−Test: p−value = 0.33

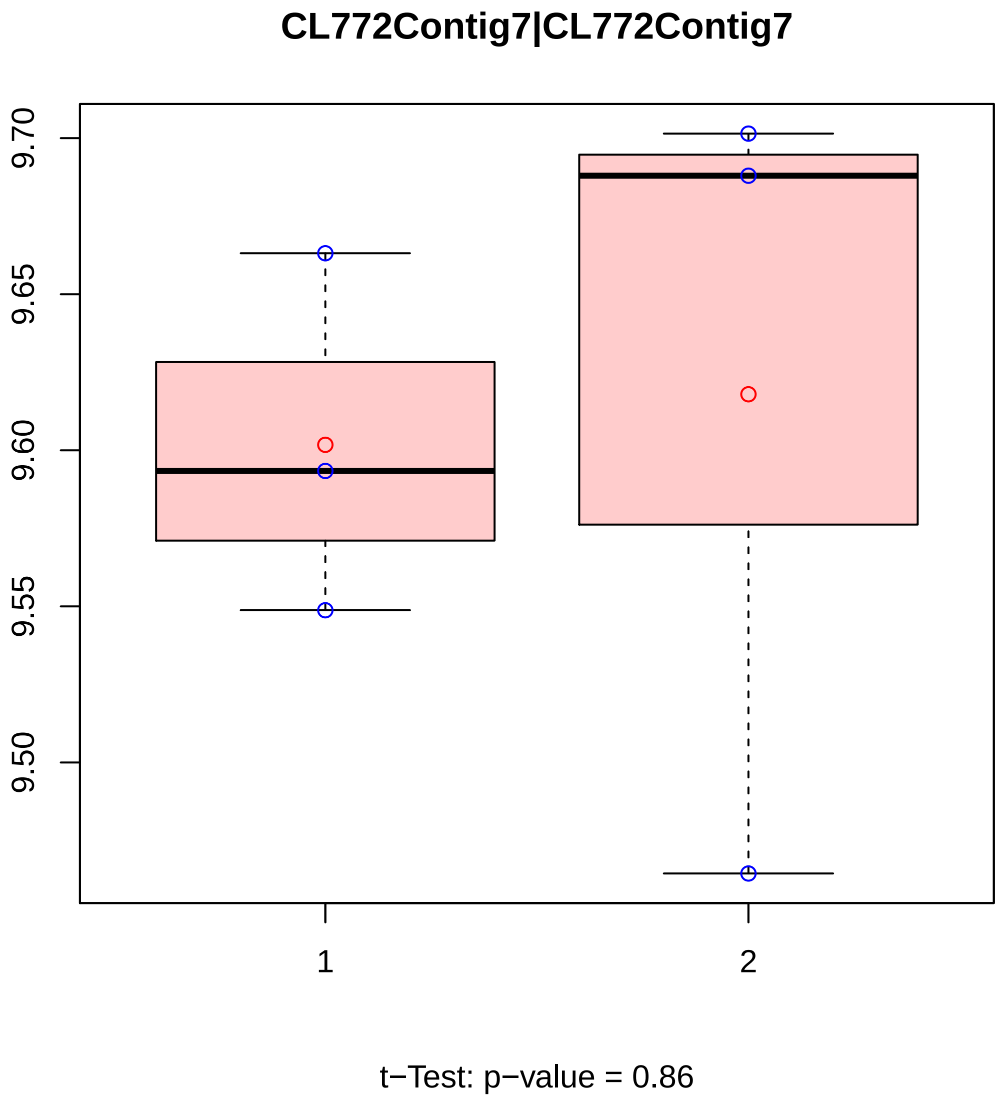

CL772Contig7|CL772Contig7
9.70
9.65
9.60
9.55
9.50
1
2
t−Test: p−value = 0.86

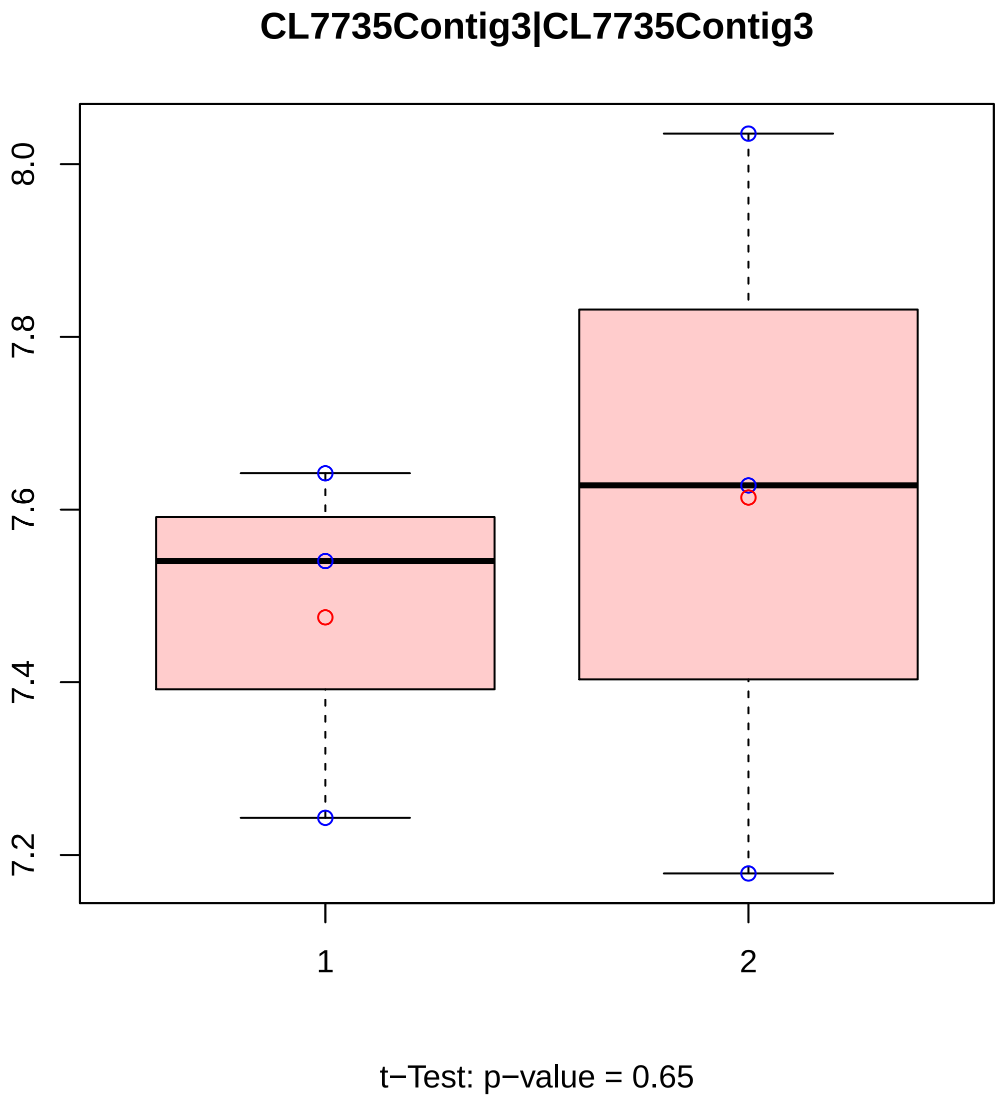

CL7735Contig3|CL7735Contig3
8.0
7.8
7.6
7.4
7.2
1
2
t−Test: p−value = 0.65

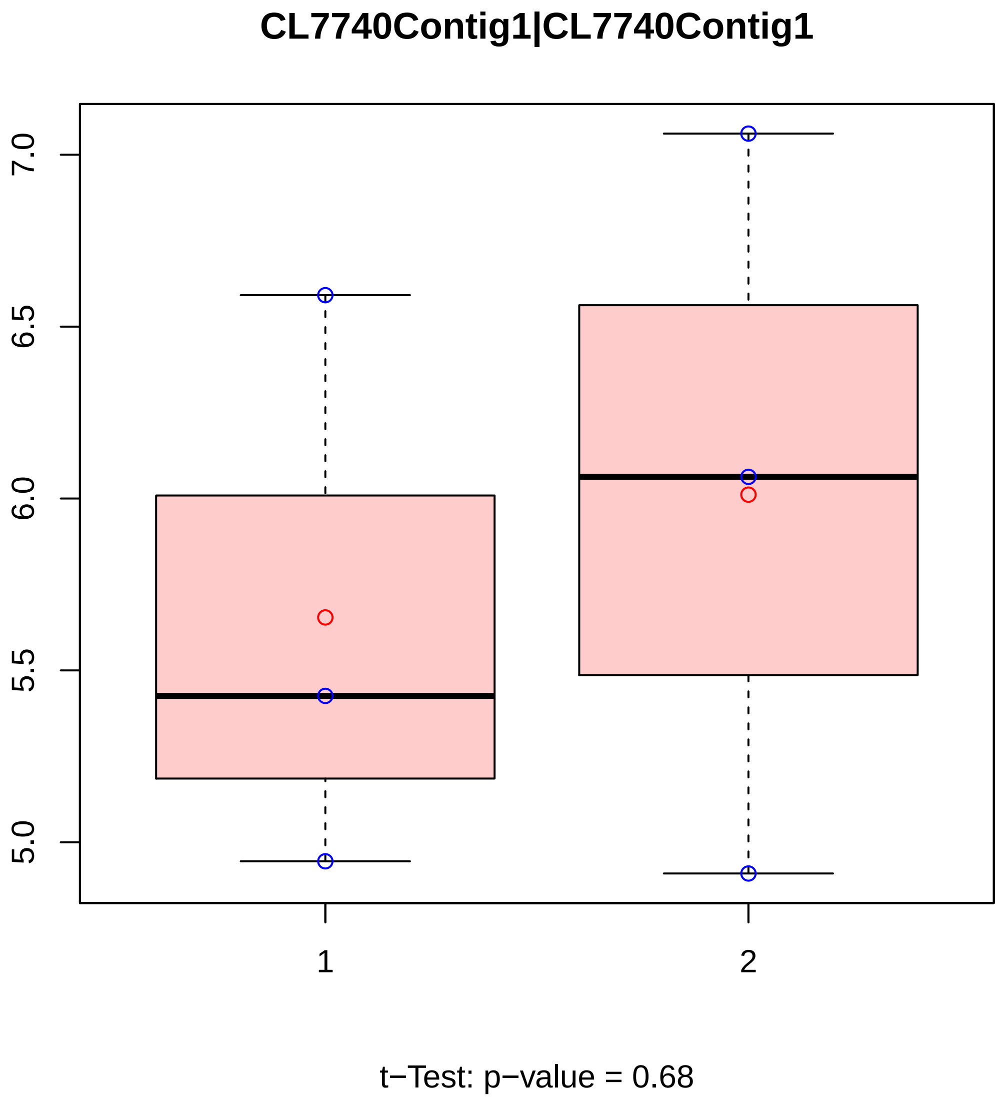

CL7740Contig1|CL7740Contig1
7.0
6.5
6.0
5.5
5.0
1
2
t−Test: p−value = 0.68

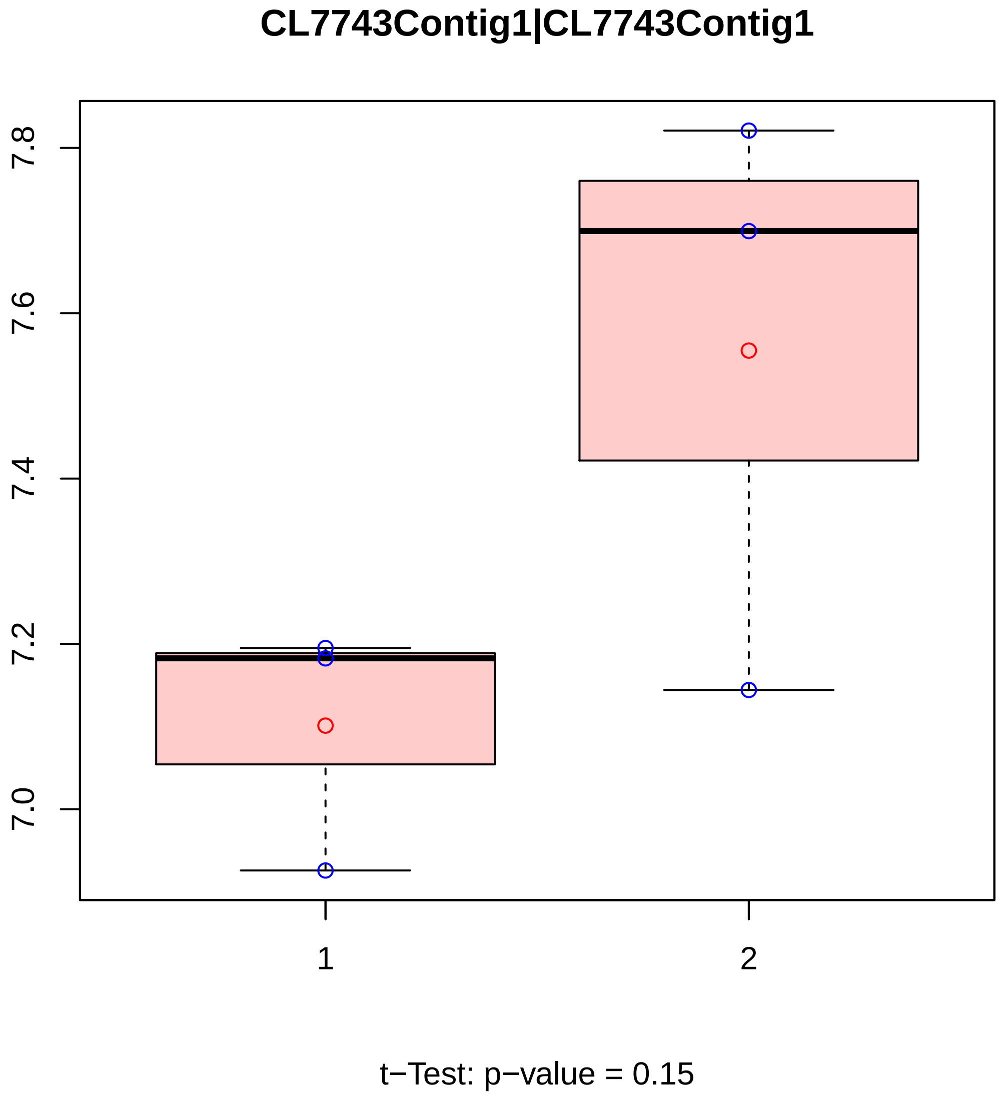
CL7743Contig1|CL7743Contig1
7.0
7.2
7.4
7.6
7.8
1
2
t−Test: p−value = 0.15

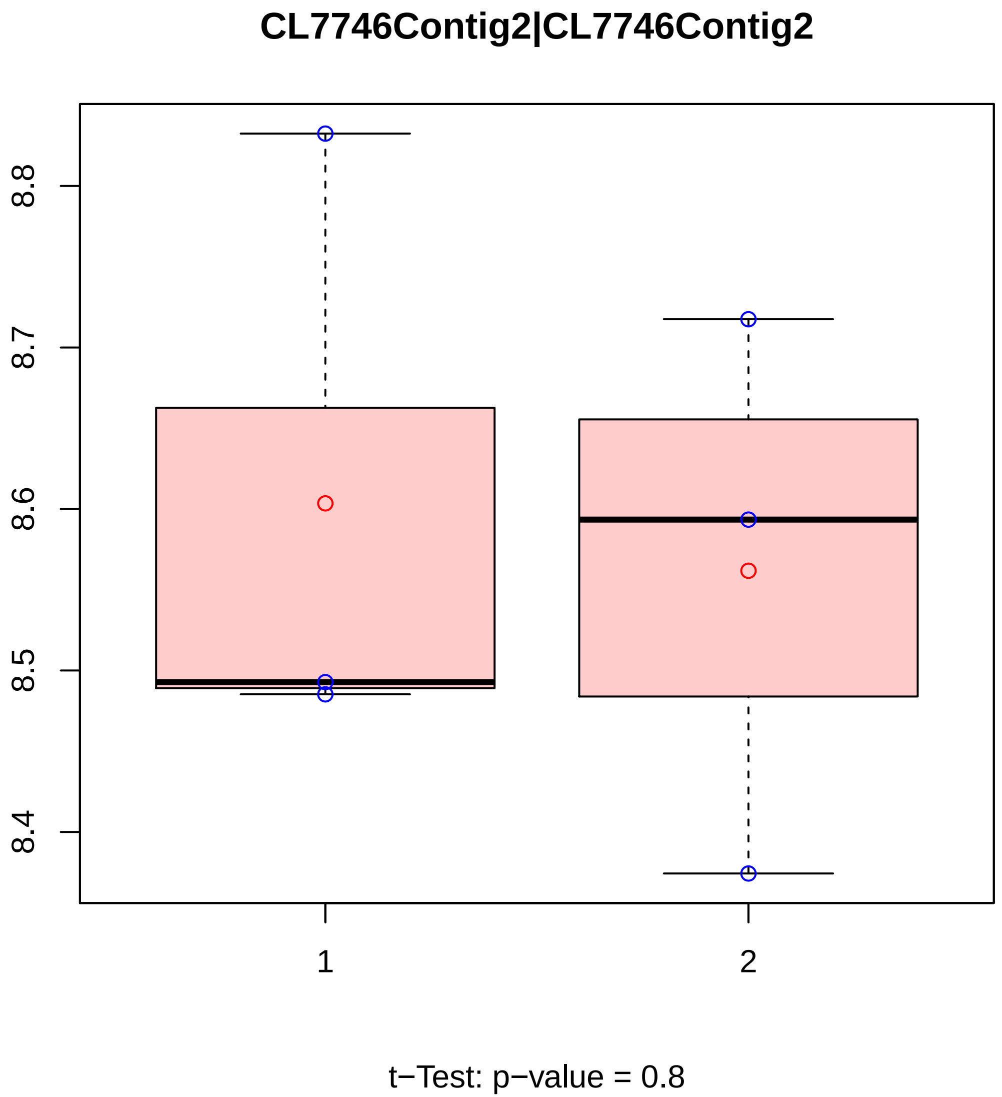
CL7746Contig2|CL7746Contig2
8.4
8.5
8.6
8.7
8.8
1
2
t−Test: p−value = 0.8

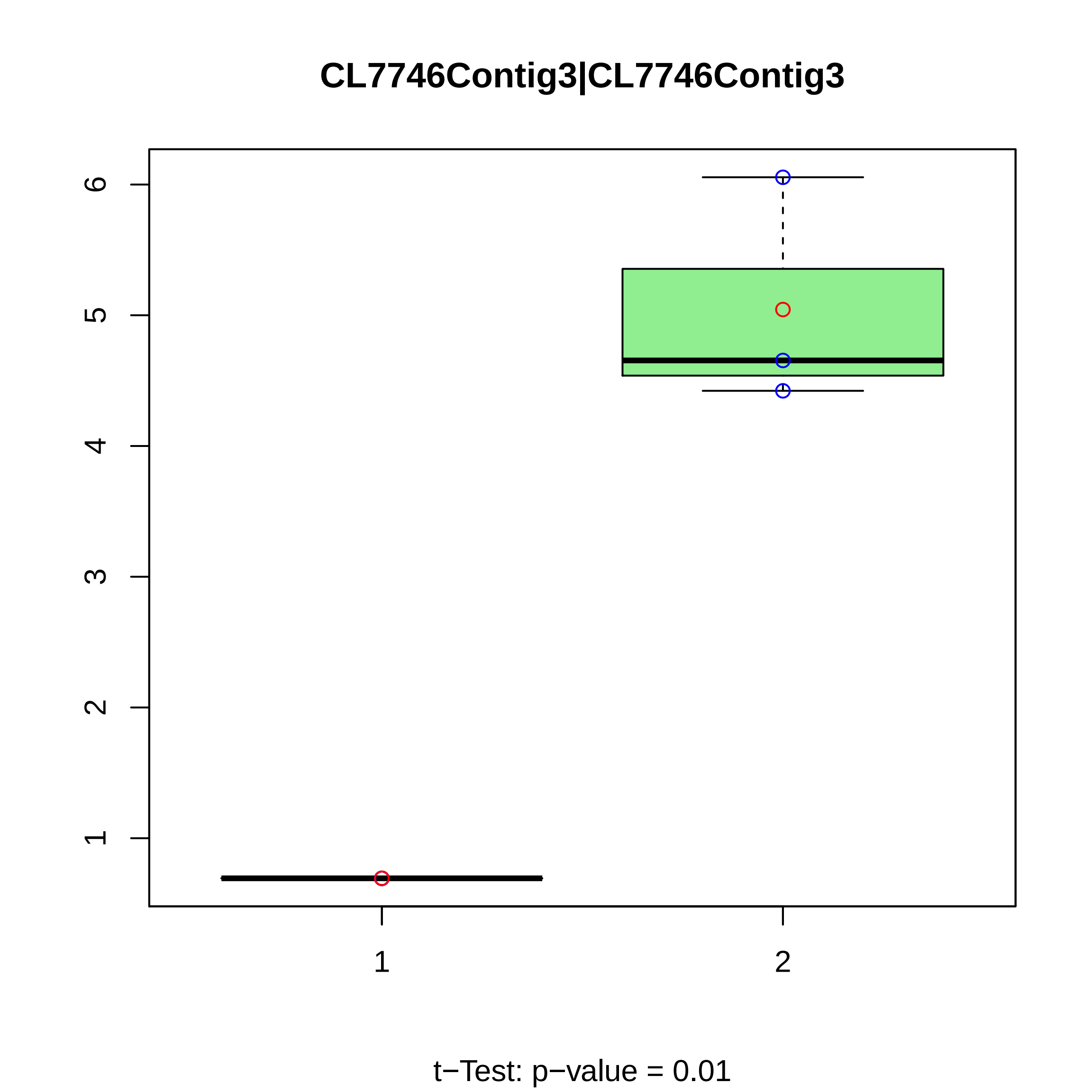

CL7746Contig3|CL7746Contig3
6
5
4
3
2
1
1
2
t−Test: p−value = 0.01

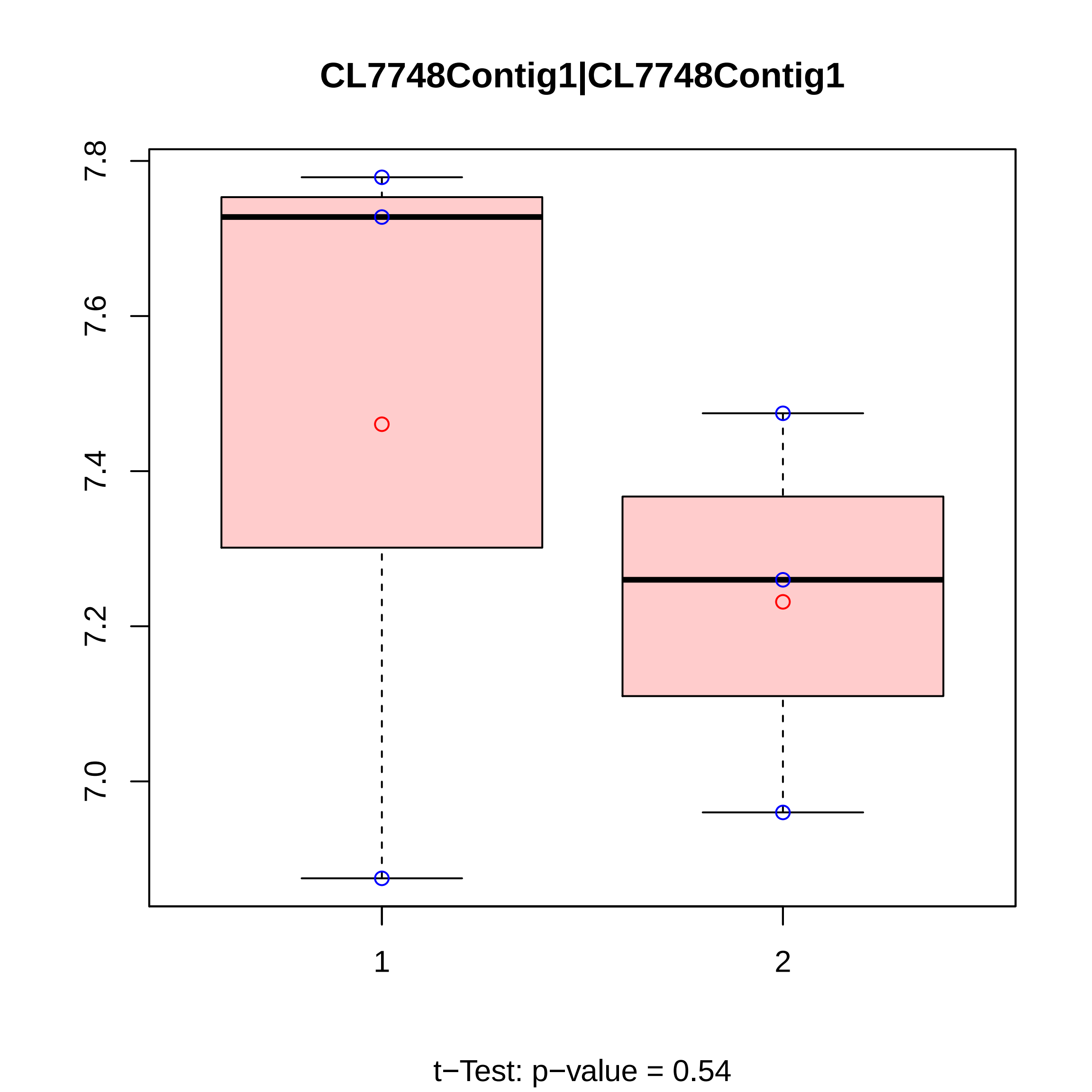
CL7748Contig1|CL7748Contig1
7.8
7.6
7.4
7.2
7.0
1
2
t−Test: p−value = 0.54

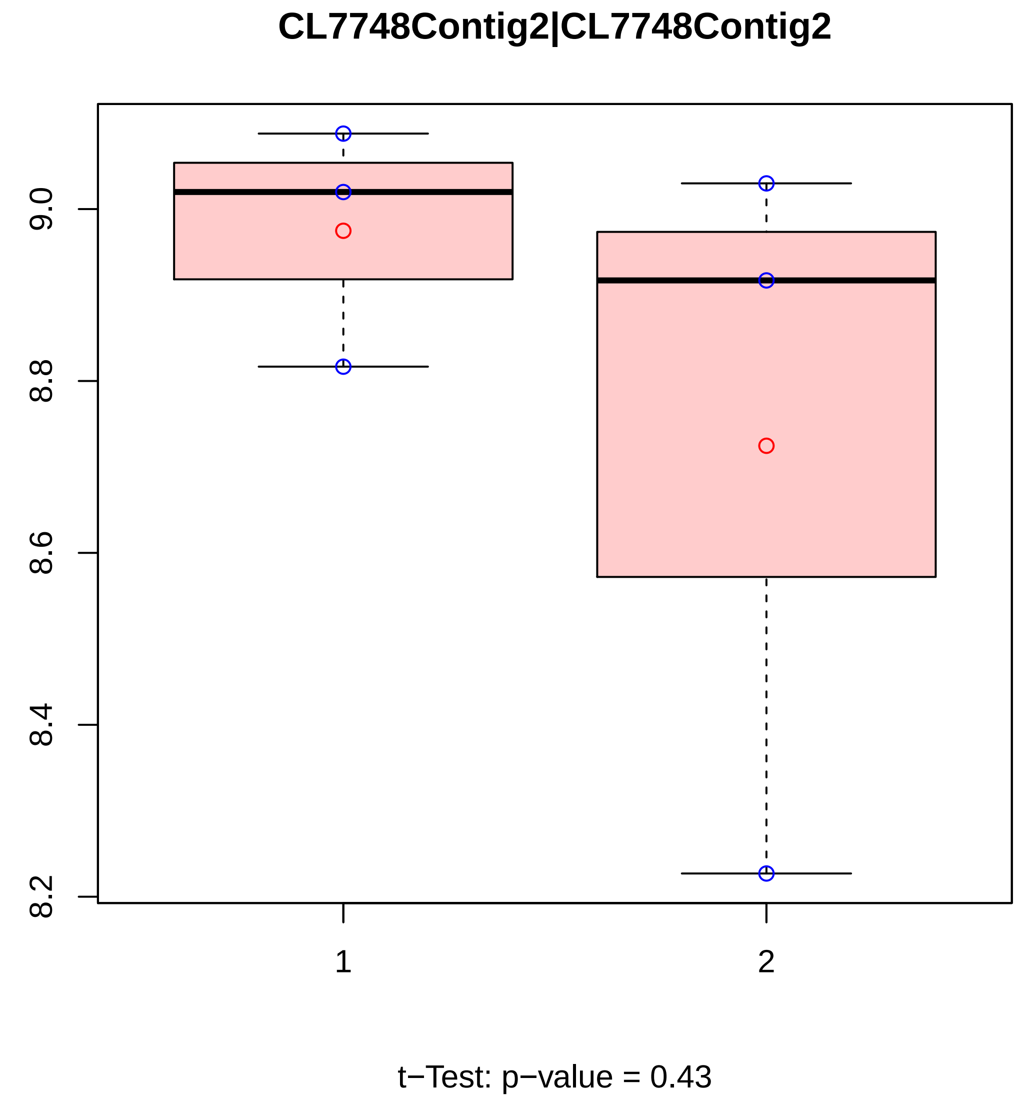
CL7748Contig2|CL7748Contig2
9.0
8.8
8.6
8.4
8.2
1
2
t−Test: p−value = 0.43

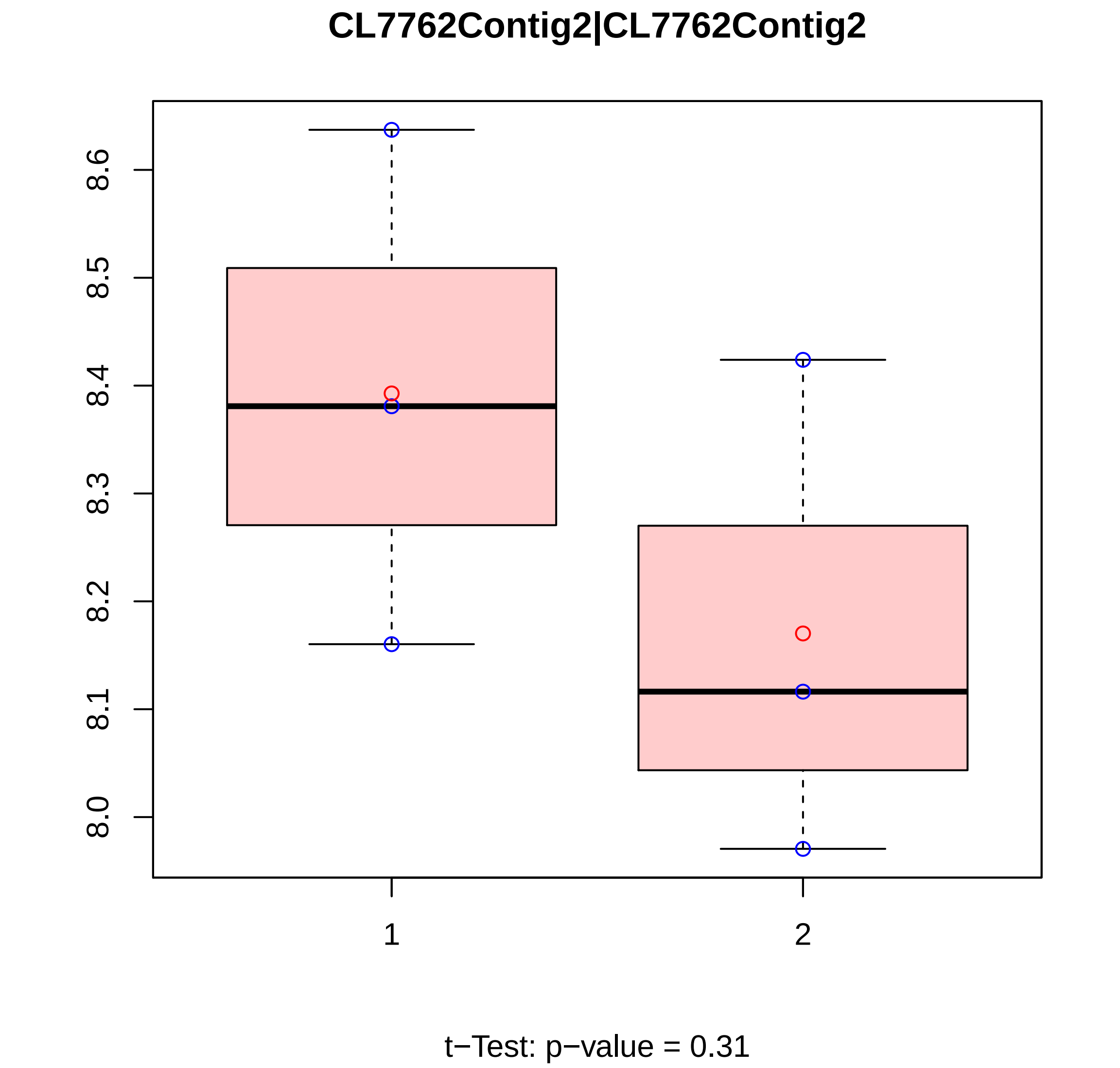
CL7762Contig2|CL7762Contig2
8.0
8.1
8.2
8.3
8.4
8.5
8.6
1
2
t−Test: p−value = 0.31

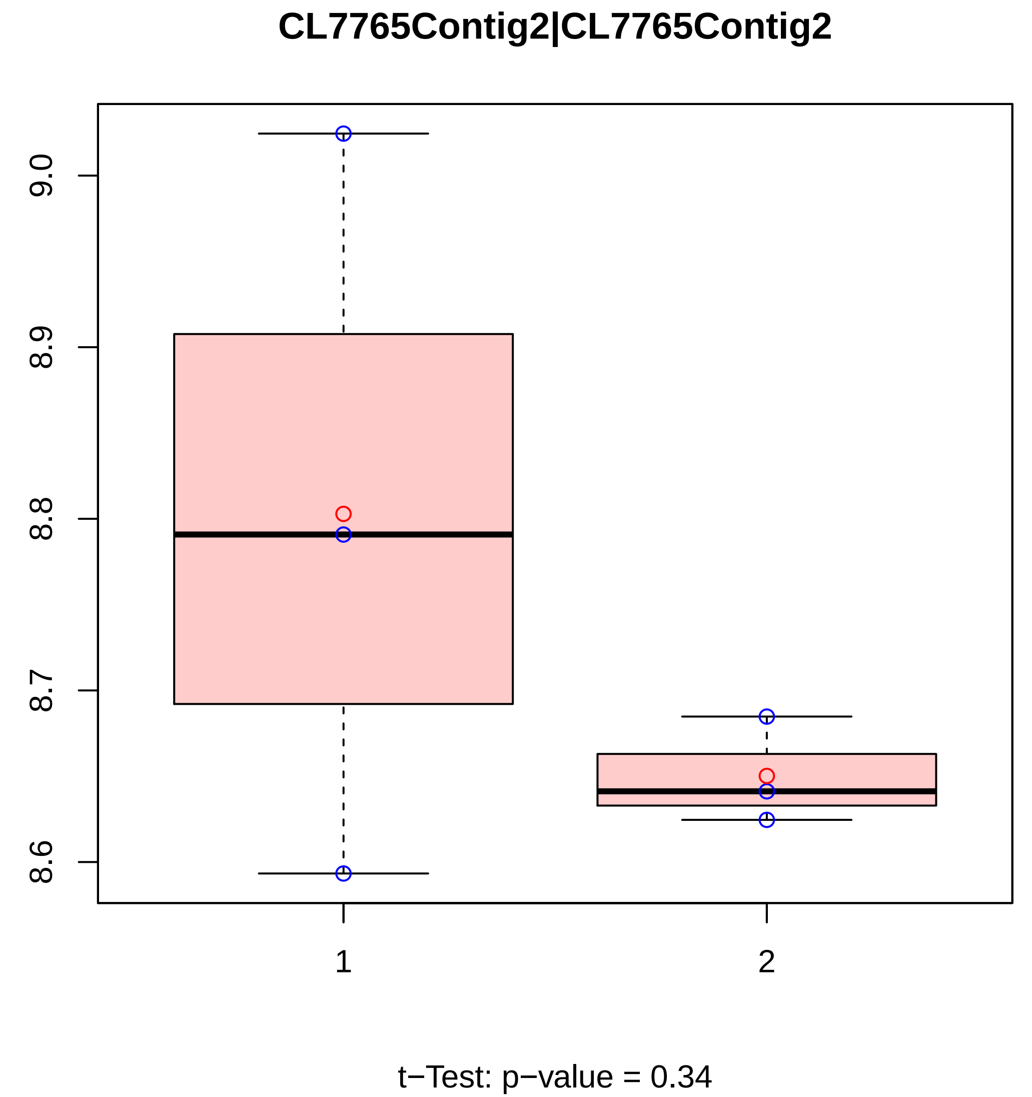

CL7765Contig2|CL7765Contig2
9.0
8.9
8.8
8.7
8.6
1
2
t−Test: p−value = 0.34

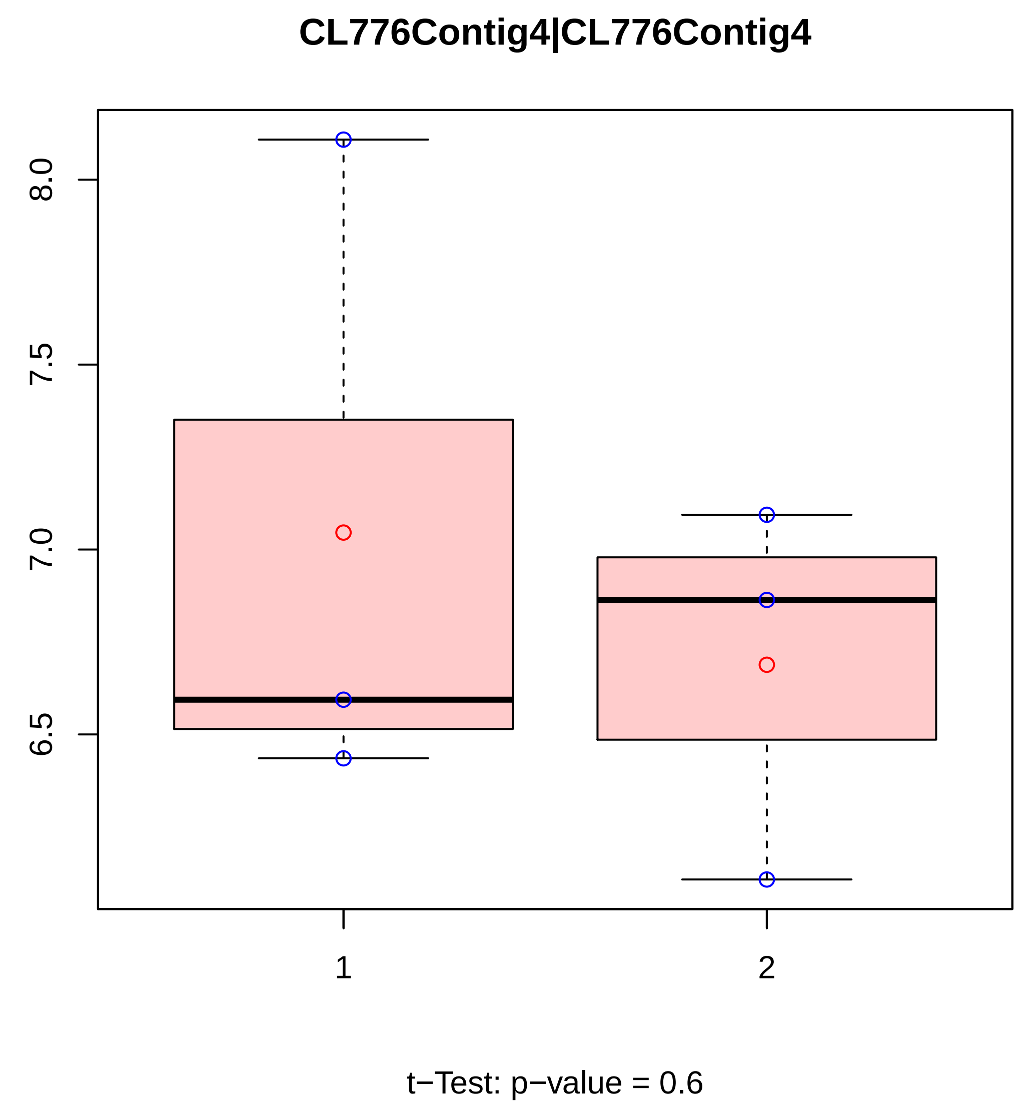
CL776Contig4|CL776Contig4
8.0
7.5
7.0
6.5
1
2
t−Test: p−value = 0.6

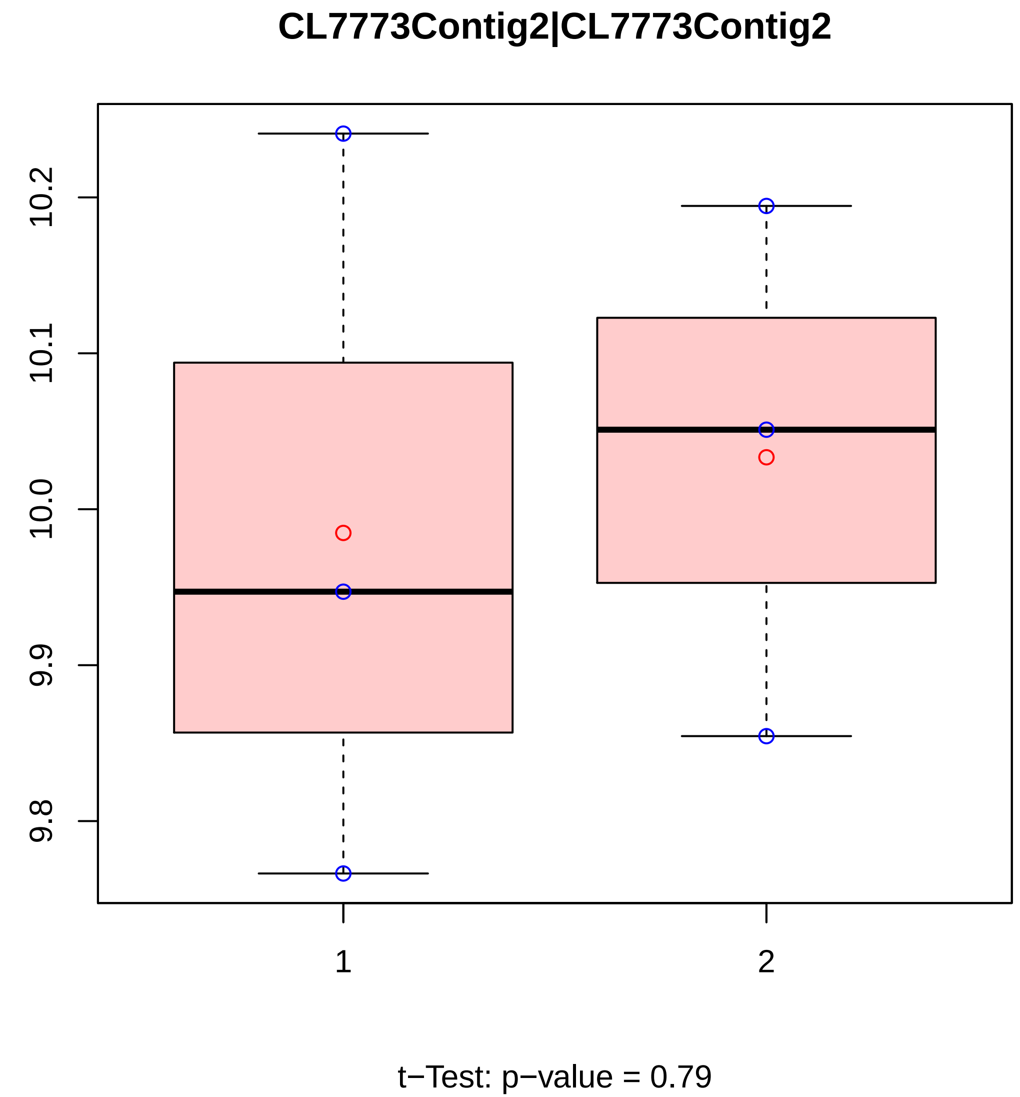
CL7773Contig2|CL7773Contig2
10.2
10.1
10.0
9.9
9.8
1
2
t−Test: p−value = 0.79

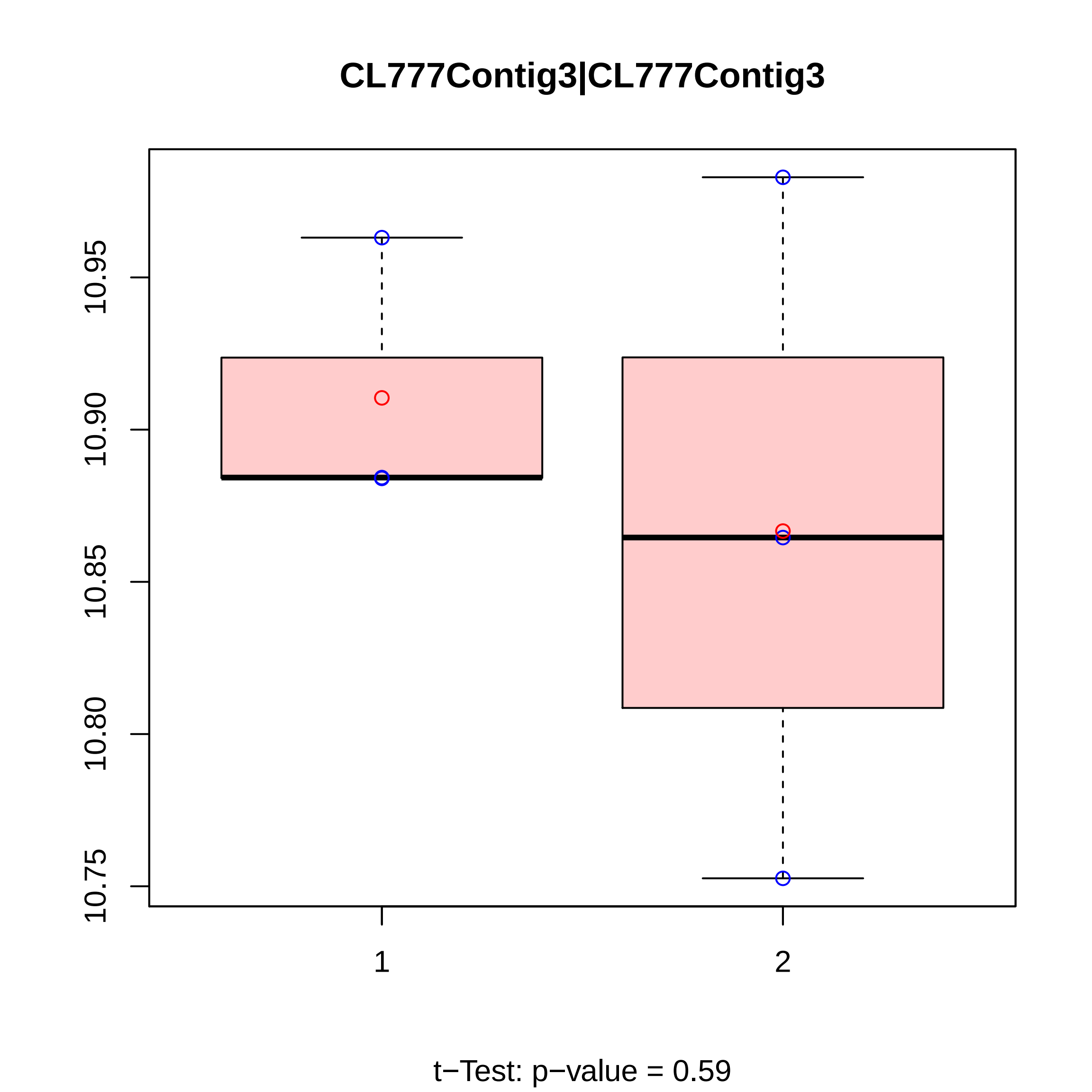

CL777Contig3|CL777Contig3
10.95
10.90
10.85
10.80
10.75
1
2
t−Test: p−value = 0.59

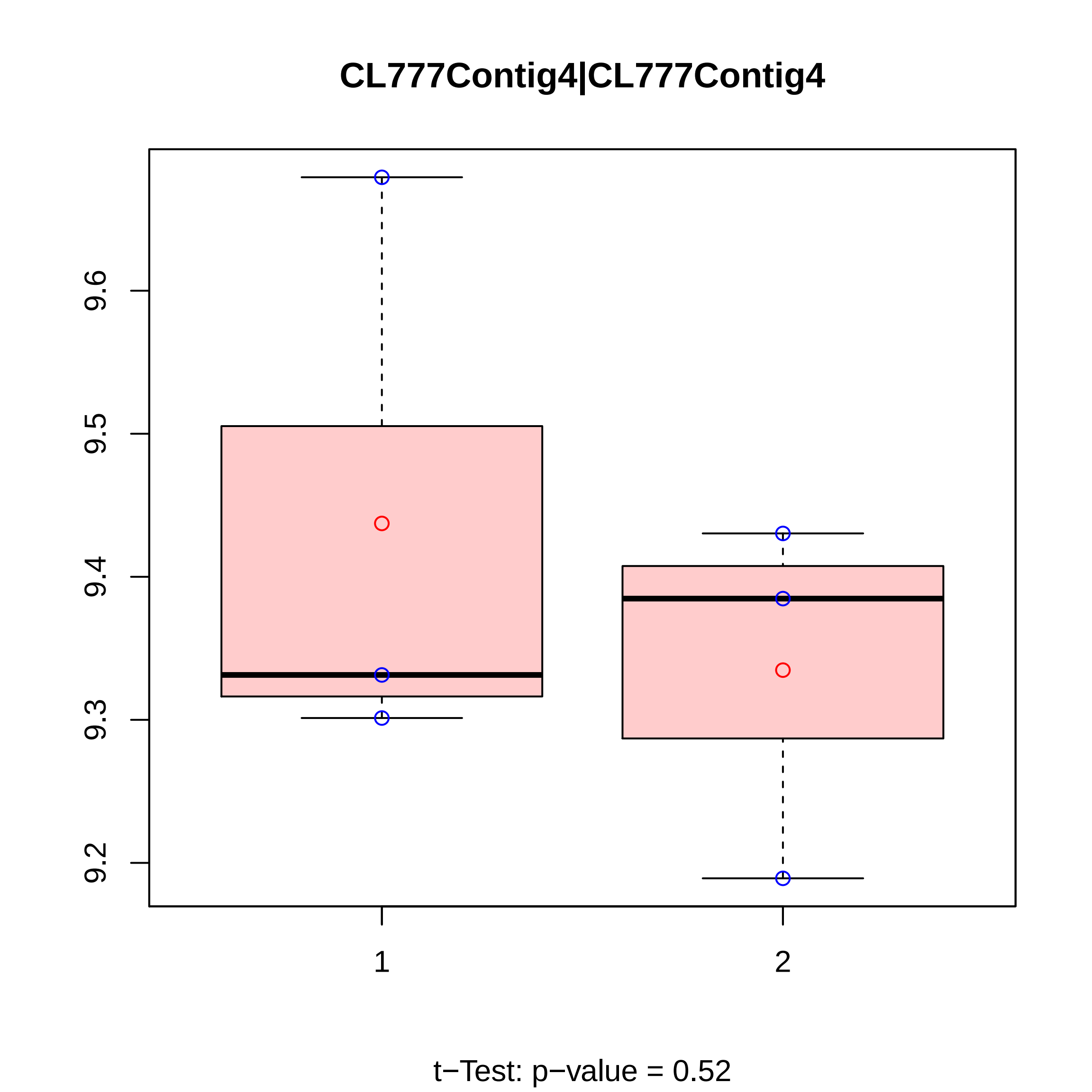
CL777Contig4|CL777Contig4
9.2
9.3
9.4
9.5
9.6
1
2
t−Test: p−value = 0.52

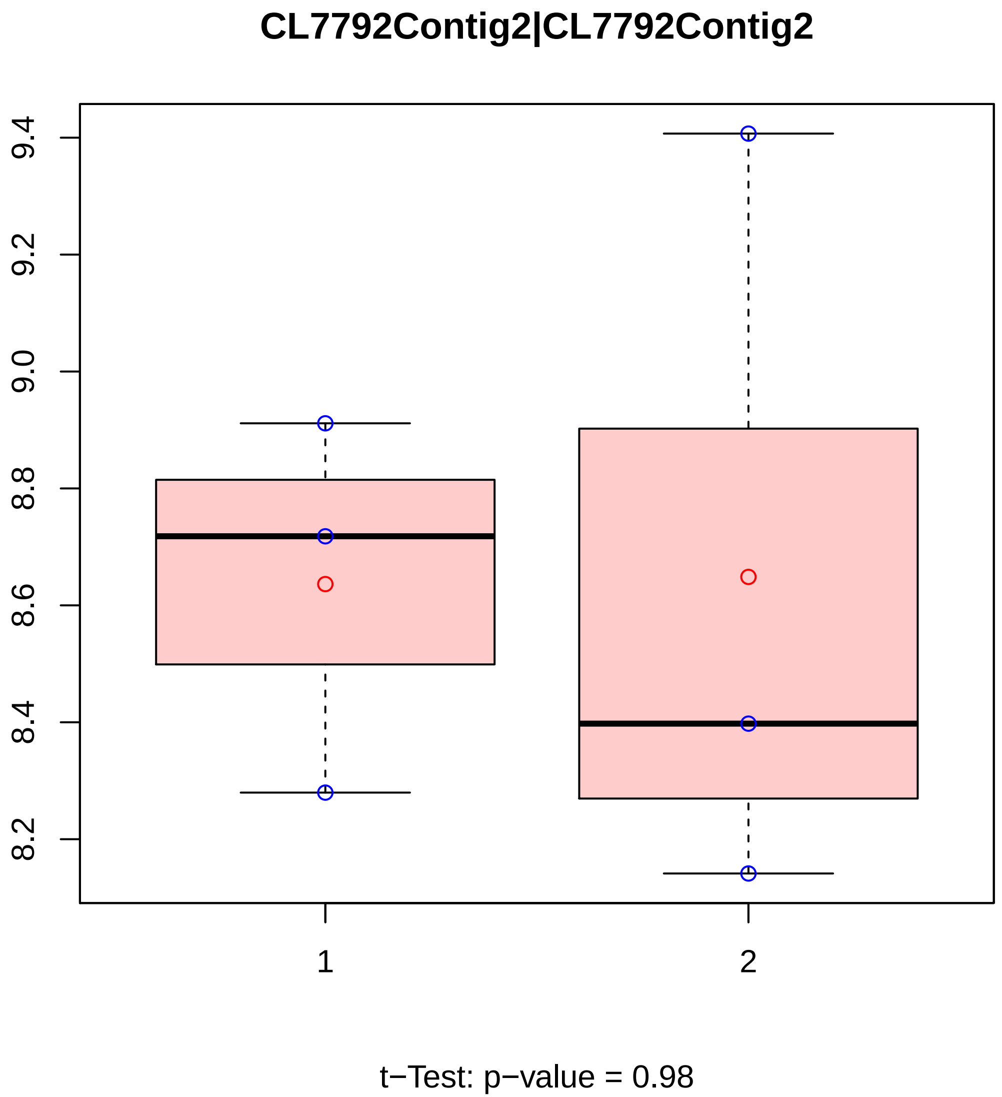
CL7792Contig2|CL7792Contig2
9.4
9.2
9.0
8.8
8.6
8.4
8.2
1
2
t−Test: p−value = 0.98

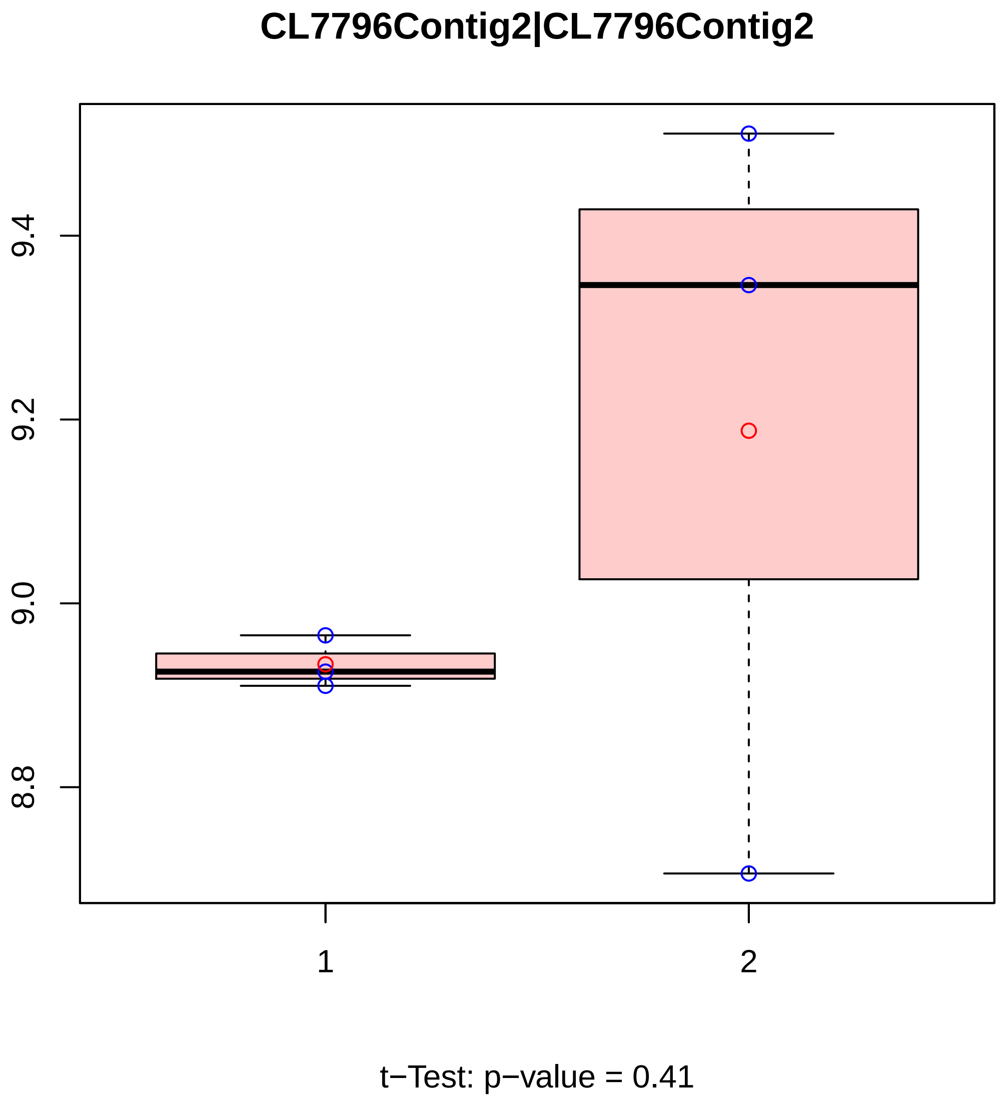
CL7796Contig2|CL7796Contig2
9.4
9.2
9.0
8.8
1
2
t−Test: p−value = 0.41

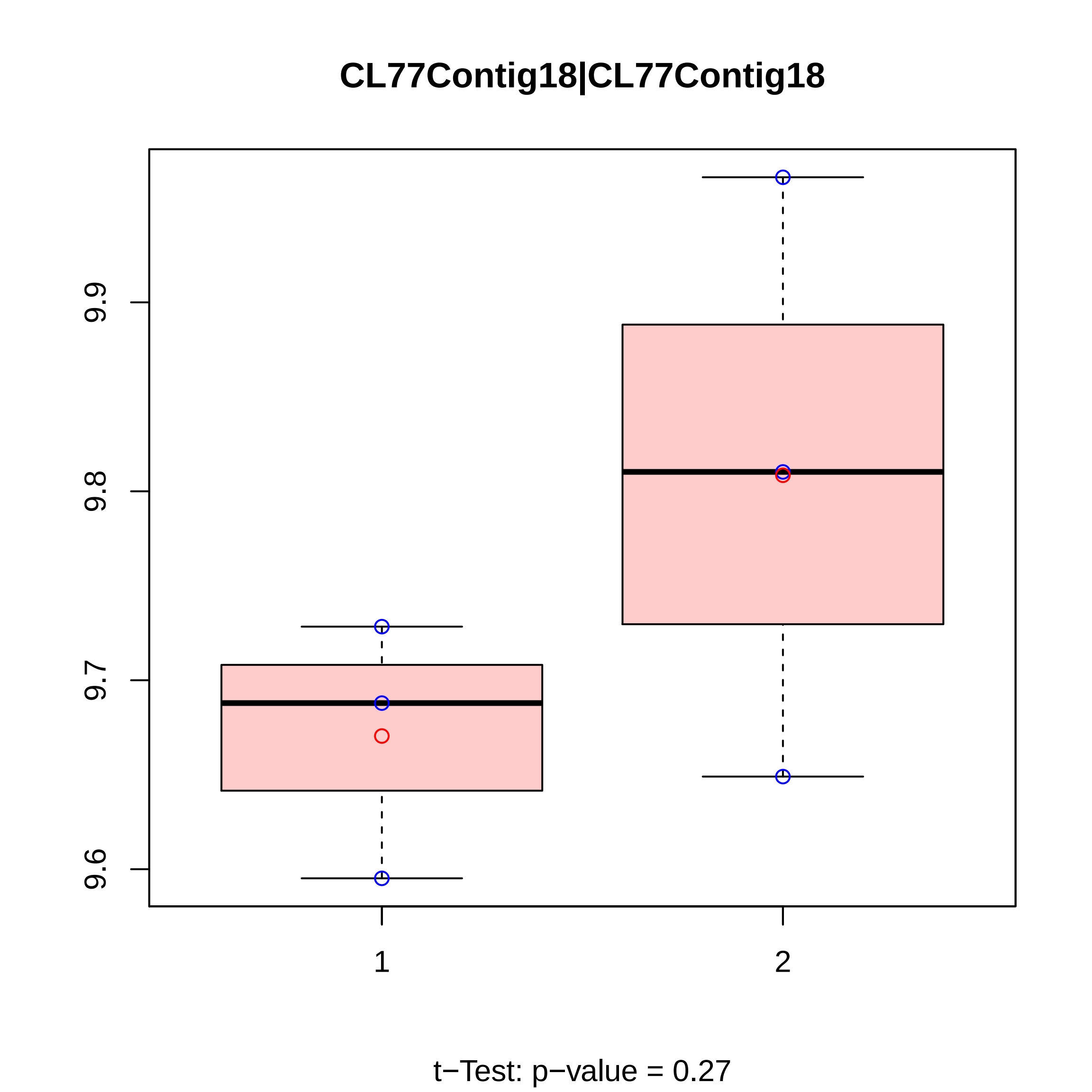
CL77Contig18|CL77Contig18
9.6
9.7
9.8
9.9
1
2
t−Test: p−value = 0.27

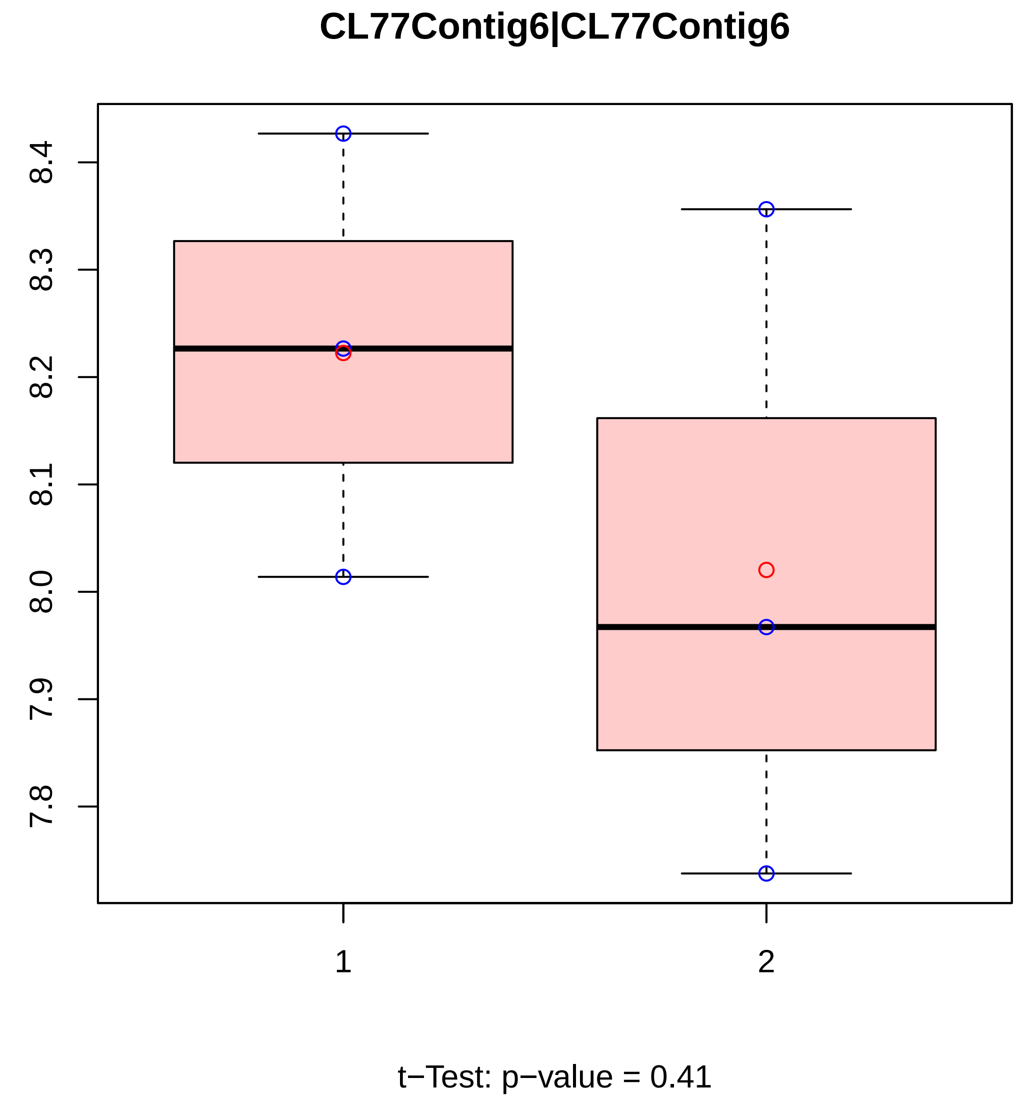

CL77Contig6|CL77Contig6
8.4
8.3
8.2
8.1
8.0
7.9
7.8
1
2
t−Test: p−value = 0.41

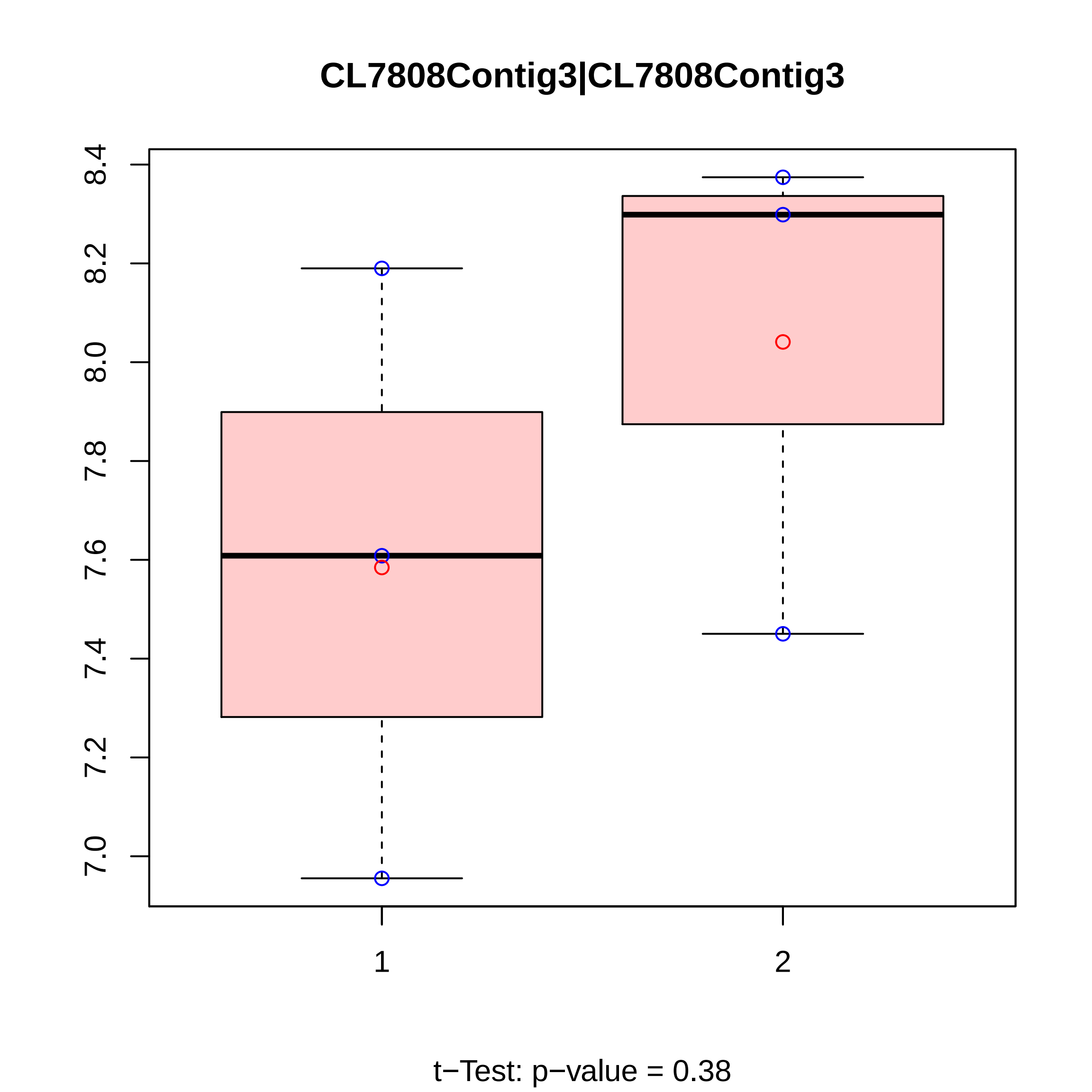
CL7808Contig3|CL7808Contig3
8.4
8.2
8.0
7.8
7.6
7.4
7.2
7.0
1
2
t−Test: p−value = 0.38

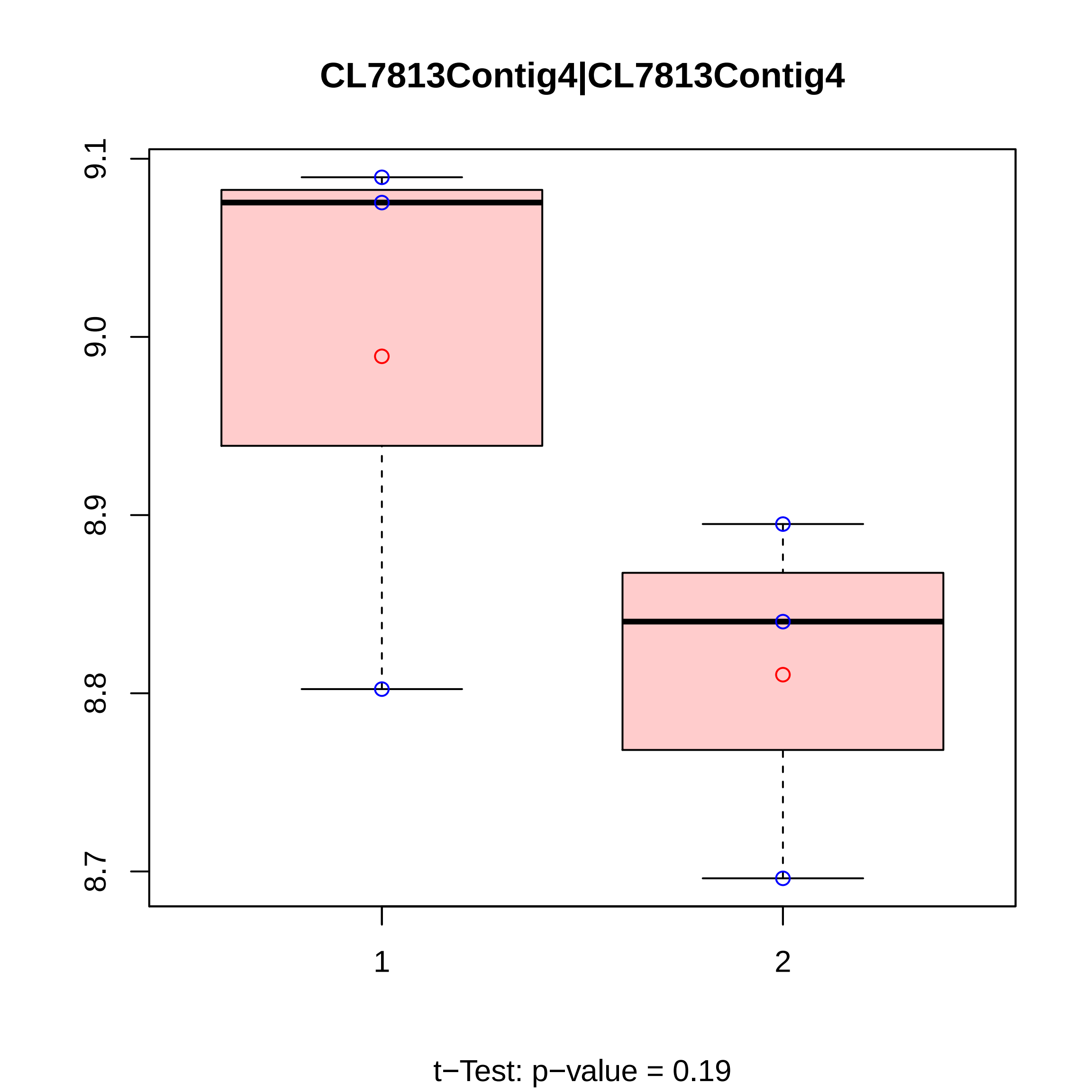

CL7813Contig4|CL7813Contig4
9.1
9.0
8.9
8.8
8.7
1
2
t−Test: p−value = 0.19

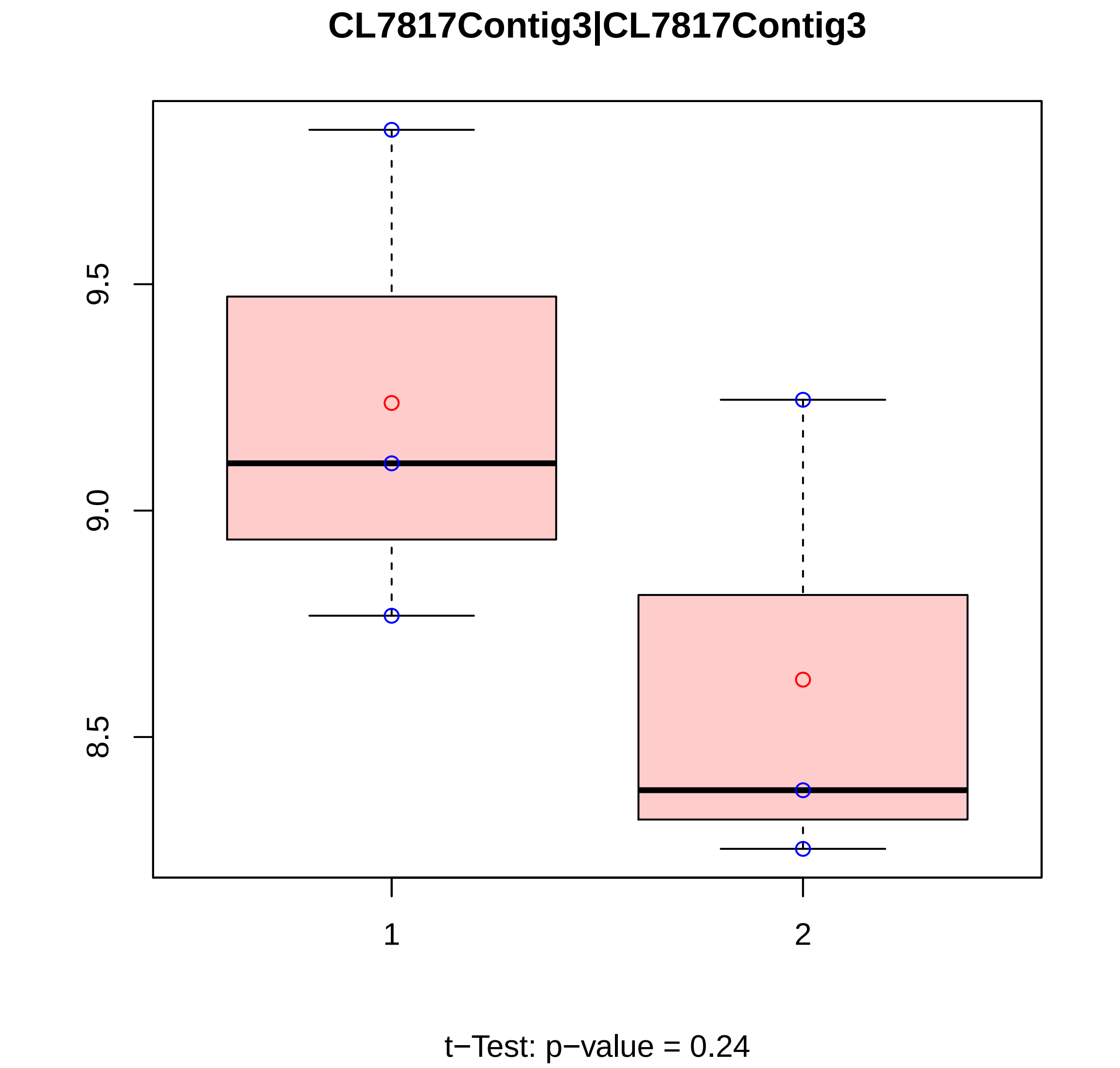

CL7817Contig3|CL7817Contig3
9.5
9.0
8.5
1
2
t−Test: p−value = 0.24

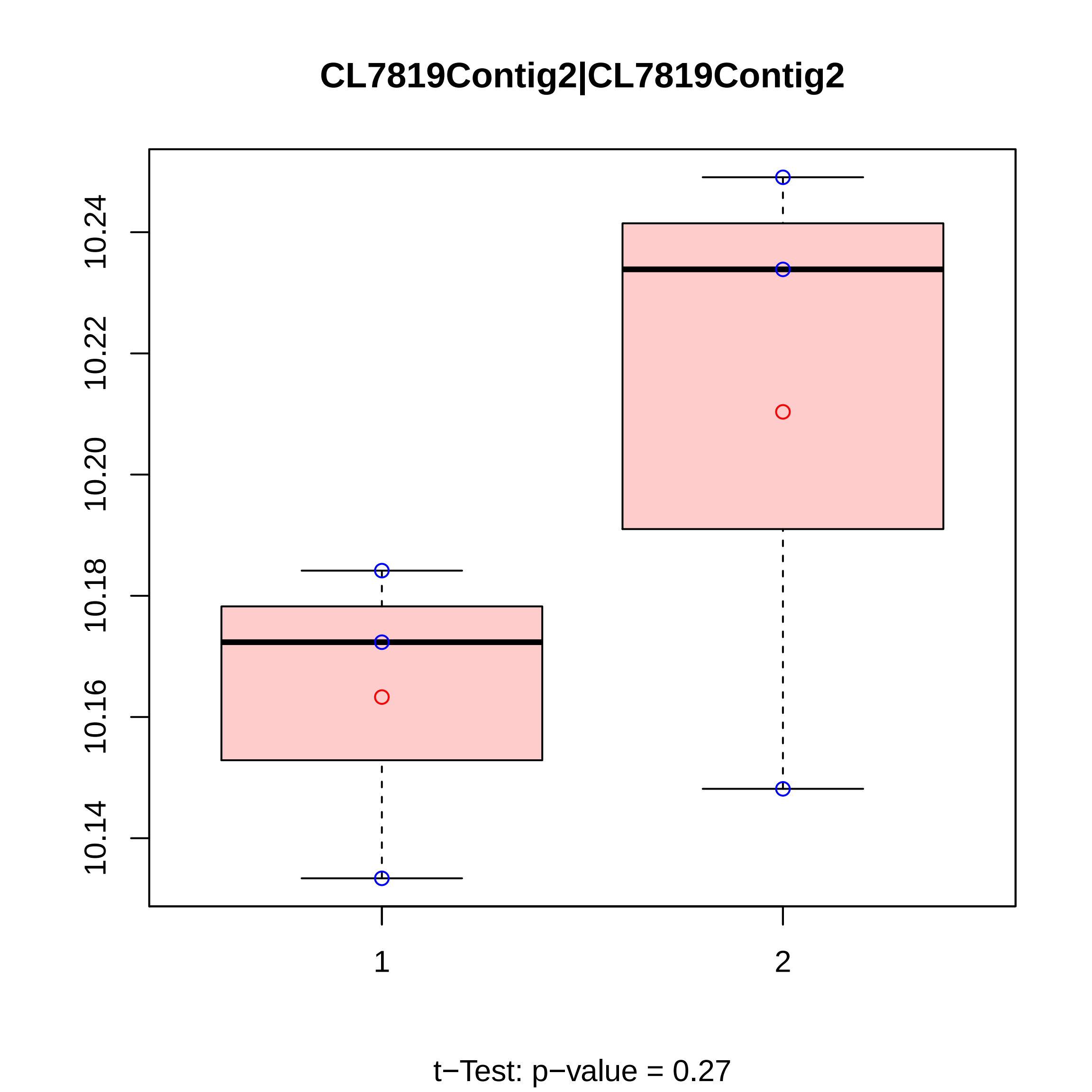

CL7819Contig2|CL7819Contig2
10.14
10.16
10.18
10.20
10.22
10.24
1
2
t−Test: p−value = 0.27

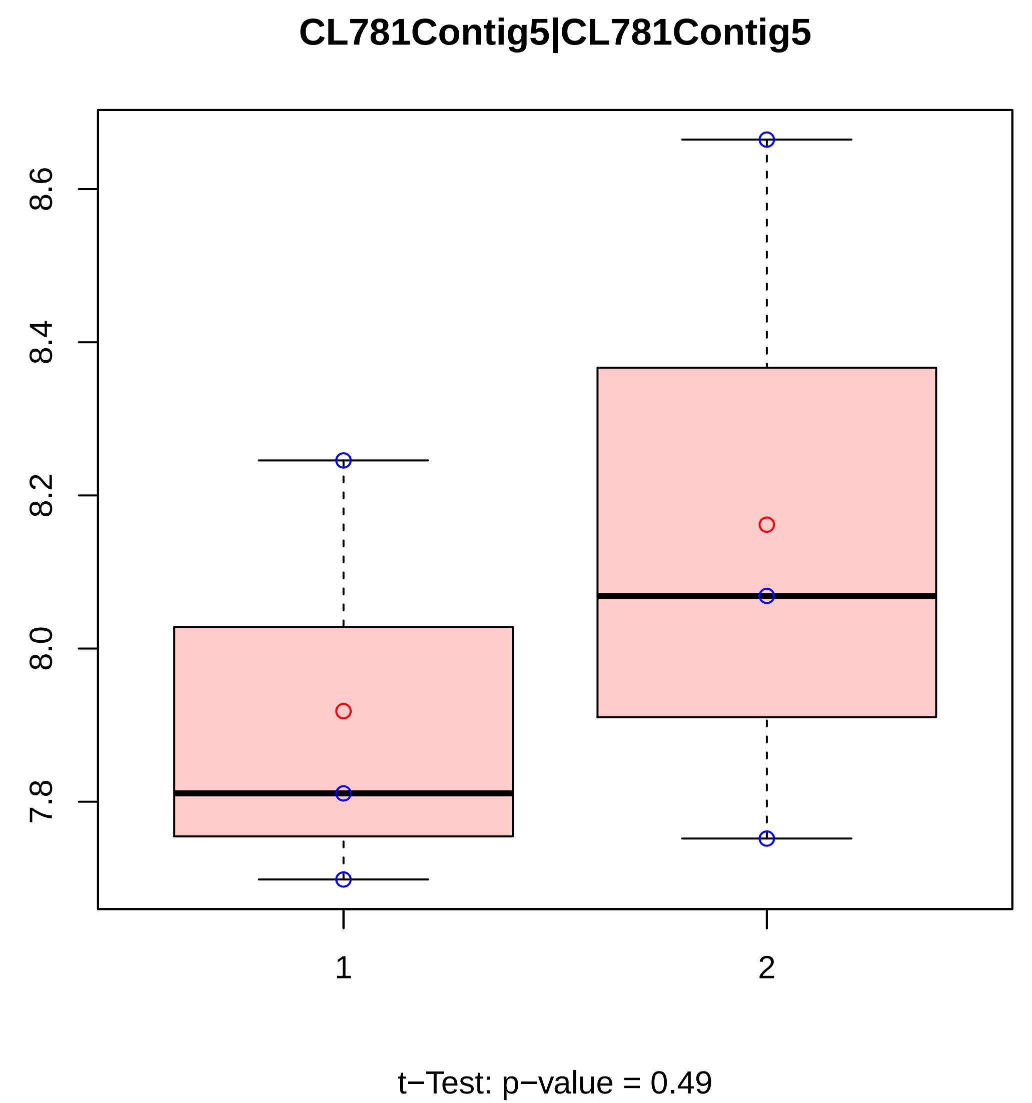
CL781Contig5|CL781Contig5
8.6
8.4
8.2
8.0
7.8
1
2
t−Test: p−value = 0.49

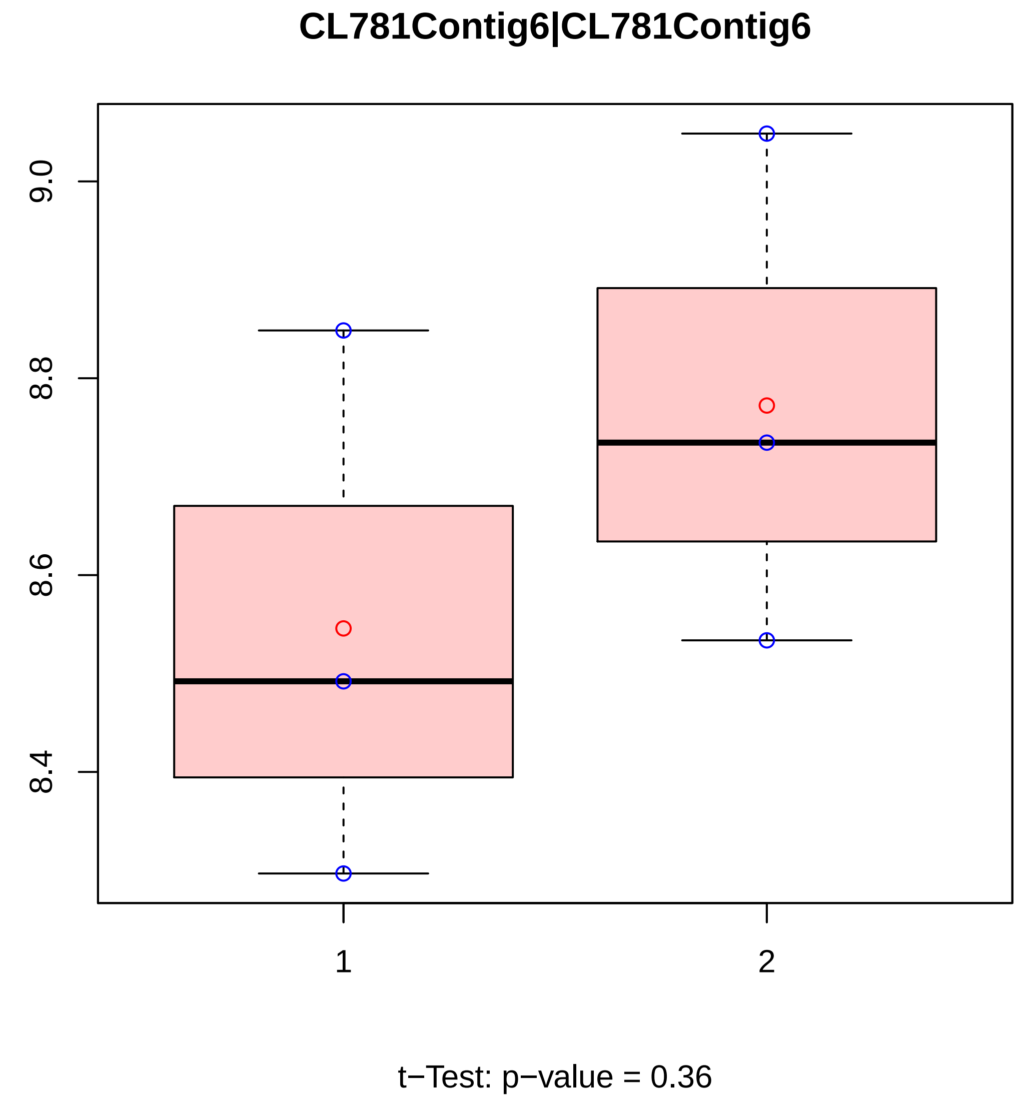
CL781Contig6|CL781Contig6
9.0
8.8
8.6
8.4
1
2
t−Test: p−value = 0.36

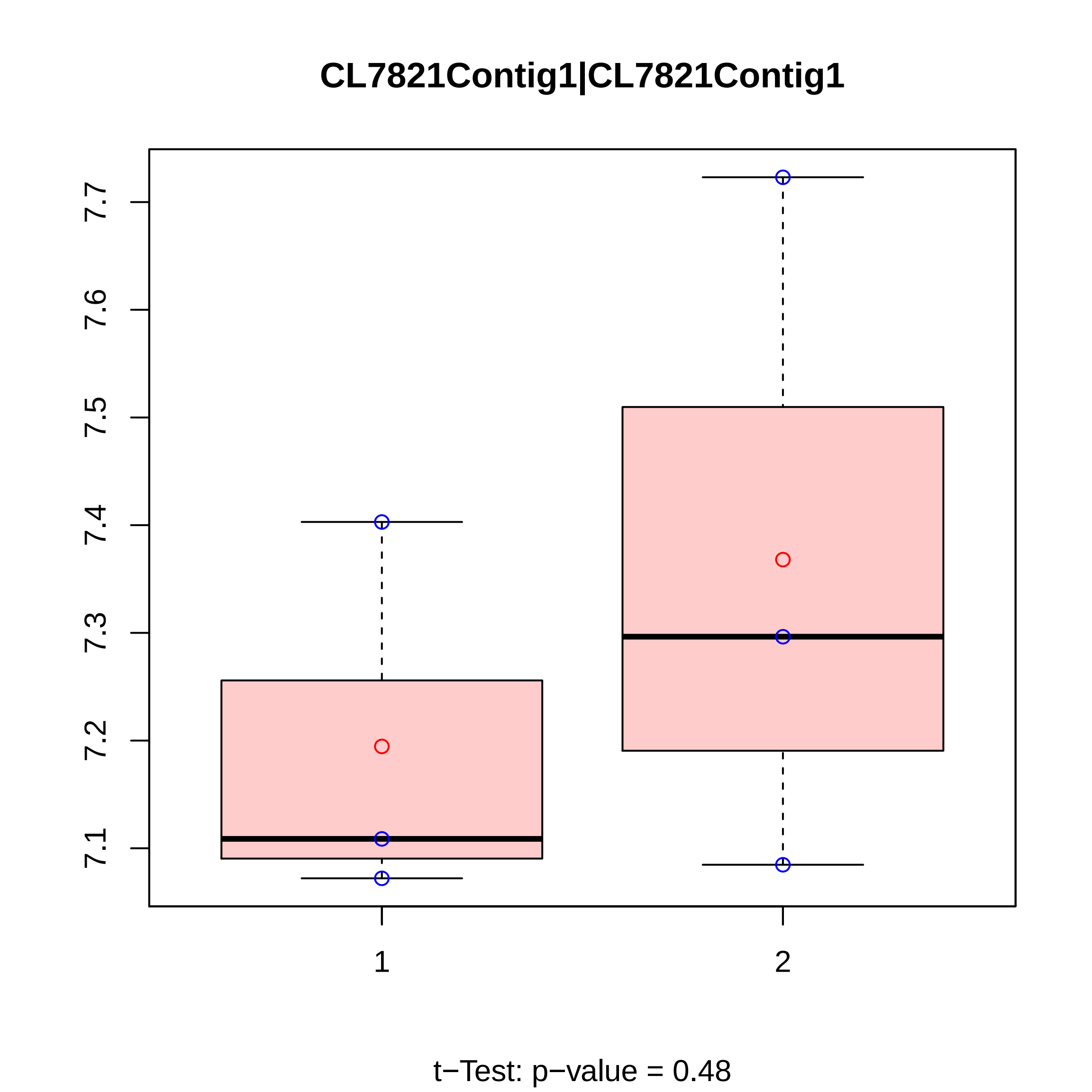

CL7821Contig1|CL7821Contig1
7.1
7.2
7.3
7.4
7.5
7.6
7.7
1
2
t−Test: p−value = 0.48

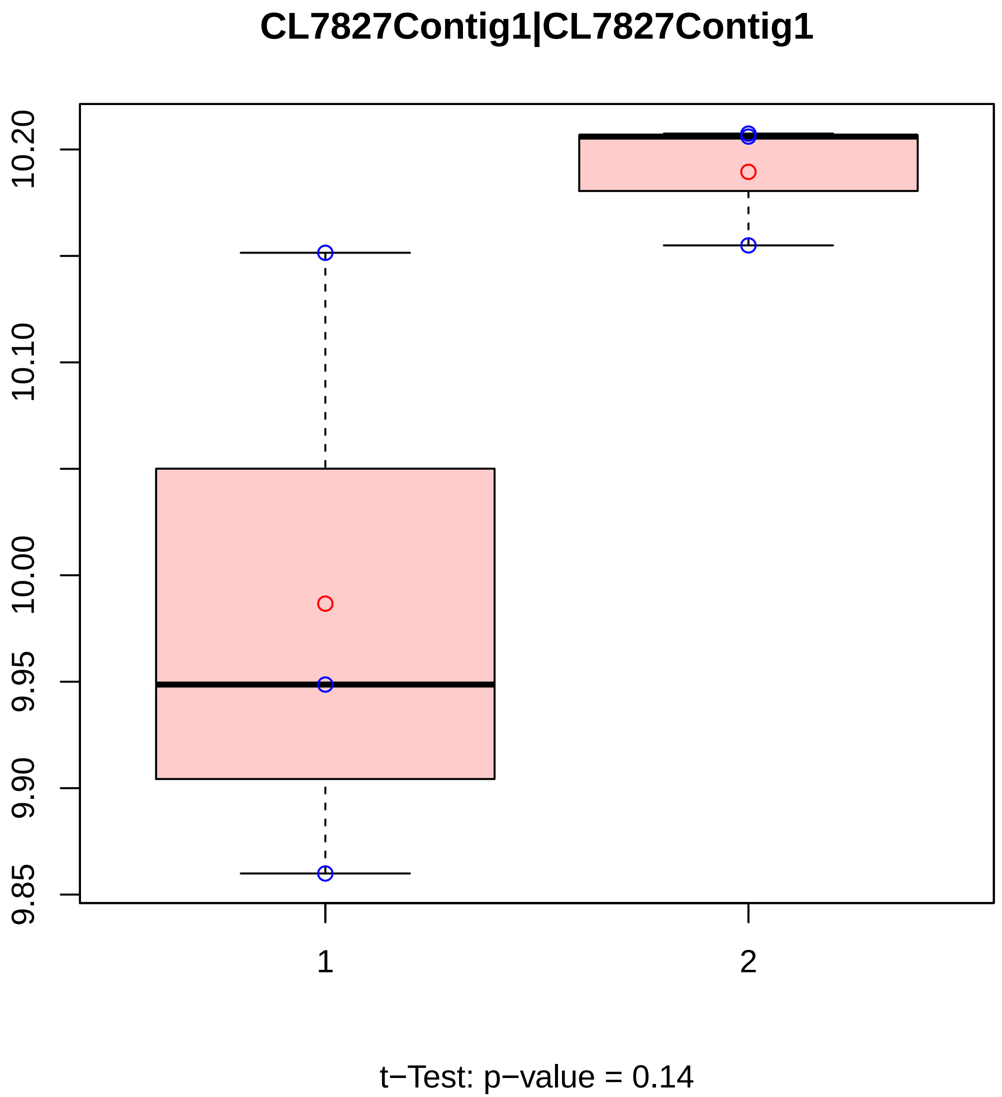
CL7827Contig1|CL7827Contig1
10.20
10.10
10.00
9.95
9.90
9.85
1
2
t−Test: p−value = 0.14

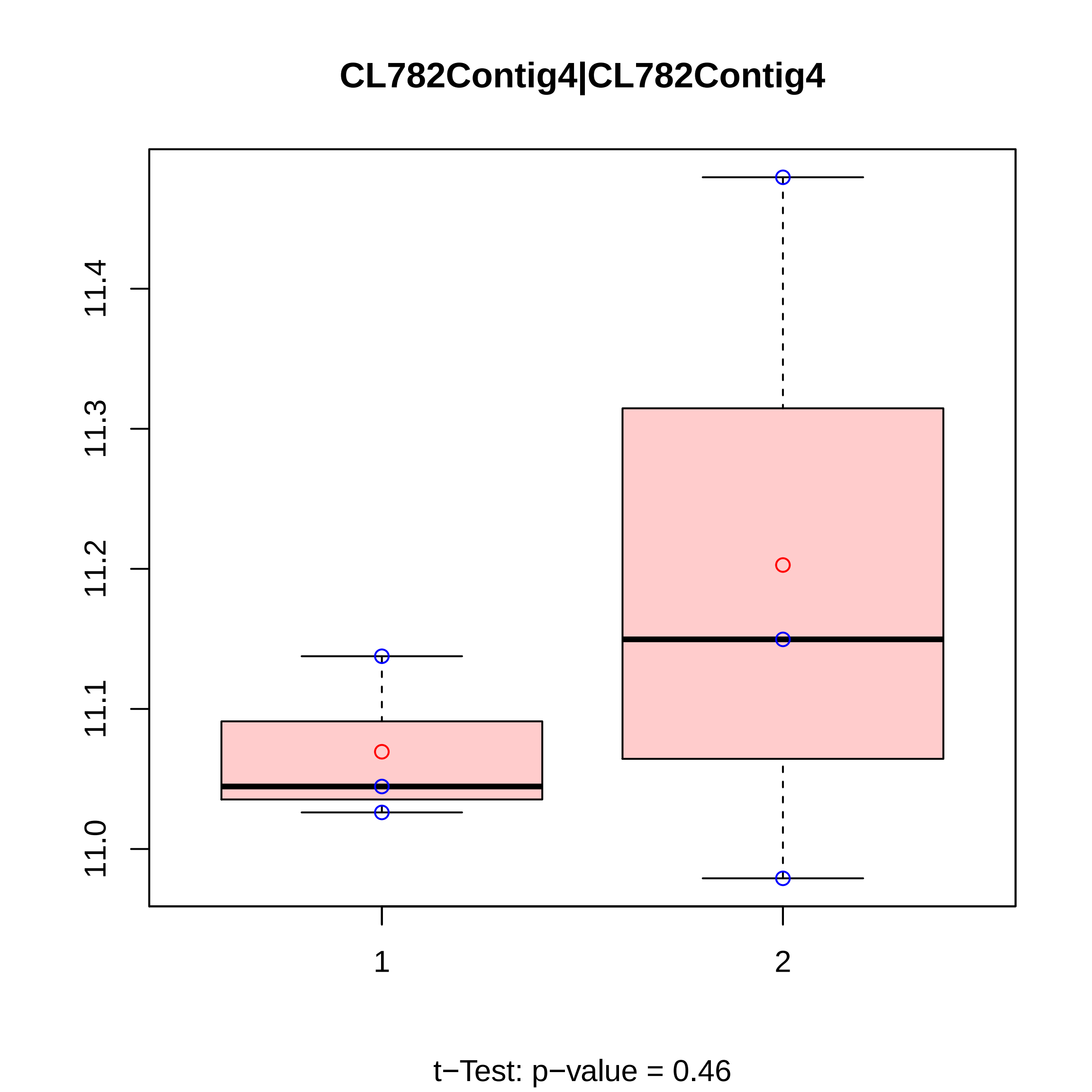

CL782Contig4|CL782Contig4
11.0
11.1
11.2
11.3
11.4
1
2
t−Test: p−value = 0.46

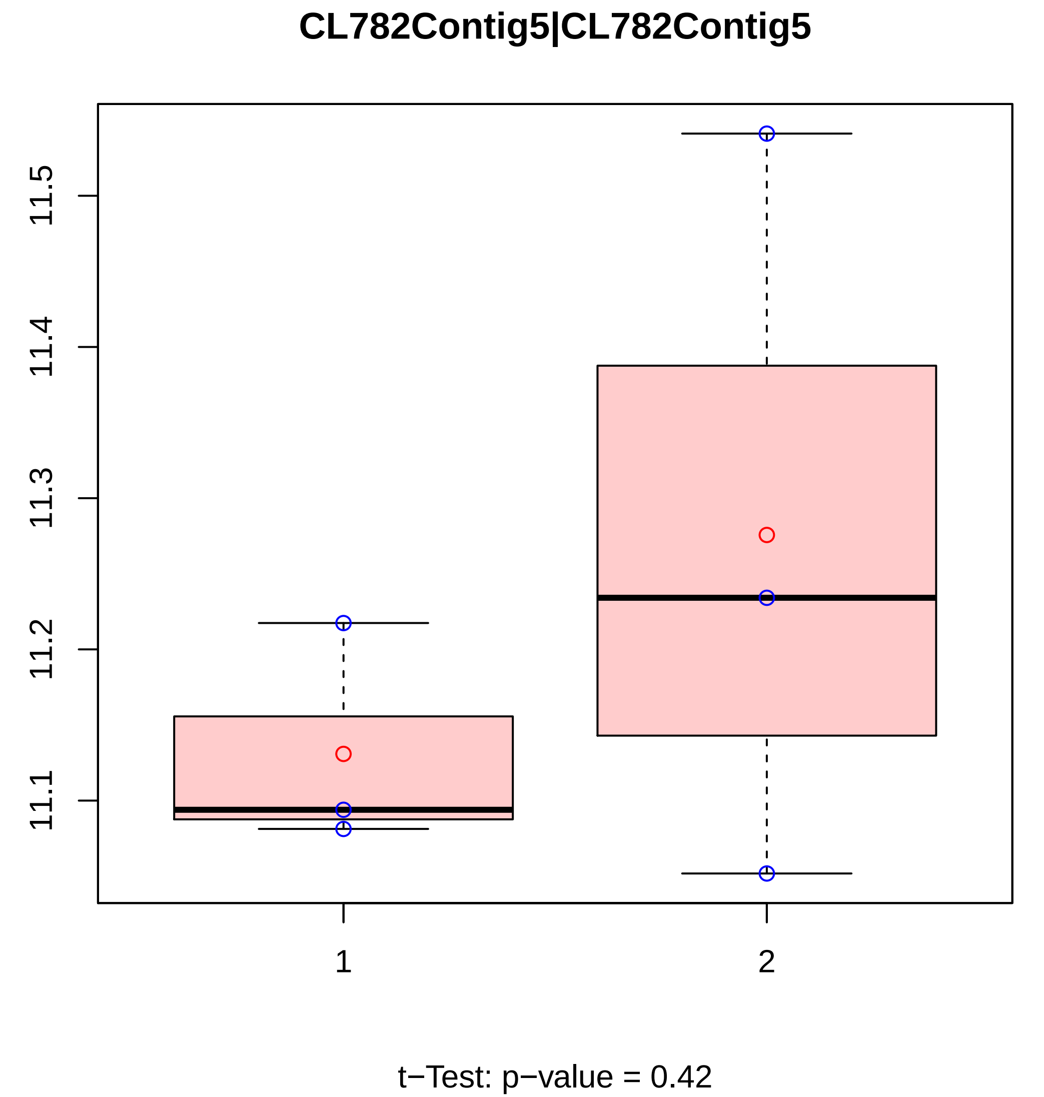
CL782Contig5|CL782Contig5
11.1
11.2
11.3
11.4
11.5
1
2
t−Test: p−value = 0.42

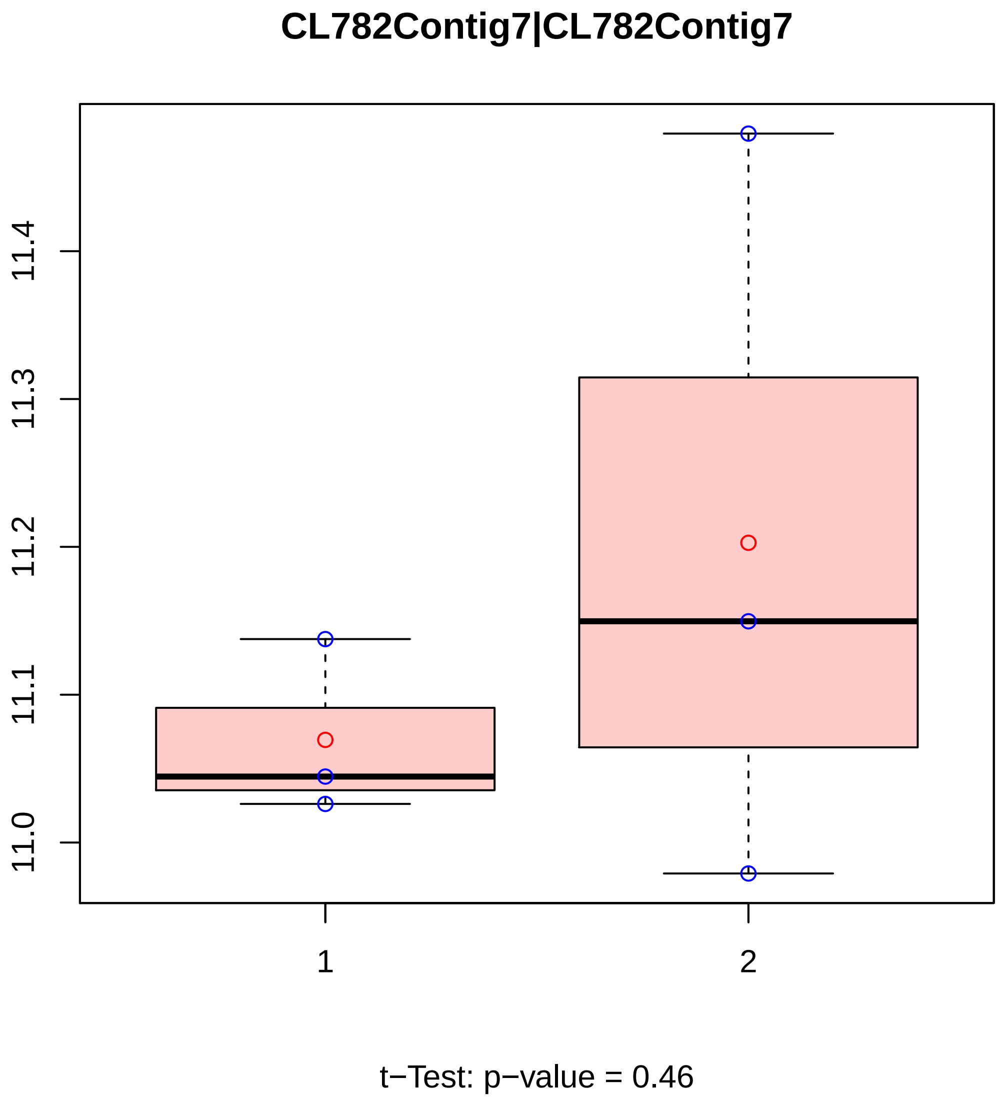
CL782Contig7|CL782Contig7
11.0
11.1
11.2
11.3
11.4
1
2
t−Test: p−value = 0.46

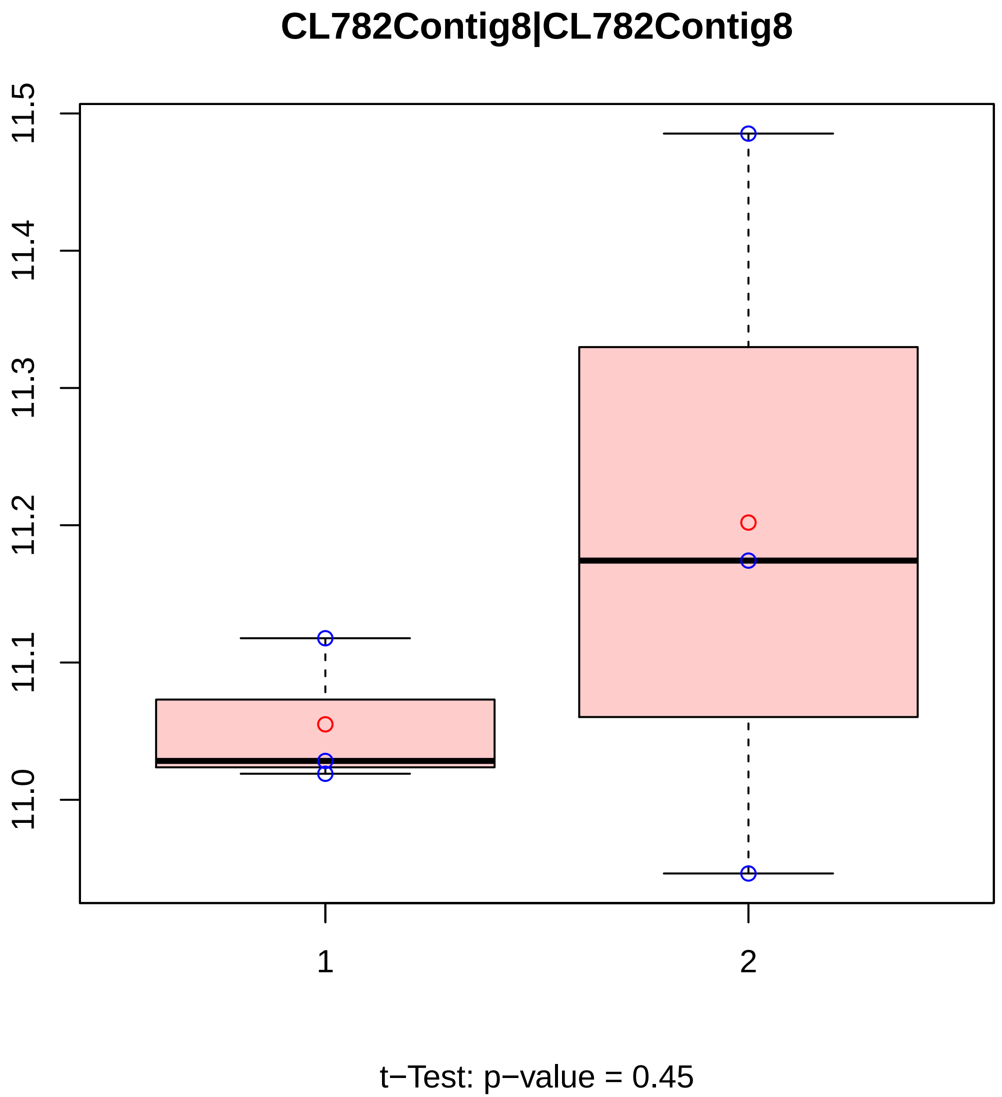

CL782Contig8|CL782Contig8
11.0
11.1
11.2
11.3
11.4
11.5
1
2
t−Test: p−value = 0.45

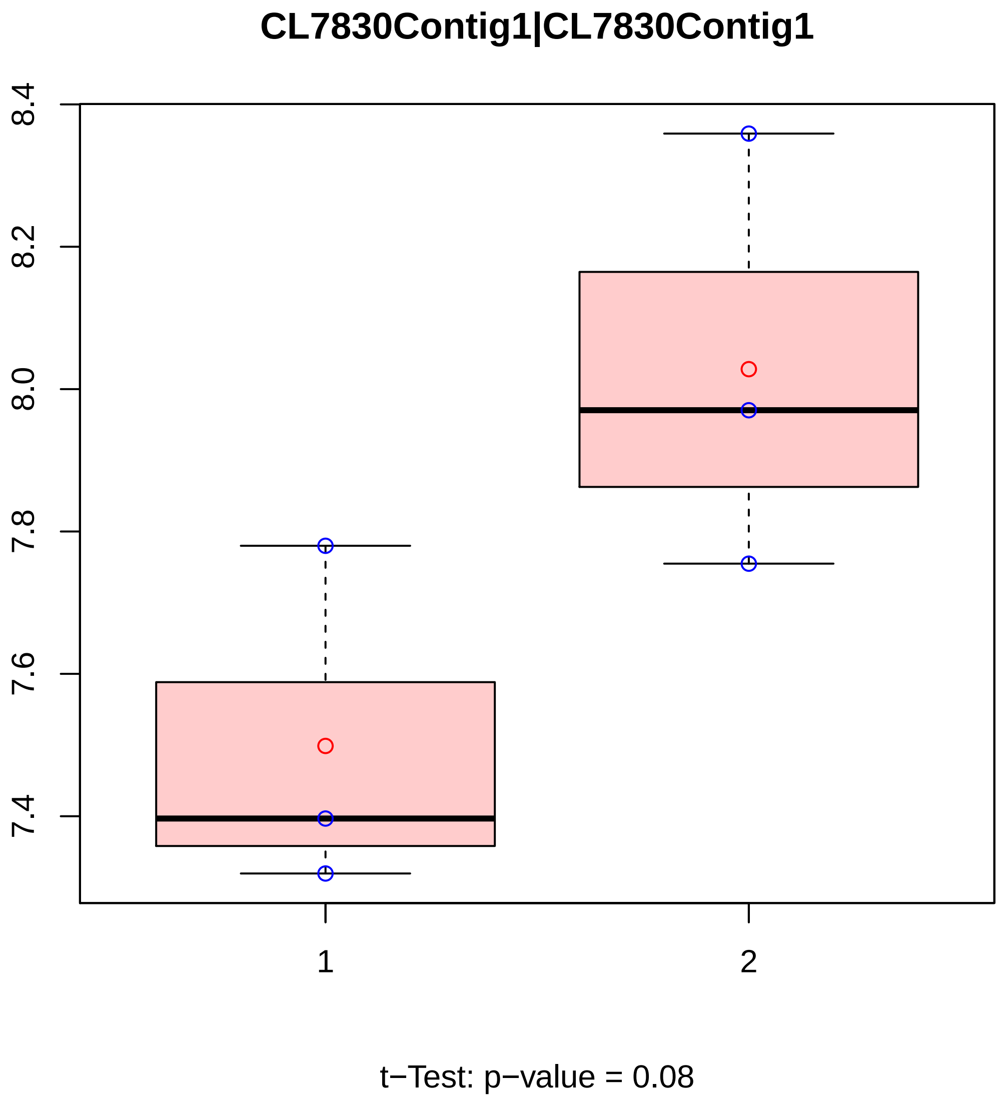
CL7830Contig1|CL7830Contig1
8.4
8.2
8.0
7.8
7.6
7.4
1
2
t−Test: p−value = 0.08

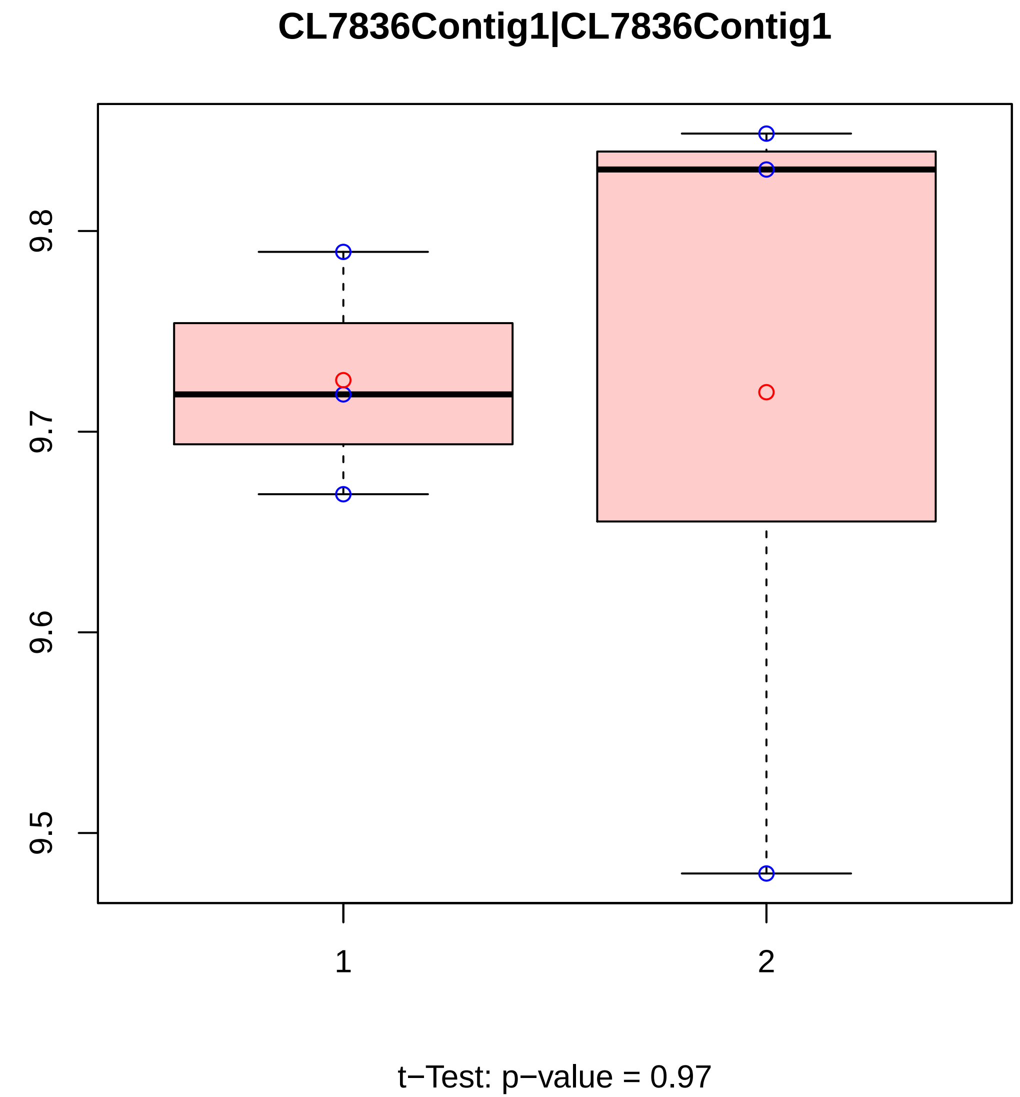
CL7836Contig1|CL7836Contig1
9.5
9.6
9.7
9.8
1
2
t−Test: p−value = 0.97

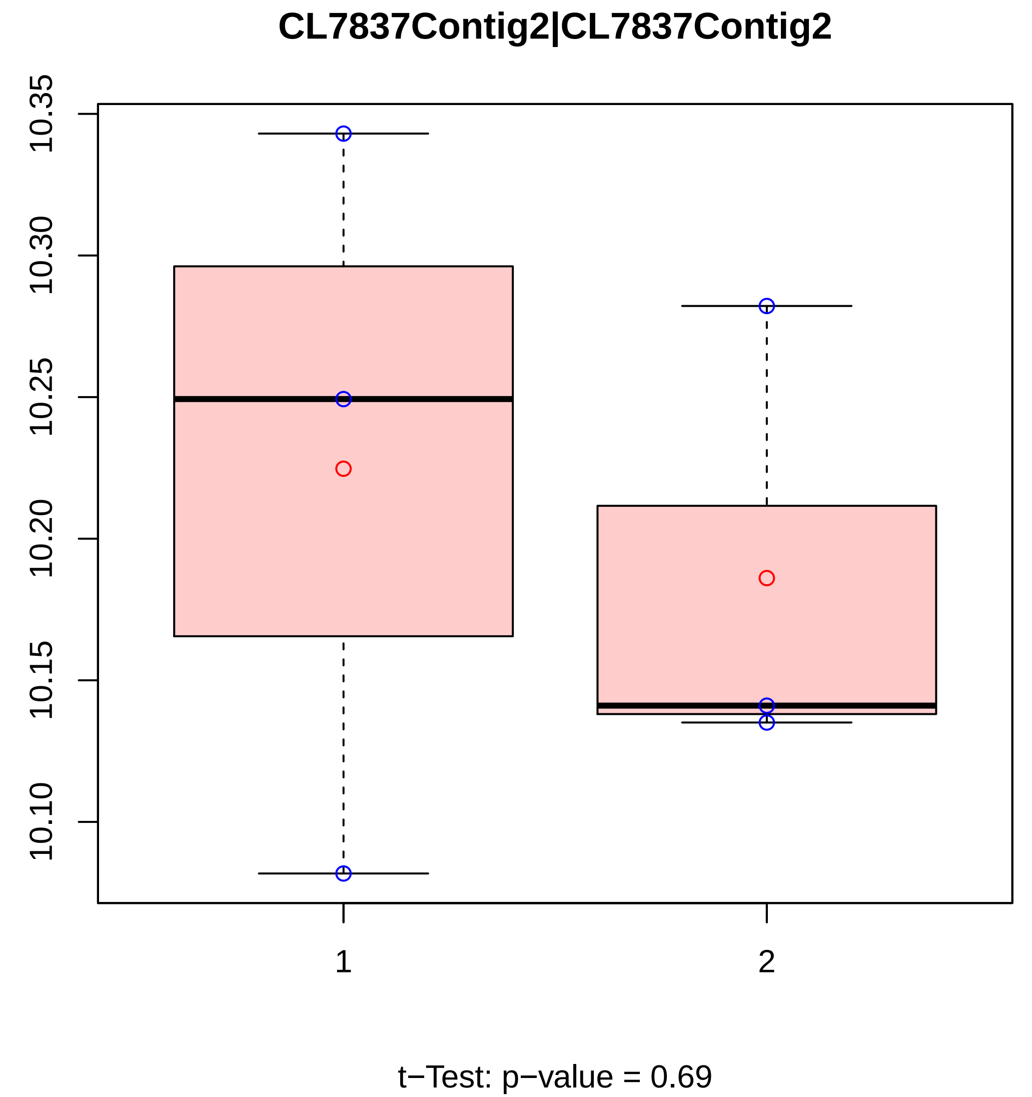
CL7837Contig2|CL7837Contig2
10.35
10.30
10.25
10.20
10.15
10.10
1
2
t−Test: p−value = 0.69

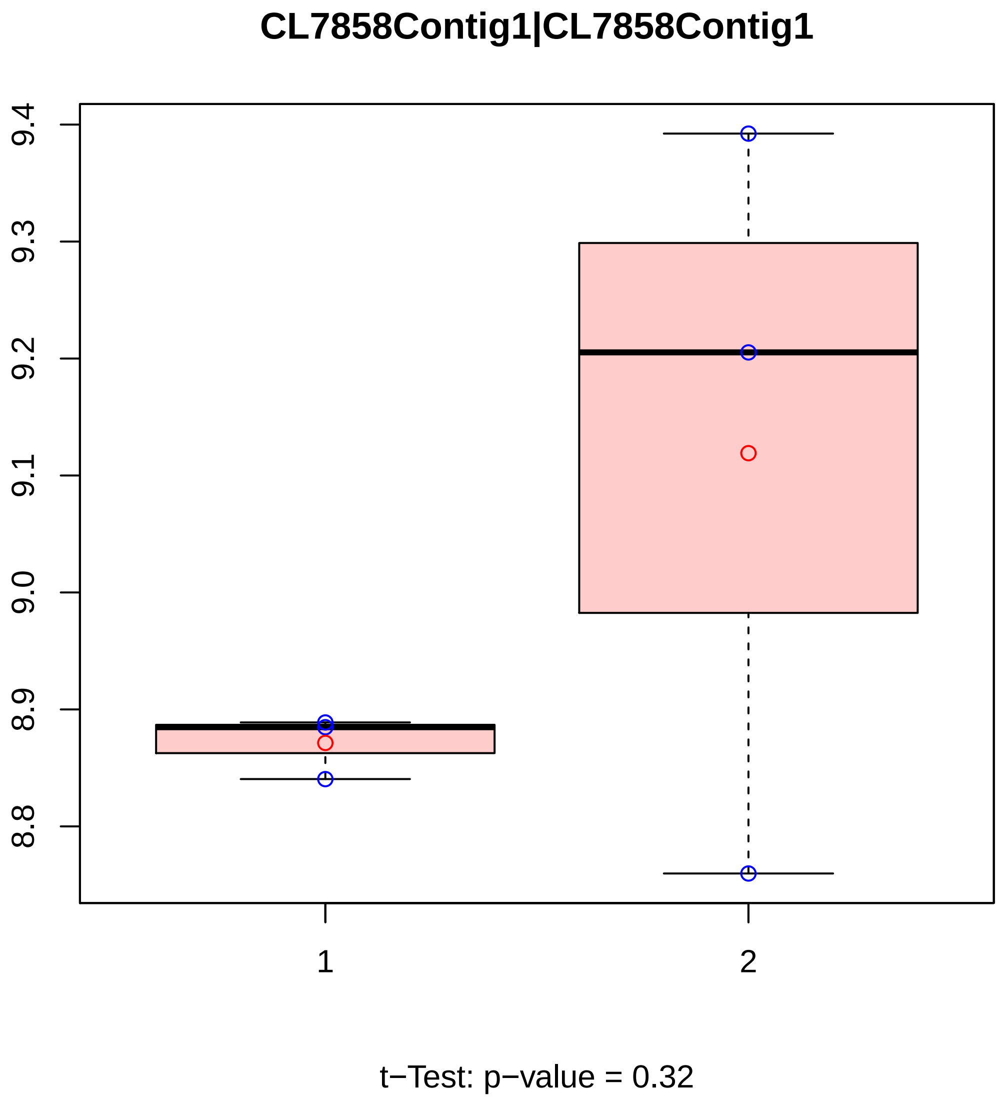
CL7858Contig1|CL7858Contig1
9.4
9.3
9.2
9.1
9.0
8.9
8.8
1
2
t−Test: p−value = 0.32

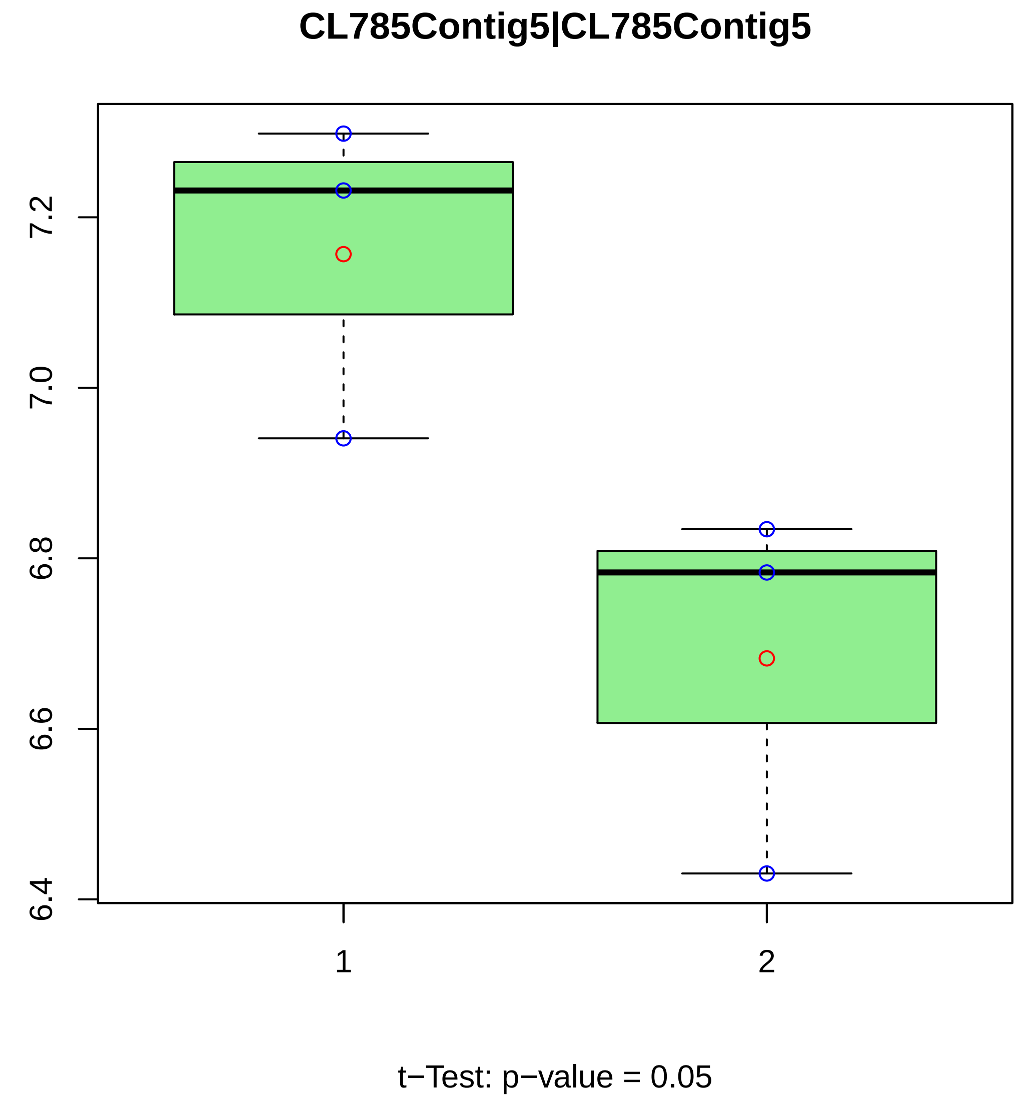
CL785Contig5|CL785Contig5
7.2
7.0
6.8
6.6
6.4
1
2
t−Test: p−value = 0.05

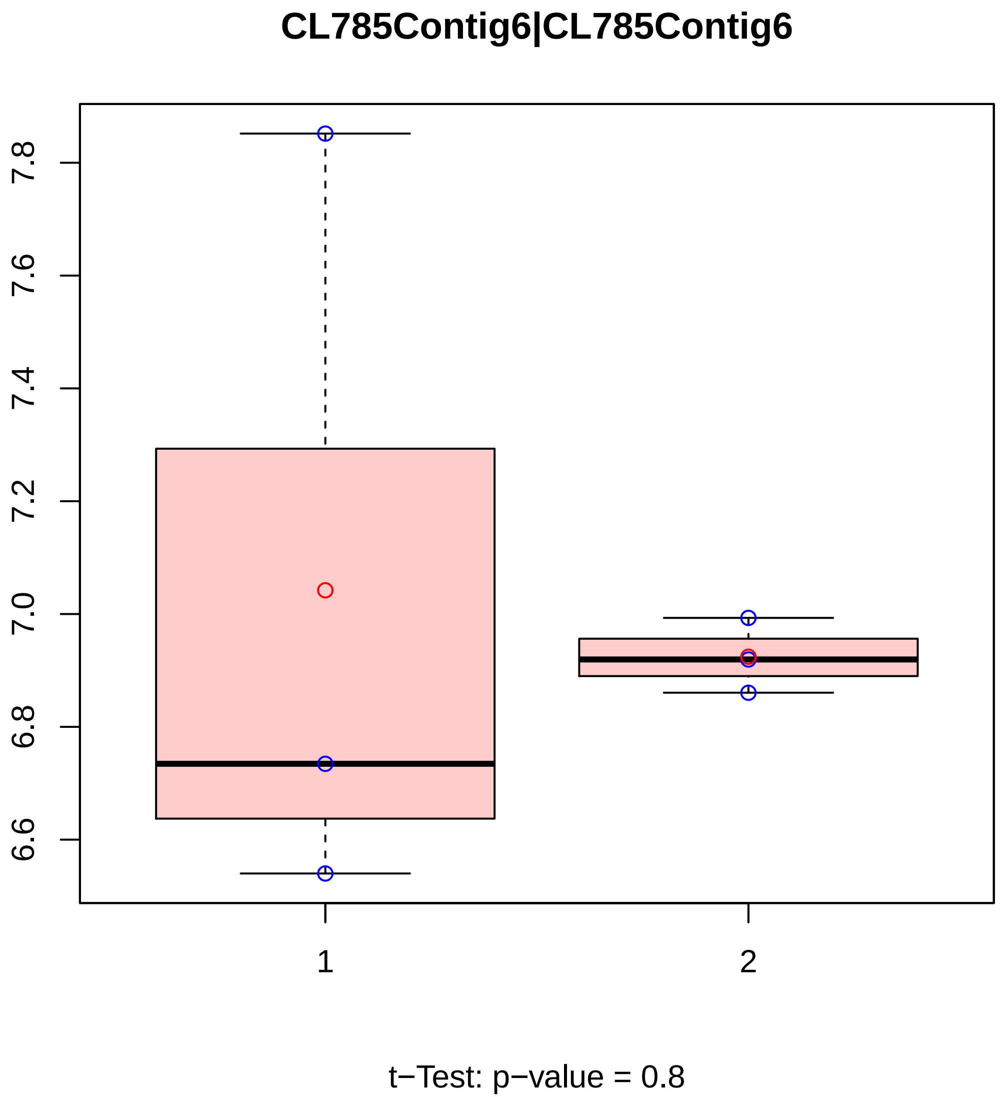
CL785Contig6|CL785Contig6
7.8
7.6
7.4
7.2
7.0
6.8
6.6
1
2
t−Test: p−value = 0.8

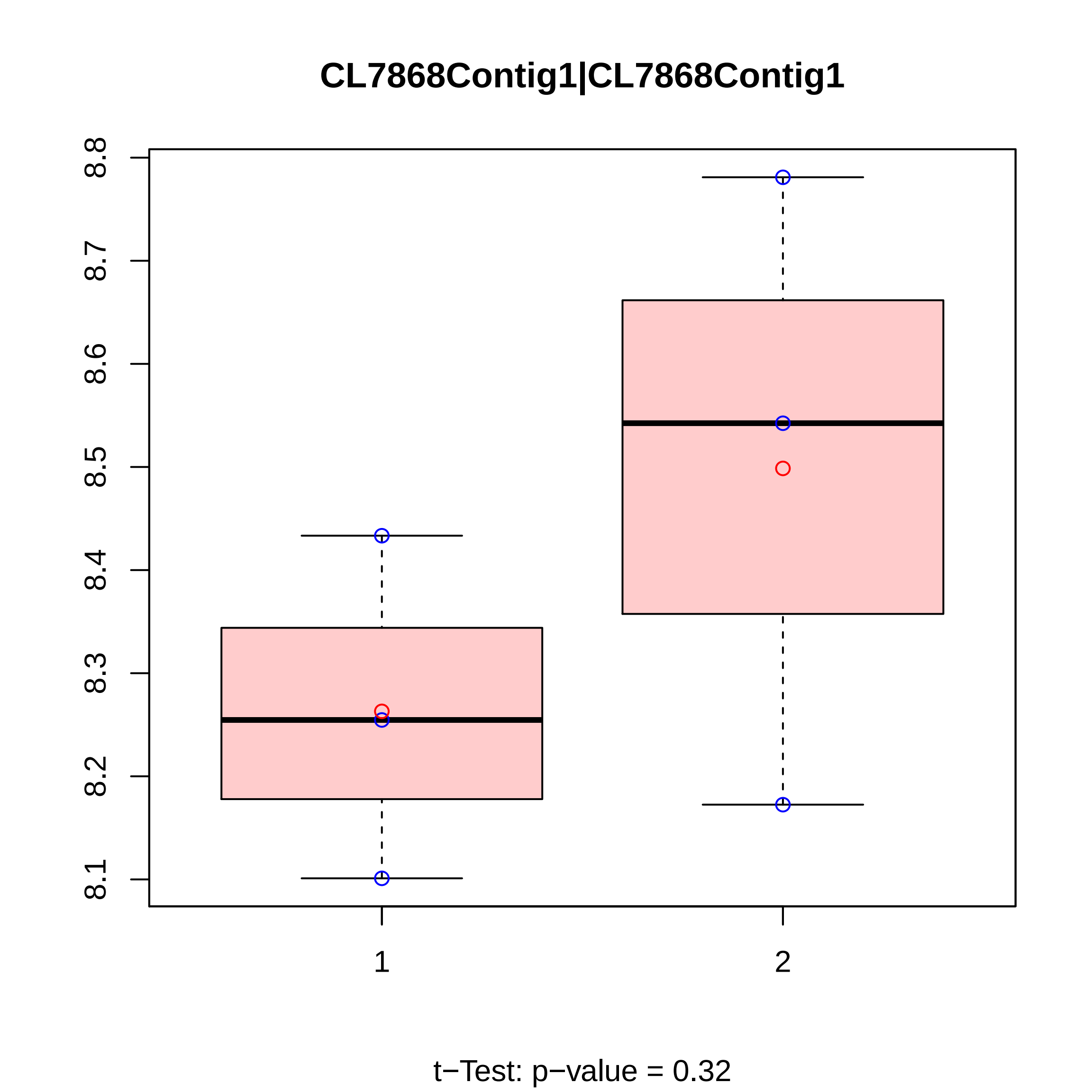
CL7868Contig1|CL7868Contig1
8.1
8.2
8.3
8.4
8.5
8.6
8.7
8.8
1
2
t−Test: p−value = 0.32

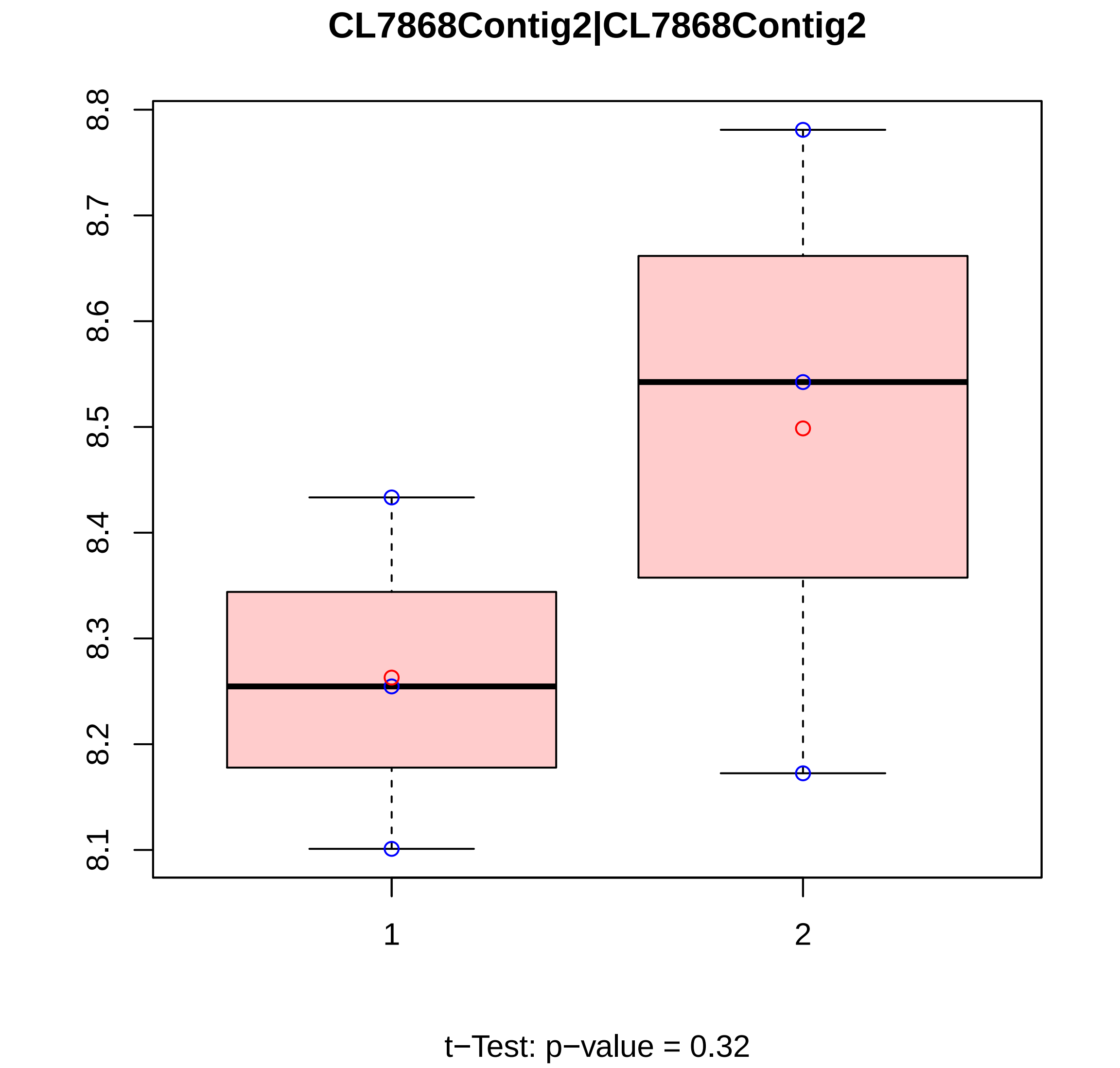

CL7868Contig2|CL7868Contig2
8.1
8.2
8.3
8.4
8.5
8.6
8.7
8.8
1
2
t−Test: p−value = 0.32

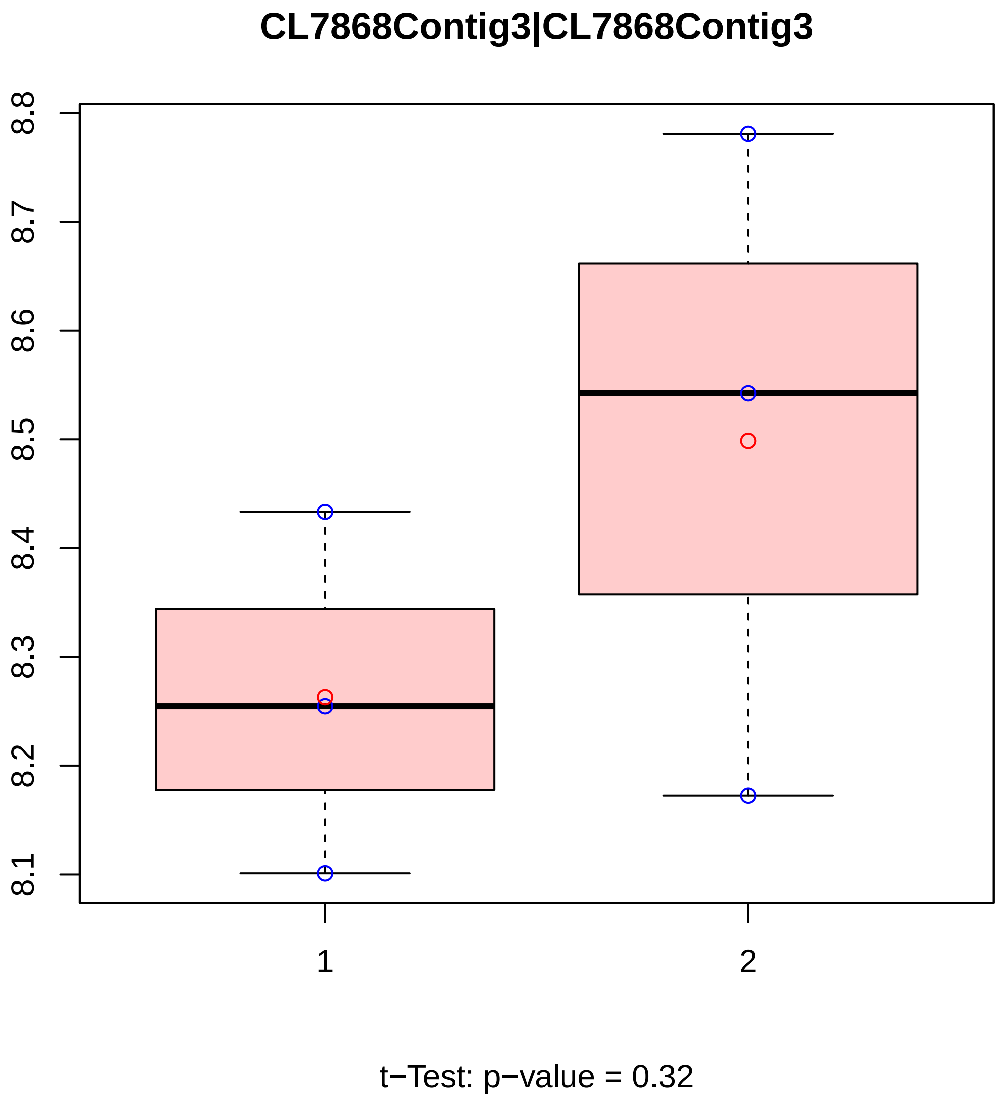
CL7868Contig3|CL7868Contig3
8.1
8.2
8.3
8.4
8.5
8.6
8.7
8.8
1
2
t−Test: p−value = 0.32

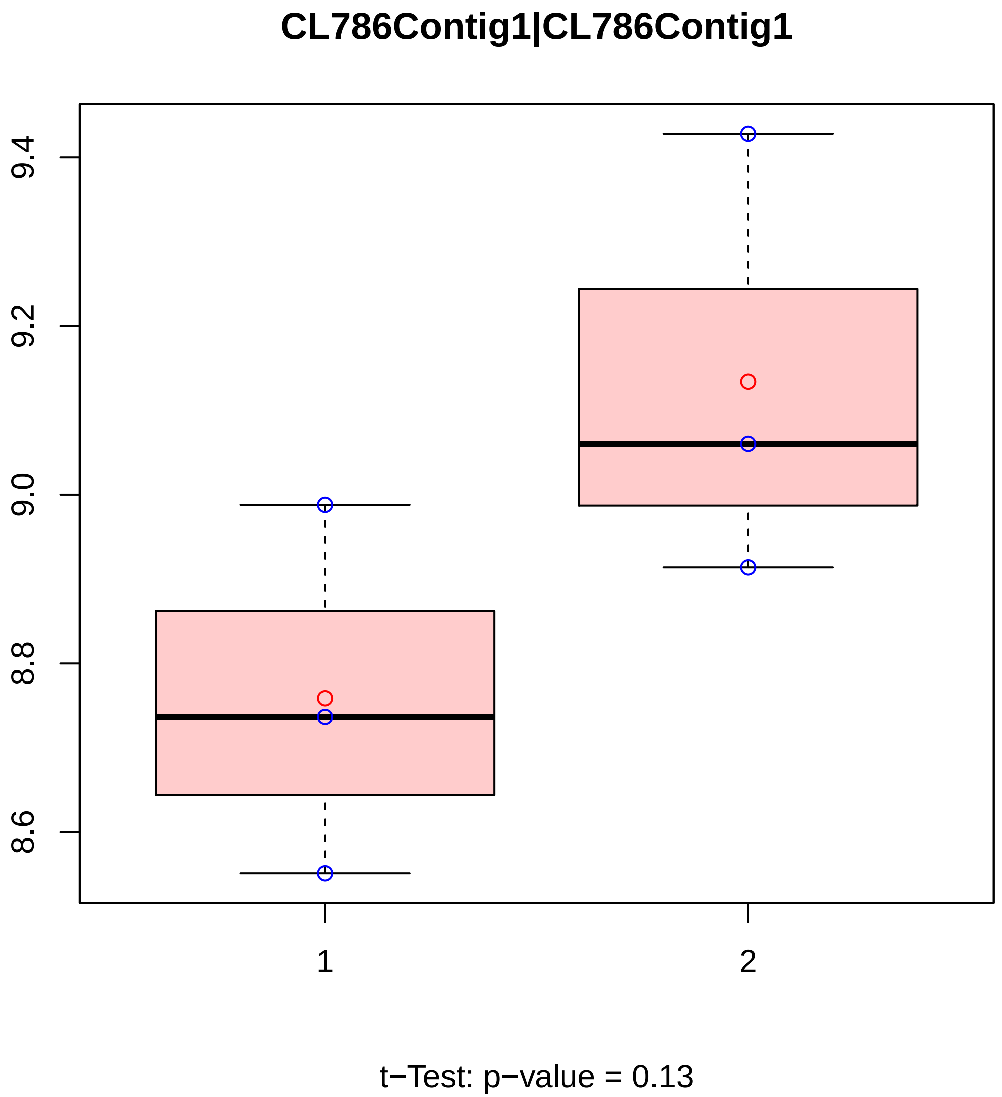

CL786Contig1|CL786Contig1
9.4
9.2
9.0
8.8
8.6
1
2
t−Test: p−value = 0.13

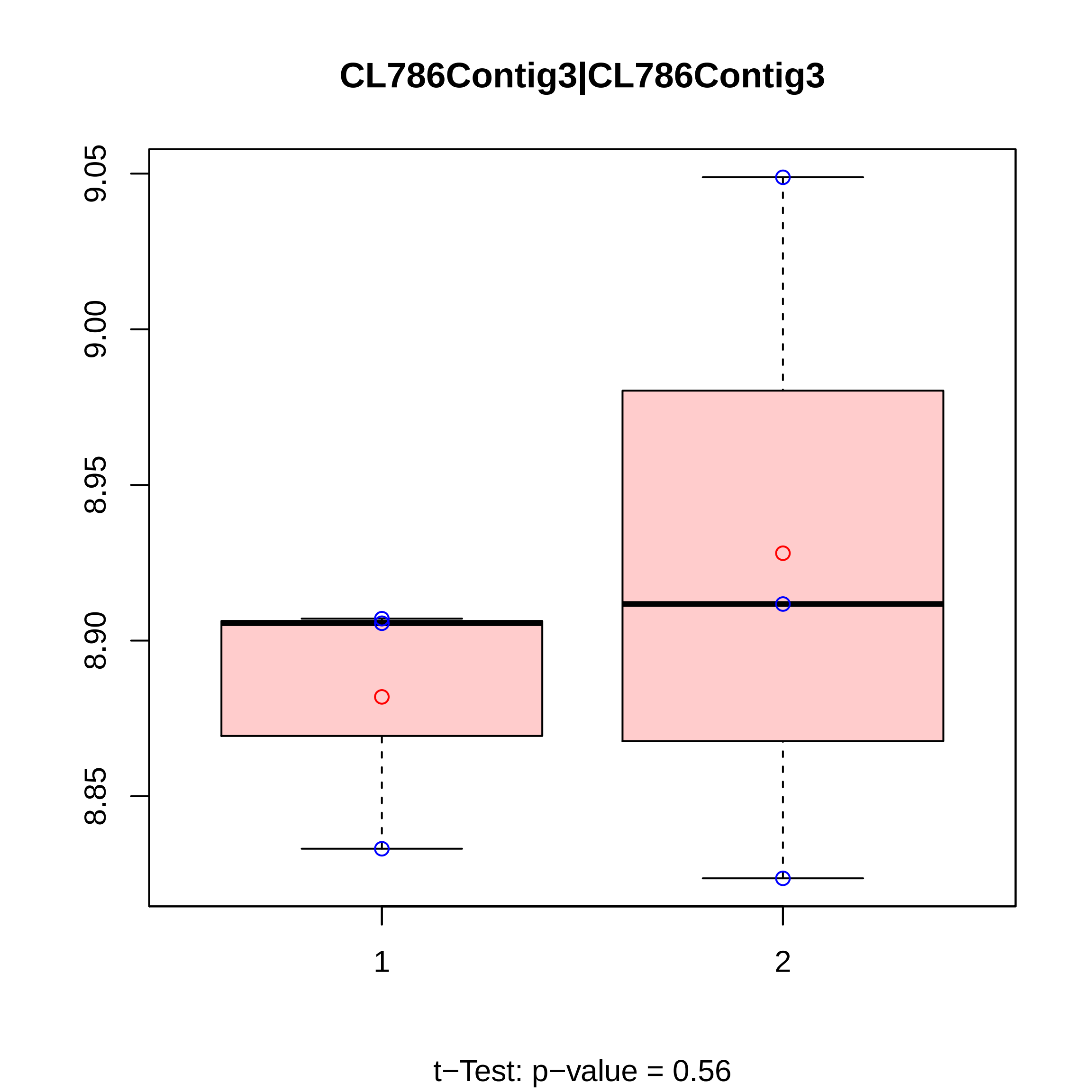

CL786Contig3|CL786Contig3
9.05
9.00
8.95
8.90
8.85
1
2
t−Test: p−value = 0.56

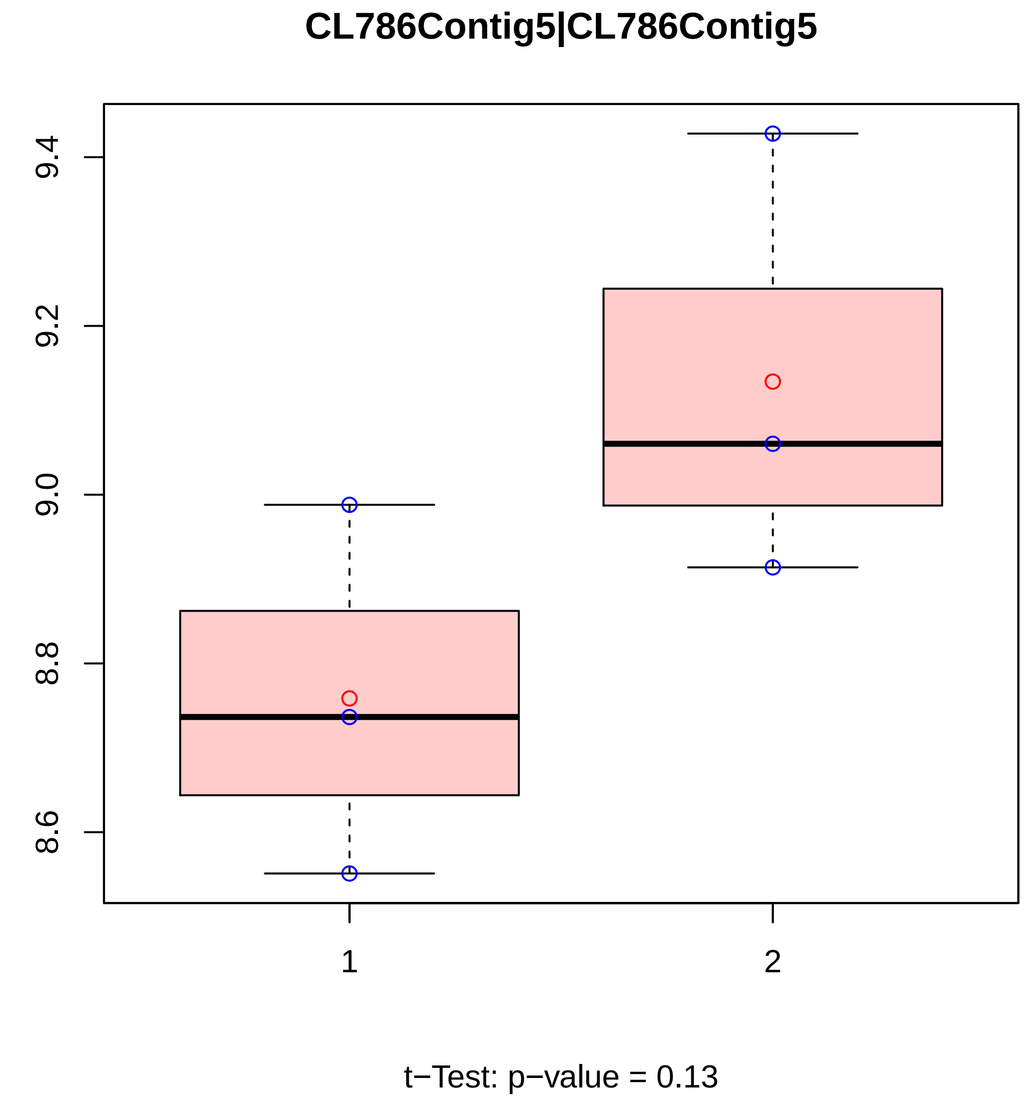
CL786Contig5|CL786Contig5
9.4
9.2
9.0
8.8
8.6
1
2
t−Test: p−value = 0.13

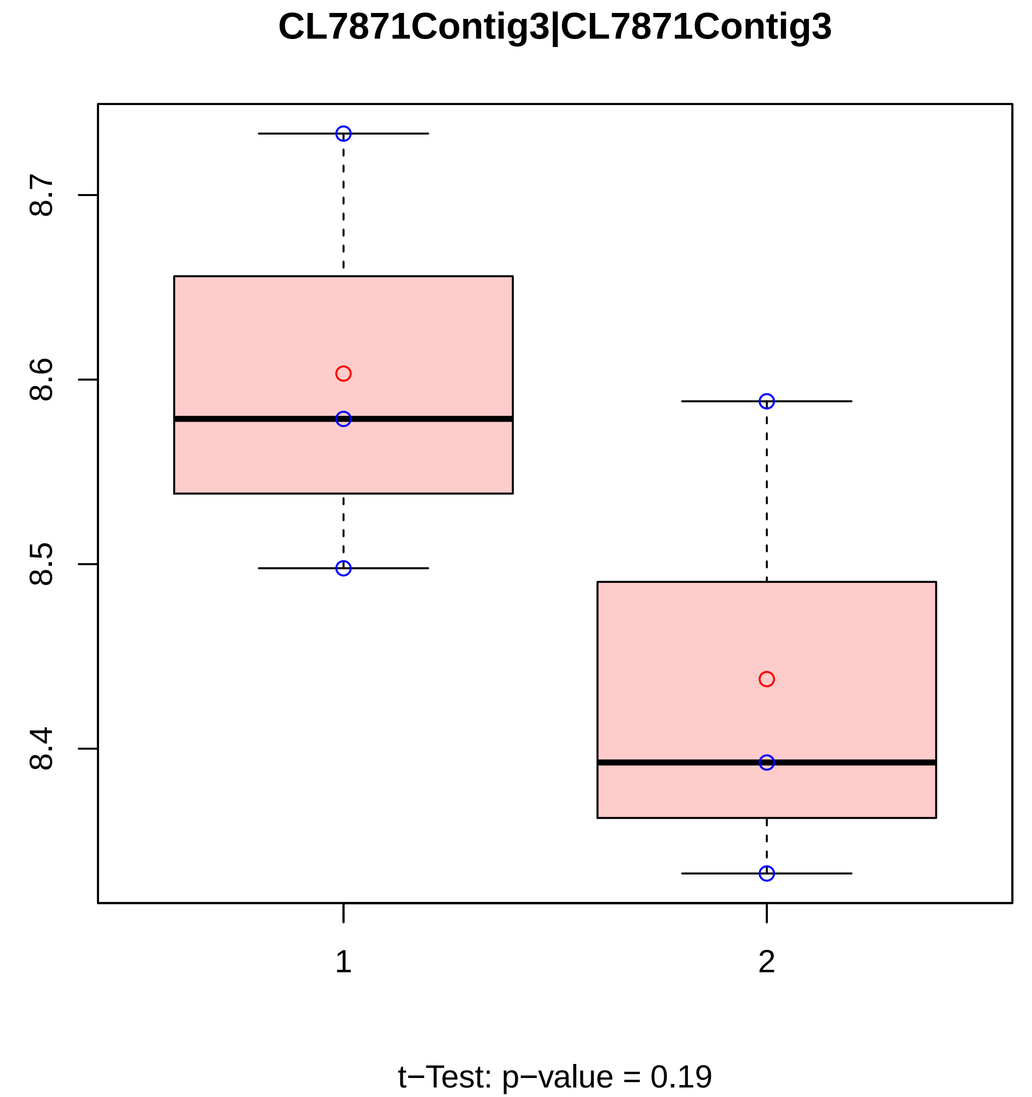

CL7871Contig3|CL7871Contig3
8.7
8.6
8.5
8.4
1
2
t−Test: p−value = 0.19

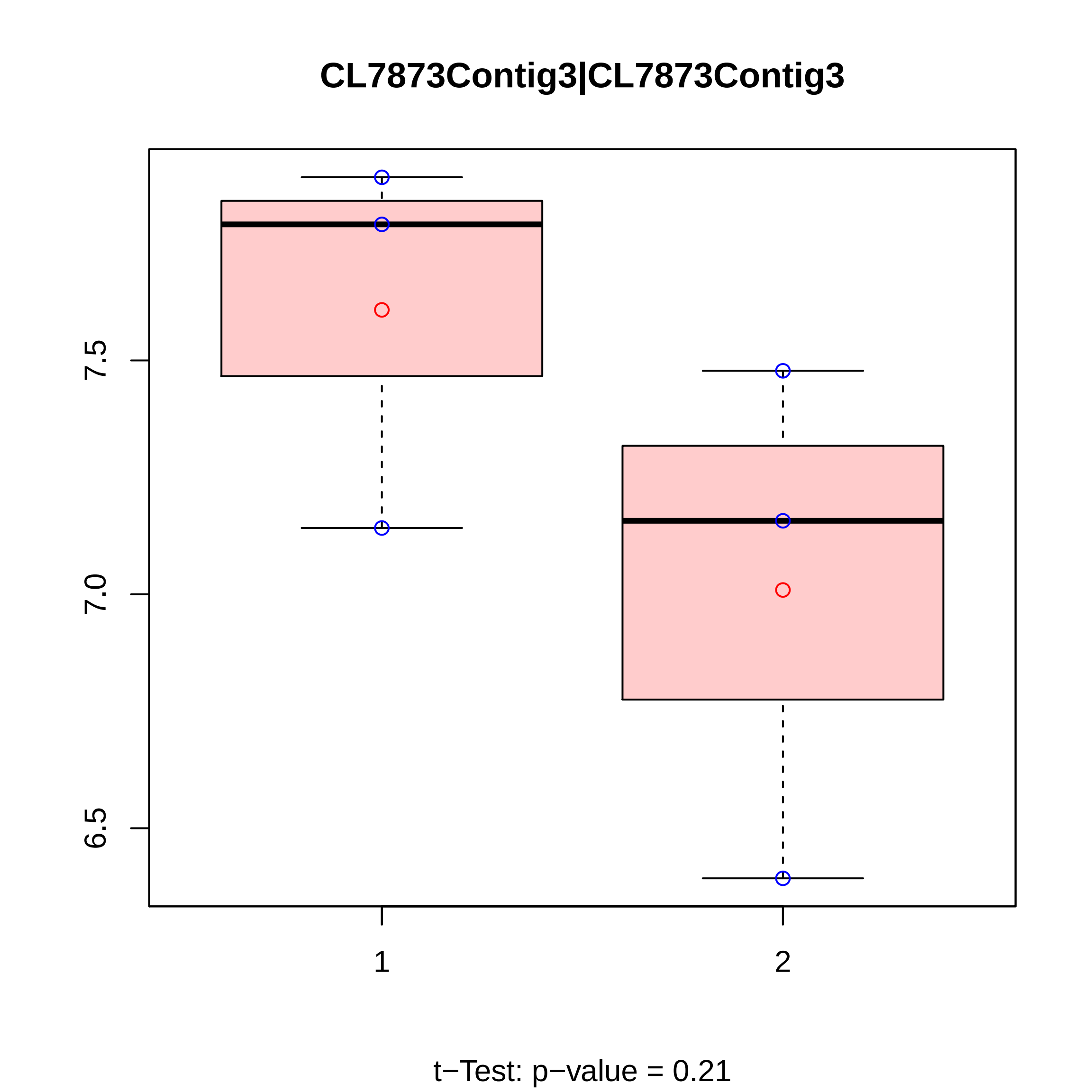
CL7873Contig3|CL7873Contig3
7.5
7.0
6.5
1
2
t−Test: p−value = 0.21

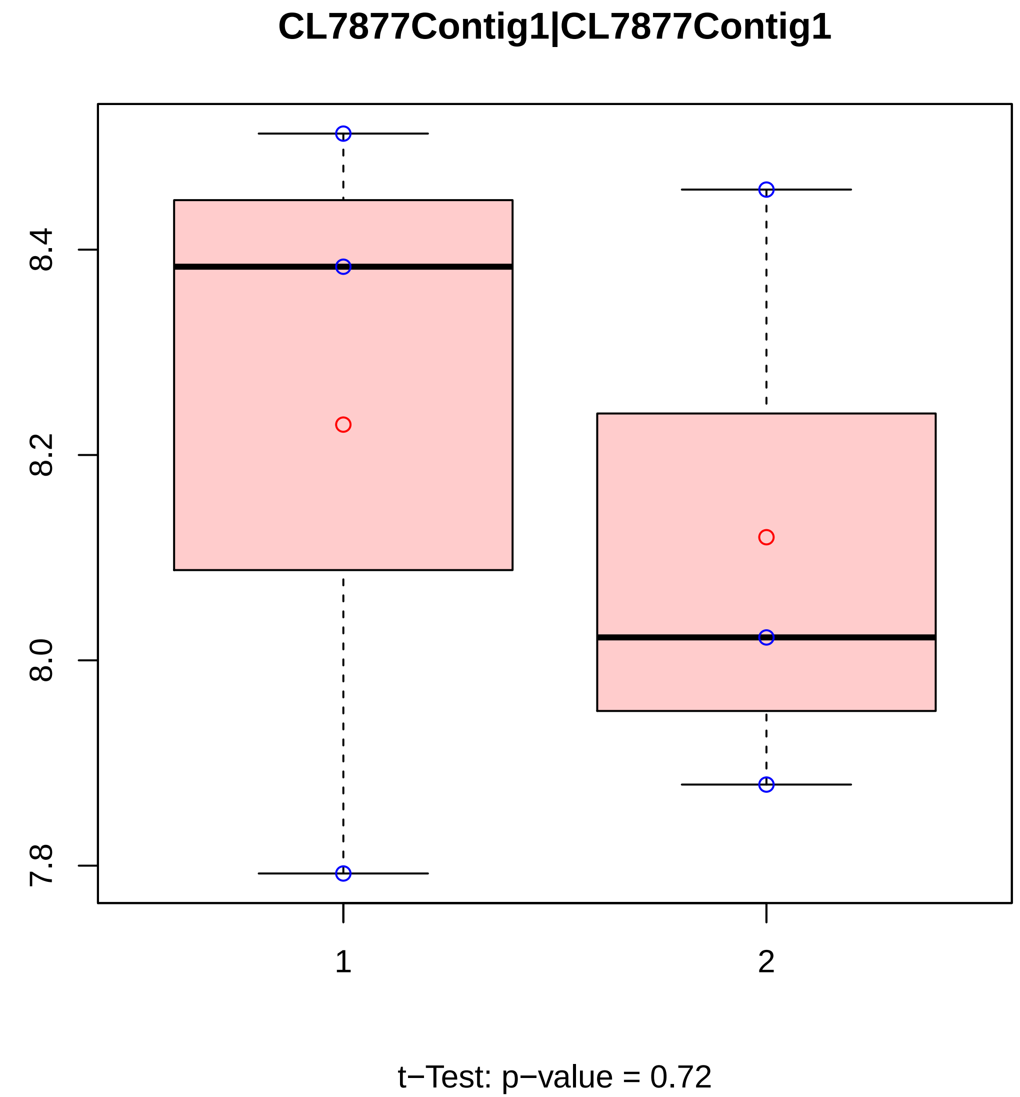
CL7877Contig1|CL7877Contig1
8.4
8.2
8.0
7.8
1
2
t−Test: p−value = 0.72

# CL7885Contig1|CL7885Contig1

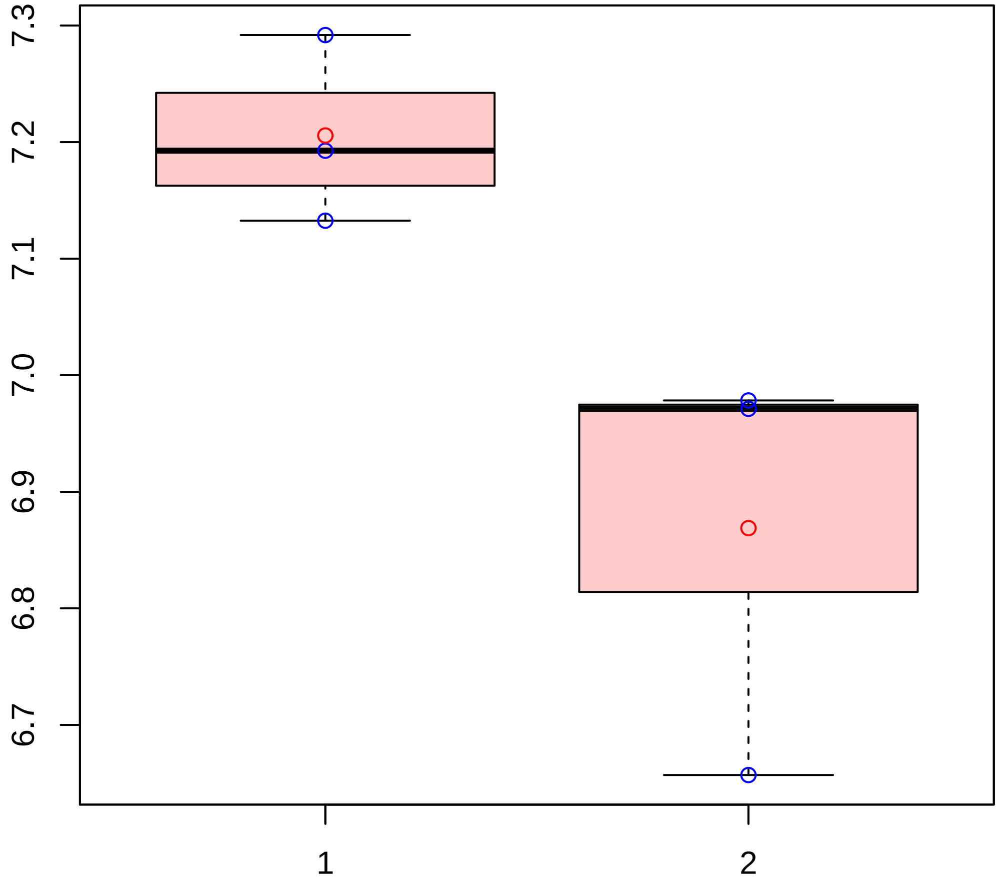


t−Test: p−value = 0.07

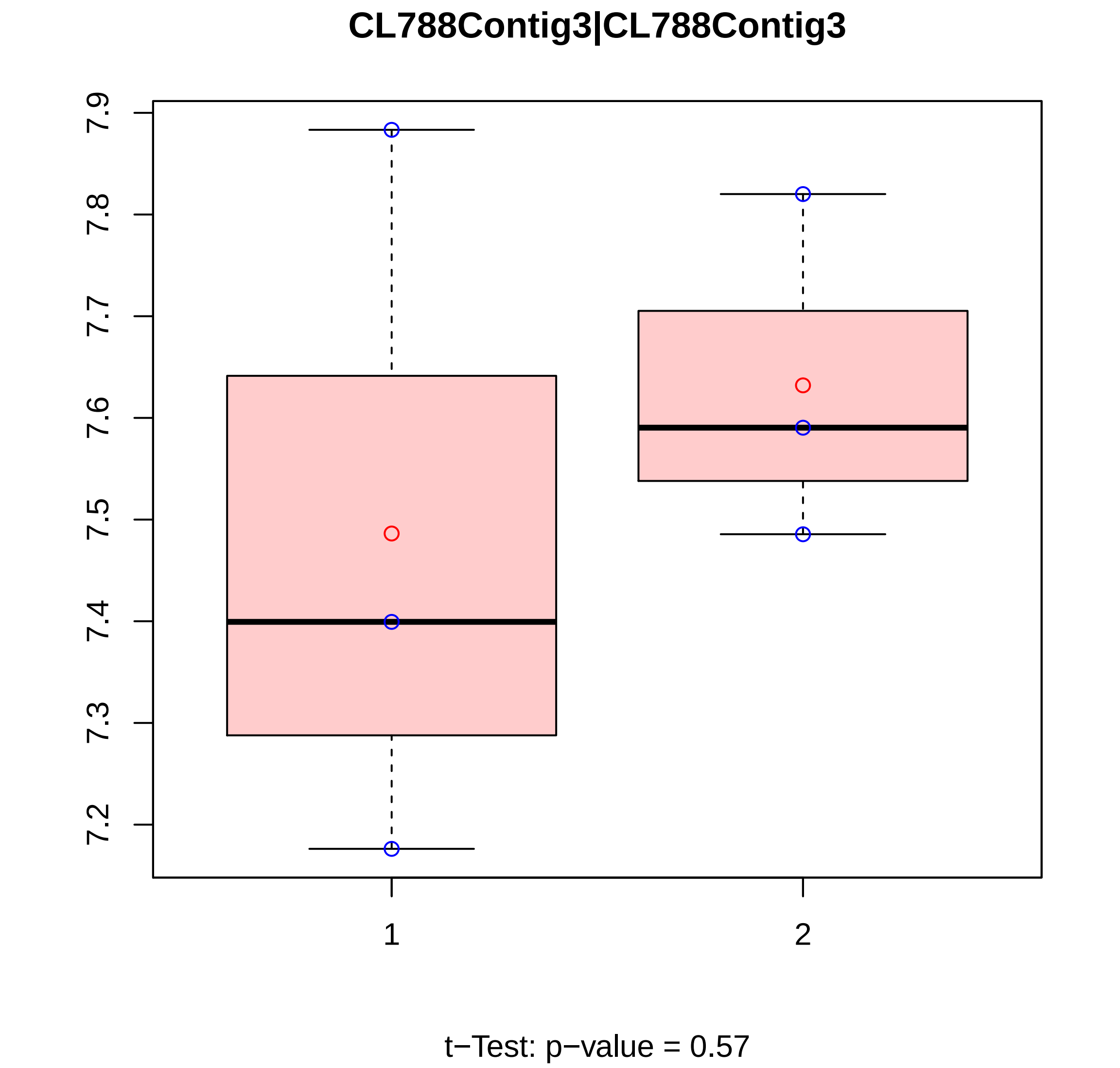

CL788Contig3|CL788Contig3
7.2
7.3
7.4
7.5
7.6
7.7
7.8
7.9
1
2
t−Test: p−value = 0.57

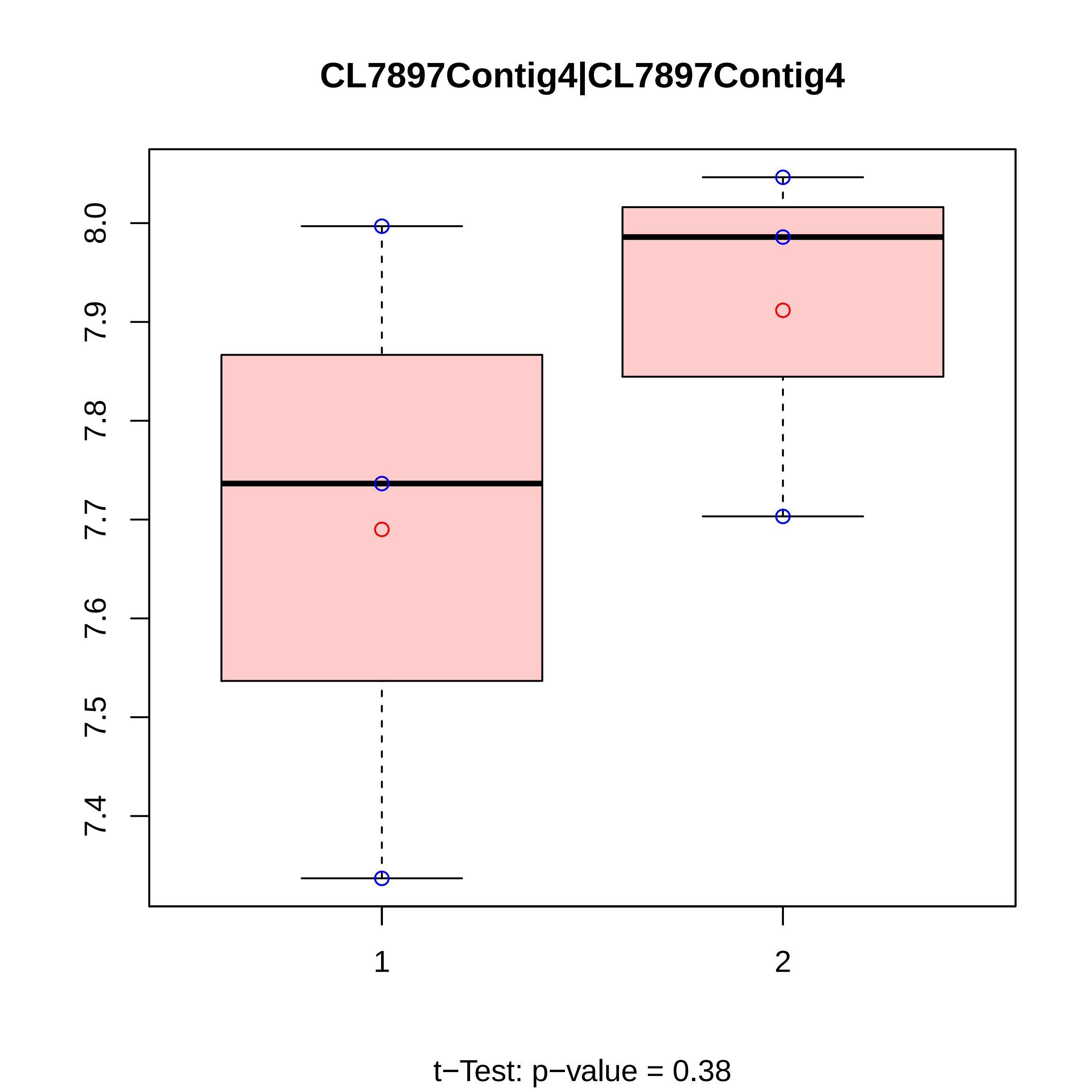

CL7897Contig4|CL7897Contig4
8.0
7.9
7.8
7.7
7.6
7.5
7.4
1
2
t−Test: p−value = 0.38

## CL7898Contig1|CL7898Contig1

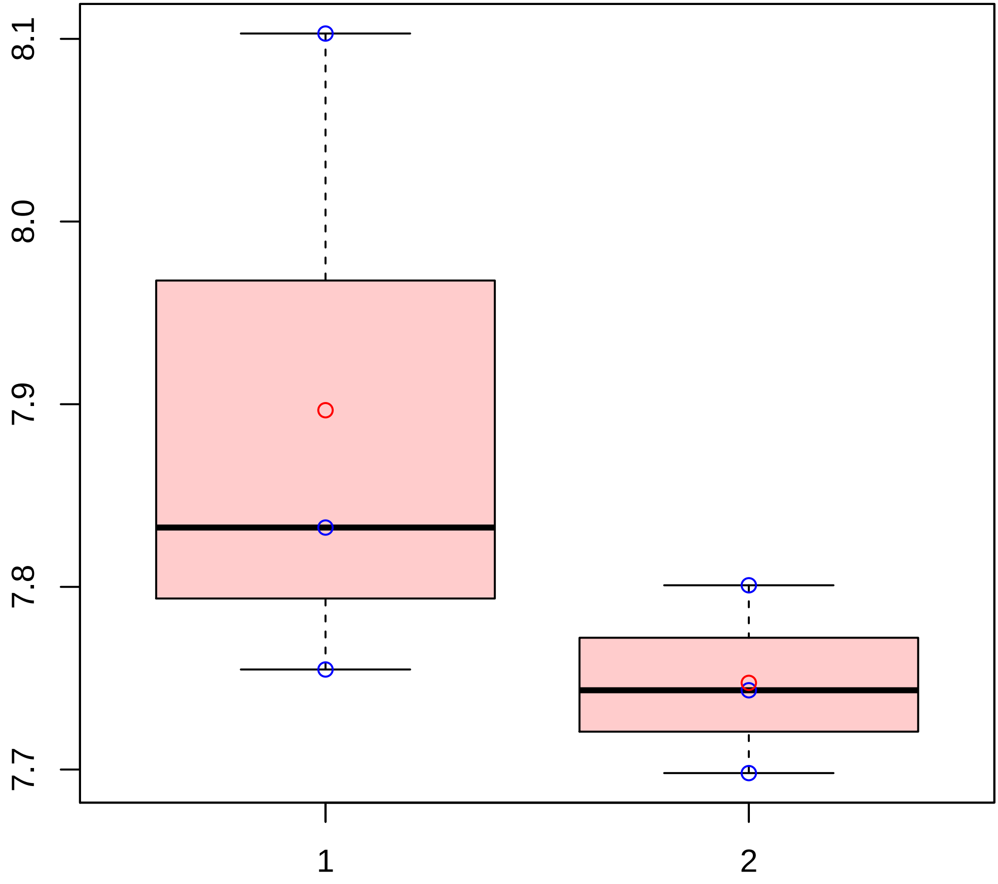


t−Test: p−value = 0.29

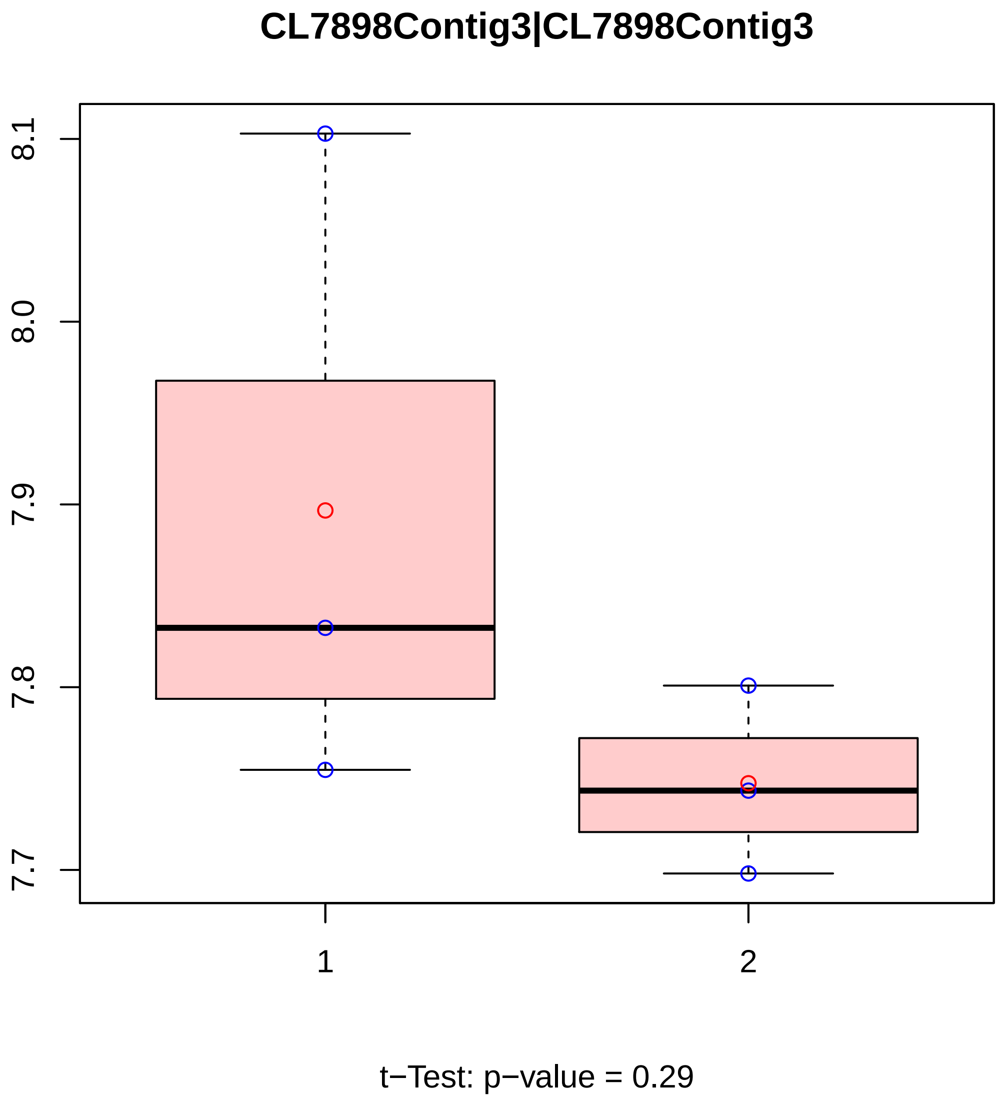
CL7898Contig3|CL7898Contig3
8.1
8.0
7.9
7.8
7.7
1
2
t−Test: p−value = 0.29

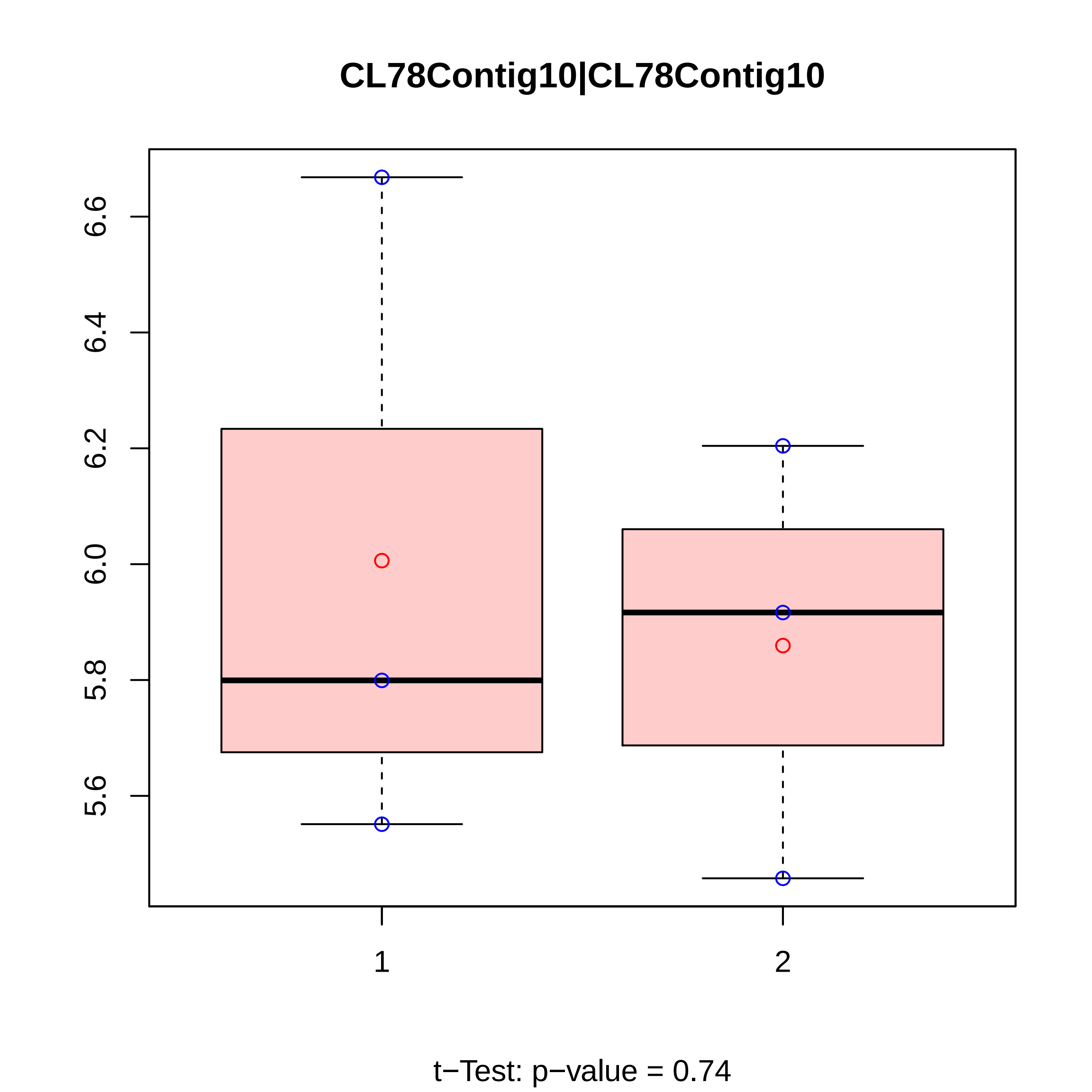
CL78Contig10|CL78Contig10
6.6
6.4
6.2
6.0
5.8
5.6
1
2
t−Test: p−value = 0.74

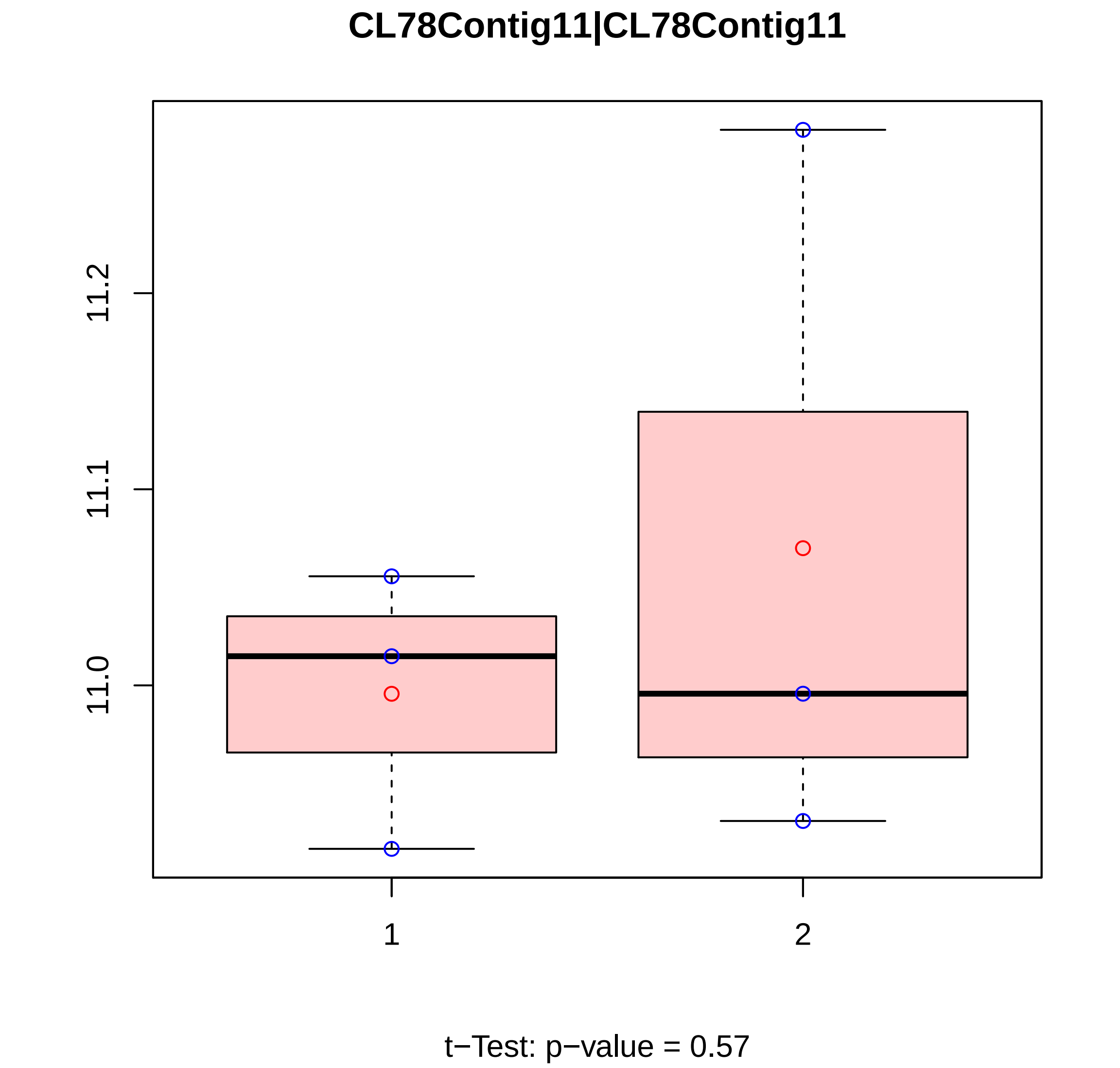
CL78Contig11|CL78Contig11
11.0
11.1
11.2
1
2
t−Test: p−value = 0.57

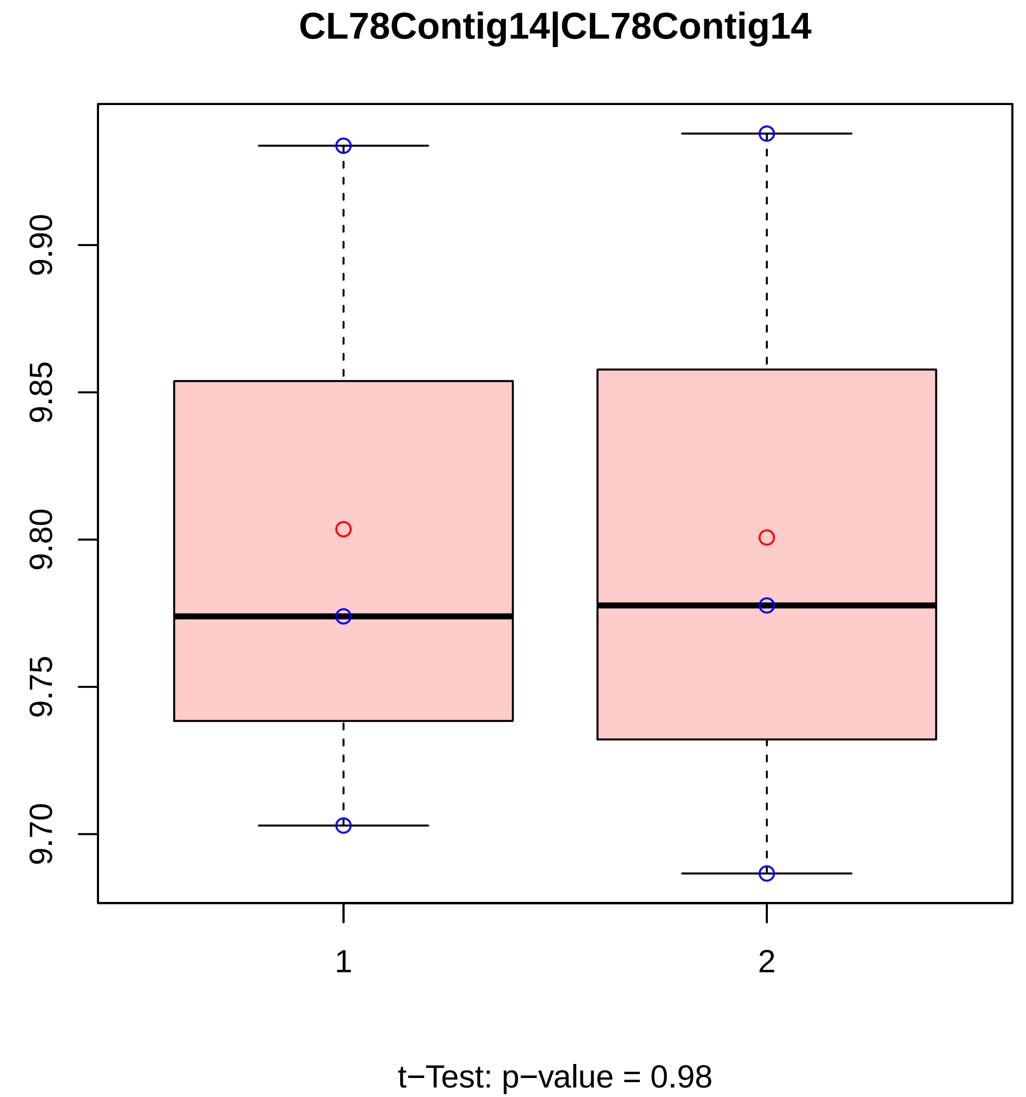
CL78Contig14|CL78Contig14
9.70
9.75
9.80
9.85
9.90
1
2
t−Test: p−value = 0.98

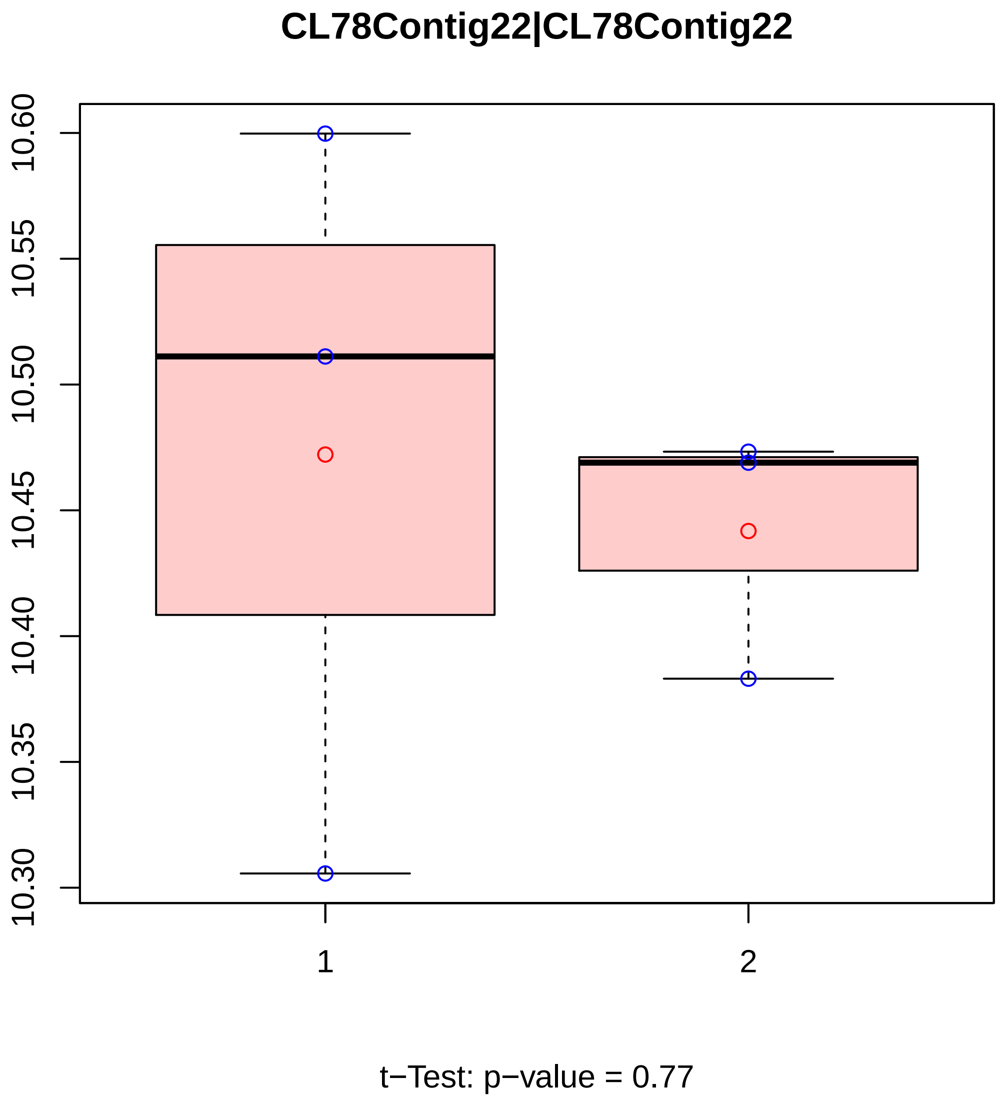

CL78Contig22|CL78Contig22
10.30
10.35
10.40
10.45
10.50
10.55
10.60
1
2
t−Test: p−value = 0.77

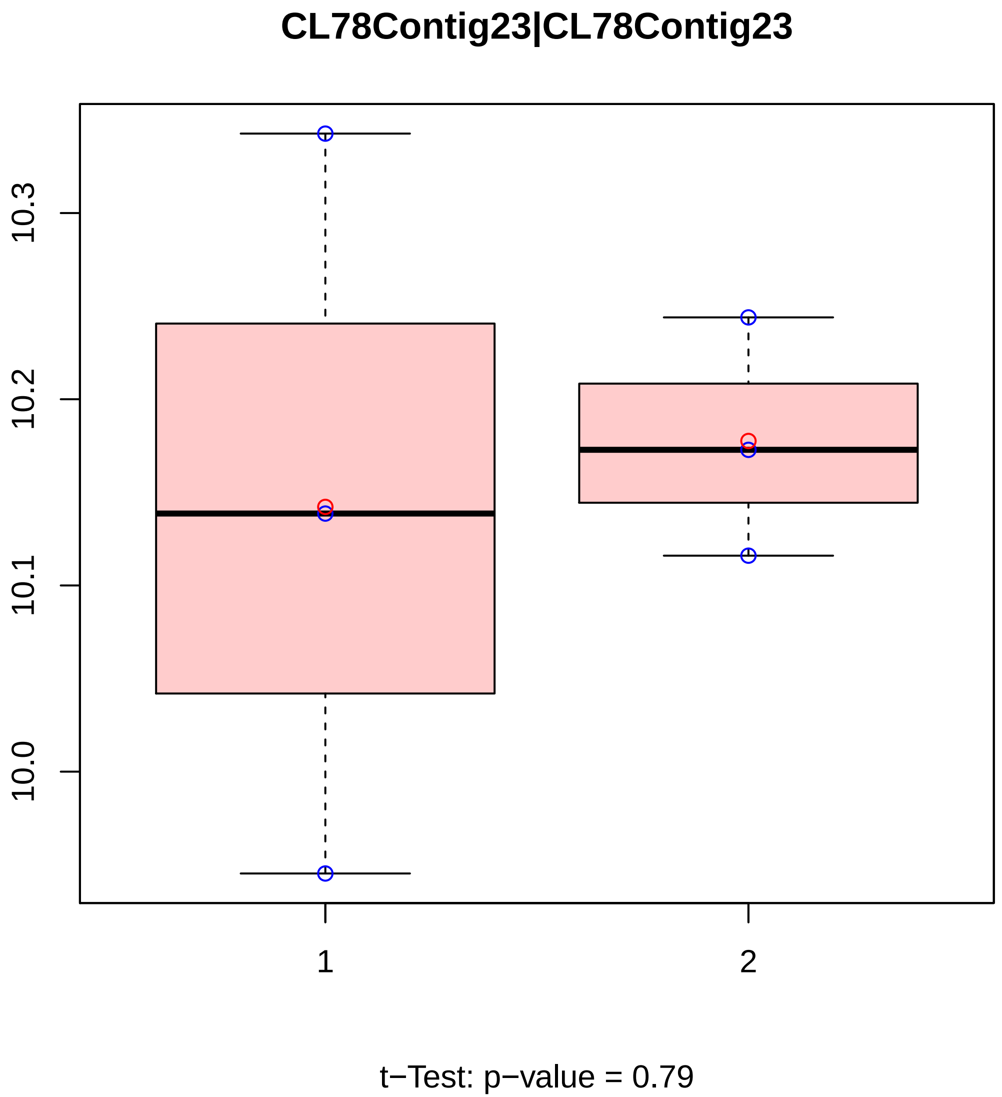
CL78Contig23|CL78Contig23
10.0
10.1
10.2
10.3
1
2
t−Test: p−value = 0.79

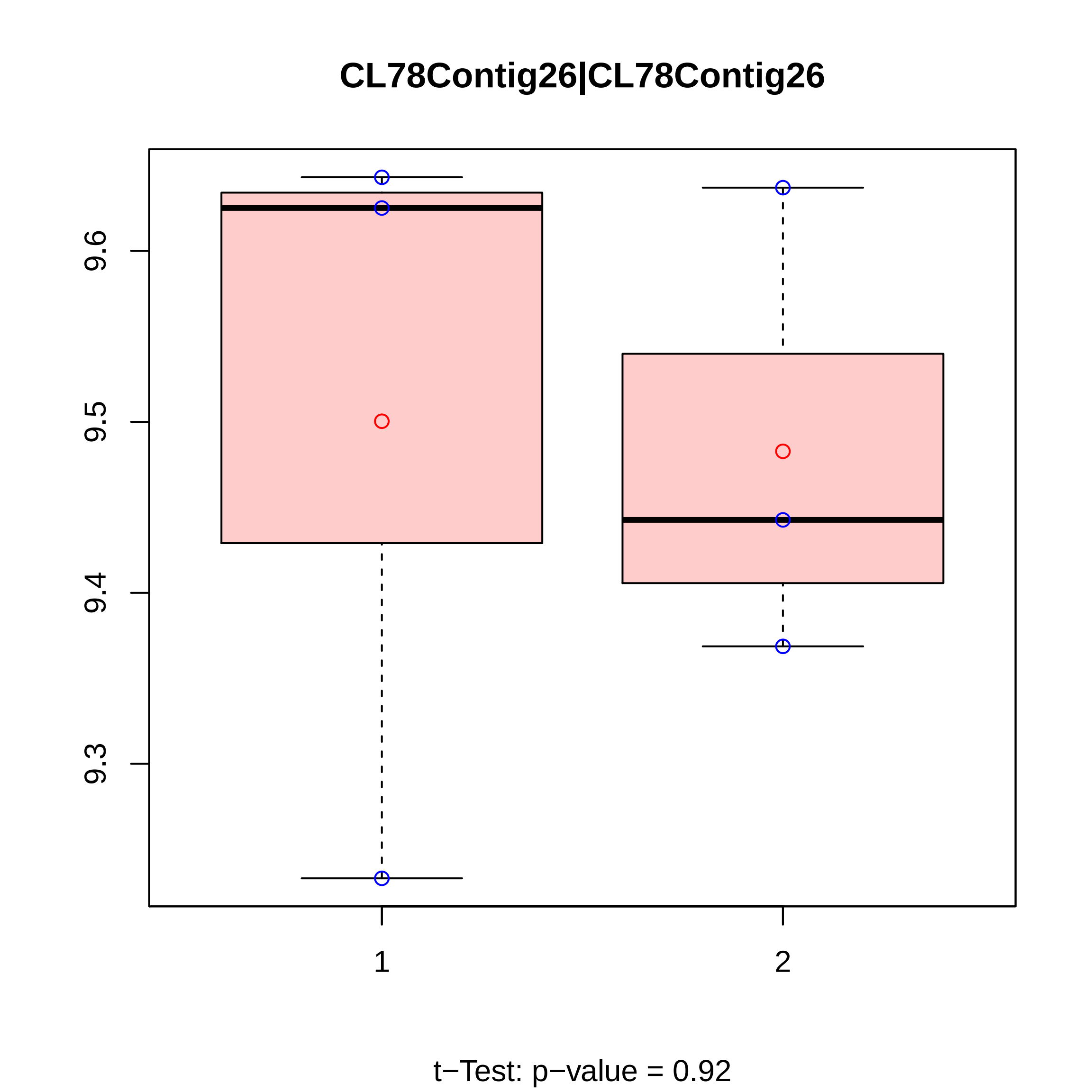
CL78Contig26|CL78Contig26
9.3
9.4
9.5
9.6
1
2
t−Test: p−value = 0.92

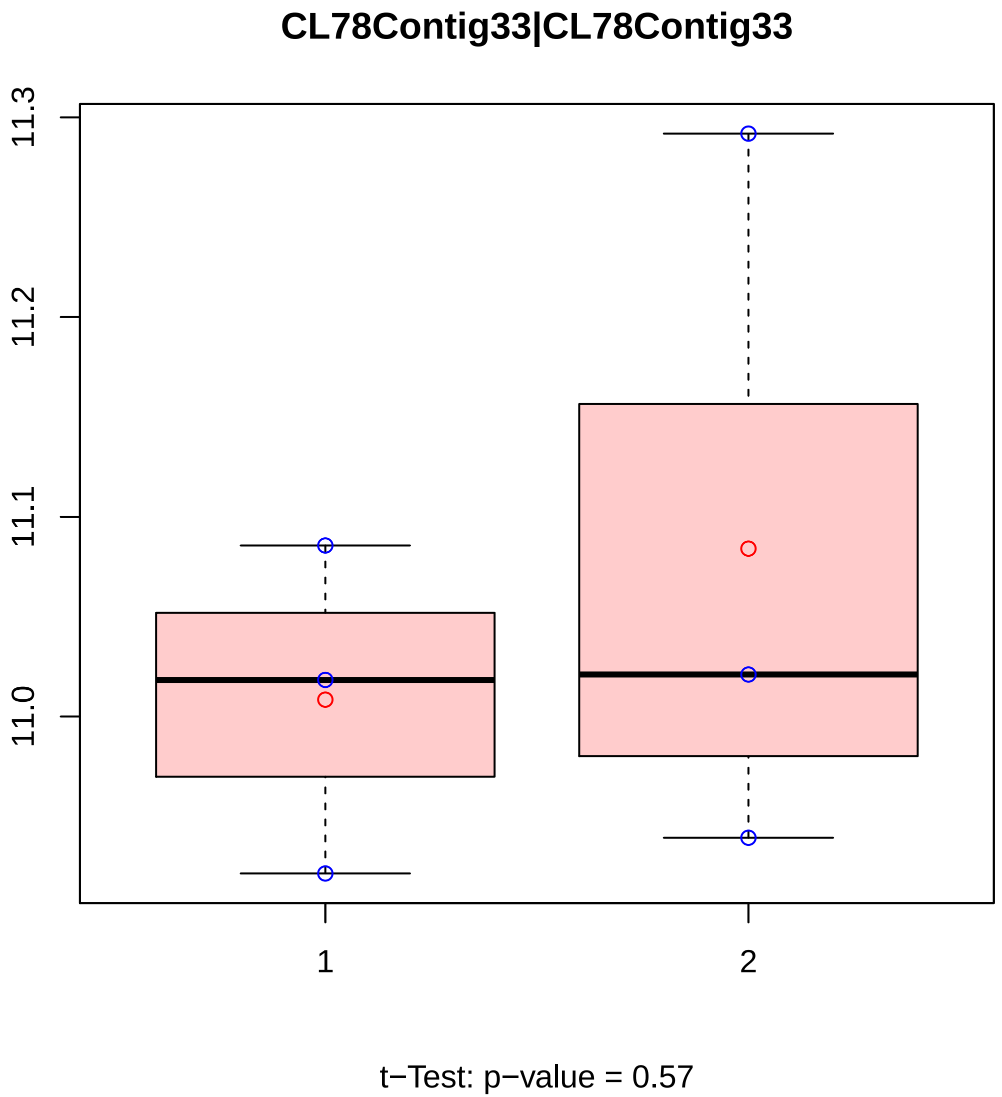
CL78Contig33|CL78Contig33
11.3
11.2
11.1
11.0
1
2
t−Test: p−value = 0.57

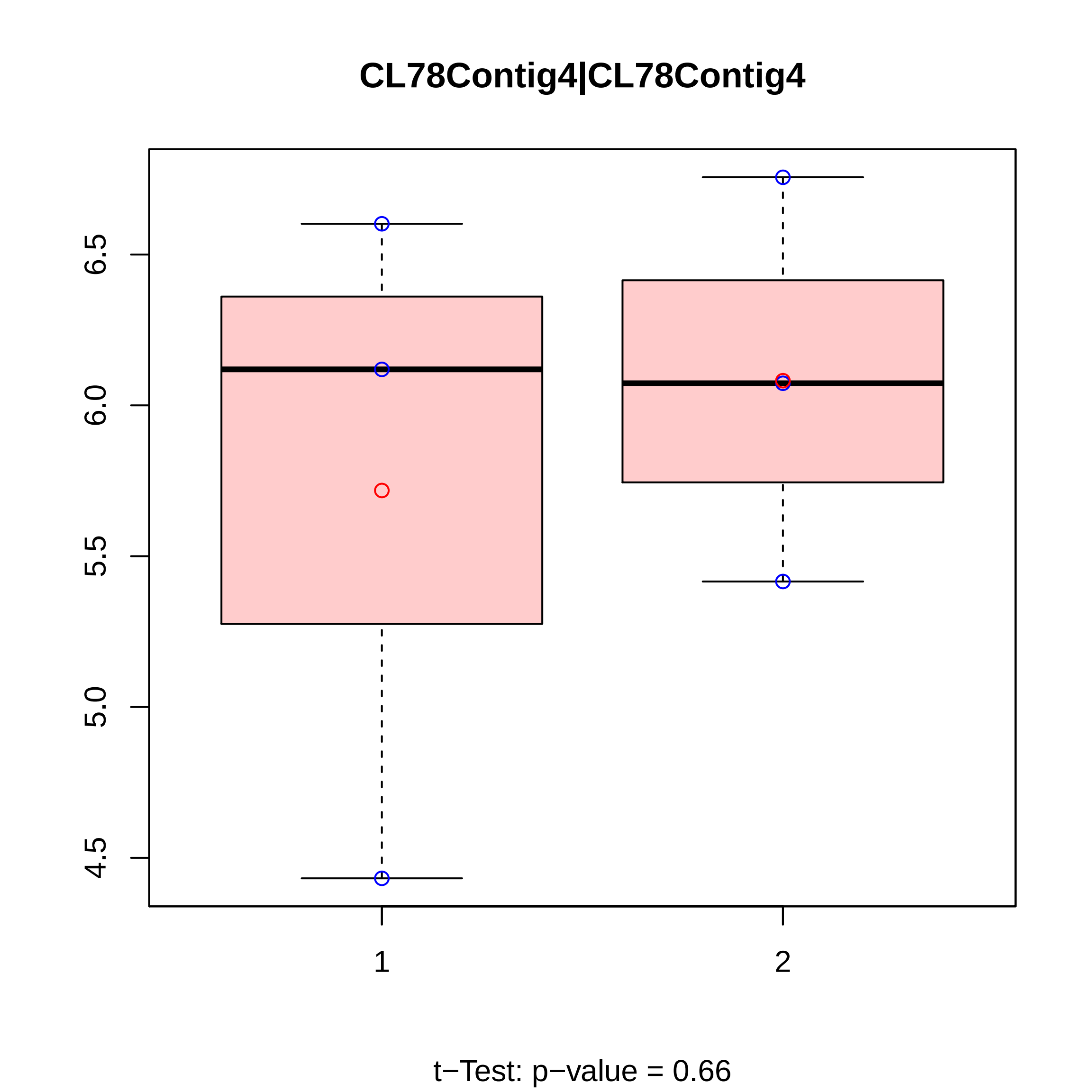

CL78Contig4|CL78Contig4
6.5
6.0
5.5
5.0
4.5
1
2
t−Test: p−value = 0.66

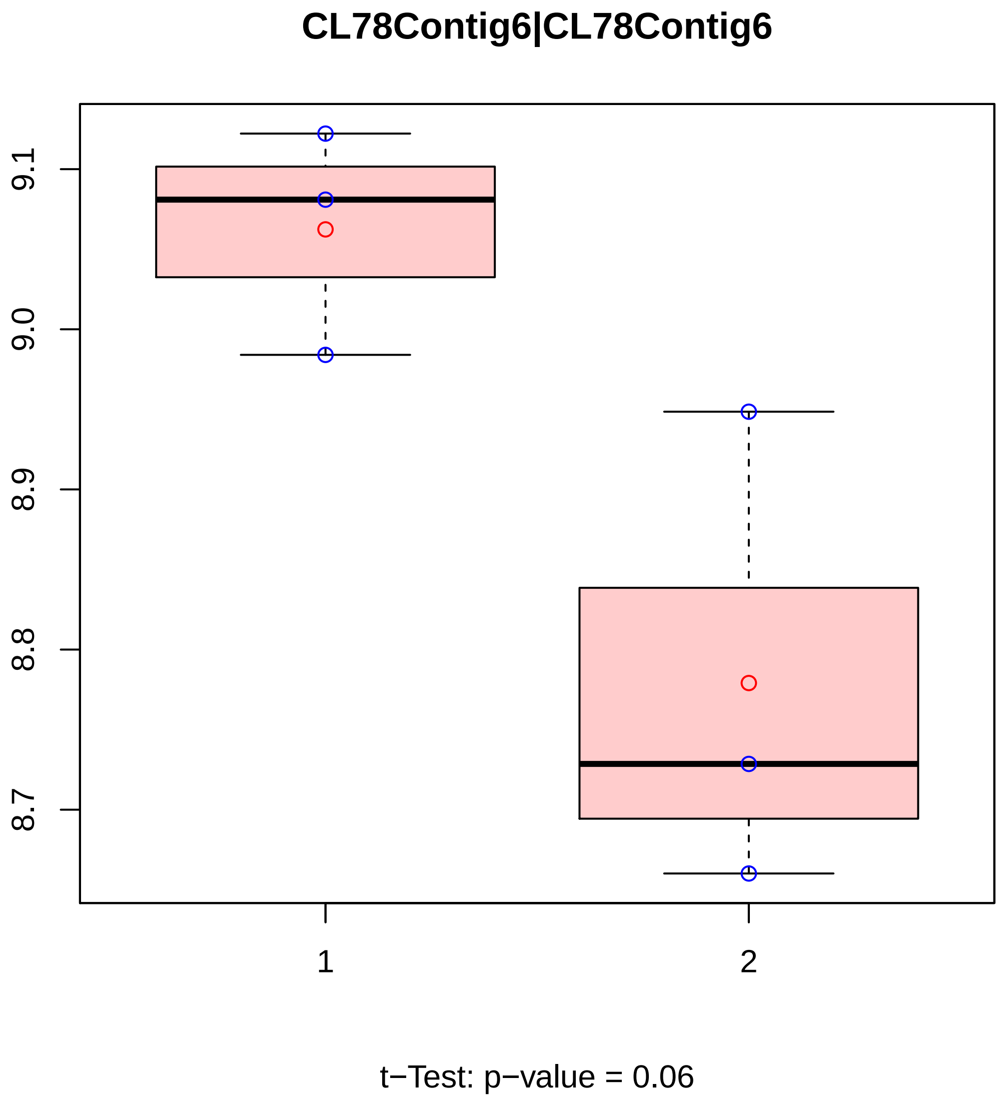
CL78Contig6|CL78Contig6
9.1
9.0
8.9
8.8
8.7
1
2
t−Test: p−value = 0.06

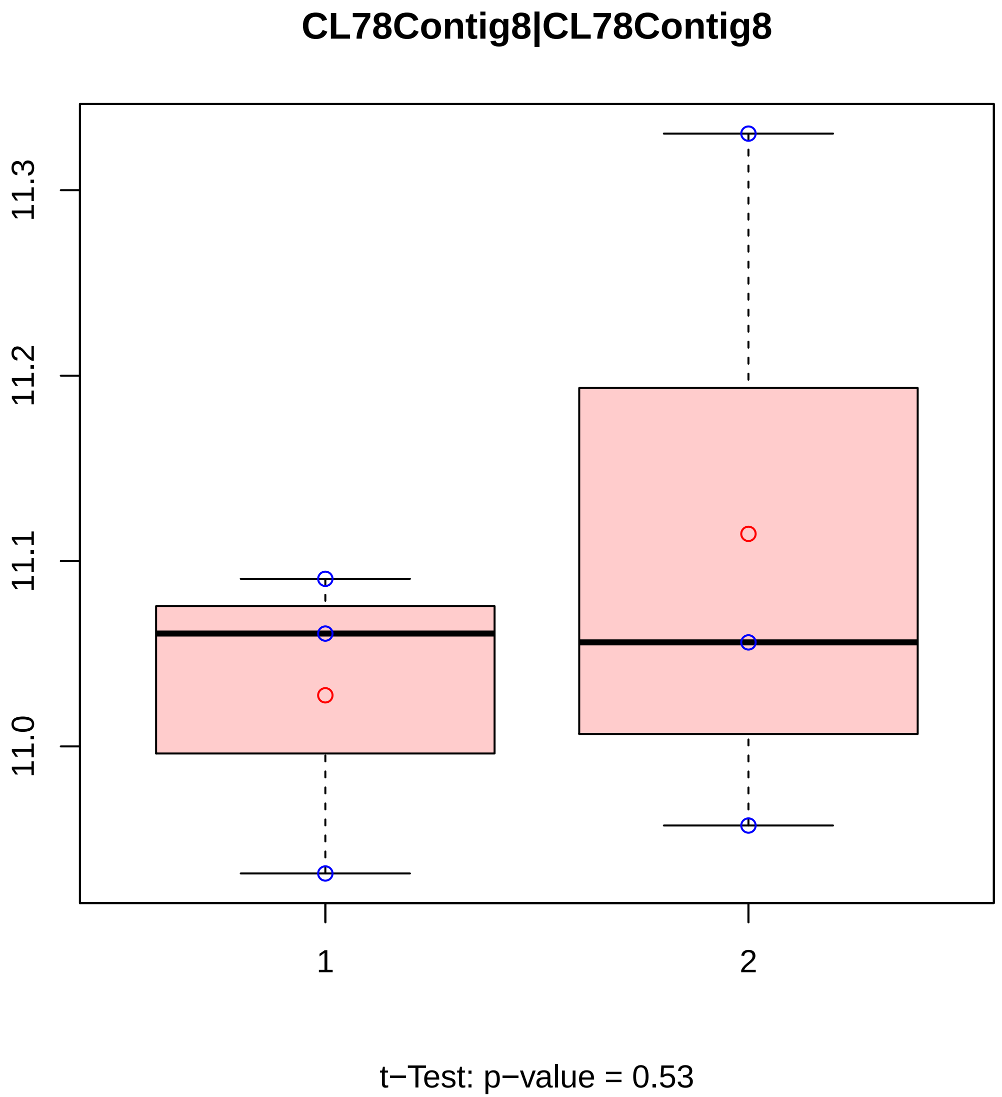

CL78Contig8|CL78Contig8
11.0
11.1
11.2
11.3
1
2
t−Test: p−value = 0.53

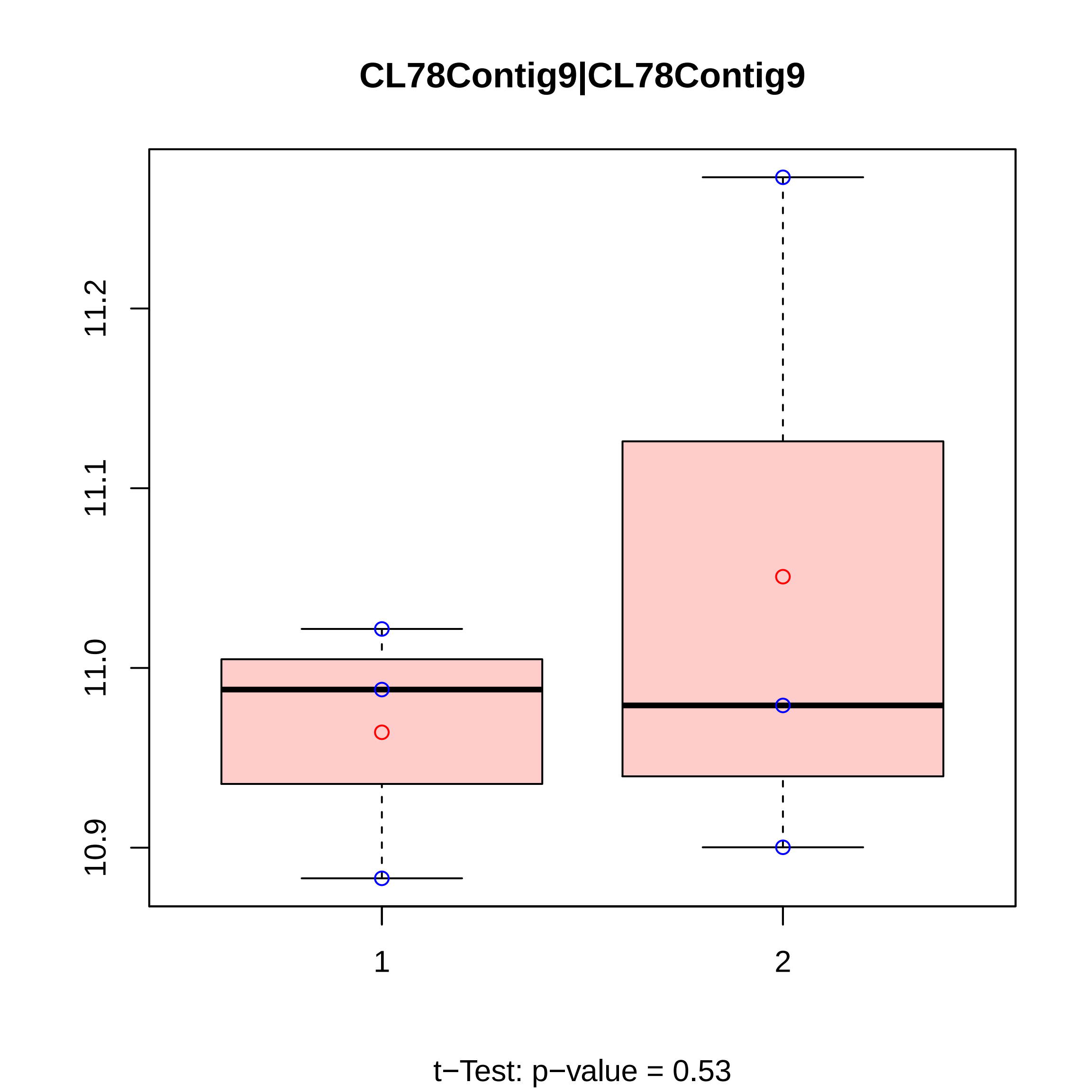
CL78Contig9|CL78Contig9
11.2
11.1
11.0
10.9
1
2
t−Test: p−value = 0.53

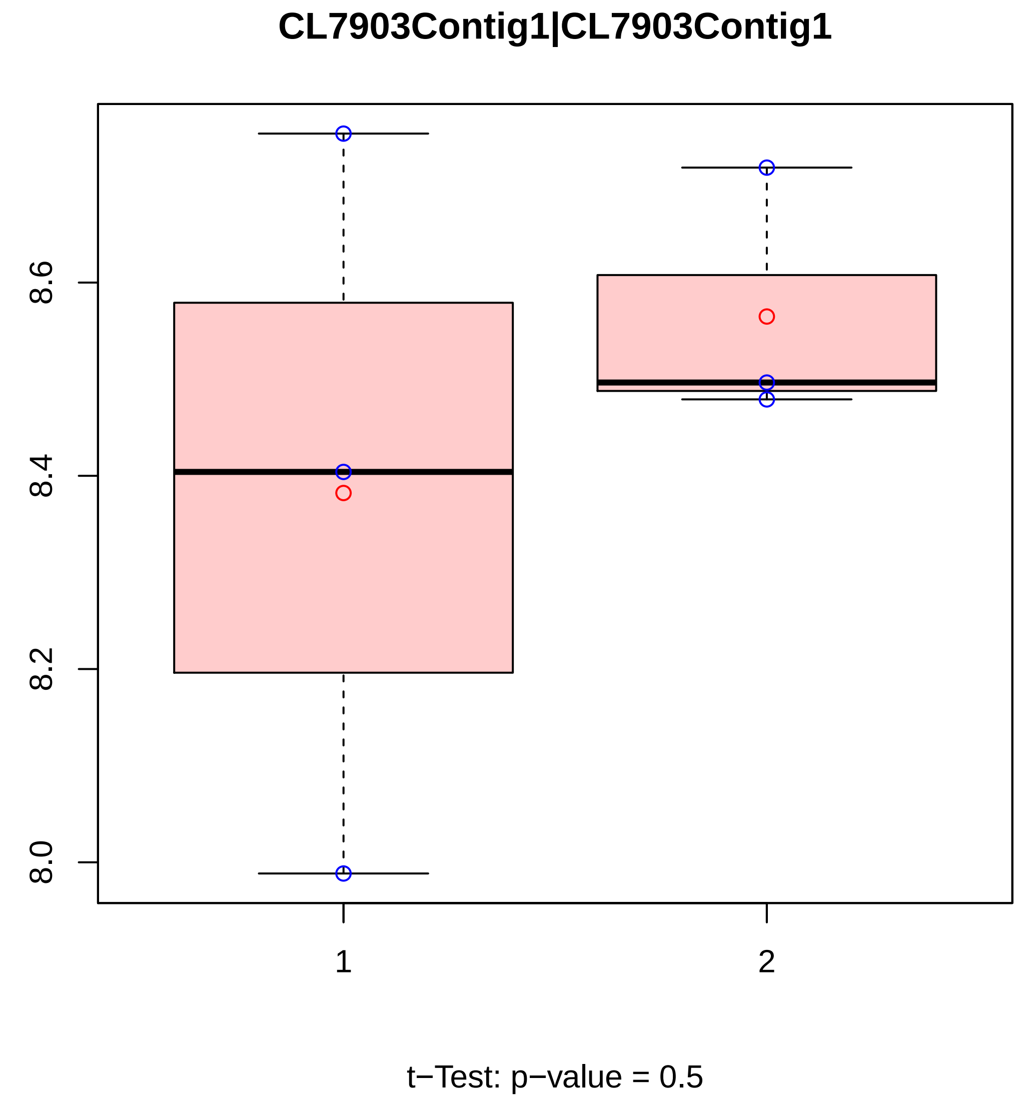
CL7903Contig1|CL7903Contig1
8.0
8.2
8.4
8.6
1
2
t−Test: p−value = 0.5

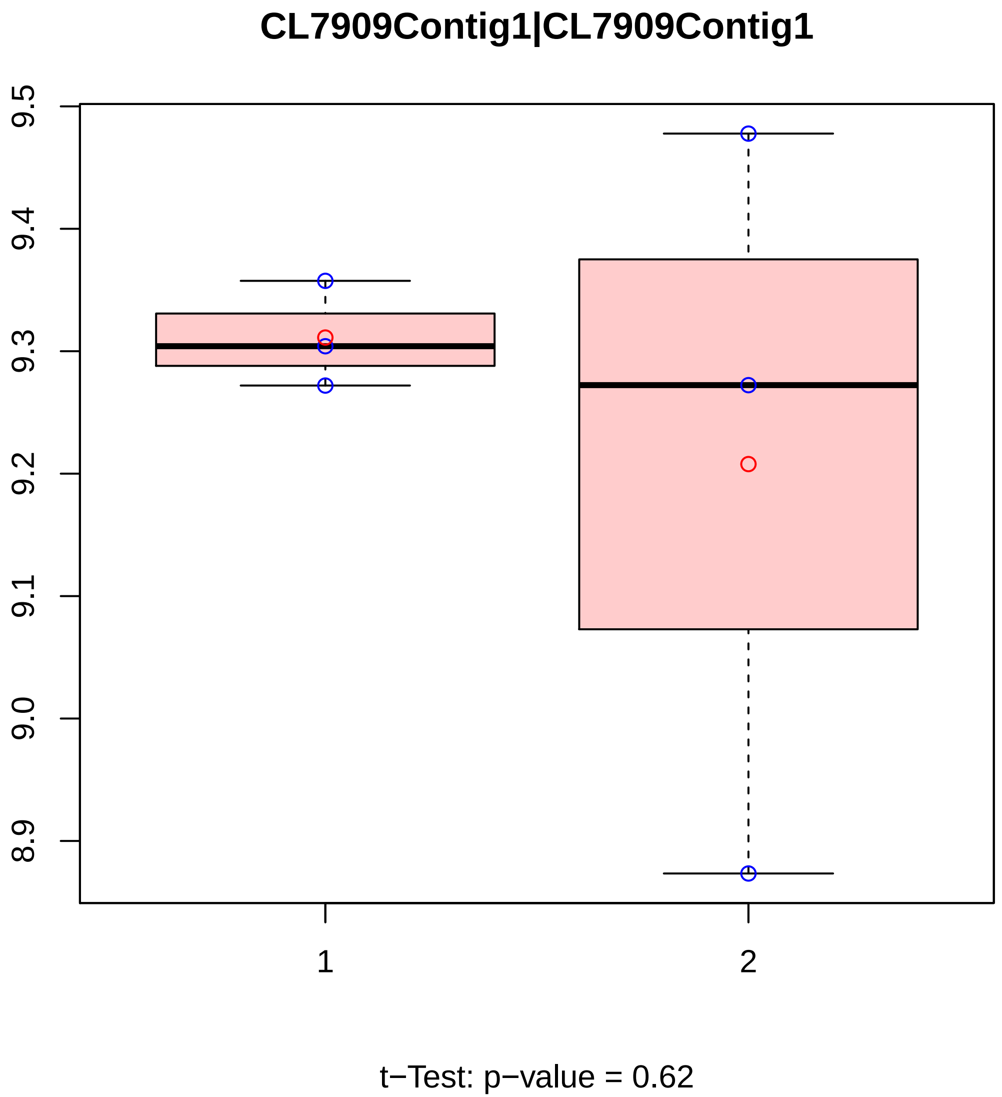

CL7909Contig1|CL7909Contig1
9.5
9.4
9.3
9.2
9.1
9.0
8.9
1
2
t−Test: p−value = 0.62

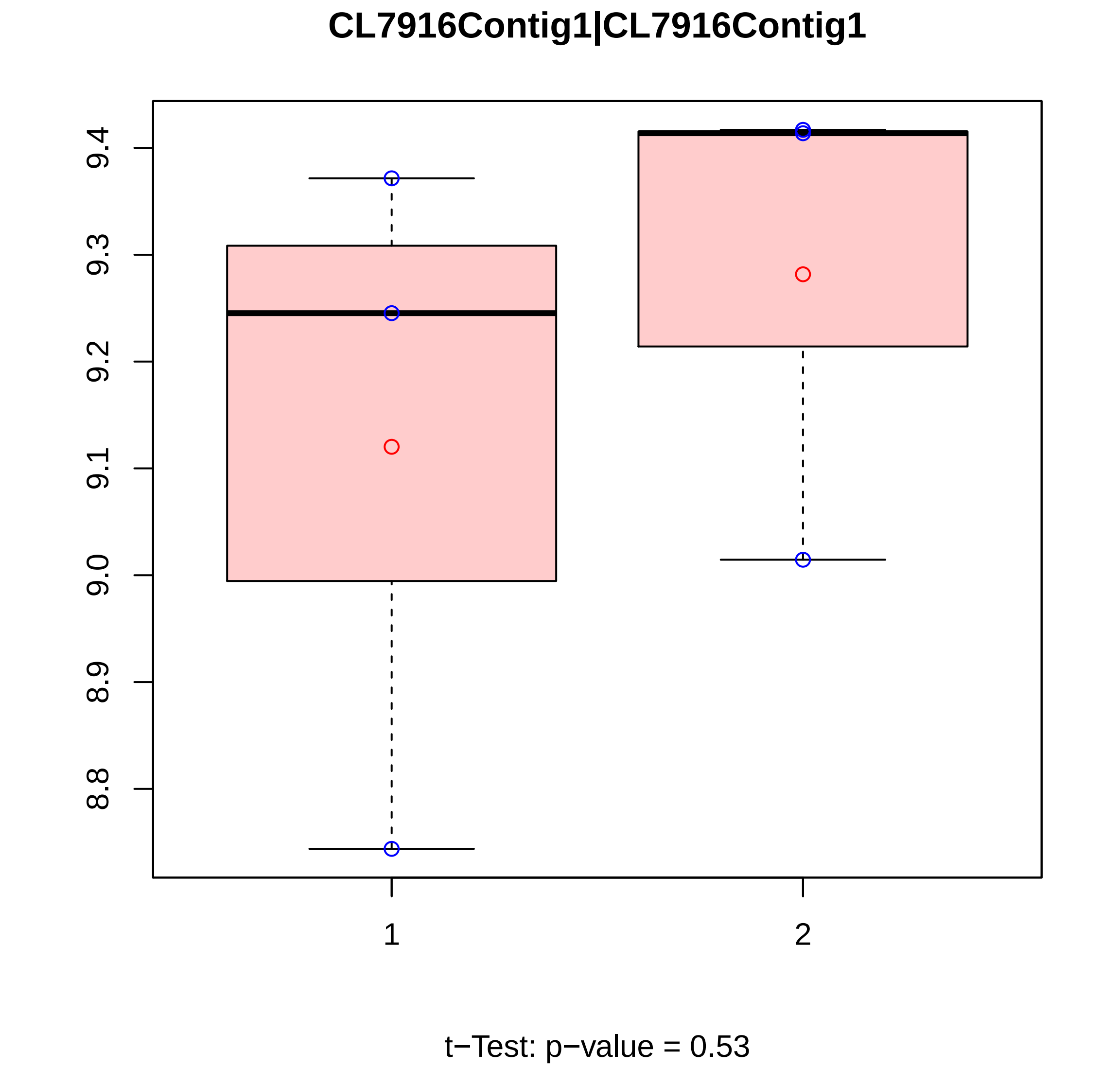

CL7916Contig1|CL7916Contig1
9.4
9.3
9.2
9.1
9.0
8.9
8.8
1
2
t−Test: p−value = 0.53

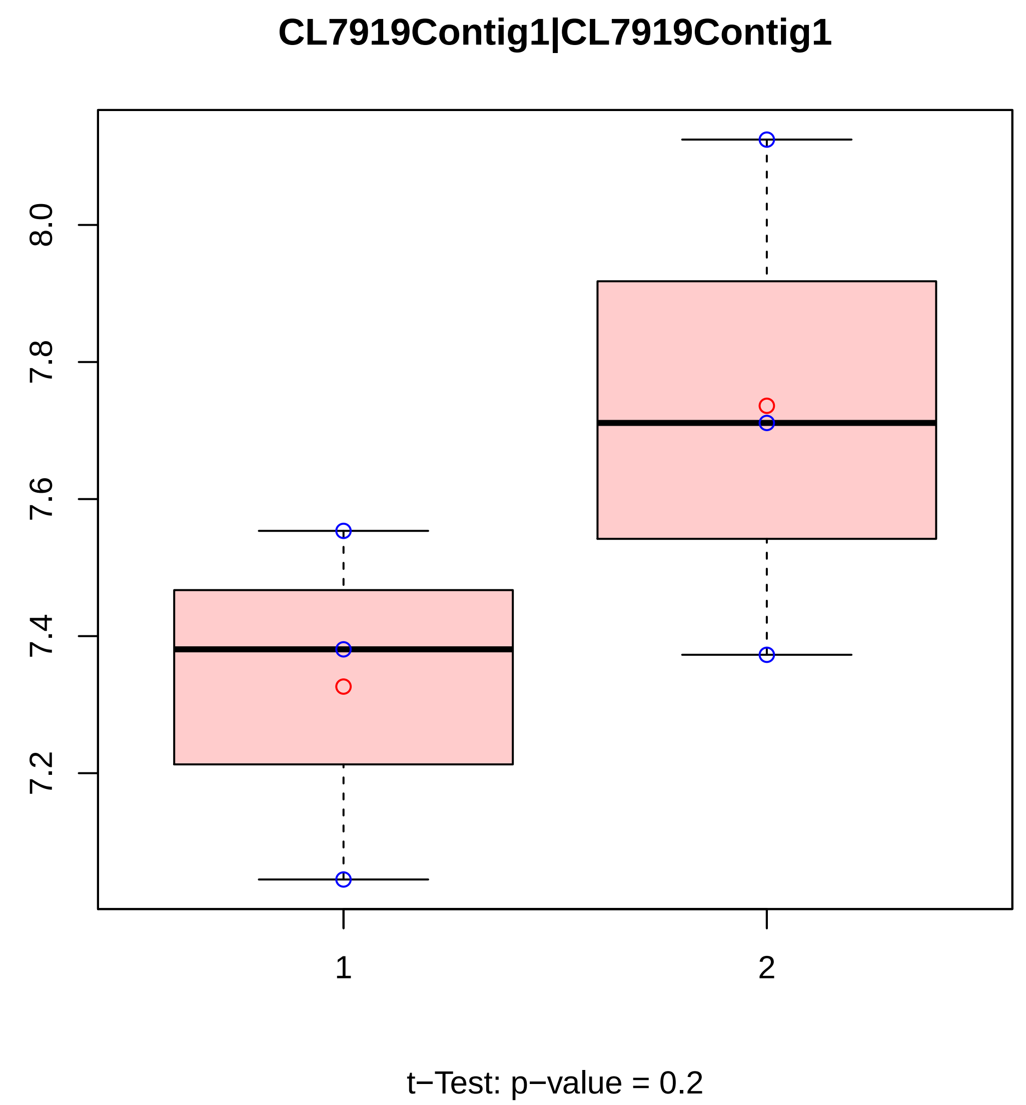
CL7919Contig1|CL7919Contig1
8.0
7.8
7.6
7.4
7.2
1
2
t−Test: p−value = 0.2

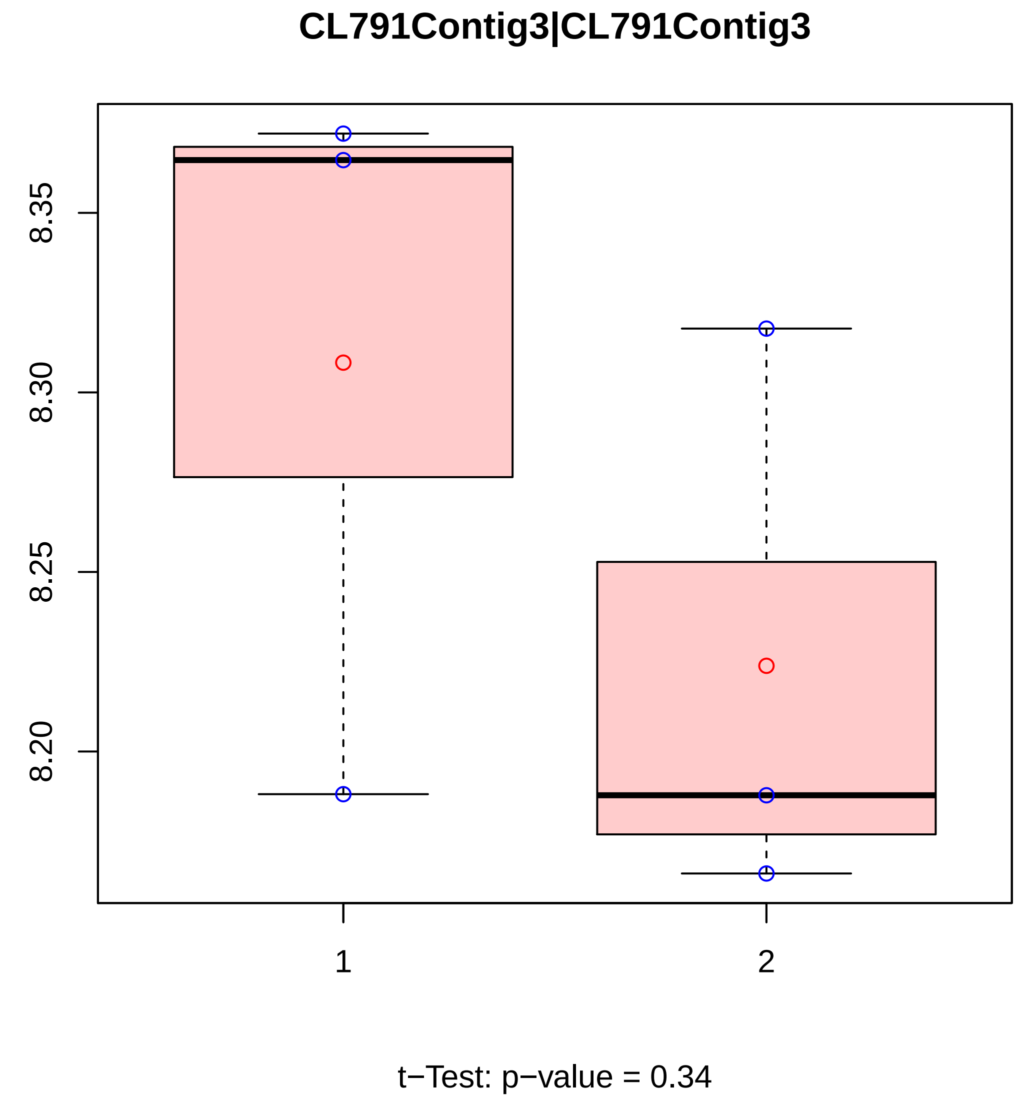

CL791Contig3|CL791Contig3
8.20
8.25
8.30
8.35
1
2
t−Test: p−value = 0.34

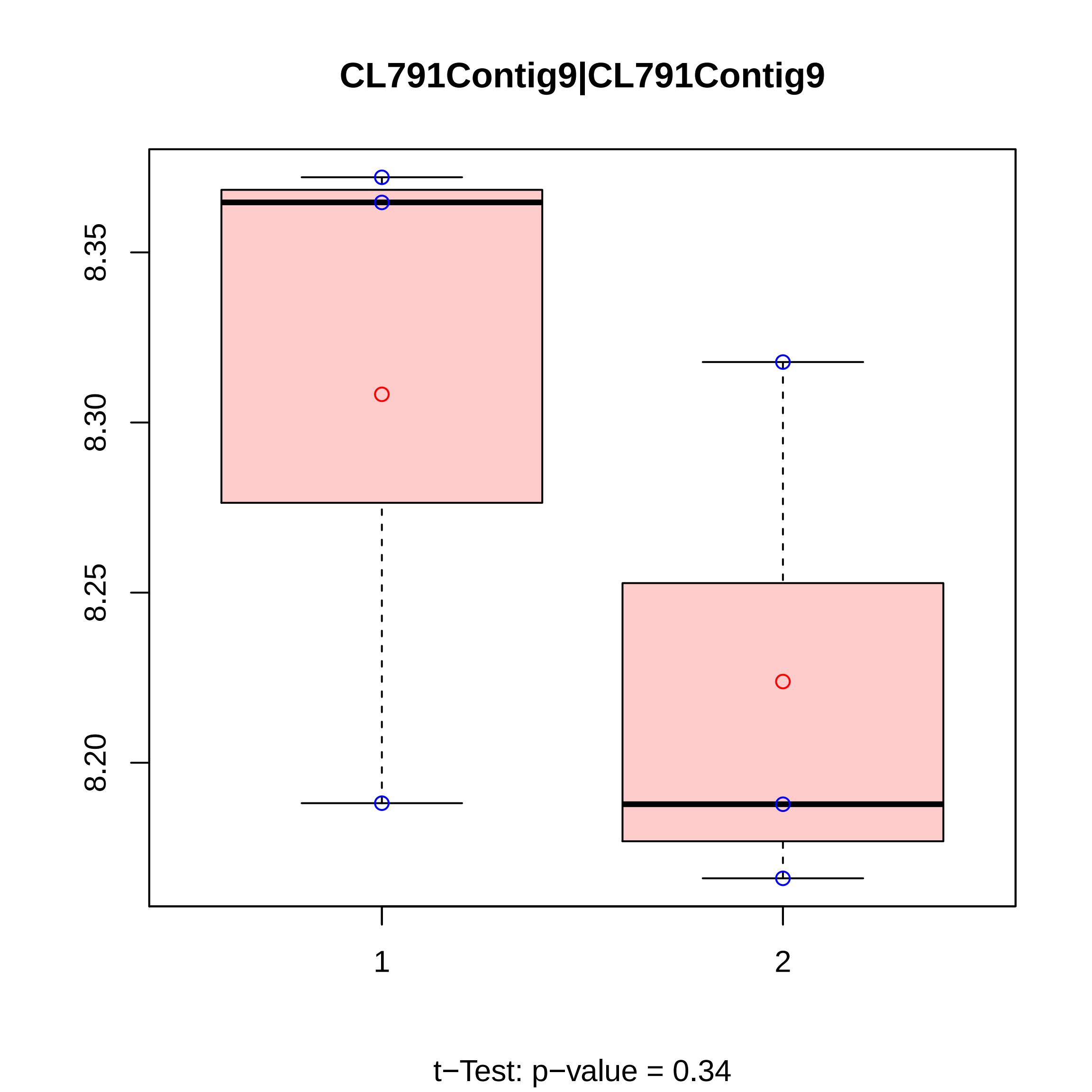
CL791Contig9|CL791Contig9
8.35
8.30
8.25
8.20
1
2
t−Test: p−value = 0.34

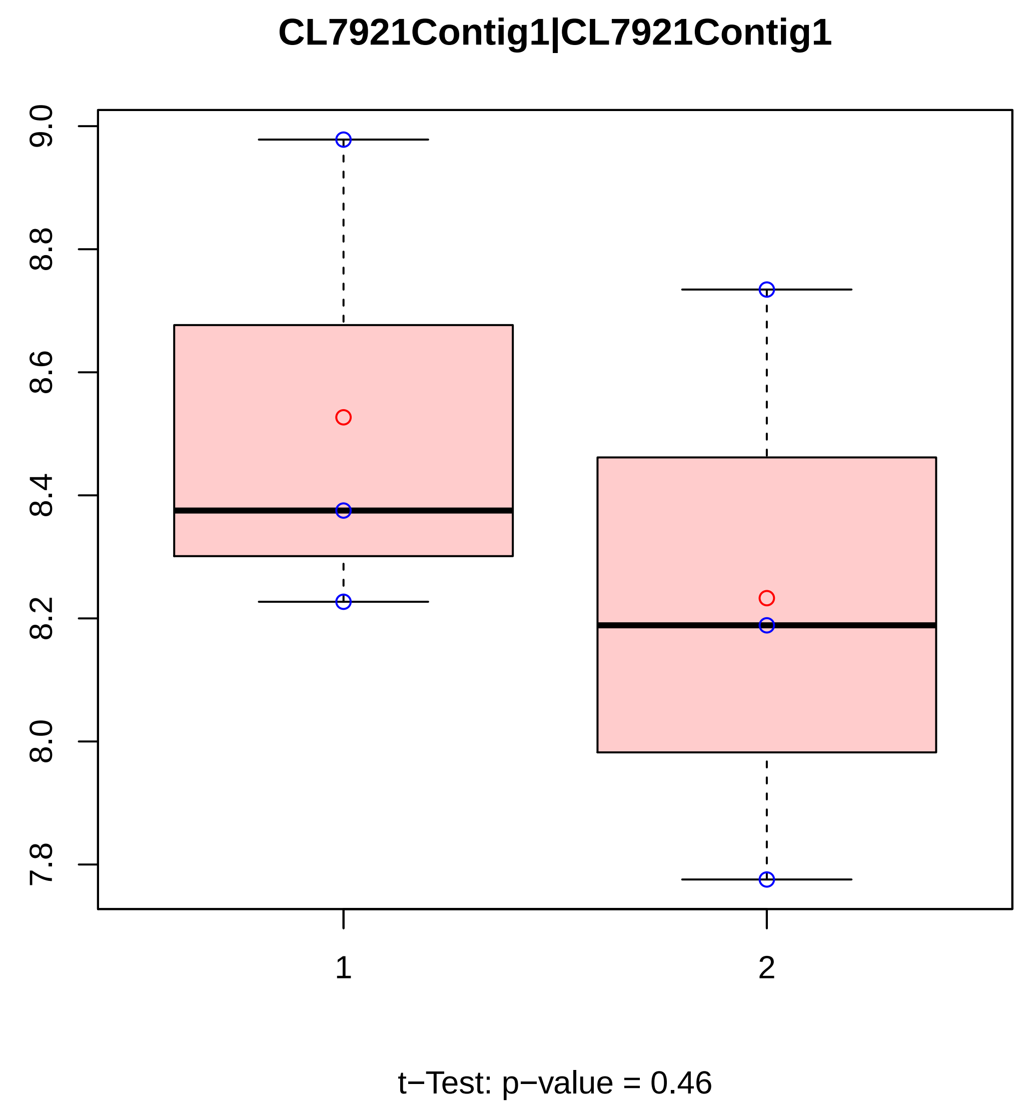
CL7921Contig1|CL7921Contig1
9.0
8.8
8.6
8.4
8.2
8.0
7.8
1
2
t−Test: p−value = 0.46

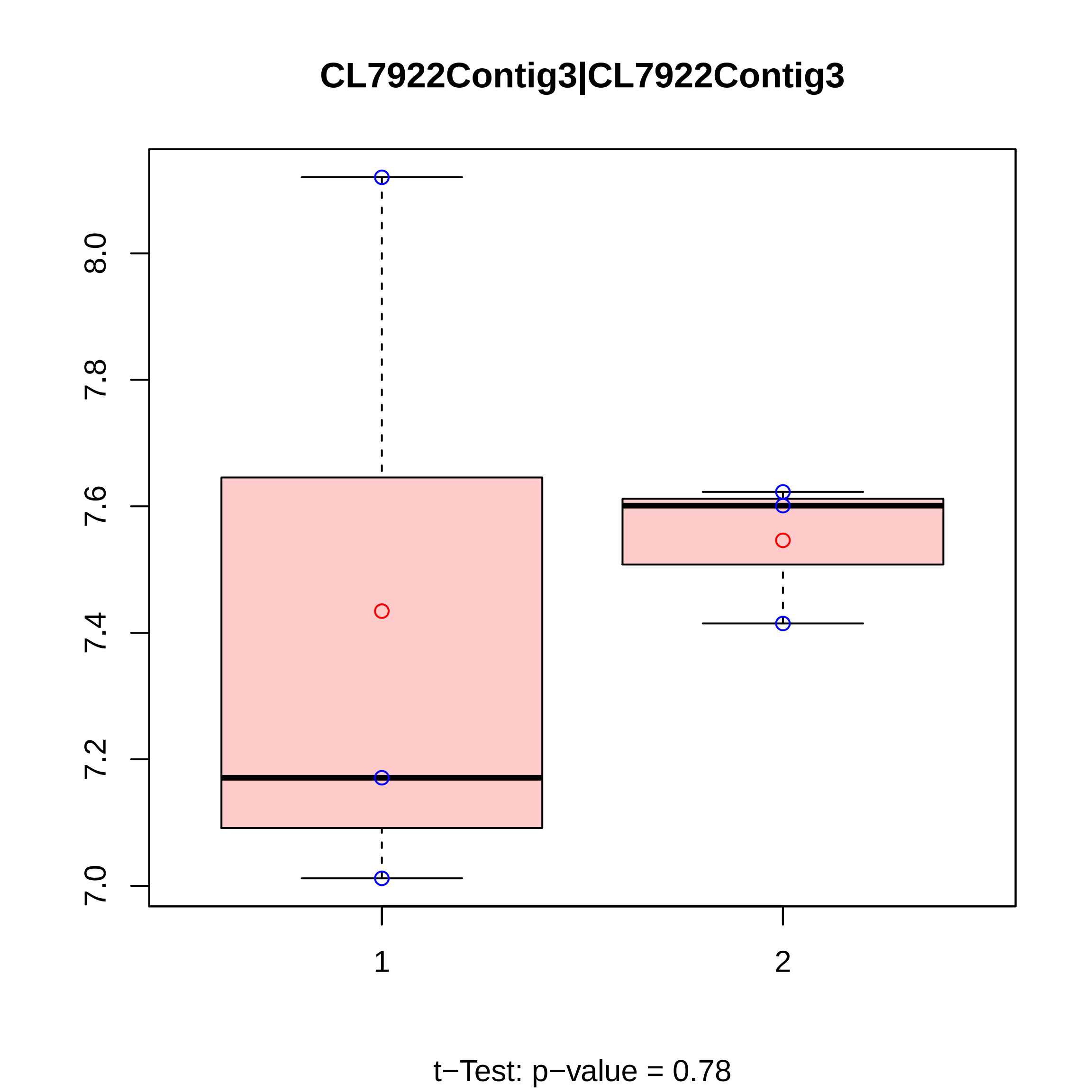
CL7922Contig3|CL7922Contig3
8.0
7.8
7.6
7.4
7.2
7.0
1
2
t−Test: p−value = 0.78

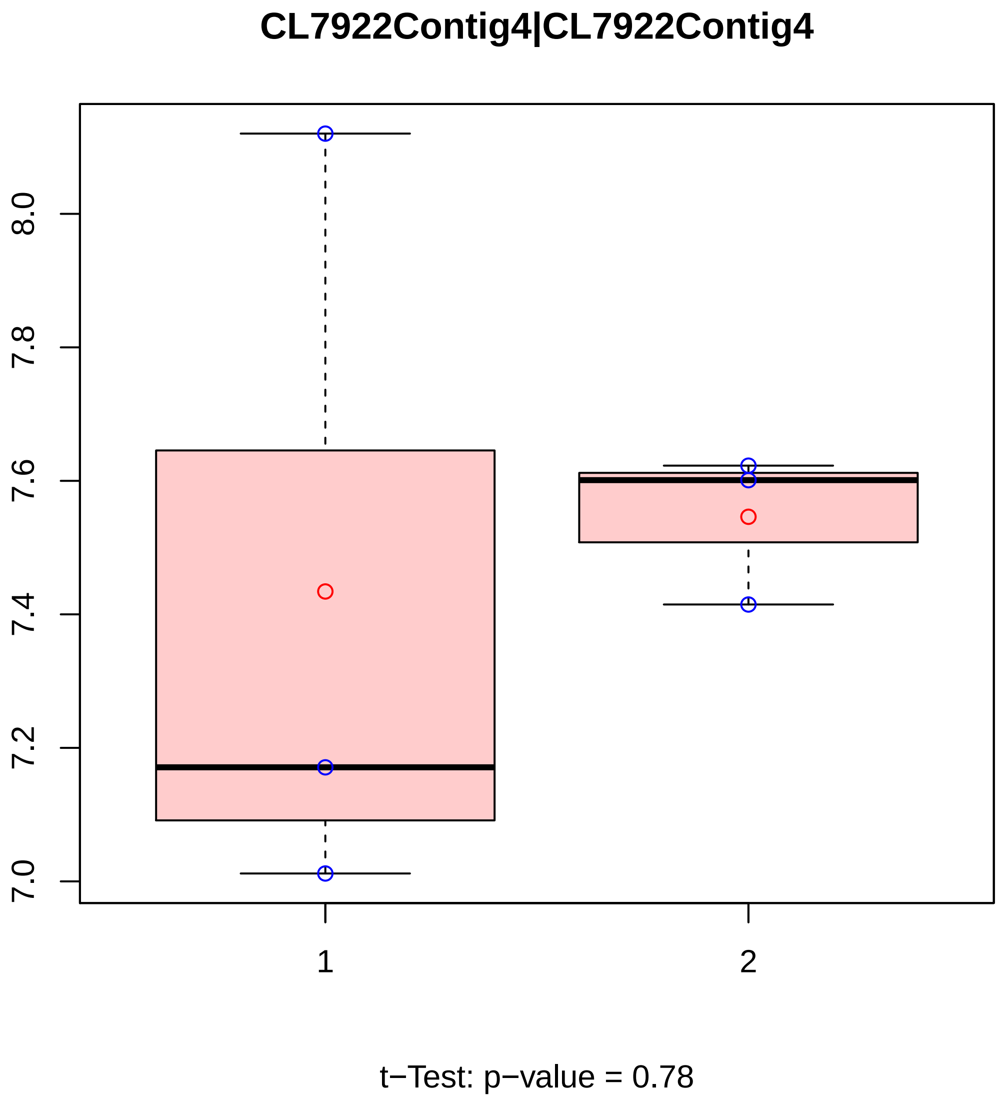
CL7922Contig4|CL7922Contig4
8.0
7.8
7.6
7.4
7.2
7.0
1
2
t−Test: p−value = 0.78

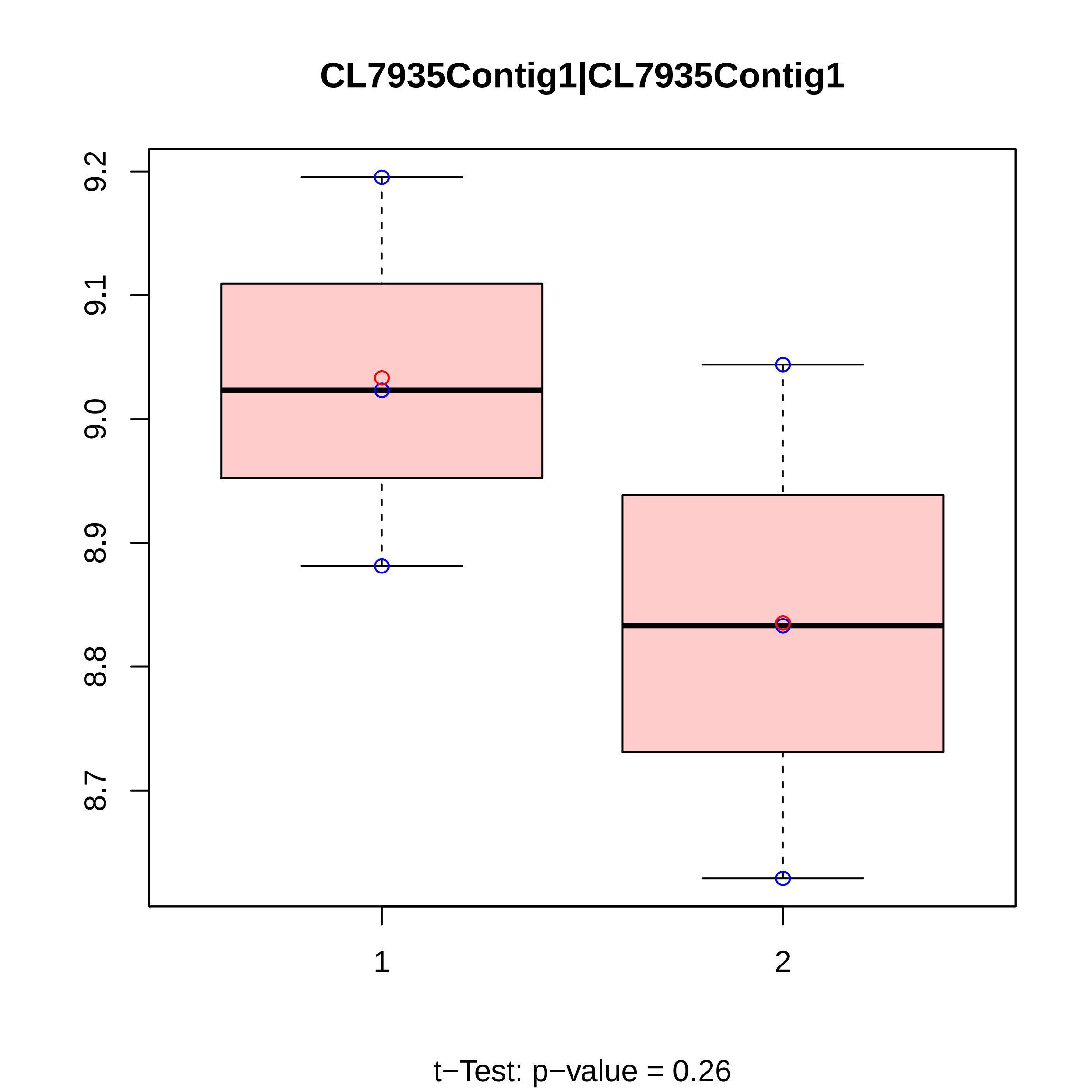
CL7935Contig1|CL7935Contig1
9.2
9.1
9.0
8.9
8.8
8.7
1
2
t−Test: p−value = 0.26

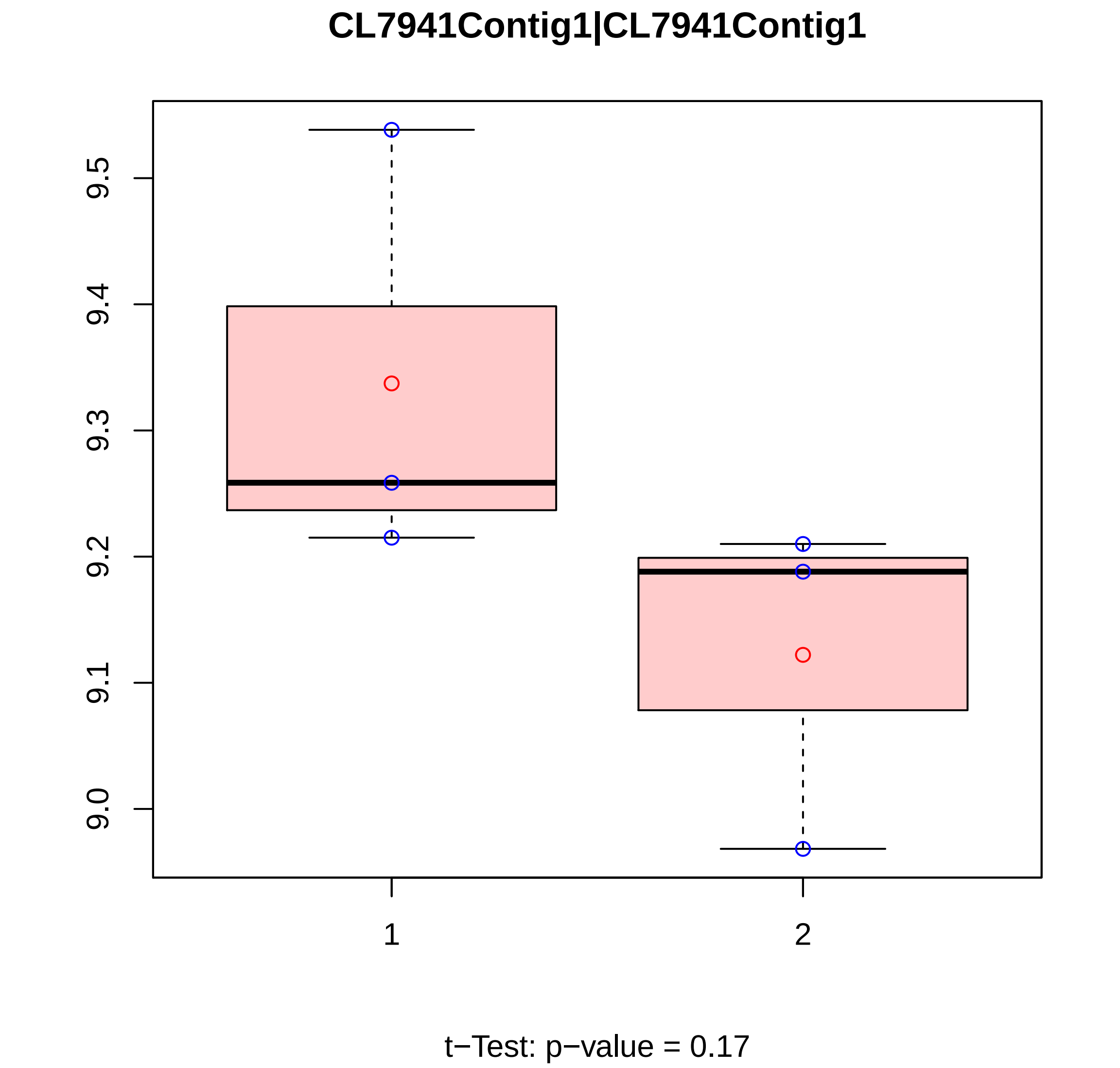

CL7941Contig1|CL7941Contig1
9.0
9.1
9.2
9.3
9.4
9.5
1
2
t−Test: p−value = 0.17

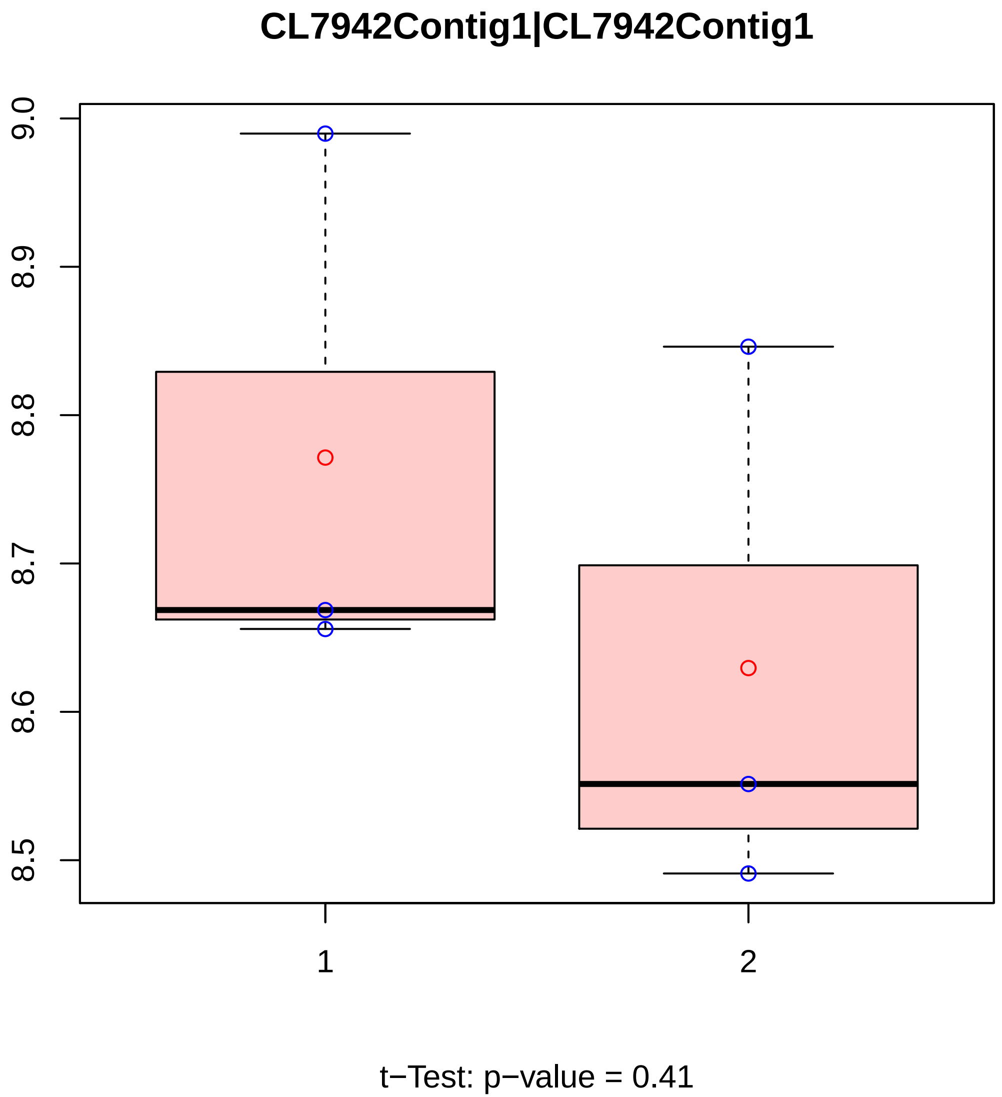
CL7942Contig1|CL7942Contig1
9.0
8.9
8.8
8.7
8.6
8.5
1
2
t−Test: p−value = 0.41

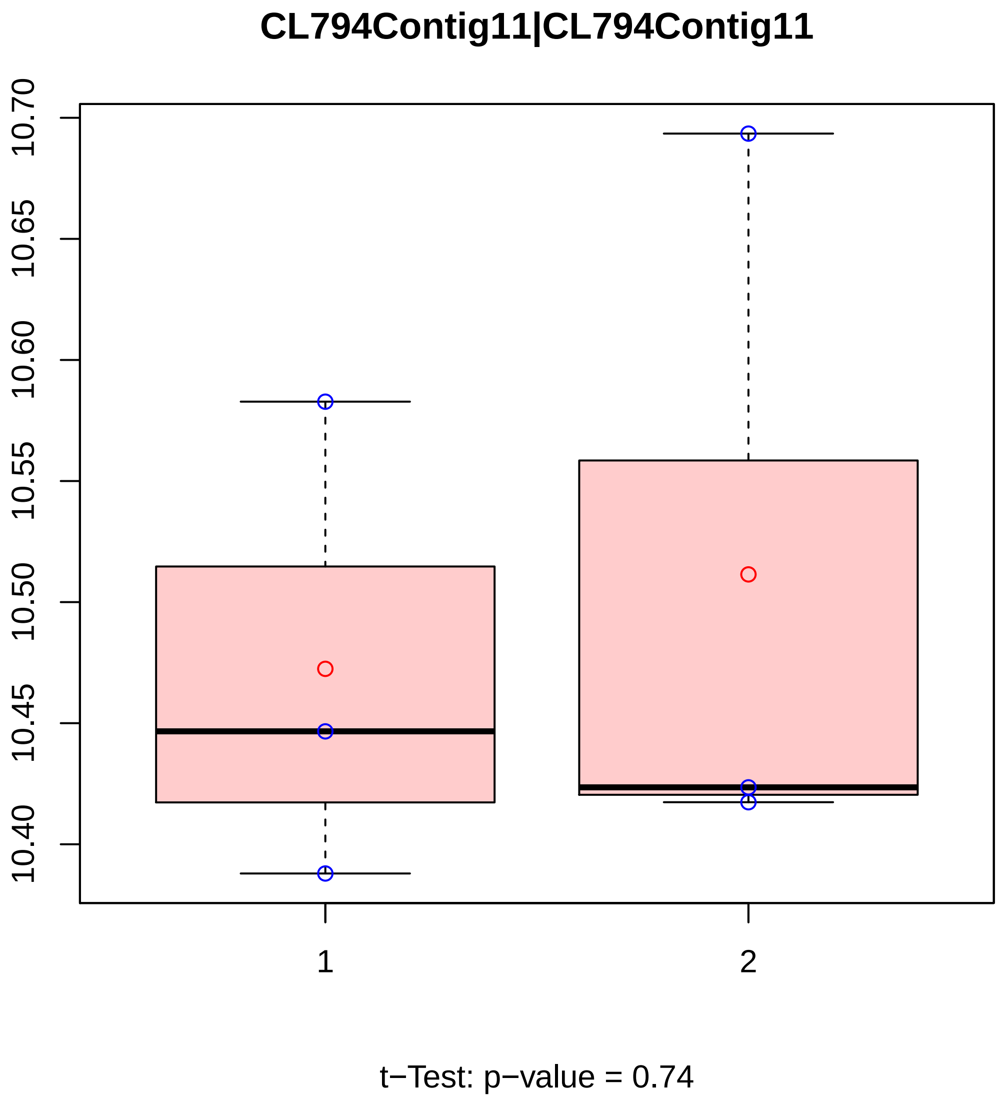

CL794Contig11|CL794Contig11
10.40
10.45
10.50
10.55
10.60
10.65
10.70
1
2
t−Test: p−value = 0.74

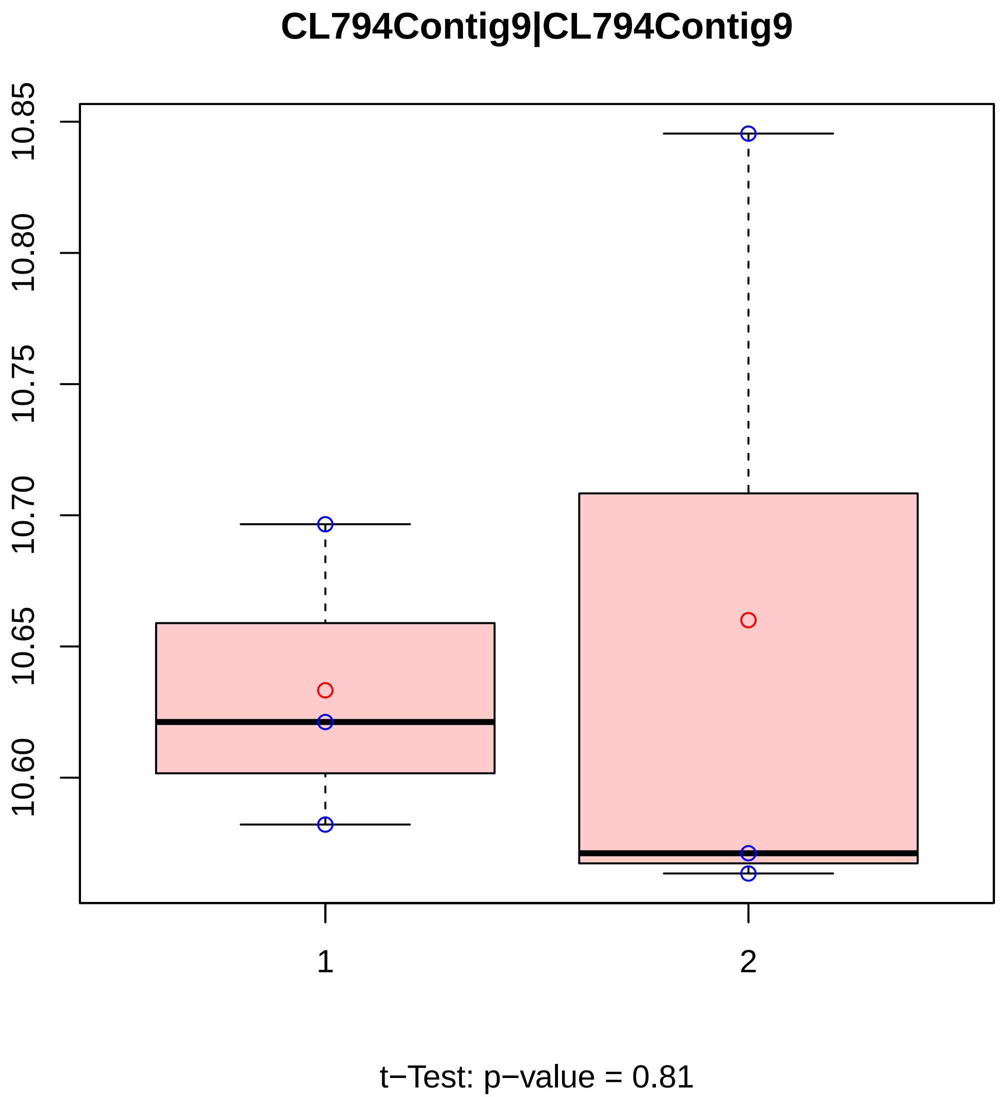

CL794Contig9|CL794Contig9
10.60
10.65
10.70
10.75
10.80
10.85
1
2
t−Test: p−value = 0.81

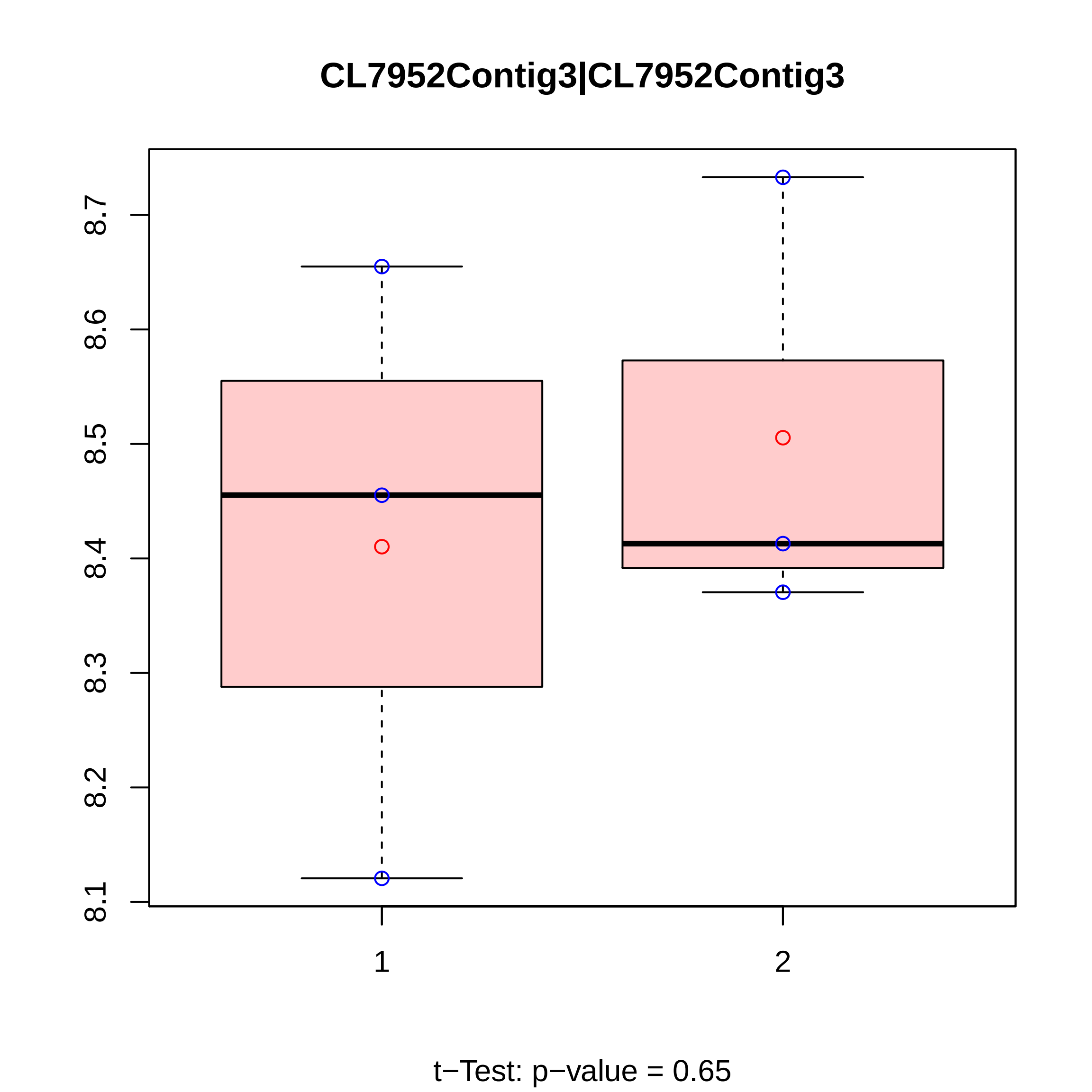

CL7952Contig3|CL7952Contig3
8.1
8.2
8.3
8.4
8.5
8.6
8.7
1
2
t−Test: p−value = 0.65

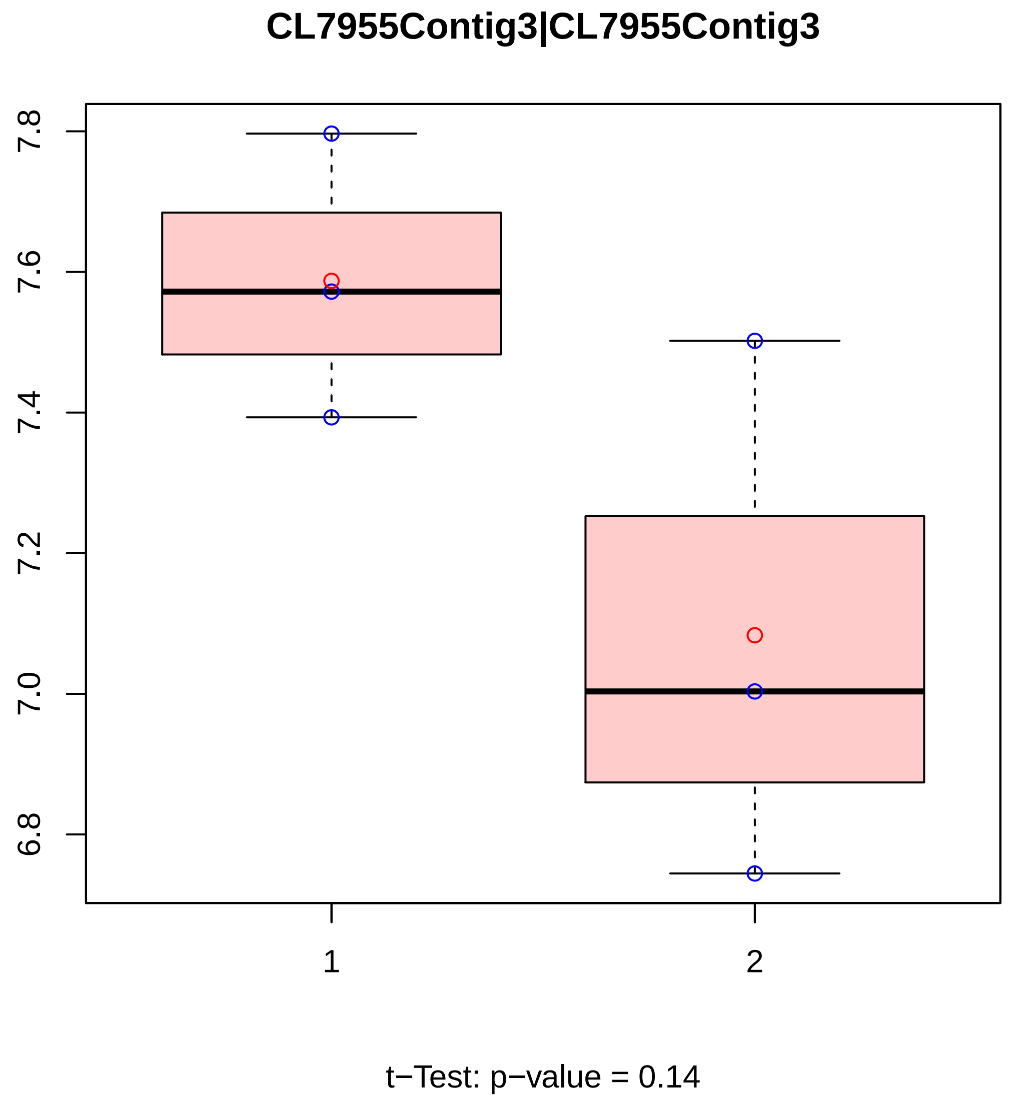
CL7955Contig3|CL7955Contig3
7.8
7.6
7.4
7.2
7.0
6.8
1
2
t−Test: p−value = 0.14

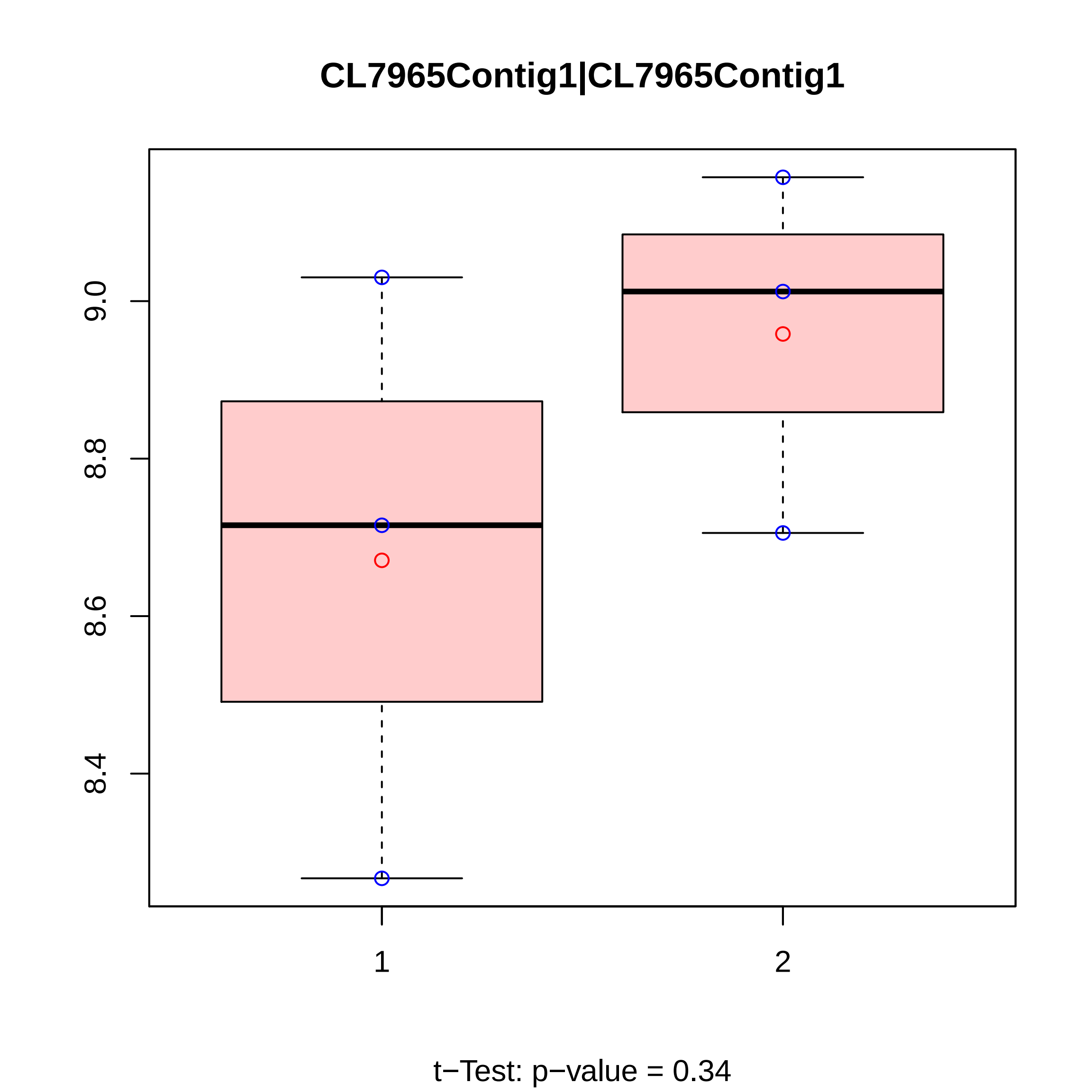
CL7965Contig1|CL7965Contig1
9.0
8.8
8.6
8.4
1
2
t−Test: p−value = 0.34

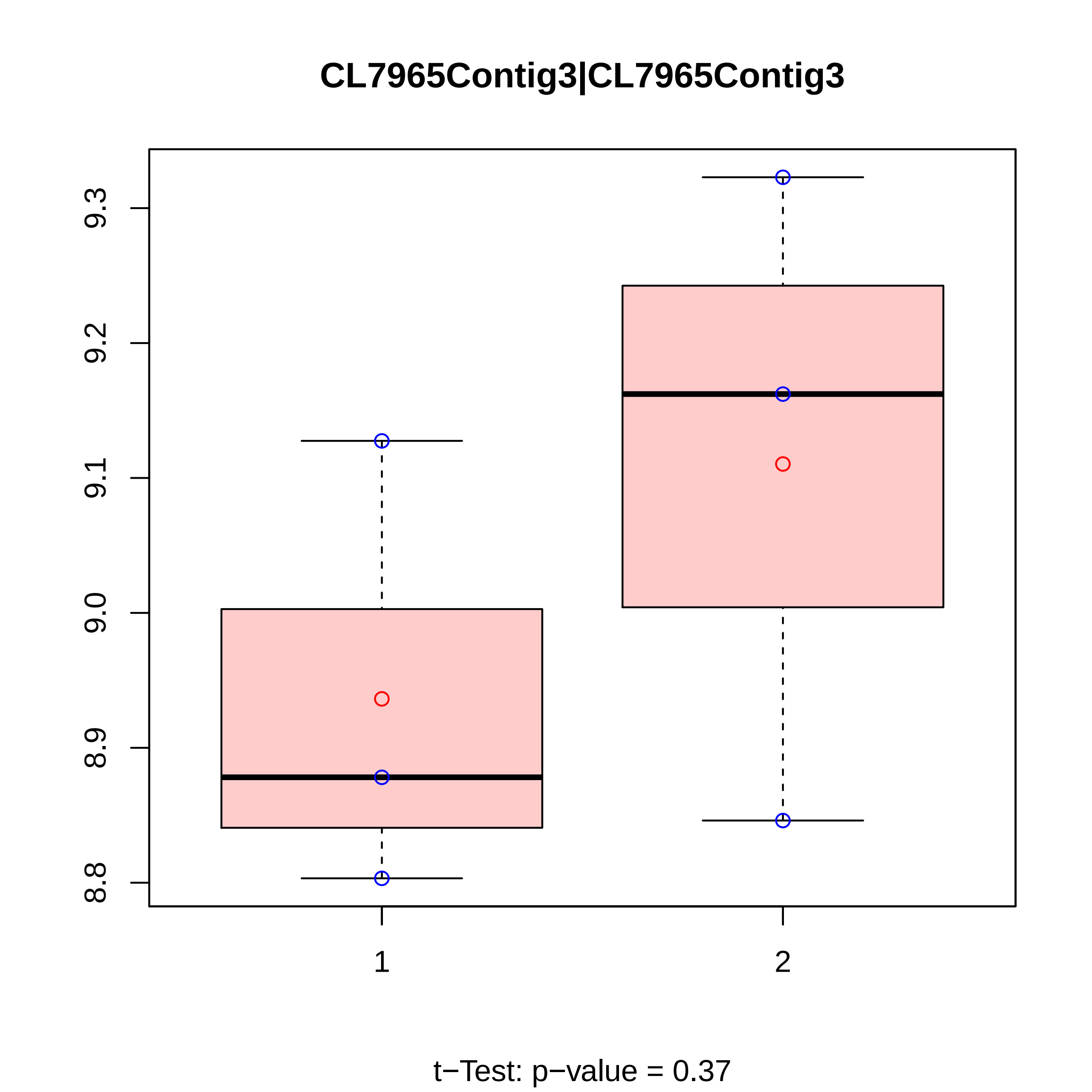
CL7965Contig3|CL7965Contig3
9.3
9.2
9.1
9.0
8.9
8.8
1
2
t−Test: p−value = 0.37

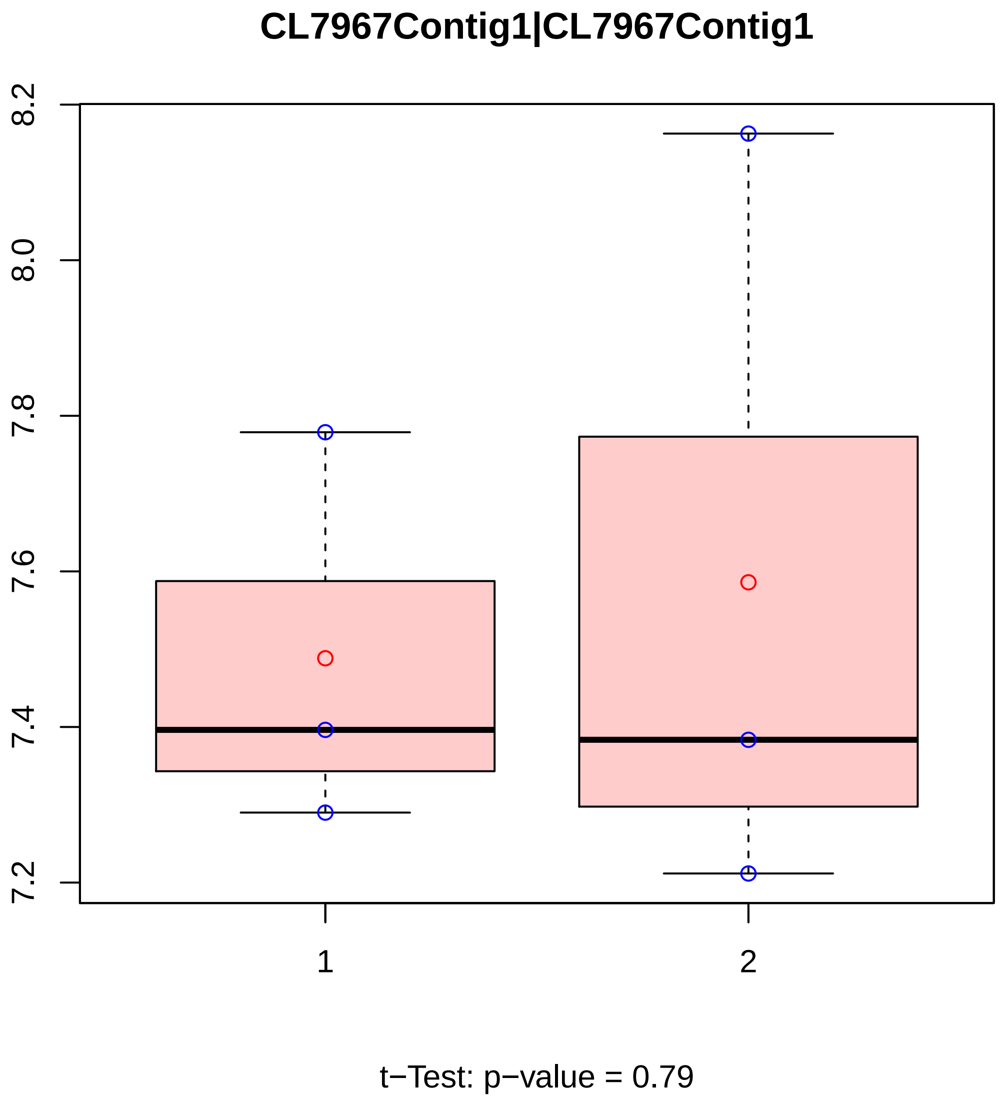
CL7967Contig1|CL7967Contig1
8.2
8.0
7.8
7.6
7.4
7.2
1
2
t−Test: p−value = 0.79

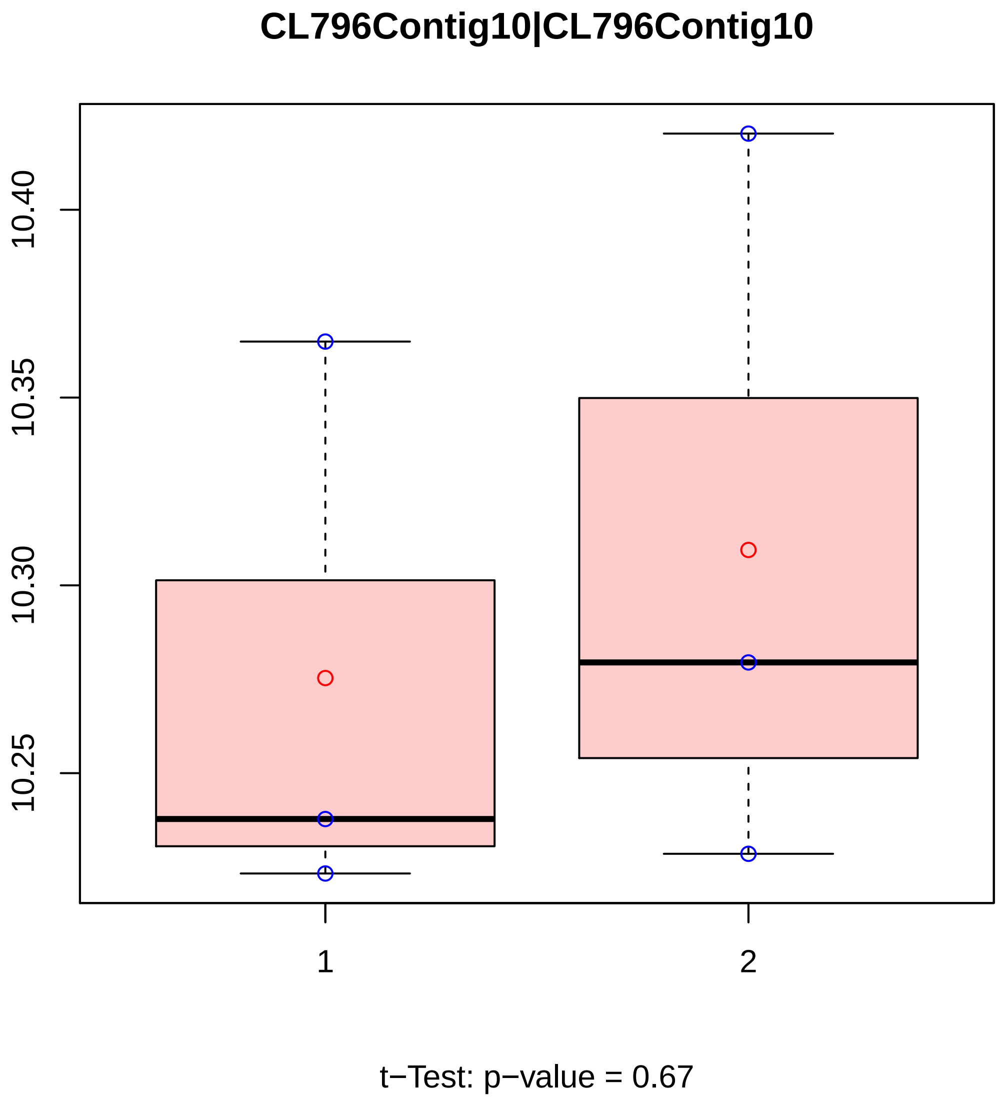

CL796Contig10|CL796Contig10
10.25
10.30
10.35
10.40
1
2
t−Test: p−value = 0.67

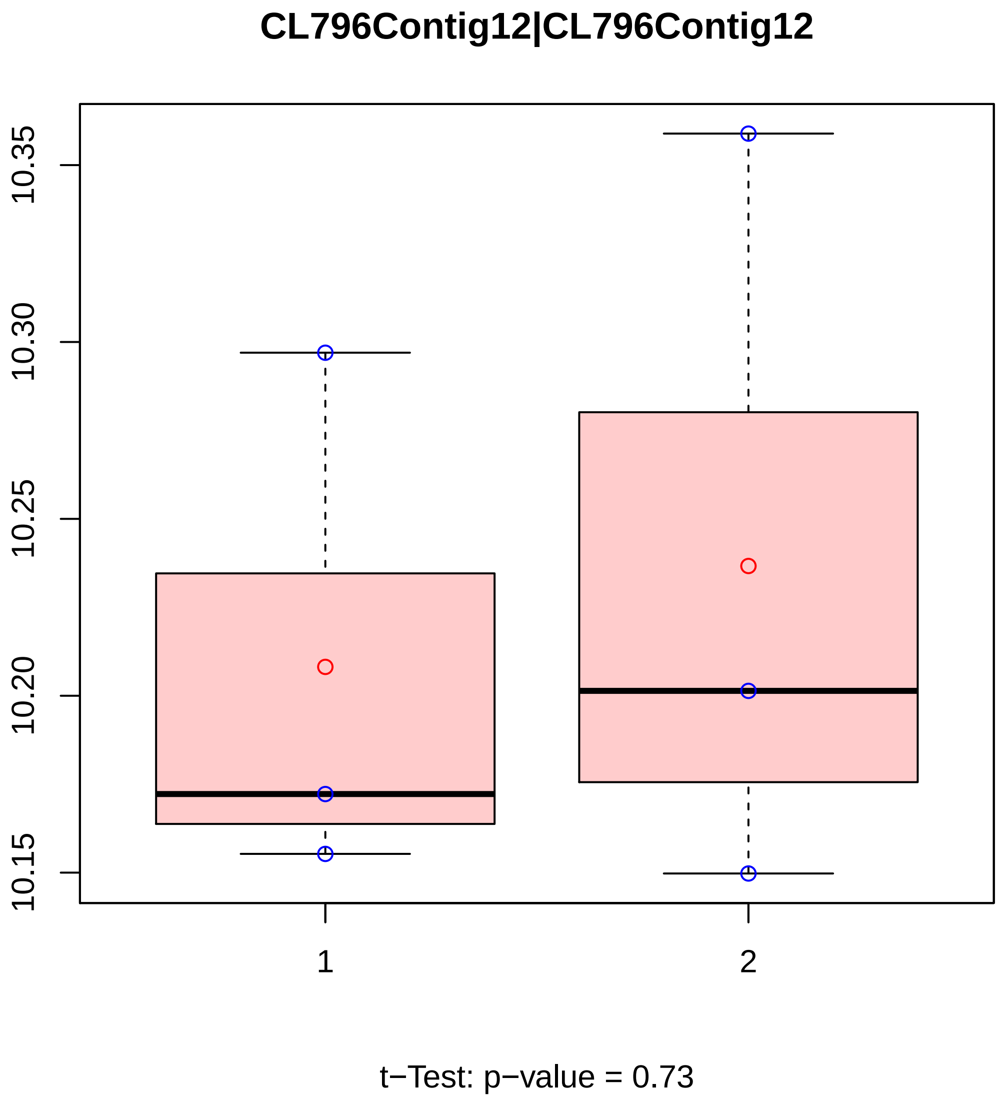
CL796Contig12|CL796Contig12
10.15
10.20
10.25
10.30
10.35
1
2
t−Test: p−value = 0.73

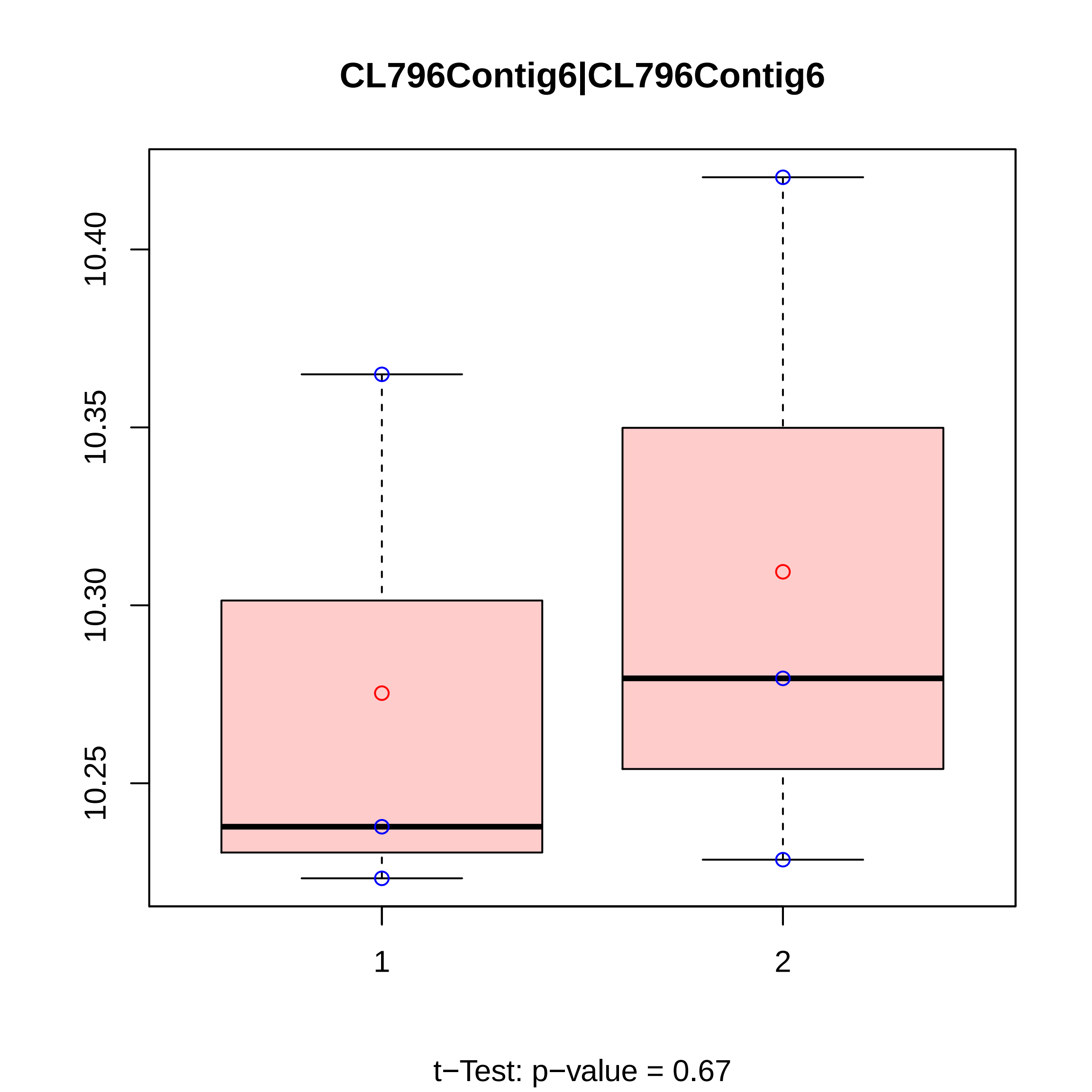

CL796Contig6|CL796Contig6
10.40
10.35
10.30
10.25
1
2
t−Test: p−value = 0.67

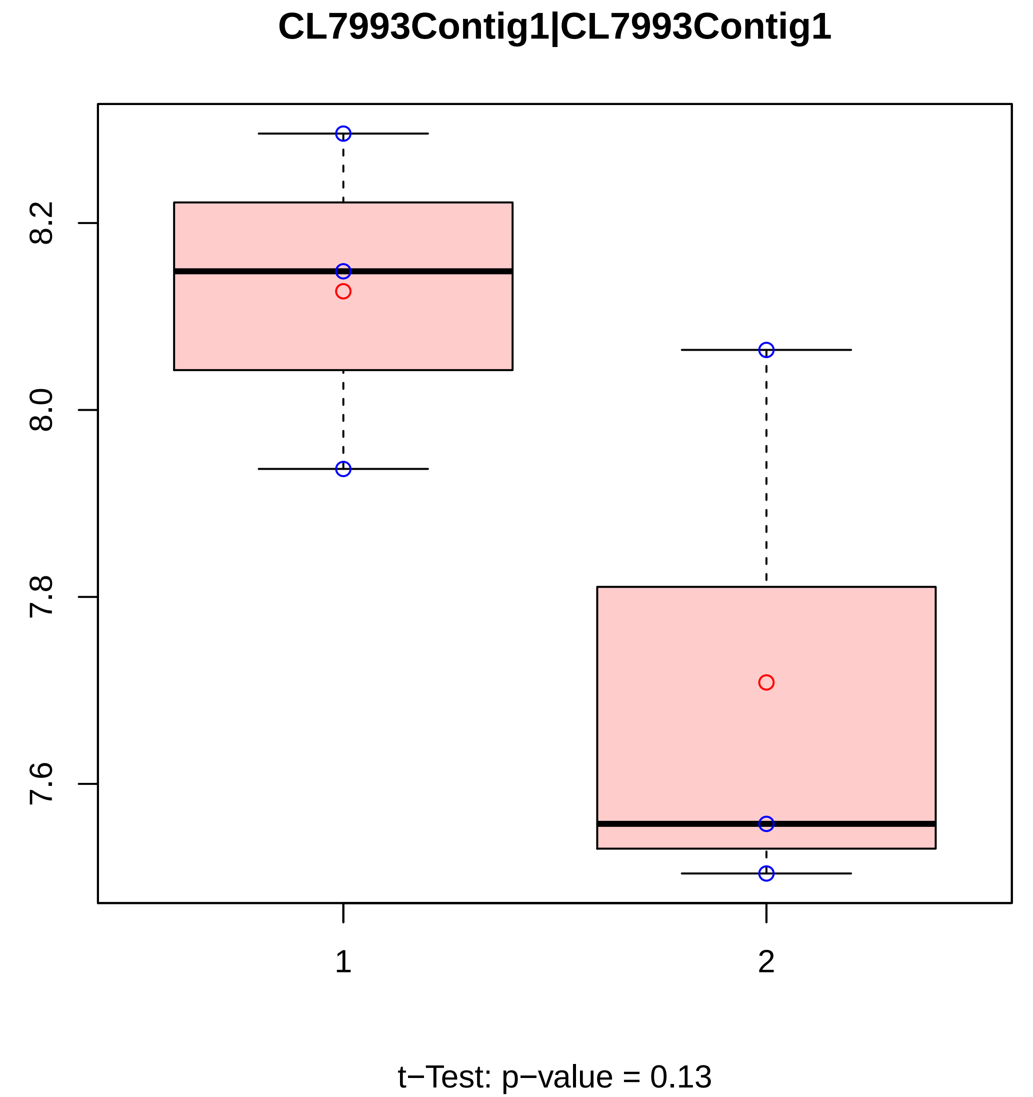
CL7993Contig1|CL7993Contig1
8.2
8.0
7.8
7.6
1
2
t−Test: p−value = 0.13

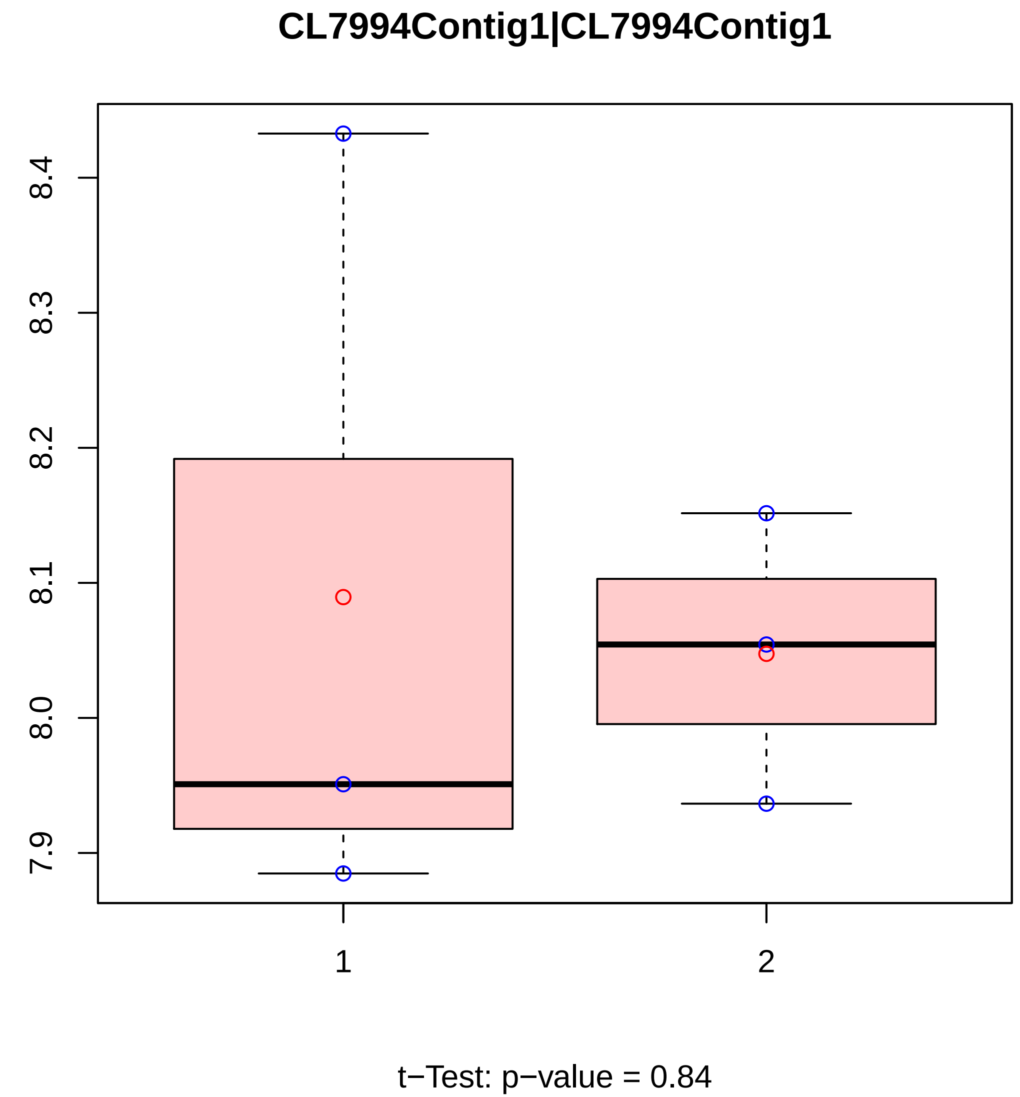

CL7994Contig1|CL7994Contig1
8.4
8.3
8.2
8.1
8.0
7.9
1
2
t−Test: p−value = 0.84

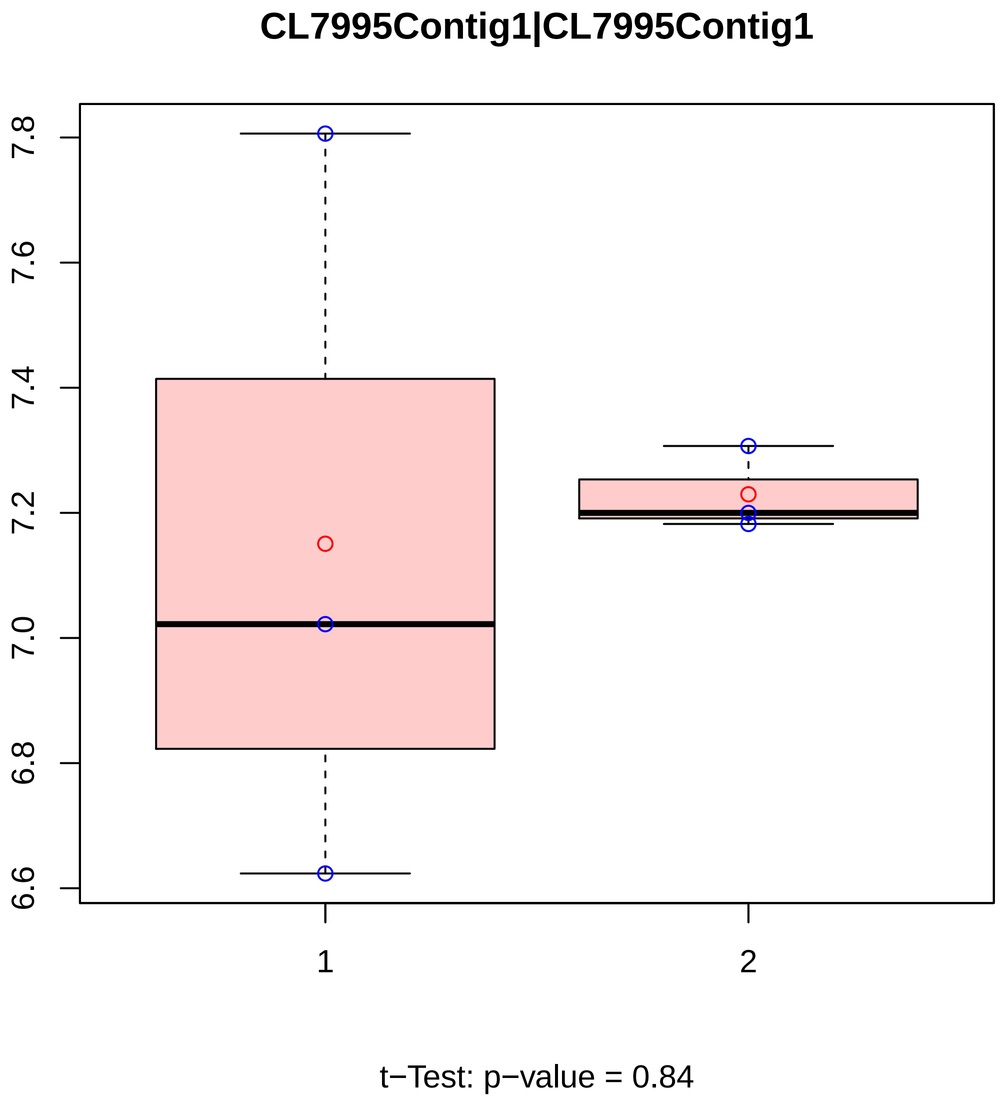
CL7995Contig1|CL7995Contig1
7.8
7.6
7.4
7.2
7.0
6.8
6.6
1
2
t−Test: p−value = 0.84

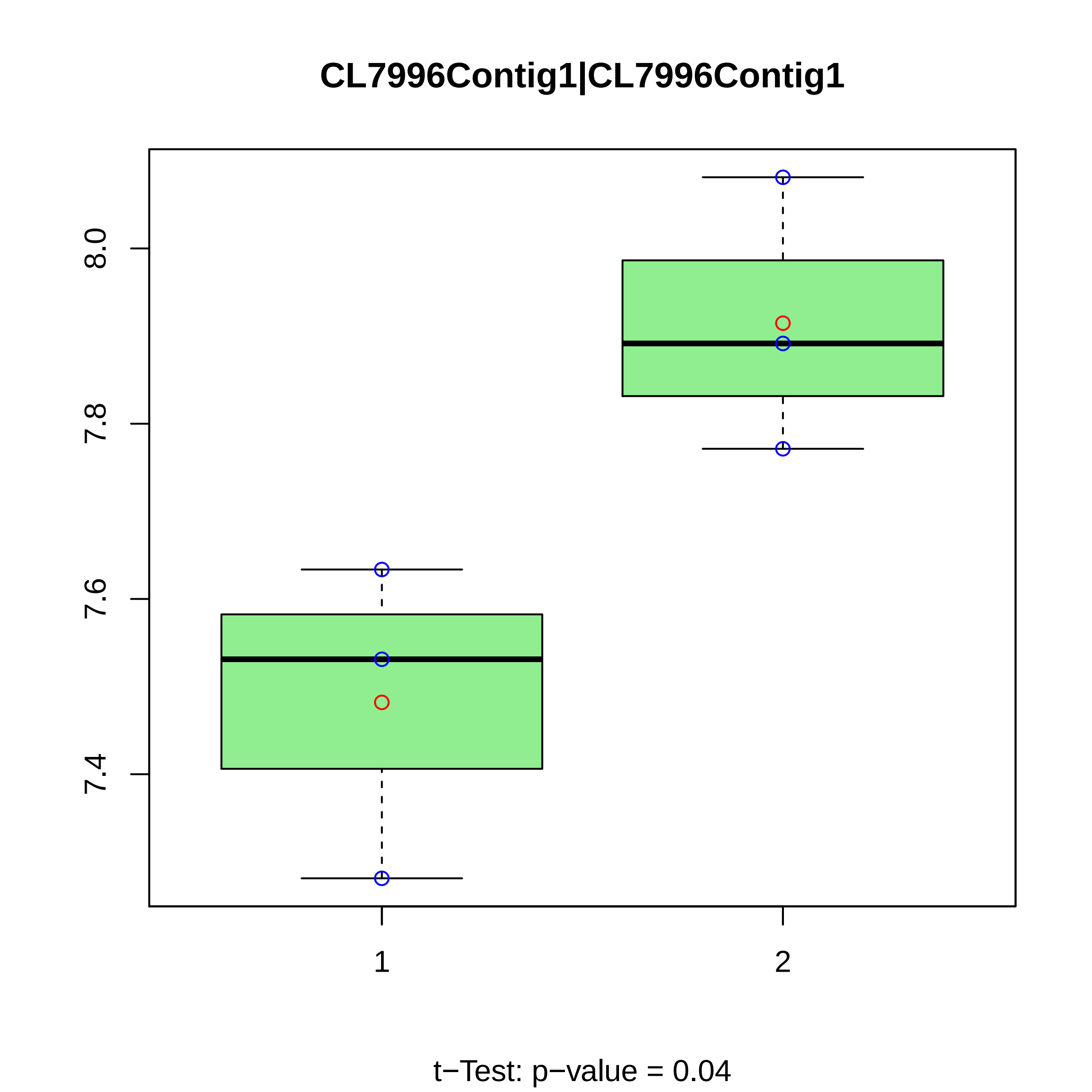

CL7996Contig1|CL7996Contig1
8.0
7.8
7.6
7.4
1
2
t−Test: p−value = 0.04

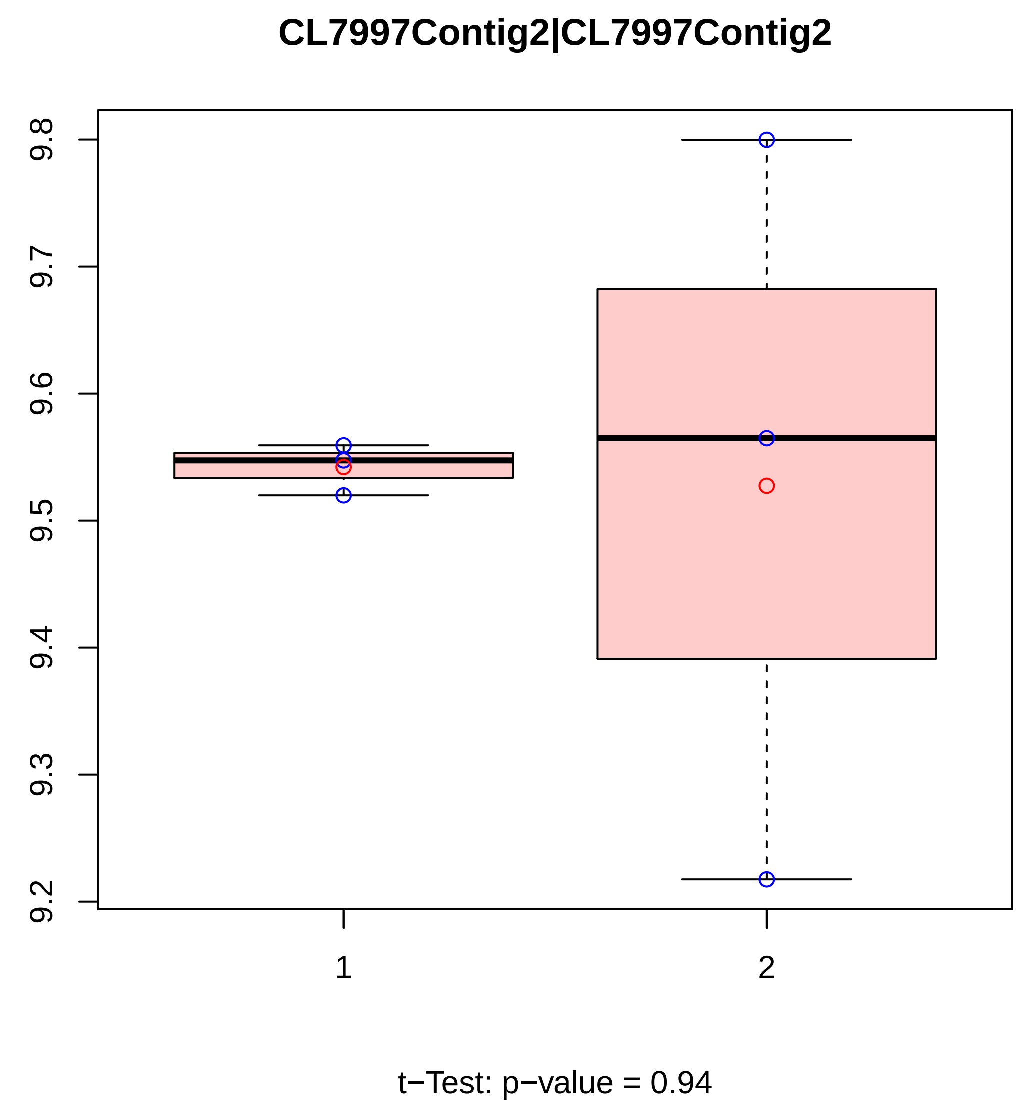
CL7997Contig2|CL7997Contig2
9.2
9.3
9.4
9.5
9.6
9.7
9.8
1
2
t−Test: p−value = 0.94

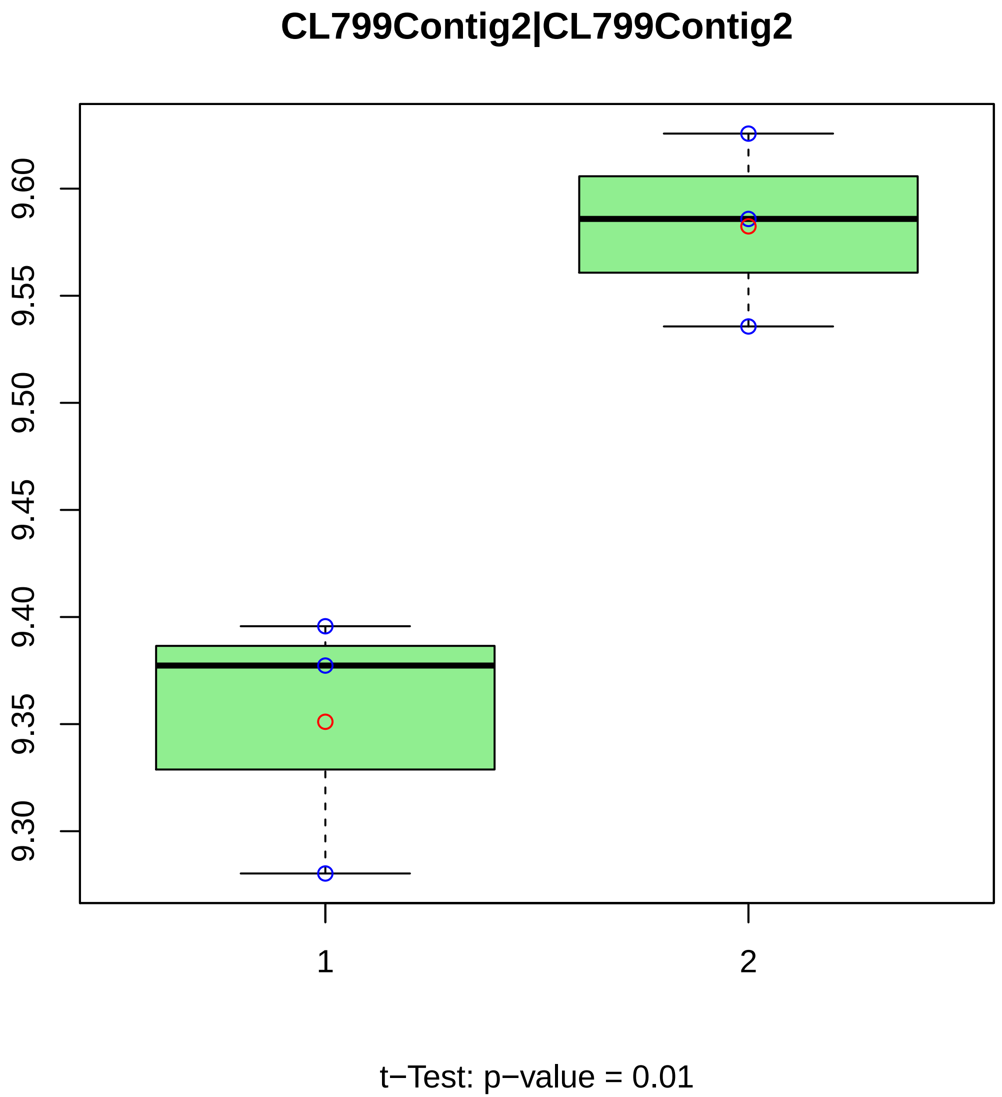
CL799Contig2|CL799Contig2
9.30
9.35
9.40
9.45
9.50
9.55
9.60
1
2
t−Test: p−value = 0.01

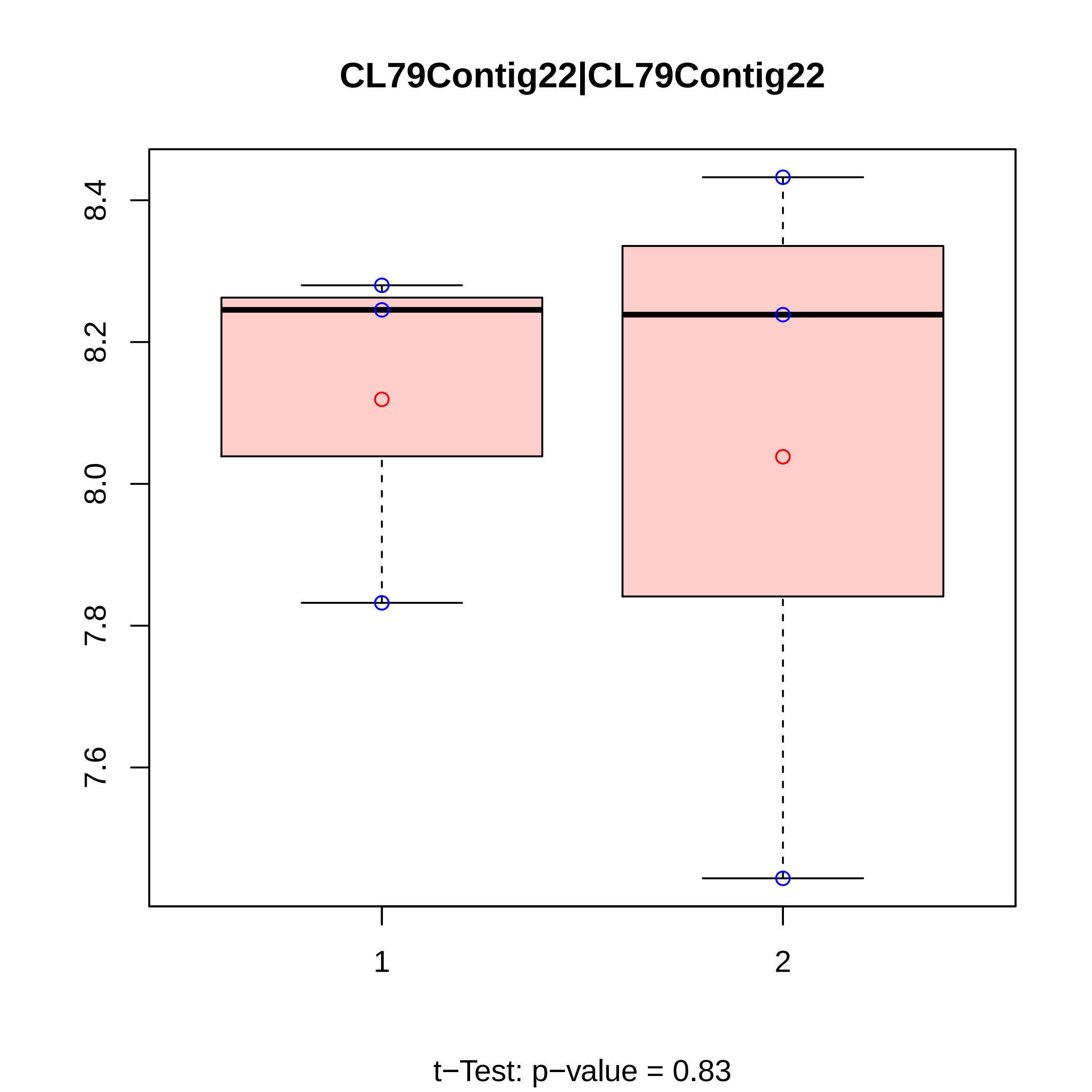

CL79Contig22|CL79Contig22
8.4
8.2
8.0
7.8
7.6
1
2
t−Test: p−value = 0.83

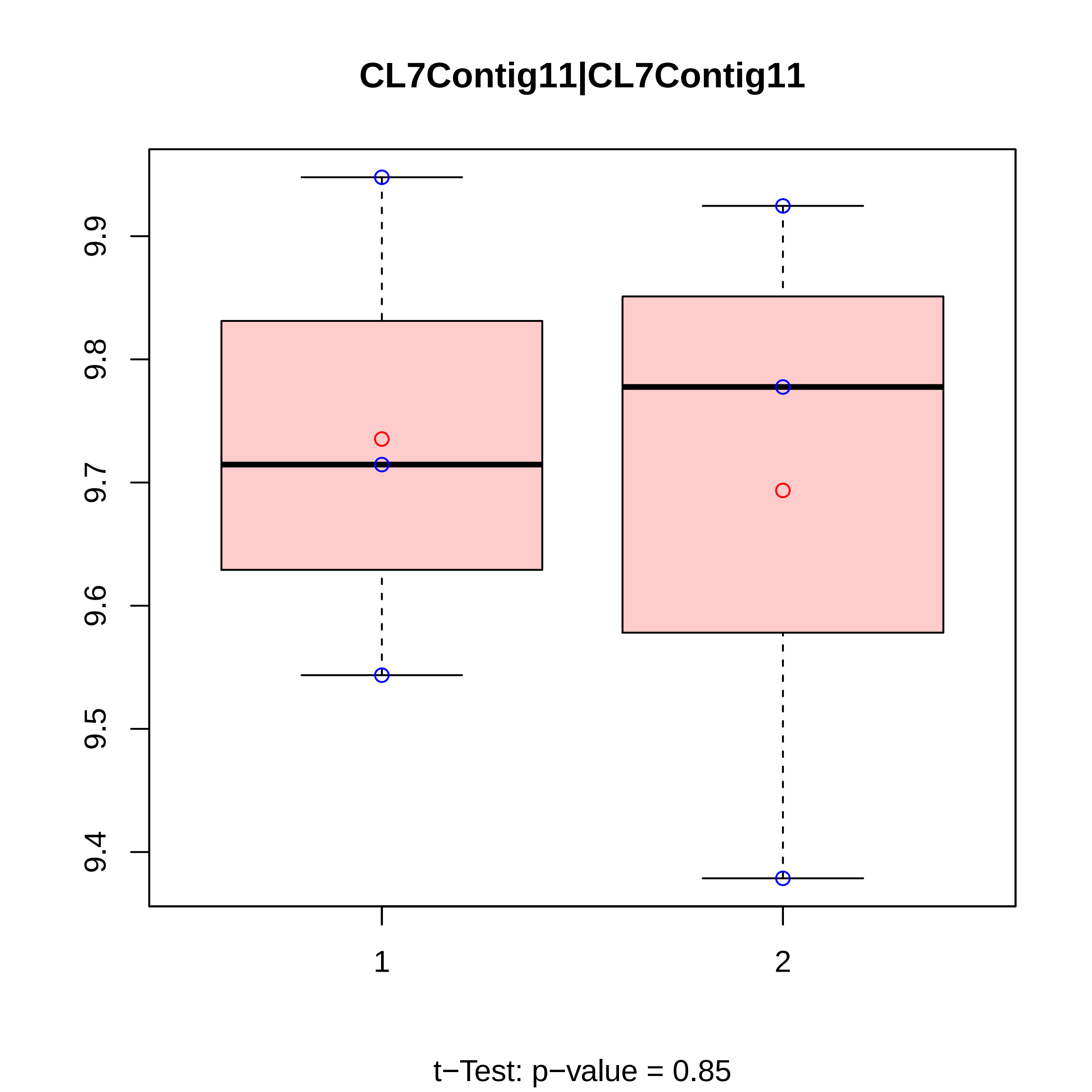

CL7Contig11|CL7Contig11
9.4
9.5
9.6
9.7
9.8
9.9
1
2
t−Test: p−value = 0.85

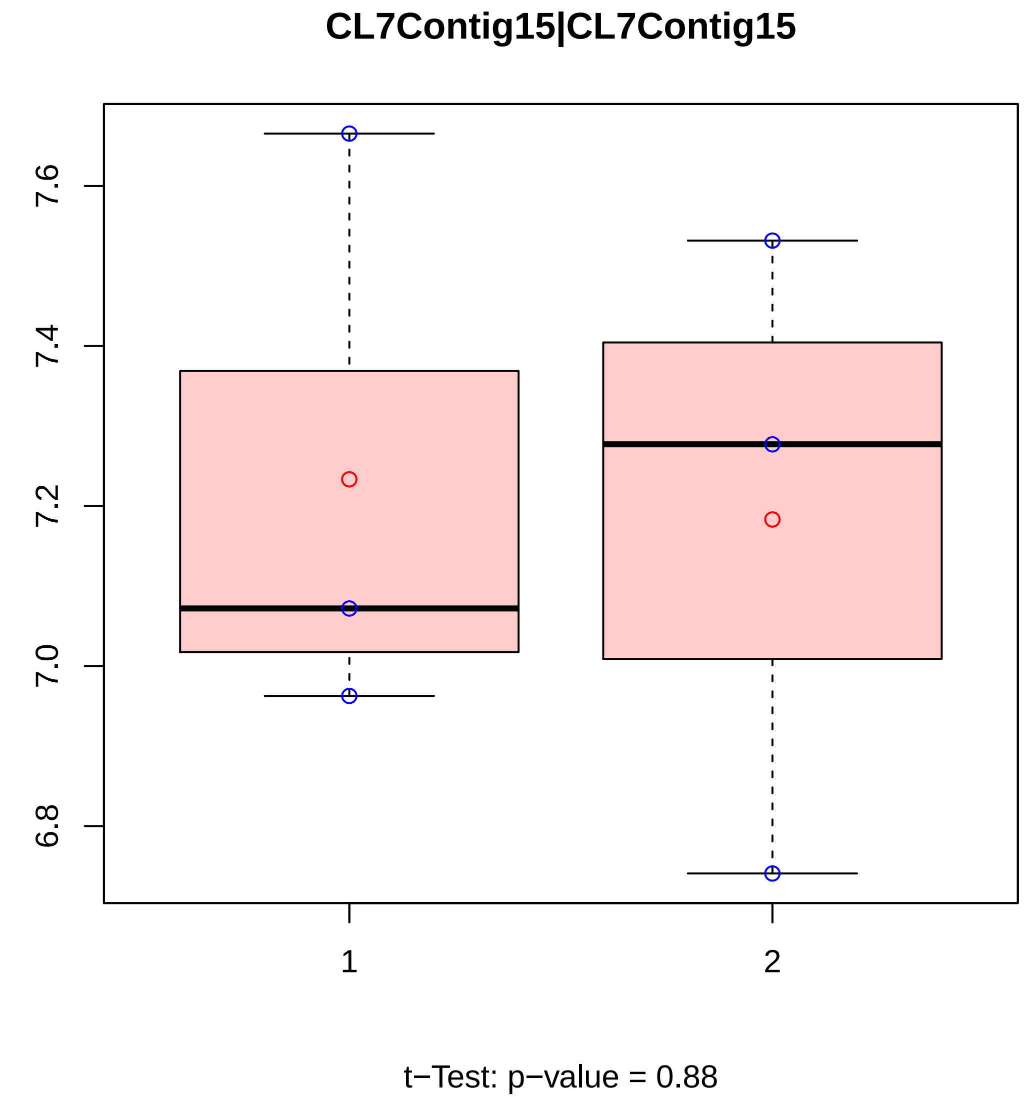

CL7Contig15|CL7Contig15
7.6
7.4
7.2
7.0
6.8
1
2
t−Test: p−value = 0.88

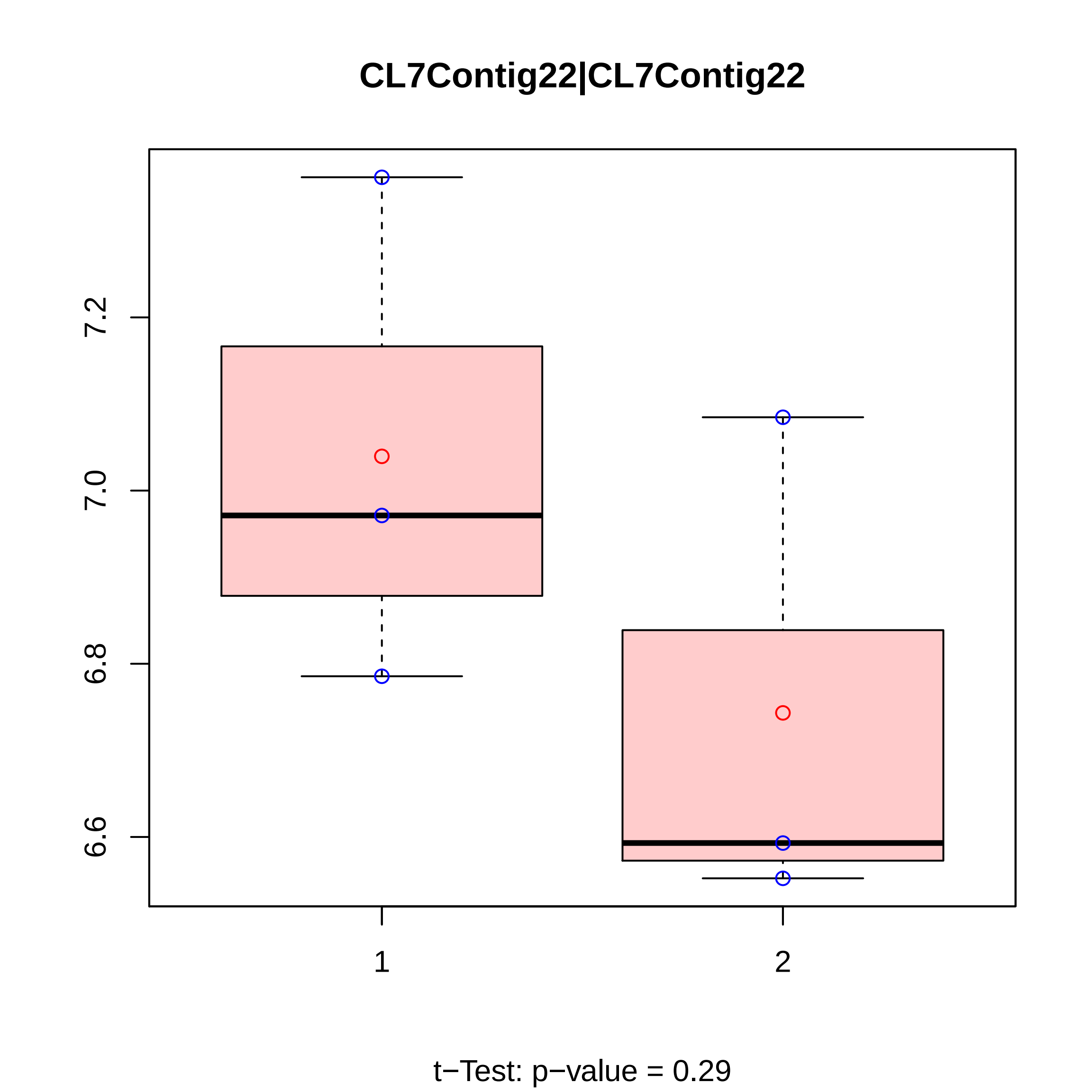

CL7Contig22|CL7Contig22
7.2
7.0
6.8
6.6
1
2
t−Test: p−value = 0.29

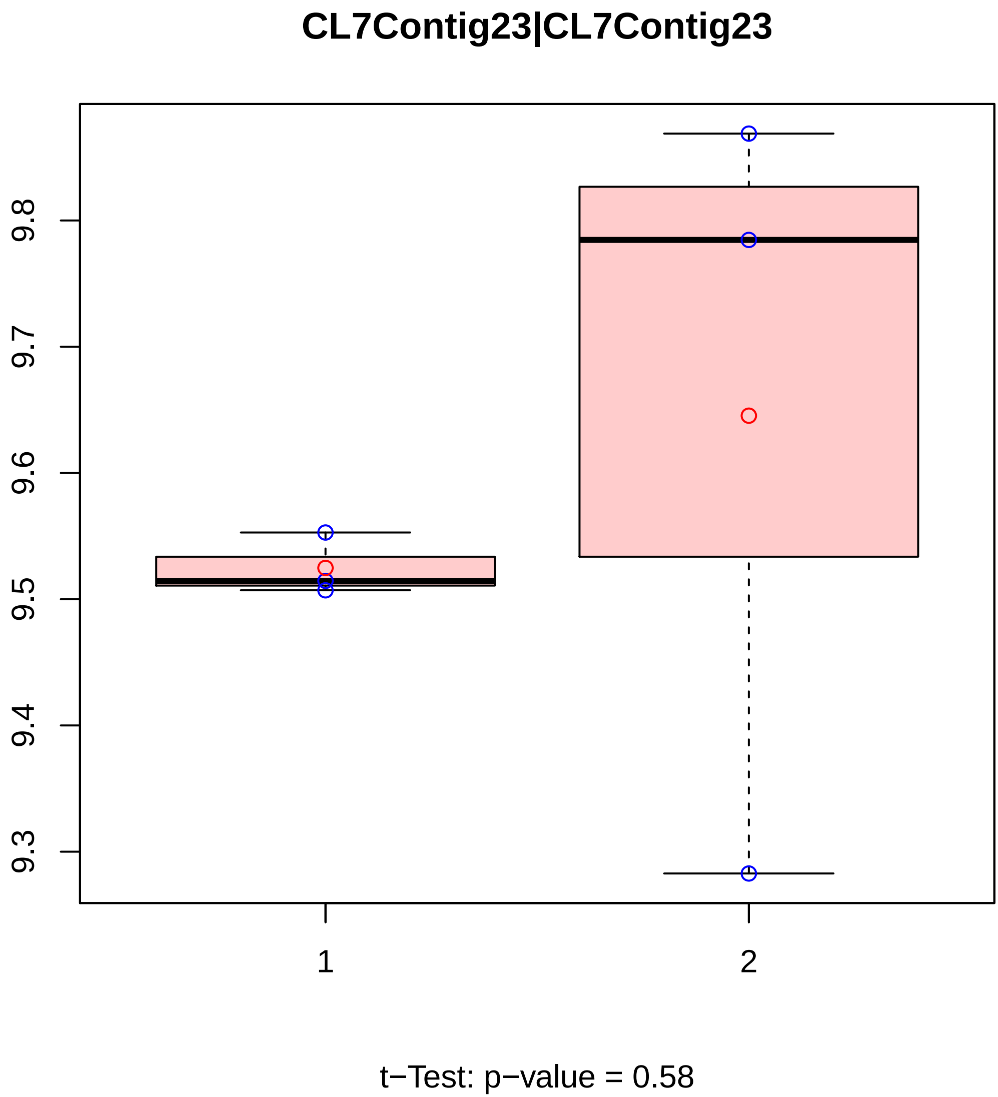
CL7Contig23|CL7Contig23
9.3
9.4
9.5
9.6
9.7
9.8
1
2
t−Test: p−value = 0.58

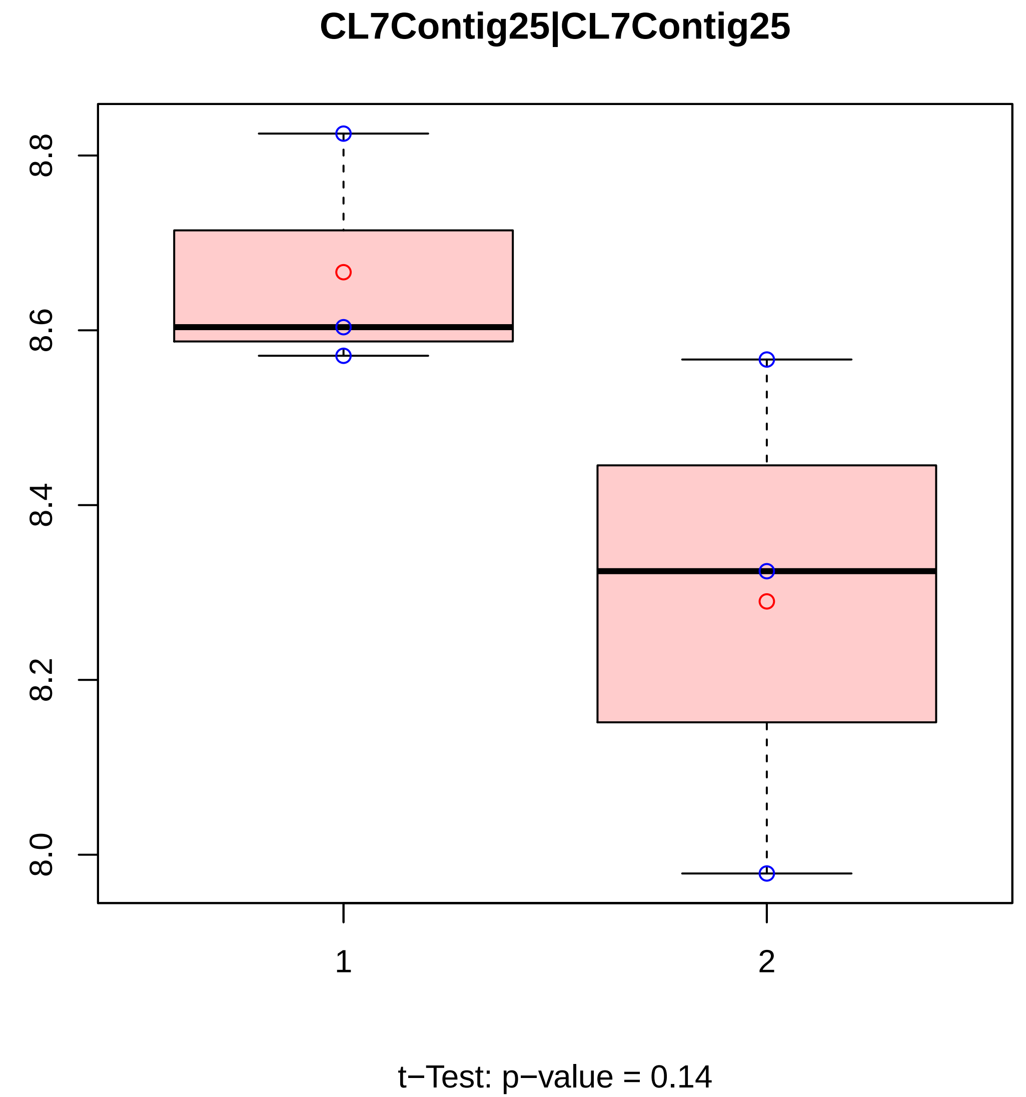
CL7Contig25|CL7Contig25
8.0
8.2
8.4
8.6
8.8
1
2
t−Test: p−value = 0.14

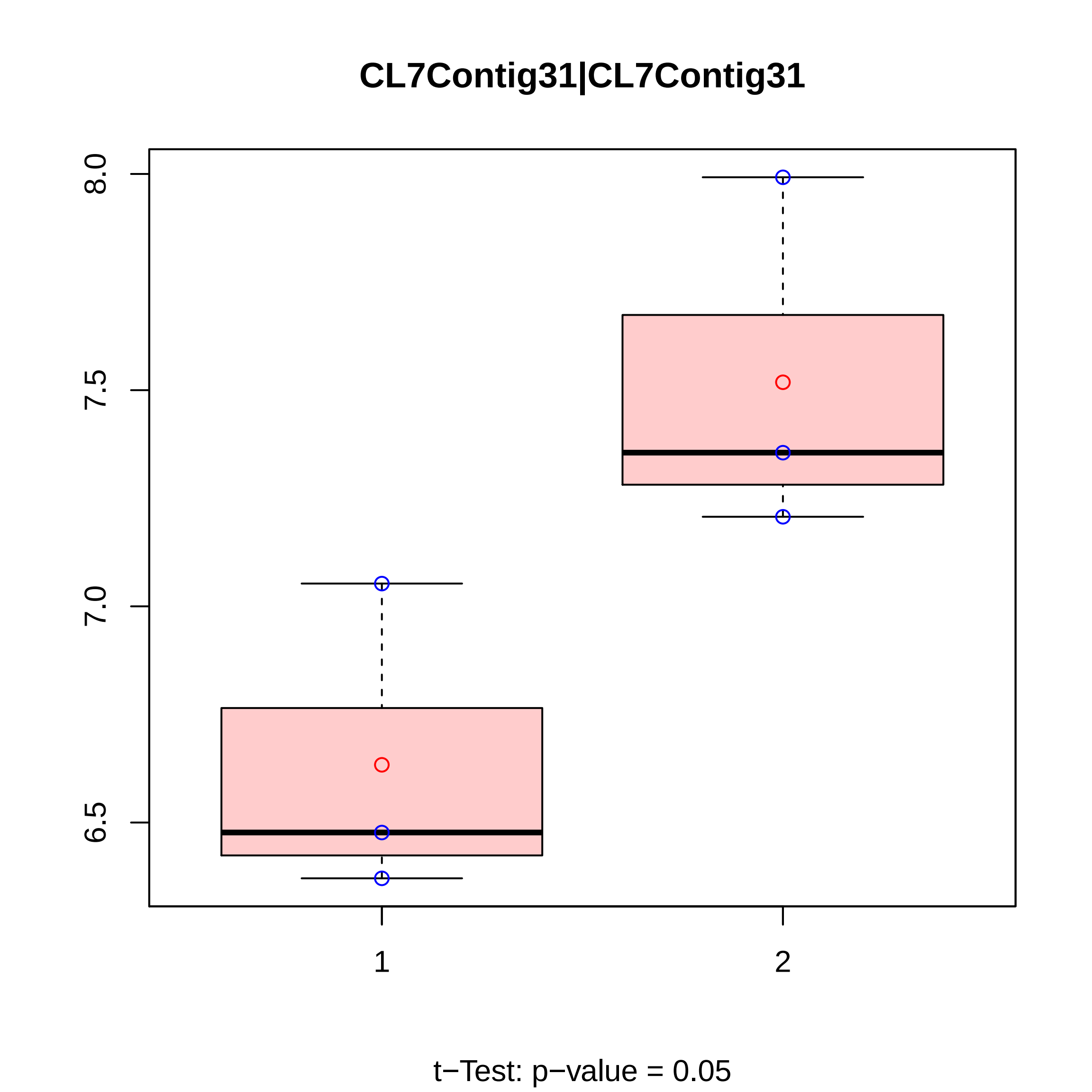
CL7Contig31|CL7Contig31
8.0
7.5
7.0
6.5
1
2
t−Test: p−value = 0.05

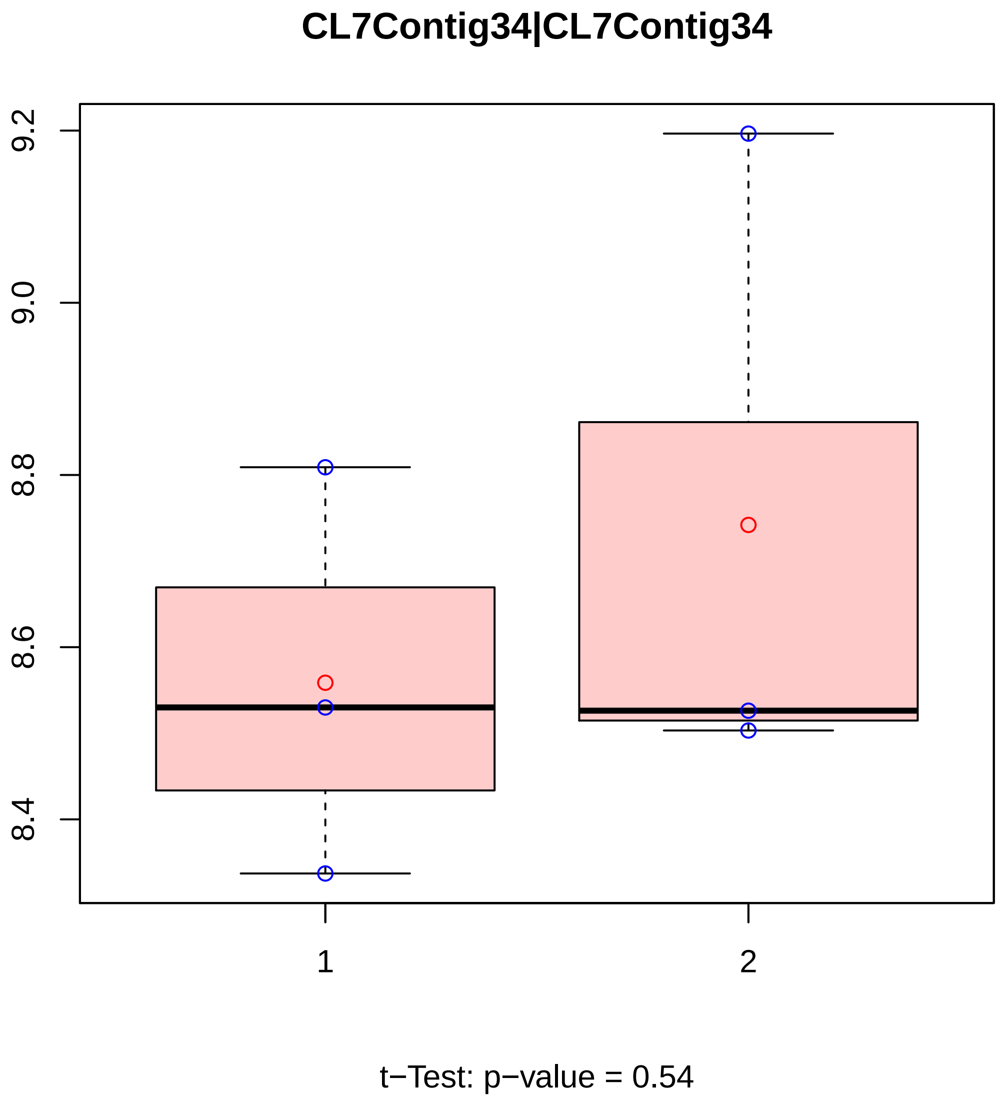

CL7Contig34|CL7Contig34
9.2
9.0
8.8
8.6
8.4
1
2
t−Test: p−value = 0.54

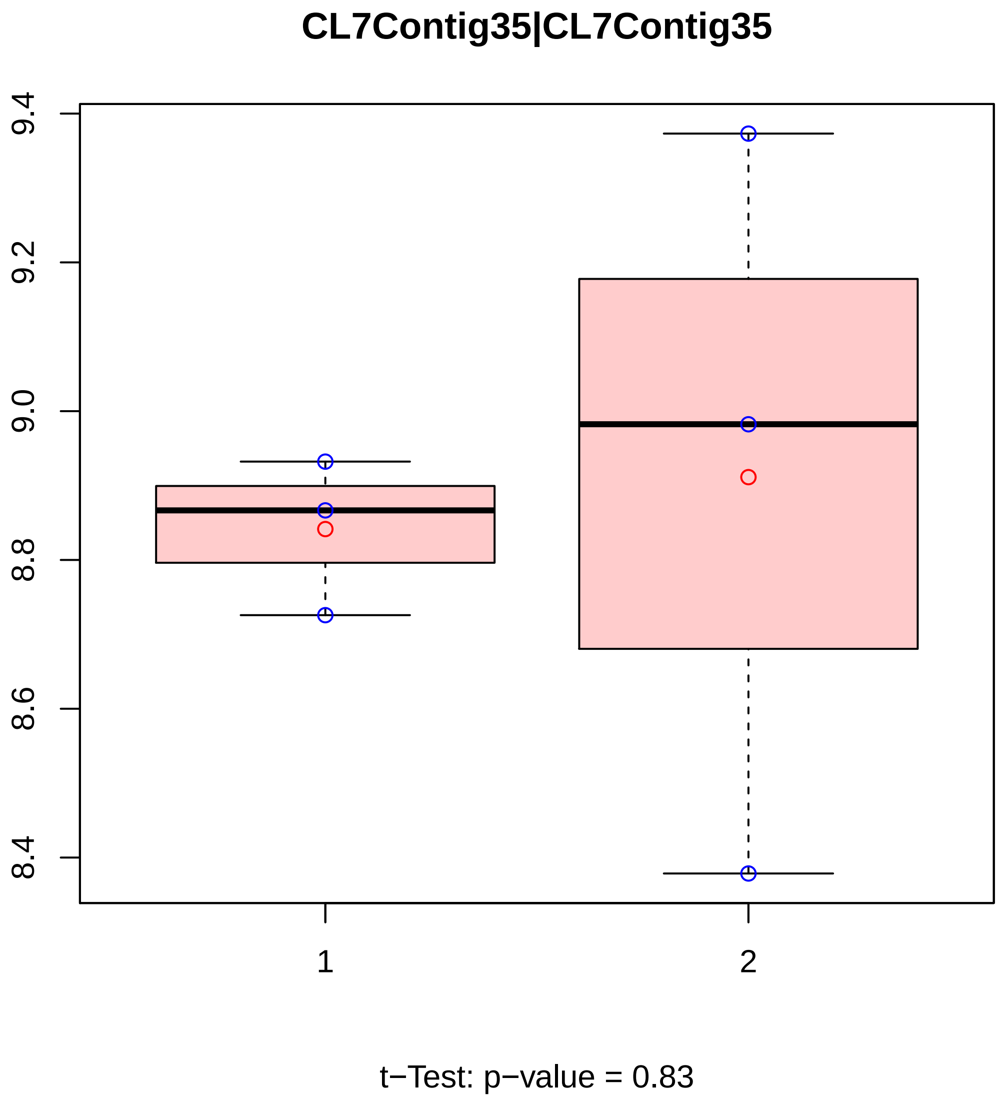
CL7Contig35|CL7Contig35
9.4
9.2
9.0
8.8
8.6
8.4
1
2
t−Test: p−value = 0.83

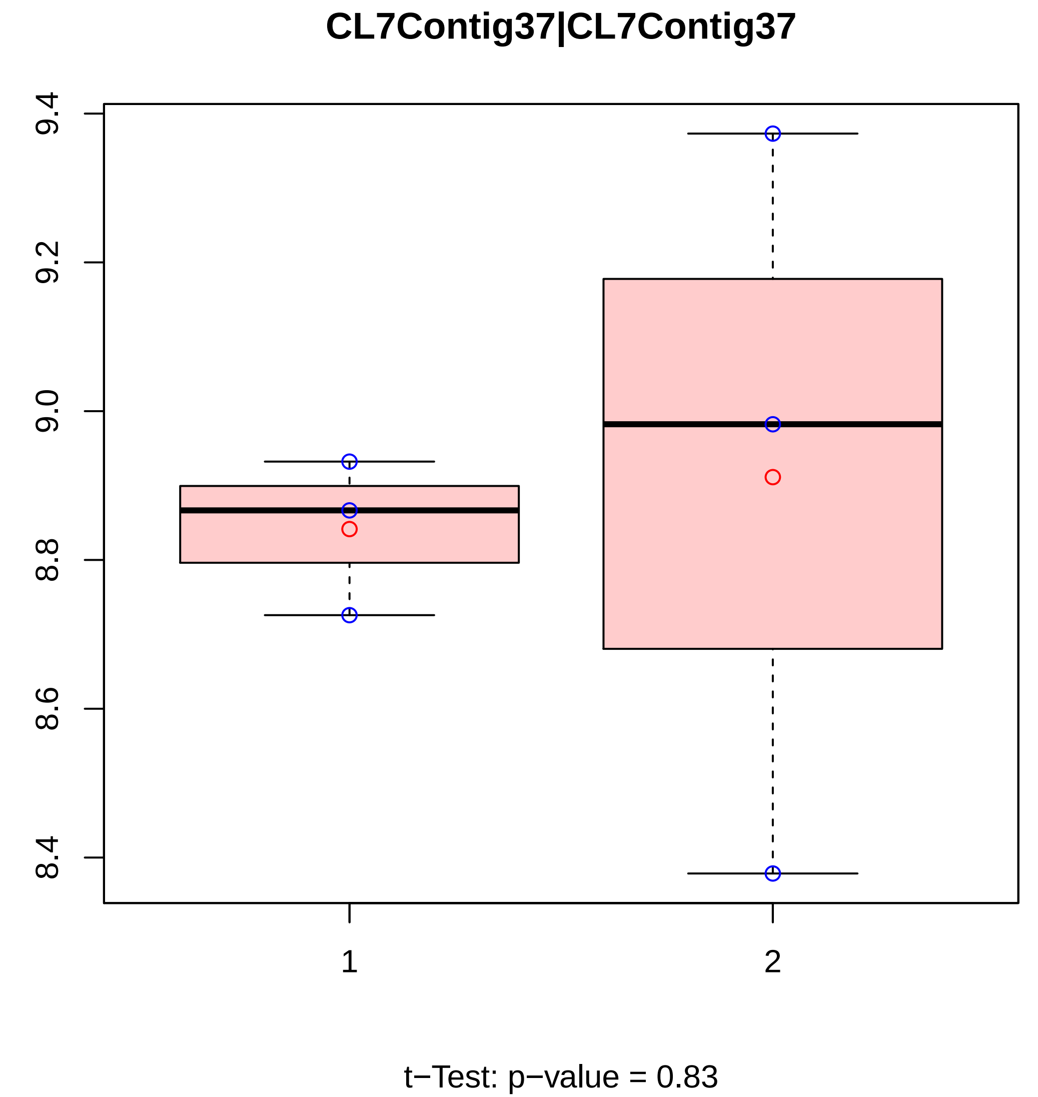
CL7Contig37|CL7Contig37
9.4
9.2
9.0
8.8
8.6
8.4
1
2
t−Test: p−value = 0.83

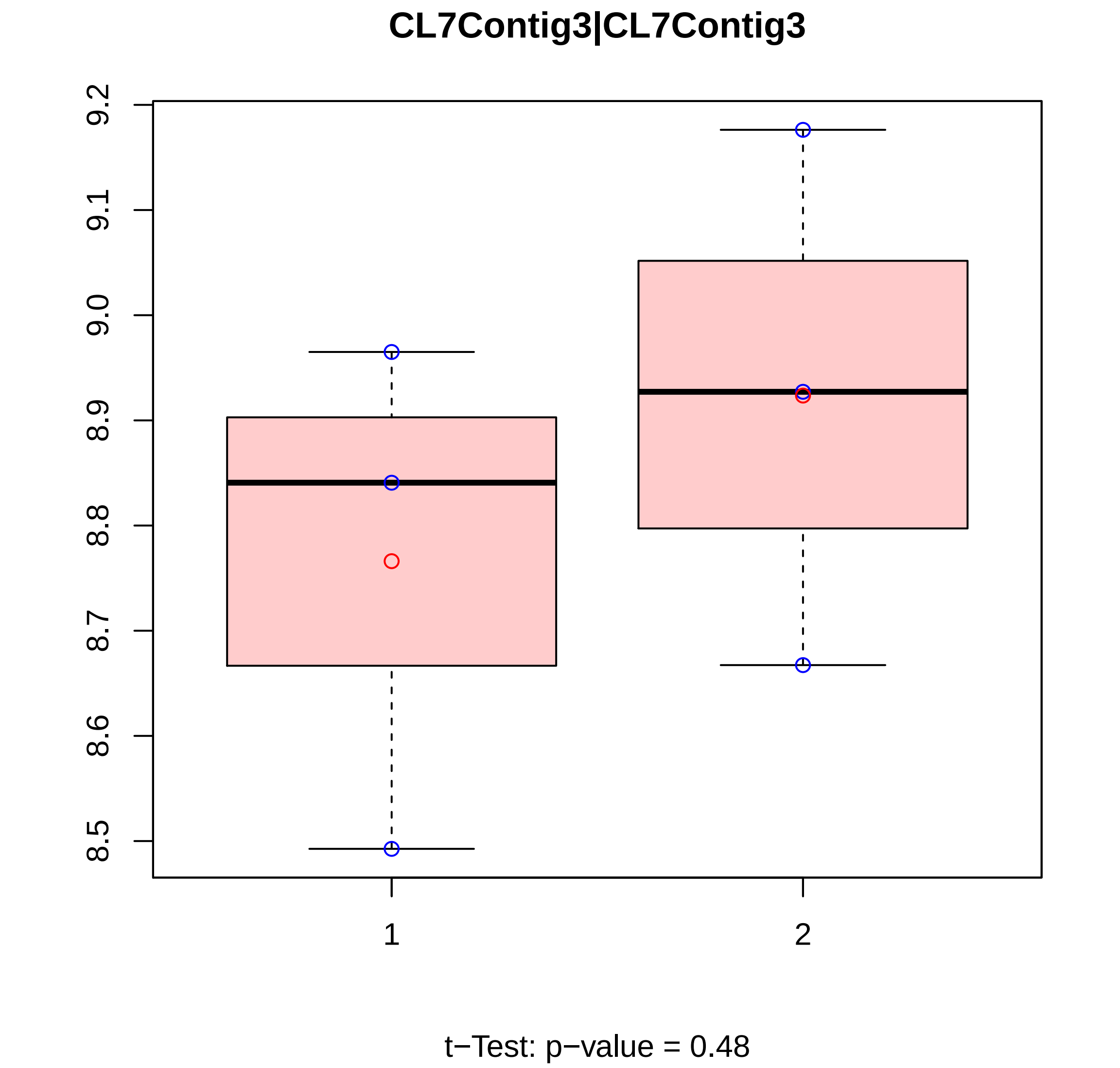

CL7Contig3|CL7Contig3
9.2
9.1
9.0
8.9
8.8
8.7
8.6
8.5
1
2
t−Test: p−value = 0.48

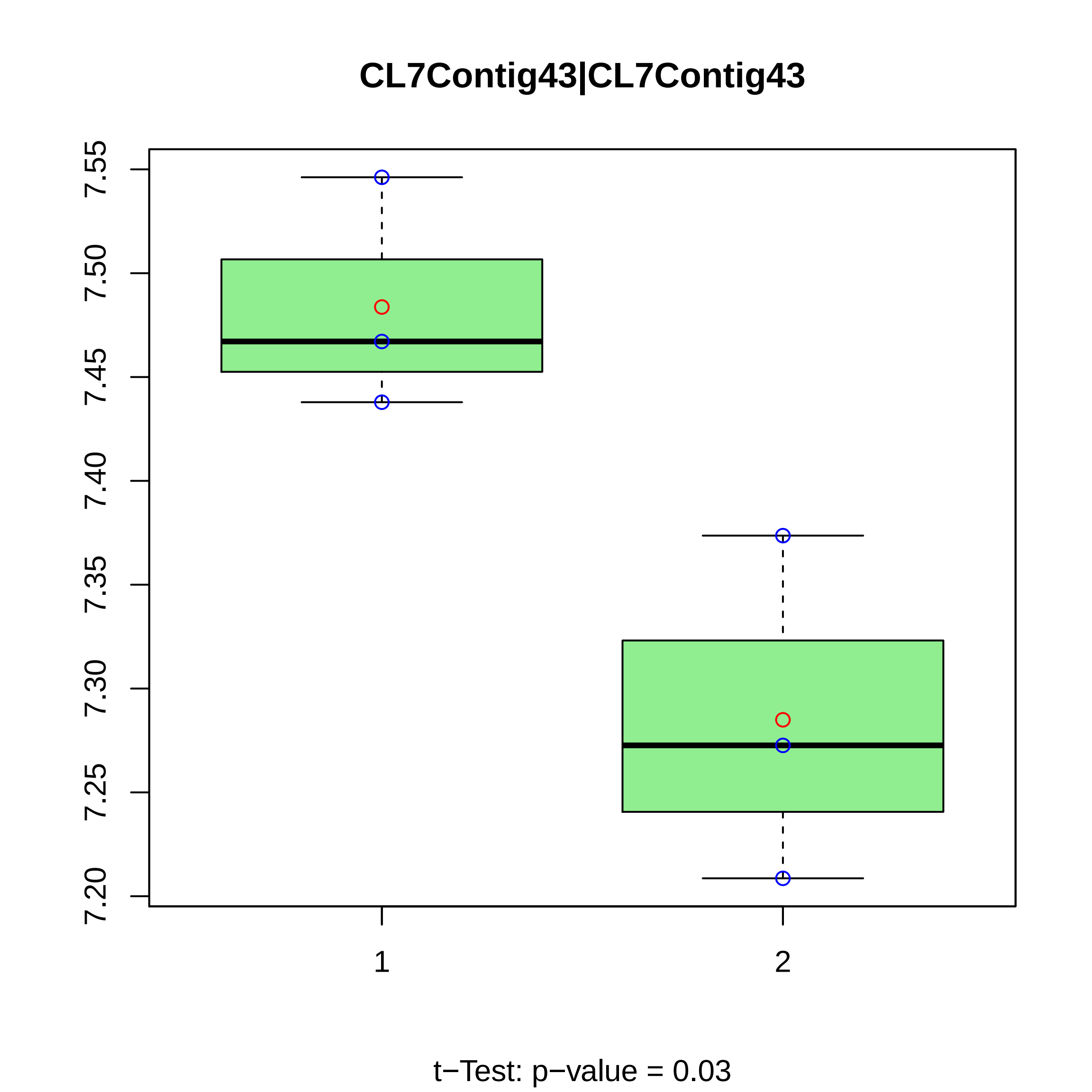
CL7Contig43|CL7Contig43
7.20
7.25
7.30
7.35
7.40
7.45
7.50
7.55
1
2
t−Test: p−value = 0.03

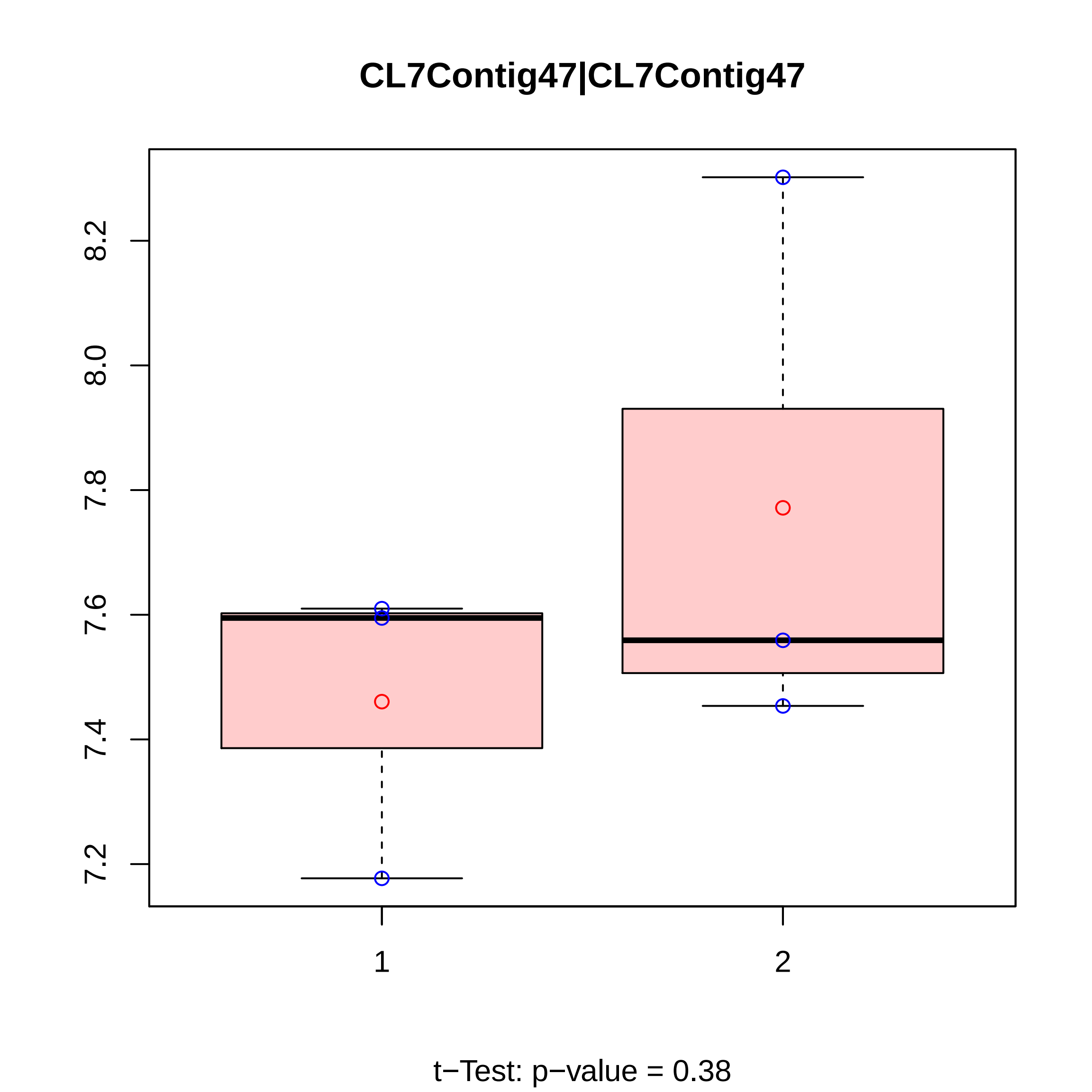
CL7Contig47|CL7Contig47
8.2
8.0
7.8
7.6
7.4
7.2
1
2
t−Test: p−value = 0.38

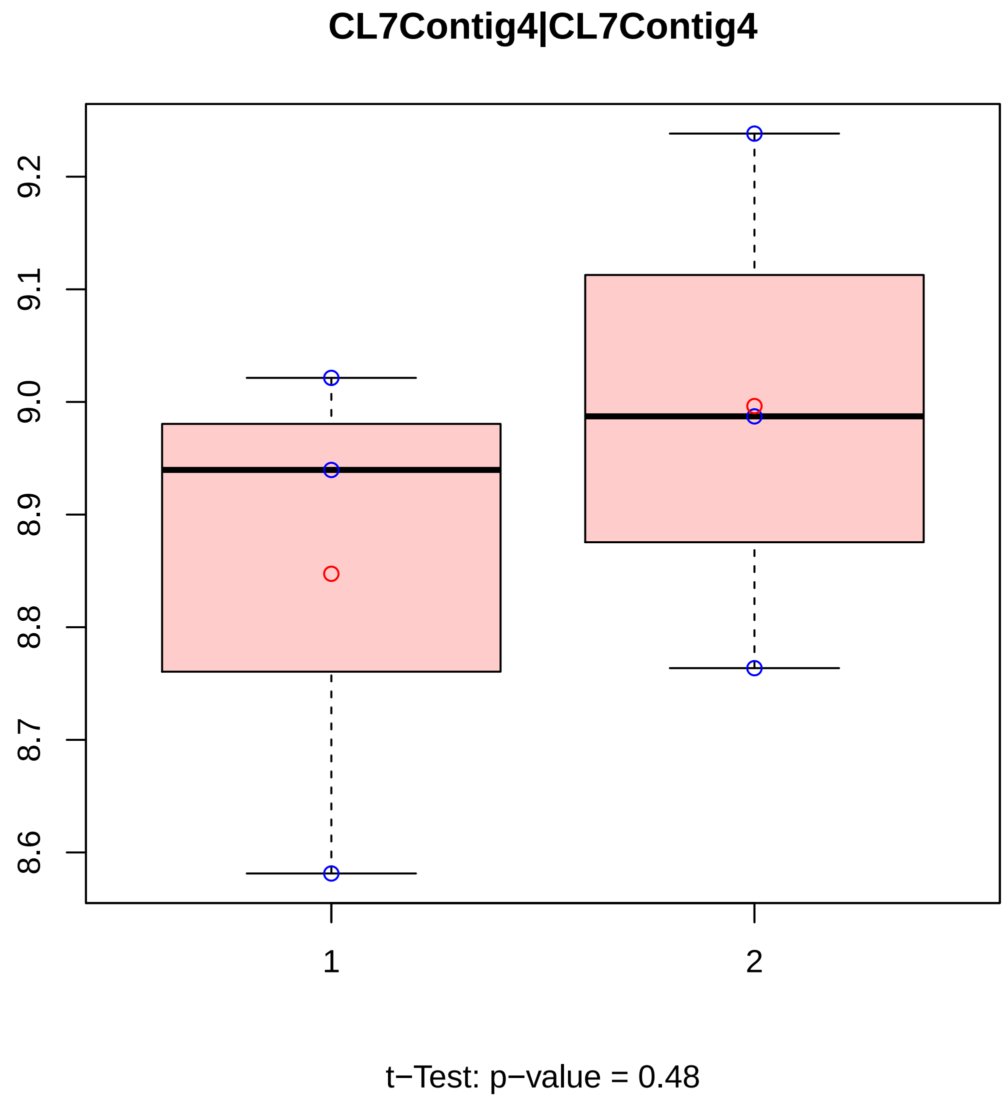

CL7Contig4|CL7Contig4
9.2
9.1
9.0
8.9
8.8
8.7
8.6
1
2
t−Test: p−value = 0.48

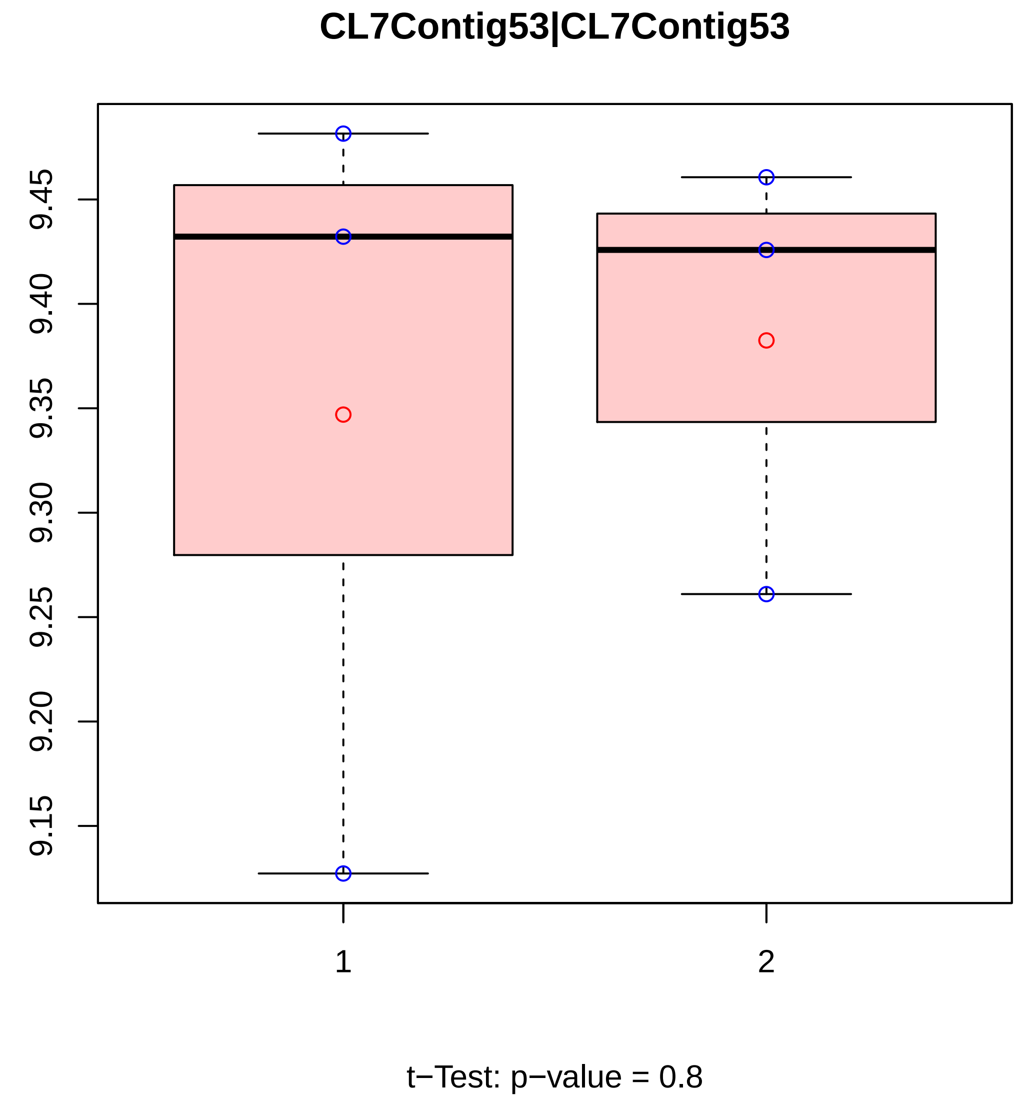
CL7Contig53|CL7Contig53
9.15
9.20
9.25
9.30
9.35
9.40
9.45
1
2
t−Test: p−value = 0.8

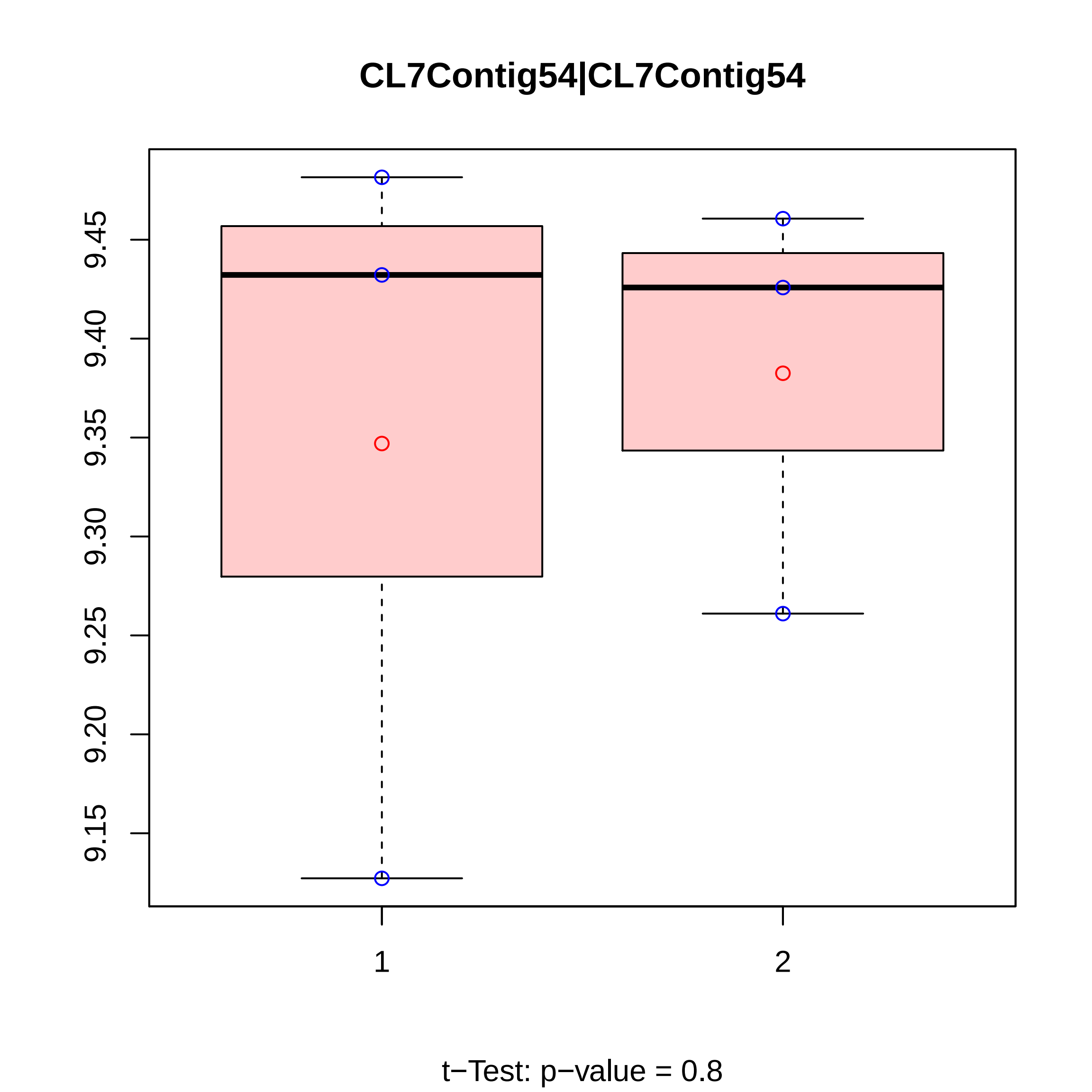
CL7Contig54|CL7Contig54
9.15
9.20
9.25
9.30
9.35
9.40
9.45
1
2
t−Test: p−value = 0.8

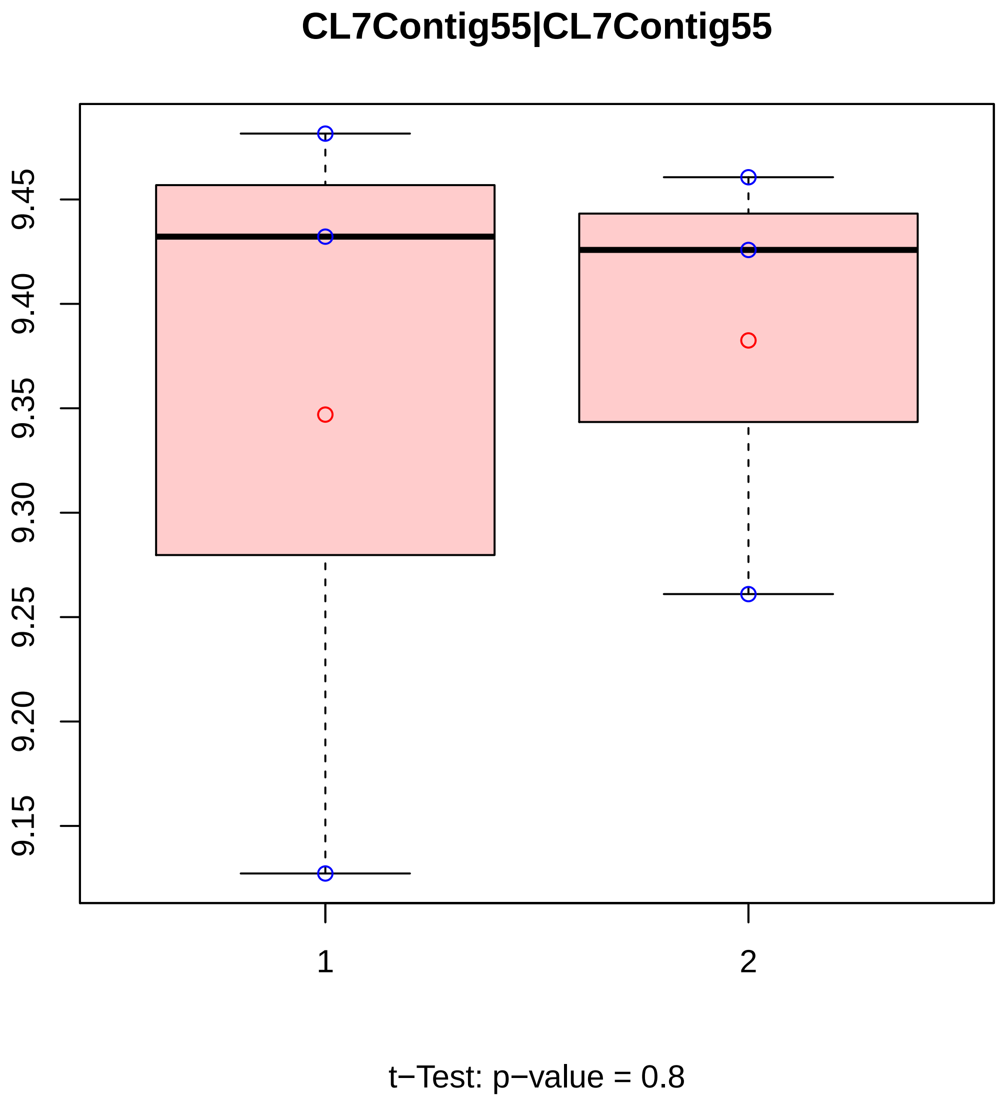

CL7Contig55|CL7Contig55
9.15
9.20
9.25
9.30
9.35
9.40
9.45
1
2
t−Test: p−value = 0.8

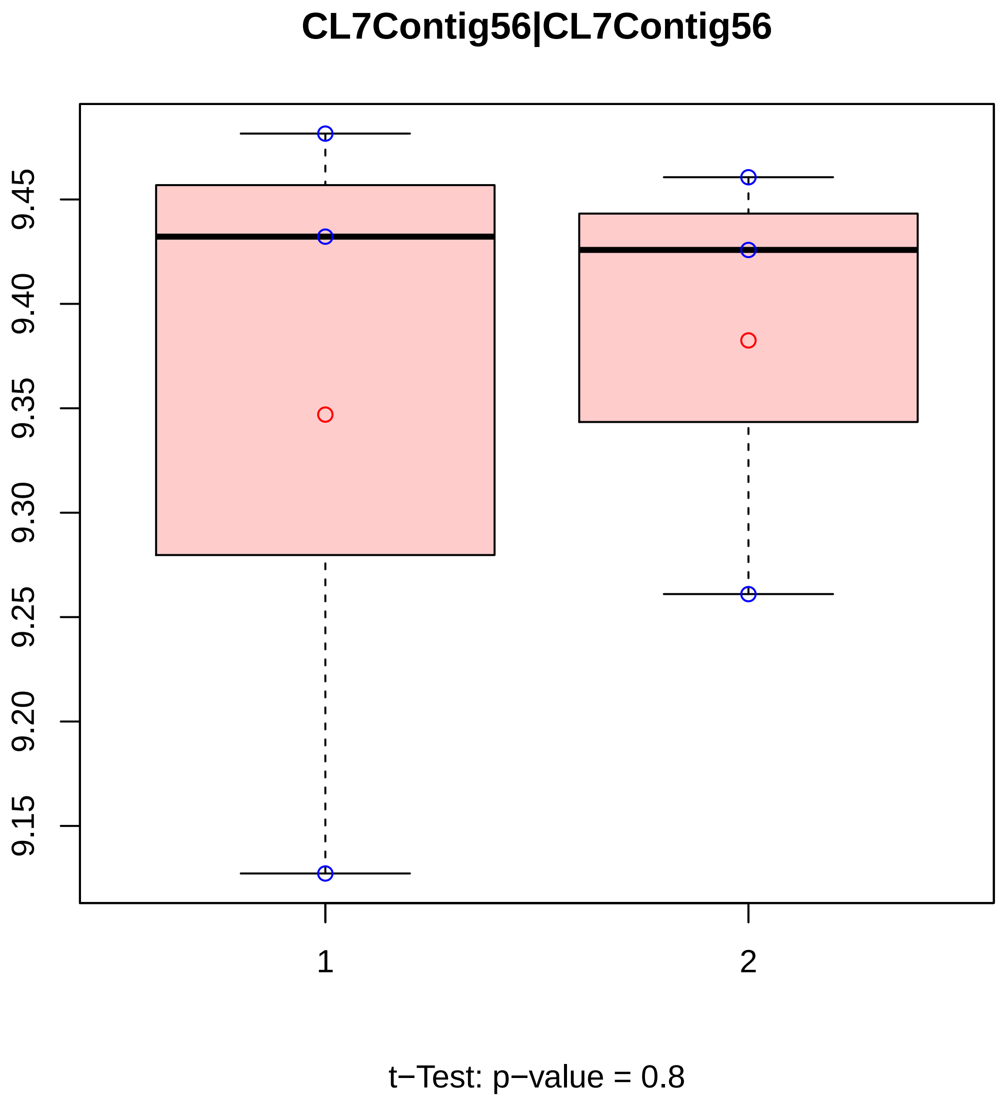
CL7Contig56|CL7Contig56
9.15
9.20
9.25
9.30
9.35
9.40
9.45
1
2
t−Test: p−value = 0.8

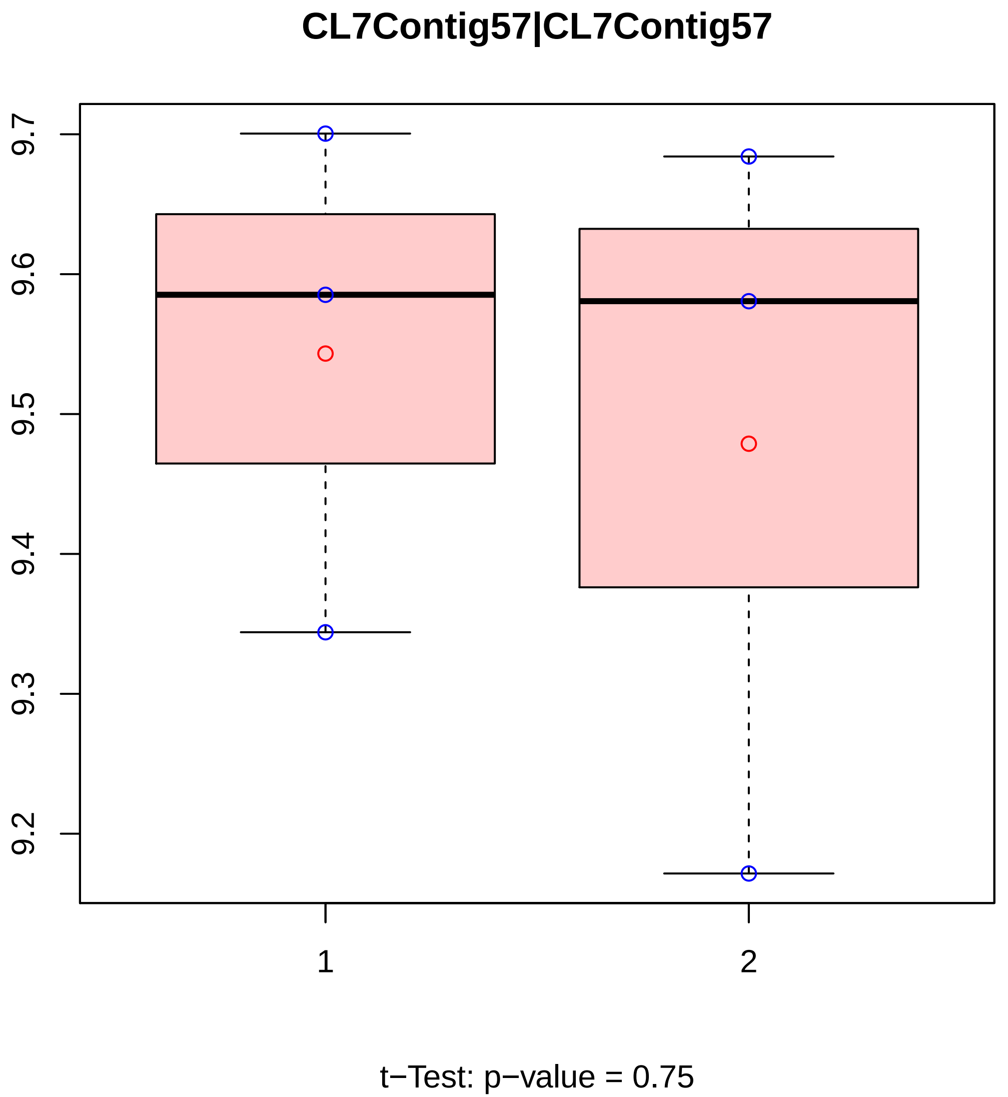
CL7Contig57|CL7Contig57
9.7
9.6
9.5
9.4
9.3
9.2
1
2
t−Test: p−value = 0.75

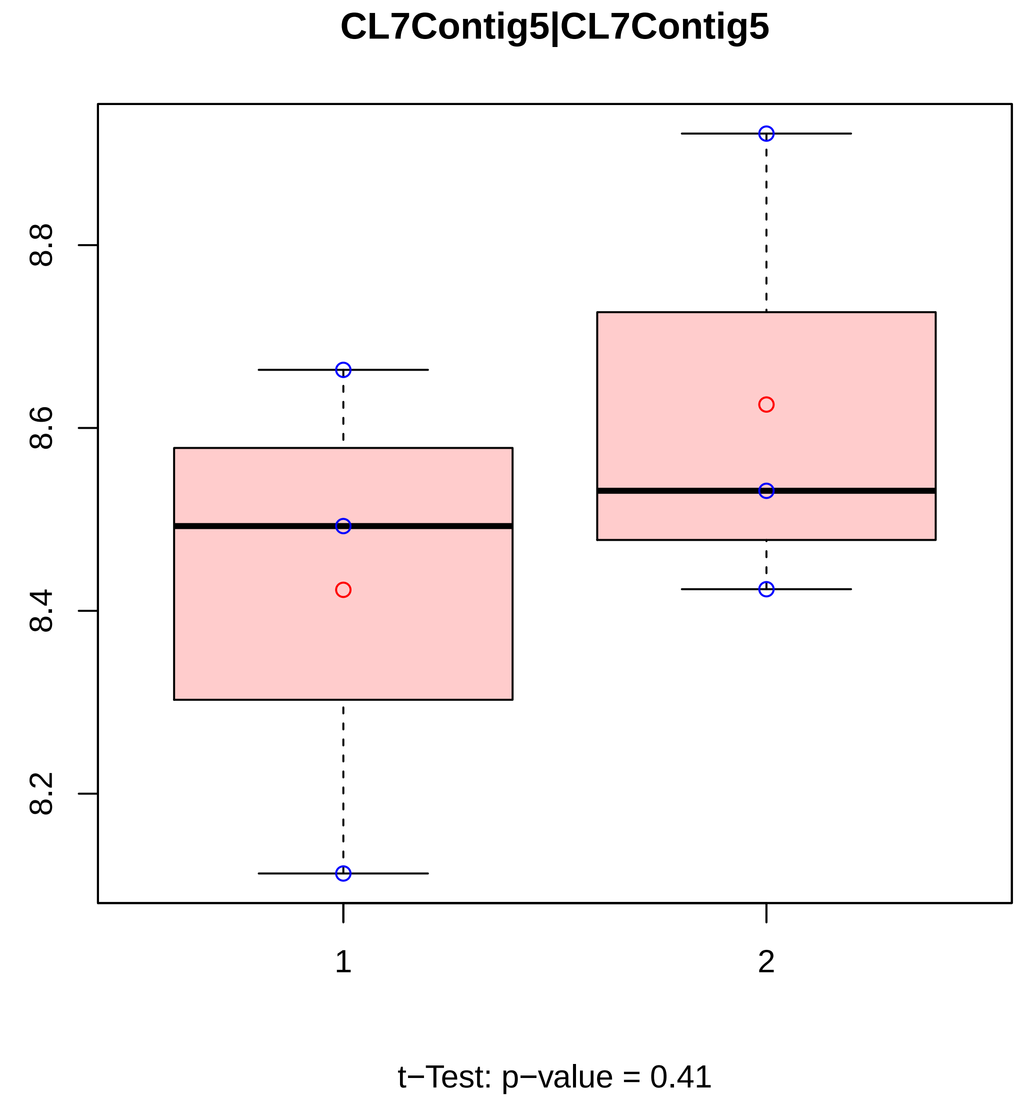

CL7Contig5|CL7Contig5
8.2
8.4
8.6
8.8
1
2
t−Test: p−value = 0.41

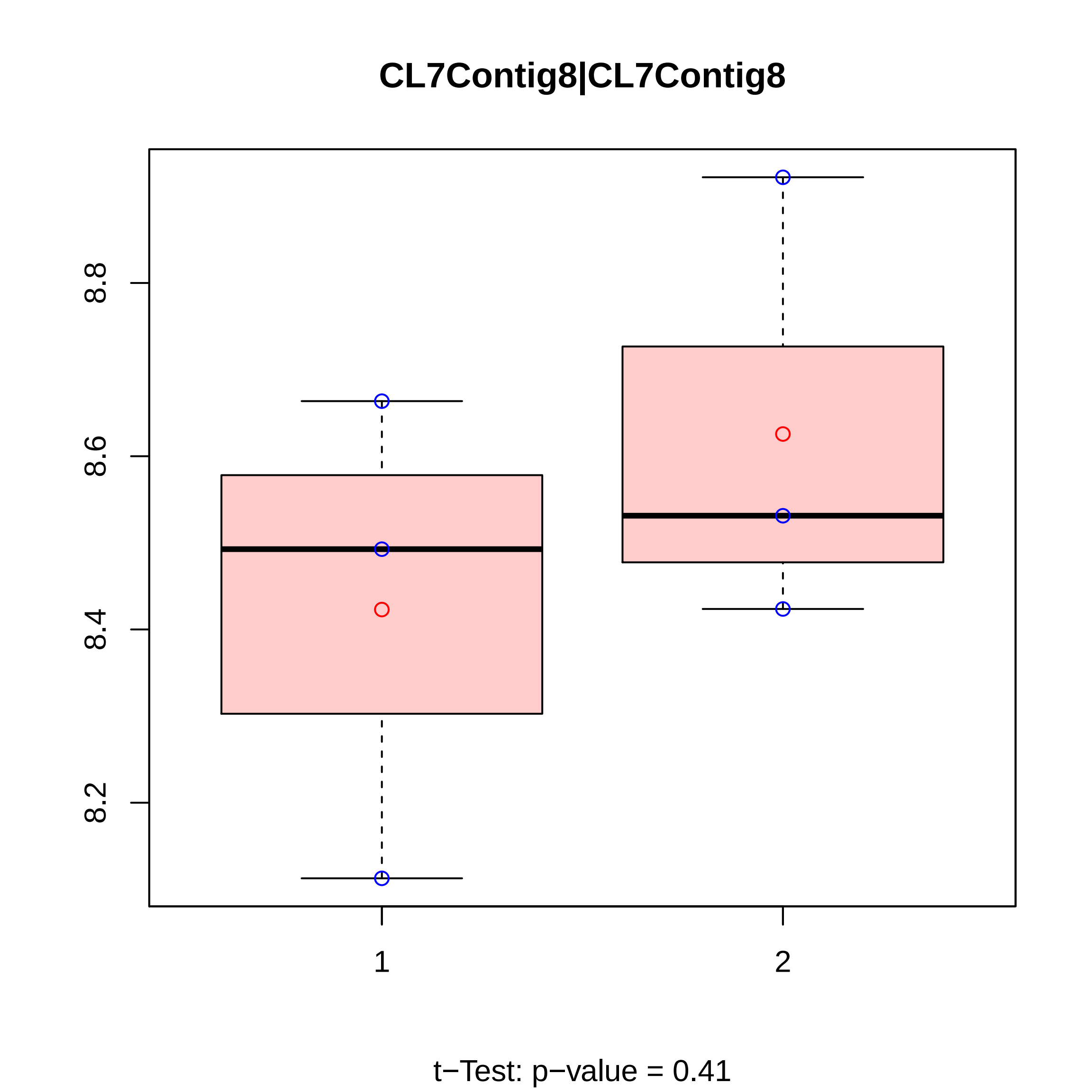
CL7Contig8|CL7Contig8
8.2
8.4
8.6
8.8
1
2
t−Test: p−value = 0.41

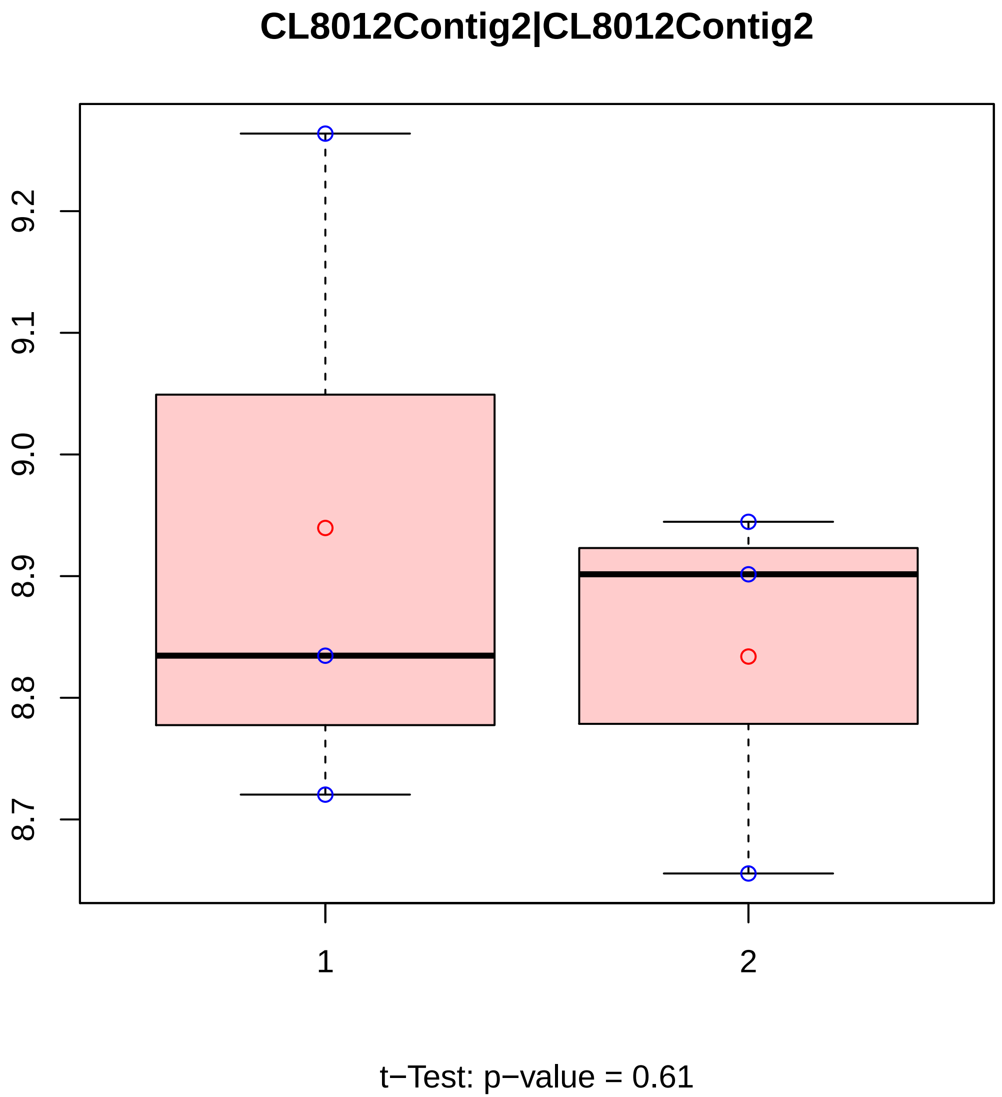
CL8012Contig2|CL8012Contig2
9.2
9.1
9.0
8.9
8.8
8.7
1
2
t−Test: p−value = 0.61

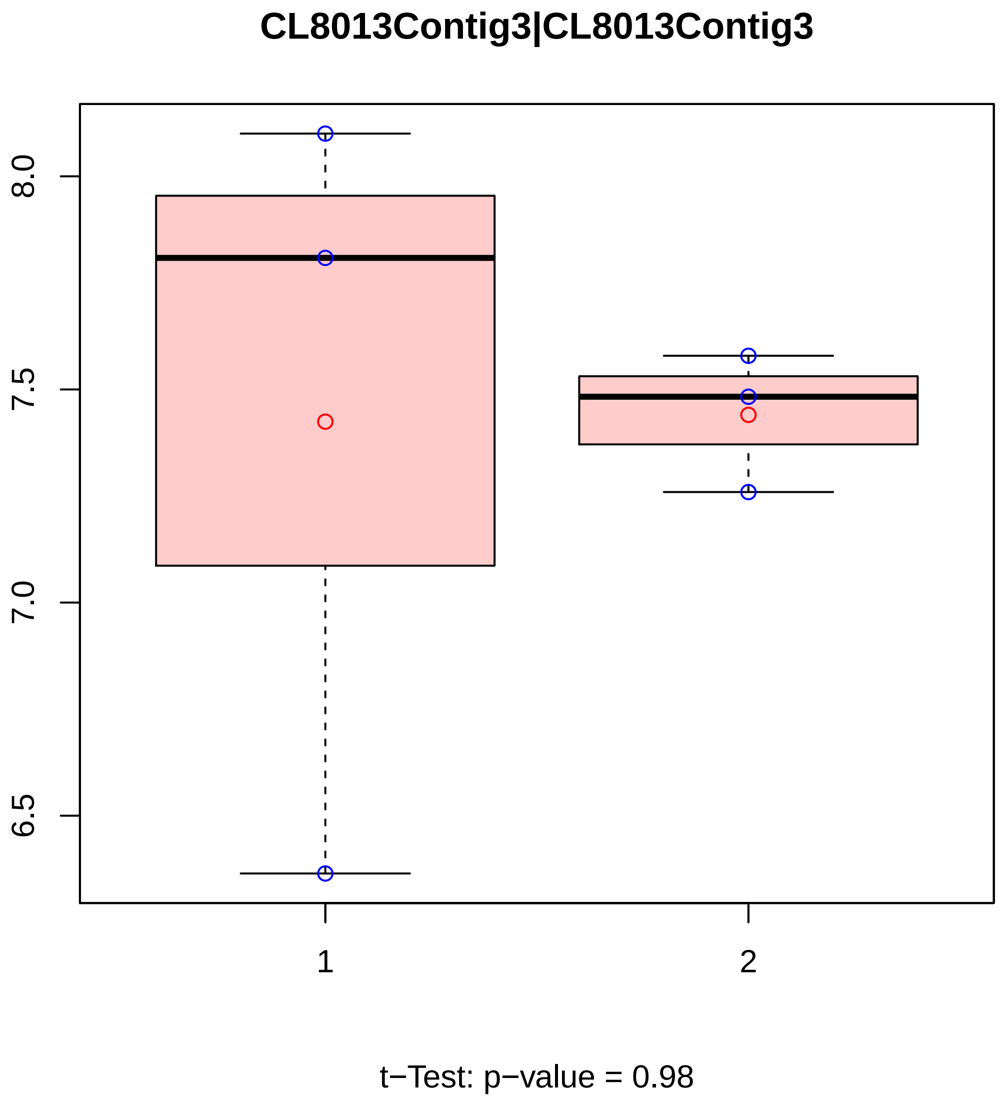
CL8013Contig3|CL8013Contig3
8.0
7.5
7.0
6.5
1
2
t−Test: p−value = 0.98

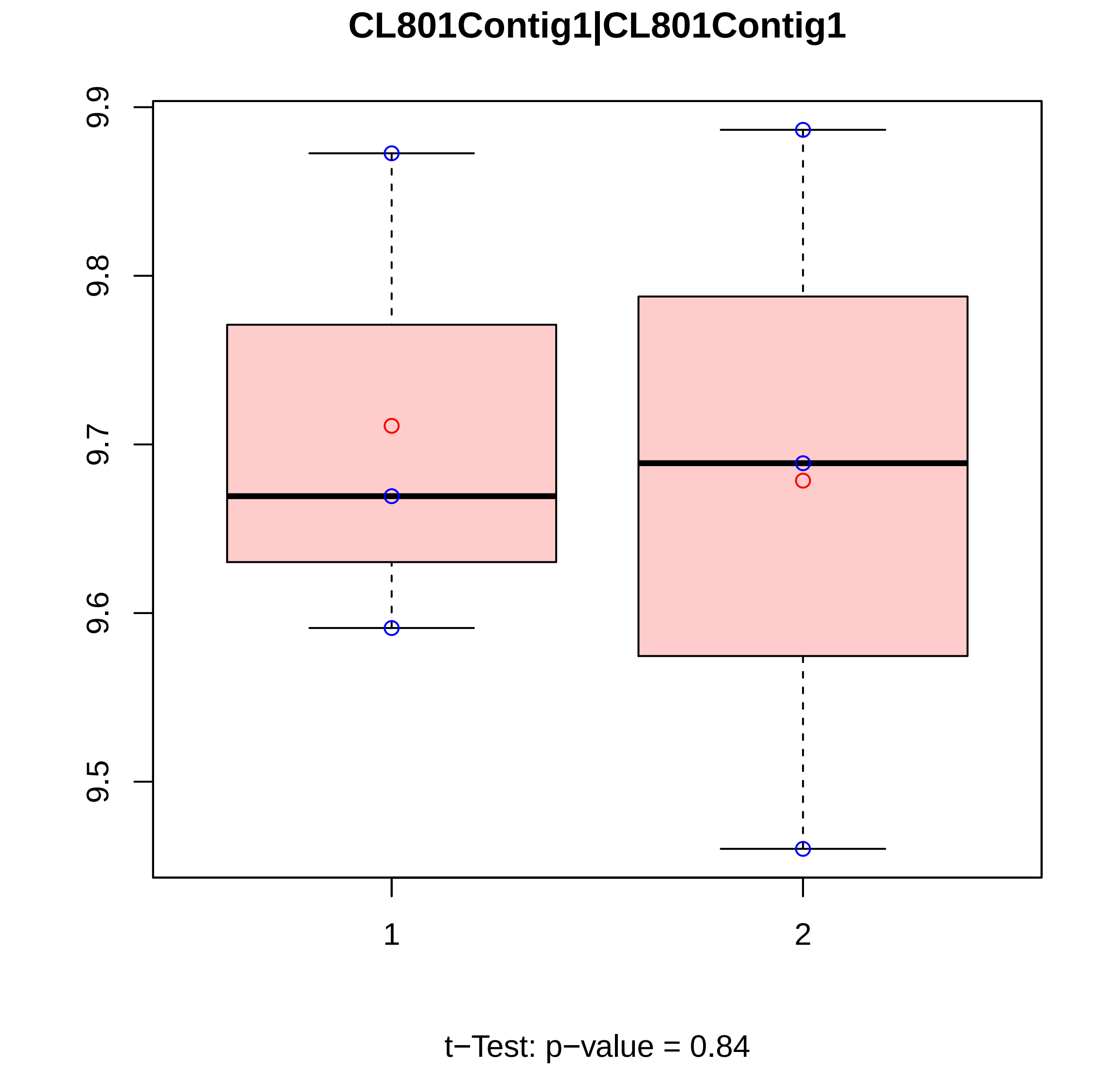
CL801Contig1|CL801Contig1
9.9
9.8
9.7
9.6
9.5
1
2
t−Test: p−value = 0.84

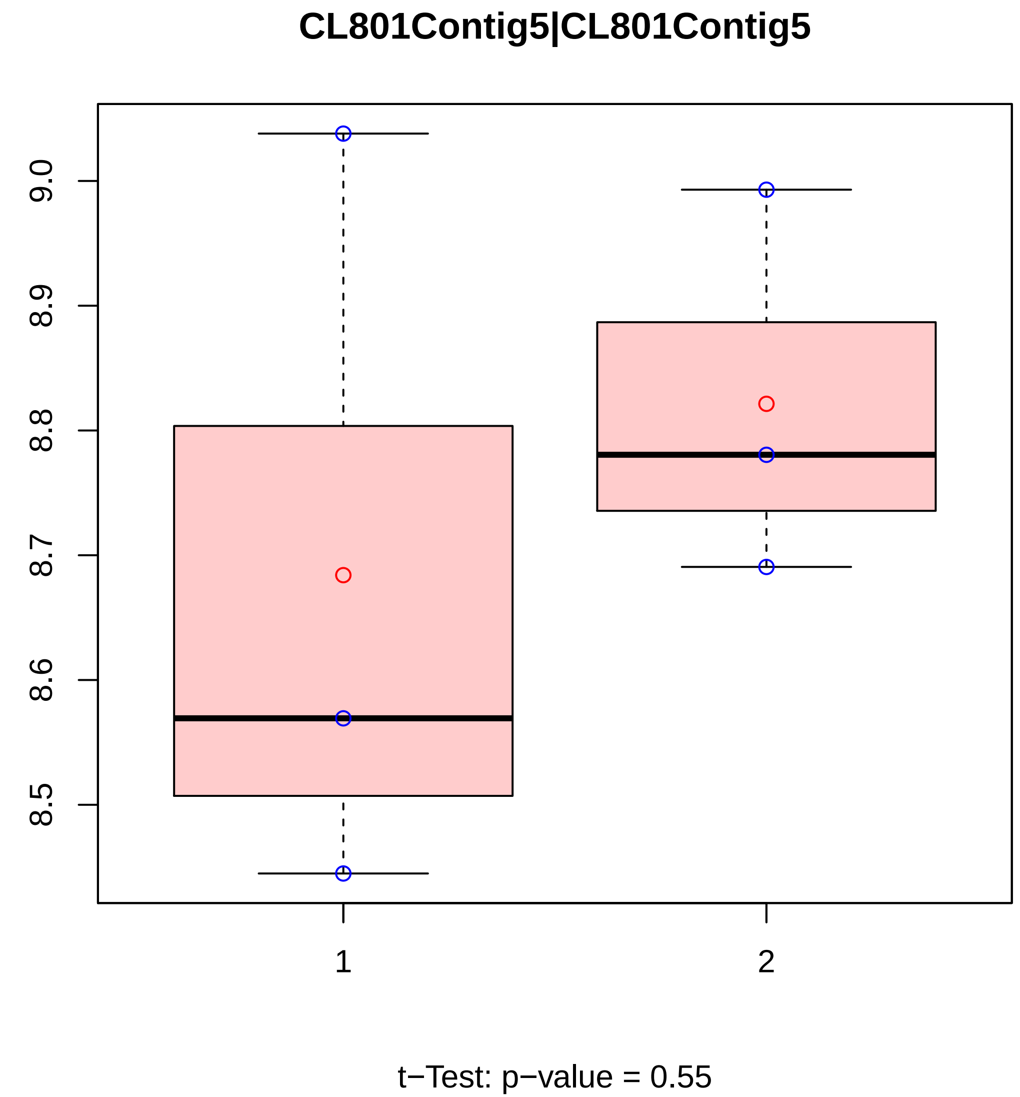
CL801Contig5|CL801Contig5
9.0
8.9
8.8
8.7
8.6
8.5
1
2
t−Test: p−value = 0.55

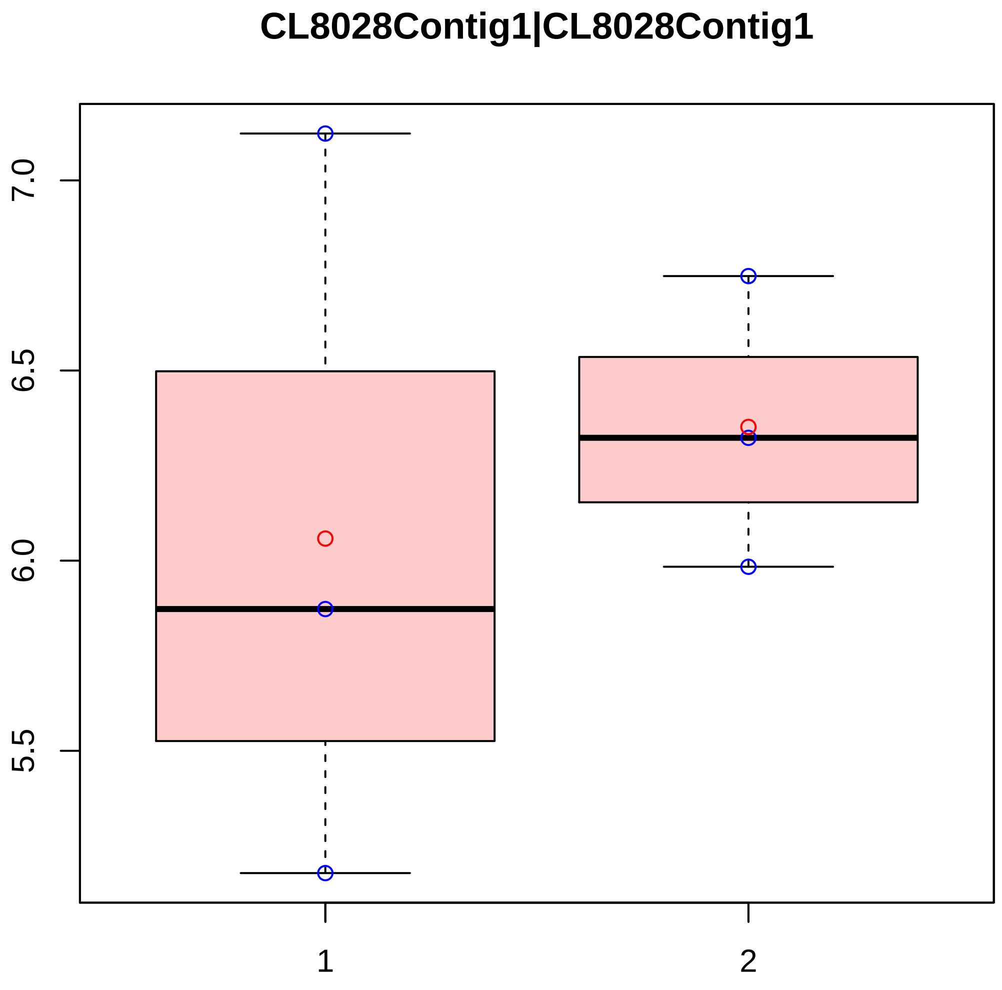


t−Test: p−value = 0.67

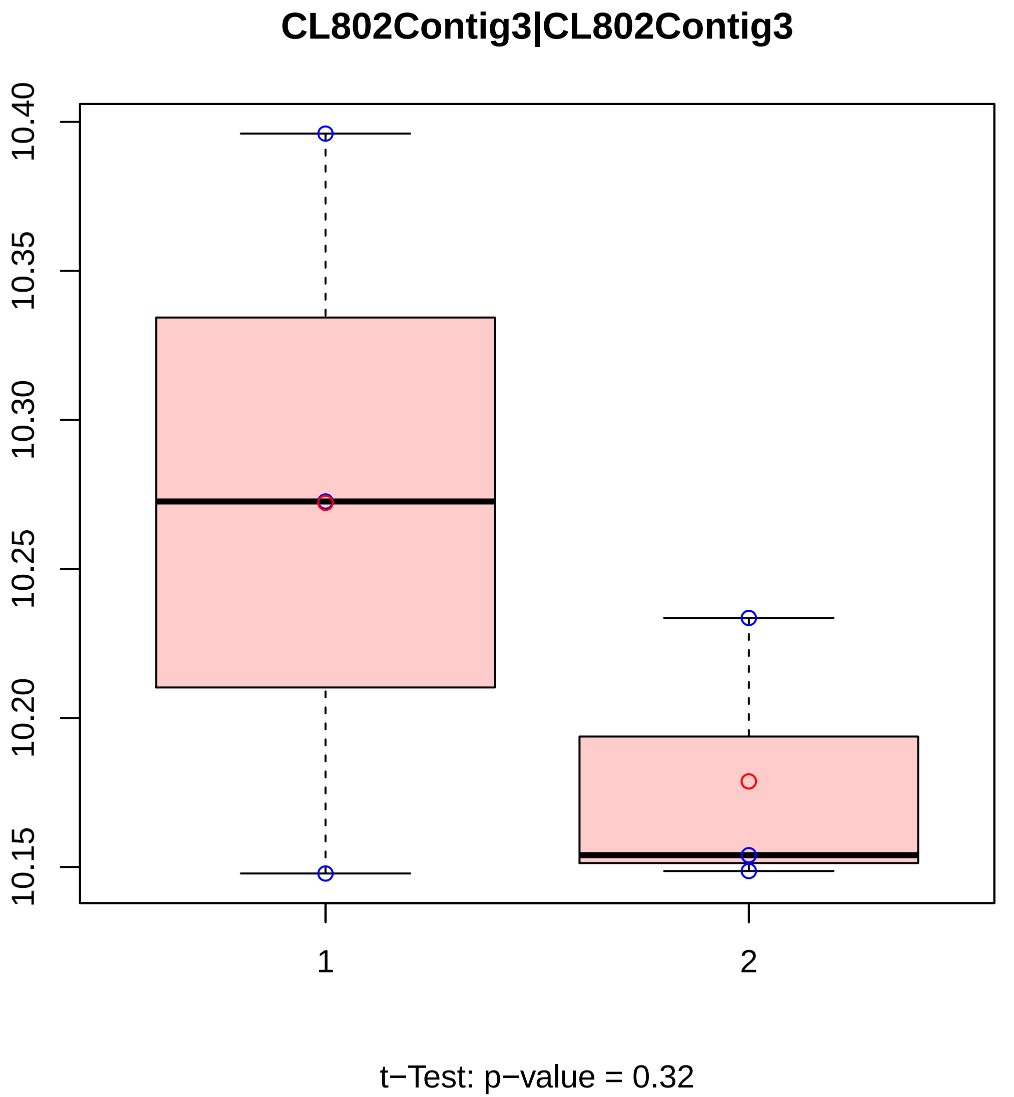
CL802Contig3|CL802Contig3
10.40
10.35
10.30
10.25
10.20
10.15
1
2
t−Test: p−value = 0.32

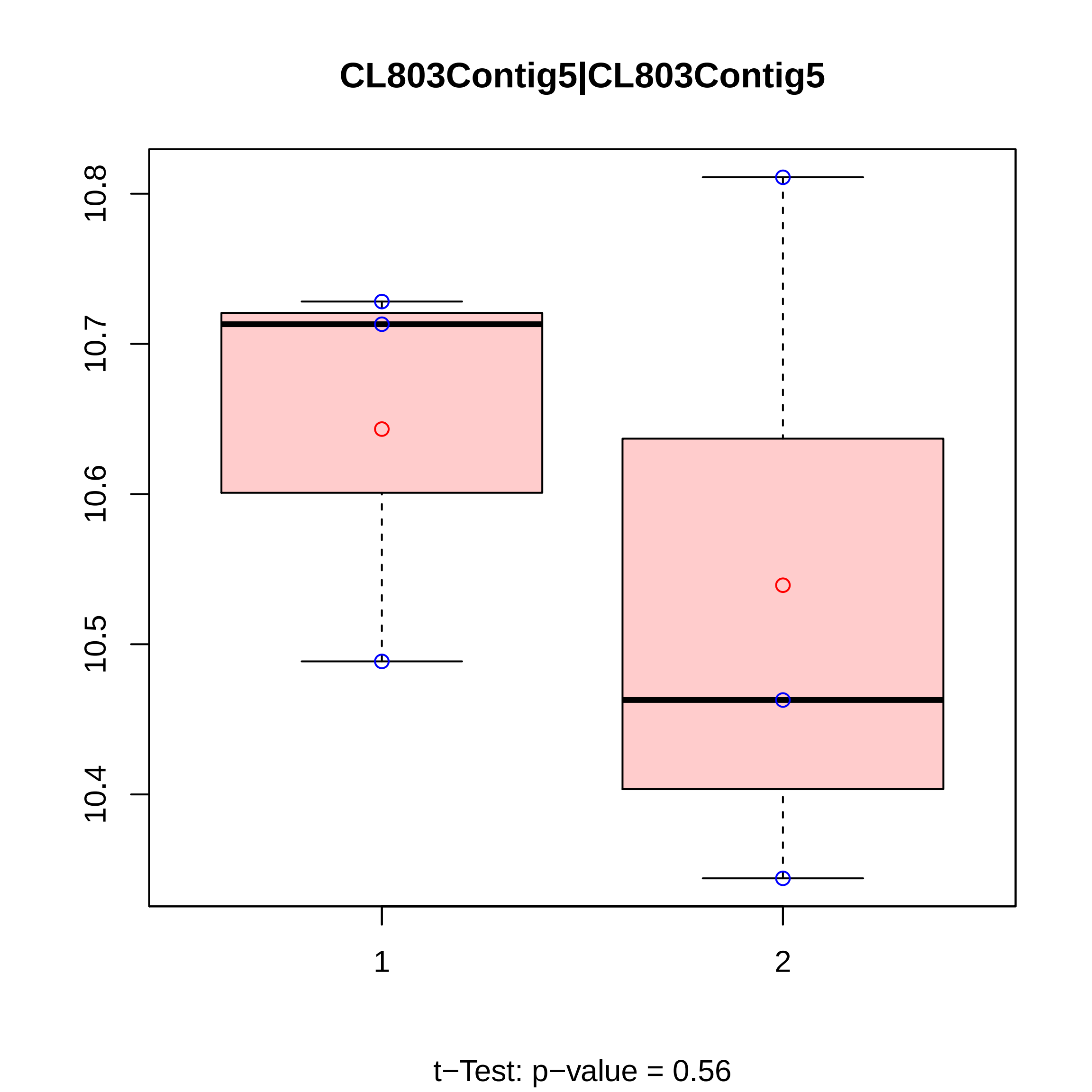
CL803Contig5|CL803Contig5
10.4
10.5
10.6
10.7
10.8
1
2
t−Test: p−value = 0.56

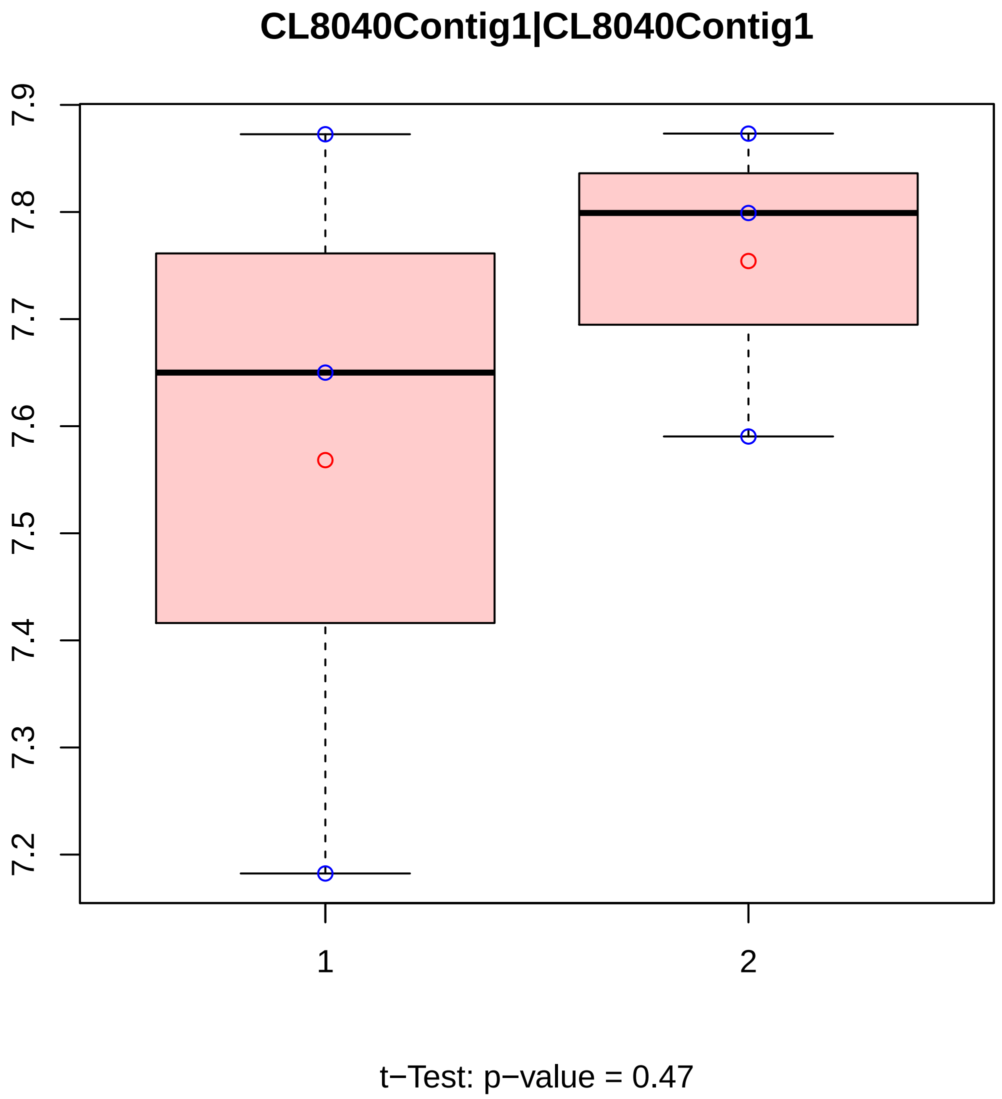

CL8040Contig1|CL8040Contig1
7.2
7.3
7.4
7.5
7.6
7.7
7.8
7.9
1
2
t−Test: p−value = 0.47

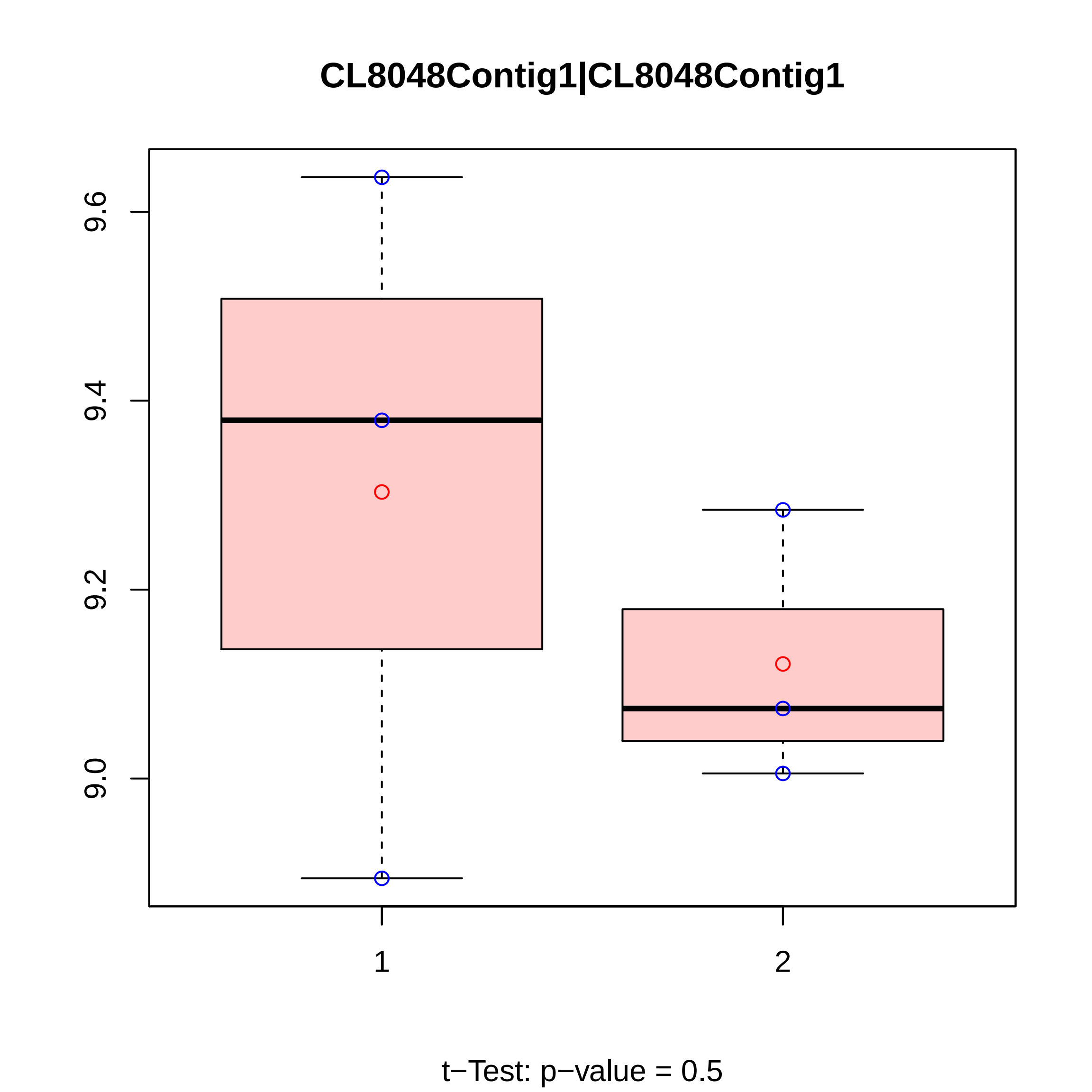

CL8048Contig1|CL8048Contig1
9.0
9.2
9.4
9.6
1
2
t−Test: p−value = 0.5

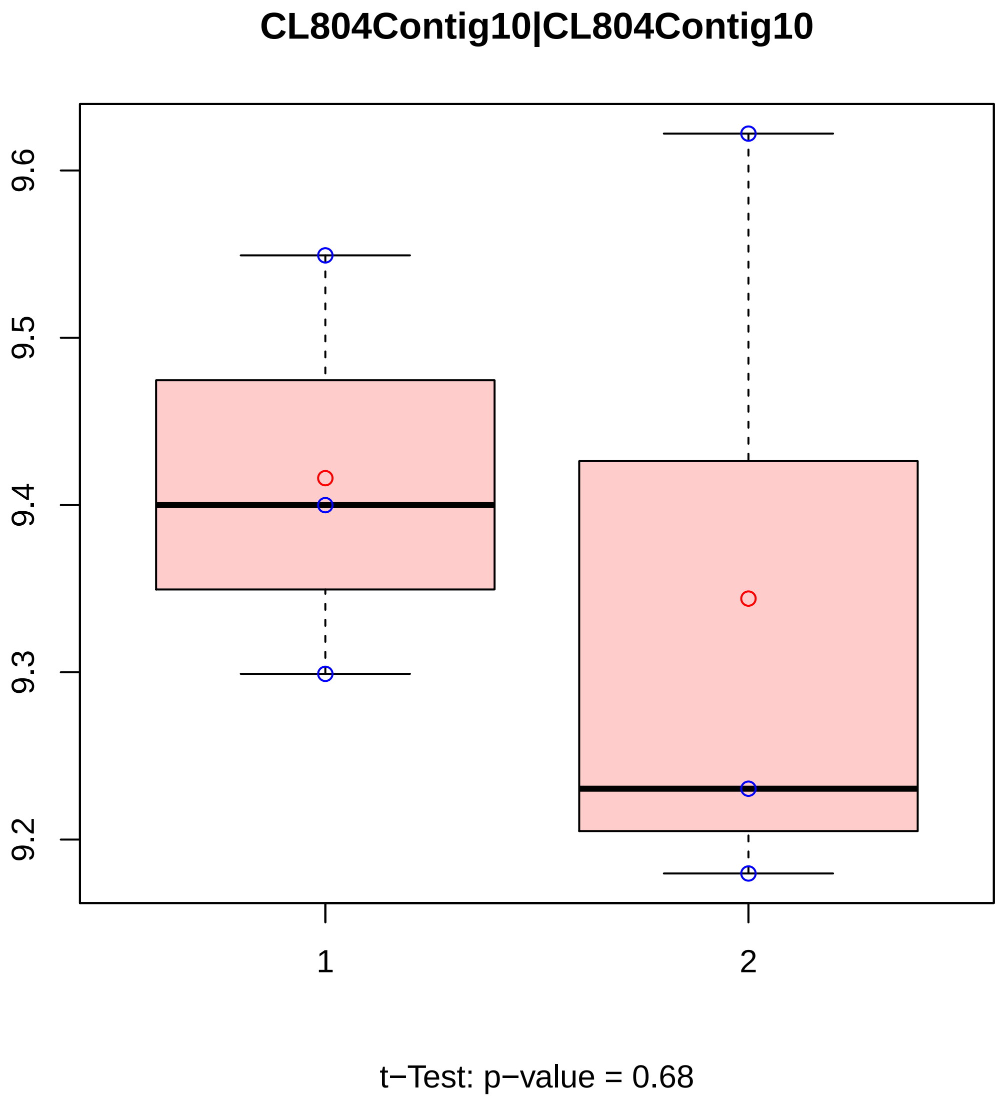
CL804Contig10|CL804Contig10
9.2
9.3
9.4
9.5
9.6
1
2
t−Test: p−value = 0.68

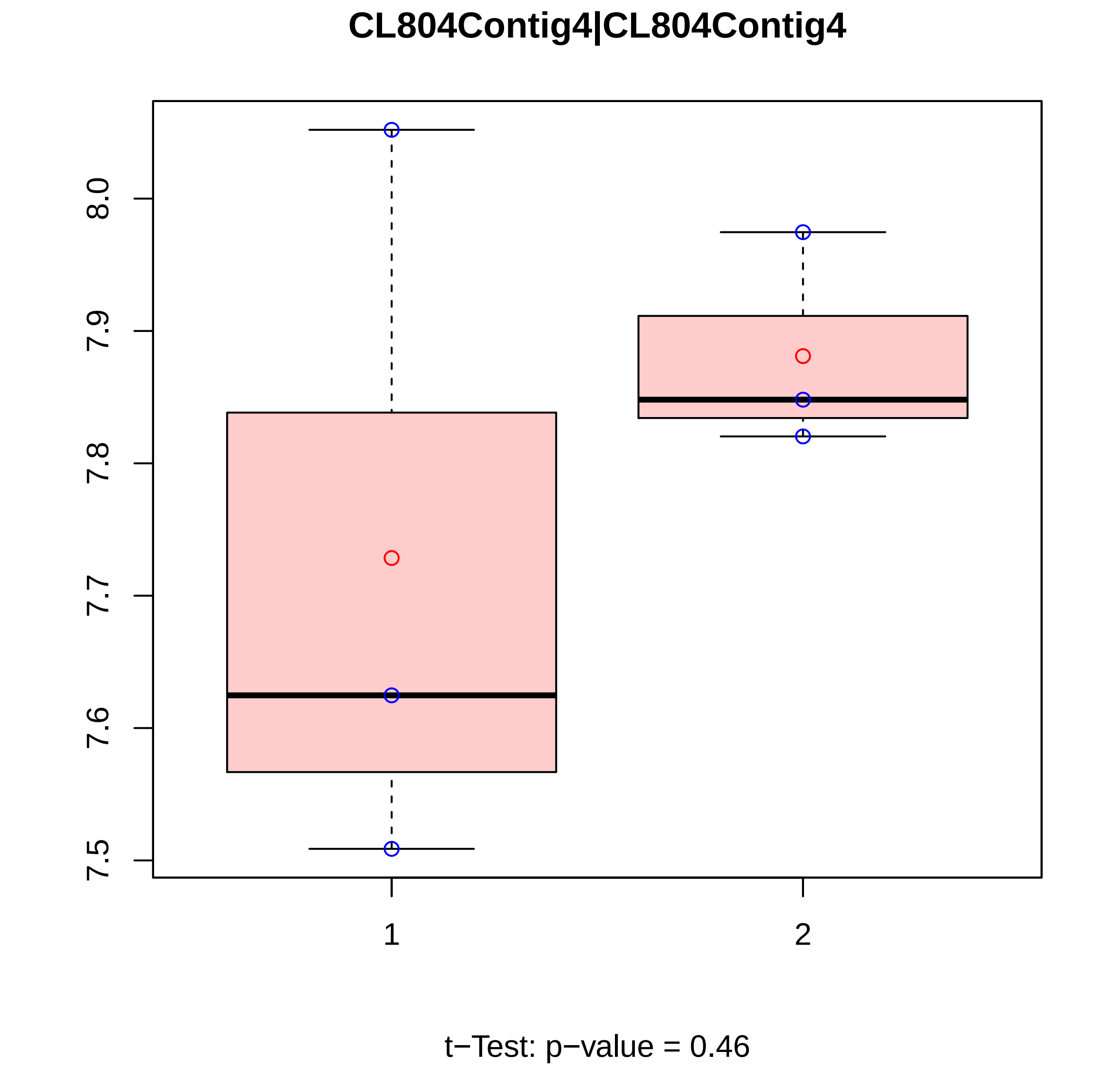
CL804Contig4|CL804Contig4
8.0
7.9
7.8
7.7
7.6
7.5
1
2
t−Test: p−value = 0.46

## CL8050Contig1|CL8050Contig1

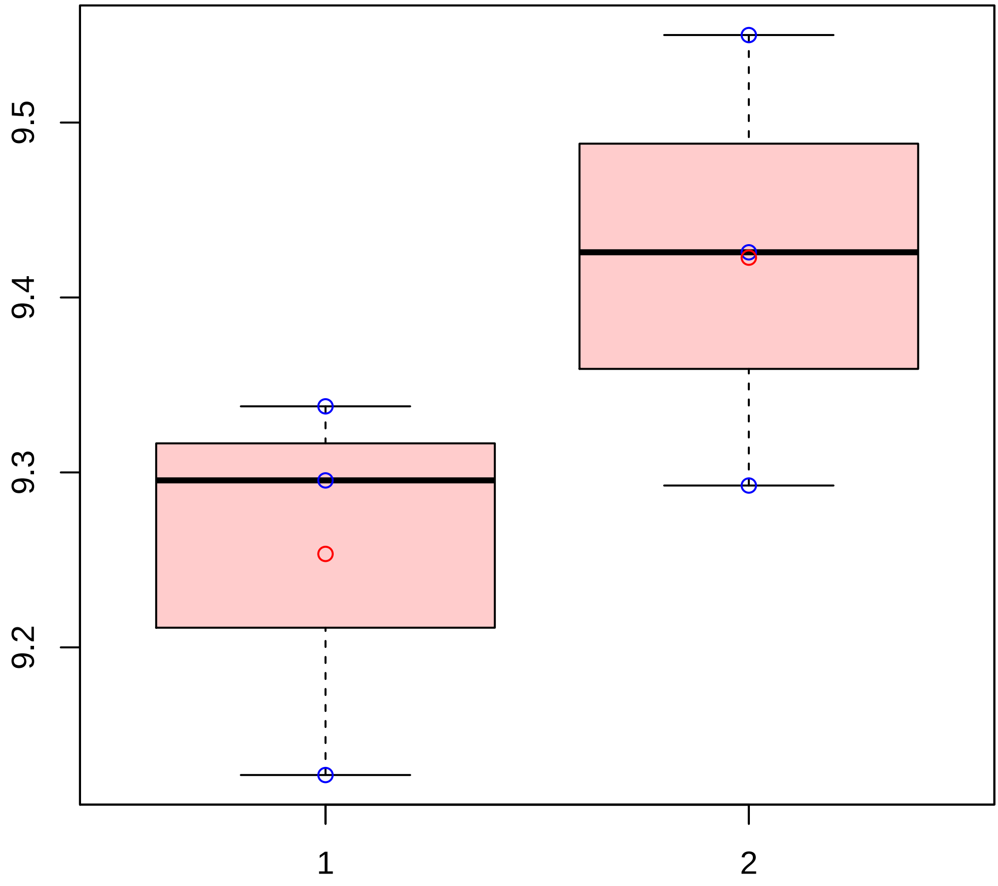


t−Test: p−value = 0.16

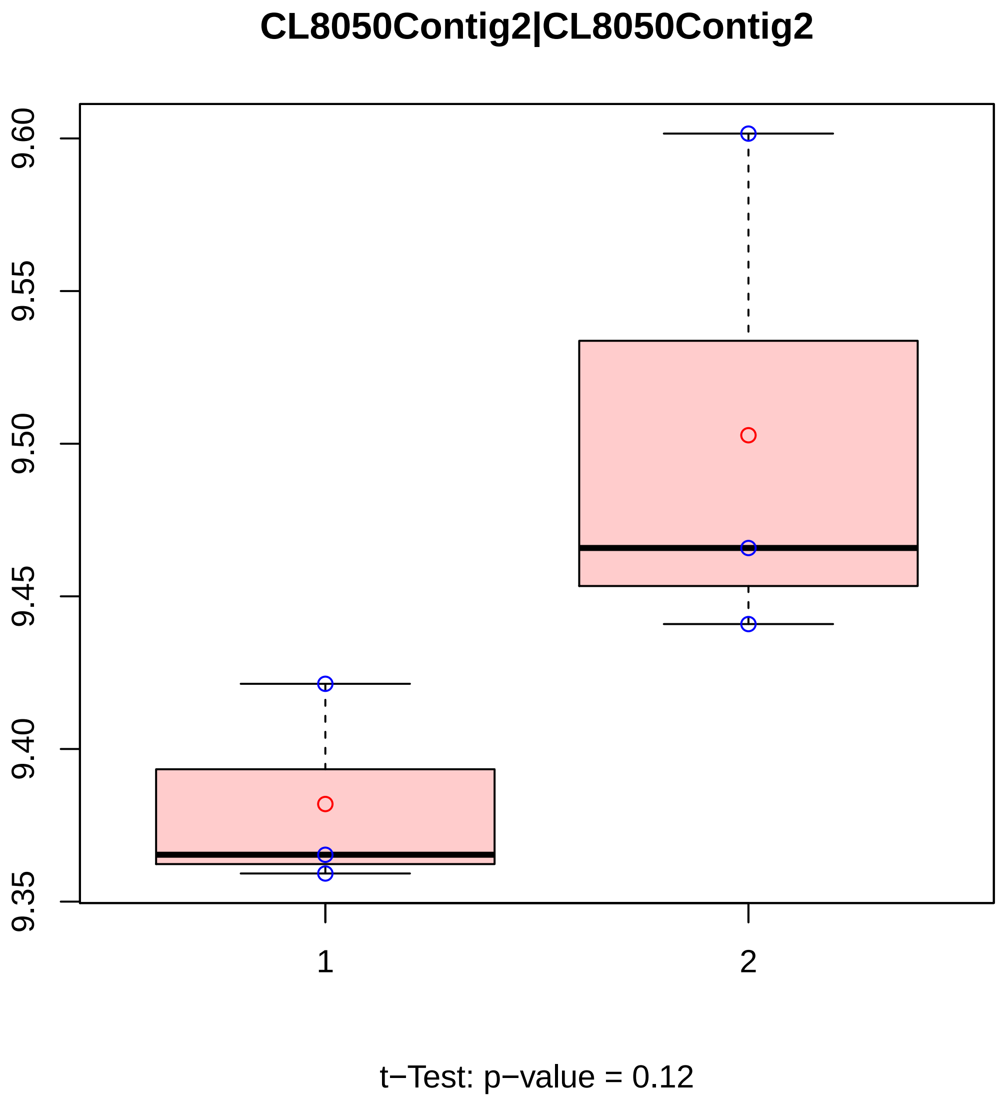
CL8050Contig2|CL8050Contig2
9.35
9.40
9.45
9.50
9.55
9.60
1
2
t−Test: p−value = 0.12

# CL8056Contig1|CL8056Contig1

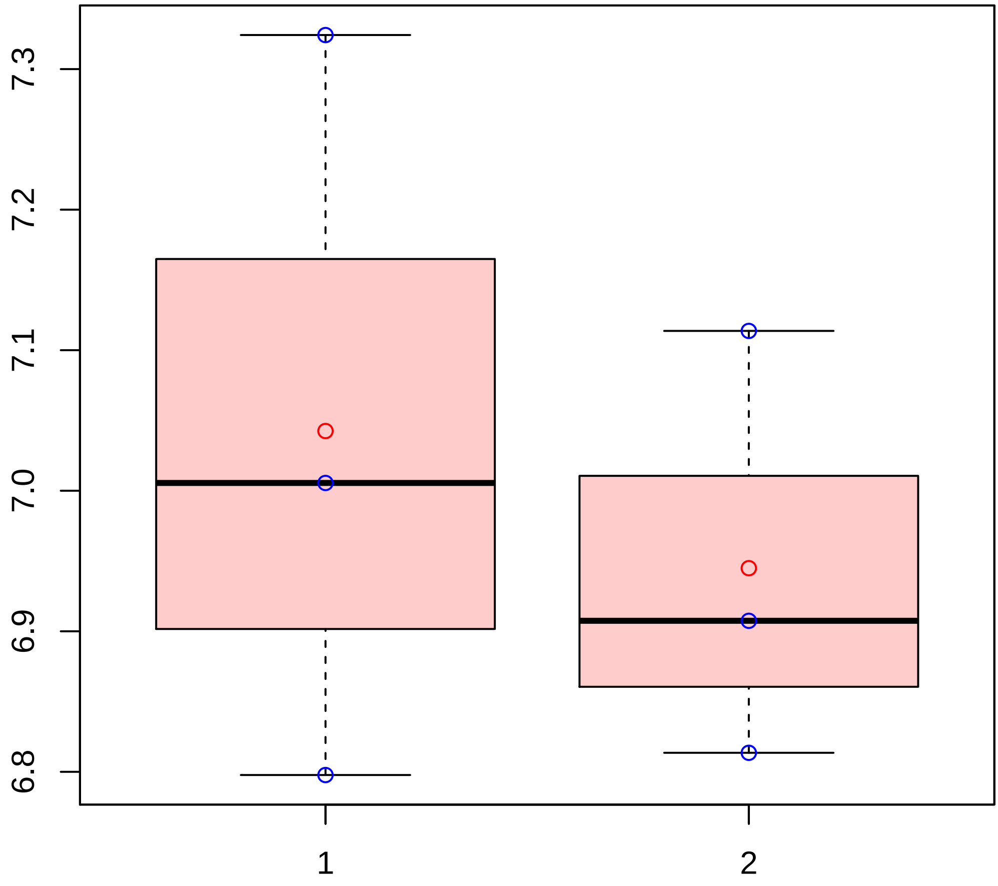


t−Test: p−value = 0.62

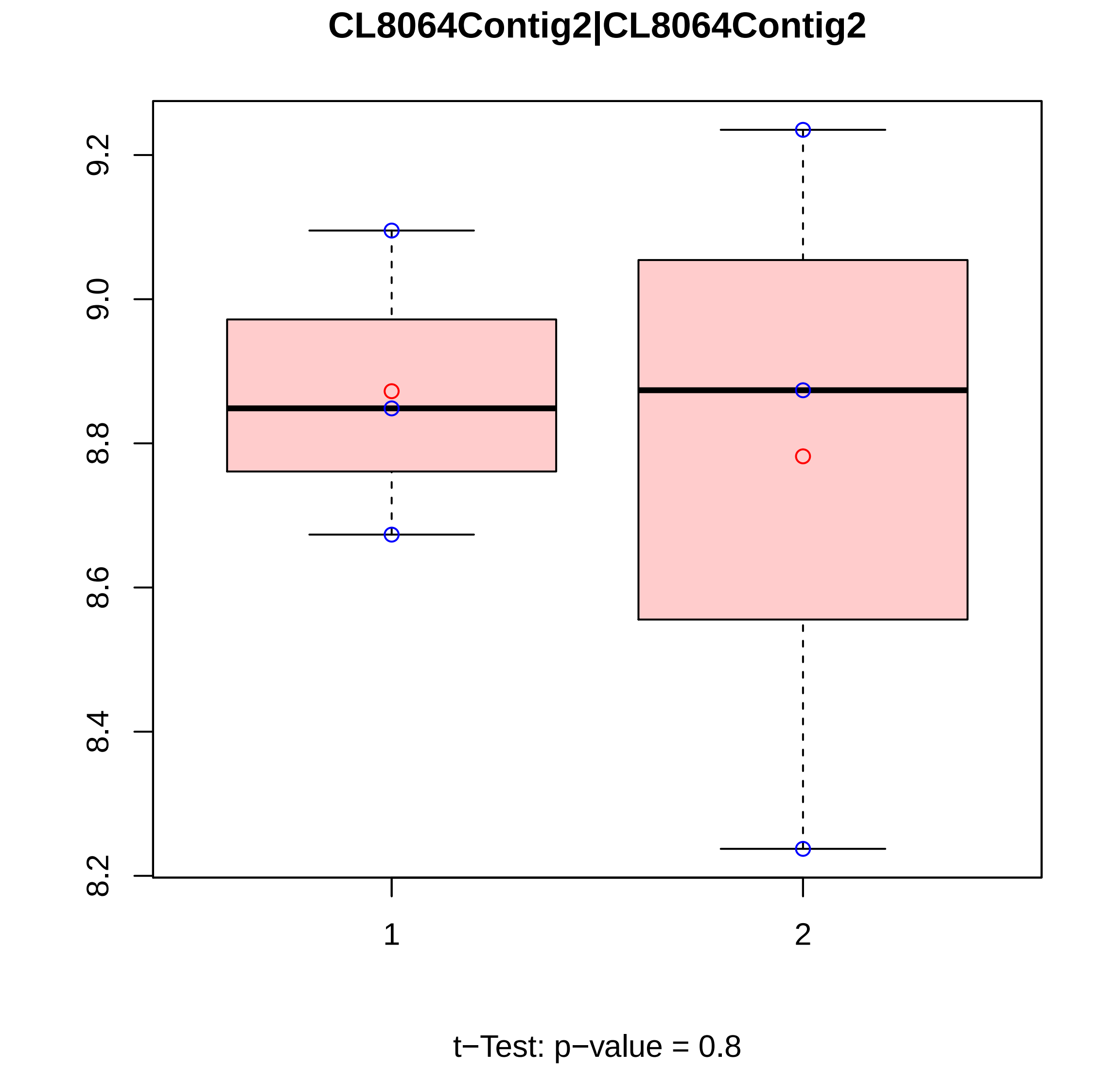
CL8064Contig2|CL8064Contig2
9.2
9.0
8.8
8.6
8.4
8.2
1
2
t−Test: p−value = 0.8

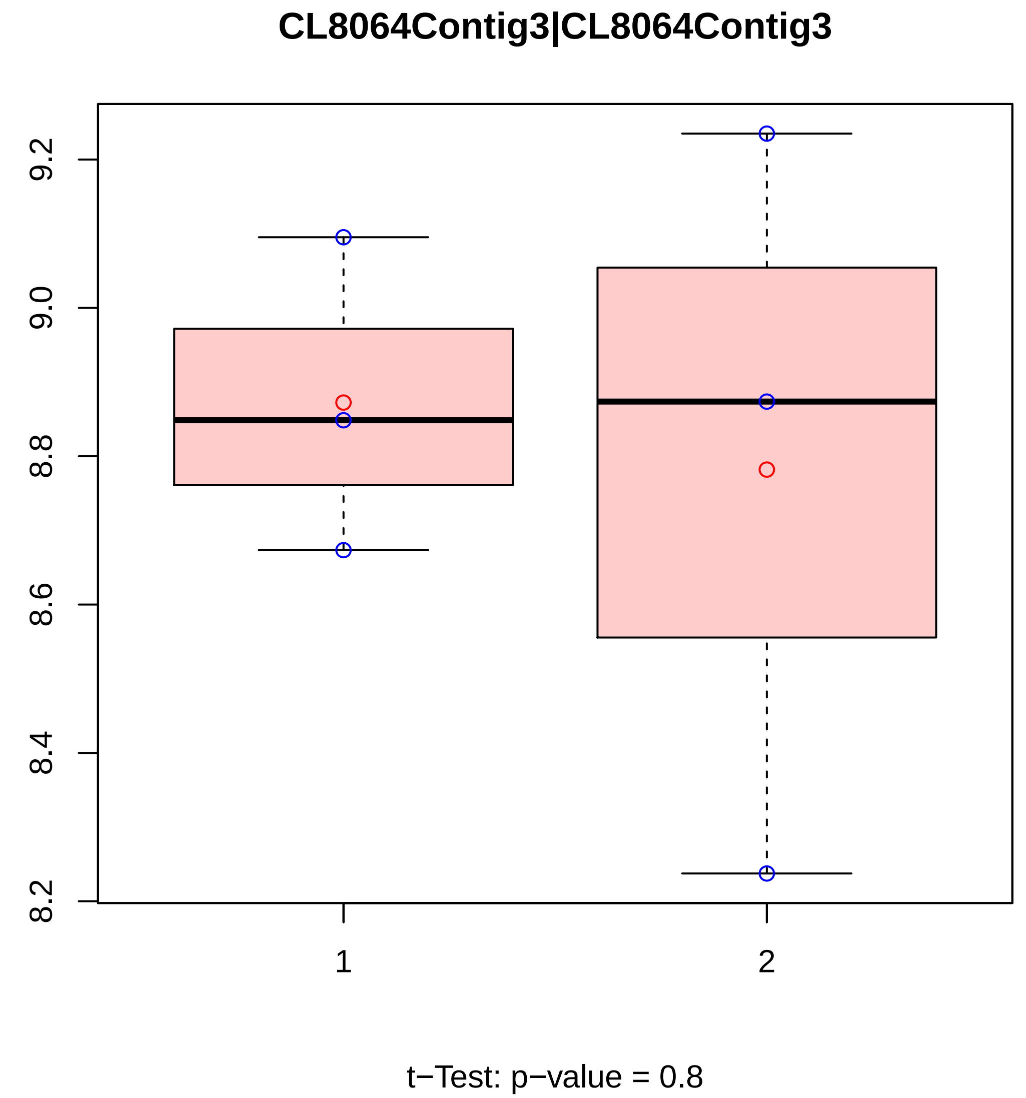
CL8064Contig3|CL8064Contig3
9.2
9.0
8.8
8.6
8.4
8.2
1
2
t−Test: p−value = 0.8

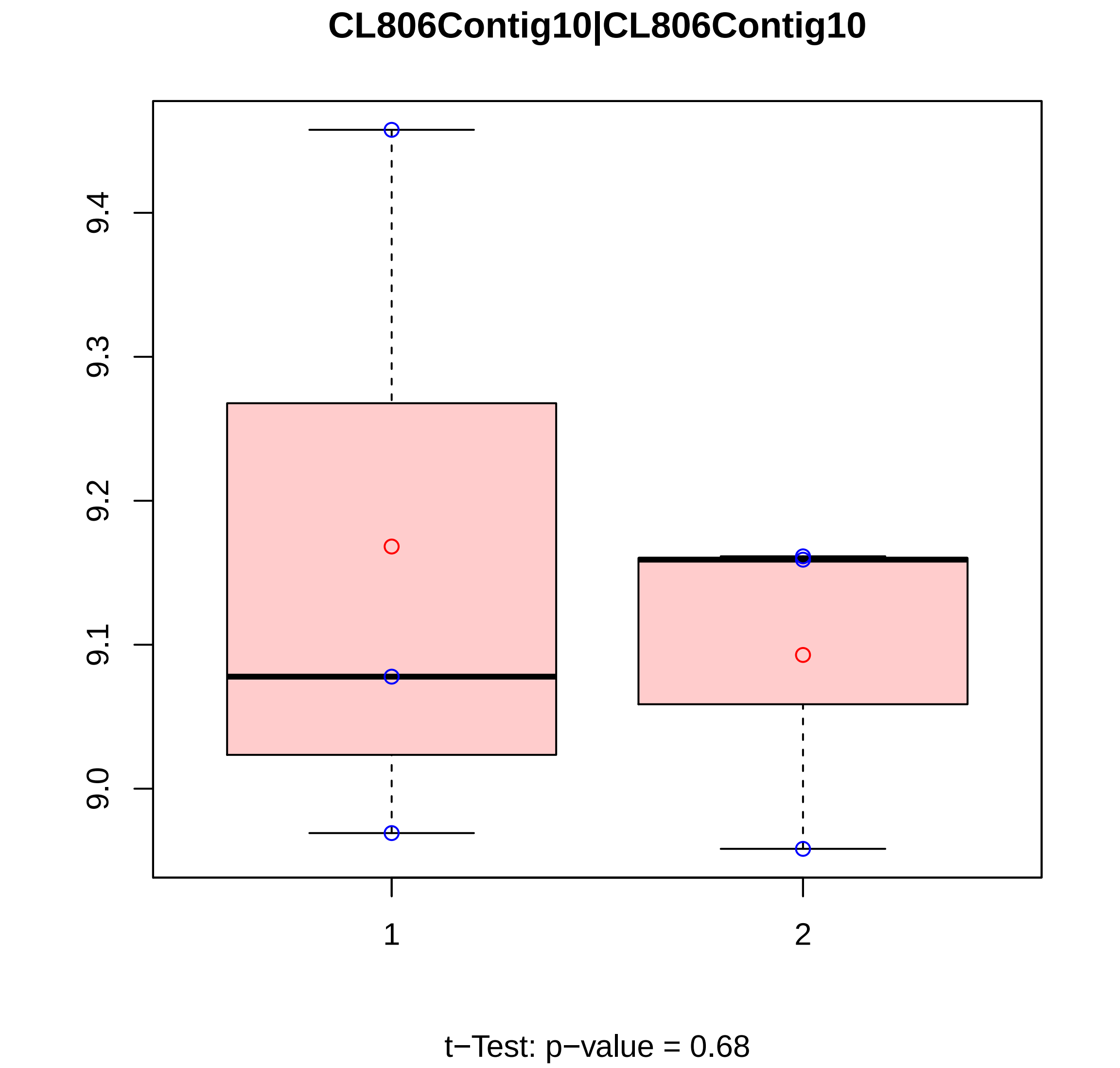
CL806Contig10|CL806Contig10
9.0
9.1
9.2
9.3
9.4
1
2
t−Test: p−value = 0.68

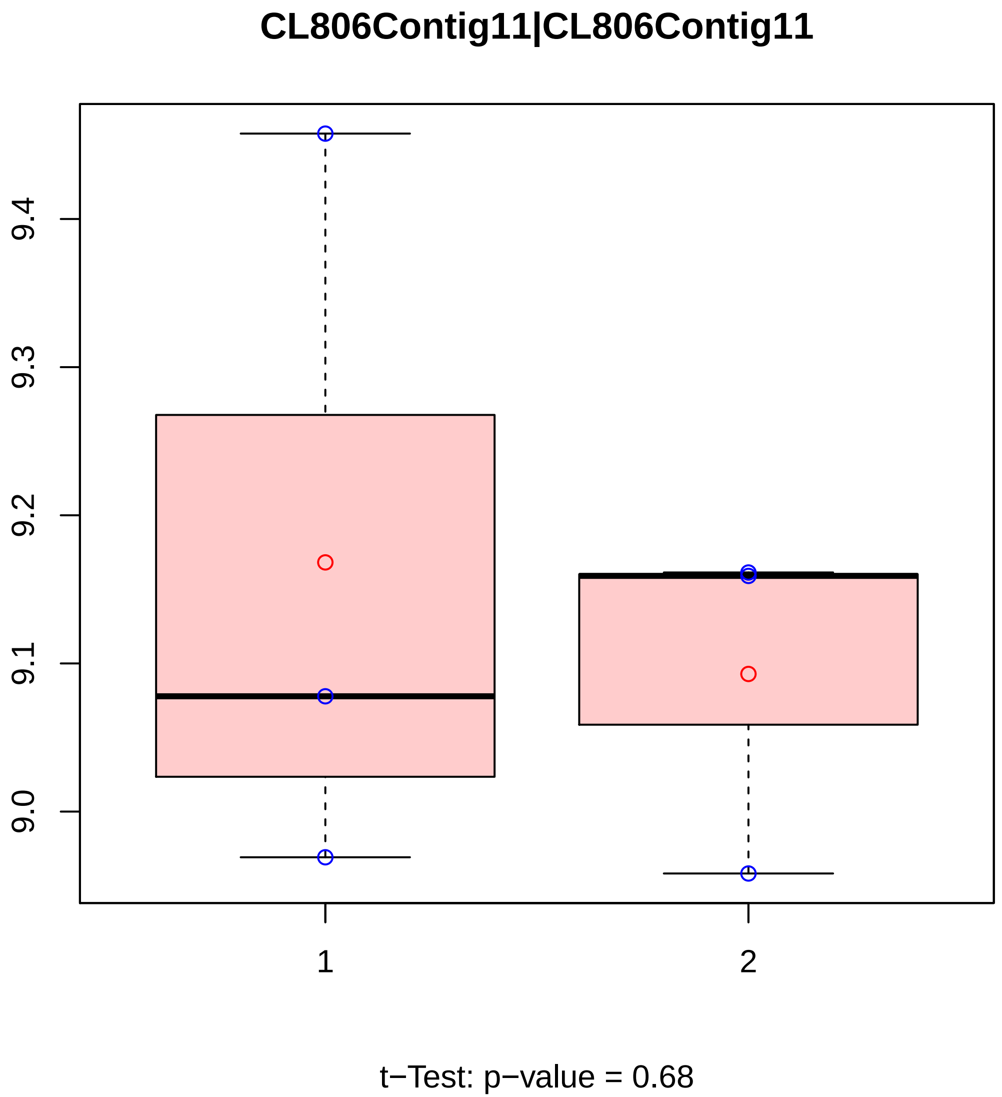

CL806Contig11|CL806Contig11
9.0
9.1
9.2
9.3
9.4
1
2
t−Test: p−value = 0.68

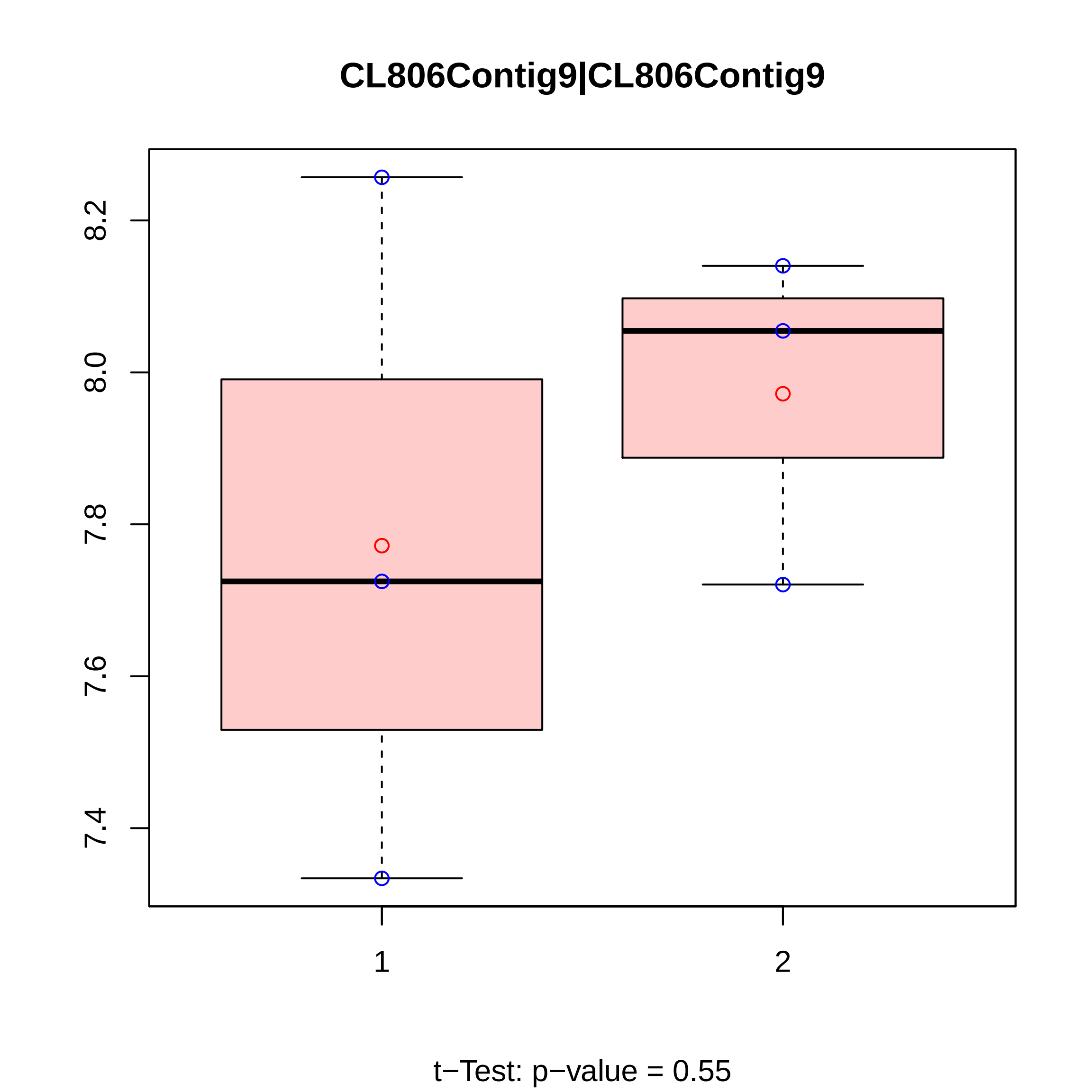
CL806Contig9|CL806Contig9
8.2
8.0
7.8
7.6
7.4
1
2
t−Test: p−value = 0.55

## CL8072Contig2|CL8072Contig2

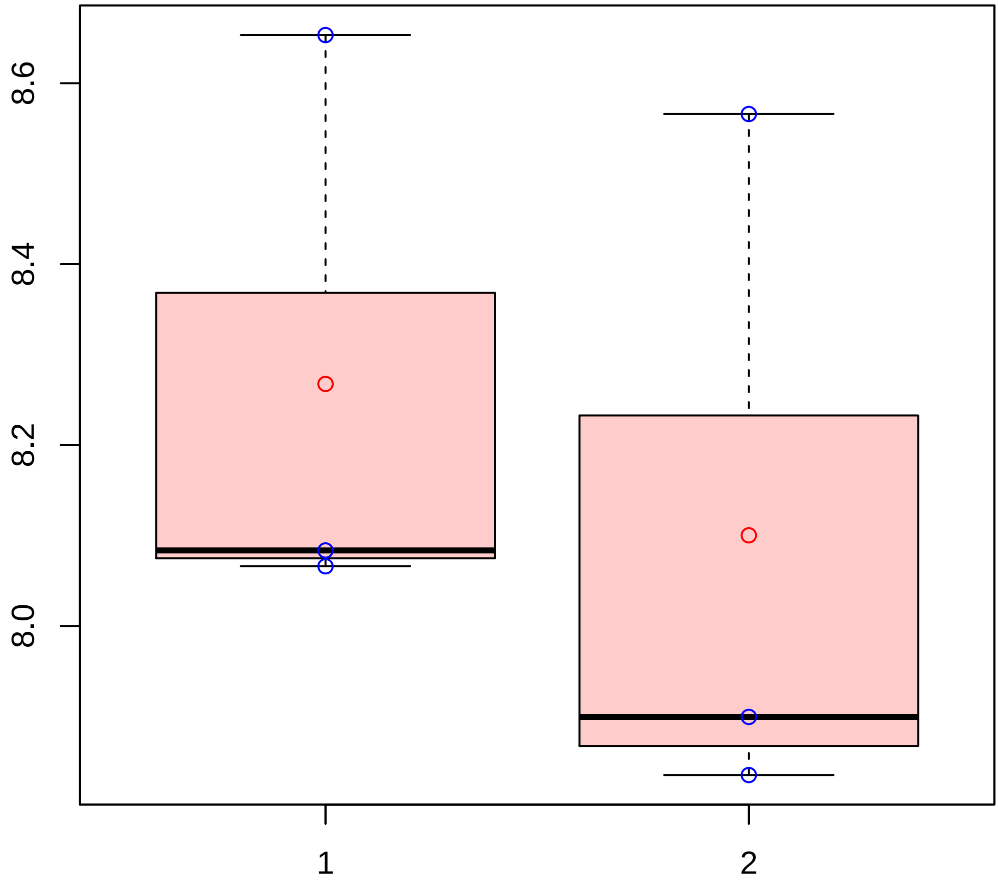


t−Test: p−value = 0.61

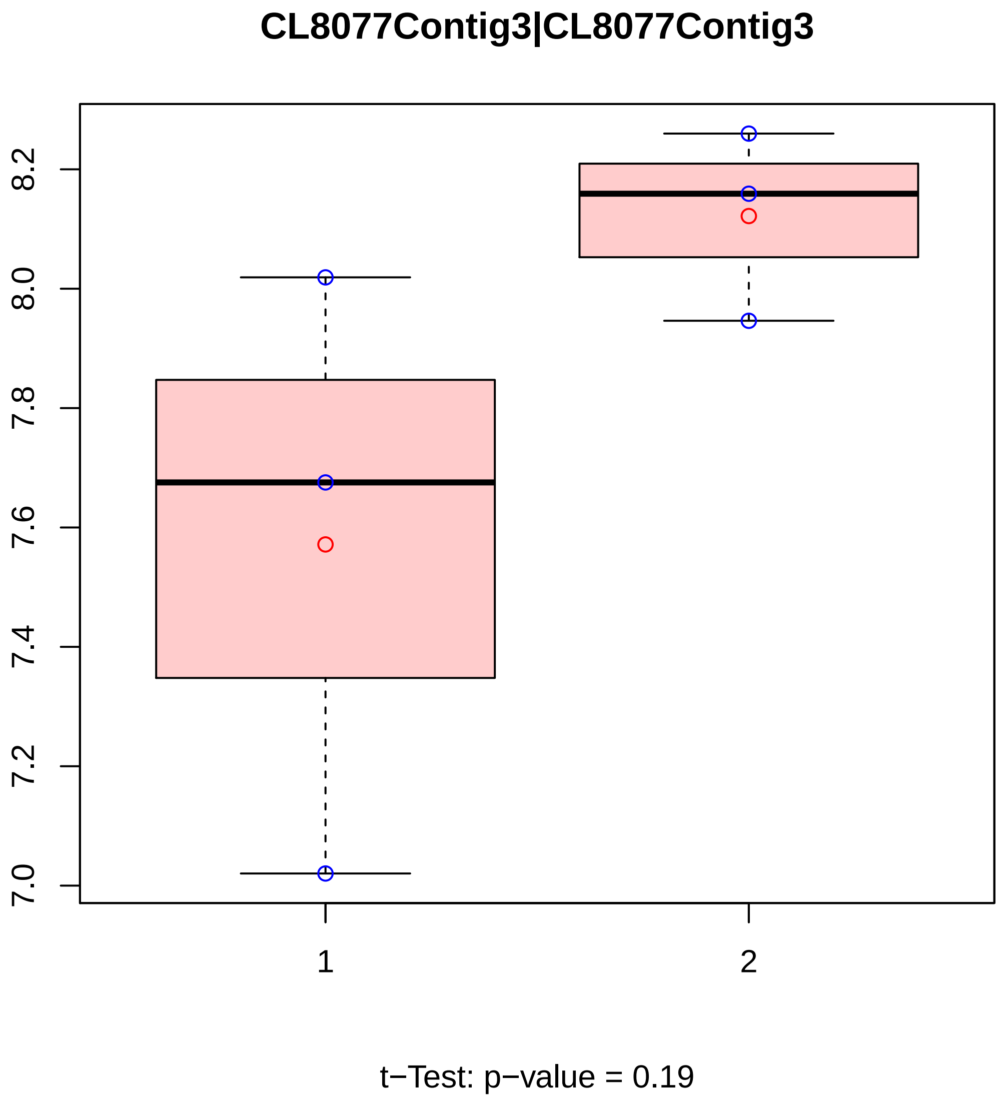
CL8077Contig3|CL8077Contig3
8.2
8.0
7.8
7.6
7.4
7.2
7.0
1
2
t−Test: p−value = 0.19

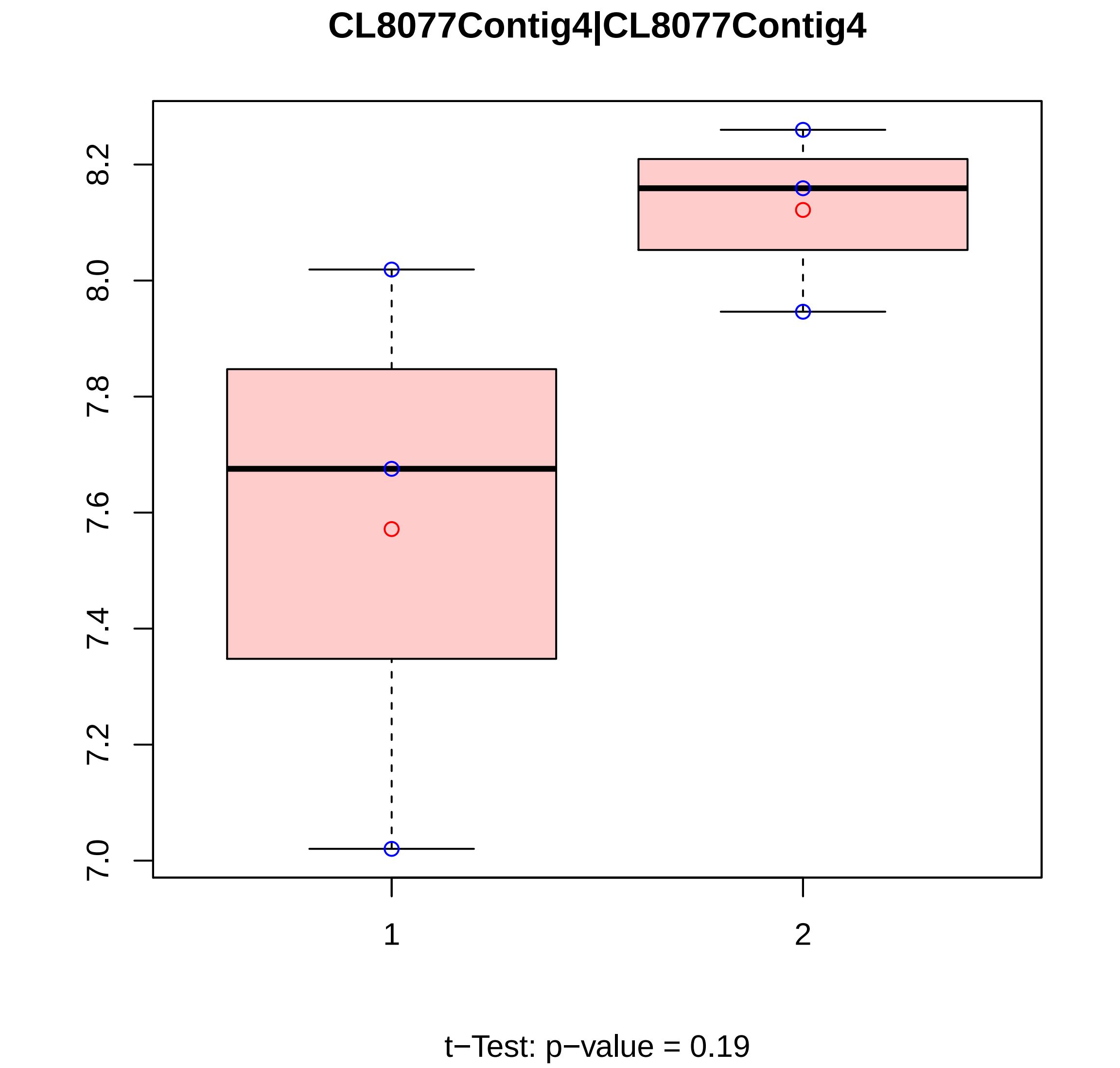

CL8077Contig4|CL8077Contig4
8.2
8.0
7.8
7.6
7.4
7.2
7.0
1
2
t−Test: p−value = 0.19

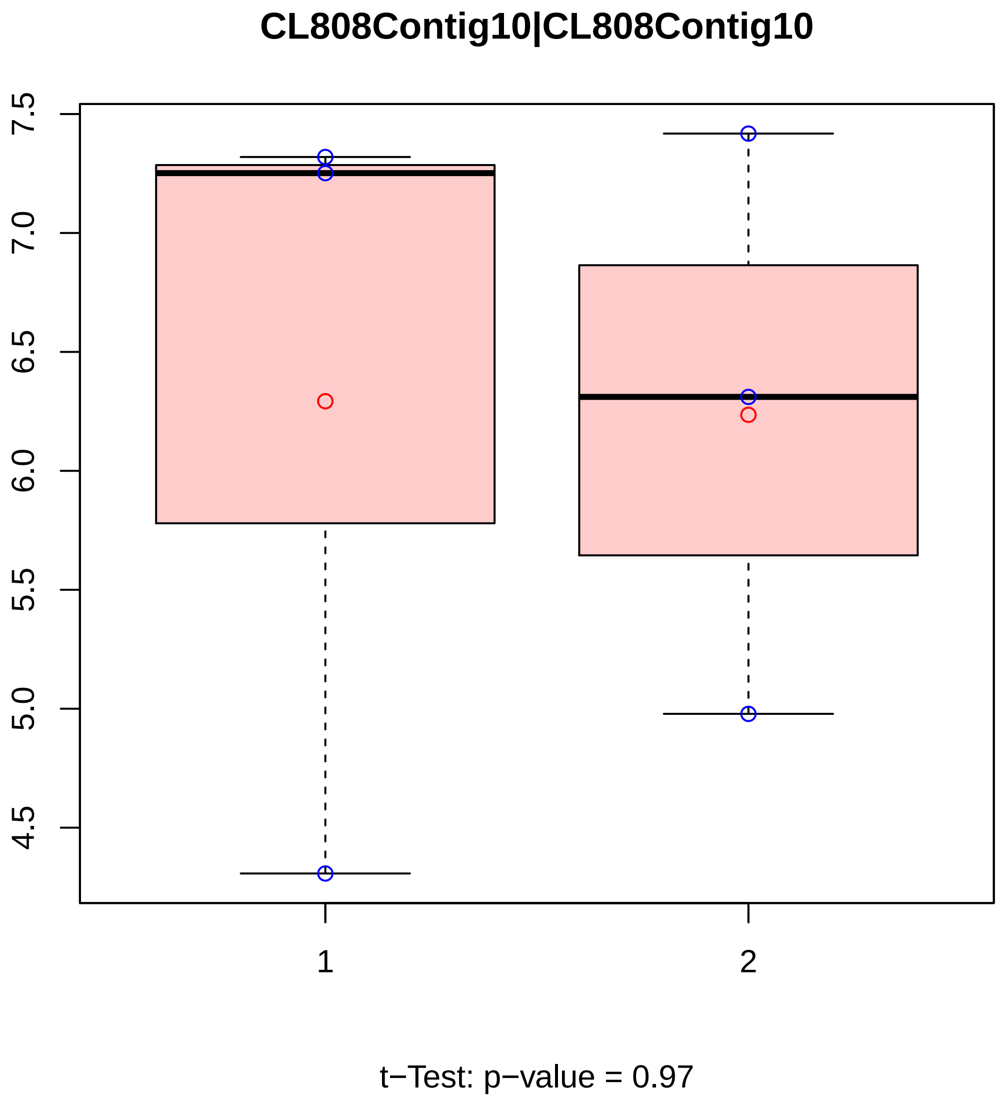
CL808Contig10|CL808Contig10
7.5
7.0
6.5
6.0
5.5
5.0
4.5
1
2
t−Test: p−value = 0.97

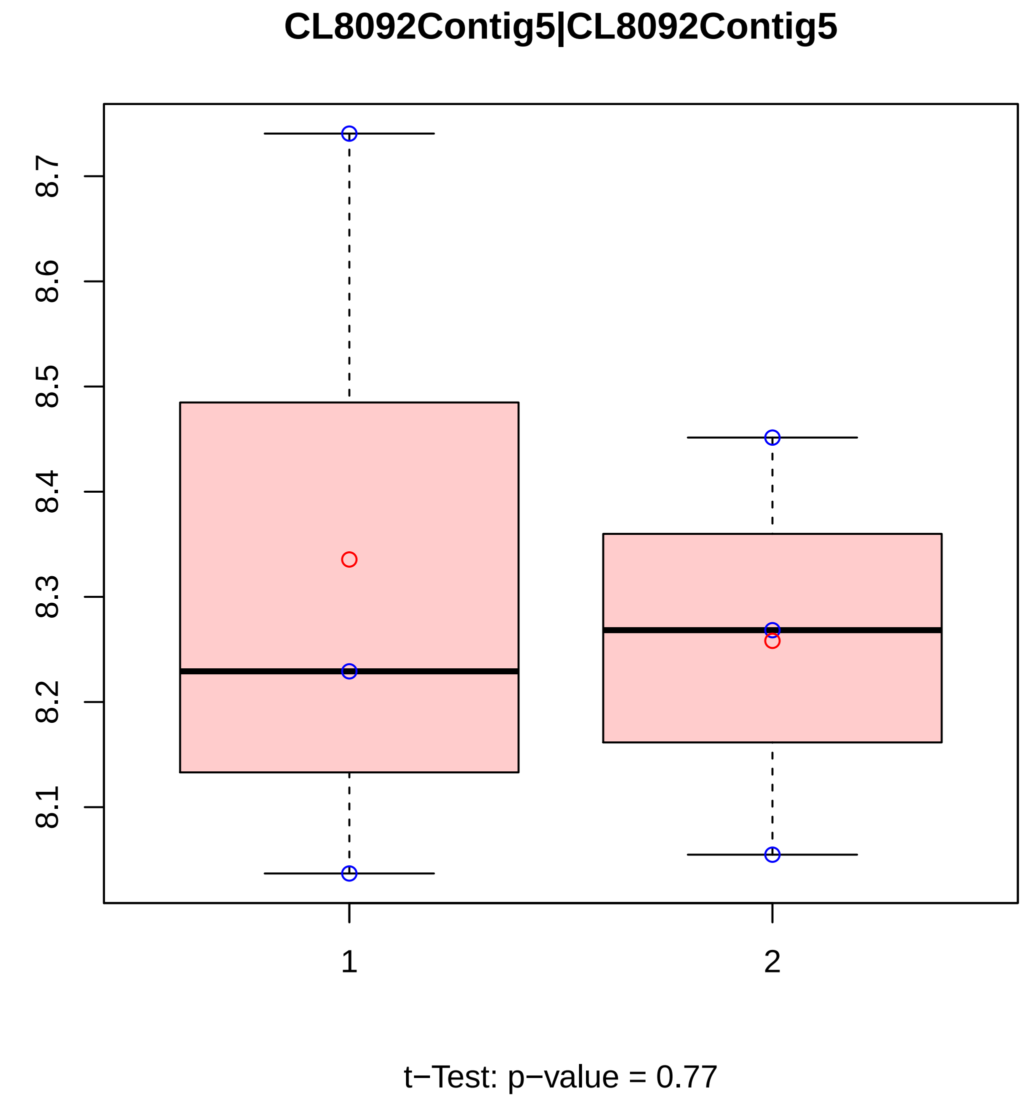
CL8092Contig5|CL8092Contig5
8.1
8.2
8.3
8.4
8.5
8.6
8.7
1
2
t−Test: p−value = 0.77

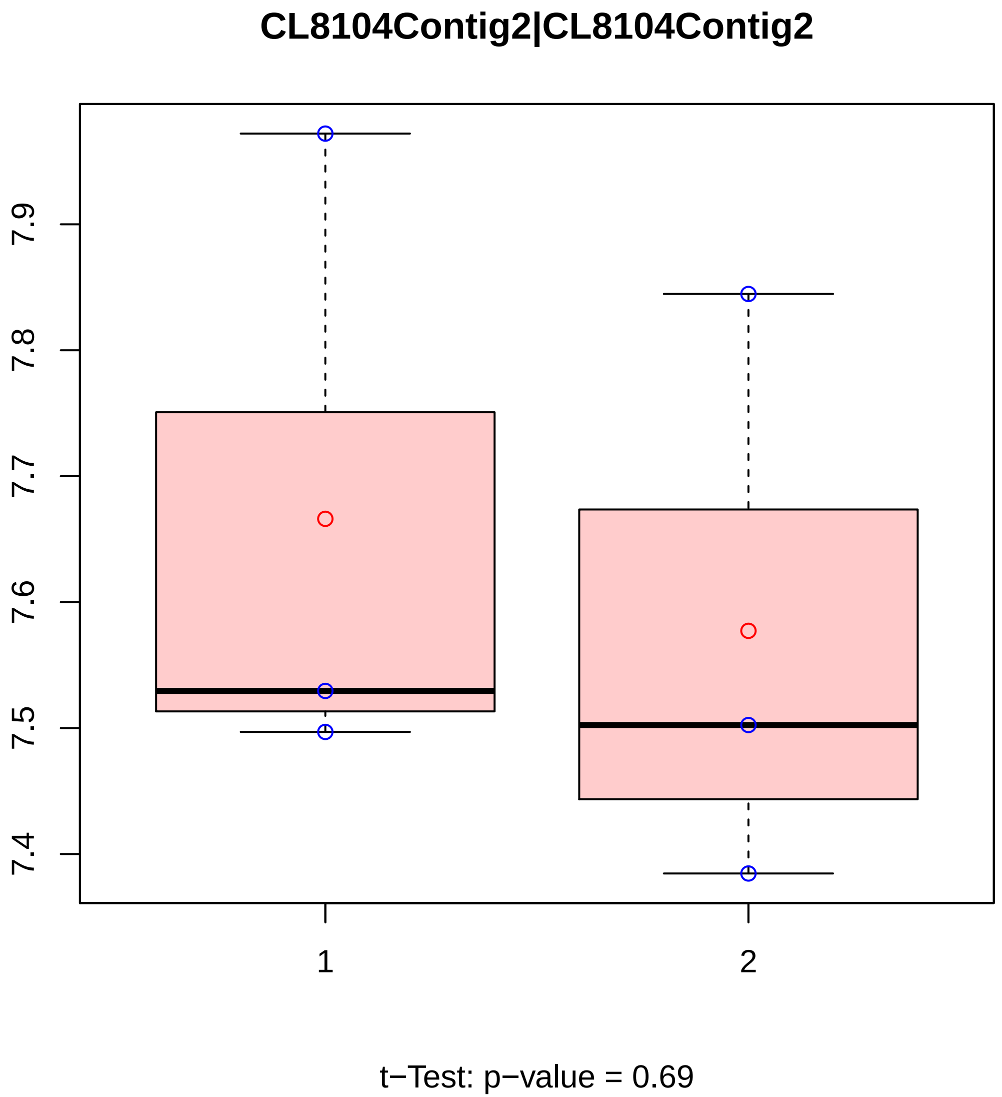
CL8104Contig2|CL8104Contig2
7.4
7.5
7.6
7.7
7.8
7.9
1
2
t−Test: p−value = 0.69

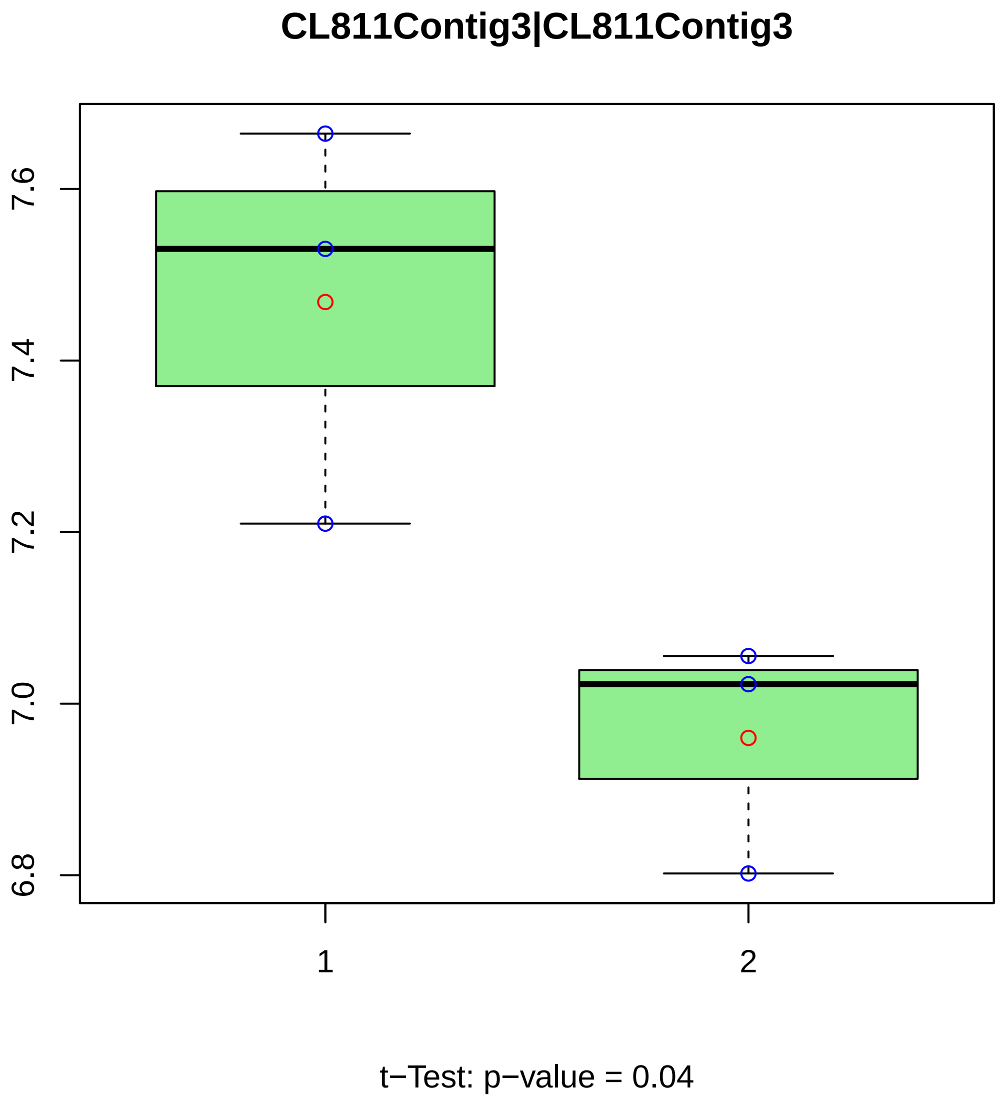

CL811Contig3|CL811Contig3
7.6
7.4
7.2
7.0
6.8
1
2
t−Test: p−value = 0.04

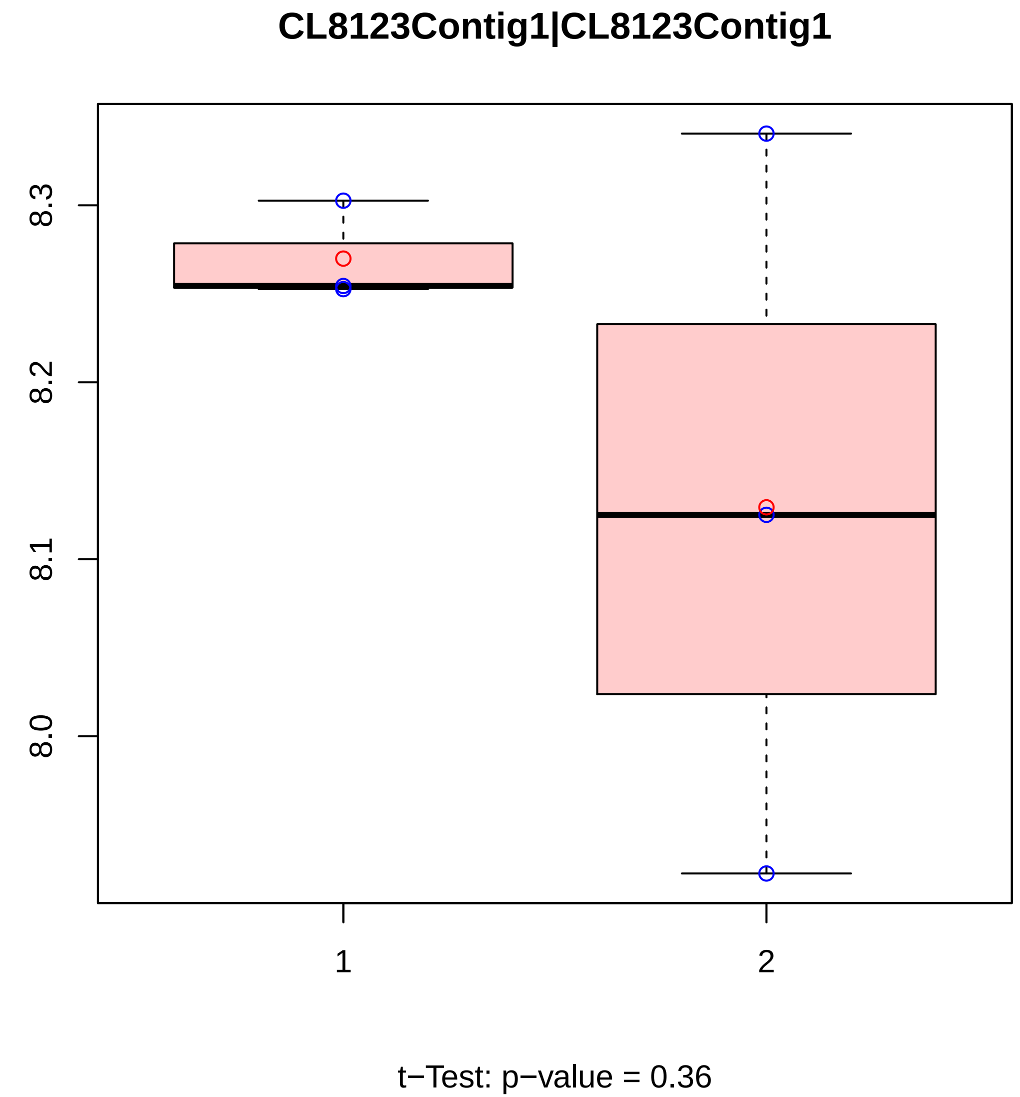
CL8123Contig1|CL8123Contig1
8.3
8.2
8.1
8.0
1
2
t−Test: p−value = 0.36

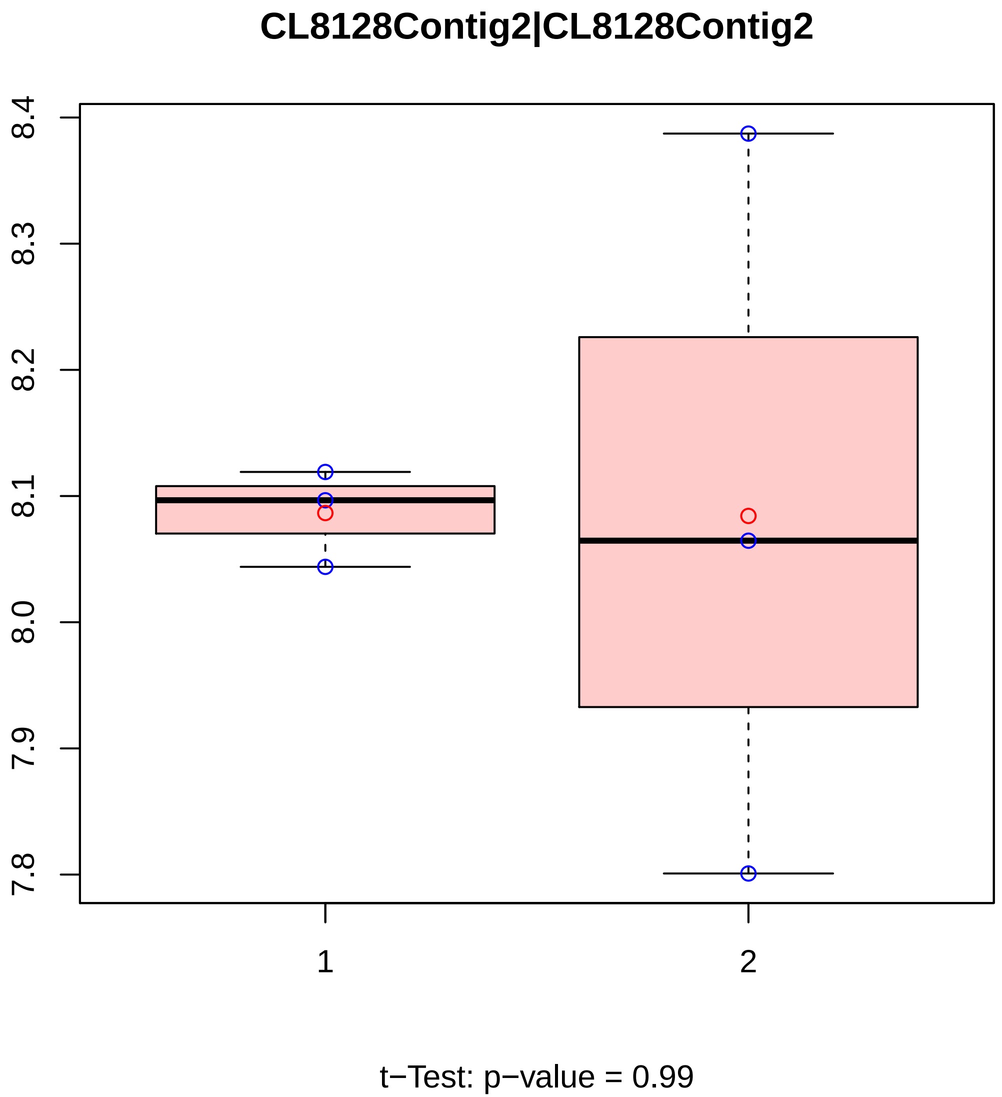
CL8128Contig2|CL8128Contig2
8.4
8.3
8.2
8.1
8.0
7.9
7.8
1
2
t−Test: p−value = 0.99

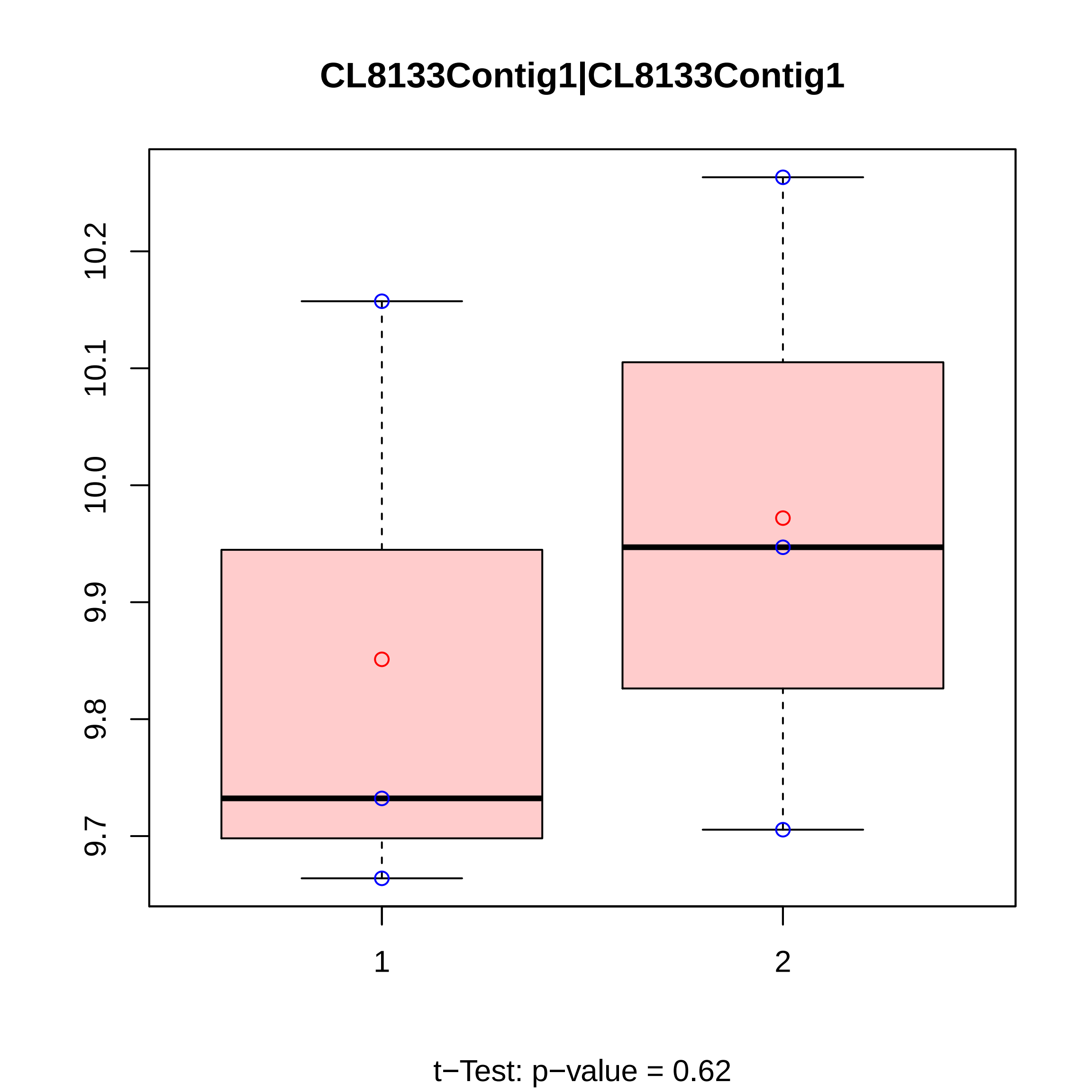
CL8133Contig1|CL8133Contig1
10.2
10.1
10.0
9.9
9.8
9.7
1
2
t−Test: p−value = 0.62

# CL8133Contig2|CL8133Contig2

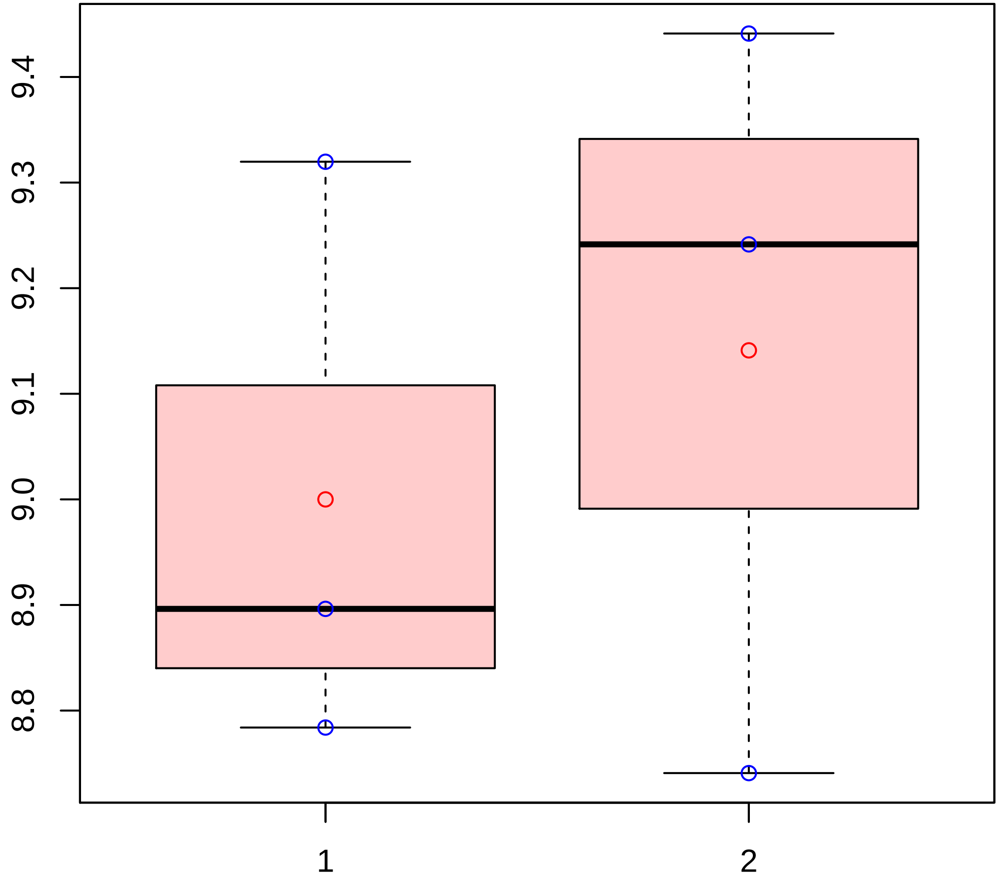


t−Test: p−value = 0.62

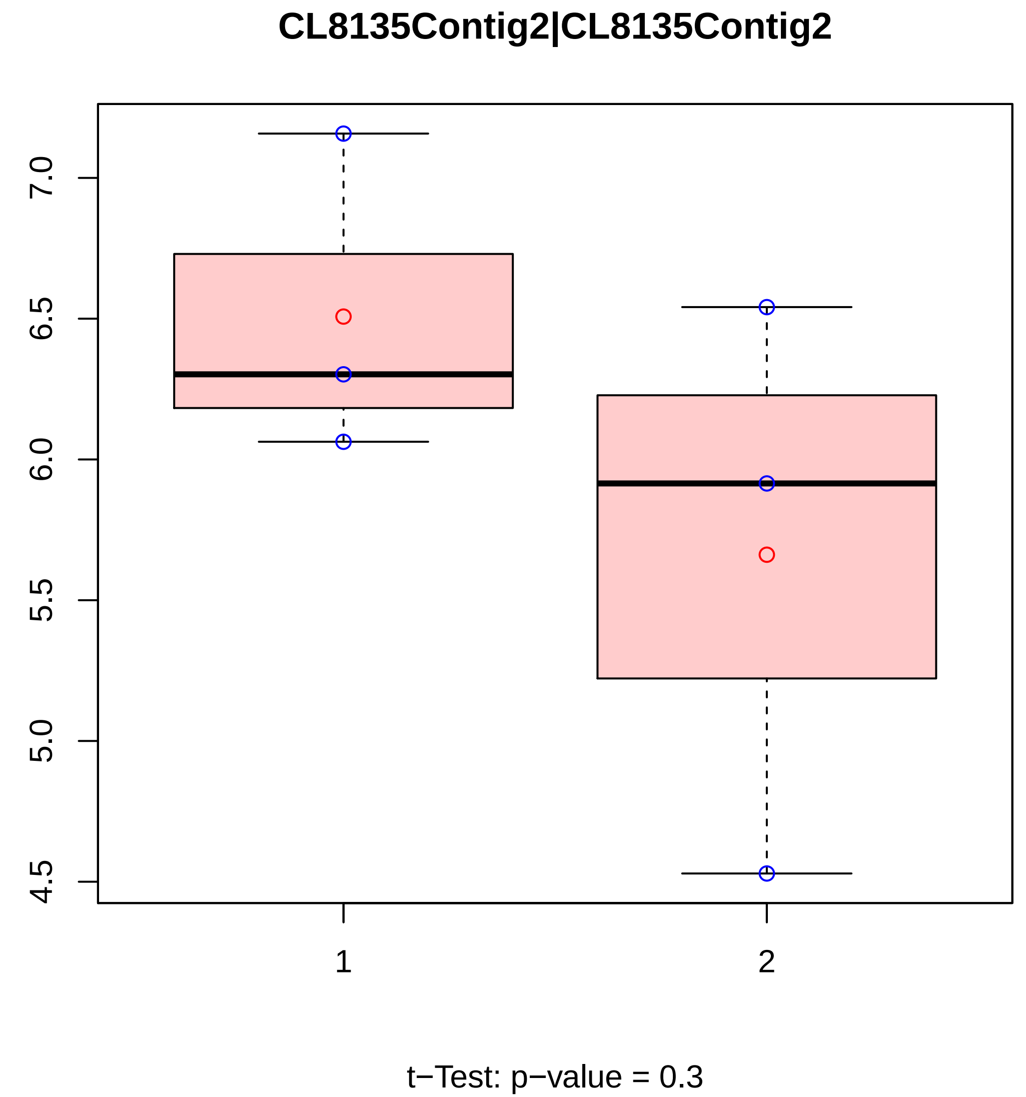
CL8135Contig2|CL8135Contig2
7.0
6.5
6.0
5.5
5.0
4.5
1
2
t−Test: p−value = 0.3

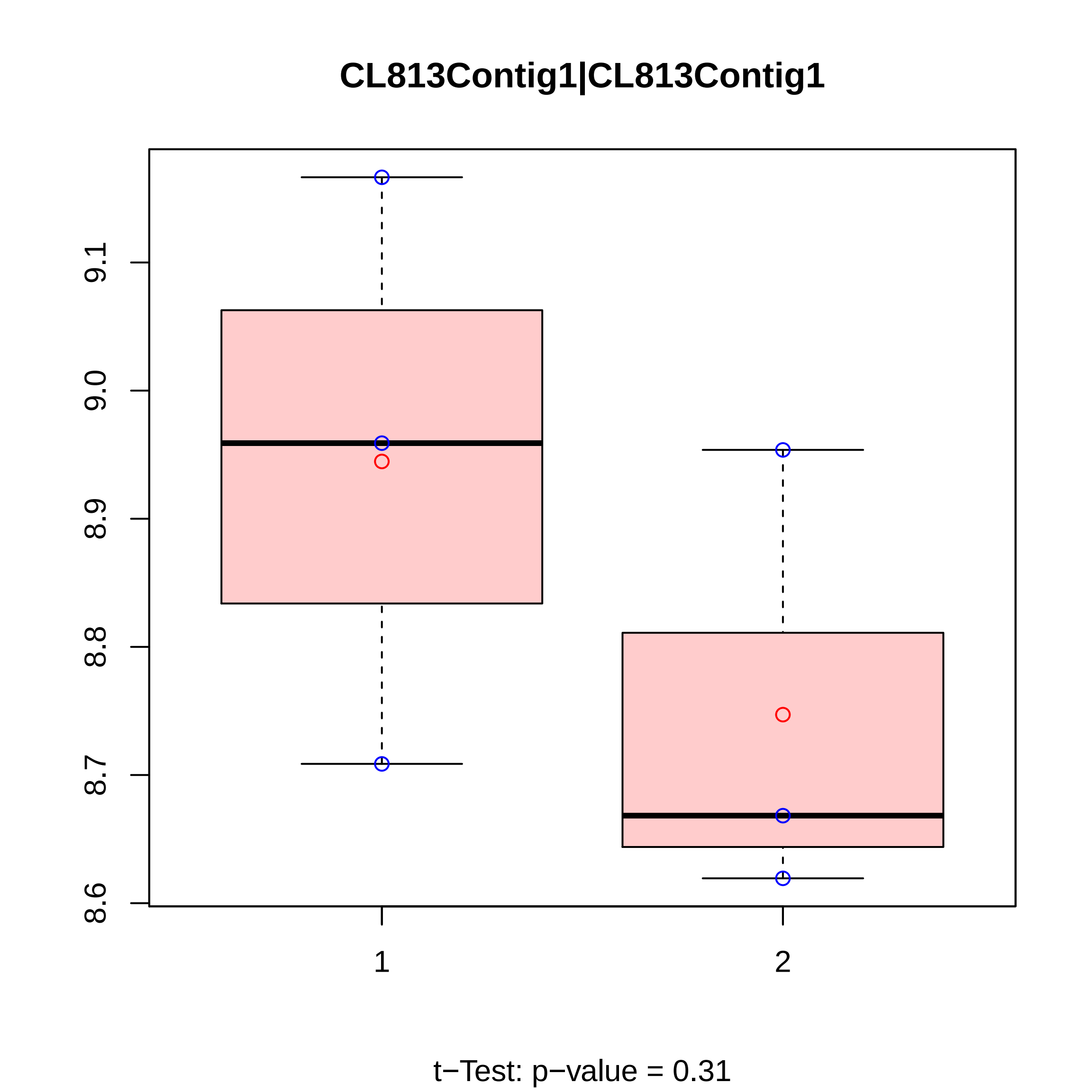

CL813Contig1|CL813Contig1
9.1
9.0
8.9
8.8
8.7
8.6
1
2
t−Test: p−value = 0.31

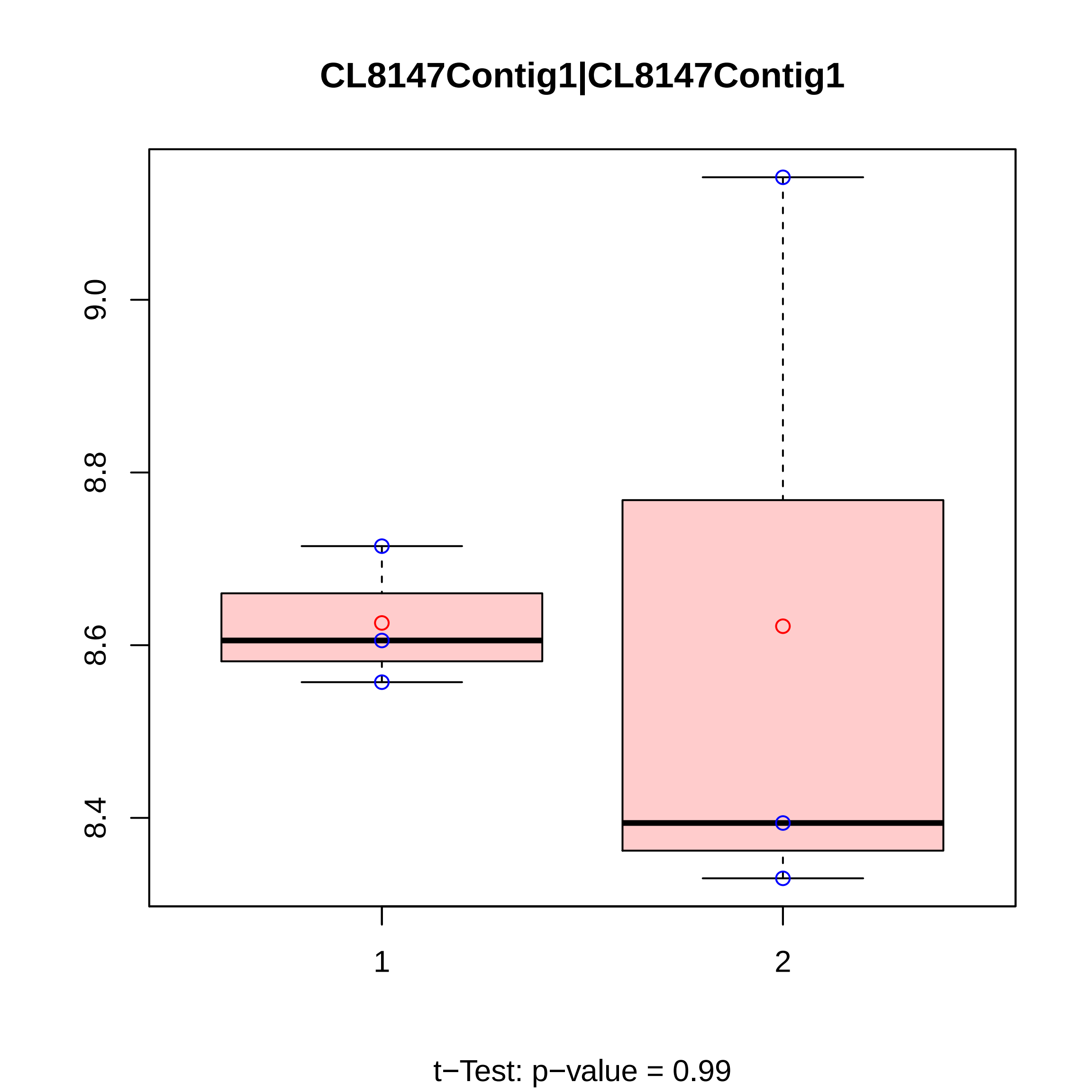

CL8147Contig1|CL8147Contig1
9.0
8.8
8.6
8.4
1
2
t−Test: p−value = 0.99

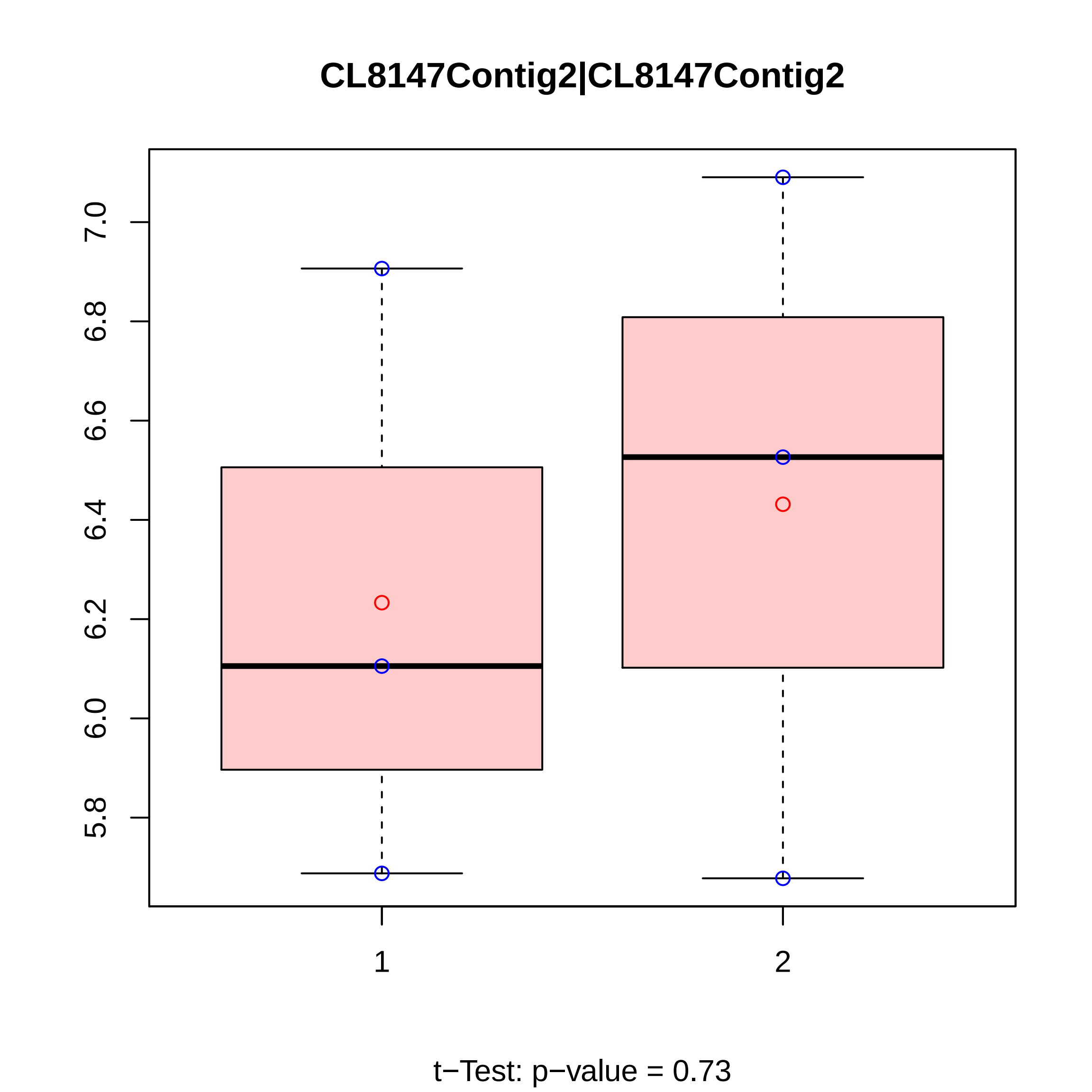
CL8147Contig2|CL8147Contig2
7.0
6.8
6.6
6.4
6.2
6.0
5.8
1
2
t−Test: p−value = 0.73

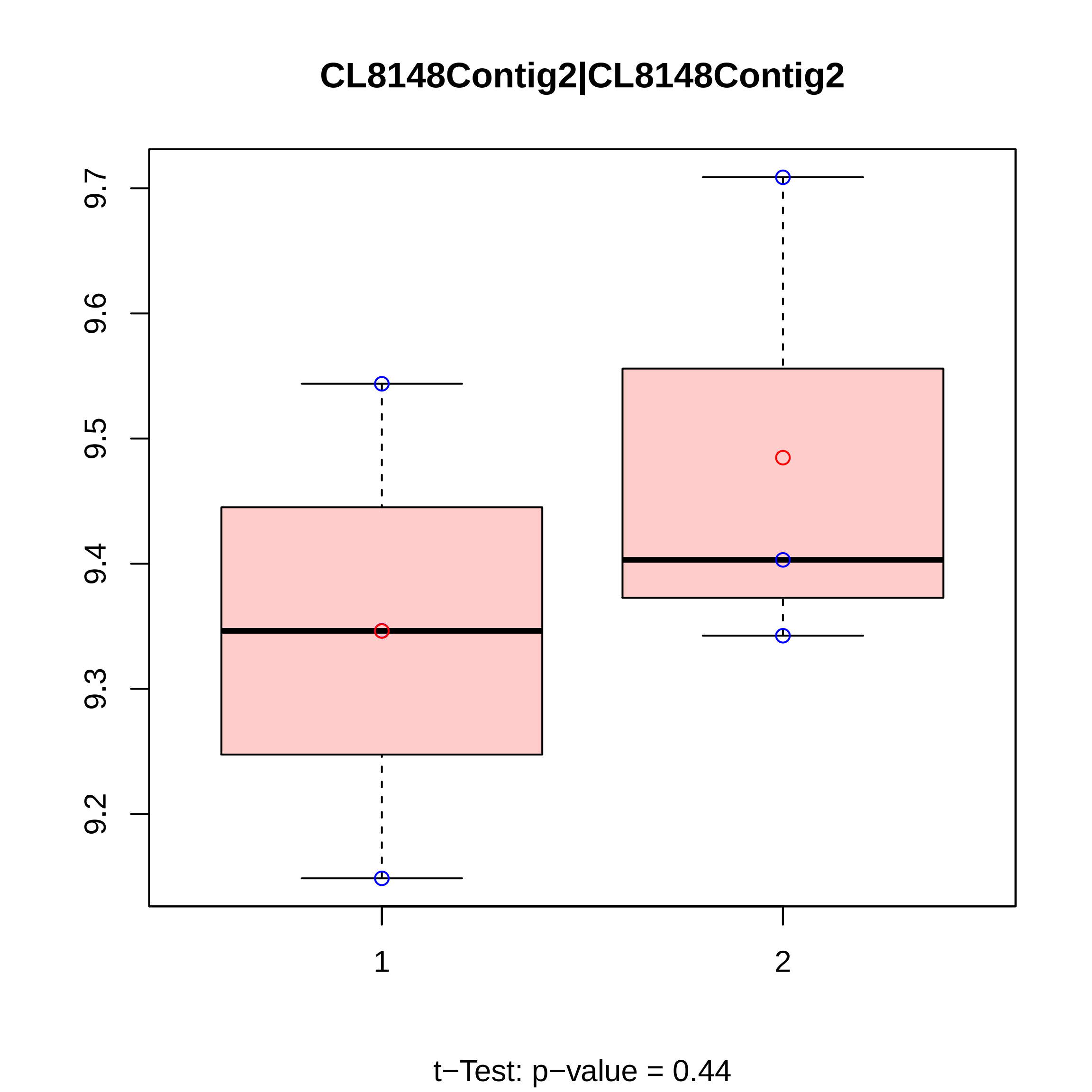

CL8148Contig2|CL8148Contig2
9.7
9.6
9.5
9.4
9.3
9.2
1
2
t−Test: p−value = 0.44

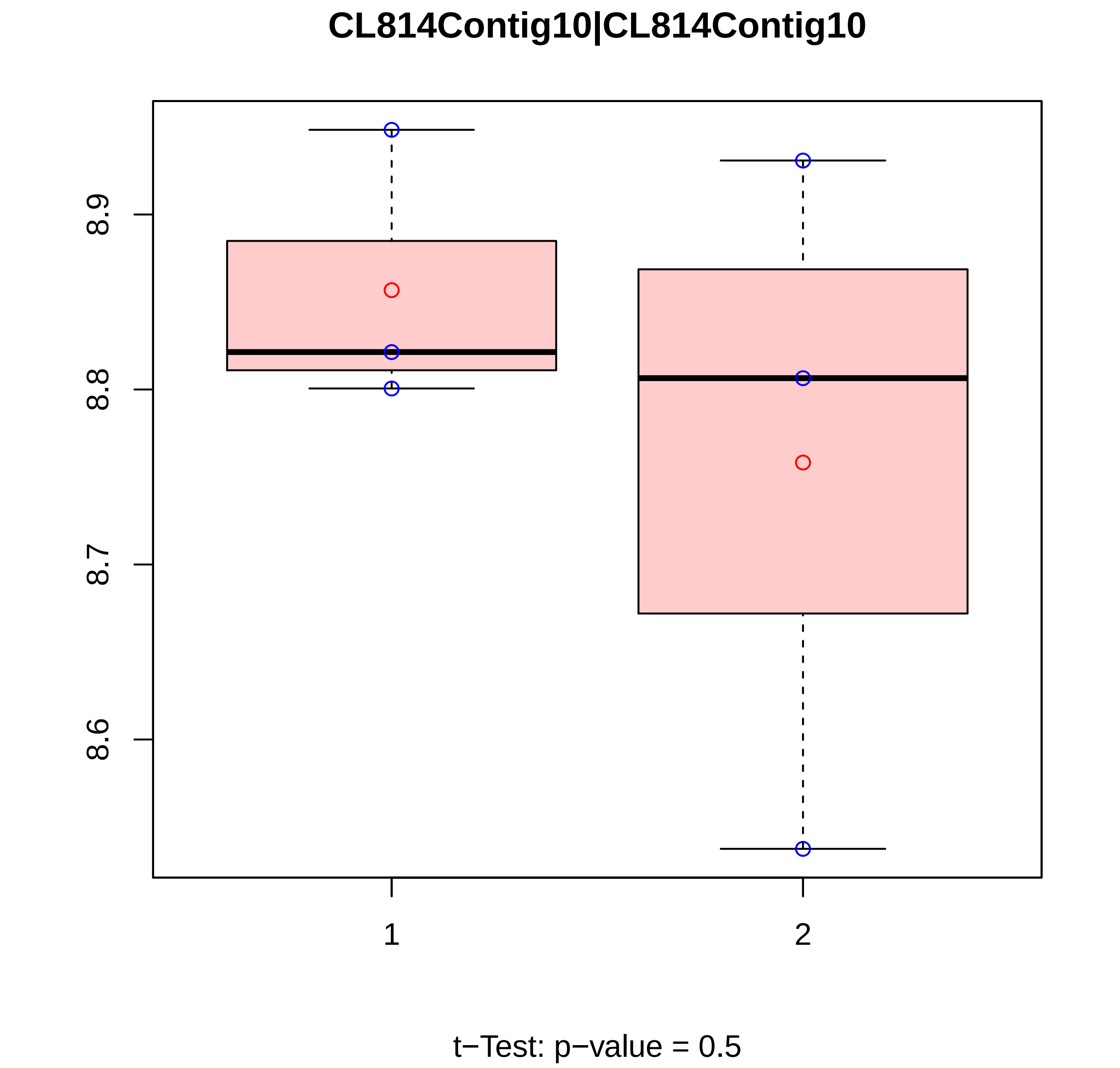
CL814Contig10|CL814Contig10
8.6
8.7
8.8
8.9
1
2
t−Test: p−value = 0.5

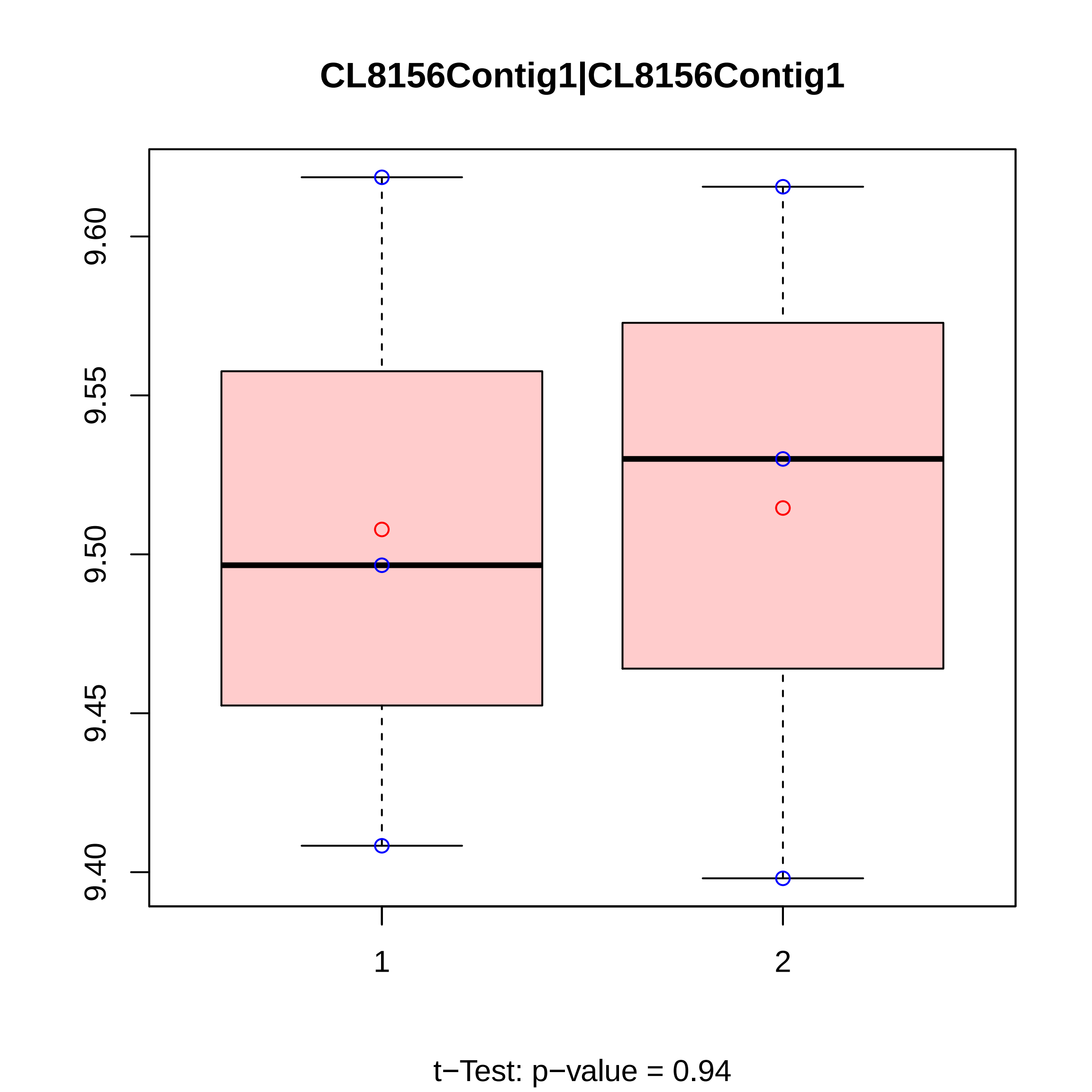

CL8156Contig1|CL8156Contig1
9.40
9.45
9.50
9.55
9.60
1
2
t−Test: p−value = 0.94

# CL8162Contig6|CL8162Contig6

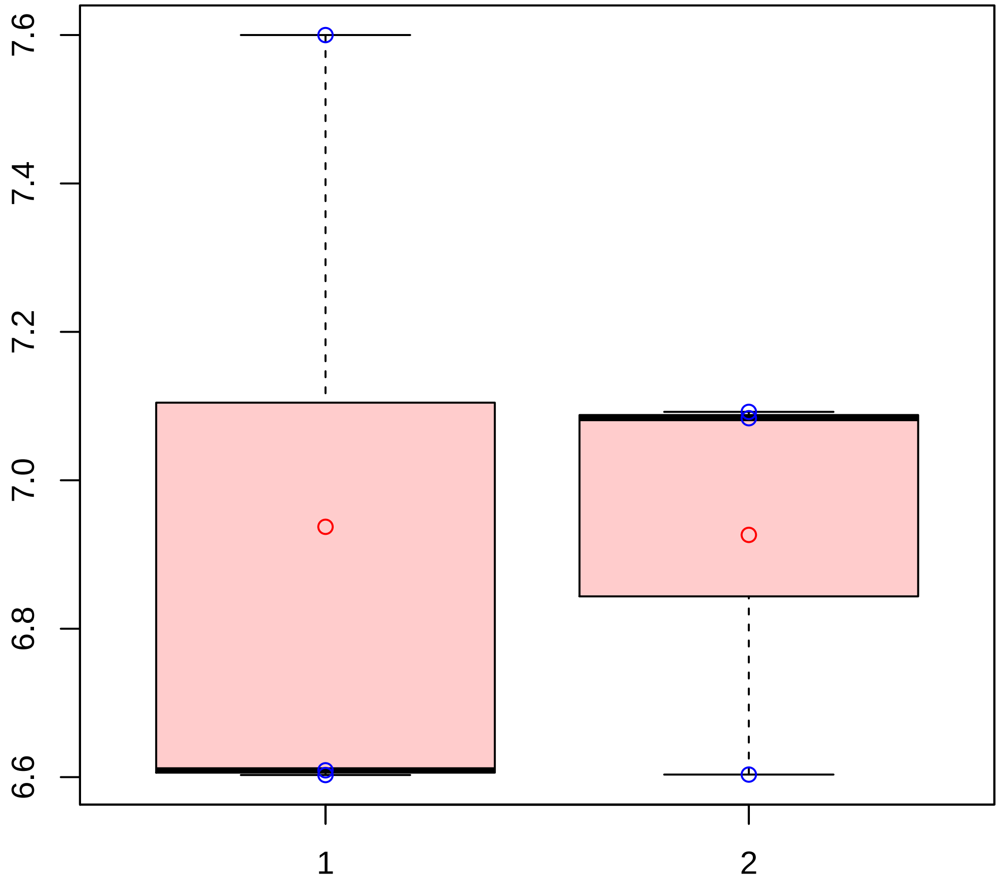


t−Test: p−value = 0.98

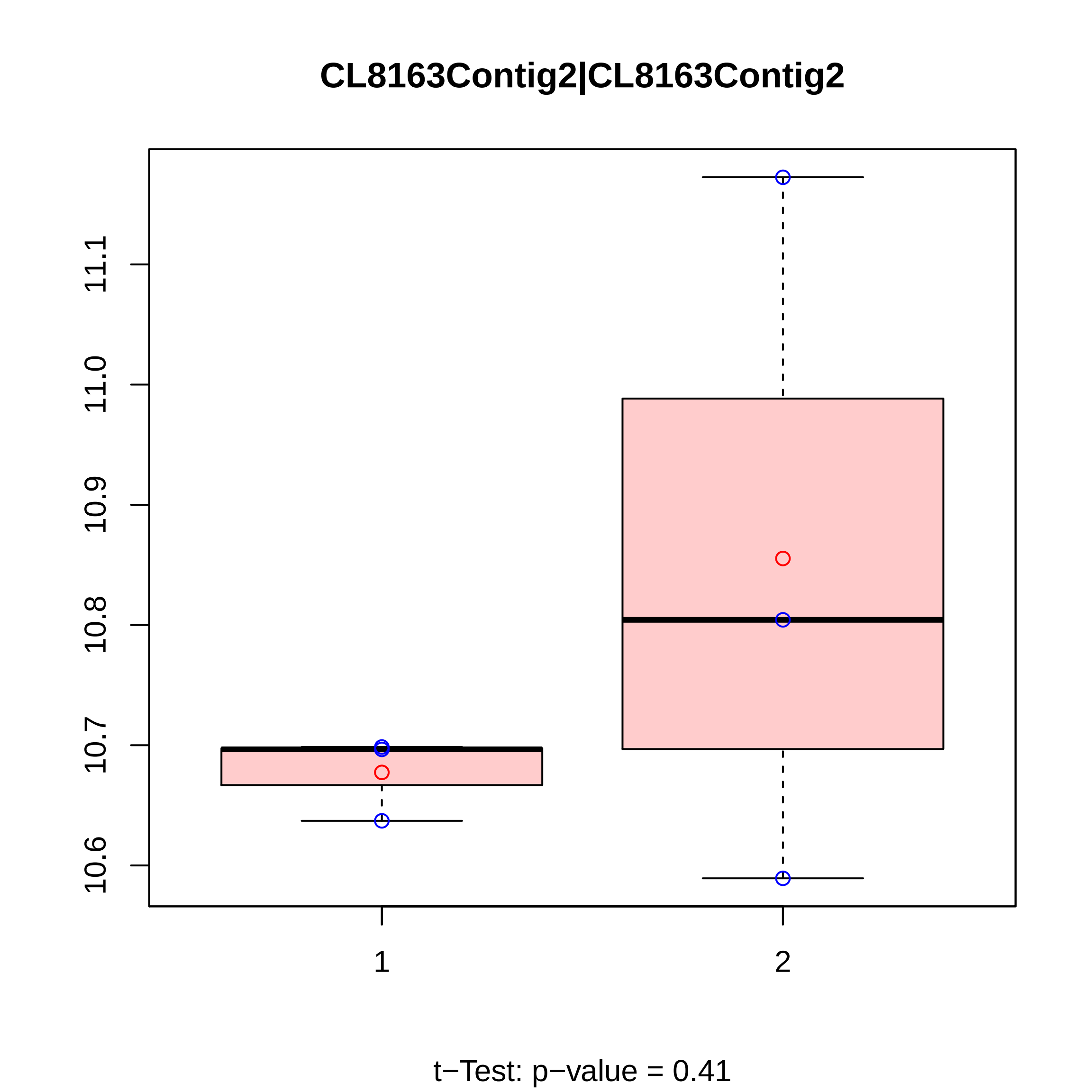

CL8163Contig2|CL8163Contig2
10.6
10.7
10.8
10.9
11.0
11.1
1
2
t−Test: p−value = 0.41

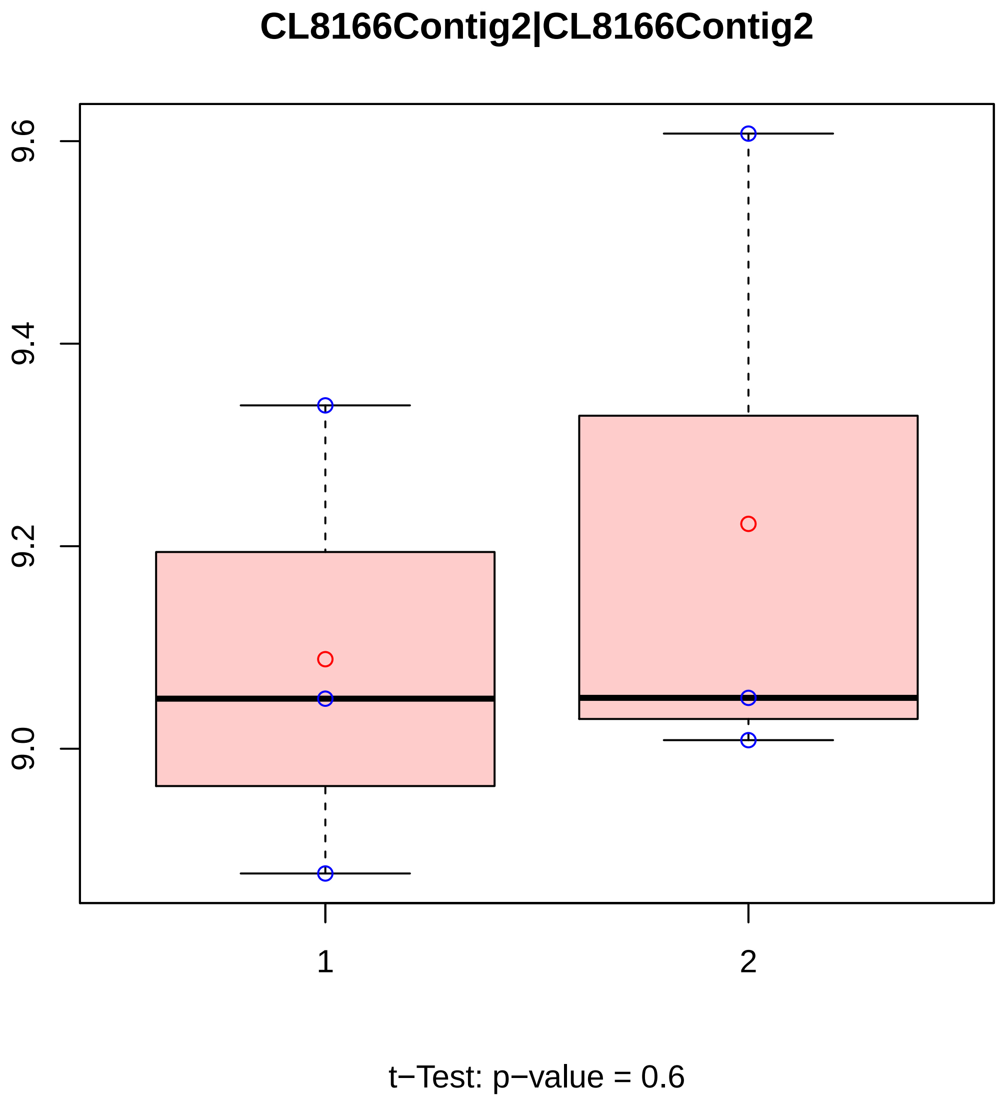
CL8166Contig2|CL8166Contig2
9.0
9.2
9.4
9.6
1
2
t−Test: p−value = 0.6

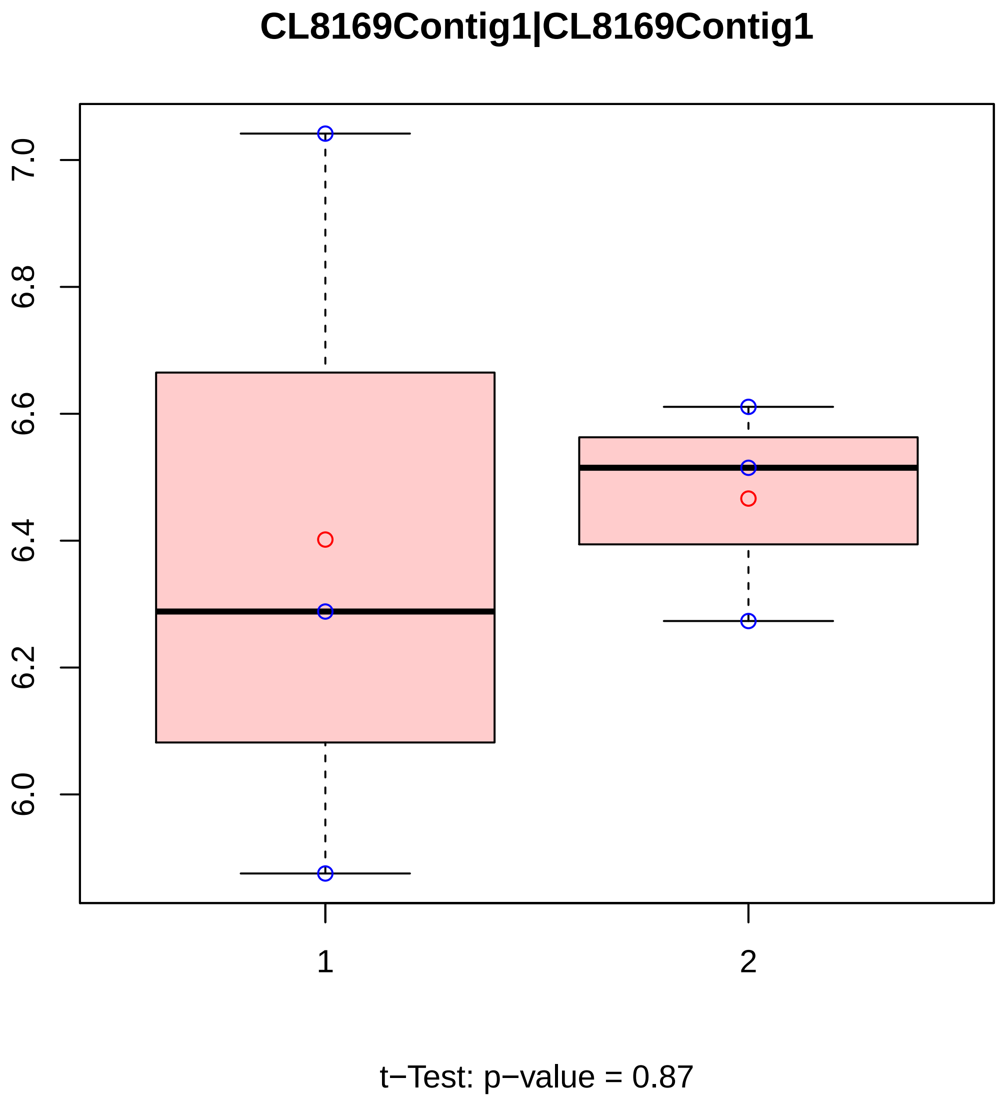
CL8169Contig1|CL8169Contig1
7.0
6.8
6.6
6.4
6.2
6.0
1
2
t−Test: p−value = 0.87

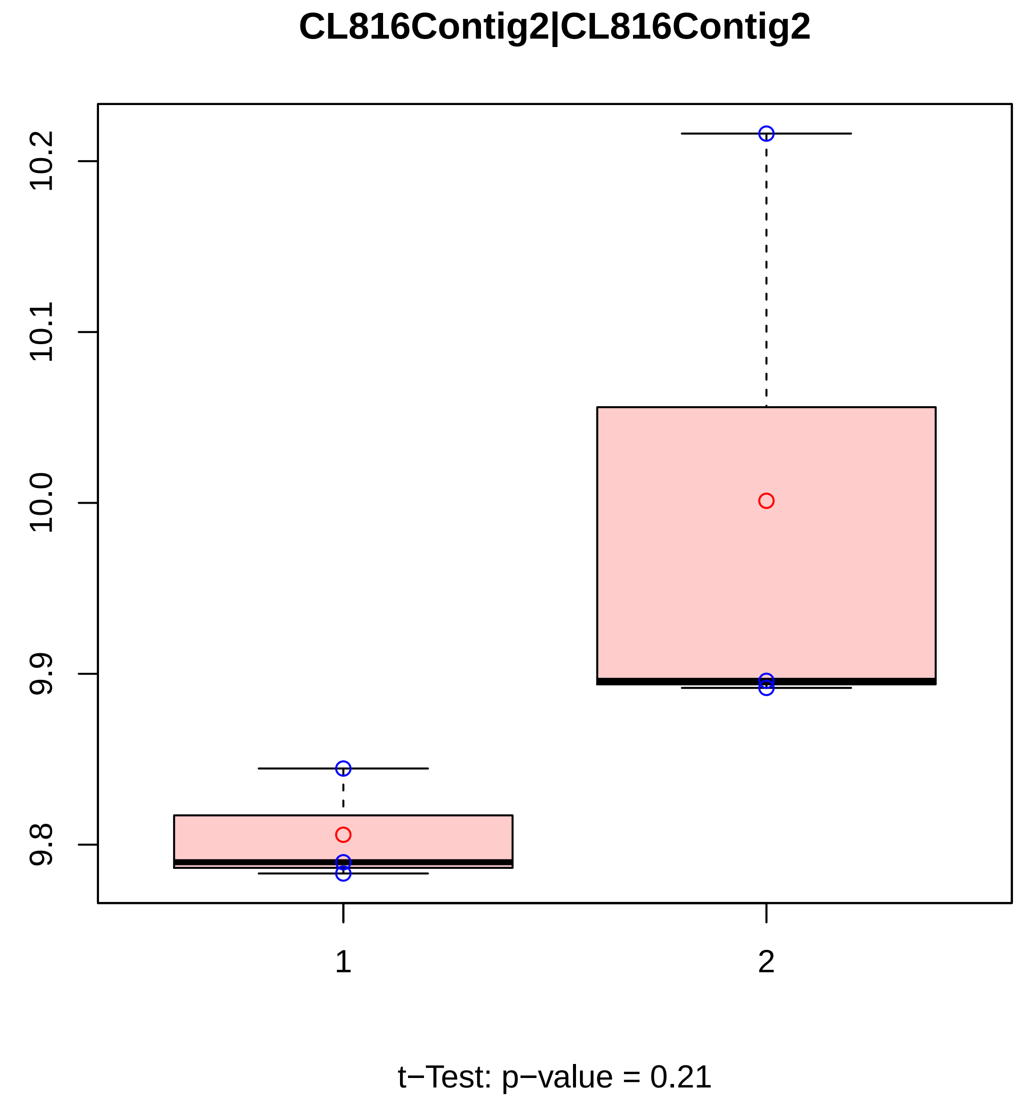
CL816Contig2|CL816Contig2
10.2
10.1
10.0
9.9
9.8
1
2
t−Test: p−value = 0.21

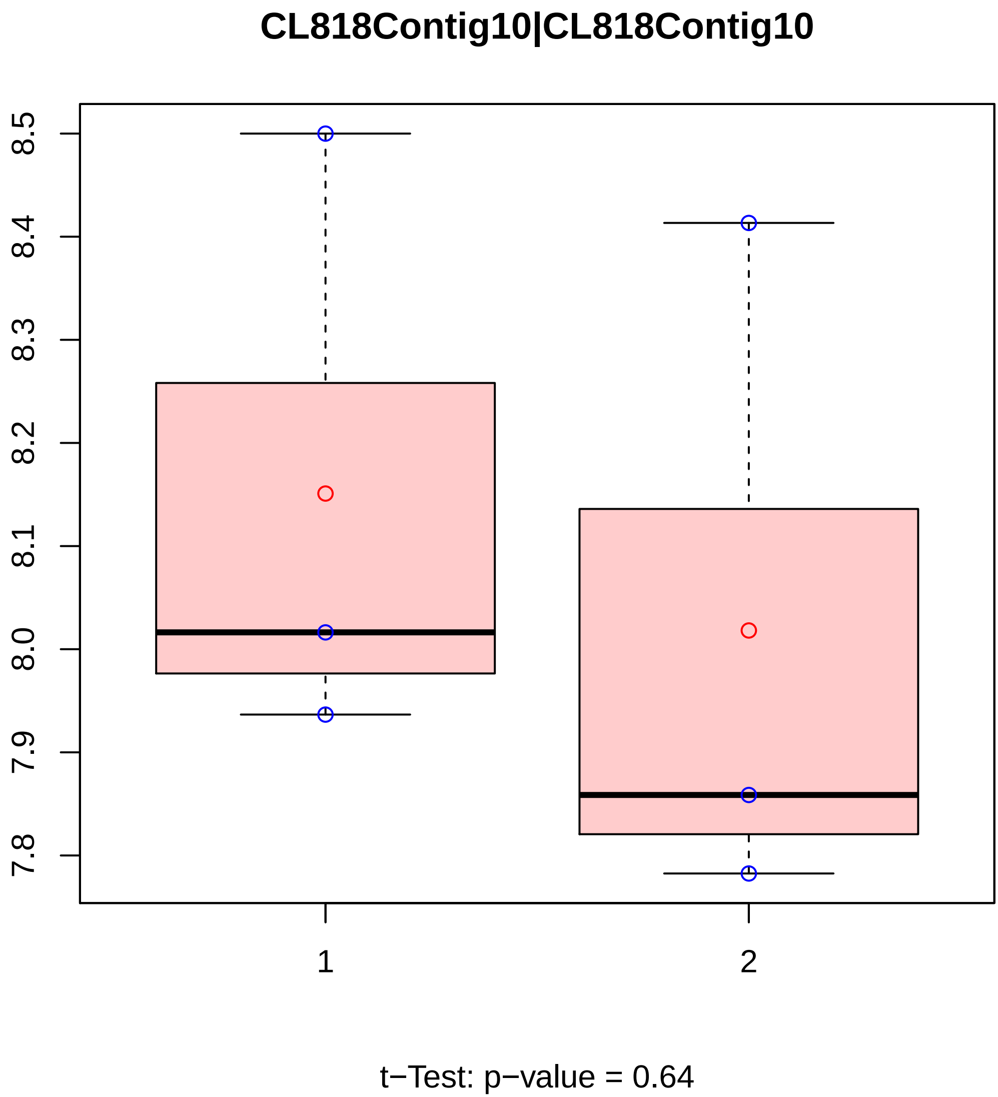

CL818Contig10|CL818Contig10
8.5
8.4
8.3
8.2
8.1
8.0
7.9
7.8
1
2
t−Test: p−value = 0.64

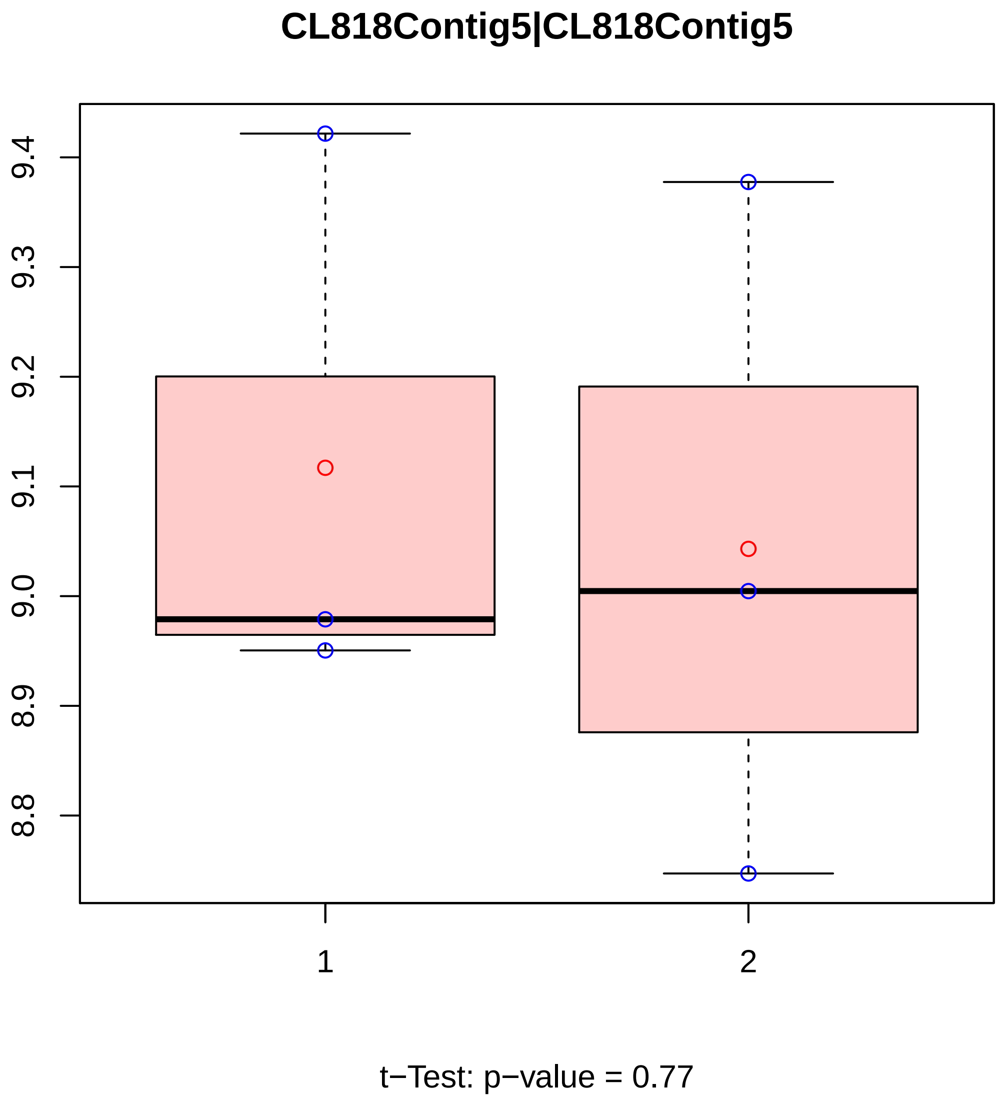

CL818Contig5|CL818Contig5
9.4
9.3
9.2
9.1
9.0
8.9
8.8
1
2
t−Test: p−value = 0.77

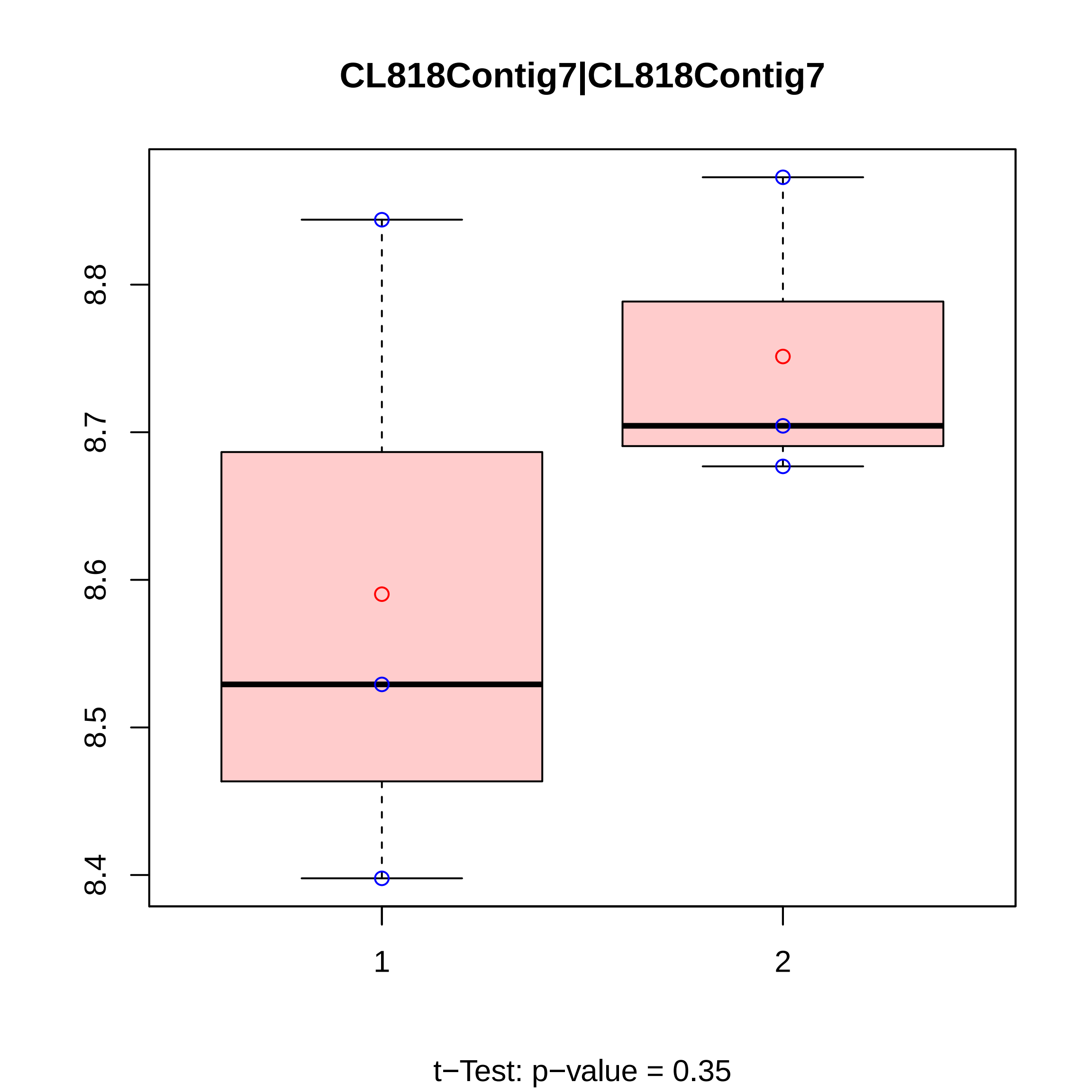

CL818Contig7|CL818Contig7
8.4
8.5
8.6
8.7
8.8
1
2
t−Test: p−value = 0.35

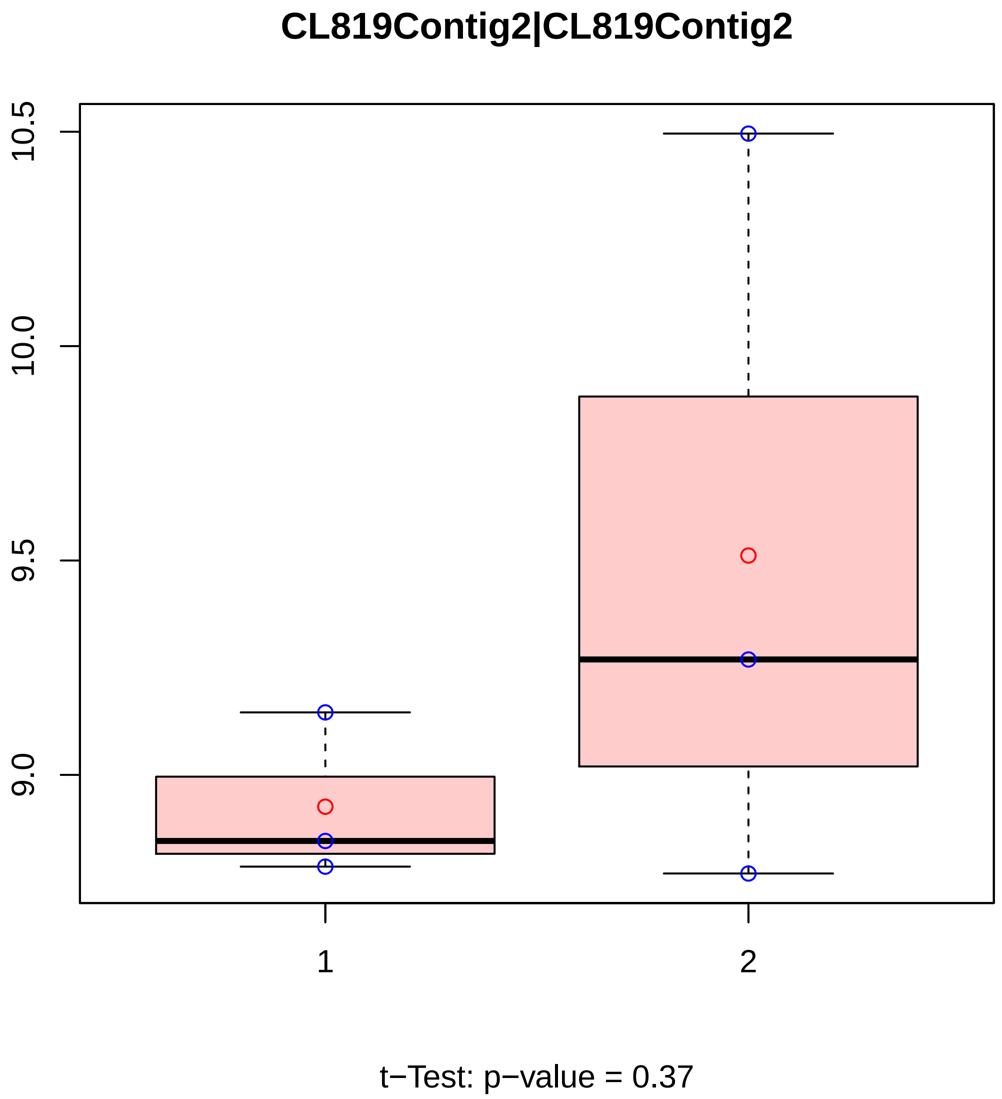

CL819Contig2|CL819Contig2
10.5
10.0
9.5
9.0
1
2
t−Test: p−value = 0.37

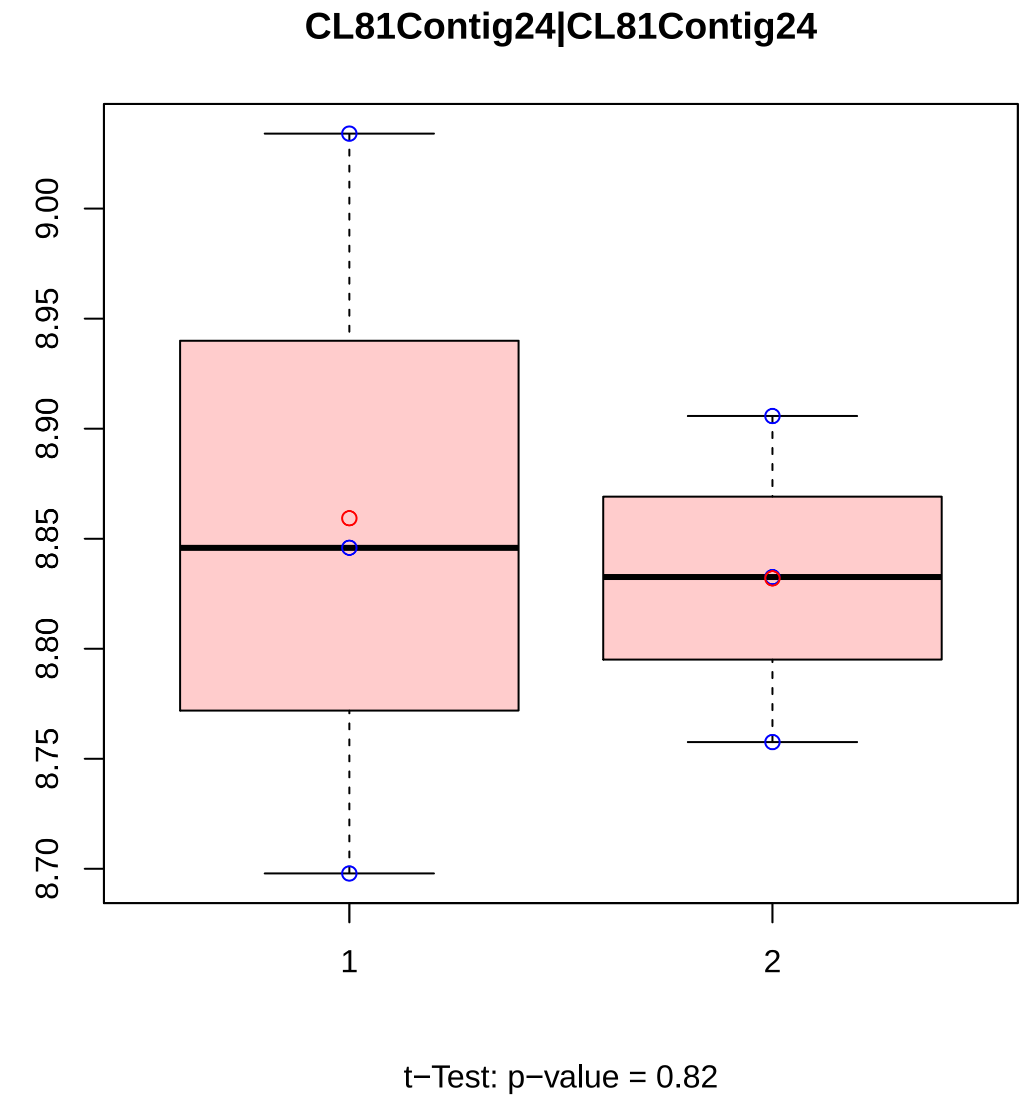

CL81Contig24|CL81Contig24
9.00
8.95
8.90
8.85
8.80
8.75
8.70
1
2
t−Test: p−value = 0.82

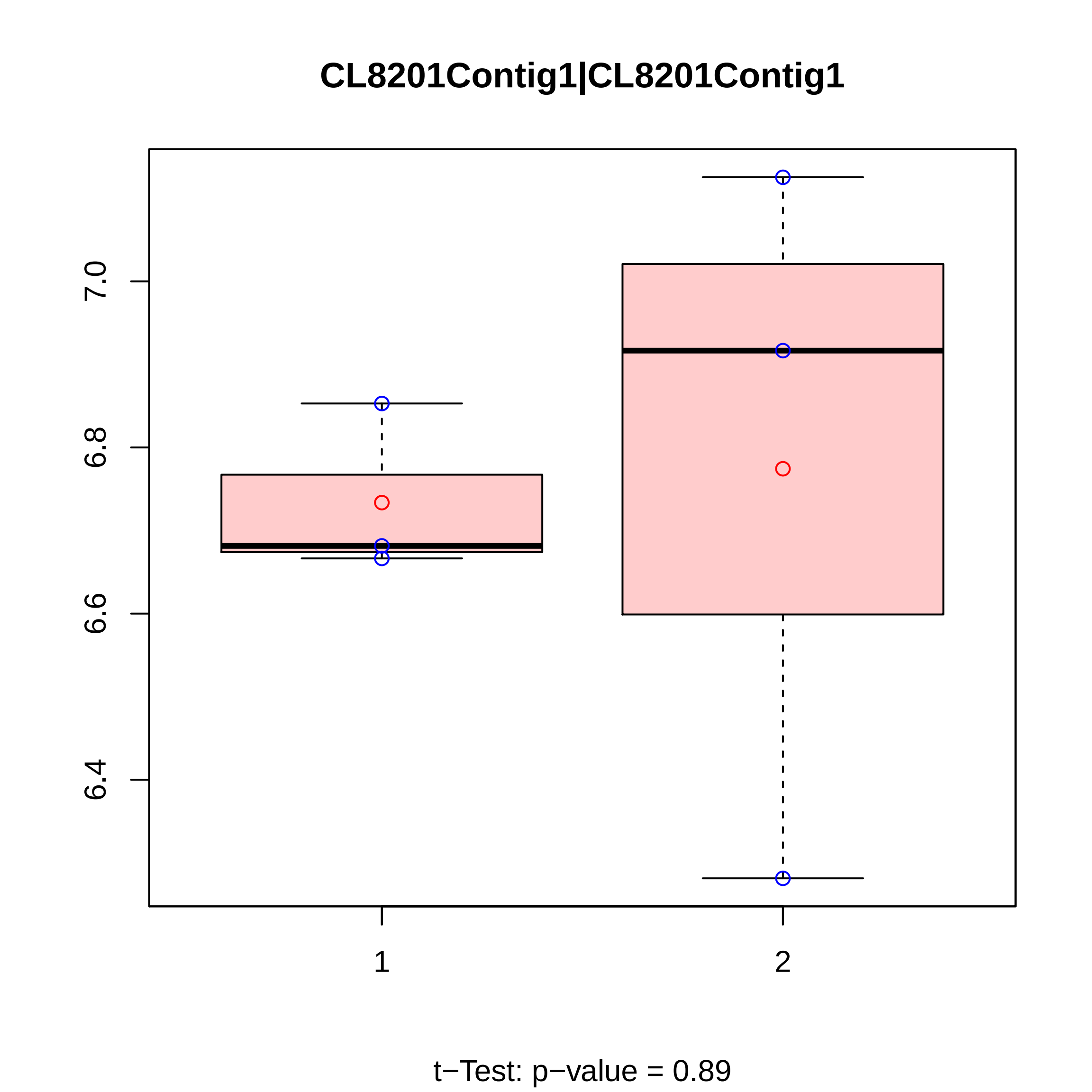
CL8201Contig1|CL8201Contig1
7.0
6.8
6.6
6.4
1
2
t−Test: p−value = 0.89

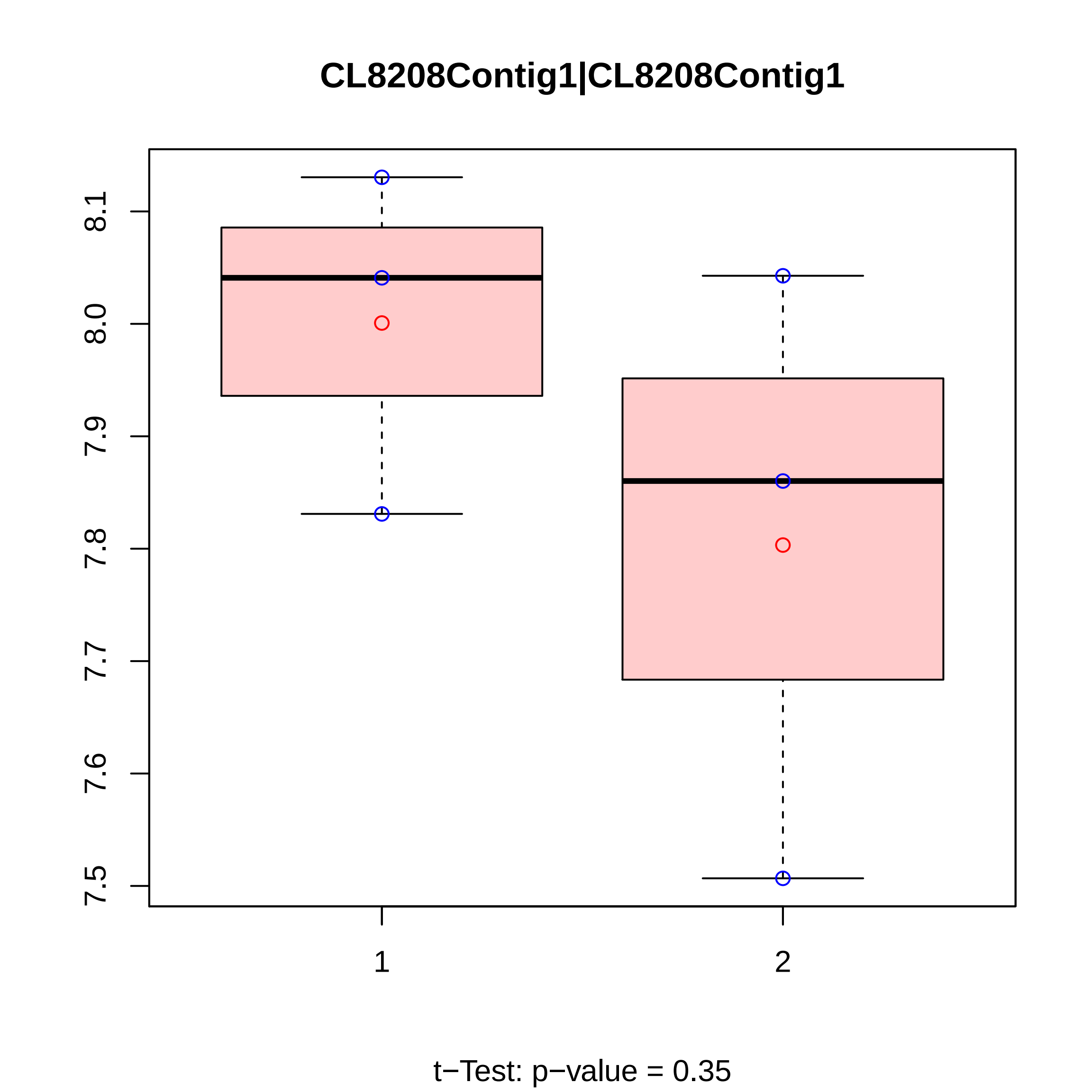
CL8208Contig1|CL8208Contig1
8.1
8.0
7.9
7.8
7.7
7.6
7.5
1
2
t−Test: p−value = 0.35

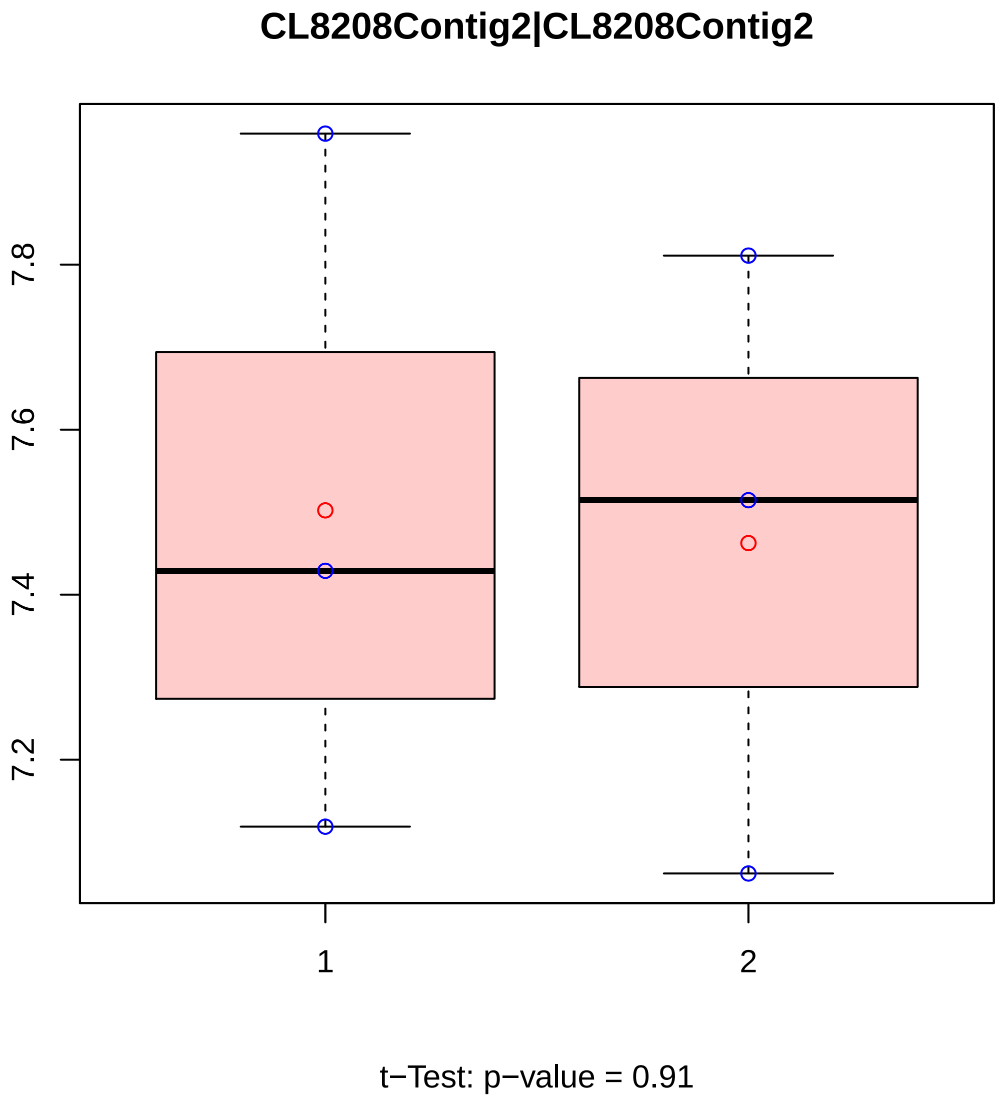
CL8208Contig2|CL8208Contig2
7.2
7.4
7.6
7.8
1
2
t−Test: p−value = 0.91

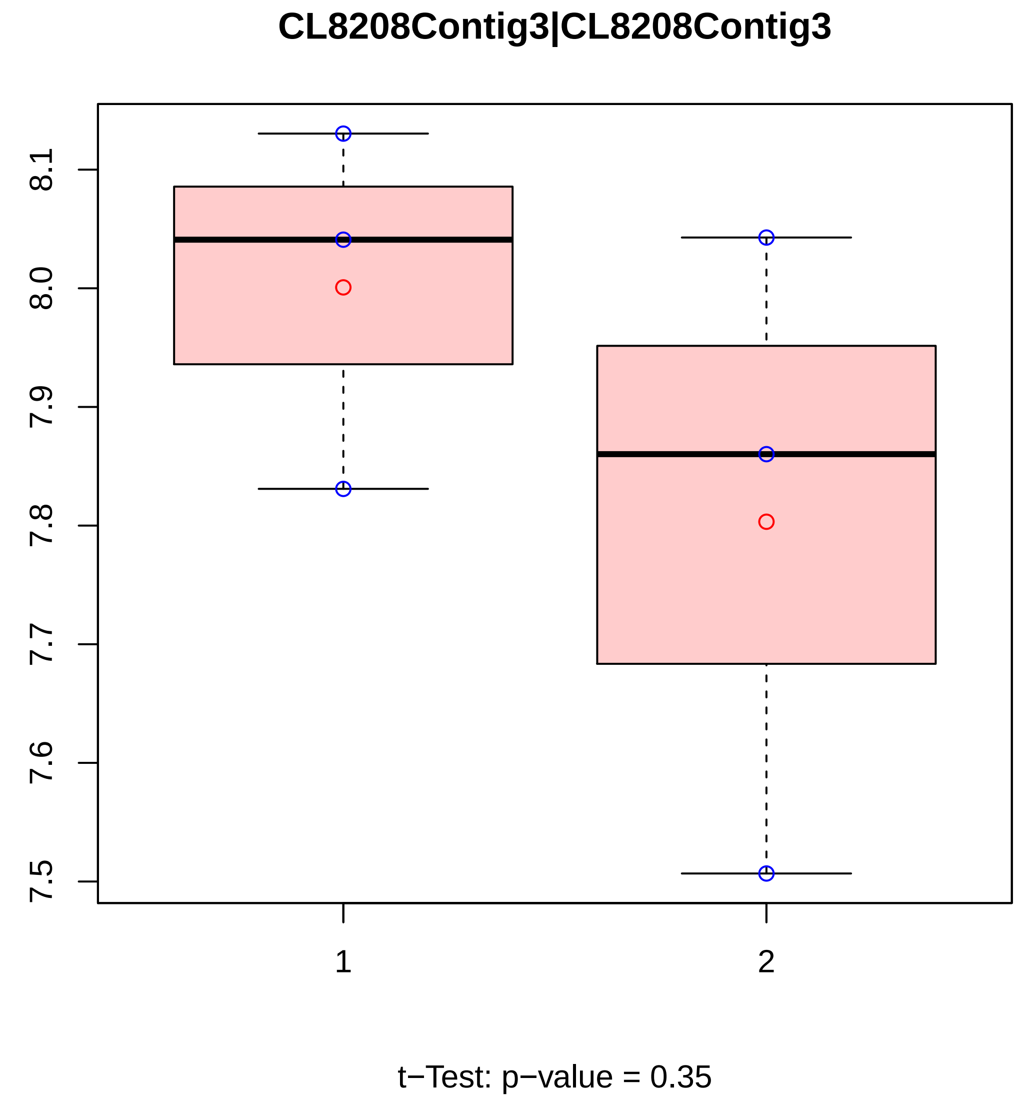
CL8208Contig3|CL8208Contig3
8.1
8.0
7.9
7.8
7.7
7.6
7.5
1
2
t−Test: p−value = 0.35

## CL820Contig1|CL820Contig1

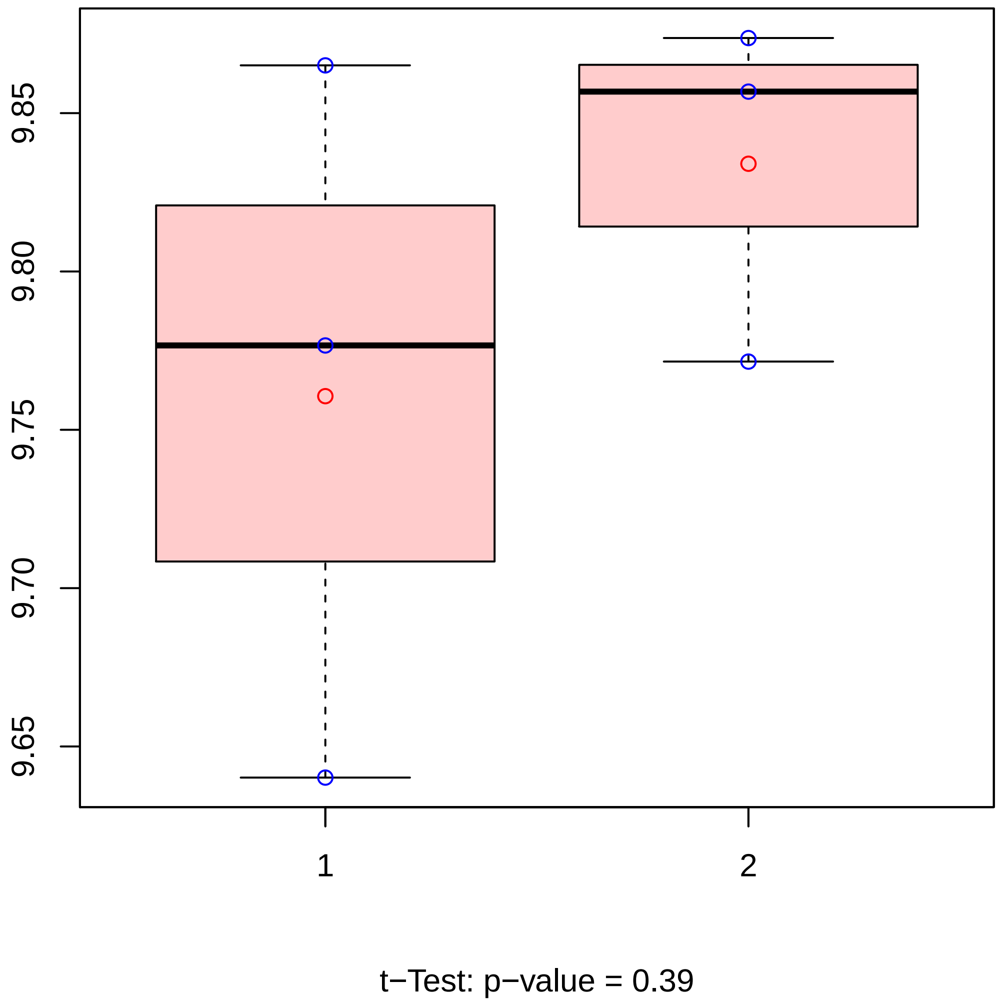

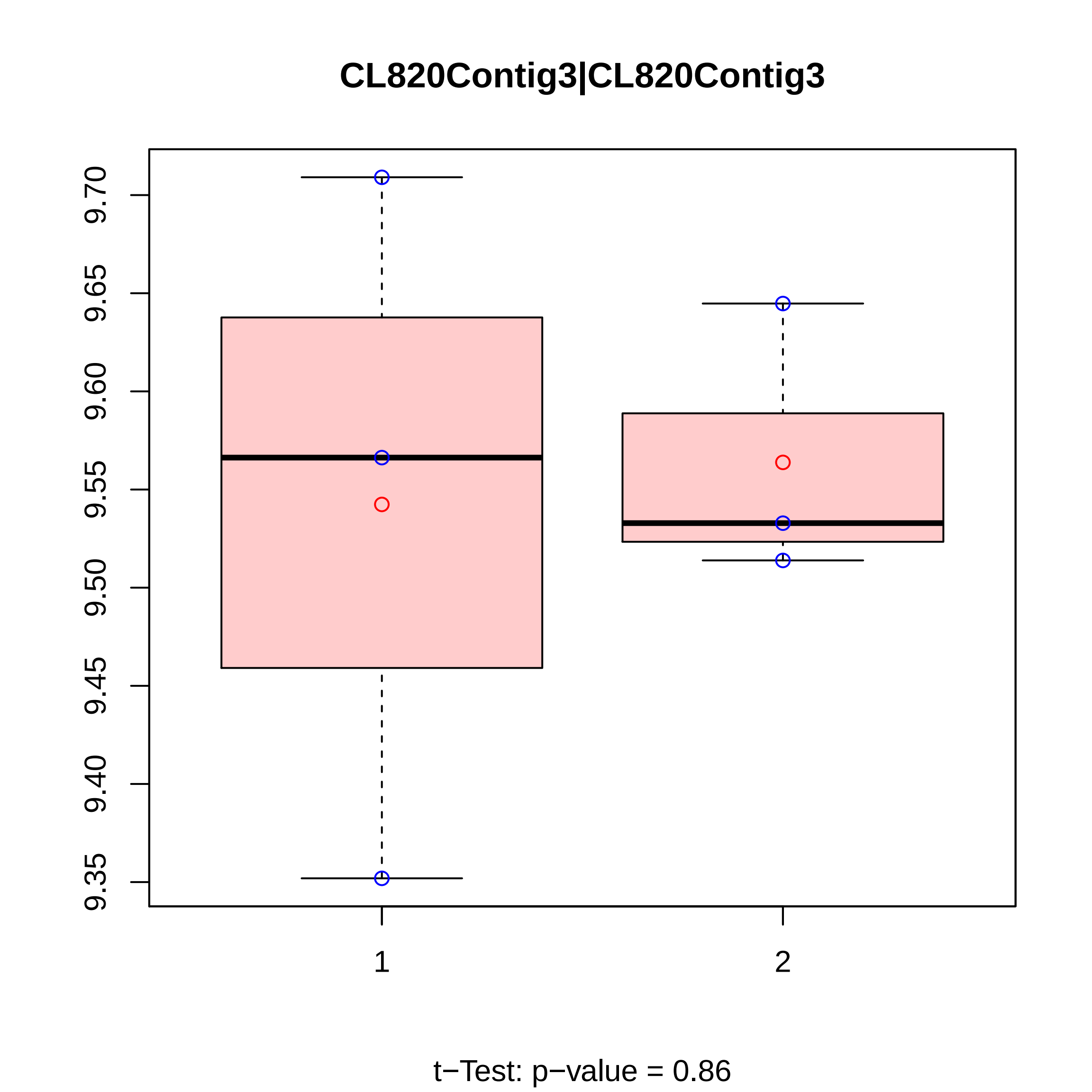

CL820Contig3|CL820Contig3
9.35
9.40
9.45
9.50
9.55
9.60
9.65
9.70
1
2
t−Test: p−value = 0.86

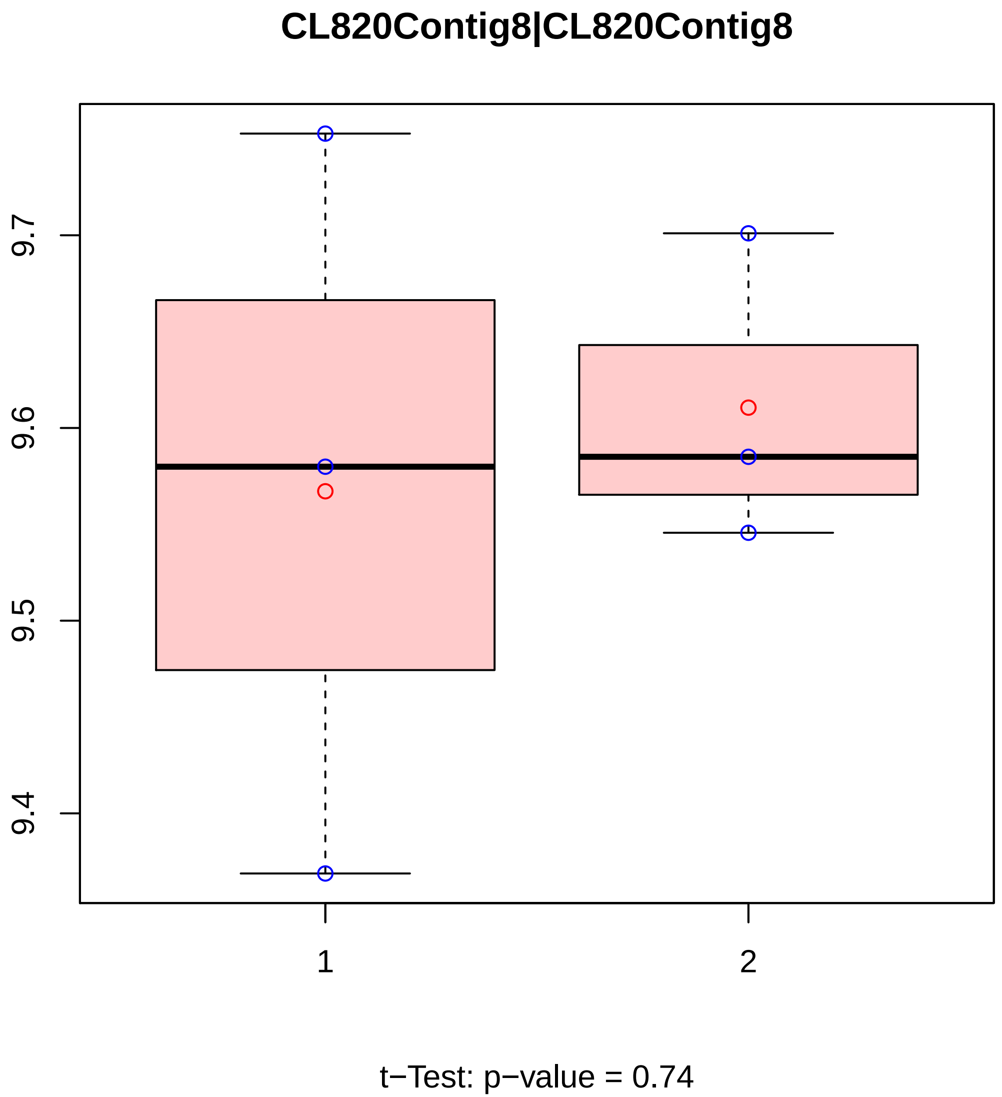
CL820Contig8|CL820Contig8
9.4
9.5
9.6
9.7
1
2
t−Test: p−value = 0.74

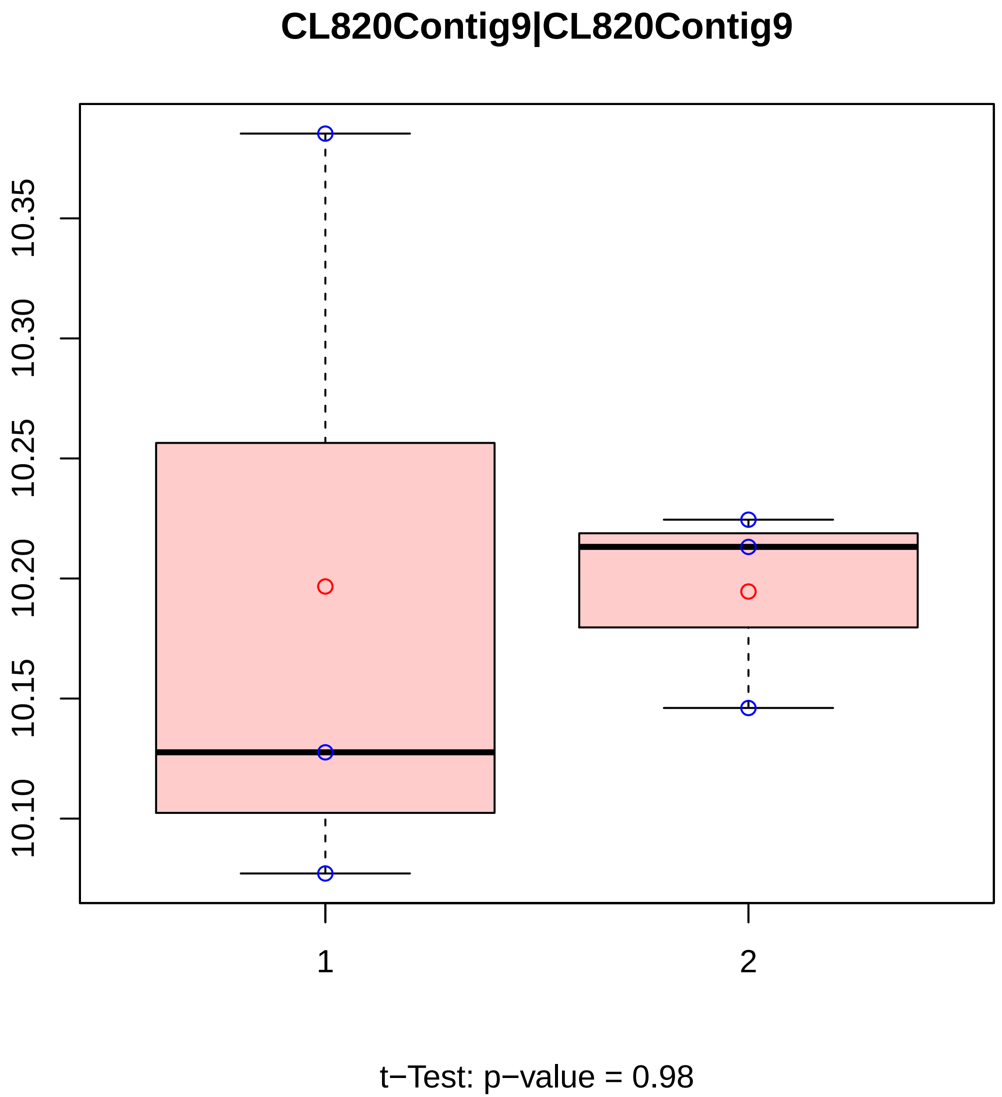

CL820Contig9|CL820Contig9
10.10
10.15
10.20
10.25
10.30
10.35
1
2
t−Test: p−value = 0.98

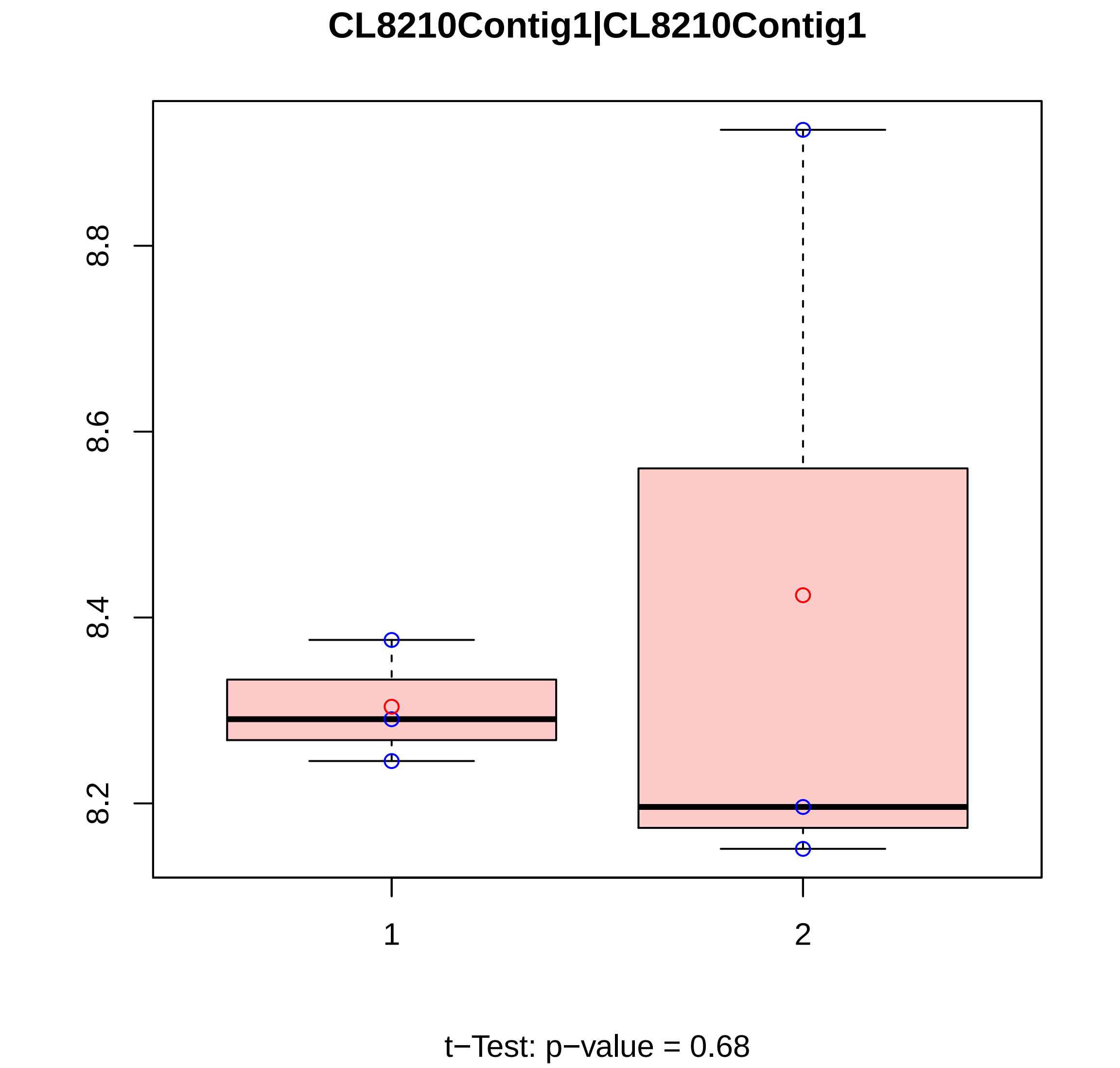

CL8210Contig1|CL8210Contig1
8.2
8.4
8.6
8.8
1
2
t−Test: p−value = 0.68

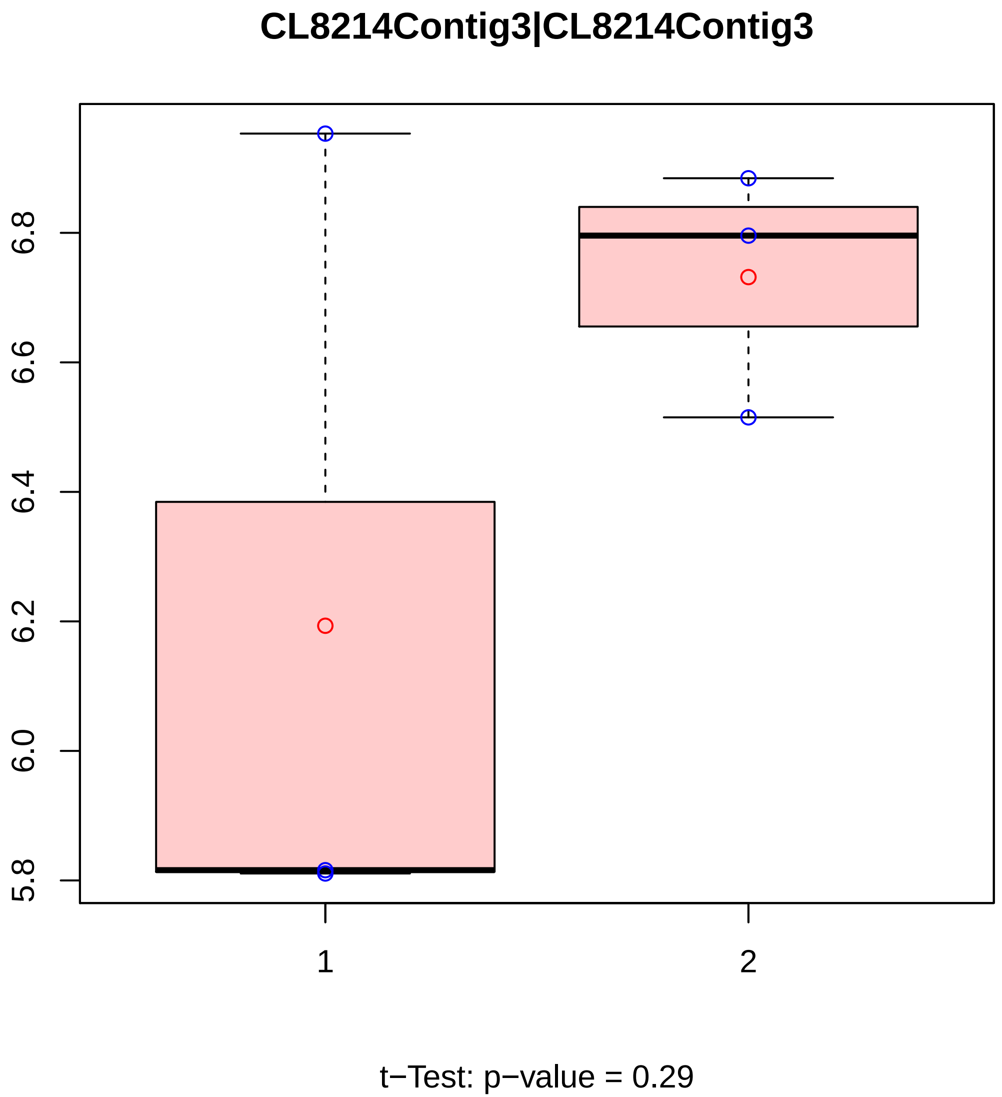
CL8214Contig3|CL8214Contig3
6.8
6.6
6.4
6.2
6.0
5.8
1
2
t−Test: p−value = 0.29

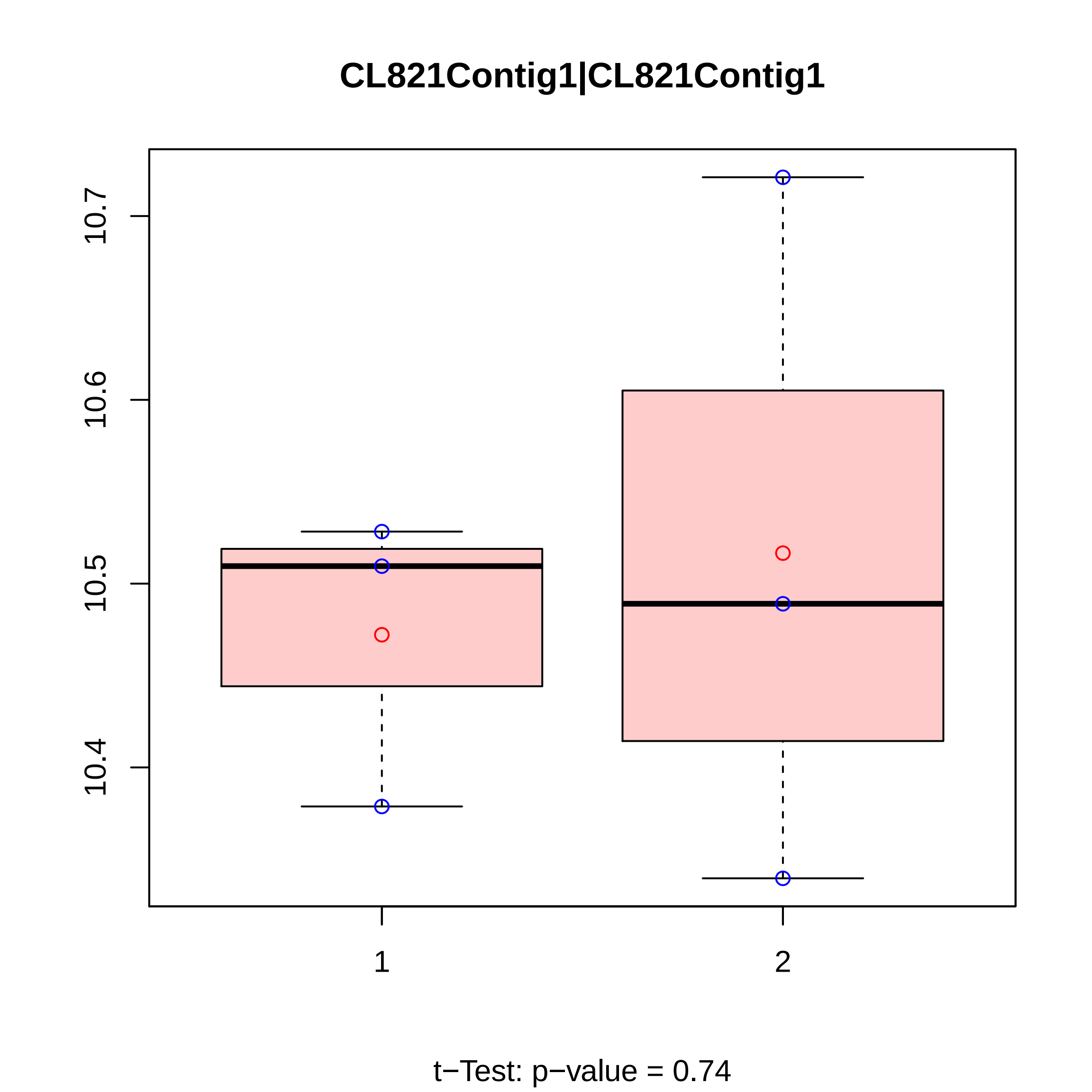
CL821Contig1|CL821Contig1
10.4
10.5
10.6
10.7
1
2
t−Test: p−value = 0.74

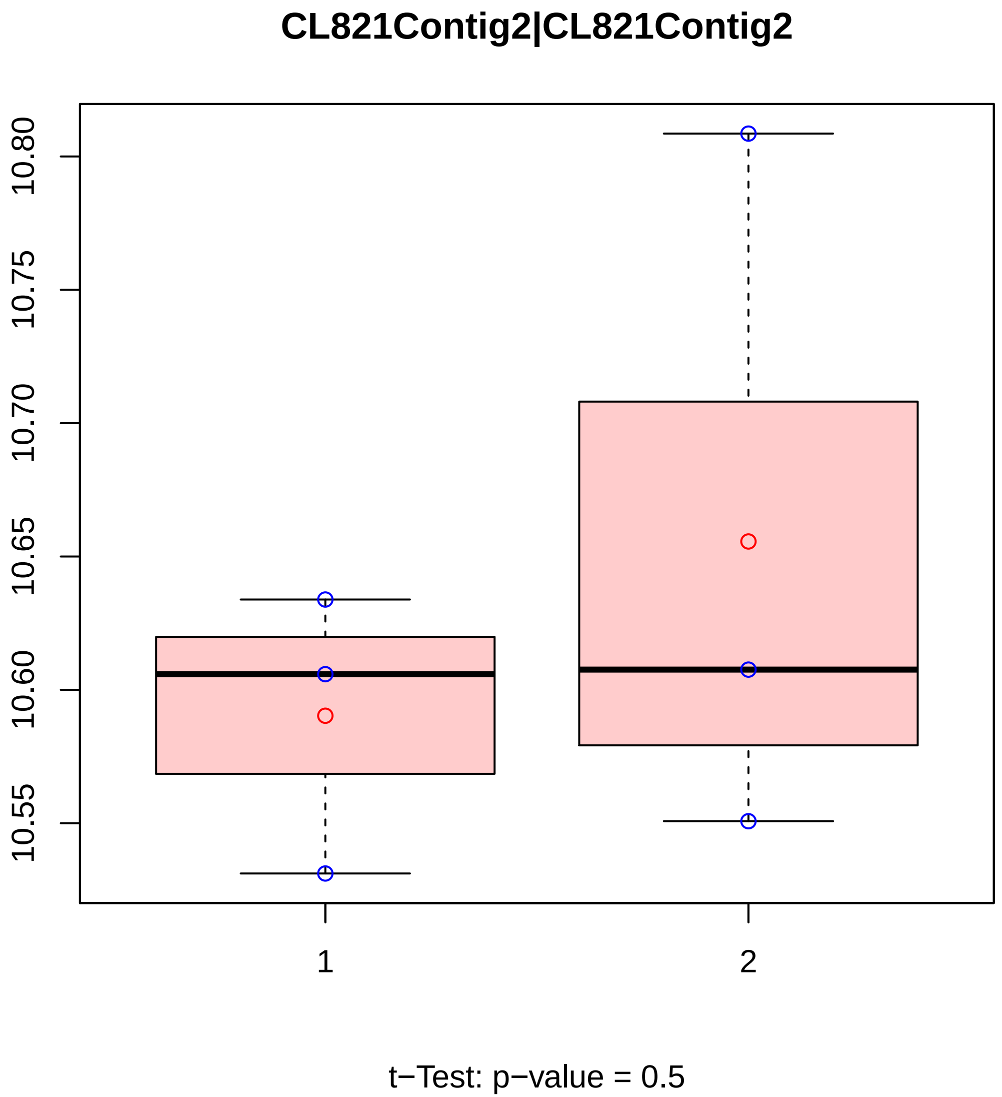
CL821Contig2|CL821Contig2
10.80
10.75
10.70
10.65
10.60
10.55
1
2
t−Test: p−value = 0.5

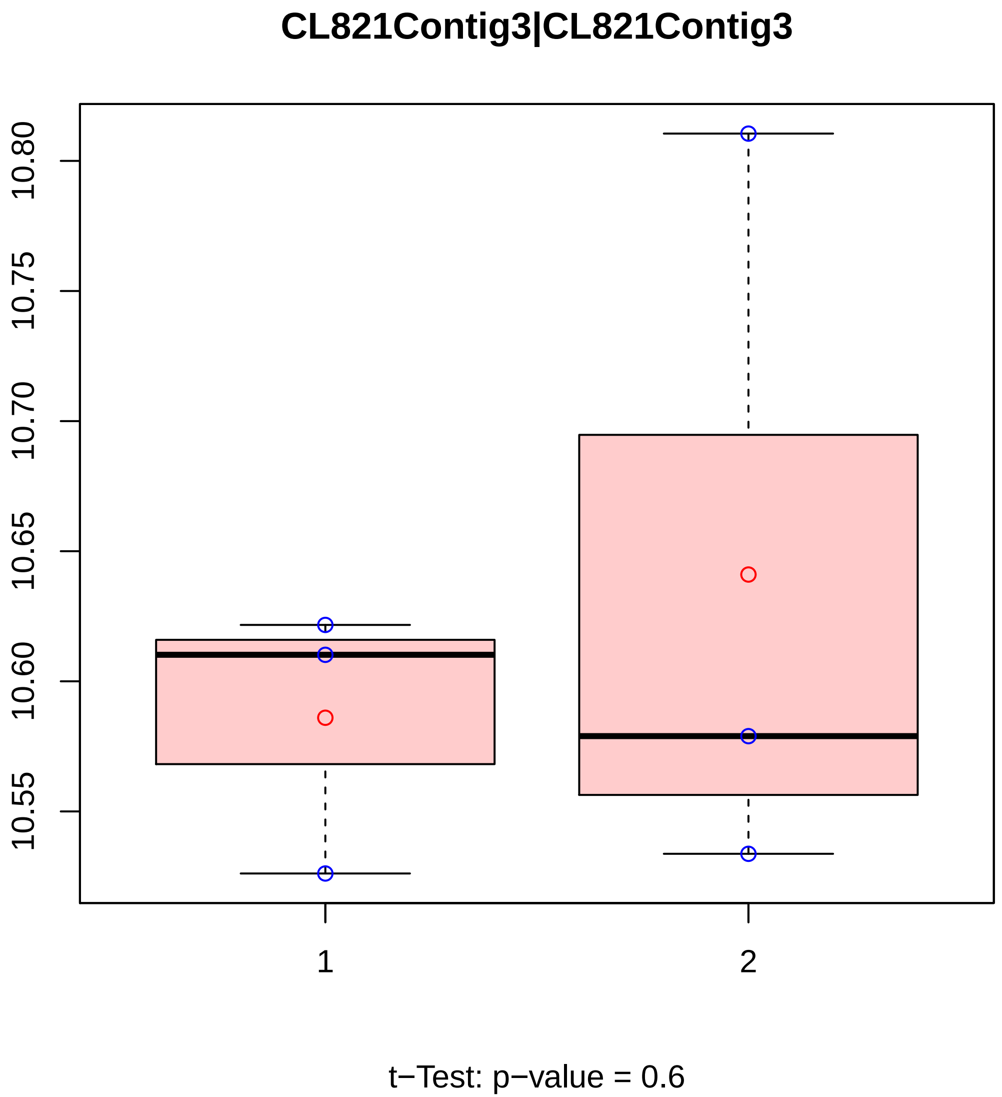

CL821Contig3|CL821Contig3
10.55
10.60
10.65
10.70
10.75
10.80
1
2
t−Test: p−value = 0.6

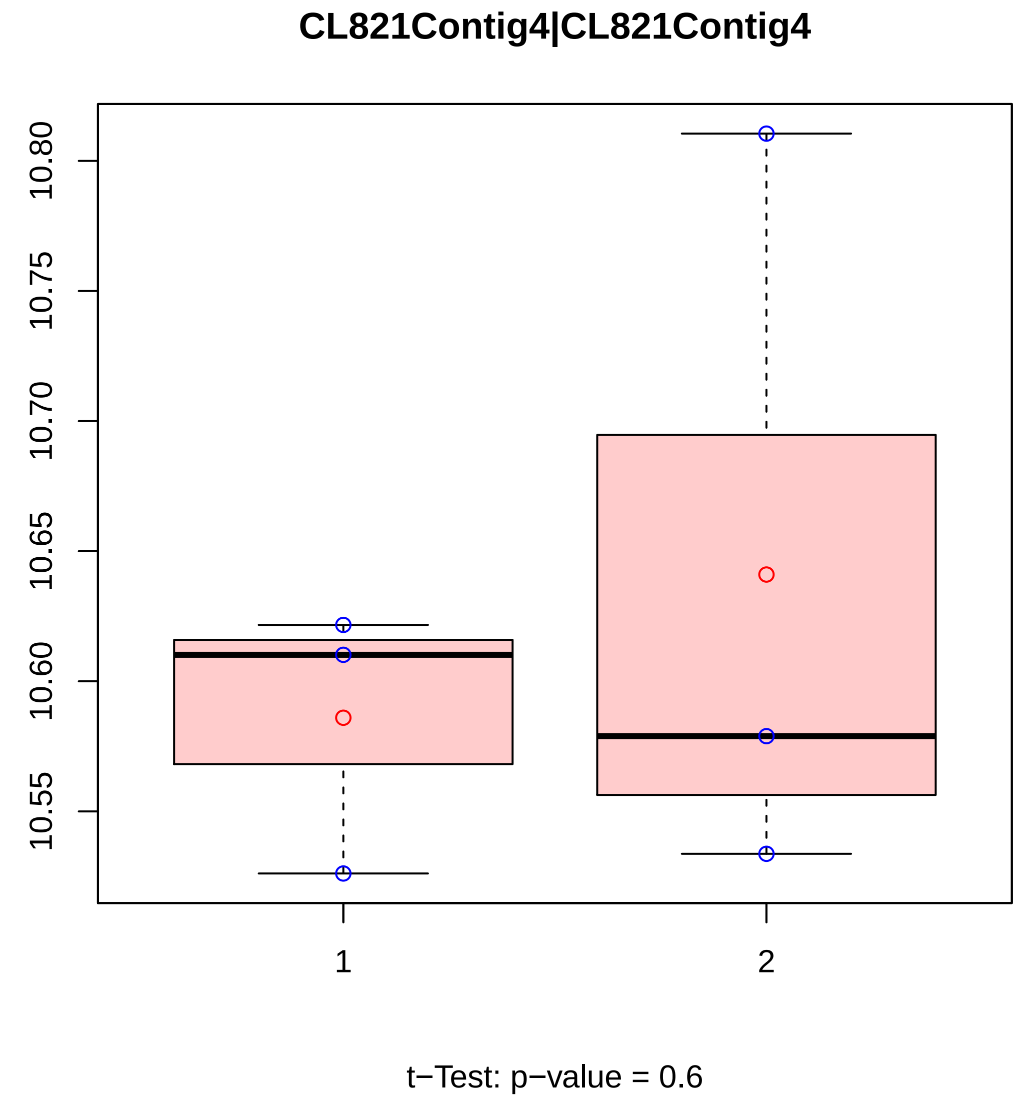

CL821Contig4|CL821Contig4
10.80
10.75
10.70
10.65
10.60
10.55
1
2
t−Test: p−value = 0.6

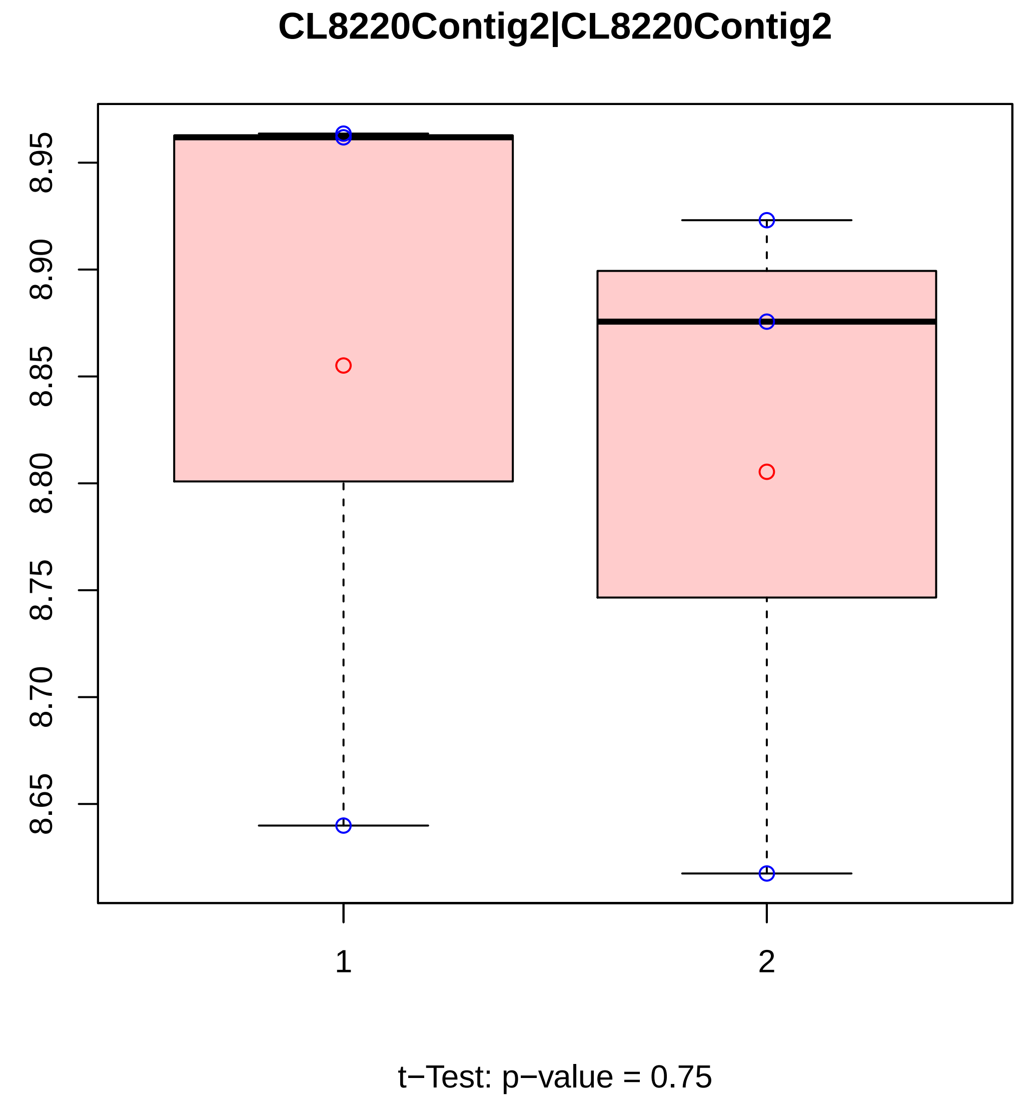
CL8220Contig2|CL8220Contig2
8.65
8.70
8.75
8.80
8.85
8.90
8.95
1
2
t−Test: p−value = 0.75

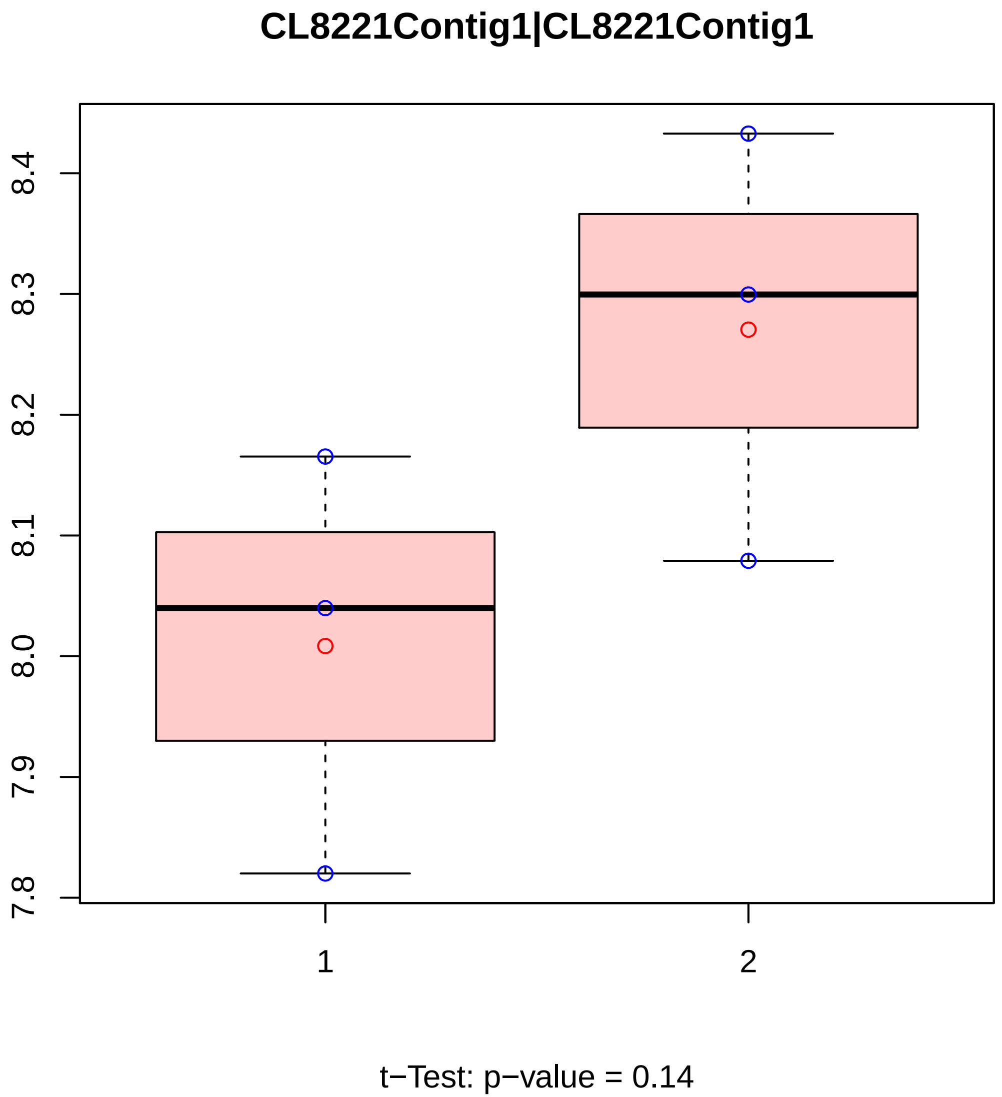

CL8221Contig1|CL8221Contig1
8.4
8.3
8.2
8.1
8.0
7.9
7.8
1
2
t−Test: p−value = 0.14

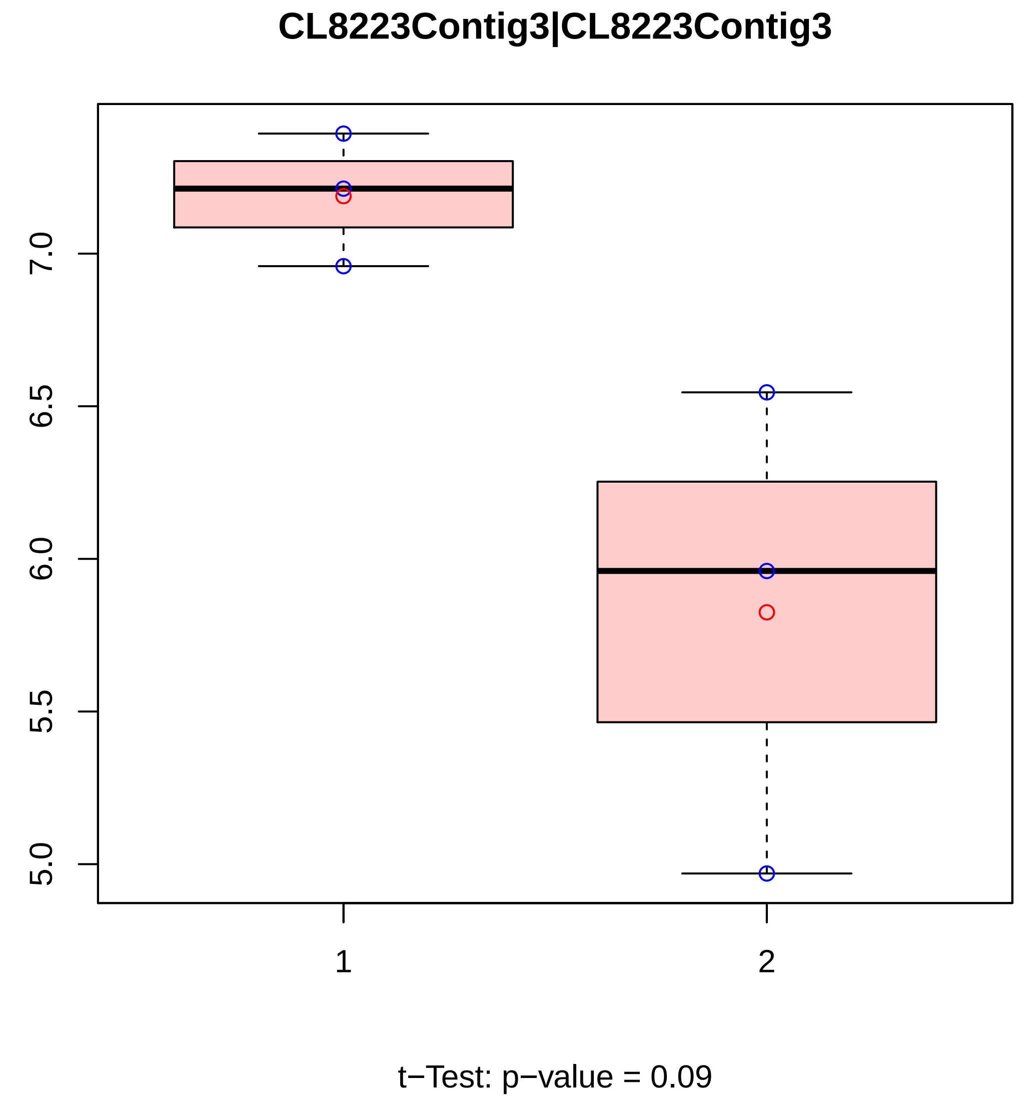

CL8223Contig3|CL8223Contig3
7.0
6.5
6.0
5.5
5.0
1
2
t−Test: p−value = 0.09

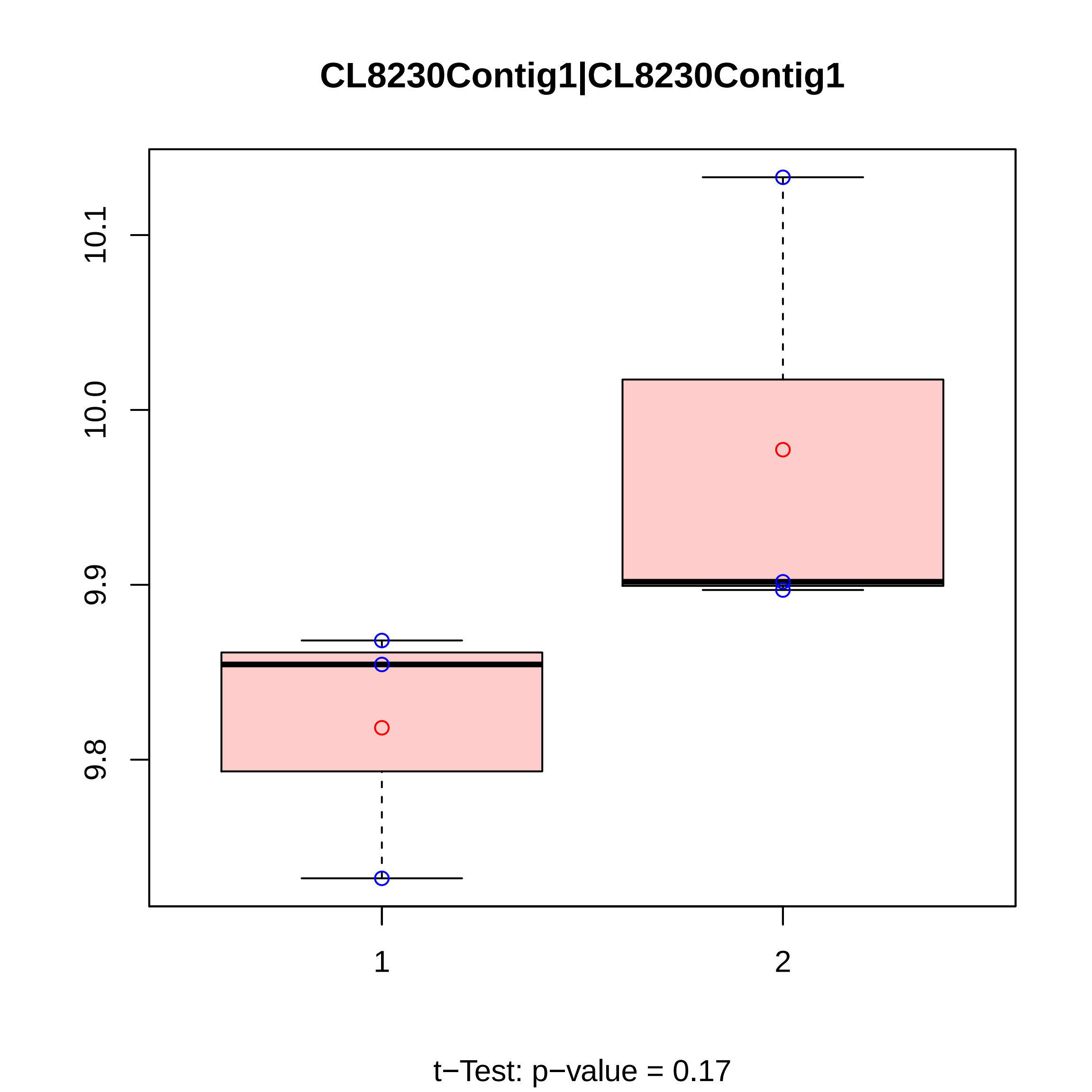
CL8230Contig1|CL8230Contig1
10.1
10.0
9.9
9.8
1
2
t−Test: p−value = 0.17

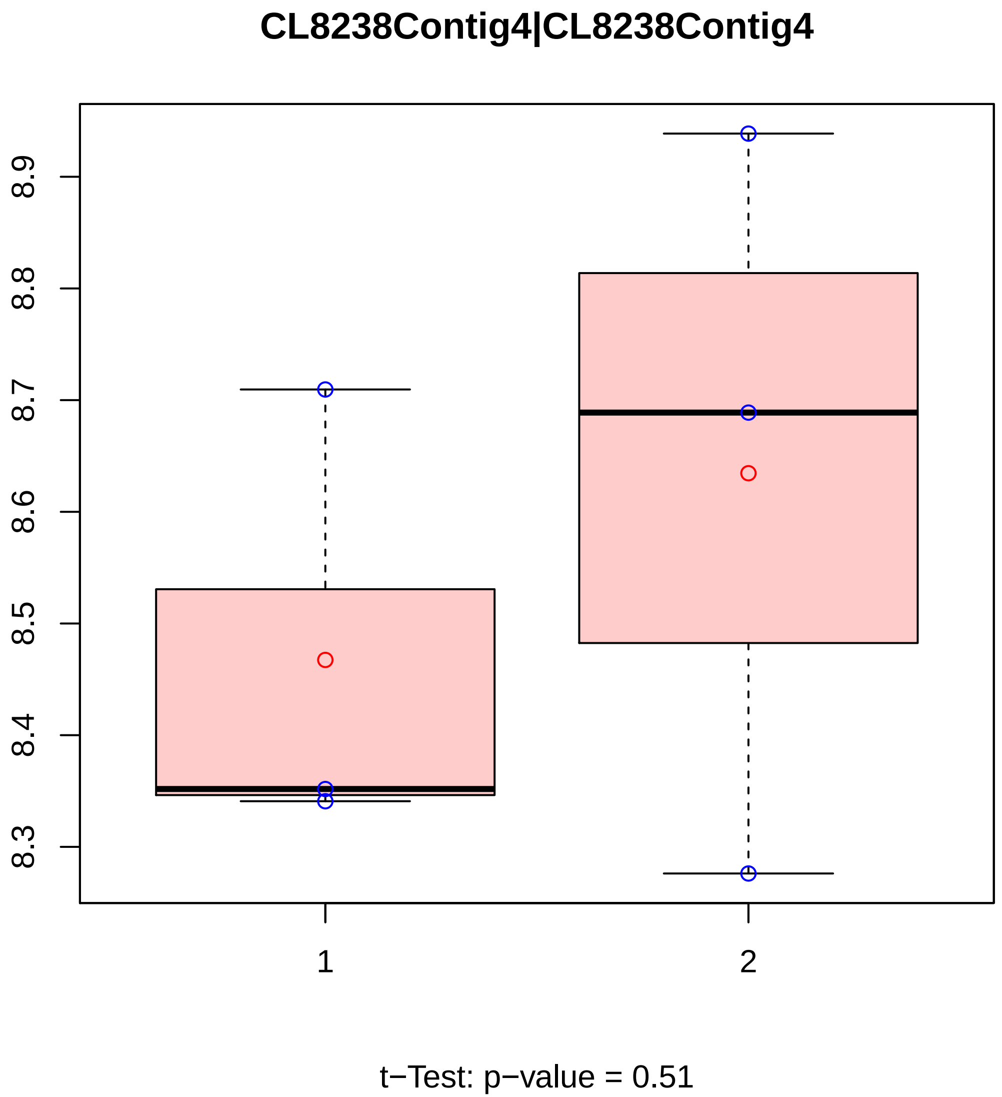
CL8238Contig4|CL8238Contig4
8.3
8.4
8.5
8.6
8.7
8.8
8.9
1
2
t−Test: p−value = 0.51

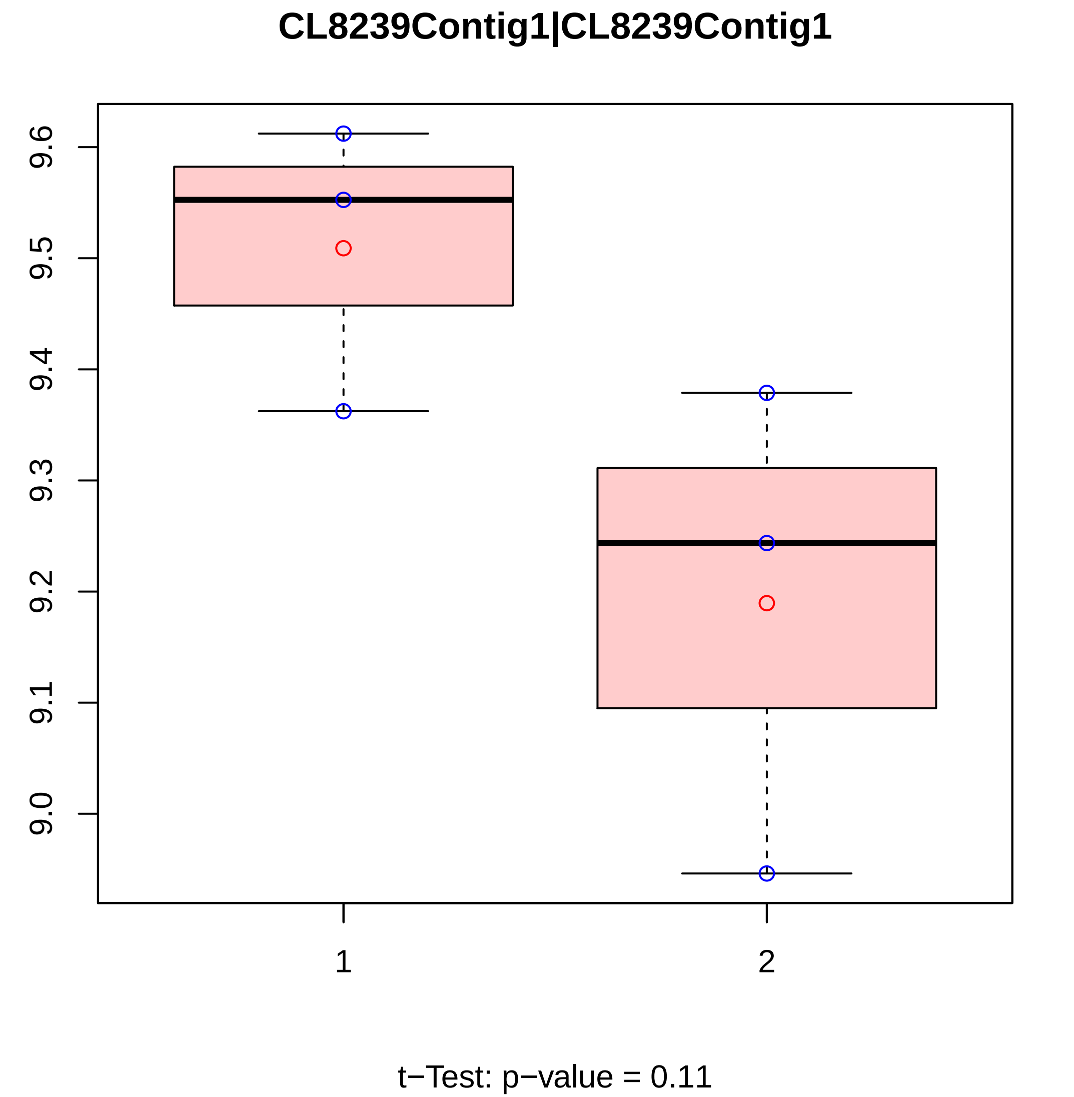
CL8239Contig1|CL8239Contig1
9.0
9.1
9.2
9.3
9.4
9.5
9.6
1
2
t−Test: p−value = 0.11

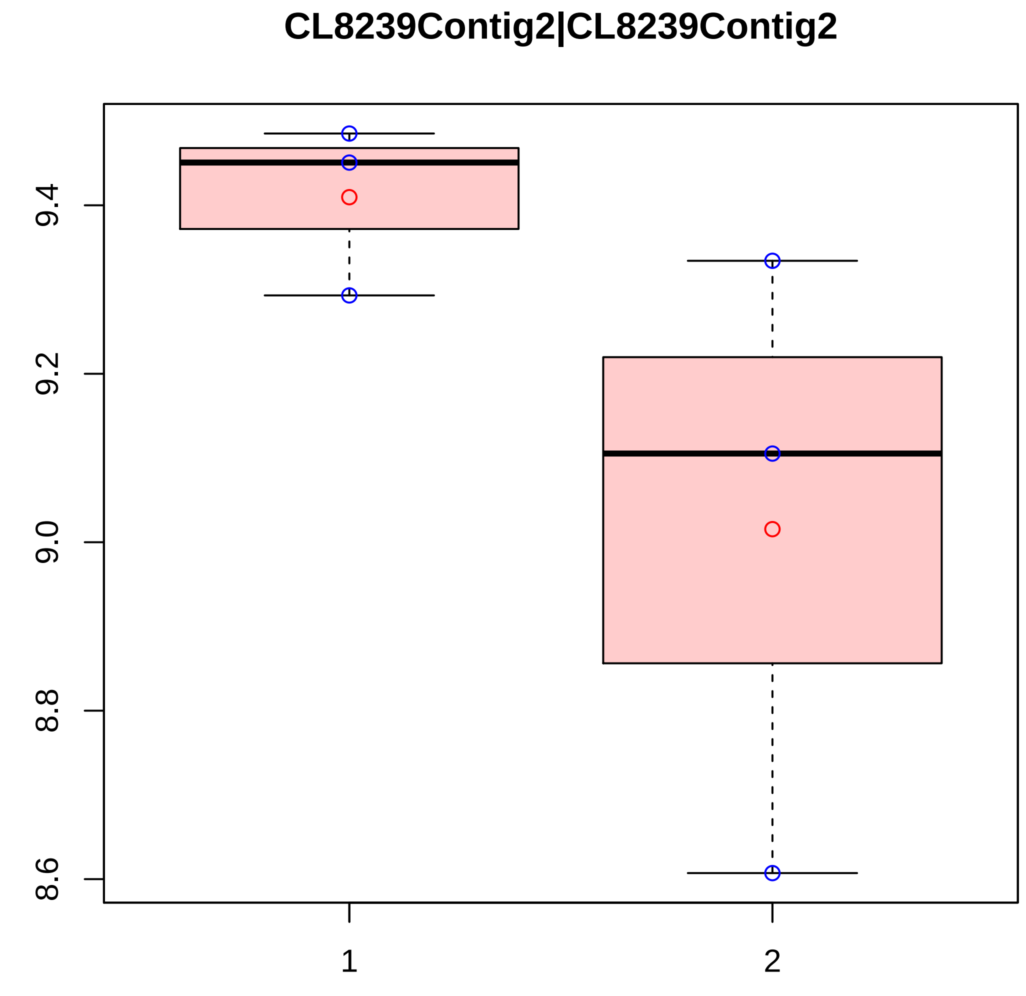


t−Test: p−value = 0.2

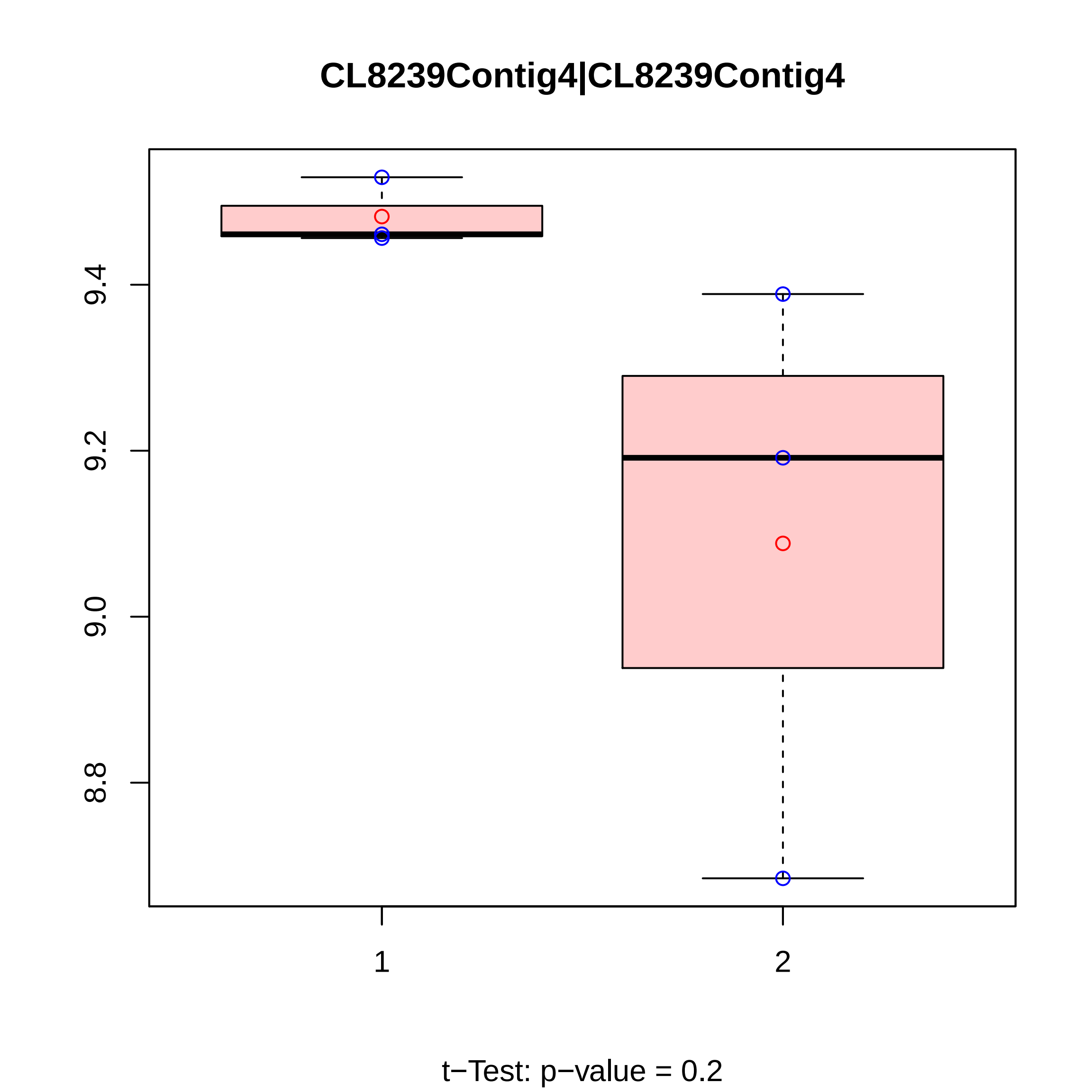

CL8239Contig4|CL8239Contig4
9.4
9.2
9.0
8.8
1
2
t−Test: p−value = 0.2

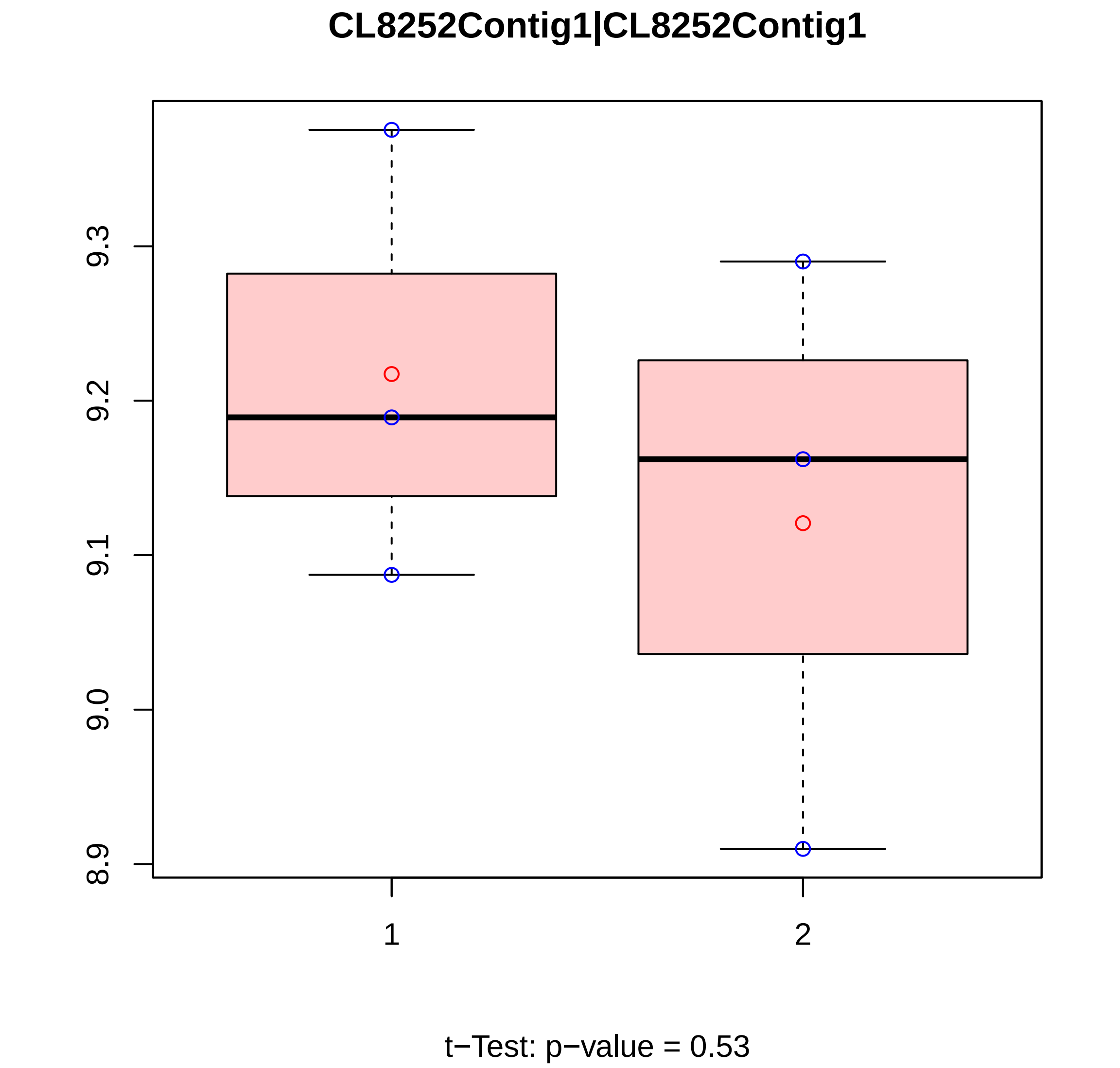

CL8252Contig1|CL8252Contig1
9.3
9.2
9.1
9.0
8.9
1
2
t−Test: p−value = 0.53

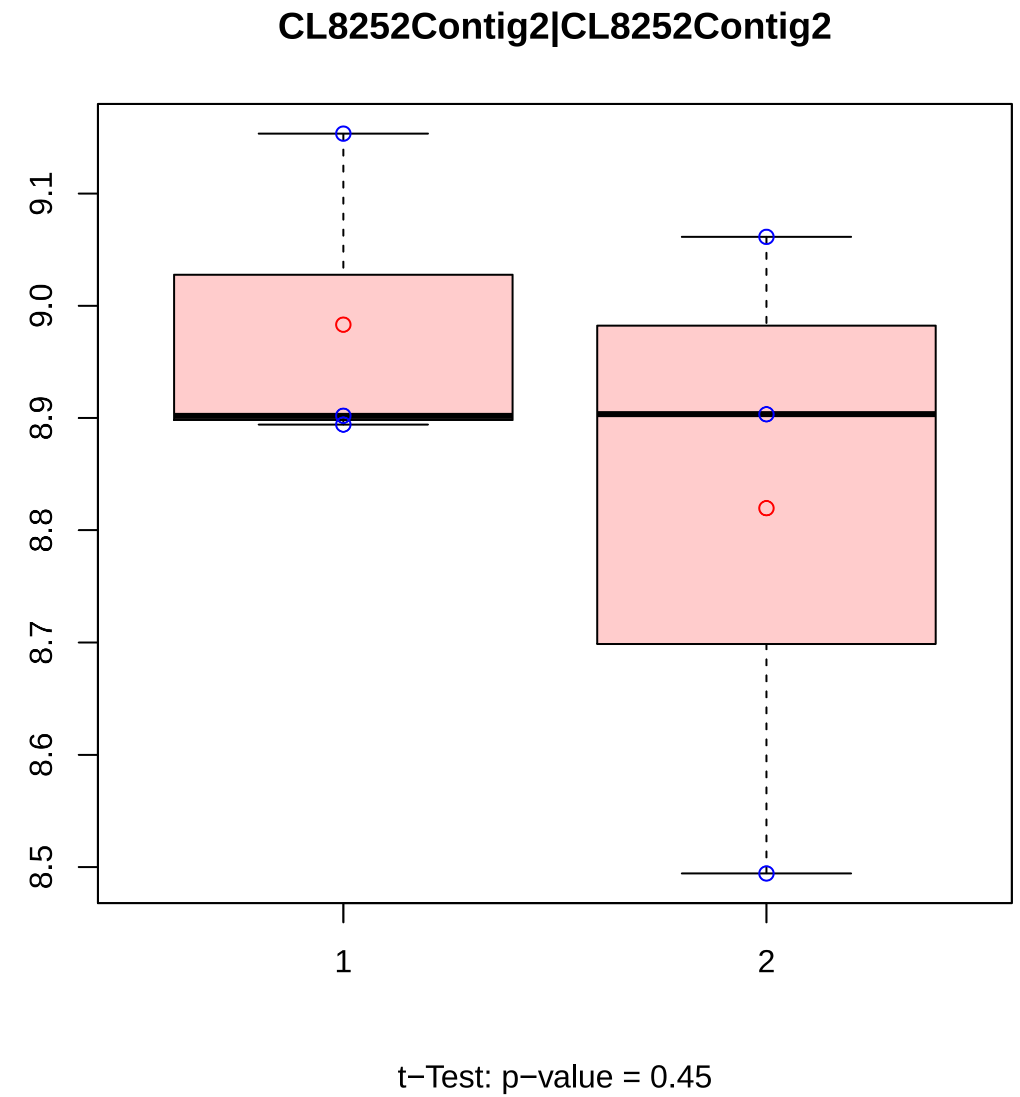
CL8252Contig2|CL8252Contig2
9.1
9.0
8.9
8.8
8.7
8.6
8.5
1
2
t−Test: p−value = 0.45

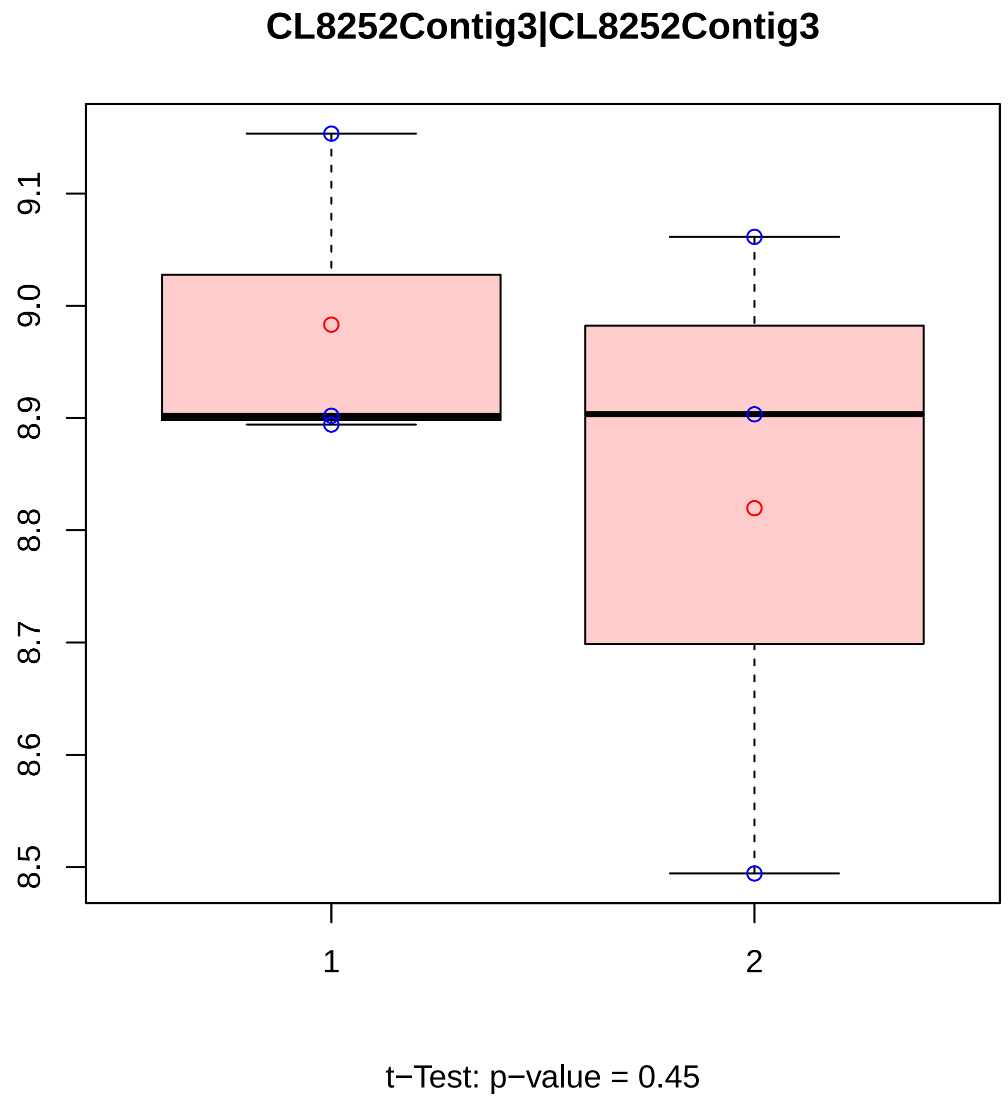

CL8252Contig3|CL8252Contig3
9.1
9.0
8.9
8.8
8.7
8.6
8.5
1
2
t−Test: p−value = 0.45

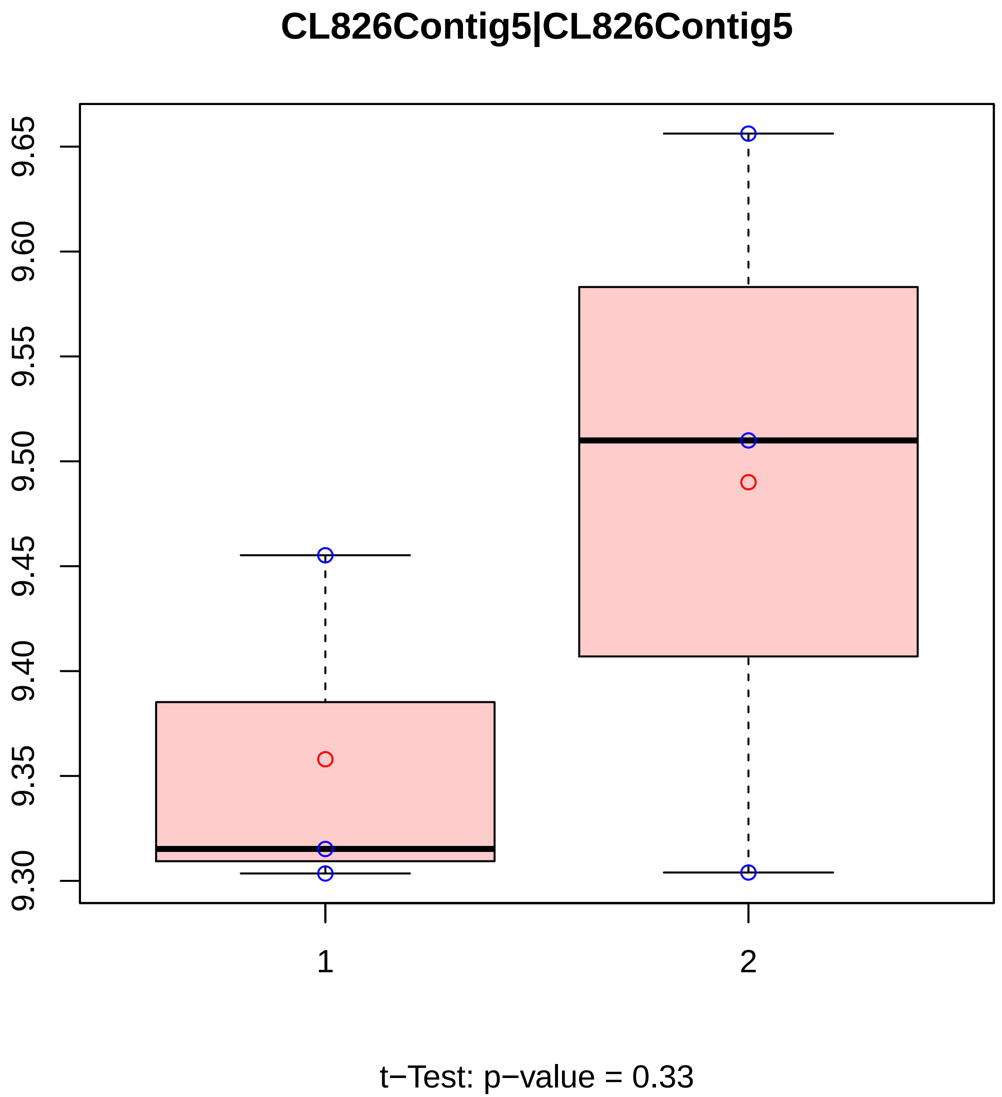

CL826Contig5|CL826Contig5
9.30
9.35
9.40
9.45
9.50
9.55
9.60
9.65
1
2
t−Test: p−value = 0.33

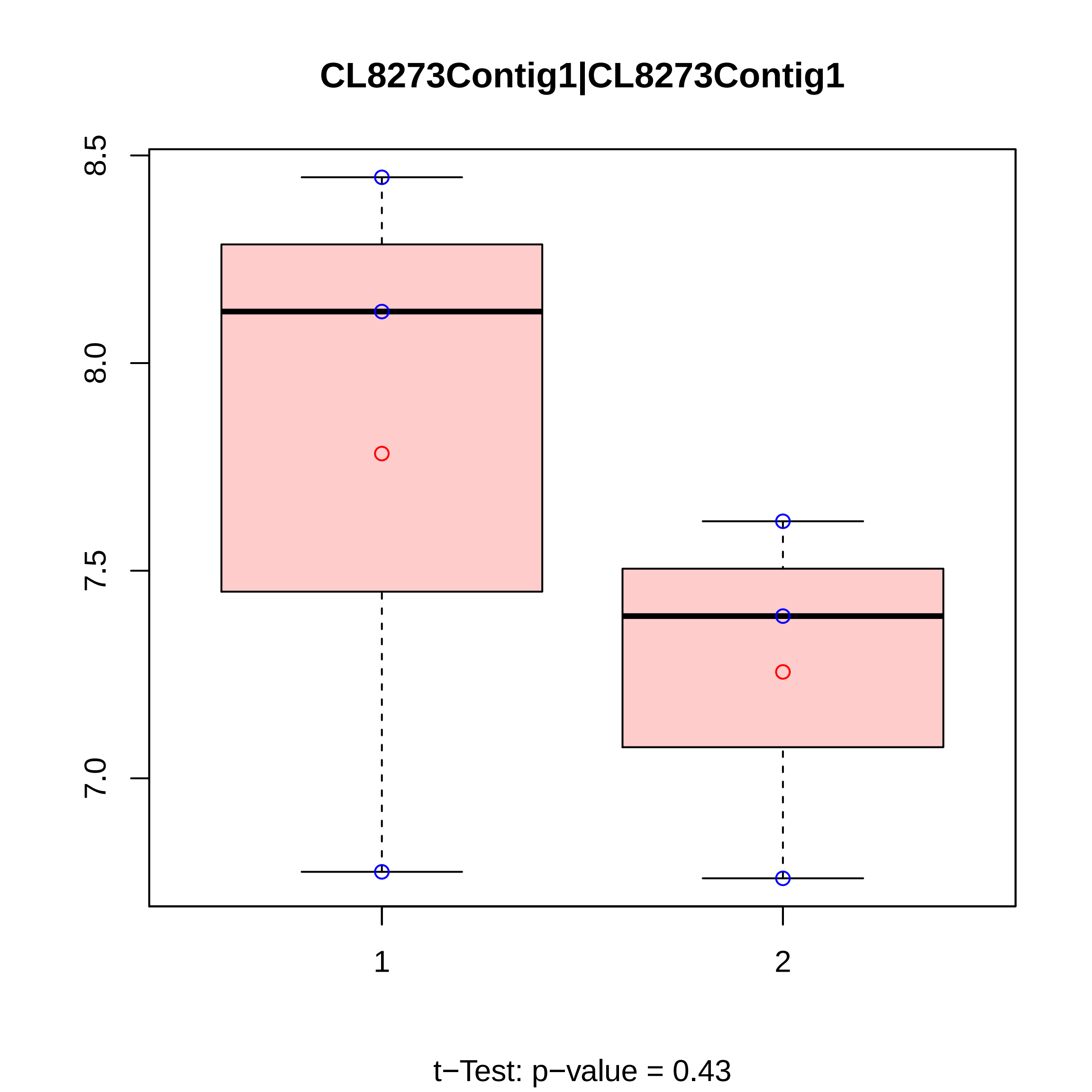
CL8273Contig1|CL8273Contig1
8.5
8.0
7.5
7.0
1
2
t−Test: p−value = 0.43

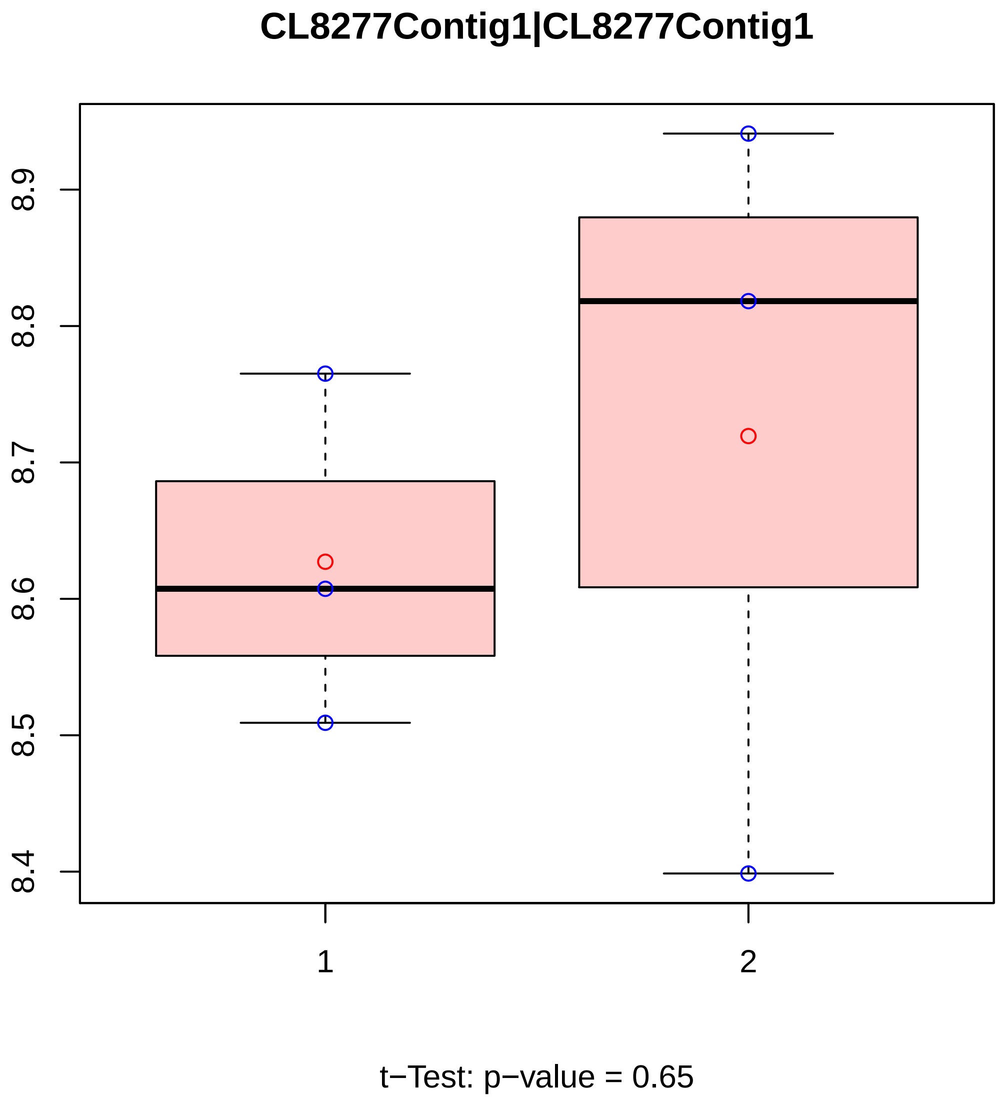
CL8277Contig1|CL8277Contig1
8.4
8.5
8.6
8.7
8.8
8.9
1
2
t−Test: p−value = 0.65

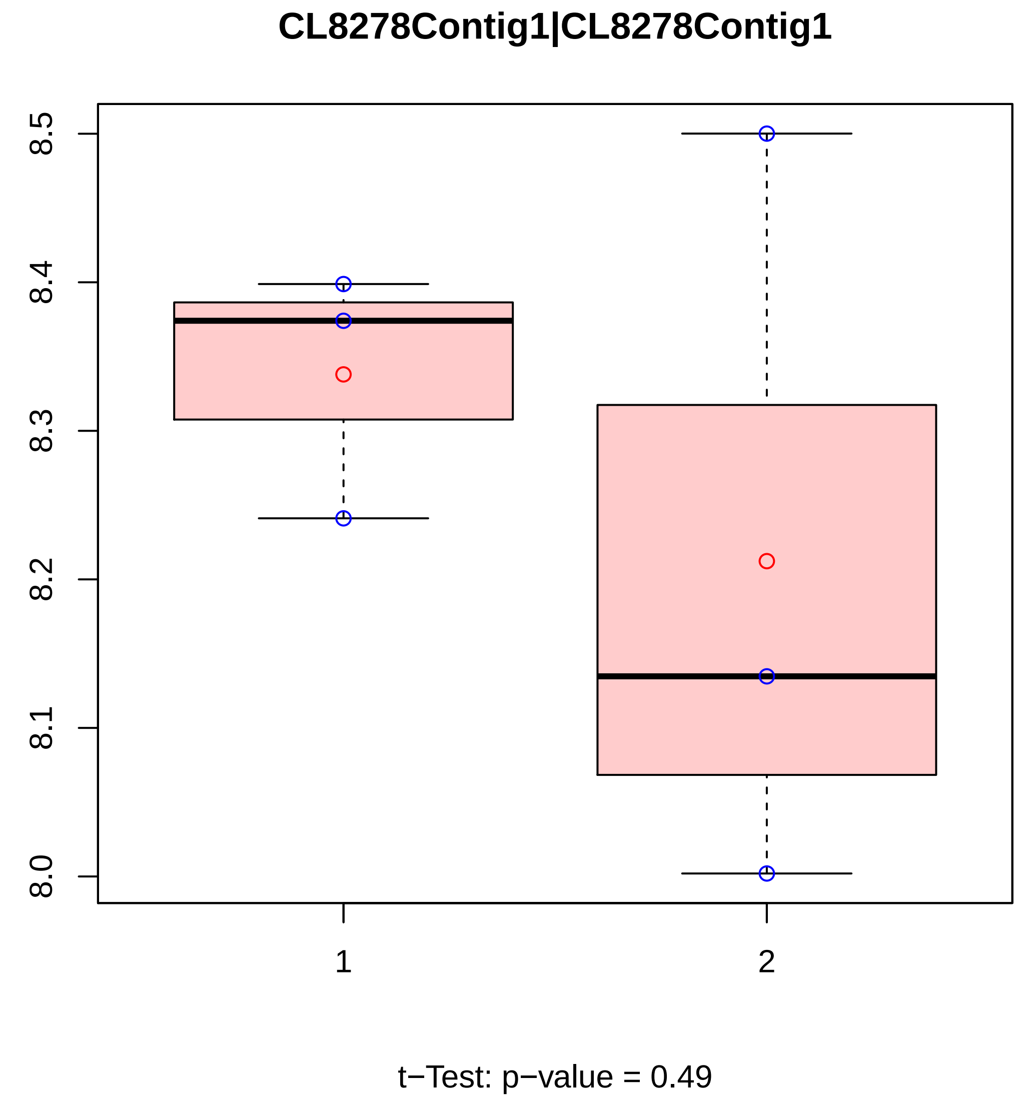
CL8278Contig1|CL8278Contig1
8.0
8.1
8.2
8.3
8.4
8.5
1
2
t−Test: p−value = 0.49

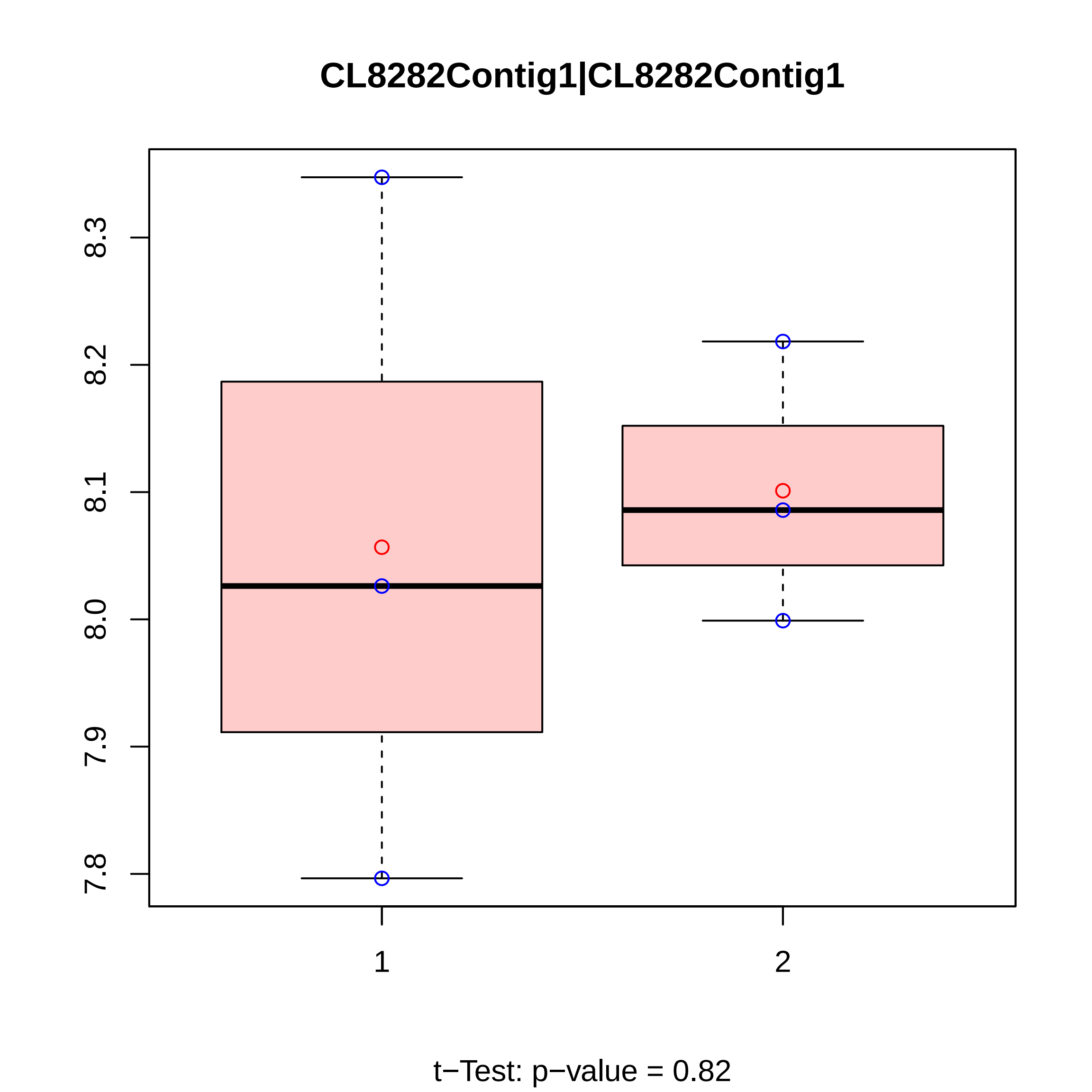

CL8282Contig1|CL8282Contig1
8.3
8.2
8.1
8.0
7.9
7.8
1
2
t−Test: p−value = 0.82

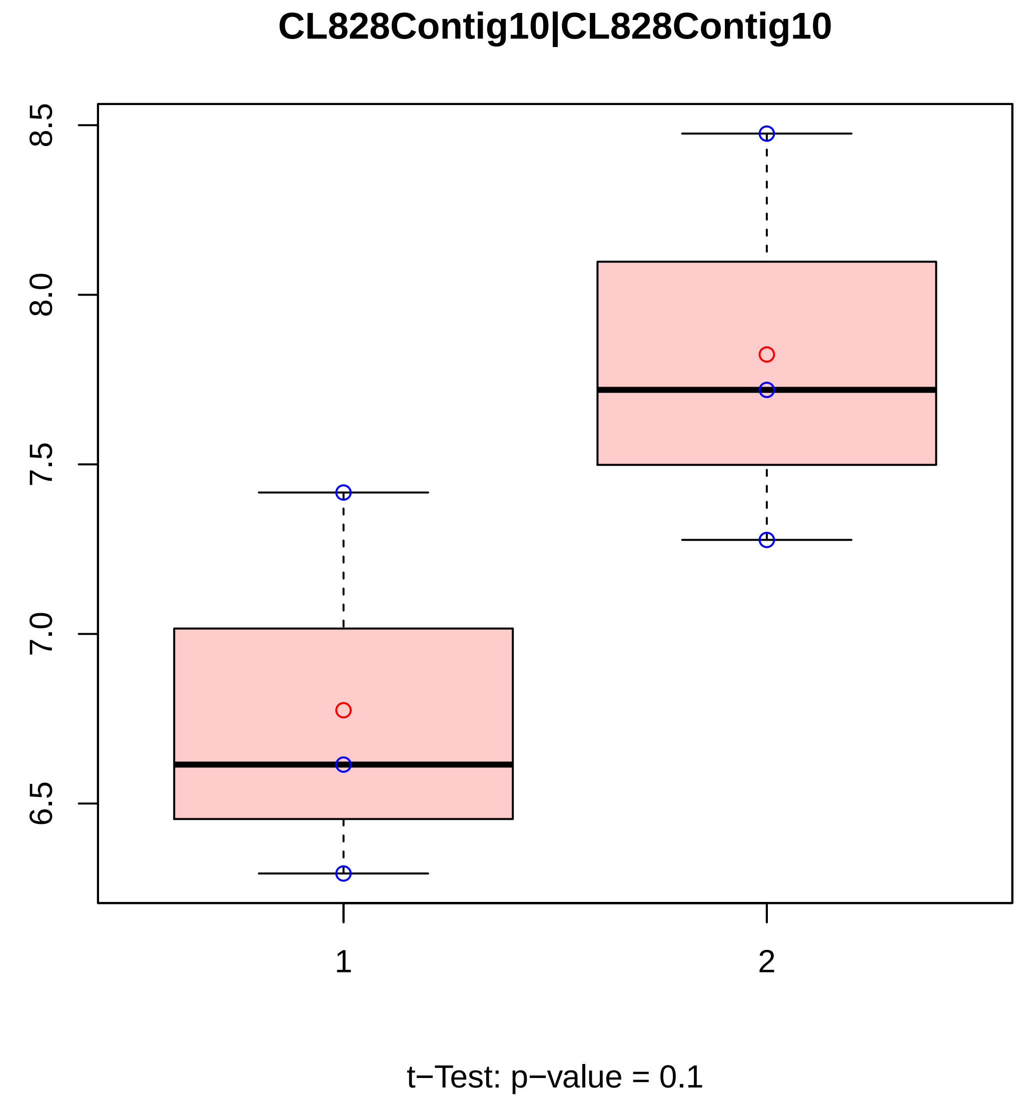
CL828Contig10|CL828Contig10
8.5
8.0
7.5
7.0
6.5
1
2
t−Test: p−value = 0.1

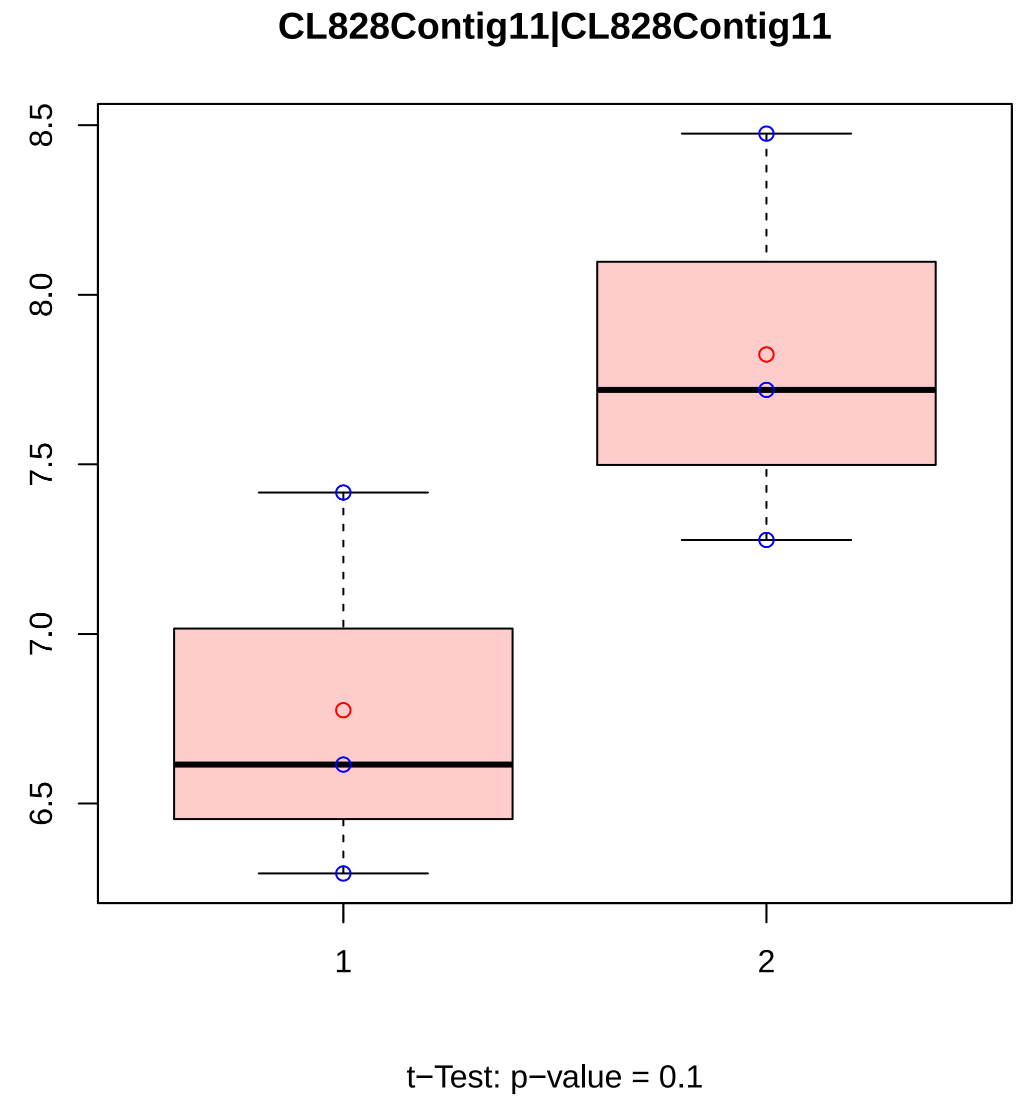
CL828Contig11|CL828Contig11
8.5
8.0
7.5
7.0
6.5
1
2
t−Test: p−value = 0.1

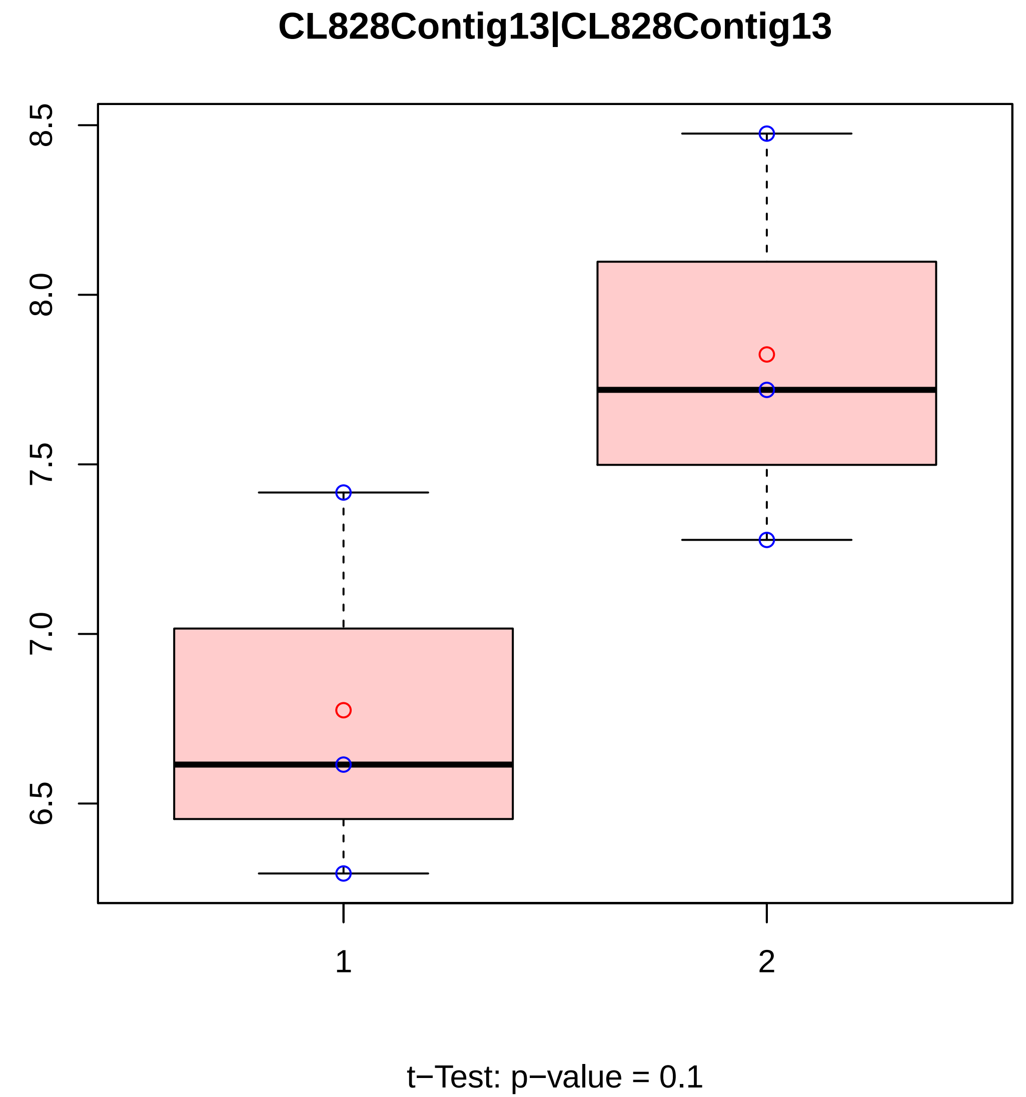
CL828Contig13|CL828Contig13
8.5
8.0
7.5
7.0
6.5
1
2
t−Test: p−value = 0.1

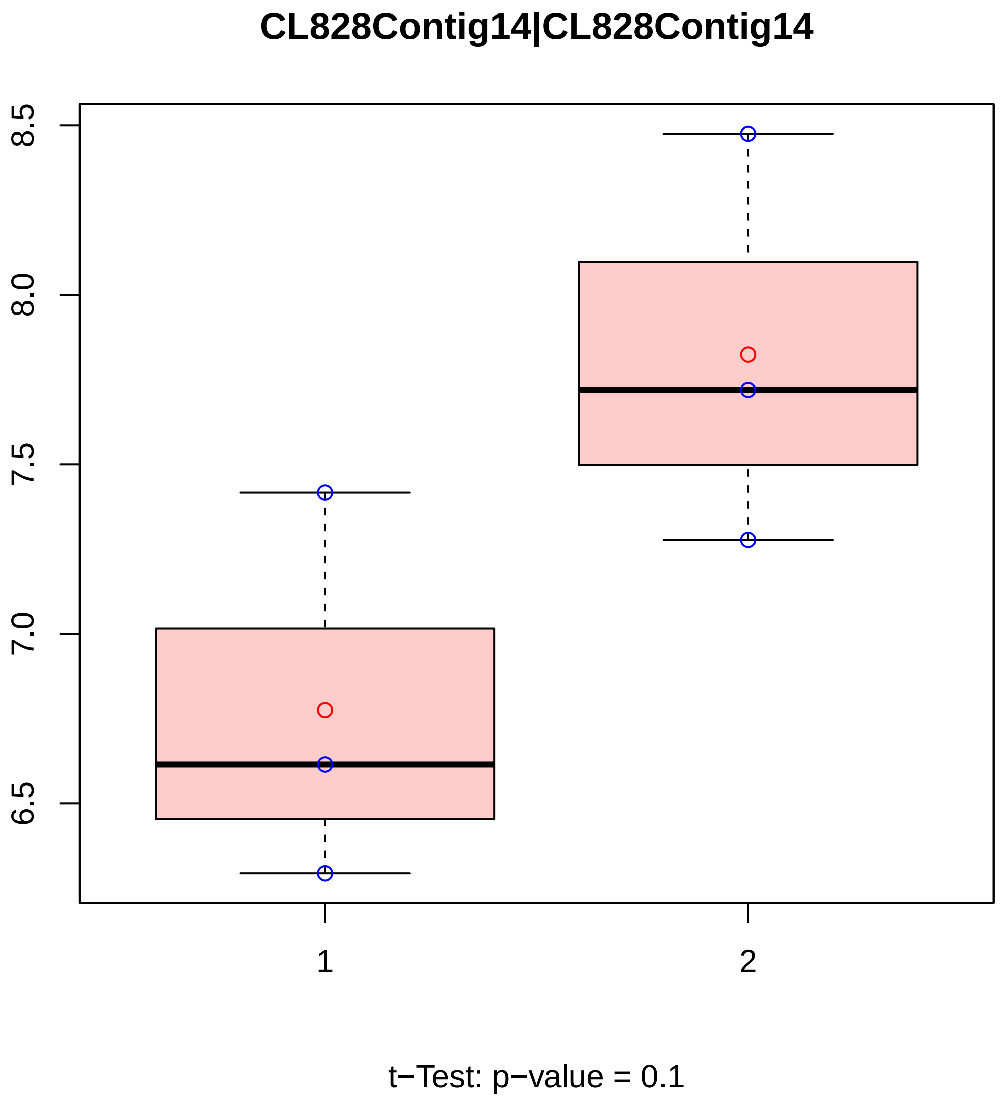
CL828Contig14|CL828Contig14
8.5
8.0
7.5
7.0
6.5
1
2
t−Test: p−value = 0.1

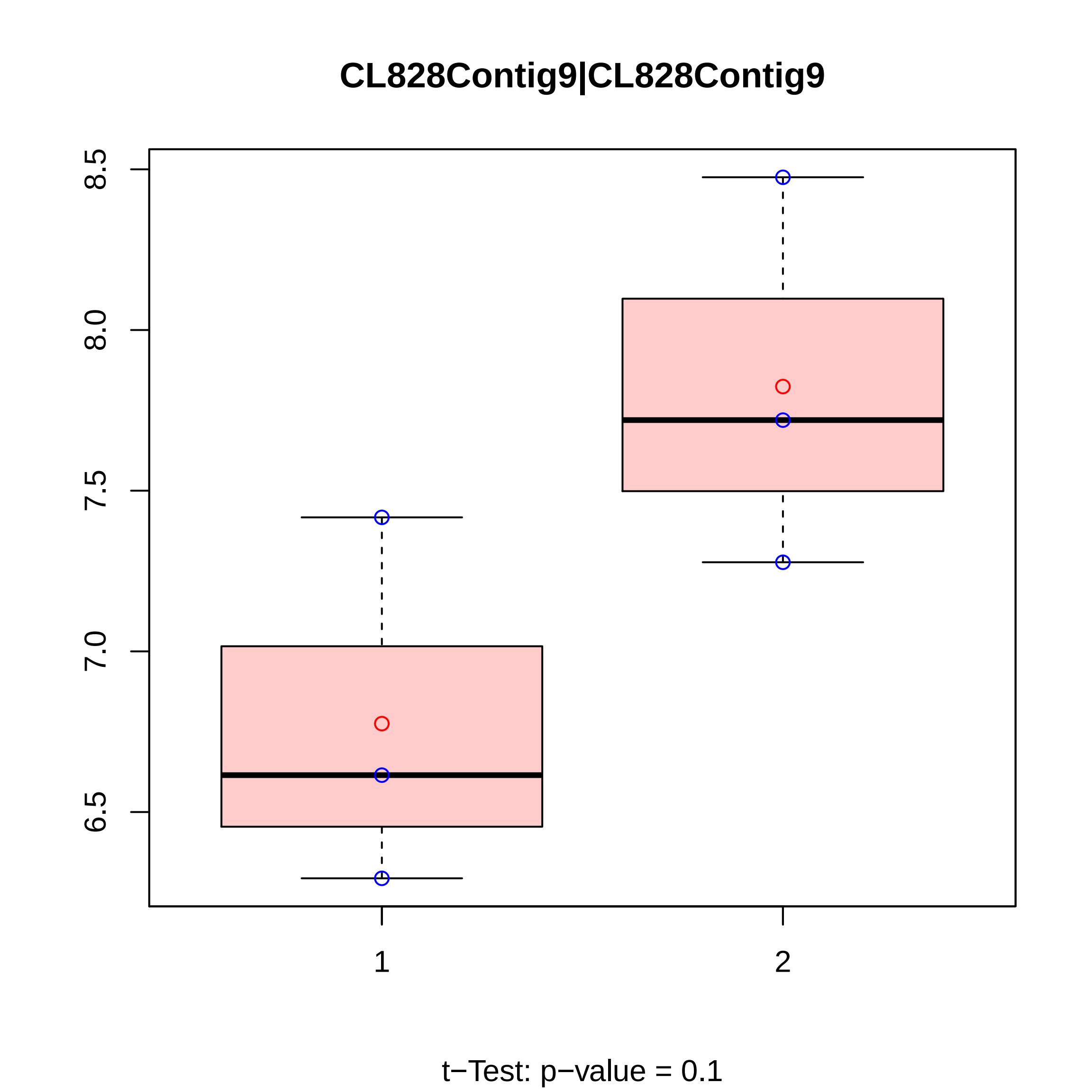

CL828Contig9|CL828Contig9
8.5
8.0
7.5
7.0
6.5
1
2
t−Test: p−value = 0.1

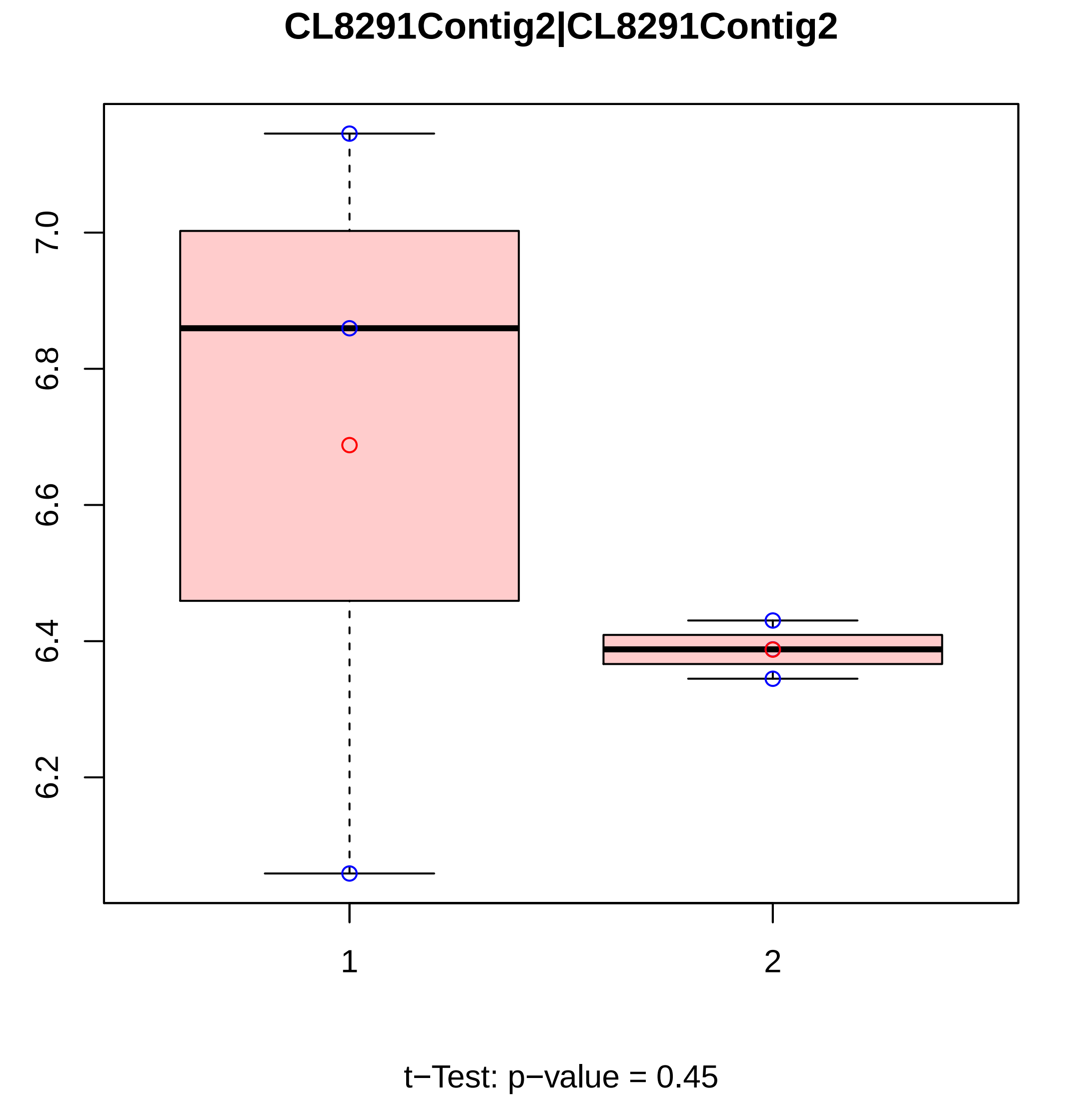
CL8291Contig2|CL8291Contig2
7.0
6.8
6.6
6.4
6.2
1
2
t−Test: p−value = 0.45

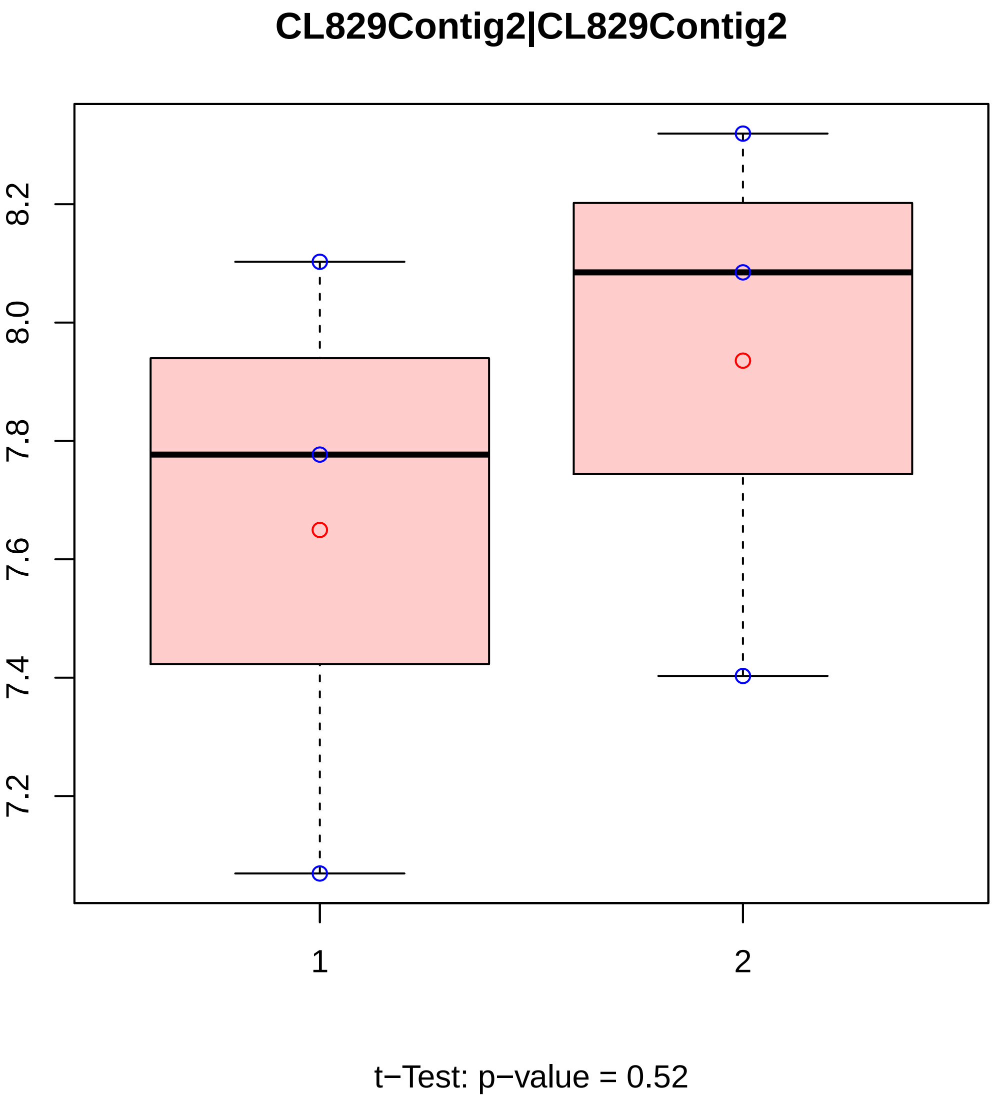

CL829Contig2|CL829Contig2
8.2
8.0
7.8
7.6
7.4
7.2
1
2
t−Test: p−value = 0.52

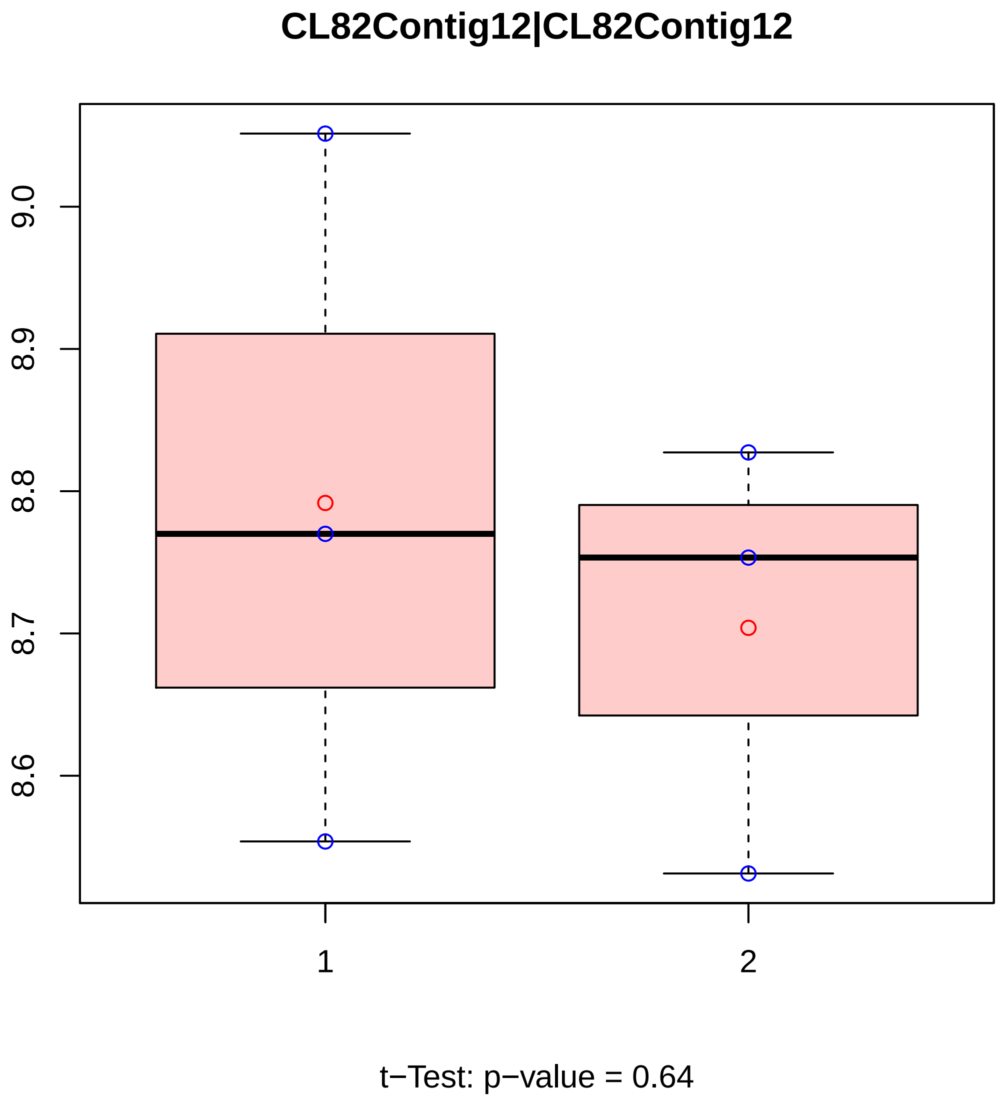
CL82Contig12|CL82Contig12
9.0
8.9
8.8
8.7
8.6
1
2
t−Test: p−value = 0.64

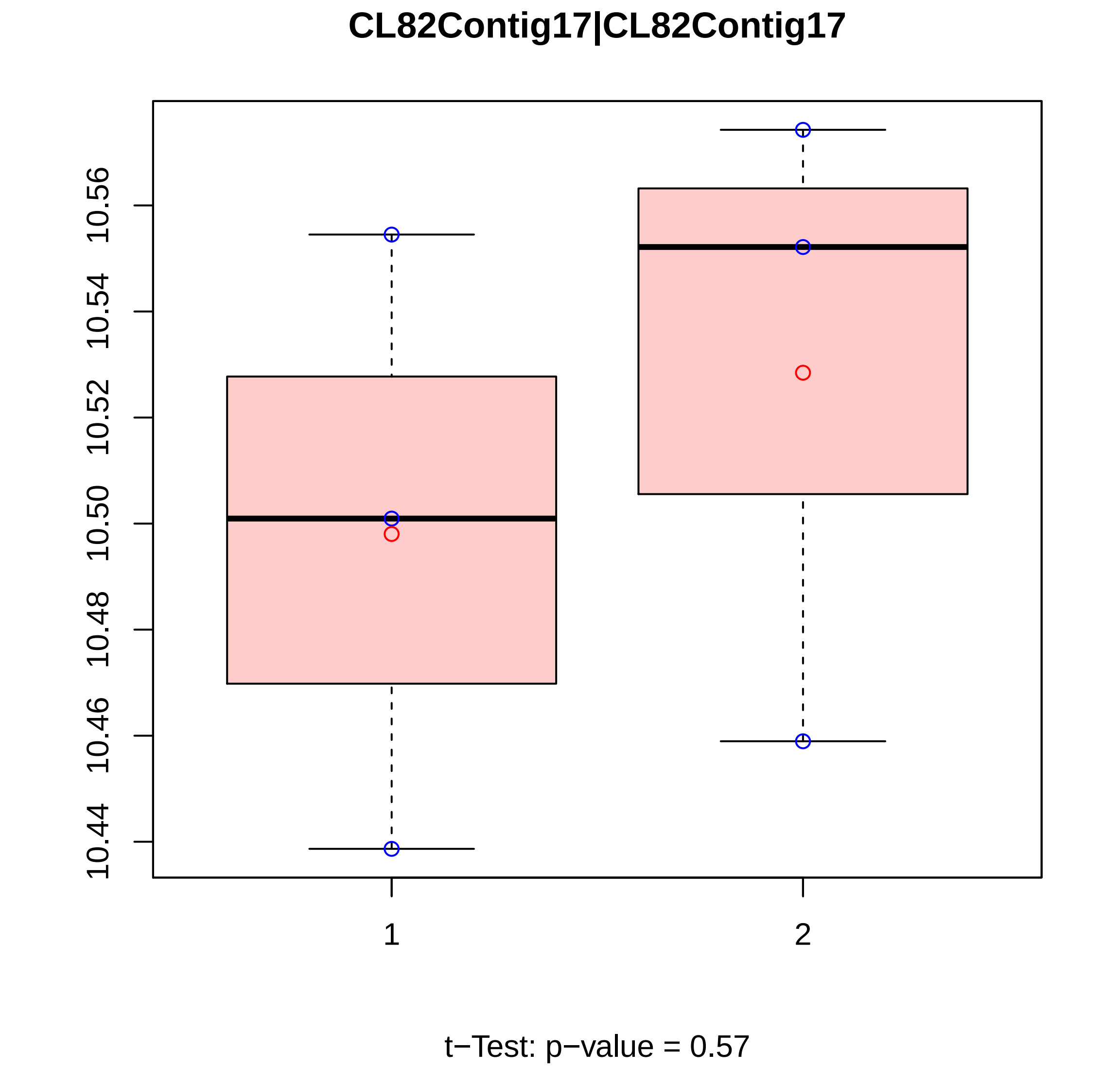

CL82Contig17|CL82Contig17
10.44
10.46
10.48
10.50
10.52
10.54
10.56
1
2
t−Test: p−value = 0.57

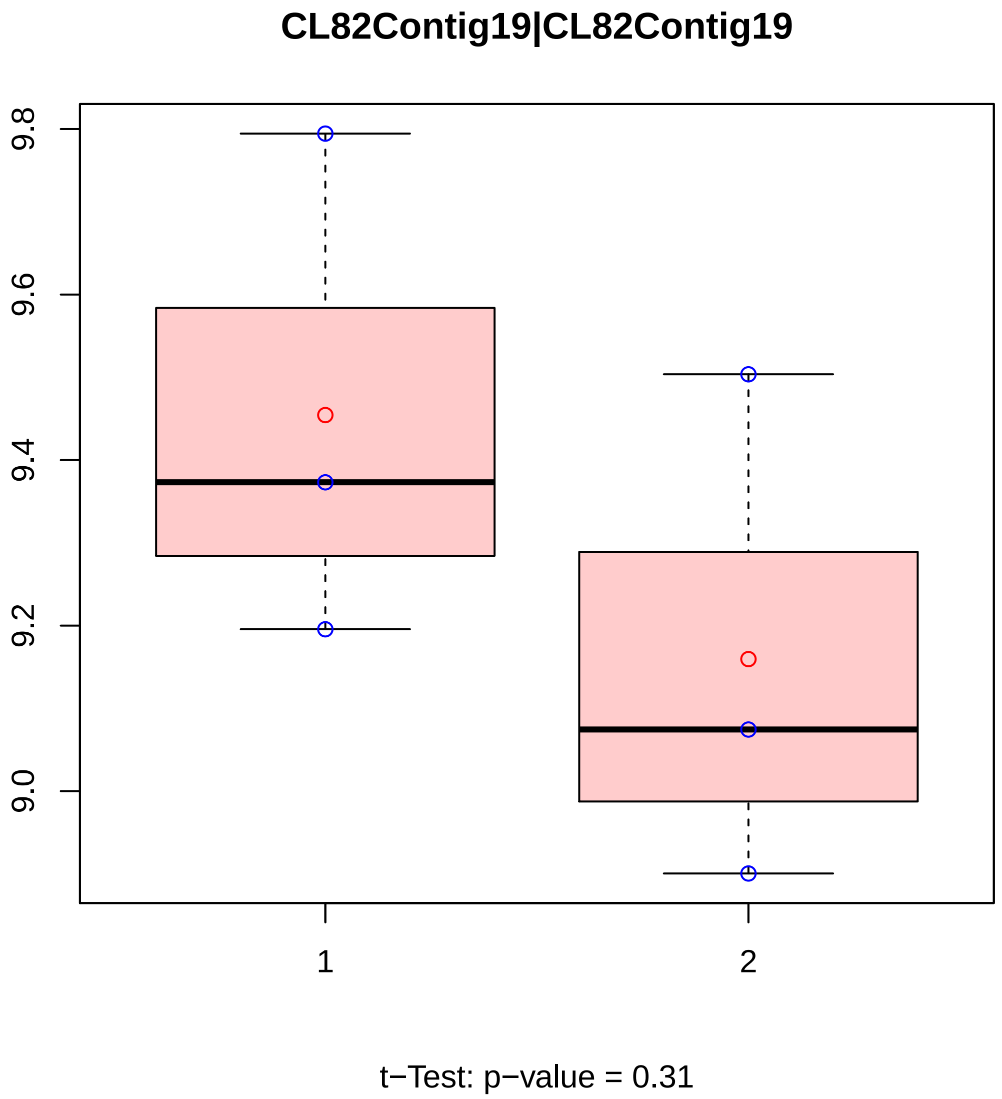
CL82Contig19|CL82Contig19
9.8
9.6
9.4
9.2
9.0
1
2
t−Test: p−value = 0.31

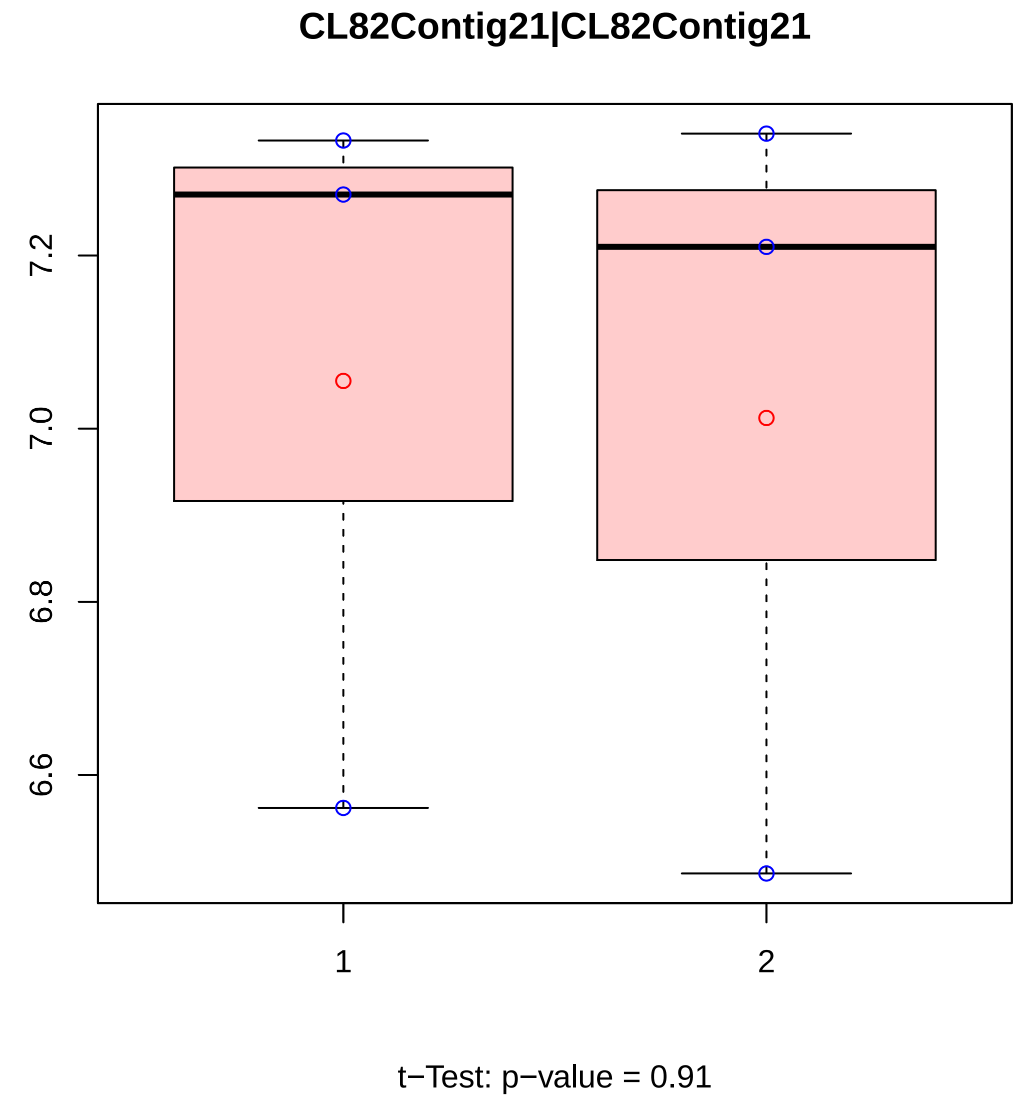
CL82Contig21|CL82Contig21
7.2
7.0
6.8
6.6
1
2
t−Test: p−value = 0.91

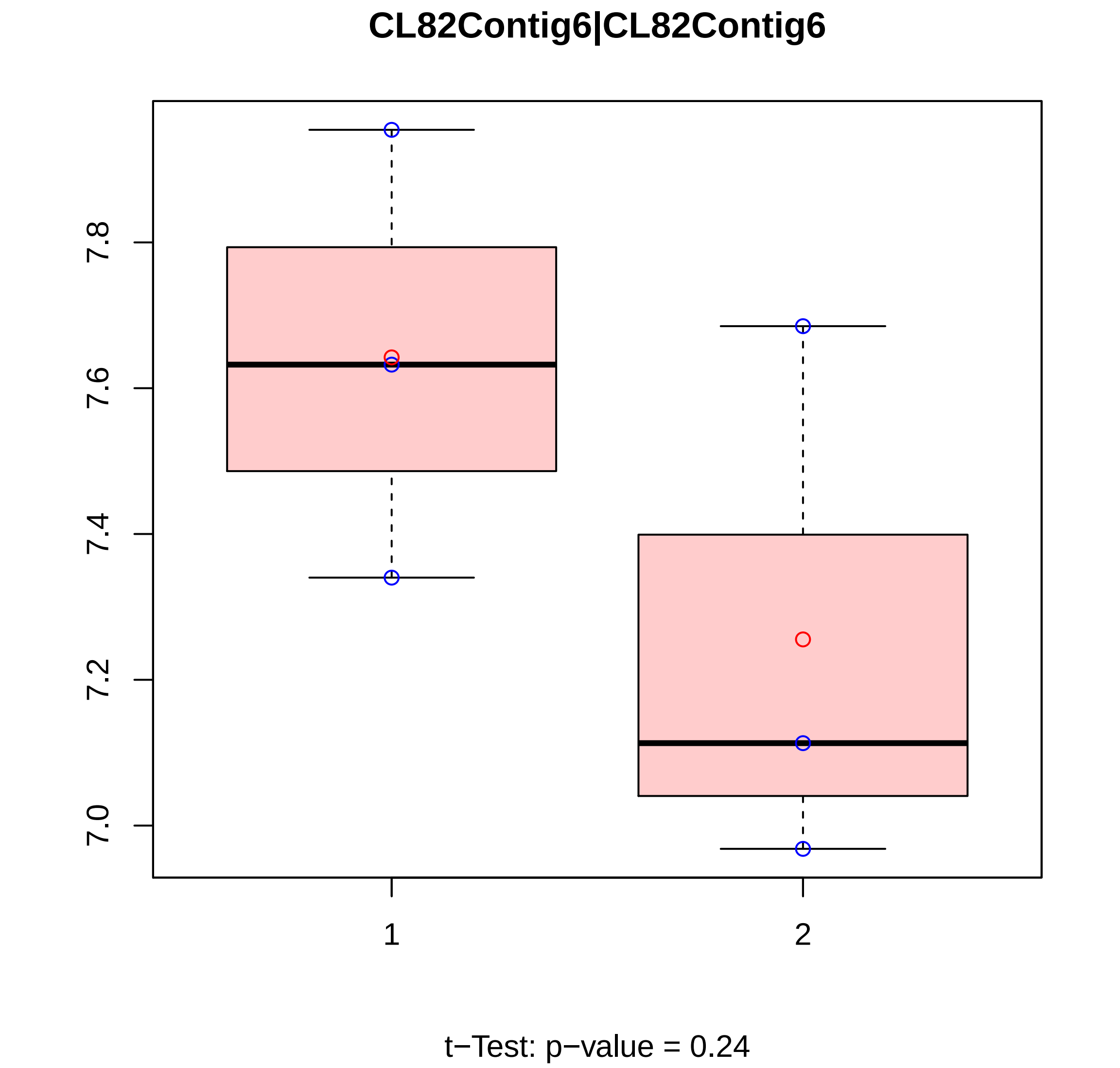
CL82Contig6|CL82Contig6
7.0
7.2
7.4
7.6
7.8
1
2
t−Test: p−value = 0.24

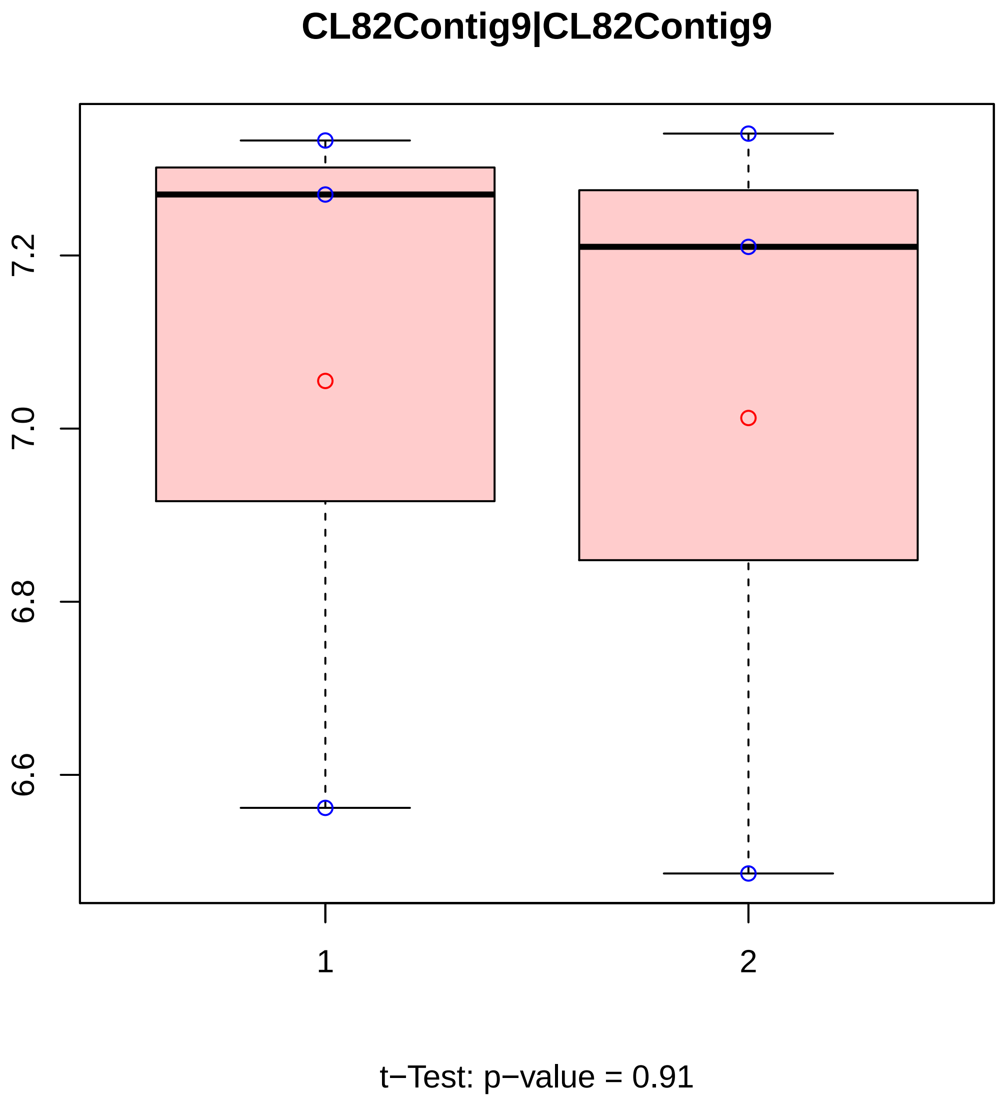
CL82Contig9|CL82Contig9
7.2
7.0
6.8
6.6
1
2
t−Test: p−value = 0.91

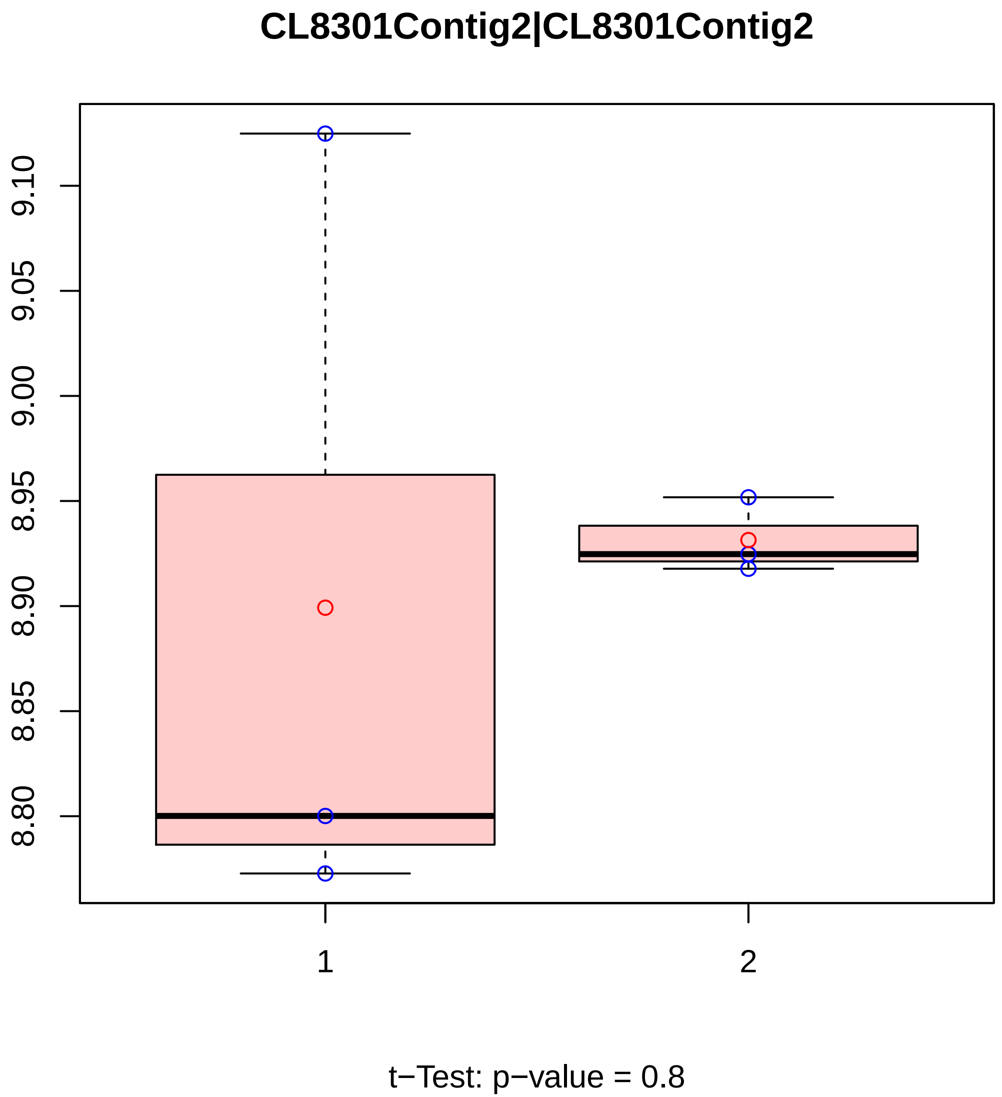
CL8301Contig2|CL8301Contig2
9.10
9.05
9.00
8.95
8.90
8.85
8.80
1
2
t−Test: p−value = 0.8

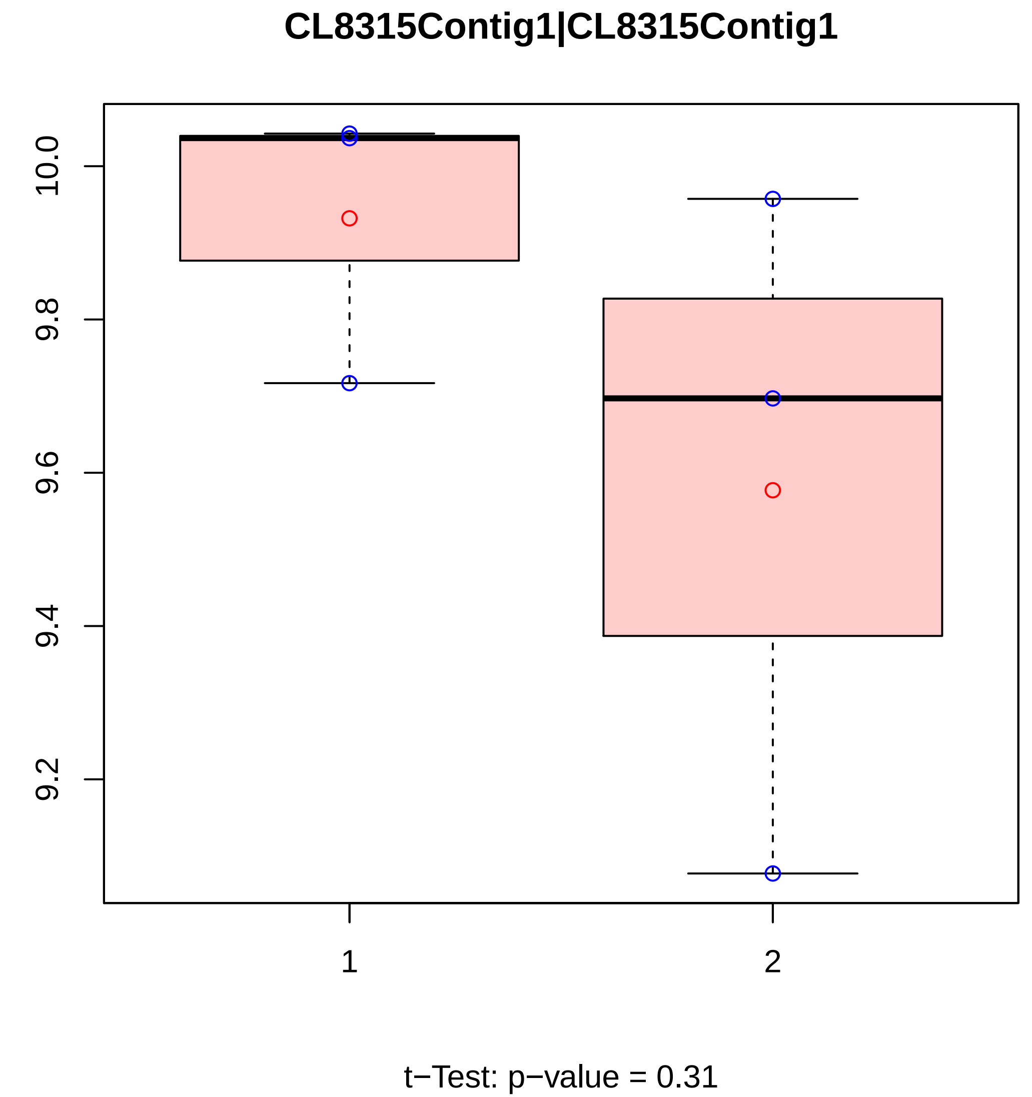

CL8315Contig1|CL8315Contig1
10.0
9.8
9.6
9.4
9.2
1
2
t−Test: p−value = 0.31

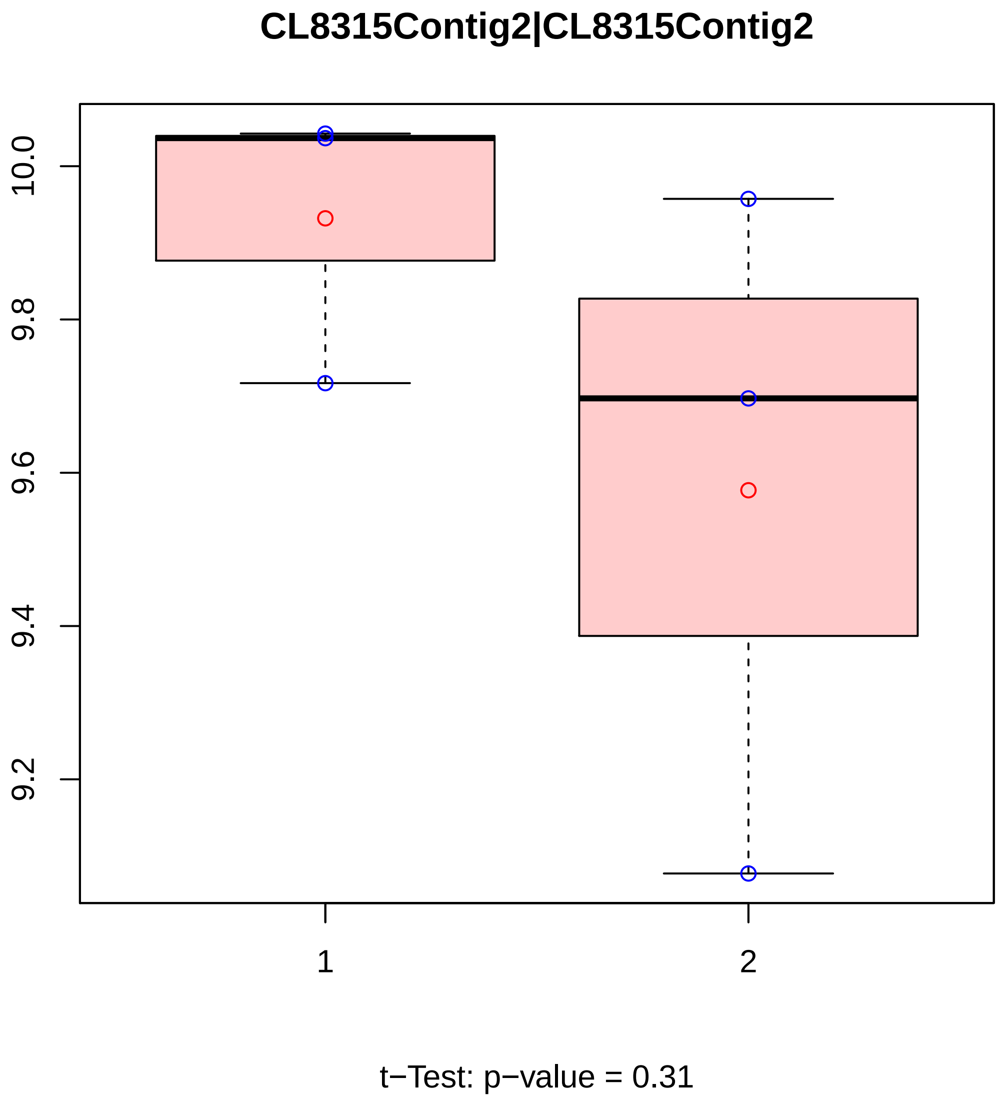
CL8315Contig2|CL8315Contig2
10.0
9.8
9.6
9.4
9.2
1
2
t−Test: p−value = 0.31

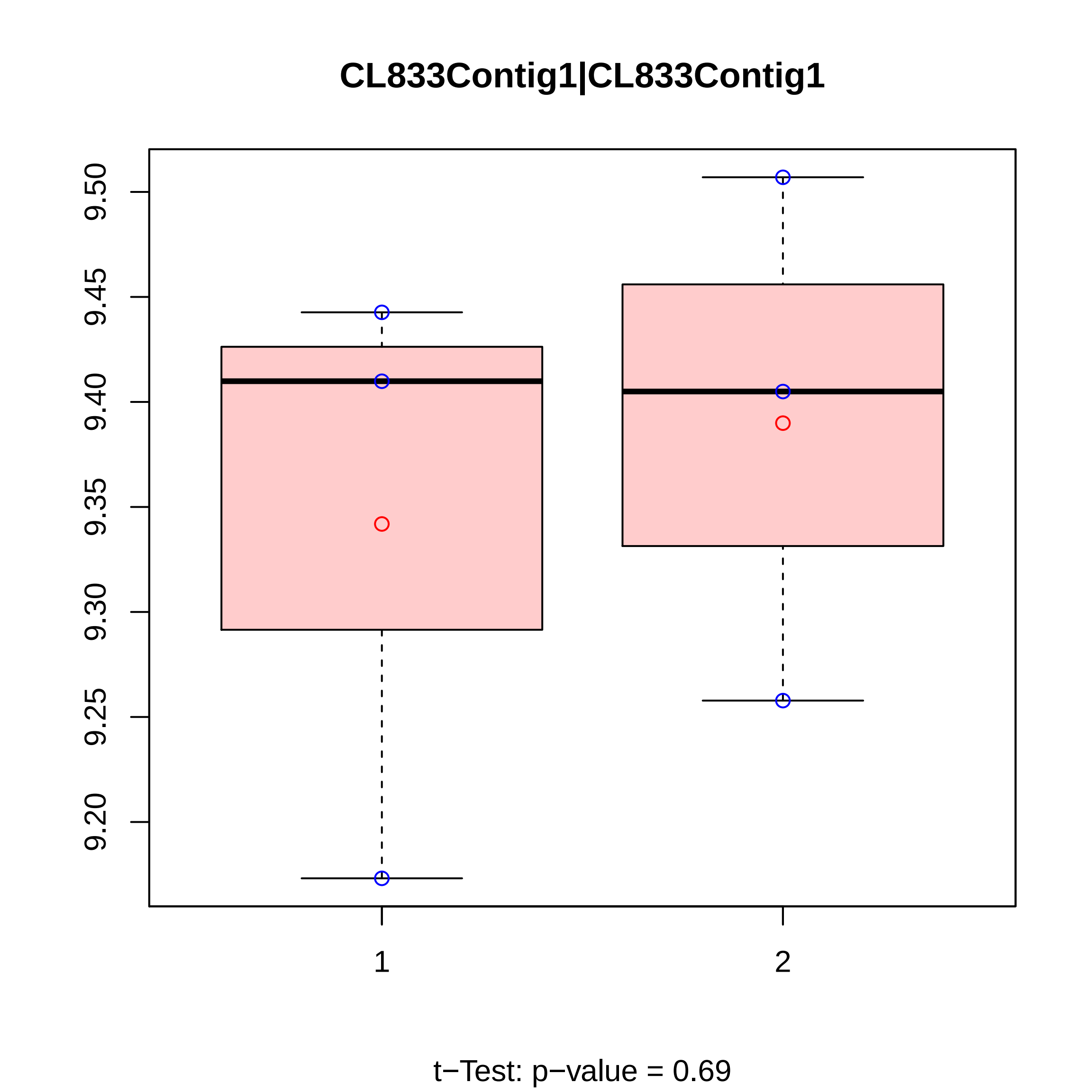
CL833Contig1|CL833Contig1
9.50
9.45
9.40
9.35
9.30
9.25
9.20
1
2
t−Test: p−value = 0.69

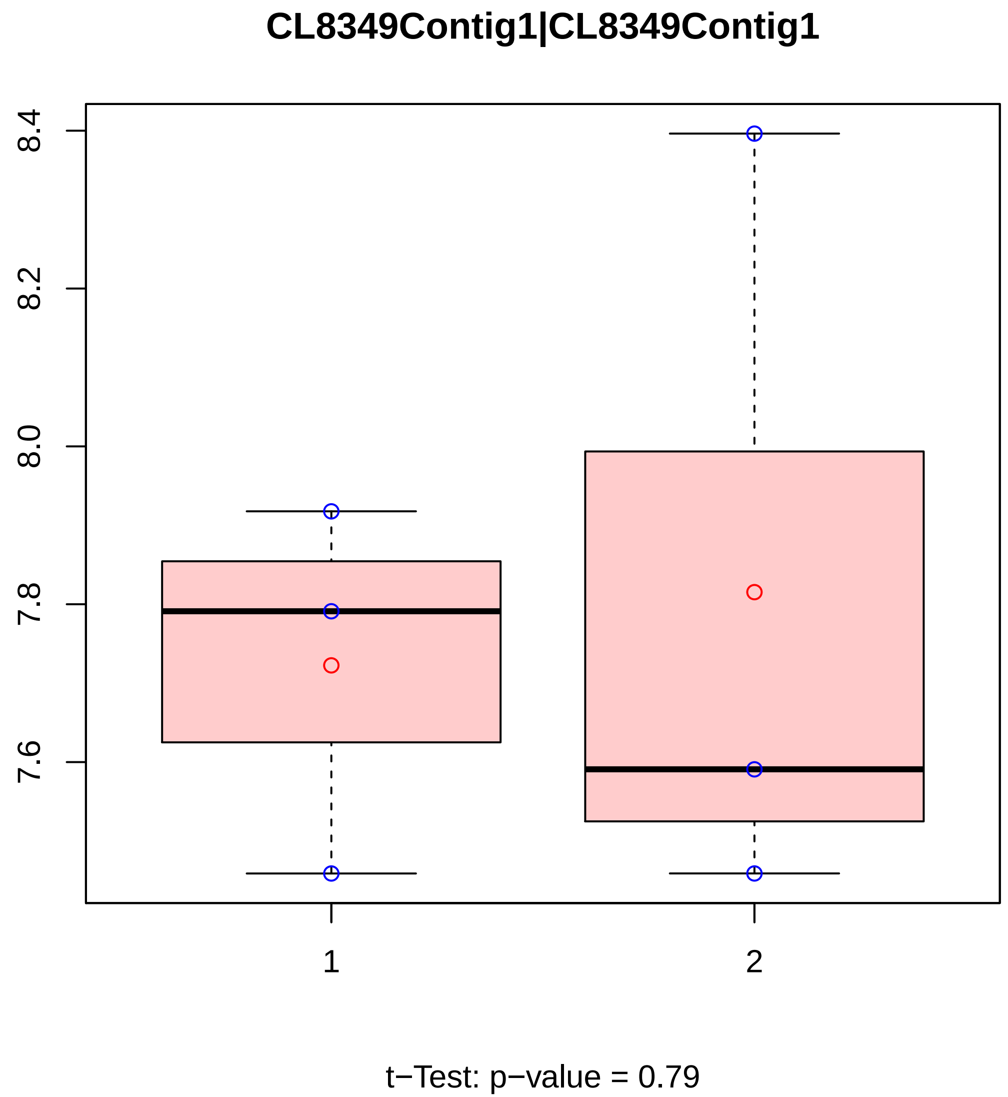
CL8349Contig1|CL8349Contig1
8.4
8.2
8.0
7.8
7.6
1
2
t−Test: p−value = 0.79

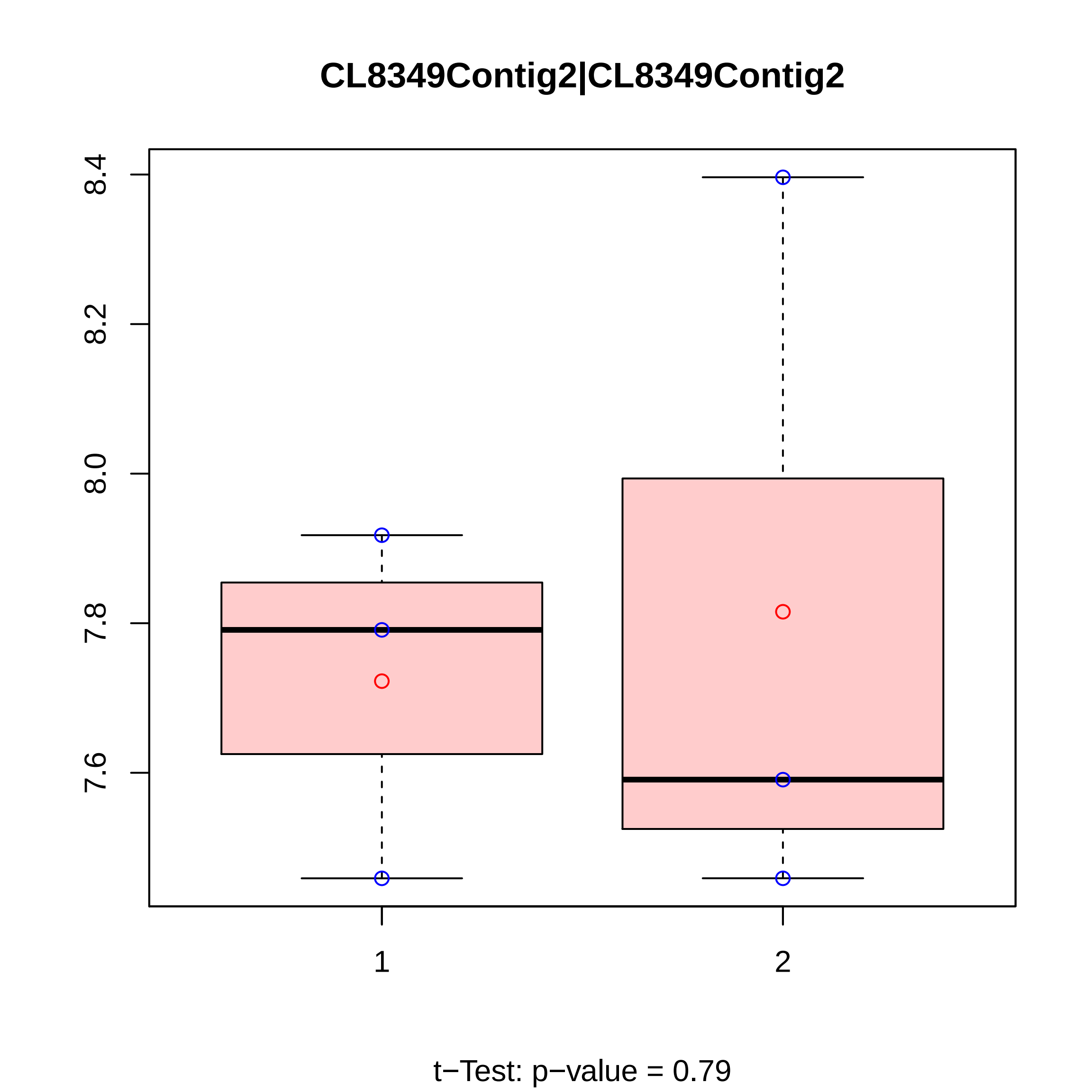
CL8349Contig2|CL8349Contig2
8.4
8.2
8.0
7.8
7.6
1
2
t−Test: p−value = 0.79

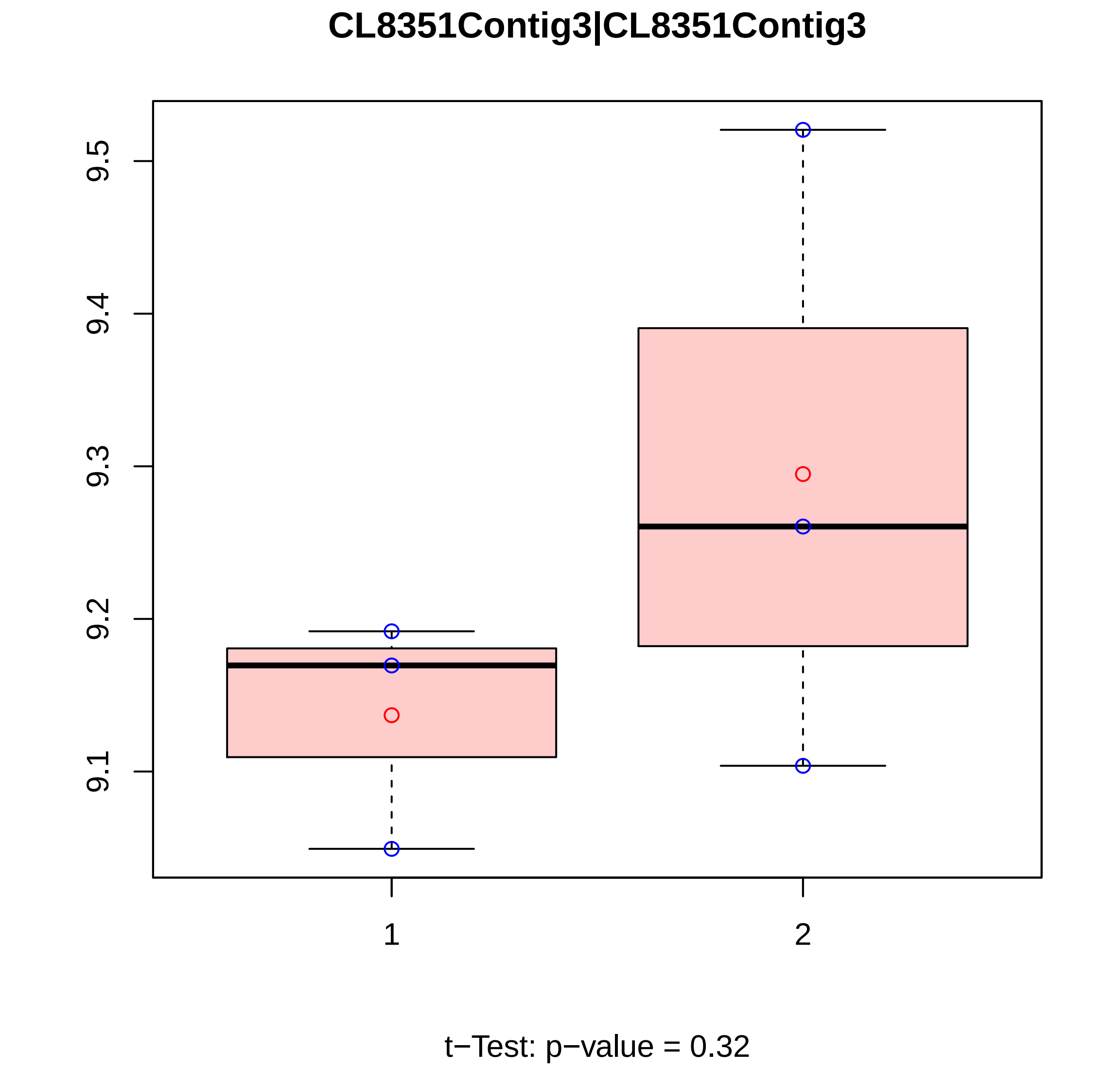
CL8351Contig3|CL8351Contig3
9.1
9.2
9.3
9.4
9.5
1
2
t−Test: p−value = 0.32

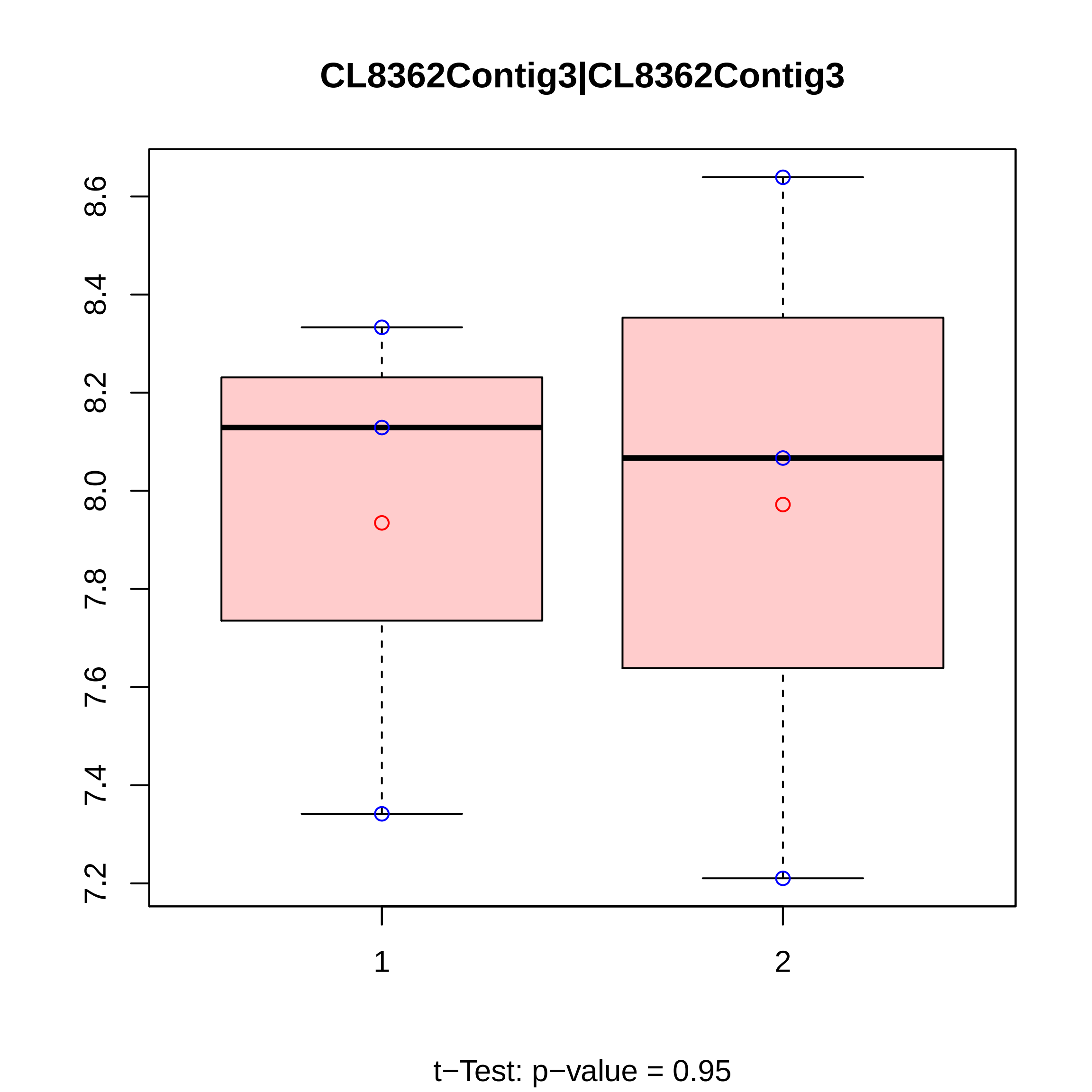

CL8362Contig3|CL8362Contig3
8.6
8.4
8.2
8.0
7.8
7.6
7.4
7.2
1
2
t−Test: p−value = 0.95

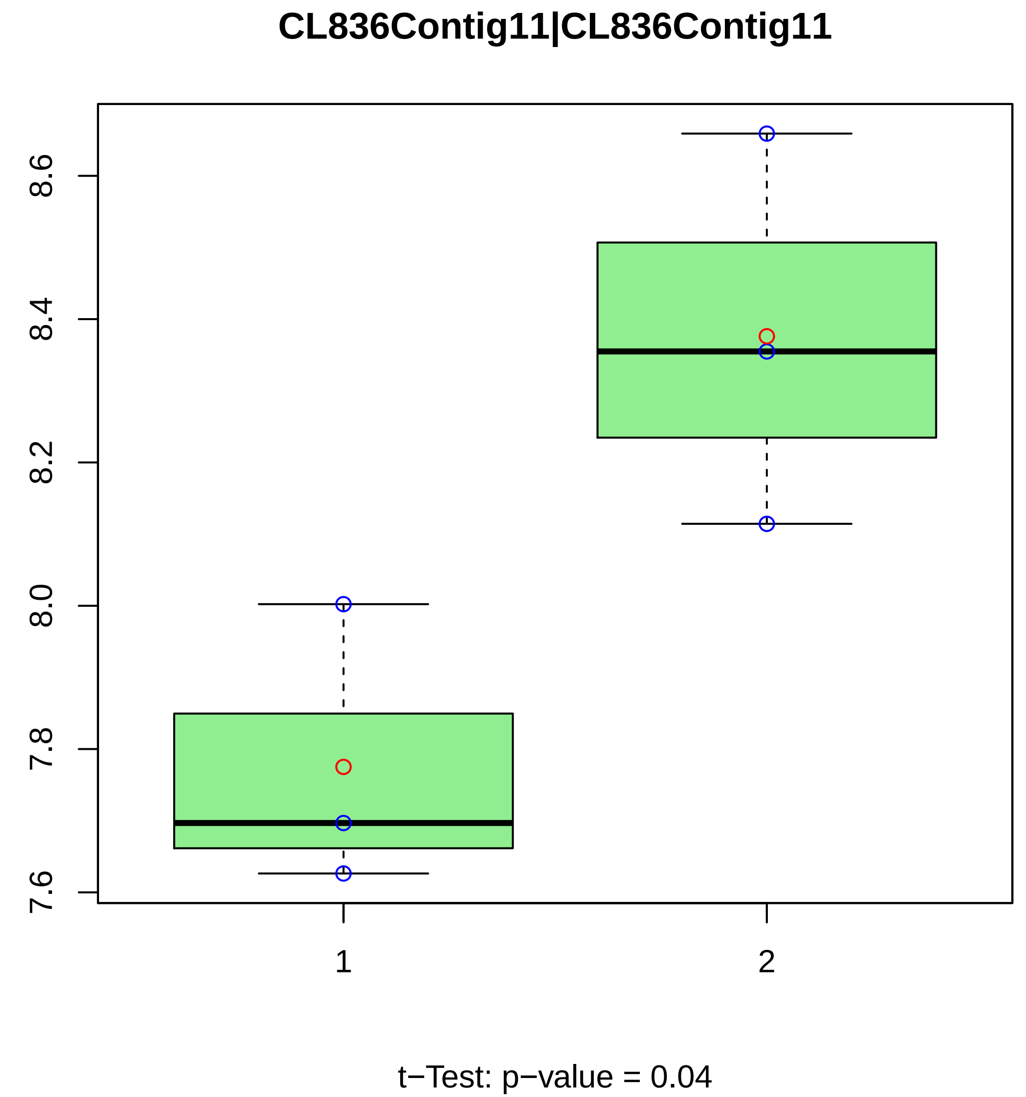
CL836Contig11|CL836Contig11
8.6
8.4
8.2
8.0
7.8
7.6
1
2
t−Test: p−value = 0.04

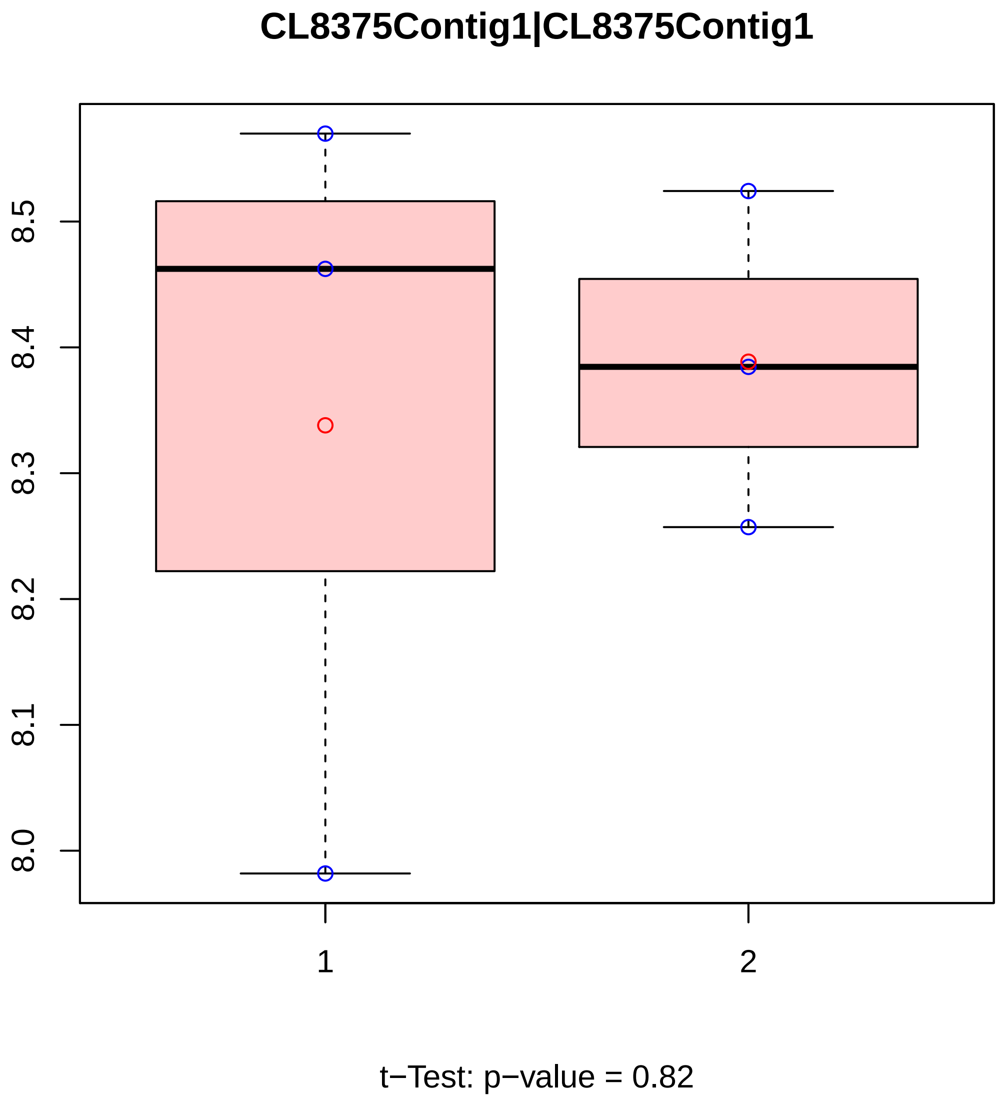

CL8375Contig1|CL8375Contig1
8.0
8.1
8.2
8.3
8.4
8.5
1
2
t−Test: p−value = 0.82

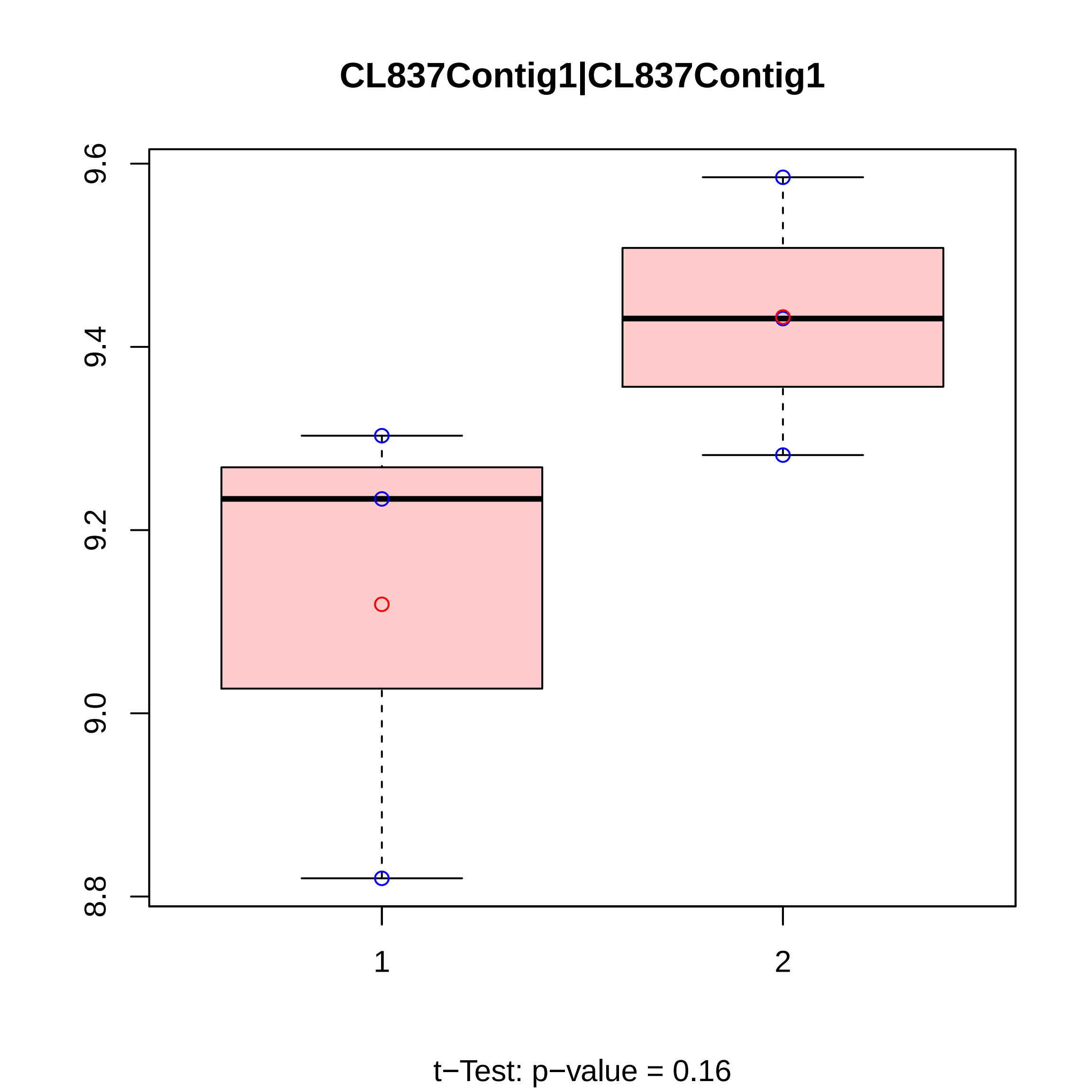
CL837Contig1|CL837Contig1
9.6
9.4
9.2
9.0
8.8
1
2
t−Test: p−value = 0.16

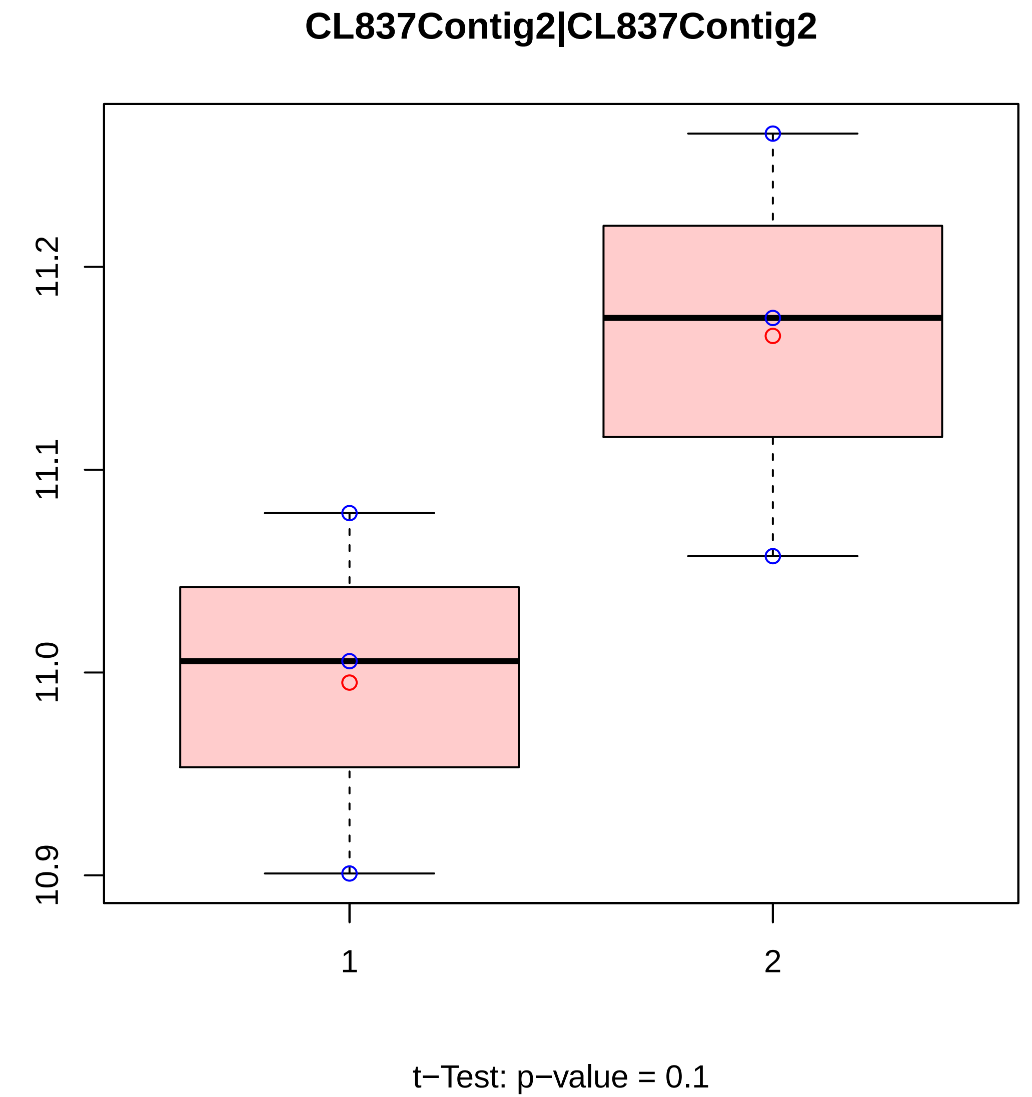
CL837Contig2|CL837Contig2
11.2
11.1
11.0
10.9
1
2
t−Test: p−value = 0.1

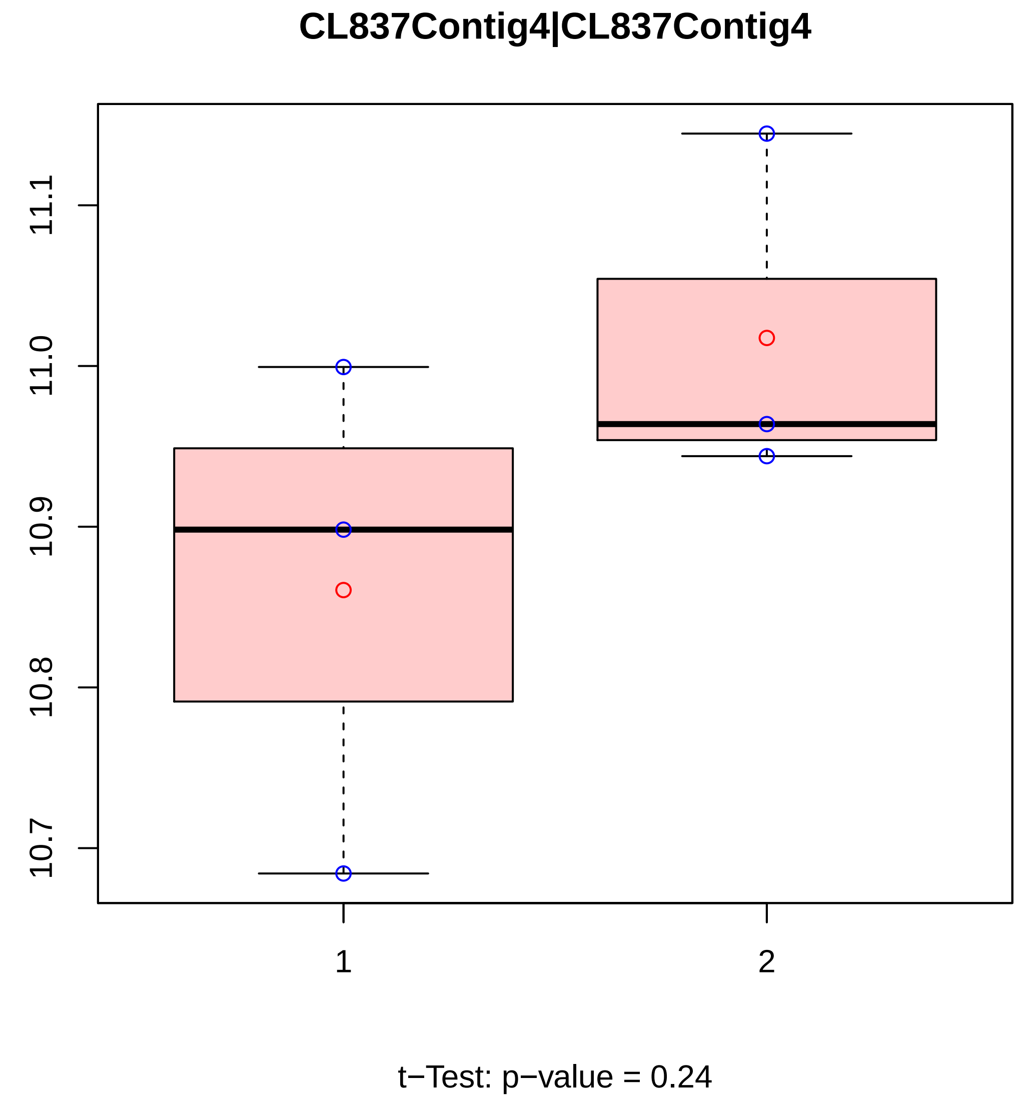
CL837Contig4|CL837Contig4
11.1
11.0
10.9
10.8
10.7
1
2
t−Test: p−value = 0.24

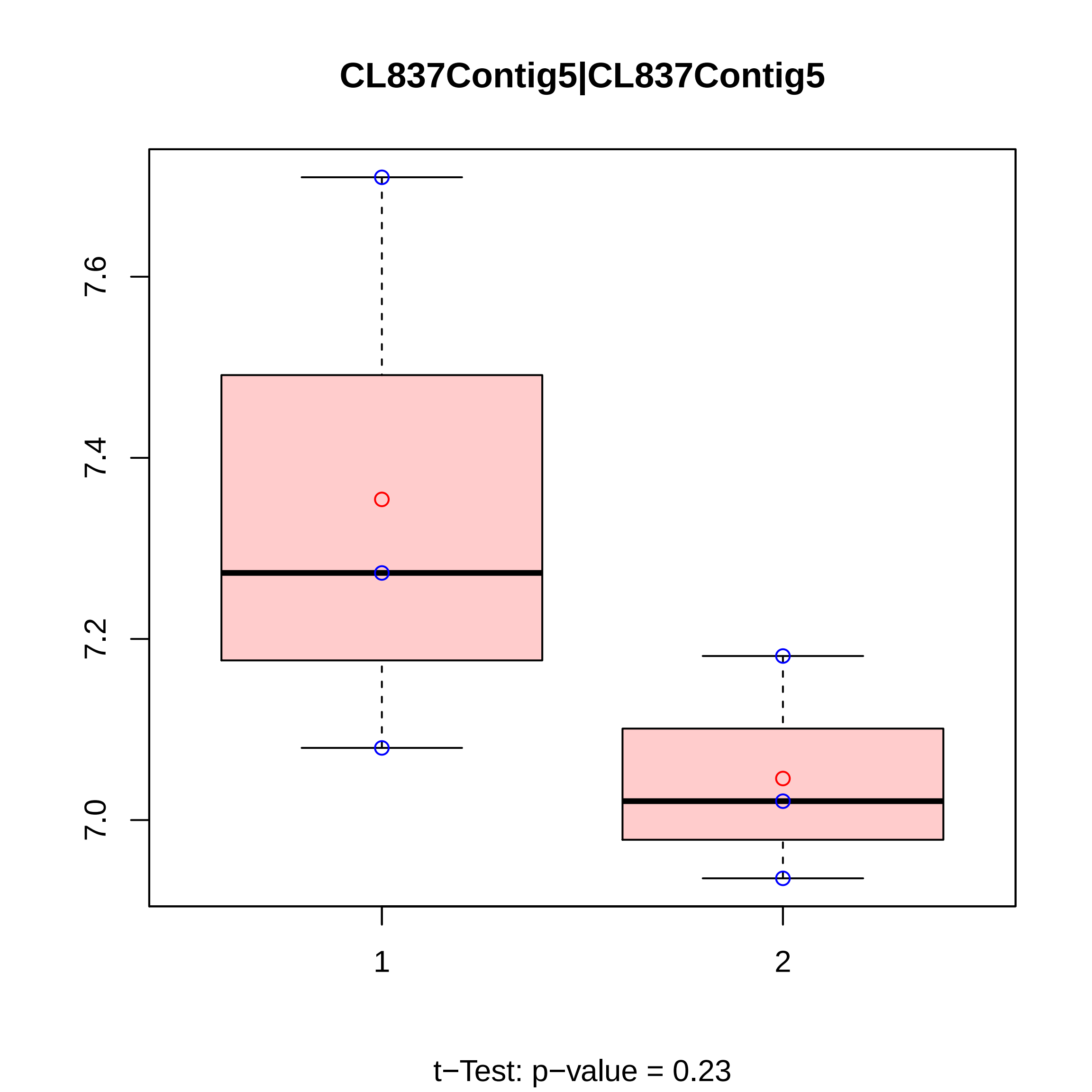
CL837Contig5|CL837Contig5
7.0
7.2
7.4
7.6
1
2
t−Test: p−value = 0.23

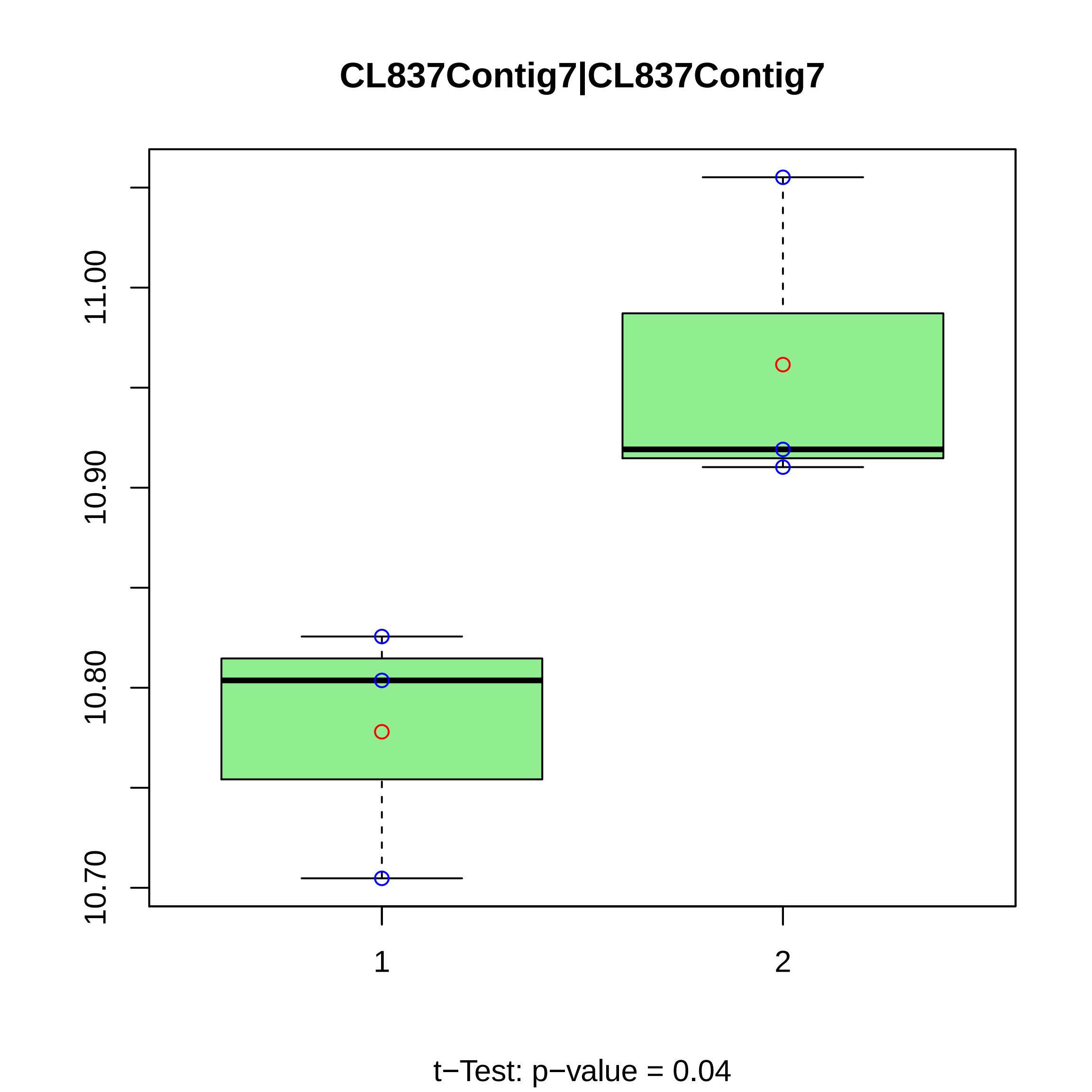
CL837Contig7|CL837Contig7
11.00
10.90
10.80
10.70
1
2
t−Test: p−value = 0.04

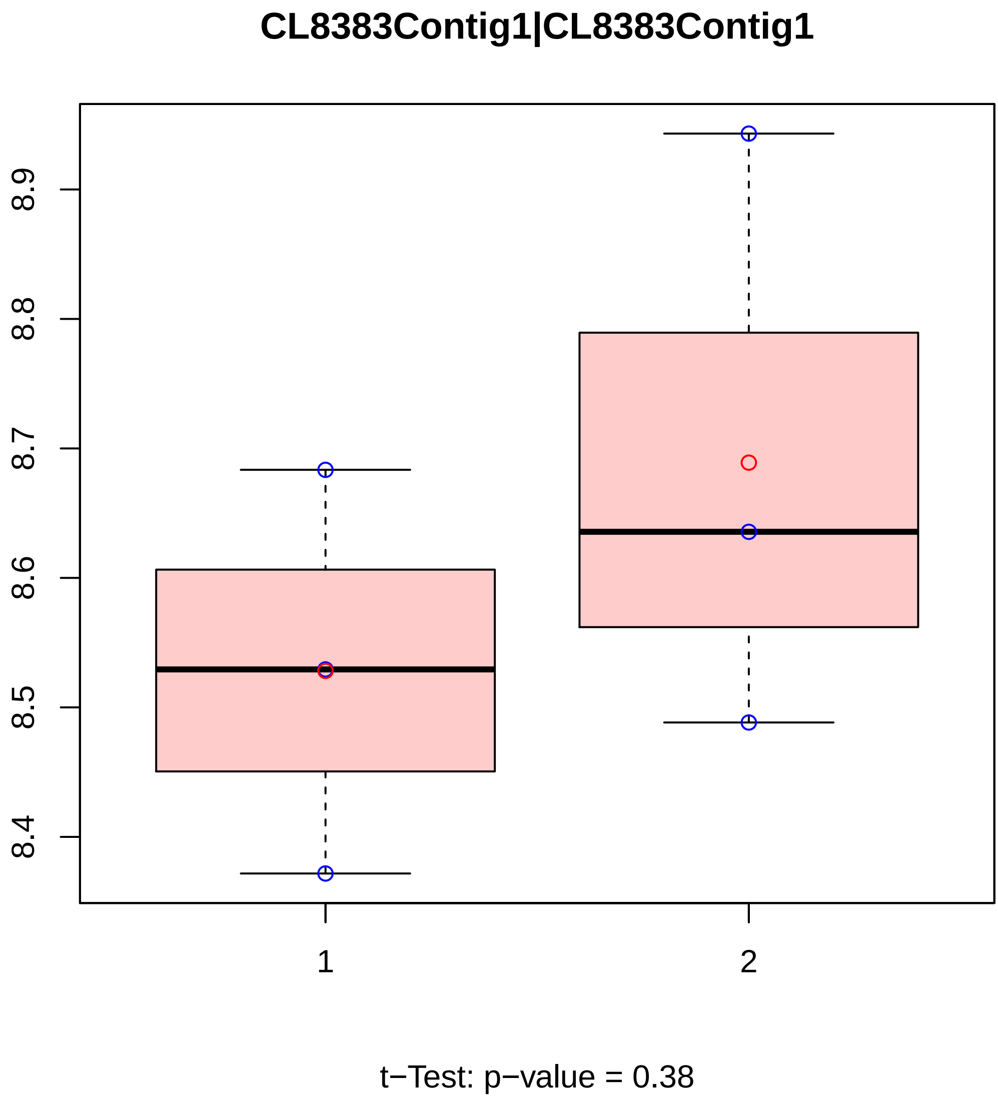
CL8383Contig1|CL8383Contig1
8.4
8.5
8.6
8.7
8.8
8.9
1
2
t−Test: p−value = 0.38

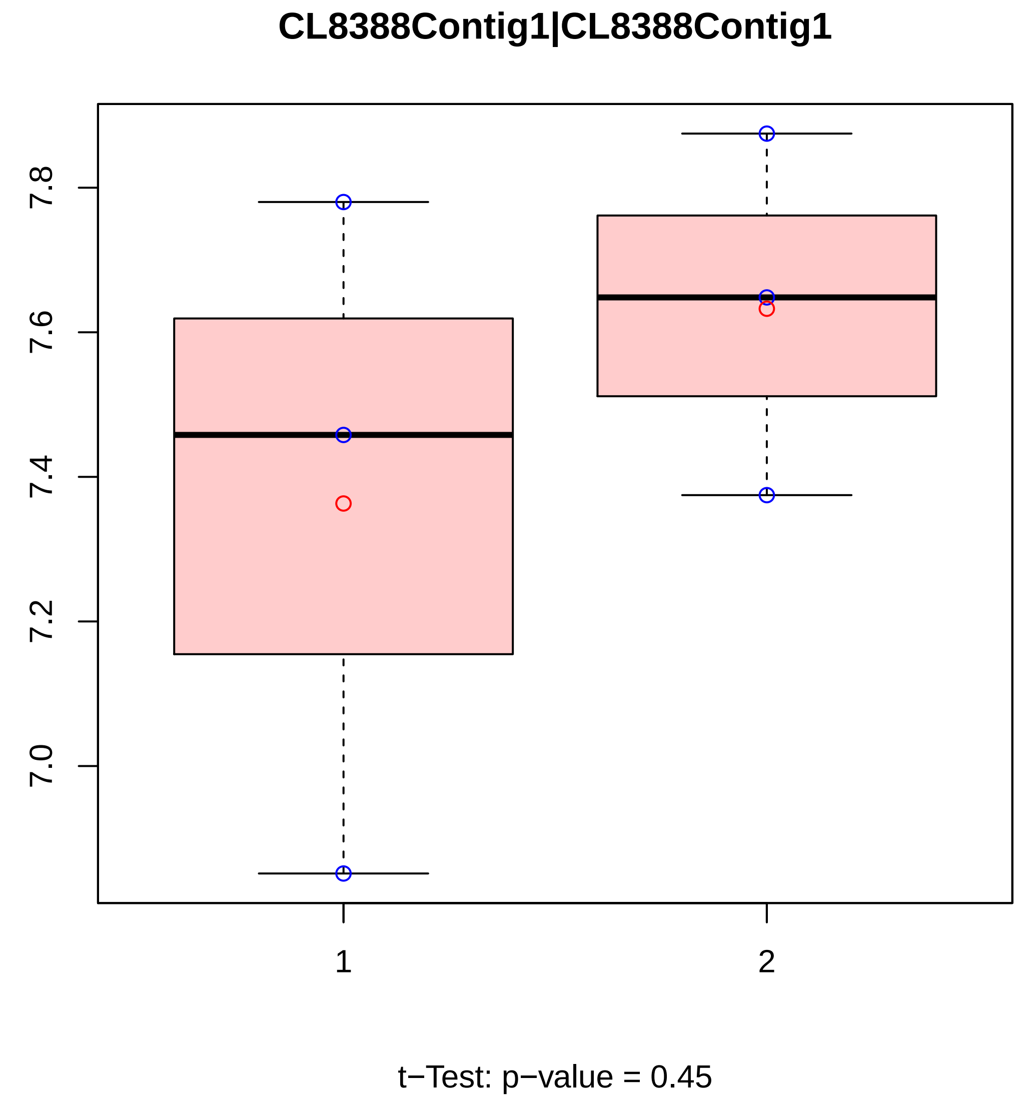
CL8388Contig1|CL8388Contig1
7.0
7.2
7.4
7.6
7.8
1
2
t−Test: p−value = 0.45

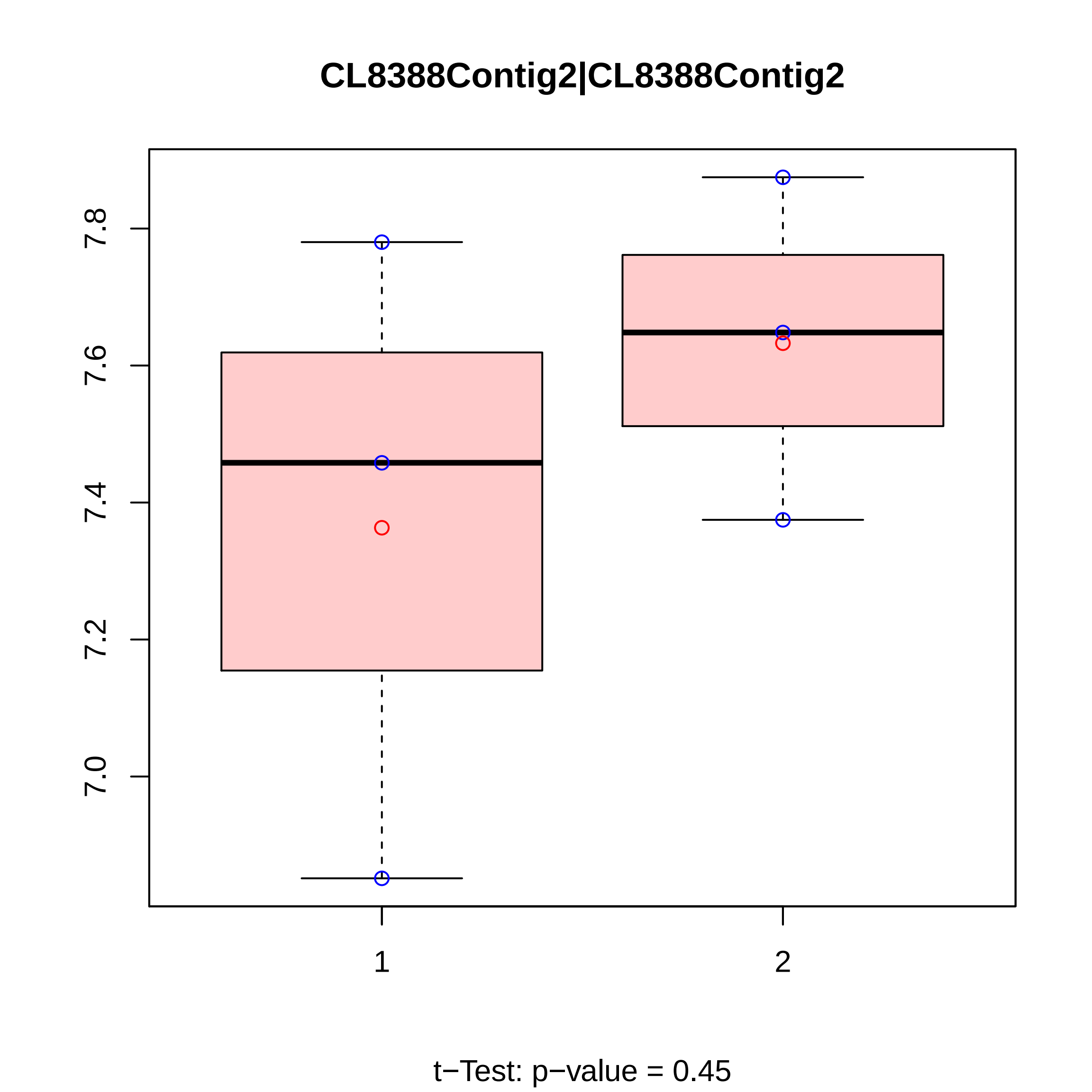
CL8388Contig2|CL8388Contig2
7.0
7.2
7.4
7.6
7.8
1
2
t−Test: p−value = 0.45

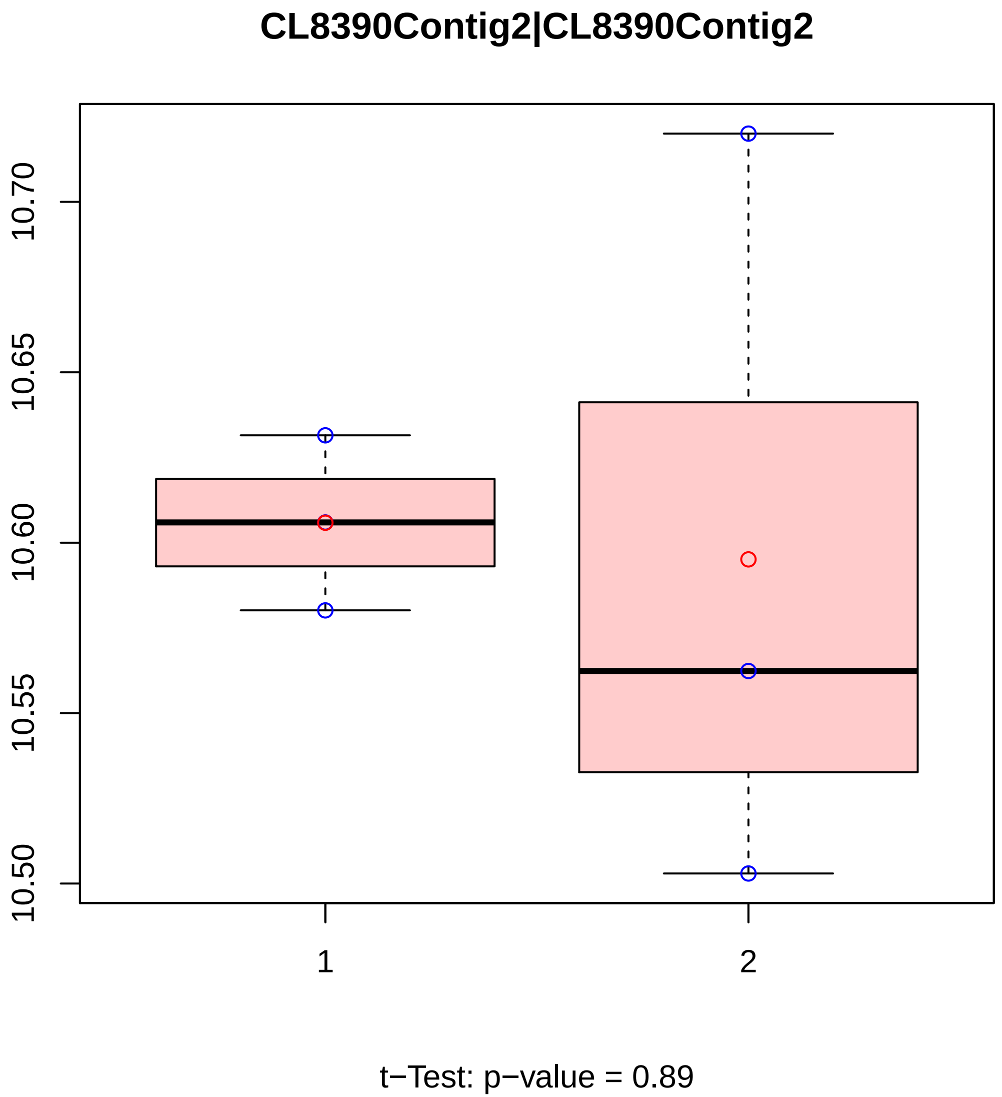
CL8390Contig2|CL8390Contig2
10.50
10.55
10.60
10.65
10.70
1
2
t−Test: p−value = 0.89

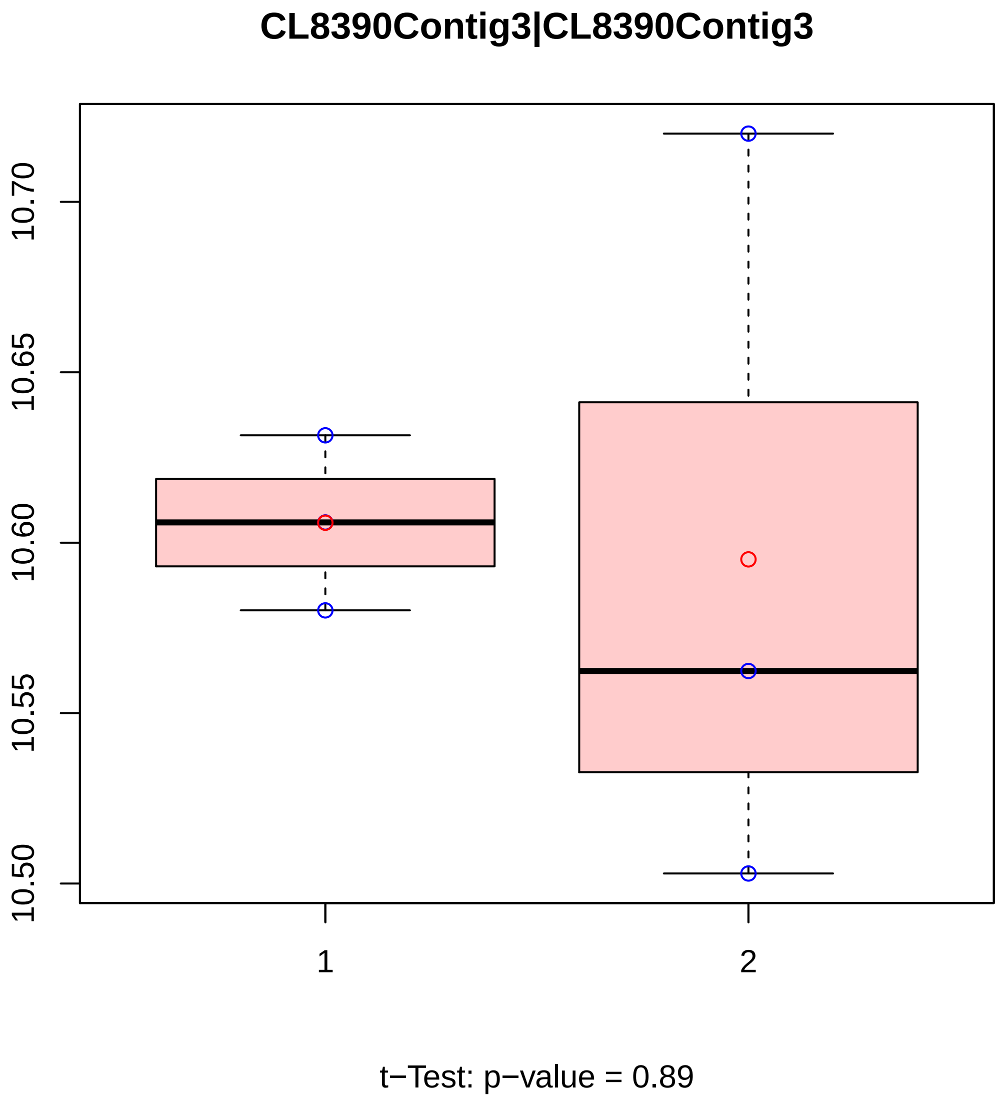

CL8390Contig3|CL8390Contig3
10.50
10.55
10.60
10.65
10.70
1
2
t−Test: p−value = 0.89

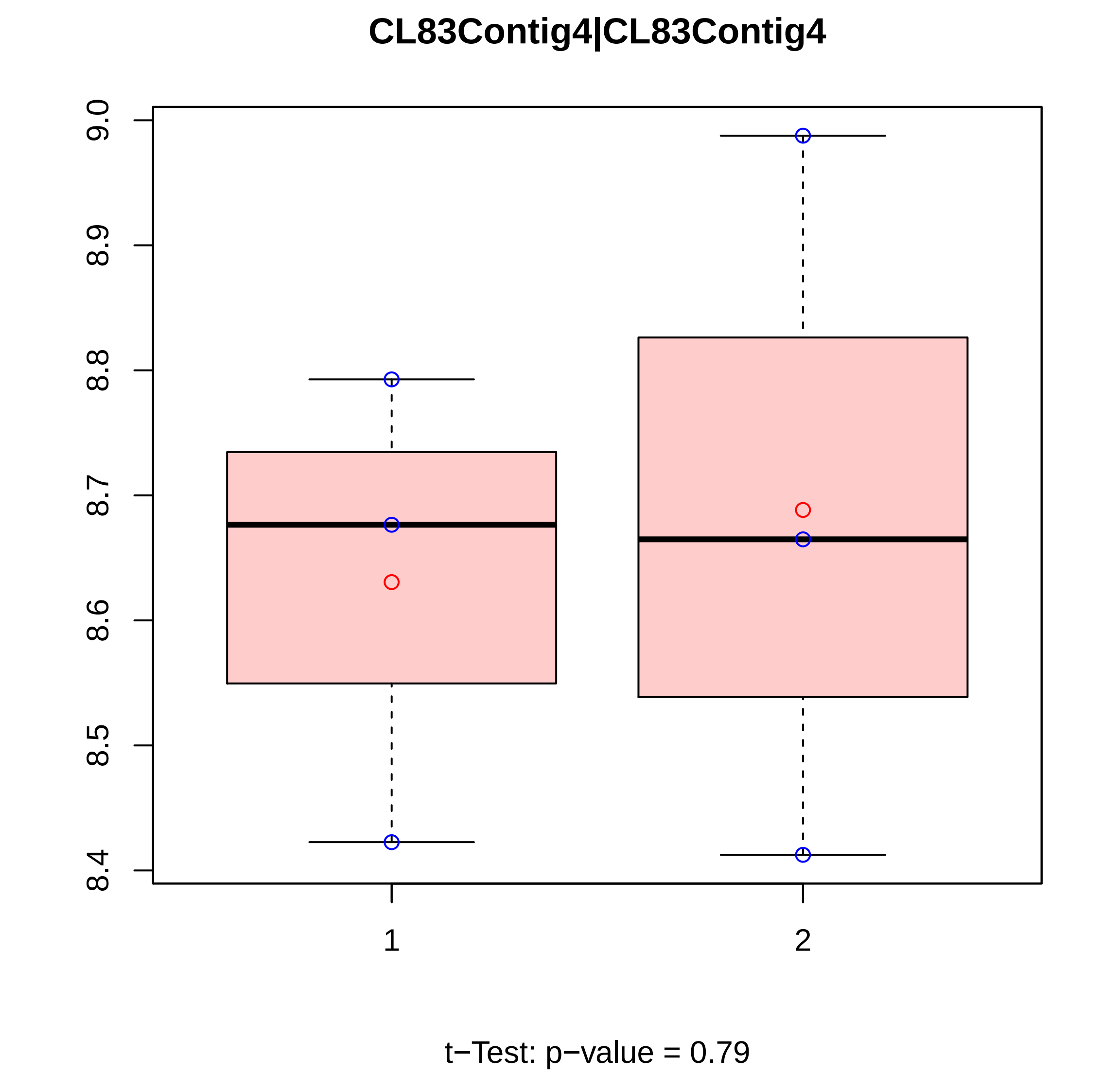
CL83Contig4|CL83Contig4
9.0
8.9
8.8
8.7
8.6
8.5
8.4
1
2
t−Test: p−value = 0.79

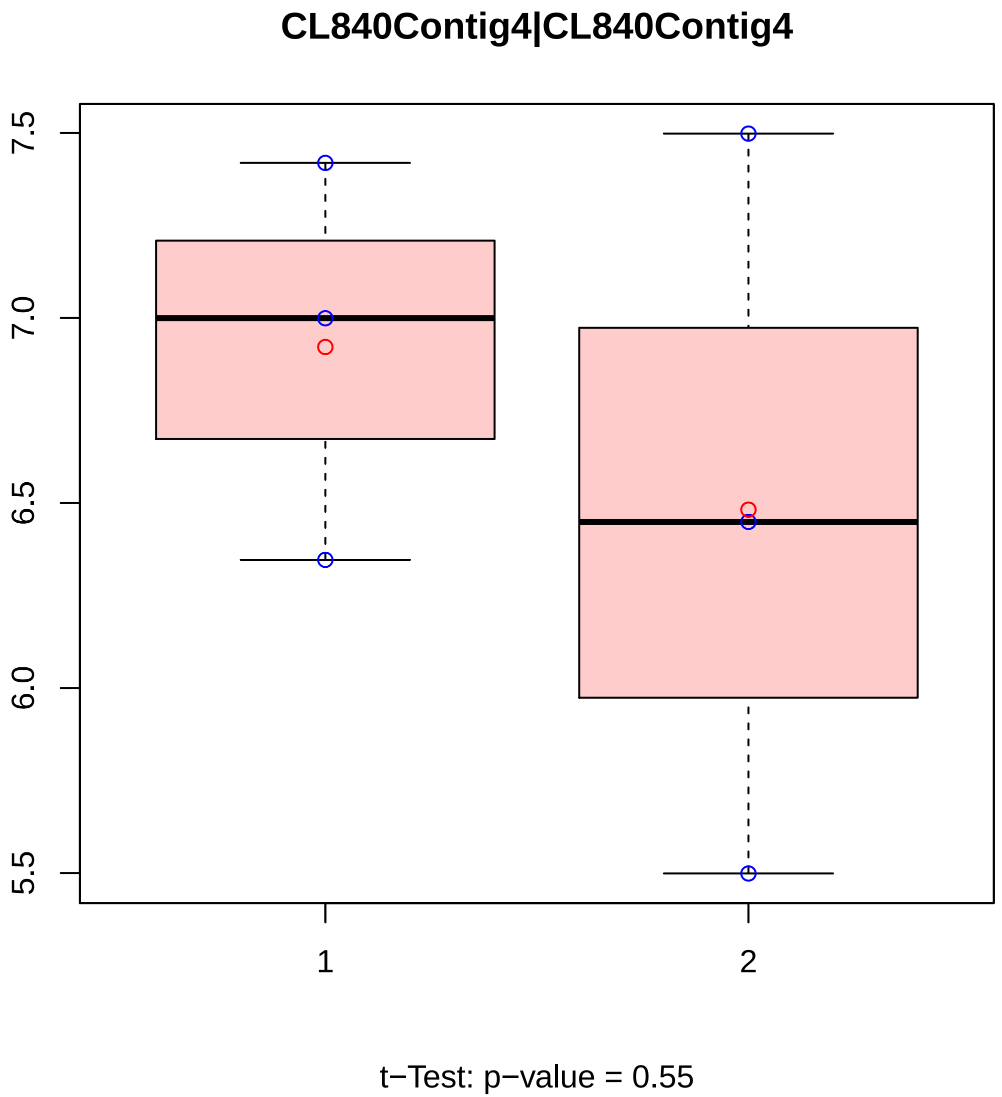

CL840Contig4|CL840Contig4
7.5
7.0
6.5
6.0
5.5
1
2
t−Test: p−value = 0.55

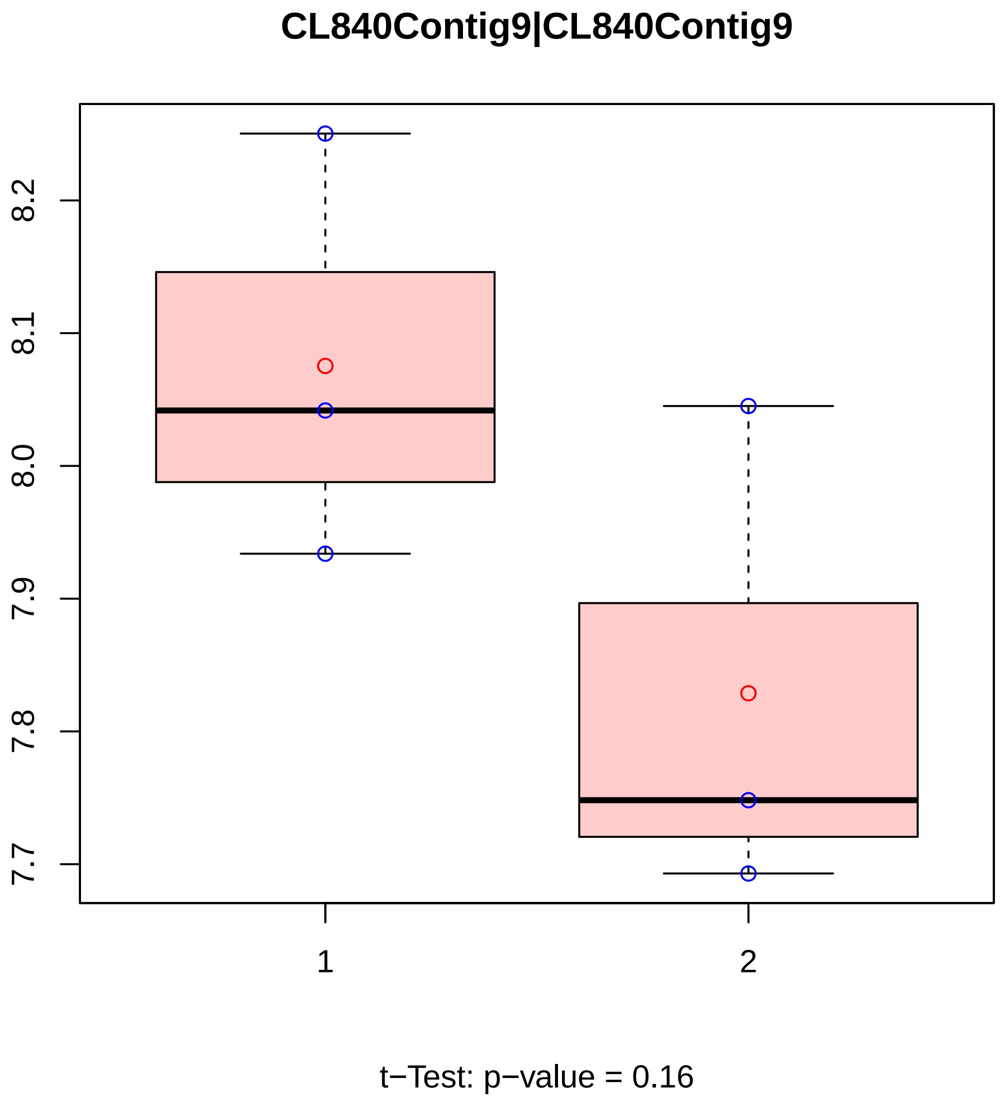

CL840Contig9|CL840Contig9
8.2
8.1
8.0
7.9
7.8
7.7
1
2
t−Test: p−value = 0.16

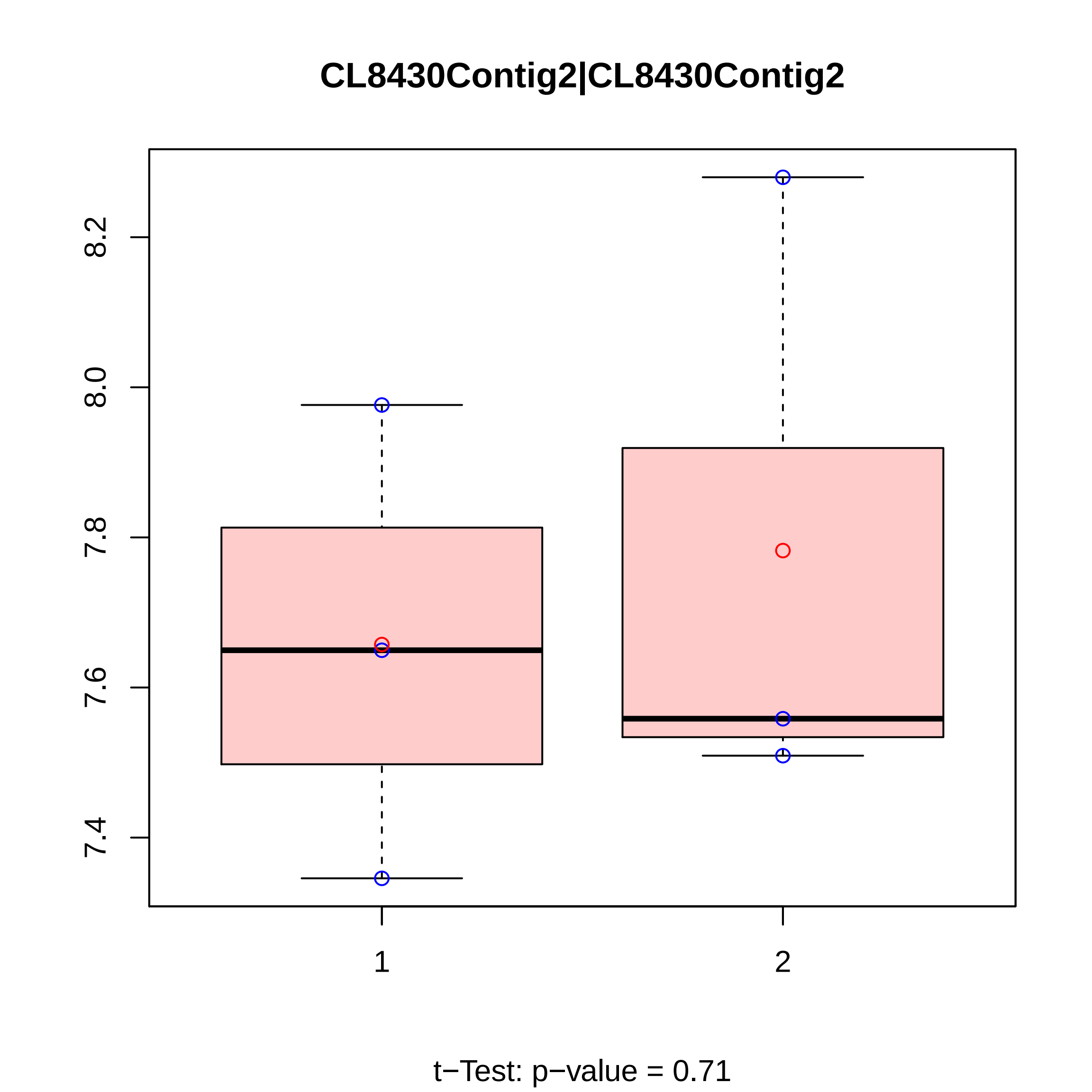
CL8430Contig2|CL8430Contig2
8.2
8.0
7.8
7.6
7.4
1
2
t−Test: p−value = 0.71

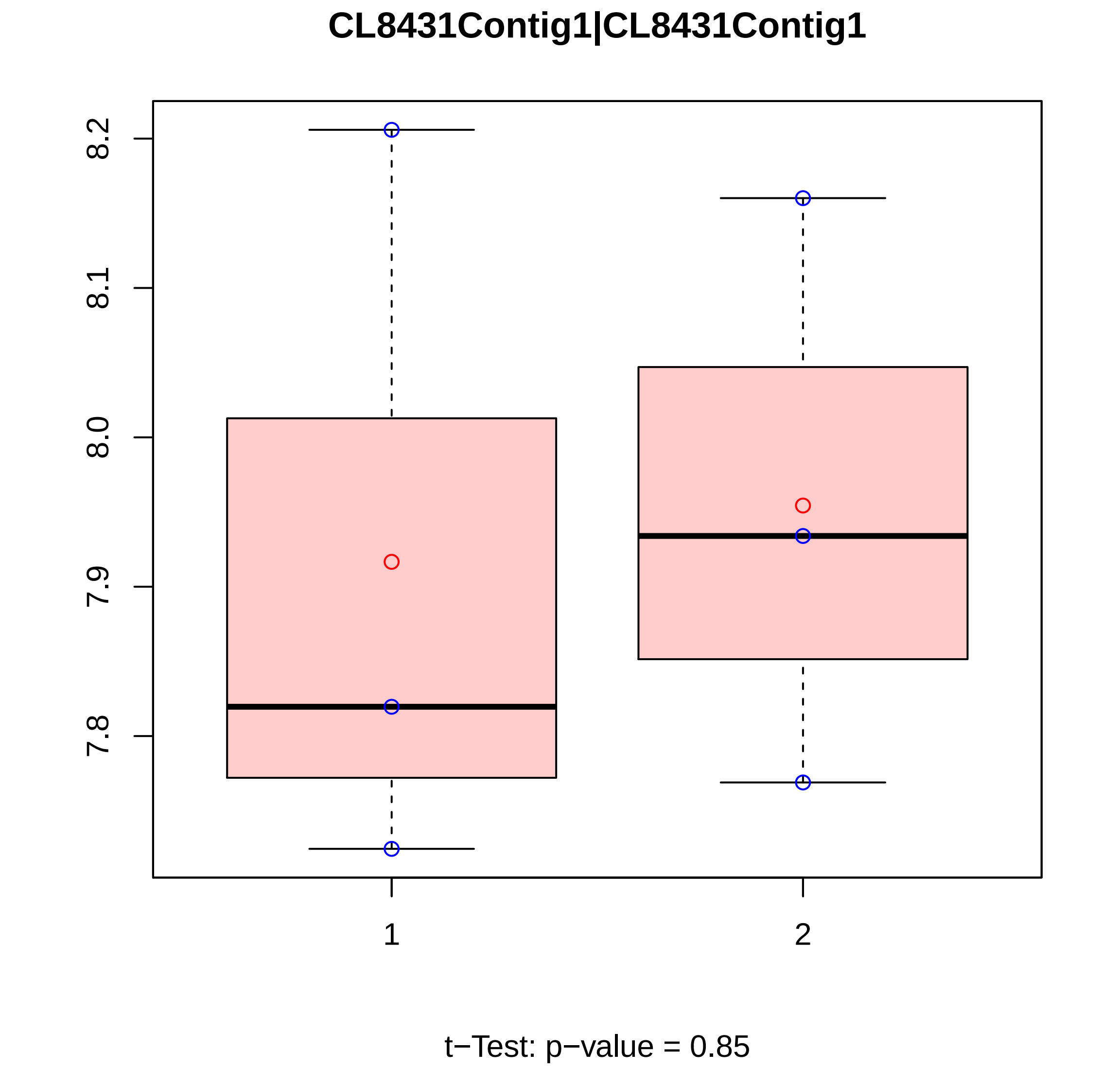

CL8431Contig1|CL8431Contig1
8.2
8.1
8.0
7.9
7.8
1
2
t−Test: p−value = 0.85

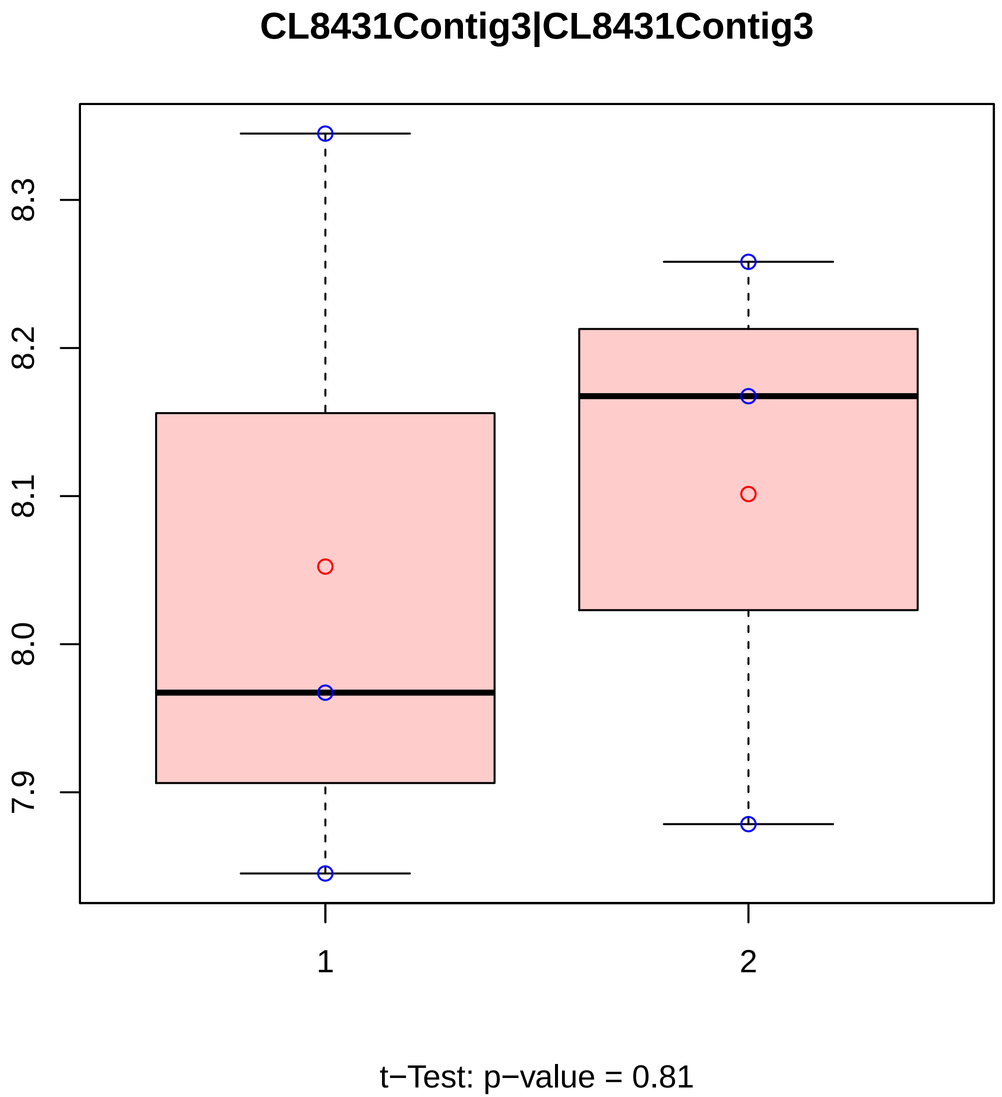

CL8431Contig3|CL8431Contig3
7.9
8.0
8.1
8.2
8.3
1
2
t−Test: p−value = 0.81

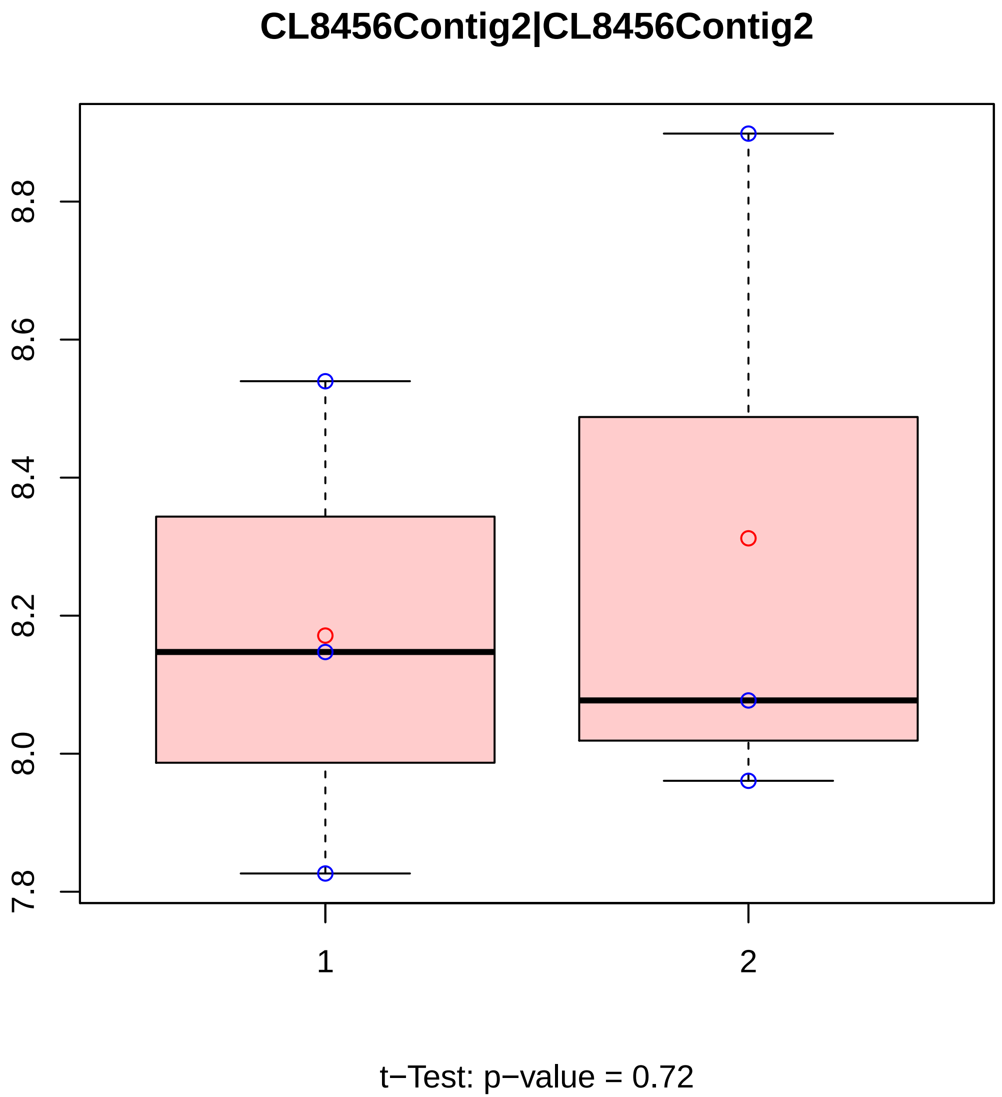
CL8456Contig2|CL8456Contig2
7.8
8.0
8.2
8.4
8.6
8.8
1
2
t−Test: p−value = 0.72

# CL846Contig15|CL846Contig15

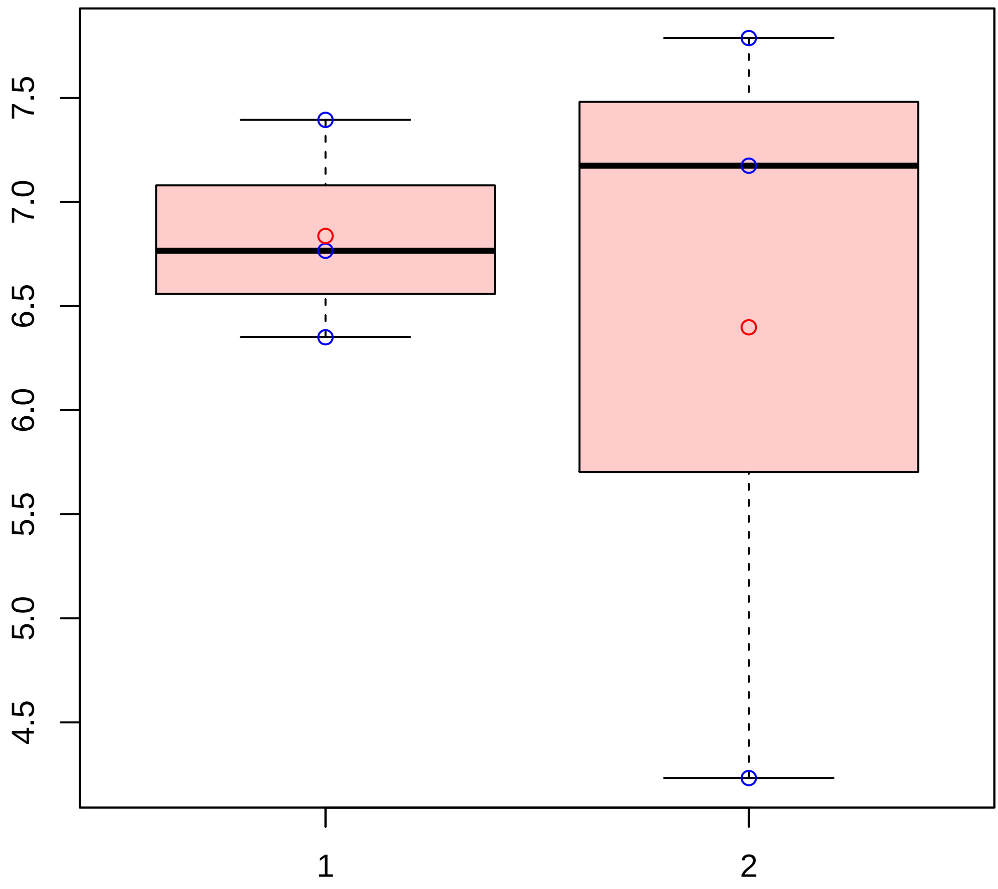


t−Test: p−value = 0.73

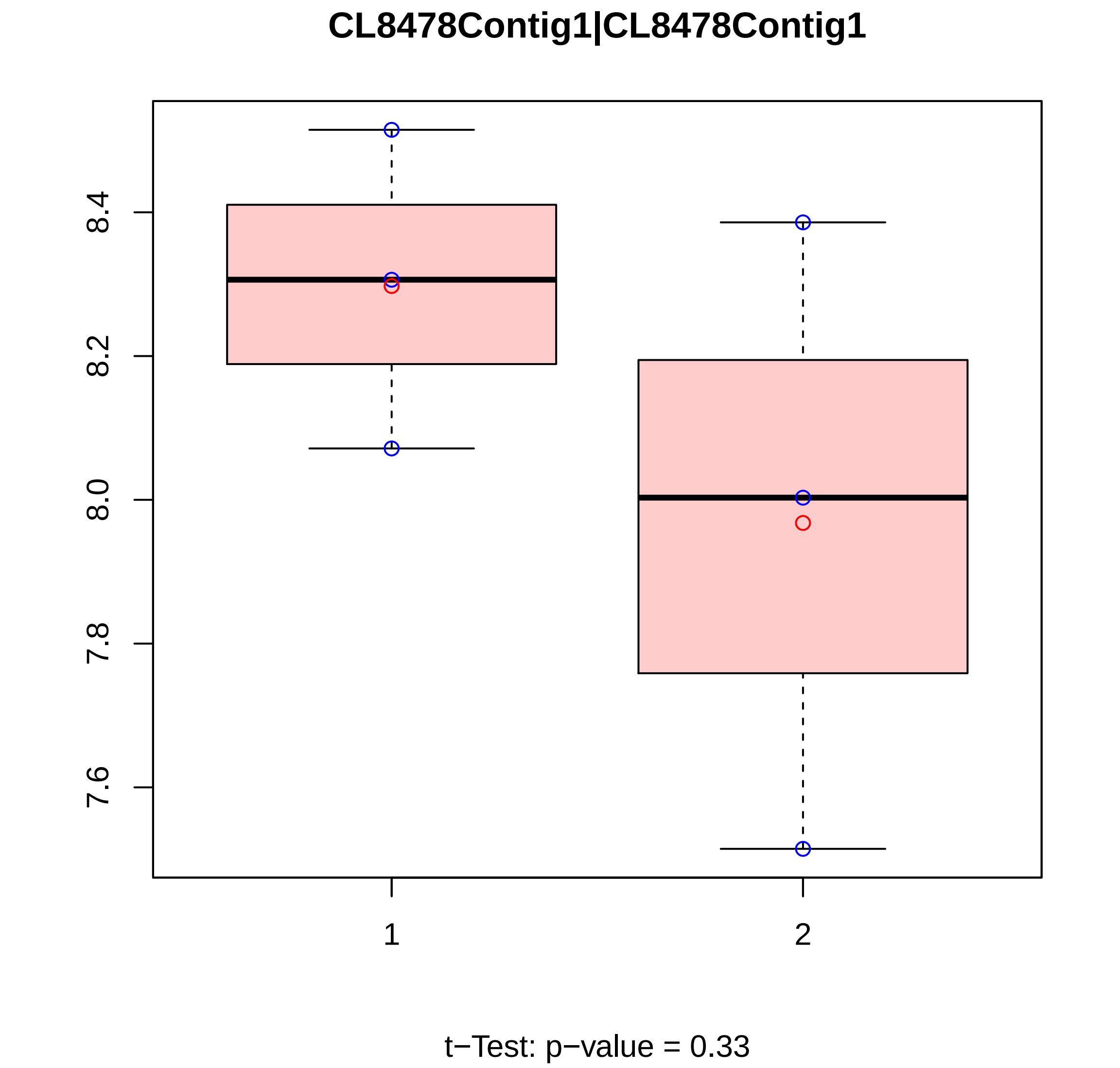
CL8478Contig1|CL8478Contig1
8.4
8.2
8.0
7.8
7.6
1
2
t−Test: p−value = 0.33

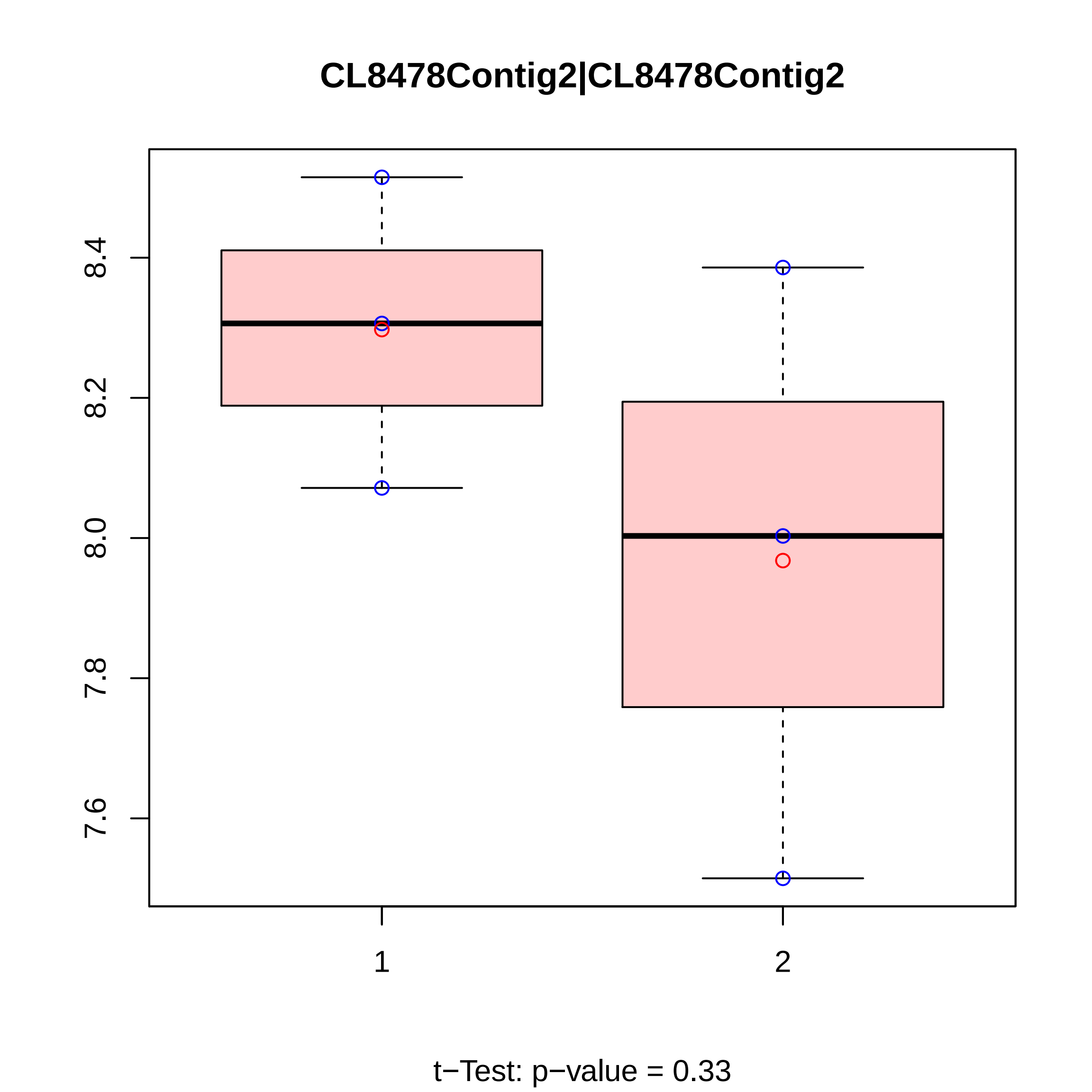
CL8478Contig2|CL8478Contig2
8.4
8.2
8.0
7.8
7.6
1
2
t−Test: p−value = 0.33

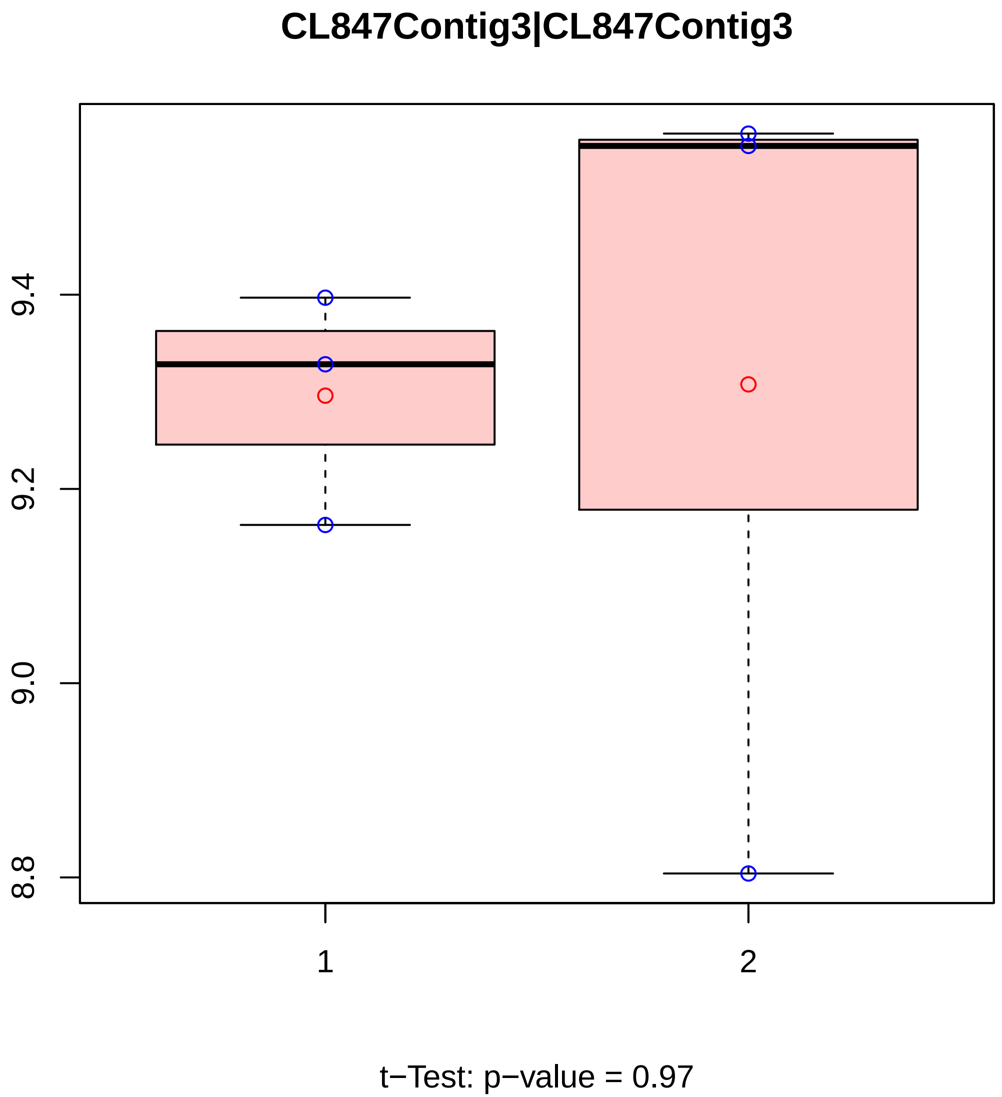
CL847Contig3|CL847Contig3
9.4
9.2
9.0
8.8
1
2
t−Test: p−value = 0.97

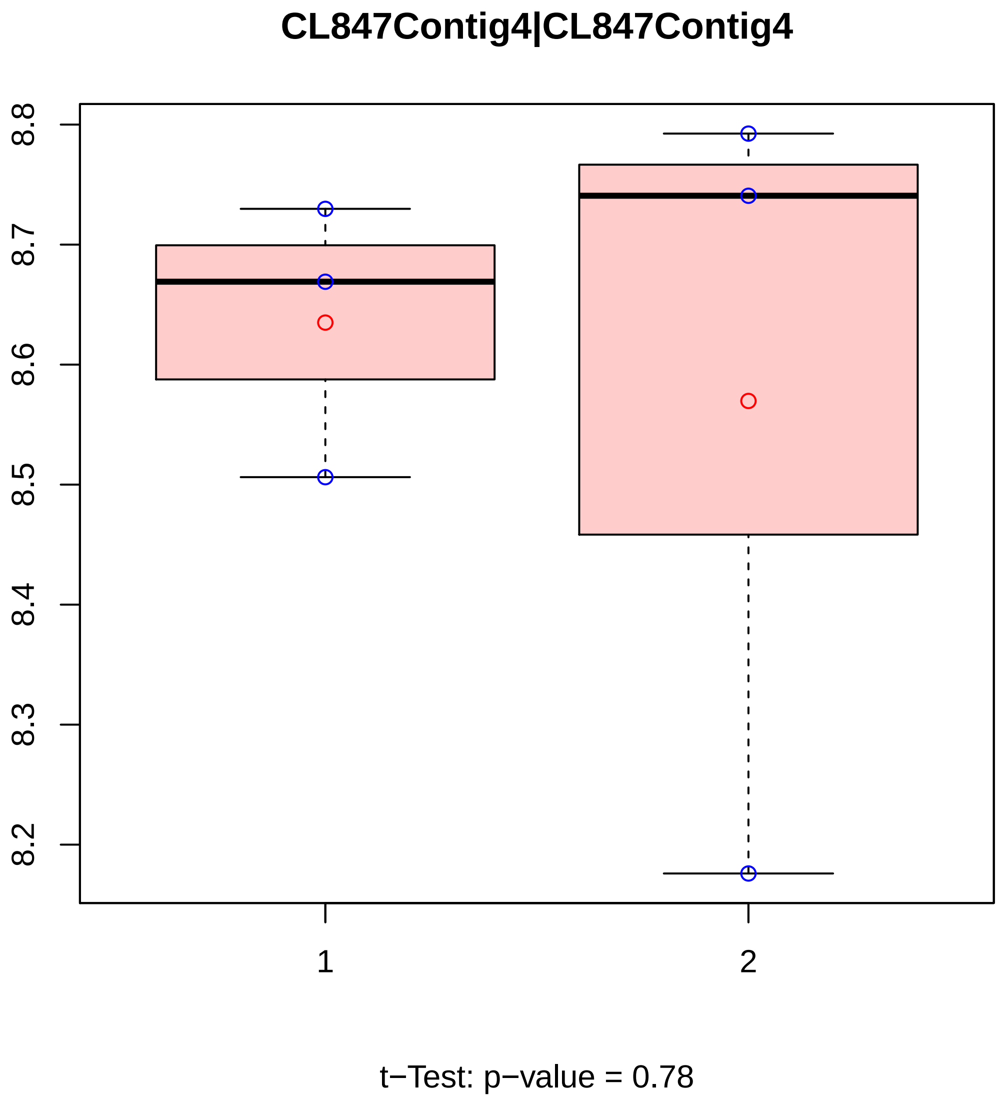

CL847Contig4|CL847Contig4
8.8
8.7
8.6
8.5
8.4
8.3
8.2
1
2
t−Test: p−value = 0.78

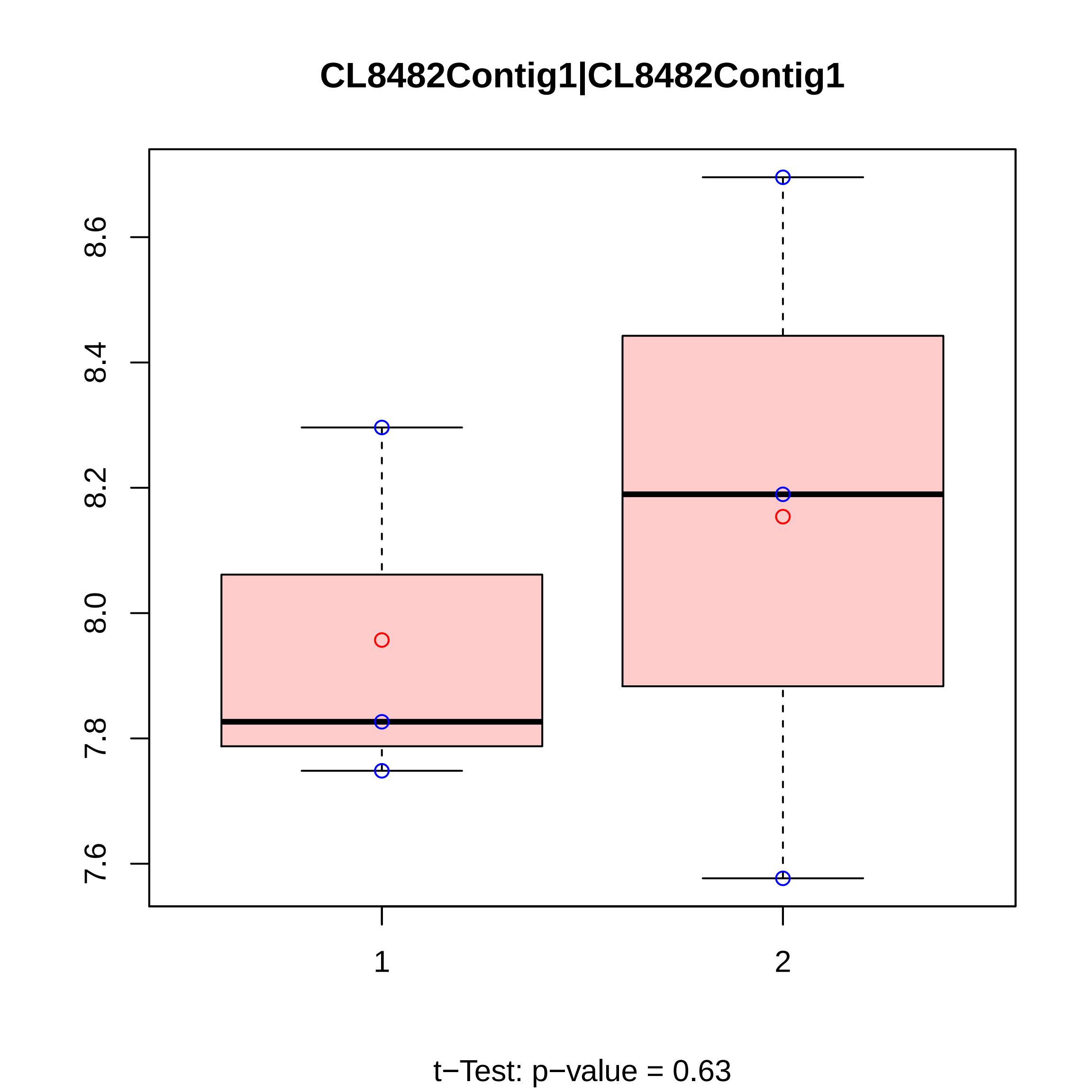

CL8482Contig1|CL8482Contig1
8.6
8.4
8.2
8.0
7.8
7.6
1
2
t−Test: p−value = 0.63

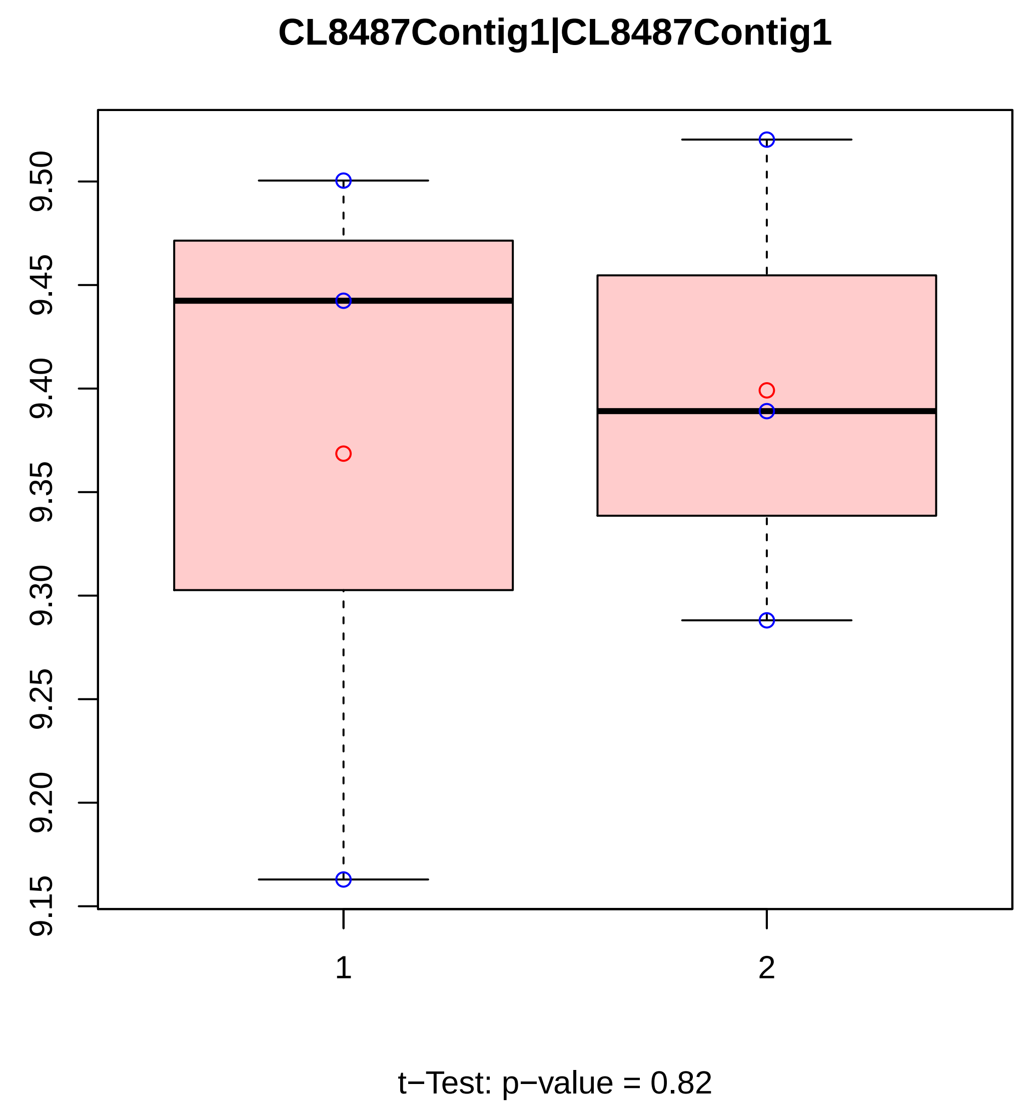

CL8487Contig1|CL8487Contig1
9.15
9.20
9.25
9.30
9.35
9.40
9.45
9.50
1
2
t−Test: p−value = 0.82

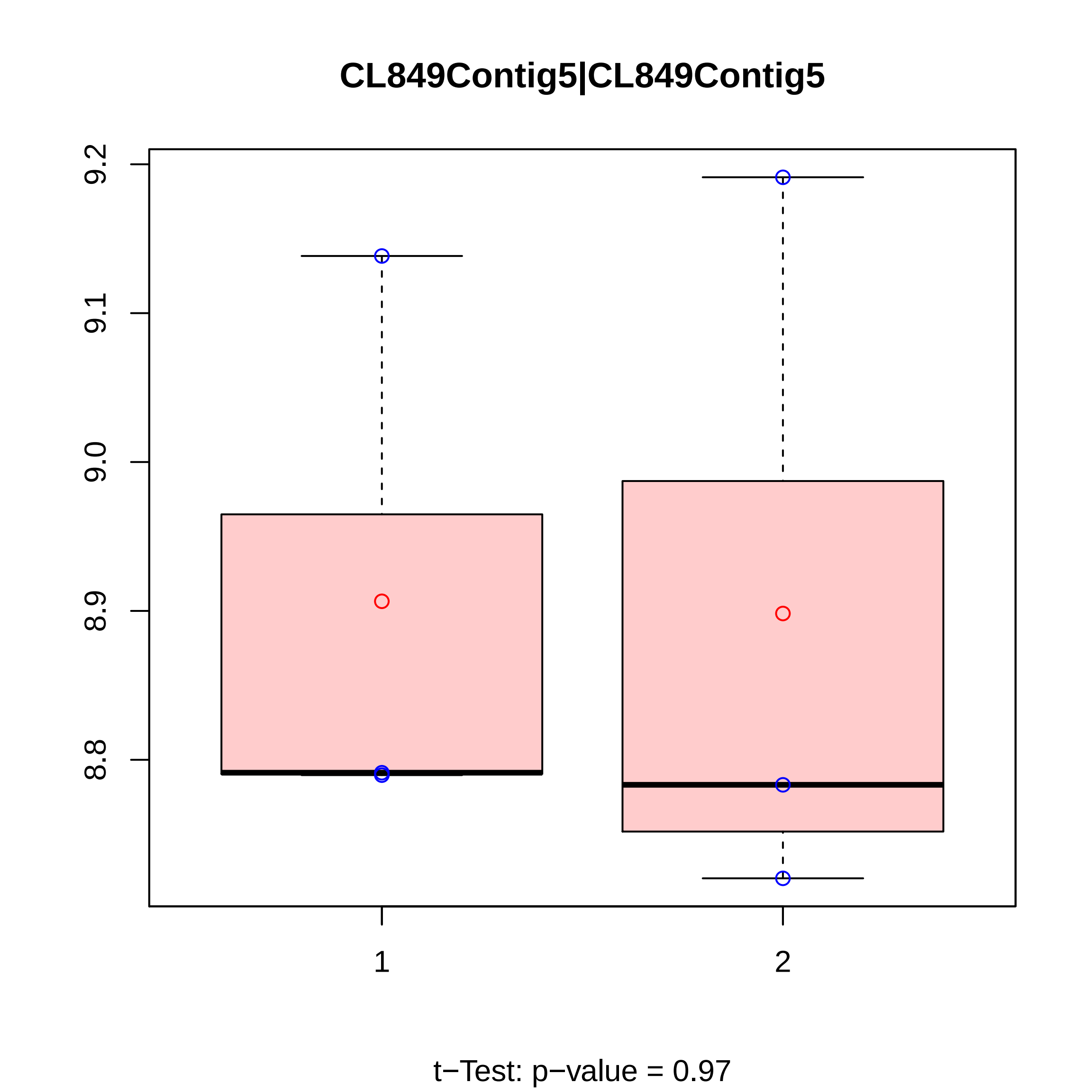
CL849Contig5|CL849Contig5
9.2
9.1
9.0
8.9
8.8
1
2
t−Test: p−value = 0.97

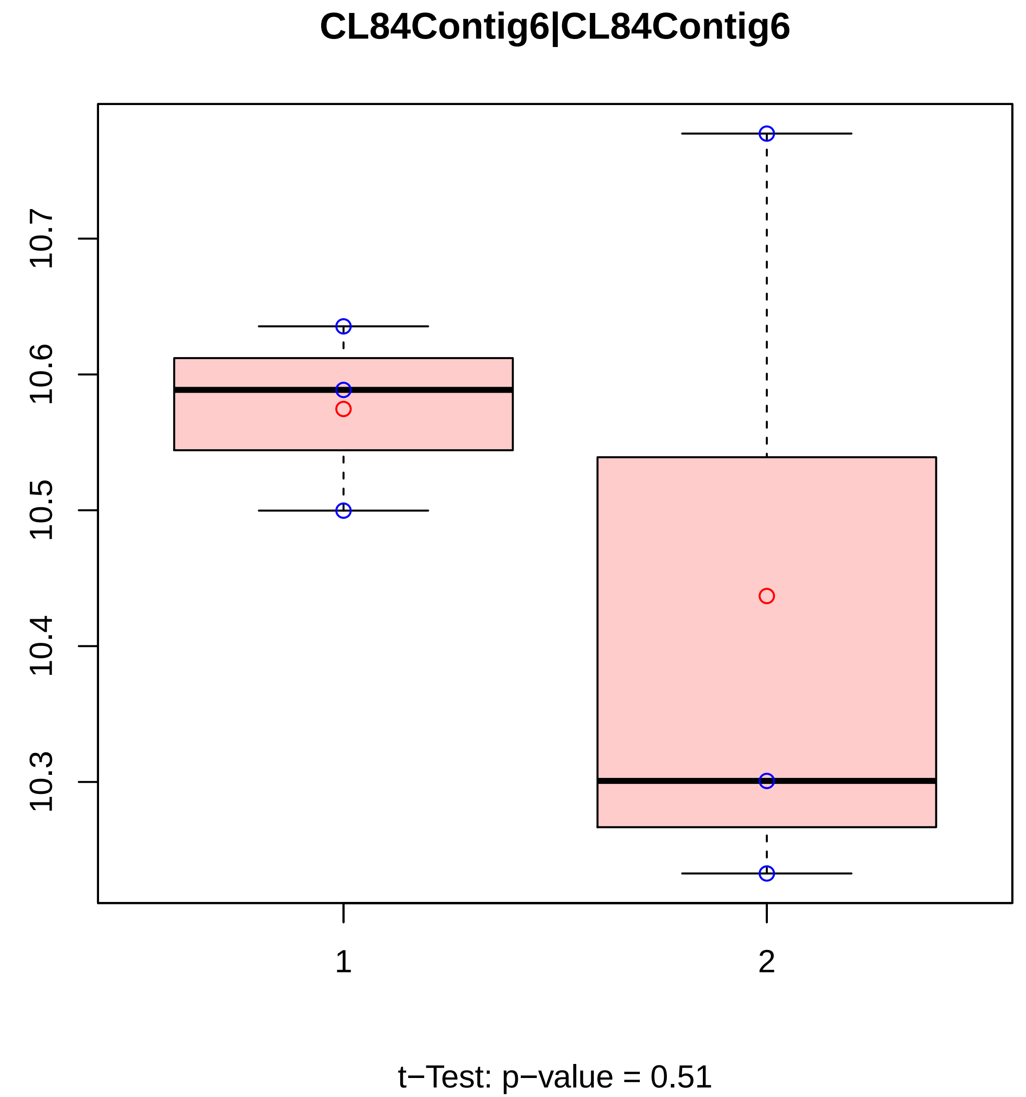
CL84Contig6|CL84Contig6
10.7
10.6
10.5
10.4
10.3
1
2
t−Test: p−value = 0.51

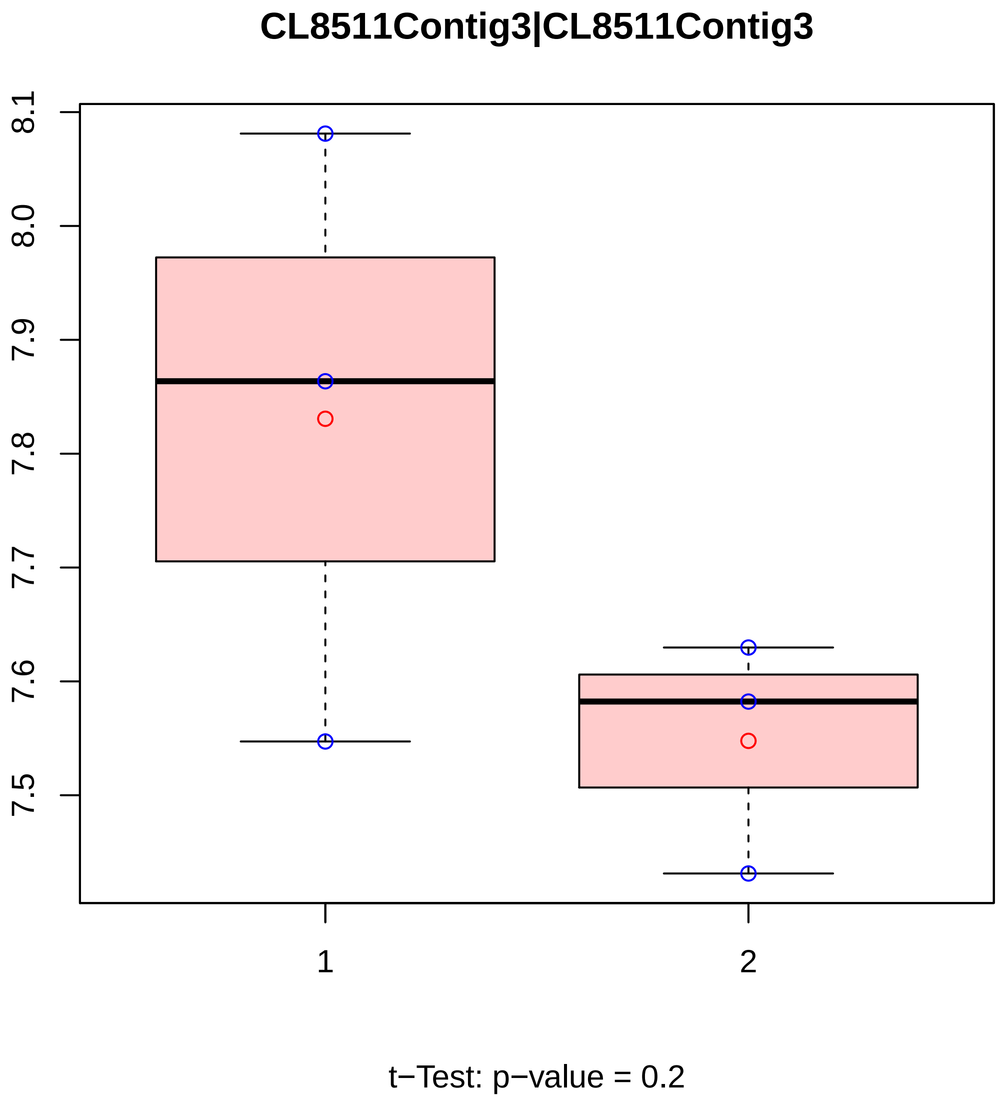
CL8511Contig3|CL8511Contig3
8.1
8.0
7.9
7.8
7.7
7.6
7.5
1
2
t−Test: p−value = 0.2

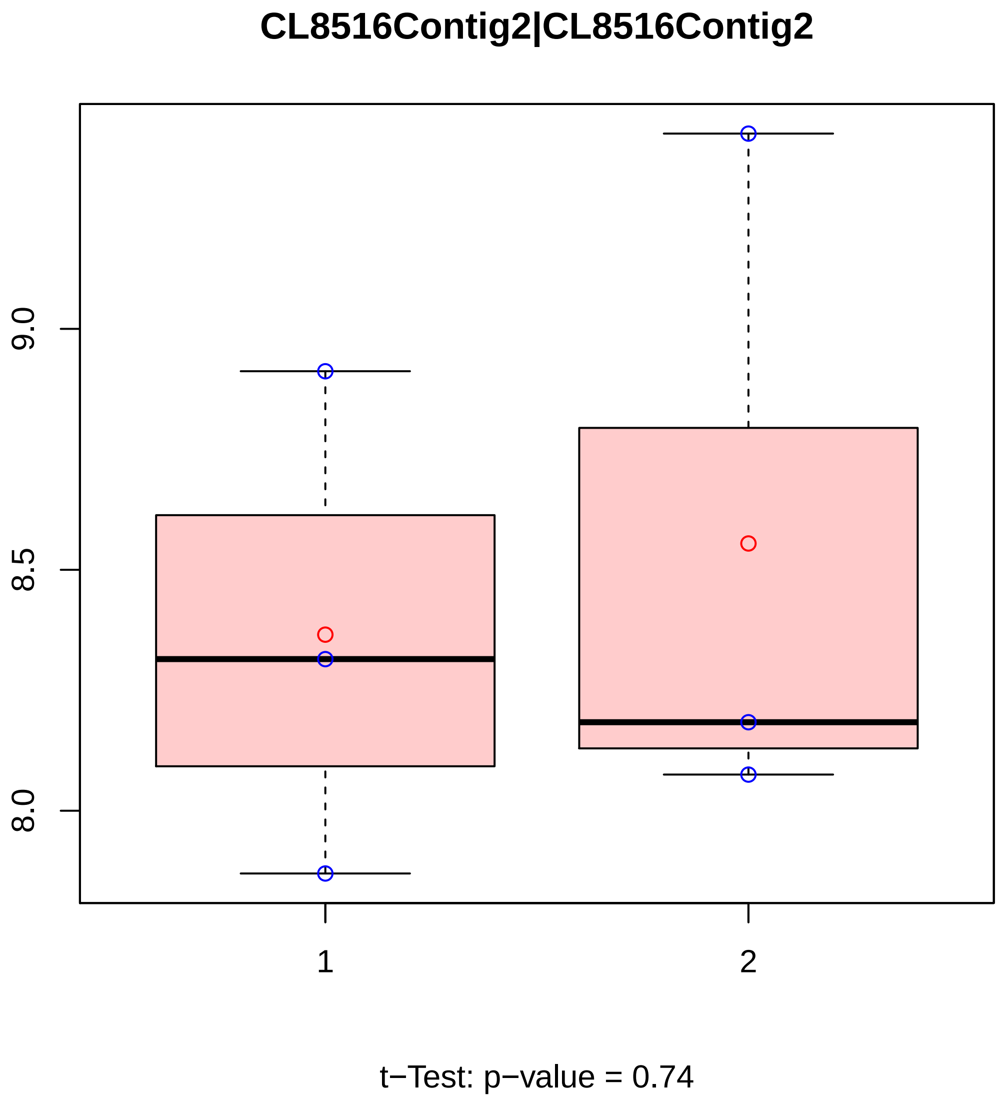
CL8516Contig2|CL8516Contig2
9.0
8.5
8.0
1
2
t−Test: p−value = 0.74

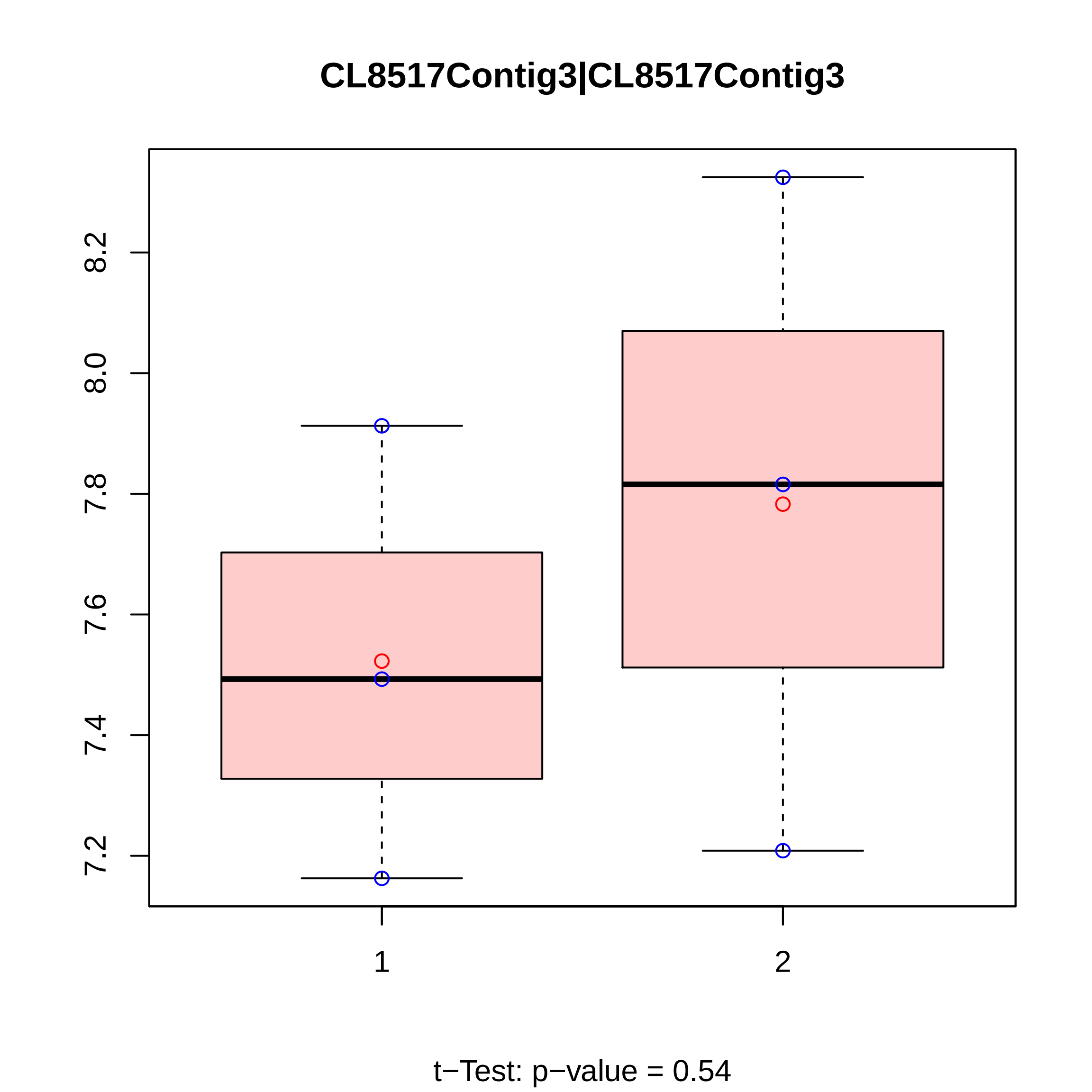
CL8517Contig3|CL8517Contig3
8.2
8.0
7.8
7.6
7.4
7.2
1
2
t−Test: p−value = 0.54

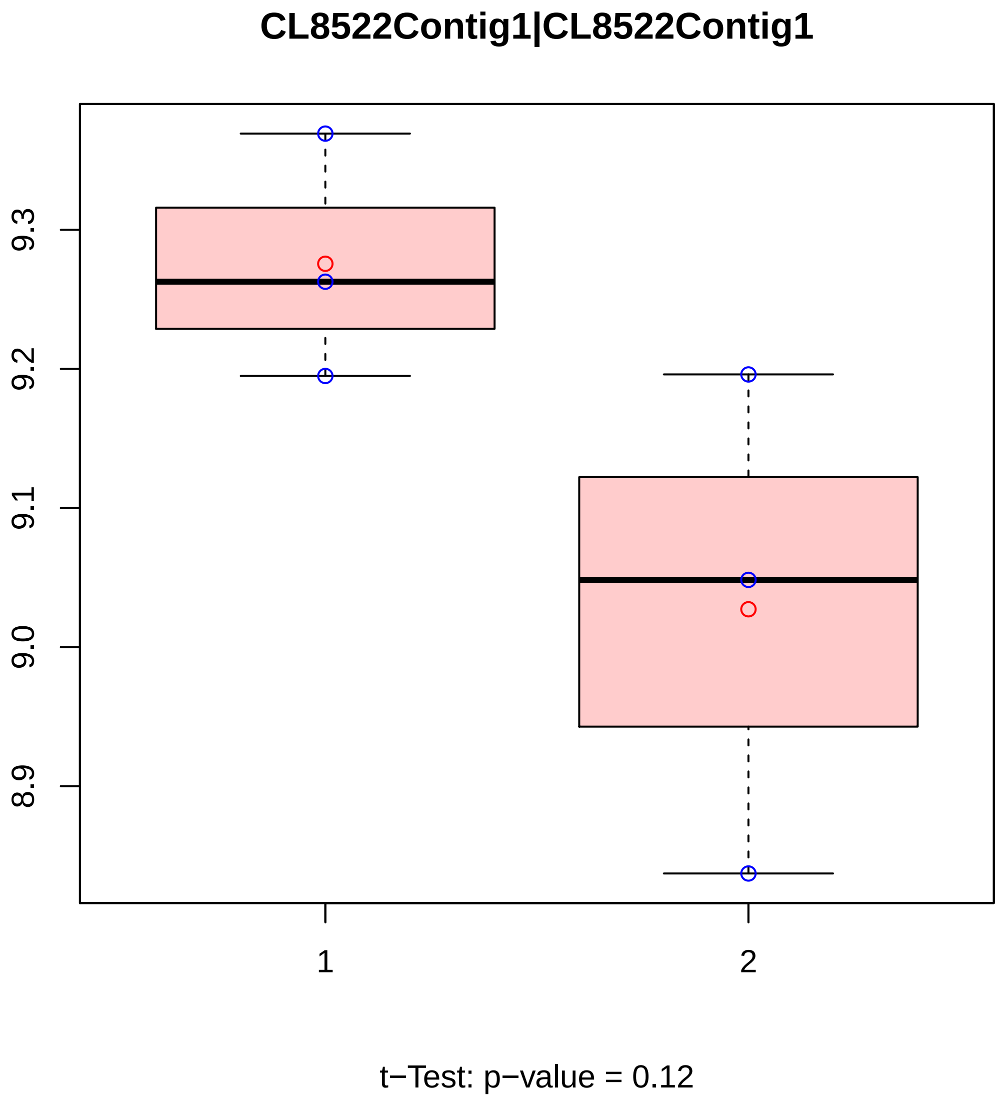
CL8522Contig1|CL8522Contig1
9.3
9.2
9.1
9.0
8.9
1
2
t−Test: p−value = 0.12

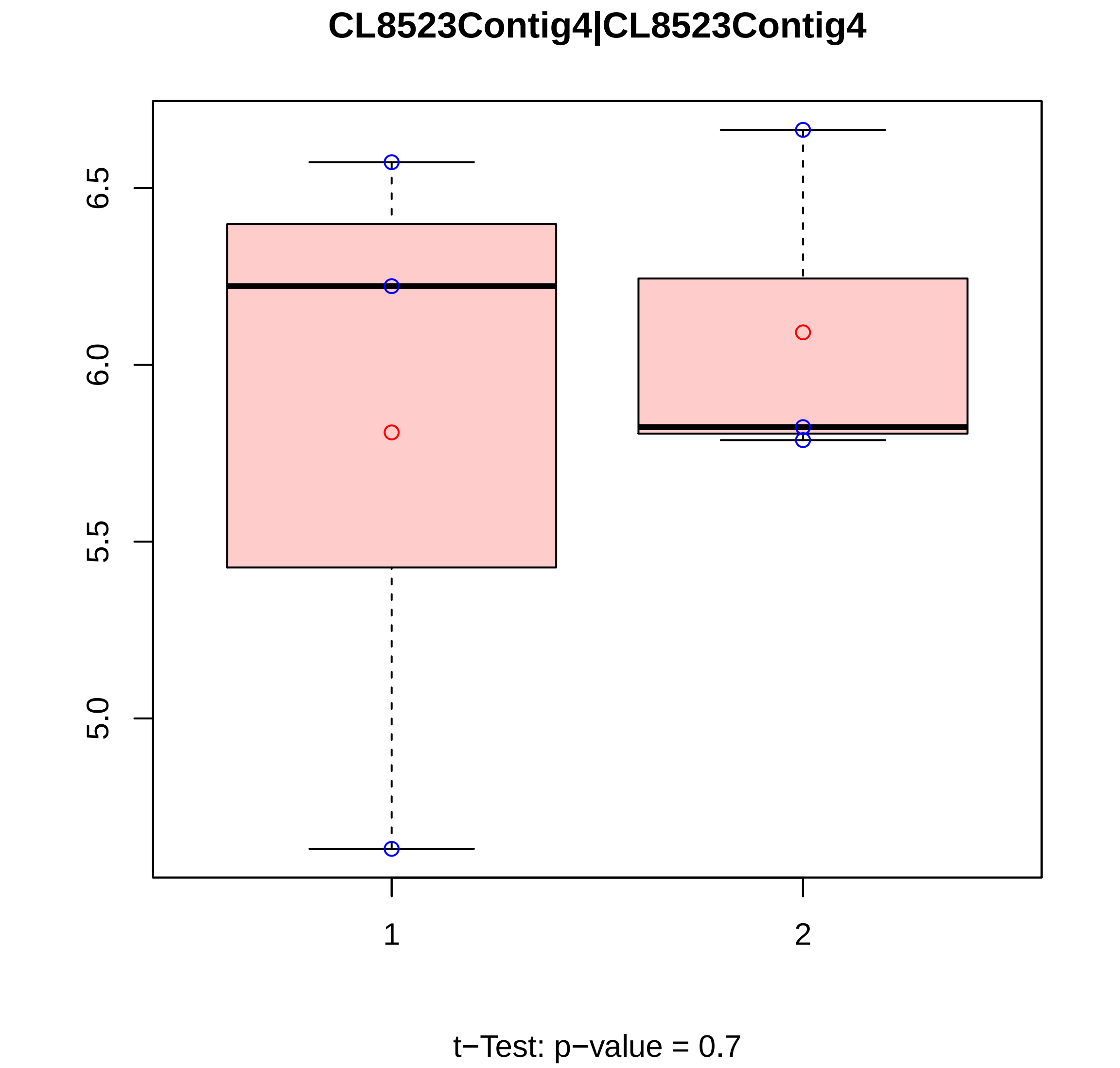
CL8523Contig4|CL8523Contig4
6.5
6.0
5.5
5.0
1
2
t−Test: p−value = 0.7

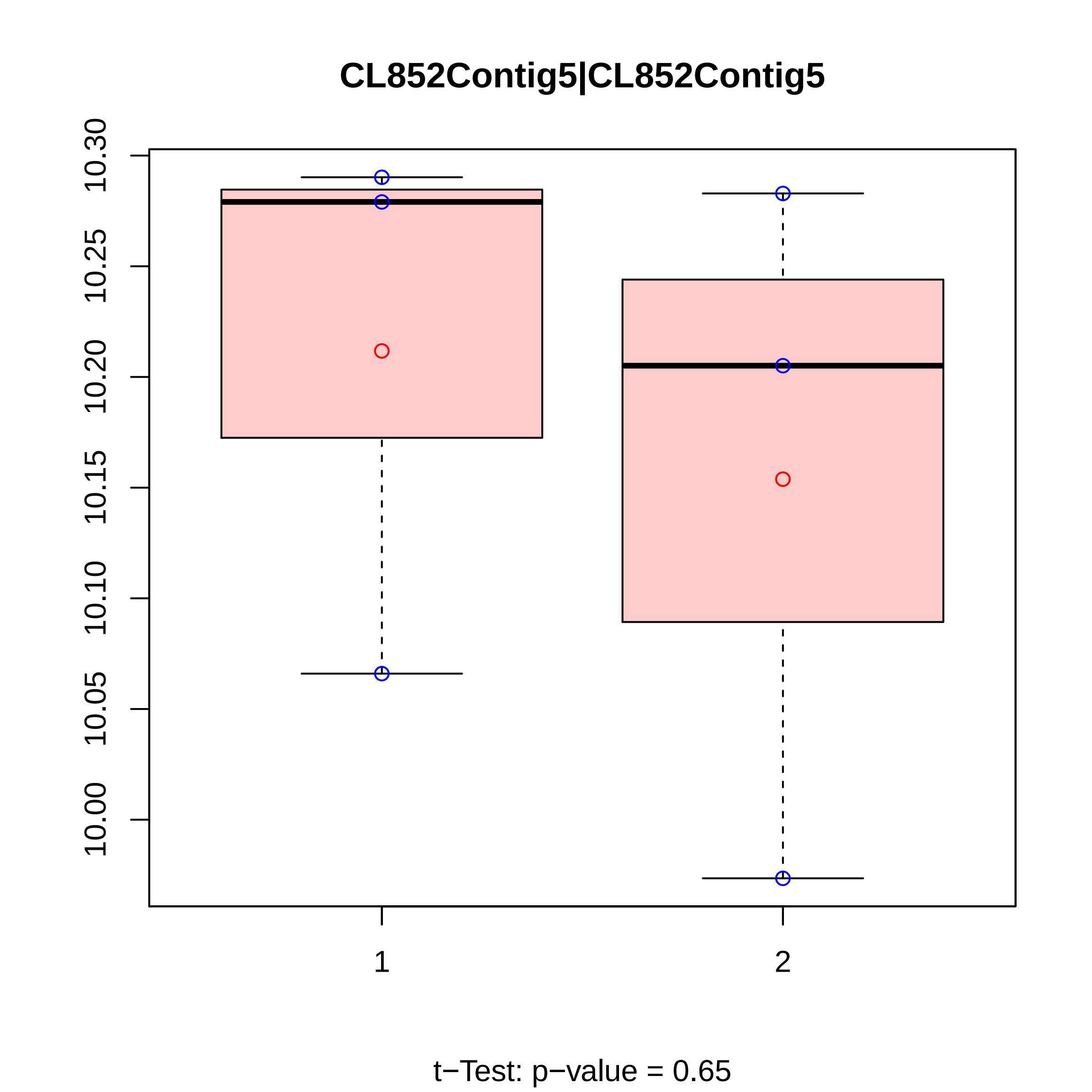
CL852Contig5|CL852Contig5
10.30
10.25
10.20
10.15
10.10
10.05
10.00
1
2
t−Test: p−value = 0.65

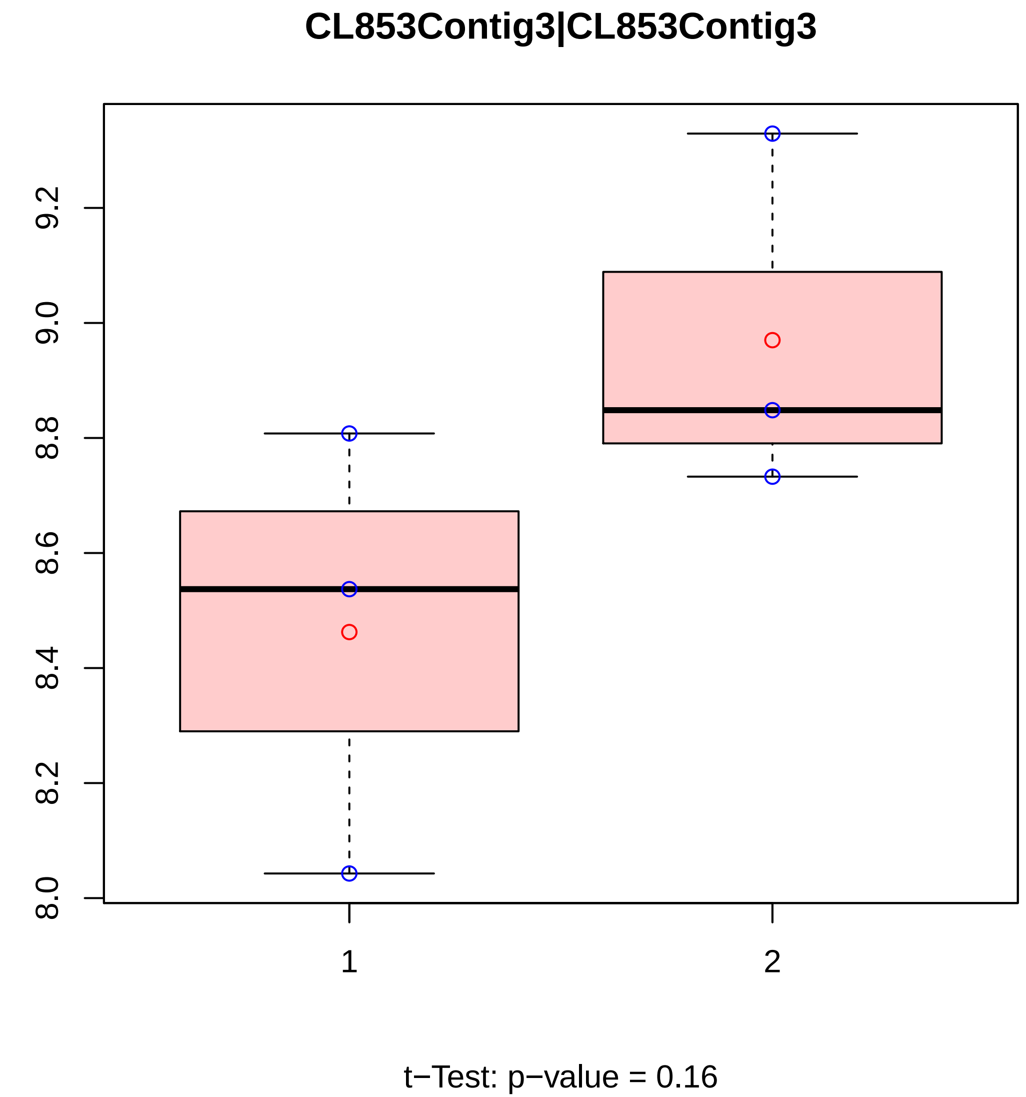
CL853Contig3|CL853Contig3
9.2
9.0
8.8
8.6
8.4
8.2
8.0
1
2
t−Test: p−value = 0.16

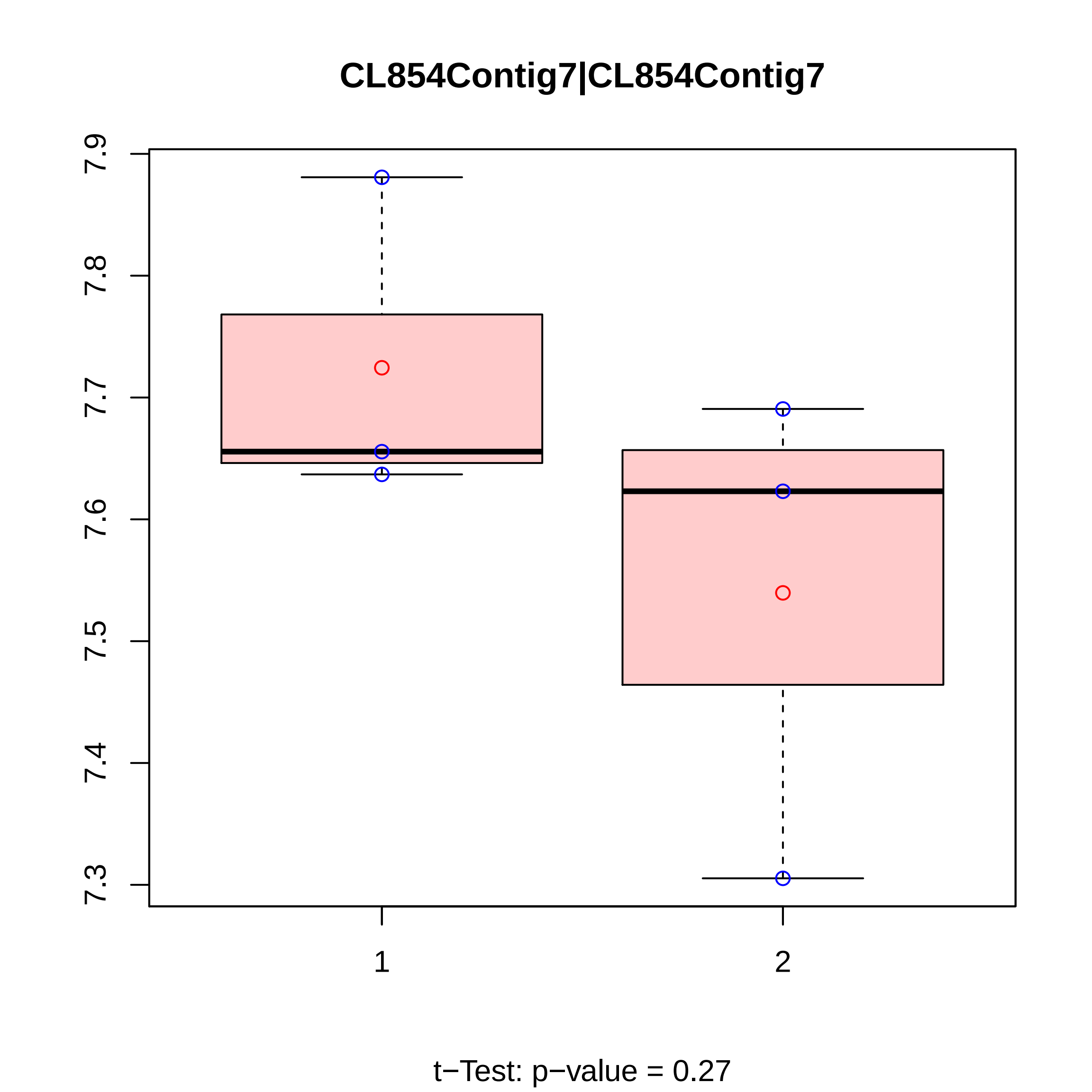

CL854Contig7|CL854Contig7
7.9
7.8
7.7
7.6
7.5
7.4
7.3
1
2
t−Test: p−value = 0.27

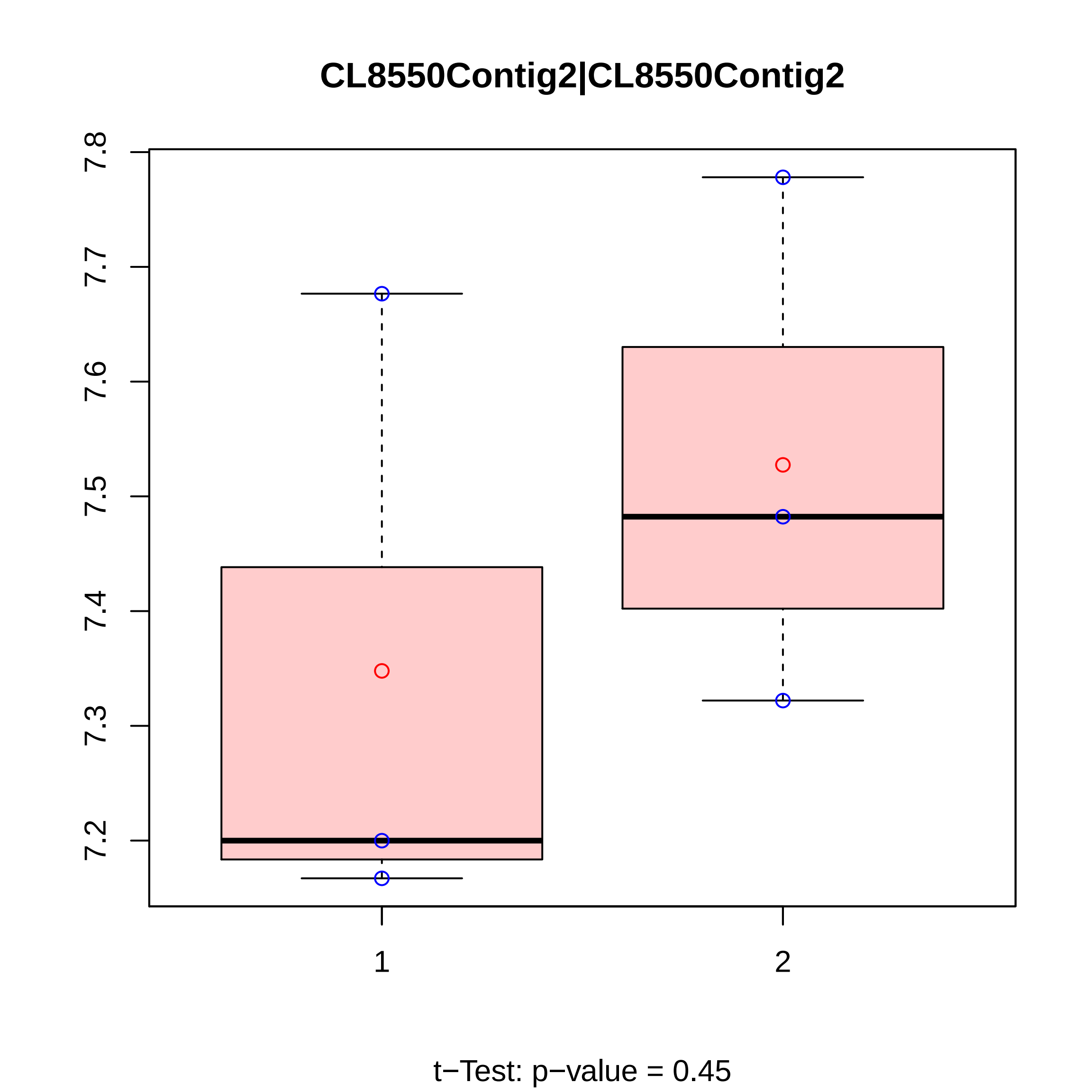
CL8550Contig2|CL8550Contig2
7.2
7.3
7.4
7.5
7.6
7.7
7.8
1
2
t−Test: p−value = 0.45

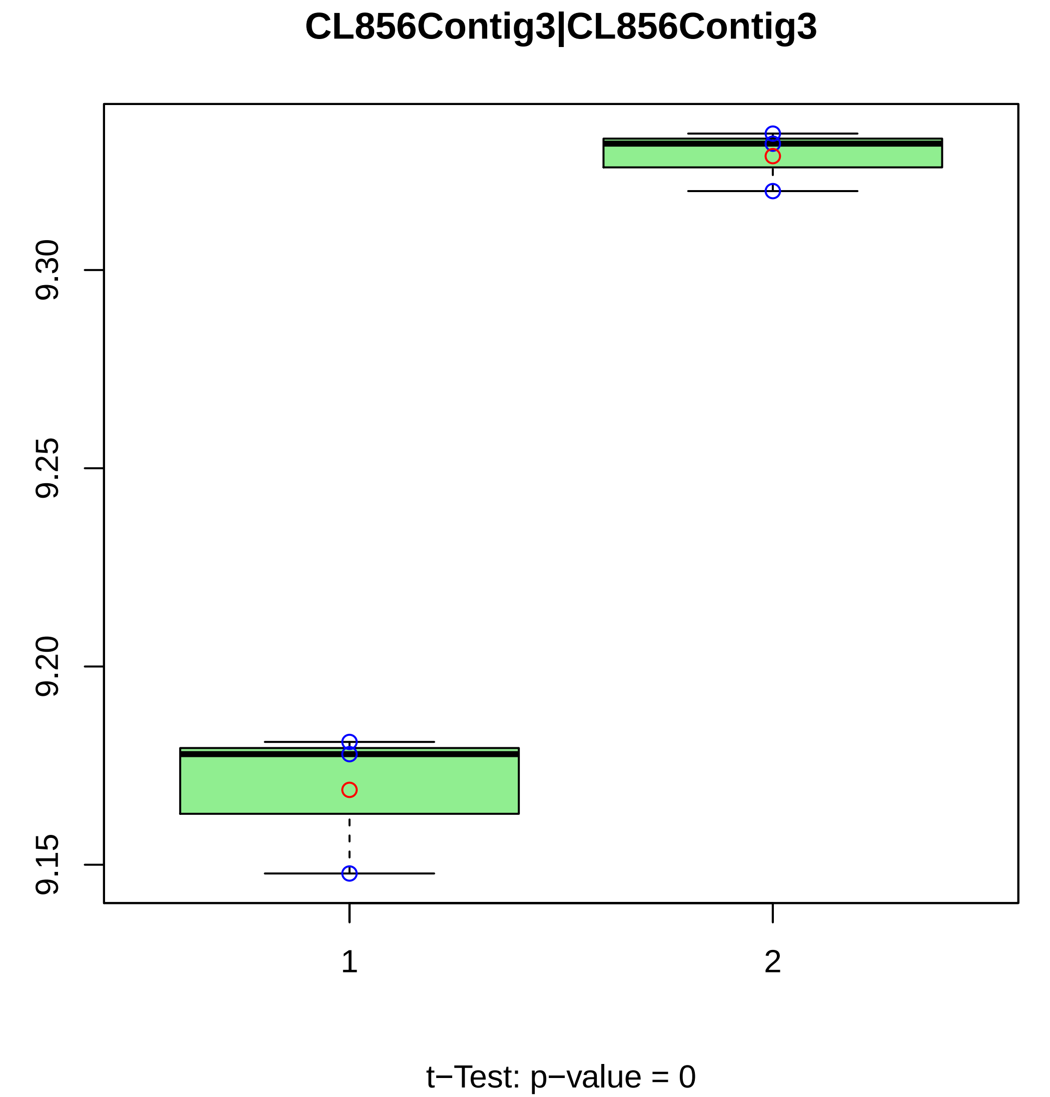

CL856Contig3|CL856Contig3
9.30
9.25
9.20
9.15
1
2
t−Test: p−value = 0

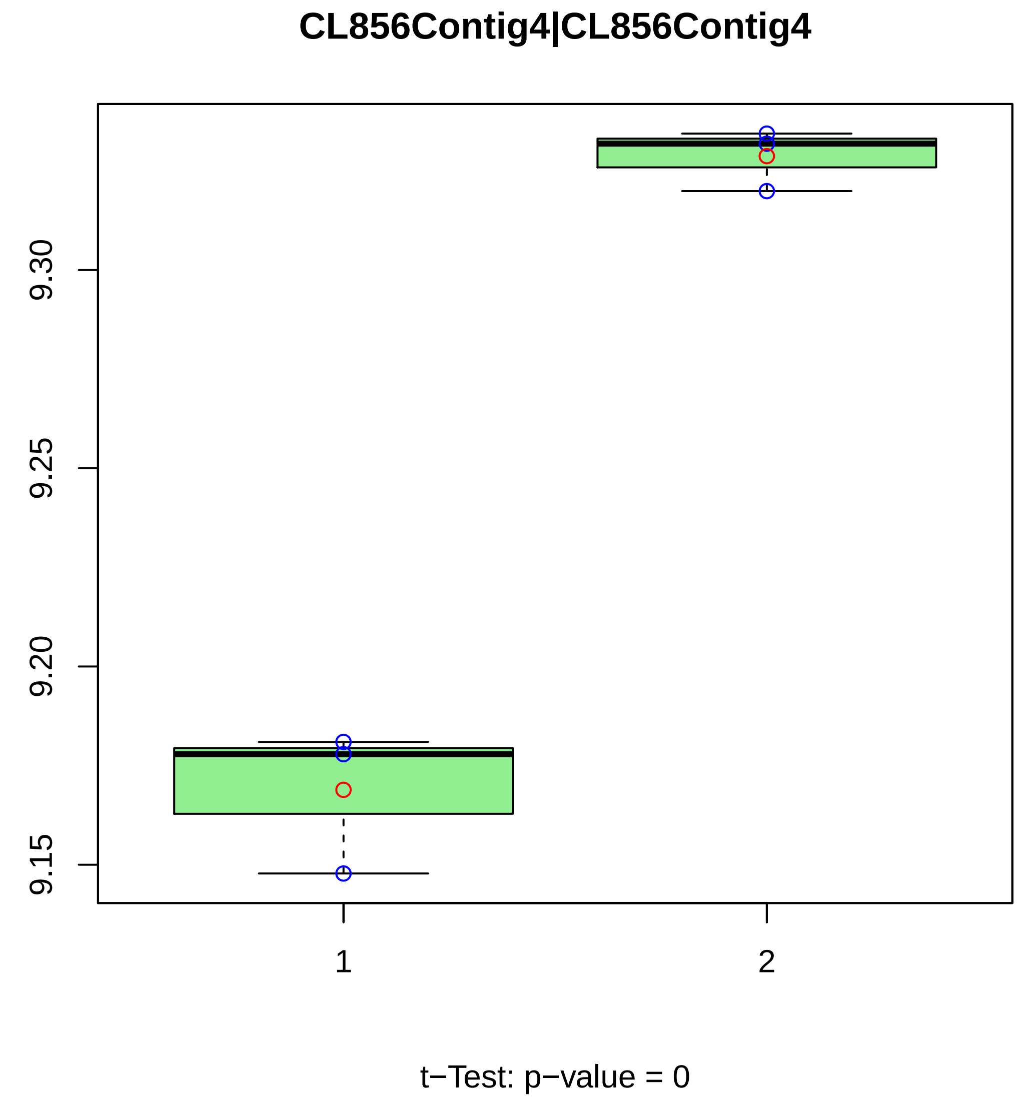
CL856Contig4|CL856Contig4
9.30
9.25
9.20
9.15
1
2
t−Test: p−value = 0

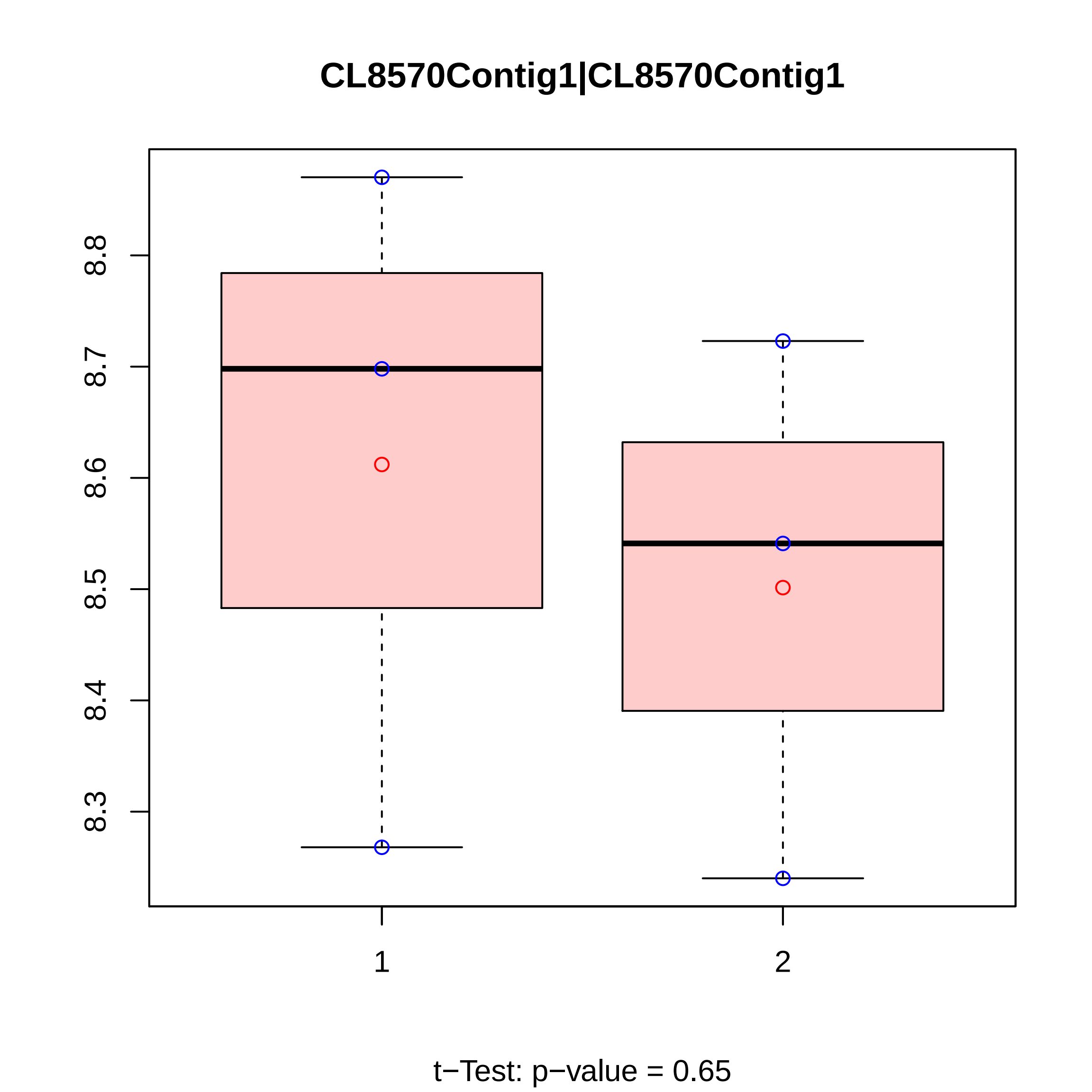
CL8570Contig1|CL8570Contig1
8.3
8.4
8.5
8.6
8.7
8.8
1
2
t−Test: p−value = 0.65

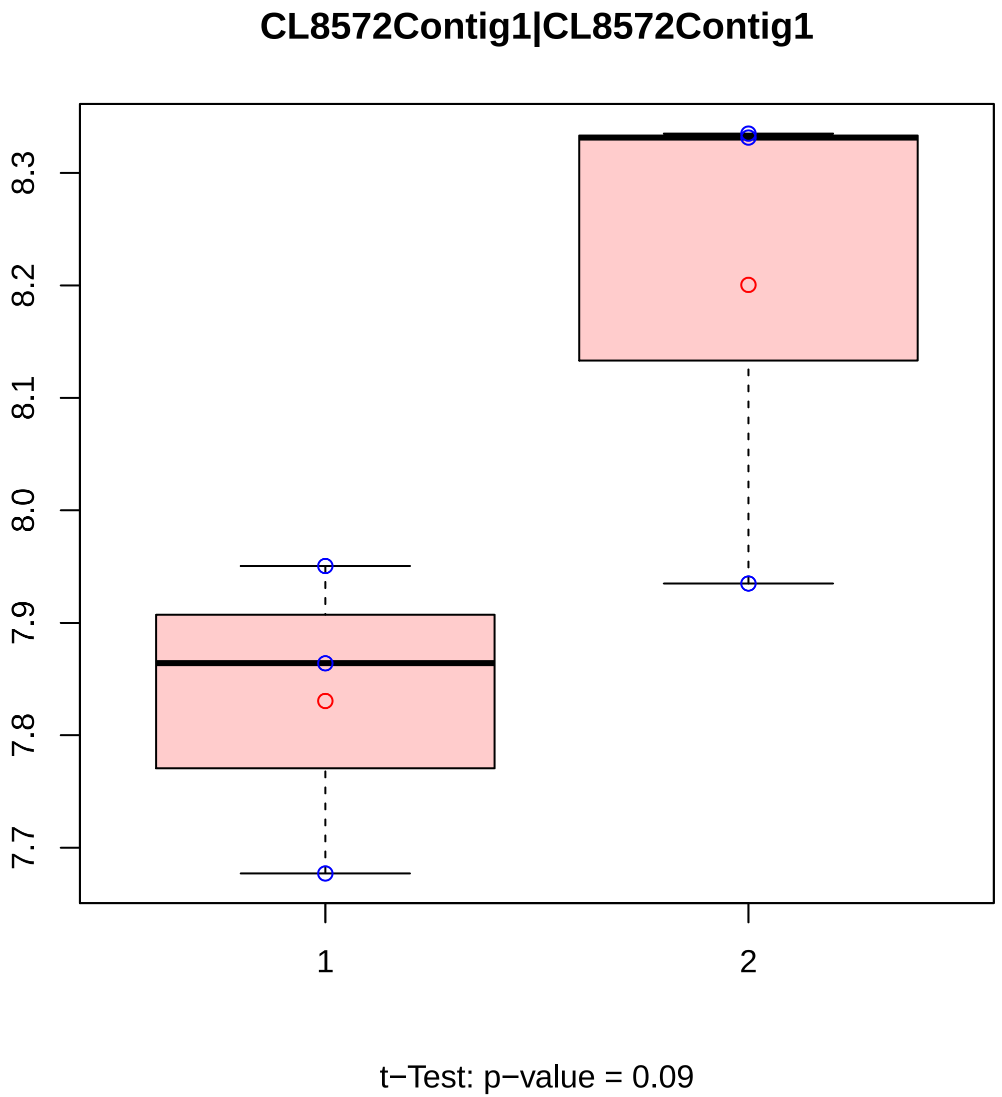

CL8572Contig1|CL8572Contig1
8.3
8.2
8.1
8.0
7.9
7.8
7.7
1
2
t−Test: p−value = 0.09

# CL857Contig8|CL857Contig8

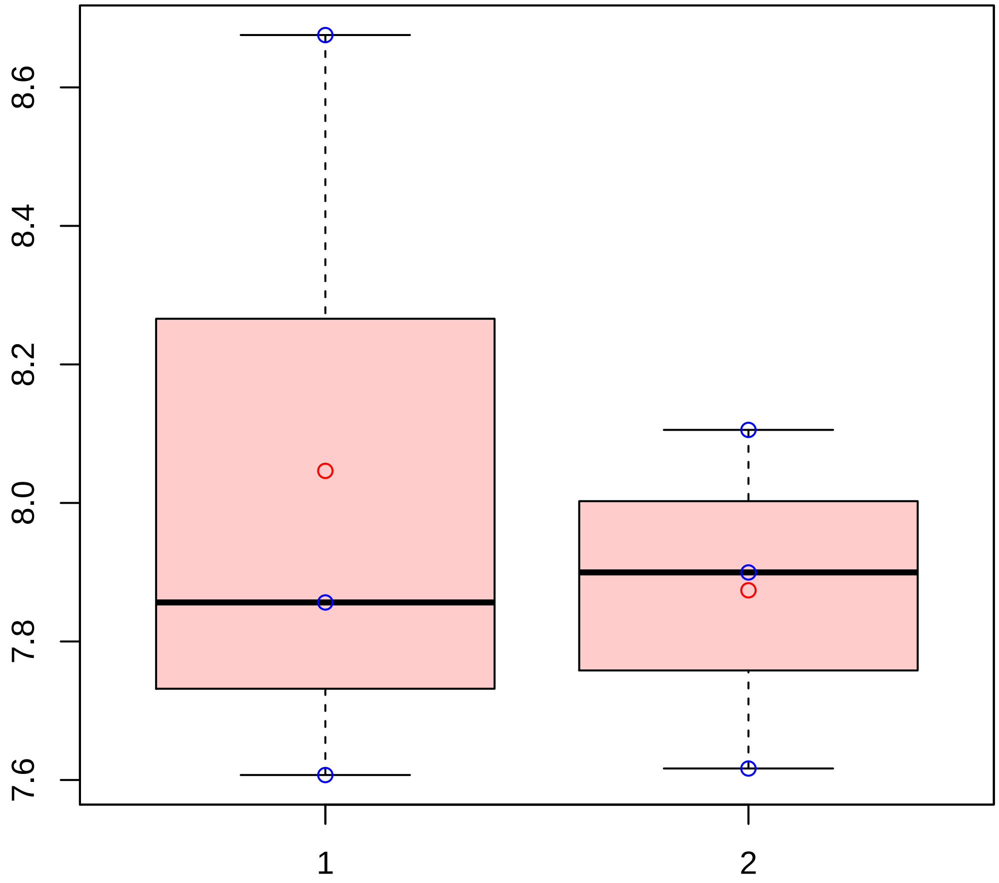


t−Test: p−value = 0.66

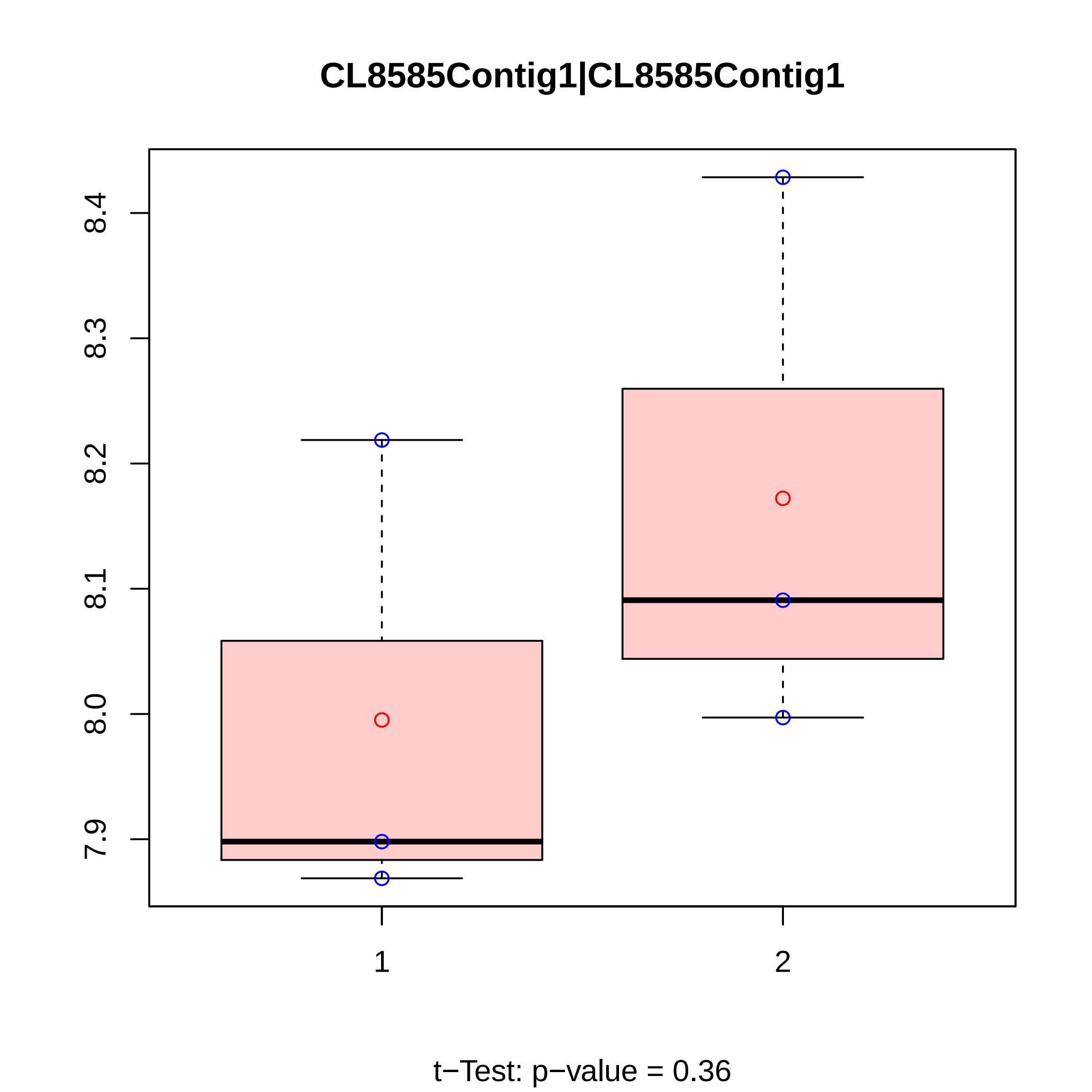

CL8585Contig1|CL8585Contig1
8.4
8.3
8.2
8.1
8.0
7.9
1
2
t−Test: p−value = 0.36

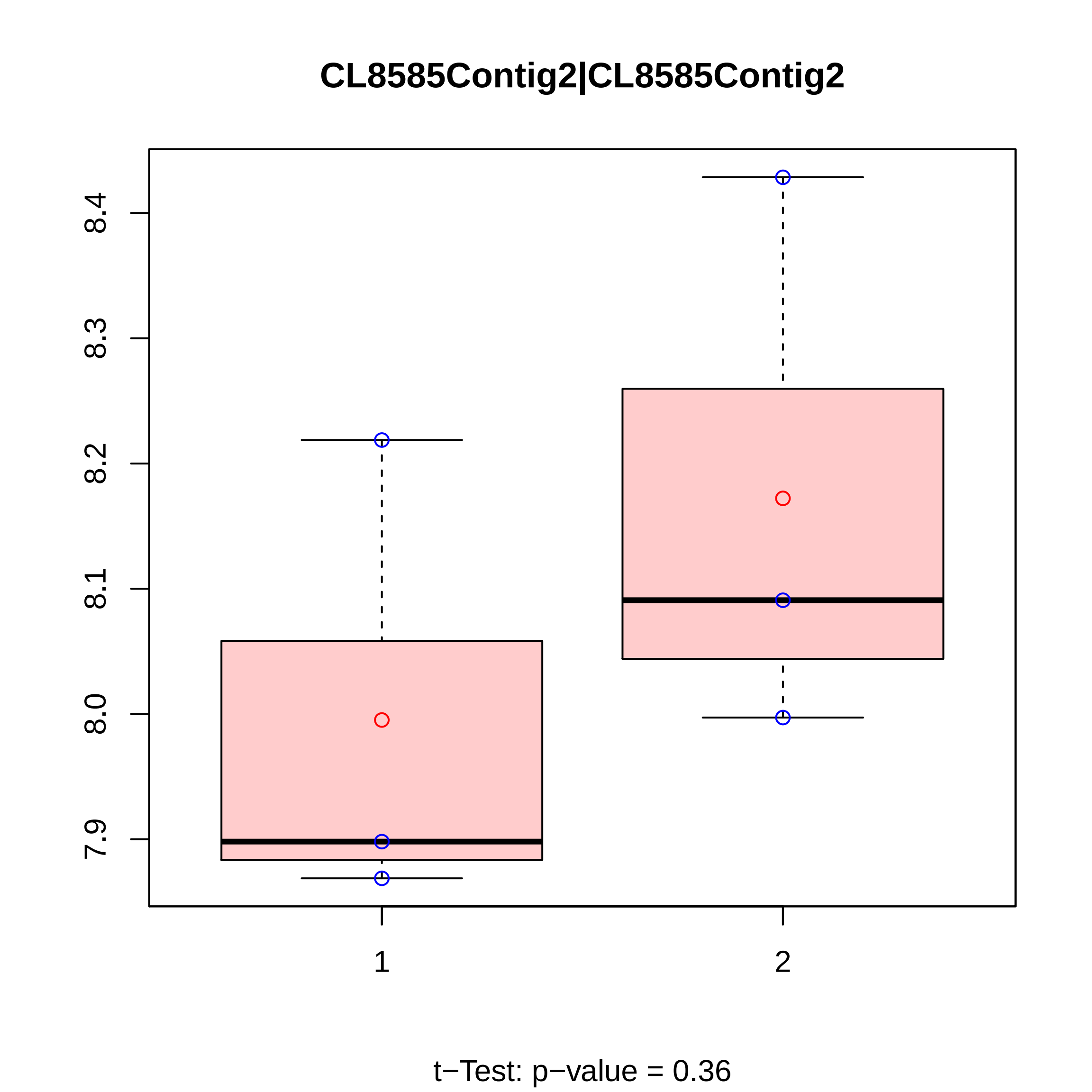
CL8585Contig2|CL8585Contig2
8.4
8.3
8.2
8.1
8.0
7.9
1
2
t−Test: p−value = 0.36

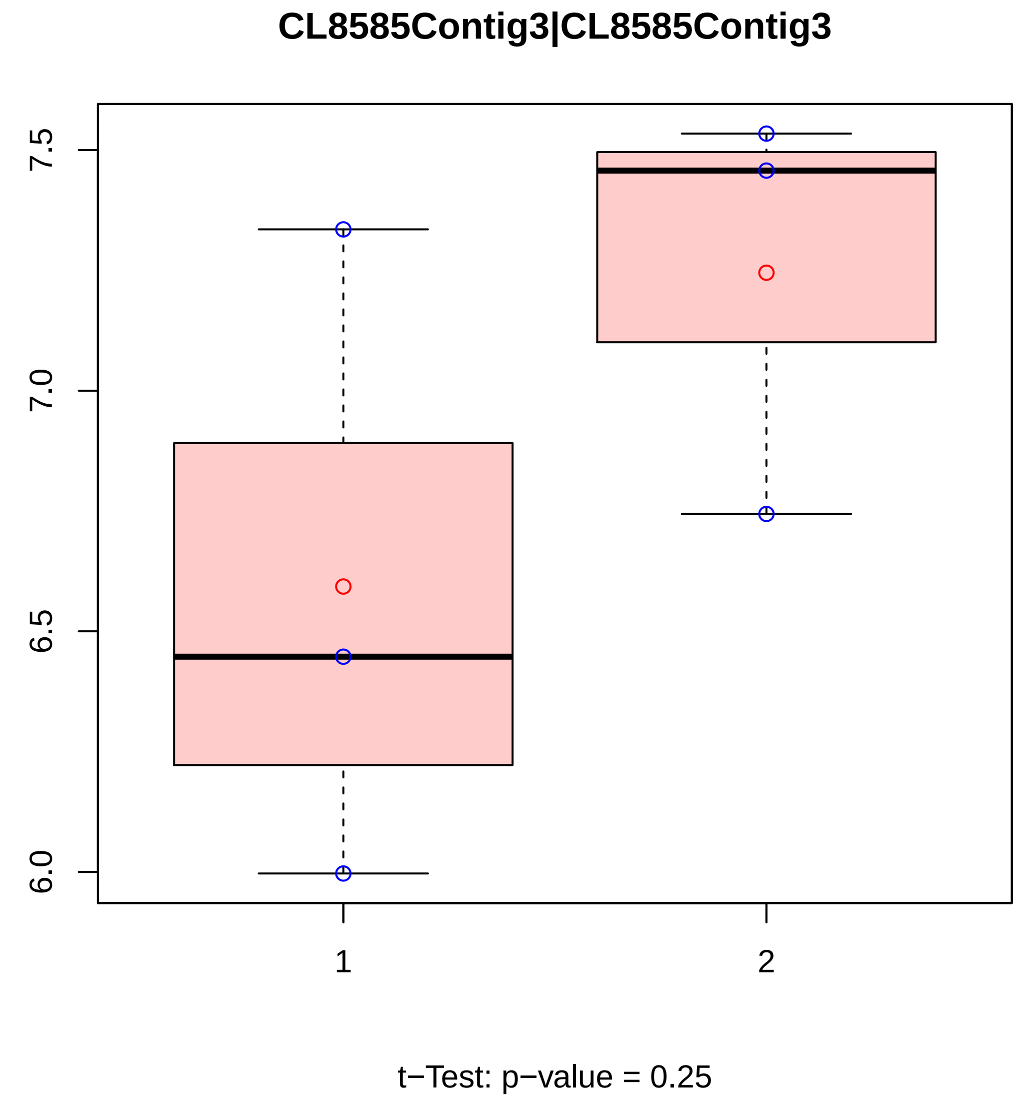
CL8585Contig3|CL8585Contig3
7.5
7.0
6.5
6.0
1
2
t−Test: p−value = 0.25

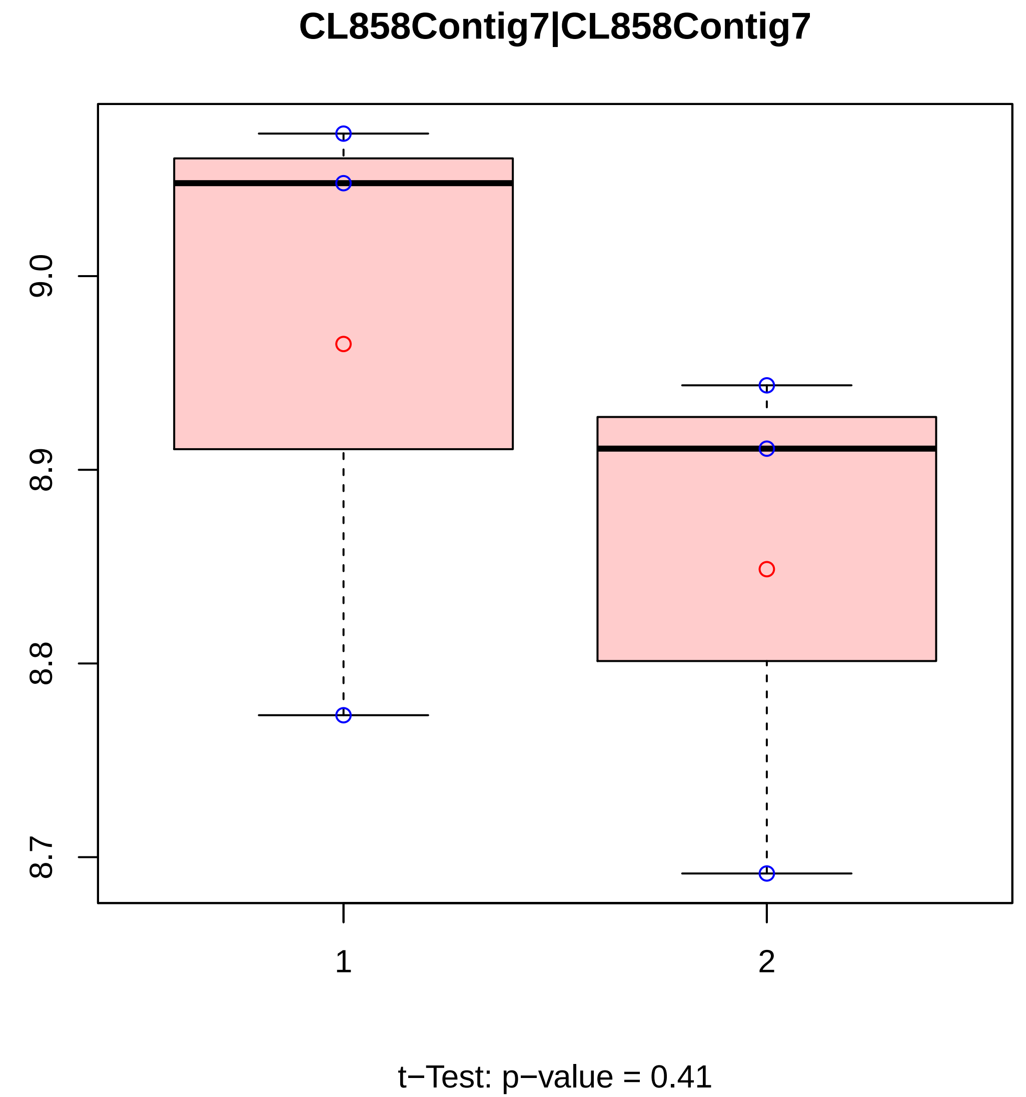

CL858Contig7|CL858Contig7
9.0
8.9
8.8
8.7
1
2
t−Test: p−value = 0.41

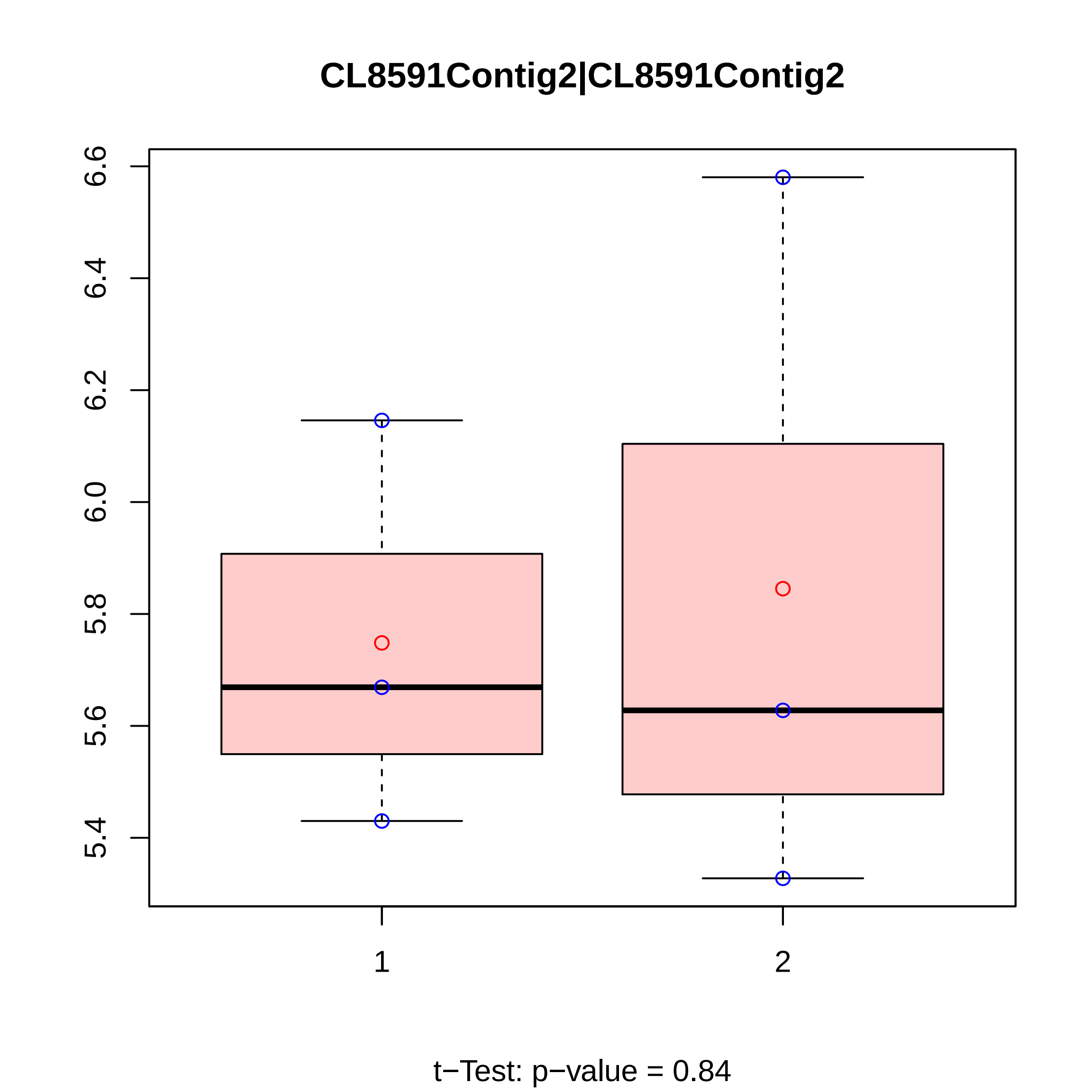
CL8591Contig2|CL8591Contig2
6.6
6.4
6.2
6.0
5.8
5.6
5.4
1
2
t−Test: p−value = 0.84

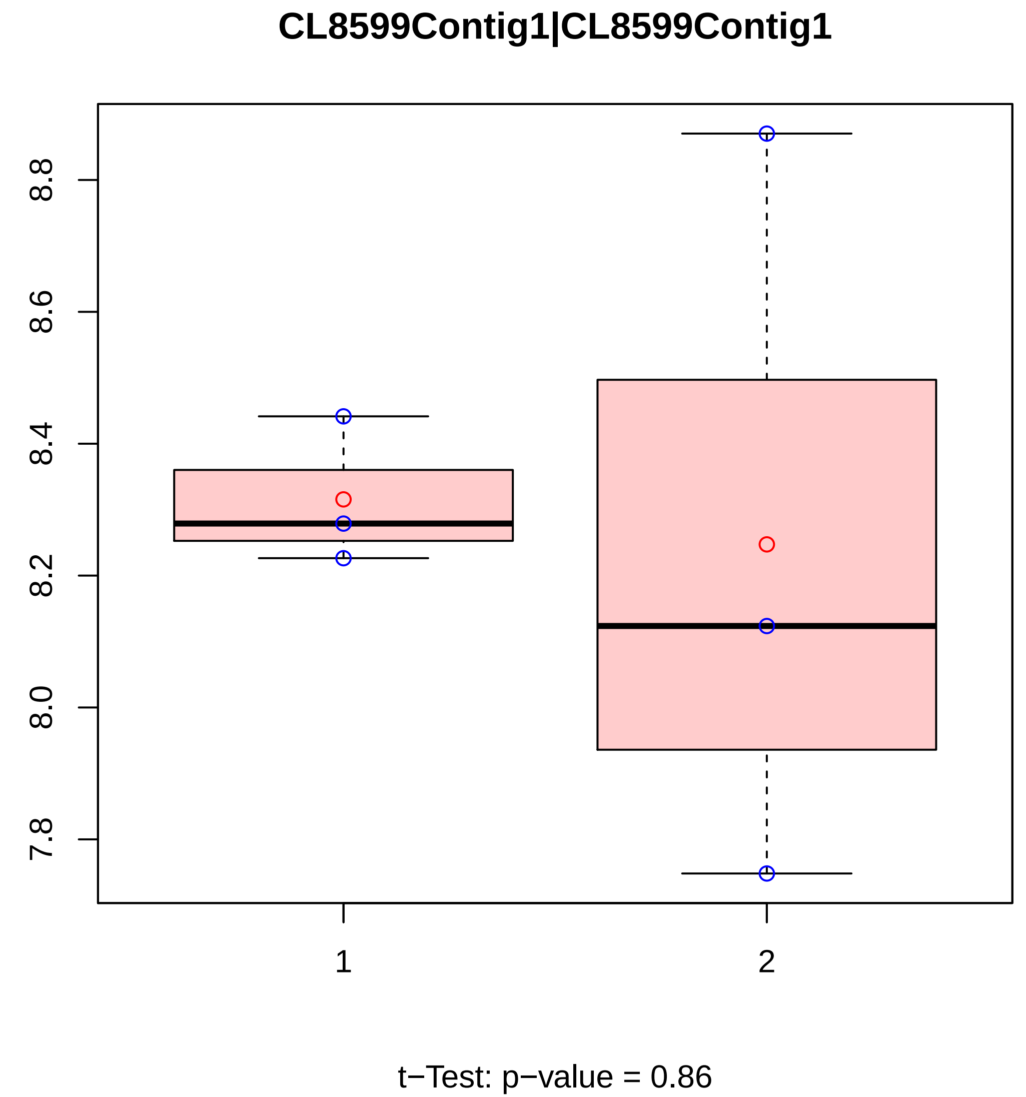
CL8599Contig1|CL8599Contig1
8.8
8.6
8.4
8.2
8.0
7.8
1
2
t−Test: p−value = 0.86

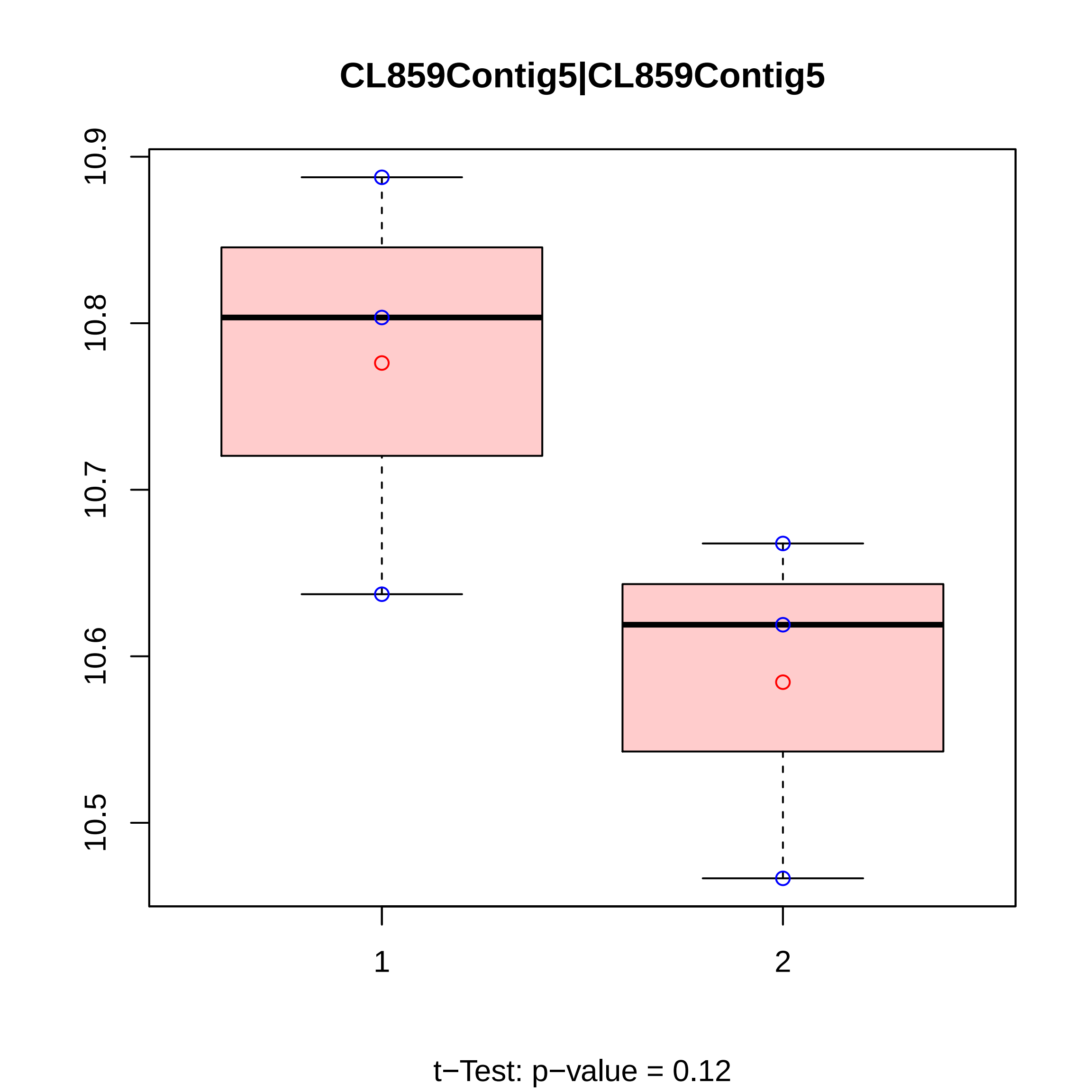

CL859Contig5|CL859Contig5
10.9
10.8
10.7
10.6
10.5
1
2
t−Test: p−value = 0.12

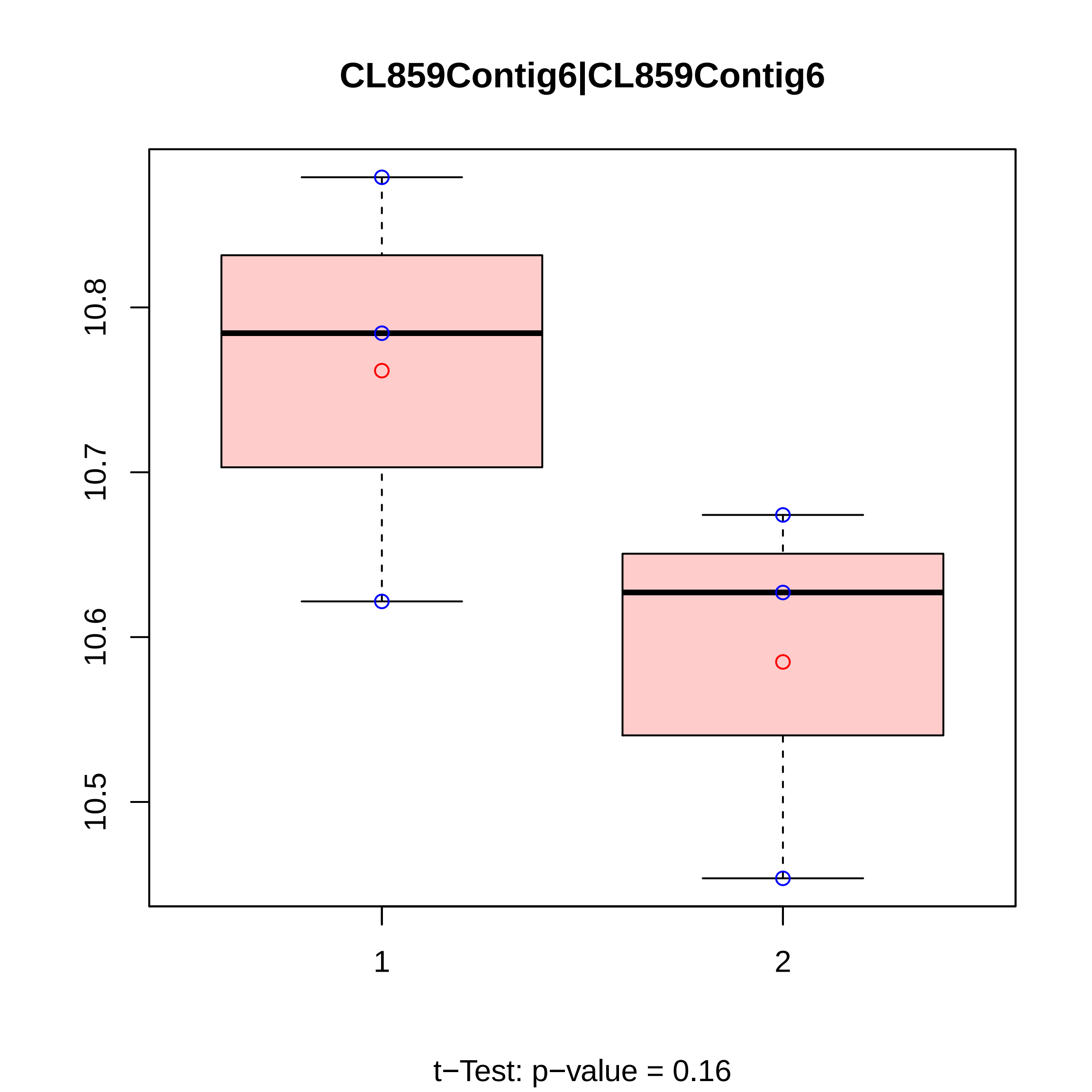
CL859Contig6|CL859Contig6
10.5
10.6
10.7
10.8
1
2
t−Test: p−value = 0.16

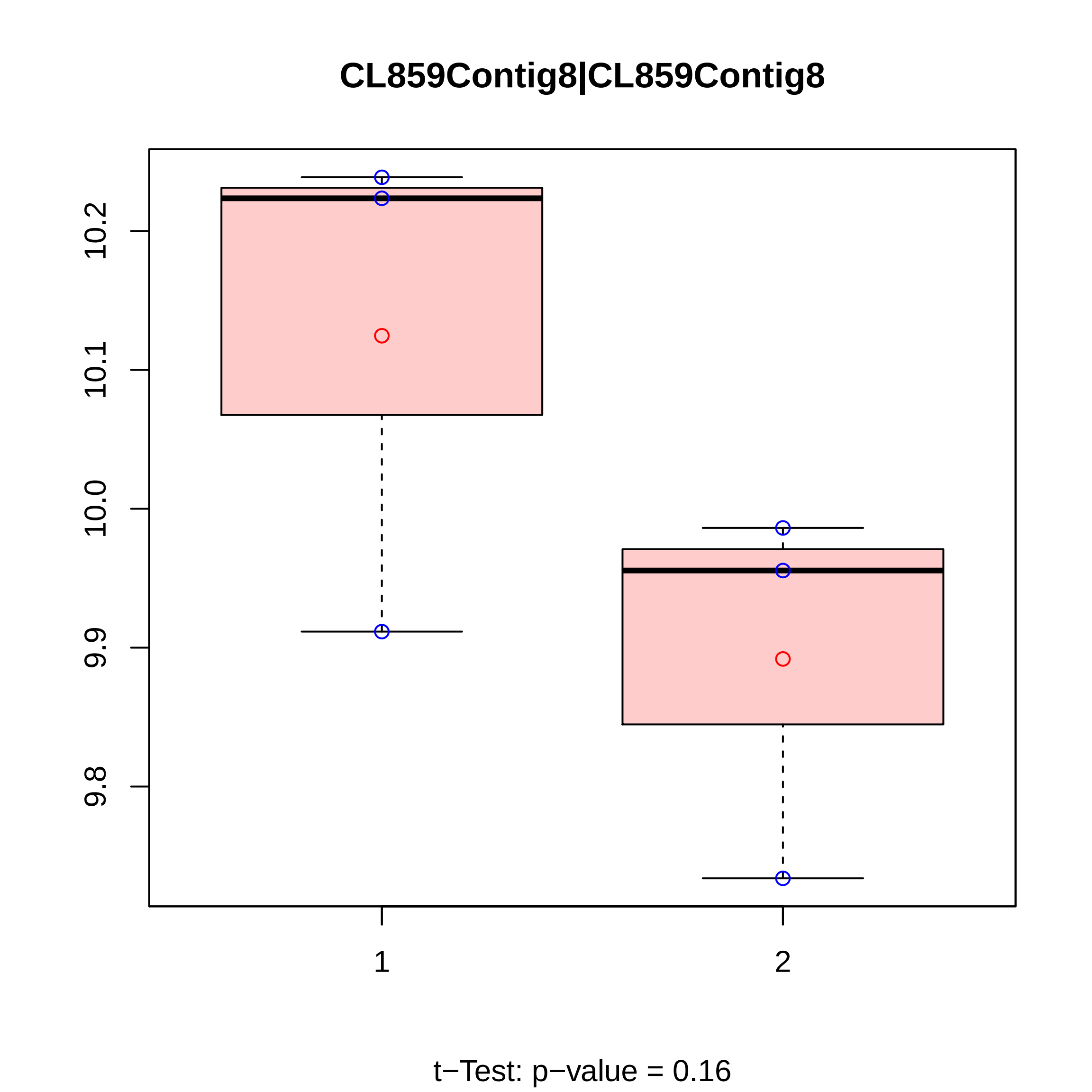
CL859Contig8|CL859Contig8
10.2
10.1
10.0
9.9
9.8
1
2
t−Test: p−value = 0.16

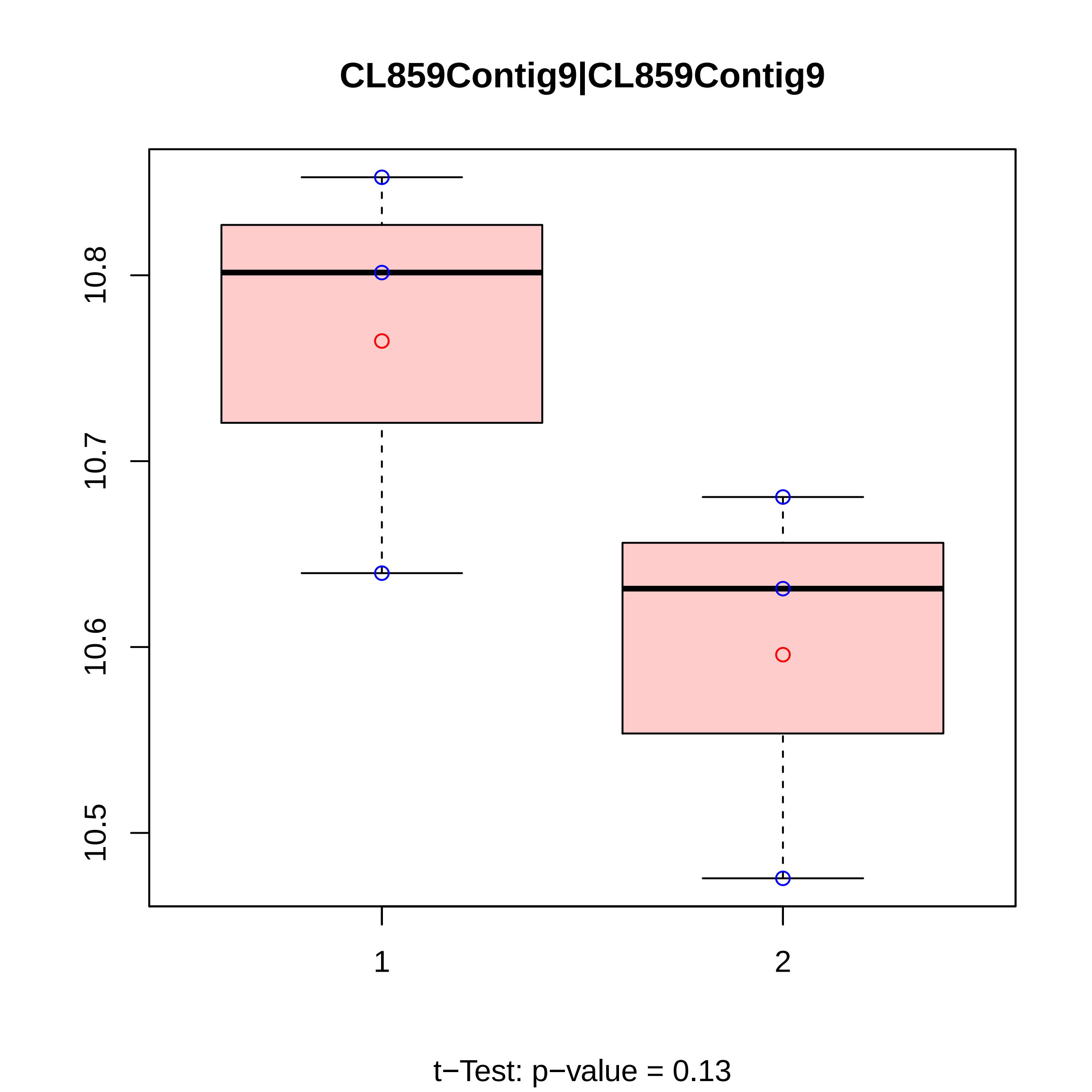

CL859Contig9|CL859Contig9
10.5
10.6
10.7
10.8
1
2
t−Test: p−value = 0.13

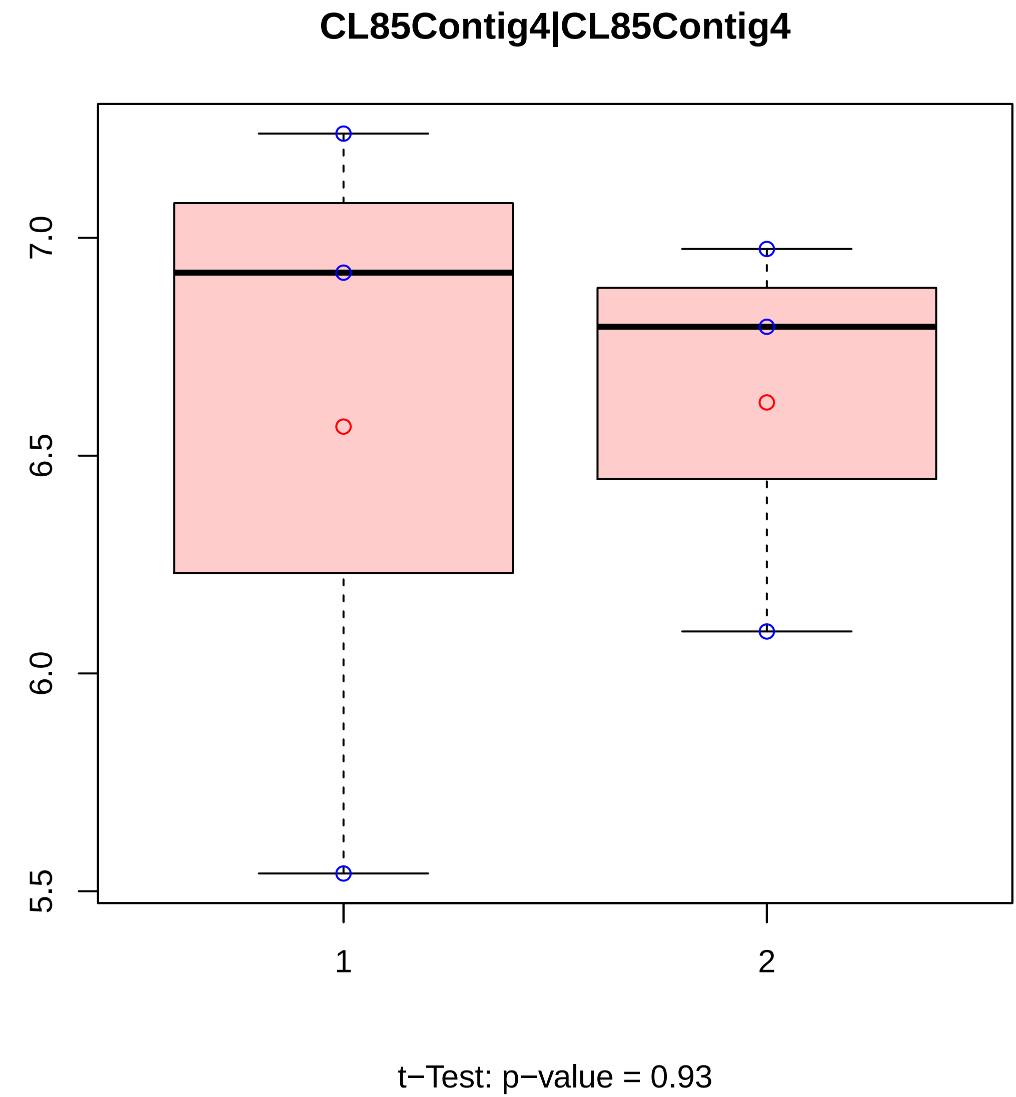
CL85Contig4|CL85Contig4
7.0
6.5
6.0
5.5
1
2
t−Test: p−value = 0.93

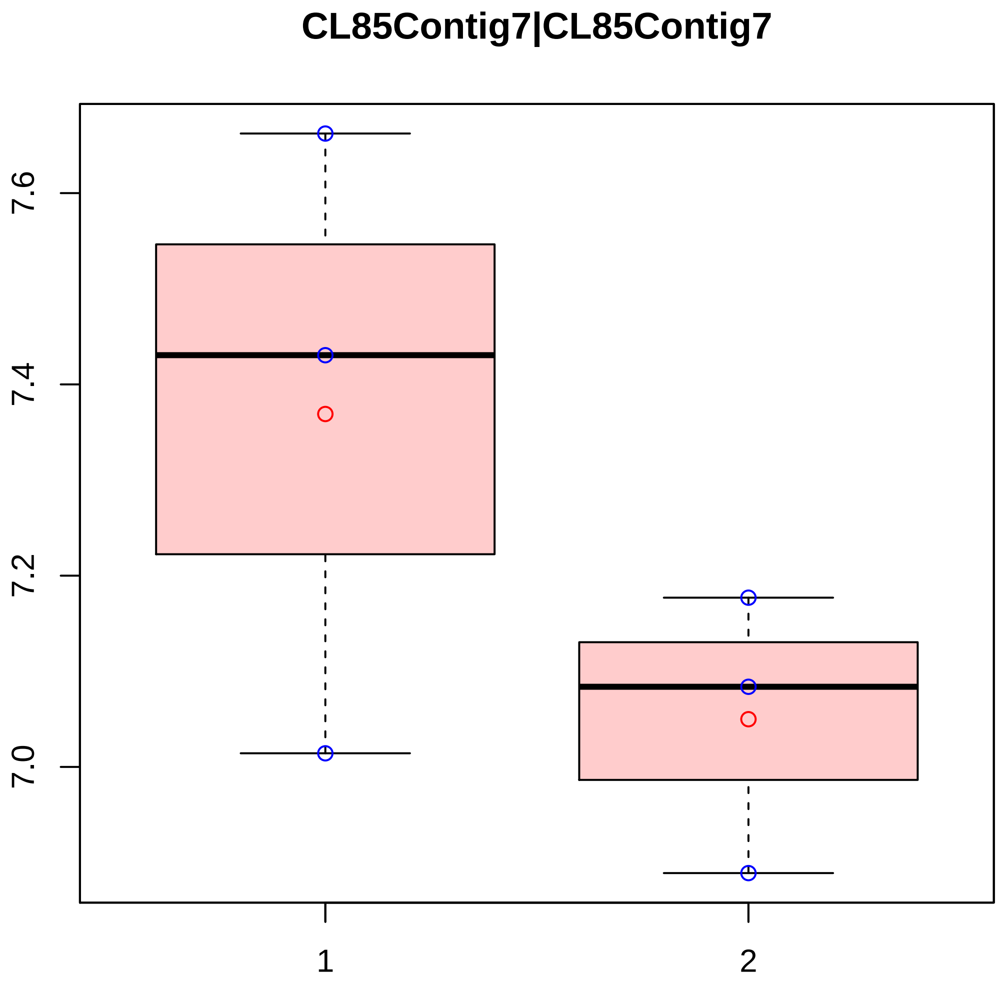


t−Test: p−value = 0.23

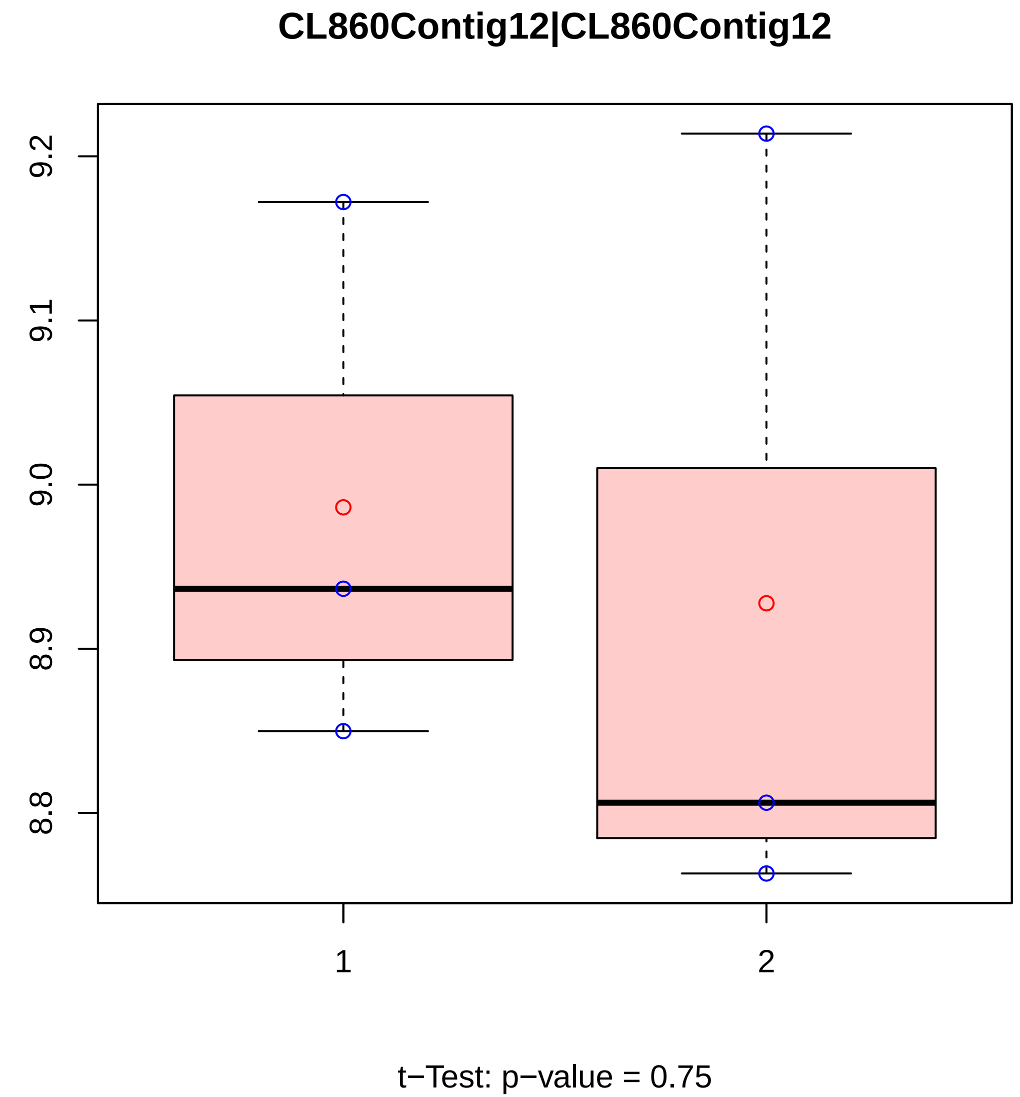
CL860Contig12|CL860Contig12
9.2
9.1
9.0
8.9
8.8
1
2
t−Test: p−value = 0.75

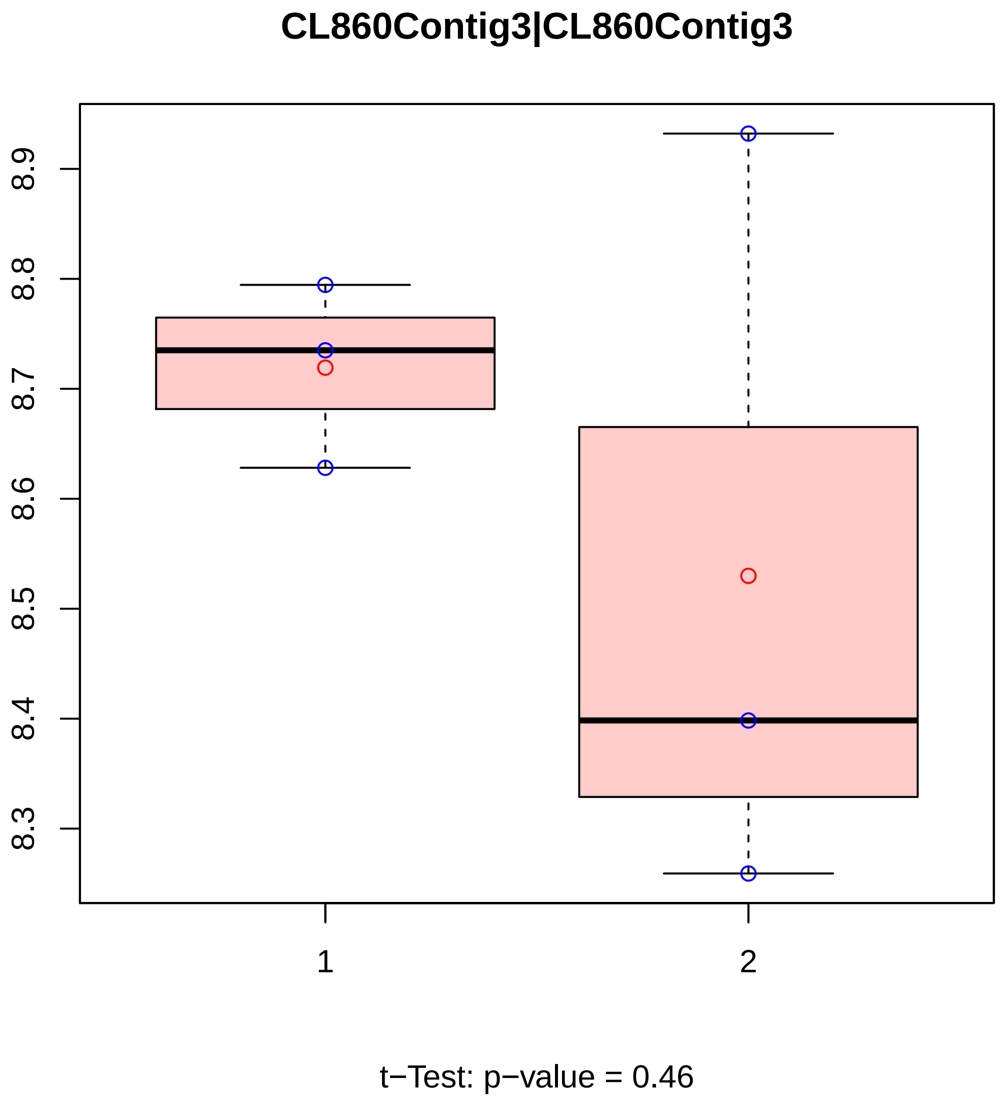
CL860Contig3|CL860Contig3
8.3
8.4
8.5
8.6
8.7
8.8
8.9
1
2
t−Test: p−value = 0.46

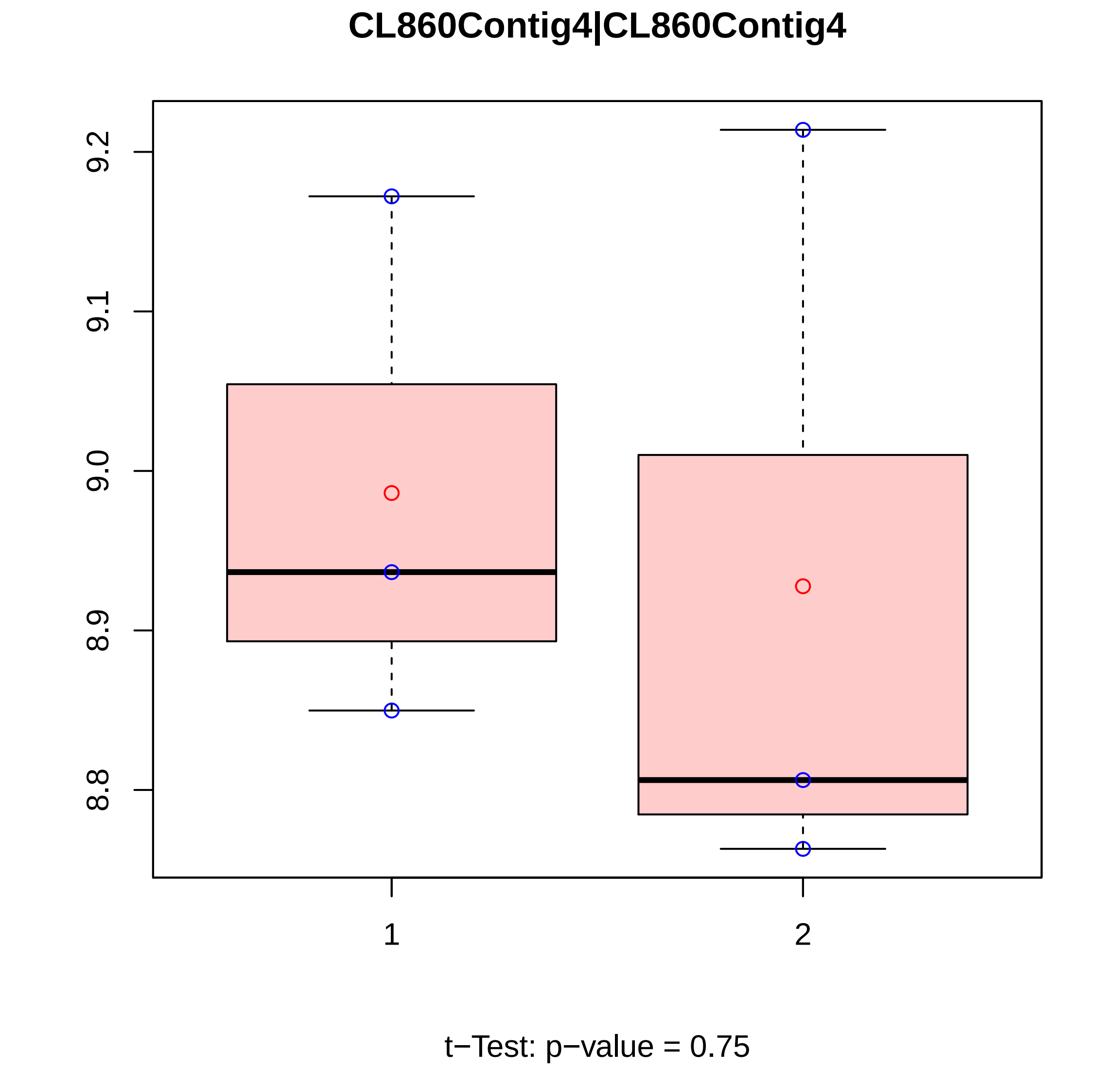

CL860Contig4|CL860Contig4
9.2
9.1
9.0
8.9
8.8
1
2
t−Test: p−value = 0.75

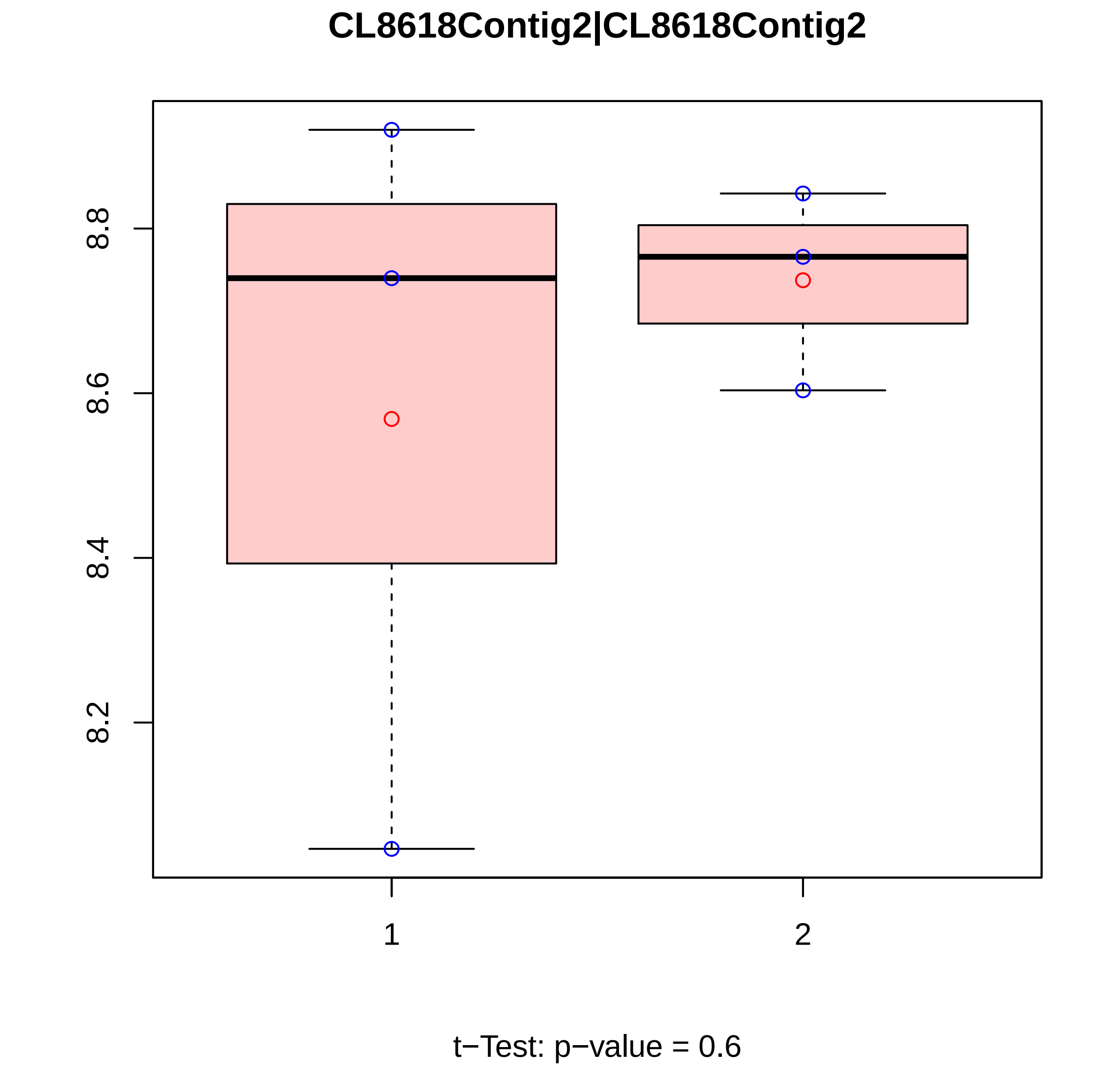
CL8618Contig2|CL8618Contig2
8.2
8.4
8.6
8.8
1
2
t−Test: p−value = 0.6

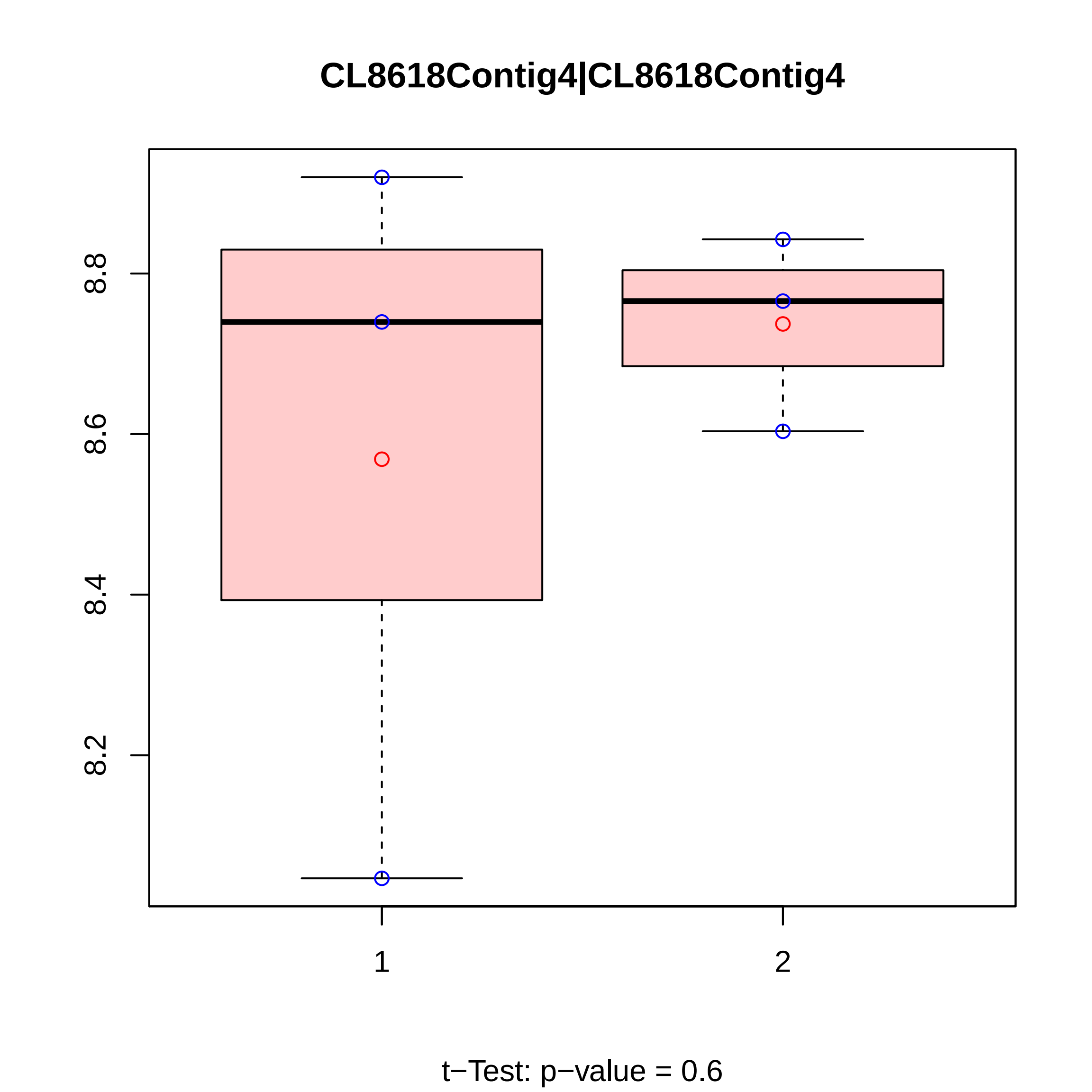

CL8618Contig4|CL8618Contig4
8.2
8.4
8.6
8.8
1
2
t−Test: p−value = 0.6

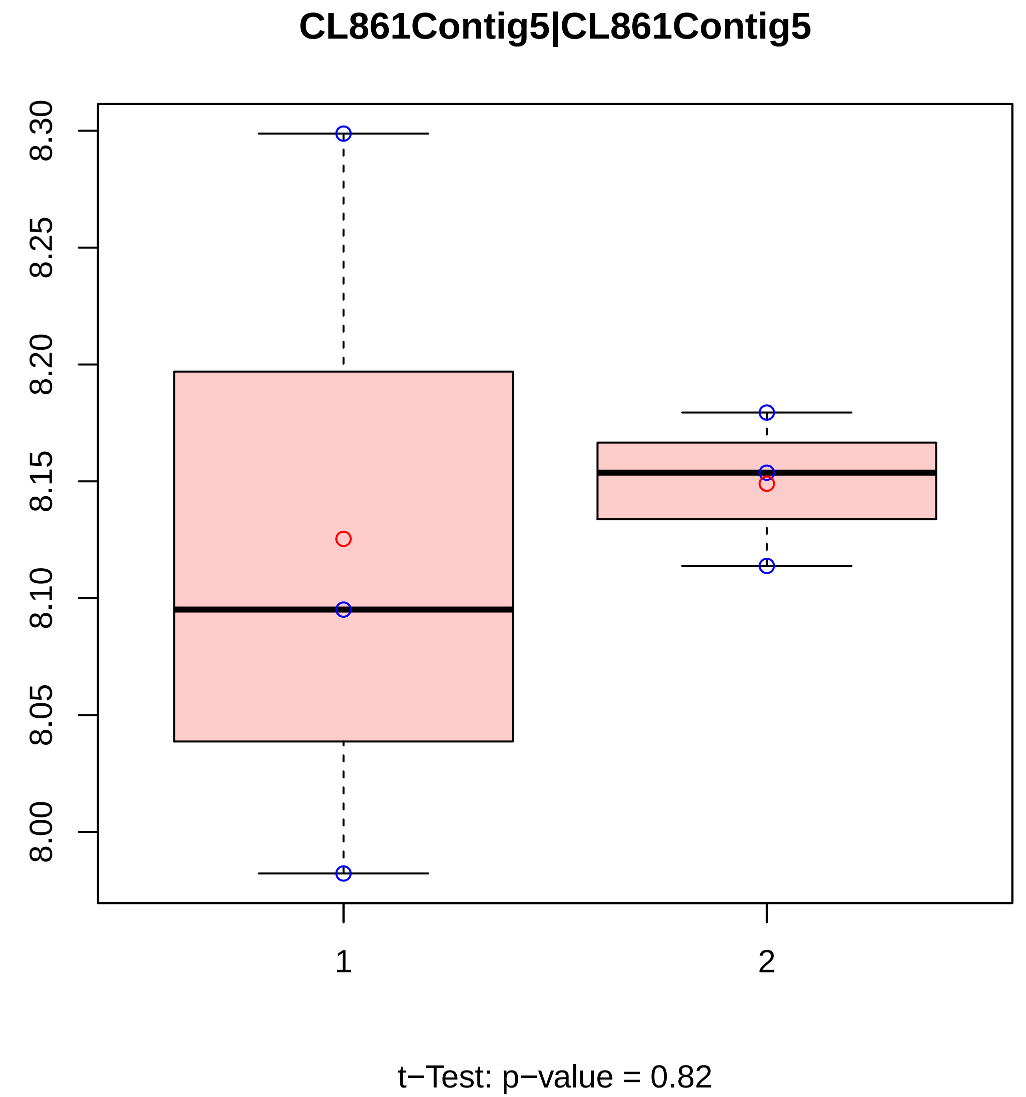
CL861Contig5|CL861Contig5
8.30
8.25
8.20
8.15
8.10
8.05
8.00
1
2
t−Test: p−value = 0.82

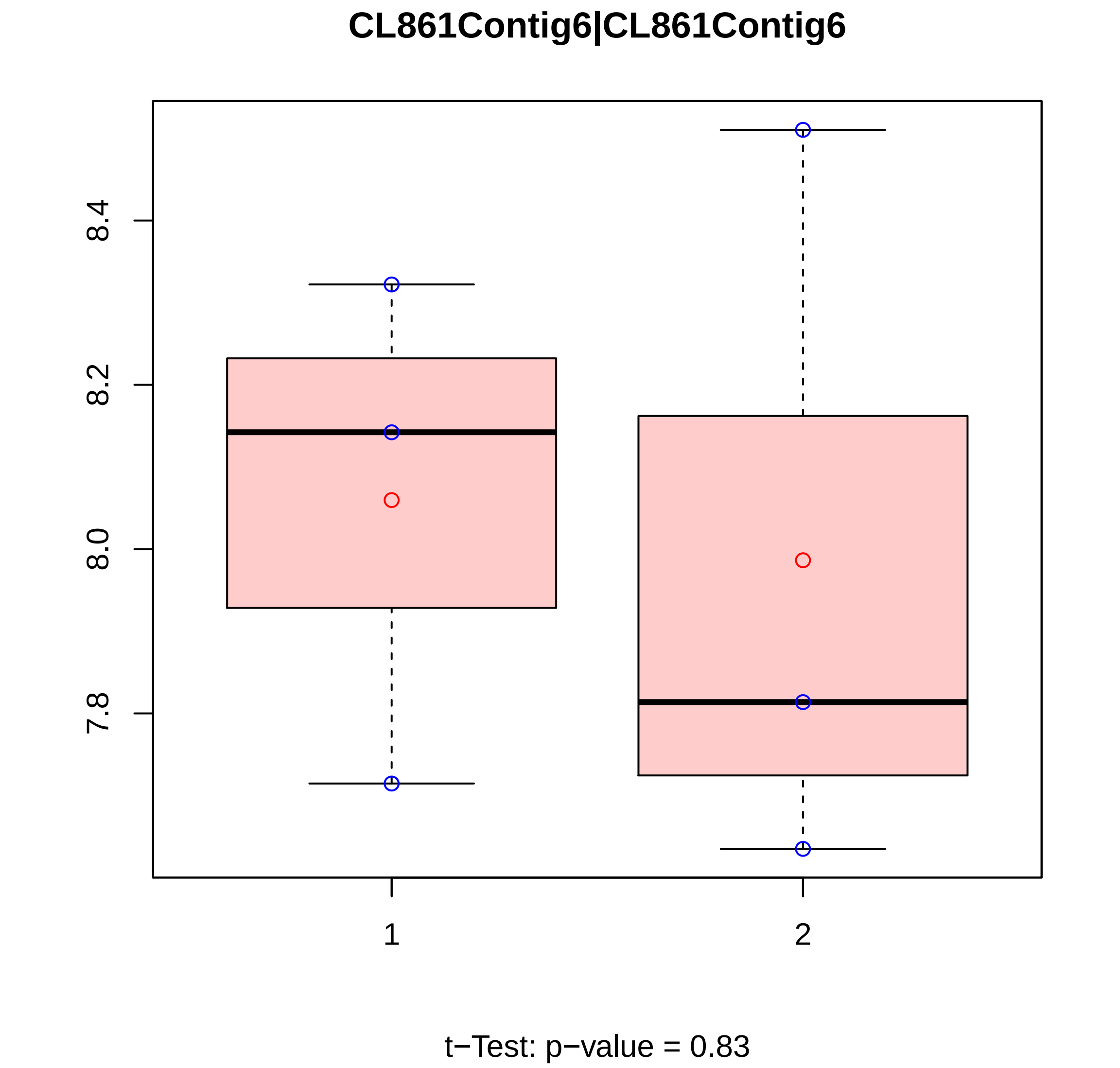
CL861Contig6|CL861Contig6
8.4
8.2
8.0
7.8
1
2
t−Test: p−value = 0.83

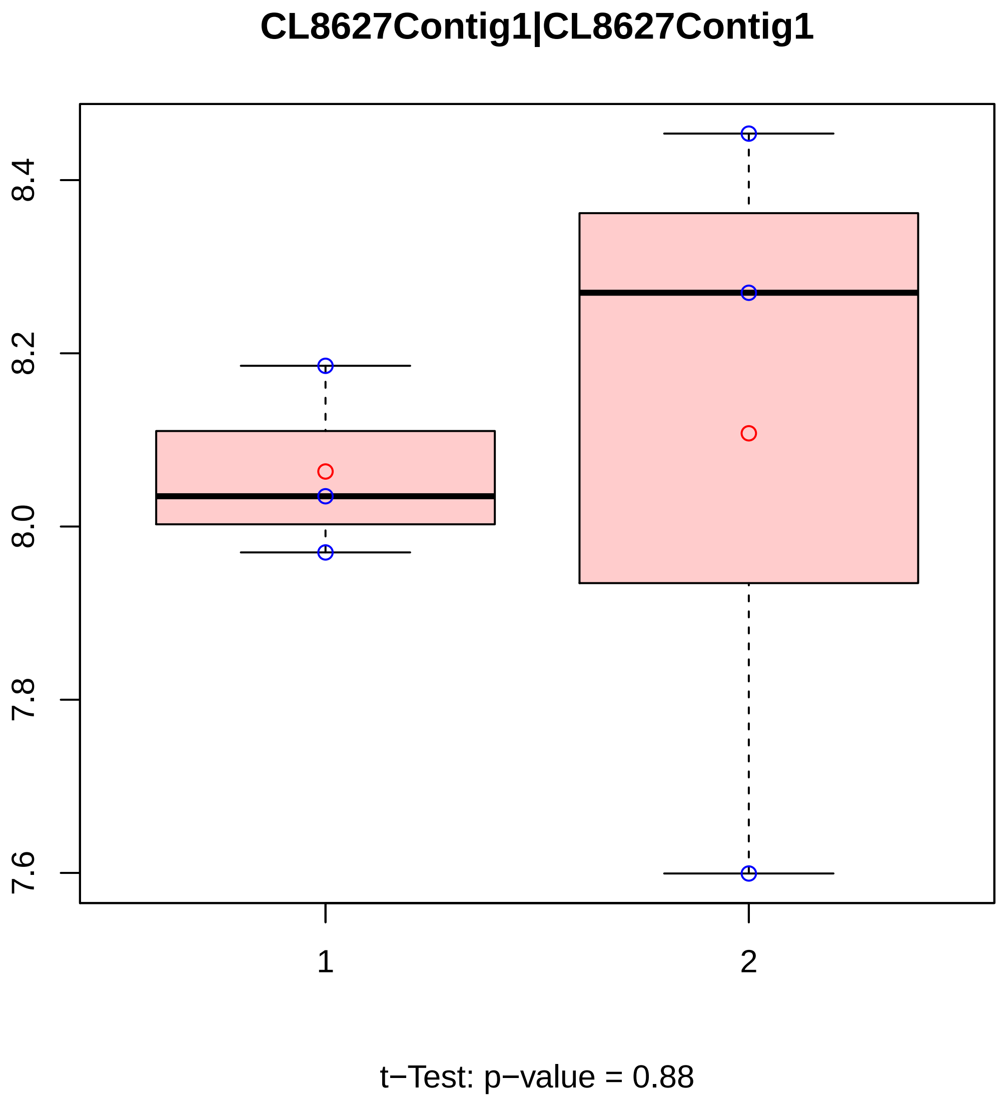

CL8627Contig1|CL8627Contig1
8.4
8.2
8.0
7.8
7.6
1
2
t−Test: p−value = 0.88

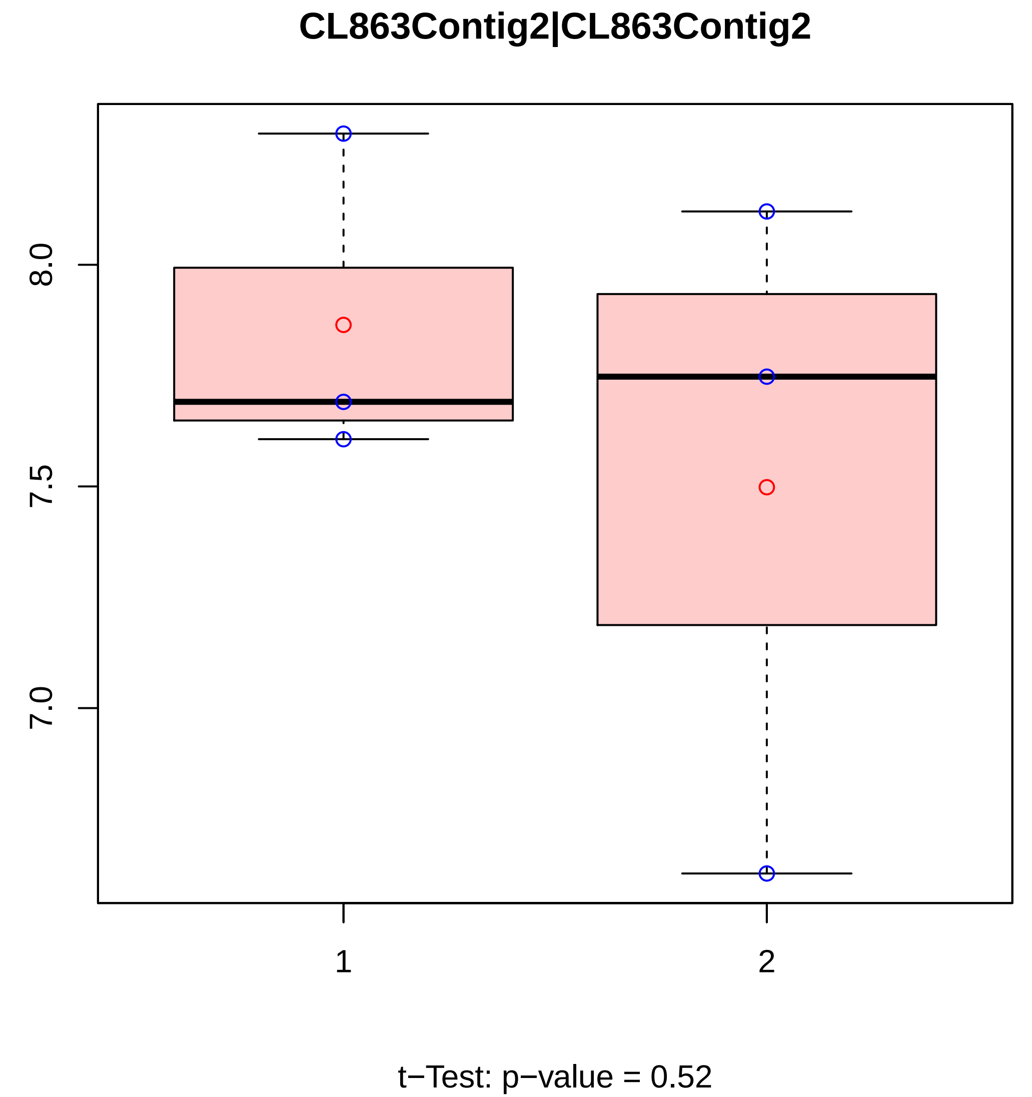
CL863Contig2|CL863Contig2
8.0
7.5
7.0
1
2
t−Test: p−value = 0.52

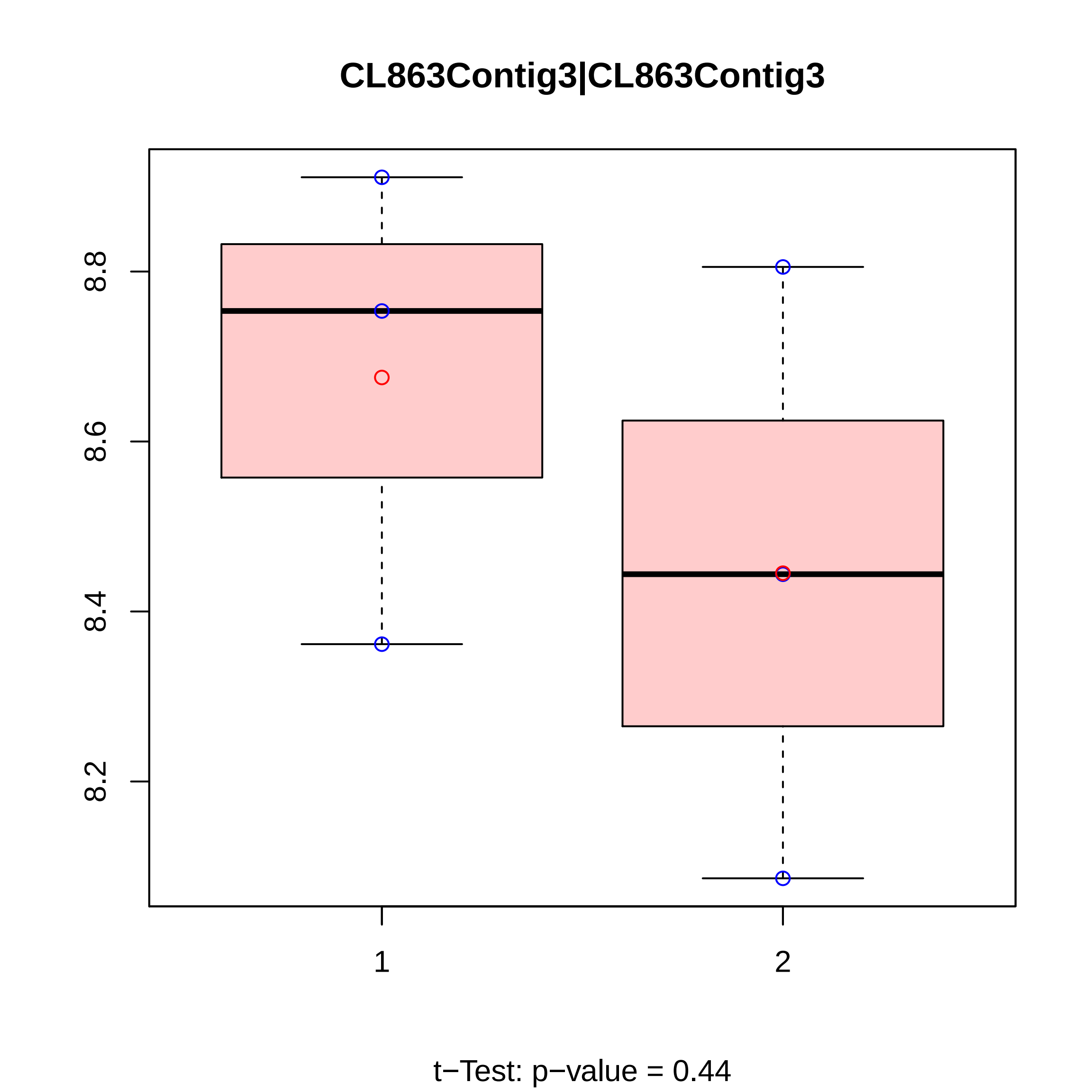

CL863Contig3|CL863Contig3
8.2
8.4
8.6
8.8
1
2
t−Test: p−value = 0.44

# CL863Contig4|CL863Contig4

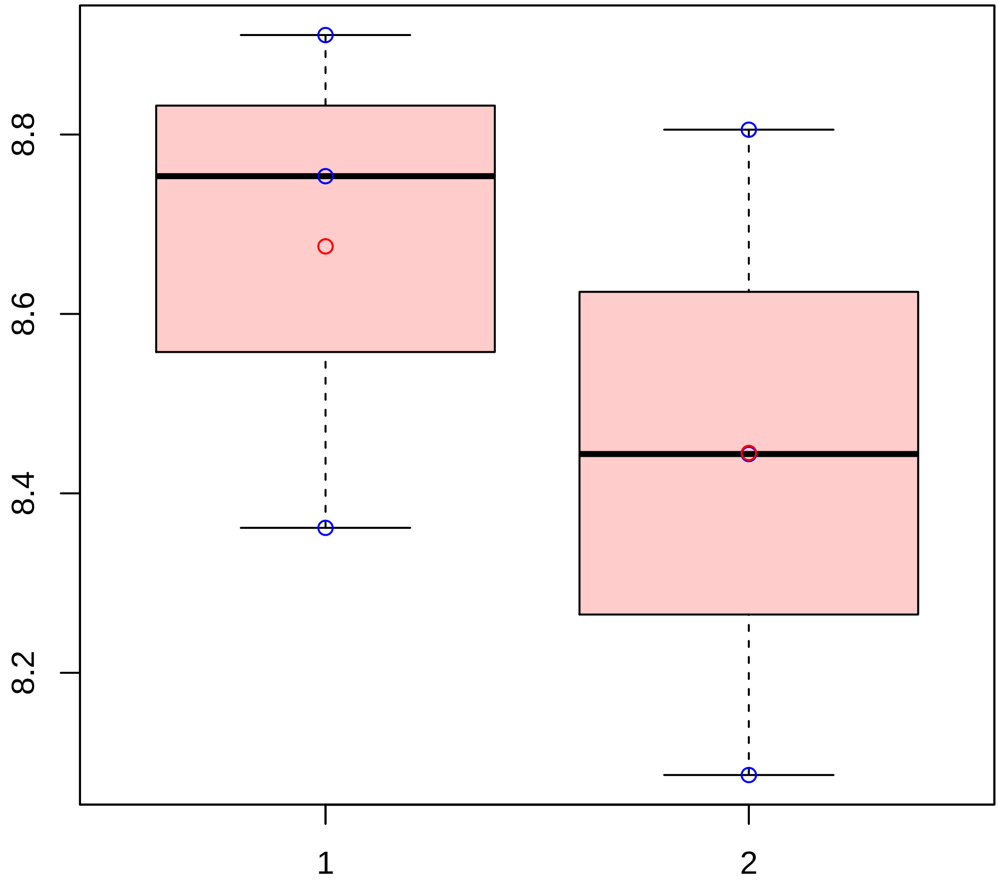


t−Test: p−value = 0.44

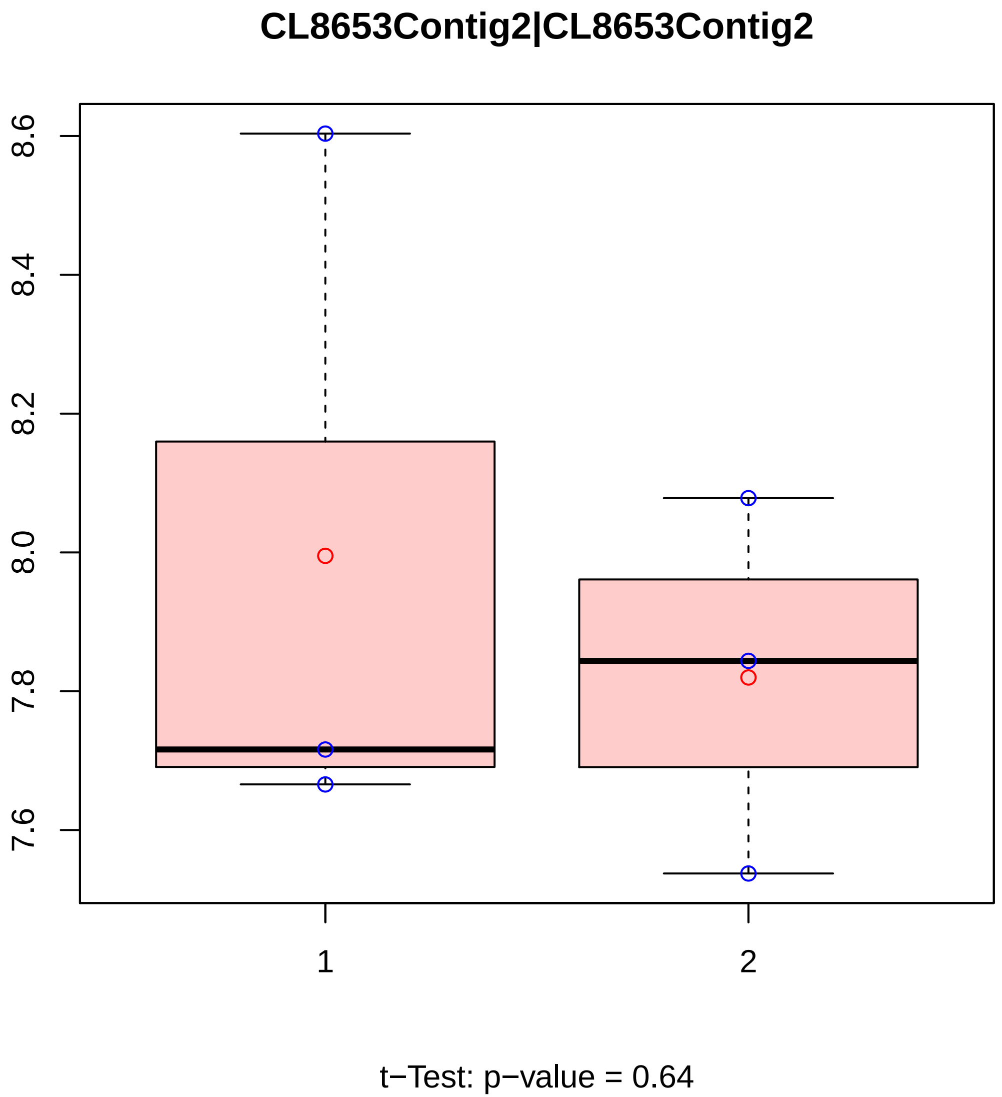
CL8653Contig2|CL8653Contig2
8.6
8.4
8.2
8.0
7.8
7.6
1
2
t−Test: p−value = 0.64

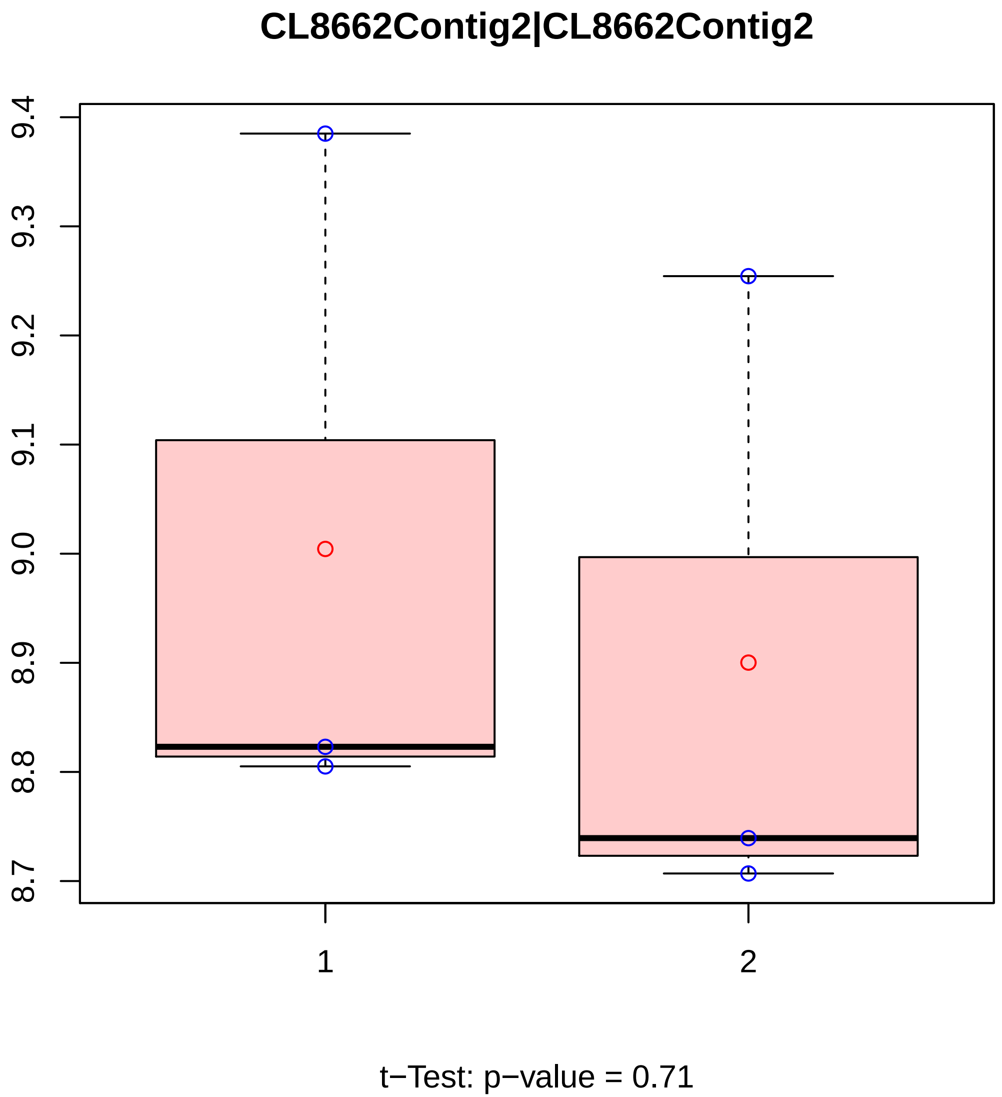
CL8662Contig2|CL8662Contig2
9.4
9.3
9.2
9.1
9.0
8.9
8.8
8.7
1
2
t−Test: p−value = 0.71

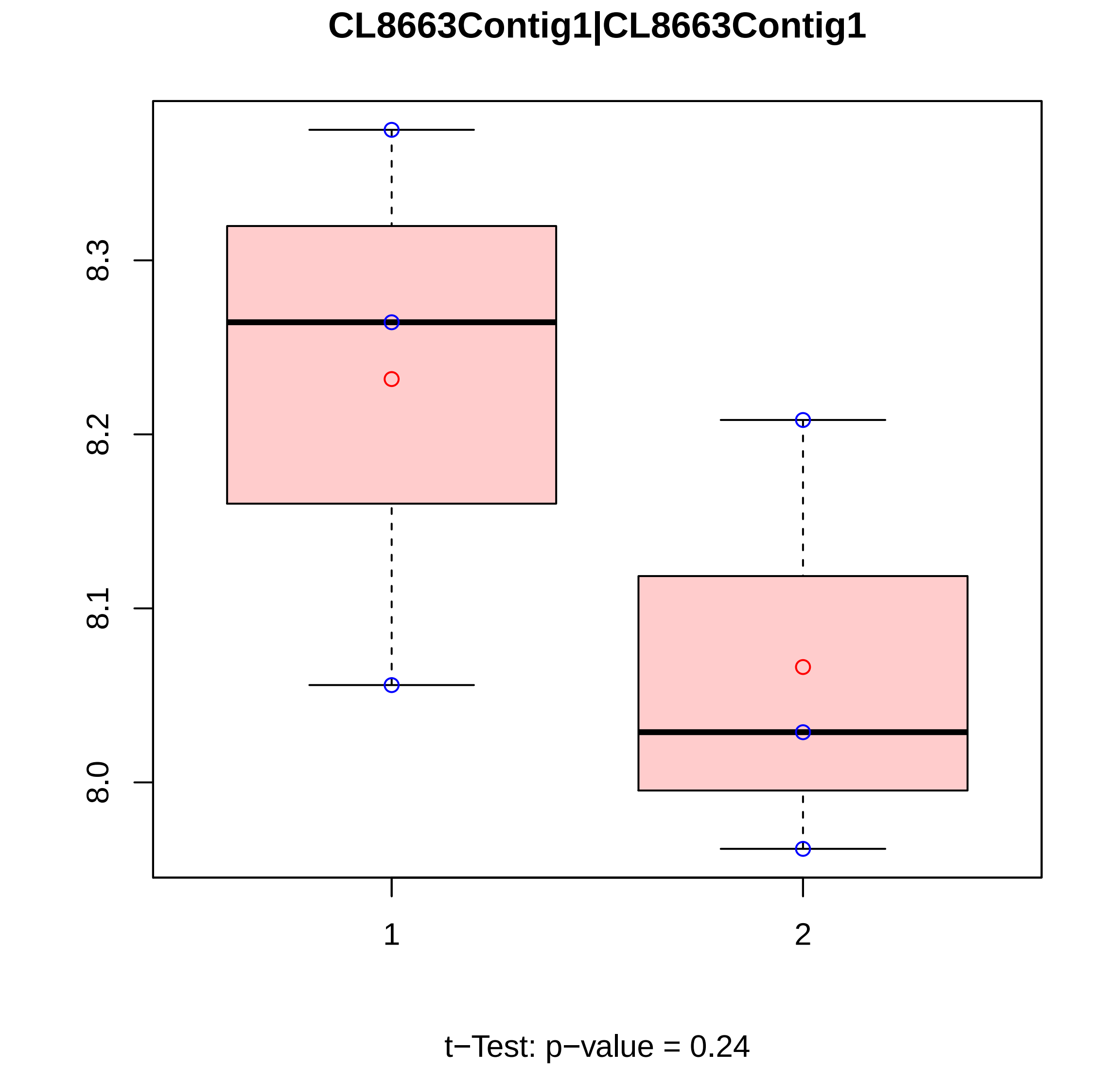

CL8663Contig1|CL8663Contig1
8.0
8.1
8.2
8.3
1
2
t−Test: p−value = 0.24

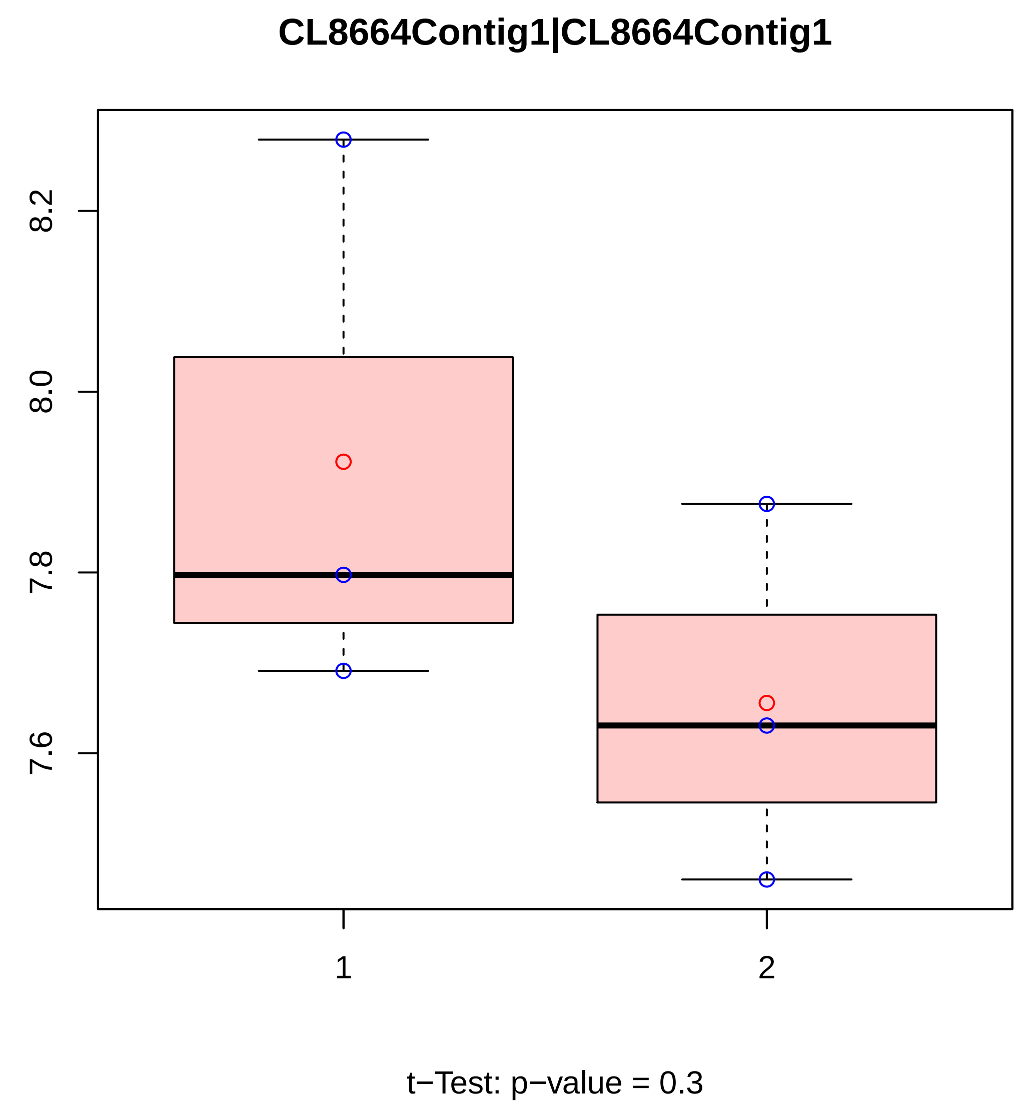
CL8664Contig1|CL8664Contig1
8.2
8.0
7.8
7.6
1
2
t−Test: p−value = 0.3

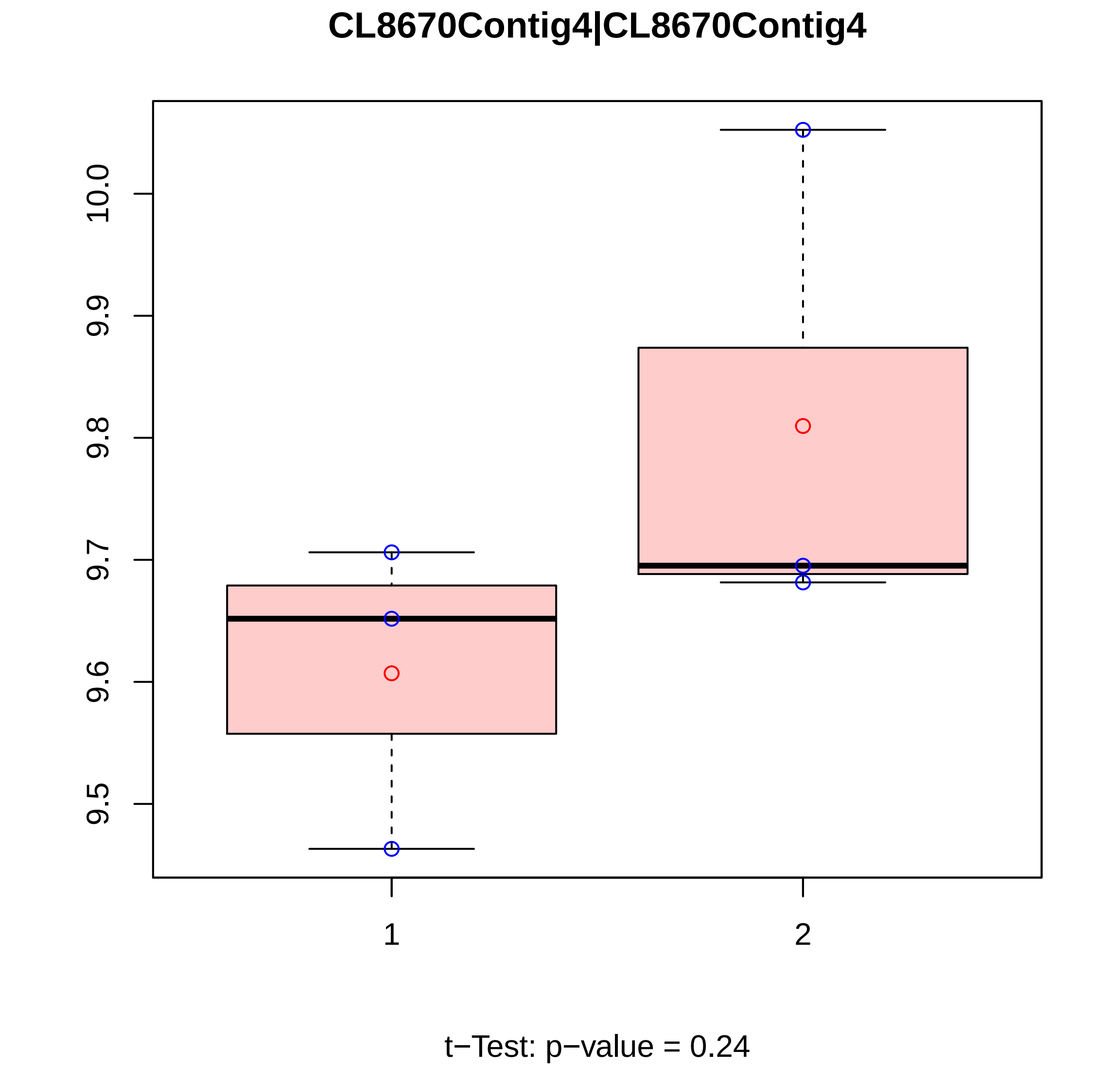
CL8670Contig4|CL8670Contig4
10.0
9.9
9.8
9.7
9.6
9.5
1
2
t−Test: p−value = 0.24

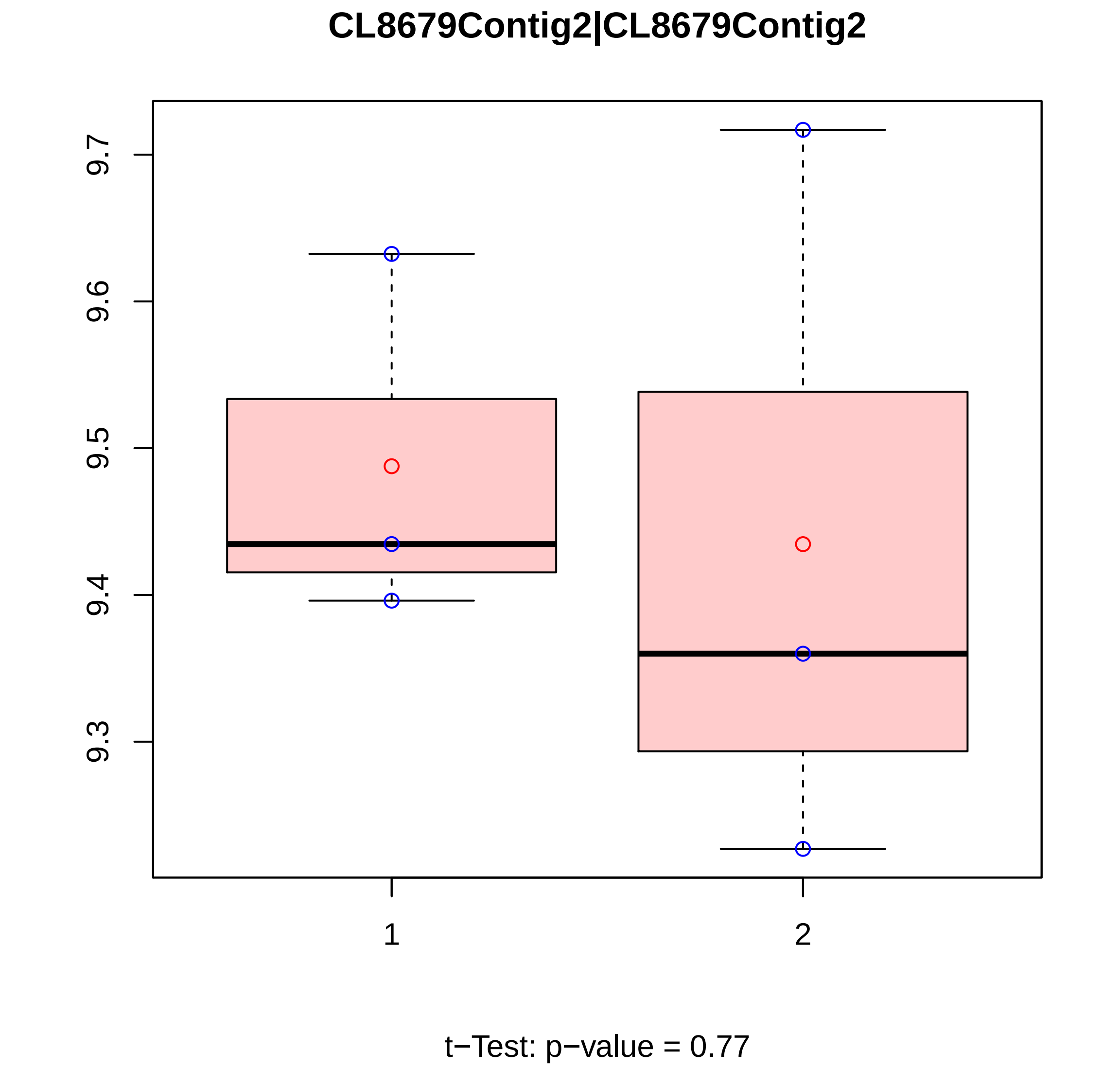

CL8679Contig2|CL8679Contig2
9.3
9.4
9.5
9.6
9.7
1
2
t−Test: p−value = 0.77

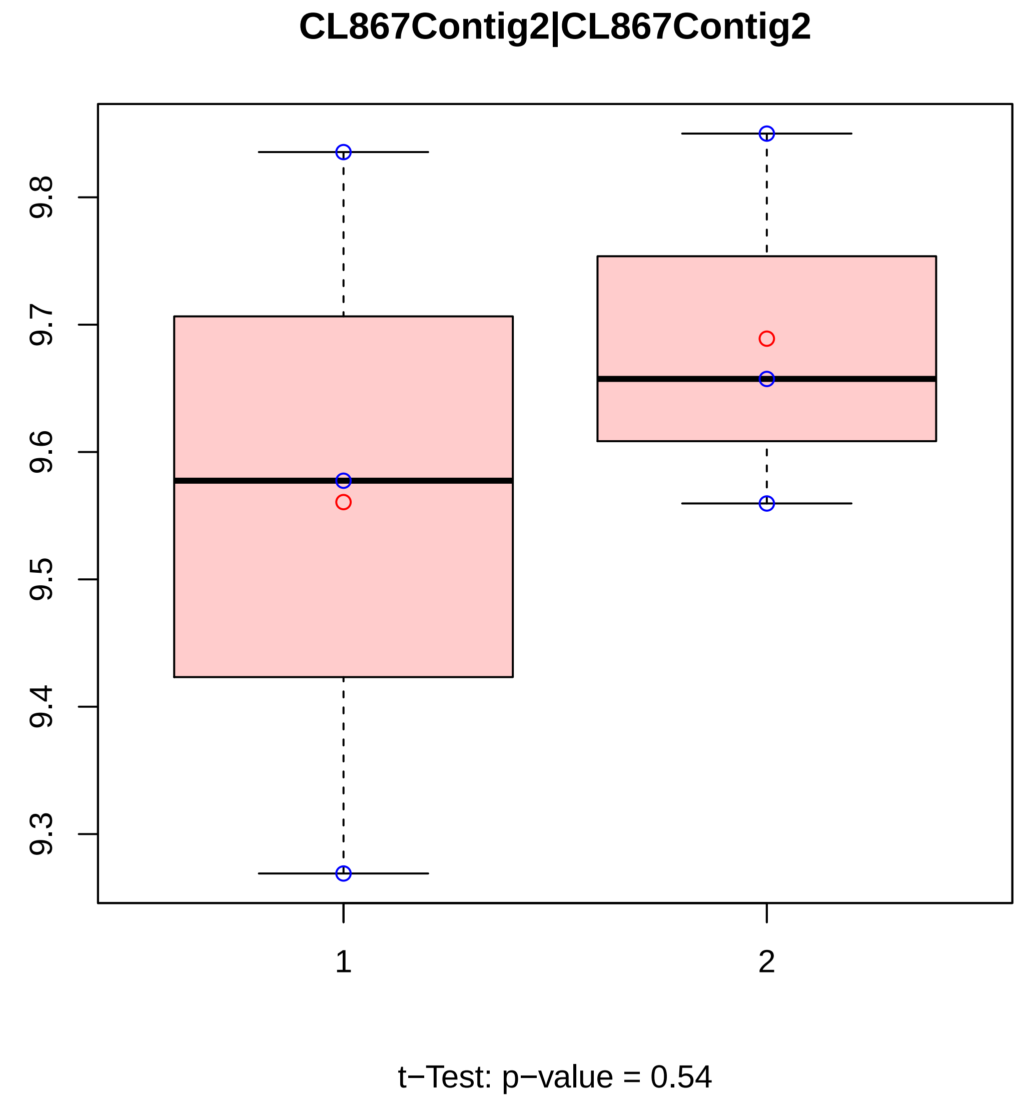

CL867Contig2|CL867Contig2
9.3
9.4
9.5
9.6
9.7
9.8
1
2
t−Test: p−value = 0.54

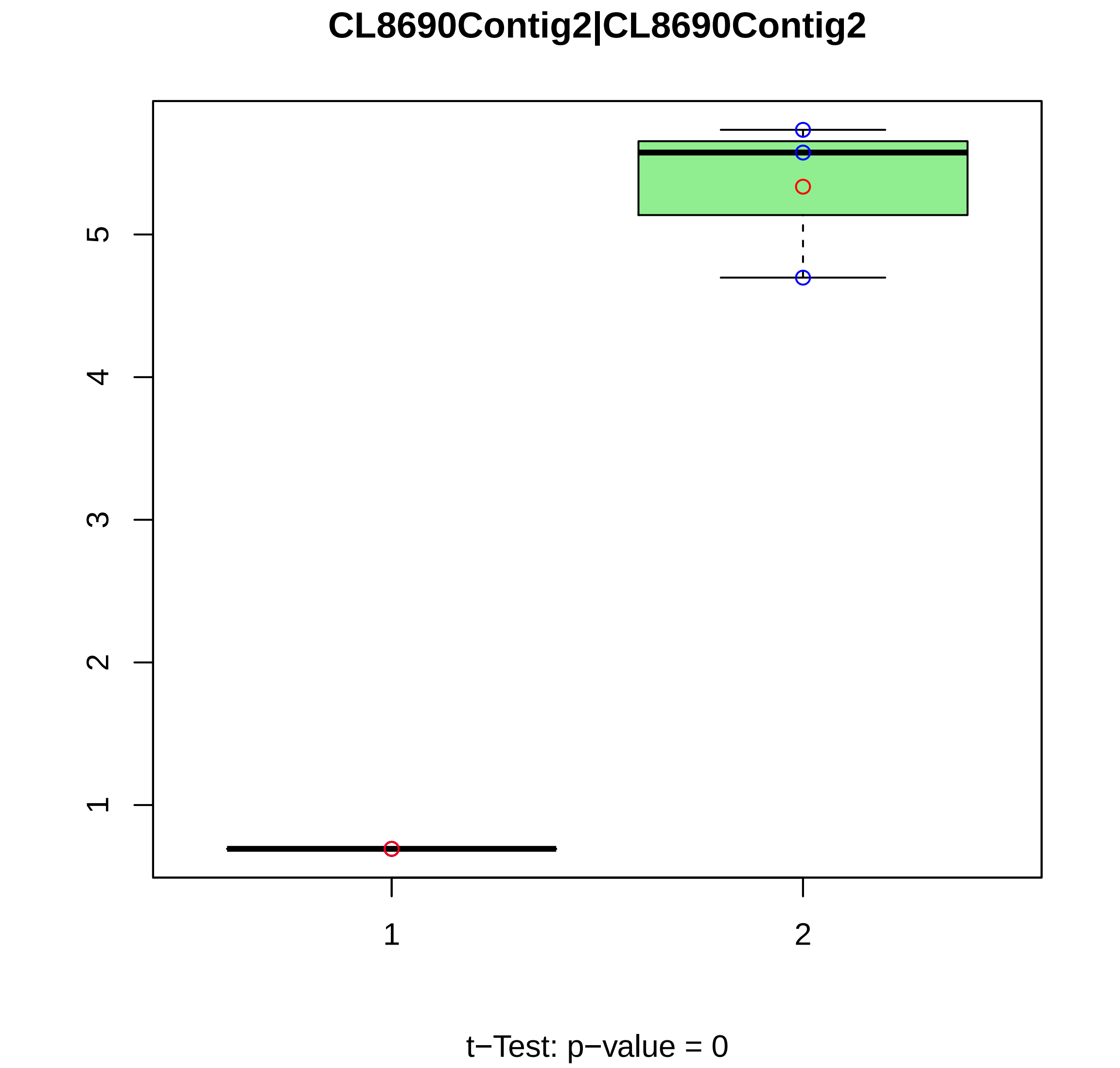
CL8690Contig2|CL8690Contig2
1
2
3
4
5
1
2
t−Test: p−value = 0

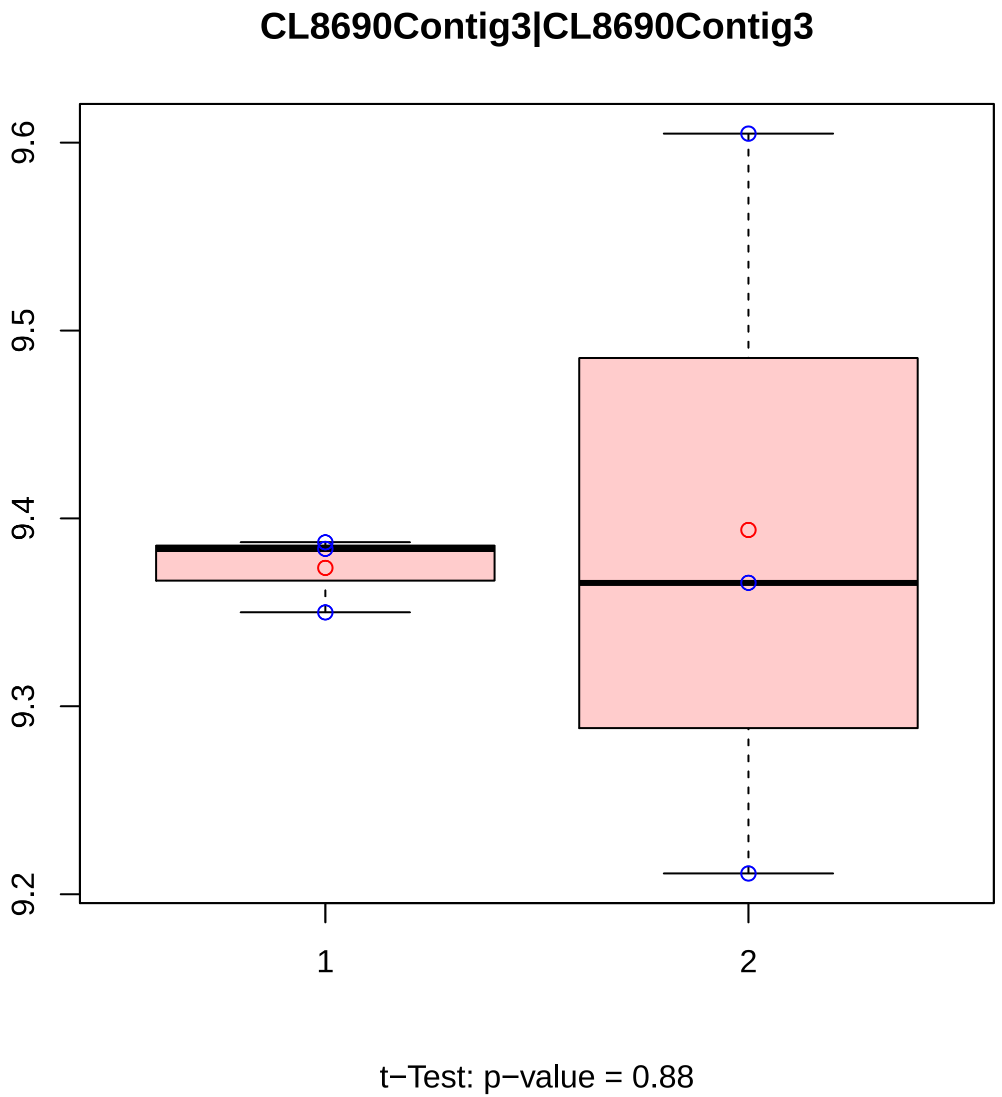

CL8690Contig3|CL8690Contig3
9.6
9.5
9.4
9.3
9.2
1
2
t−Test: p−value = 0.88

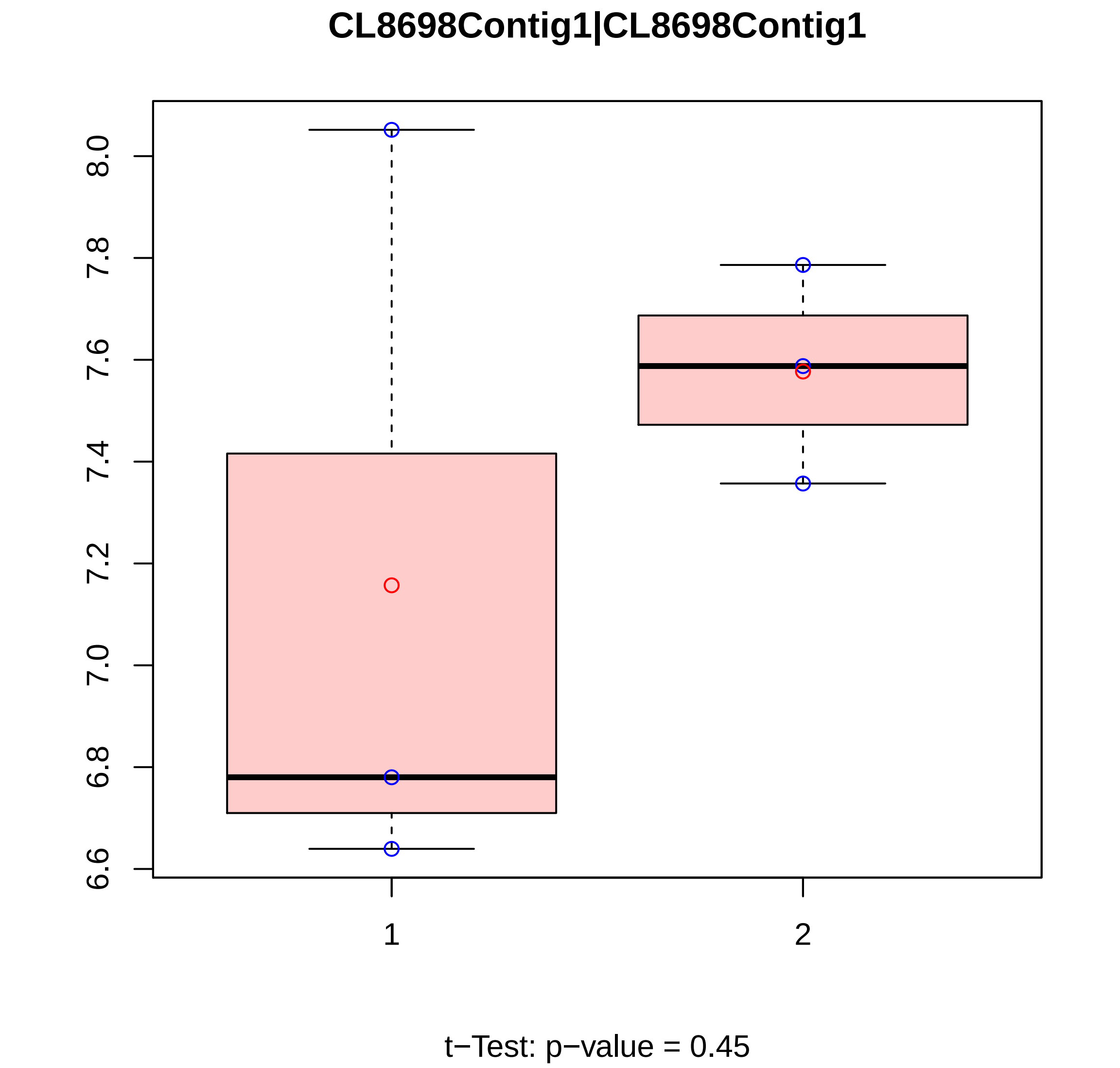

CL8698Contig1|CL8698Contig1
8.0
7.8
7.6
7.4
7.2
7.0
6.8
6.6
1
2
t−Test: p−value = 0.45

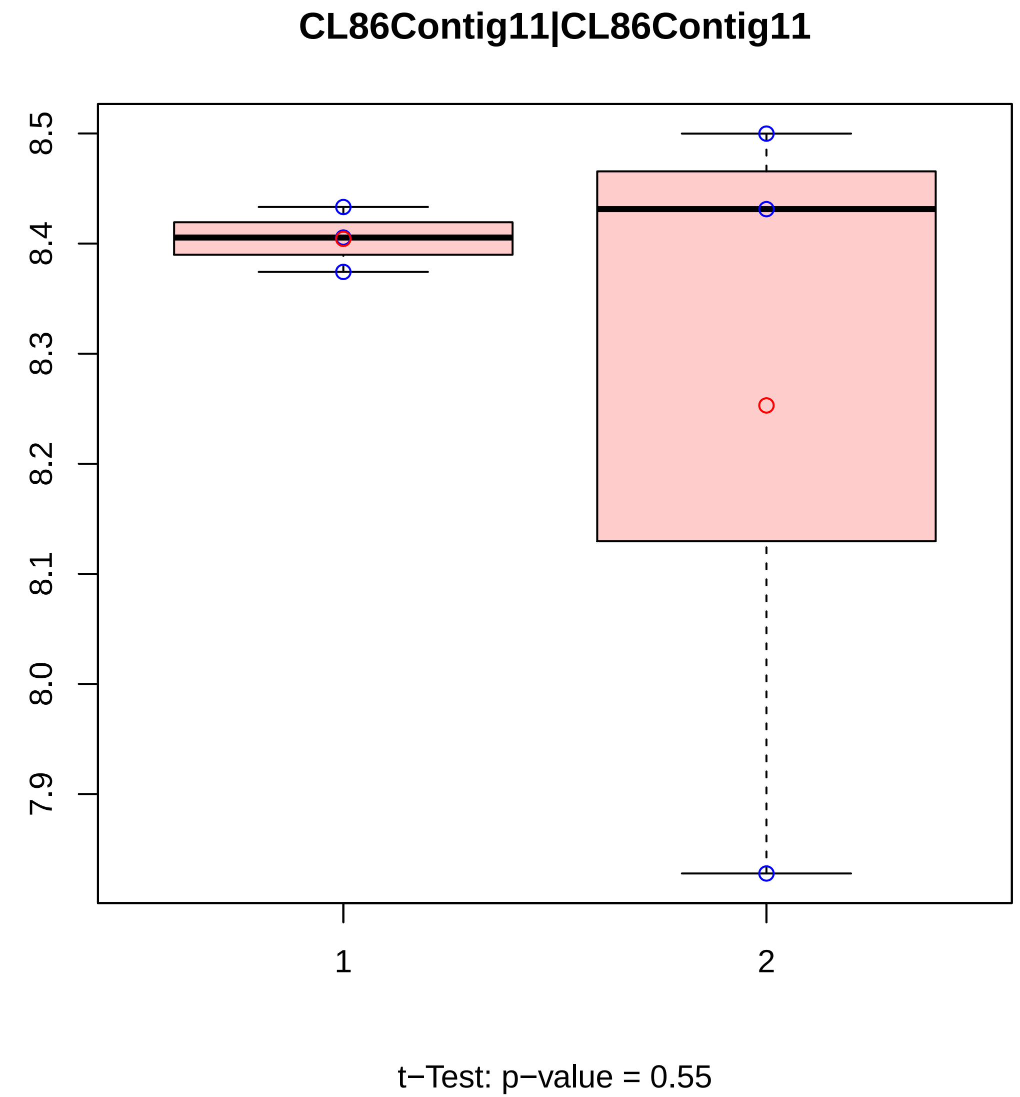
CL86Contig11|CL86Contig11
8.5
8.4
8.3
8.2
8.1
8.0
7.9
1
2
t−Test: p−value = 0.55

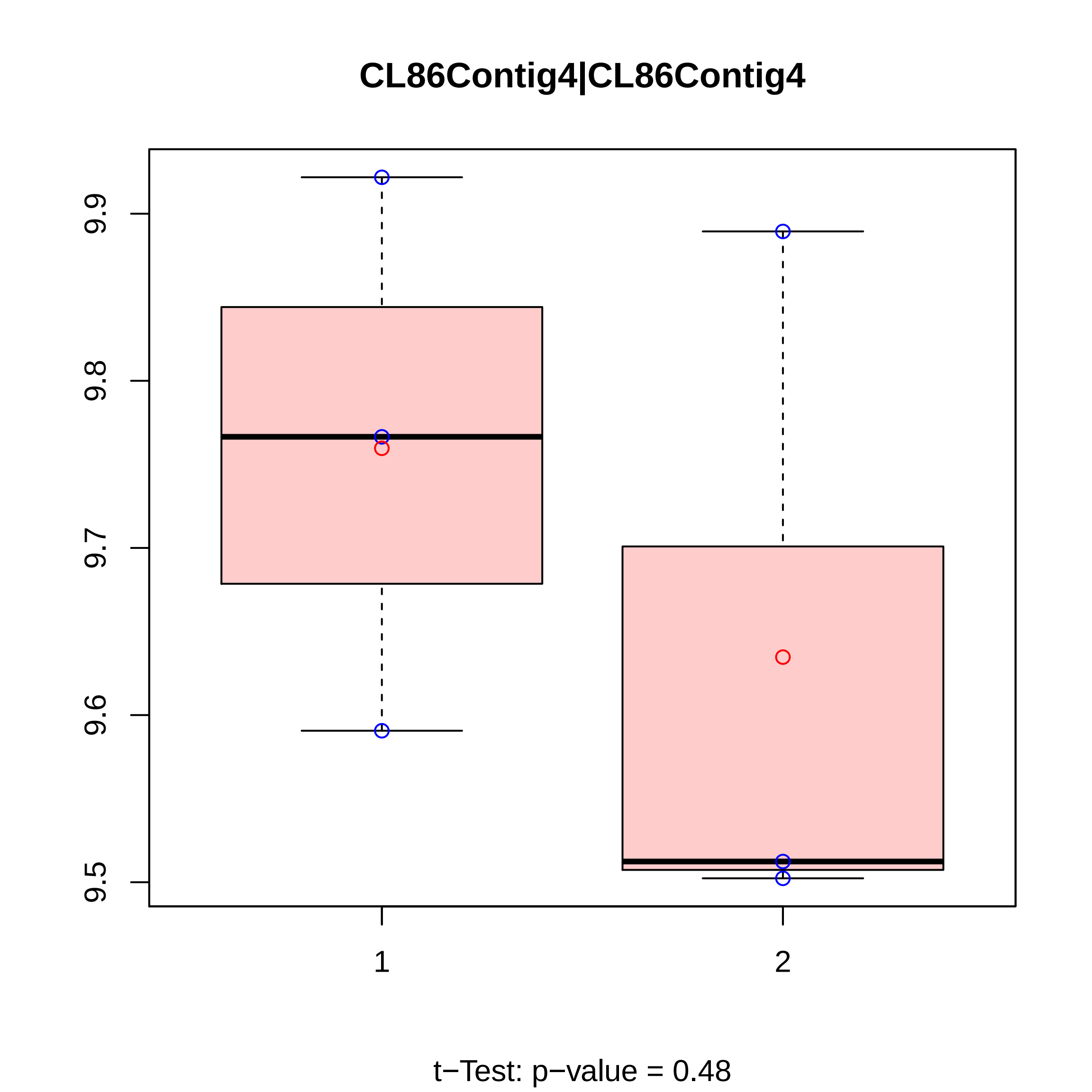
CL86Contig4|CL86Contig4
9.5
9.6
9.7
9.8
9.9
1
2
t−Test: p−value = 0.48

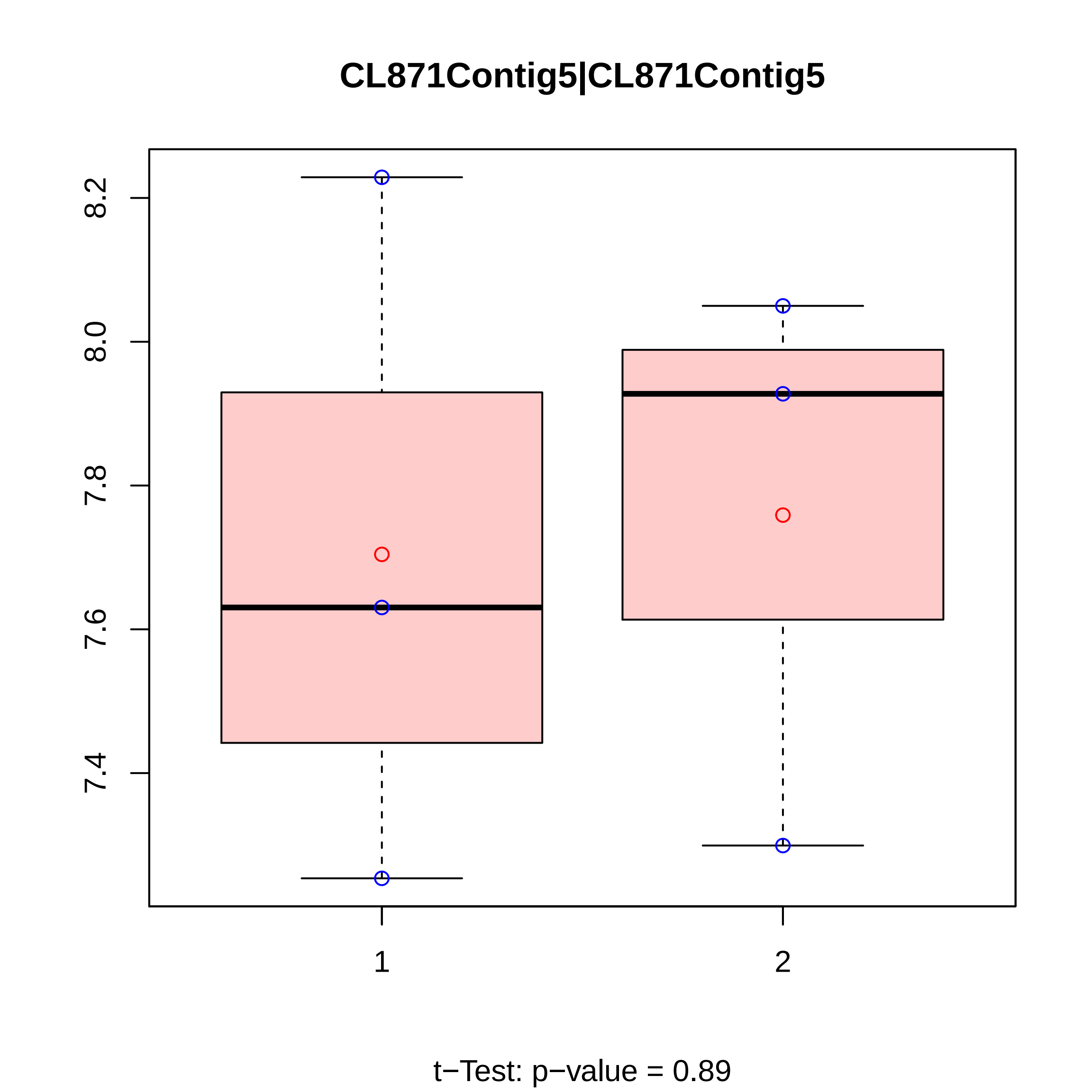
CL871Contig5|CL871Contig5
8.2
8.0
7.8
7.6
7.4
1
2
t−Test: p−value = 0.89

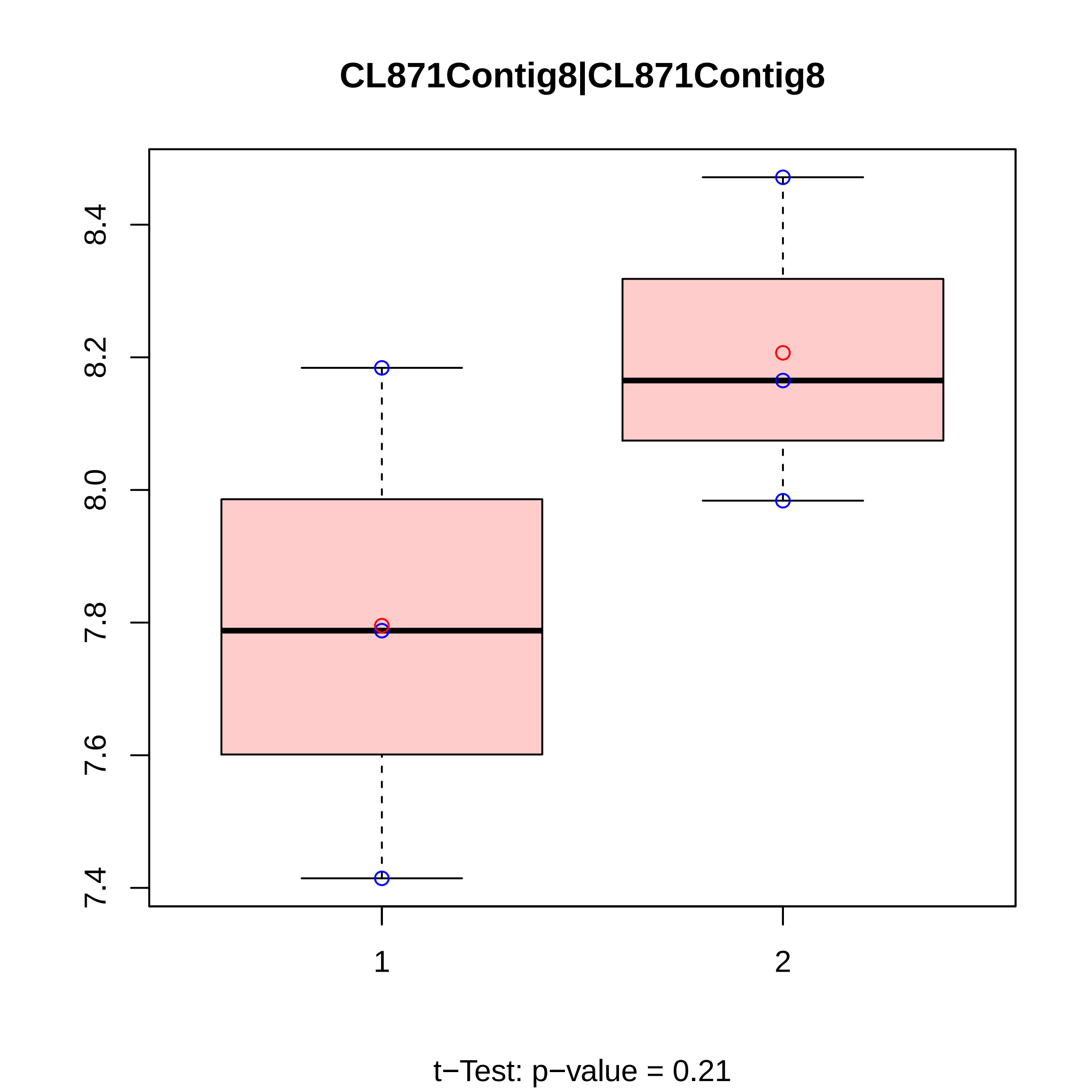
CL871Contig8|CL871Contig8
8.4
8.2
8.0
7.8
7.6
7.4
1
2
t−Test: p−value = 0.21

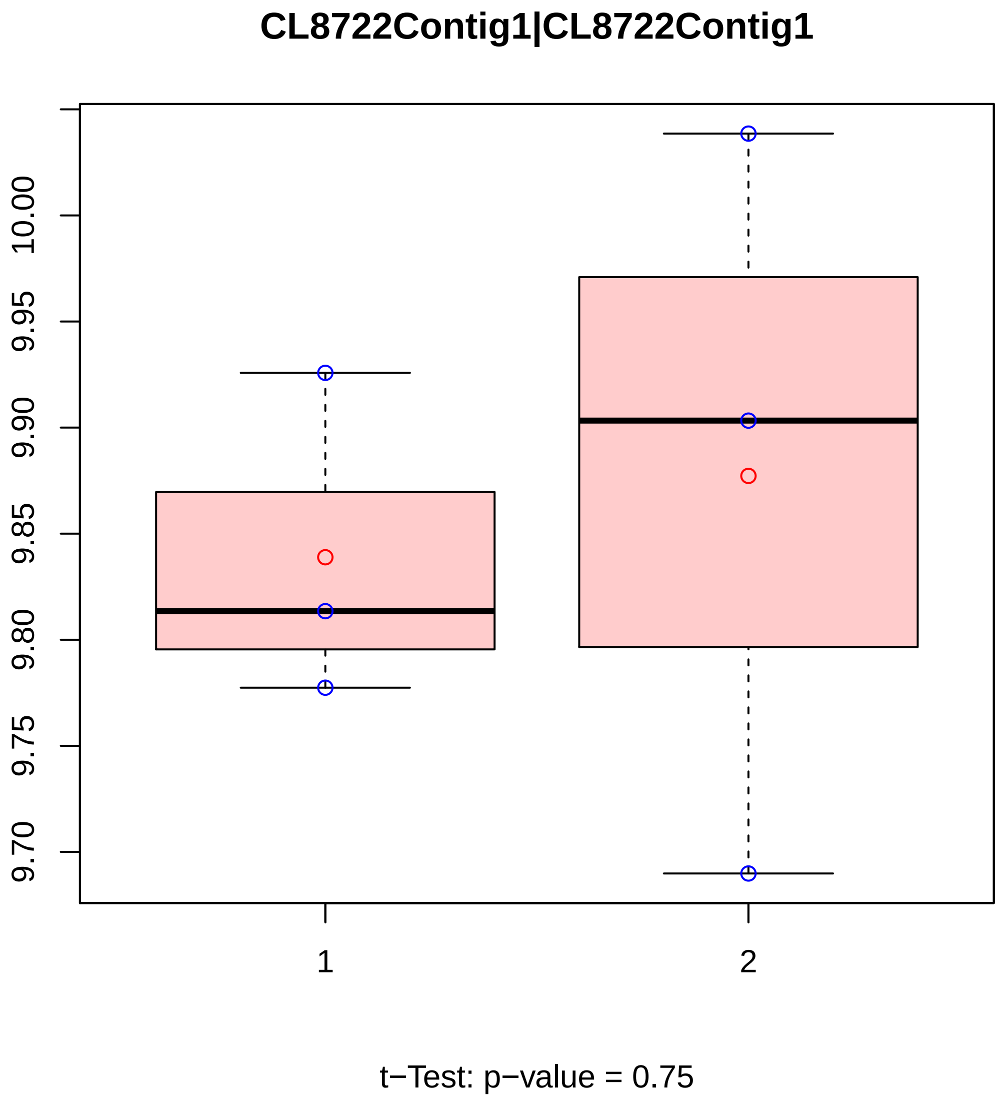
CL8722Contig1|CL8722Contig1
10.00
9.95
9.90
9.85
9.80
9.75
9.70
1
2
t−Test: p−value = 0.75

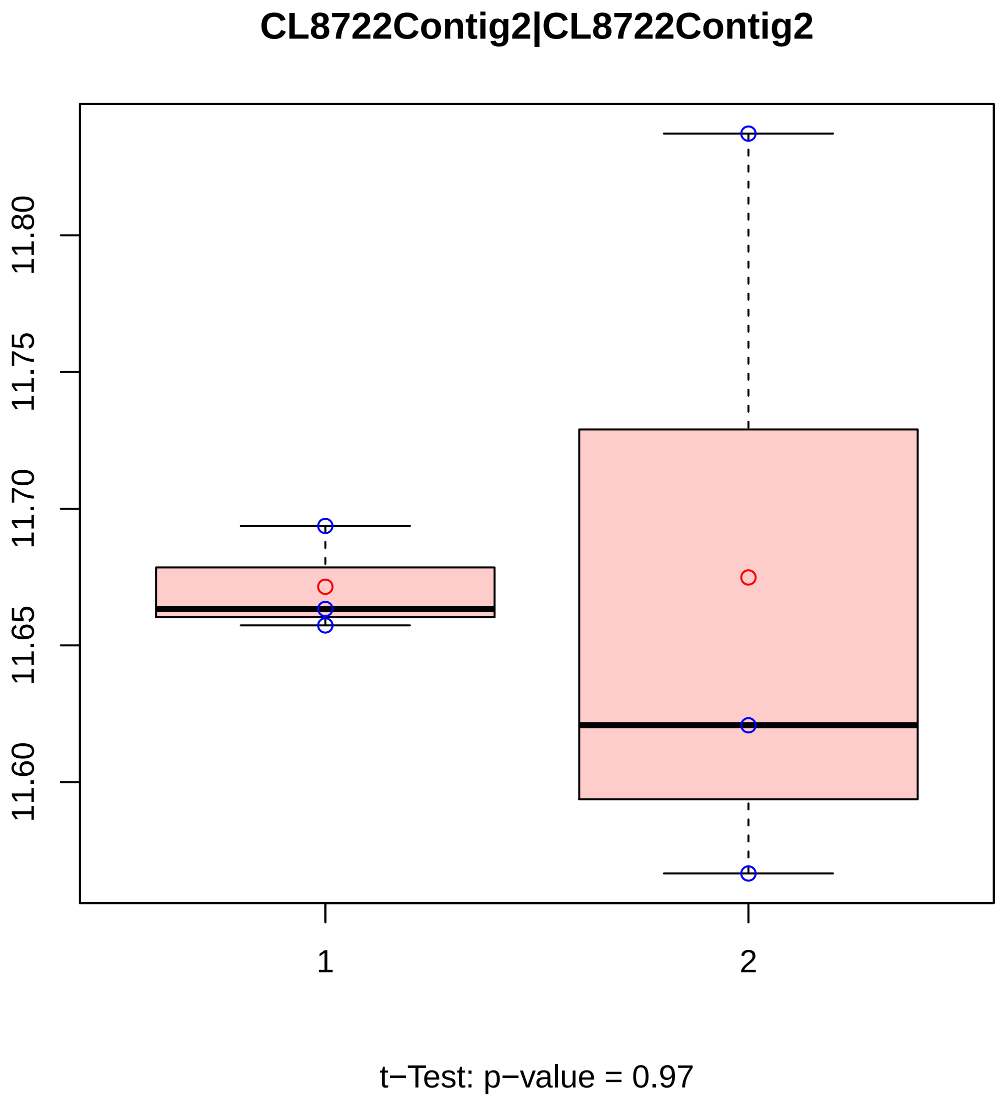
CL8722Contig2|CL8722Contig2
11.60
11.65
11.70
11.75
11.80
1
2
t−Test: p−value = 0.97

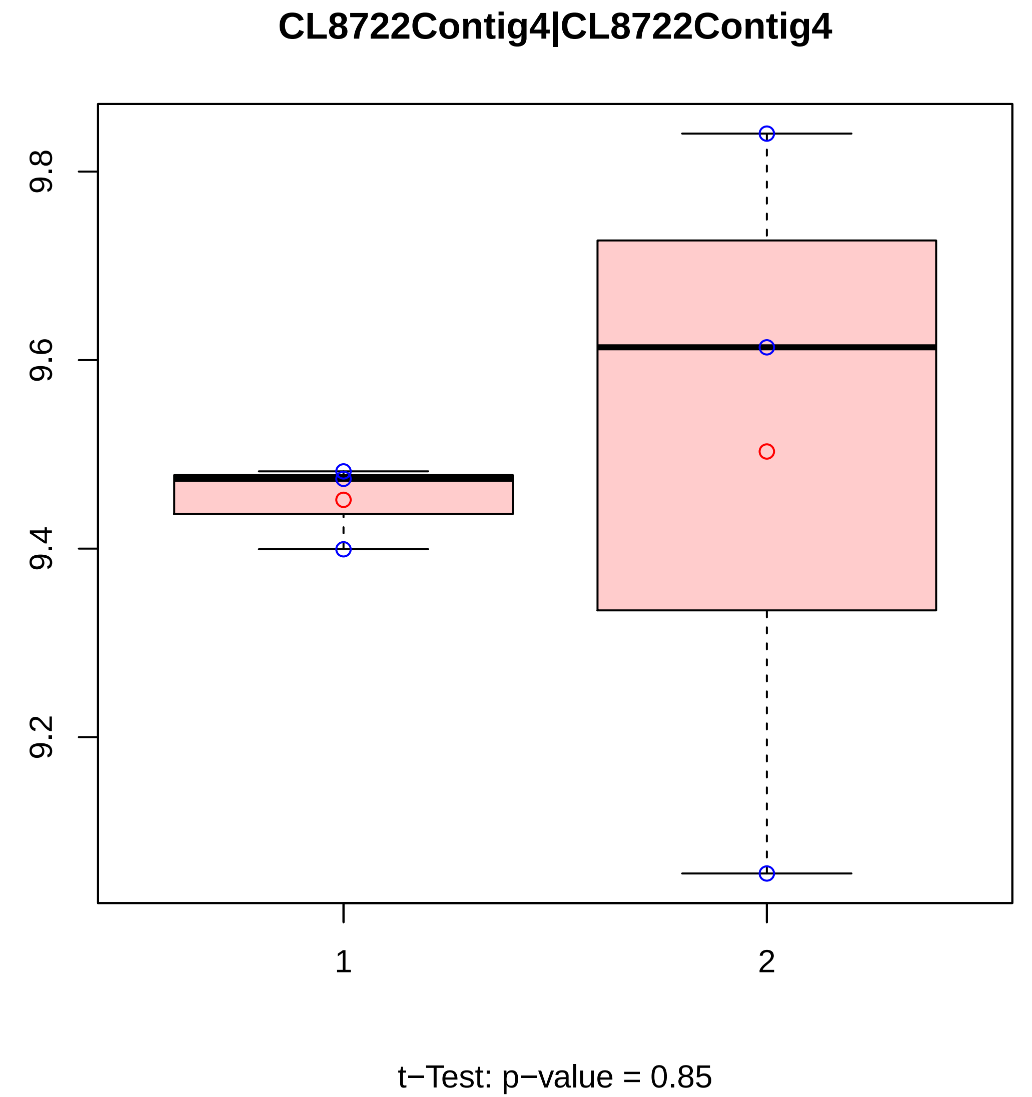
CL8722Contig4|CL8722Contig4
9.8
9.6
9.4
9.2
1
2
t−Test: p−value = 0.85

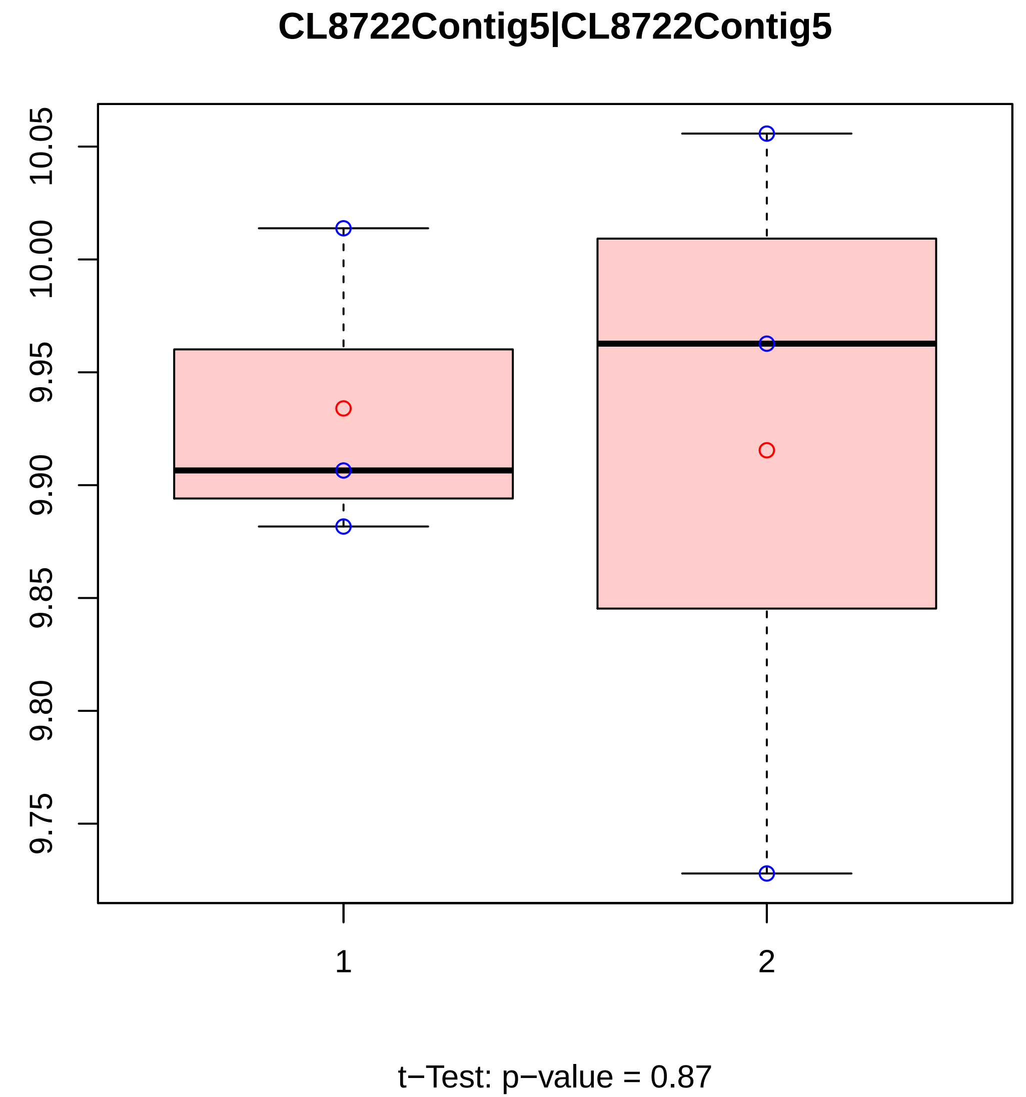
CL8722Contig5|CL8722Contig5
10.05
10.00
9.95
9.90
9.85
9.80
9.75
1
2
t−Test: p−value = 0.87

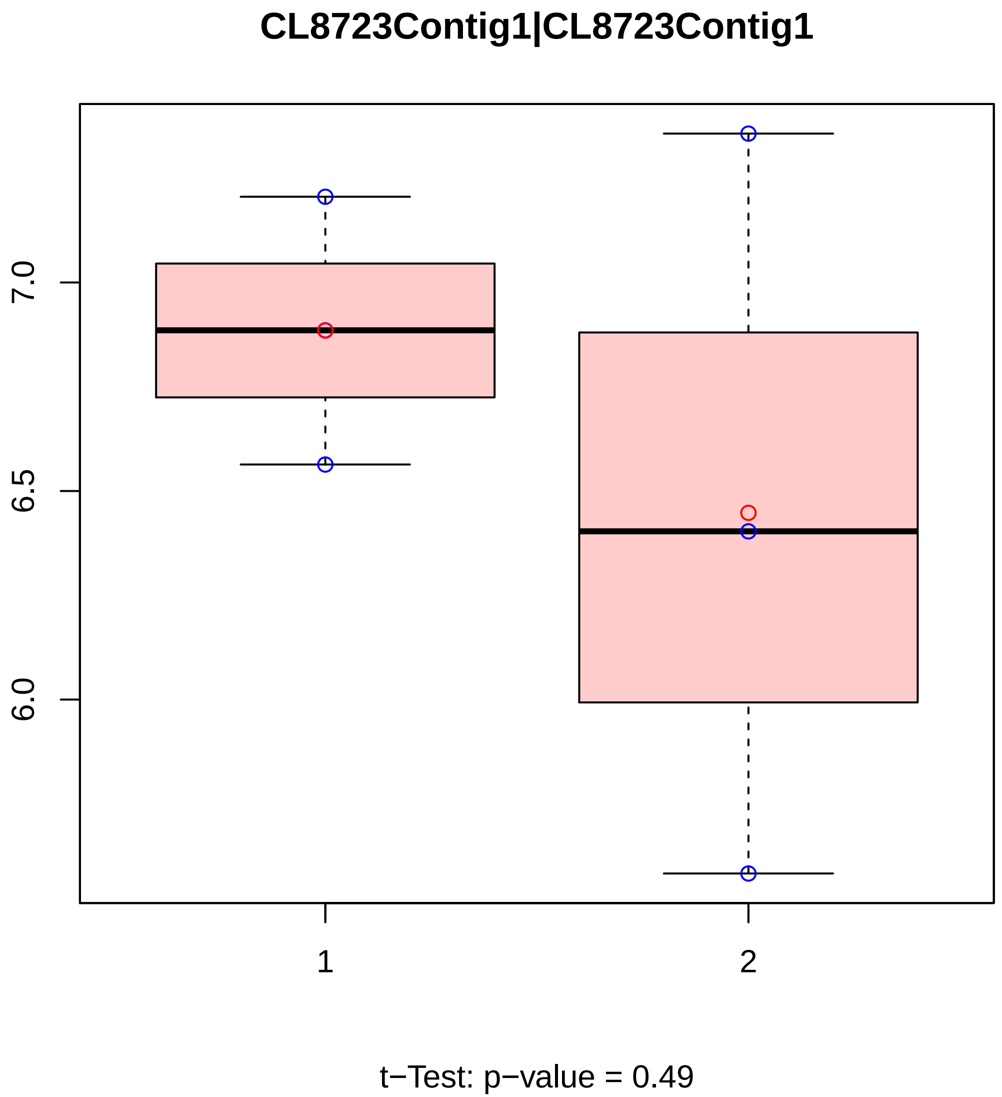
CL8723Contig1|CL8723Contig1
7.0
6.5
6.0
1
2
t−Test: p−value = 0.49

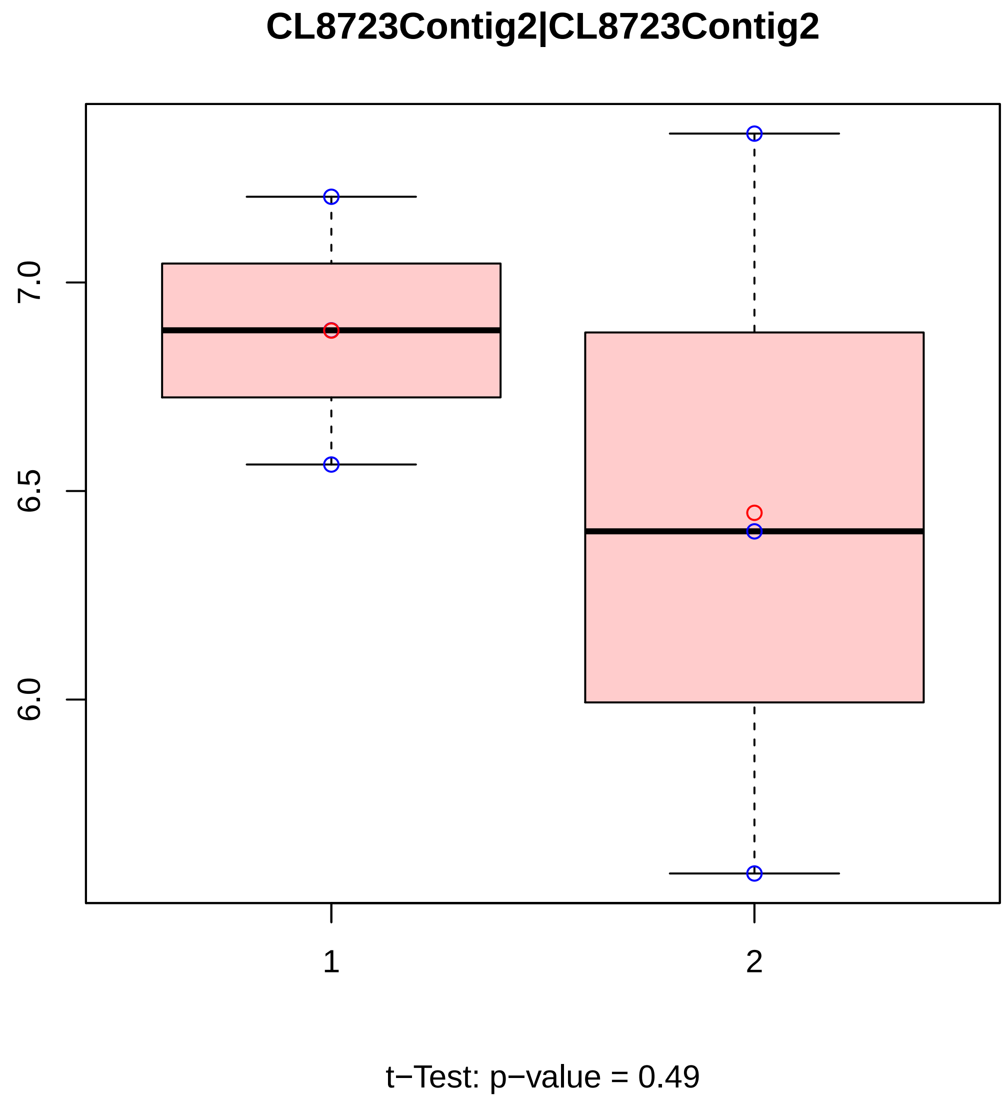

CL8723Contig2|CL8723Contig2
7.0
6.5
6.0
1
2
t−Test: p−value = 0.49

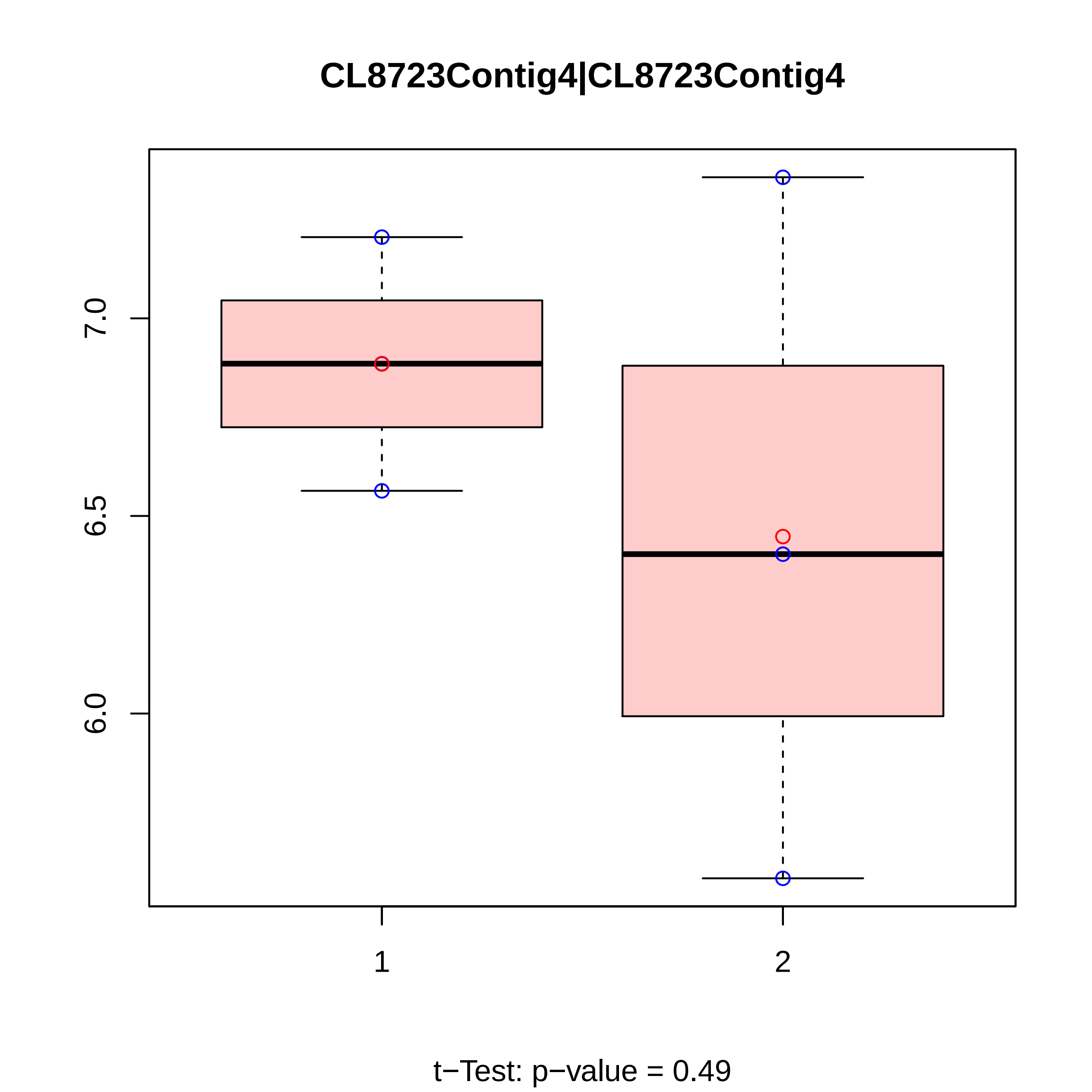
CL8723Contig4|CL8723Contig4
7.0
6.5
6.0
1
2
t−Test: p−value = 0.49

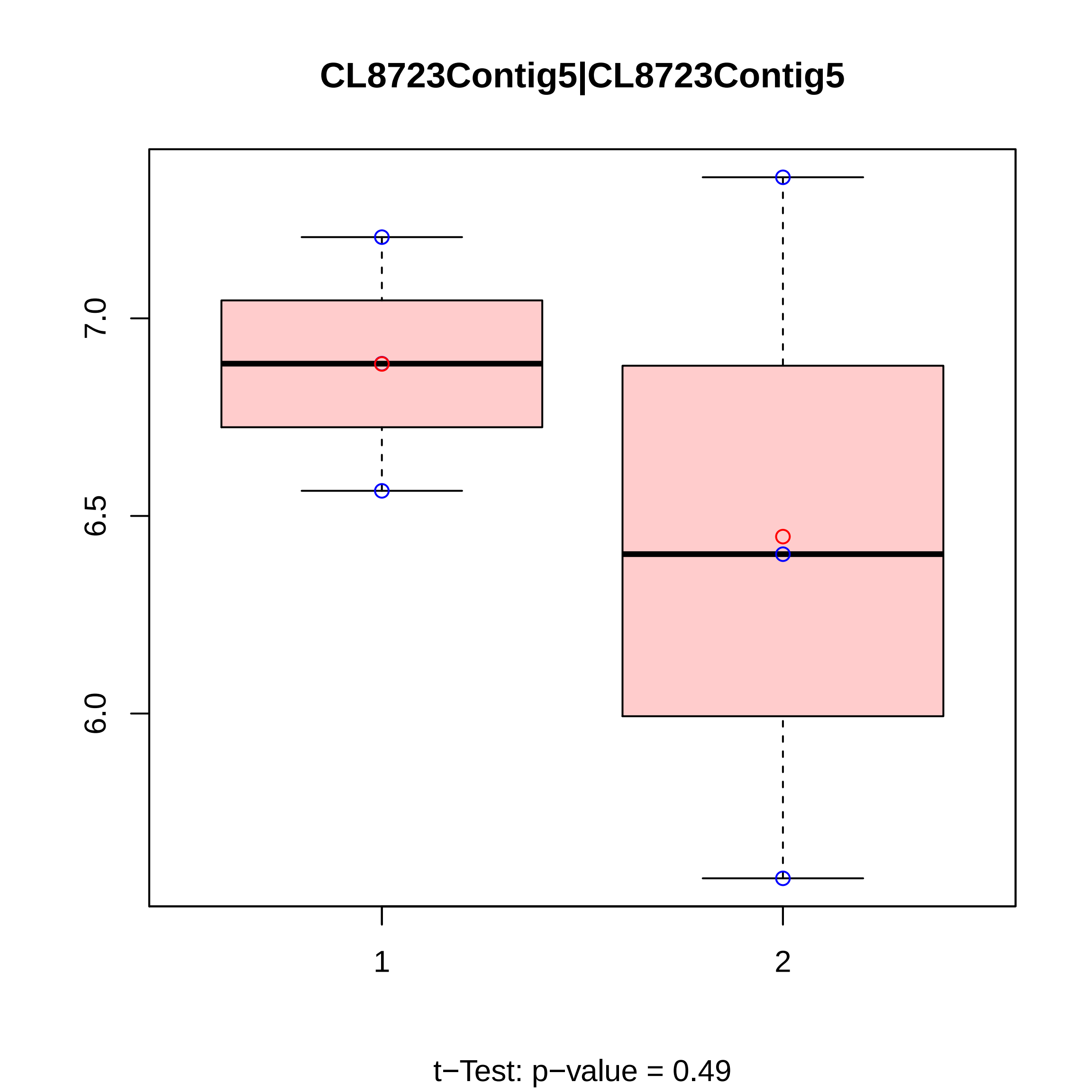
CL8723Contig5|CL8723Contig5
7.0
6.5
6.0
1
2
t−Test: p−value = 0.49

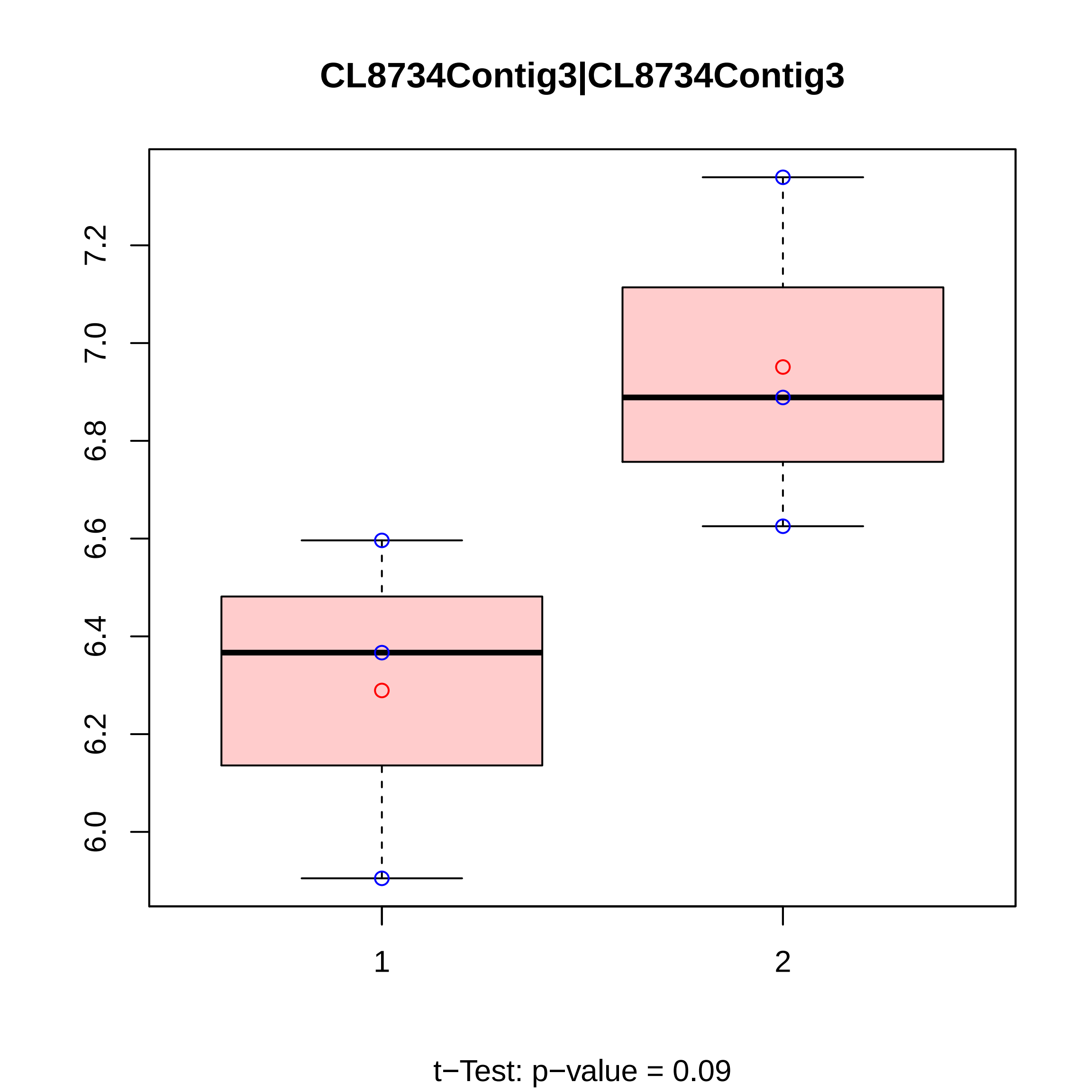

CL8734Contig3|CL8734Contig3
7.2
7.0
6.8
6.6
6.4
6.2
6.0
1
2
t−Test: p−value = 0.09

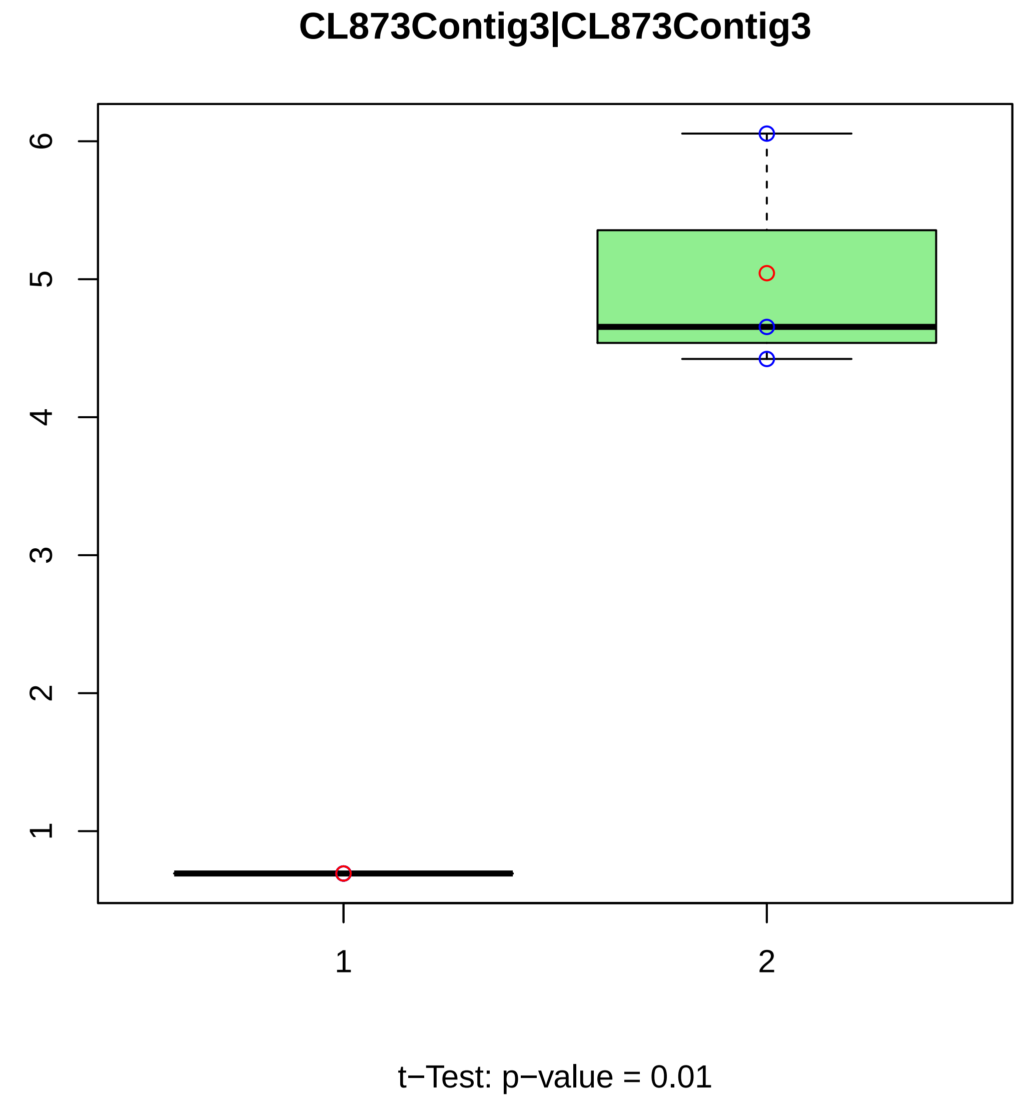
CL873Contig3|CL873Contig3
6
5
4
3
2
1
1
2
t−Test: p−value = 0.01

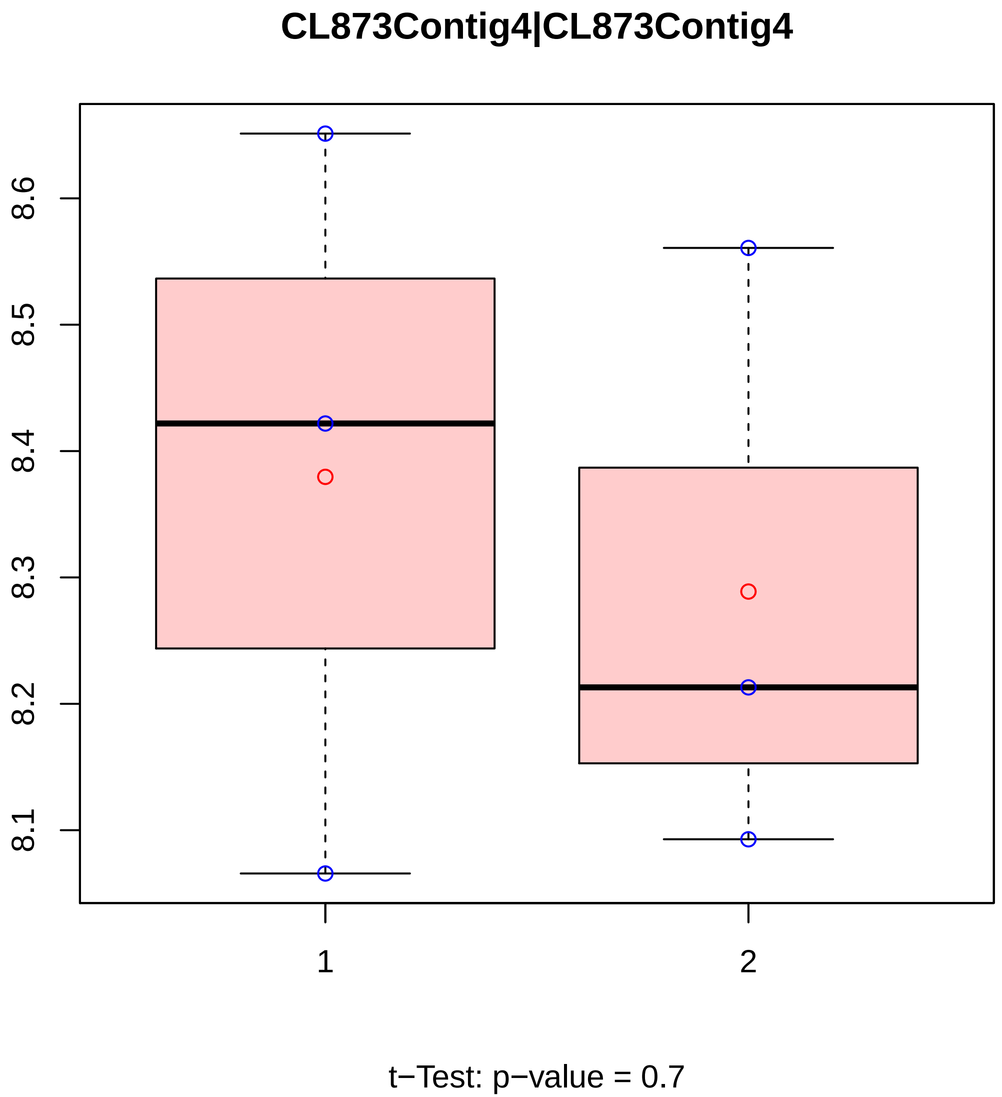
CL873Contig4|CL873Contig4
8.1
8.2
8.3
8.4
8.5
8.6
1
2
t−Test: p−value = 0.7

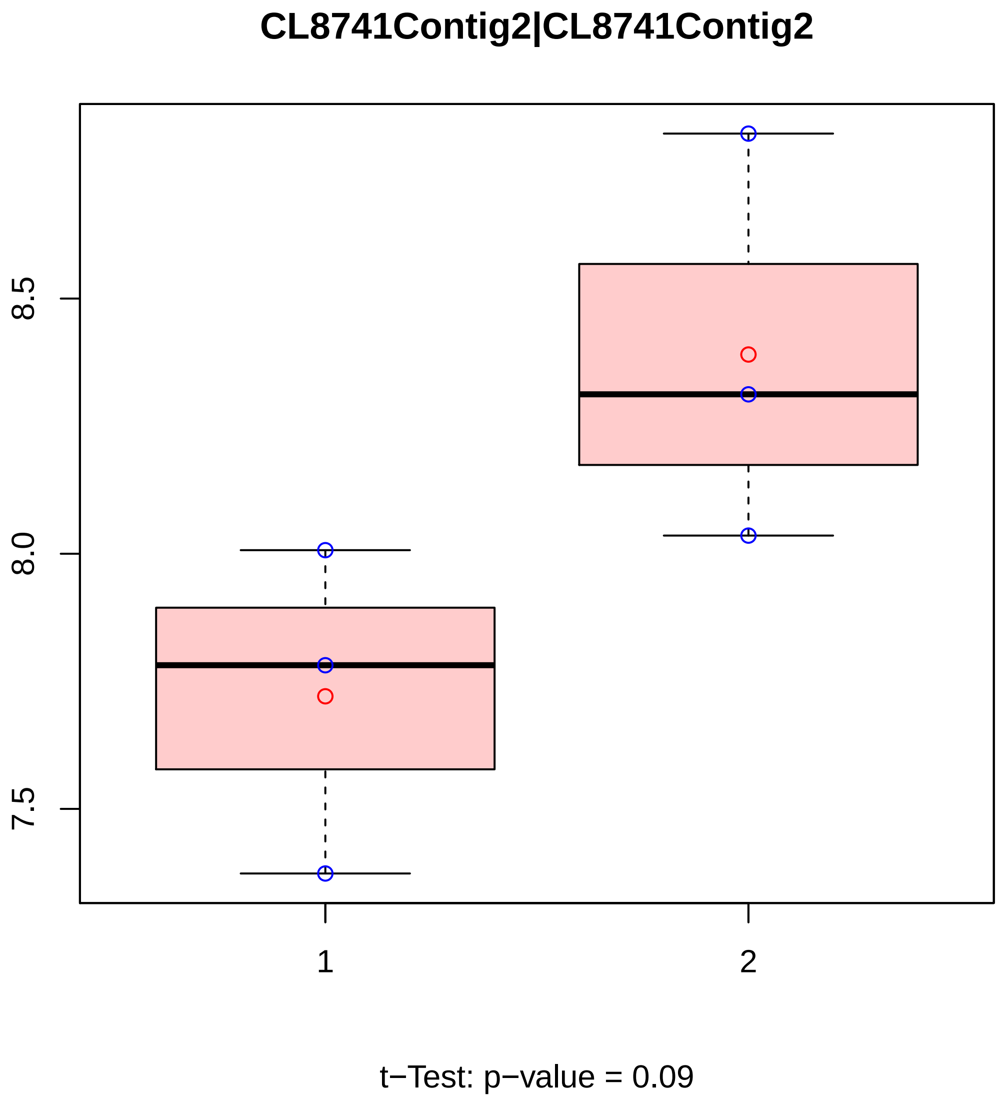
CL8741Contig2|CL8741Contig2
8.5
8.0
7.5
1
2
t−Test: p−value = 0.09

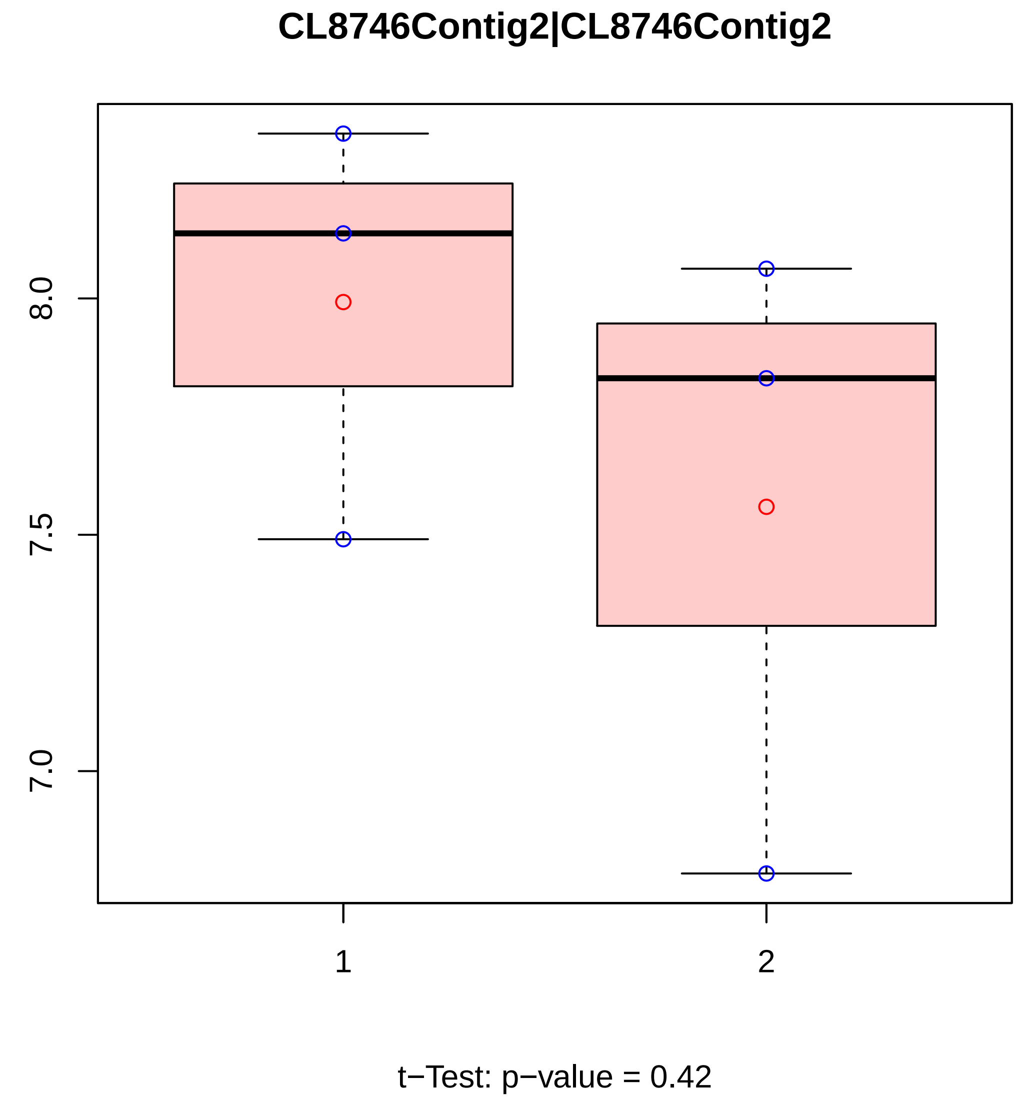
CL8746Contig2|CL8746Contig2
8.0
7.5
7.0
1
2
t−Test: p−value = 0.42

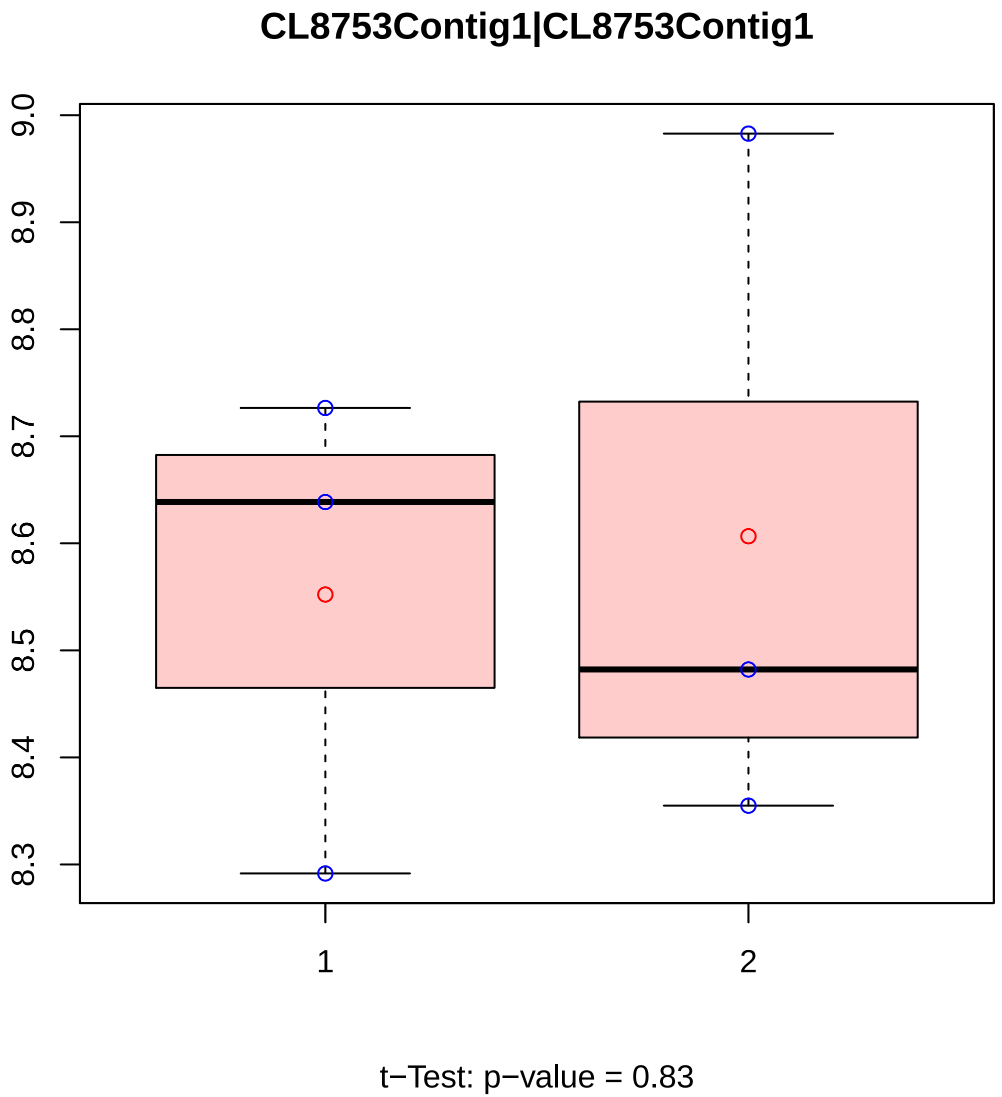
CL8753Contig1|CL8753Contig1
9.0
8.9
8.8
8.7
8.6
8.5
8.4
8.3
1
2
t−Test: p−value = 0.83

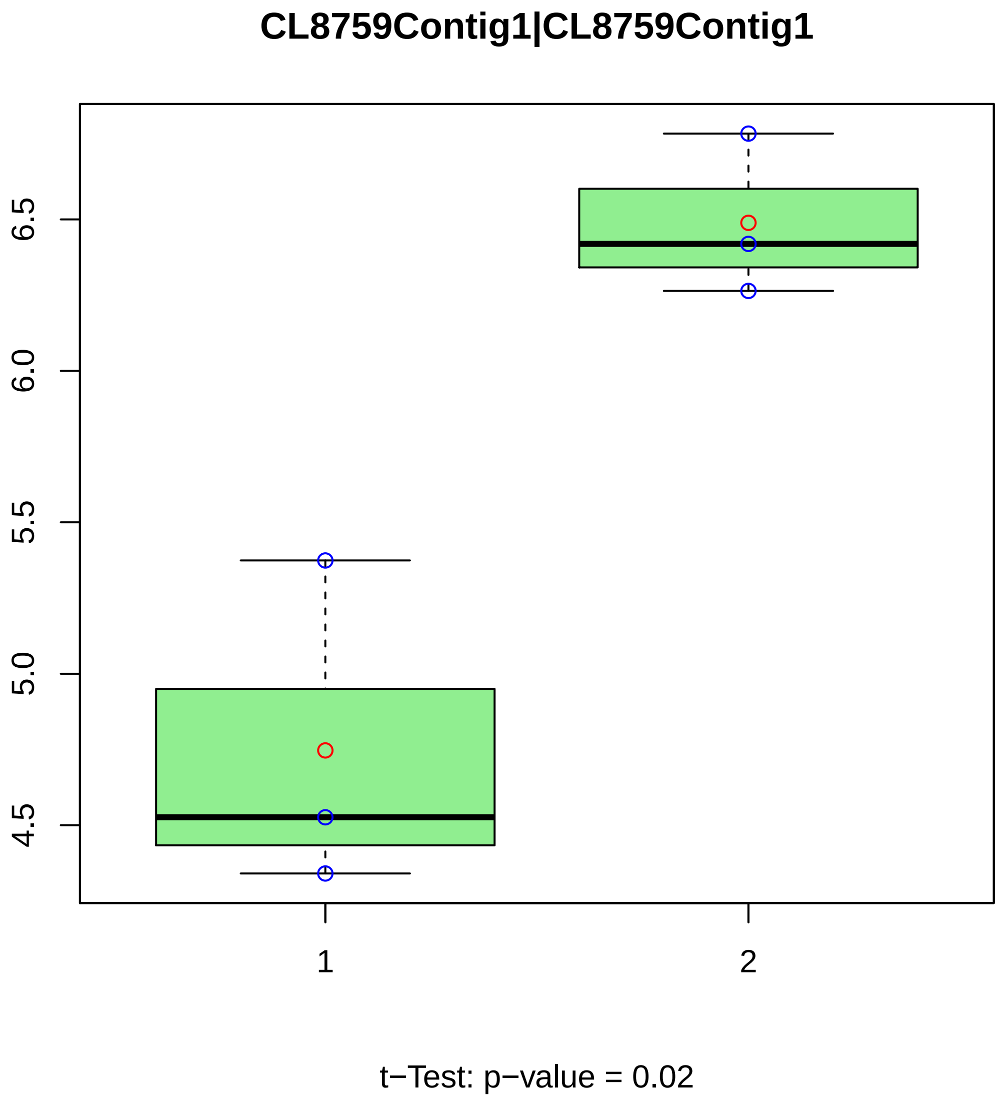

CL8759Contig1|CL8759Contig1
6.5
6.0
5.5
5.0
4.5
1
2
t−Test: p−value = 0.02

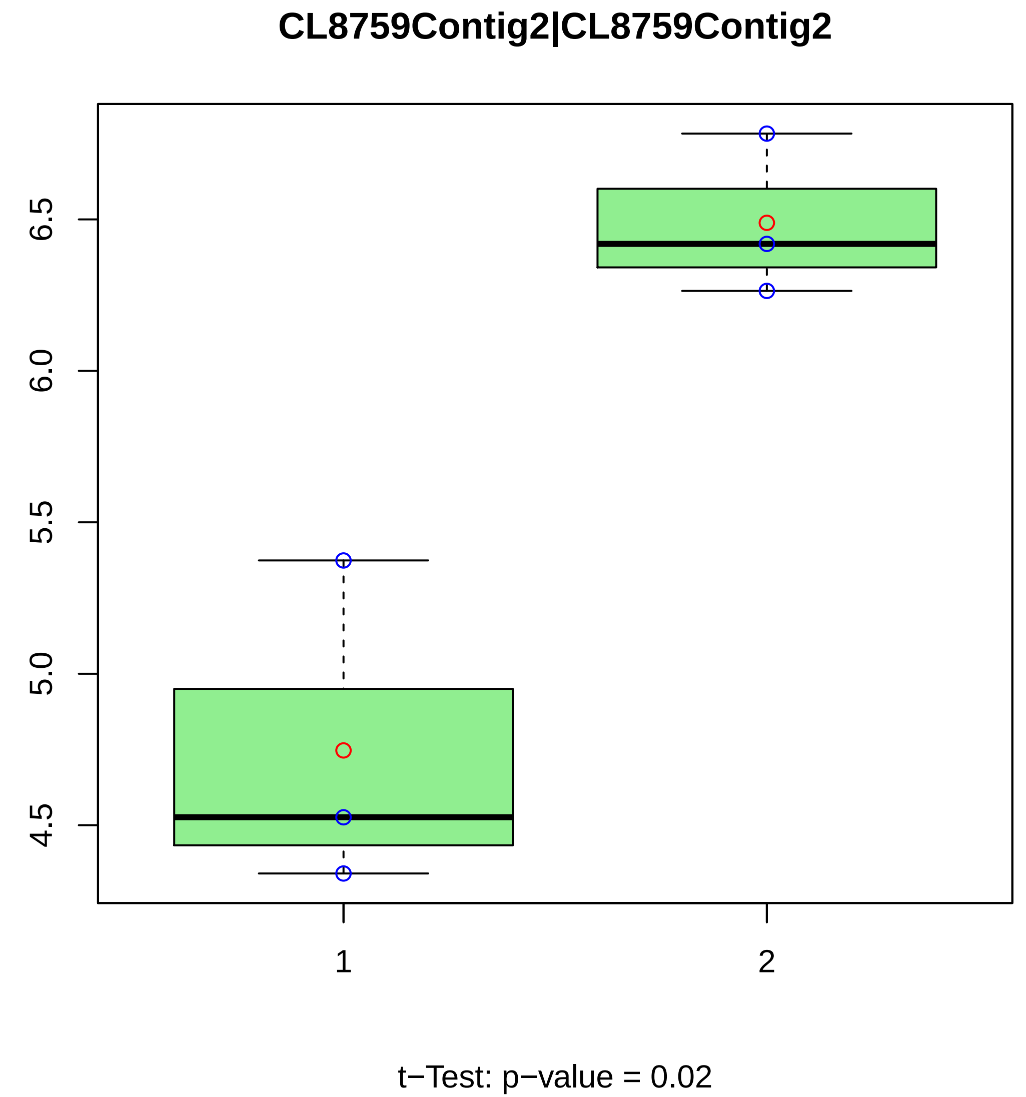
CL8759Contig2|CL8759Contig2
6.5
6.0
5.5
5.0
4.5
1
2
t−Test: p−value = 0.02

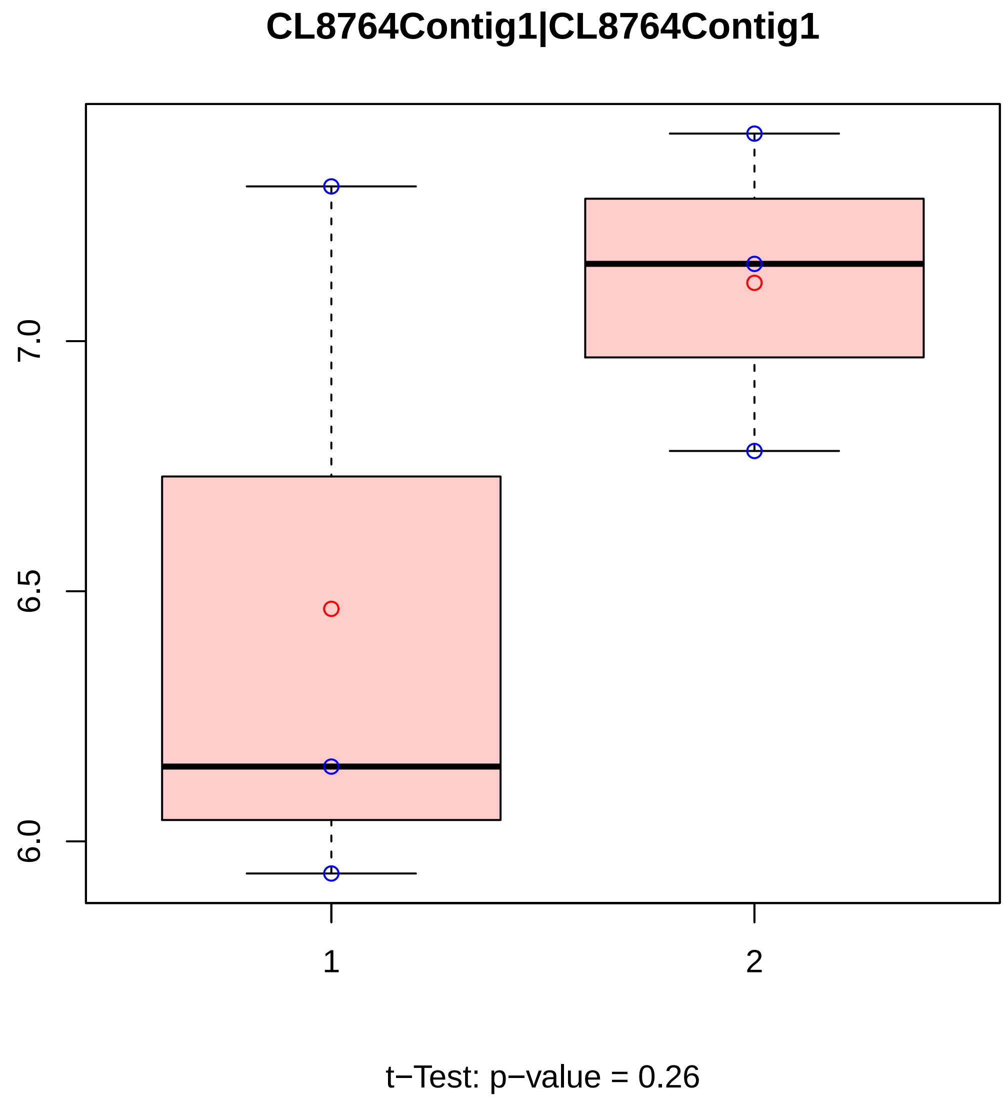

CL8764Contig1|CL8764Contig1
7.0
6.5
6.0
1
2
t−Test: p−value = 0.26

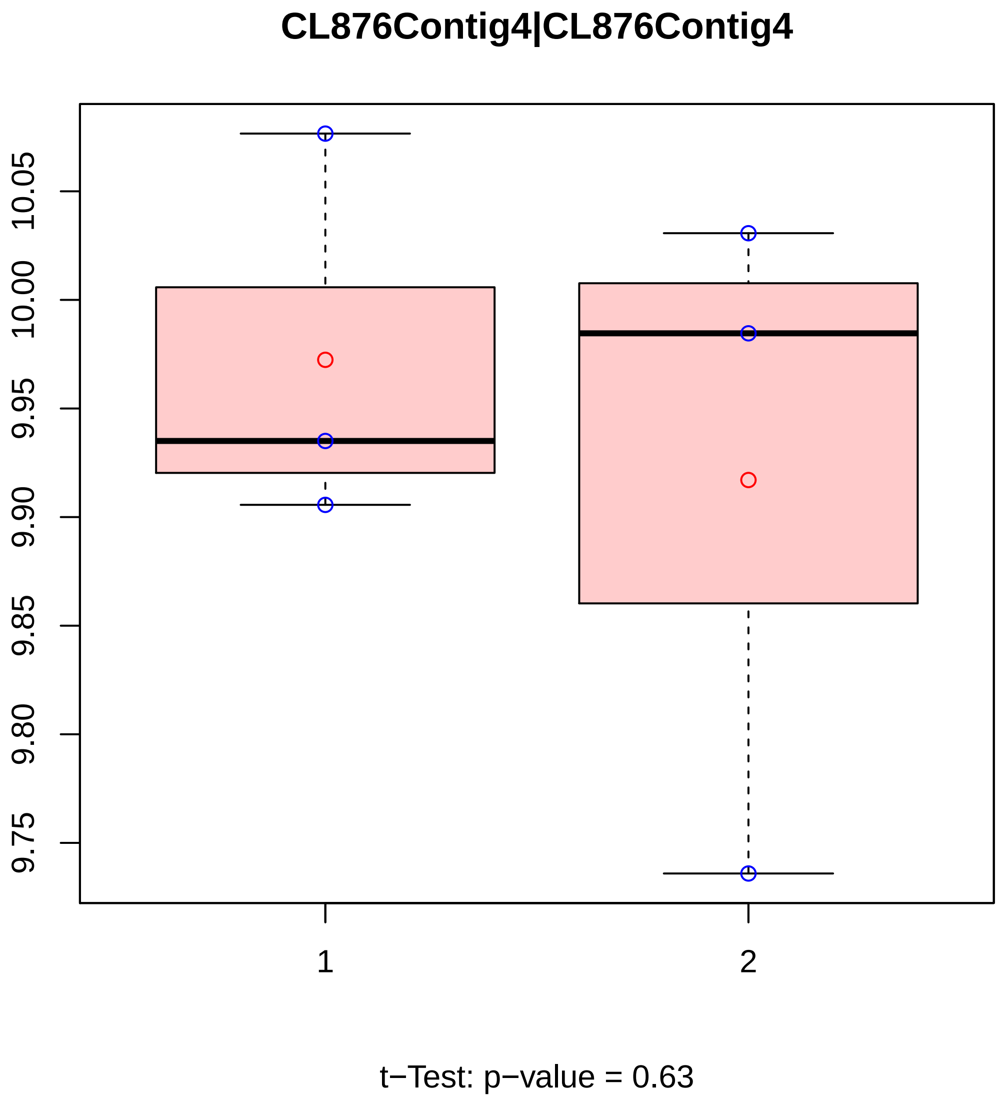
CL876Contig4|CL876Contig4
10.05
10.00
9.95
9.90
9.85
9.80
9.75
1
2
t−Test: p−value = 0.63

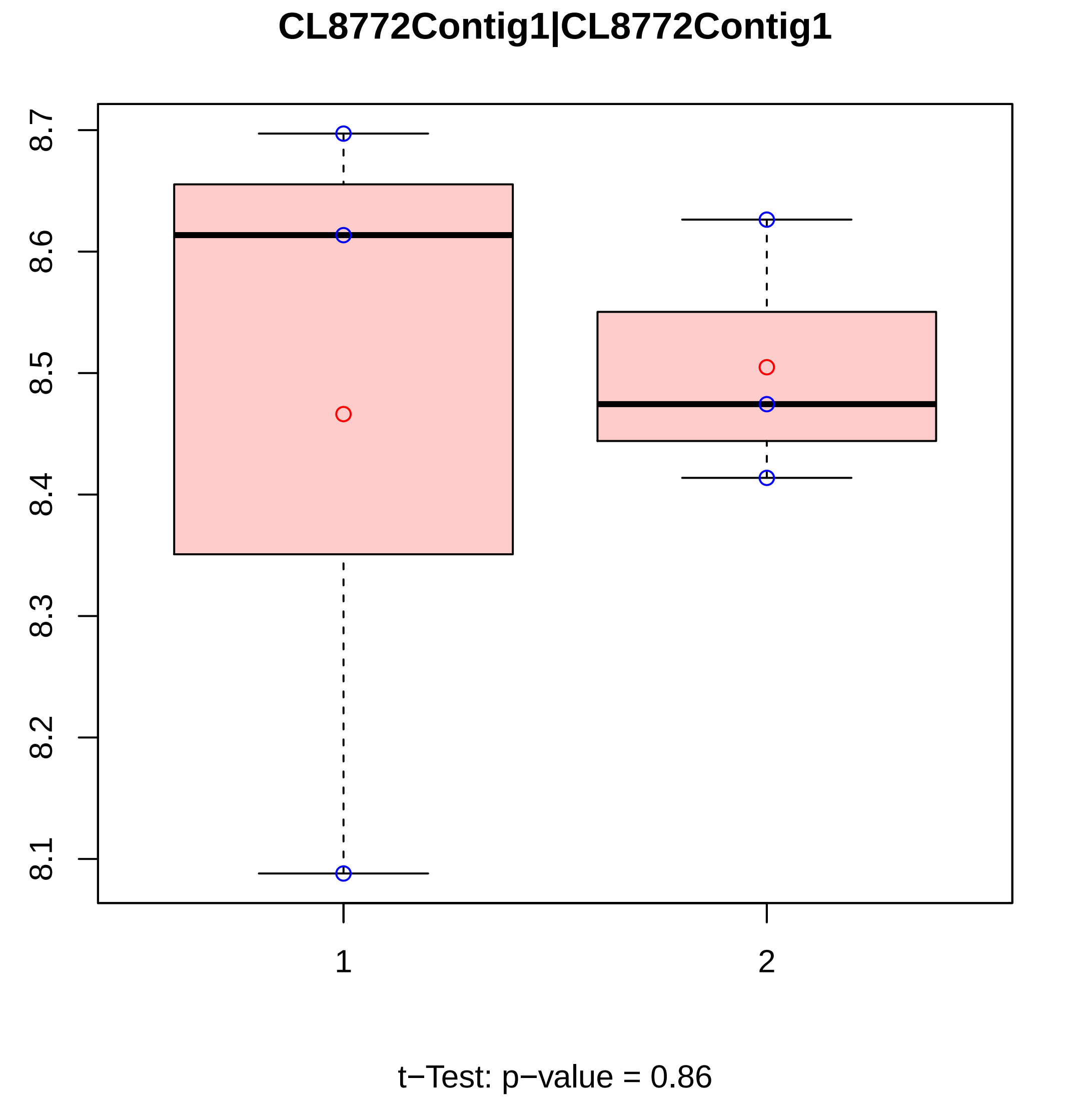
CL8772Contig1|CL8772Contig1
8.1
8.2
8.3
8.4
8.5
8.6
8.7
1
2
t−Test: p−value = 0.86

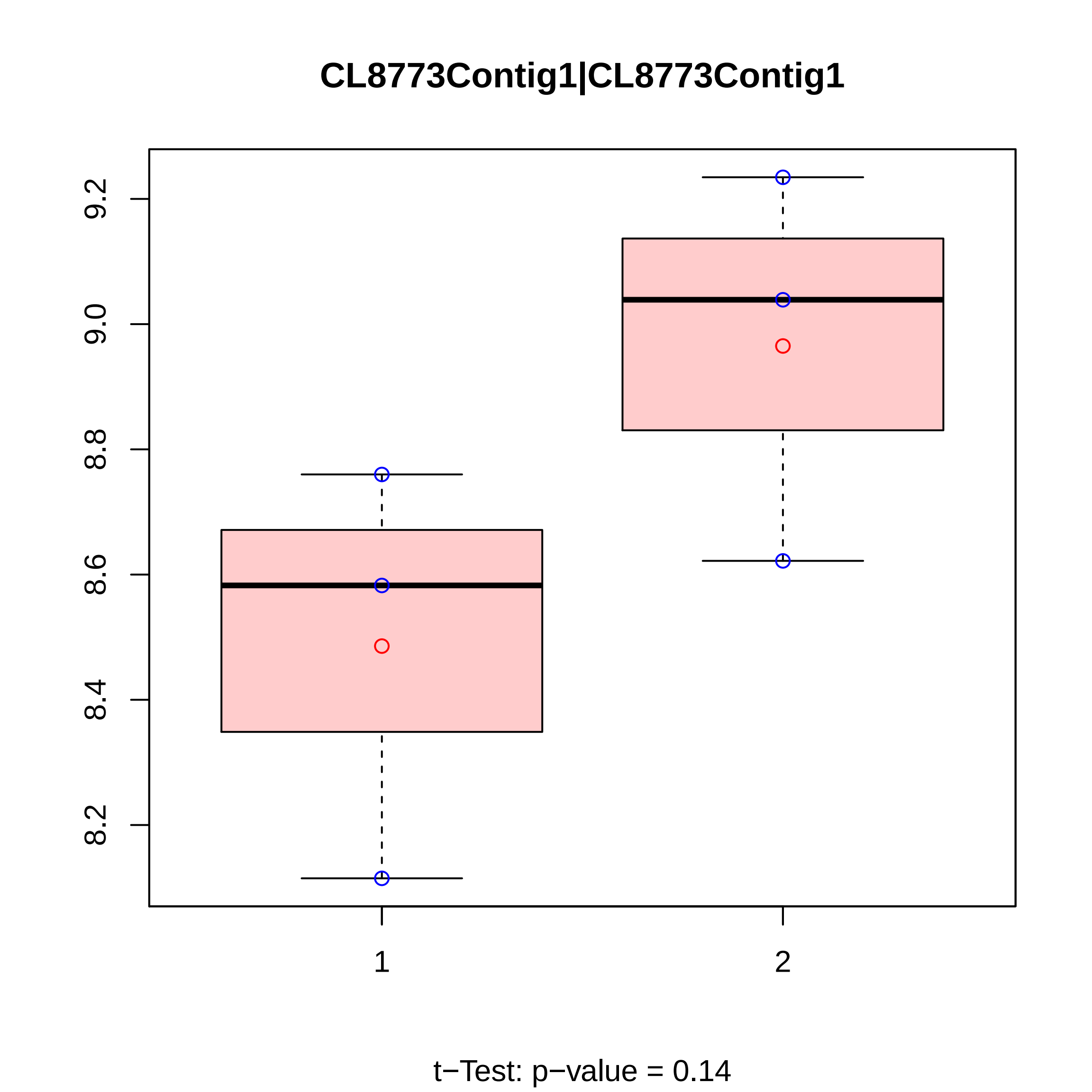
CL8773Contig1|CL8773Contig1
9.2
9.0
8.8
8.6
8.4
8.2
1
2
t−Test: p−value = 0.14

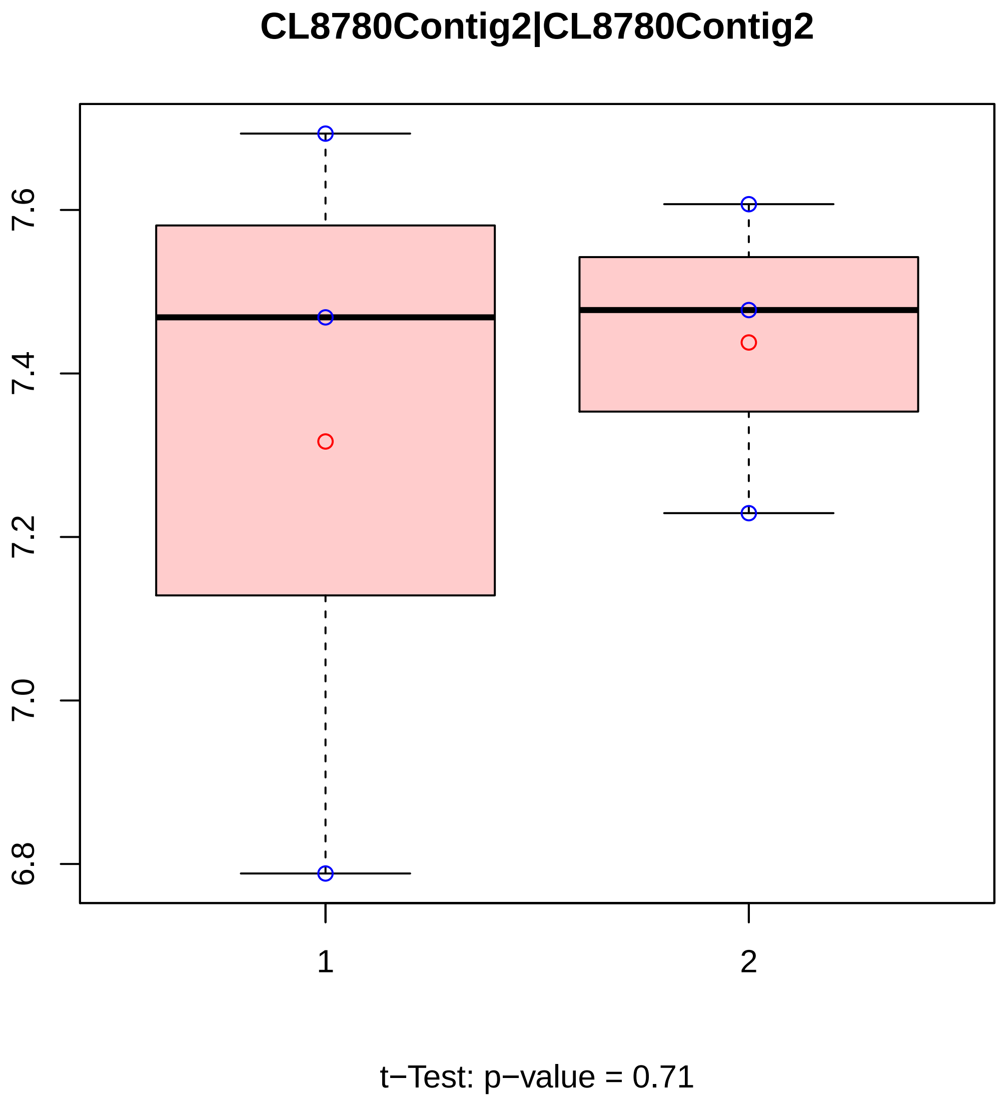

CL8780Contig2|CL8780Contig2
7.6
7.4
7.2
7.0
6.8
1
2
t−Test: p−value = 0.71

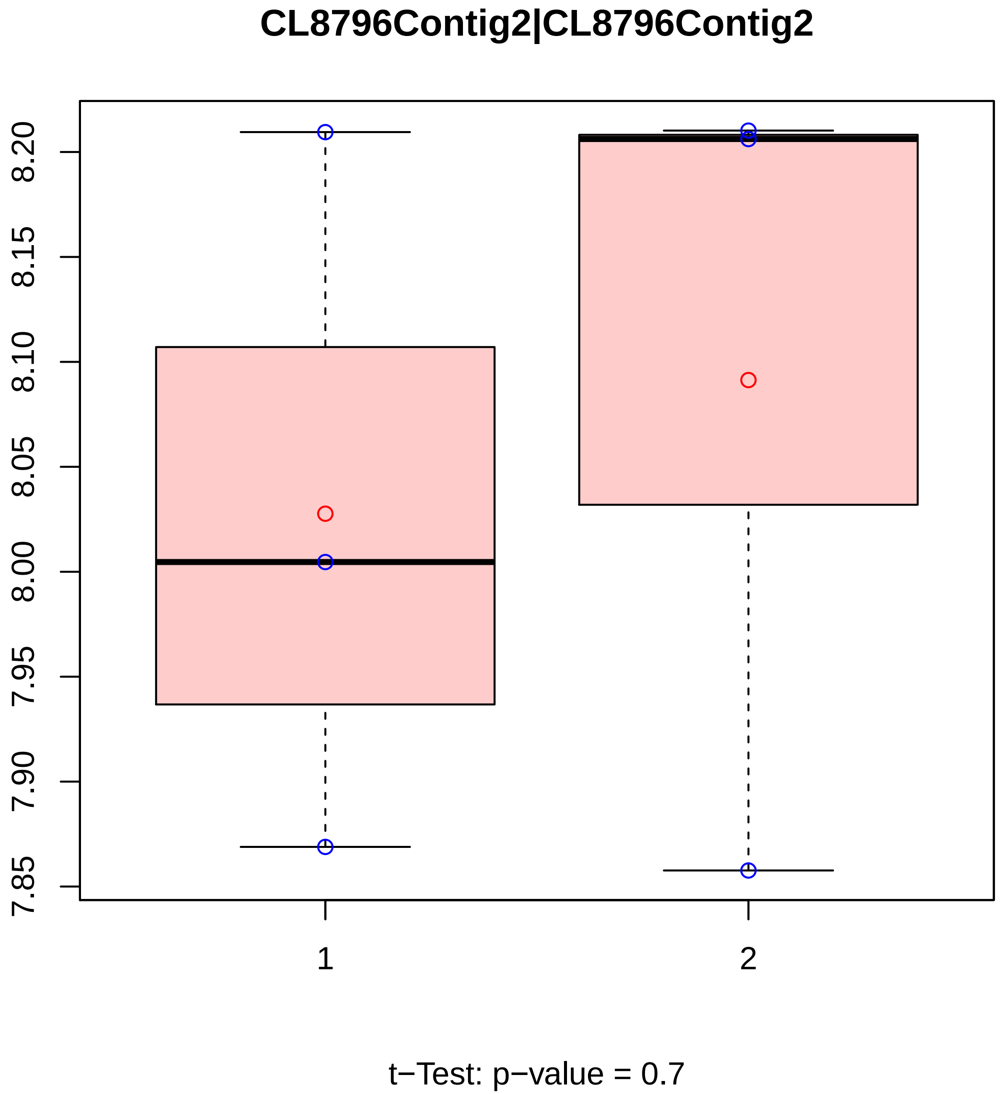

CL8796Contig2|CL8796Contig2
8.20
8.15
8.10
8.05
8.00
7.95
7.90
7.85
1
2
t−Test: p−value = 0.7

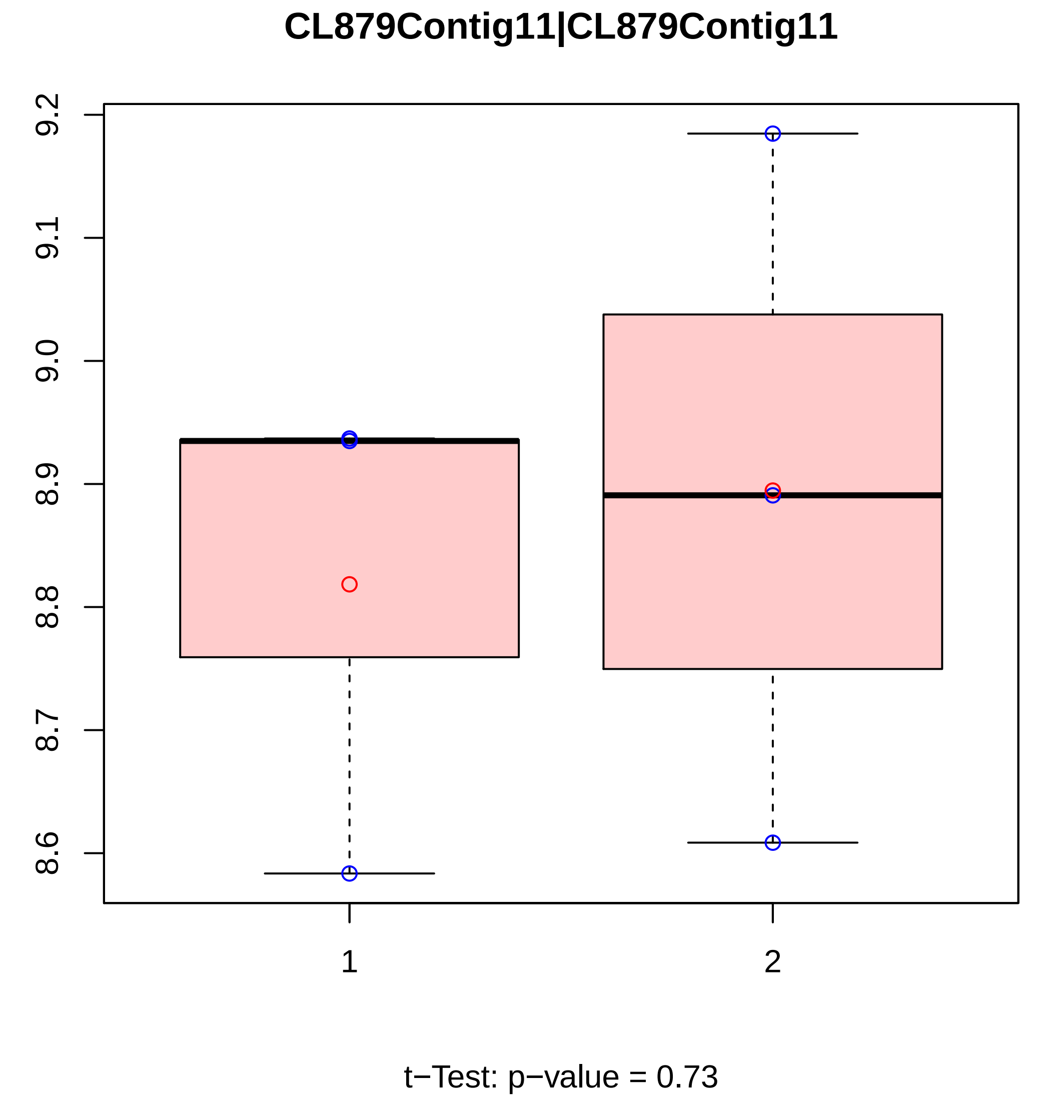
CL879Contig11|CL879Contig11
9.2
9.1
9.0
8.9
8.8
8.7
8.6
1
2
t−Test: p−value = 0.73

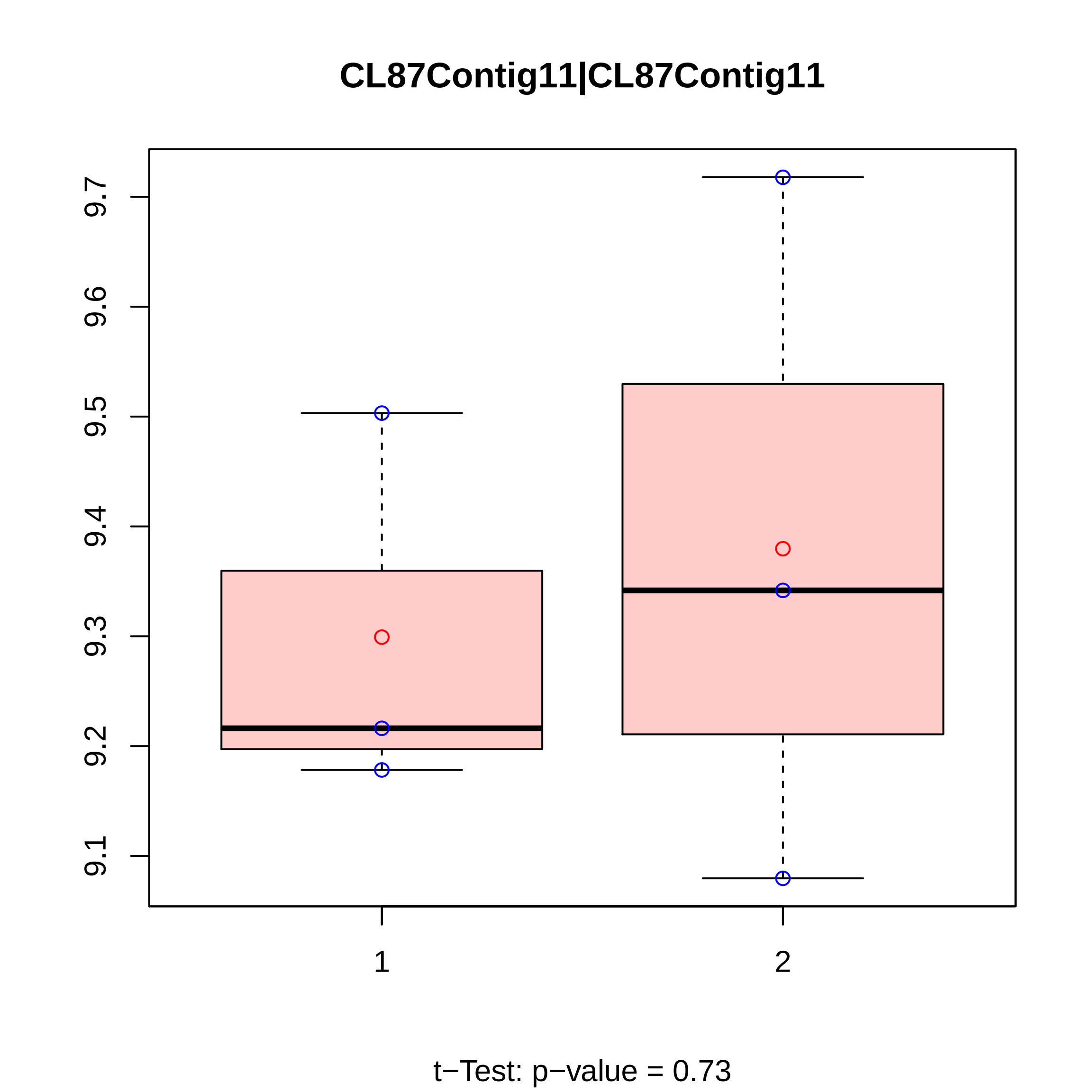

CL87Contig11|CL87Contig11
9.1
9.2
9.3
9.4
9.5
9.6
9.7
1
2
t−Test: p−value = 0.73

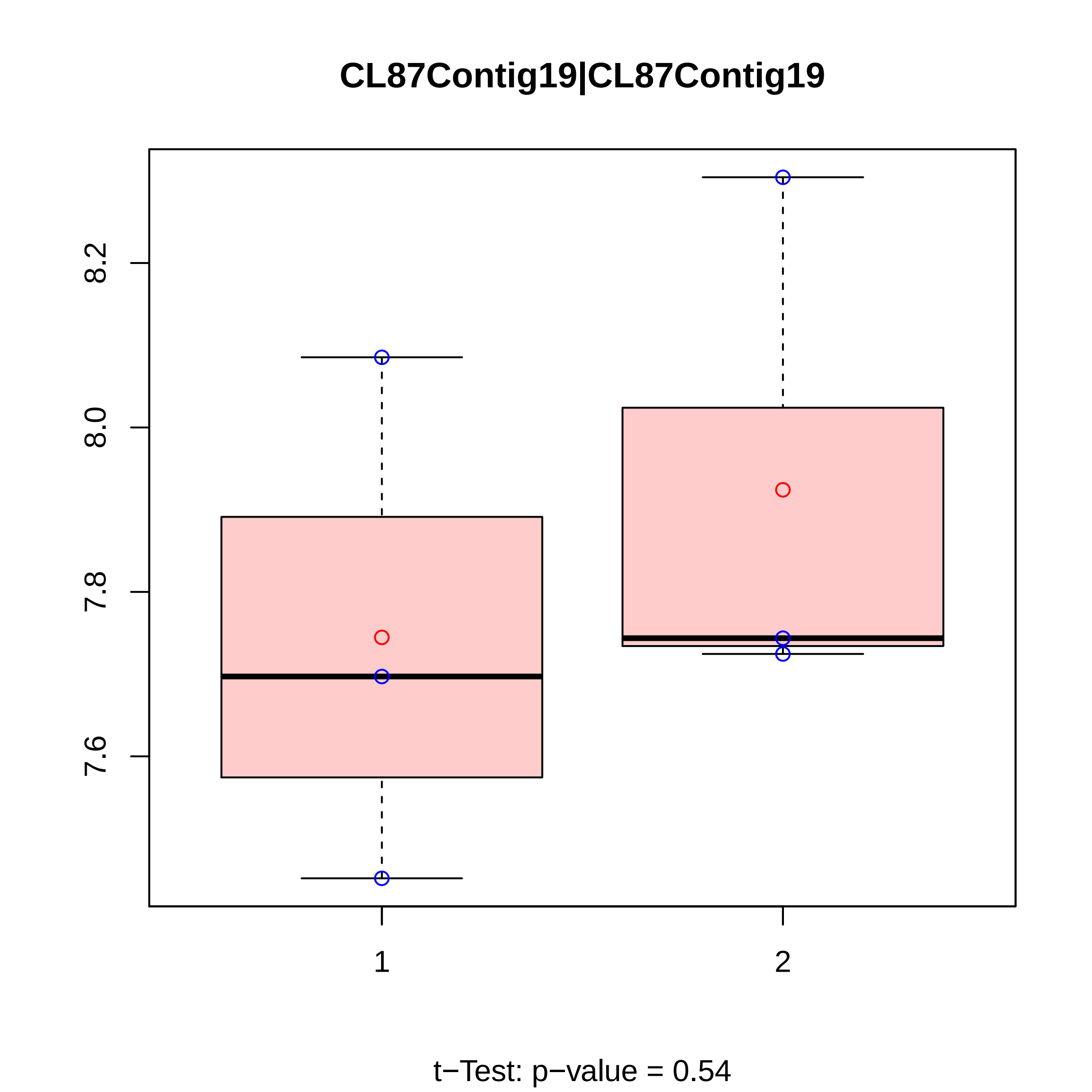

CL87Contig19|CL87Contig19
8.2
8.0
7.8
7.6
1
2
t−Test: p−value = 0.54

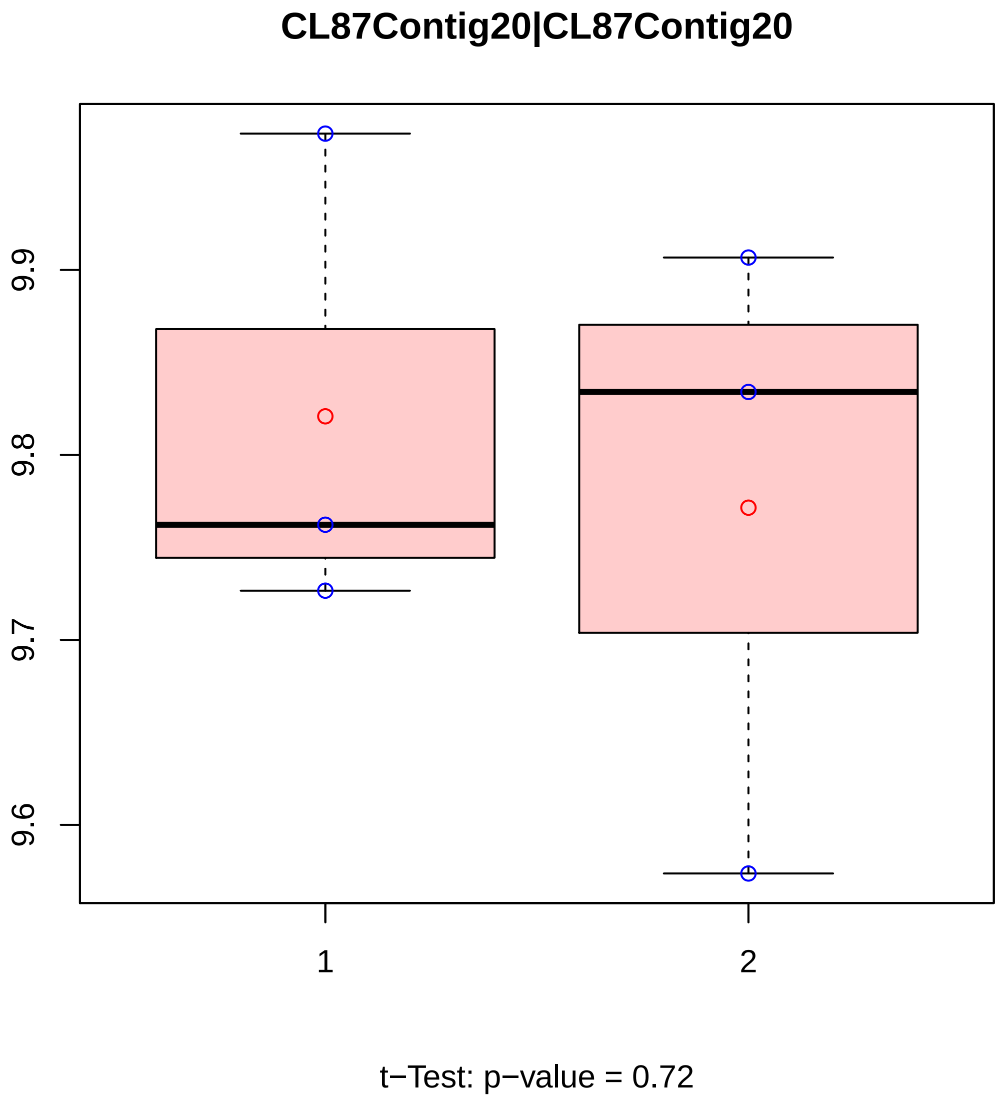
CL87Contig20|CL87Contig20
9.6
9.7
9.8
9.9
1
2
t−Test: p−value = 0.72

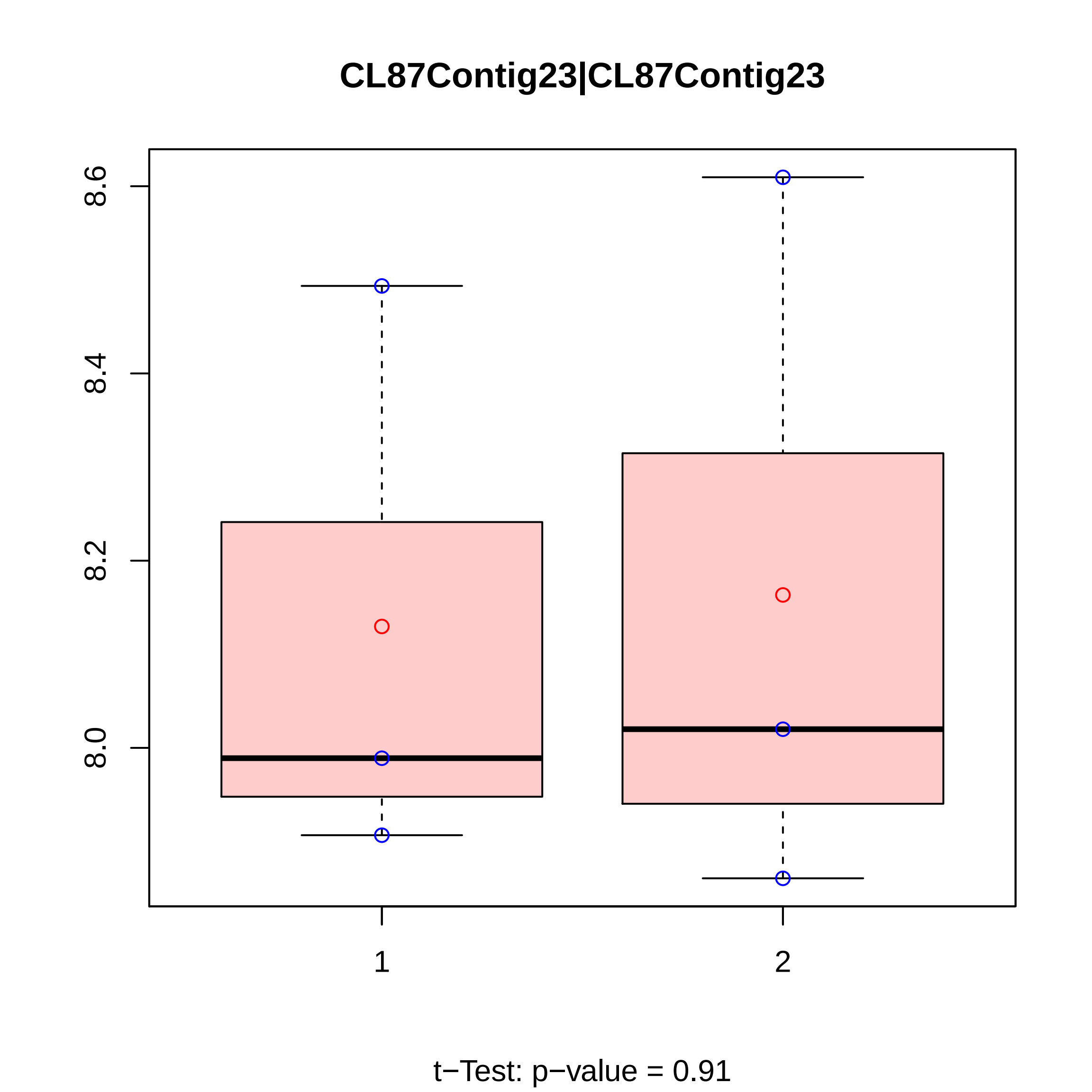
CL87Contig23|CL87Contig23
8.6
8.4
8.2
8.0
1
2
t−Test: p−value = 0.91

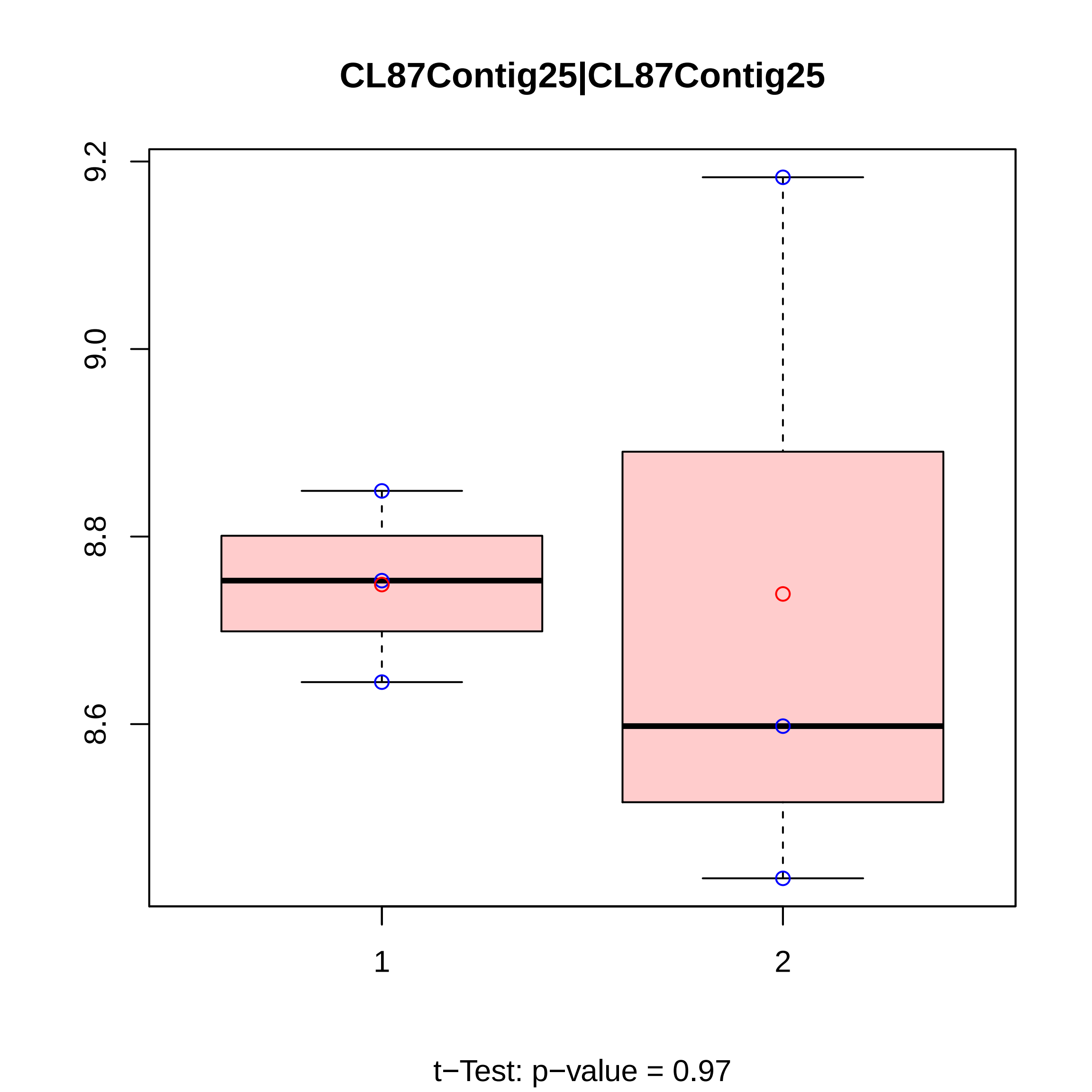

CL87Contig25|CL87Contig25
9.2
9.0
8.8
8.6
1
2
t−Test: p−value = 0.97

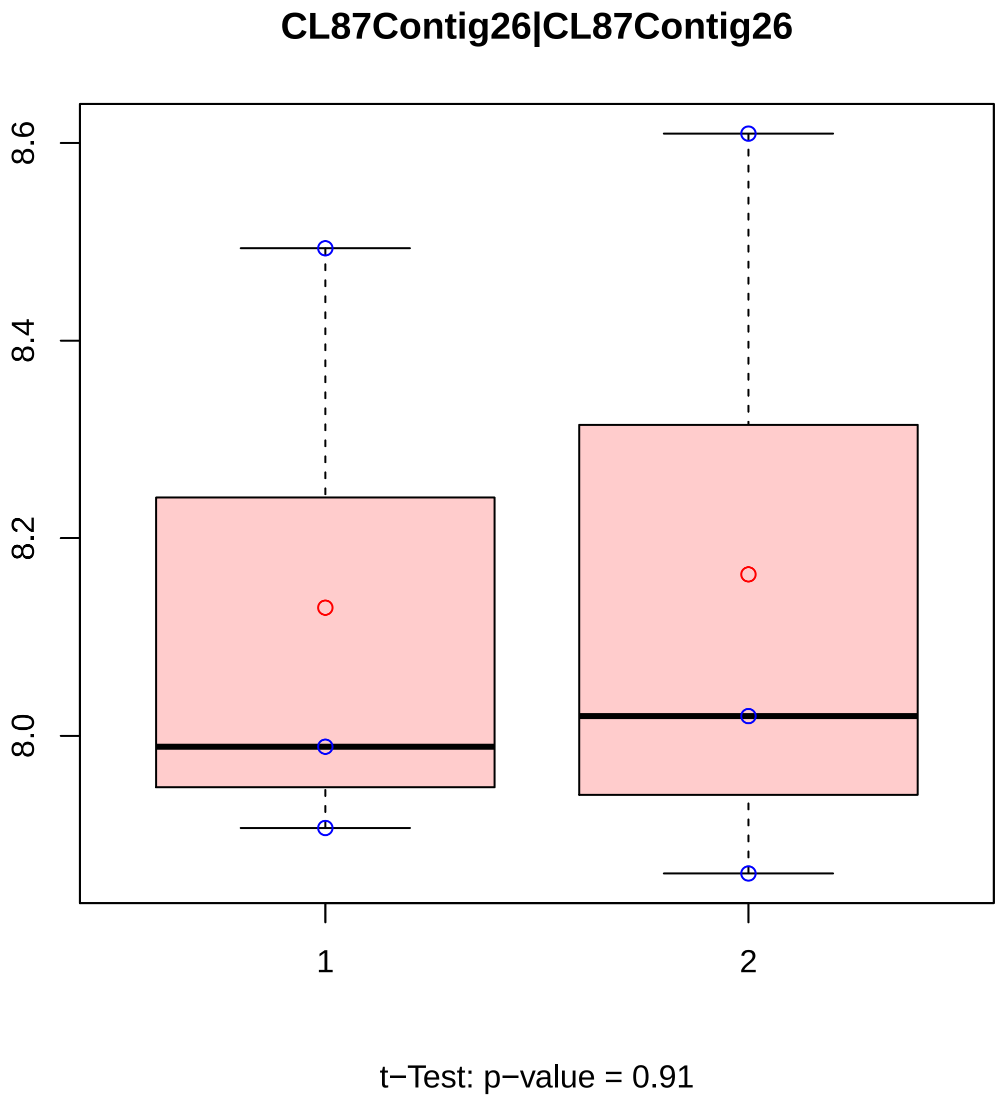

CL87Contig26|CL87Contig26
8.0
8.2
8.4
8.6
1
2
t−Test: p−value = 0.91

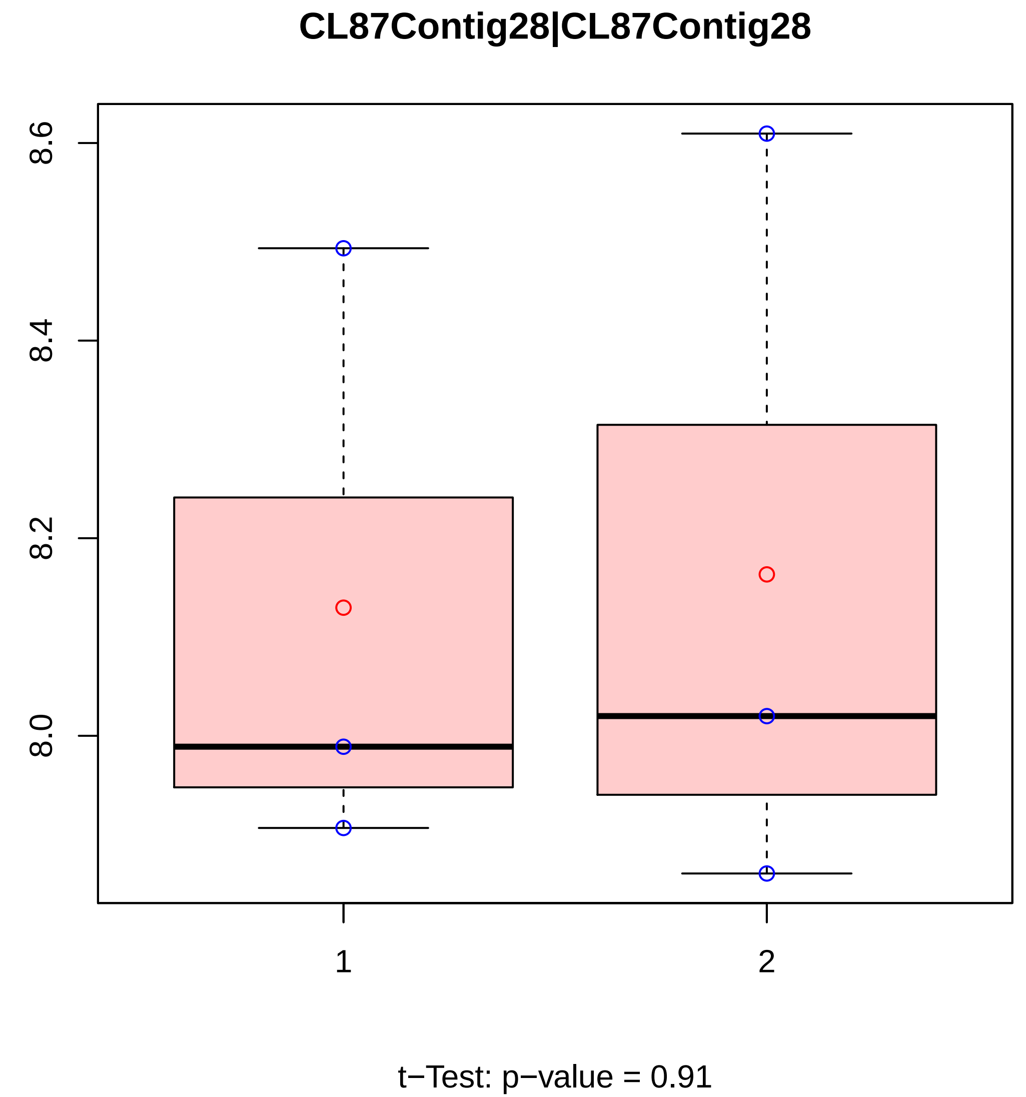
CL87Contig28|CL87Contig28
8.6
8.4
8.2
8.0
1
2
t−Test: p−value = 0.91

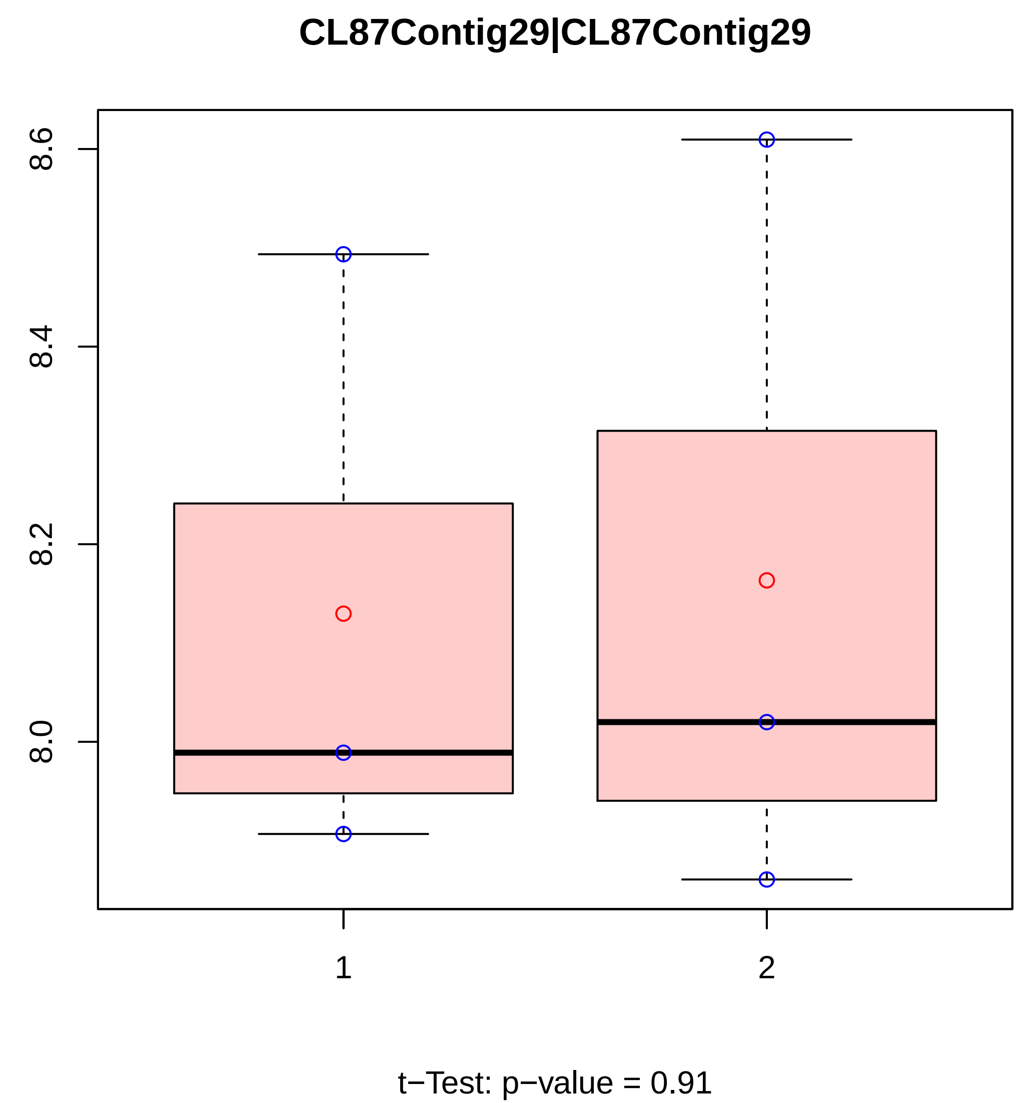
CL87Contig29|CL87Contig29
8.6
8.4
8.2
8.0
1
2
t−Test: p−value = 0.91

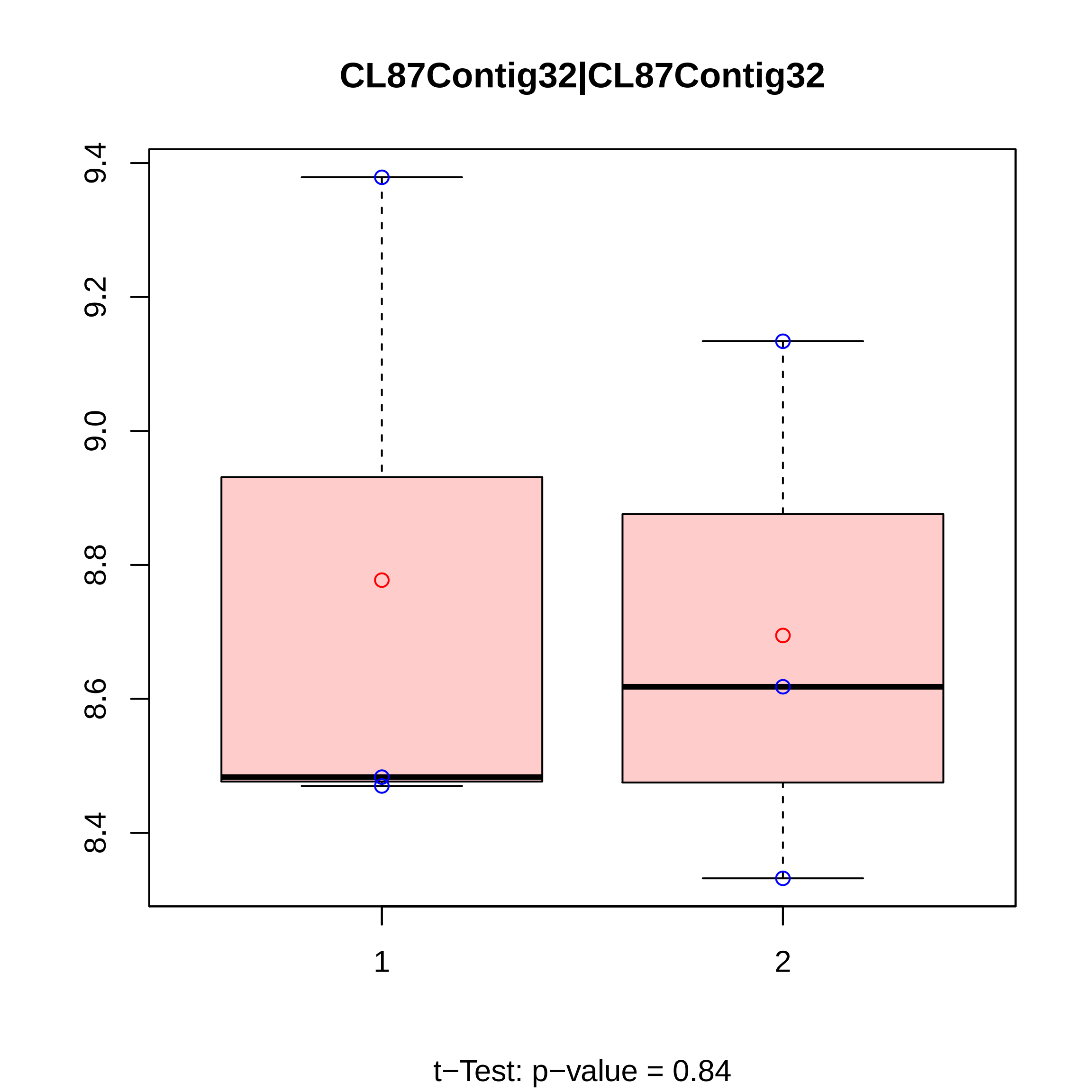
CL87Contig32|CL87Contig32
9.4
9.2
9.0
8.8
8.6
8.4
1
2
t−Test: p−value = 0.84

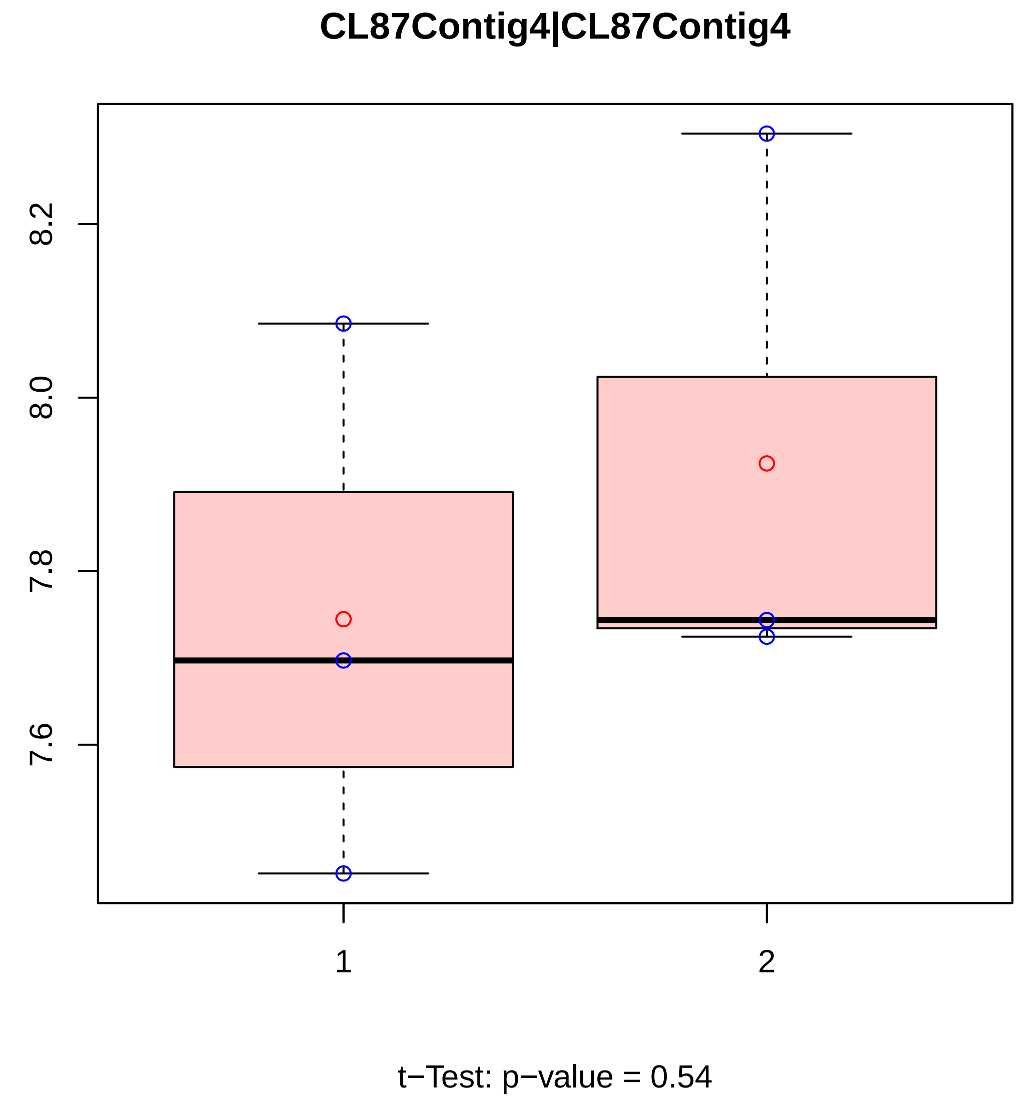
CL87Contig4|CL87Contig4
8.2
8.0
7.8
7.6
1
2
t−Test: p−value = 0.54

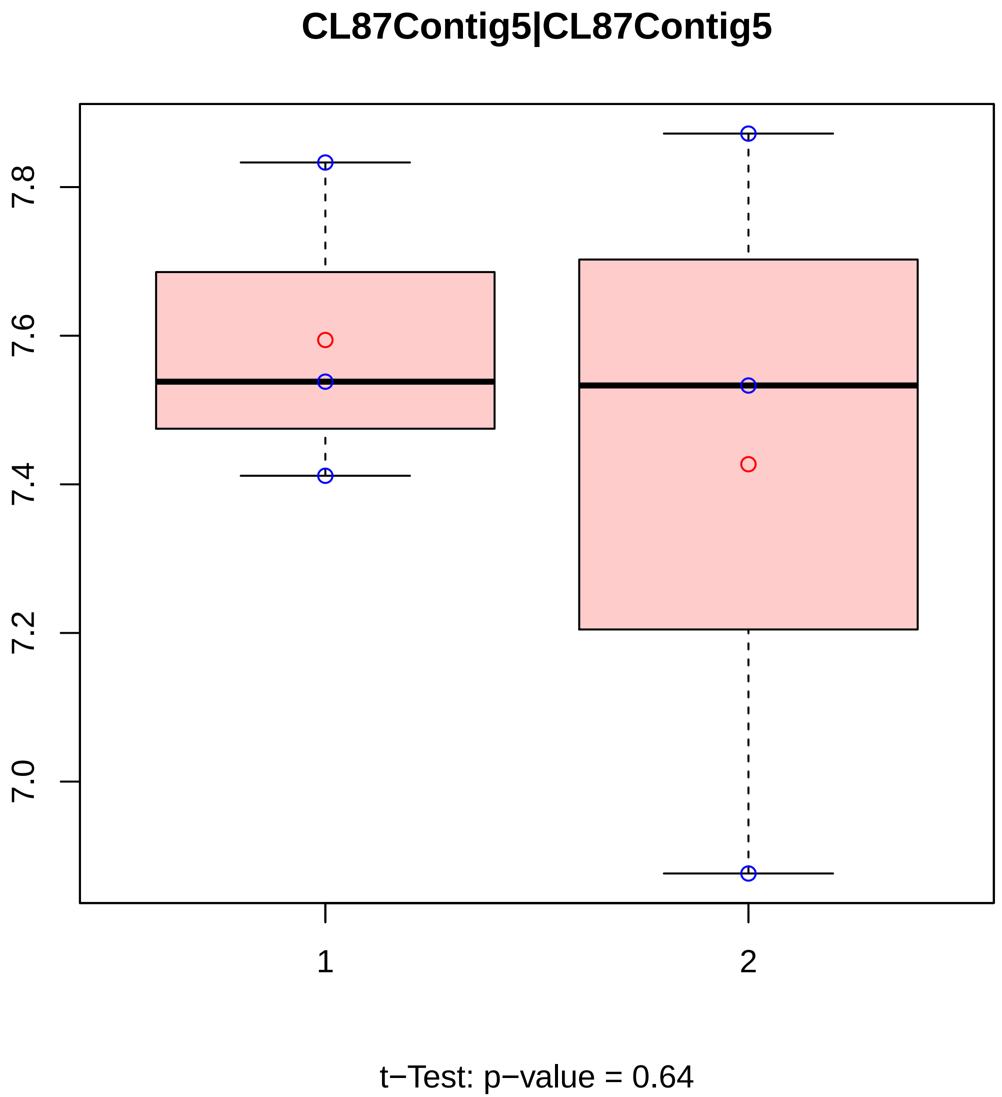
CL87Contig5|CL87Contig5
7.0
7.2
7.4
7.6
7.8
1
2
t−Test: p−value = 0.64

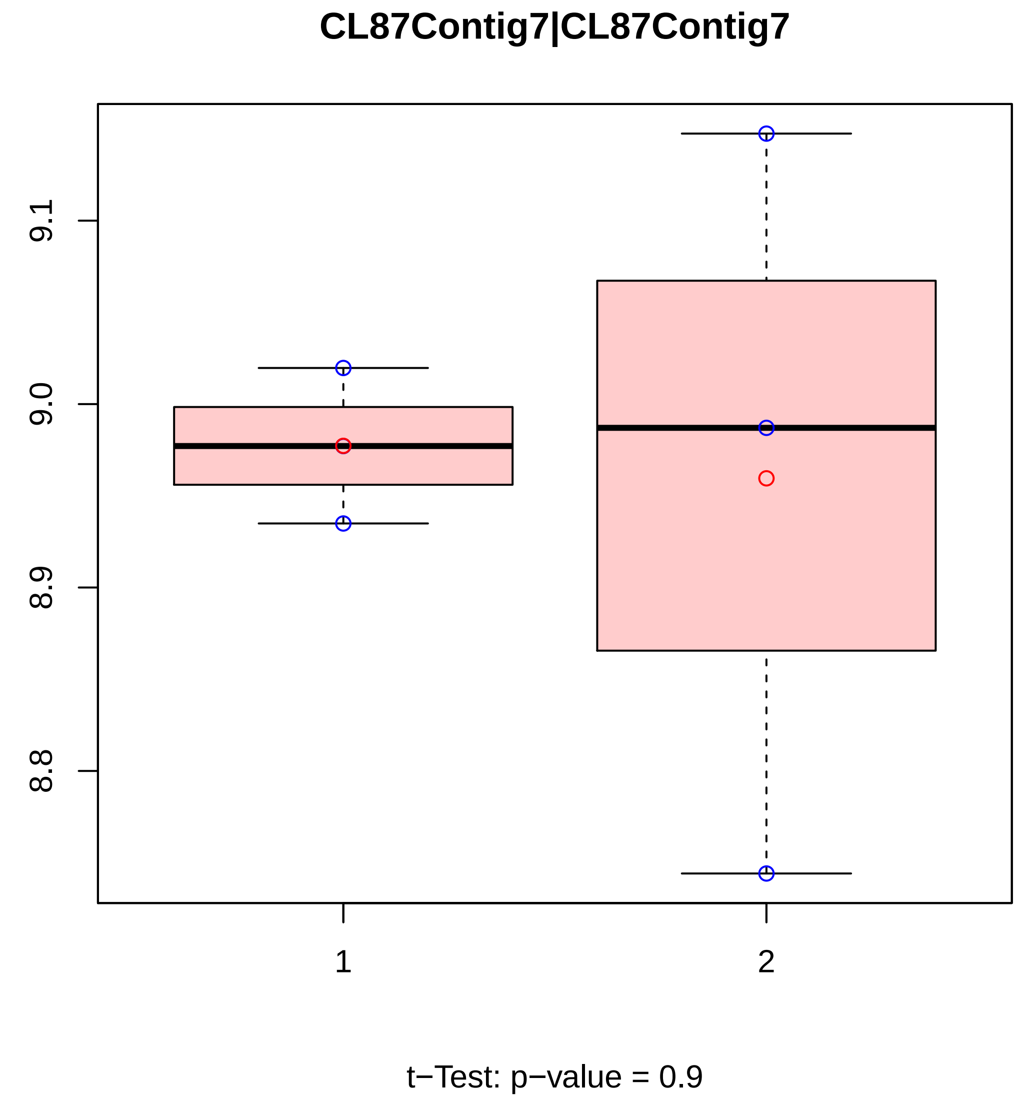
CL87Contig7|CL87Contig7
9.1
9.0
8.9
8.8
1
2
t−Test: p−value = 0.9

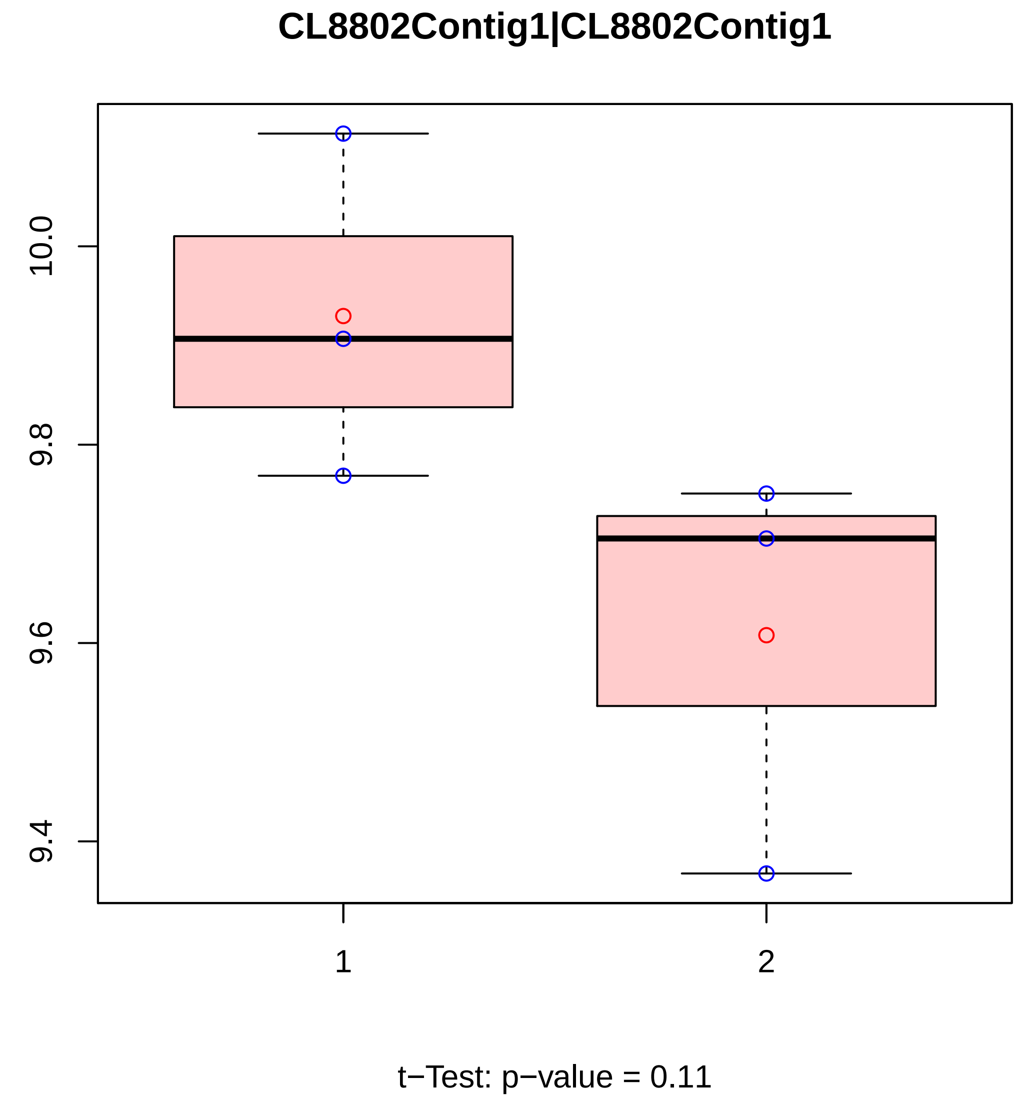

CL8802Contig1|CL8802Contig1
10.0
9.8
9.6
9.4
1
2
t−Test: p−value = 0.11

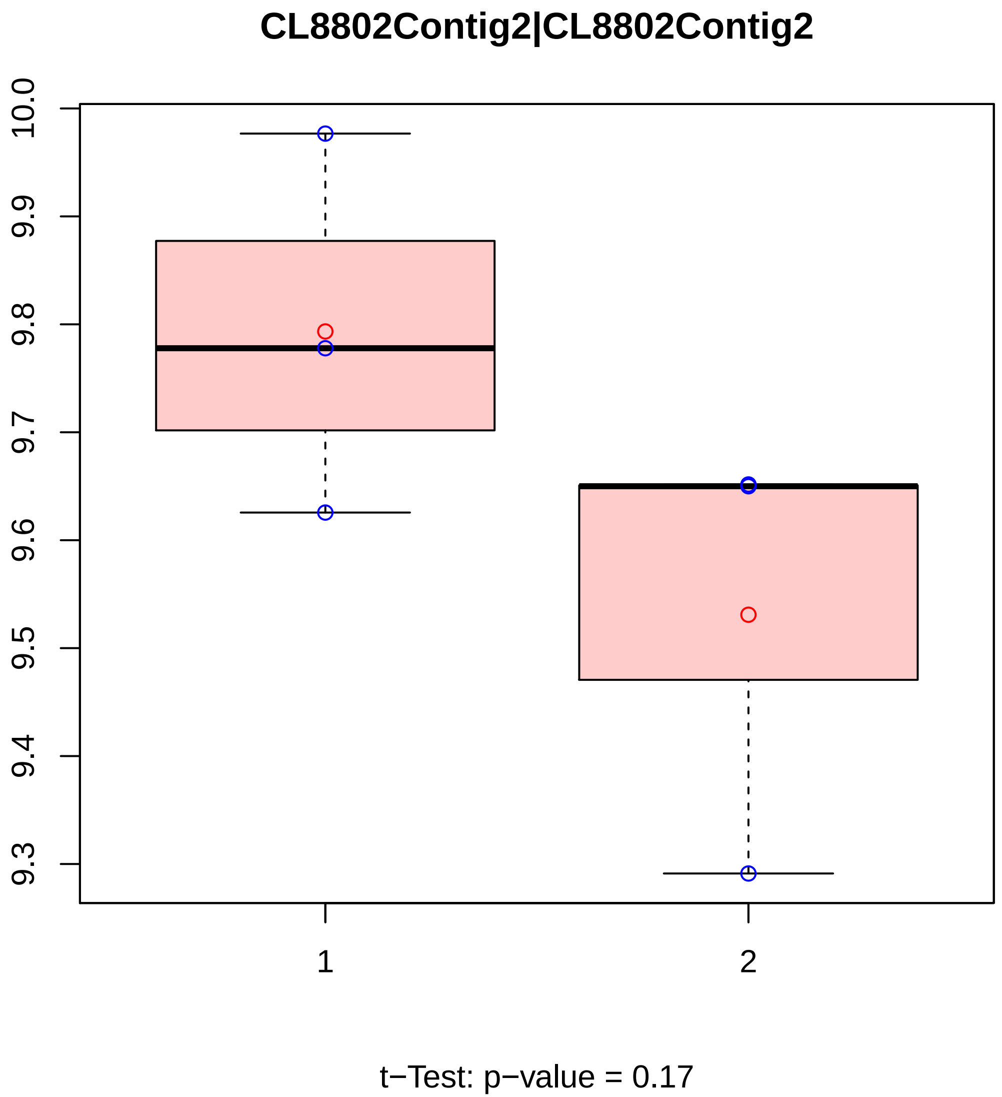
CL8802Contig2|CL8802Contig2
10.0
9.9
9.8
9.7
9.6
9.5
9.4
9.3
1
2
t−Test: p−value = 0.17

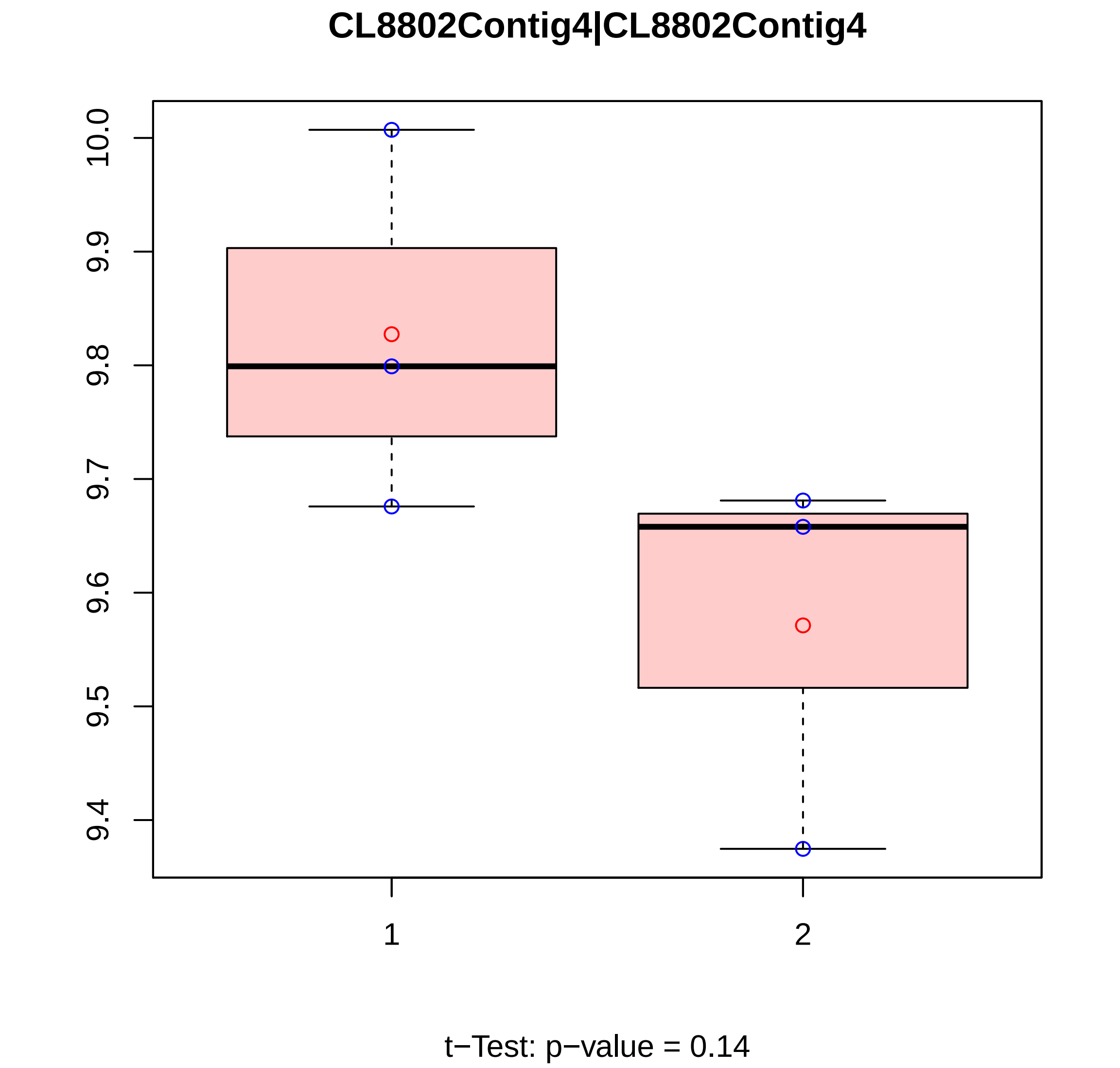
CL8802Contig4|CL8802Contig4
10.0
9.9
9.8
9.7
9.6
9.5
9.4
1
2
t−Test: p−value = 0.14

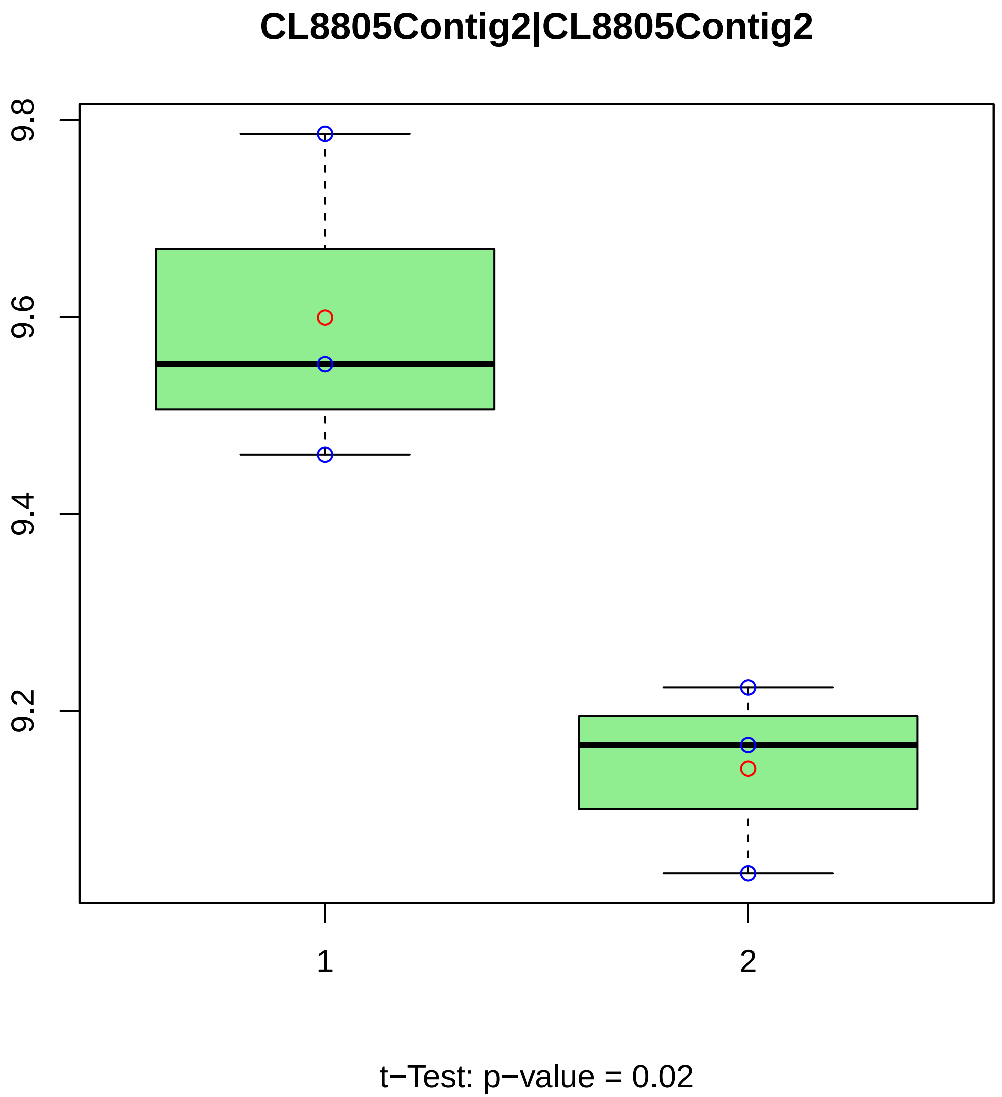

CL8805Contig2|CL8805Contig2
9.8
9.6
9.4
9.2
1
2
t−Test: p−value = 0.02

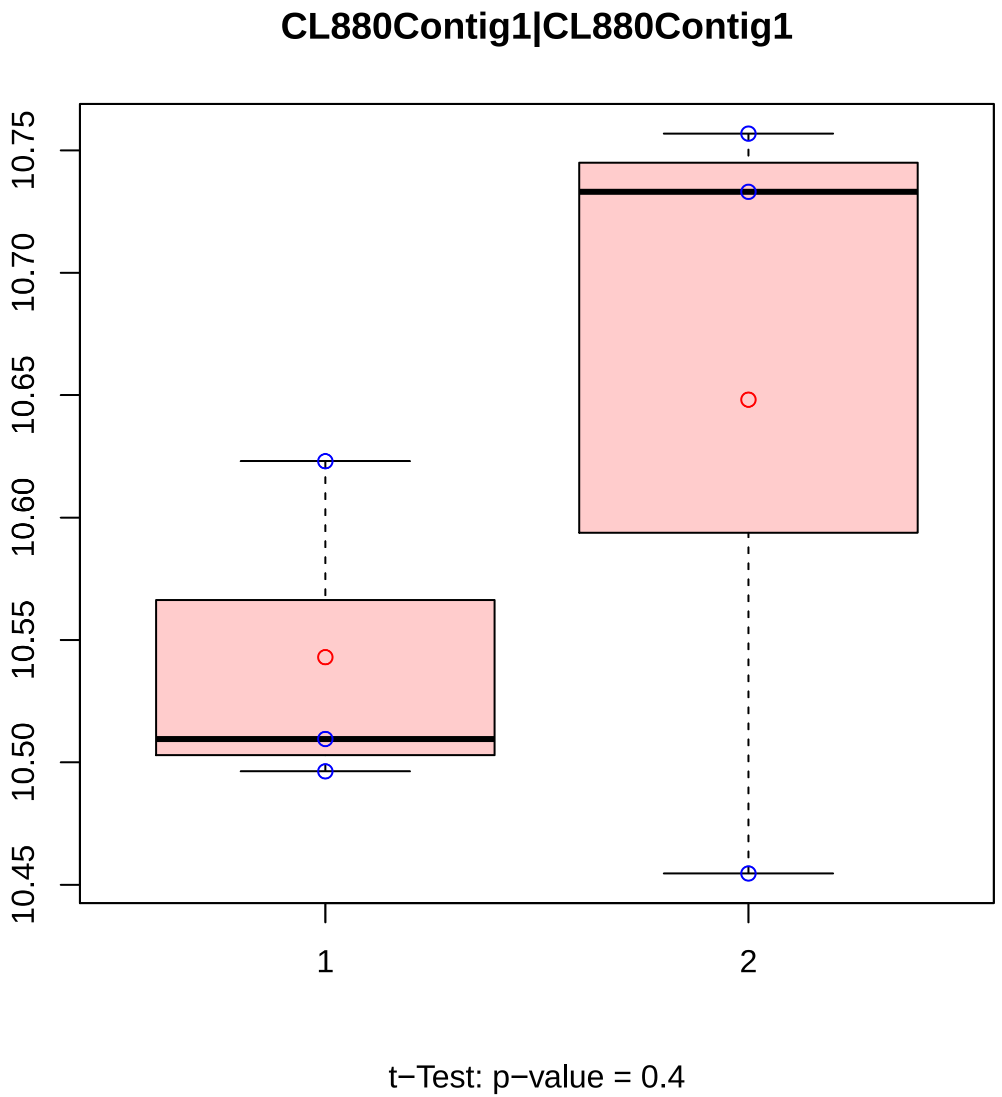
CL880Contig1|CL880Contig1
10.45
10.50
10.55
10.60
10.65
10.70
10.75
1
2
t−Test: p−value = 0.4

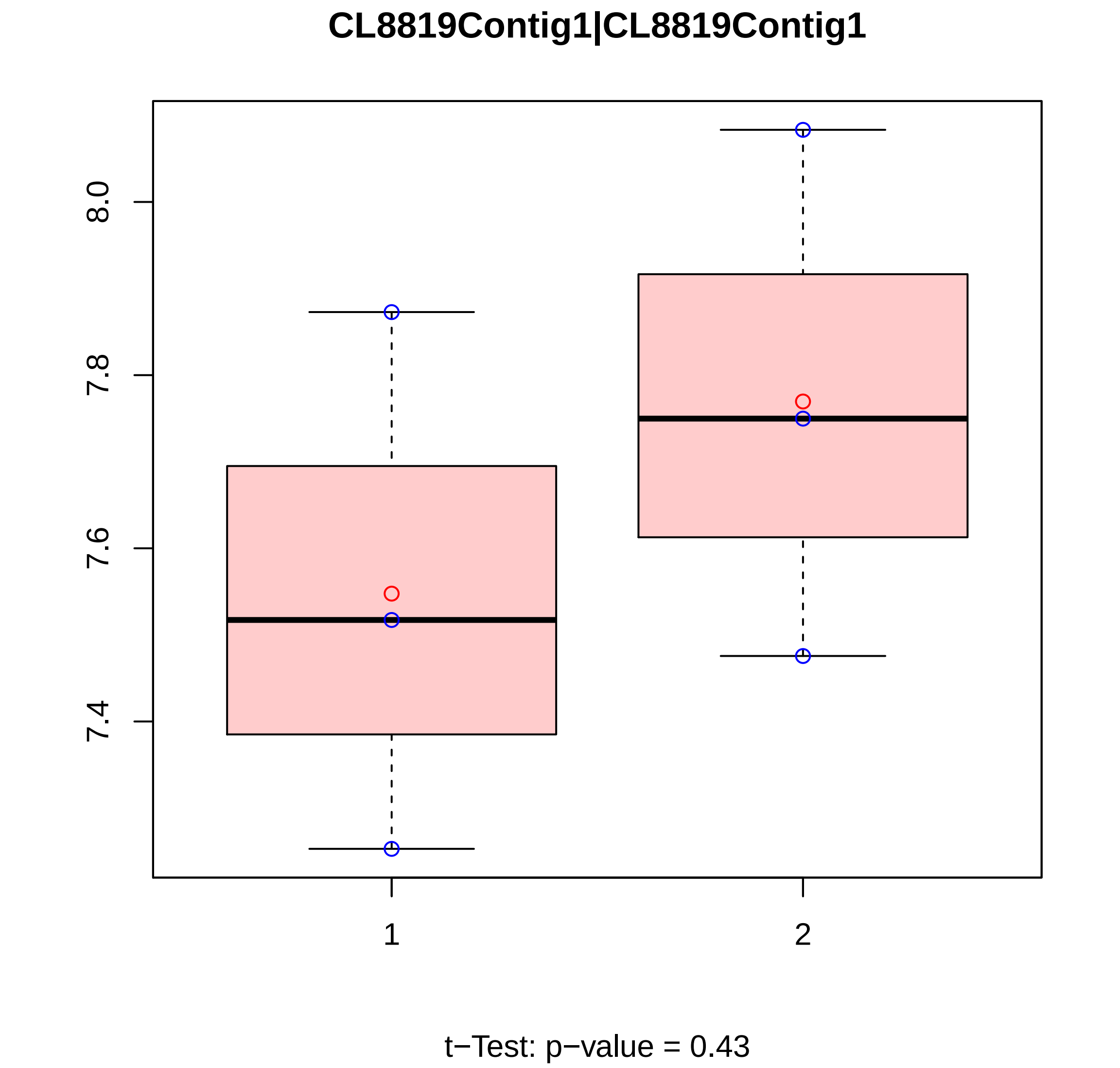
CL8819Contig1|CL8819Contig1
8.0
7.8
7.6
7.4
1
2
t−Test: p−value = 0.43

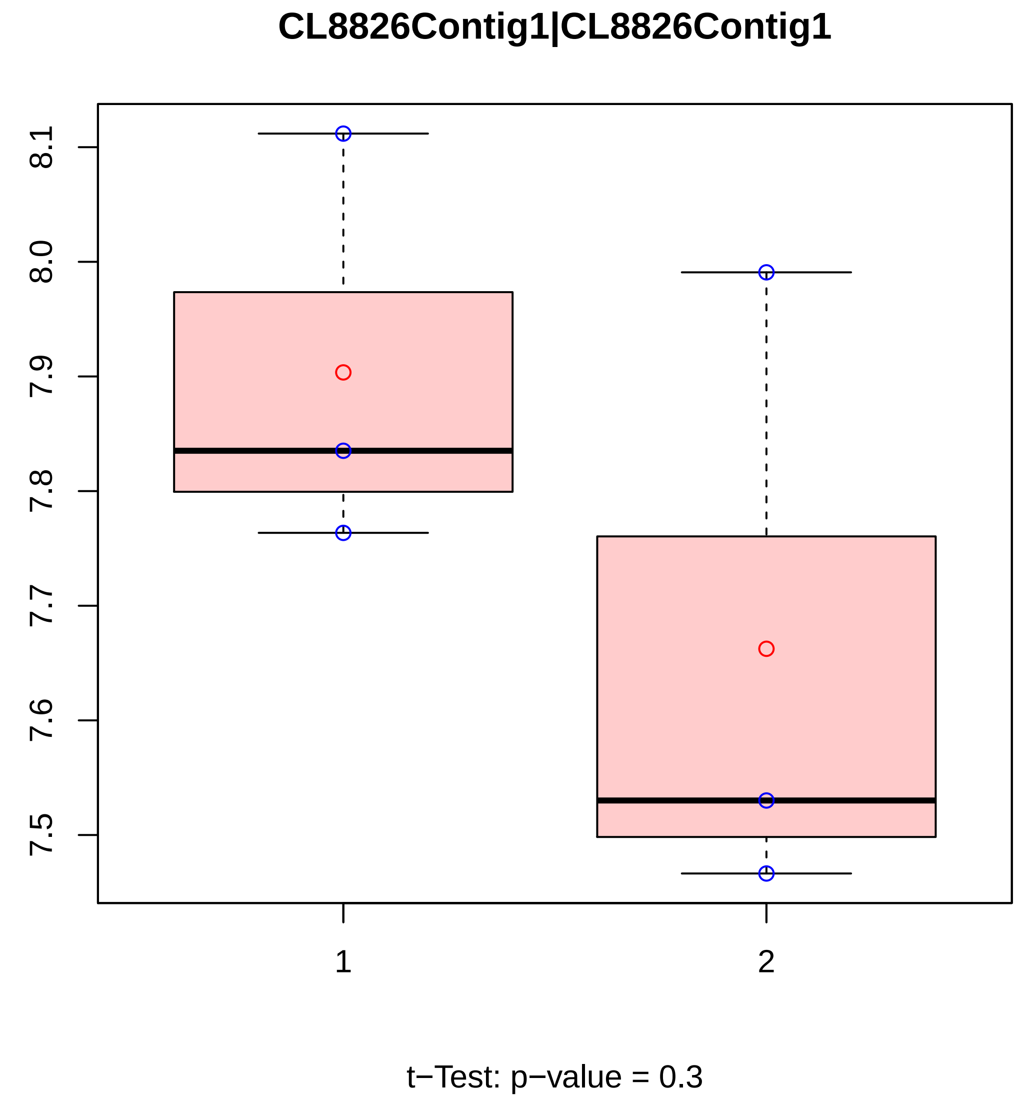
CL8826Contig1|CL8826Contig1
8.1
8.0
7.9
7.8
7.7
7.6
7.5
1
2
t−Test: p−value = 0.3

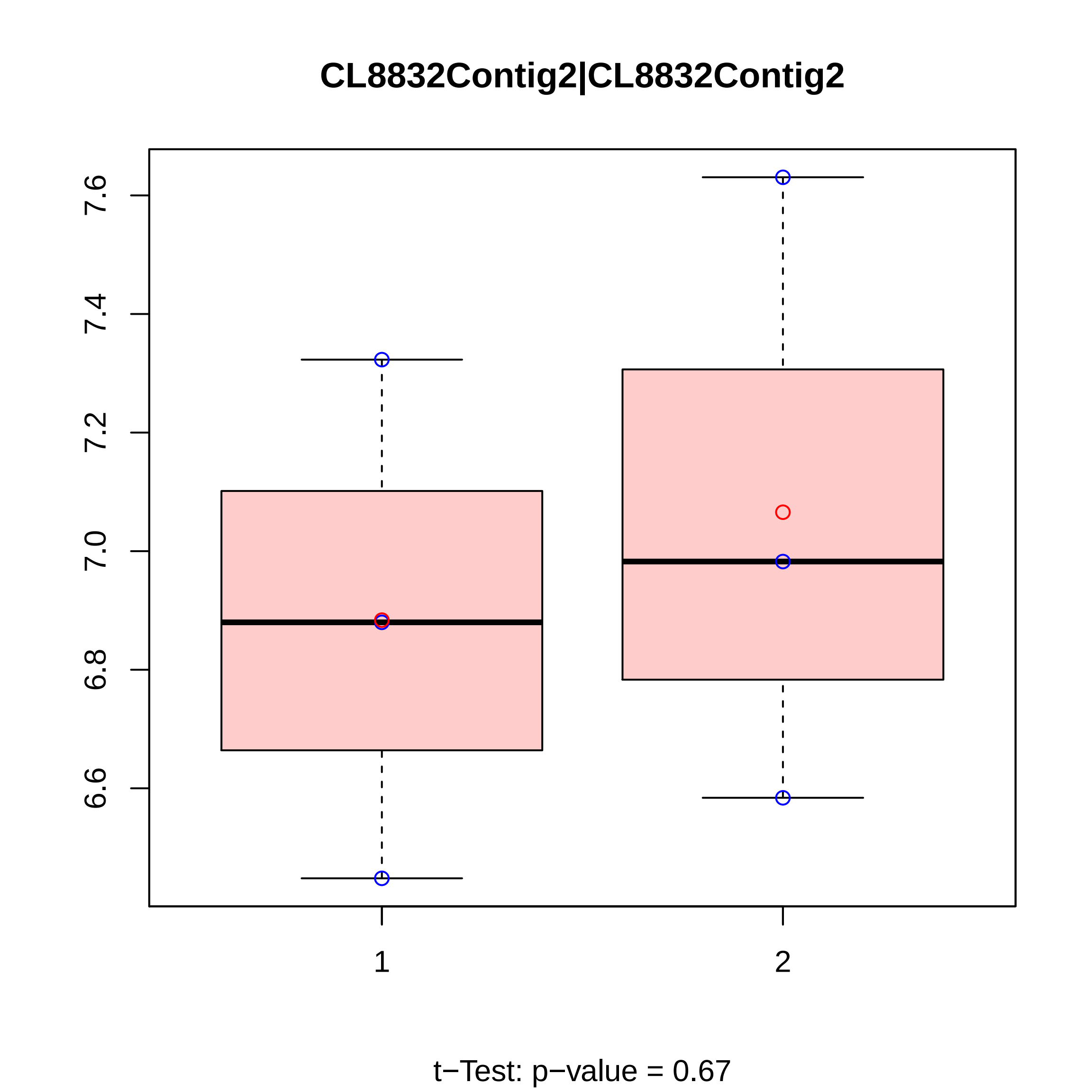

CL8832Contig2|CL8832Contig2
7.6
7.4
7.2
7.0
6.8
6.6
1
2
t−Test: p−value = 0.67

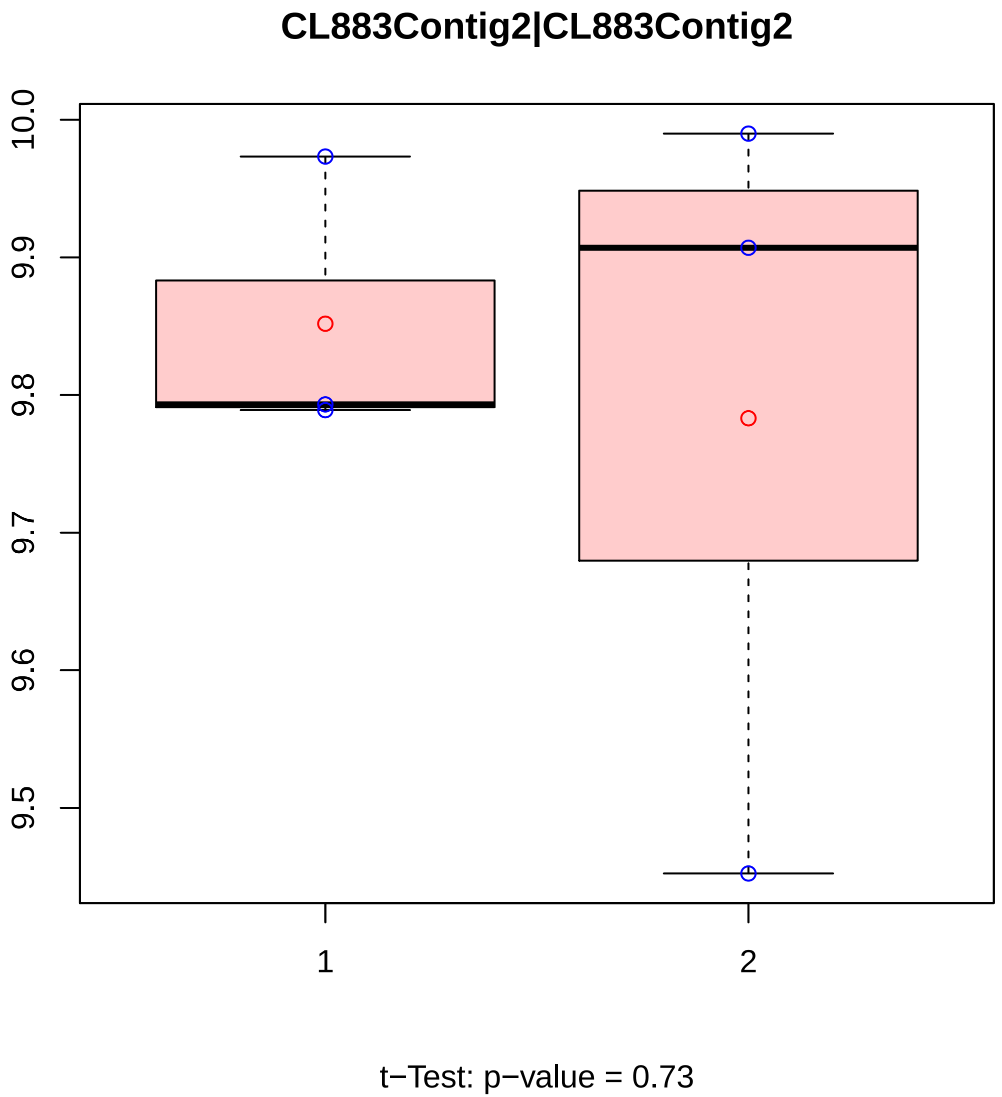
CL883Contig2|CL883Contig2
10.0
9.9
9.8
9.7
9.6
9.5
1
2
t−Test: p−value = 0.73

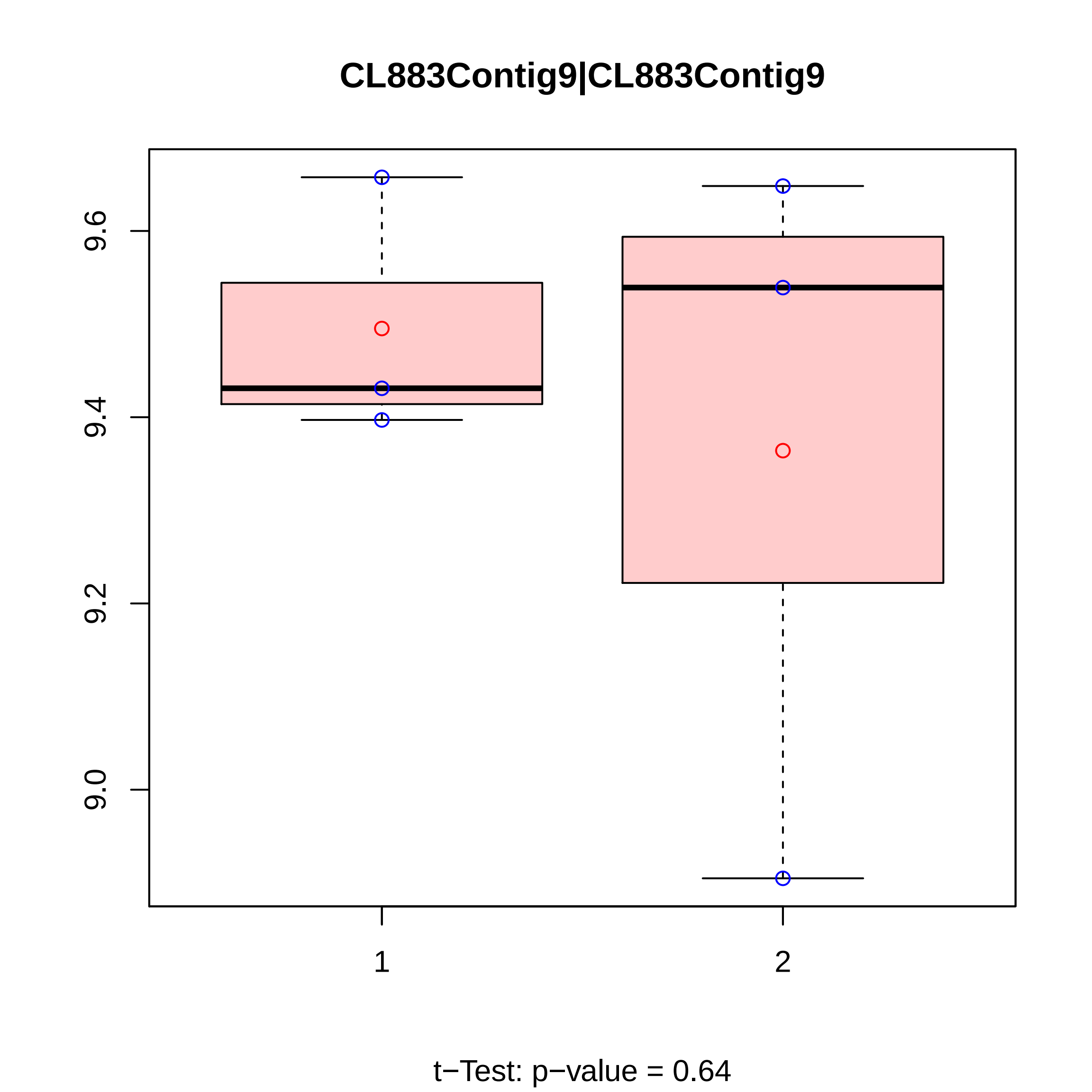

CL883Contig9|CL883Contig9
9.6
9.4
9.2
9.0
1
2
t−Test: p−value = 0.64

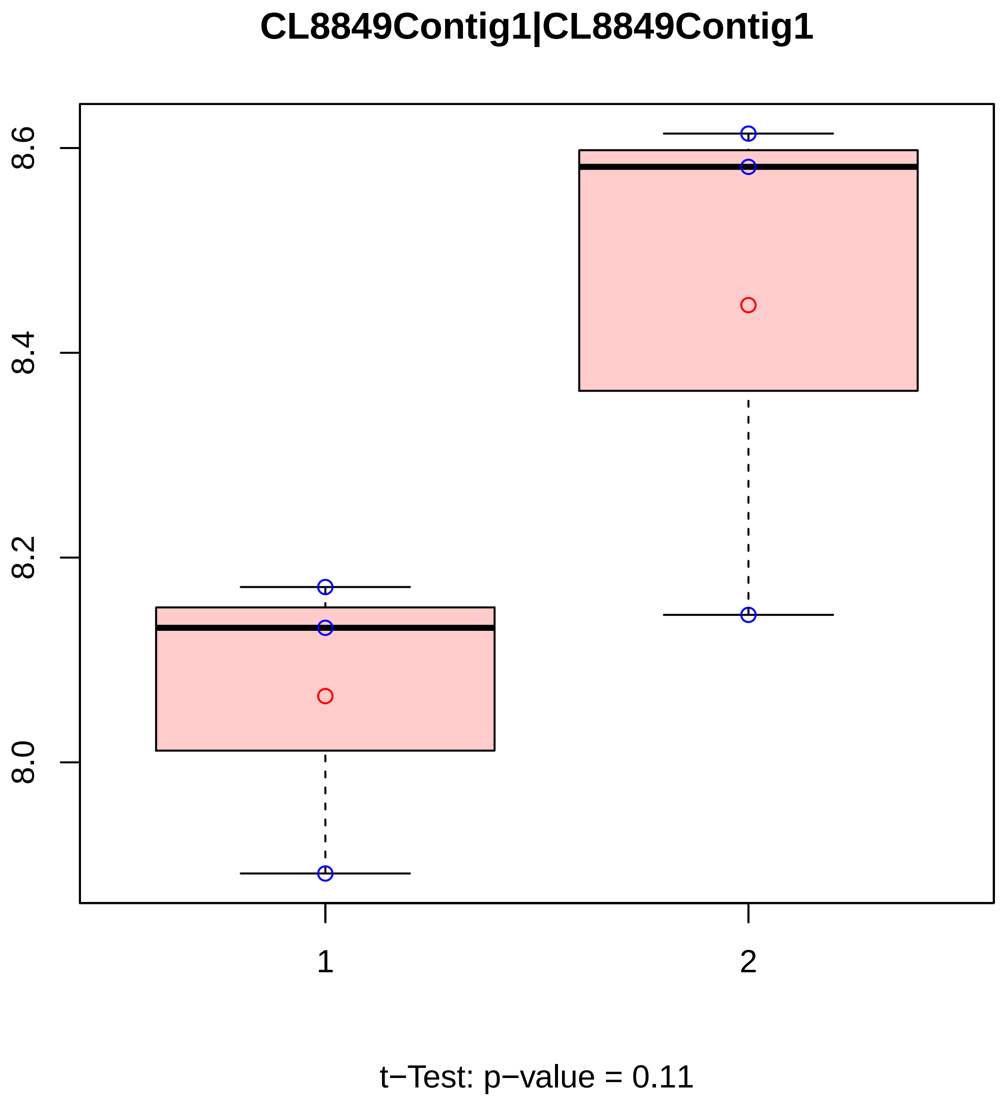
CL8849Contig1|CL8849Contig1
8.6
8.4
8.2
8.0
1
2
t−Test: p−value = 0.11

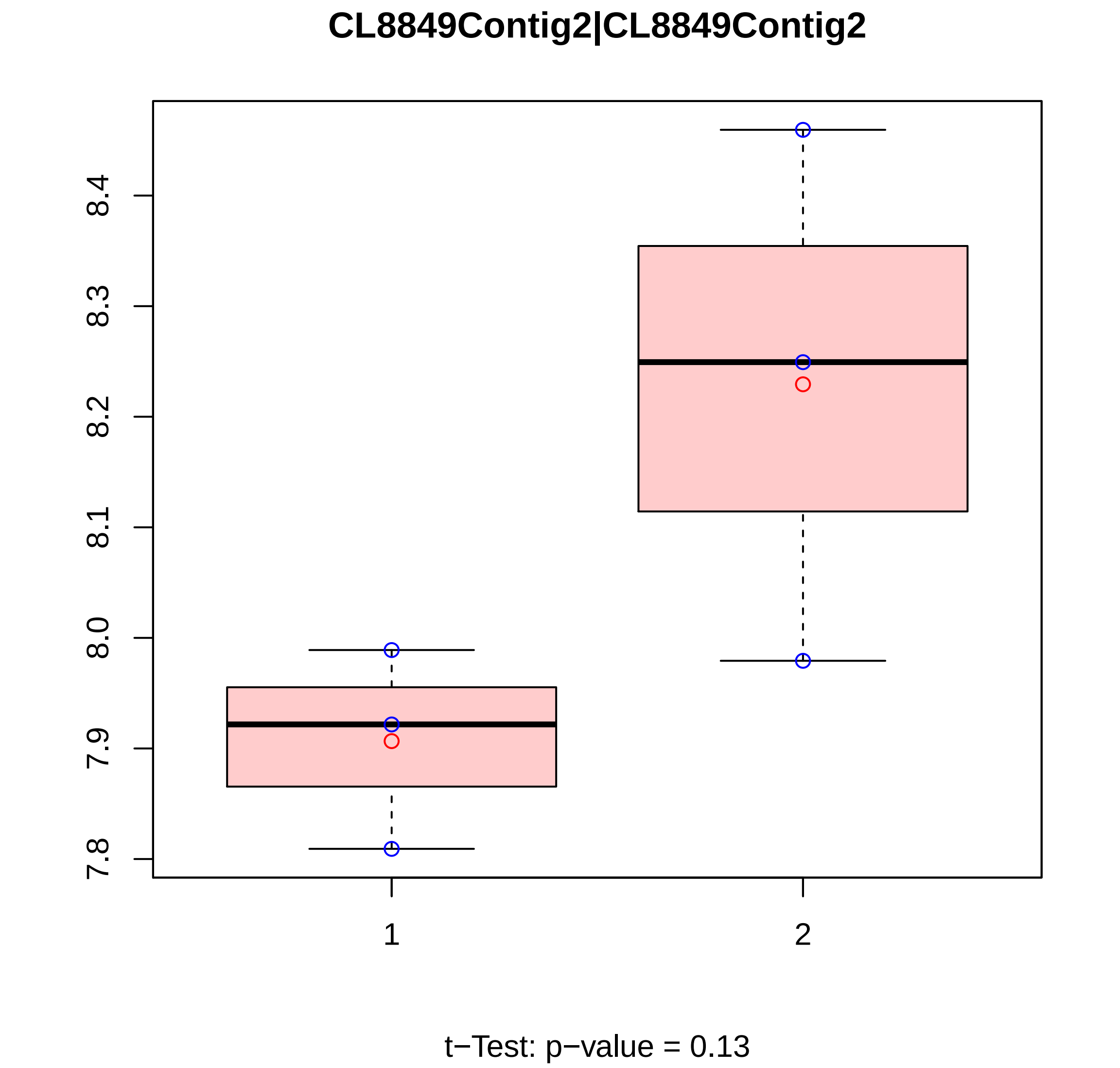
CL8849Contig2|CL8849Contig2
8.4
8.3
8.2
8.1
8.0
7.9
7.8
1
2
t−Test: p−value = 0.13

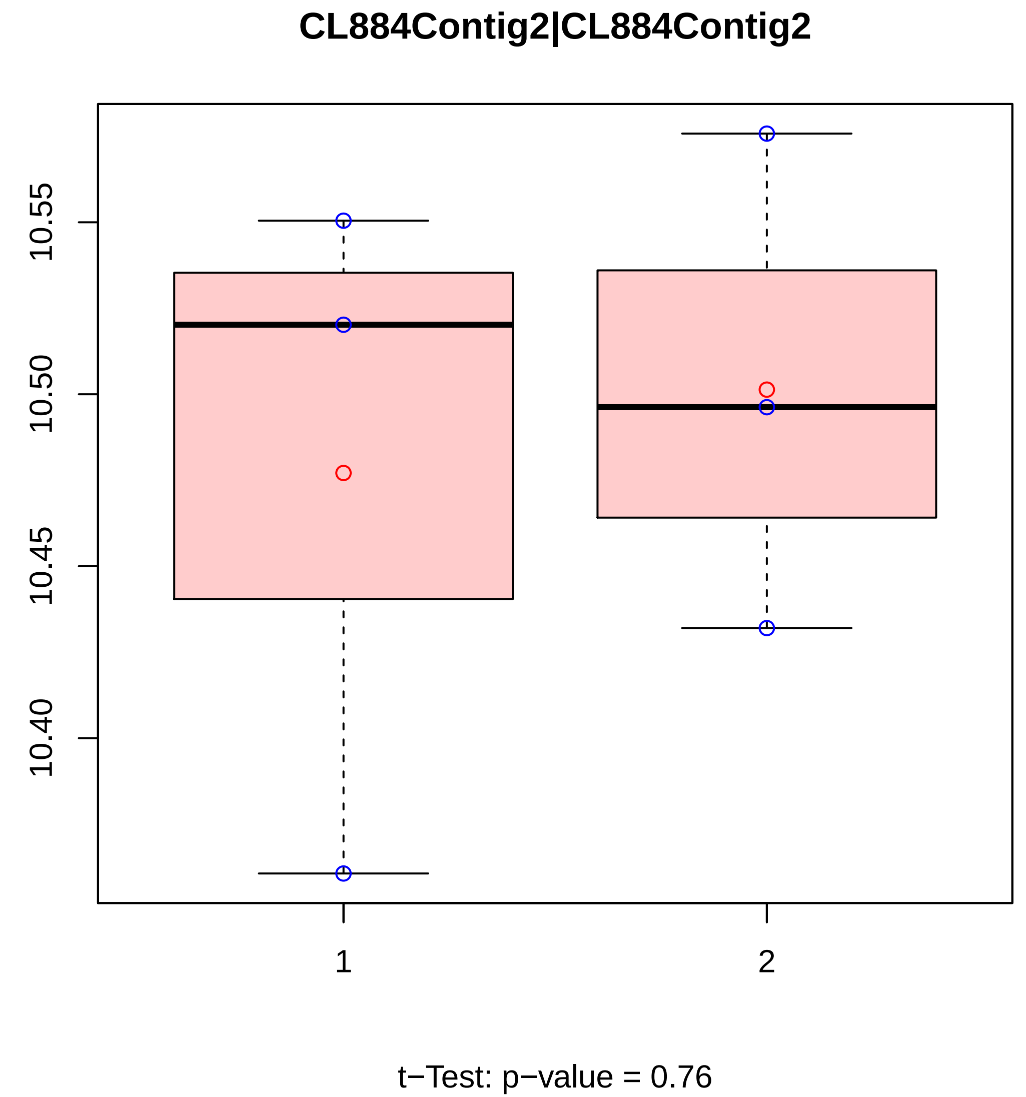
CL884Contig2|CL884Contig2
10.40
10.45
10.50
10.55
1
2
t−Test: p−value = 0.76

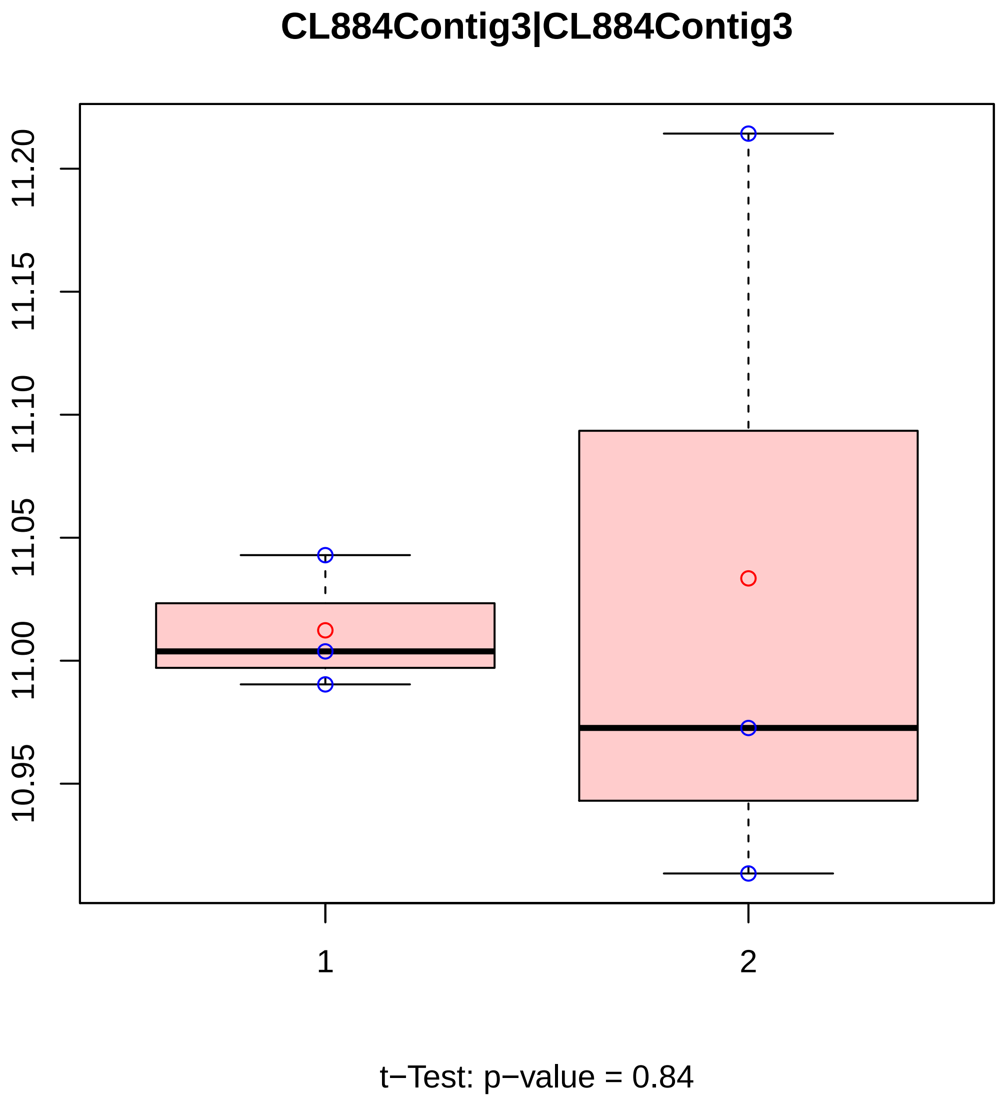

CL884Contig3|CL884Contig3
11.20
11.15
11.10
11.05
11.00
10.95
1
2
t−Test: p−value = 0.84

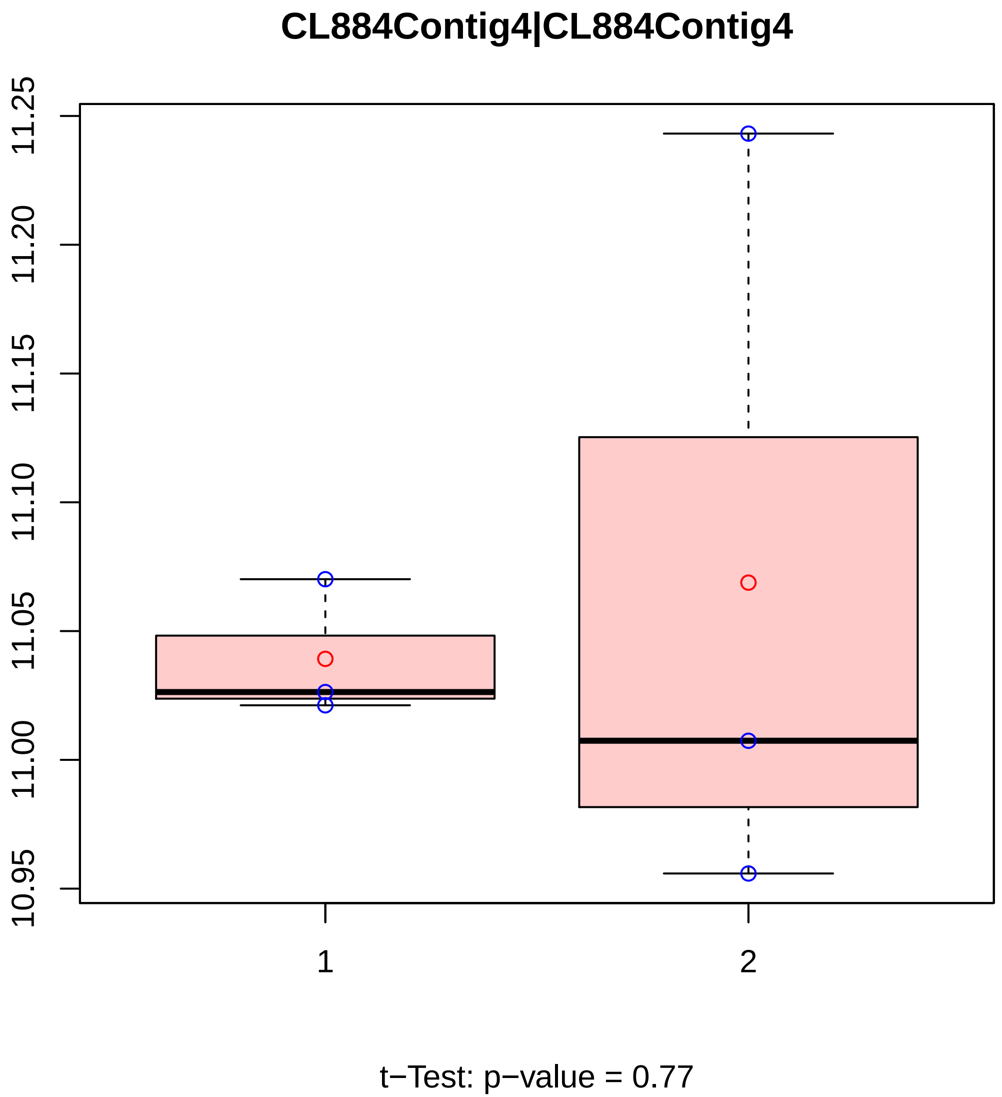

CL884Contig4|CL884Contig4
11.25
11.20
11.15
11.10
11.05
11.00
10.95
1
2
t−Test: p−value = 0.77

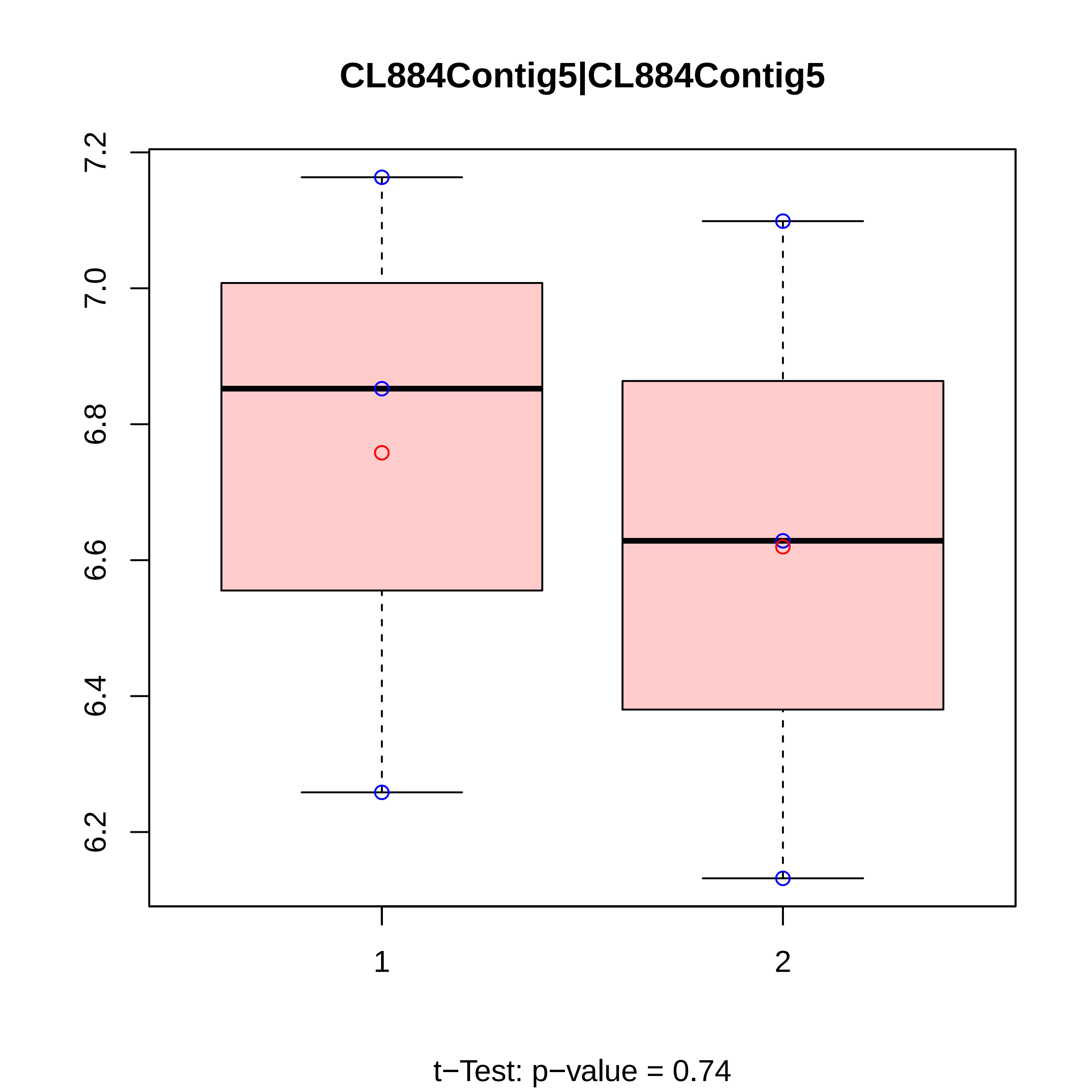

CL884Contig5|CL884Contig5
7.2
7.0
6.8
6.6
6.4
6.2
1
2
t−Test: p−value = 0.74

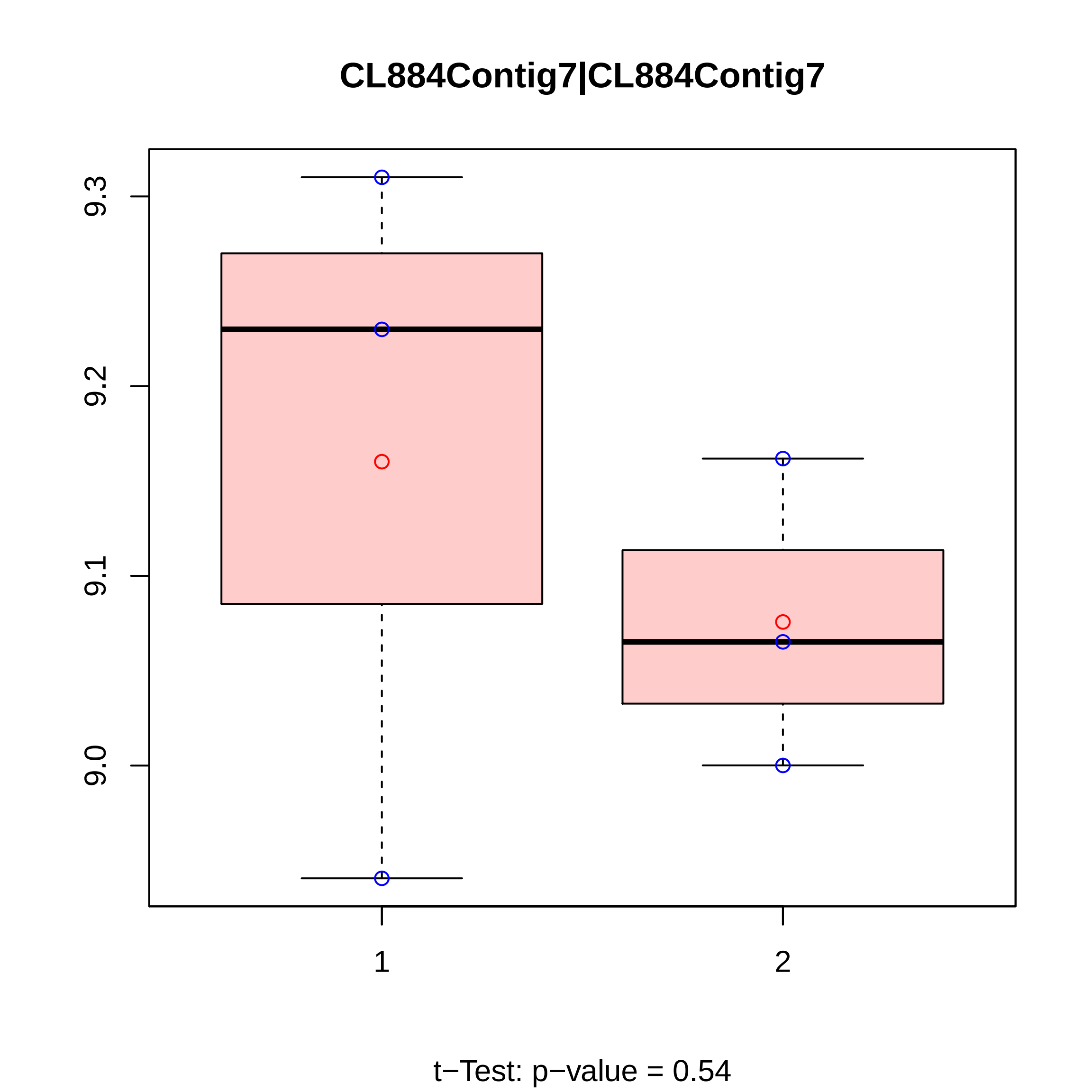
CL884Contig7|CL884Contig7
9.0
9.1
9.2
9.3
1
2
t−Test: p−value = 0.54

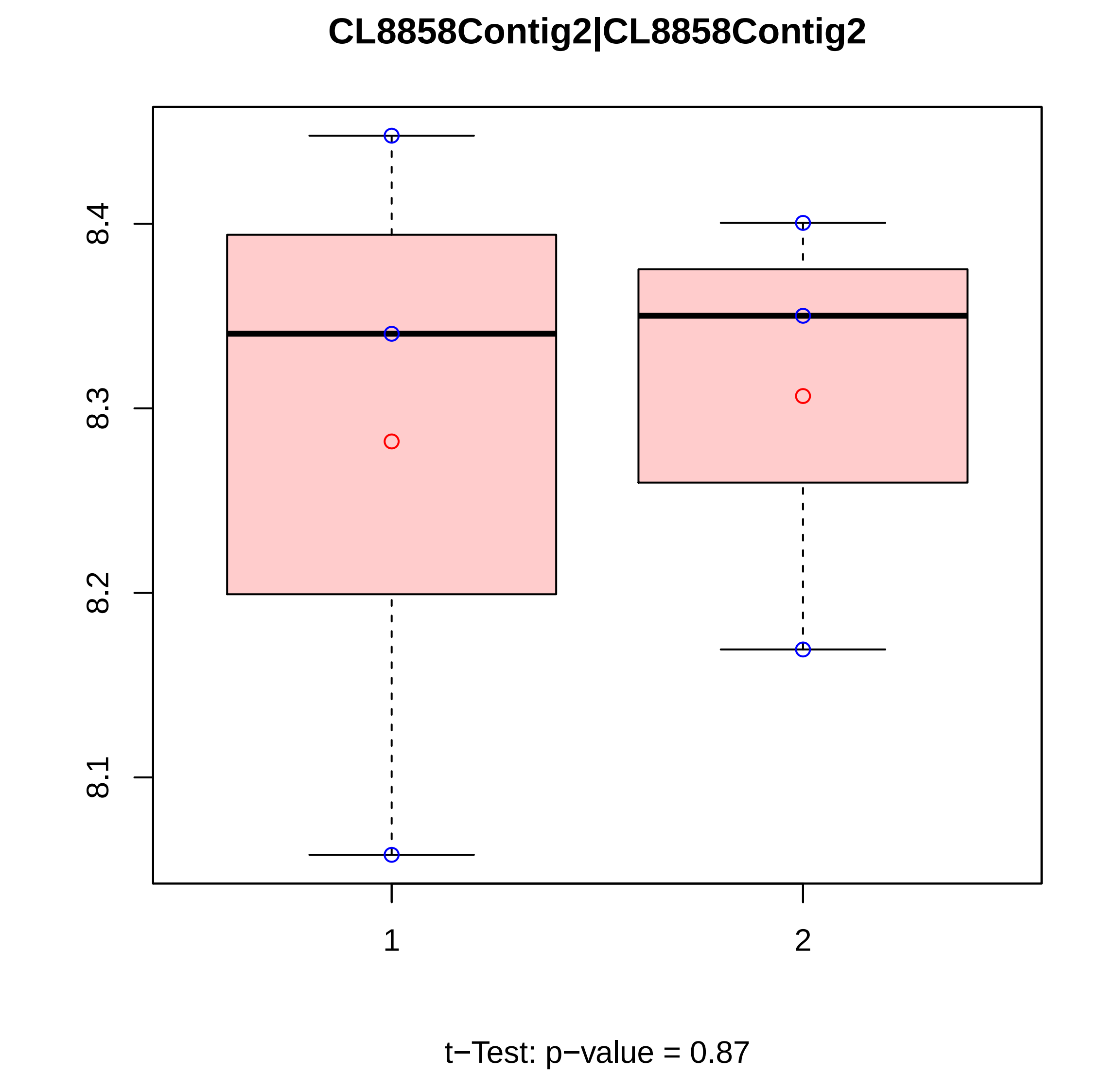

CL8858Contig2|CL8858Contig2
8.1
8.2
8.3
8.4
1
2
t−Test: p−value = 0.87

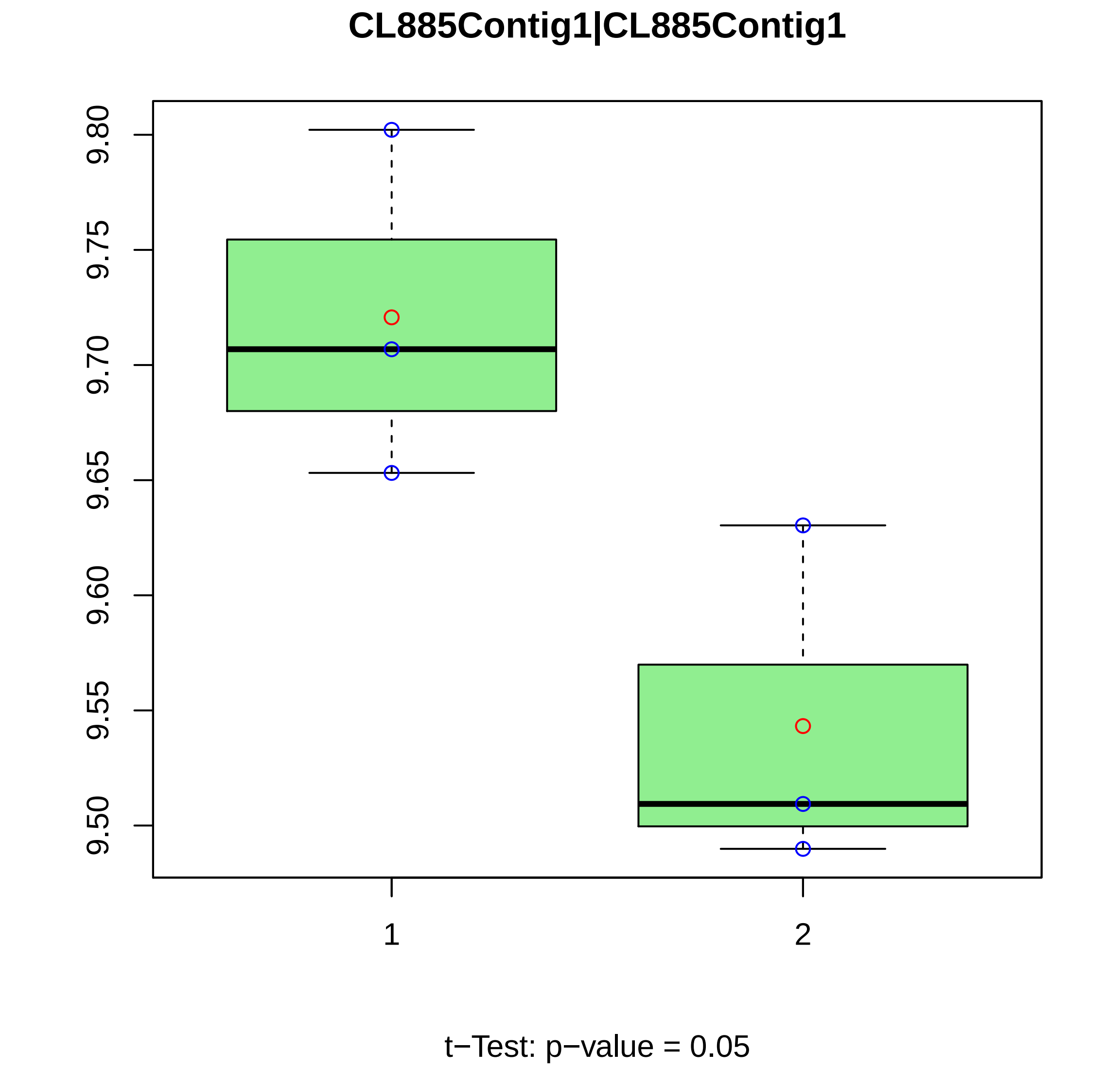
CL885Contig1|CL885Contig1
9.80
9.75
9.70
9.65
9.60
9.55
9.50
1
2
t−Test: p−value = 0.05

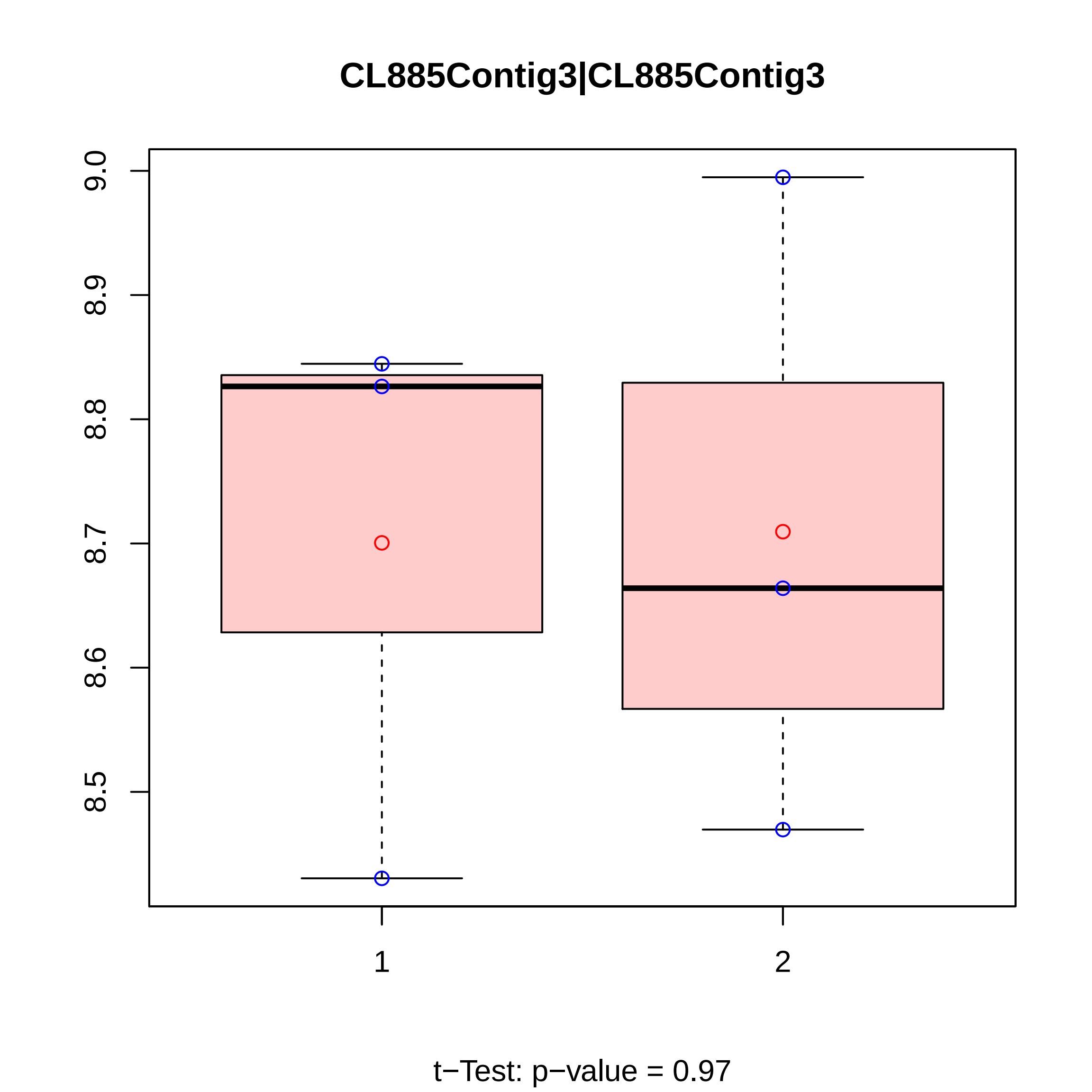

CL885Contig3|CL885Contig3
9.0
8.9
8.8
8.7
8.6
8.5
1
2
t−Test: p−value = 0.97

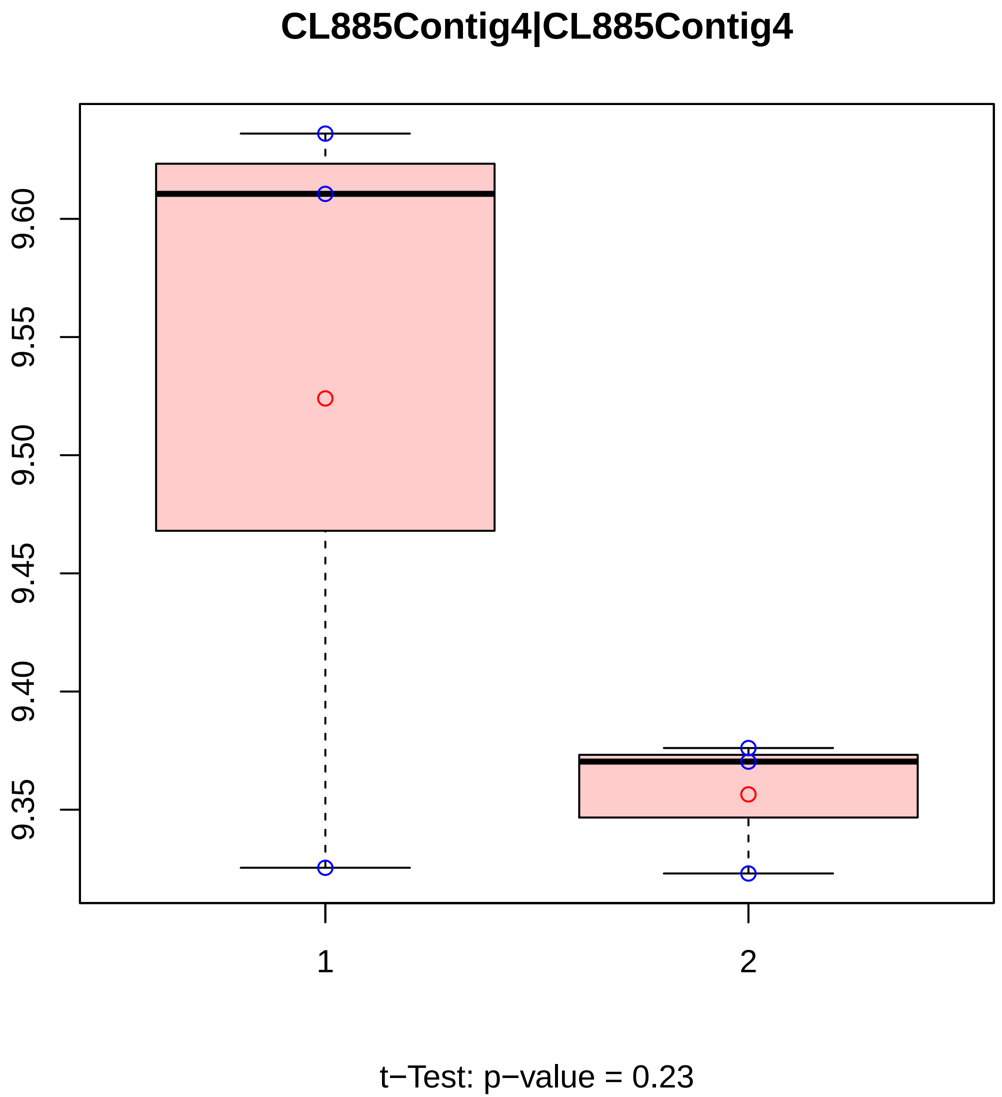

CL885Contig4|CL885Contig4
9.35
9.40
9.45
9.50
9.55
9.60
1
2
t−Test: p−value = 0.23

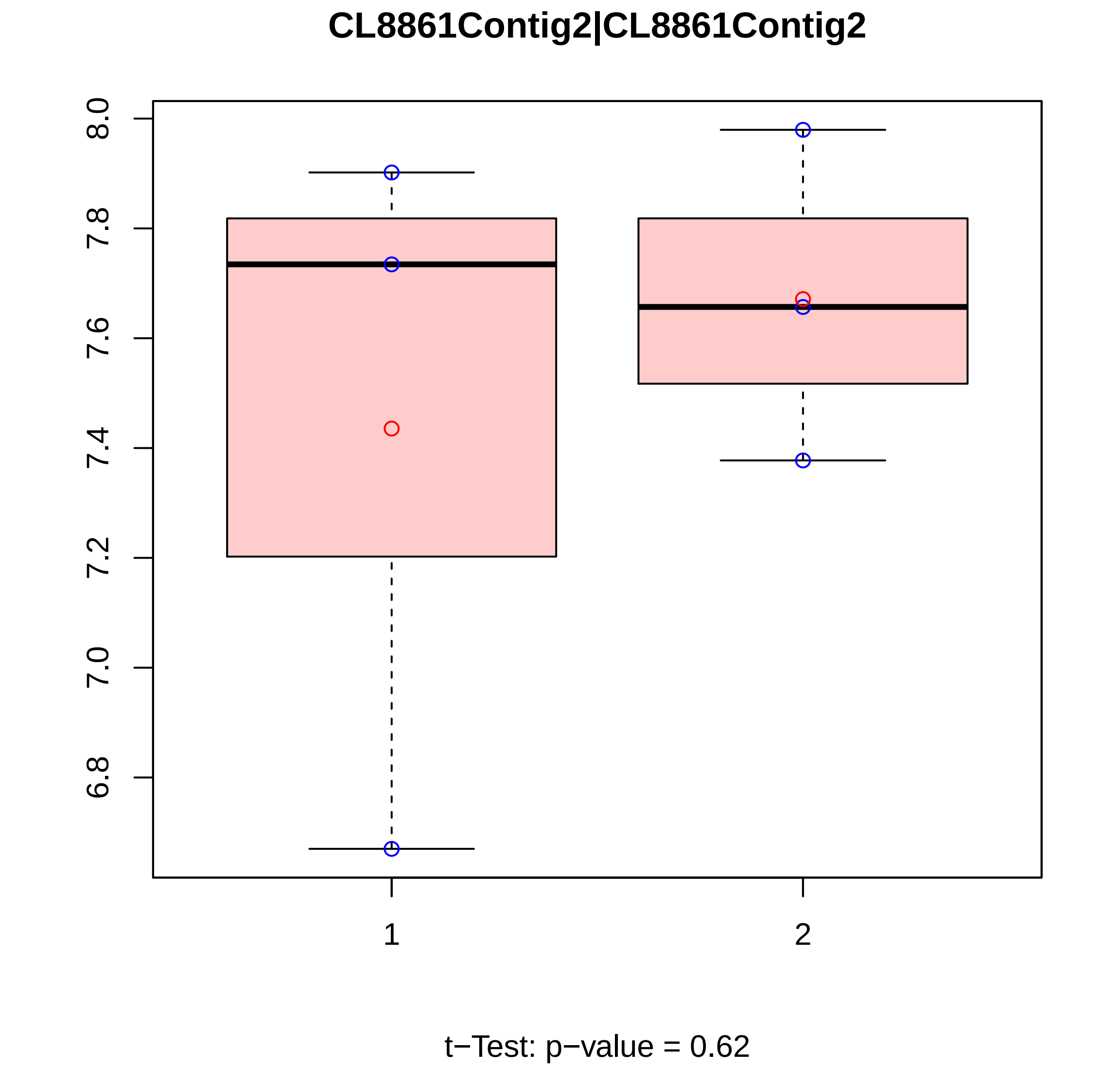
CL8861Contig2|CL8861Contig2
8.0
7.8
7.6
7.4
7.2
7.0
6.8
1
2
t−Test: p−value = 0.62

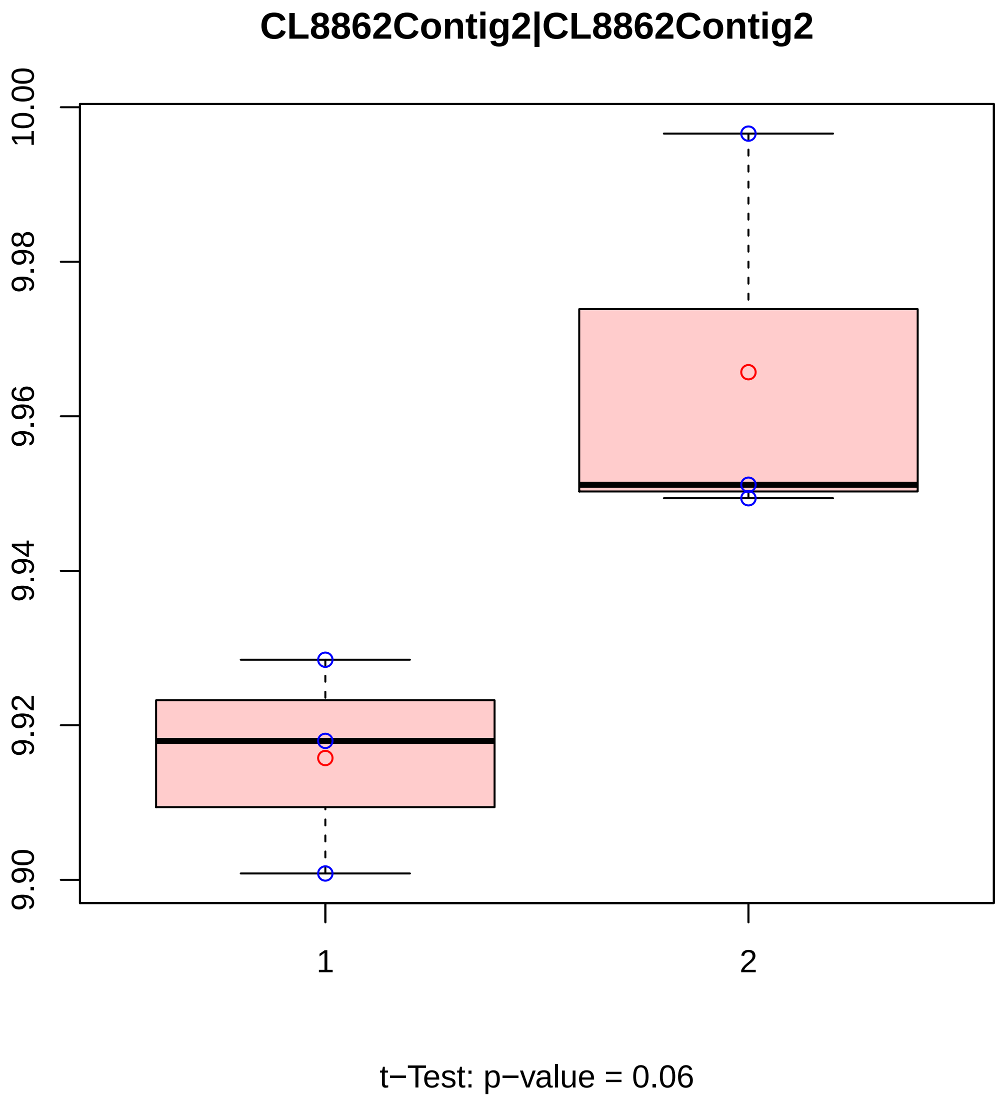
CL8862Contig2|CL8862Contig2
10.00
9.98
9.96
9.94
9.92
9.90
1
2
t−Test: p−value = 0.06

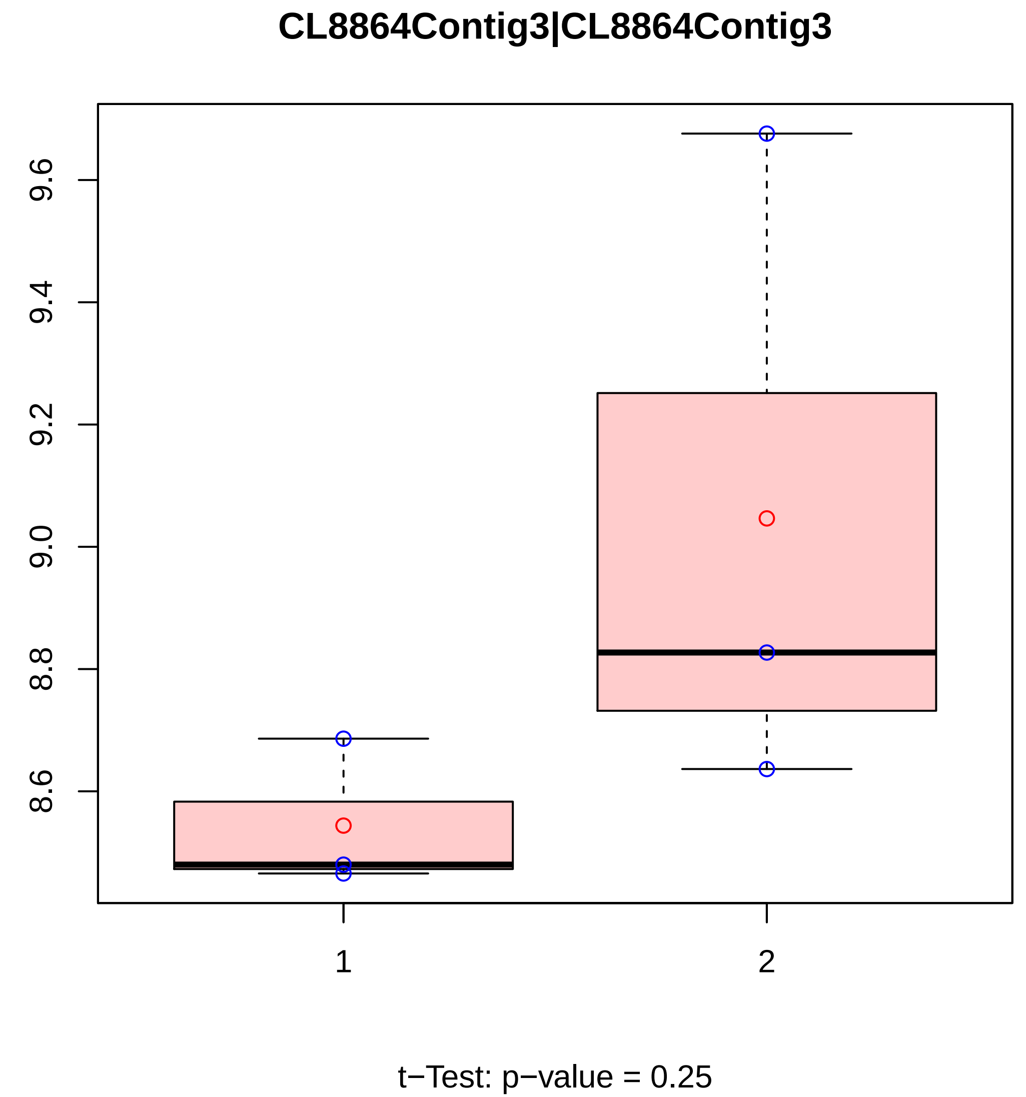

CL8864Contig3|CL8864Contig3
9.6
9.4
9.2
9.0
8.8
8.6
1
2
t−Test: p−value = 0.25

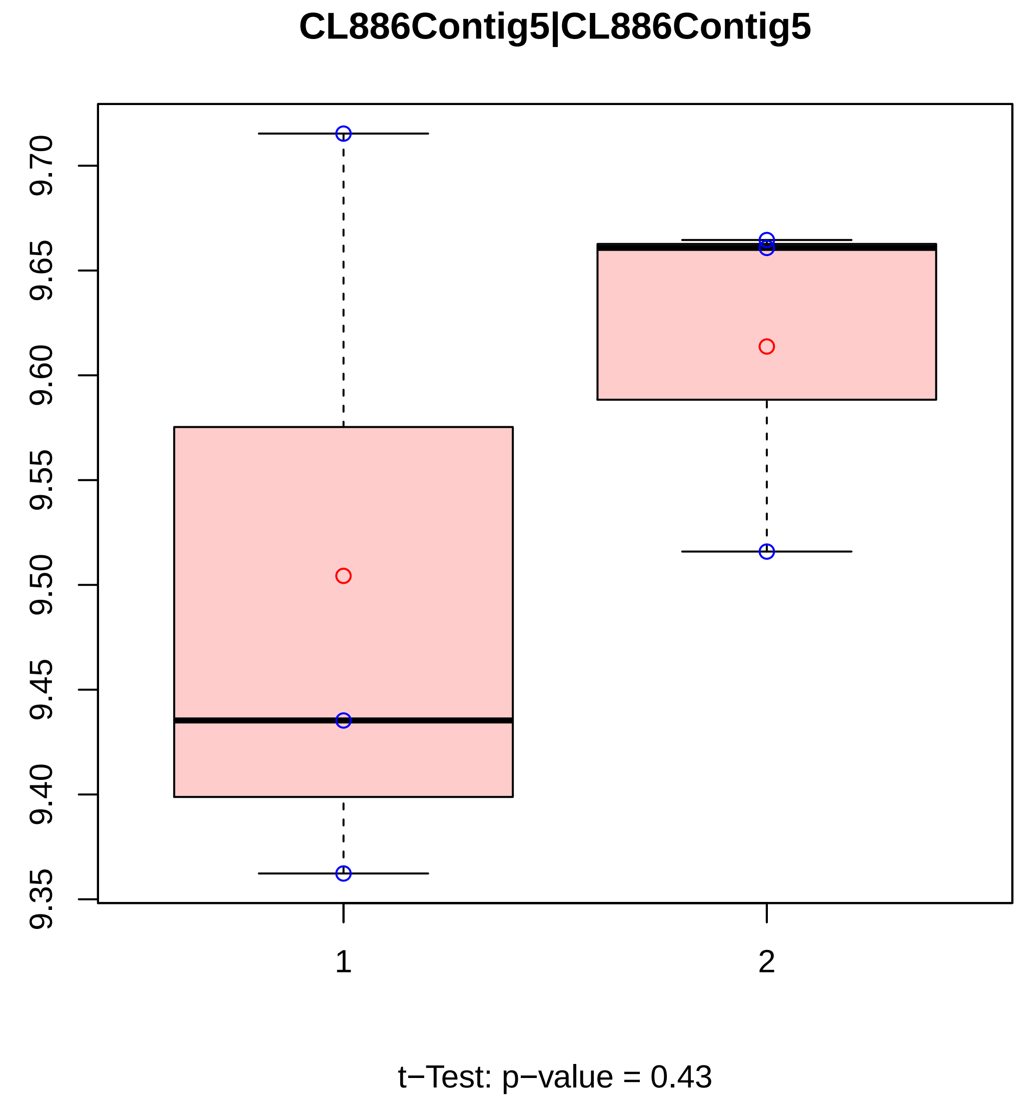
CL886Contig5|CL886Contig5
9.35
9.40
9.45
9.50
9.55
9.60
9.65
9.70
1
2
t−Test: p−value = 0.43

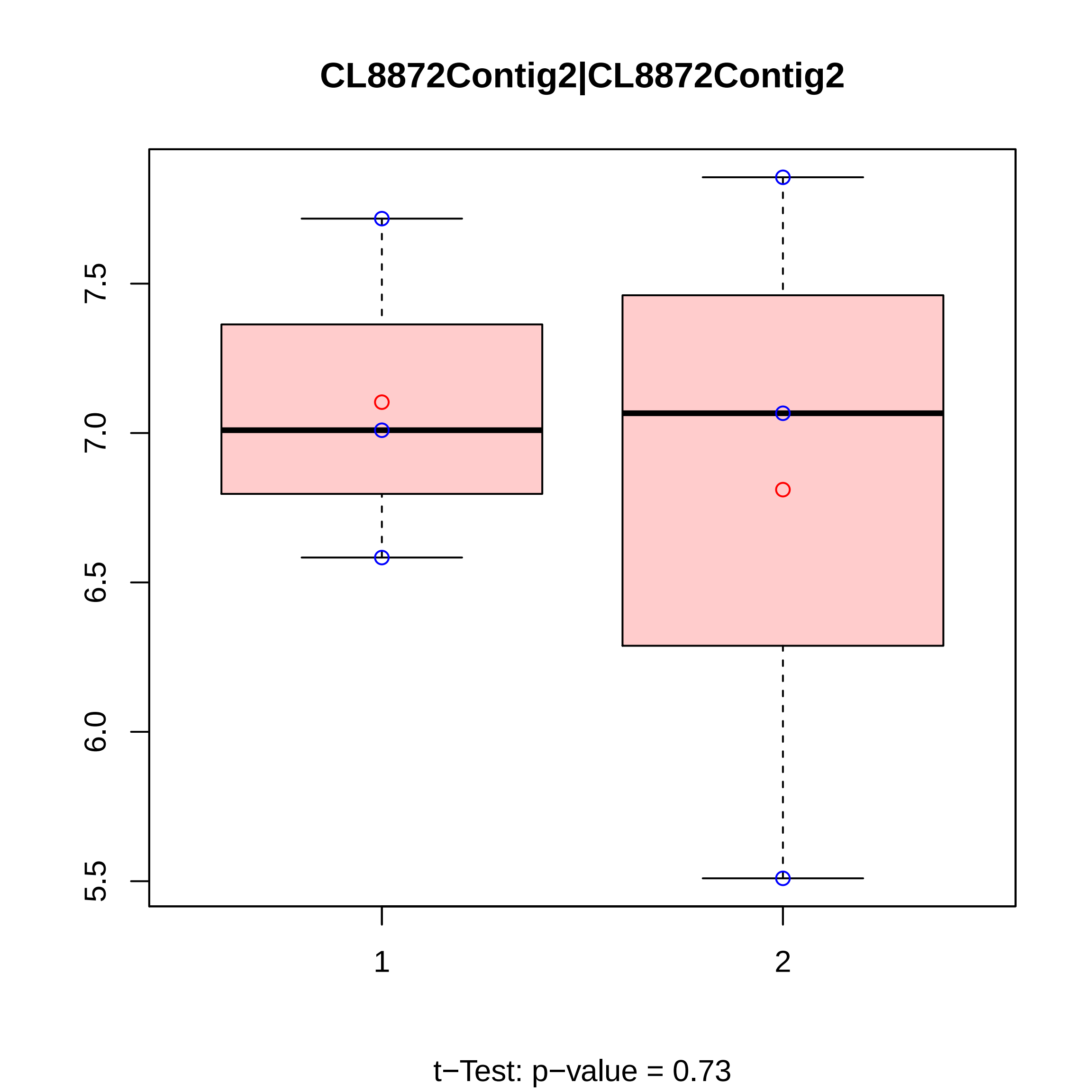
CL8872Contig2|CL8872Contig2
7.5
7.0
6.5
6.0
5.5
1
2
t−Test: p−value = 0.73

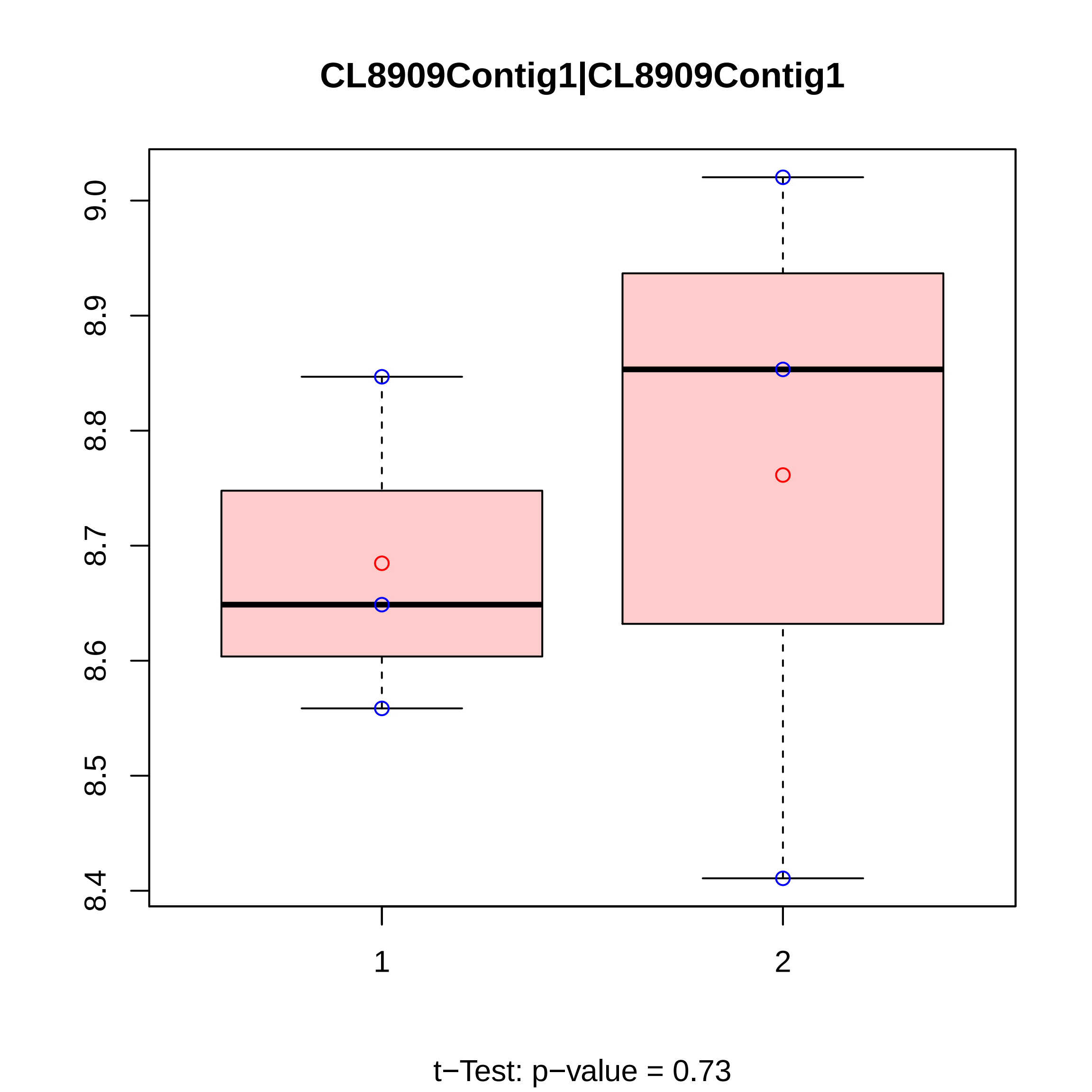
CL8909Contig1|CL8909Contig1
9.0
8.9
8.8
8.7
8.6
8.5
8.4
1
2
t−Test: p−value = 0.73

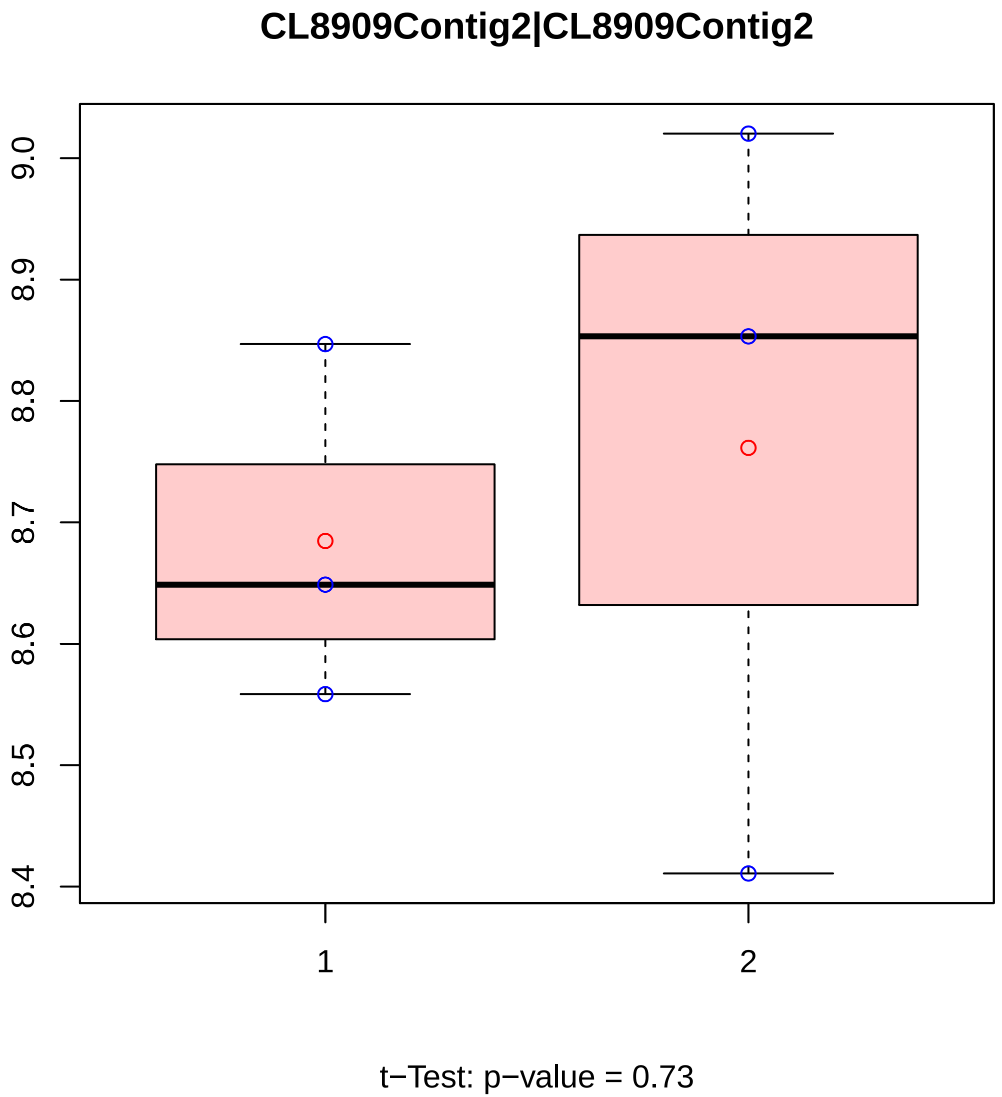
CL8909Contig2|CL8909Contig2
9.0
8.9
8.8
8.7
8.6
8.5
8.4
1
2
t−Test: p−value = 0.73

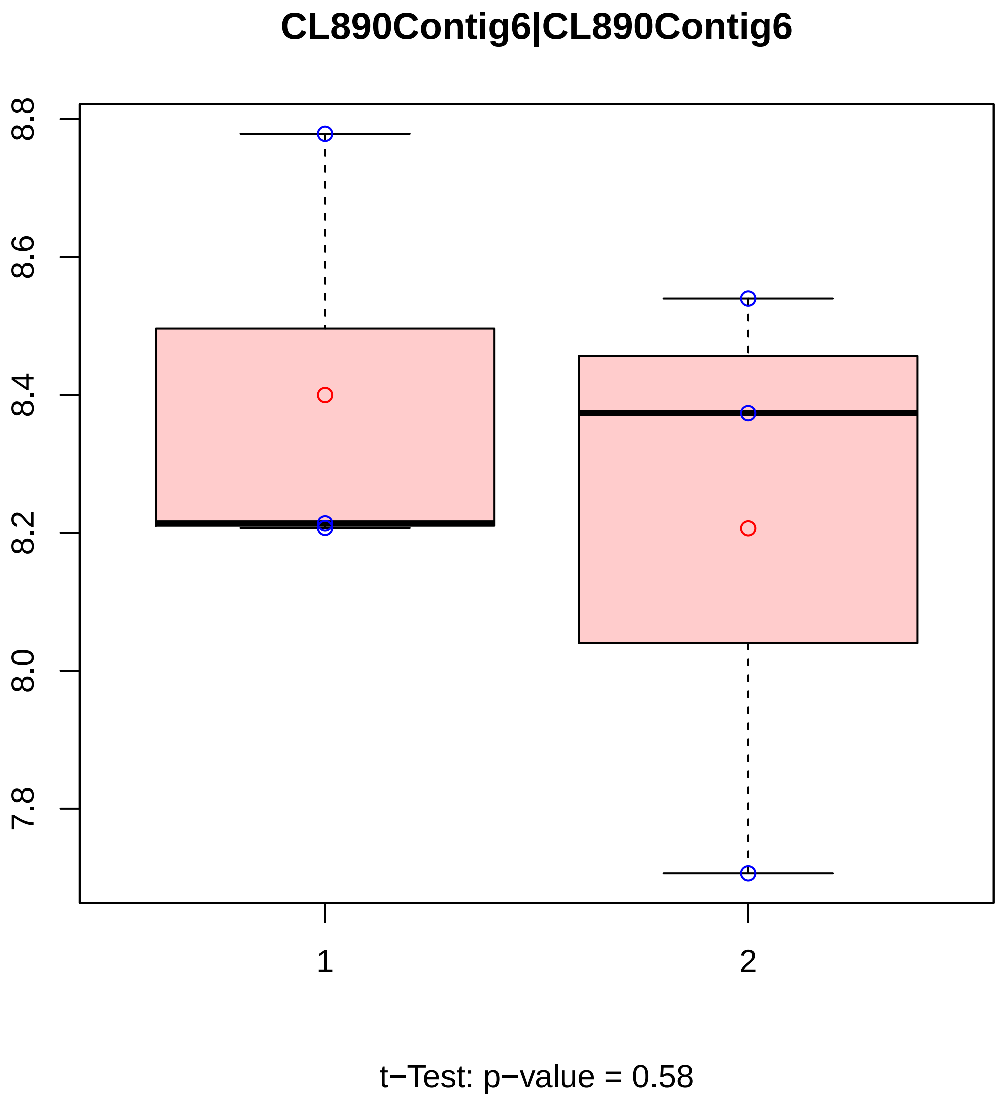

CL890Contig6|CL890Contig6
8.8
8.6
8.4
8.2
8.0
7.8
1
2
t−Test: p−value = 0.58

CL8917Contig2|CL8917Contig2

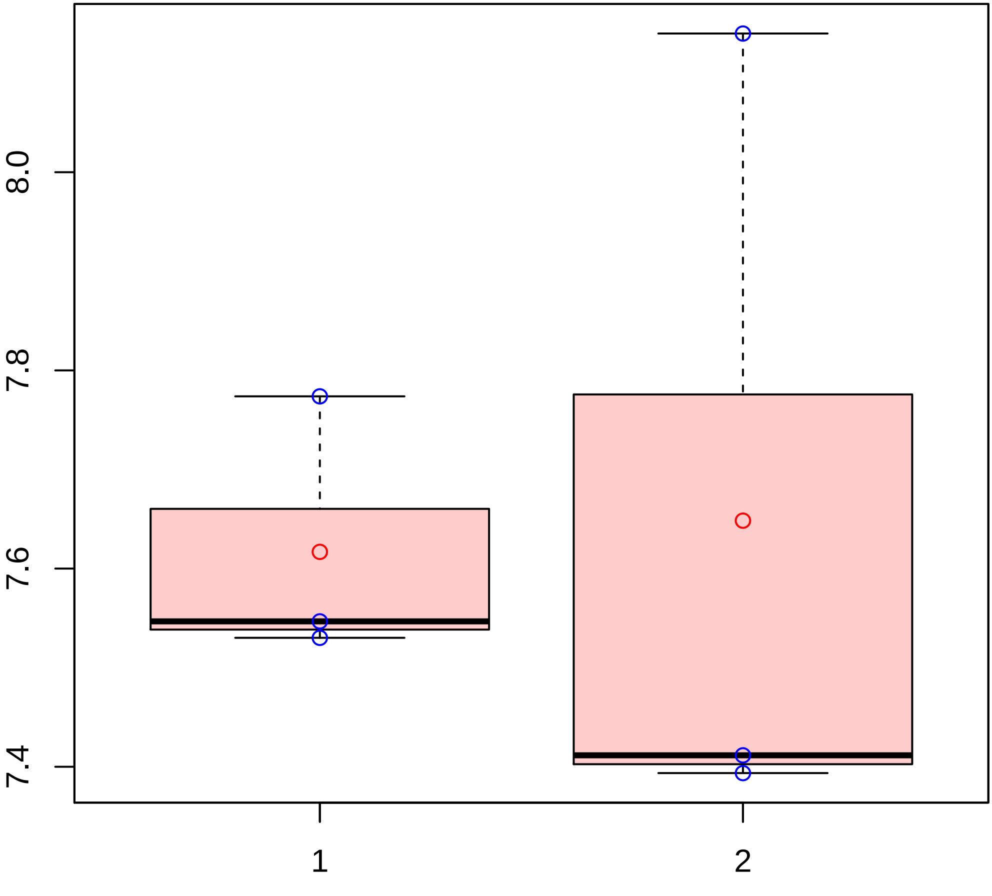


t−Test: p−value = 0.91

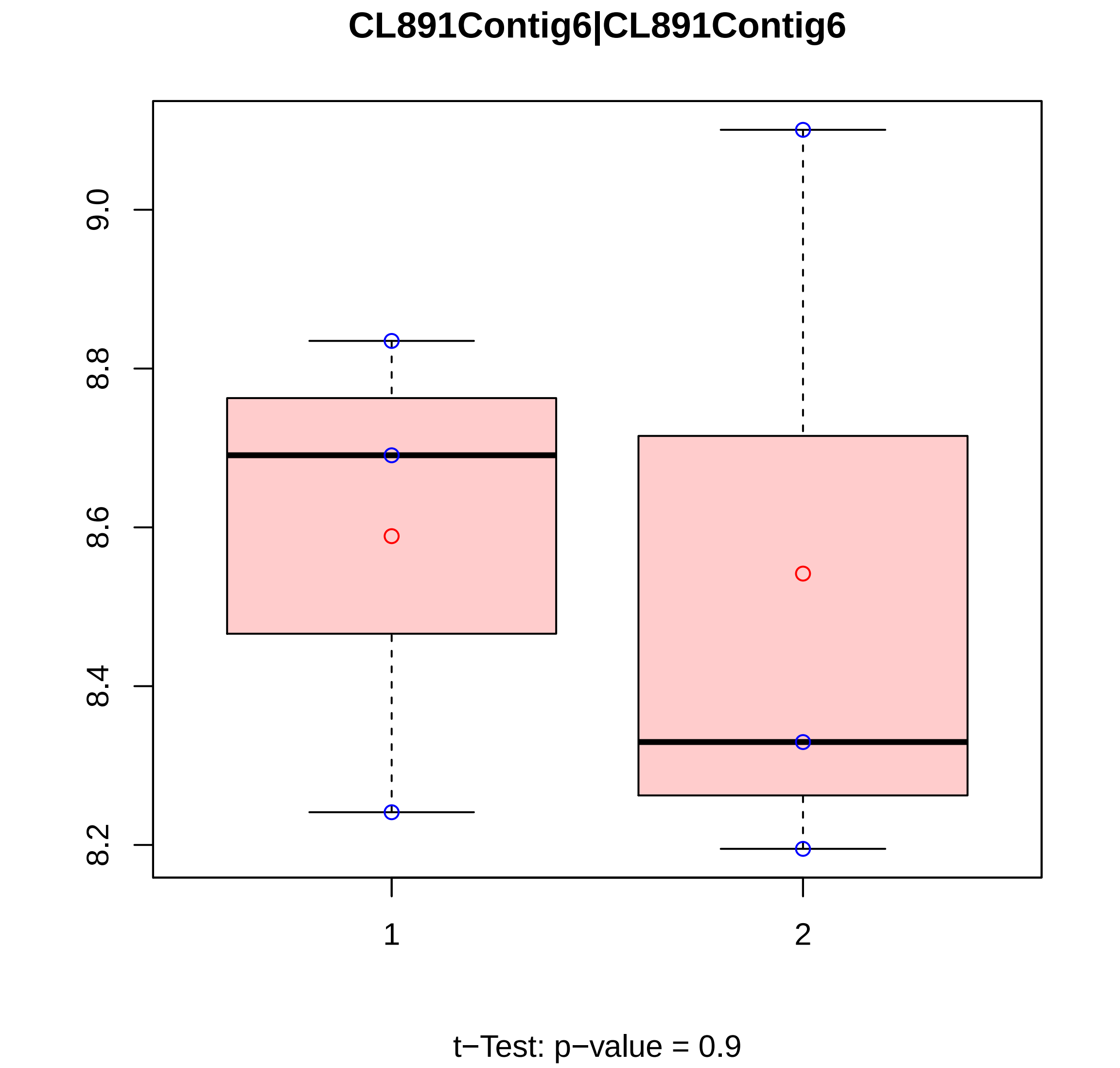
CL891Contig6|CL891Contig6
9.0
8.8
8.6
8.4
8.2
1
2
t−Test: p−value = 0.9

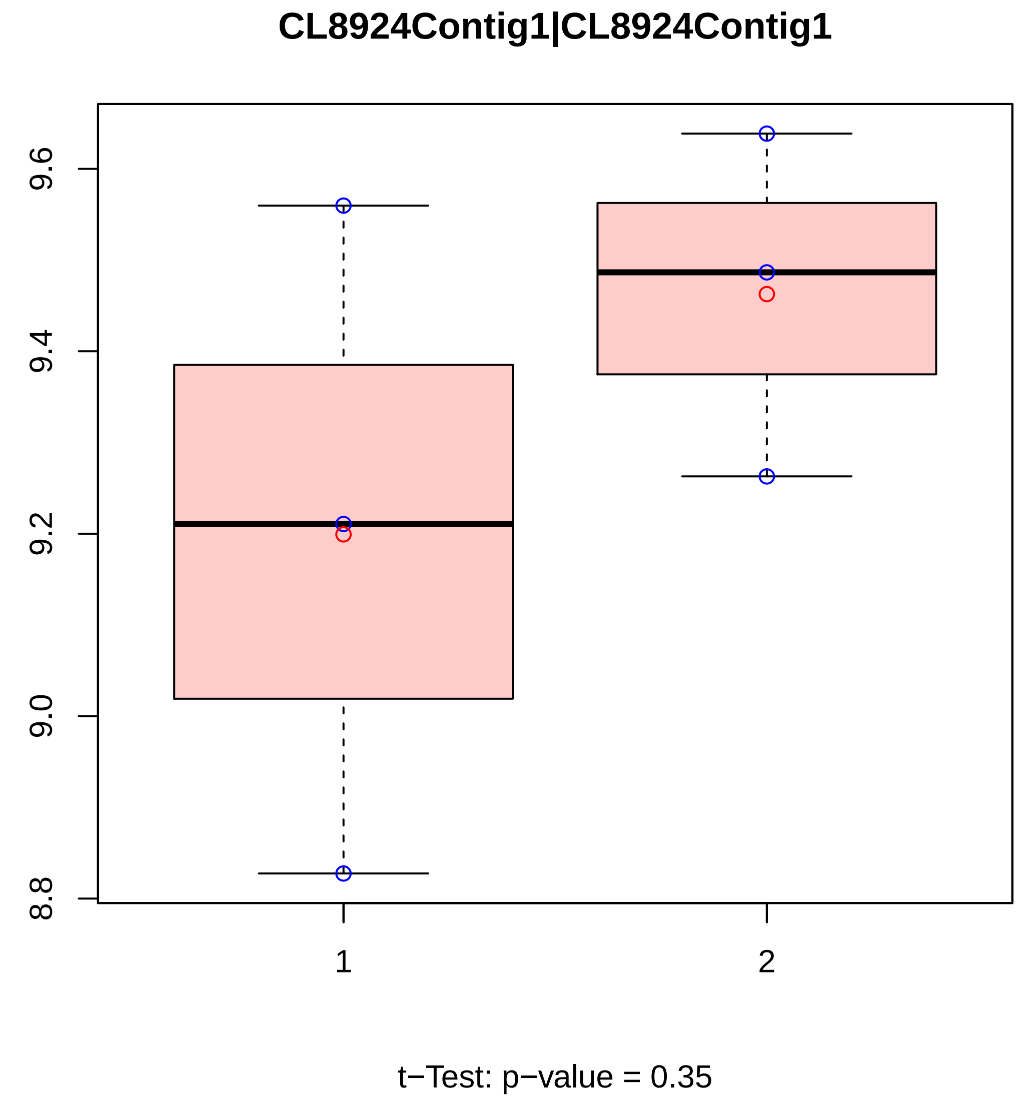
CL8924Contig1|CL8924Contig1
9.6
9.4
9.2
9.0
8.8
1
2
t−Test: p−value = 0.35

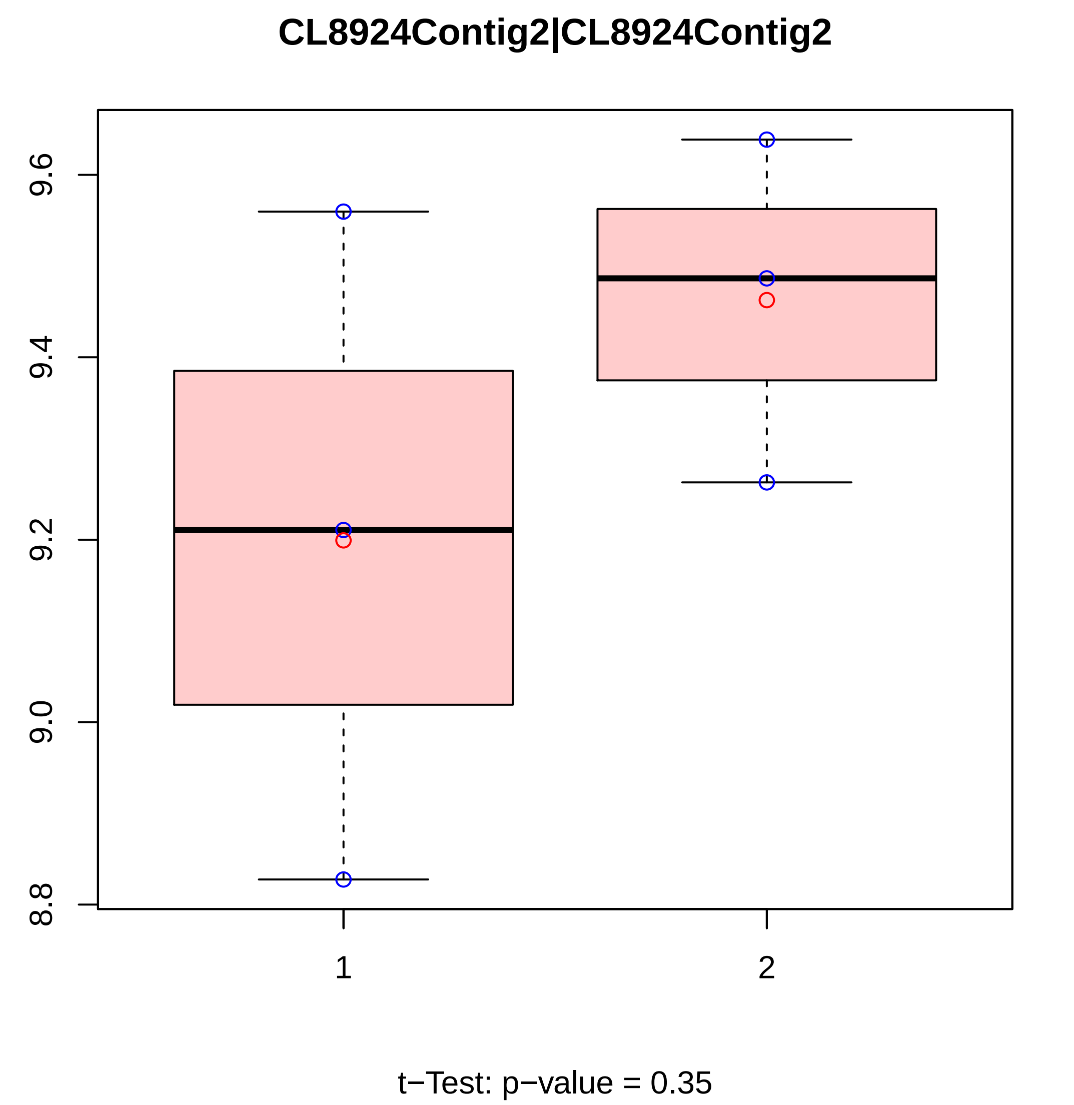

CL8924Contig2|CL8924Contig2
9.6
9.4
9.2
9.0
8.8
1
2
t−Test: p−value = 0.35

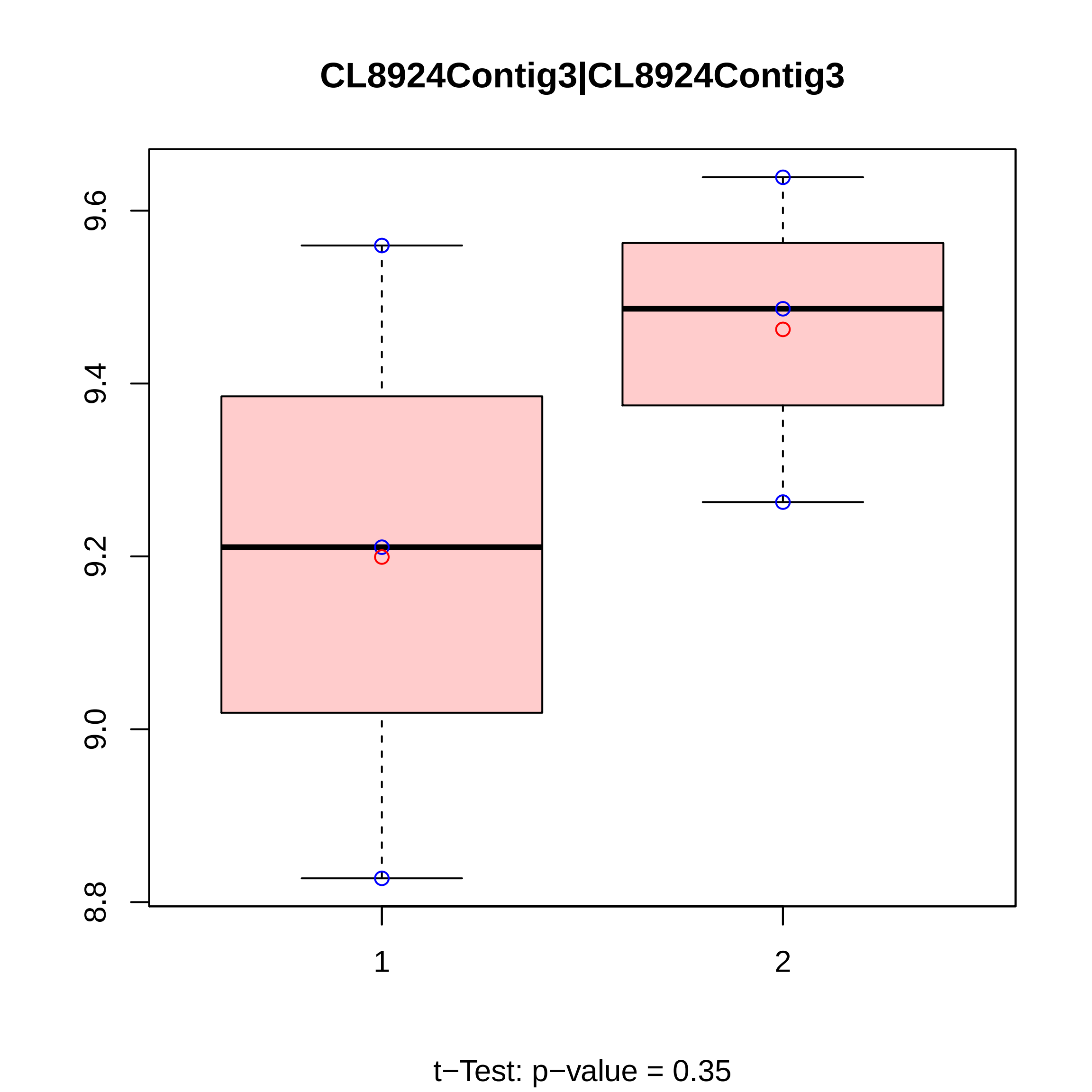
CL8924Contig3|CL8924Contig3
9.6
9.4
9.2
9.0
8.8
1
2
t−Test: p−value = 0.35

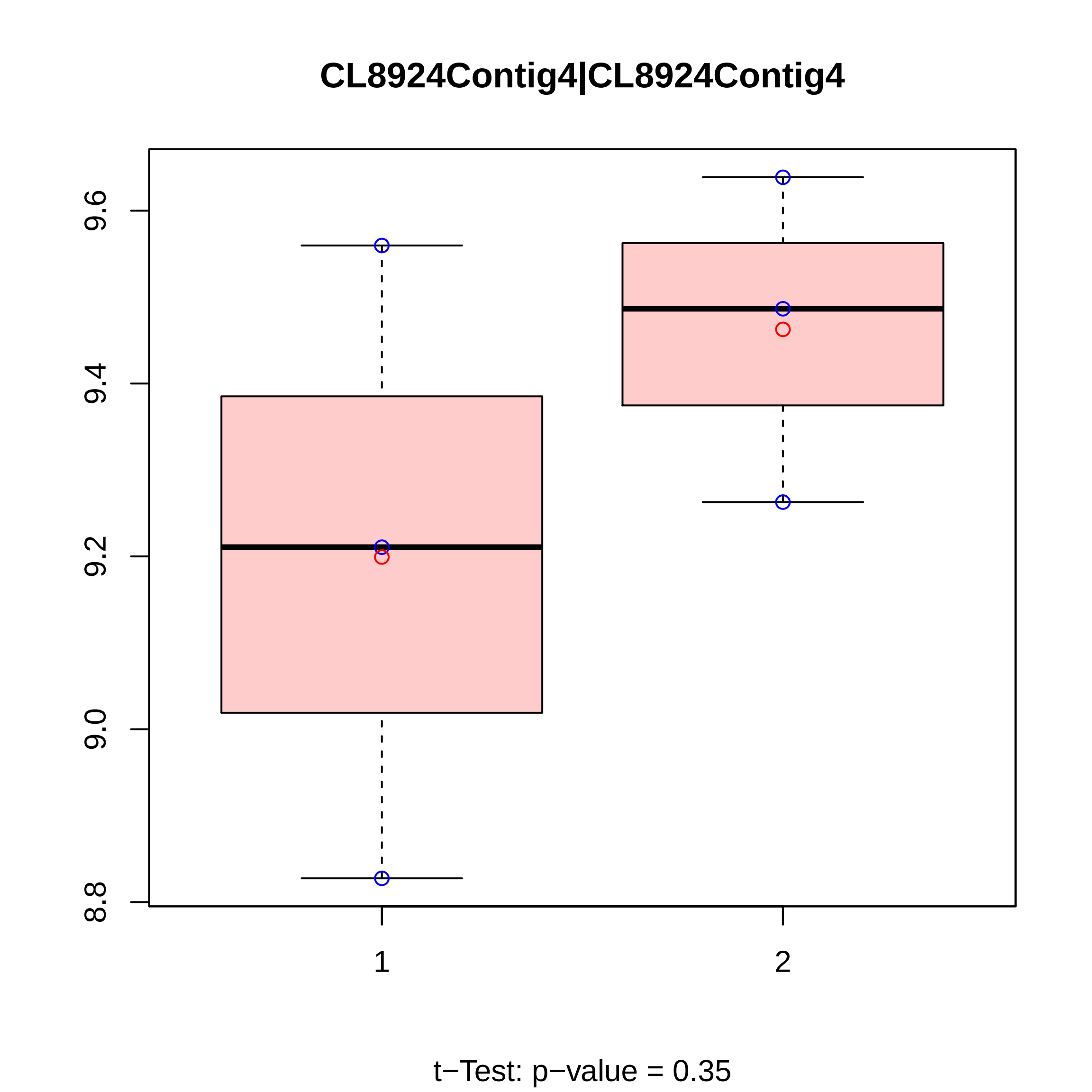

CL8924Contig4|CL8924Contig4
9.6
9.4
9.2
9.0
8.8
1
2
t−Test: p−value = 0.35

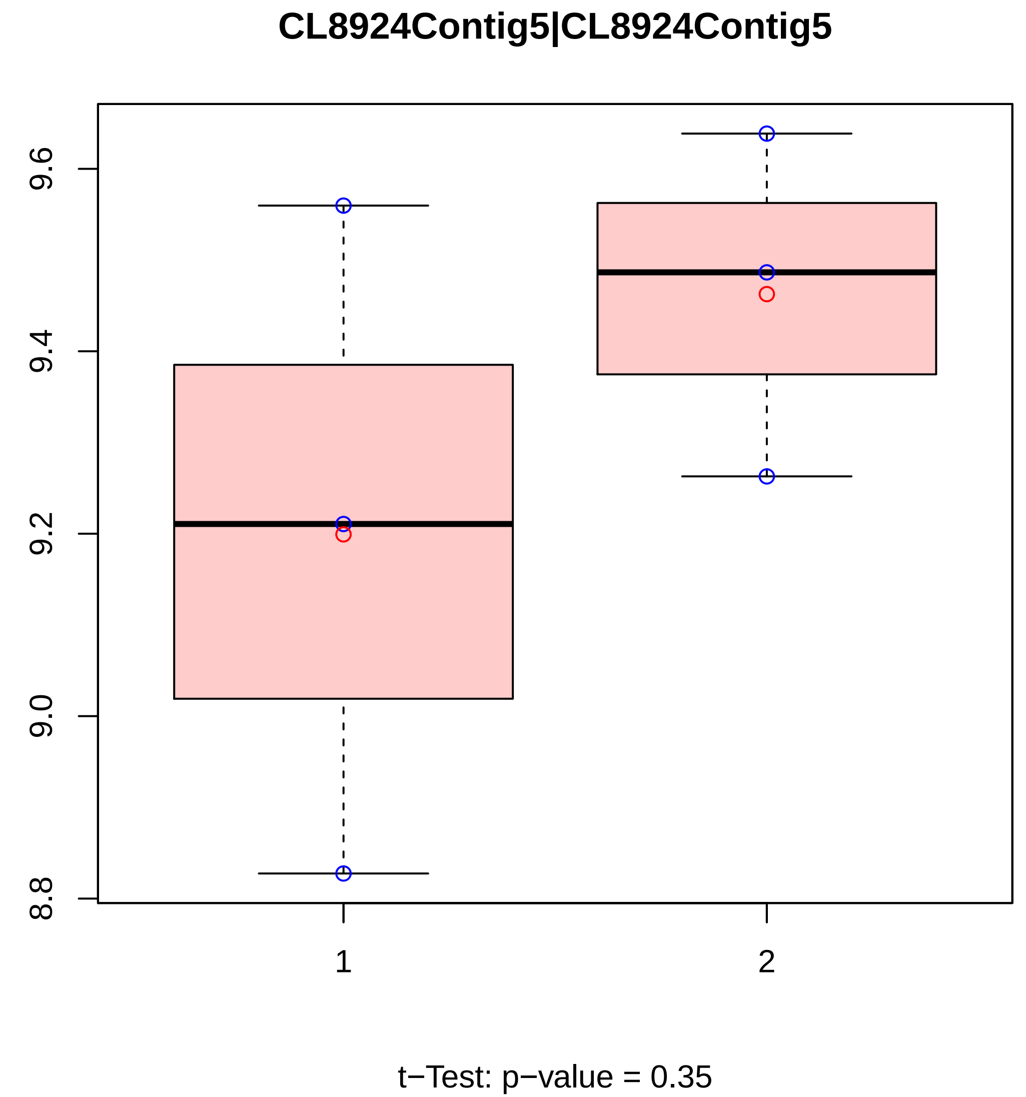

CL8924Contig5|CL8924Contig5
9.6
9.4
9.2
9.0
8.8
1
2
t−Test: p−value = 0.35

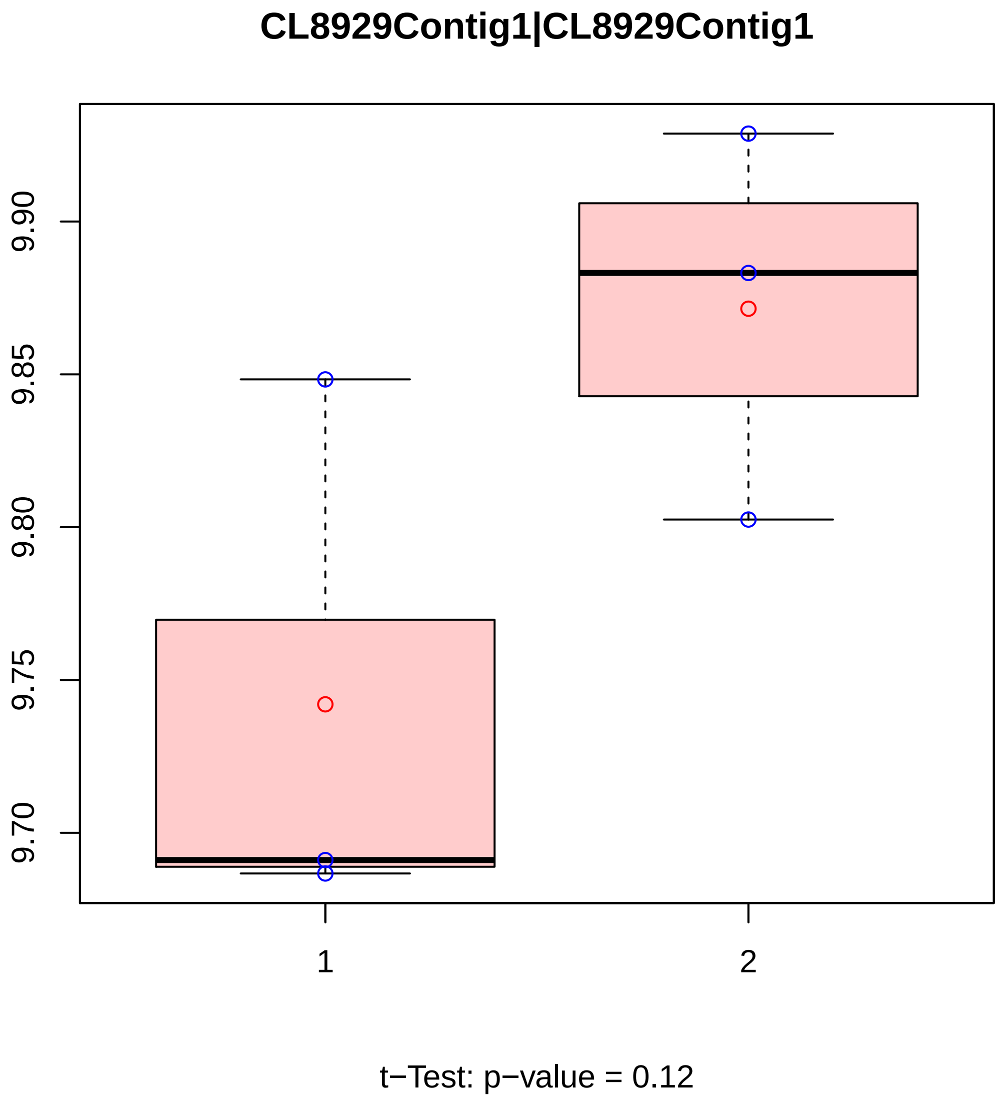
CL8929Contig1|CL8929Contig1
9.70
9.75
9.80
9.85
9.90
1
2
t−Test: p−value = 0.12

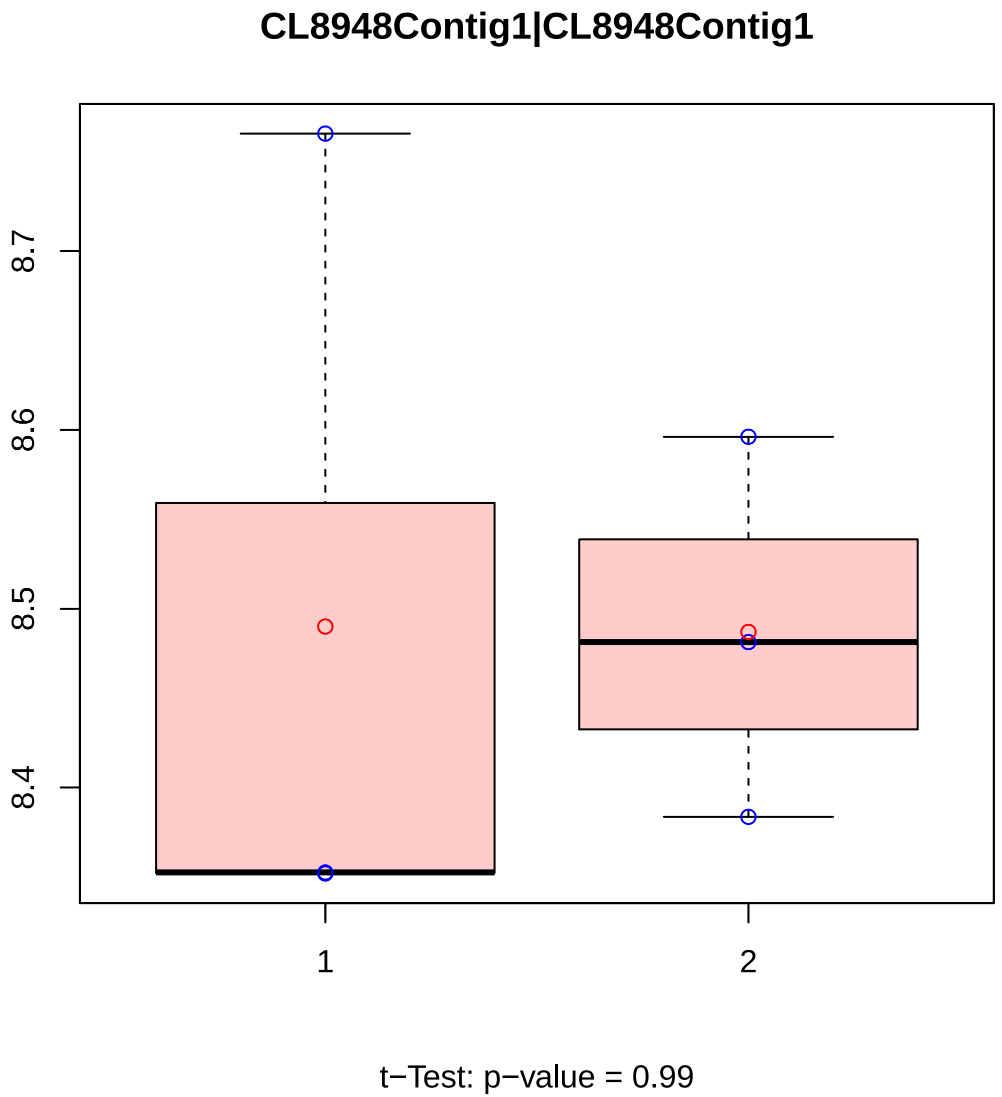
CL8948Contig1|CL8948Contig1
8.4
8.5
8.6
8.7
1
2
t−Test: p−value = 0.99

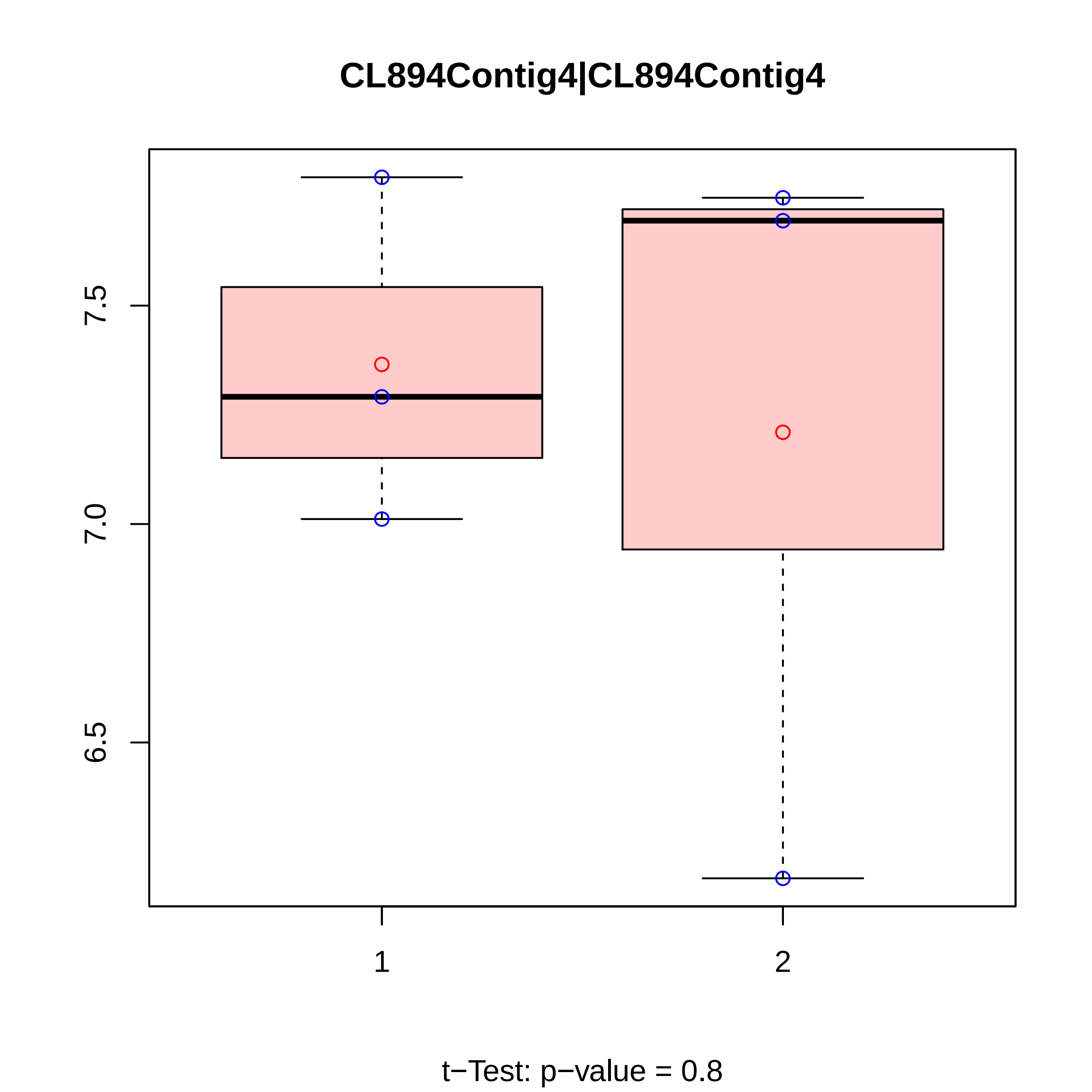

CL894Contig4|CL894Contig4
7.5
7.0
6.5
1
2
t−Test: p−value = 0.8

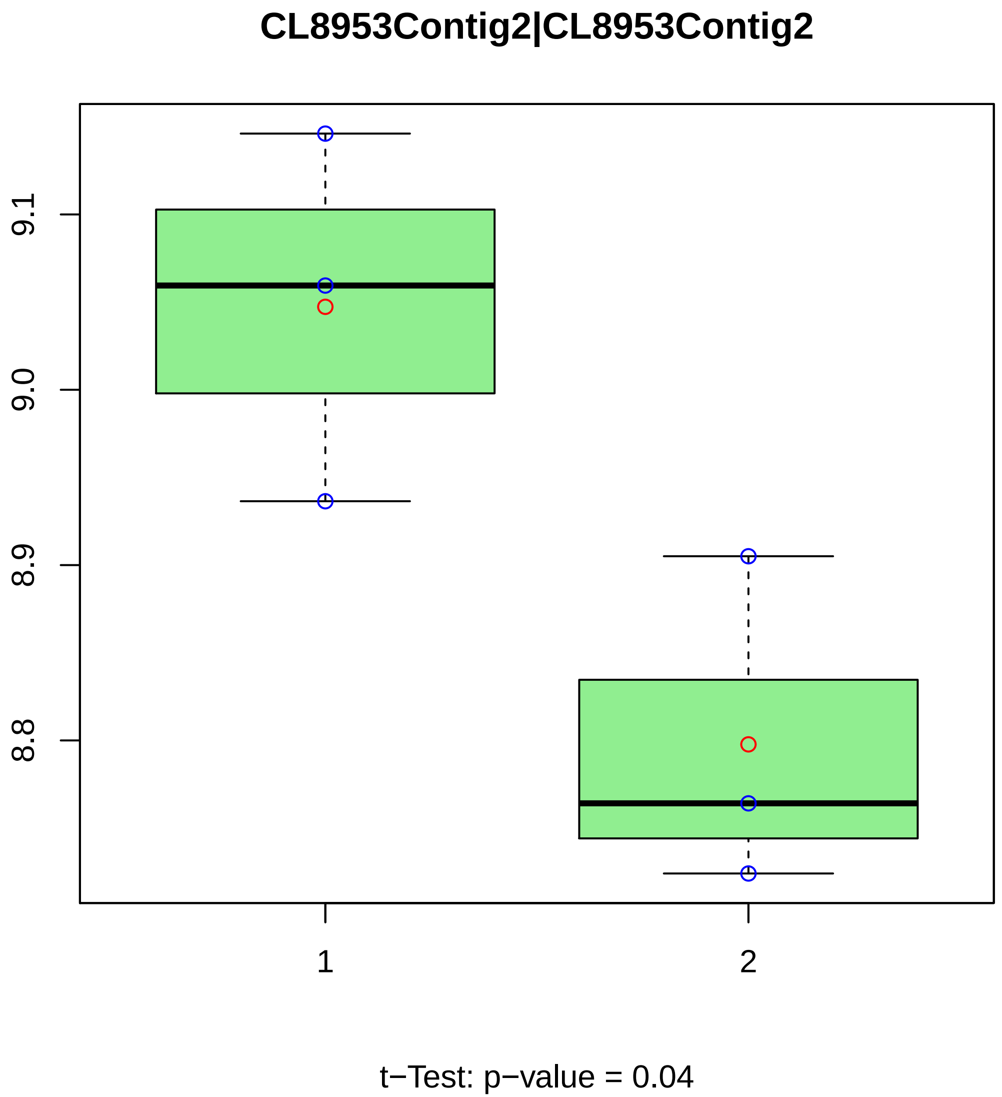
CL8953Contig2|CL8953Contig2
9.1
9.0
8.9
8.8
1
2
t−Test: p−value = 0.04

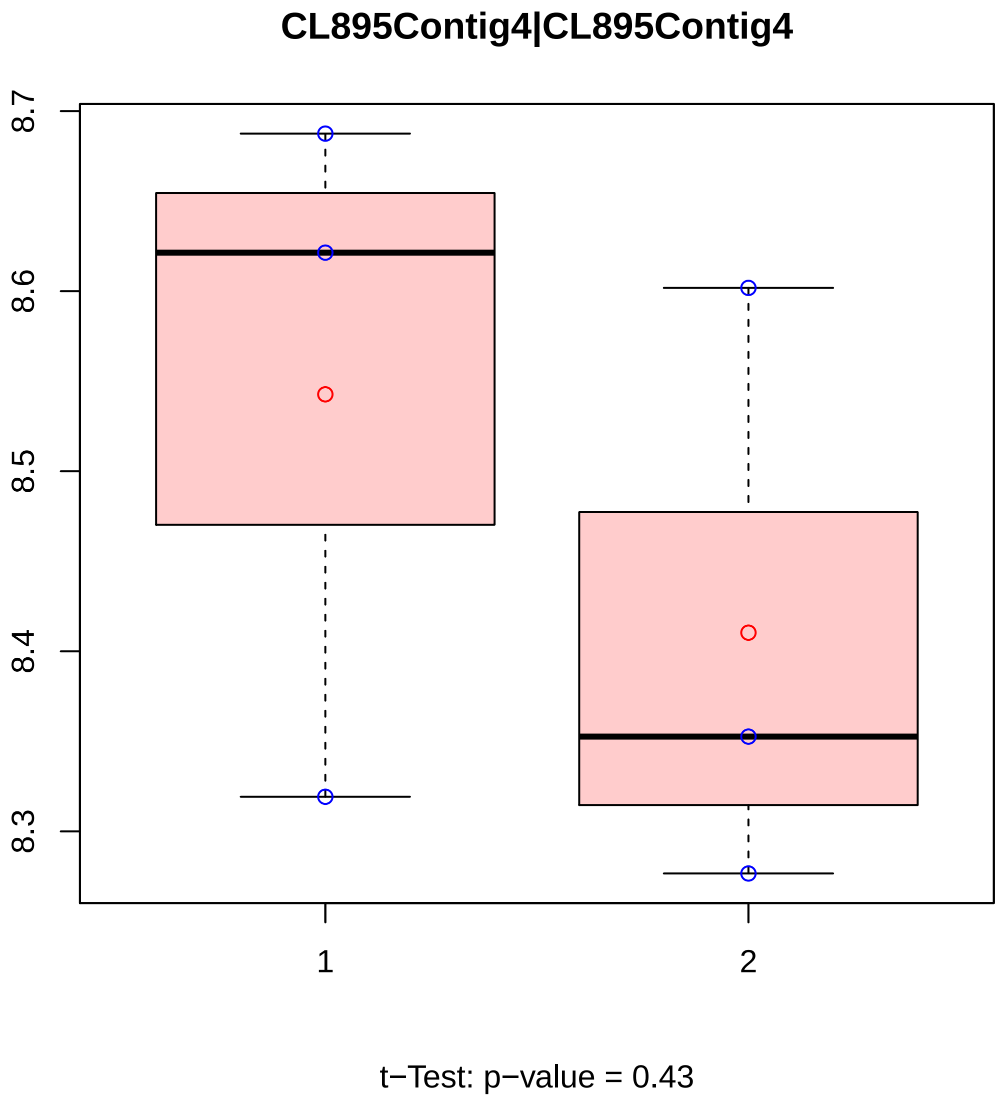
CL895Contig4|CL895Contig4
8.7
8.6
8.5
8.4
8.3
1
2
t−Test: p−value = 0.43

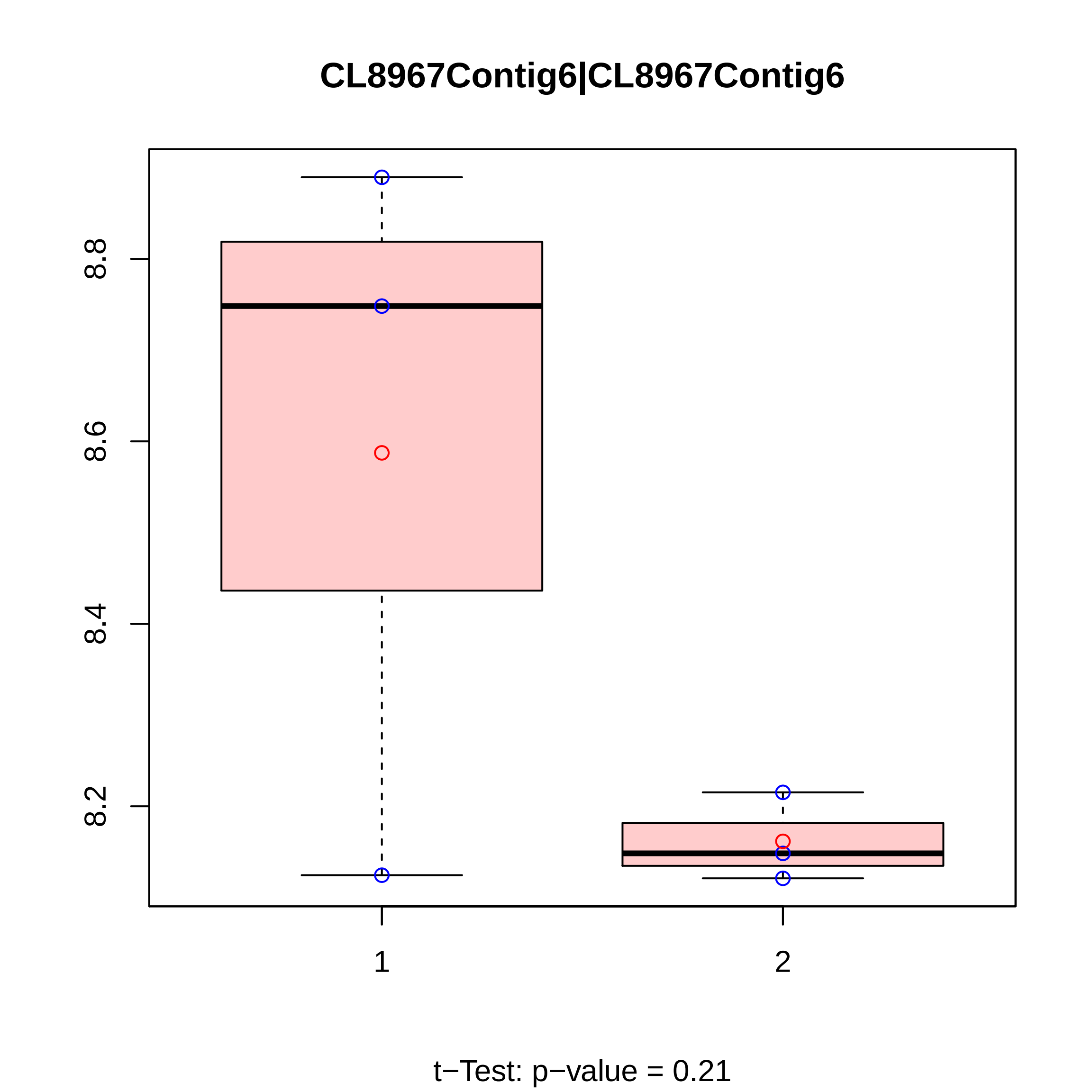

CL8967Contig6|CL8967Contig6
8.2
8.4
8.6
8.8
1
2
t−Test: p−value = 0.21

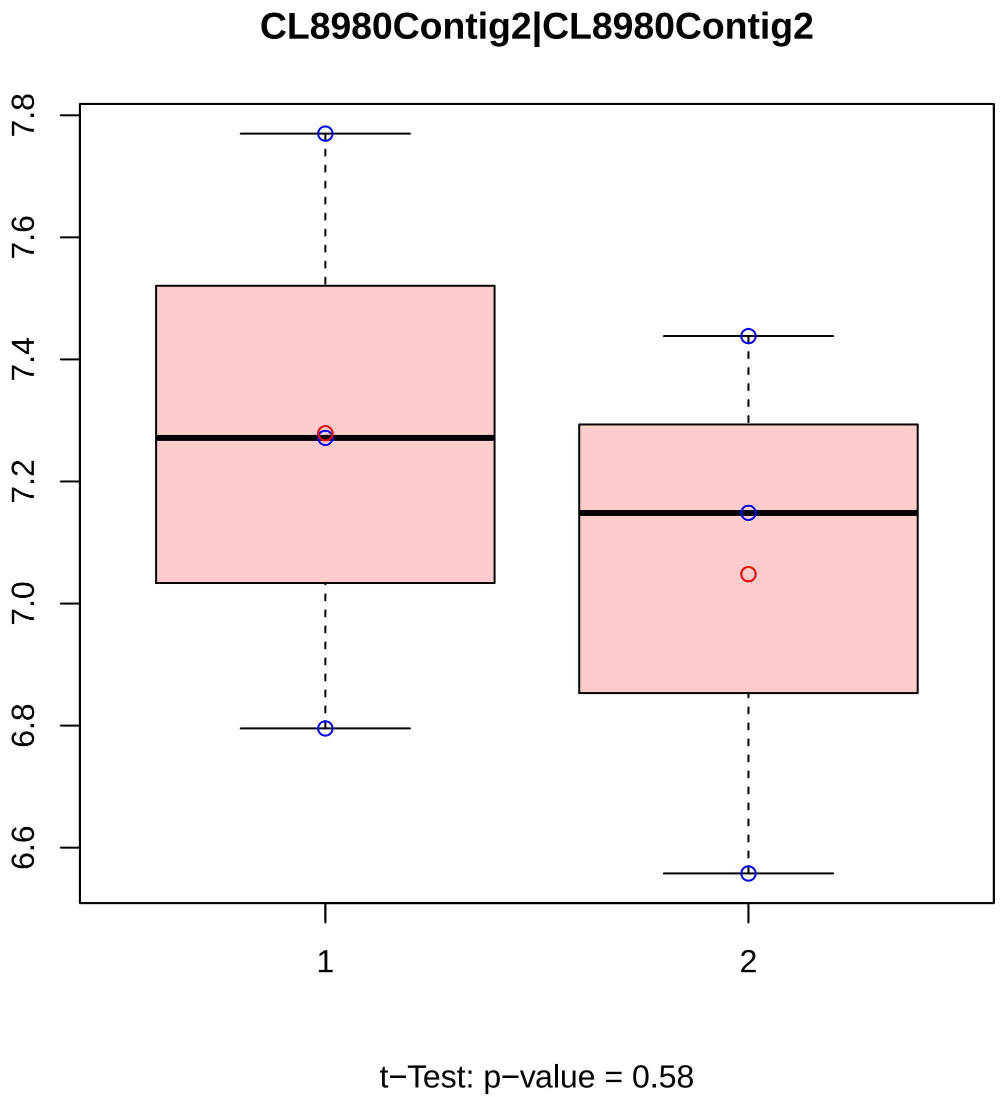
CL8980Contig2|CL8980Contig2
7.8
7.6
7.4
7.2
7.0
6.8
6.6
1
2
t−Test: p−value = 0.58

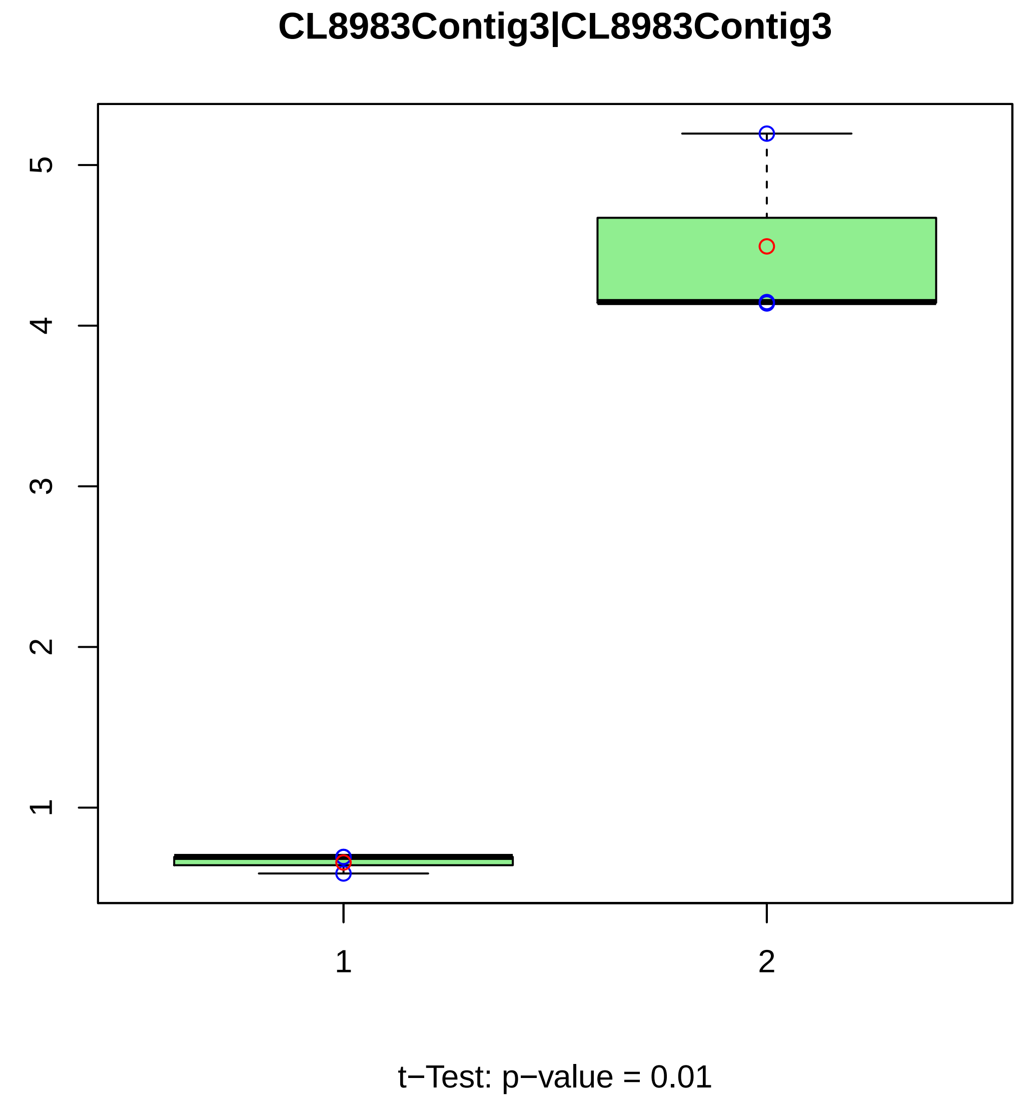
CL8983Contig3|CL8983Contig3
5
4
3
2
1
1
2
t−Test: p−value = 0.01

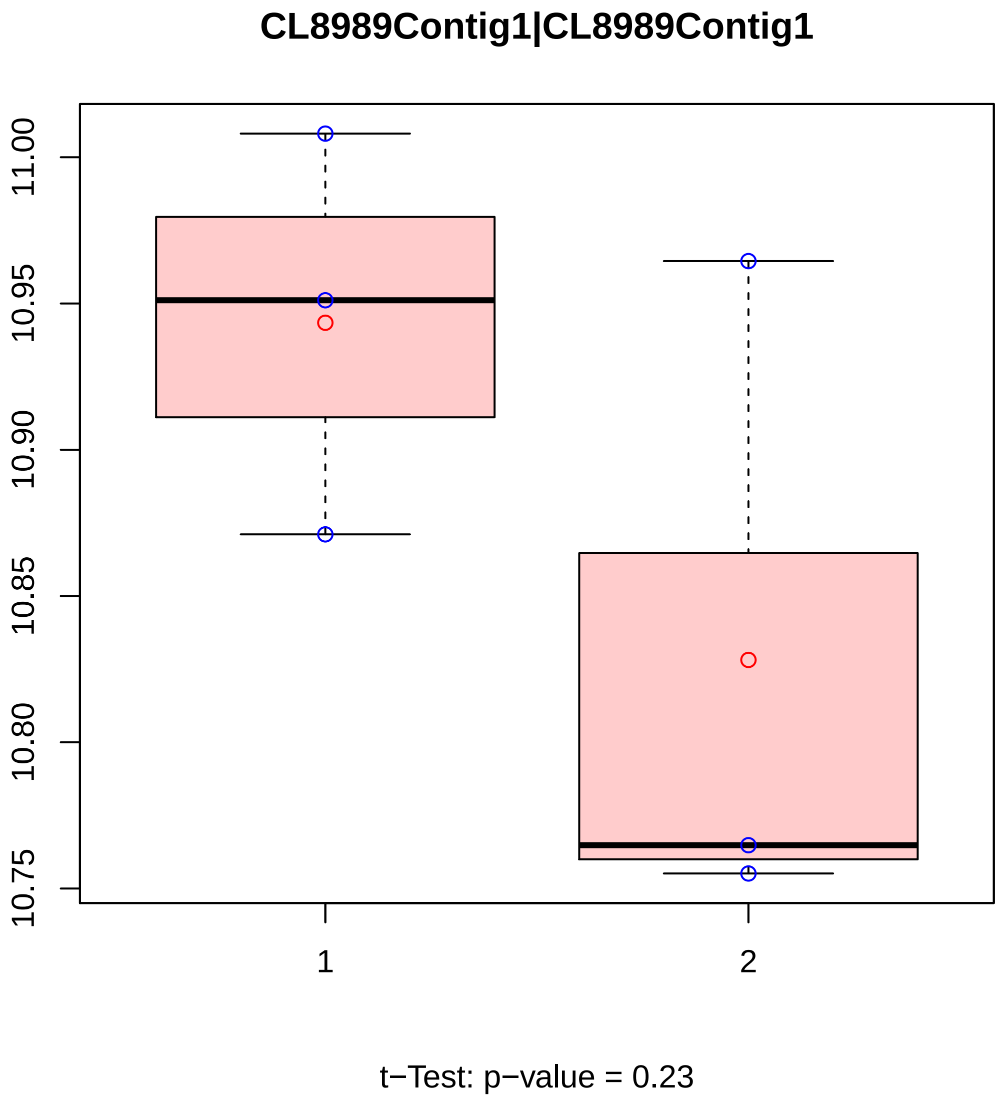

CL8989Contig1|CL8989Contig1
11.00
10.95
10.90
10.85
10.80
10.75
1
2
t−Test: p−value = 0.23

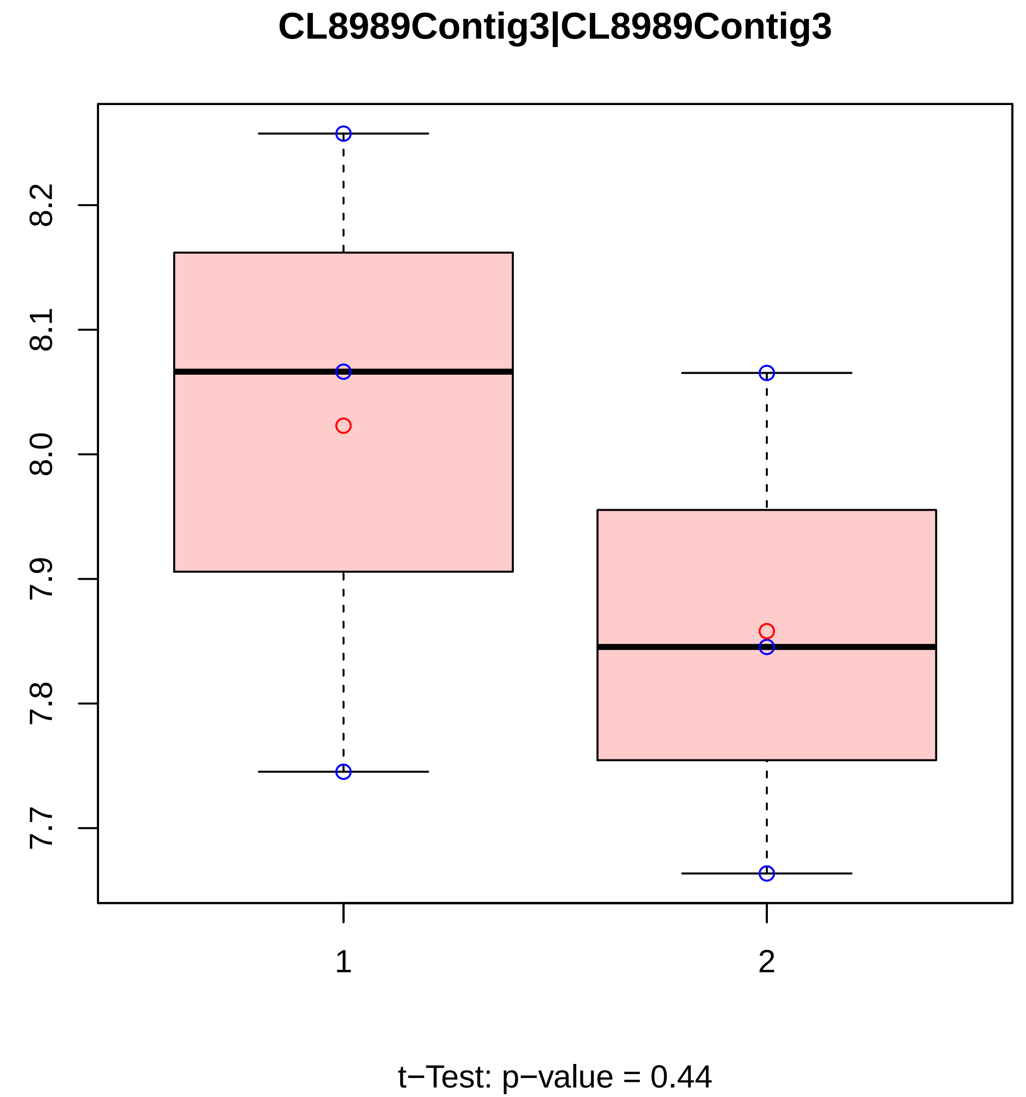
CL8989Contig3|CL8989Contig3
8.2
8.1
8.0
7.9
7.8
7.7
1
2
t−Test: p−value = 0.44

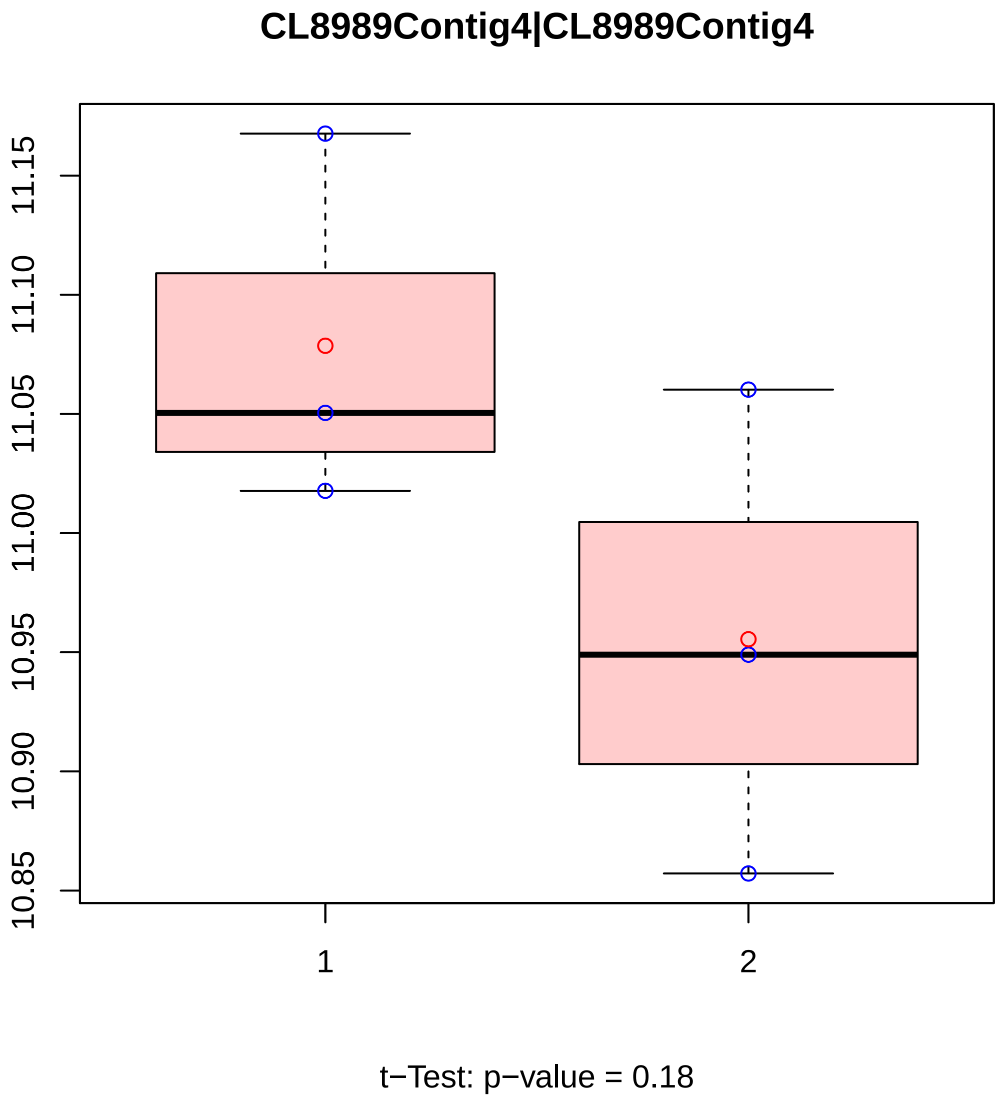

CL8989Contig4|CL8989Contig4
11.15
11.10
11.05
11.00
10.95
10.90
10.85
1
2
t−Test: p−value = 0.18

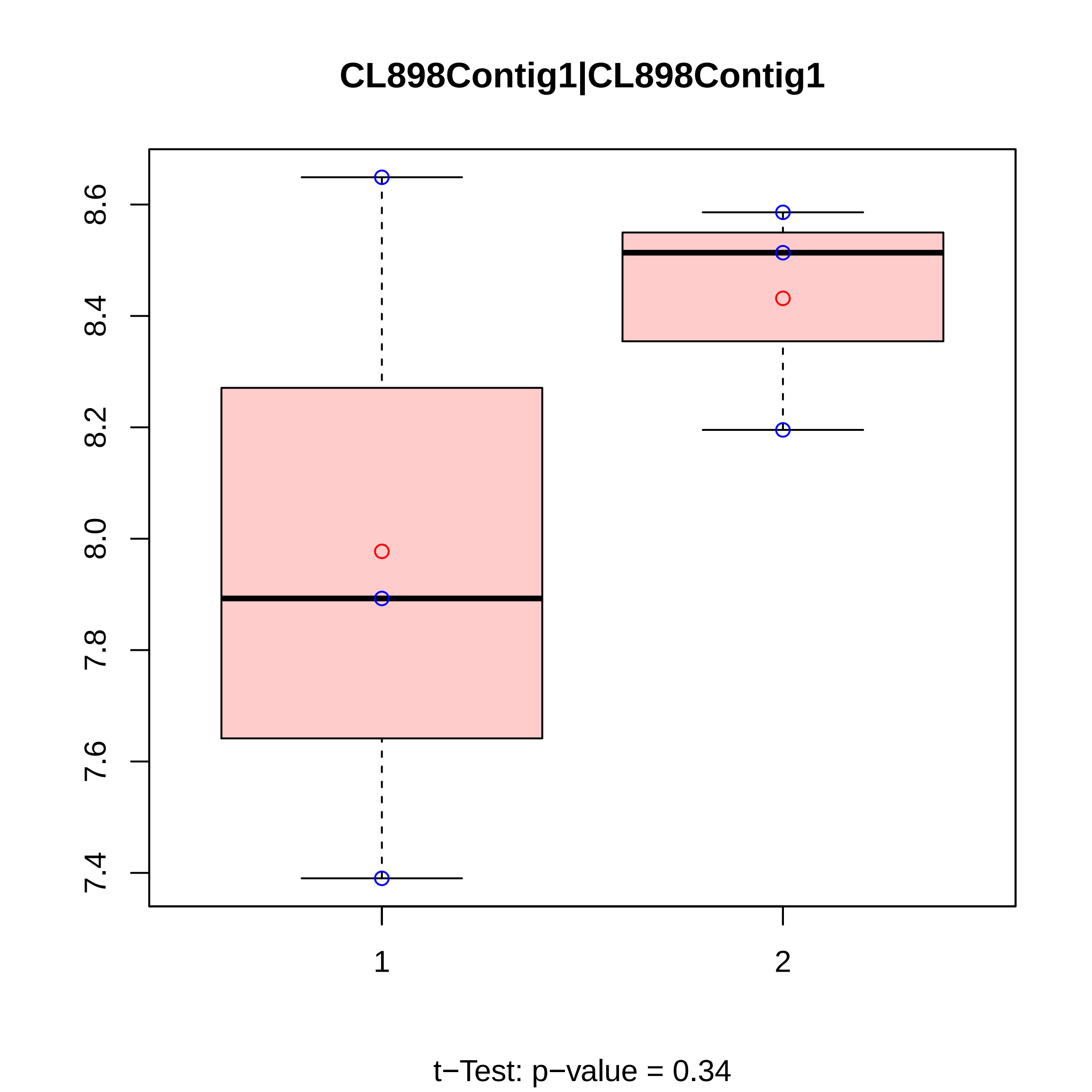

CL898Contig1|CL898Contig1
8.6
8.4
8.2
8.0
7.8
7.6
7.4
1
2
t−Test: p−value = 0.34

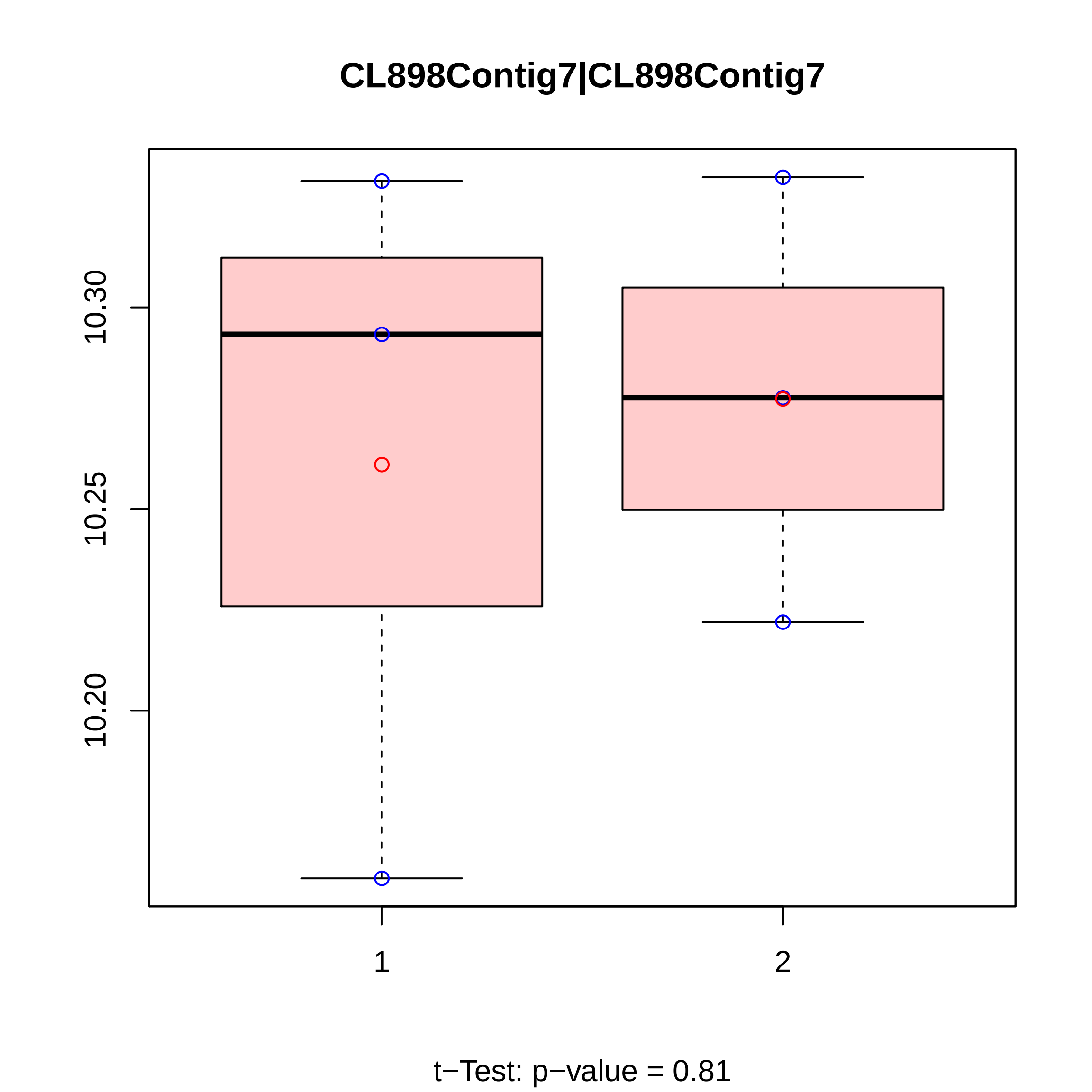

CL898Contig7|CL898Contig7
10.30
10.25
10.20
1
2
t−Test: p−value = 0.81

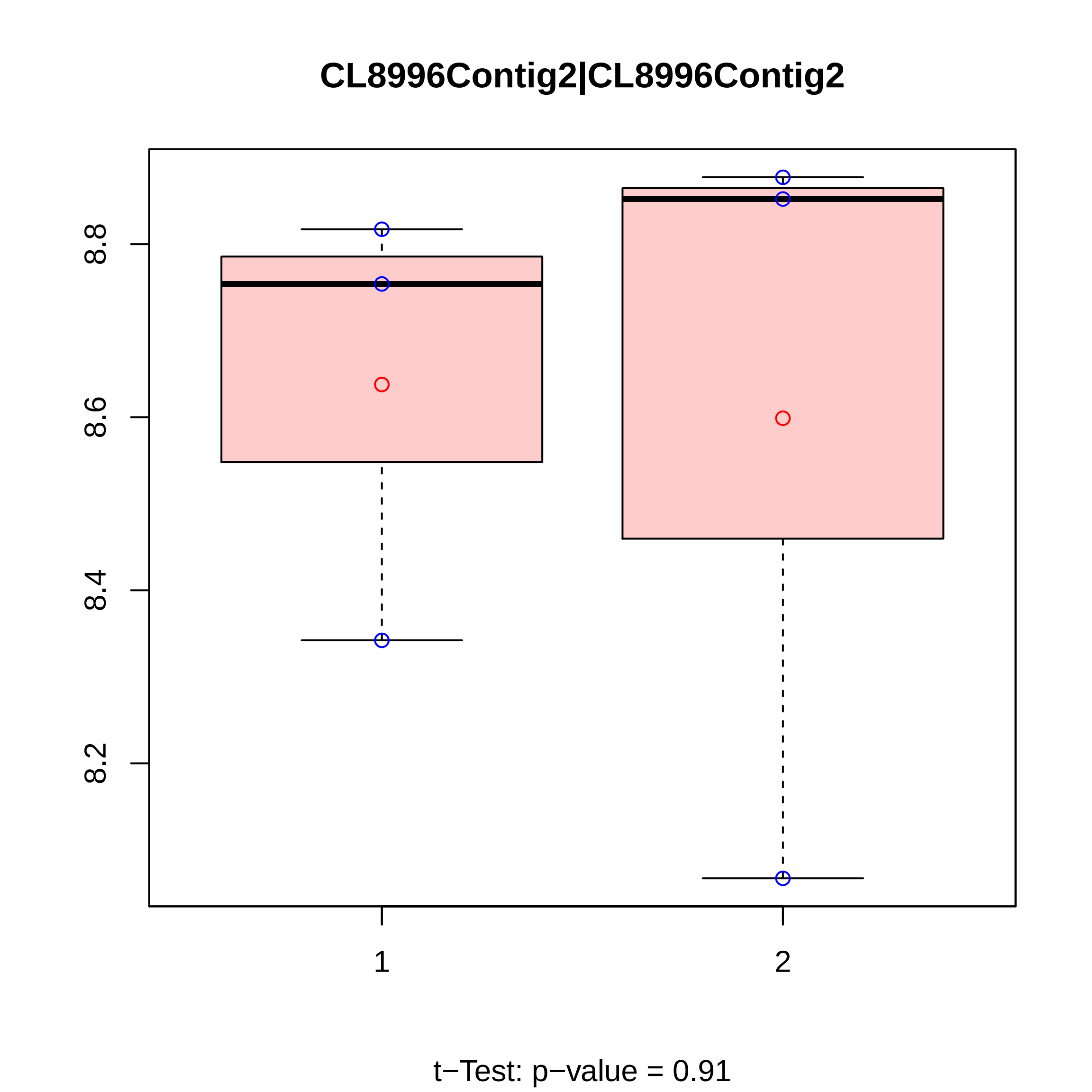

CL8996Contig2|CL8996Contig2
8.2
8.4
8.6
8.8
1
2
t−Test: p−value = 0.91

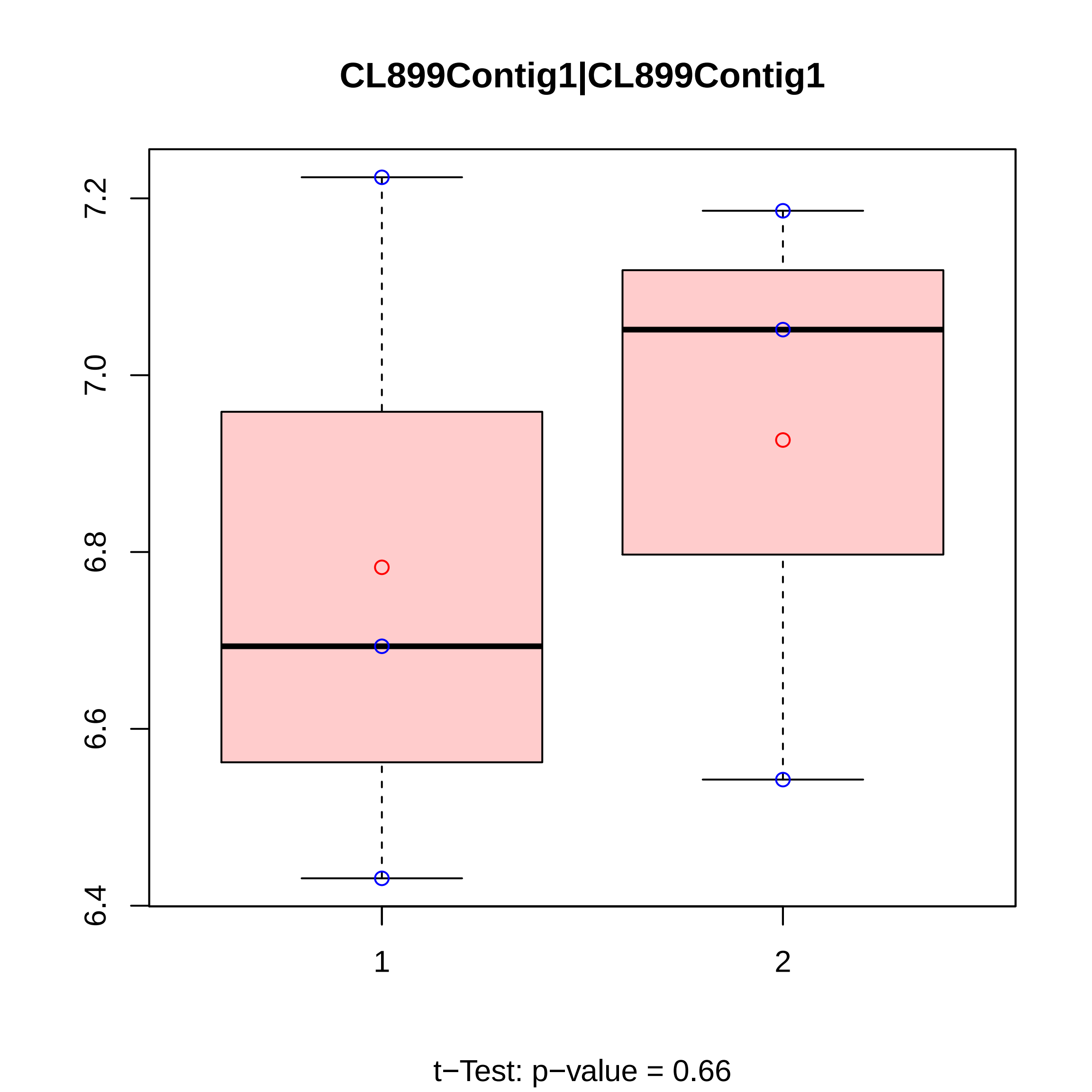
CL899Contig1|CL899Contig1
7.2
7.0
6.8
6.6
6.4
1
2
t−Test: p−value = 0.66

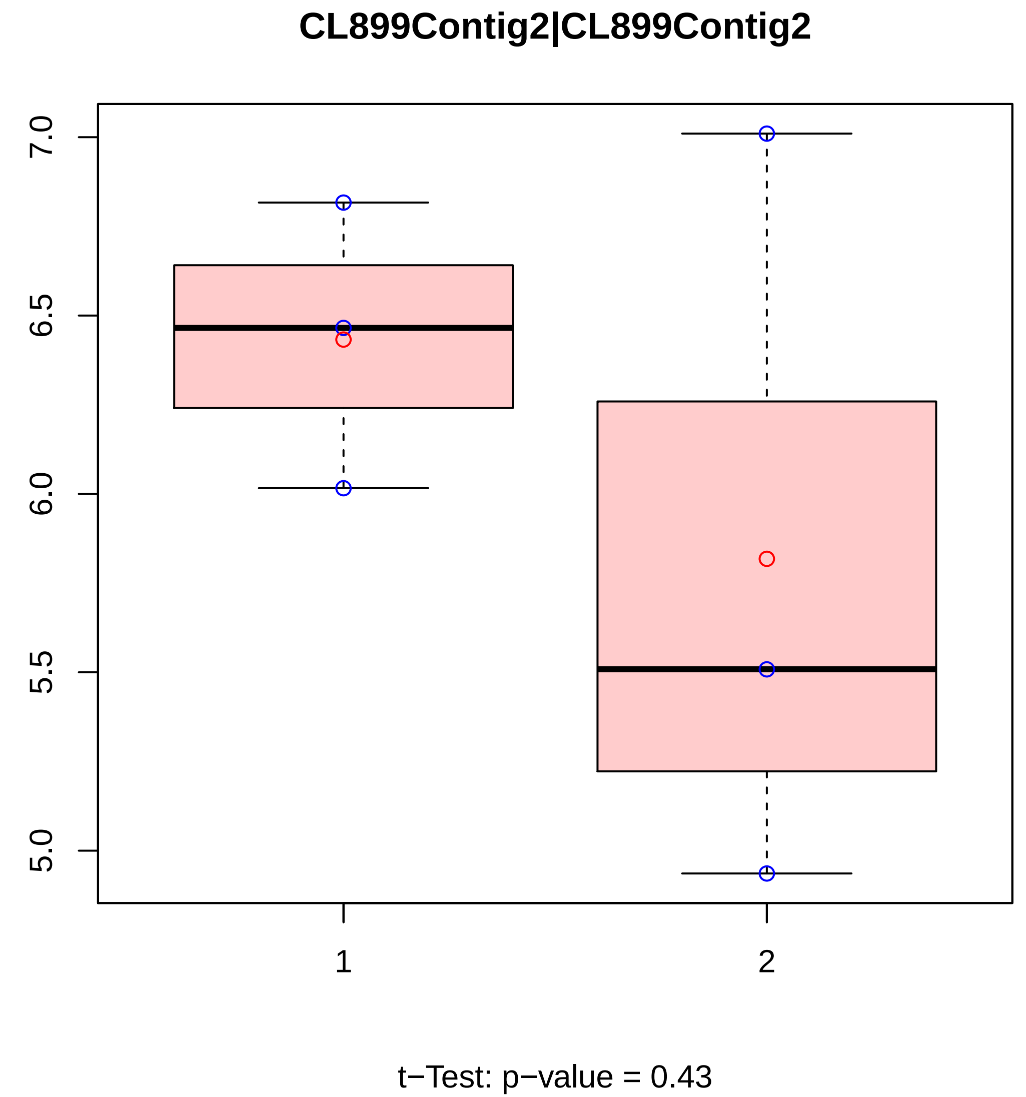
CL899Contig2|CL899Contig2
7.0
6.5
6.0
5.5
5.0
1
2
t−Test: p−value = 0.43

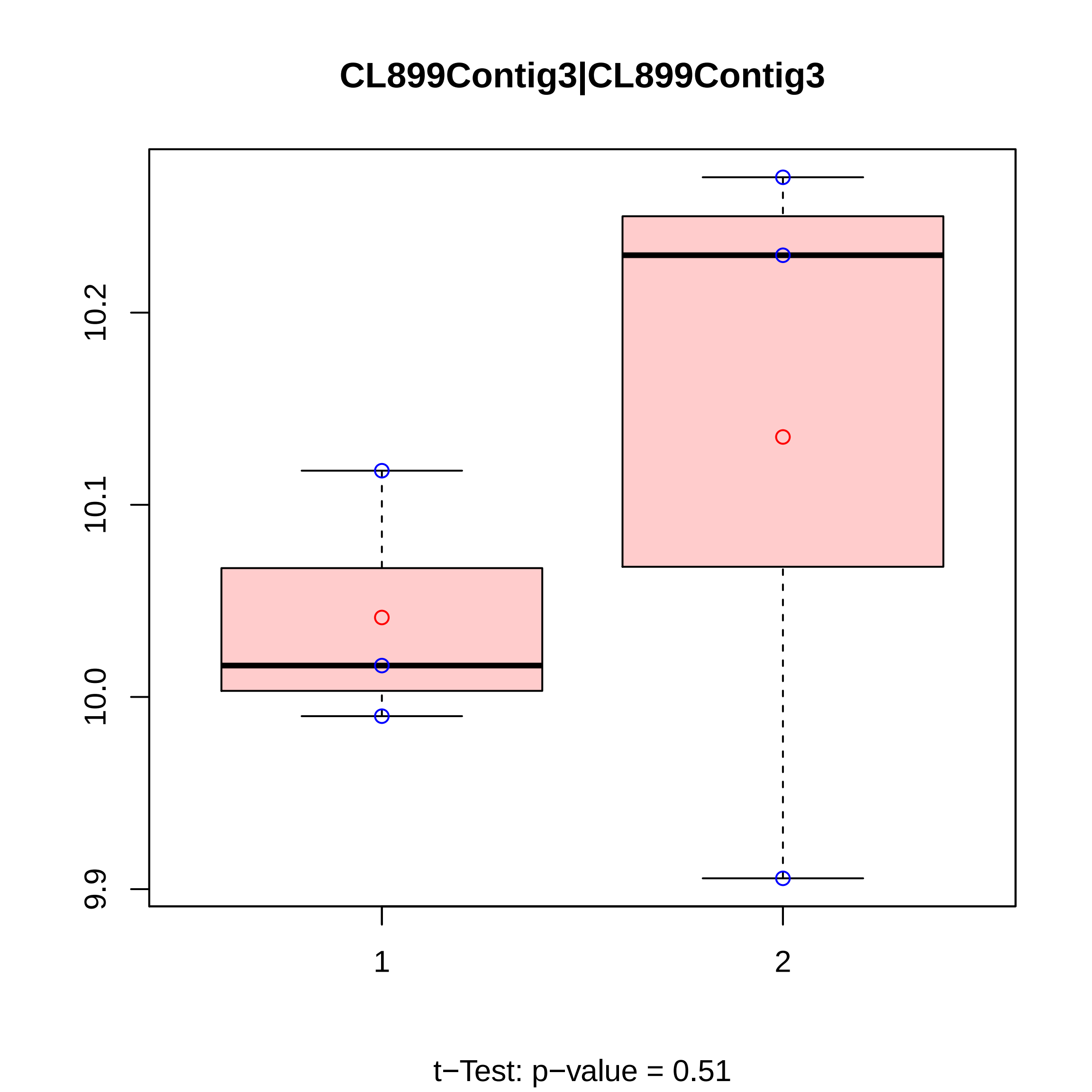

CL899Contig3|CL899Contig3
10.2
10.1
10.0
9.9
1
2
t−Test: p−value = 0.51

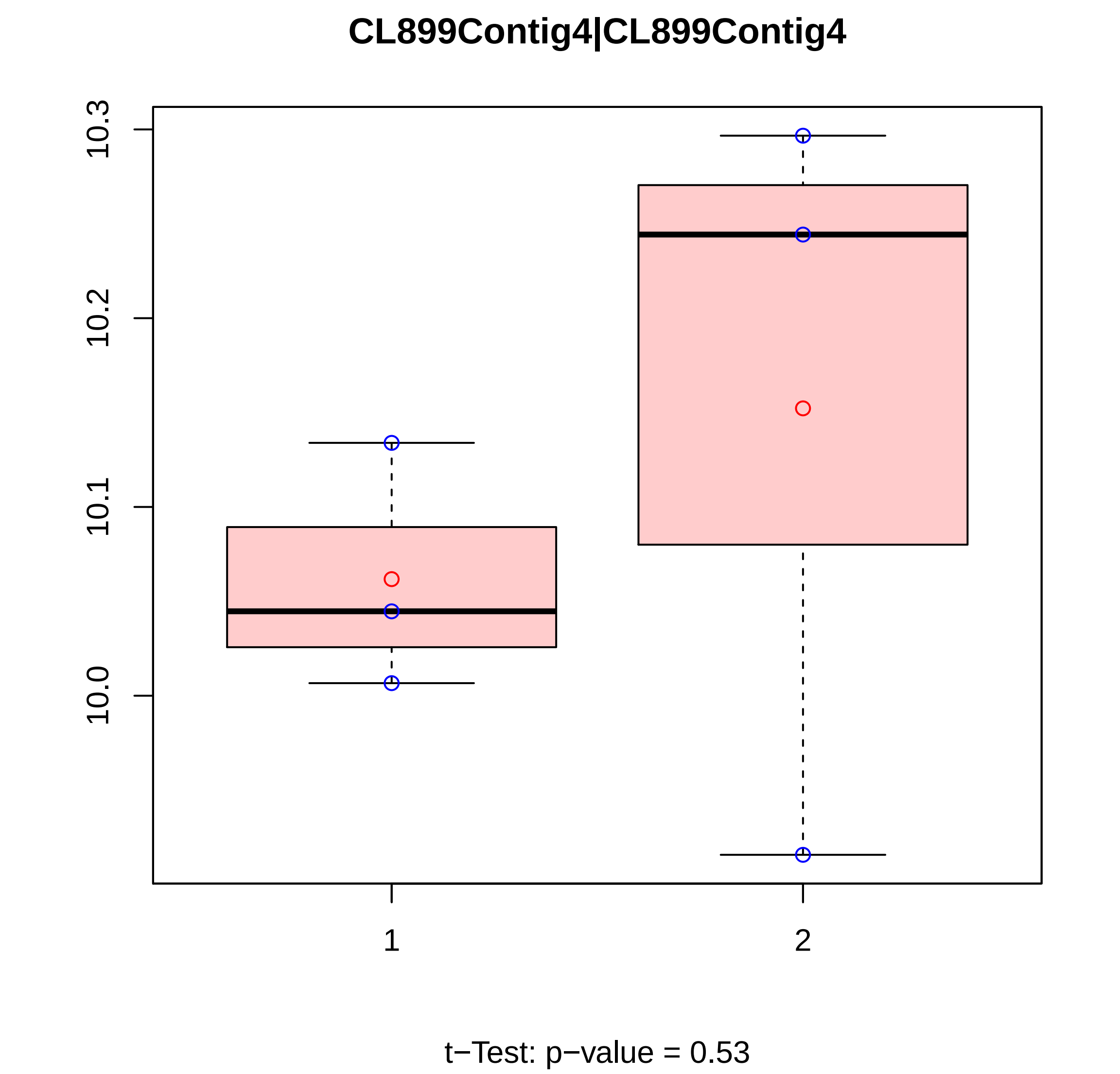
CL899Contig4|CL899Contig4
10.3
10.2
10.1
10.0
1
2
t−Test: p−value = 0.53

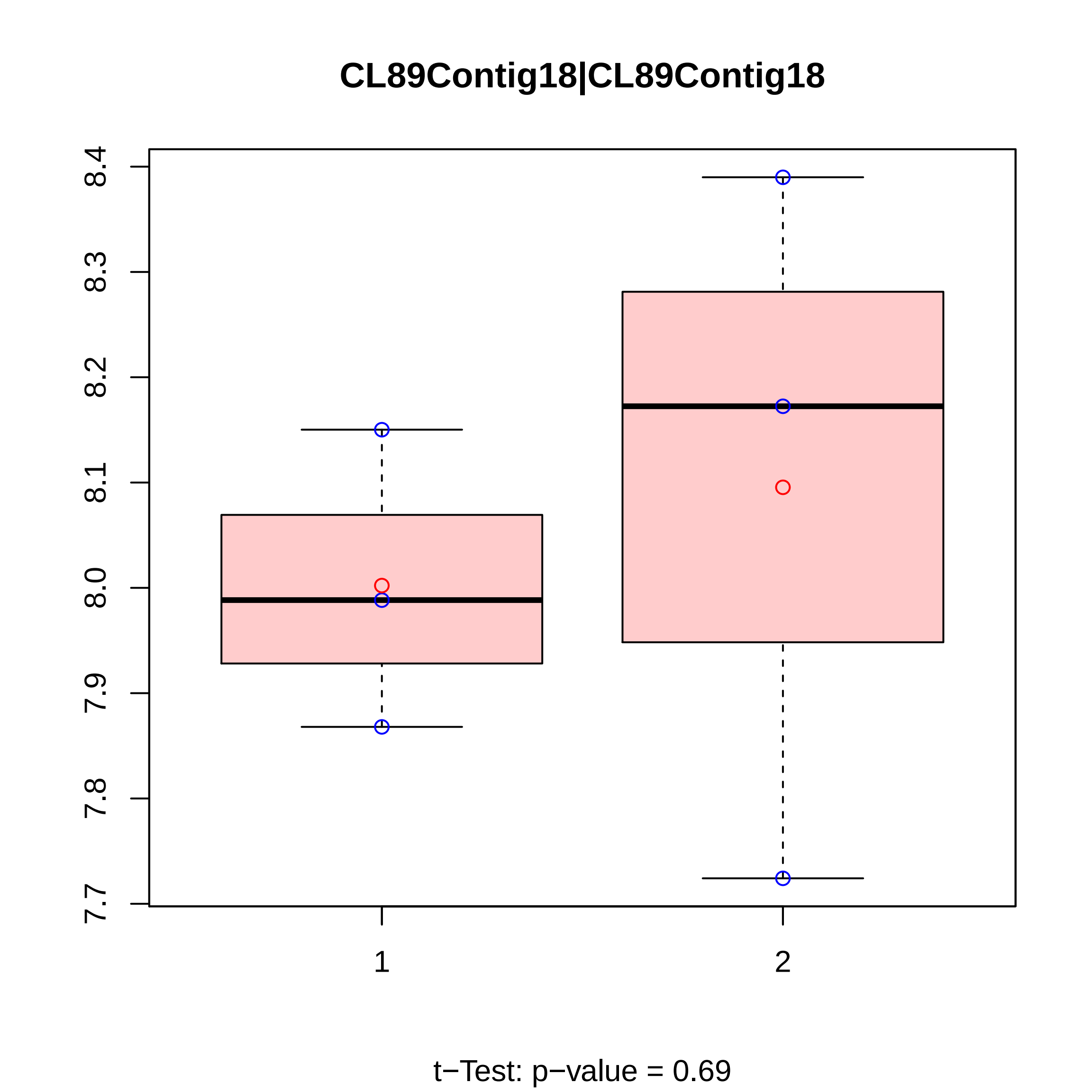

CL89Contig18|CL89Contig18
8.4
8.3
8.2
8.1
8.0
7.9
7.8
7.7
1
2
t−Test: p−value = 0.69

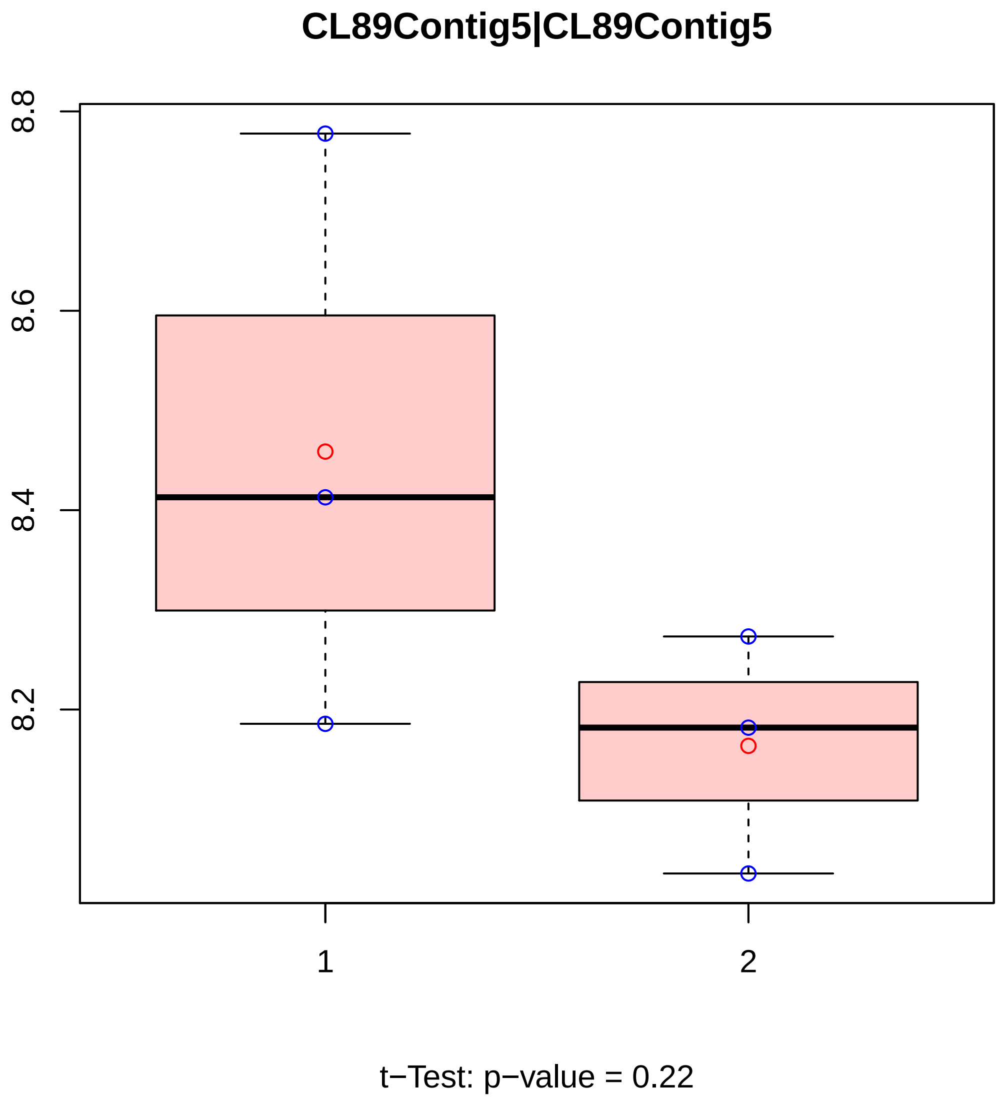

CL89Contig5|CL89Contig5
8.8
8.6
8.4
8.2
1
2
t−Test: p−value = 0.22

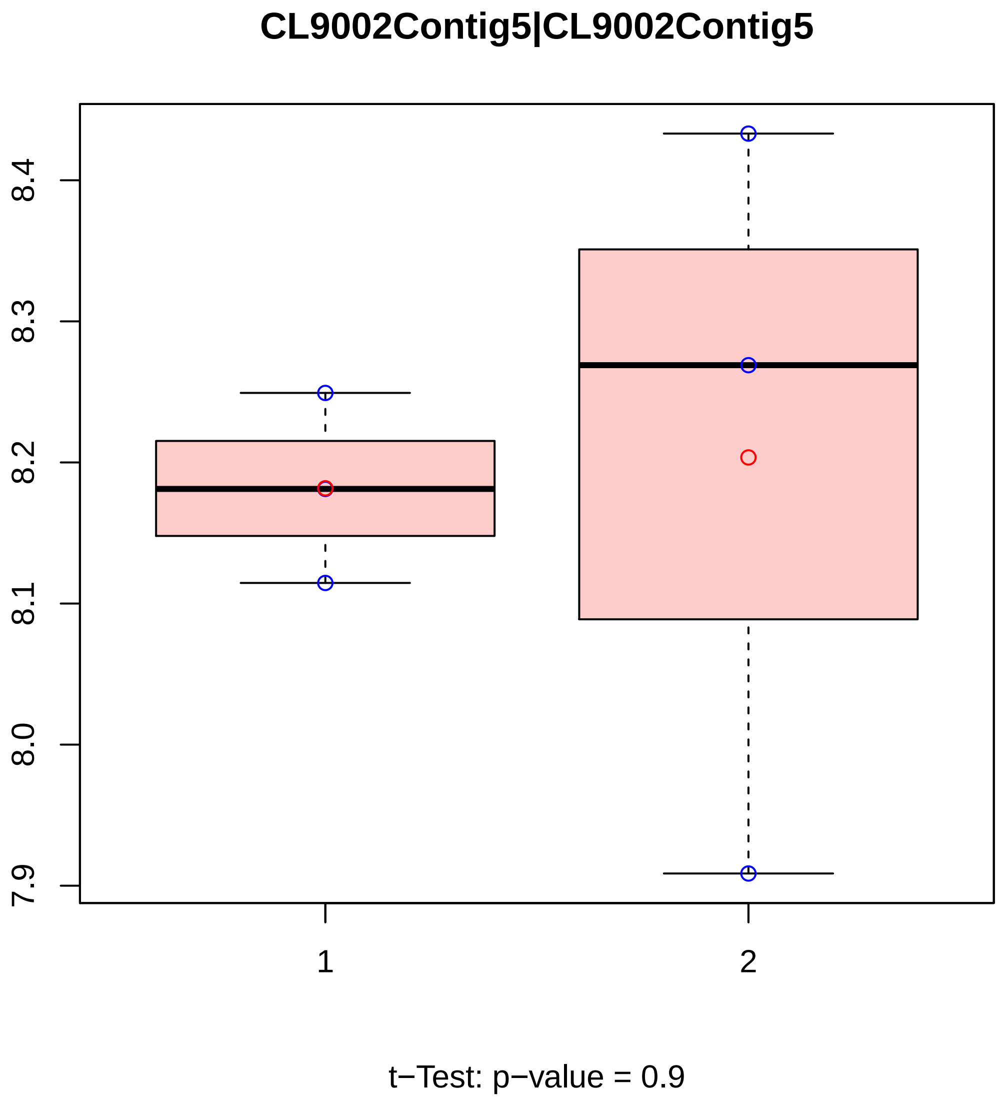
CL9002Contig5|CL9002Contig5
8.4
8.3
8.2
8.1
8.0
7.9
1
2
t−Test: p−value = 0.9

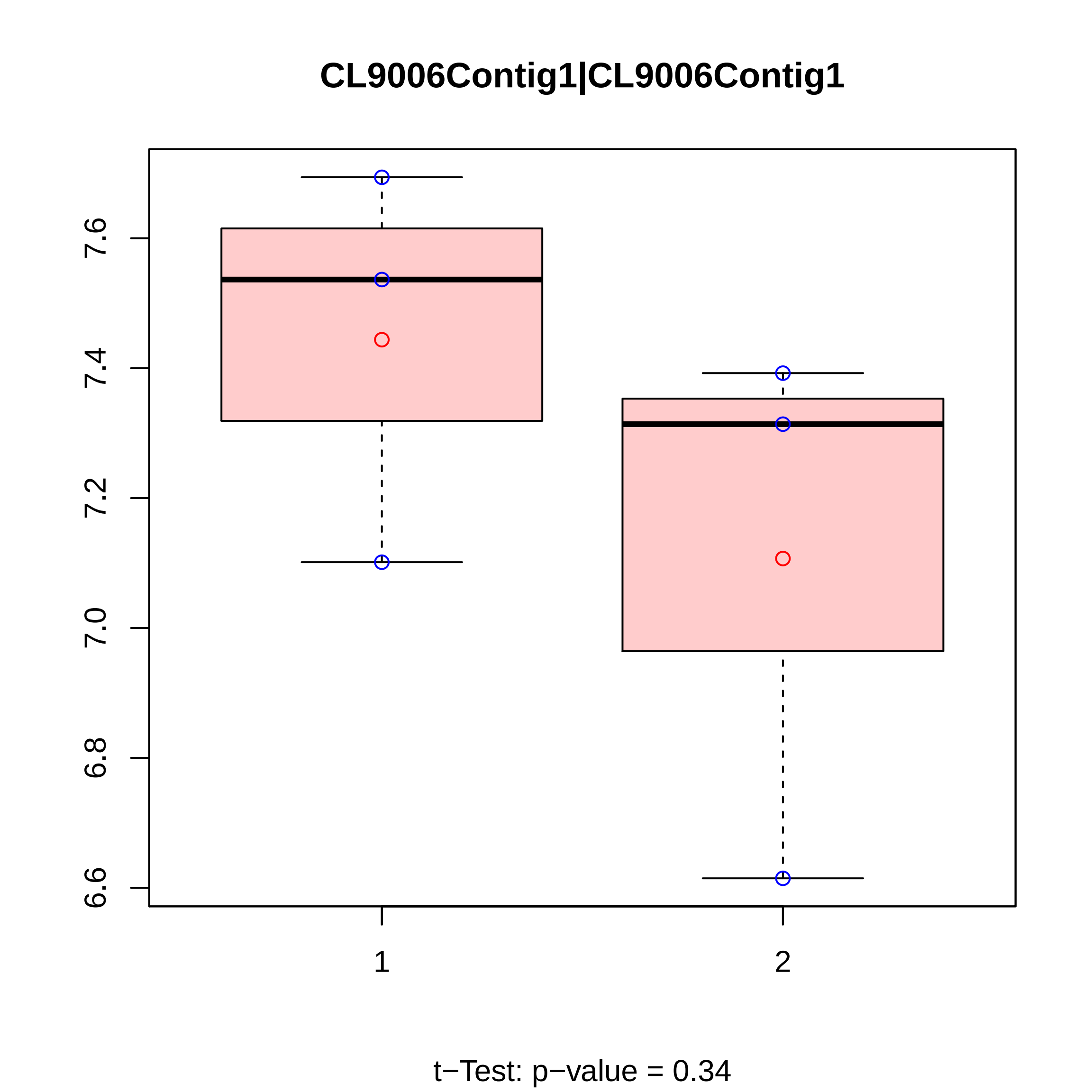

CL9006Contig1|CL9006Contig1
7.6
7.4
7.2
7.0
6.8
6.6
1
2
t−Test: p−value = 0.34

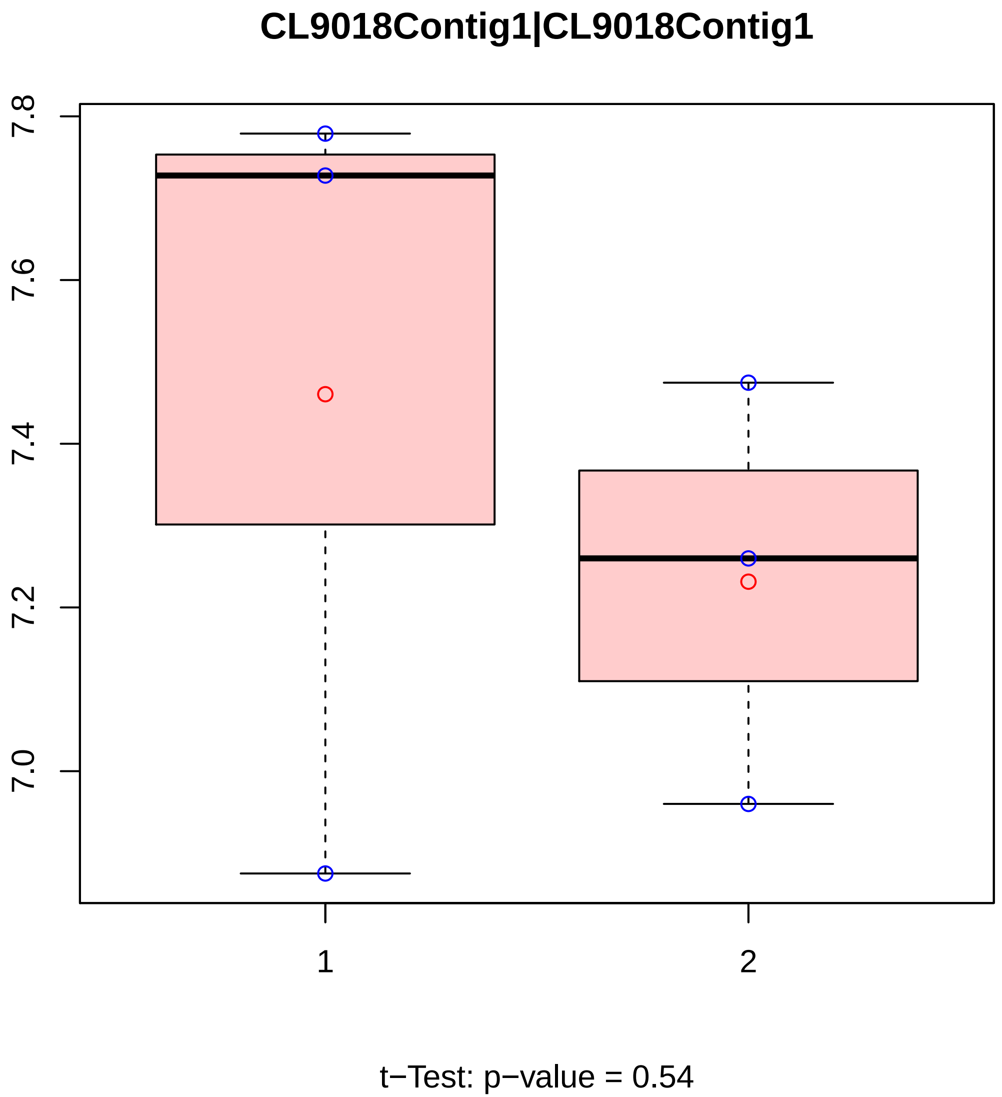

CL9018Contig1|CL9018Contig1
7.8
7.6
7.4
7.2
7.0
1
2
t−Test: p−value = 0.54

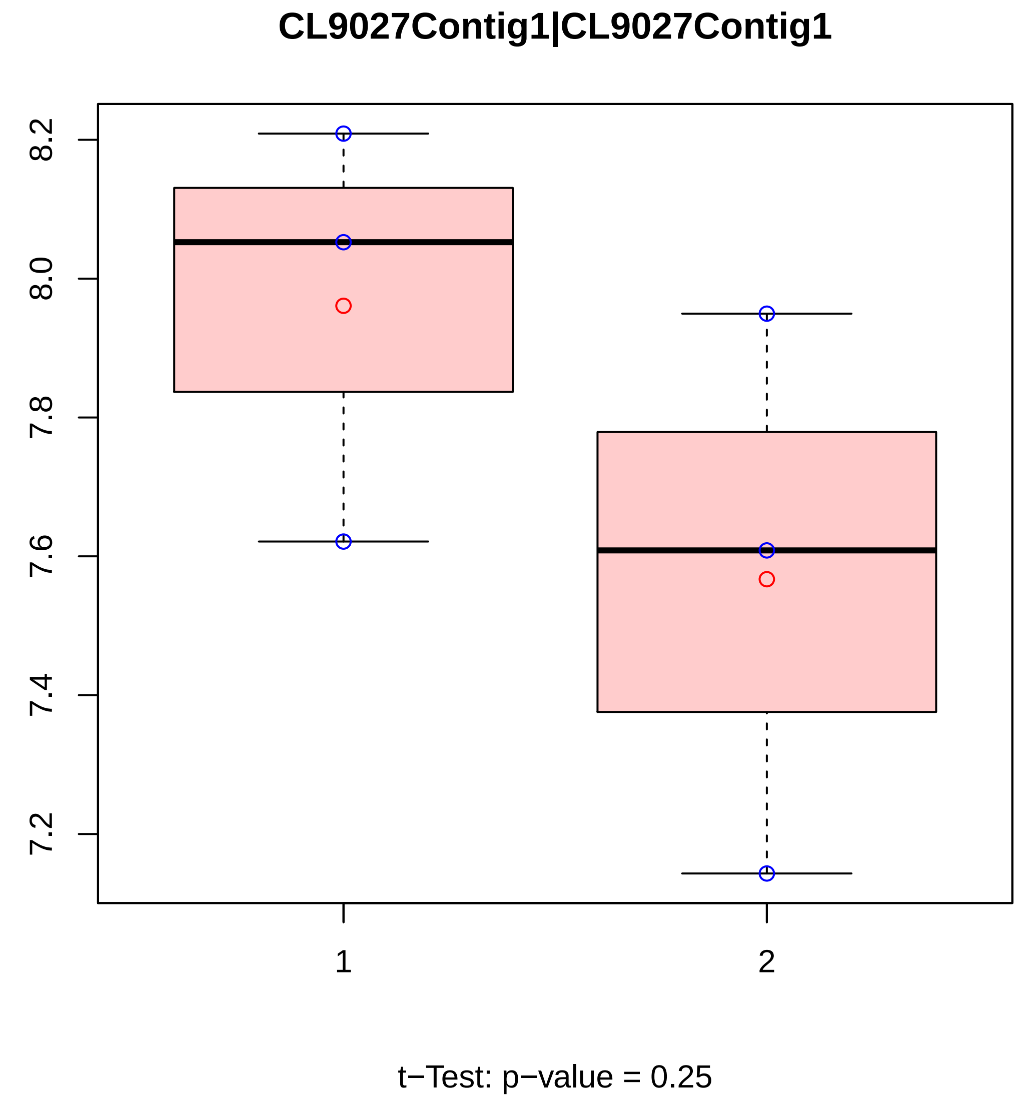
CL9027Contig1|CL9027Contig1
8.2
8.0
7.8
7.6
7.4
7.2
1
2
t−Test: p−value = 0.25

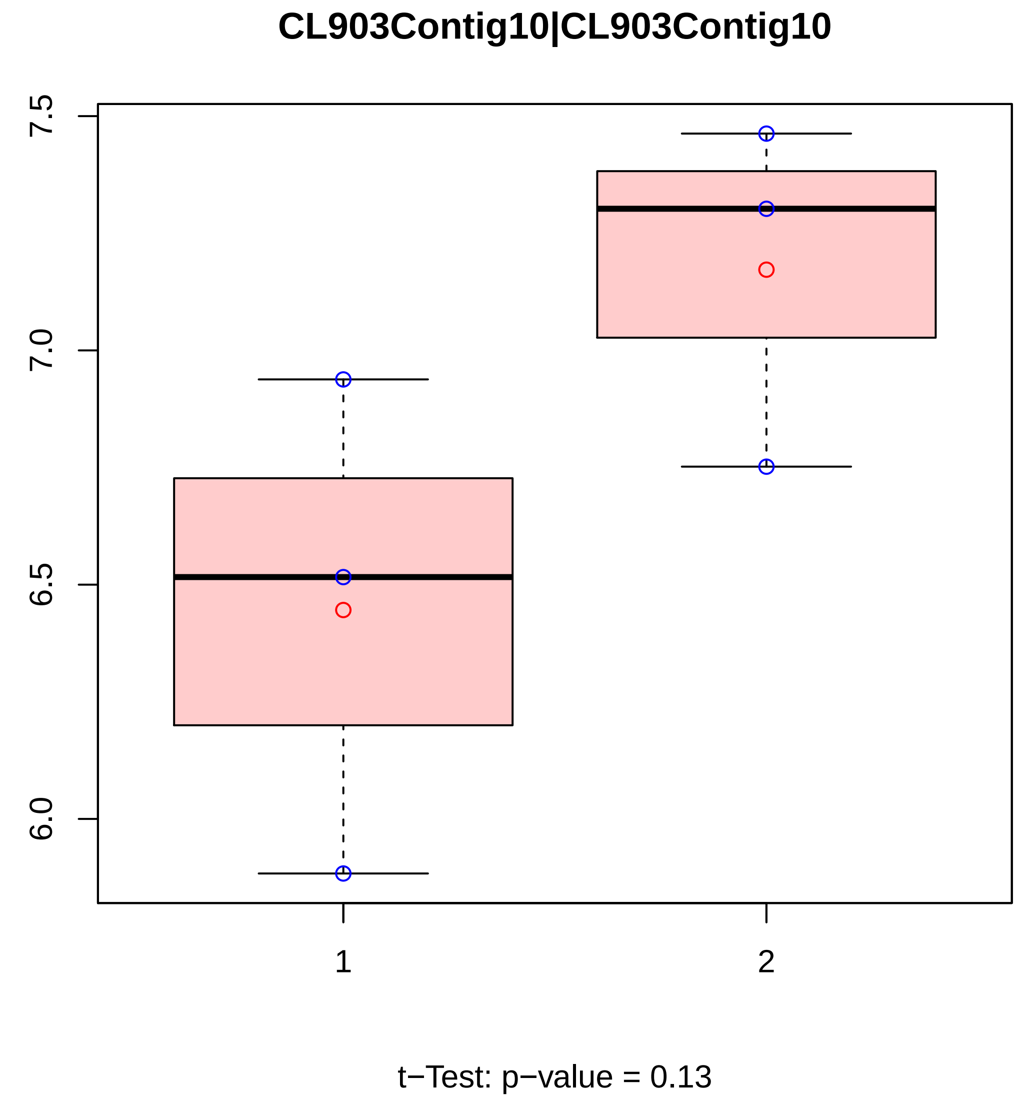
CL903Contig10|CL903Contig10
7.5
7.0
6.5
6.0
1
2
t−Test: p−value = 0.13

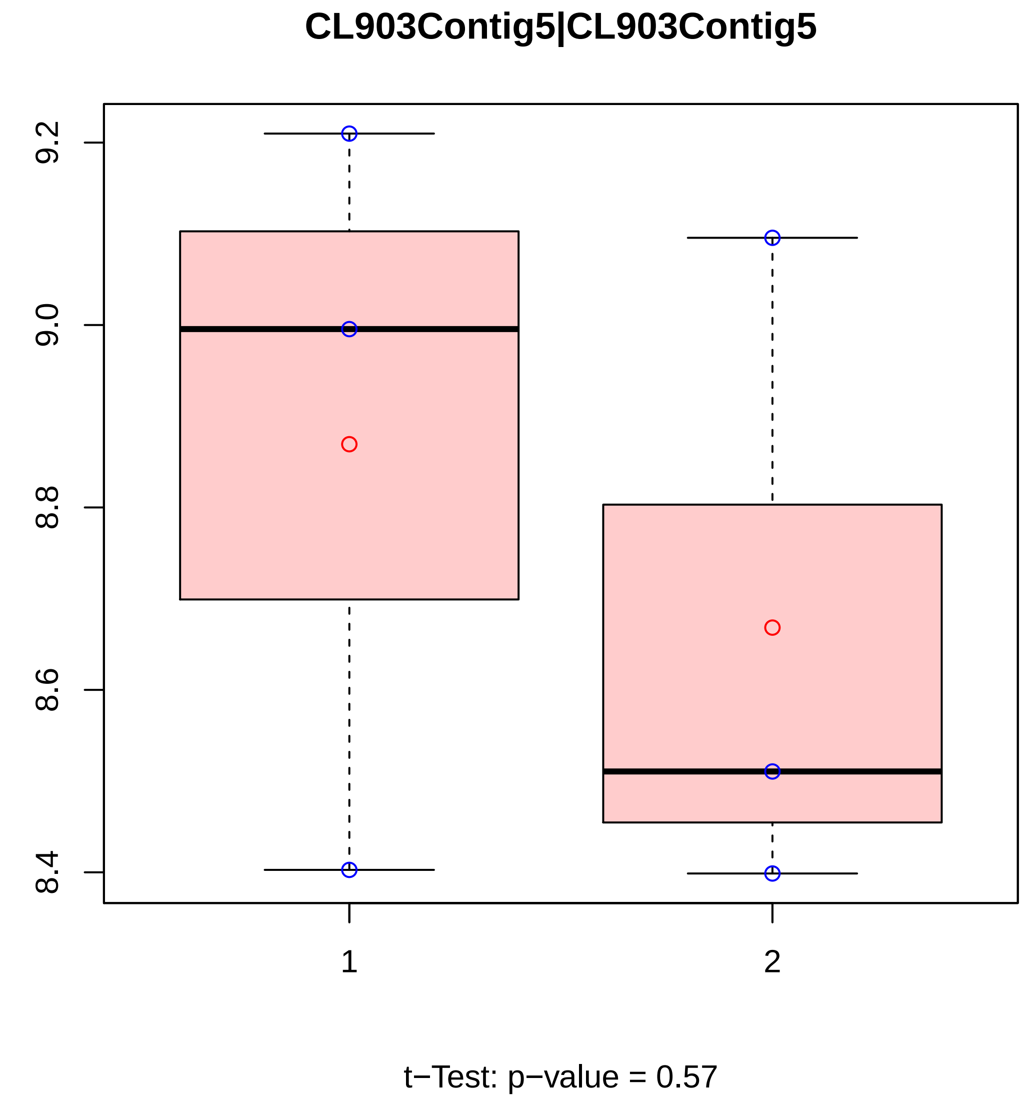
CL903Contig5|CL903Contig5
9.2
9.0
8.8
8.6
8.4
1
2
t−Test: p−value = 0.57

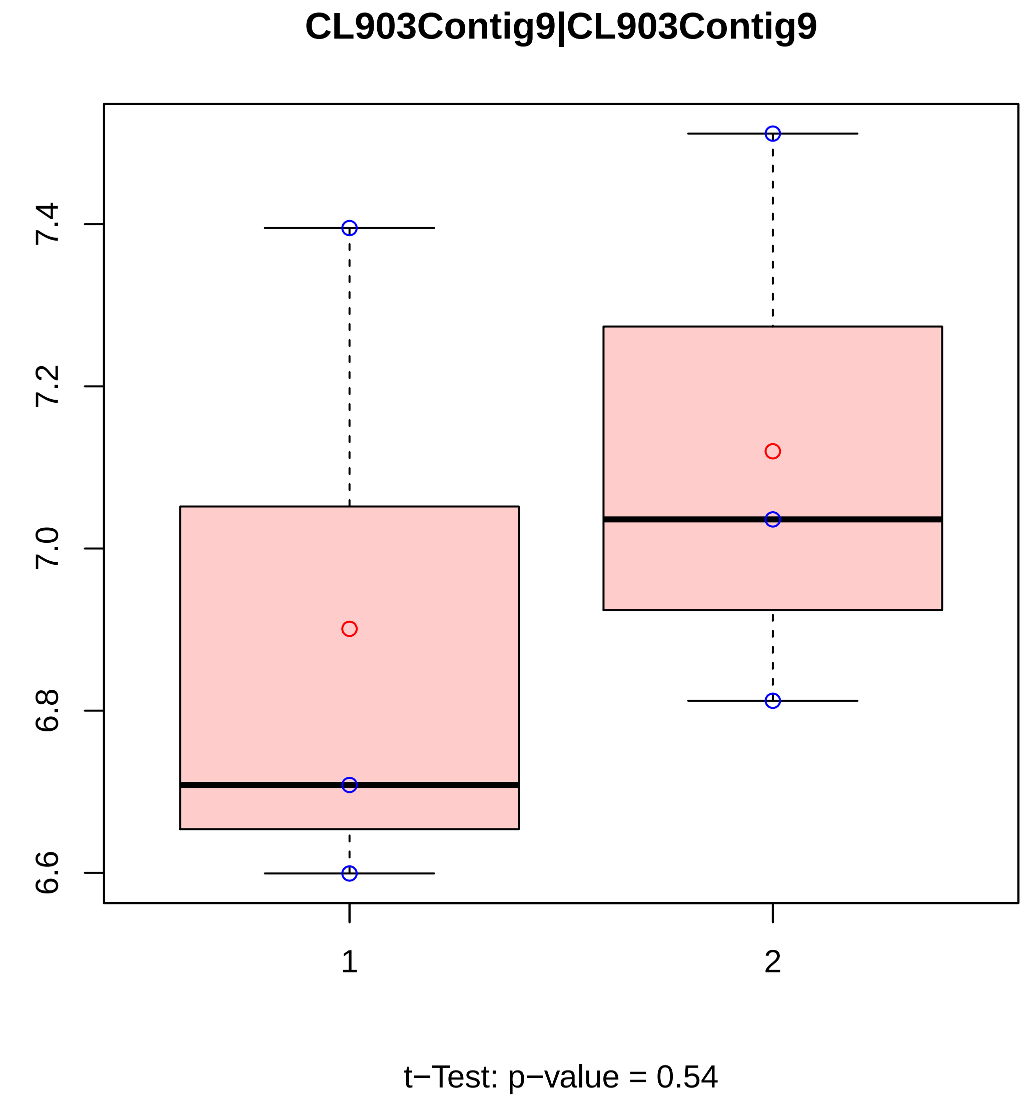

CL903Contig9|CL903Contig9
7.4
7.2
7.0
6.8
6.6
1
2
t−Test: p−value = 0.54

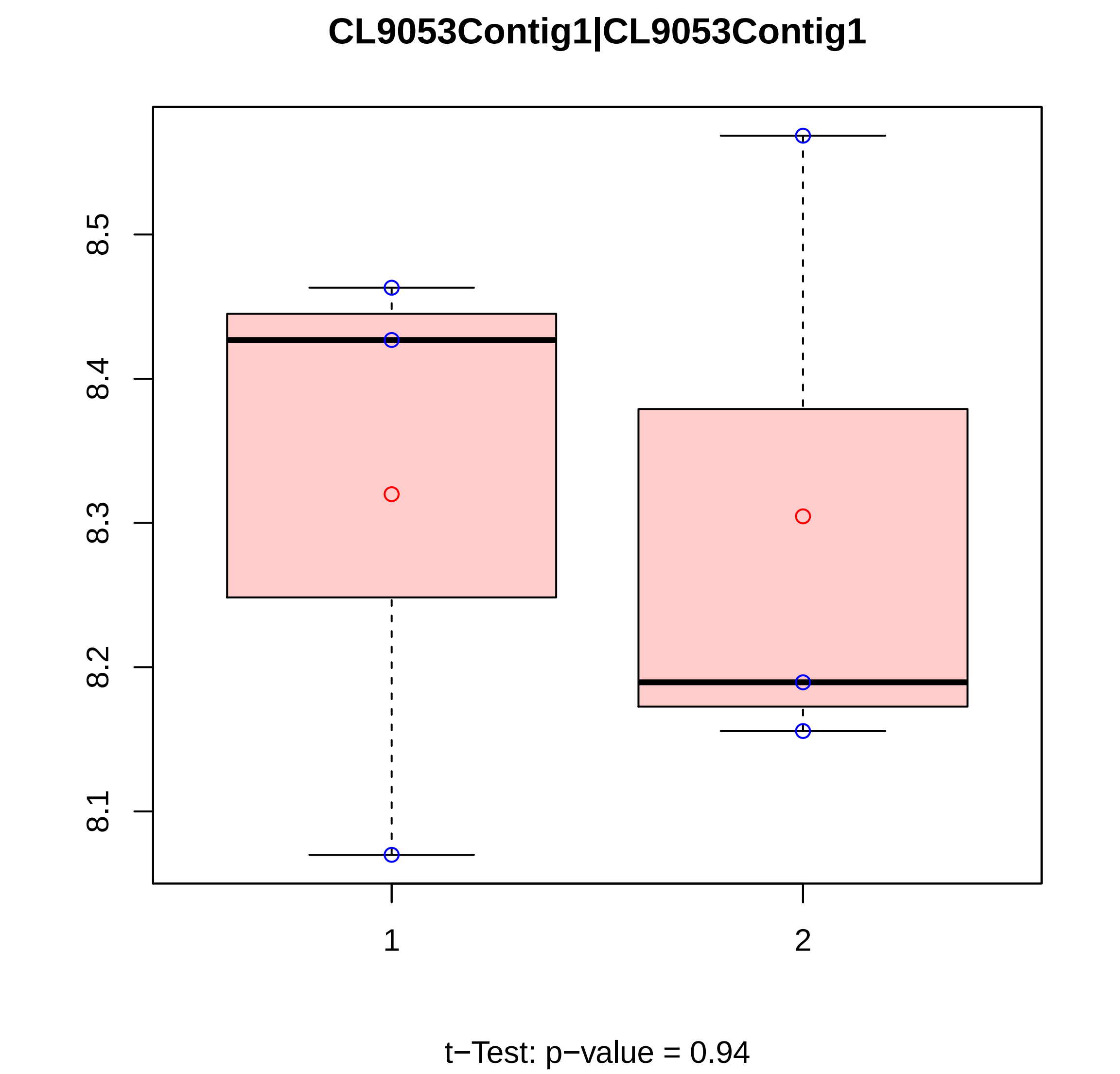

CL9053Contig1|CL9053Contig1
8.1
8.2
8.3
8.4
8.5
1
2
t−Test: p−value = 0.94

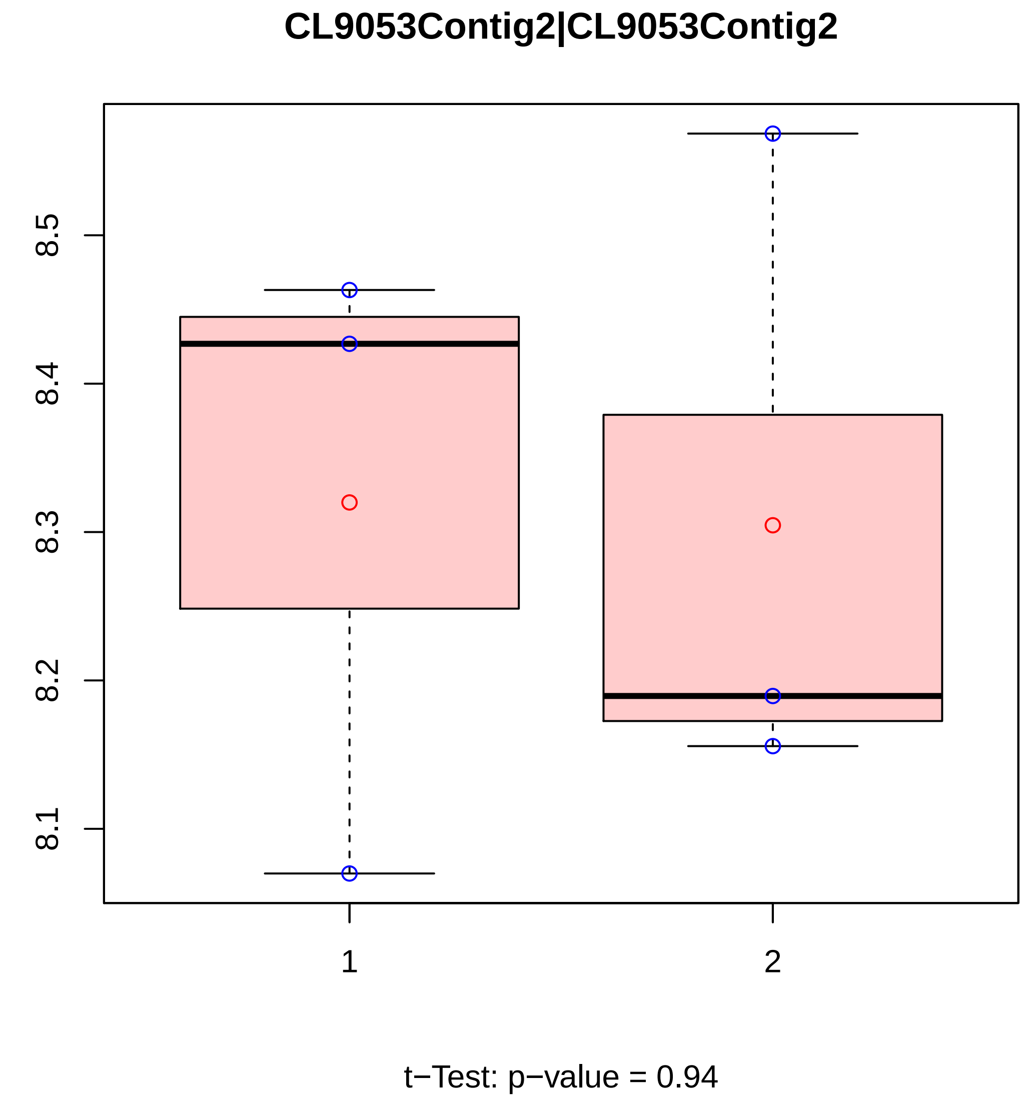
CL9053Contig2|CL9053Contig2
8.1
8.2
8.3
8.4
8.5
1
2
t−Test: p−value = 0.94

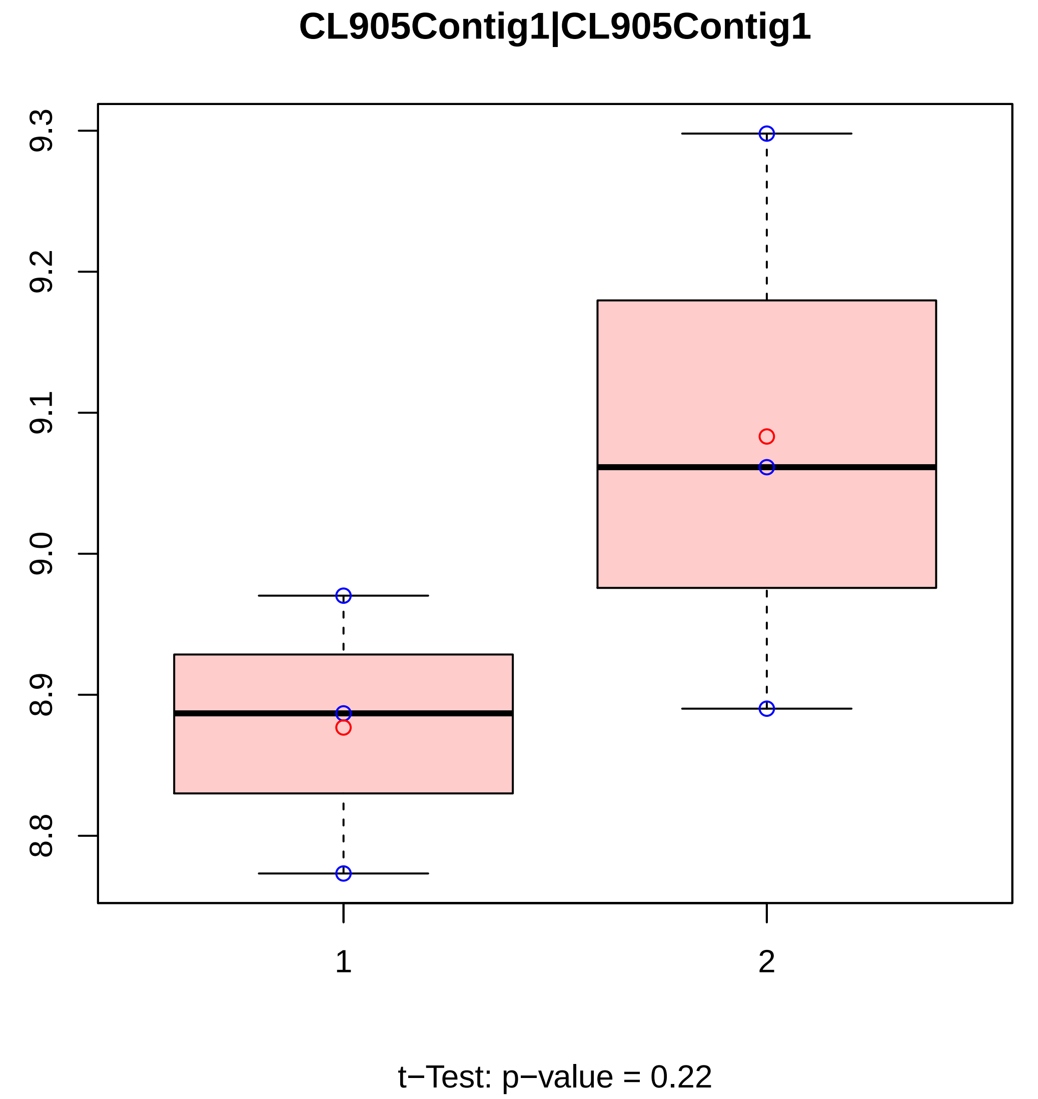
CL905Contig1|CL905Contig1
9.3
9.2
9.1
9.0
8.9
8.8
1
2
t−Test: p−value = 0.22

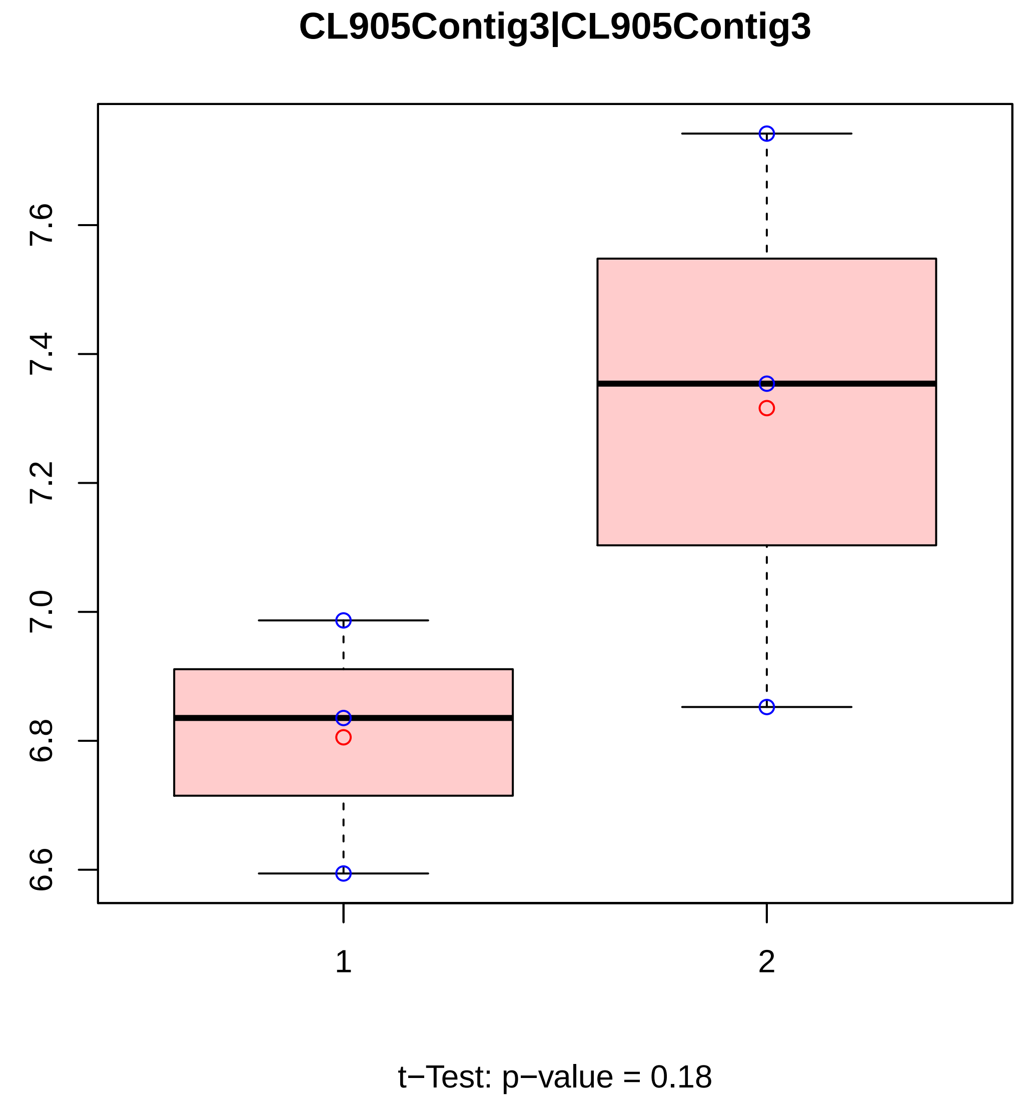
CL905Contig3|CL905Contig3
6.6
6.8
7.0
7.2
7.4
7.6
1
2
t−Test: p−value = 0.18

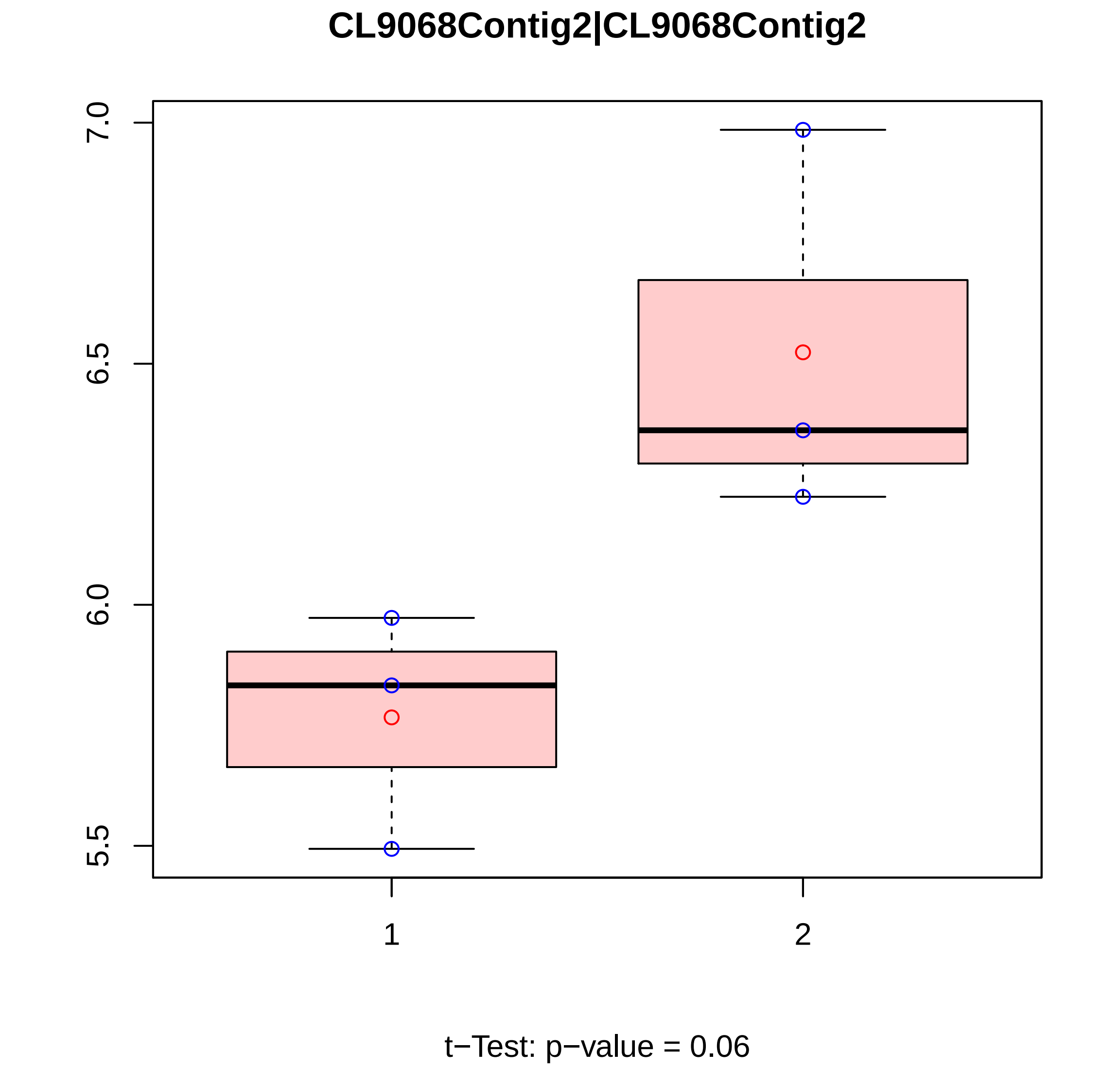
CL9068Contig2|CL9068Contig2
7.0
6.5
6.0
5.5
1
2
t−Test: p−value = 0.06

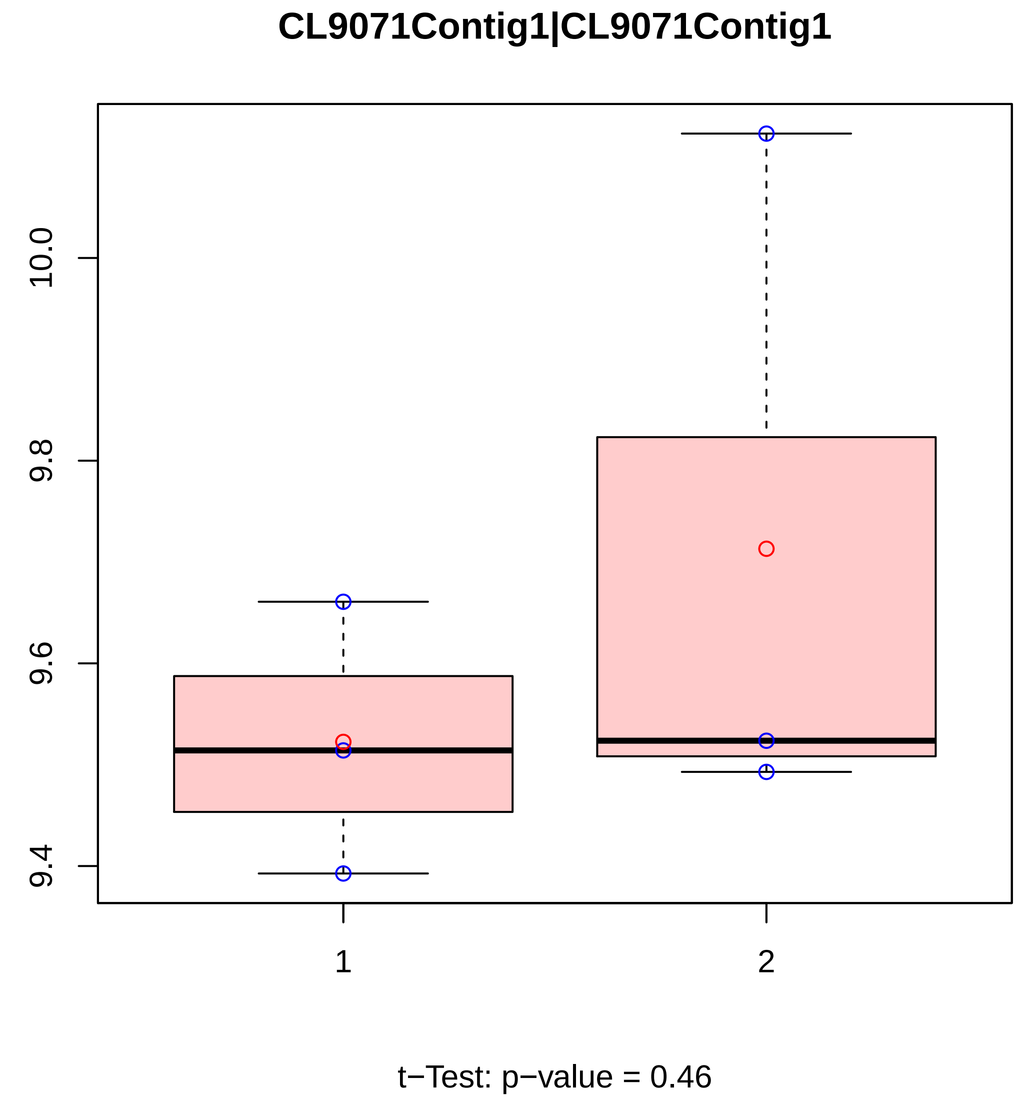

CL9071Contig1|CL9071Contig1
10.0
9.8
9.6
9.4
1
2
t−Test: p−value = 0.46

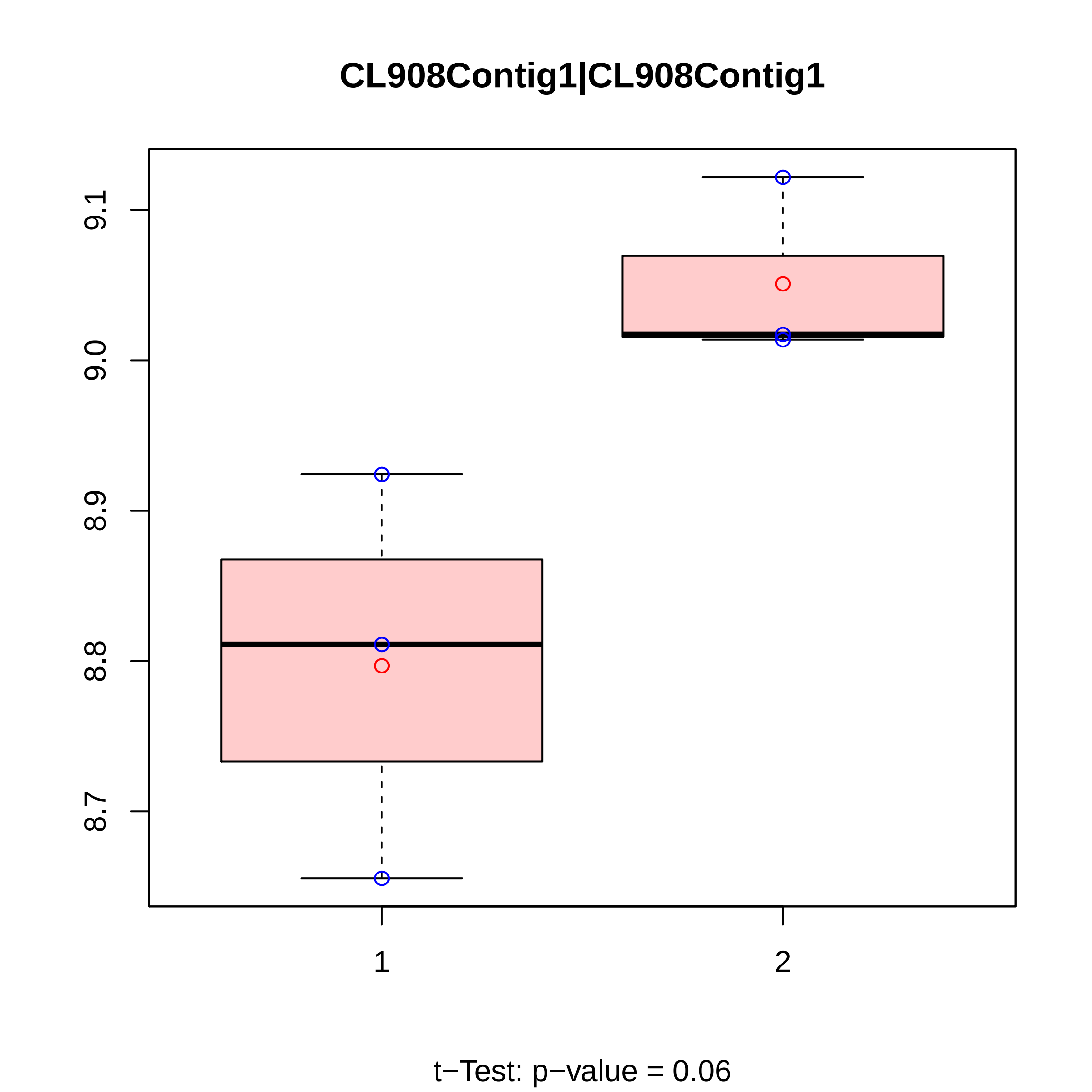
CL908Contig1|CL908Contig1
9.1
9.0
8.9
8.8
8.7
1
2
t−Test: p−value = 0.06

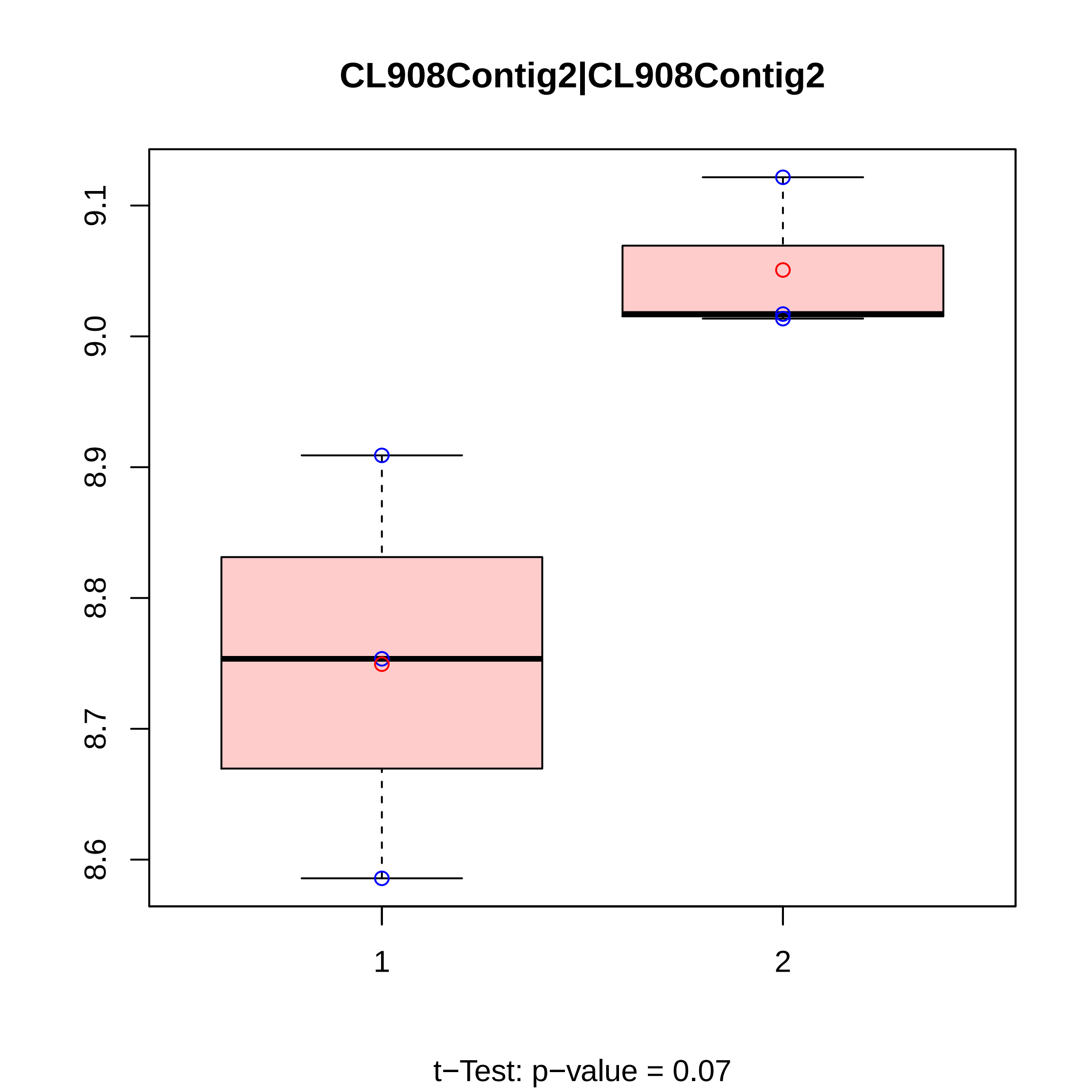
CL908Contig2|CL908Contig2
9.1
9.0
8.9
8.8
8.7
8.6
1
2
t−Test: p−value = 0.07

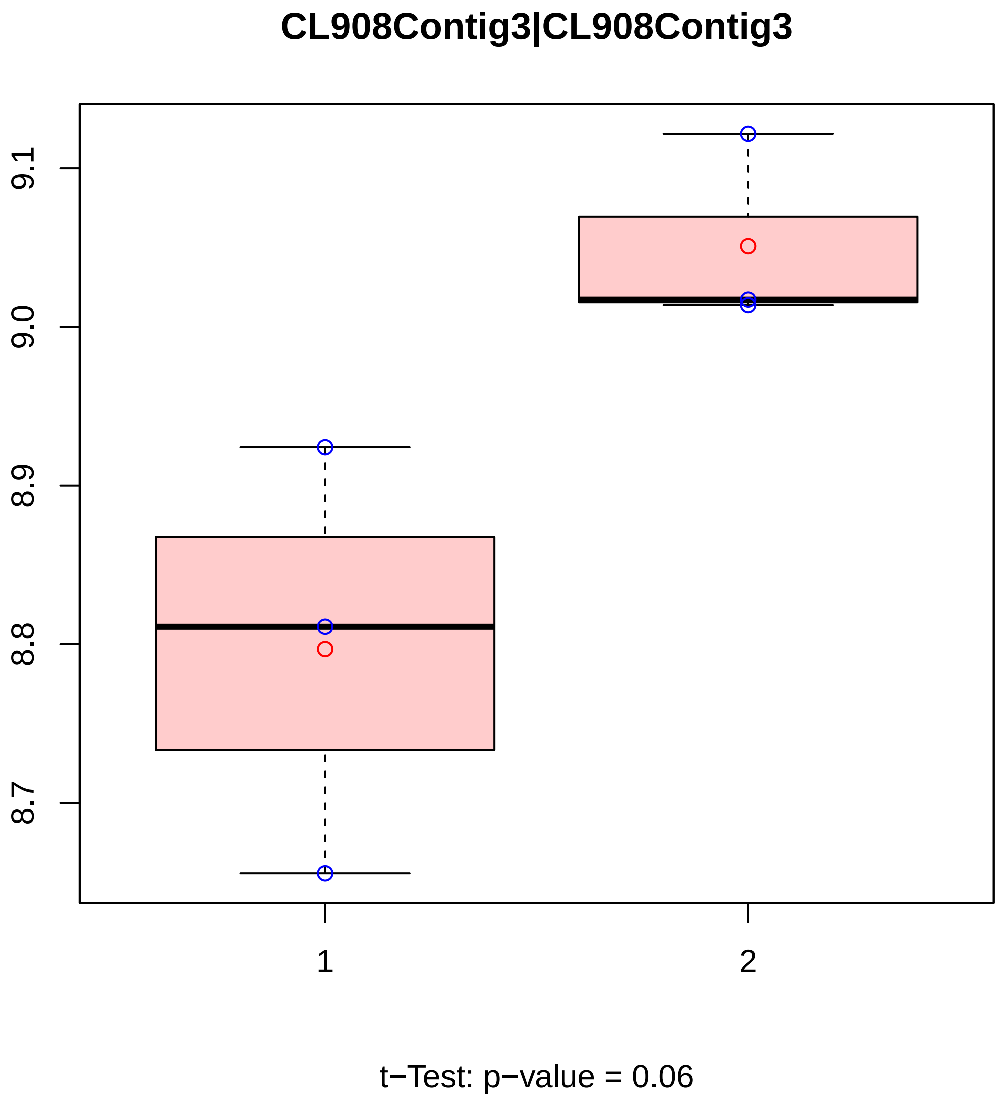
CL908Contig3|CL908Contig3
9.1
9.0
8.9
8.8
8.7
1
2
t−Test: p−value = 0.06

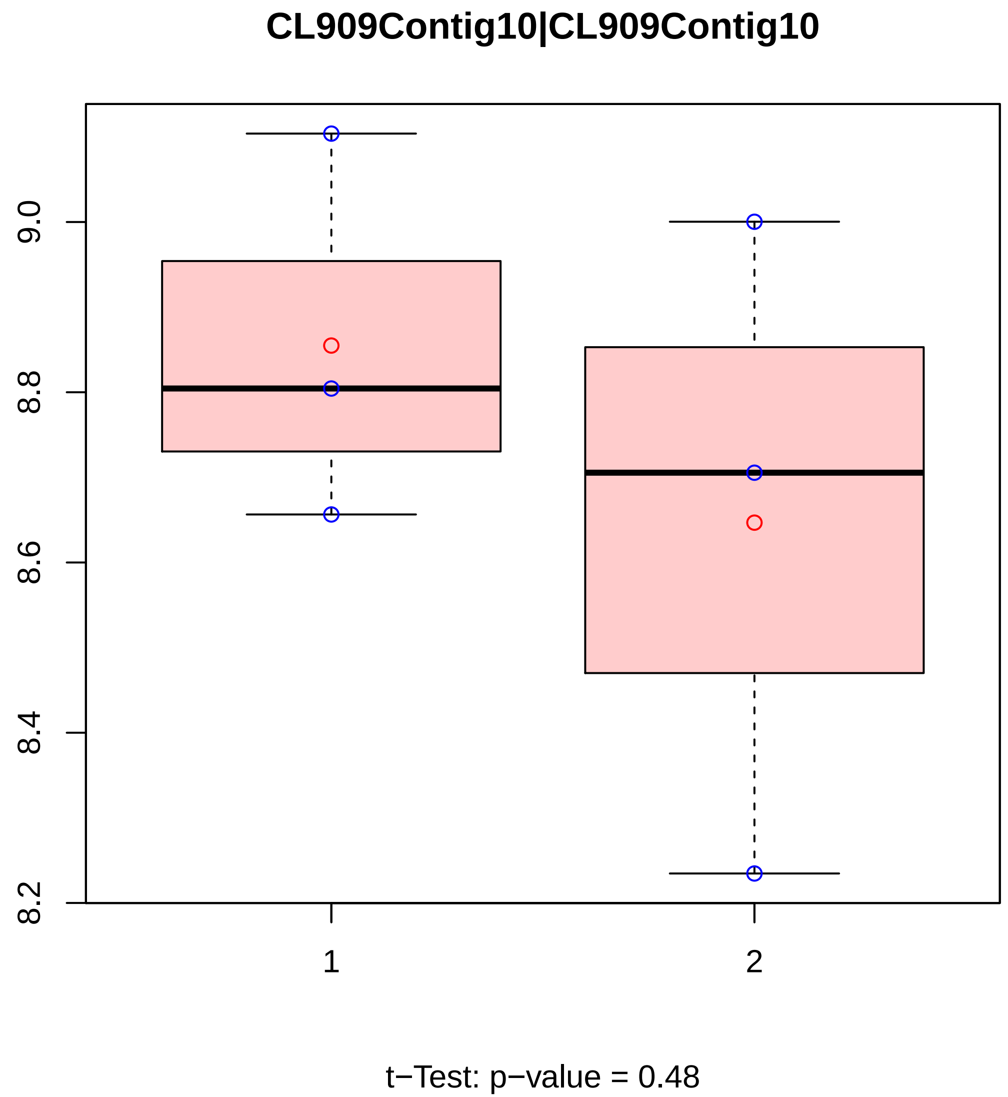
CL909Contig10|CL909Contig10
9.0
8.8
8.6
8.4
8.2
1
2
t−Test: p−value = 0.48

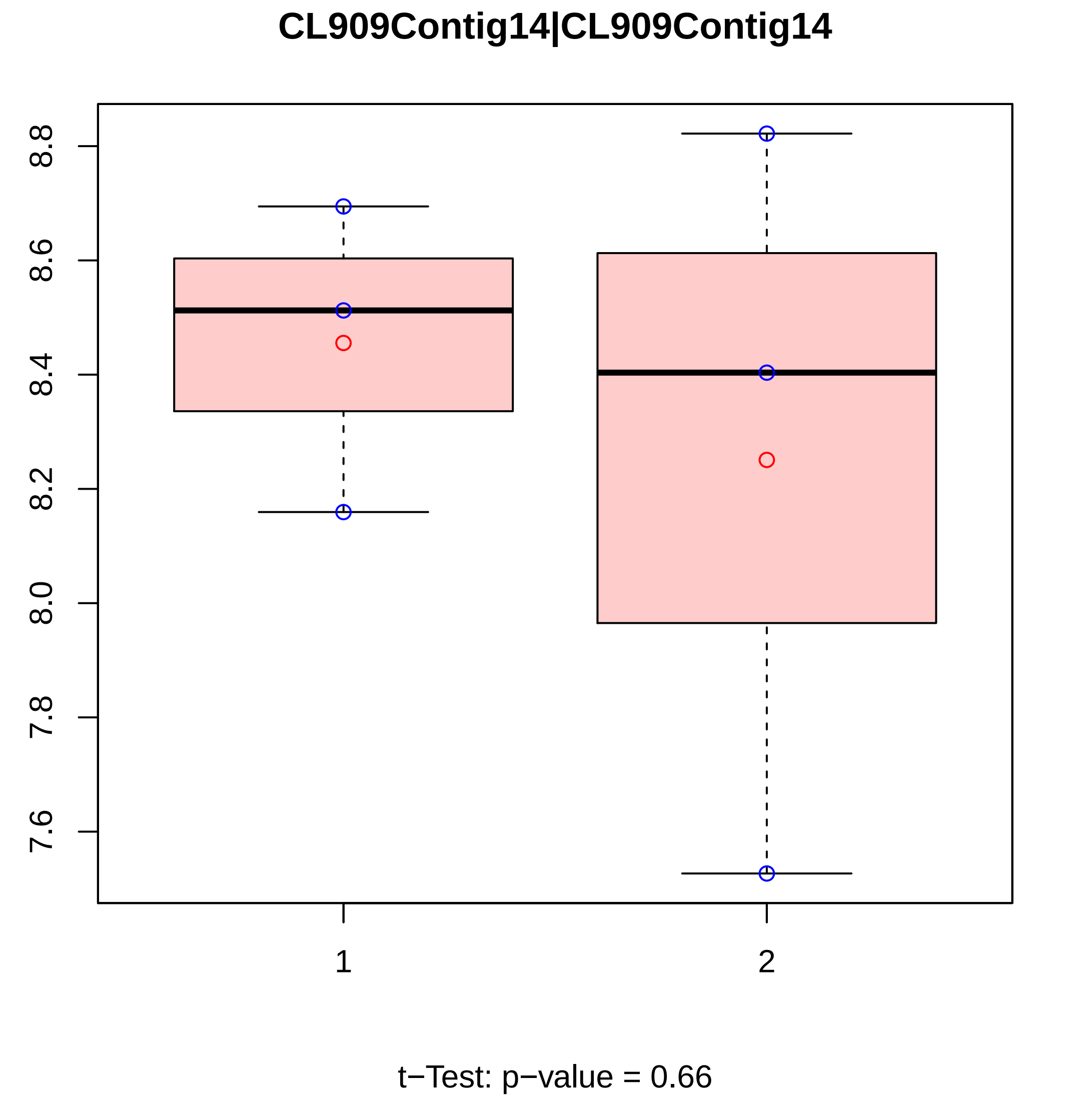
CL909Contig14|CL909Contig14
8.8
8.6
8.4
8.2
8.0
7.8
7.6
1
2
t−Test: p−value = 0.66

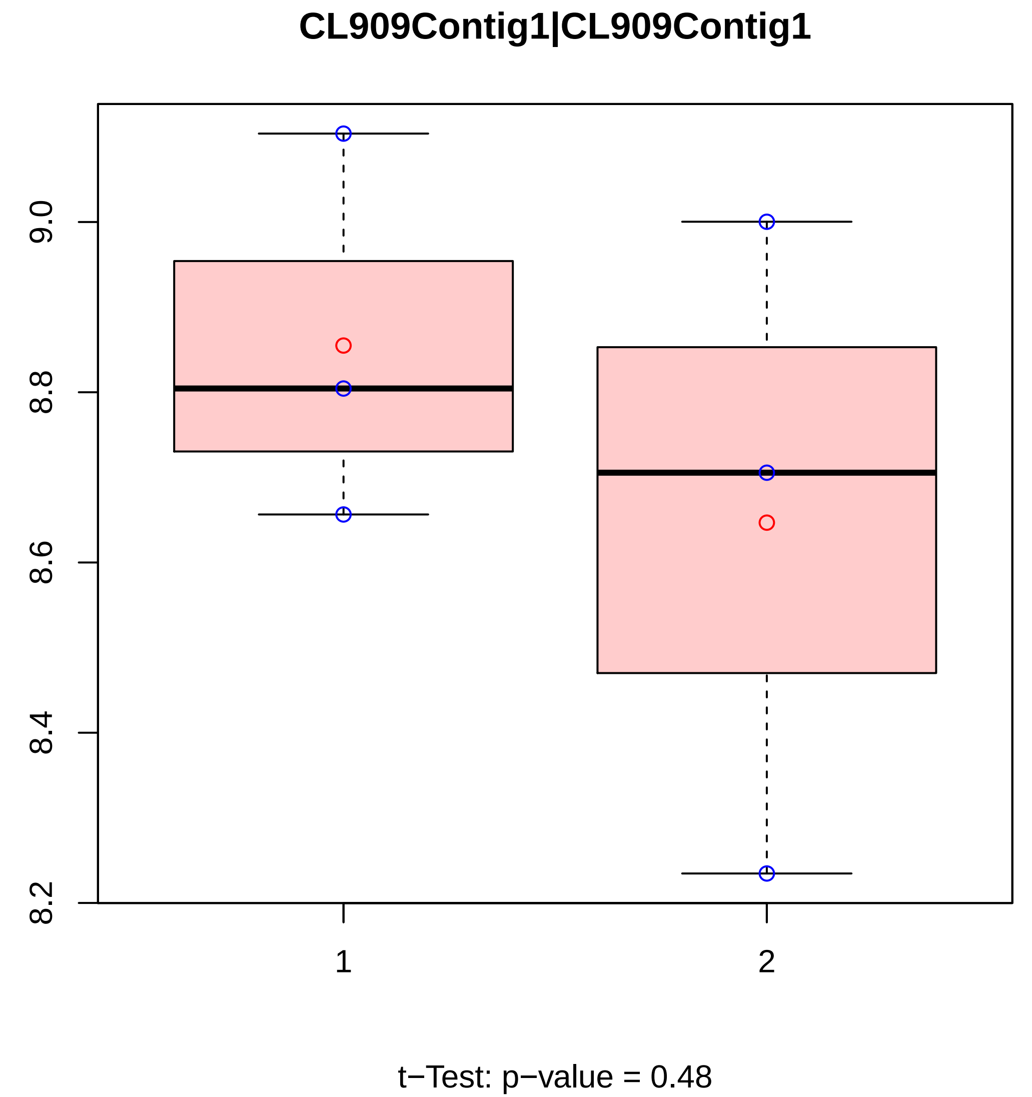

CL909Contig1|CL909Contig1
9.0
8.8
8.6
8.4
8.2
1
2
t−Test: p−value = 0.48

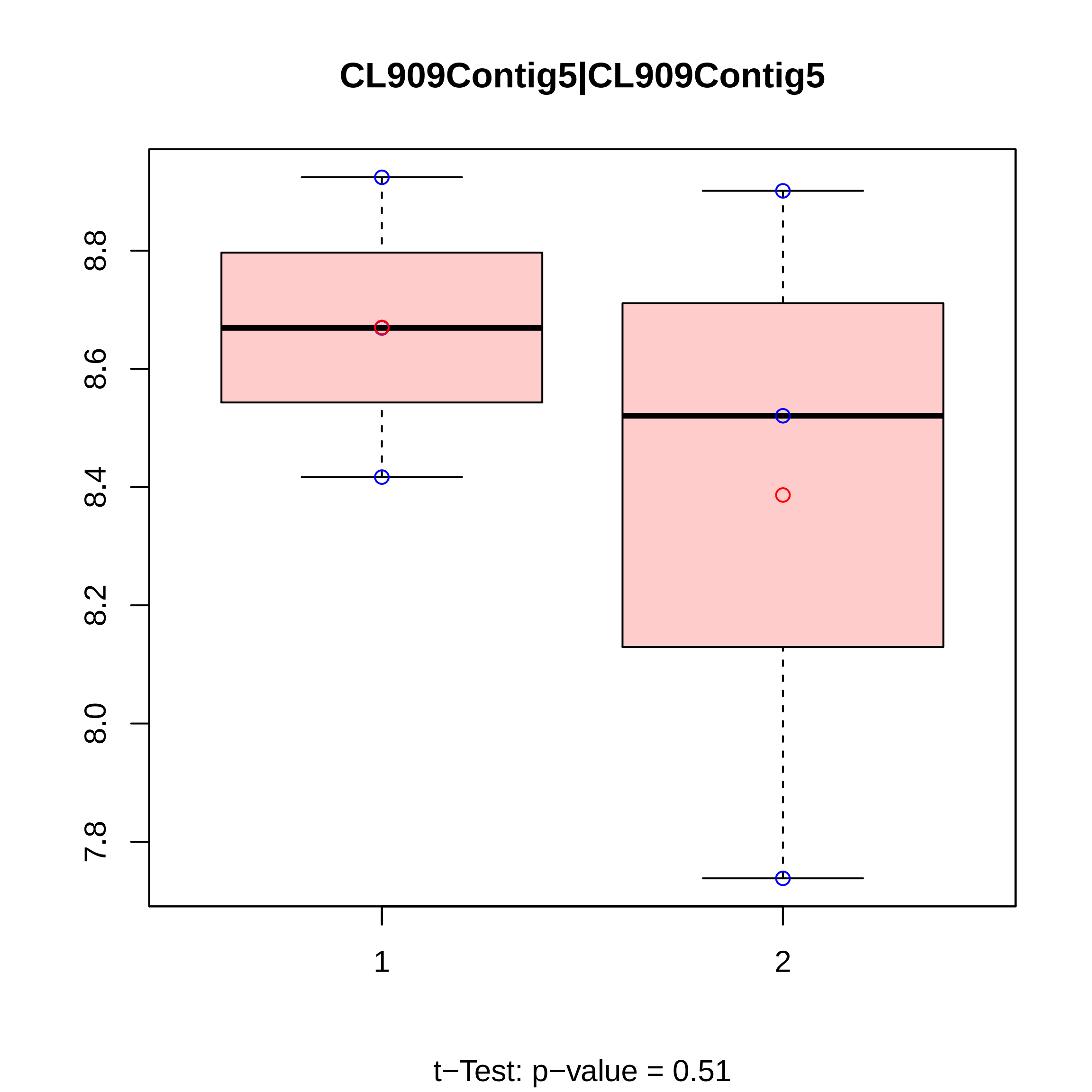

CL909Contig5|CL909Contig5
8.8
8.6
8.4
8.2
8.0
7.8
1
2
t−Test: p−value = 0.51

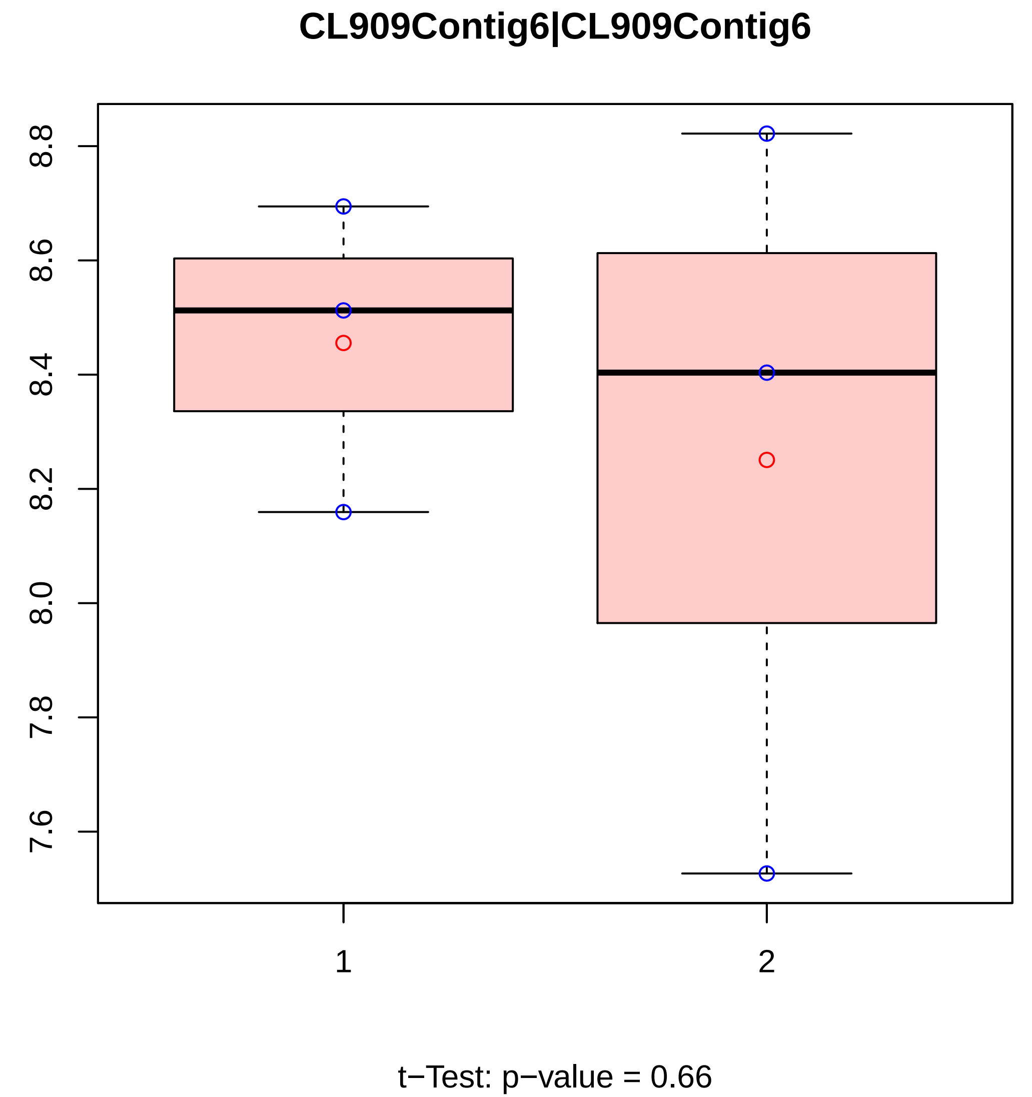
CL909Contig6|CL909Contig6
8.8
8.6
8.4
8.2
8.0
7.8
7.6
1
2
t−Test: p−value = 0.66

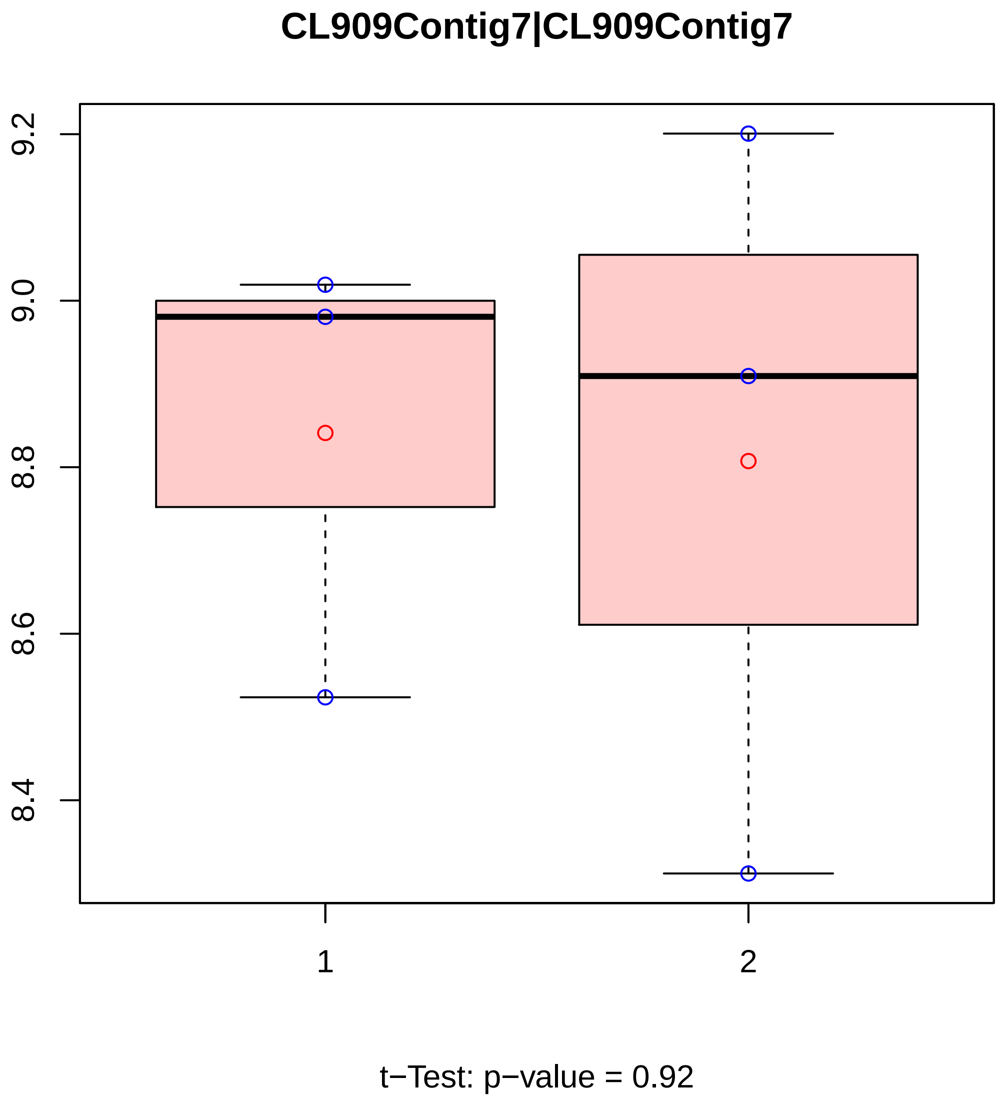
CL909Contig7|CL909Contig7
9.2
9.0
8.8
8.6
8.4
1
2
t−Test: p−value = 0.92

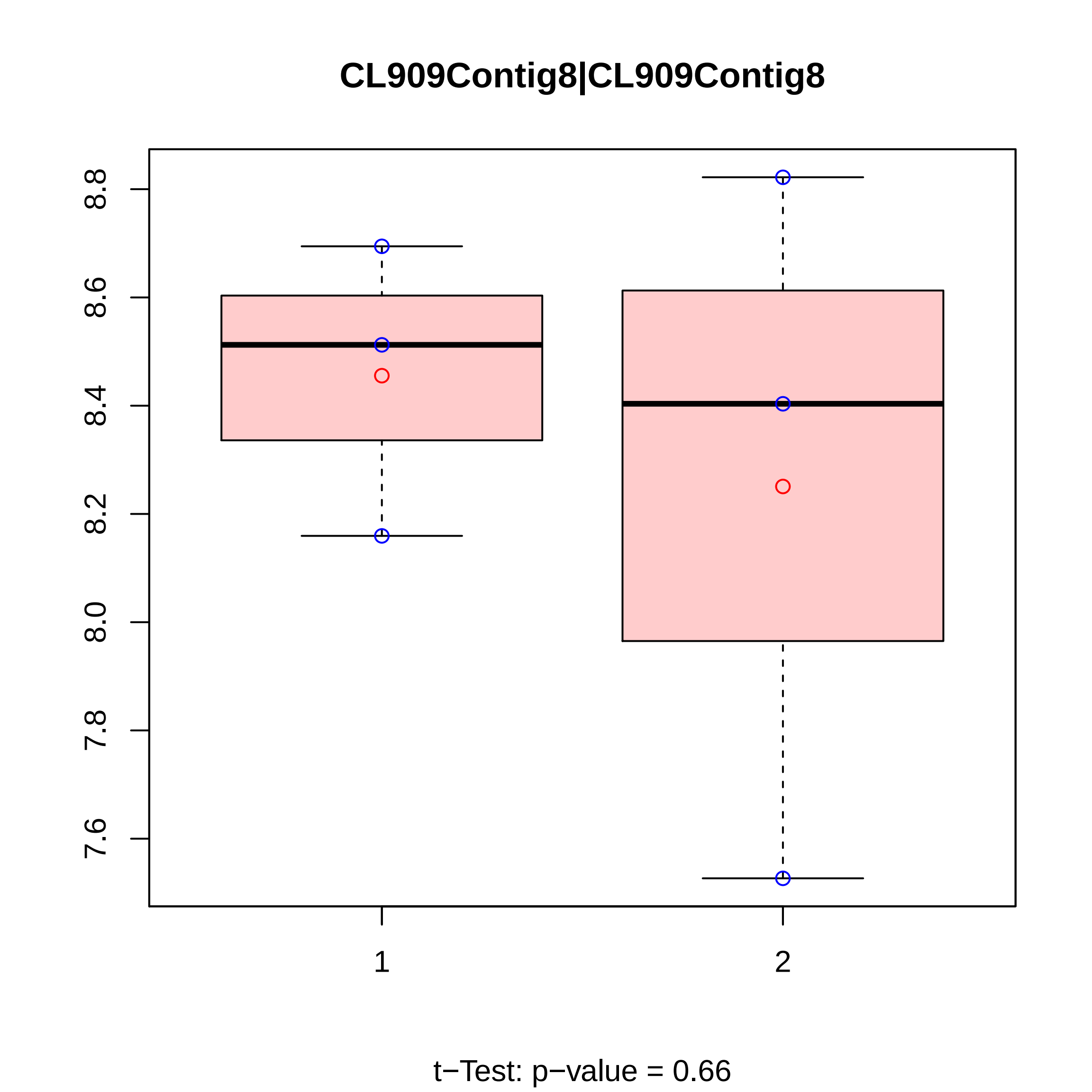

CL909Contig8|CL909Contig8
8.8
8.6
8.4
8.2
8.0
7.8
7.6
1
2
t−Test: p−value = 0.66

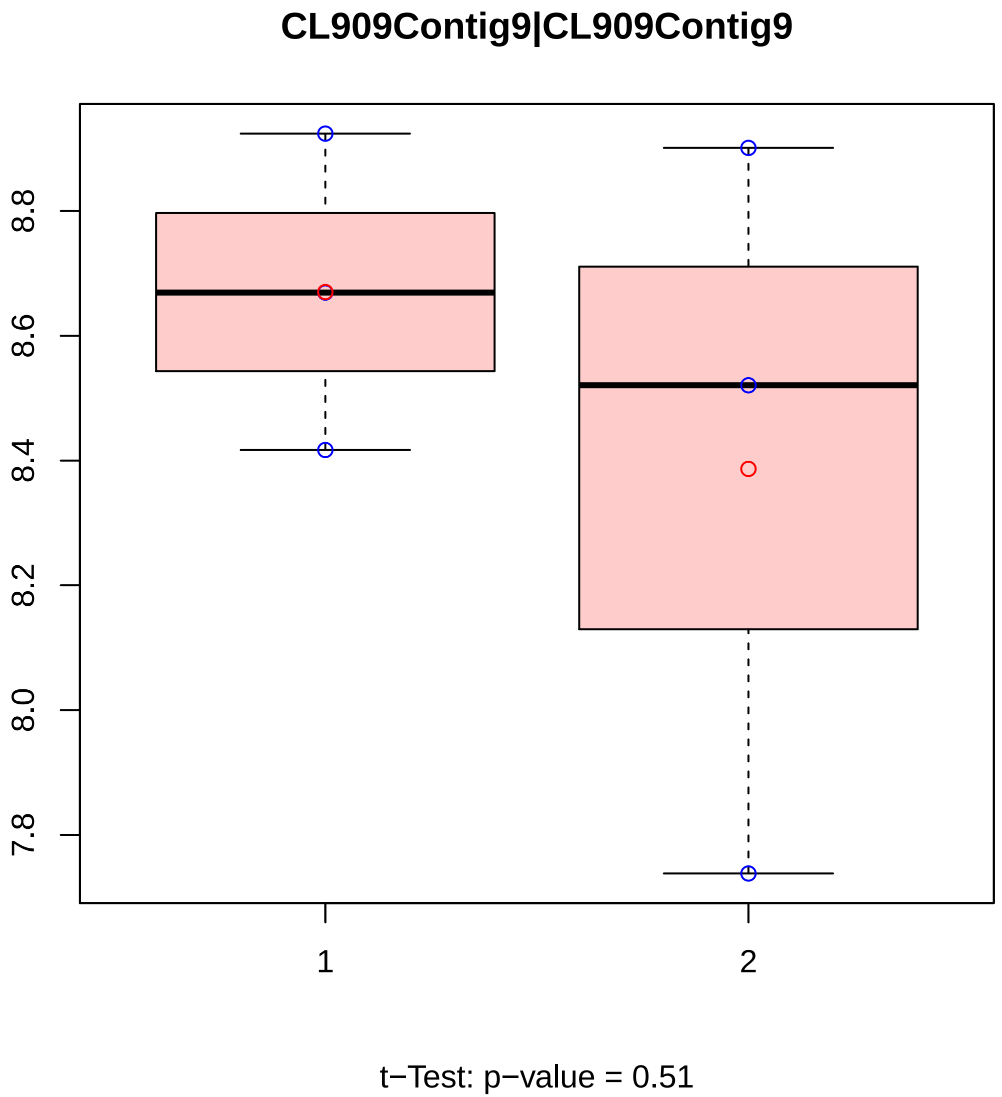

CL909Contig9|CL909Contig9
8.8
8.6
8.4
8.2
8.0
7.8
1
2
t−Test: p−value = 0.51

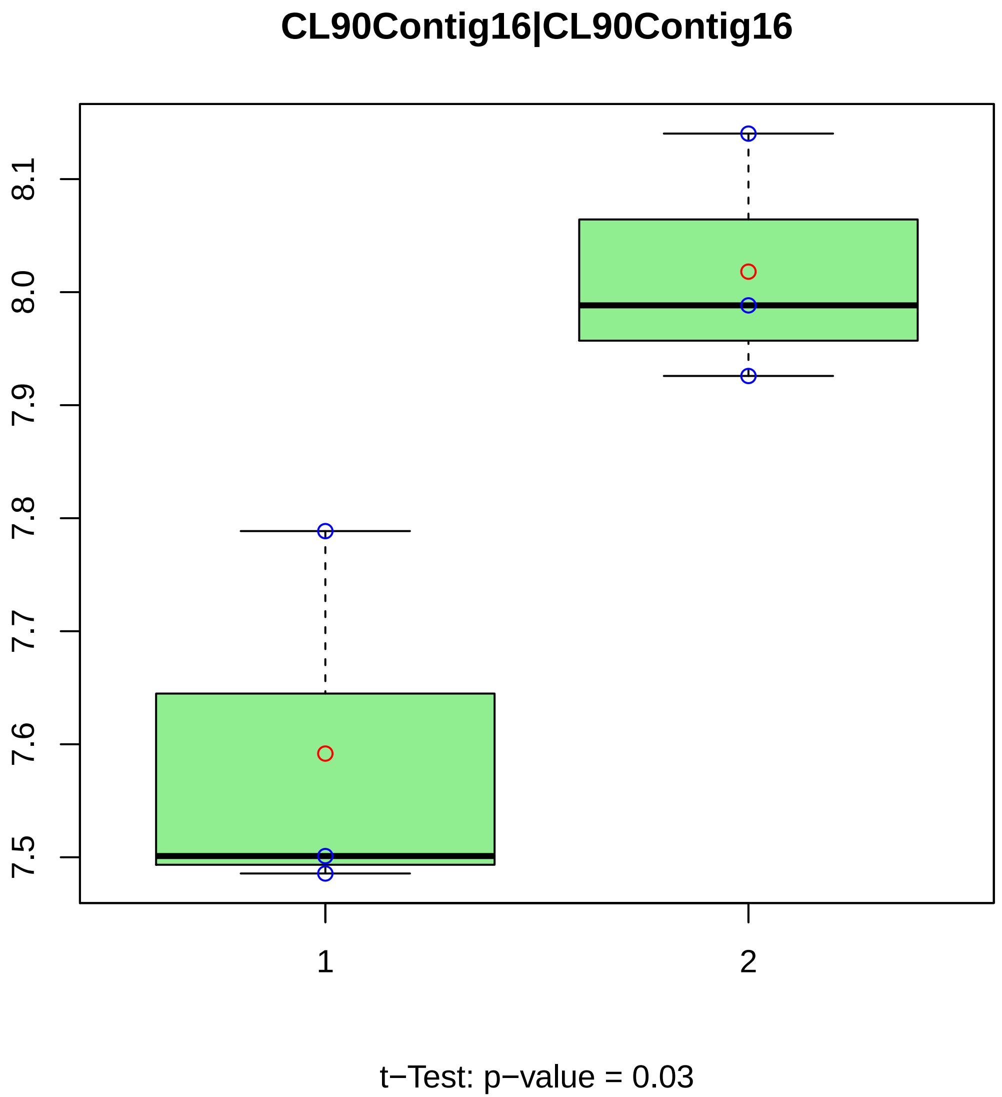

CL90Contig16|CL90Contig16
8.1
8.0
7.9
7.8
7.7
7.6
7.5
1
2
t−Test: p−value = 0.03

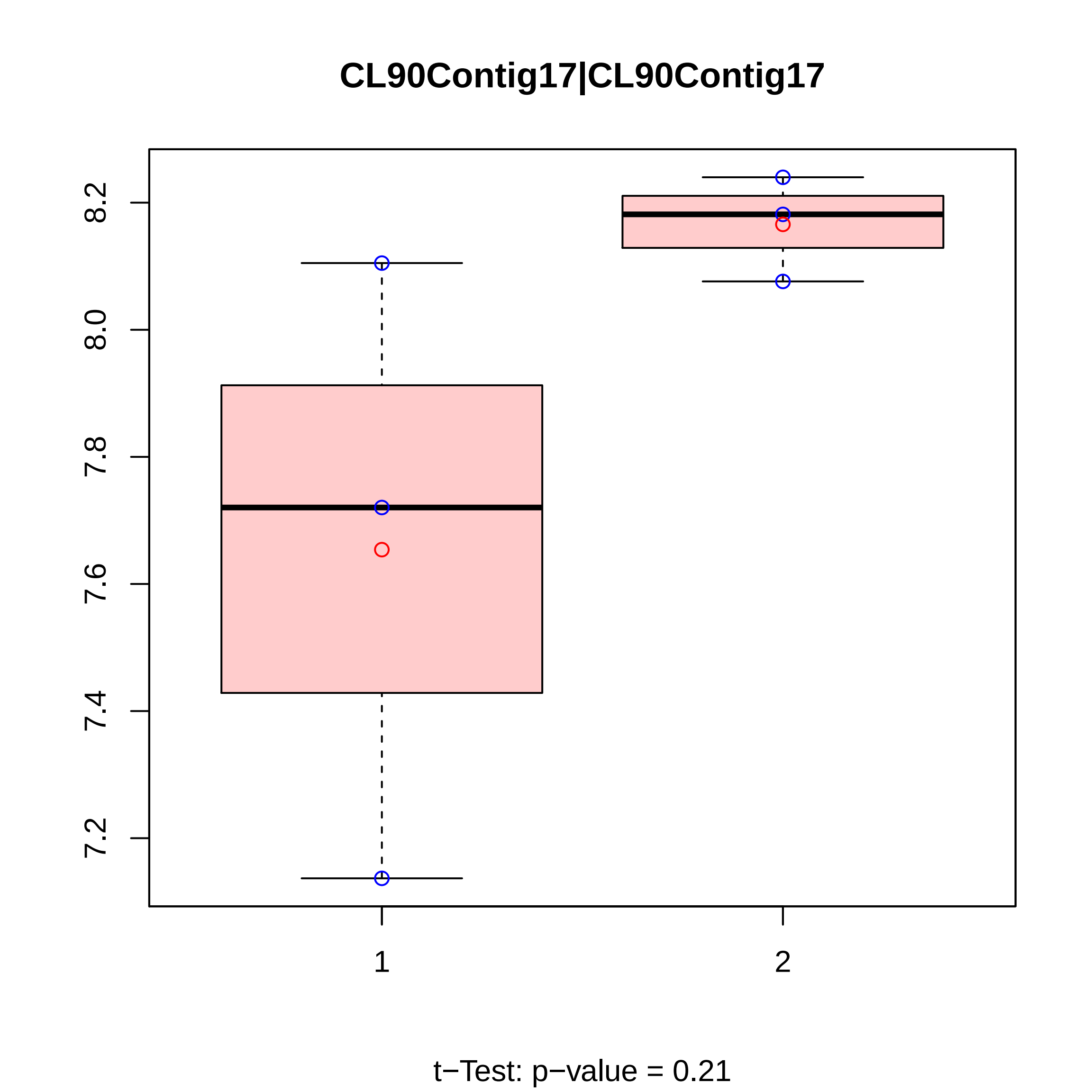
CL90Contig17|CL90Contig17
8.2
8.0
7.8
7.6
7.4
7.2
1
2
t−Test: p−value = 0.21

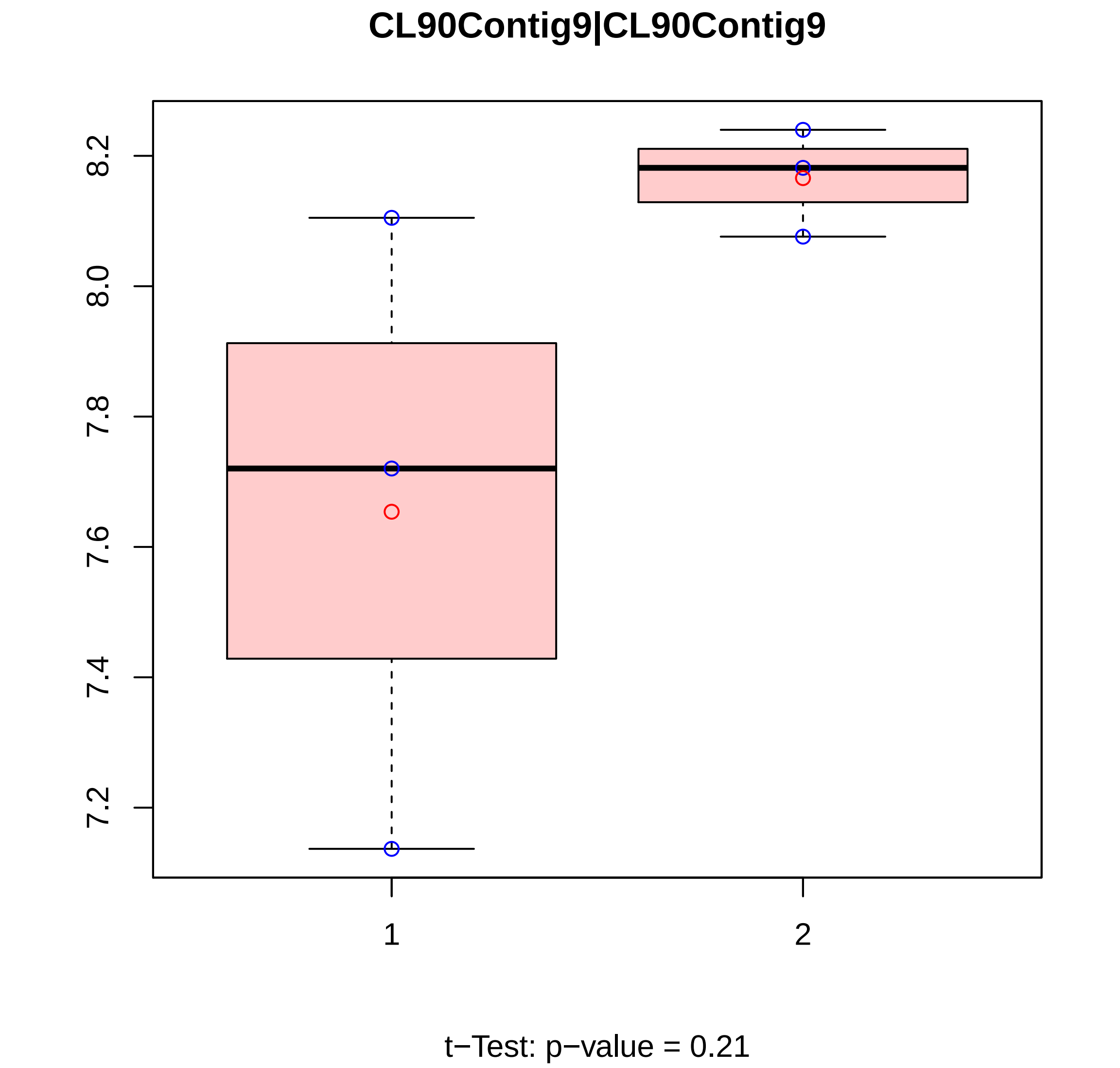
CL90Contig9|CL90Contig9
8.2
8.0
7.8
7.6
7.4
7.2
1
2
t−Test: p−value = 0.21

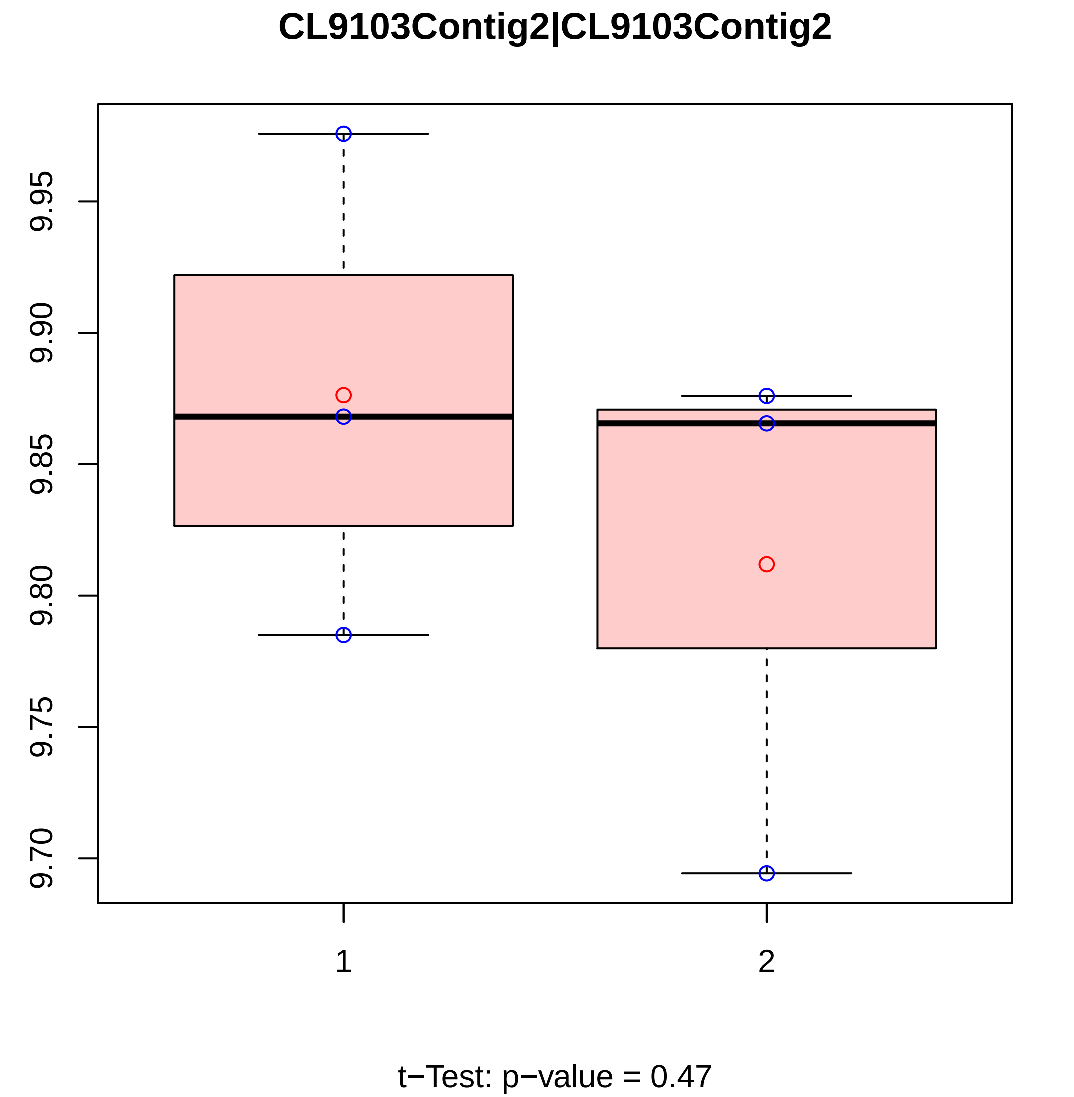
CL9103Contig2|CL9103Contig2
9.70
9.75
9.80
9.85
9.90
9.95
1
2
t−Test: p−value = 0.47

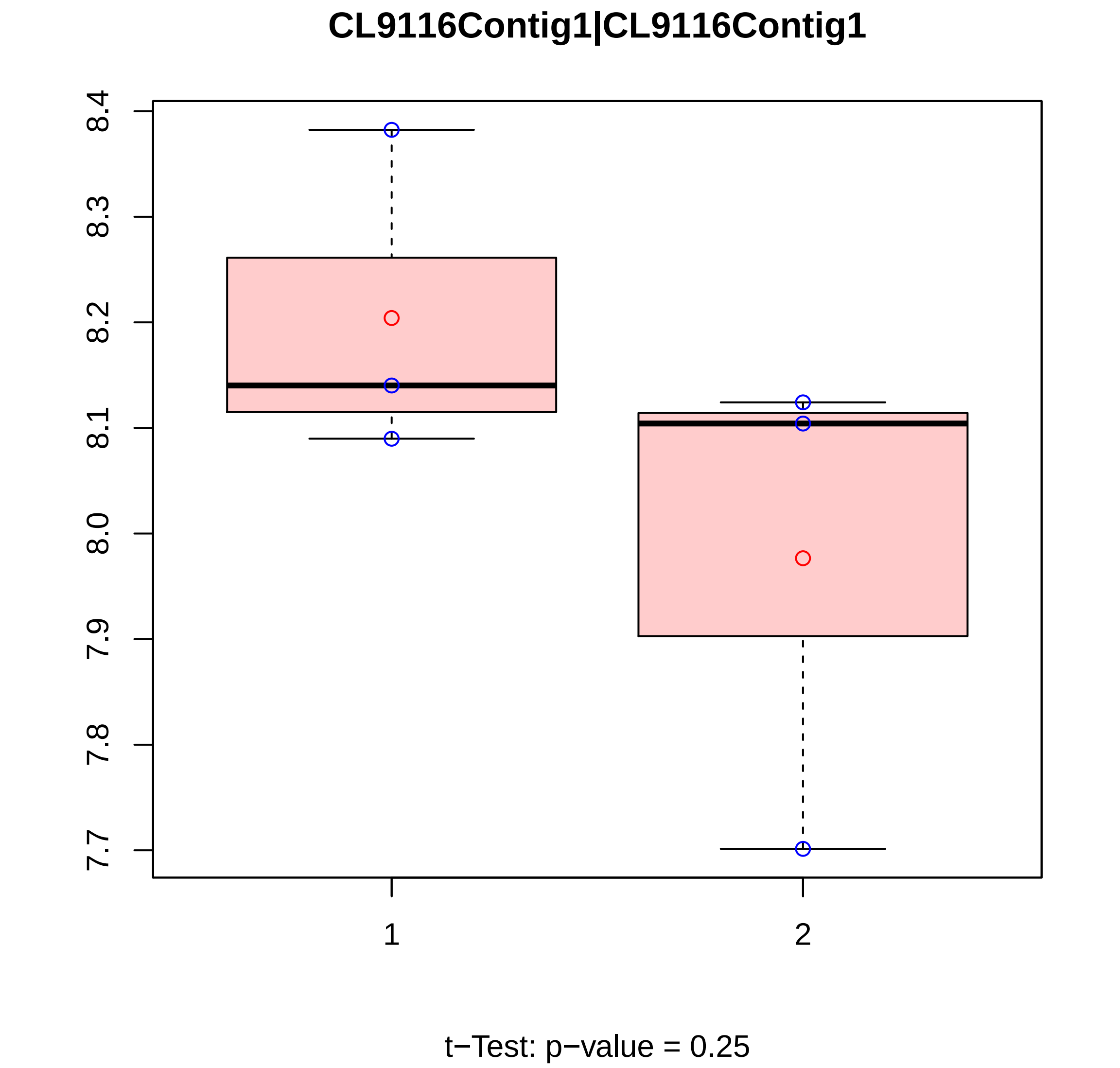
CL9116Contig1|CL9116Contig1
8.4
8.3
8.2
8.1
8.0
7.9
7.8
7.7
1
2
t−Test: p−value = 0.25

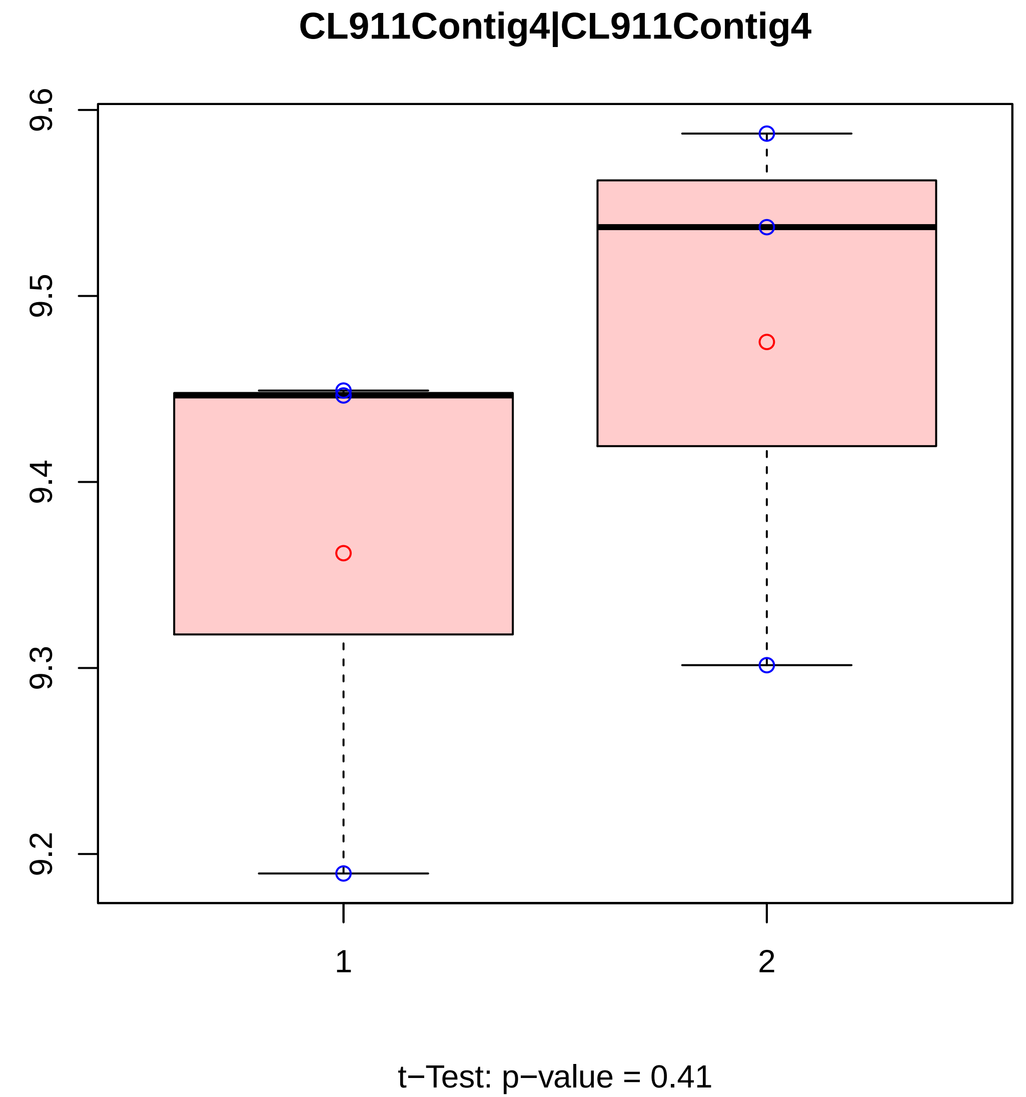
CL911Contig4|CL911Contig4
9.6
9.5
9.4
9.3
9.2
1
2
t−Test: p−value = 0.41

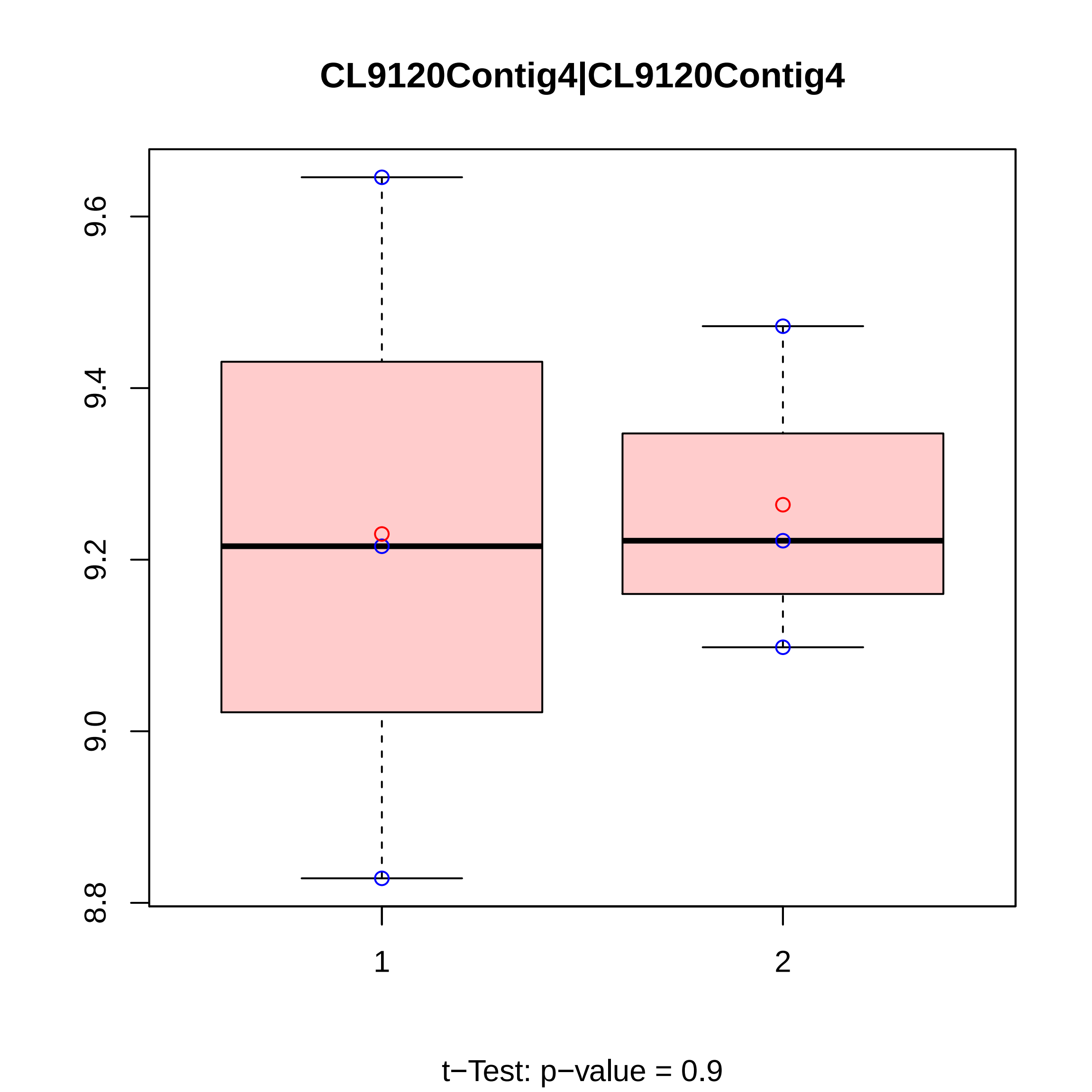
CL9120Contig4|CL9120Contig4
9.6
9.4
9.2
9.0
8.8
1
2
t−Test: p−value = 0.9

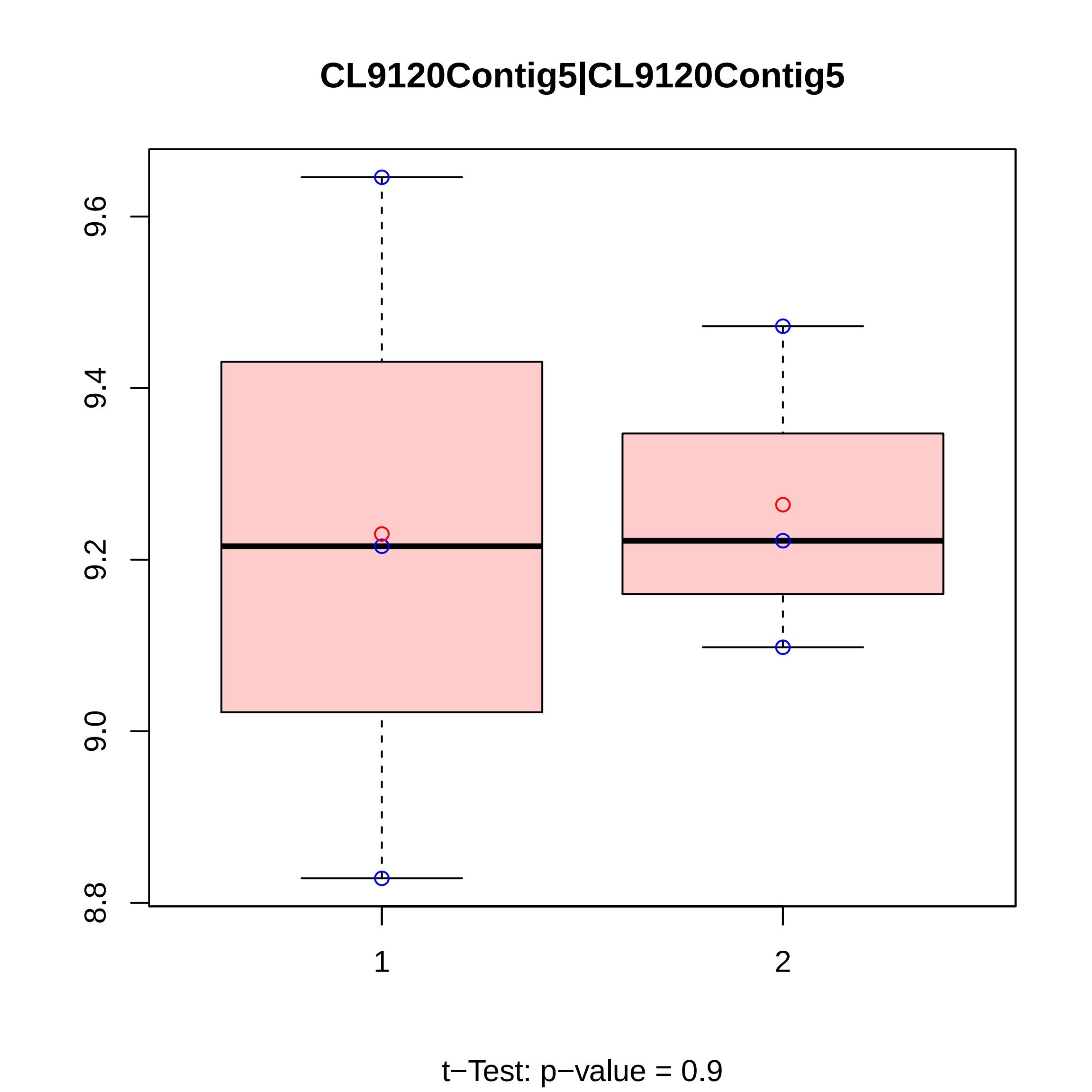

CL9120Contig5|CL9120Contig5
9.6
9.4
9.2
9.0
8.8
1
2
t−Test: p−value = 0.9

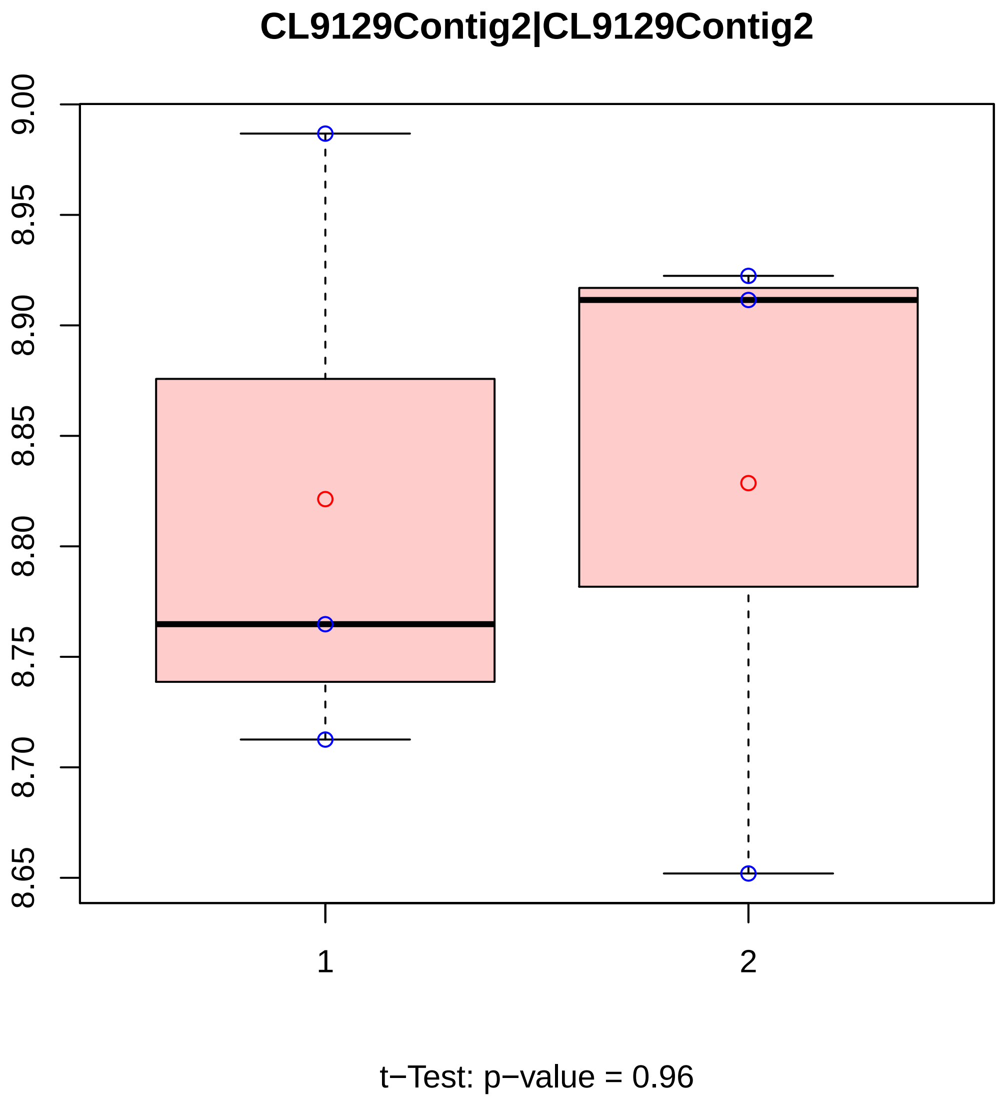

CL9129Contig2|CL9129Contig2
9.00
8.95
8.90
8.85
8.80
8.75
8.70
8.65
1
2
t−Test: p−value = 0.96

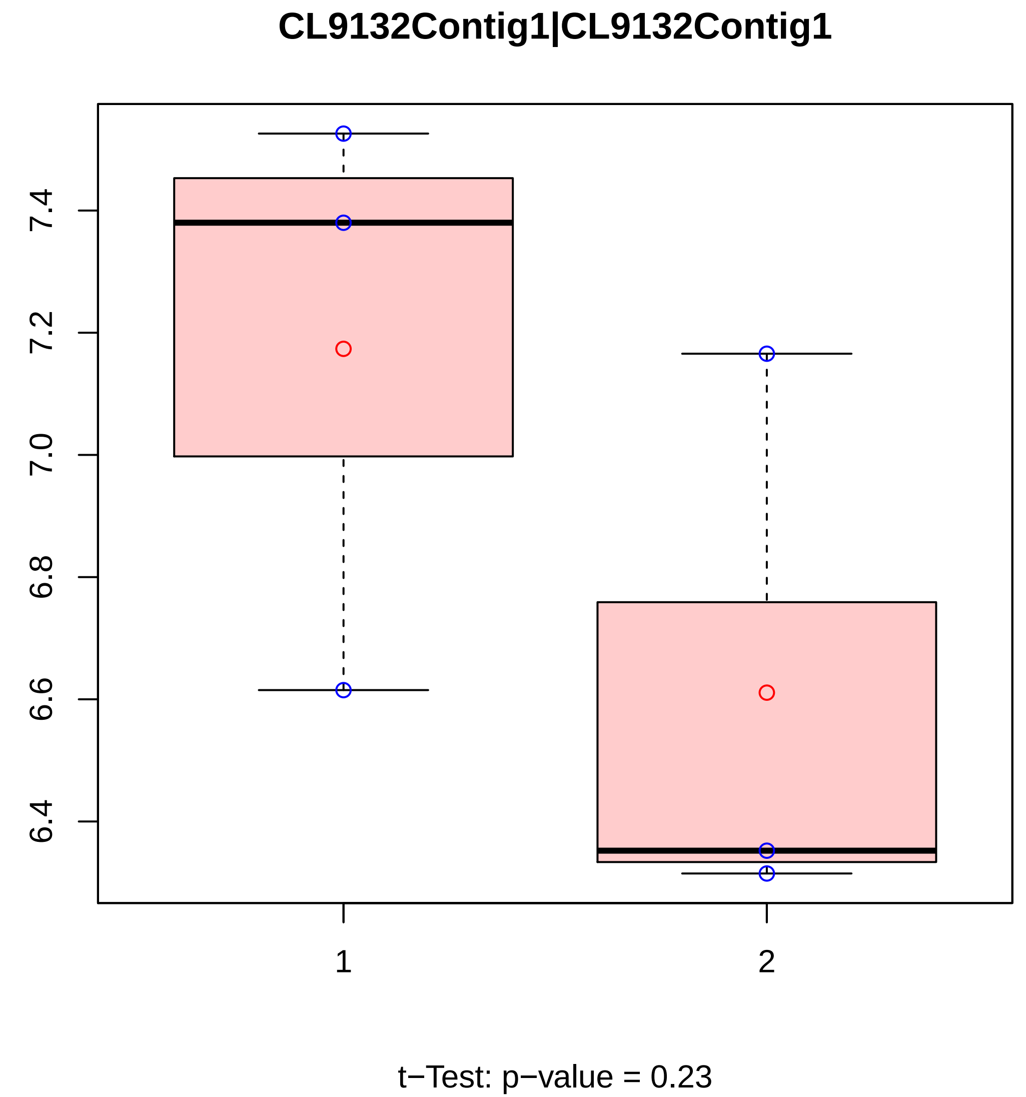

CL9132Contig1|CL9132Contig1
7.4
7.2
7.0
6.8
6.6
6.4
1
2
t−Test: p−value = 0.23

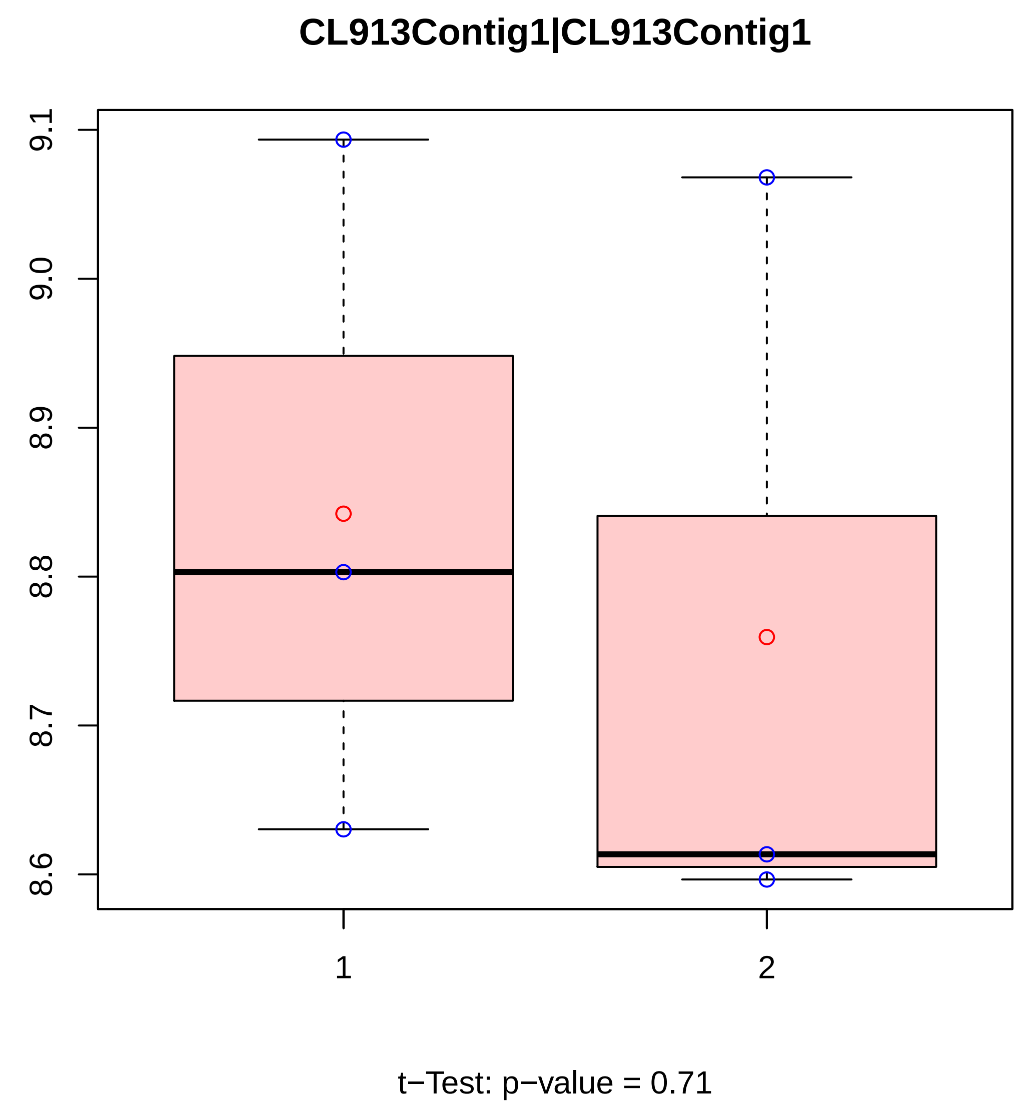
CL913Contig1|CL913Contig1
9.1
9.0
8.9
8.8
8.7
8.6
1
2
t−Test: p−value = 0.71

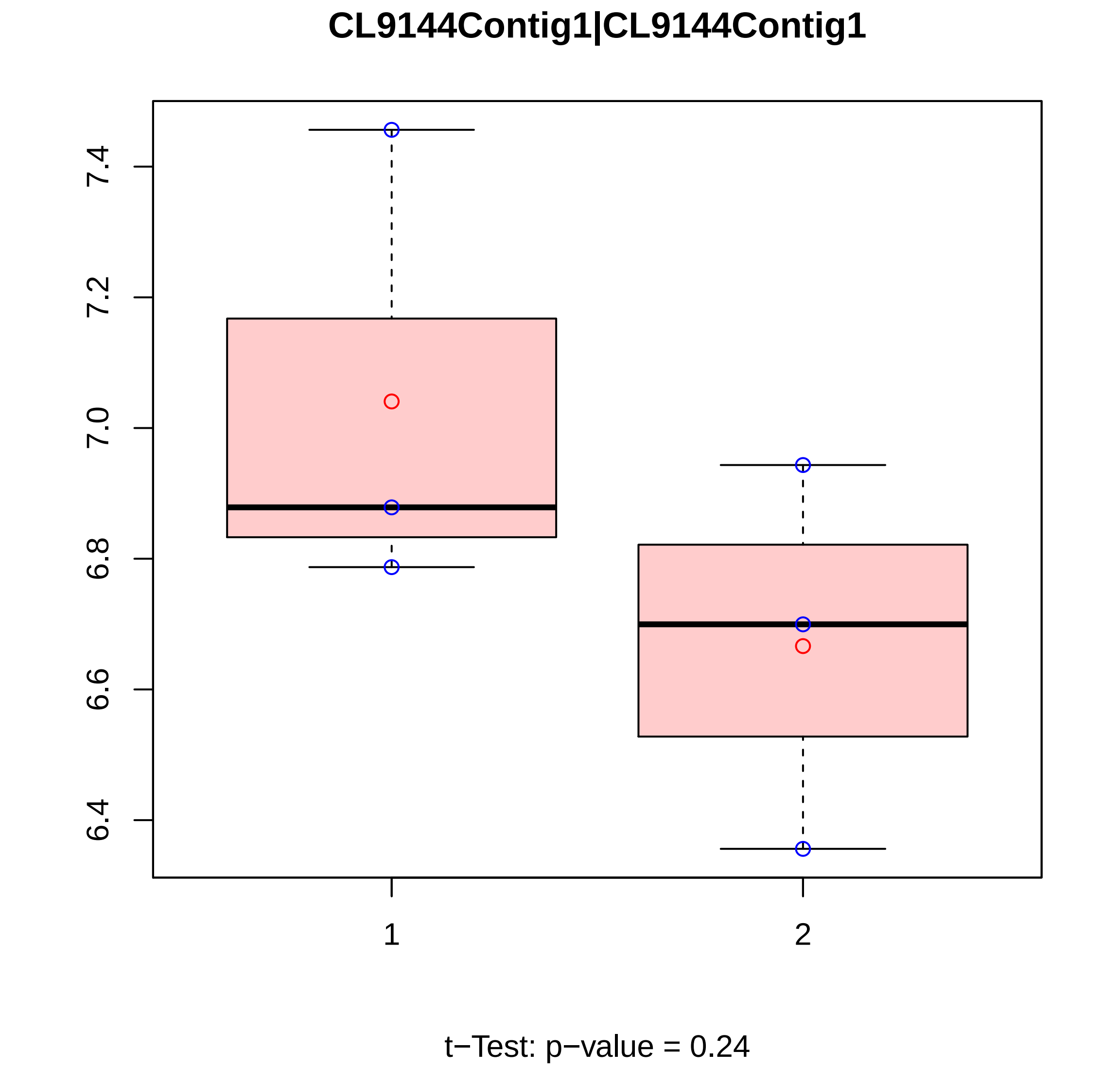
CL9144Contig1|CL9144Contig1
7.4
7.2
7.0
6.8
6.6
6.4
1
2
t−Test: p−value = 0.24

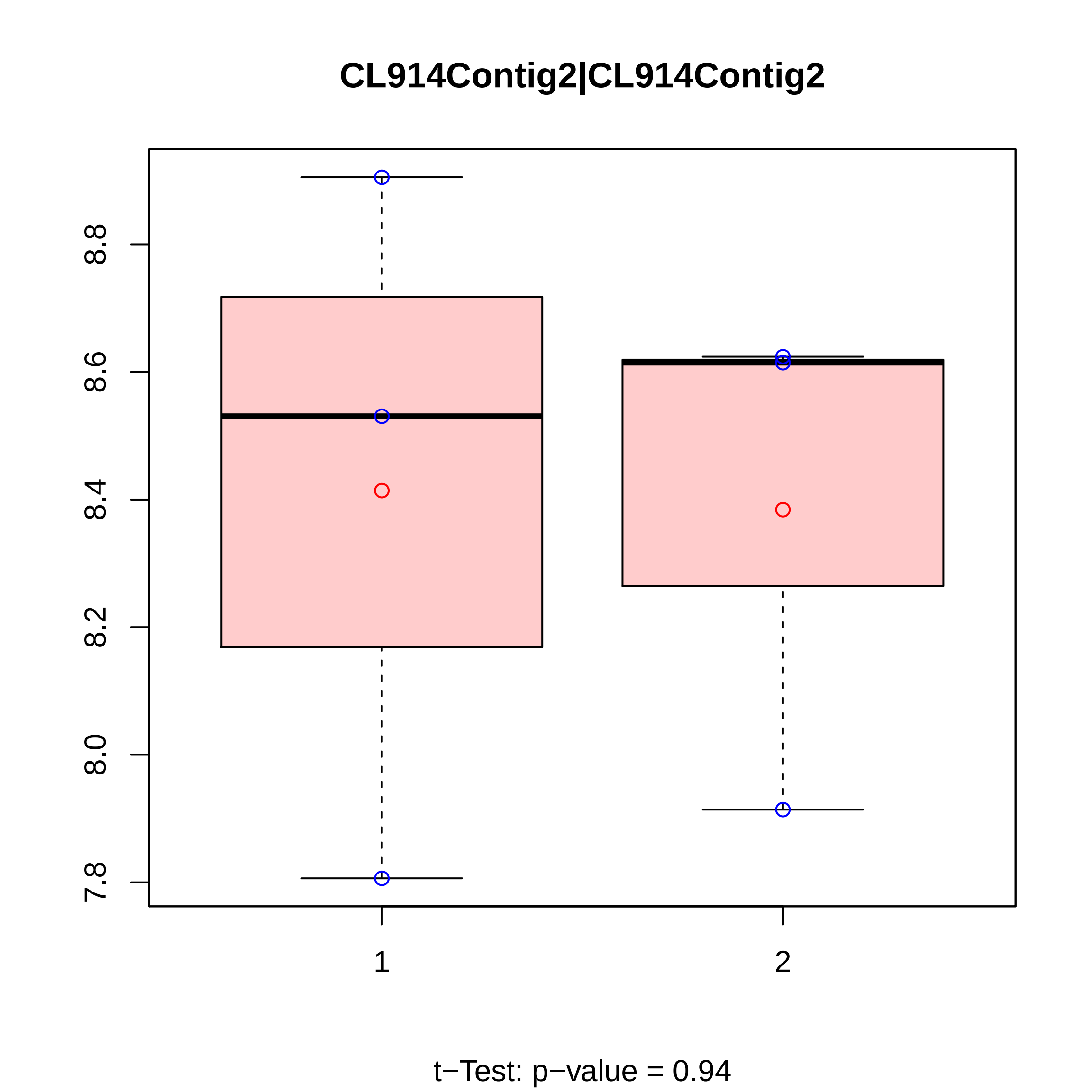
CL914Contig2|CL914Contig2
8.8
8.6
8.4
8.2
8.0
7.8
1
2
t−Test: p−value = 0.94

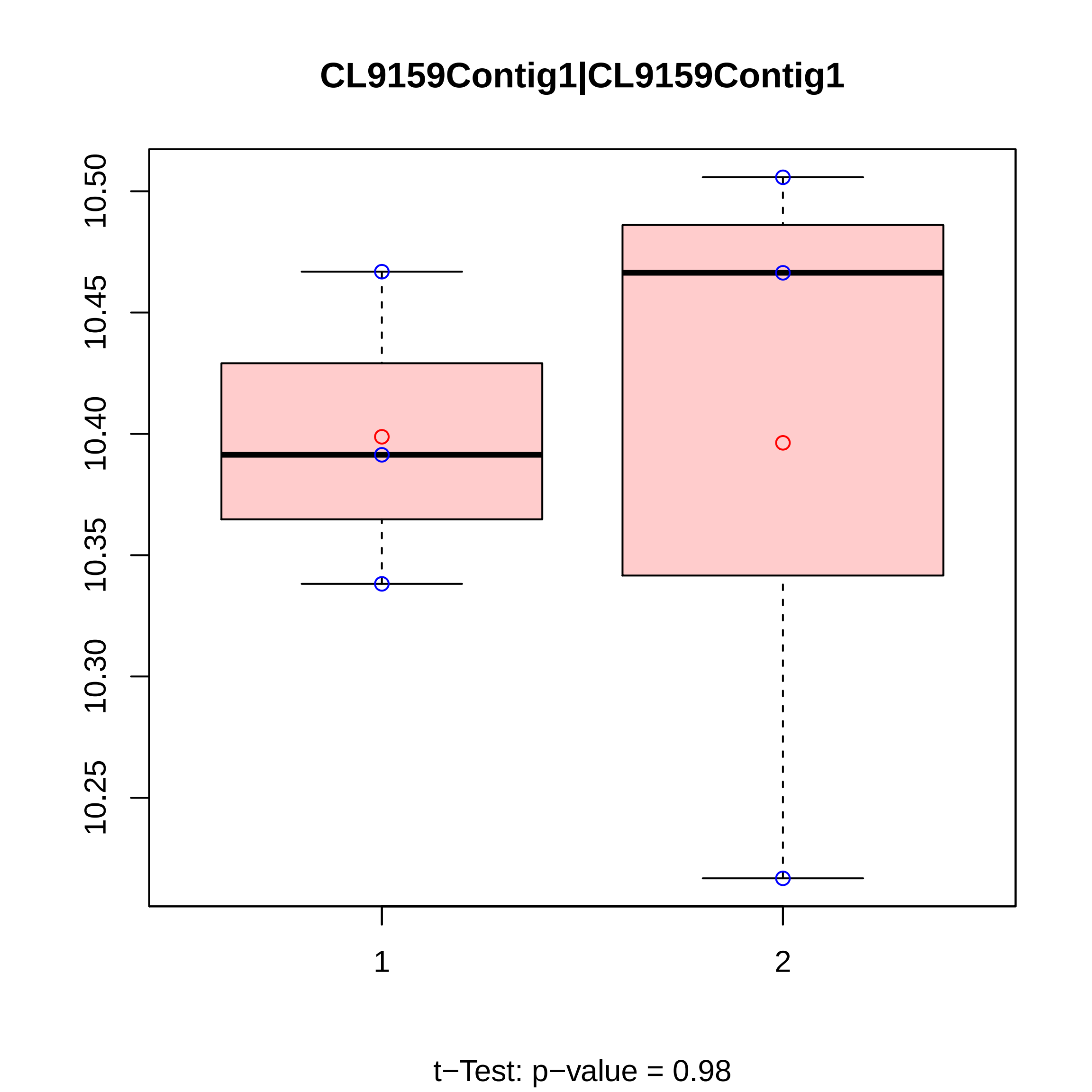

CL9159Contig1|CL9159Contig1
10.50
10.45
10.40
10.35
10.30
10.25
1
2
t−Test: p−value = 0.98

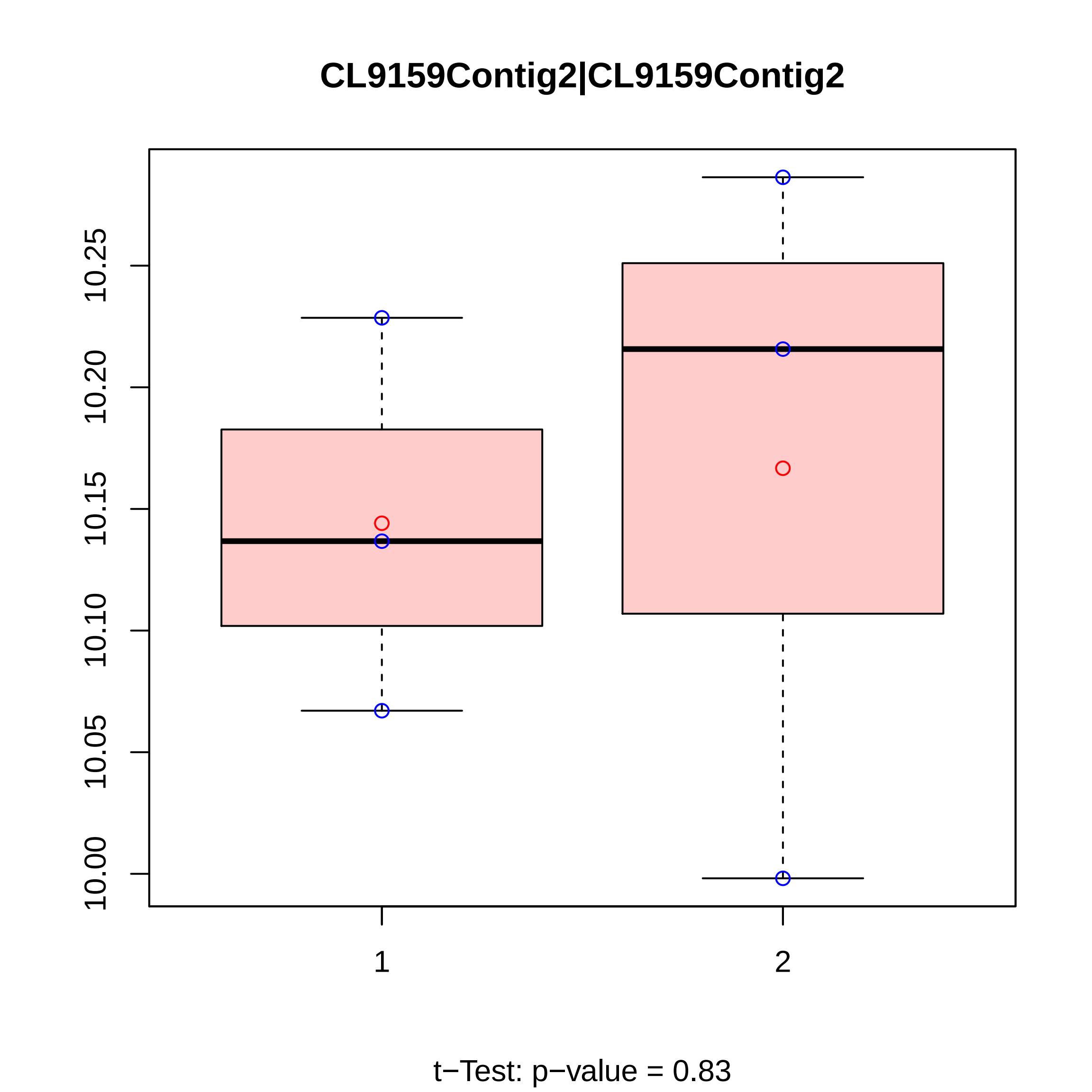
CL9159Contig2|CL9159Contig2
10.00
10.05
10.10
10.15
10.20
10.25
1
2
t−Test: p−value = 0.83

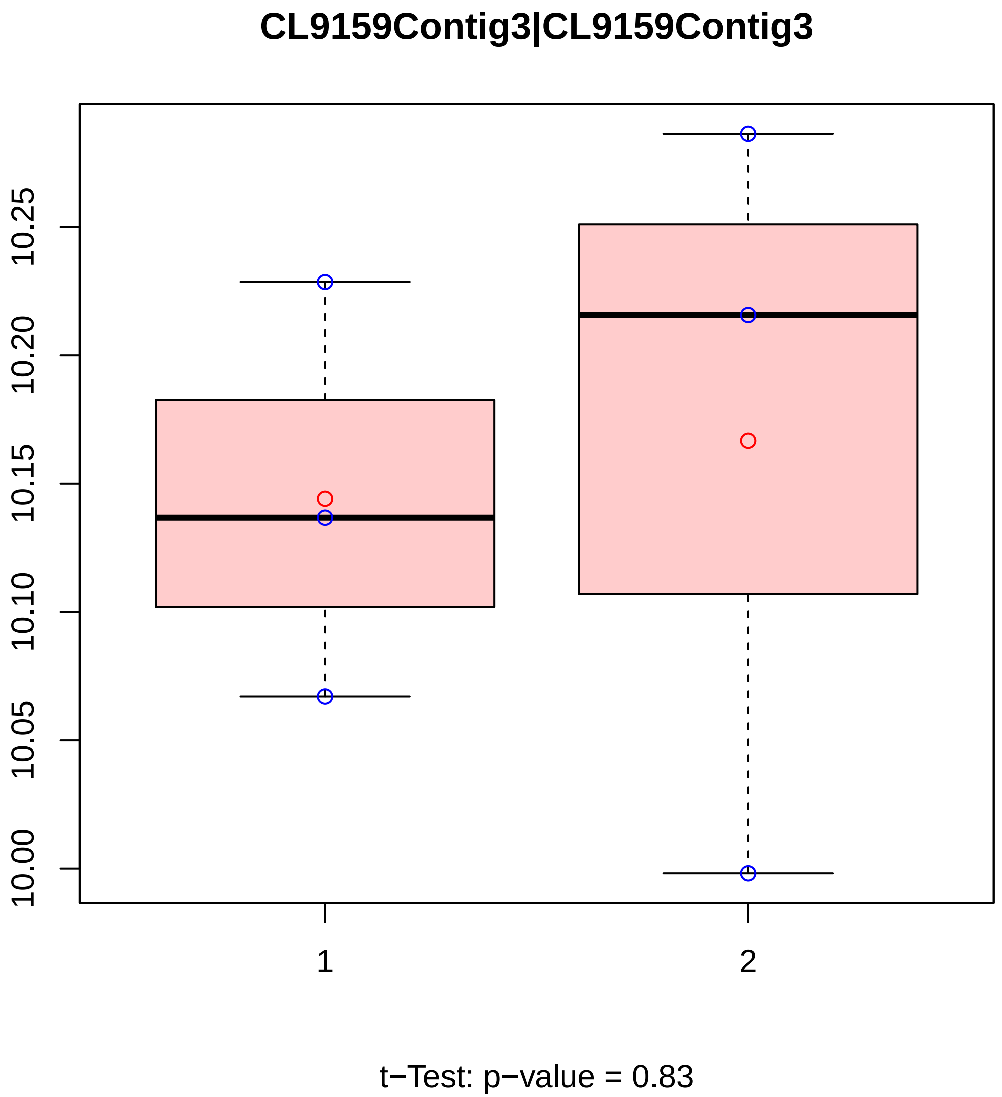
CL9159Contig3|CL9159Contig3
10.00
10.05
10.10
10.15
10.20
10.25
1
2
t−Test: p−value = 0.83

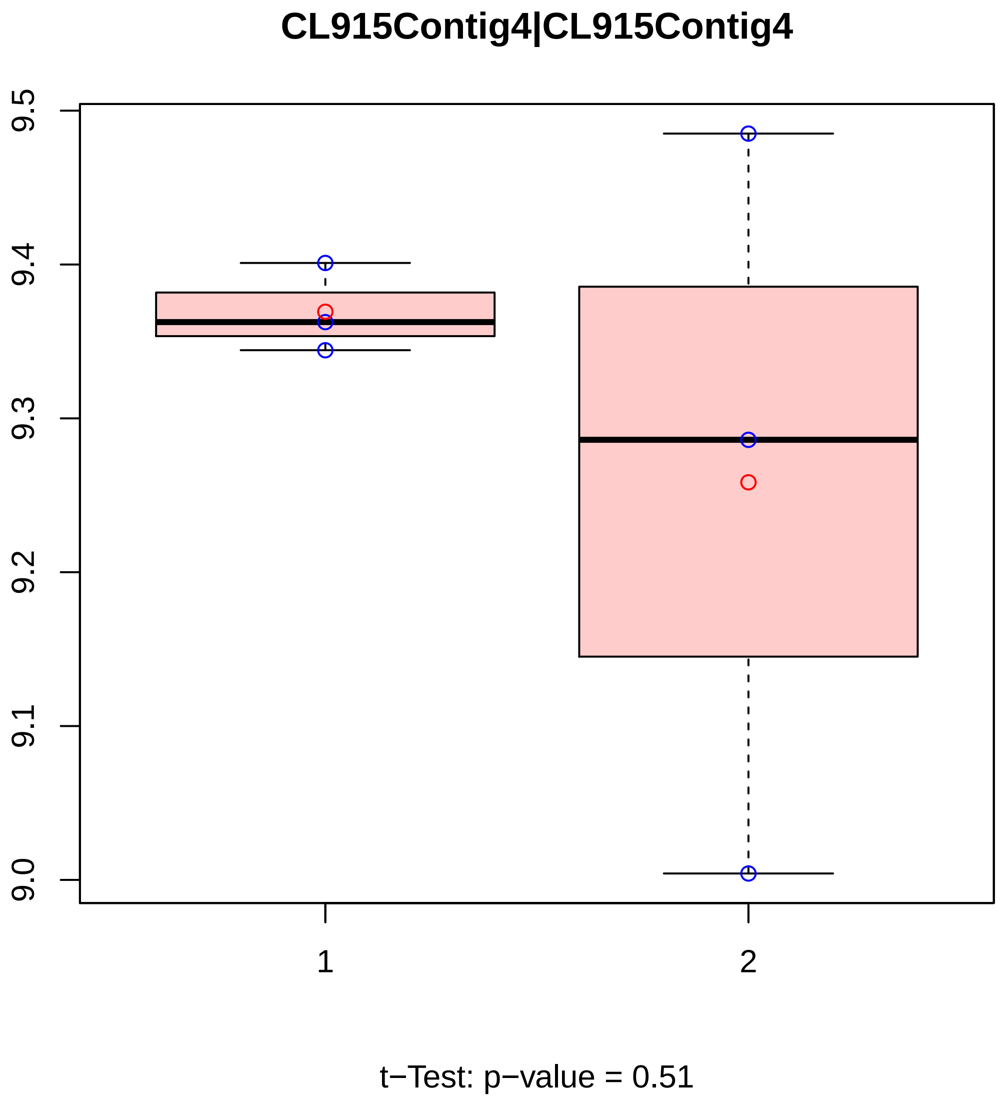
CL915Contig4|CL915Contig4
9.0
9.1
9.2
9.3
9.4
9.5
1
2
t−Test: p−value = 0.51

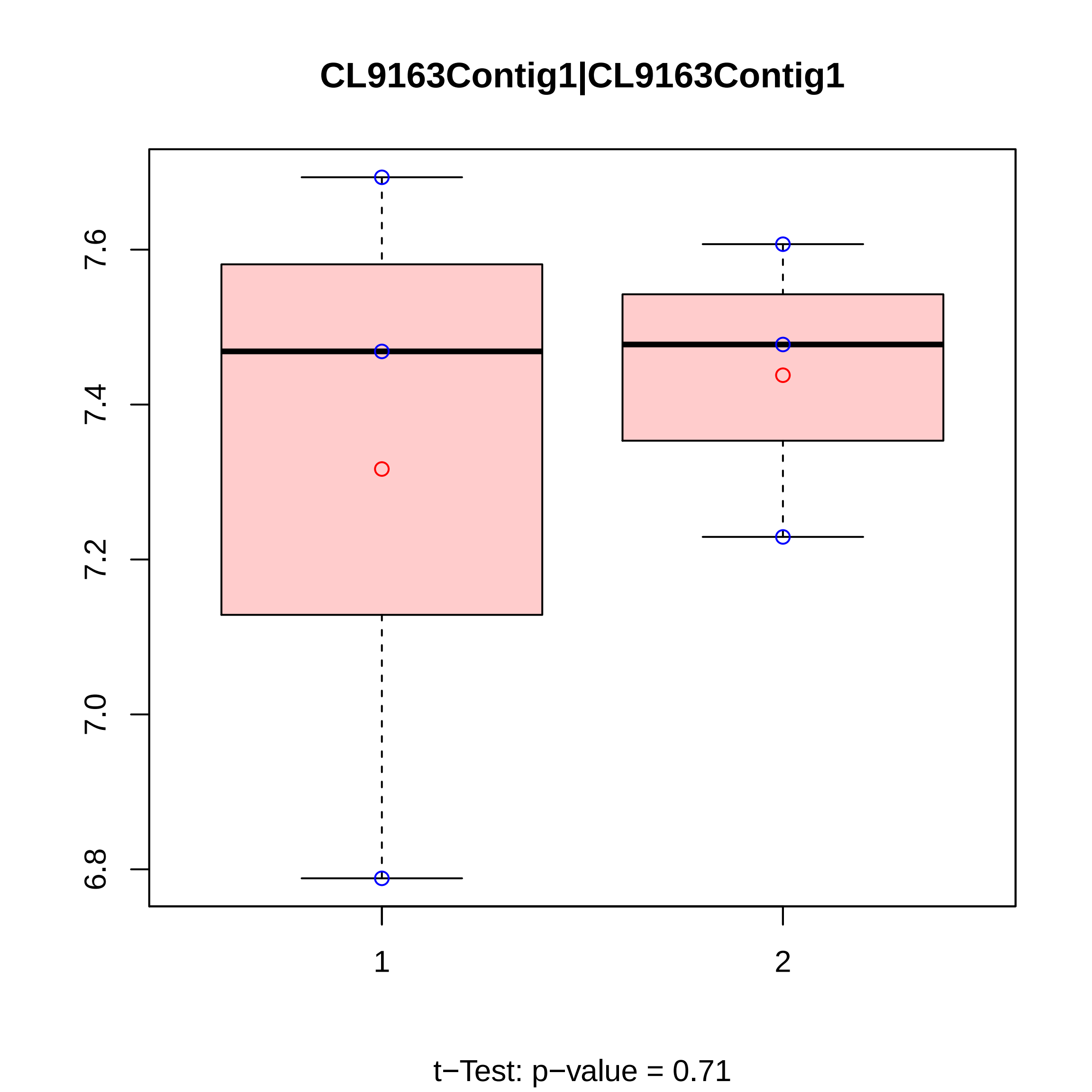
CL9163Contig1|CL9163Contig1
7.6
7.4
7.2
7.0
6.8
1
2
t−Test: p−value = 0.71

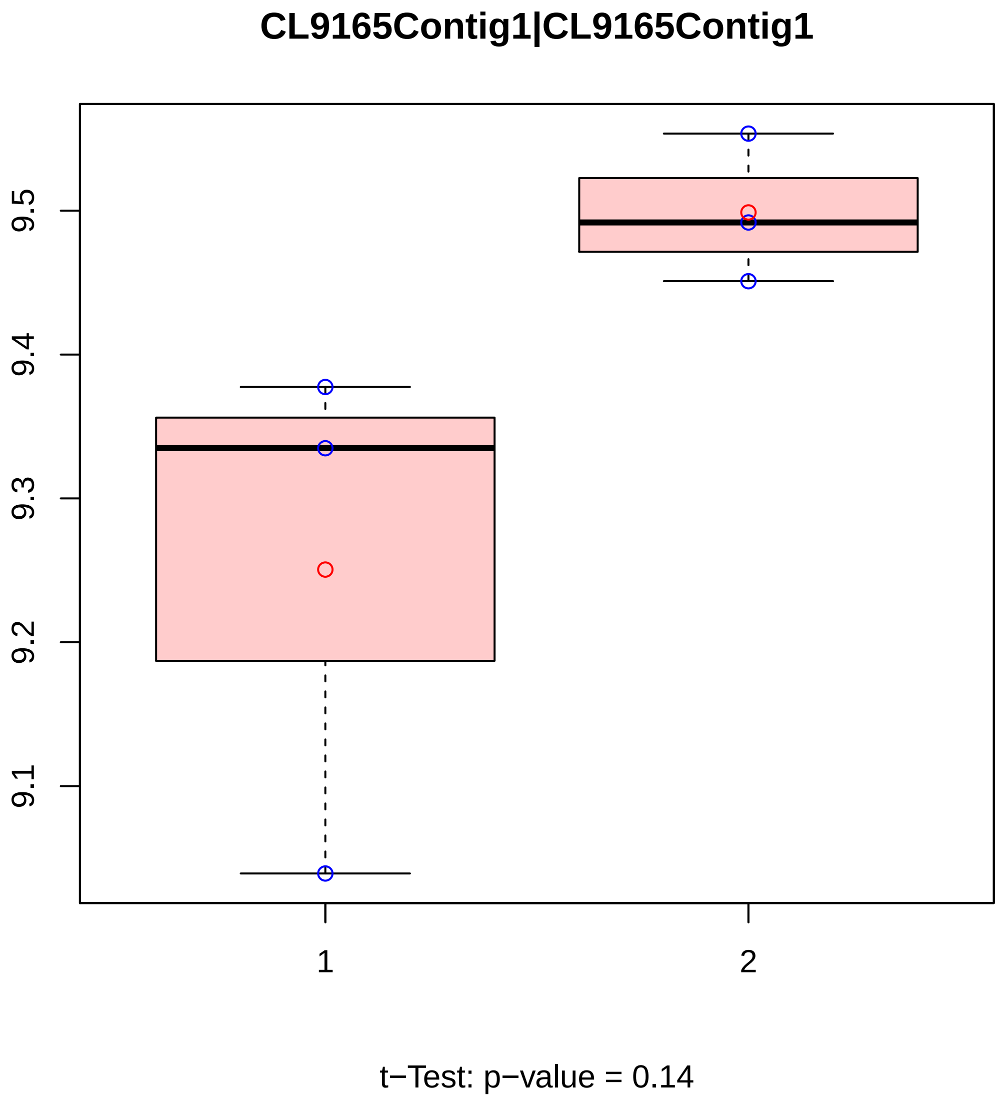
CL9165Contig1|CL9165Contig1
9.1
9.2
9.3
9.4
9.5
1
2
t−Test: p−value = 0.14

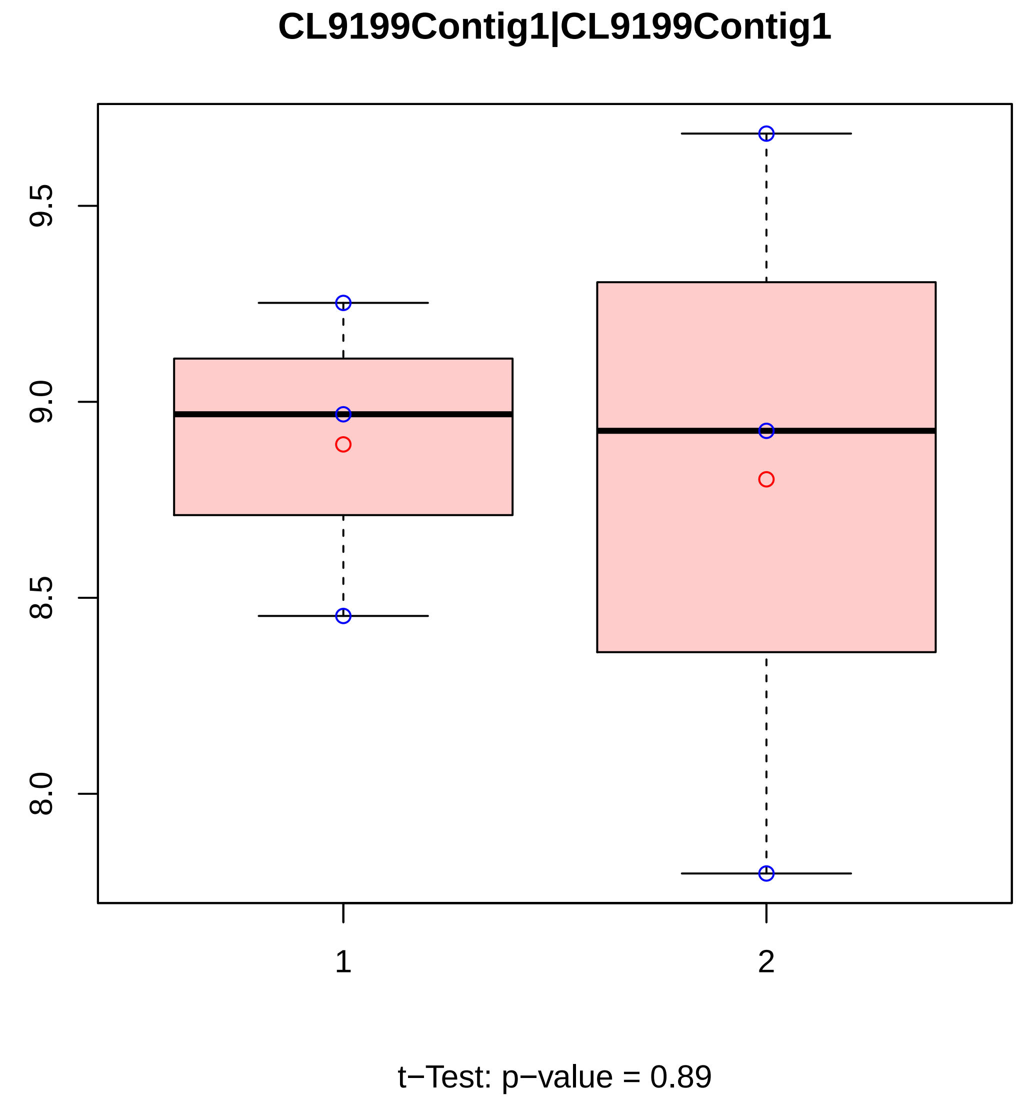
CL9199Contig1|CL9199Contig1
9.5
9.0
8.5
8.0
1
2
t−Test: p−value = 0.89

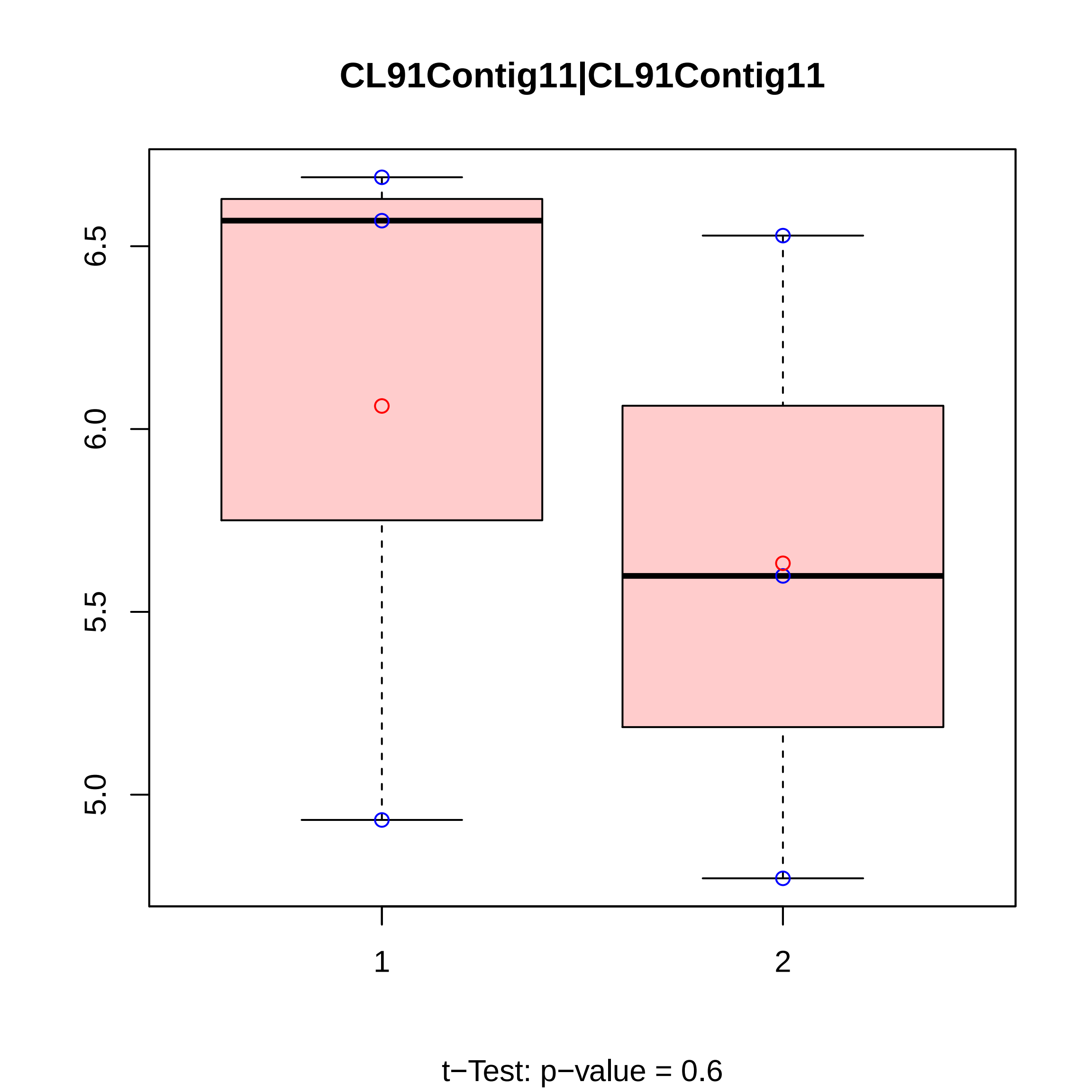

CL91Contig11|CL91Contig11
6.5
6.0
5.5
5.0
1
2
t−Test: p−value = 0.6

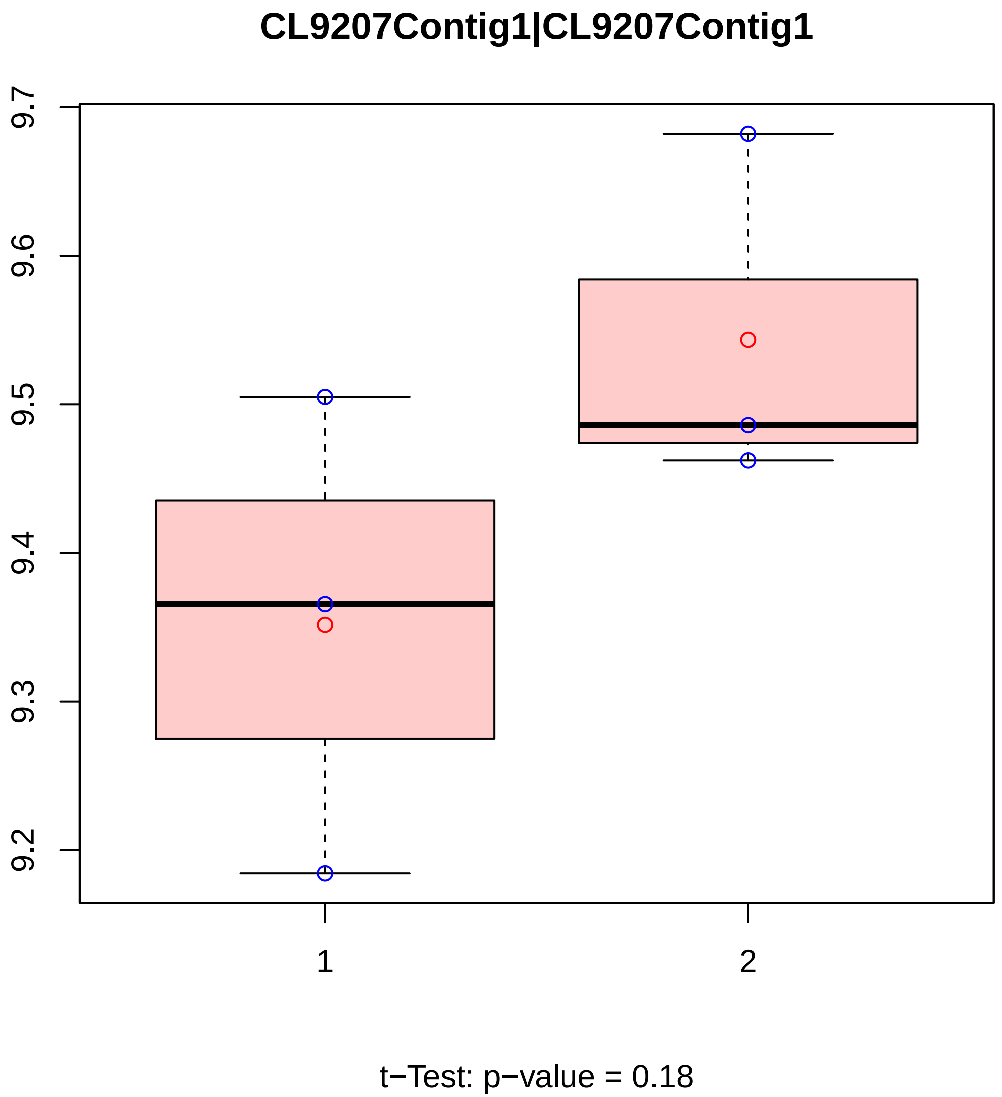
CL9207Contig1|CL9207Contig1
9.7
9.6
9.5
9.4
9.3
9.2
1
2
t−Test: p−value = 0.18

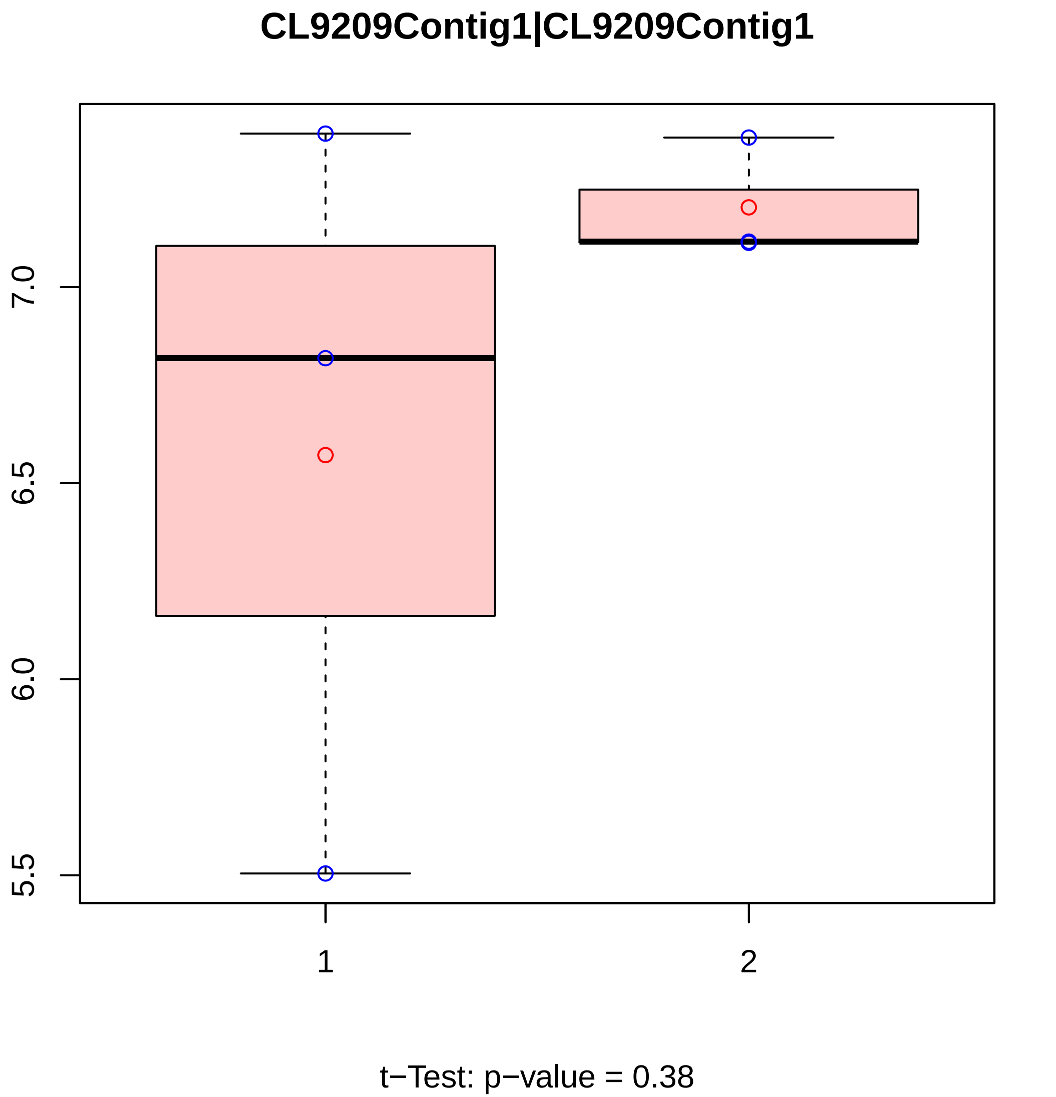
CL9209Contig1|CL9209Contig1
7.0
6.5
6.0
5.5
1
2
t−Test: p−value = 0.38

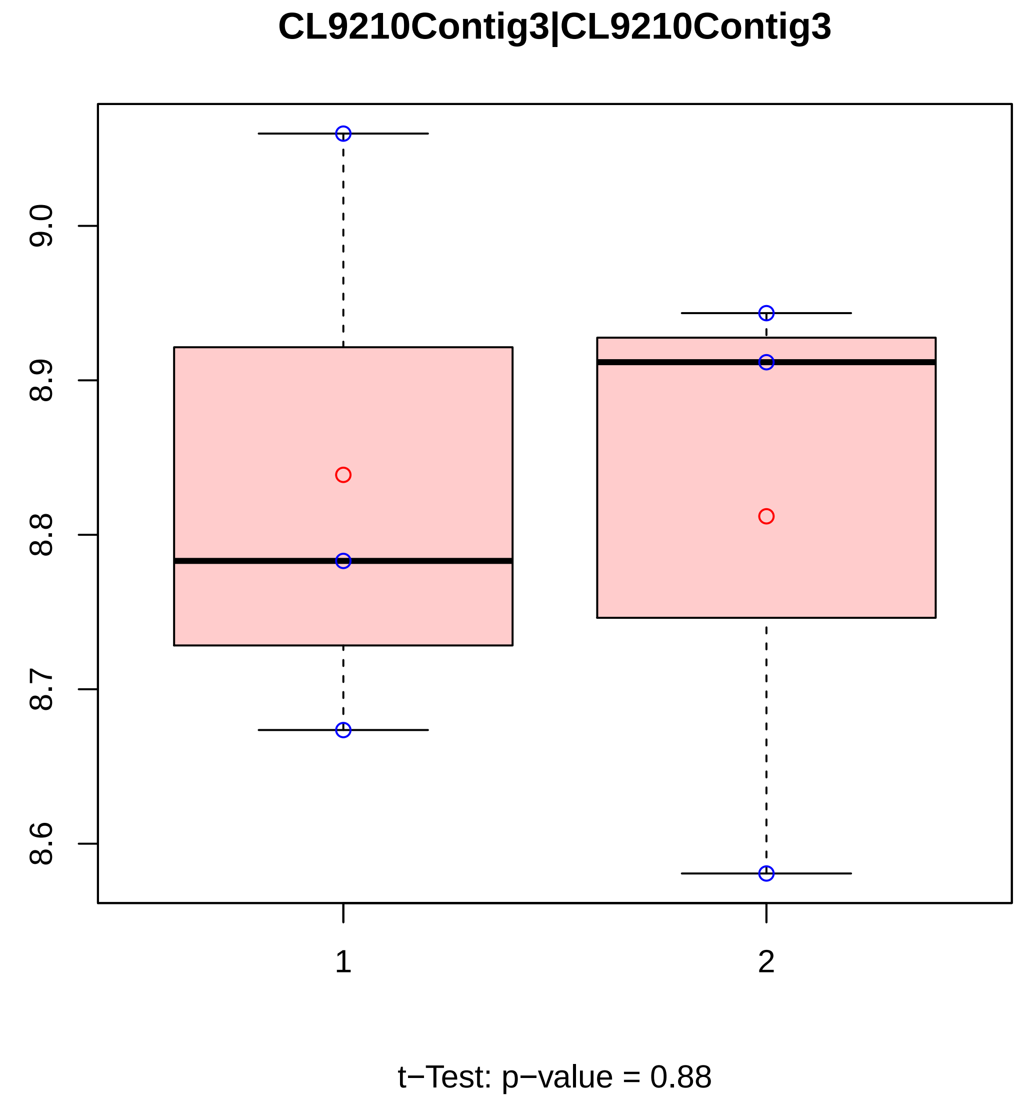
CL9210Contig3|CL9210Contig3
9.0
8.9
8.8
8.7
8.6
1
2
t−Test: p−value = 0.88

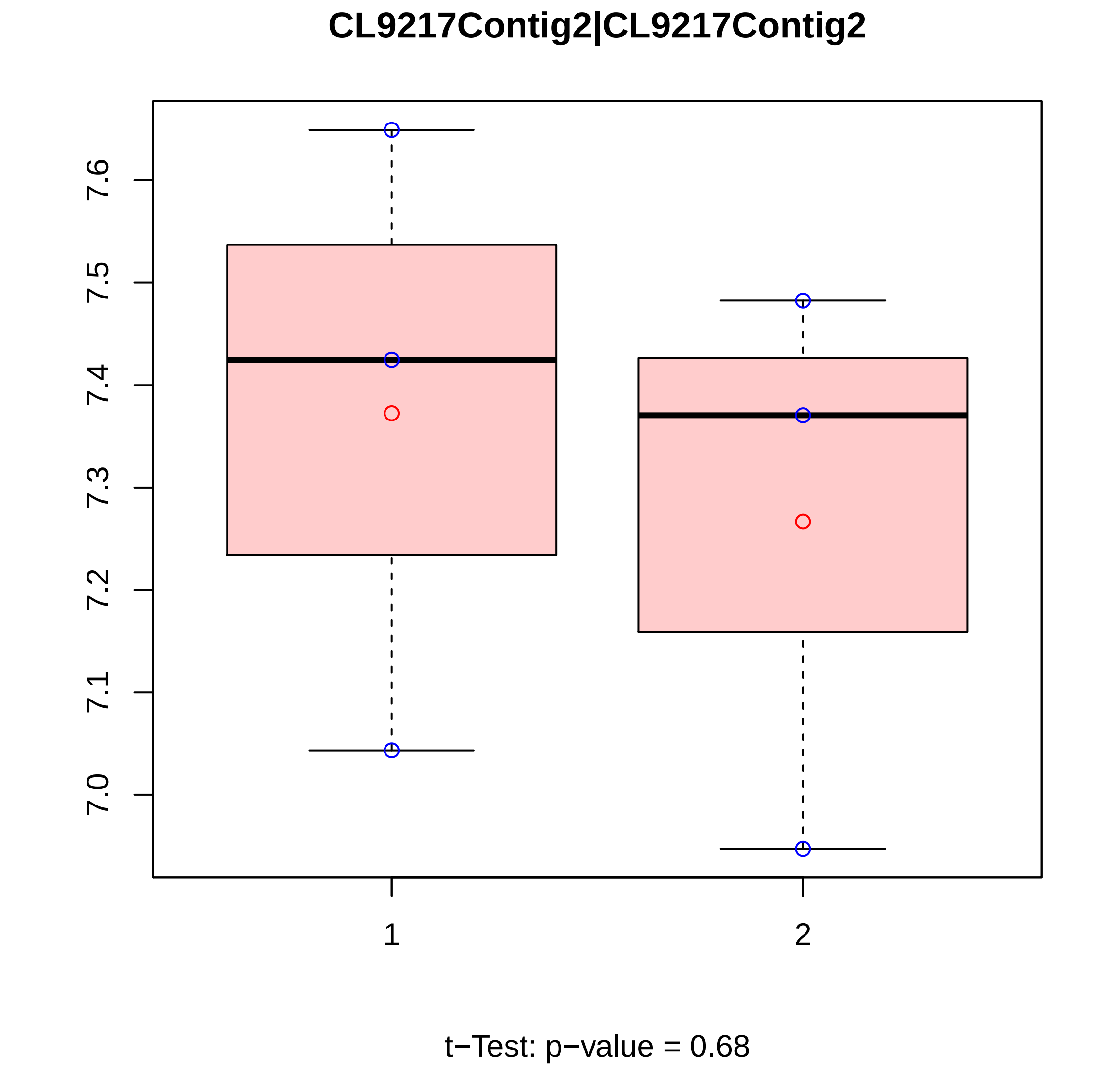

CL9217Contig2|CL9217Contig2
7.0
7.1
7.2
7.3
7.4
7.5
7.6
1
2
t−Test: p−value = 0.68

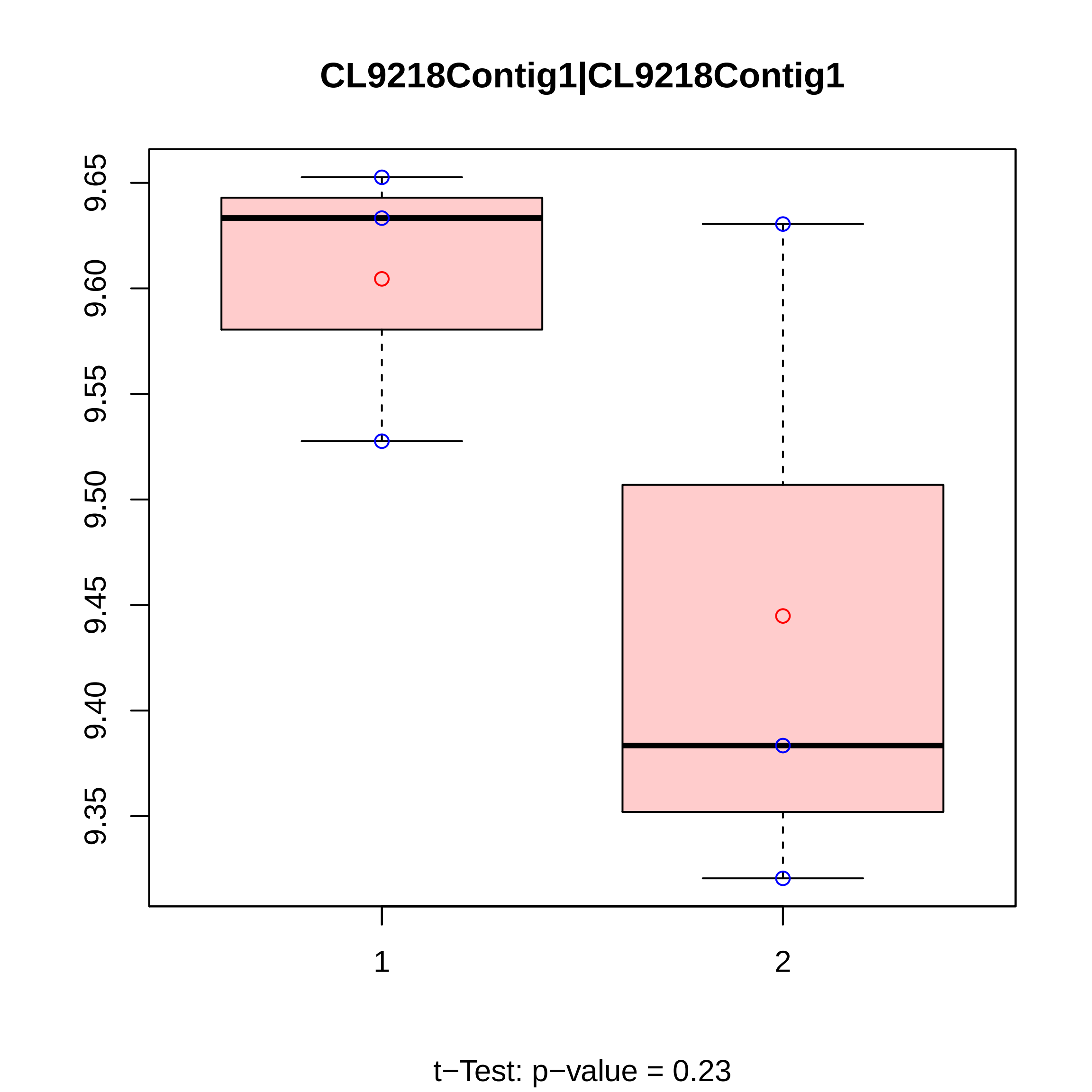

CL9218Contig1|CL9218Contig1
9.35
9.40
9.45
9.50
9.55
9.60
9.65
1
2
t−Test: p−value = 0.23

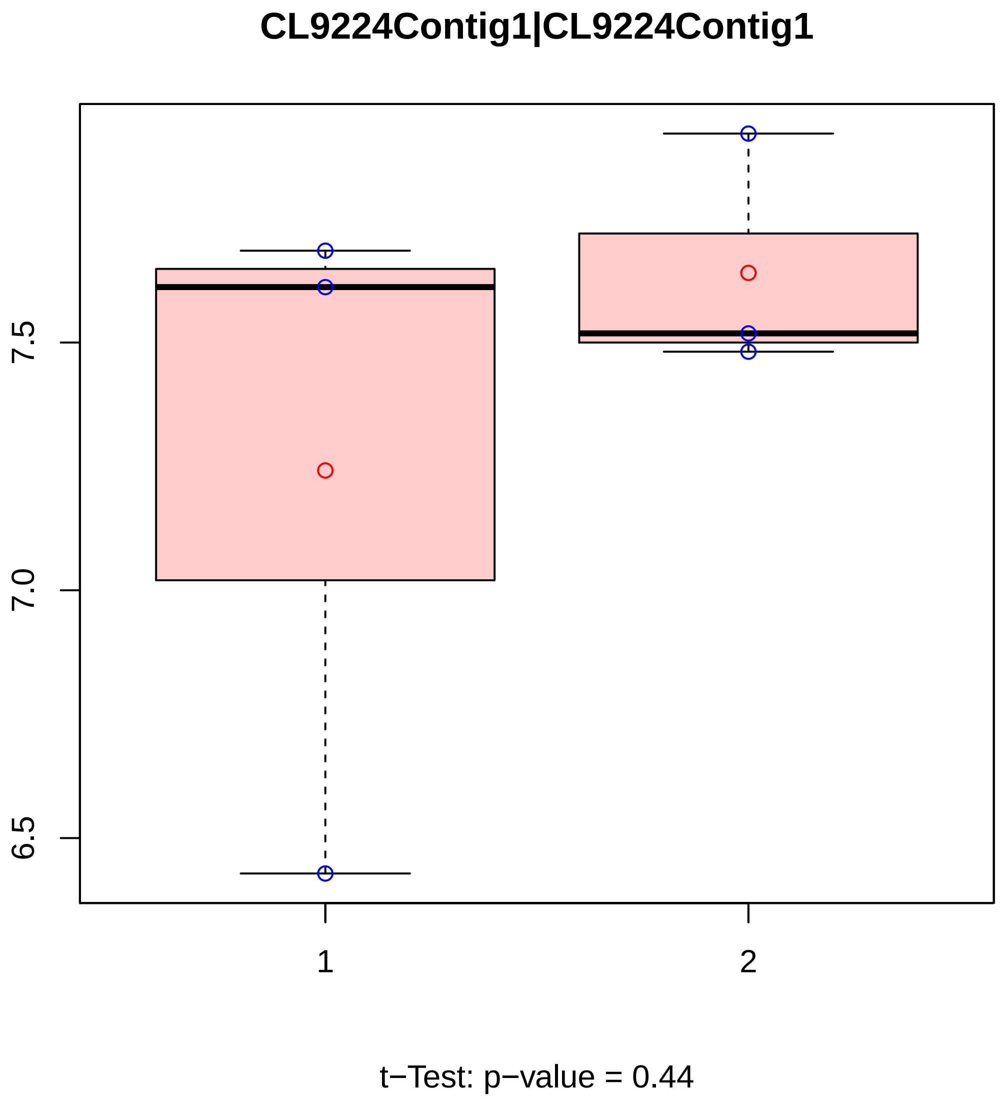
CL9224Contig1|CL9224Contig1
7.5
7.0
6.5
1
2
t−Test: p−value = 0.44

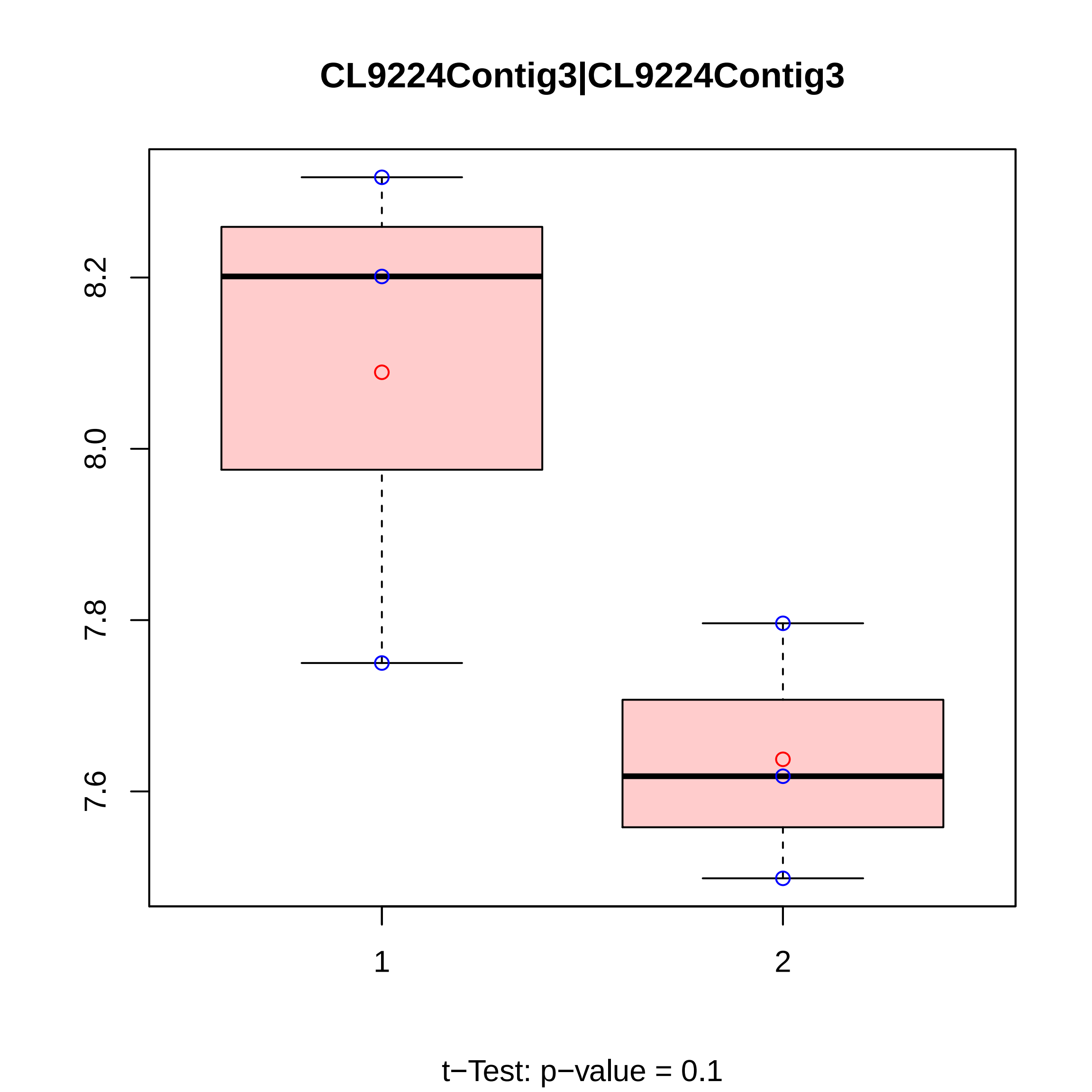
CL9224Contig3|CL9224Contig3
8.2
8.0
7.8
7.6
1
2
t−Test: p−value = 0.1

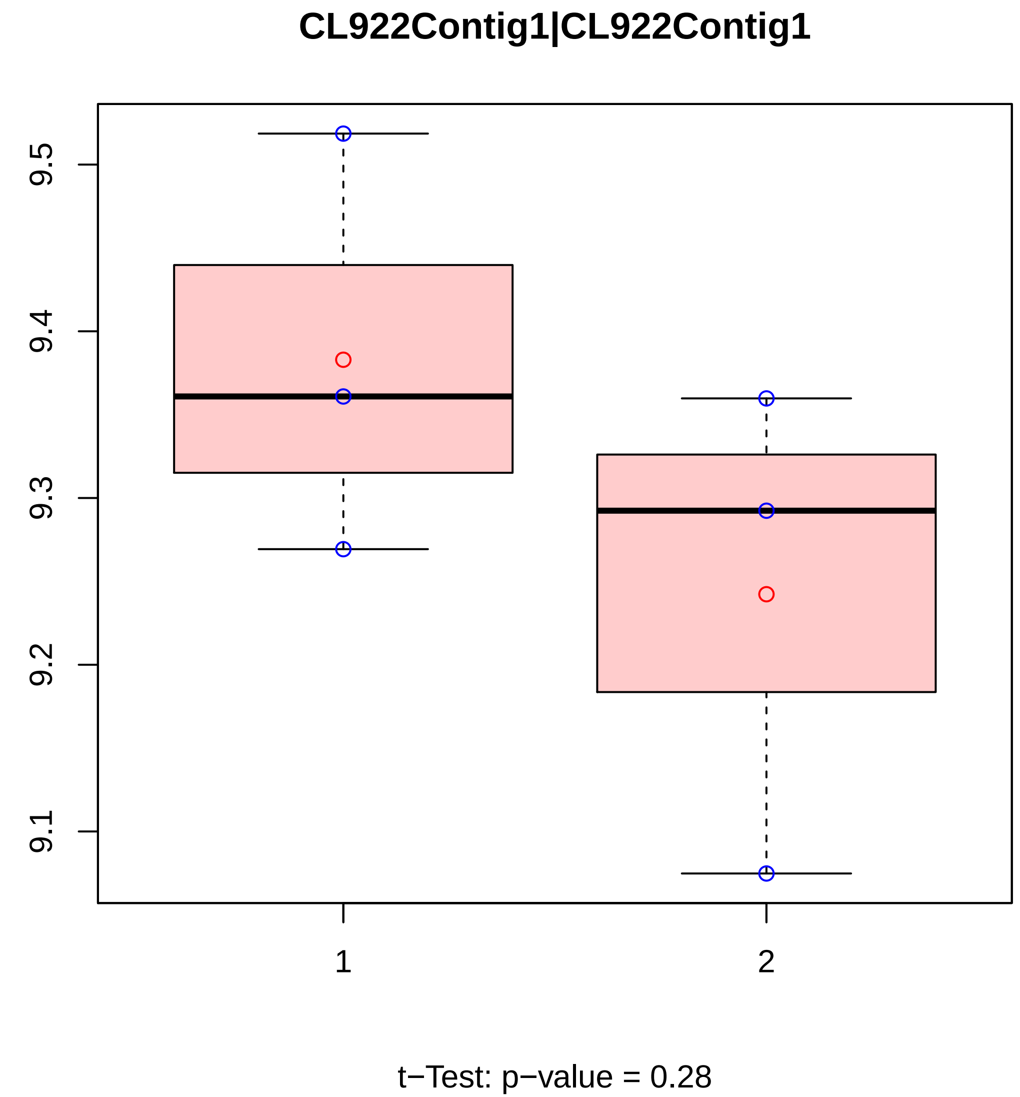

CL922Contig1|CL922Contig1
9.5
9.4
9.3
9.2
9.1
1
2
t−Test: p−value = 0.28

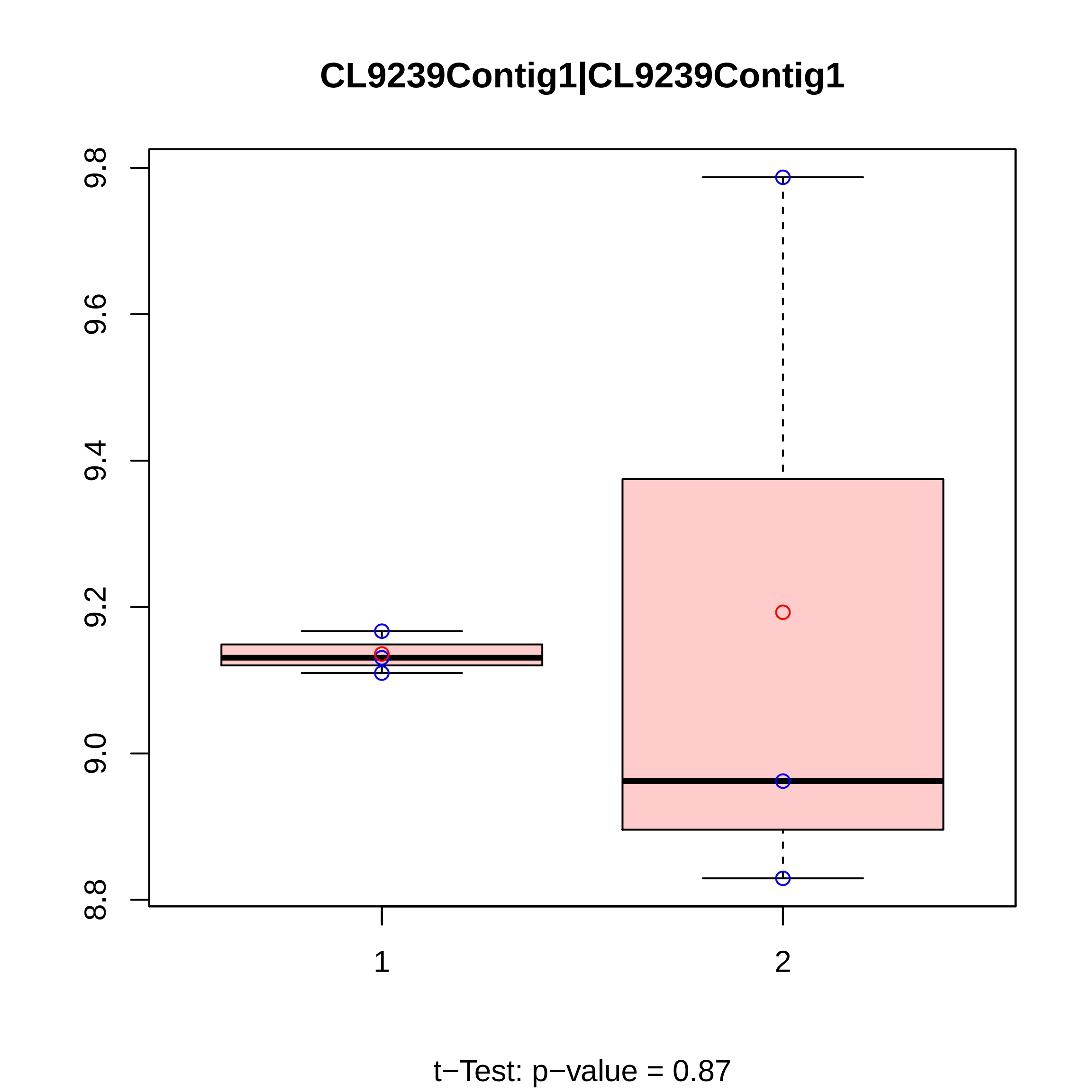

CL9239Contig1|CL9239Contig1
9.8
9.6
9.4
9.2
9.0
8.8
1
2
t−Test: p−value = 0.87

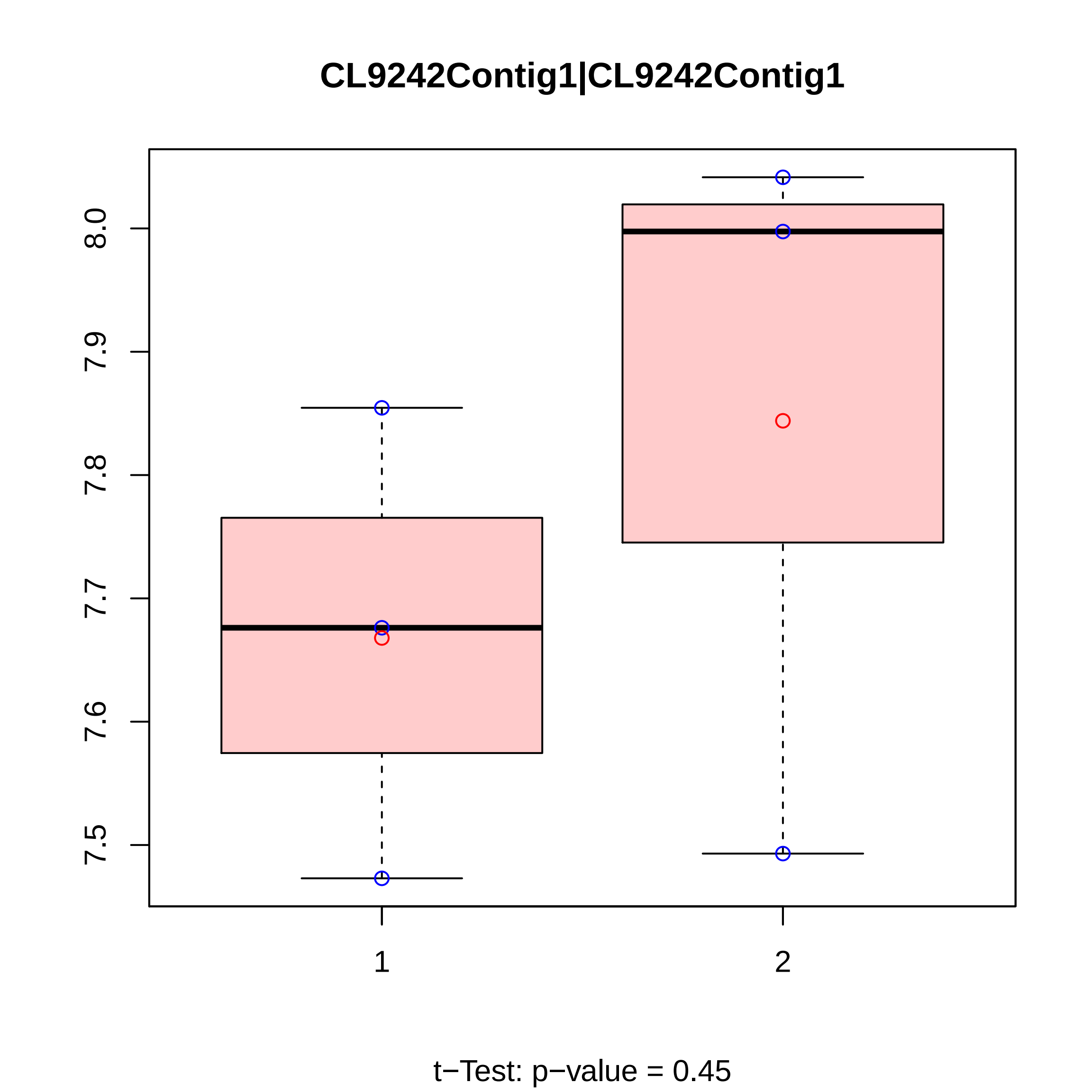
CL9242Contig1|CL9242Contig1
8.0
7.9
7.8
7.7
7.6
7.5
1
2
t−Test: p−value = 0.45

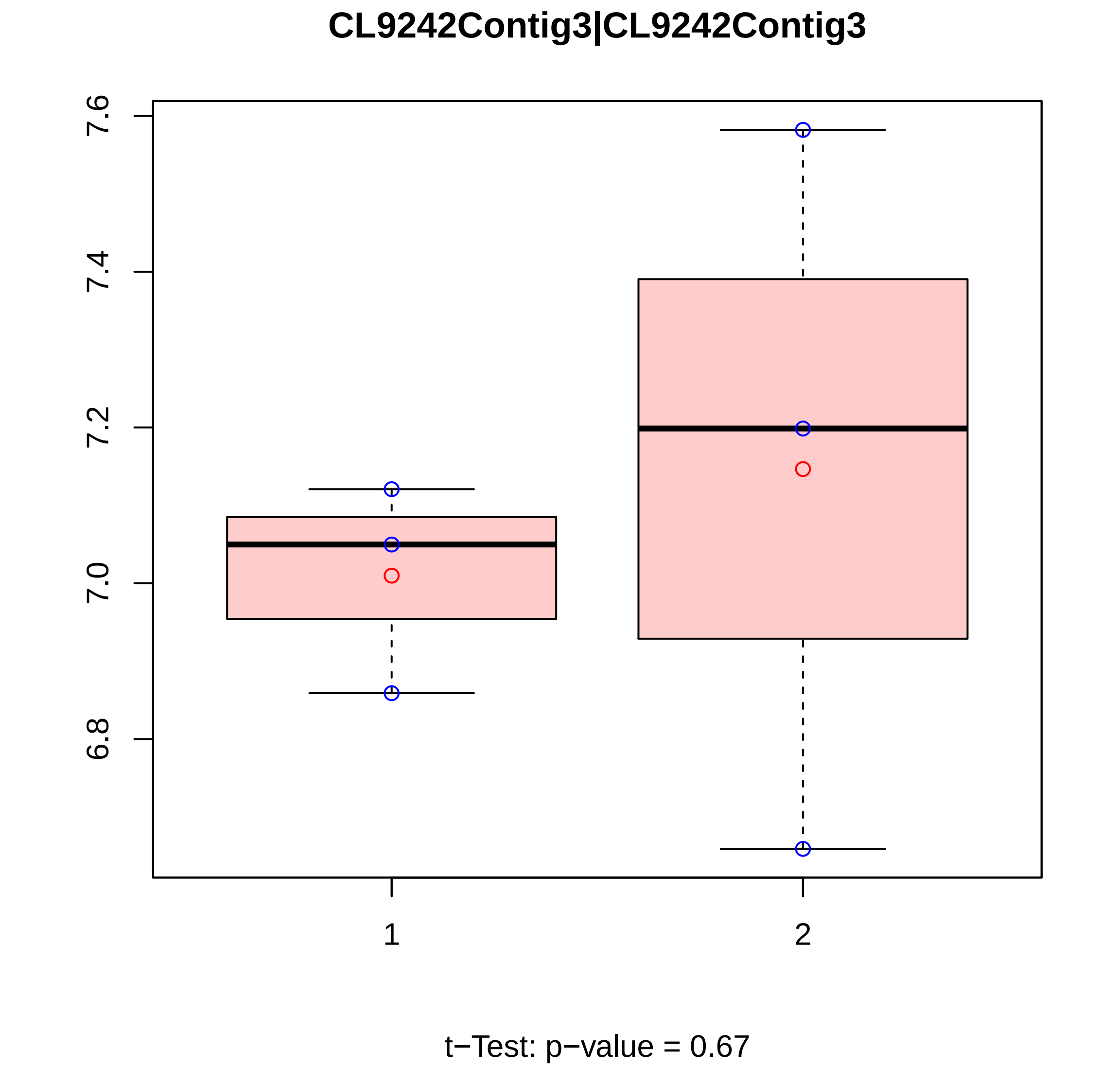
CL9242Contig3|CL9242Contig3
7.6
7.4
7.2
7.0
6.8
1
2
t−Test: p−value = 0.67

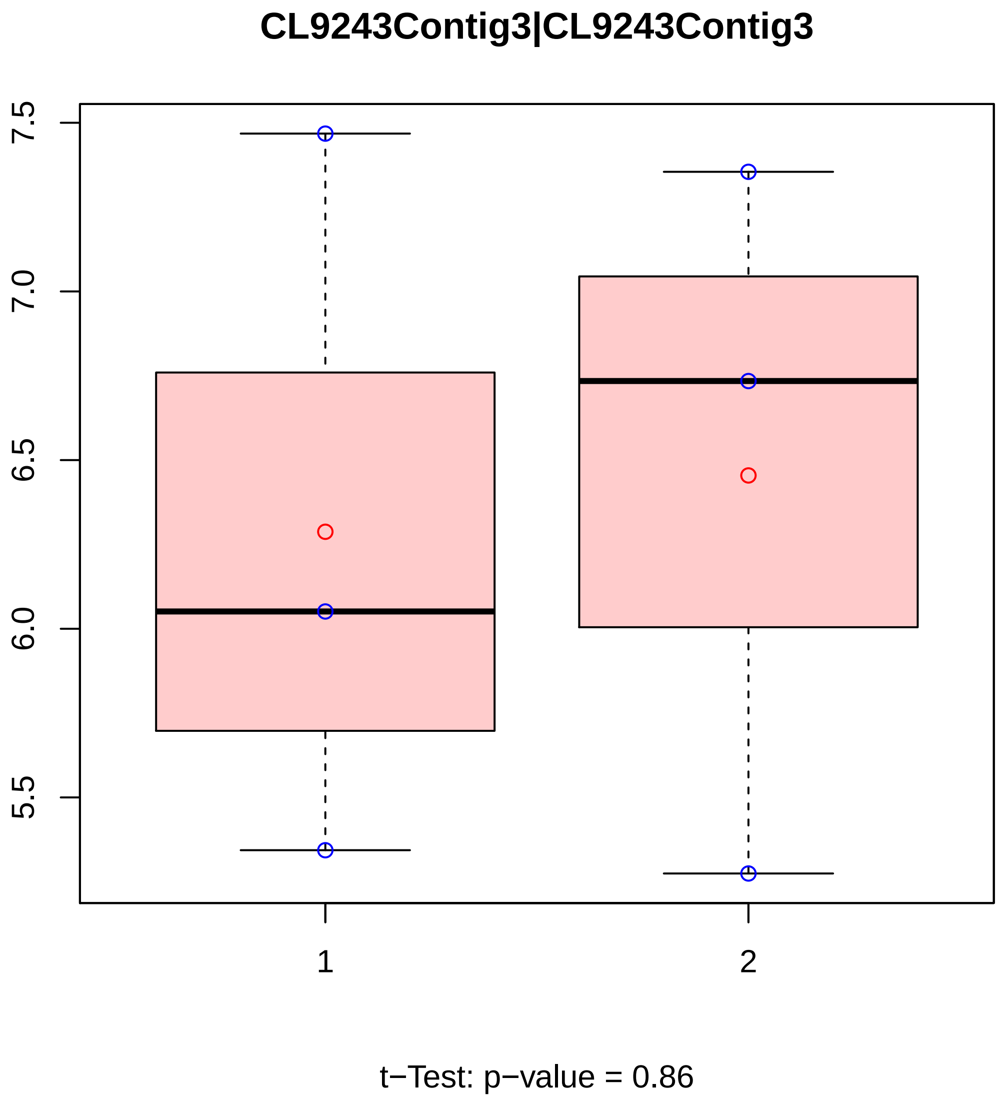
CL9243Contig3|CL9243Contig3
7.5
7.0
6.5
6.0
5.5
1
2
t−Test: p−value = 0.86

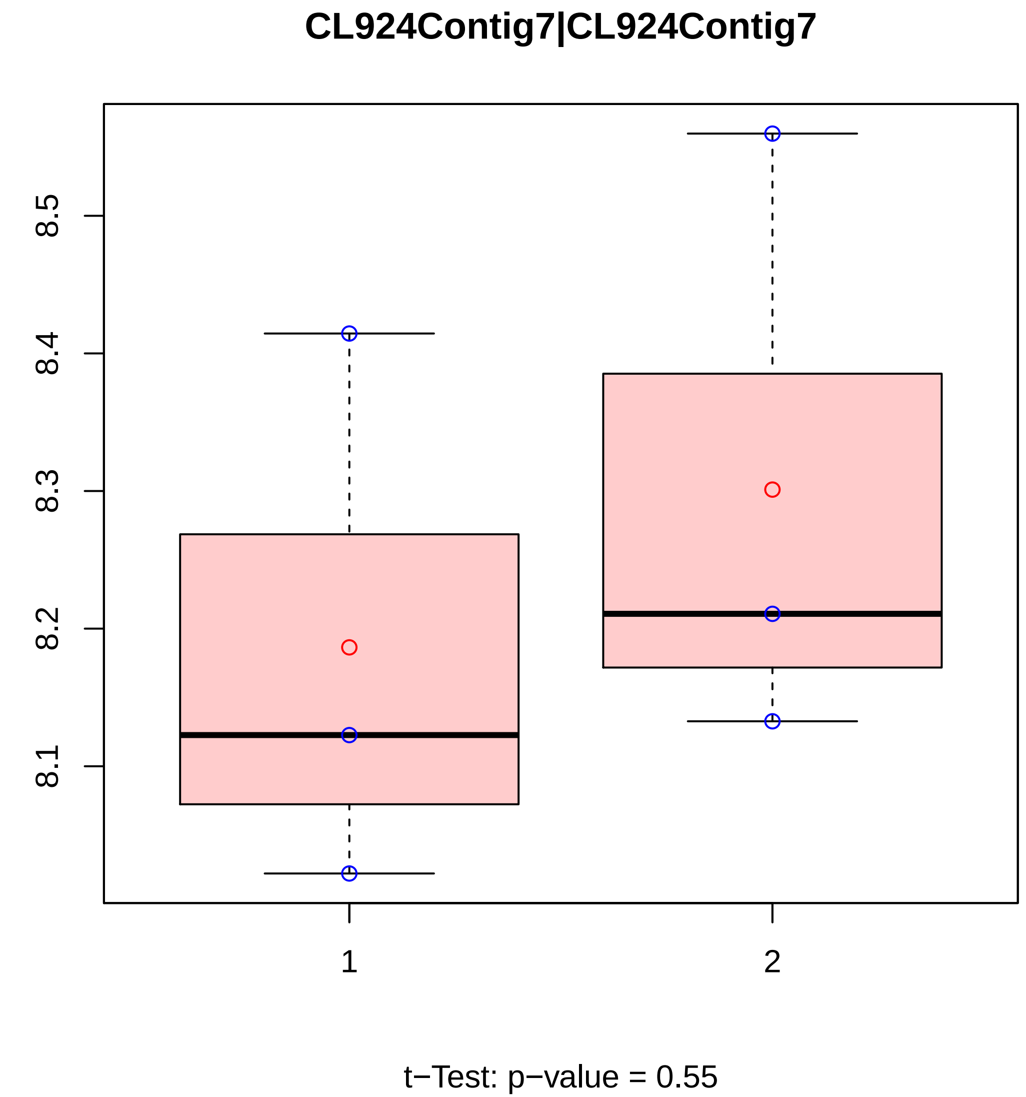

CL924Contig7|CL924Contig7
8.1
8.2
8.3
8.4
8.5
1
2
t−Test: p−value = 0.55

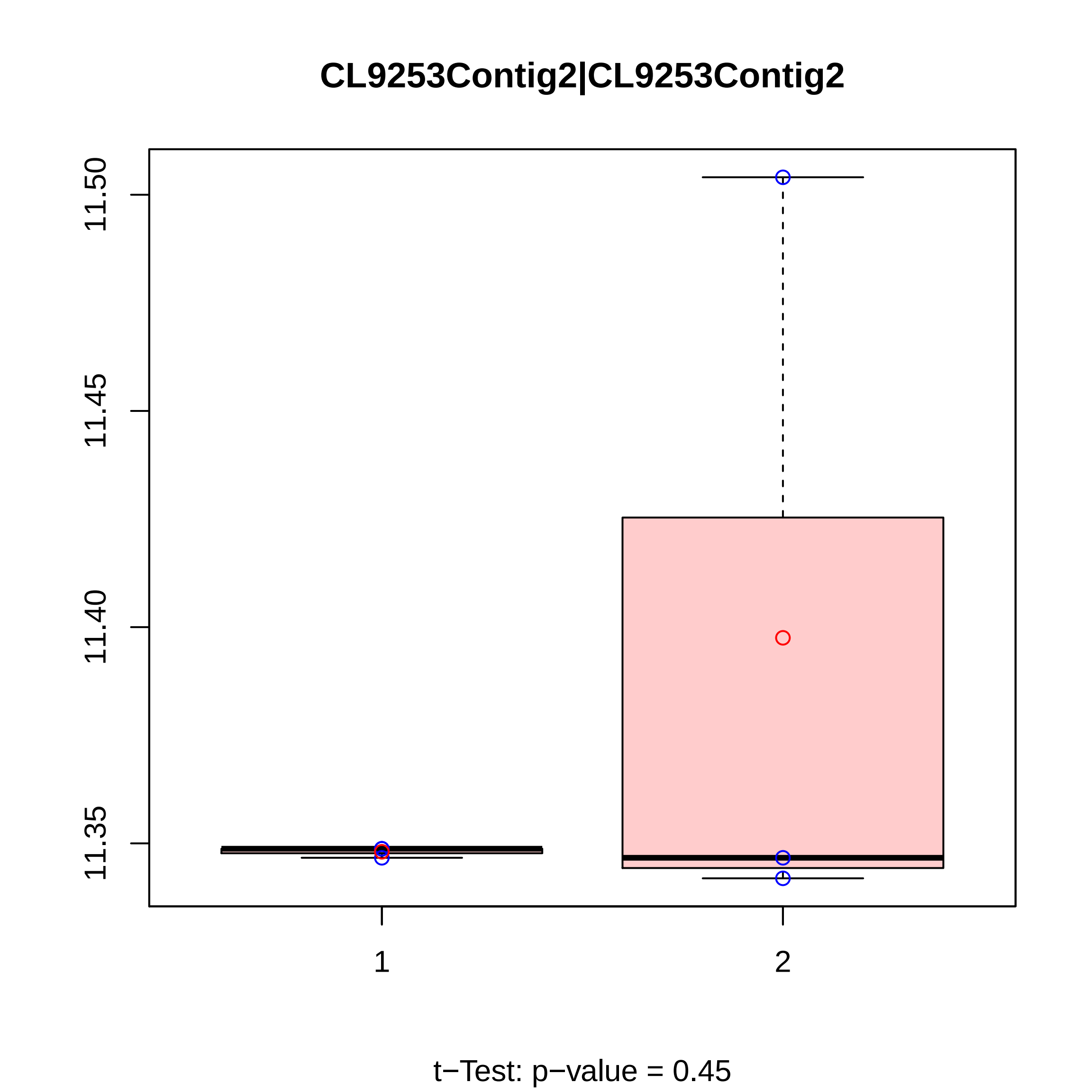

CL9253Contig2|CL9253Contig2
11.35
11.40
11.45
11.50
1
2
t−Test: p−value = 0.45

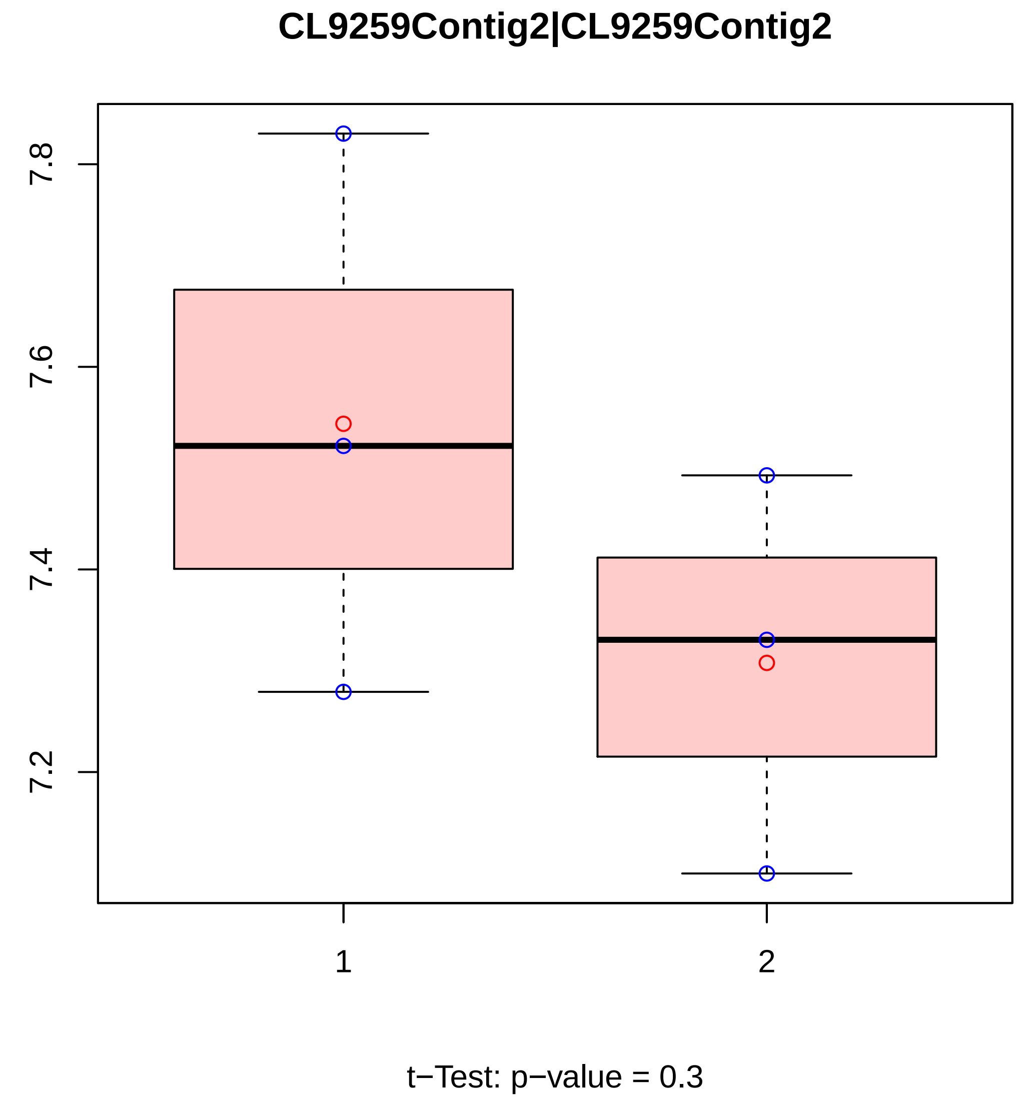

CL9259Contig2|CL9259Contig2
7.2
7.4
7.6
7.8
1
2
t−Test: p−value = 0.3

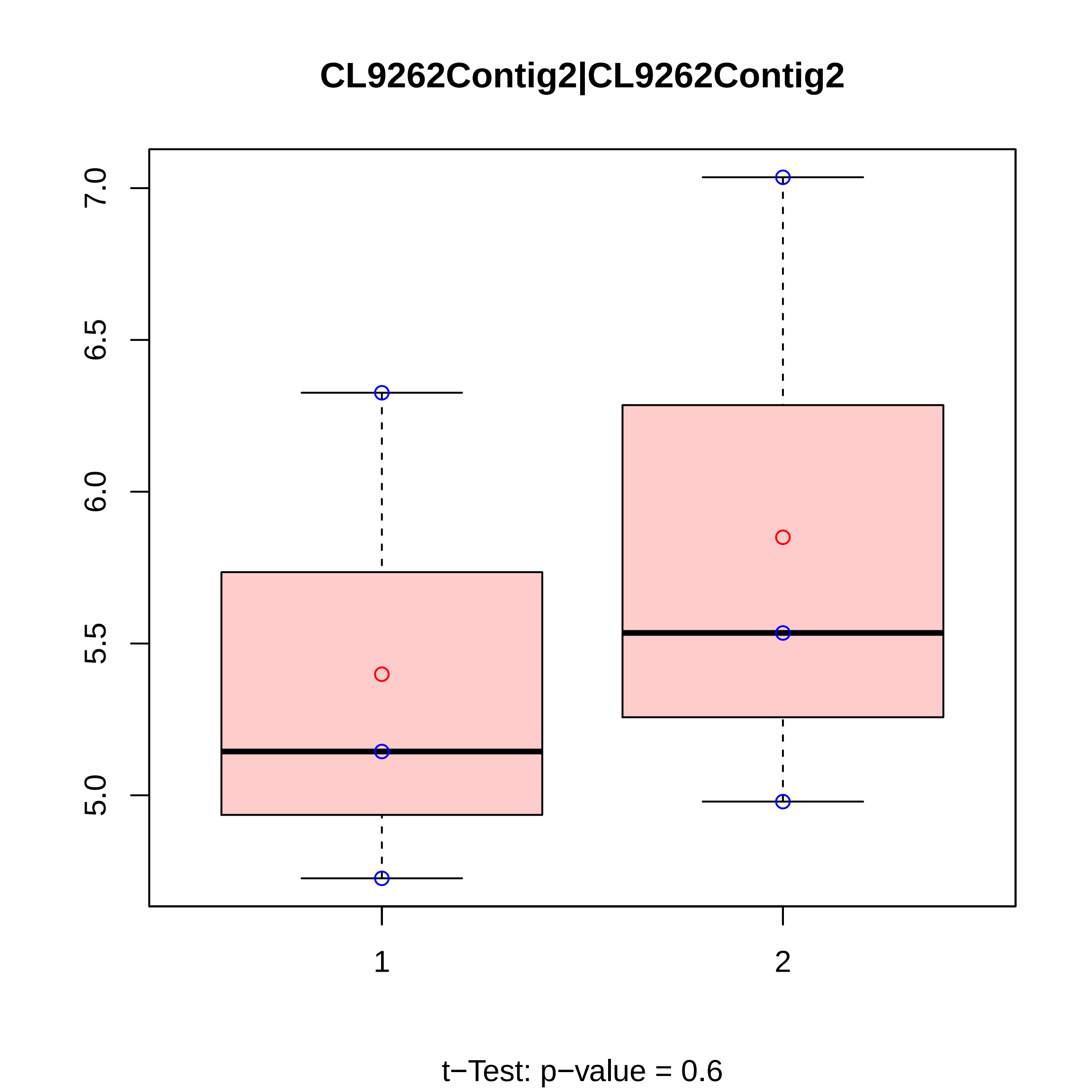
CL9262Contig2|CL9262Contig2
7.0
6.5
6.0
5.5
5.0
1
2
t−Test: p−value = 0.6

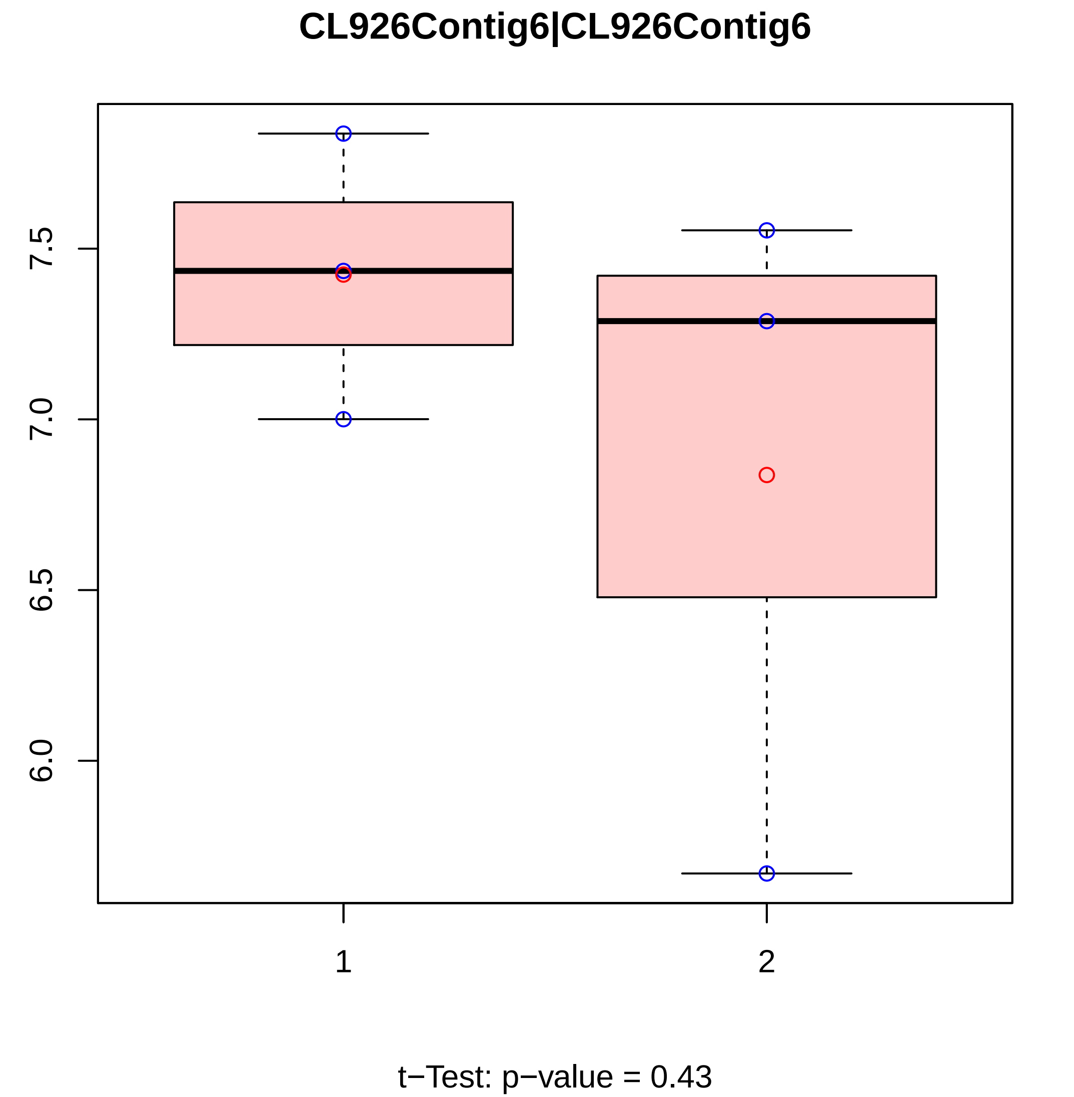
CL926Contig6|CL926Contig6
7.5
7.0
6.5
6.0
1
2
t−Test: p−value = 0.43

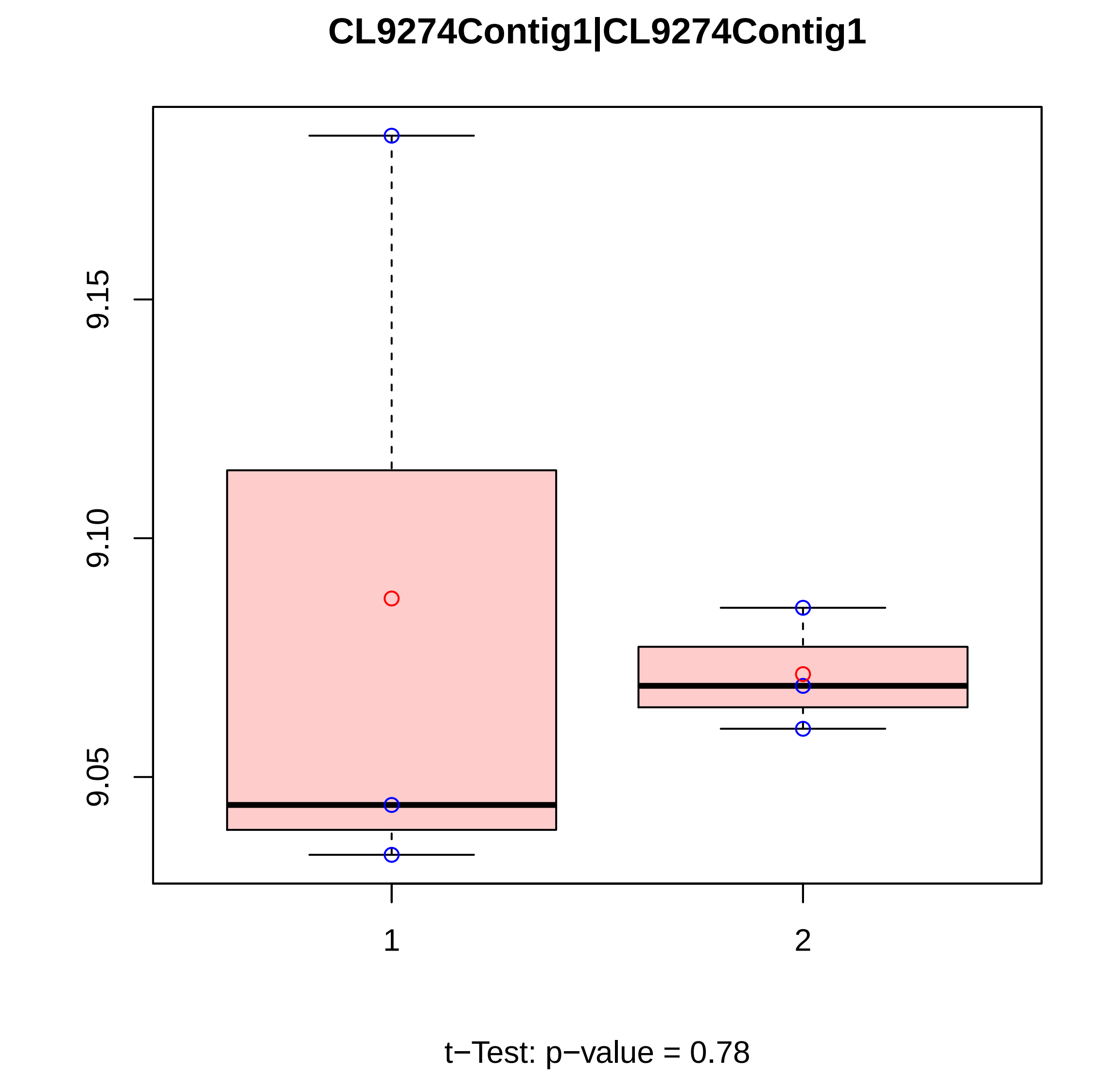

CL9274Contig1|CL9274Contig1
9.15
9.10
9.05
1
2
t−Test: p−value = 0.78

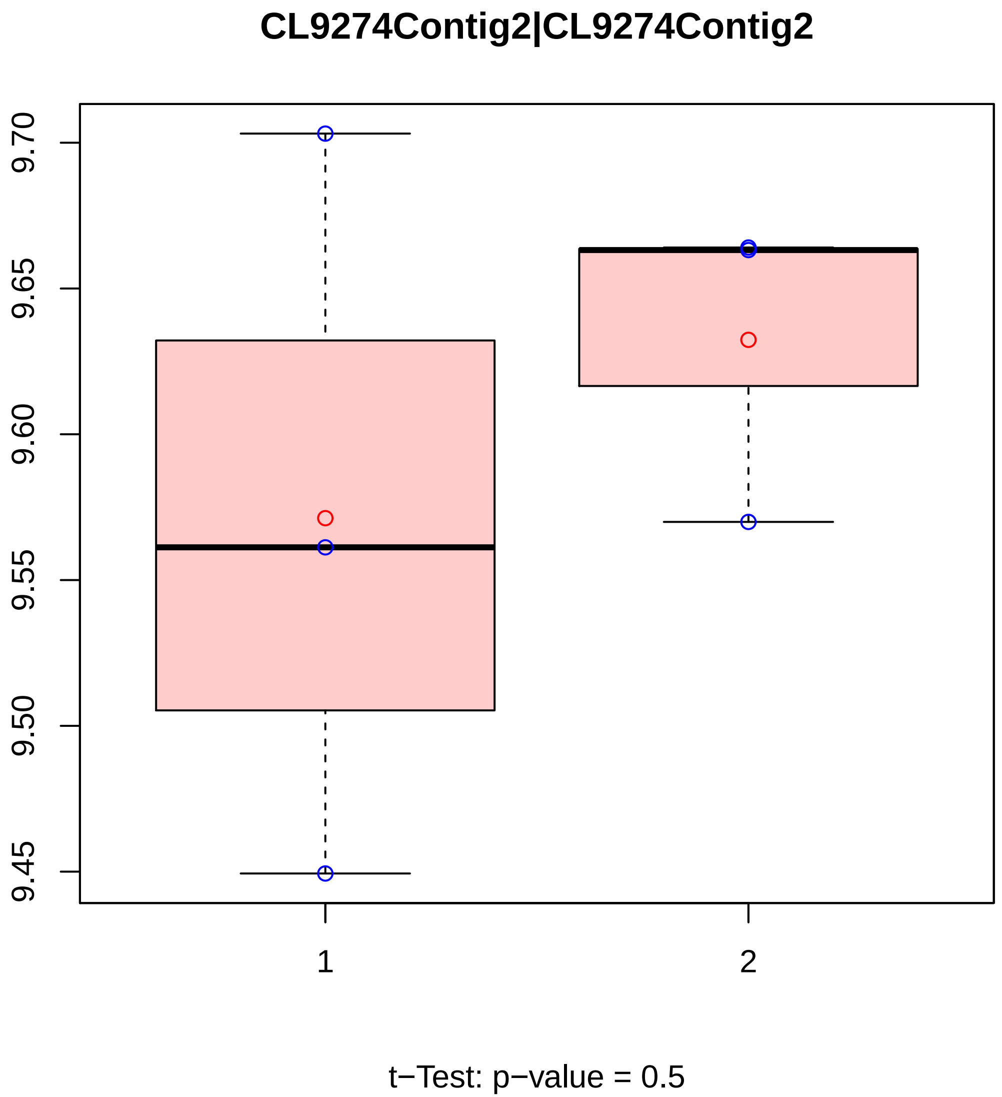
CL9274Contig2|CL9274Contig2
9.70
9.65
9.60
9.55
9.50
9.45
1
2
t−Test: p−value = 0.5

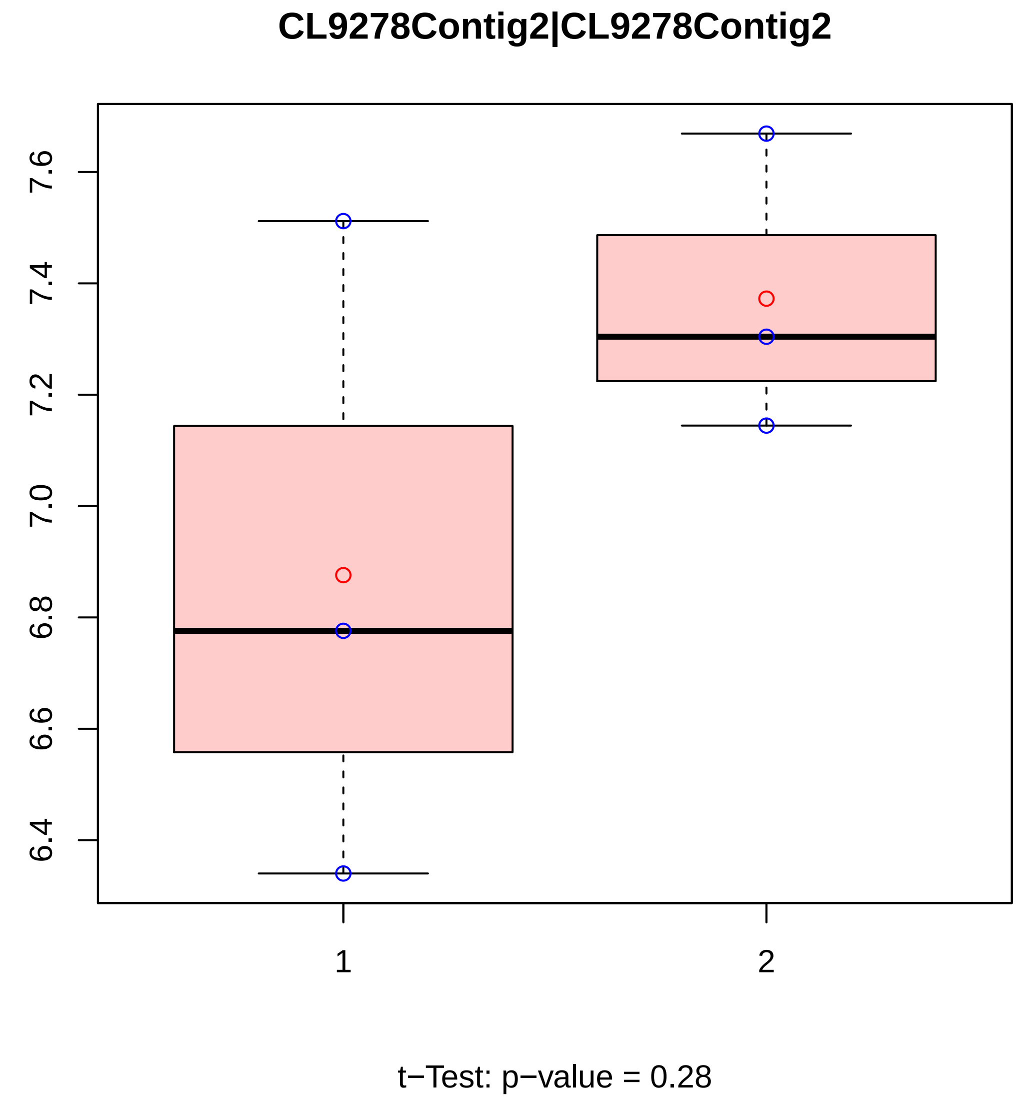
CL9278Contig2|CL9278Contig2
7.6
7.4
7.2
7.0
6.8
6.6
6.4
1
2
t−Test: p−value = 0.28

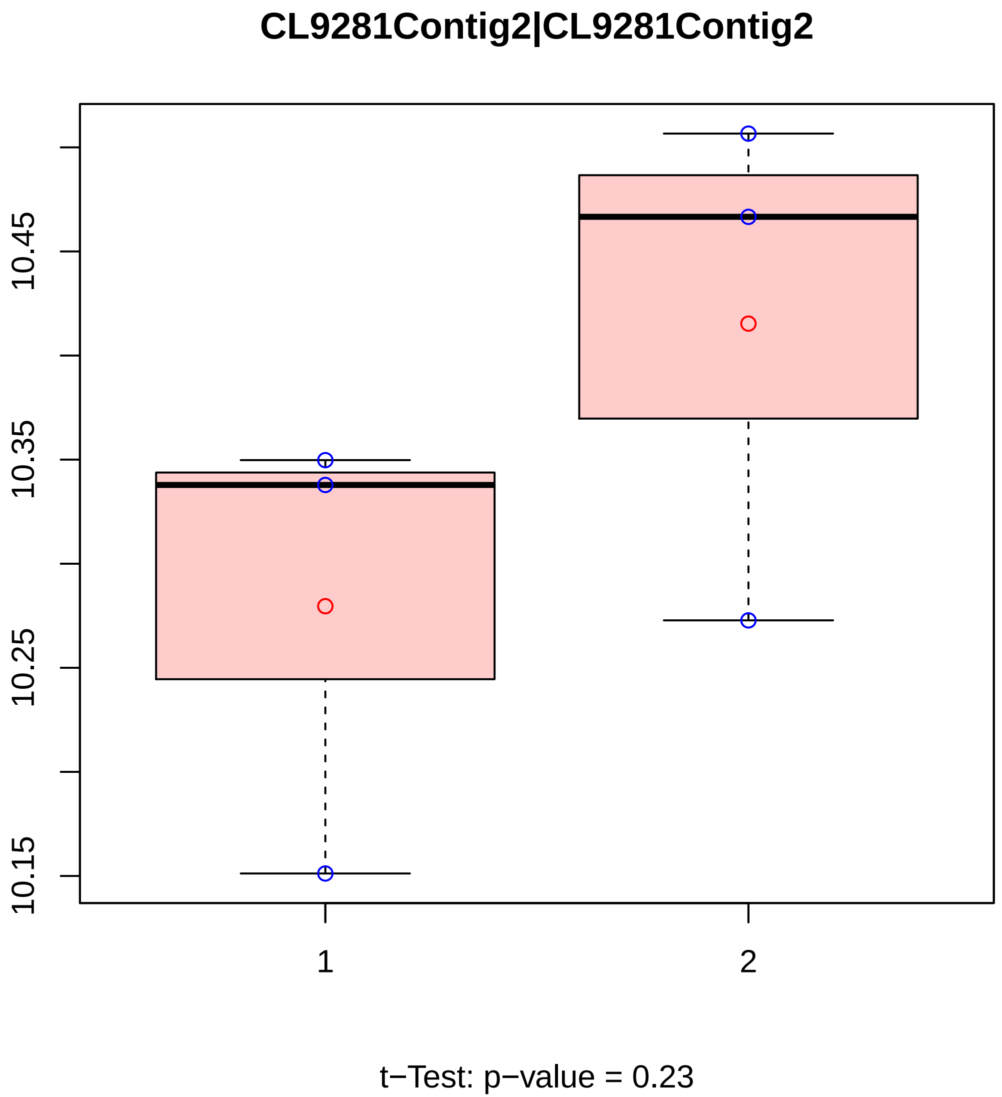
CL9281Contig2|CL9281Contig2
10.15
10.25
10.35
10.45
1
2
t−Test: p−value = 0.23

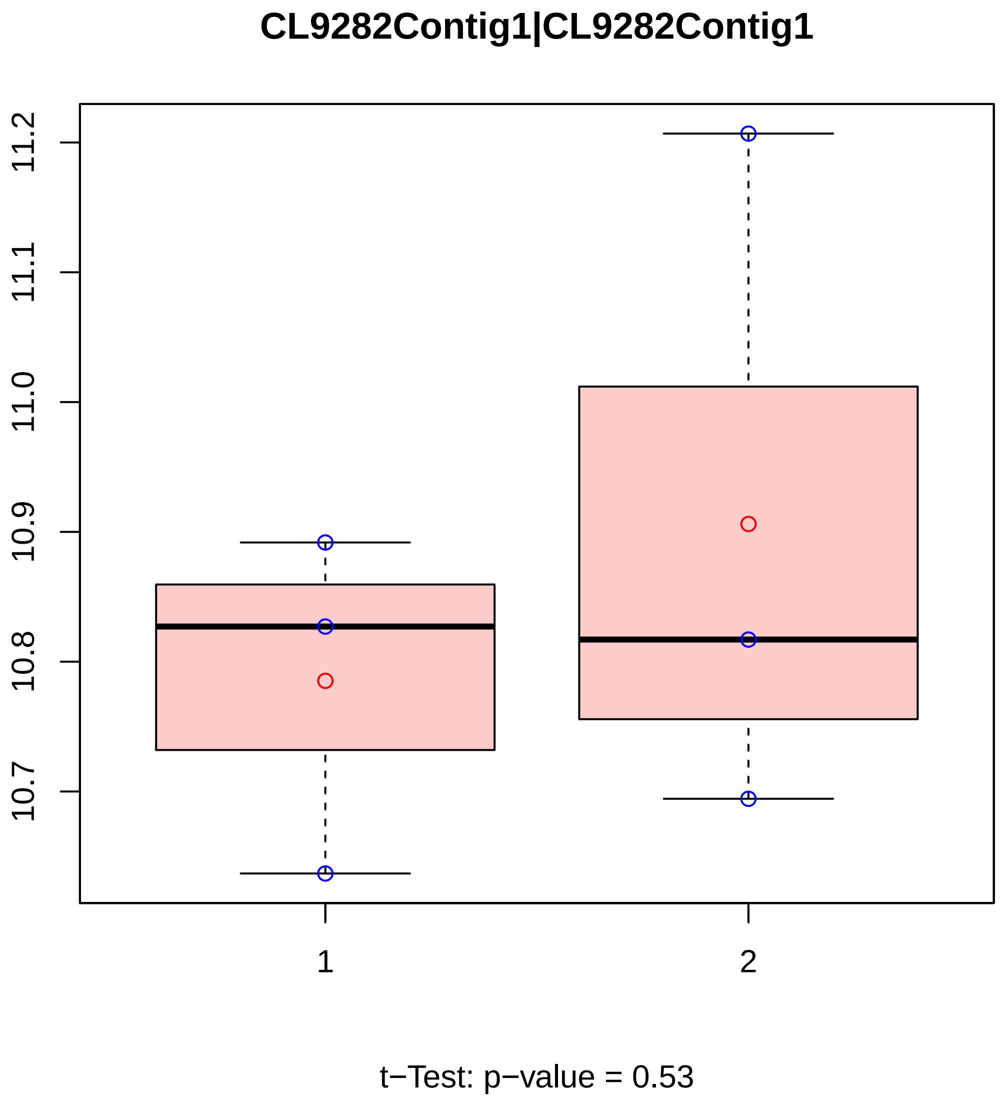
CL9282Contig1|CL9282Contig1
11.2
11.1
11.0
10.9
10.8
10.7
1
2
t−Test: p−value = 0.53

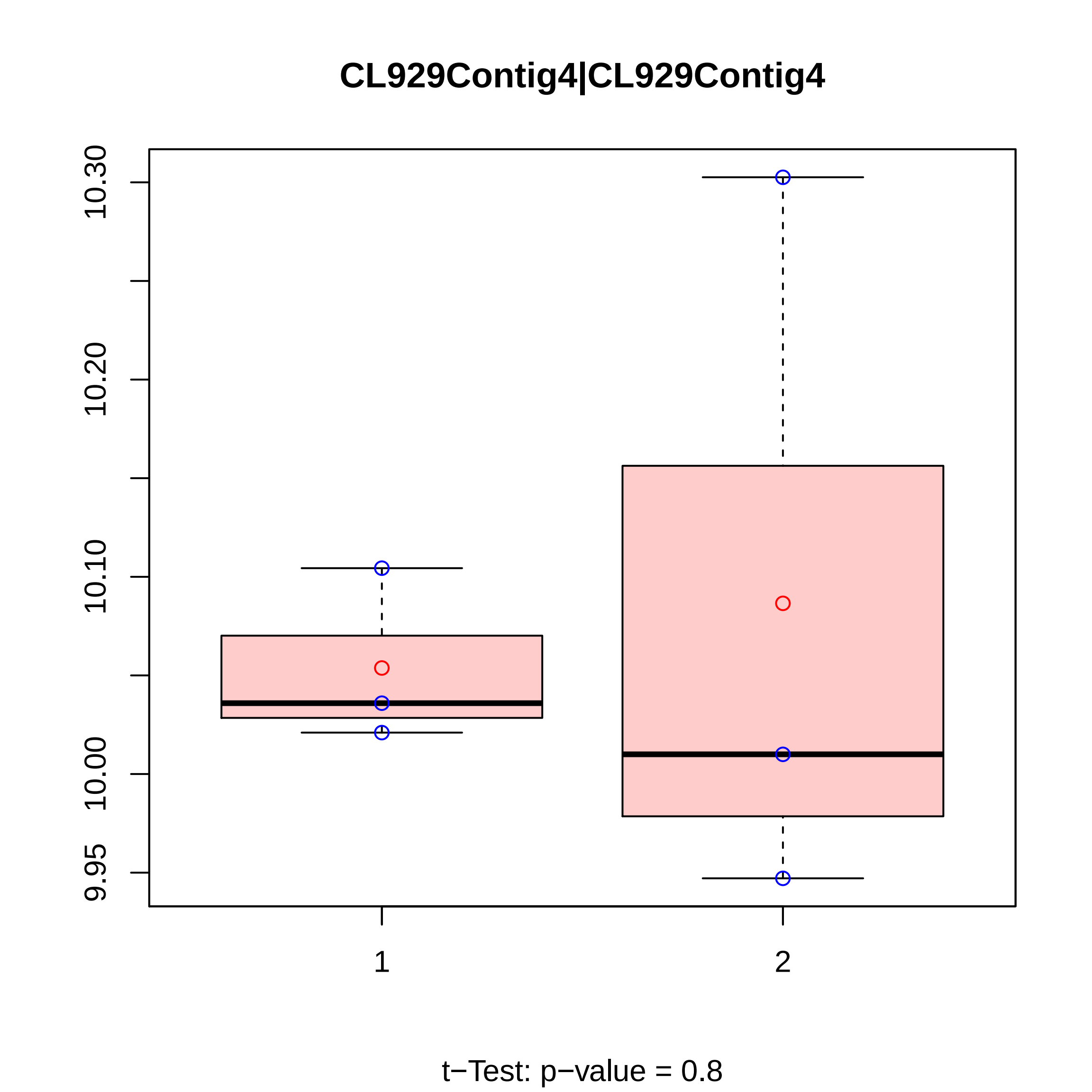

CL929Contig4|CL929Contig4
10.30
10.20
10.10
10.00
9.95
1
2
t−Test: p−value = 0.8

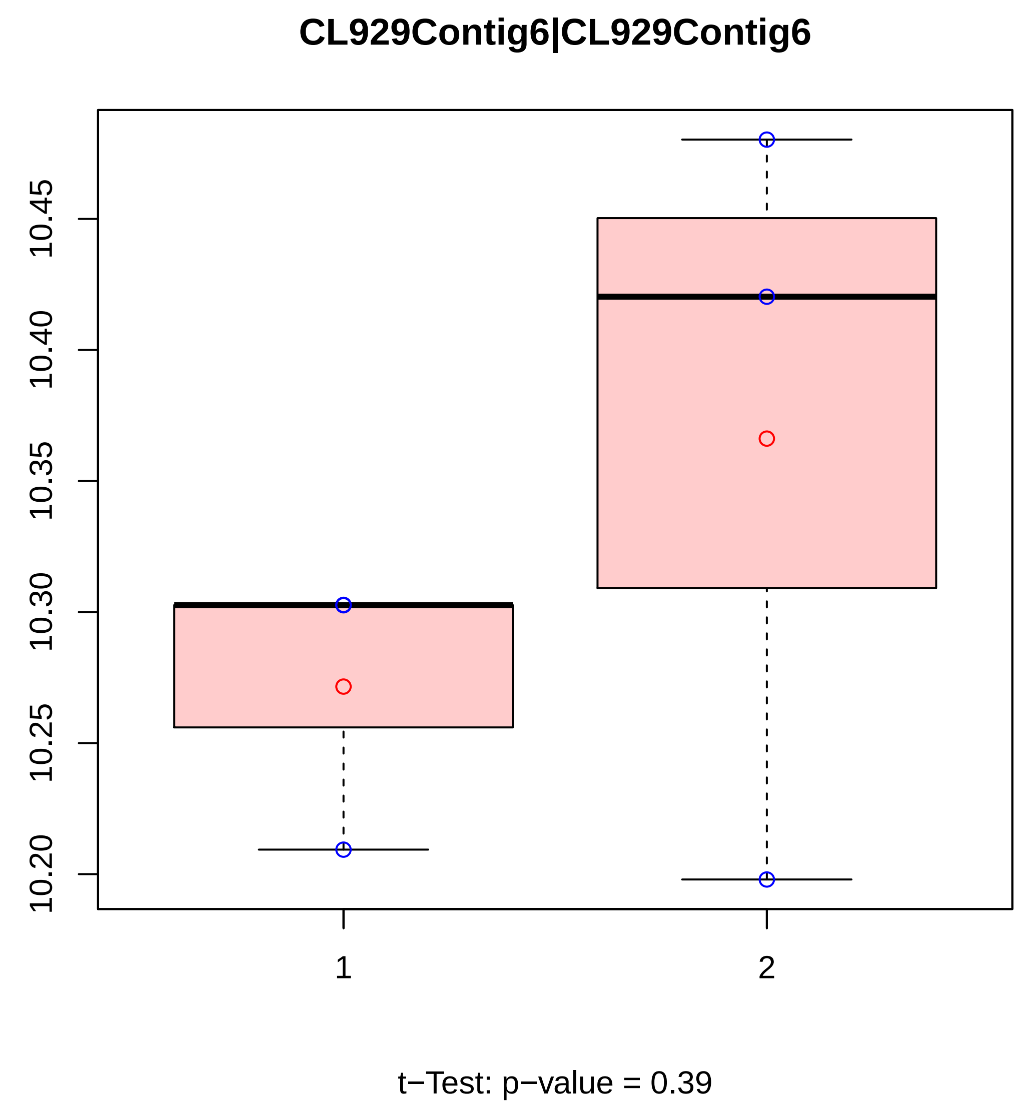
CL929Contig6|CL929Contig6
10.20
10.25
10.30
10.35
10.40
10.45
1
2
t−Test: p−value = 0.39

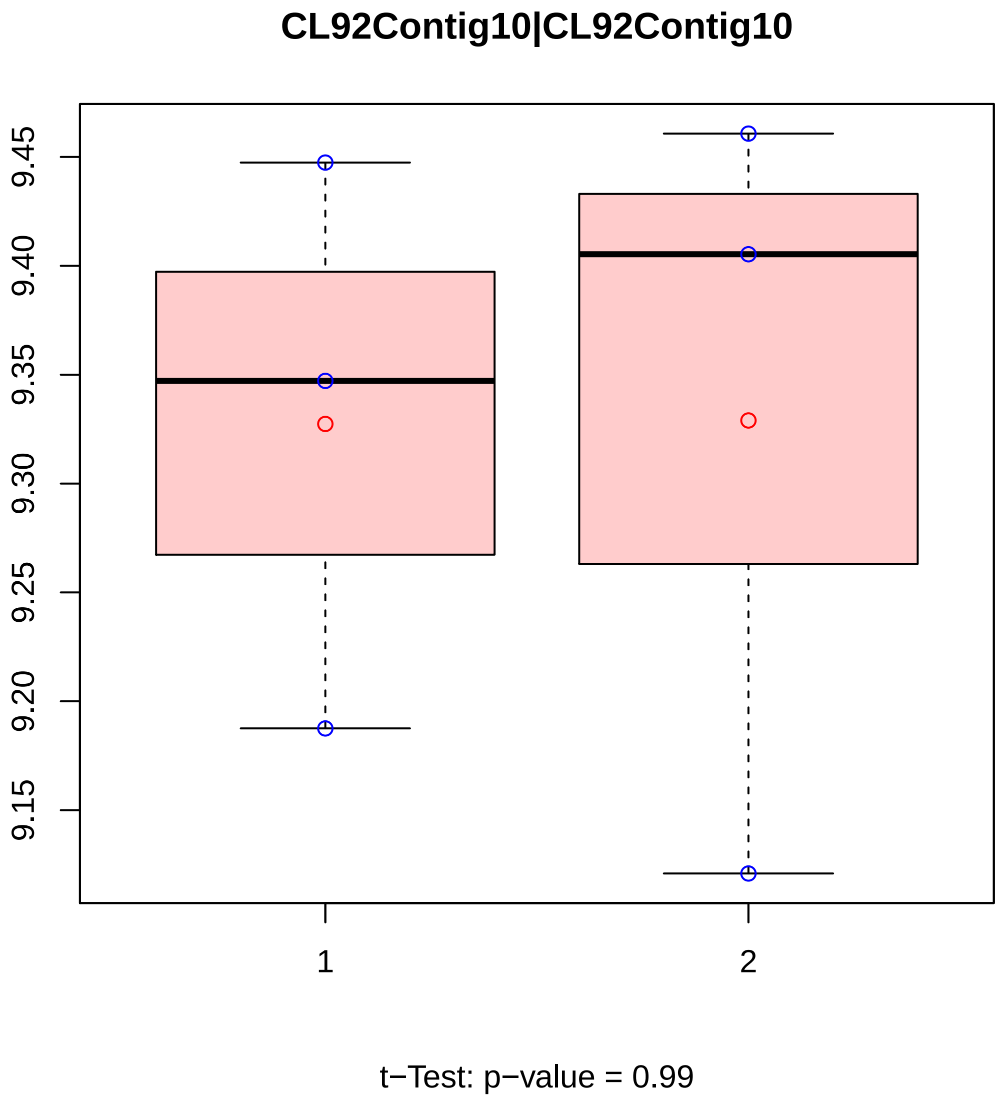
CL92Contig10|CL92Contig10
9.15
9.20
9.25
9.30
9.35
9.40
9.45
1
2
t−Test: p−value = 0.99

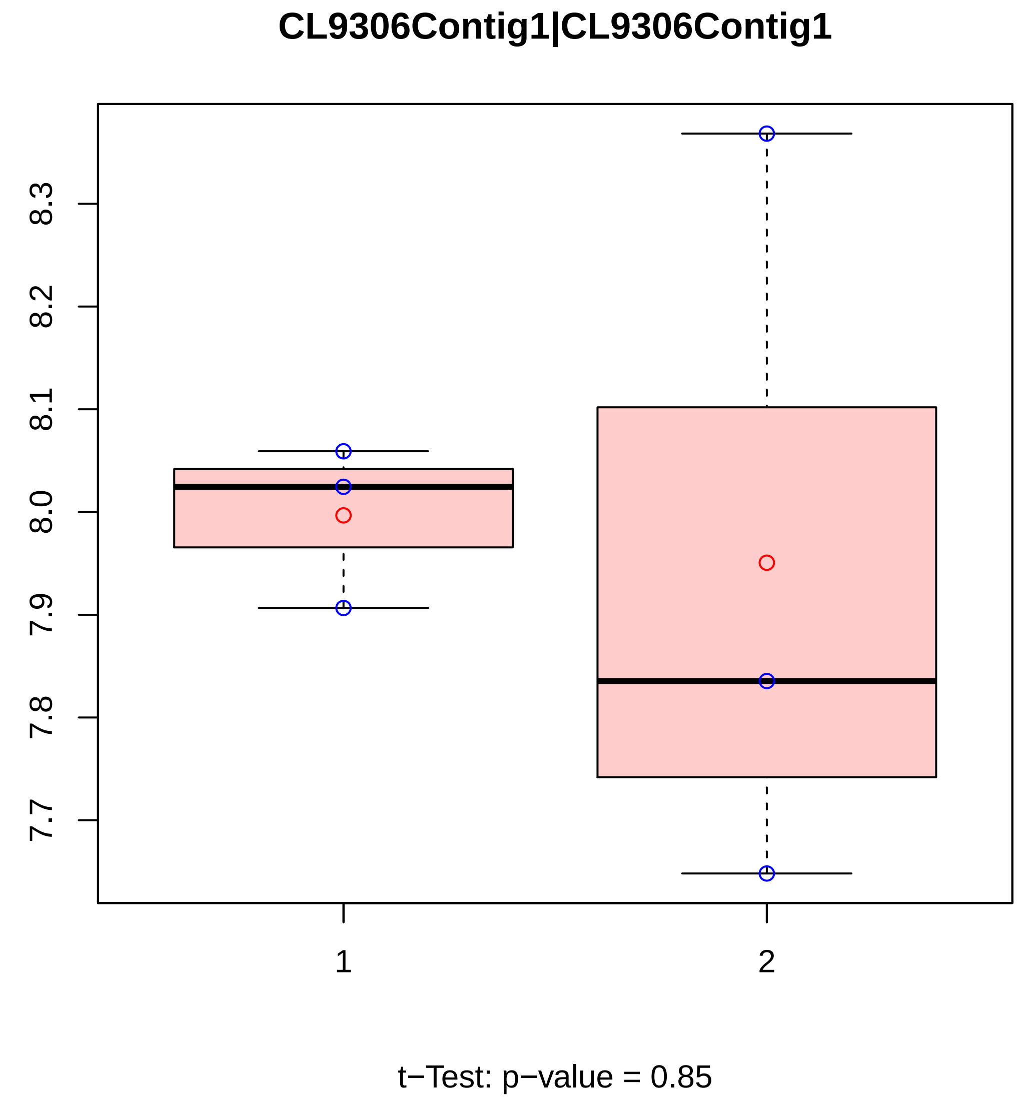
CL9306Contig1|CL9306Contig1
8.3
8.2
8.1
8.0
7.9
7.8
7.7
1
2
t−Test: p−value = 0.85

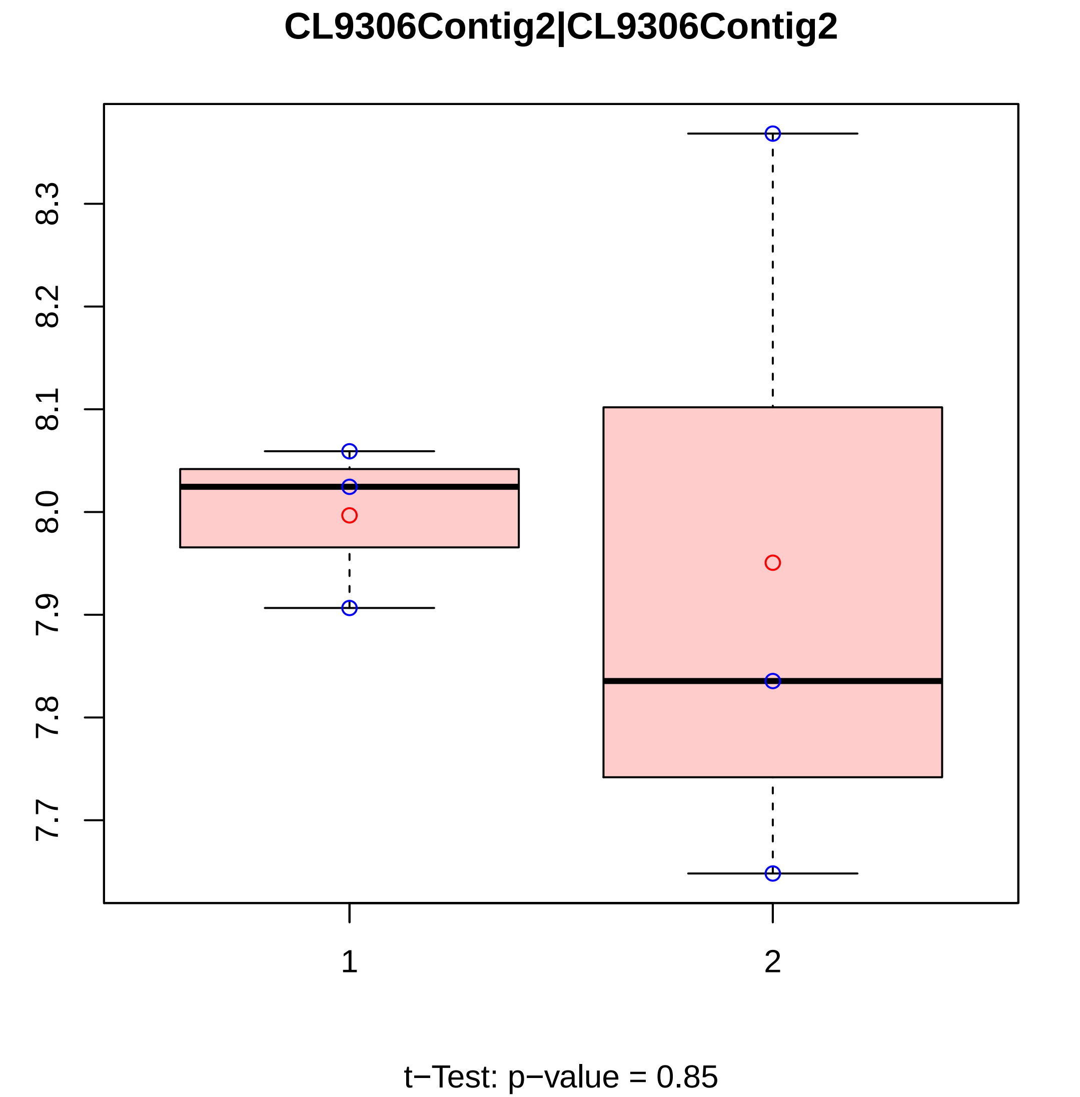
CL9306Contig2|CL9306Contig2
8.3
8.2
8.1
8.0
7.9
7.8
7.7
1
2
t−Test: p−value = 0.85

# CL9307Contig1|CL9307Contig1

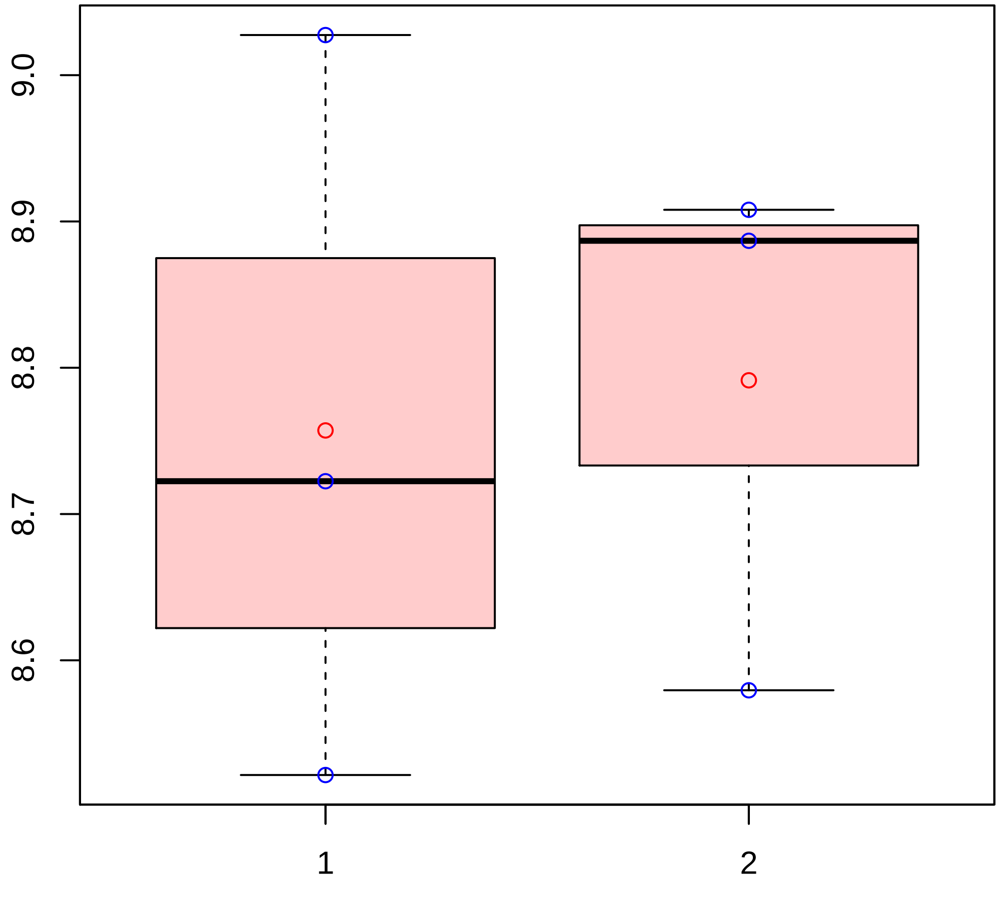


t−Test: p−value = 0.86

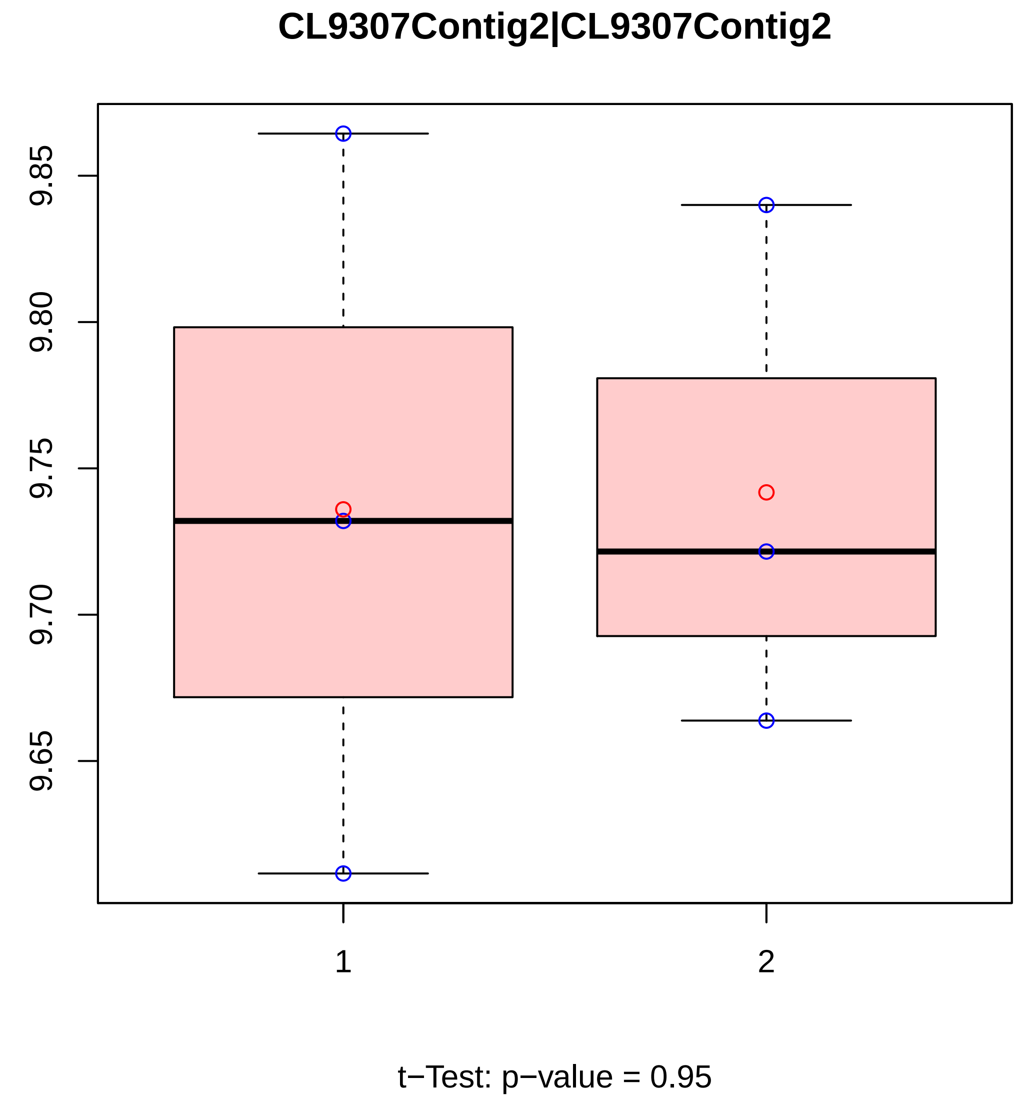
CL9307Contig2|CL9307Contig2
9.65
9.70
9.75
9.80
9.85
1
2
t−Test: p−value = 0.95

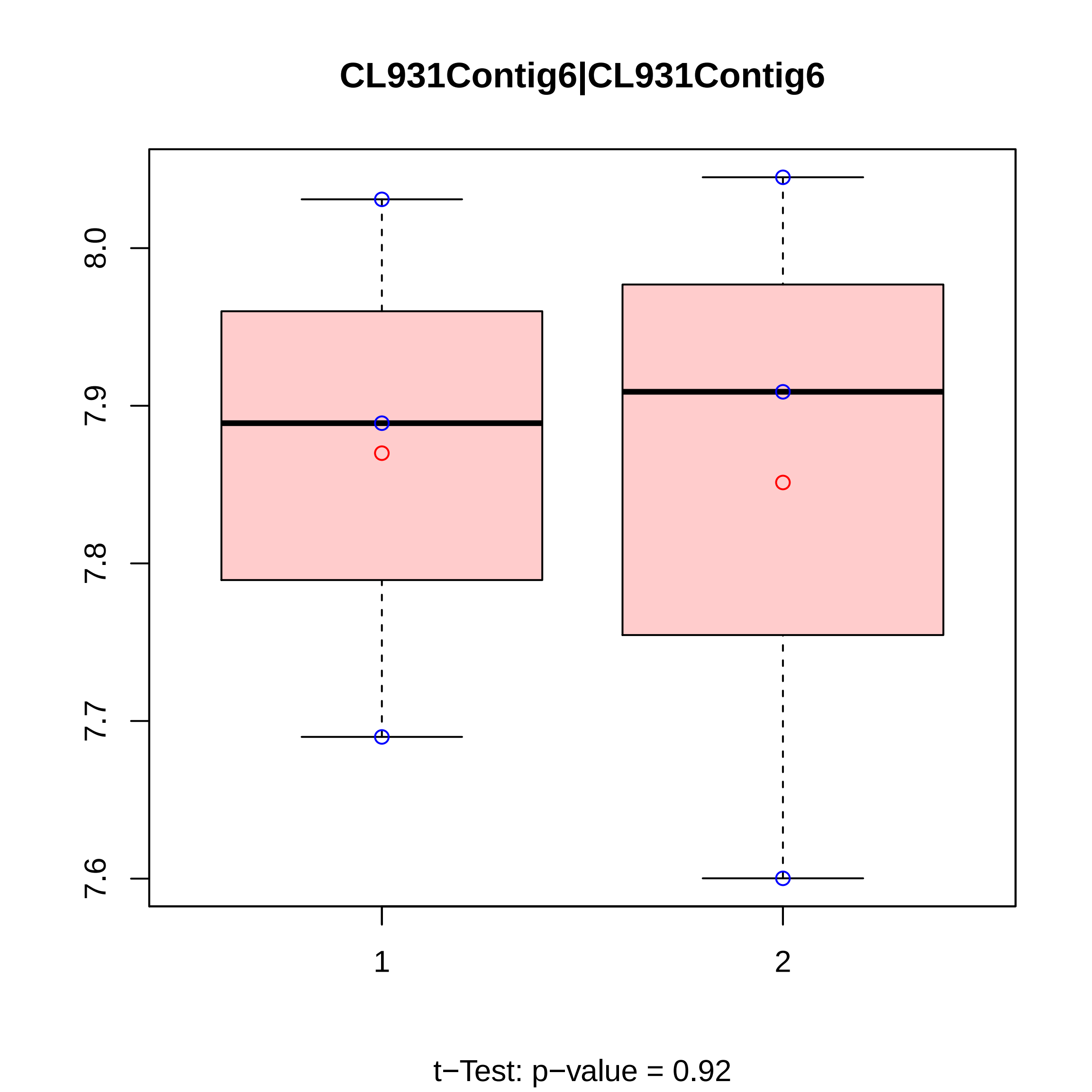

CL931Contig6|CL931Contig6
8.0
7.9
7.8
7.7
7.6
1
2
t−Test: p−value = 0.92

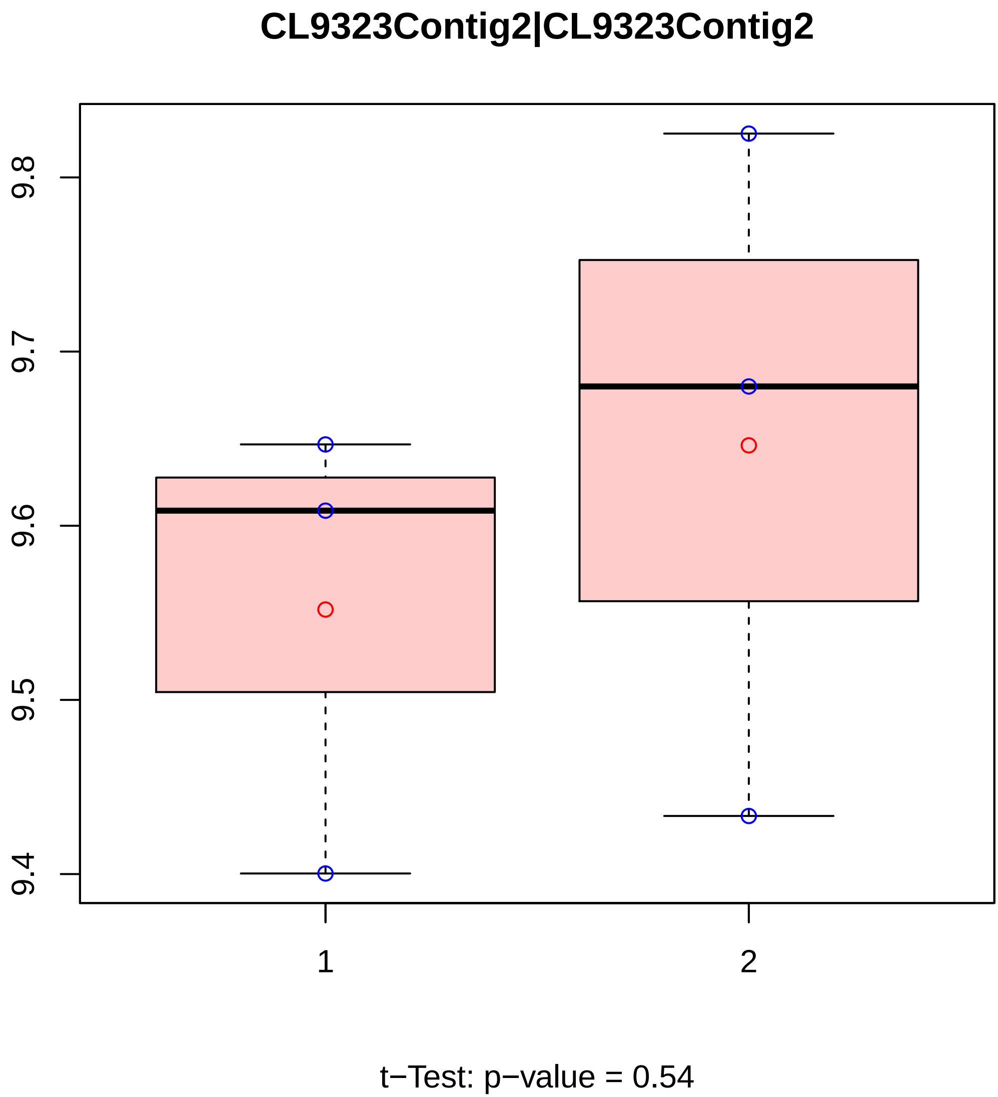
CL9323Contig2|CL9323Contig2
9.4
9.5
9.6
9.7
9.8
1
2
t−Test: p−value = 0.54

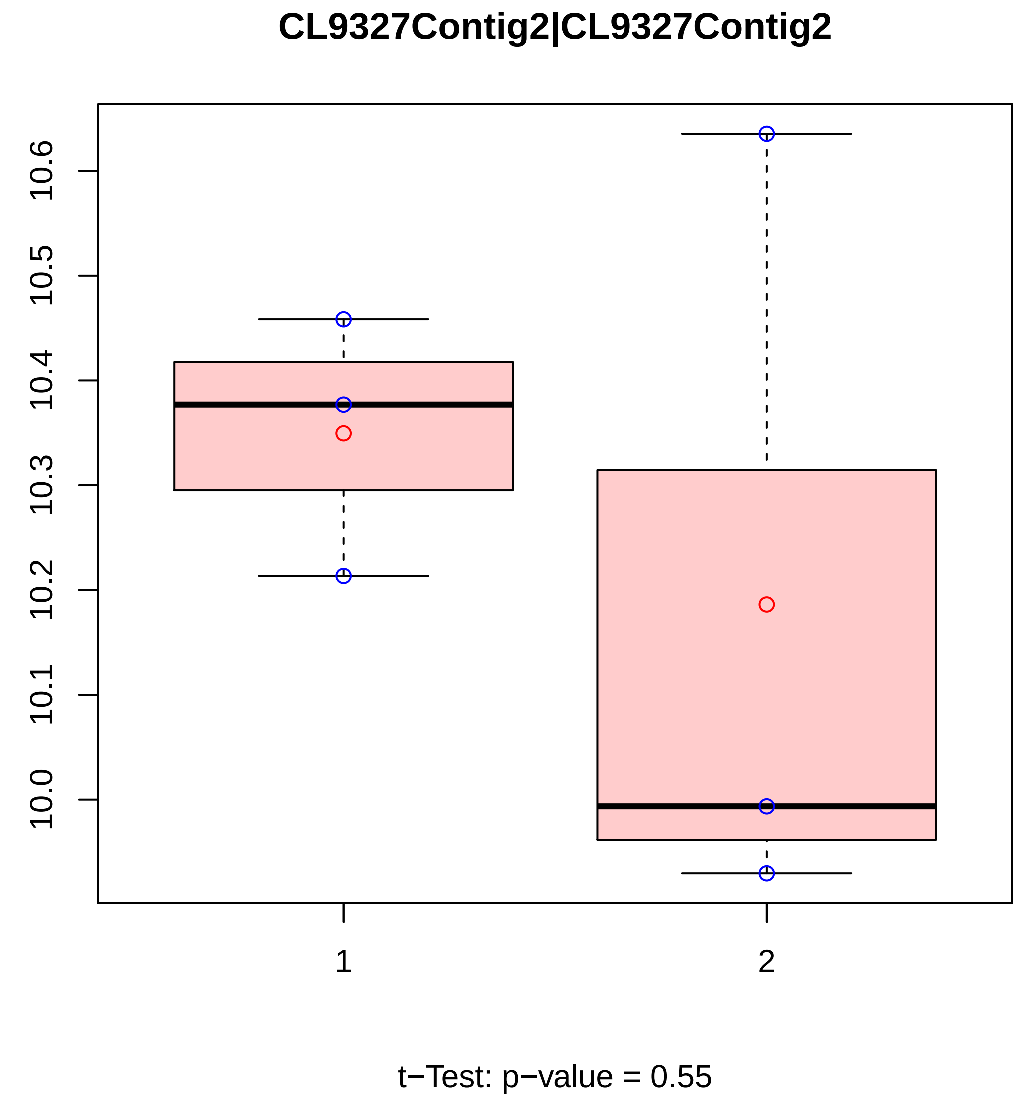

CL9327Contig2|CL9327Contig2
10.0
10.1
10.2
10.3
10.4
10.5
10.6
1
2
t−Test: p−value = 0.55

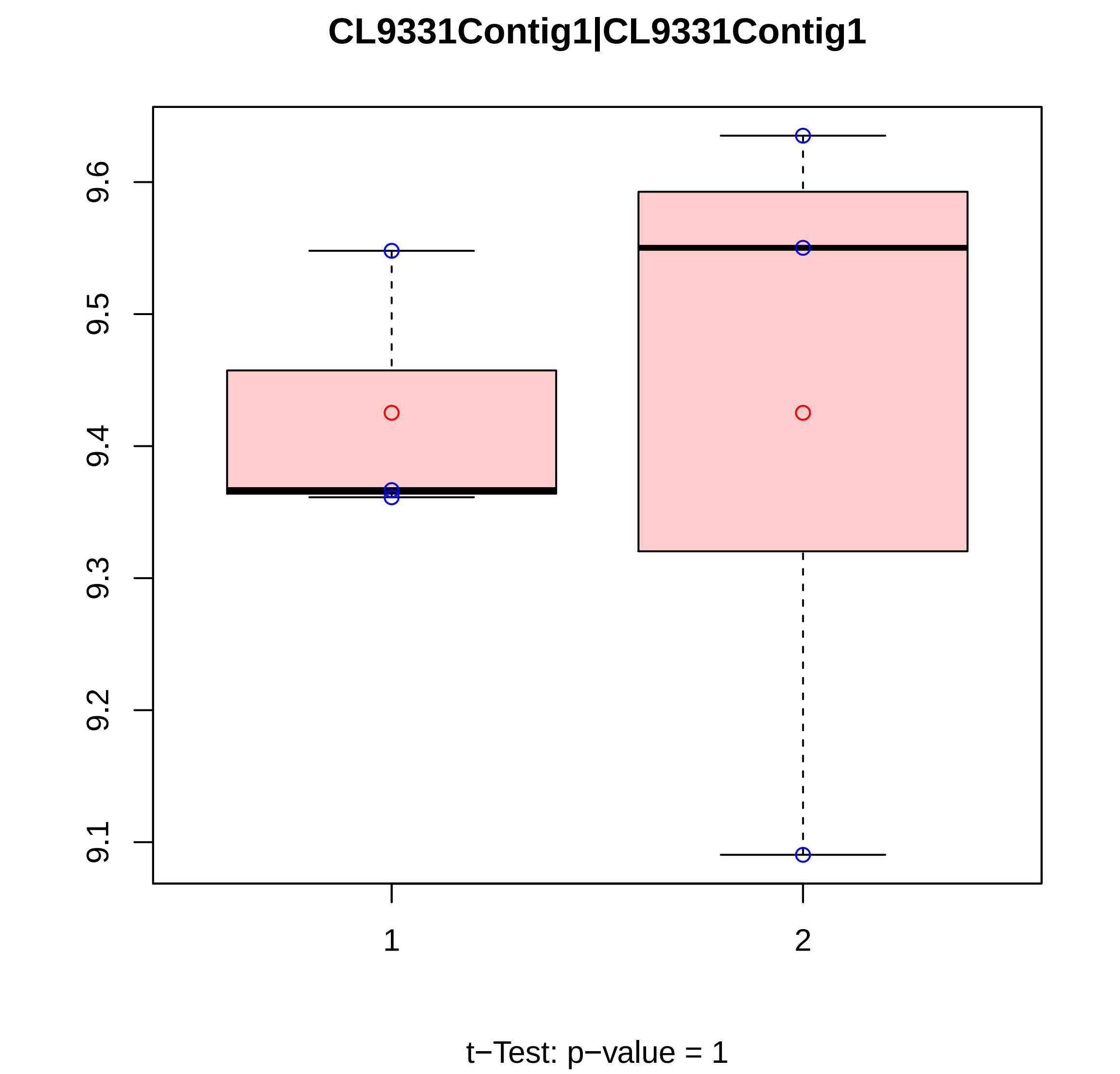
CL9331Contig1|CL9331Contig1
9.1
9.2
9.3
9.4
9.5
9.6
1
2
t−Test: p−value = 1

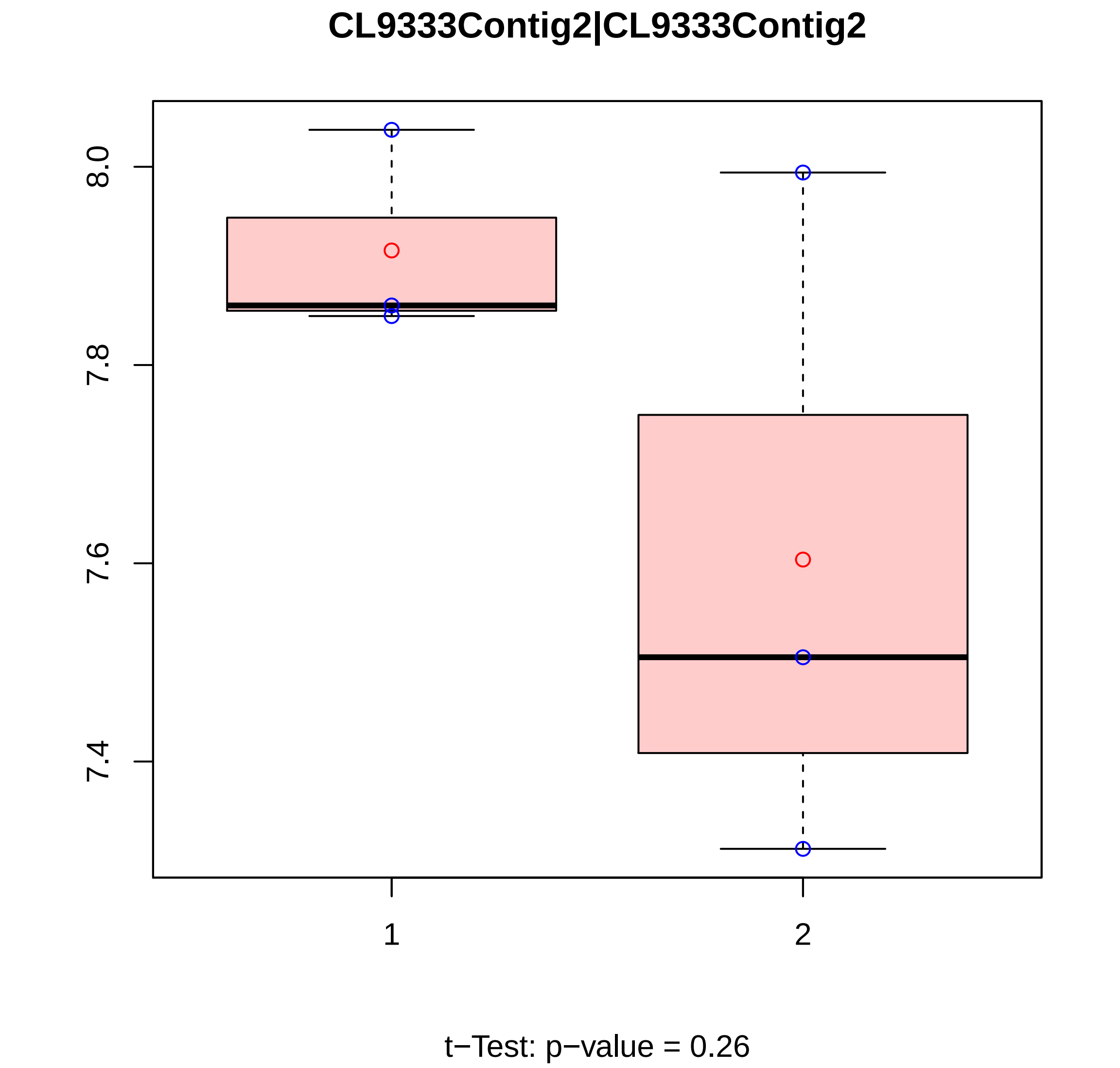

CL9333Contig2|CL9333Contig2
8.0
7.8
7.6
7.4
1
2
t−Test: p−value = 0.26

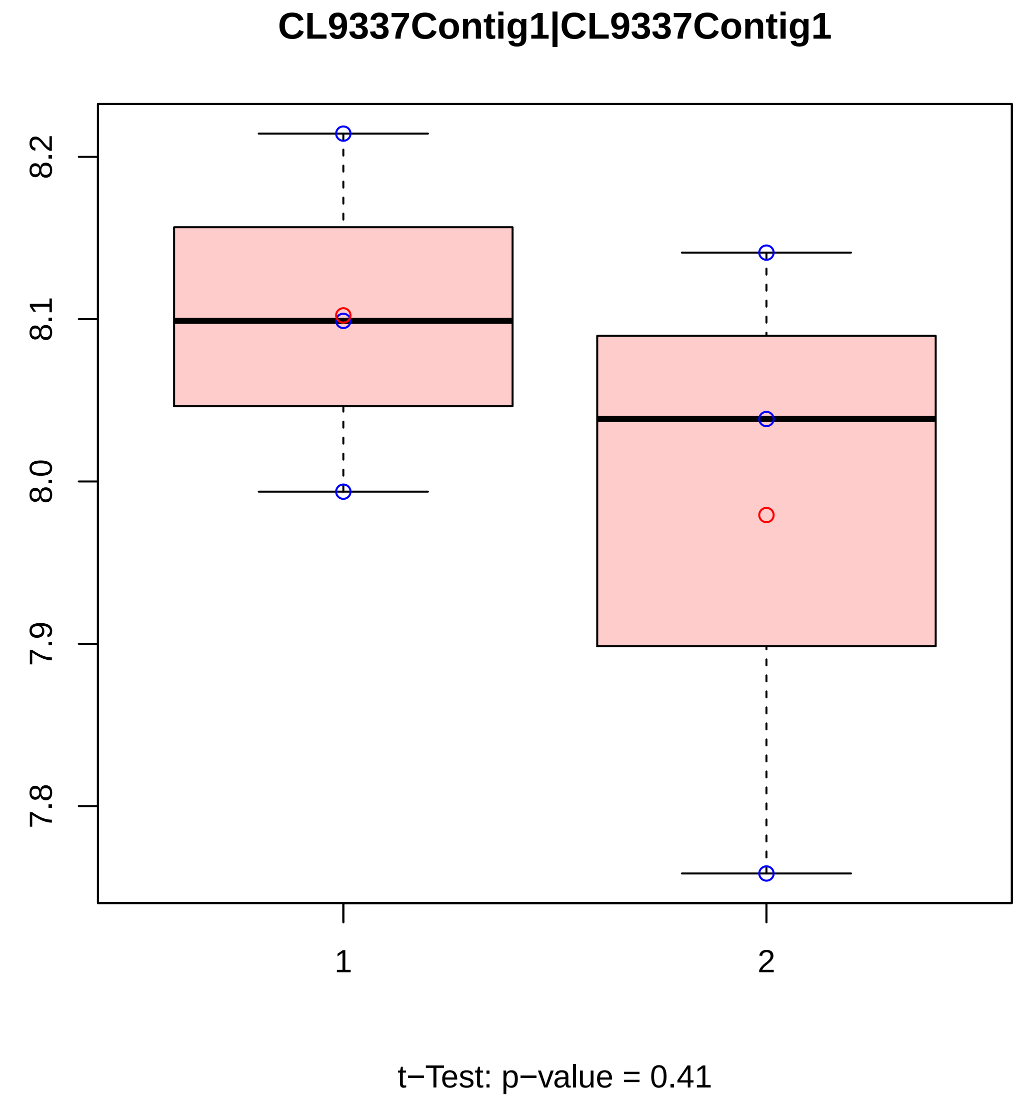
CL9337Contig1|CL9337Contig1
8.2
8.1
8.0
7.9
7.8
1
2
t−Test: p−value = 0.41

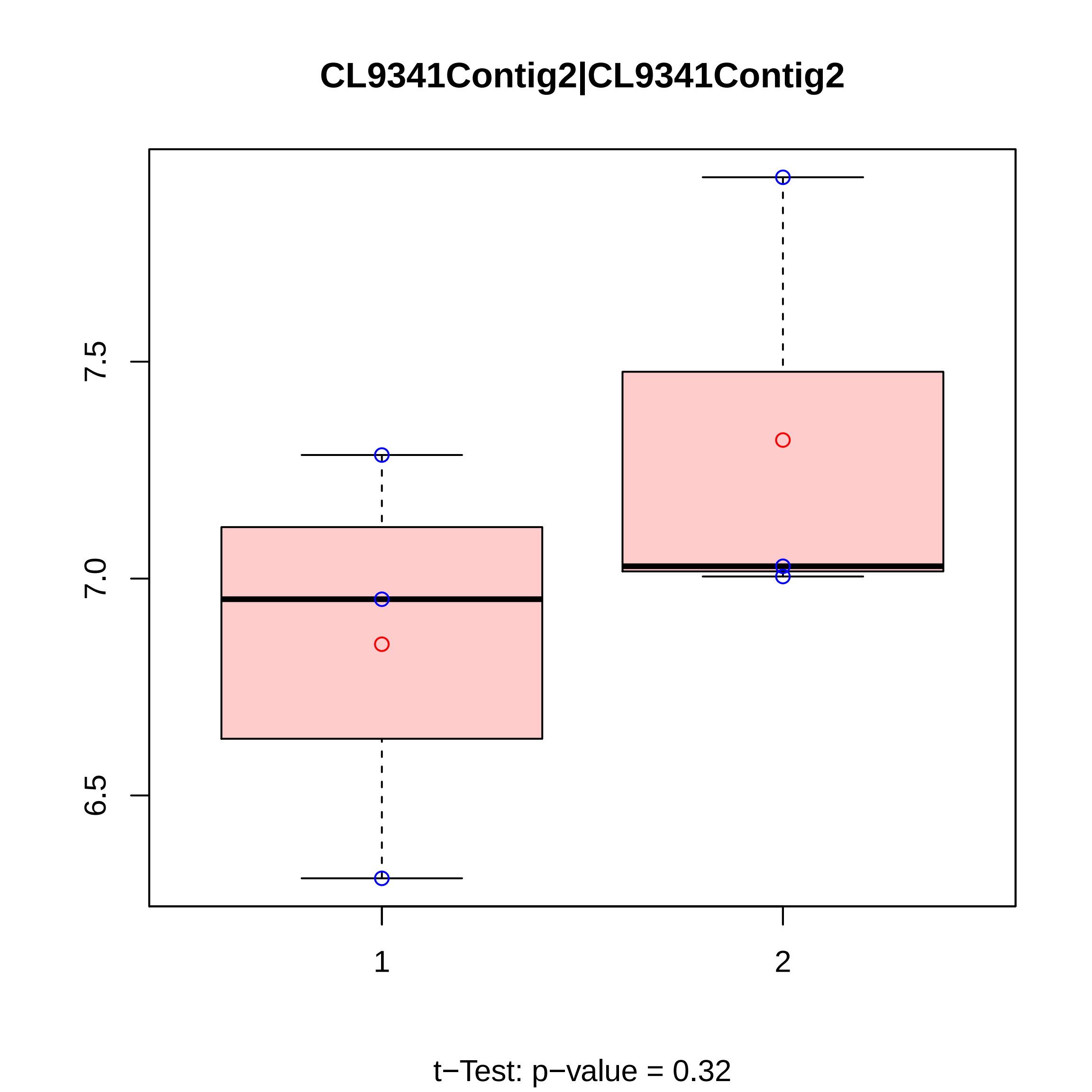
CL9341Contig2|CL9341Contig2
7.5
7.0
6.5
1
2
t−Test: p−value = 0.32

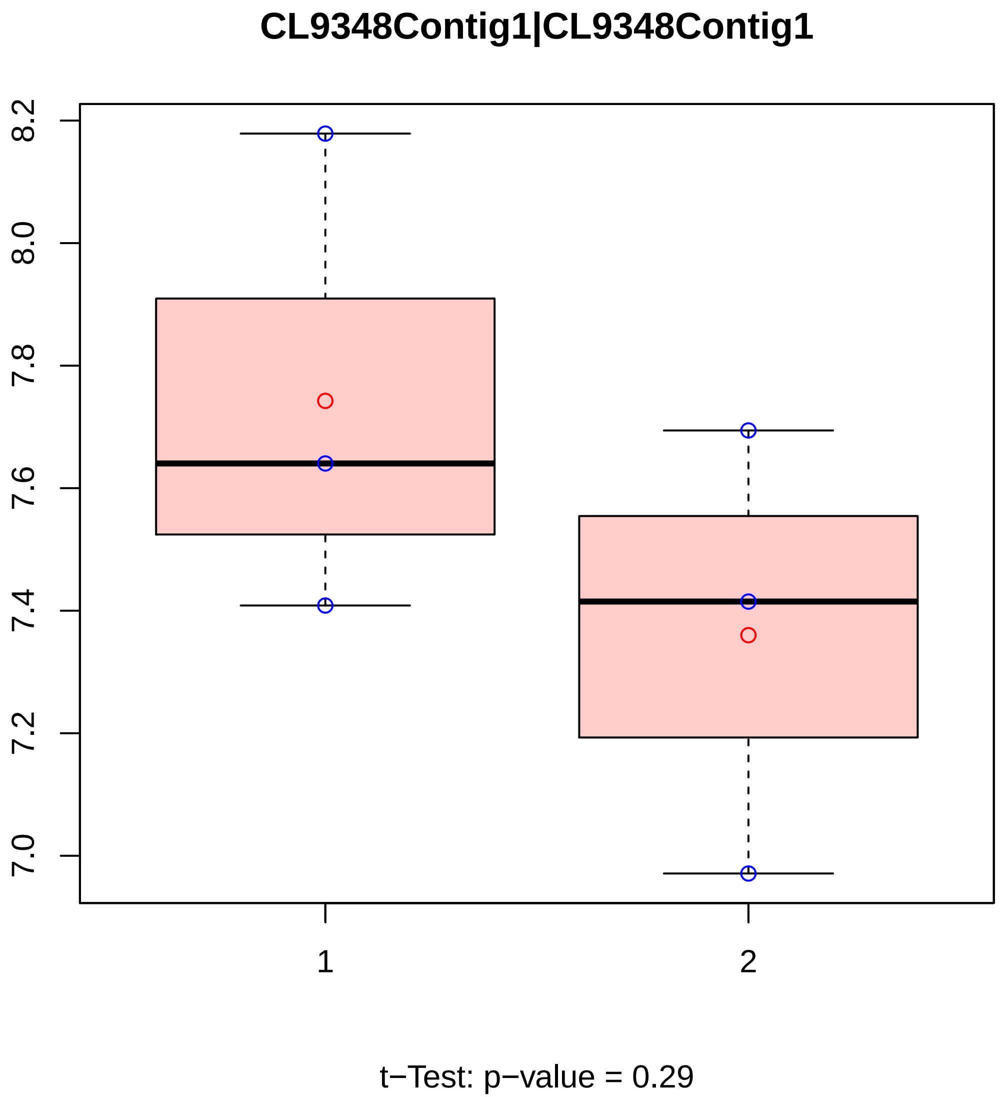

CL9348Contig1|CL9348Contig1
8.2
8.0
7.8
7.6
7.4
7.2
7.0
1
2
t−Test: p−value = 0.29

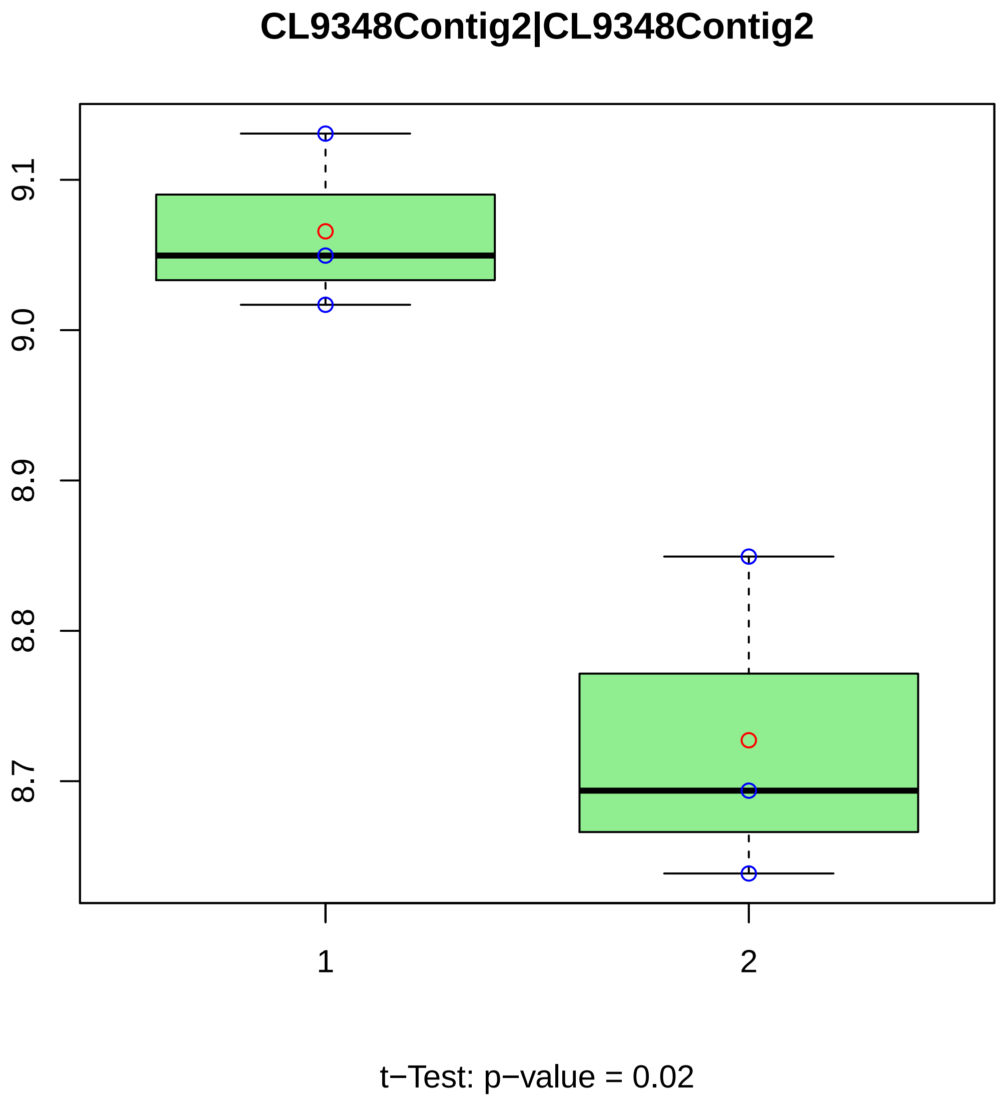

CL9348Contig2|CL9348Contig2
9.1
9.0
8.9
8.8
8.7
1
2
t−Test: p−value = 0.02

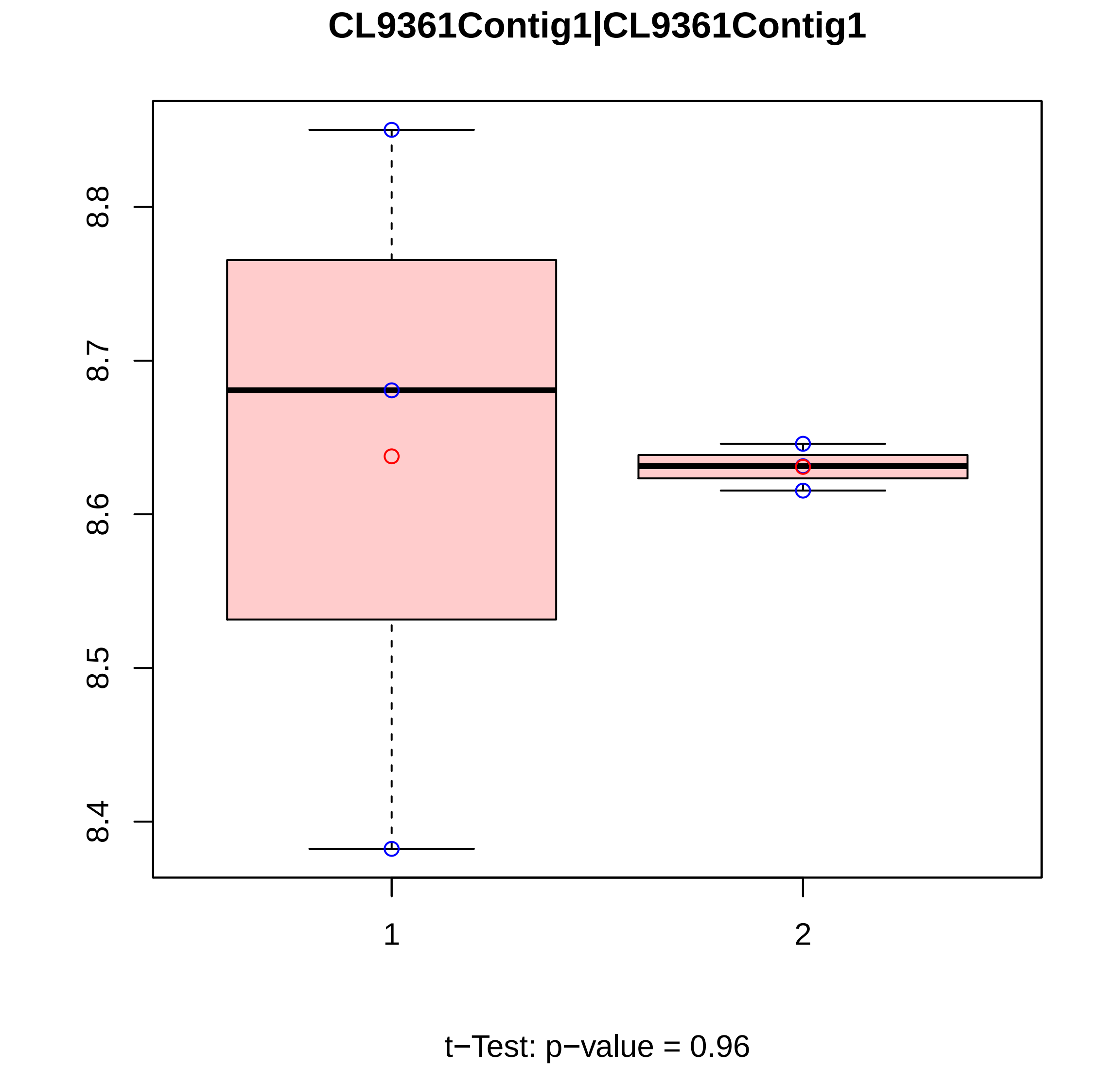

CL9361Contig1|CL9361Contig1
8.4
8.5
8.6
8.7
8.8
1
2
t−Test: p−value = 0.96

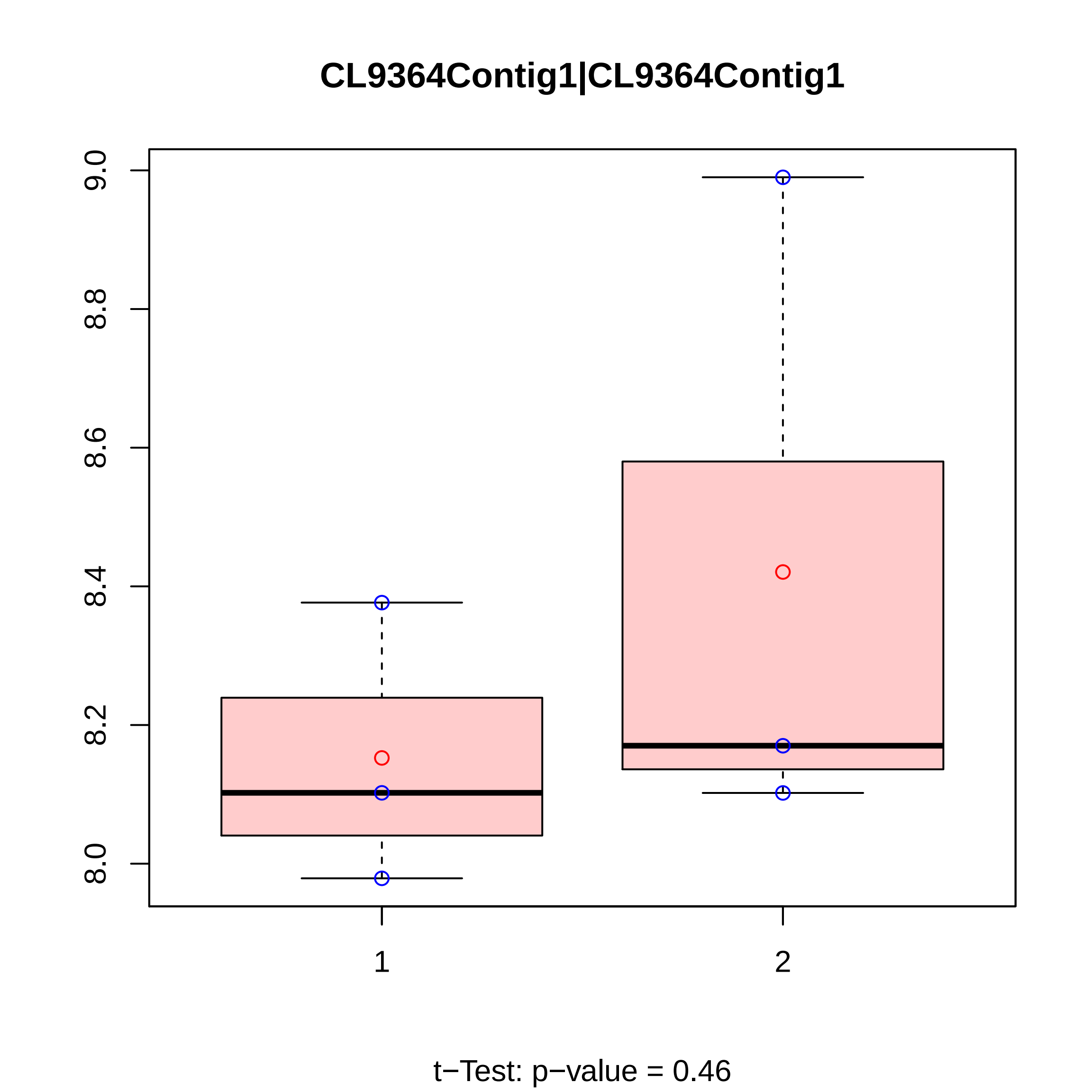
CL9364Contig1|CL9364Contig1
9.0
8.8
8.6
8.4
8.2
8.0
1
2
t−Test: p−value = 0.46

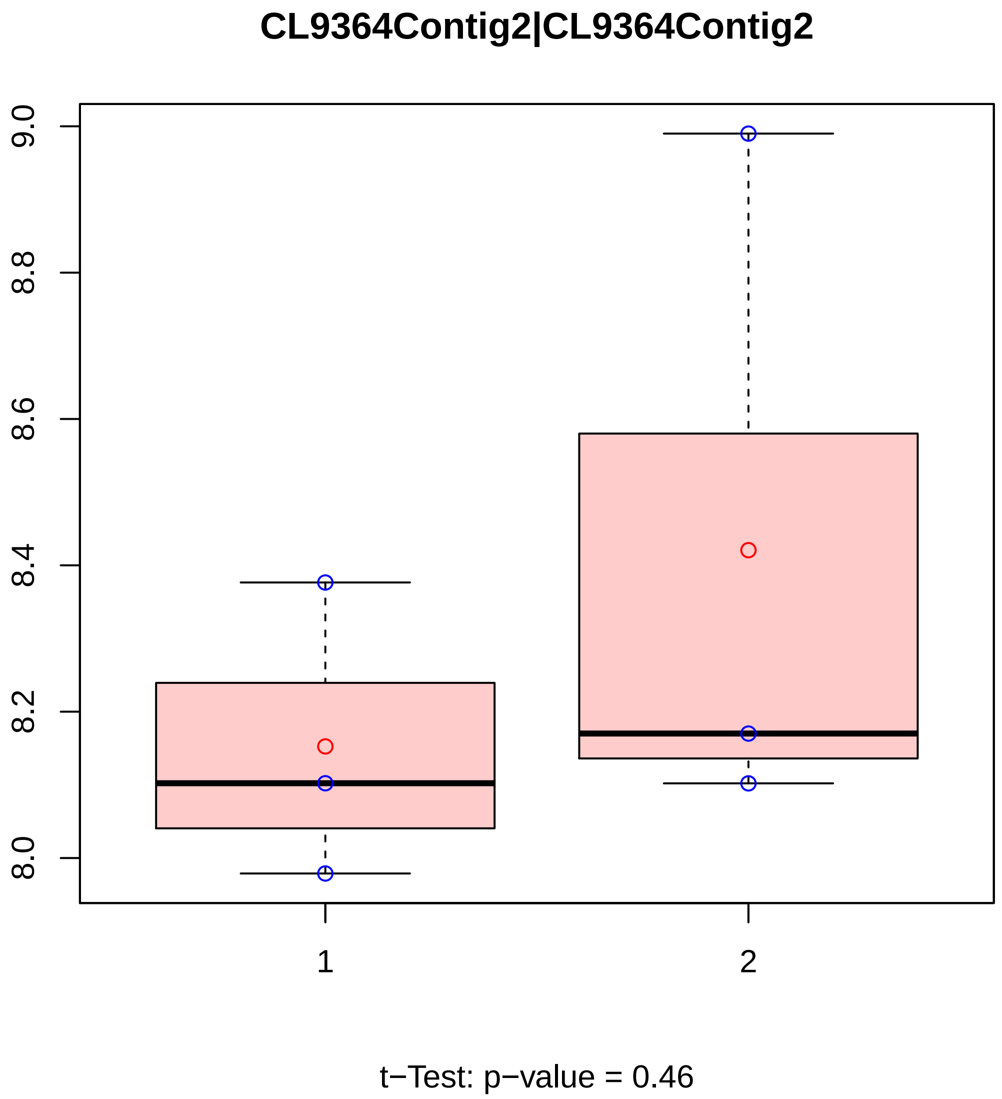

CL9364Contig2|CL9364Contig2
9.0
8.8
8.6
8.4
8.2
8.0
1
2
t−Test: p−value = 0.46

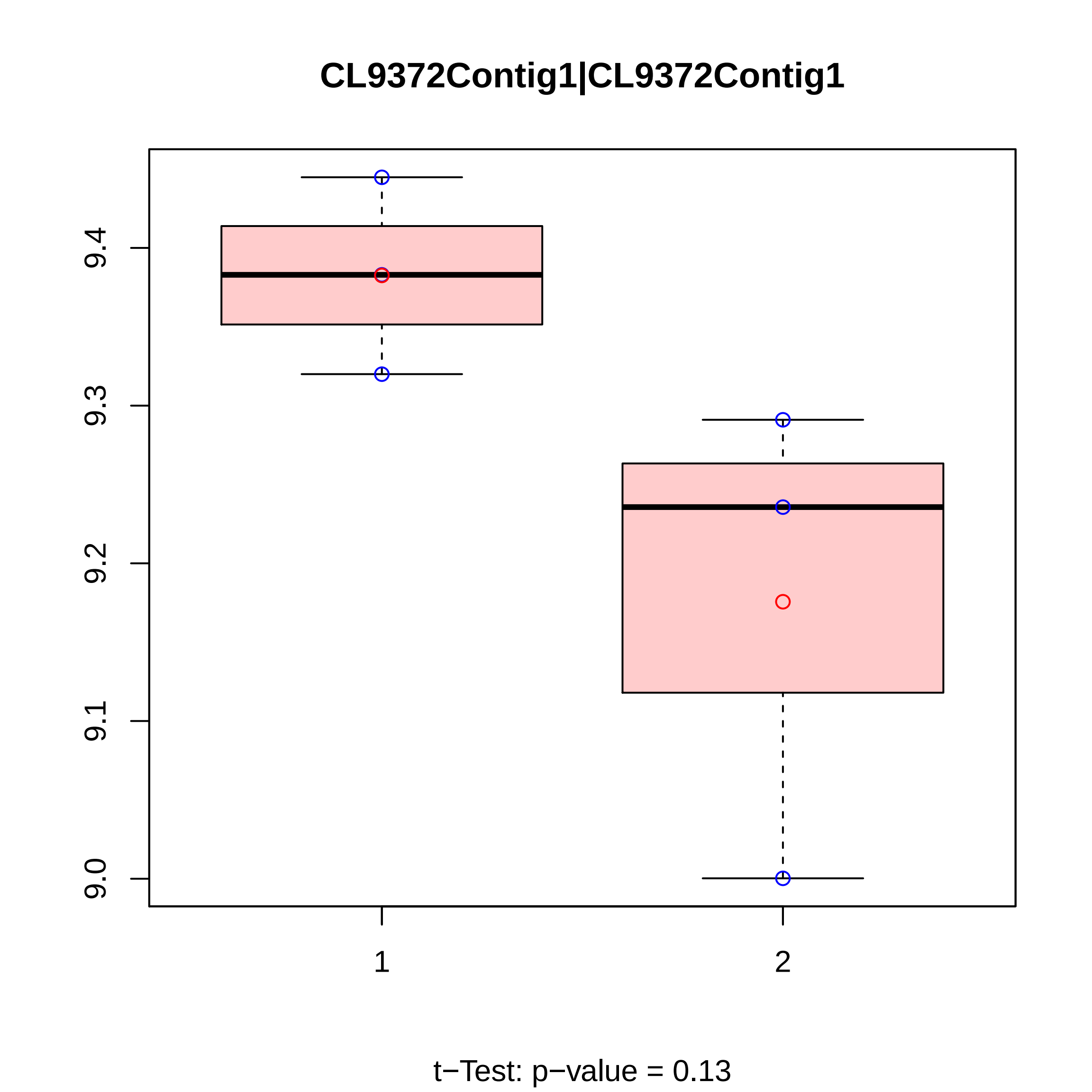
CL9372Contig1|CL9372Contig1
9.0
9.1
9.2
9.3
9.4
1
2
t−Test: p−value = 0.13

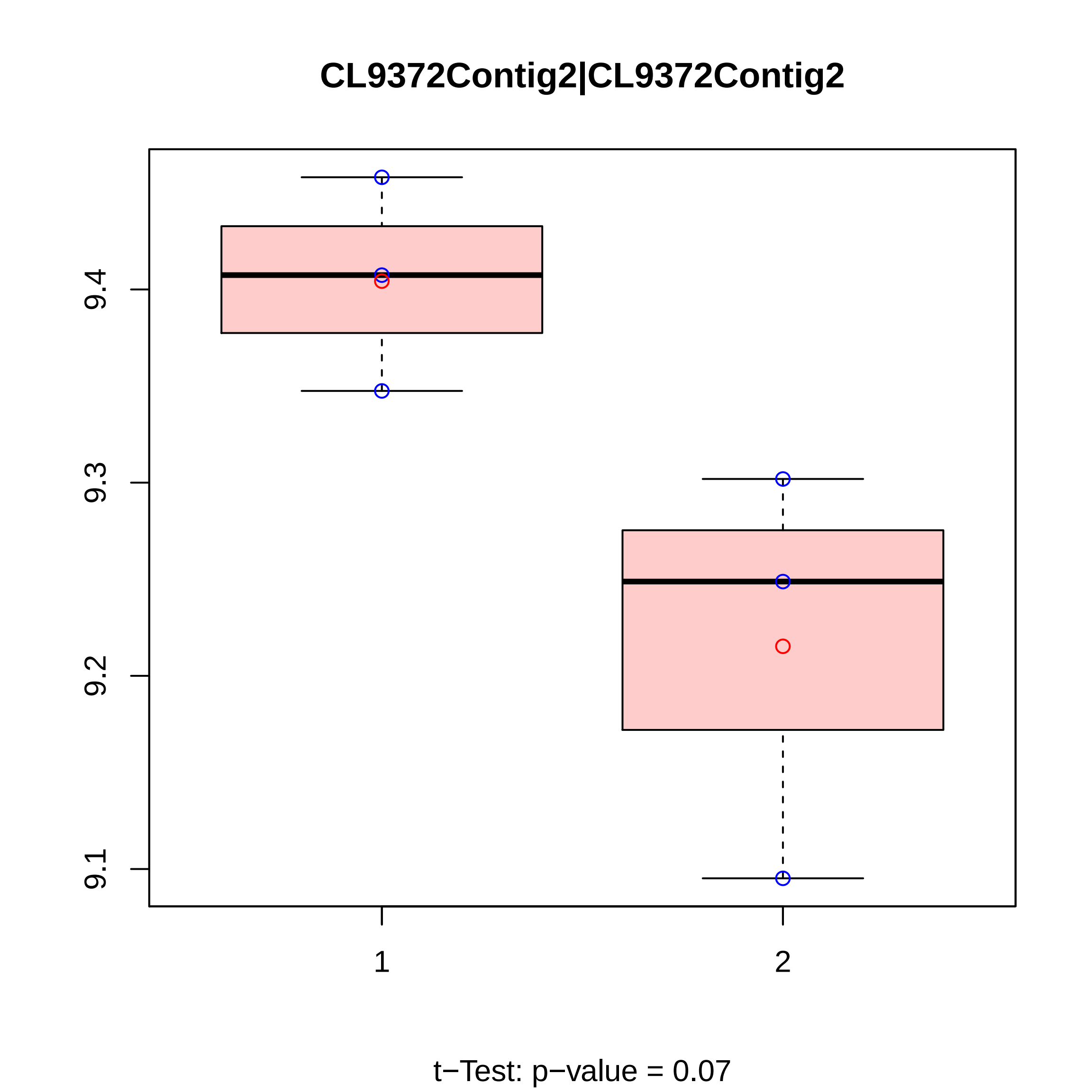
CL9372Contig2|CL9372Contig2
9.1
9.2
9.3
9.4
1
2
t−Test: p−value = 0.07

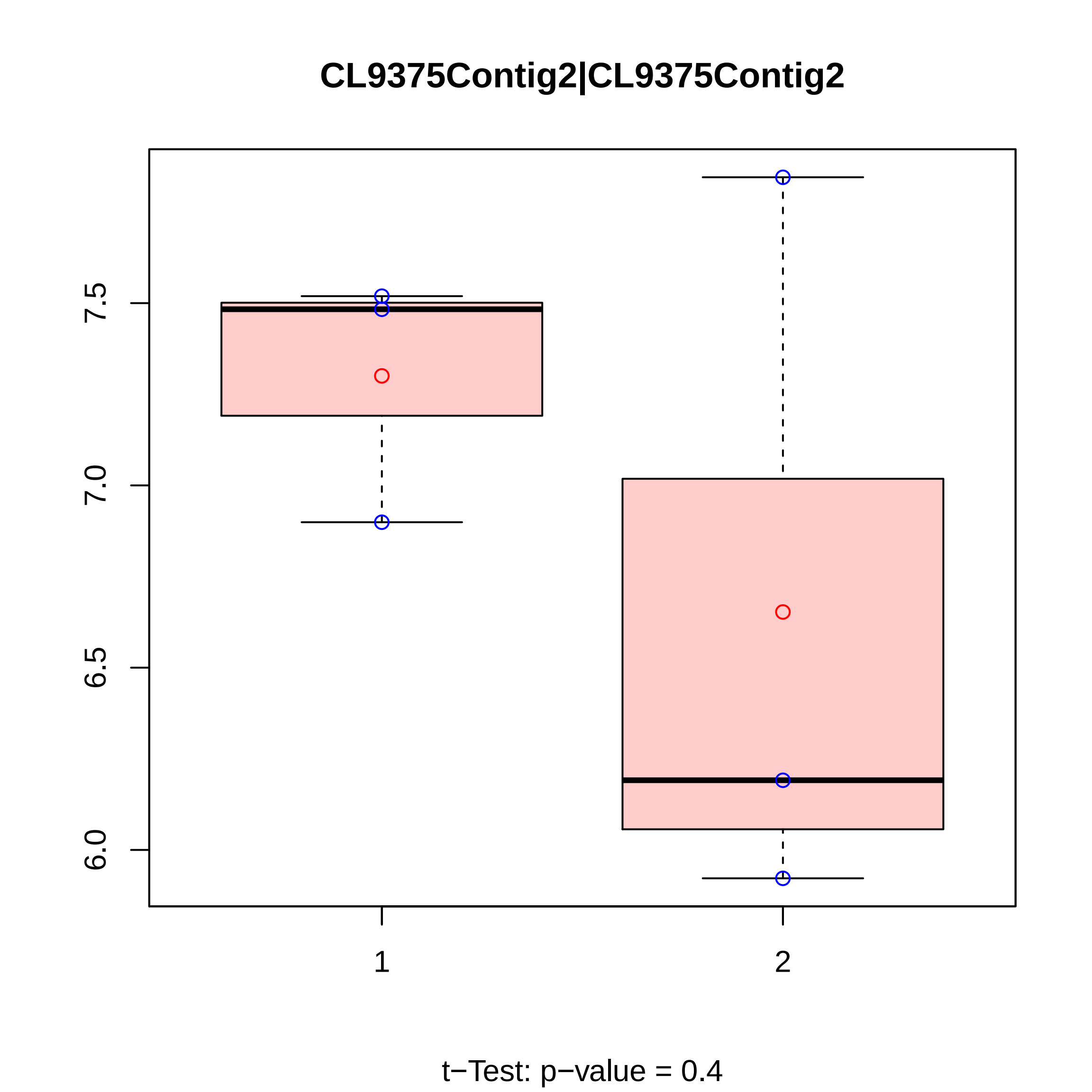
CL9375Contig2|CL9375Contig2
7.5
7.0
6.5
6.0
1
2
t−Test: p−value = 0.4

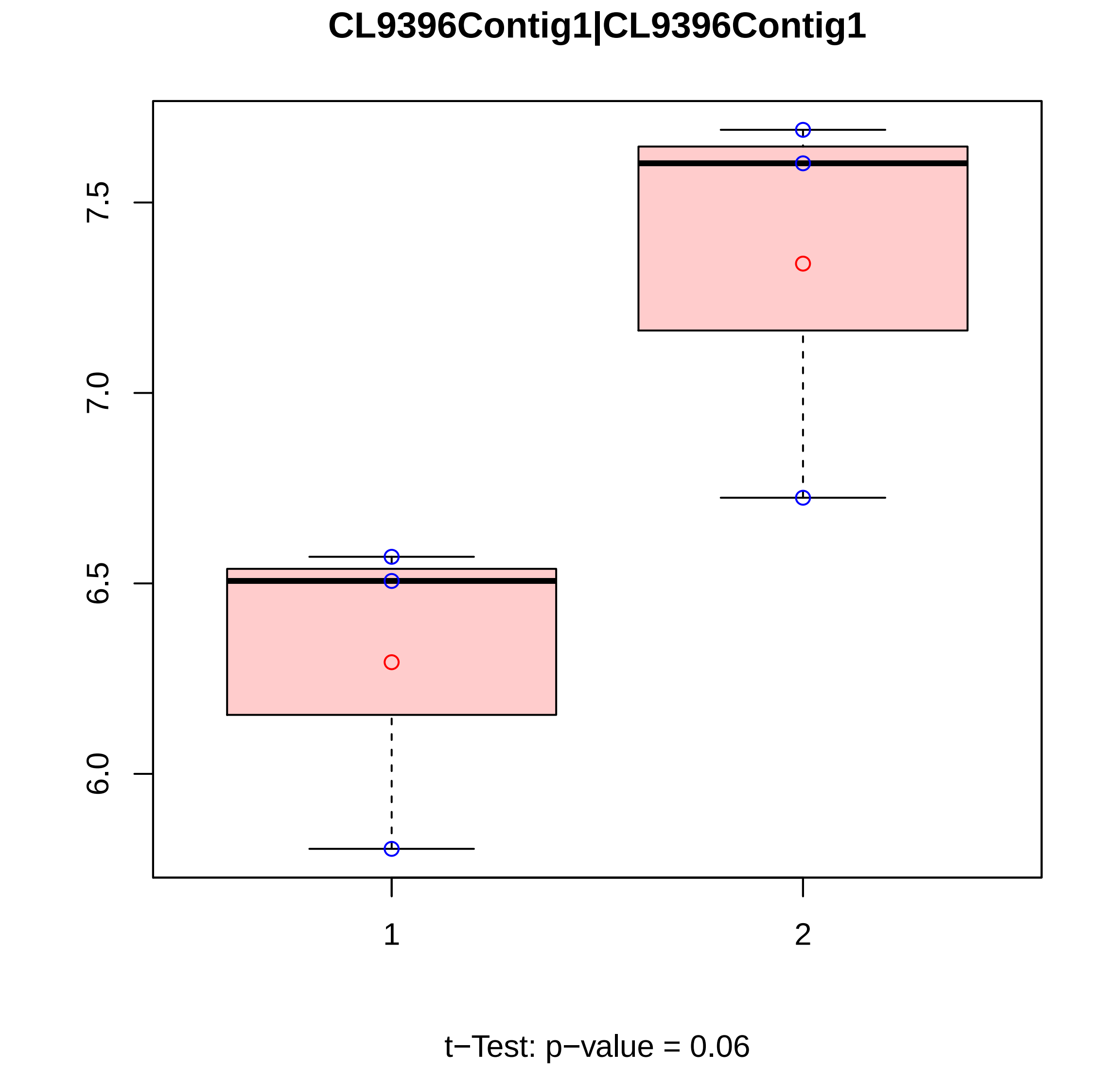

CL9396Contig1|CL9396Contig1
7.5
7.0
6.5
6.0
1
2
t−Test: p−value = 0.06

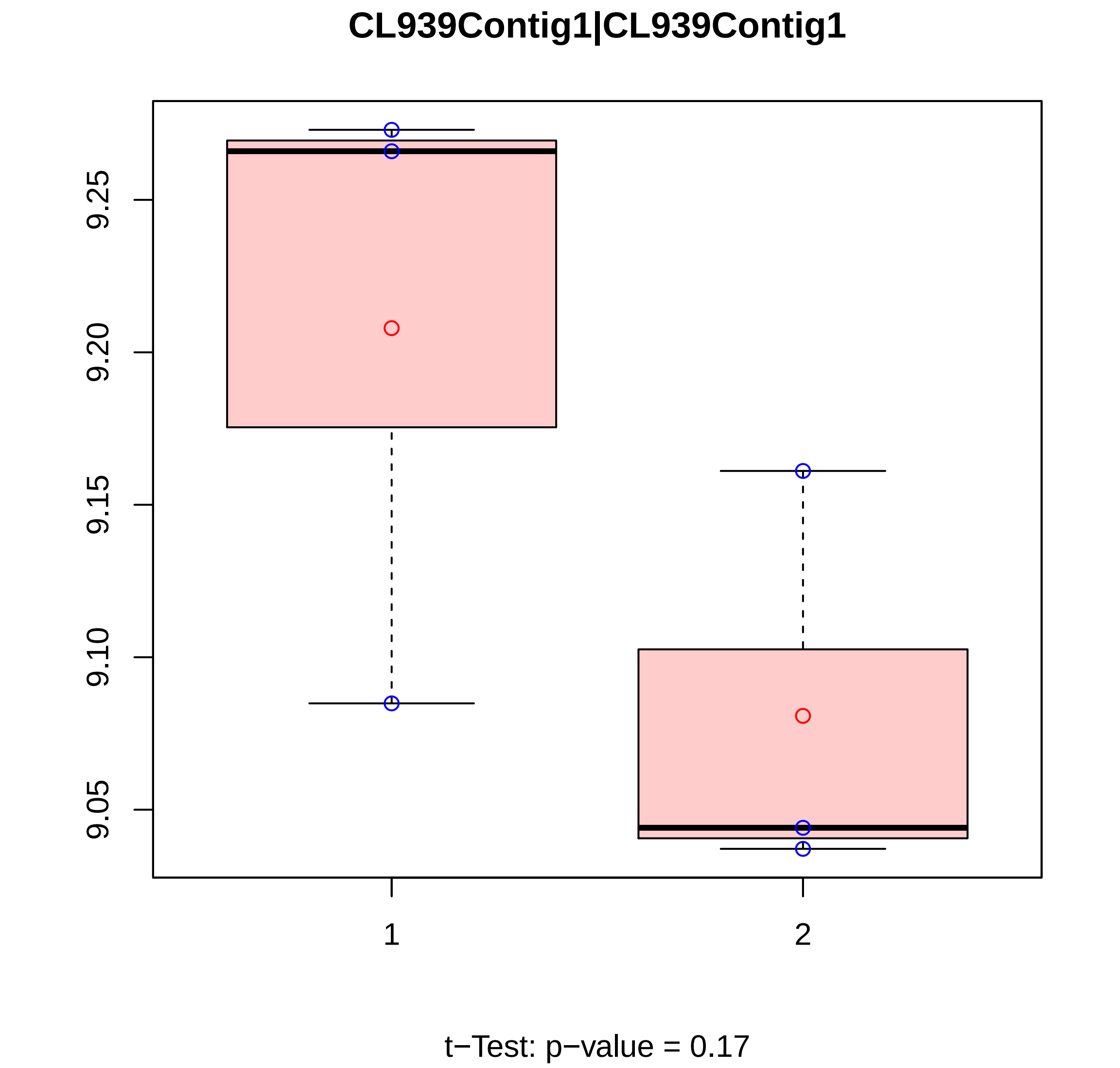

CL939Contig1|CL939Contig1
9.25
9.20
9.15
9.10
9.05
1
2
t−Test: p−value = 0.17

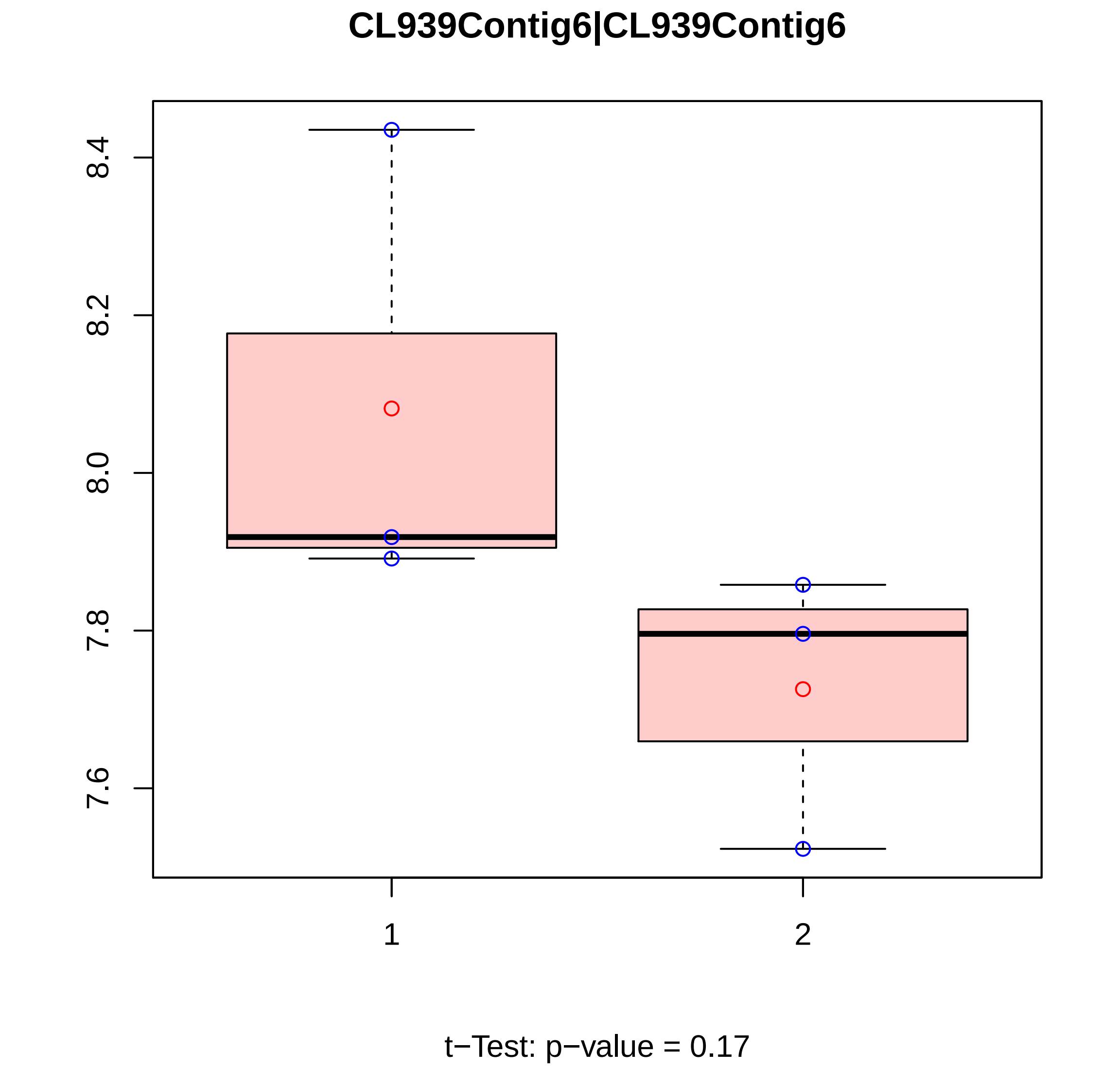
CL939Contig6|CL939Contig6
8.4
8.2
8.0
7.8
7.6
1
2
t−Test: p−value = 0.17

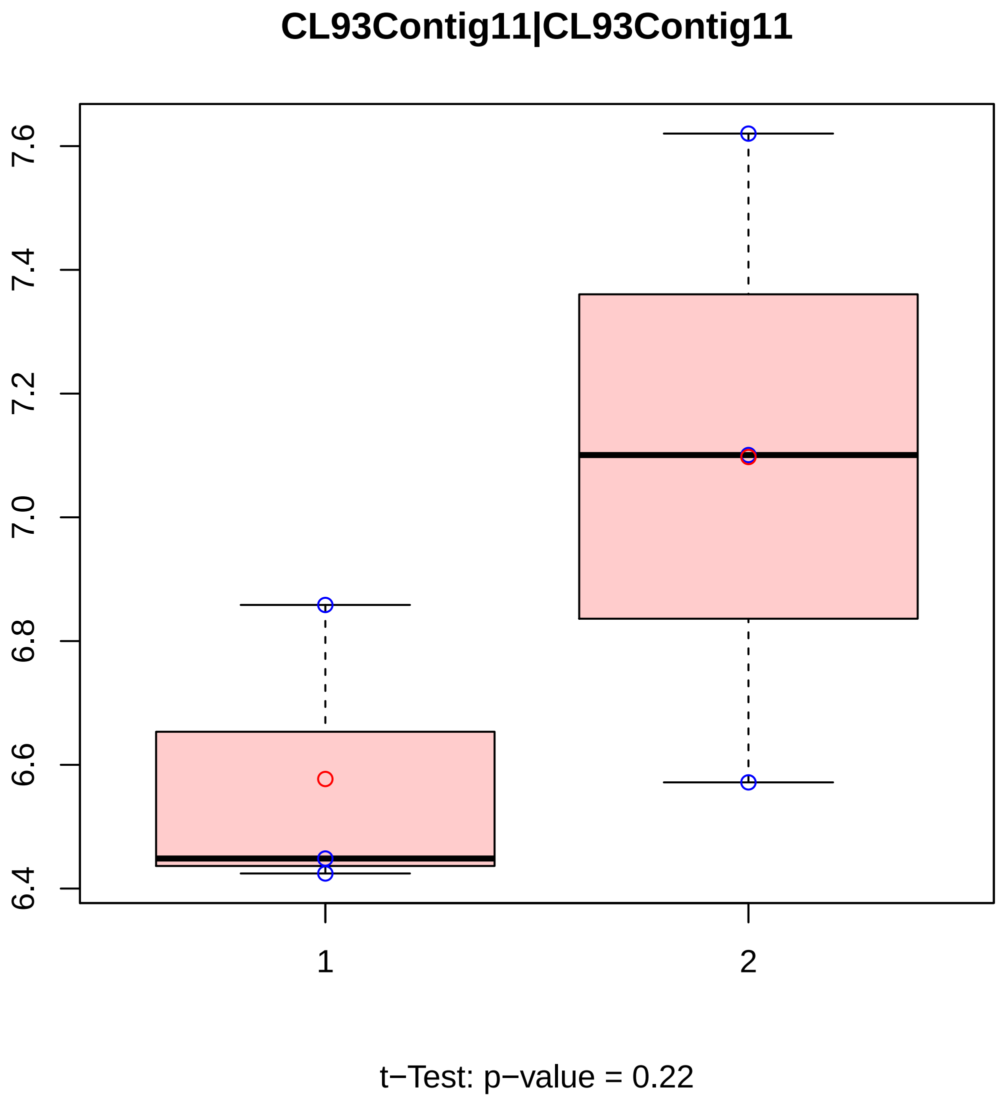
CL93Contig11|CL93Contig11
6.4
6.6
6.8
7.0
7.2
7.4
7.6
1
2
t−Test: p−value = 0.22

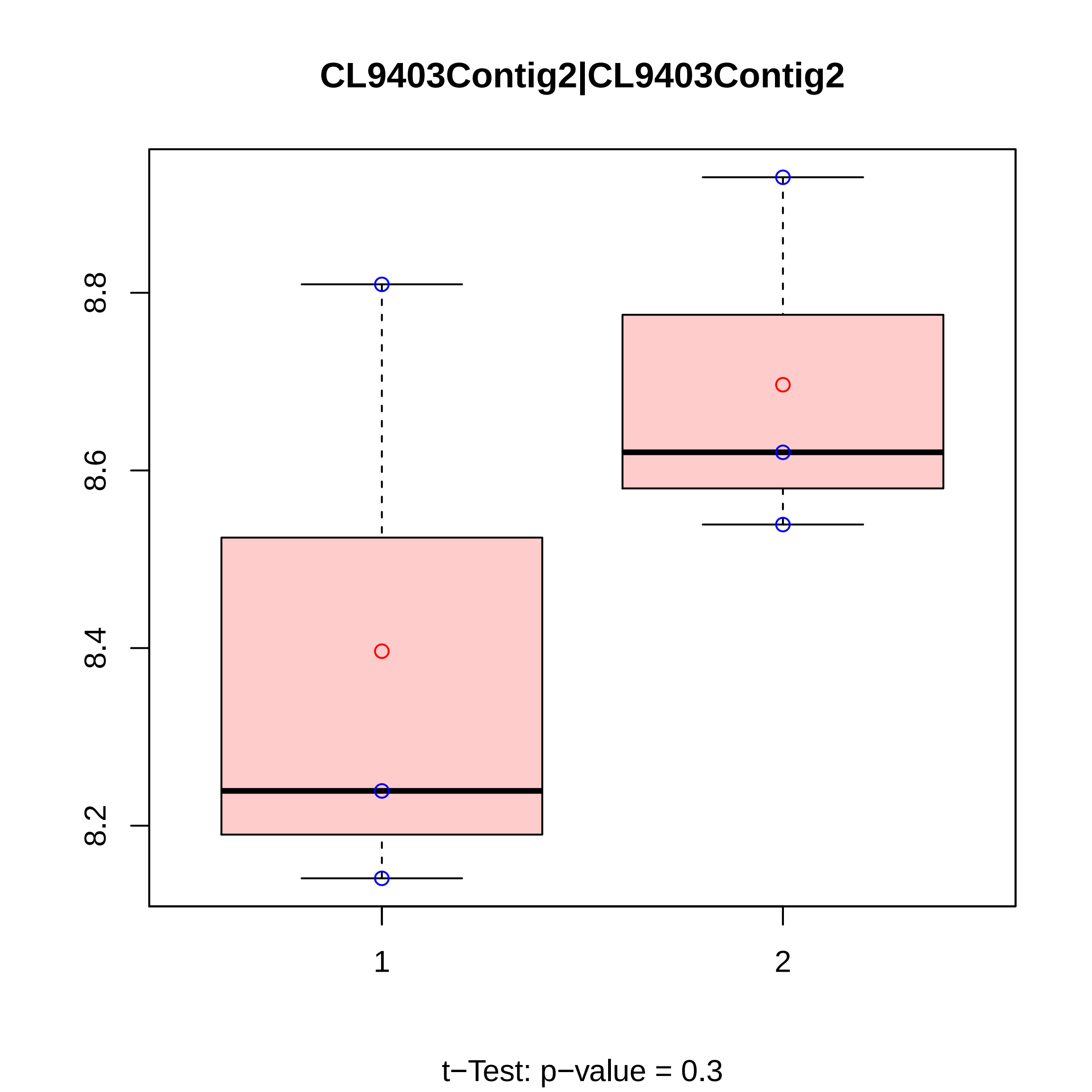

CL9403Contig2|CL9403Contig2
8.2
8.4
8.6
8.8
1
2
t−Test: p−value = 0.3

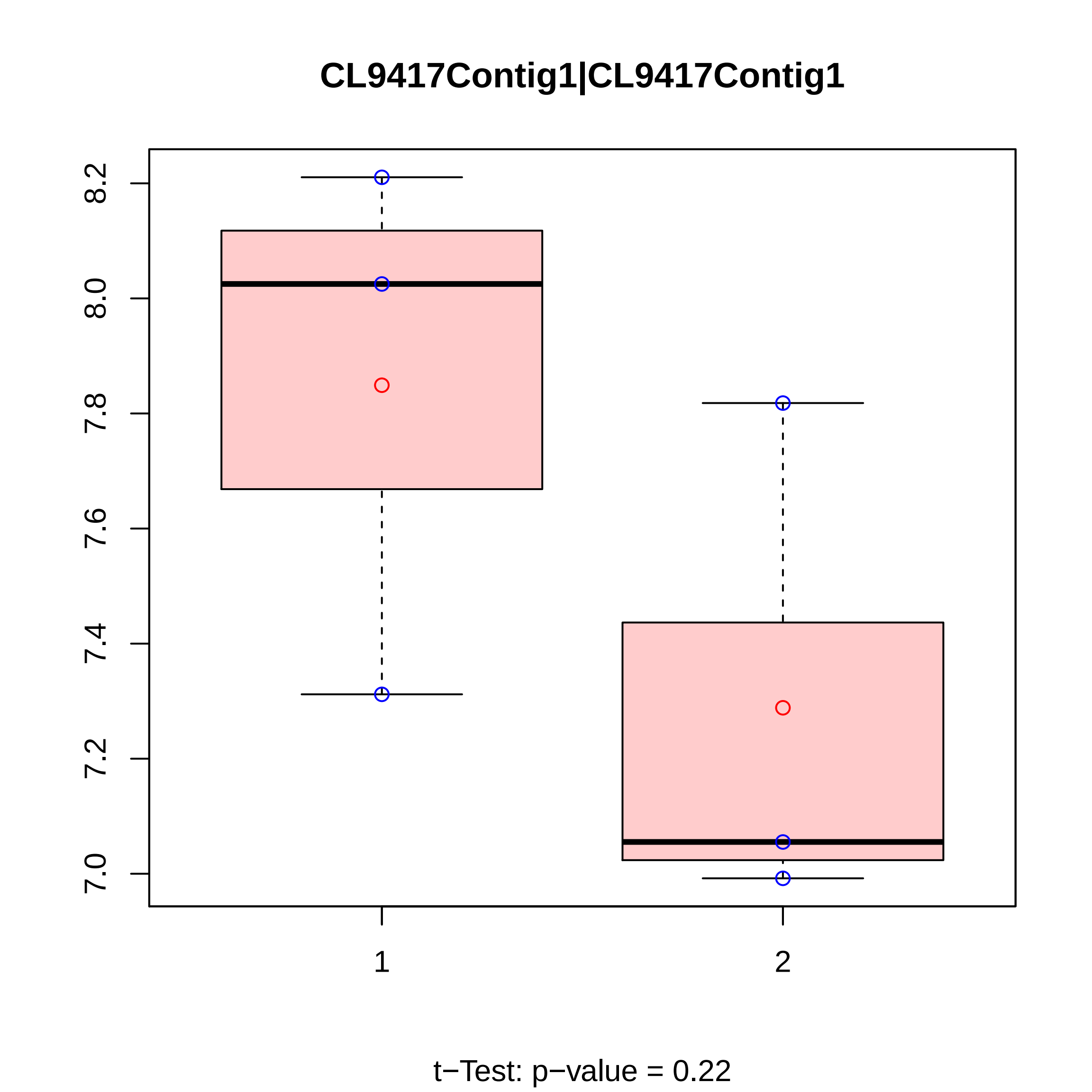
CL9417Contig1|CL9417Contig1
8.2
8.0
7.8
7.6
7.4
7.2
7.0
1
2
t−Test: p−value = 0.22

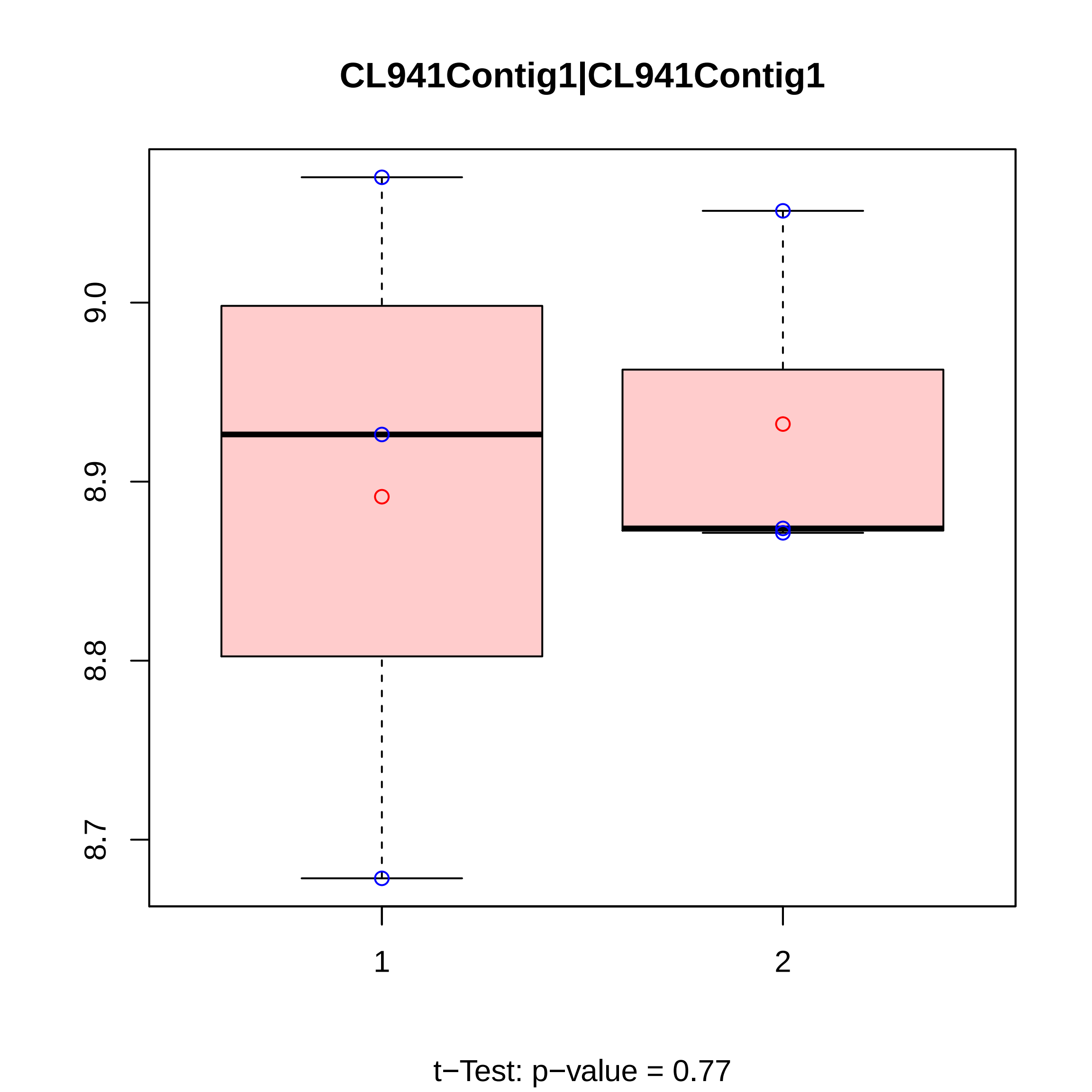

CL941Contig1|CL941Contig1
9.0
8.9
8.8
8.7
1
2
t−Test: p−value = 0.77

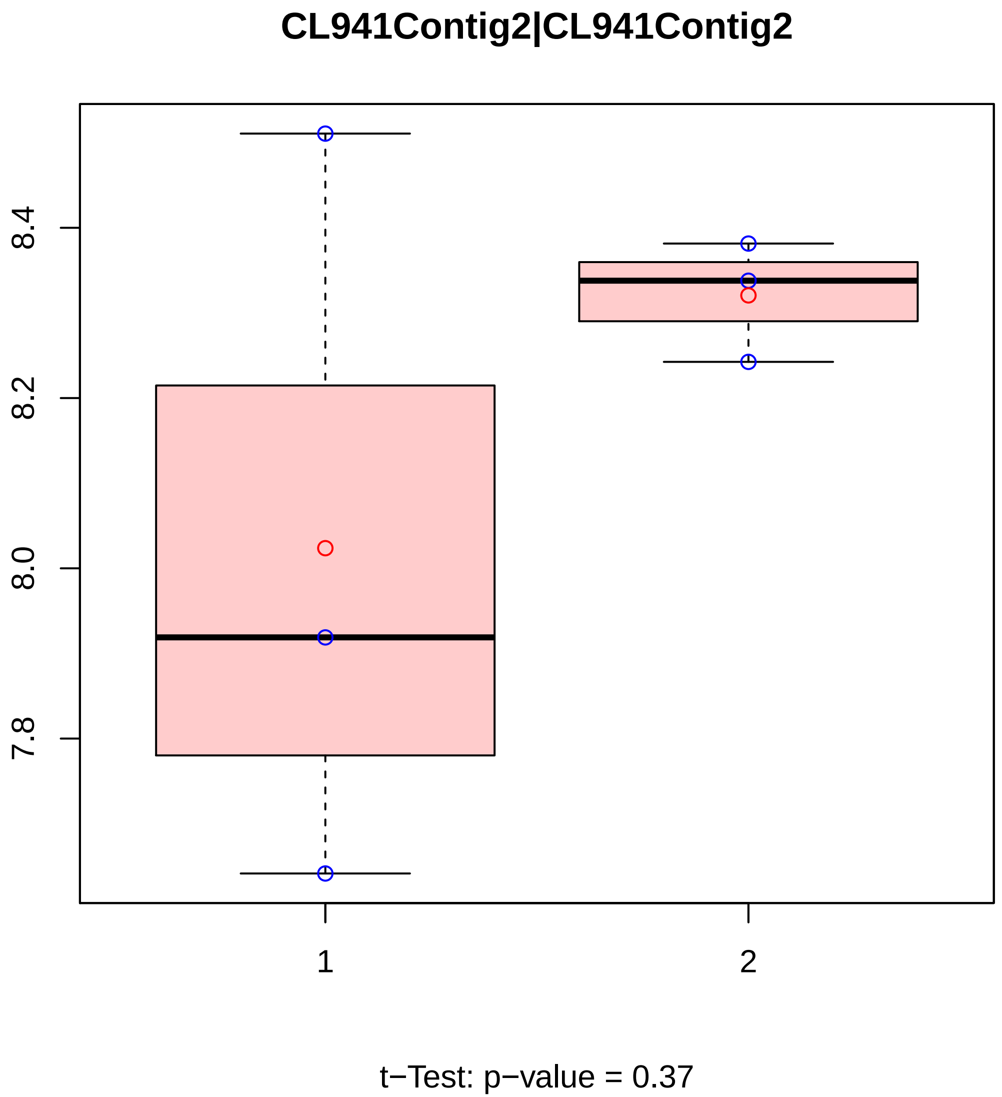

CL941Contig2|CL941Contig2
8.4
8.2
8.0
7.8
1
2
t−Test: p−value = 0.37

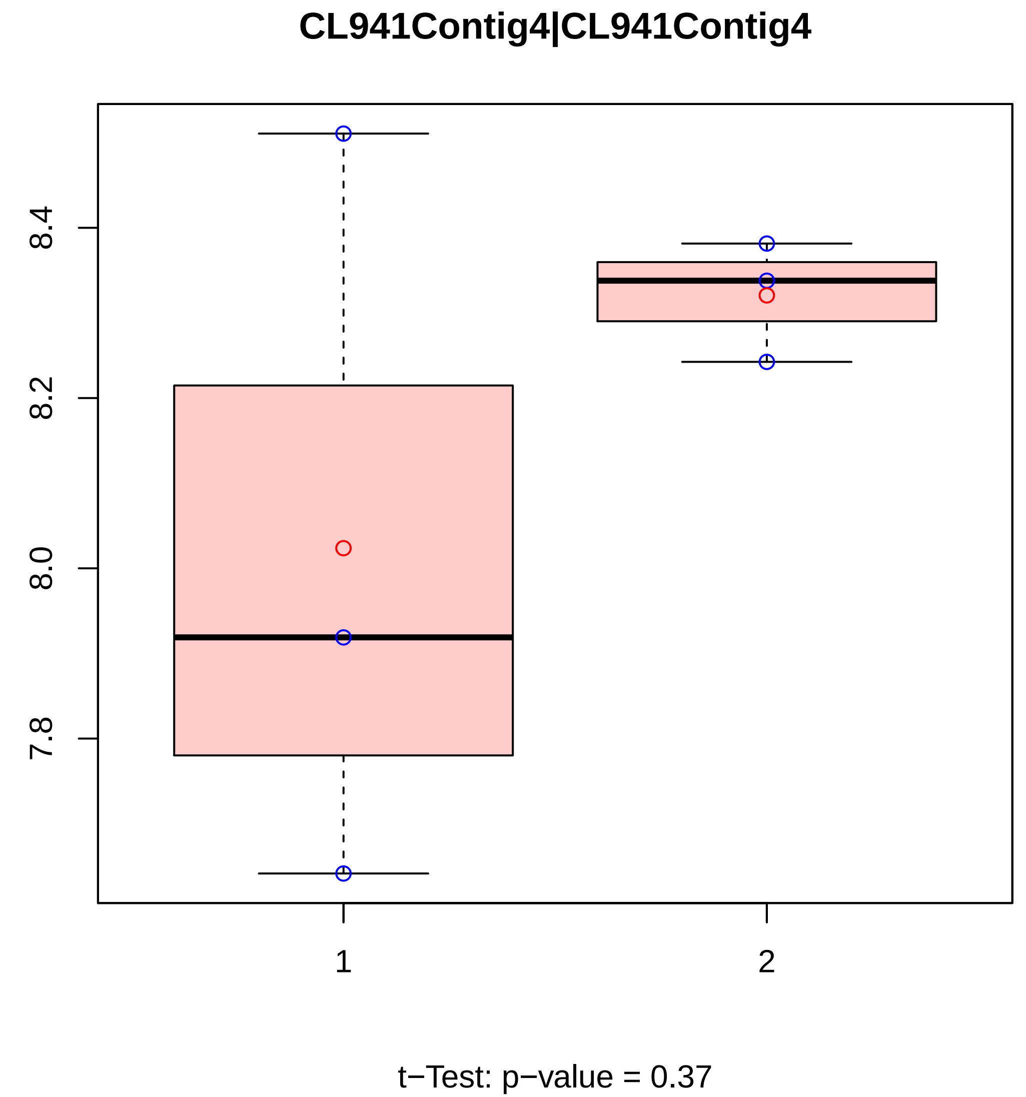
CL941Contig4|CL941Contig4
8.4
8.2
8.0
7.8
1
2
t−Test: p−value = 0.37

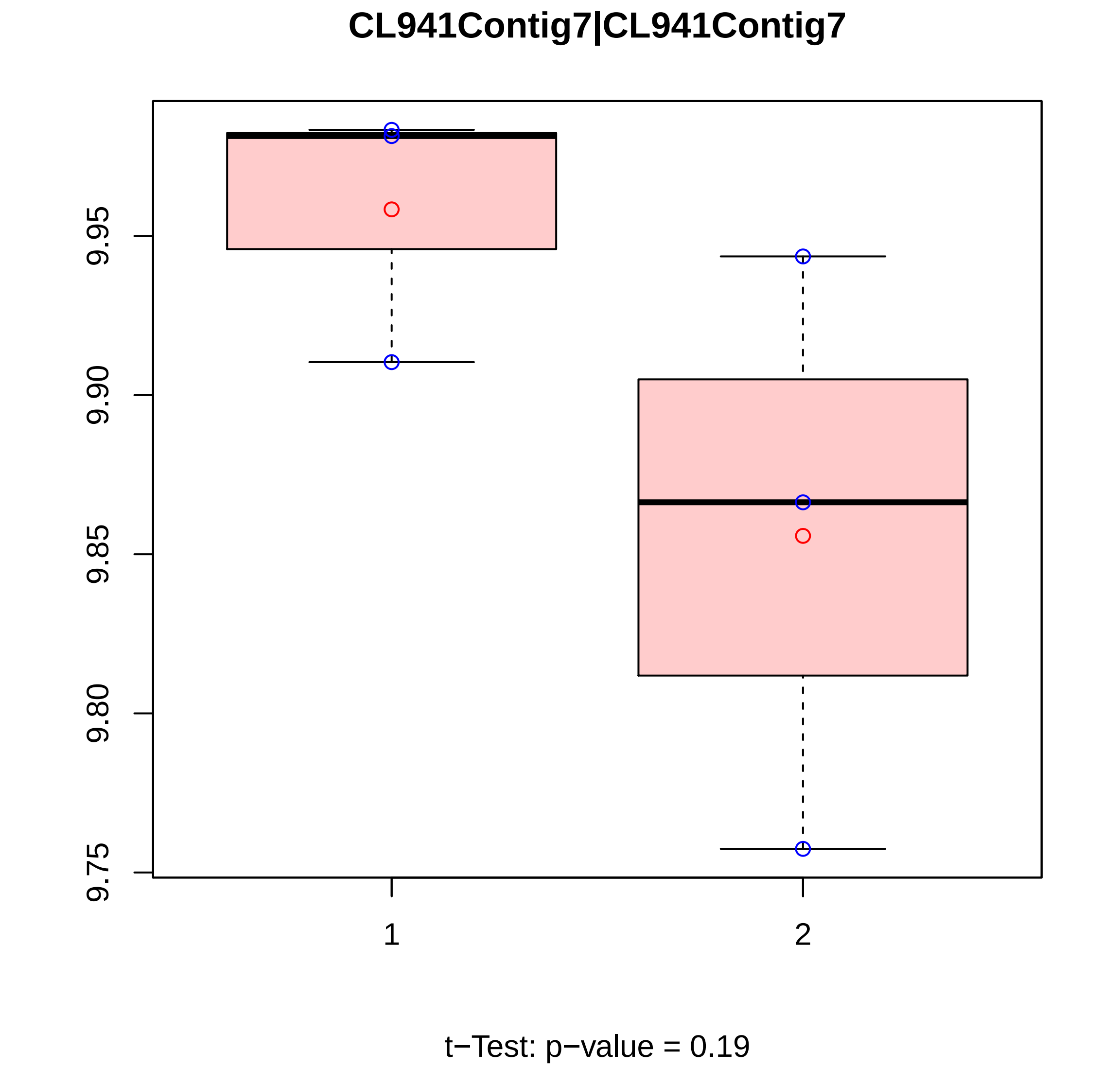
CL941Contig7|CL941Contig7
9.75
9.80
9.85
9.90
9.95
1
2
t−Test: p−value = 0.19

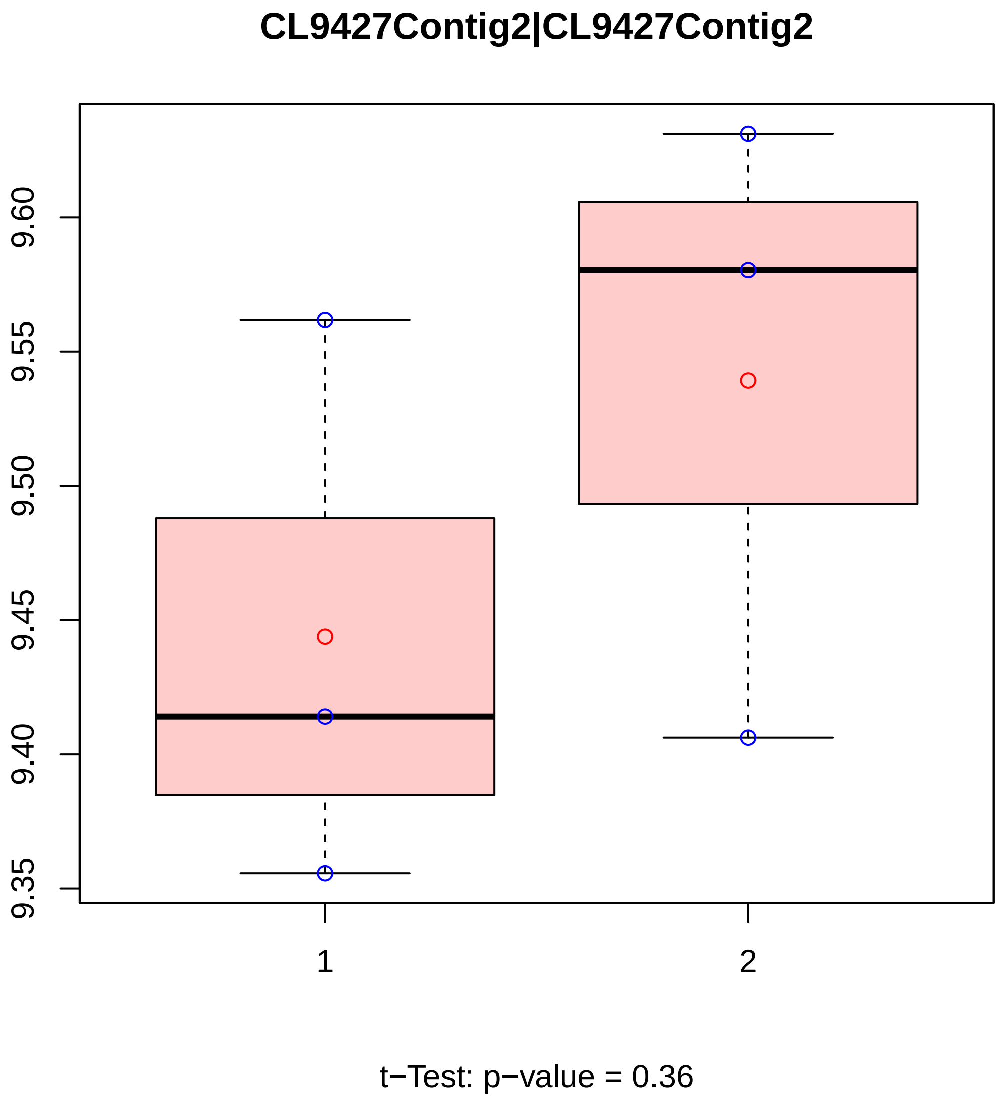

CL9427Contig2|CL9427Contig2
9.35
9.40
9.45
9.50
9.55
9.60
1
2
t−Test: p−value = 0.36

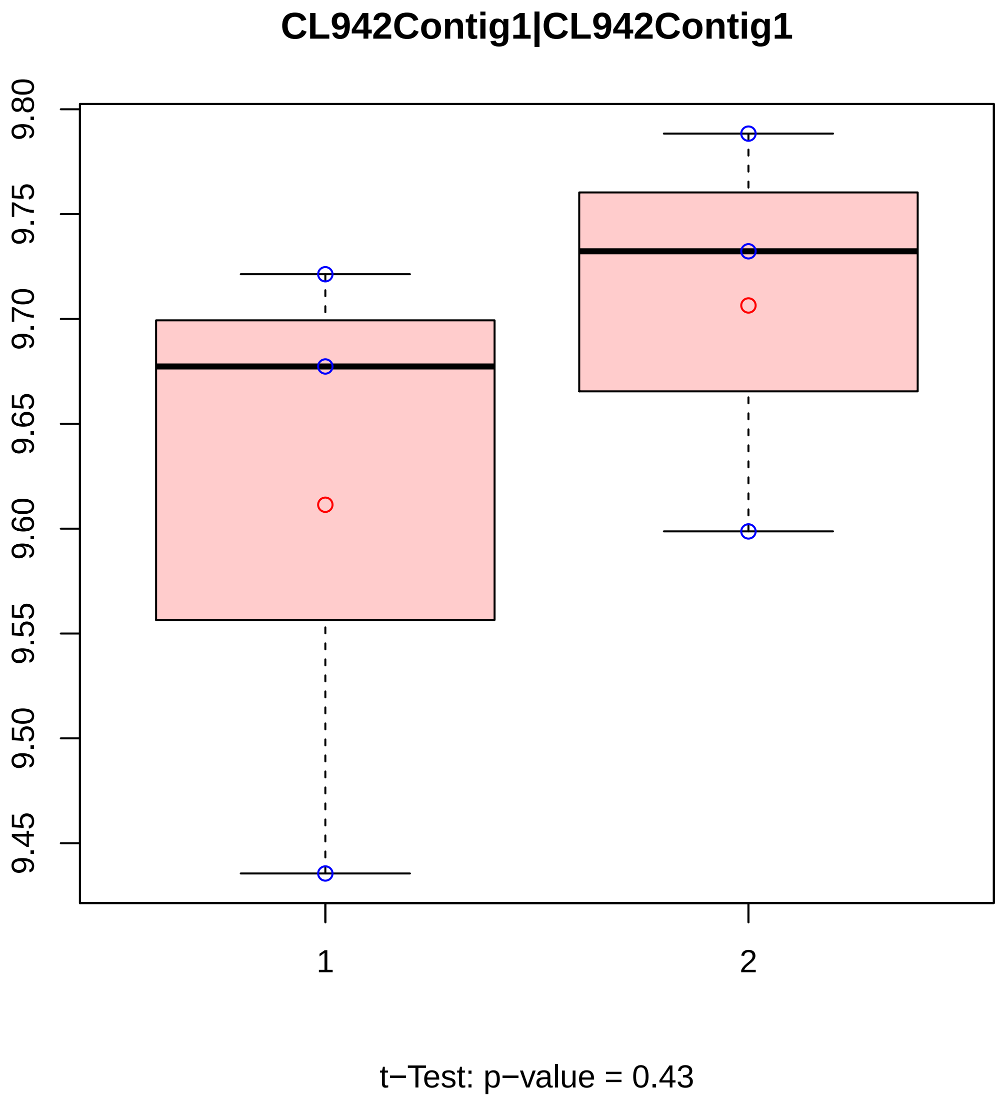
CL942Contig1|CL942Contig1
9.45
9.50
9.55
9.60
9.65
9.70
9.75
9.80
1
2
t−Test: p−value = 0.43

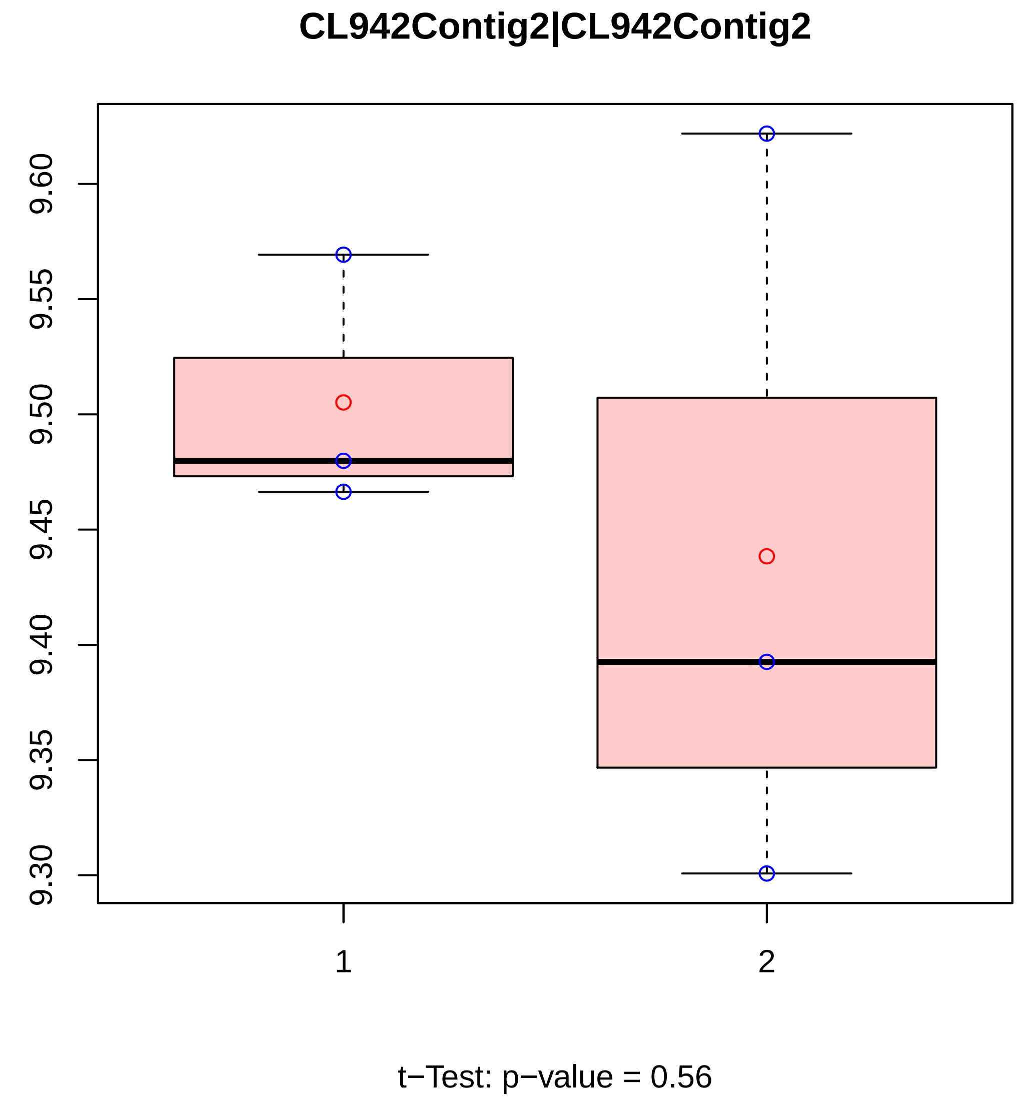

CL942Contig2|CL942Contig2
9.30
9.35
9.40
9.45
9.50
9.55
9.60
1
2
t−Test: p−value = 0.56

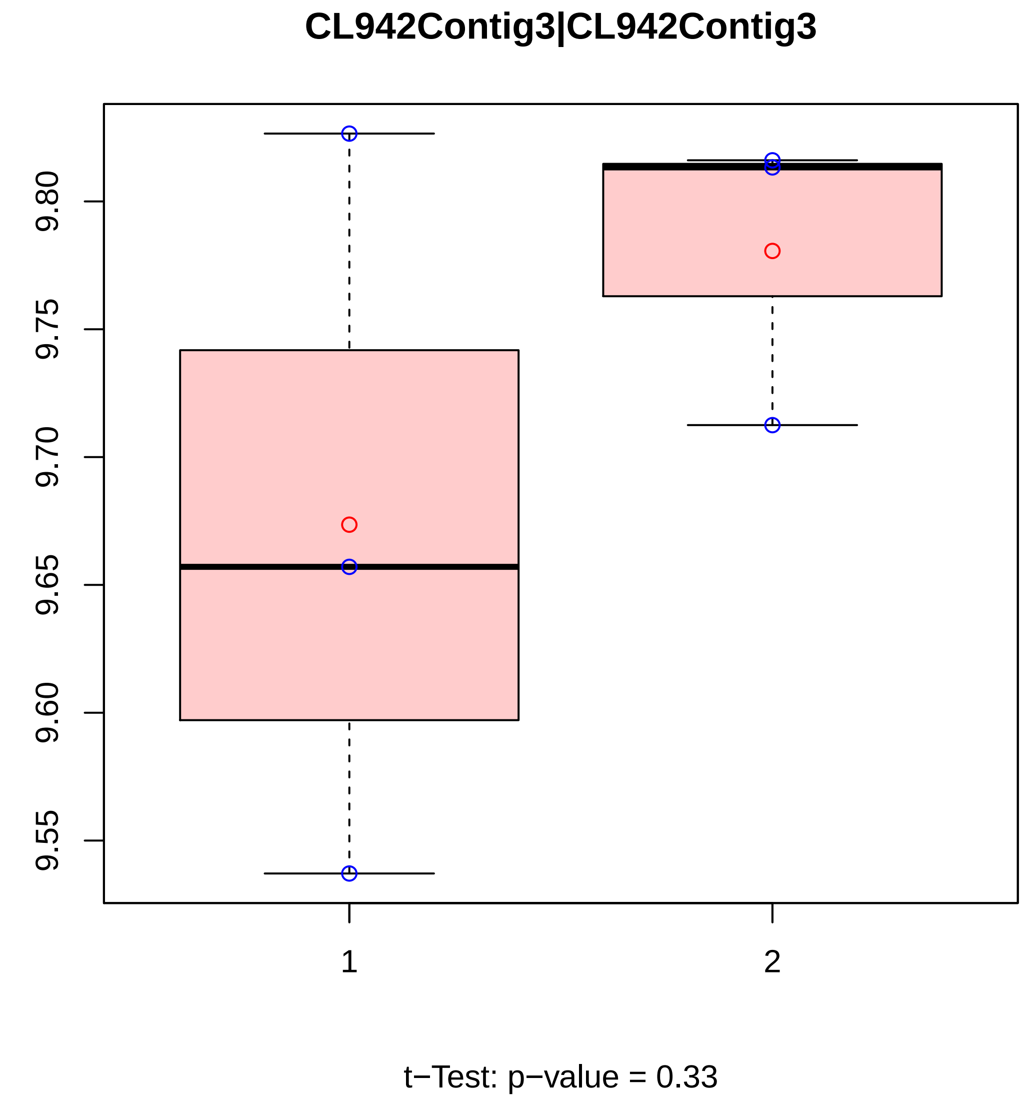
CL942Contig3|CL942Contig3
9.55
9.60
9.65
9.70
9.75
9.80
1
2
t−Test: p−value = 0.33

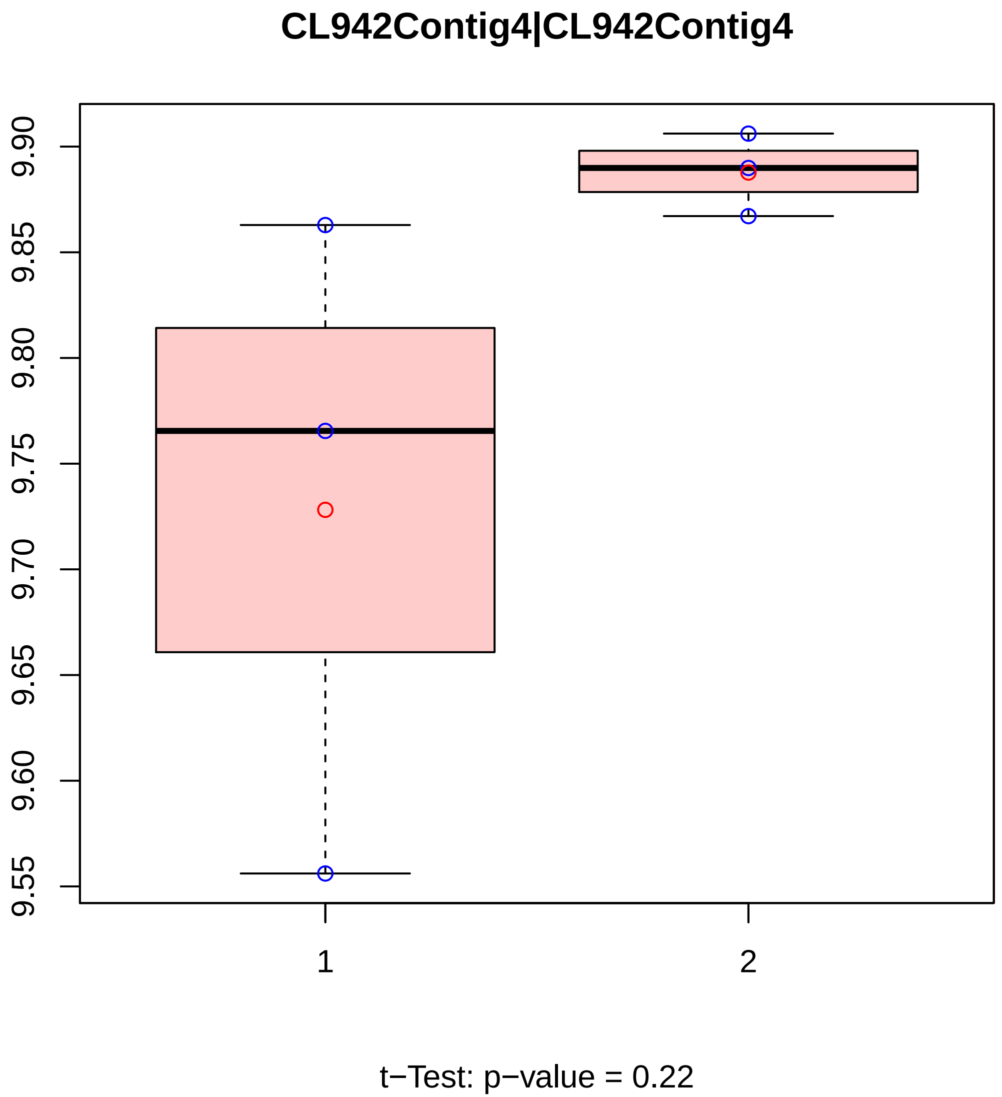

CL942Contig4|CL942Contig4
9.55
9.60
9.65
9.70
9.75
9.80
9.85
9.90
1
2
t−Test: p−value = 0.22

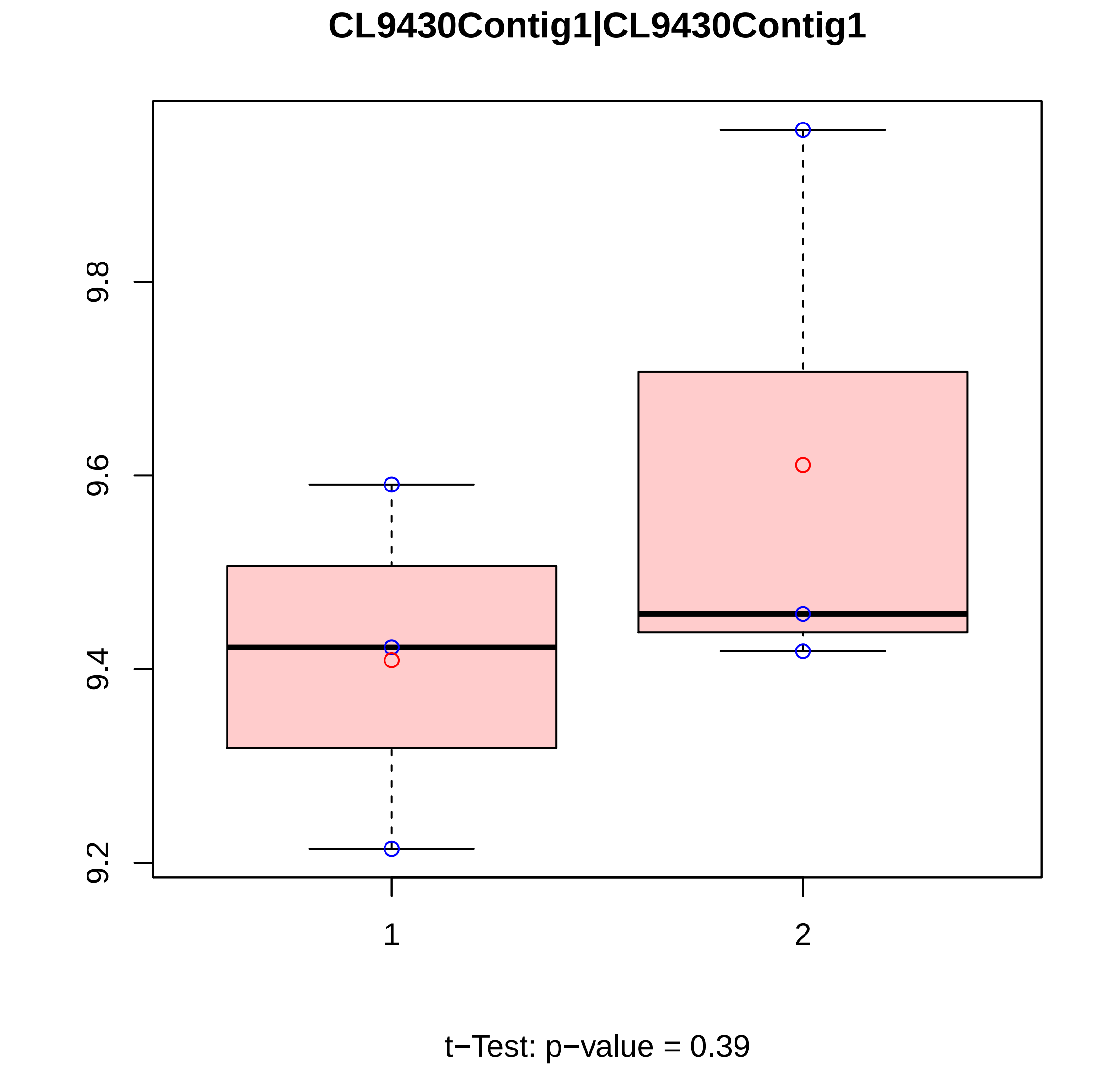
CL9430Contig1|CL9430Contig1
9.2
9.4
9.6
9.8
1
2
t−Test: p−value = 0.39

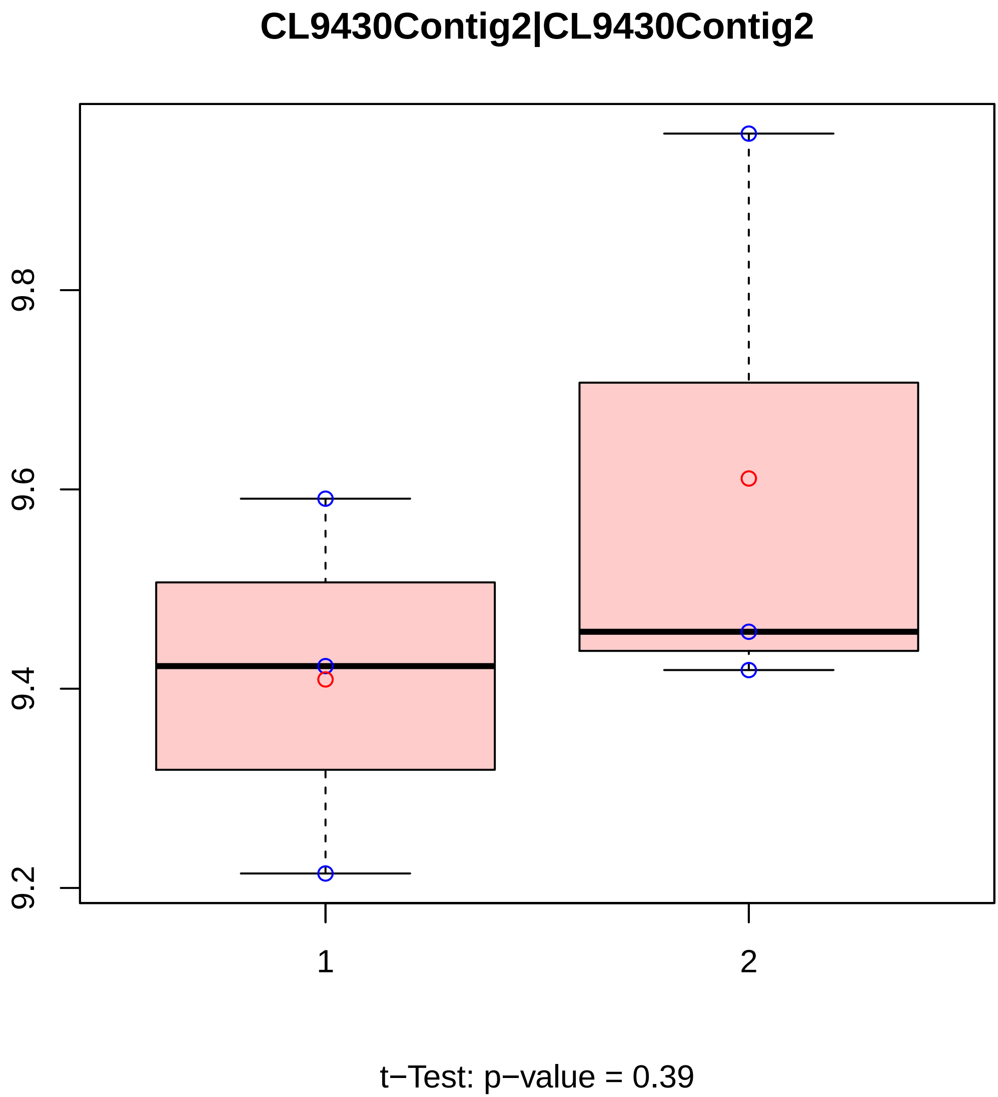
CL9430Contig2|CL9430Contig2
9.2
9.4
9.6
9.8
1
2
t−Test: p−value = 0.39

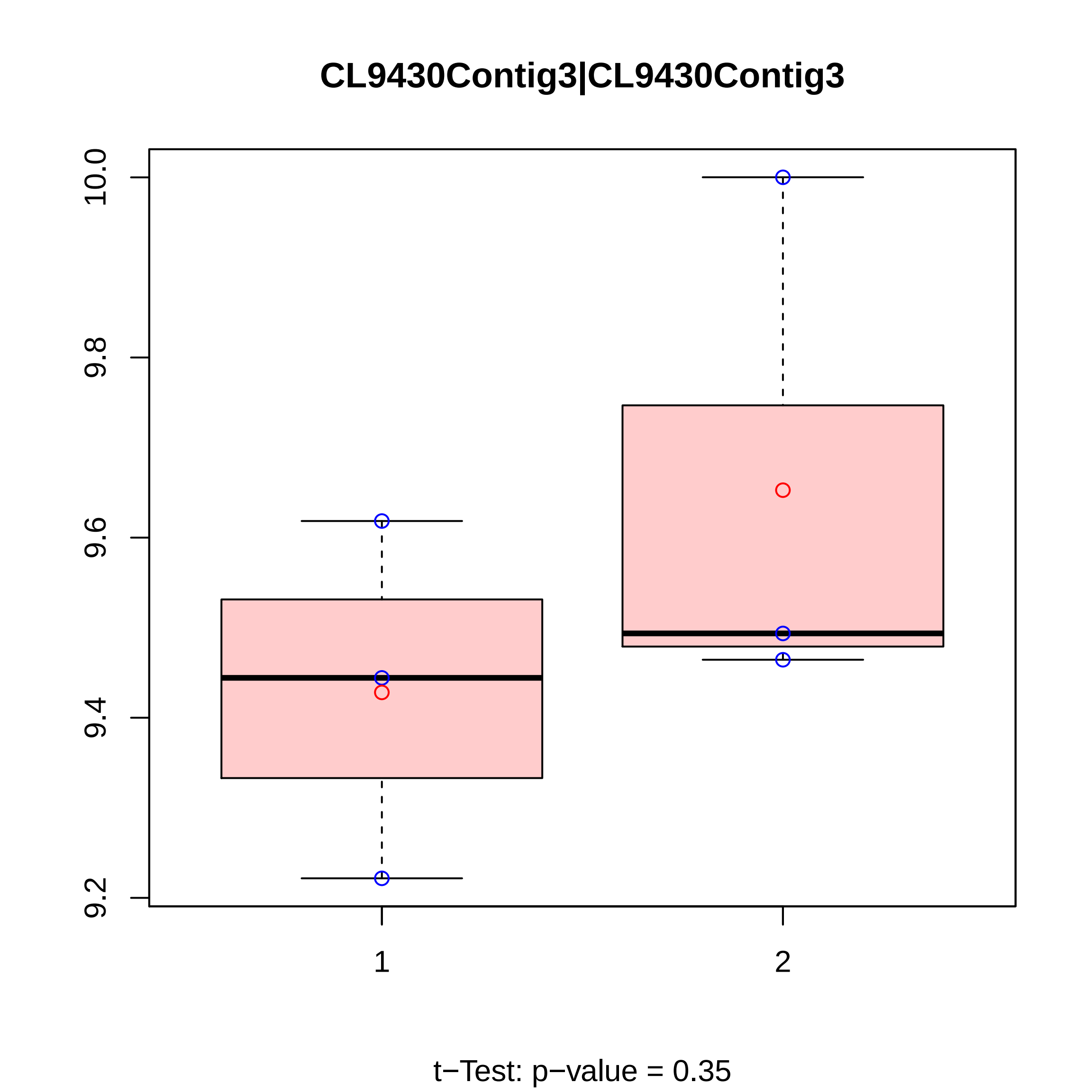

CL9430Contig3|CL9430Contig3
10.0
9.8
9.6
9.4
9.2
1
2
t−Test: p−value = 0.35

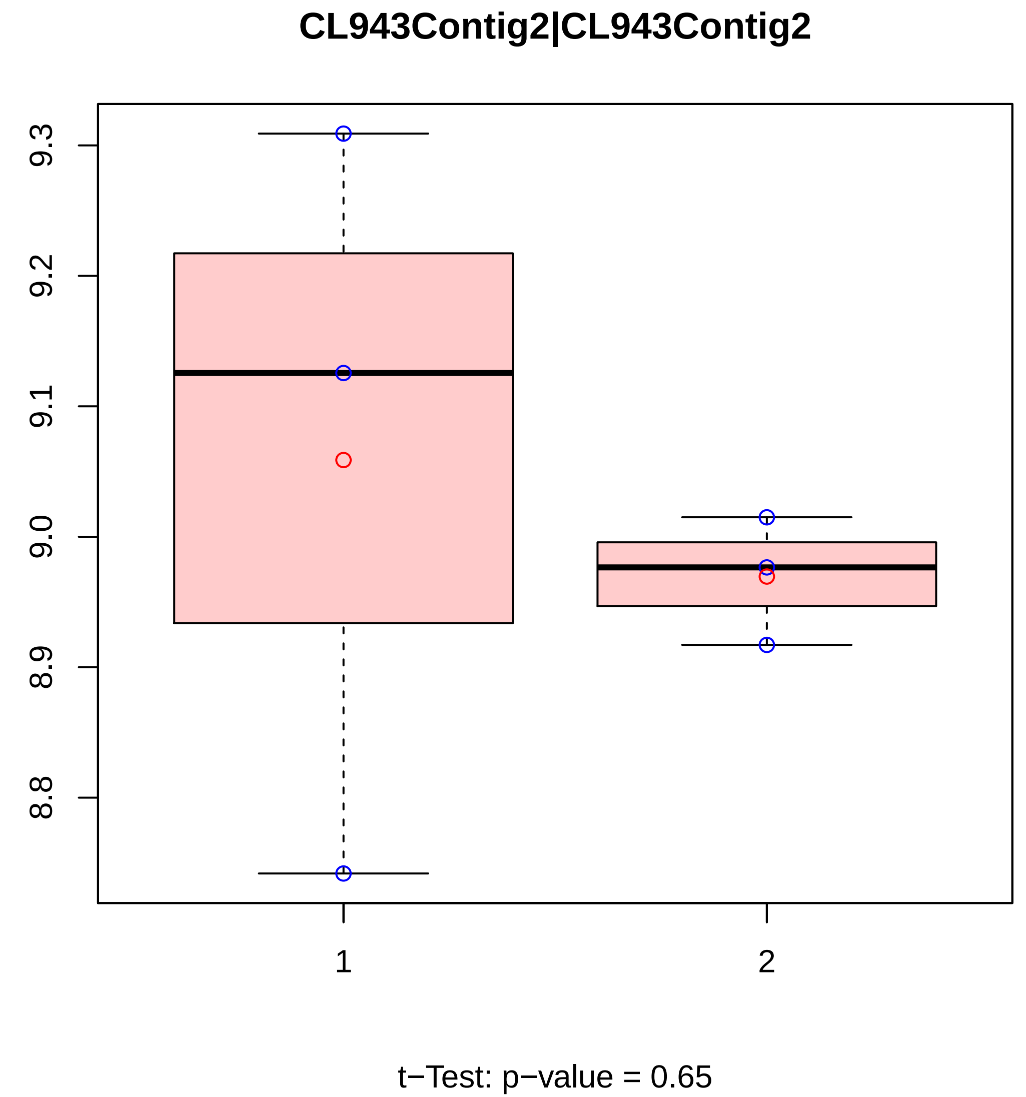
CL943Contig2|CL943Contig2
9.3
9.2
9.1
9.0
8.9
8.8
1
2
t−Test: p−value = 0.65

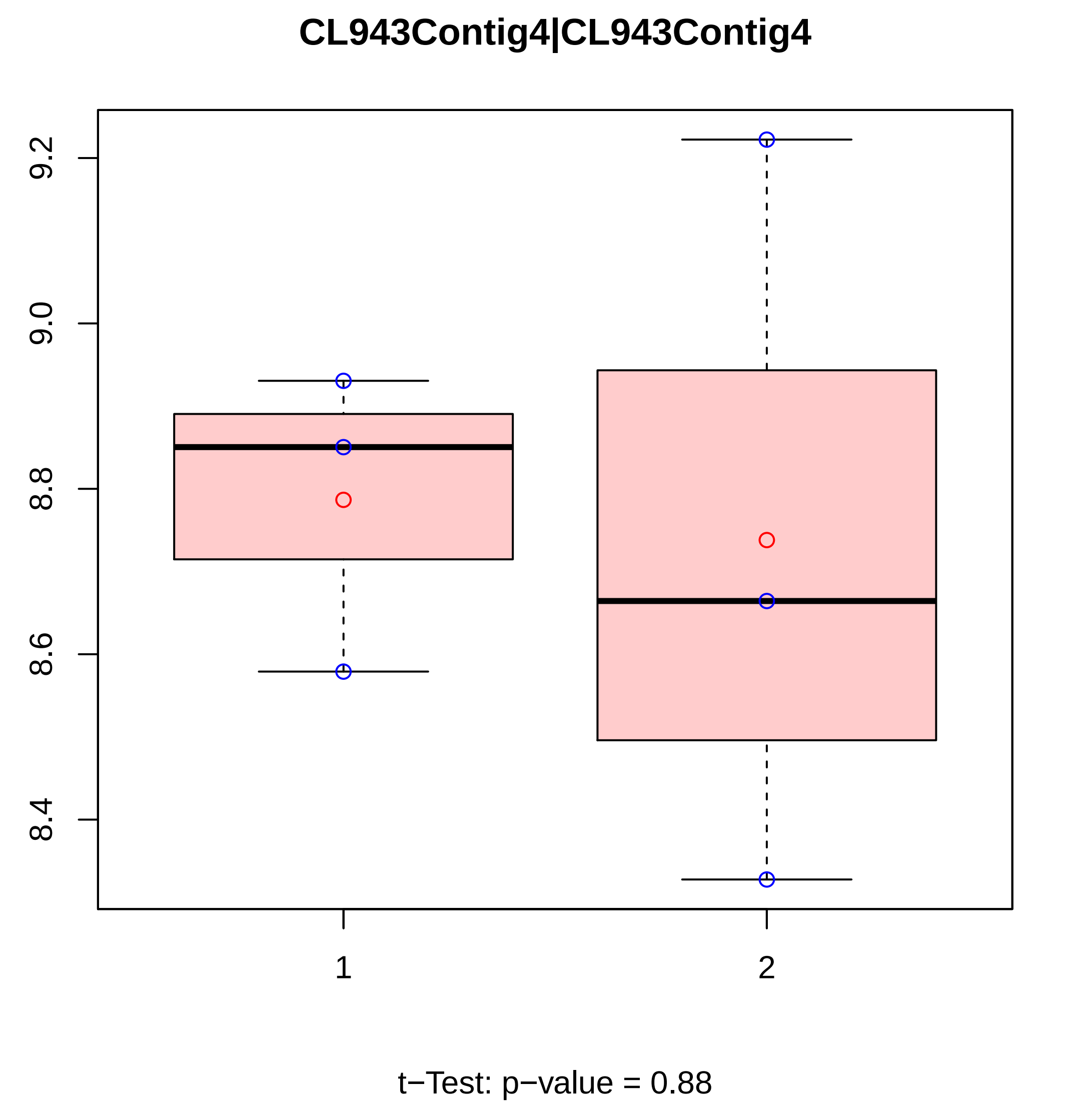
CL943Contig4|CL943Contig4
9.2
9.0
8.8
8.6
8.4
1
2
t−Test: p−value = 0.88

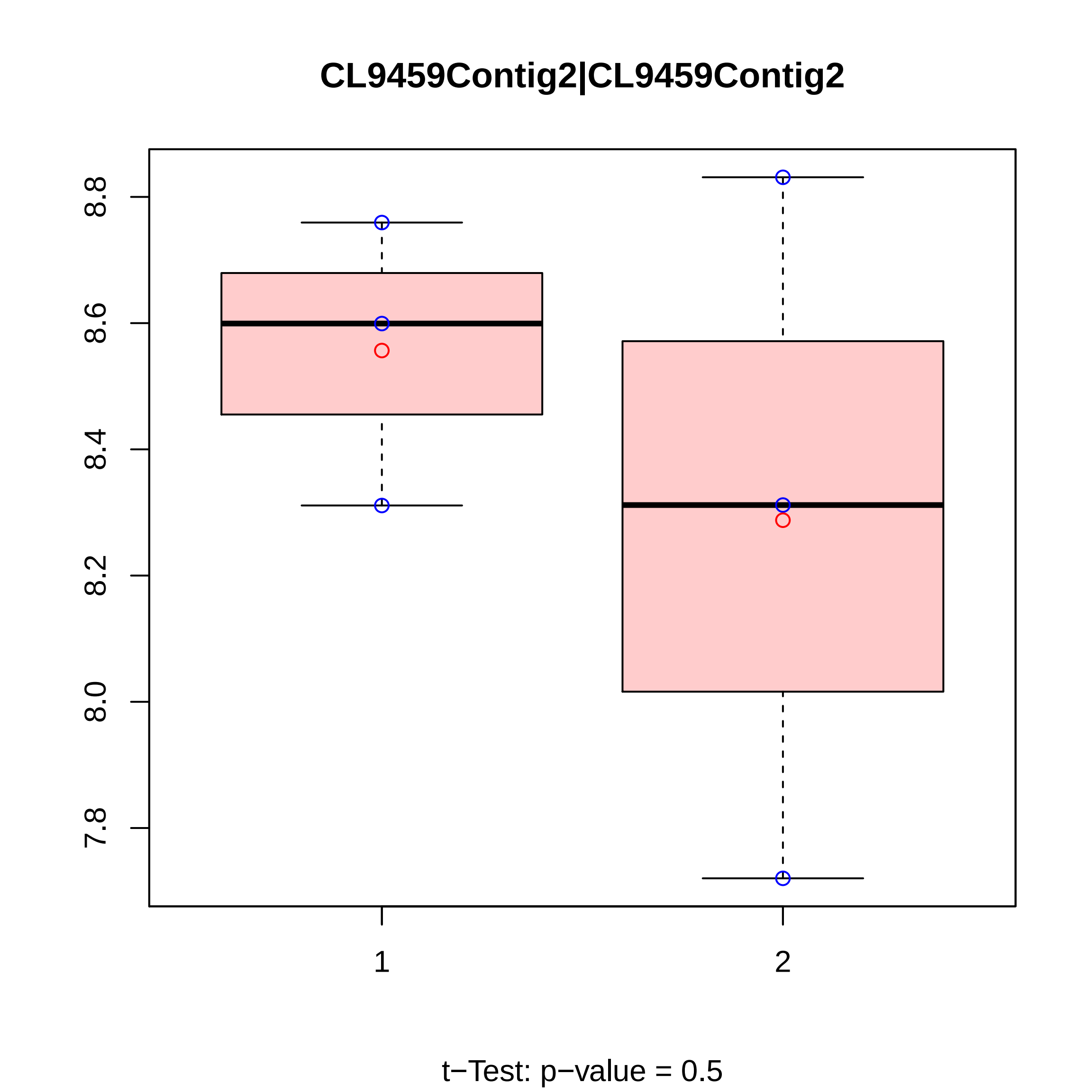
CL9459Contig2|CL9459Contig2
8.8
8.6
8.4
8.2
8.0
7.8
1
2
t−Test: p−value = 0.5

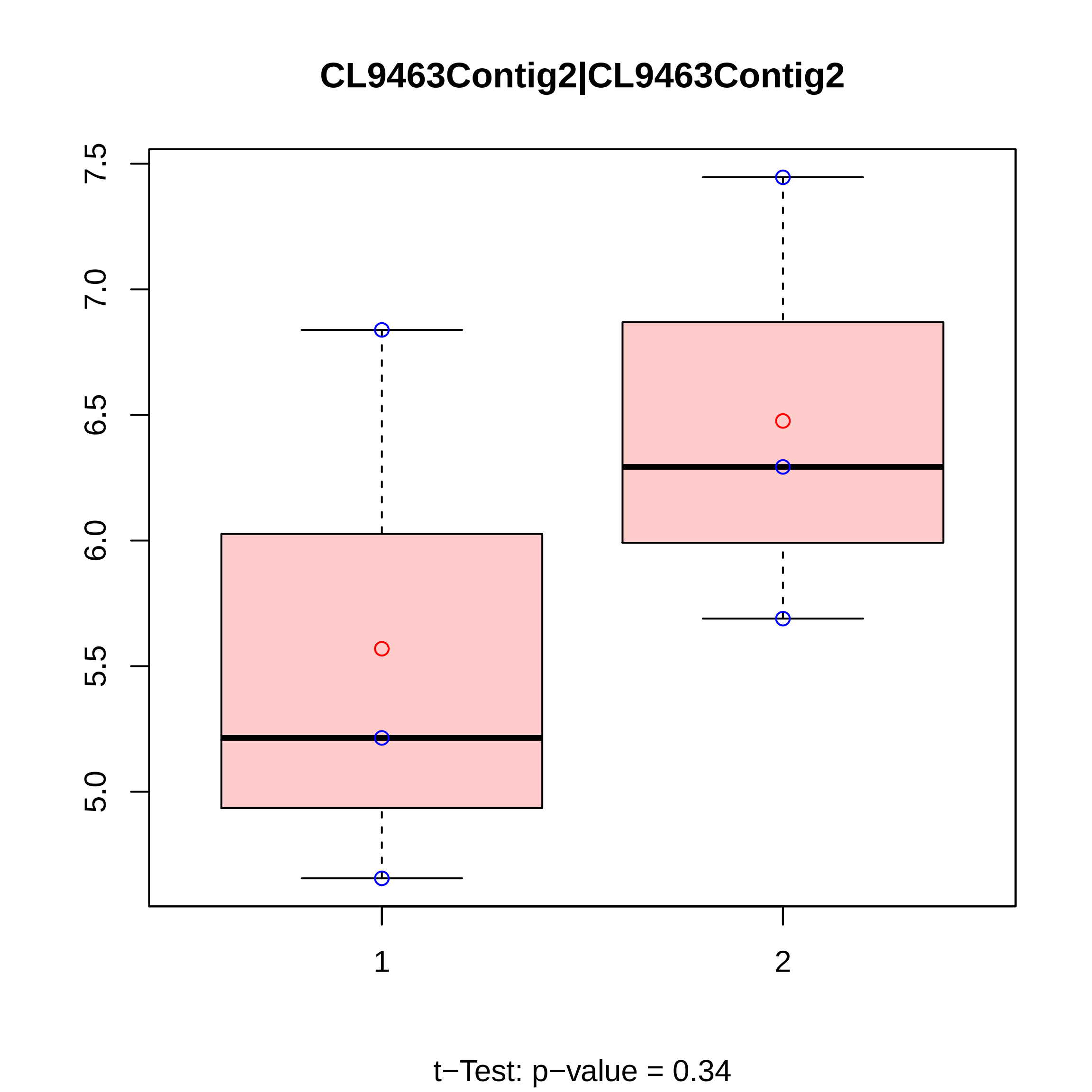
CL9463Contig2|CL9463Contig2
7.5
7.0
6.5
6.0
5.5
5.0
1
2
t−Test: p−value = 0.34

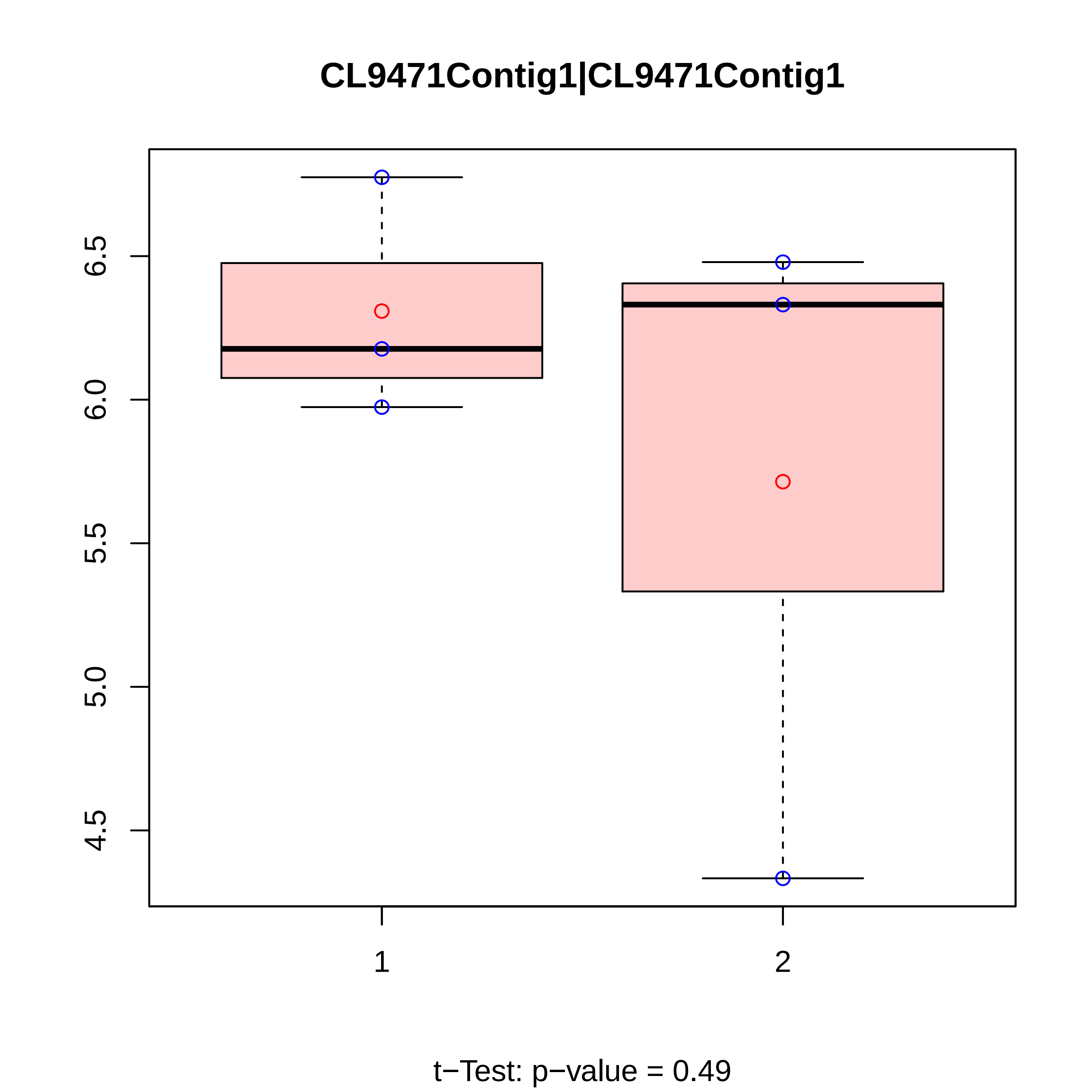
CL9471Contig1|CL9471Contig1
6.5
6.0
5.5
5.0
4.5
1
2
t−Test: p−value = 0.49

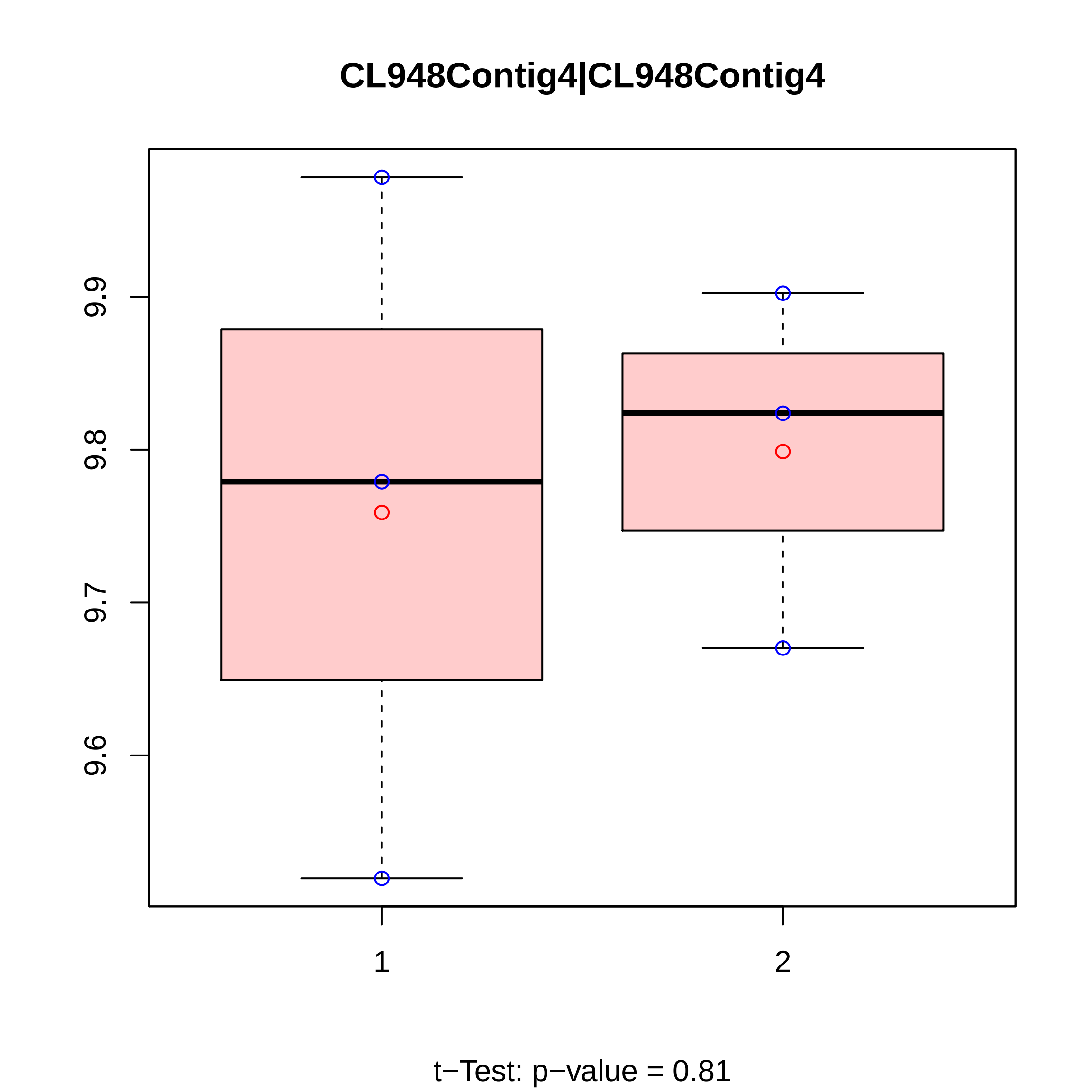

CL948Contig4|CL948Contig4
9.6
9.7
9.8
9.9
1
2
t−Test: p−value = 0.81

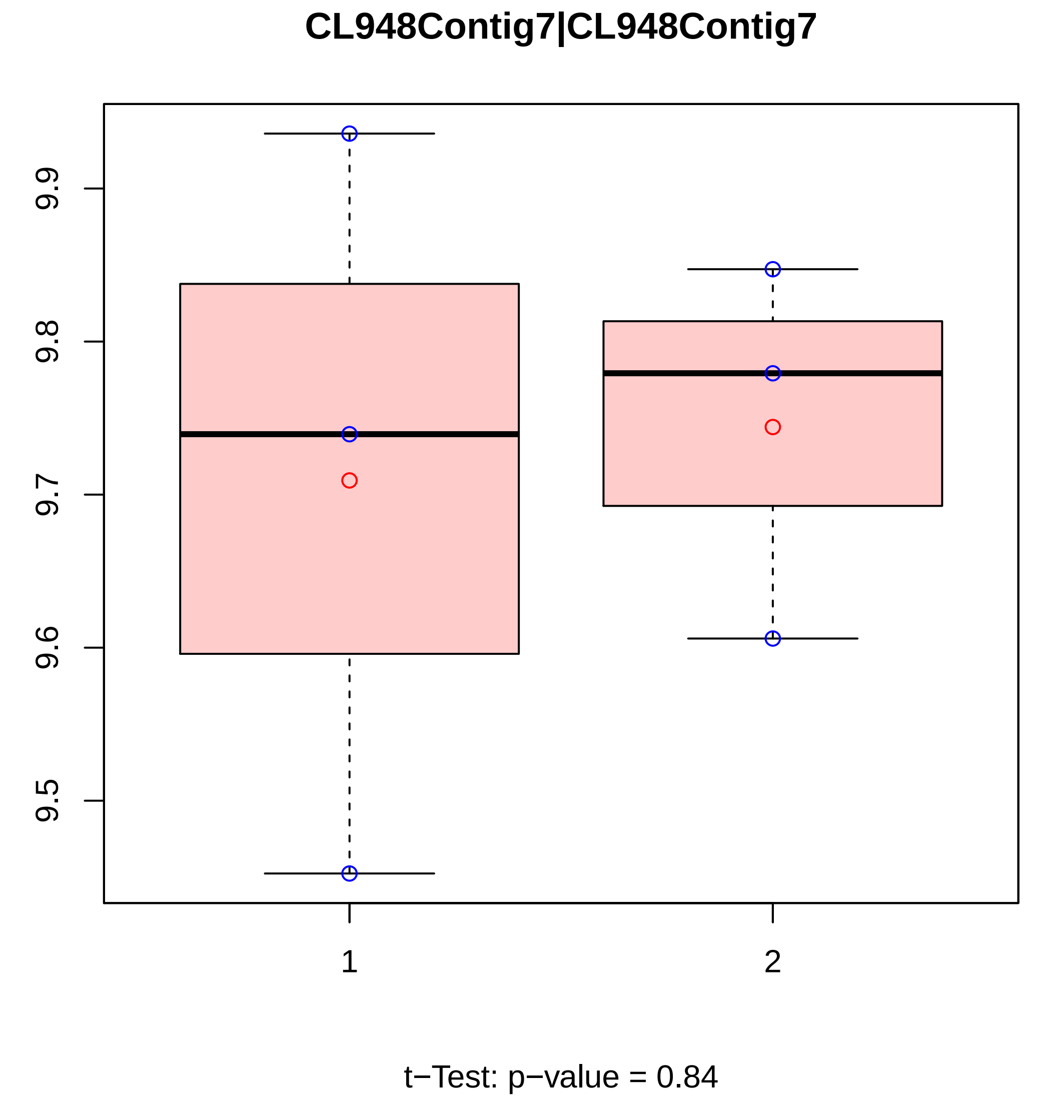
CL948Contig7|CL948Contig7
9.5
9.6
9.7
9.8
9.9
1
2
t−Test: p−value = 0.84

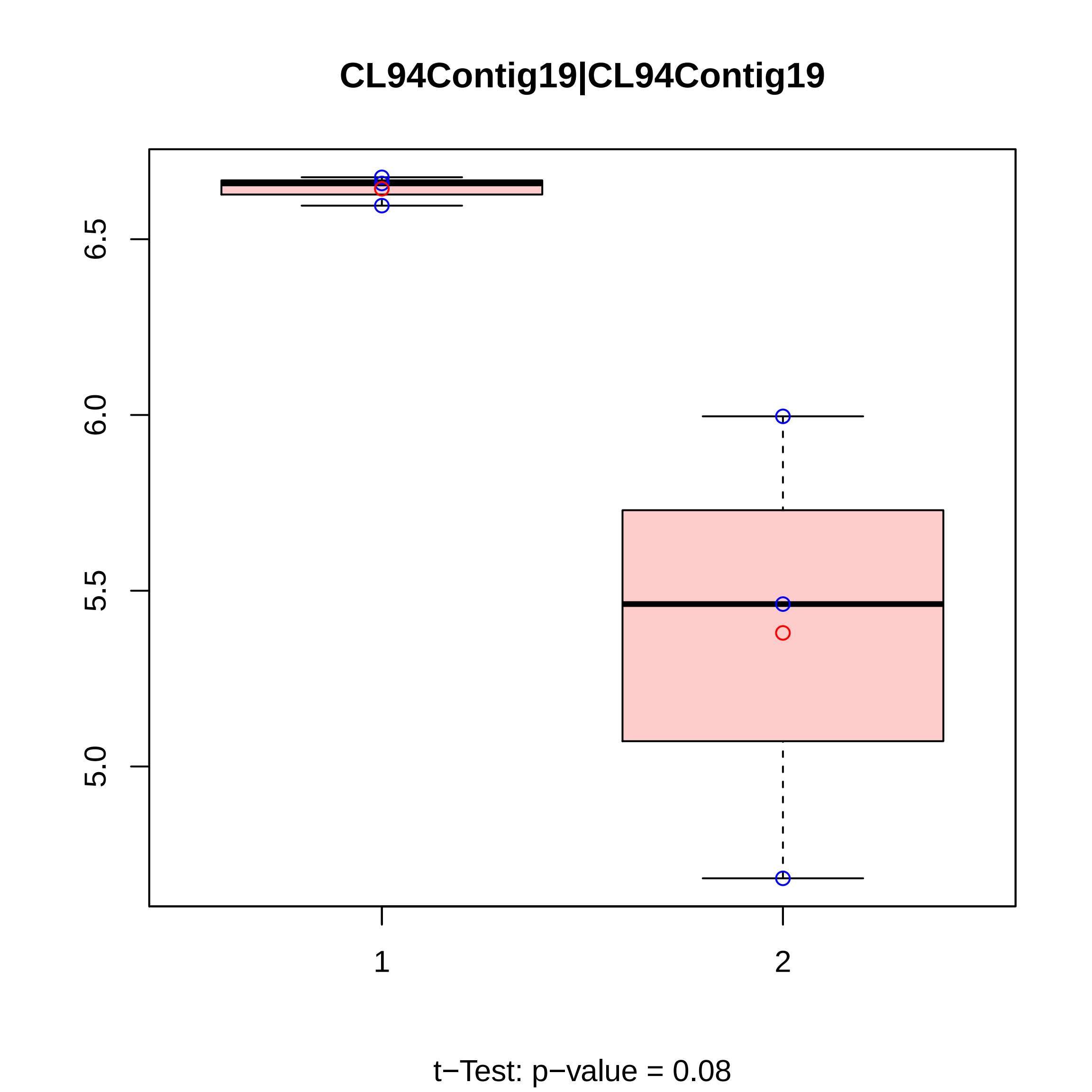
CL94Contig19|CL94Contig19
6.5
6.0
5.5
5.0
1
2
t−Test: p−value = 0.08

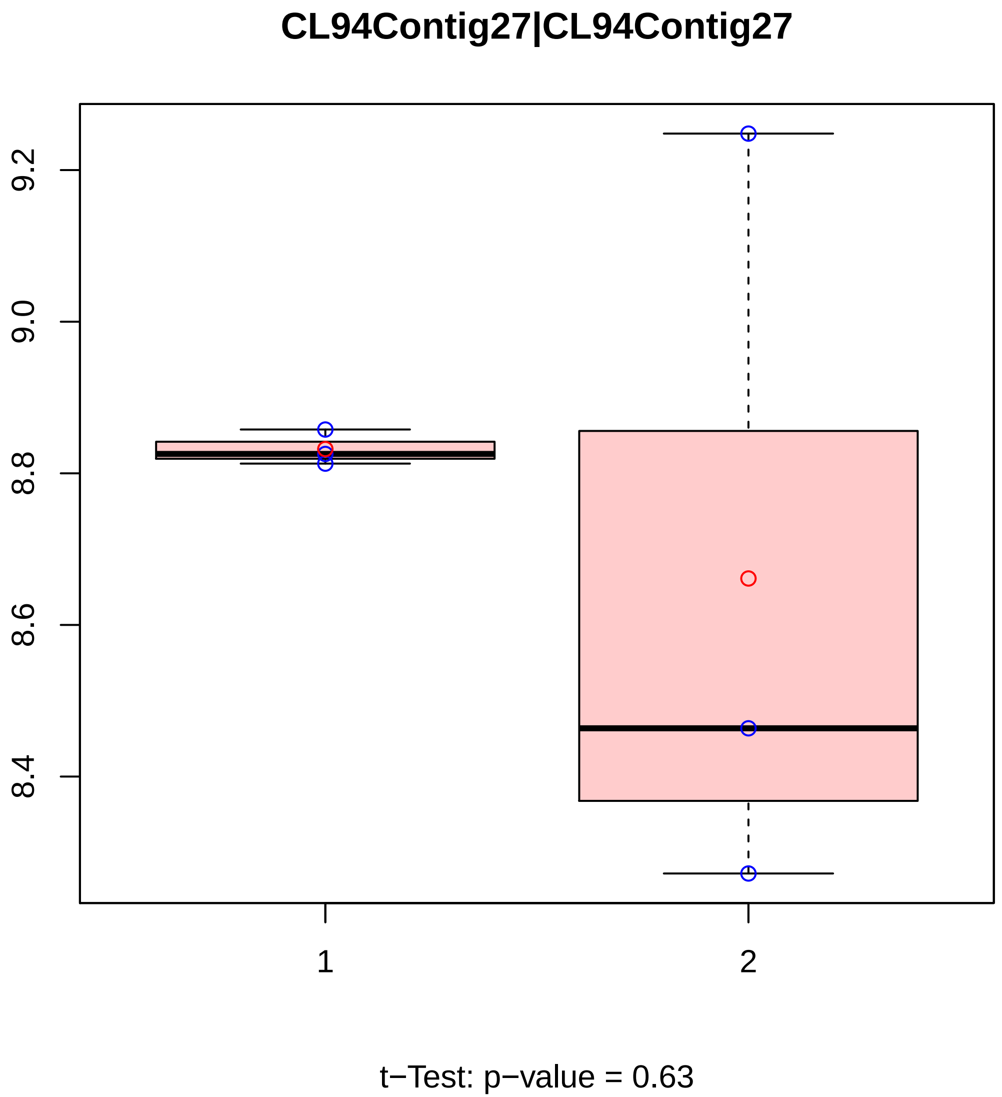

CL94Contig27|CL94Contig27
9.2
9.0
8.8
8.6
8.4
1
2
t−Test: p−value = 0.63

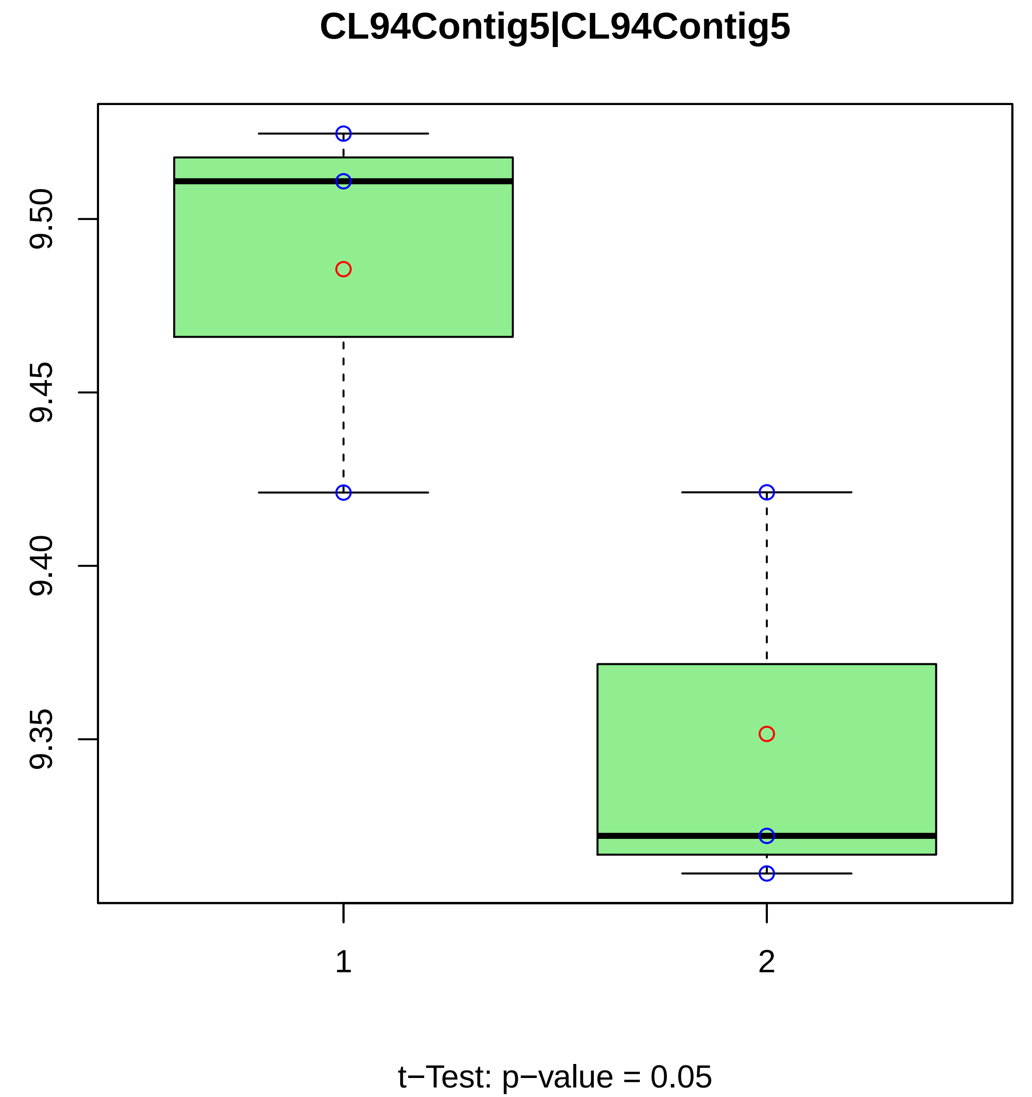

CL94Contig5|CL94Contig5
9.50
9.45
9.40
9.35
1
2
t−Test: p−value = 0.05

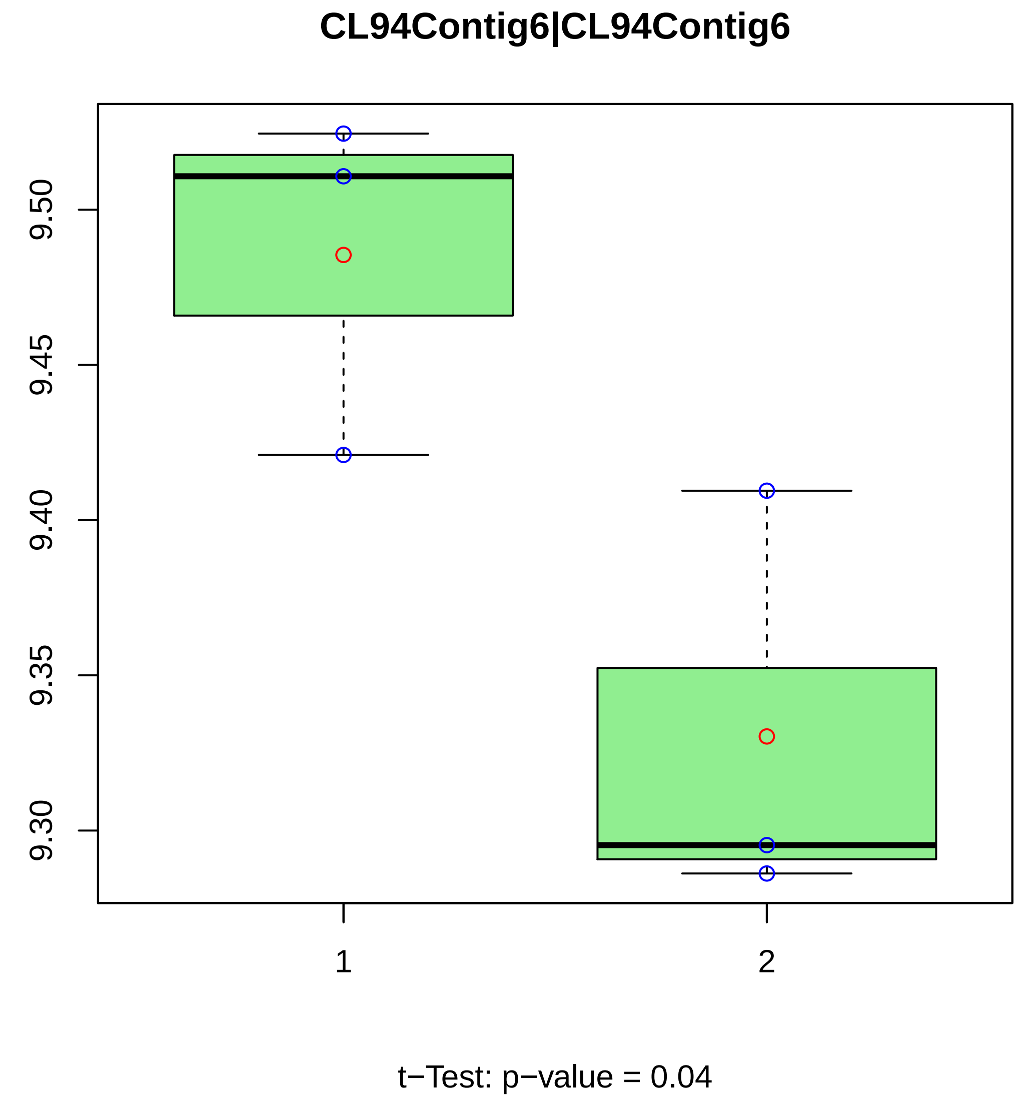

CL94Contig6|CL94Contig6
9.30
9.35
9.40
9.45
9.50
1
2
t−Test: p−value = 0.04

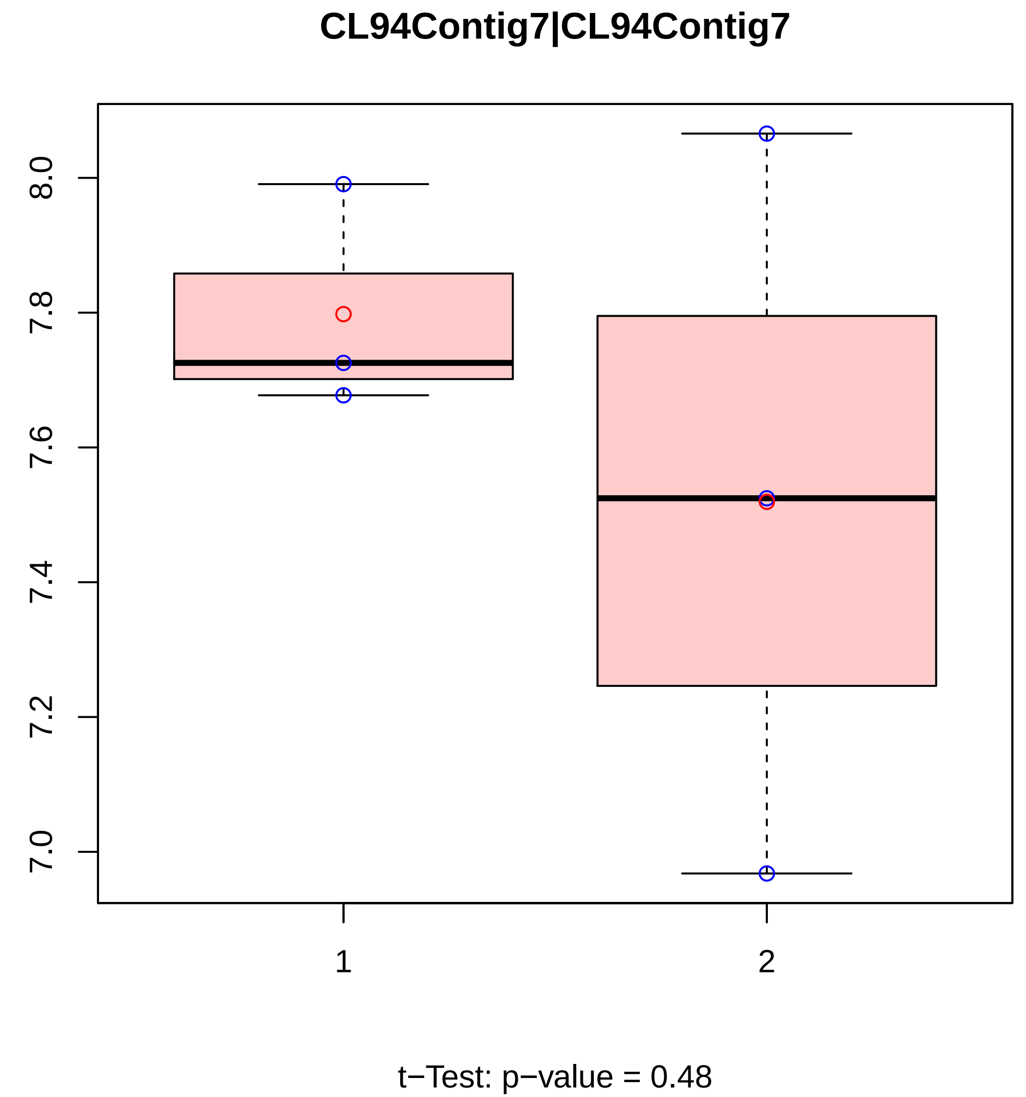
CL94Contig7|CL94Contig7
8.0
7.8
7.6
7.4
7.2
7.0
1
2
t−Test: p−value = 0.48

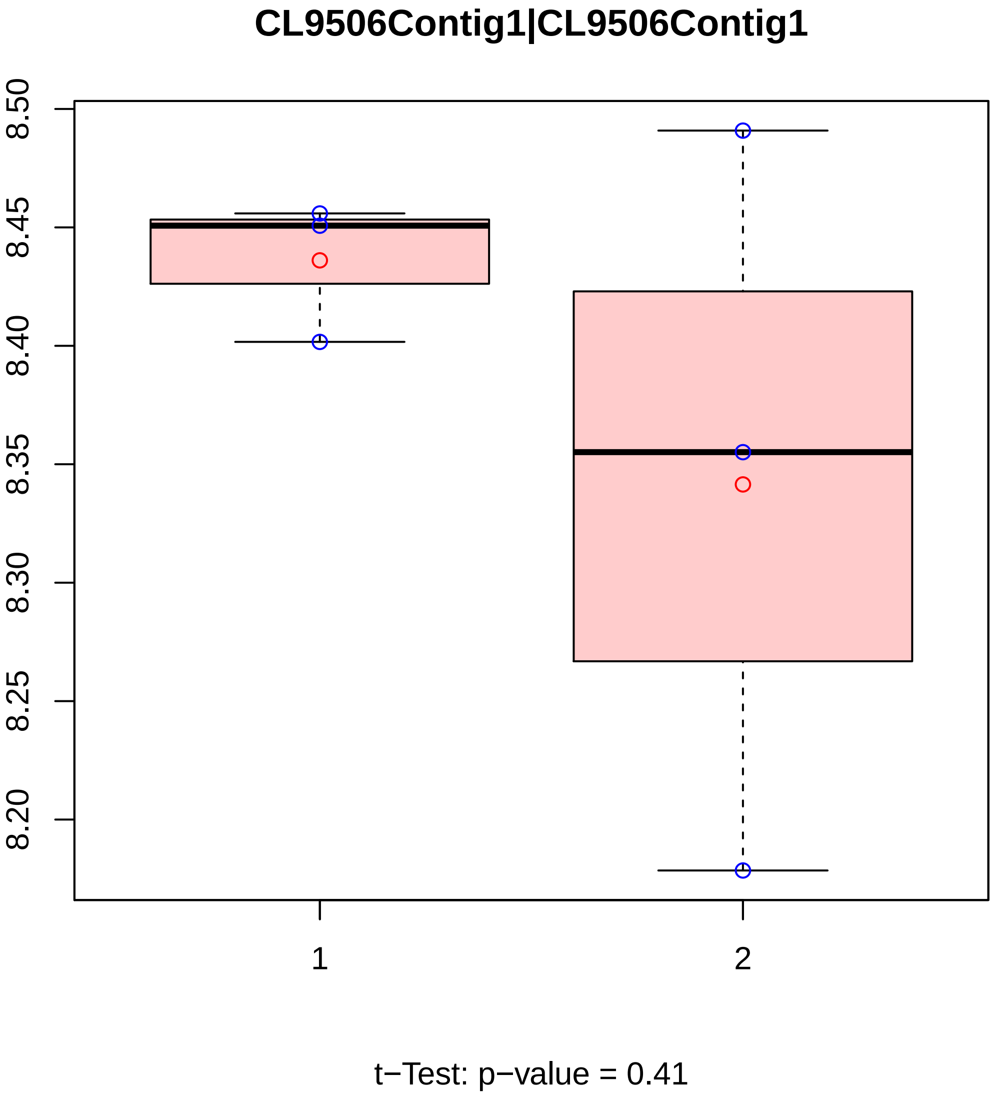

CL9506Contig1|CL9506Contig1
8.50
8.45
8.40
8.35
8.30
8.25
8.20
1
2
t−Test: p−value = 0.41

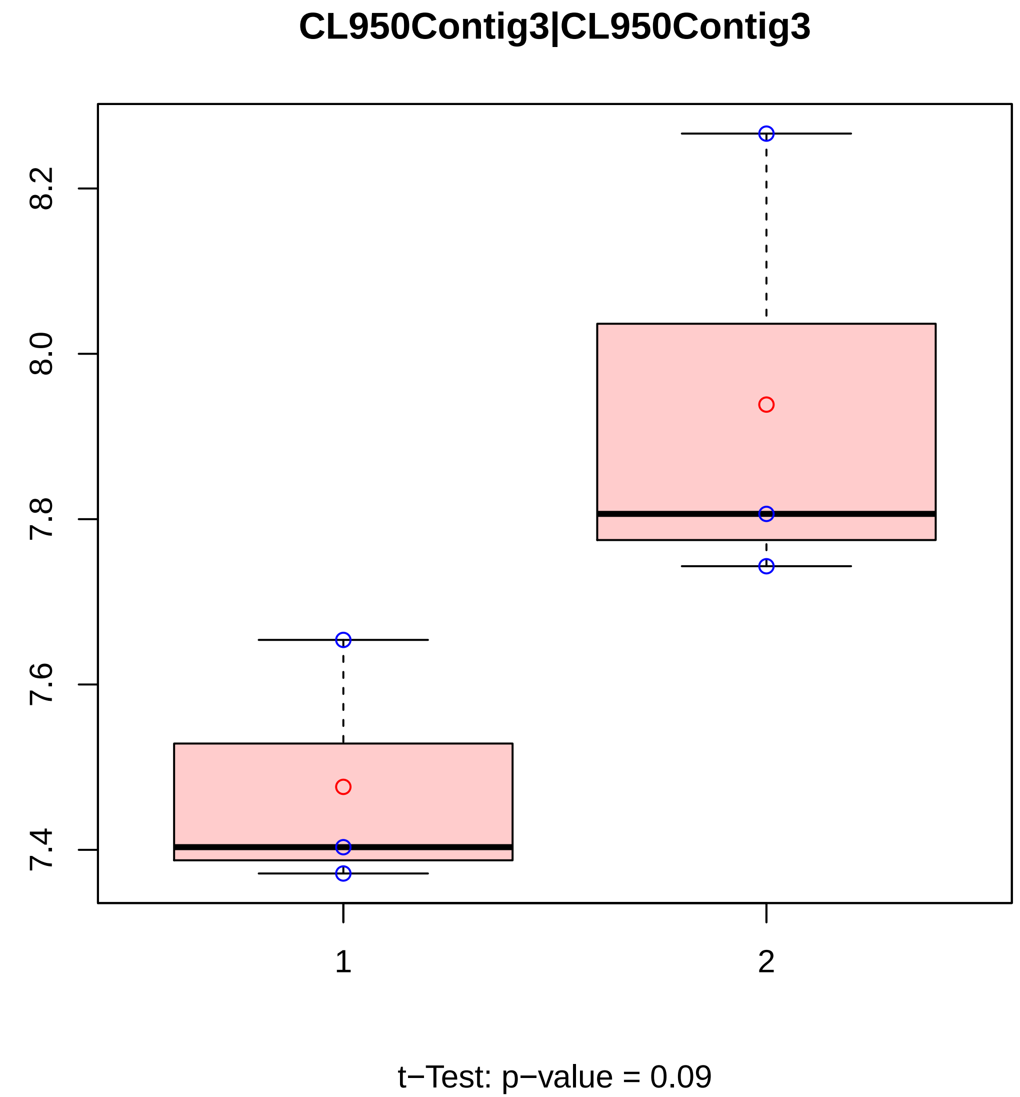

CL950Contig3|CL950Contig3
8.2
8.0
7.8
7.6
7.4
1
2
t−Test: p−value = 0.09

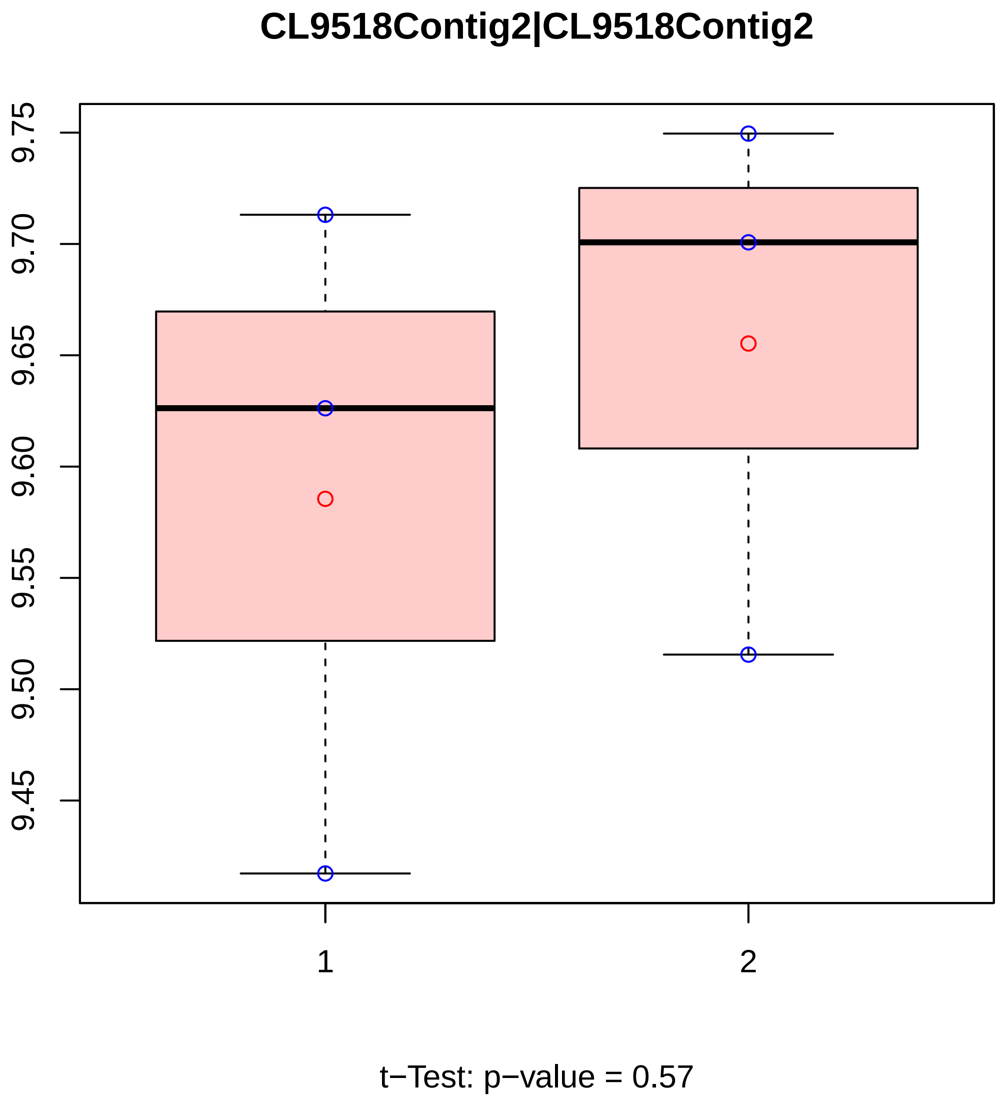

CL9518Contig2|CL9518Contig2
9.45
9.50
9.55
9.60
9.65
9.70
9.75
1
2
t−Test: p−value = 0.57

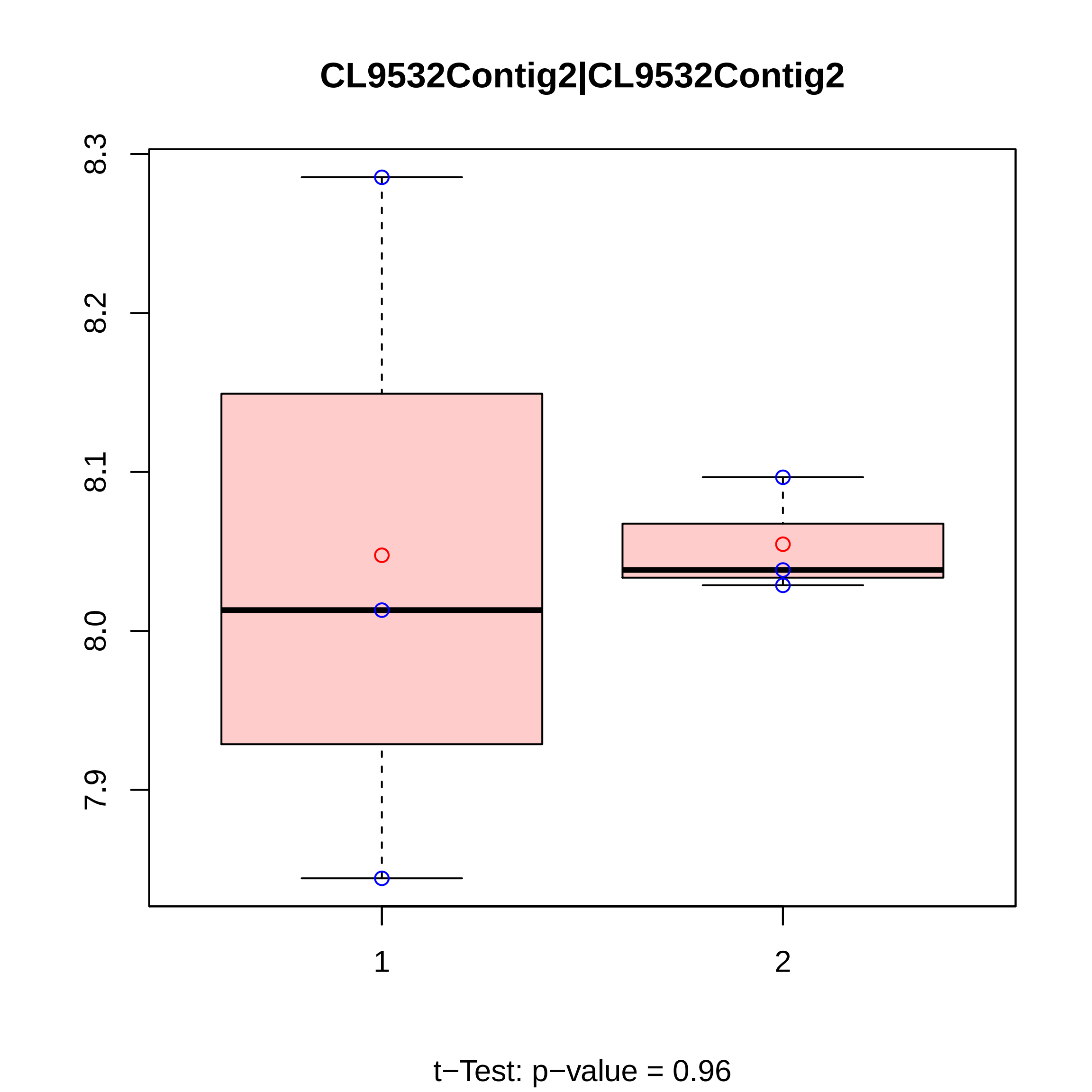
CL9532Contig2|CL9532Contig2
8.3
8.2
8.1
8.0
7.9
1
2
t−Test: p−value = 0.96

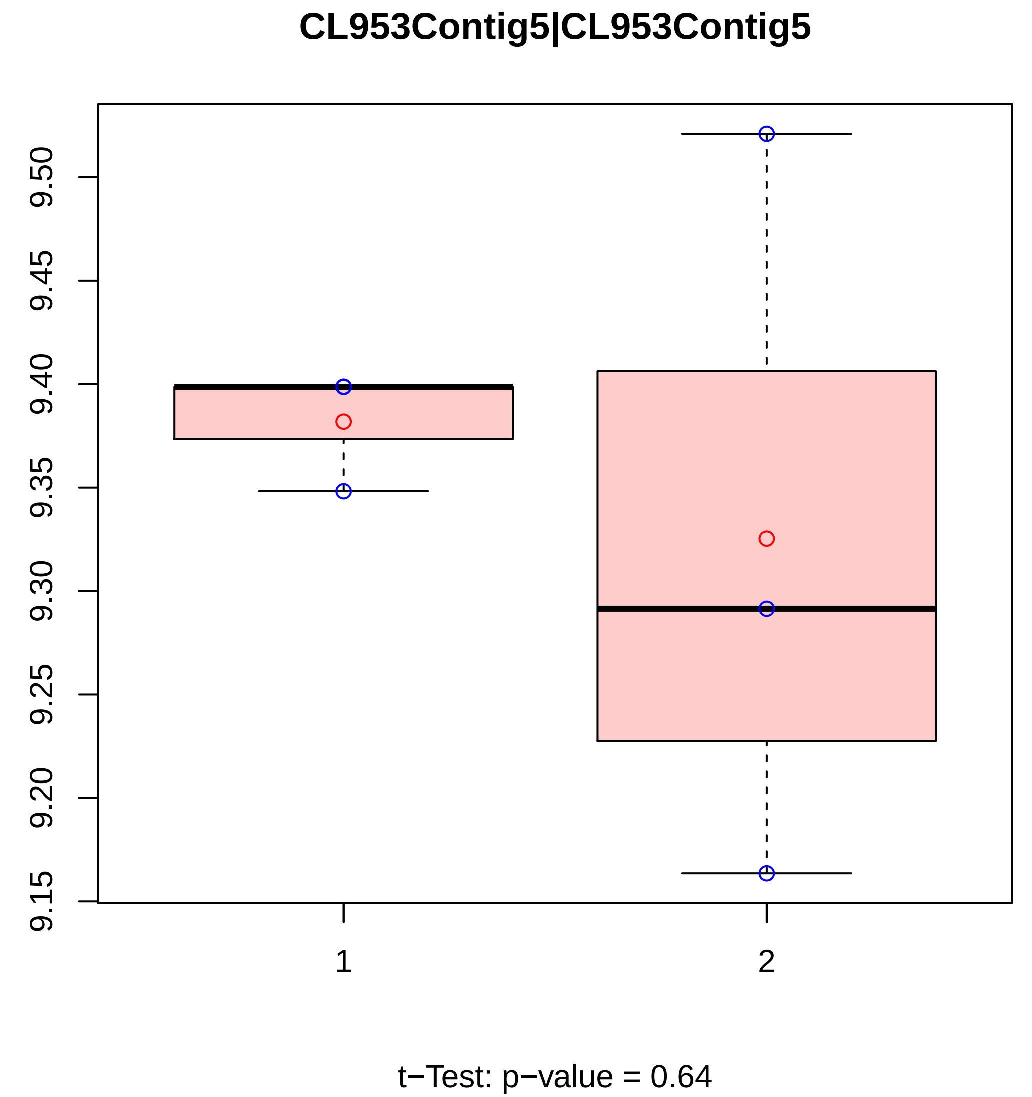
CL953Contig5|CL953Contig5
9.15
9.20
9.25
9.30
9.35
9.40
9.45
9.50
1
2
t−Test: p−value = 0.64

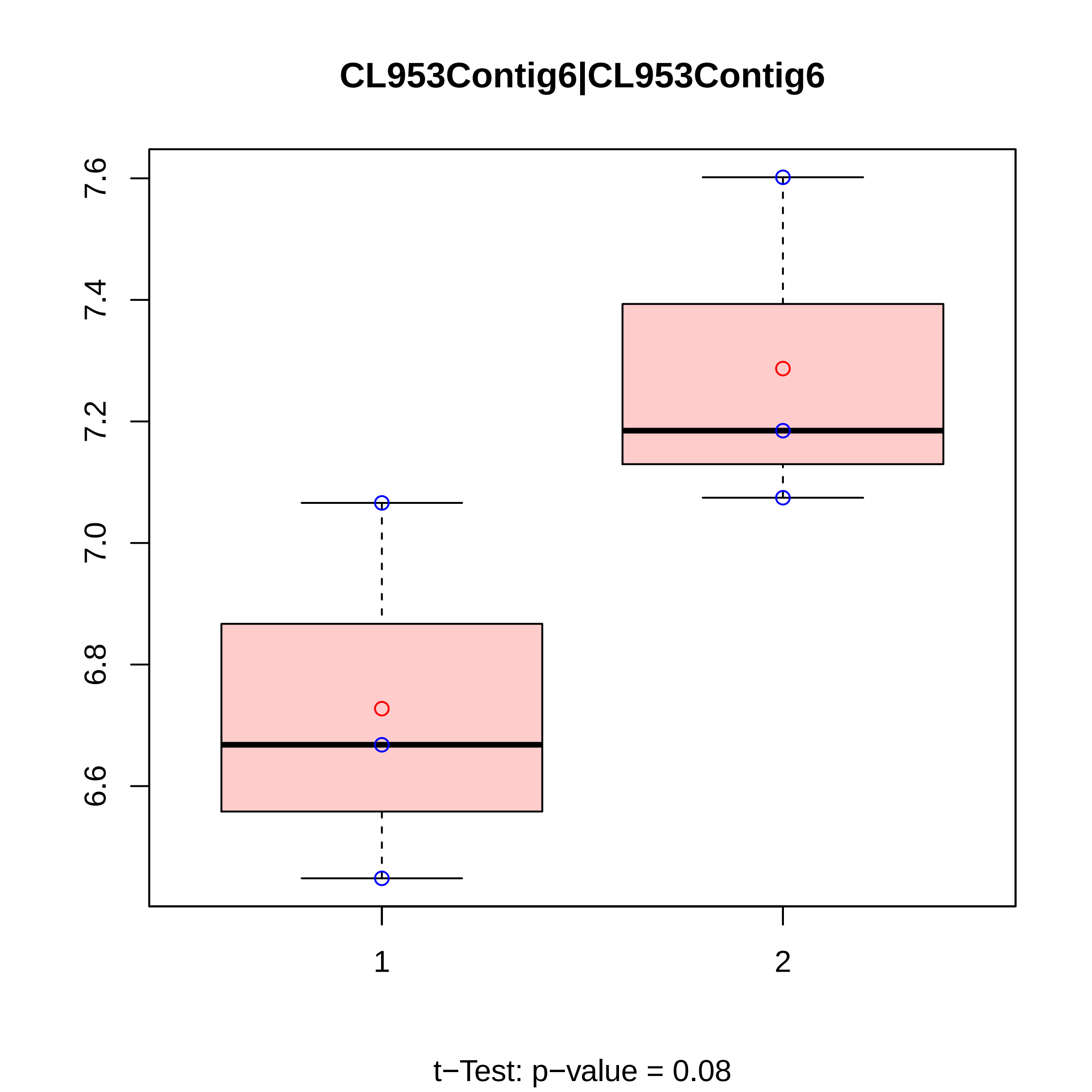

CL953Contig6|CL953Contig6
7.6
7.4
7.2
7.0
6.8
6.6
1
2
t−Test: p−value = 0.08

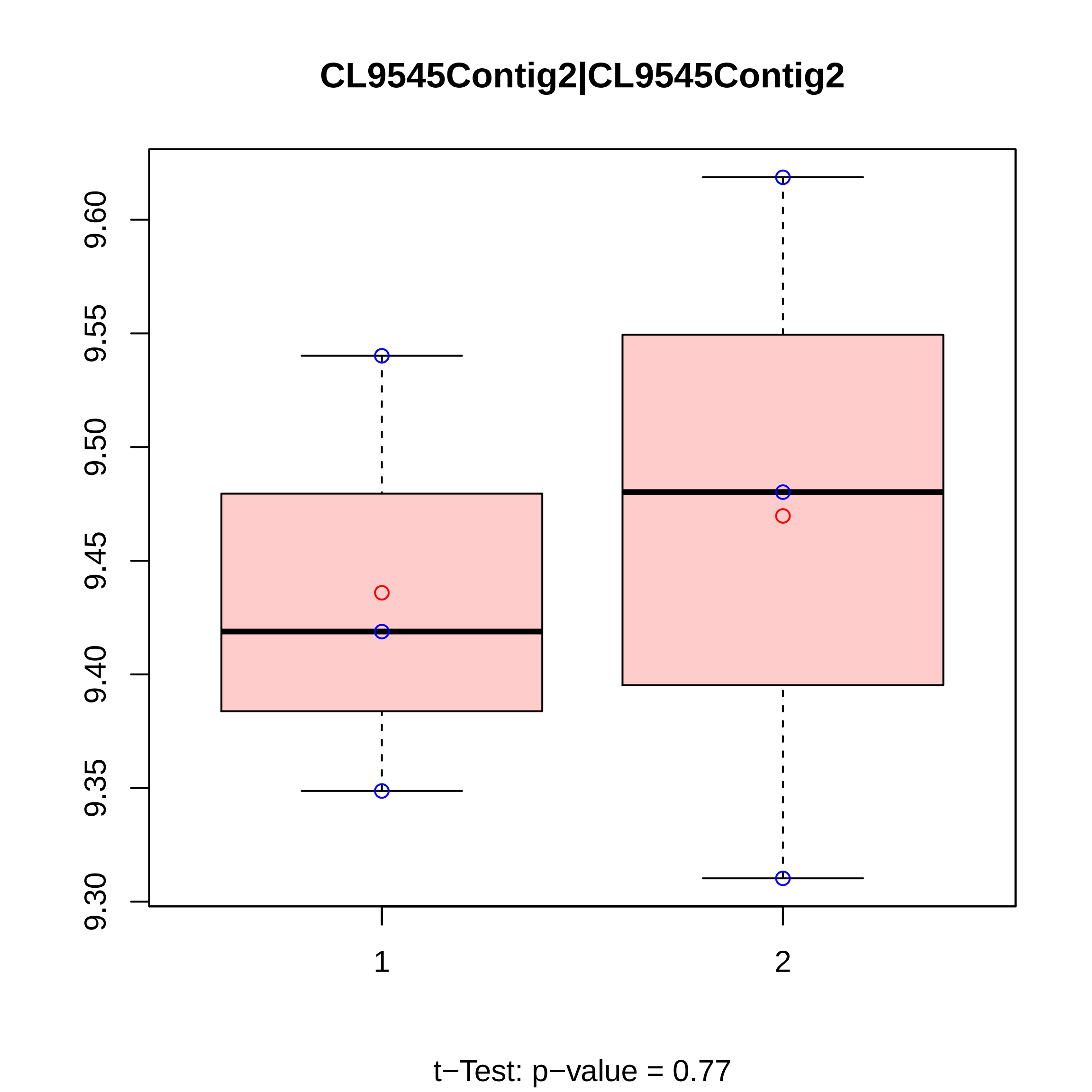
CL9545Contig2|CL9545Contig2
9.30
9.35
9.40
9.45
9.50
9.55
9.60
1
2
t−Test: p−value = 0.77

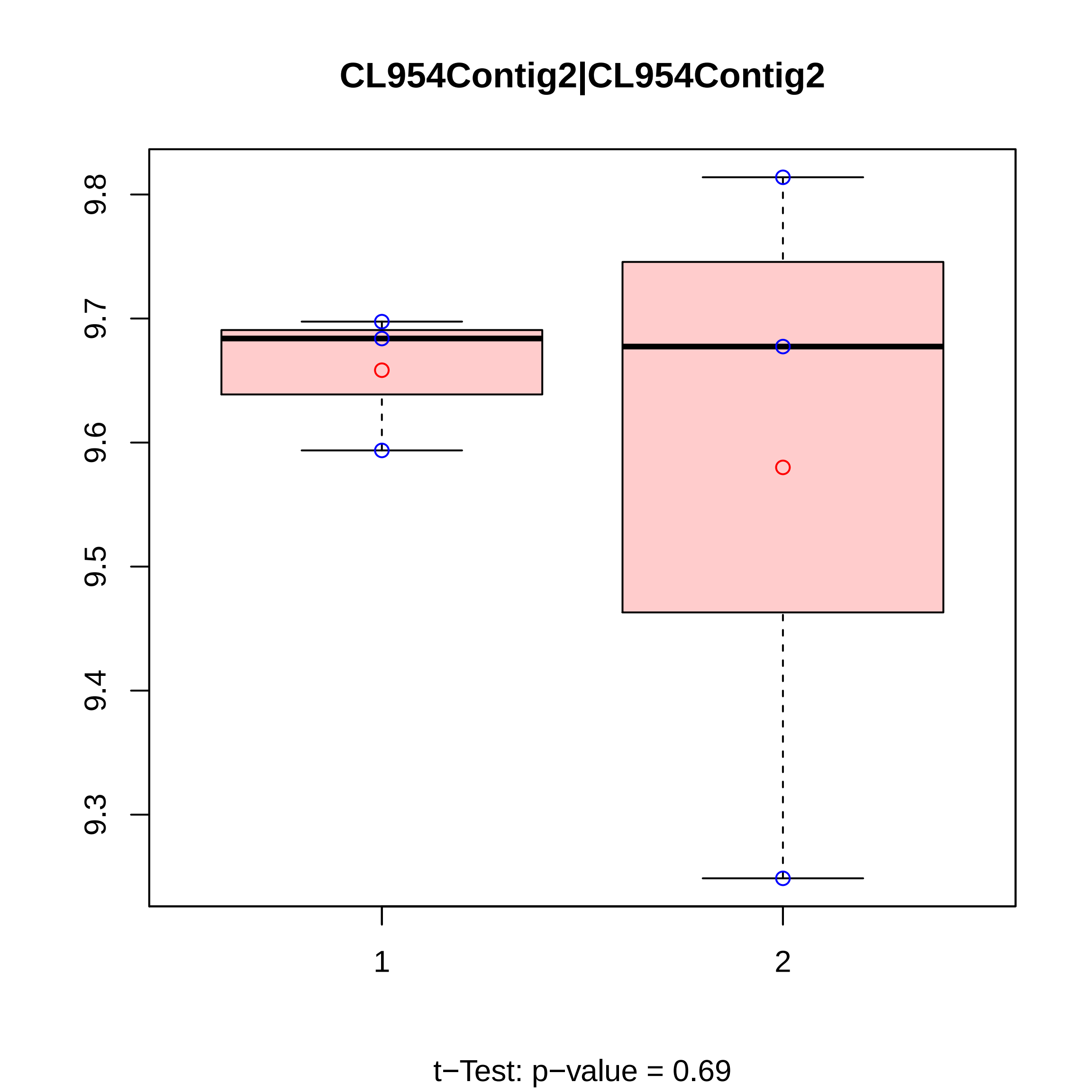

CL954Contig2|CL954Contig2
9.3
9.4
9.5
9.6
9.7
9.8
1
2
t−Test: p−value = 0.69

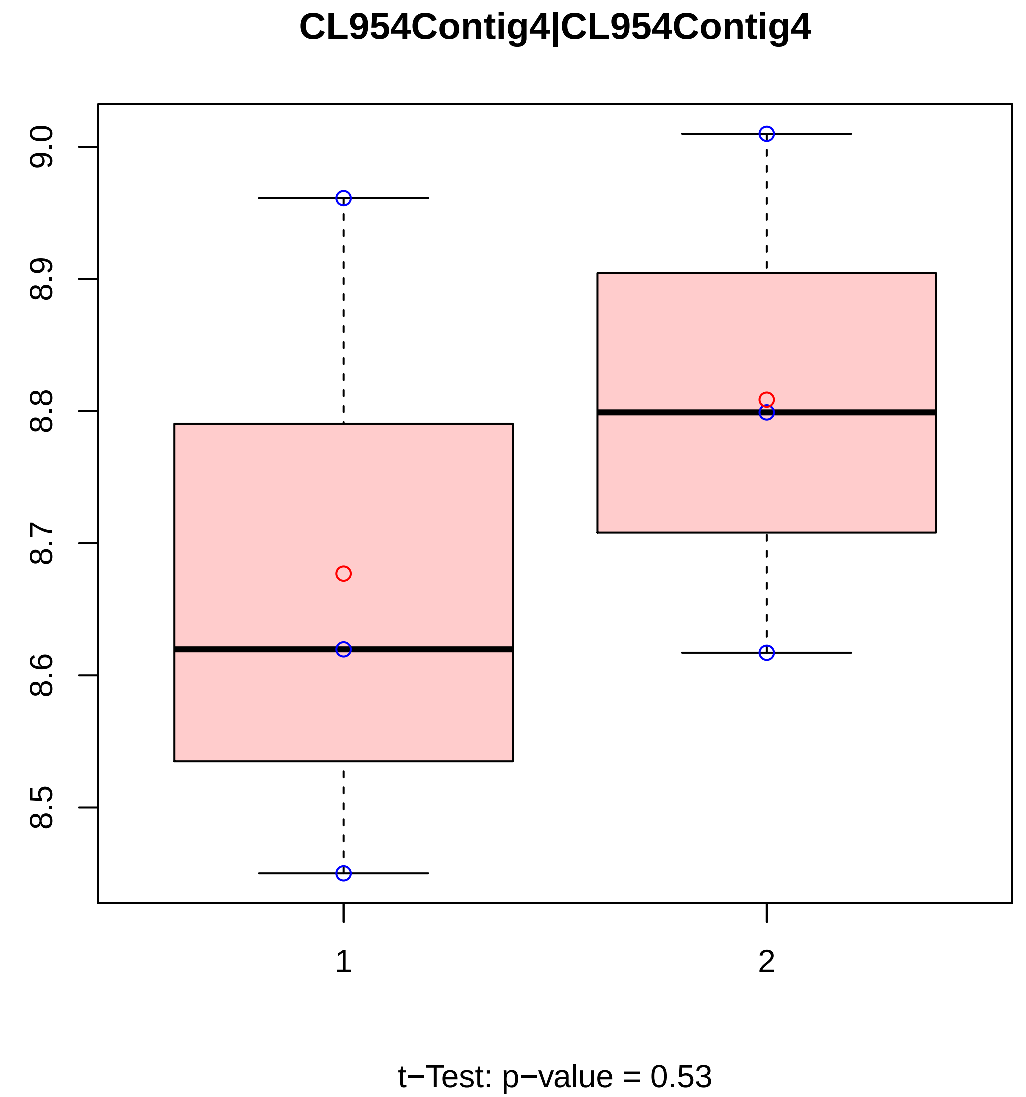

CL954Contig4|CL954Contig4
9.0
8.9
8.8
8.7
8.6
8.5
1
2
t−Test: p−value = 0.53

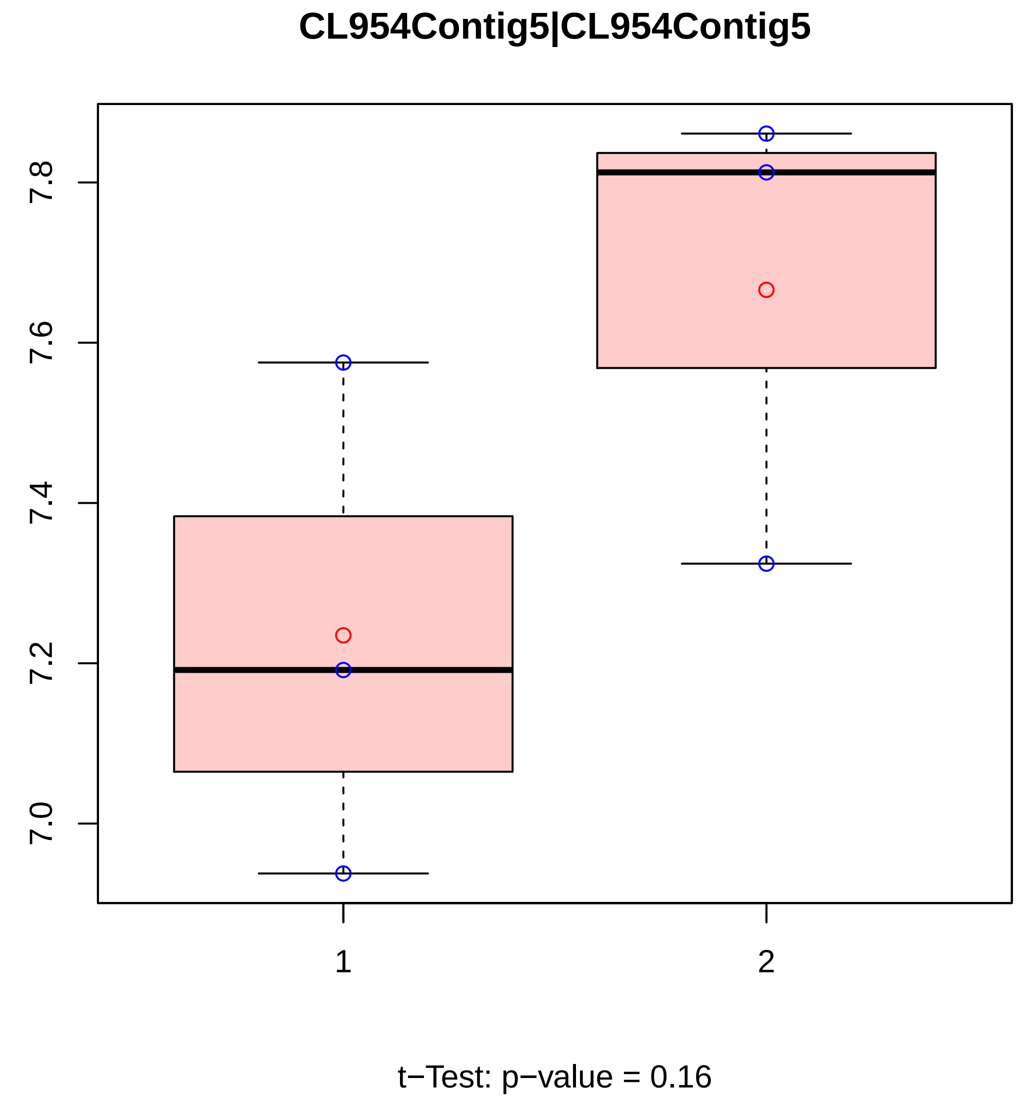
CL954Contig5|CL954Contig5
7.0
7.2
7.4
7.6
7.8
1
2
t−Test: p−value = 0.16

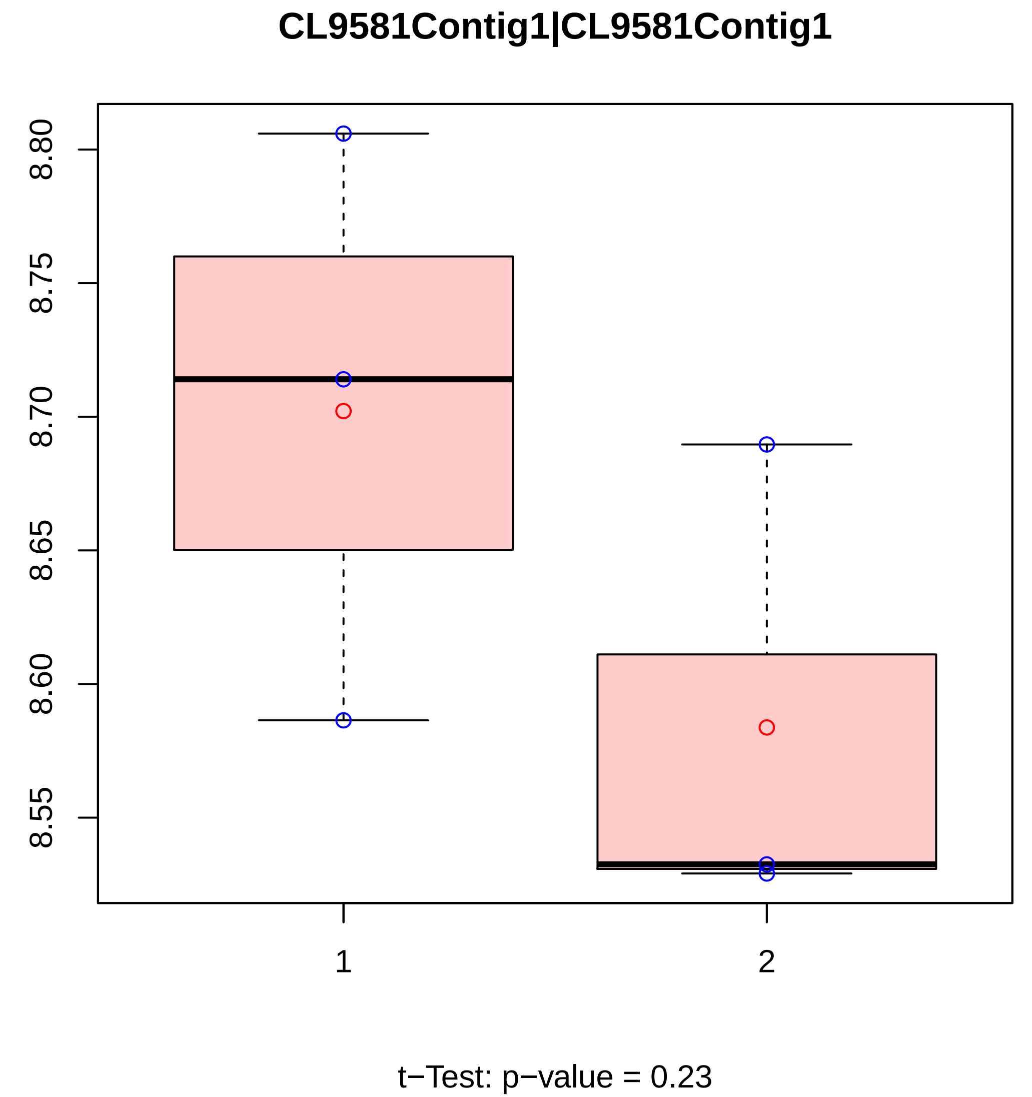
CL9581Contig1|CL9581Contig1
8.80
8.75
8.70
8.65
8.60
8.55
1
2
t−Test: p−value = 0.23

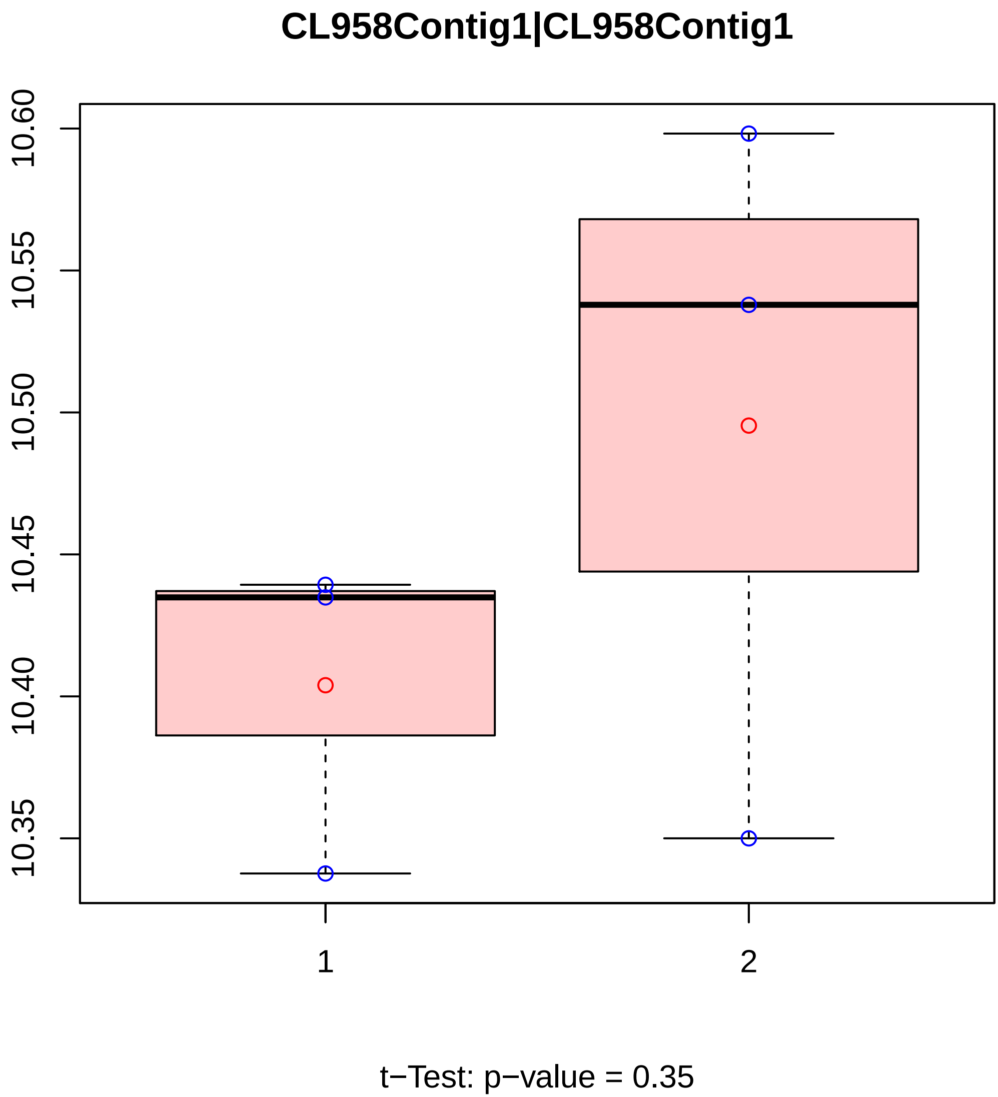

CL958Contig1|CL958Contig1
10.35
10.40
10.45
10.50
10.55
10.60
1
2
t−Test: p−value = 0.35

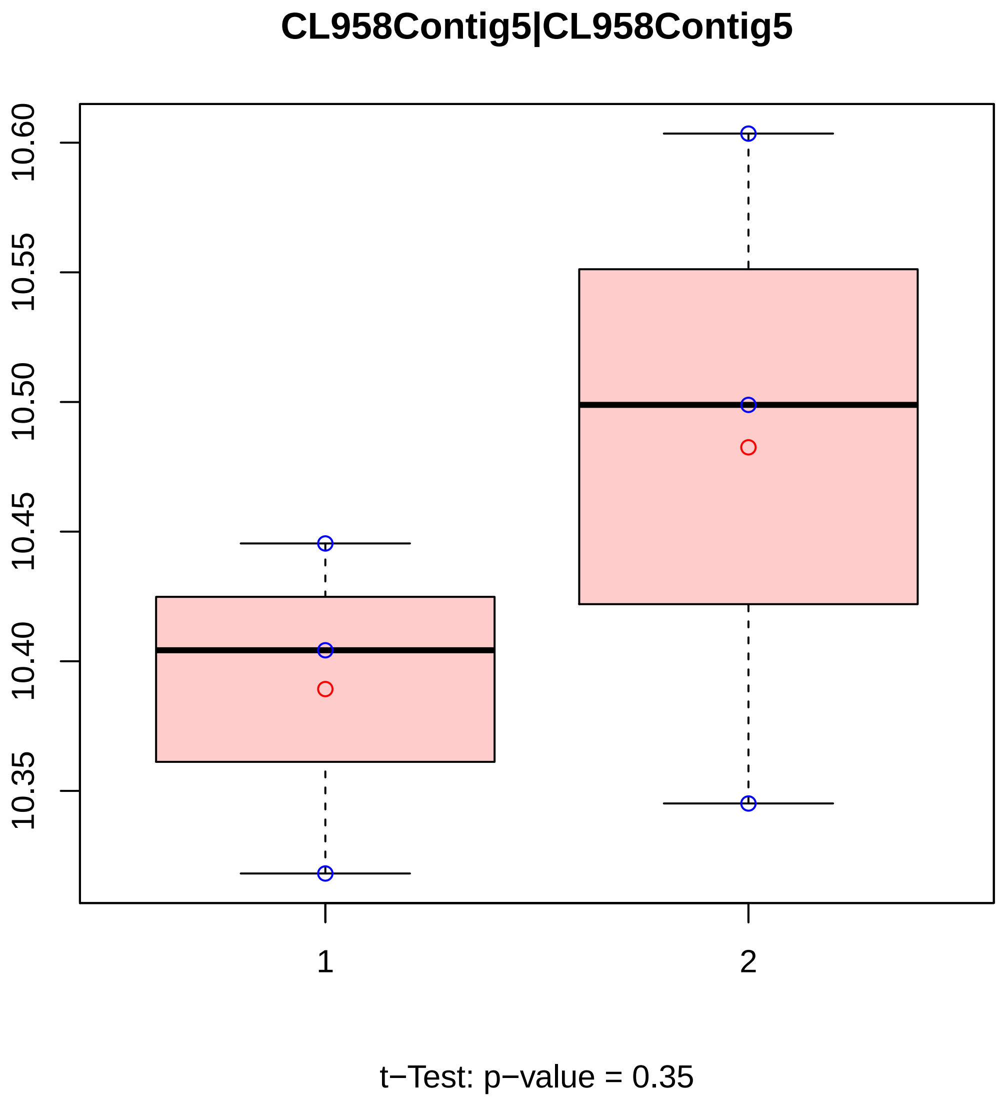
CL958Contig5|CL958Contig5
10.60
10.55
10.50
10.45
10.40
10.35
1
2
t−Test: p−value = 0.35

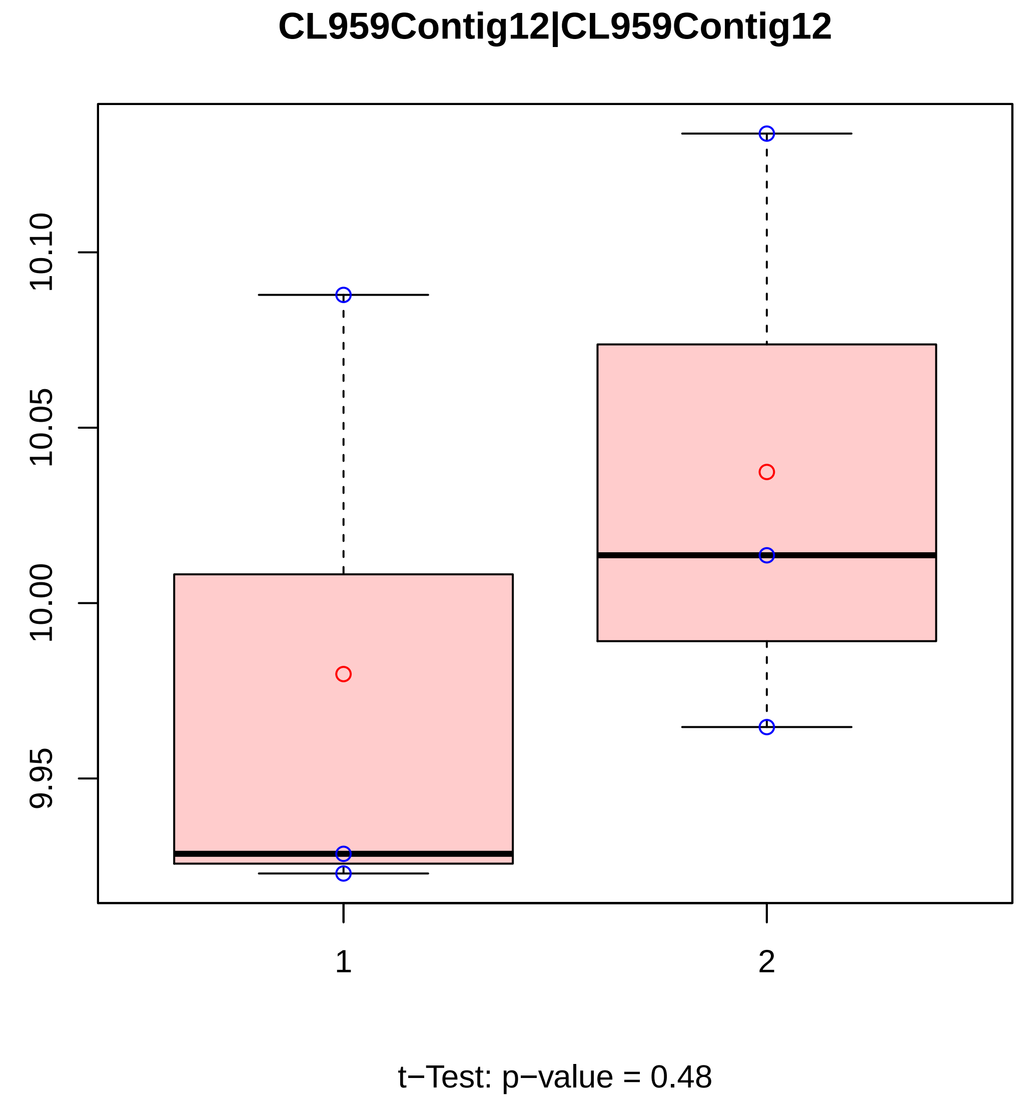

CL959Contig12|CL959Contig12
10.10
10.05
10.00
9.95
1
2
t−Test: p−value = 0.48

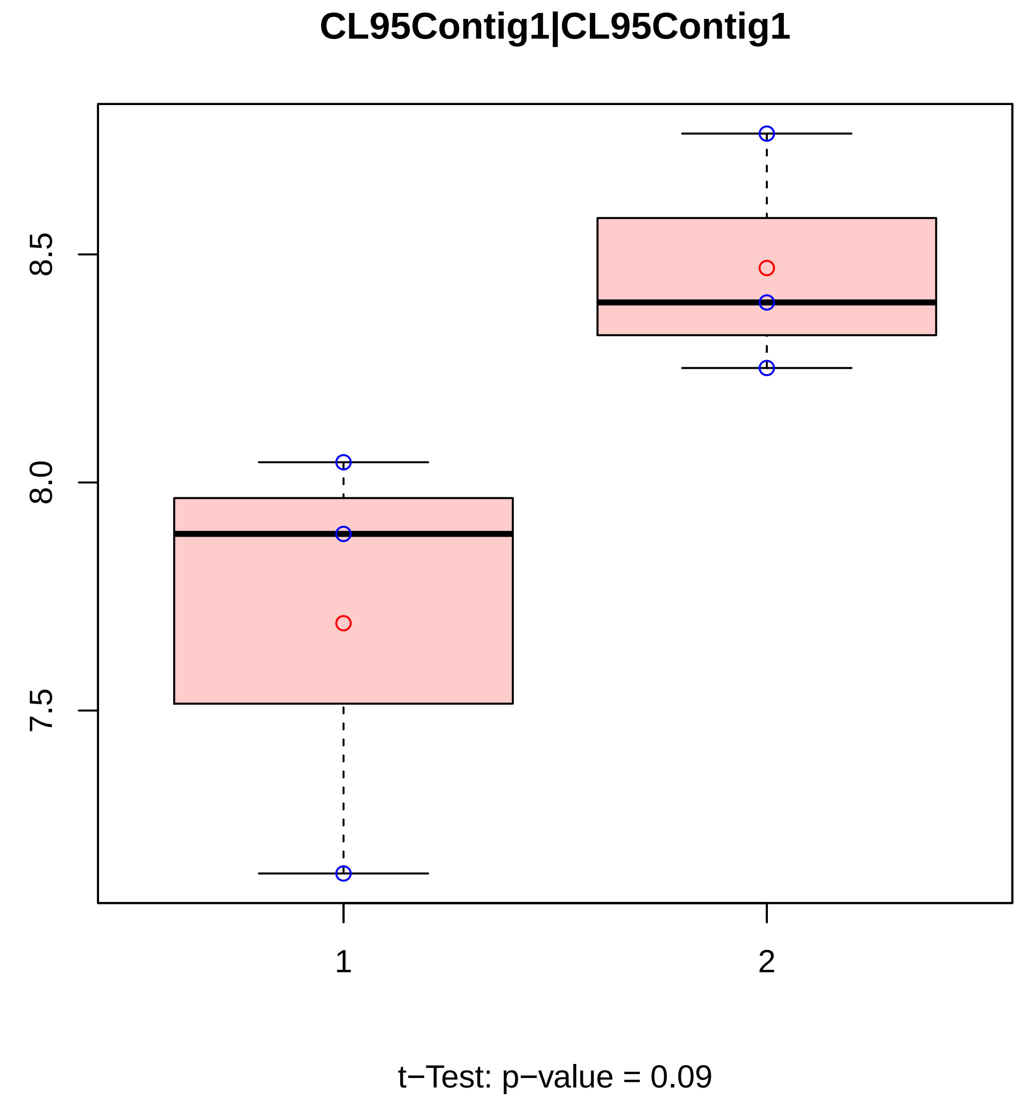
CL95Contig1|CL95Contig1
8.5
8.0
7.5
1
2
t−Test: p−value = 0.09

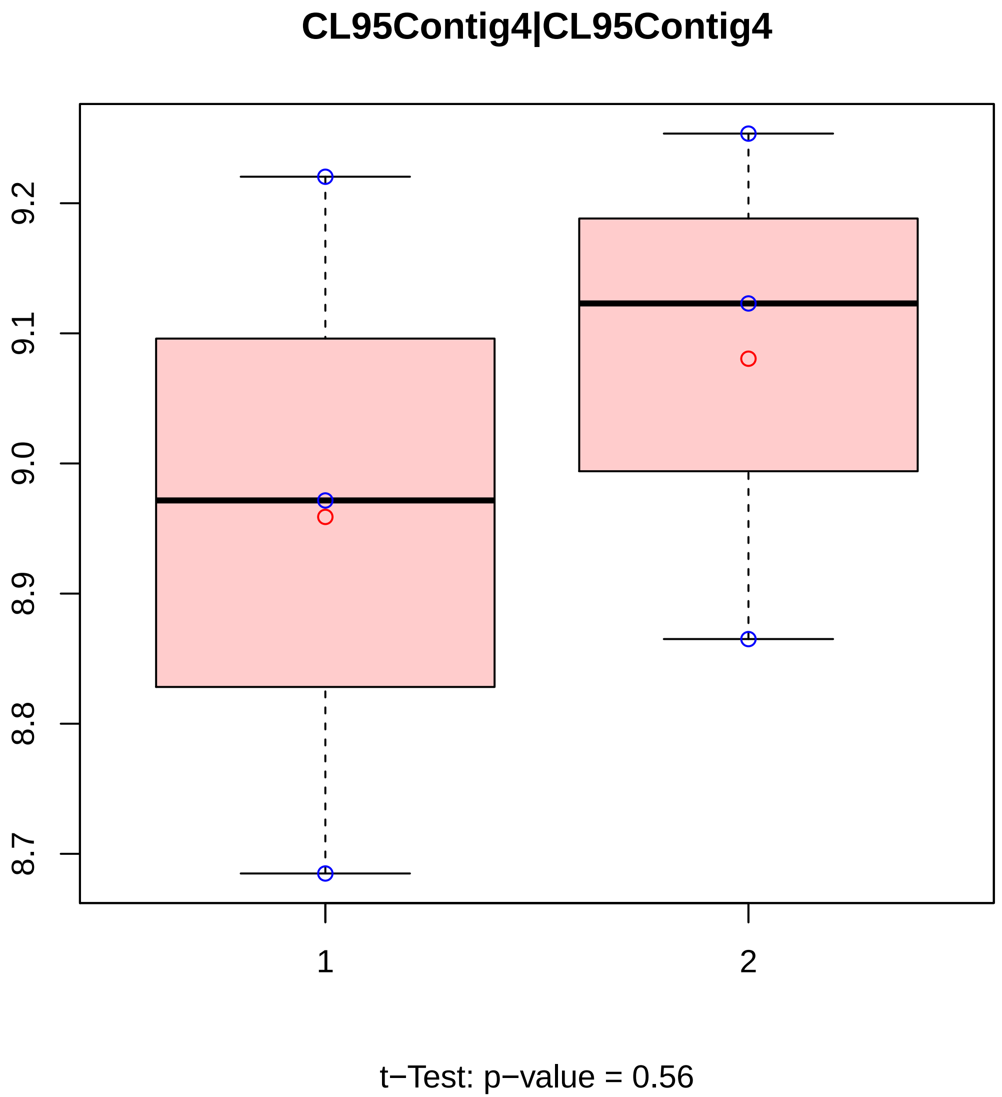
CL95Contig4|CL95Contig4
9.2
9.1
9.0
8.9
8.8
8.7
1
2
t−Test: p−value = 0.56

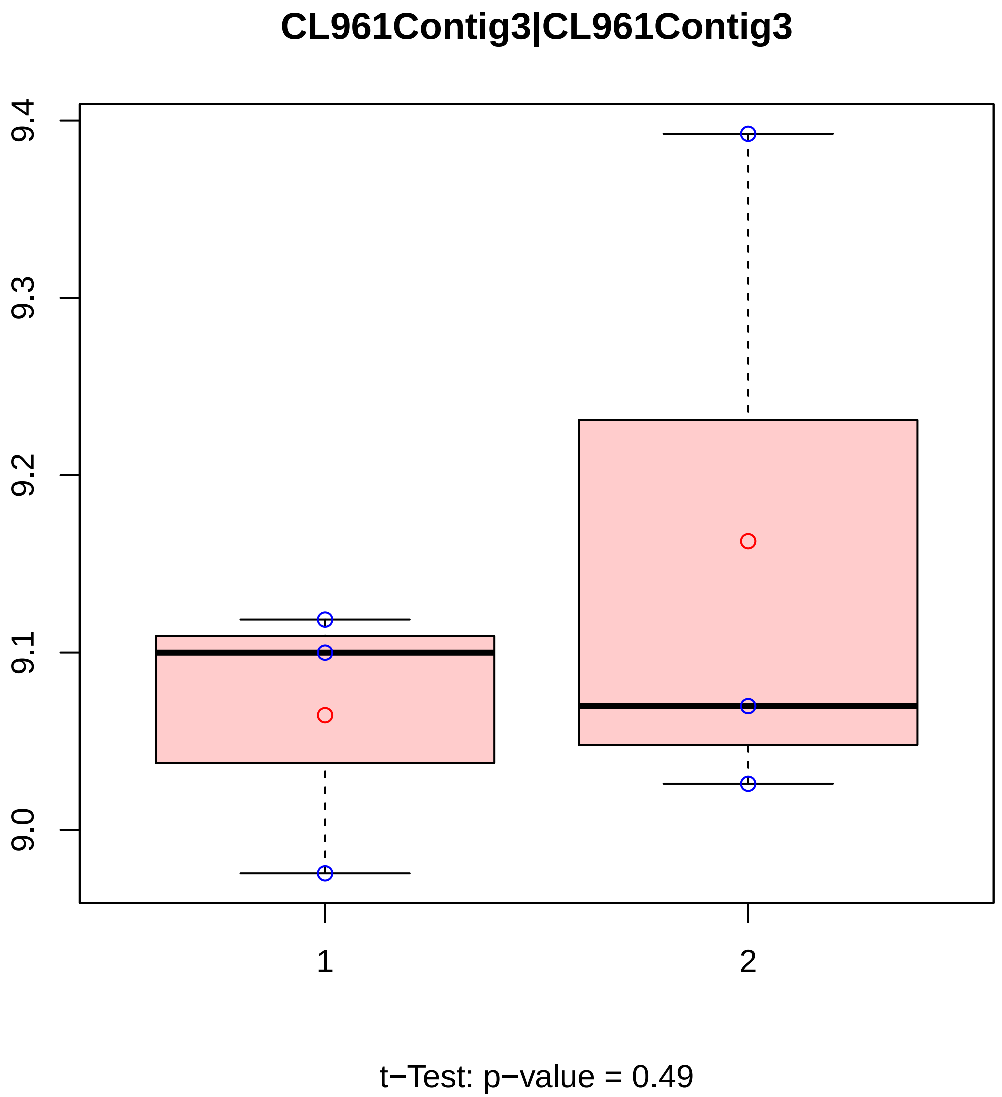
CL961Contig3|CL961Contig3
9.0
9.1
9.2
9.3
9.4
1
2
t−Test: p−value = 0.49

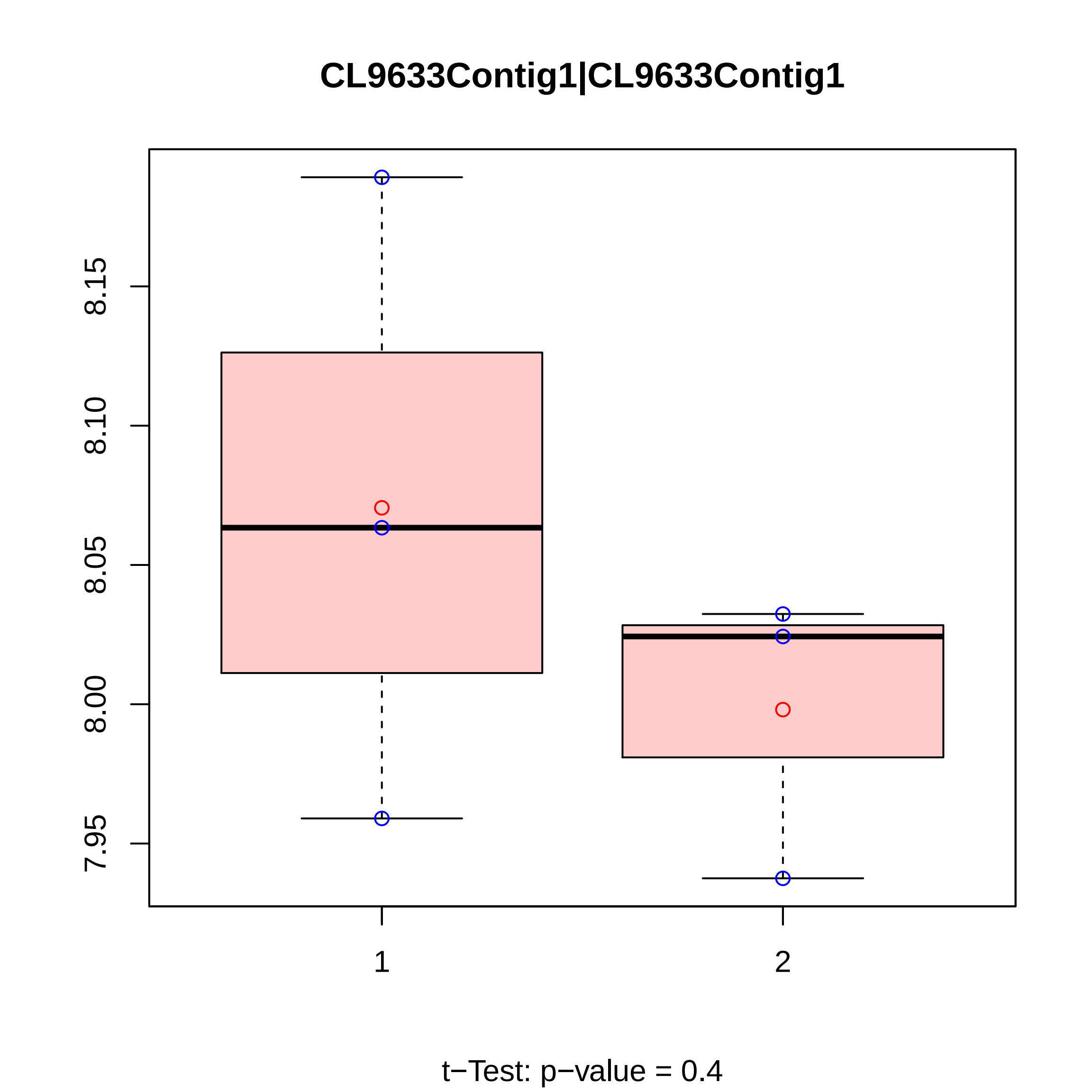

CL9633Contig1|CL9633Contig1
8.15
8.10
8.05
8.00
7.95
1
2
t−Test: p−value = 0.4

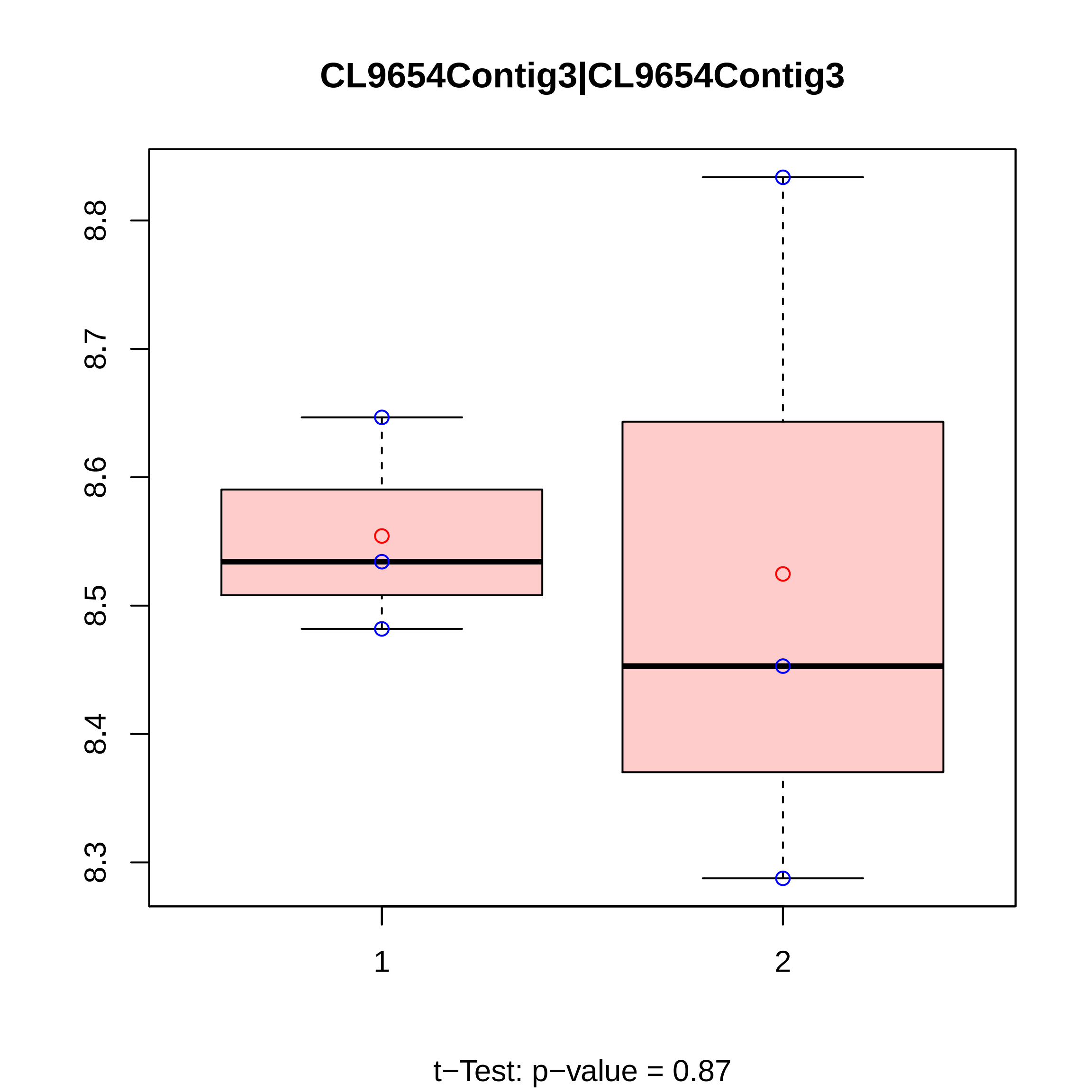
CL9654Contig3|CL9654Contig3
8.3
8.4
8.5
8.6
8.7
8.8
1
2
t−Test: p−value = 0.87

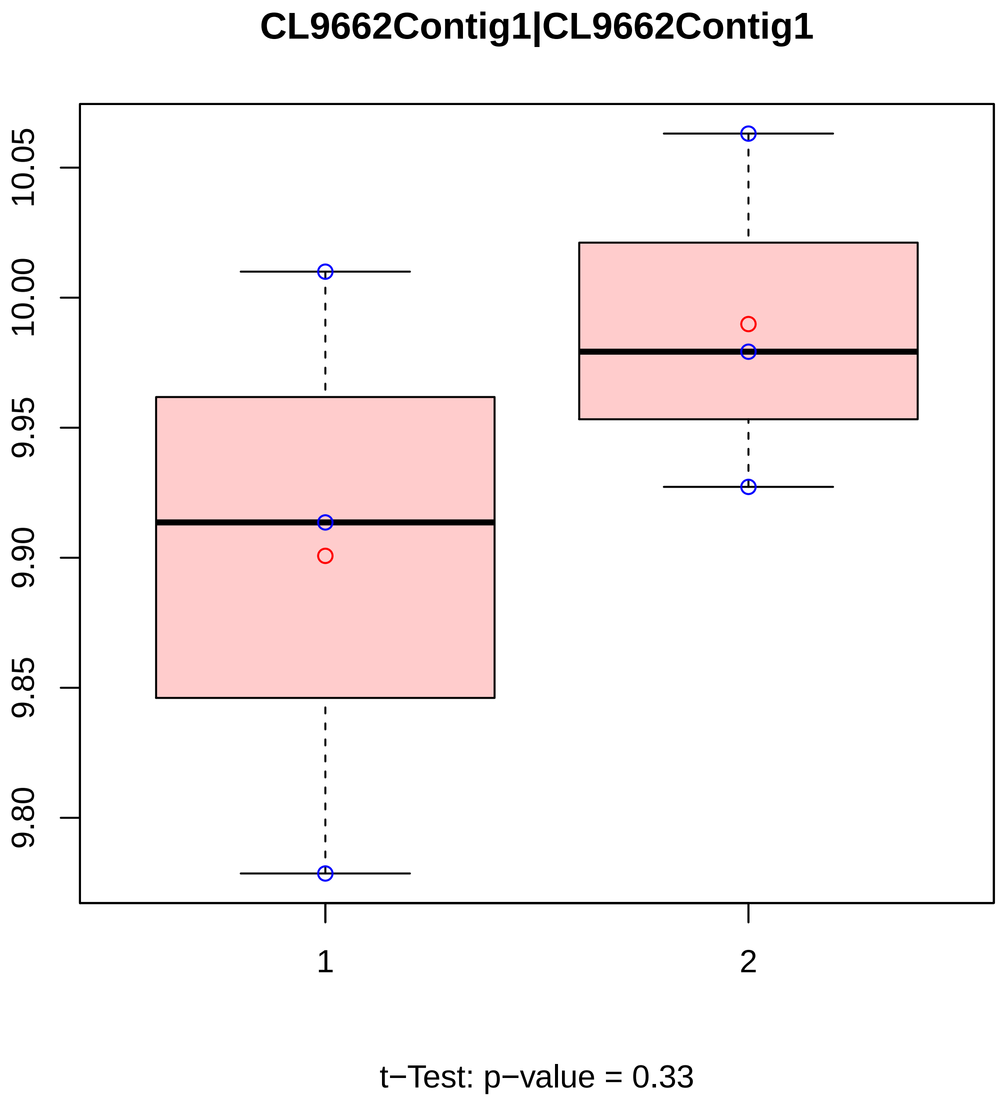
CL9662Contig1|CL9662Contig1
10.05
10.00
9.95
9.90
9.85
9.80
1
2
t−Test: p−value = 0.33

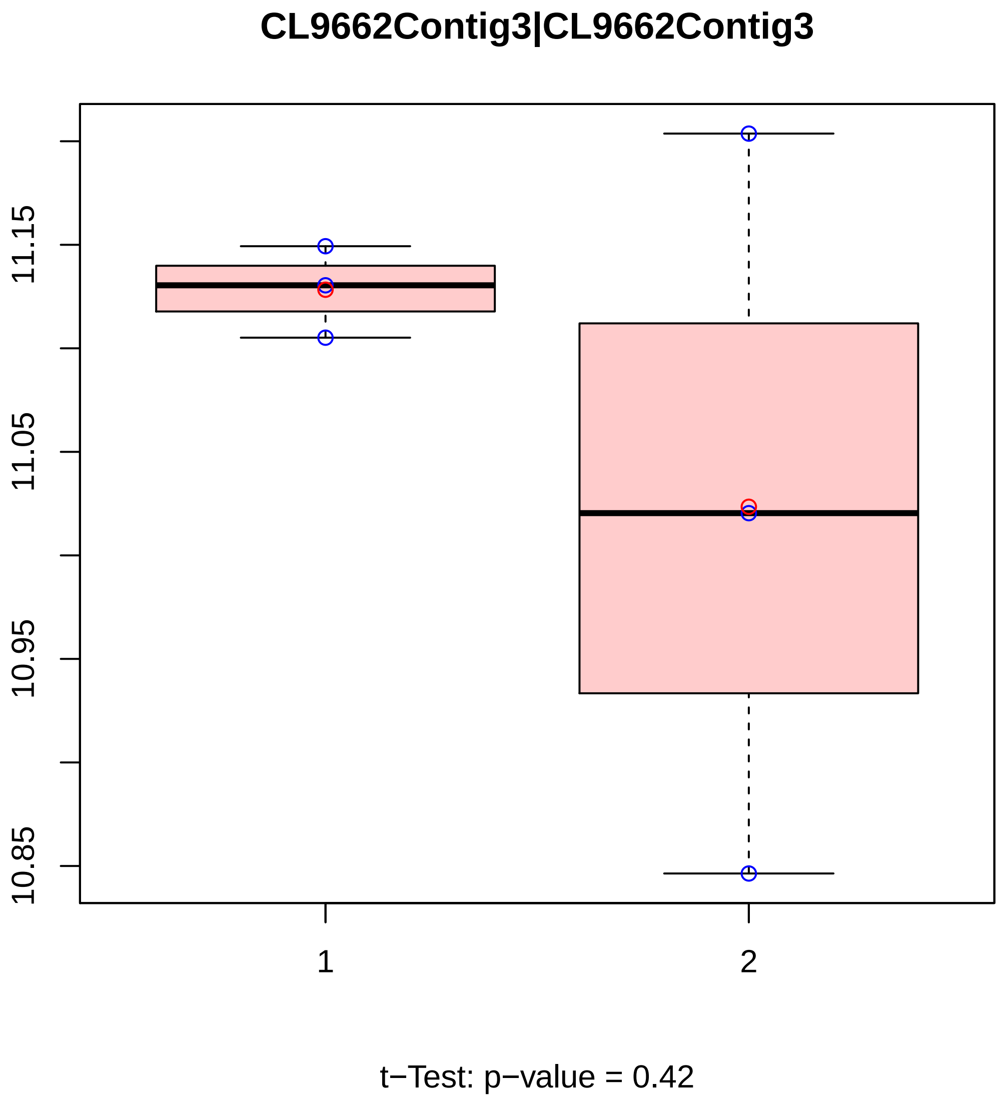

CL9662Contig3|CL9662Contig3
11.15
11.05
10.95
10.85
1
2
t−Test: p−value = 0.42

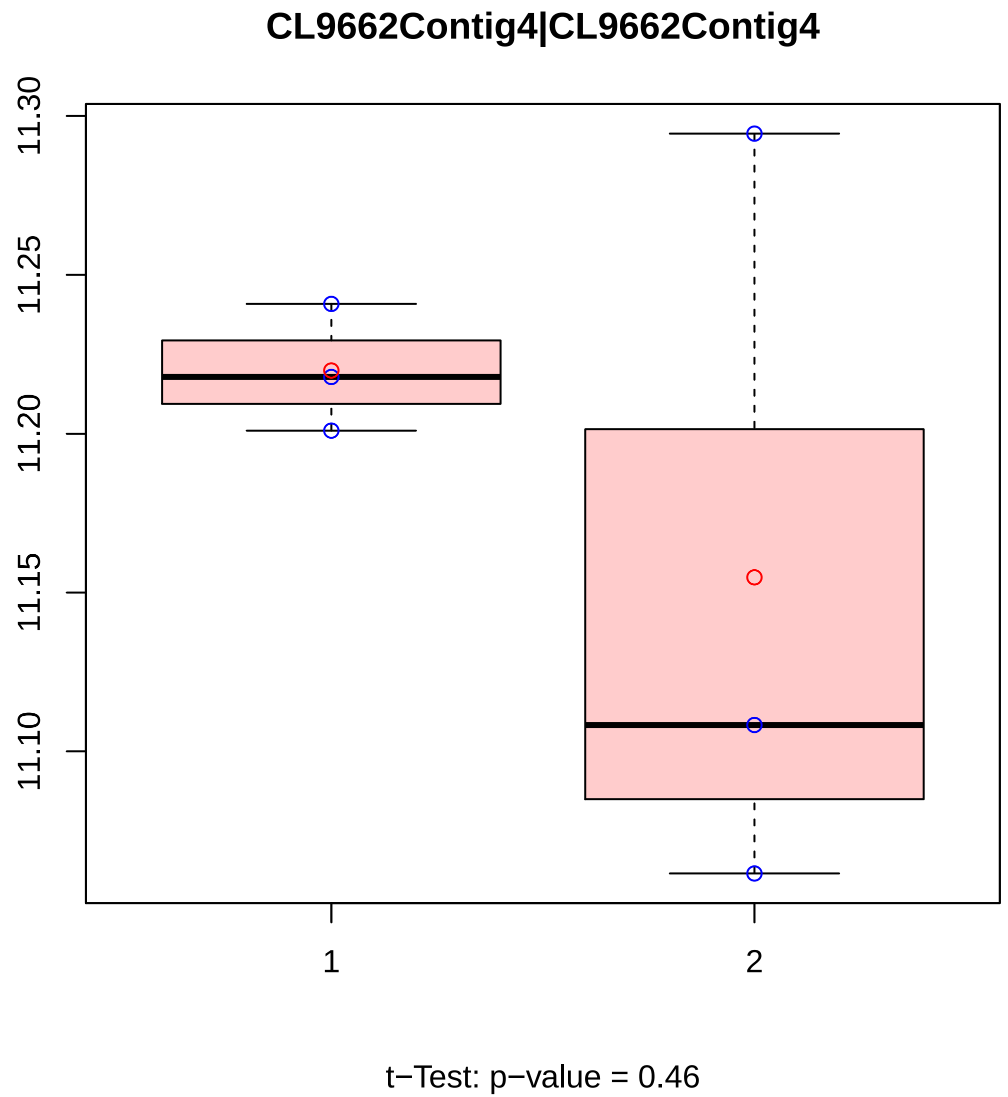

CL9662Contig4|CL9662Contig4
11.30
11.25
11.20
11.15
11.10
1
2
t−Test: p−value = 0.46

# CL9662Contig5|CL9662Contig5

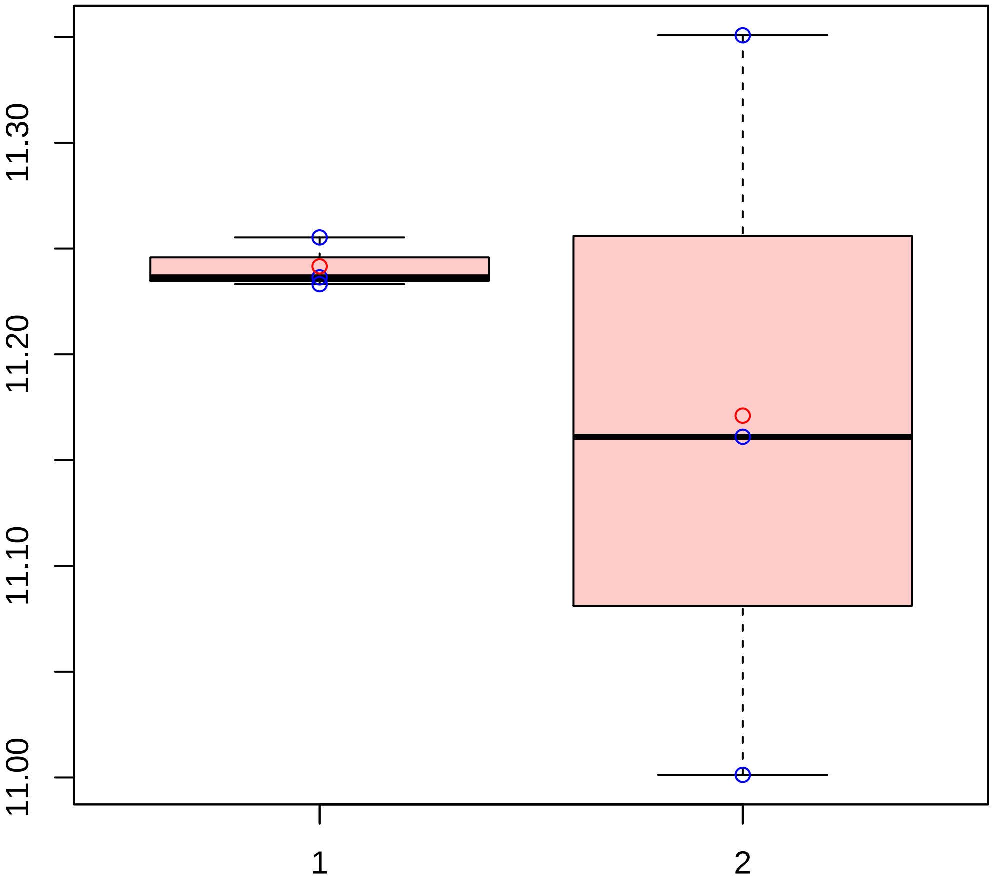


t−Test: p−value = 0.56

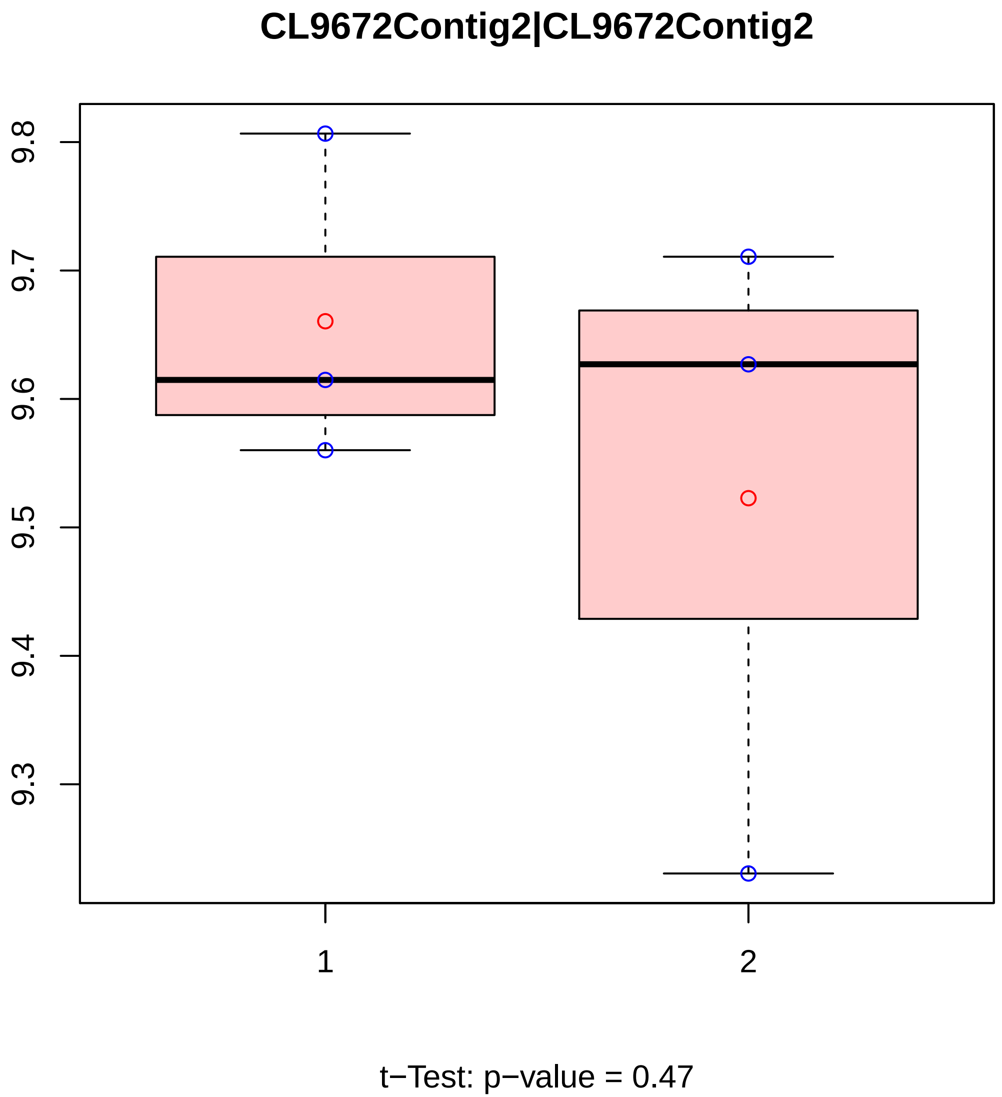
CL9672Contig2|CL9672Contig2
9.3
9.4
9.5
9.6
9.7
9.8
1
2
t−Test: p−value = 0.47

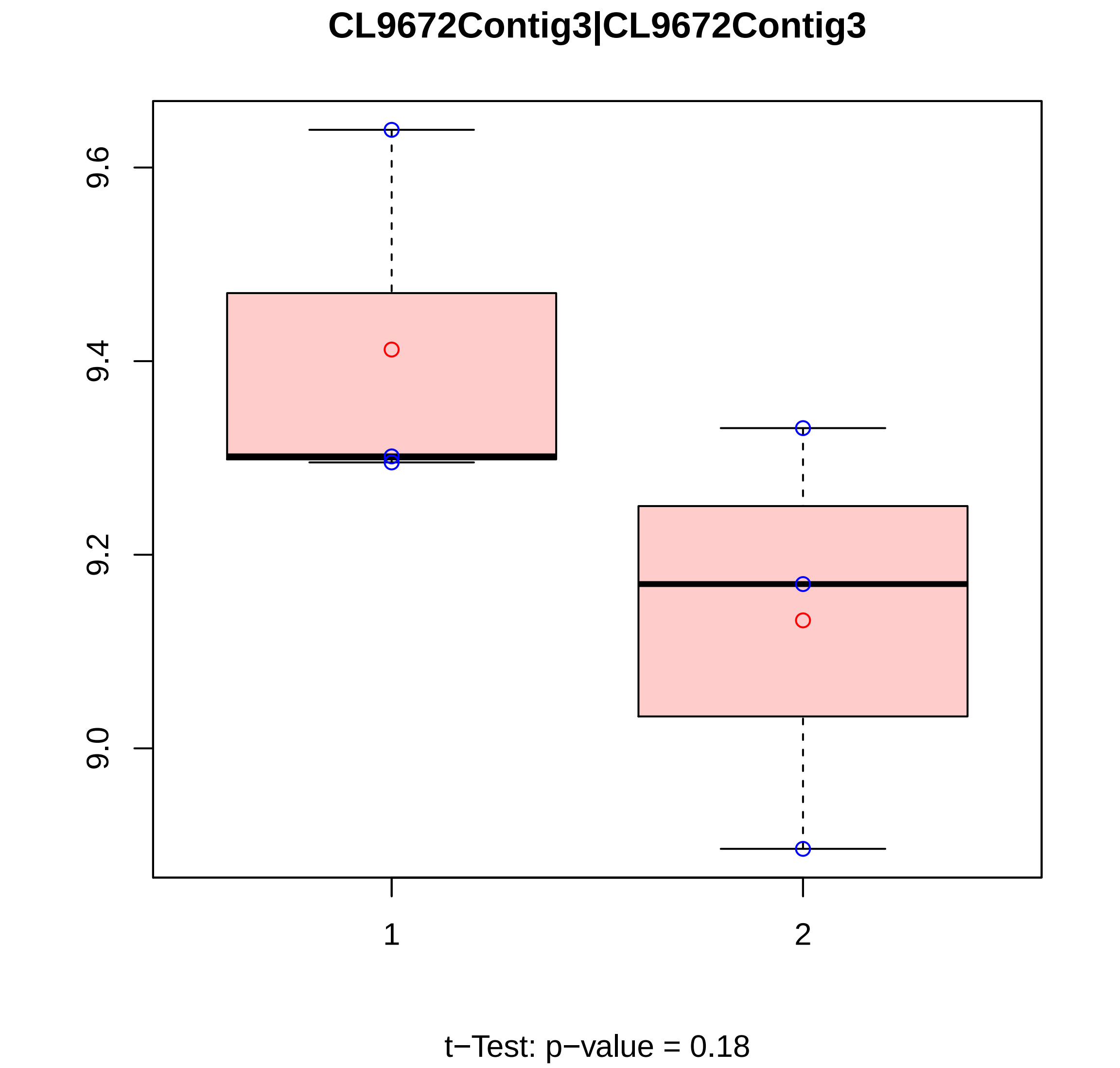

CL9672Contig3|CL9672Contig3
9.0
9.2
9.4
9.6
1
2
t−Test: p−value = 0.18

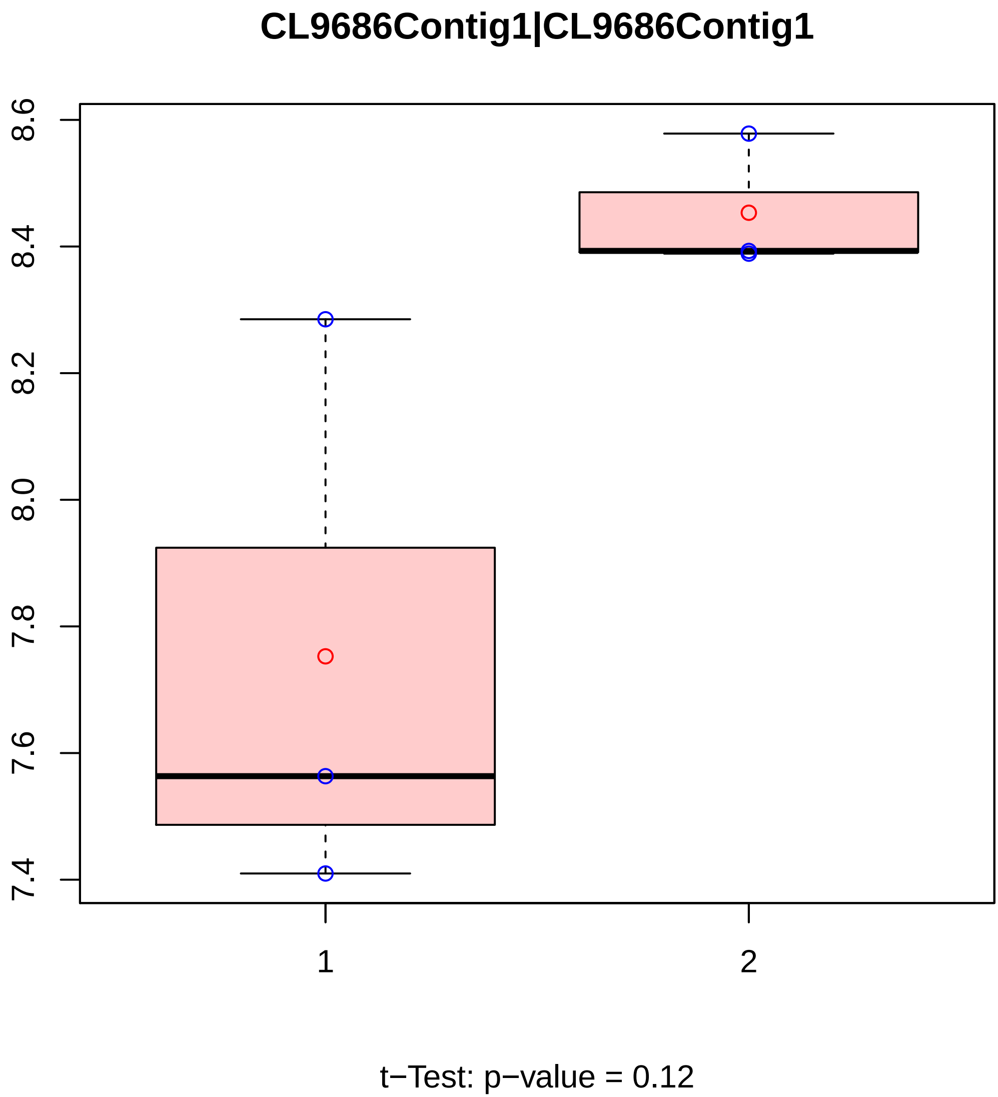
CL9686Contig1|CL9686Contig1
8.6
8.4
8.2
8.0
7.8
7.6
7.4
1
2
t−Test: p−value = 0.12

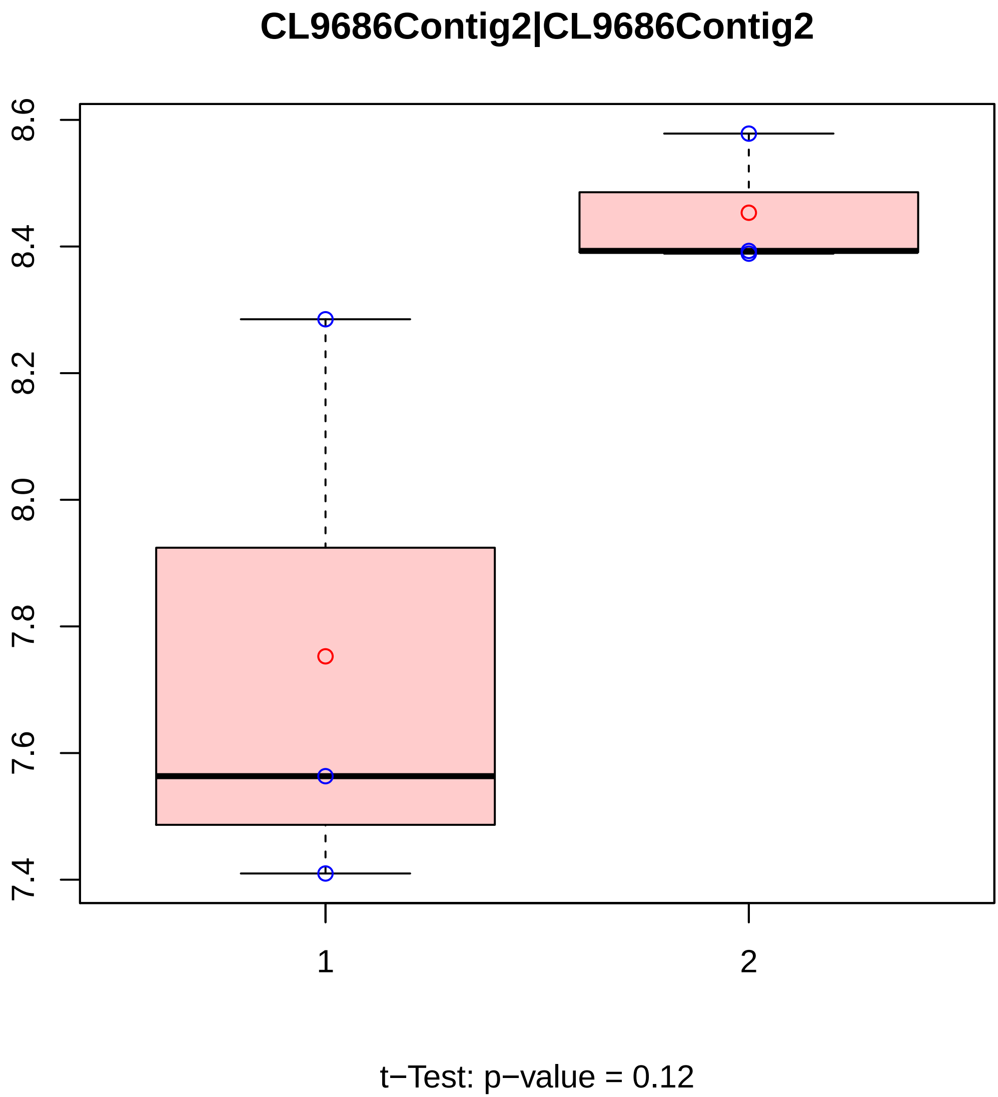

CL9686Contig2|CL9686Contig2
8.6
8.4
8.2
8.0
7.8
7.6
7.4
1
2
t−Test: p−value = 0.12

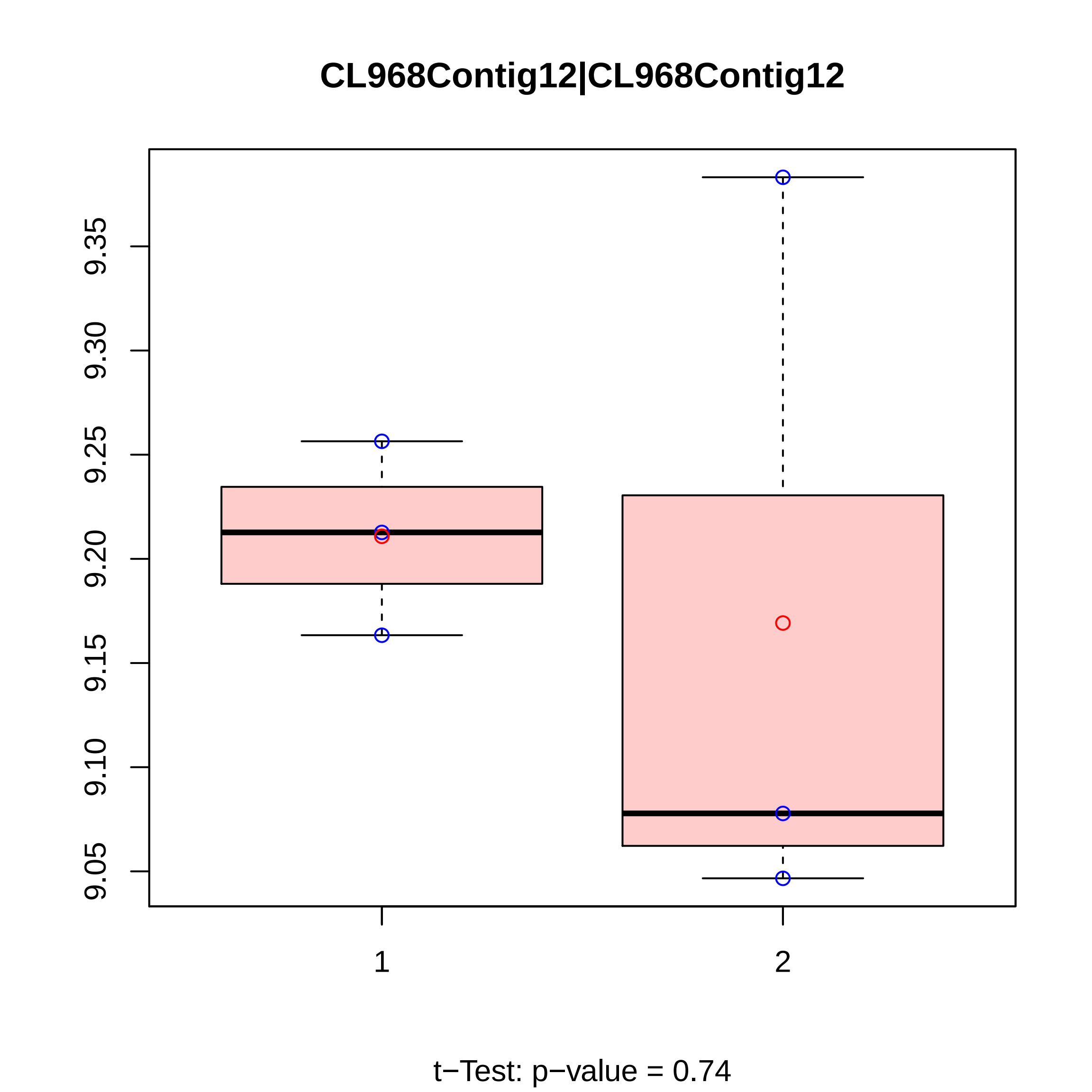
CL968Contig12|CL968Contig12
9.05
9.10
9.15
9.20
9.25
9.30
9.35
1
2
t−Test: p−value = 0.74

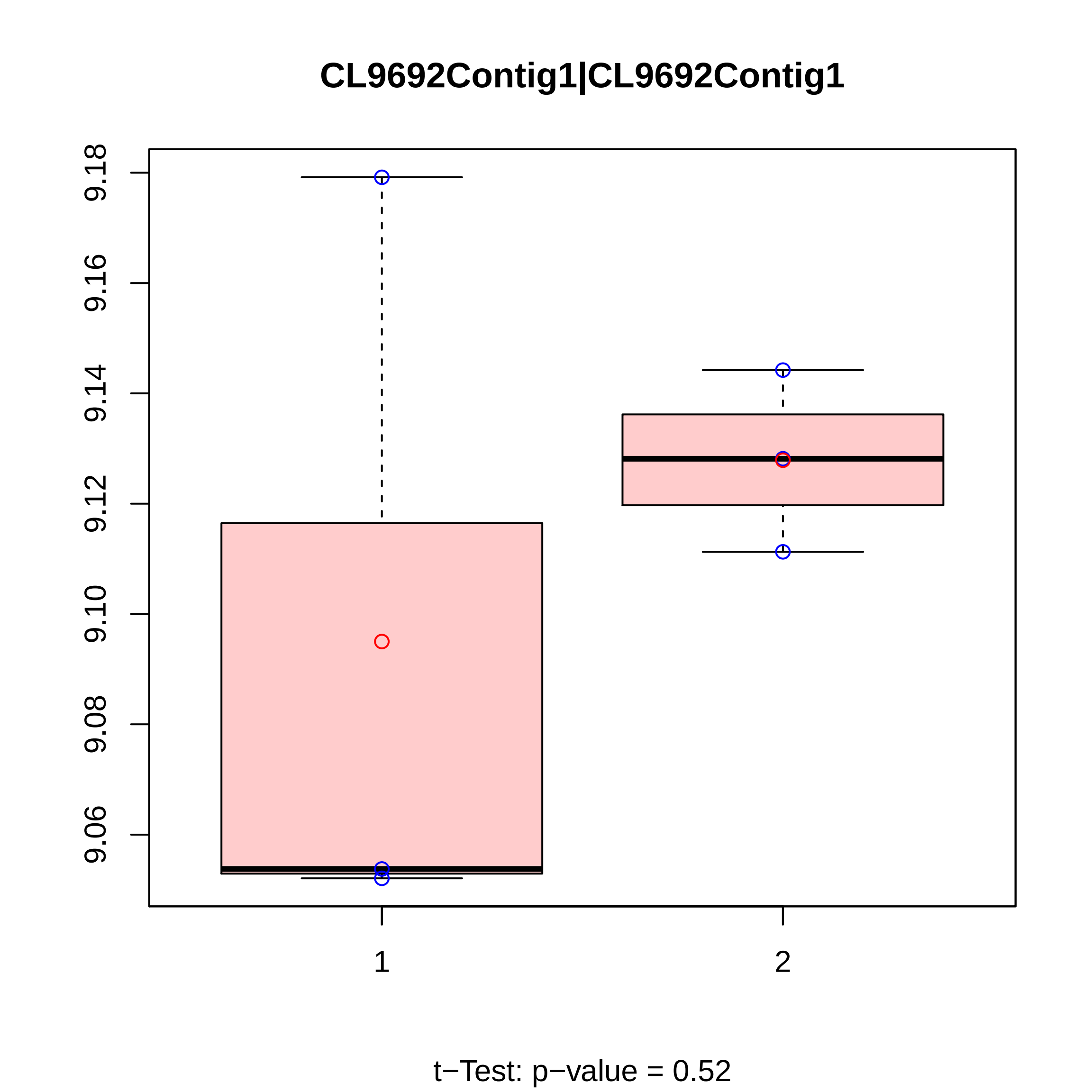
CL9692Contig1|CL9692Contig1
9.18
9.16
9.14
9.12
9.10
9.08
9.06
1
2
t−Test: p−value = 0.52

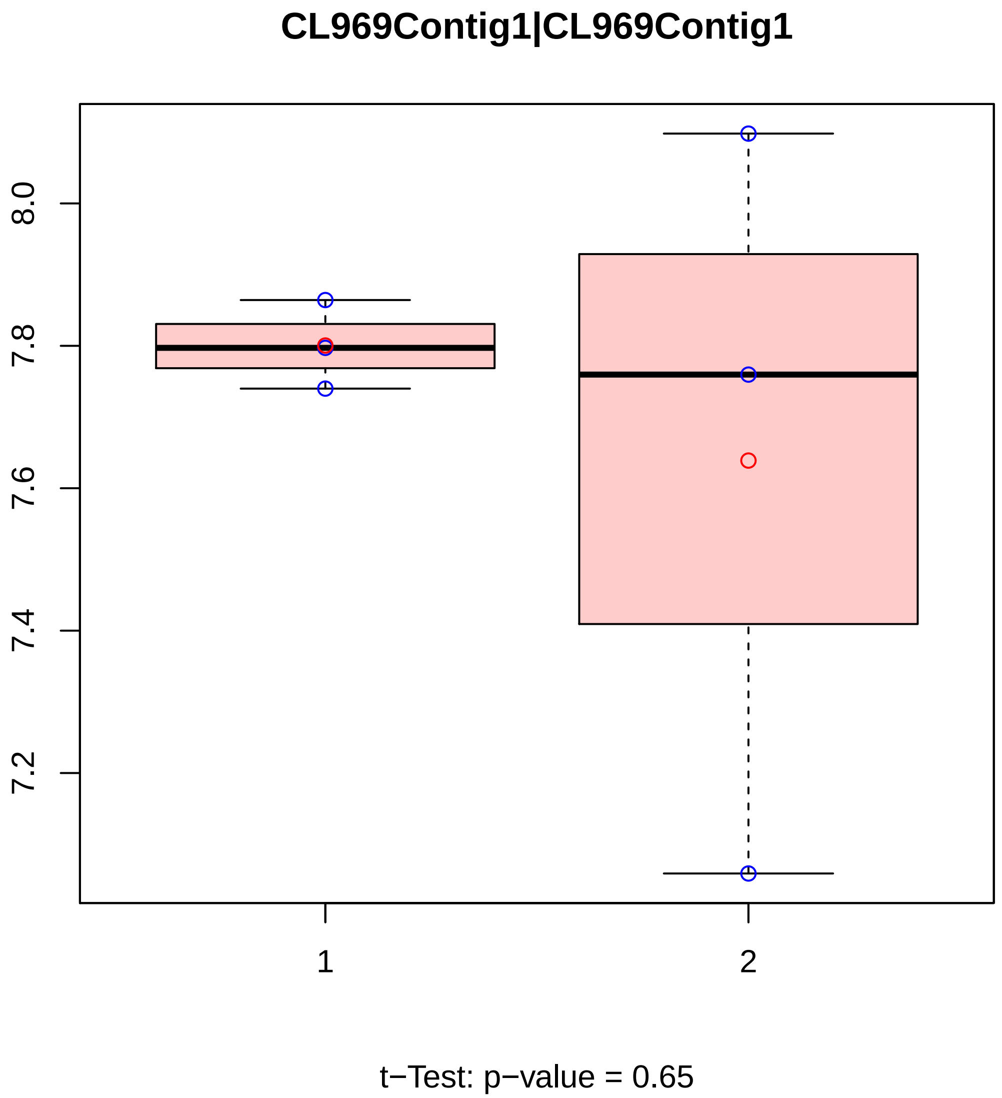
CL969Contig1|CL969Contig1
8.0
7.8
7.6
7.4
7.2
1
2
t−Test: p−value = 0.65

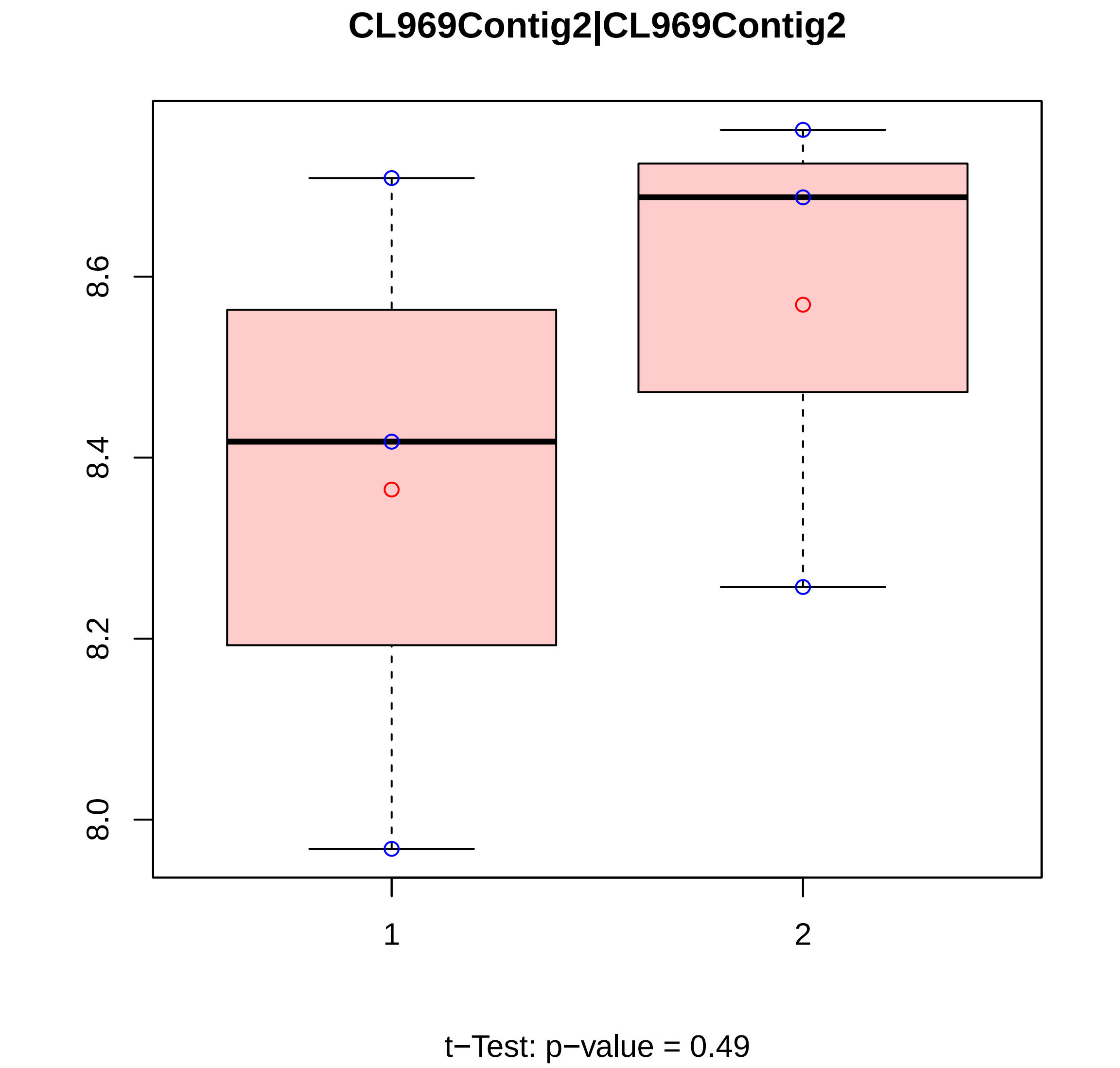
CL969Contig2|CL969Contig2
8.0
8.2
8.4
8.6
1
2
t−Test: p−value = 0.49

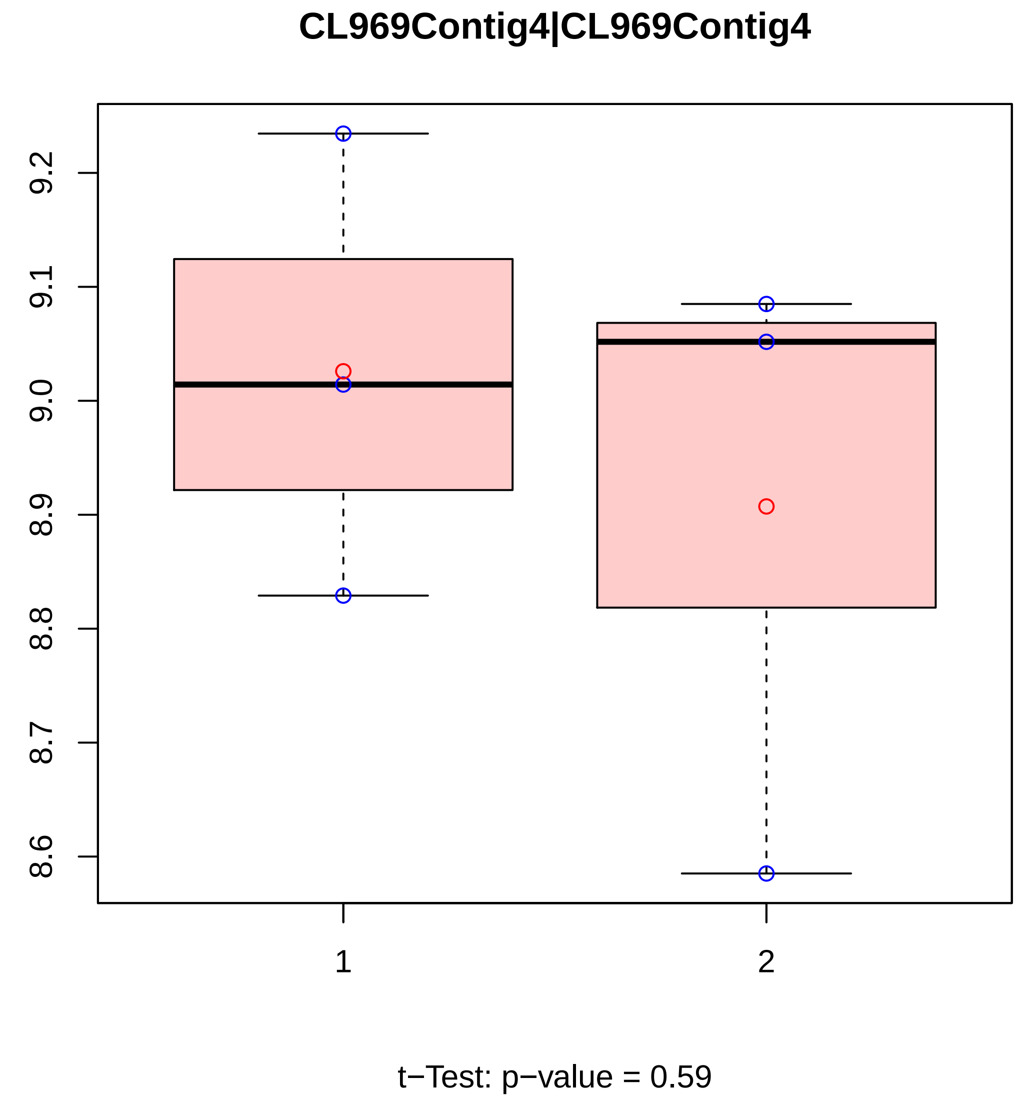
CL969Contig4|CL969Contig4
9.2
9.1
9.0
8.9
8.8
8.7
8.6
1
2
t−Test: p−value = 0.59

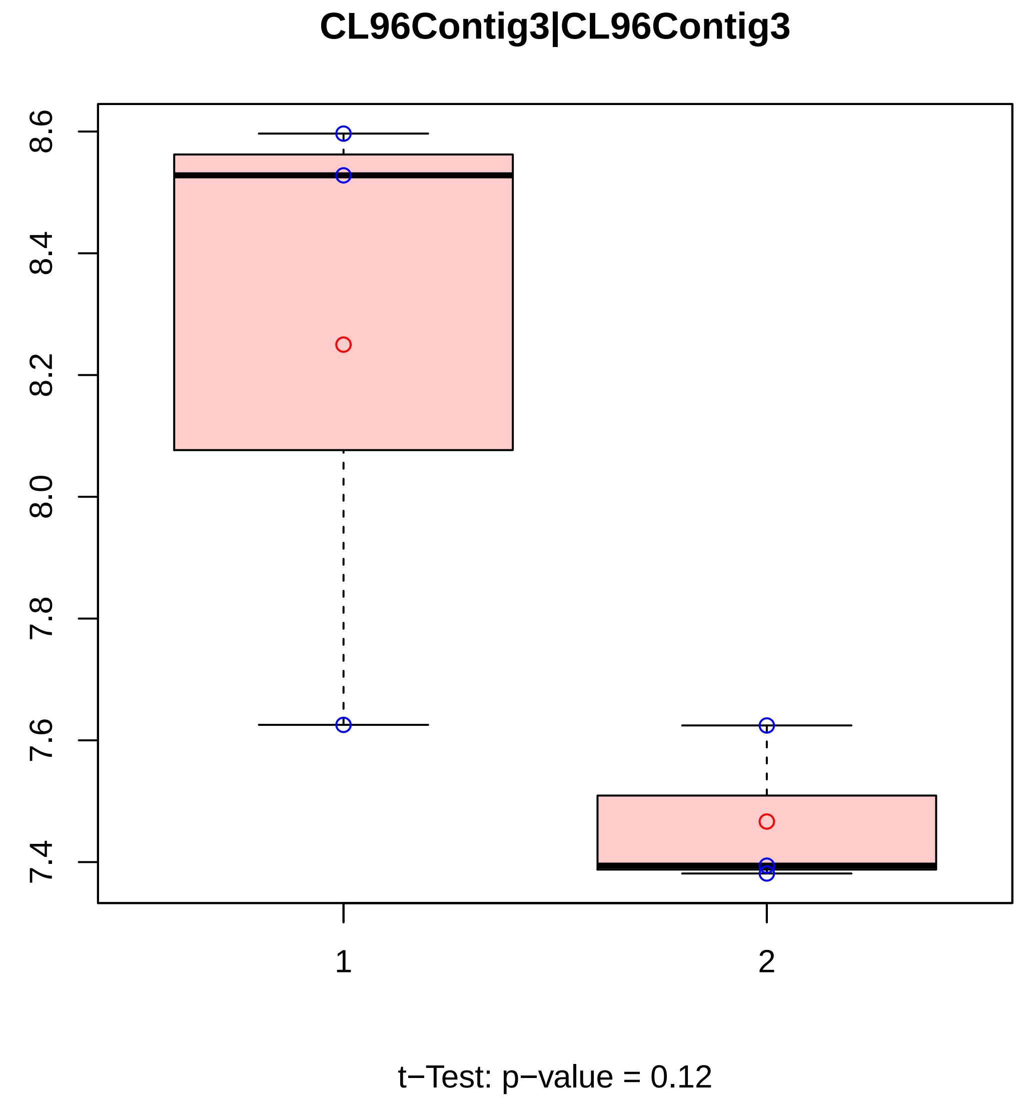
CL96Contig3|CL96Contig3
8.6
8.4
8.2
8.0
7.8
7.6
7.4
1
2
t−Test: p−value = 0.12

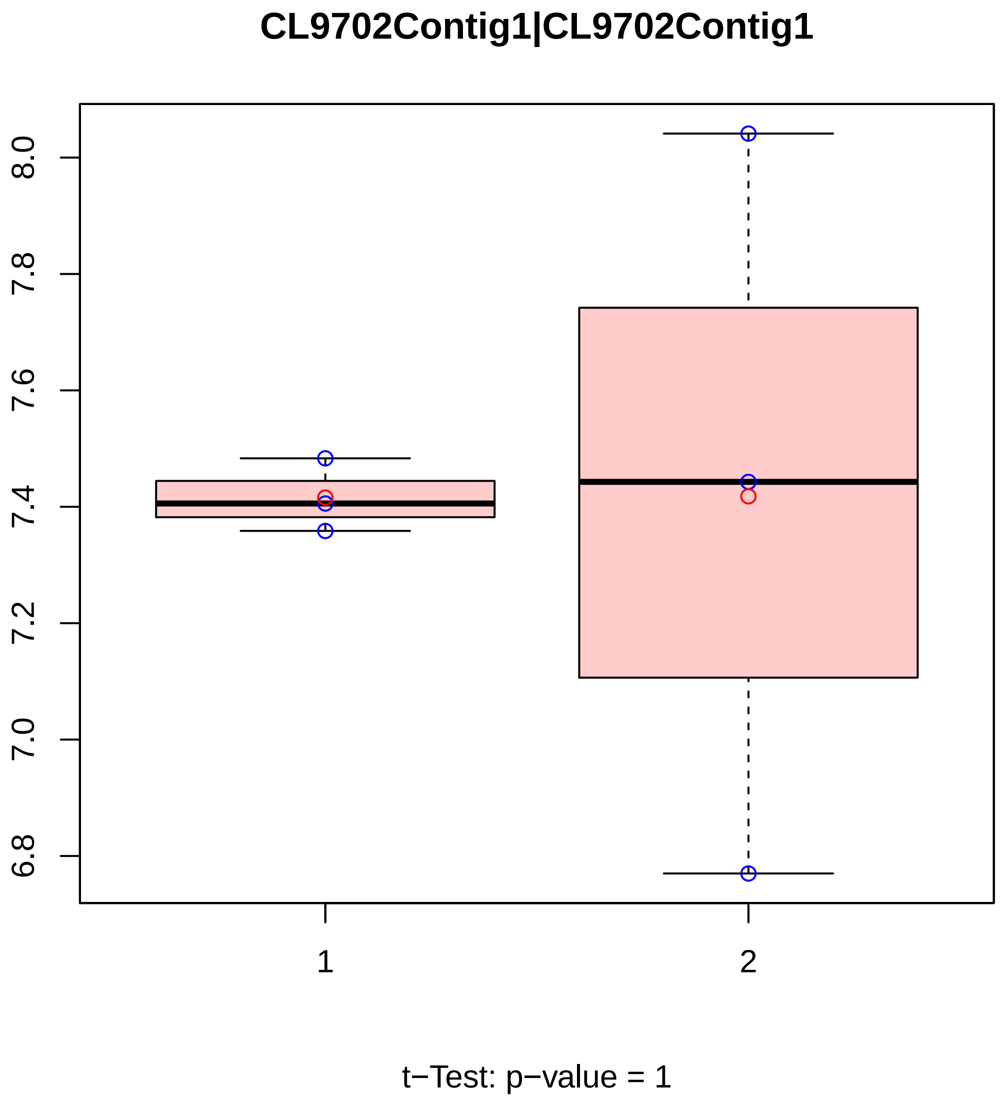
CL9702Contig1|CL9702Contig1
8.0
7.8
7.6
7.4
7.2
7.0
6.8
1
2
t−Test: p−value = 1

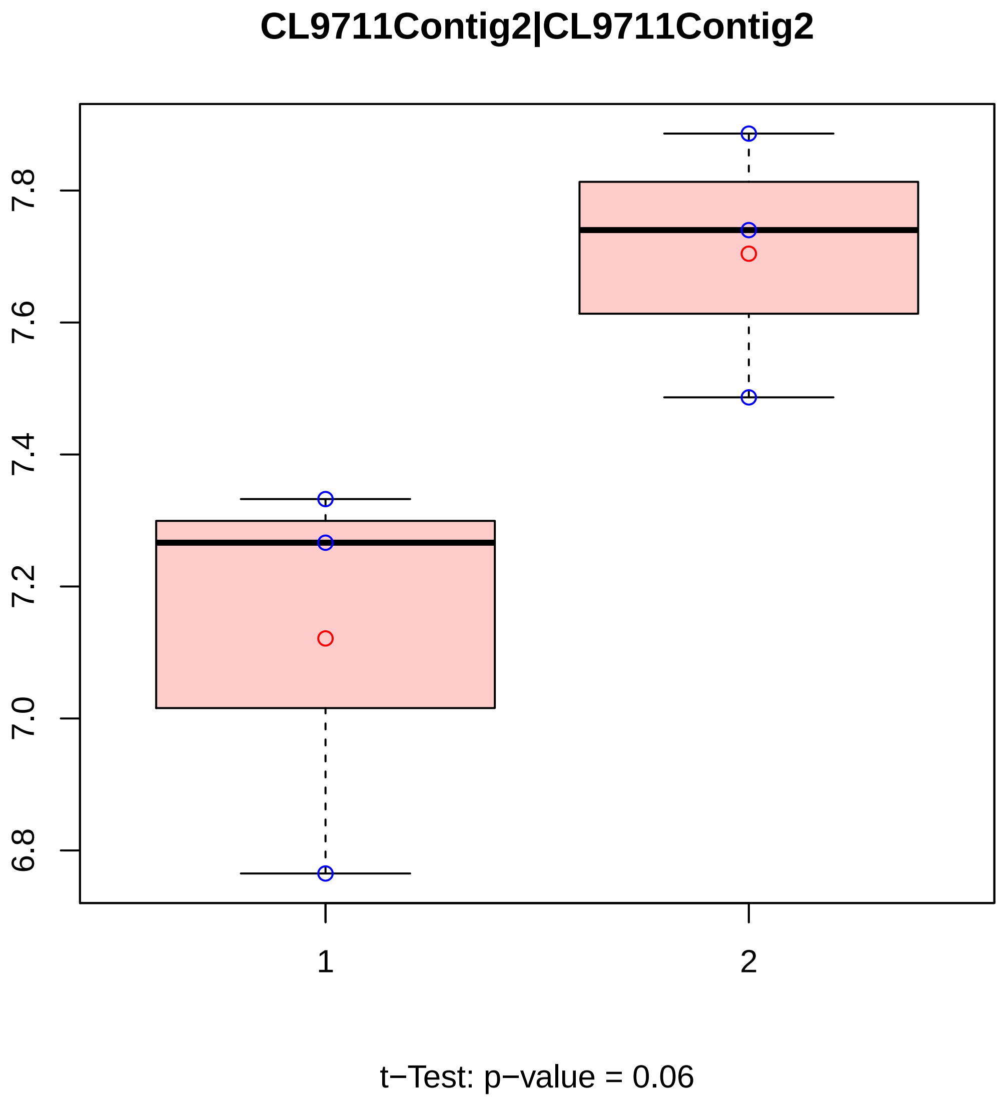
CL9711Contig2|CL9711Contig2
7.8
7.6
7.4
7.2
7.0
6.8
1
2
t−Test: p−value = 0.06

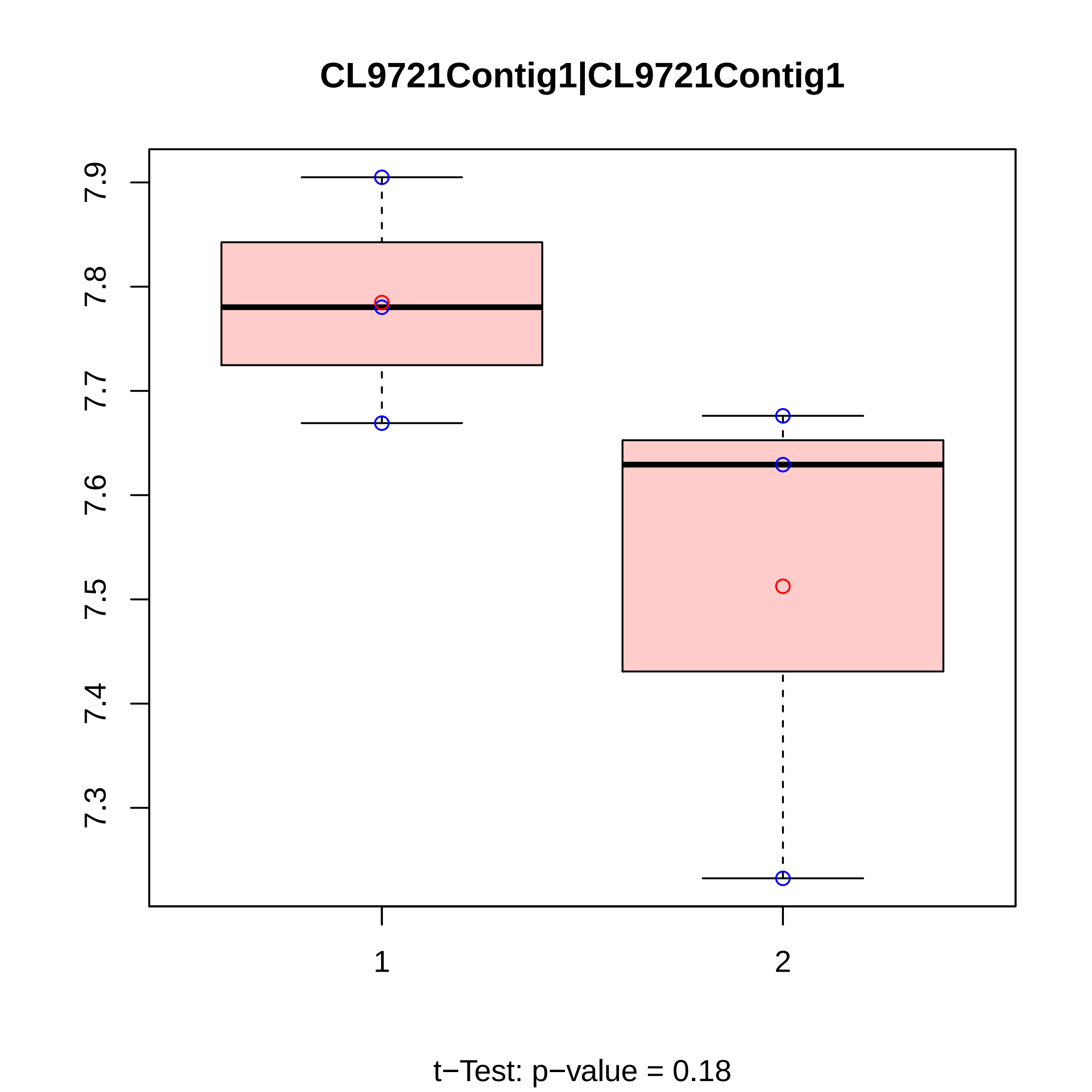

CL9721Contig1|CL9721Contig1
7.3
7.4
7.5
7.6
7.7
7.8
7.9
1
2
t−Test: p−value = 0.18

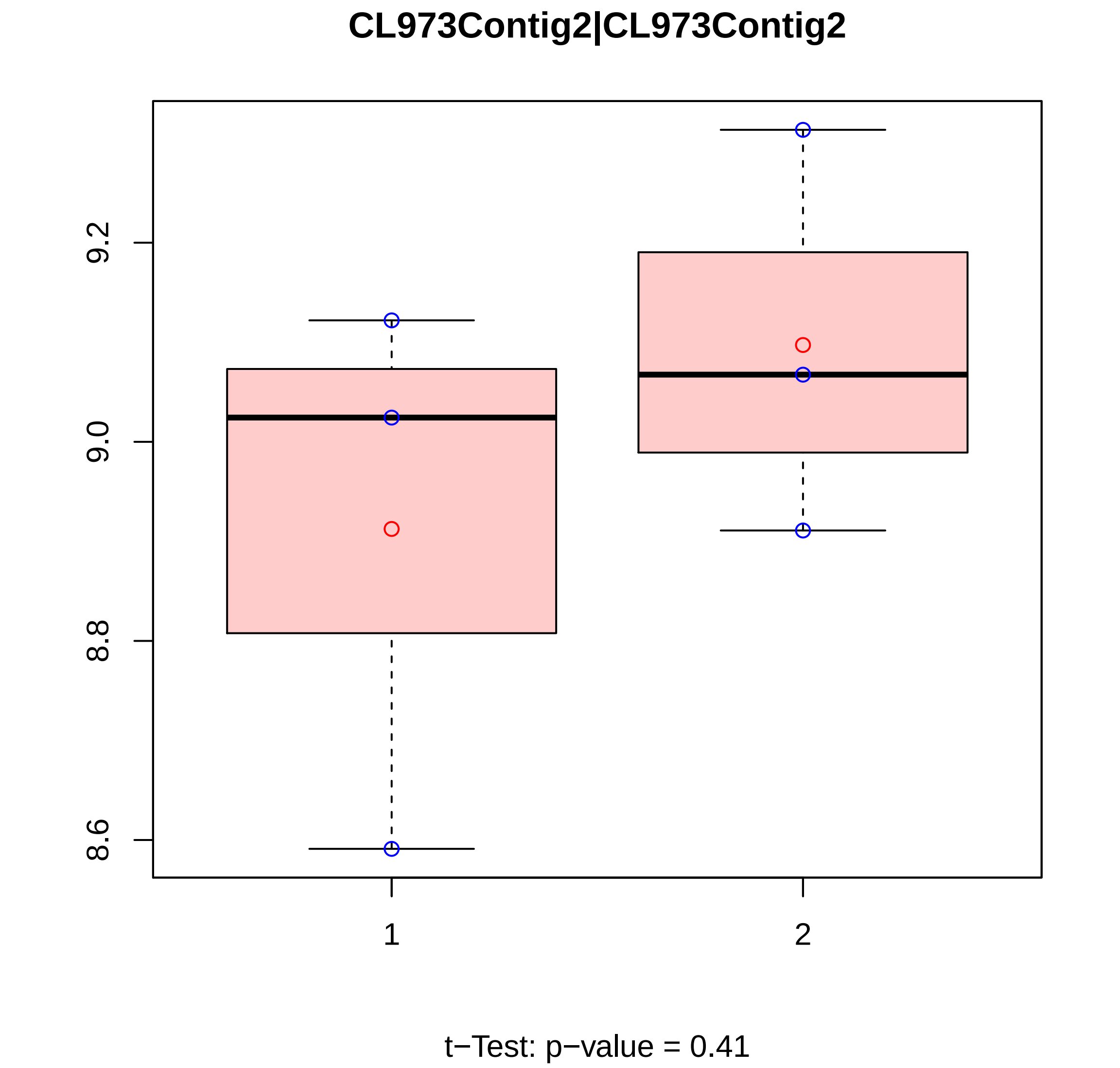
CL973Contig2|CL973Contig2
9.2
9.0
8.8
8.6
1
2
t−Test: p−value = 0.41

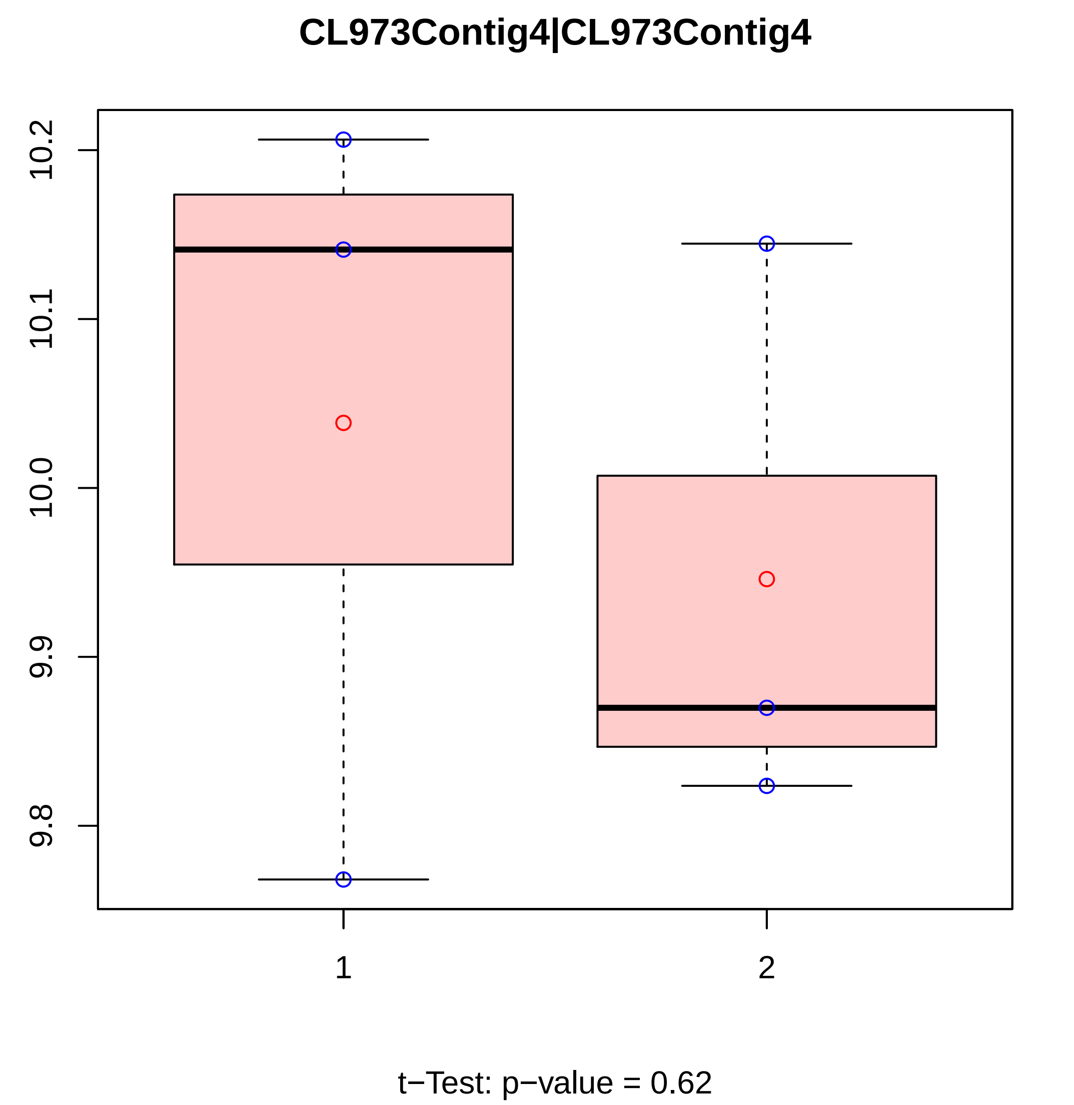
CL973Contig4|CL973Contig4
10.2
10.1
10.0
9.9
9.8
1
2
t−Test: p−value = 0.62

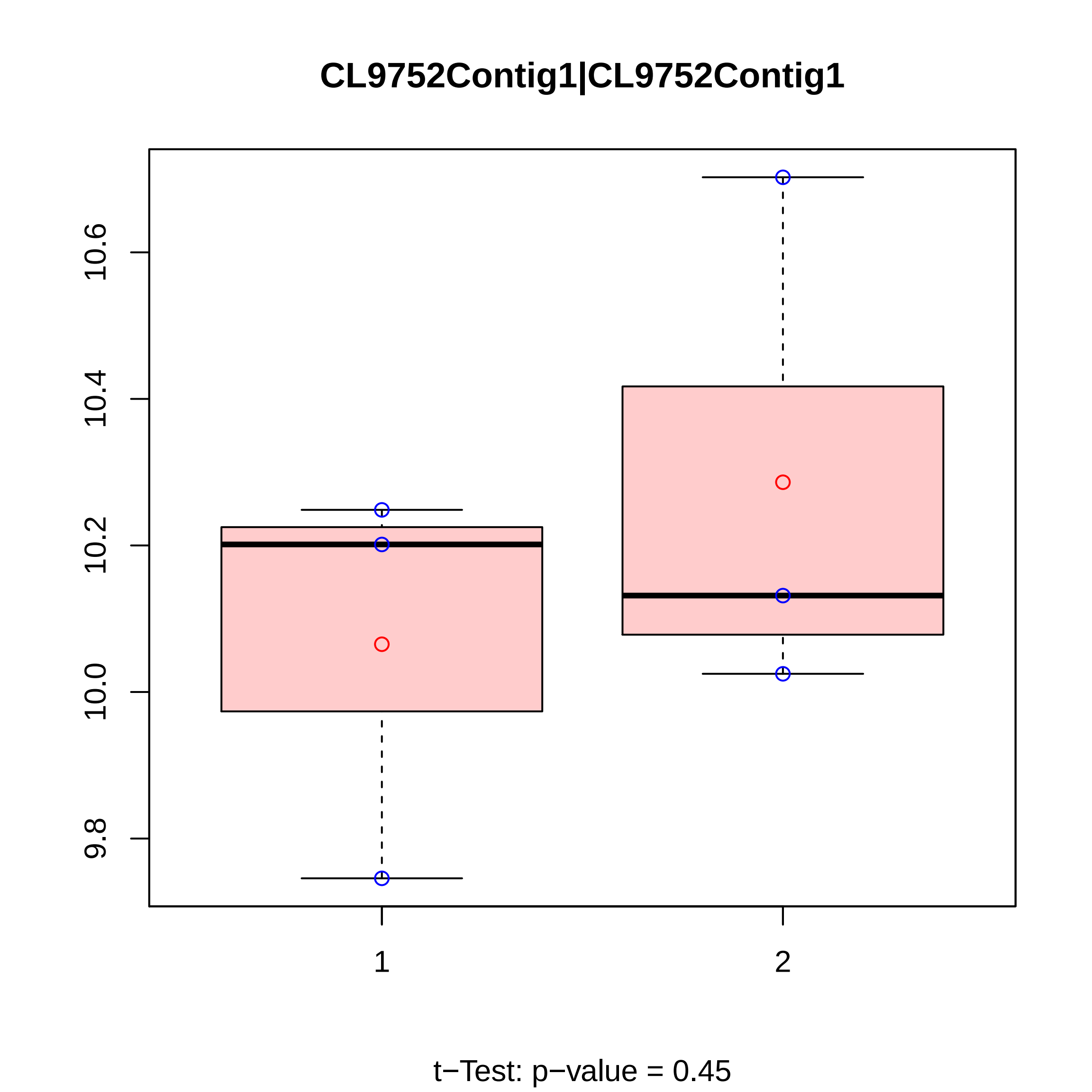

CL9752Contig1|CL9752Contig1
9.8
10.0
10.2
10.4
10.6
1
2
t−Test: p−value = 0.45

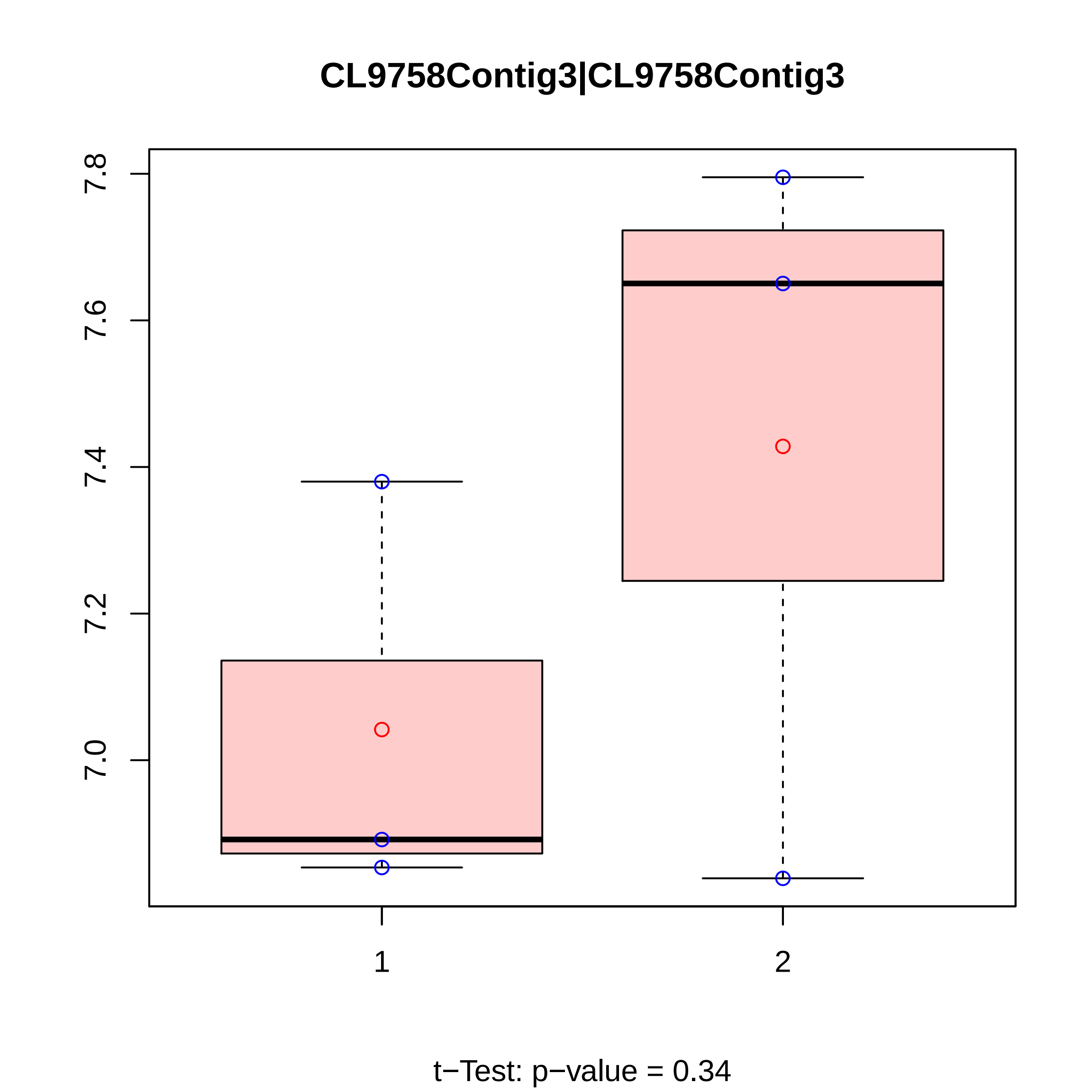
CL9758Contig3|CL9758Contig3
7.0
7.2
7.4
7.6
7.8
1
2
t−Test: p−value = 0.34

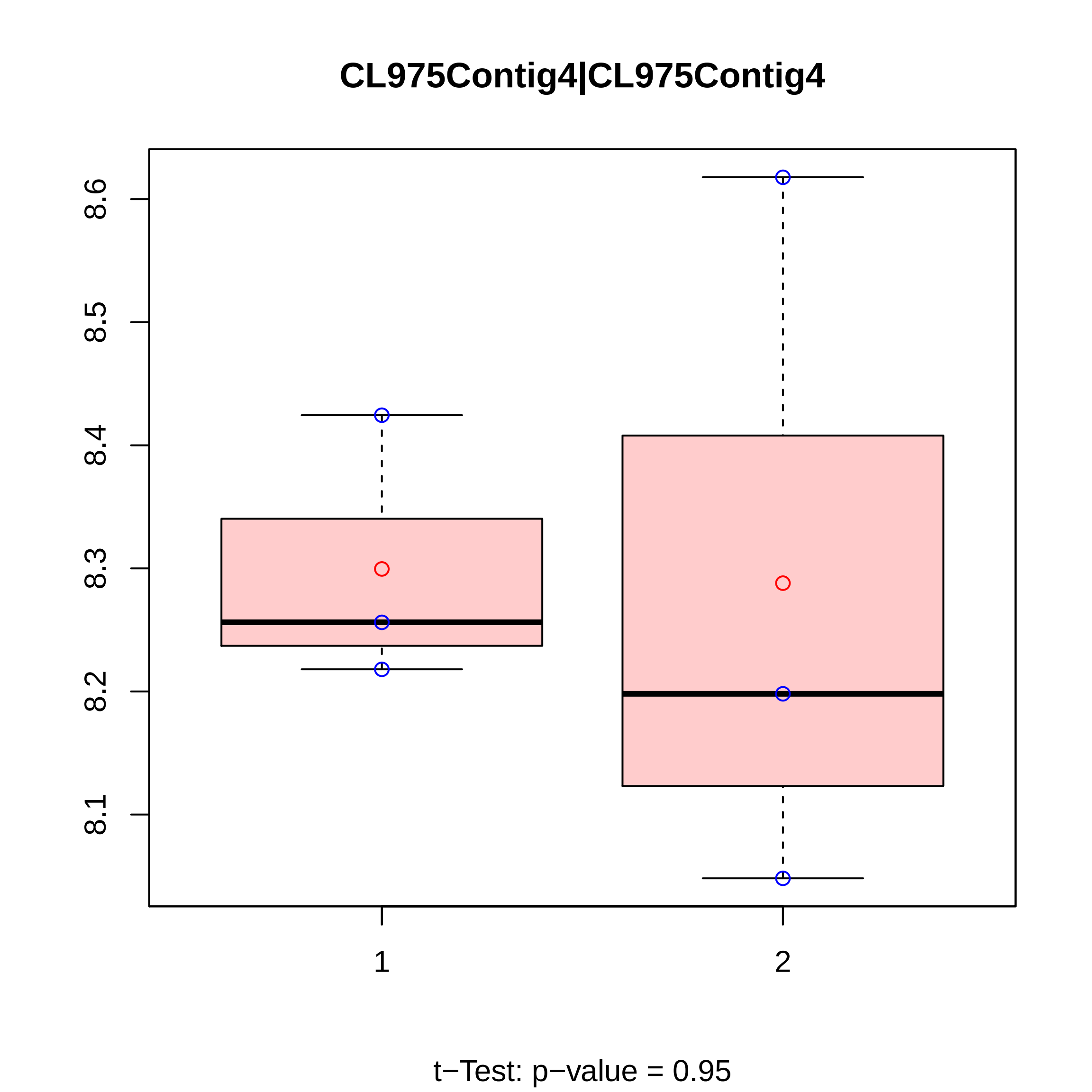
CL975Contig4|CL975Contig4
8.1
8.2
8.3
8.4
8.5
8.6
1
2
t−Test: p−value = 0.95

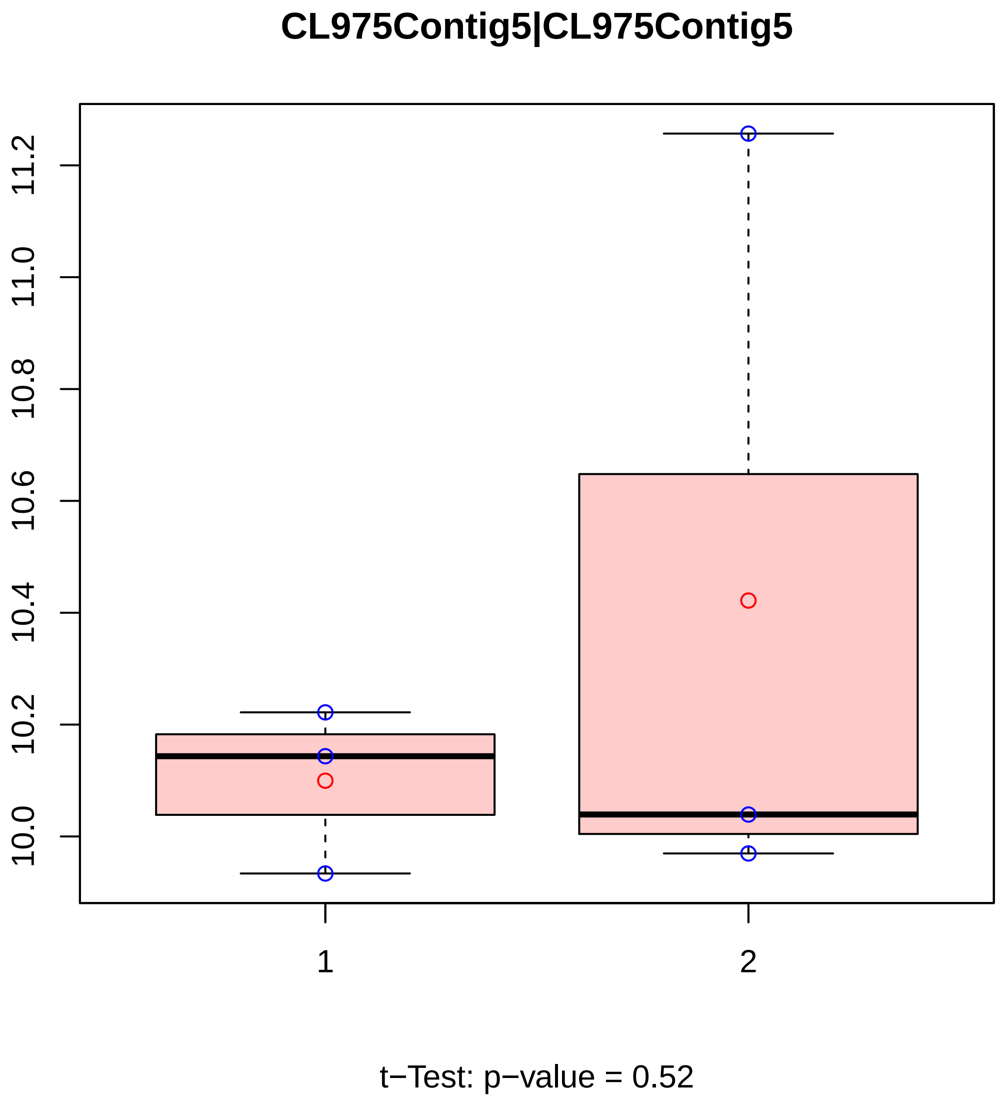

CL975Contig5|CL975Contig5
11.2
11.0
10.8
10.6
10.4
10.2
10.0
1
2
t−Test: p−value = 0.52

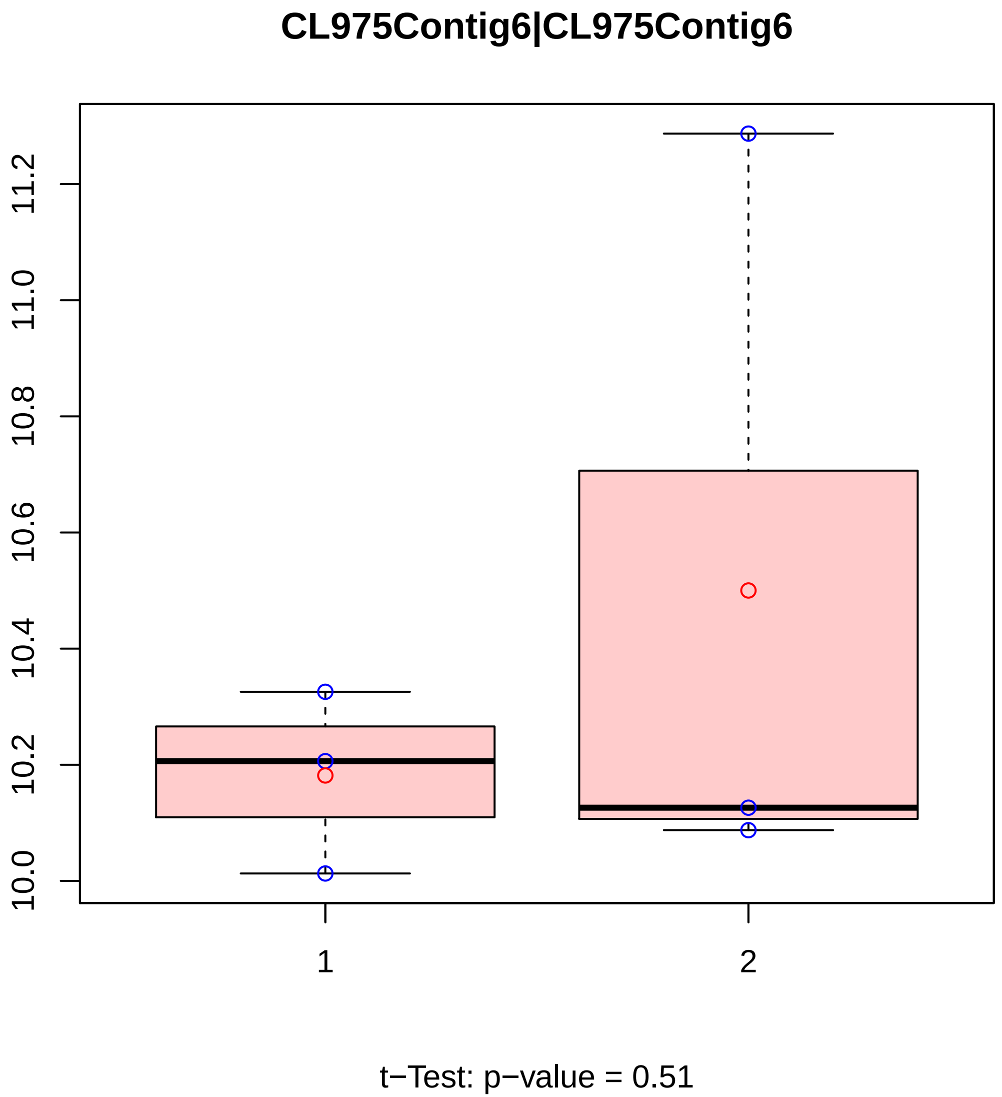
CL975Contig6|CL975Contig6
11.2
11.0
10.8
10.6
10.4
10.2
10.0
1
2
t−Test: p−value = 0.51

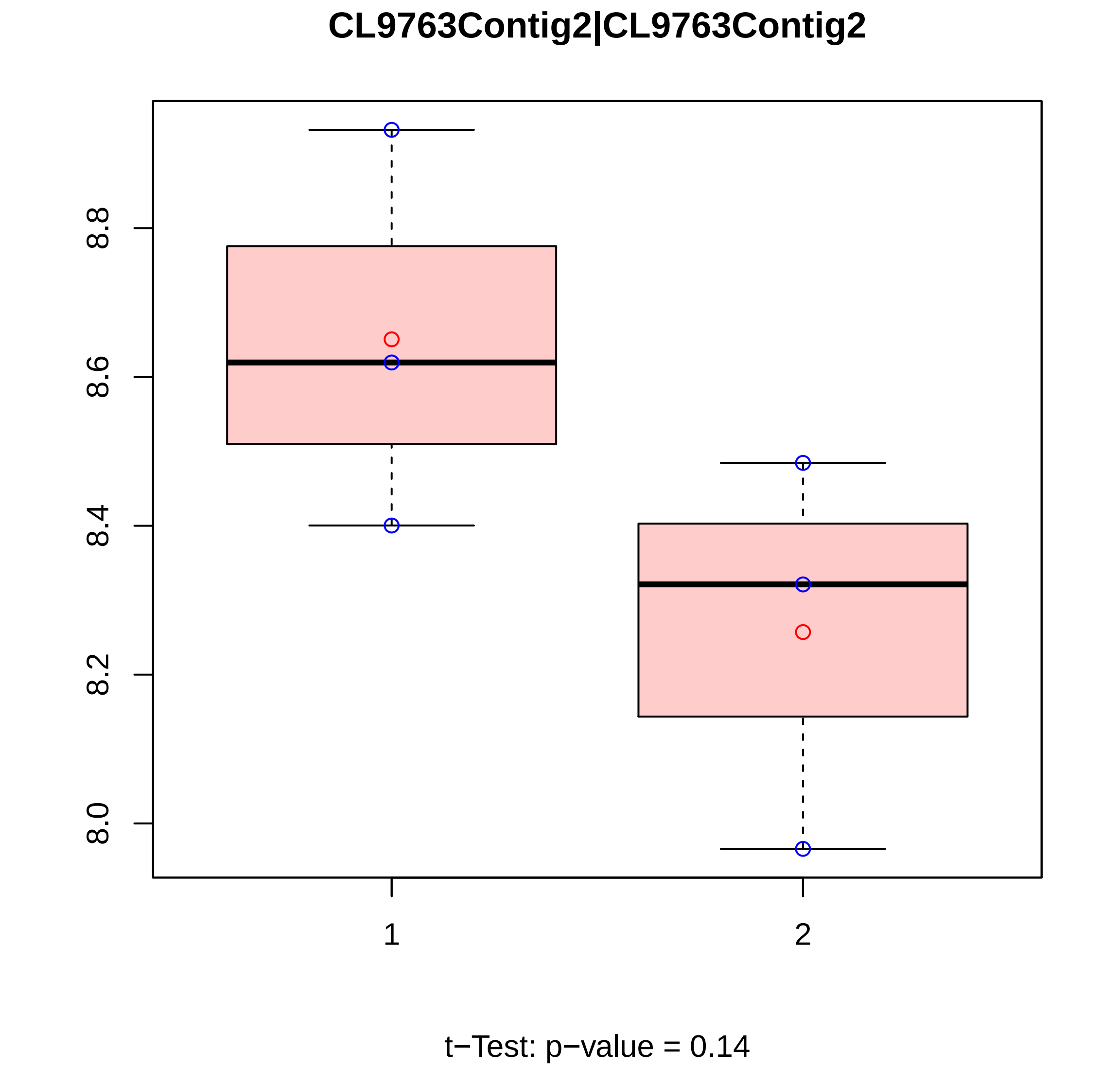

CL9763Contig2|CL9763Contig2
8.0
8.2
8.4
8.6
8.8
1
2
t−Test: p−value = 0.14

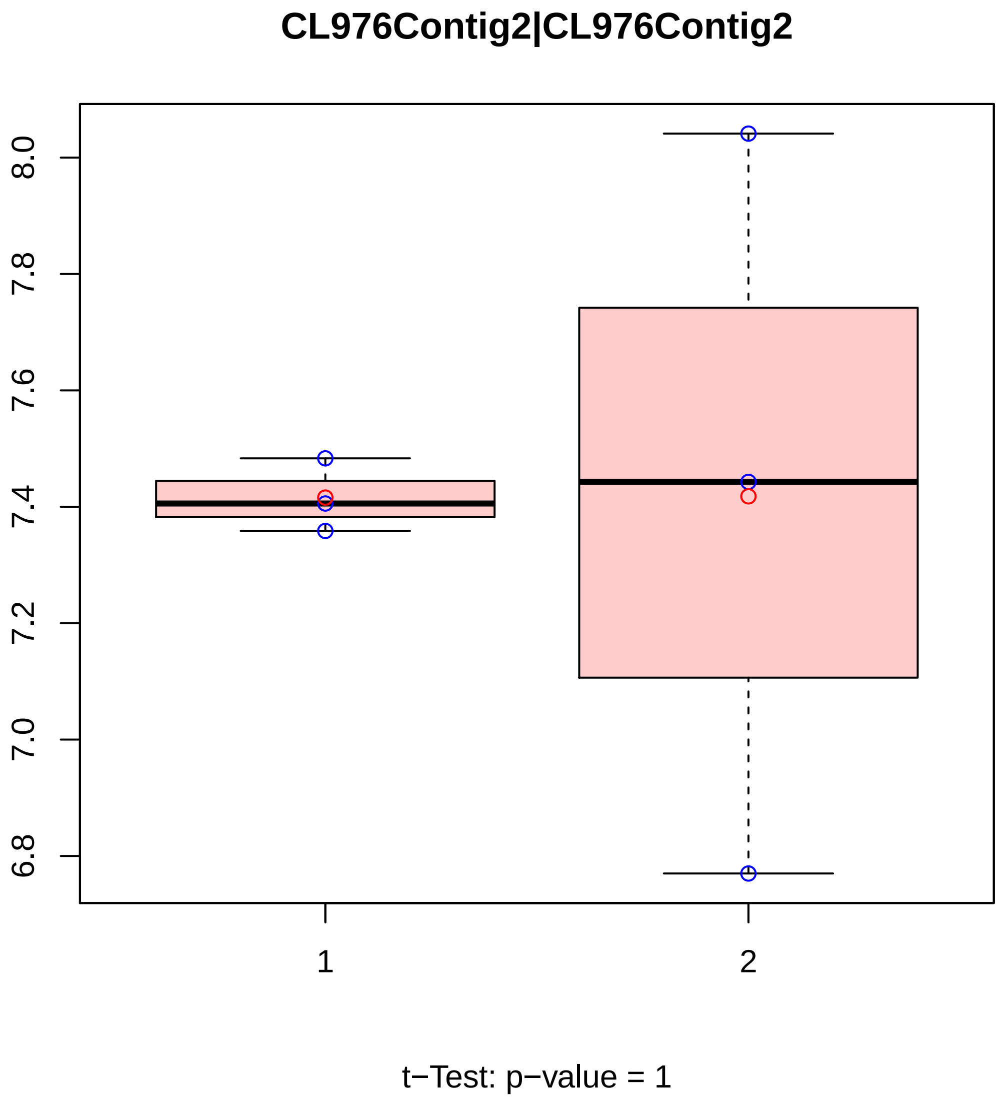

CL976Contig2|CL976Contig2
8.0
7.8
7.6
7.4
7.2
7.0
6.8
1
2
t−Test: p−value = 1

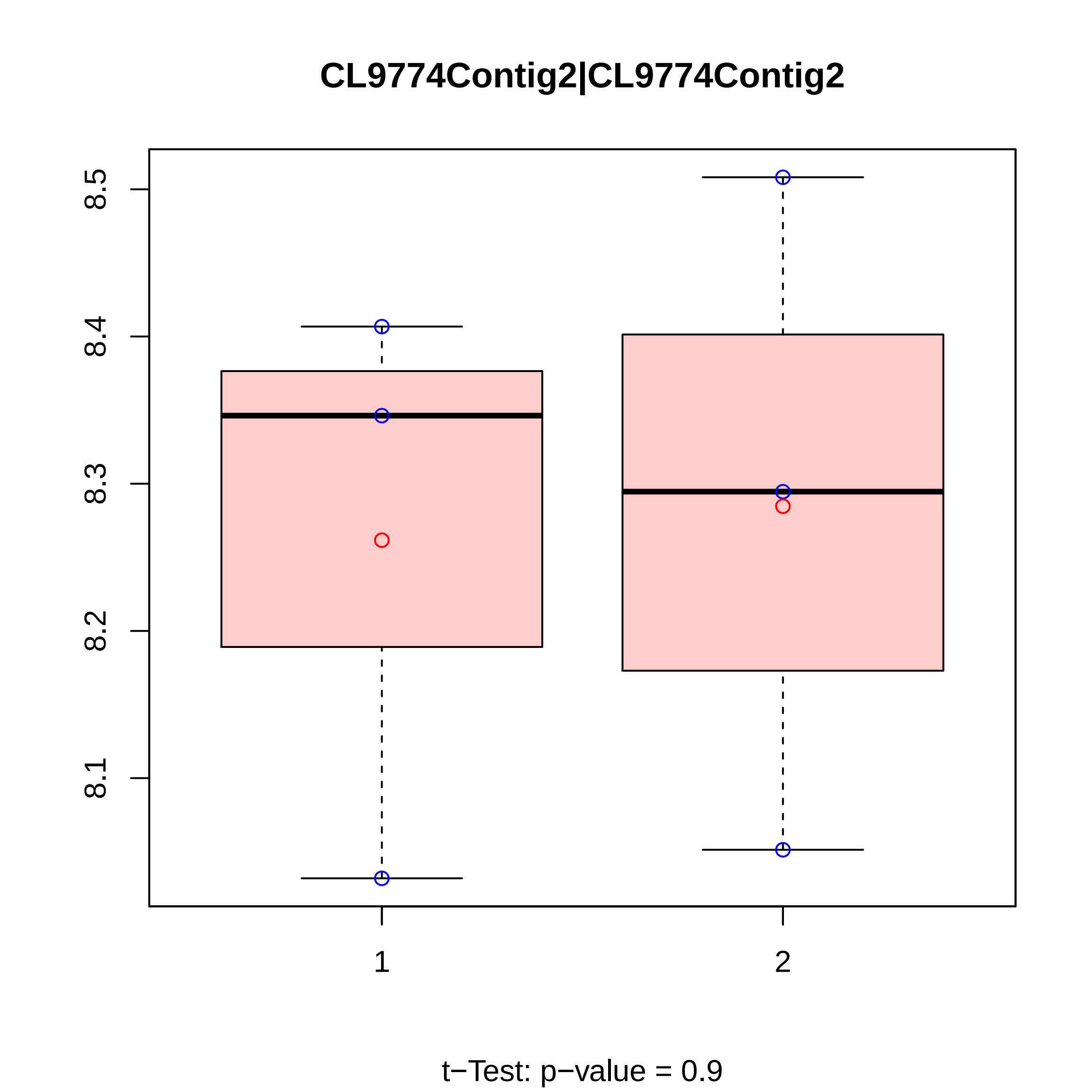

CL9774Contig2|CL9774Contig2
8.1
8.2
8.3
8.4
8.5
1
2
t−Test: p−value = 0.9

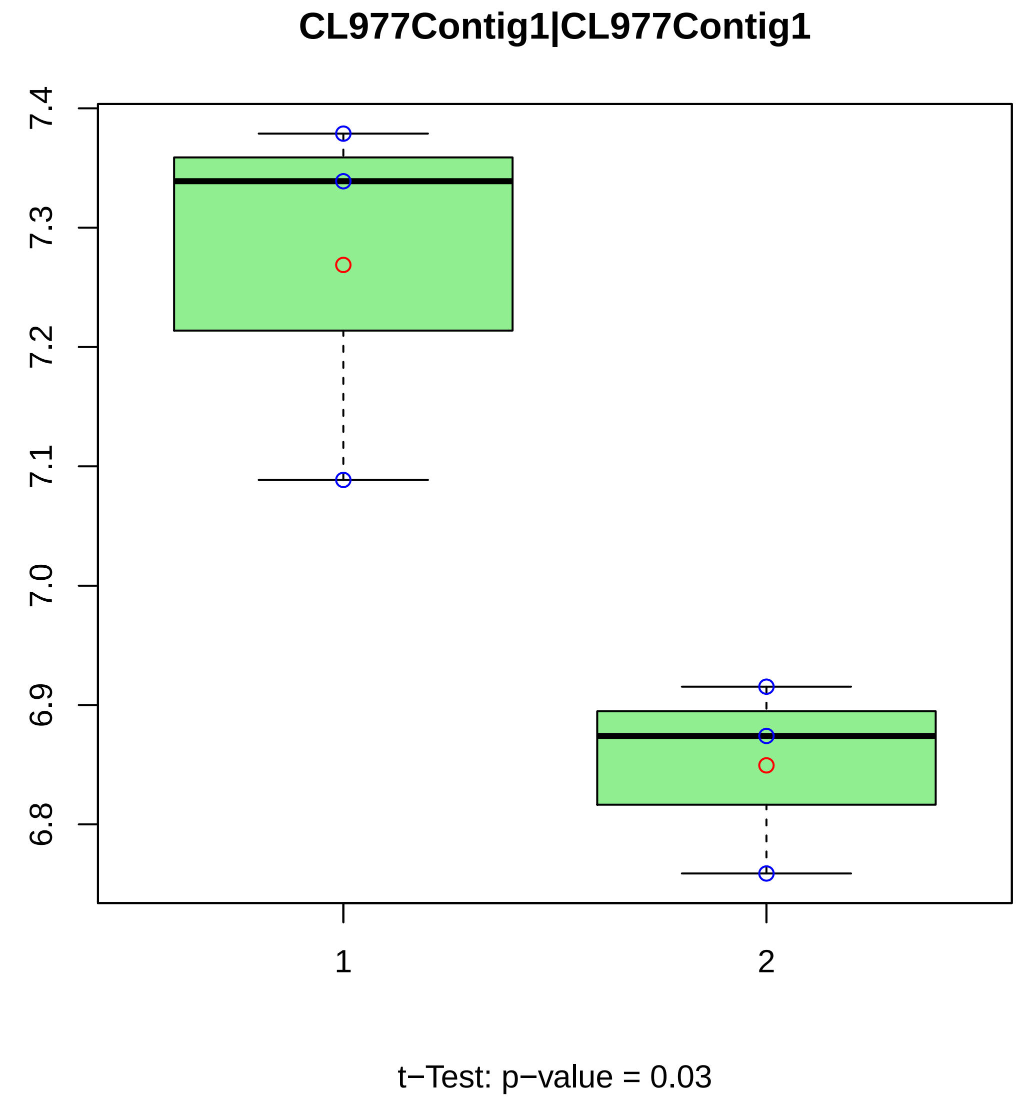
CL977Contig1|CL977Contig1
7.4
7.3
7.2
7.1
7.0
6.9
6.8
1
2
t−Test: p−value = 0.03

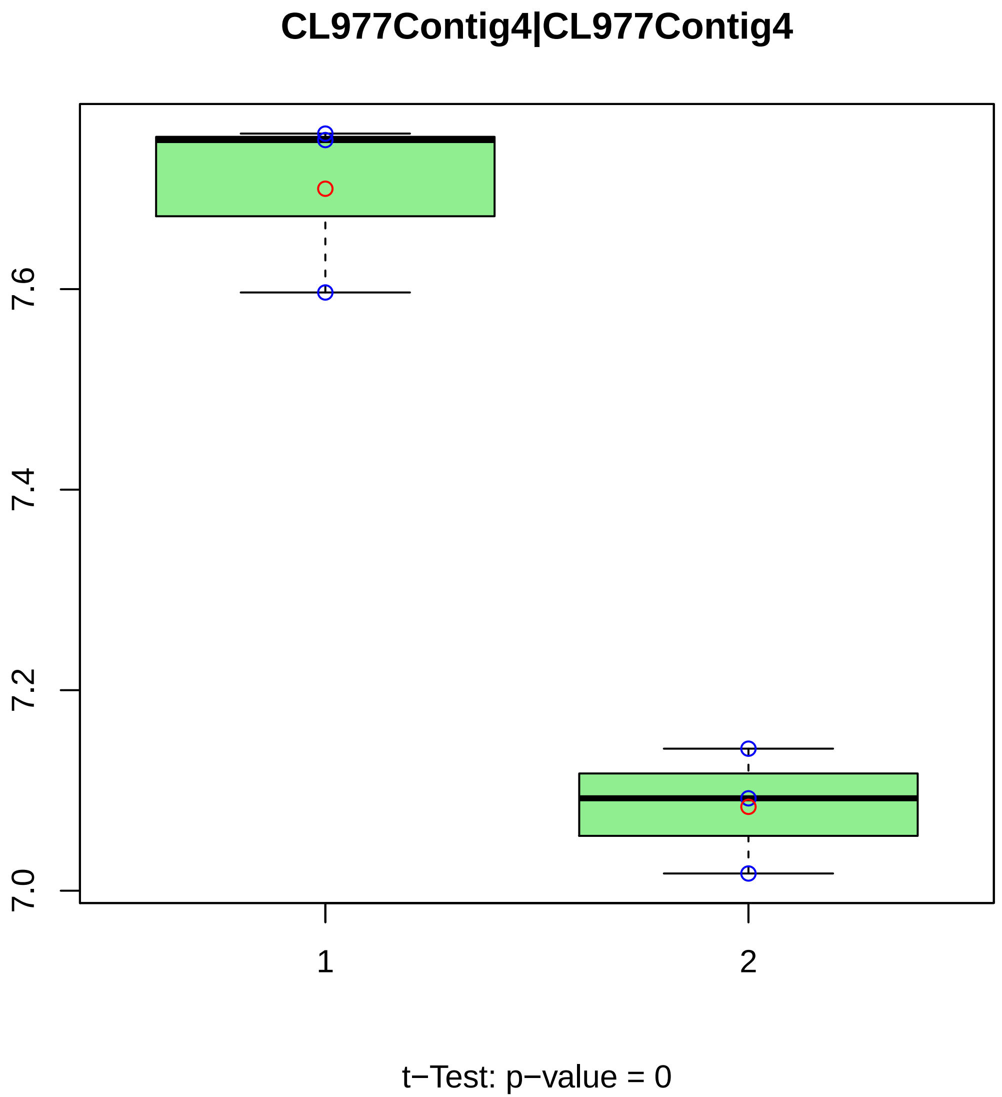
CL977Contig4|CL977Contig4
7.0
7.2
7.4
7.6
1
2
t−Test: p−value = 0

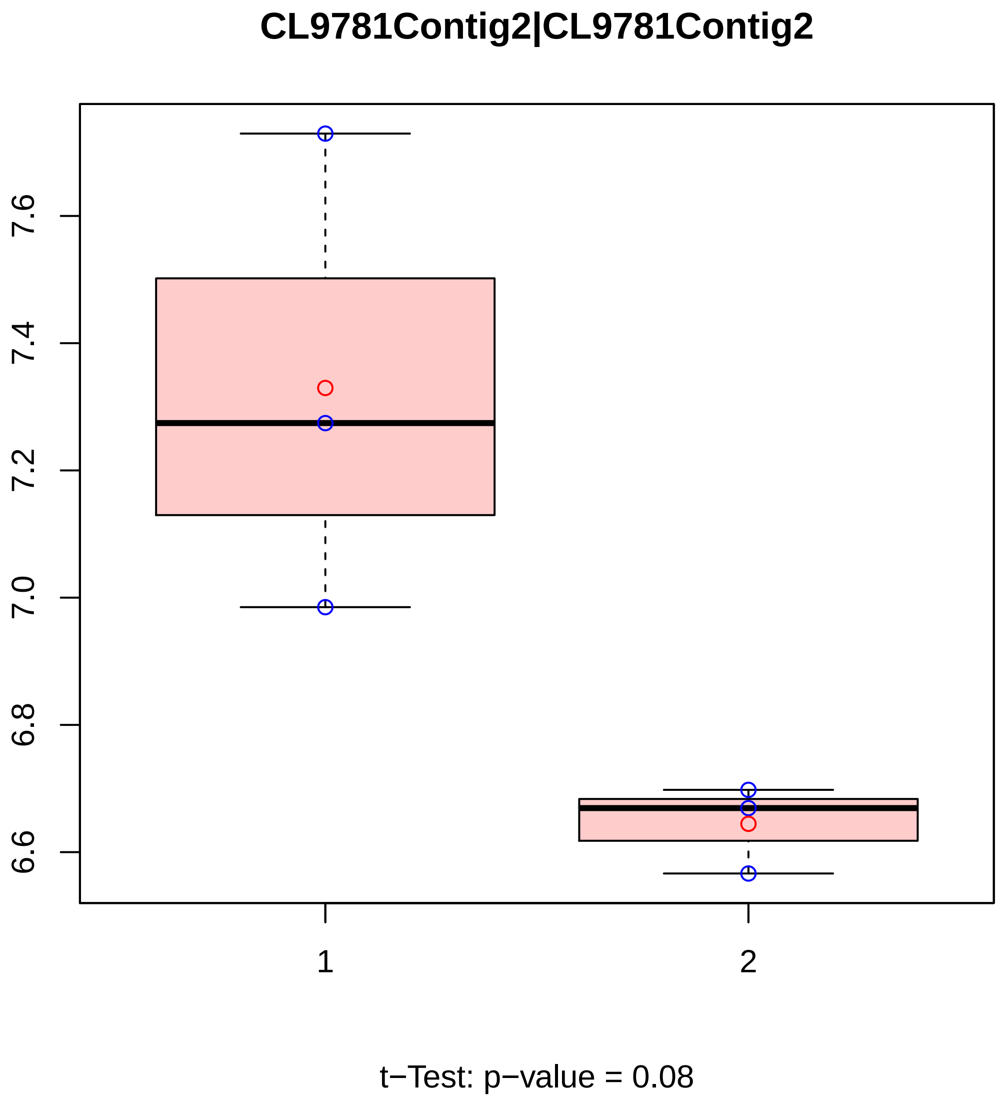
CL9781Contig2|CL9781Contig2
7.6
7.4
7.2
7.0
6.8
6.6
1
2
t−Test: p−value = 0.08

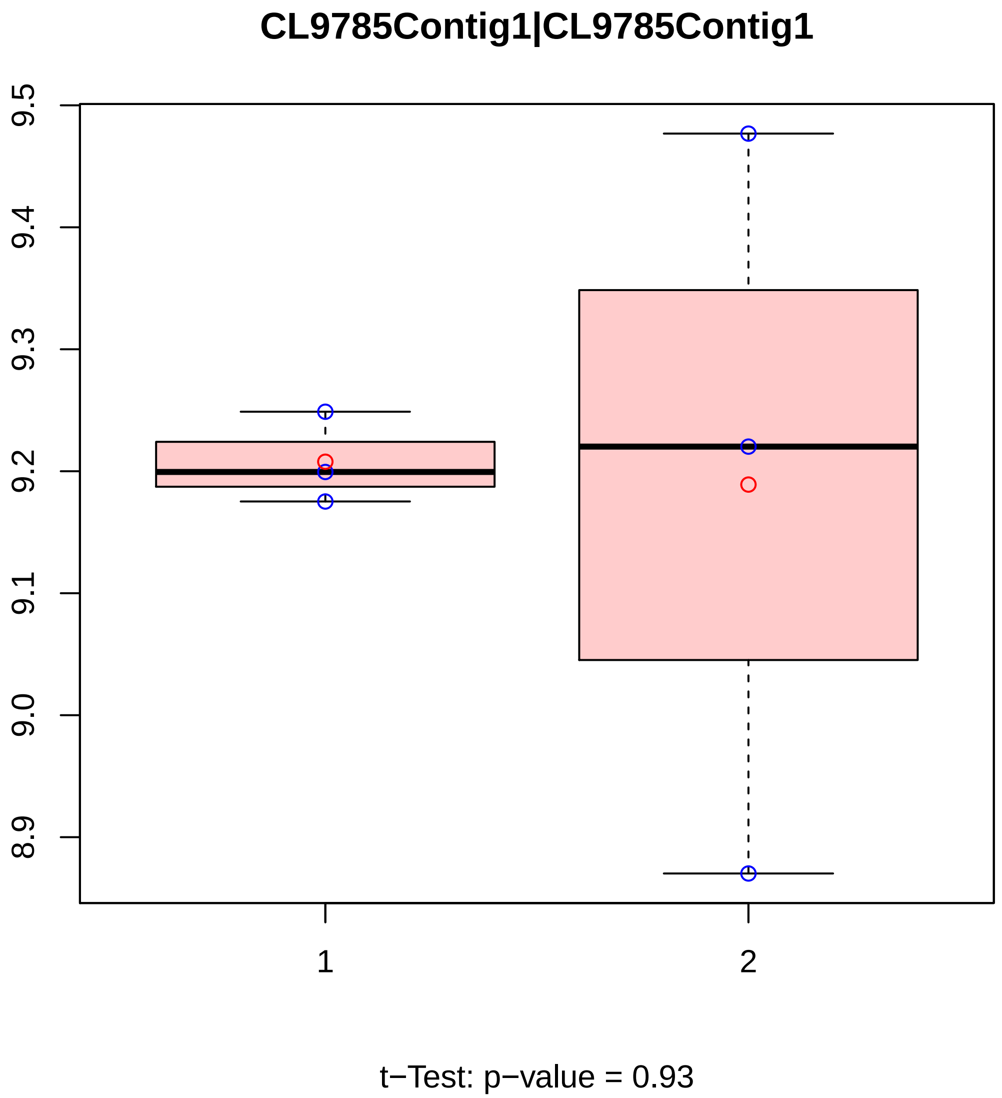

CL9785Contig1|CL9785Contig1
9.5
9.4
9.3
9.2
9.1
9.0
8.9
1
2
t−Test: p−value = 0.93

# CL978Contig1|CL978Contig1

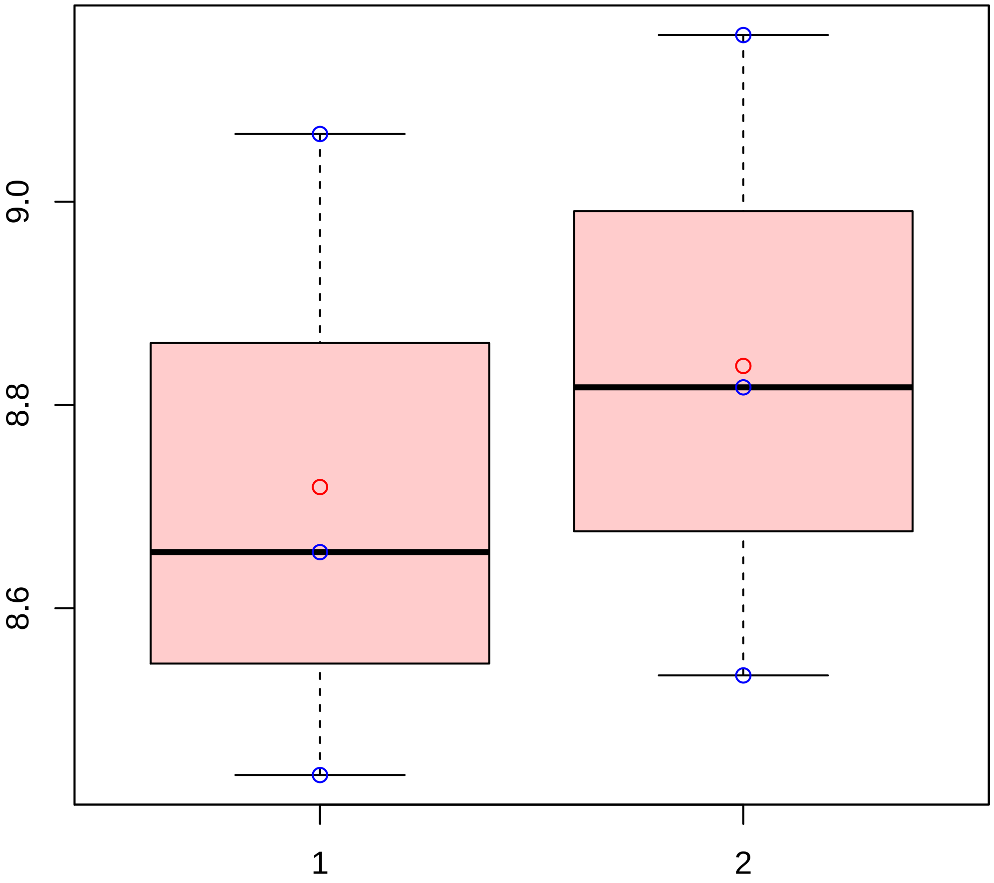


t−Test: p−value = 0.67

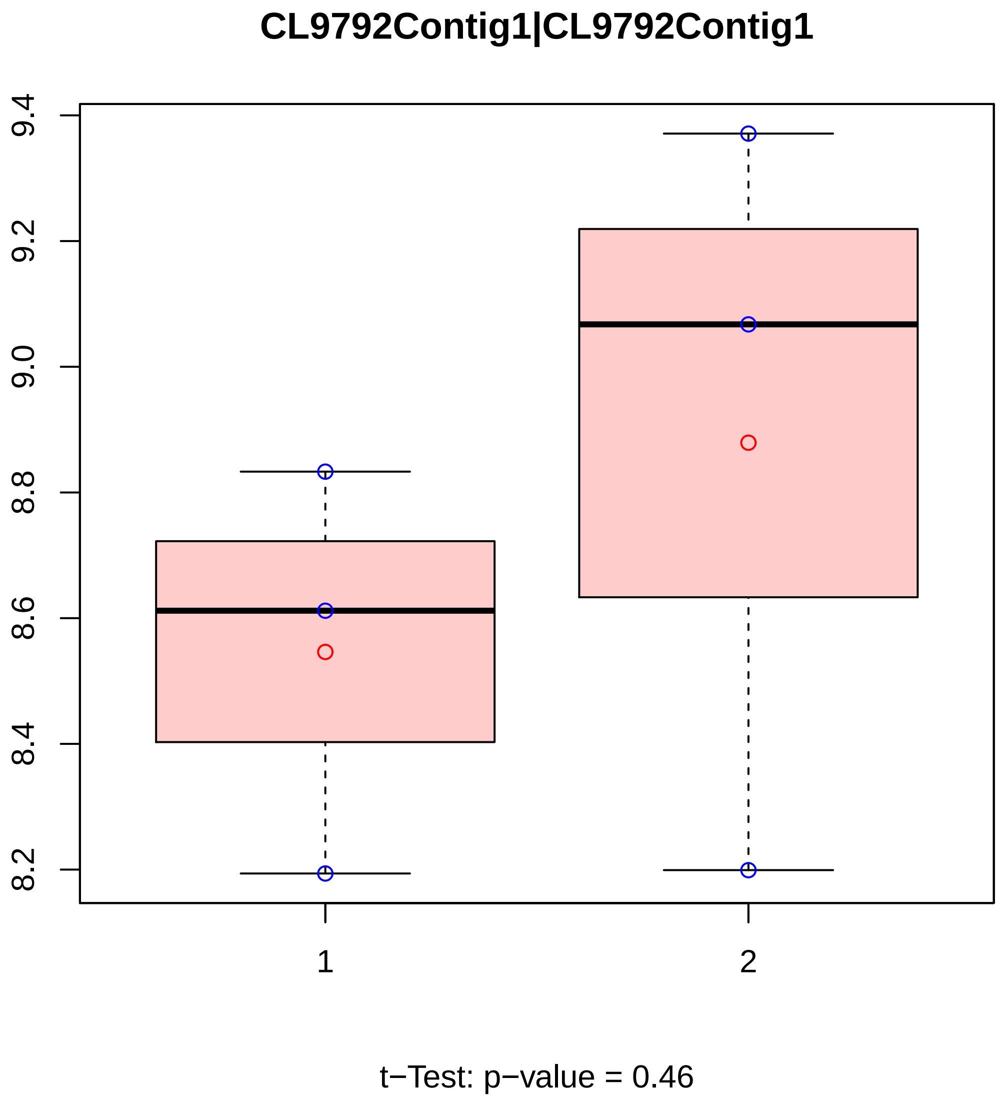
CL9792Contig1|CL9792Contig1
9.4
9.2
9.0
8.8
8.6
8.4
8.2
1
2
t−Test: p−value = 0.46

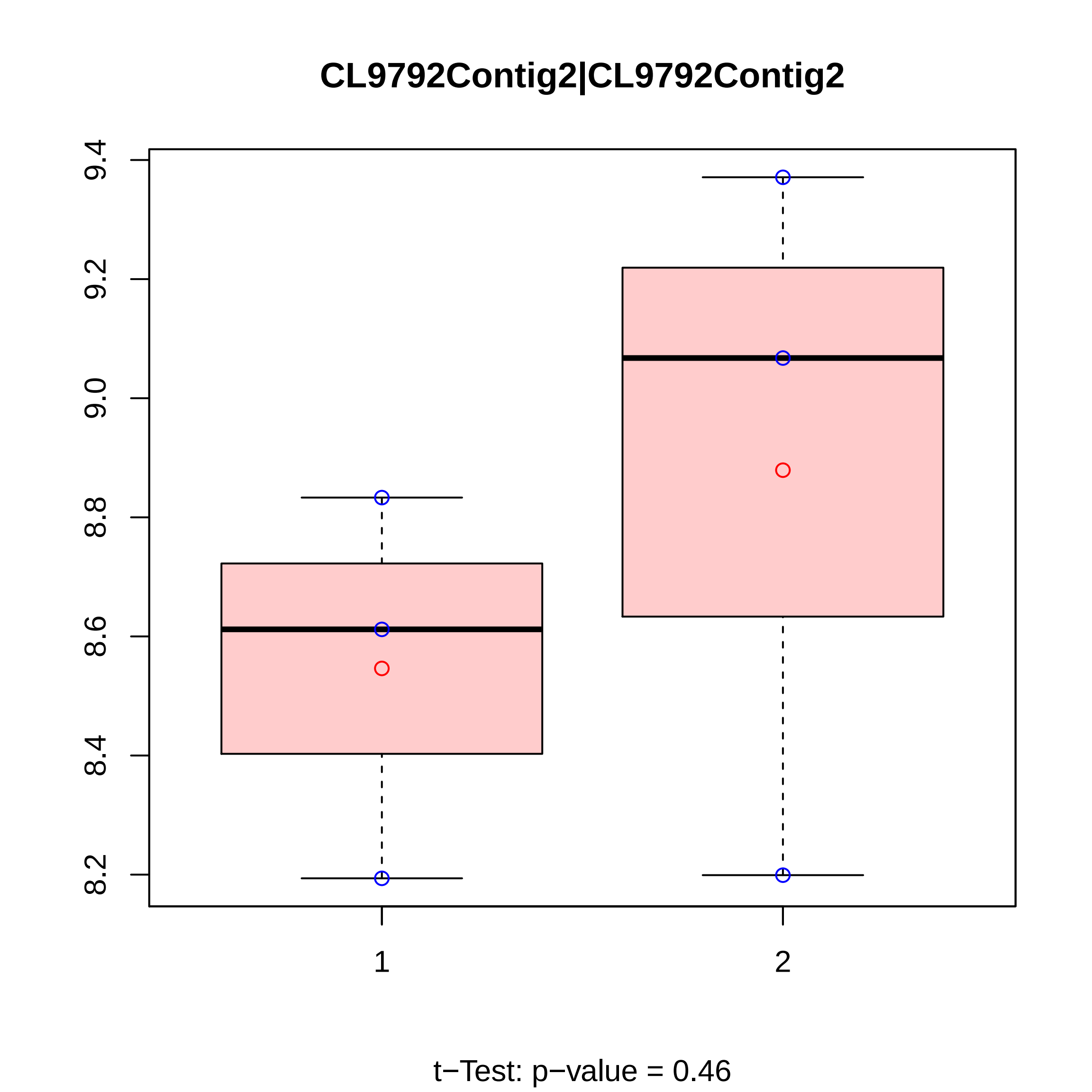

CL9792Contig2|CL9792Contig2
9.4
9.2
9.0
8.8
8.6
8.4
8.2
1
2
t−Test: p−value = 0.46

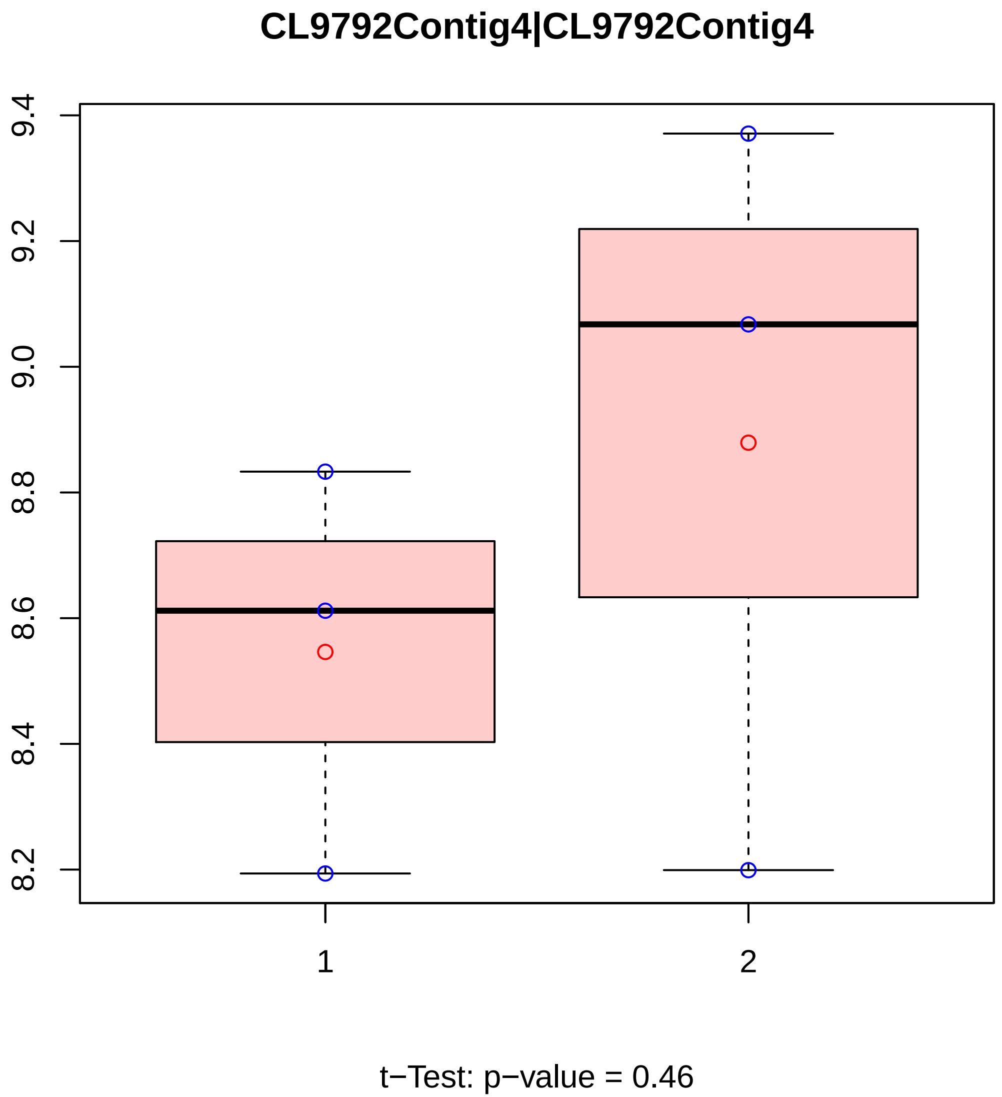
CL9792Contig4|CL9792Contig4
9.4
9.2
9.0
8.8
8.6
8.4
8.2
1
2
t−Test: p−value = 0.46

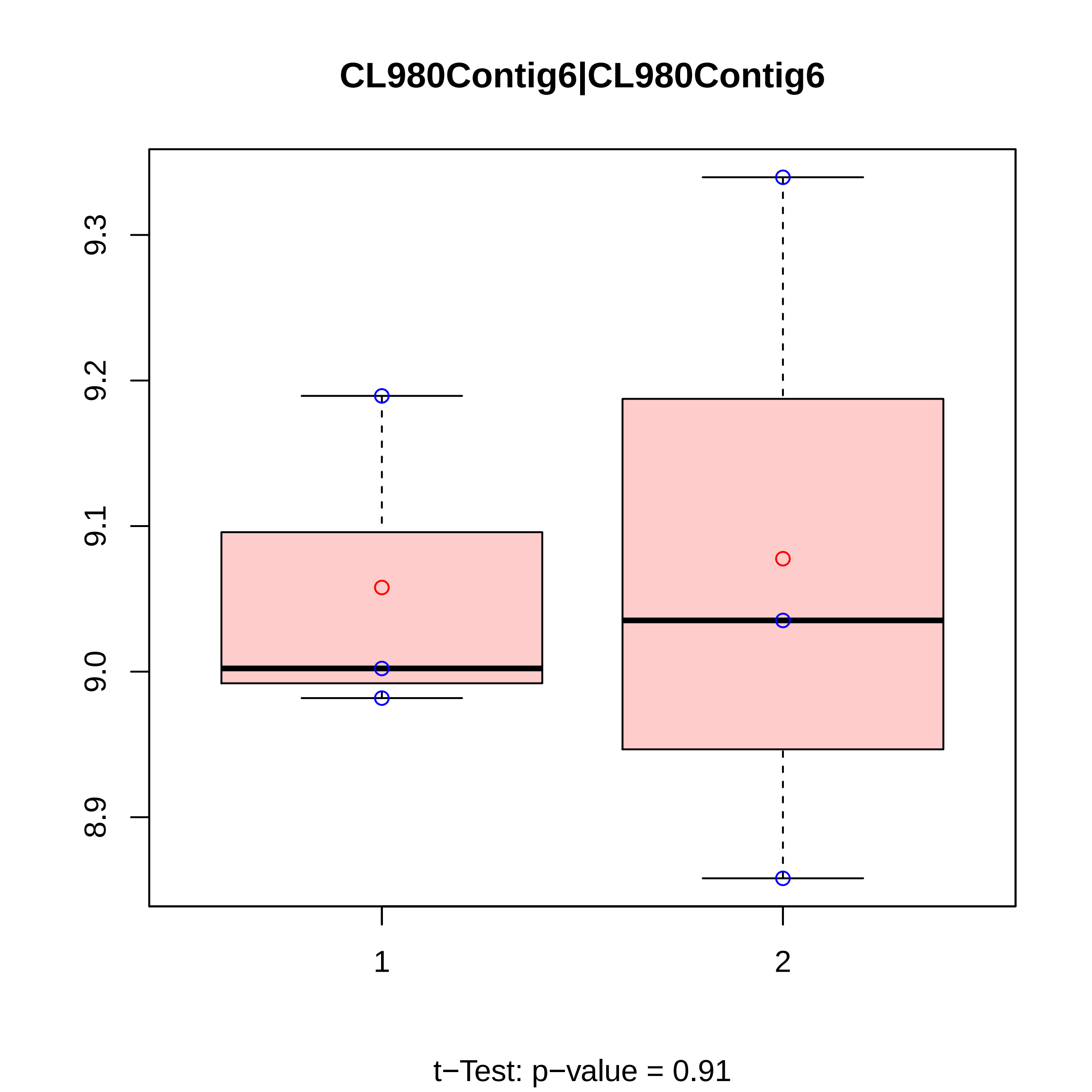

CL980Contig6|CL980Contig6
9.3
9.2
9.1
9.0
8.9
1
2
t−Test: p−value = 0.91

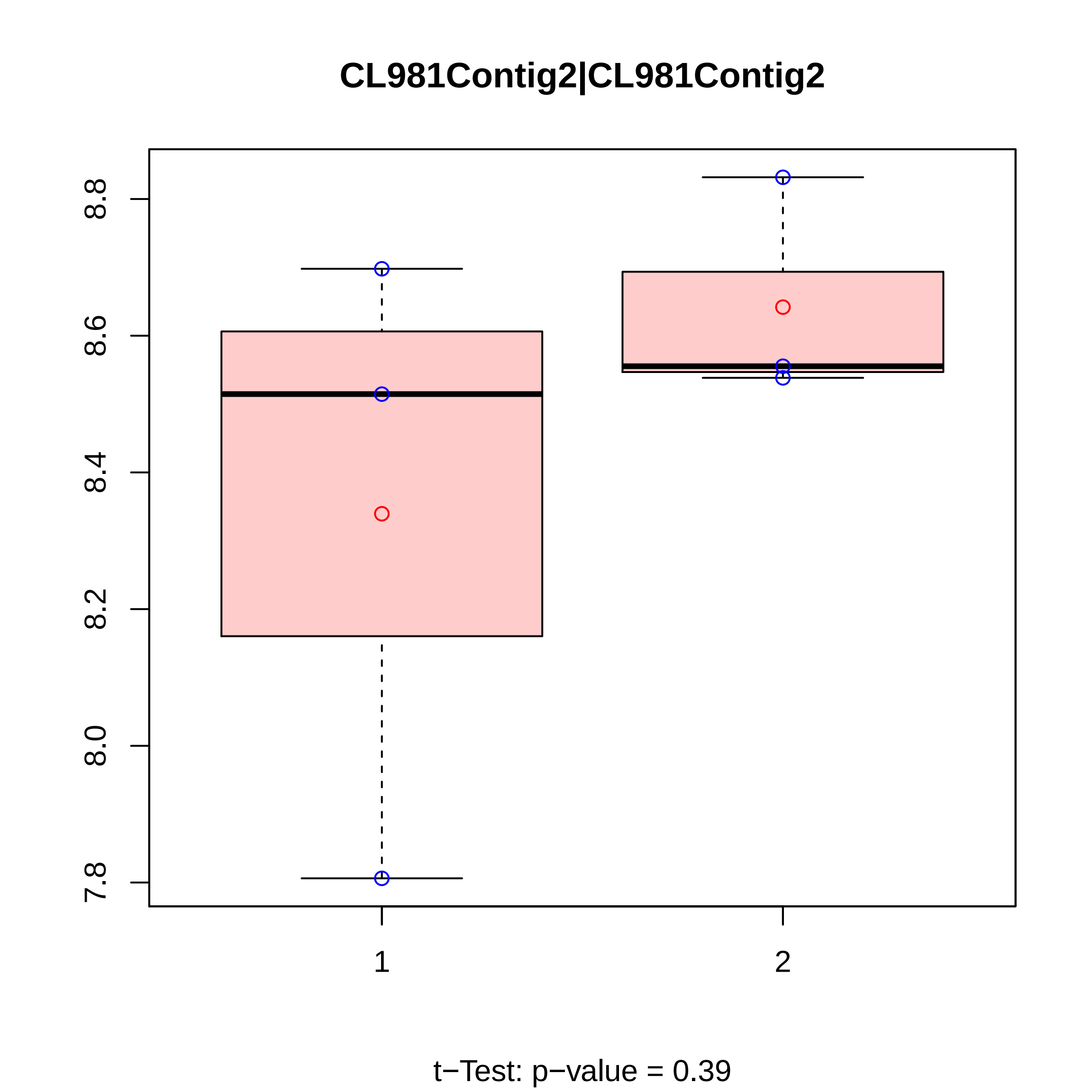
CL981Contig2|CL981Contig2
8.8
8.6
8.4
8.2
8.0
7.8
1
2
t−Test: p−value = 0.39

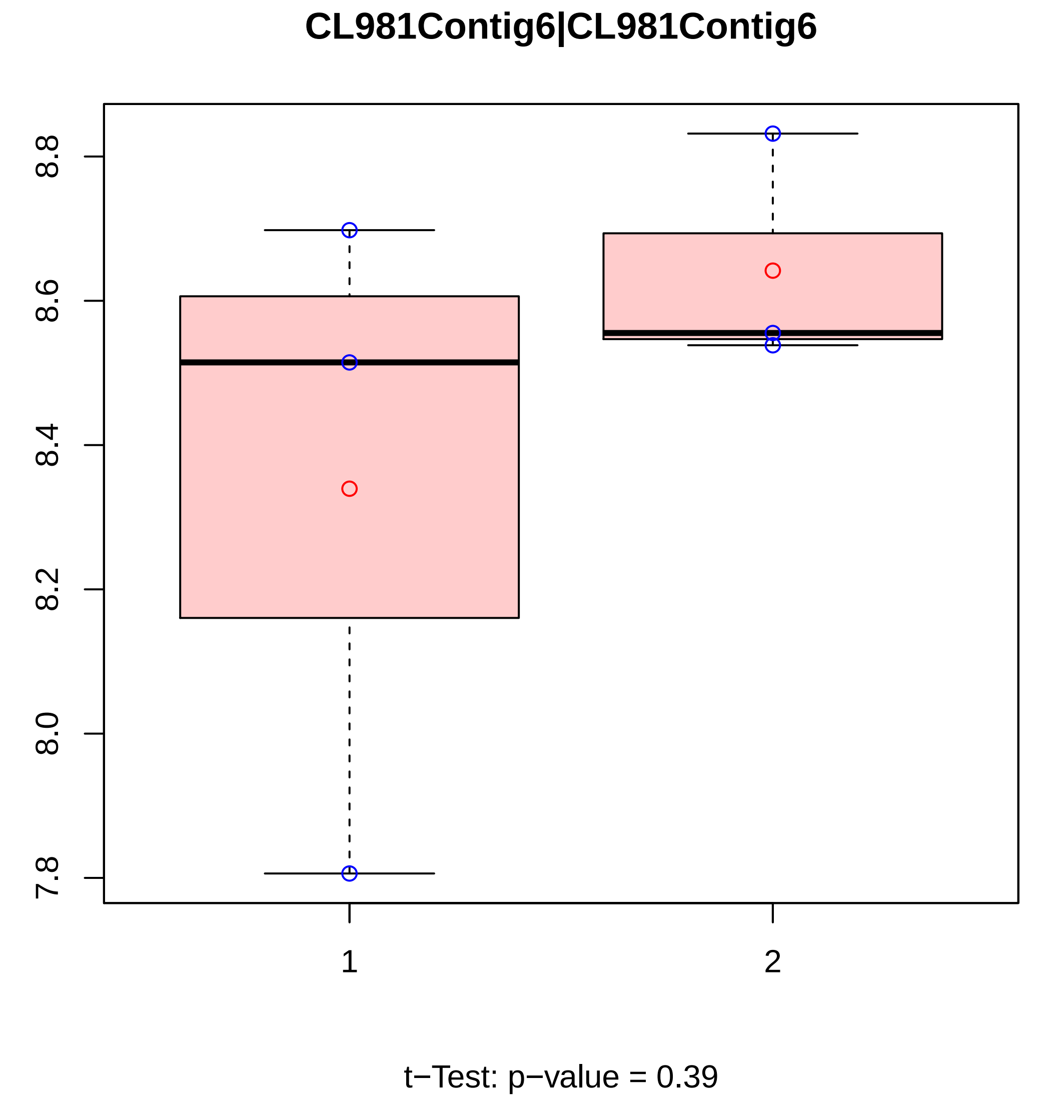

CL981Contig6|CL981Contig6
8.8
8.6
8.4
8.2
8.0
7.8
1
2
t−Test: p−value = 0.39

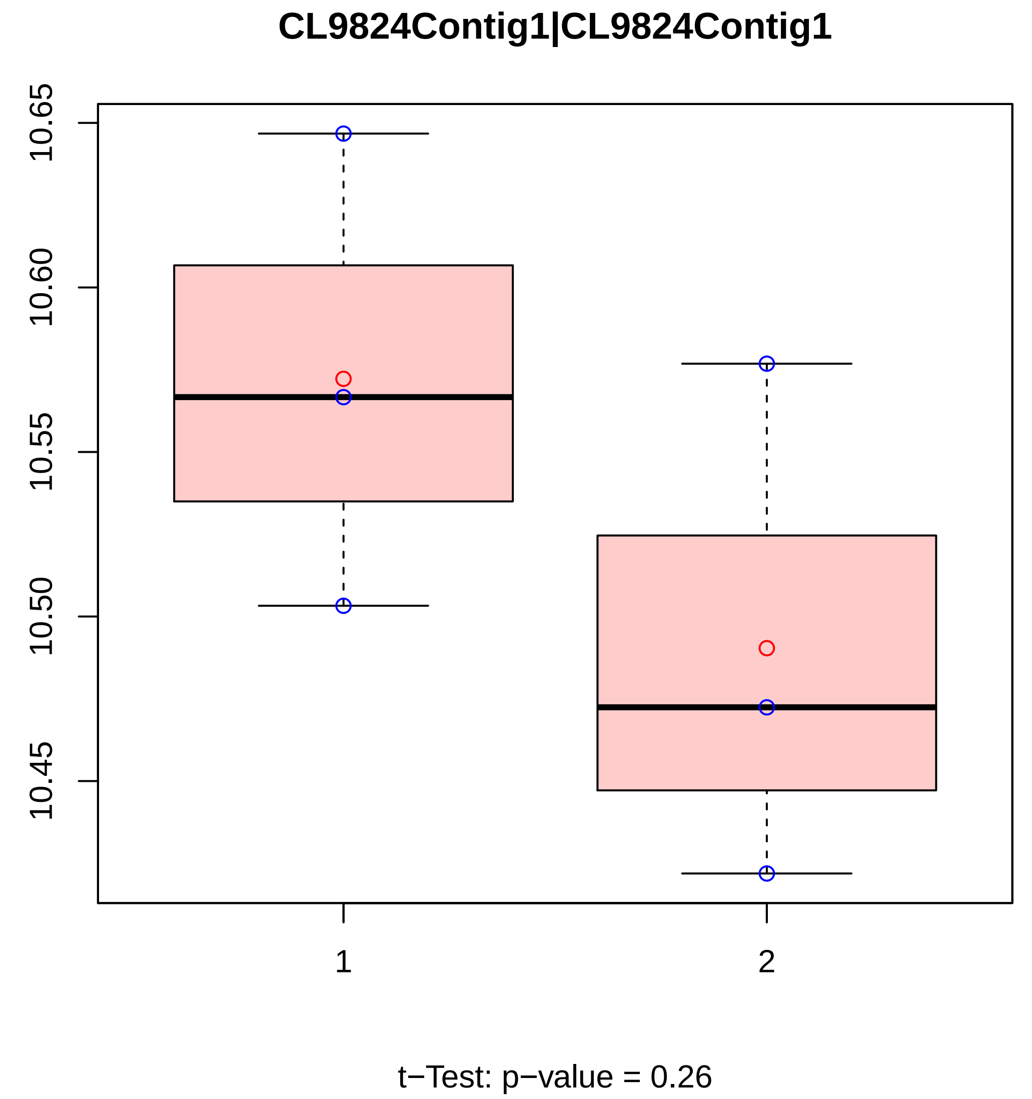
CL9824Contig1|CL9824Contig1
10.65
10.60
10.55
10.50
10.45
1
2
t−Test: p−value = 0.26

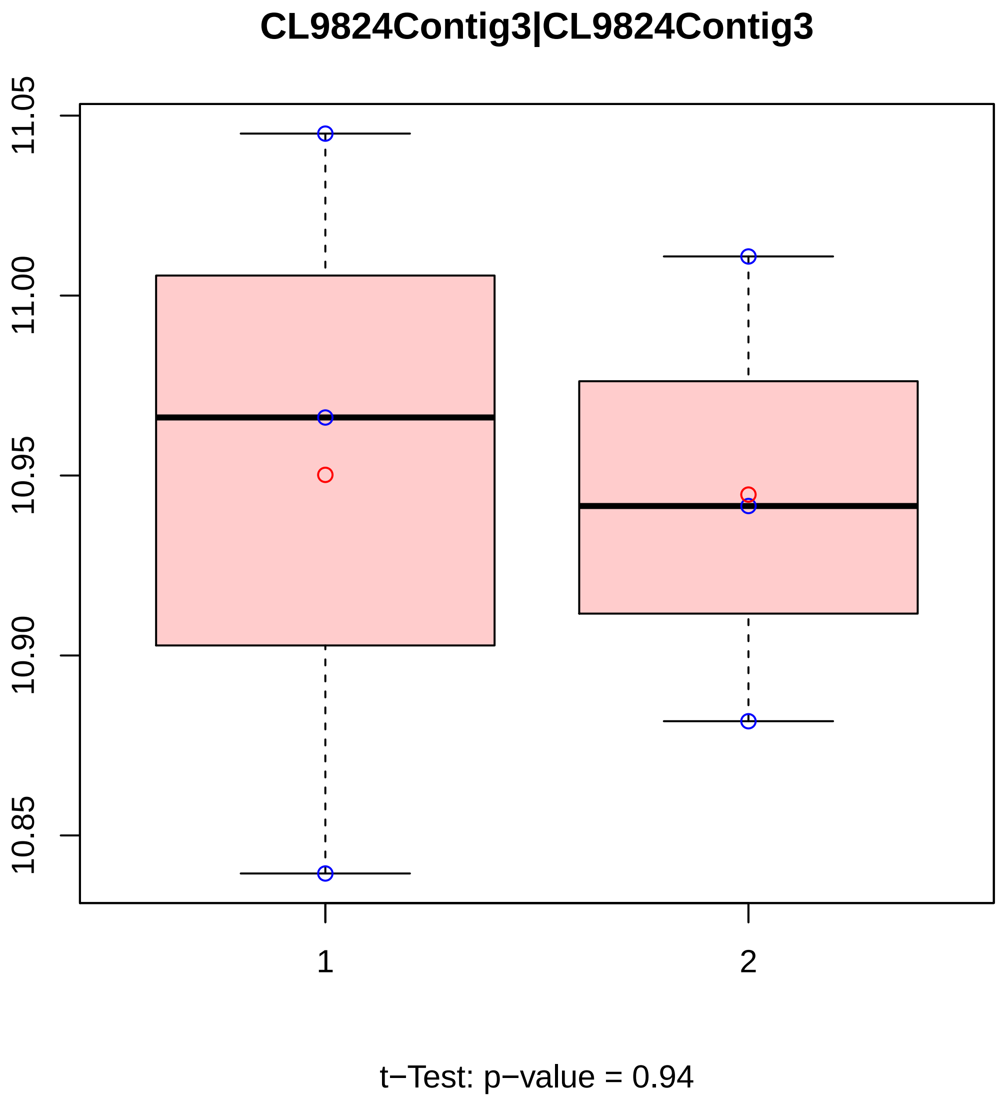

CL9824Contig3|CL9824Contig3
11.05
11.00
10.95
10.90
10.85
1
2
t−Test: p−value = 0.94

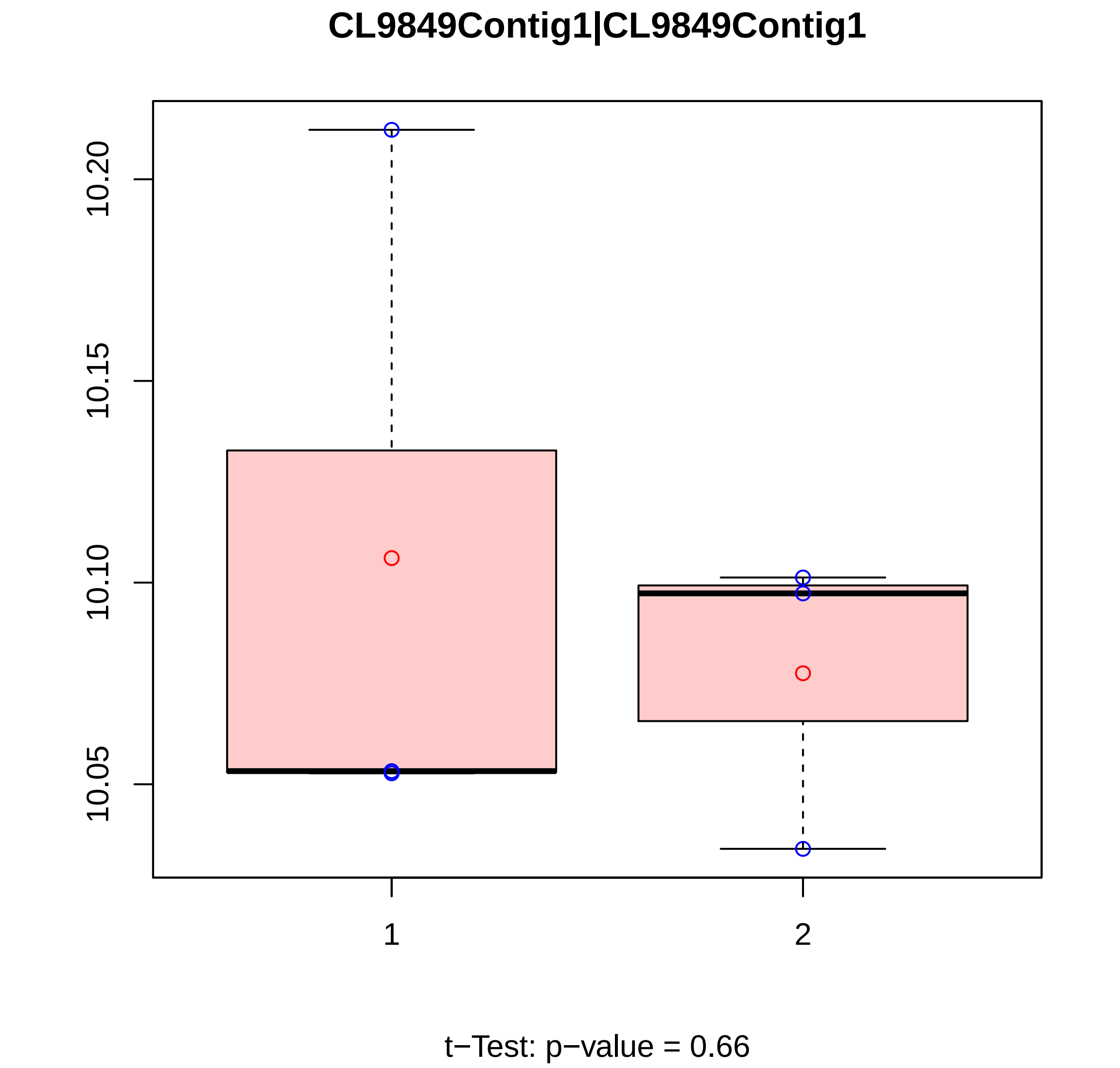
CL9849Contig1|CL9849Contig1
10.20
10.15
10.10
10.05
1
2
t−Test: p−value = 0.66

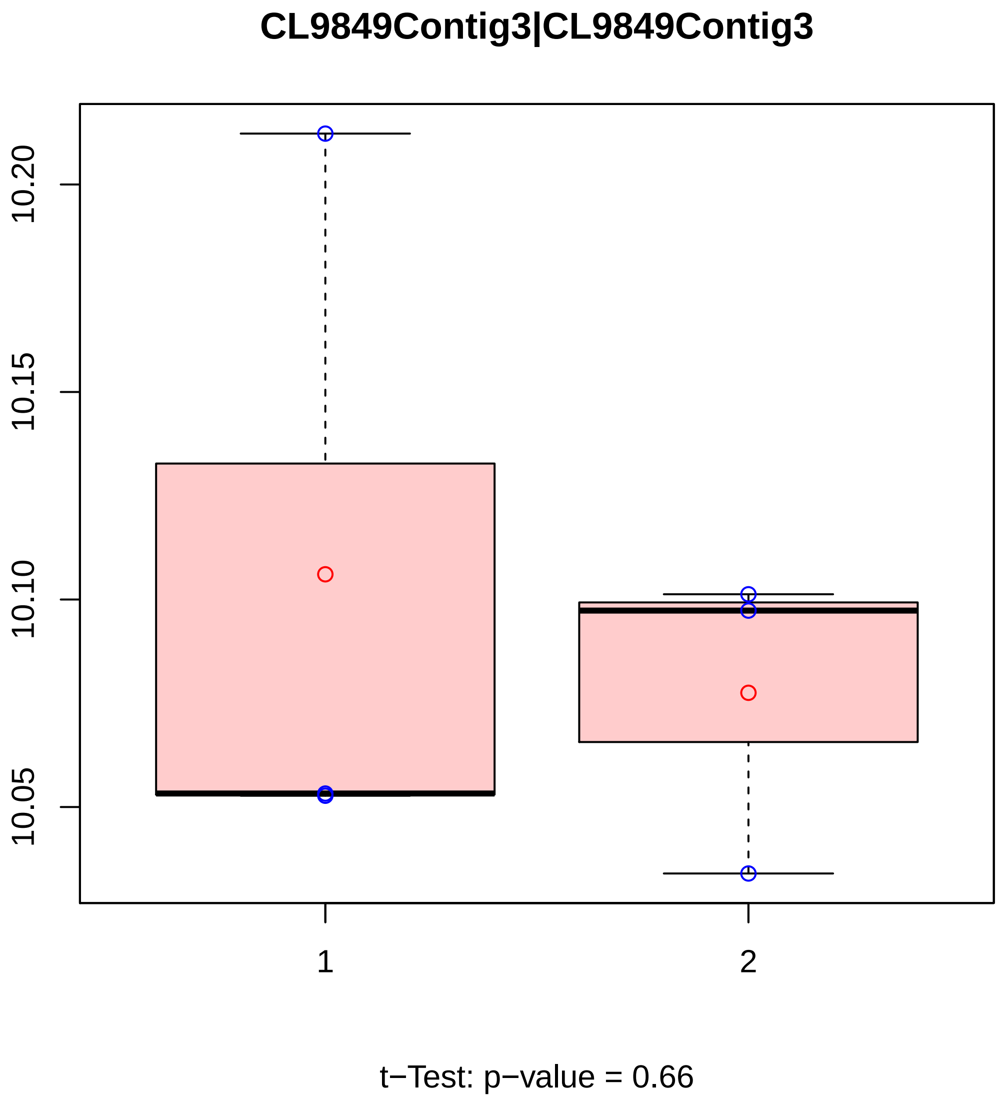

CL9849Contig3|CL9849Contig3
10.05
10.10
10.15
10.20
1
2
t−Test: p−value = 0.66

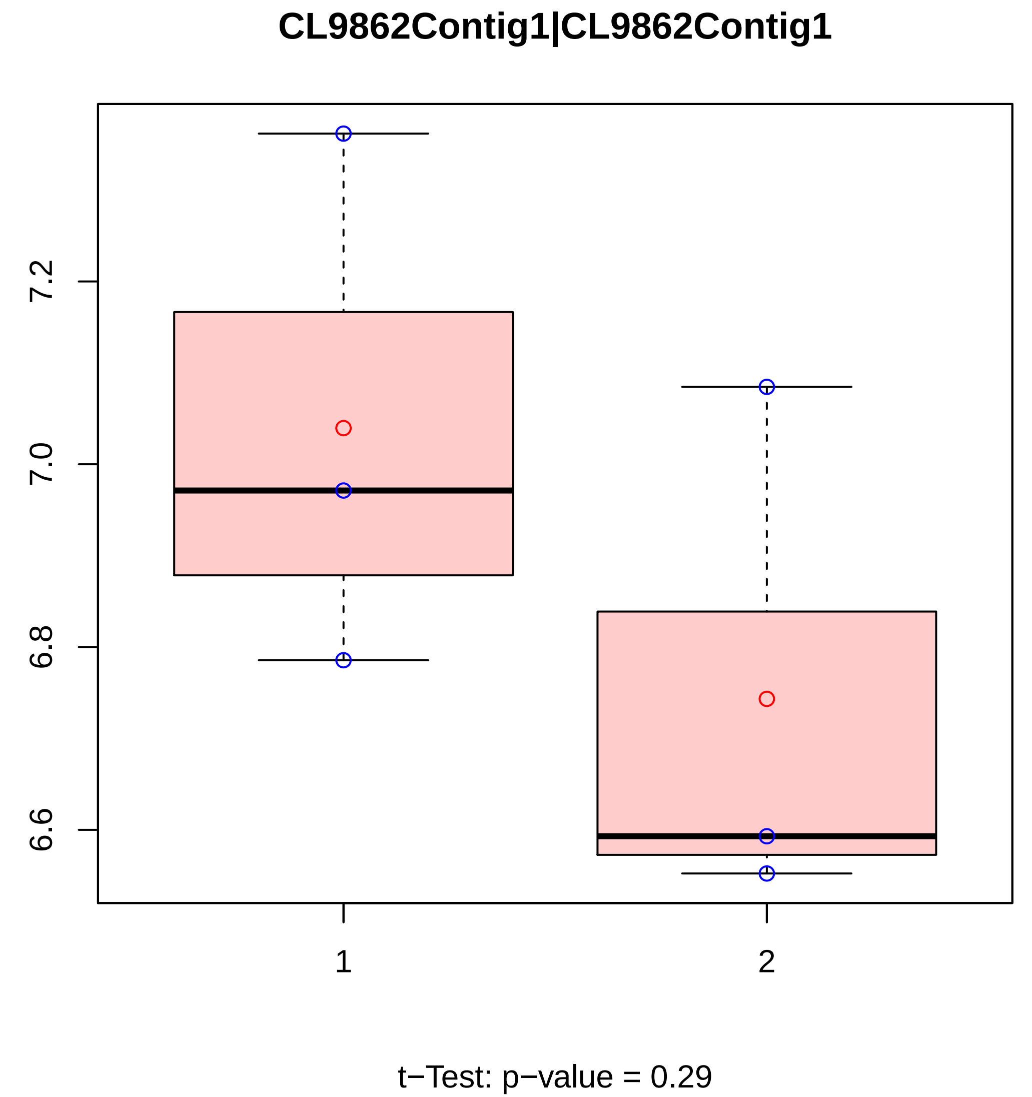
CL9862Contig1|CL9862Contig1
7.2
7.0
6.8
6.6
1
2
t−Test: p−value = 0.29

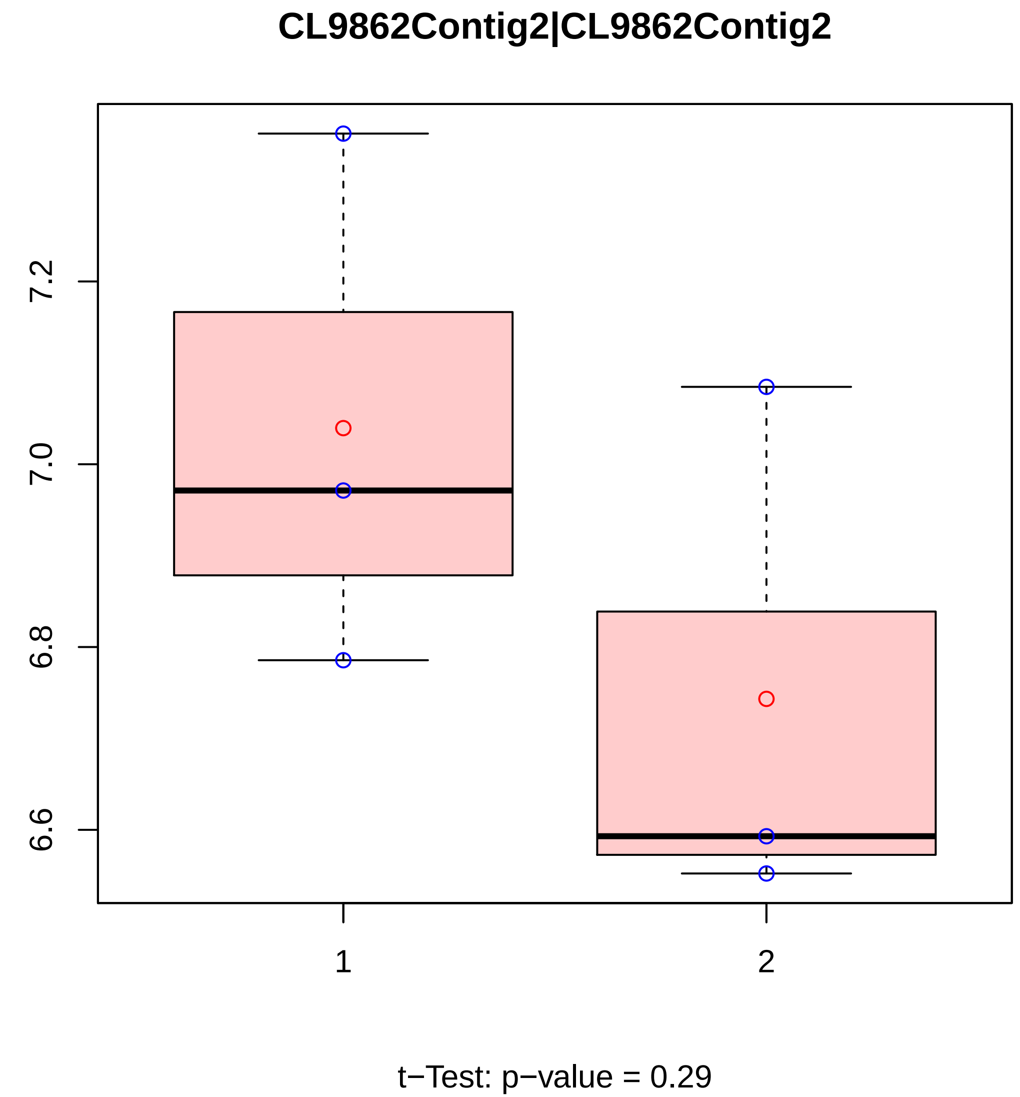
CL9862Contig2|CL9862Contig2
7.2
7.0
6.8
6.6
1
2
t−Test: p−value = 0.29

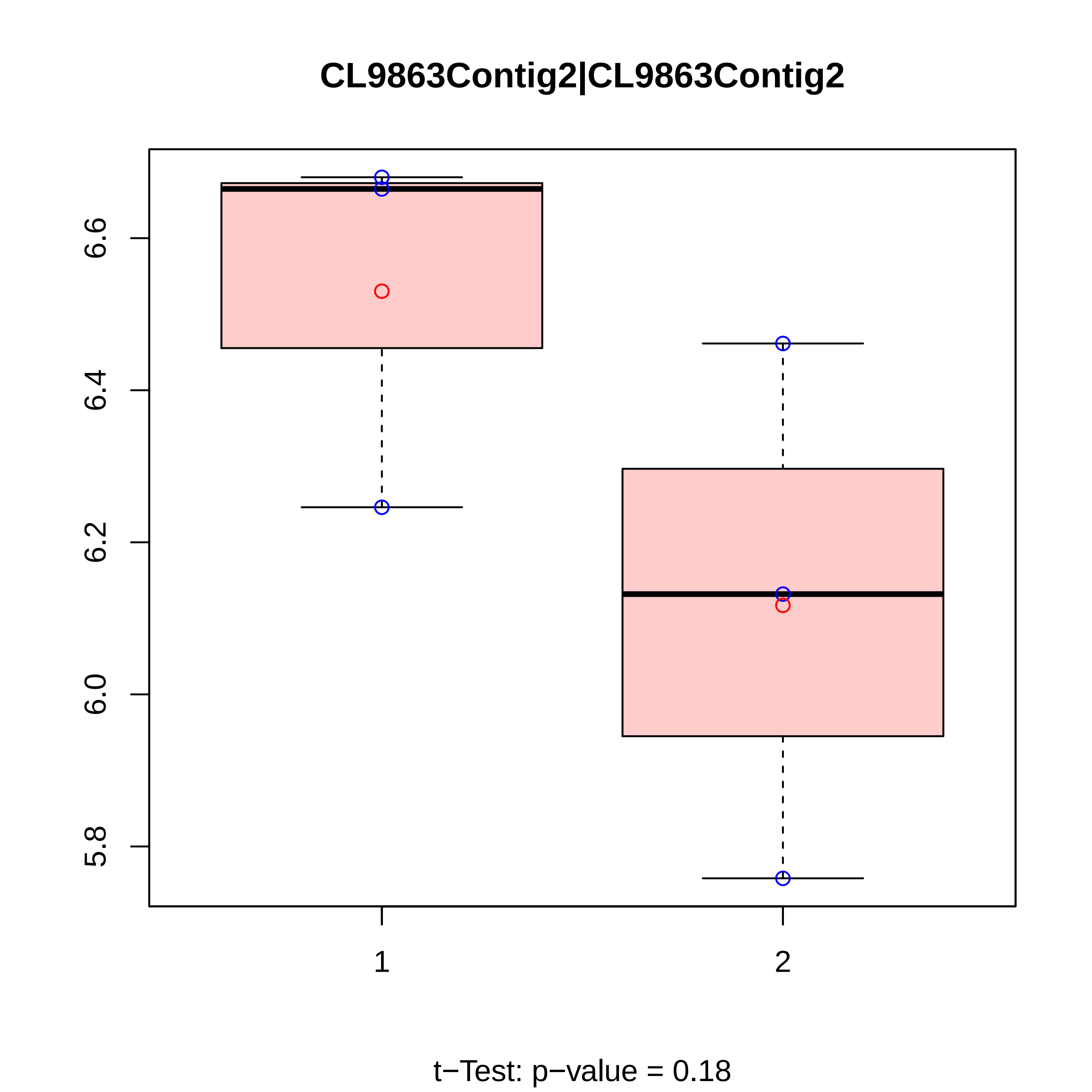
CL9863Contig2|CL9863Contig2
6.6
6.4
6.2
6.0
5.8
1
2
t−Test: p−value = 0.18

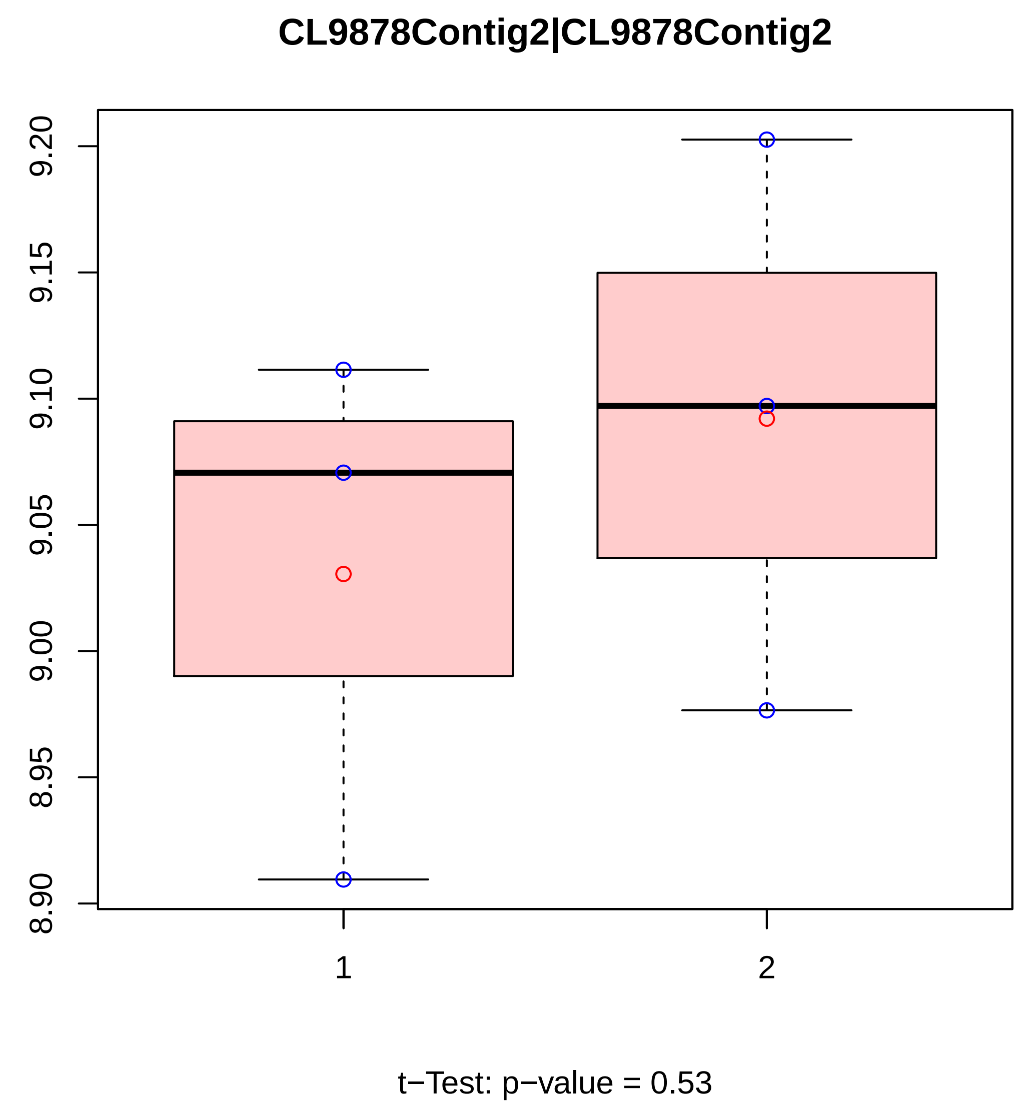
CL9878Contig2|CL9878Contig2
9.20
9.15
9.10
9.05
9.00
8.95
8.90
1
2
t−Test: p−value = 0.53

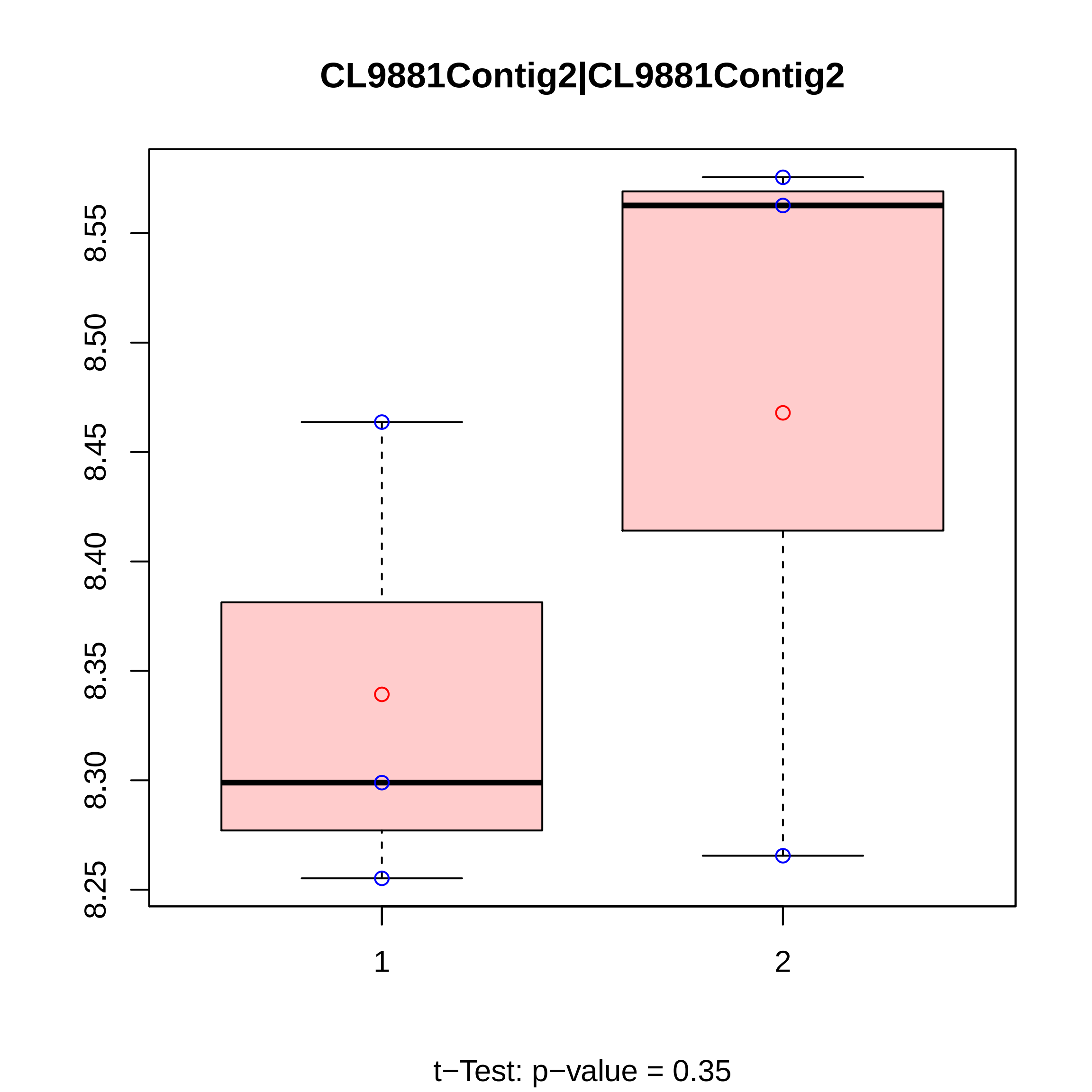
CL9881Contig2|CL9881Contig2
8.25
8.30
8.35
8.40
8.45
8.50
8.55
1
2
t−Test: p−value = 0.35

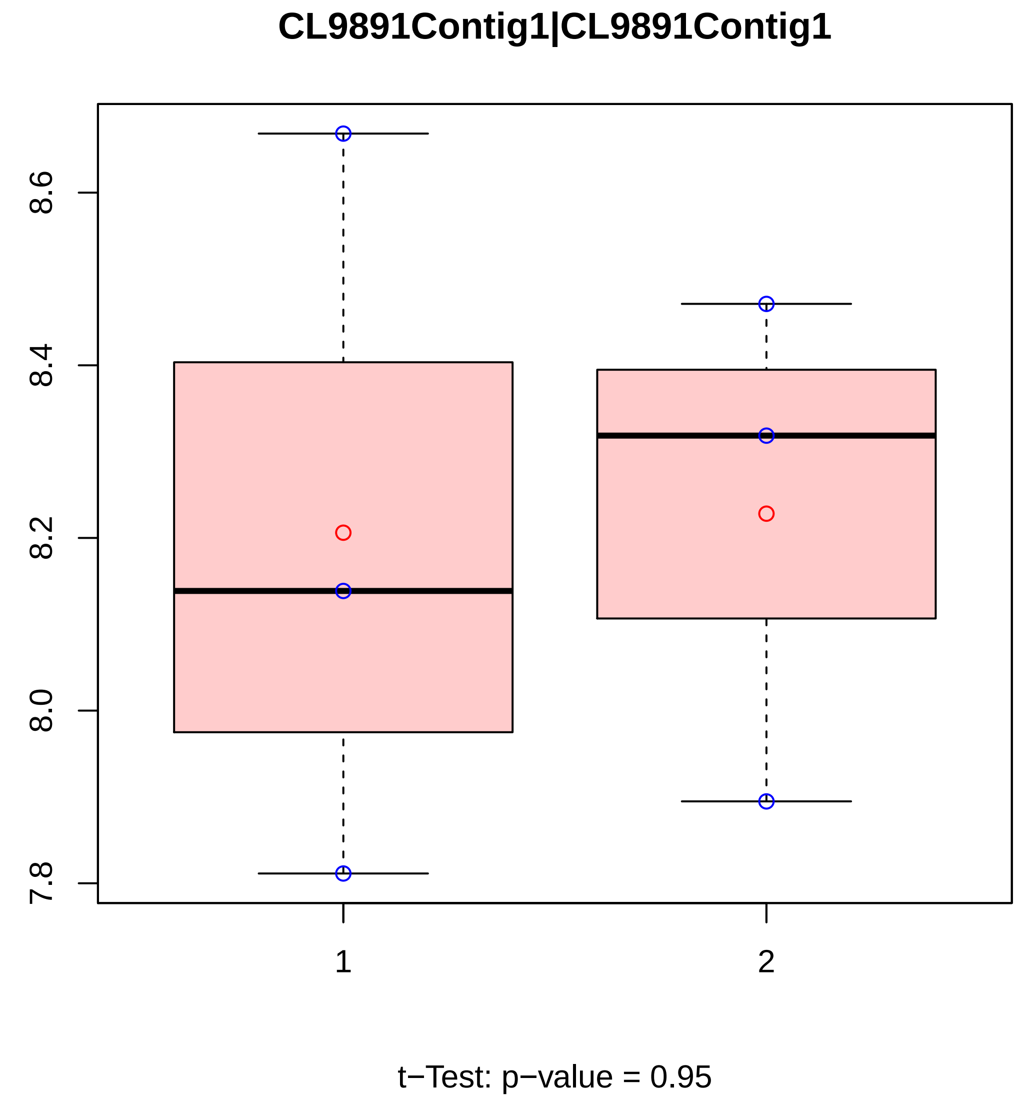

CL9891Contig1|CL9891Contig1
8.6
8.4
8.2
8.0
7.8
1
2
t−Test: p−value = 0.95

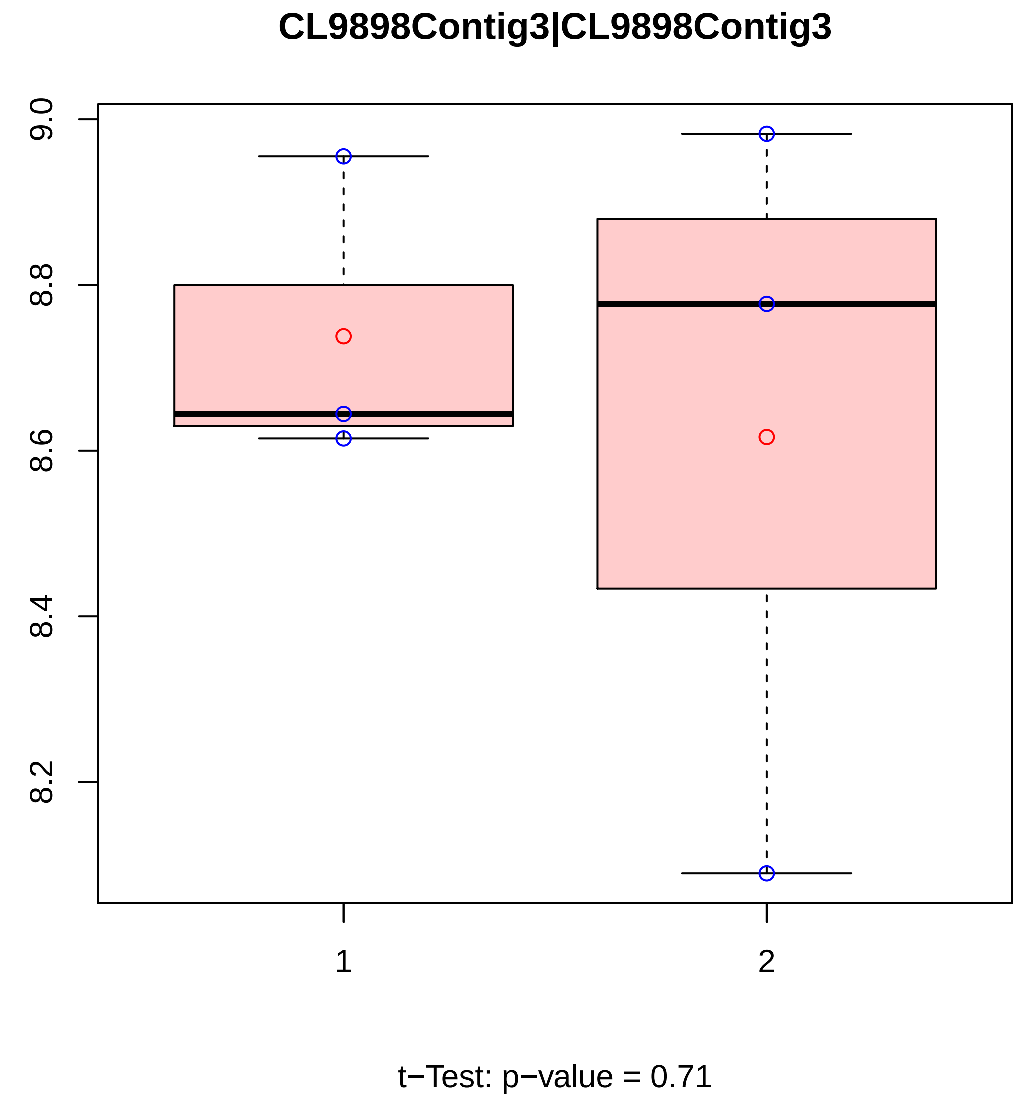
CL9898Contig3|CL9898Contig3
9.0
8.8
8.6
8.4
8.2
1
2
t−Test: p−value = 0.71

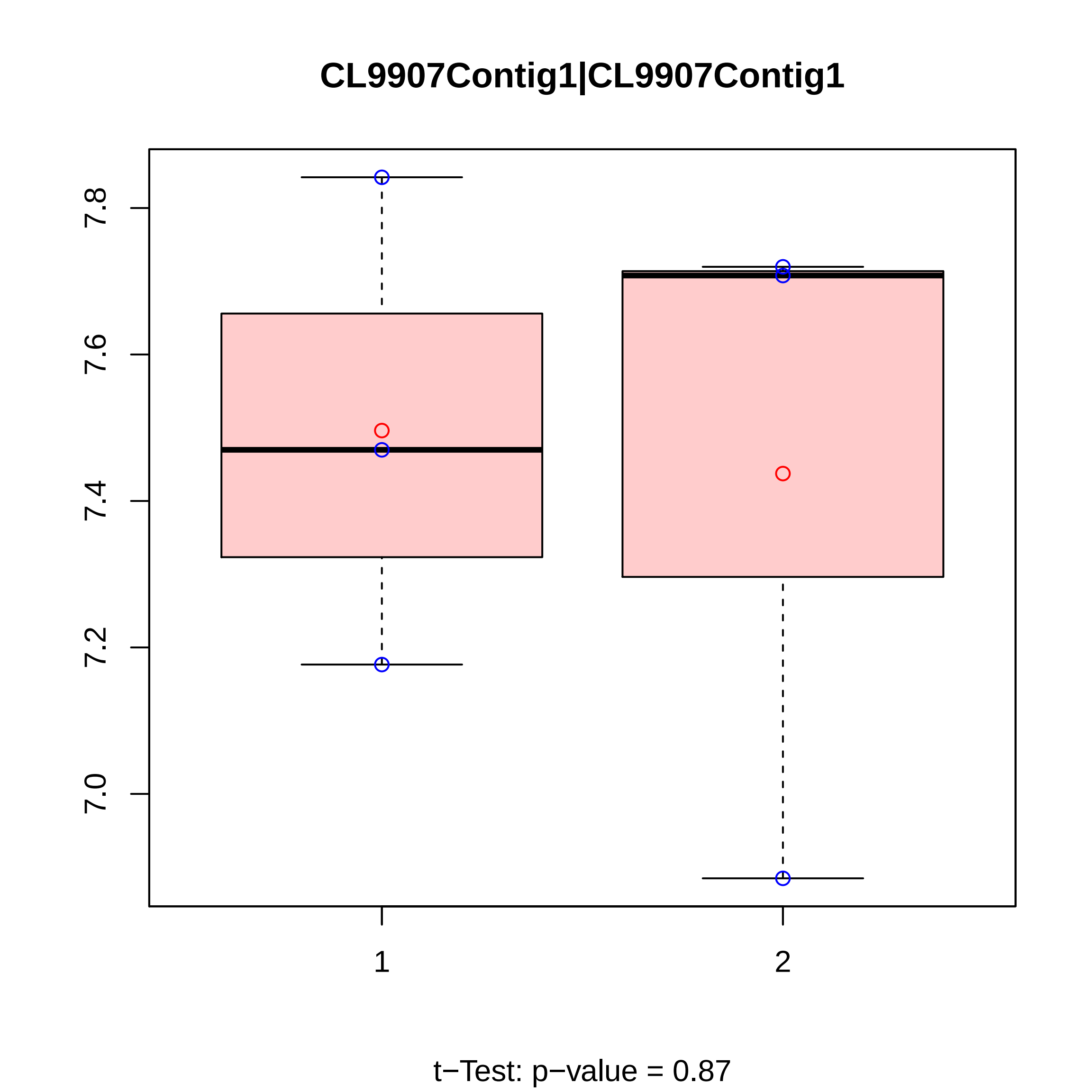

CL9907Contig1|CL9907Contig1
7.0
7.2
7.4
7.6
7.8
1
2
t−Test: p−value = 0.87

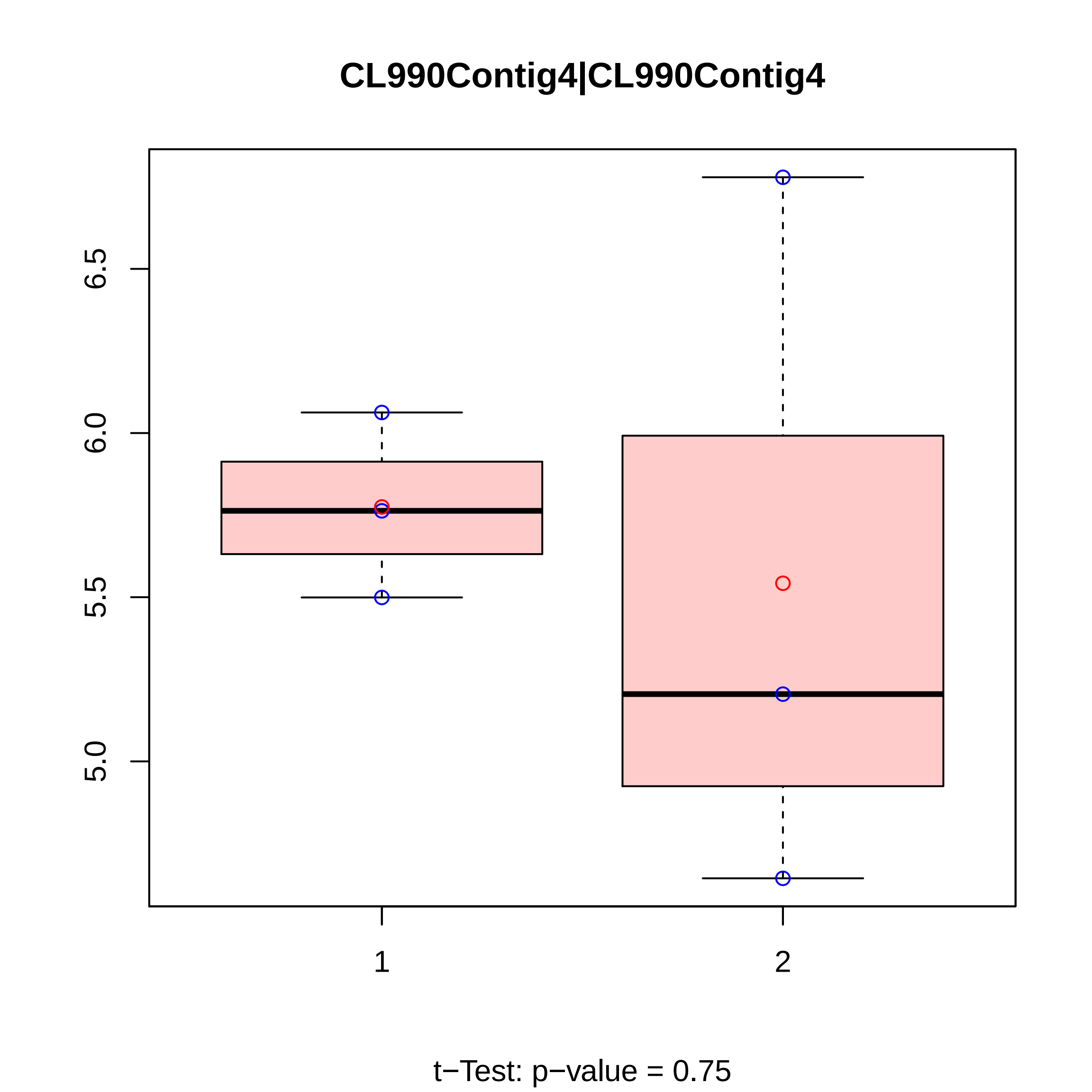
CL990Contig4|CL990Contig4
6.5
6.0
5.5
5.0
1
2
t−Test: p−value = 0.75

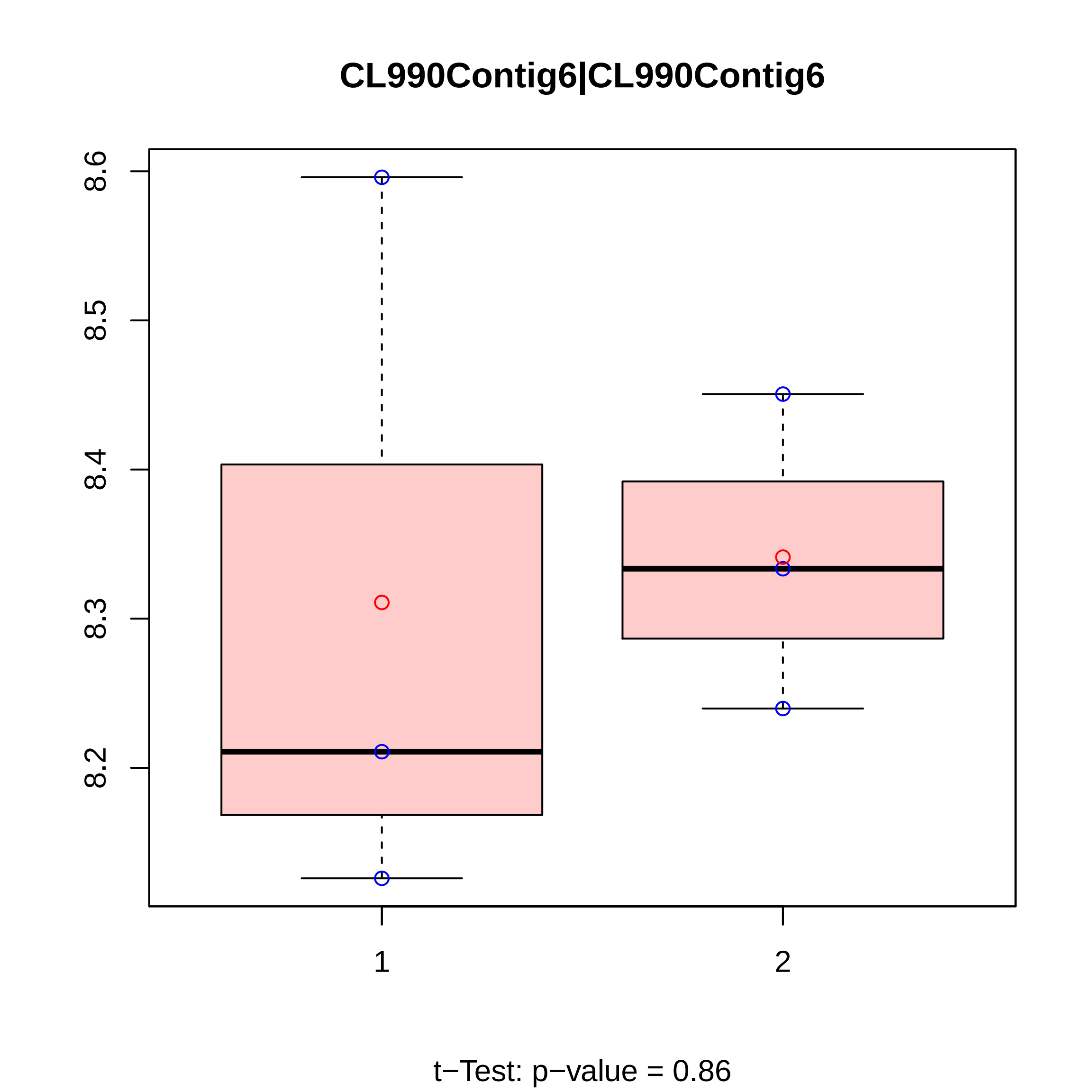
CL990Contig6|CL990Contig6
8.2
8.3
8.4
8.5
8.6
1
2
t−Test: p−value = 0.86

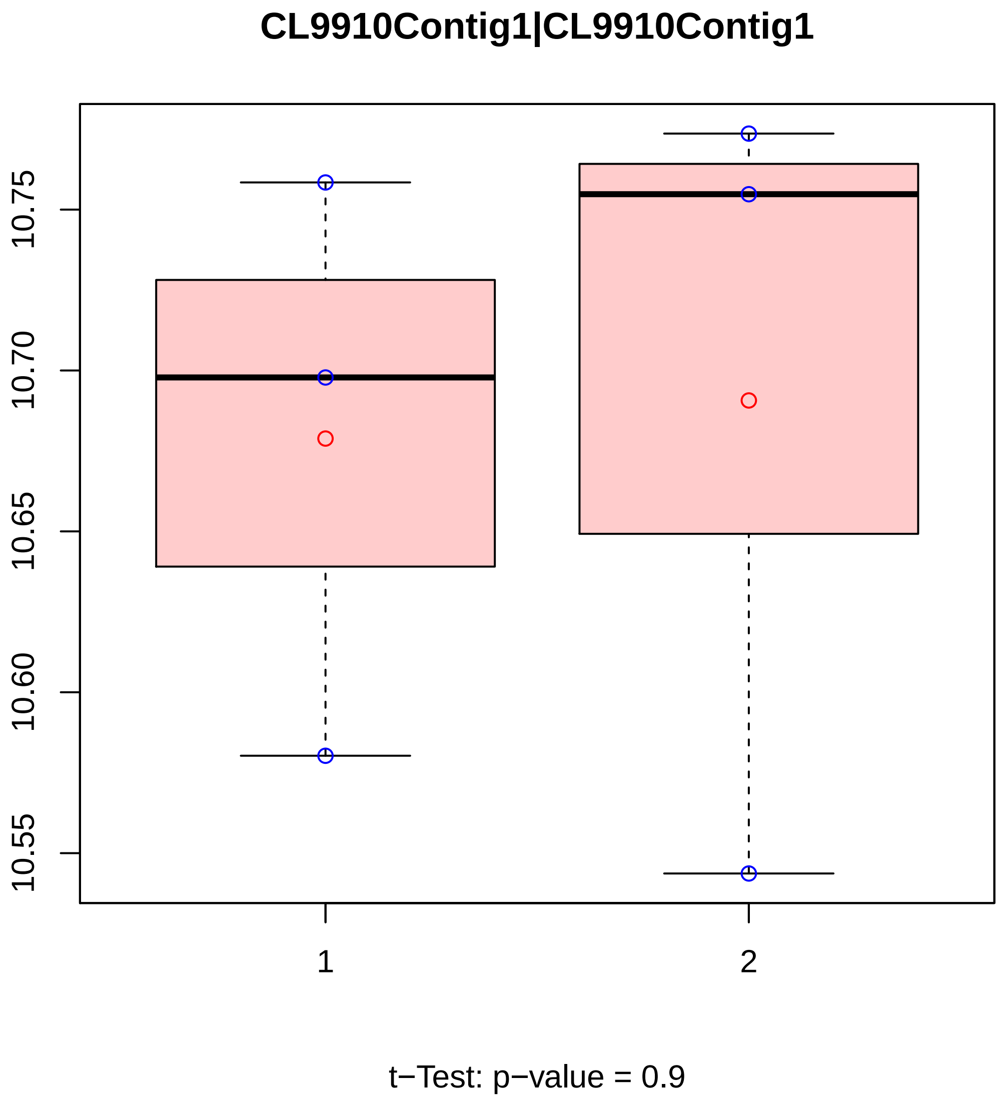
CL9910Contig1|CL9910Contig1
10.55
10.60
10.65
10.70
10.75
1
2
t−Test: p−value = 0.9

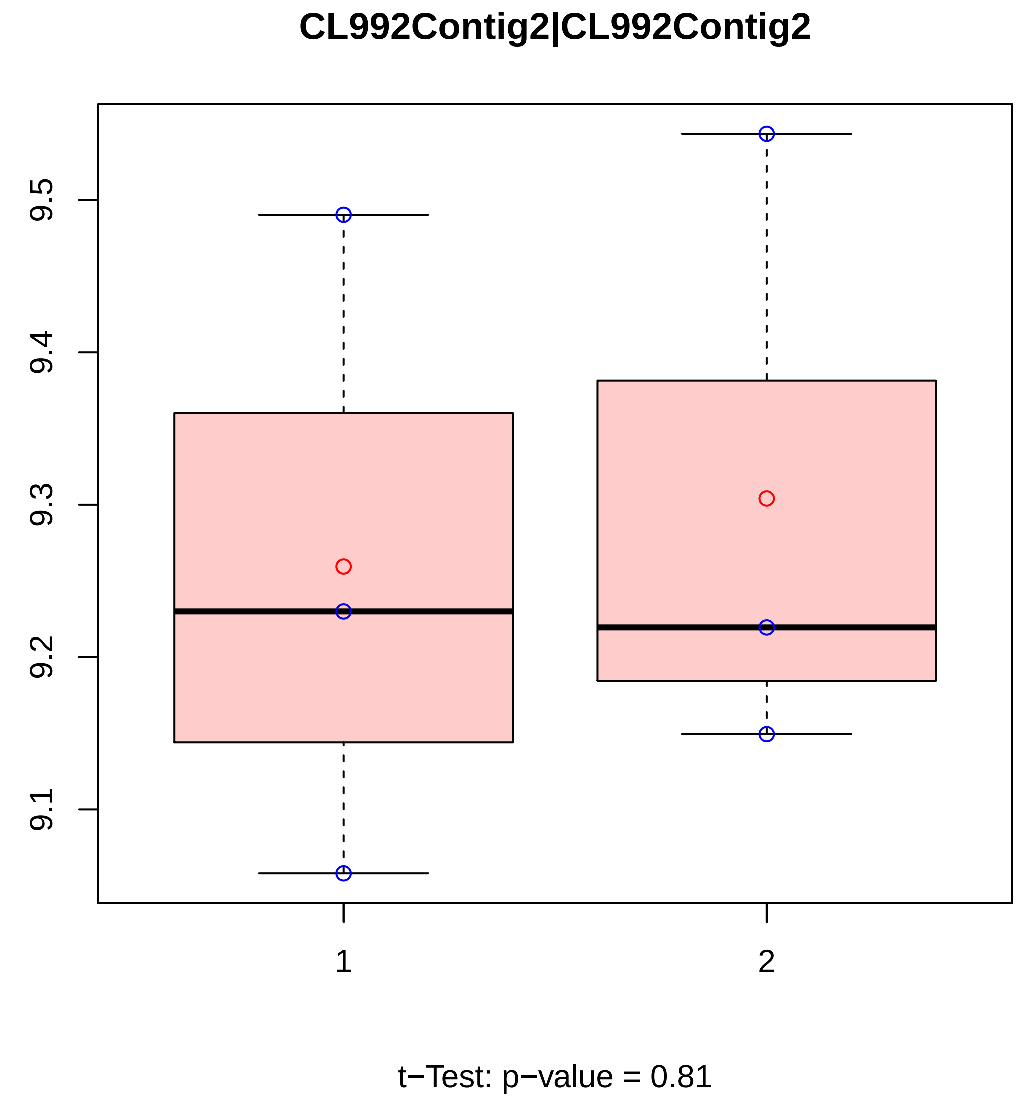
CL992Contig2|CL992Contig2
9.1
9.2
9.3
9.4
9.5
1
2
t−Test: p−value = 0.81

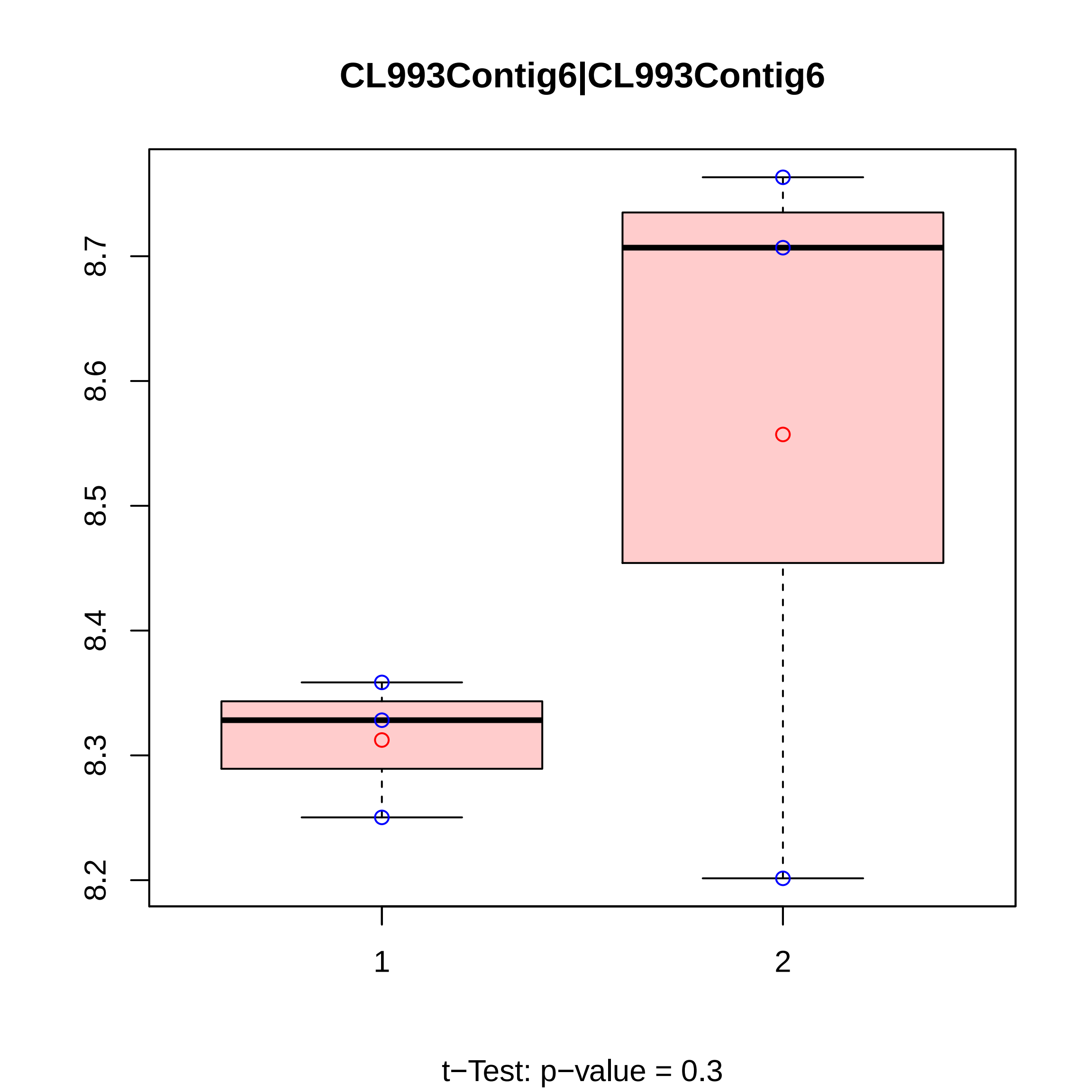
CL993Contig6|CL993Contig6
8.2
8.3
8.4
8.5
8.6
8.7
1
2
t−Test: p−value = 0.3

# CL993Contig9|CL993Contig9

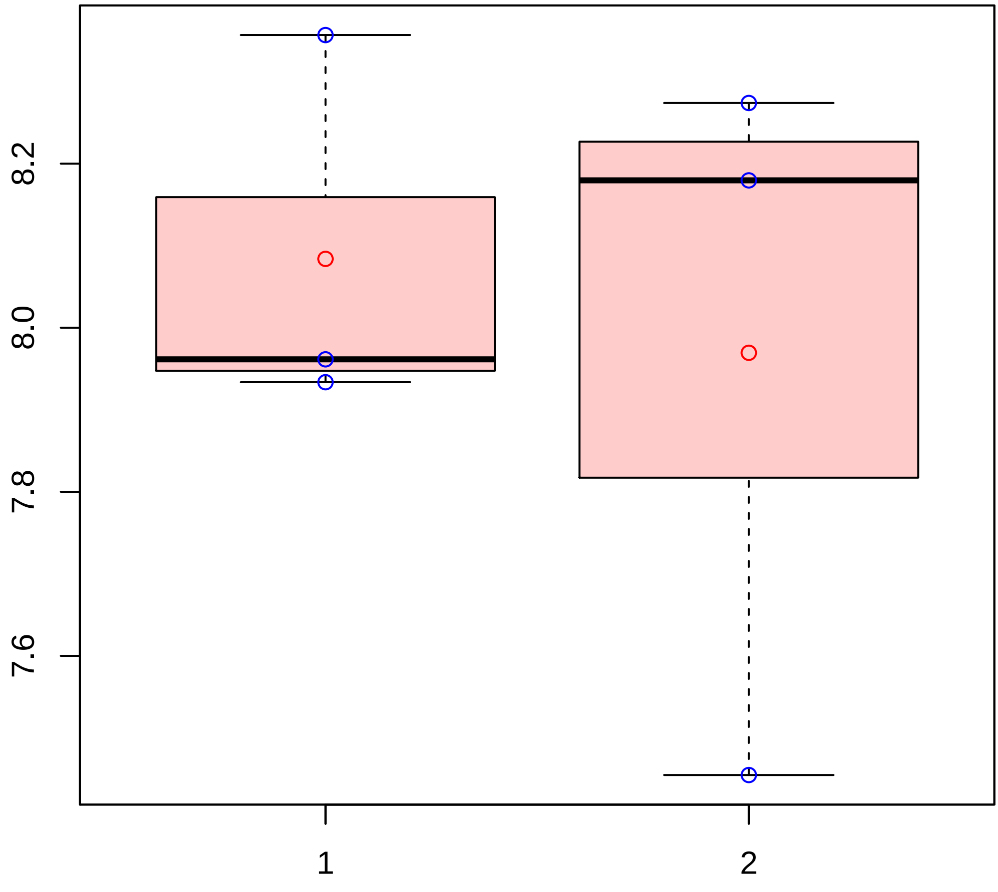


t−Test: p−value = 0.72

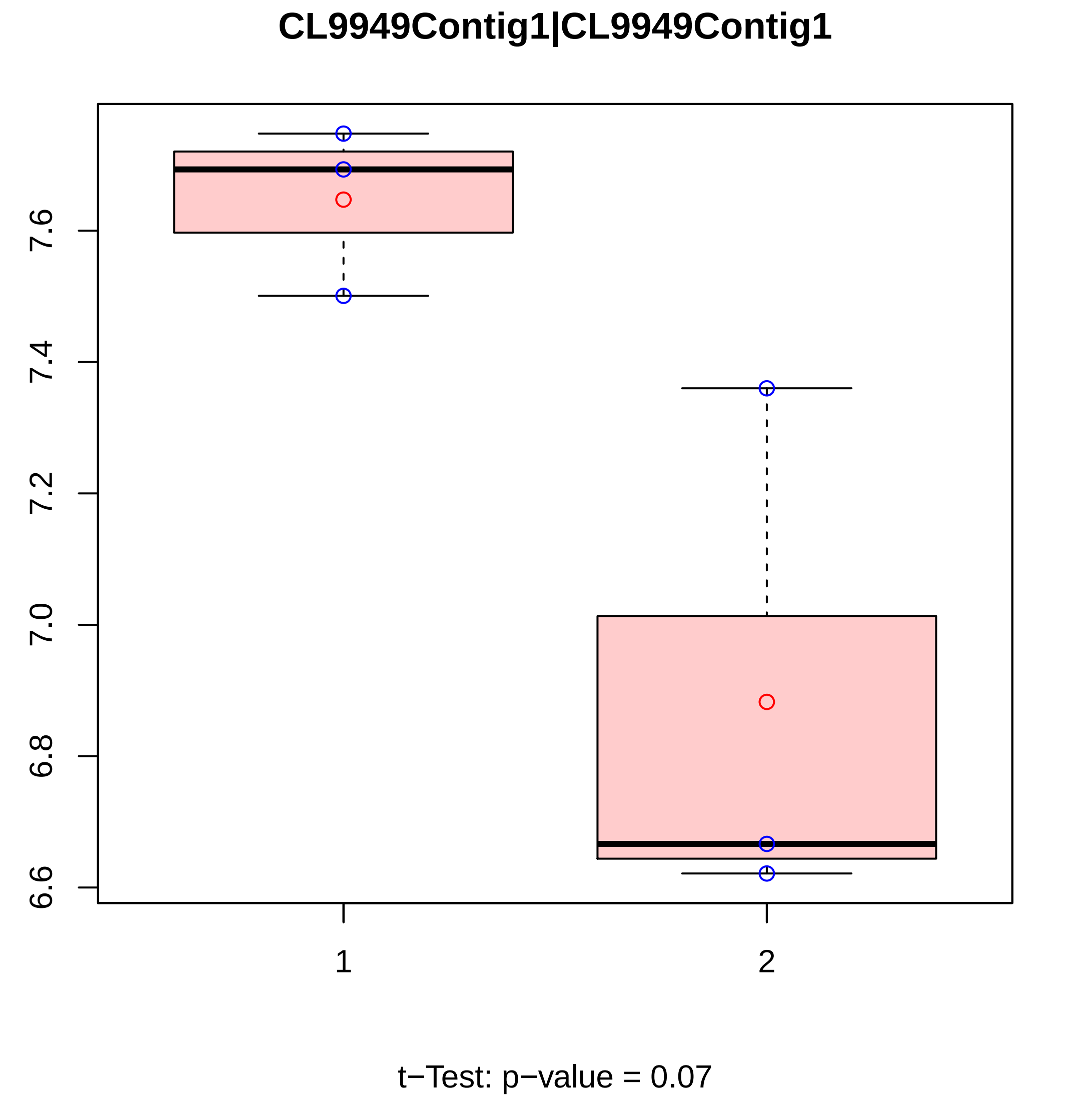

CL9949Contig1|CL9949Contig1
7.6
7.4
7.2
7.0
6.8
6.6
1
2
t−Test: p−value = 0.07

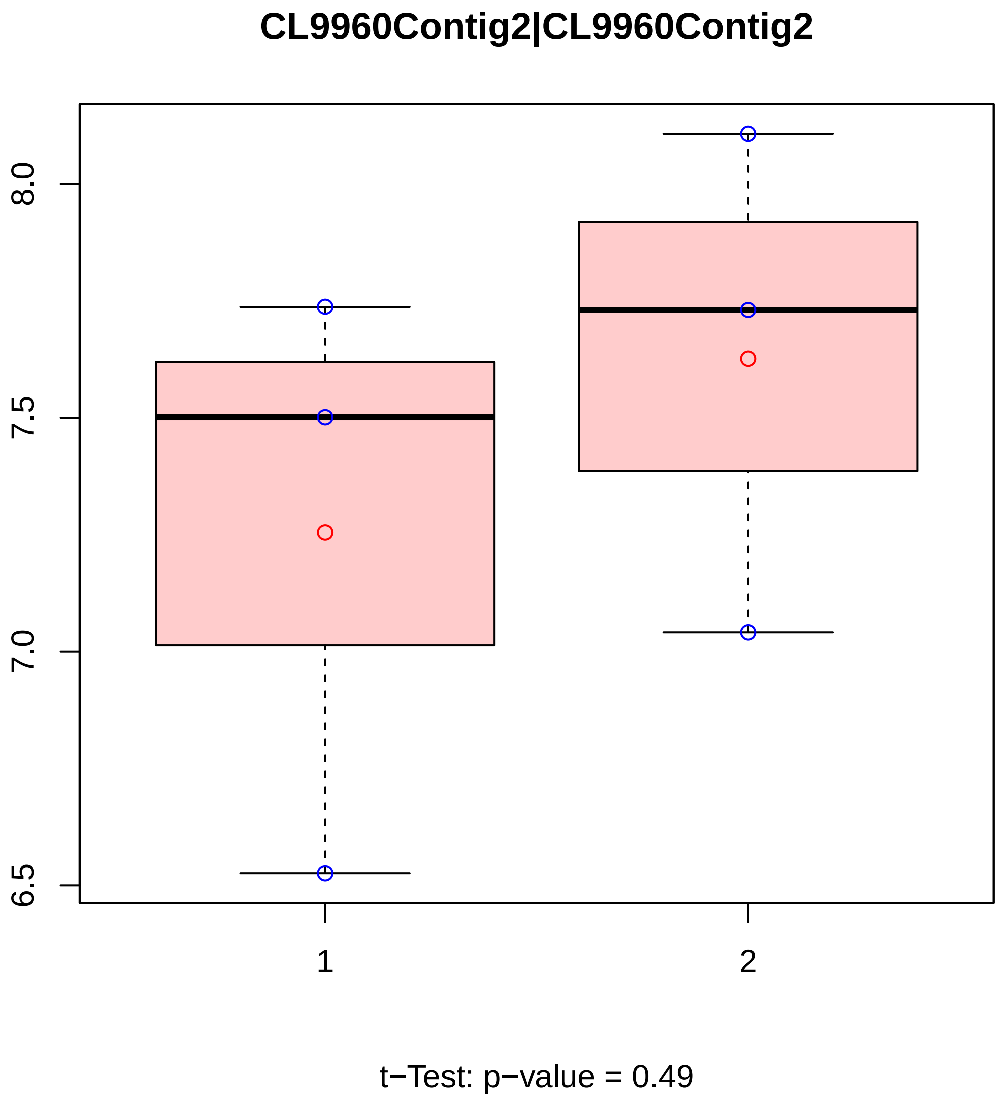
CL9960Contig2|CL9960Contig2
8.0
7.5
7.0
6.5
1
2
t−Test: p−value = 0.49

# CL9963Contig1|CL9963Contig1

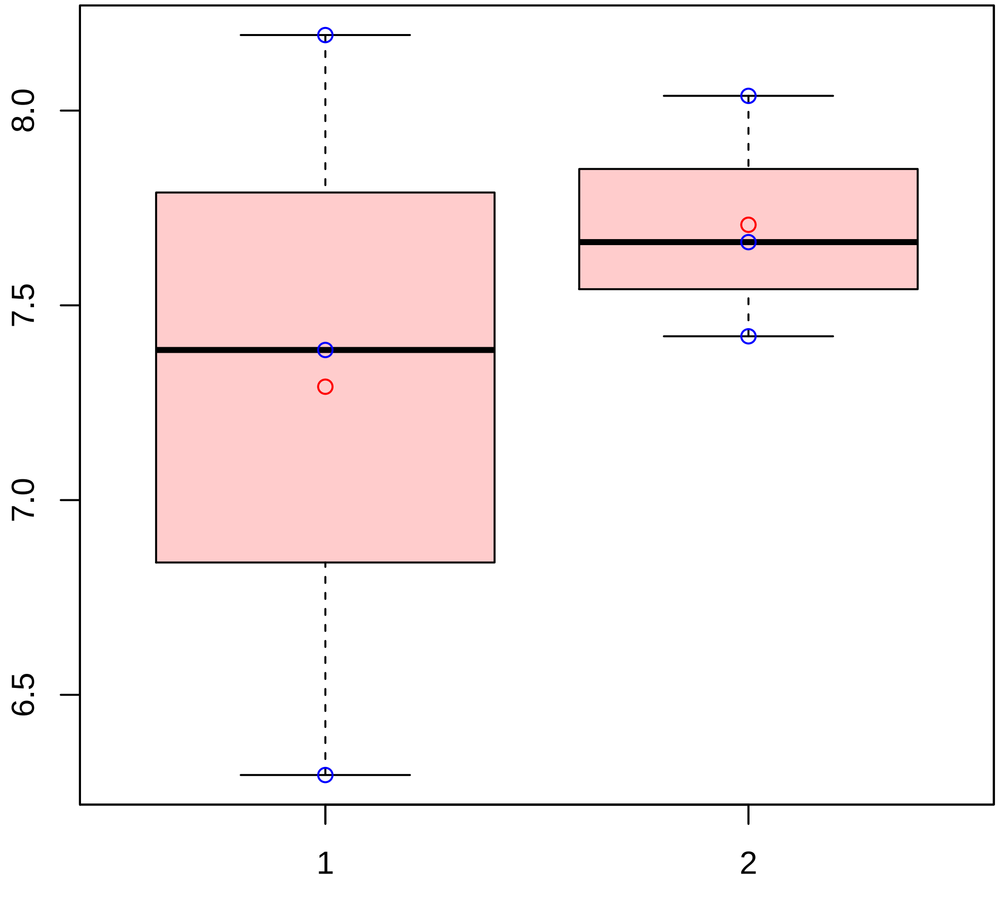


t−Test: p−value = 0.54

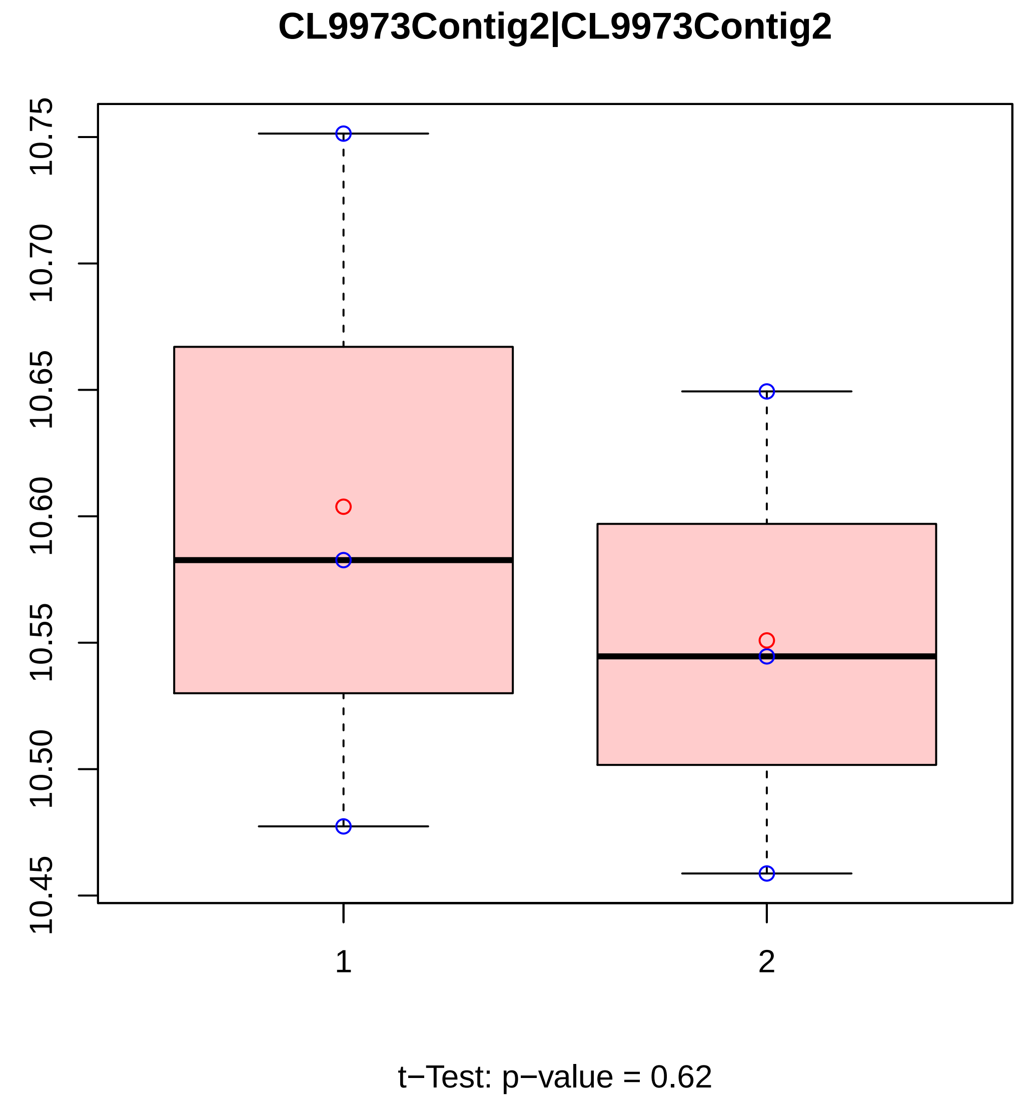
CL9973Contig2|CL9973Contig2
10.45
10.50
10.55
10.60
10.65
10.70
10.75
1
2
t−Test: p−value = 0.62

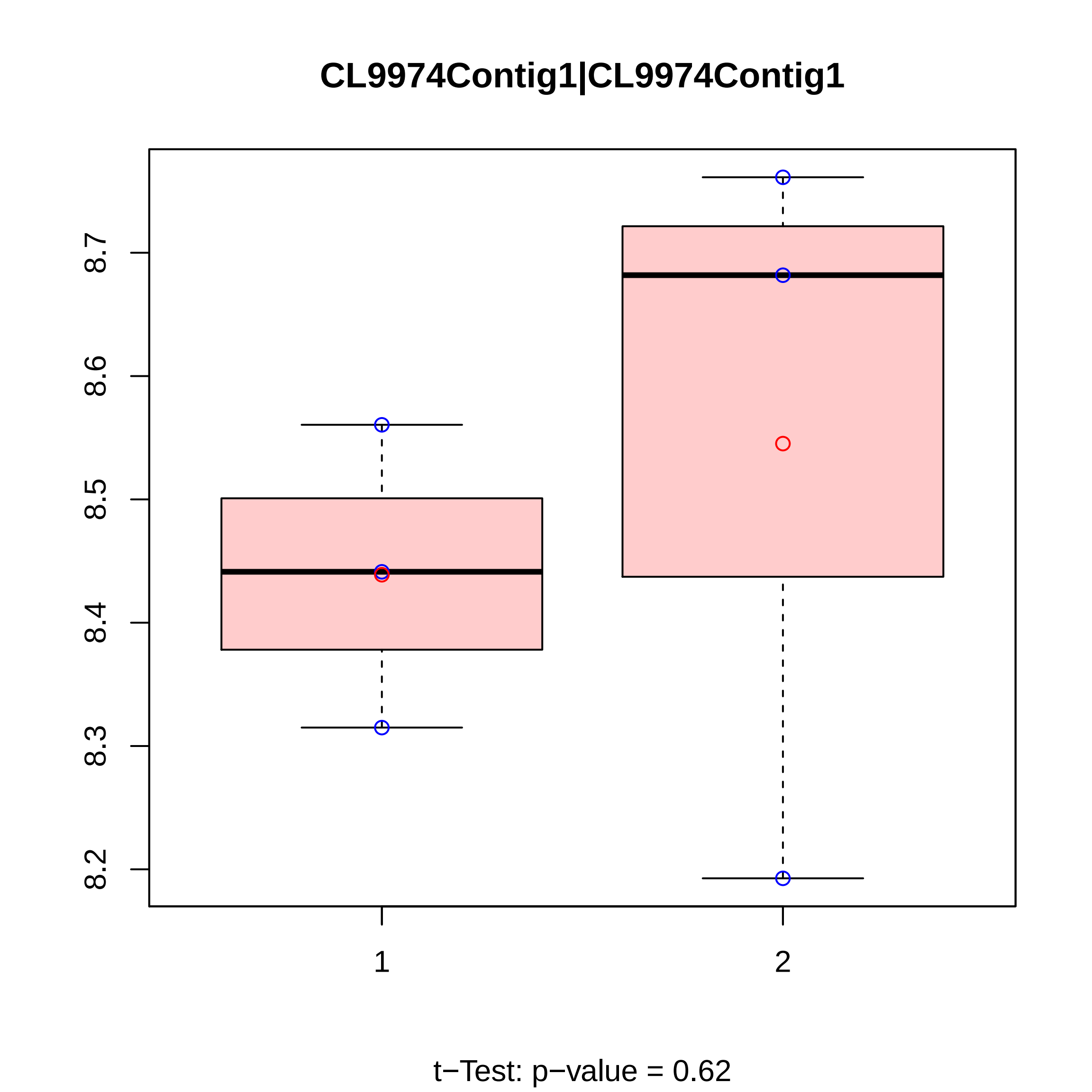
CL9974Contig1|CL9974Contig1
8.2
8.3
8.4
8.5
8.6
8.7
1
2
t−Test: p−value = 0.62

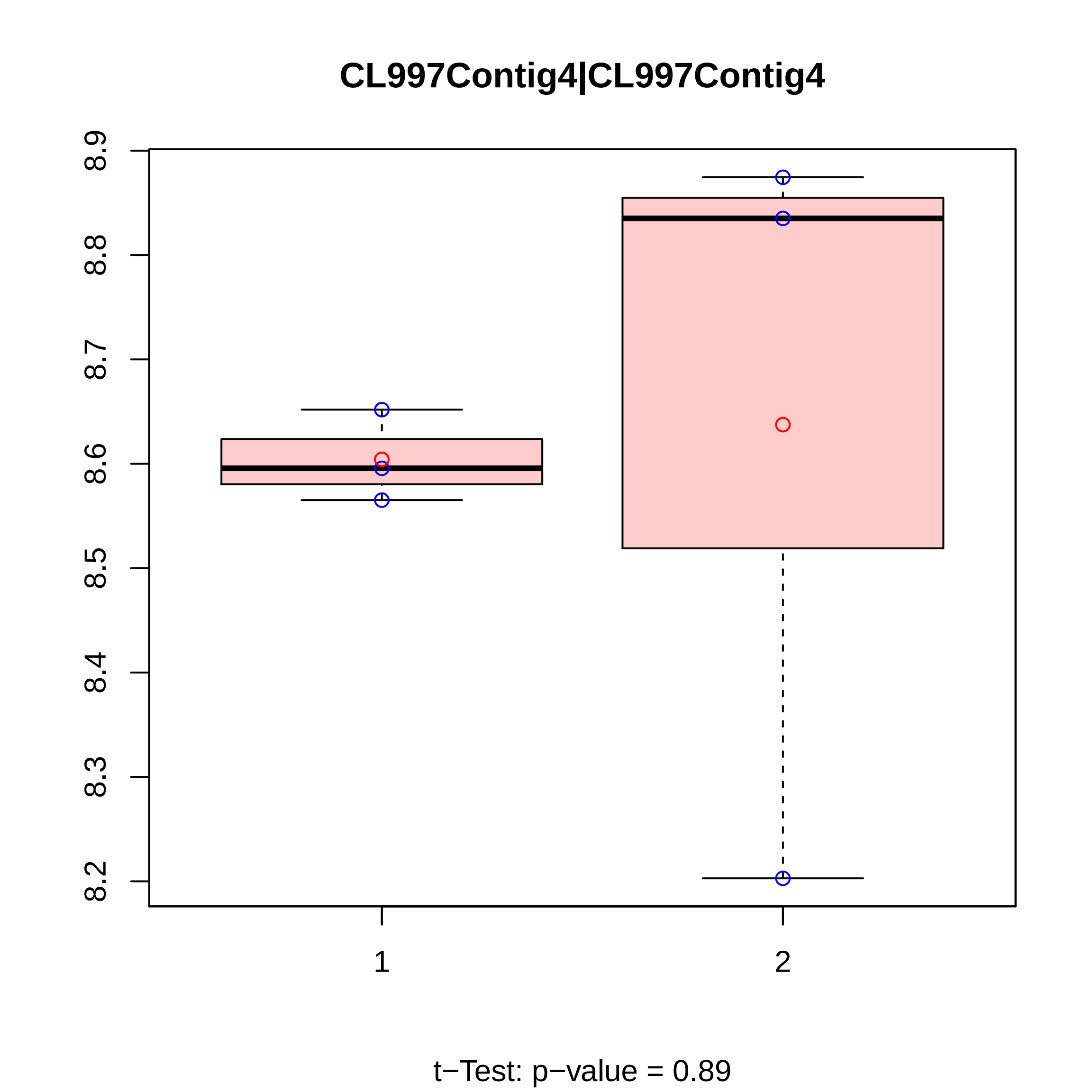
CL997Contig4|CL997Contig4
8.2
8.3
8.4
8.5
8.6
8.7
8.8
8.9
1
2
t−Test: p−value = 0.89

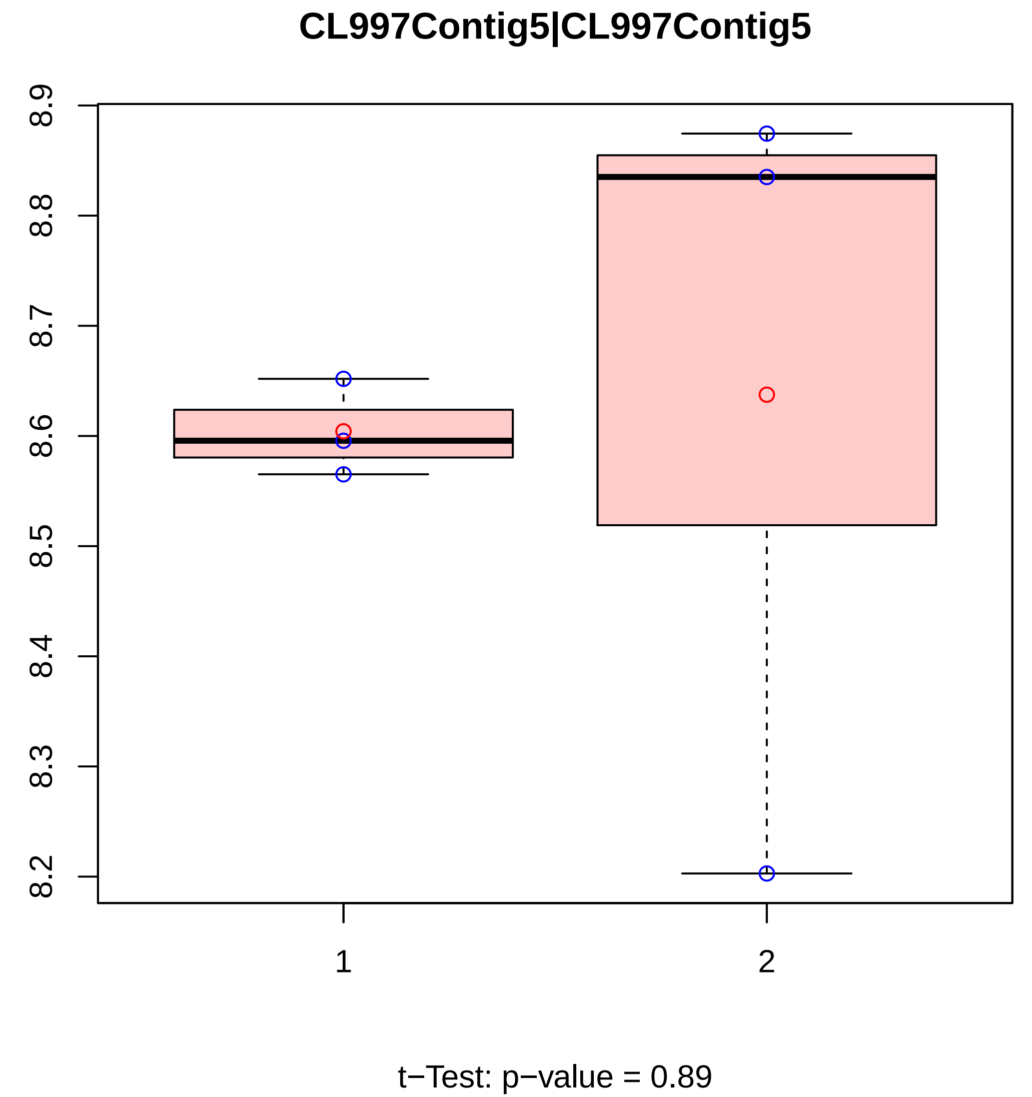
CL997Contig5|CL997Contig5
8.2
8.3
8.4
8.5
8.6
8.7
8.8
8.9
1
2
t−Test: p−value = 0.89

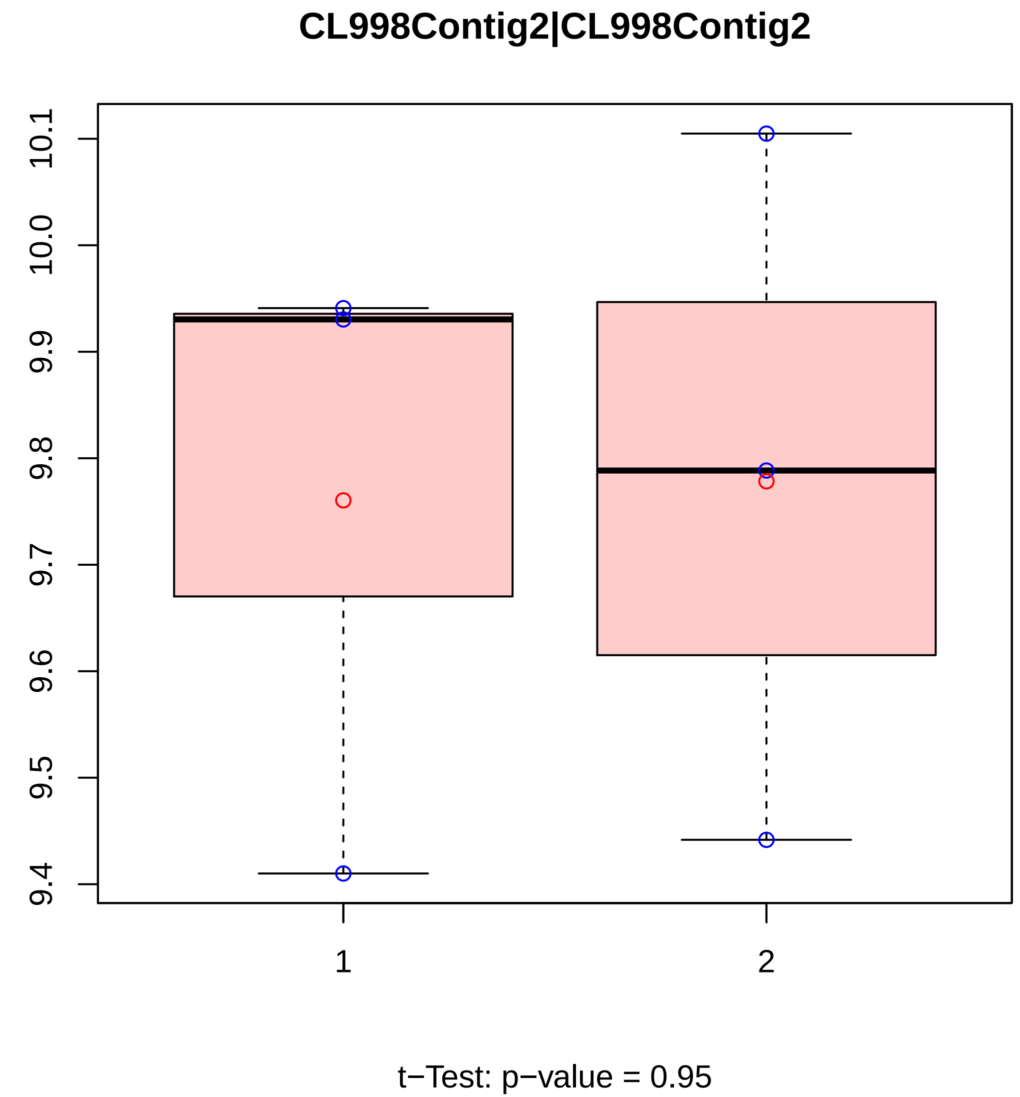
CL998Contig2|CL998Contig2
10.1
10.0
9.9
9.8
9.7
9.6
9.5
9.4
1
2
t−Test: p−value = 0.95

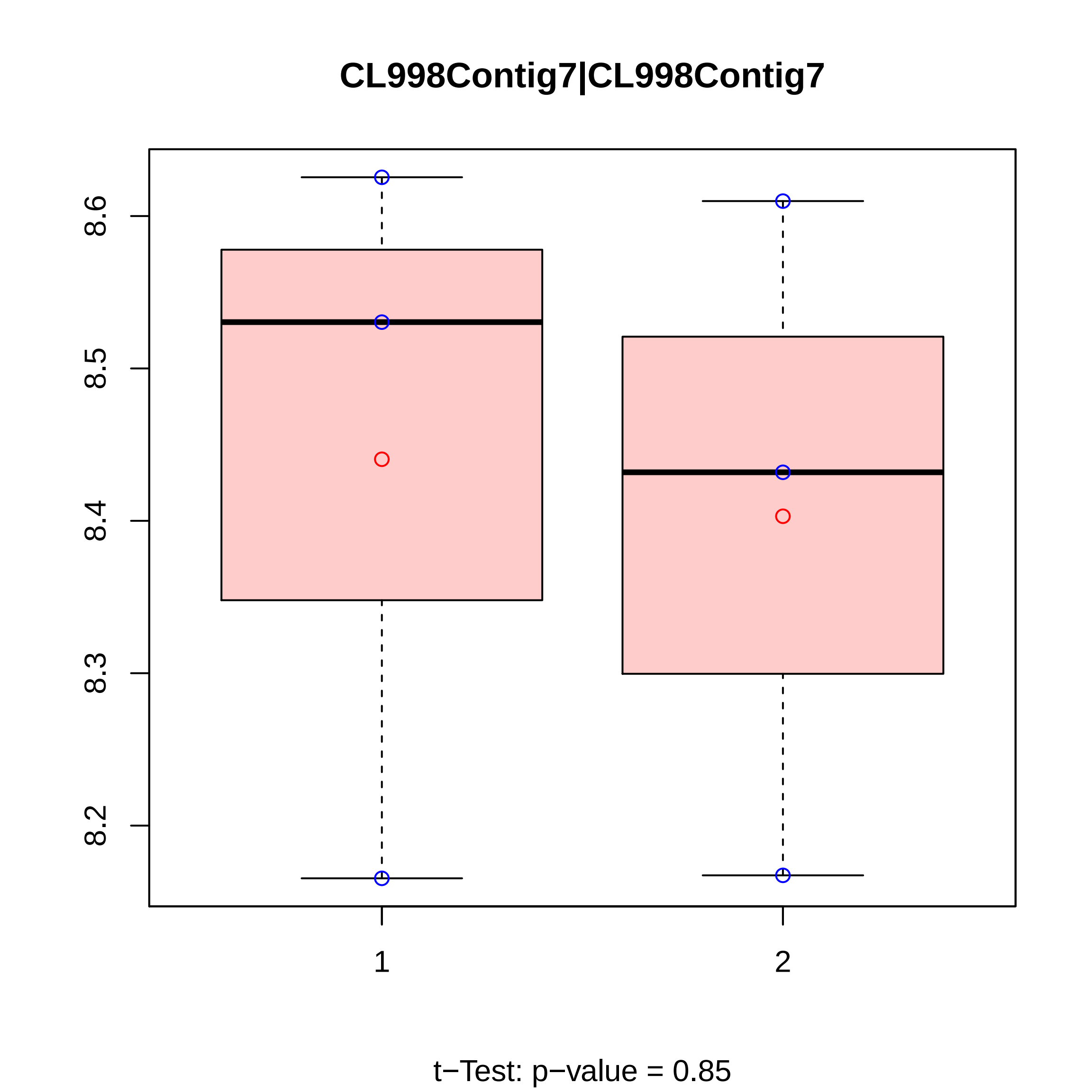

CL998Contig7|CL998Contig7
8.2
8.3
8.4
8.5
8.6
1
2
t−Test: p−value = 0.85

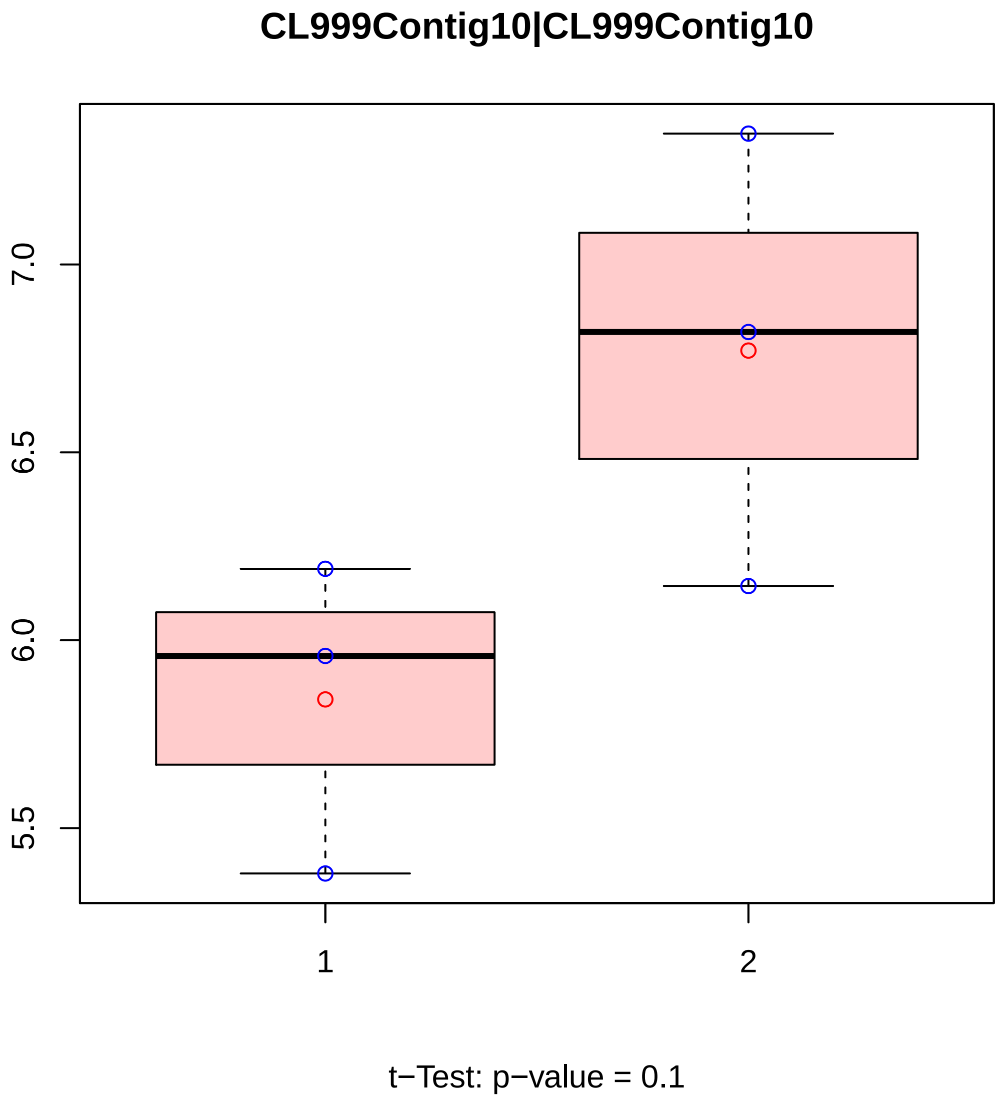
CL999Contig10|CL999Contig10
7.0
6.5
6.0
5.5
1
2
t−Test: p−value = 0.1

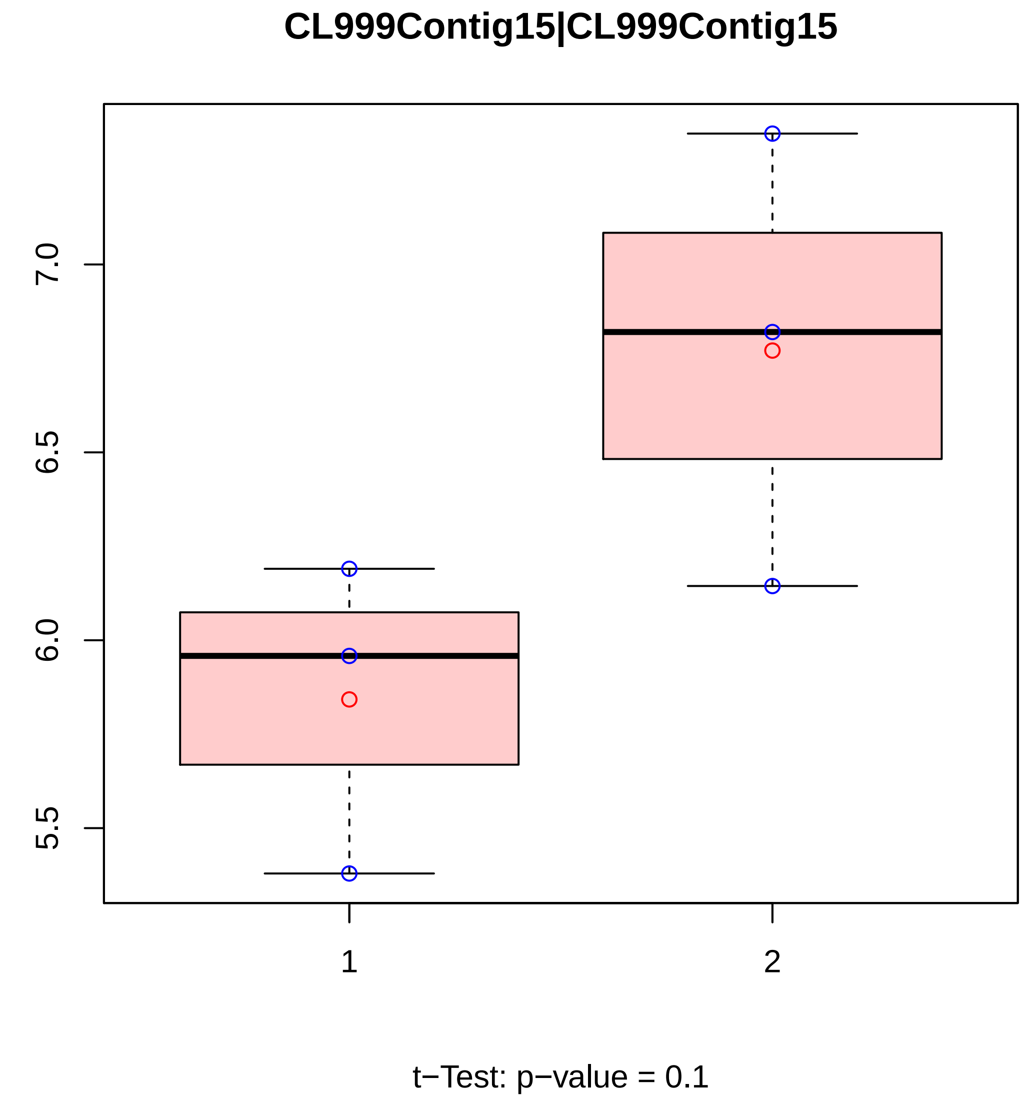
CL999Contig15|CL999Contig15
7.0
6.5
6.0
5.5
1
2
t−Test: p−value = 0.1

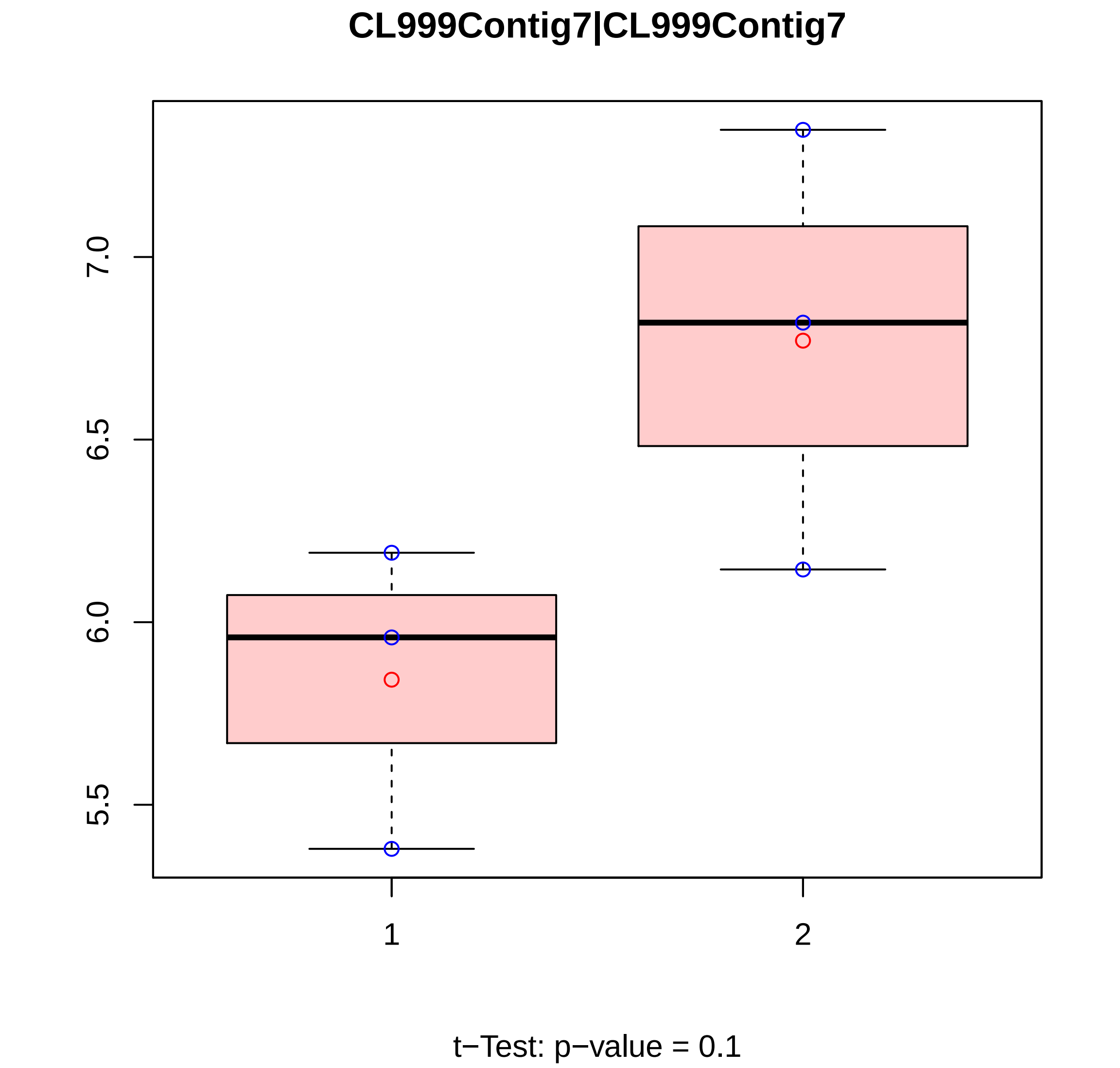
CL999Contig7|CL999Contig7
7.0
6.5
6.0
5.5
1
2
t−Test: p−value = 0.1

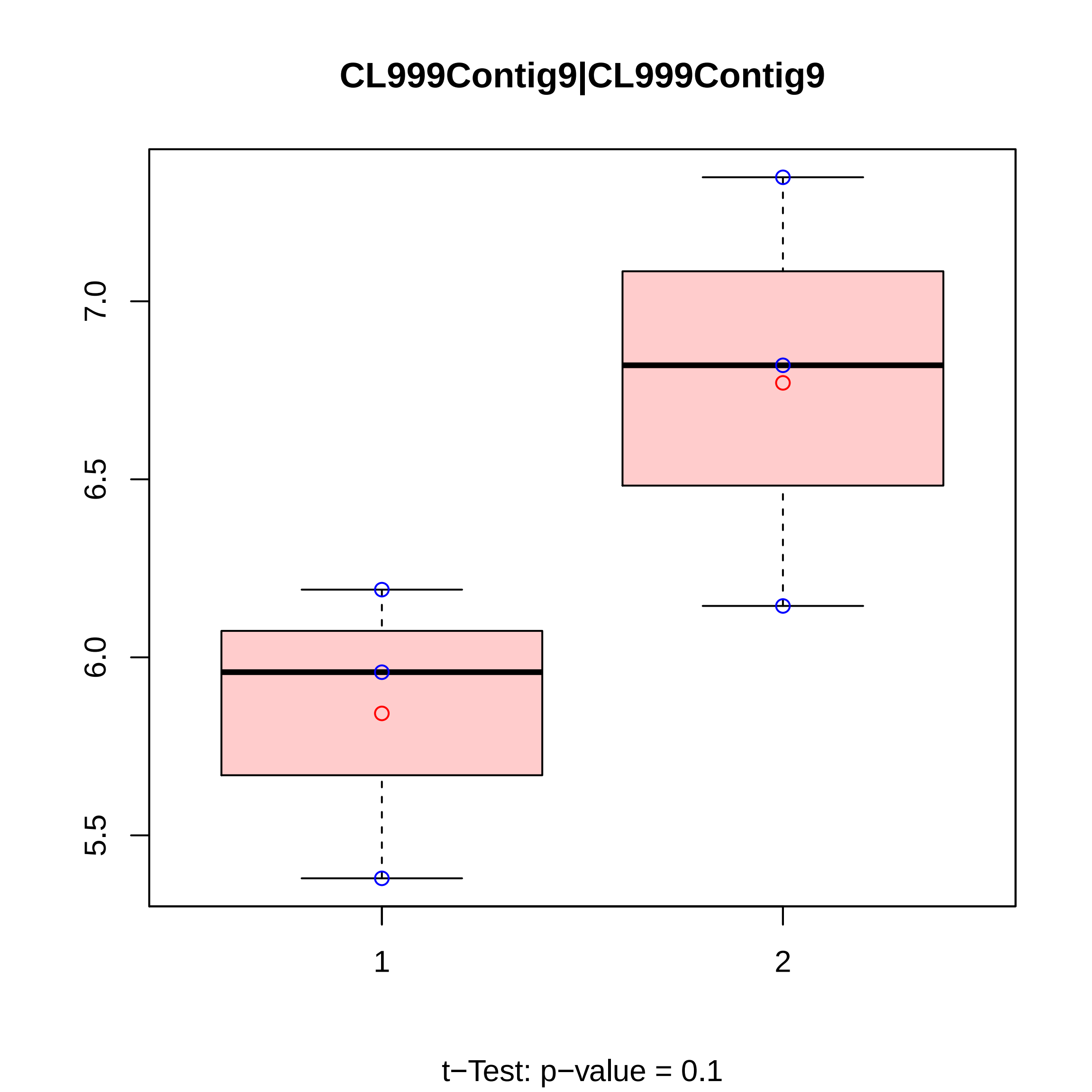
CL999Contig9|CL999Contig9
7.0
6.5
6.0
5.5
1
2
t−Test: p−value = 0.1

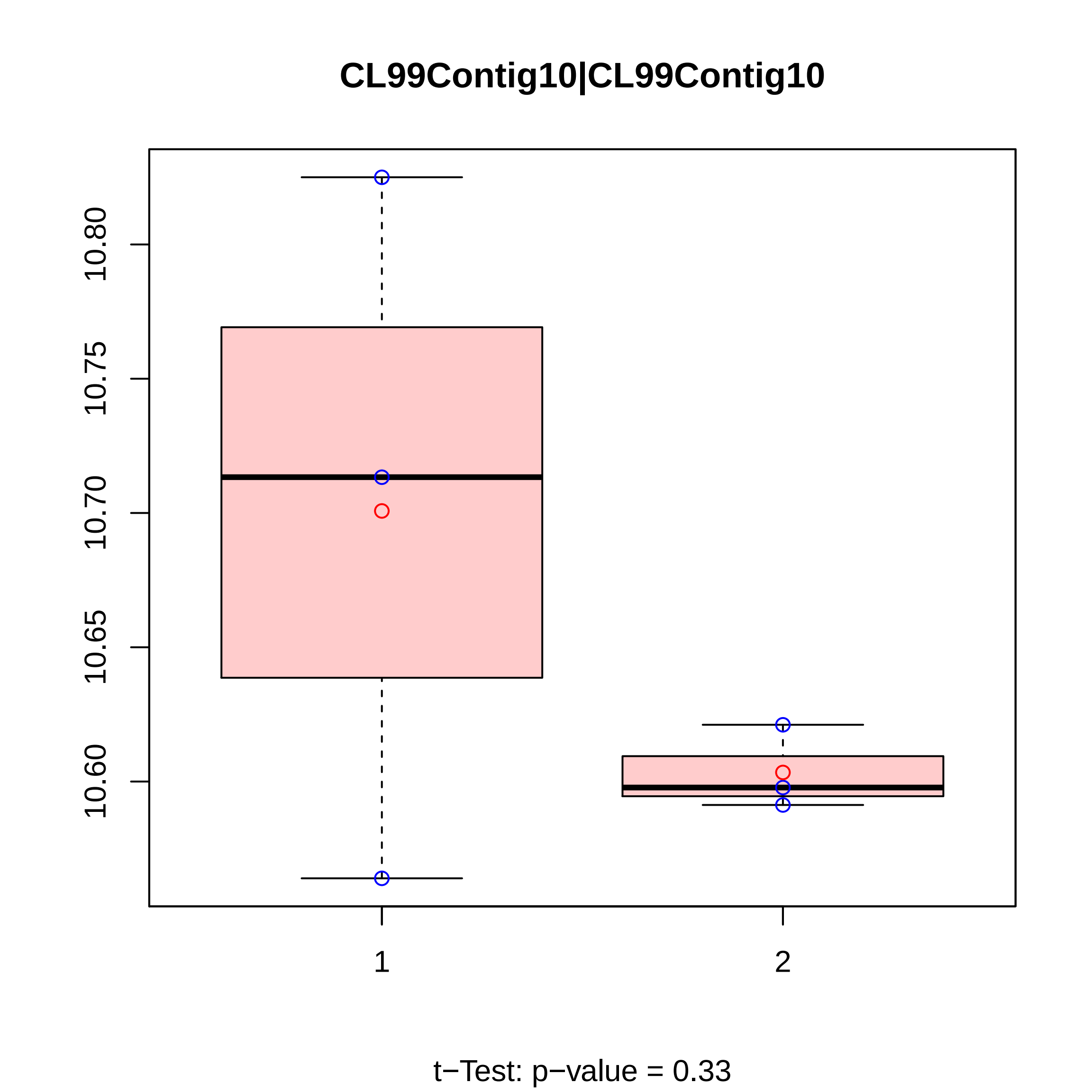

CL99Contig10|CL99Contig10
10.60
10.65
10.70
10.75
10.80
1
2
t−Test: p−value = 0.33

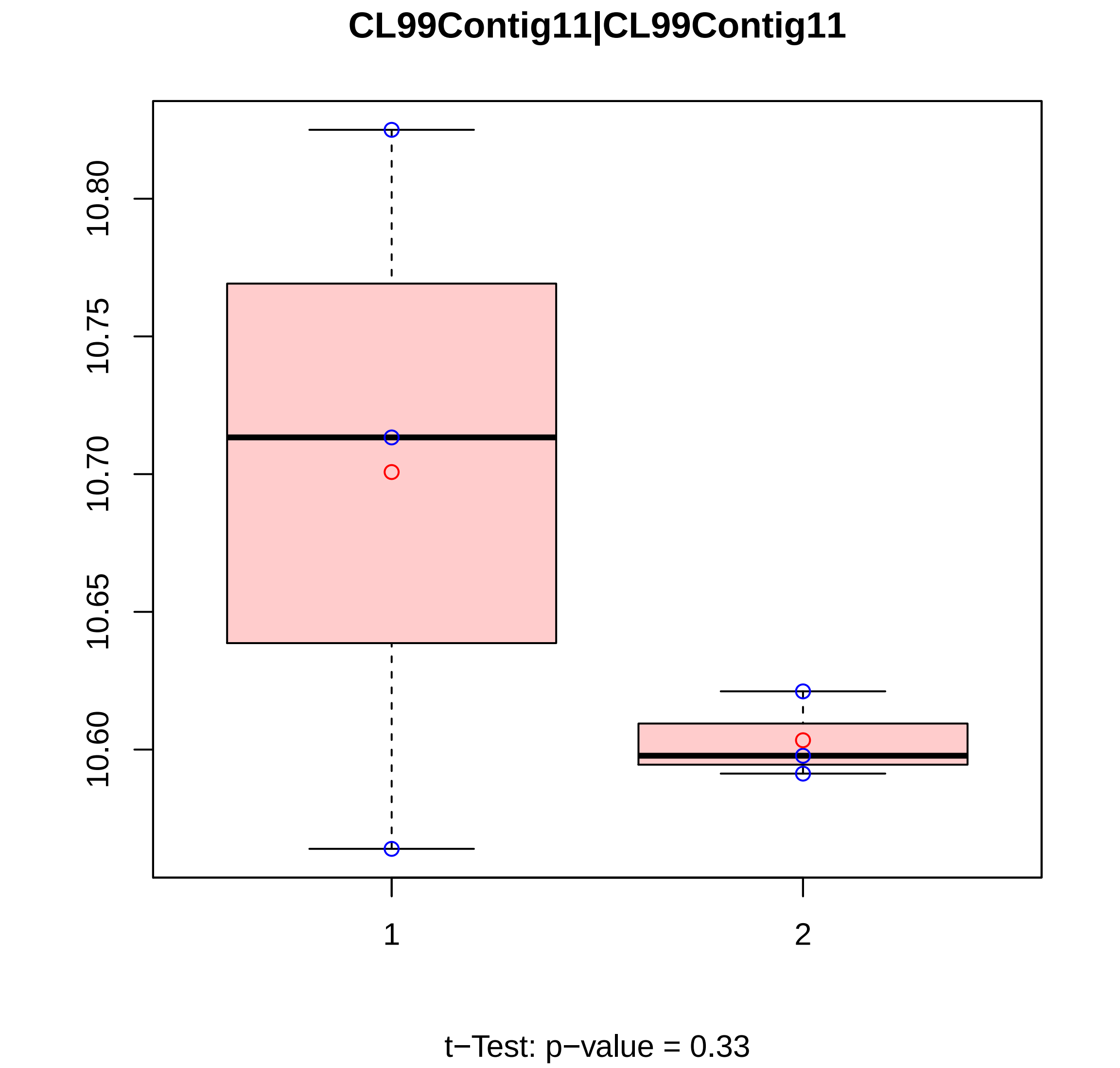
CL99Contig11|CL99Contig11
10.60
10.65
10.70
10.75
10.80
1
2
t−Test: p−value = 0.33

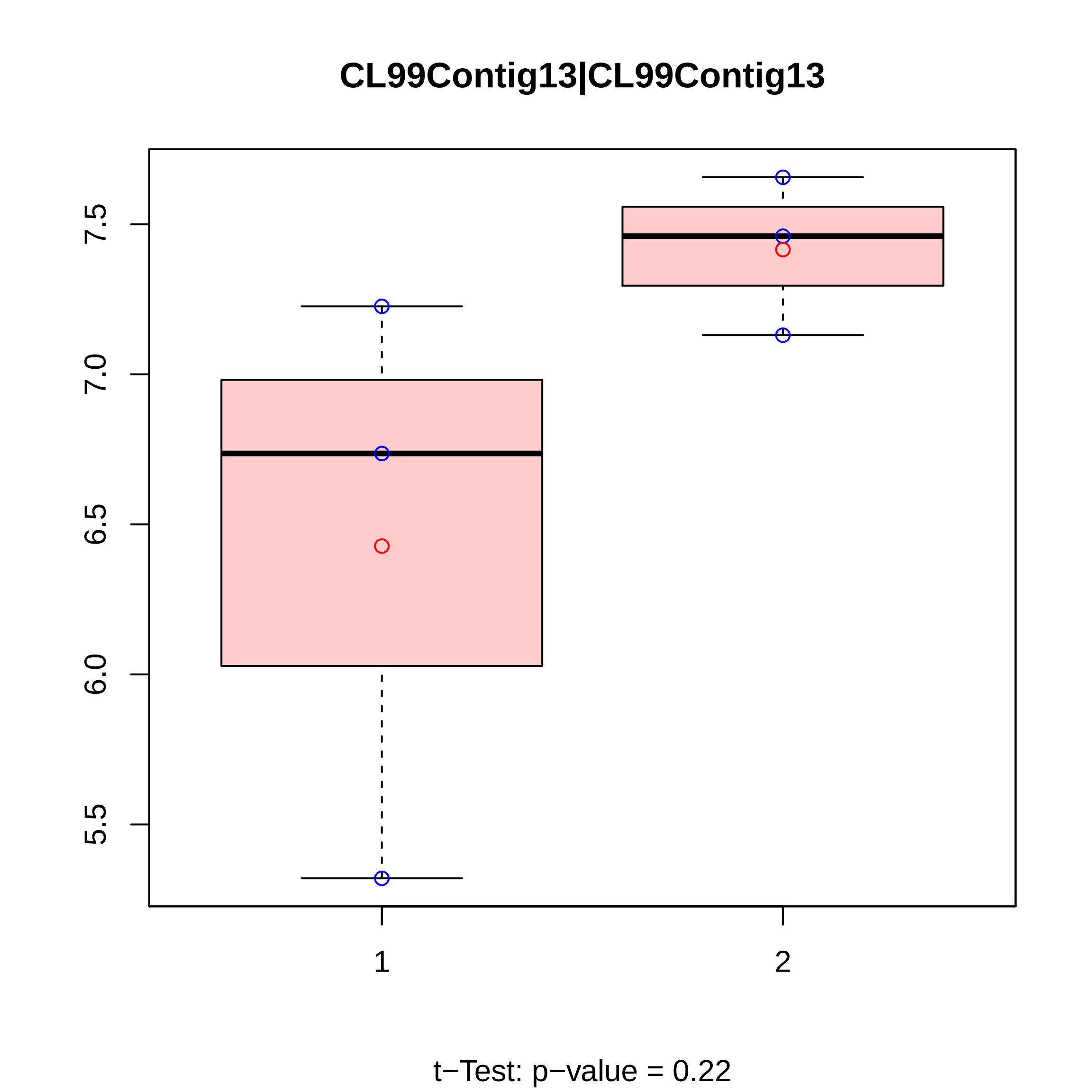

CL99Contig13|CL99Contig13
7.5
7.0
6.5
6.0
5.5
1
2
t−Test: p−value = 0.22

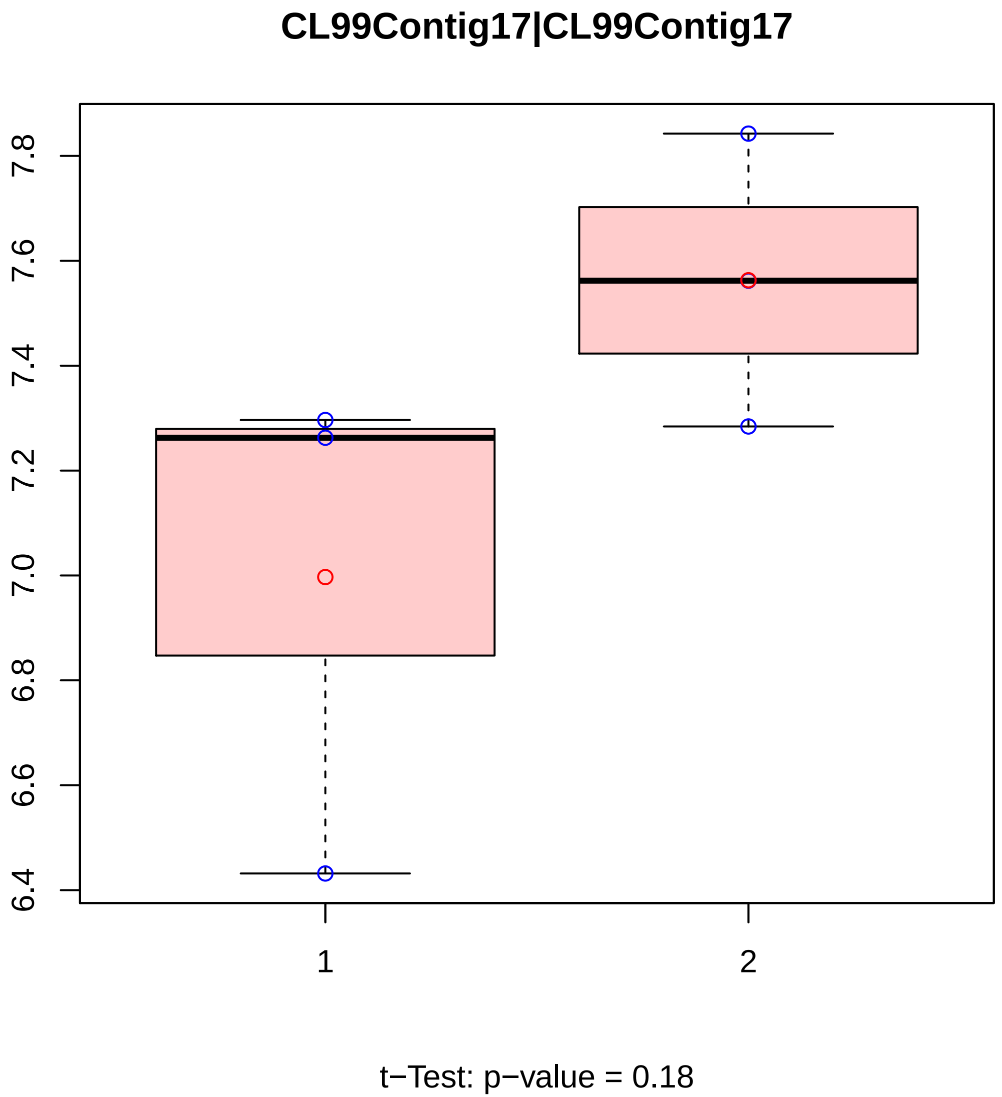

CL99Contig17|CL99Contig17
7.8
7.6
7.4
7.2
7.0
6.8
6.6
6.4
1
2
t−Test: p−value = 0.18

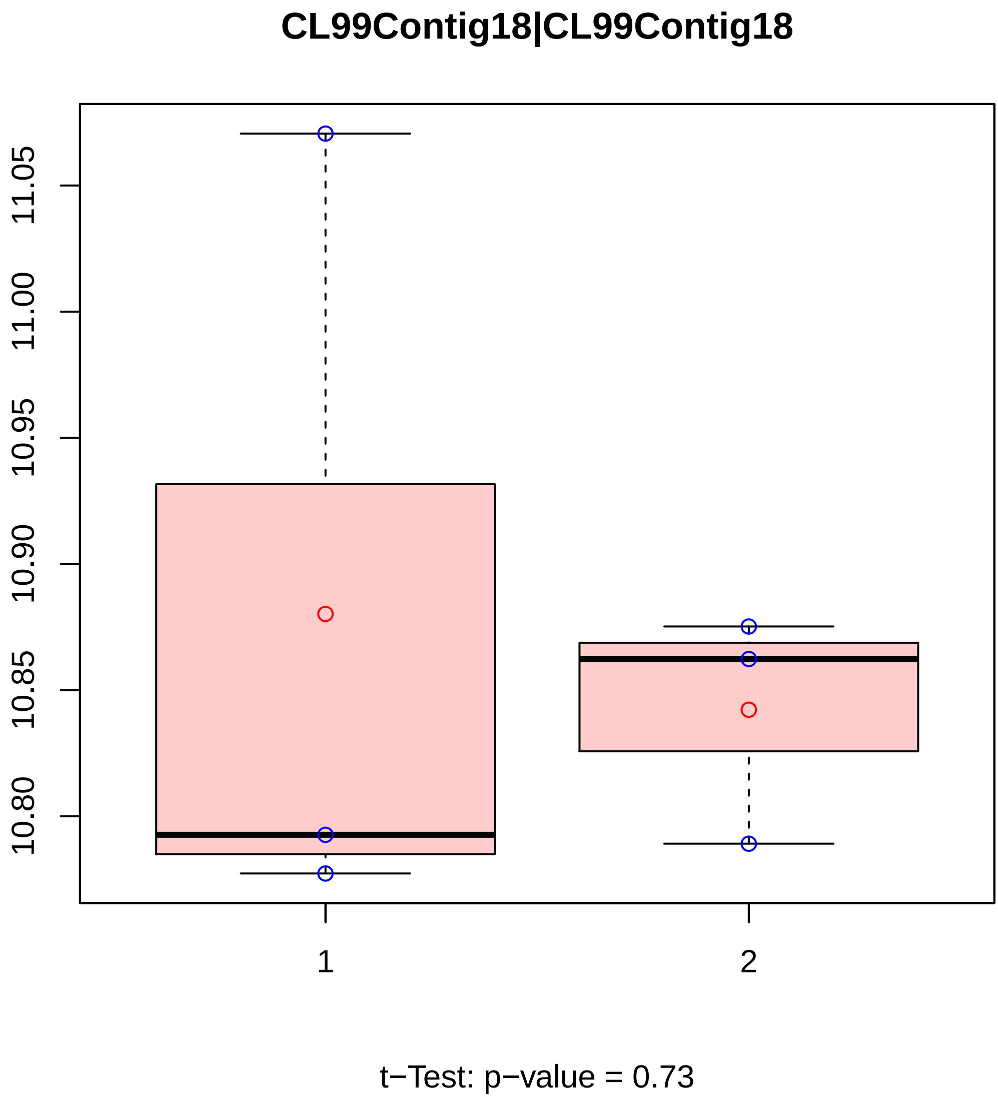
CL99Contig18|CL99Contig18
10.80
10.85
10.90
10.95
11.00
11.05
1
2
t−Test: p−value = 0.73

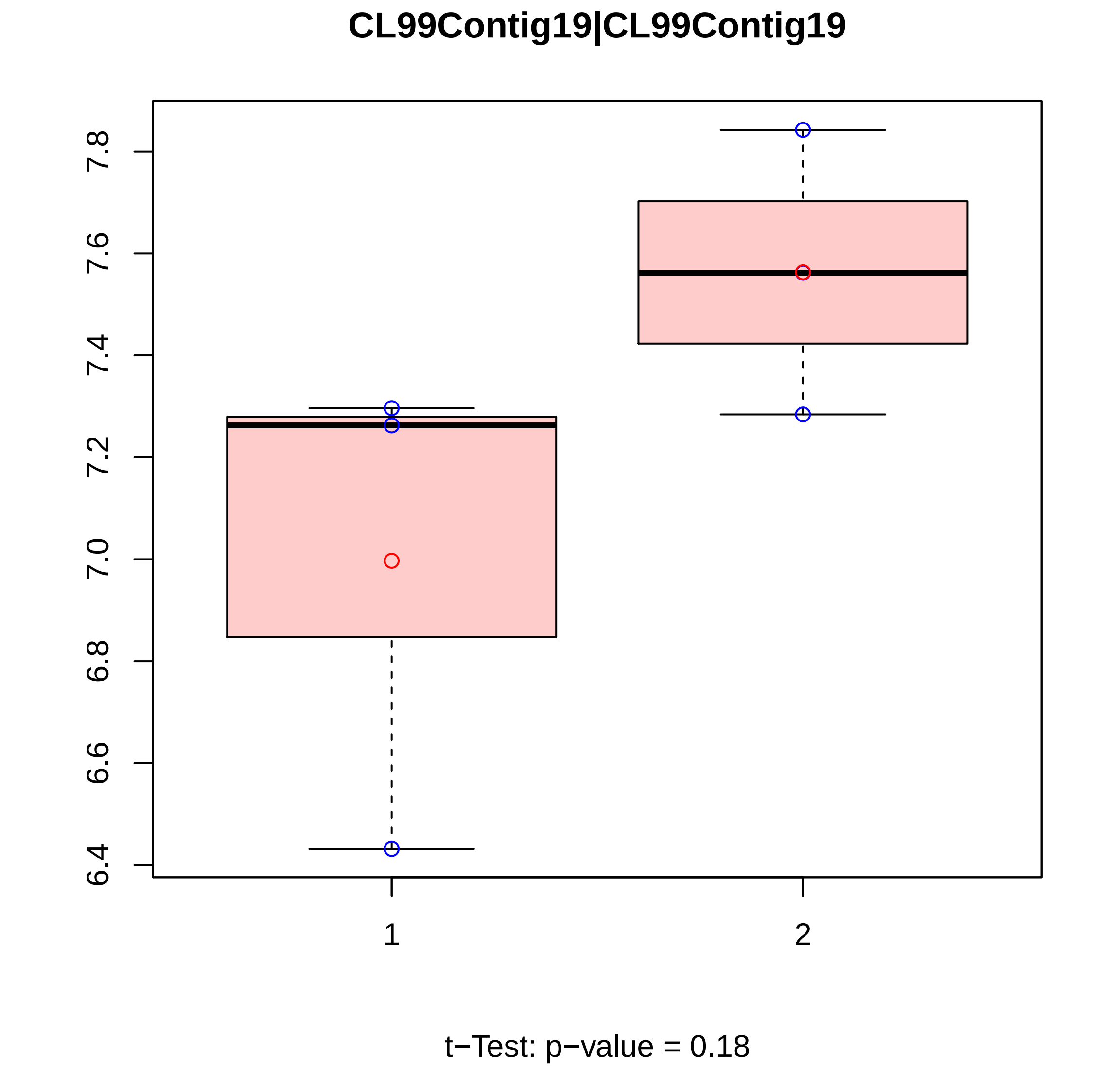
CL99Contig19|CL99Contig19
7.8
7.6
7.4
7.2
7.0
6.8
6.6
6.4
1
2
t−Test: p−value = 0.18

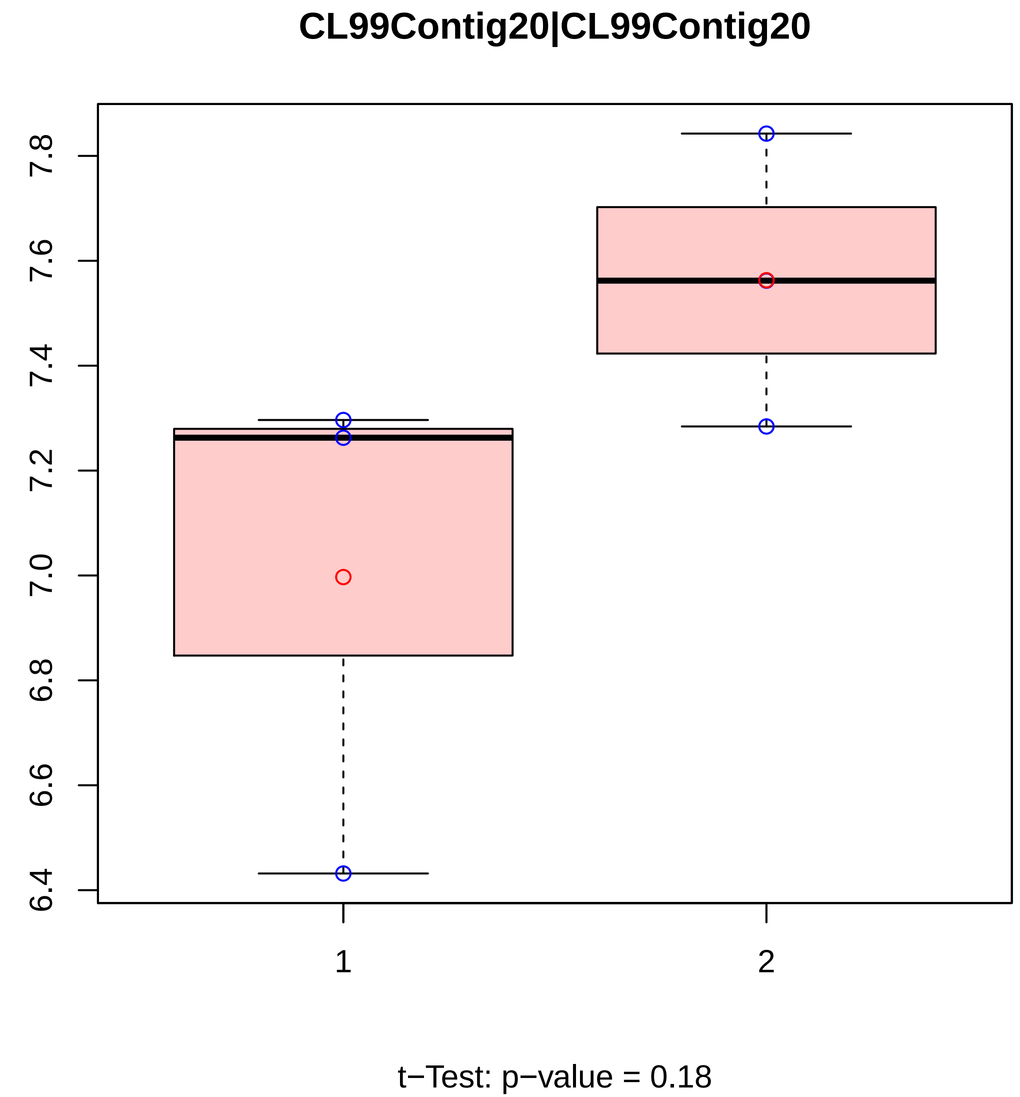
CL99Contig20|CL99Contig20
7.8
7.6
7.4
7.2
7.0
6.8
6.6
6.4
1
2
t−Test: p−value = 0.18

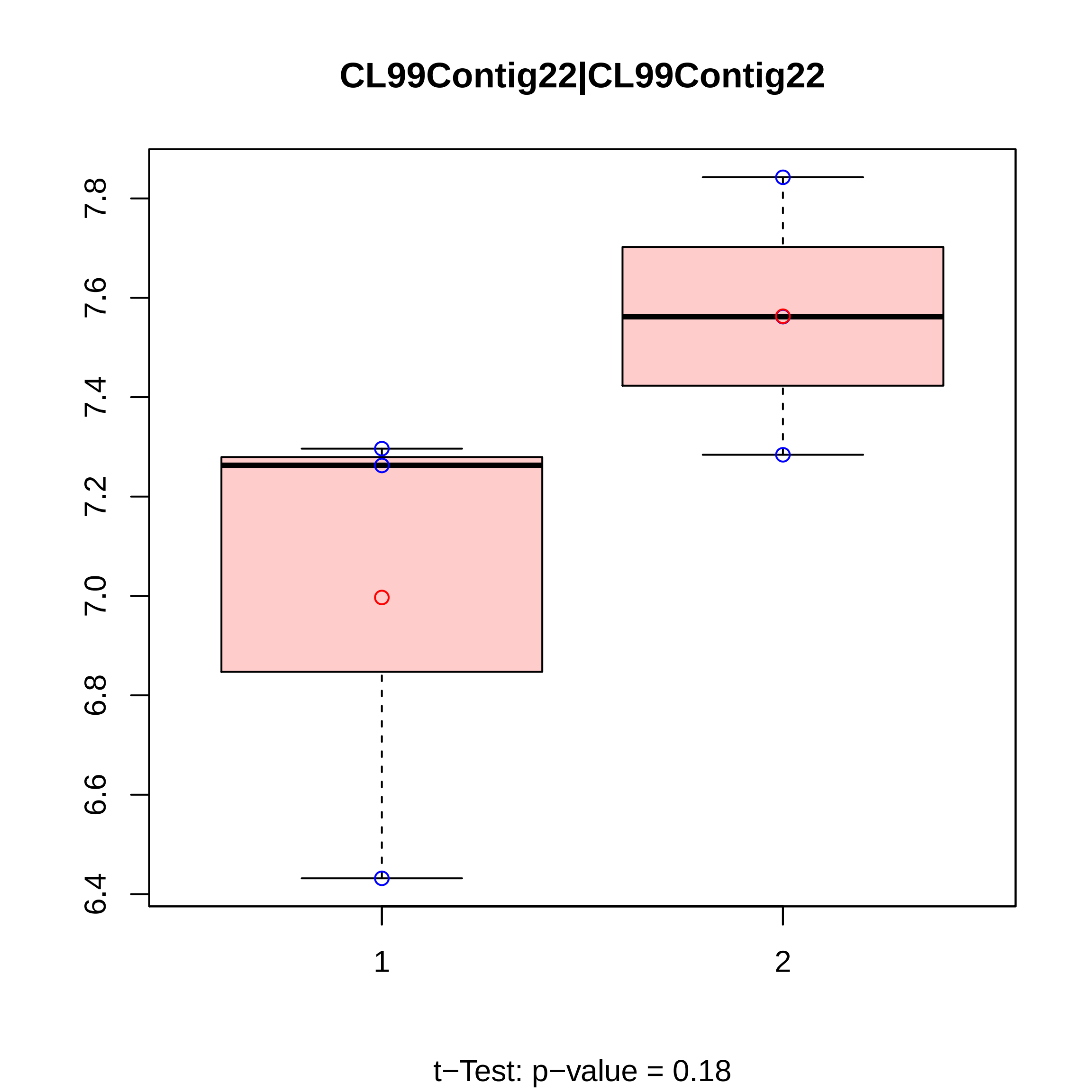
CL99Contig22|CL99Contig22
7.8
7.6
7.4
7.2
7.0
6.8
6.6
6.4
1
2
t−Test: p−value = 0.18

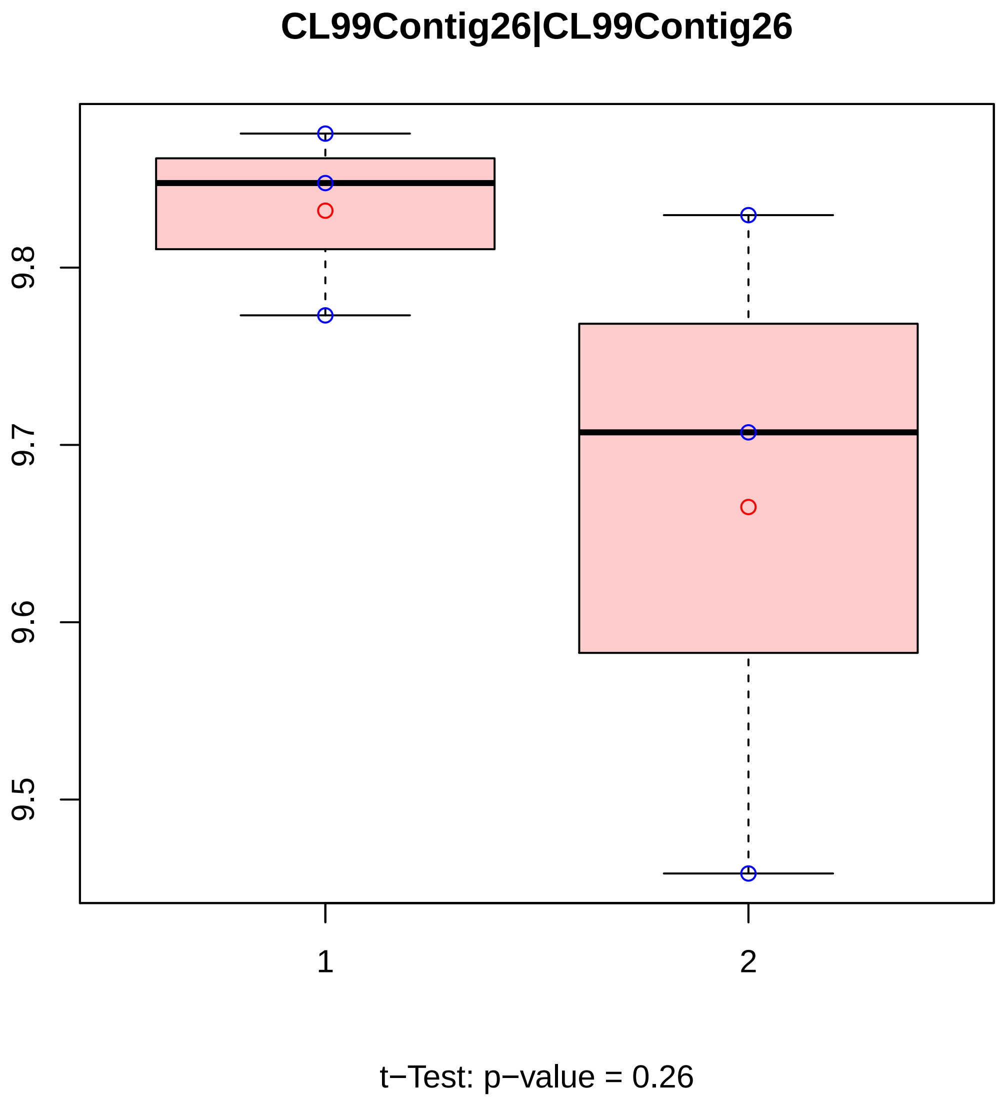
CL99Contig26|CL99Contig26
9.8
9.7
9.6
9.5
1
2
t−Test: p−value = 0.26

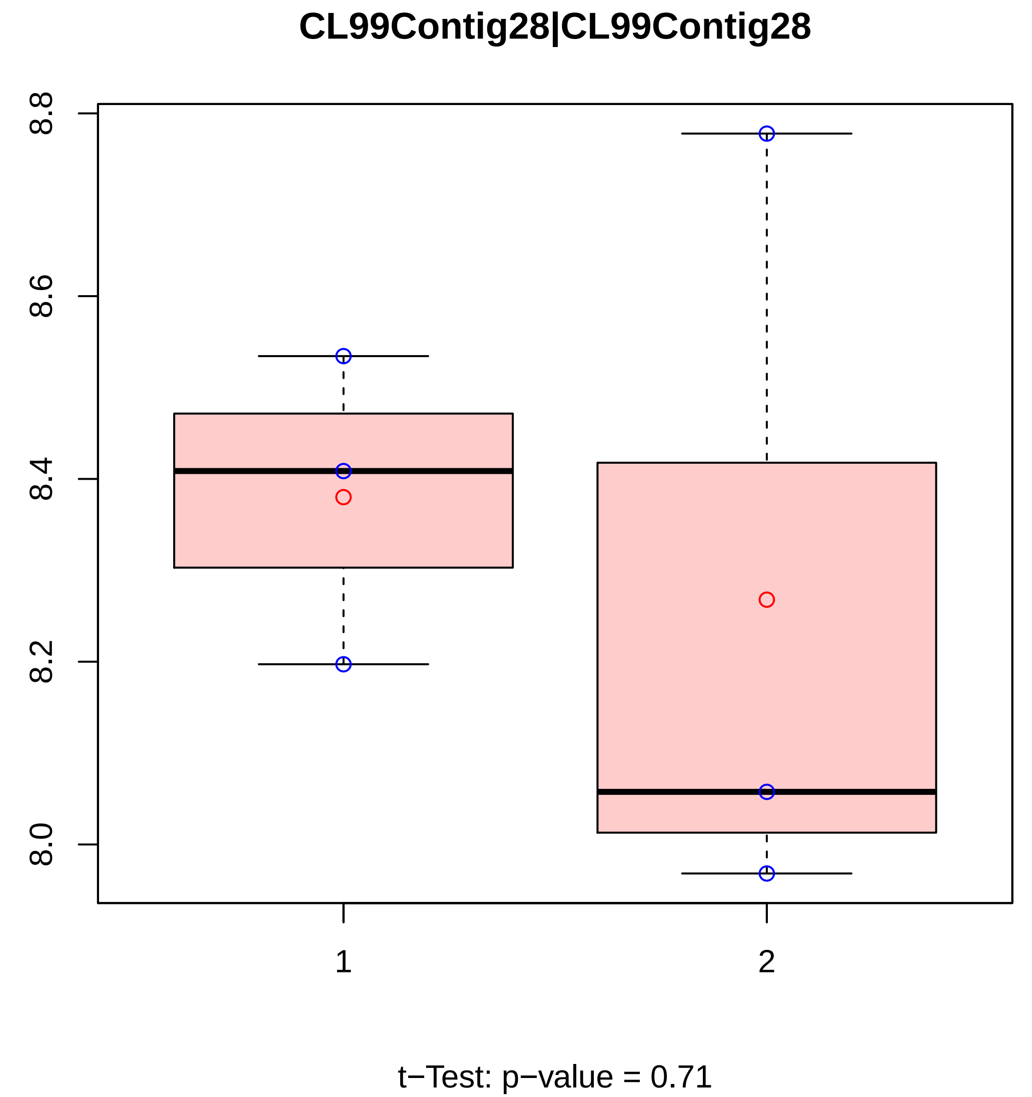

CL99Contig28|CL99Contig28
8.0
8.2
8.4
8.6
8.8
1
2
t−Test: p−value = 0.71

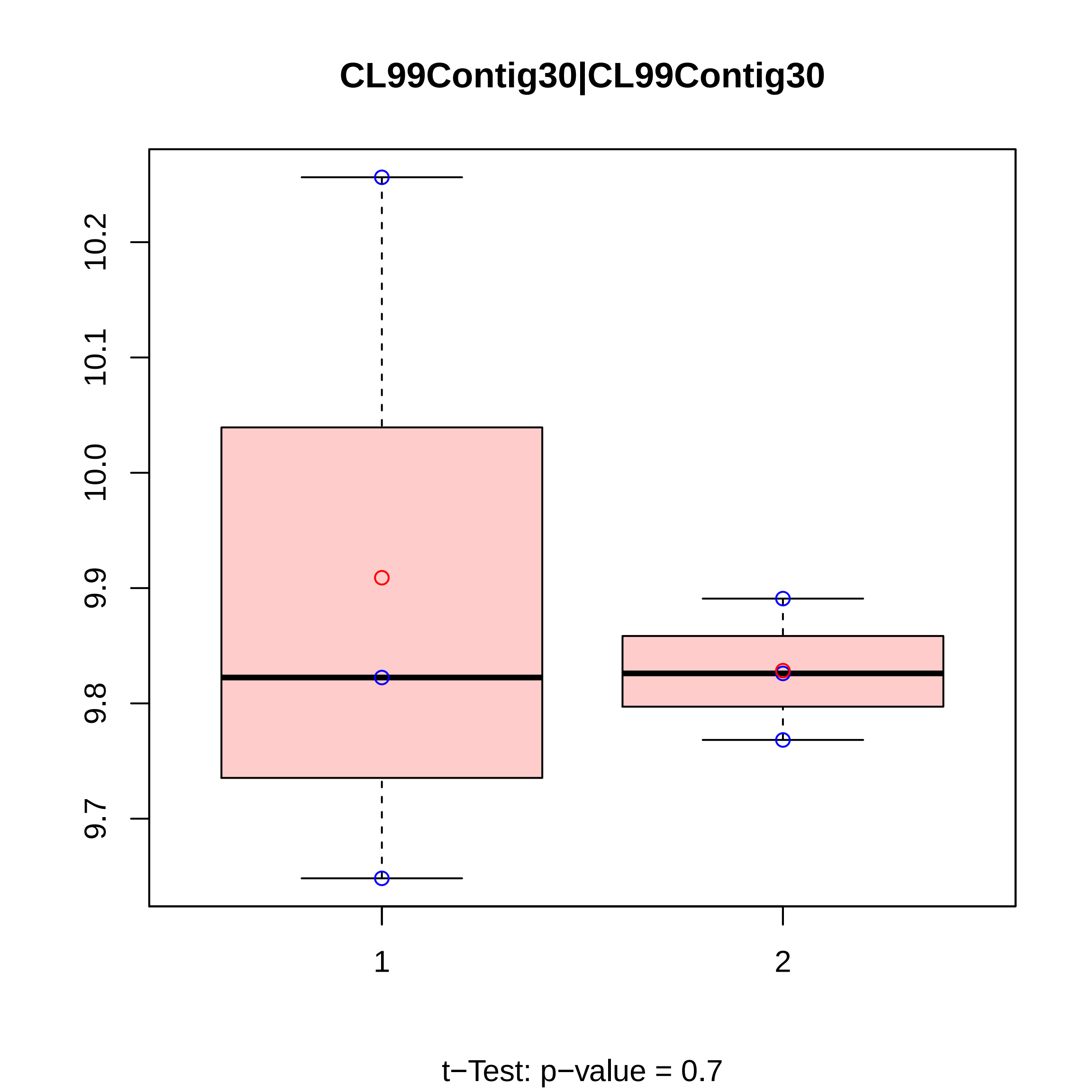
CL99Contig30|CL99Contig30
10.2
10.1
10.0
9.9
9.8
9.7
1
2
t−Test: p−value = 0.7

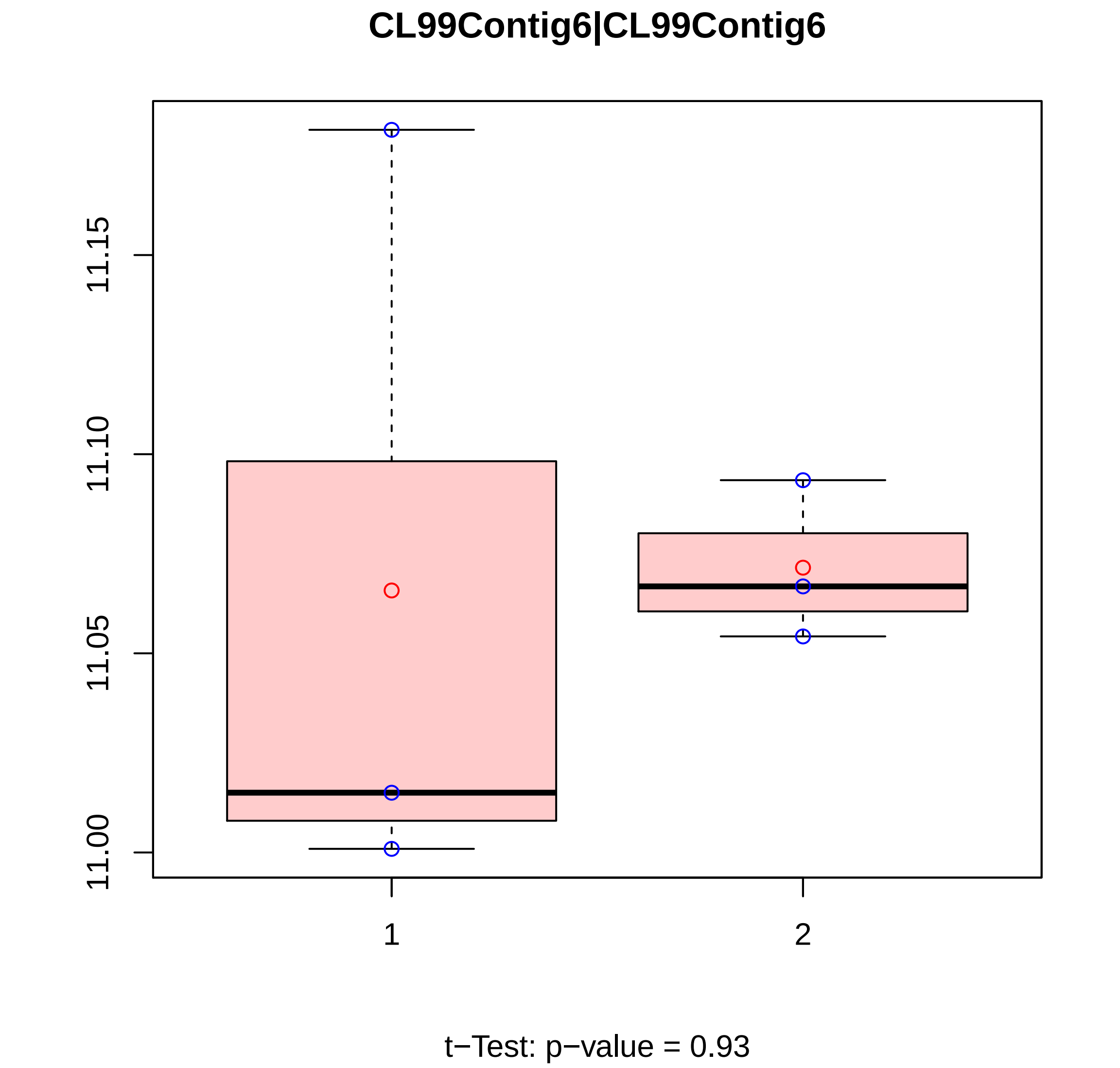

CL99Contig6|CL99Contig6
11.00
11.05
11.10
11.15
1
2
t−Test: p−value = 0.93

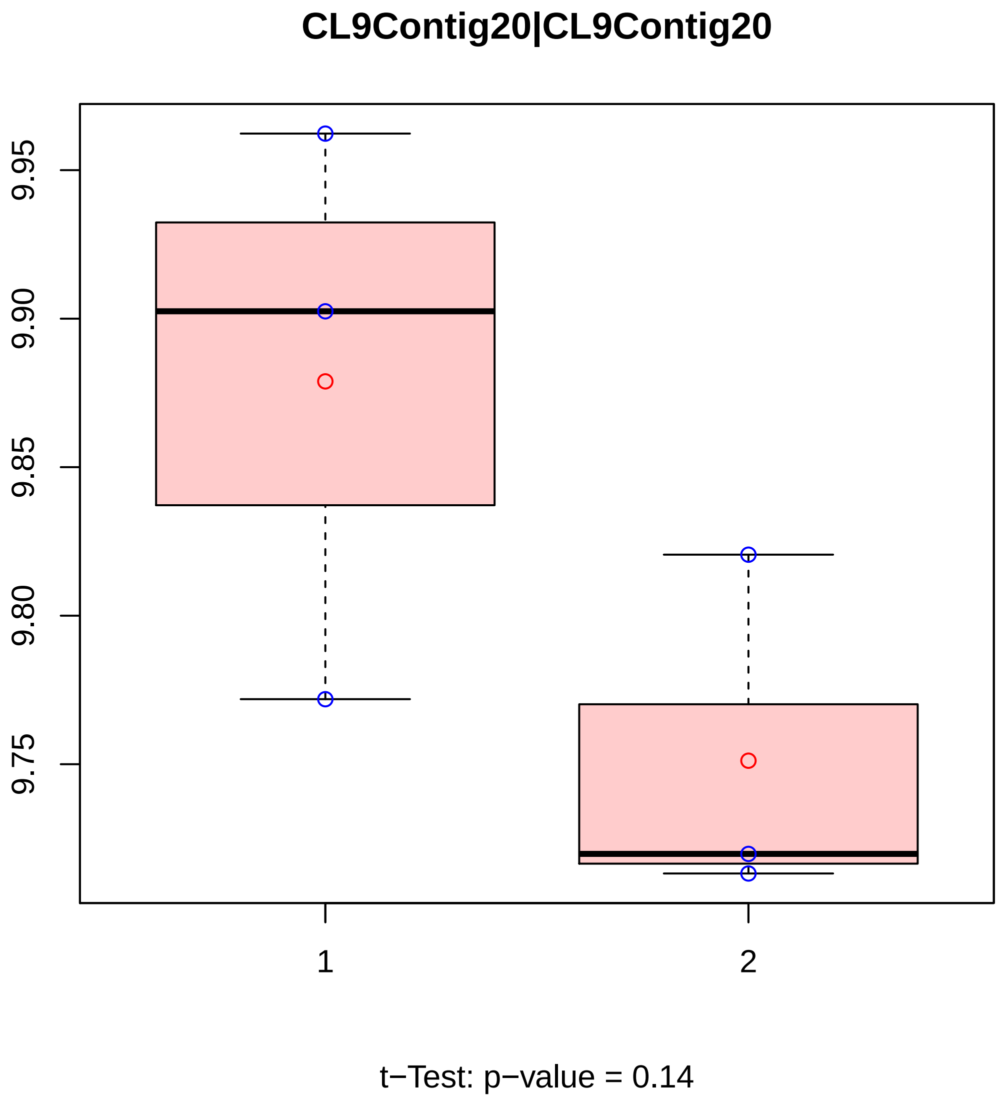

CL9Contig20|CL9Contig20
9.75
9.80
9.85
9.90
9.95
1
2
t−Test: p−value = 0.14

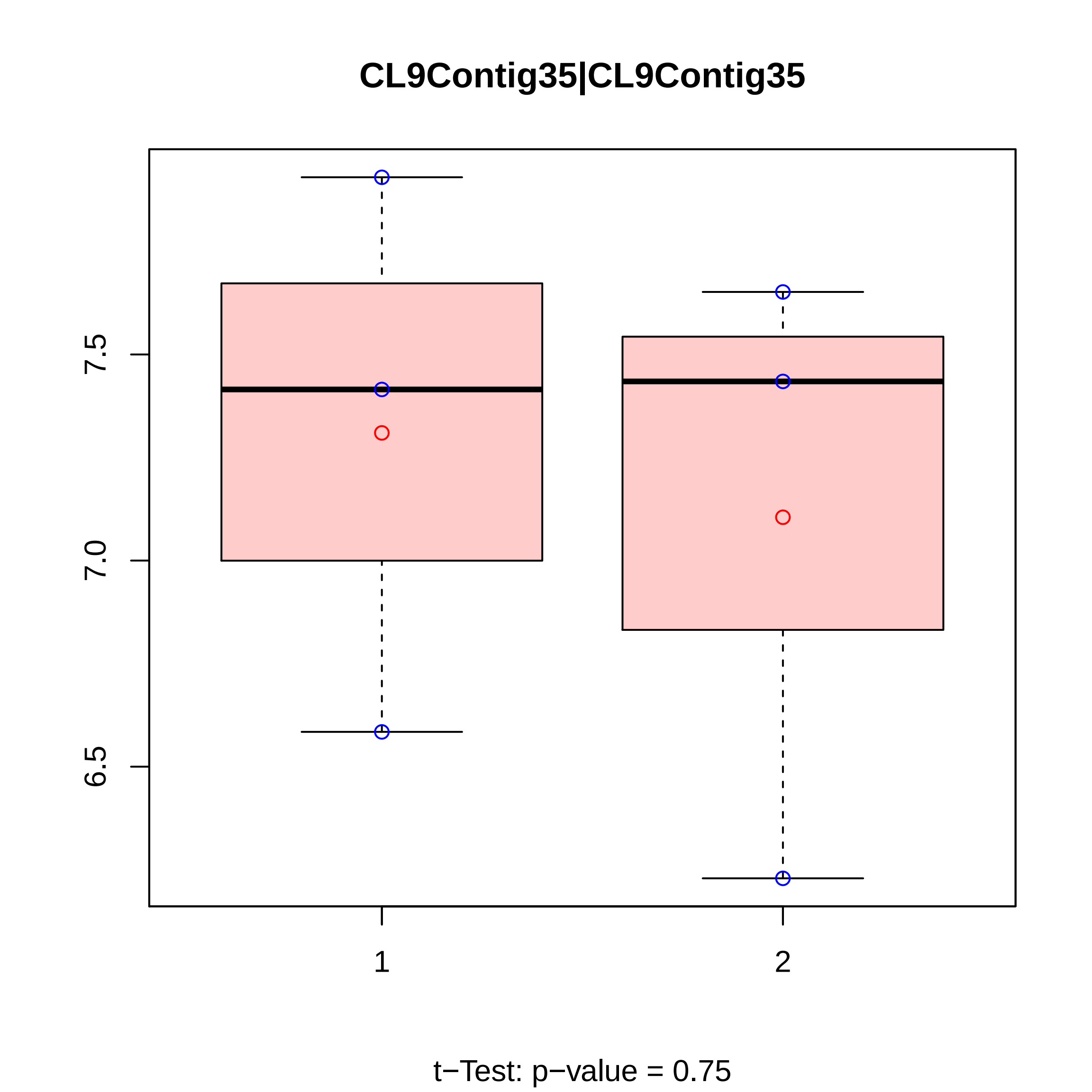

CL9Contig35|CL9Contig35
7.5
7.0
6.5
1
2
t−Test: p−value = 0.75

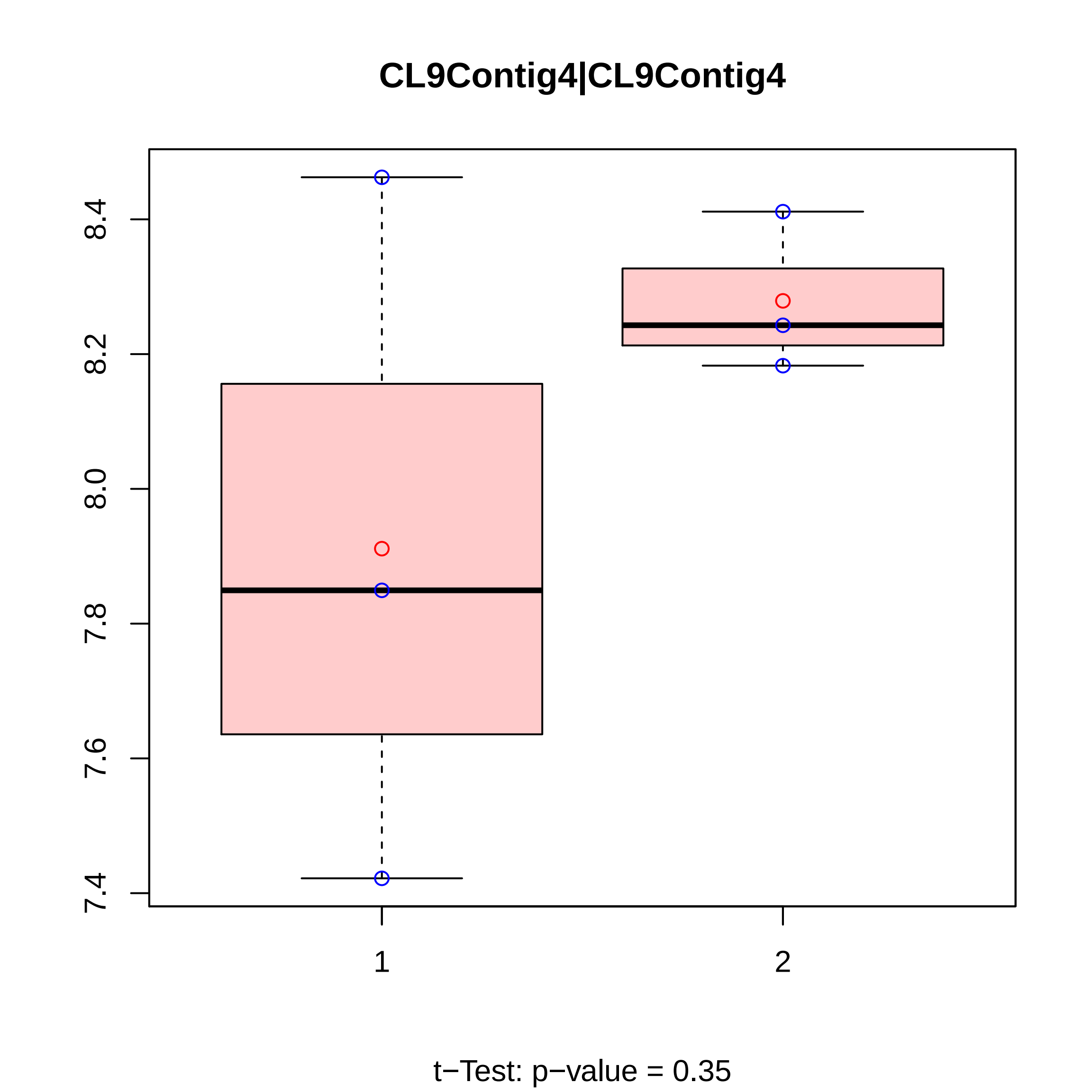

CL9Contig4|CL9Contig4
8.4
8.2
8.0
7.8
7.6
7.4
1
2
t−Test: p−value = 0.35

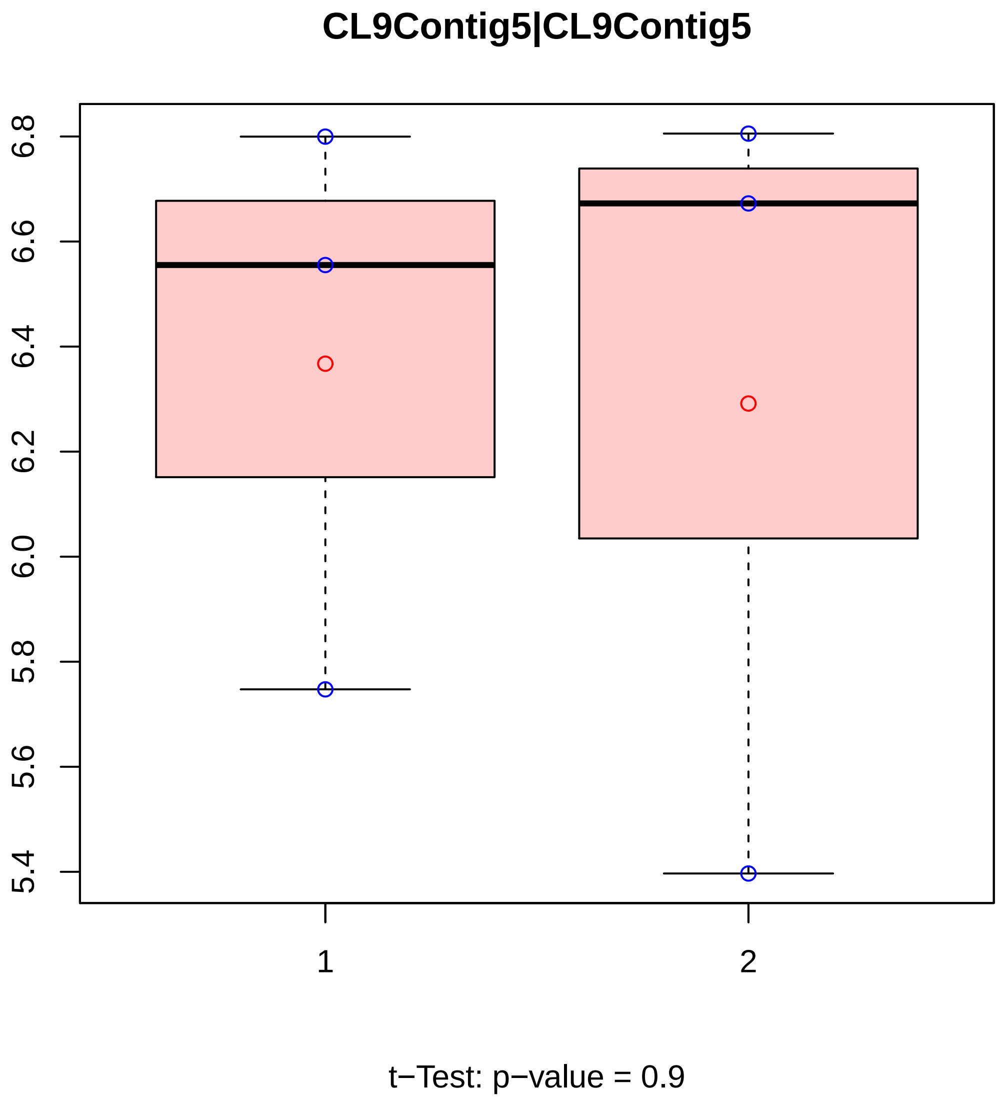
CL9Contig5|CL9Contig5
6.8
6.6
6.4
6.2
6.0
5.8
5.6
5.4
1
2
t−Test: p−value = 0.9

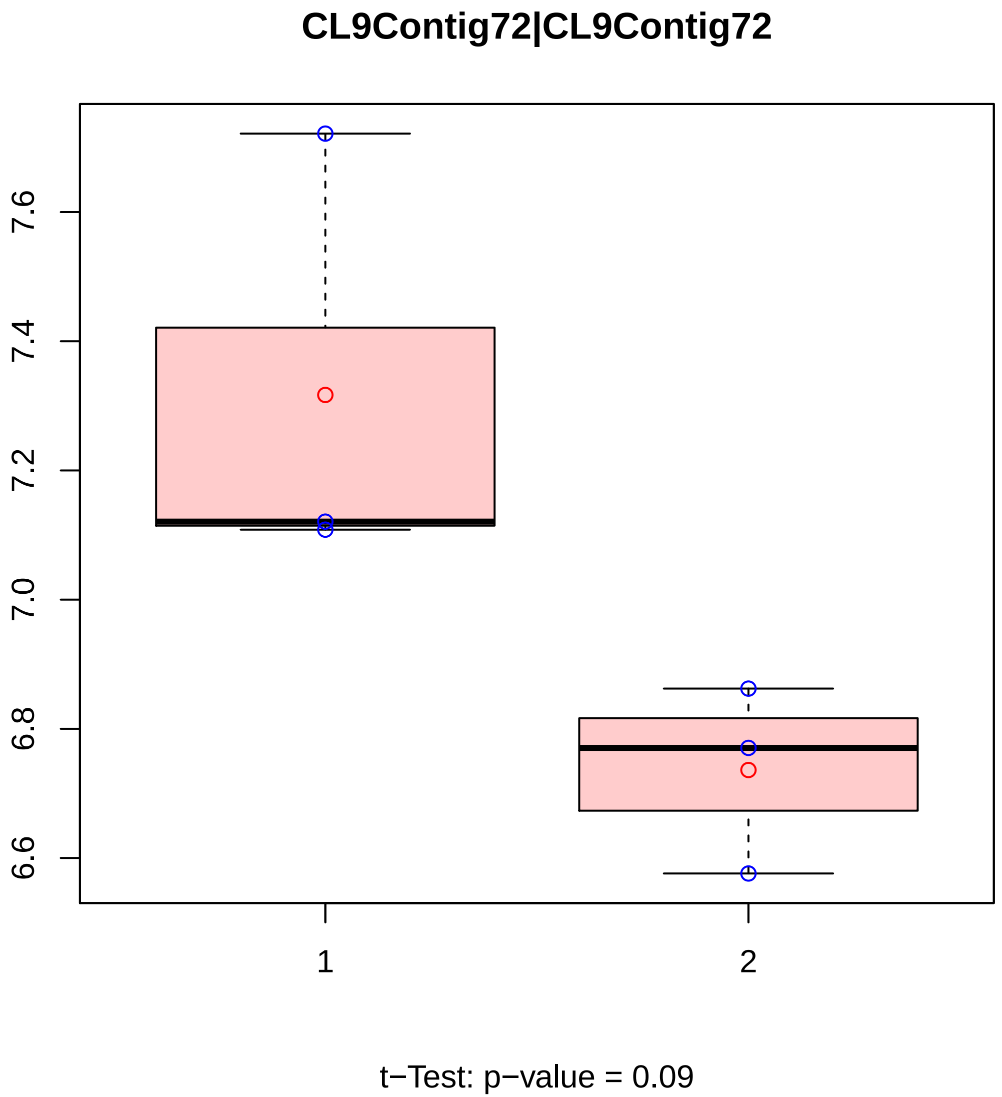
CL9Contig72|CL9Contig72
7.6
7.4
7.2
7.0
6.8
6.6
1
2
t−Test: p−value = 0.09

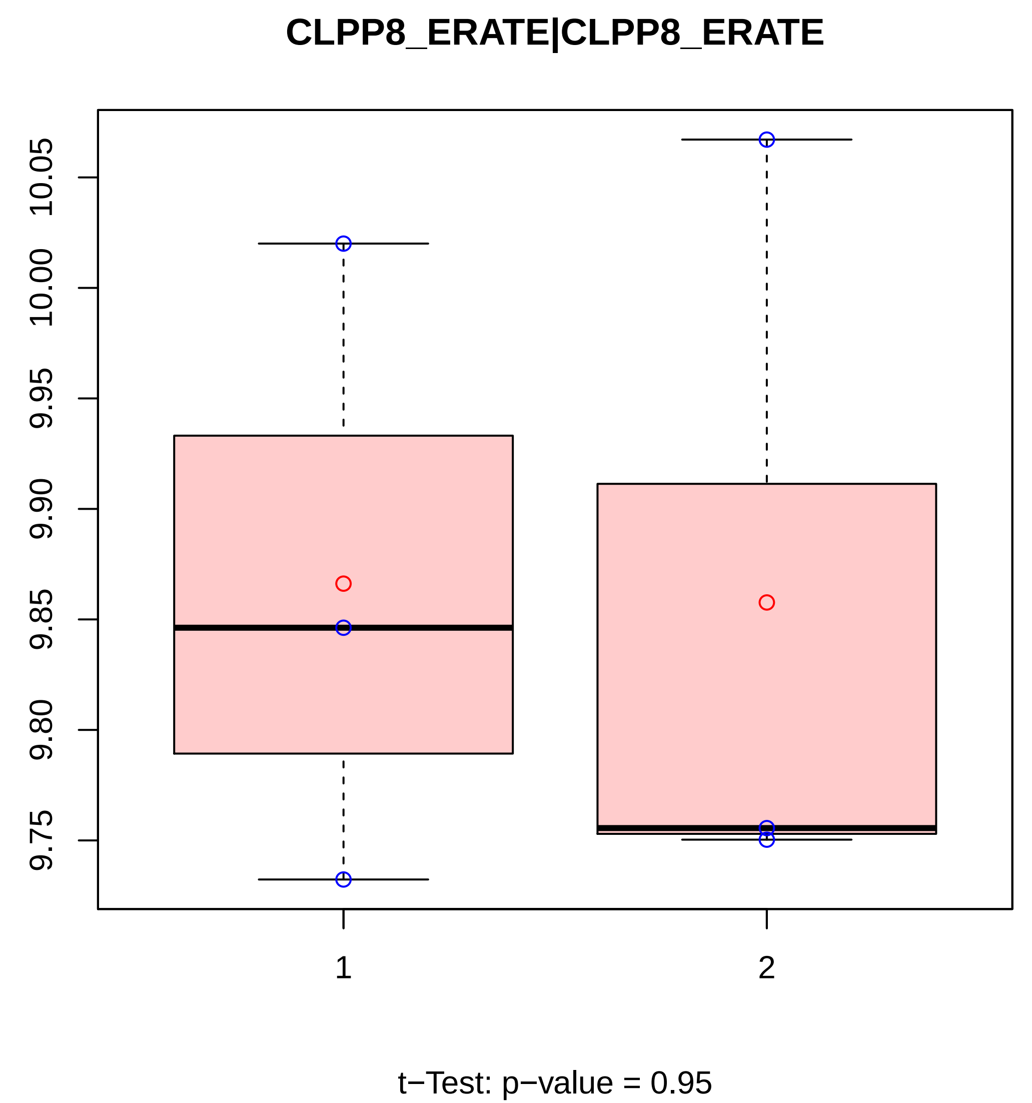
CLPP8_ERATE|CLPP8_ERATE
10.05
10.00
9.95
9.90
9.85
9.80
9.75
1
2
t−Test: p−value = 0.95

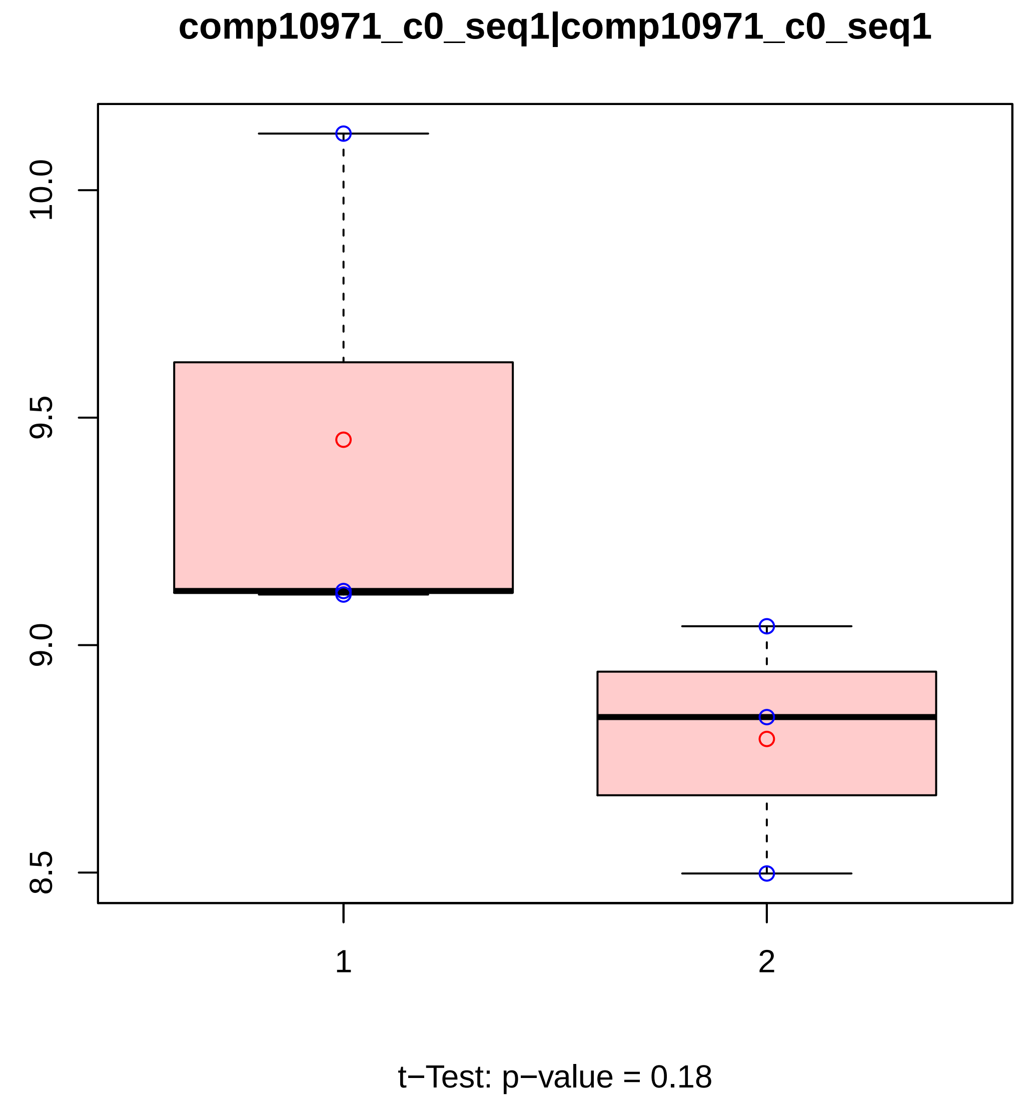
comp10971_c0_seq1|comp10971_c0_seq1
10.0
9.5
9.0
8.5
1
2
t−Test: p−value = 0.18

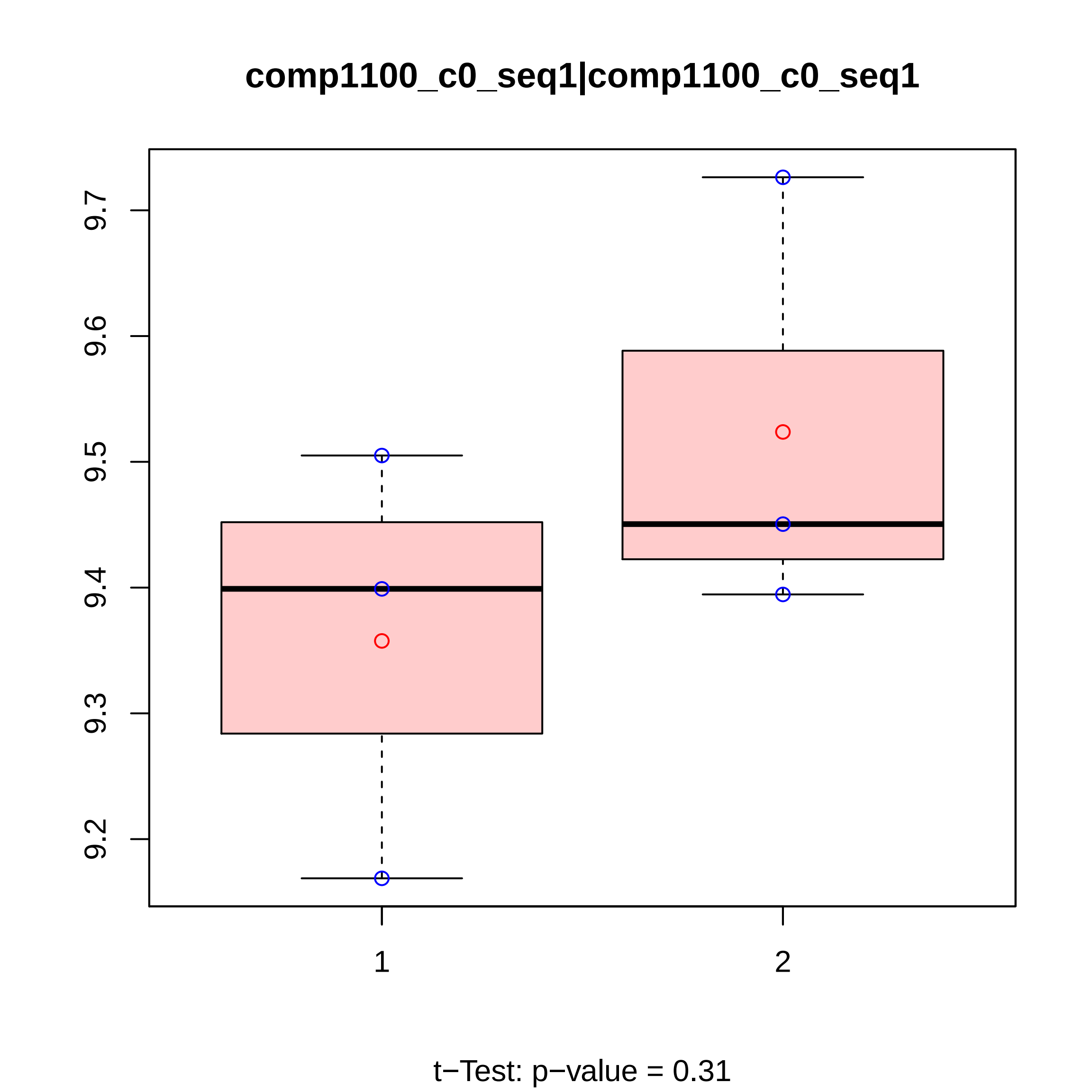

comp1100_c0_seq1|comp1100_c0_seq1
9.2
9.3
9.4
9.5
9.6
9.7
1
2
t−Test: p−value = 0.31

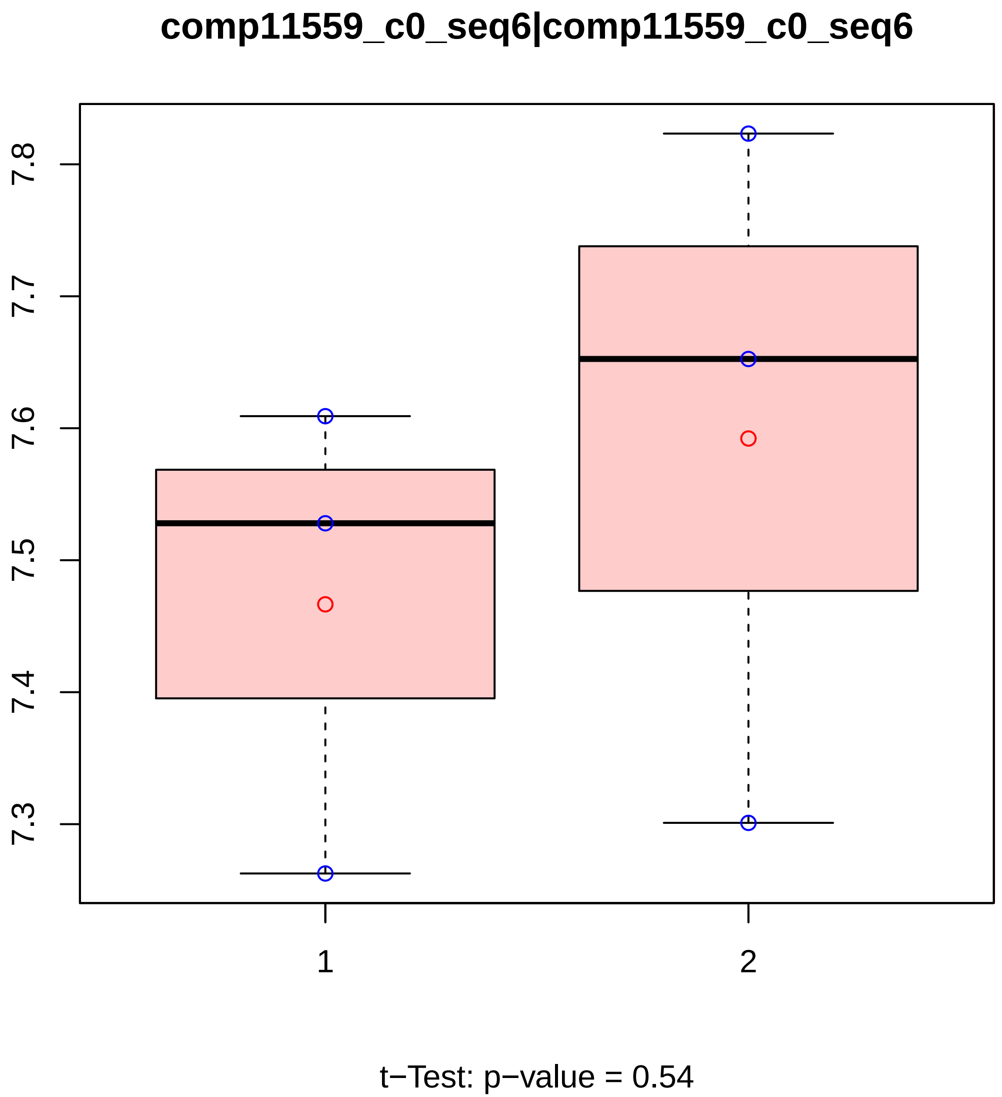
comp11559_c0_seq6|comp11559_c0_seq6
7.3
7.4
7.5
7.6
7.7
7.8
1
2
t−Test: p−value = 0.54

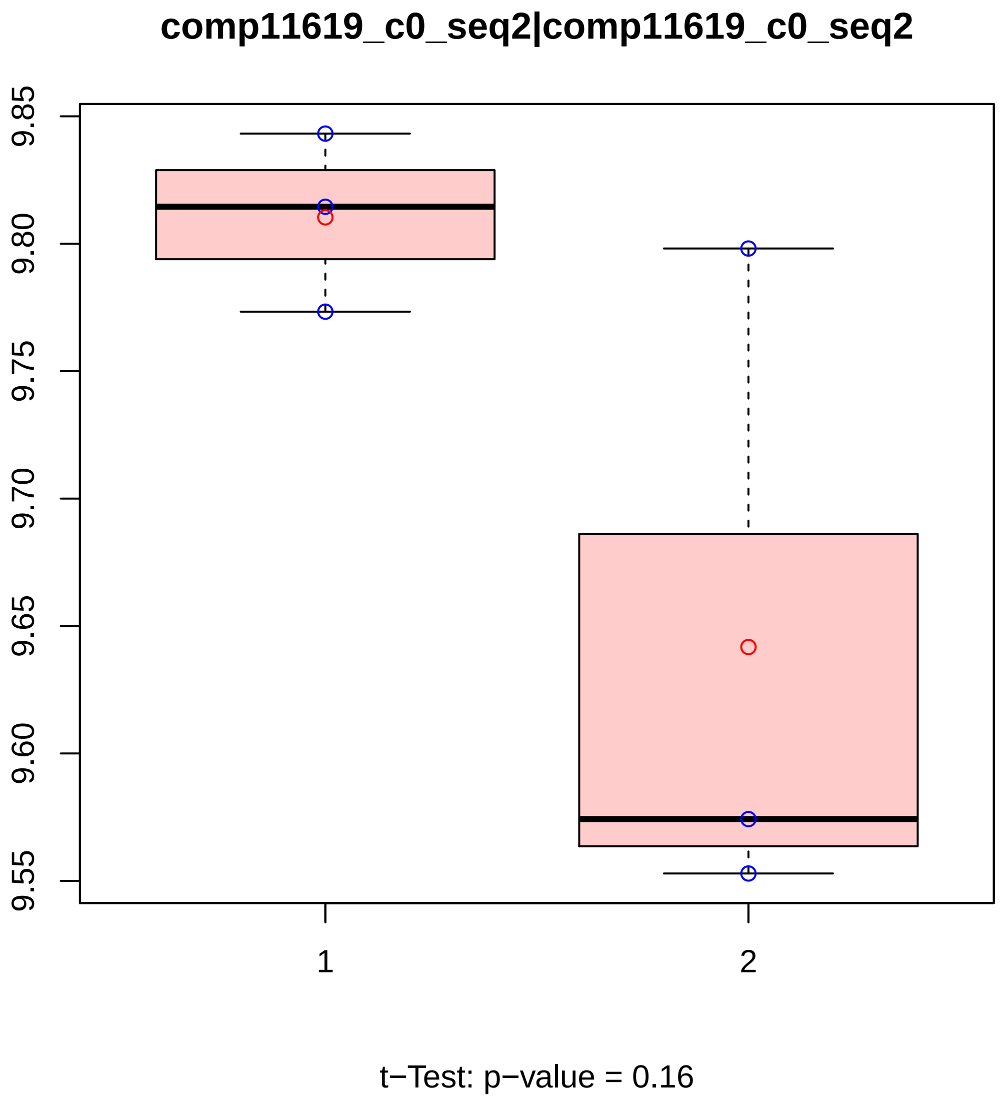
comp11619_c0_seq2|comp11619_c0_seq2
9.85
9.80
9.75
9.70
9.65
9.60
9.55
1
2
t−Test: p−value = 0.16

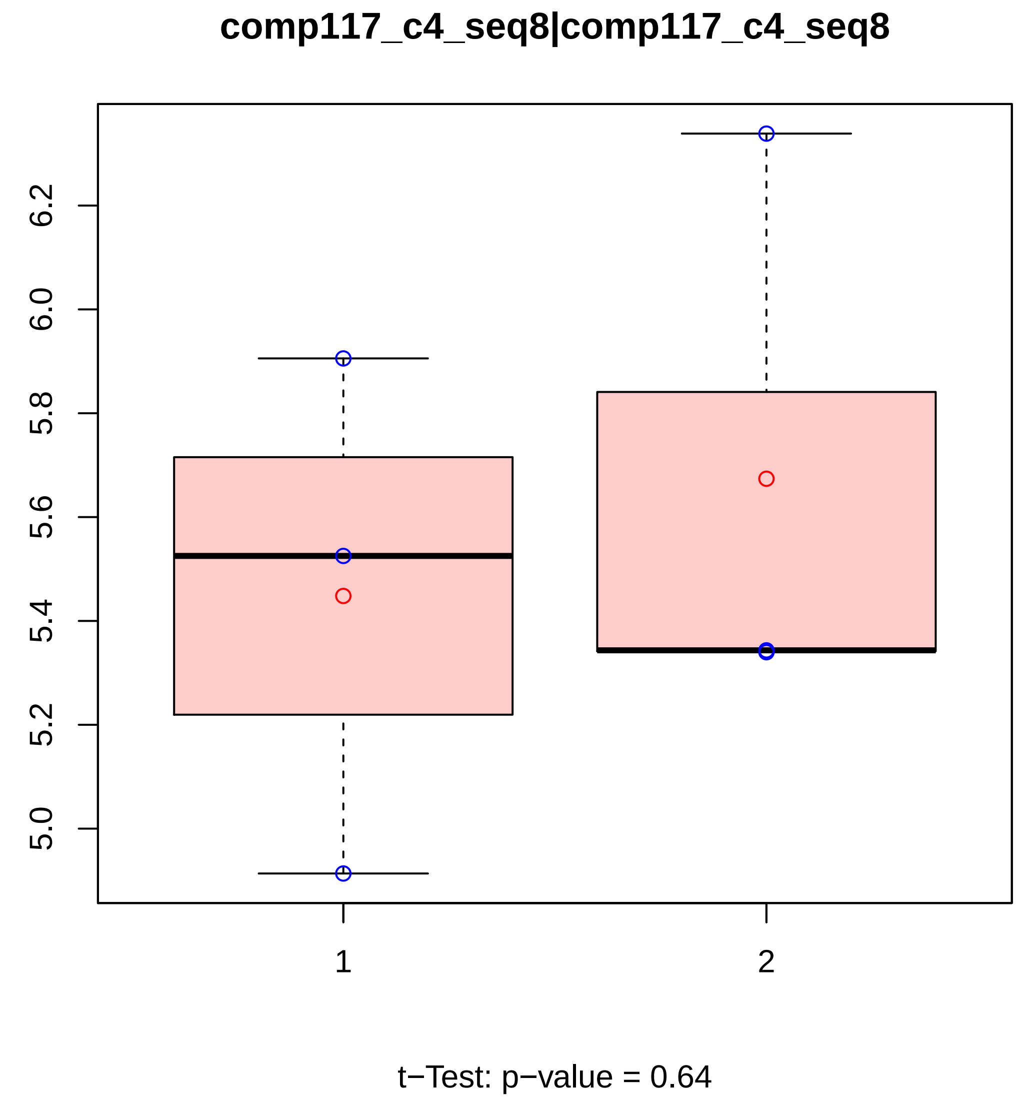

comp117_c4_seq8|comp117_c4_seq8
5.0
5.2
5.4
5.6
5.8
6.0
6.2
1
2
t−Test: p−value = 0.64

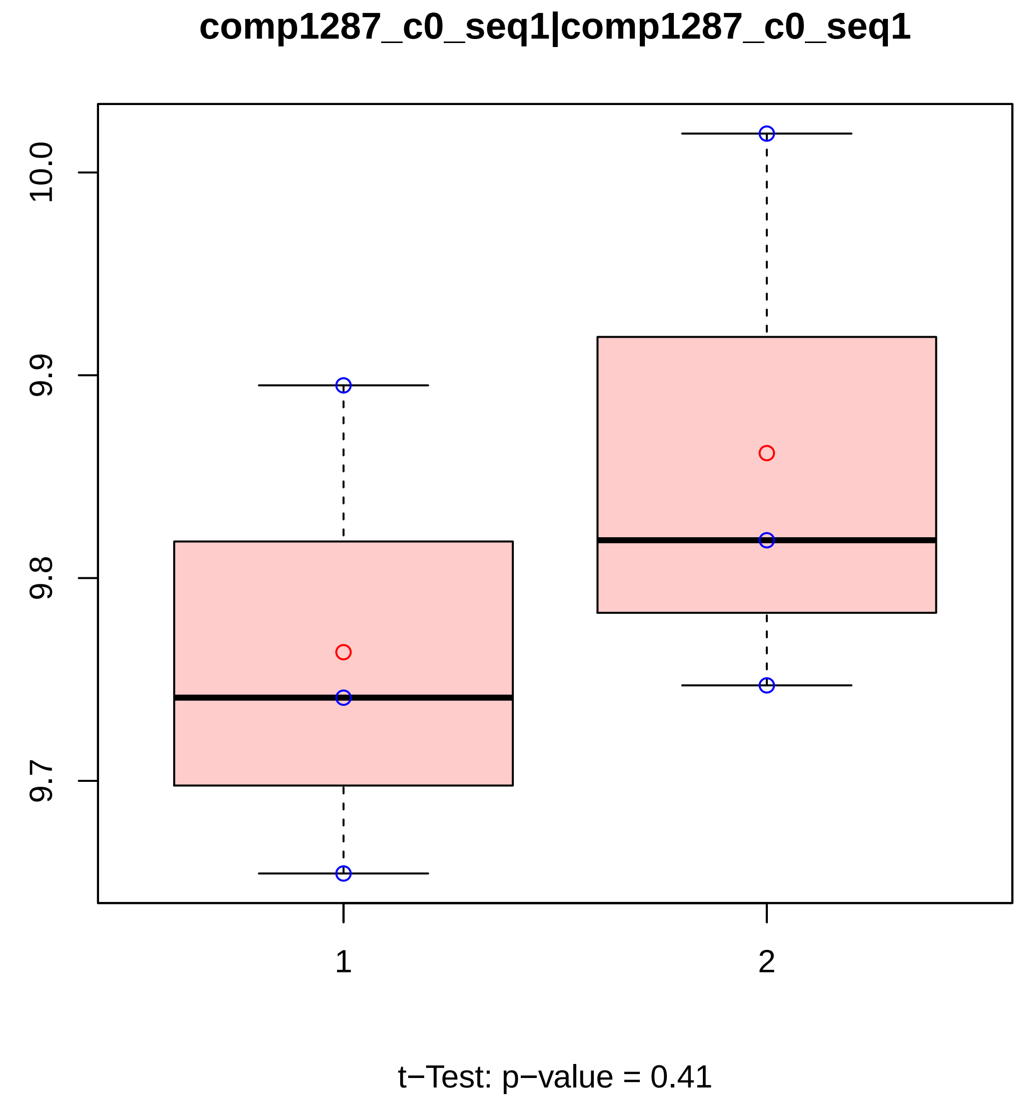
comp1287_c0_seq1|comp1287_c0_seq1
10.0
9.9
9.8
9.7
1
2
t−Test: p−value = 0.41

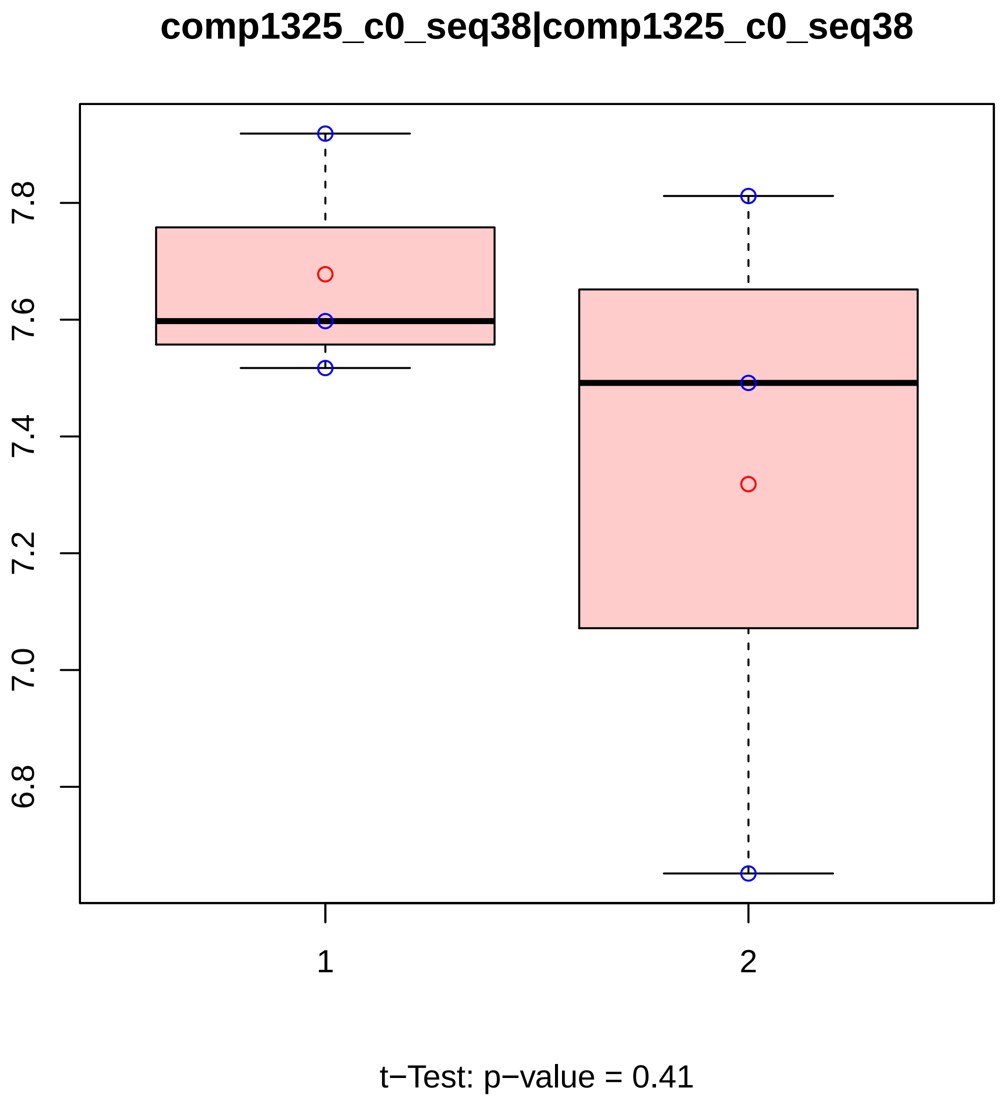

comp1325_c0_seq38|comp1325_c0_seq38
7.8
7.6
7.4
7.2
7.0
6.8
1
2
t−Test: p−value = 0.41

# comp1386_c0_seq1|comp1386_c0_seq1

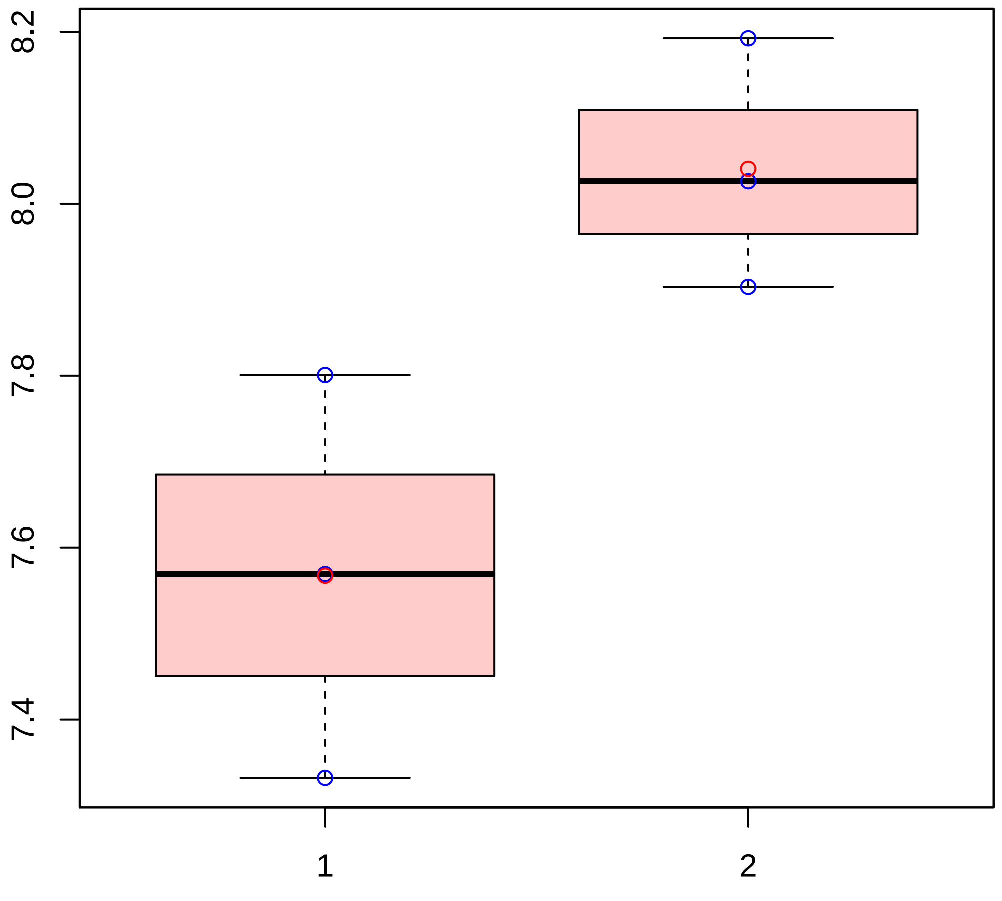


t−Test: p−value = 0.05

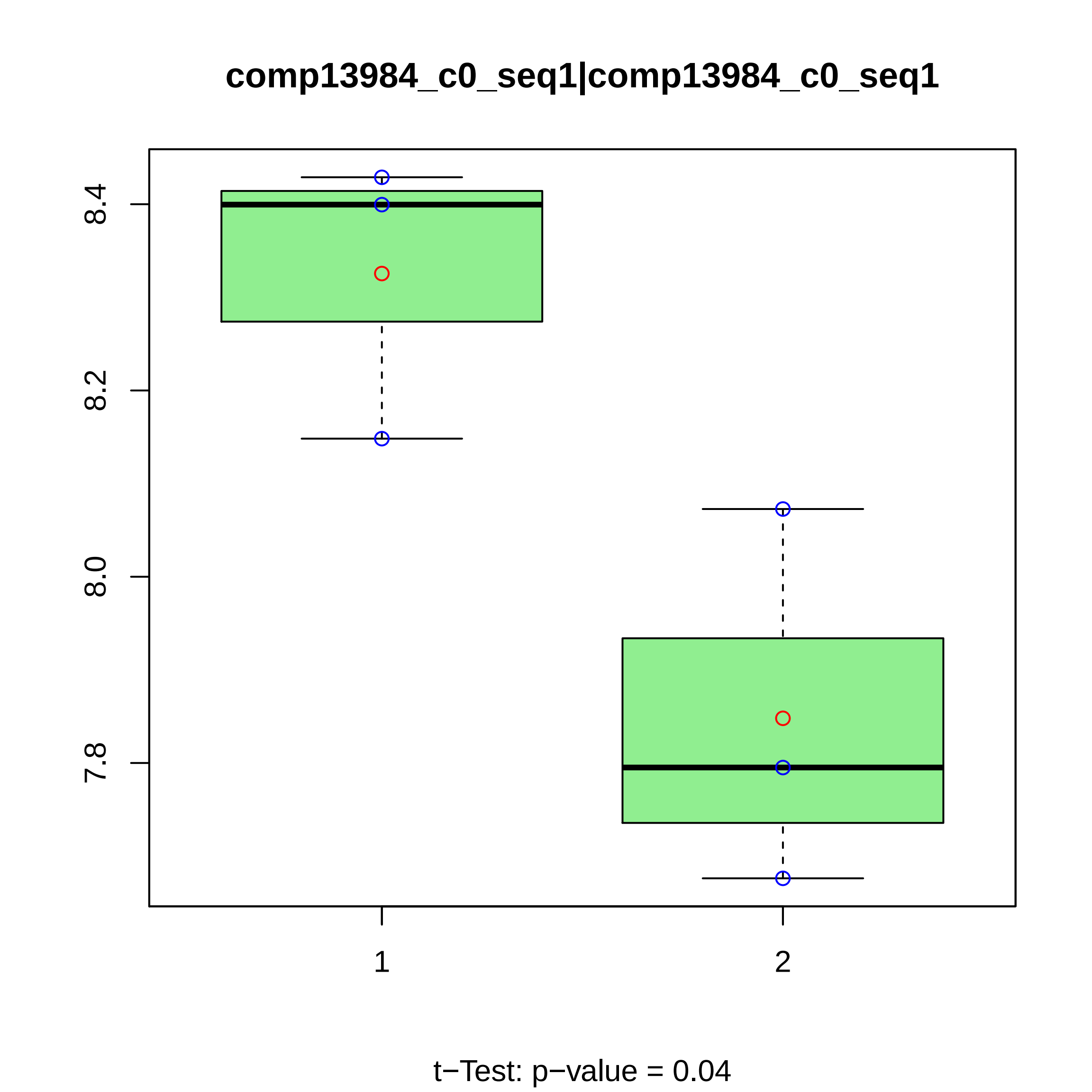
comp13984_c0_seq1|comp13984_c0_seq1
8.4
8.2
8.0
7.8
1
2
t−Test: p−value = 0.04

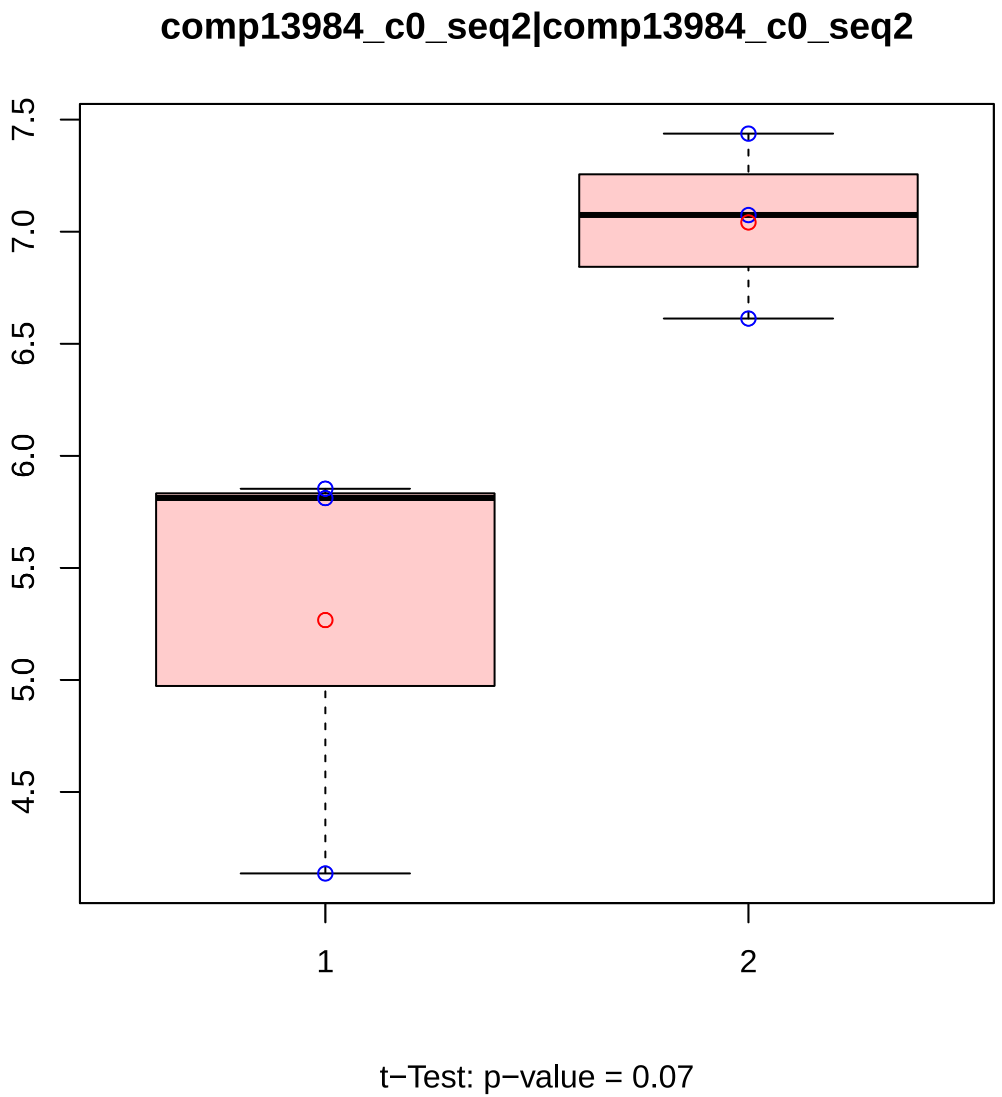

comp13984_c0_seq2|comp13984_c0_seq2
7.5
7.0
6.5
6.0
5.5
5.0
4.5
1
2
t−Test: p−value = 0.07

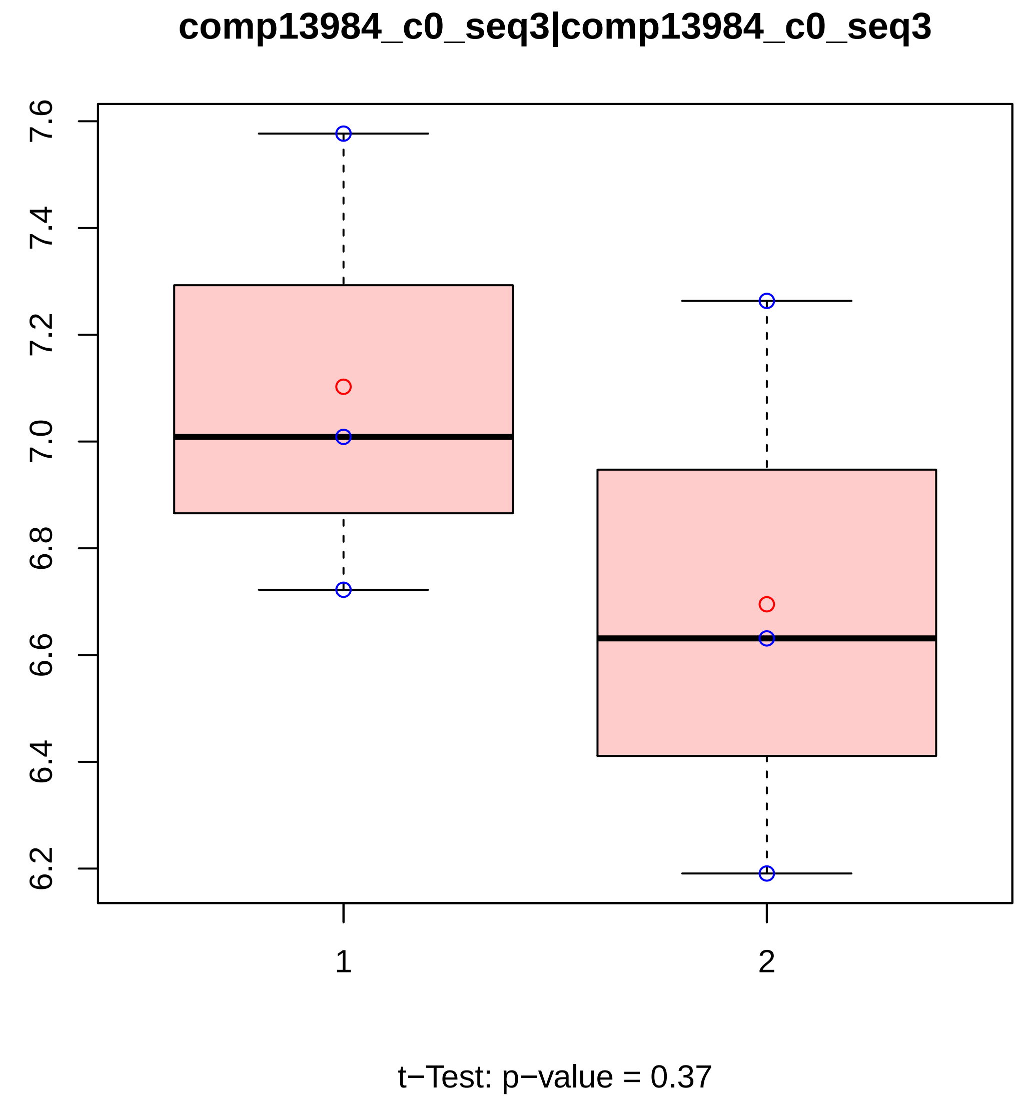
comp13984_c0_seq3|comp13984_c0_seq3
7.6
7.4
7.2
7.0
6.8
6.6
6.4
6.2
1
2
t−Test: p−value = 0.37

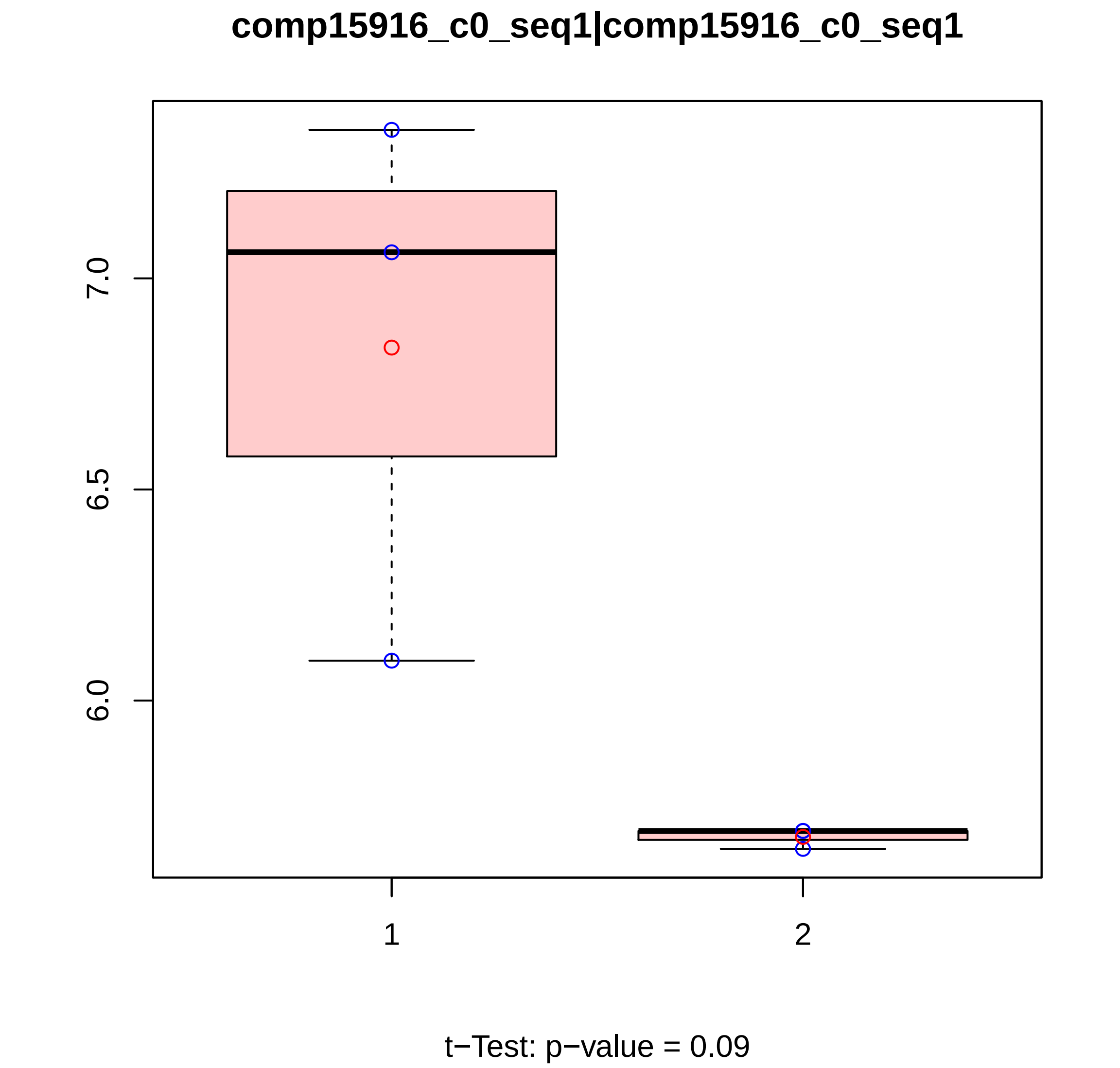
comp15916_c0_seq1|comp15916_c0_seq1
7.0
6.5
6.0
1
2
t−Test: p−value = 0.09

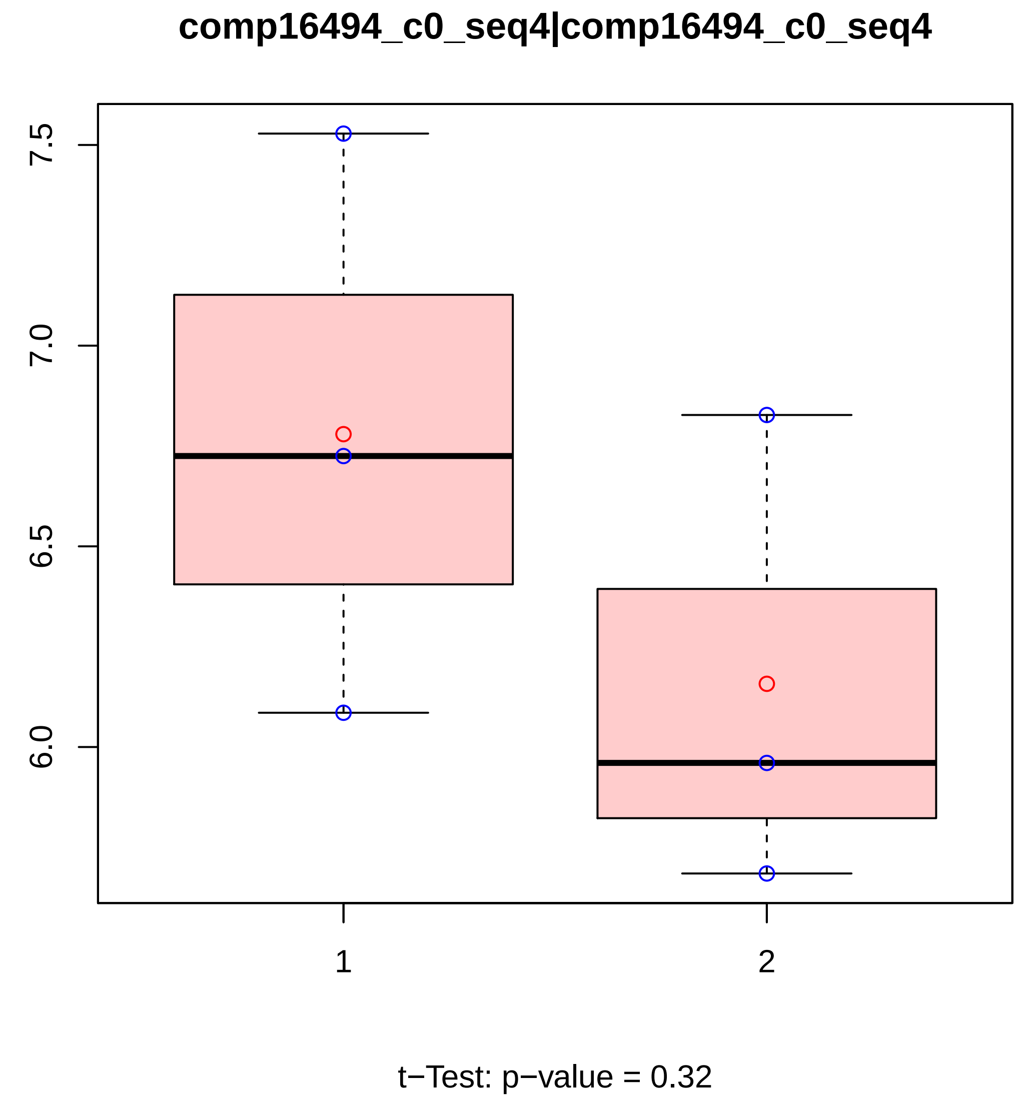
comp16494_c0_seq4|comp16494_c0_seq4
7.5
7.0
6.5
6.0
1
2
t−Test: p−value = 0.32

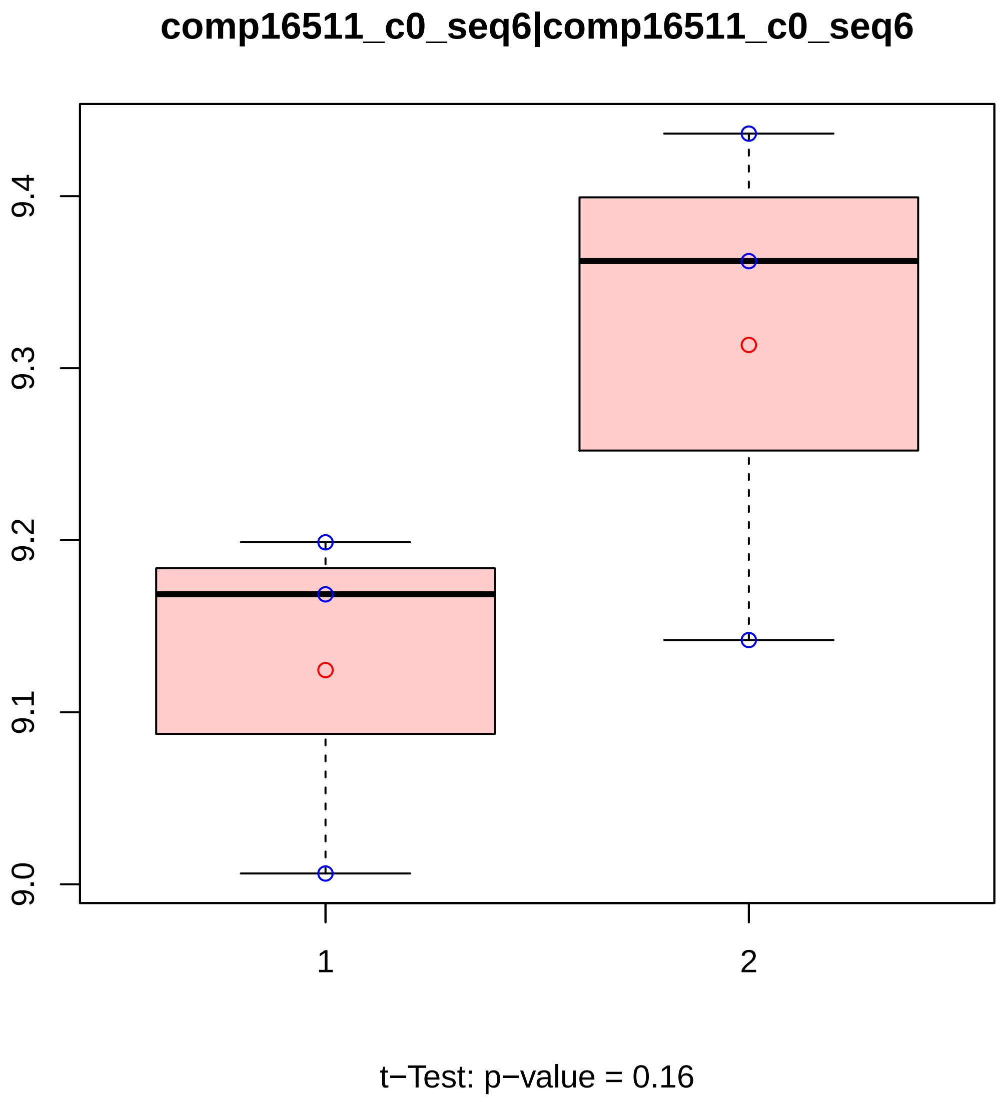
comp16511_c0_seq6|comp16511_c0_seq6
9.0
9.1
9.2
9.3
9.4
1
2
t−Test: p−value = 0.16

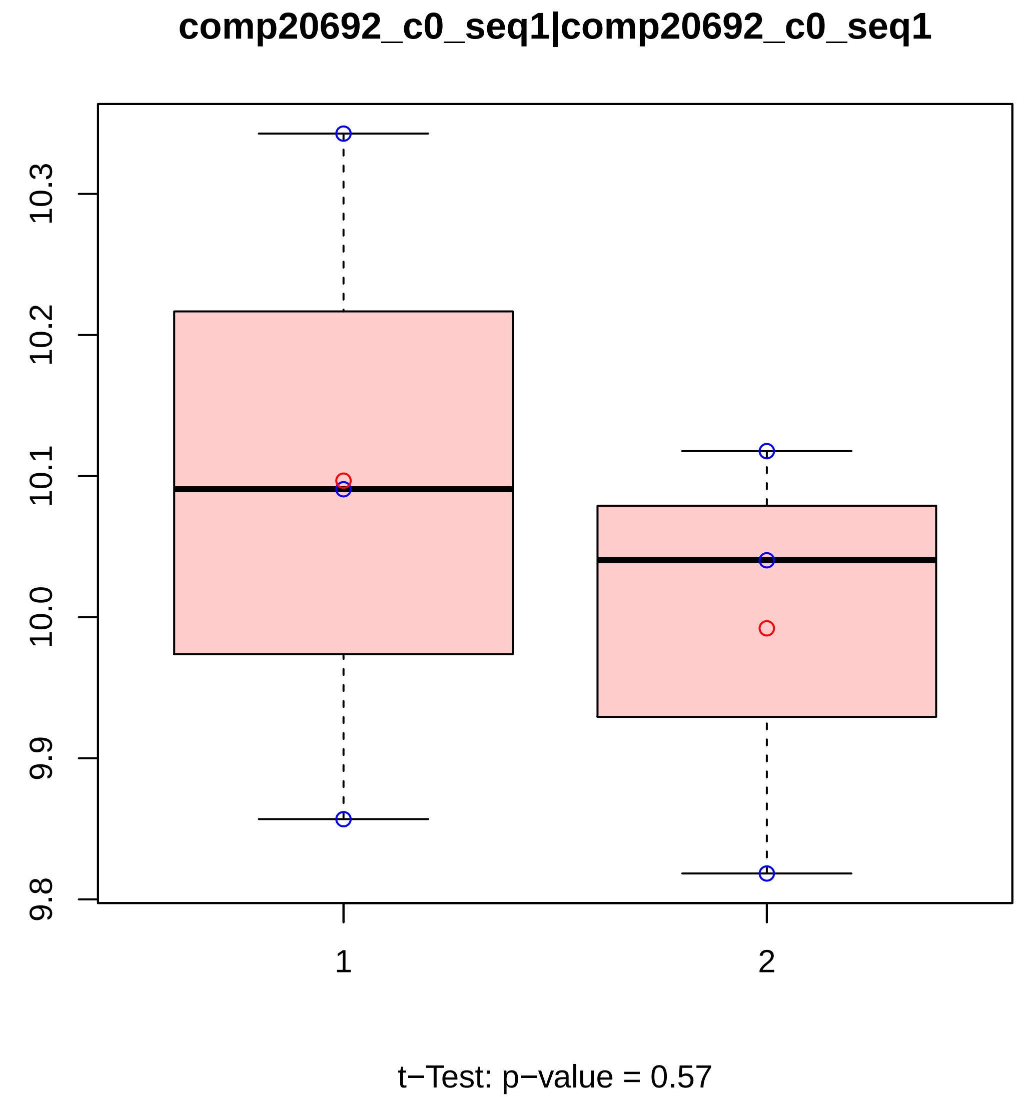

comp20692_c0_seq1|comp20692_c0_seq1
10.3
10.2
10.1
10.0
9.9
9.8
1
2
t−Test: p−value = 0.57

**comp21315_c0_seq2|comp21315_c0_seq2**

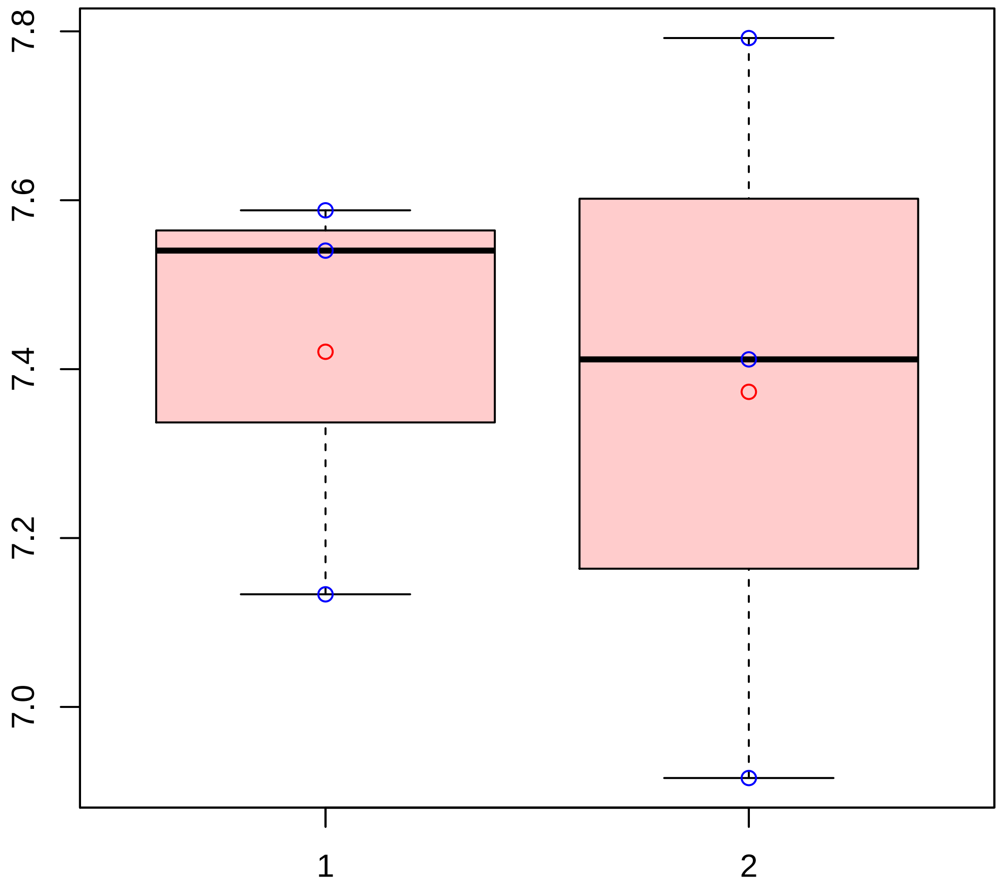


t−Test: p−value = 0.88

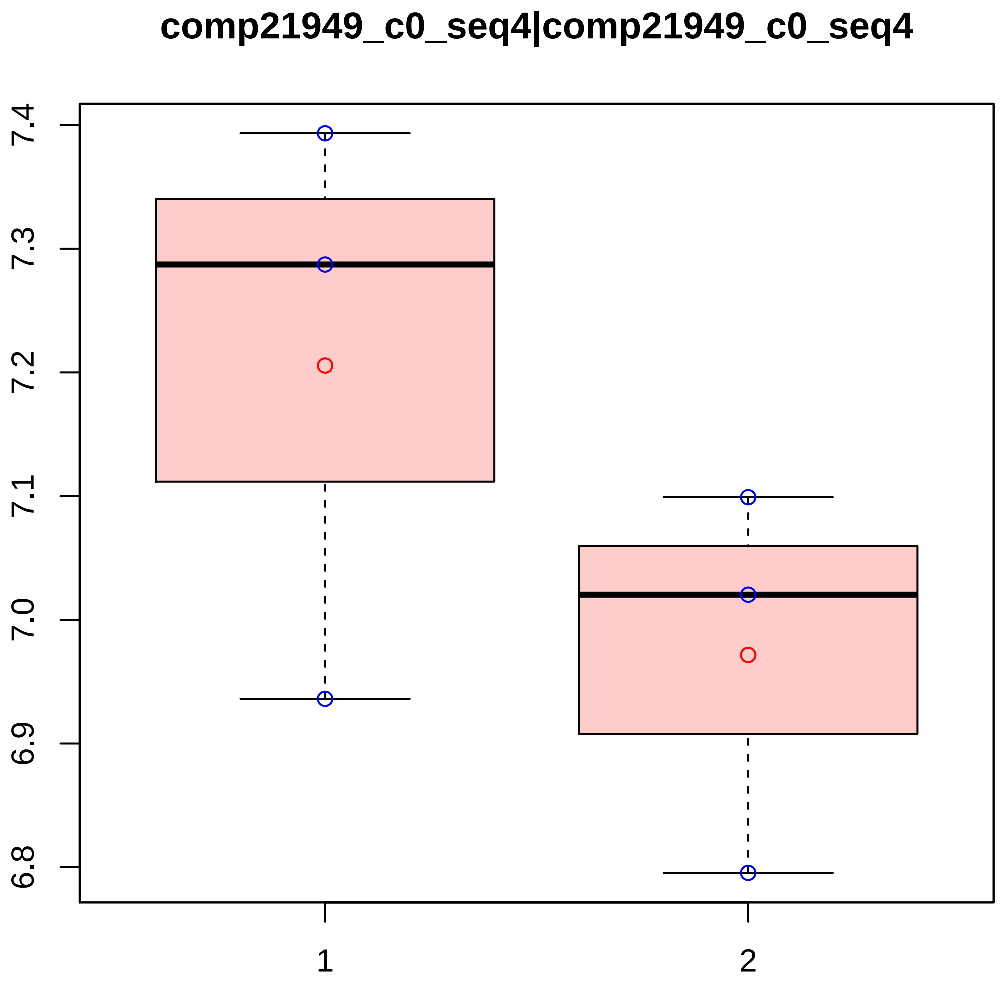


t−Test: p−value = 0.24

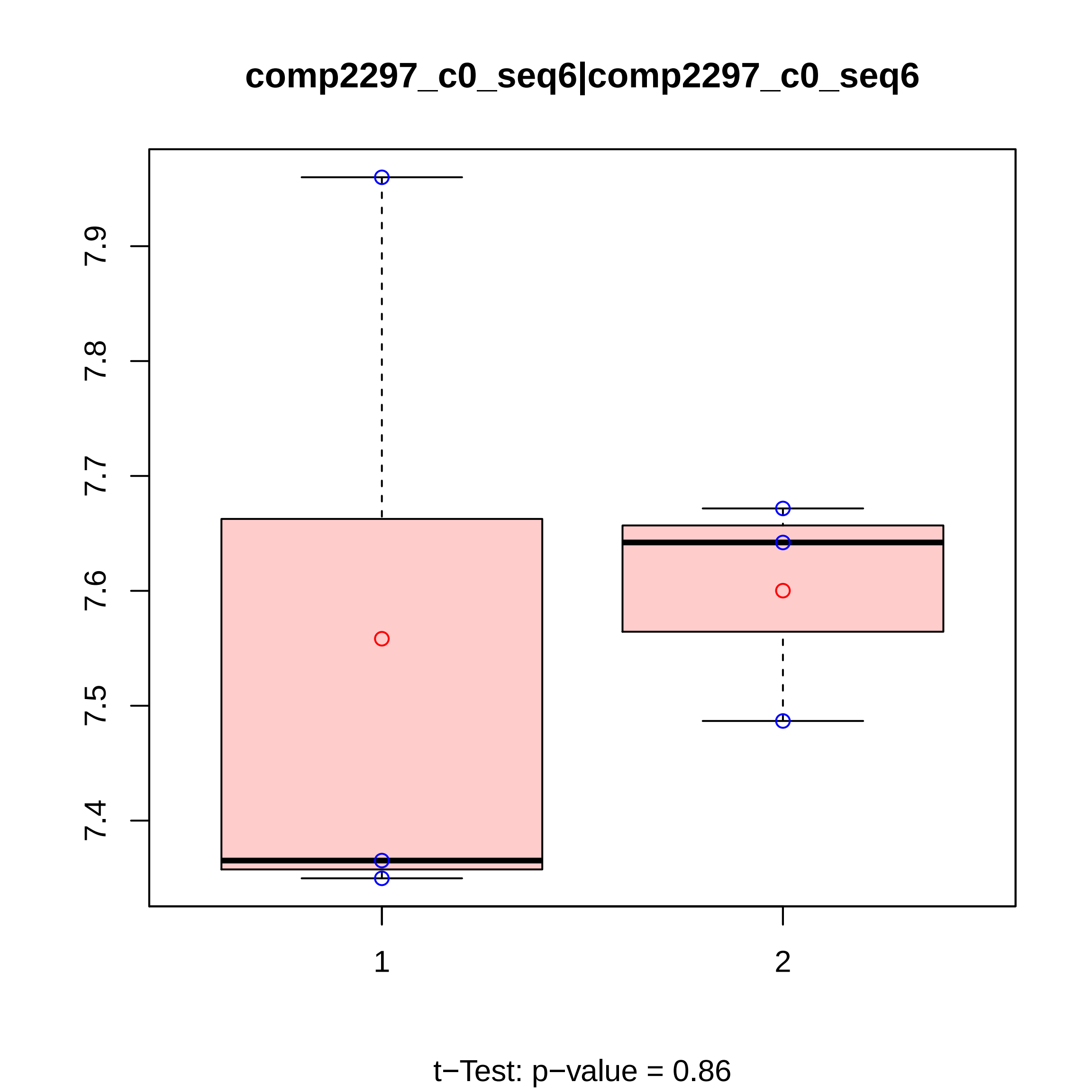
comp2297_c0_seq6|comp2297_c0_seq6
7.4
7.5
7.6
7.7
7.8
7.9
1
2
t−Test: p−value = 0.86

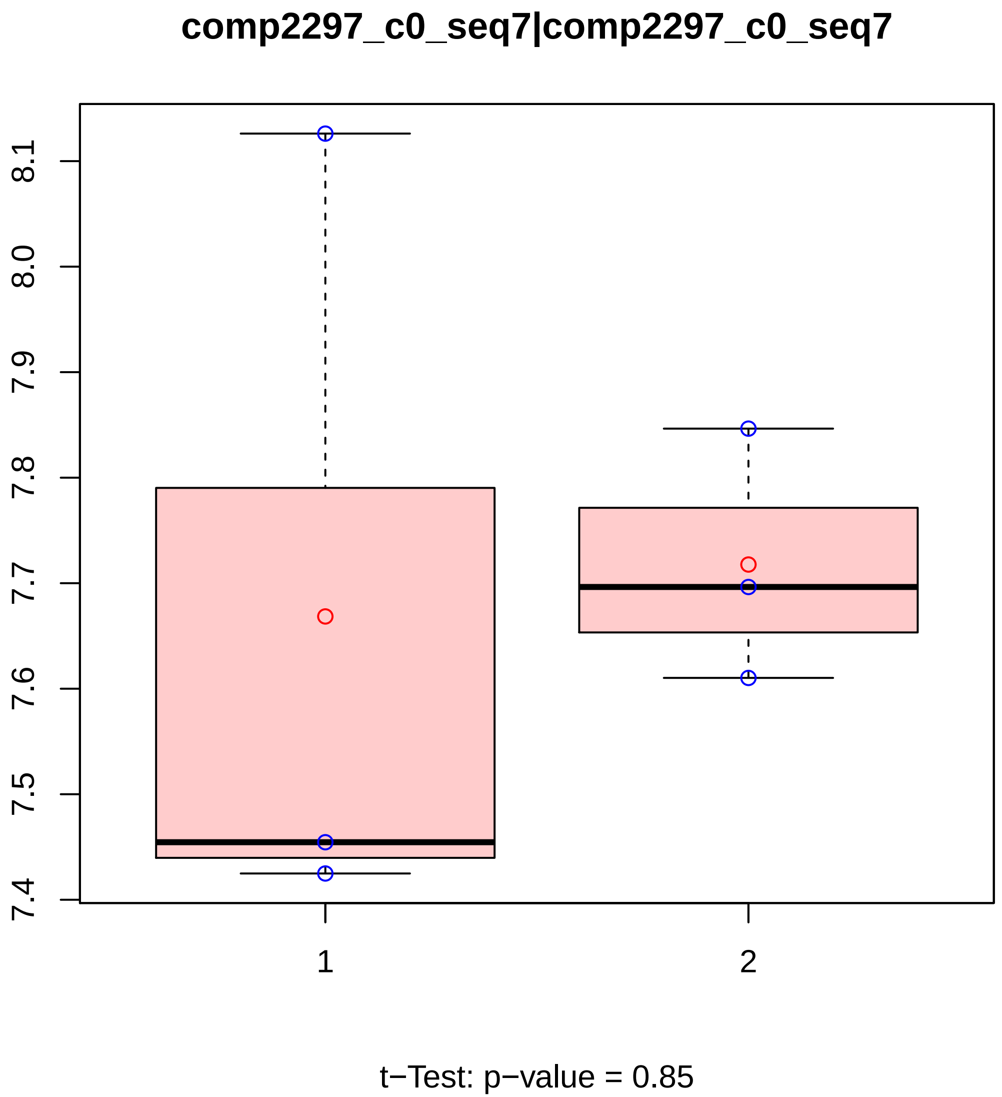
comp2297_c0_seq7|comp2297_c0_seq7
8.1
8.0
7.9
7.8
7.7
7.6
7.5
7.4
1
2
t−Test: p−value = 0.85

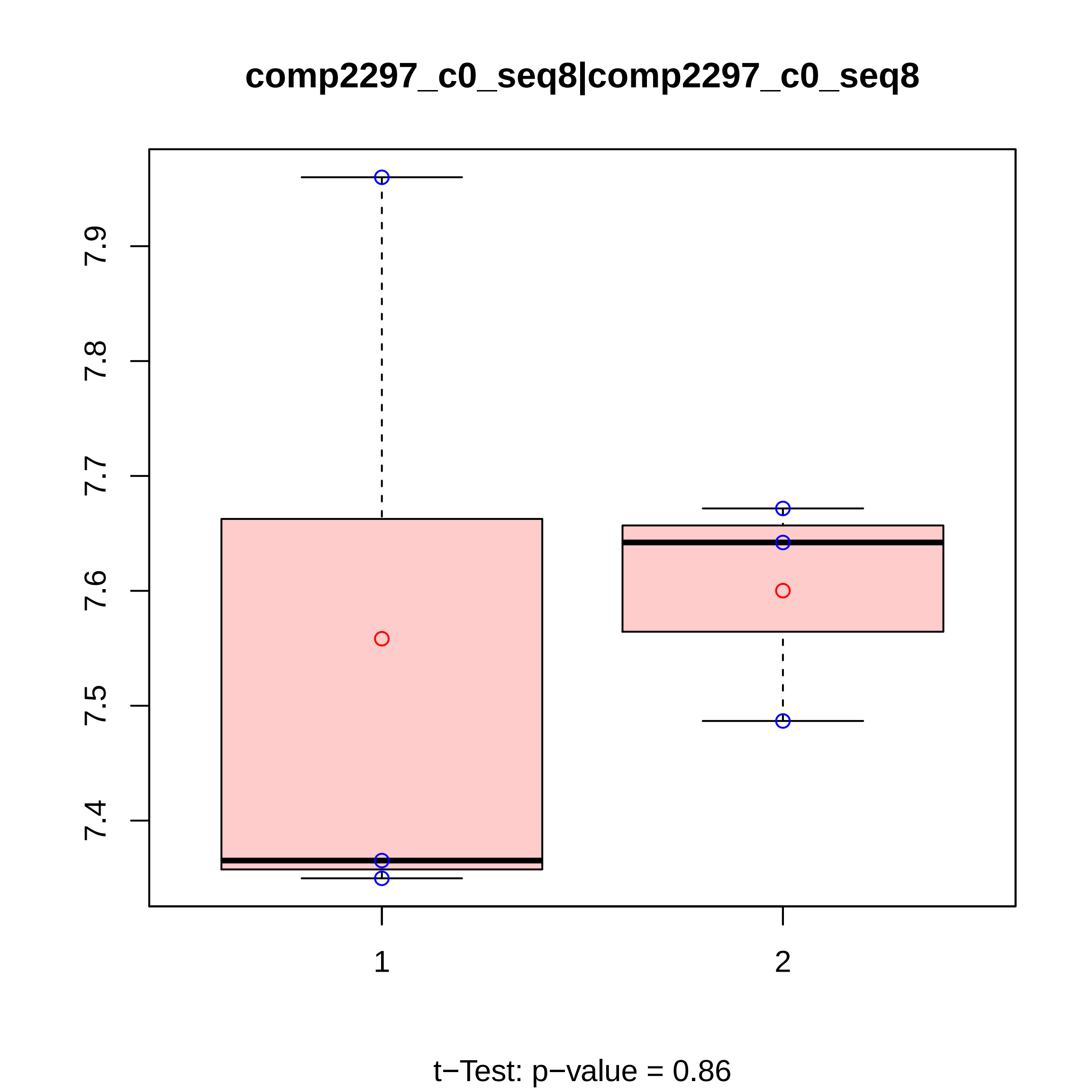
comp2297_c0_seq8|comp2297_c0_seq8
7.4
7.5
7.6
7.7
7.8
7.9
1
2
t−Test: p−value = 0.86

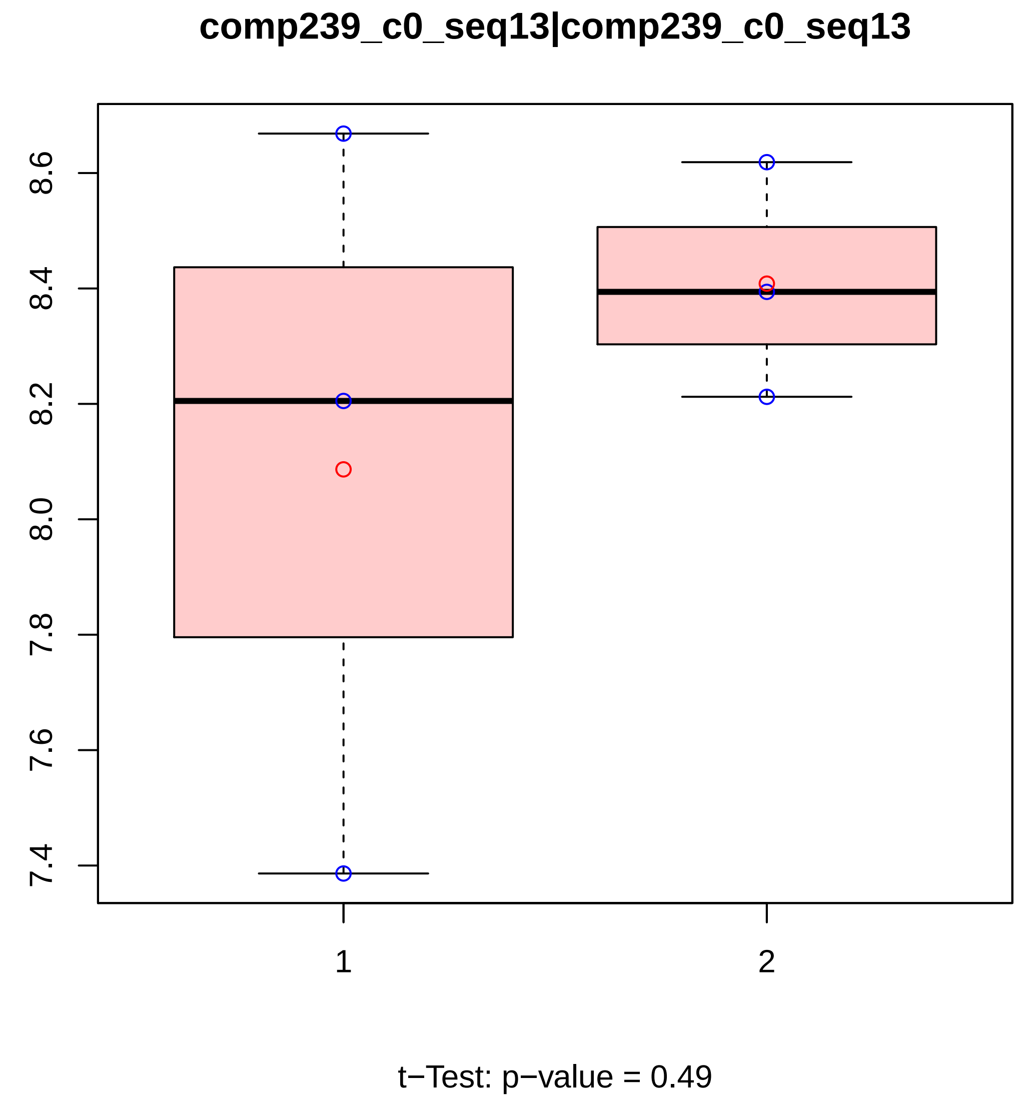
comp239_c0_seq13|comp239_c0_seq13
8.6
8.4
8.2
8.0
7.8
7.6
7.4
1
2
t−Test: p−value = 0.49

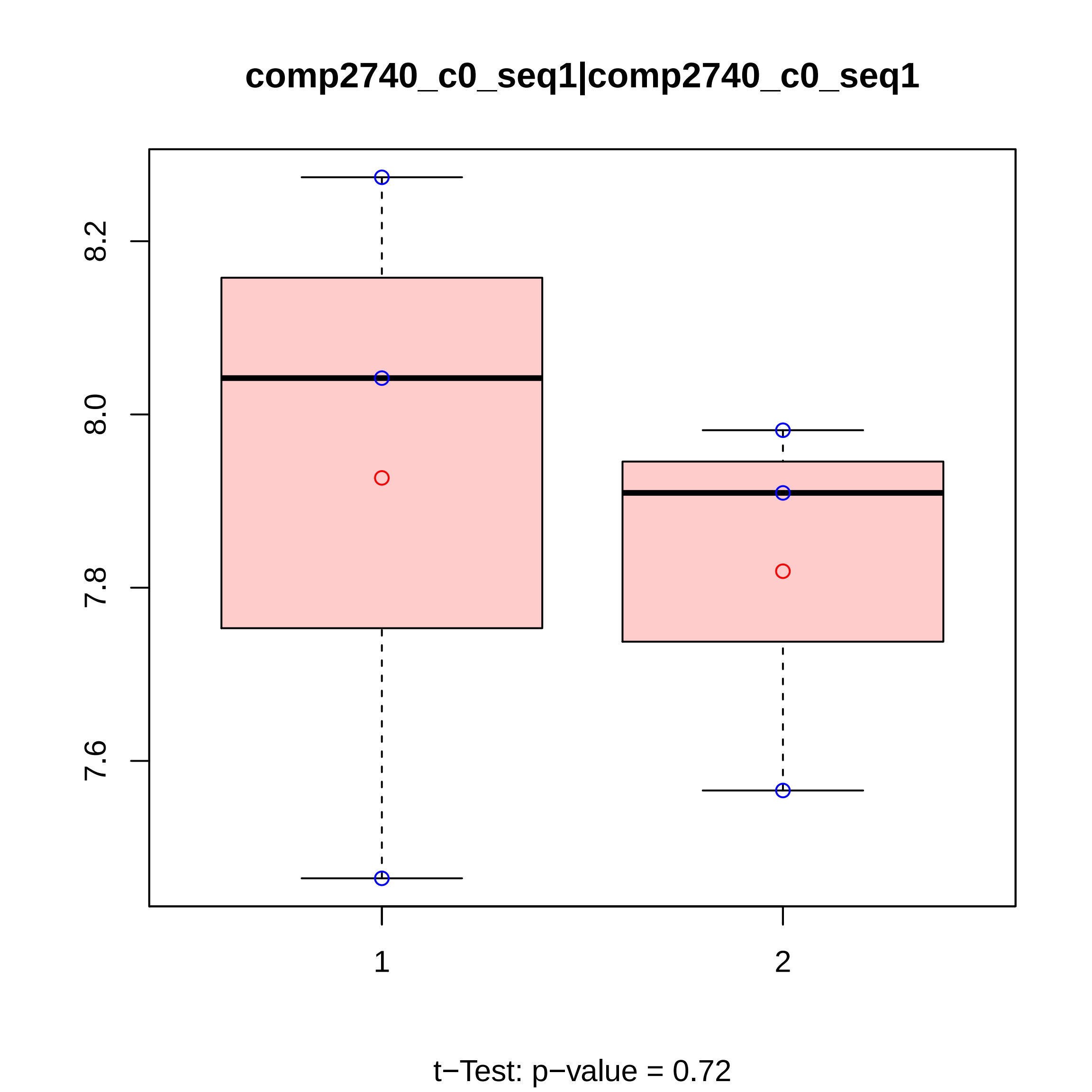
comp2740_c0_seq1|comp2740_c0_seq1
8.2
8.0
7.8
7.6
1
2
t−Test: p−value = 0.72

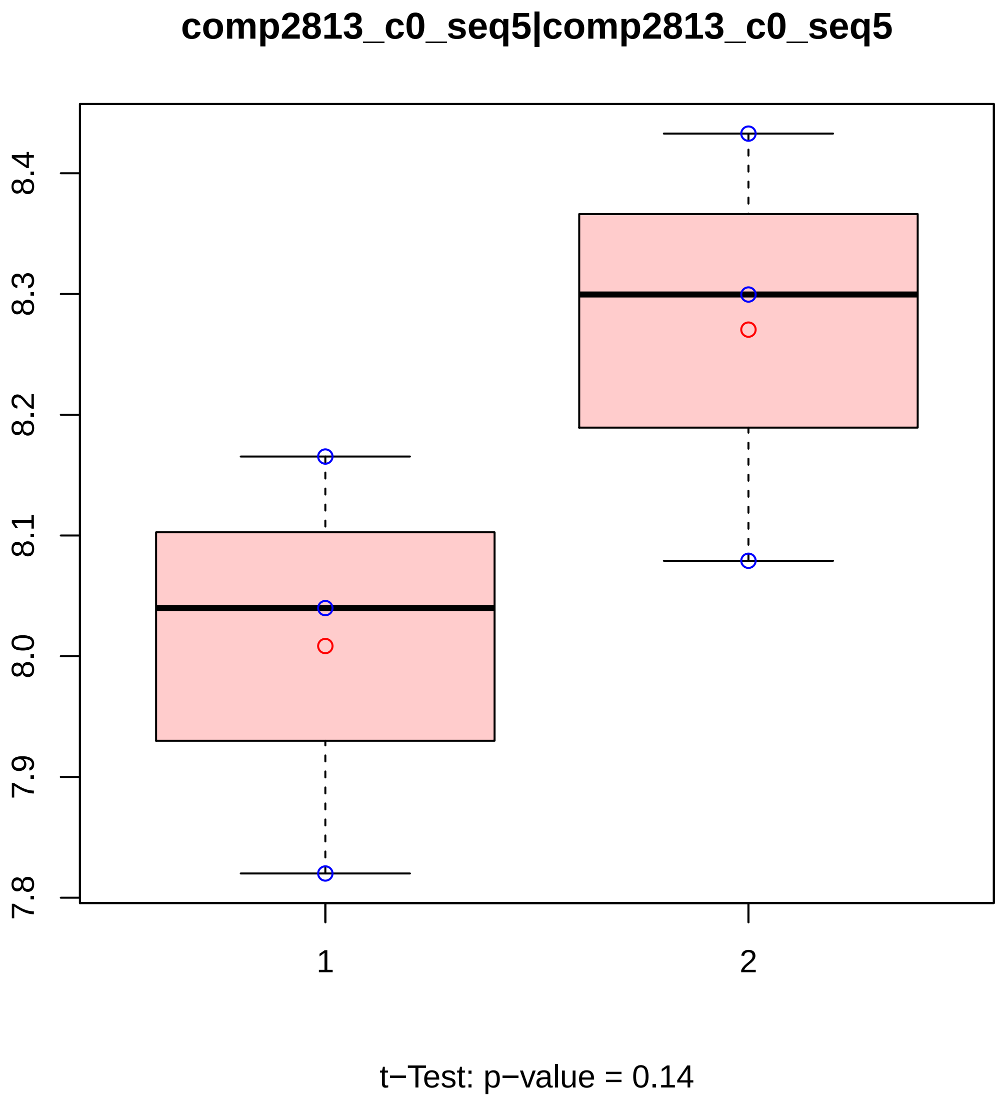
comp2813_c0_seq5|comp2813_c0_seq5
8.4
8.3
8.2
8.1
8.0
7.9
7.8
1
2
t−Test: p−value = 0.14

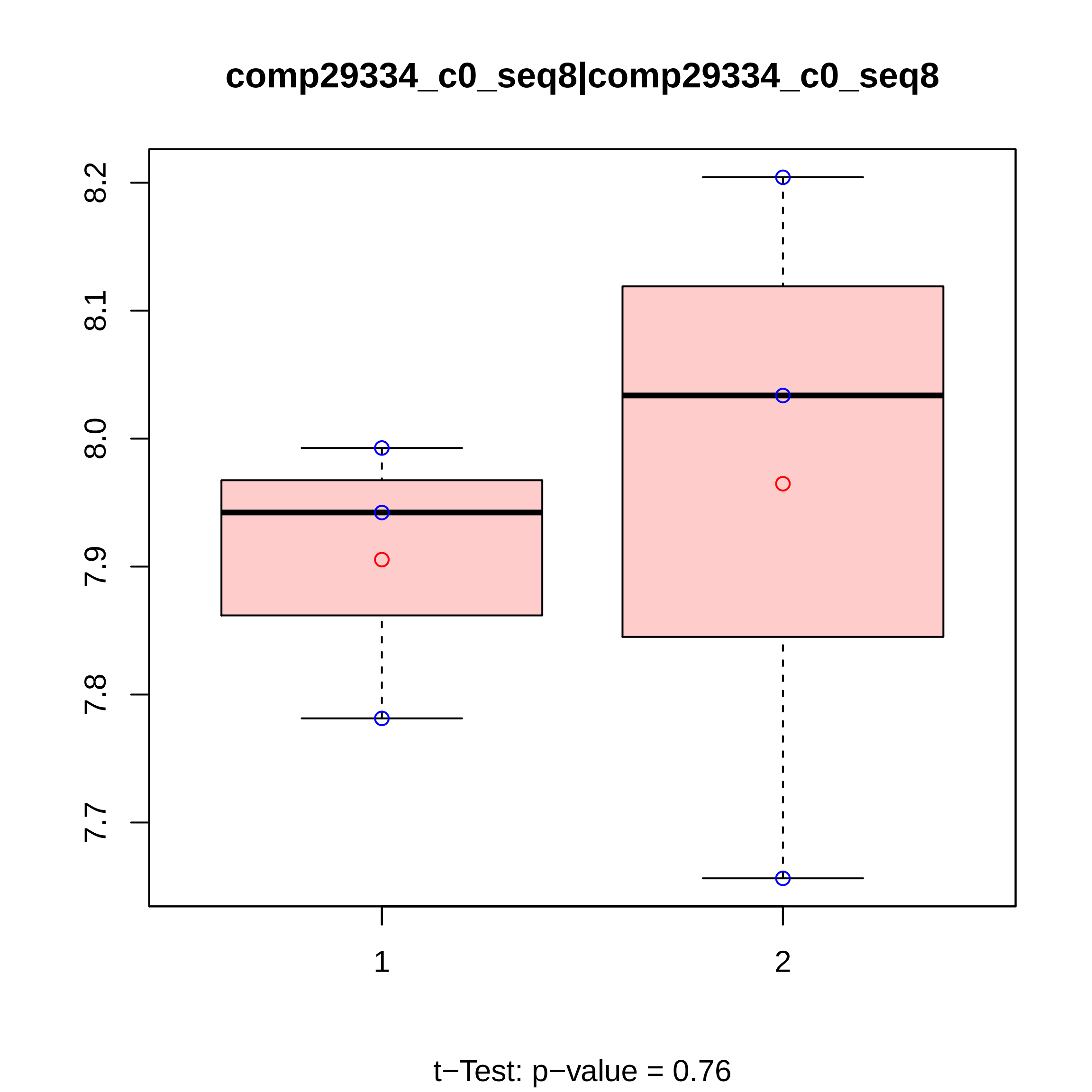
comp29334_c0_seq8|comp29334_c0_seq8
8.2
8.1
8.0
7.9
7.8
7.7
1
2
t−Test: p−value = 0.76

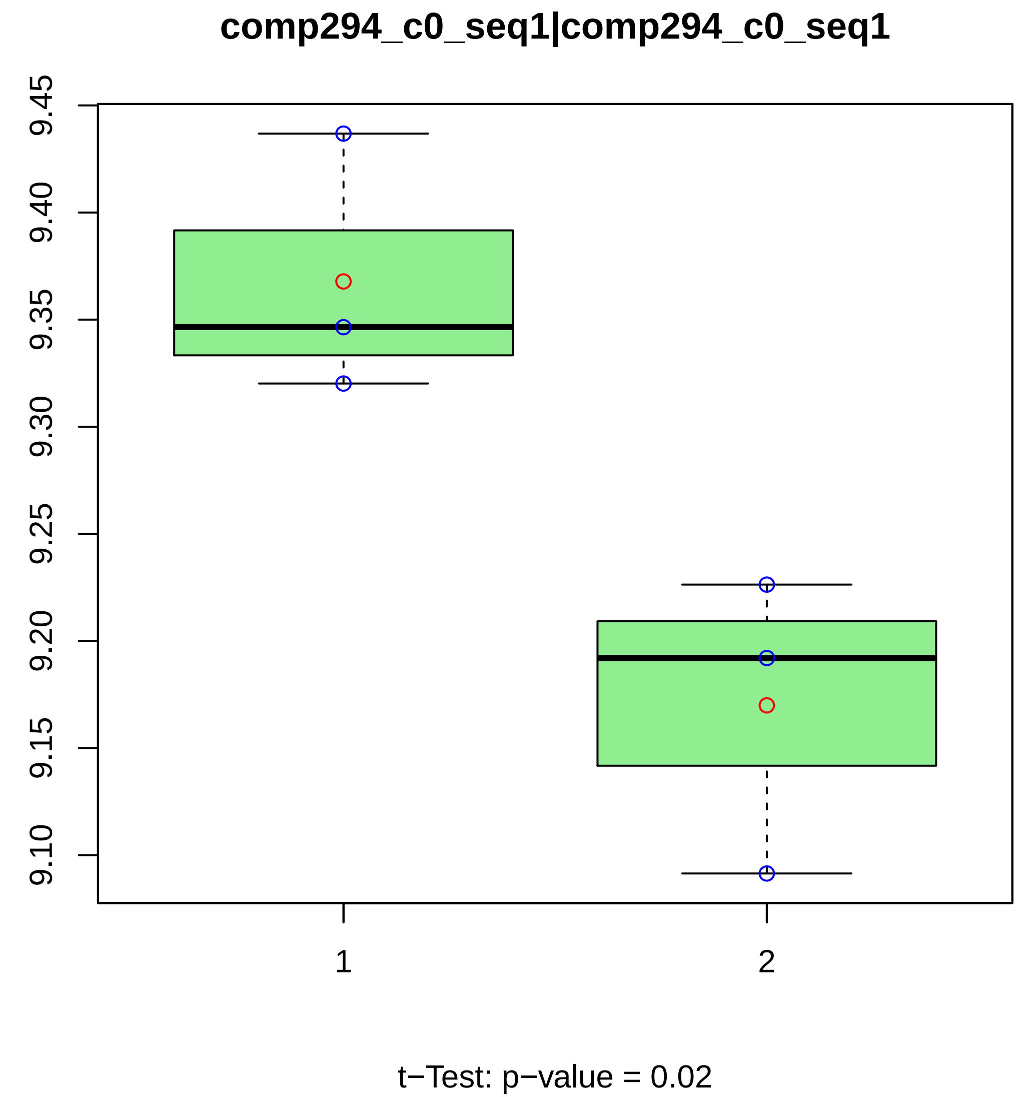

comp294_c0_seq1|comp294_c0_seq1
9.45
9.40
9.35
9.30
9.25
9.20
9.15
9.10
1
2
t−Test: p−value = 0.02

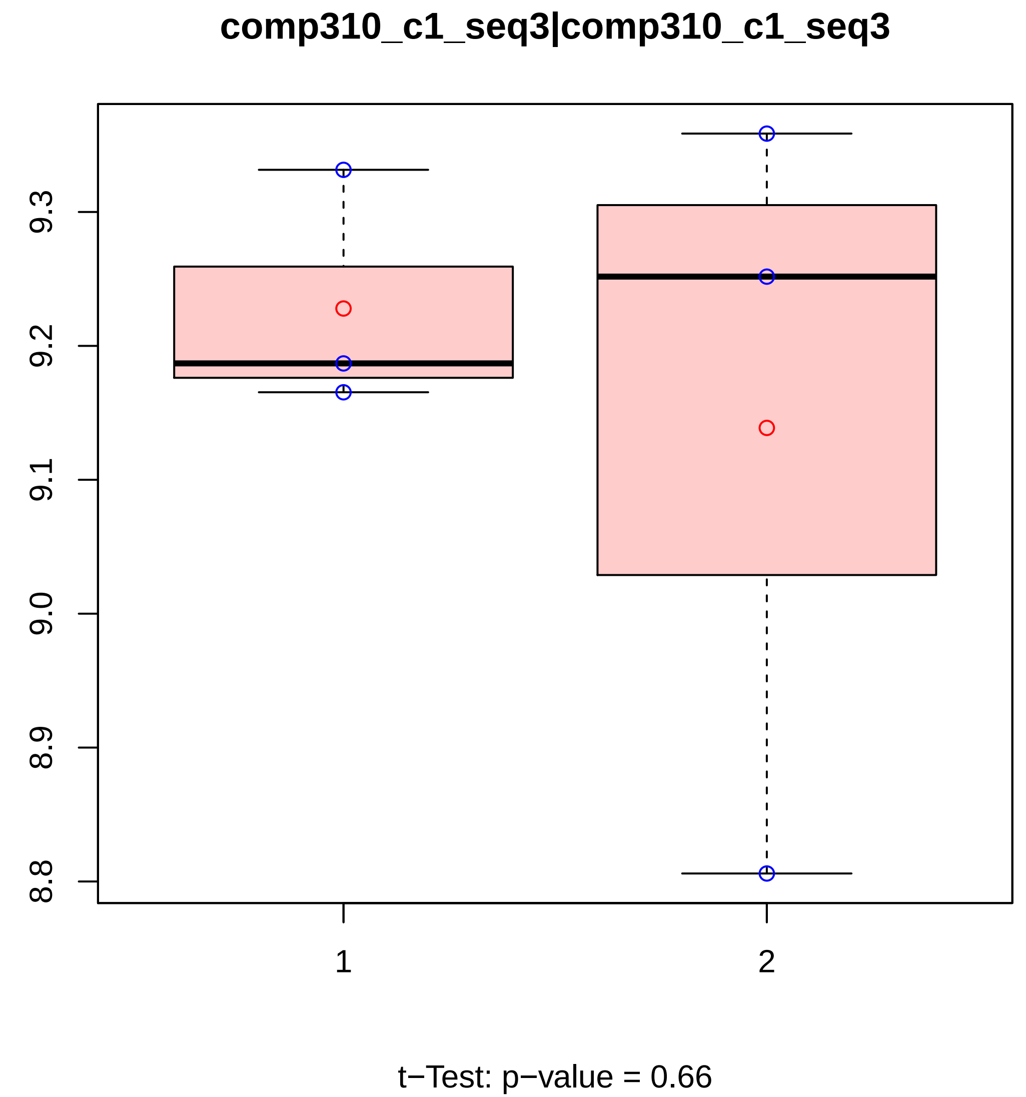
comp310_c1_seq3|comp310_c1_seq3
9.3
9.2
9.1
9.0
8.9
8.8
1
2
t−Test: p−value = 0.66

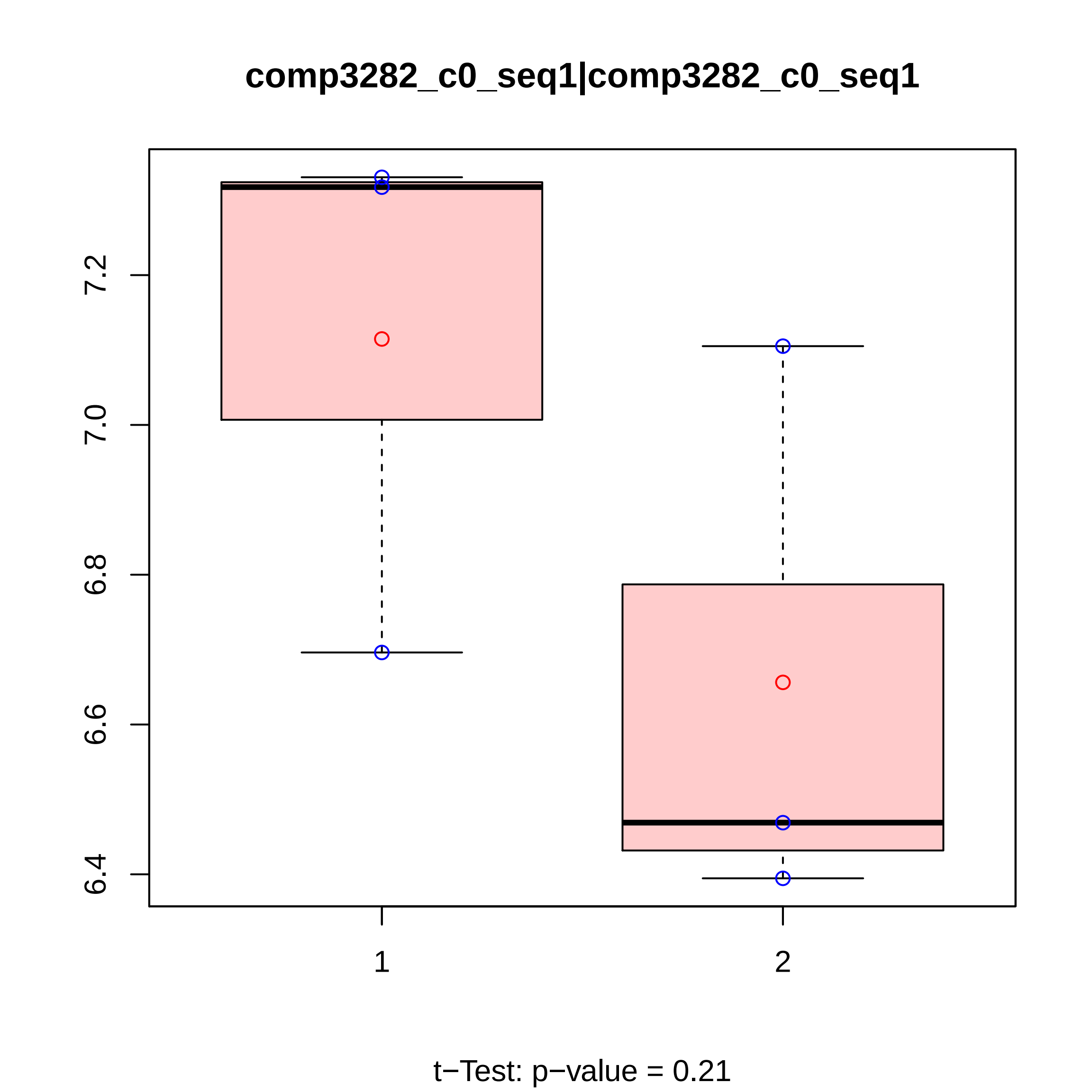
comp3282_c0_seq1|comp3282_c0_seq1
7.2
7.0
6.8
6.6
6.4
1
2
t−Test: p−value = 0.21

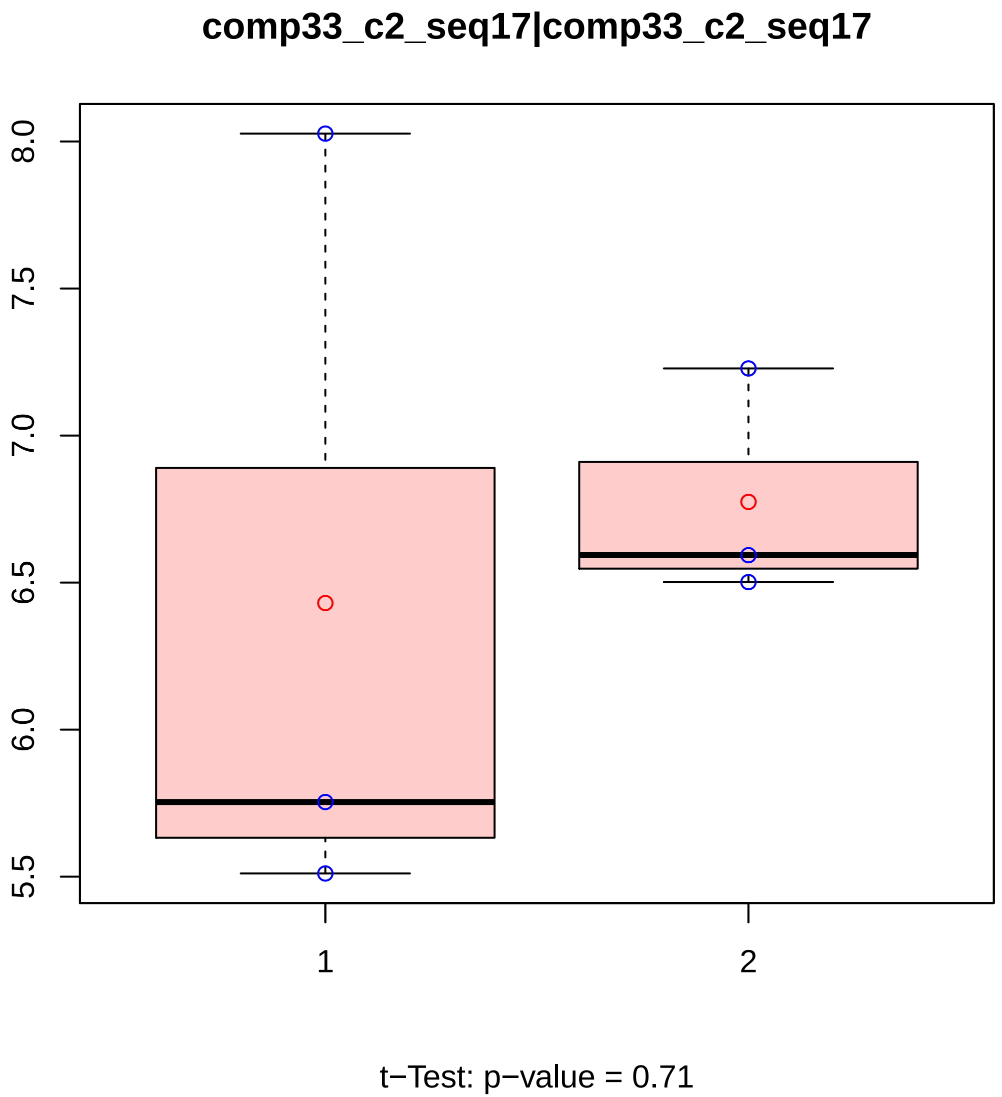
comp33_c2_seq17|comp33_c2_seq17
8.0
7.5
7.0
6.5
6.0
5.5
1
2
t−Test: p−value = 0.71

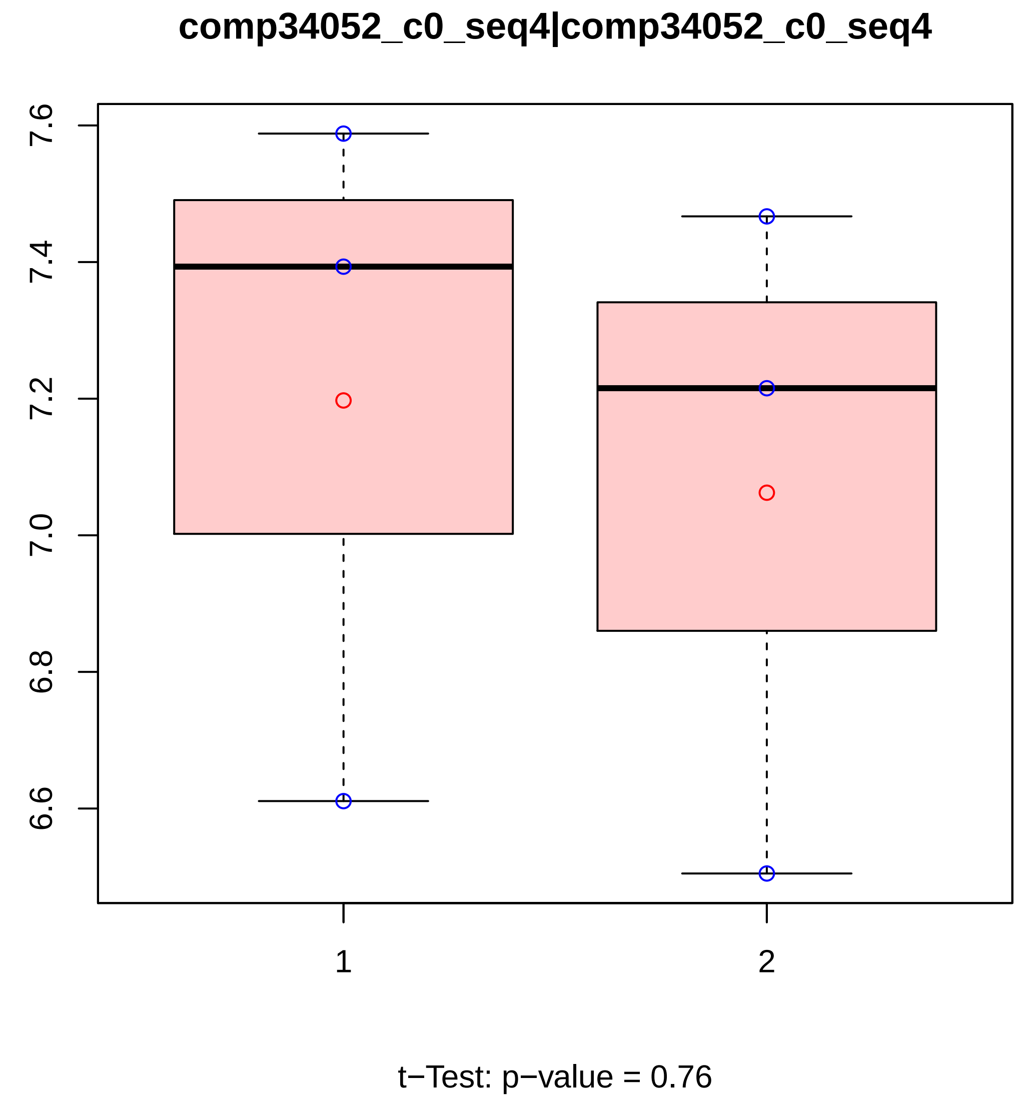
comp34052_c0_seq4|comp34052_c0_seq4
7.6
7.4
7.2
7.0
6.8
6.6
1
2
t−Test: p−value = 0.76

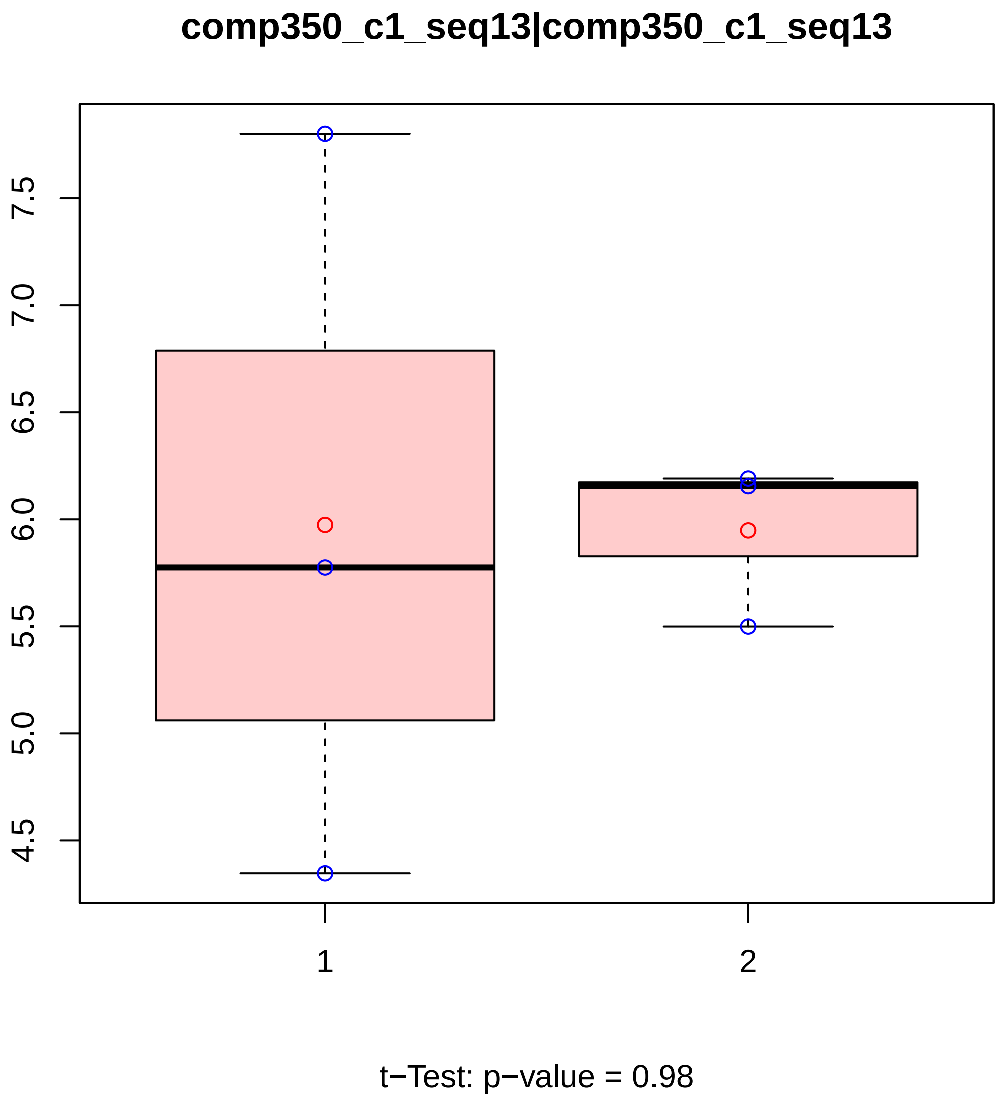
comp350_c1_seq13|comp350_c1_seq13
4.5
5.0
5.5
6.0
6.5
7.0
7.5
1
2
t−Test: p−value = 0.98

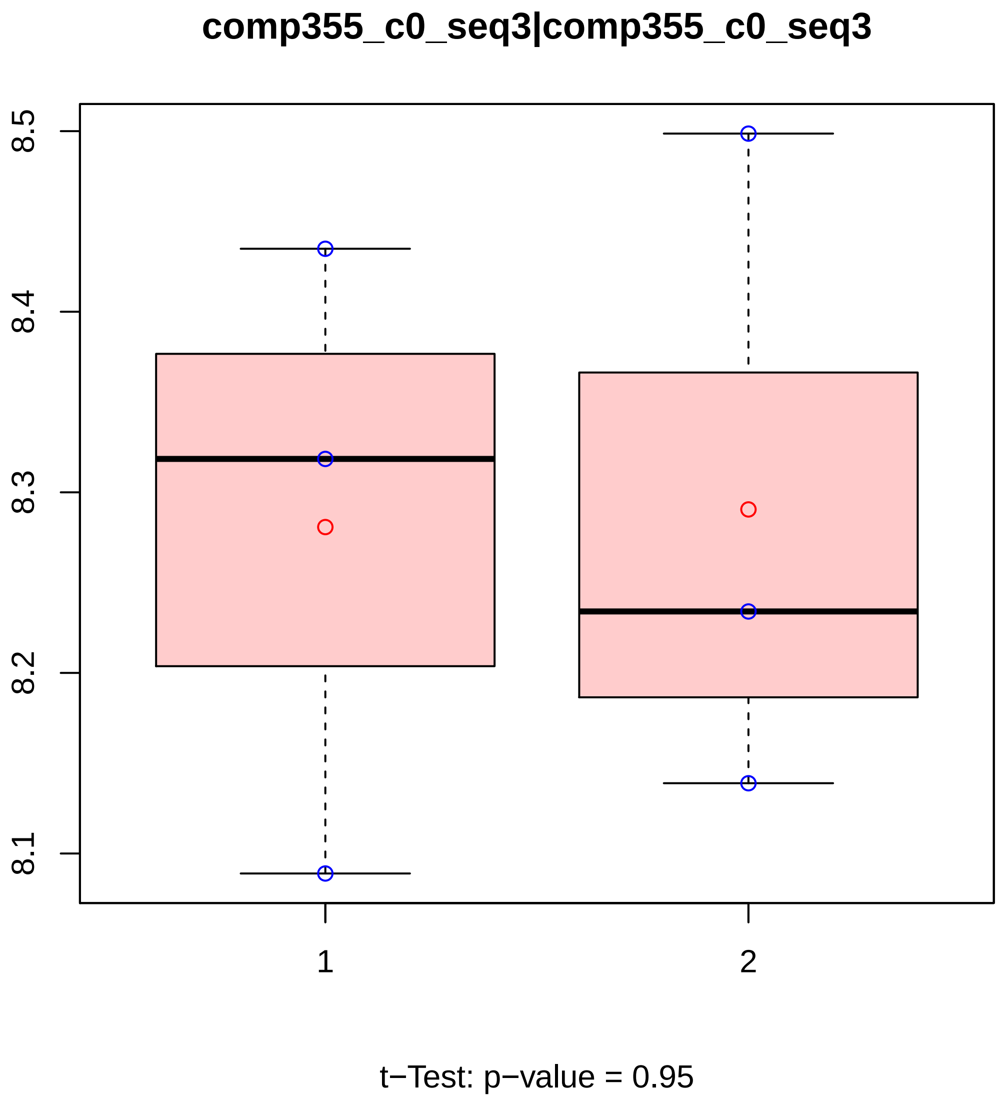

comp355_c0_seq3|comp355_c0_seq3
8.1
8.2
8.3
8.4
8.5
1
2
t−Test: p−value = 0.95

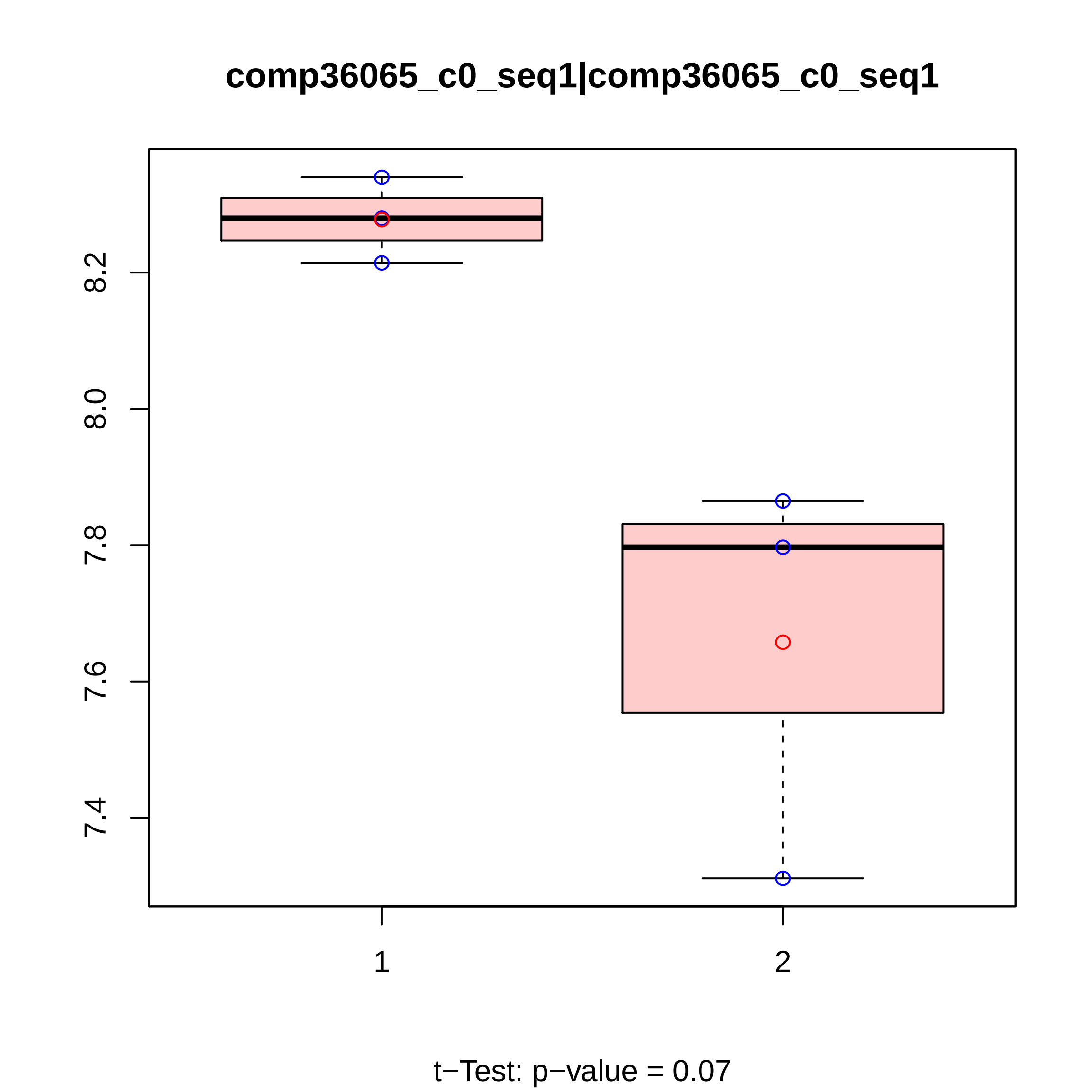

comp36065_c0_seq1|comp36065_c0_seq1
8.2
8.0
7.8
7.6
7.4
1
2
t−Test: p−value = 0.07

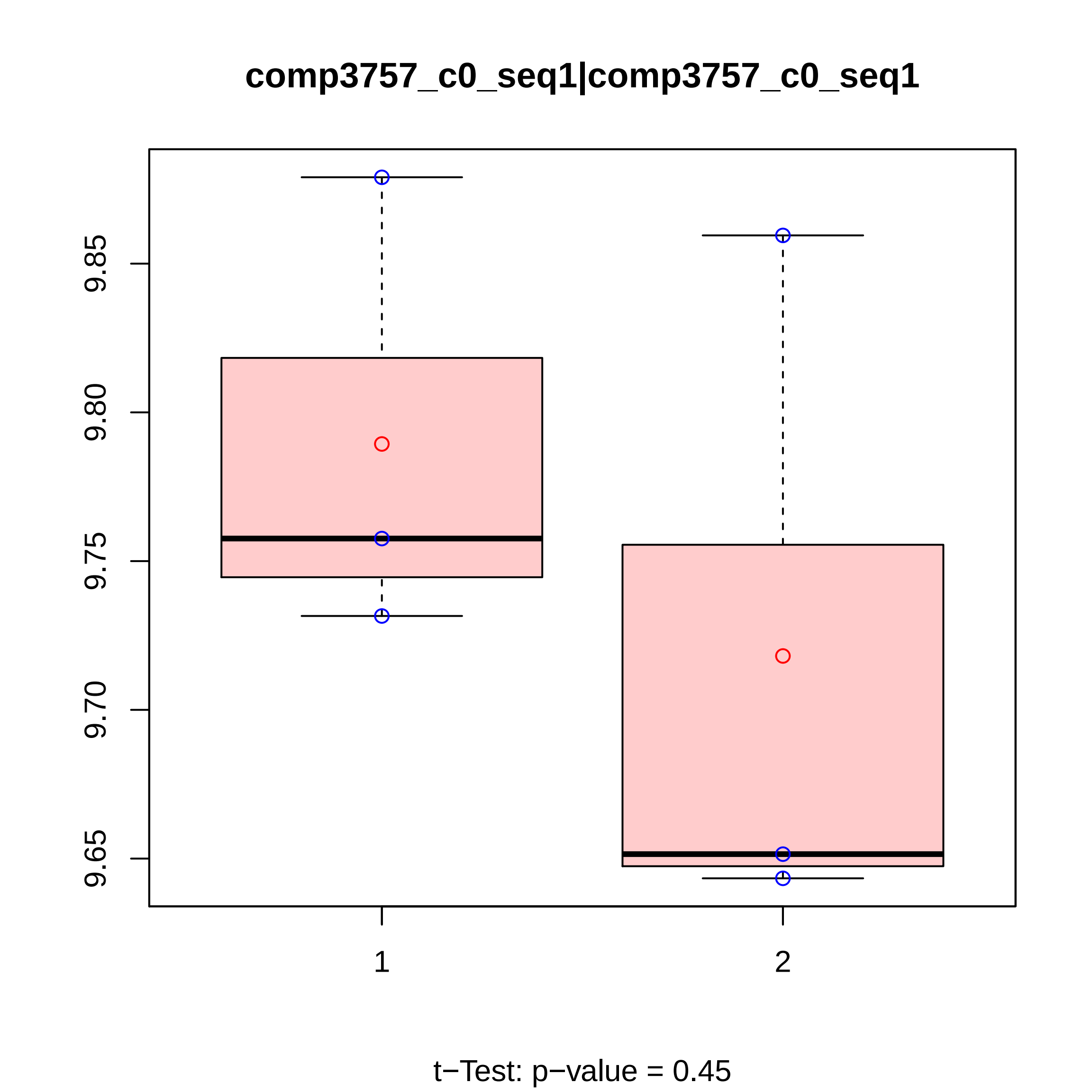

comp3757_c0_seq1|comp3757_c0_seq1
9.85
9.80
9.75
9.70
9.65
1
2
t−Test: p−value = 0.45

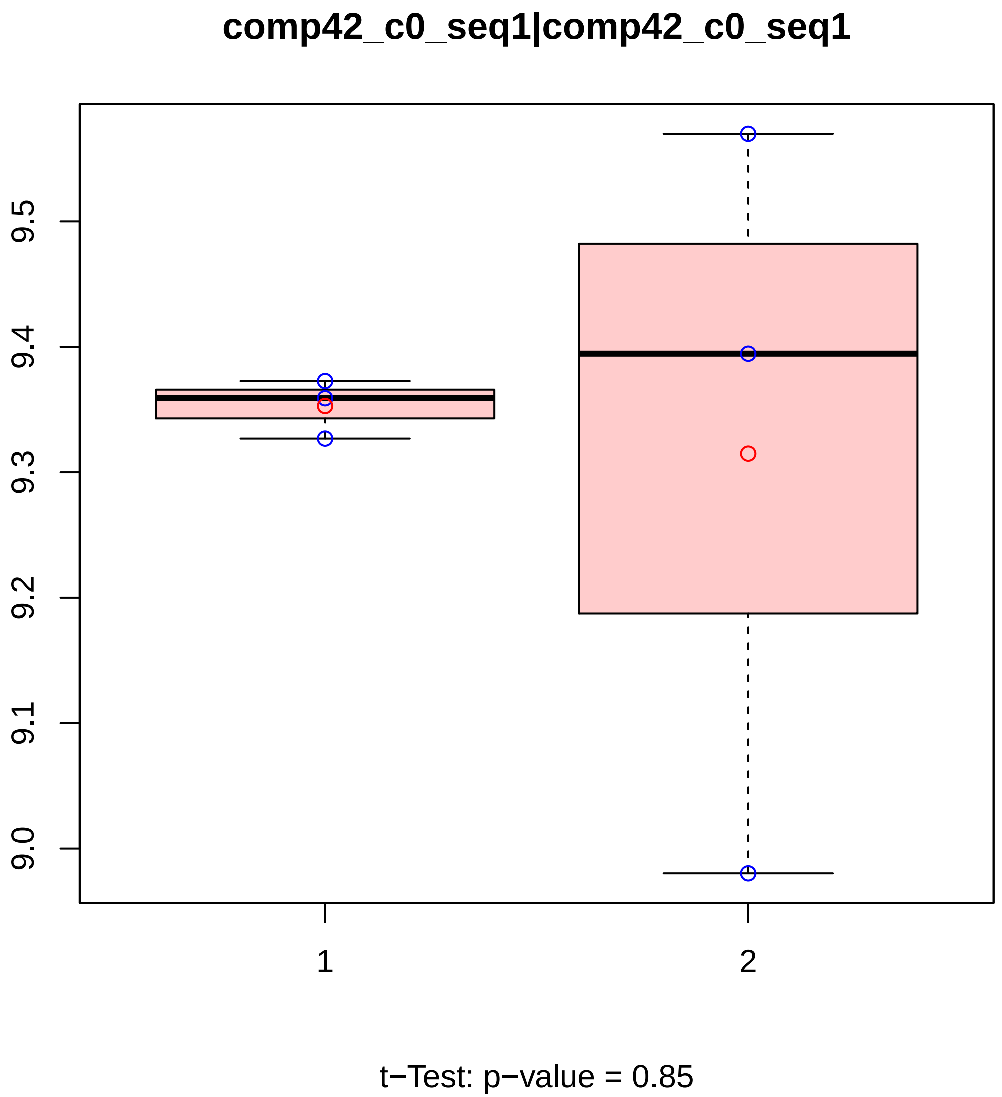

comp42_c0_seq1|comp42_c0_seq1
9.0
9.1
9.2
9.3
9.4
9.5
1
2
t−Test: p−value = 0.85

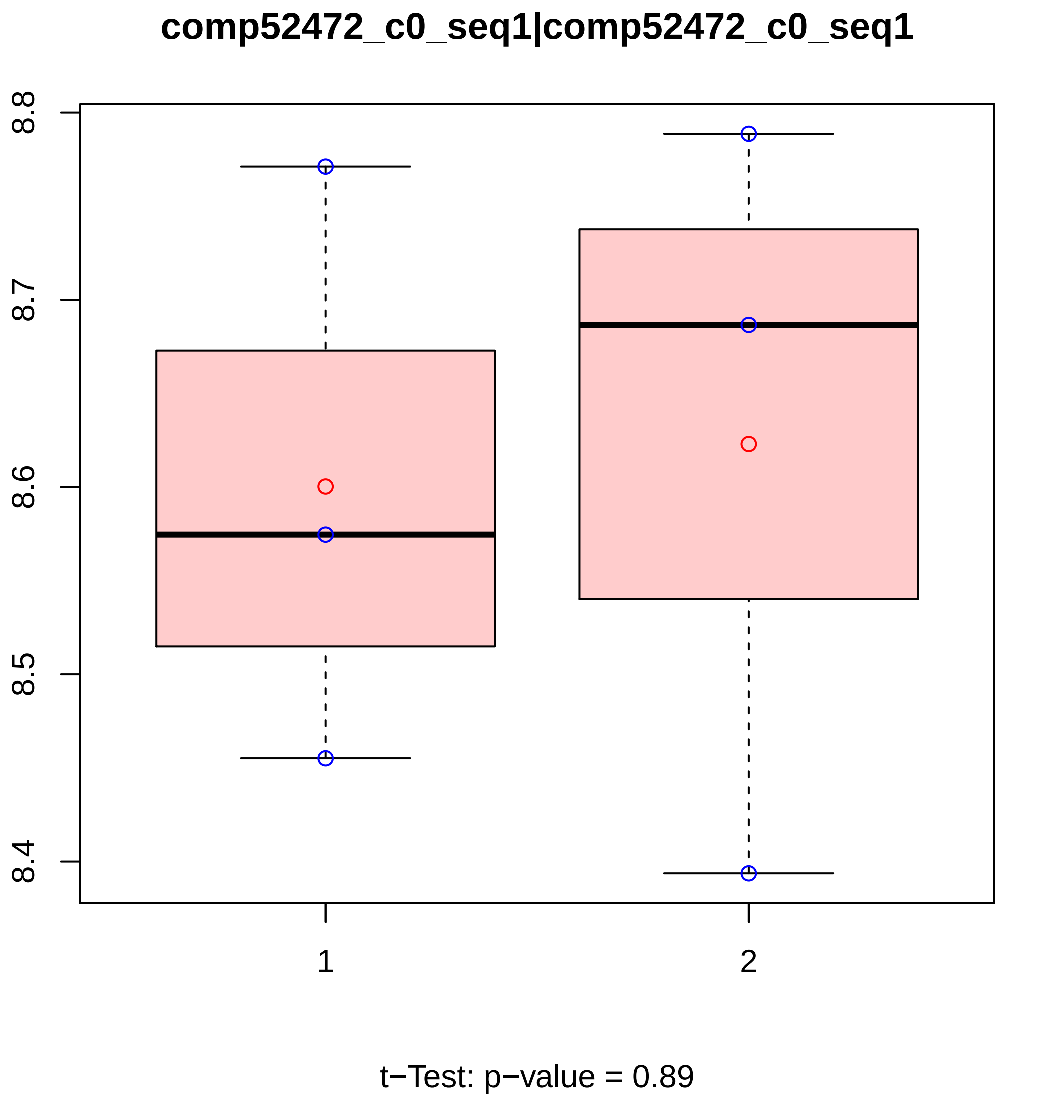

comp52472_c0_seq1|comp52472_c0_seq1
8.8
8.7
8.6
8.5
8.4
1
2
t−Test: p−value = 0.89

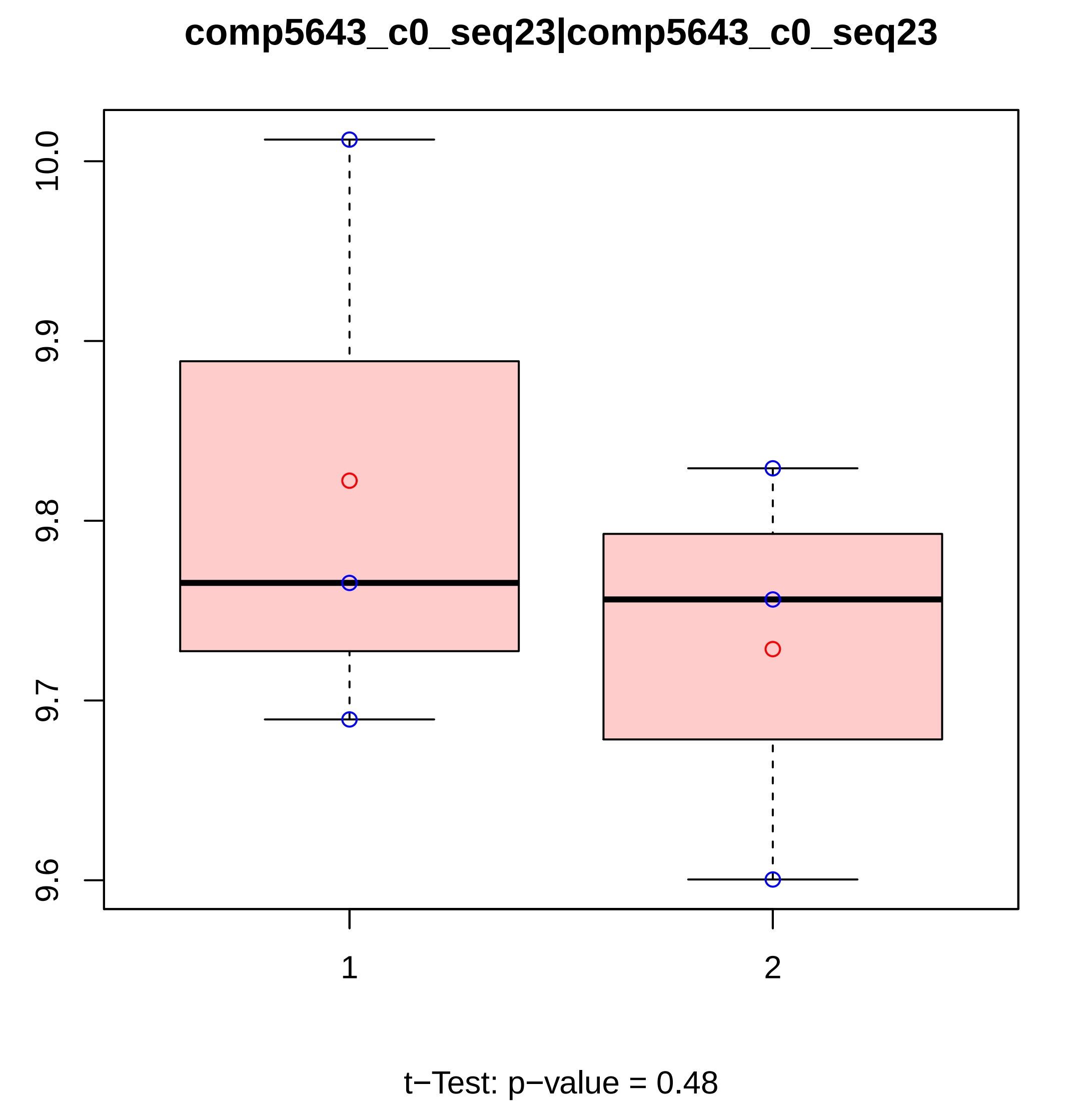

comp5643_c0_seq23|comp5643_c0_seq23
10.0
9.9
9.8
9.7
9.6
1
2
t−Test: p−value = 0.48

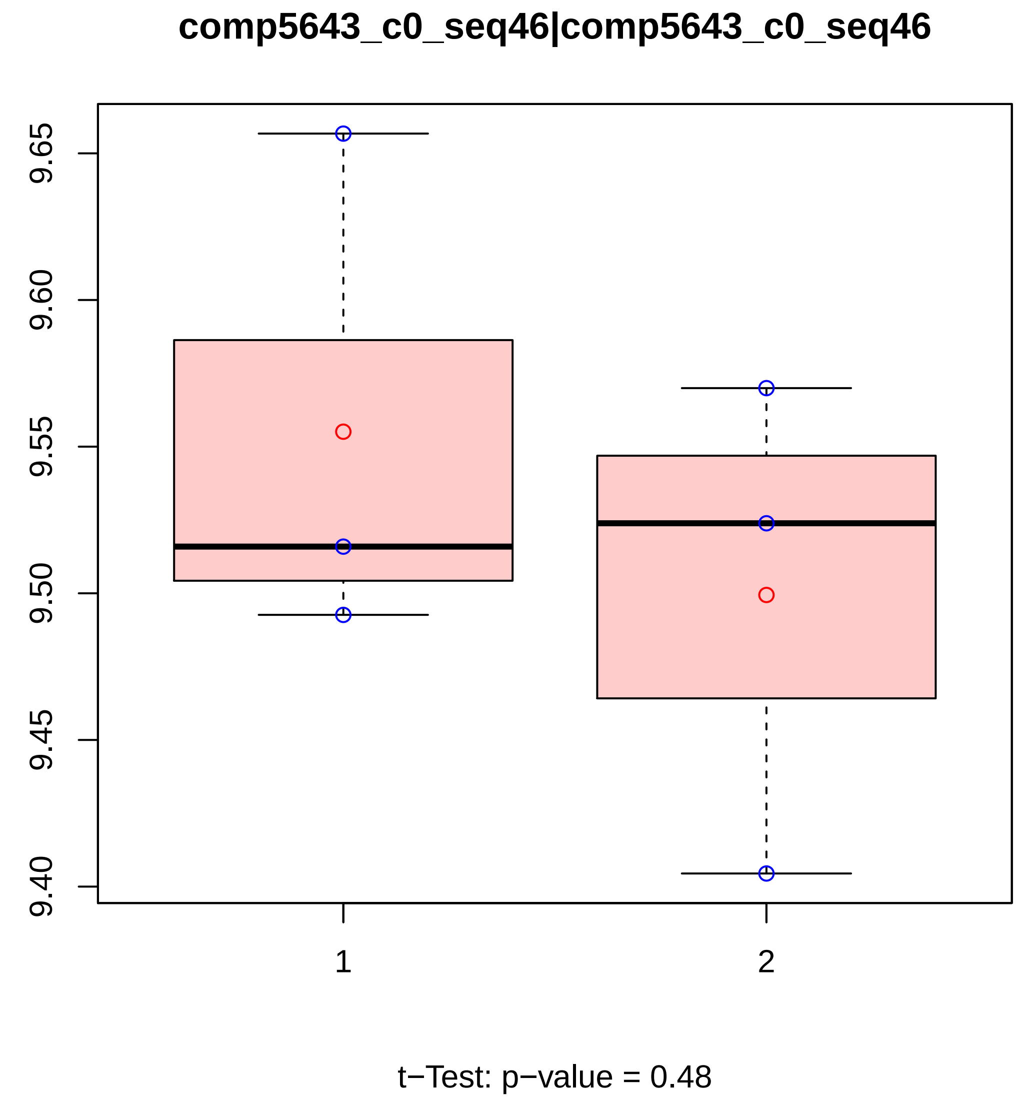

comp5643_c0_seq46|comp5643_c0_seq46
9.65
9.60
9.55
9.50
9.45
9.40
1
2
t−Test: p−value = 0.48

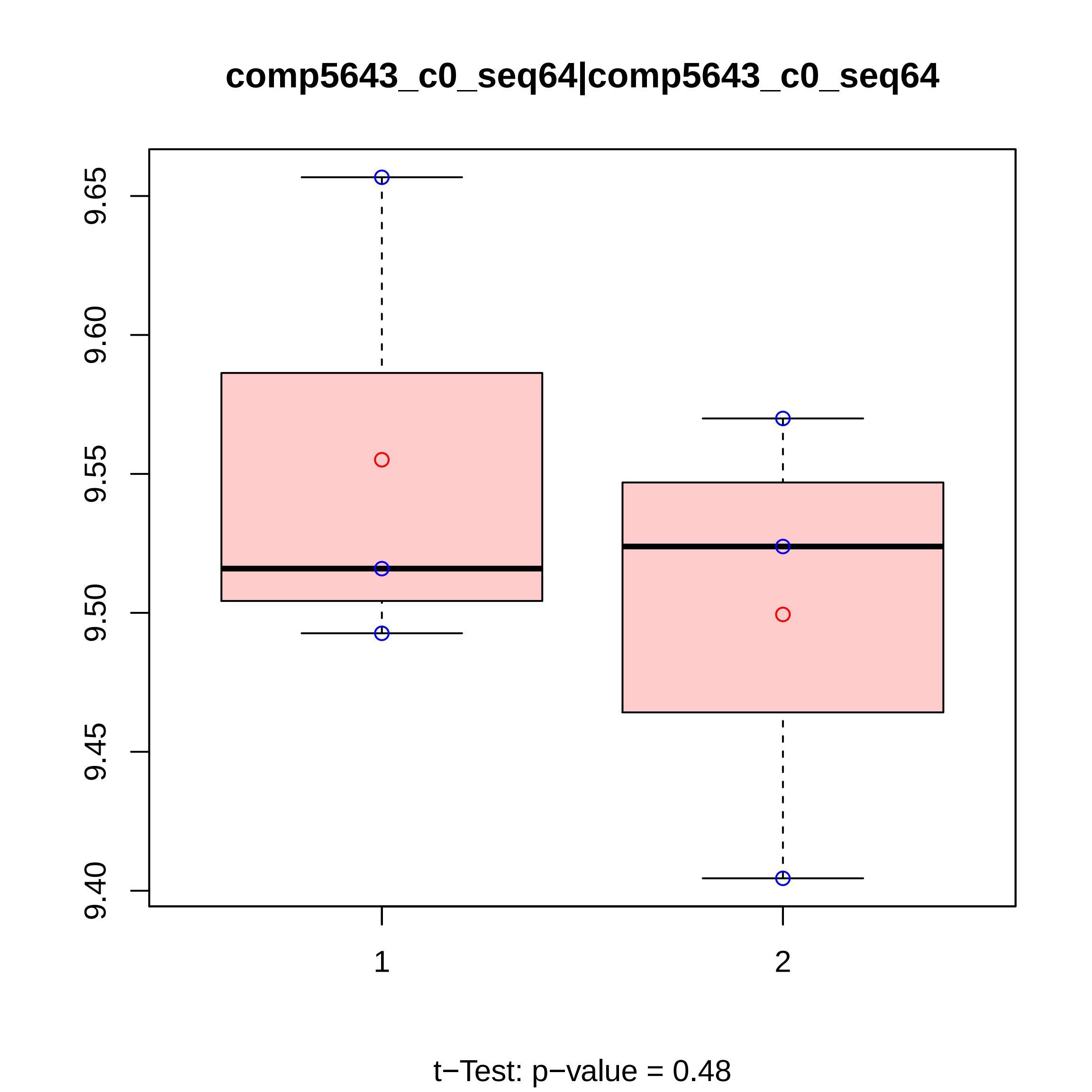

comp5643_c0_seq64|comp5643_c0_seq64
9.40
9.45
9.50
9.55
9.60
9.65
1
2
t−Test: p−value = 0.48

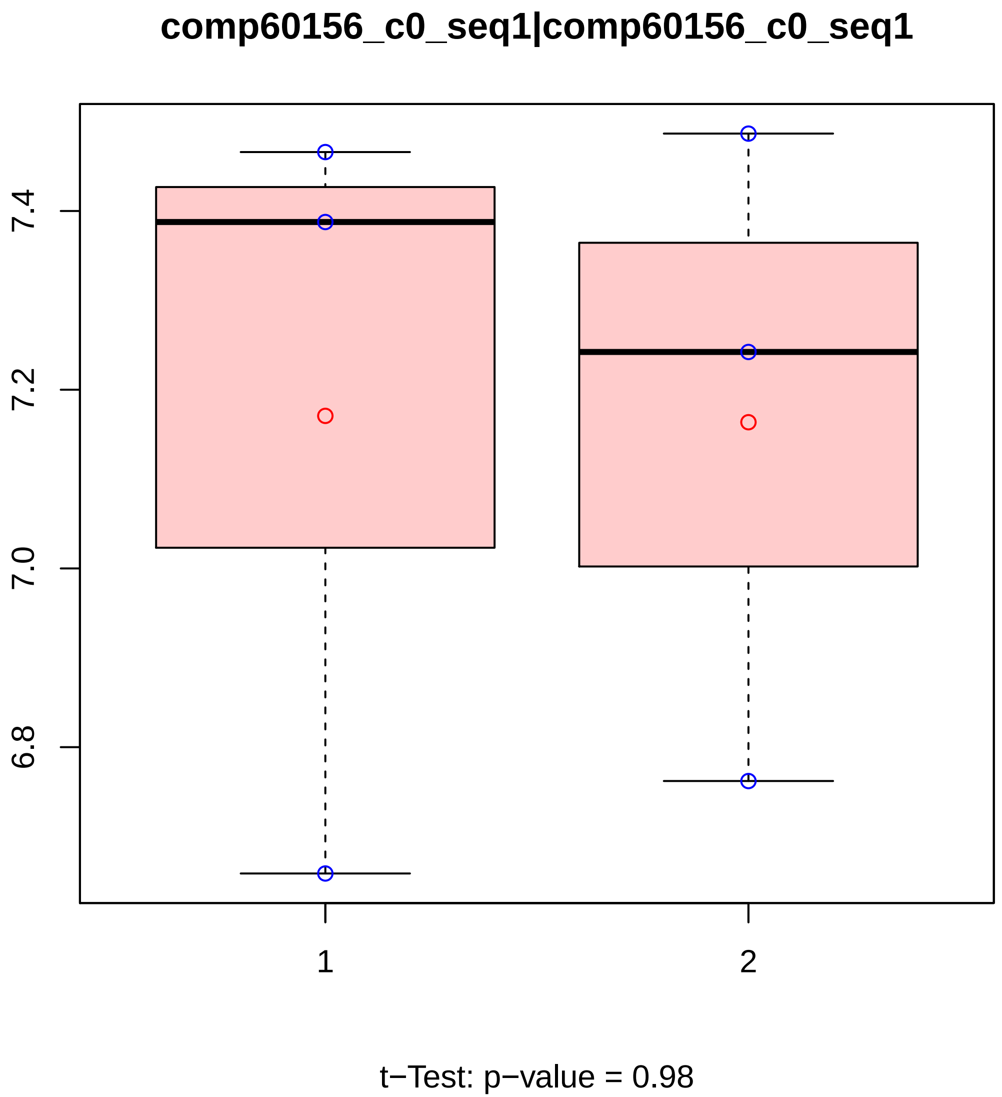

comp60156_c0_seq1|comp60156_c0_seq1
7.4
7.2
7.0
6.8
1
2
t−Test: p−value = 0.98

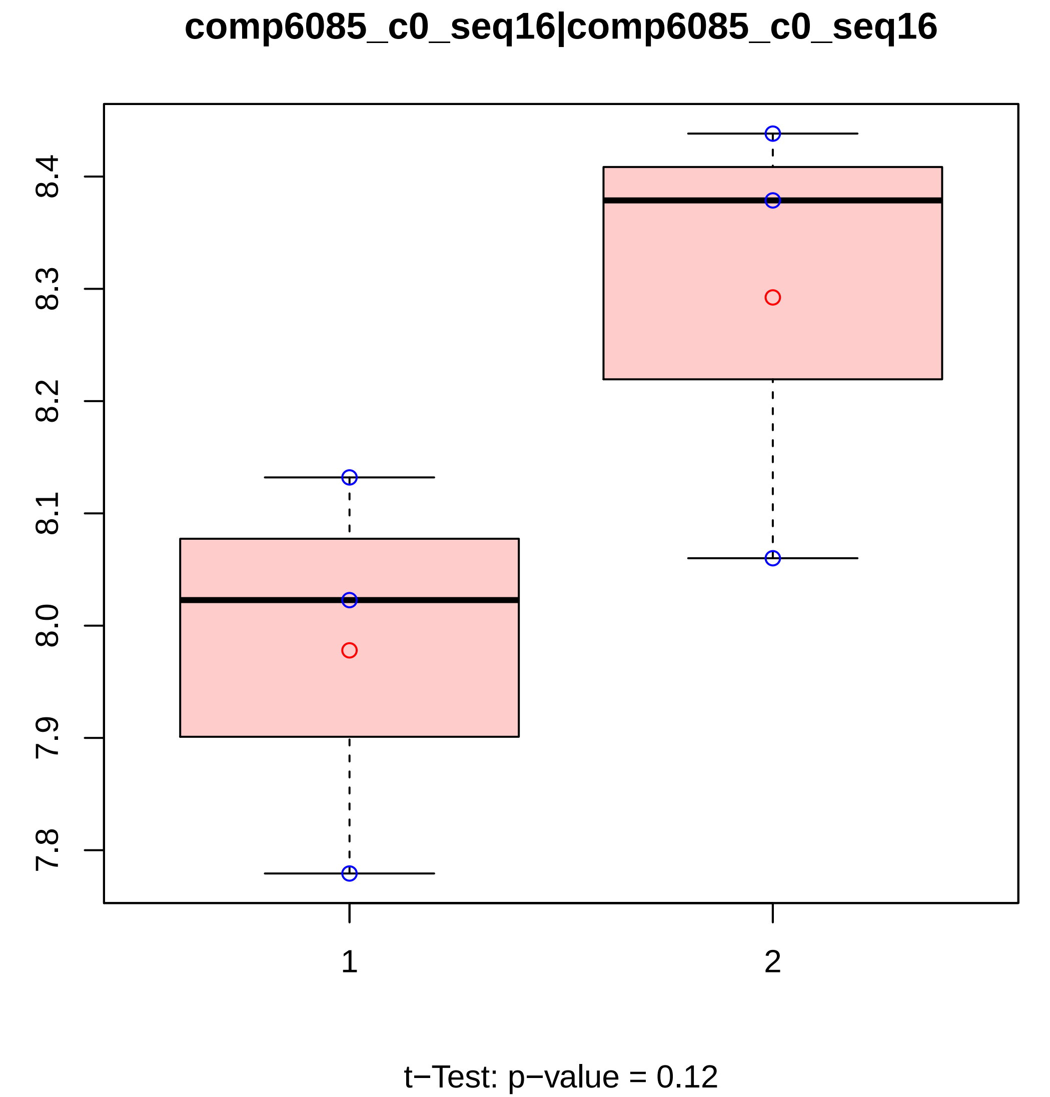
comp6085_c0_seq16|comp6085_c0_seq16
8.4
8.3
8.2
8.1
8.0
7.9
7.8
1
2
t−Test: p−value = 0.12

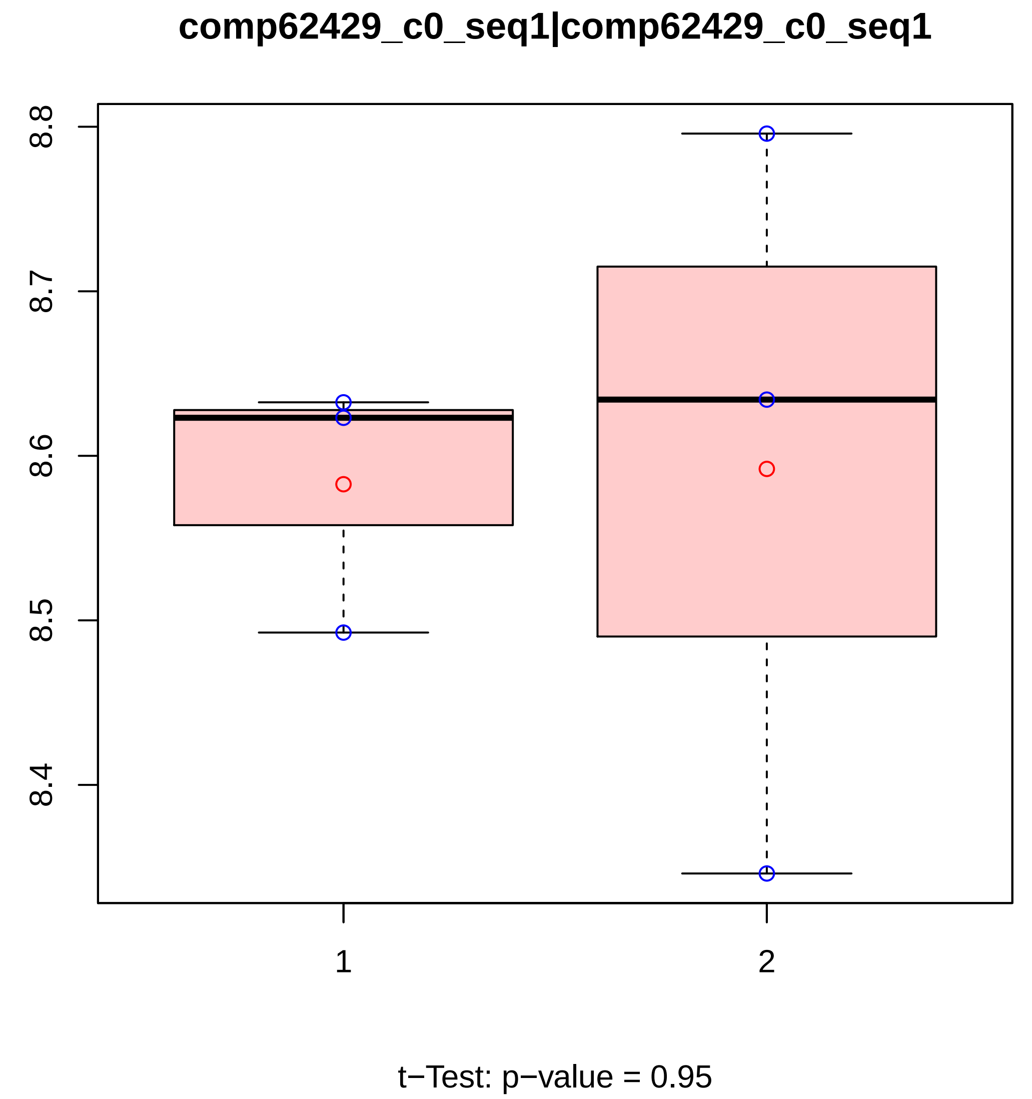
comp62429_c0_seq1|comp62429_c0_seq1
8.8
8.7
8.6
8.5
8.4
1
2
t−Test: p−value = 0.95

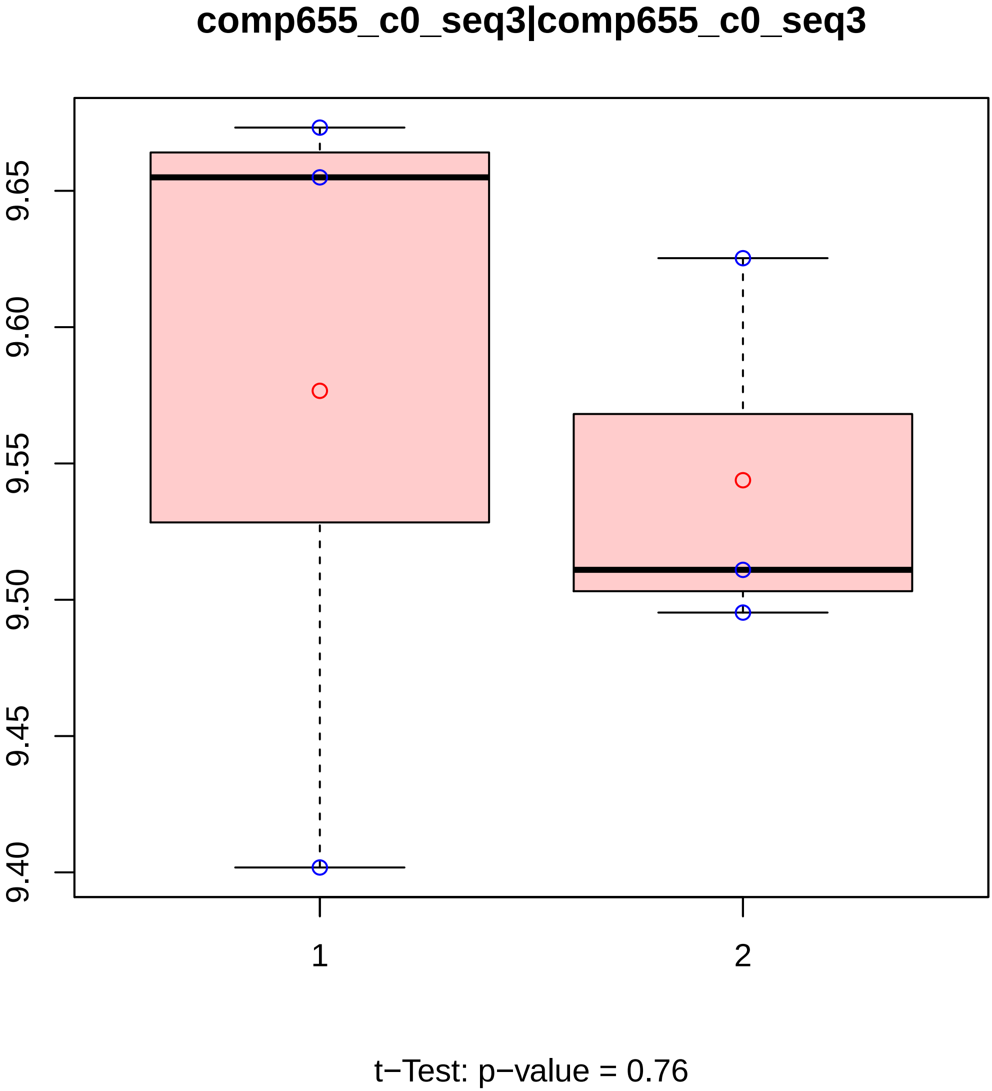
comp655_c0_seq3|comp655_c0_seq3
9.40
9.45
9.50
9.55
9.60
9.65
1
2
t−Test: p−value = 0.76

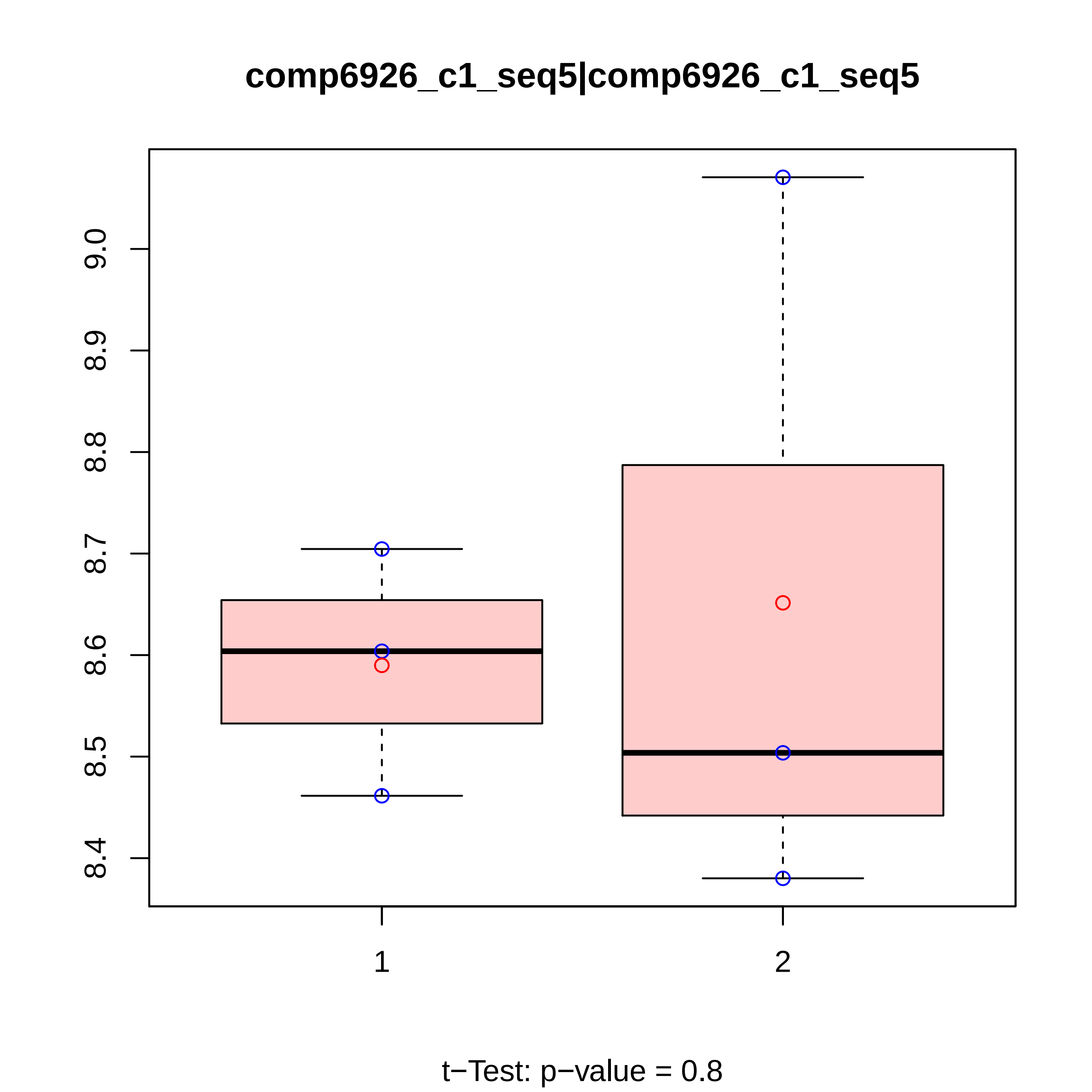

comp6926_c1_seq5|comp6926_c1_seq5
9.0
8.9
8.8
8.7
8.6
8.5
8.4
1
2
t−Test: p−value = 0.8

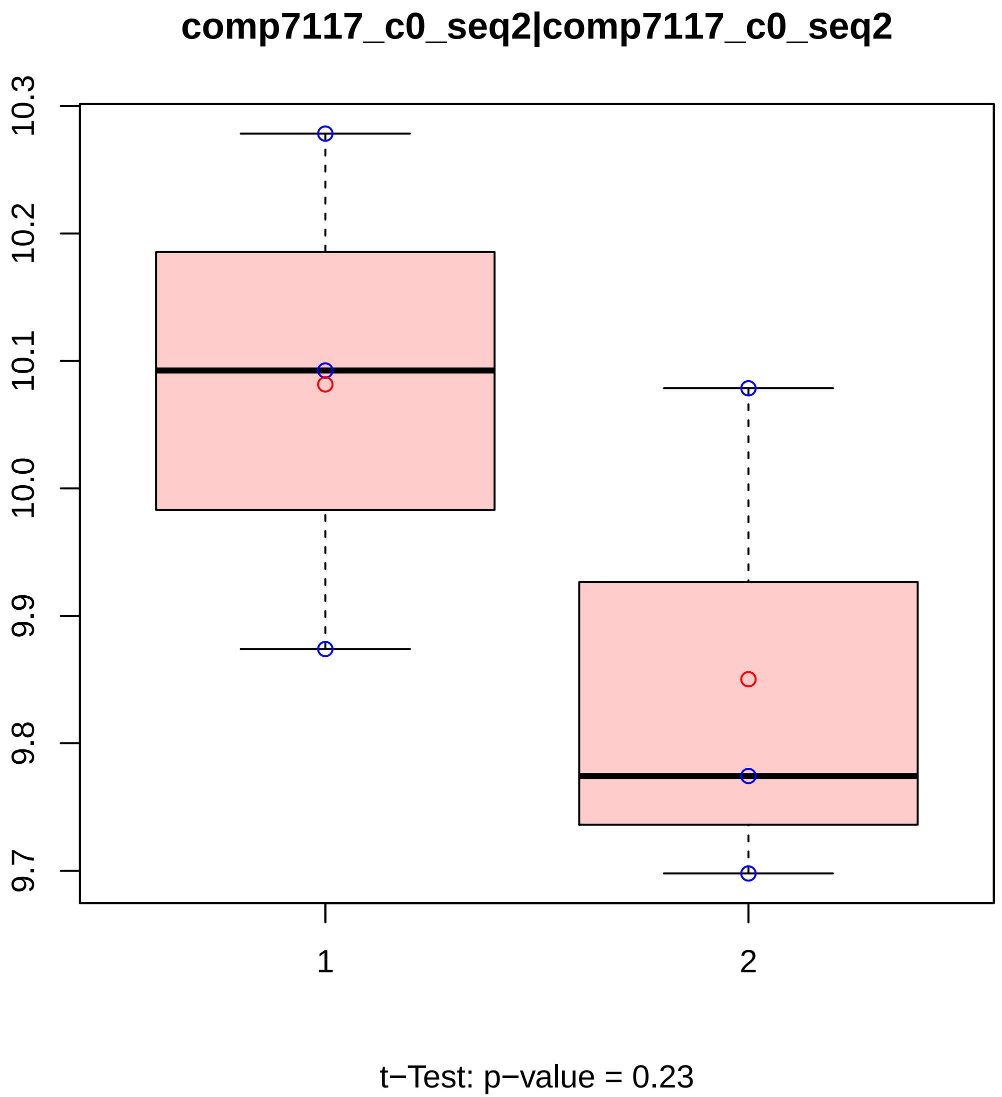
comp7117_c0_seq2|comp7117_c0_seq2
10.3
10.2
10.1
10.0
9.9
9.8
9.7
1
2
t−Test: p−value = 0.23

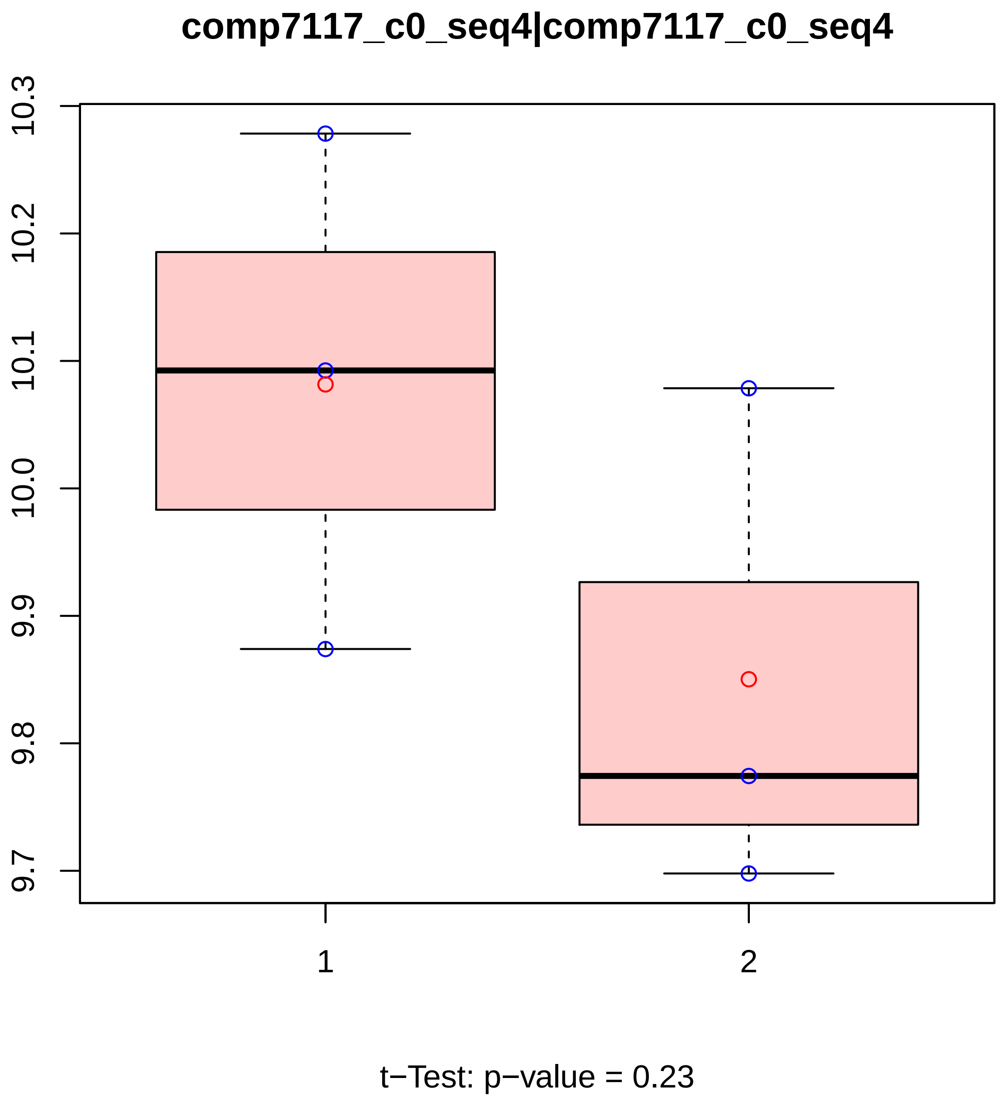

comp7117_c0_seq4|comp7117_c0_seq4
10.3
10.2
10.1
10.0
9.9
9.8
9.7
1
2
t−Test: p−value = 0.23

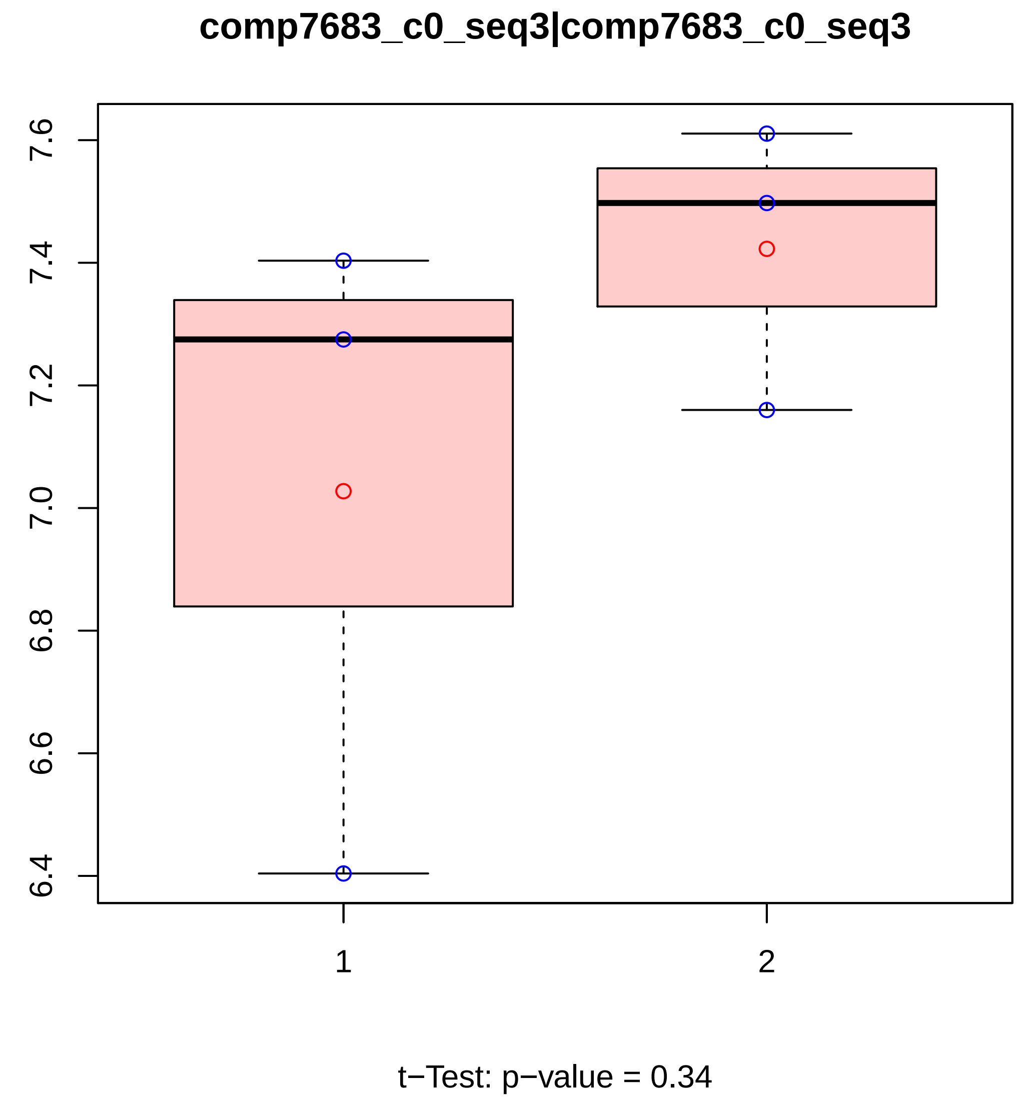
comp7683_c0_seq3|comp7683_c0_seq3
7.6
7.4
7.2
7.0
6.8
6.6
6.4
1
2
t−Test: p−value = 0.34

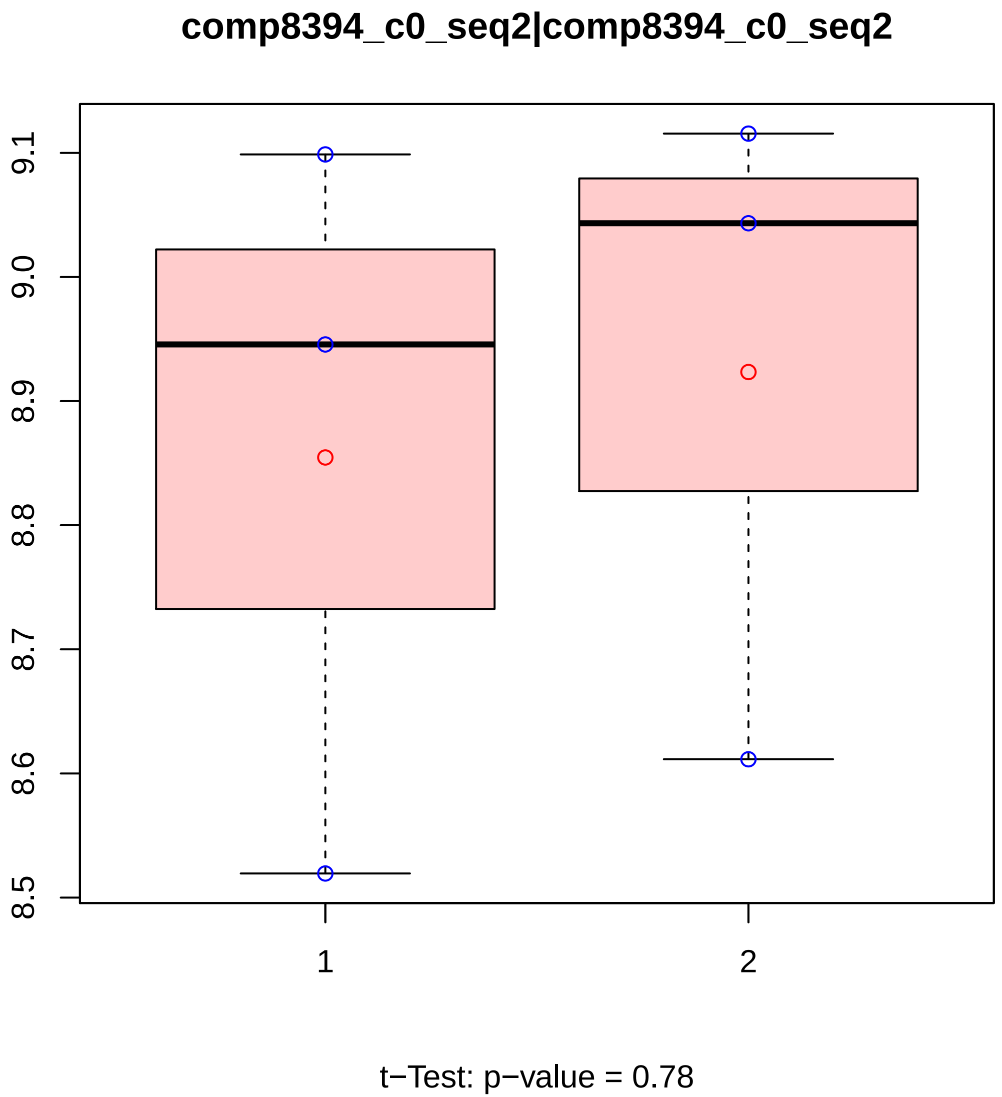

comp8394_c0_seq2|comp8394_c0_seq2
9.1
9.0
8.9
8.8
8.7
8.6
8.5
1
2
t−Test: p−value = 0.78

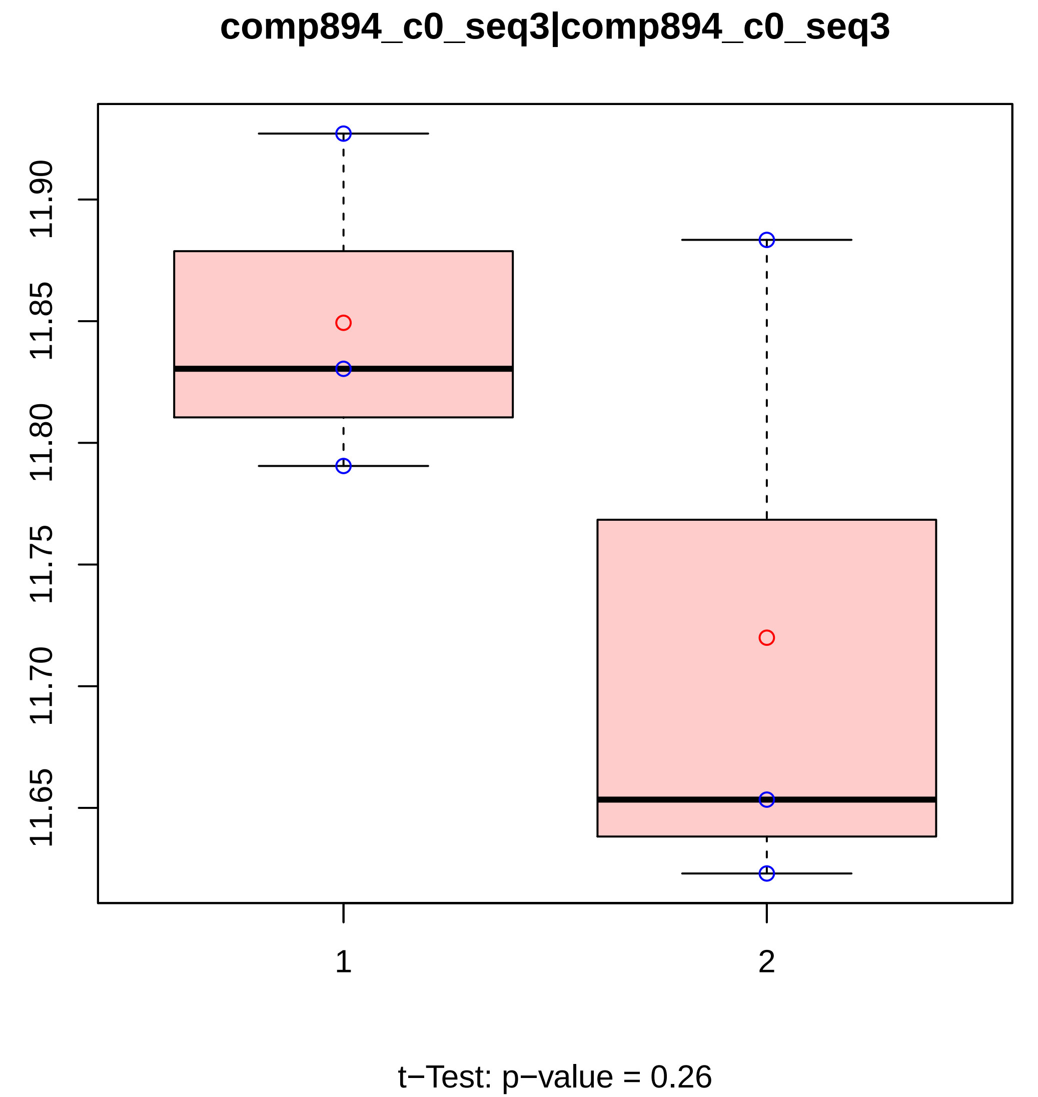
comp894_c0_seq3|comp894_c0_seq3
11.90
11.85
11.80
11.75
11.70
11.65
1
2
t−Test: p−value = 0.26

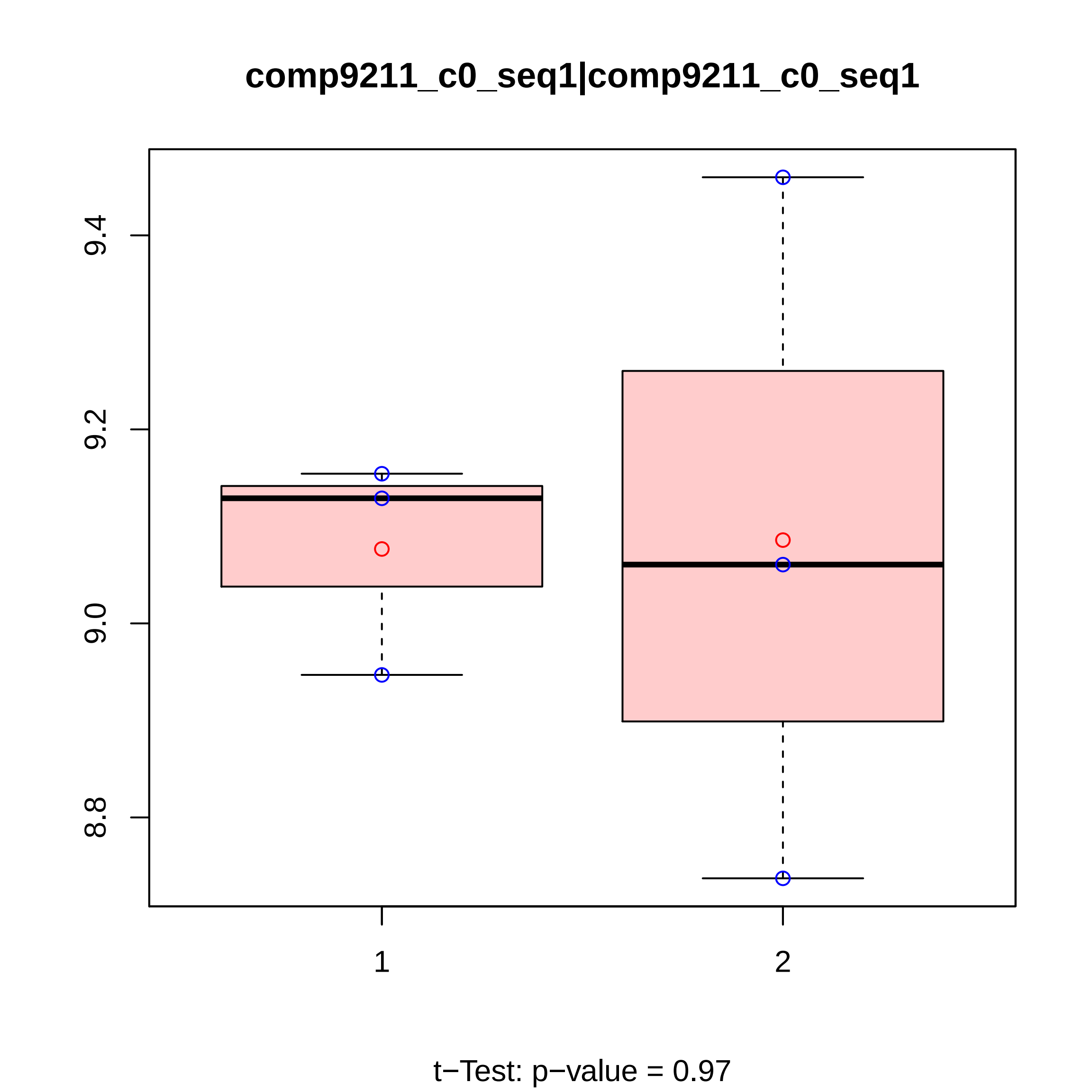
comp9211_c0_seq1|comp9211_c0_seq1
9.4
9.2
9.0
8.8
1
2
t−Test: p−value = 0.97

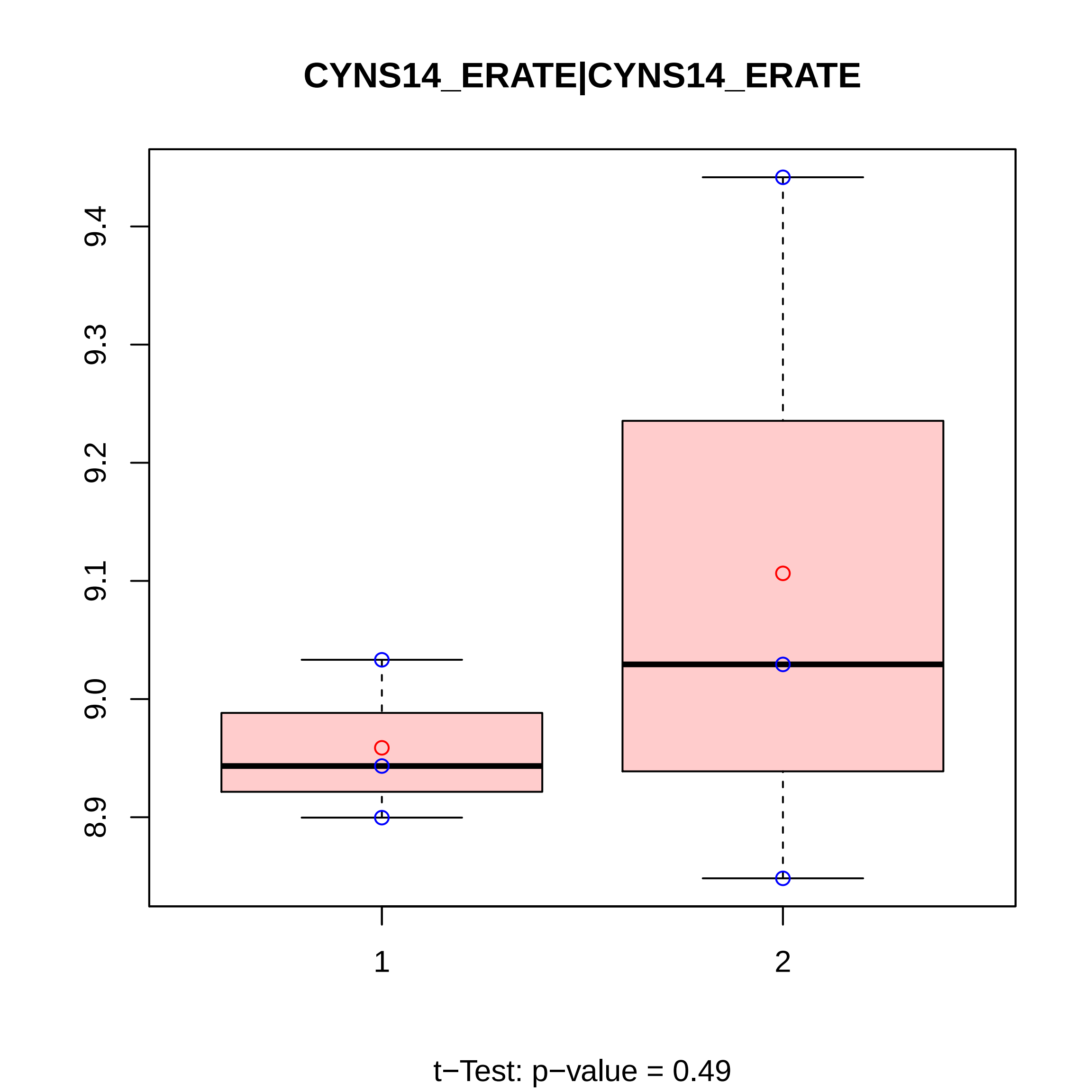

CYNS14_ERATE|CYNS14_ERATE
9.4
9.3
9.2
9.1
9.0
8.9
1
2
t−Test: p−value = 0.49

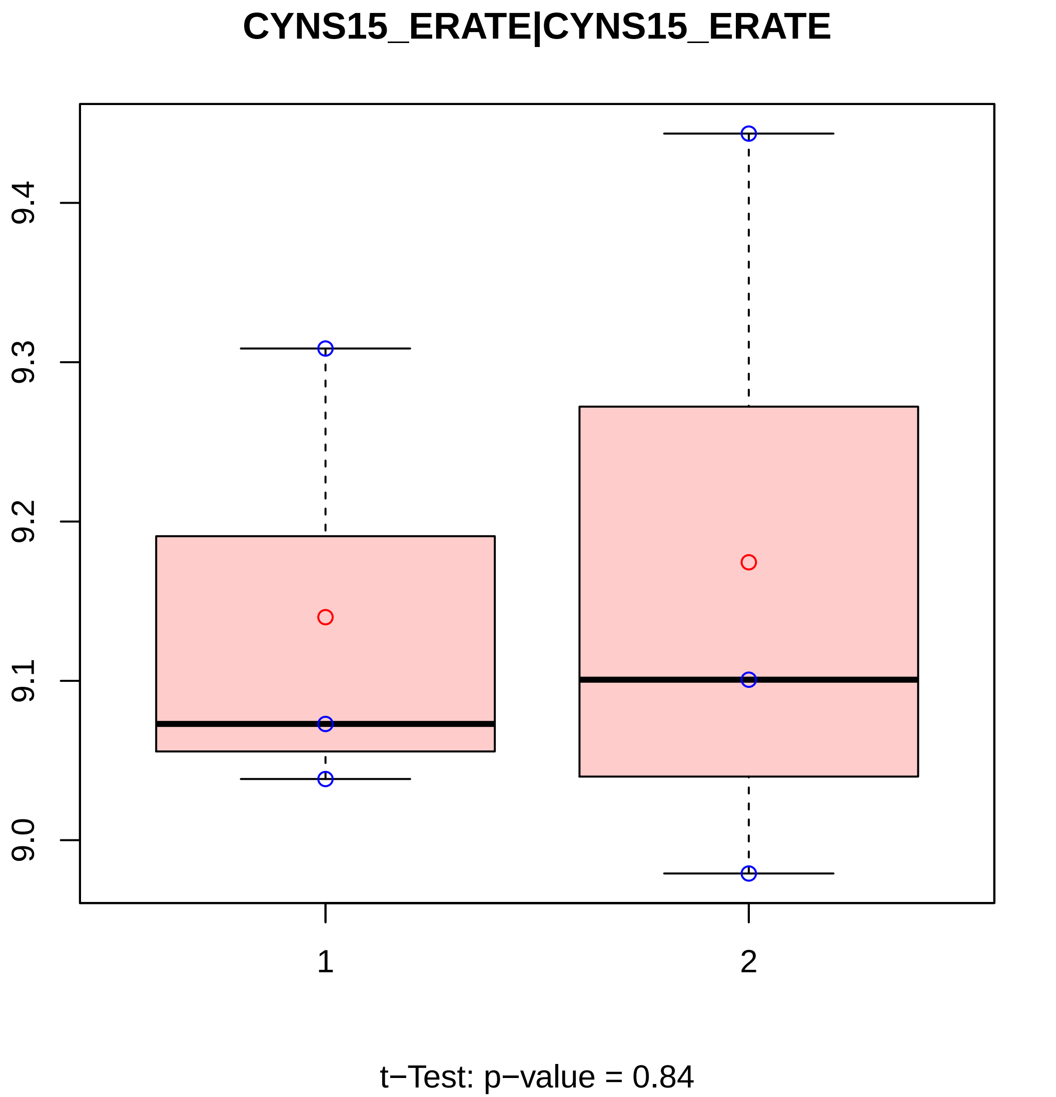
CYNS15_ERATE|CYNS15_ERATE
9.0
9.1
9.2
9.3
9.4
1
2
t−Test: p−value = 0.84

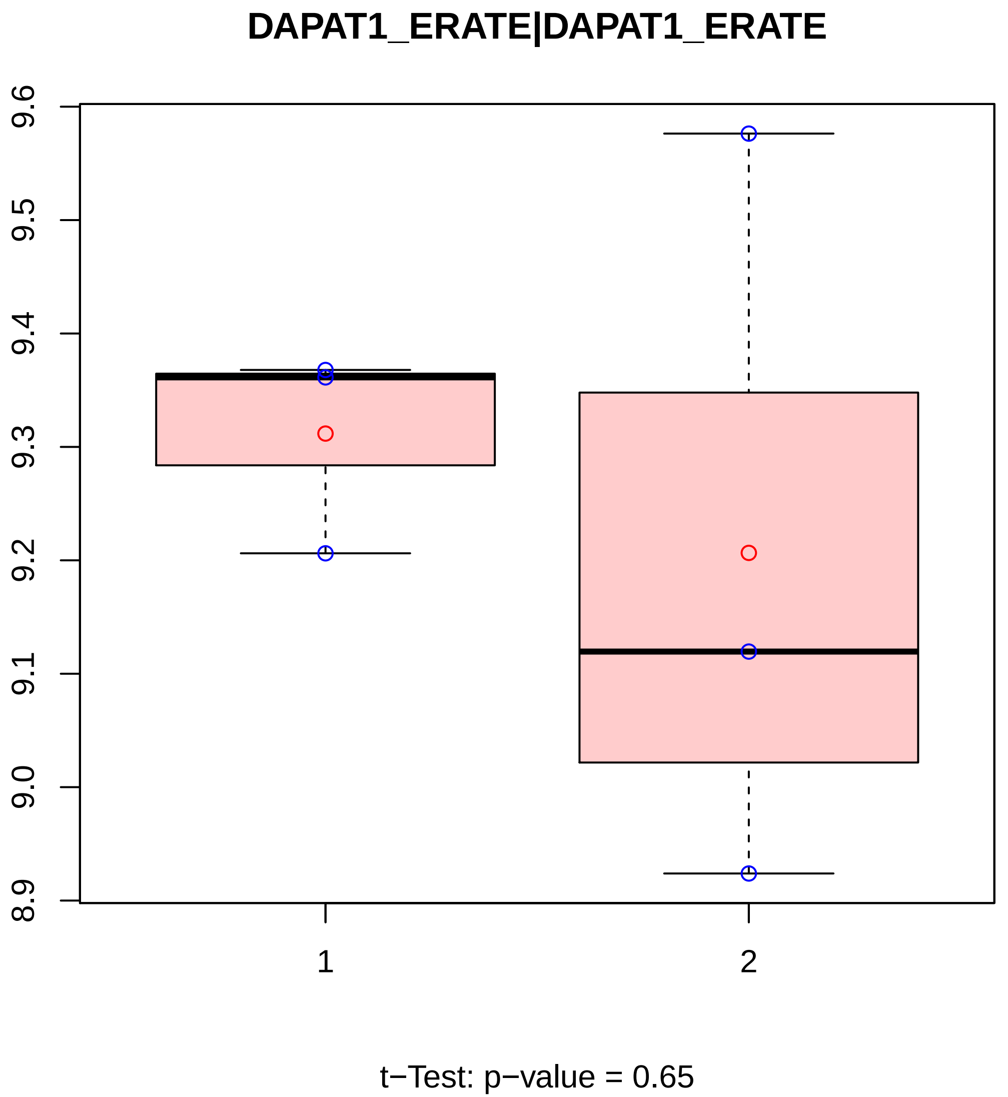
DAPAT1_ERATE|DAPAT1_ERATE
9.6
9.5
9.4
9.3
9.2
9.1
9.0
8.9
1
2
t−Test: p−value = 0.65

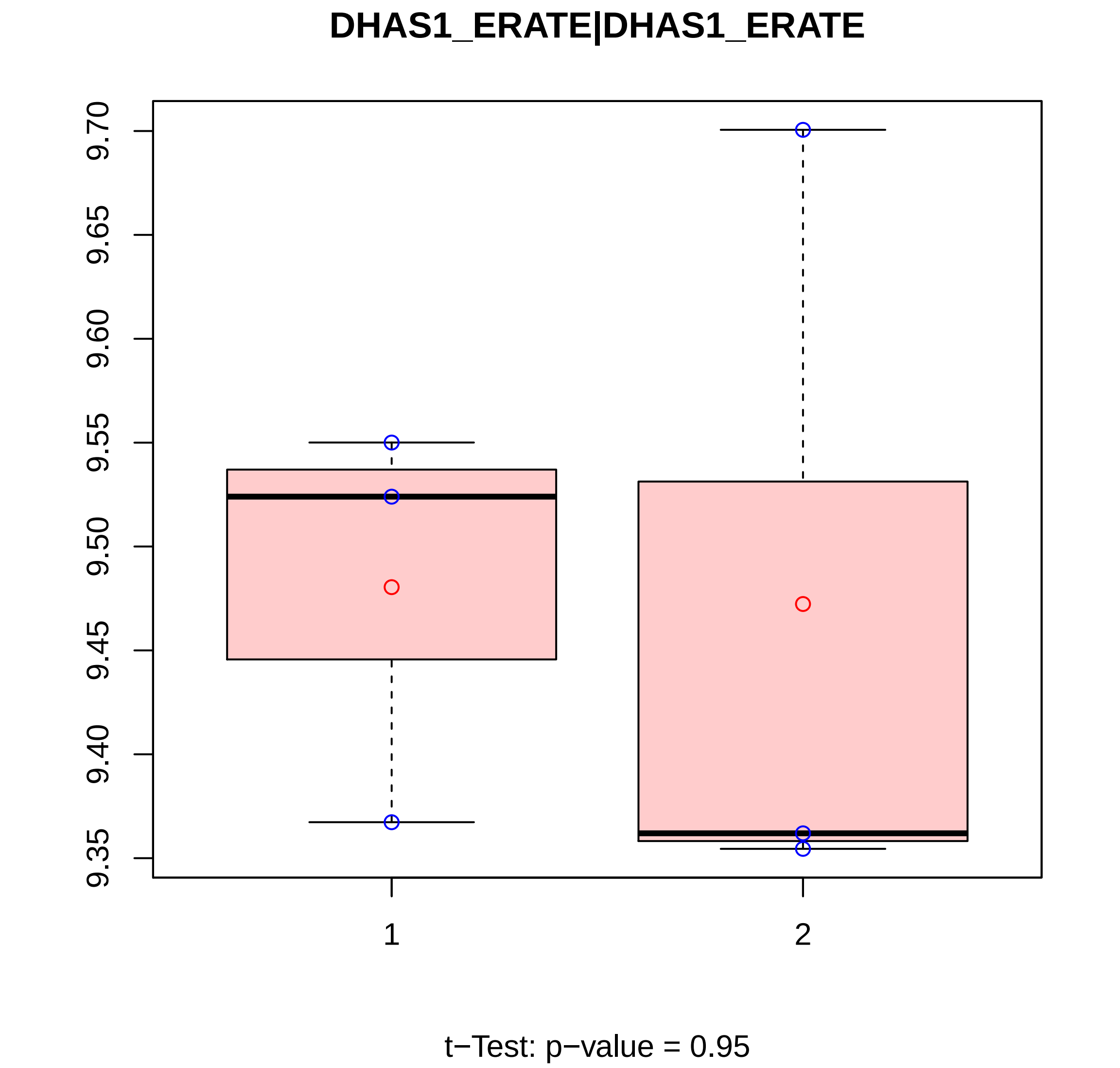

DHAS1_ERATE|DHAS1_ERATE
9.70
9.65
9.60
9.55
9.50
9.45
9.40
9.35
1
2
t−Test: p−value = 0.95

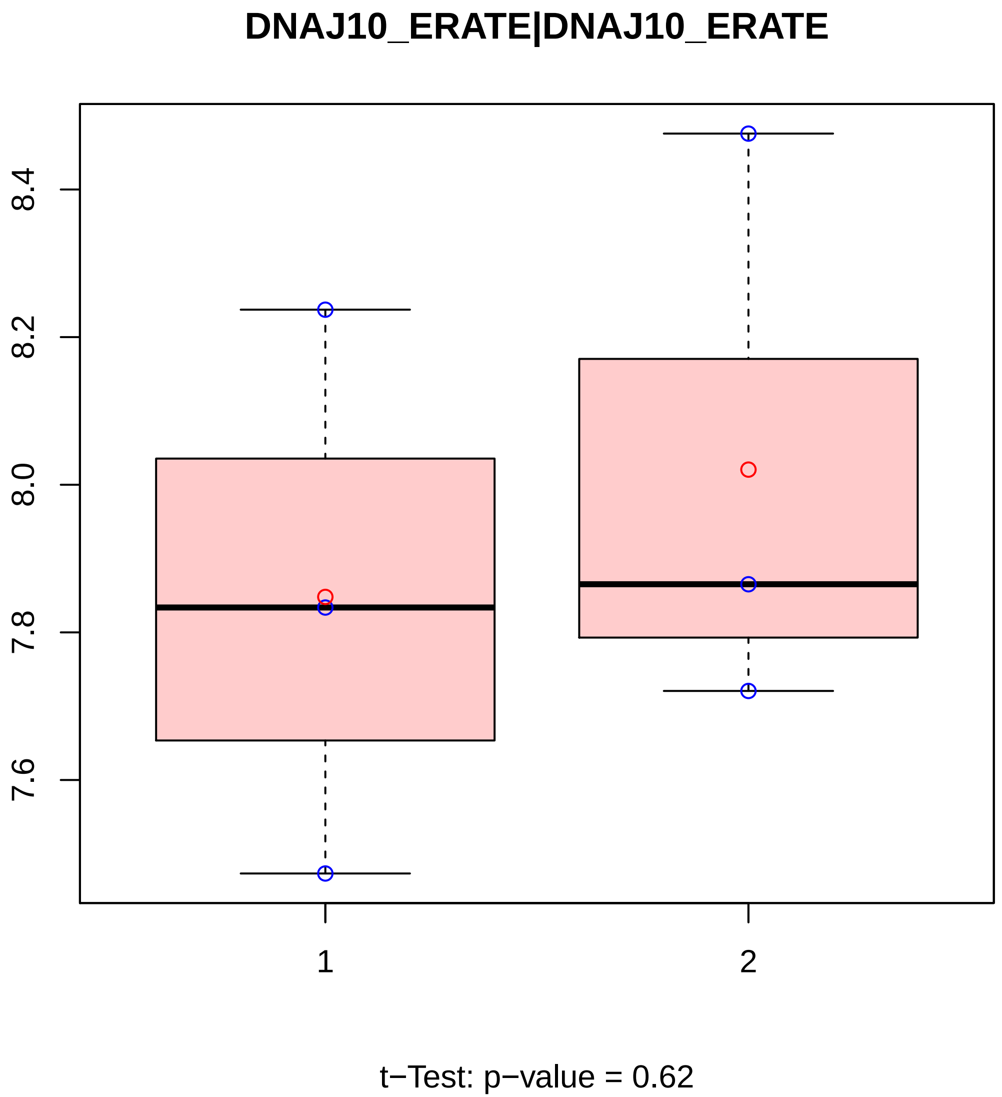
DNAJ10_ERATE|DNAJ10_ERATE
8.4
8.2
8.0
7.8
7.6
1
2
t−Test: p−value = 0.62

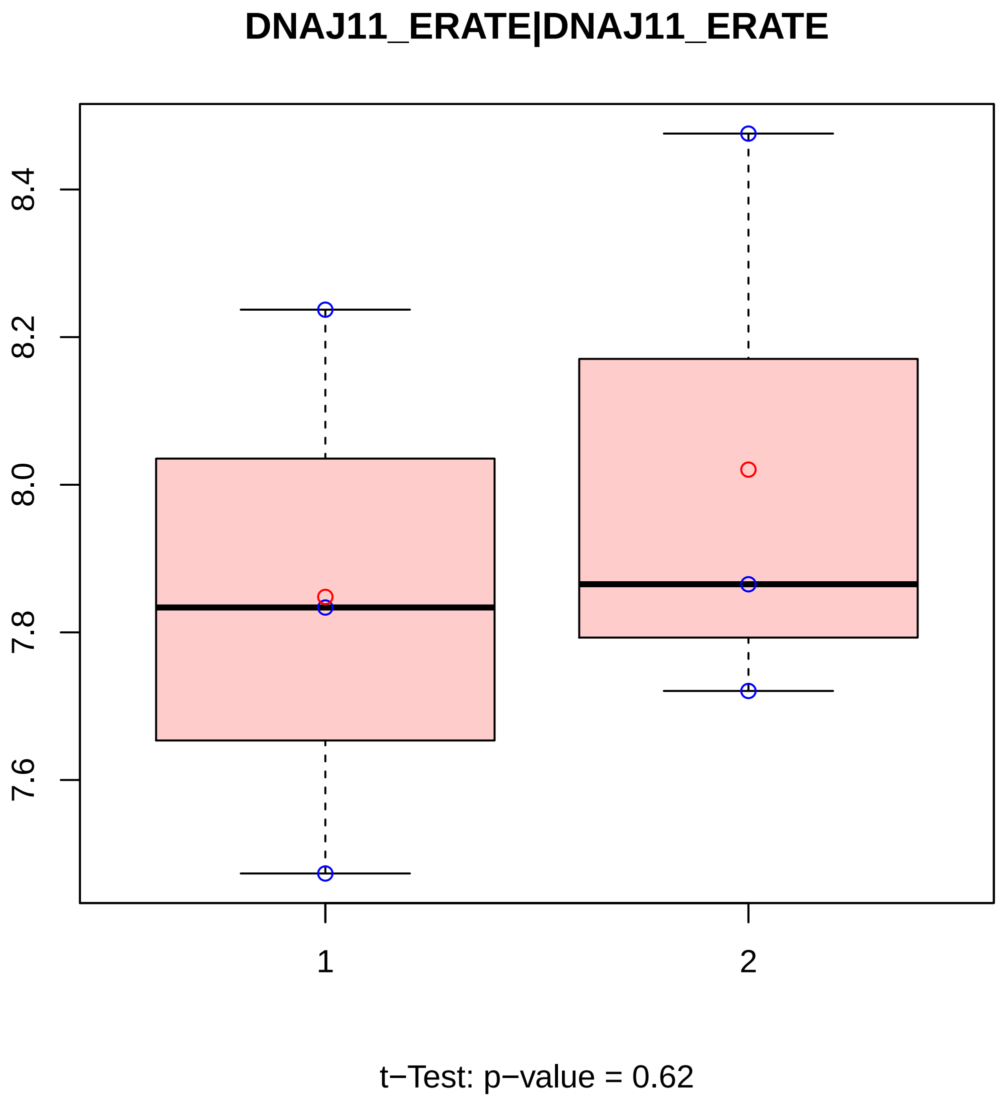

DNAJ11_ERATE|DNAJ11_ERATE
8.4
8.2
8.0
7.8
7.6
1
2
t−Test: p−value = 0.62

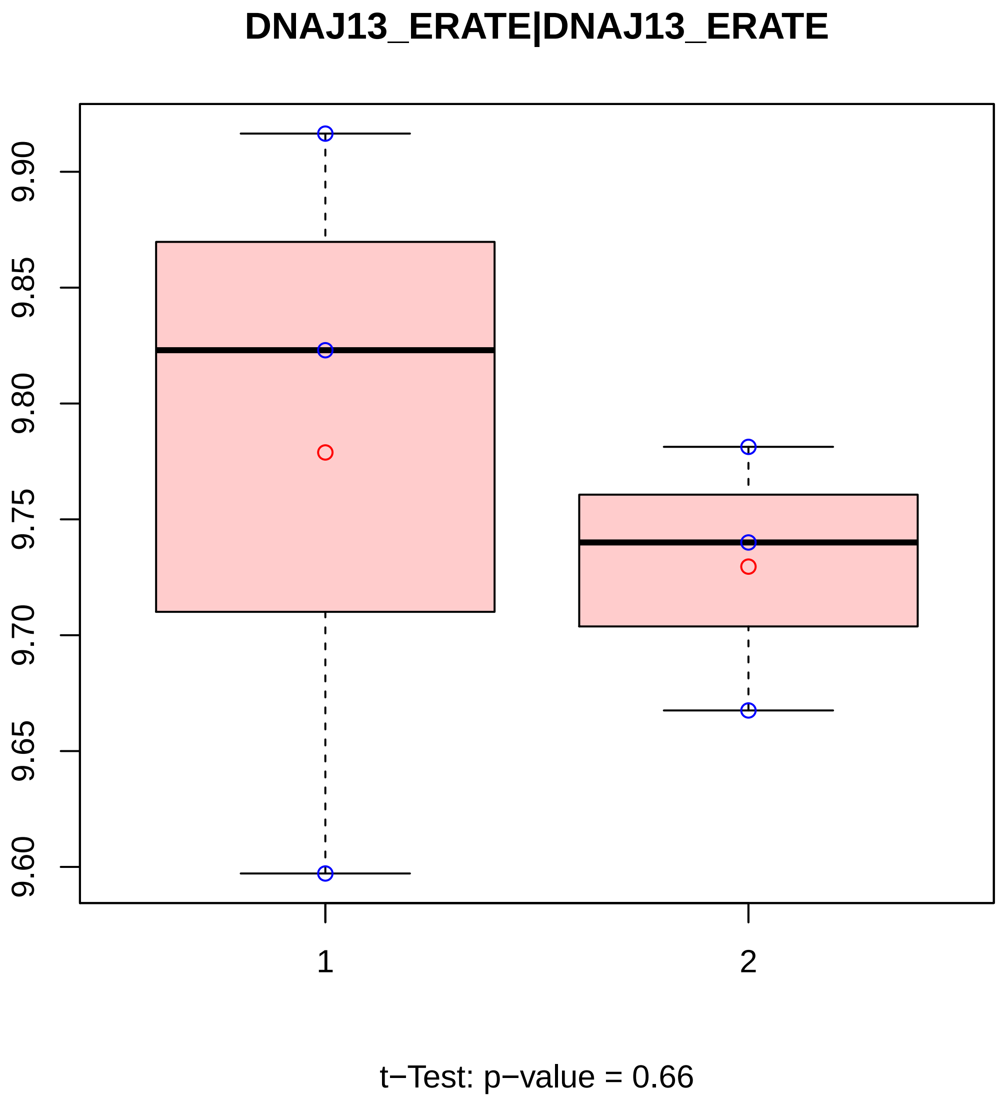
DNAJ13_ERATE|DNAJ13_ERATE
9.60
9.65
9.70
9.75
9.80
9.85
9.90
1
2
t−Test: p−value = 0.66

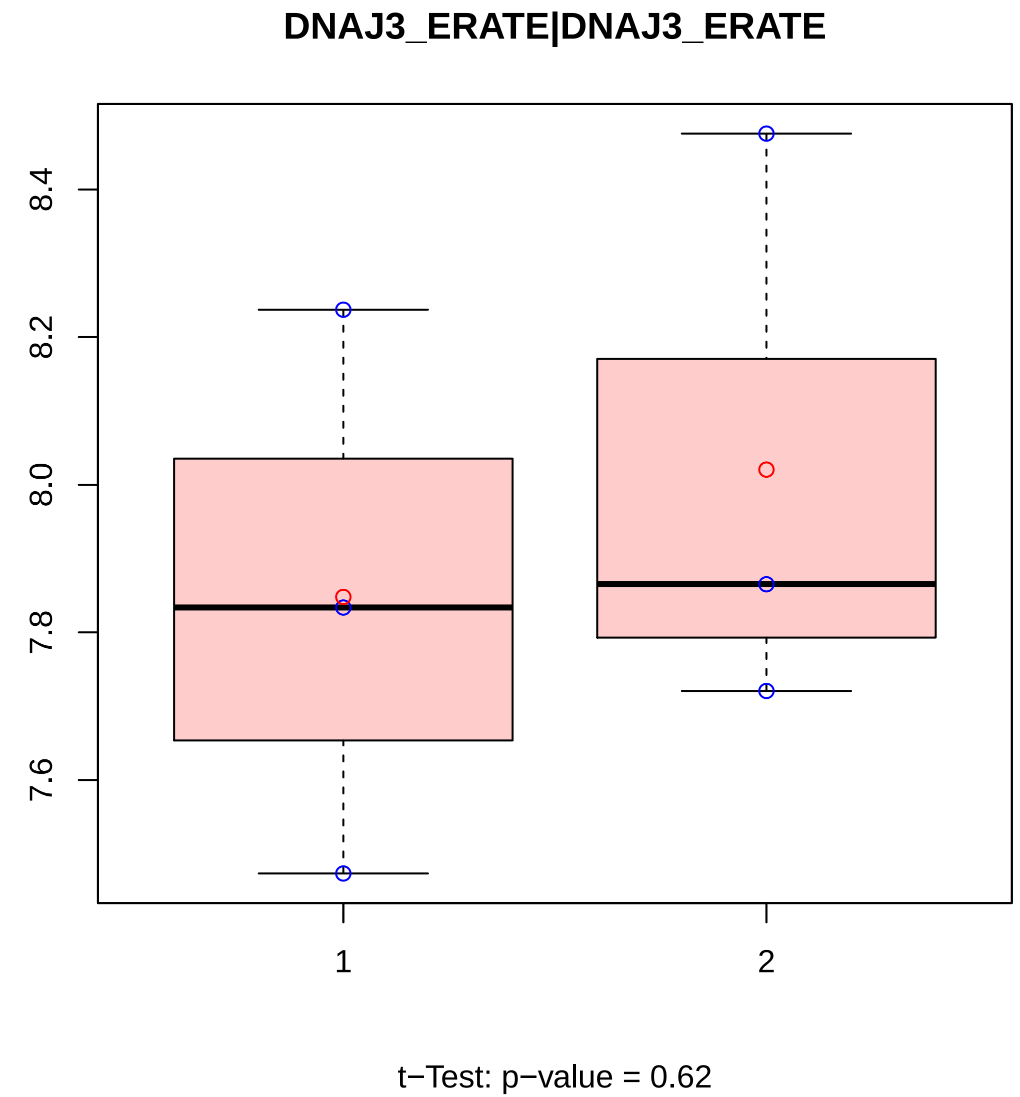
DNAJ3_ERATE|DNAJ3_ERATE
8.4
8.2
8.0
7.8
7.6
1
2
t−Test: p−value = 0.62

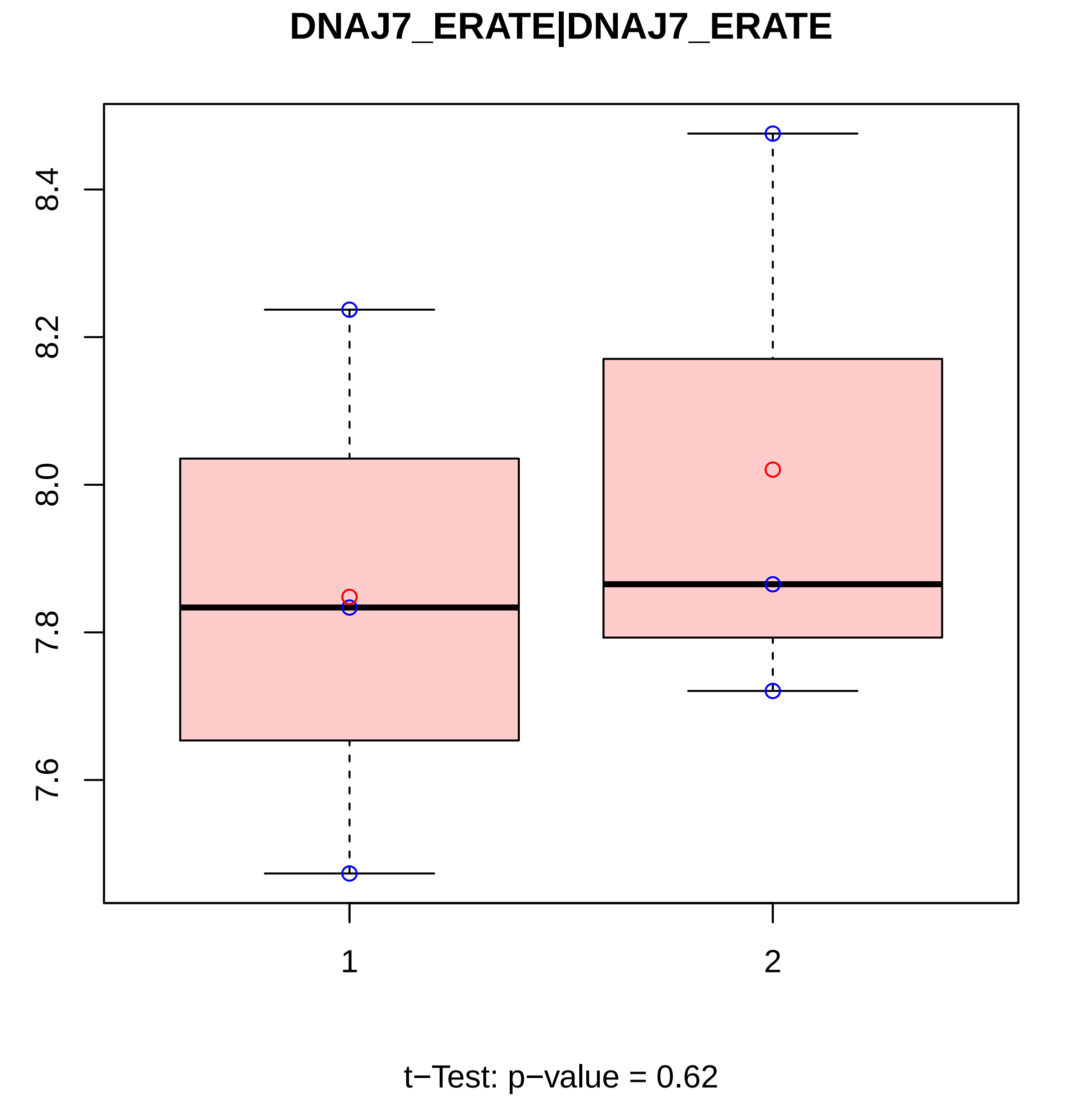
DNAJ7_ERATE|DNAJ7_ERATE
8.4
8.2
8.0
7.8
7.6
1
2
t−Test: p−value = 0.62

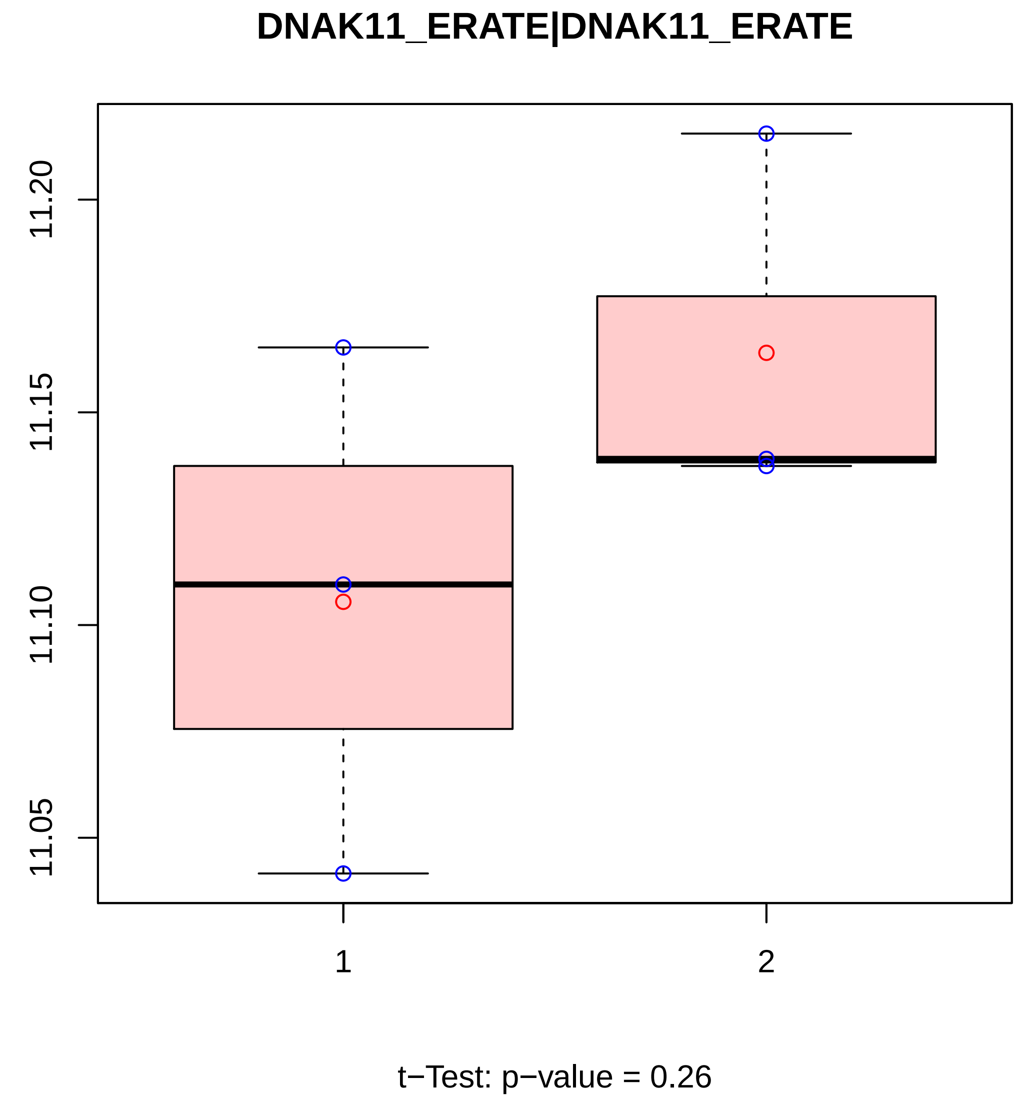

DNAK11_ERATE|DNAK11_ERATE
11.05
11.10
11.15
11.20
1
2
t−Test: p−value = 0.26

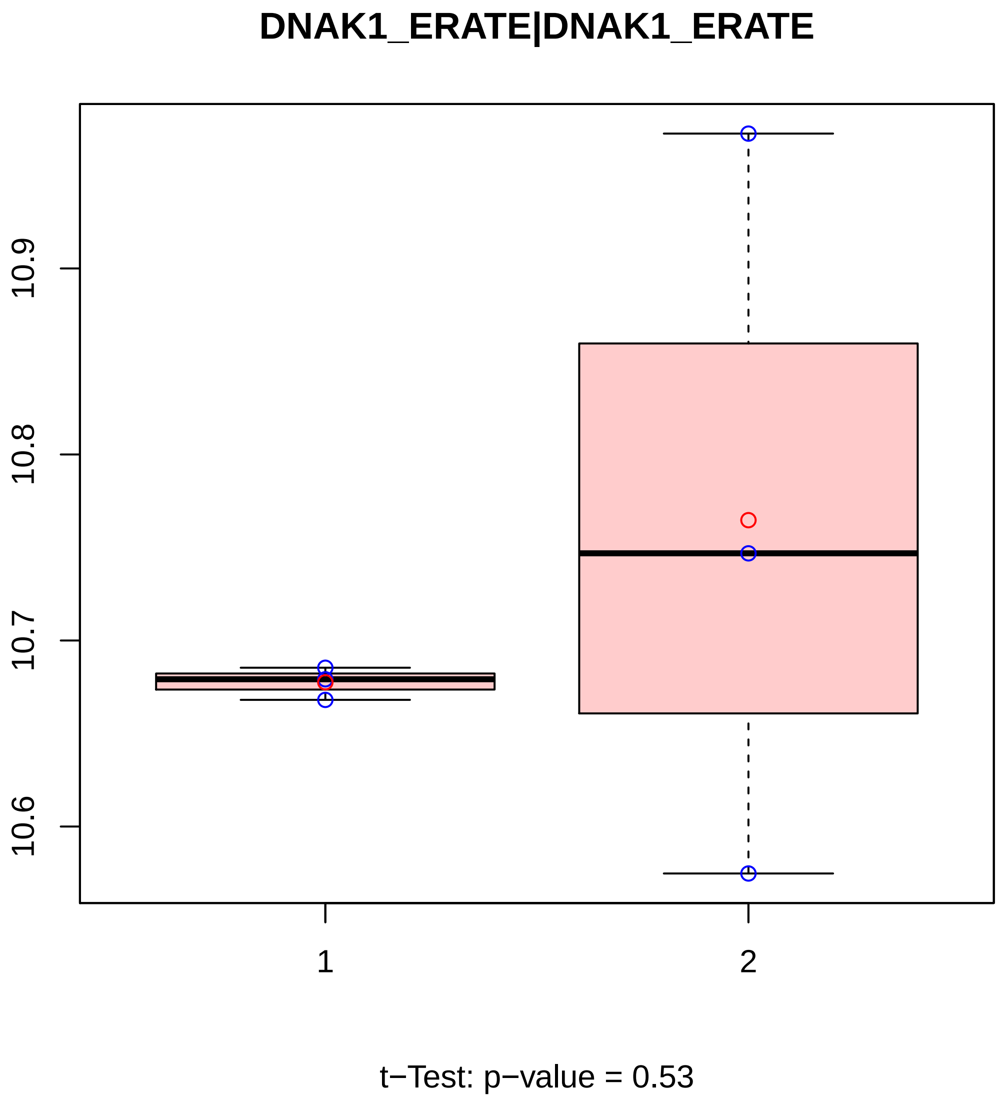

DNAK1_ERATE|DNAK1_ERATE
10.6
10.7
10.8
10.9
1
2
t−Test: p−value = 0.53

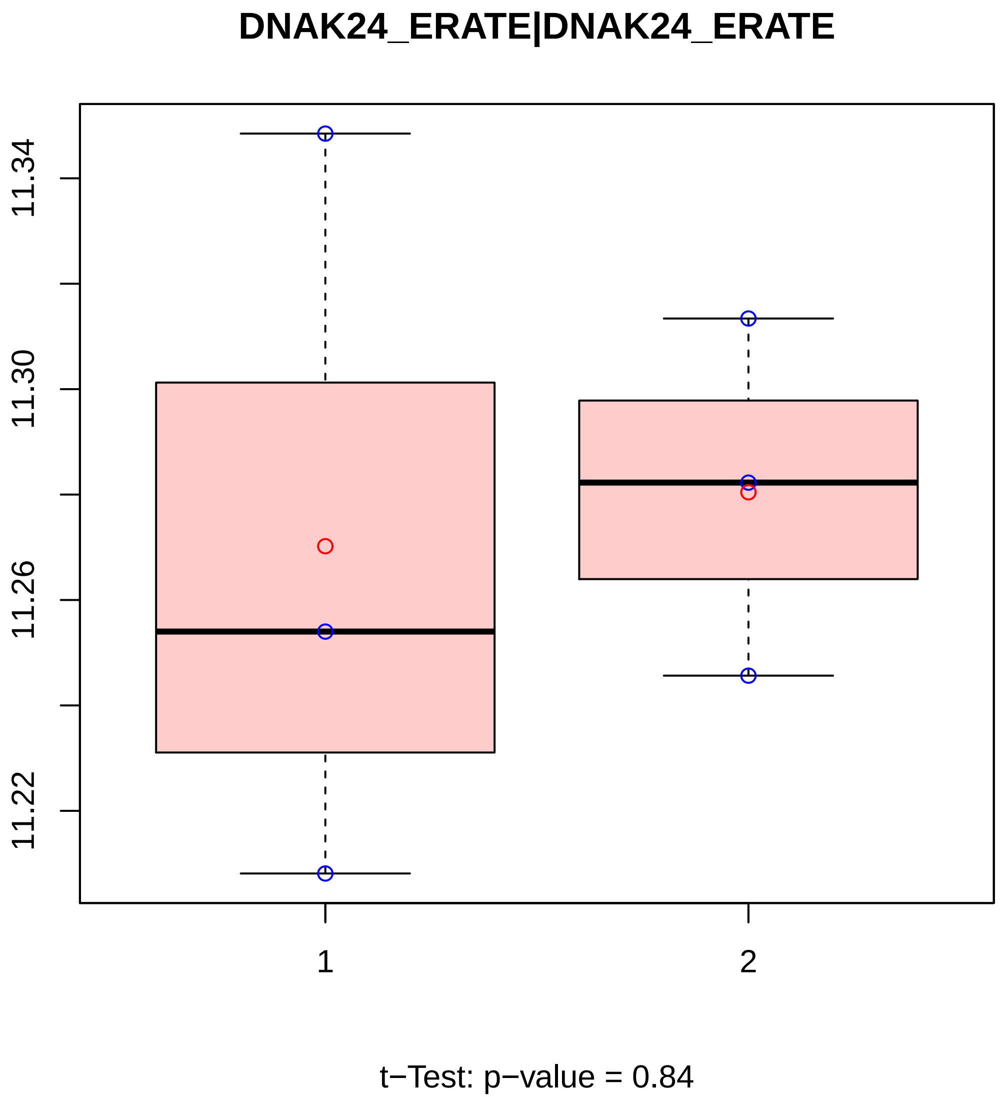

DNAK24_ERATE|DNAK24_ERATE
11.34
11.30
11.26
11.22
1
2
t−Test: p−value = 0.84

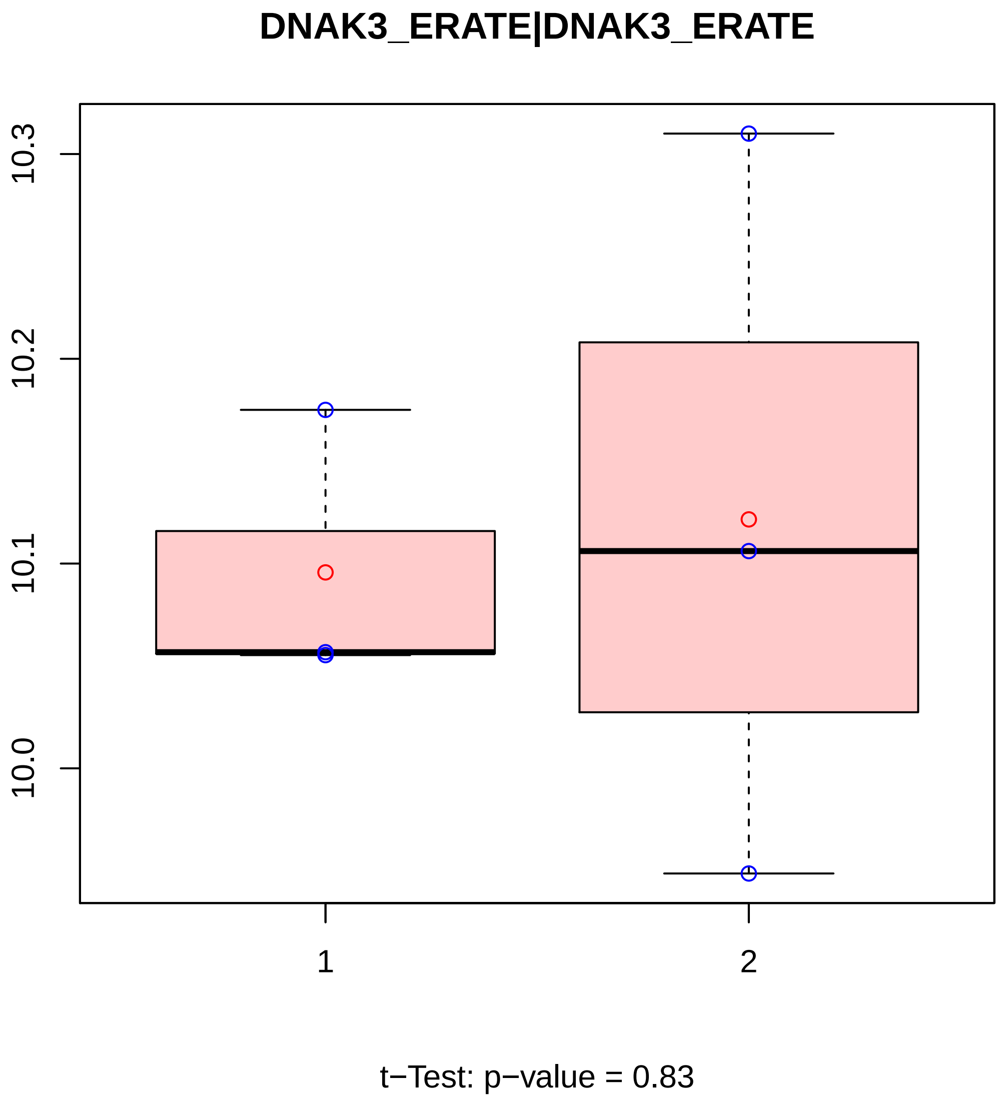

DNAK3_ERATE|DNAK3_ERATE
10.3
10.2
10.1
10.0
1
2
t−Test: p−value = 0.83

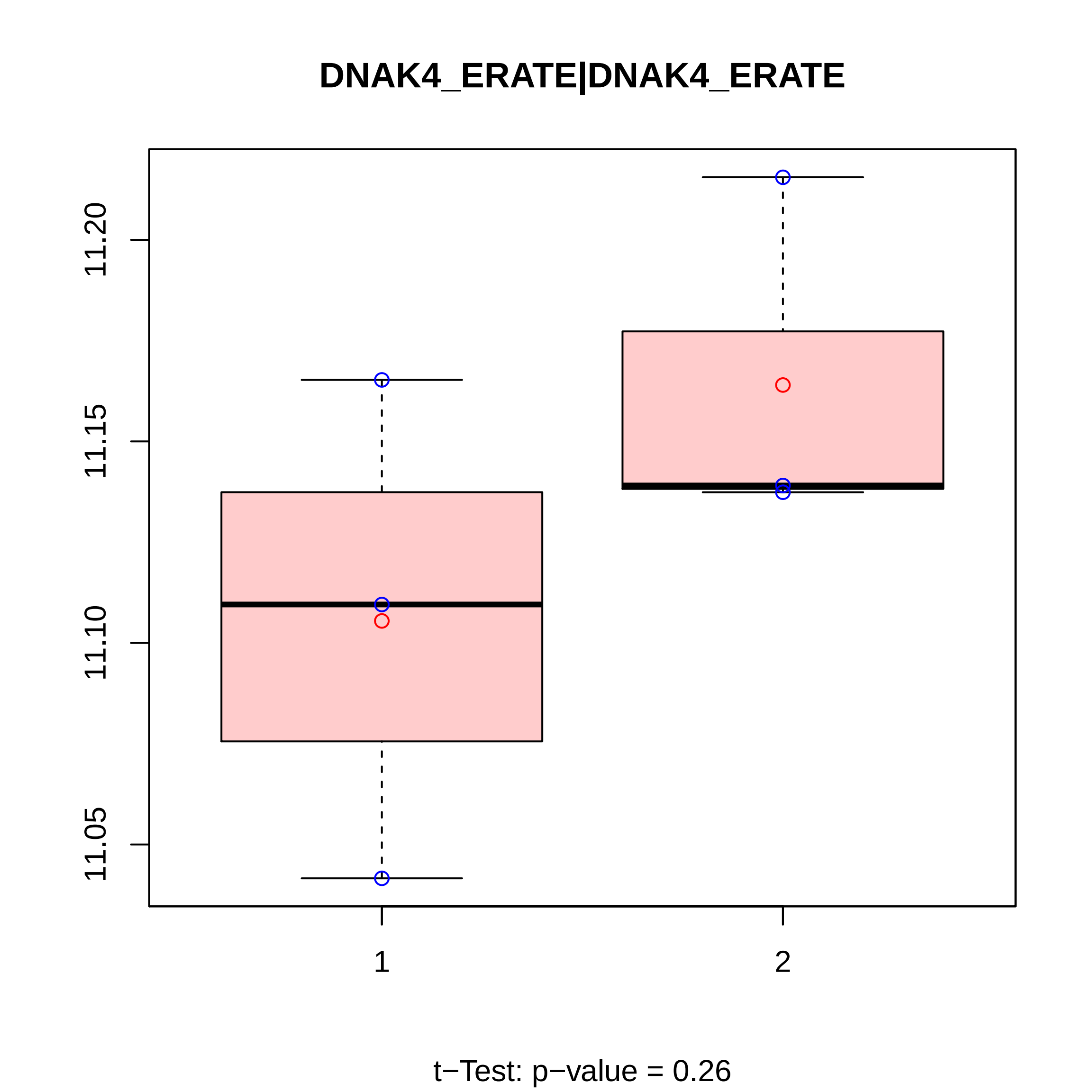
DNAK4_ERATE|DNAK4_ERATE
11.20
11.15
11.10
11.05
1
2
t−Test: p−value = 0.26

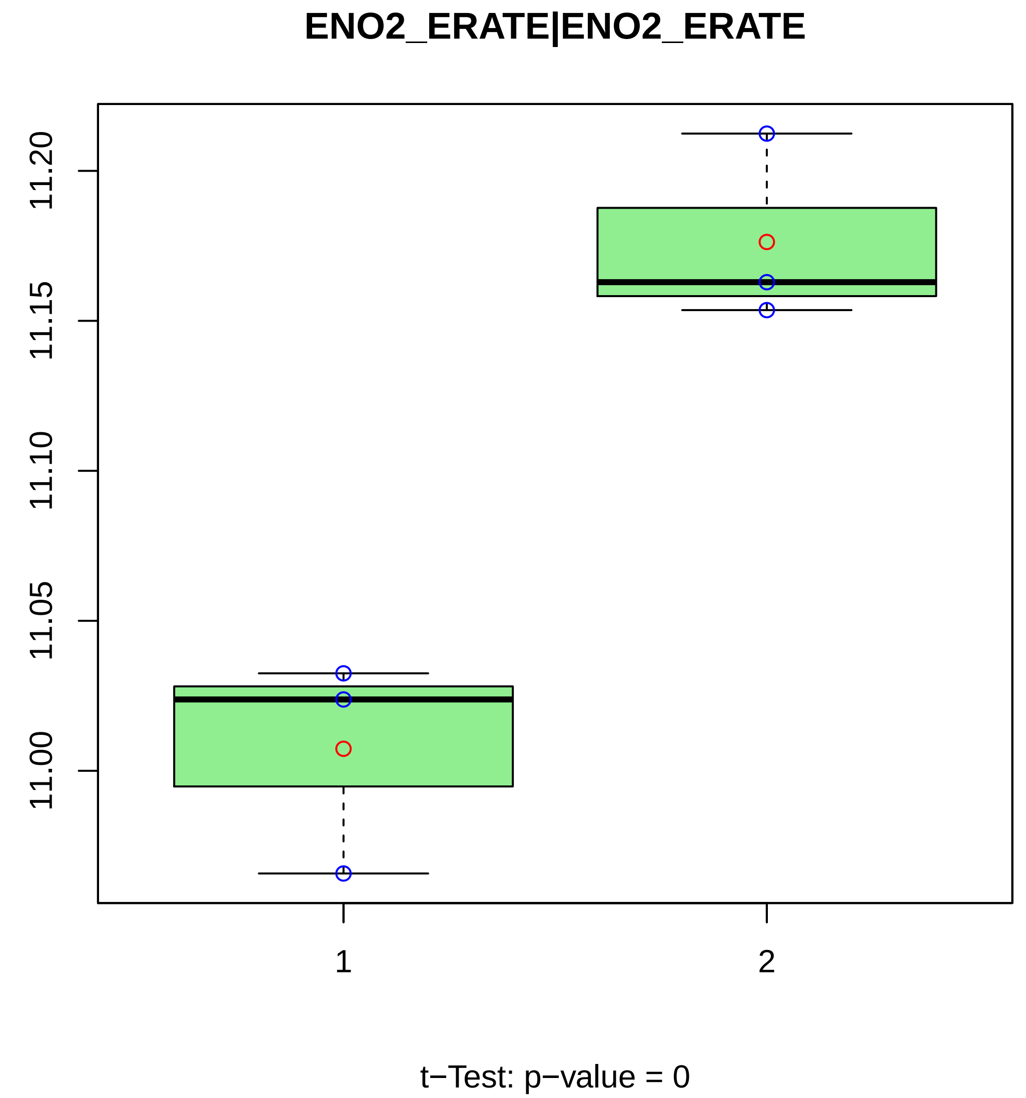

ENO2_ERATE|ENO2_ERATE
11.00
11.05
11.10
11.15
11.20
1
2
t−Test: p−value = 0

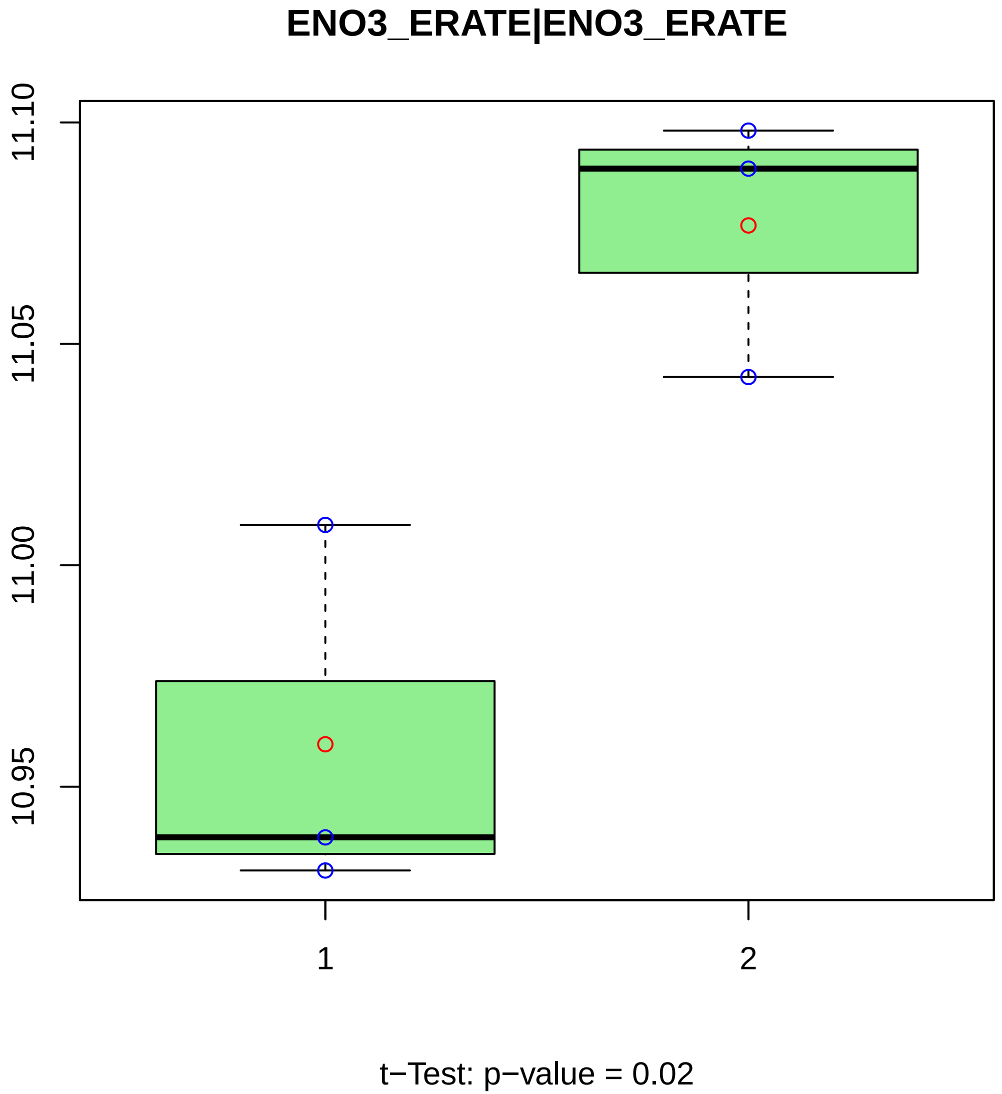
ENO3_ERATE|ENO3_ERATE
11.10
11.05
11.00
10.95
1
2
t−Test: p−value = 0.02

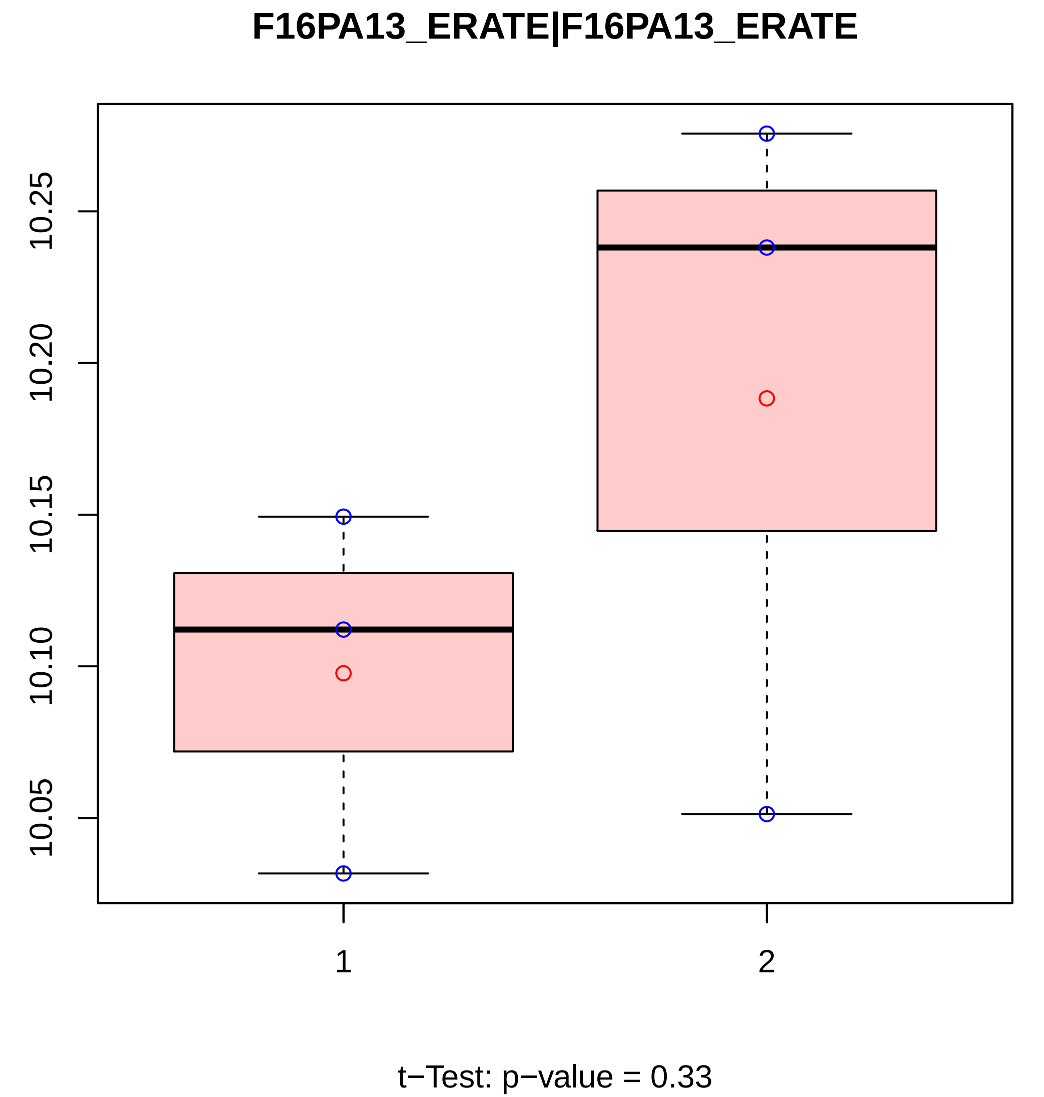
F16PA13_ERATE|F16PA13_ERATE
10.05
10.10
10.15
10.20
10.25
1
2
t−Test: p−value = 0.33

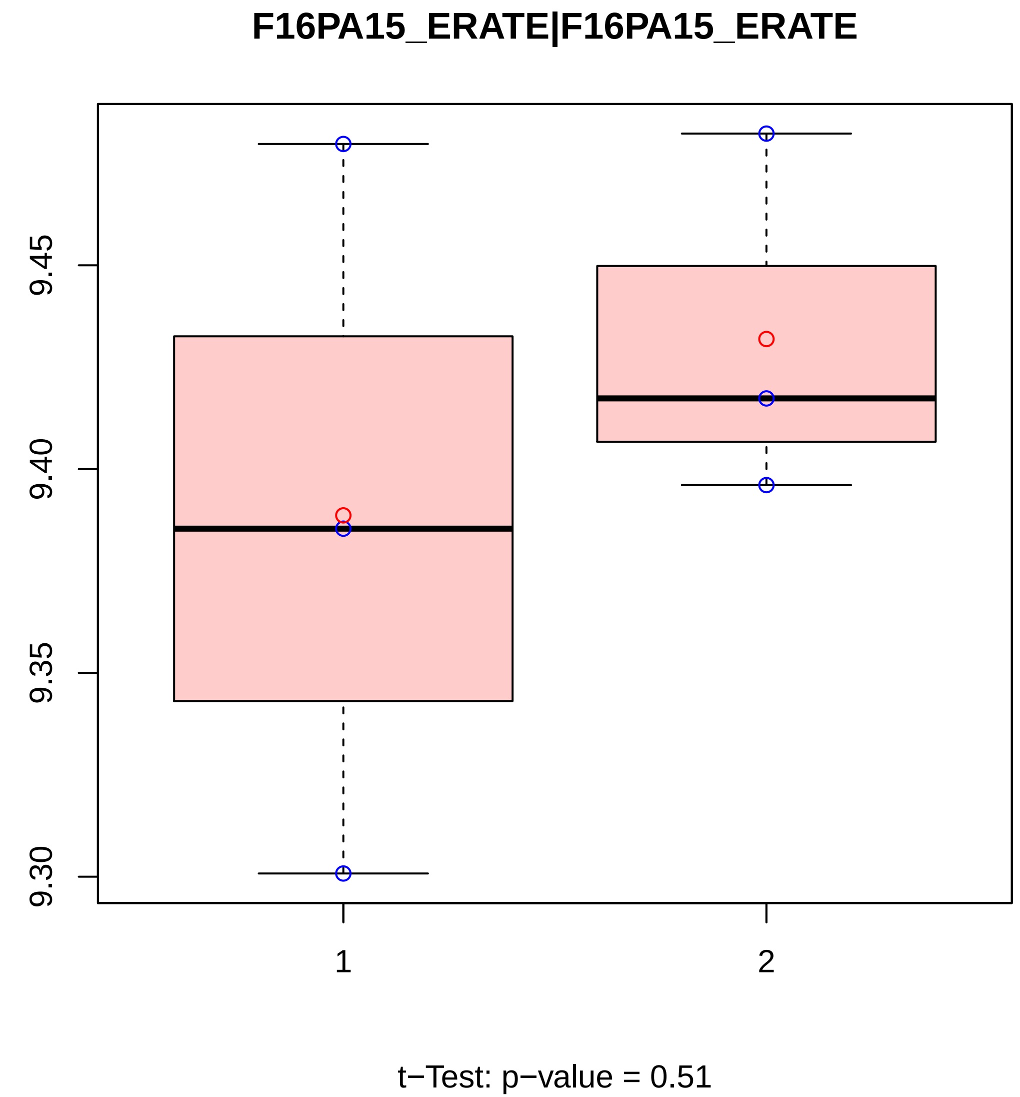

F16PA15_ERATE|F16PA15_ERATE
9.30
9.35
9.40
9.45
1
2
t−Test: p−value = 0.51

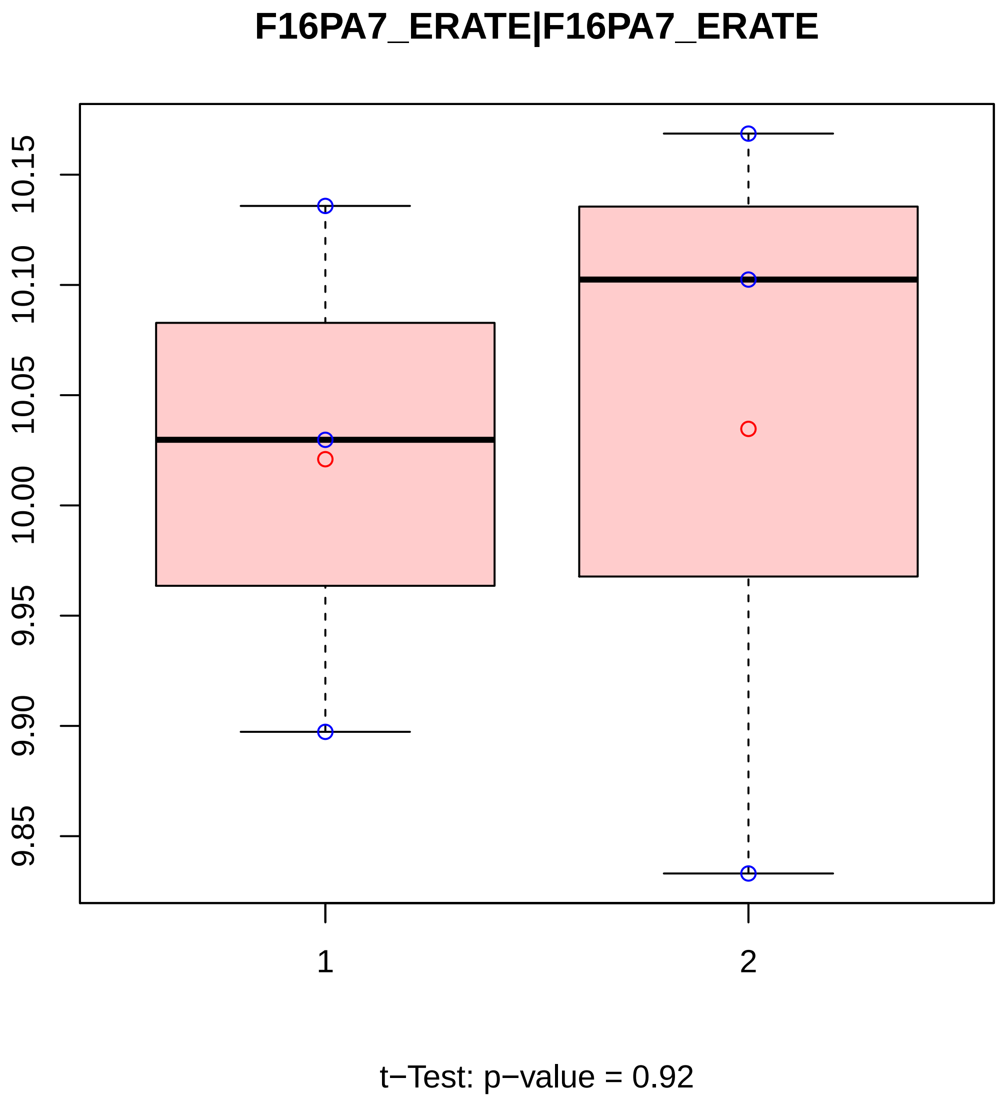
F16PA7_ERATE|F16PA7_ERATE
10.15
10.10
10.05
10.00
9.95
9.90
9.85
1
2
t−Test: p−value = 0.92

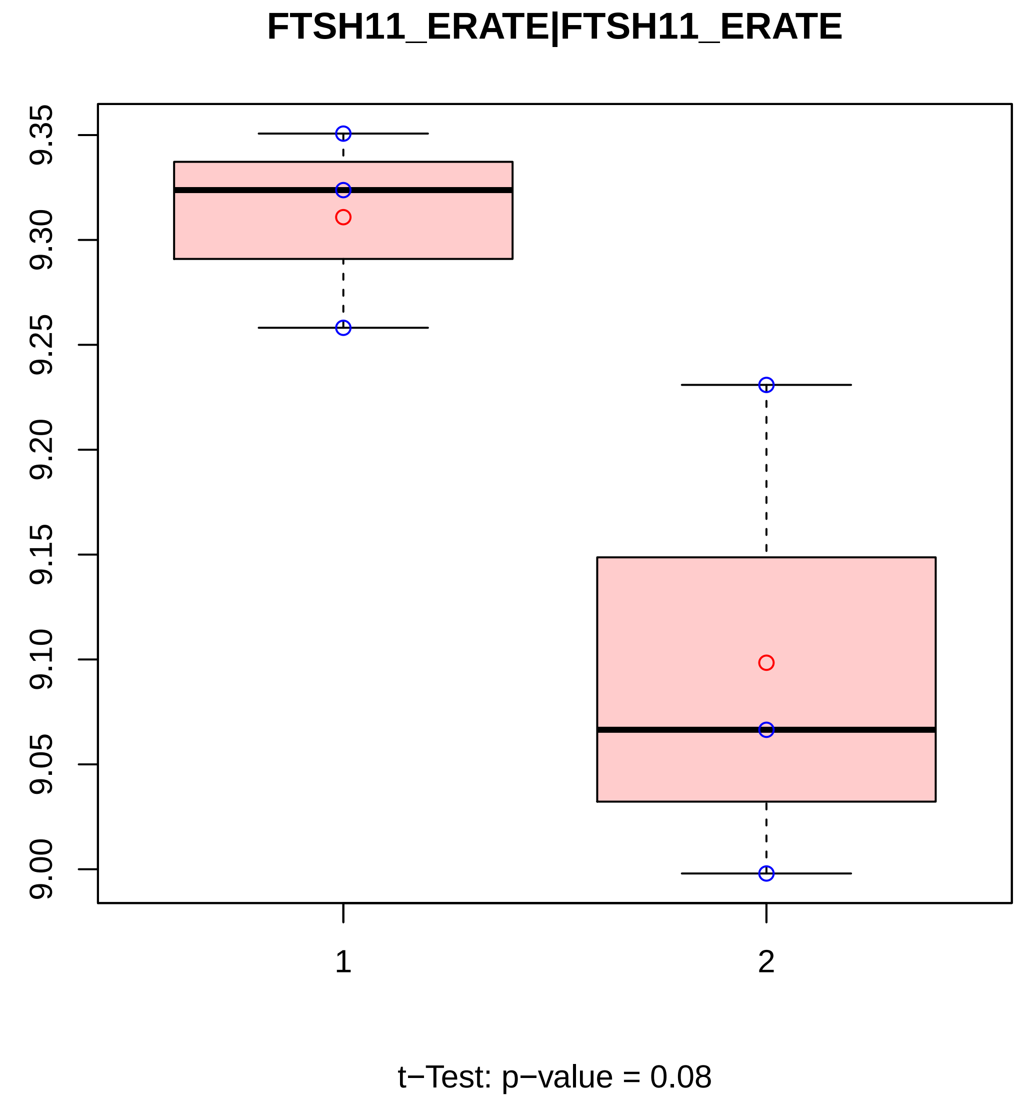

FTSH11_ERATE|FTSH11_ERATE
9.35
9.30
9.25
9.20
9.15
9.10
9.05
9.00
1
2
t−Test: p−value = 0.08

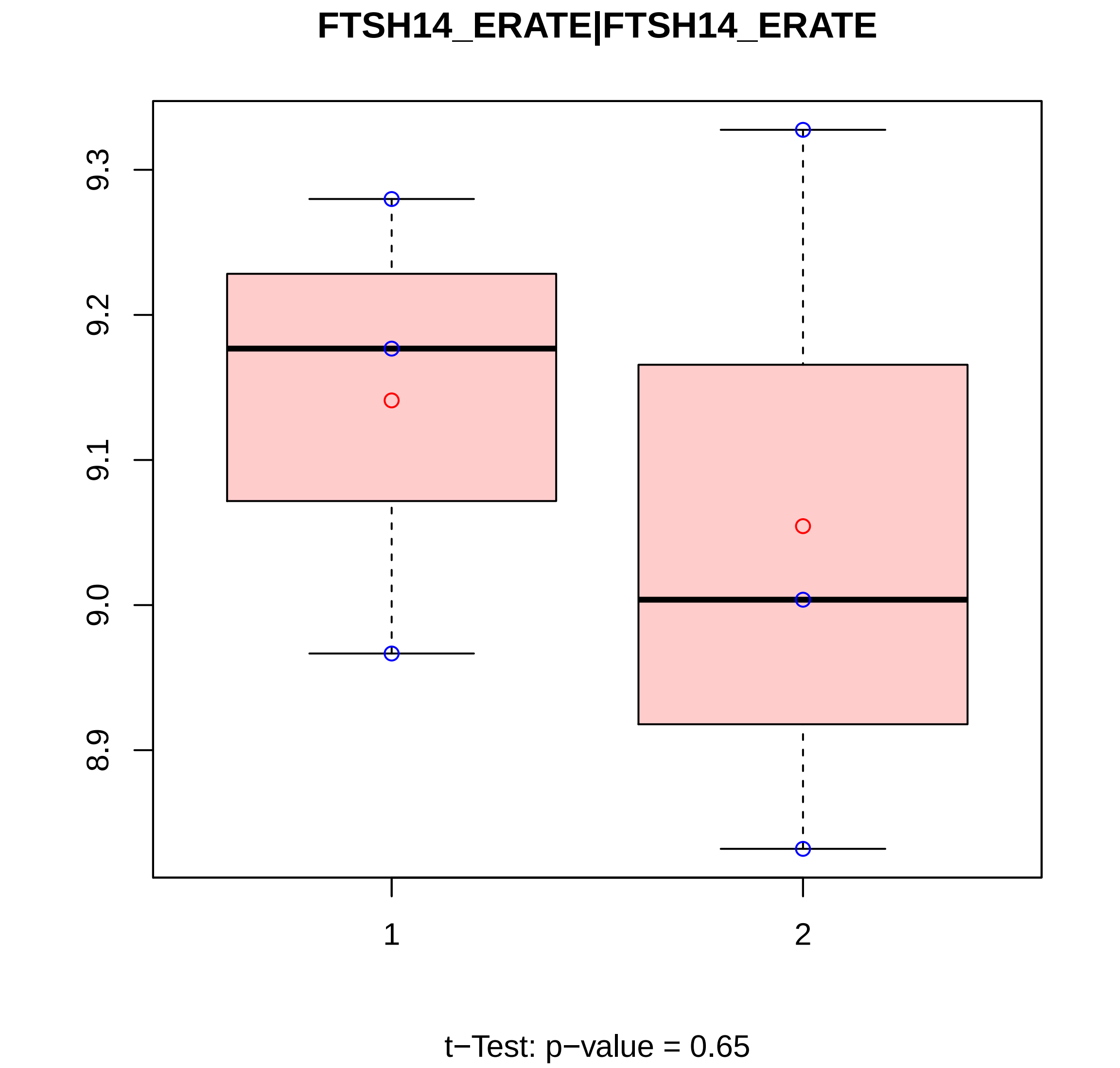

FTSH14_ERATE|FTSH14_ERATE
9.3
9.2
9.1
9.0
8.9
1
2
t−Test: p−value = 0.65

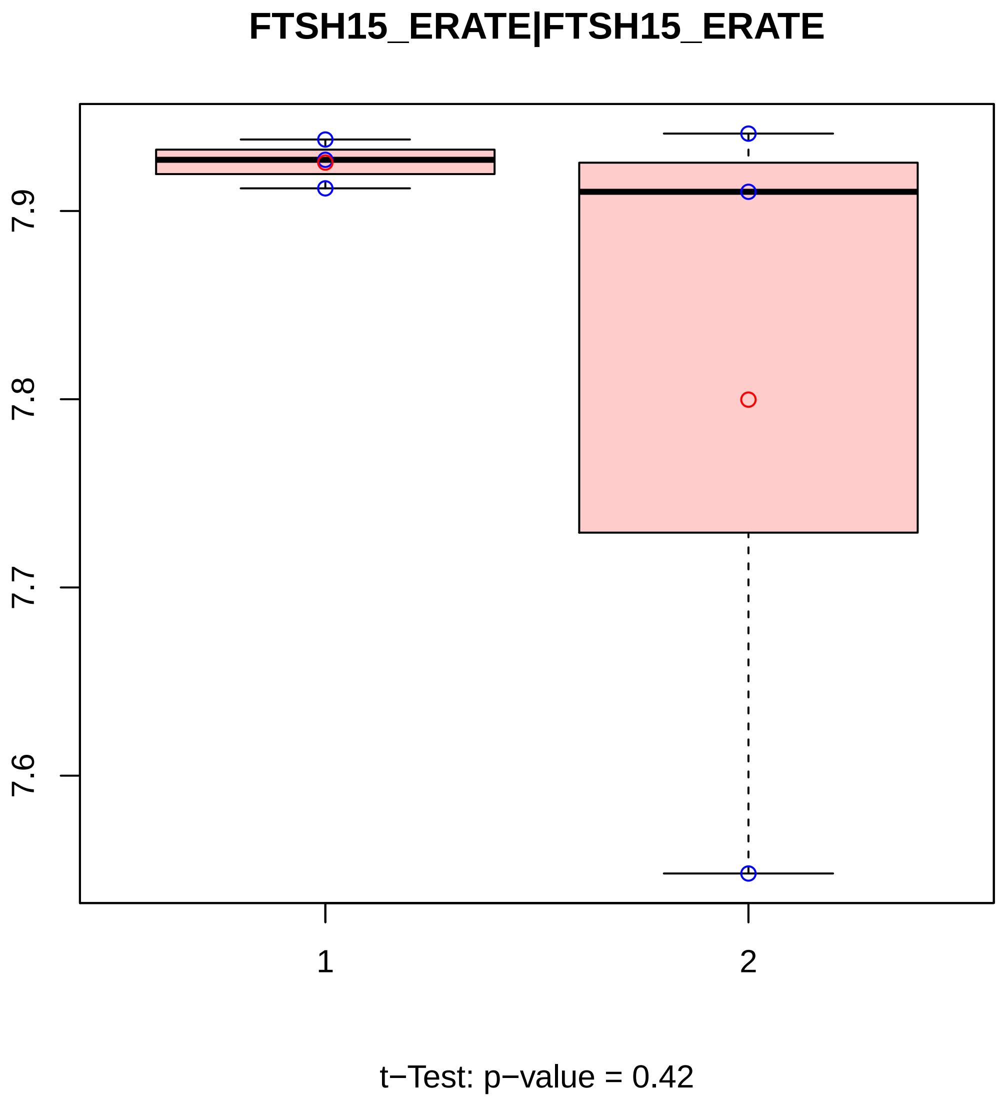

FTSH15_ERATE|FTSH15_ERATE
7.9
7.8
7.7
7.6
1
2
t−Test: p−value = 0.42

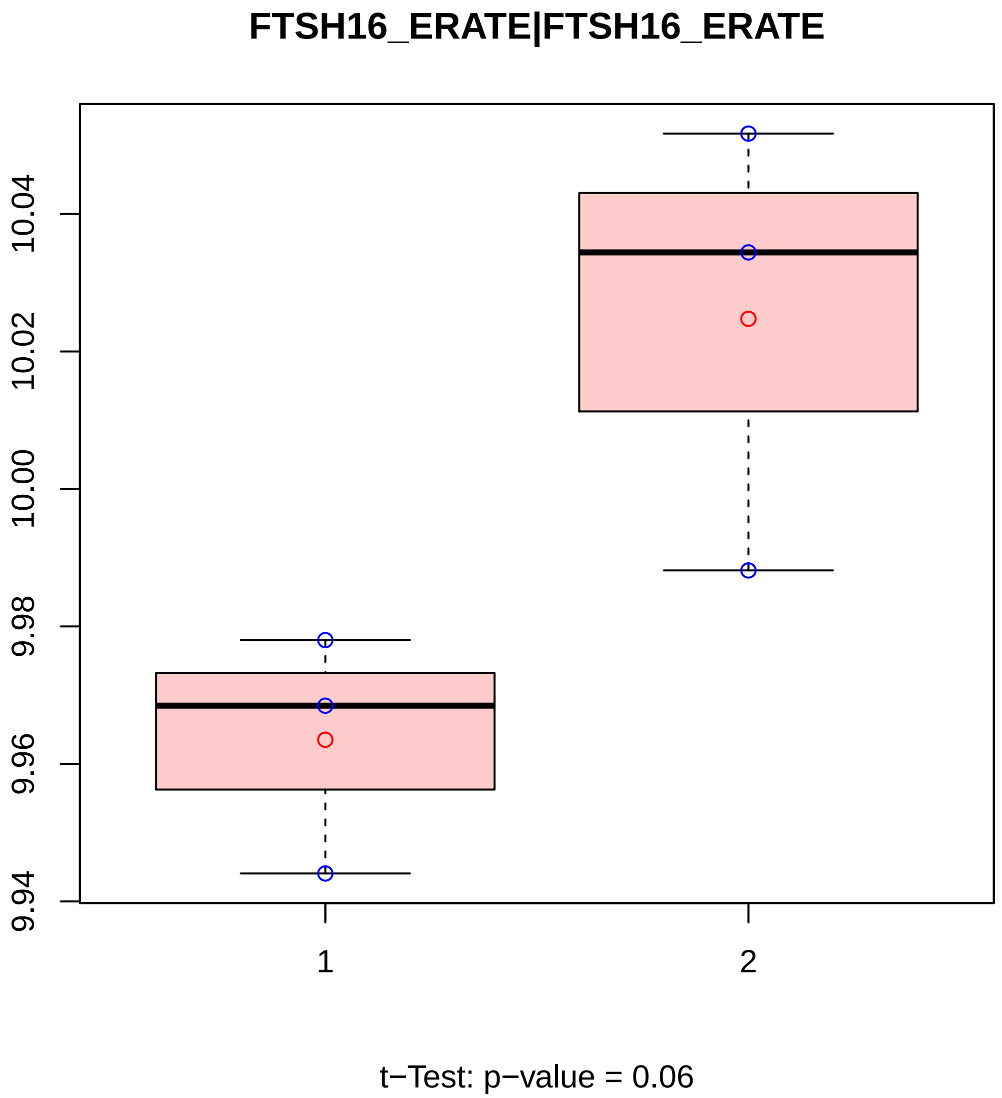

FTSH16_ERATE|FTSH16_ERATE
10.04
10.02
10.00
9.98
9.96
9.94
1
2
t−Test: p−value = 0.06

# FTSH3_ERATE|FTSH3_ERATE

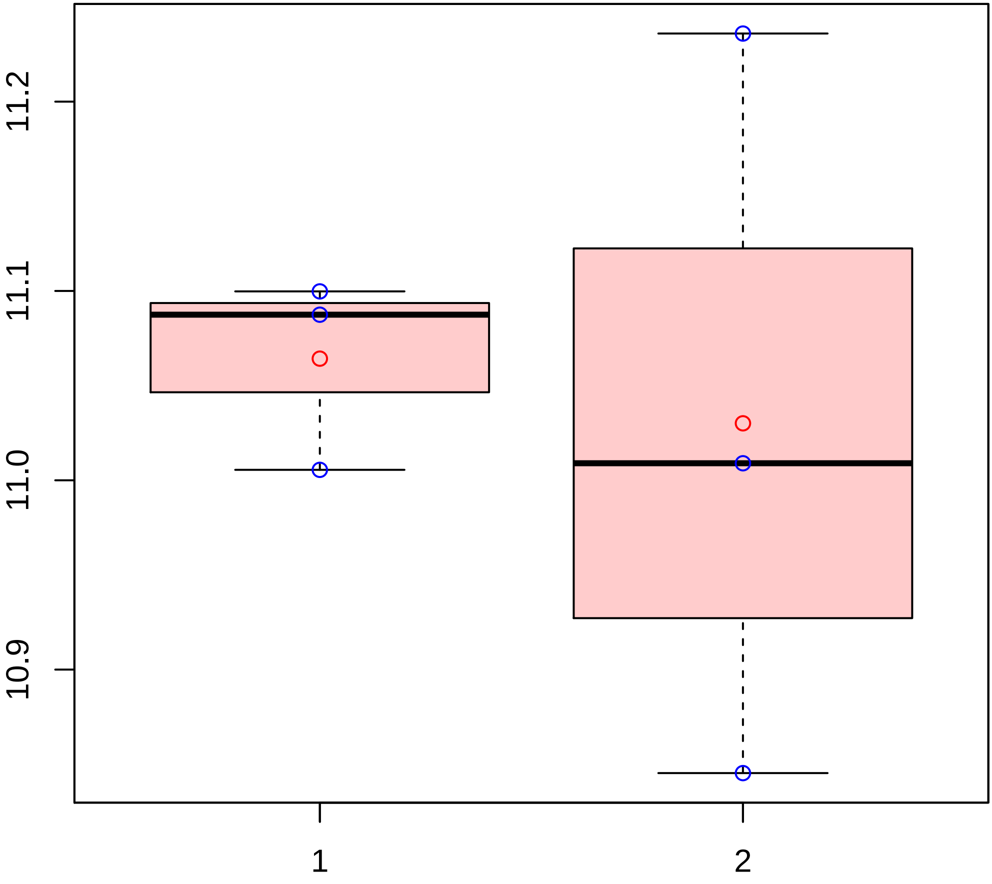


t−Test: p−value = 0.8

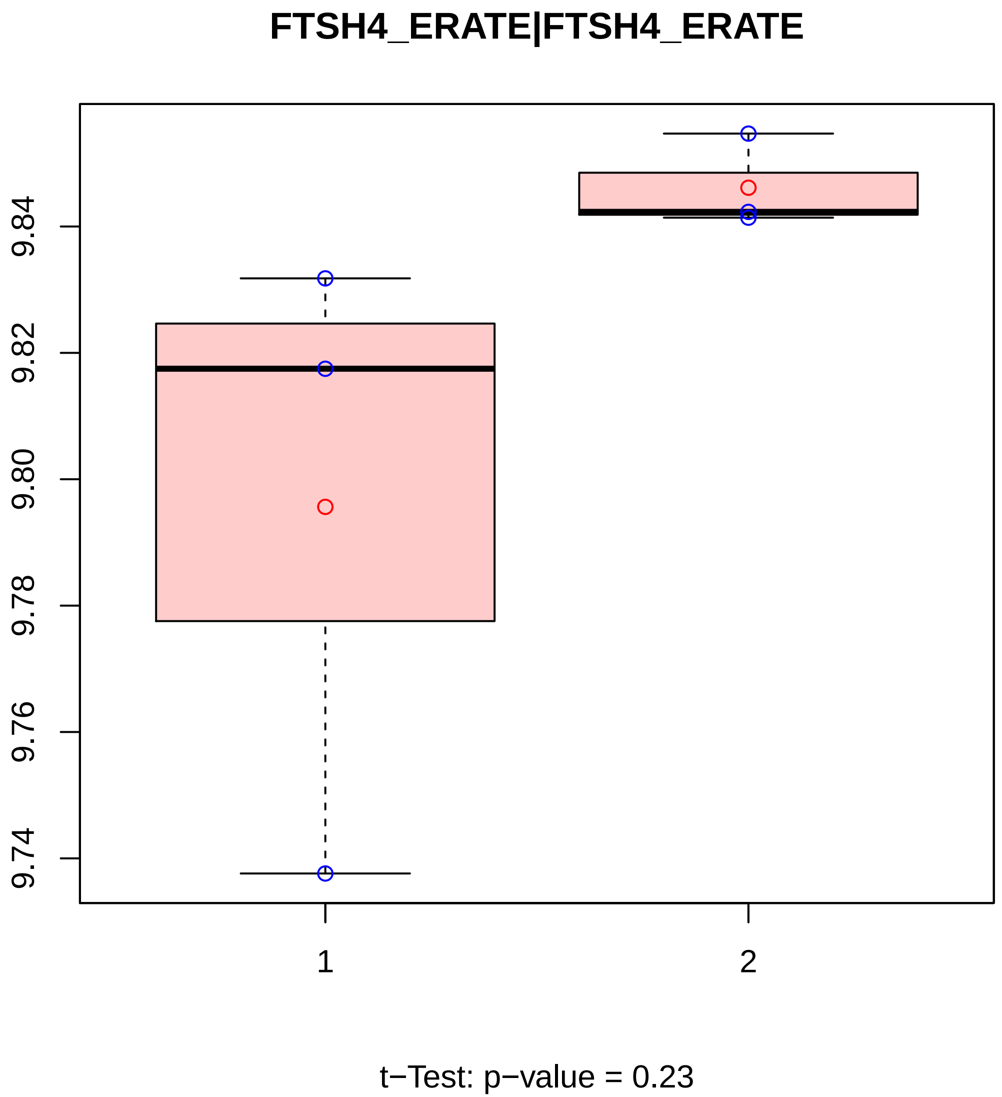
FTSH4_ERATE|FTSH4_ERATE
9.74
9.76
9.78
9.80
9.82
9.84
1
2
t−Test: p−value = 0.23

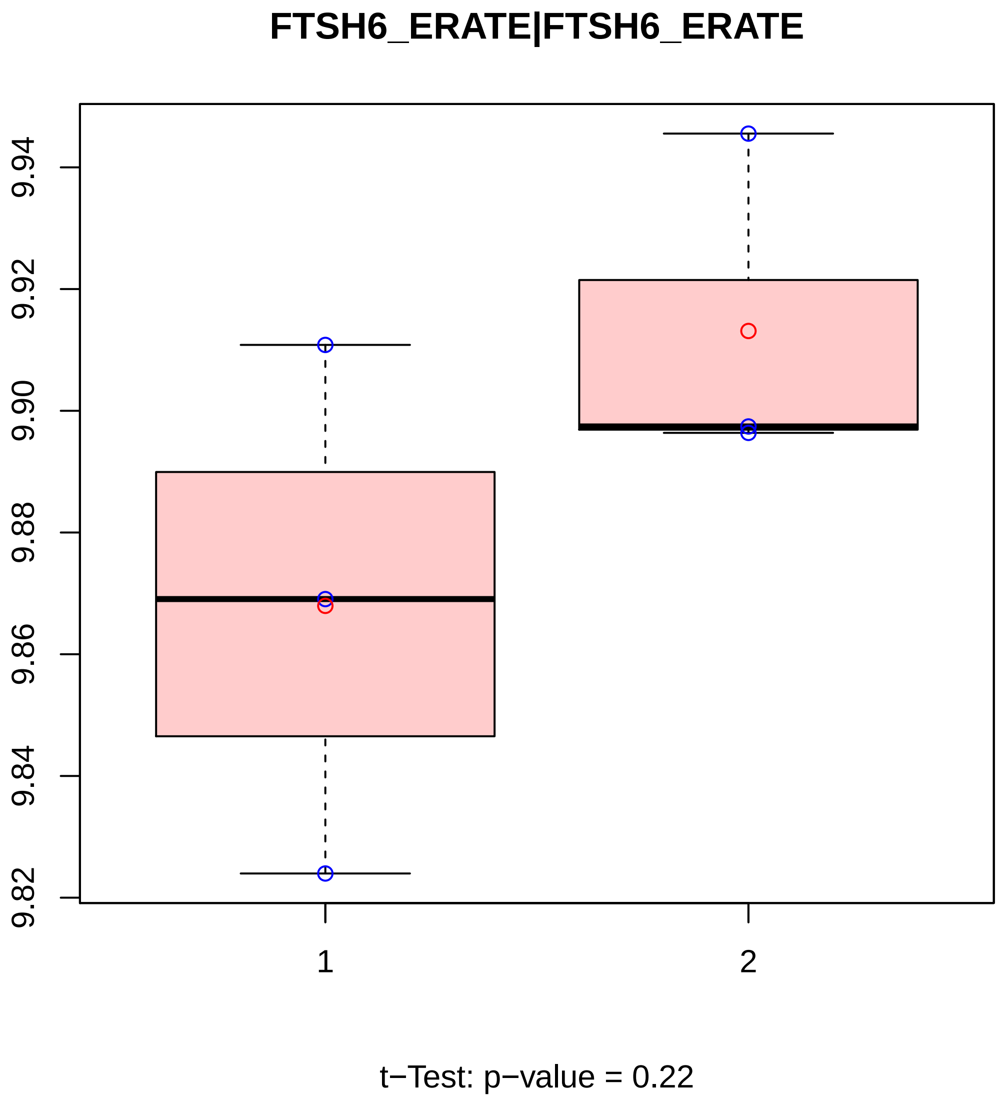

FTSH6_ERATE|FTSH6_ERATE
9.82
9.84
9.86
9.88
9.90
9.92
9.94
1
2
t−Test: p−value = 0.22

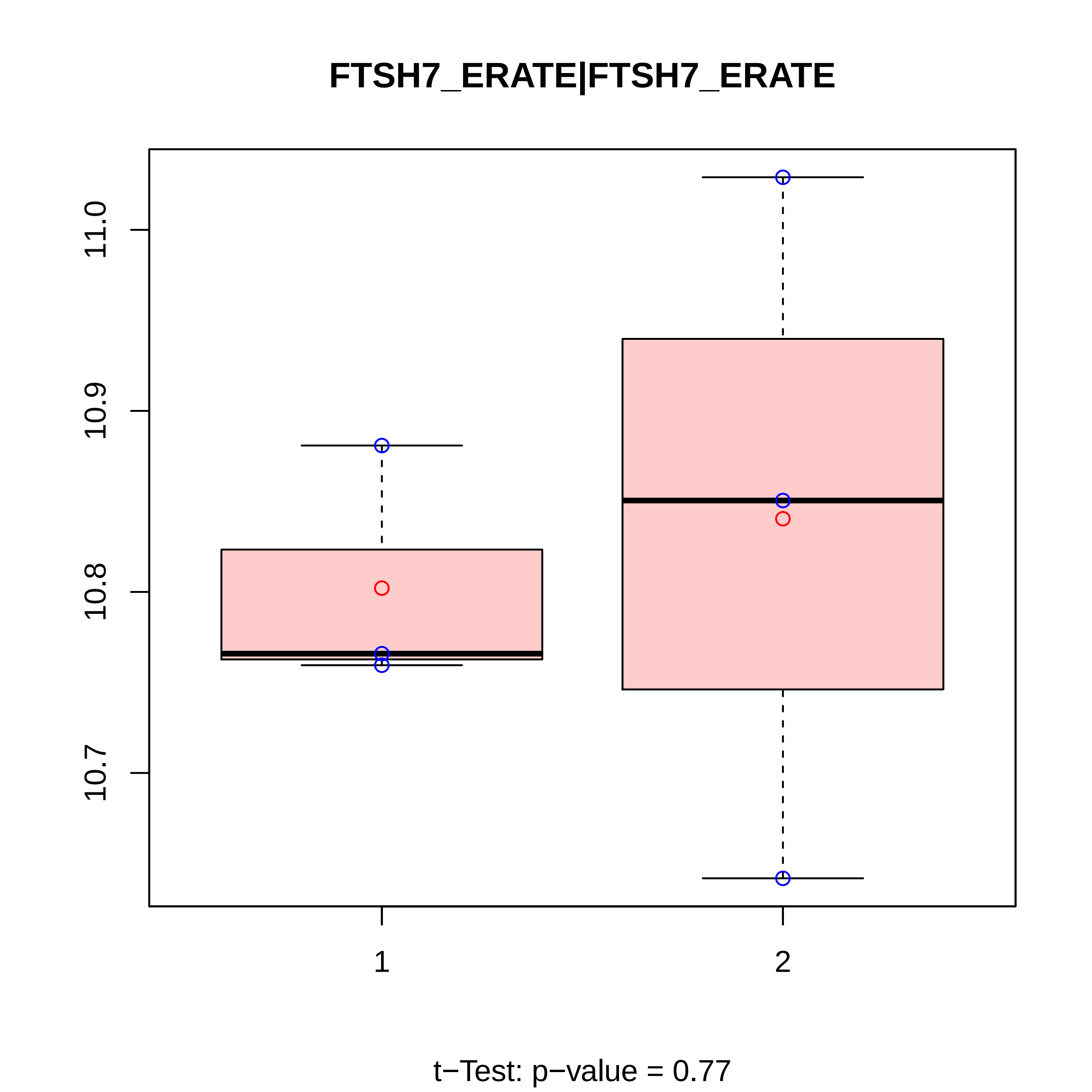
FTSH7_ERATE|FTSH7_ERATE
11.0
10.9
10.8
10.7
1
2
t−Test: p−value = 0.77

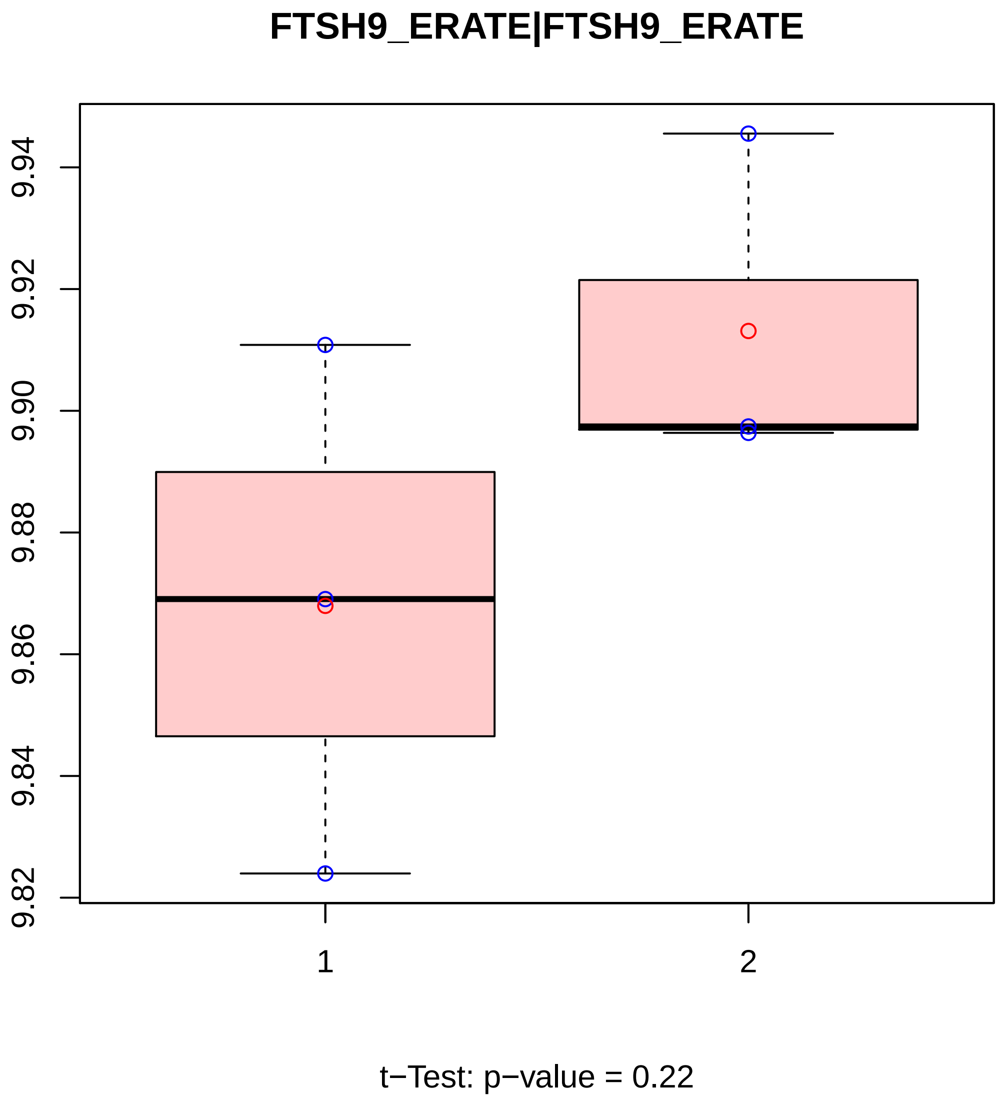

FTSH9_ERATE|FTSH9_ERATE
9.94
9.92
9.90
9.88
9.86
9.84
9.82
1
2
t−Test: p−value = 0.22

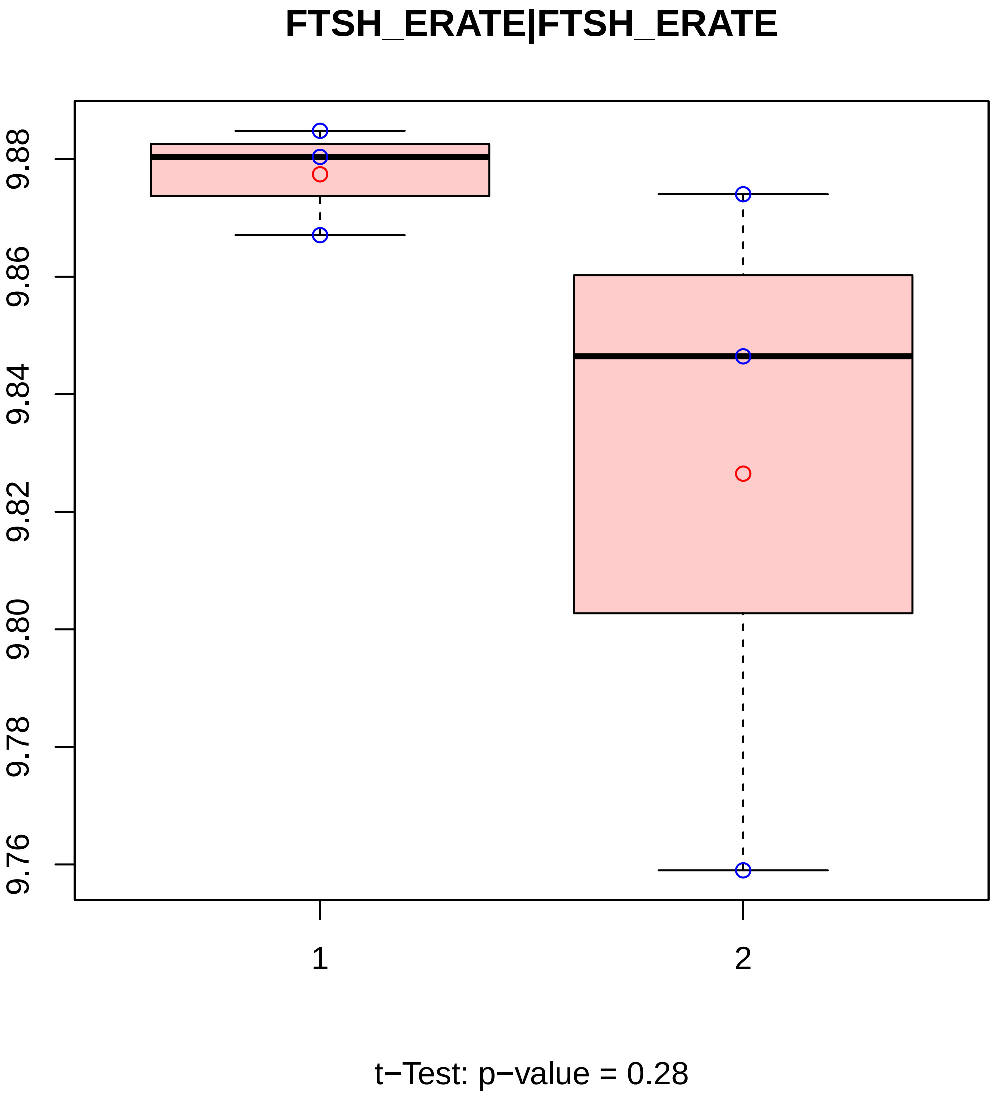
FTSH_ERATE|FTSH_ERATE
9.76
9.78
9.80
9.82
9.84
9.86
9.88
1
2
t−Test: p−value = 0.28

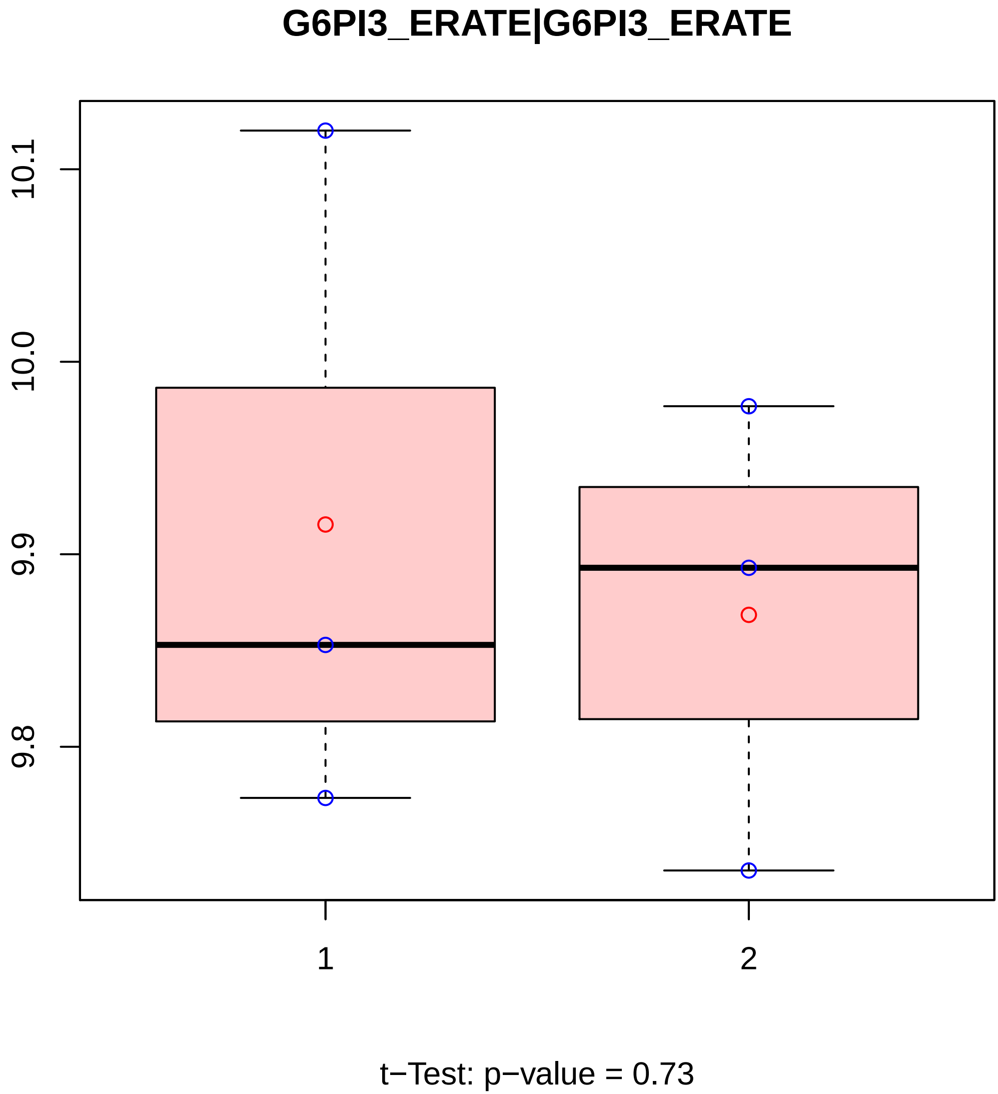
G6PI3_ERATE|G6PI3_ERATE
10.1
10.0
9.9
9.8
1
2
t−Test: p−value = 0.73

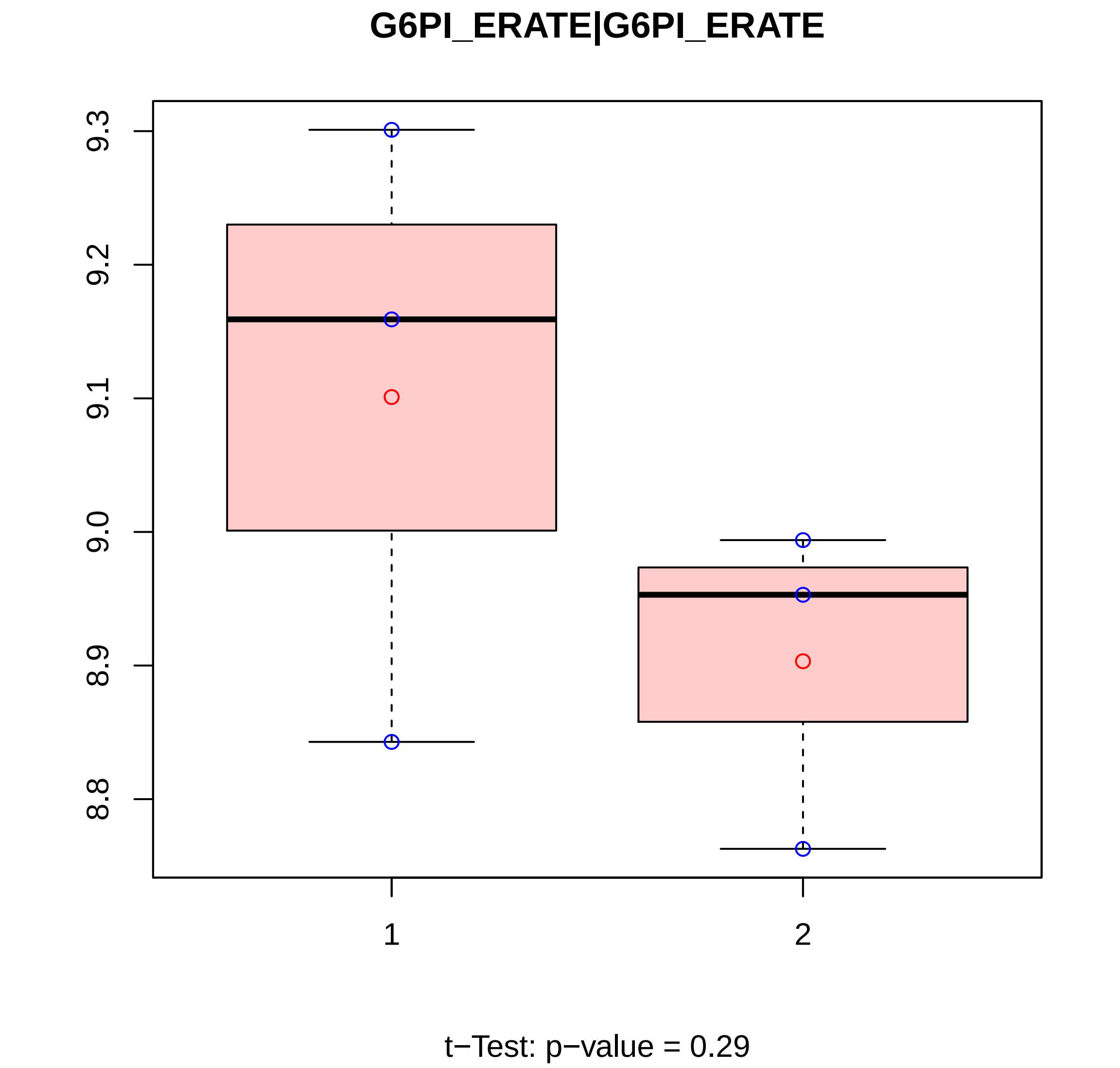

G6PI_ERATE|G6PI_ERATE
9.3
9.2
9.1
9.0
8.9
8.8
1
2
t−Test: p−value = 0.29

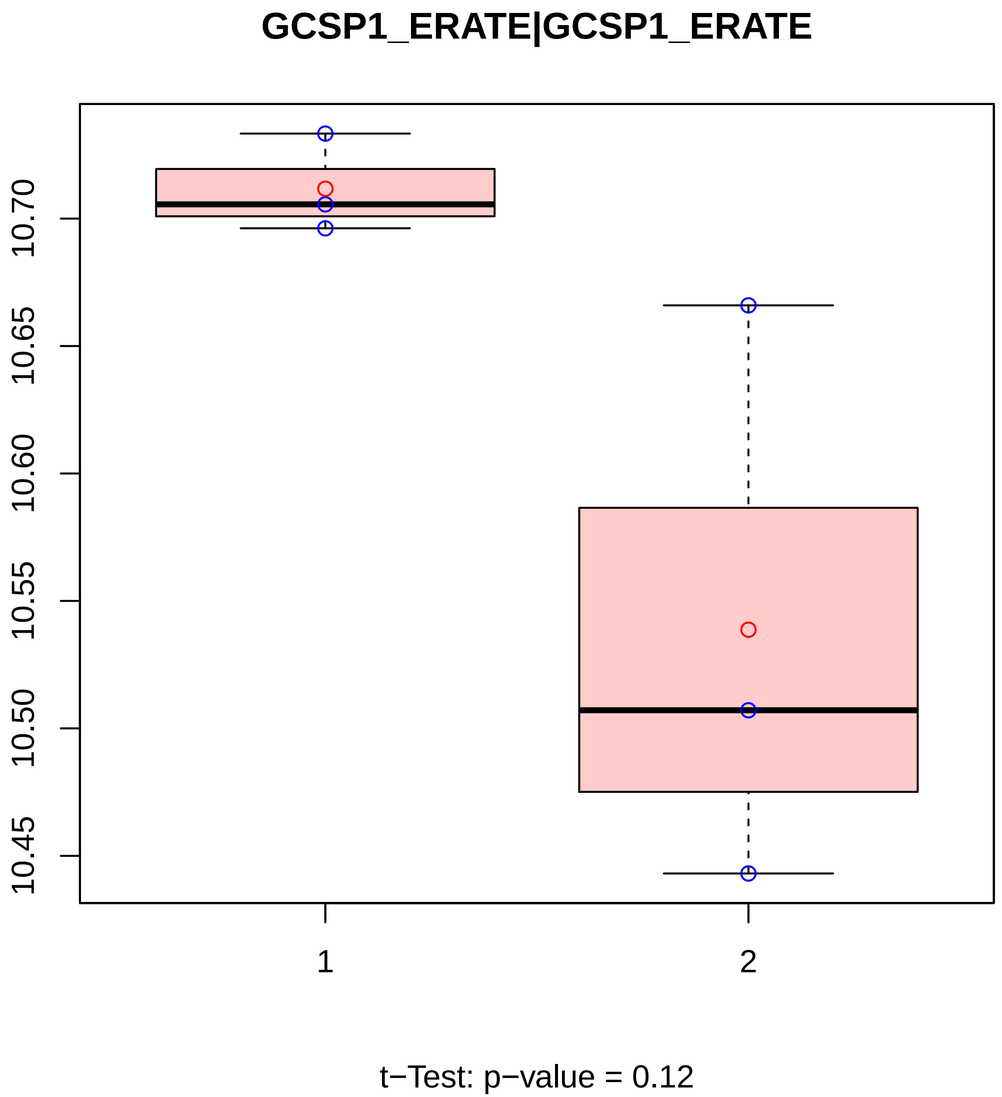
GCSP1_ERATE|GCSP1_ERATE
10.45
10.50
10.55
10.60
10.65
10.70
1
2
t−Test: p−value = 0.12

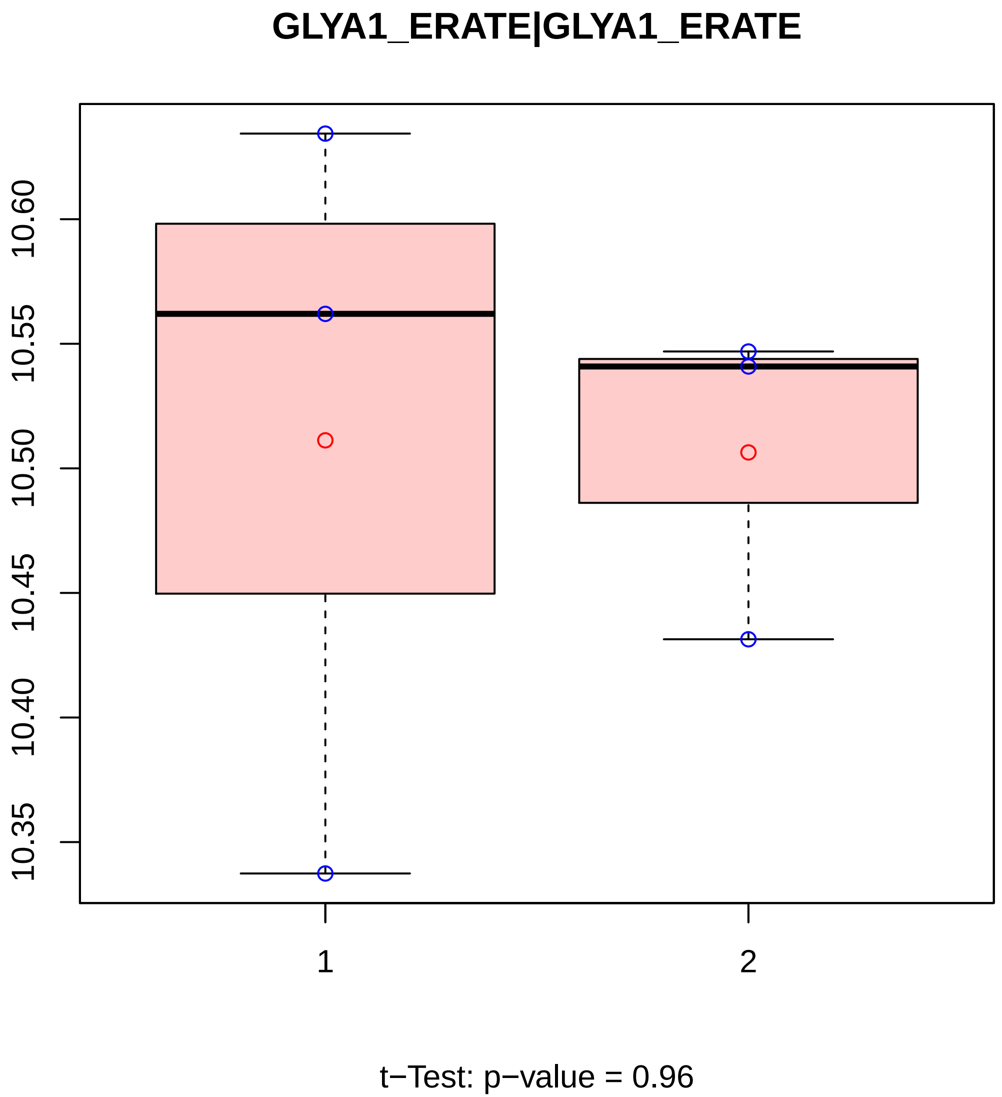

GLYA1_ERATE|GLYA1_ERATE
10.35
10.40
10.45
10.50
10.55
10.60
1
2
t−Test: p−value = 0.96

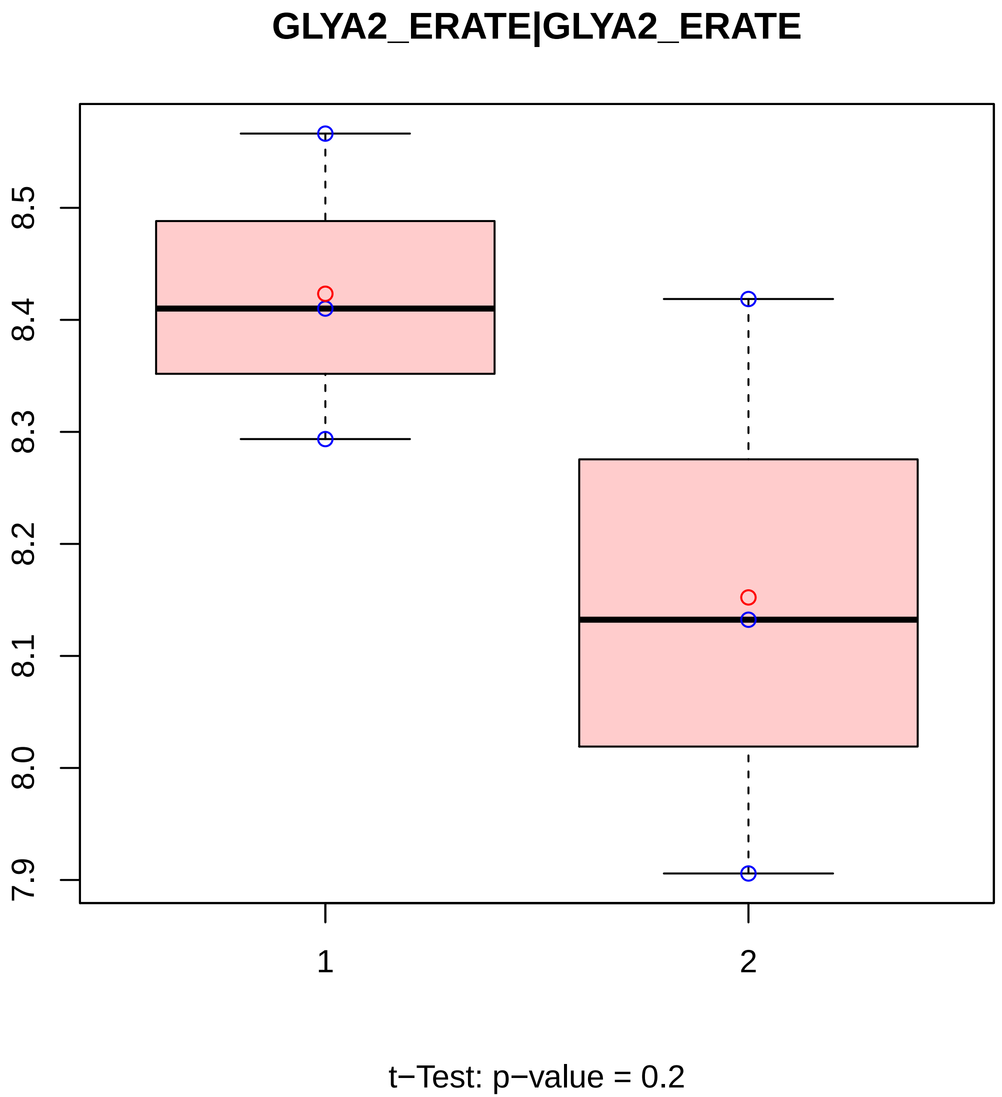
GLYA2_ERATE|GLYA2_ERATE
8.5
8.4
8.3
8.2
8.1
8.0
7.9
1
2
t−Test: p−value = 0.2

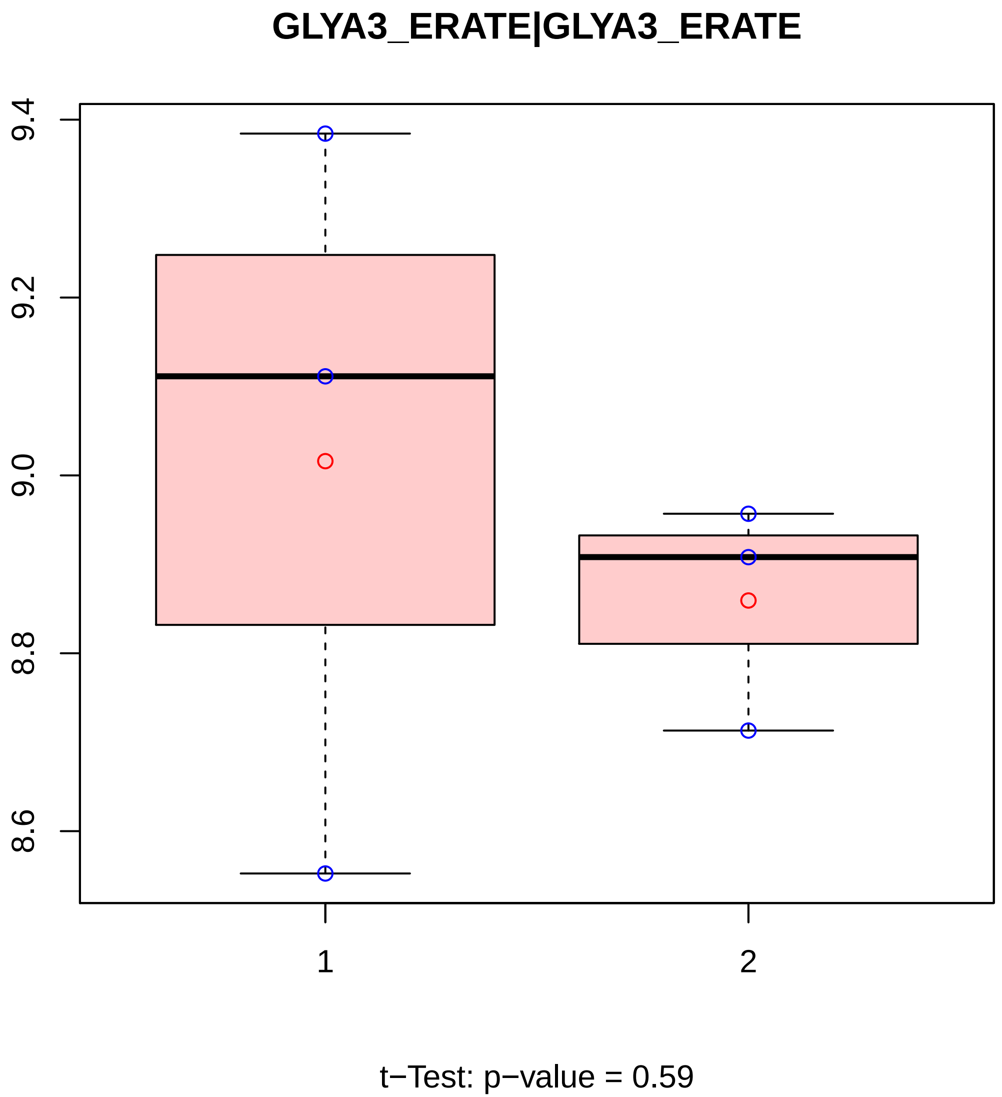
GLYA3_ERATE|GLYA3_ERATE
9.4
9.2
9.0
8.8
8.6
1
2
t−Test: p−value = 0.59

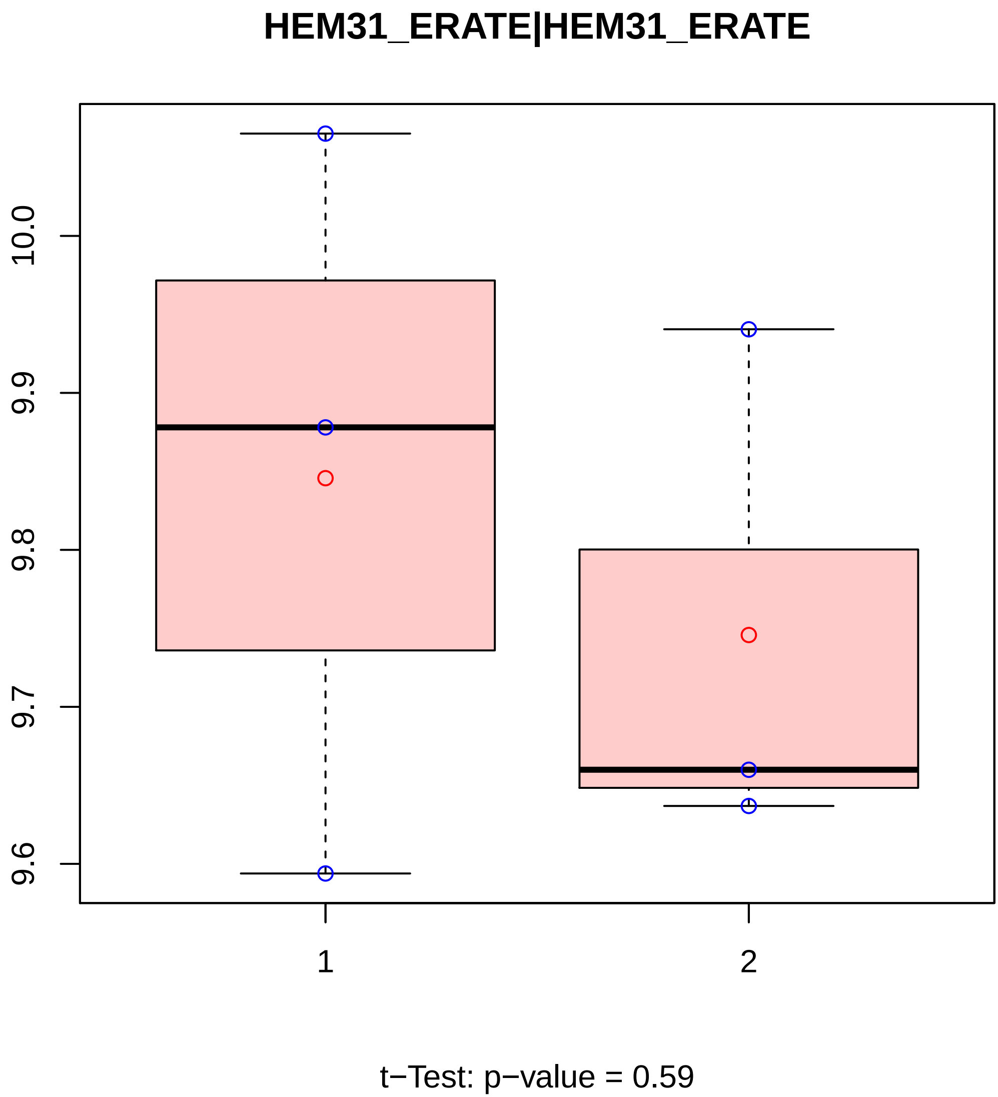
HEM31_ERATE|HEM31_ERATE
10.0
9.9
9.8
9.7
9.6
1
2
t−Test: p−value = 0.59

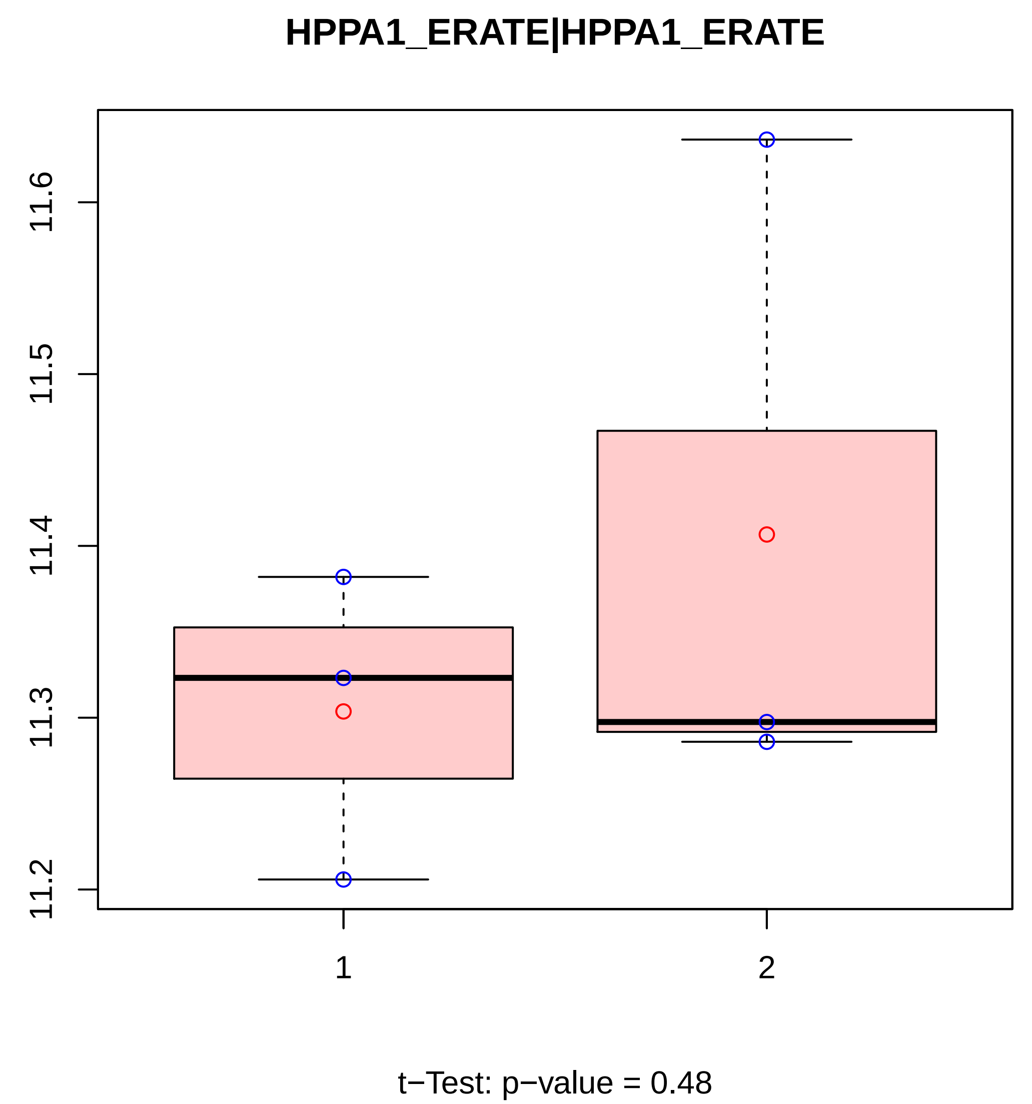
HPPA1_ERATE|HPPA1_ERATE
11.2
11.3
11.4
11.5
11.6
1
2
t−Test: p−value = 0.48

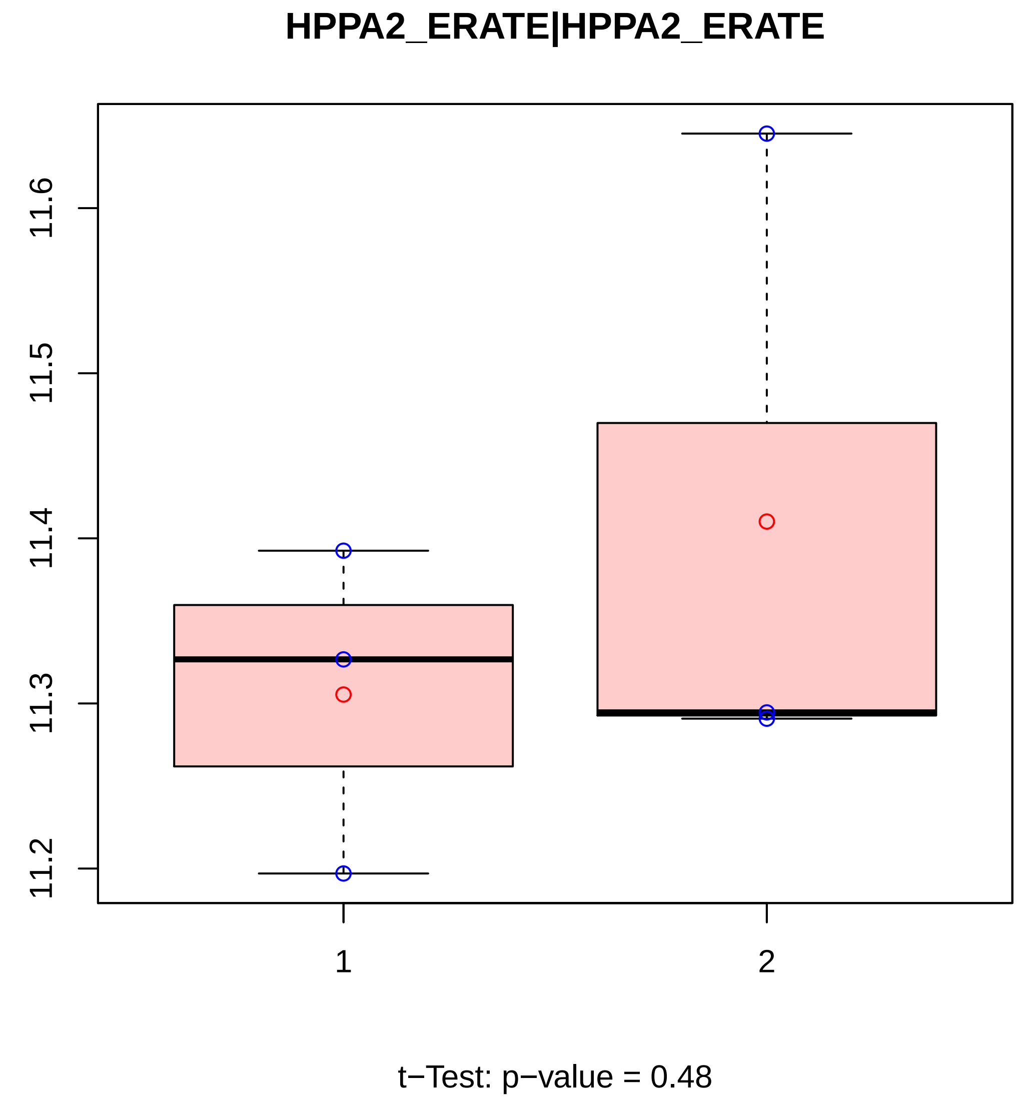

HPPA2_ERATE|HPPA2_ERATE
11.2
11.3
11.4
11.5
11.6
1
2
t−Test: p−value = 0.48

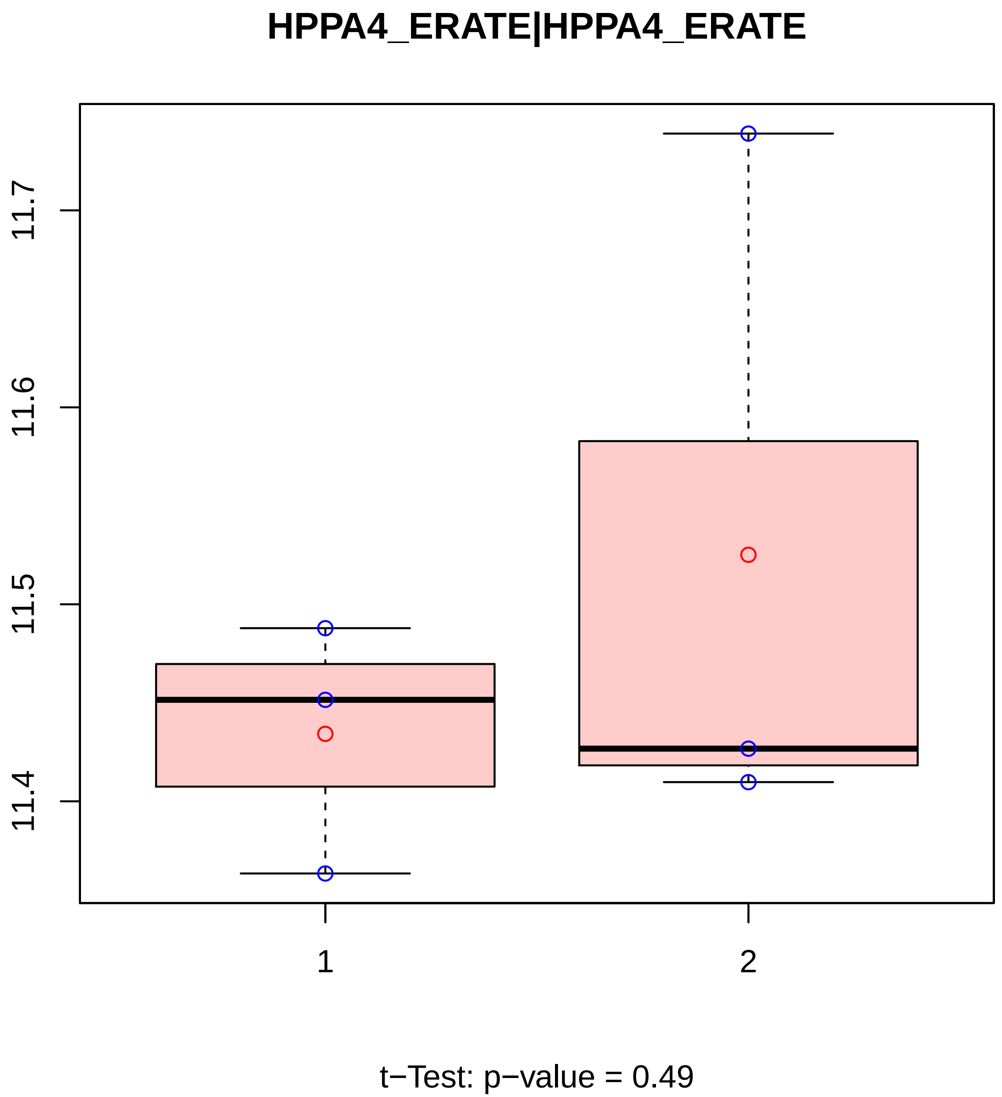

HPPA4_ERATE|HPPA4_ERATE
11.4
11.5
11.6
11.7
1
2
t−Test: p−value = 0.49

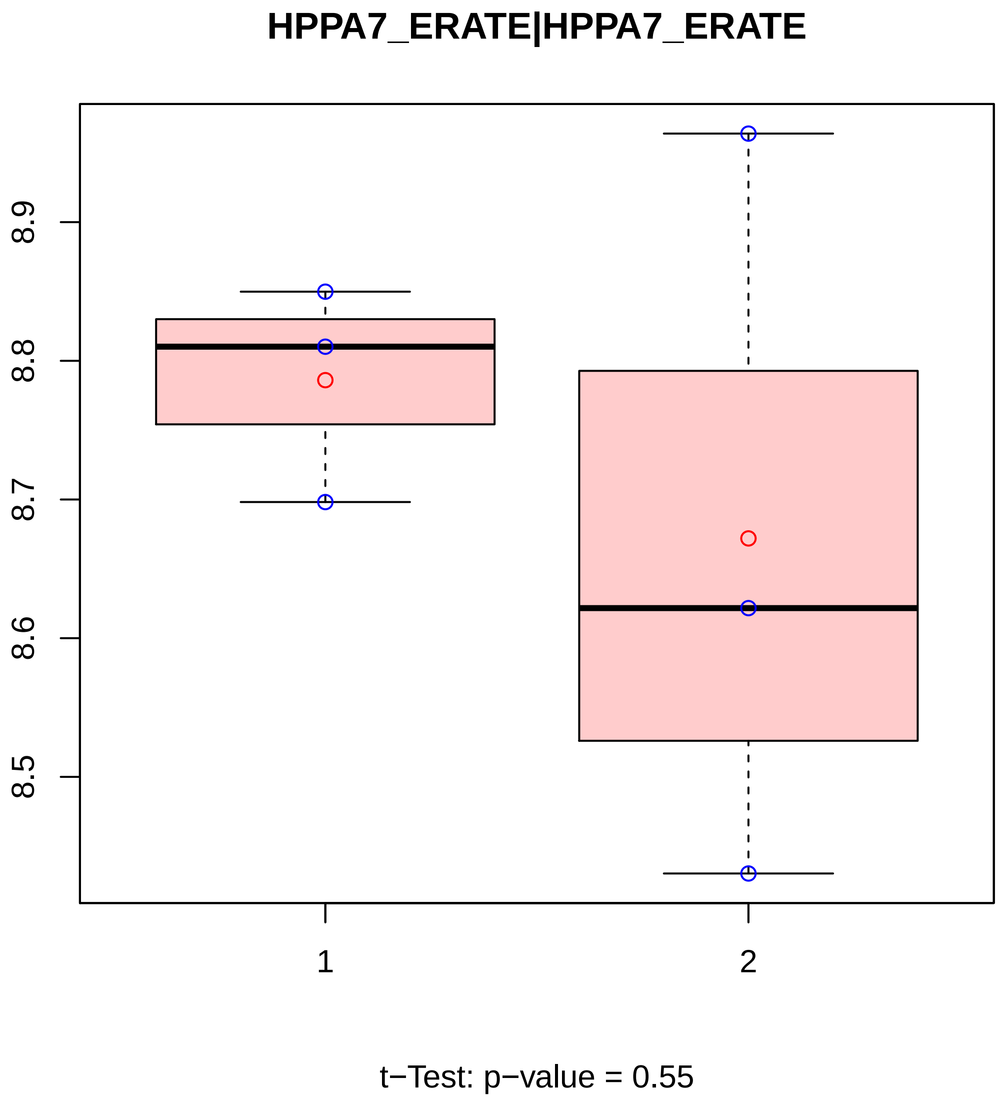

HPPA7_ERATE|HPPA7_ERATE
8.5
8.6
8.7
8.8
8.9
1
2
t−Test: p−value = 0.55

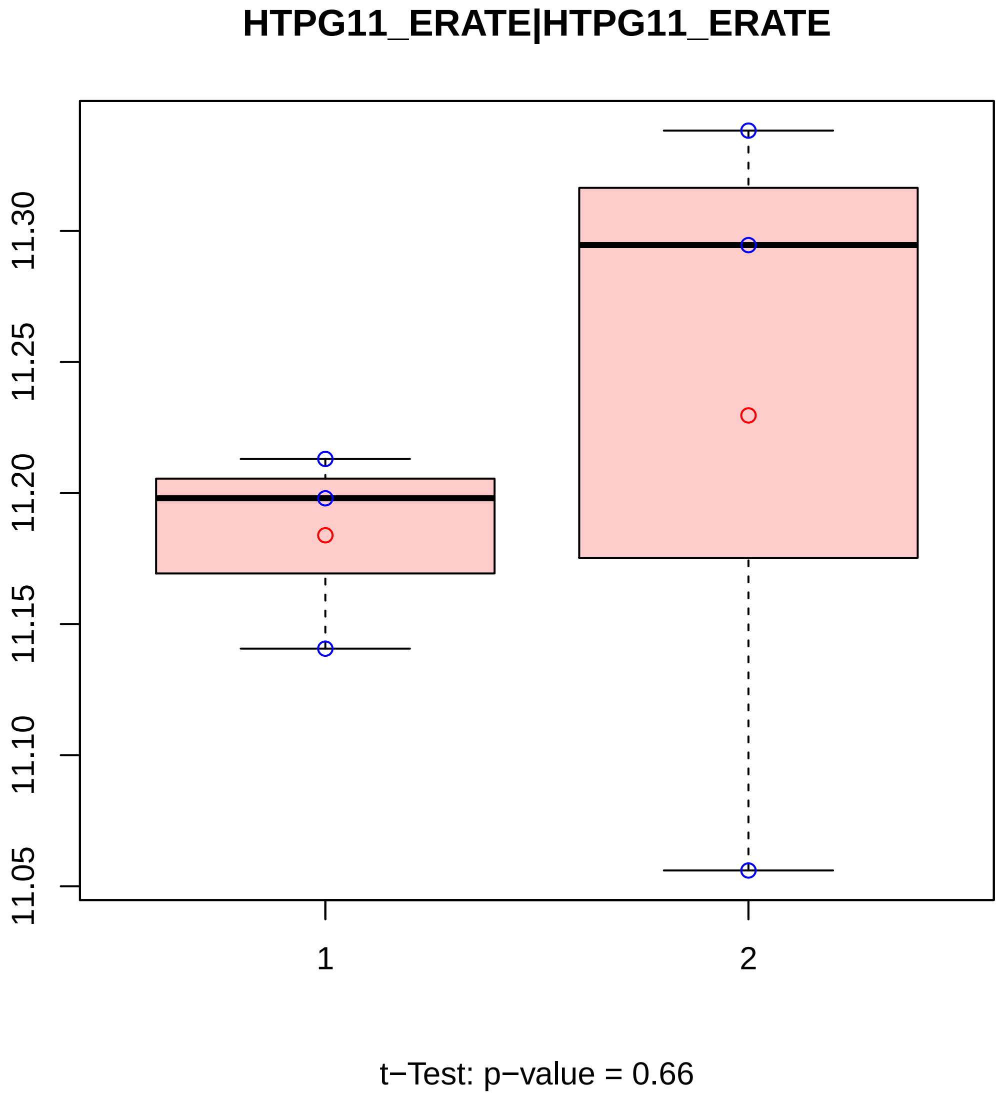

HTPG11_ERATE|HTPG11_ERATE
11.05
11.10
11.15
11.20
11.25
11.30
1
2
t−Test: p−value = 0.66

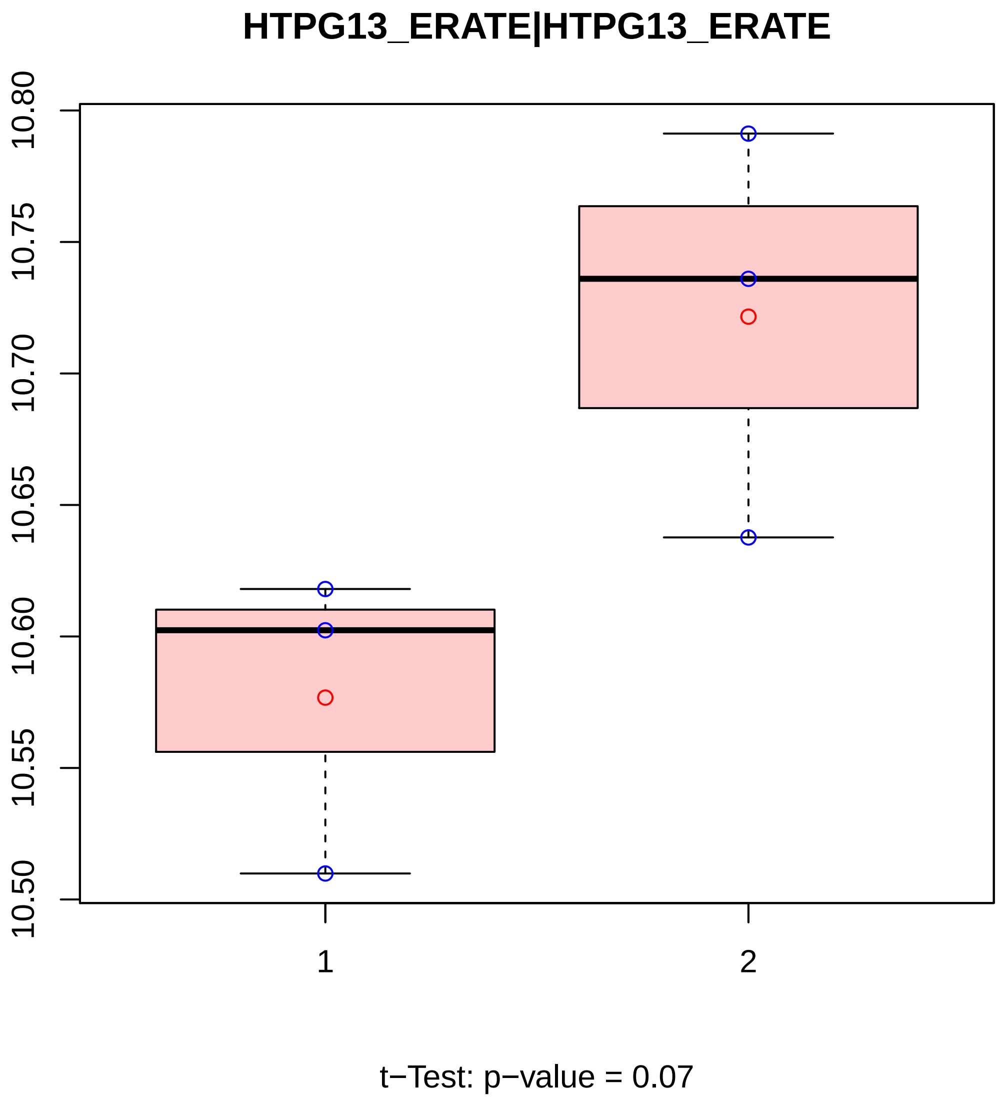
HTPG13_ERATE|HTPG13_ERATE
10.50
10.55
10.60
10.65
10.70
10.75
10.80
1
2
t−Test: p−value = 0.07

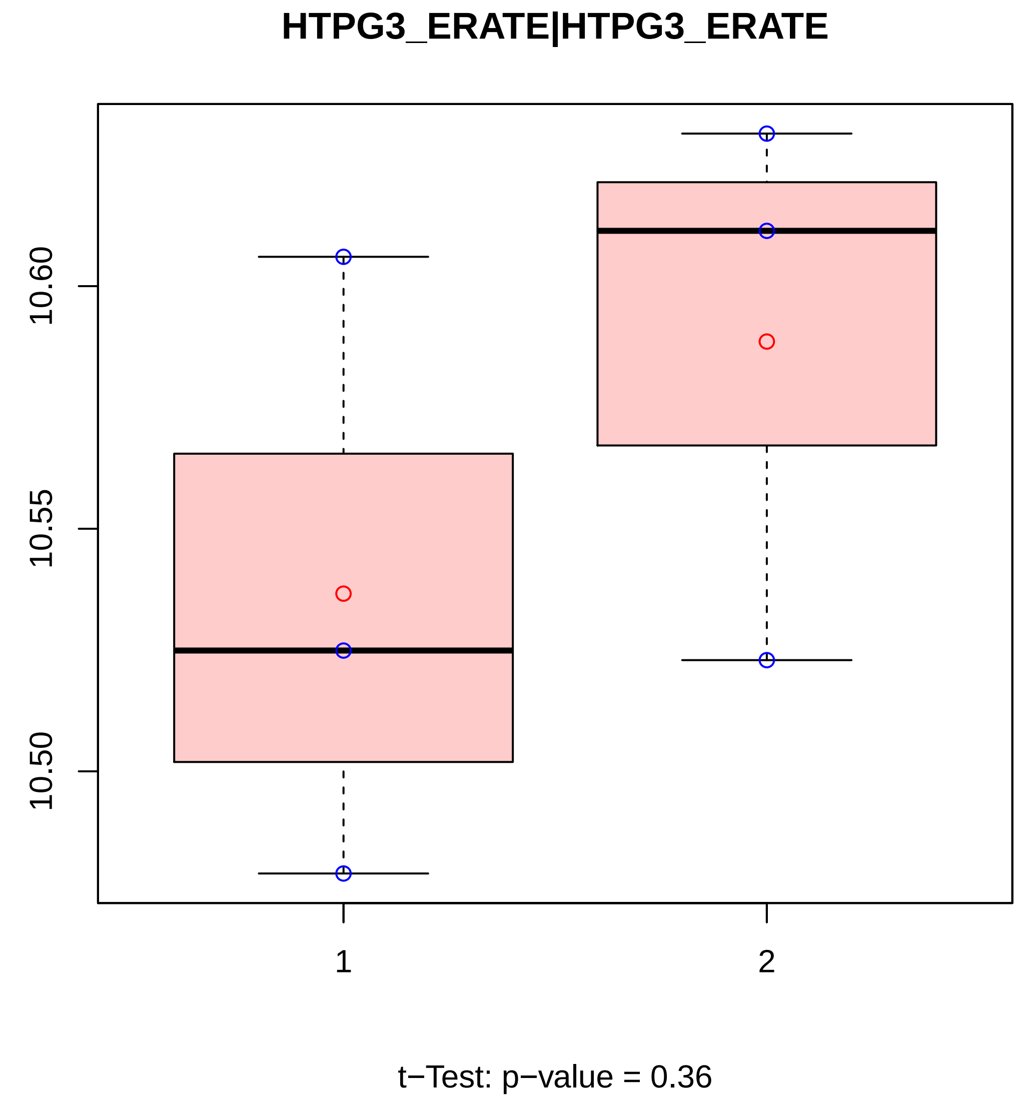
HTPG3_ERATE|HTPG3_ERATE
10.60
10.55
10.50
1
2
t−Test: p−value = 0.36

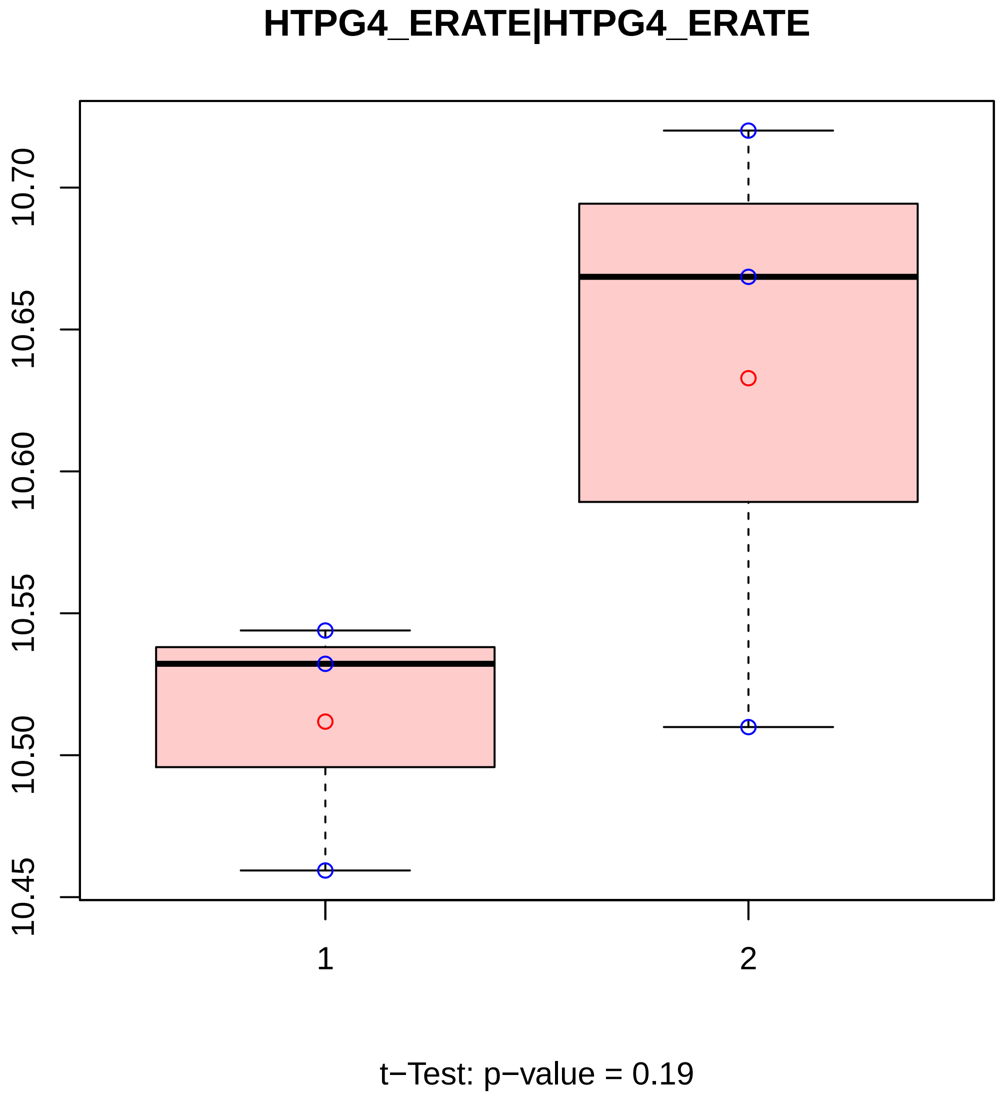

HTPG4_ERATE|HTPG4_ERATE
10.45
10.50
10.55
10.60
10.65
10.70
1
2
t−Test: p−value = 0.19

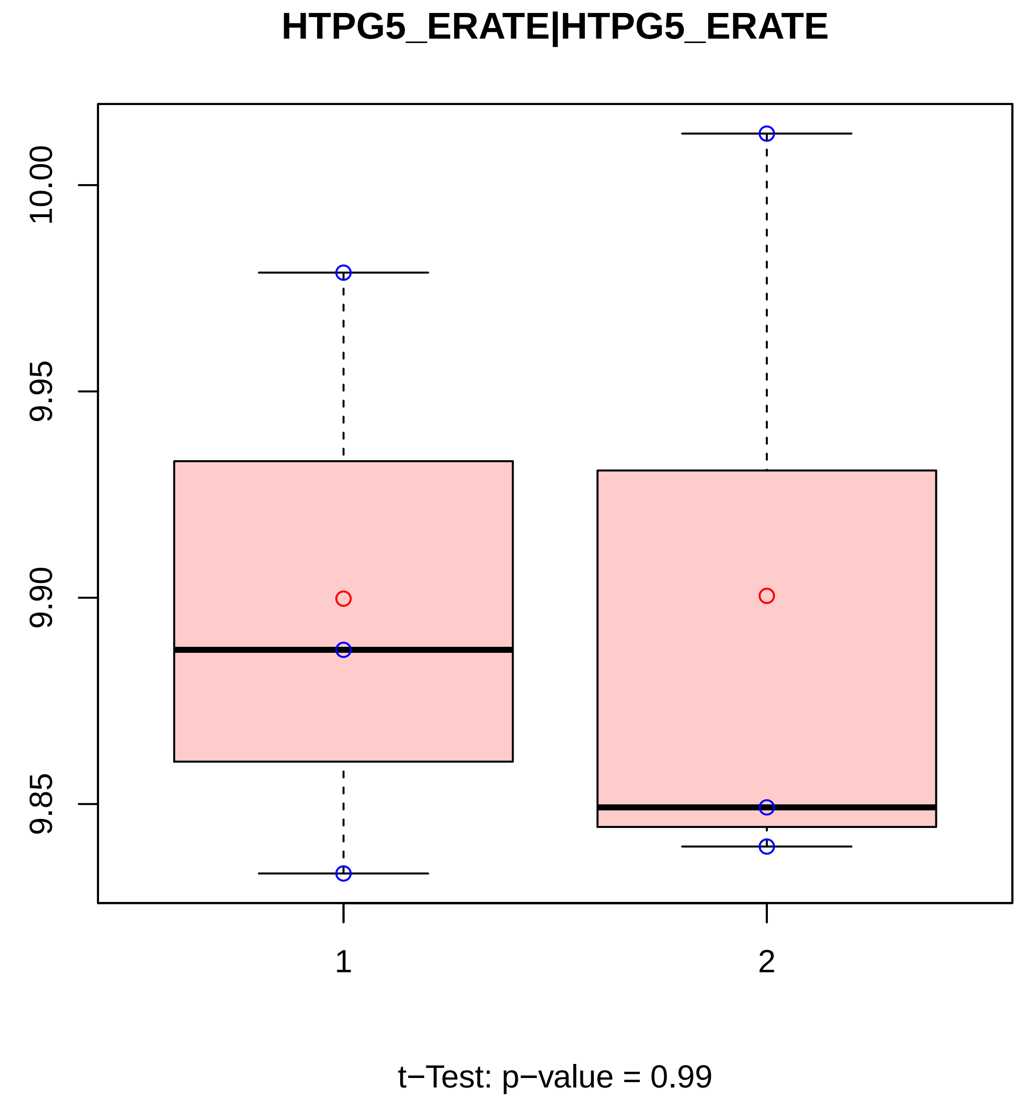
HTPG5_ERATE|HTPG5_ERATE
10.00
9.95
9.90
9.85
1
2
t−Test: p−value = 0.99

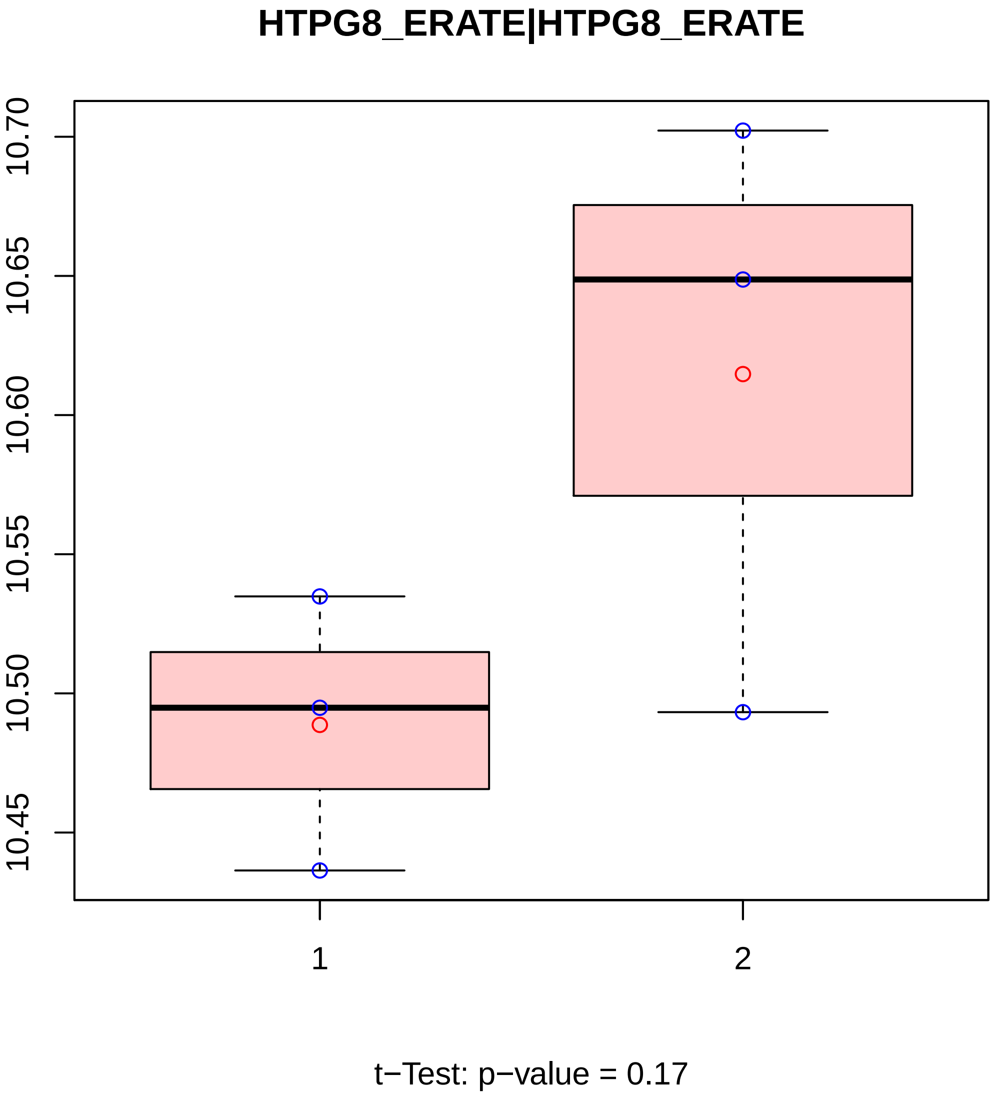
HTPG8_ERATE|HTPG8_ERATE
10.45
10.50
10.55
10.60
10.65
10.70
1
2
t−Test: p−value = 0.17

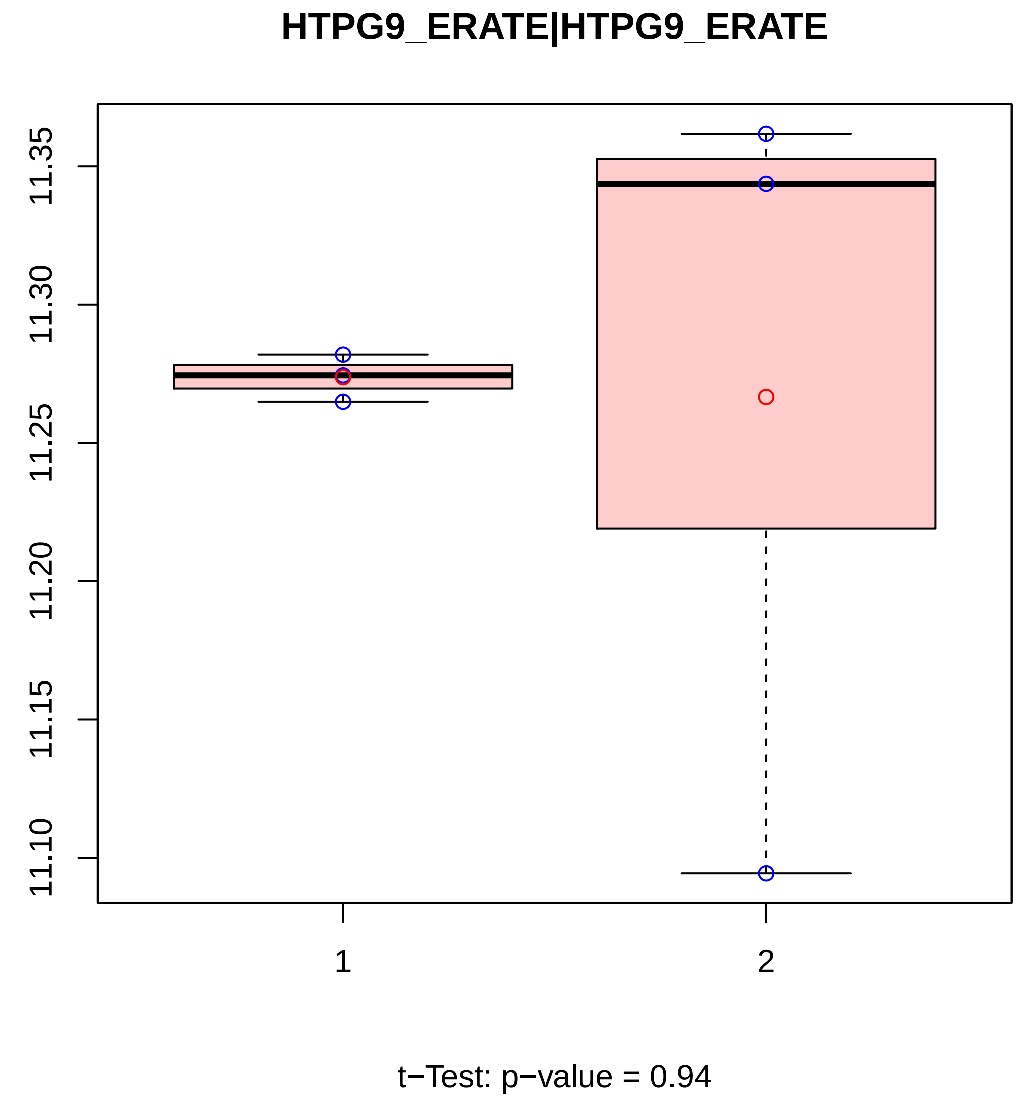

HTPG9_ERATE|HTPG9_ERATE
11.10
11.15
11.20
11.25
11.30
11.35
1
2
t−Test: p−value = 0.94

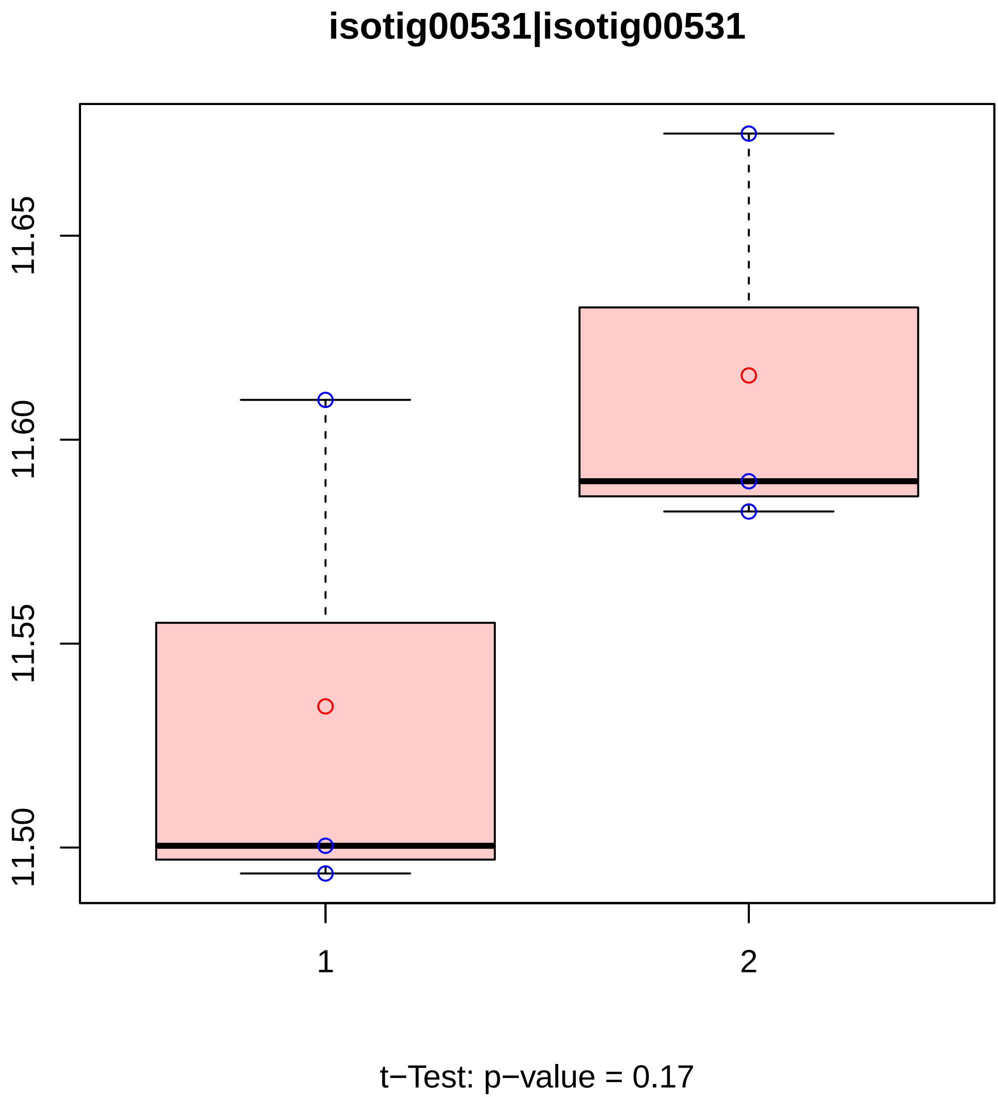

isotig00531|isotig00531
11.50
11.55
11.60
11.65
1
2
t−Test: p−value = 0.17

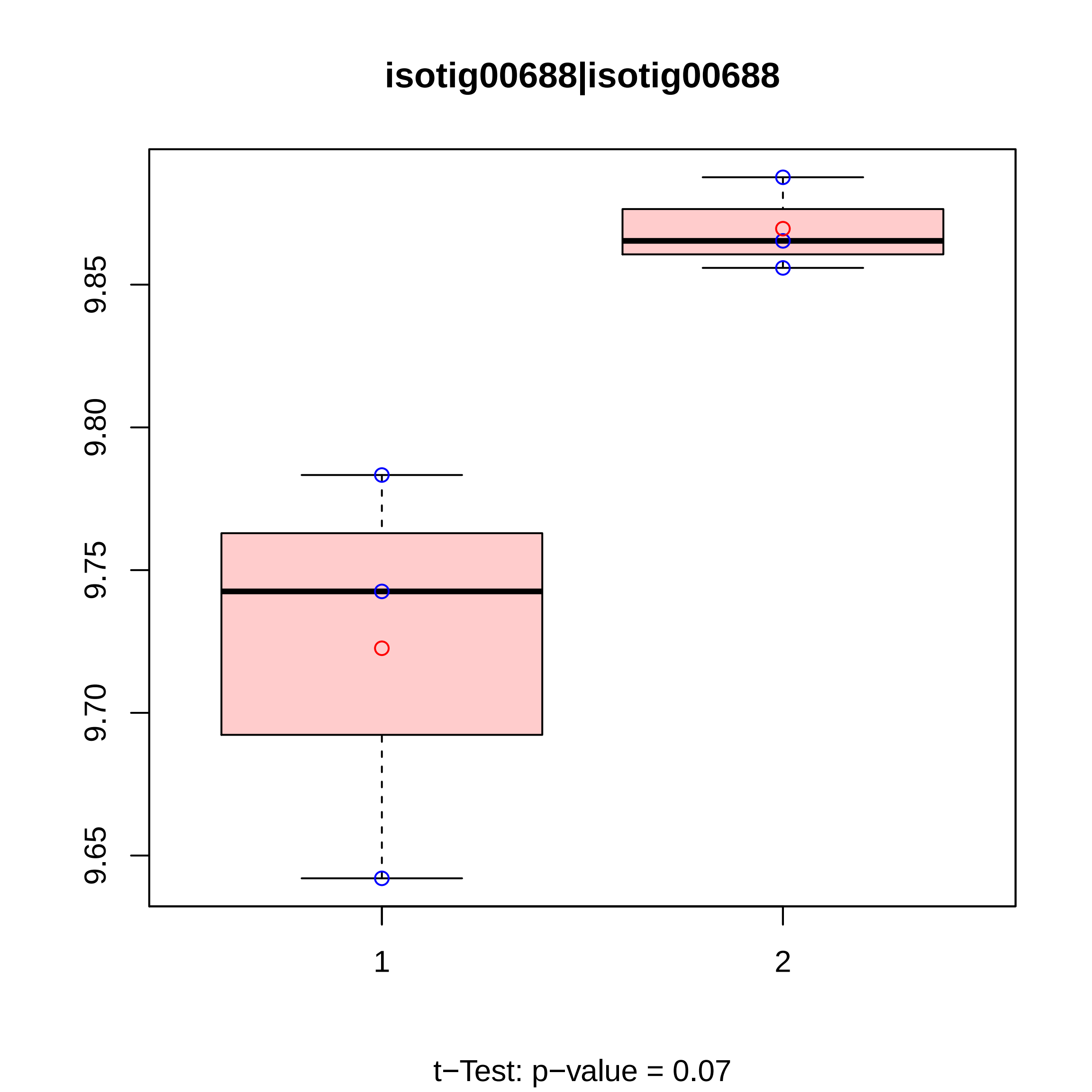

isotig00688|isotig00688
9.65
9.70
9.75
9.80
9.85
1
2
t−Test: p−value = 0.07

**isotig00697|isotig00697**

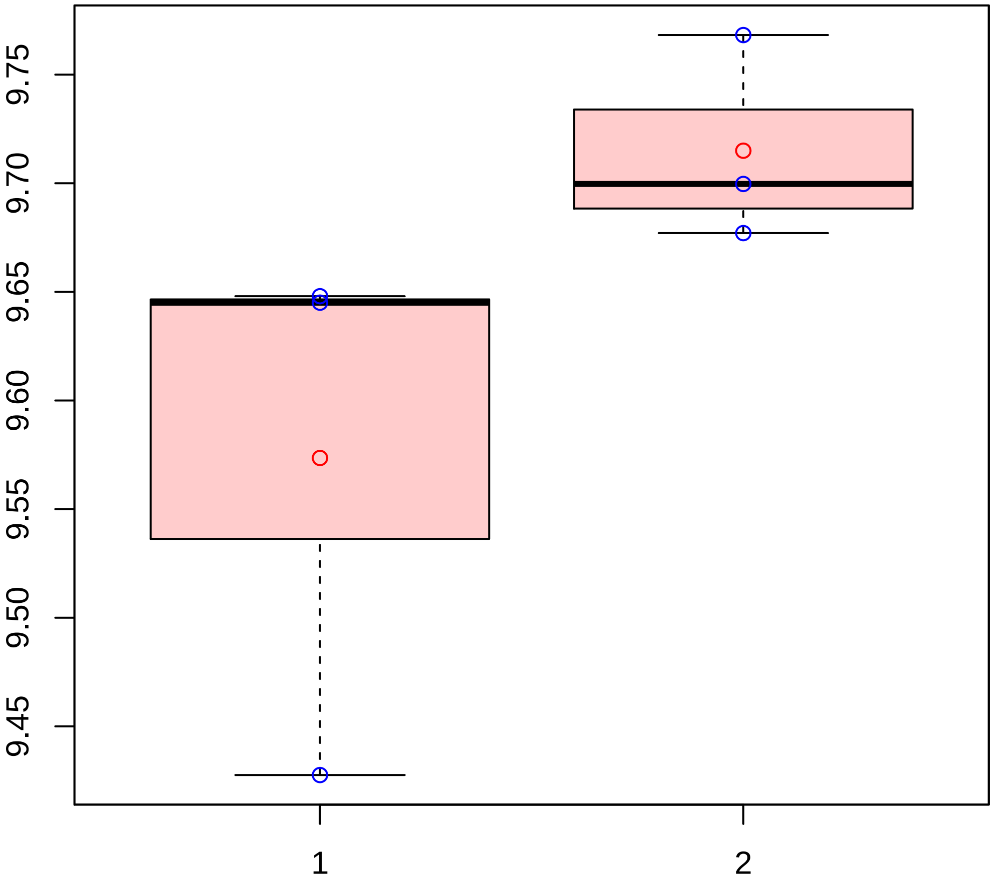


t−Test: p−value = 0.18

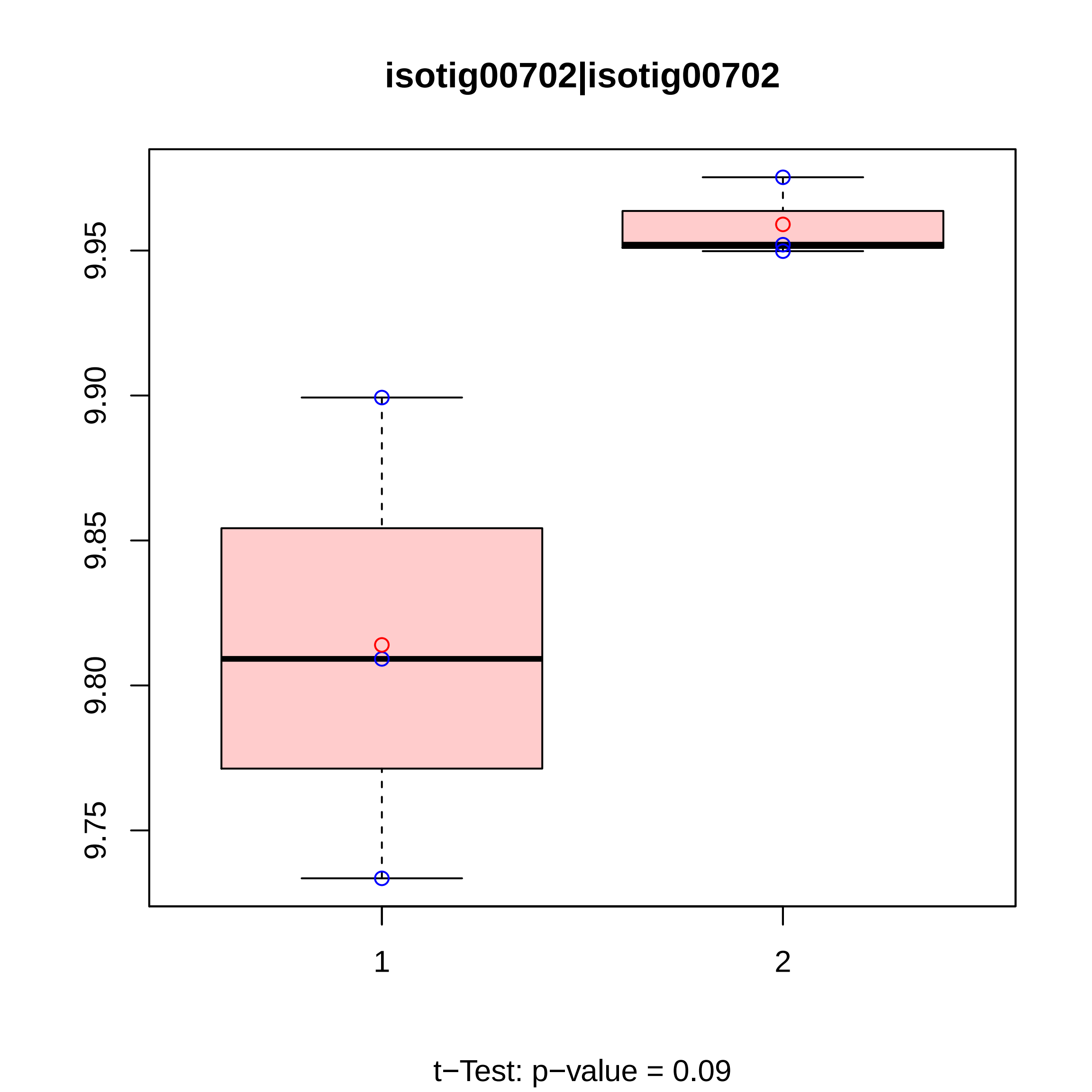

isotig00702|isotig00702
9.75
9.80
9.85
9.90
9.95
1
2
t−Test: p−value = 0.09

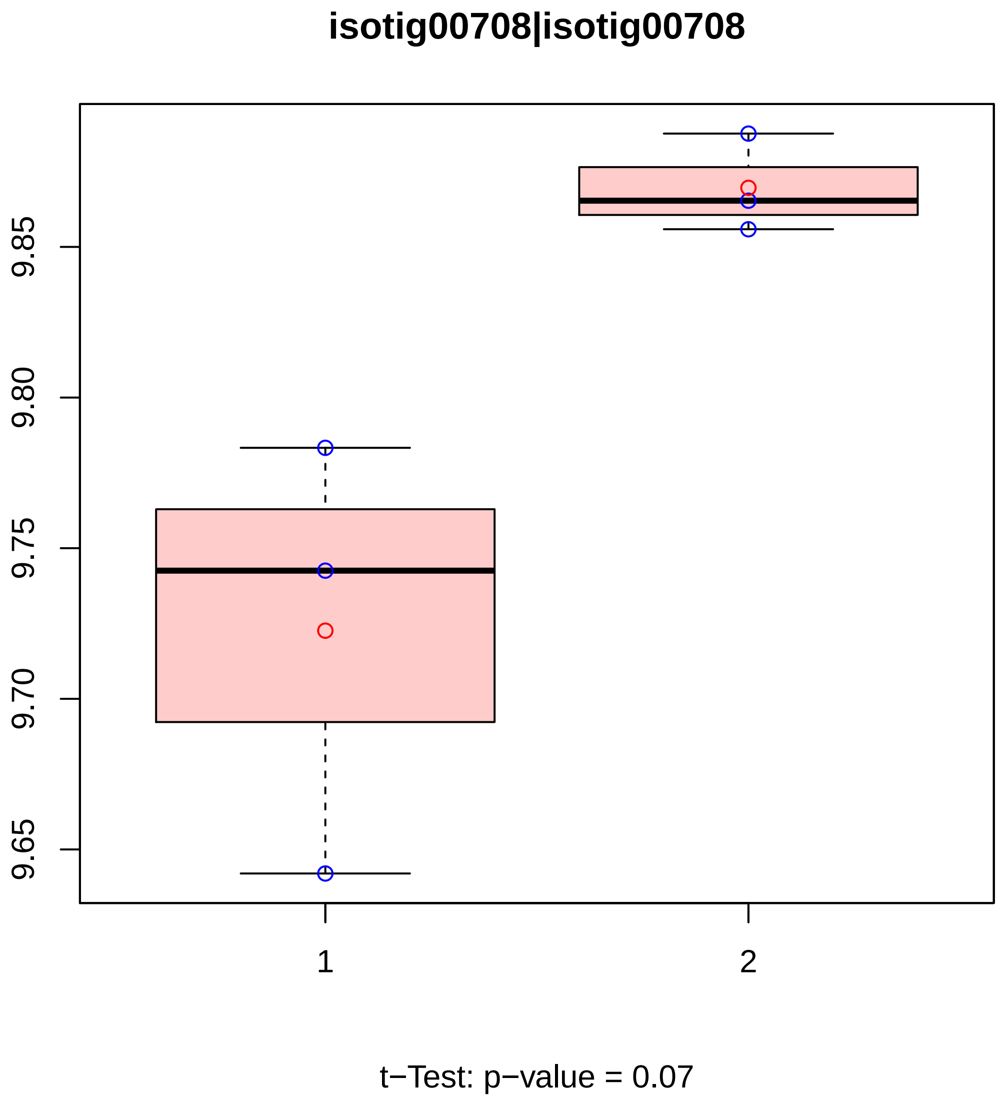
isotig00708|isotig00708
9.65
9.70
9.75
9.80
9.85
1
2
t−Test: p−value = 0.07

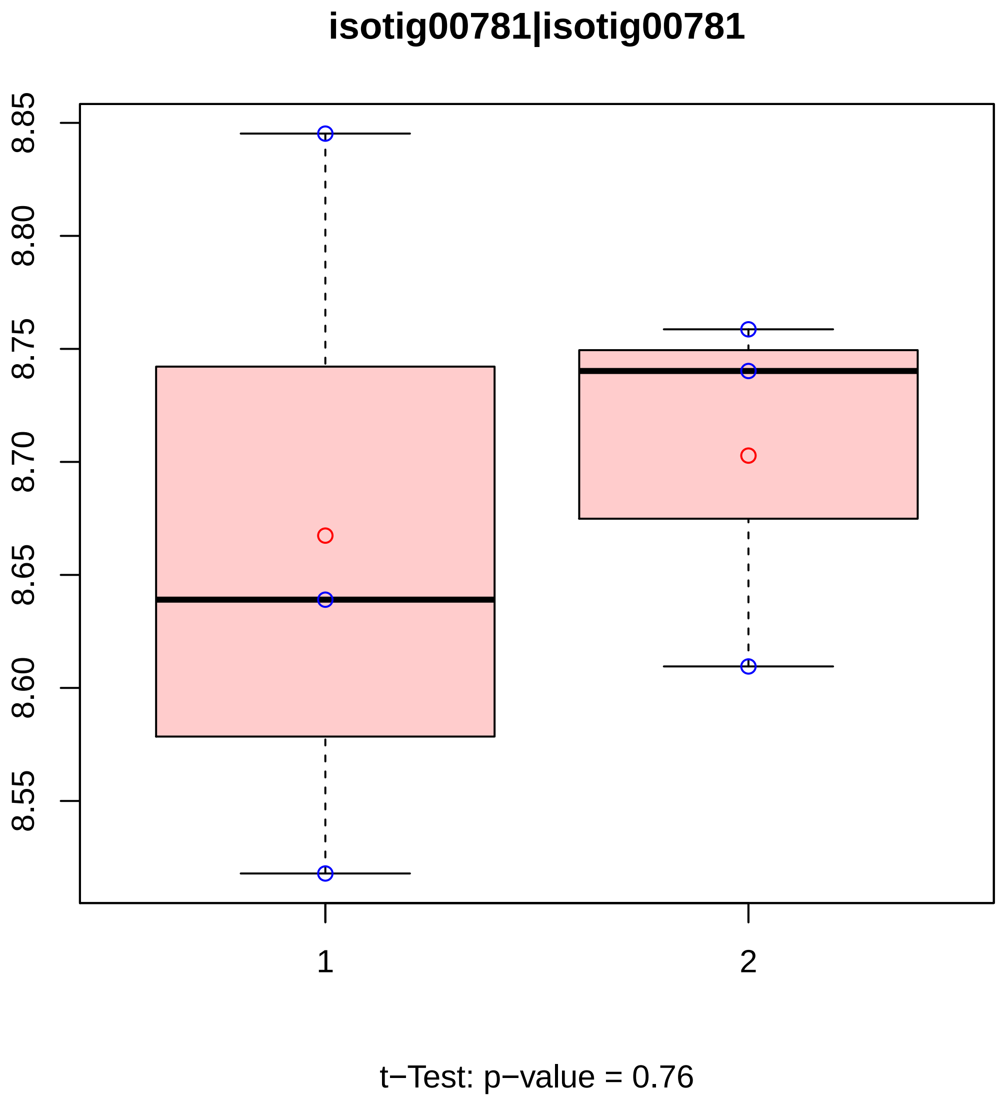
isotig00781|isotig00781
8.85
8.80
8.75
8.70
8.65
8.60
8.55
1
2
t−Test: p−value = 0.76

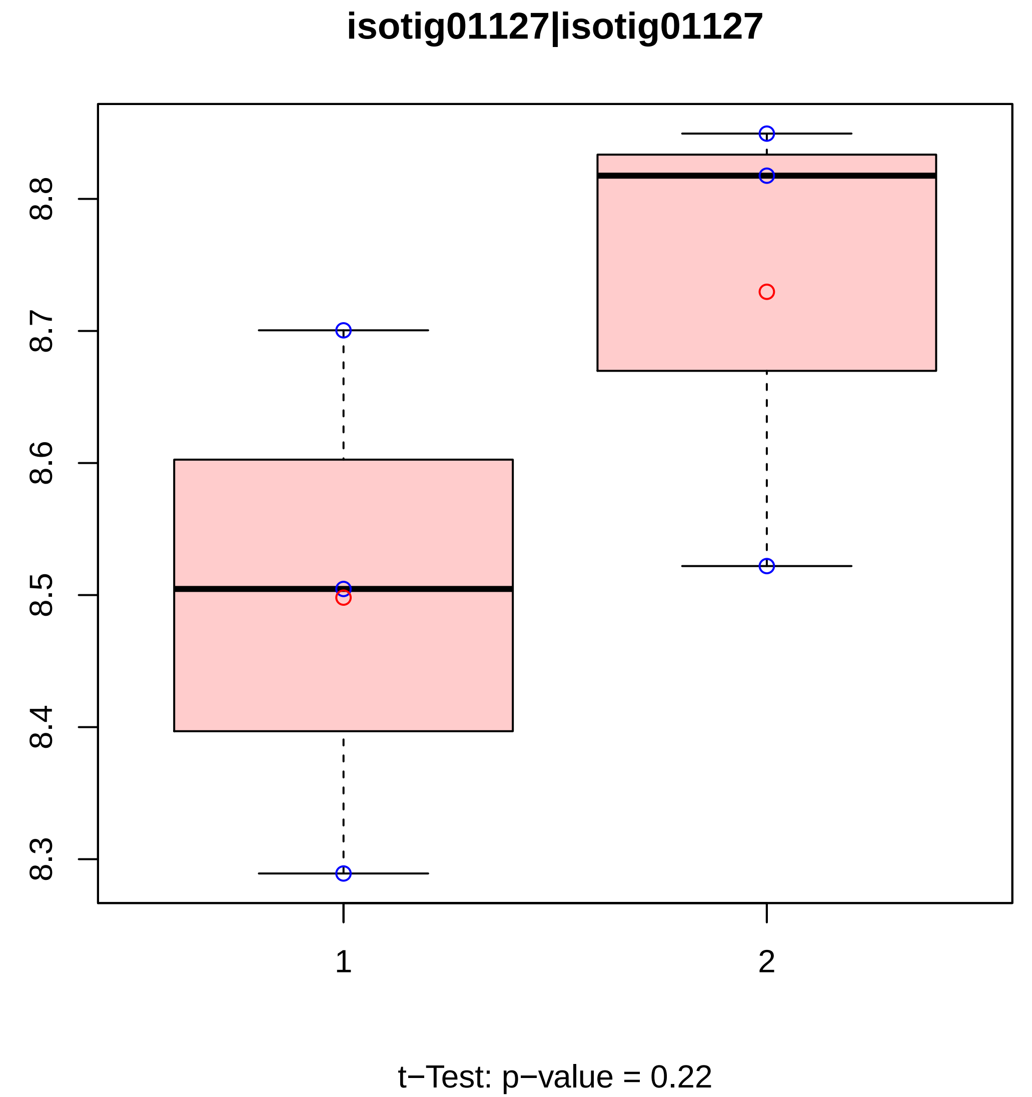

isotig01127|isotig01127
8.3
8.4
8.5
8.6
8.7
8.8
1
2
t−Test: p−value = 0.22

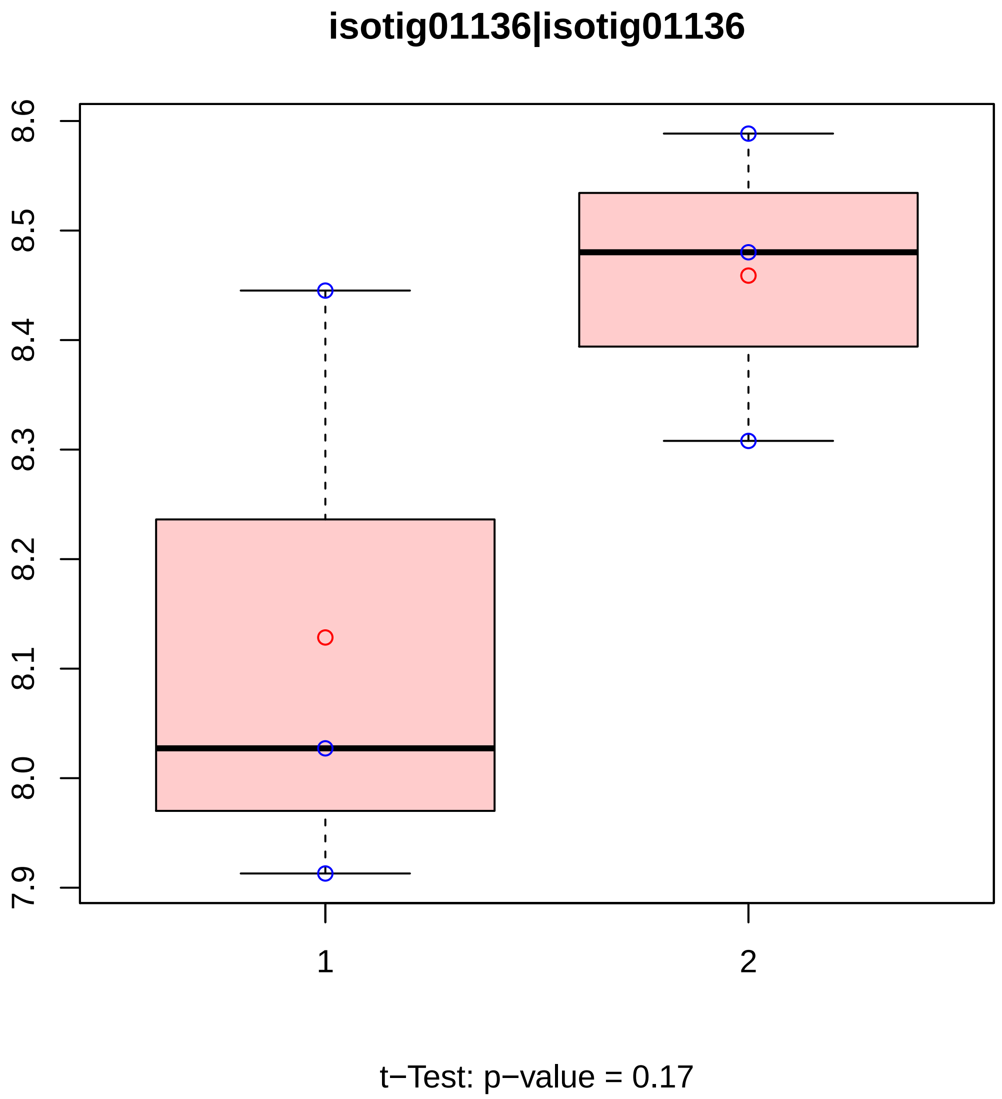
isotig01136|isotig01136
8.6
8.5
8.4
8.3
8.2
8.1
8.0
7.9
1
2
t−Test: p−value = 0.17

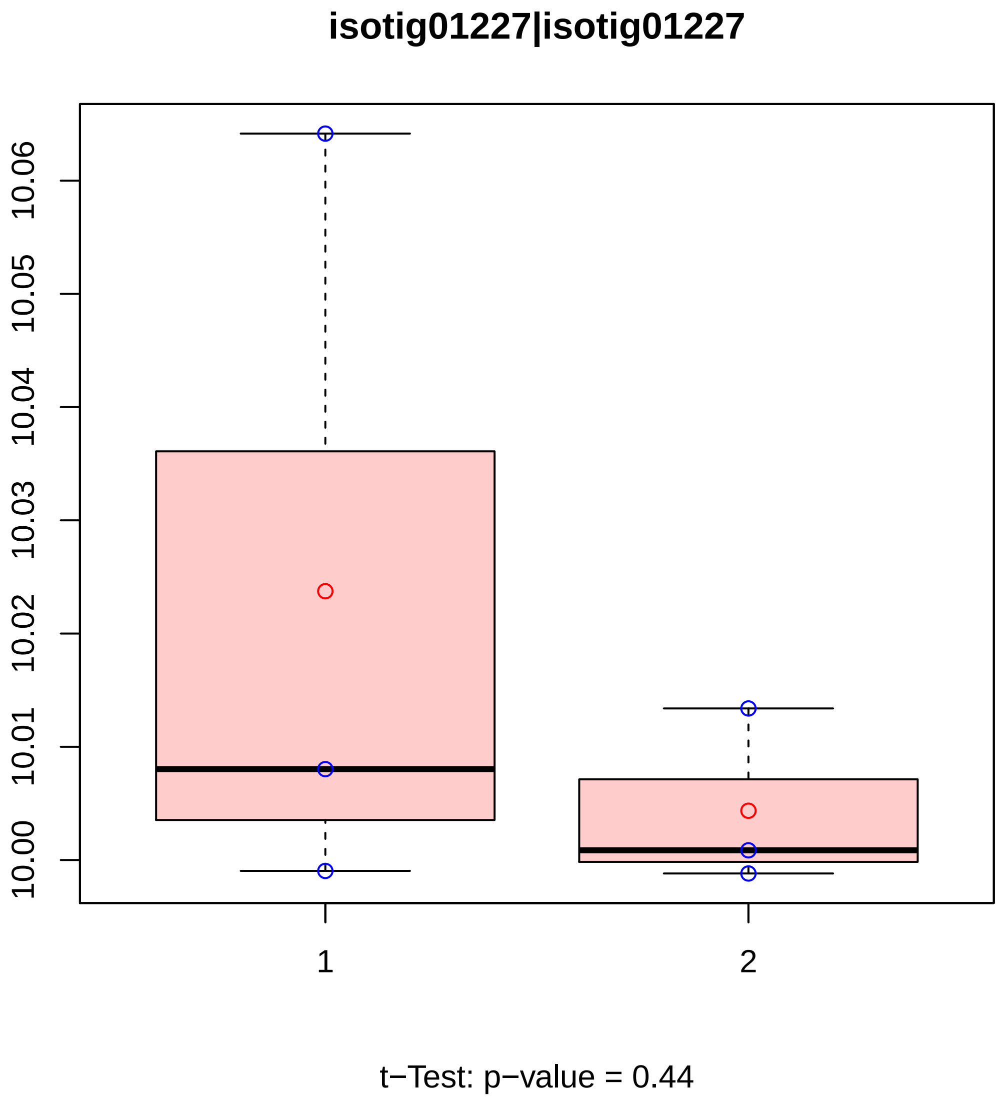
isotig01227|isotig01227
10.00
10.01
10.02
10.03
10.04
10.05
10.06
1
2
t−Test: p−value = 0.44

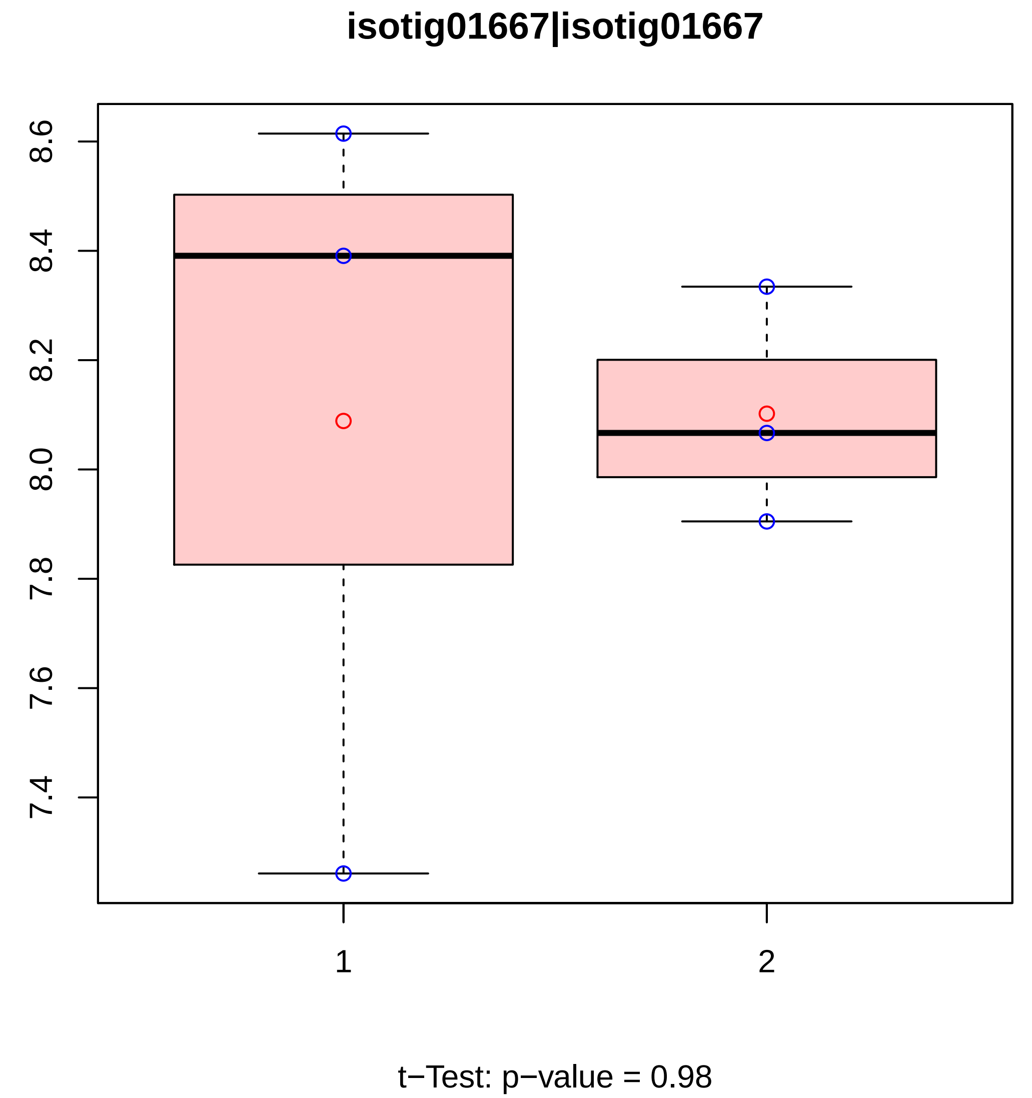
isotig01667|isotig01667
8.6
8.4
8.2
8.0
7.8
7.6
7.4
1
2
t−Test: p−value = 0.98

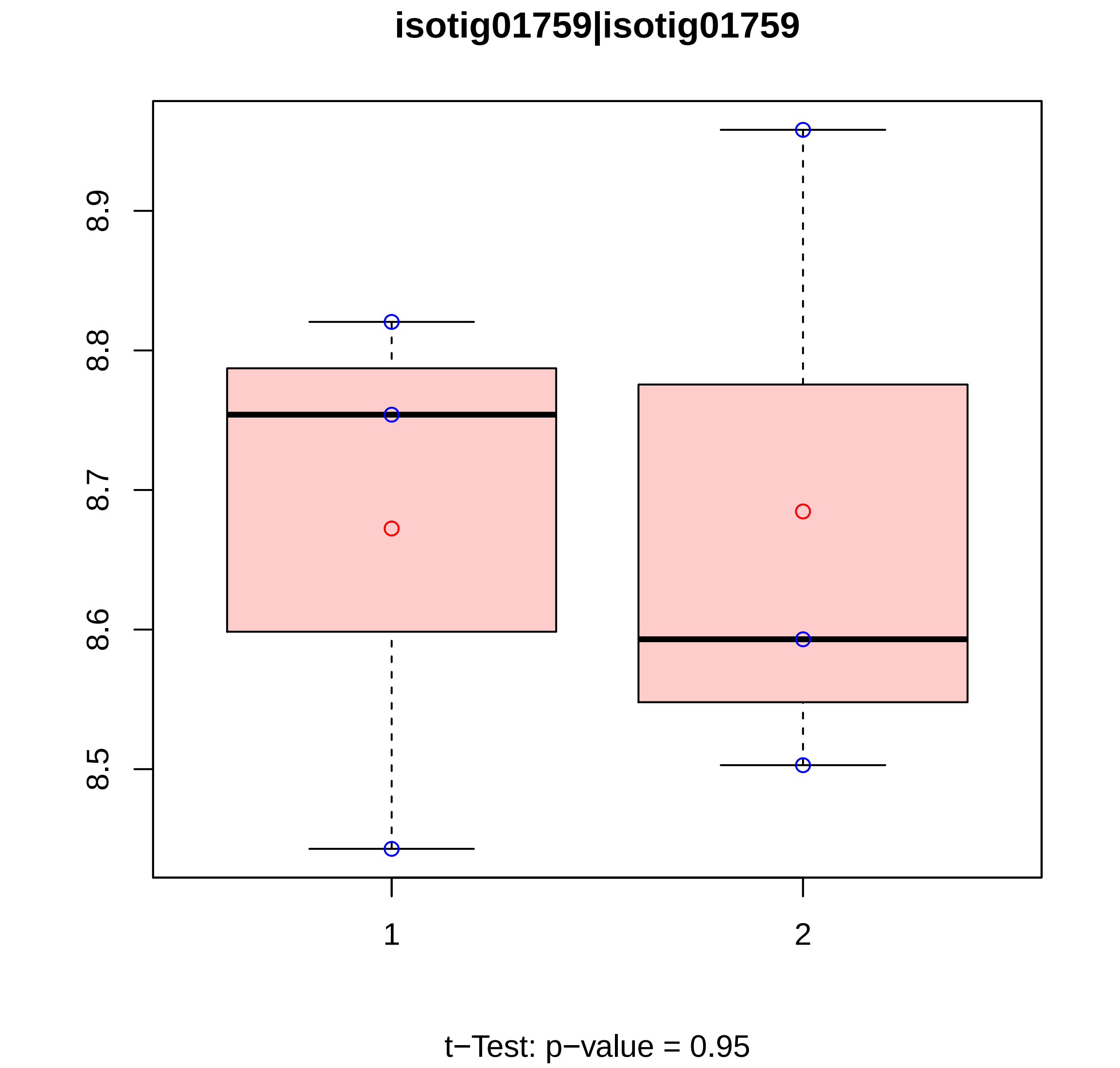

isotig01759|isotig01759
8.5
8.6
8.7
8.8
8.9
1
2
t−Test: p−value = 0.95

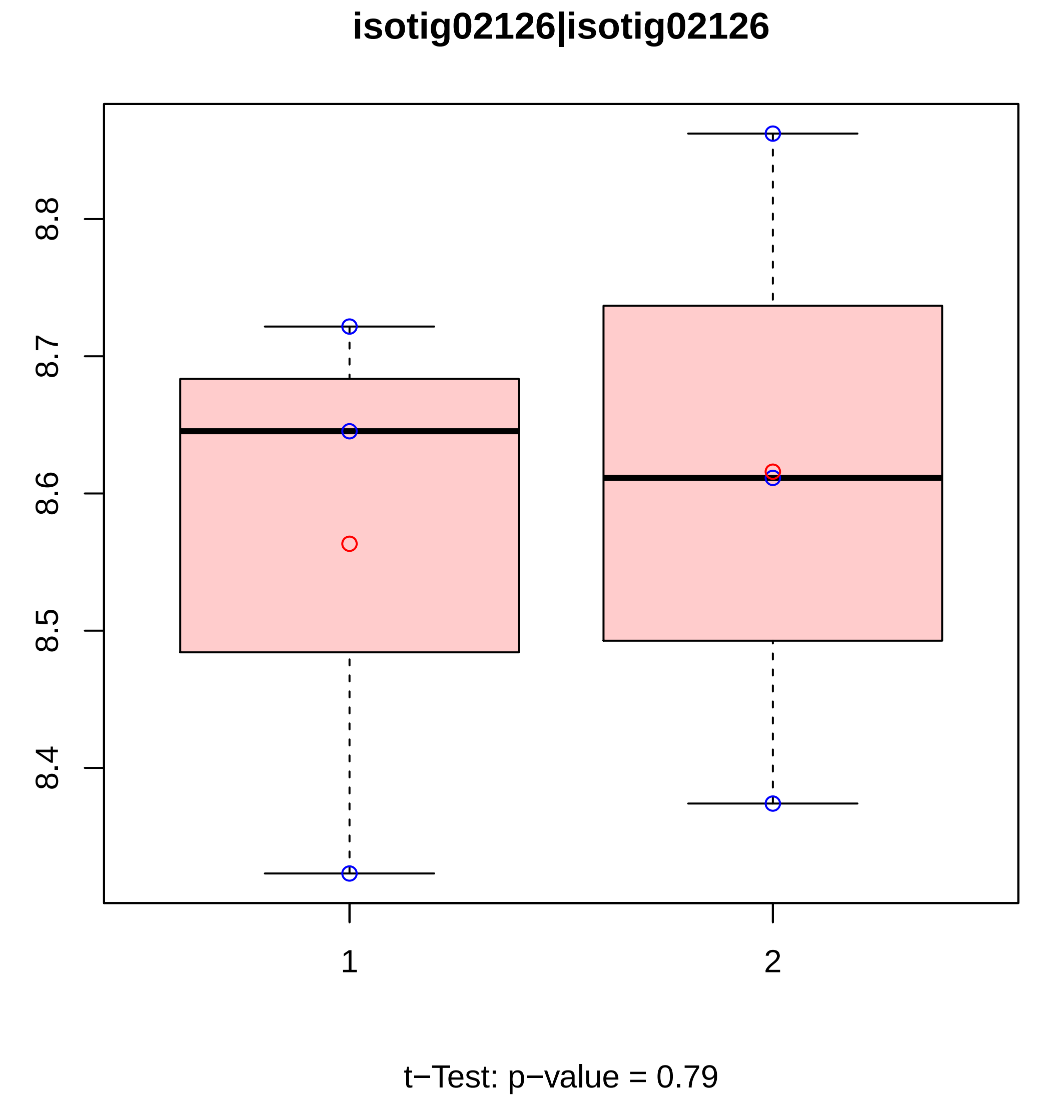
isotig02126|isotig02126
8.4
8.5
8.6
8.7
8.8
1
2
t−Test: p−value = 0.79

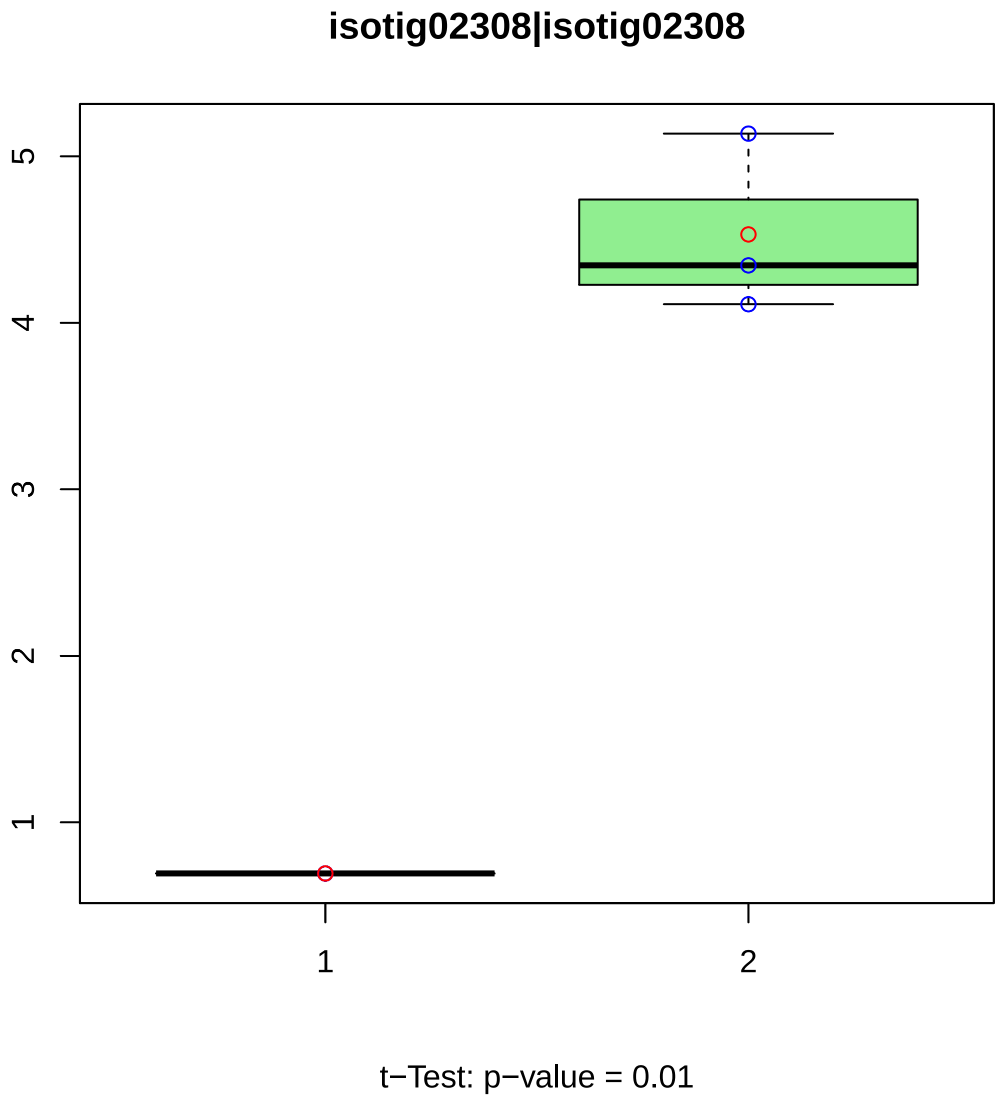
isotig02308|isotig02308
5
4
3
2
1
1
2
t−Test: p−value = 0.01

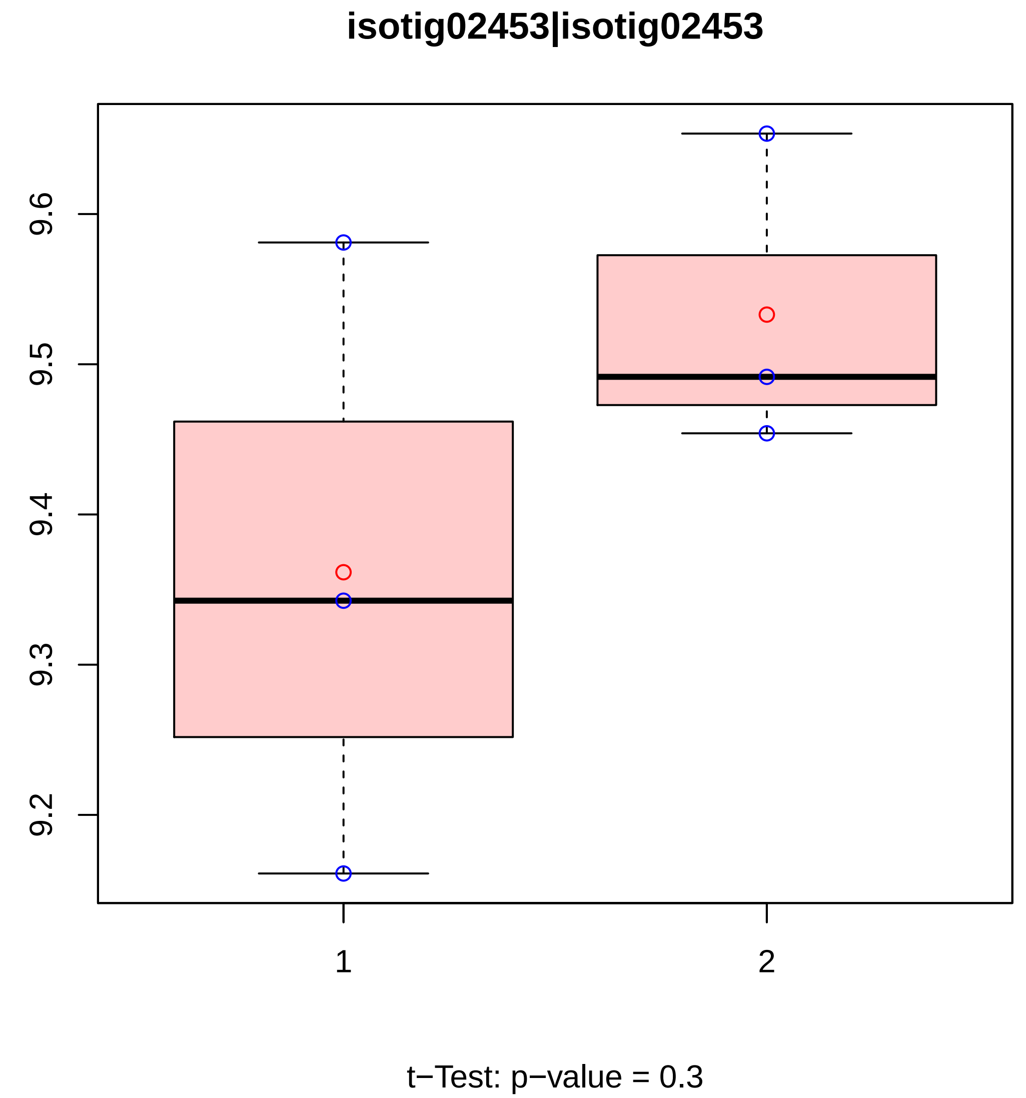
isotig02453|isotig02453
9.2
9.3
9.4
9.5
9.6
1
2
t−Test: p−value = 0.3

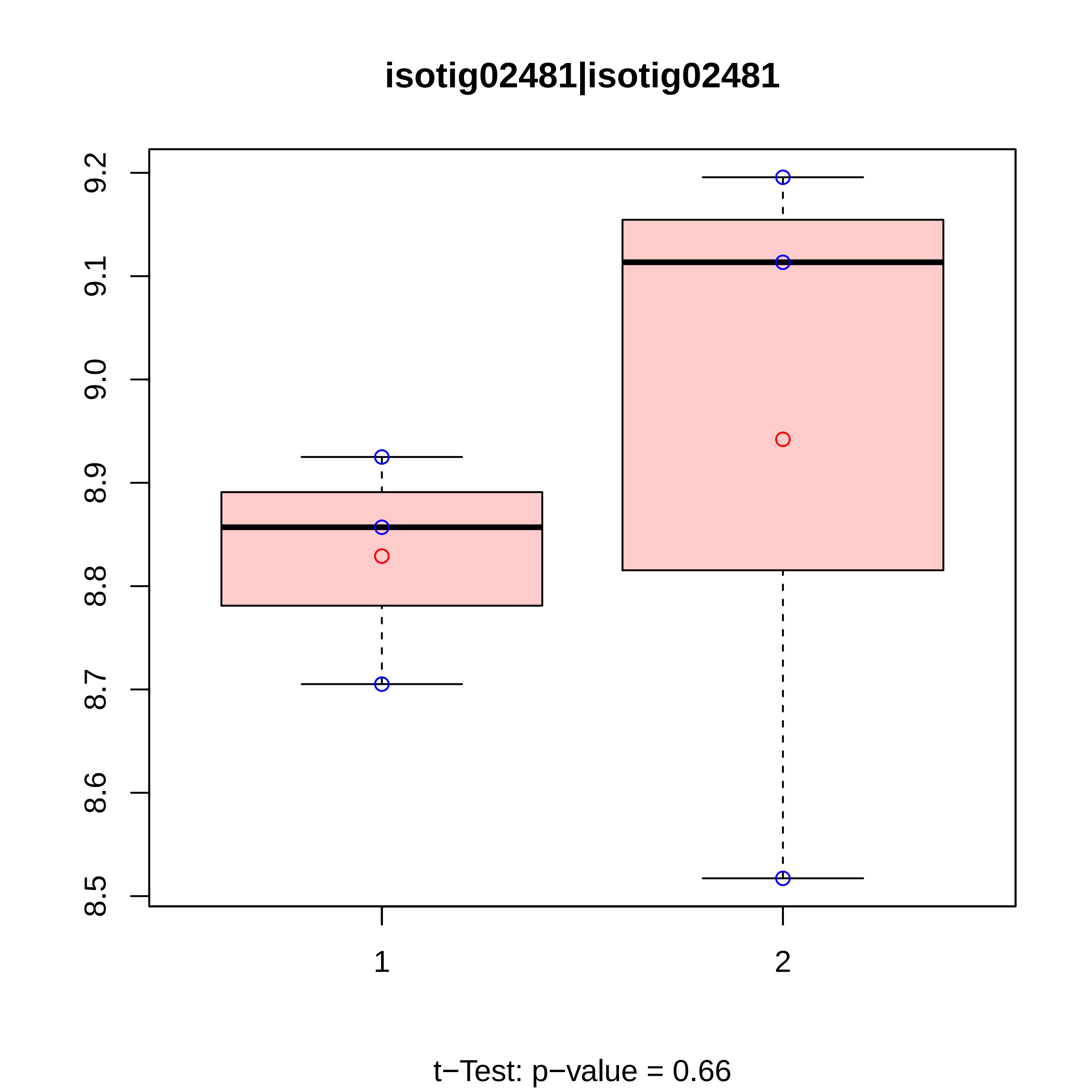

isotig02481|isotig02481
9.2
9.1
9.0
8.9
8.8
8.7
8.6
8.5
1
2
t−Test: p−value = 0.66

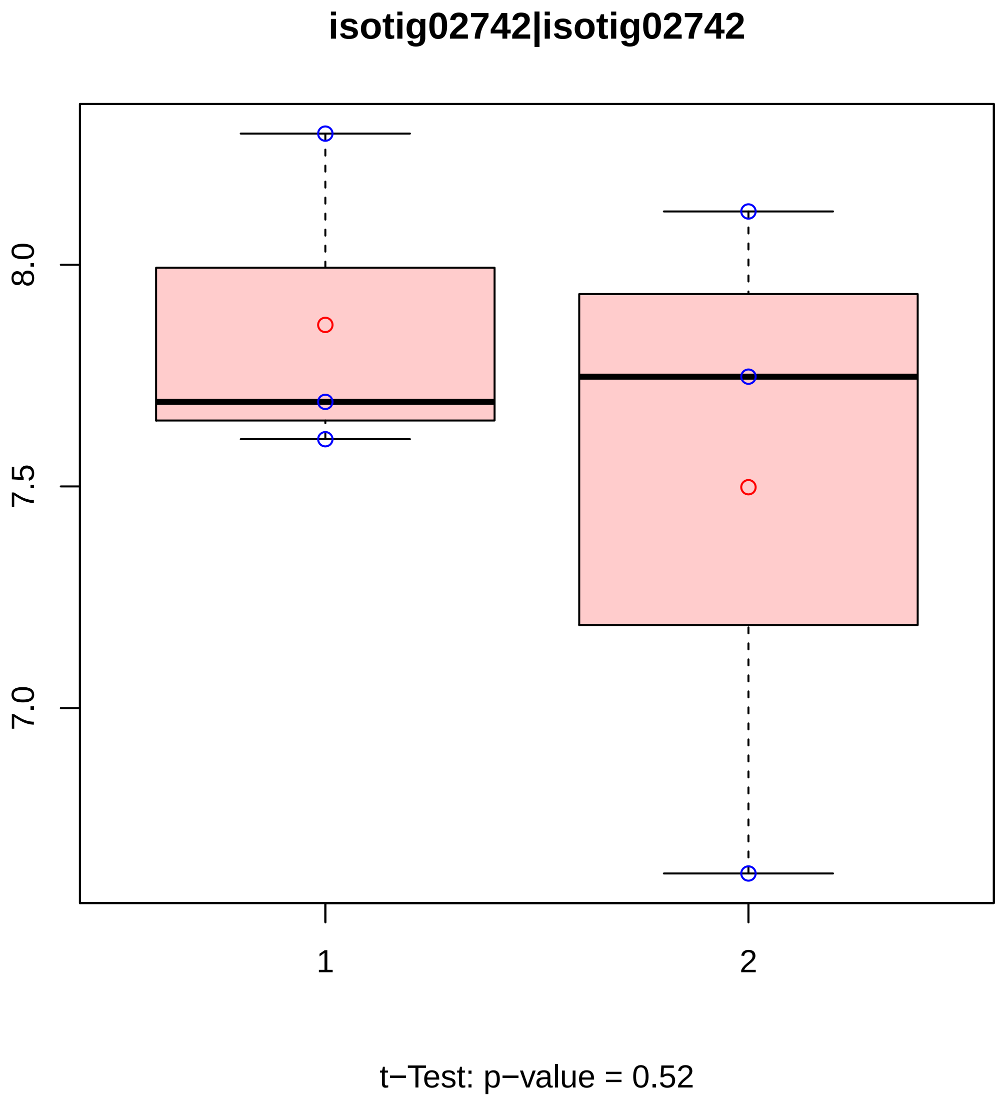
isotig02742|isotig02742
8.0
7.5
7.0
1
2
t−Test: p−value = 0.52

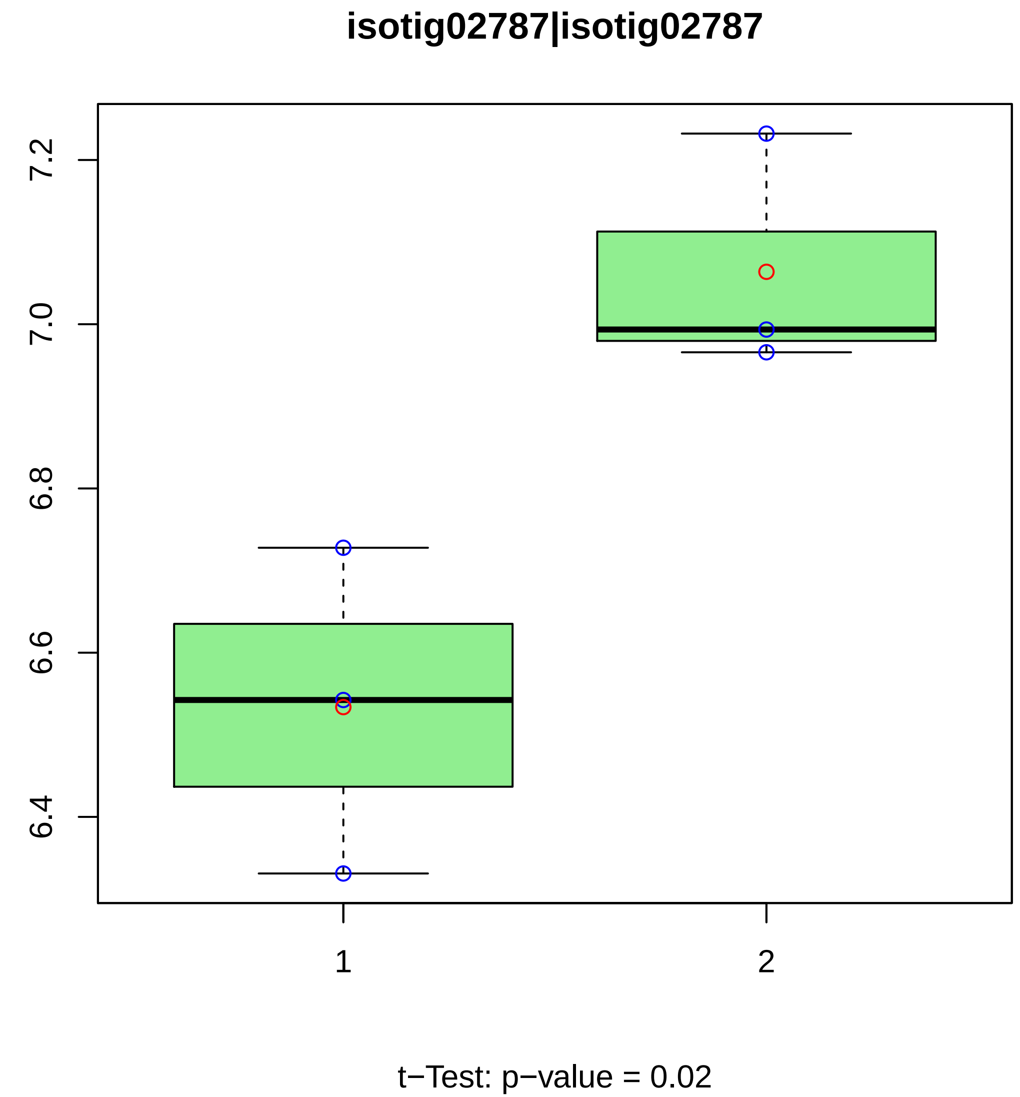

isotig02787|isotig02787
7.2
7.0
6.8
6.6
6.4
1
2
t−Test: p−value = 0.02

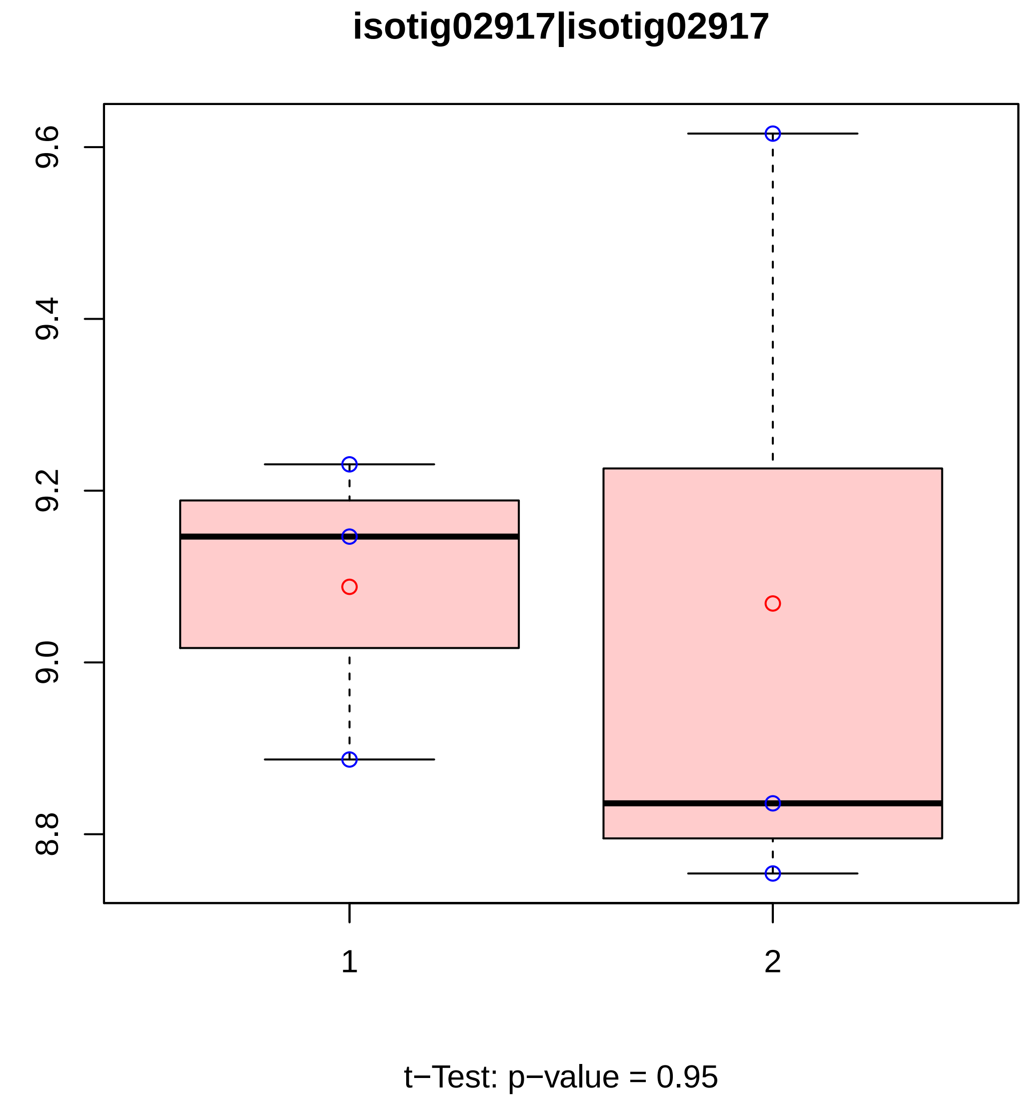

isotig02917|isotig02917
9.6
9.4
9.2
9.0
8.8
1
2
t−Test: p−value = 0.95

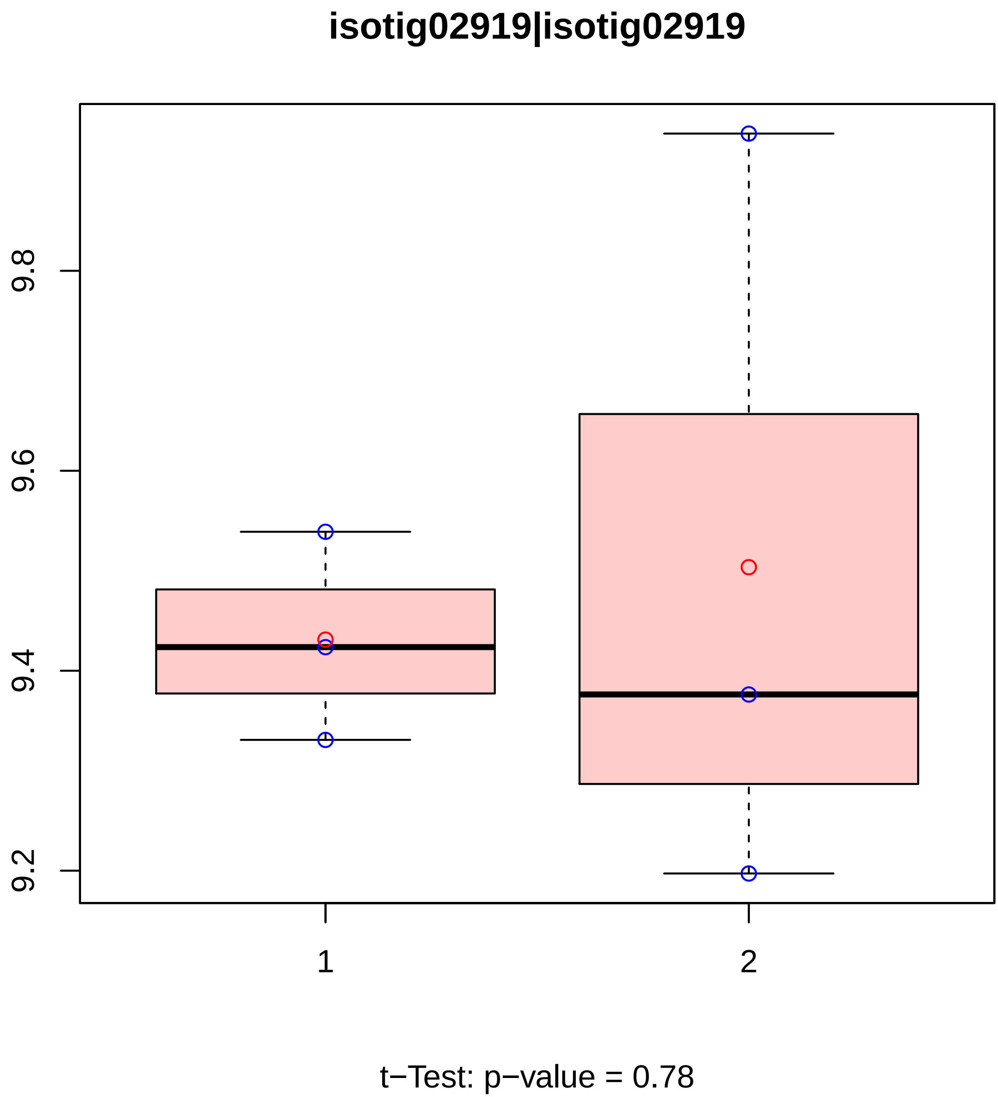
isotig02919|isotig02919
9.2
9.4
9.6
9.8
1
2
t−Test: p−value = 0.78

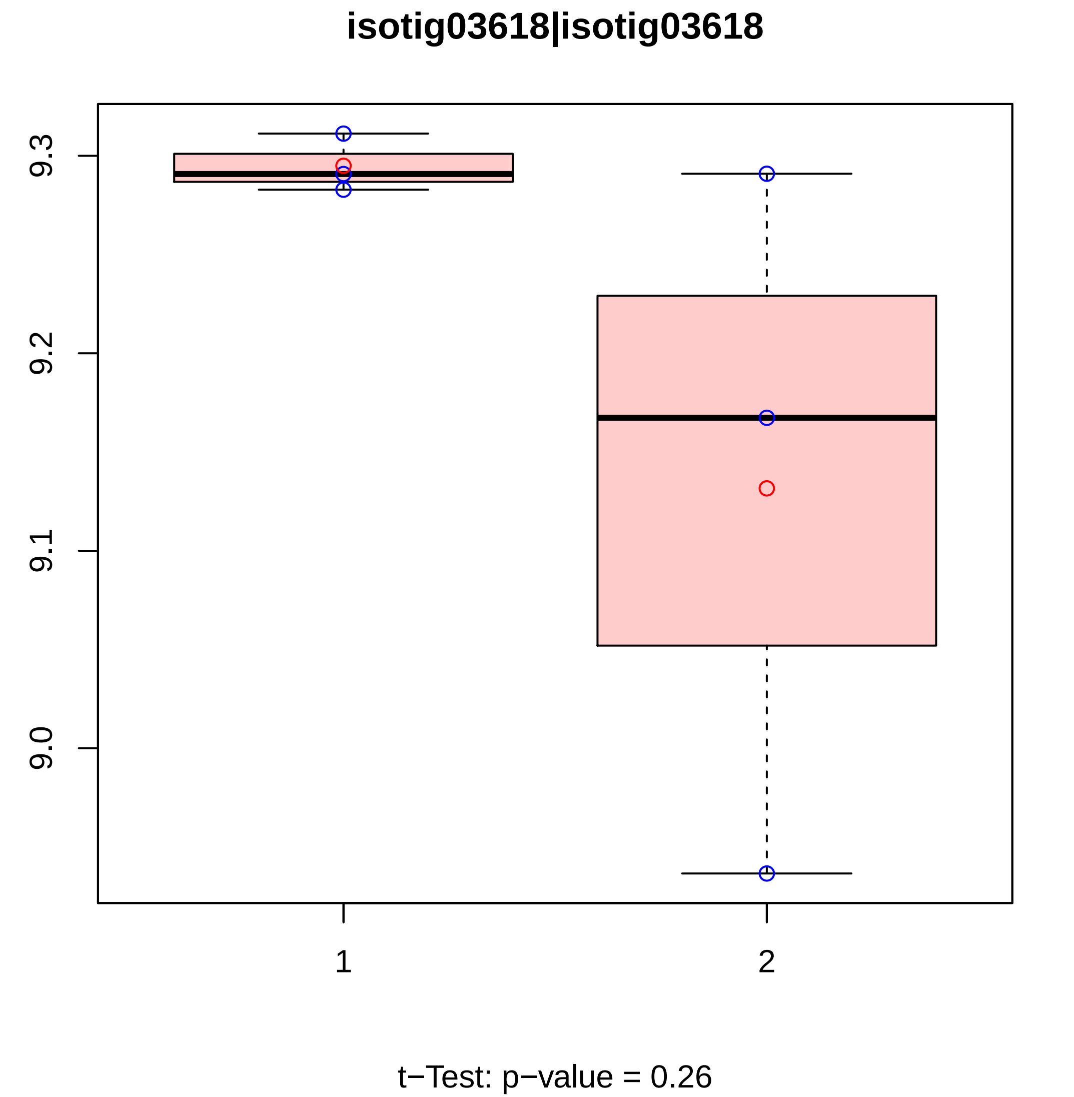
isotig03618|isotig03618
9.3
9.2
9.1
9.0
1
2
t−Test: p−value = 0.26

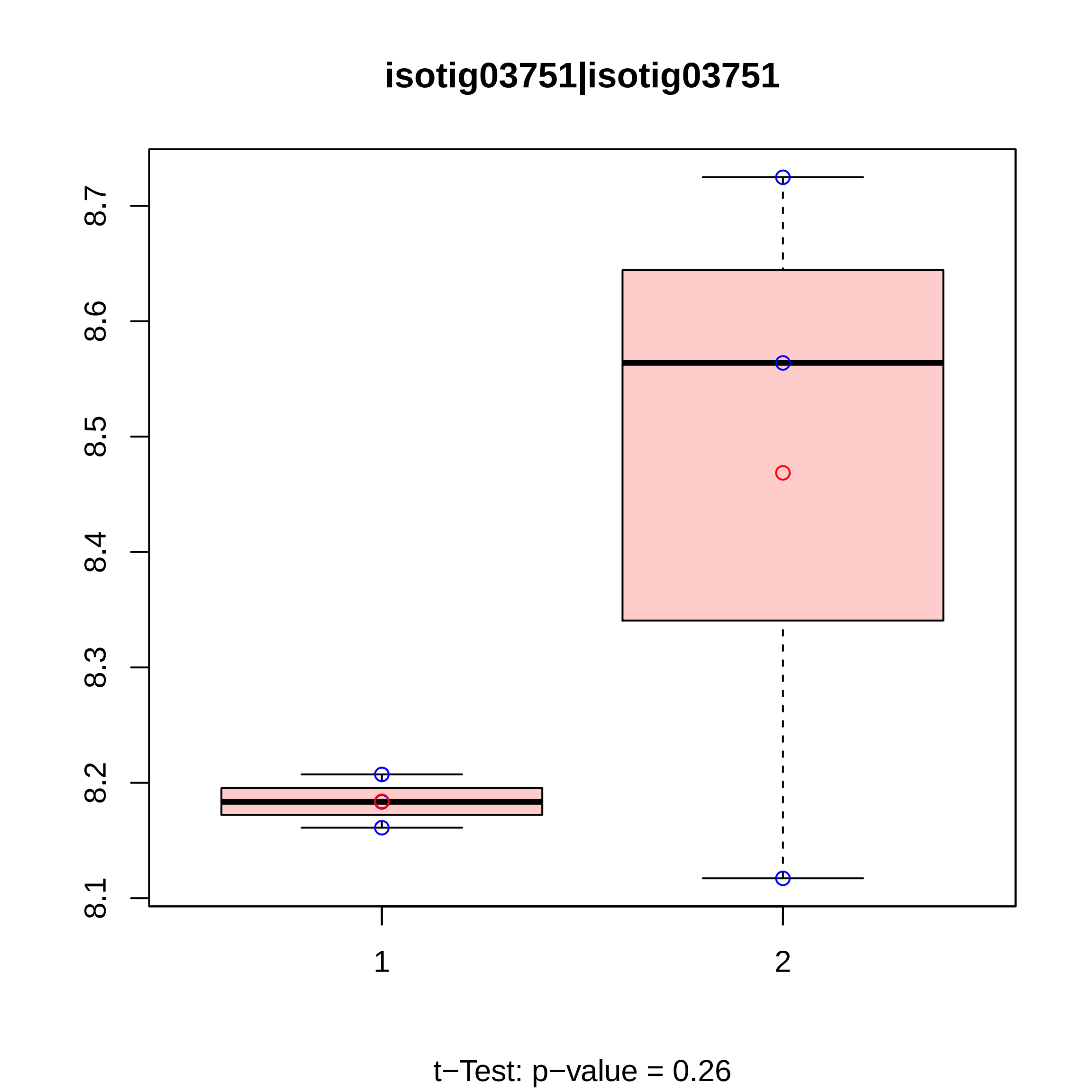

isotig03751|isotig03751
8.1
8.2
8.3
8.4
8.5
8.6
8.7
1
2
t−Test: p−value = 0.26

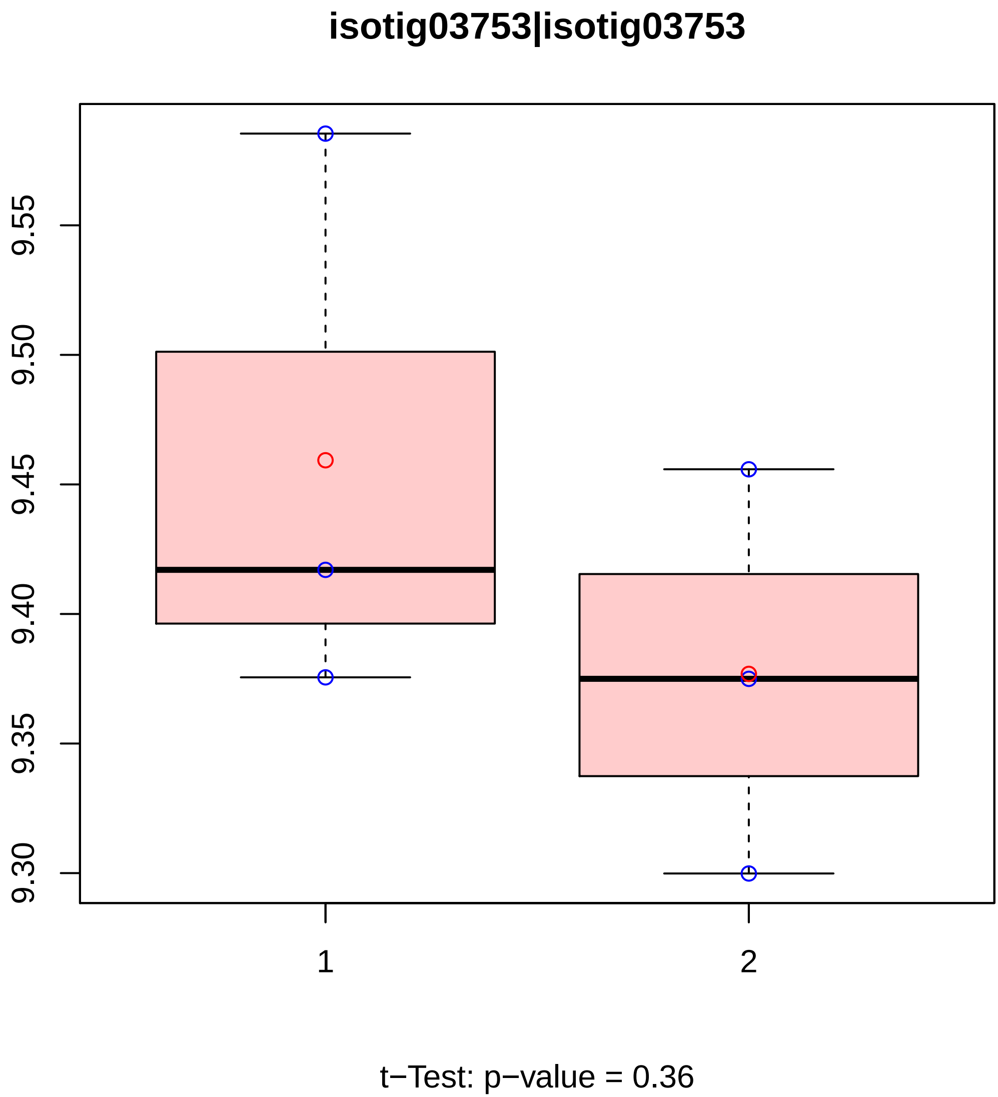
isotig03753|isotig03753
9.30
9.35
9.40
9.45
9.50
9.55
1
2
t−Test: p−value = 0.36

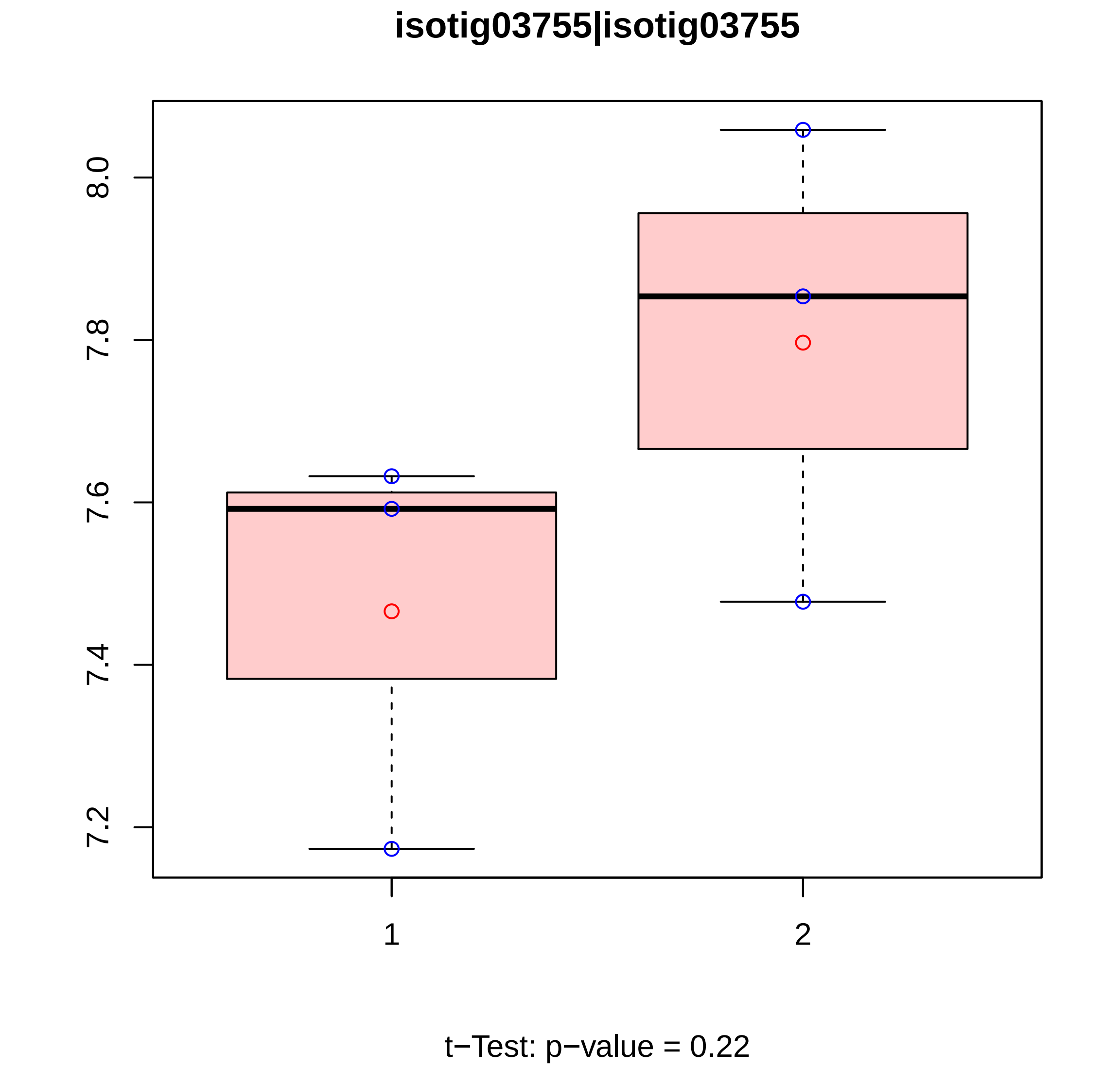

isotig03755|isotig03755
8.0
7.8
7.6
7.4
7.2
1
2
t−Test: p−value = 0.22

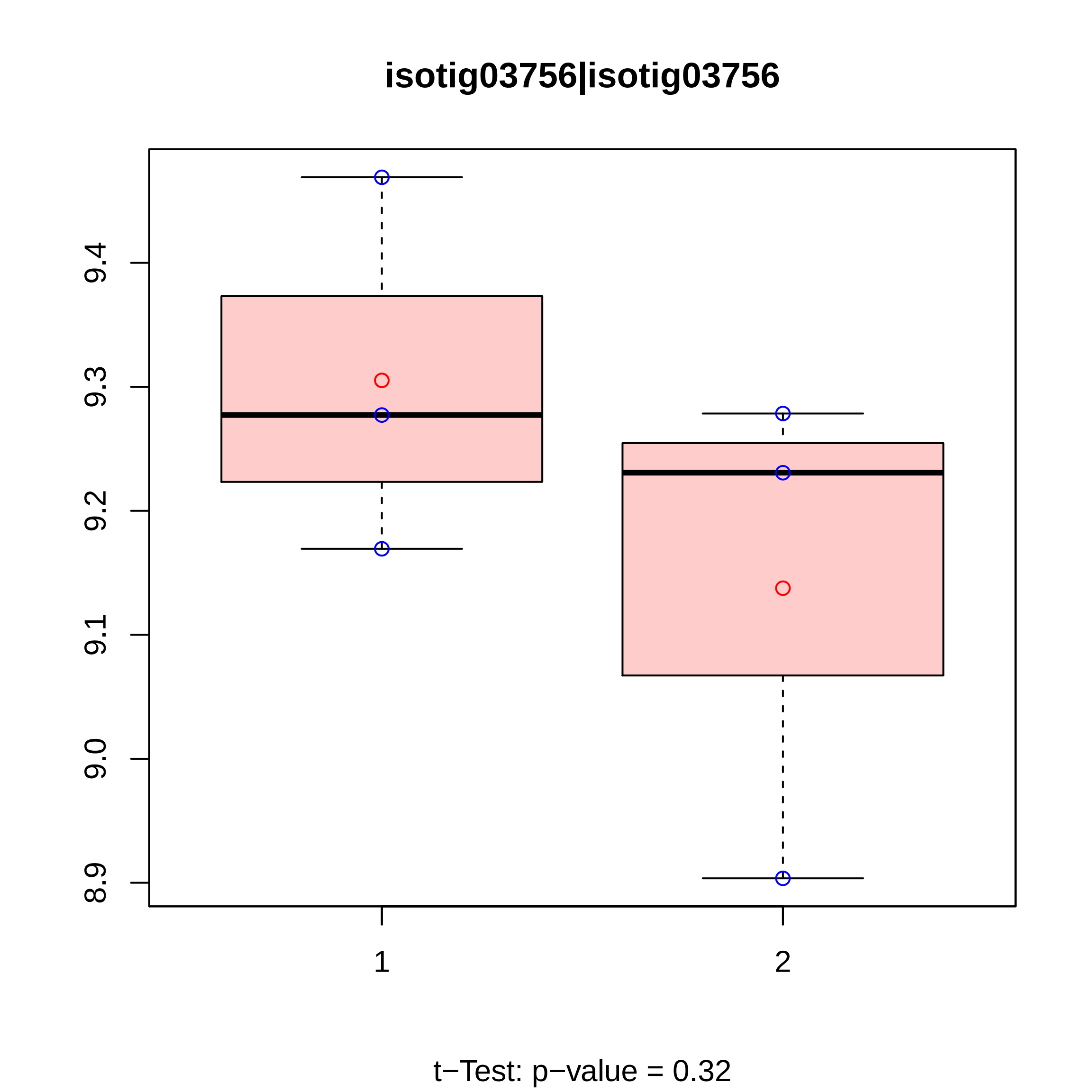

isotig03756|isotig03756
9.4
9.3
9.2
9.1
9.0
8.9
1
2
t−Test: p−value = 0.32

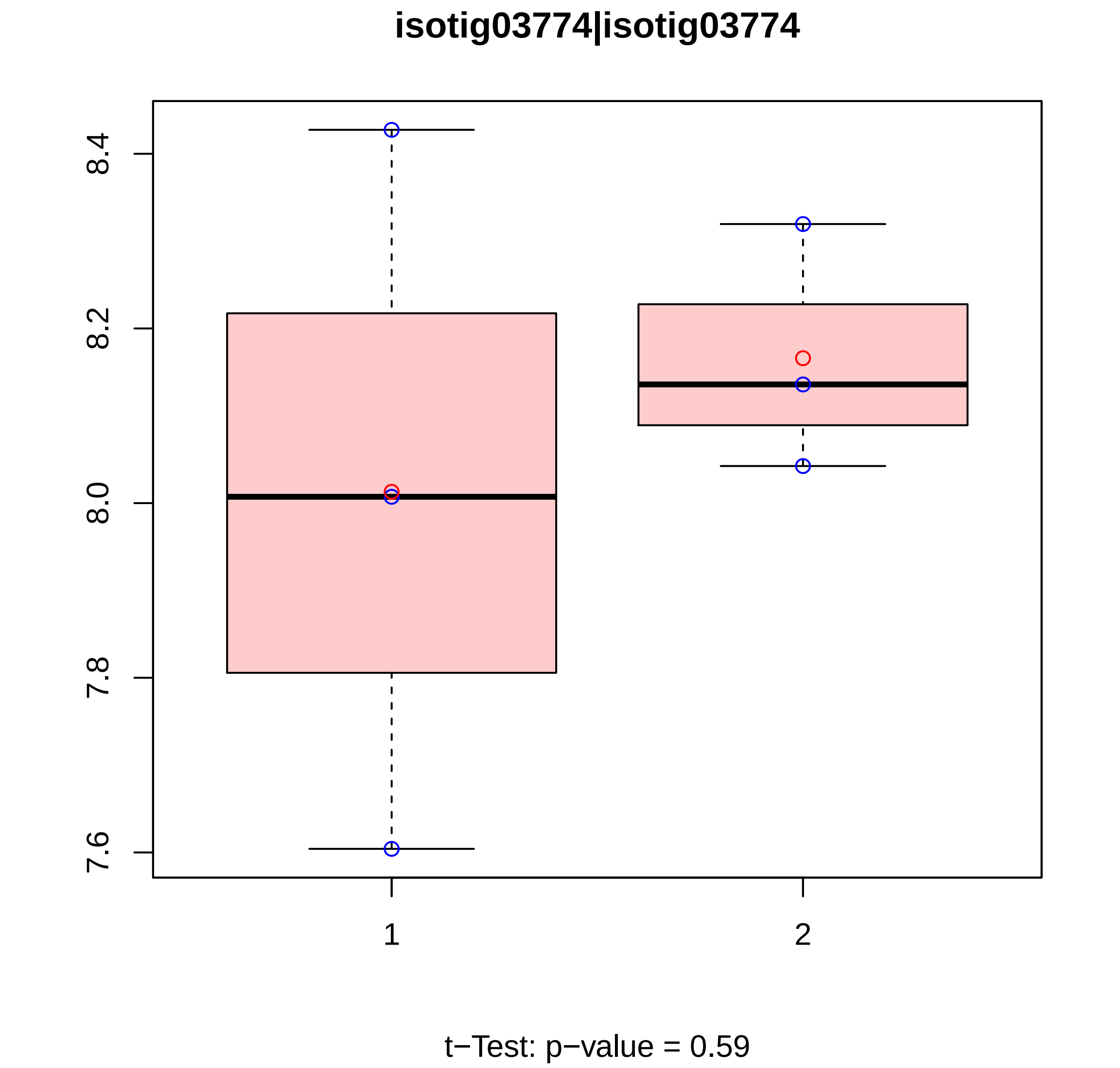
isotig03774|isotig03774
8.4
8.2
8.0
7.8
7.6
1
2
t−Test: p−value = 0.59

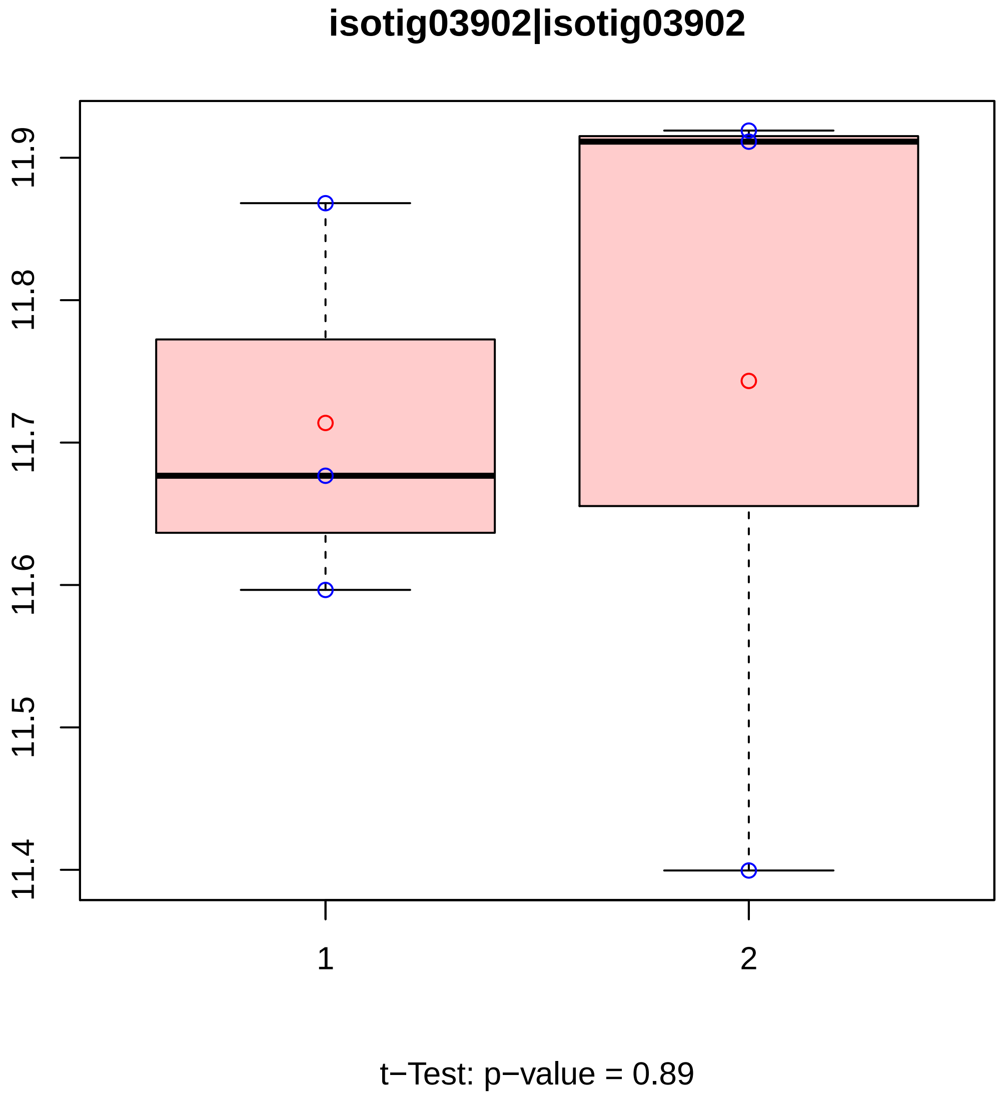

isotig03902|isotig03902
11.4
11.5
11.6
11.7
11.8
11.9
1
2
t−Test: p−value = 0.89

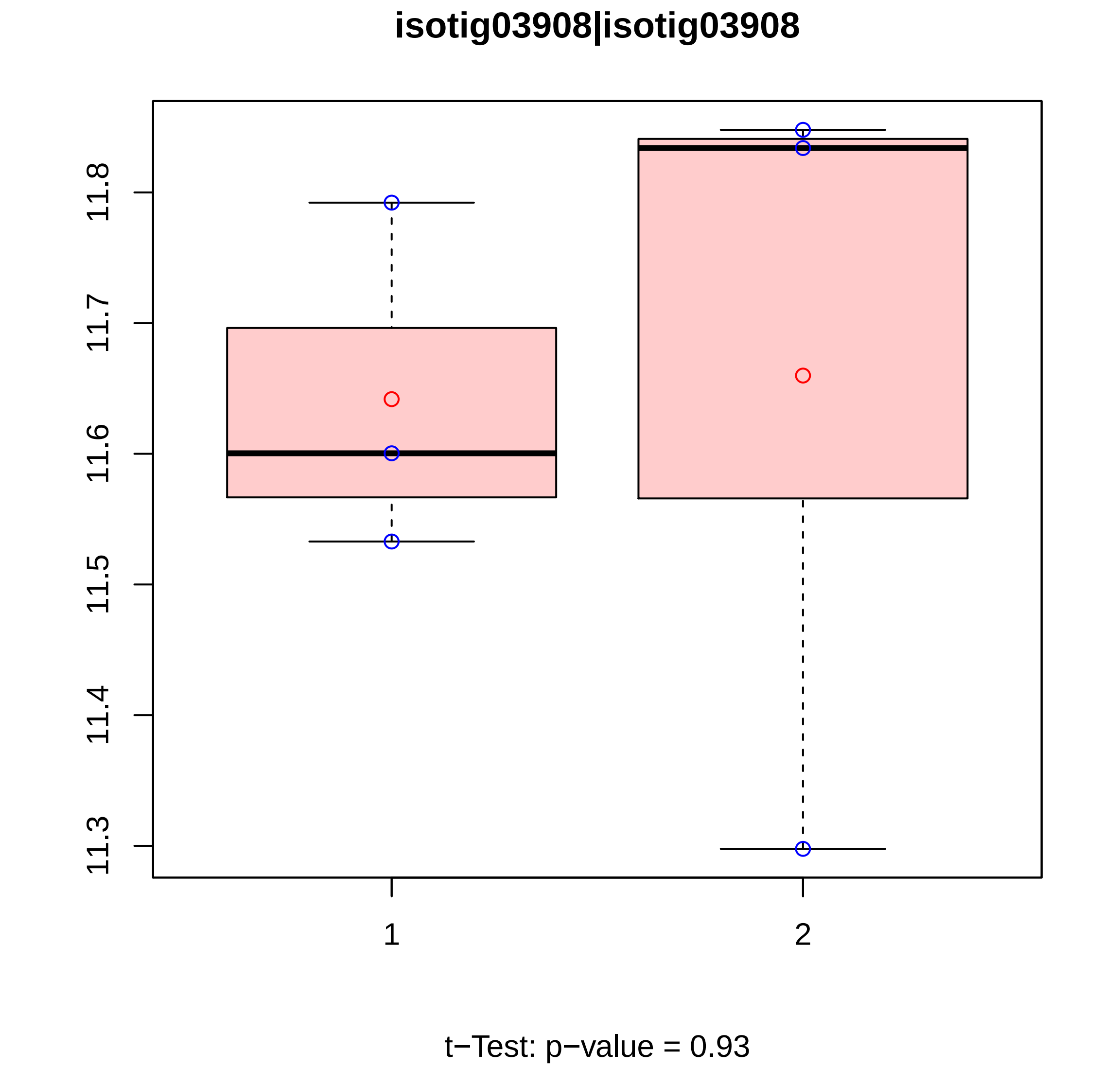
isotig03908|isotig03908
11.3
11.4
11.5
11.6
11.7
11.8
1
2
t−Test: p−value = 0.93

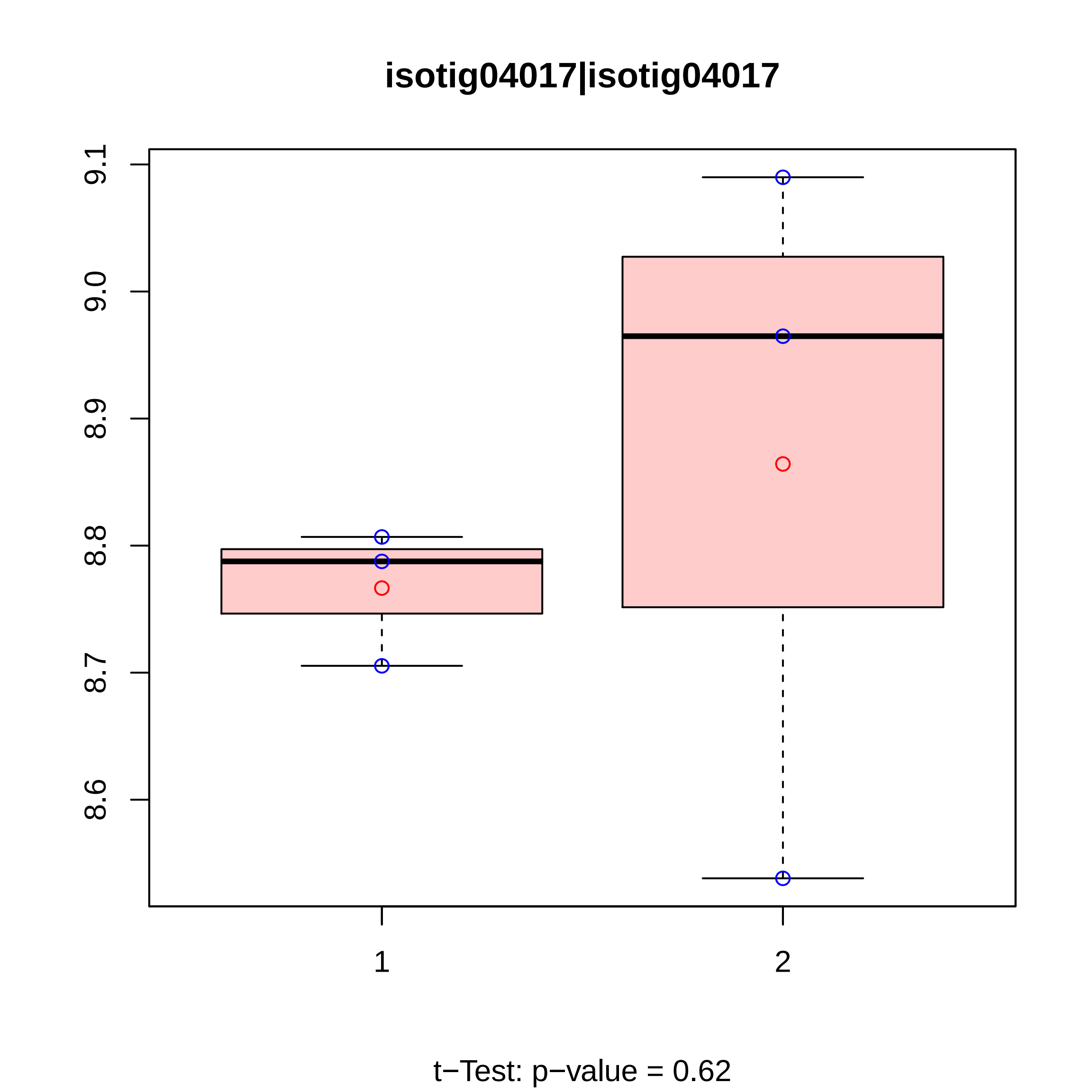

isotig04017|isotig04017
9.1
9.0
8.9
8.8
8.7
8.6
1
2
t−Test: p−value = 0.62

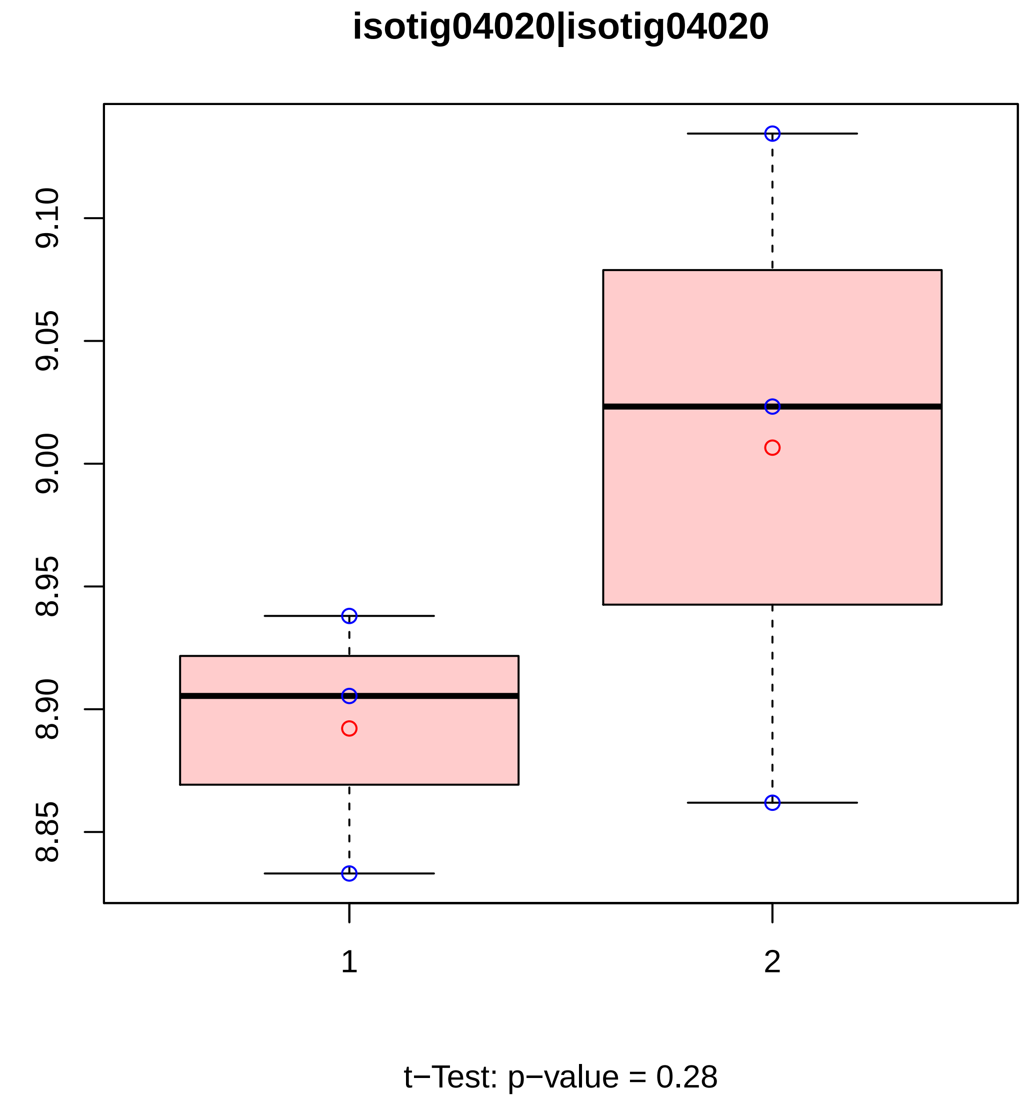
isotig04020|isotig04020
9.10
9.05
9.00
8.95
8.90
8.85
1
2
t−Test: p−value = 0.28

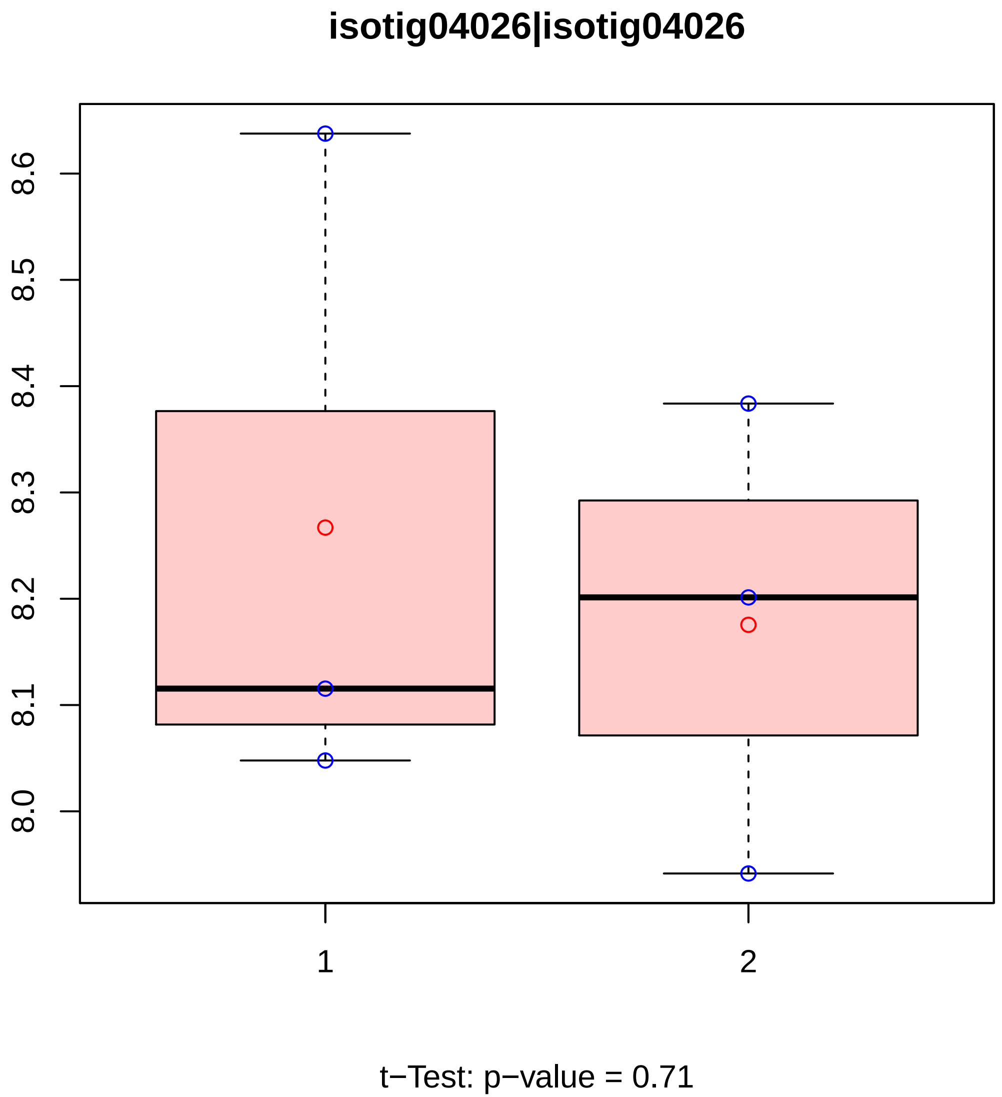
isotig04026|isotig04026
8.0
8.1
8.2
8.3
8.4
8.5
8.6
1
2
t−Test: p−value = 0.71

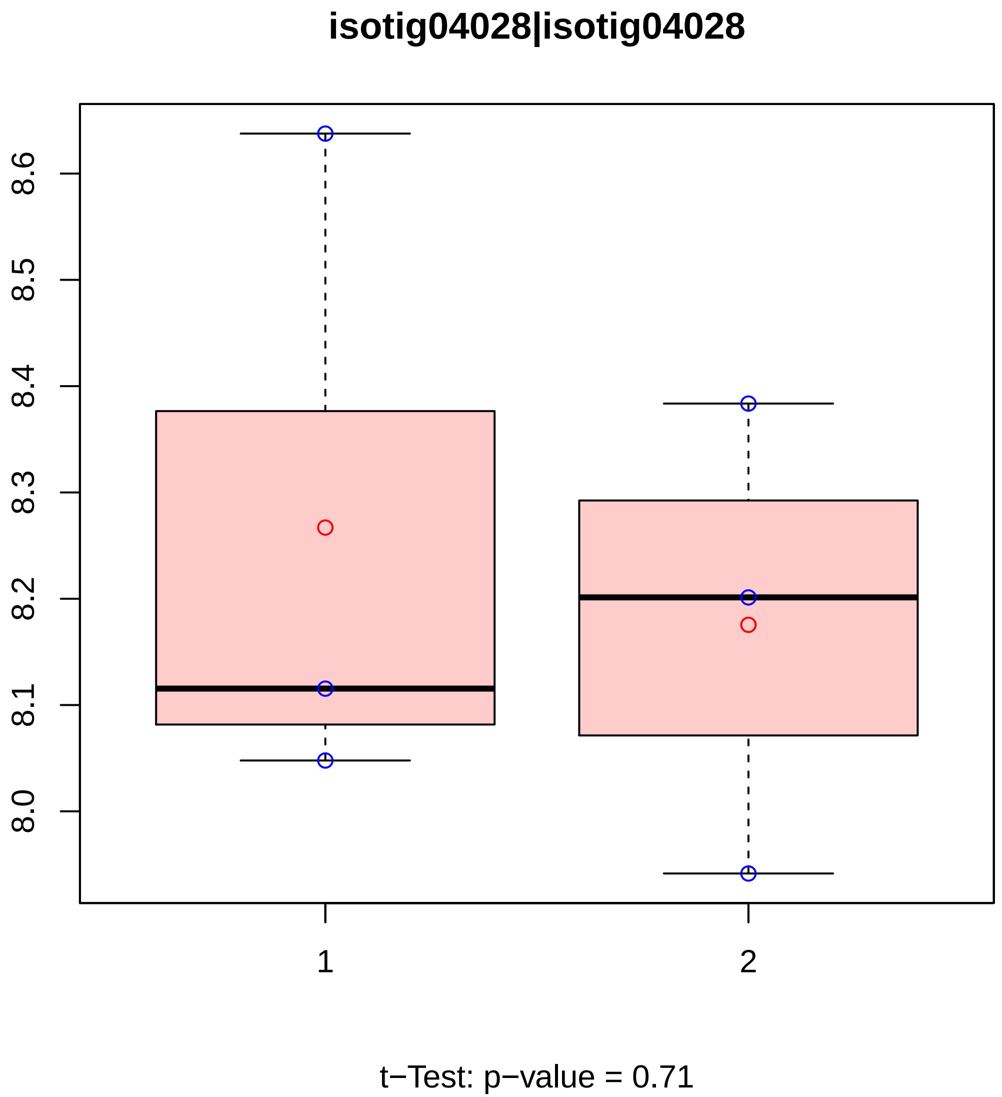
isotig04028|isotig04028
8.0
8.1
8.2
8.3
8.4
8.5
8.6
1
2
t−Test: p−value = 0.71

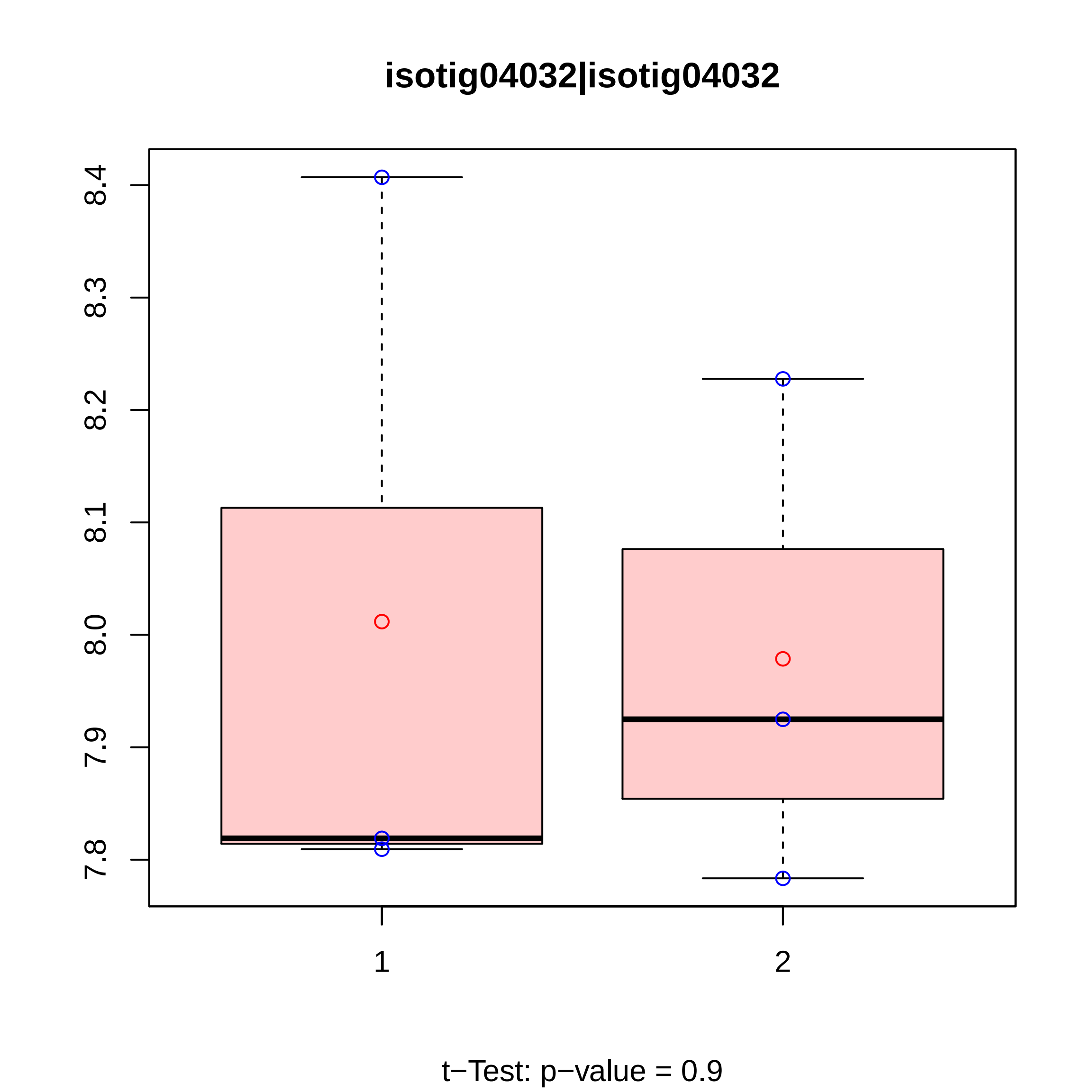
isotig04032|isotig04032
8.4
8.3
8.2
8.1
8.0
7.9
7.8
1
2
t−Test: p−value = 0.9

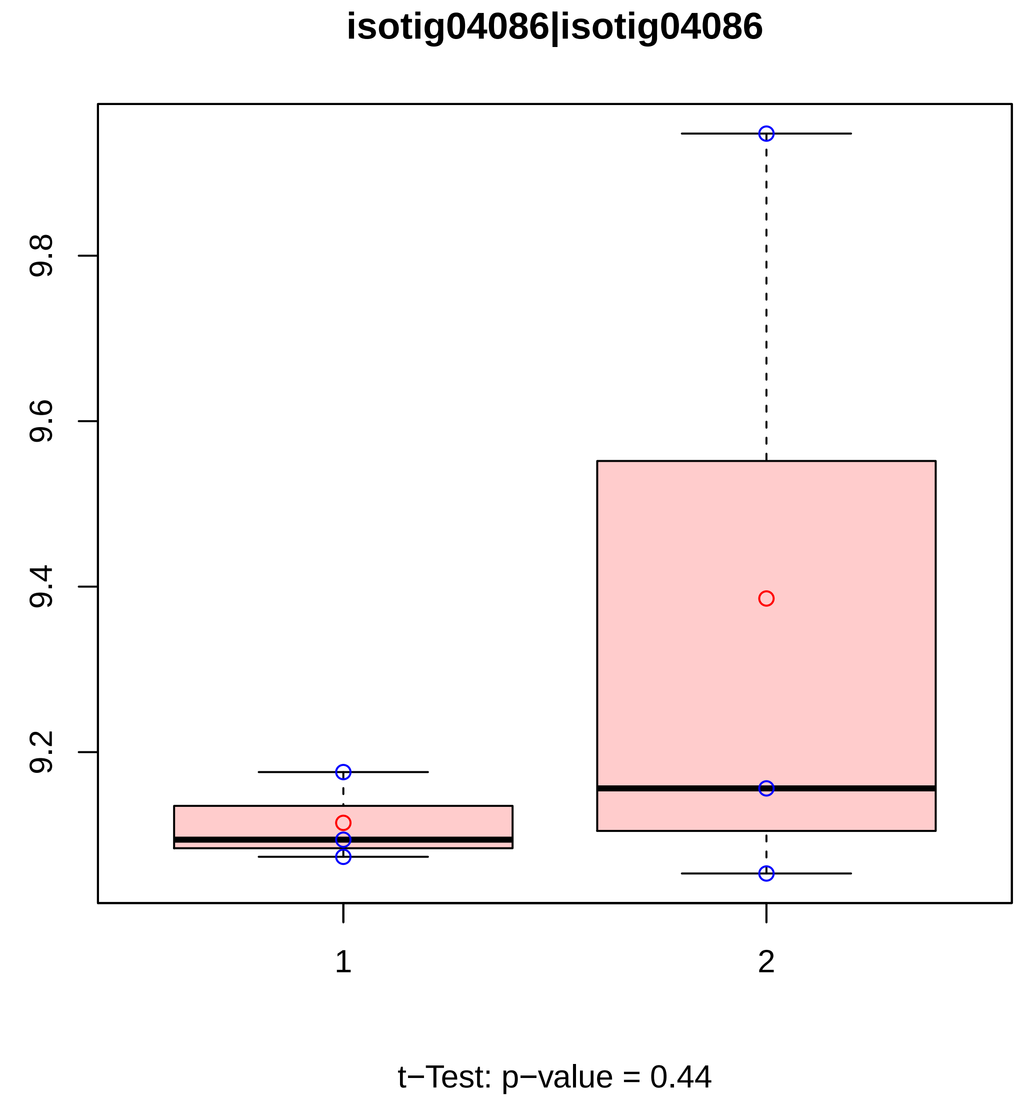
isotig04086|isotig04086
9.2
9.4
9.6
9.8
1
2
t−Test: p−value = 0.44

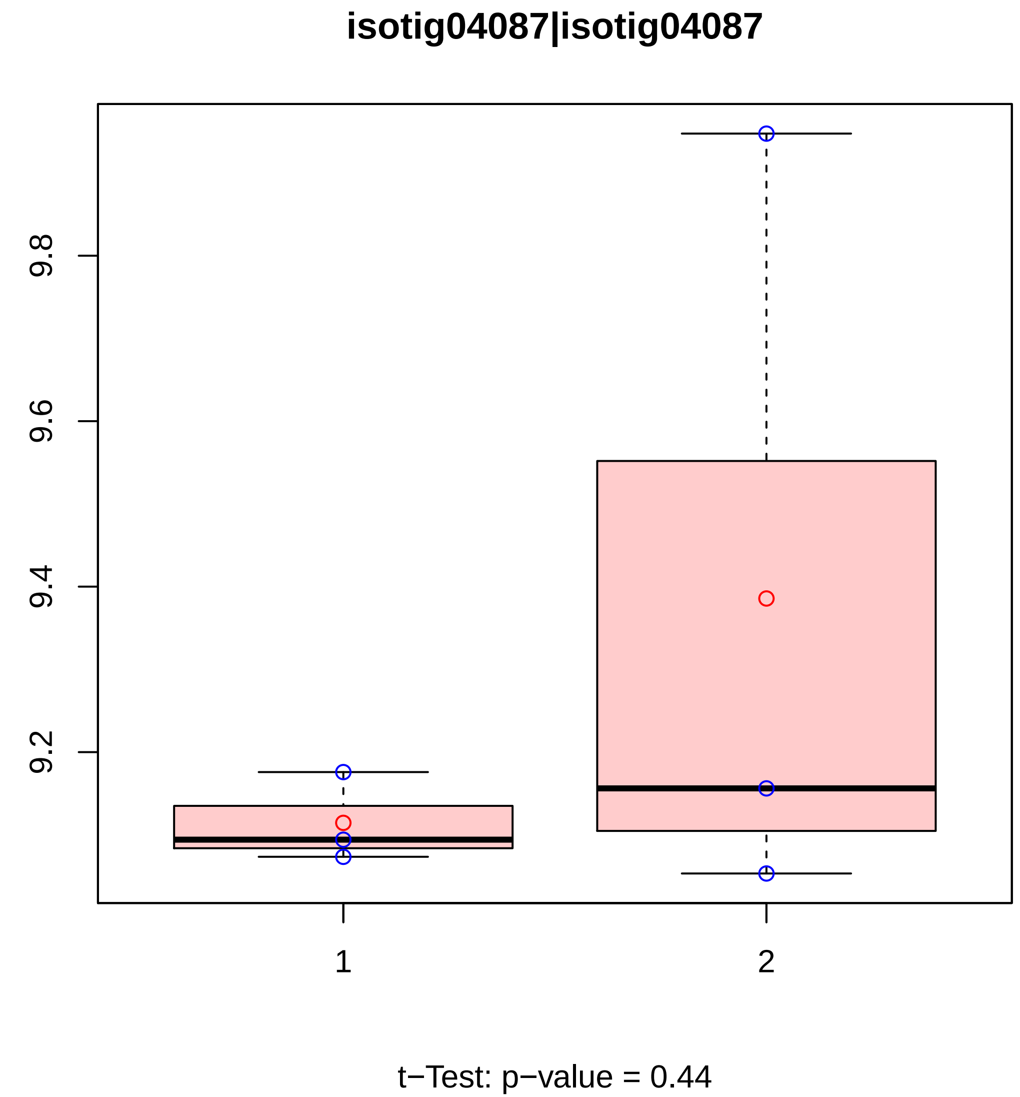

isotig04087|isotig04087
9.2
9.4
9.6
9.8
1
2
t−Test: p−value = 0.44

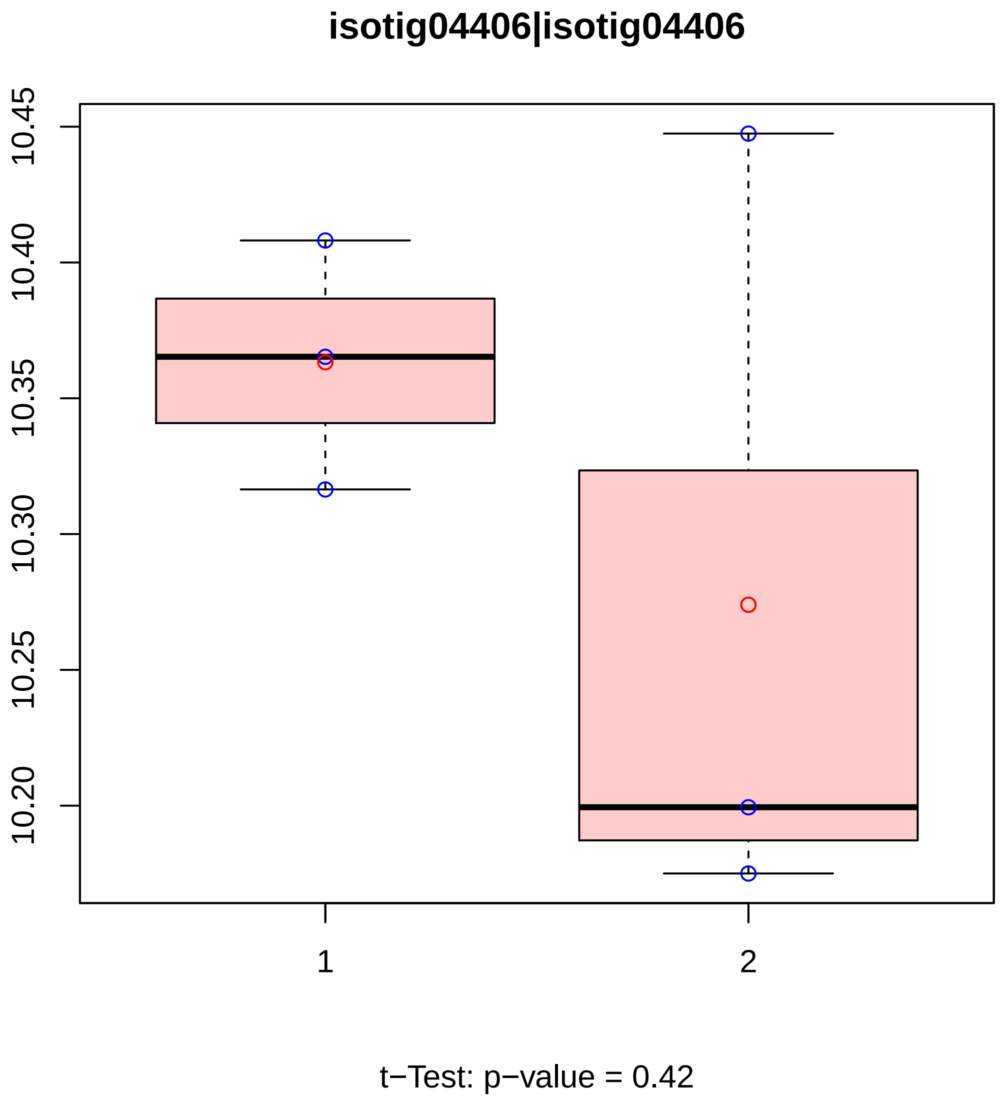
isotig04406|isotig04406
10.45
10.40
10.35
10.30
10.25
10.20
1
2
t−Test: p−value = 0.42

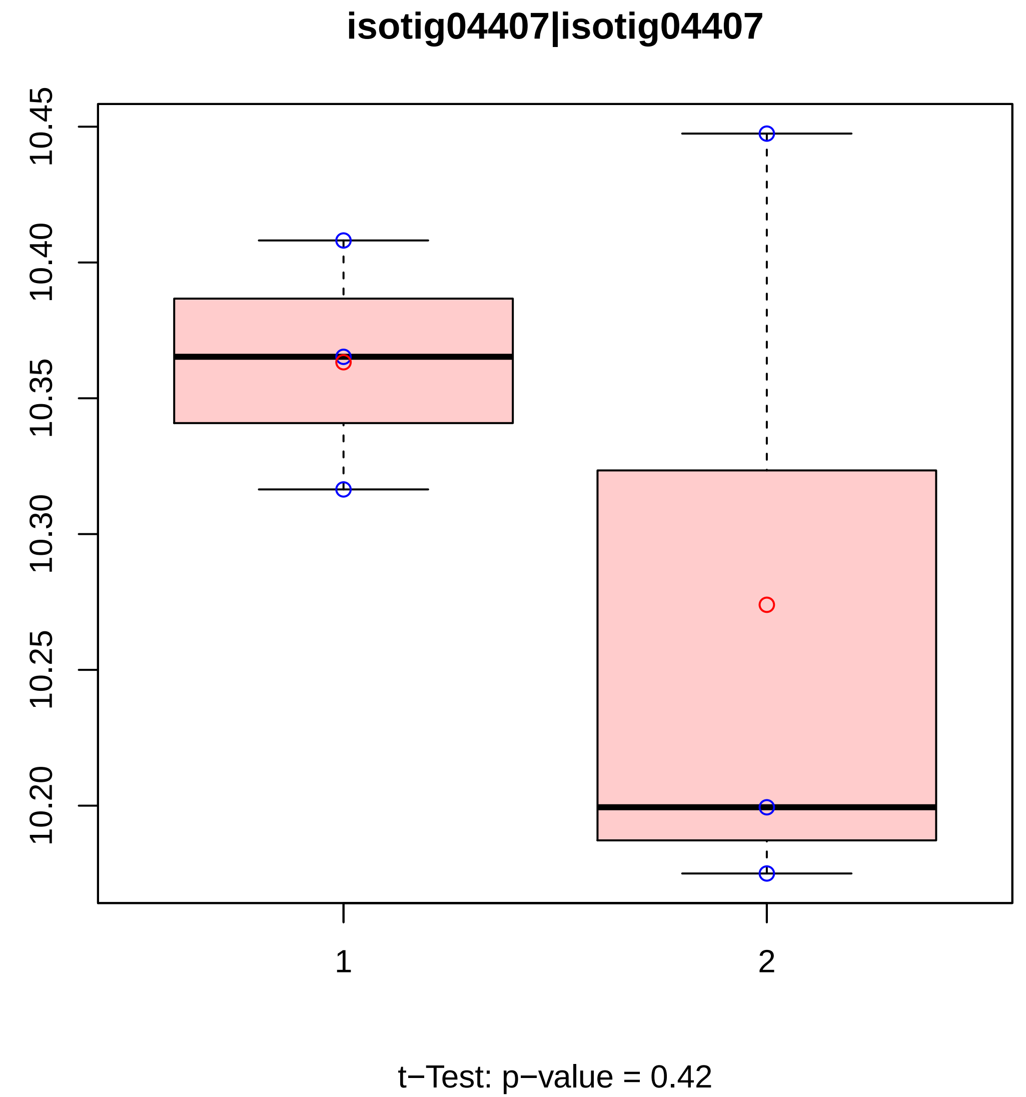

isotig04407|isotig04407
10.20
10.25
10.30
10.35
10.40
10.45
1
2
t−Test: p−value = 0.42

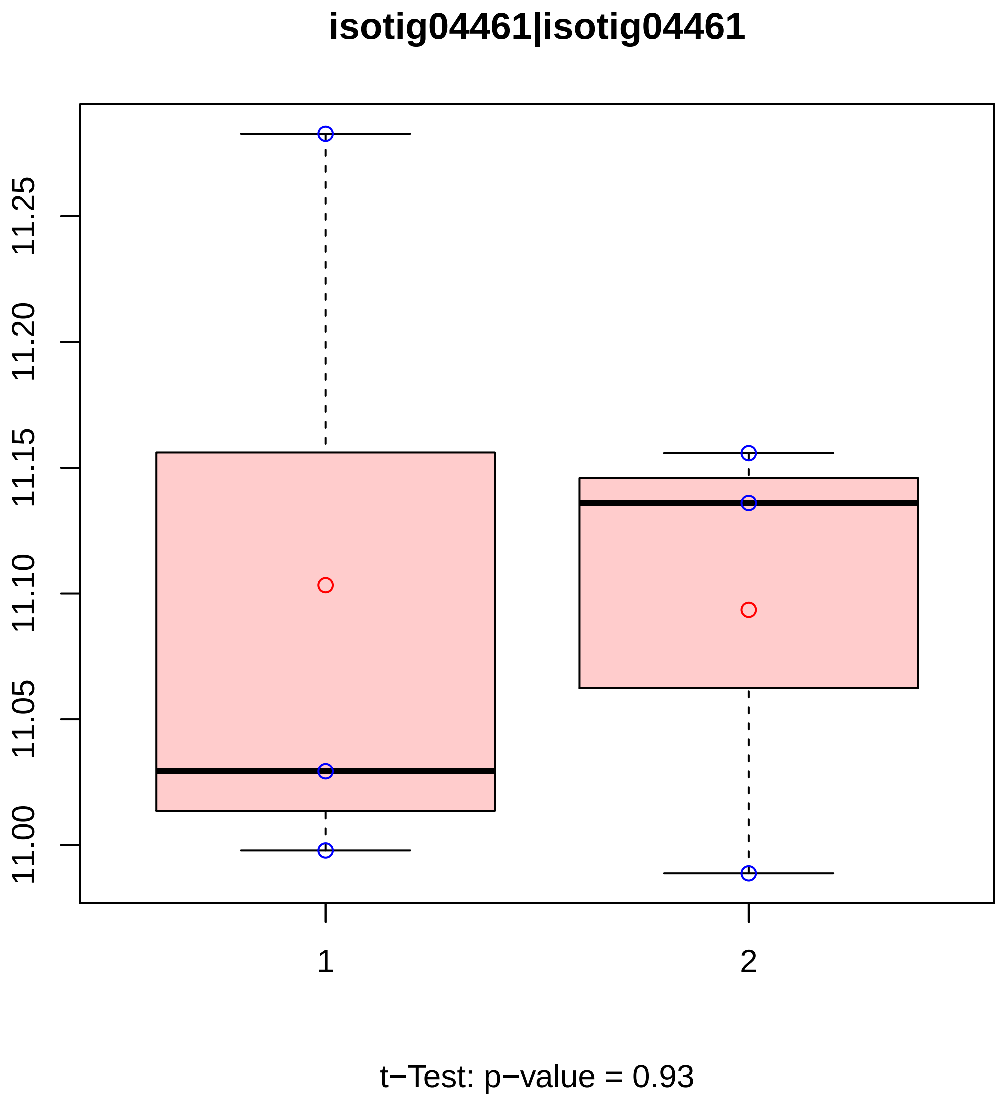
isotig04461|isotig04461
11.00
11.05
11.10
11.15
11.20
11.25
1
2
t−Test: p−value = 0.93

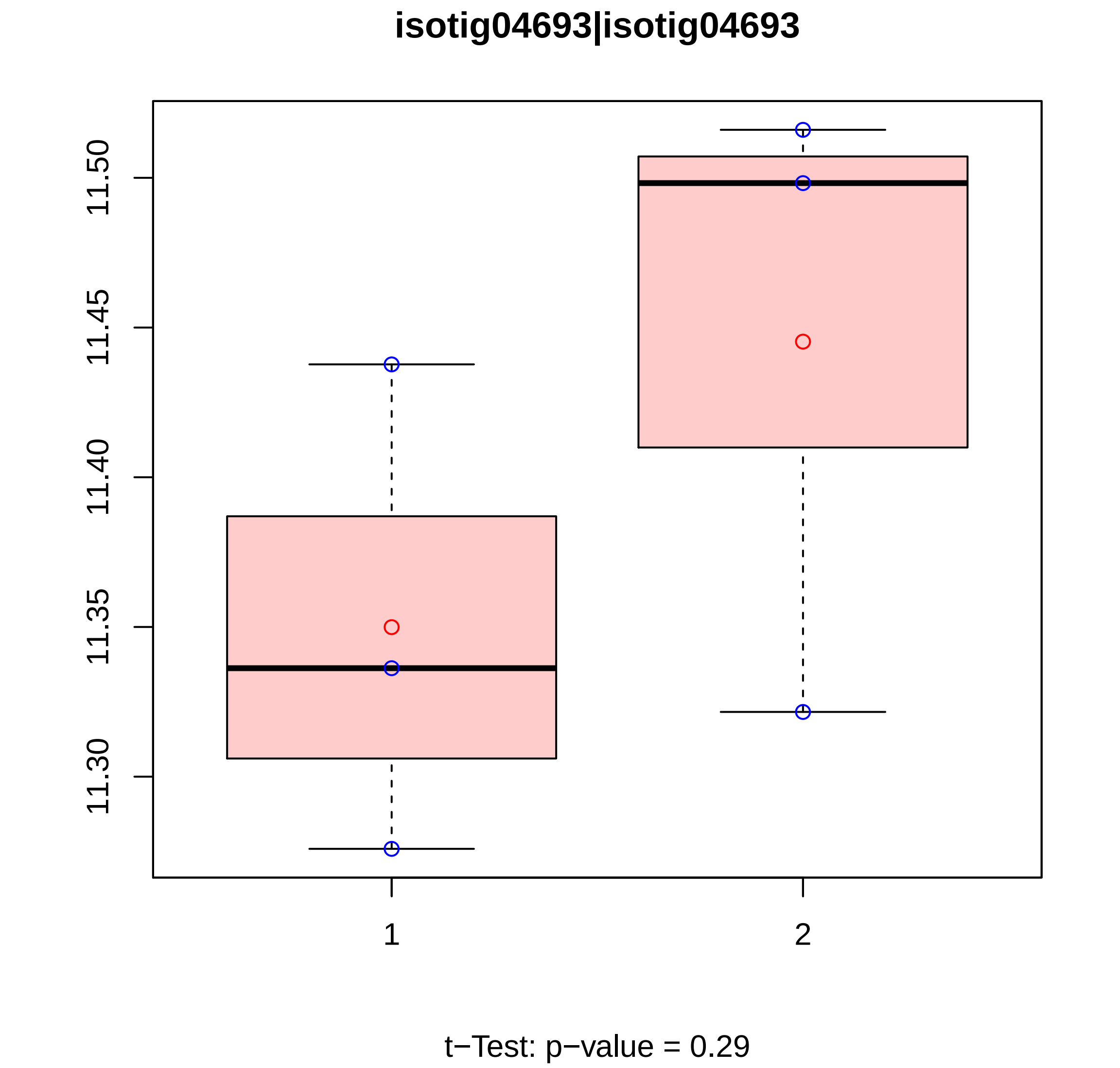

isotig04693|isotig04693
11.30
11.35
11.40
11.45
11.50
1
2
t−Test: p−value = 0.29

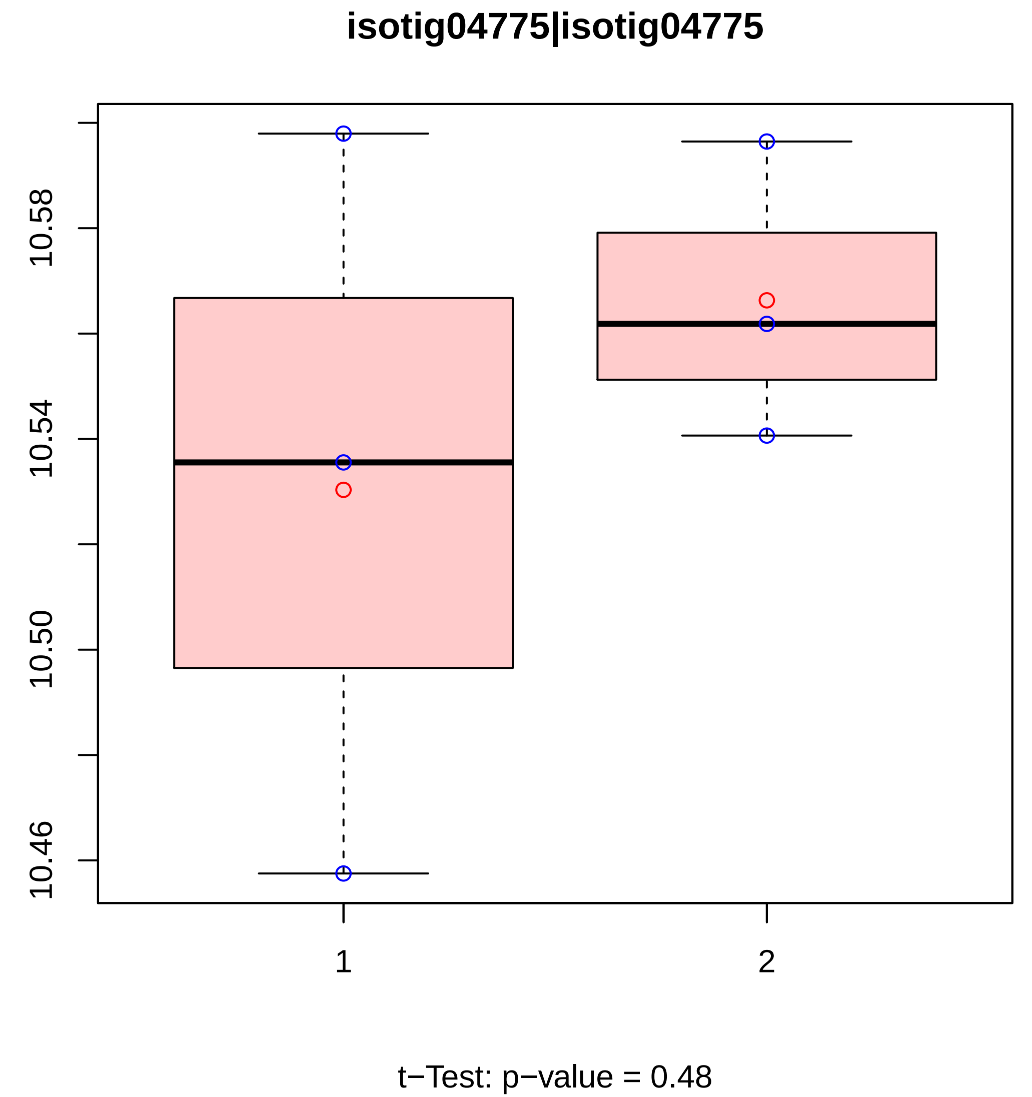
isotig04775|isotig04775
10.46
10.50
10.54
10.58
1
2
t−Test: p−value = 0.48

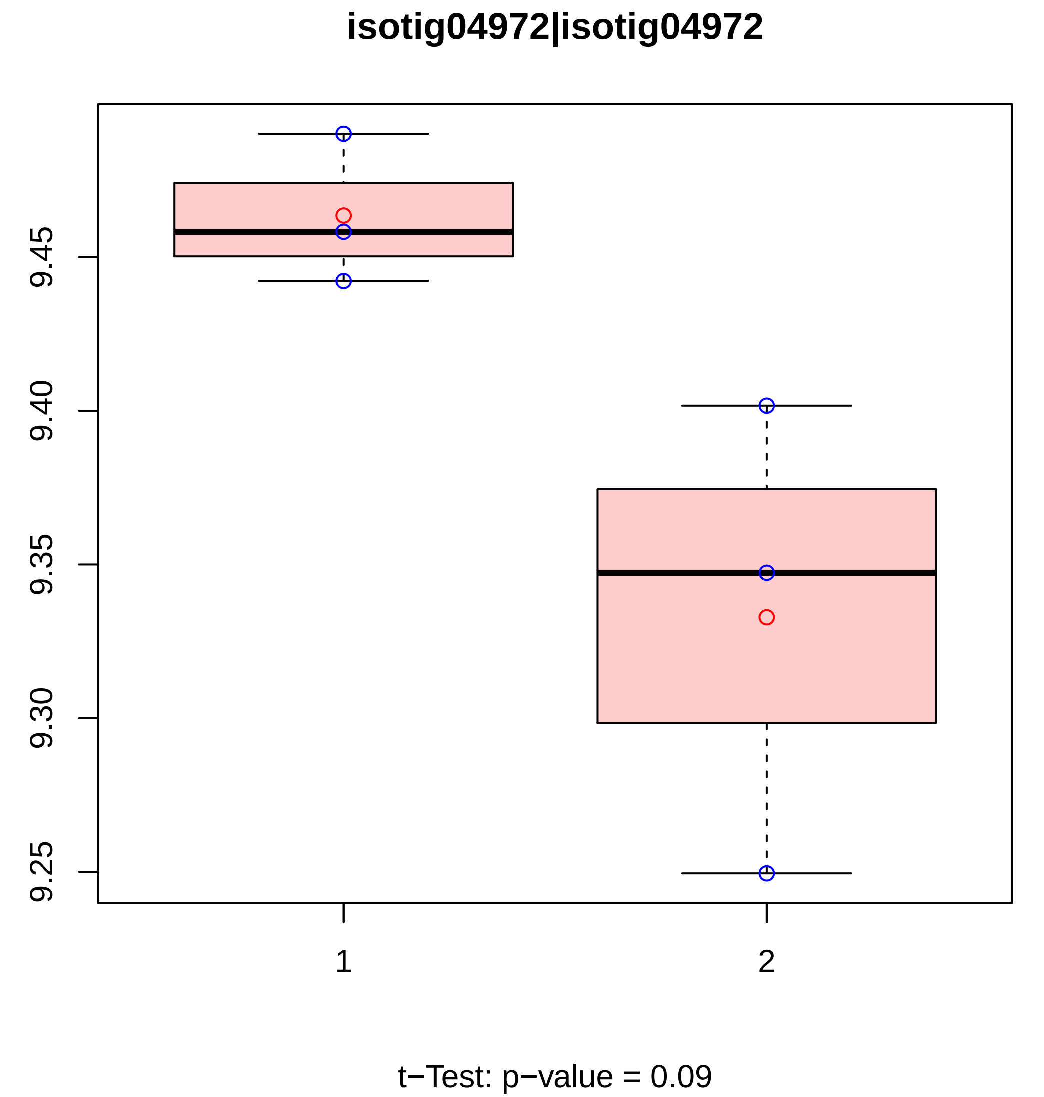

isotig04972|isotig04972
9.25
9.30
9.35
9.40
9.45
1
2
t−Test: p−value = 0.09

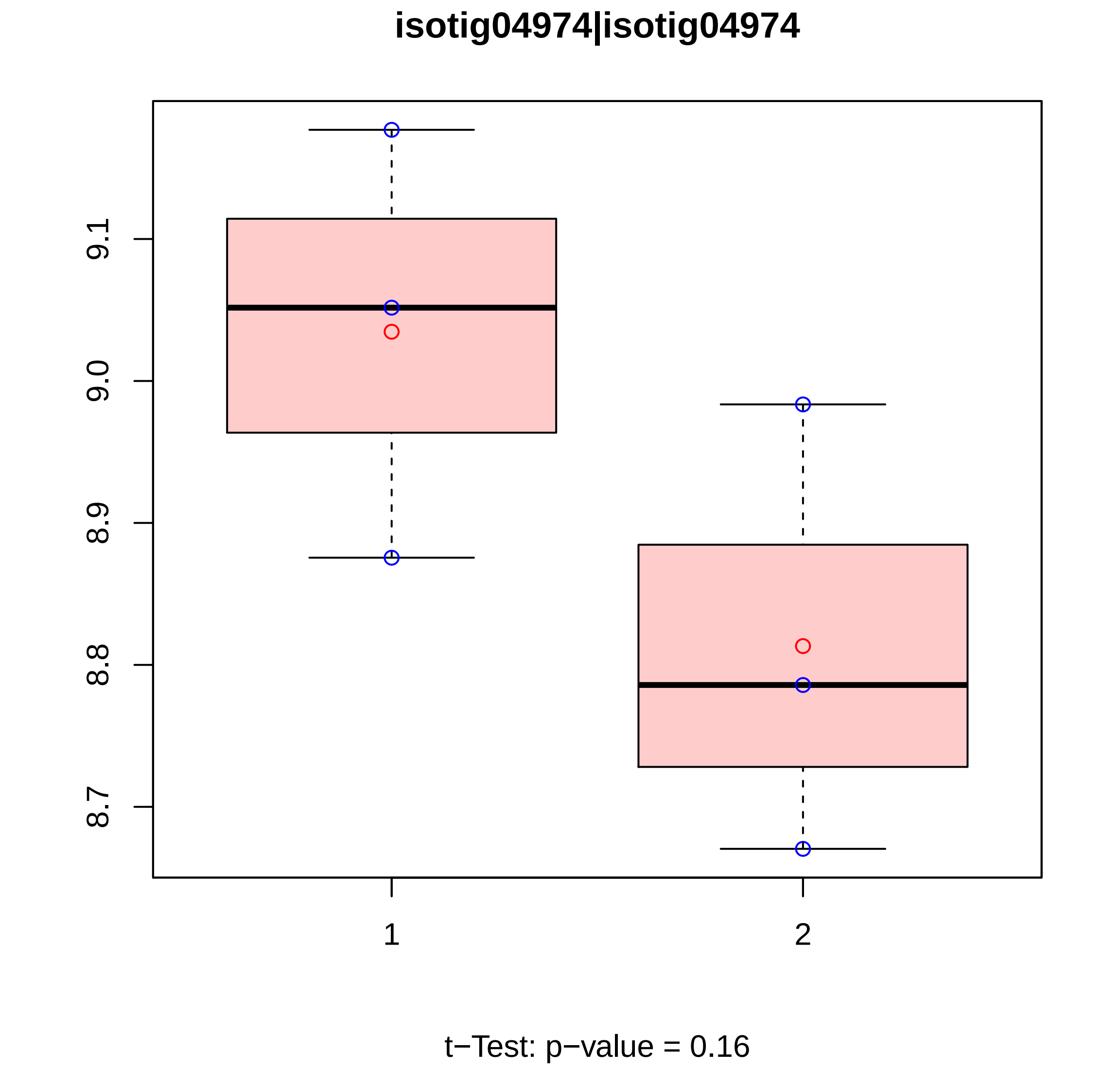
isotig04974|isotig04974
9.1
9.0
8.9
8.8
8.7
1
2
t−Test: p−value = 0.16

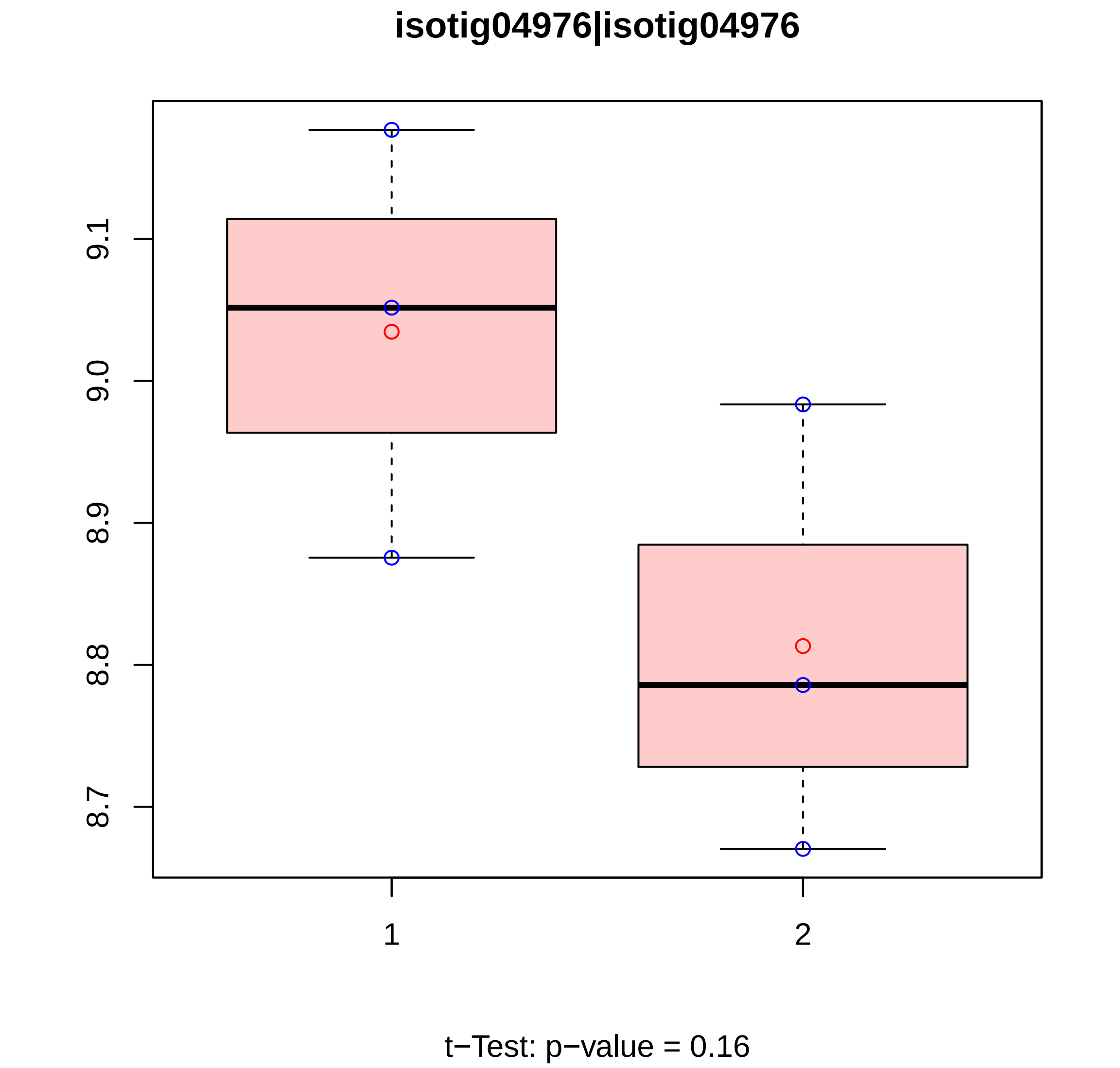
isotig04976|isotig04976
9.1
9.0
8.9
8.8
8.7
1
2
t−Test: p−value = 0.16

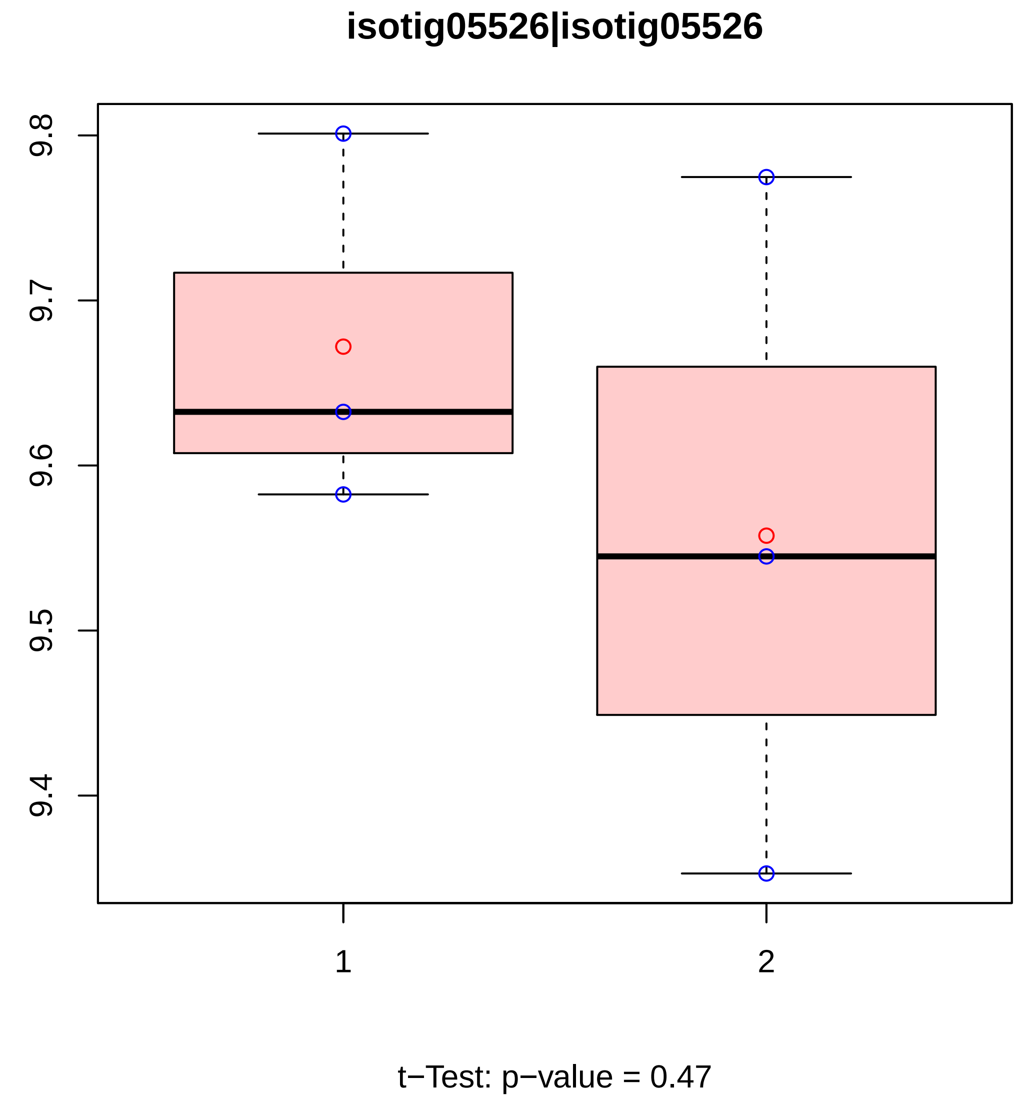
isotig05526|isotig05526
9.8
9.7
9.6
9.5
9.4
1
2
t−Test: p−value = 0.47

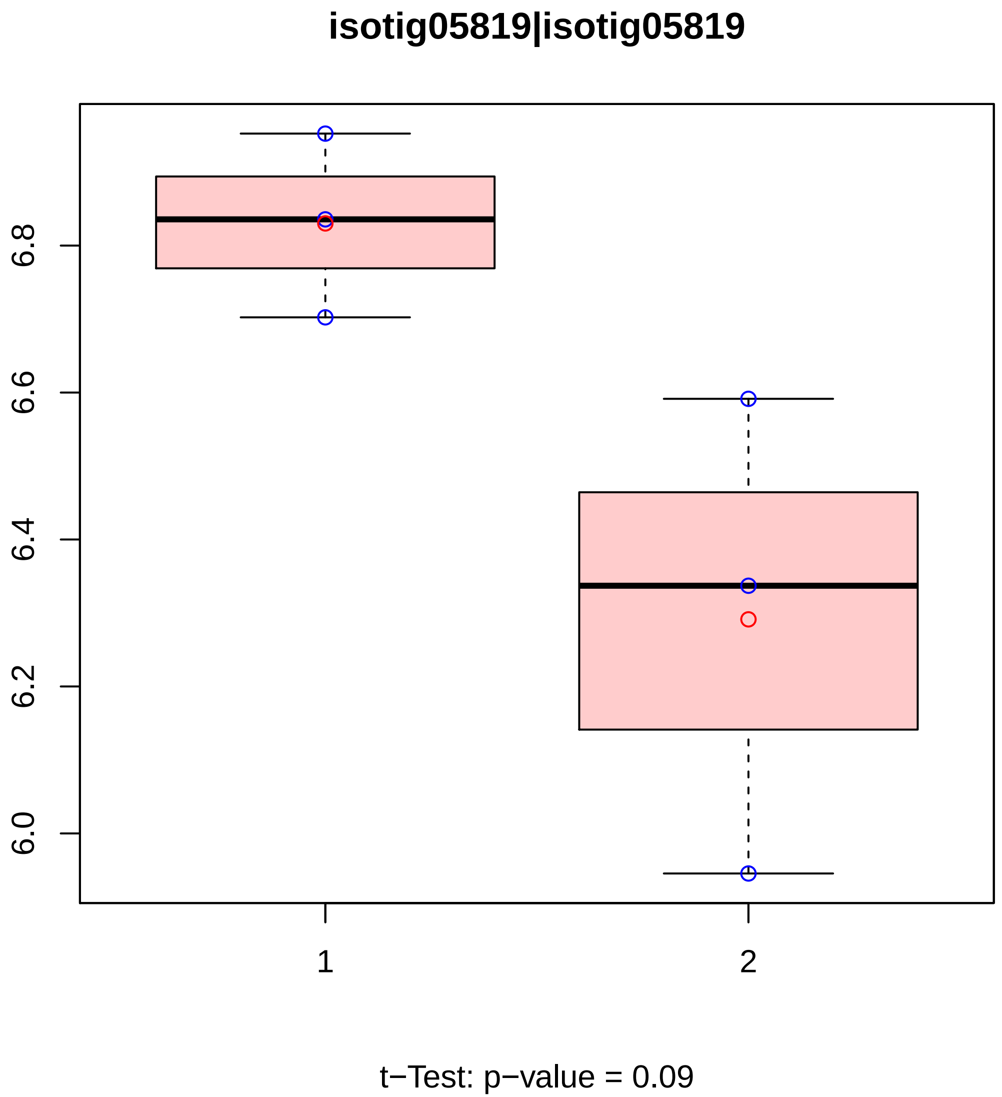

isotig05819|isotig05819
6.0
6.2
6.4
6.6
6.8
1
2
t−Test: p−value = 0.09

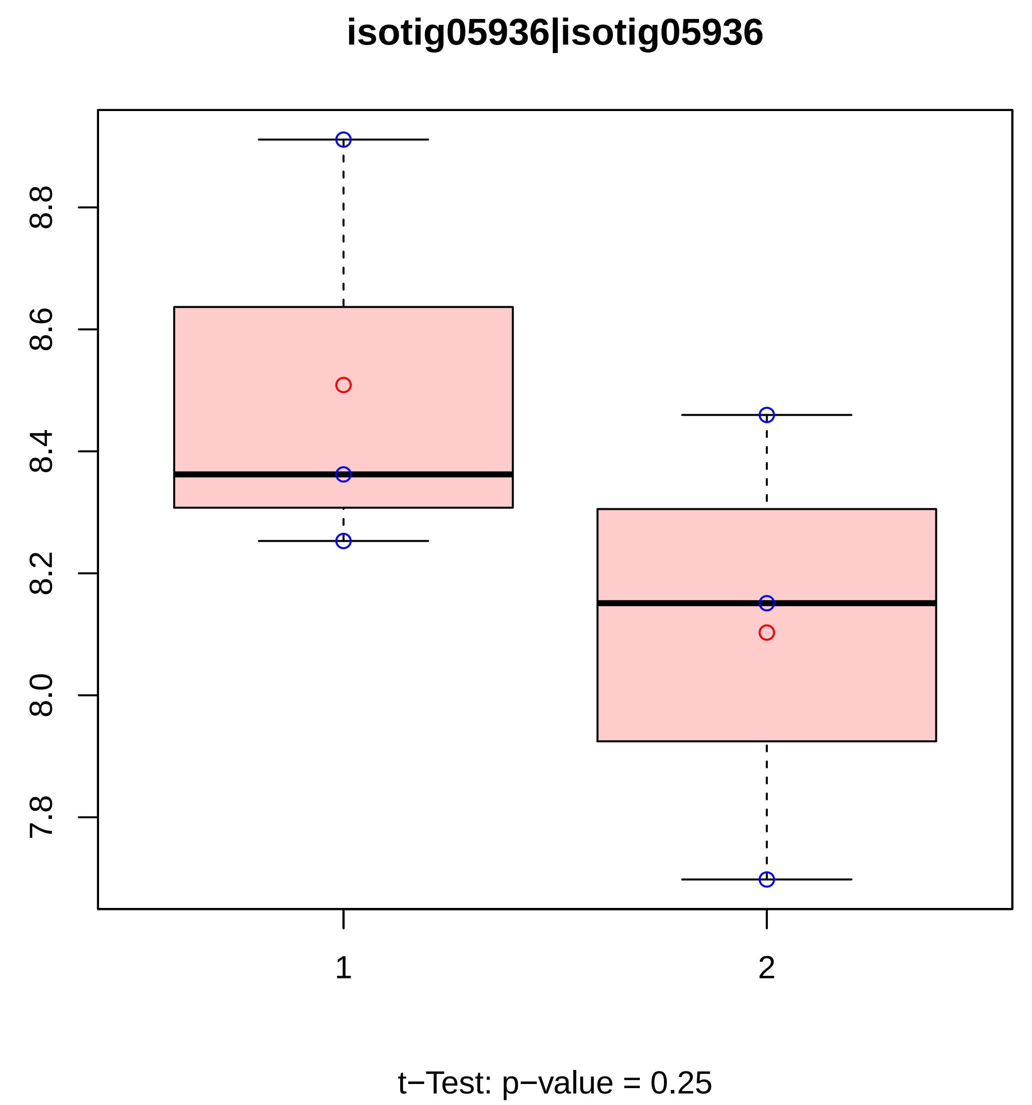

isotig05936|isotig05936
8.8
8.6
8.4
8.2
8.0
7.8
1
2
t−Test: p−value = 0.25

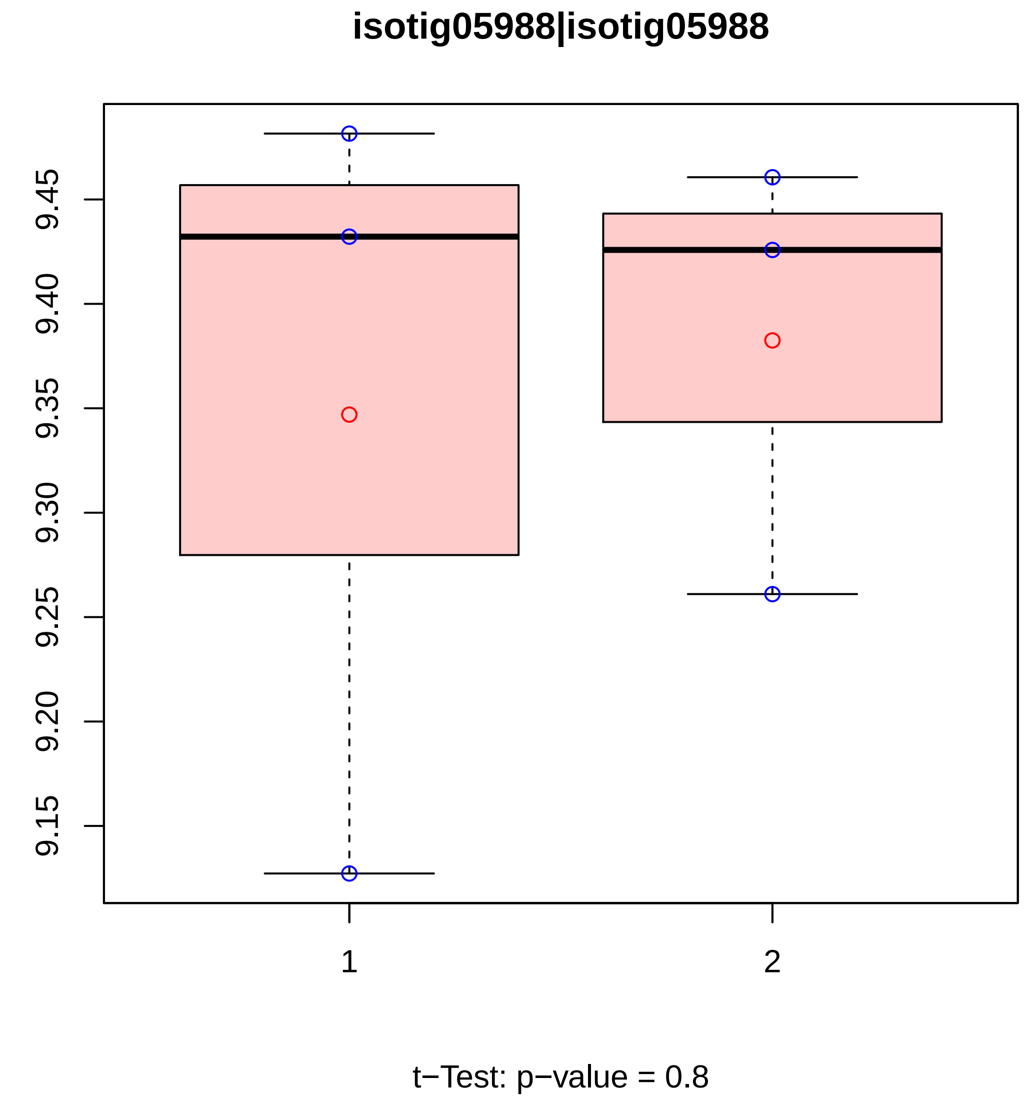

isotig05988|isotig05988
9.15
9.20
9.25
9.30
9.35
9.40
9.45
1
2
t−Test: p−value = 0.8

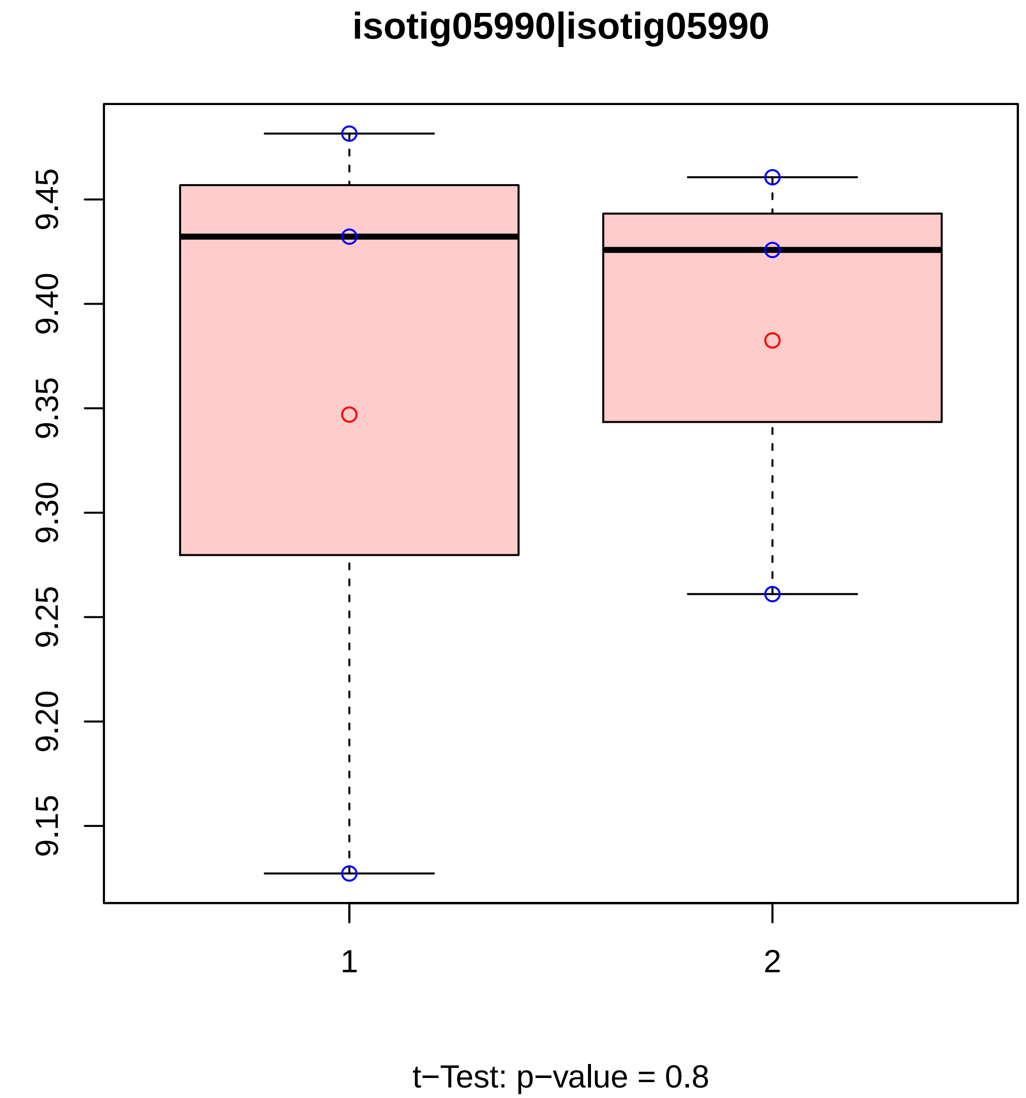
isotig05990|isotig05990
9.15
9.20
9.25
9.30
9.35
9.40
9.45
1
2
t−Test: p−value = 0.8

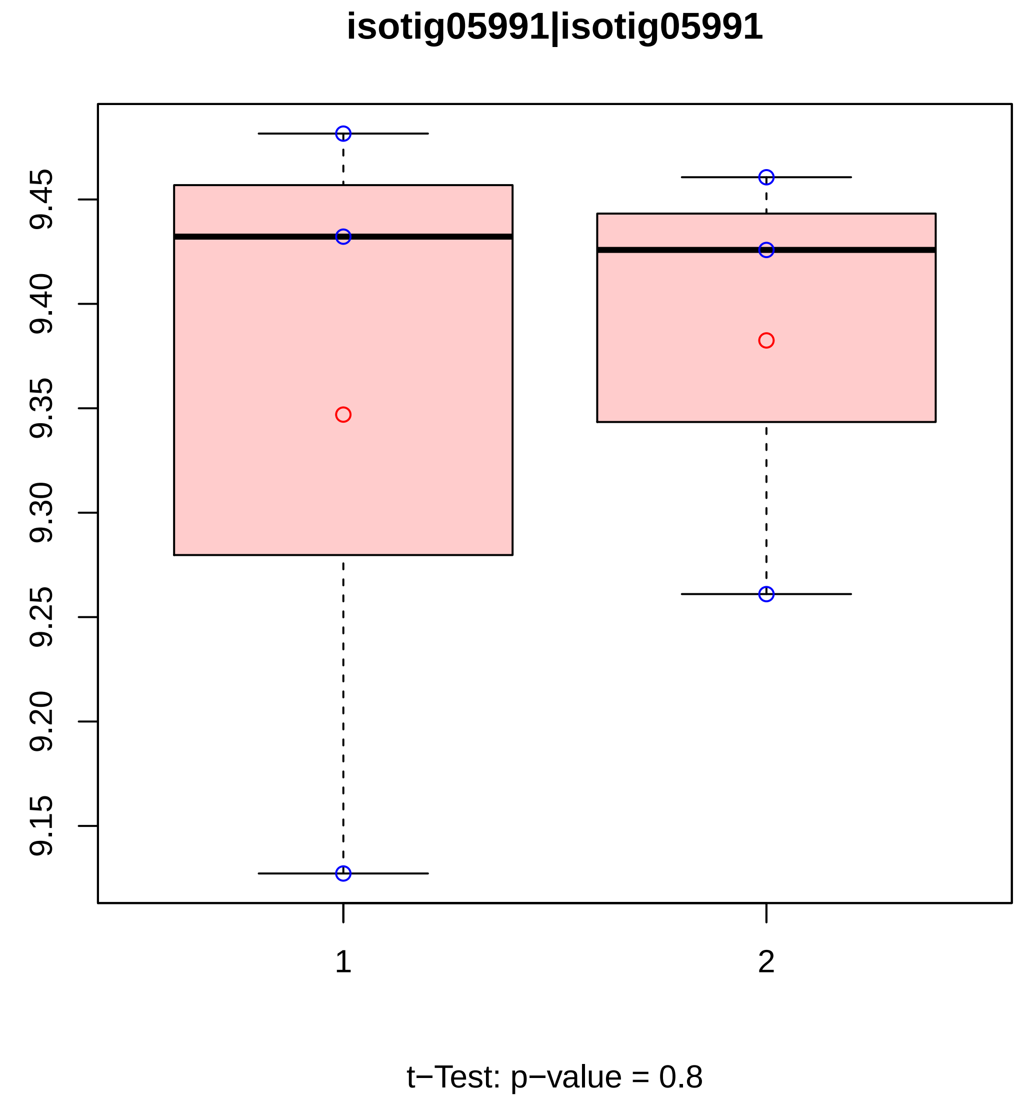

isotig05991|isotig05991
9.15
9.20
9.25
9.30
9.35
9.40
9.45
1
2
t−Test: p−value = 0.8

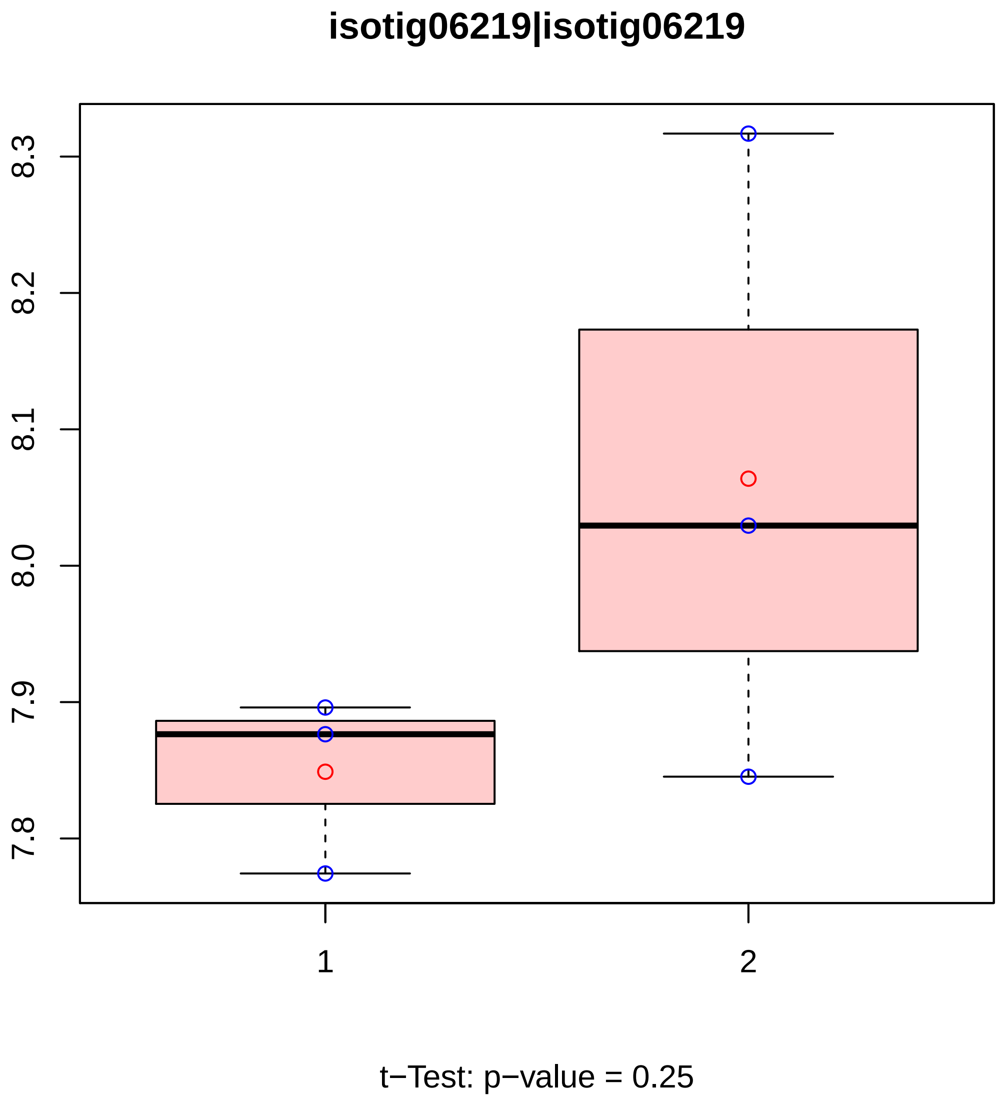
isotig06219|isotig06219
8.3
8.2
8.1
8.0
7.9
7.8
1
2
t−Test: p−value = 0.25

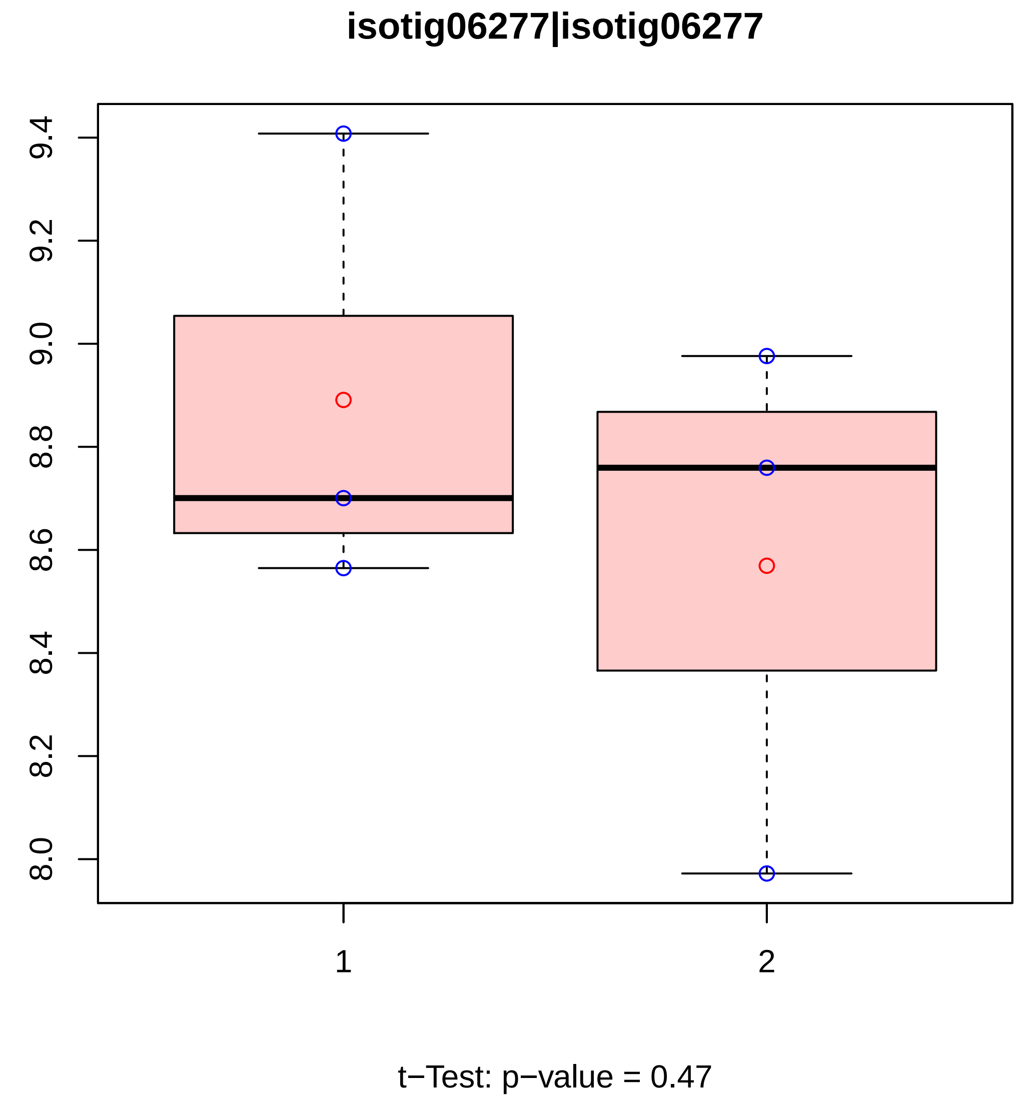
isotig06277|isotig06277
9.4
9.2
9.0
8.8
8.6
8.4
8.2
8.0
1
2
t−Test: p−value = 0.47

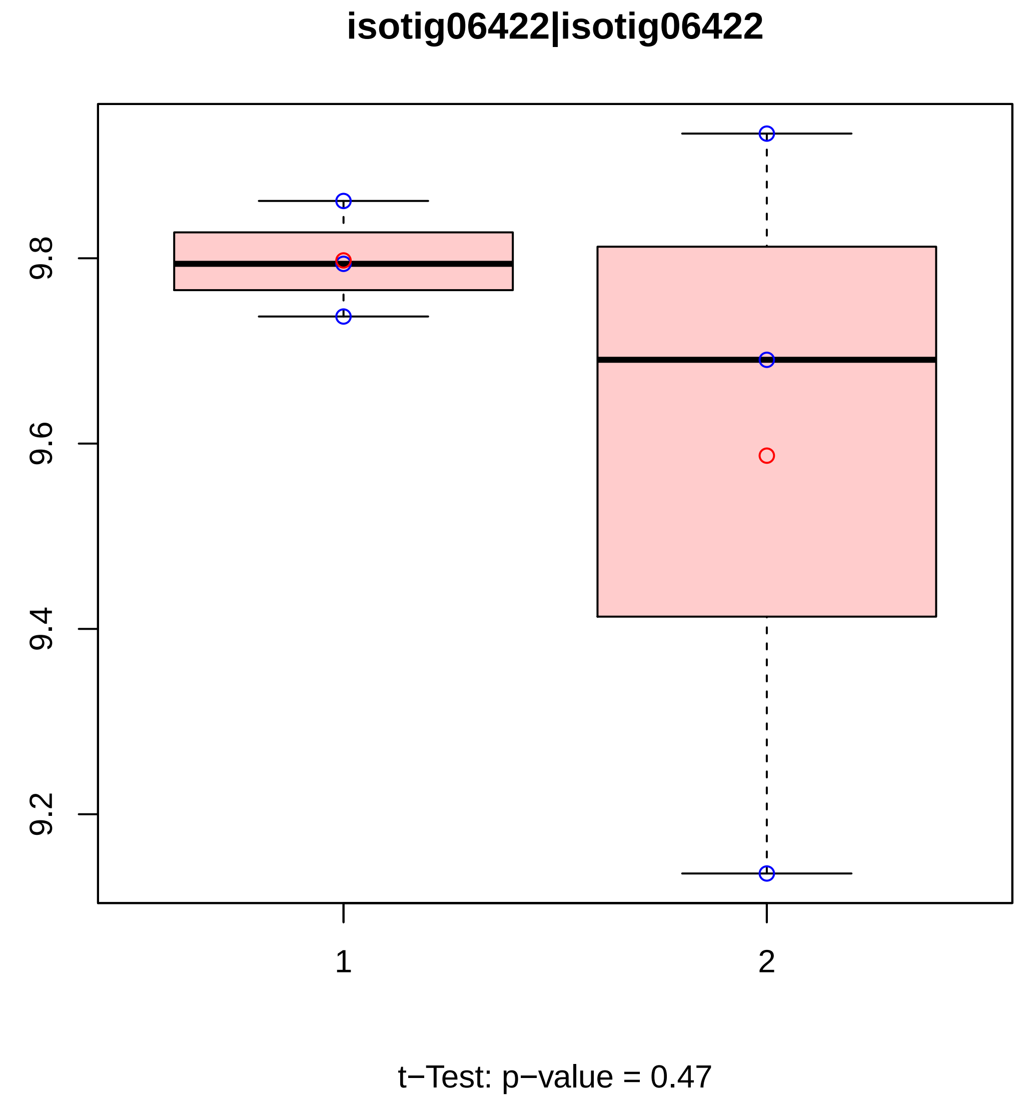
isotig06422|isotig06422
9.2
9.4
9.6
9.8
1
2
t−Test: p−value = 0.47

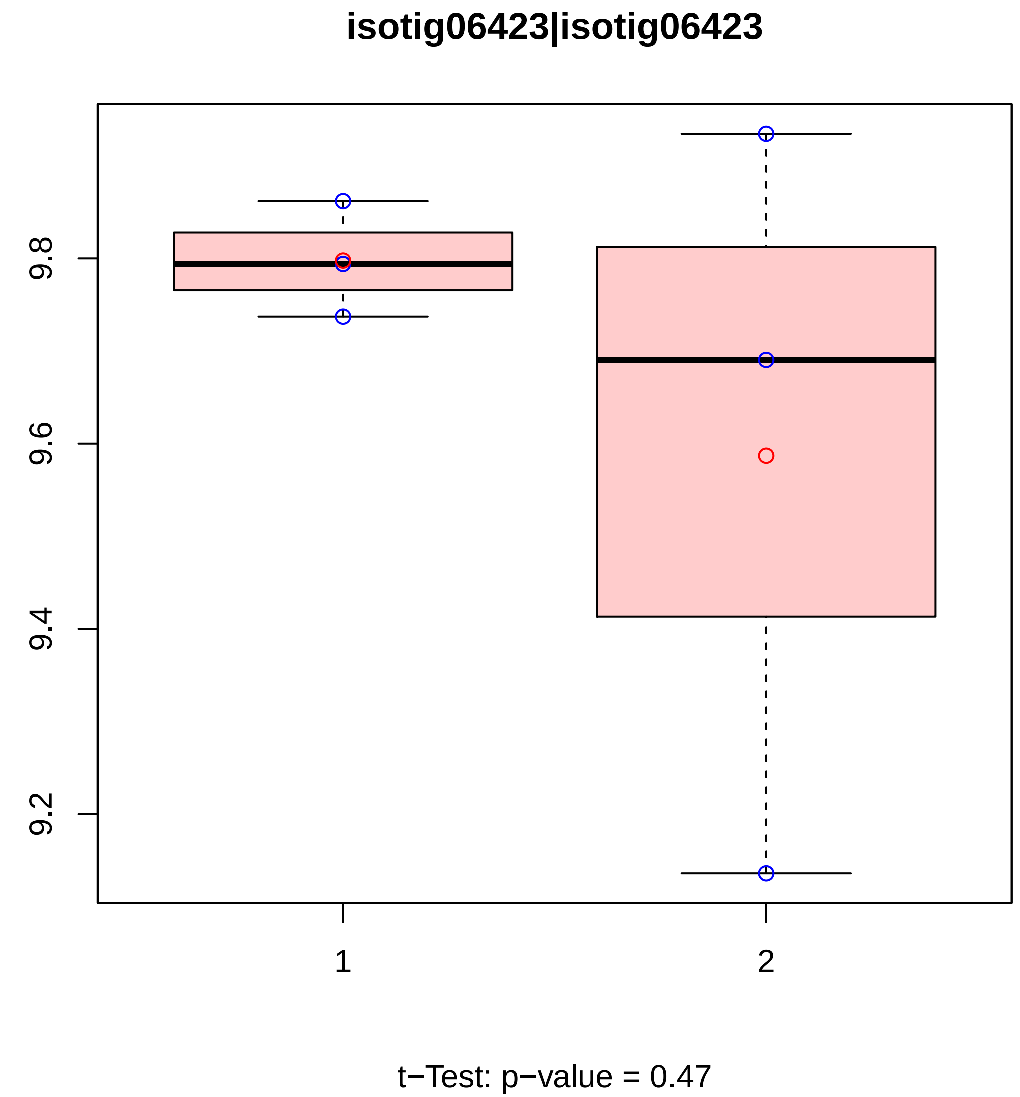
isotig06423|isotig06423
9.2
9.4
9.6
9.8
1
2
t−Test: p−value = 0.47

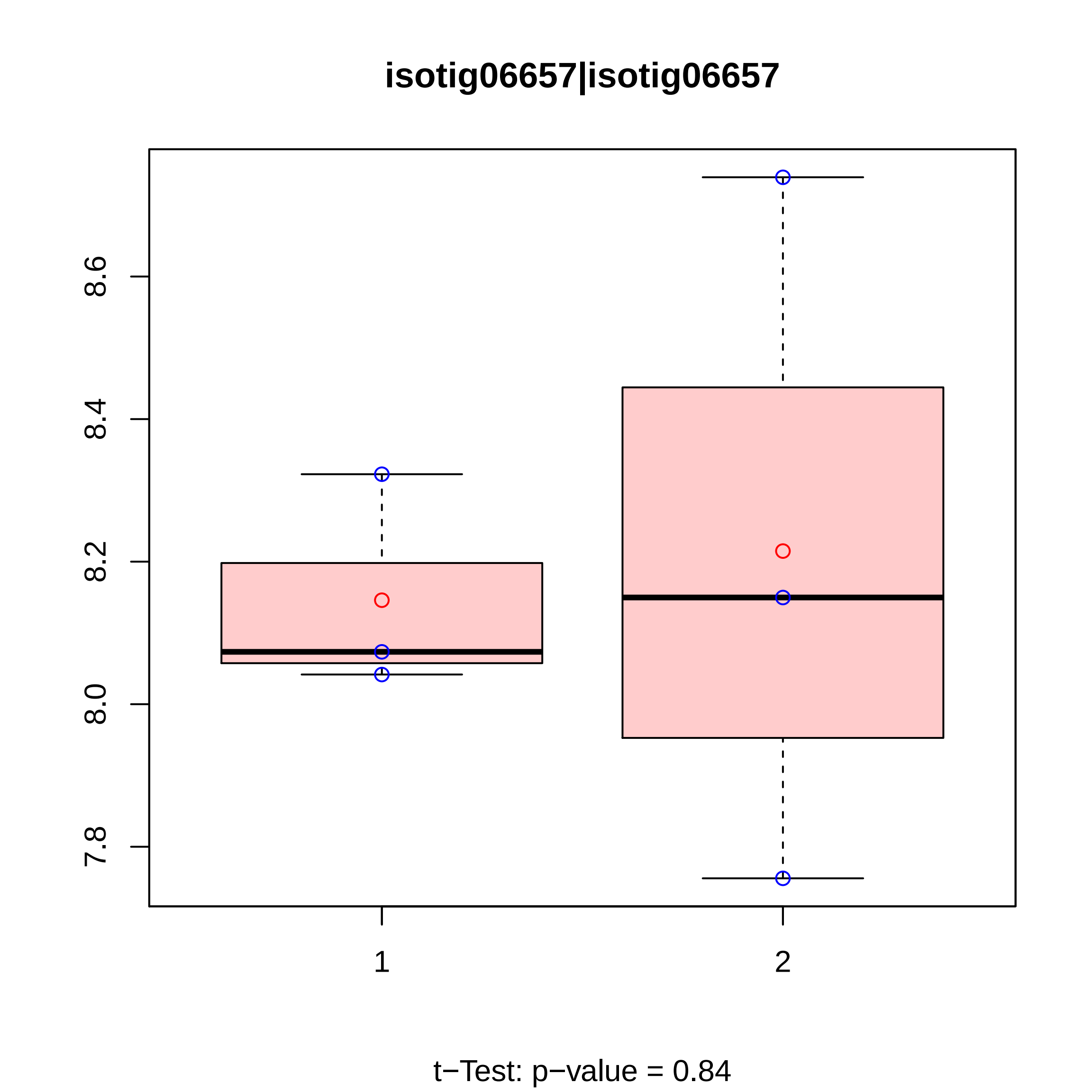
isotig06657|isotig06657
8.6
8.4
8.2
8.0
7.8
1
2
t−Test: p−value = 0.84

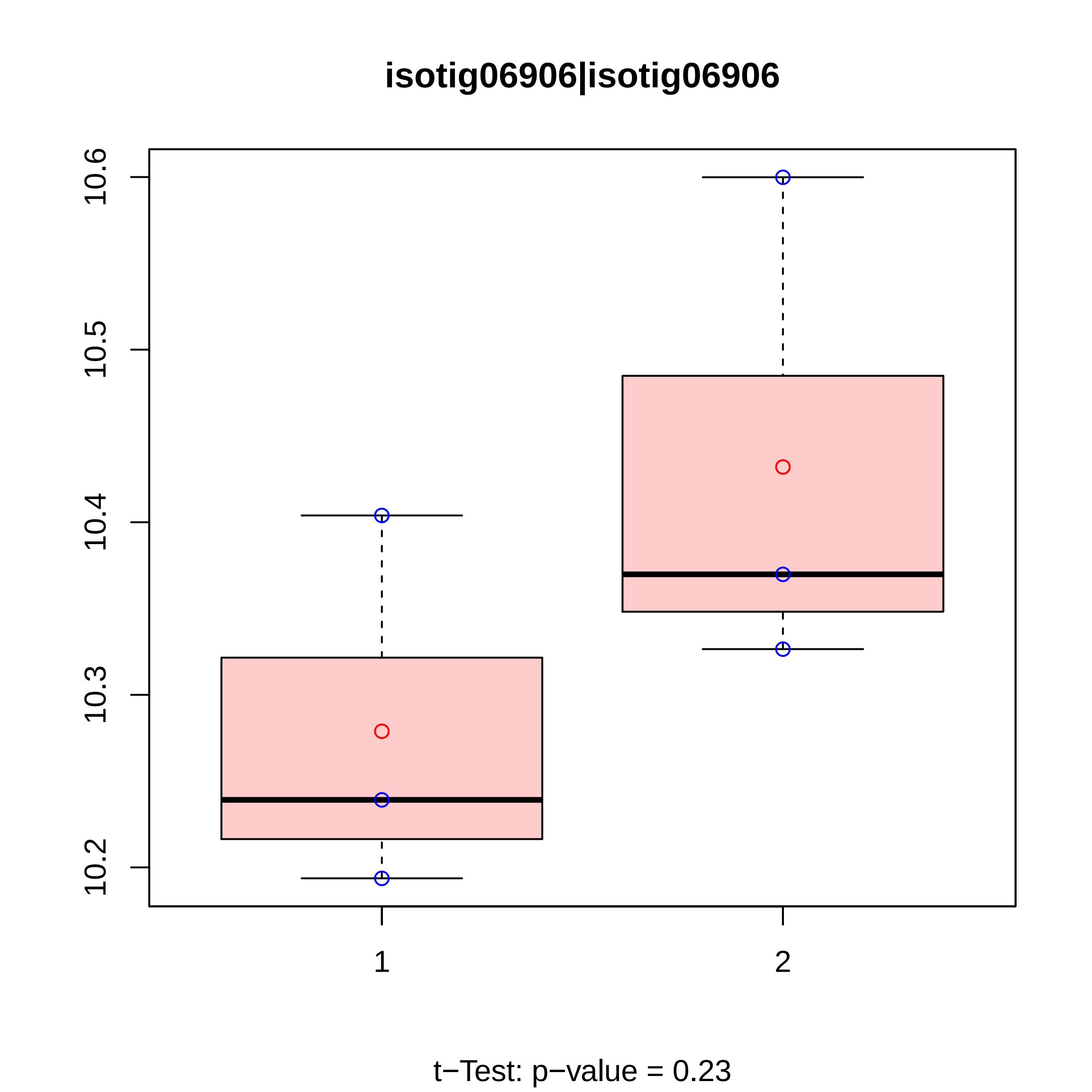
isotig06906|isotig06906
10.6
10.5
10.4
10.3
10.2
1
2
t−Test: p−value = 0.23

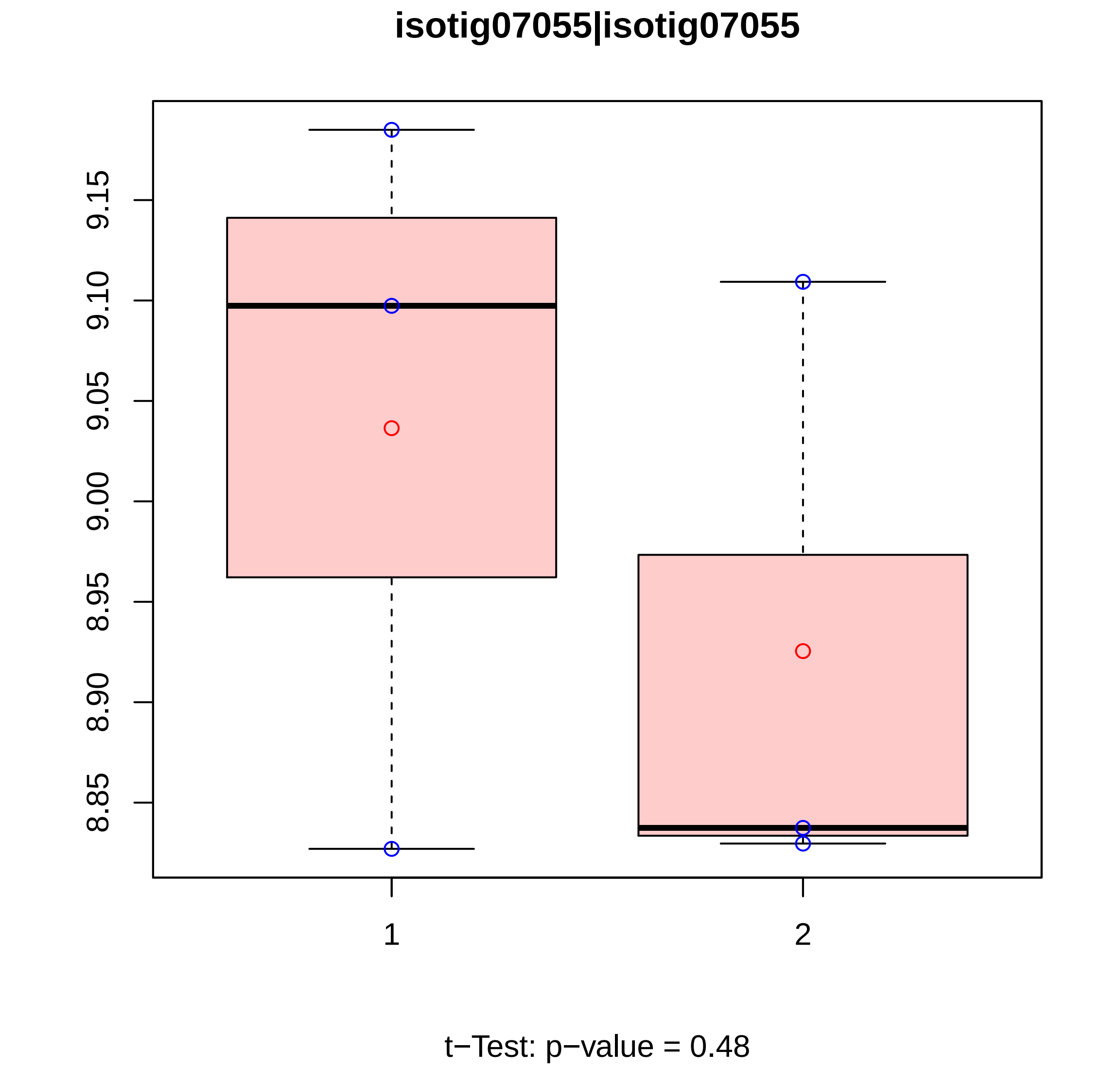
isotig07055|isotig07055
9.15
9.10
9.05
9.00
8.95
8.90
8.85
1
2
t−Test: p−value = 0.48

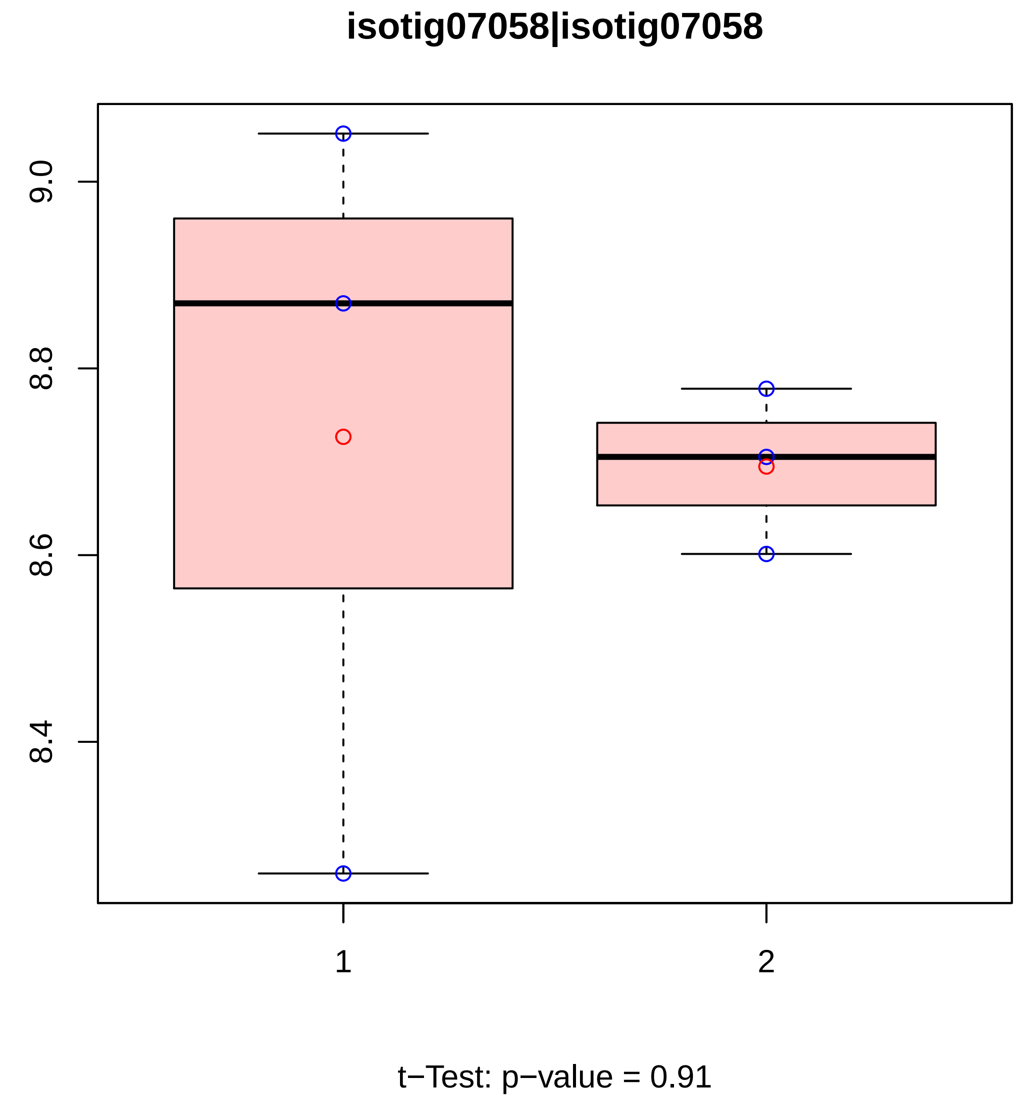
isotig07058|isotig07058
9.0
8.8
8.6
8.4
1
2
t−Test: p−value = 0.91

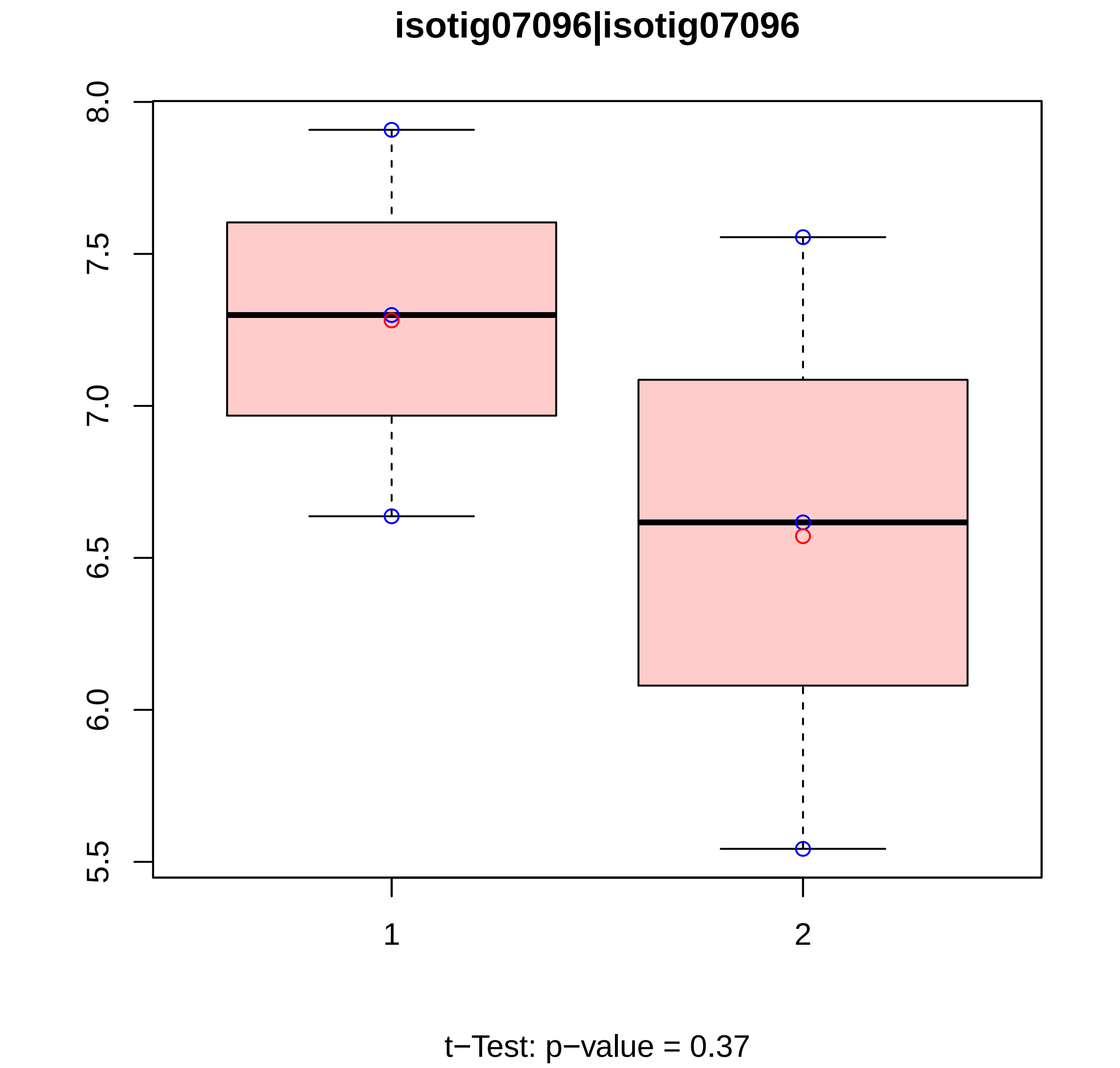
isotig07096|isotig07096
8.0
7.5
7.0
6.5
6.0
5.5
1
2
t−Test: p−value = 0.37

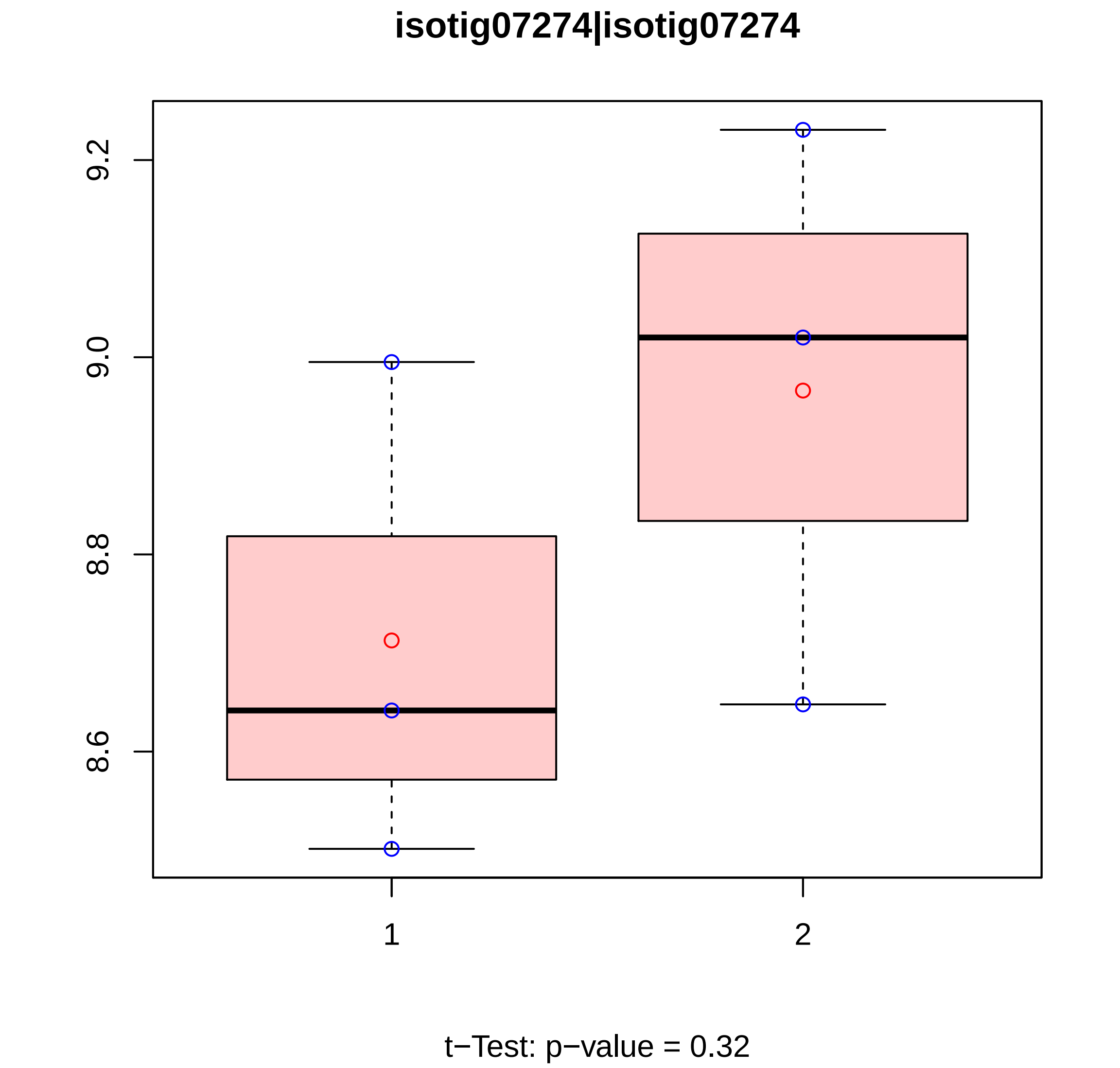

isotig07274|isotig07274
9.2
9.0
8.8
8.6
1
2
t−Test: p−value = 0.32

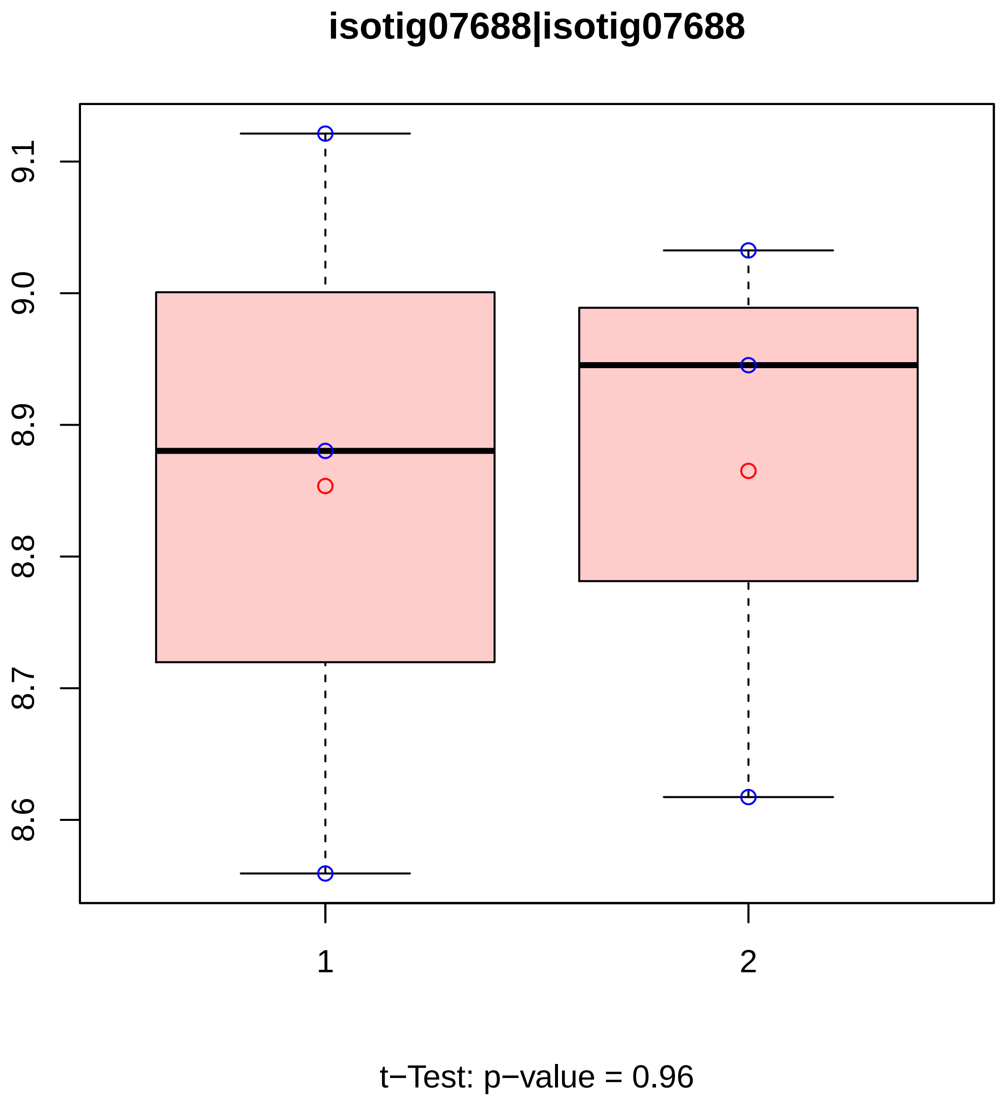
isotig07688|isotig07688
9.1
9.0
8.9
8.8
8.7
8.6
1
2
t−Test: p−value = 0.96

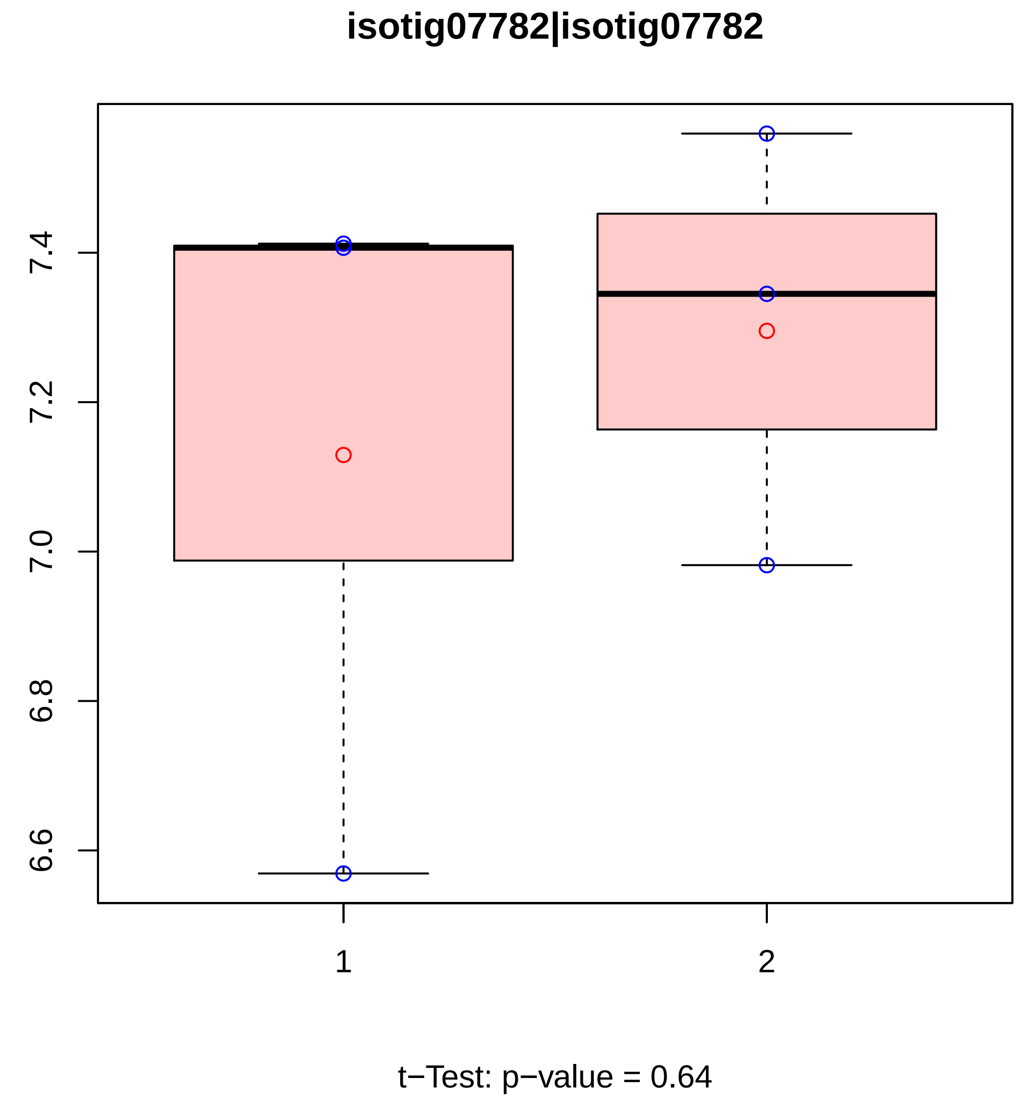
isotig07782|isotig07782
7.4
7.2
7.0
6.8
6.6
1
2
t−Test: p−value = 0.64

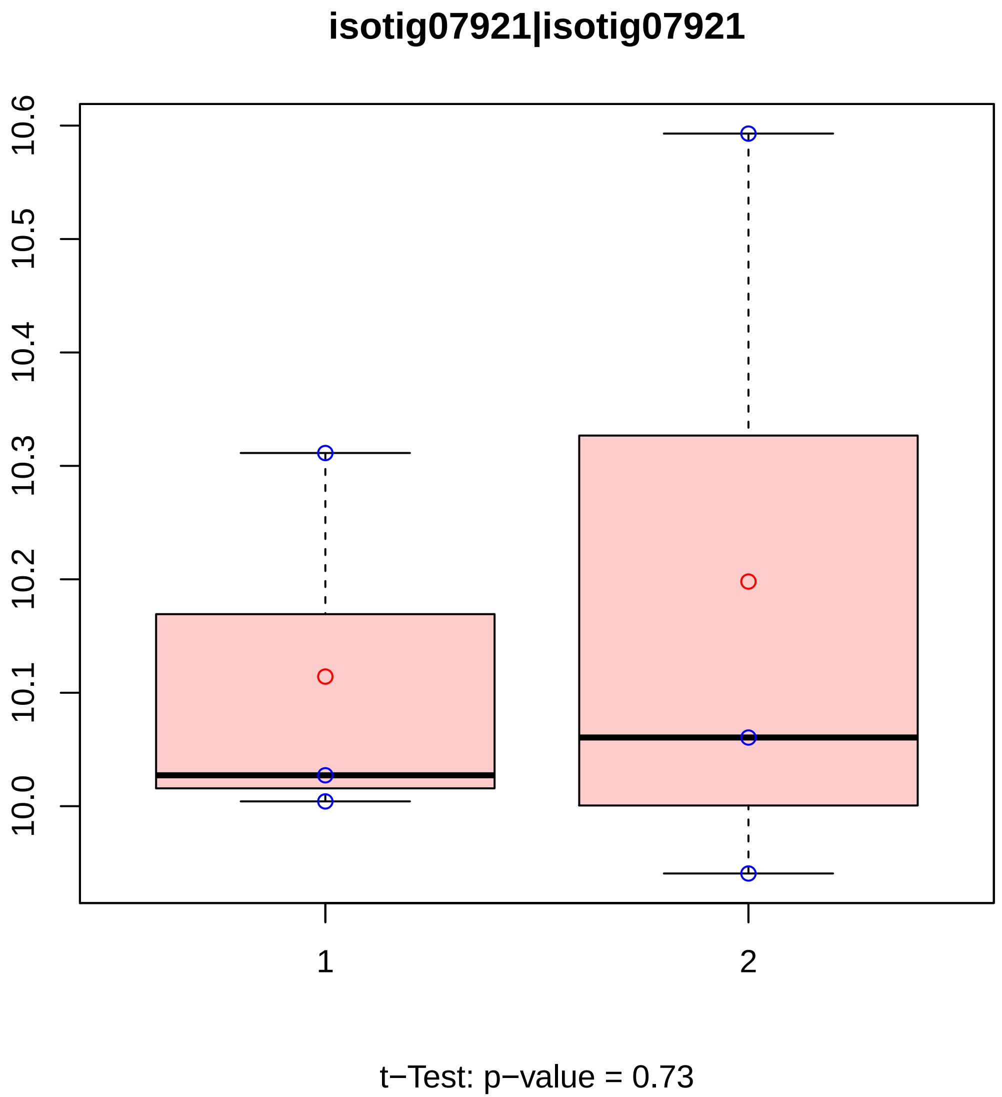

isotig07921|isotig07921
10.0
10.1
10.2
10.3
10.4
10.5
10.6
1
2
t−Test: p−value = 0.73

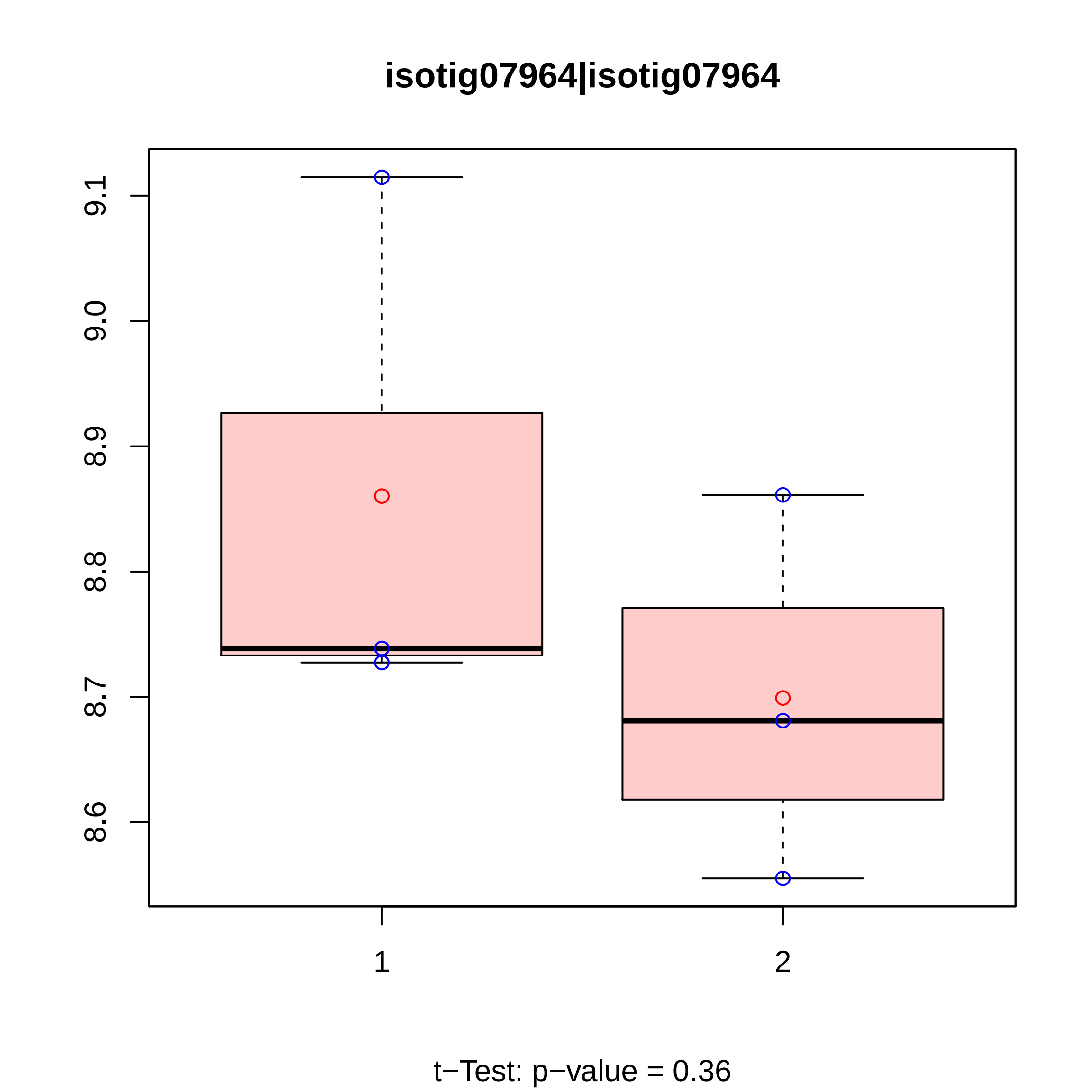
isotig07964|isotig07964
9.1
9.0
8.9
8.8
8.7
8.6
1
2
t−Test: p−value = 0.36

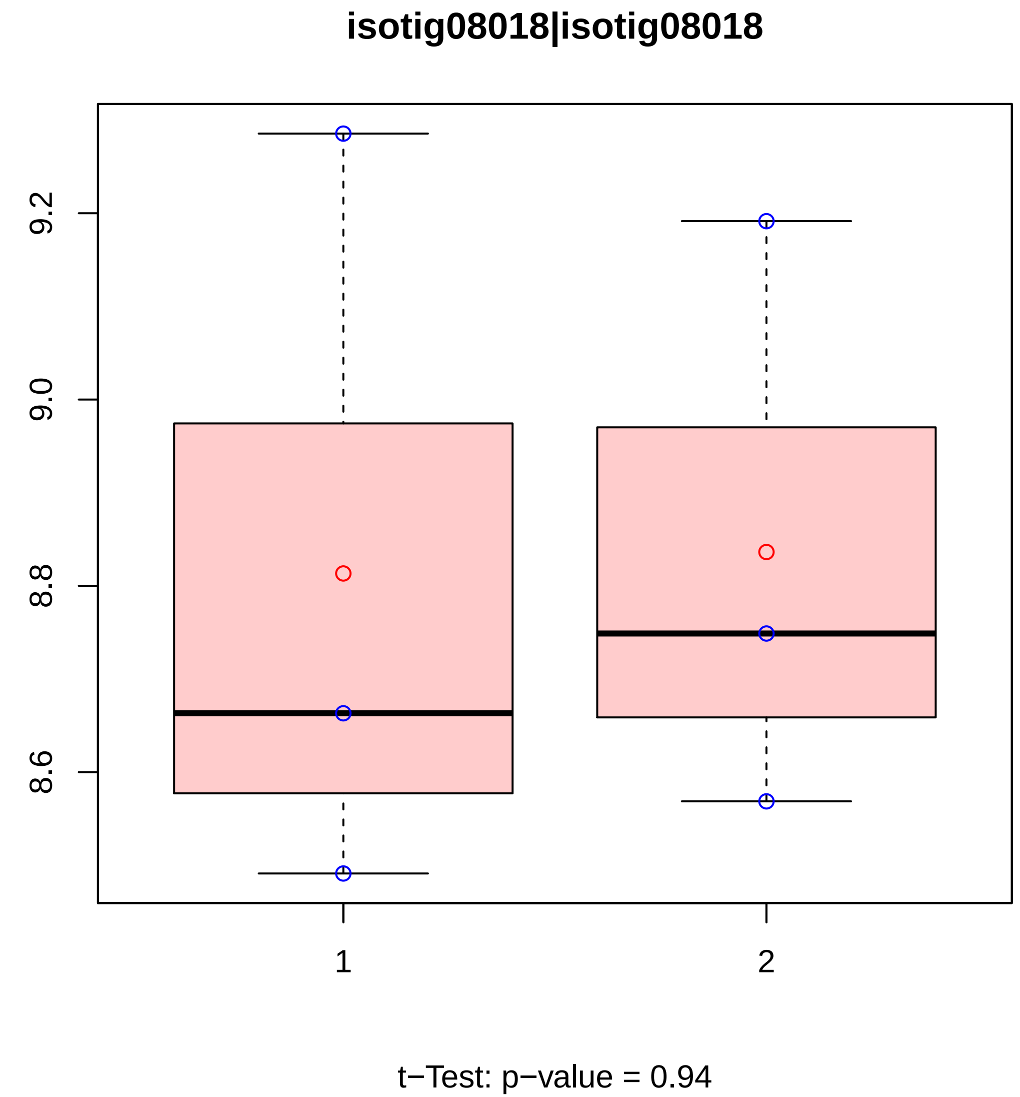

isotig08018|isotig08018
9.2
9.0
8.8
8.6
1
2
t−Test: p−value = 0.94

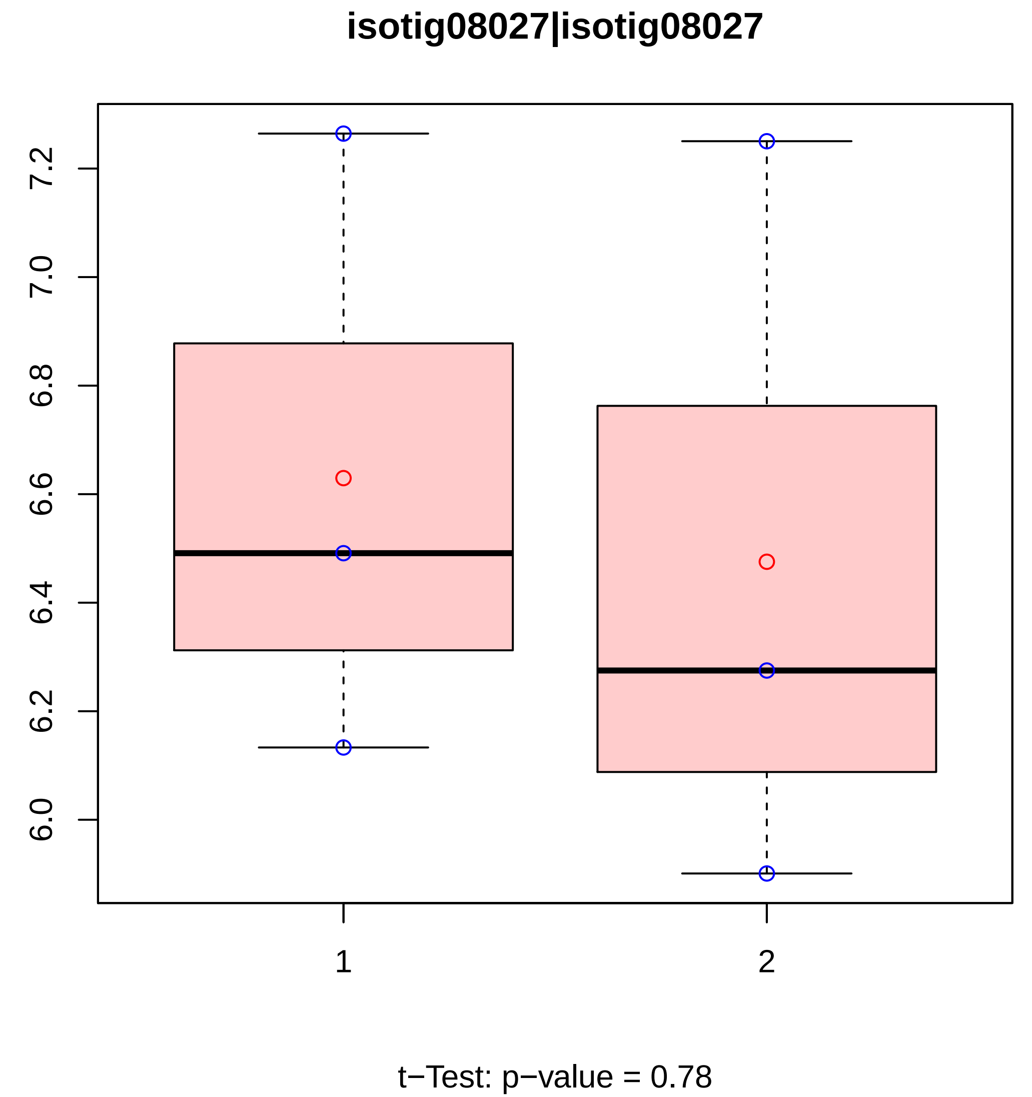
isotig08027|isotig08027
7.2
7.0
6.8
6.6
6.4
6.2
6.0
1
2
t−Test: p−value = 0.78

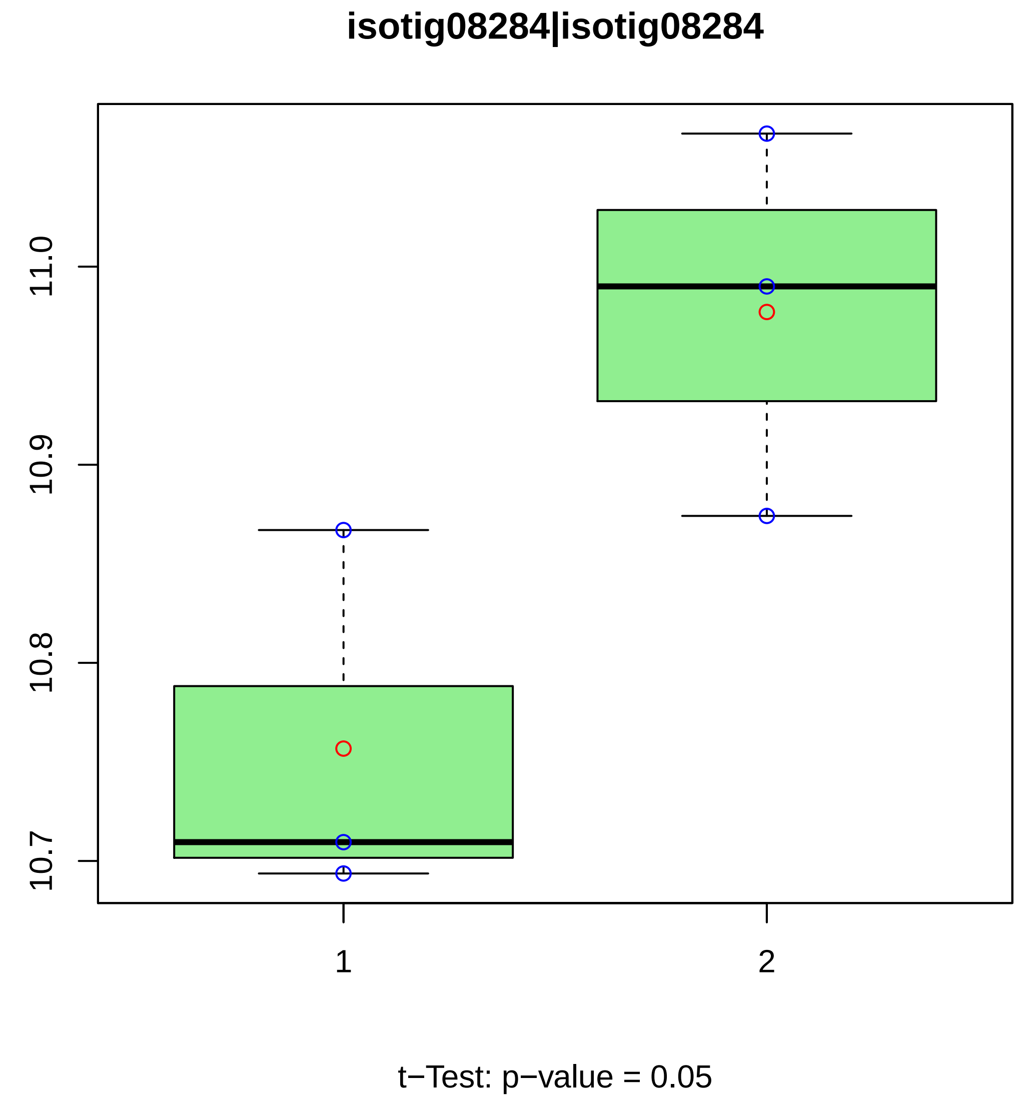
isotig08284|isotig08284
11.0
10.9
10.8
10.7
1
2
t−Test: p−value = 0.05

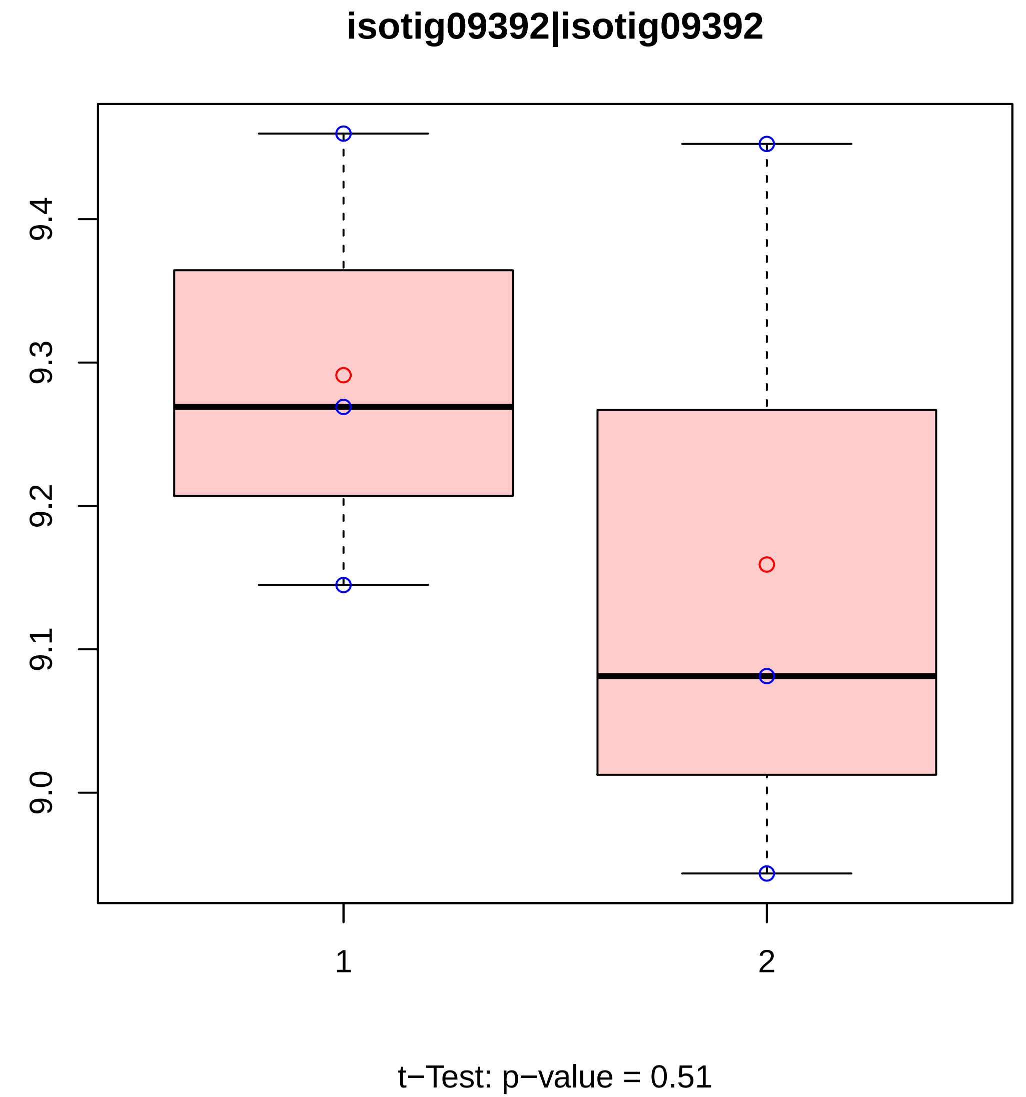
isotig09392|isotig09392
9.0
9.1
9.2
9.3
9.4
1
2
t−Test: p−value = 0.51

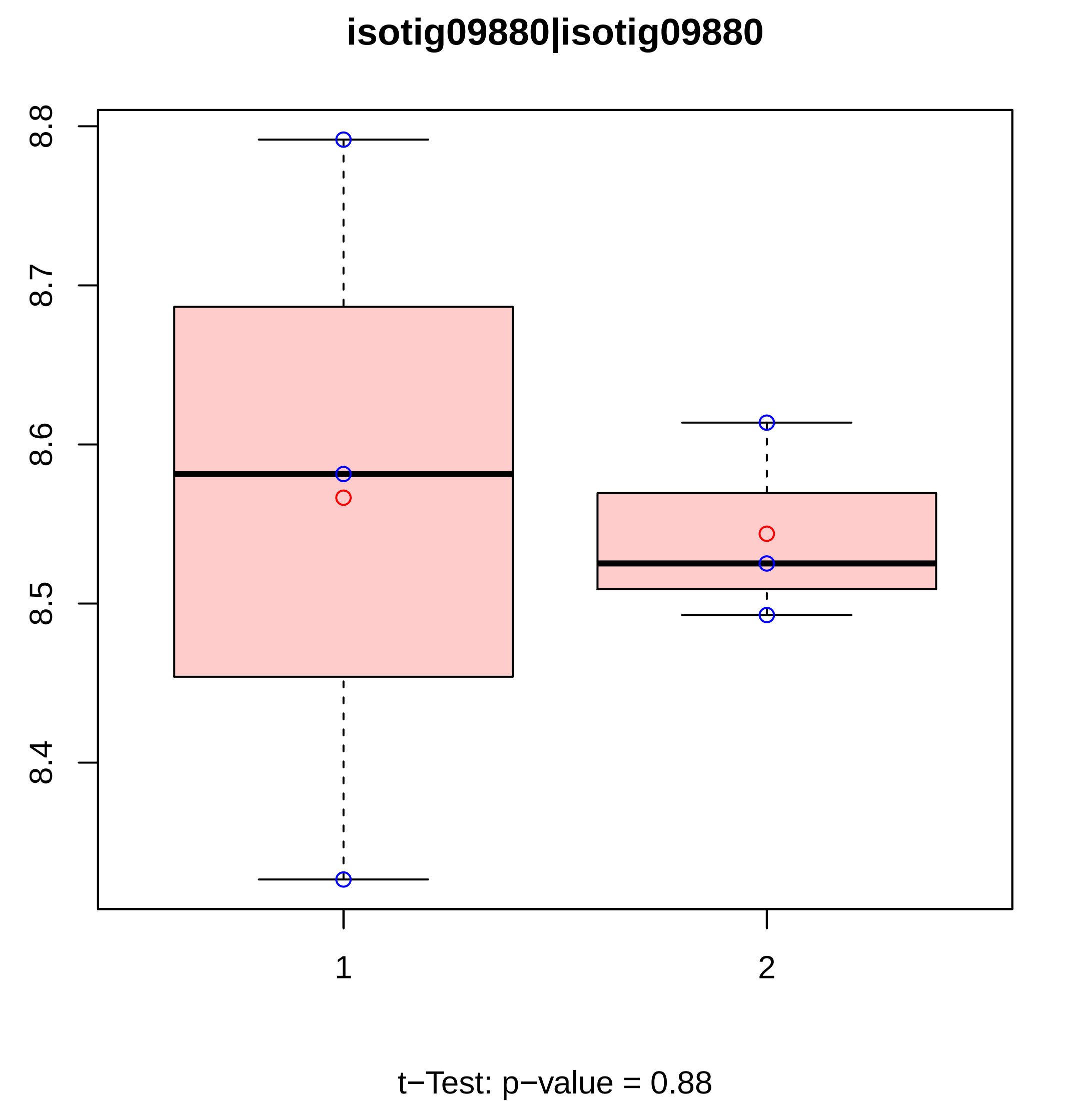
isotig09880|isotig09880
8.8
8.7
8.6
8.5
8.4
1
2
t−Test: p−value = 0.88

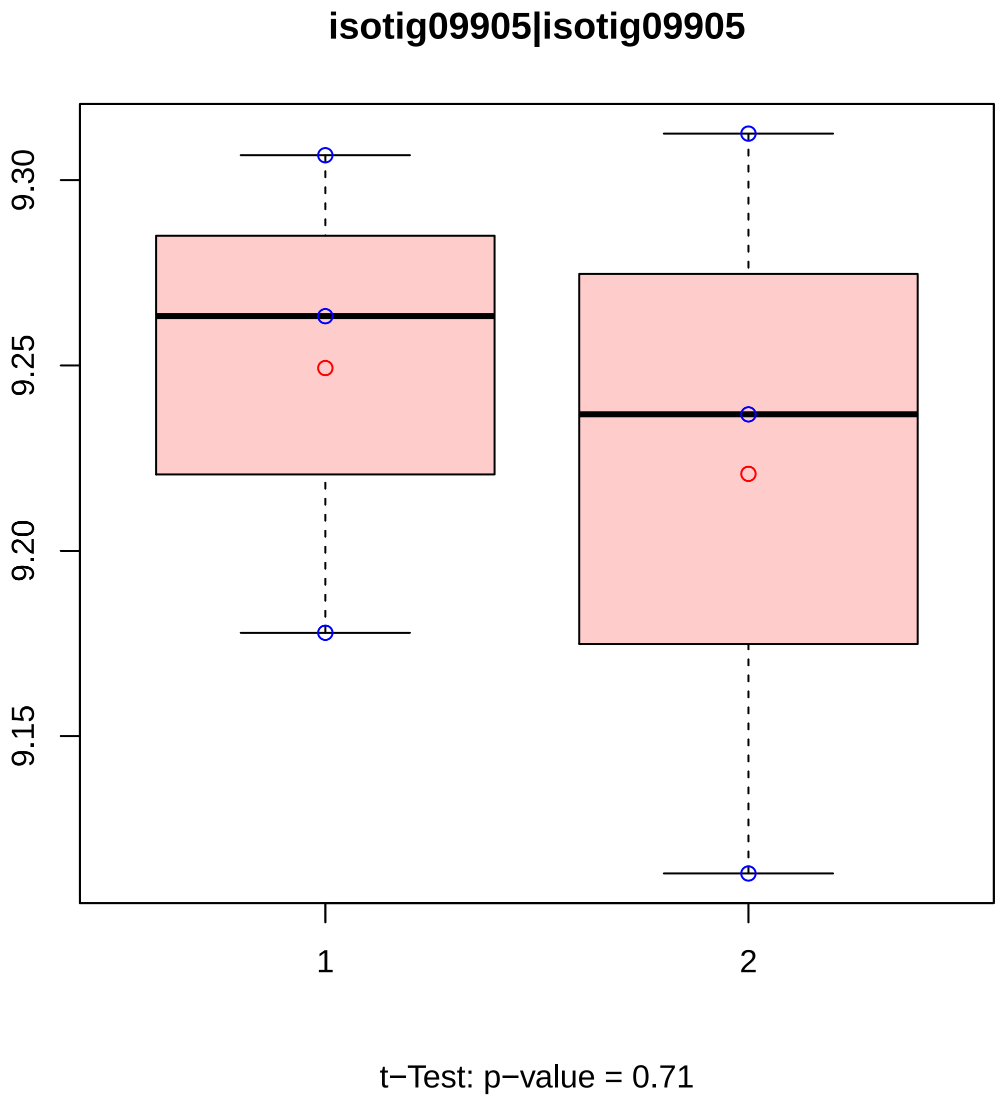
isotig09905|isotig09905
9.30
9.25
9.20
9.15
1
2
t−Test: p−value = 0.71

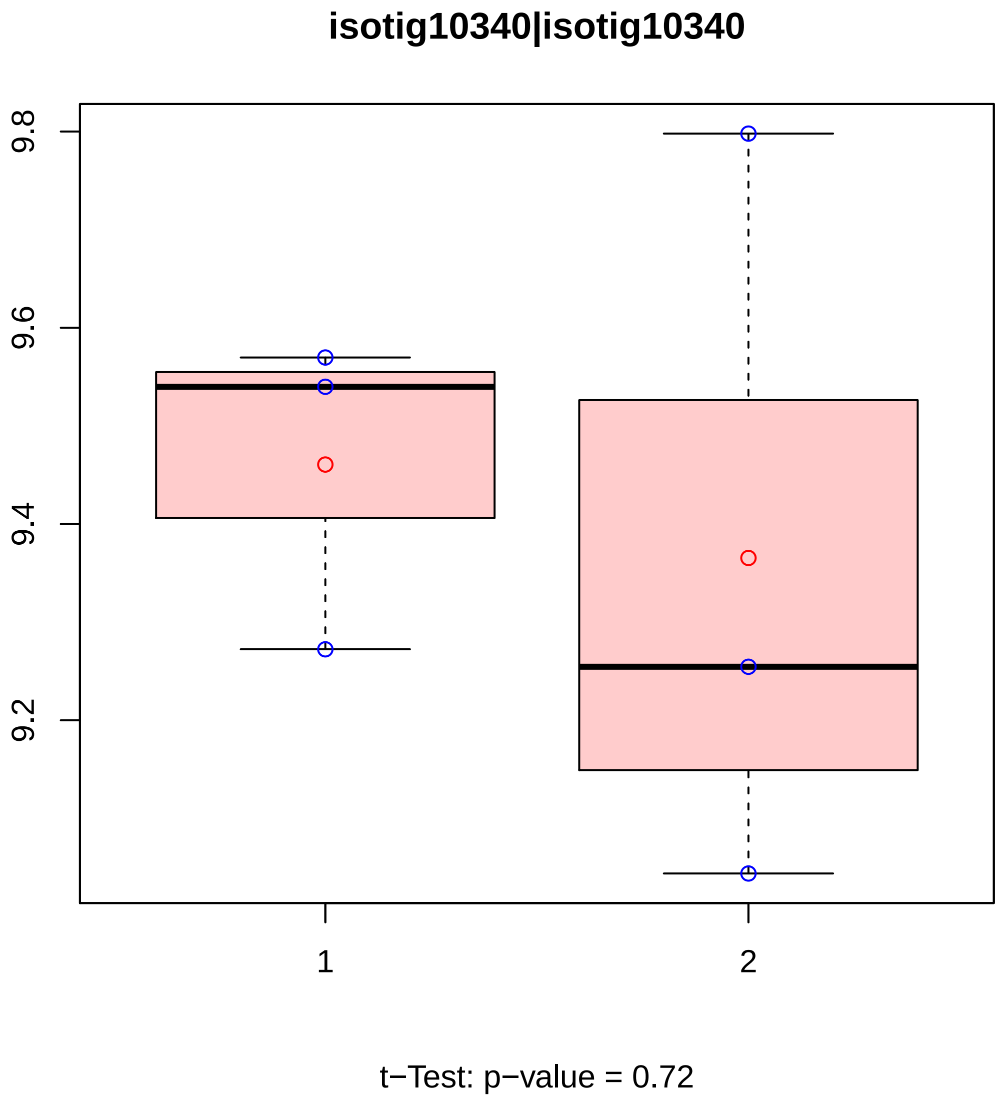
isotig10340|isotig10340
9.8
9.6
9.4
9.2
1
2
t−Test: p−value = 0.72

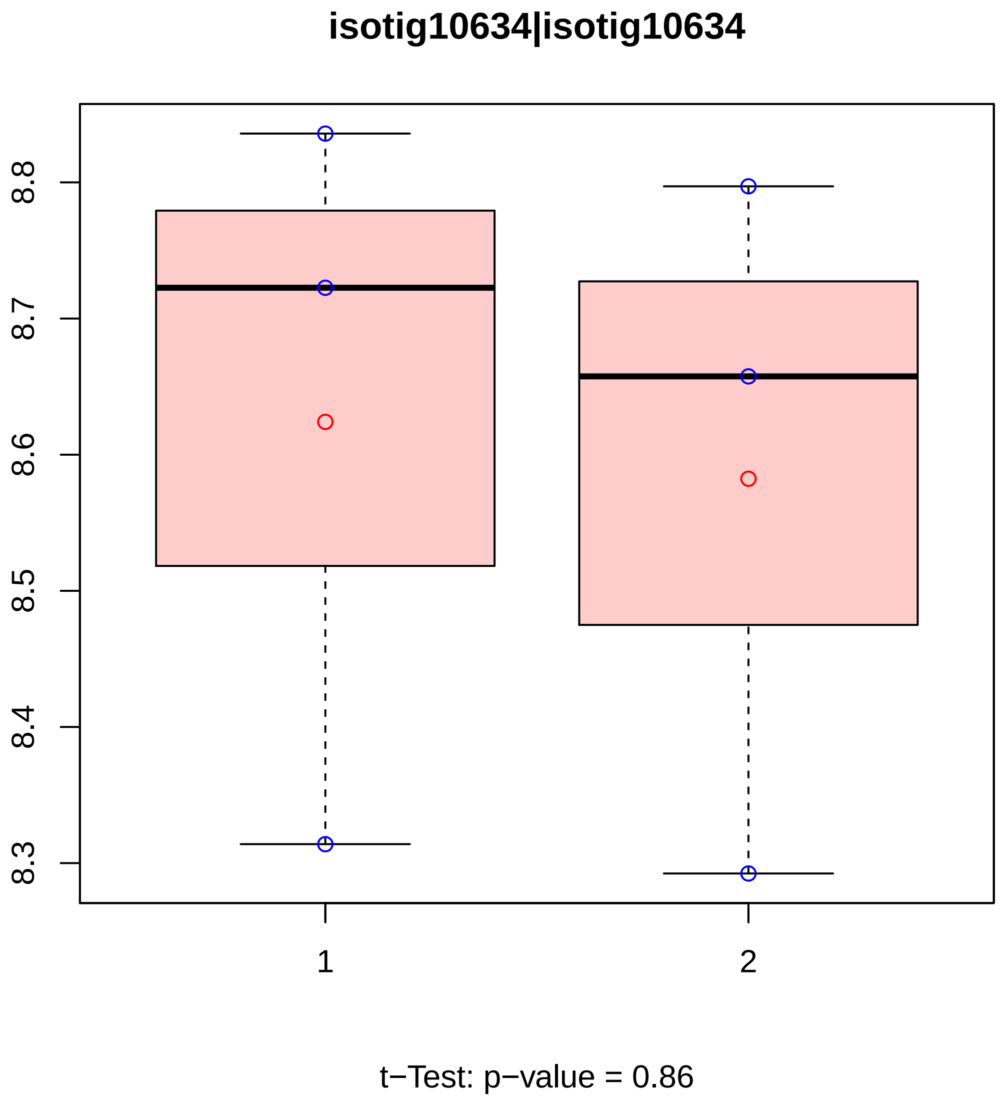
isotig10634|isotig10634
8.8
8.7
8.6
8.5
8.4
8.3
1
2
t−Test: p−value = 0.86

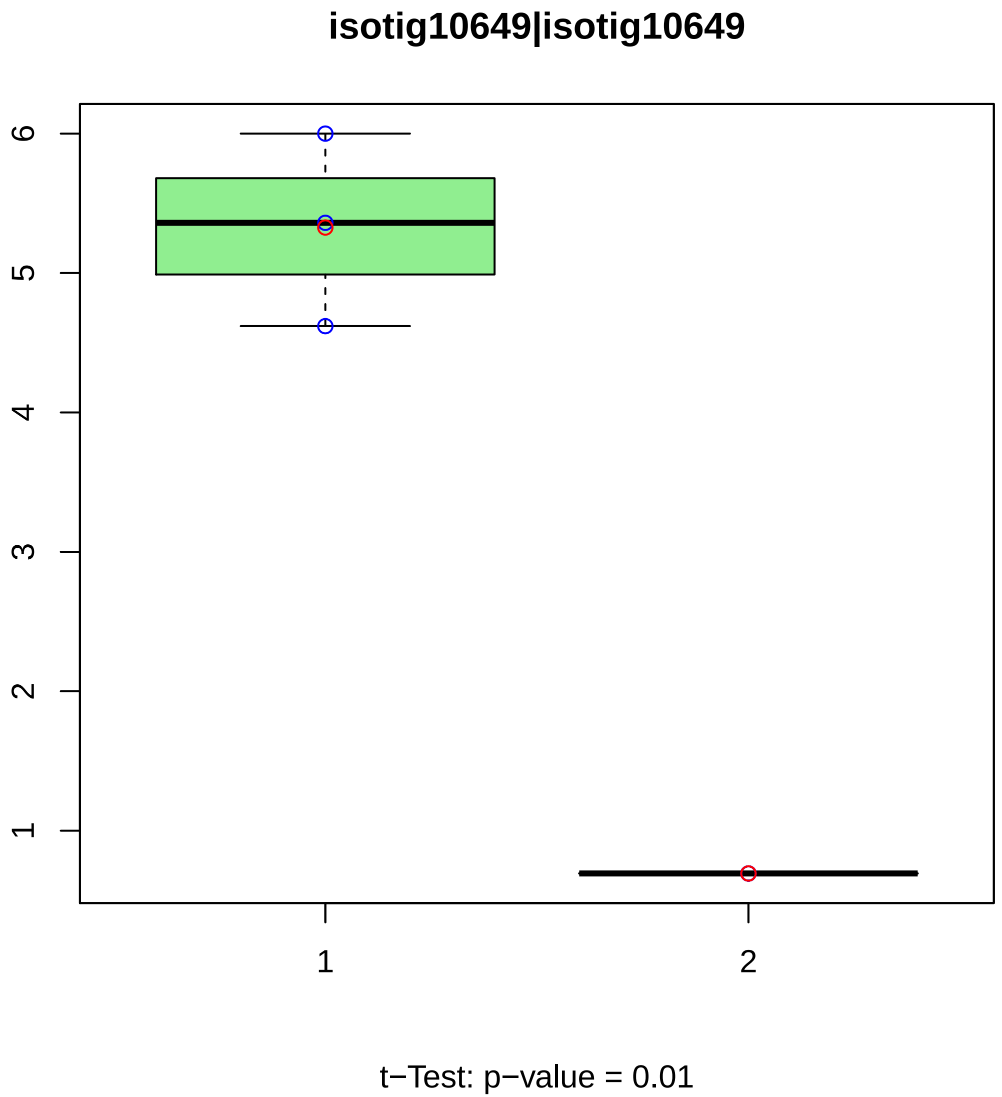

isotig10649|isotig10649
6
5
4
3
2
1
1
2
t−Test: p−value = 0.01

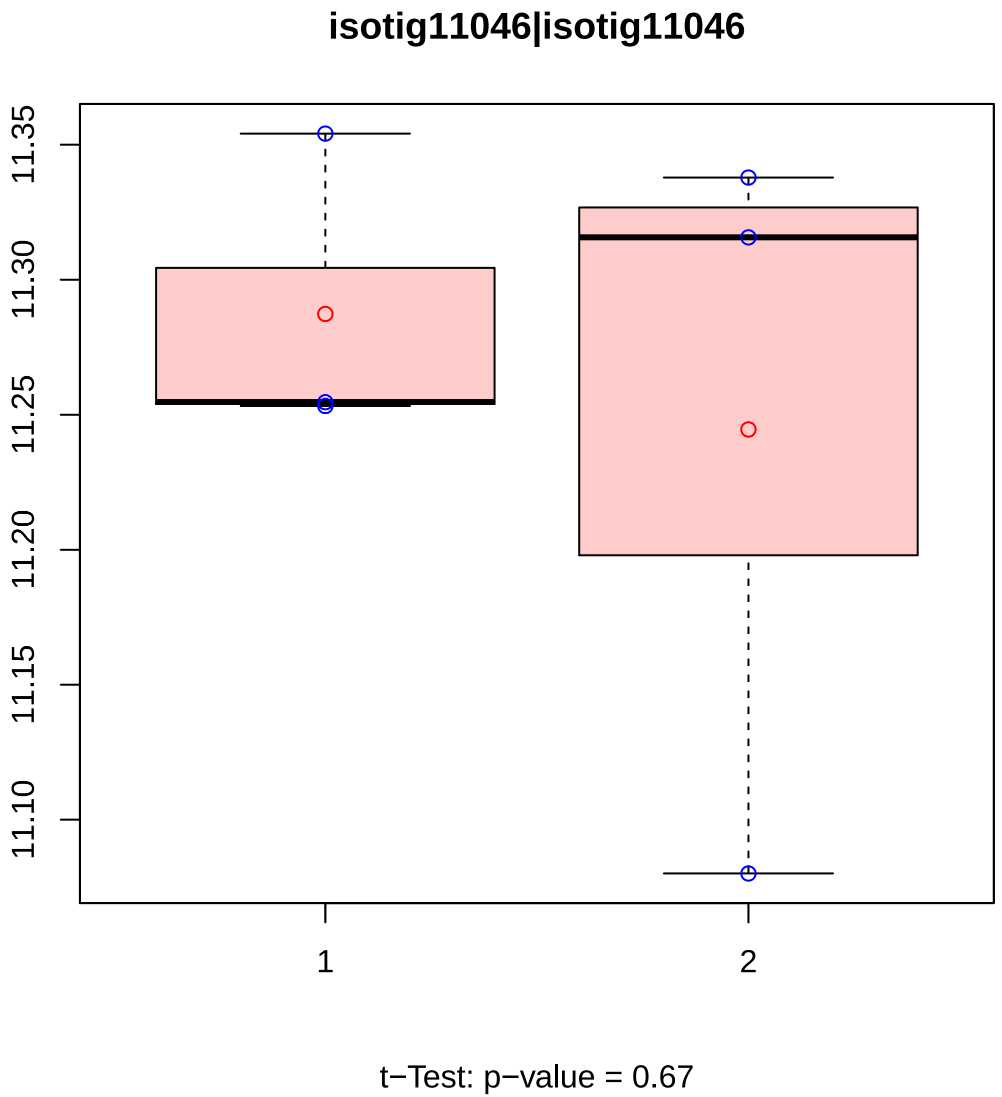
isotig11046|isotig11046
11.10
11.15
11.20
11.25
11.30
11.35
1
2
t−Test: p−value = 0.67

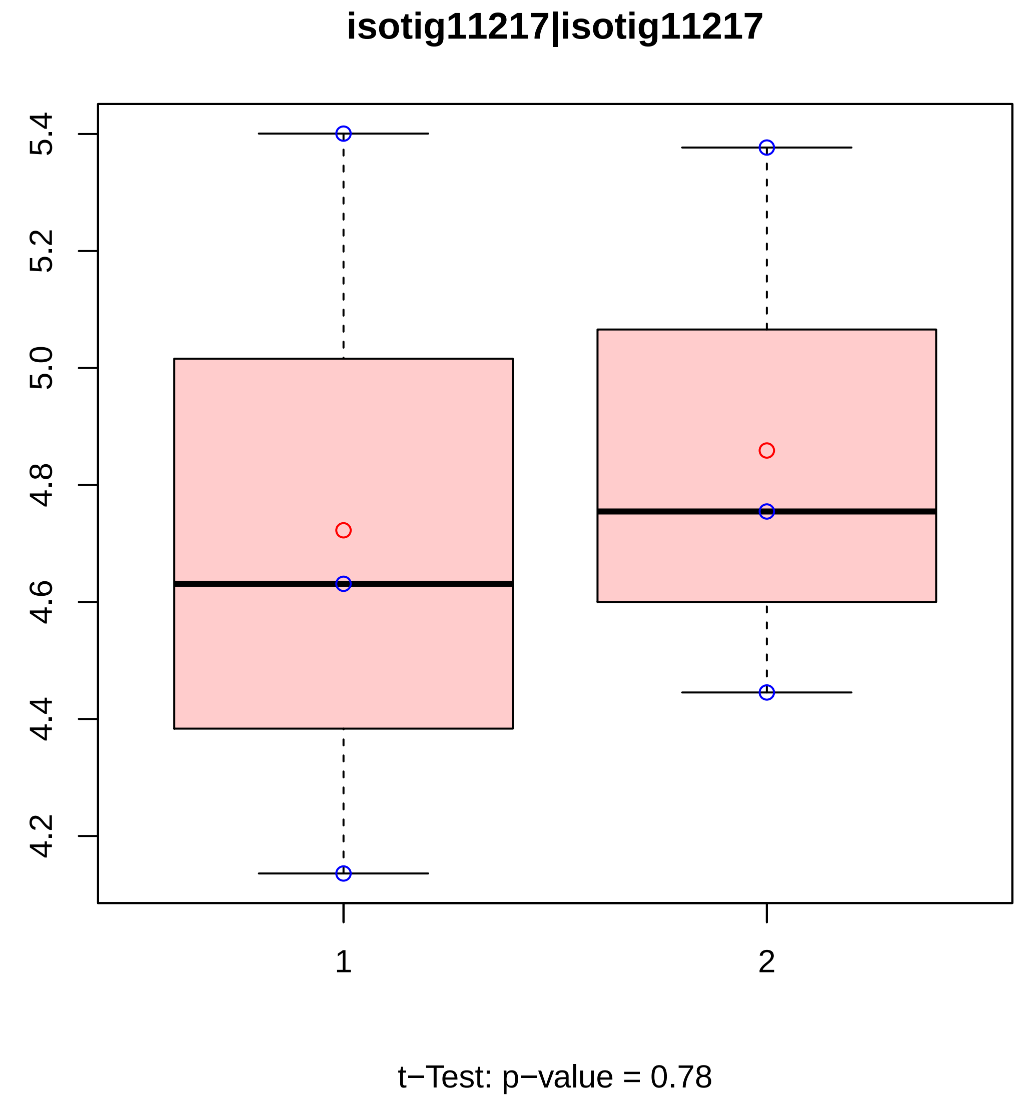
isotig11217|isotig11217
5.4
5.2
5.0
4.8
4.6
4.4
4.2
1
2
t−Test: p−value = 0.78

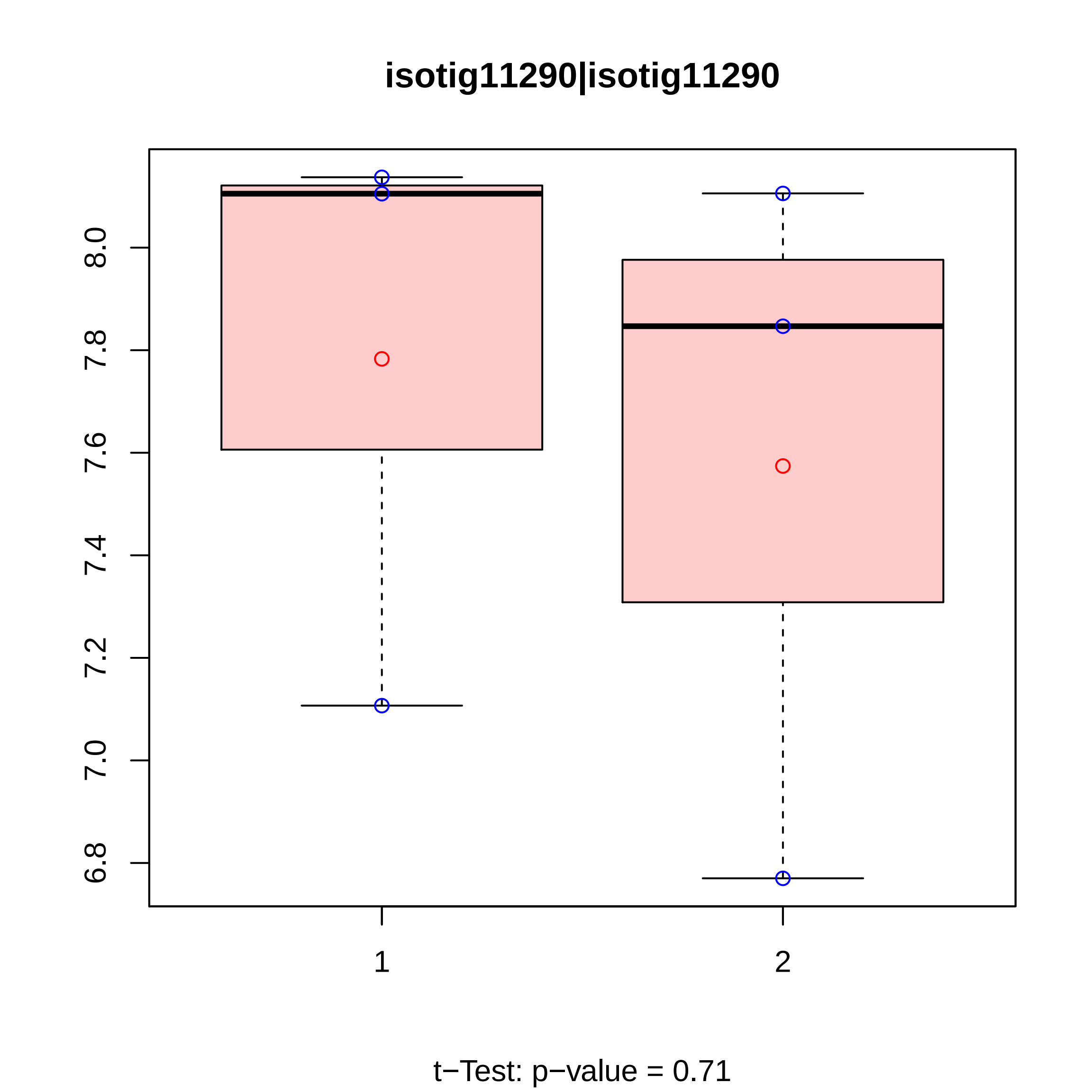
isotig11290|isotig11290
8.0
7.8
7.6
7.4
7.2
7.0
6.8
1
2
t−Test: p−value = 0.71

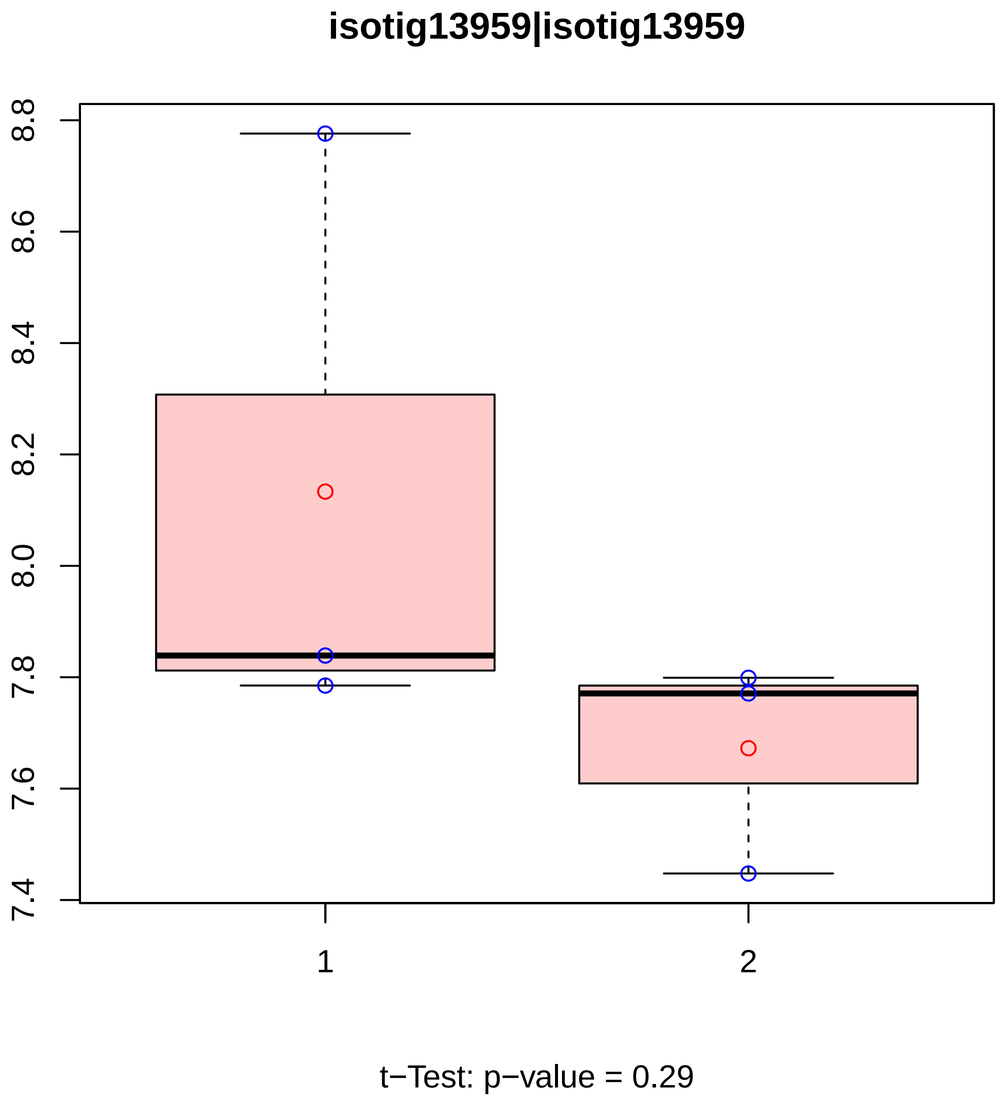
isotig13959|isotig13959
8.8
8.6
8.4
8.2
8.0
7.8
7.6
7.4
1
2
t−Test: p−value = 0.29

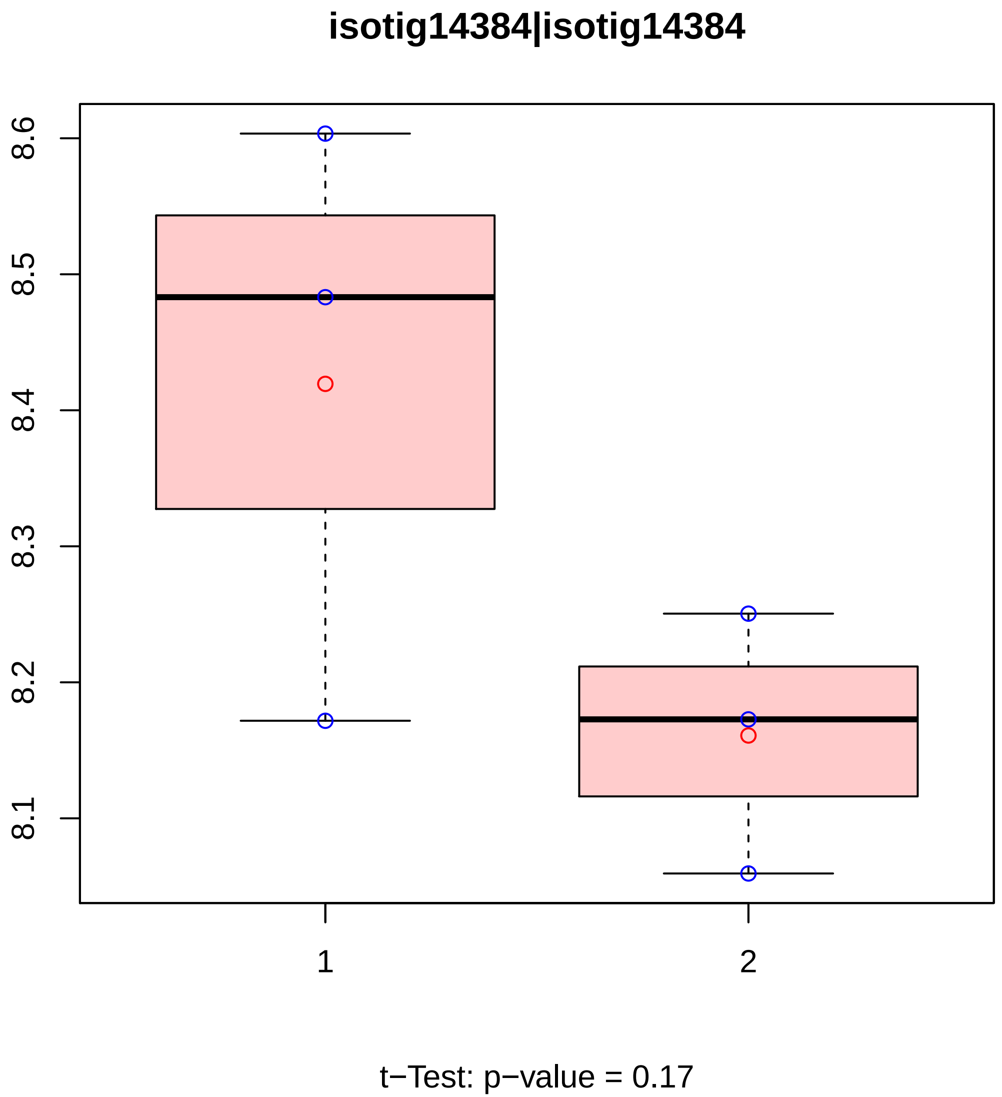
isotig14384|isotig14384
8.6
8.5
8.4
8.3
8.2
8.1
1
2
t−Test: p−value = 0.17

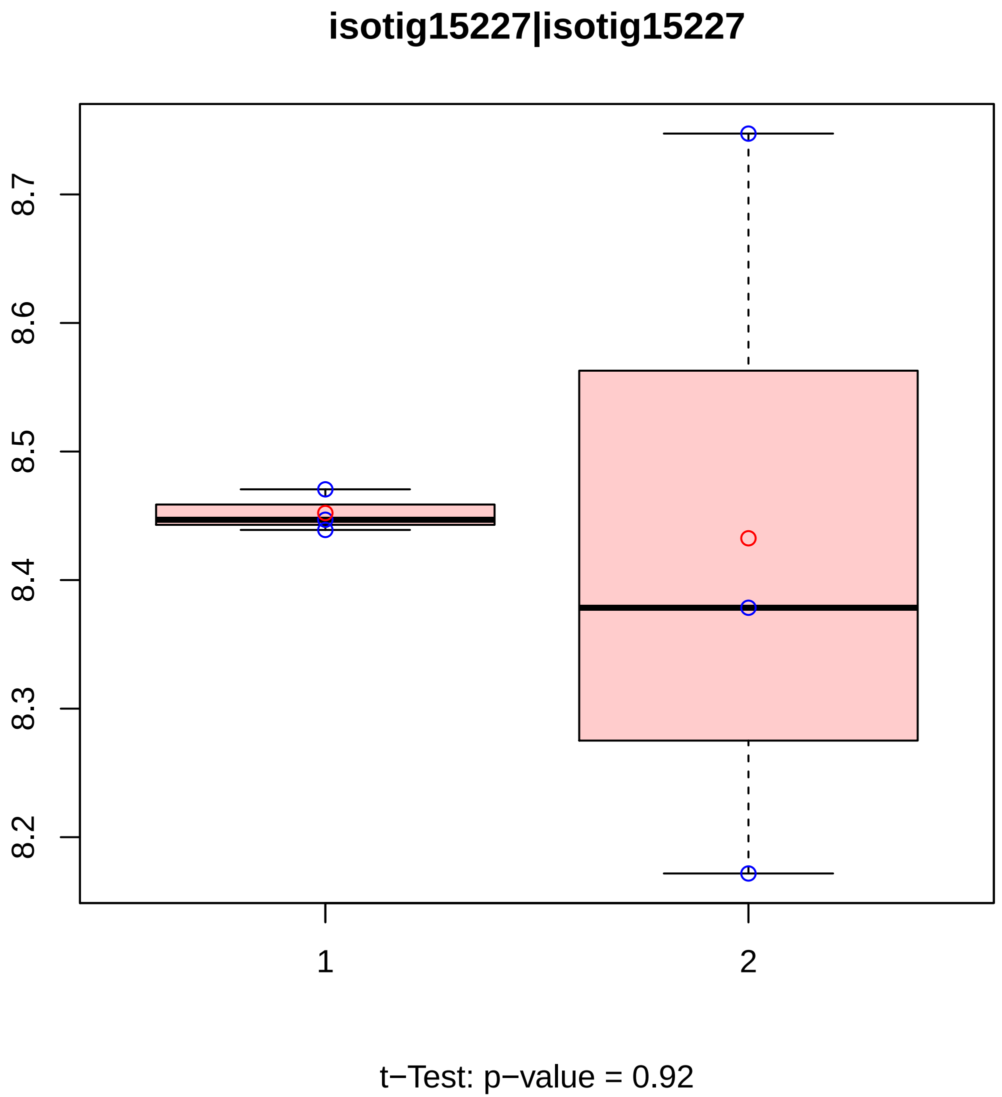

isotig15227|isotig15227
8.7
8.6
8.5
8.4
8.3
8.2
1
2
t−Test: p−value = 0.92

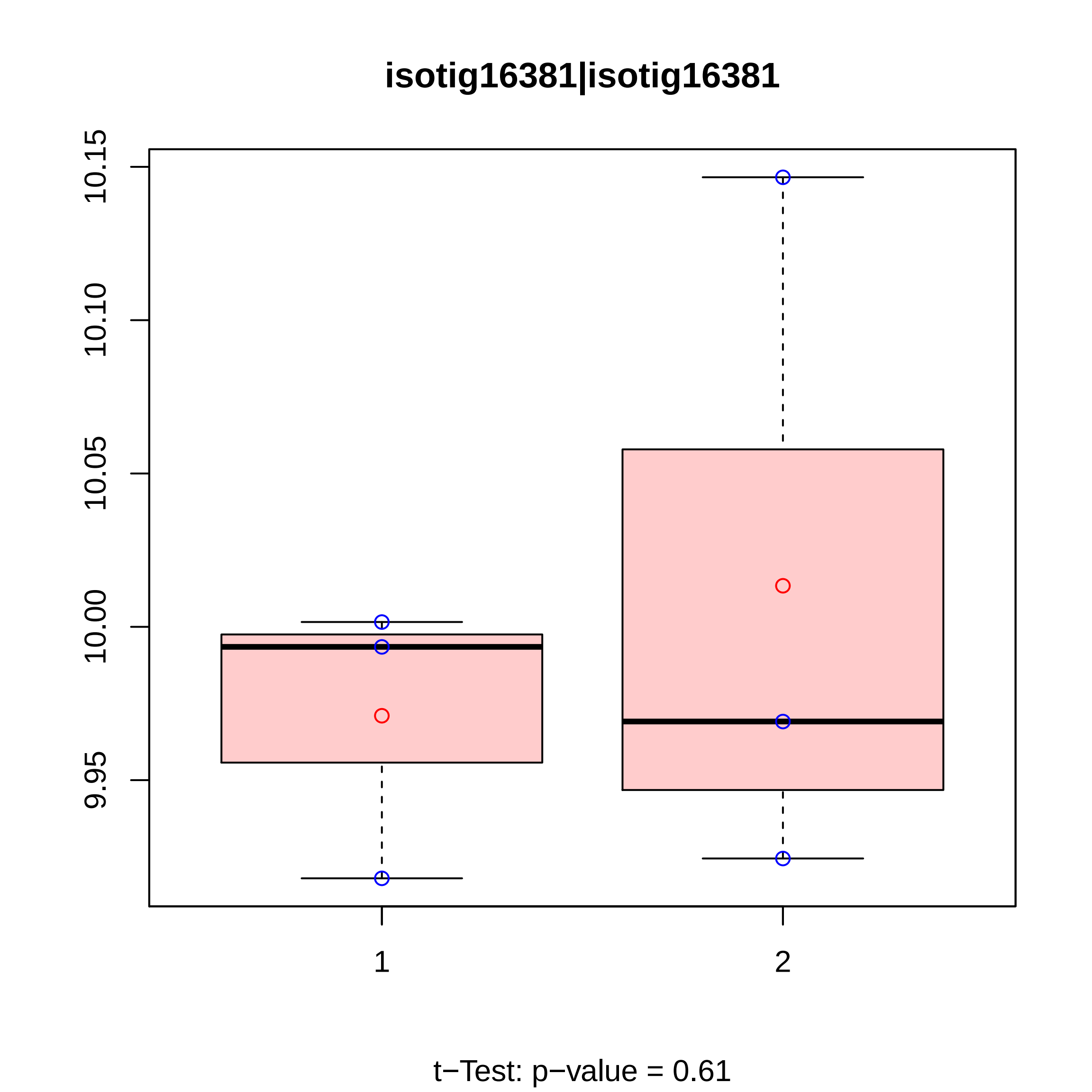
isotig16381|isotig16381
10.15
10.10
10.05
10.00
9.95
1
2
t−Test: p−value = 0.61

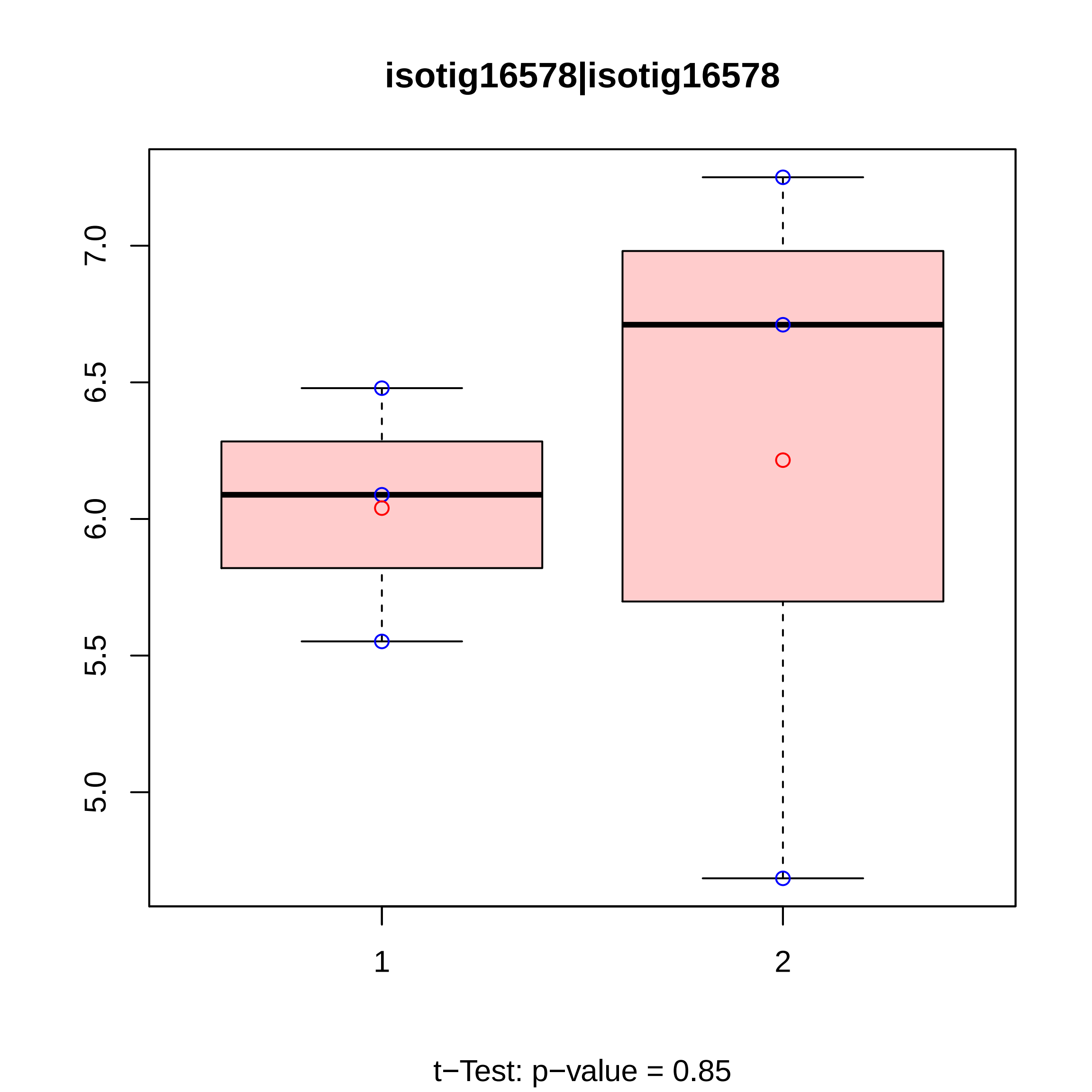
isotig16578|isotig16578
7.0
6.5
6.0
5.5
5.0
1
2
t−Test: p−value = 0.85

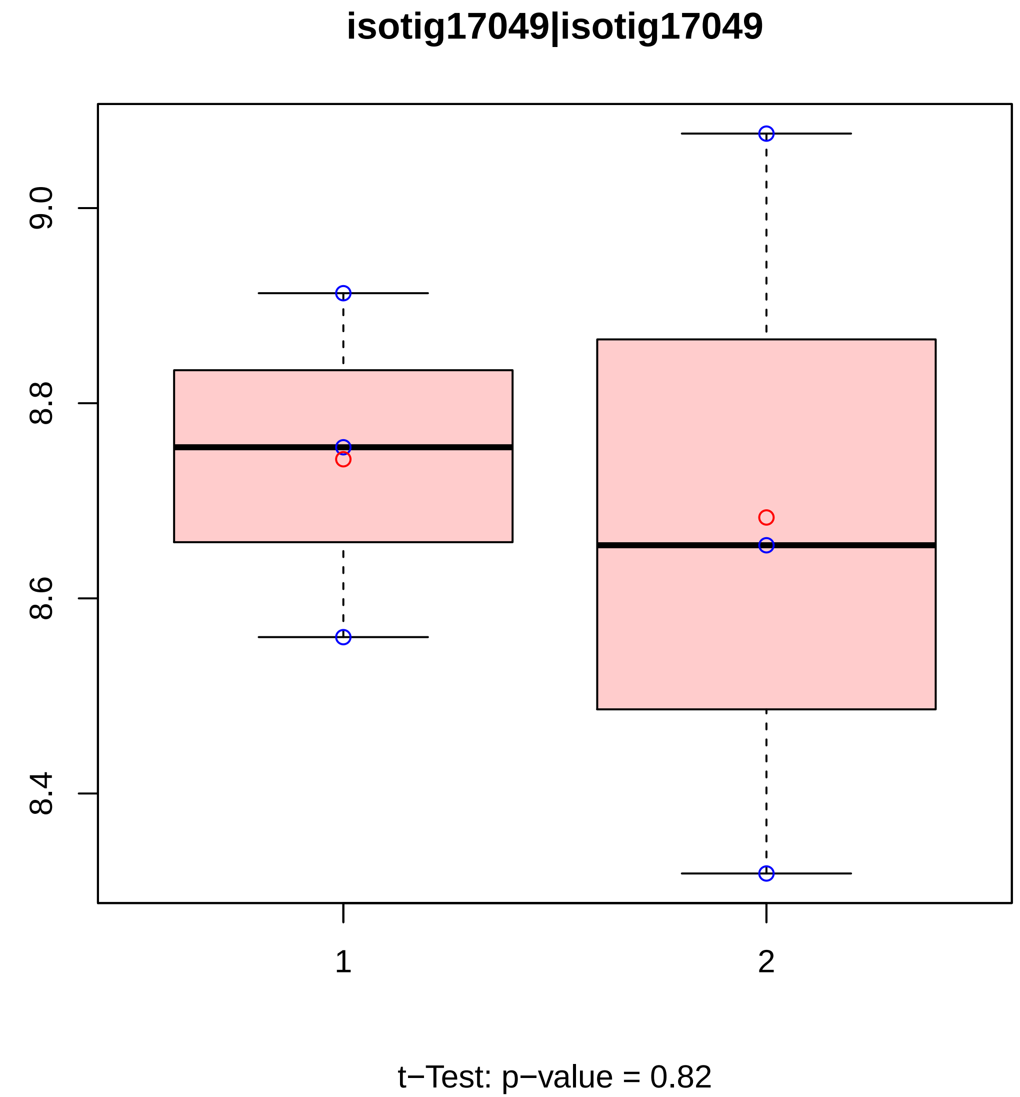
isotig17049|isotig17049
9.0
8.8
8.6
8.4
1
2
t−Test: p−value = 0.82

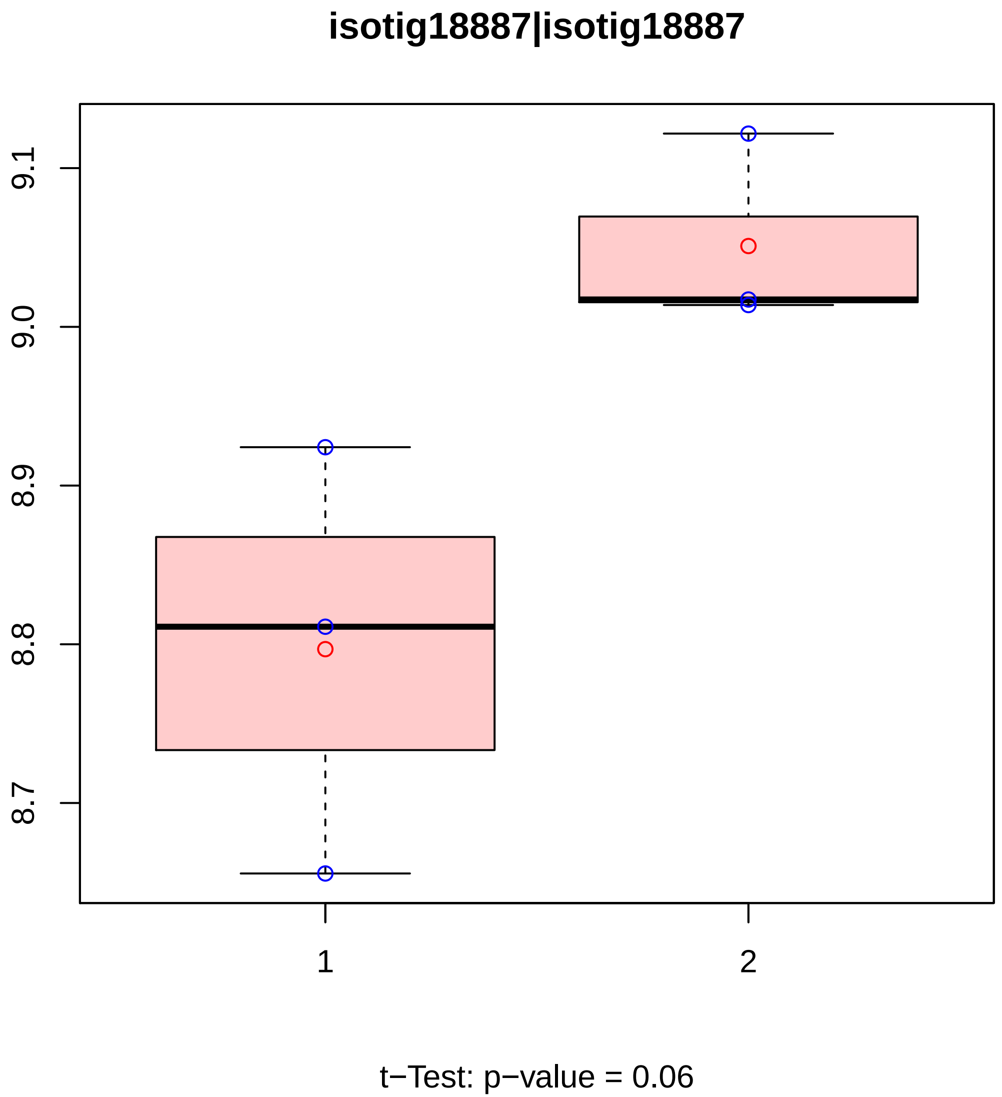
isotig18887|isotig18887
9.1
9.0
8.9
8.8
8.7
1
2
t−Test: p−value = 0.06

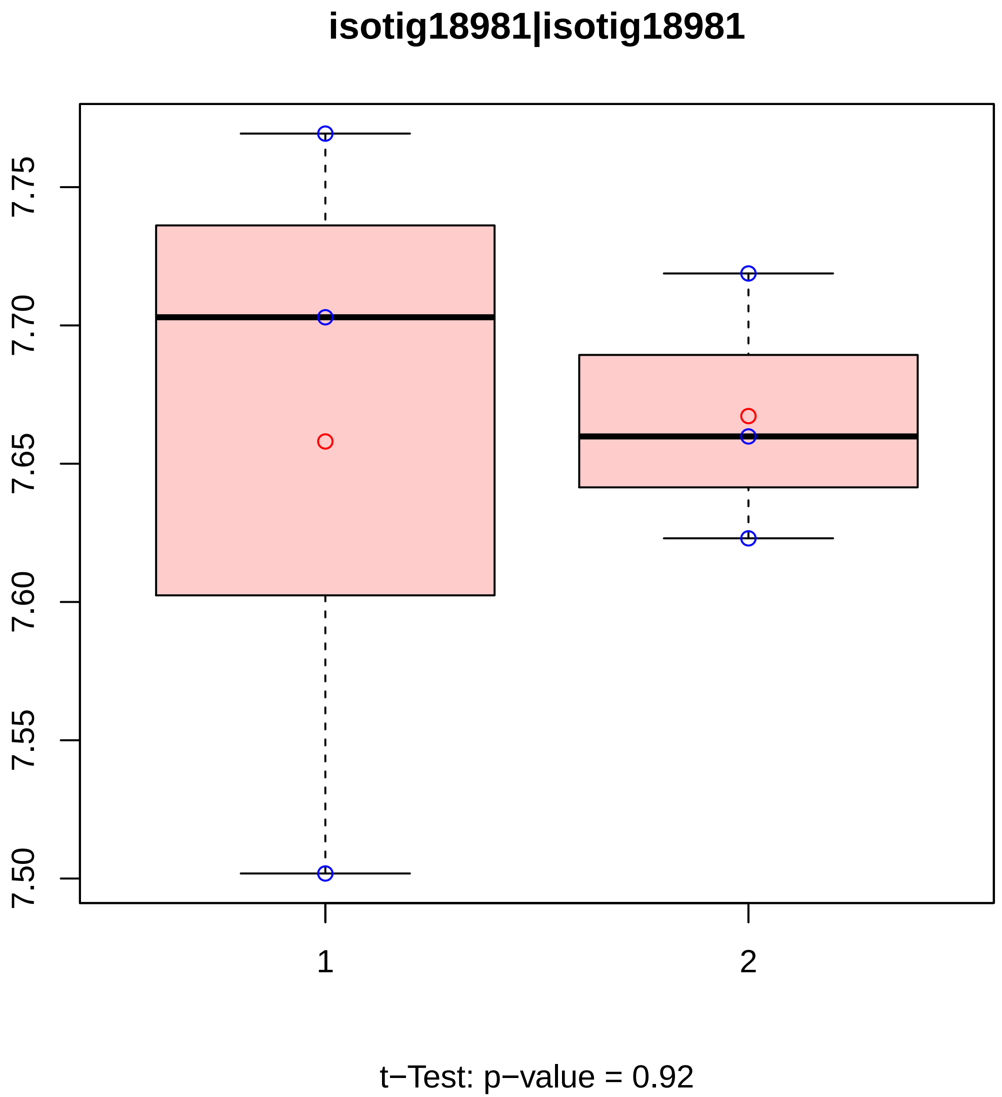

isotig18981|isotig18981
7.50
7.55
7.60
7.65
7.70
7.75
1
2
t−Test: p−value = 0.92

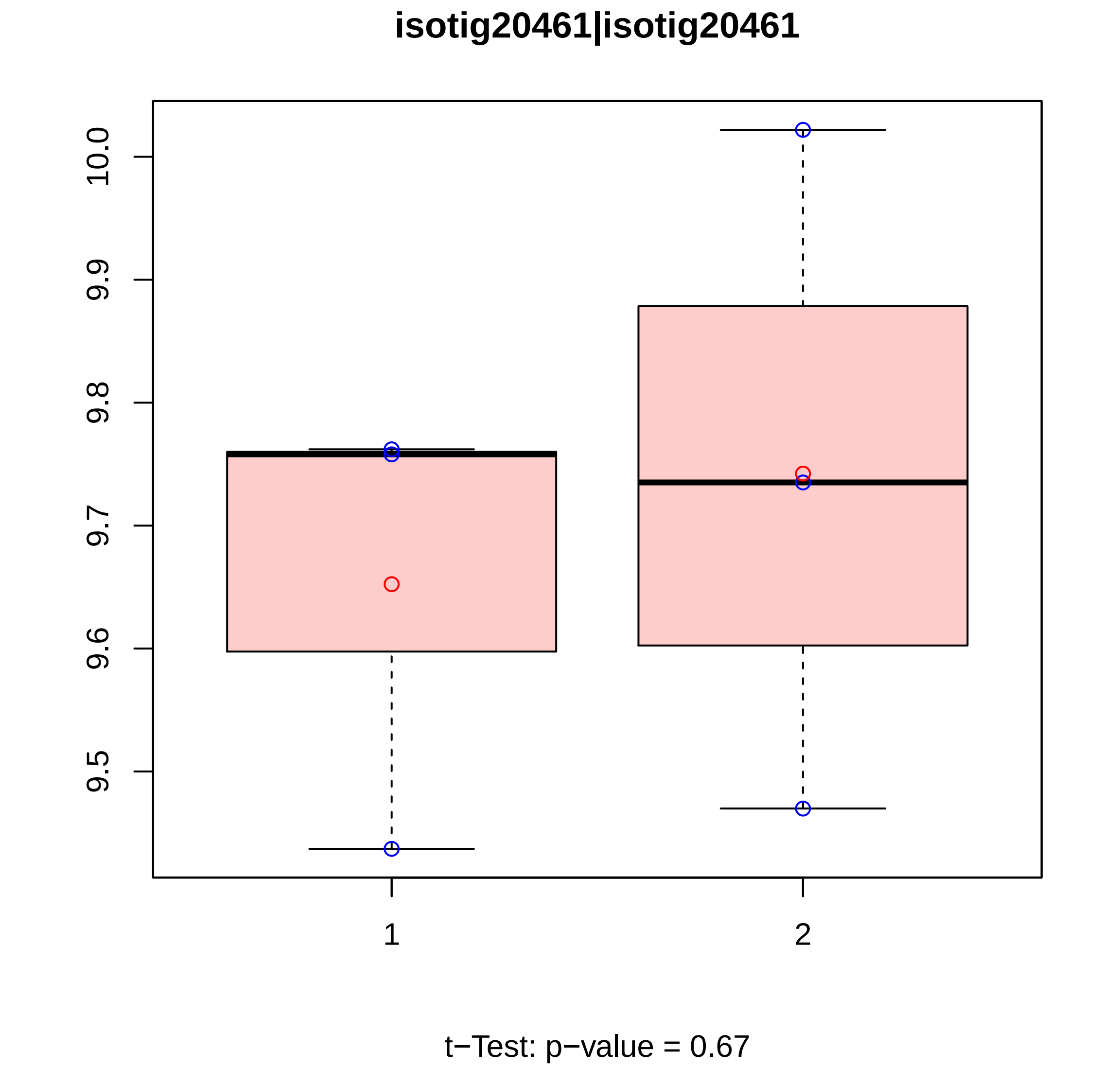

isotig20461|isotig20461
10.0
9.9
9.8
9.7
9.6
9.5
1
2
t−Test: p−value = 0.67

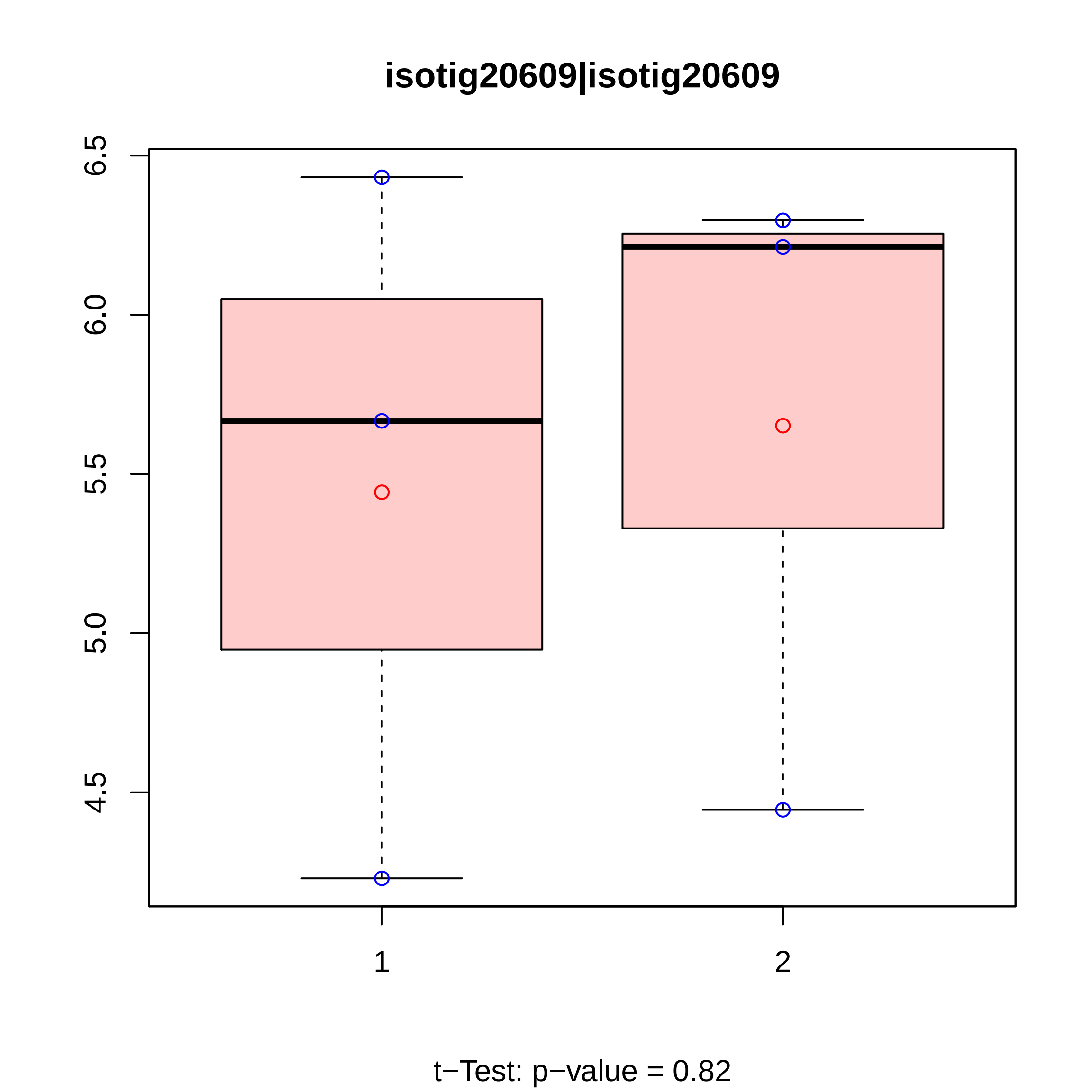
isotig20609|isotig20609
6.5
6.0
5.5
5.0
4.5
1
2
t−Test: p−value = 0.82

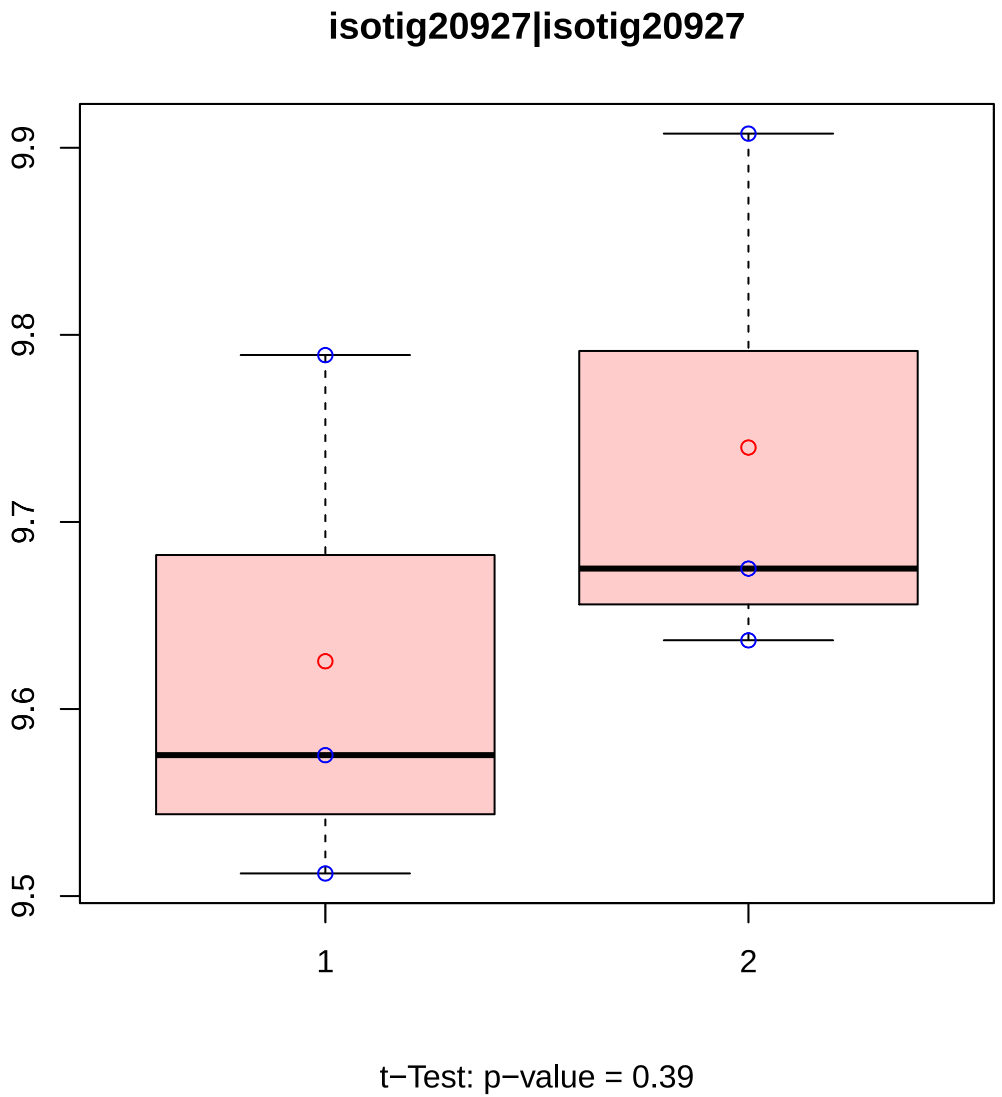
isotig20927|isotig20927
9.5
9.6
9.7
9.8
9.9
1
2
t−Test: p−value = 0.39

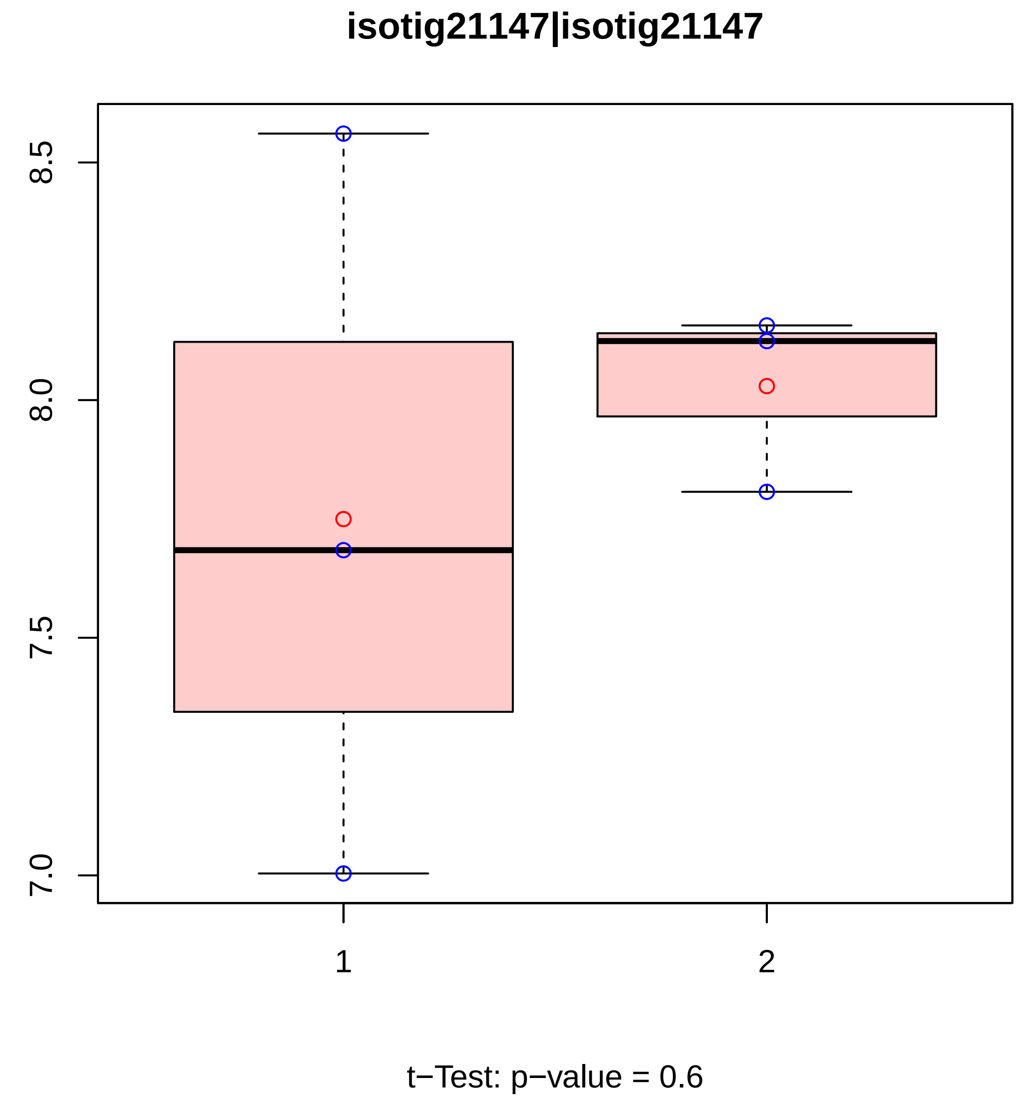
isotig21147|isotig21147
8.5
8.0
7.5
7.0
1
2
t−Test: p−value = 0.6

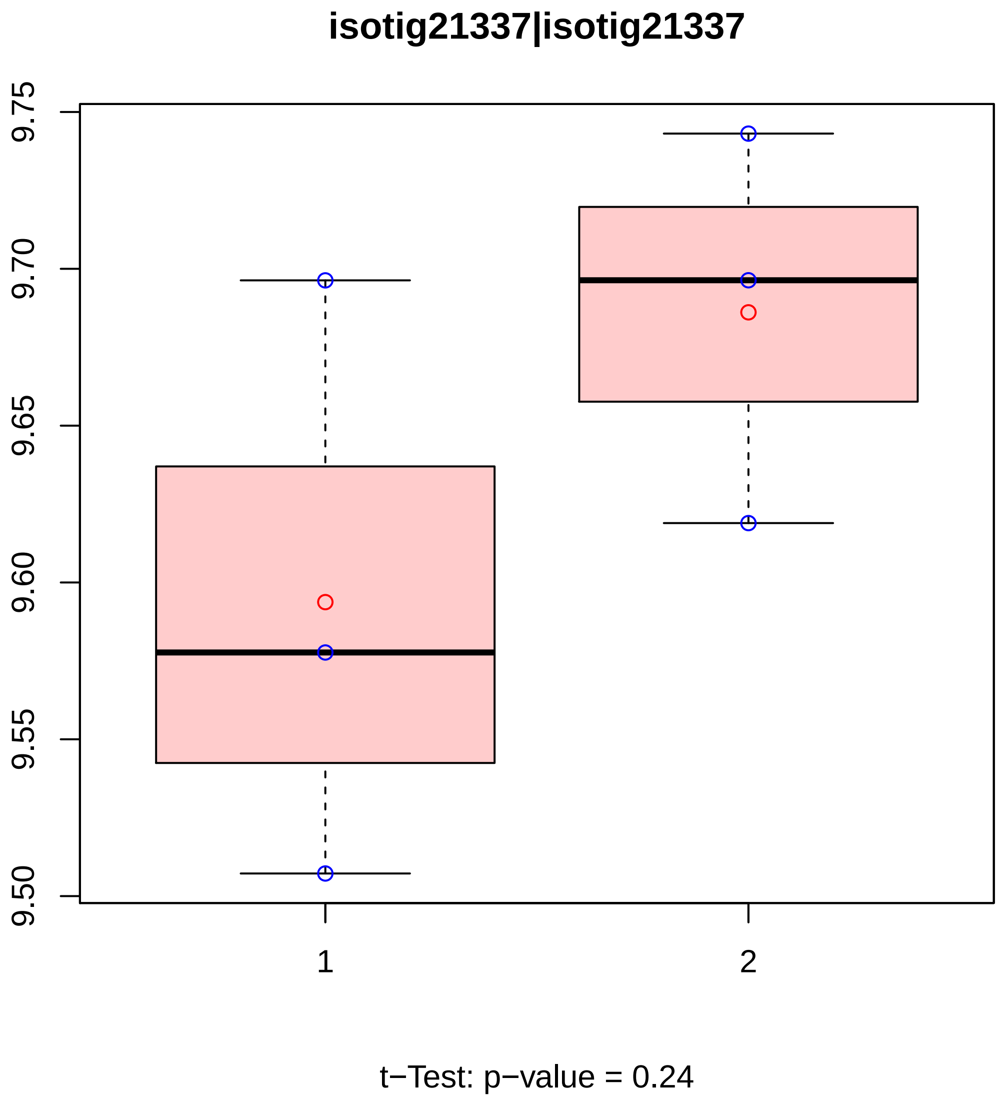
isotig21337|isotig21337
9.75
9.70
9.65
9.60
9.55
9.50
1
2
t−Test: p−value = 0.24

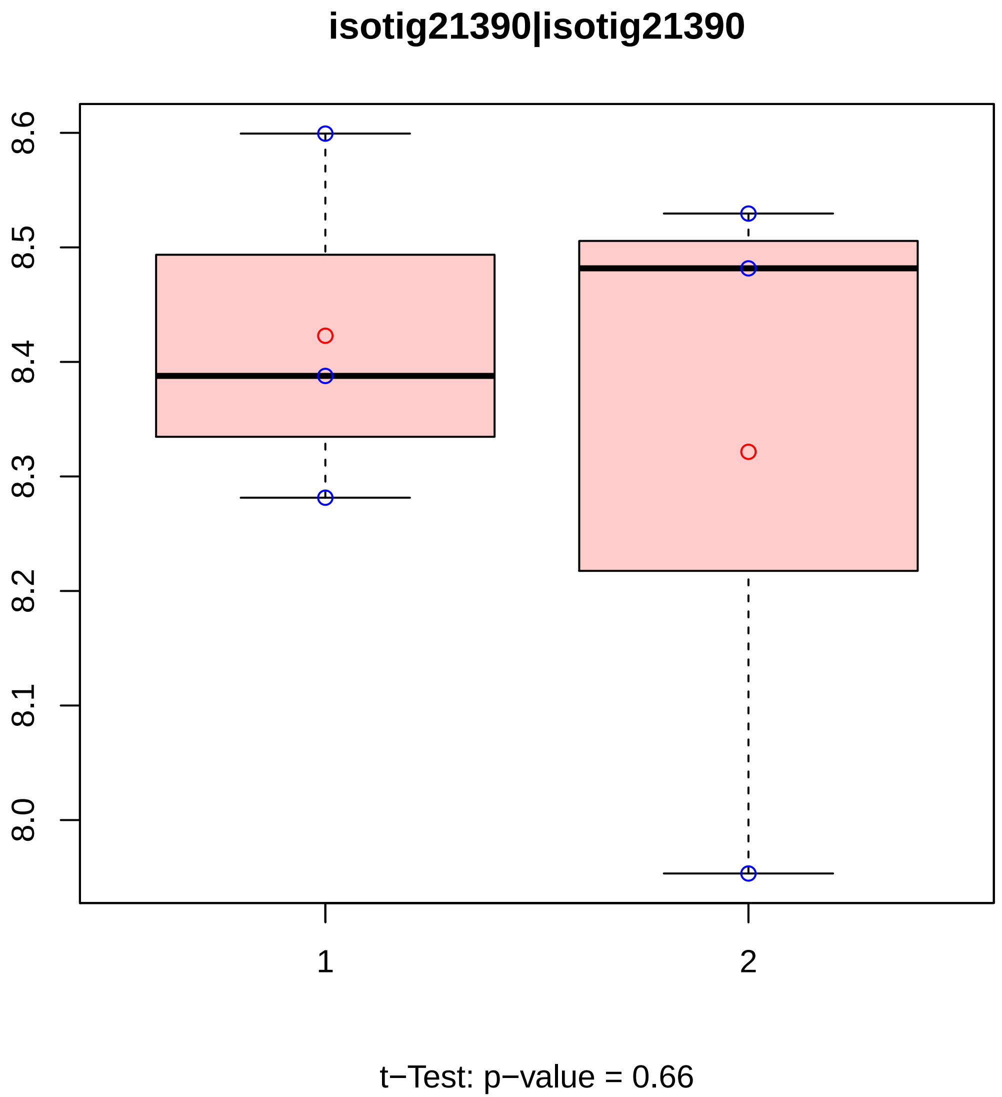
isotig21390|isotig21390
8.6
8.5
8.4
8.3
8.2
8.1
8.0
1
2
t−Test: p−value = 0.66

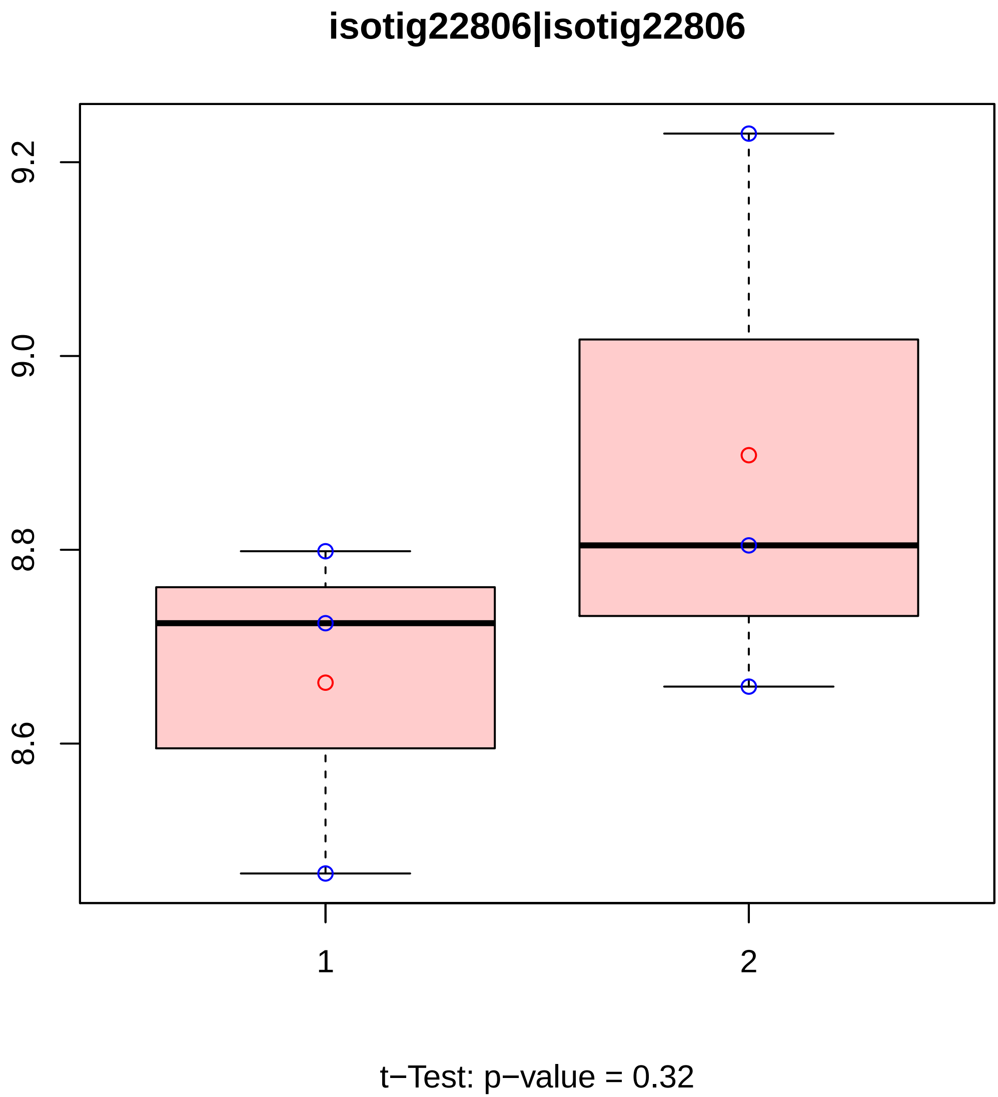
isotig22806|isotig22806
9.2
9.0
8.8
8.6
1
2
t−Test: p−value = 0.32

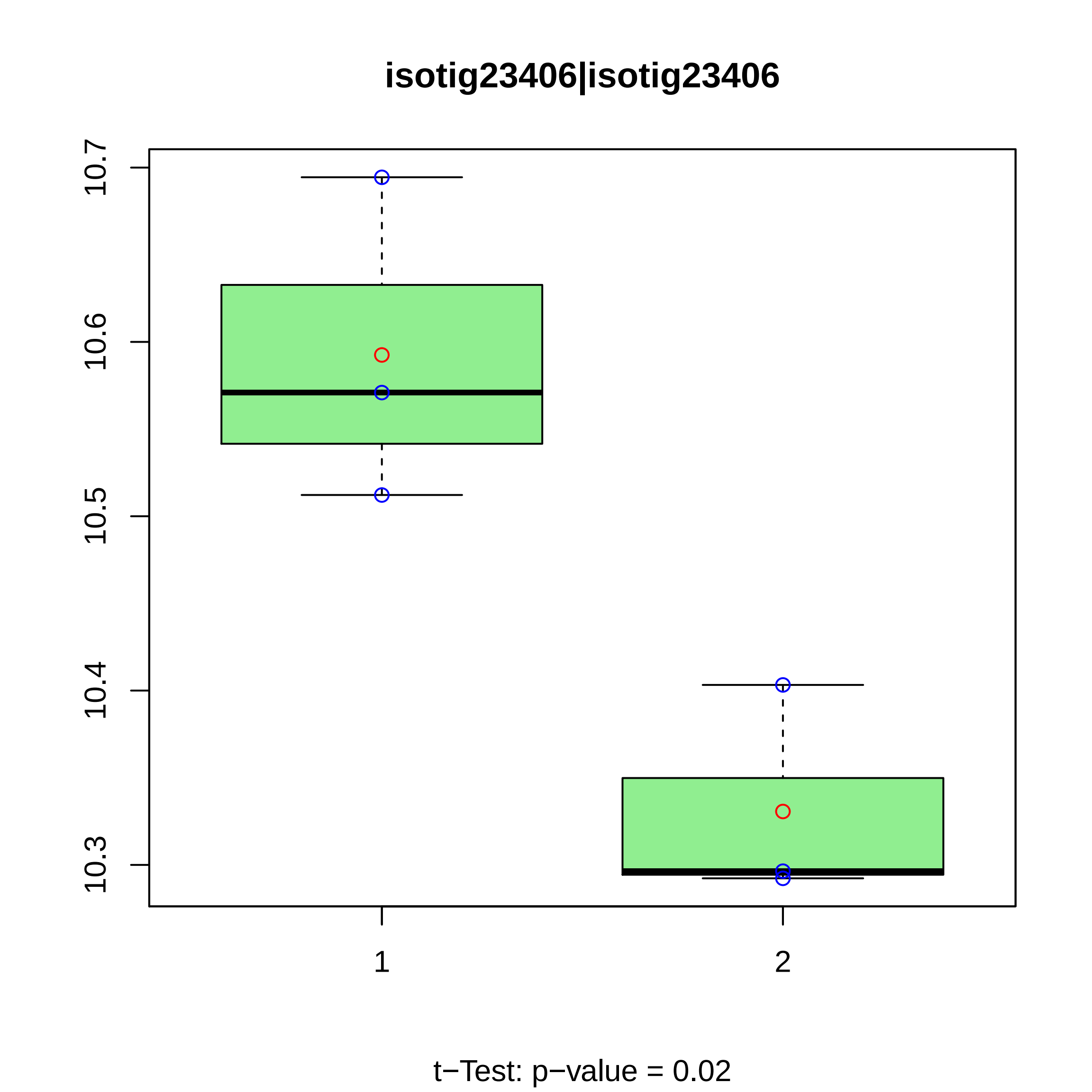
isotig23406|isotig23406
10.7
10.6
10.5
10.4
10.3
1
2
t−Test: p−value = 0.02

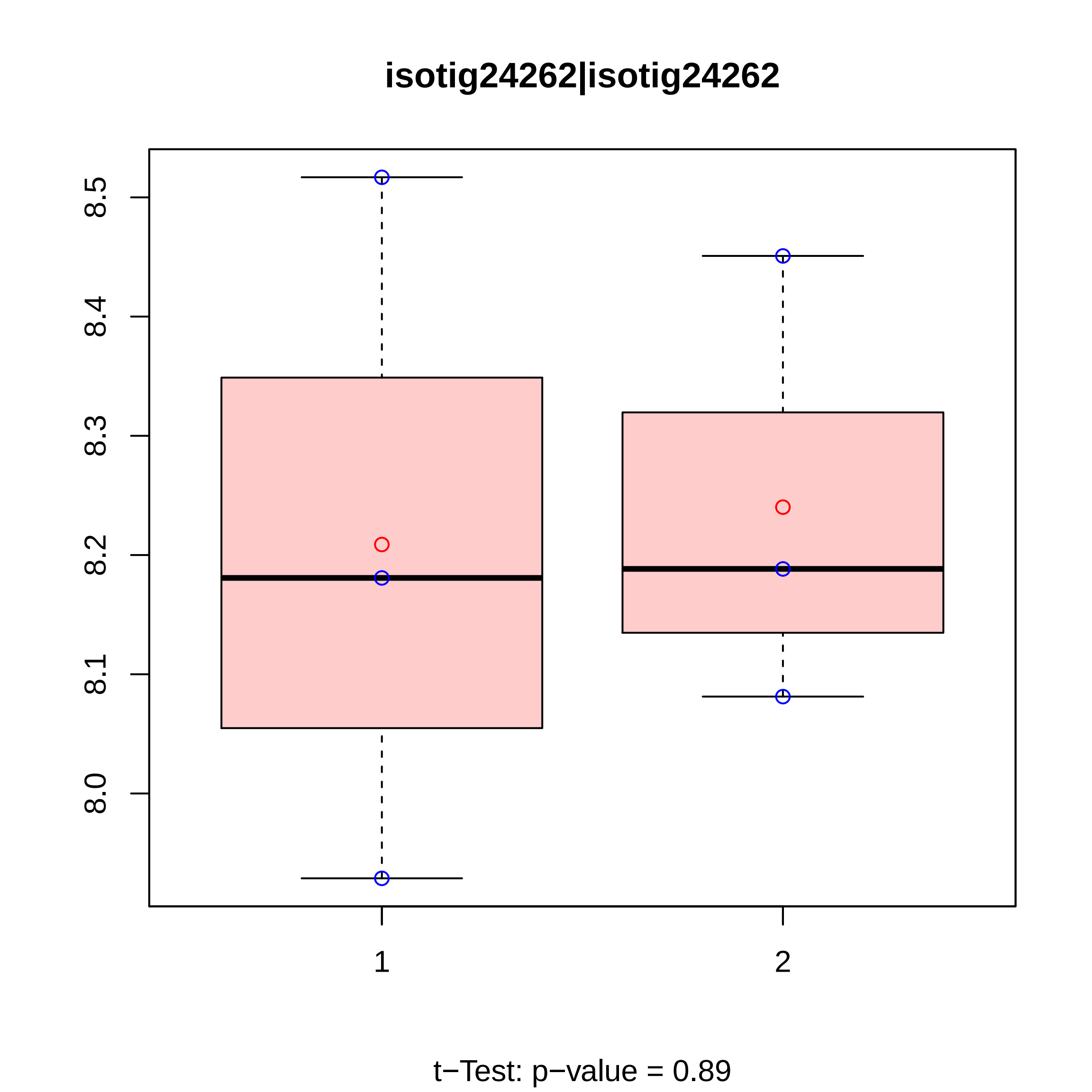

isotig24262|isotig24262
8.5
8.4
8.3
8.2
8.1
8.0
1
2
t−Test: p−value = 0.89

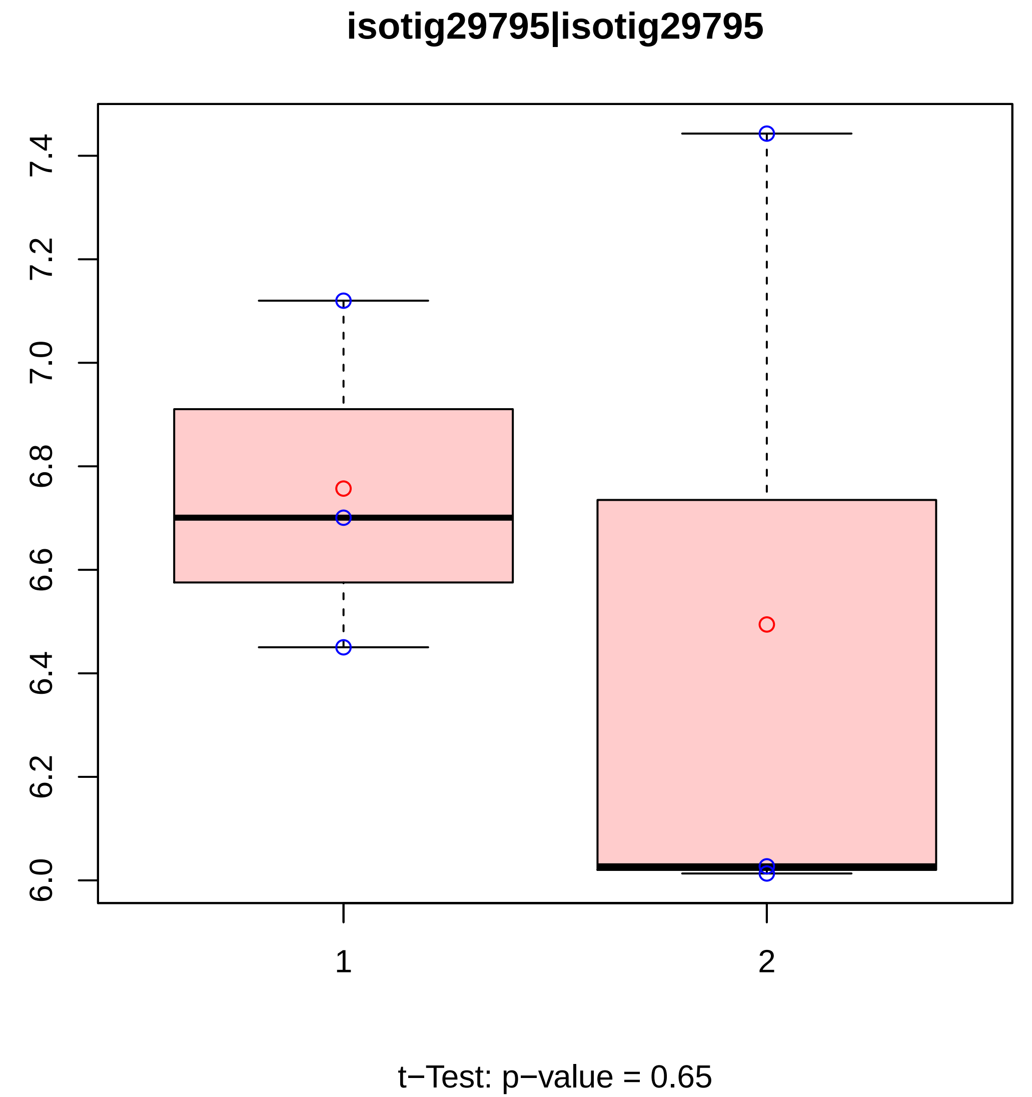
isotig29795|isotig29795
7.4
7.2
7.0
6.8
6.6
6.4
6.2
6.0
1
2
t−Test: p−value = 0.65

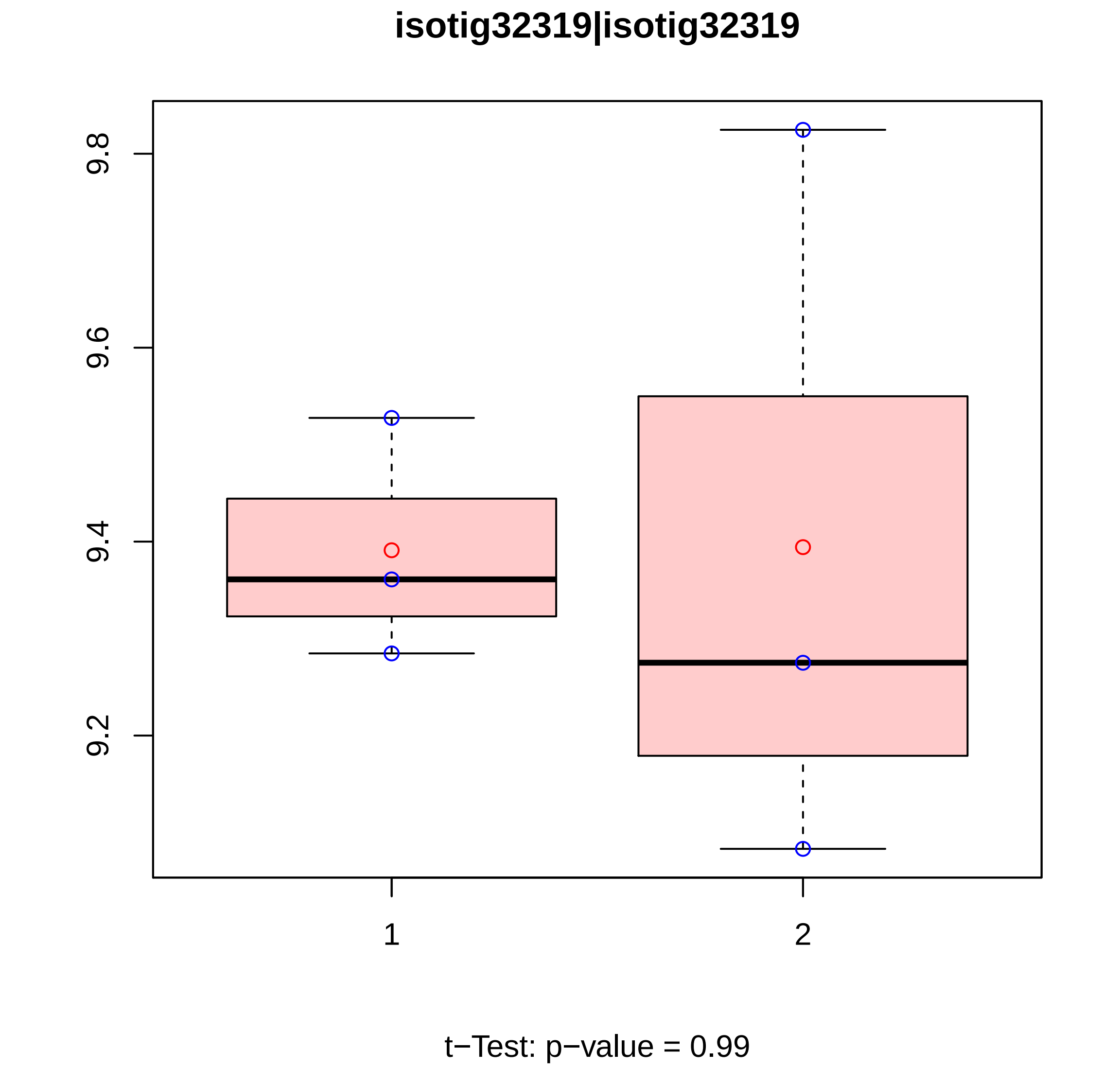
isotig32319|isotig32319
9.2
9.4
9.6
9.8
1
2
t−Test: p−value = 0.99

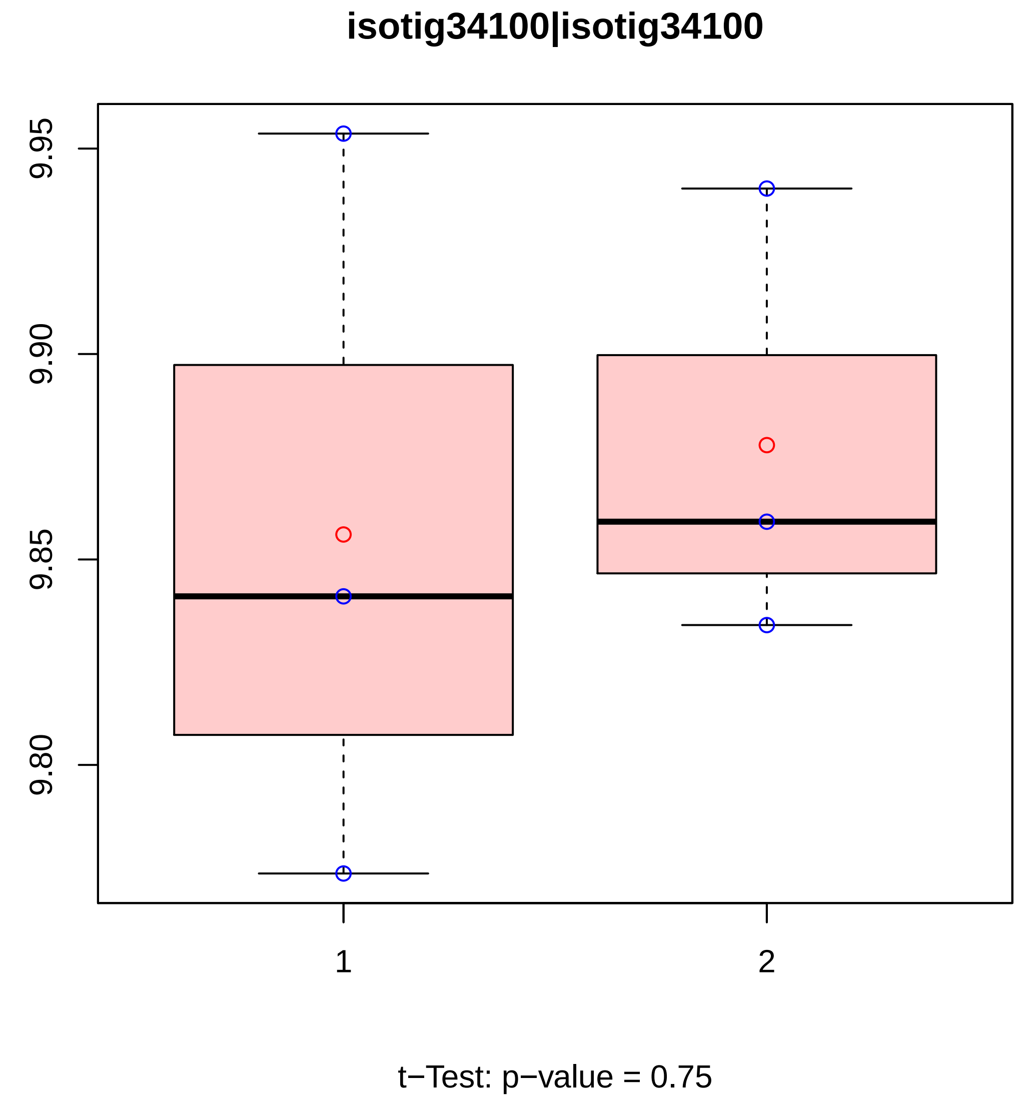
isotig34100|isotig34100
9.95
9.90
9.85
9.80
1
2
t−Test: p−value = 0.75

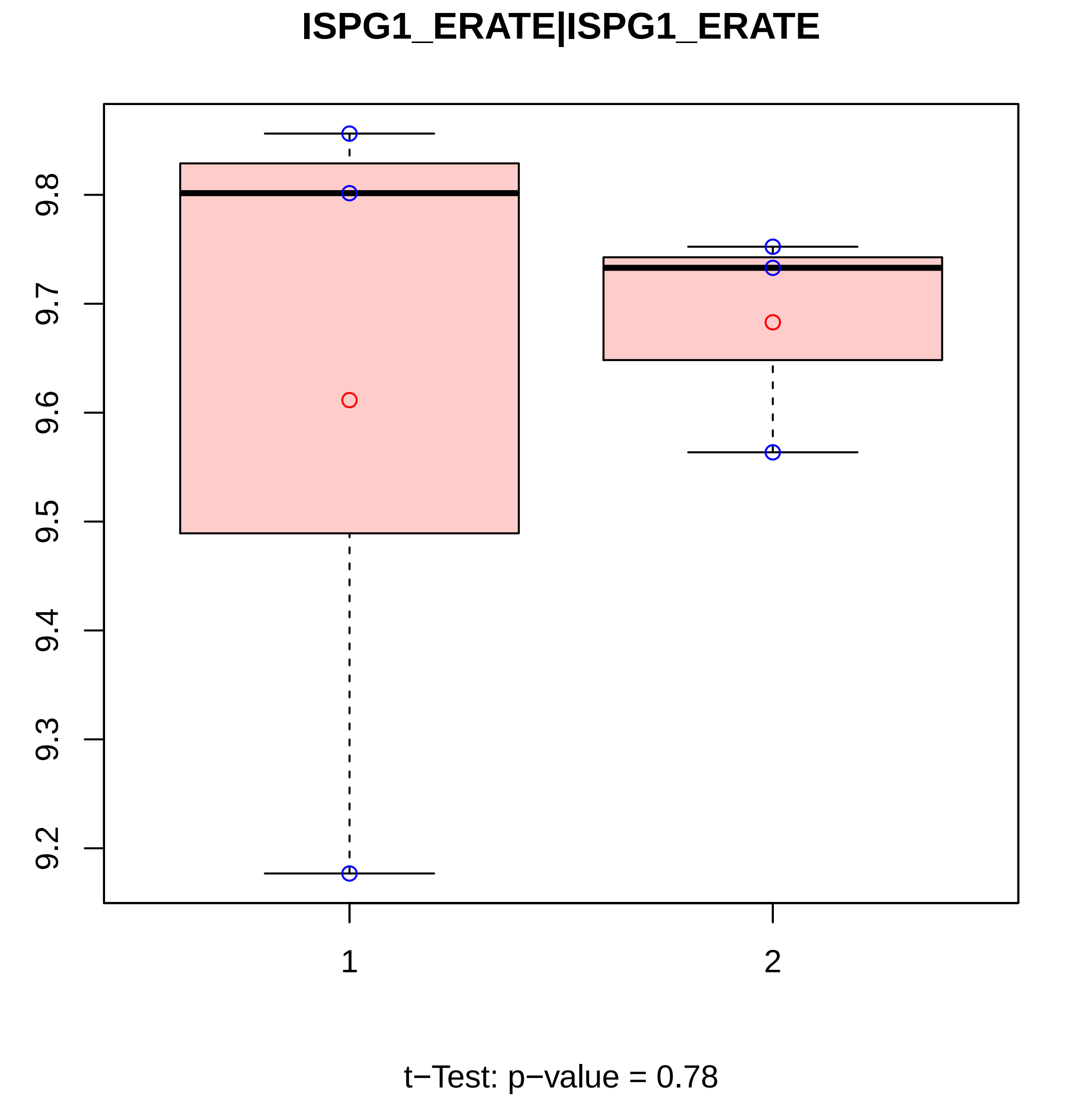
ISPG1_ERATE|ISPG1_ERATE
9.8
9.7
9.6
9.5
9.4
9.3
9.2
1
2
t−Test: p−value = 0.78

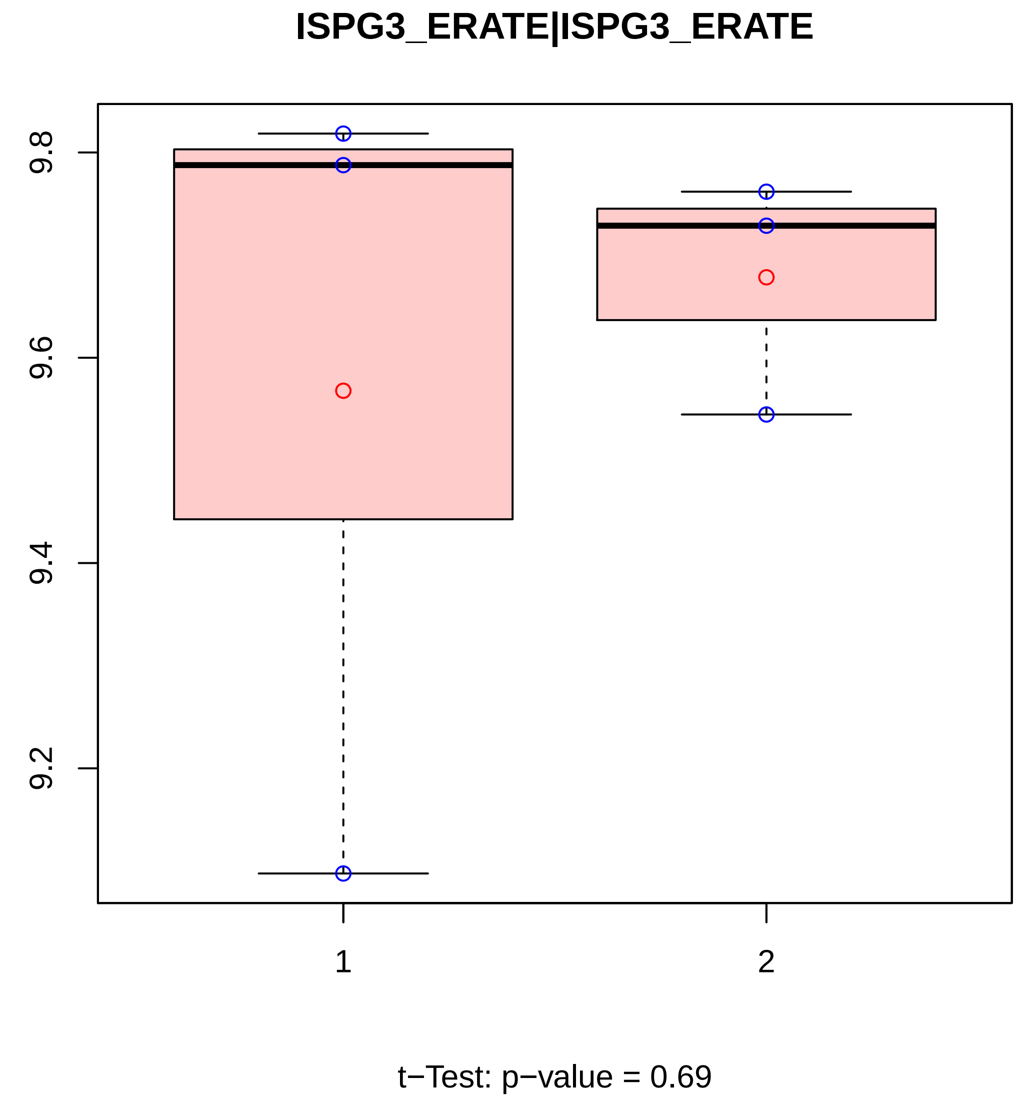
ISPG3_ERATE|ISPG3_ERATE
9.8
9.6
9.4
9.2
1
2
t−Test: p−value = 0.69

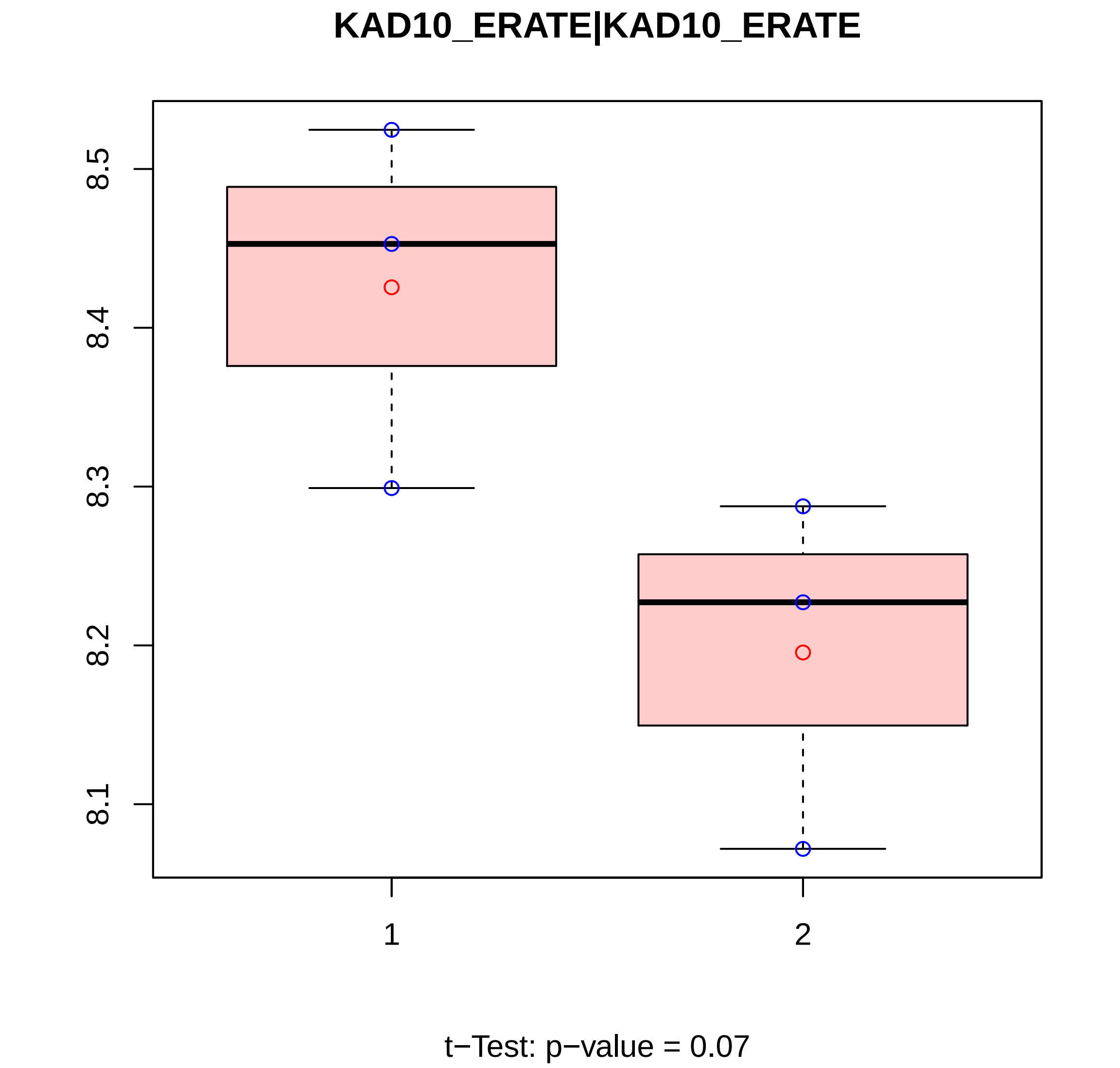
KAD10_ERATE|KAD10_ERATE
8.5
8.4
8.3
8.2
8.1
1
2
t−Test: p−value = 0.07

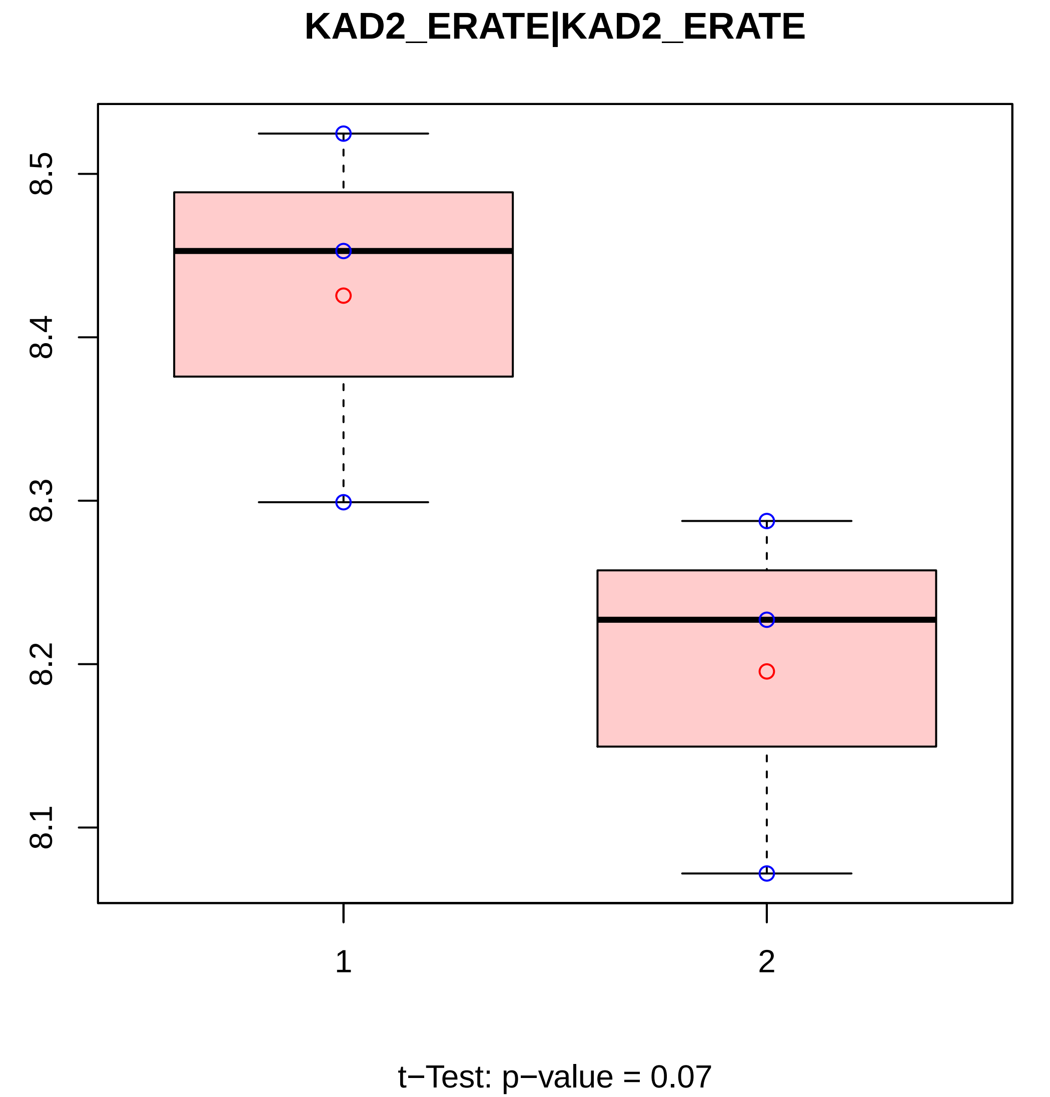
KAD2_ERATE|KAD2_ERATE
8.1
8.2
8.3
8.4
8.5
1
2
t−Test: p−value = 0.07

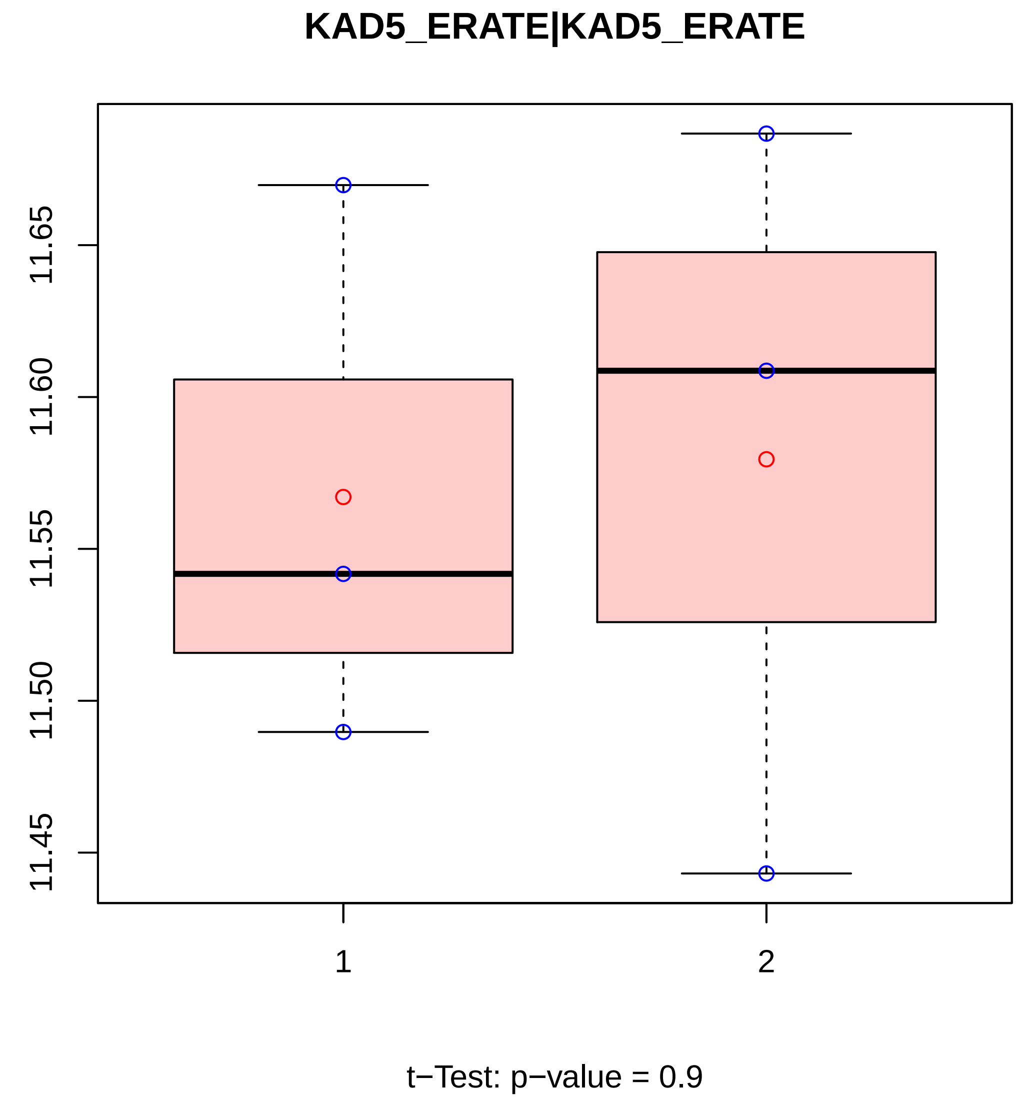

KAD5_ERATE|KAD5_ERATE
11.45
11.50
11.55
11.60
11.65
1
2
t−Test: p−value = 0.9

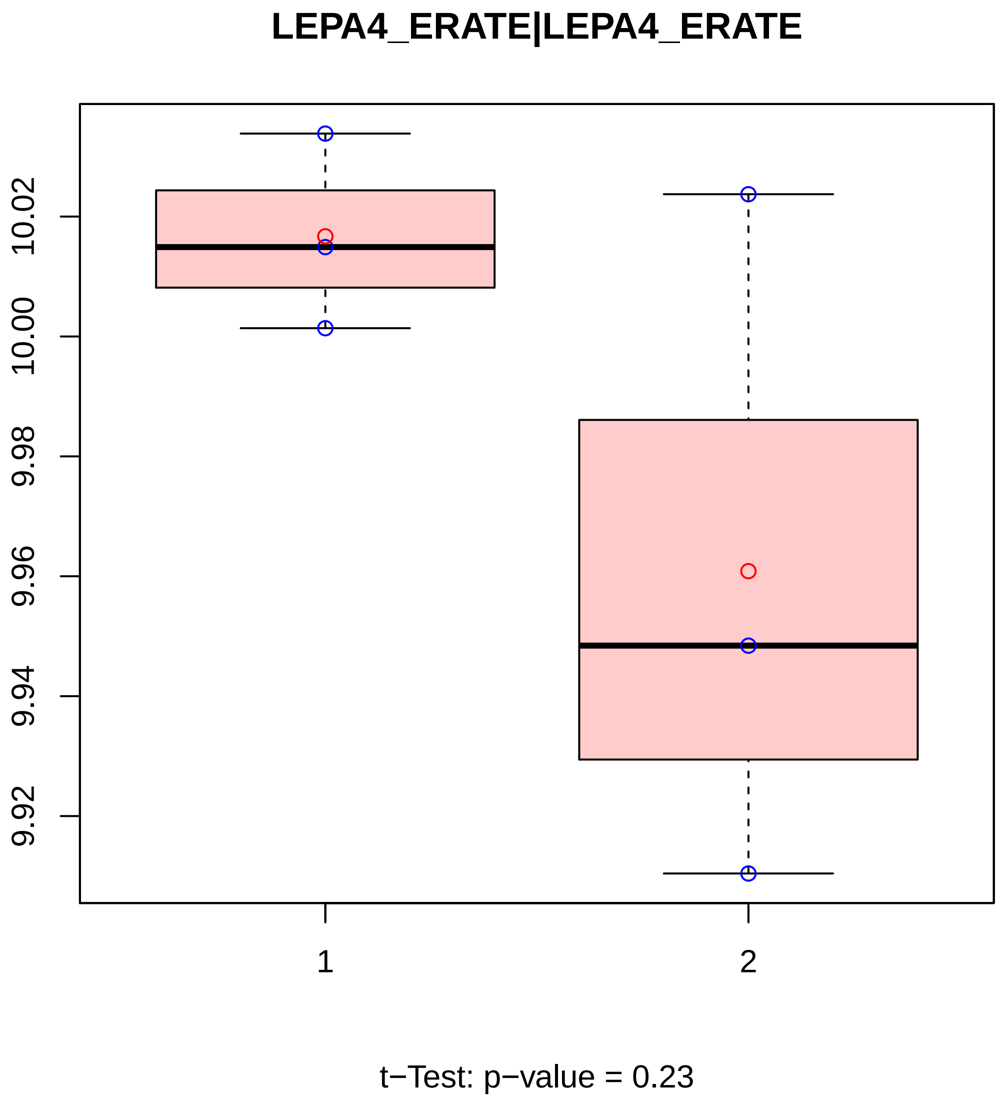

LEPA4_ERATE|LEPA4_ERATE
10.02
10.00
9.98
9.96
9.94
9.92
1
2
t−Test: p−value = 0.23

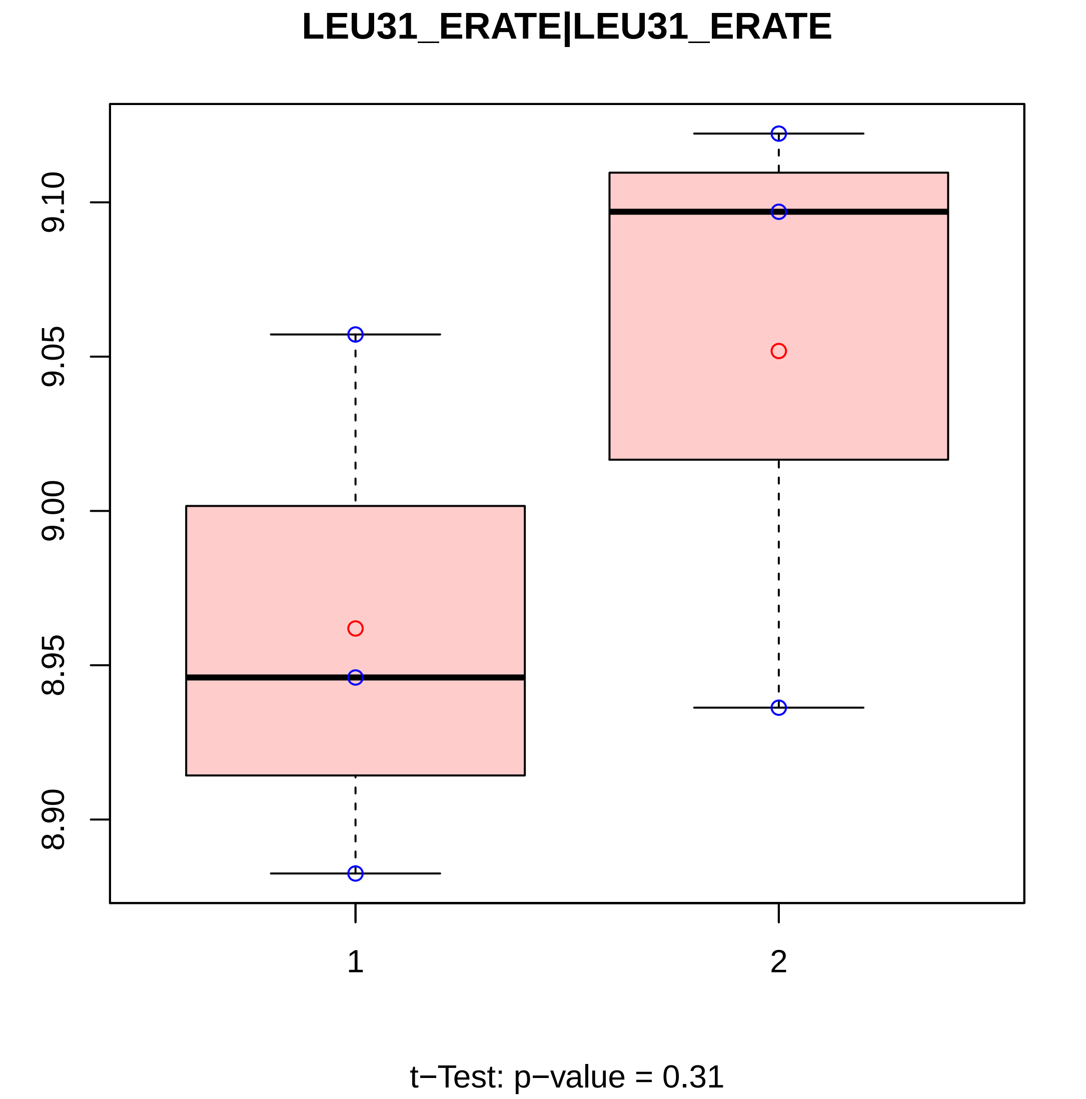

LEU31_ERATE|LEU31_ERATE
9.10
9.05
9.00
8.95
8.90
1
2
t−Test: p−value = 0.31

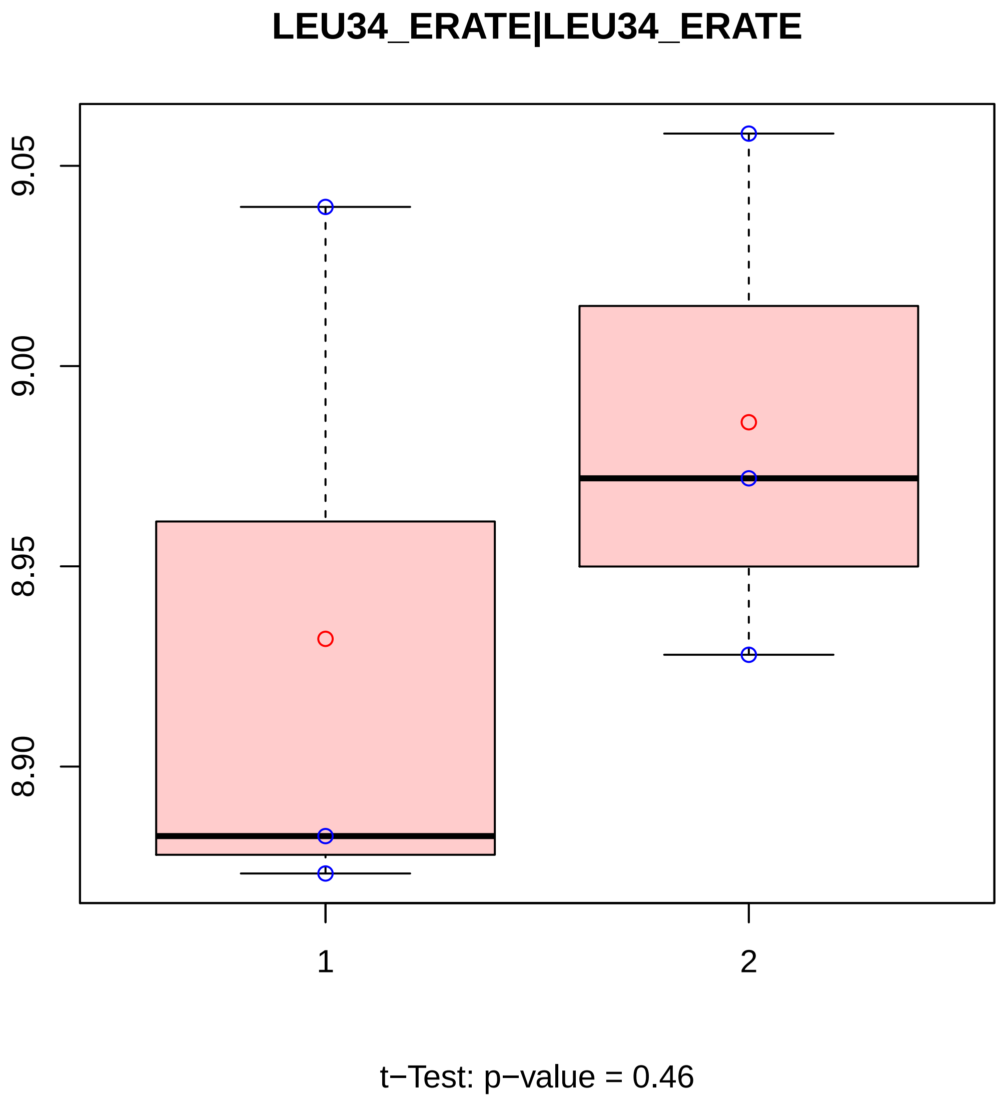

LEU34_ERATE|LEU34_ERATE
9.05
9.00
8.95
8.90
1
2
t−Test: p−value = 0.46

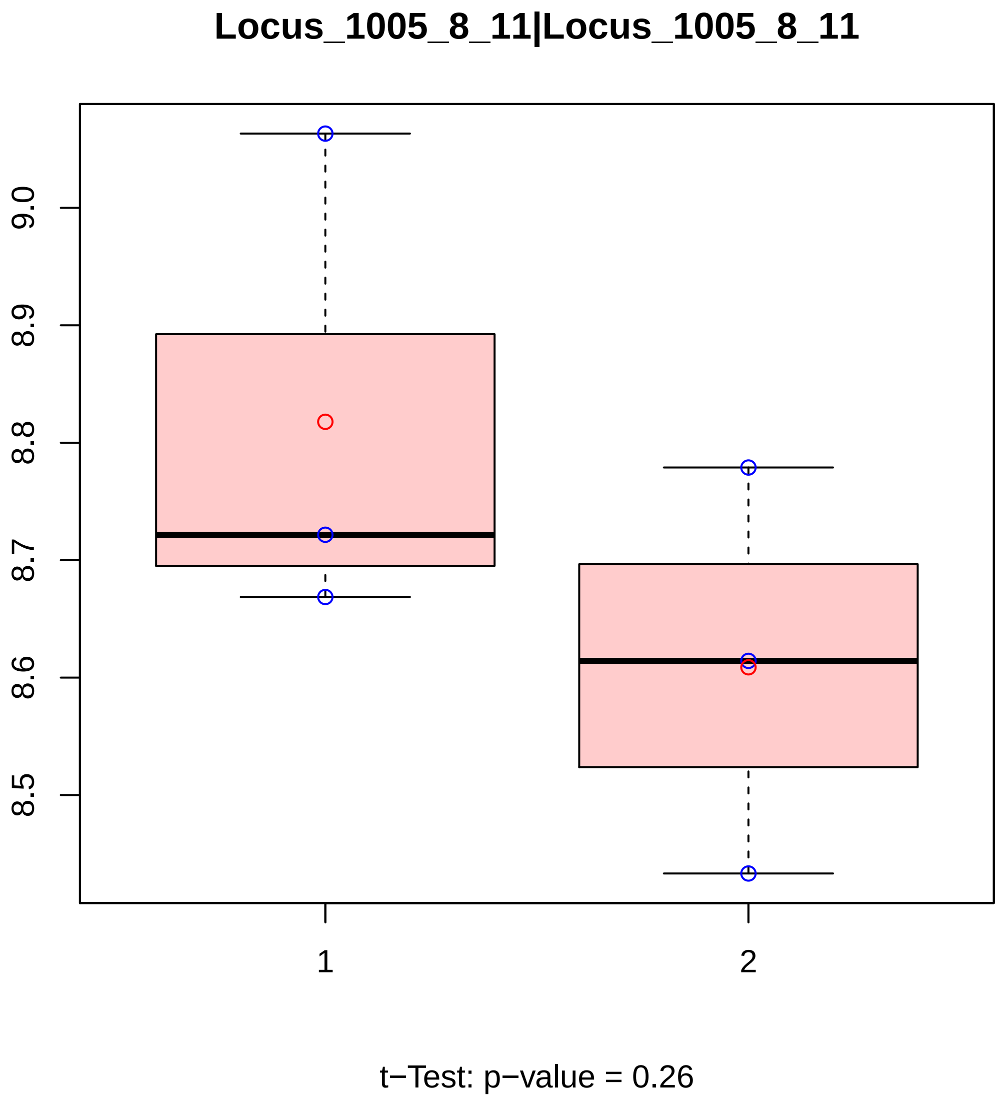

Locus_1005_8_11|Locus_1005_8_11
9.0
8.9
8.8
8.7
8.6
8.5
1
2
t−Test: p−value = 0.26

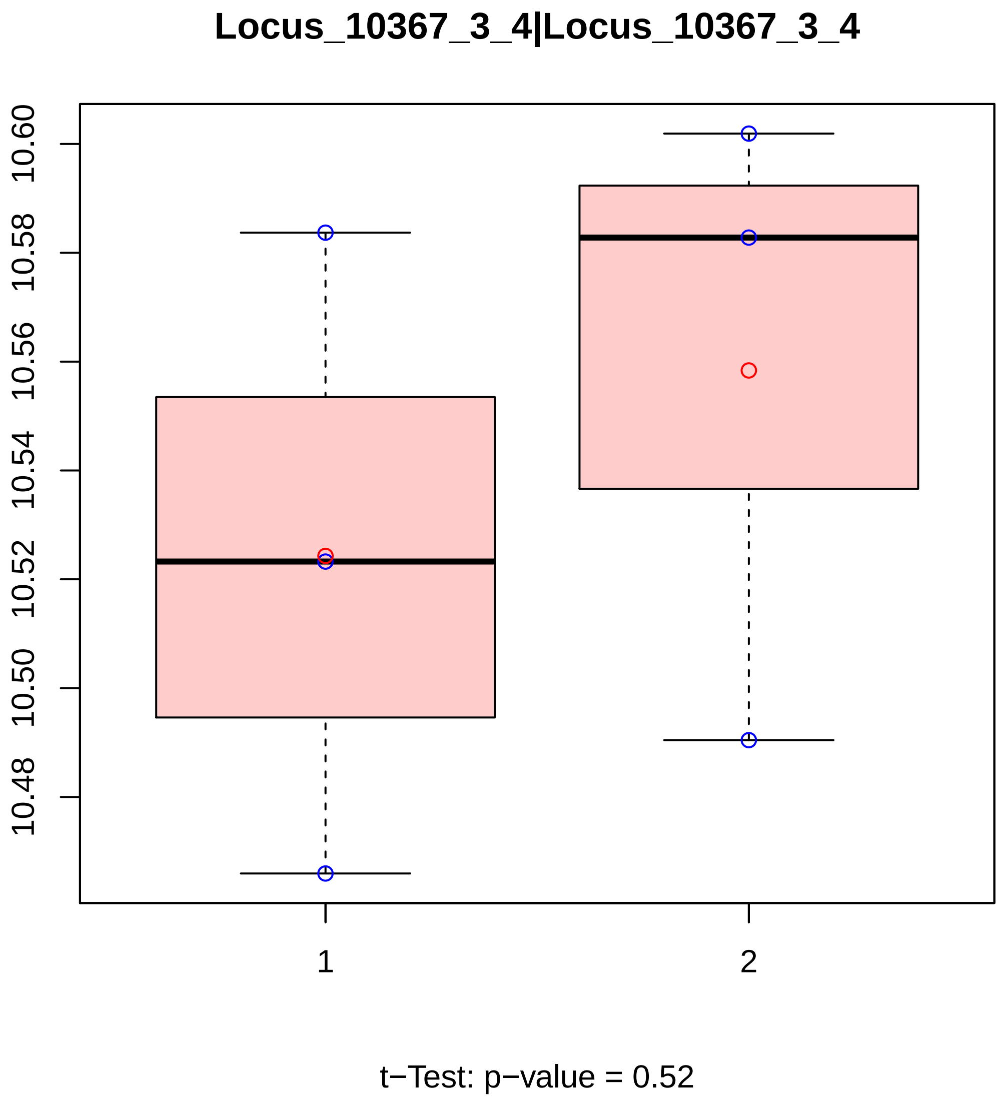

Locus_10367_3_4|Locus_10367_3_4
10.48
10.50
10.52
10.54
10.56
10.58
10.60
1
2
t−Test: p−value = 0.52

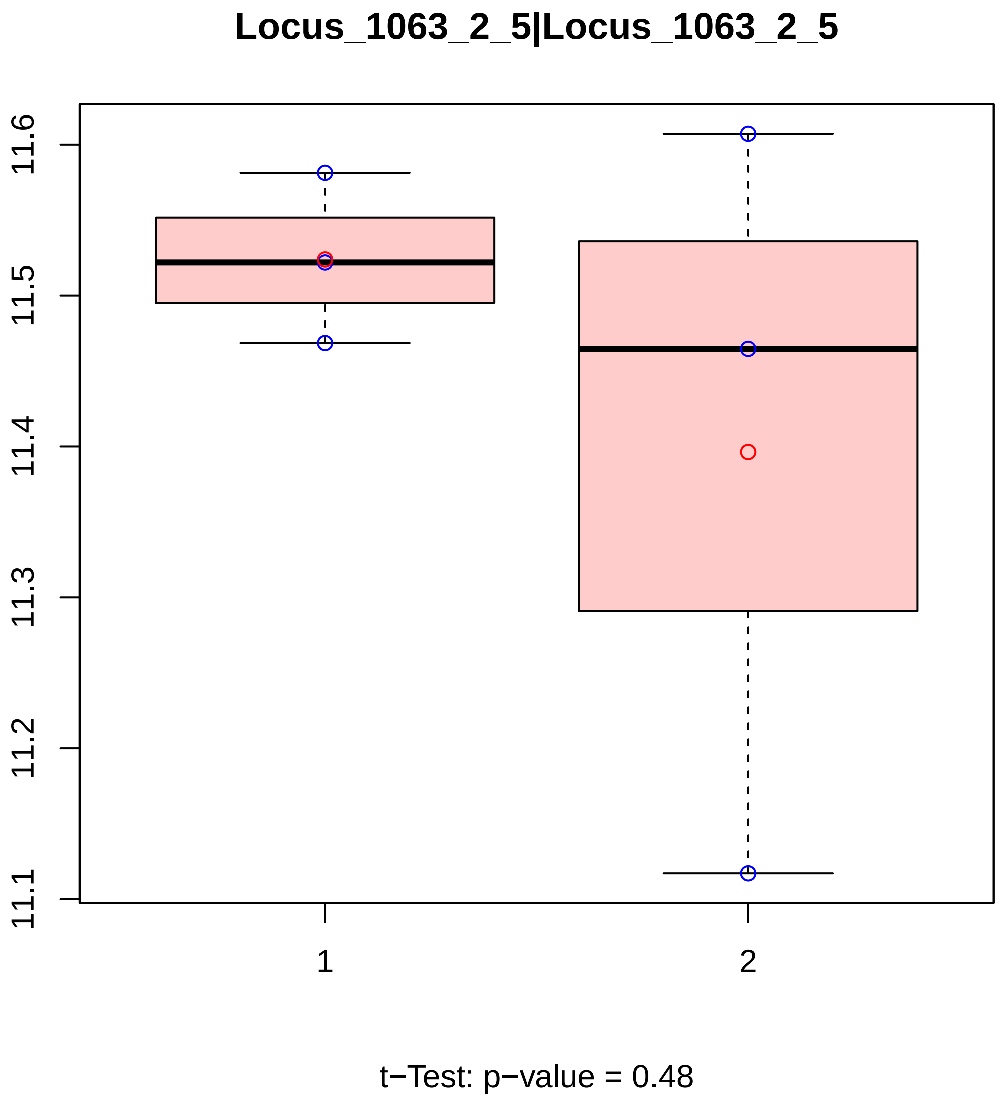

Locus_1063_2_5|Locus_1063_2_5
11.1
11.2
11.3
11.4
11.5
11.6
1
2
t−Test: p−value = 0.48

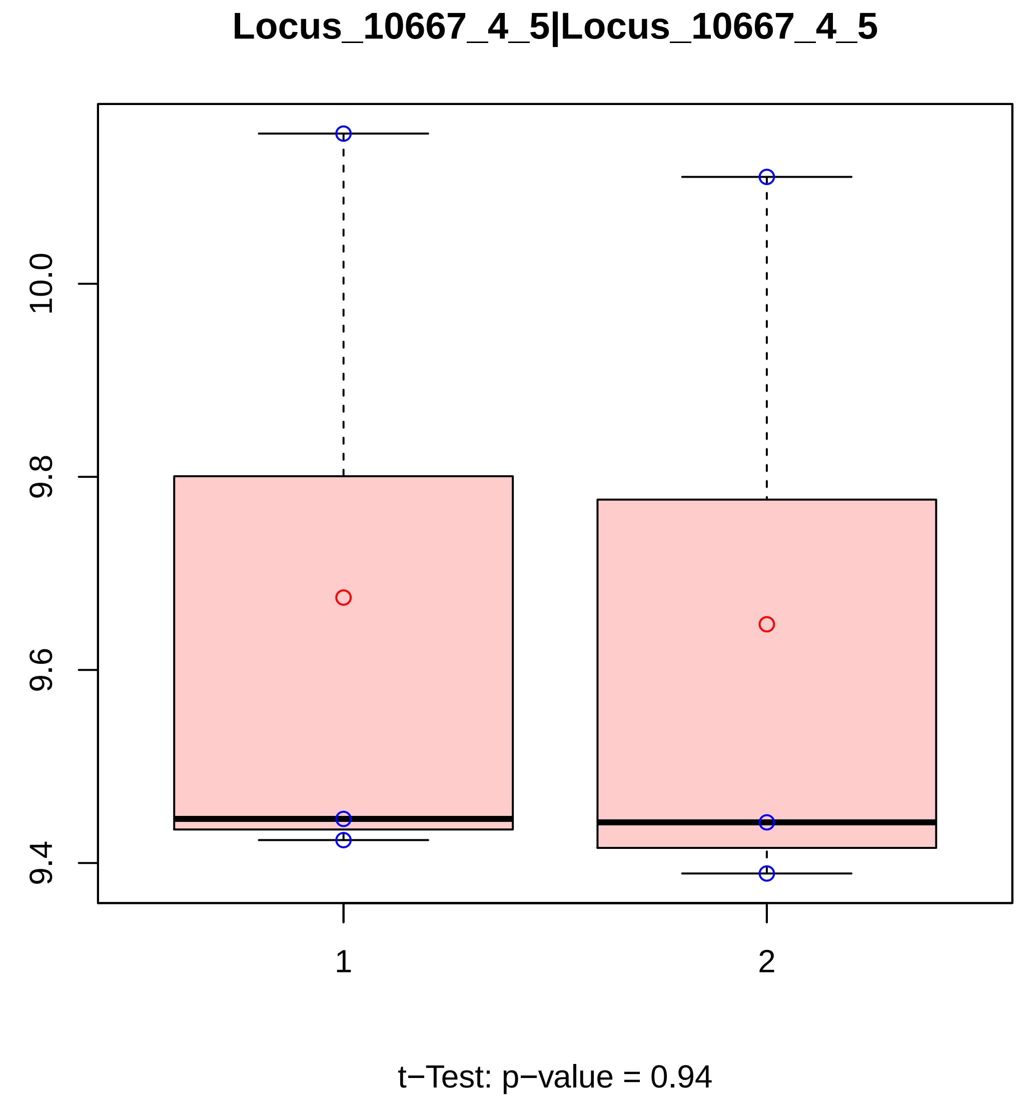

Locus_10667_4_5|Locus_10667_4_5
10.0
9.8
9.6
9.4
1
2
t−Test: p−value = 0.94

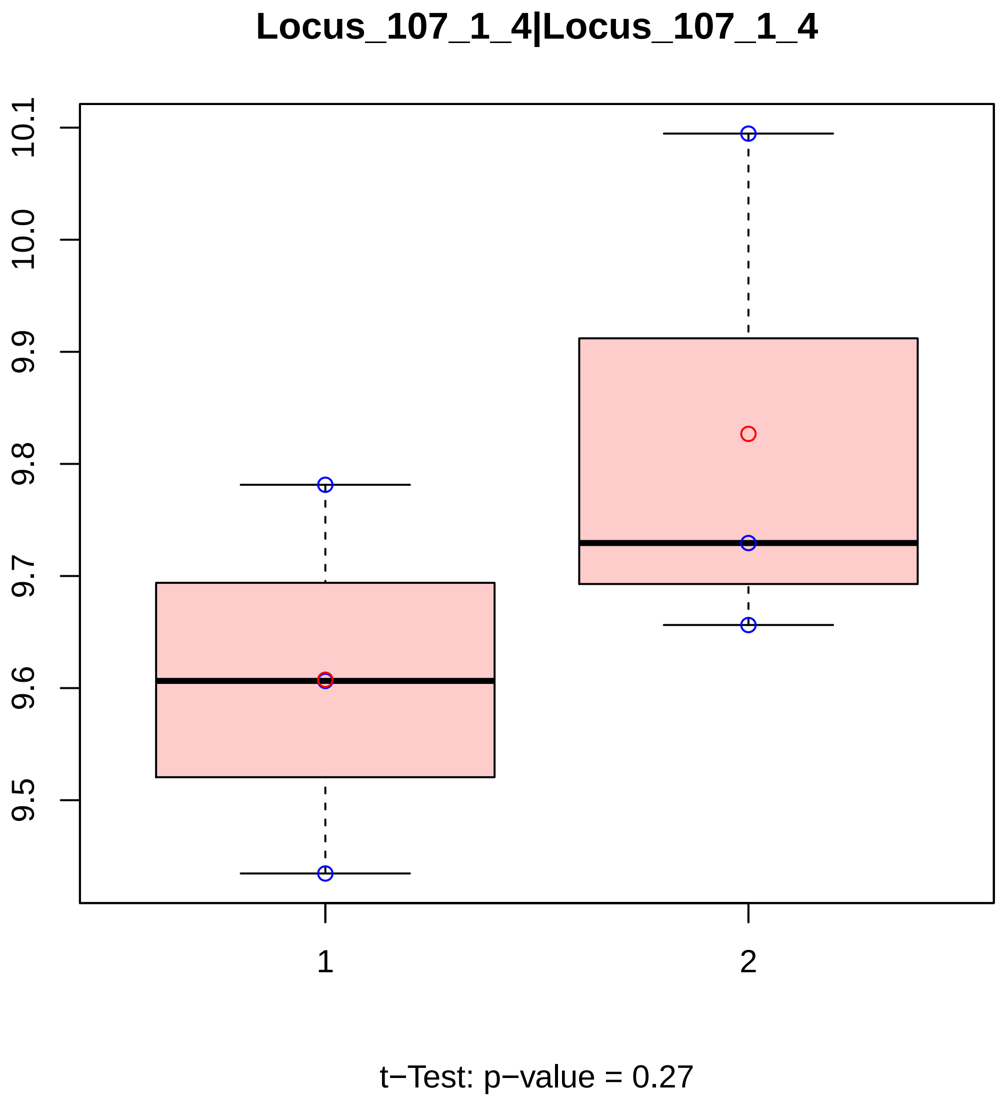
Locus_107_1_4|Locus_107_1_4
10.1
10.0
9.9
9.8
9.7
9.6
9.5
1
2
t−Test: p−value = 0.27

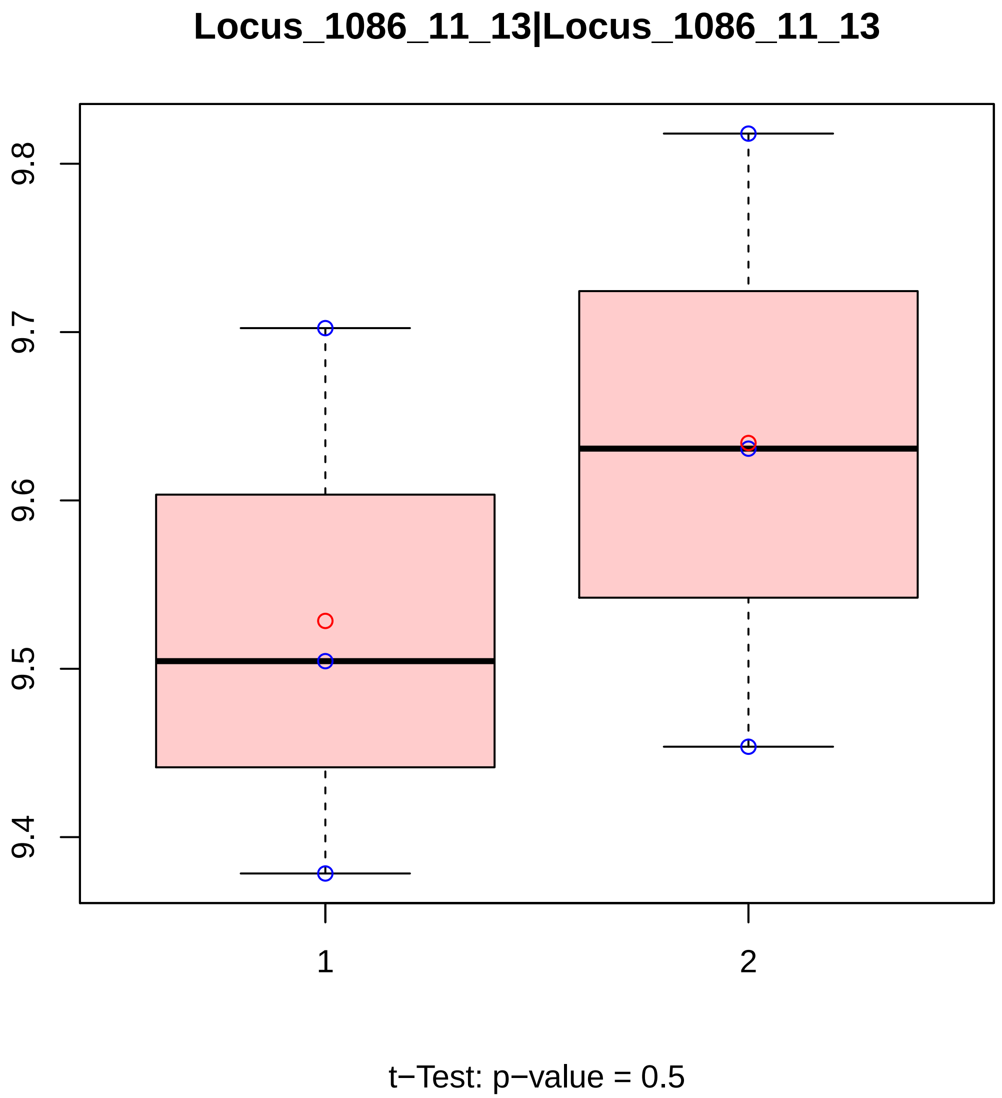

Locus_1086_11_13|Locus_1086_11_13
9.4
9.5
9.6
9.7
9.8
1
2
t−Test: p−value = 0.5

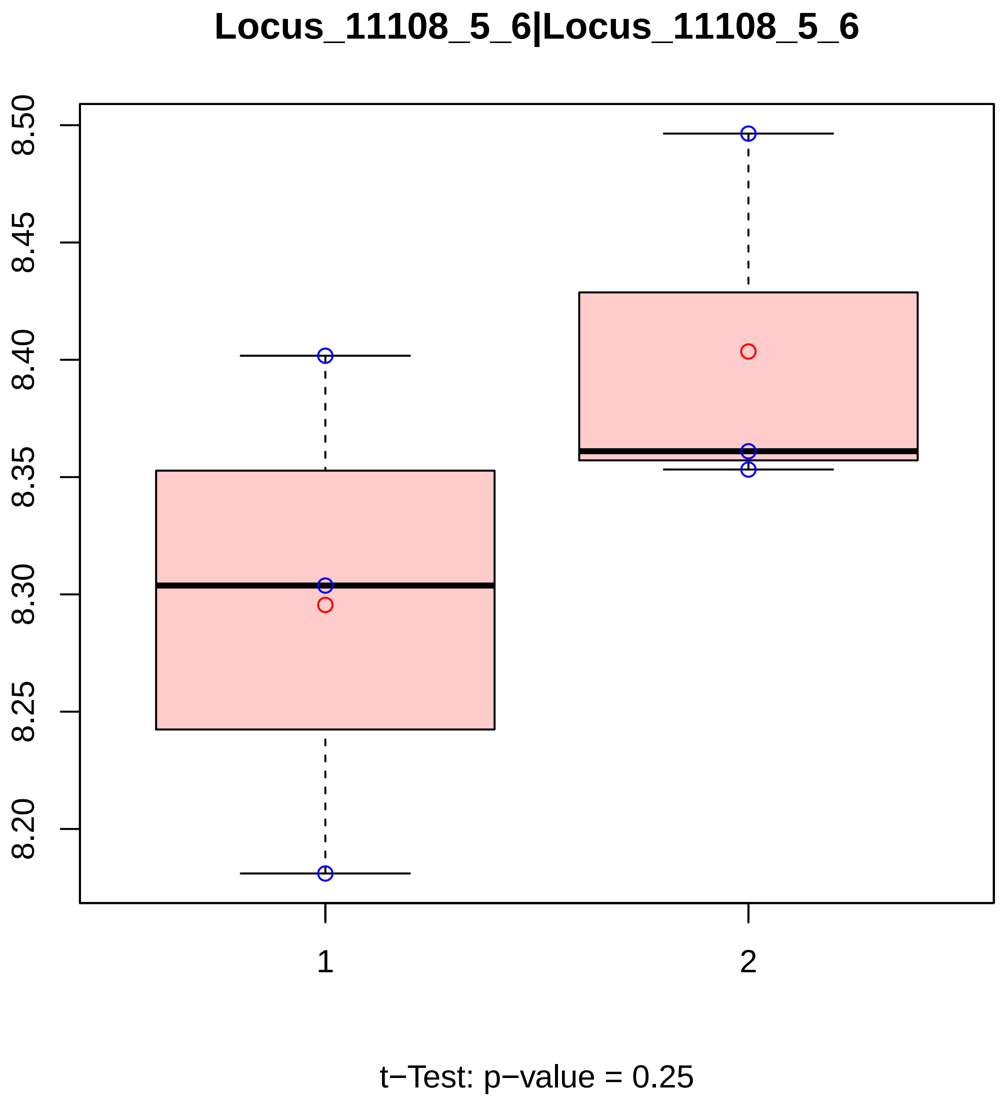

Locus_11108_5_6|Locus_11108_5_6
8.50
8.45
8.40
8.35
8.30
8.25
8.20
1
2
t−Test: p−value = 0.25

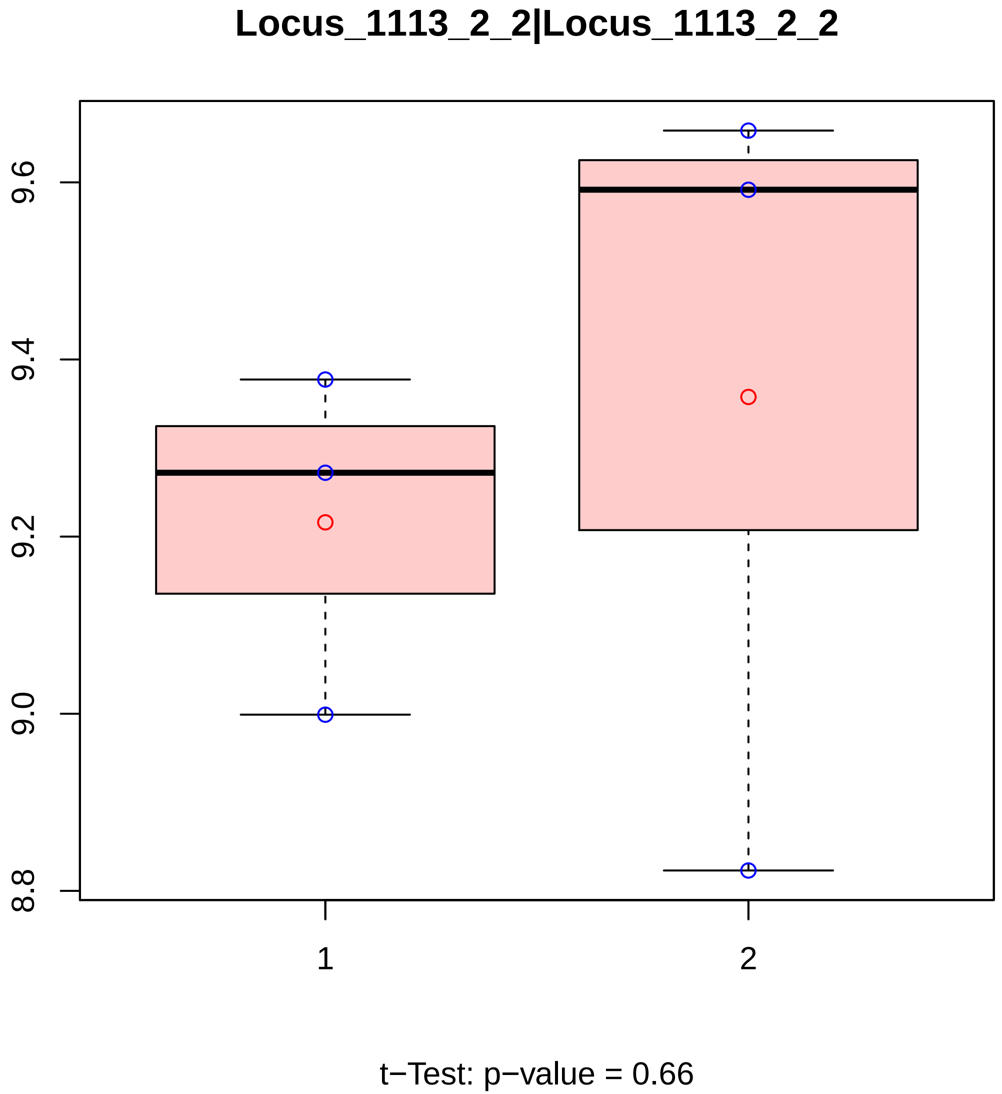
Locus_1113_2_2|Locus_1113_2_2
9.6
9.4
9.2
9.0
8.8
1
2
t−Test: p−value = 0.66

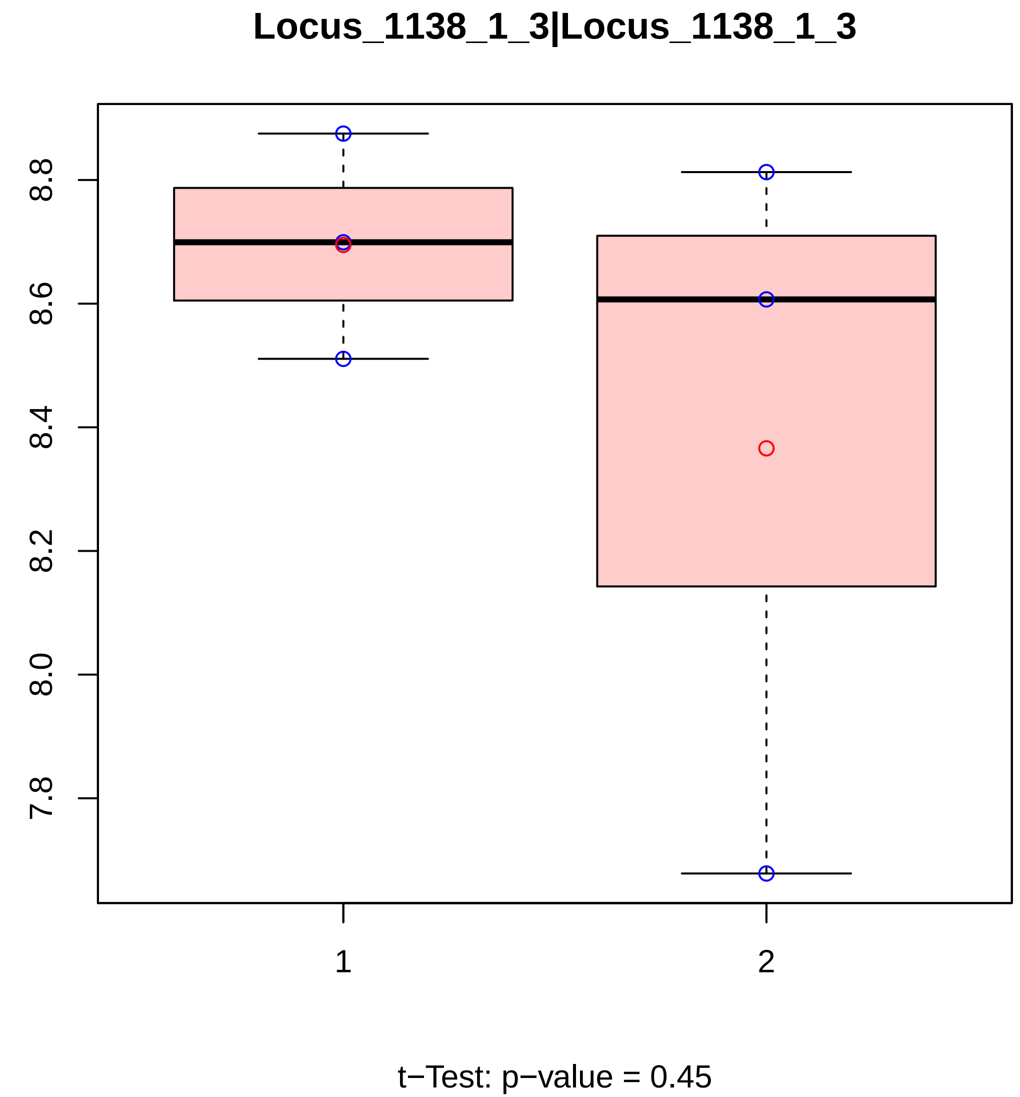

Locus_1138_1_3|Locus_1138_1_3
8.8
8.6
8.4
8.2
8.0
7.8
1
2
t−Test: p−value = 0.45

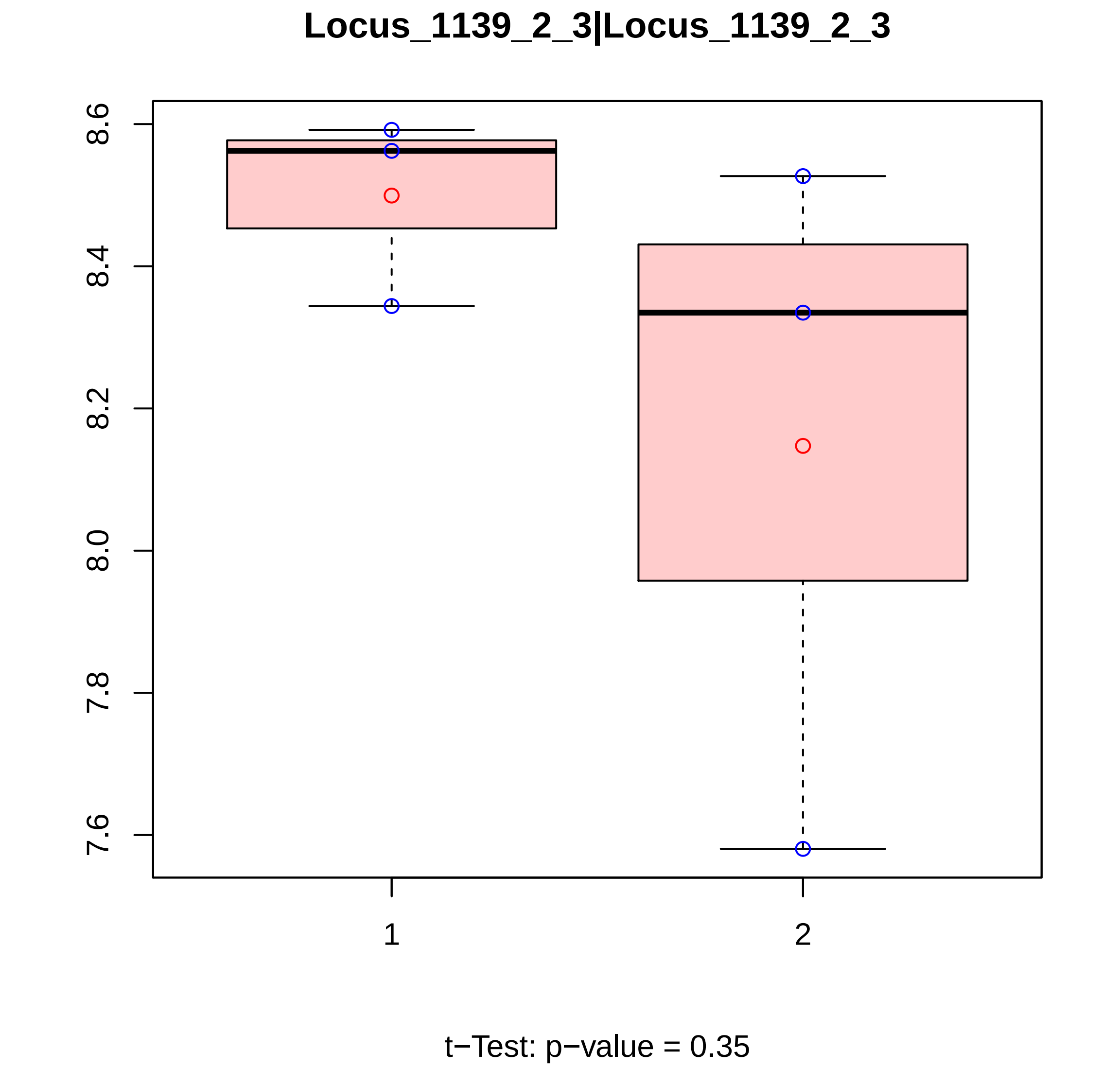
Locus_1139_2_3|Locus_1139_2_3
8.6
8.4
8.2
8.0
7.8
7.6
1
2
t−Test: p−value = 0.35

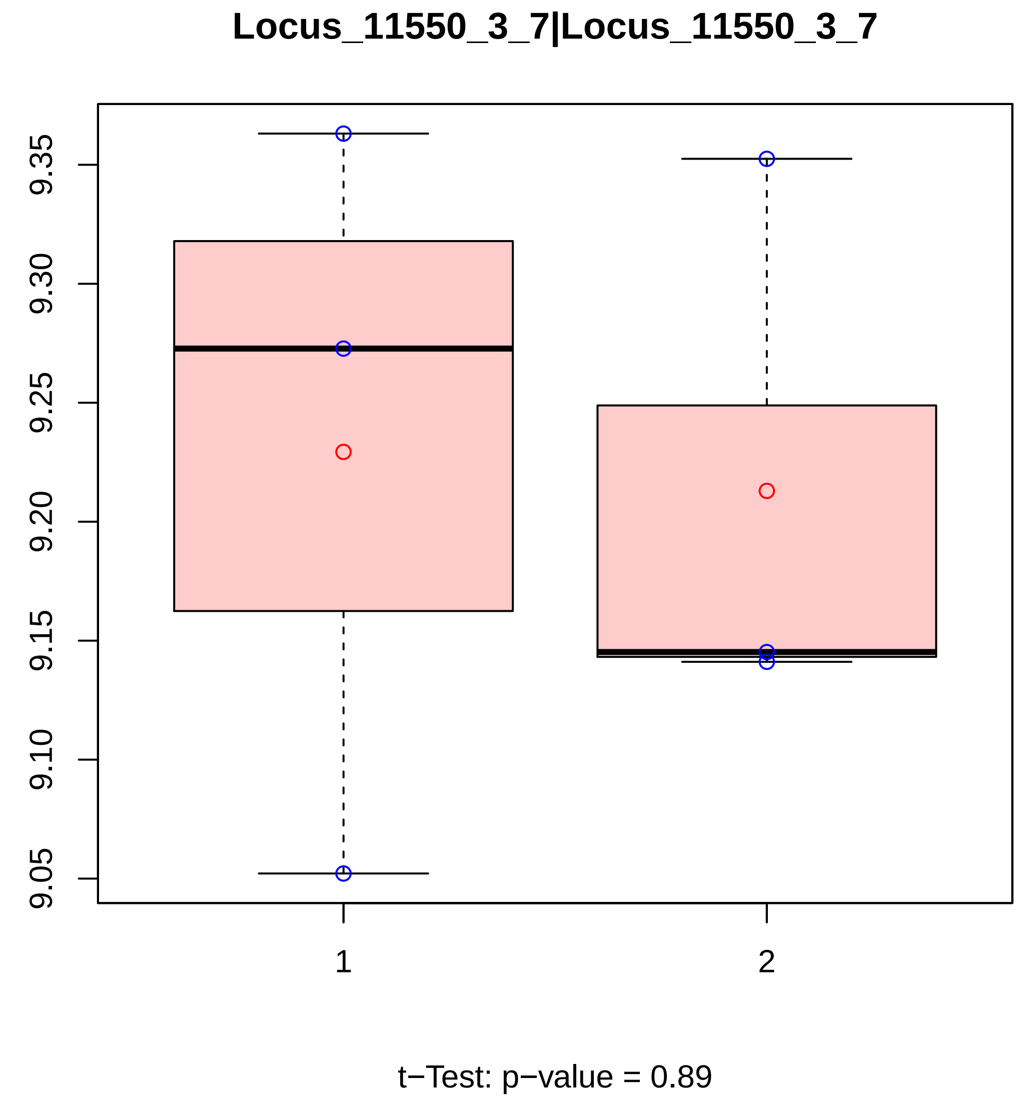
Locus_11550_3_7|Locus_11550_3_7
9.05
9.10
9.15
9.20
9.25
9.30
9.35
1
2
t−Test: p−value = 0.89

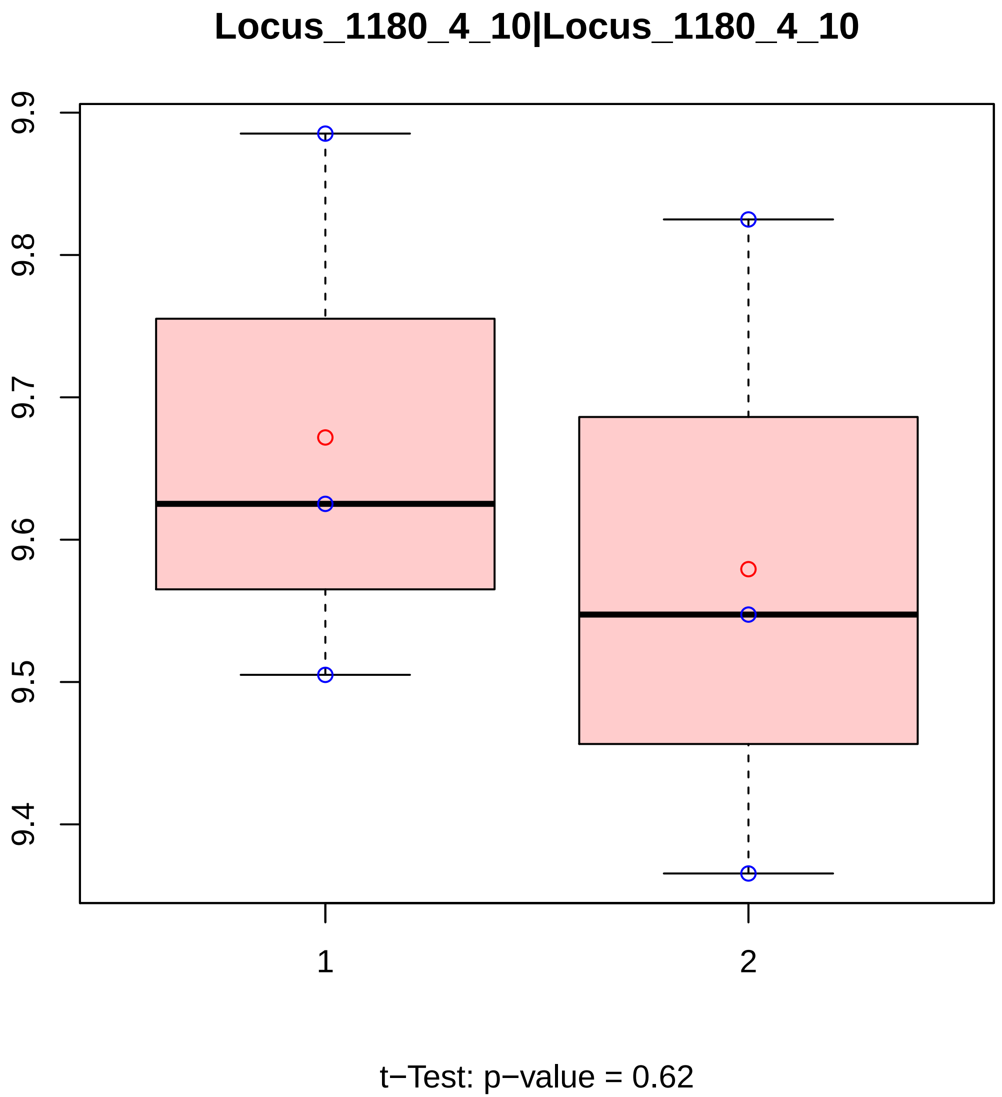

Locus_1180_4_10|Locus_1180_4_10
9.4
9.5
9.6
9.7
9.8
9.9
1
2
t−Test: p−value = 0.62

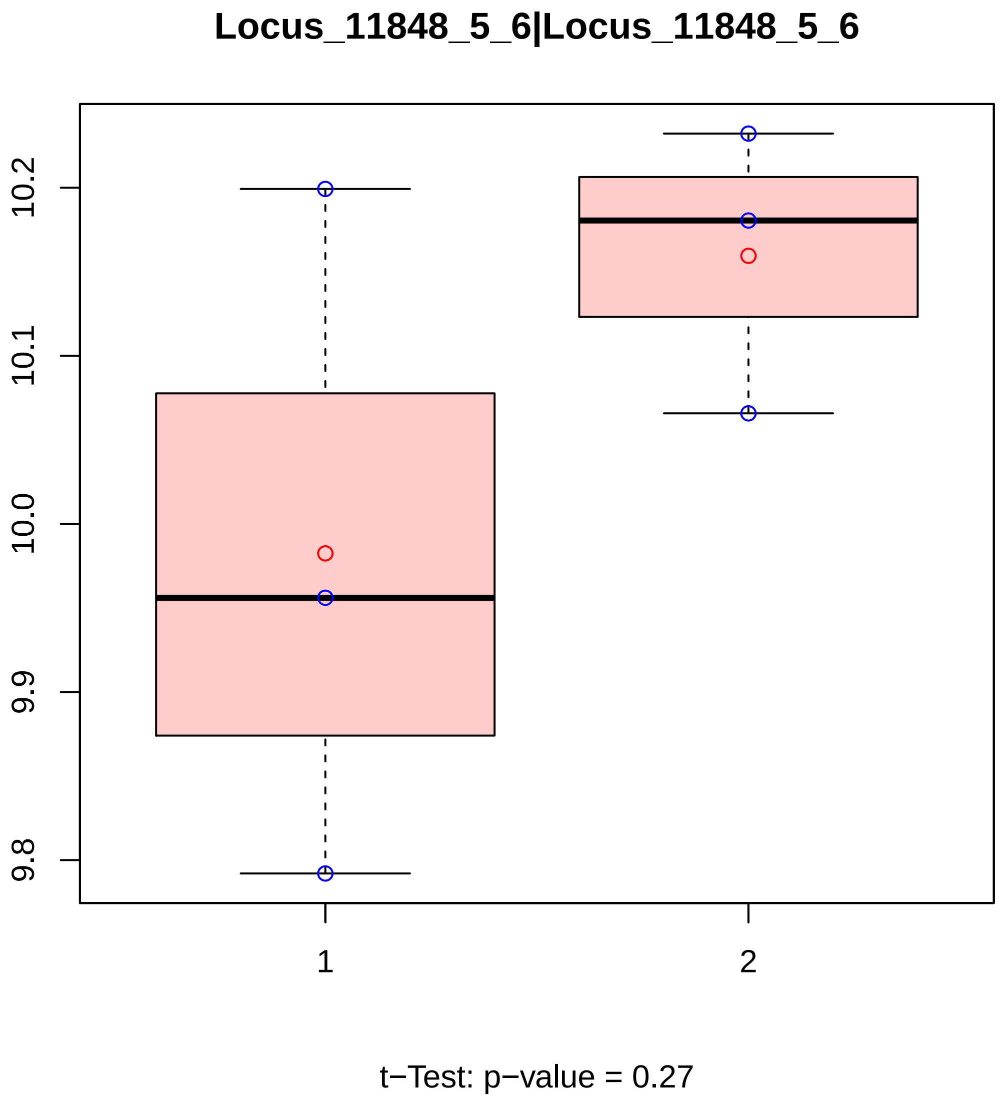
Locus_11848_5_6|Locus_11848_5_6
10.2
10.1
10.0
9.9
9.8
1
2
t−Test: p−value = 0.27

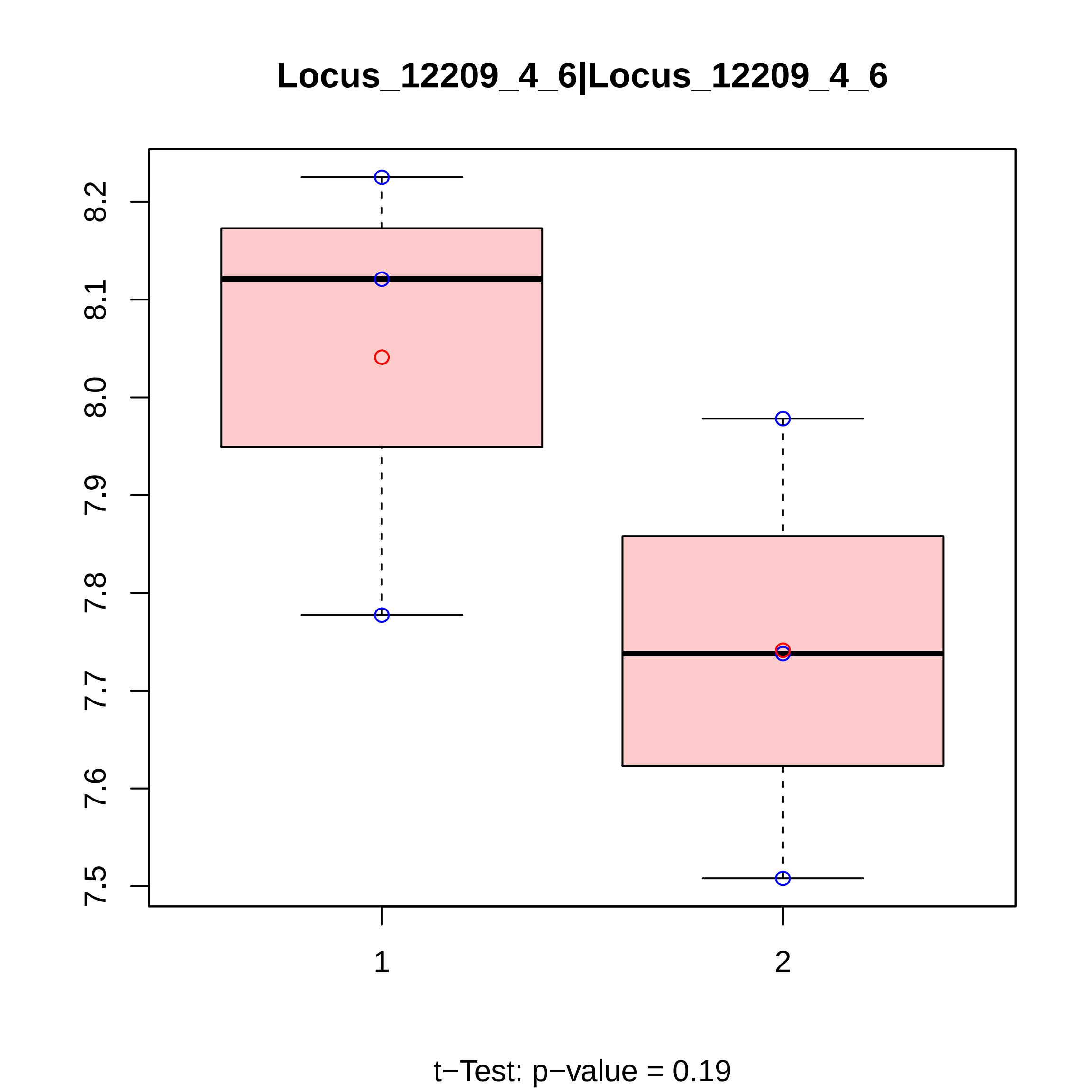
Locus_12209_4_6|Locus_12209_4_6
8.2
8.1
8.0
7.9
7.8
7.7
7.6
7.5
1
2
t−Test: p−value = 0.19

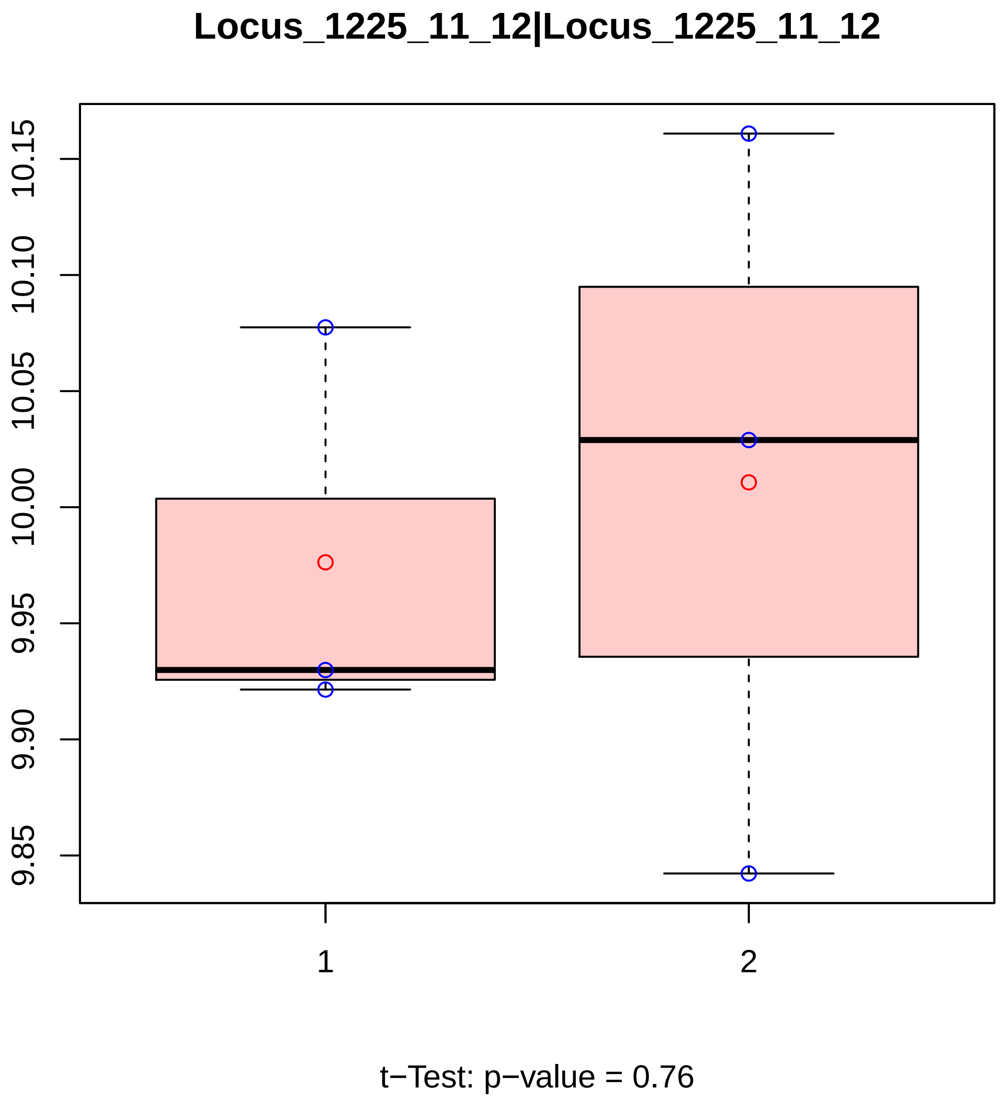

Locus_1225_11_12|Locus_1225_11_12
10.15
10.10
10.05
10.00
9.95
9.90
9.85
1
2
t−Test: p−value = 0.76

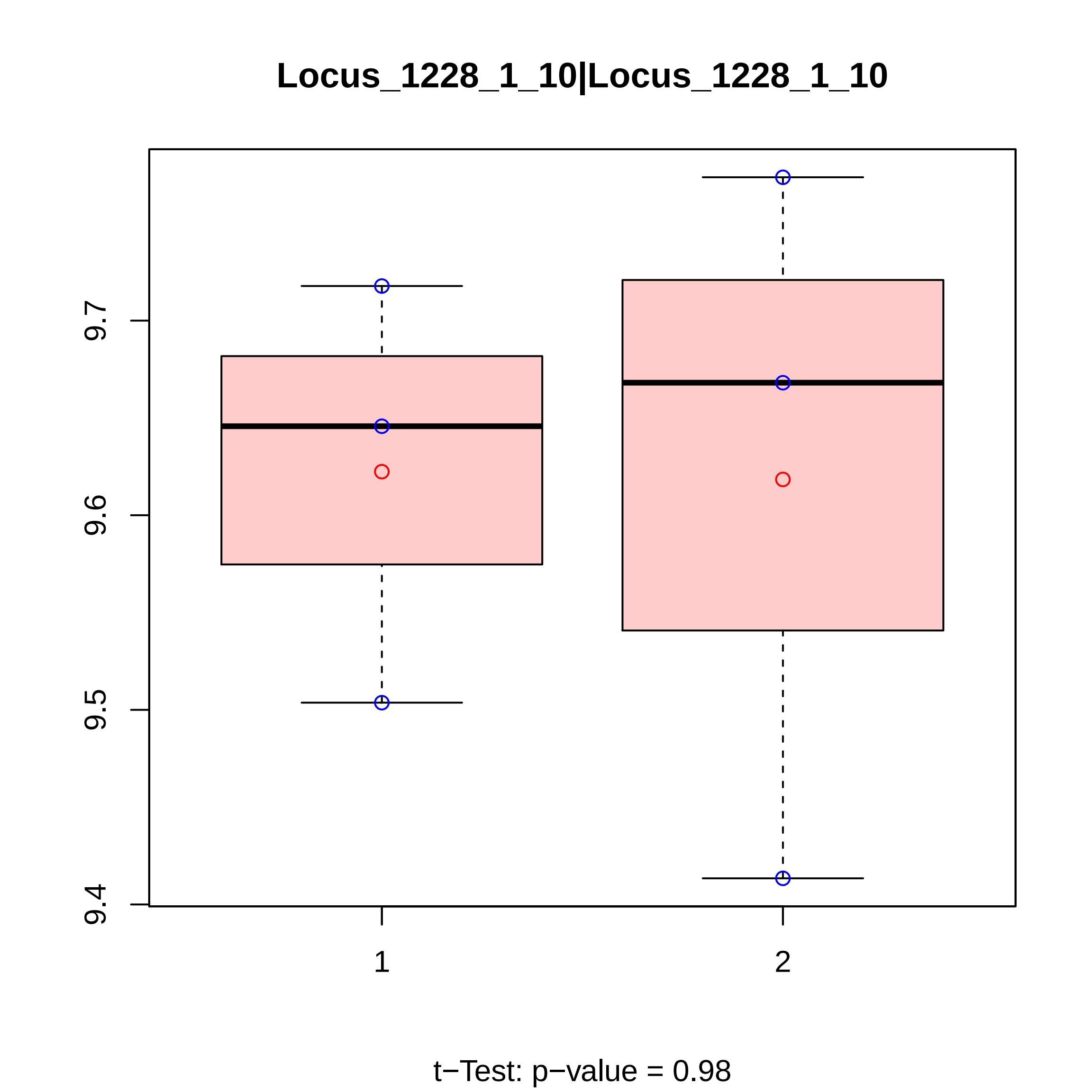

Locus_1228_1_10|Locus_1228_1_10
9.4
9.5
9.6
9.7
1
2
t−Test: p−value = 0.98

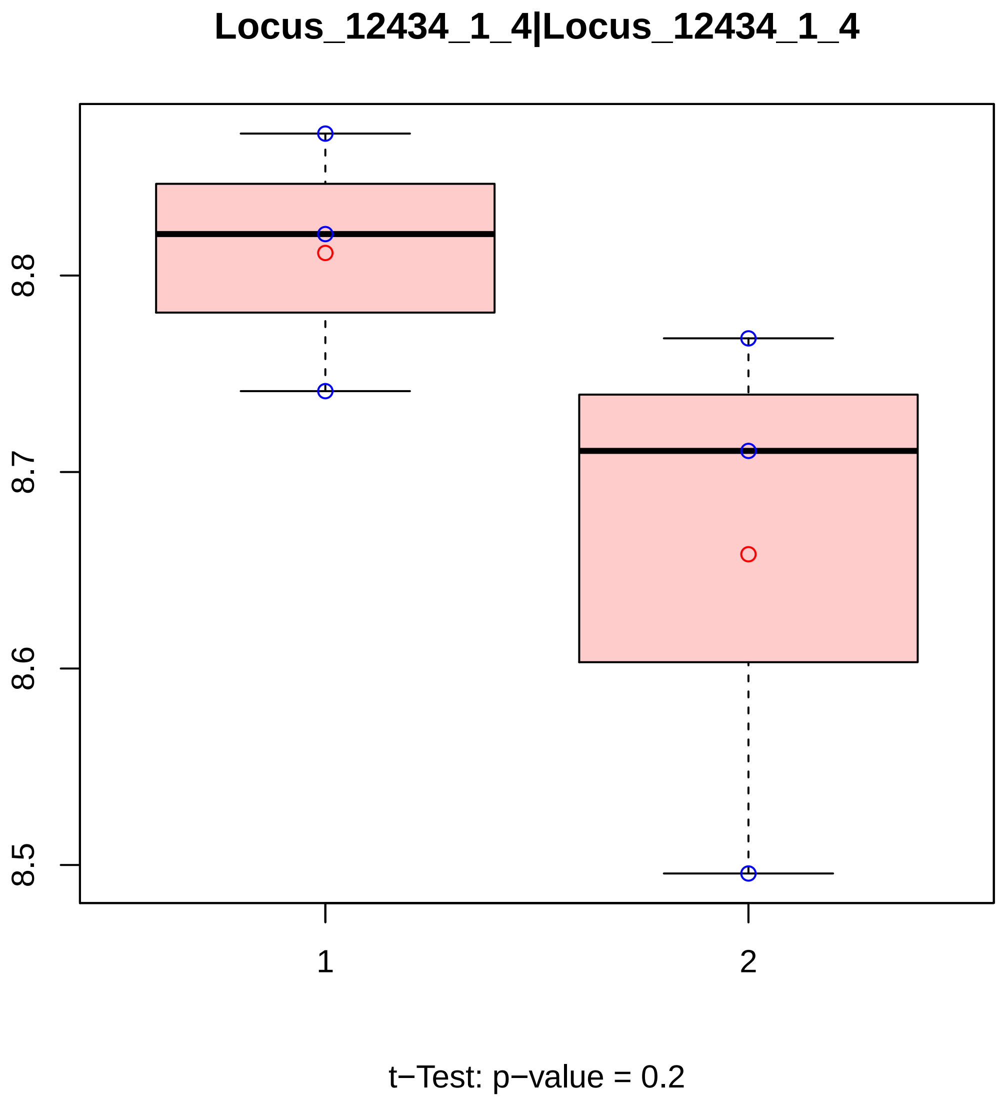

Locus_12434_1_4|Locus_12434_1_4
8.5
8.6
8.7
8.8
1
2
t−Test: p−value = 0.2

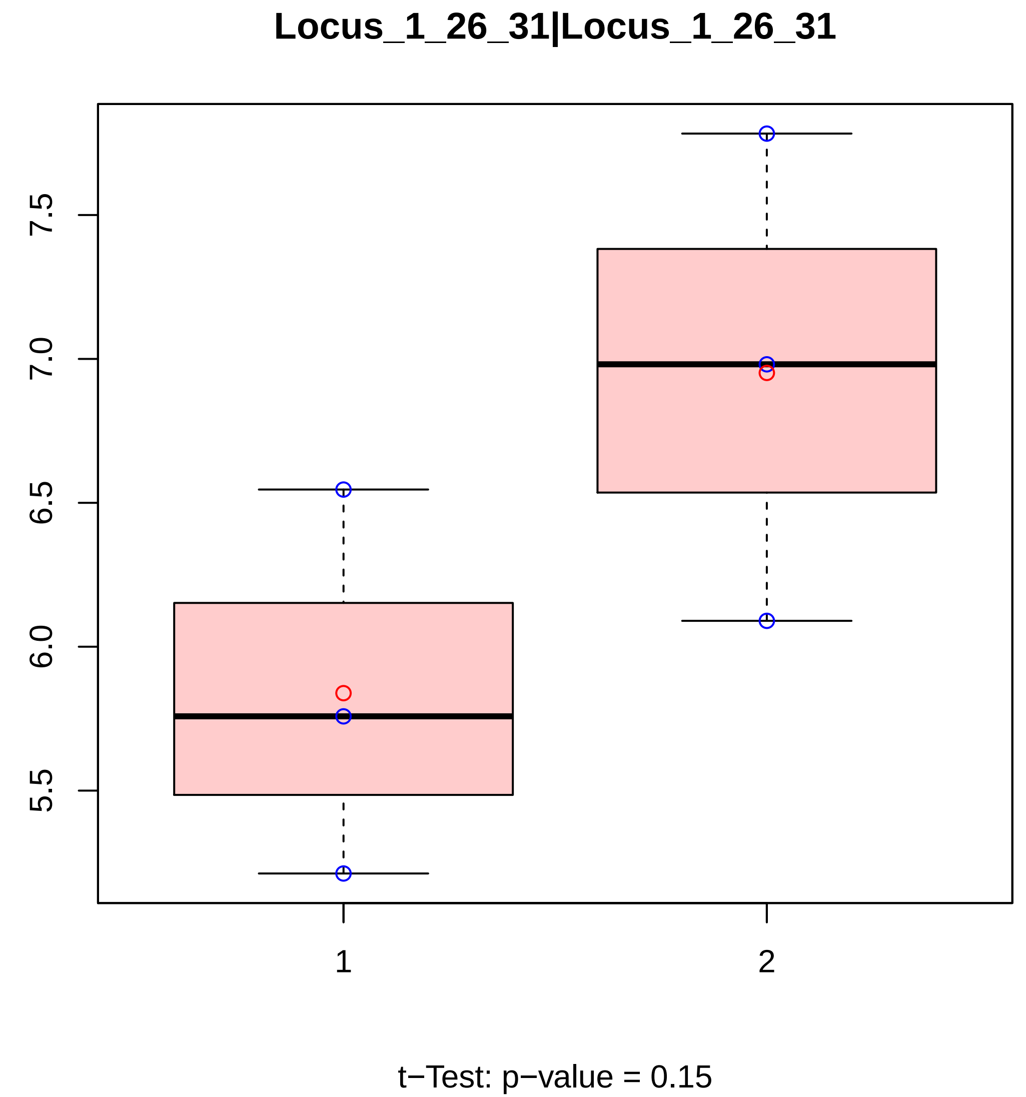
Locus_1_26_31|Locus_1_26_31
7.5
7.0
6.5
6.0
5.5
1
2
t−Test: p−value = 0.15

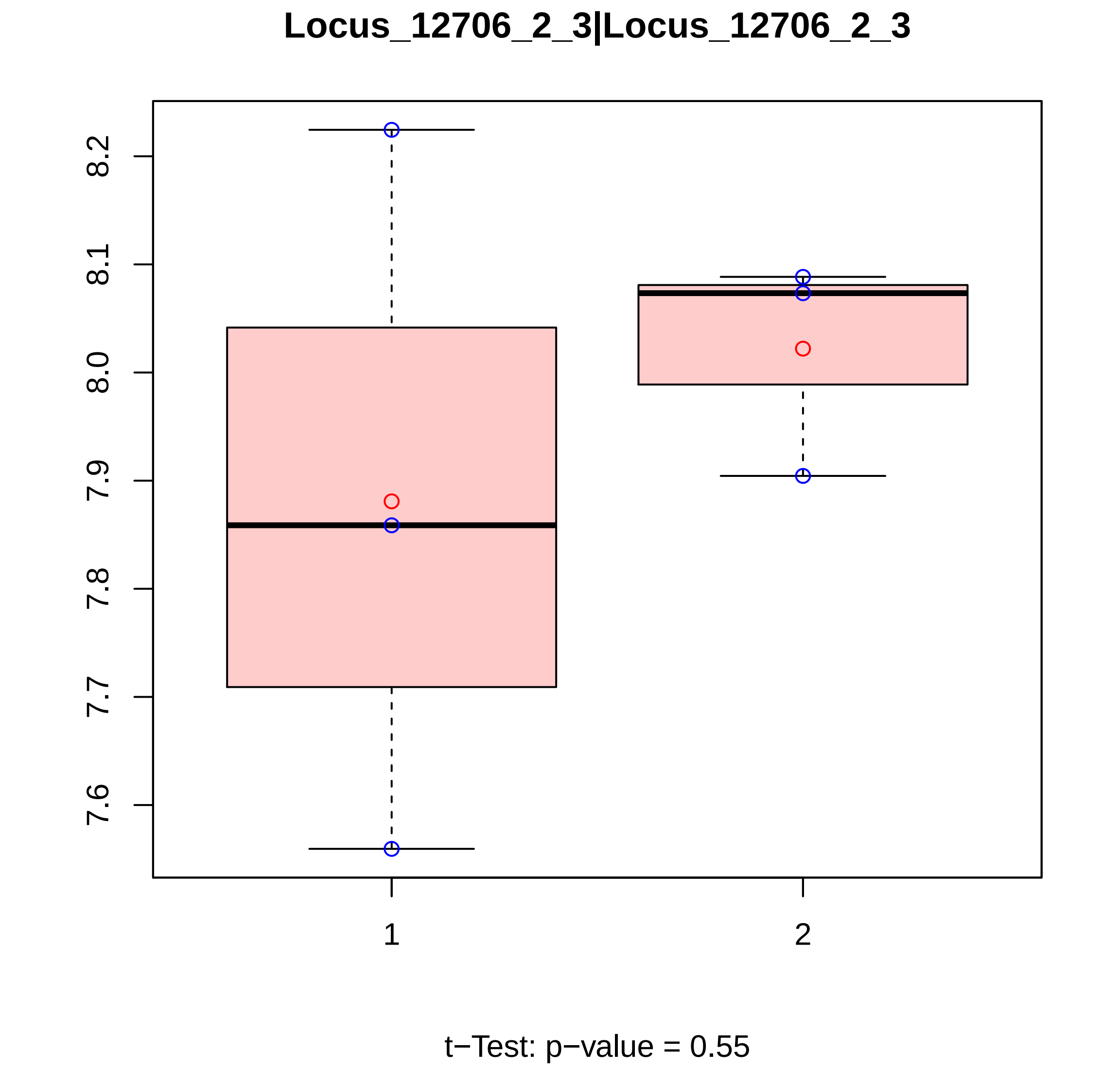
Locus_12706_2_3|Locus_12706_2_3
8.2
8.1
8.0
7.9
7.8
7.7
7.6
1
2
t−Test: p−value = 0.55

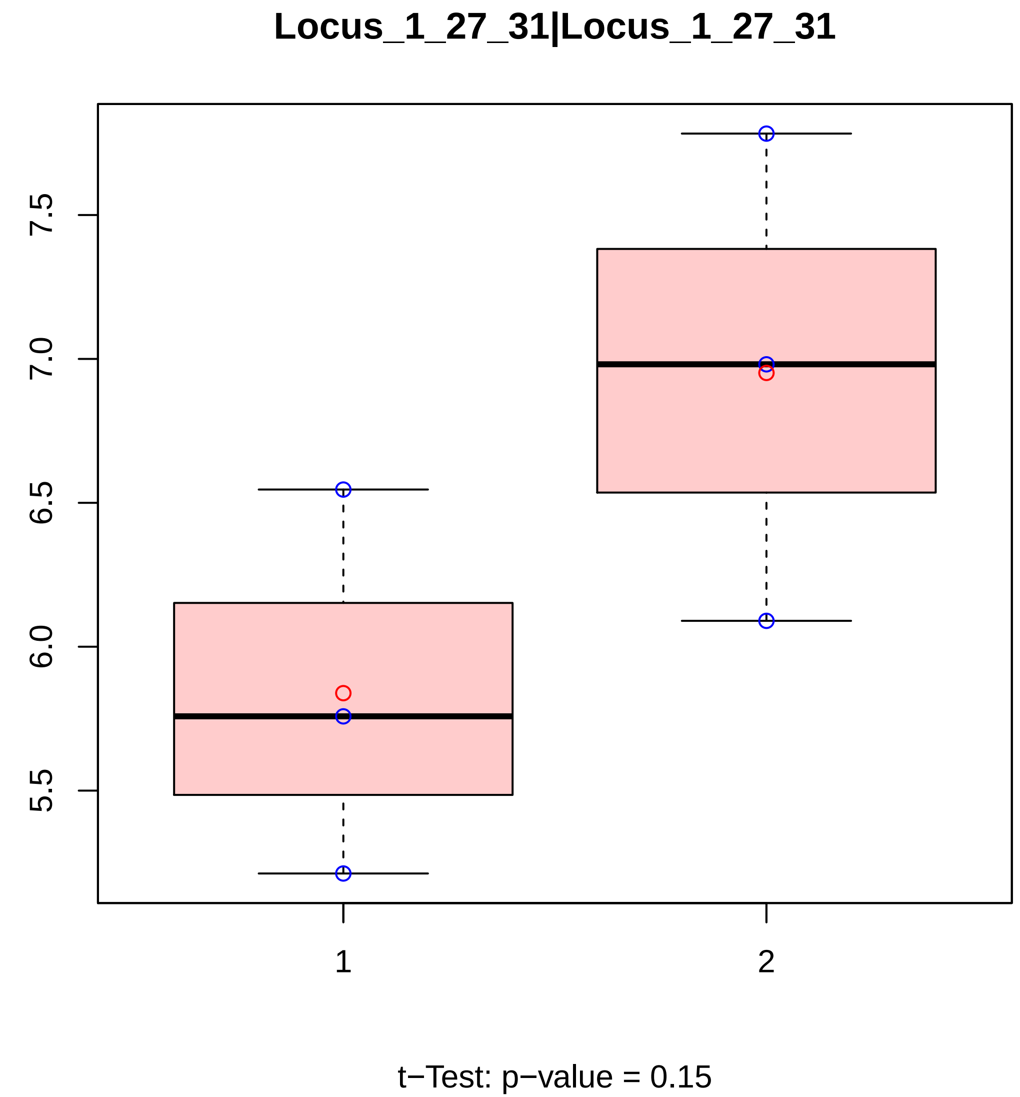

Locus_1_27_31|Locus_1_27_31
5.5
6.0
6.5
7.0
7.5
1
2
t−Test: p−value = 0.15

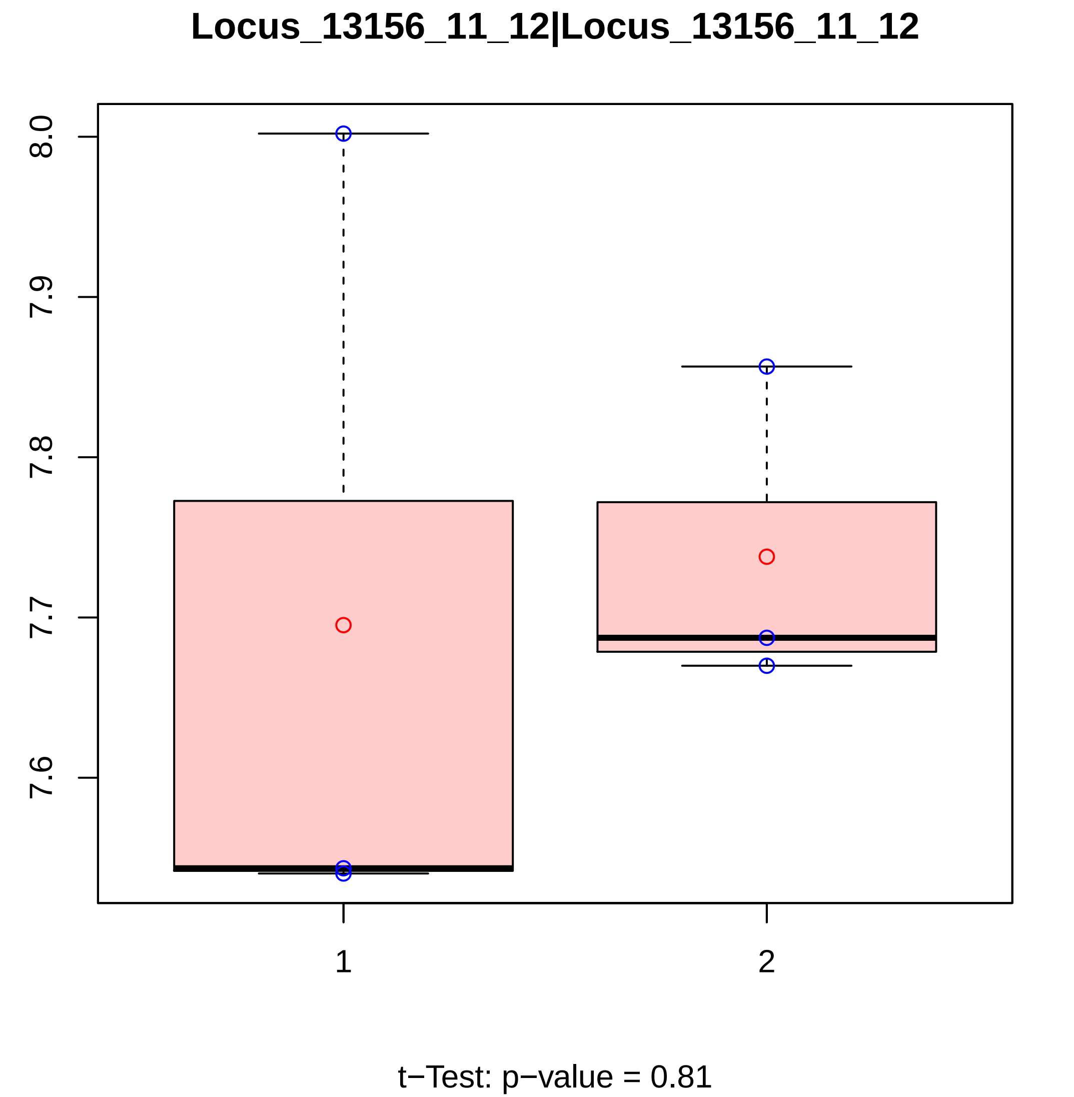

Locus_13156_11_12|Locus_13156_11_12
8.0
7.9
7.8
7.7
7.6
1
2
t−Test: p−value = 0.81

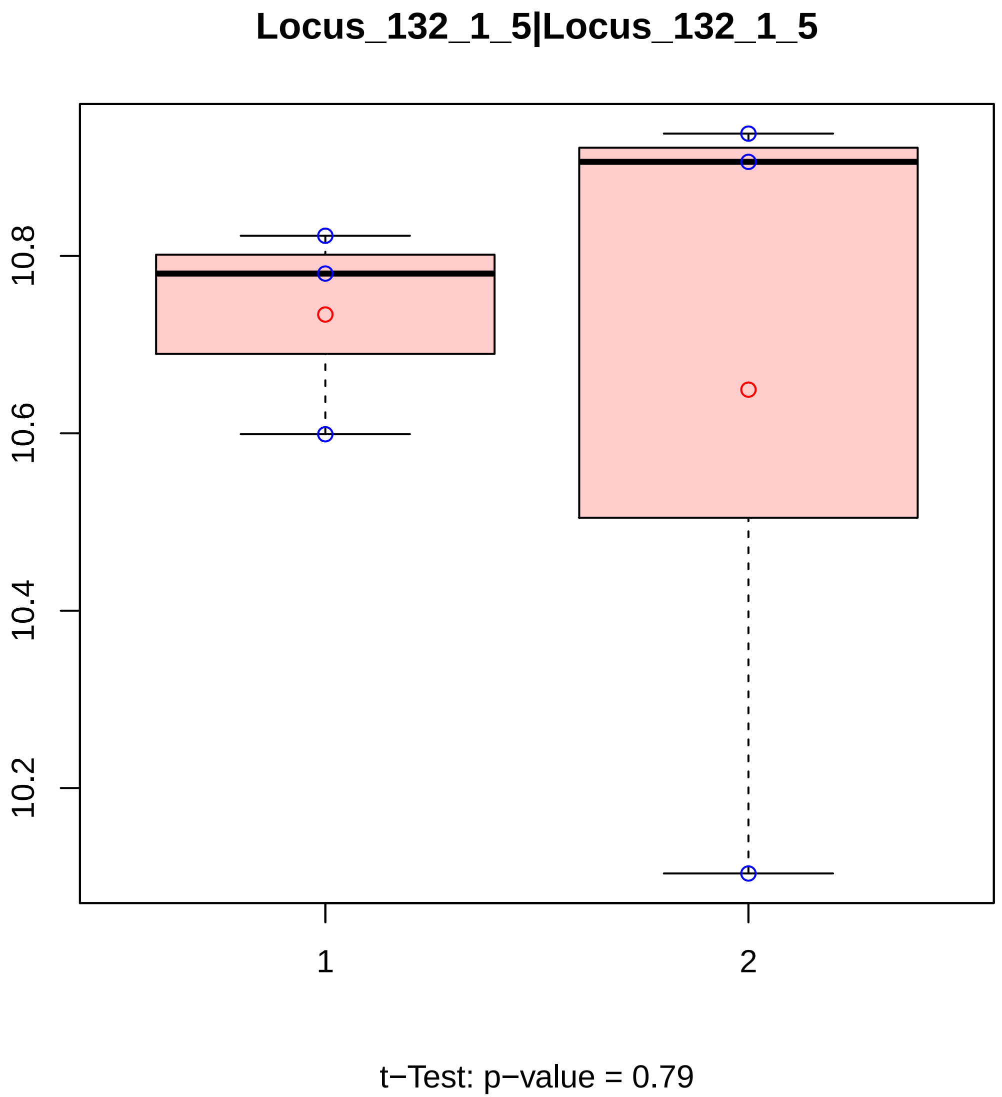

Locus_132_1_5|Locus_132_1_5
10.2
10.4
10.6
10.8
1
2
t−Test: p−value = 0.79

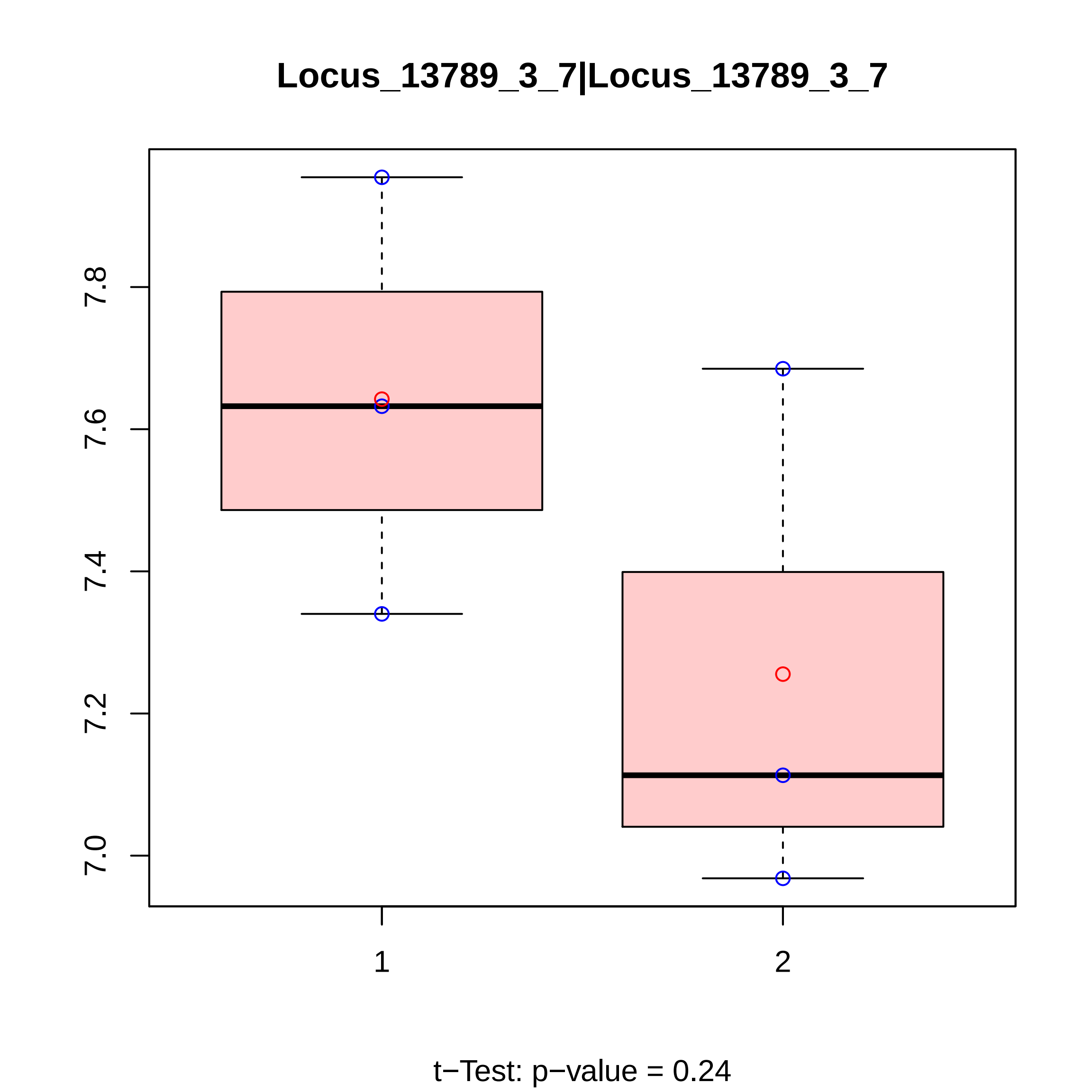
Locus_13789_3_7|Locus_13789_3_7
7.0
7.2
7.4
7.6
7.8
1
2
t−Test: p−value = 0.24

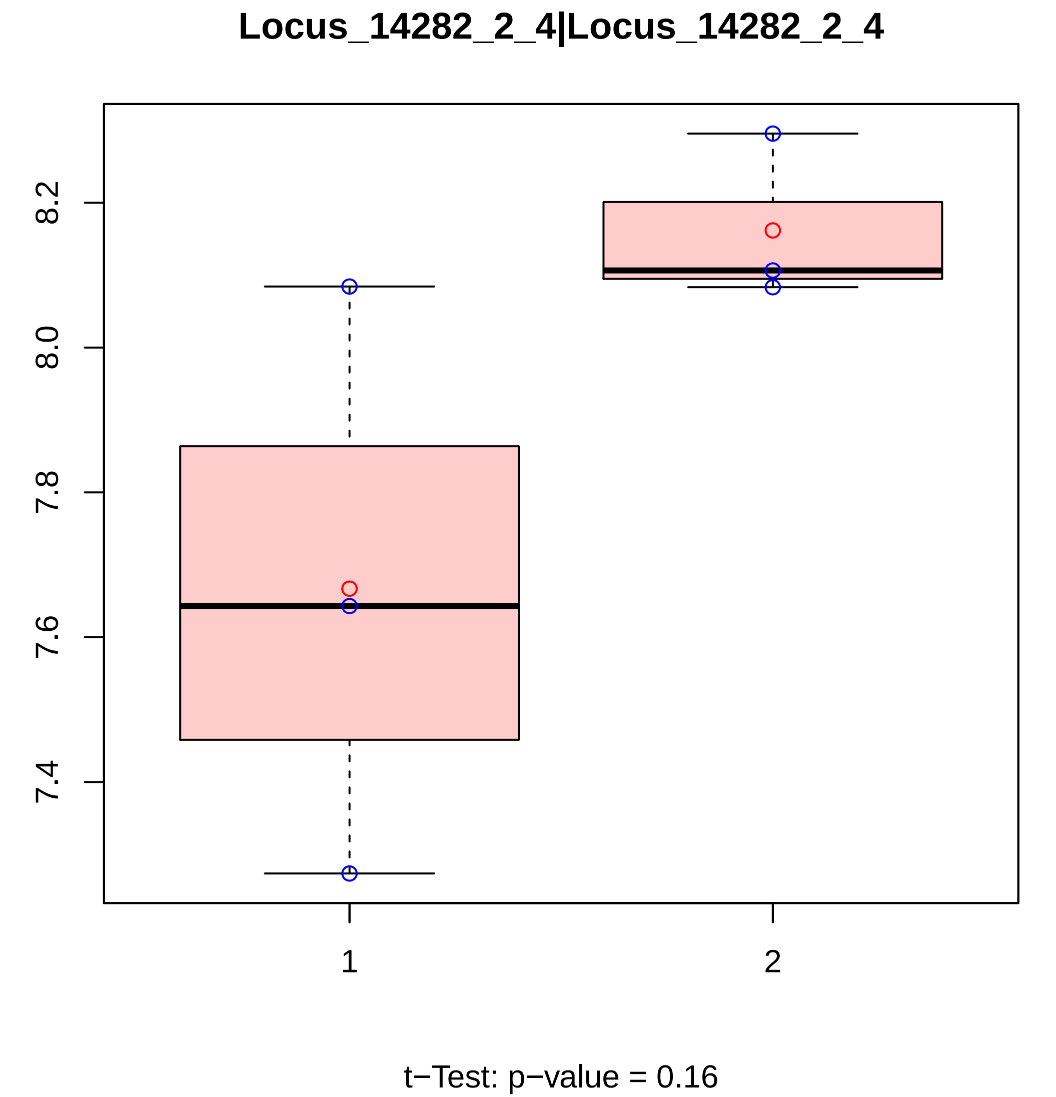

Locus_14282_2_4|Locus_14282_2_4
8.2
8.0
7.8
7.6
7.4
1
2
t−Test: p−value = 0.16

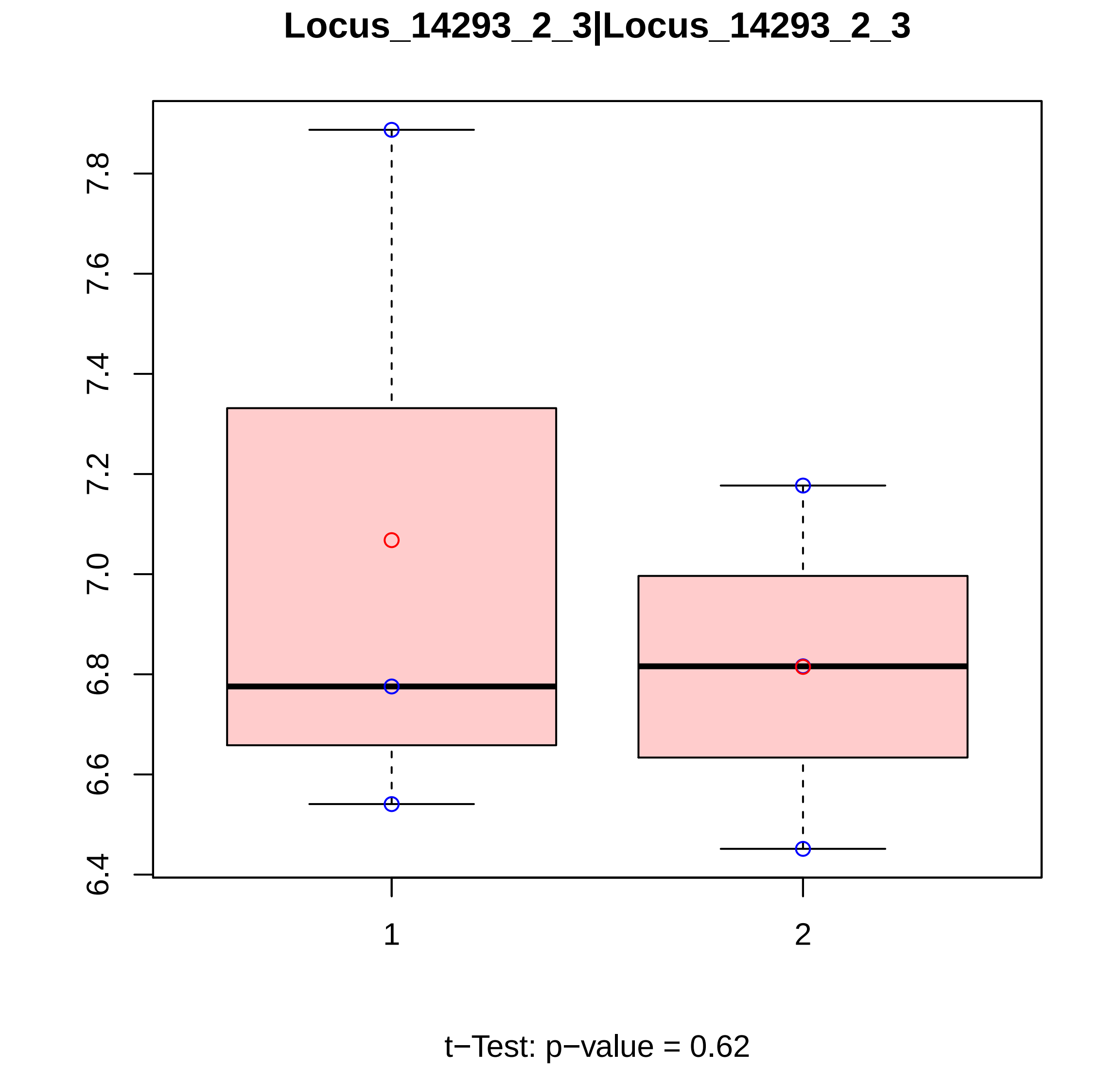

Locus_14293_2_3|Locus_14293_2_3
7.8
7.6
7.4
7.2
7.0
6.8
6.6
6.4
1
2
t−Test: p−value = 0.62

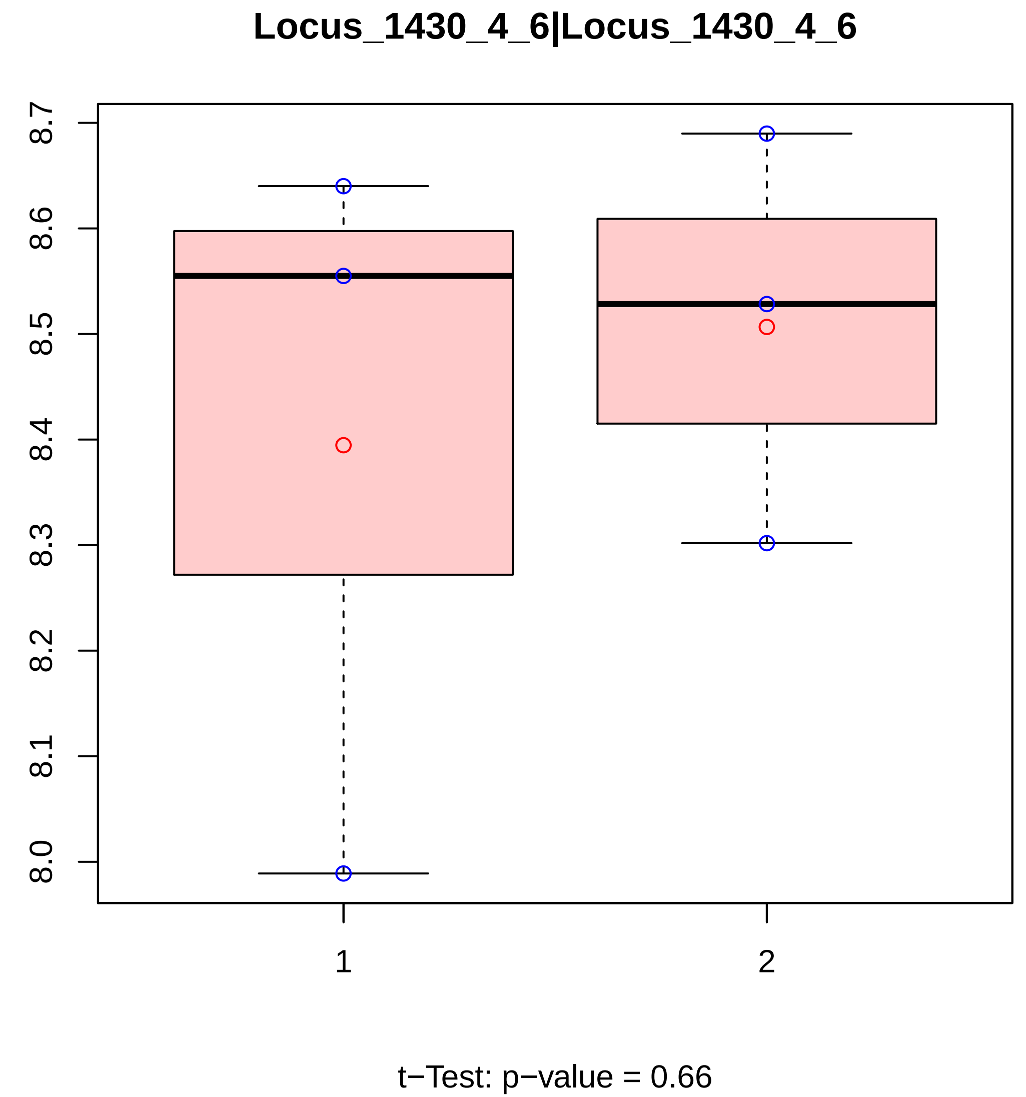

Locus_1430_4_6|Locus_1430_4_6
8.0
8.1
8.2
8.3
8.4
8.5
8.6
8.7
1
2
t−Test: p−value = 0.66

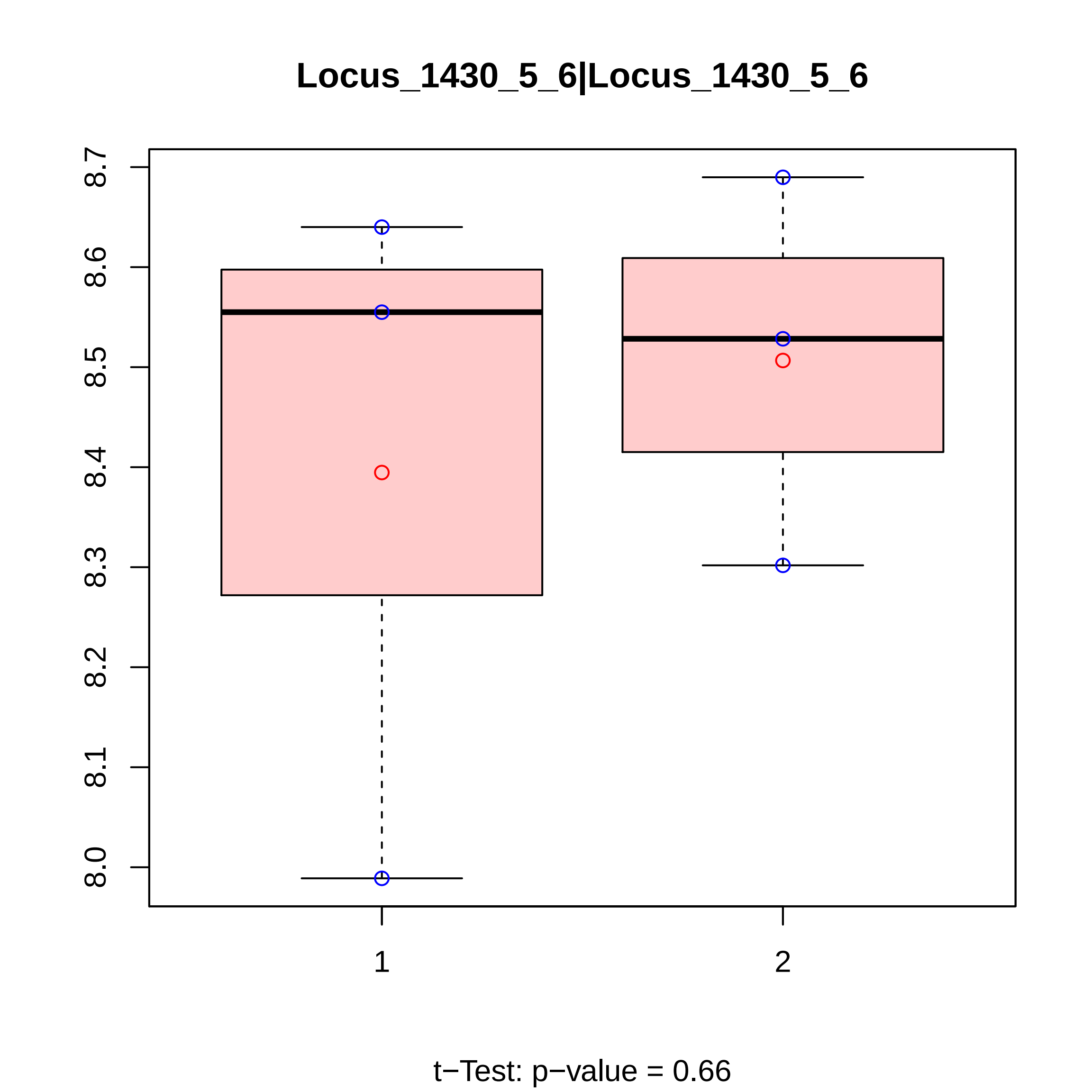
Locus_1430_5_6|Locus_1430_5_6
8.0
8.1
8.2
8.3
8.4
8.5
8.6
8.7
1
2
t−Test: p−value = 0.66

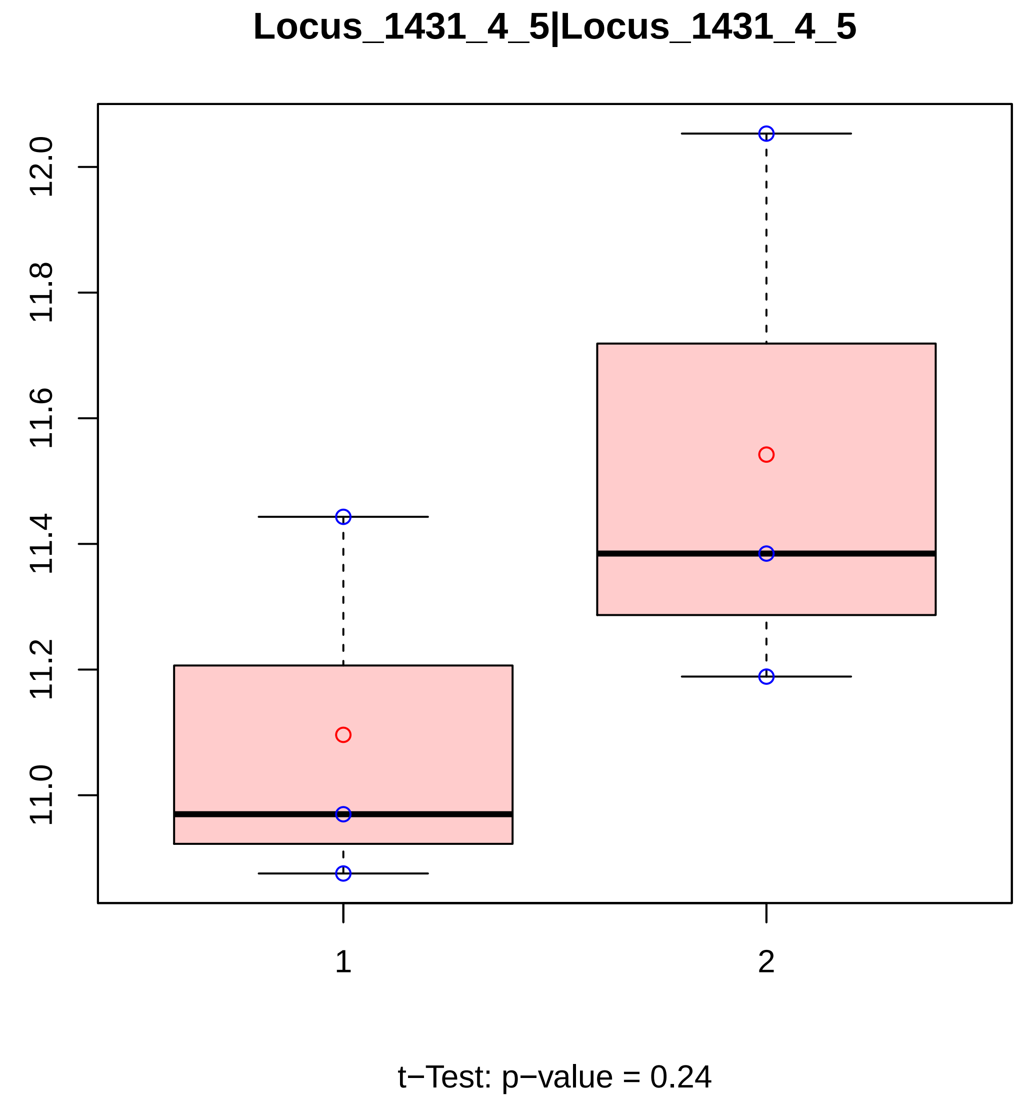
Locus_1431_4_5|Locus_1431_4_5
12.0
11.8
11.6
11.4
11.2
11.0
1
2
t−Test: p−value = 0.24

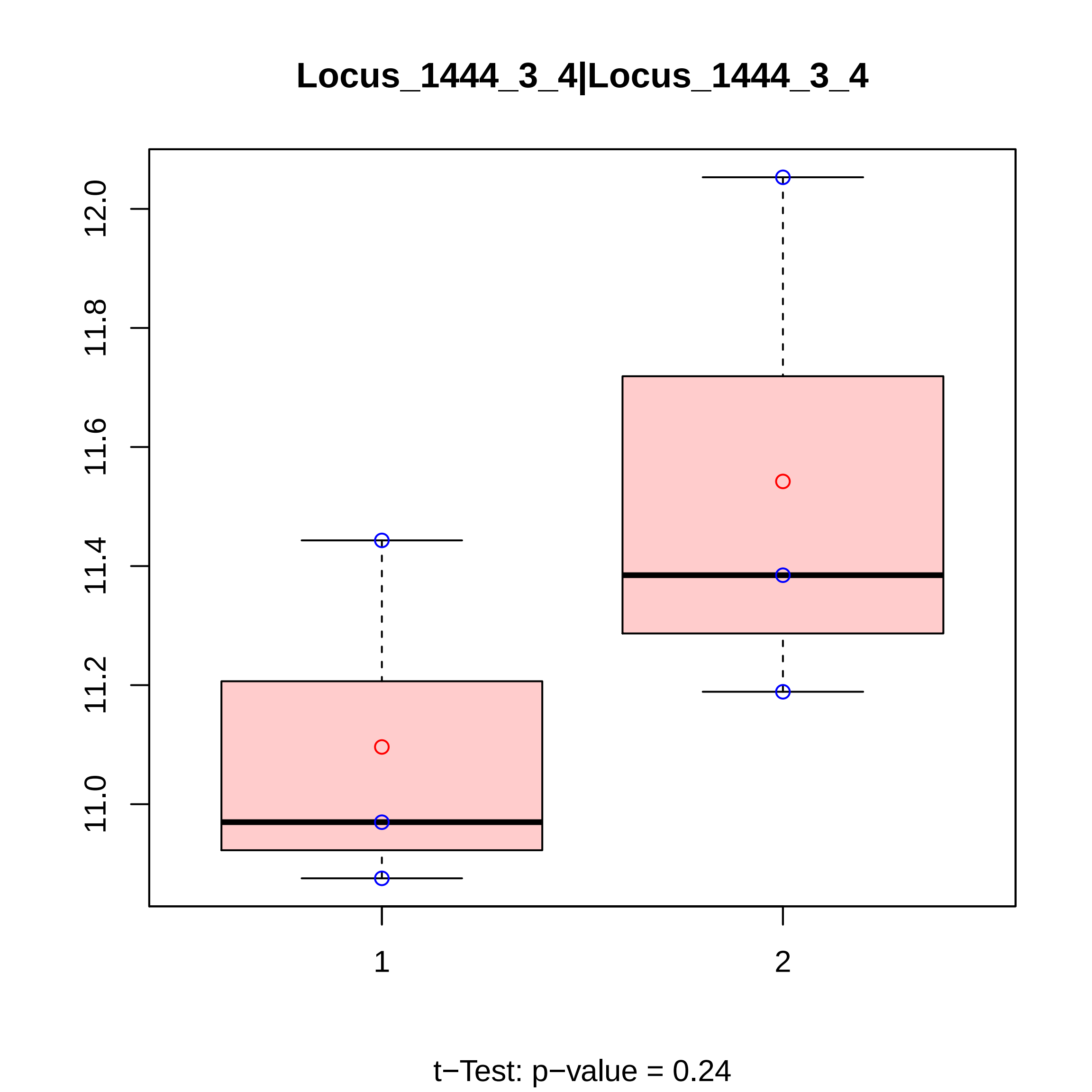

Locus_1444_3_4|Locus_1444_3_4
12.0
11.8
11.6
11.4
11.2
11.0
1
2
t−Test: p−value = 0.24

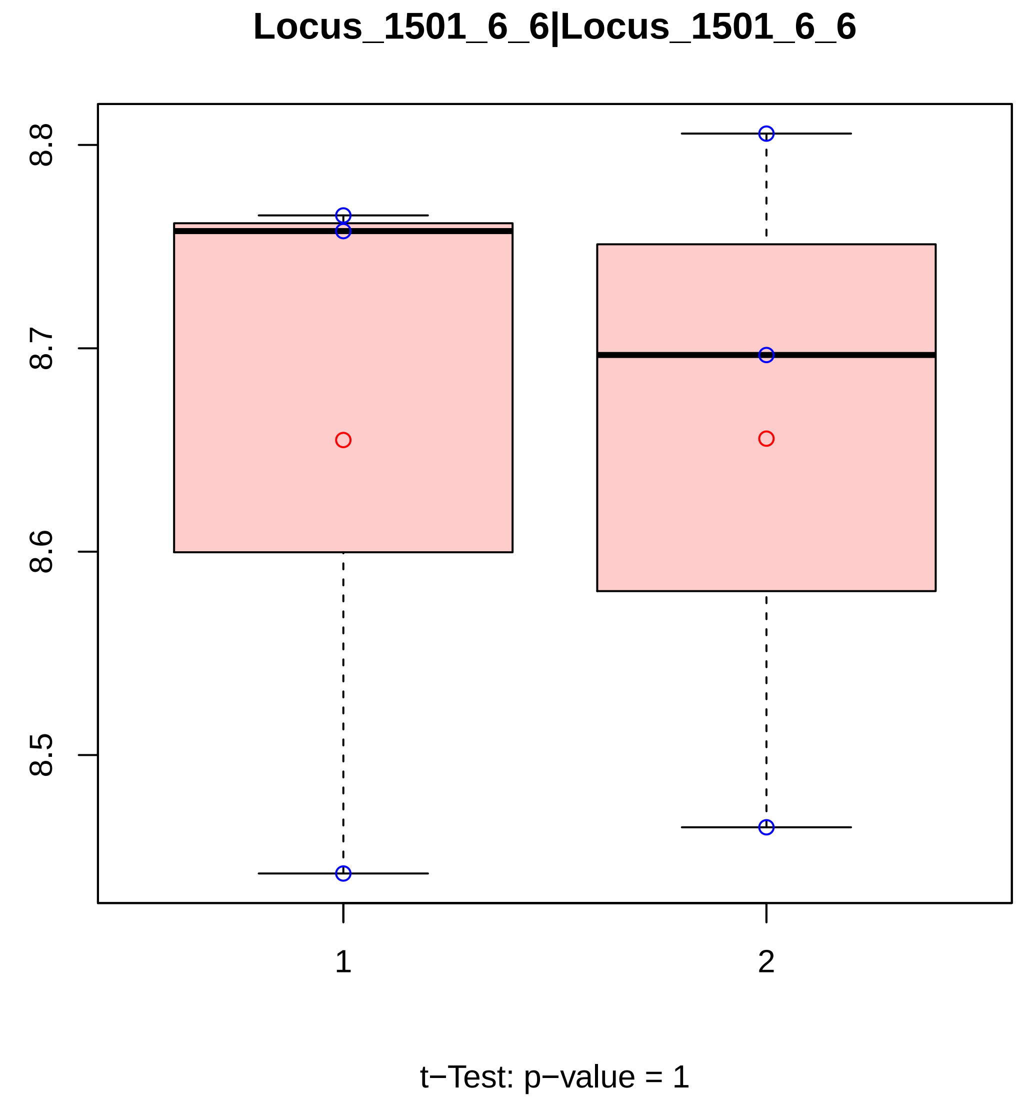
Locus_1501_6_6|Locus_1501_6_6
8.5
8.6
8.7
8.8
1
2
t−Test: p−value = 1

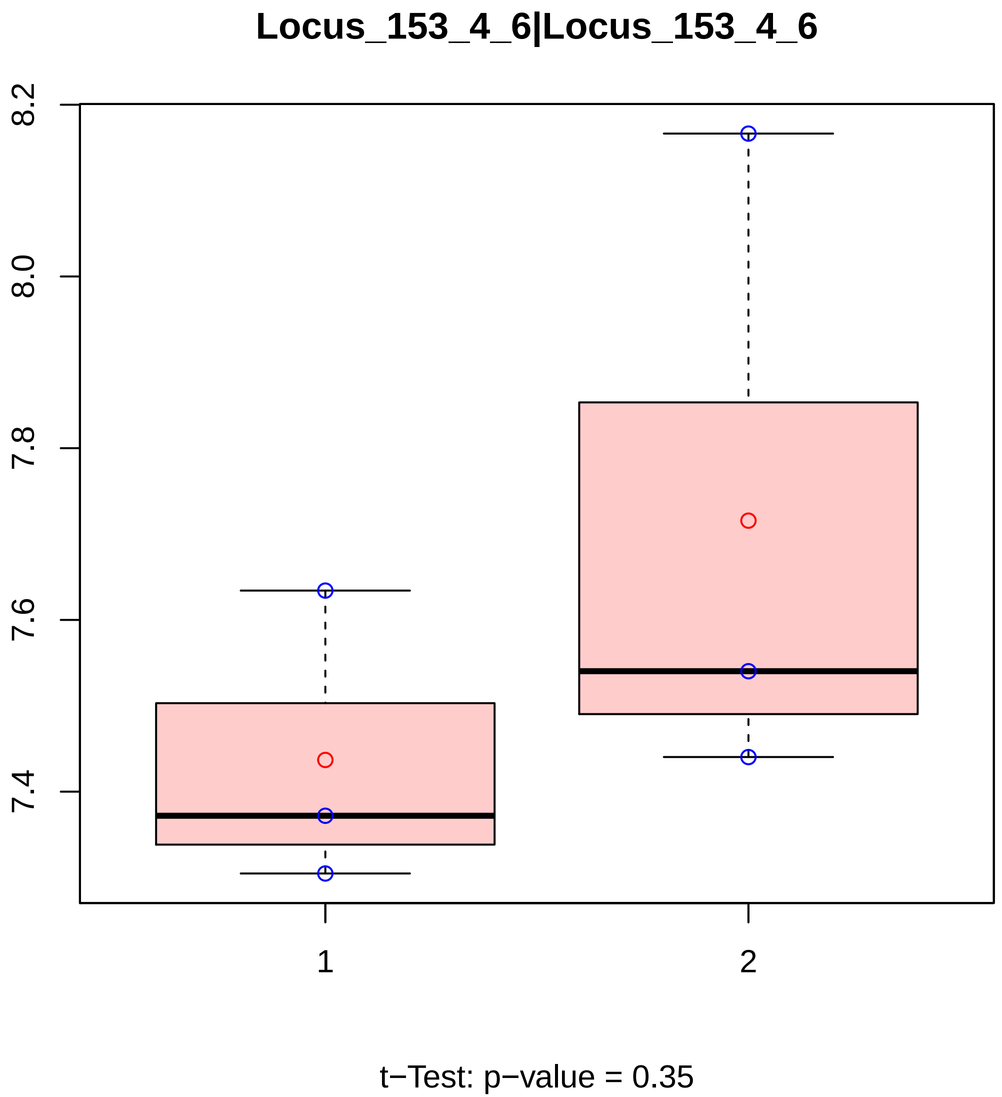
Locus_153_4_6|Locus_153_4_6
8.2
8.0
7.8
7.6
7.4
1
2
t−Test: p−value = 0.35

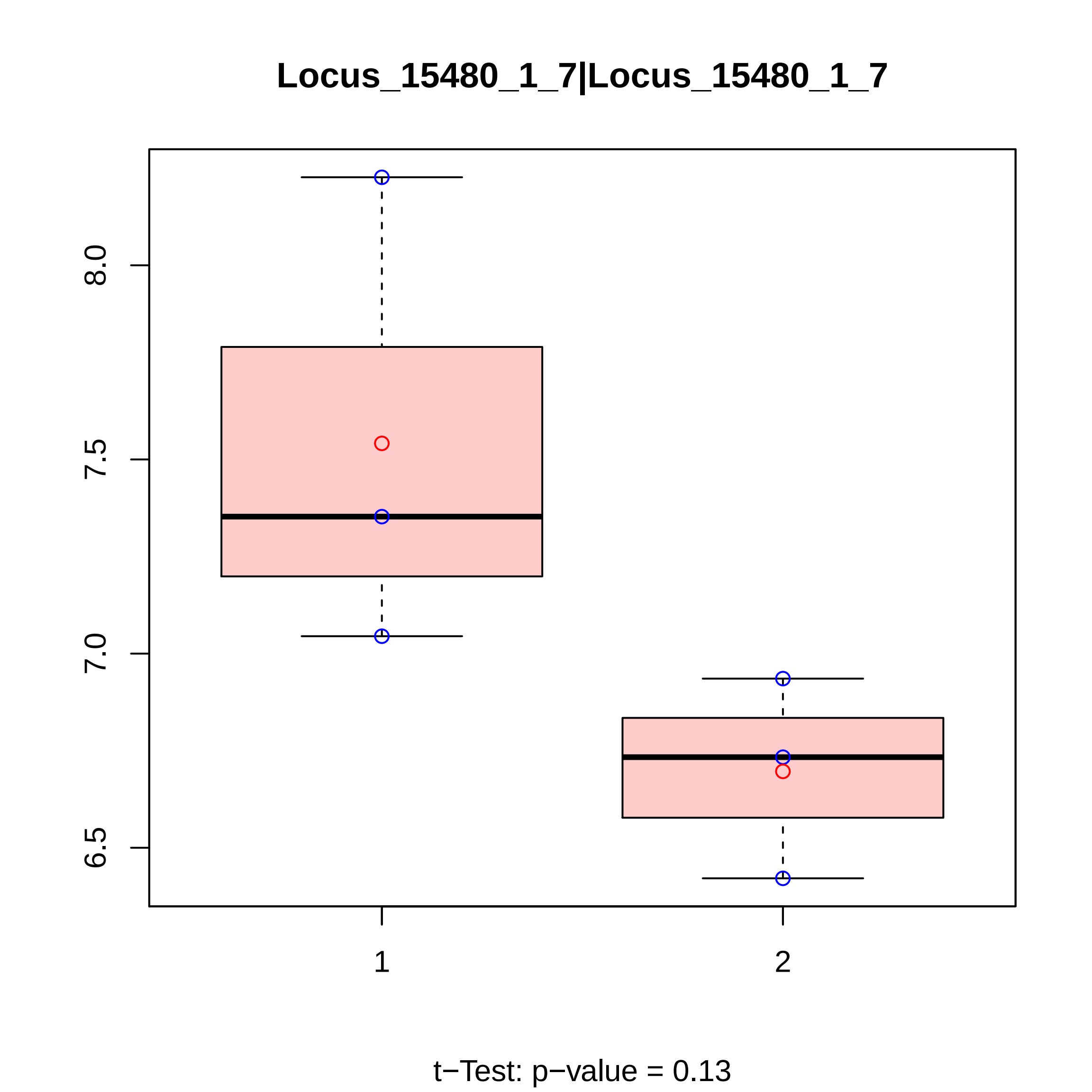

Locus_15480_1_7|Locus_15480_1_7
8.0
7.5
7.0
6.5
1
2
t−Test: p−value = 0.13

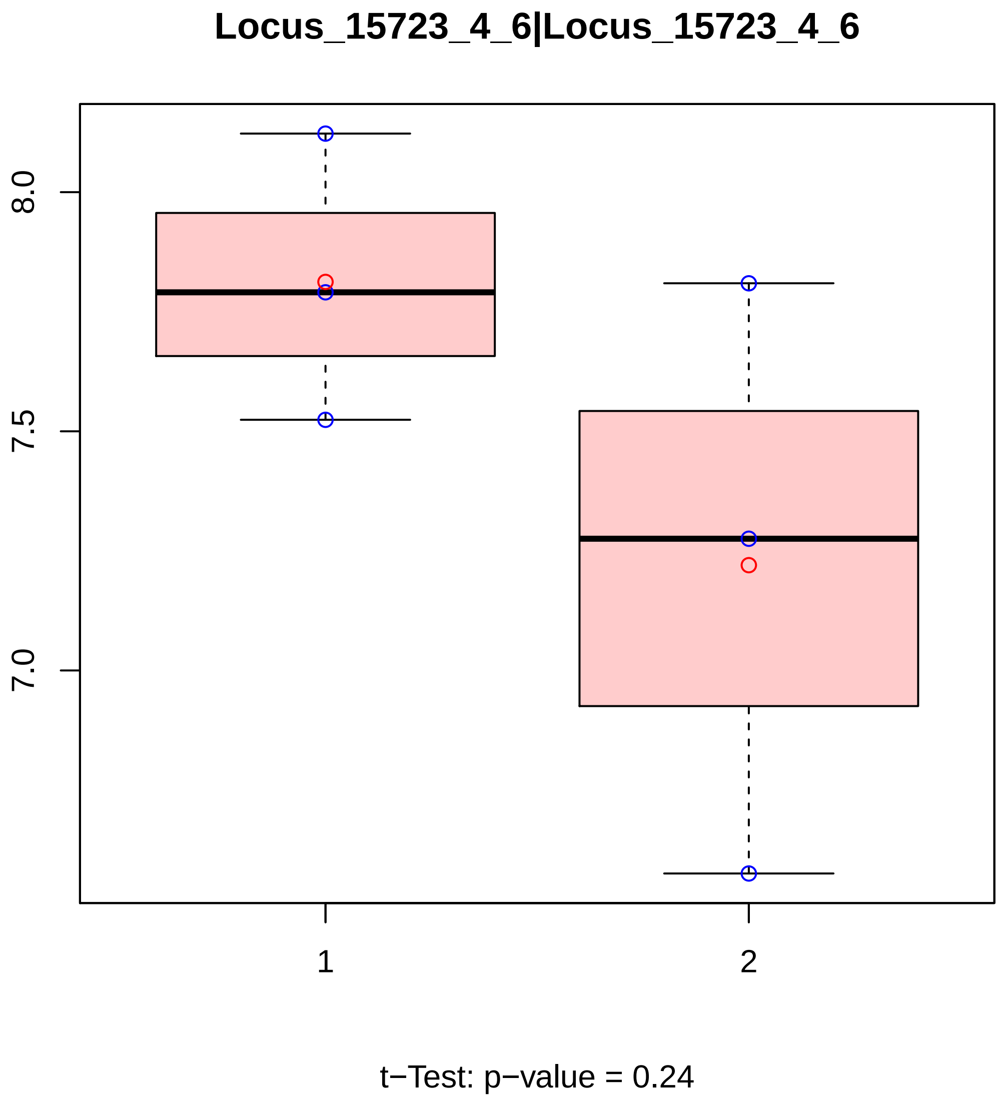
Locus_15723_4_6|Locus_15723_4_6
8.0
7.5
7.0
1
2
t−Test: p−value = 0.24

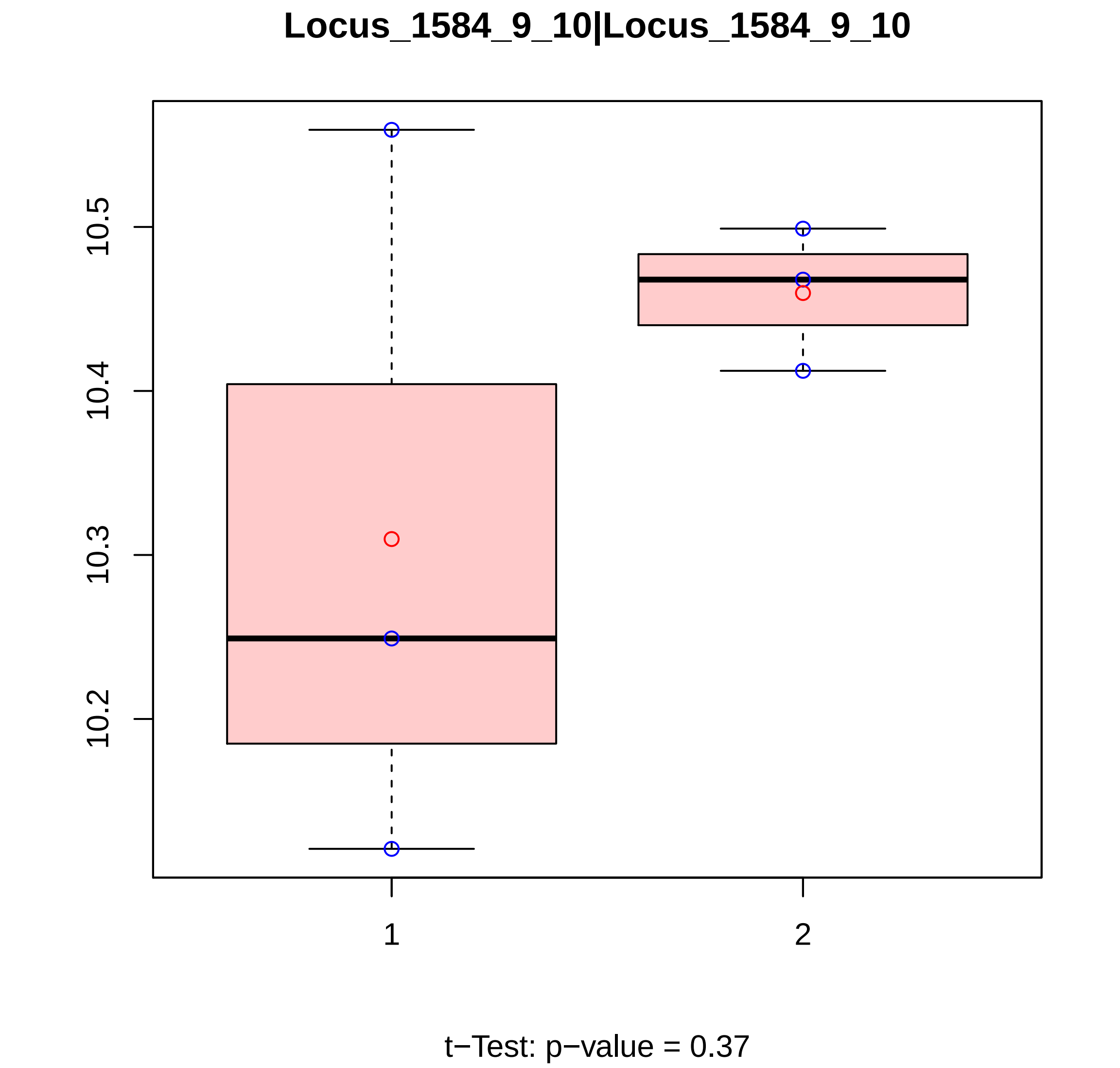
Locus_1584_9_10|Locus_1584_9_10
10.2
10.3
10.4
10.5
1
2
t−Test: p−value = 0.37

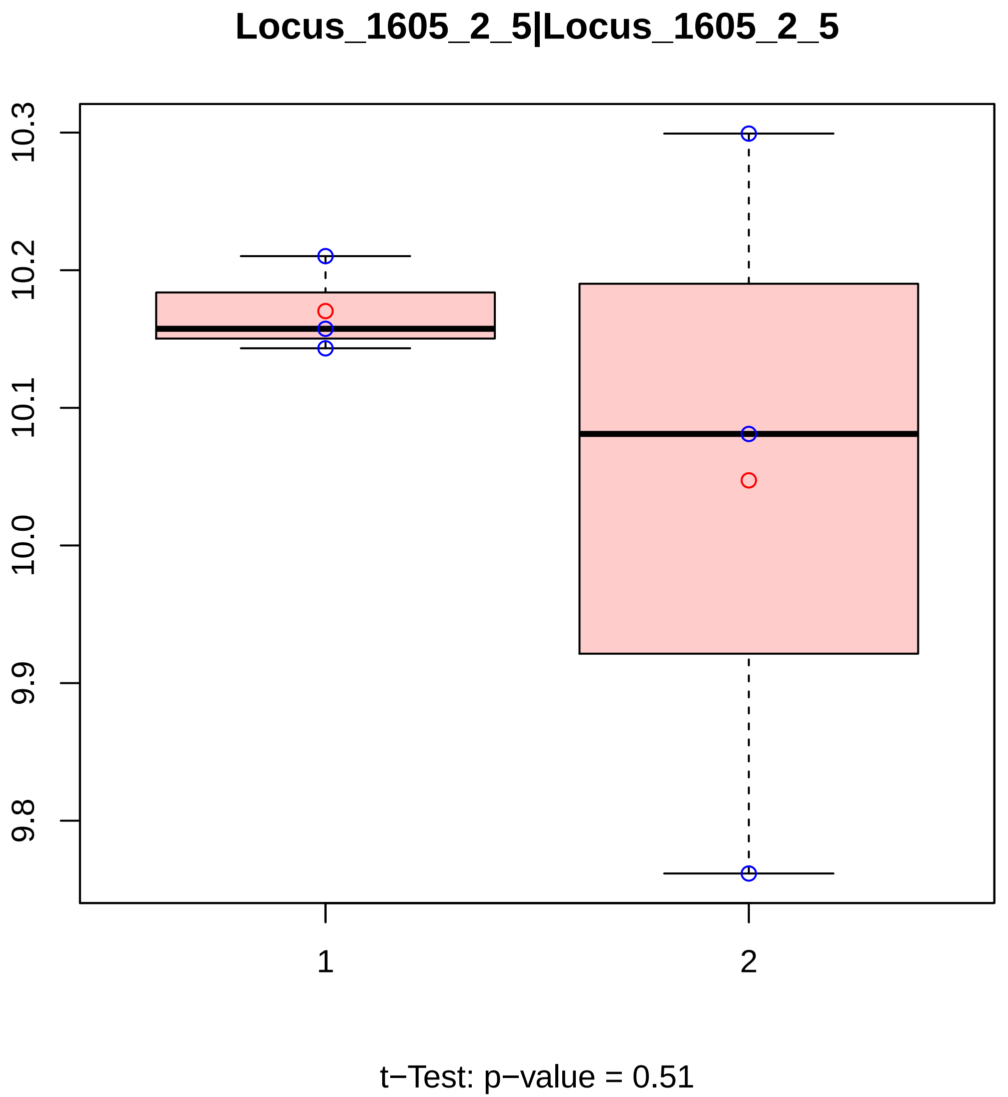
Locus_1605_2_5|Locus_1605_2_5
10.3
10.2
10.1
10.0
9.9
9.8
1
2
t−Test: p−value = 0.51

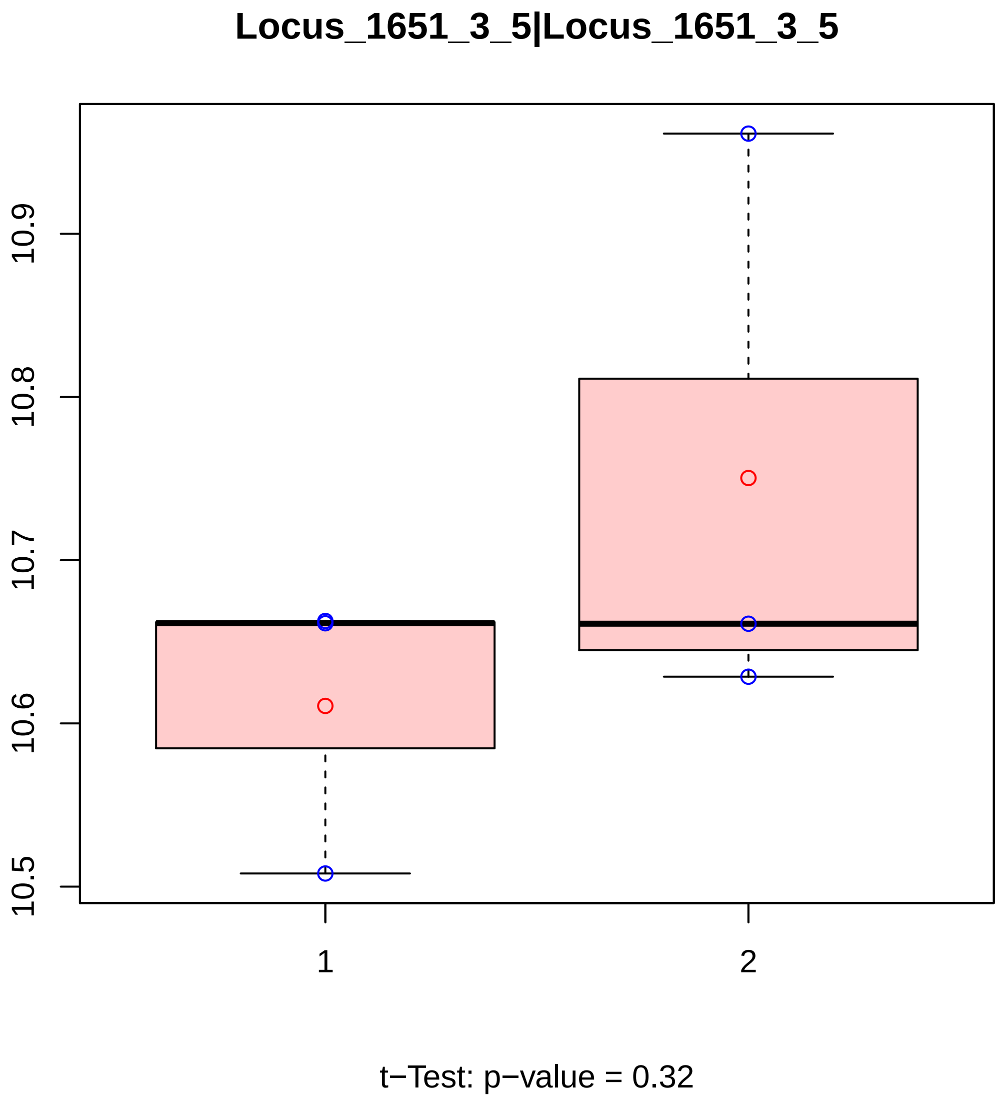

Locus_1651_3_5|Locus_1651_3_5
10.5
10.6
10.7
10.8
10.9
1
2
t−Test: p−value = 0.32

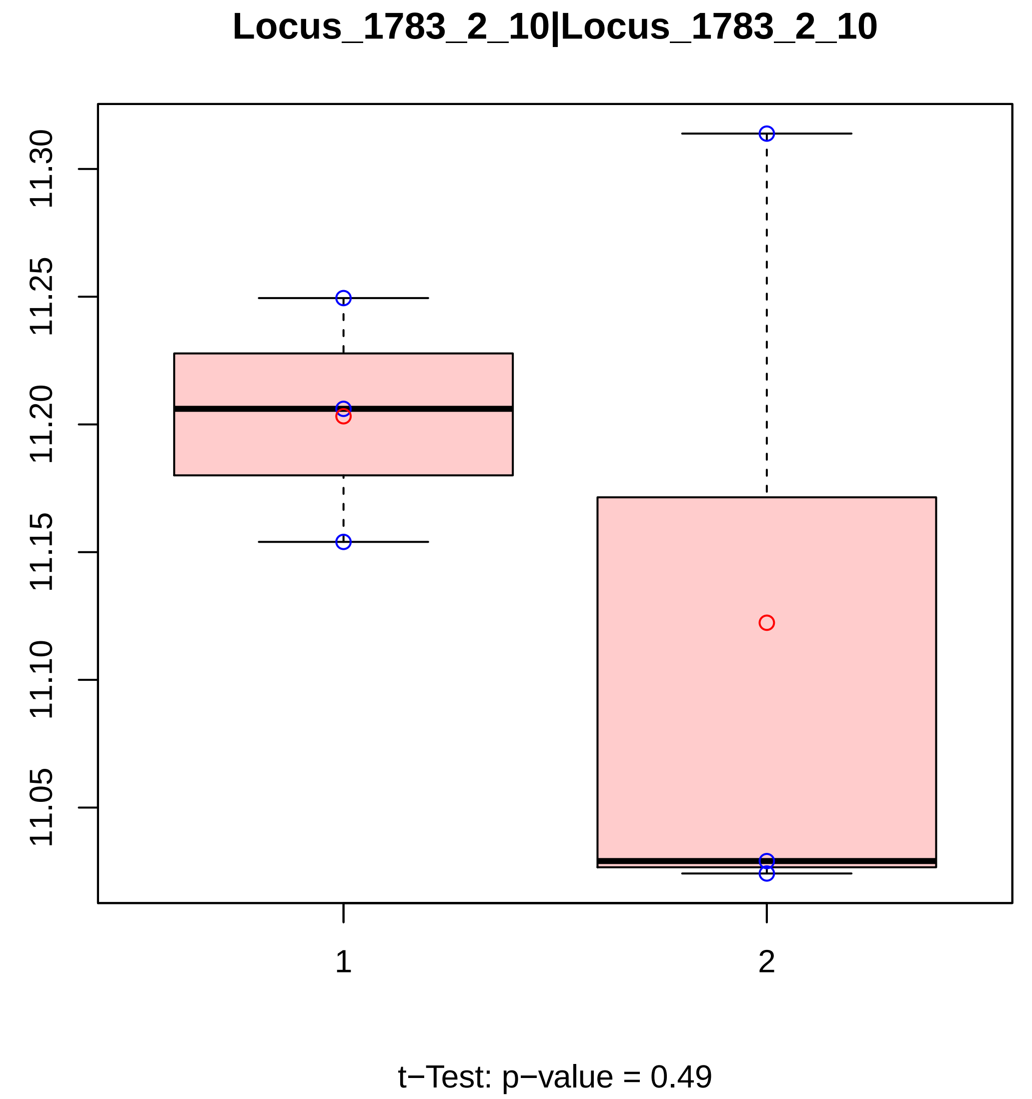

Locus_1783_2_10|Locus_1783_2_10
11.05
11.10
11.15
11.20
11.25
11.30
1
2
t−Test: p−value = 0.49

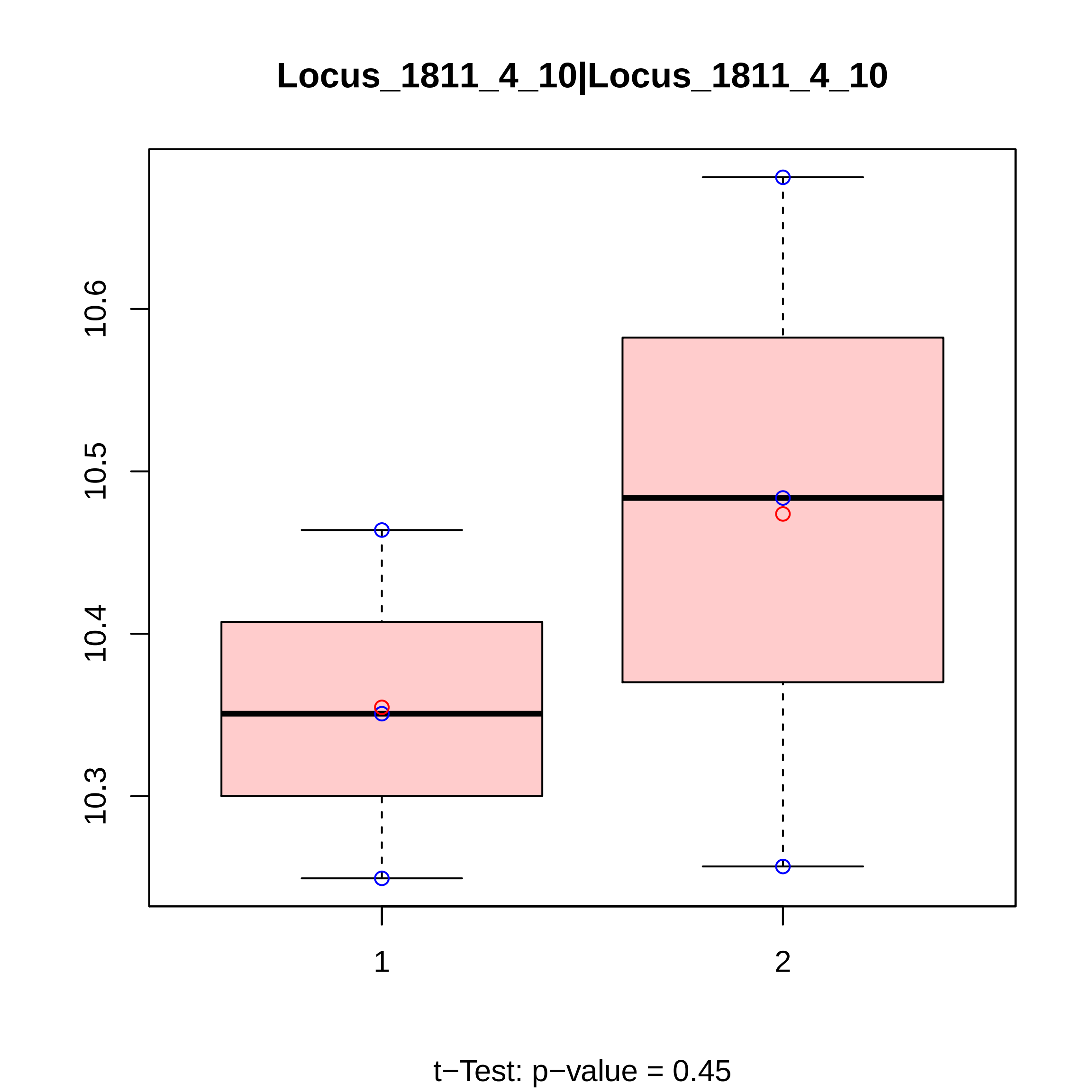

Locus_1811_4_10|Locus_1811_4_10
10.3
10.4
10.5
10.6
1
2
t−Test: p−value = 0.45

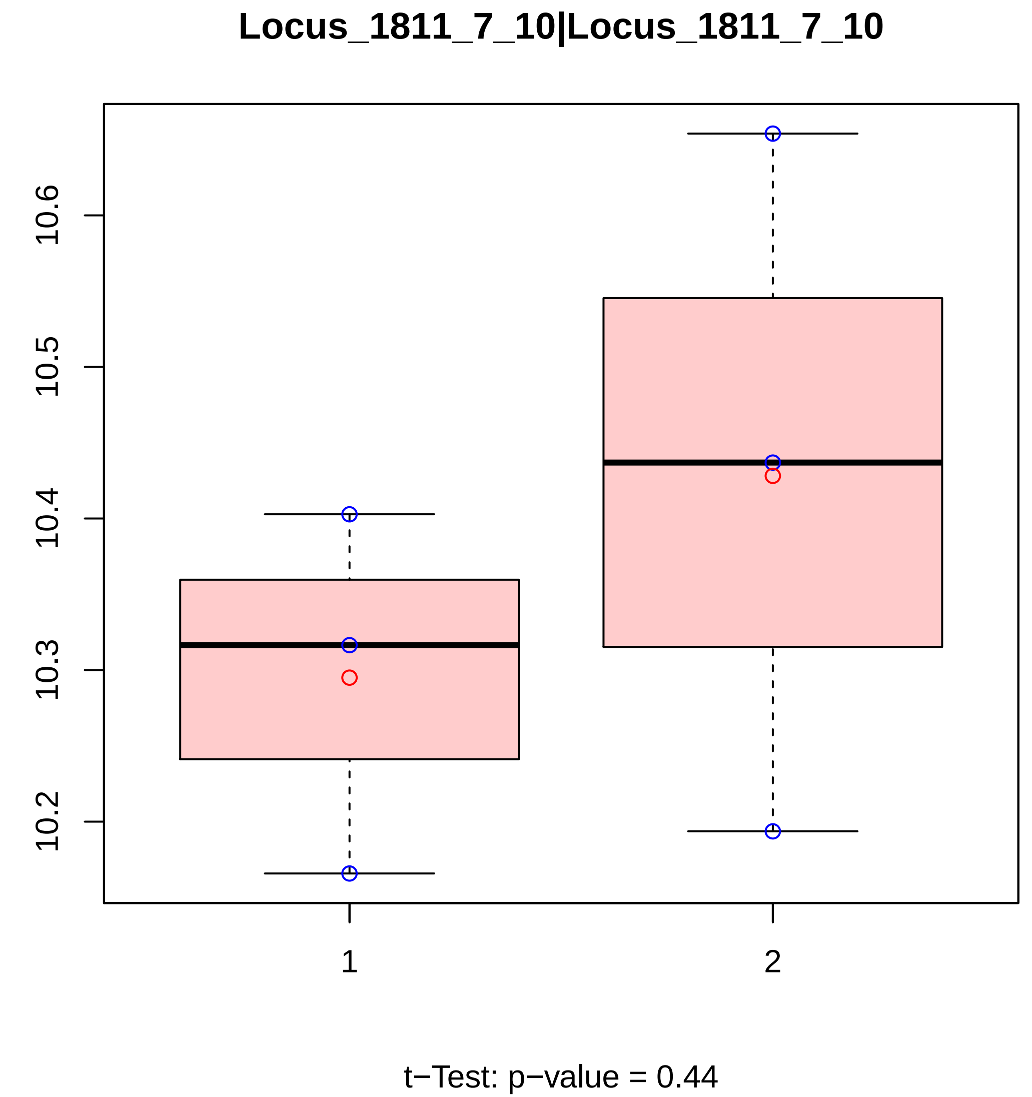
Locus_1811_7_10|Locus_1811_7_10
10.2
10.3
10.4
10.5
10.6
1
2
t−Test: p−value = 0.44

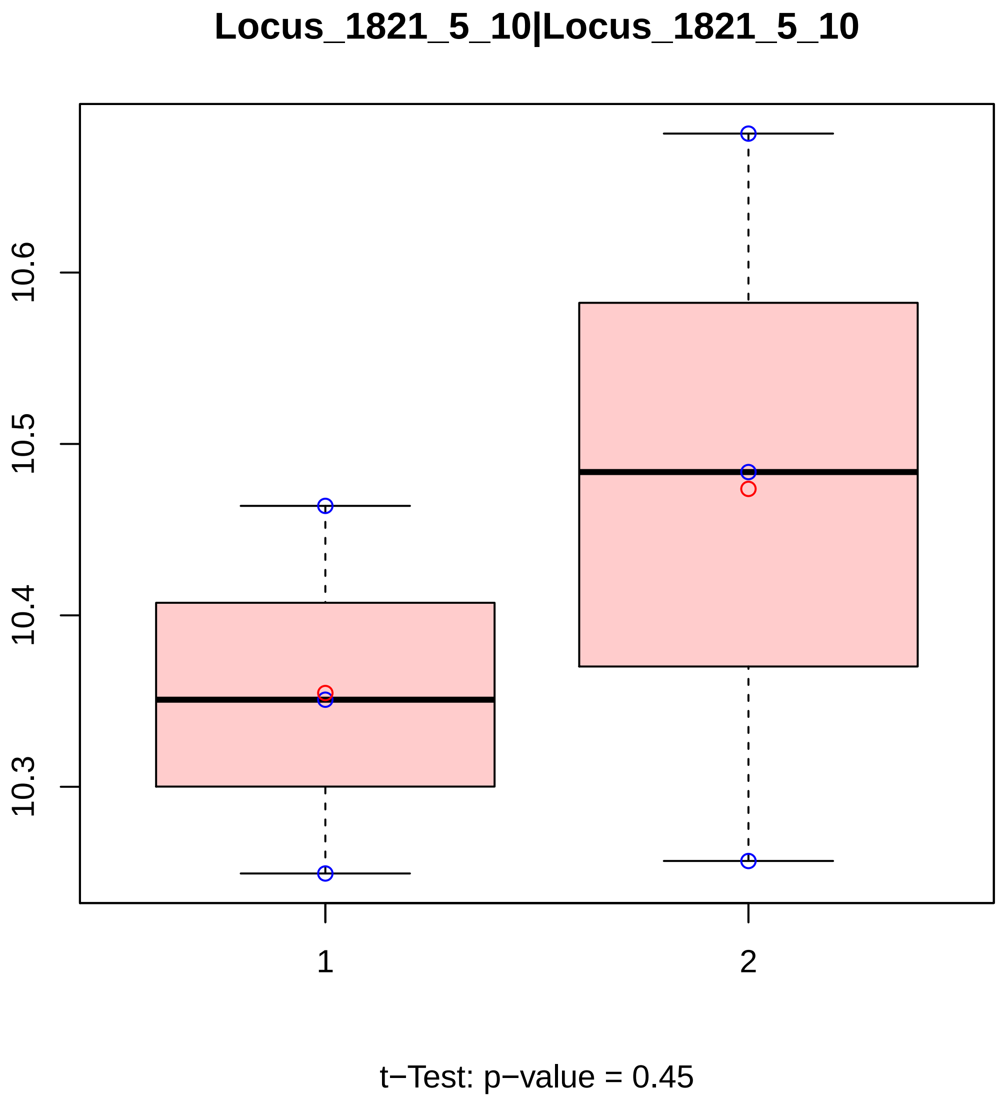
Locus_1821_5_10|Locus_1821_5_10
10.3
10.4
10.5
10.6
1
2
t−Test: p−value = 0.45

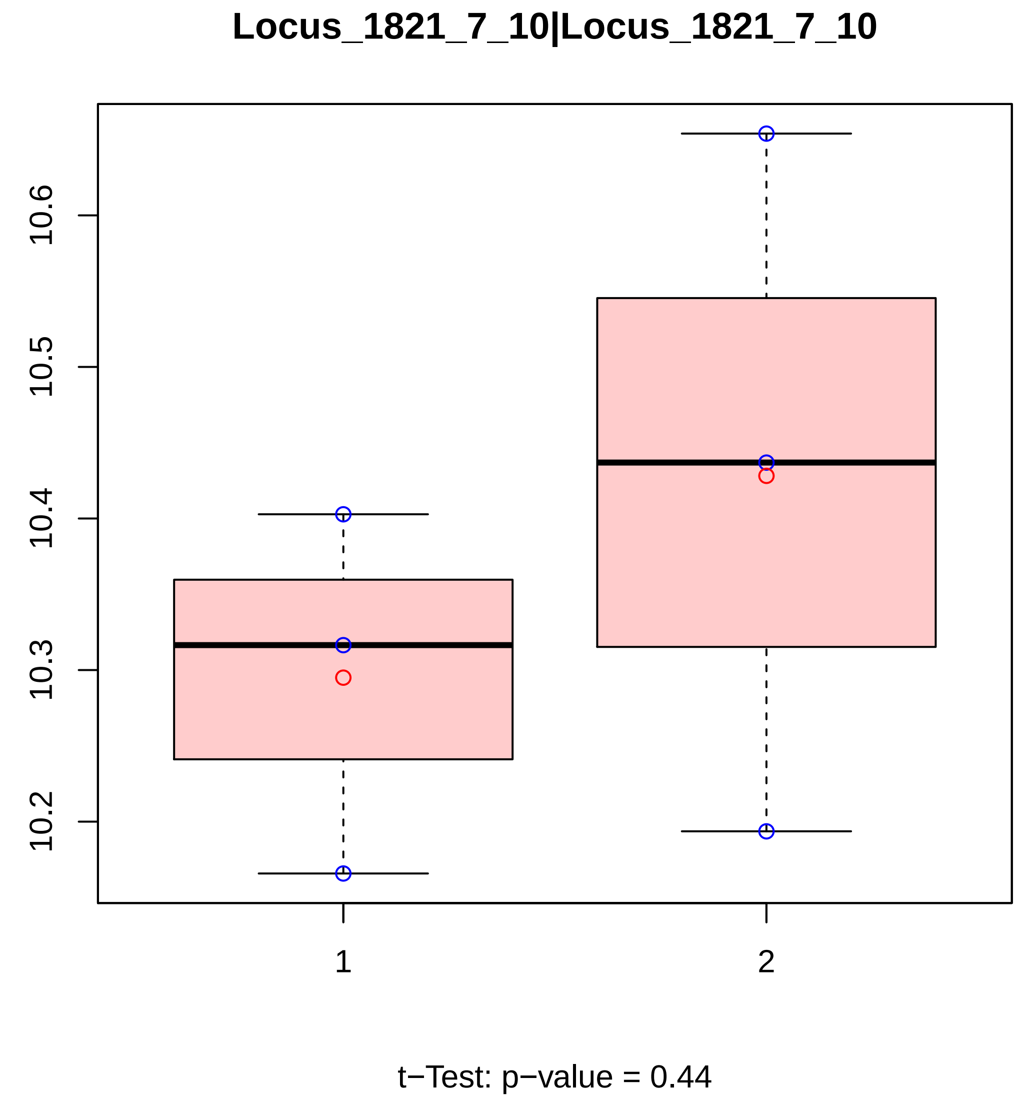

Locus_1821_7_10|Locus_1821_7_10
10.2
10.3
10.4
10.5
10.6
1
2
t−Test: p−value = 0.44

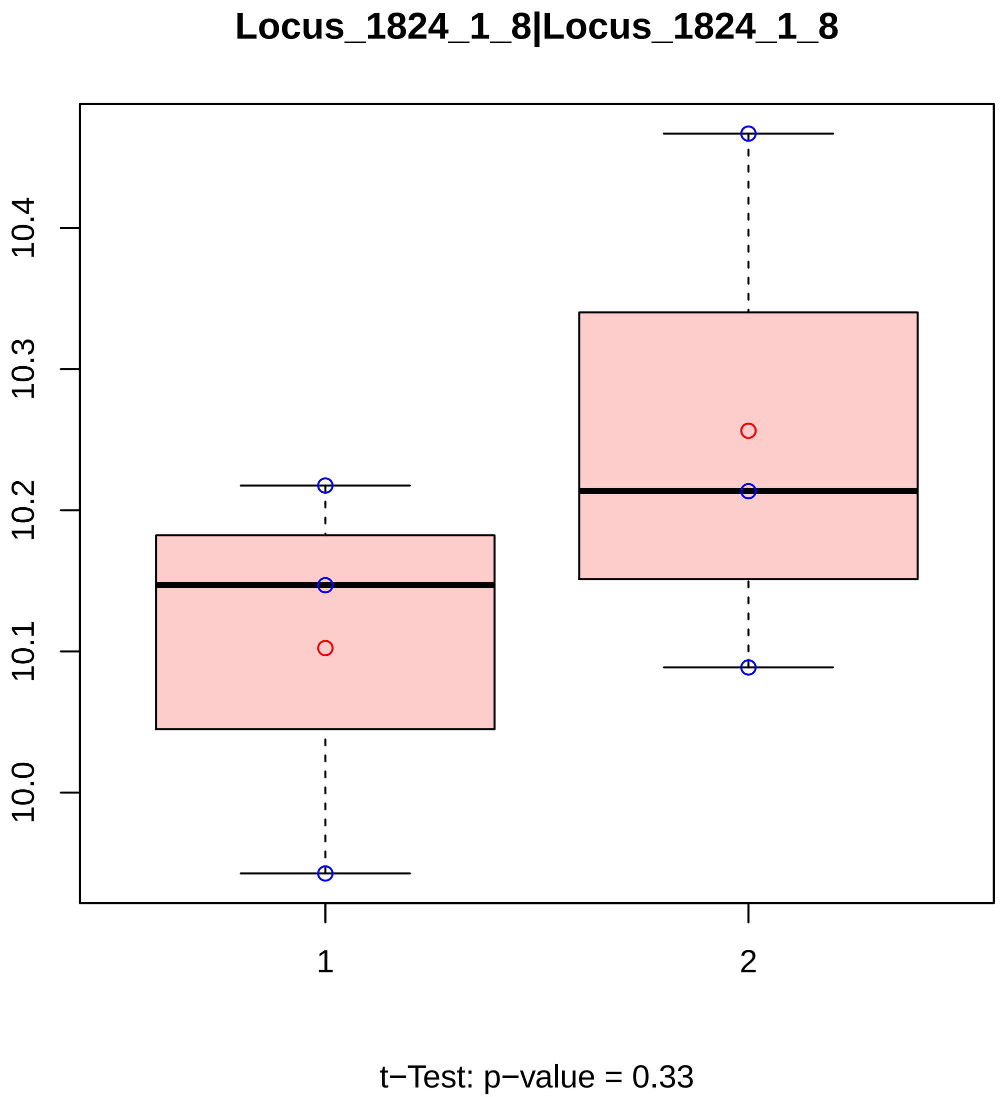
Locus_1824_1_8|Locus_1824_1_8
10.0
10.1
10.2
10.3
10.4
1
2
t−Test: p−value = 0.33

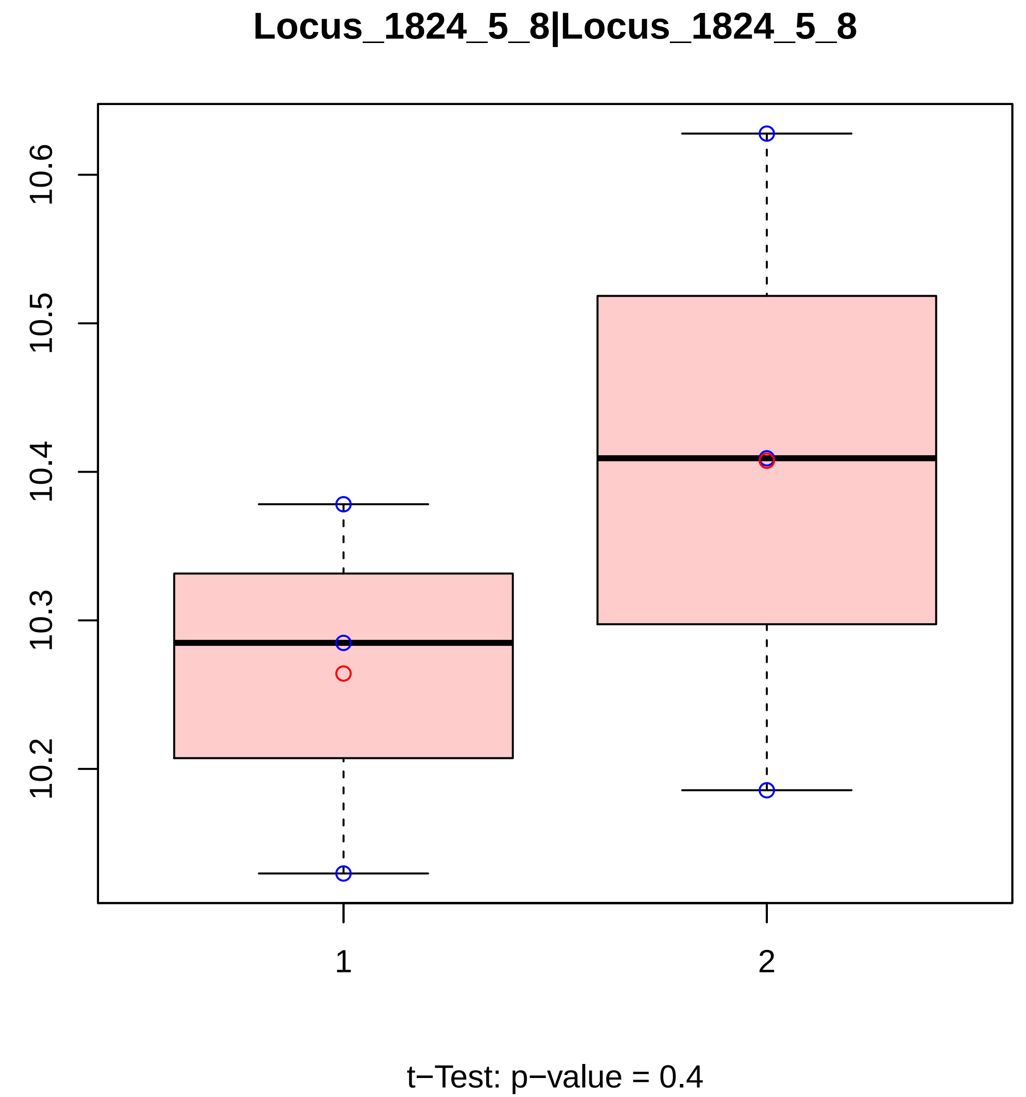

Locus_1824_5_8|Locus_1824_5_8
10.2
10.3
10.4
10.5
10.6
1
2
t−Test: p−value = 0.4

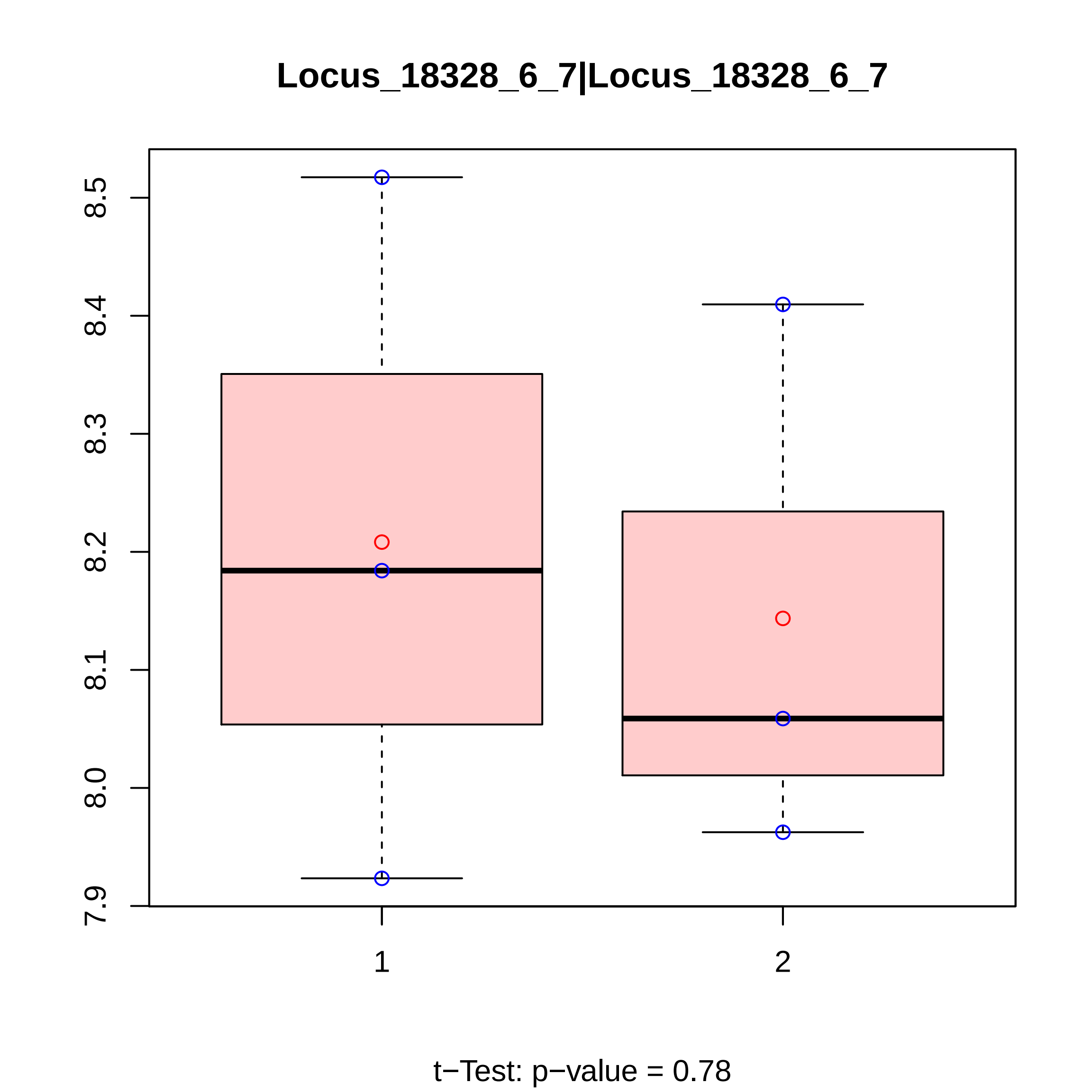

Locus_18328_6_7|Locus_18328_6_7
8.5
8.4
8.3
8.2
8.1
8.0
7.9
1
2
t−Test: p−value = 0.78

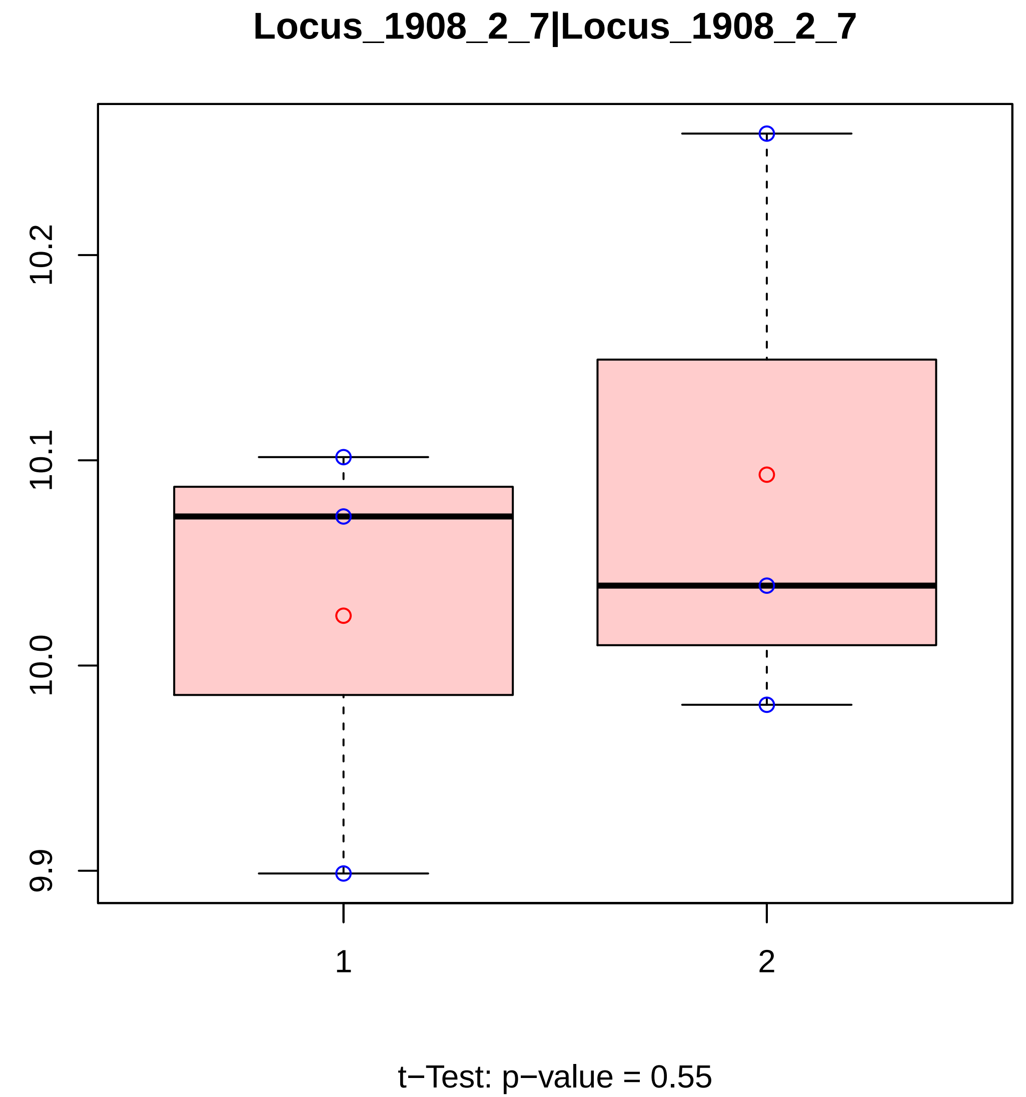
Locus_1908_2_7|Locus_1908_2_7
10.2
10.1
10.0
9.9
1
2
t−Test: p−value = 0.55

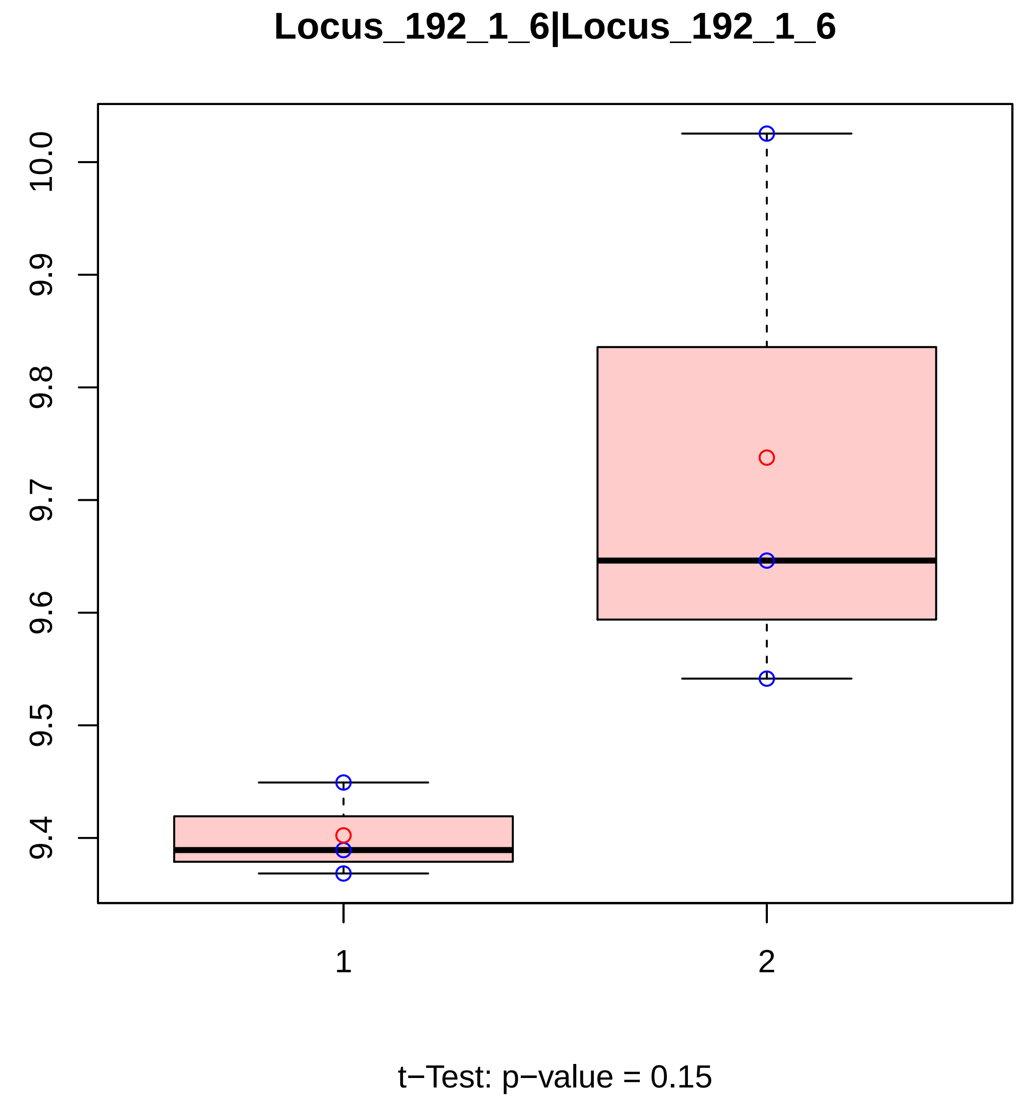

Locus_192_1_6|Locus_192_1_6
10.0
9.9
9.8
9.7
9.6
9.5
9.4
1
2
t−Test: p−value = 0.15

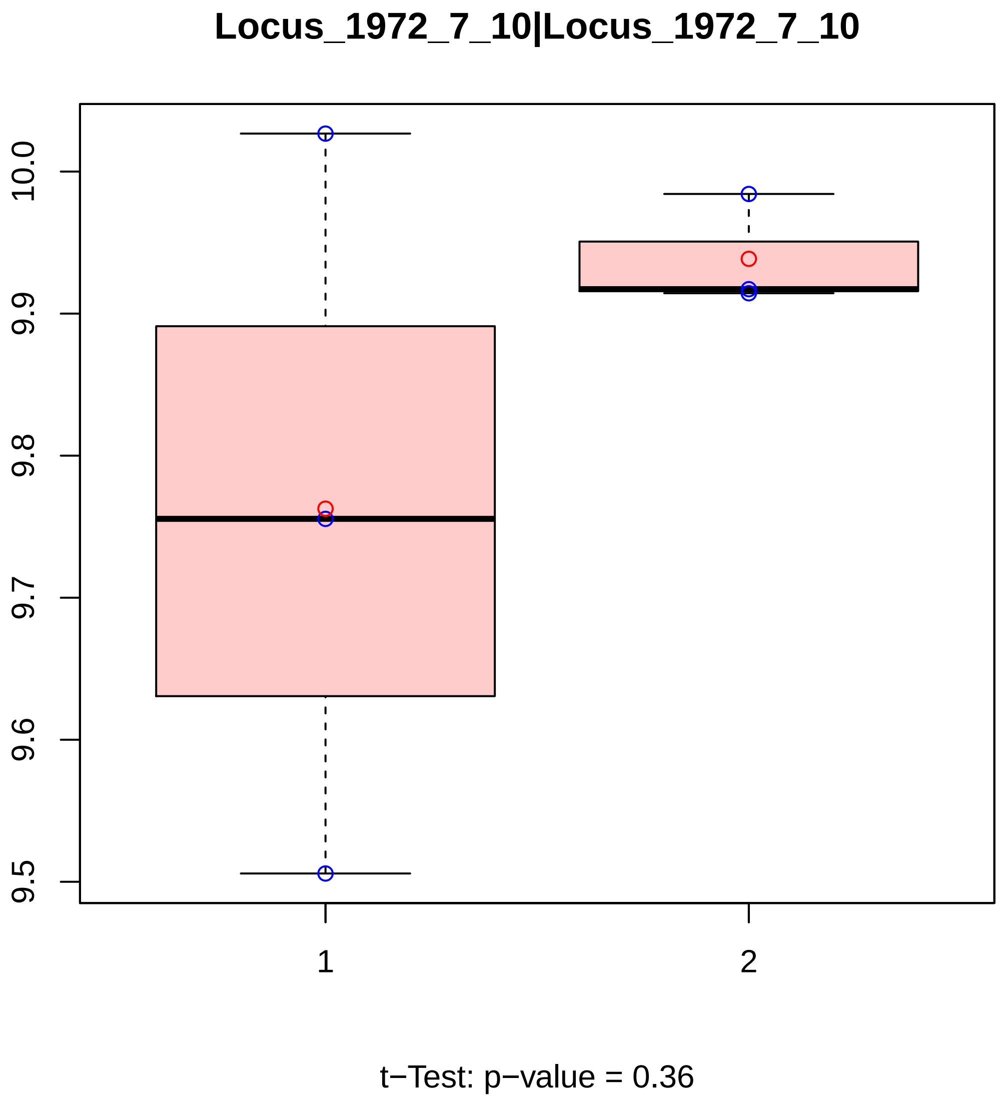

Locus_1972_7_10|Locus_1972_7_10
10.0
9.9
9.8
9.7
9.6
9.5
1
2
t−Test: p−value = 0.36

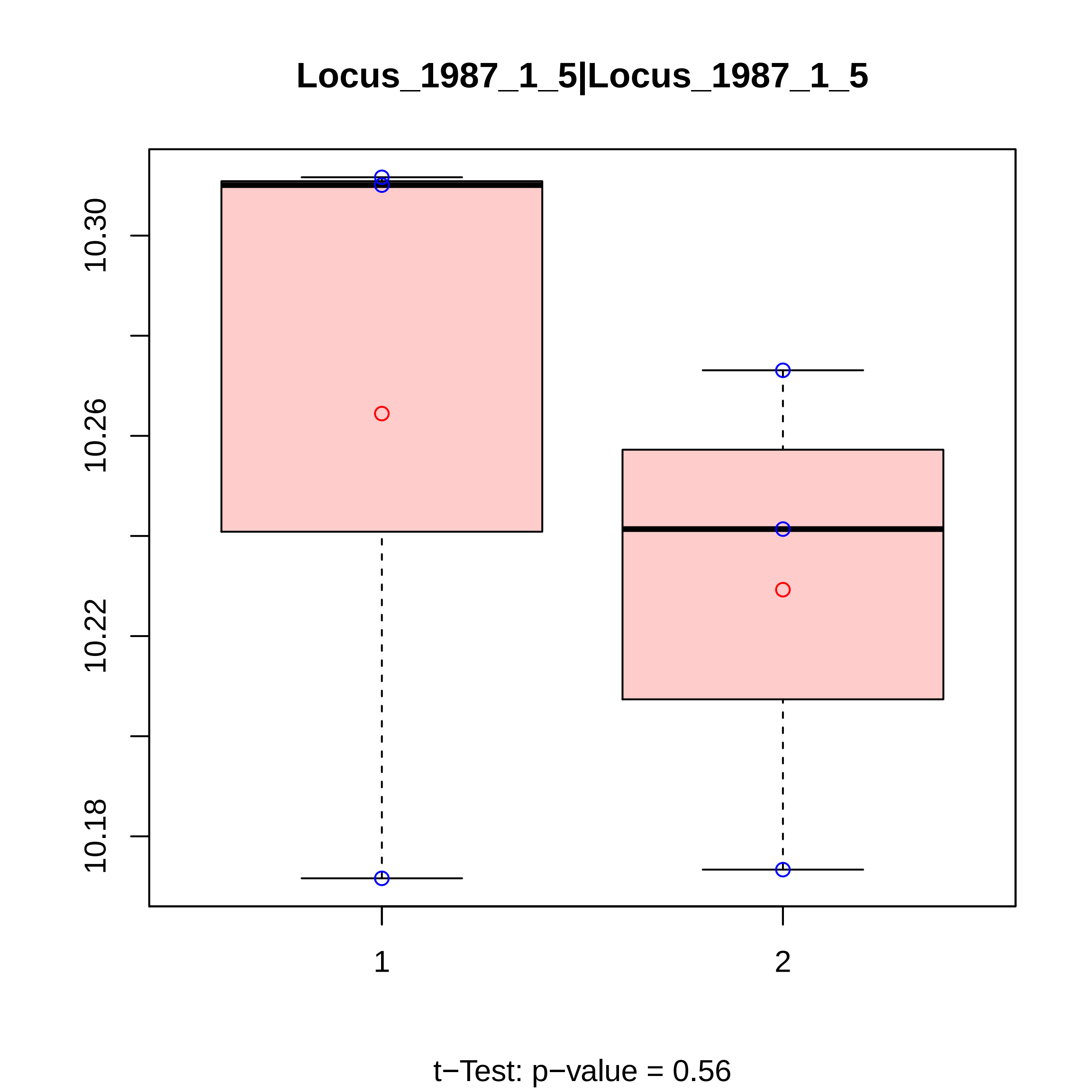
Locus_1987_1_5|Locus_1987_1_5
10.18
10.22
10.26
10.30
1
2
t−Test: p−value = 0.56

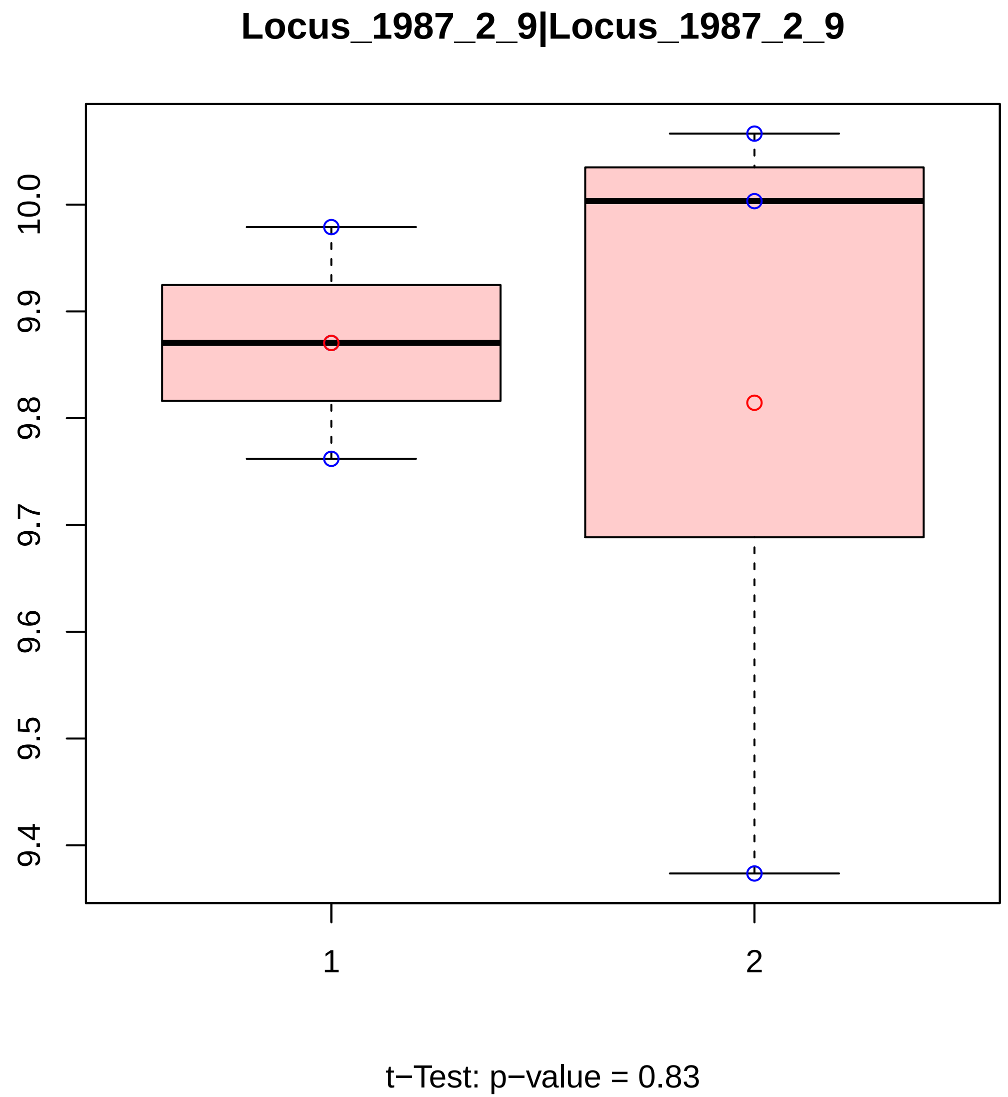
Locus_1987_2_9|Locus_1987_2_9
10.0
9.9
9.8
9.7
9.6
9.5
9.4
1
2
t−Test: p−value = 0.83

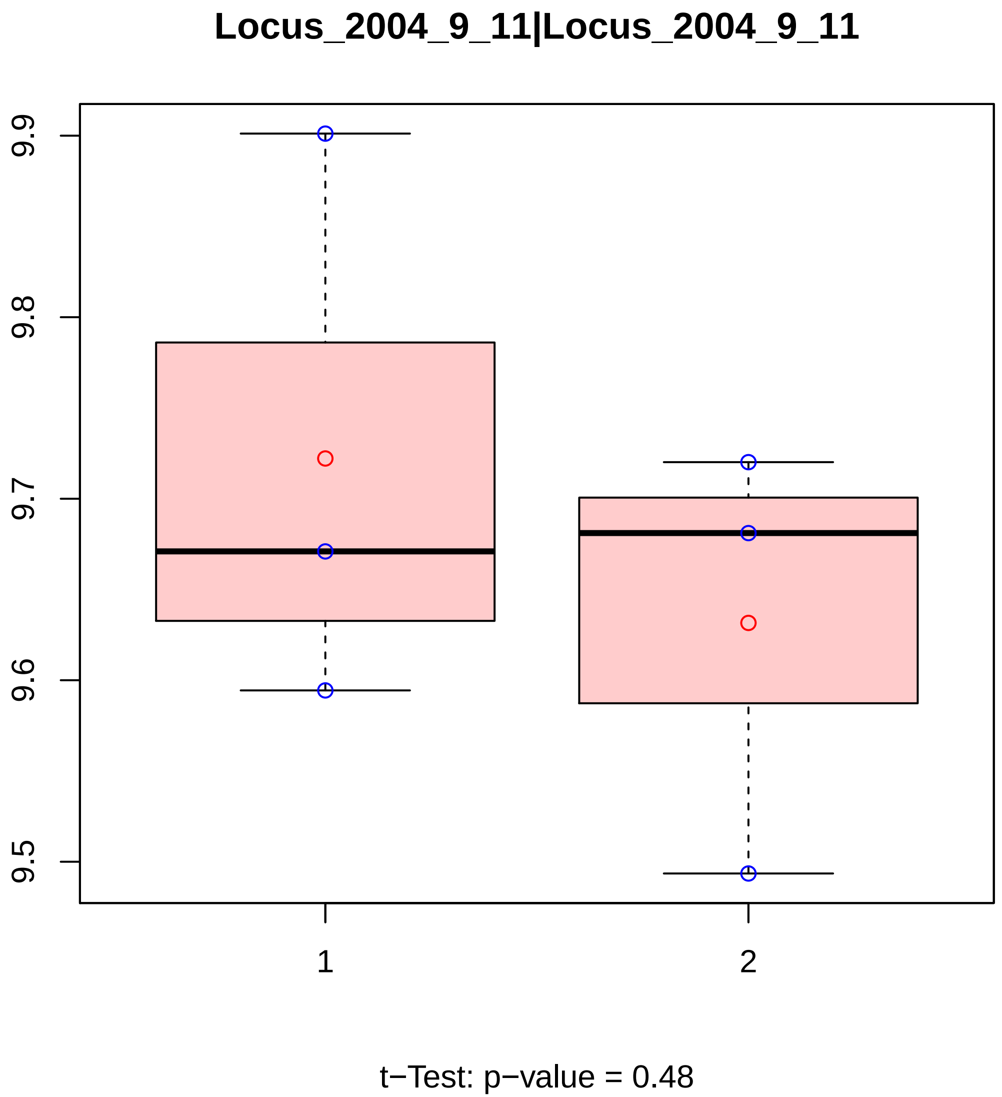

Locus_2004_9_11|Locus_2004_9_11
9.5
9.6
9.7
9.8
9.9
1
2
t−Test: p−value = 0.48

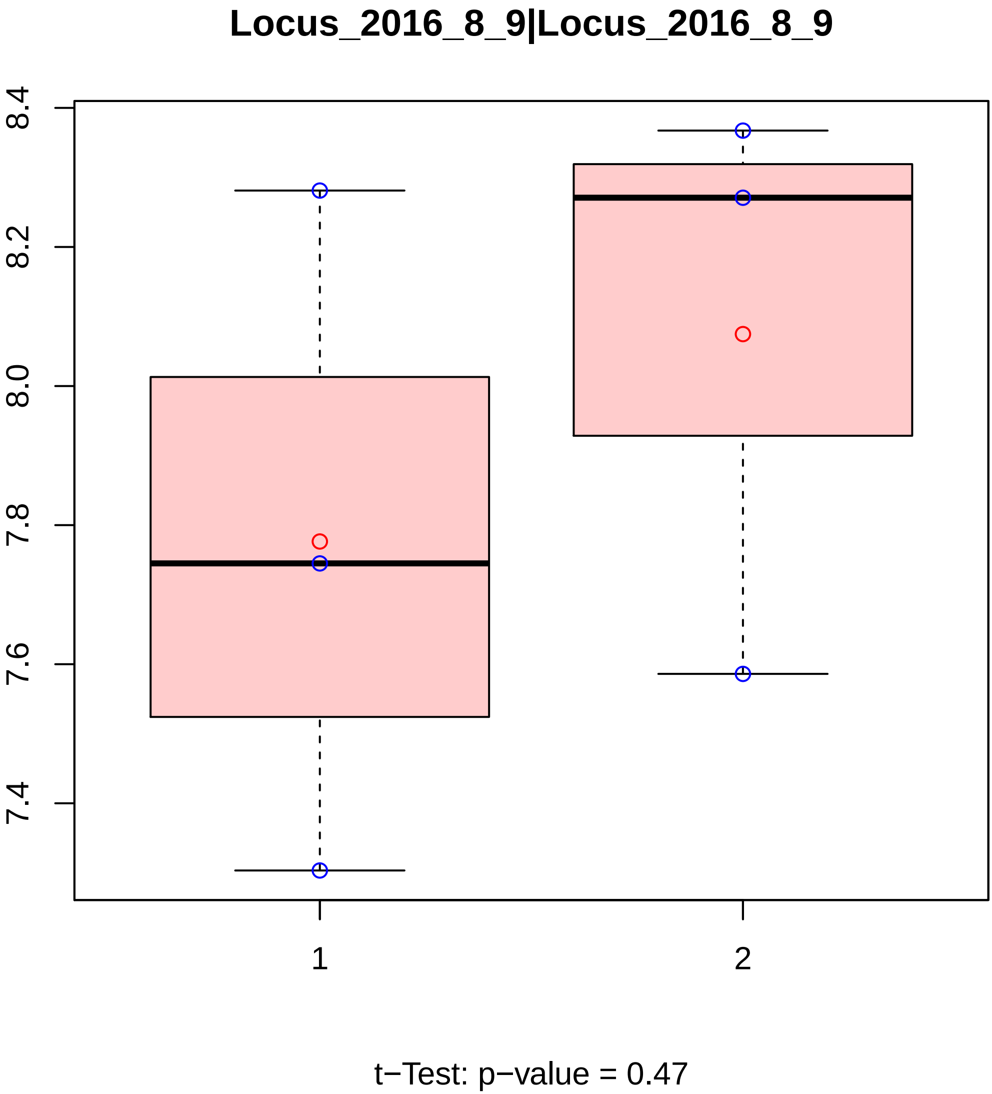

Locus_2016_8_9|Locus_2016_8_9
8.4
8.2
8.0
7.8
7.6
7.4
1
2
t−Test: p−value = 0.47

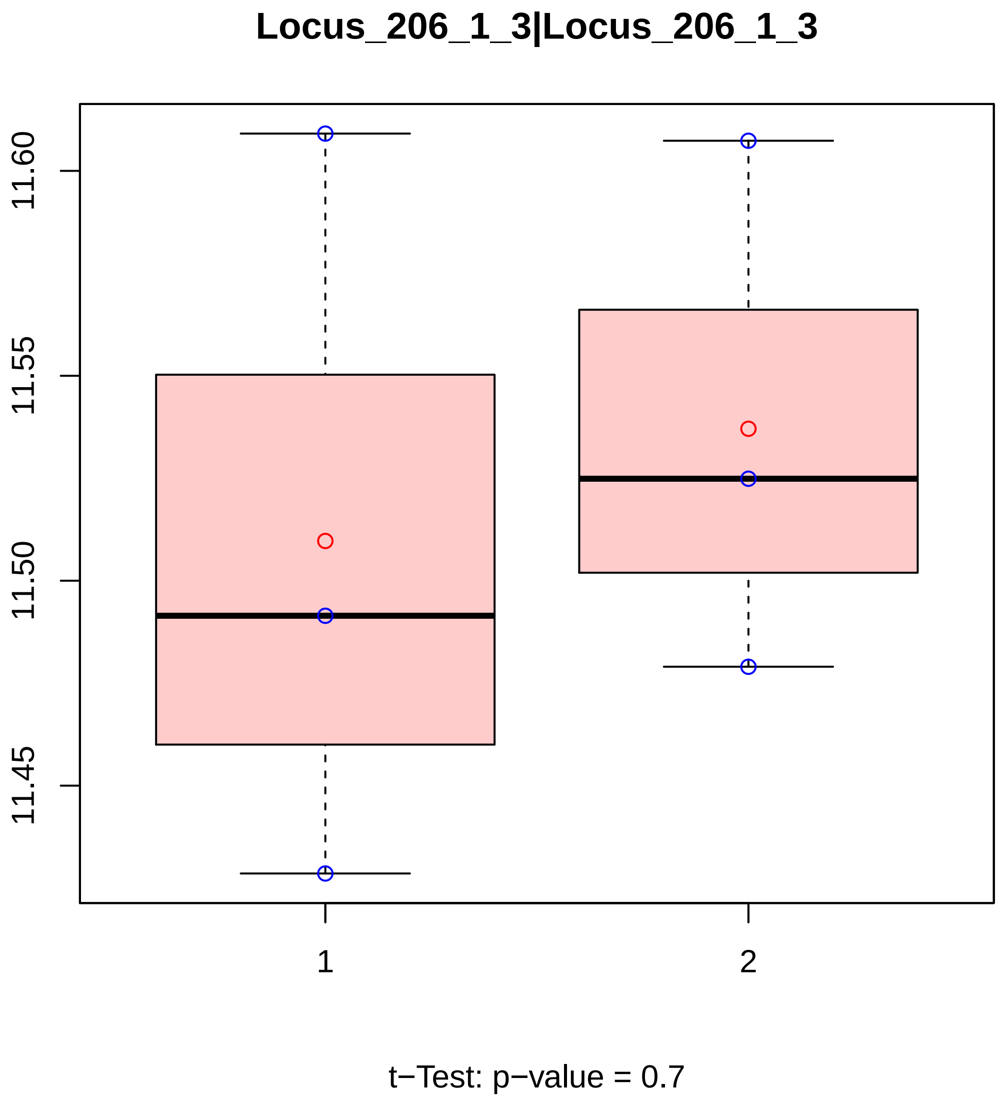

Locus_206_1_3|Locus_206_1_3
11.45
11.50
11.55
11.60
1
2
t−Test: p−value = 0.7

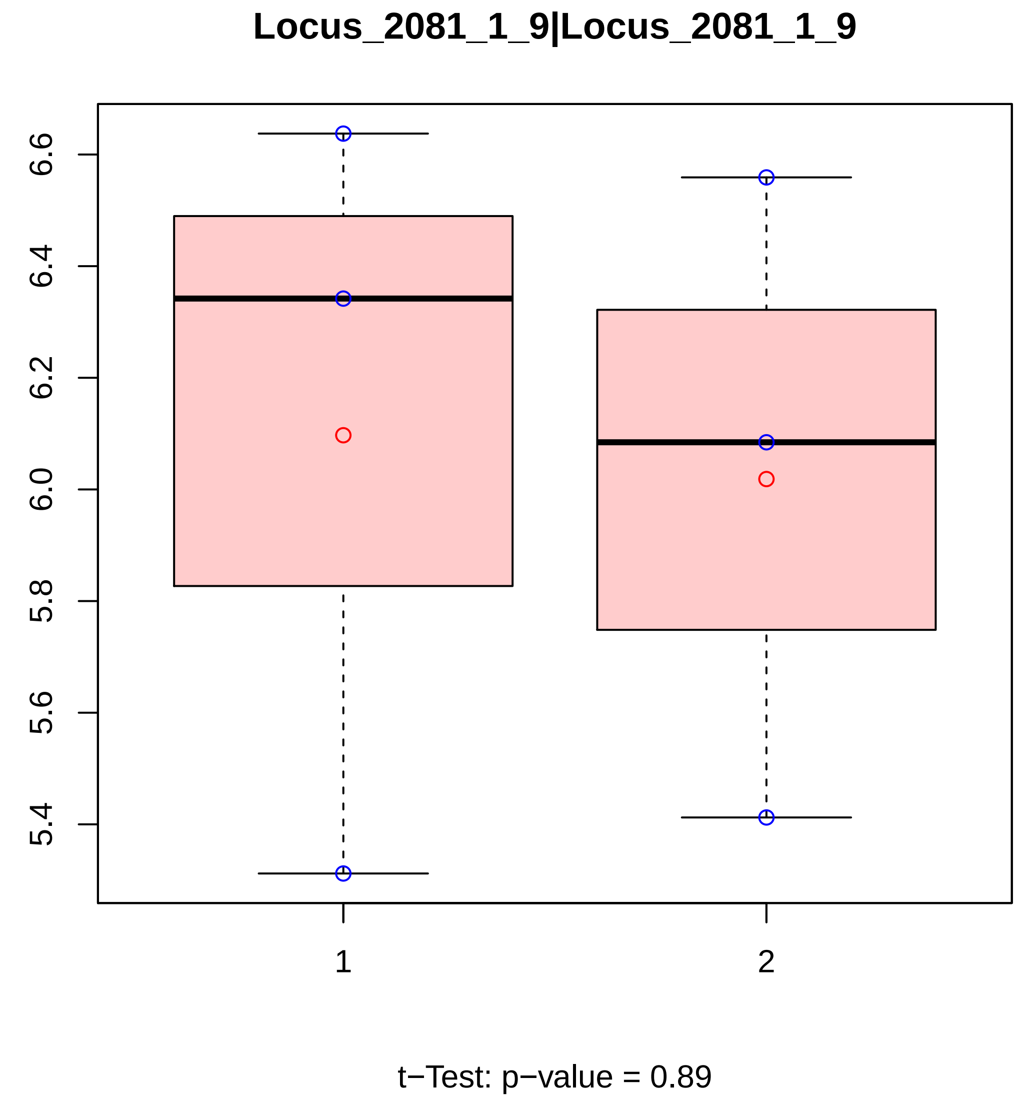

Locus_2081_1_9|Locus_2081_1_9
6.6
6.4
6.2
6.0
5.8
5.6
5.4
1
2
t−Test: p−value = 0.89

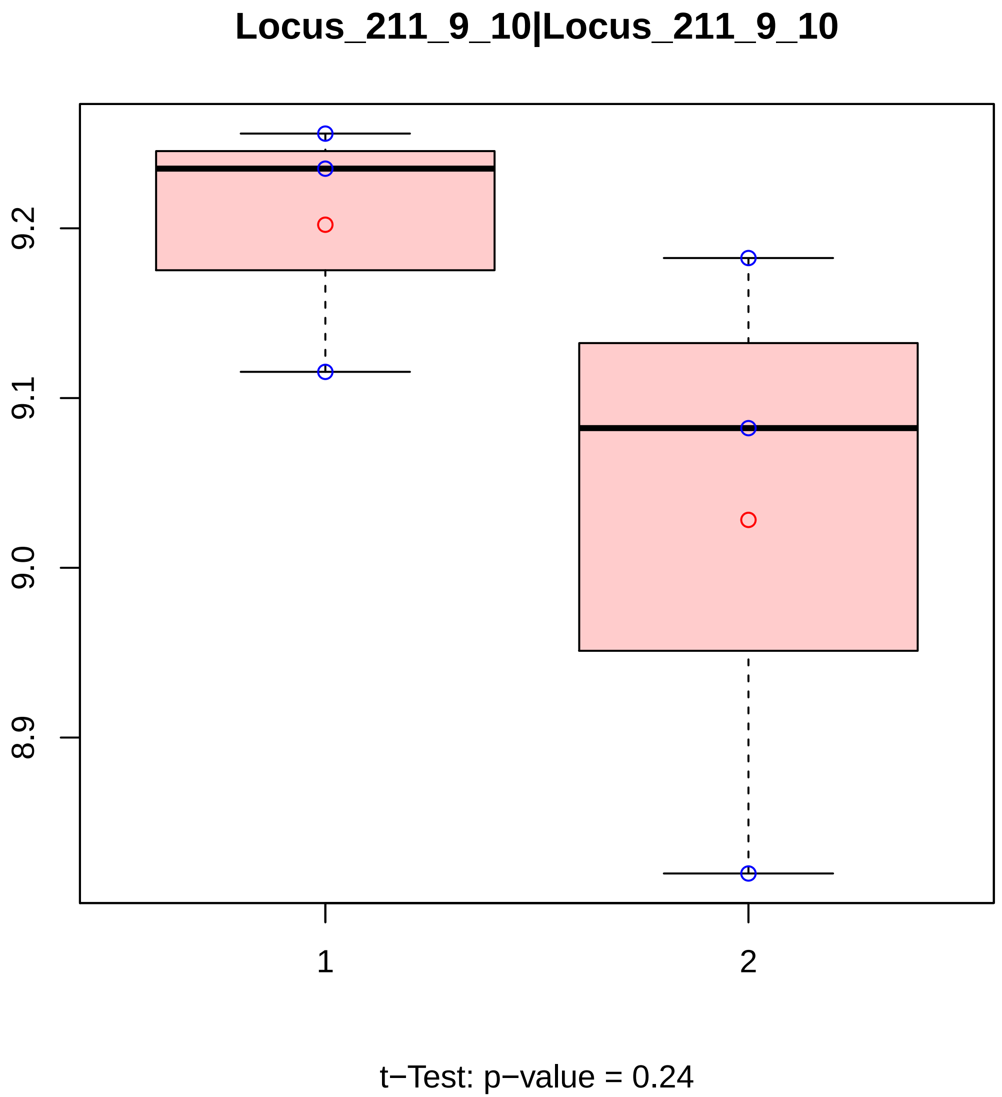

Locus_211_9_10|Locus_211_9_10
9.2
9.1
9.0
8.9
1
2
t−Test: p−value = 0.24

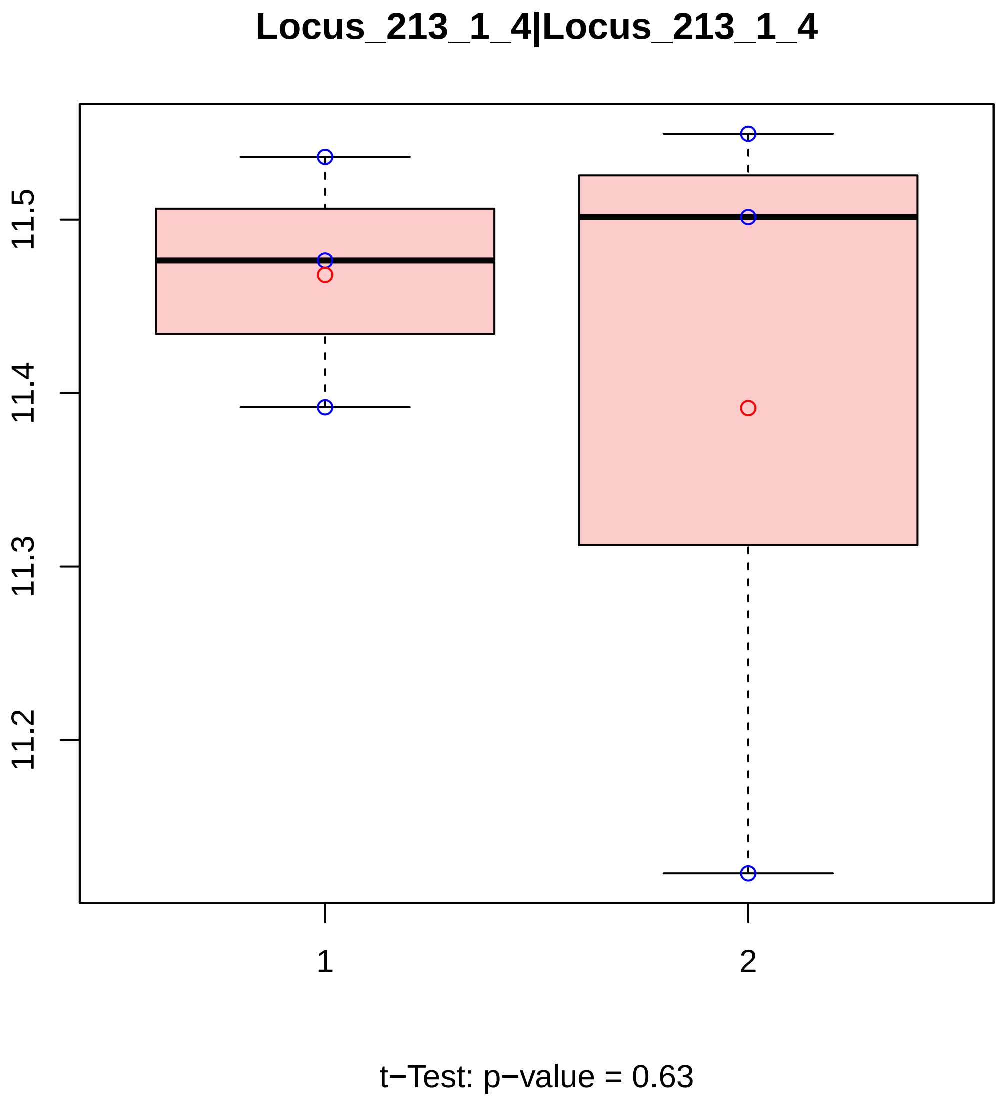
Locus_213_1_4|Locus_213_1_4
11.2
11.3
11.4
11.5
1
2
t−Test: p−value = 0.63

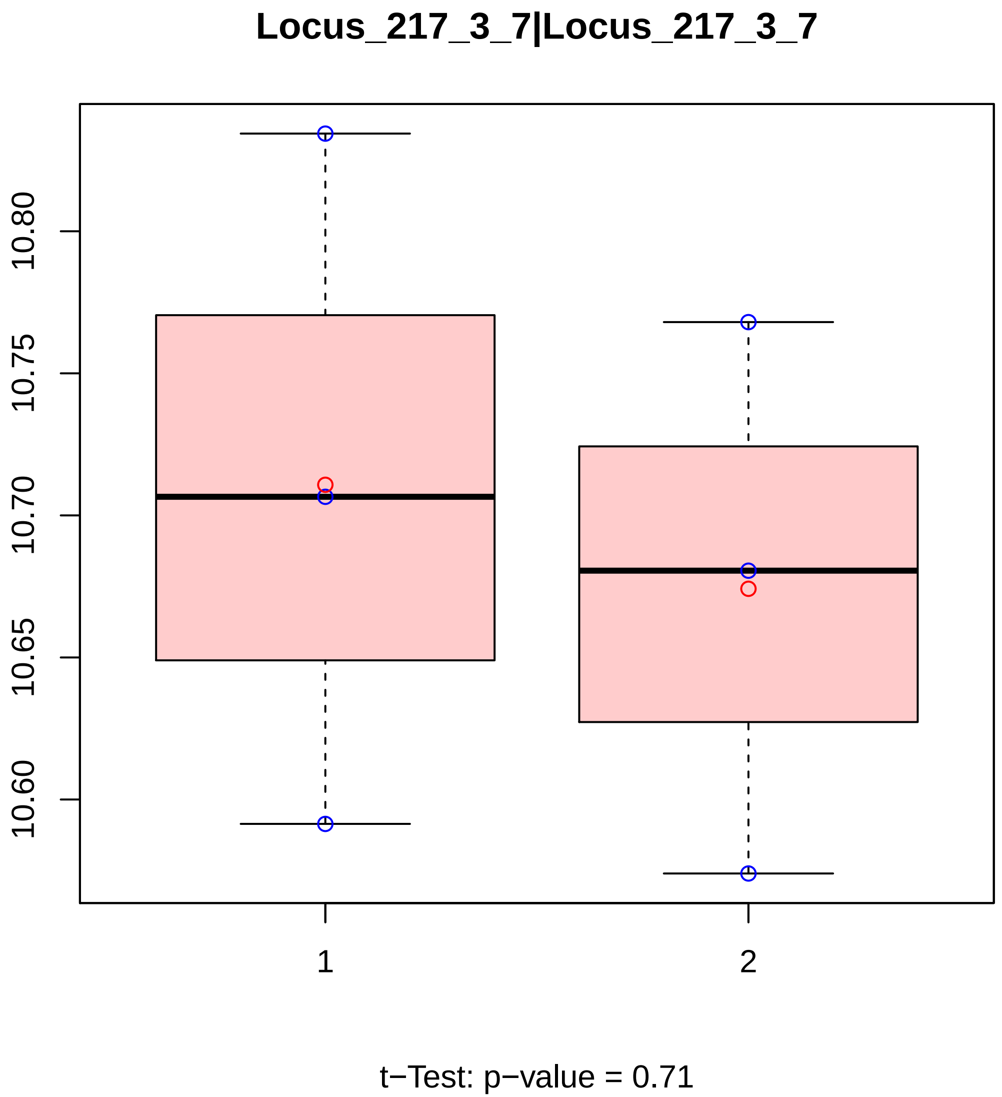
Locus_217_3_7|Locus_217_3_7
10.60
10.65
10.70
10.75
10.80
1
2
t−Test: p−value = 0.71

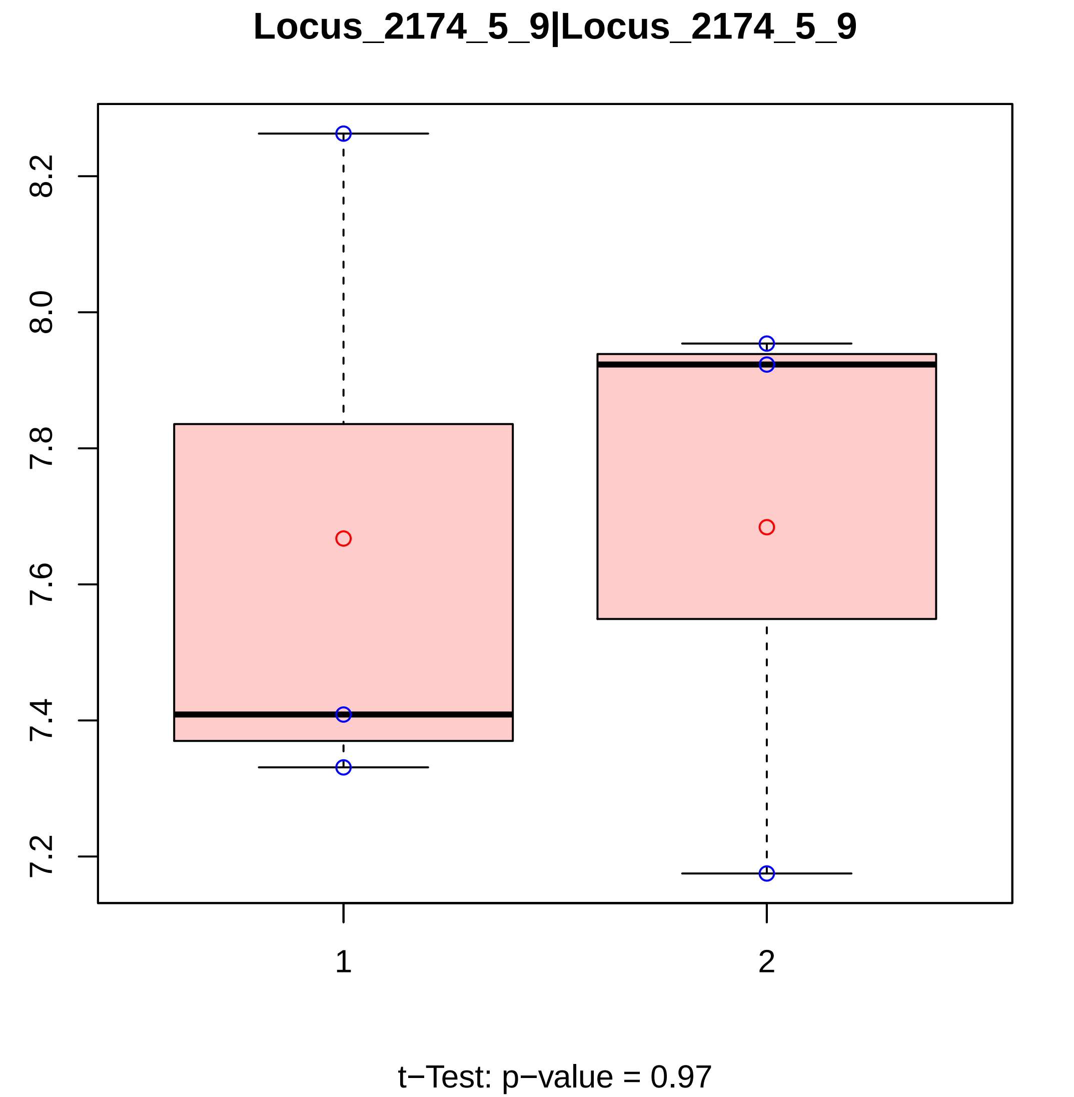
Locus_2174_5_9|Locus_2174_5_9
8.2
8.0
7.8
7.6
7.4
7.2
1
2
t−Test: p−value = 0.97

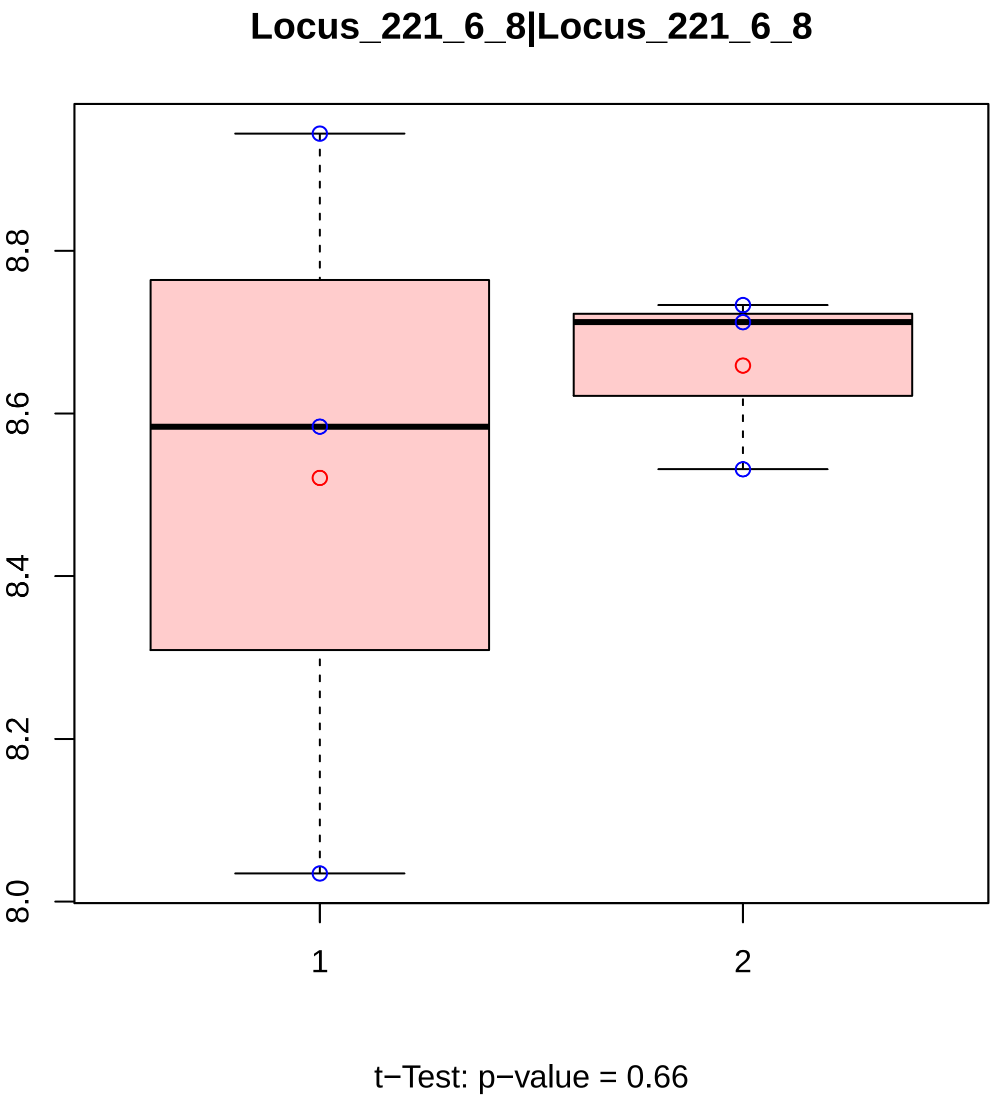
Locus_221_6_8|Locus_221_6_8
8.0
8.2
8.4
8.6
8.8
1
2
t−Test: p−value = 0.66

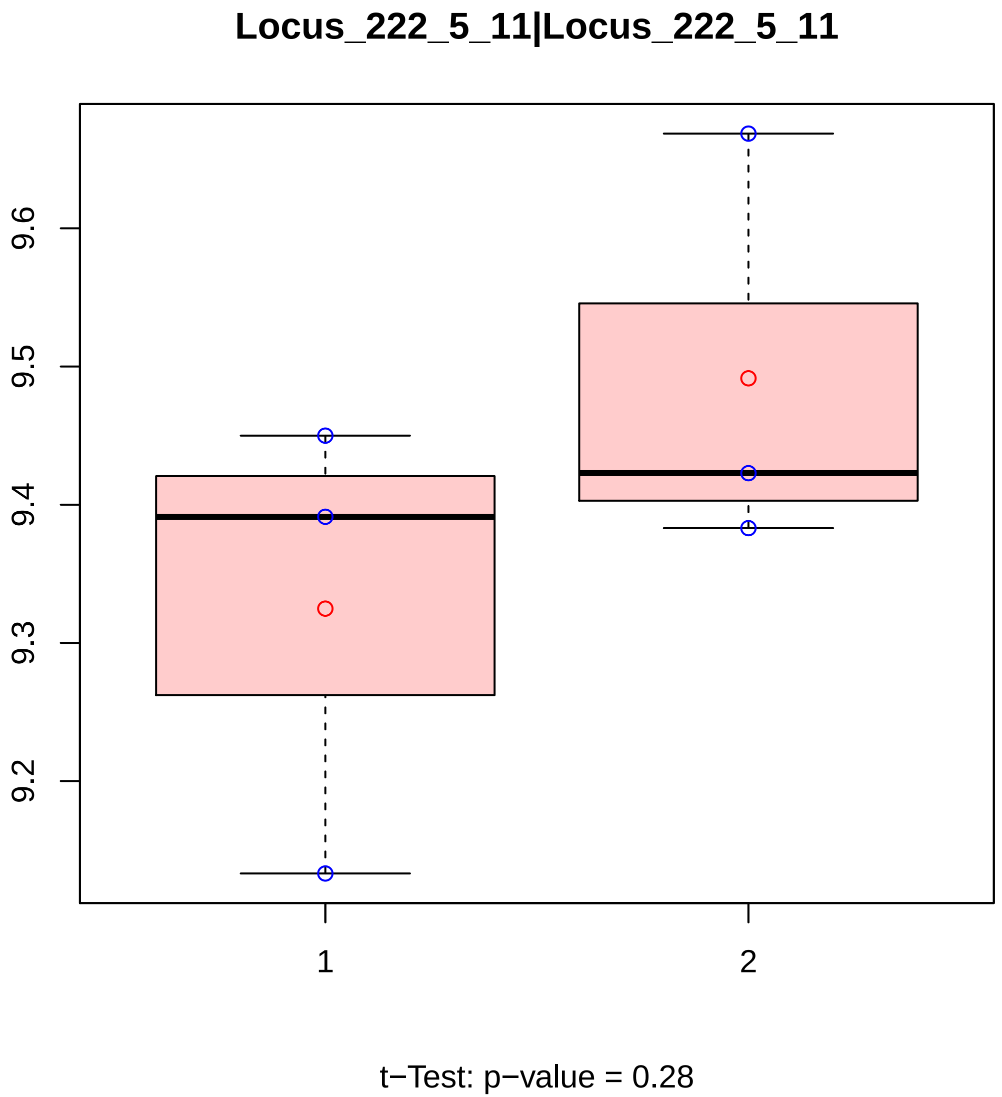
Locus_222_5_11|Locus_222_5_11
9.2
9.3
9.4
9.5
9.6
1
2
t−Test: p−value = 0.28

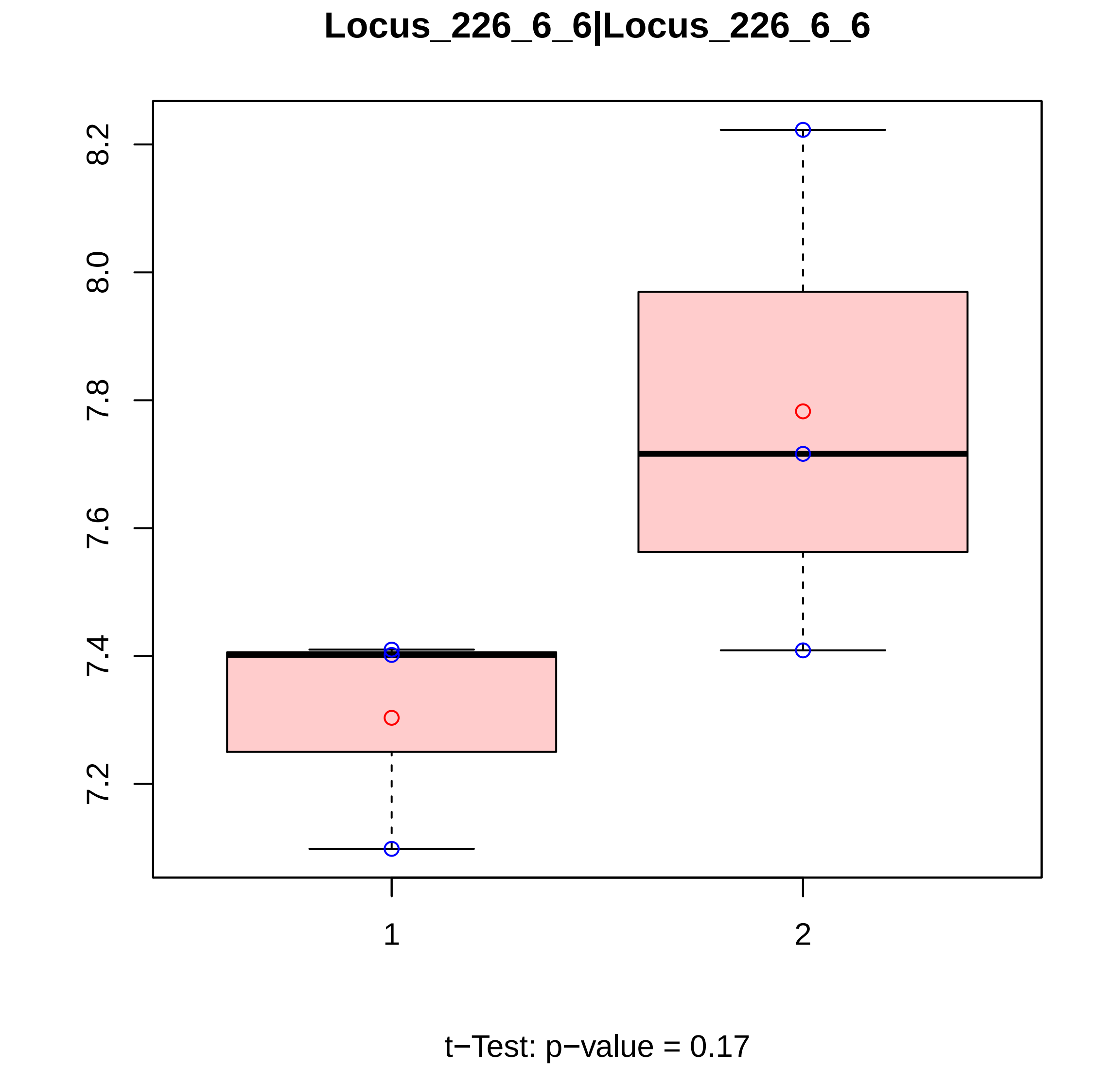

Locus_226_6_6|Locus_226_6_6
8.2
8.0
7.8
7.6
7.4
7.2
1
2
t−Test: p−value = 0.17

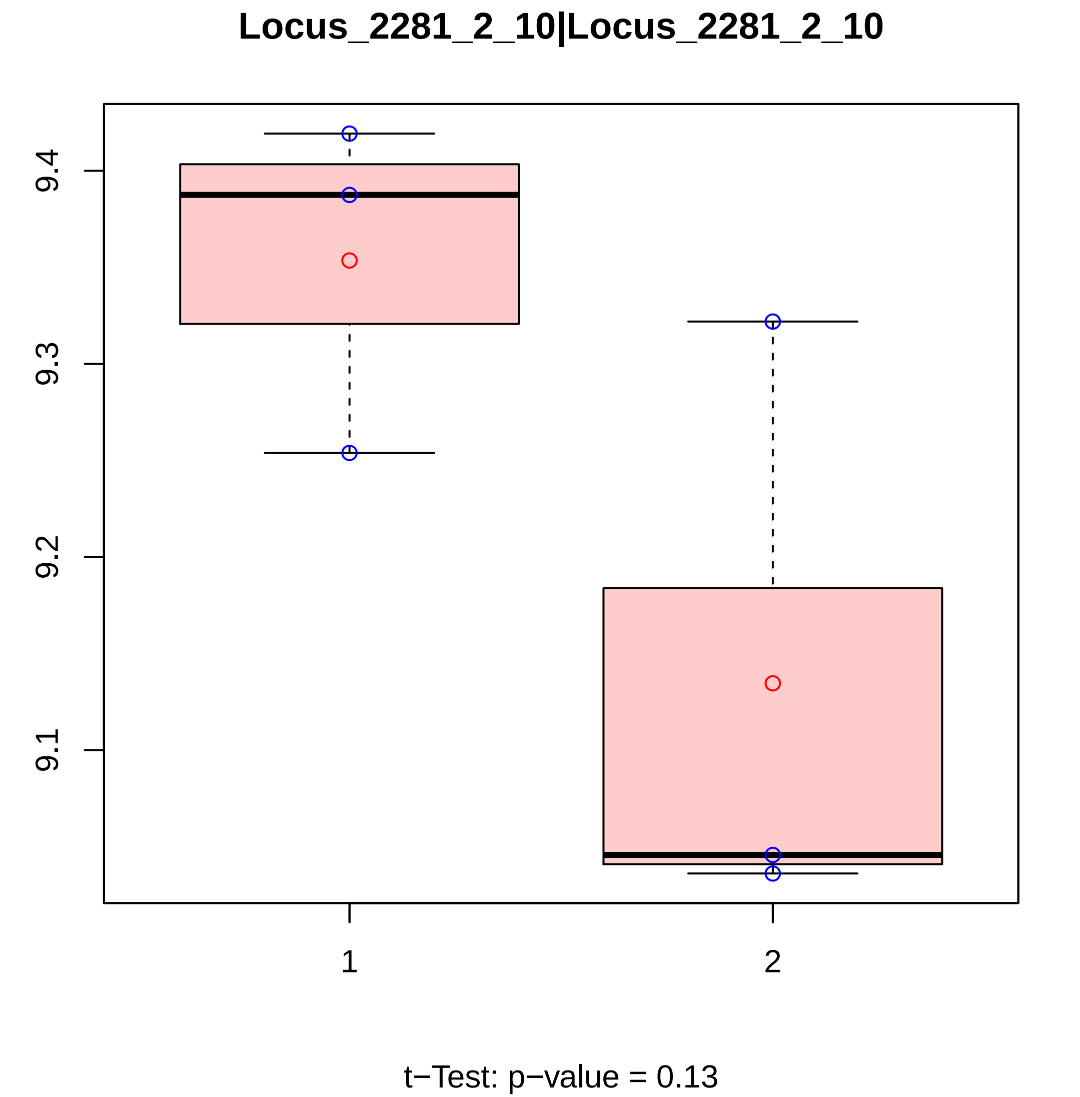
Locus_2281_2_10|Locus_2281_2_10
9.4
9.3
9.2
9.1
1
2
t−Test: p−value = 0.13

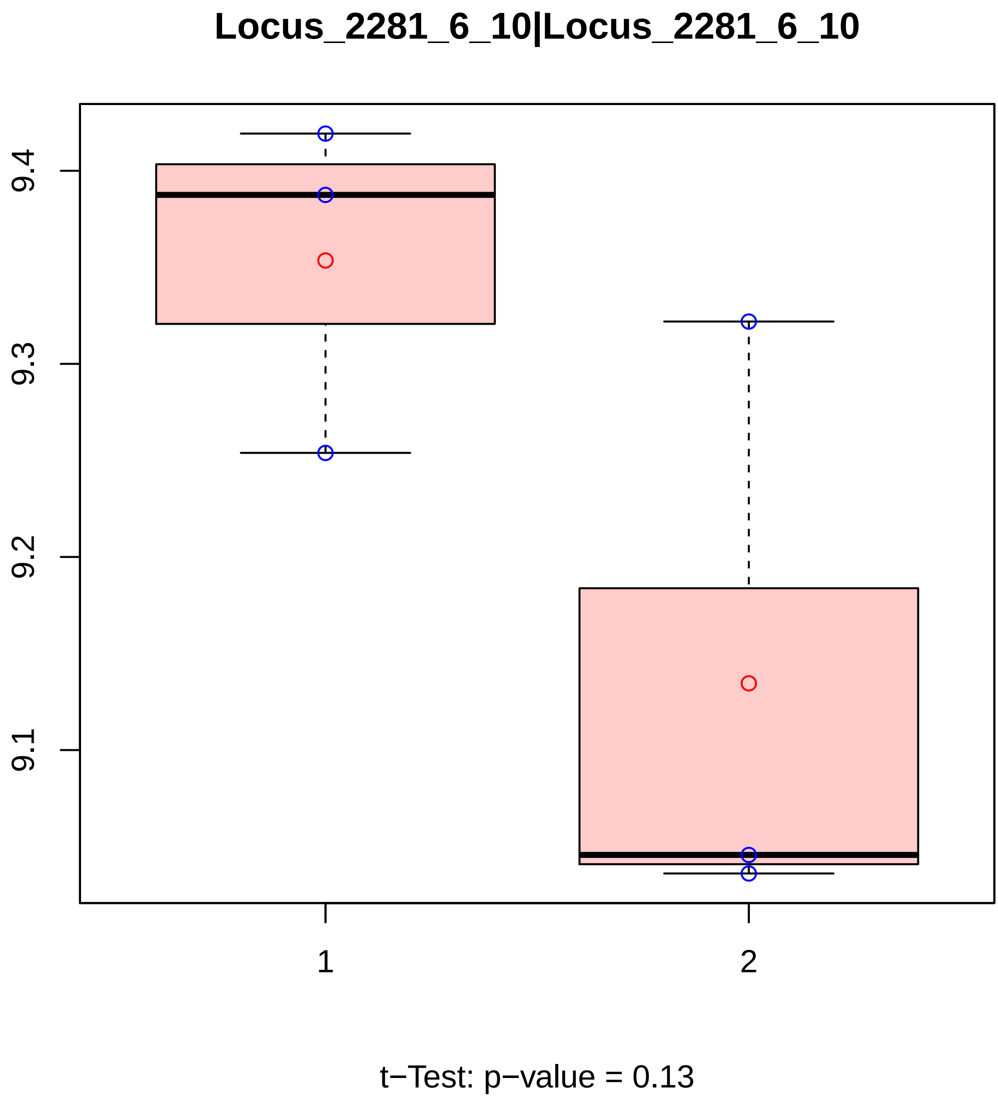

Locus_2281_6_10|Locus_2281_6_10
9.4
9.3
9.2
9.1
1
2
t−Test: p−value = 0.13

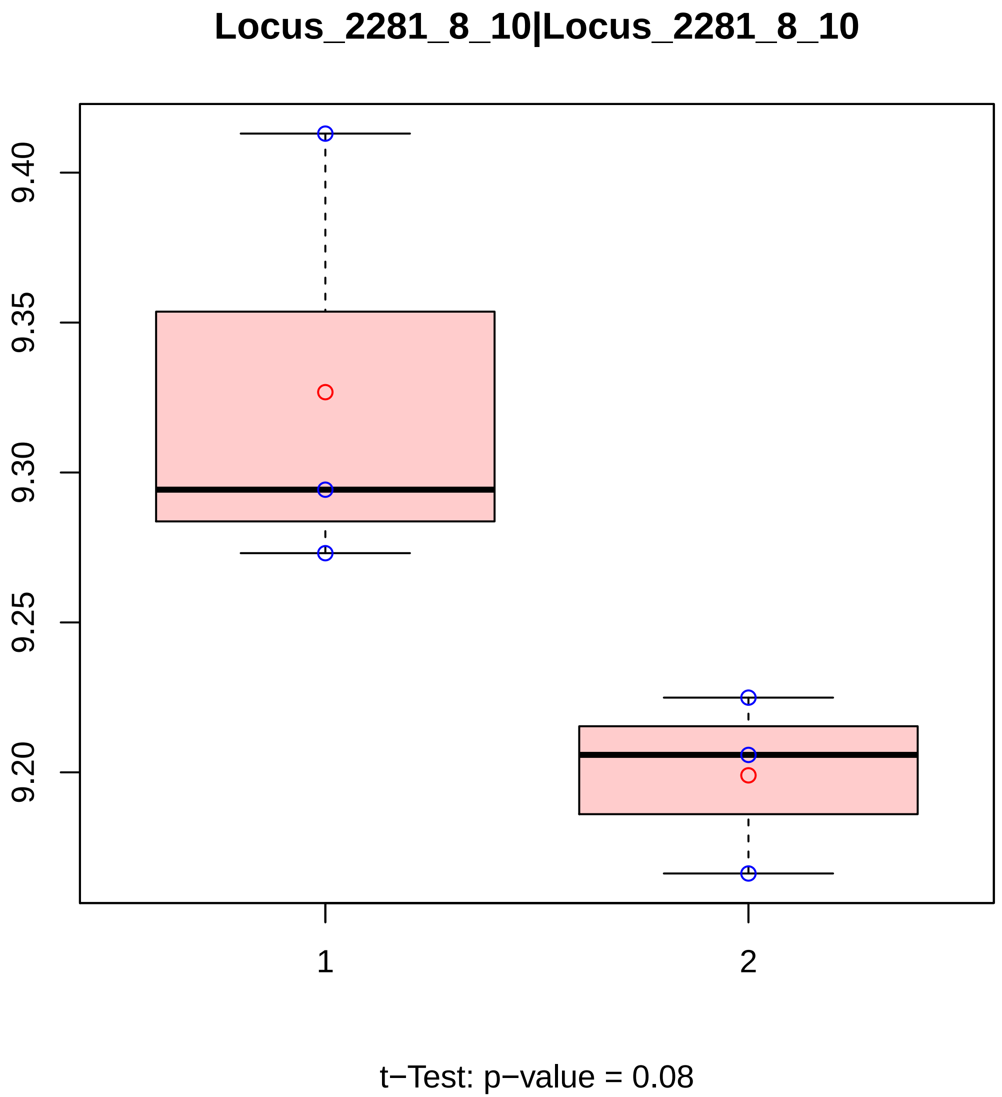
Locus_2281_8_10|Locus_2281_8_10
9.40
9.35
9.30
9.25
9.20
1
2
t−Test: p−value = 0.08

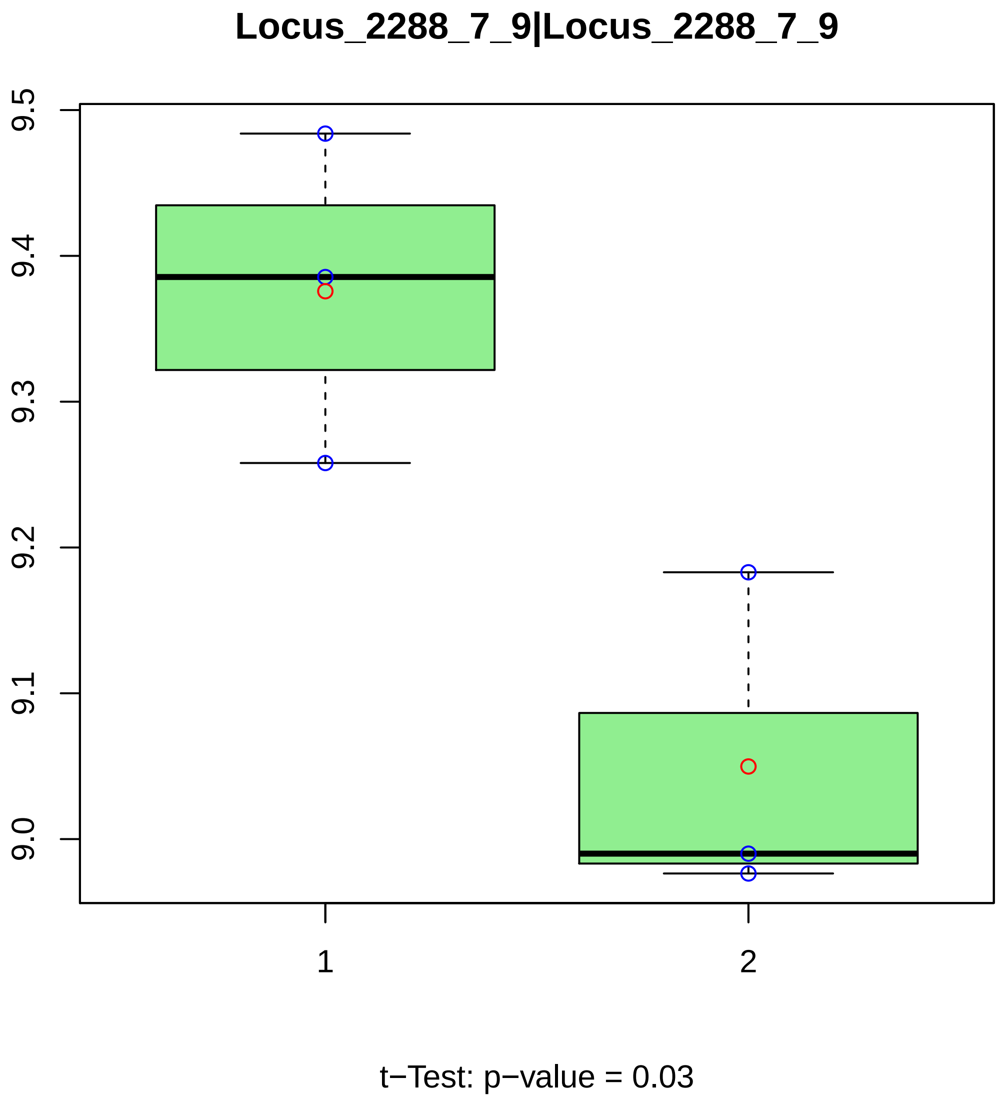

Locus_2288_7_9|Locus_2288_7_9
9.5
9.4
9.3
9.2
9.1
9.0
1
2
t−Test: p−value = 0.03

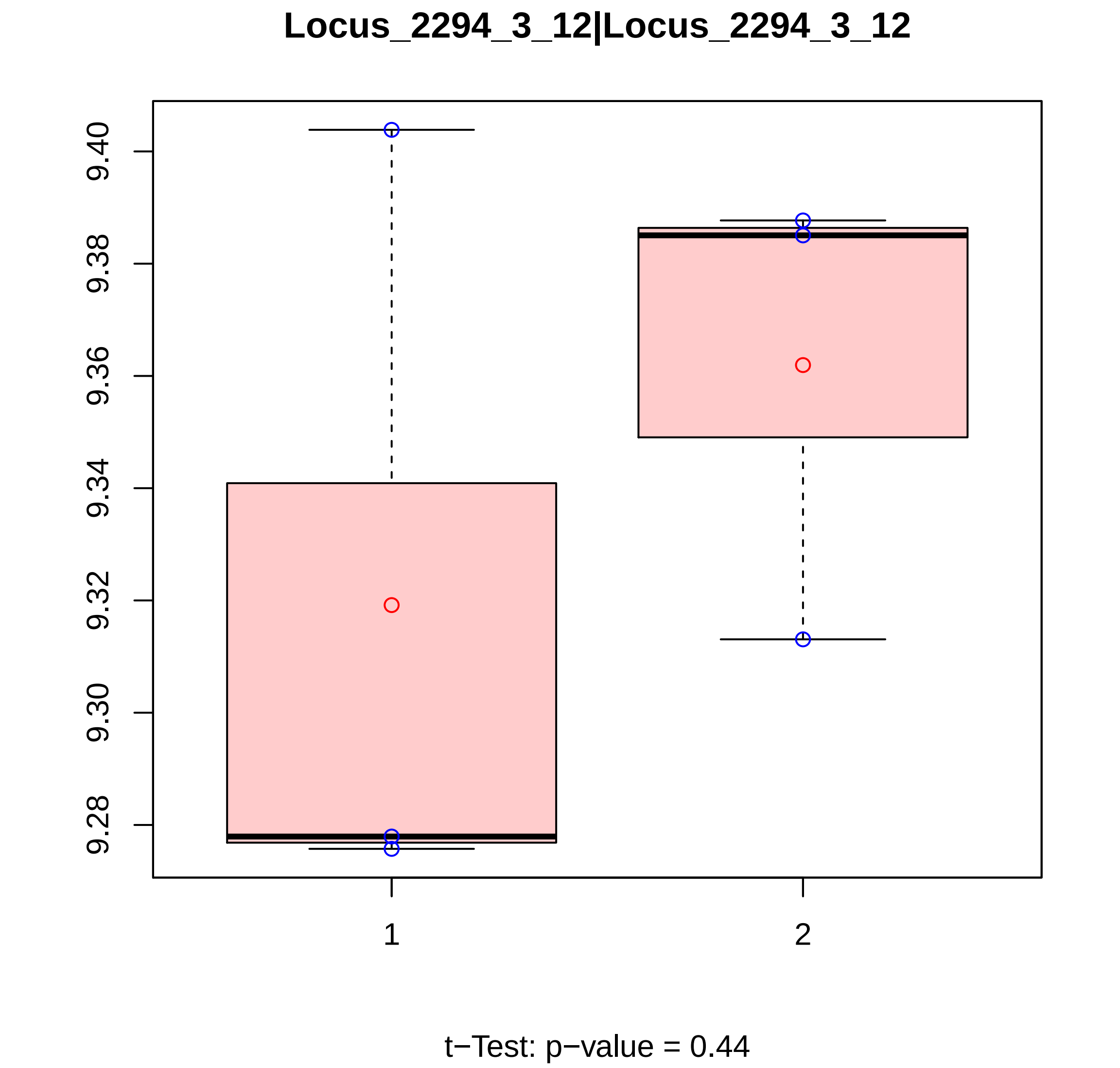

Locus_2294_3_12|Locus_2294_3_12
9.40
9.38
9.36
9.34
9.32
9.30
9.28
1
2
t−Test: p−value = 0.44

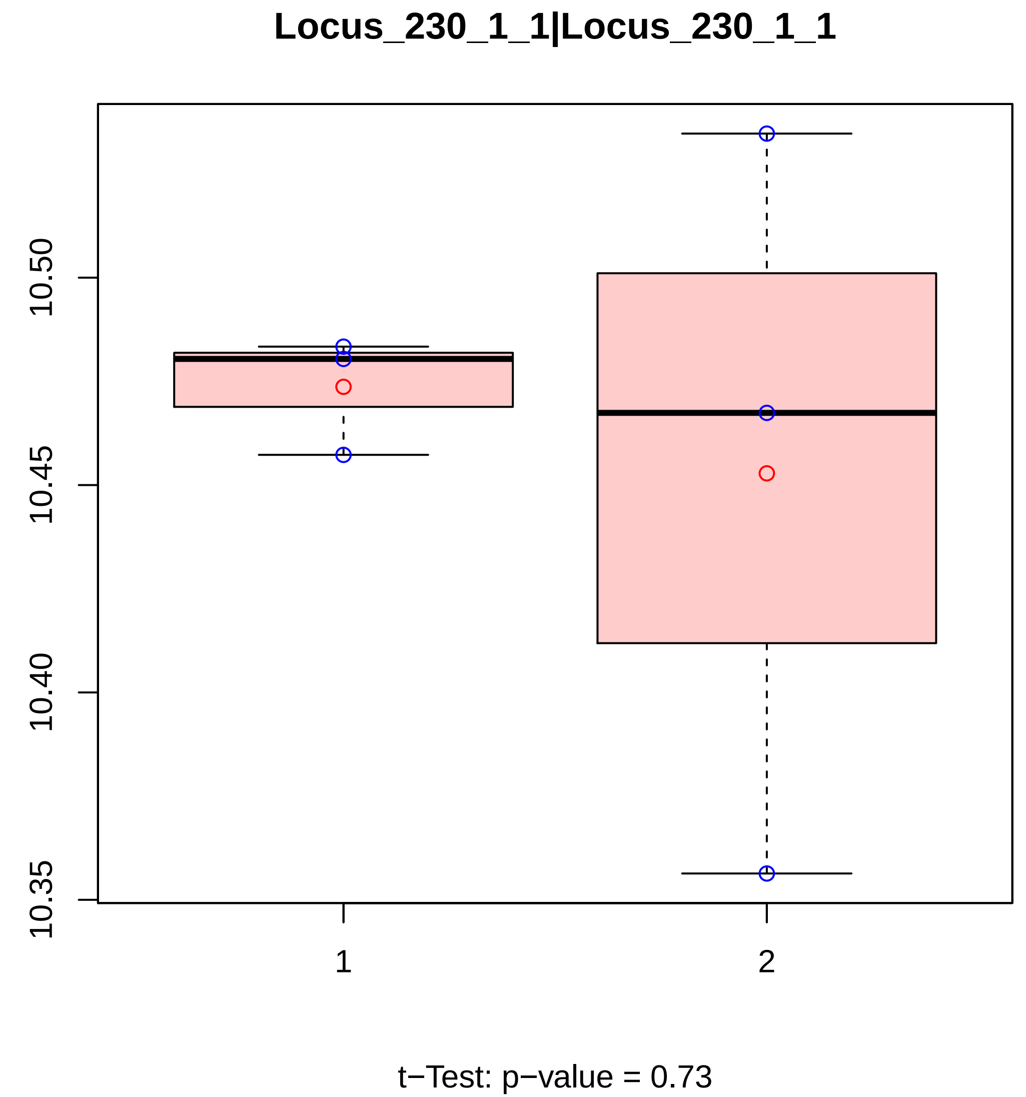
Locus_230_1_1|Locus_230_1_1
10.35
10.40
10.45
10.50
1
2
t−Test: p−value = 0.73

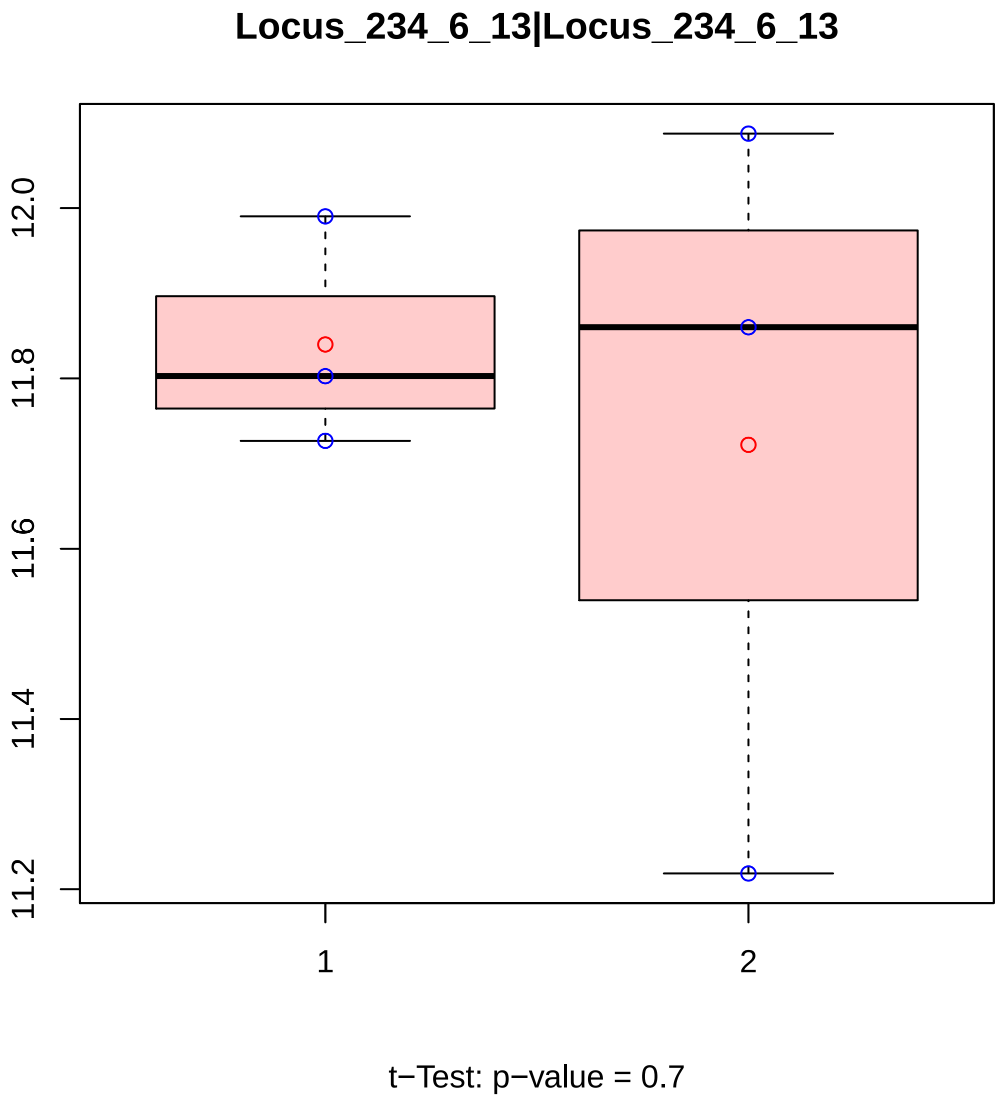
Locus_234_6_13|Locus_234_6_13
12.0
11.8
11.6
11.4
11.2
1
2
t−Test: p−value = 0.7

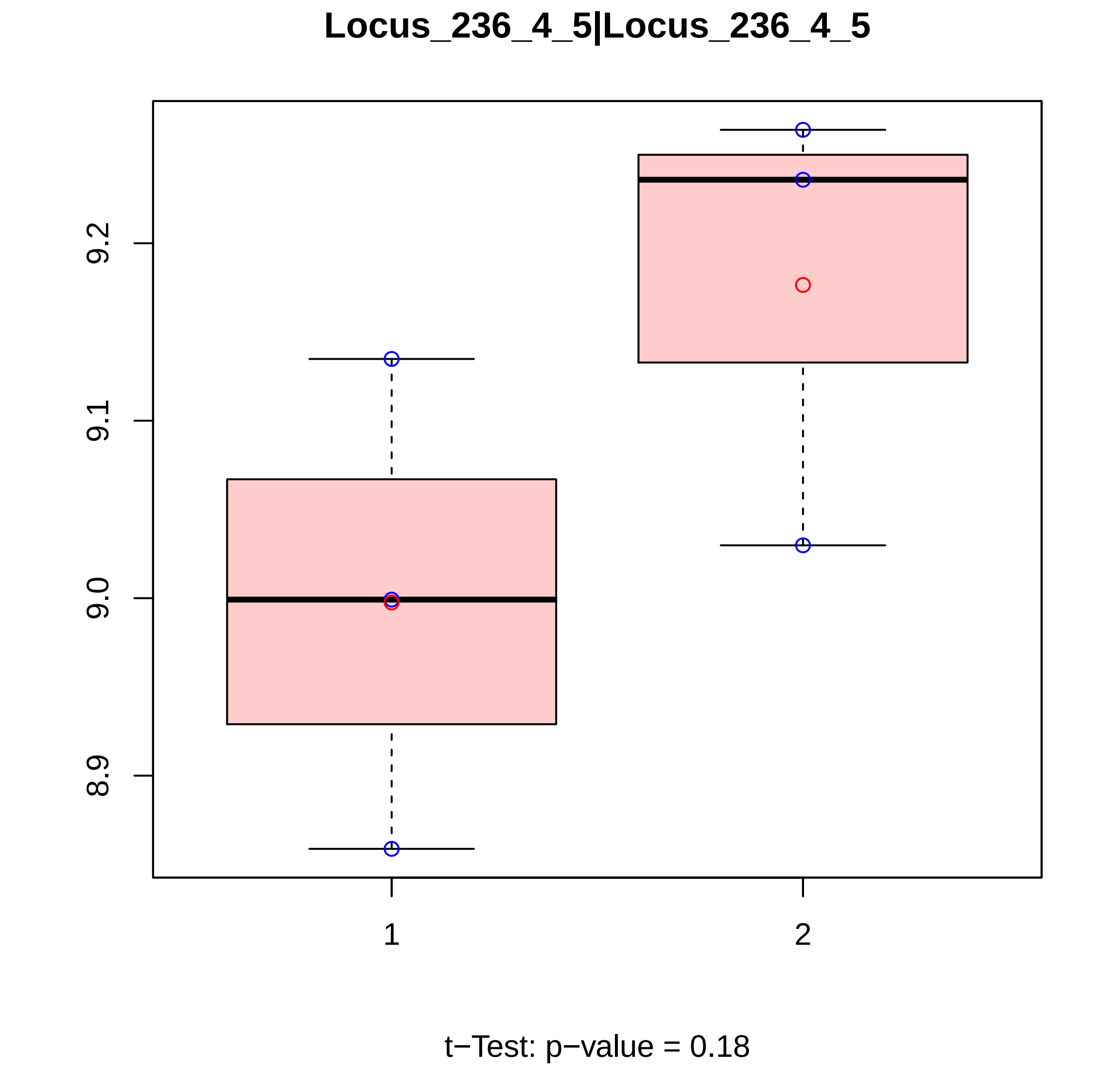
Locus_236_4_5|Locus_236_4_5
9.2
9.1
9.0
8.9
1
2
t−Test: p−value = 0.18

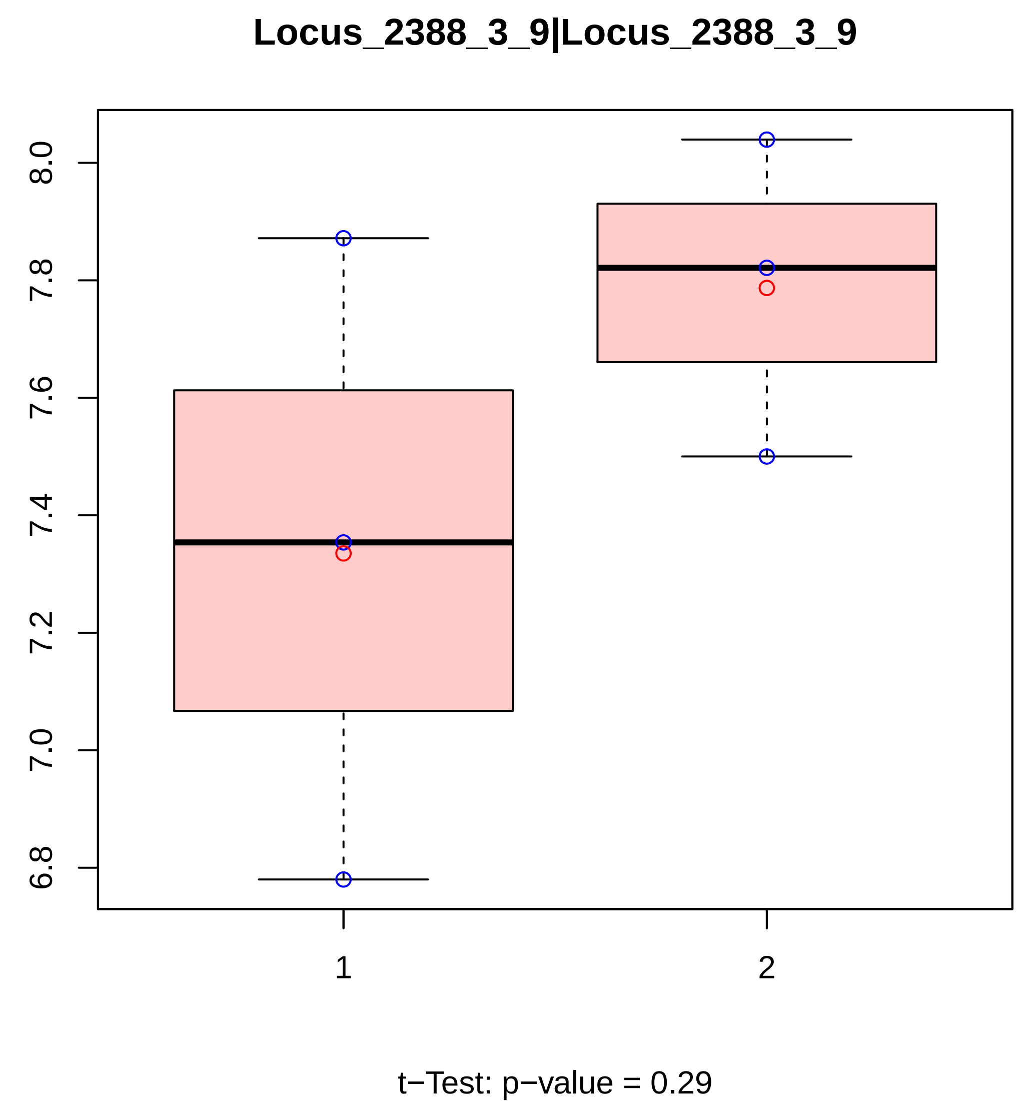

Locus_2388_3_9|Locus_2388_3_9
8.0
7.8
7.6
7.4
7.2
7.0
6.8
1
2
t−Test: p−value = 0.29

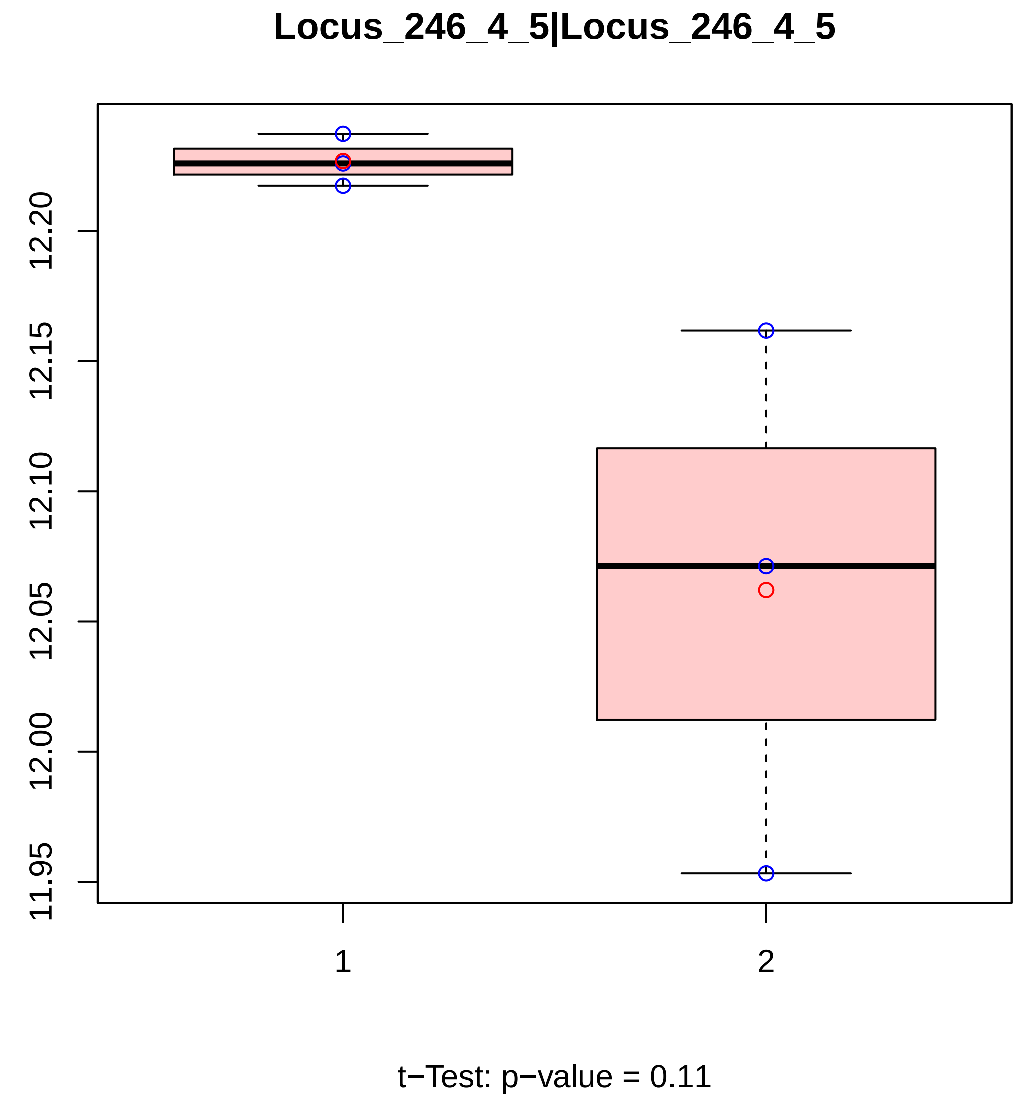
Locus_246_4_5|Locus_246_4_5
12.20
12.15
12.10
12.05
12.00
11.95
1
2
t−Test: p−value = 0.11

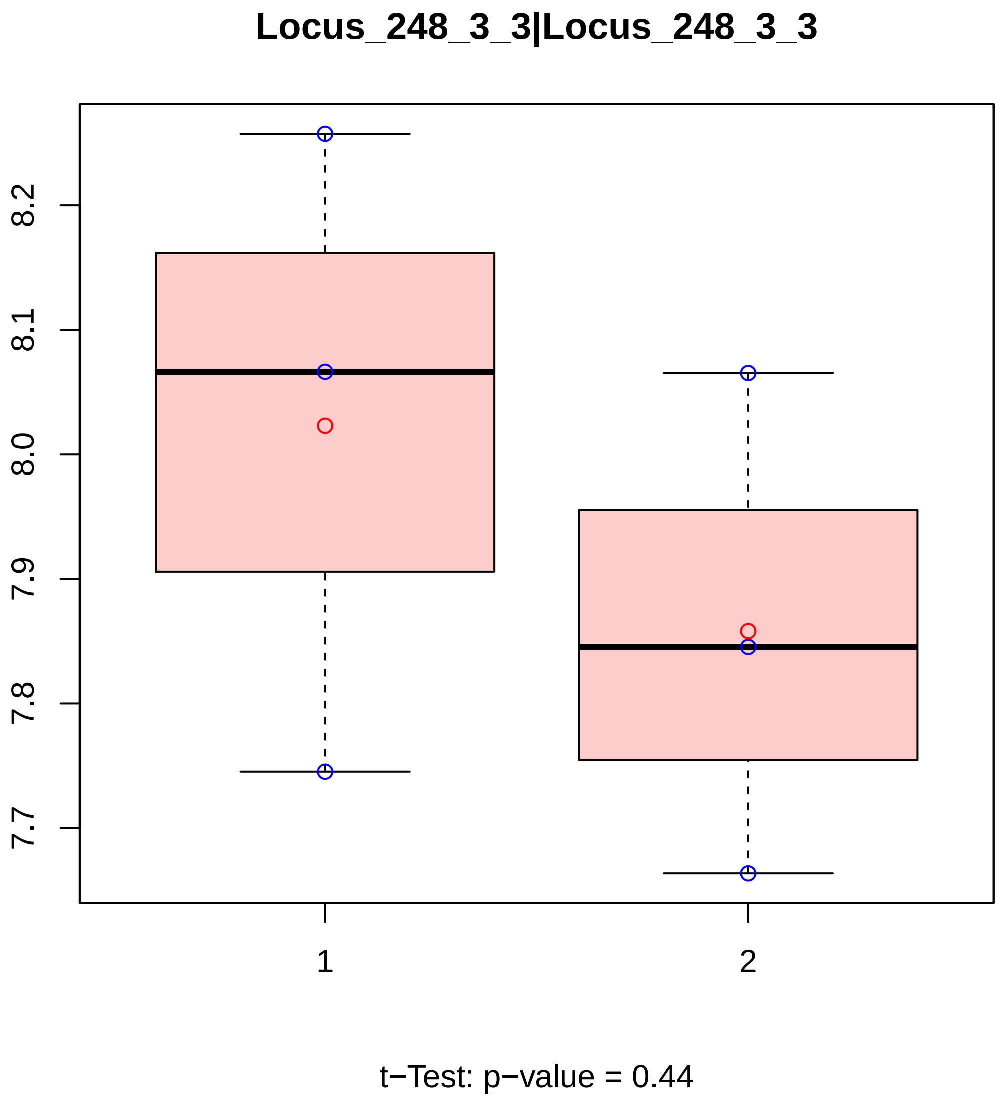
Locus_248_3_3|Locus_248_3_3
8.2
8.1
8.0
7.9
7.8
7.7
1
2
t−Test: p−value = 0.44

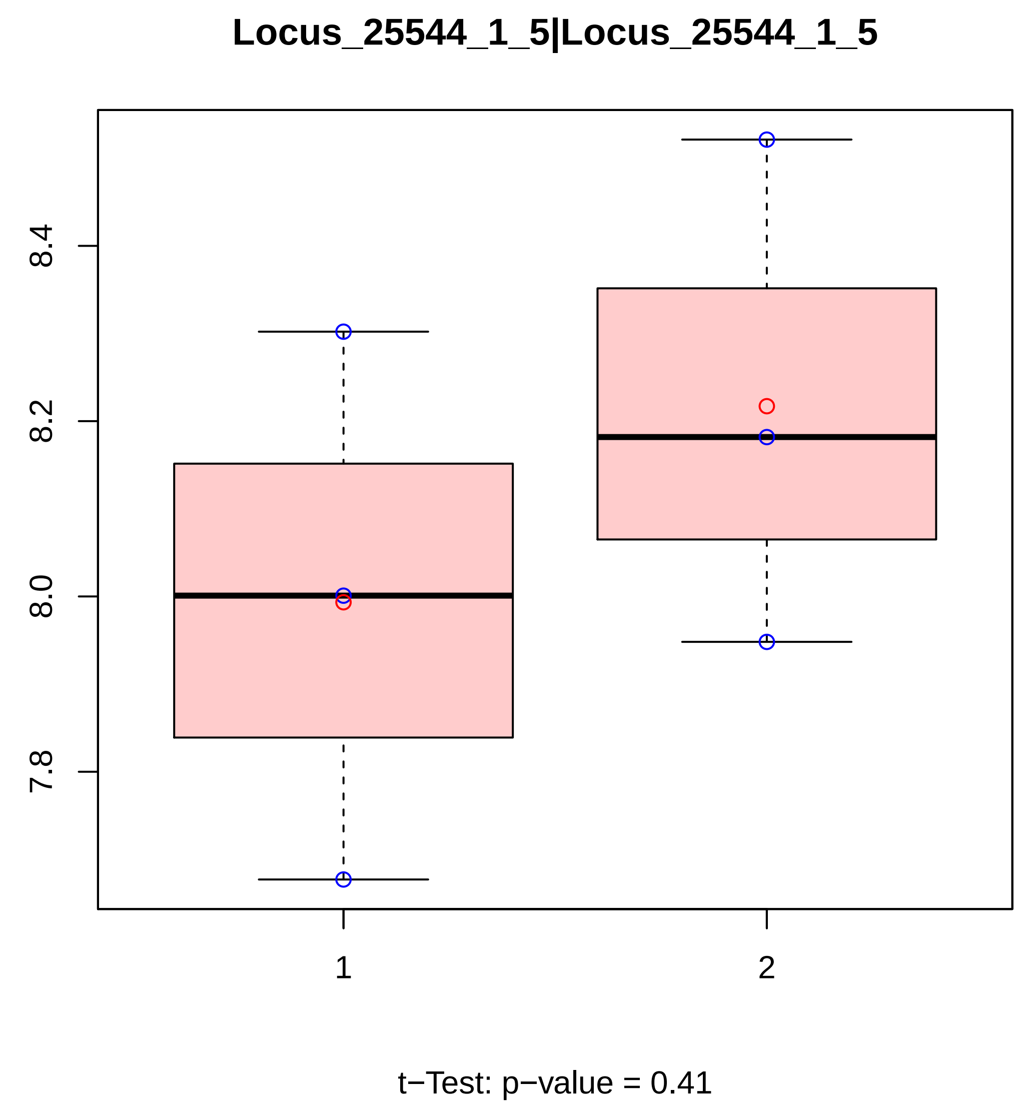
Locus_25544_1_5|Locus_25544_1_5
8.4
8.2
8.0
7.8
1
2
t−Test: p−value = 0.41

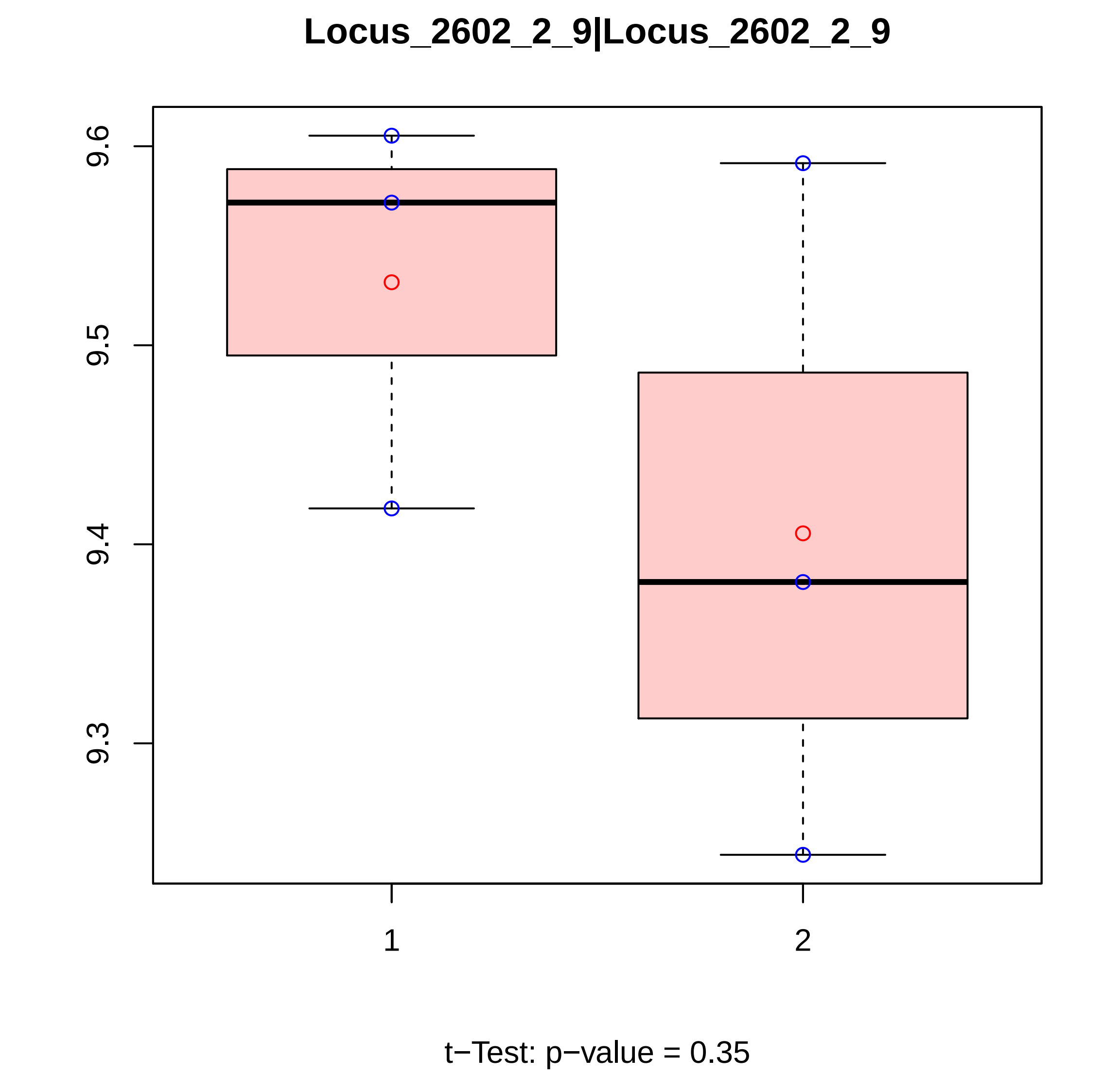
Locus_2602_2_9|Locus_2602_2_9
9.6
9.5
9.4
9.3
1
2
t−Test: p−value = 0.35

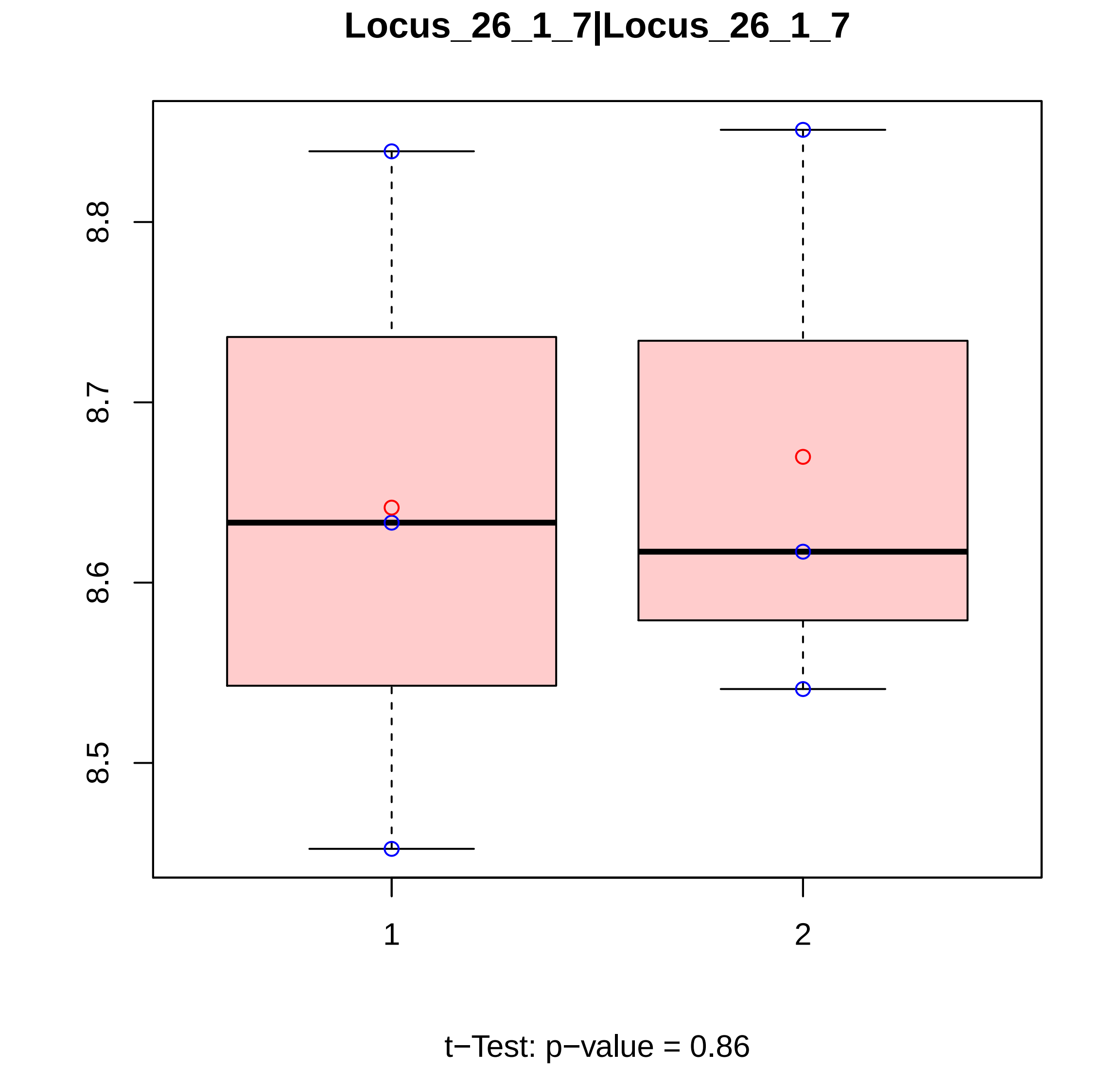

Locus_26_1_7|Locus_26_1_7
8.5
8.6
8.7
8.8
1
2
t−Test: p−value = 0.86

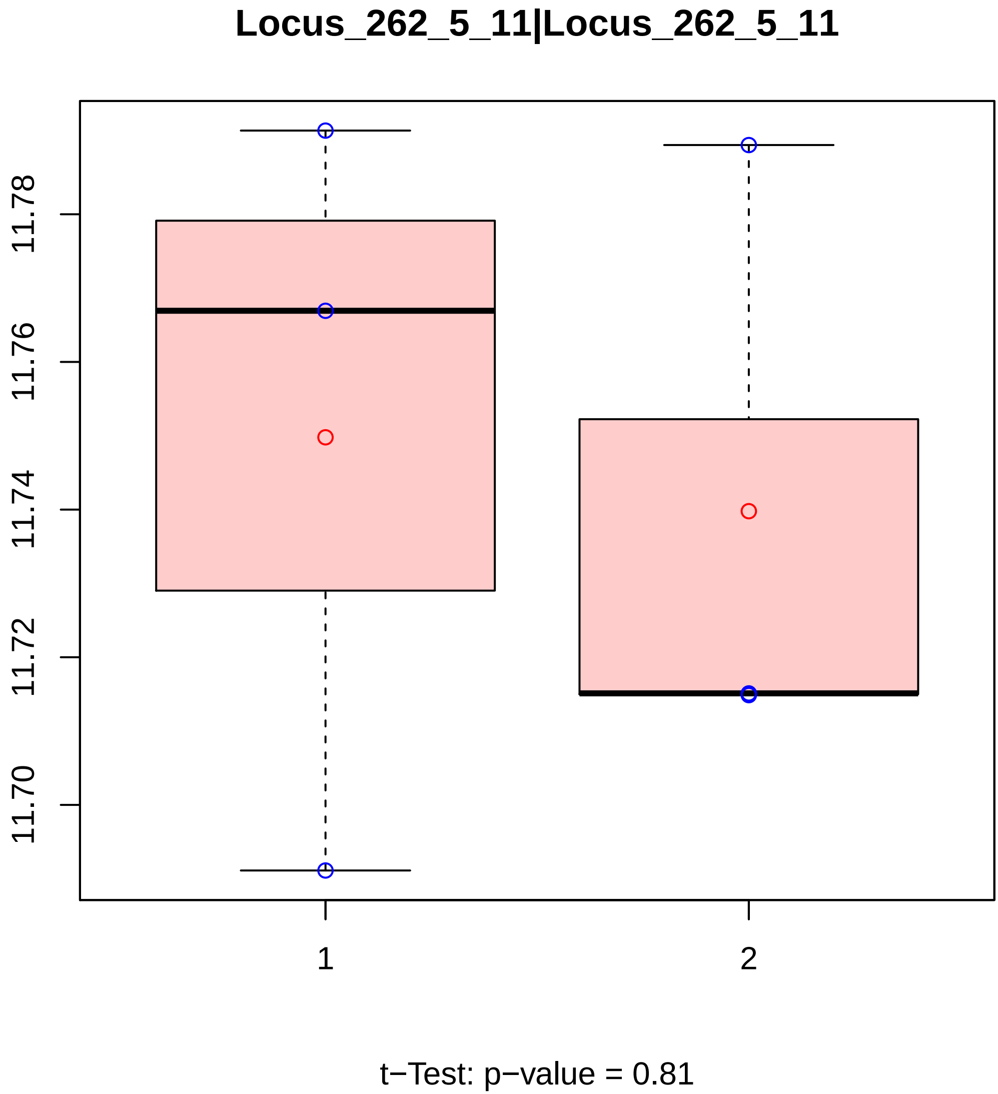
Locus_262_5_11|Locus_262_5_11
11.70
11.72
11.74
11.76
11.78
1
2
t−Test: p−value = 0.81

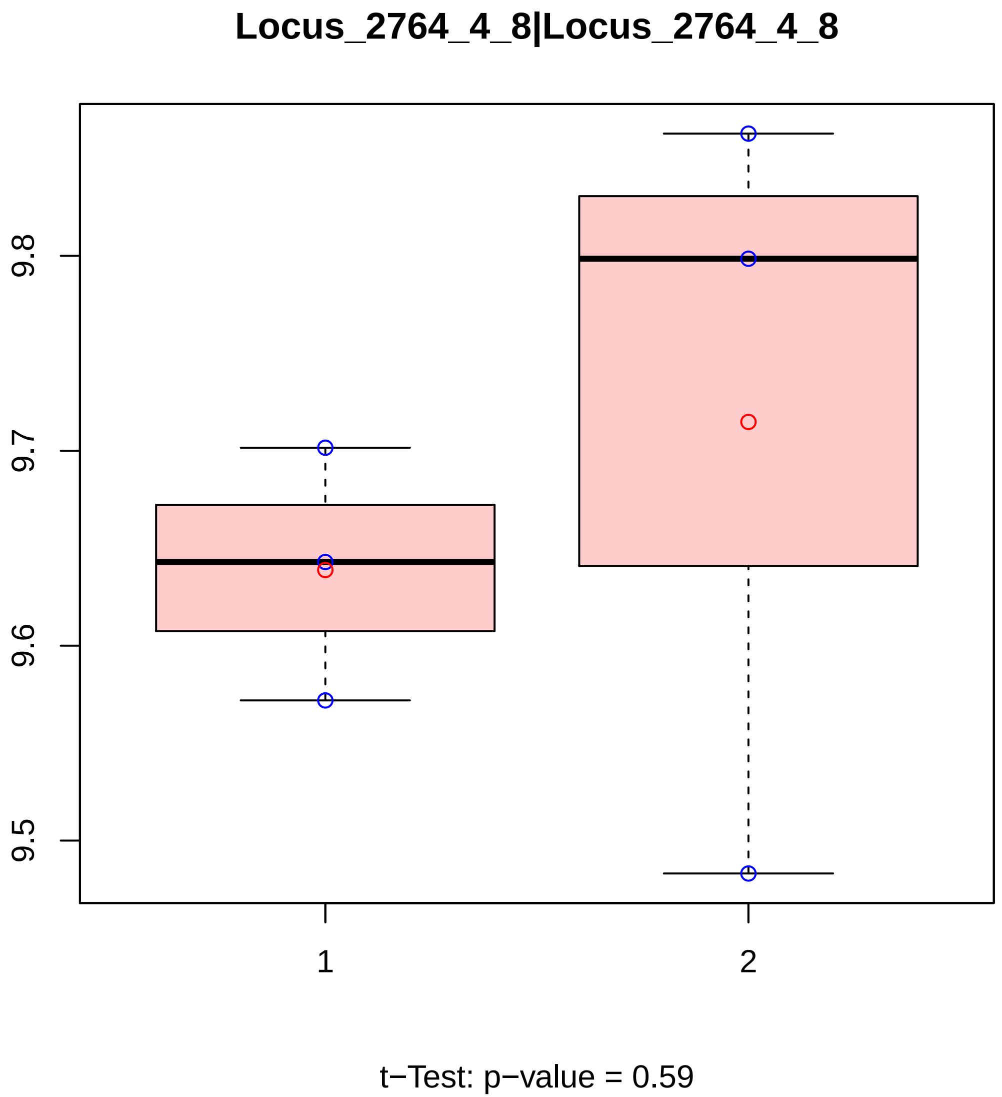

Locus_2764_4_8|Locus_2764_4_8
9.5
9.6
9.7
9.8
1
2
t−Test: p−value = 0.59

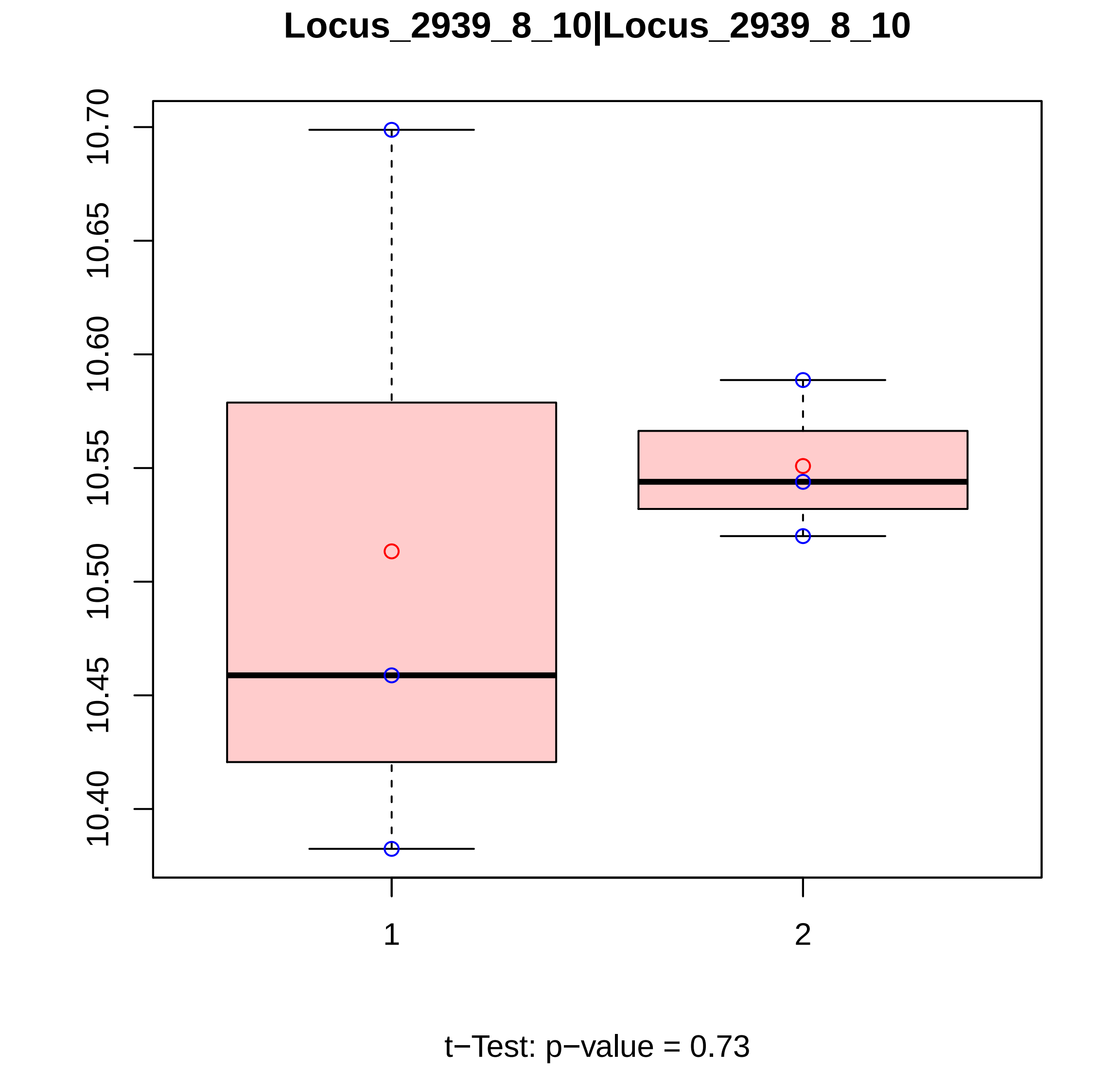

Locus_2939_8_10|Locus_2939_8_10
10.40
10.45
10.50
10.55
10.60
10.65
10.70
1
2
t−Test: p−value = 0.73

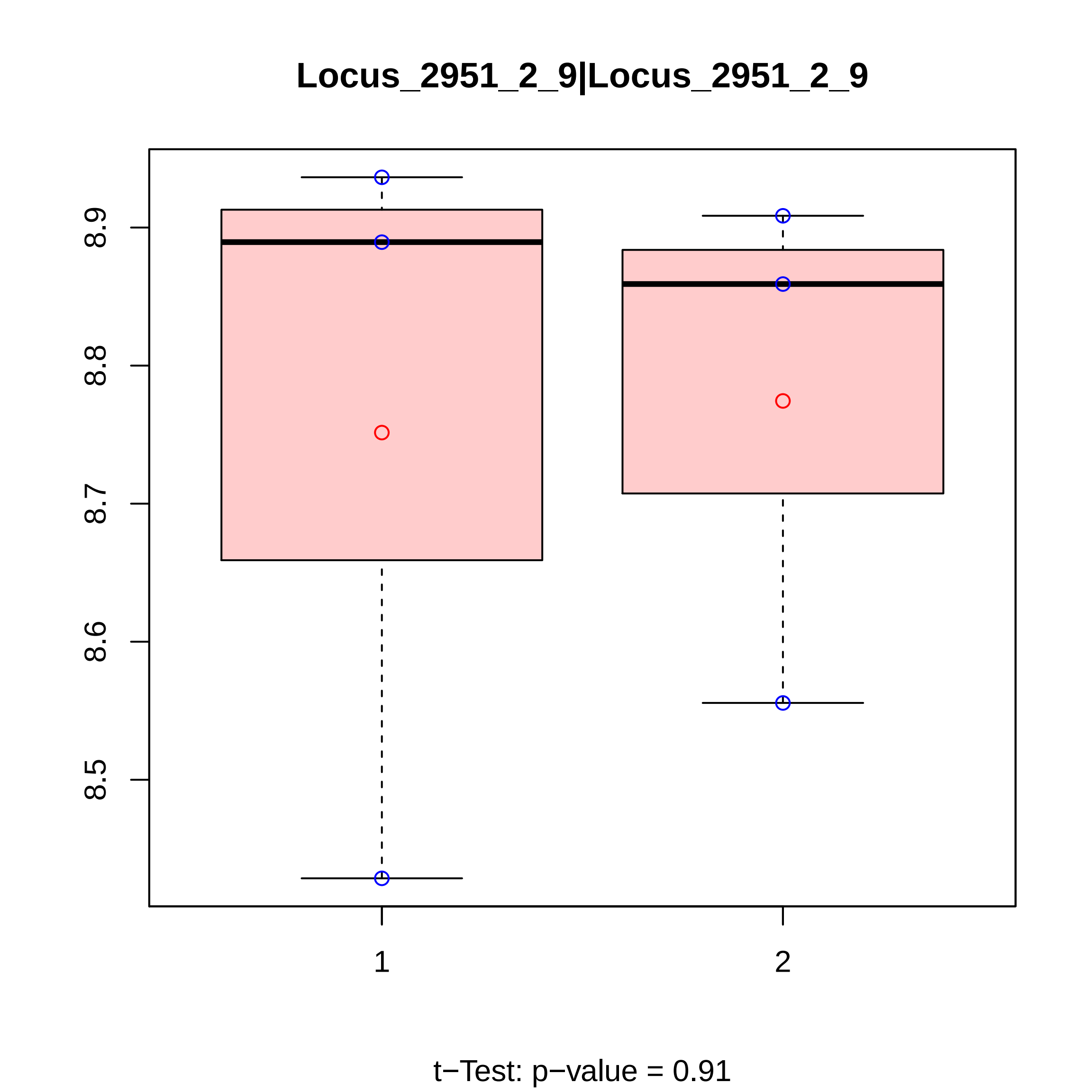

Locus_2951_2_9|Locus_2951_2_9
8.5
8.6
8.7
8.8
8.9
1
2
t−Test: p−value = 0.91

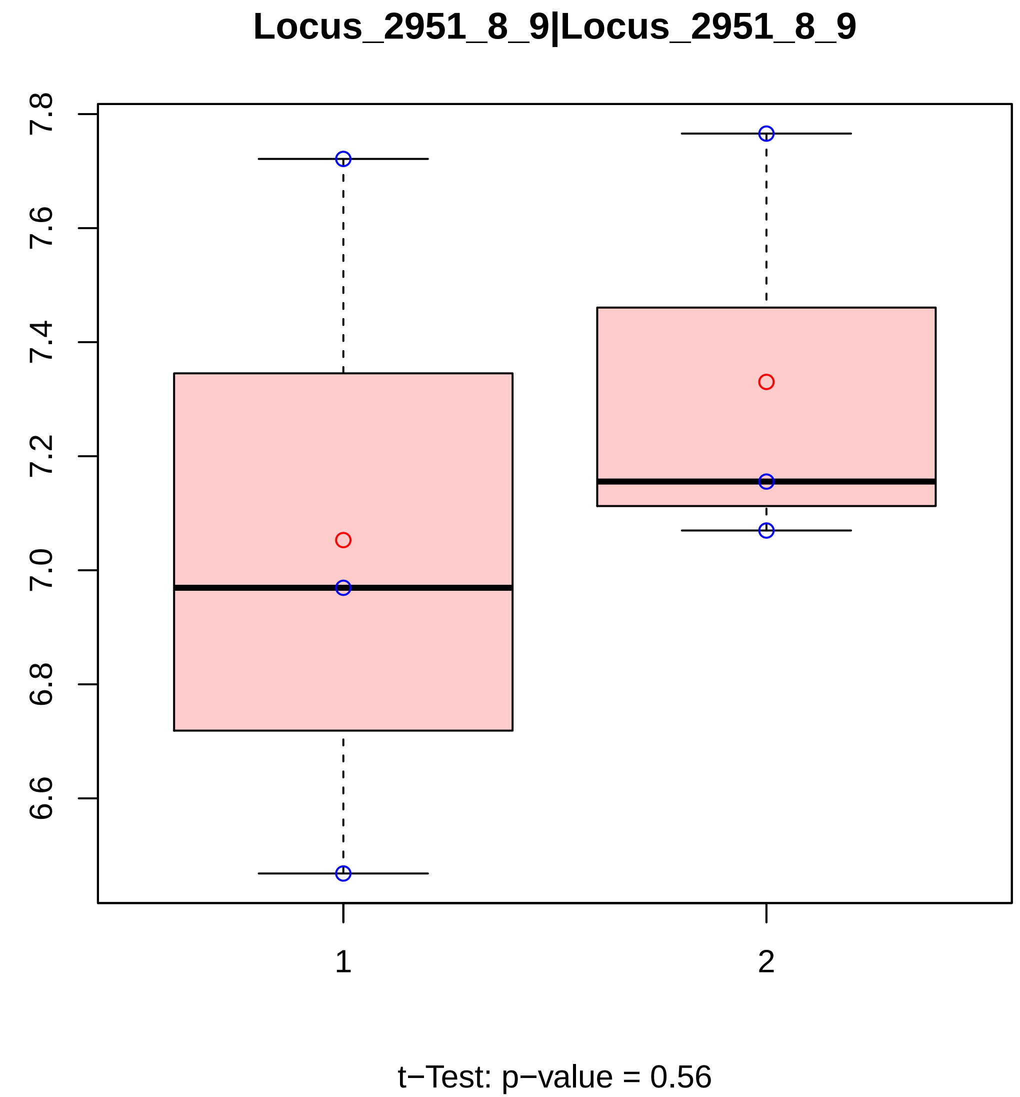

Locus_2951_8_9|Locus_2951_8_9
7.8
7.6
7.4
7.2
7.0
6.8
6.6
1
2
t−Test: p−value = 0.56

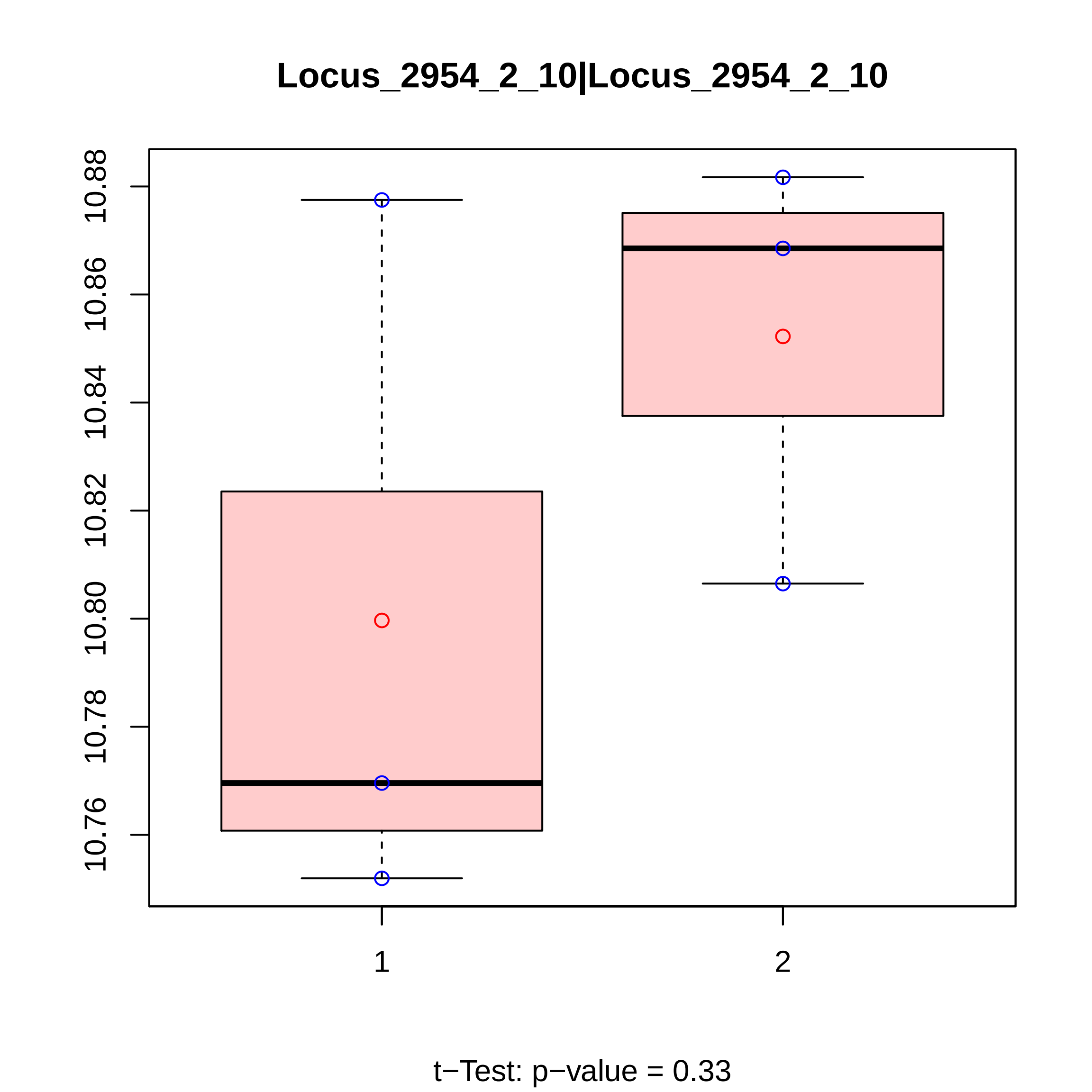
Locus_2954_2_10|Locus_2954_2_10
10.76
10.78
10.80
10.82
10.84
10.86
10.88
1
2
t−Test: p−value = 0.33

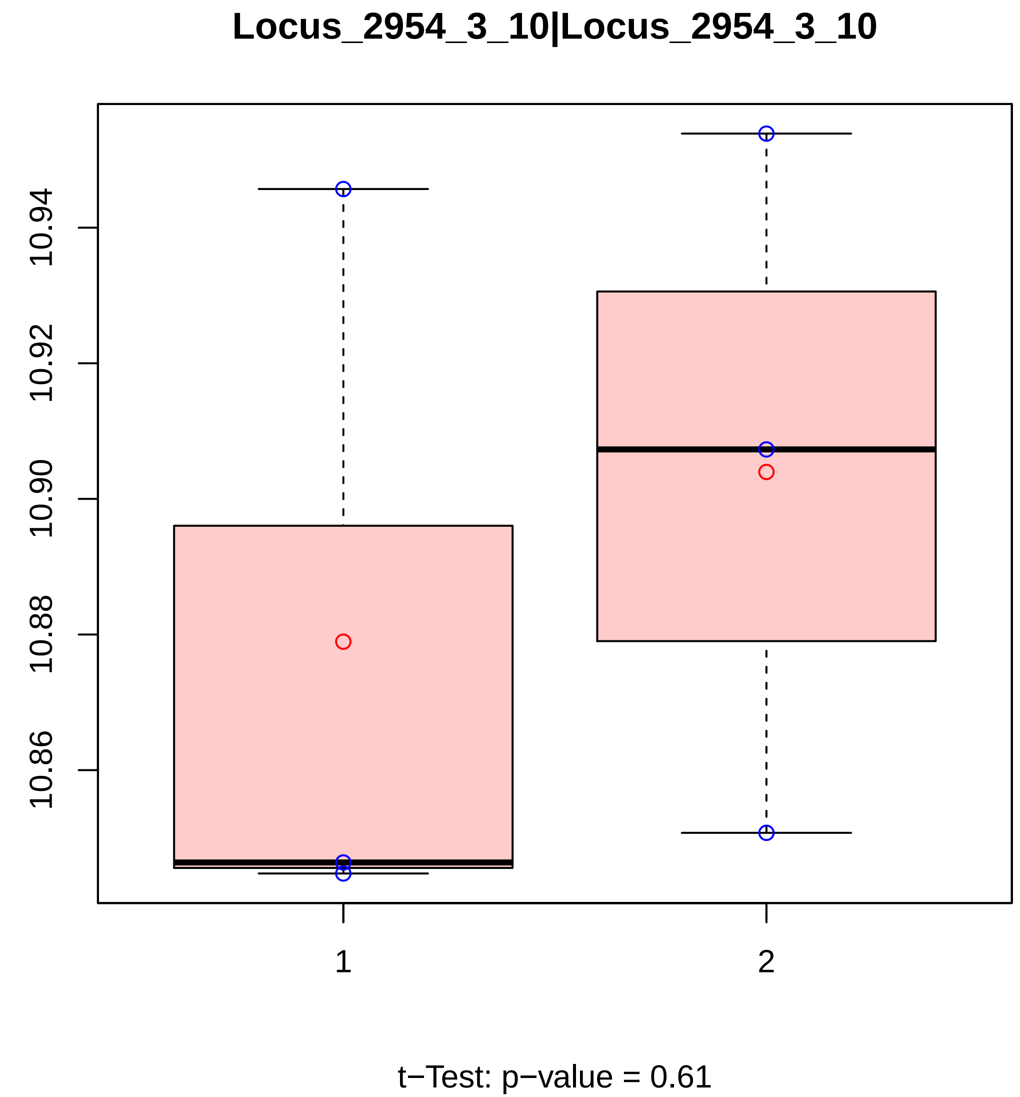

Locus_2954_3_10|Locus_2954_3_10
10.86
10.88
10.90
10.92
10.94
1
2
t−Test: p−value = 0.61

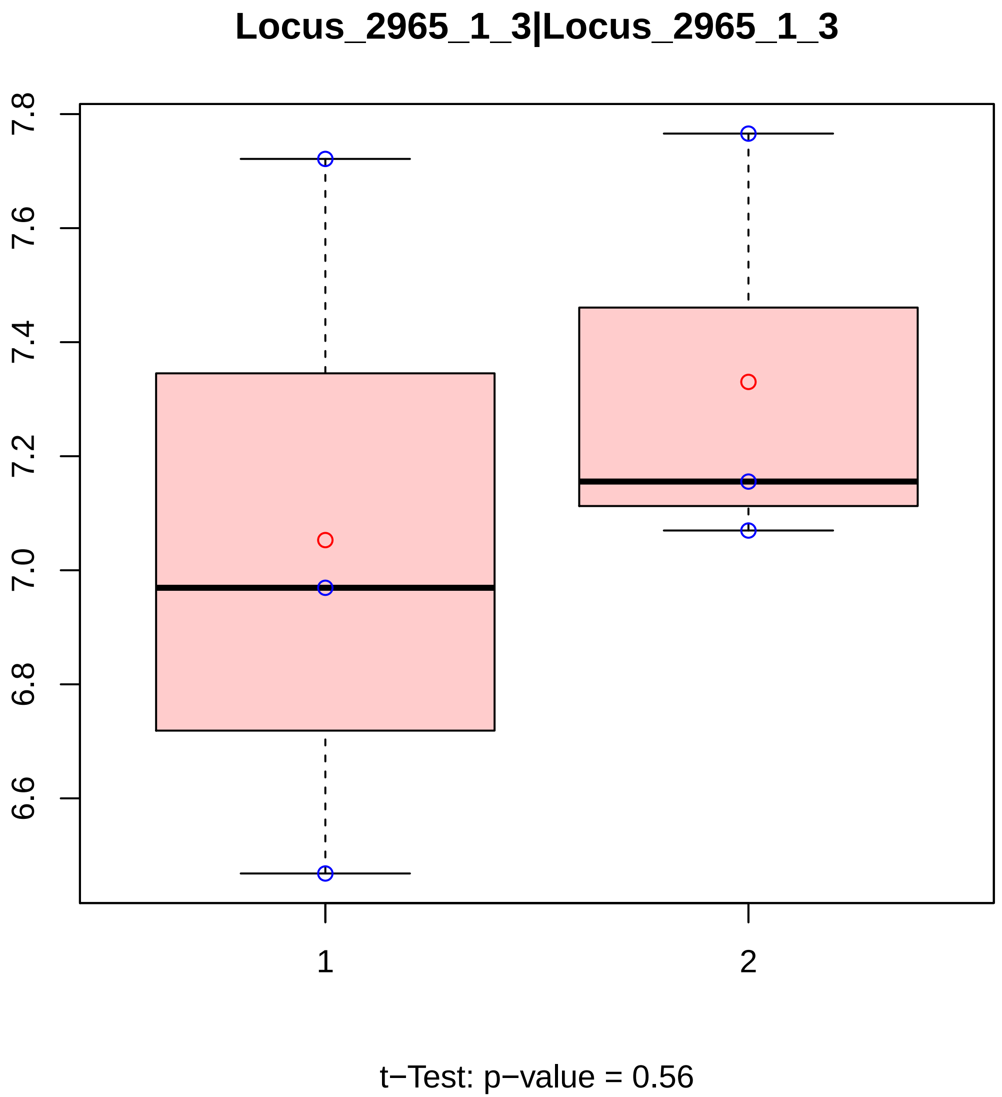

Locus_2965_1_3|Locus_2965_1_3
7.8
7.6
7.4
7.2
7.0
6.8
6.6
1
2
t−Test: p−value = 0.56

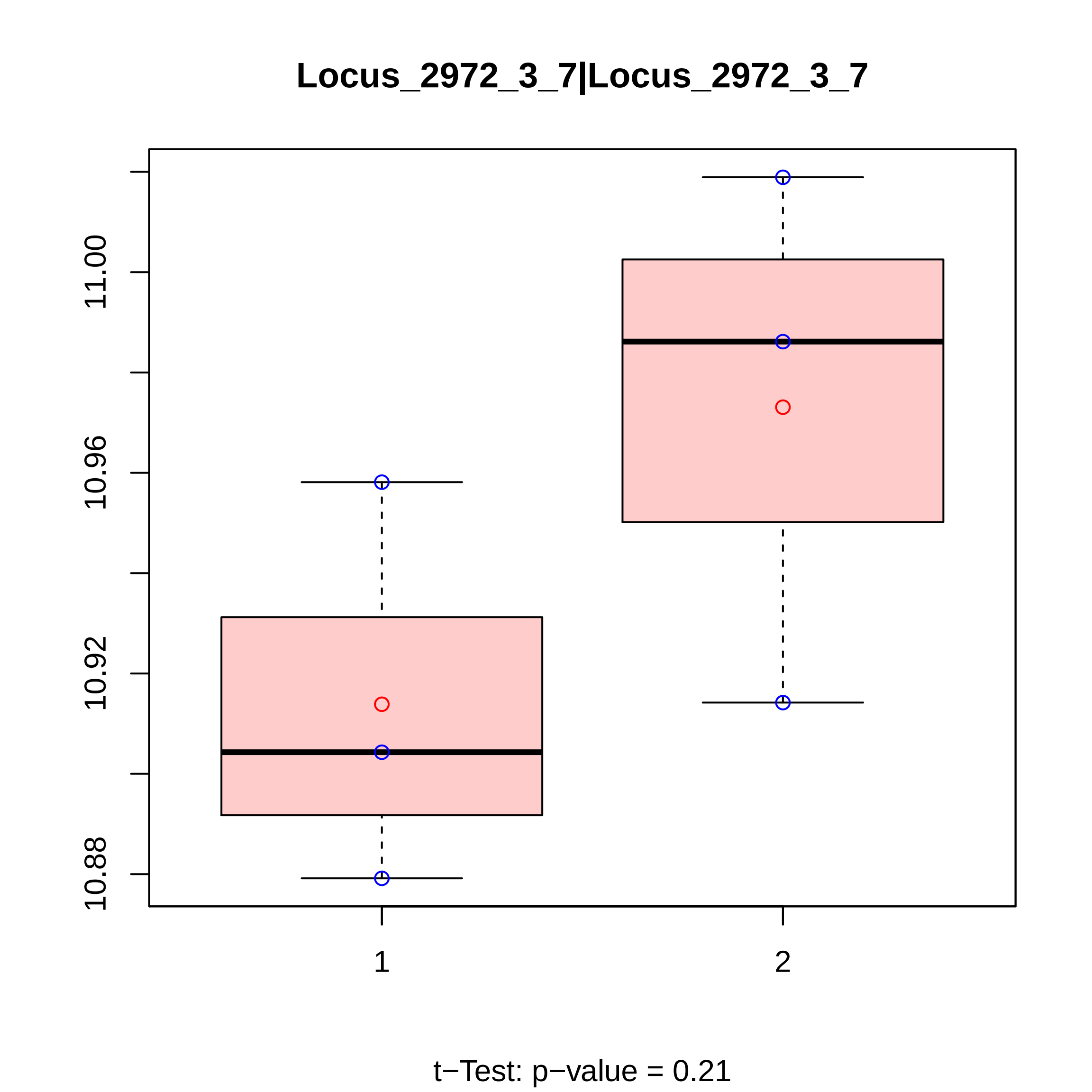

Locus_2972_3_7|Locus_2972_3_7
11.00
10.96
10.92
10.88
1
2
t−Test: p−value = 0.21

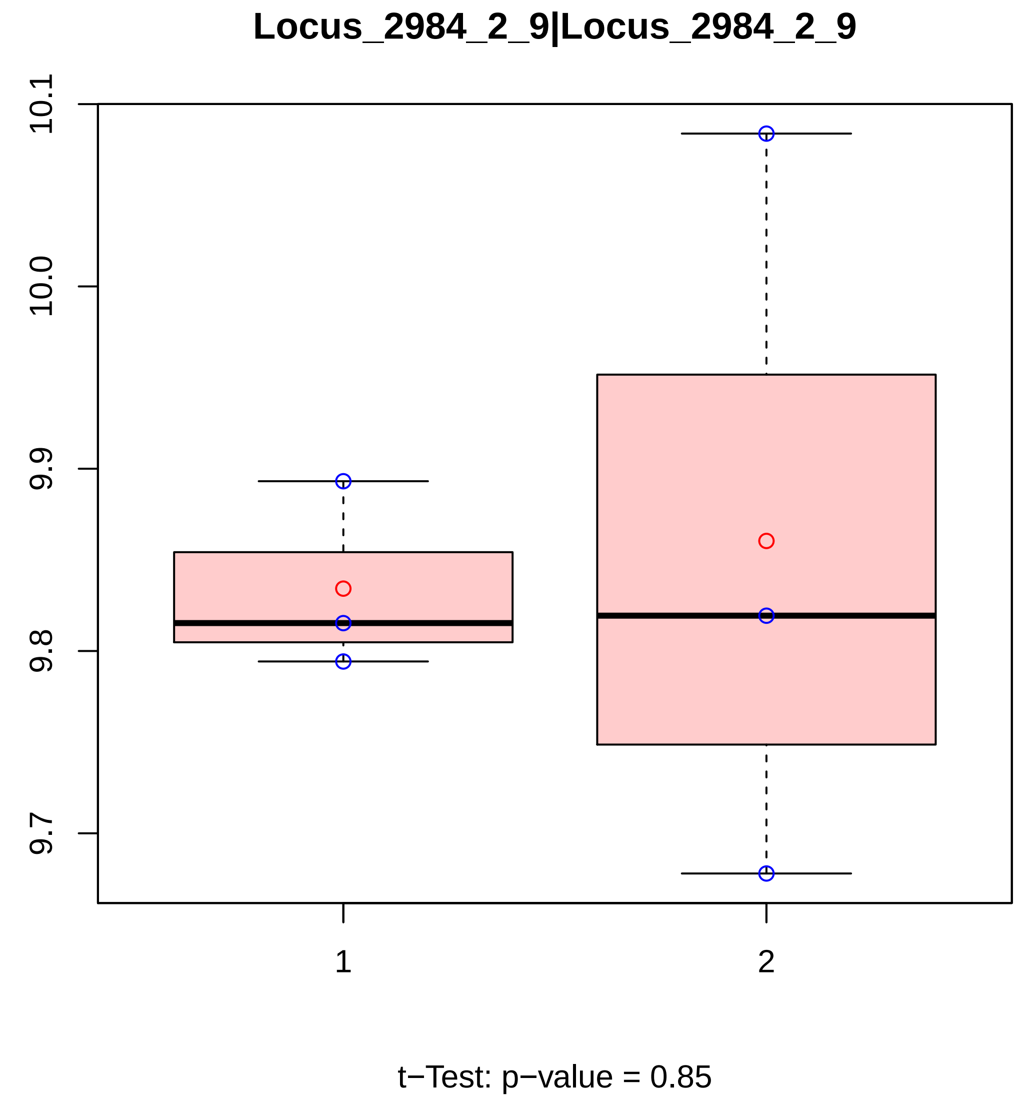
Locus_2984_2_9|Locus_2984_2_9
10.1
10.0
9.9
9.8
9.7
1
2
t−Test: p−value = 0.85

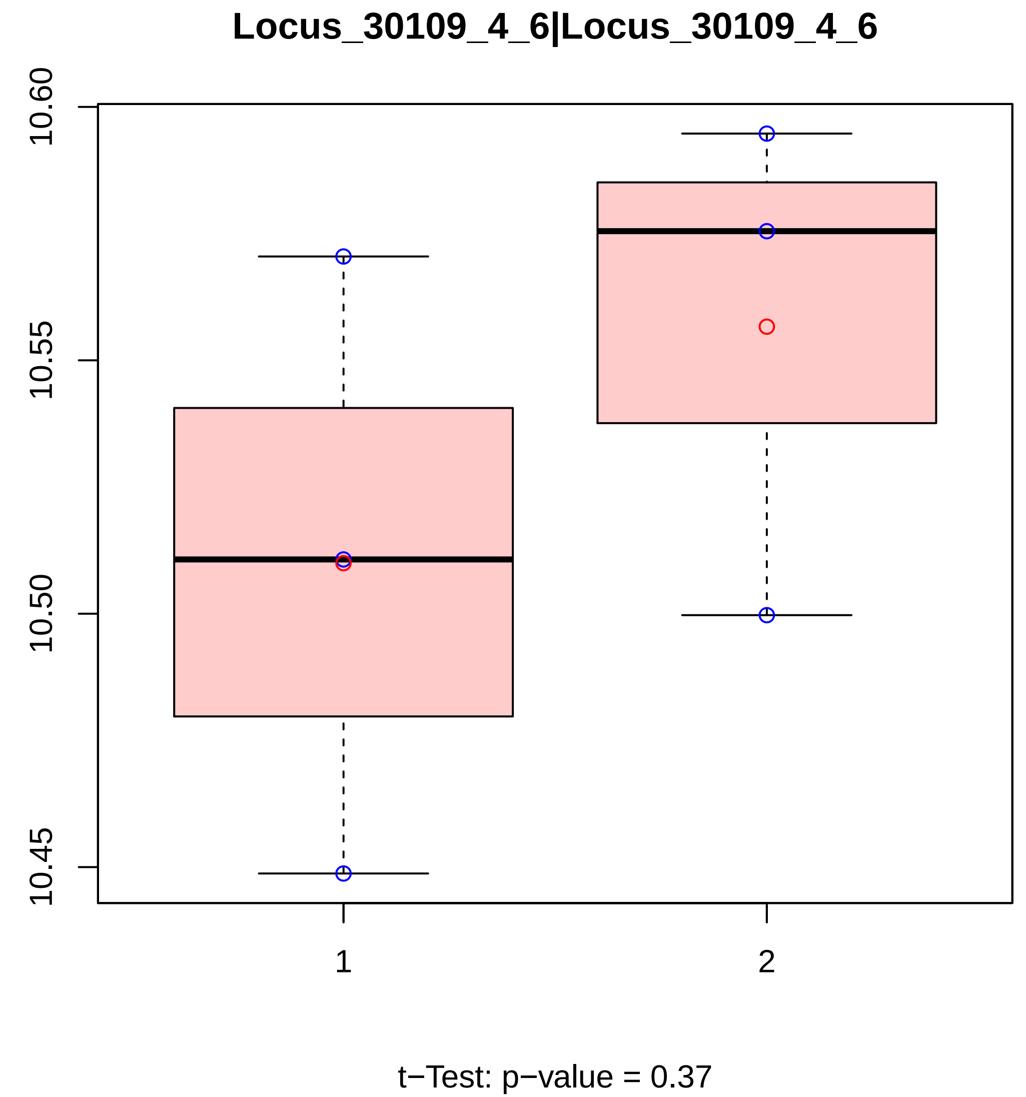
Locus_30109_4_6|Locus_30109_4_6
10.60
10.55
10.50
10.45
1
2
t−Test: p−value = 0.37

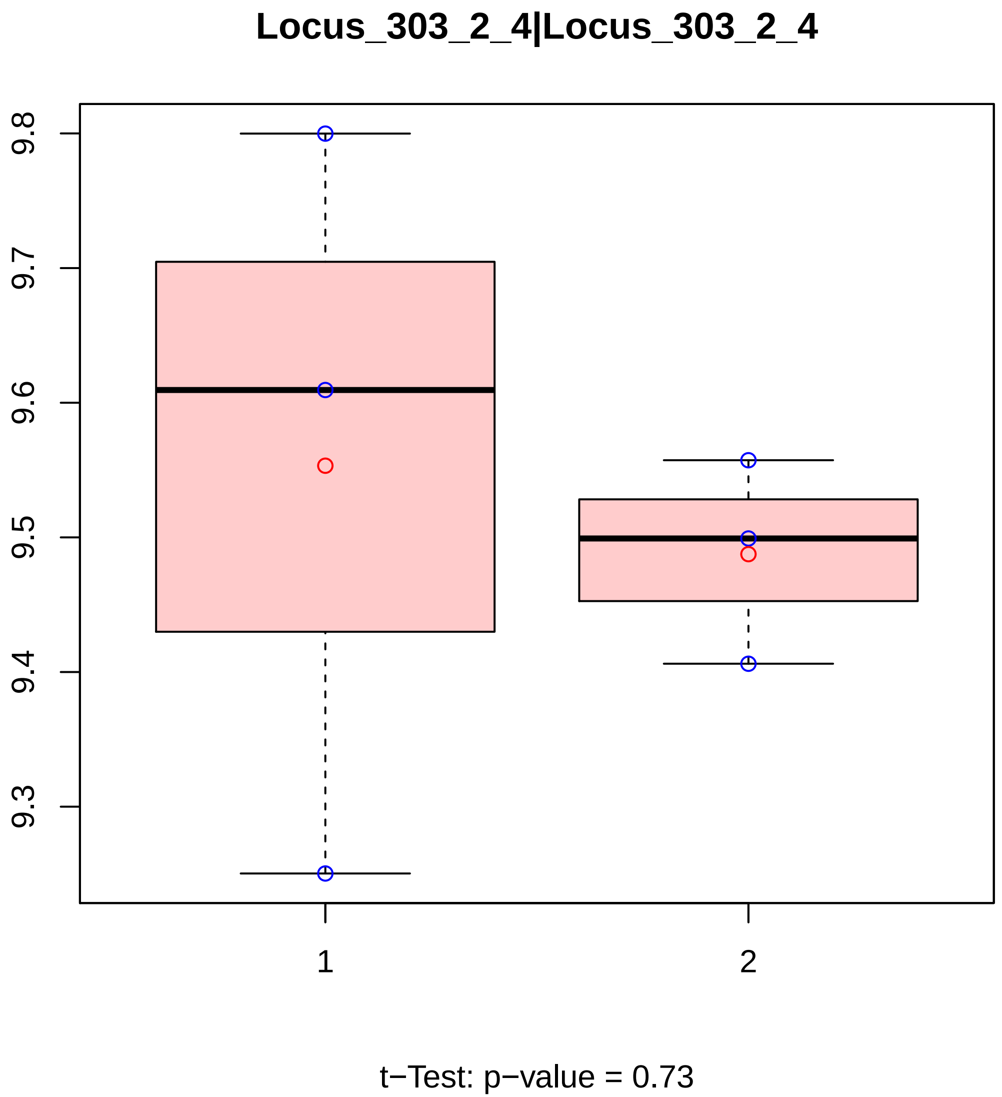
Locus_303_2_4|Locus_303_2_4
9.3
9.4
9.5
9.6
9.7
9.8
1
2
t−Test: p−value = 0.73

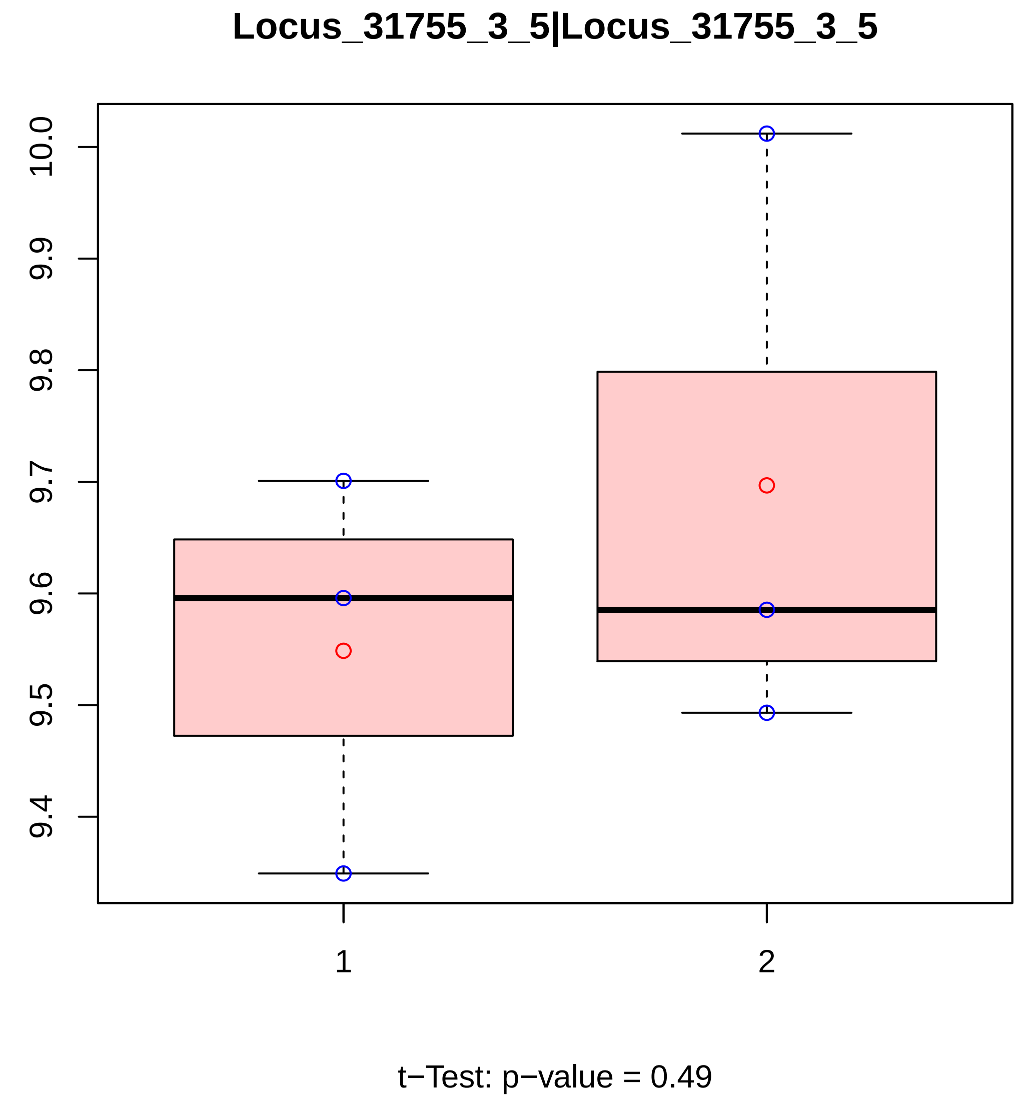
Locus_31755_3_5|Locus_31755_3_5
10.0
9.9
9.8
9.7
9.6
9.5
9.4
1
2
t−Test: p−value = 0.49

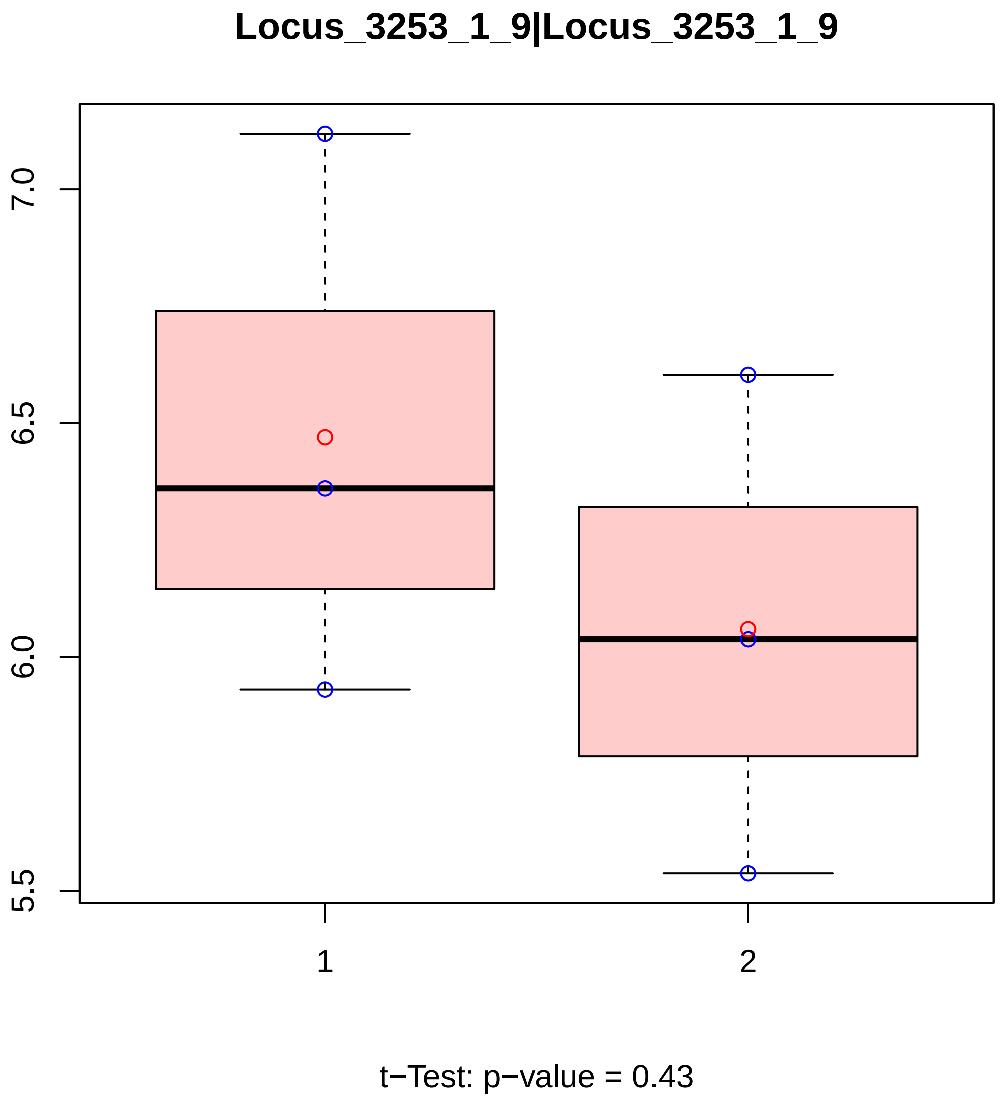

Locus_3253_1_9|Locus_3253_1_9
7.0
6.5
6.0
5.5
1
2
t−Test: p−value = 0.43

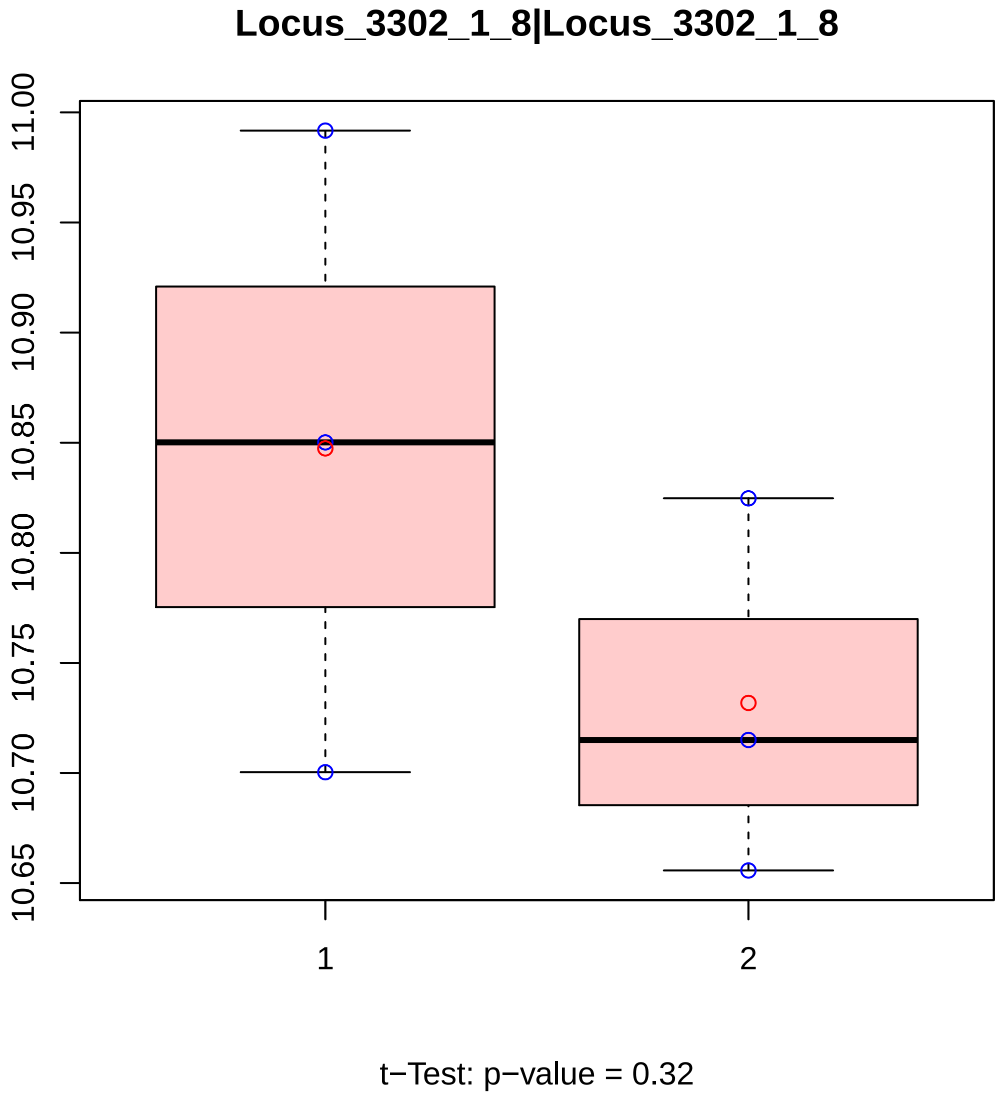
Locus_3302_1_8|Locus_3302_1_8
11.00
10.95
10.90
10.85
10.80
10.75
10.70
10.65
1
2
t−Test: p−value = 0.32

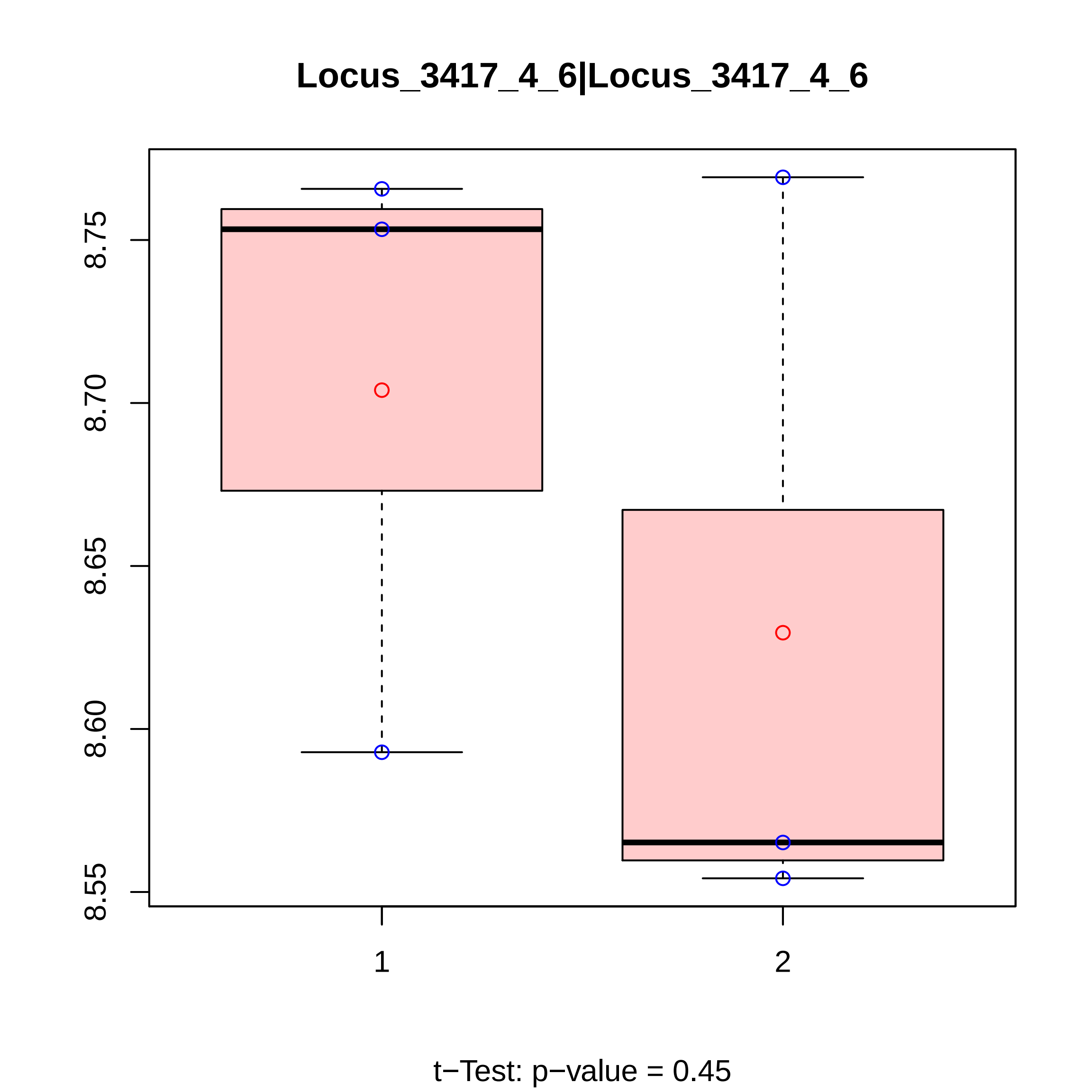

Locus_3417_4_6|Locus_3417_4_6
8.55
8.60
8.65
8.70
8.75
1
2
t−Test: p−value = 0.45

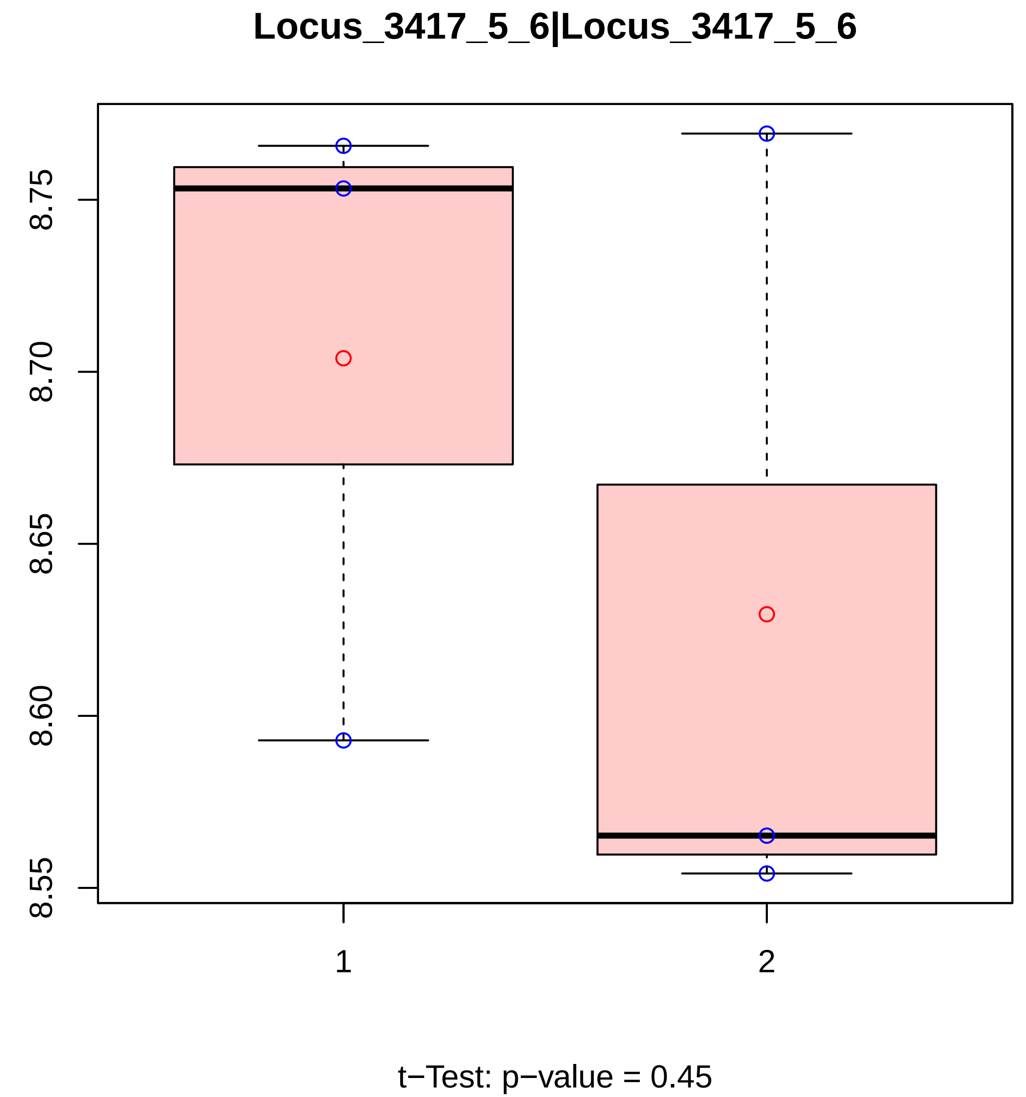
Locus_3417_5_6|Locus_3417_5_6
8.55
8.60
8.65
8.70
8.75
1
2
t−Test: p−value = 0.45

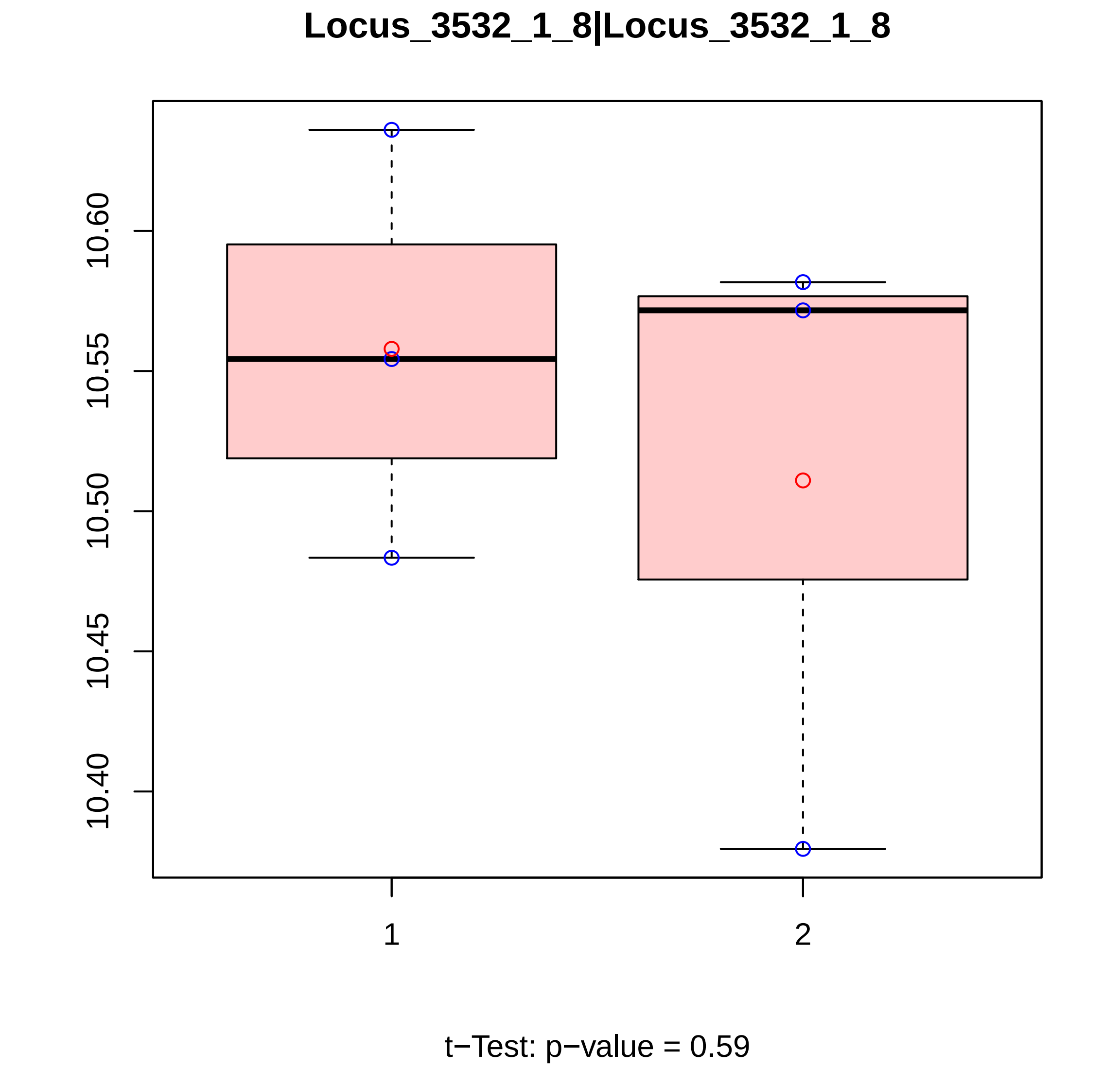

Locus_3532_1_8|Locus_3532_1_8
10.40
10.45
10.50
10.55
10.60
1
2
t−Test: p−value = 0.59

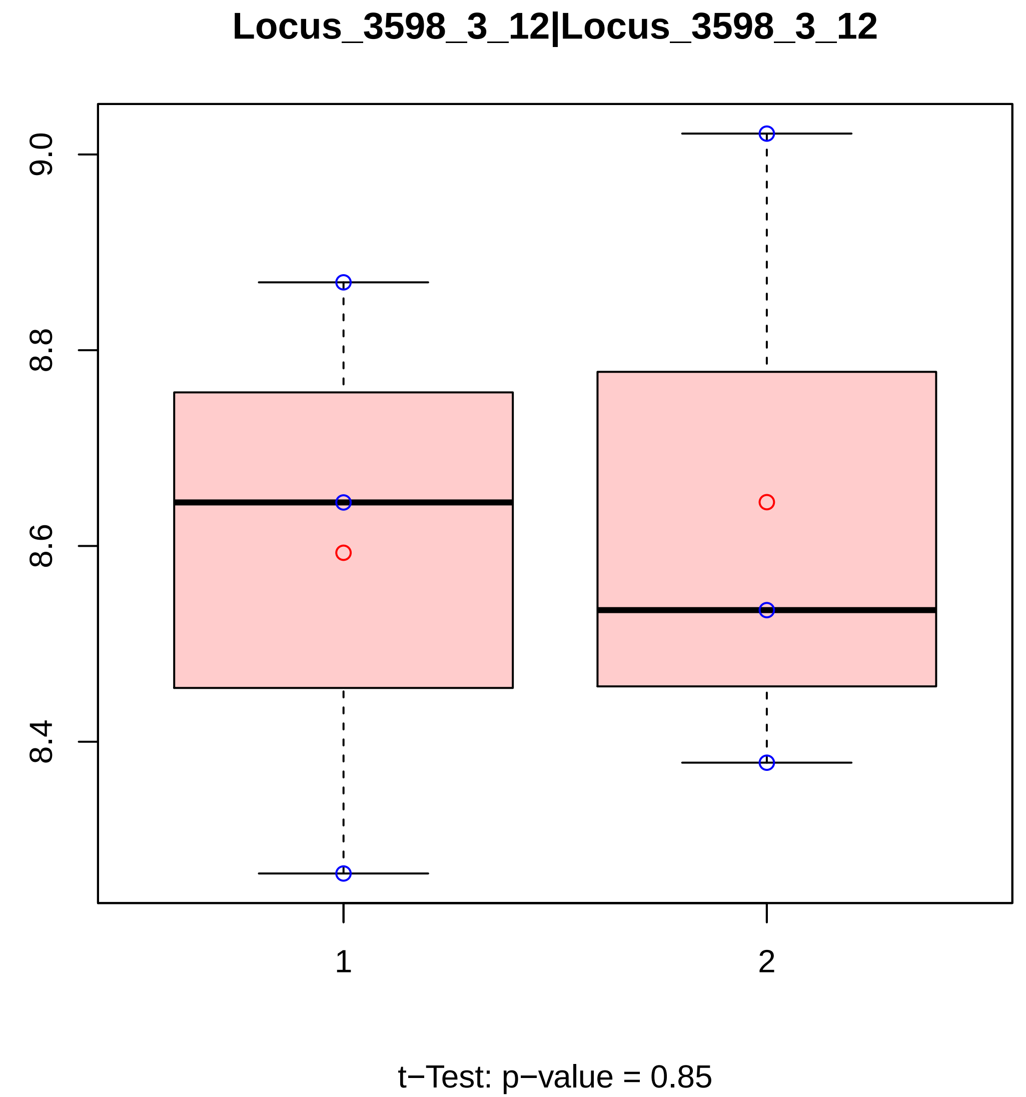
Locus_3598_3_12|Locus_3598_3_12
9.0
8.8
8.6
8.4
1
2
t−Test: p−value = 0.85

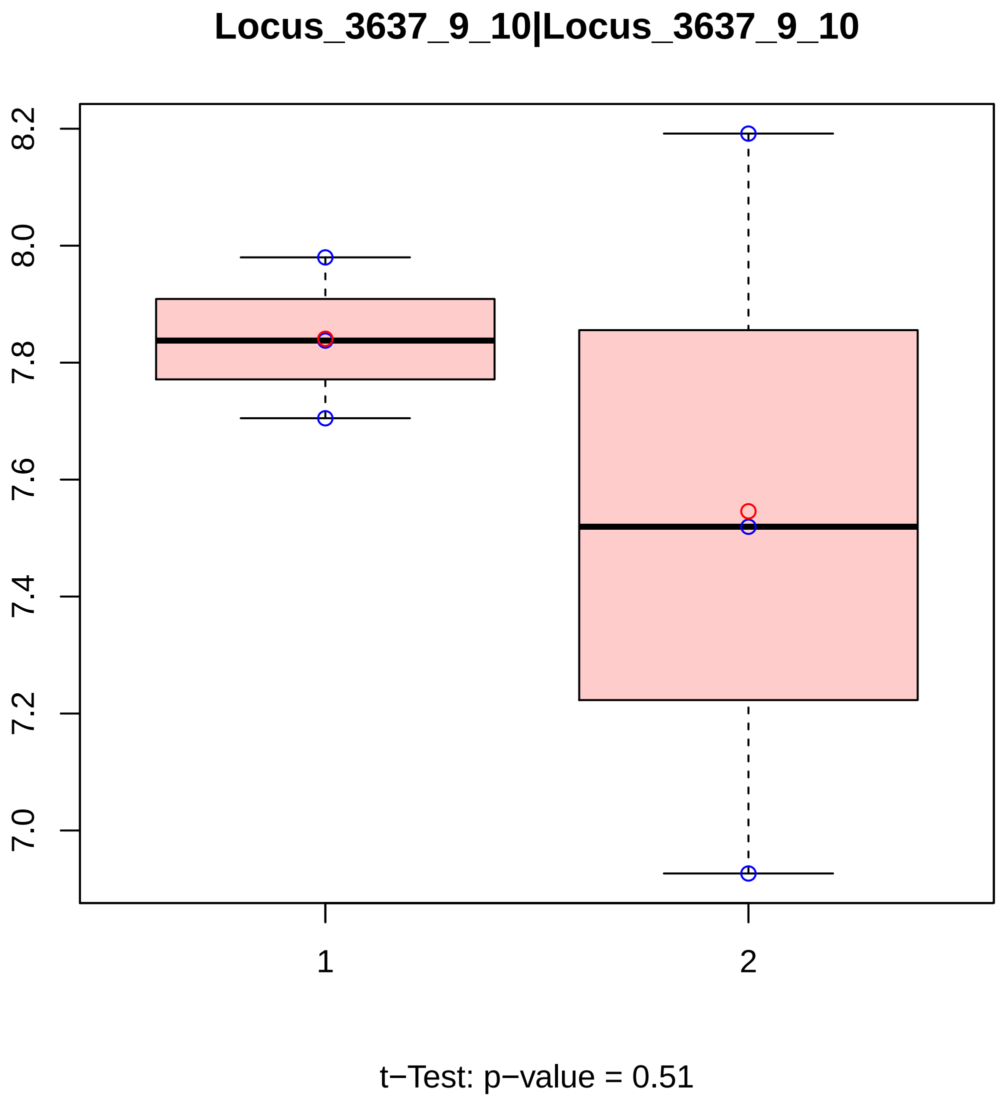

Locus_3637_9_10|Locus_3637_9_10
7.0
7.2
7.4
7.6
7.8
8.0
8.2
1
2
t−Test: p−value = 0.51

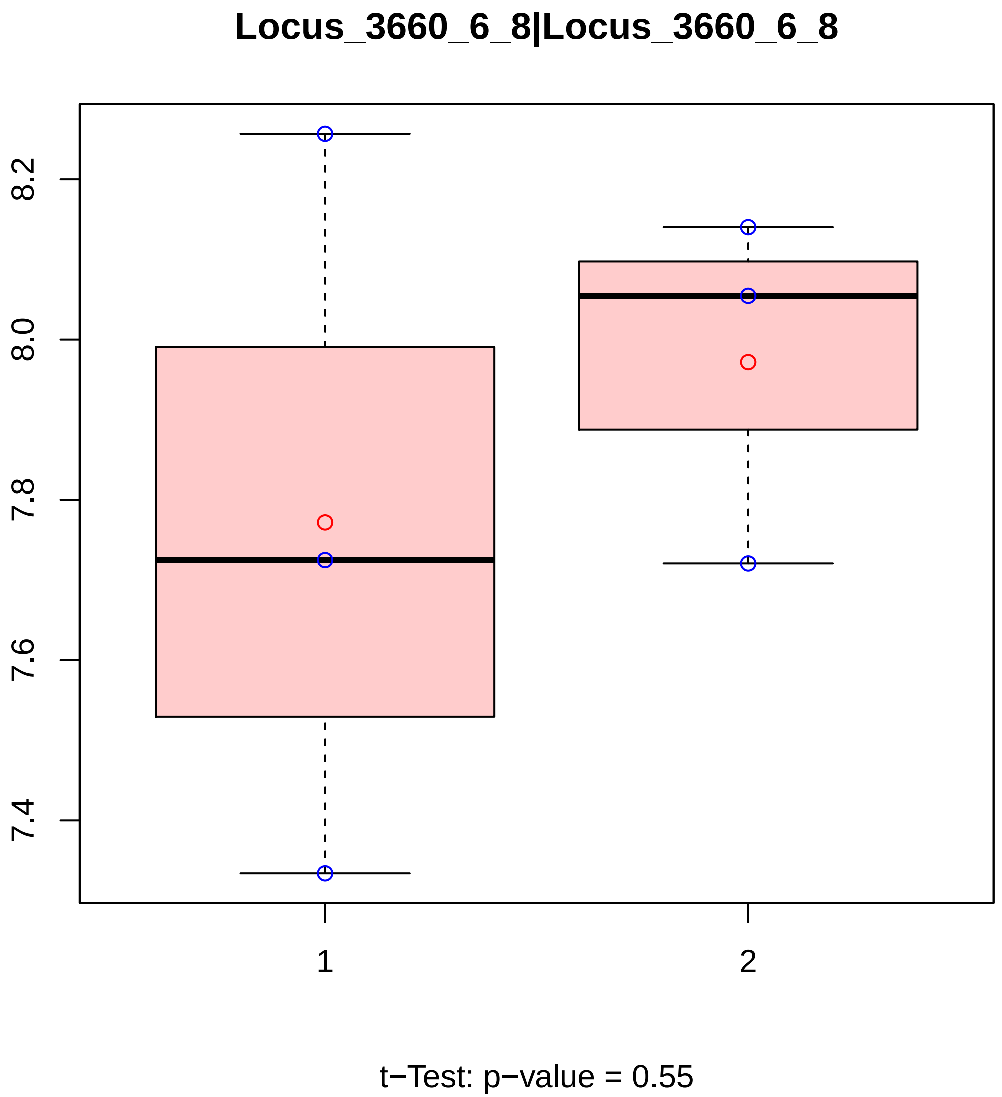

Locus_3660_6_8|Locus_3660_6_8
8.2
8.0
7.8
7.6
7.4
1
2
t−Test: p−value = 0.55

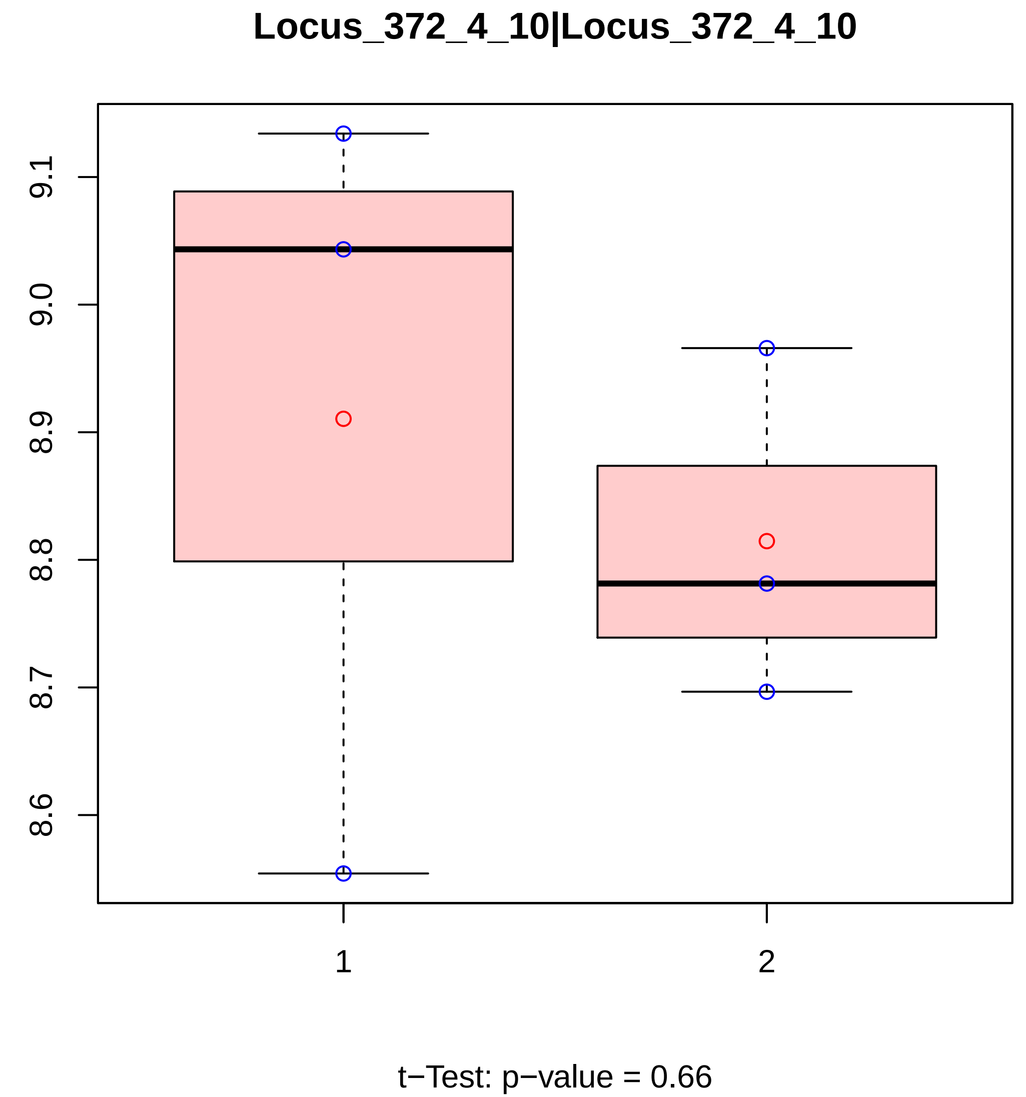
Locus_372_4_10|Locus_372_4_10
9.1
9.0
8.9
8.8
8.7
8.6
1
2
t−Test: p−value = 0.66

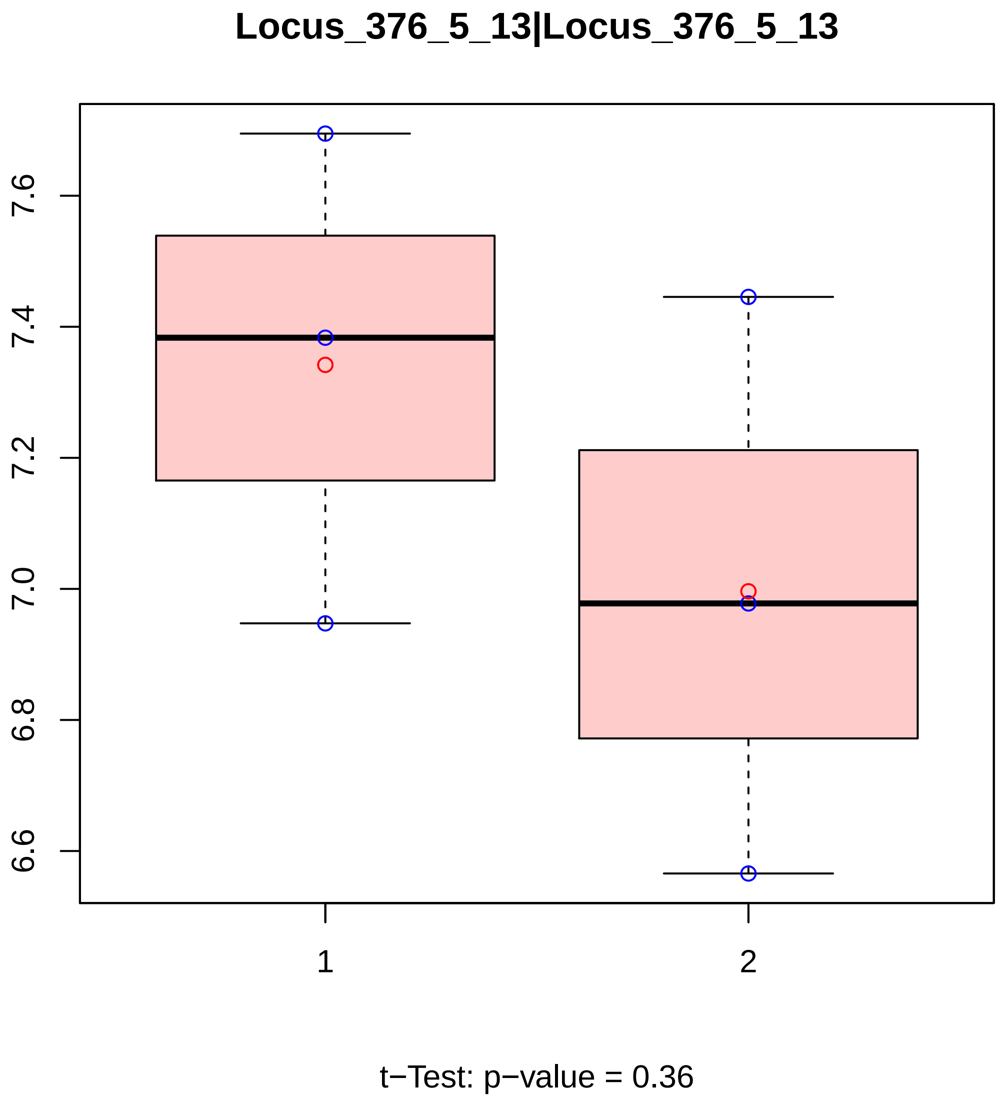
Locus_376_5_13|Locus_376_5_13
7.6
7.4
7.2
7.0
6.8
6.6
1
2
t−Test: p−value = 0.36

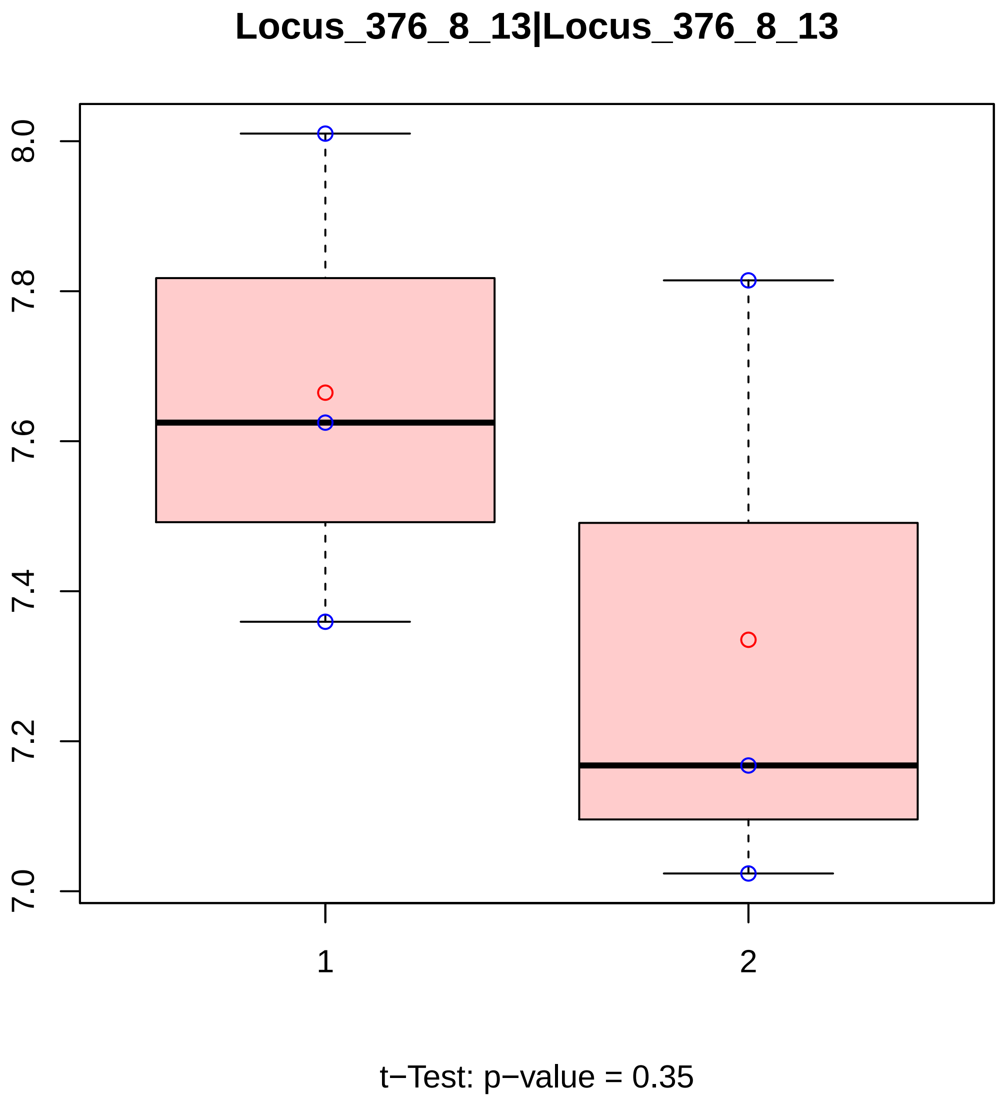
Locus_376_8_13|Locus_376_8_13
8.0
7.8
7.6
7.4
7.2
7.0
1
2
t−Test: p−value = 0.35

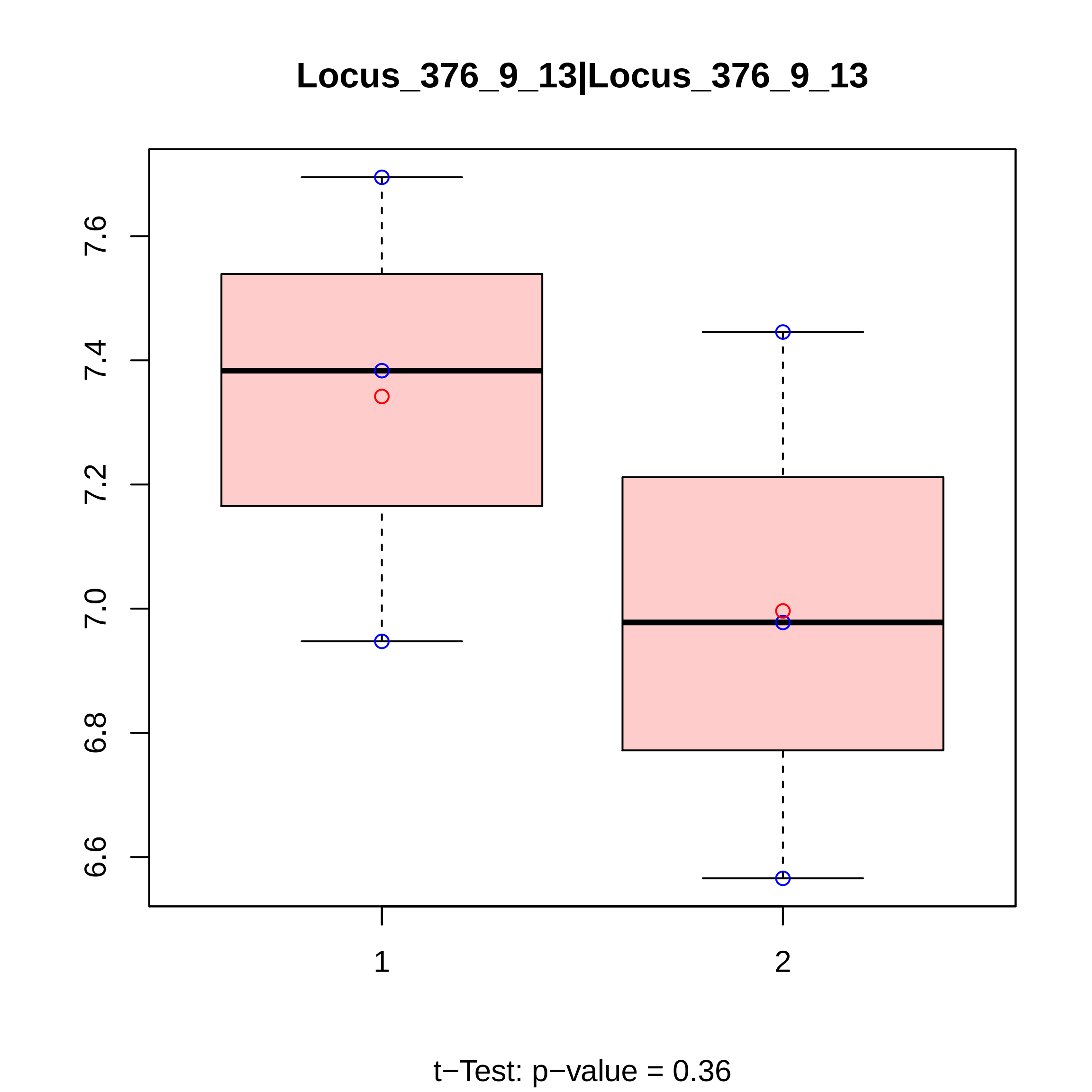

Locus_376_9_13|Locus_376_9_13
7.6
7.4
7.2
7.0
6.8
6.6
1
2
t−Test: p−value = 0.36

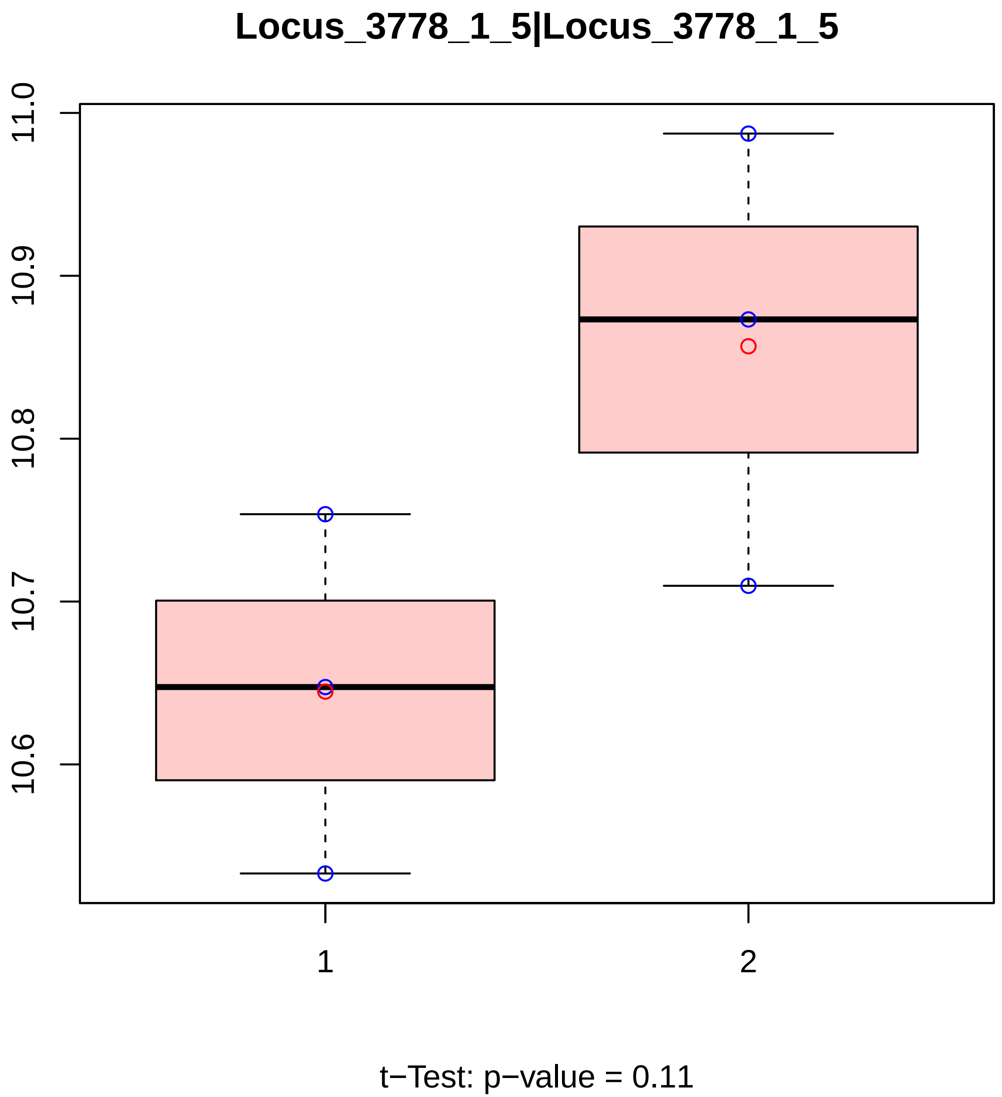

Locus_3778_1_5|Locus_3778_1_5
11.0
10.9
10.8
10.7
10.6
1
2
t−Test: p−value = 0.11

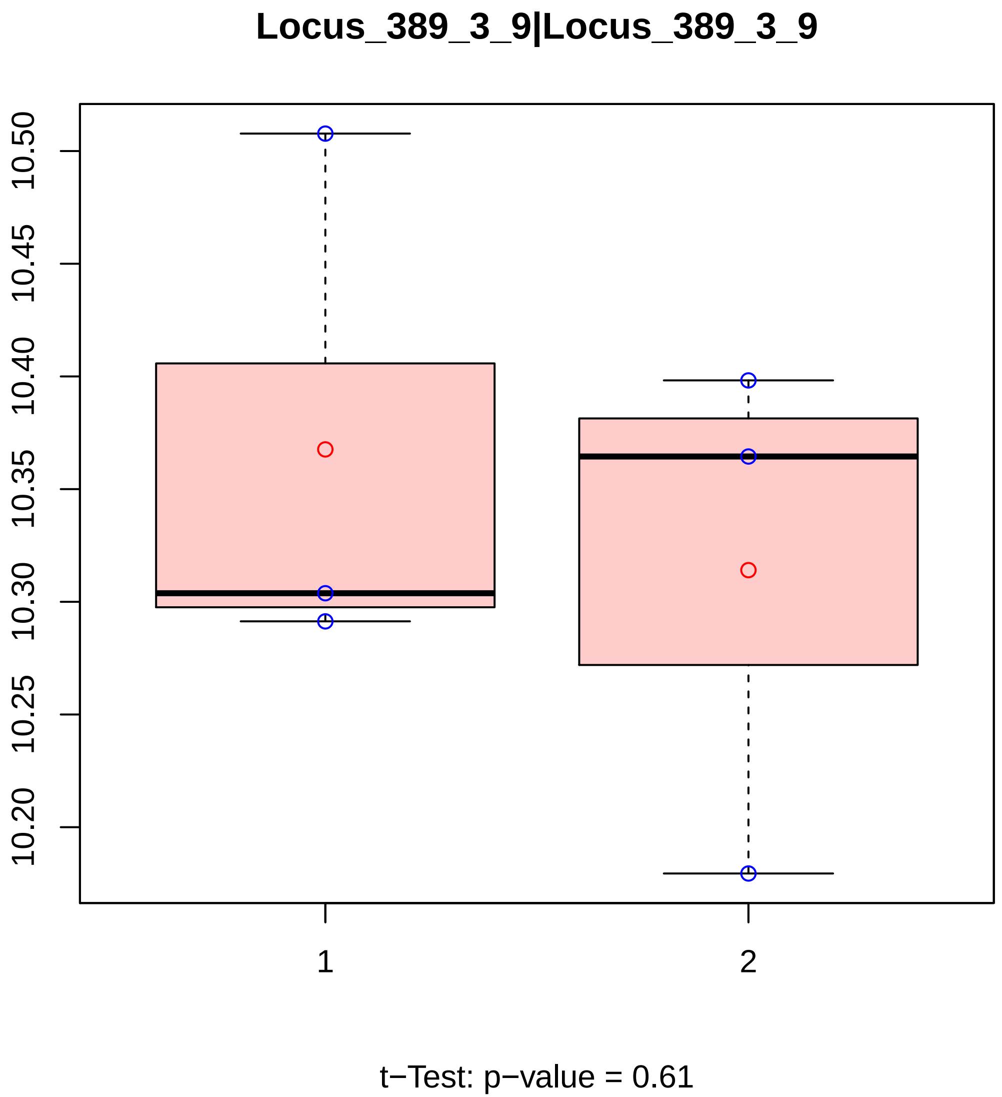
Locus_389_3_9|Locus_389_3_9
10.50
10.45
10.40
10.35
10.30
10.25
10.20
1
2
t−Test: p−value = 0.61

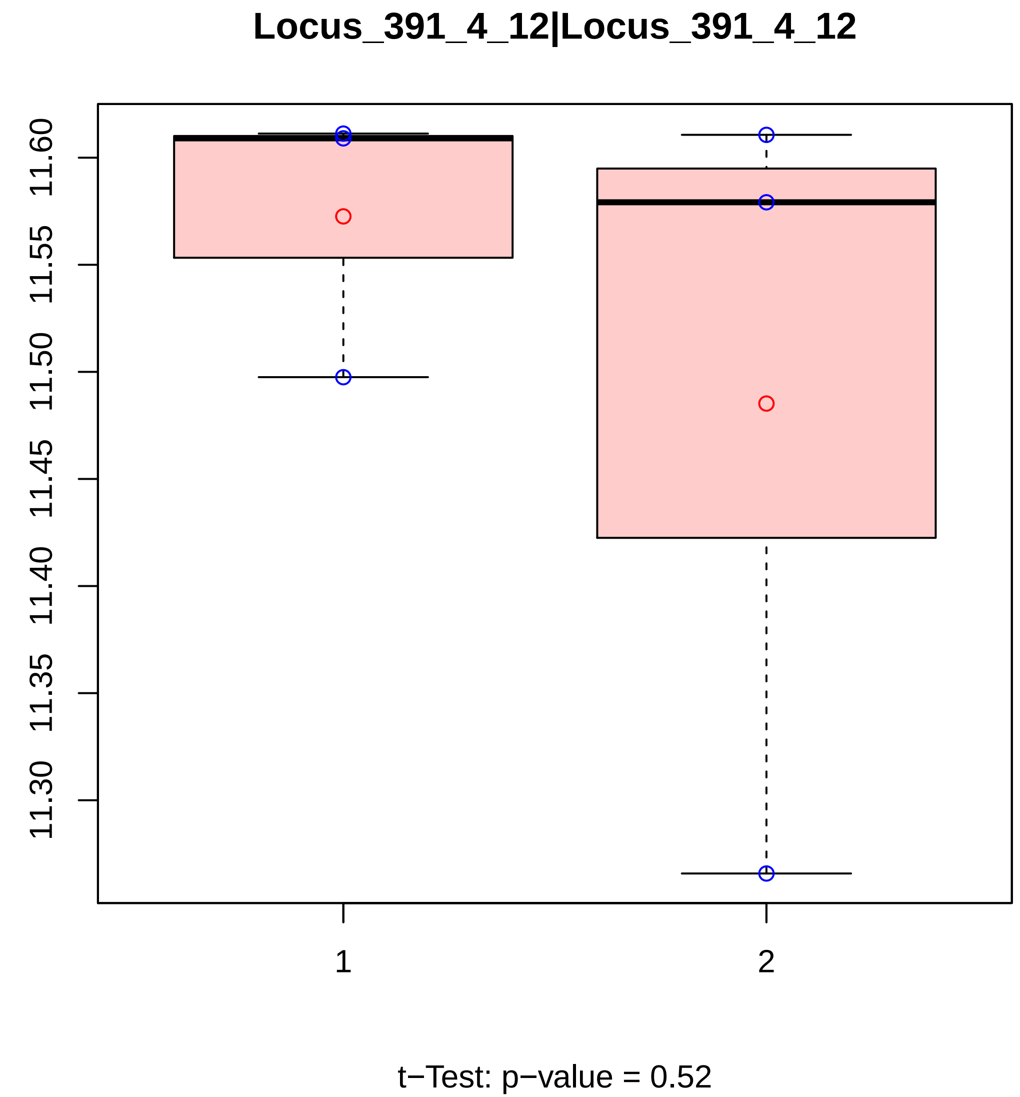

Locus_391_4_12|Locus_391_4_12
11.30
11.35
11.40
11.45
11.50
11.55
11.60
1
2
t−Test: p−value = 0.52

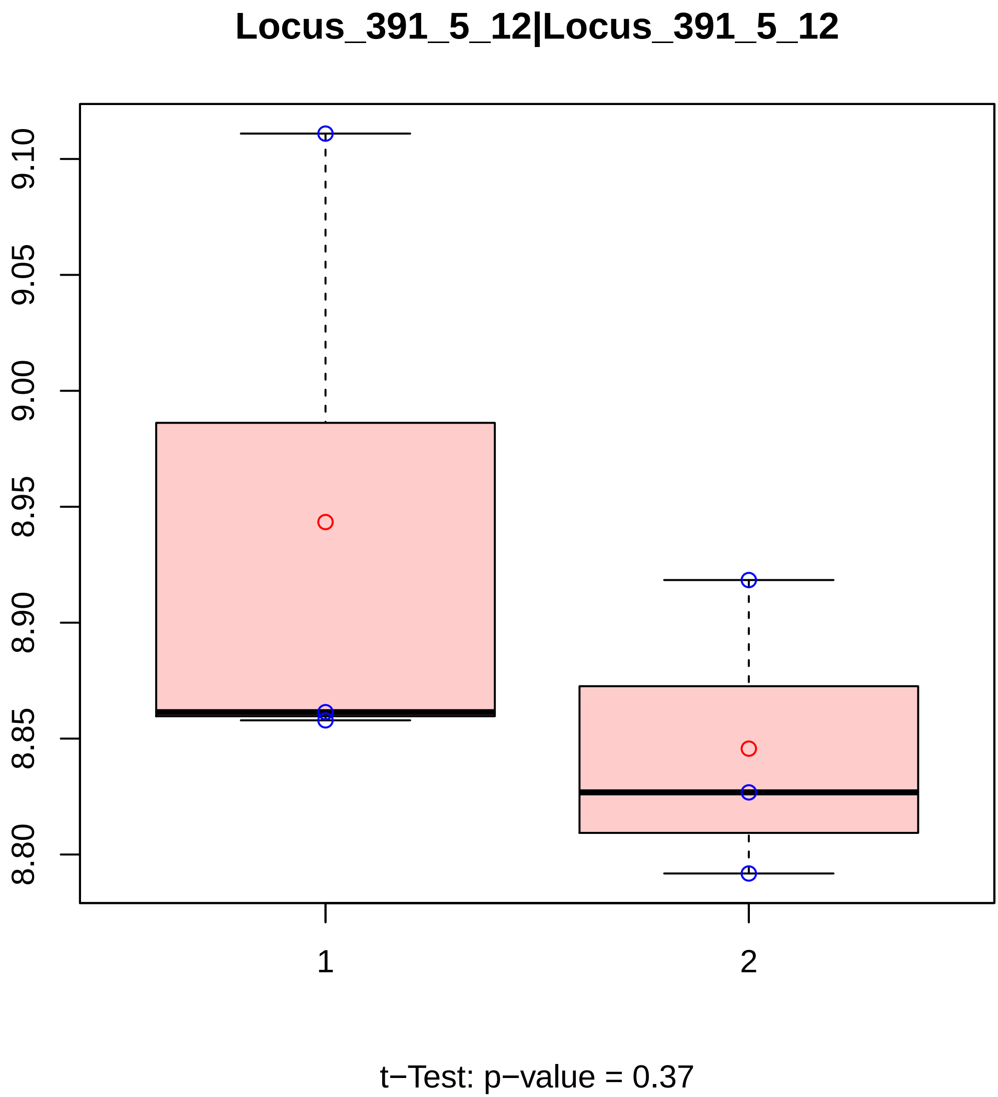
Locus_391_5_12|Locus_391_5_12
9.10
9.05
9.00
8.95
8.90
8.85
8.80
1
2
t−Test: p−value = 0.37

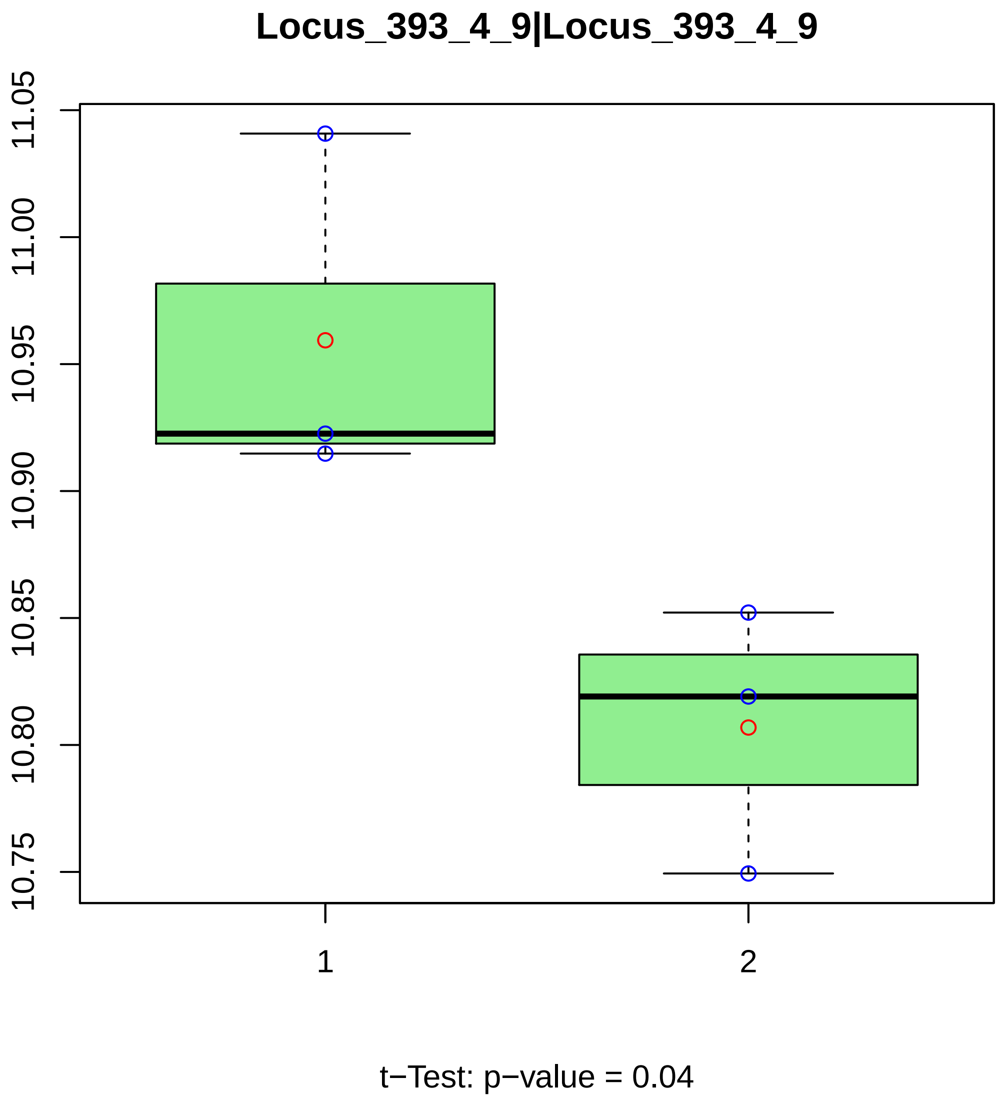

Locus_393_4_9|Locus_393_4_9
10.75
10.80
10.85
10.90
10.95
11.00
11.05
1
2
t−Test: p−value = 0.04

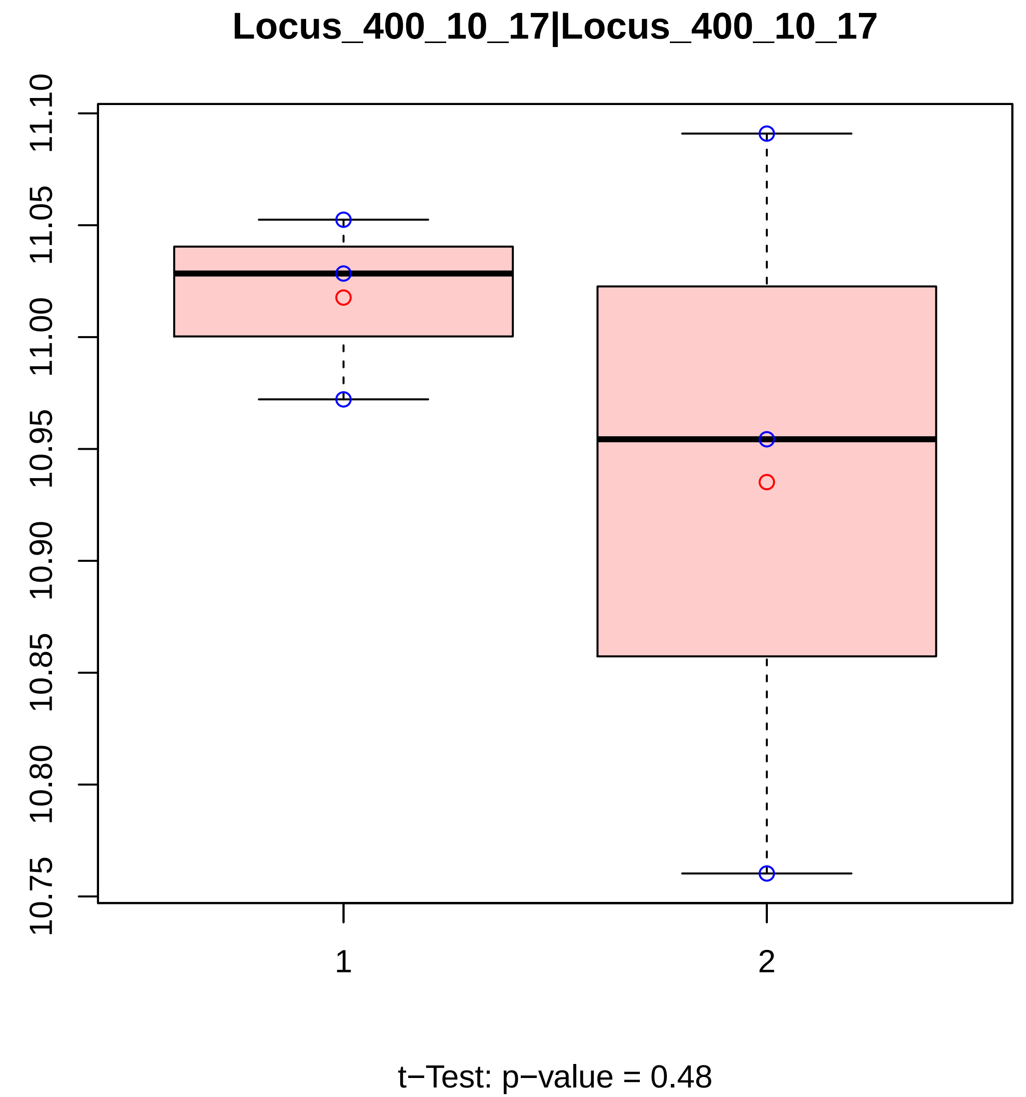

Locus_400_10_17|Locus_400_10_17
10.75
10.80
10.85
10.90
10.95
11.00
11.05
11.10
1
2
t−Test: p−value = 0.48

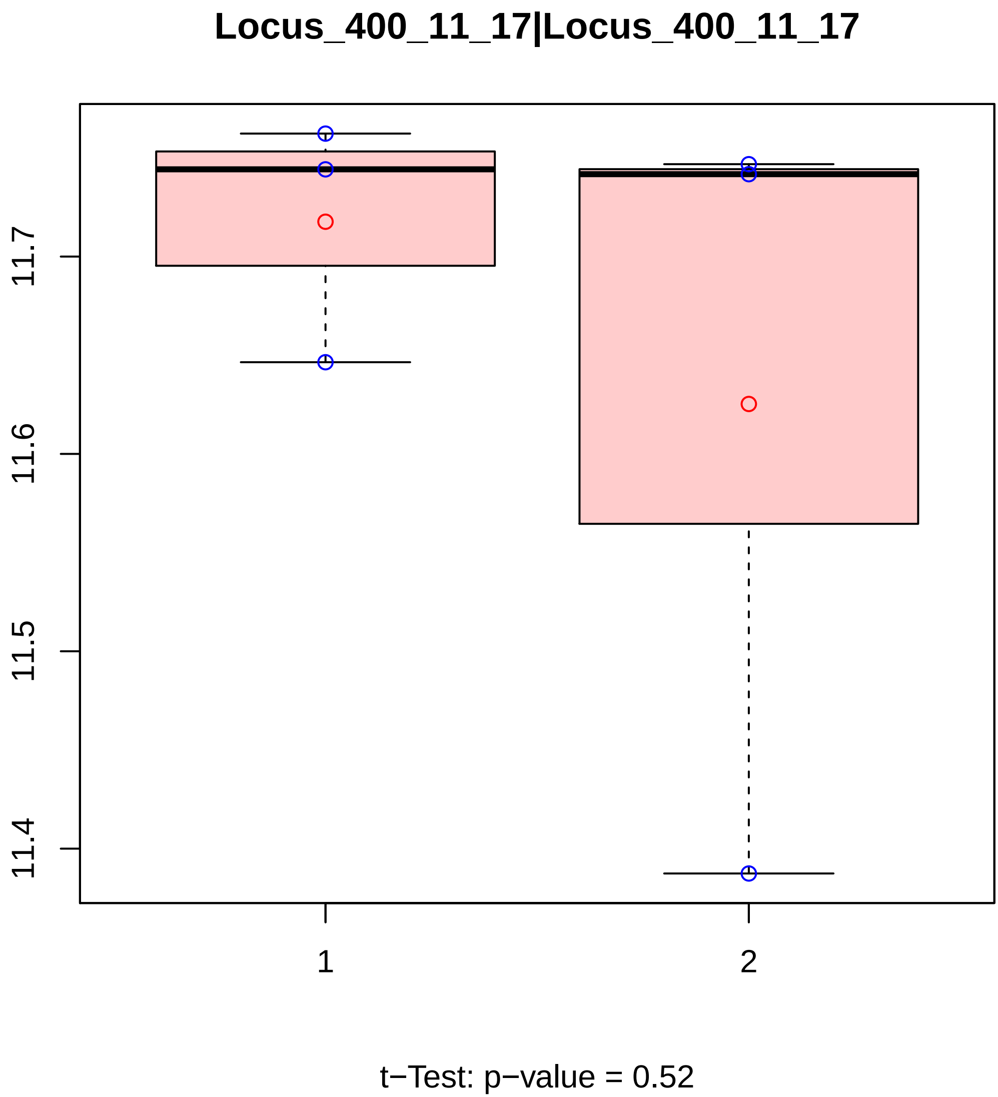

Locus_400_11_17|Locus_400_11_17
11.7
11.6
11.5
11.4
1
2
t−Test: p−value = 0.52

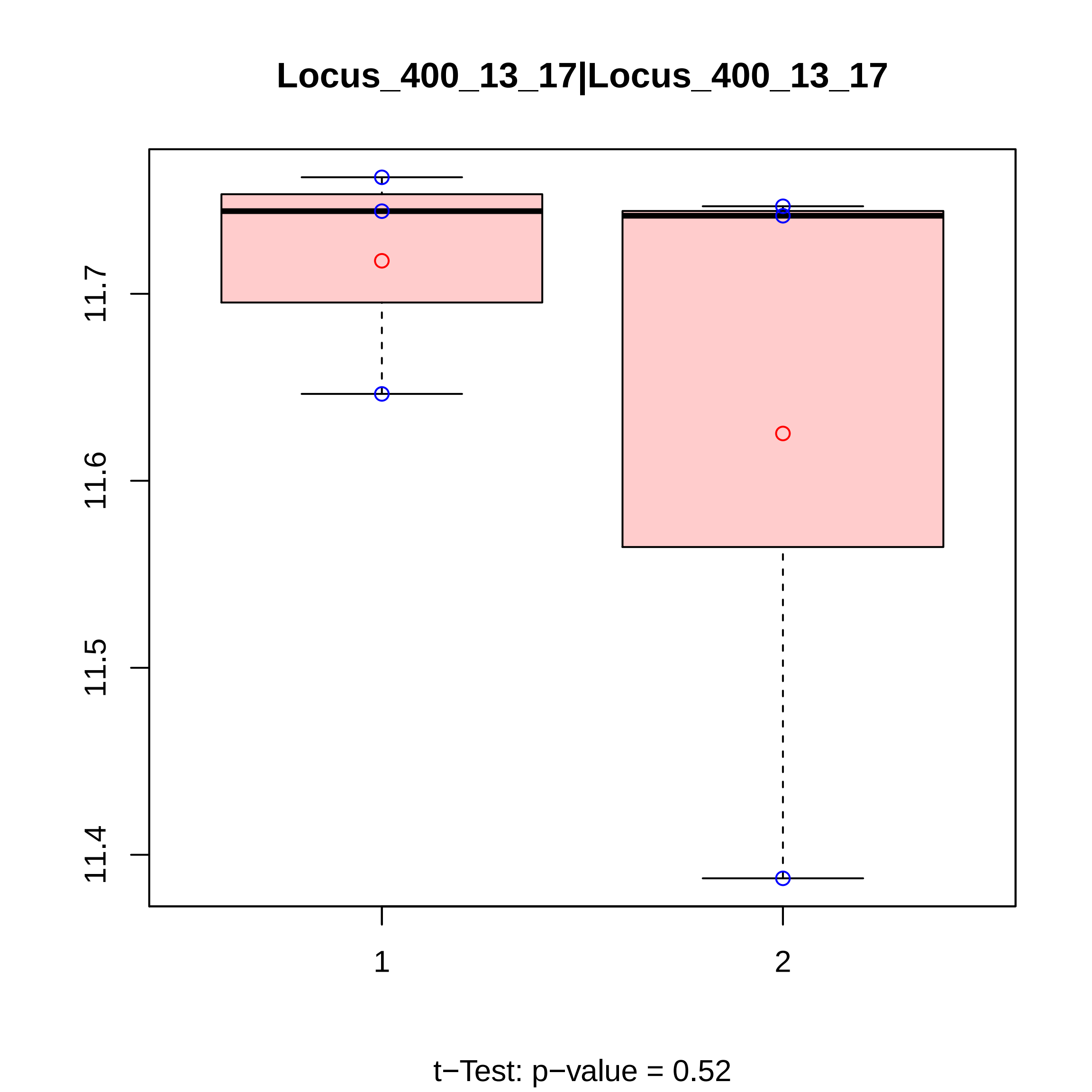

Locus_400_13_17|Locus_400_13_17
11.4
11.5
11.6
11.7
1
2
t−Test: p−value = 0.52

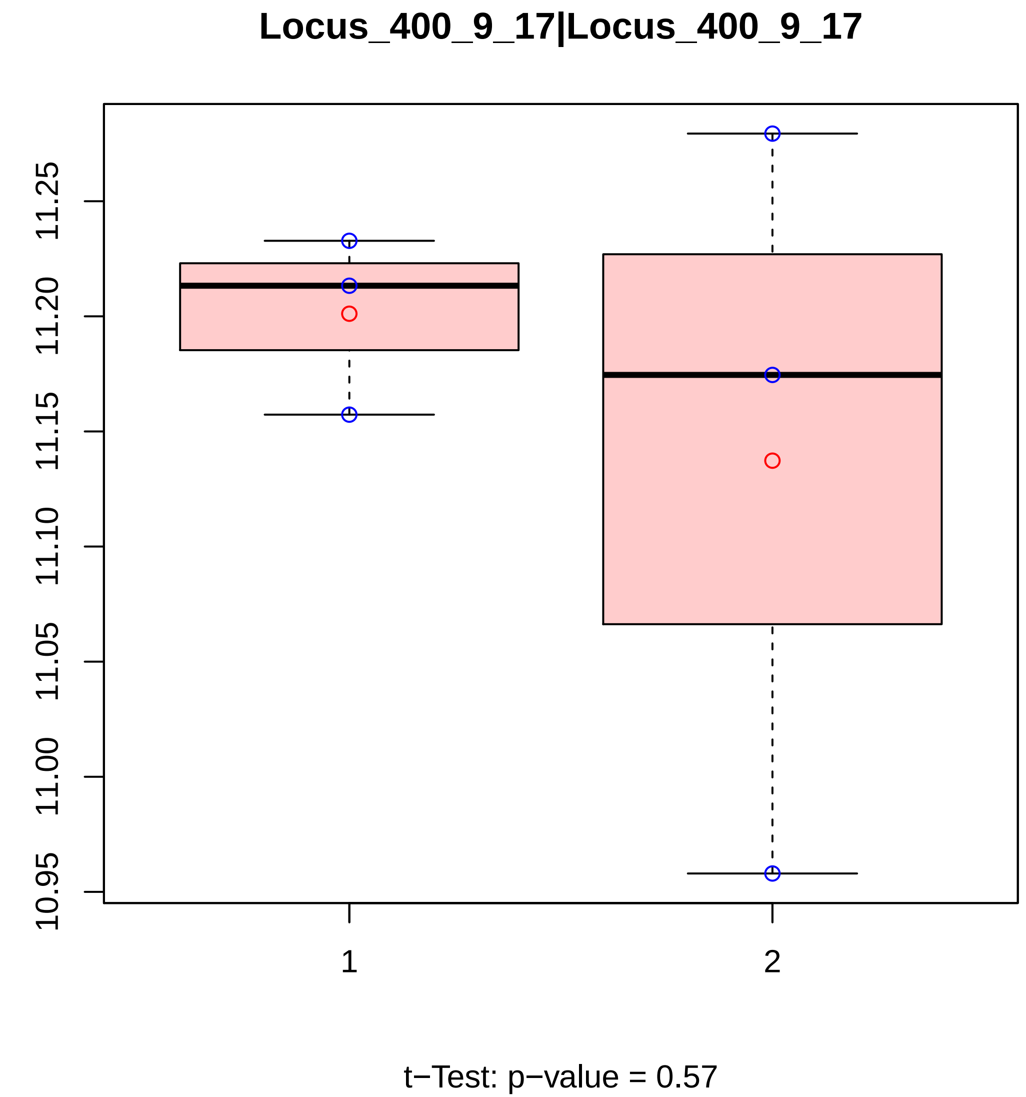

Locus_400_9_17|Locus_400_9_17
11.25
11.20
11.15
11.10
11.05
11.00
10.95
1
2
t−Test: p−value = 0.57

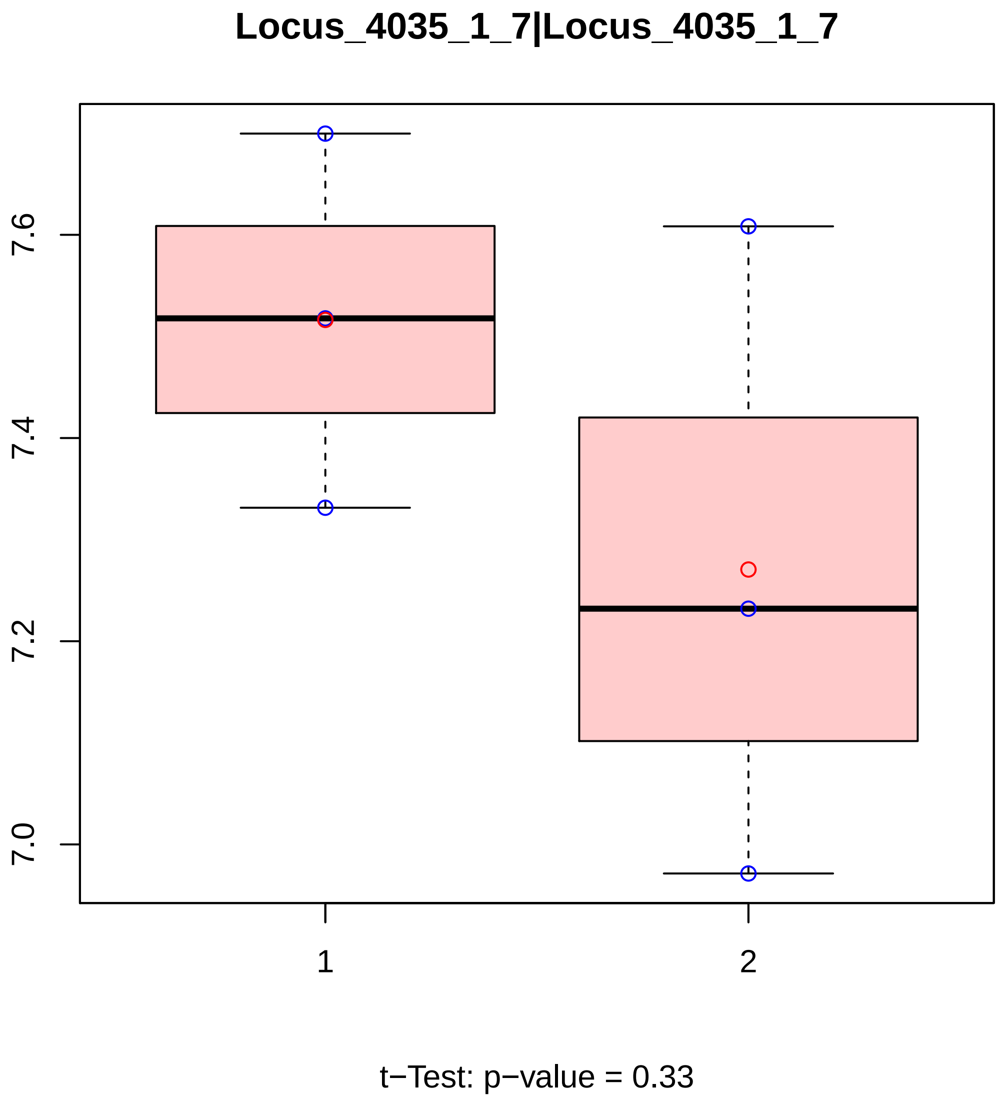

Locus_4035_1_7|Locus_4035_1_7
7.6
7.4
7.2
7.0
1
2
t−Test: p−value = 0.33

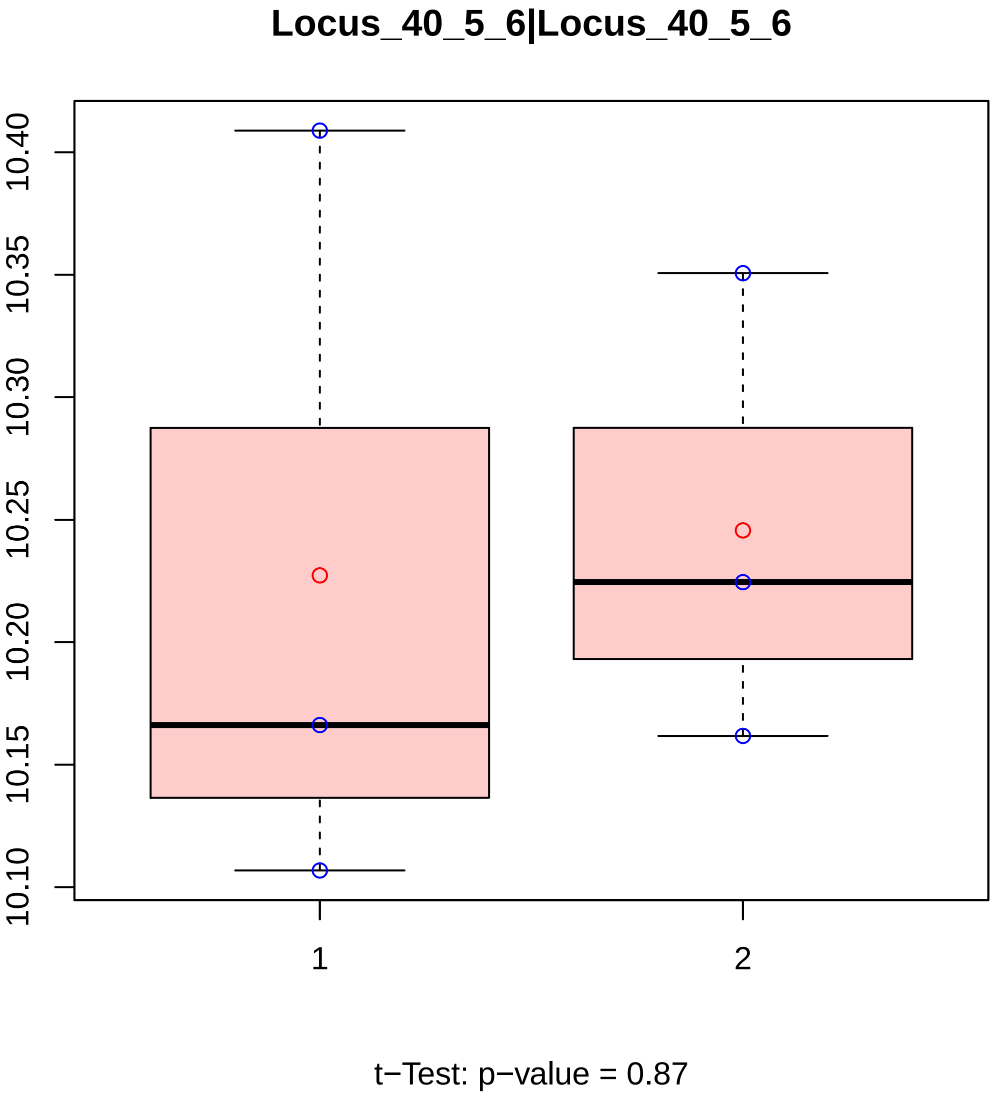
Locus_40_5_6|Locus_40_5_6
10.10
10.15
10.20
10.25
10.30
10.35
10.40
1
2
t−Test: p−value = 0.87

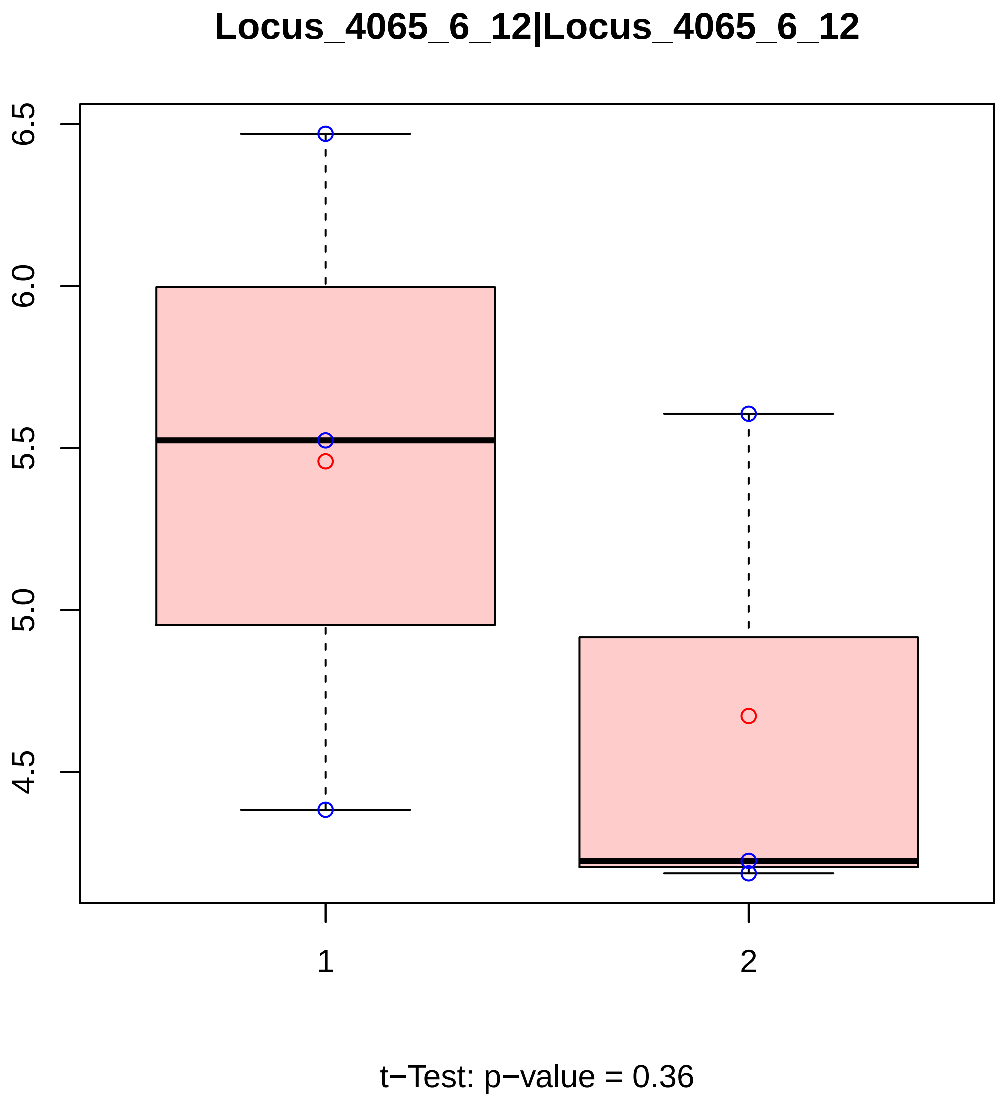

Locus_4065_6_12|Locus_4065_6_12
6.5
6.0
5.5
5.0
4.5
1
2
t−Test: p−value = 0.36

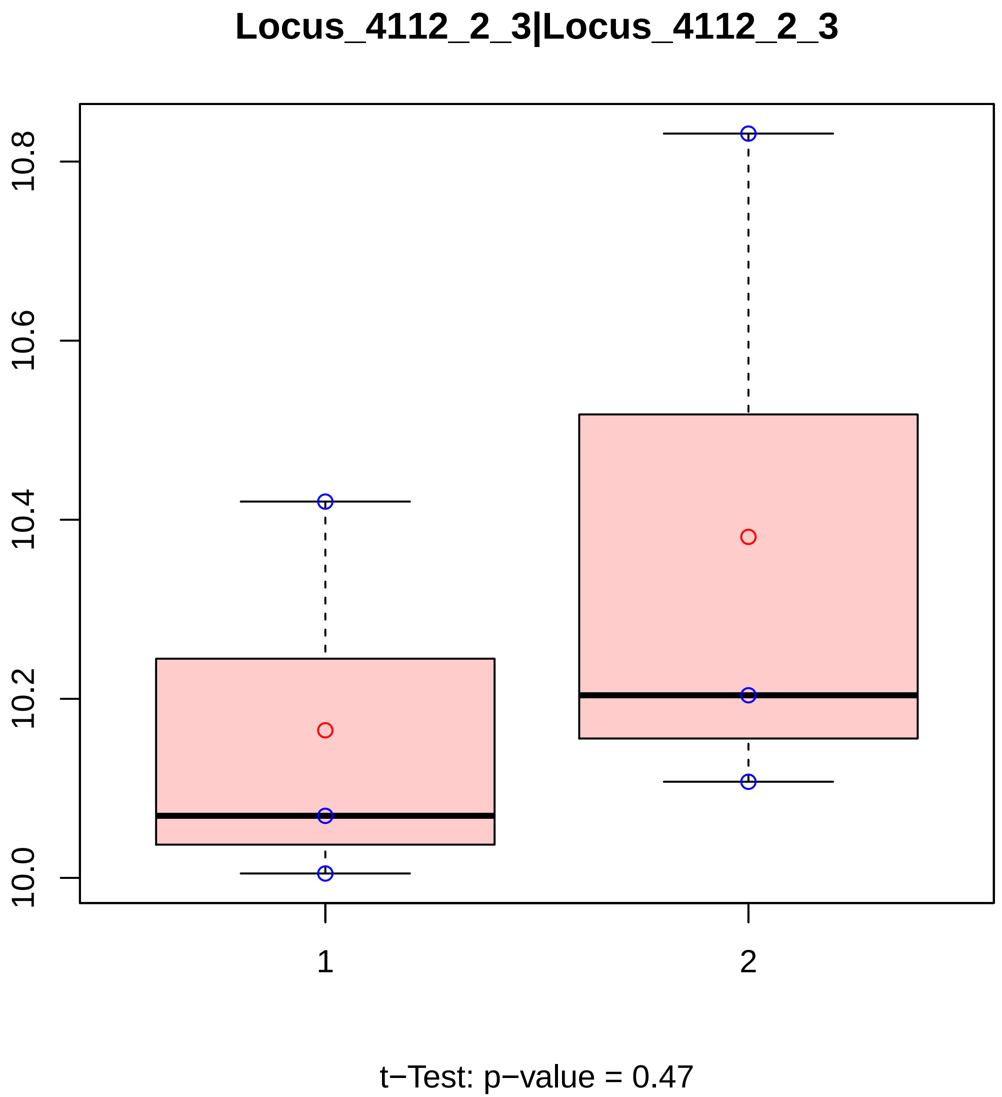
Locus_4112_2_3|Locus_4112_2_3
10.0
10.2
10.4
10.6
10.8
1
2
t−Test: p−value = 0.47

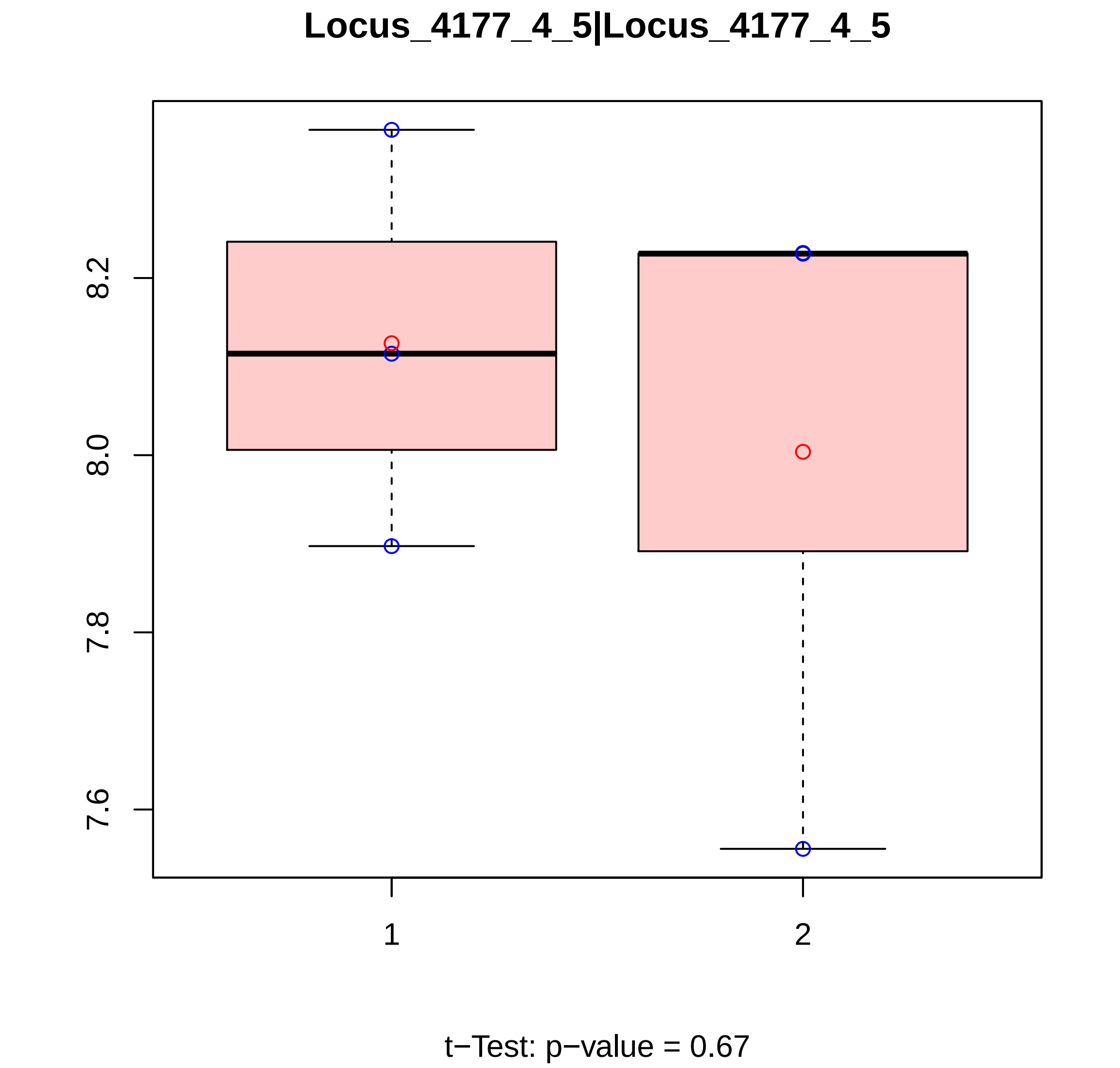

Locus_4177_4_5|Locus_4177_4_5
8.2
8.0
7.8
7.6
1
2
t−Test: p−value = 0.67

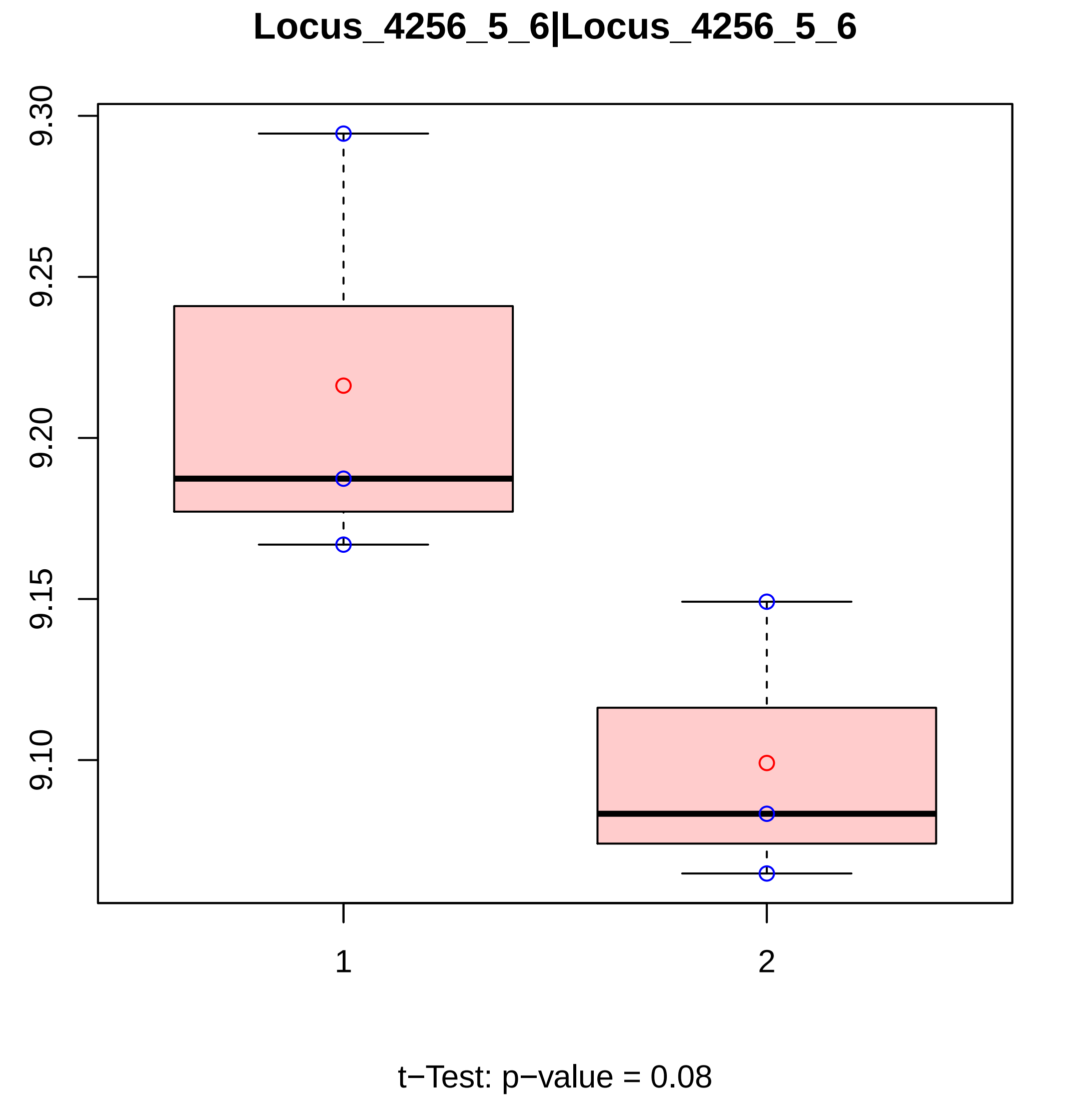

Locus_4256_5_6|Locus_4256_5_6
9.30
9.25
9.20
9.15
9.10
1
2
t−Test: p−value = 0.08

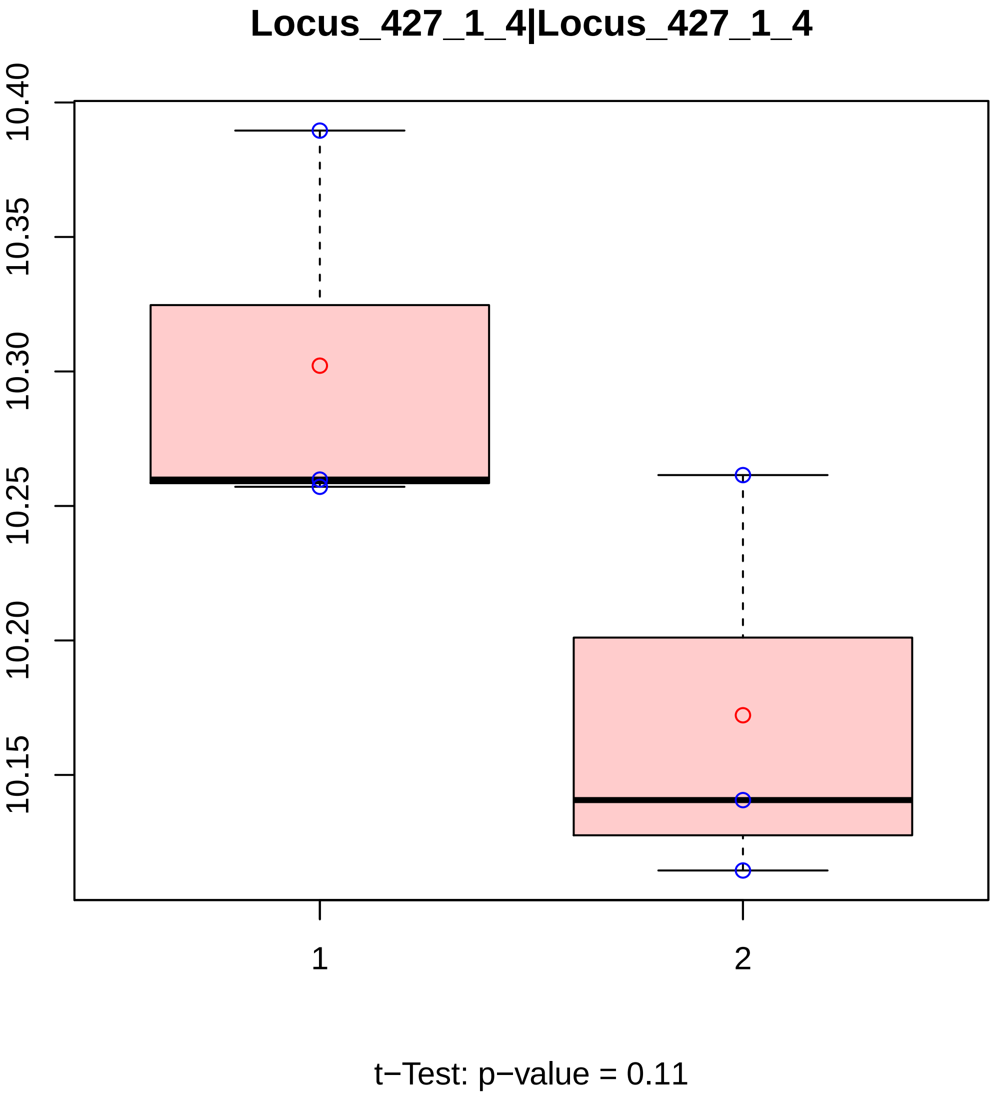

Locus_427_1_4|Locus_427_1_4
10.40
10.35
10.30
10.25
10.20
10.15
1
2
t−Test: p−value = 0.11

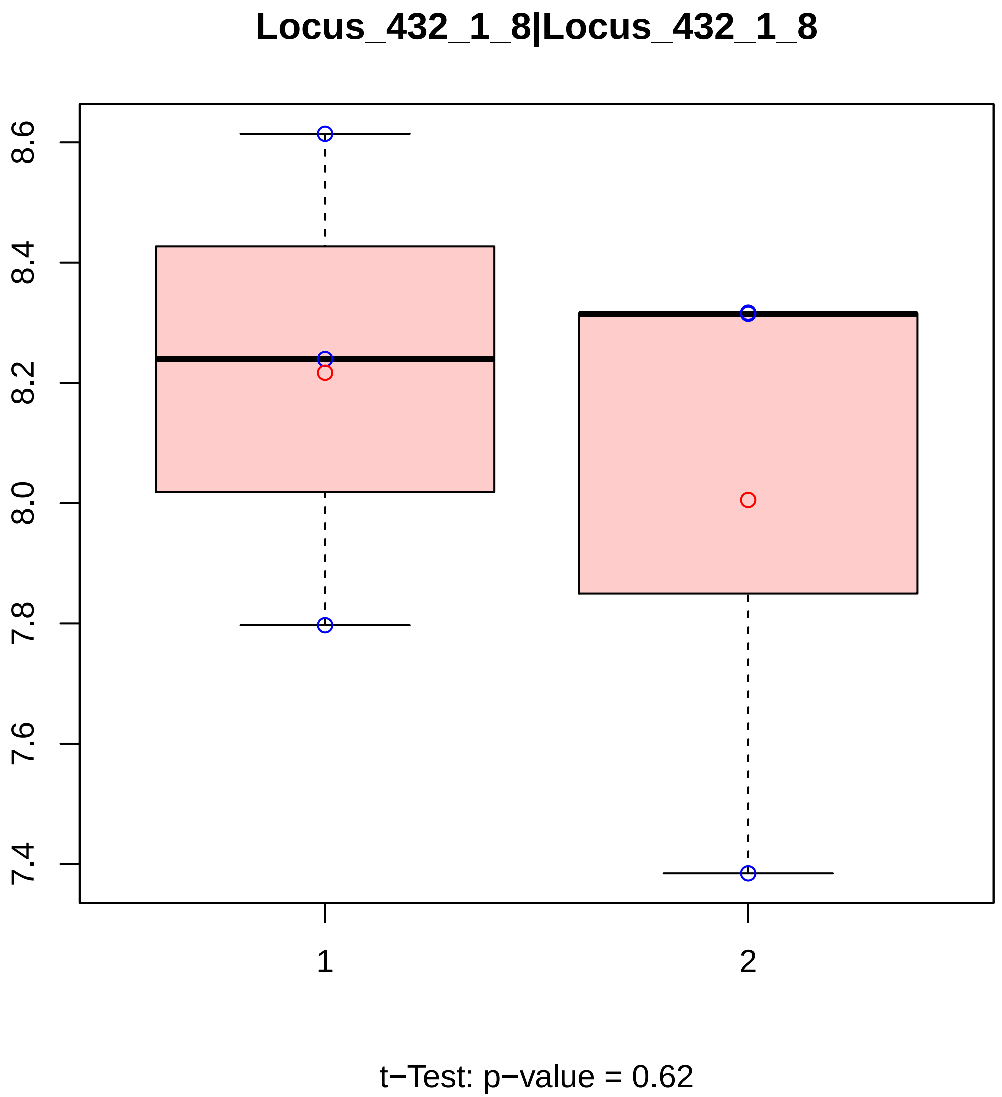
Locus_432_1_8|Locus_432_1_8
8.6
8.4
8.2
8.0
7.8
7.6
7.4
1
2
t−Test: p−value = 0.62

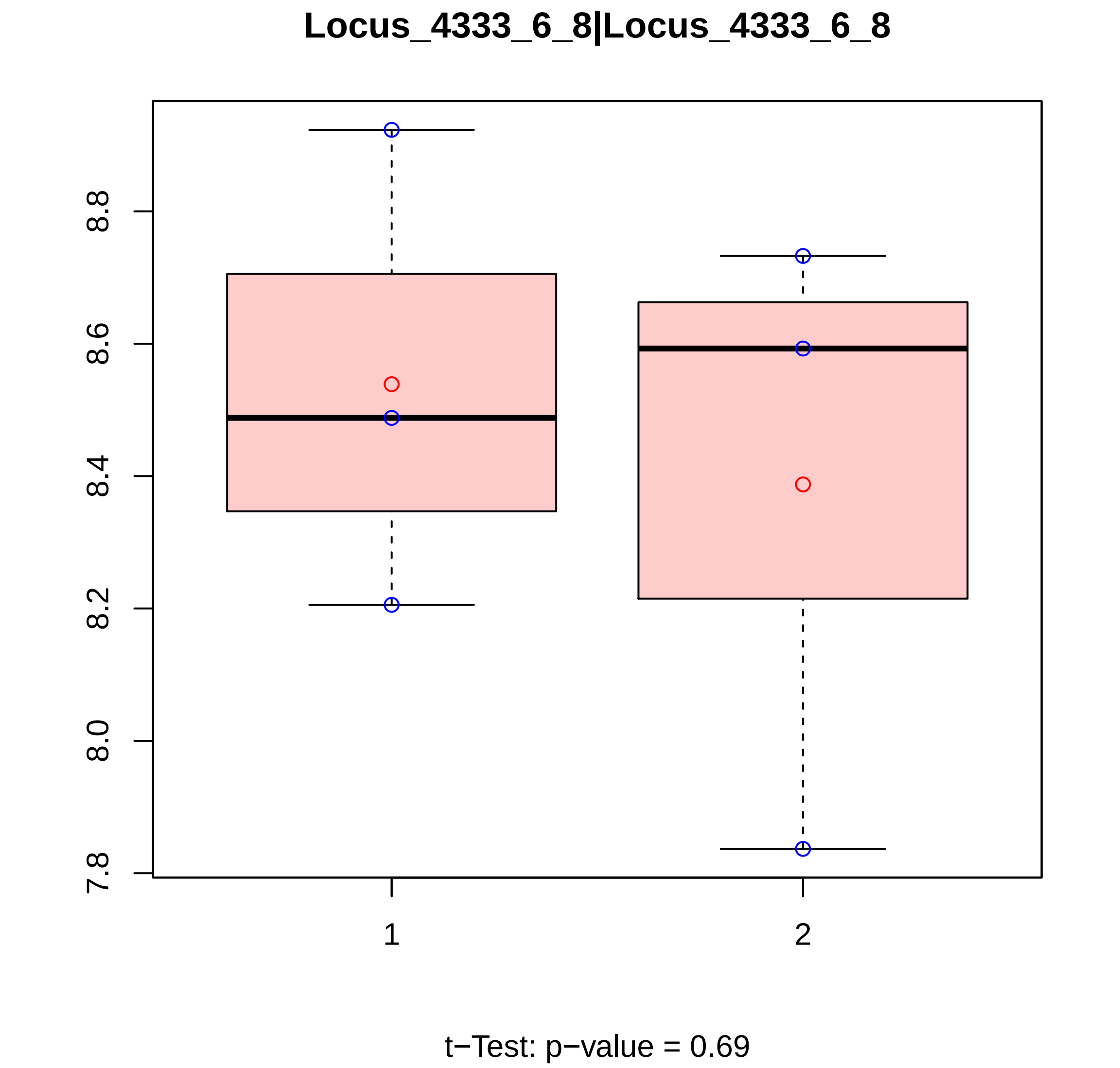
Locus_4333_6_8|Locus_4333_6_8
7.8
8.0
8.2
8.4
8.6
8.8
1
2
t−Test: p−value = 0.69

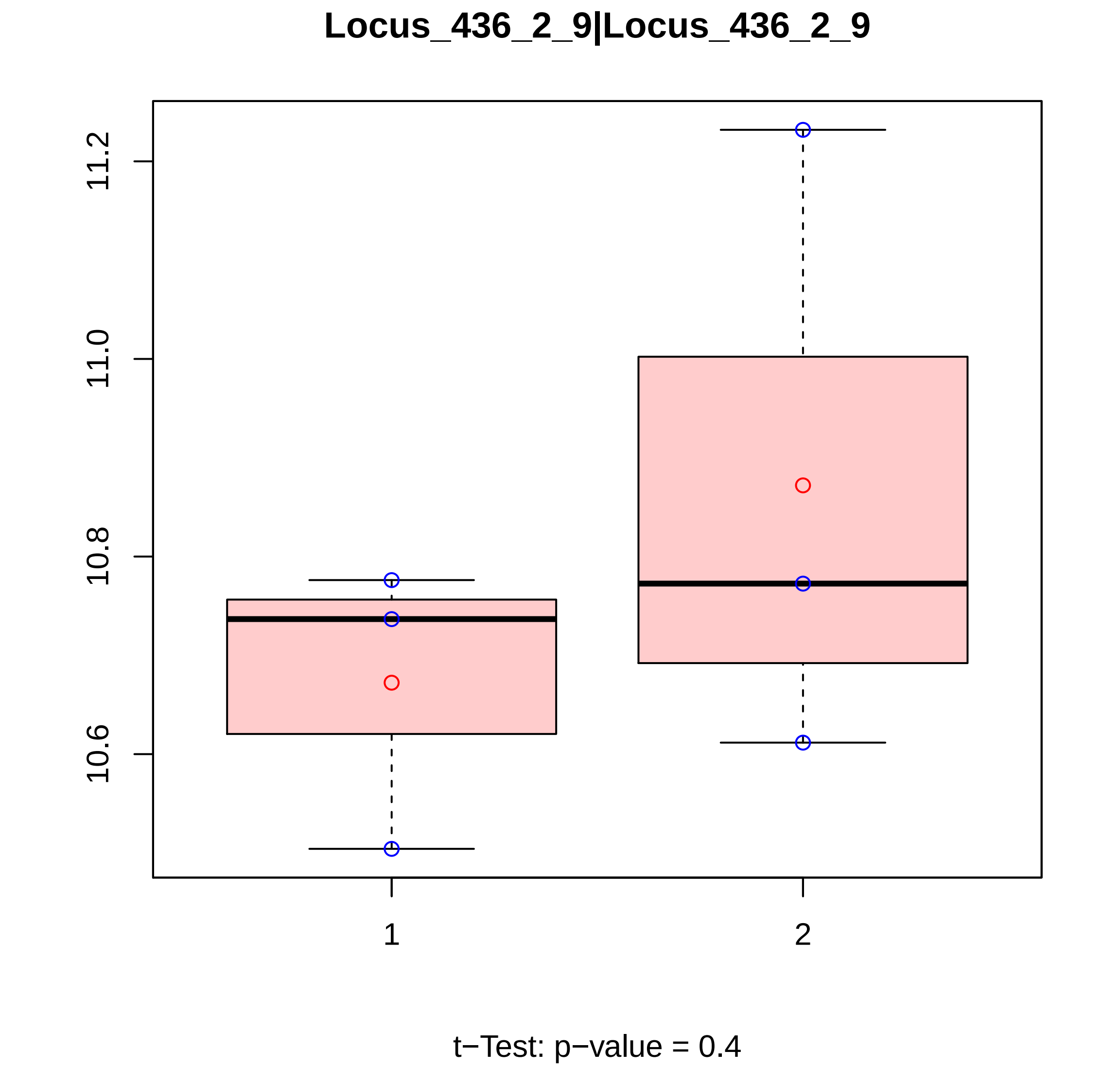
Locus_436_2_9|Locus_436_2_9
11.2
11.0
10.8
10.6
1
2
t−Test: p−value = 0.4

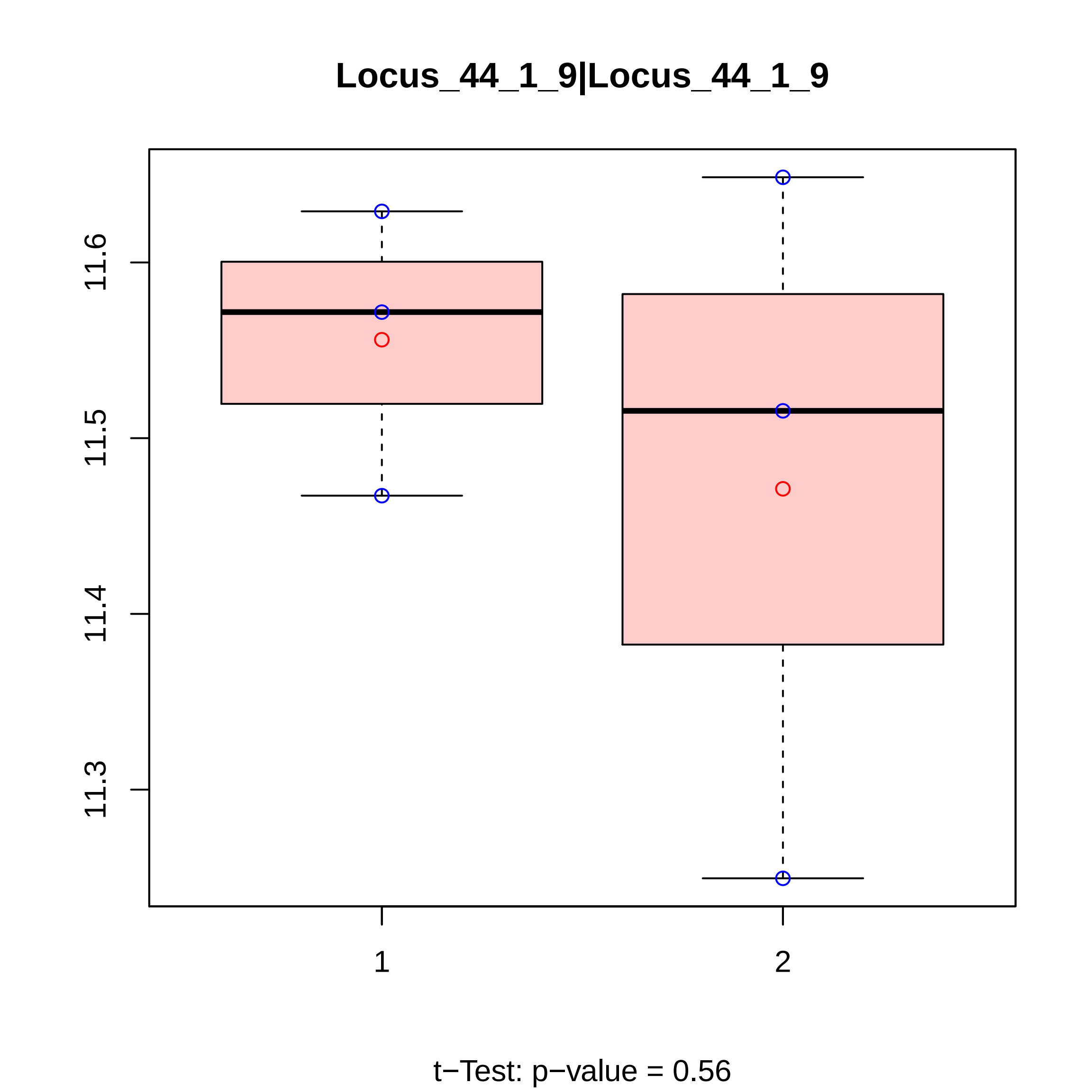
Locus_44_1_9|Locus_44_1_9
11.3
11.4
11.5
11.6
1
2
t−Test: p−value = 0.56

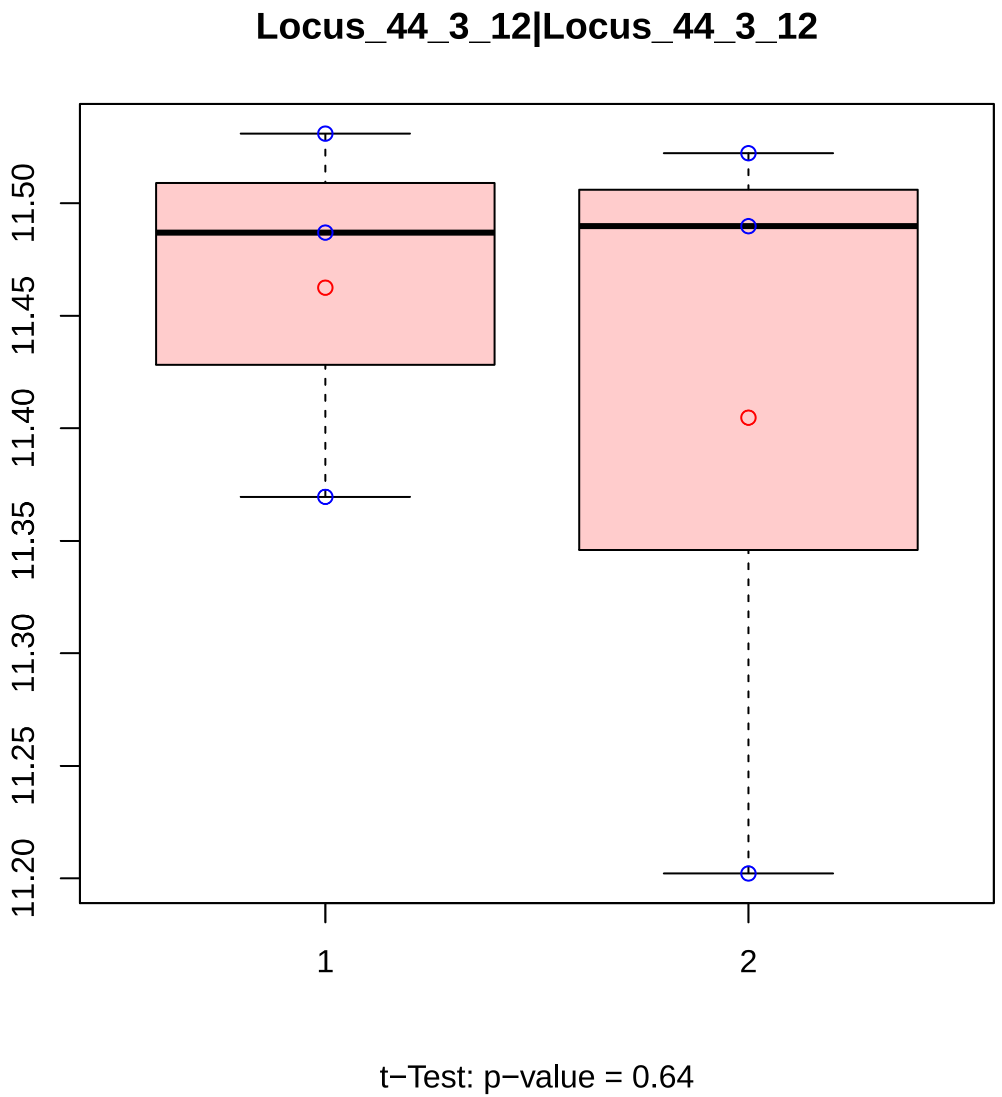
Locus_44_3_12|Locus_44_3_12
11.20
11.25
11.30
11.35
11.40
11.45
11.50
1
2
t−Test: p−value = 0.64

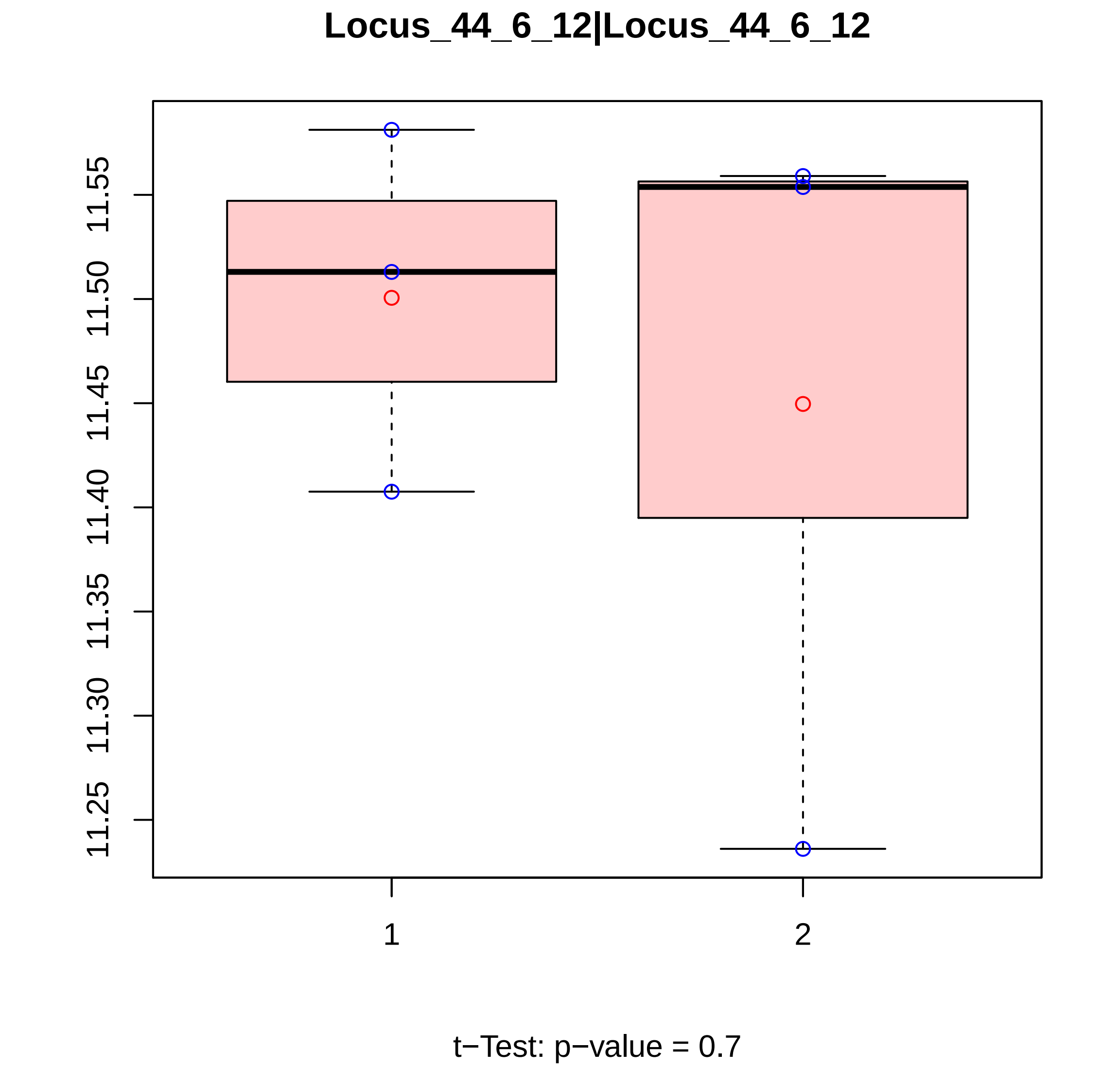

Locus_44_6_12|Locus_44_6_12
11.25
11.30
11.35
11.40
11.45
11.50
11.55
1
2
t−Test: p−value = 0.7

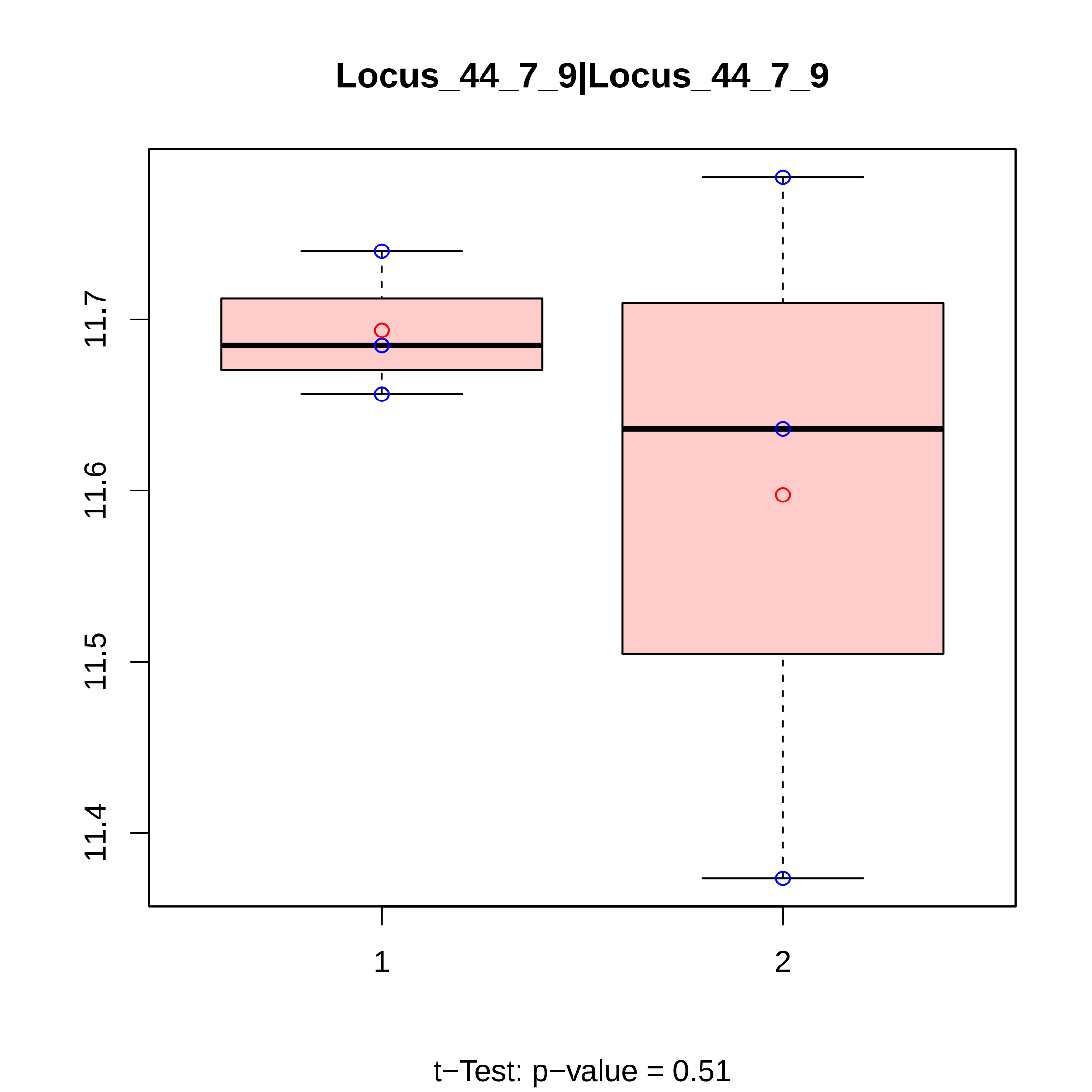
Locus_44_7_9|Locus_44_7_9
11.4
11.5
11.6
11.7
1
2
t−Test: p−value = 0.51

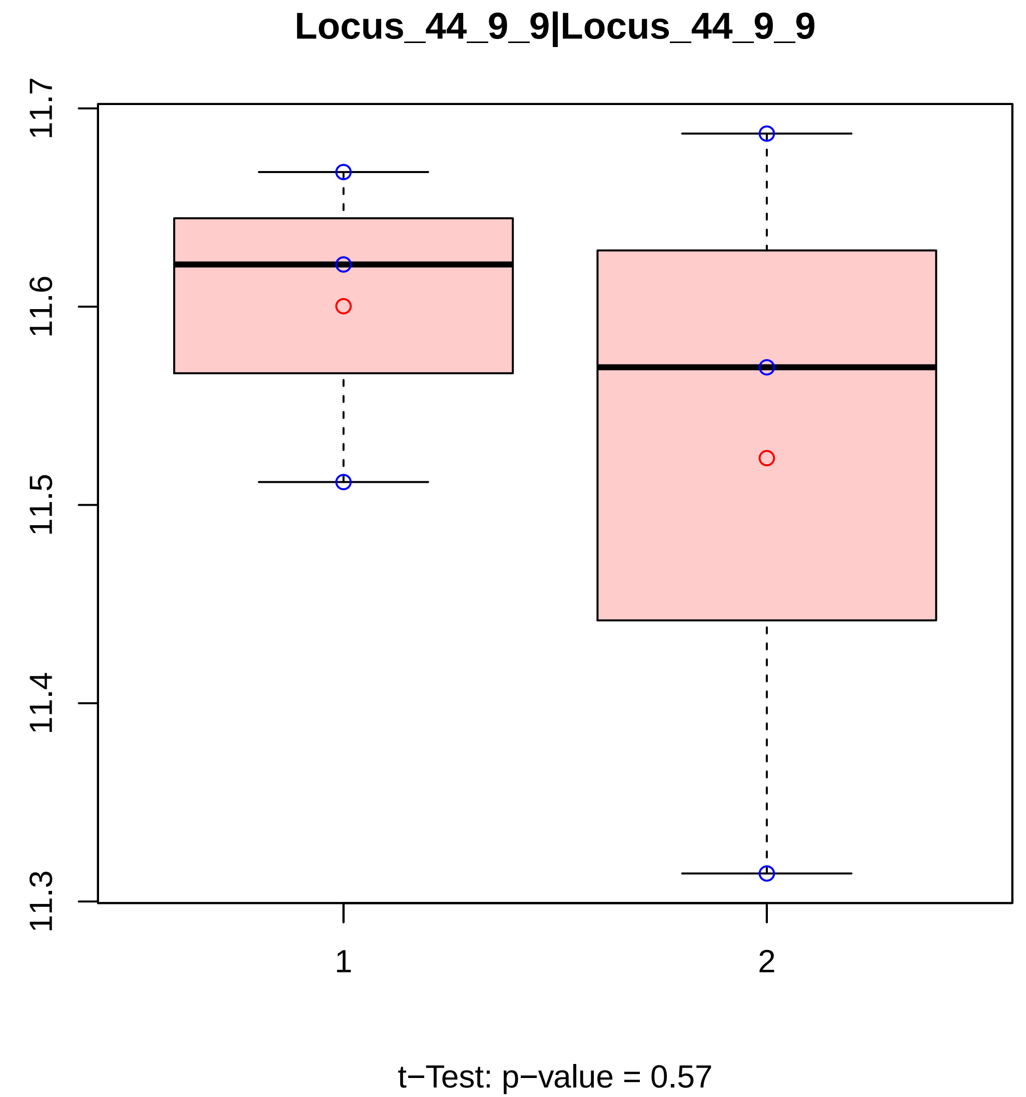

Locus_44_9_9|Locus_44_9_9
11.7
11.6
11.5
11.4
11.3
1
2
t−Test: p−value = 0.57

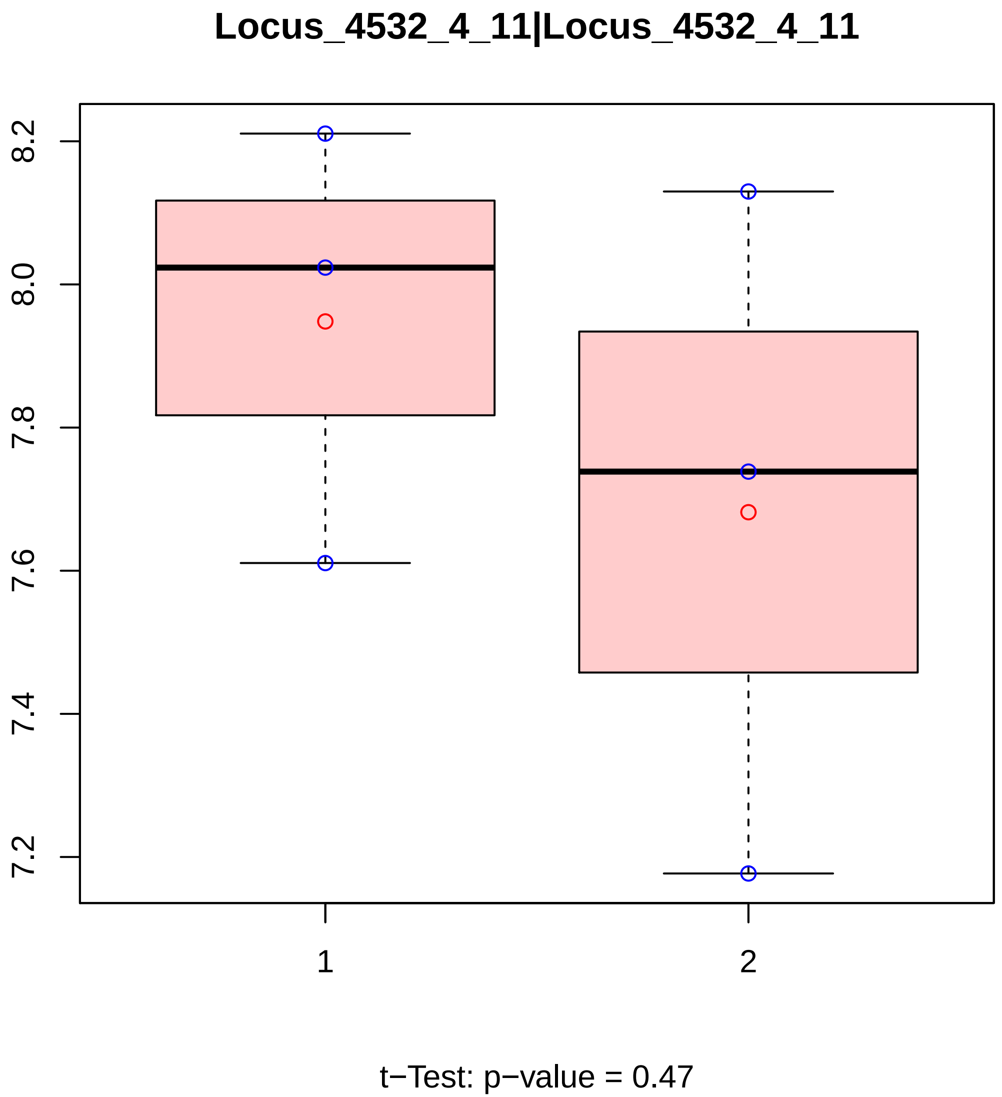
Locus_4532_4_11|Locus_4532_4_11
8.2
8.0
7.8
7.6
7.4
7.2
1
2
t−Test: p−value = 0.47

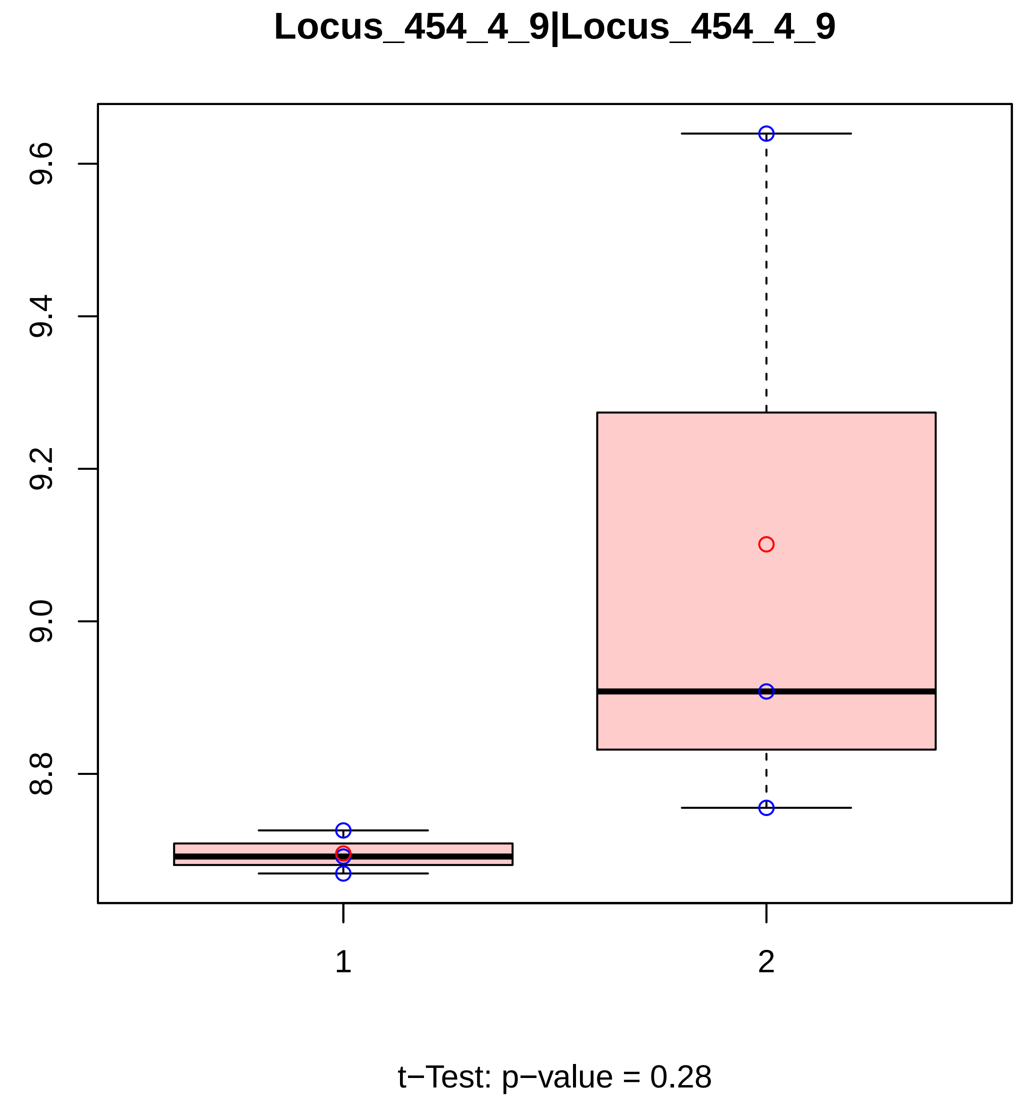

Locus_454_4_9|Locus_454_4_9
9.6
9.4
9.2
9.0
8.8
1
2
t−Test: p−value = 0.28

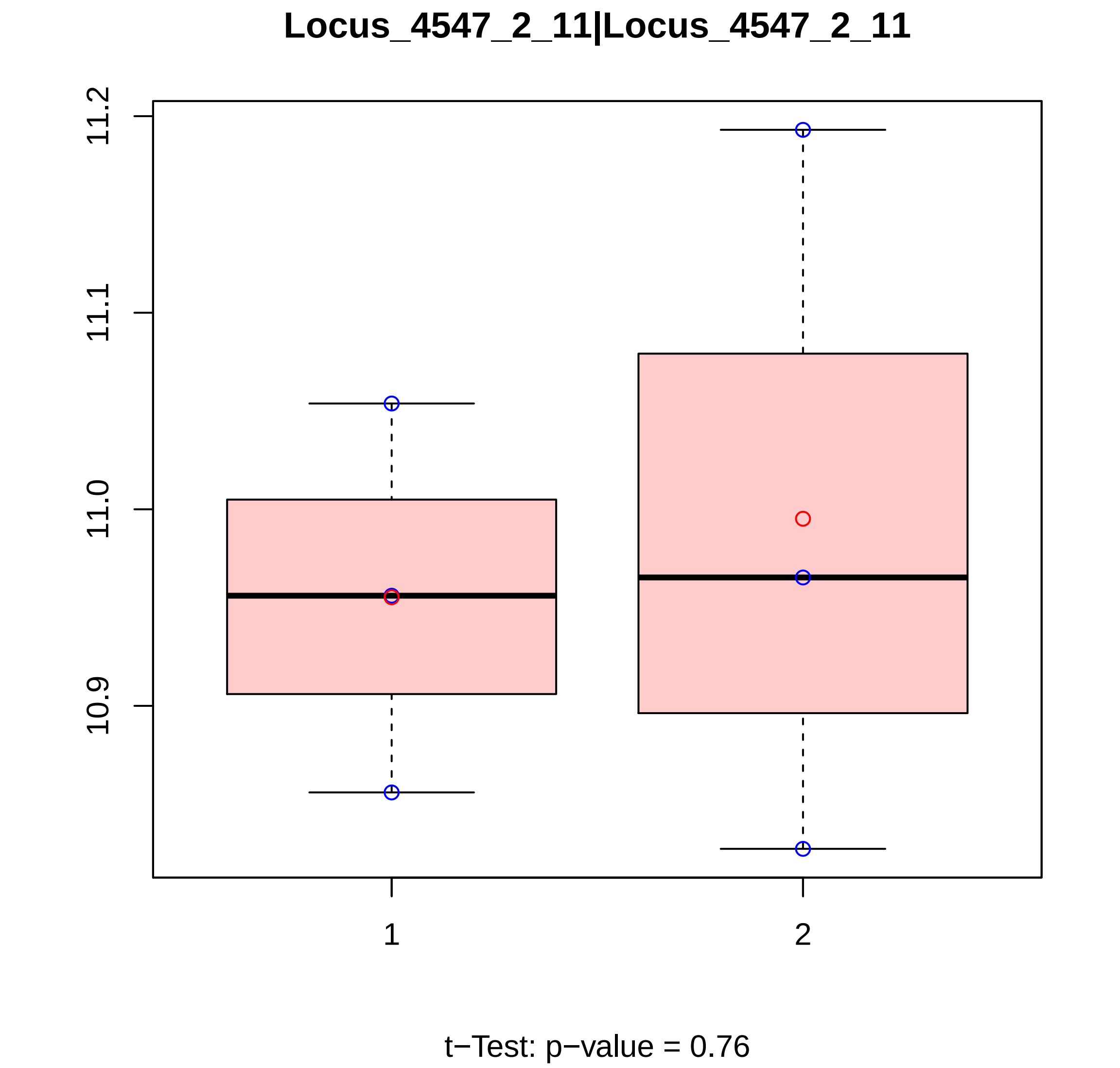

Locus_4547_2_11|Locus_4547_2_11
11.2
11.1
11.0
10.9
1
2
t−Test: p−value = 0.76

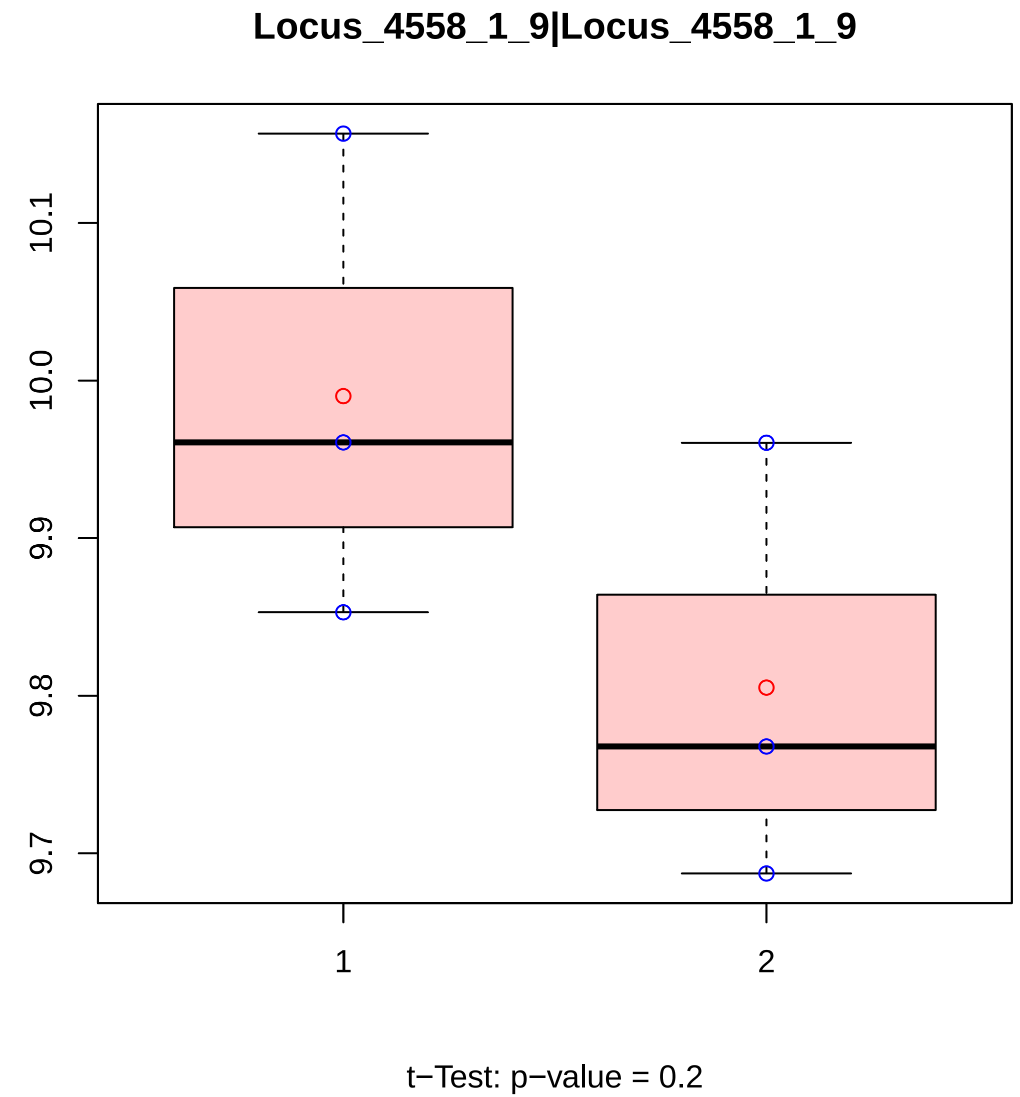

Locus_4558_1_9|Locus_4558_1_9
10.1
10.0
9.9
9.8
9.7
1
2
t−Test: p−value = 0.2

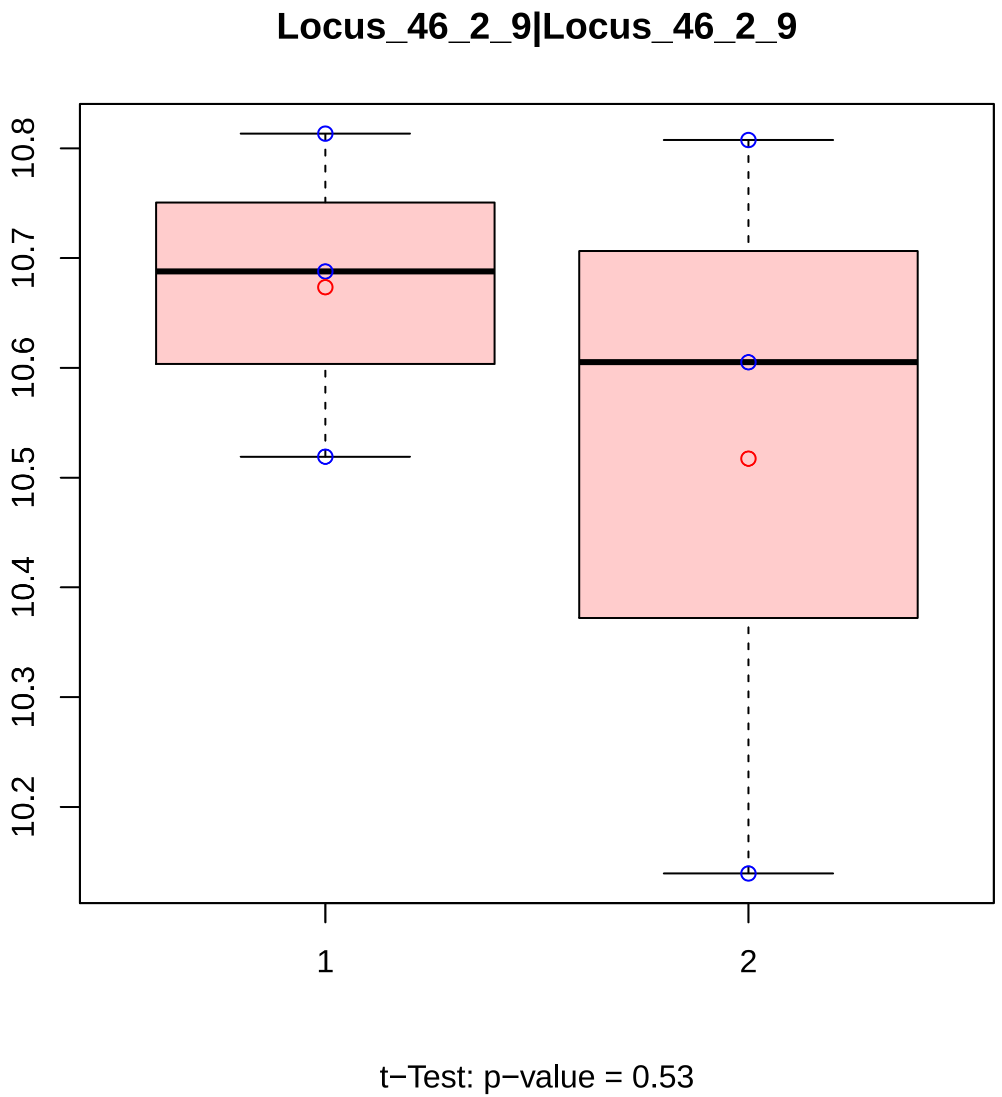

Locus_46_2_9|Locus_46_2_9
10.8
10.7
10.6
10.5
10.4
10.3
10.2
1
2
t−Test: p−value = 0.53

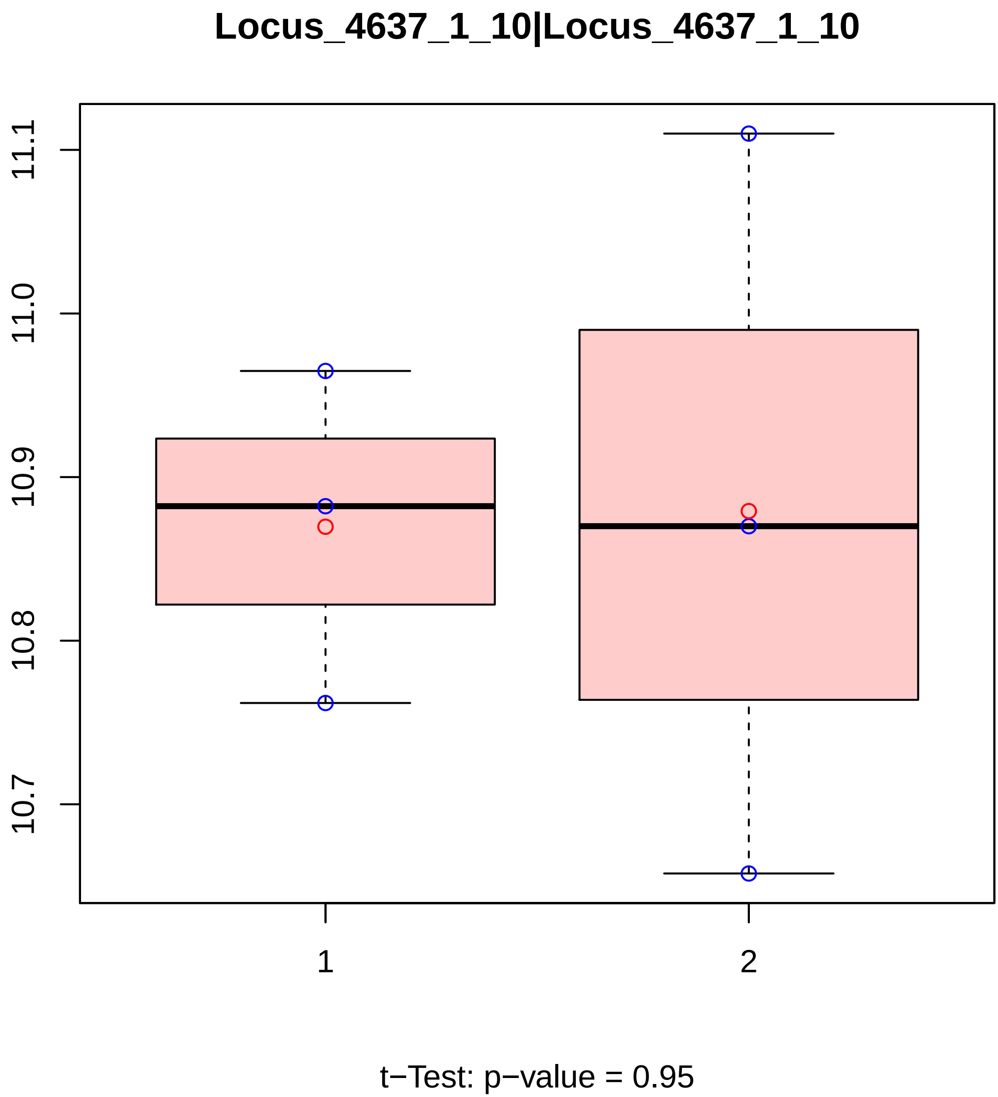

Locus_4637_1_10|Locus_4637_1_10
11.1
11.0
10.9
10.8
10.7
1
2
t−Test: p−value = 0.95

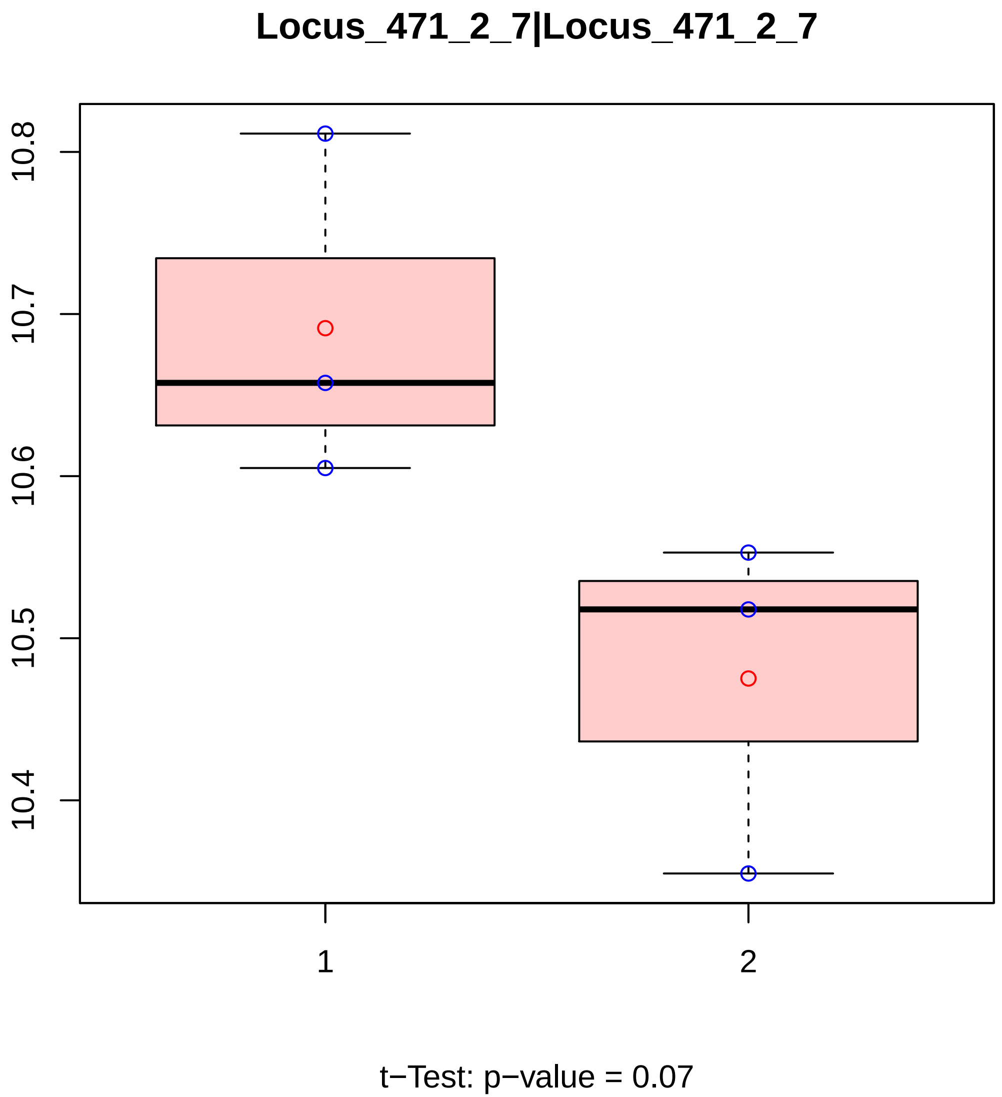
Locus_471_2_7|Locus_471_2_7
10.8
10.7
10.6
10.5
10.4
1
2
t−Test: p−value = 0.07

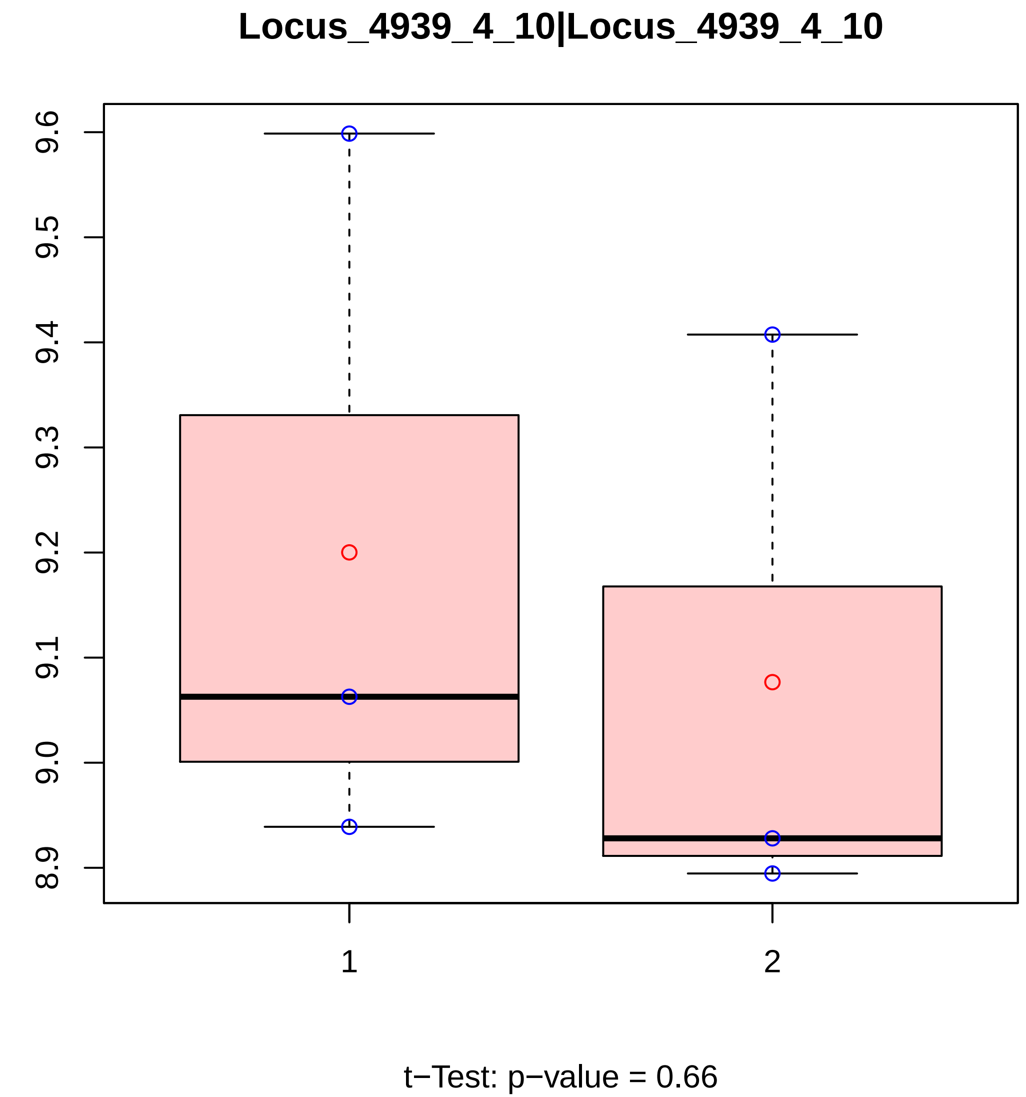
Locus_4939_4_10|Locus_4939_4_10
9.6
9.5
9.4
9.3
9.2
9.1
9.0
8.9
1
2
t−Test: p−value = 0.66

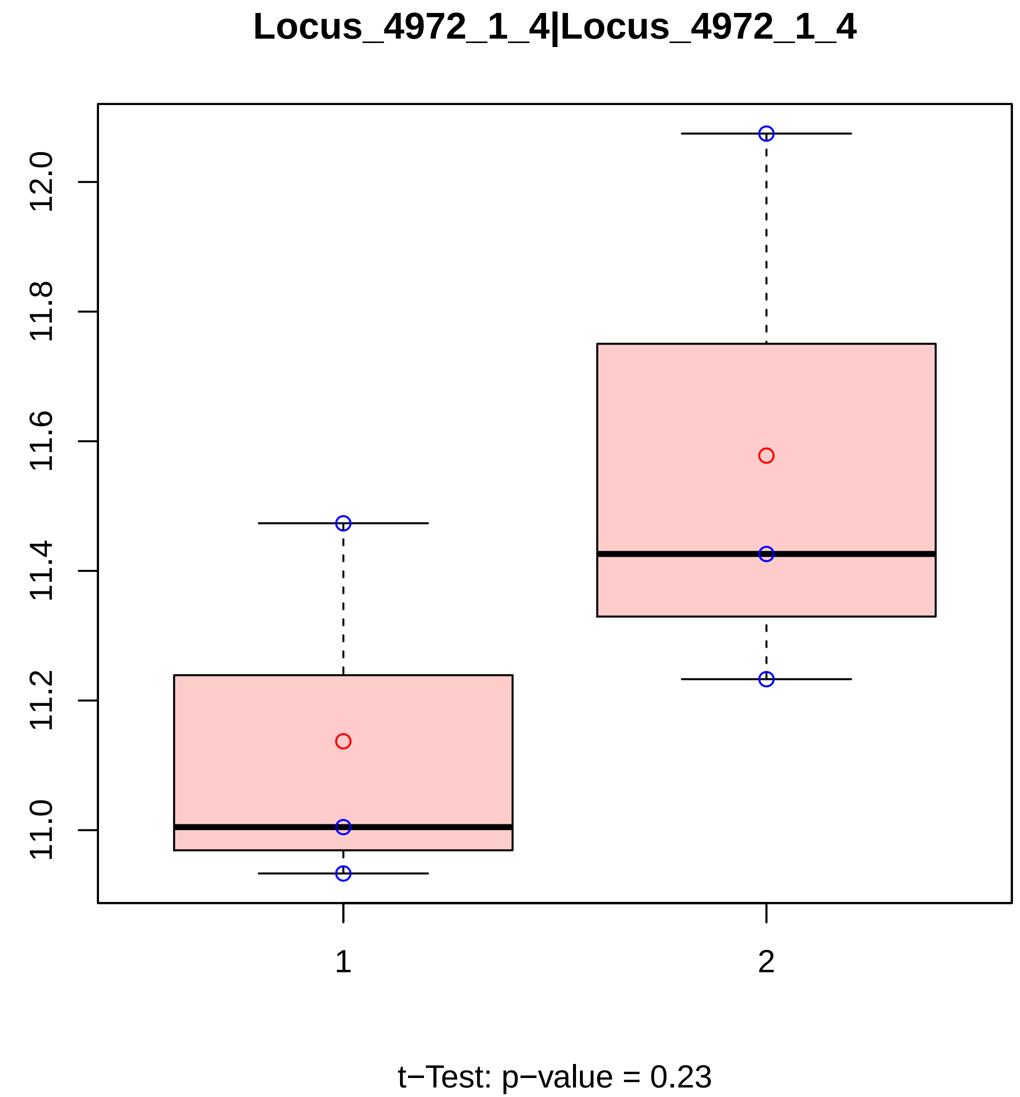

Locus_4972_1_4|Locus_4972_1_4
12.0
11.8
11.6
11.4
11.2
11.0
1
2
t−Test: p−value = 0.23

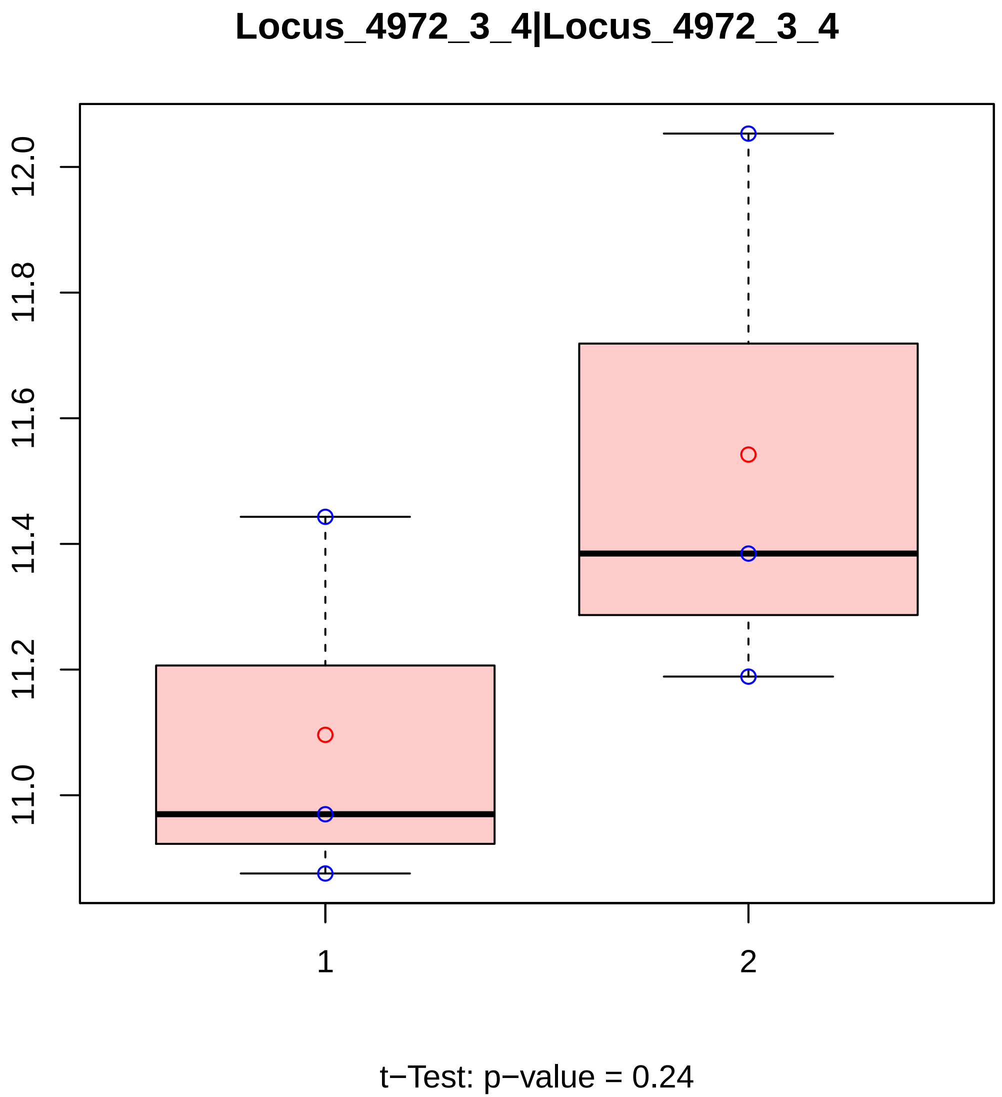

Locus_4972_3_4|Locus_4972_3_4
12.0
11.8
11.6
11.4
11.2
11.0
1
2
t−Test: p−value = 0.24

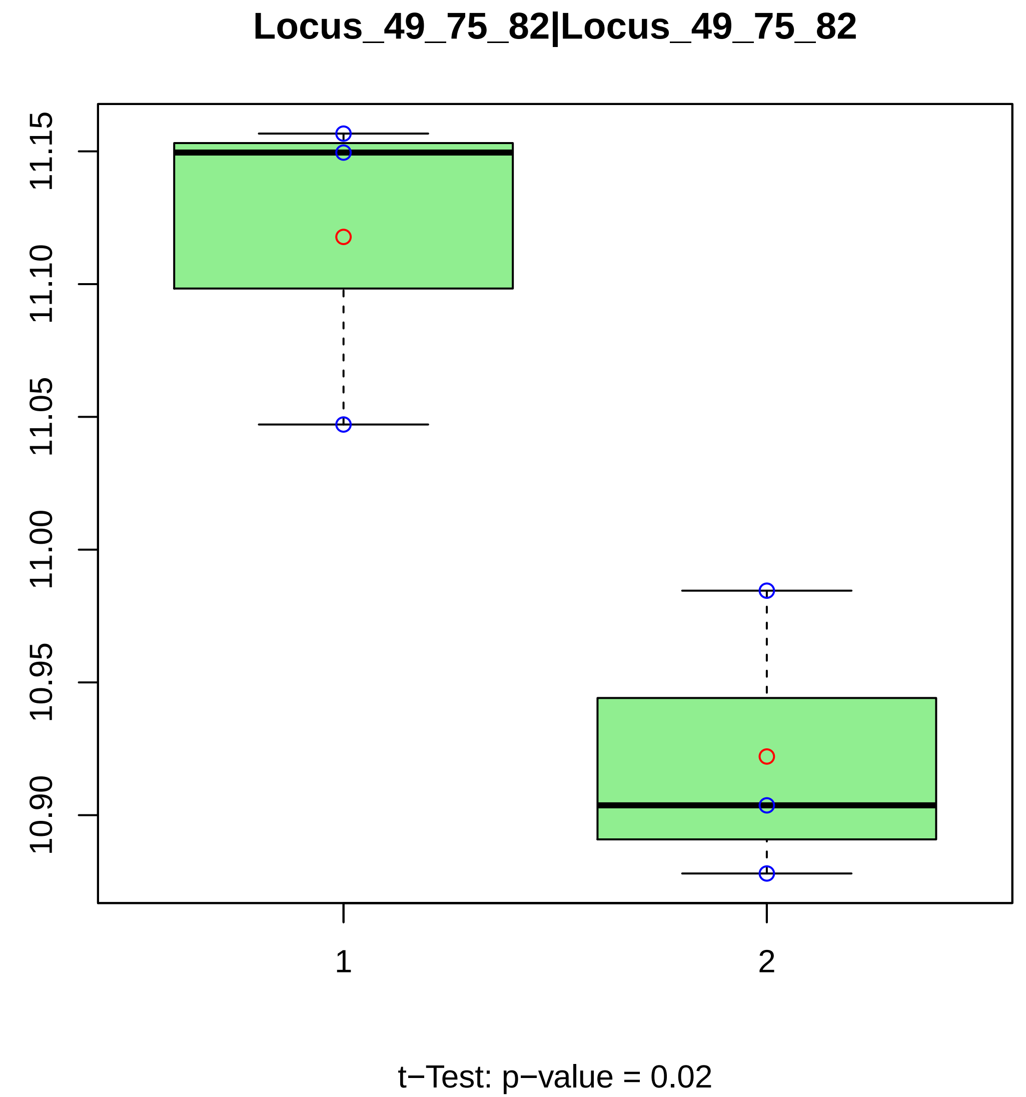

Locus_49_75_82|Locus_49_75_82
11.15
11.10
11.05
11.00
10.95
10.90
1
2
t−Test: p−value = 0.02

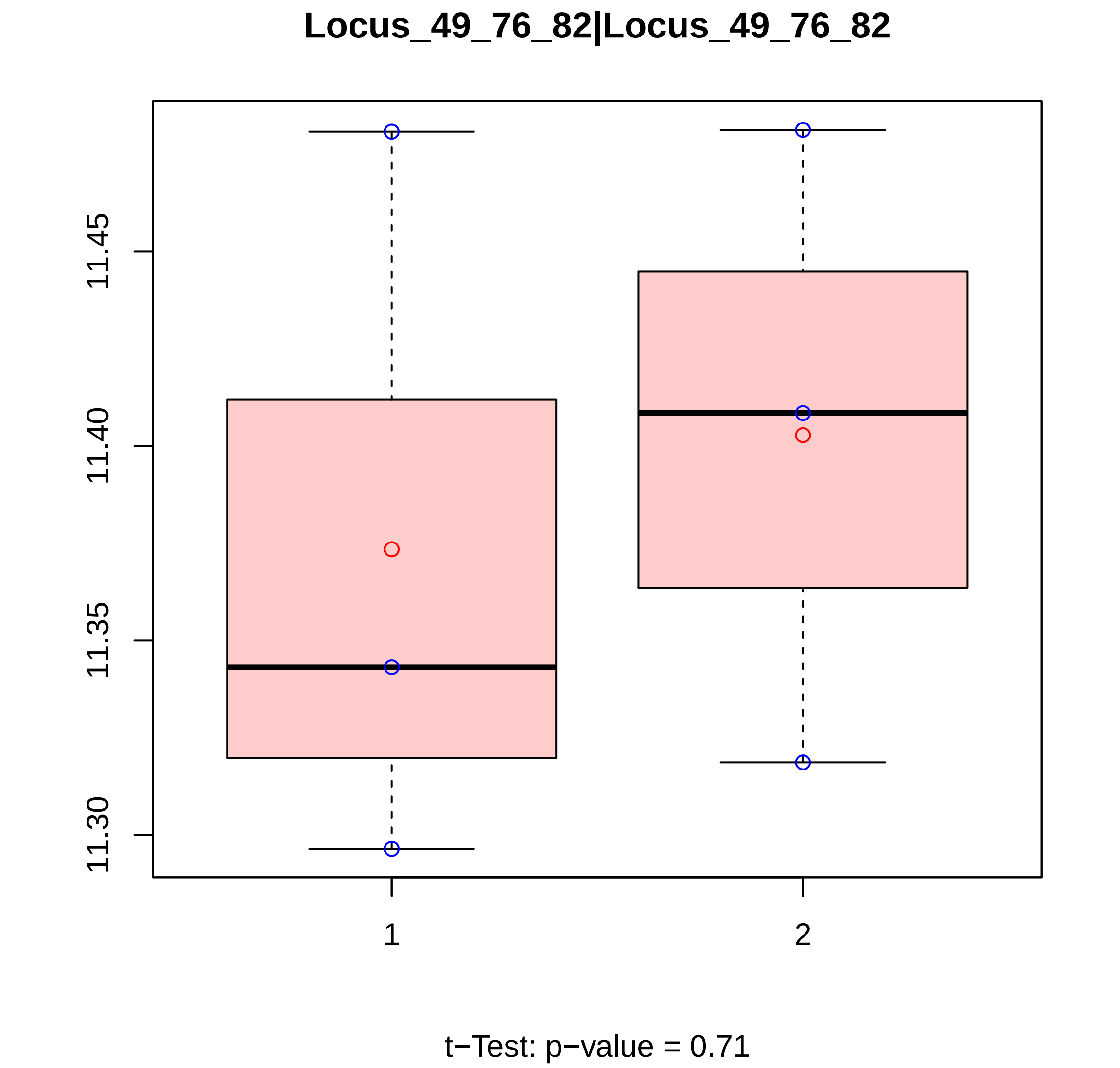
Locus_49_76_82|Locus_49_76_82
11.30
11.35
11.40
11.45
1
2
t−Test: p−value = 0.71

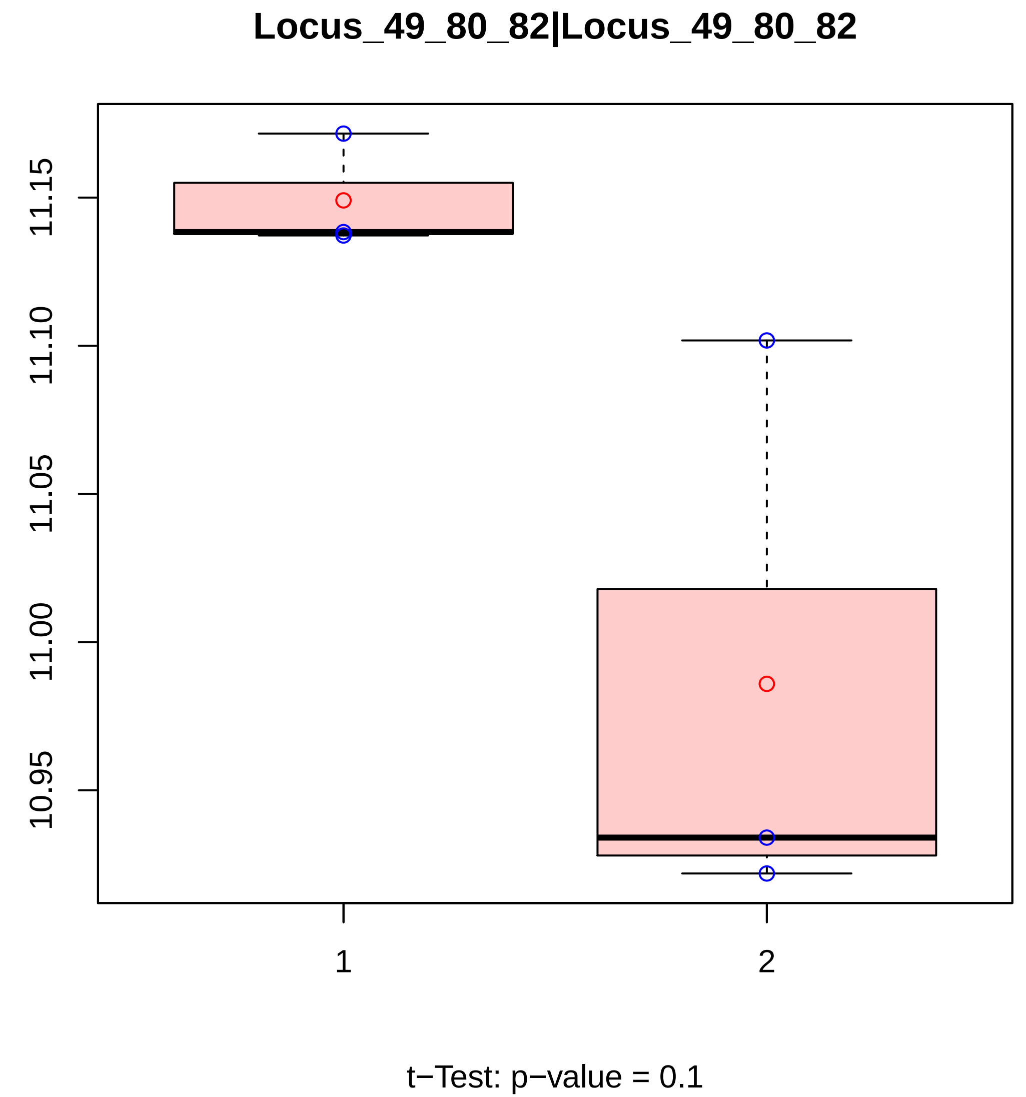
Locus_49_80_82|Locus_49_80_82
11.15
11.10
11.05
11.00
10.95
1
2
t−Test: p−value = 0.1

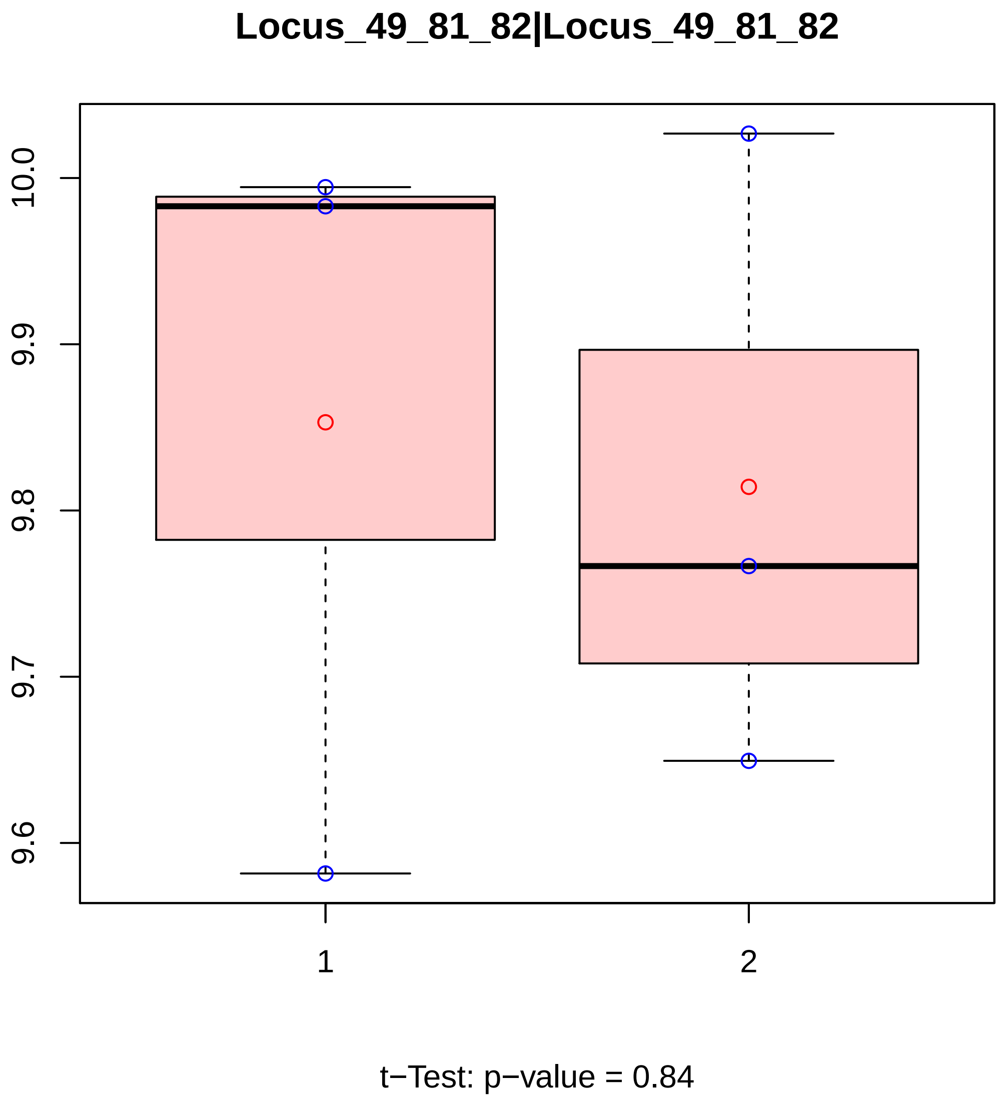

Locus_49_81_82|Locus_49_81_82
10.0
9.9
9.8
9.7
9.6
1
2
t−Test: p−value = 0.84

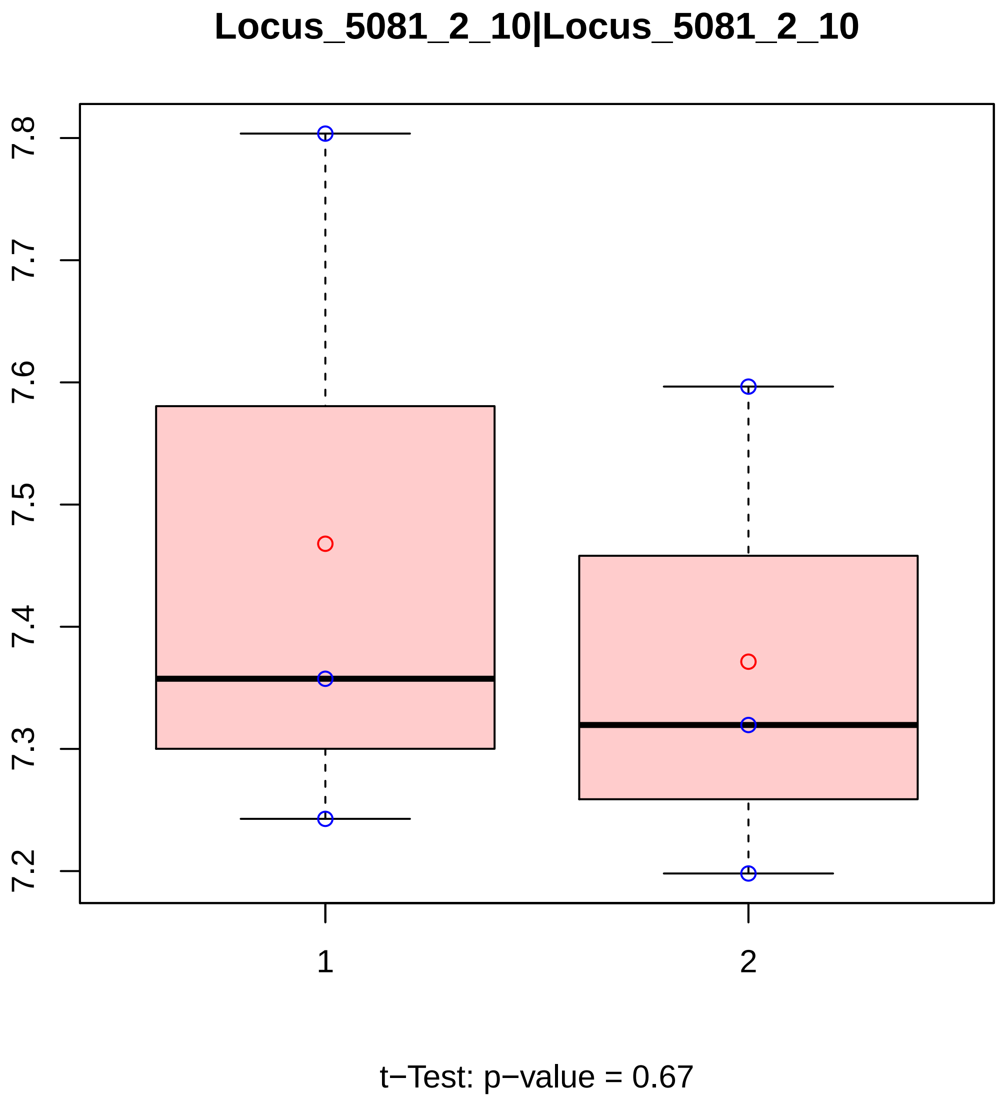

Locus_5081_2_10|Locus_5081_2_10
7.2
7.3
7.4
7.5
7.6
7.7
7.8
1
2
t−Test: p−value = 0.67

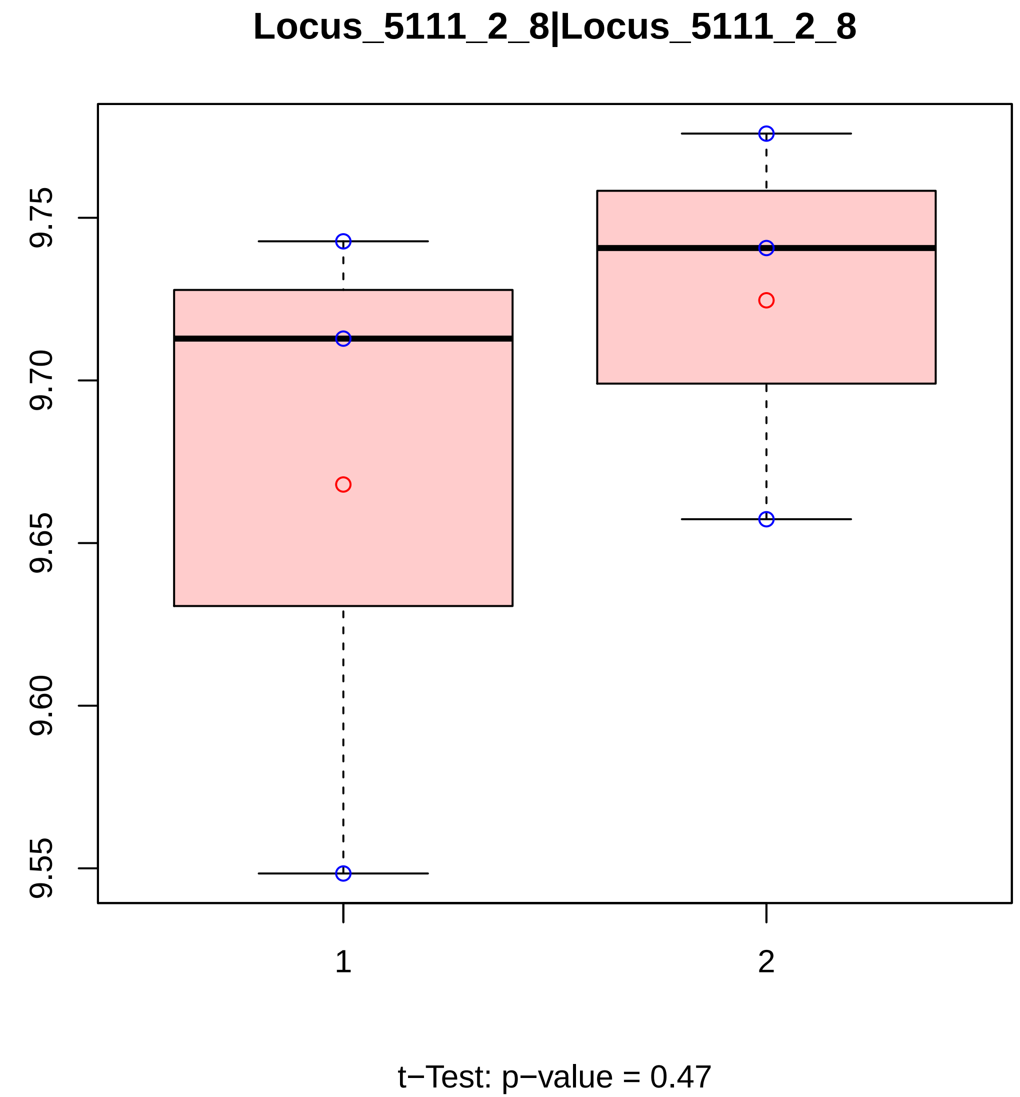

Locus_5111_2_8|Locus_5111_2_8
9.55
9.60
9.65
9.70
9.75
1
2
t−Test: p−value = 0.47

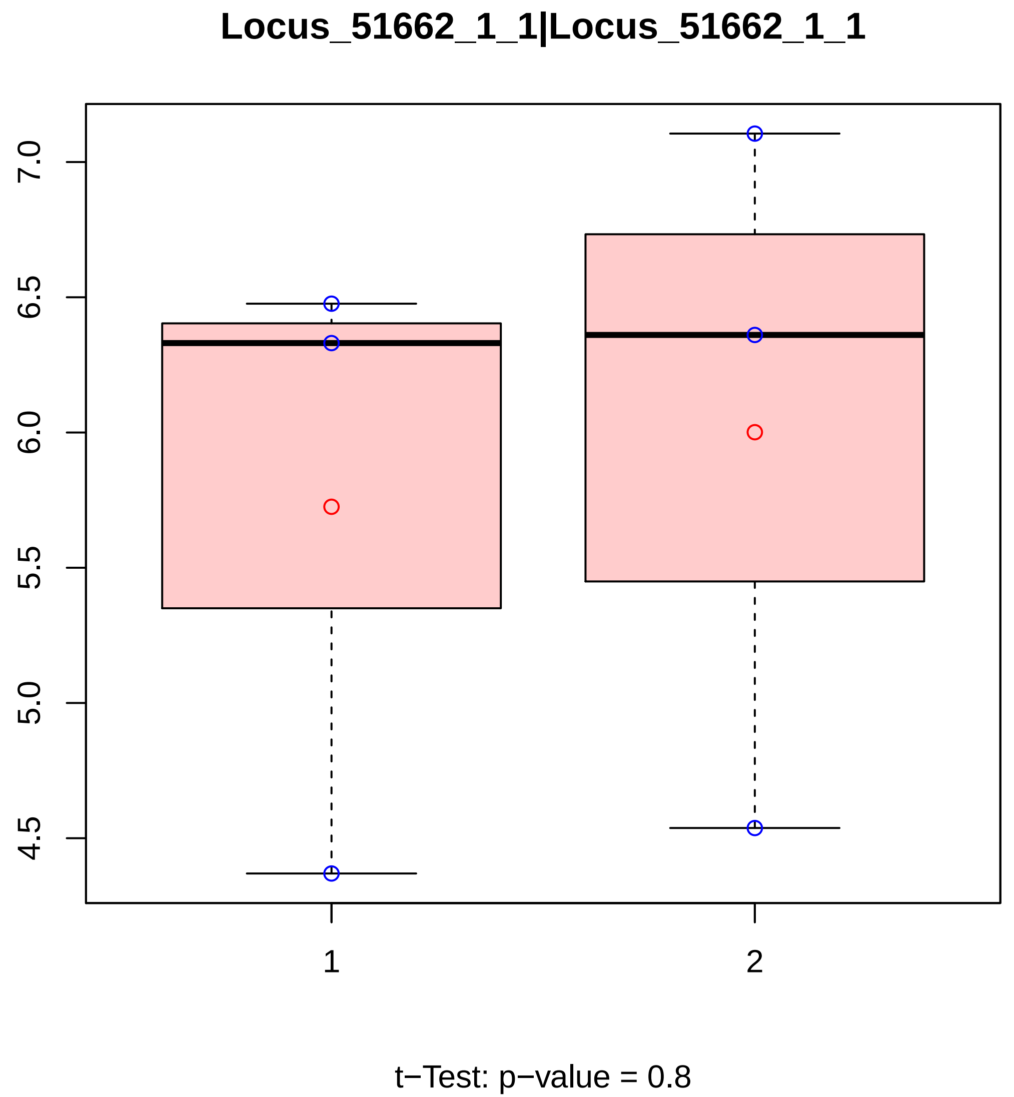

Locus_51662_1_1|Locus_51662_1_1
7.0
6.5
6.0
5.5
5.0
4.5
1
2
t−Test: p−value = 0.8

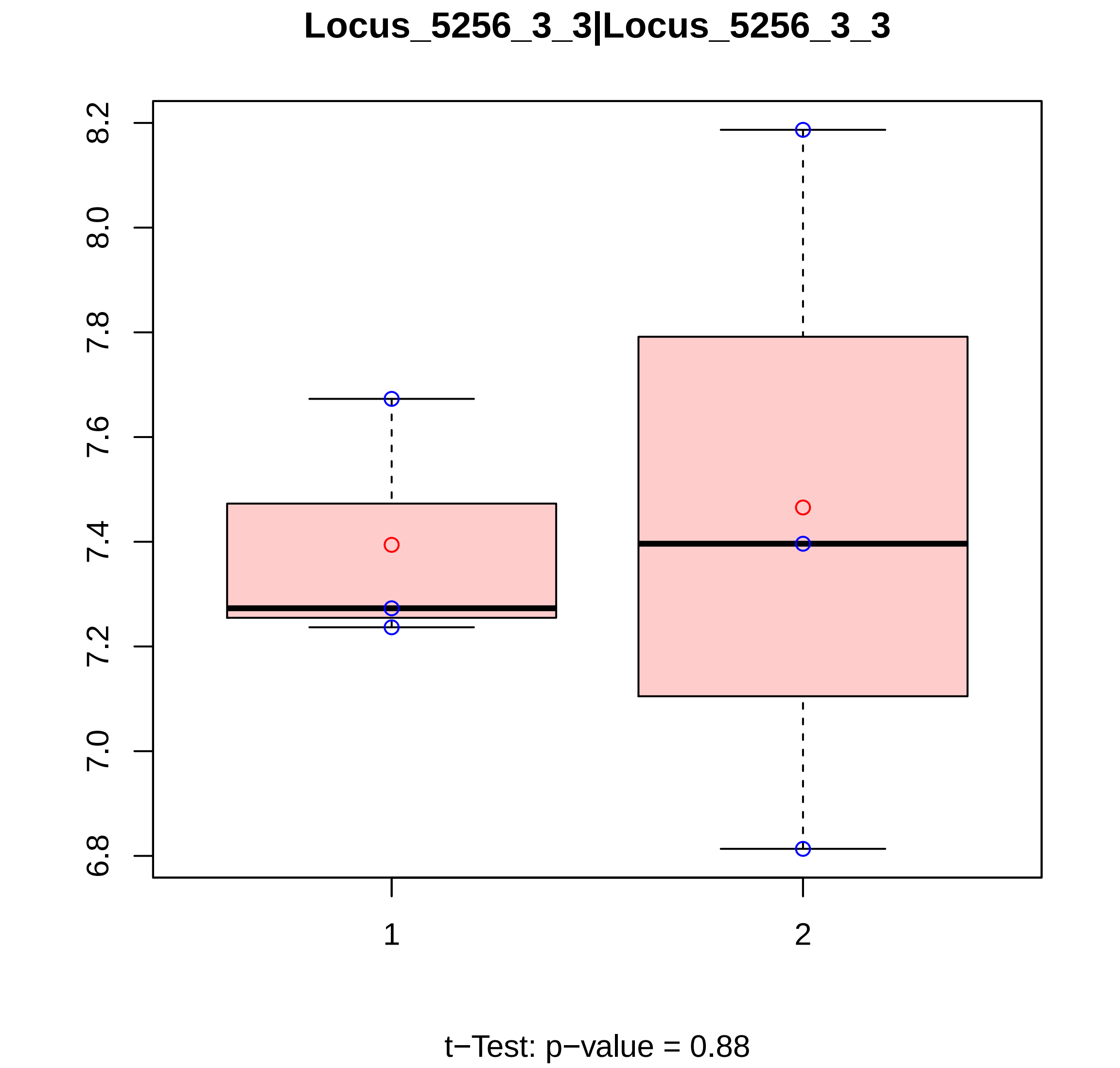
Locus_5256_3_3|Locus_5256_3_3
6.8
7.0
7.2
7.4
7.6
7.8
8.0
8.2
1
2
t−Test: p−value = 0.88

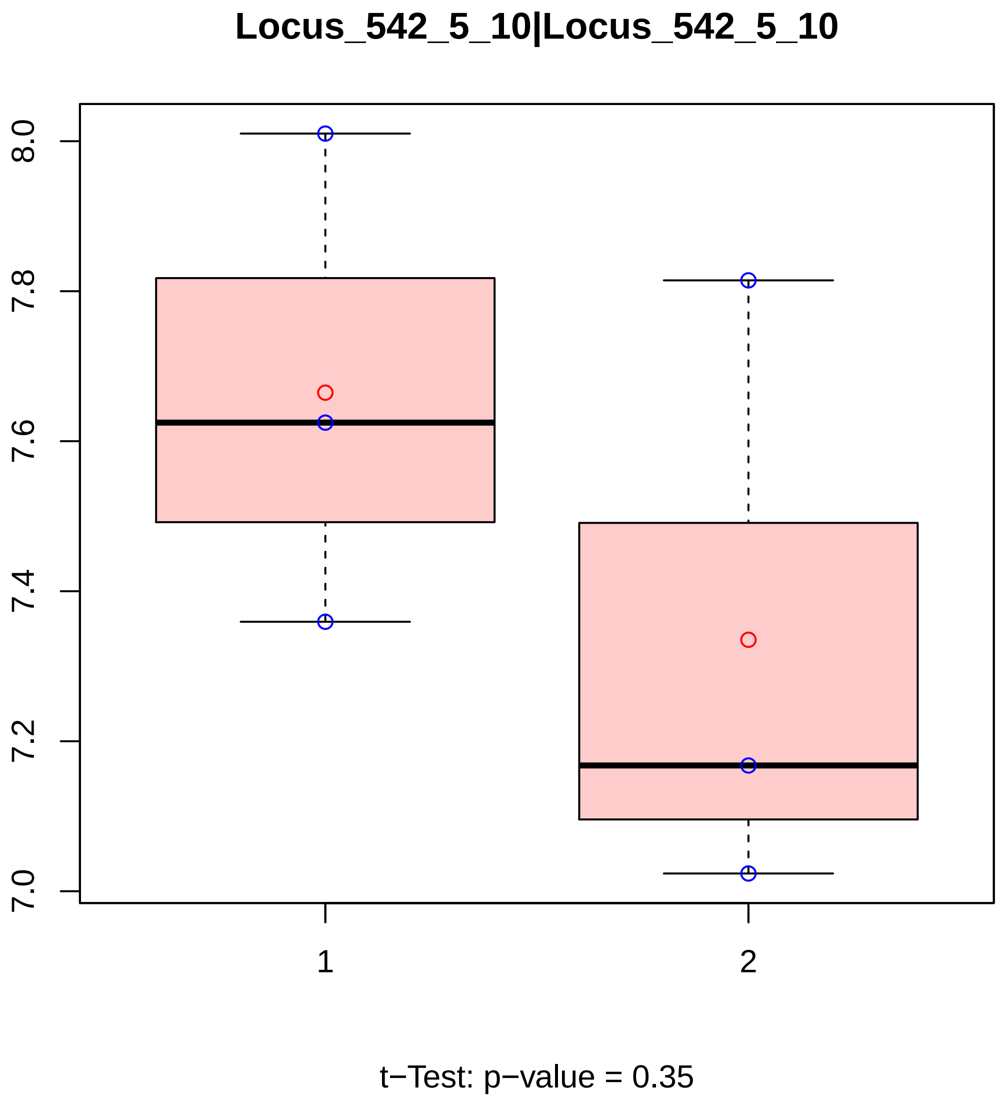

Locus_542_5_10|Locus_542_5_10
8.0
7.8
7.6
7.4
7.2
7.0
1
2
t−Test: p−value = 0.35

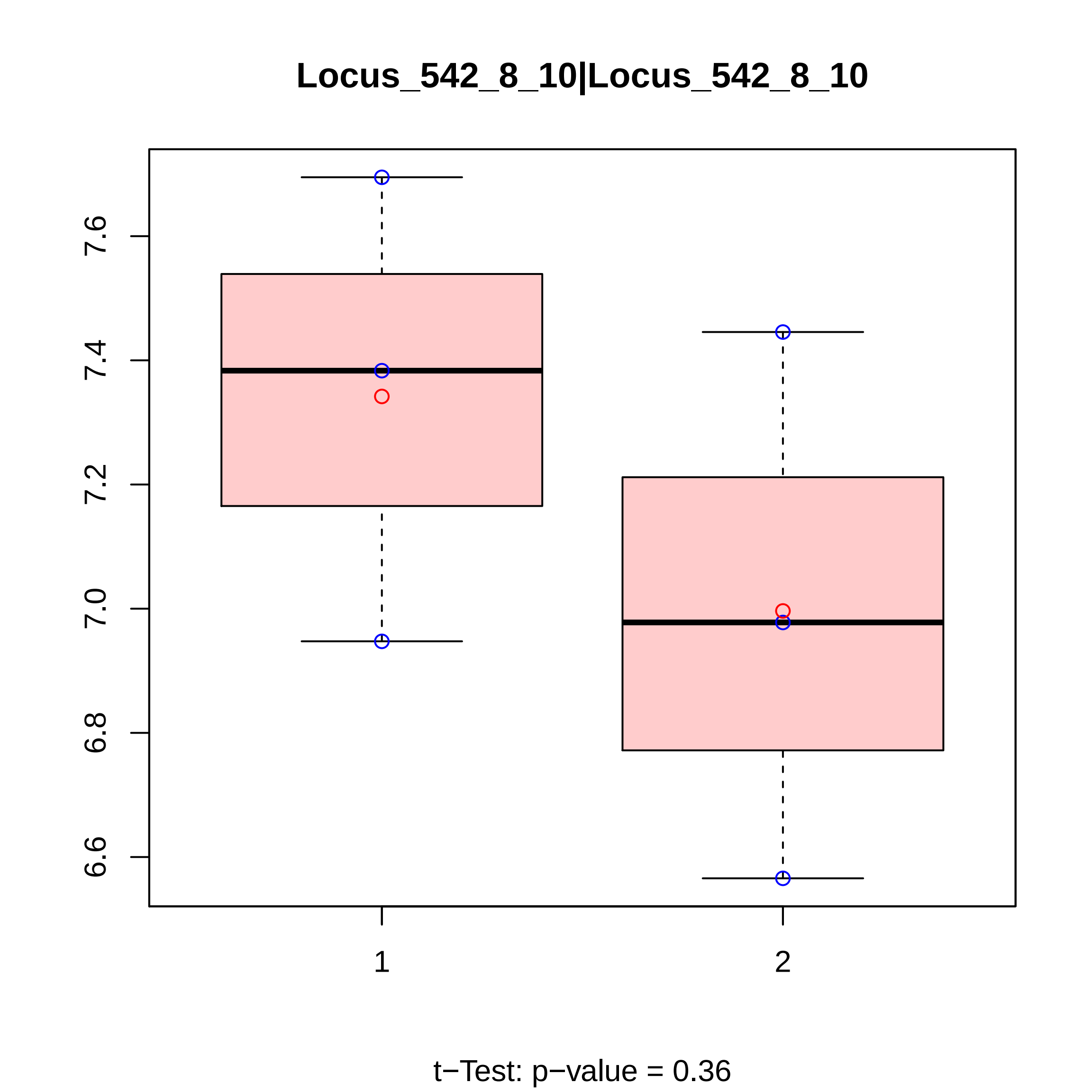

Locus_542_8_10|Locus_542_8_10
7.6
7.4
7.2
7.0
6.8
6.6
1
2
t−Test: p−value = 0.36

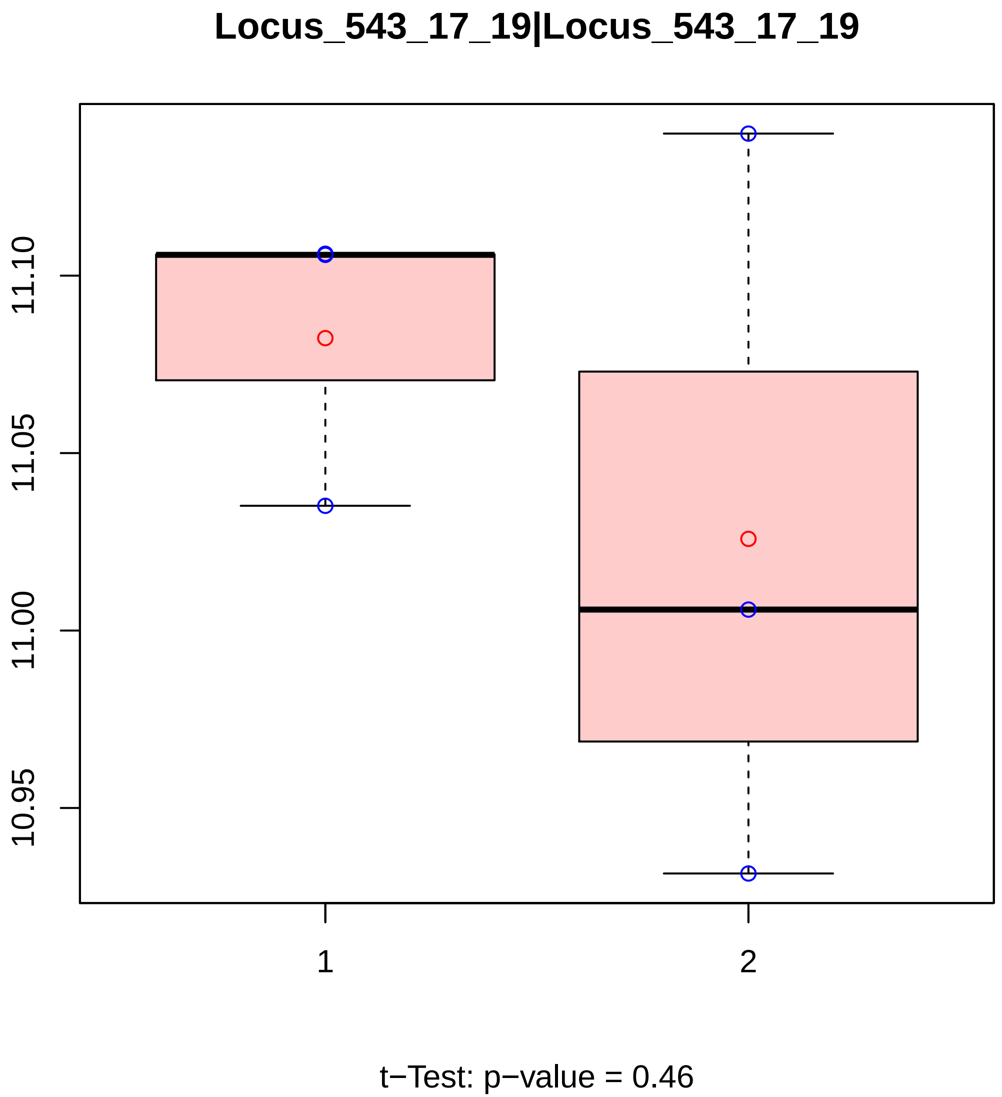
Locus_543_17_19|Locus_543_17_19
11.10
11.05
11.00
10.95
1
2
t−Test: p−value = 0.46

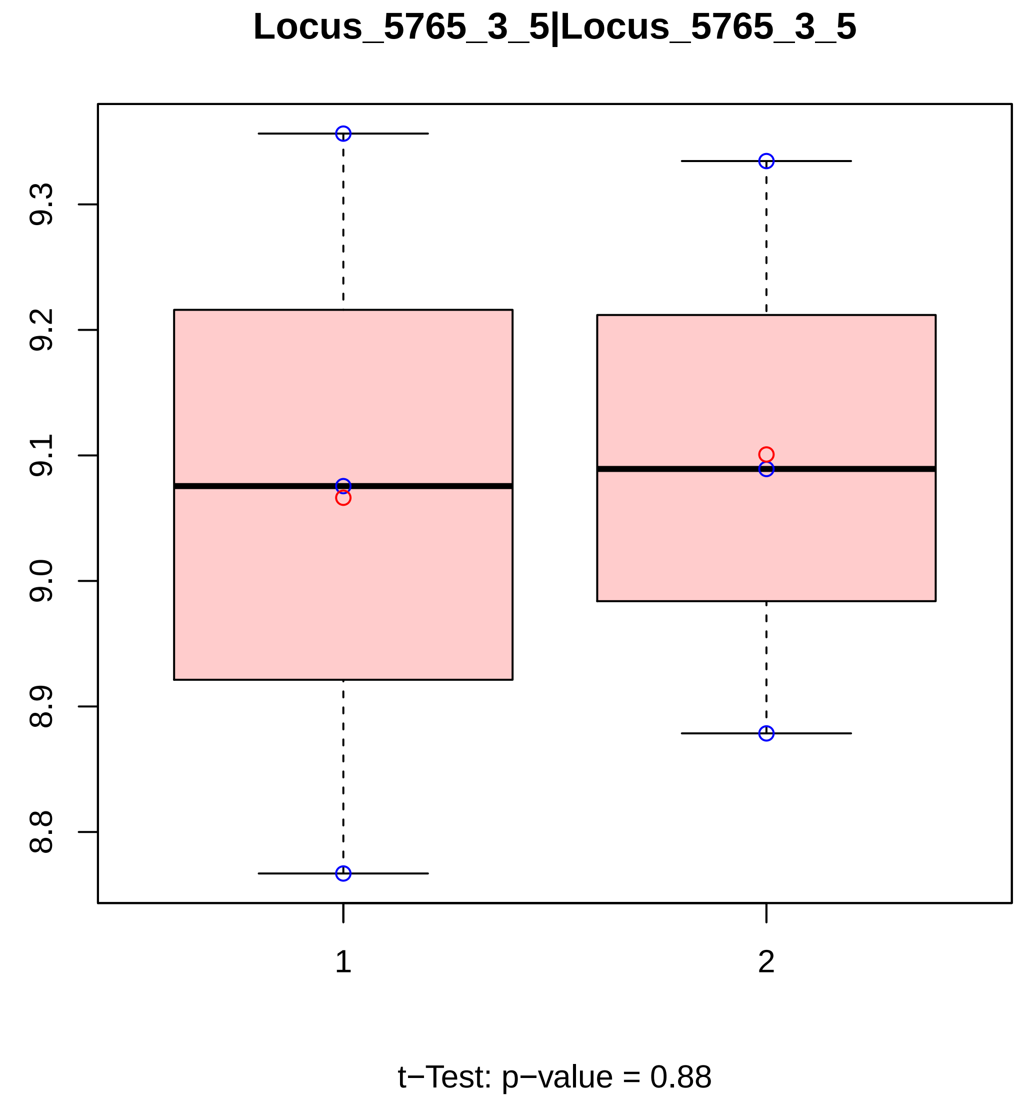
Locus_5765_3_5|Locus_5765_3_5
9.3
9.2
9.1
9.0
8.9
8.8
1
2
t−Test: p−value = 0.88

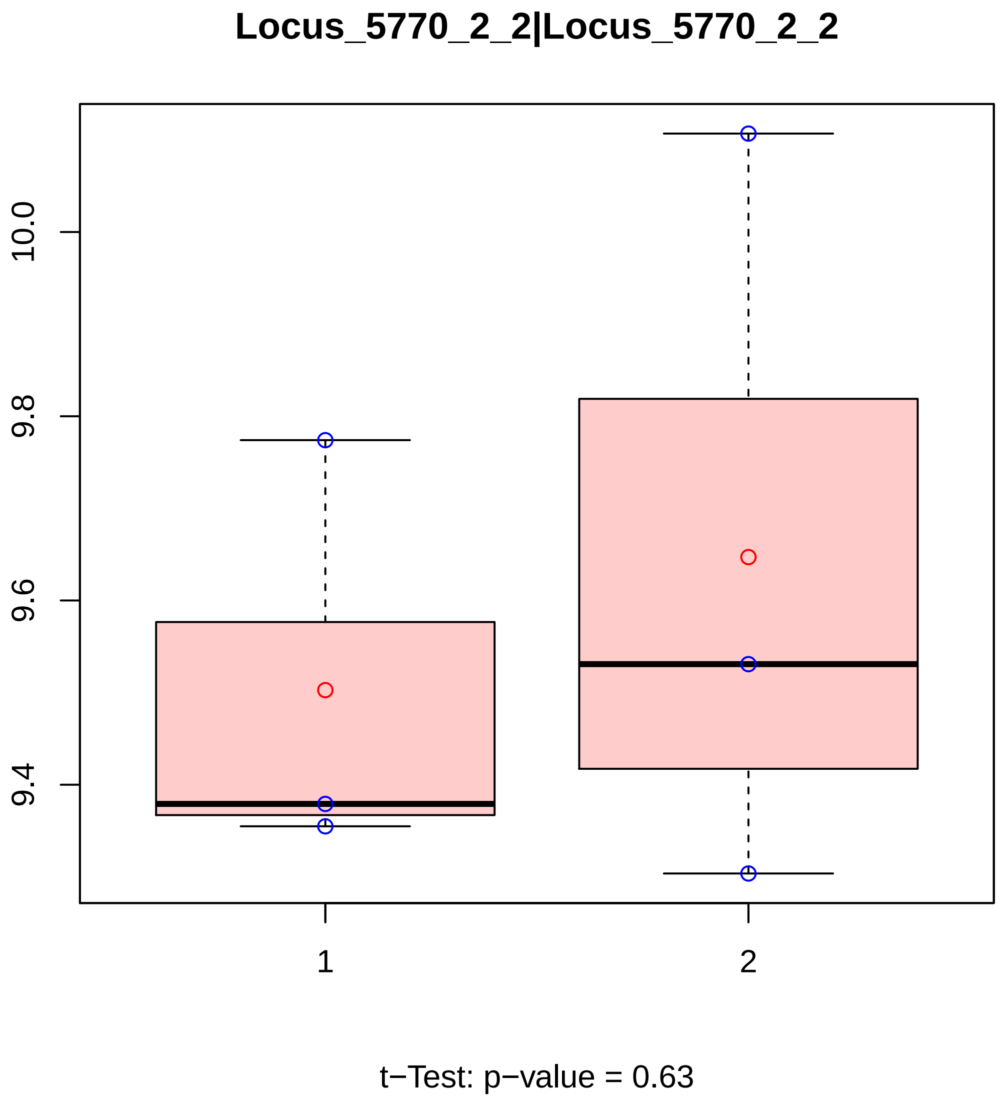
Locus_5770_2_2|Locus_5770_2_2
10.0
9.8
9.6
9.4
1
2
t−Test: p−value = 0.63

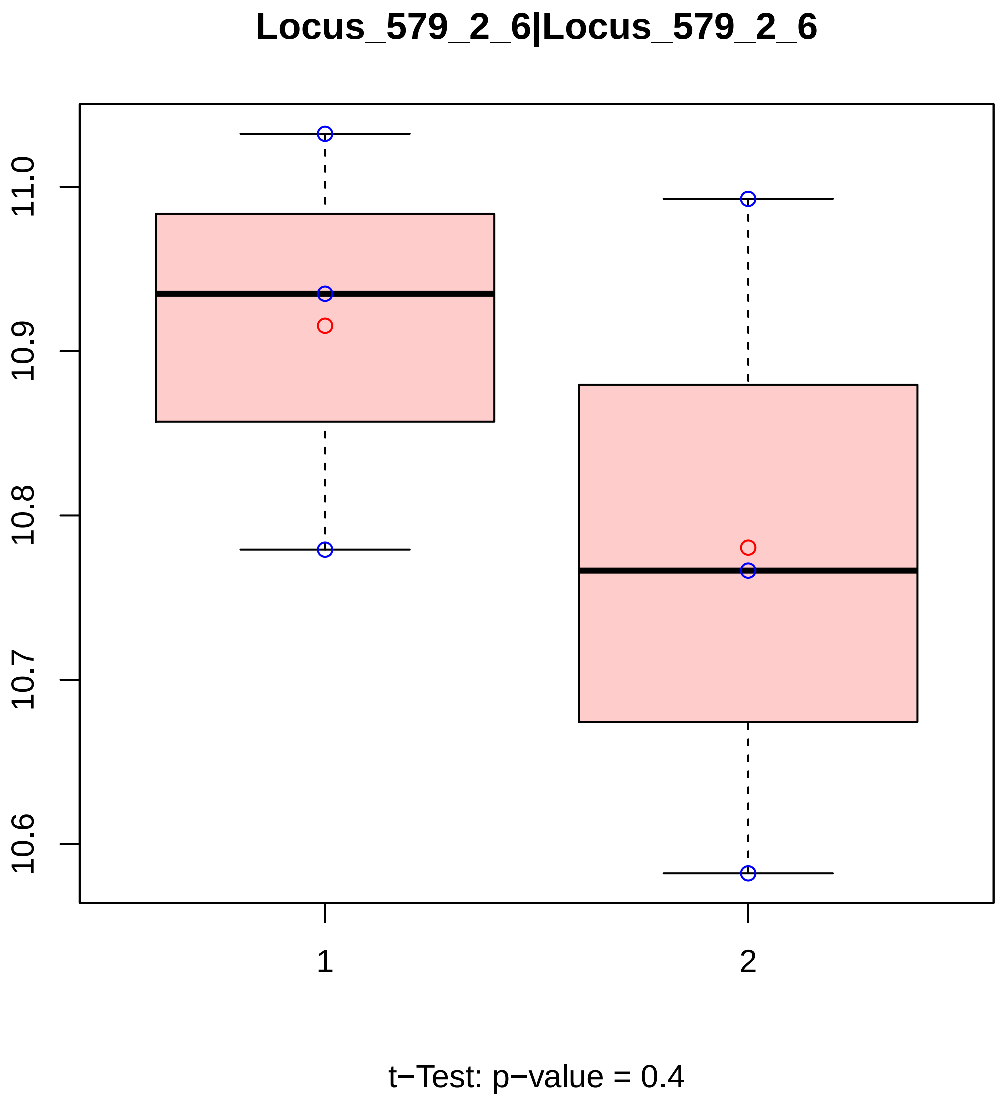

Locus_579_2_6|Locus_579_2_6
11.0
10.9
10.8
10.7
10.6
1
2
t−Test: p−value = 0.4

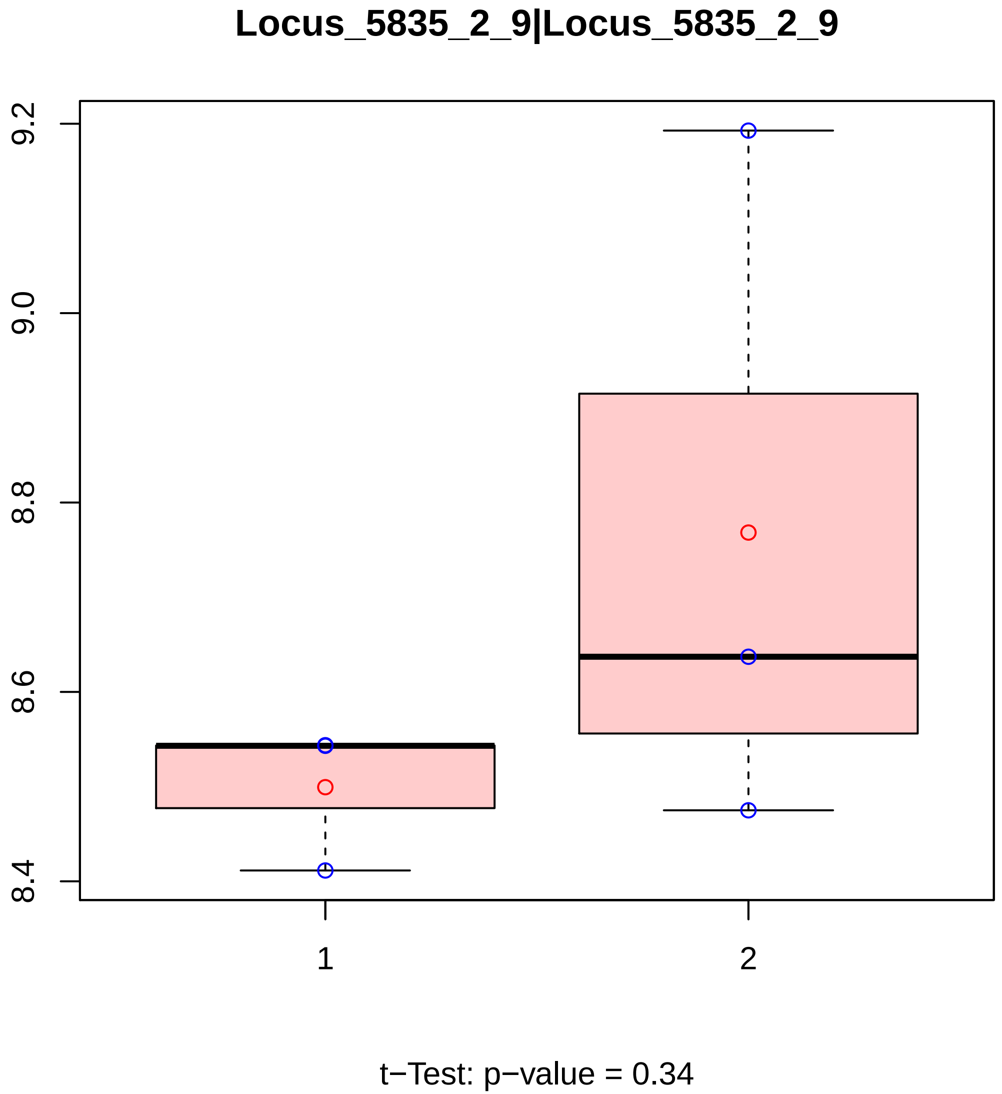
Locus_5835_2_9|Locus_5835_2_9
9.2
9.0
8.8
8.6
8.4
1
2
t−Test: p−value = 0.34

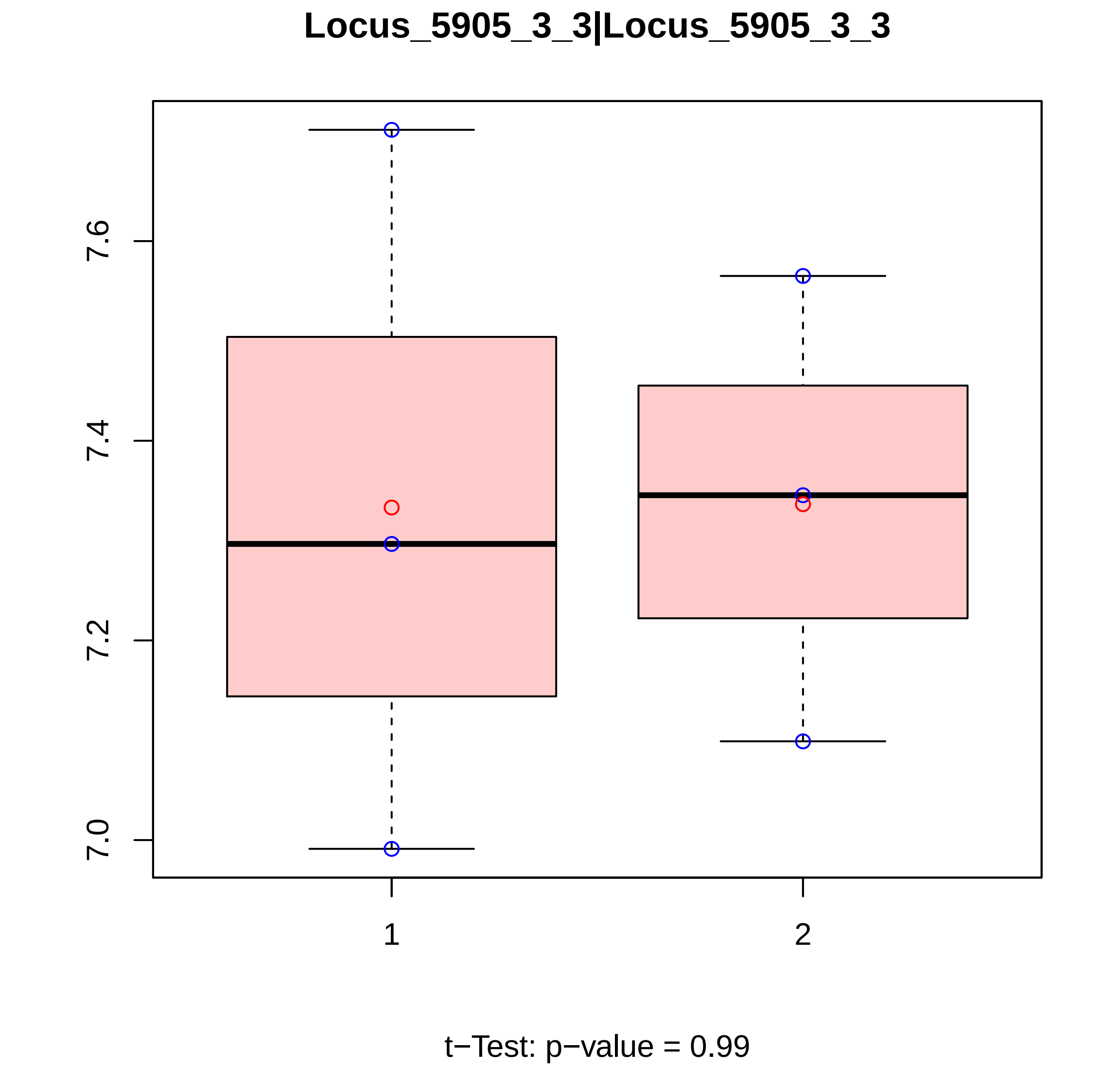

Locus_5905_3_3|Locus_5905_3_3
7.0
7.2
7.4
7.6
1
2
t−Test: p−value = 0.99

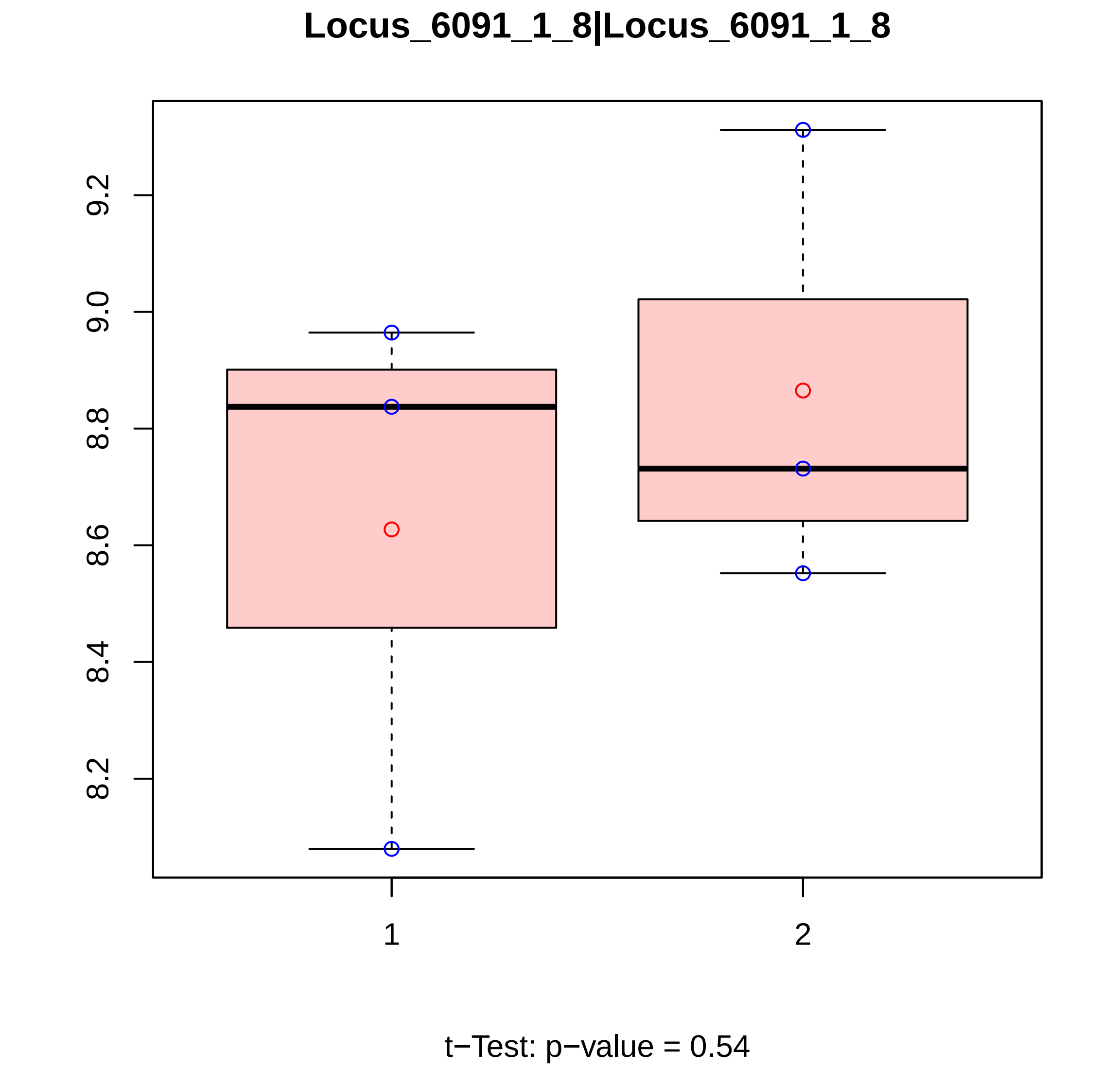
Locus_6091_1_8|Locus_6091_1_8
9.2
9.0
8.8
8.6
8.4
8.2
1
2
t−Test: p−value = 0.54

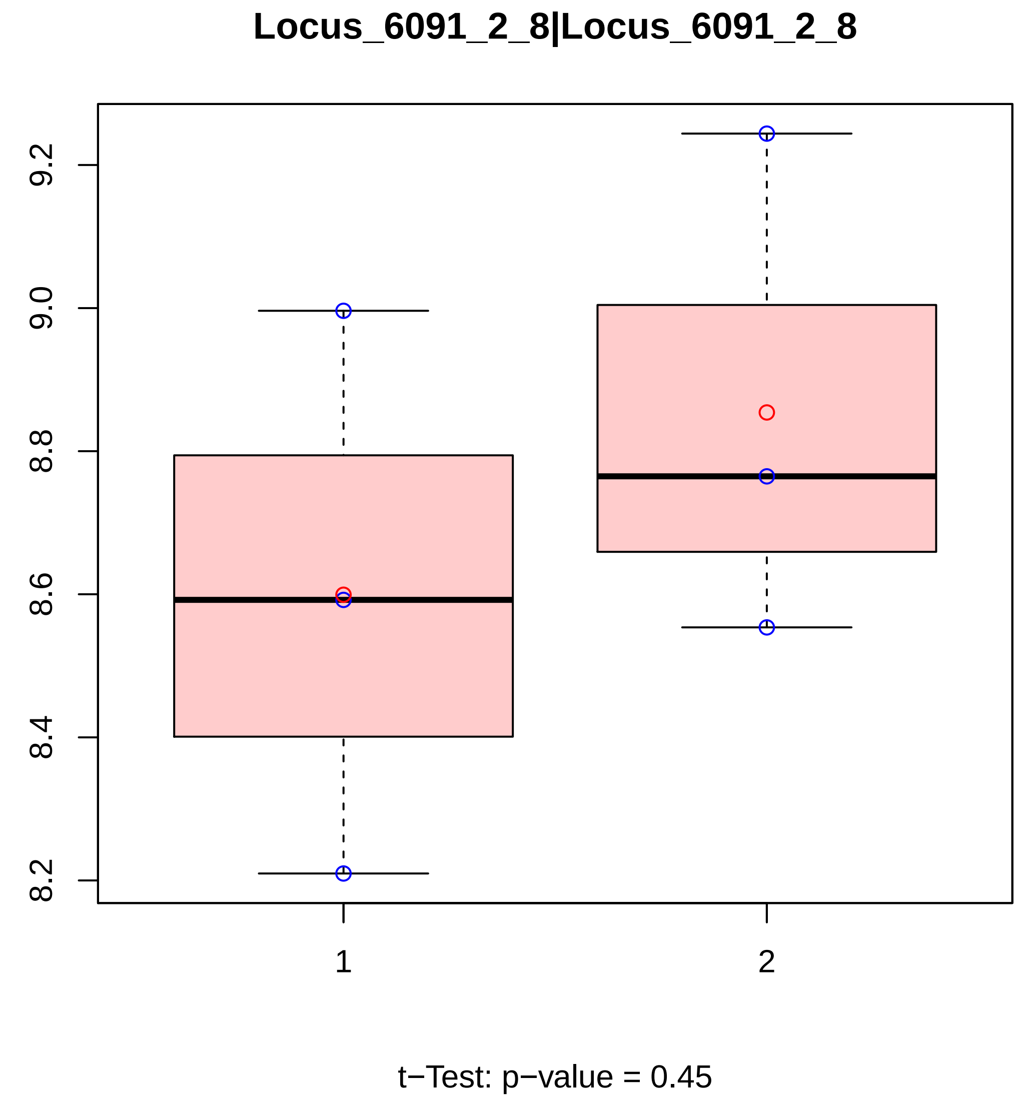
Locus_6091_2_8|Locus_6091_2_8
9.2
9.0
8.8
8.6
8.4
8.2
1
2
t−Test: p−value = 0.45

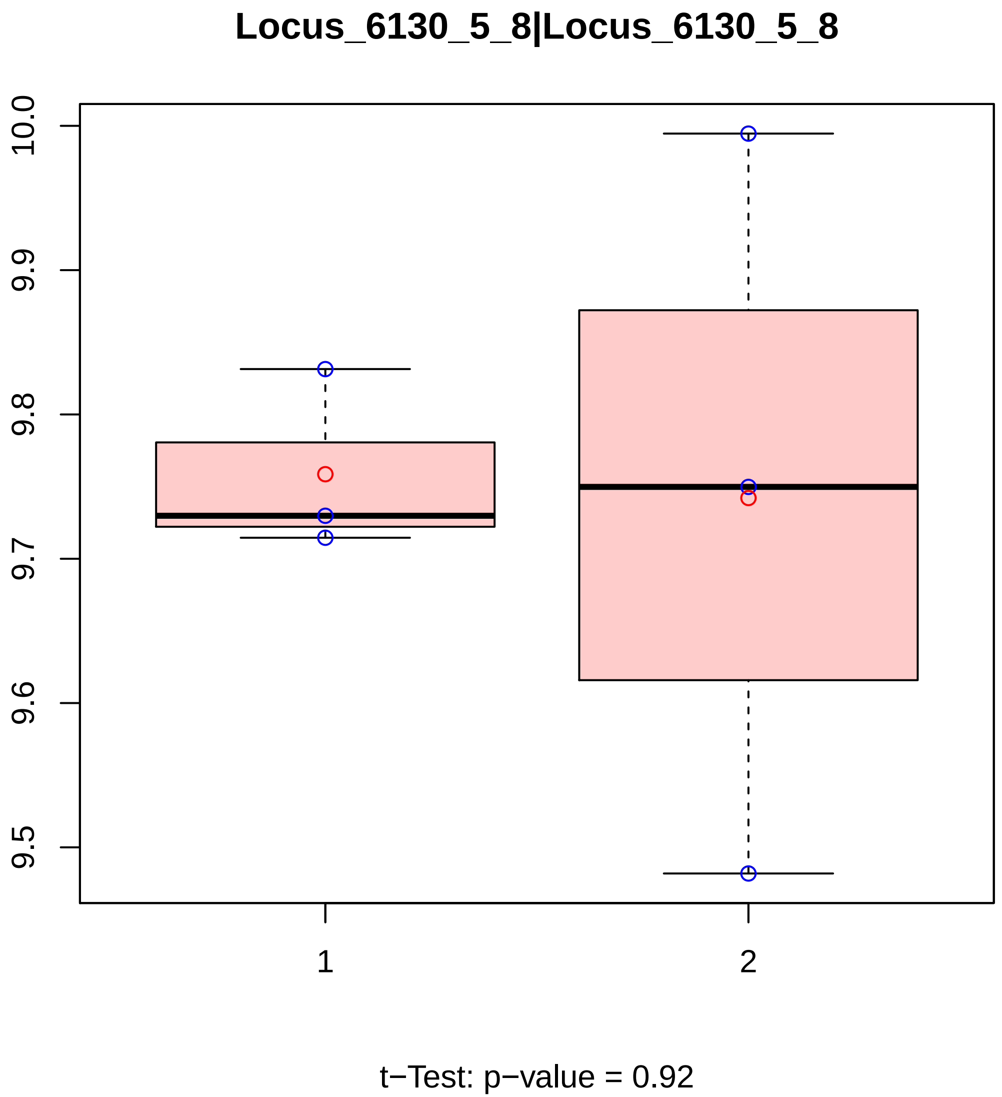
Locus_6130_5_8|Locus_6130_5_8
10.0
9.9
9.8
9.7
9.6
9.5
1
2
t−Test: p−value = 0.92

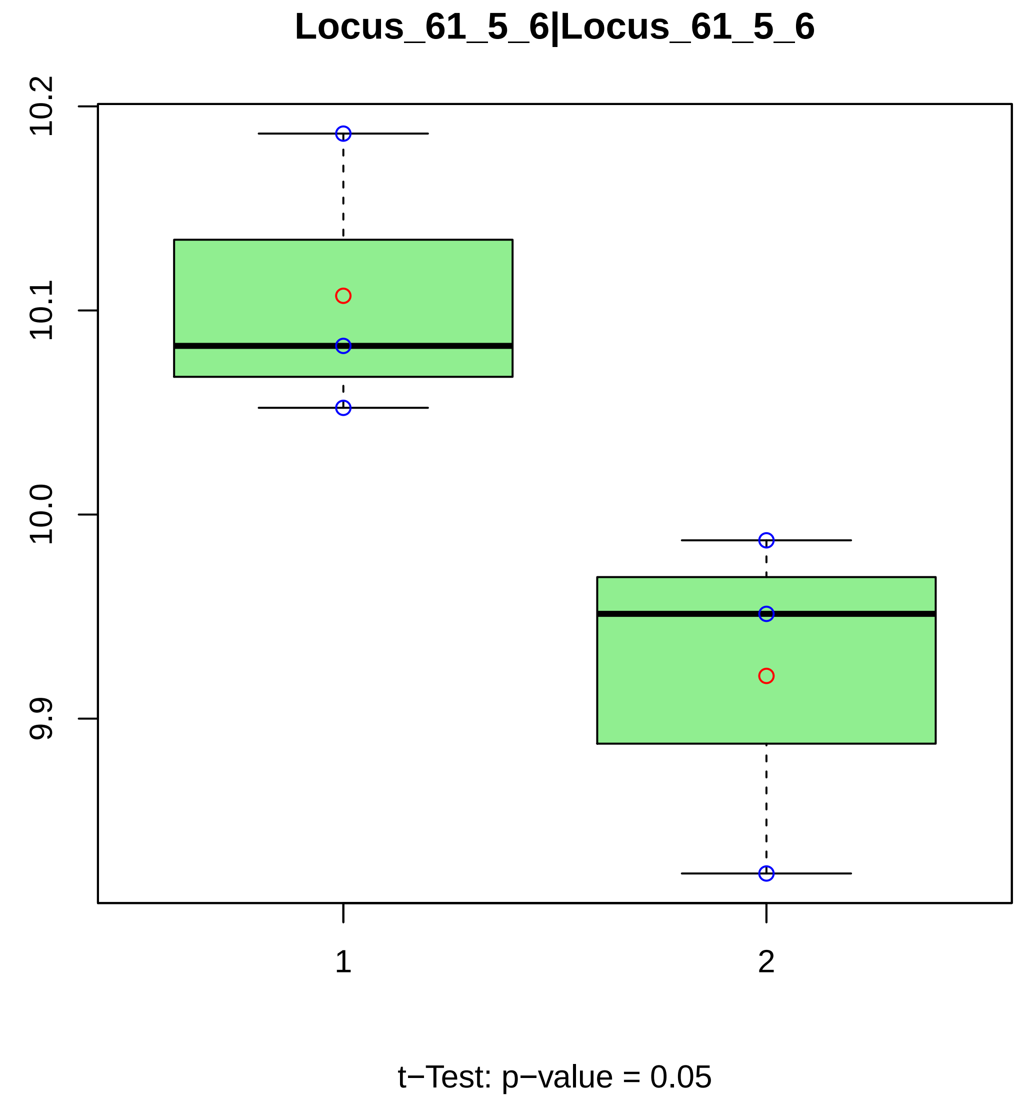

Locus_61_5_6|Locus_61_5_6
10.2
10.1
10.0
9.9
1
2
t−Test: p−value = 0.05

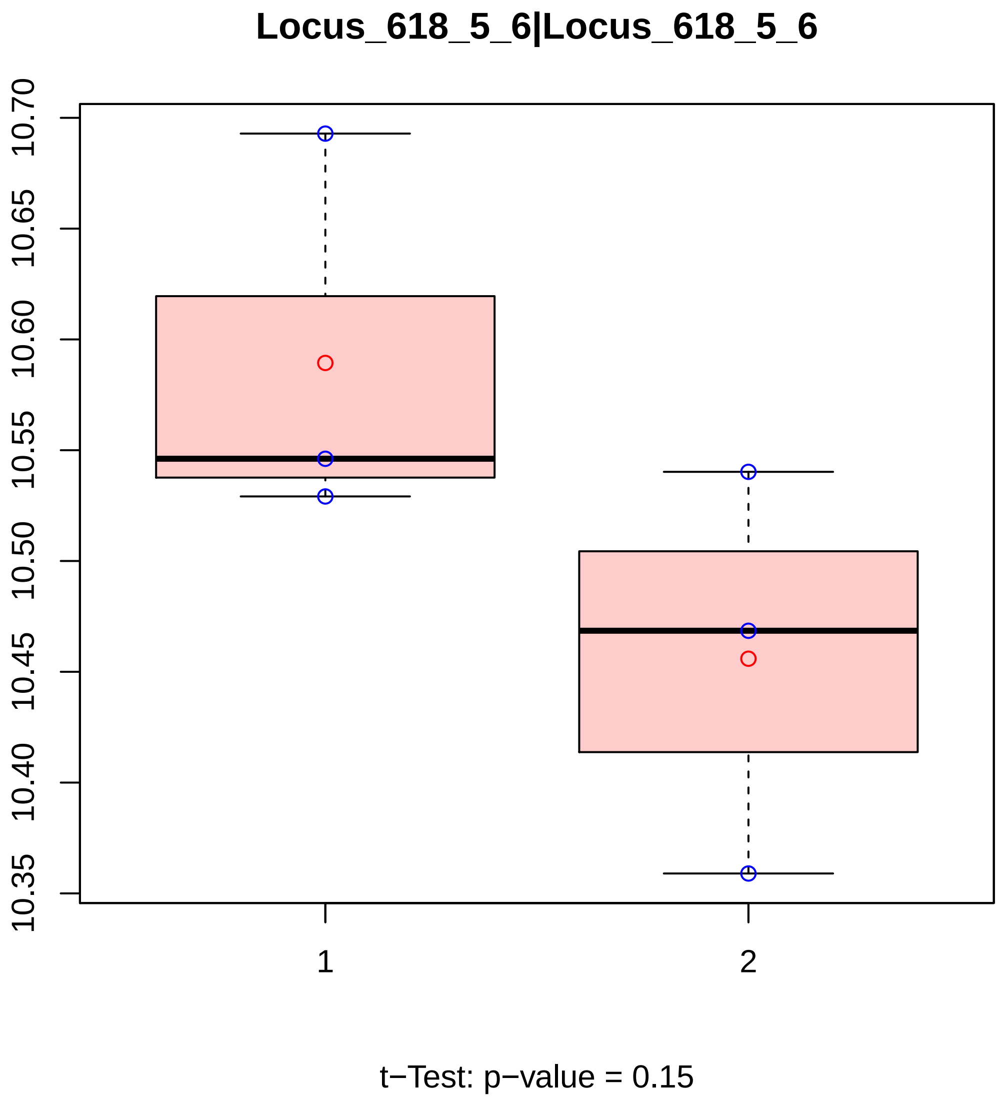

Locus_618_5_6|Locus_618_5_6
10.35
10.40
10.45
10.50
10.55
10.60
10.65
10.70
1
2
t−Test: p−value = 0.15

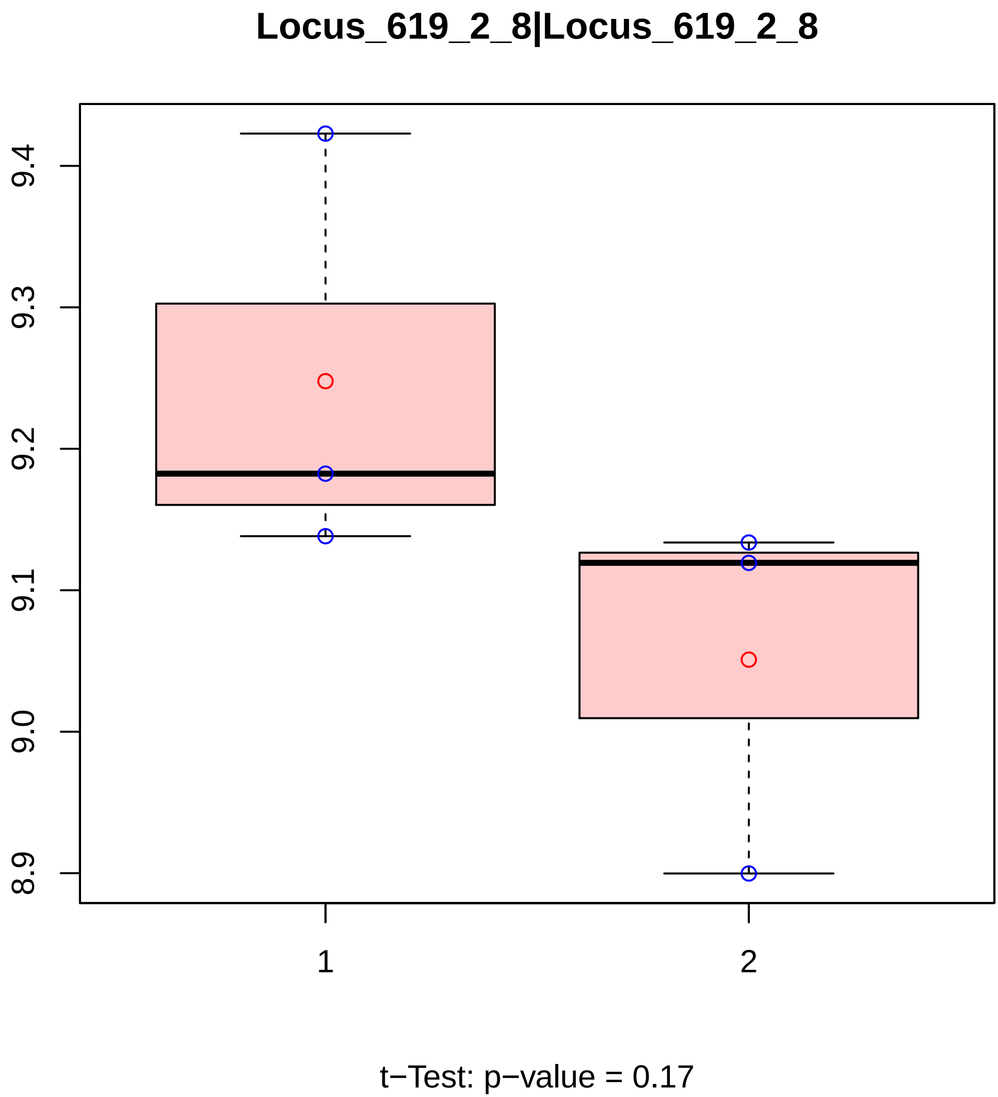

Locus_619_2_8|Locus_619_2_8
9.4
9.3
9.2
9.1
9.0
8.9
1
2
t−Test: p−value = 0.17

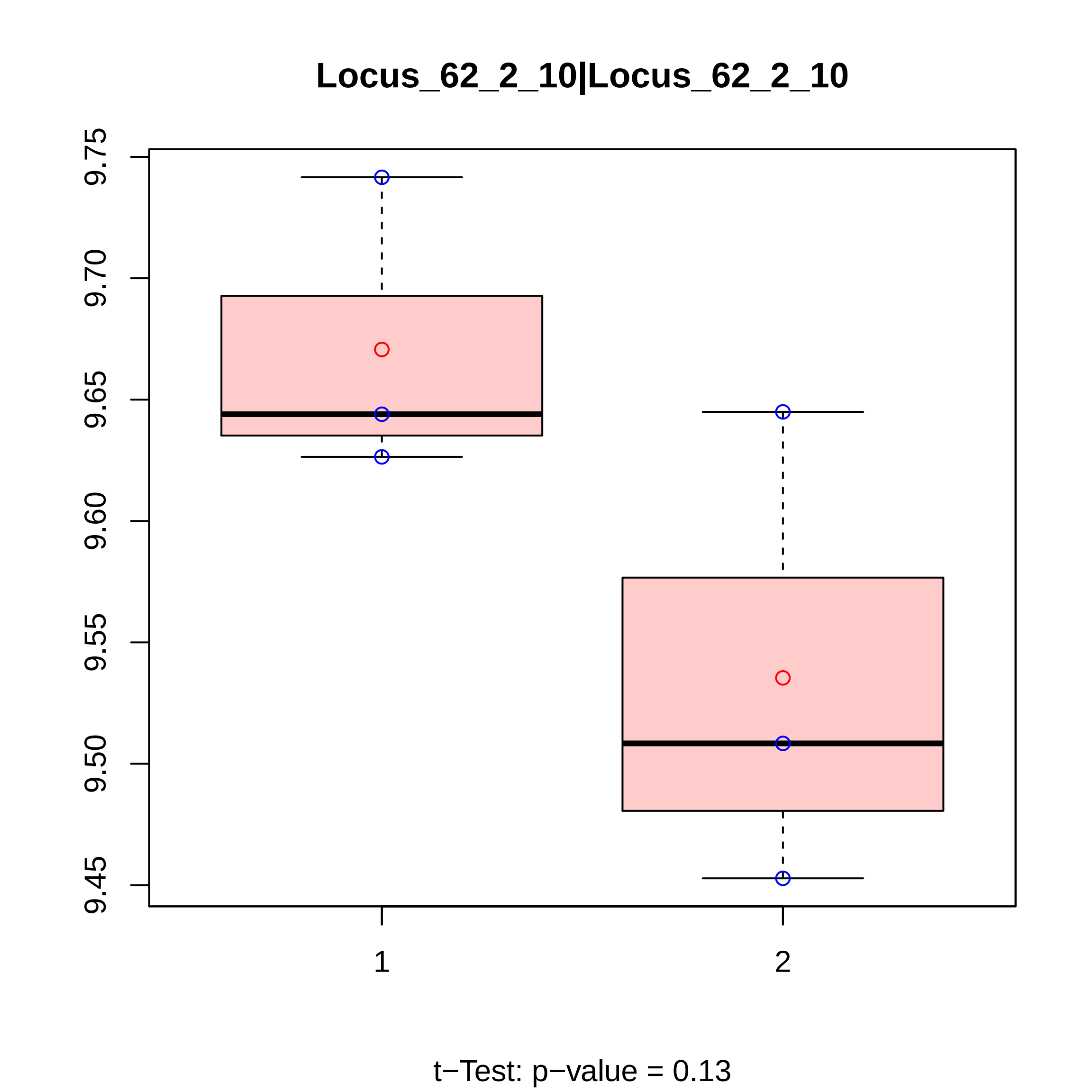

Locus_62_2_10|Locus_62_2_10
9.75
9.70
9.65
9.60
9.55
9.50
9.45
1
2
t−Test: p−value = 0.13

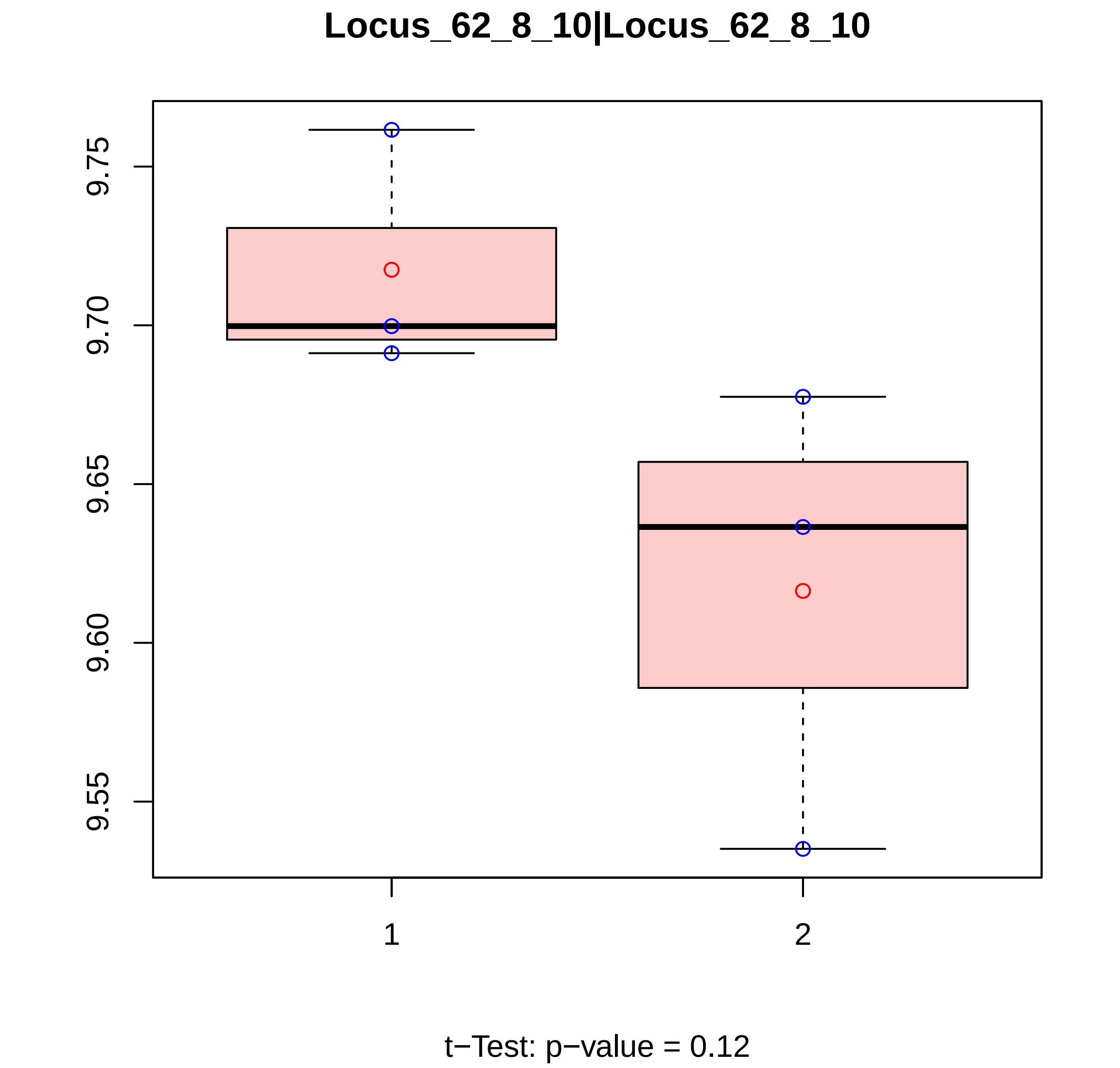
Locus_62_8_10|Locus_62_8_10
9.75
9.70
9.65
9.60
9.55
1
2
t−Test: p−value = 0.12

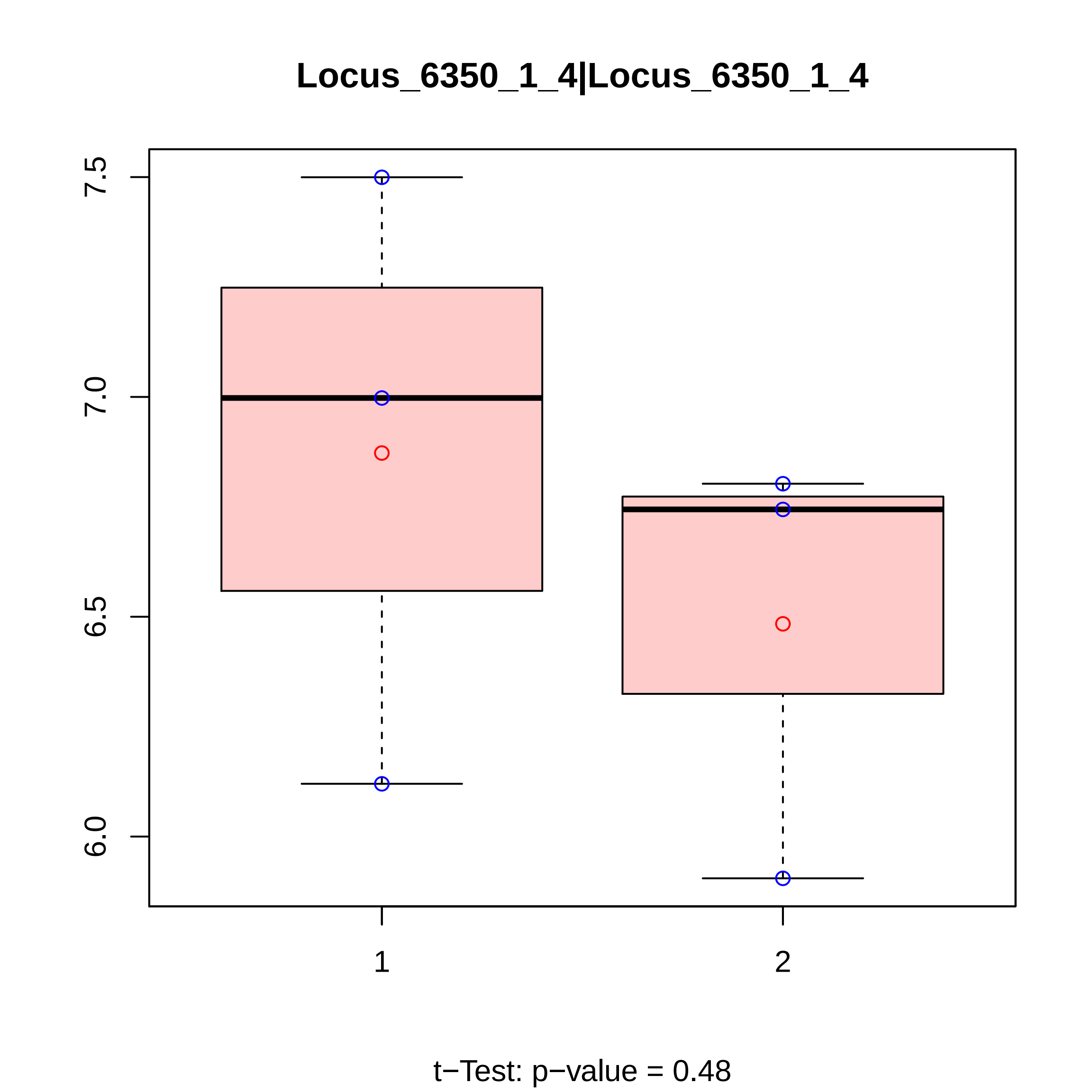
Locus_6350_1_4|Locus_6350_1_4
7.5
7.0
6.5
6.0
1
2
t−Test: p−value = 0.48

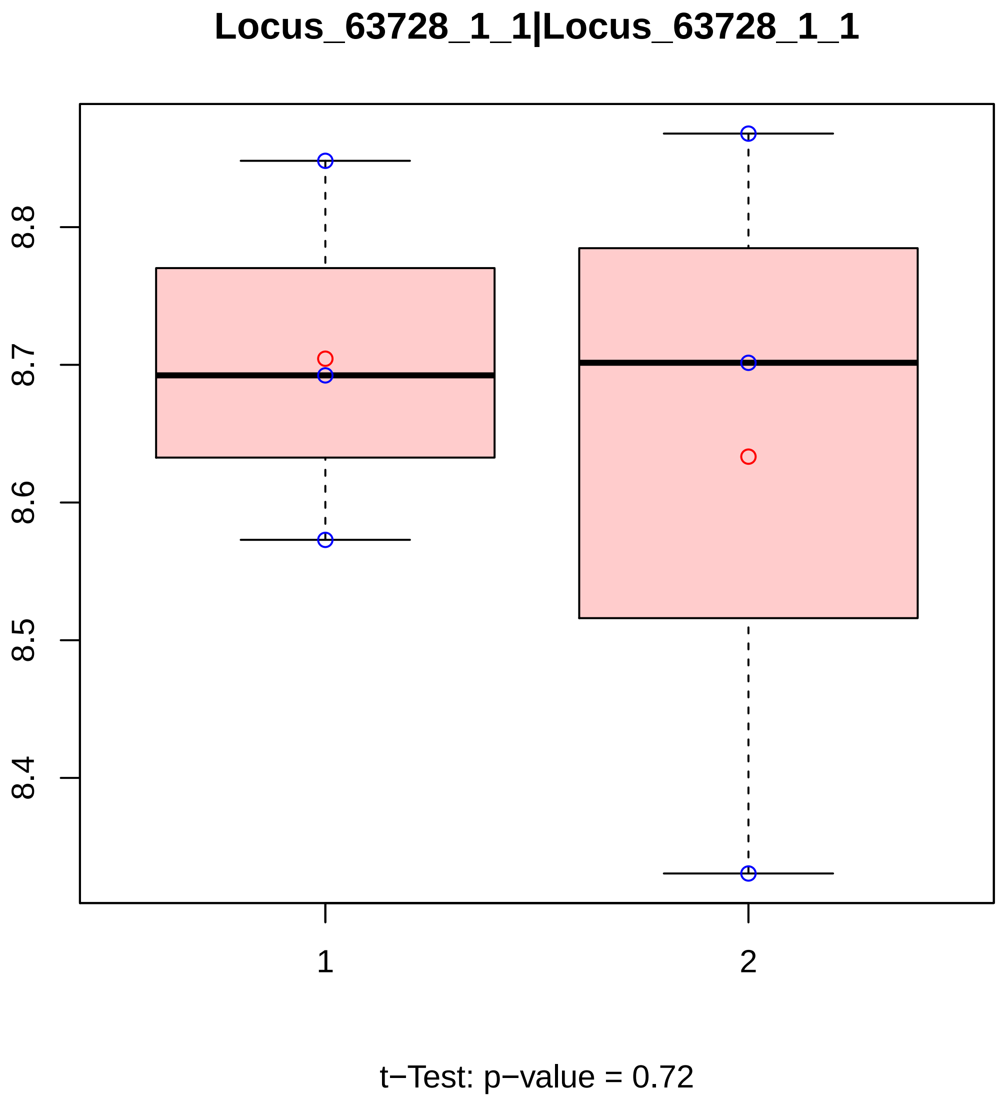

Locus_63728_1_1|Locus_63728_1_1
8.4
8.5
8.6
8.7
8.8
1
2
t−Test: p−value = 0.72

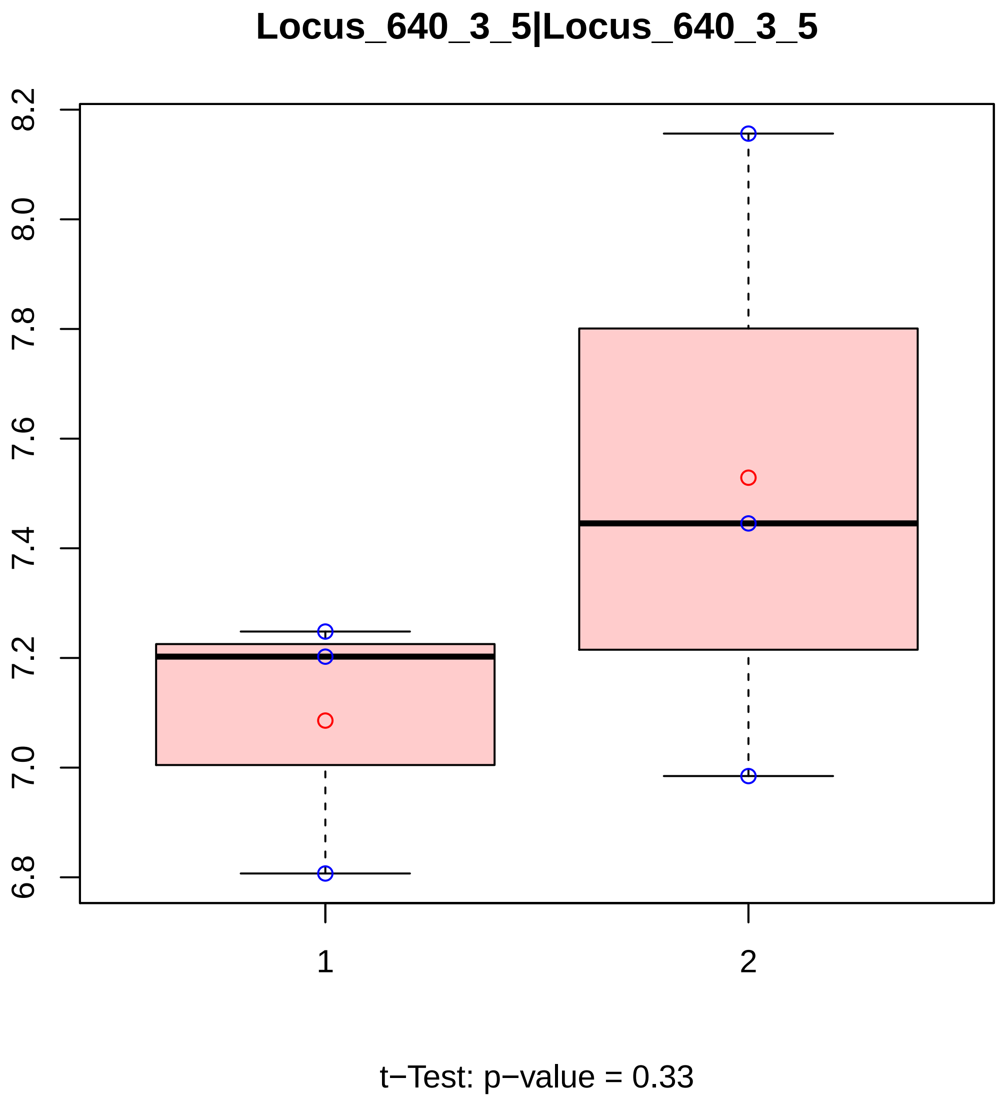
Locus_640_3_5|Locus_640_3_5
8.2
8.0
7.8
7.6
7.4
7.2
7.0
6.8
1
2
t−Test: p−value = 0.33

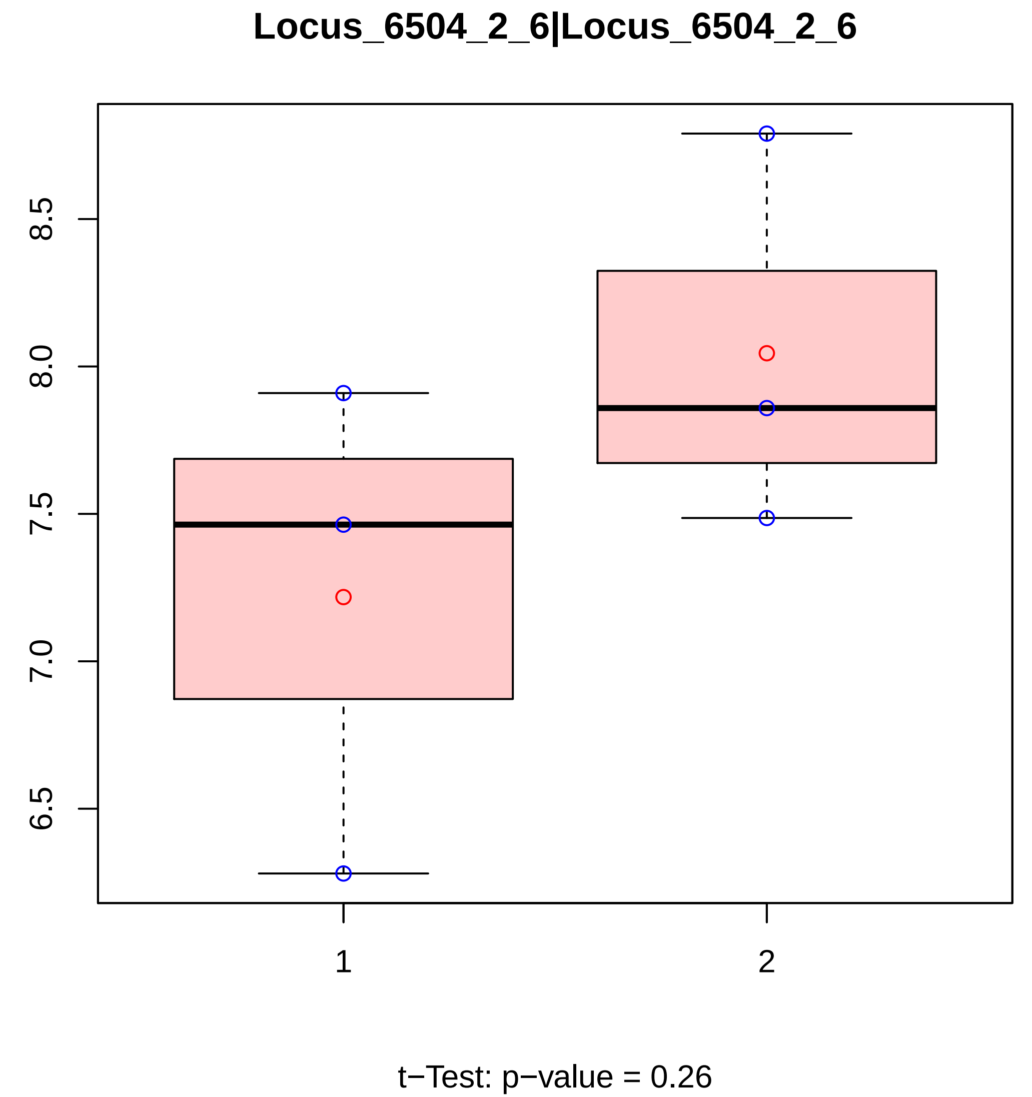

Locus_6504_2_6|Locus_6504_2_6
6.5
7.0
7.5
8.0
8.5
1
2
t−Test: p−value = 0.26

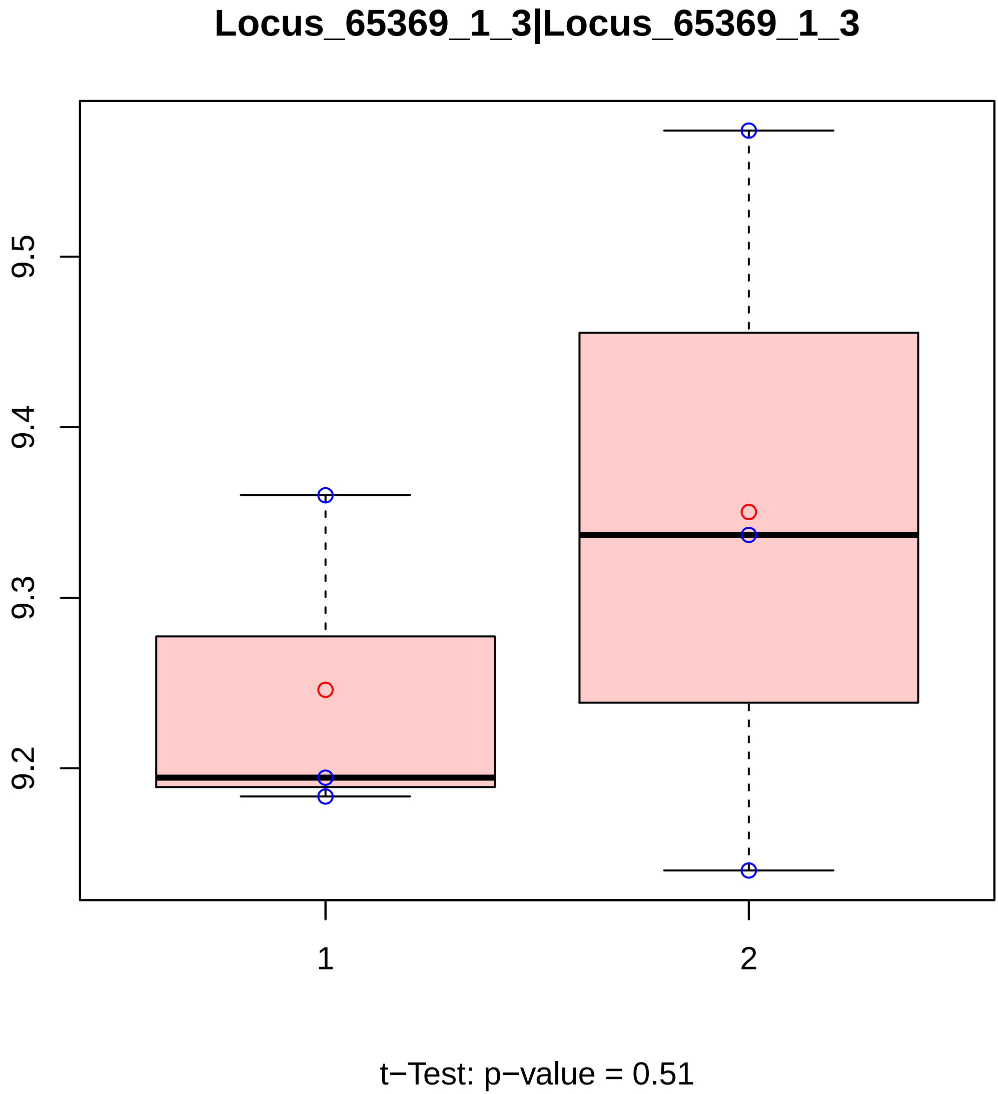
Locus_65369_1_3|Locus_65369_1_3
9.2
9.3
9.4
9.5
1
2
t−Test: p−value = 0.51

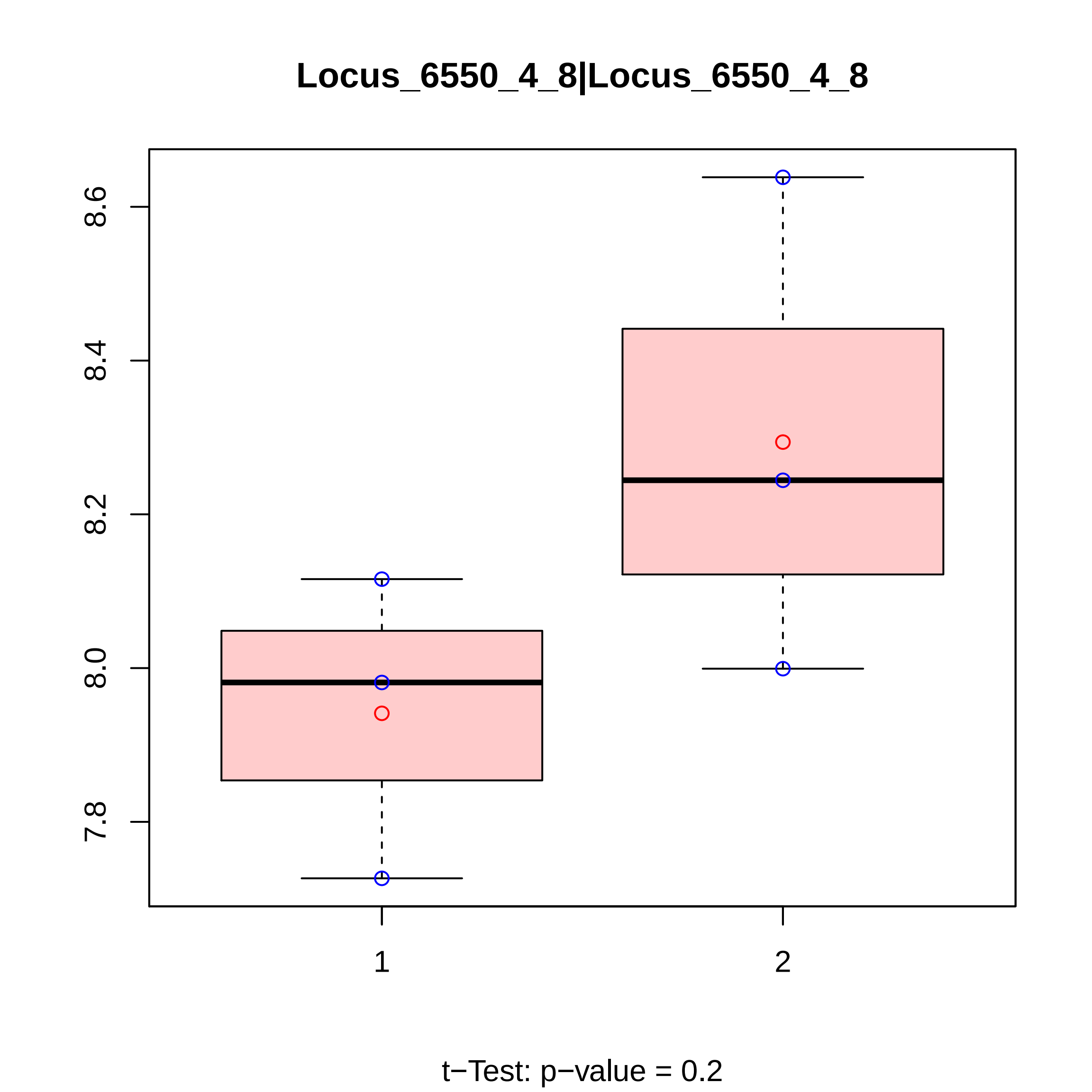
Locus_6550_4_8|Locus_6550_4_8
8.6
8.4
8.2
8.0
7.8
1
2
t−Test: p−value = 0.2

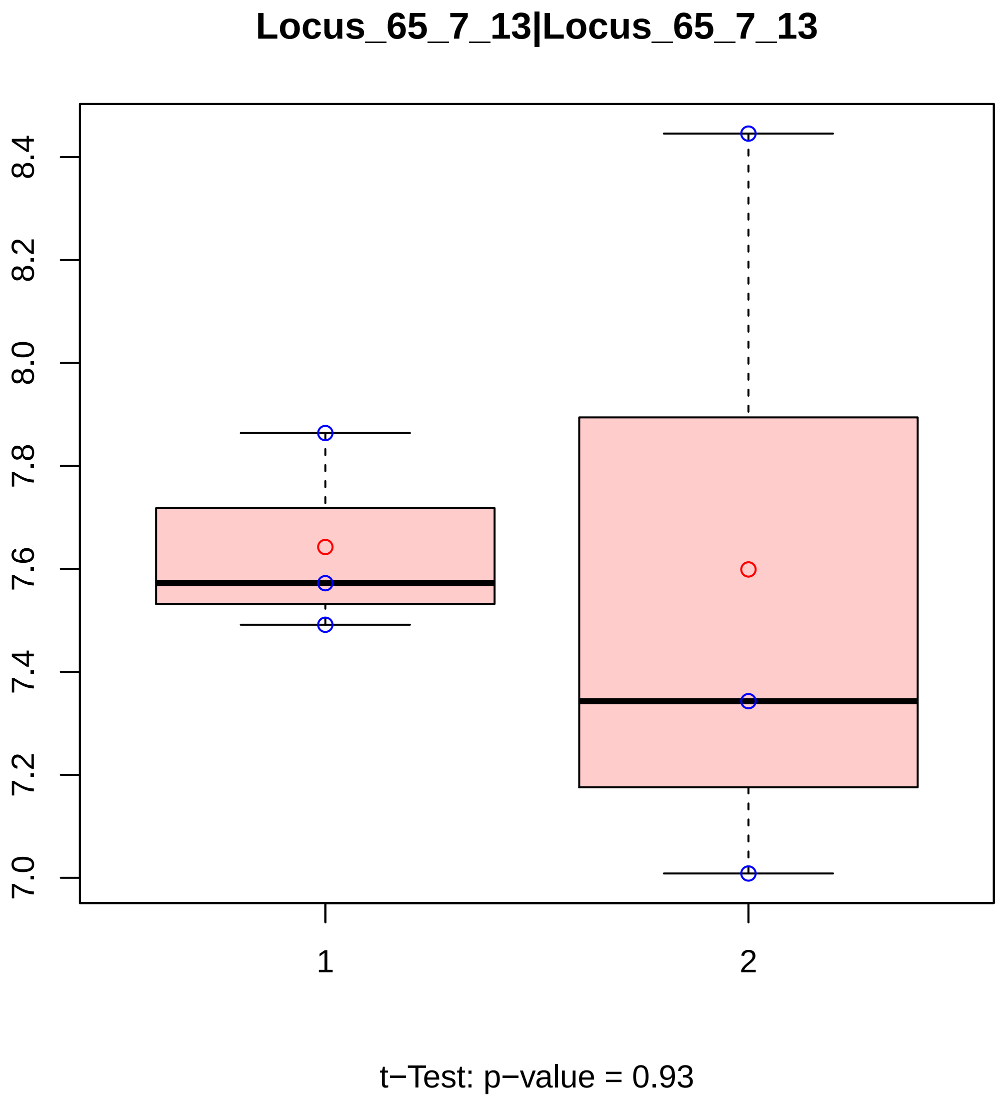

Locus_65_7_13|Locus_65_7_13
8.4
8.2
8.0
7.8
7.6
7.4
7.2
7.0
1
2
t−Test: p−value = 0.93

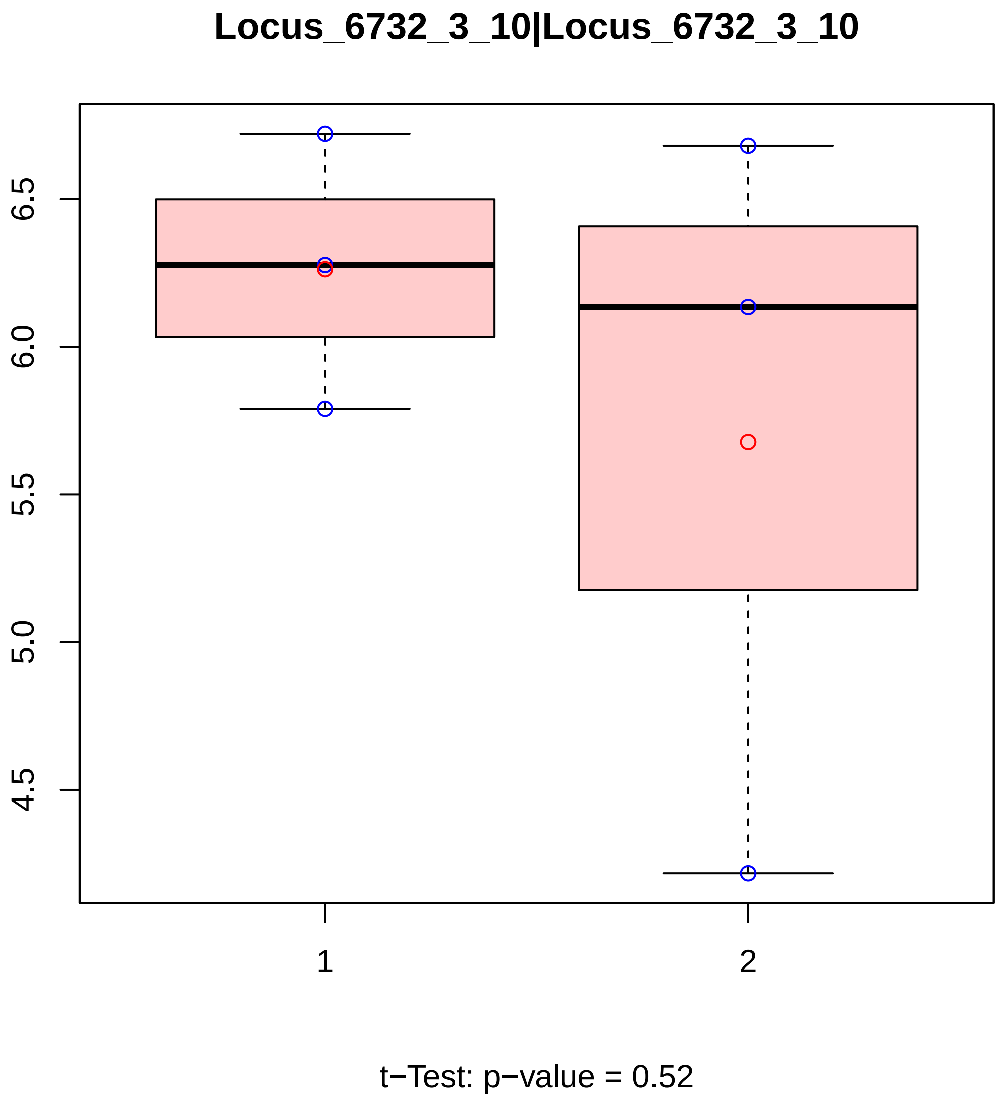

Locus_6732_3_10|Locus_6732_3_10
6.5
6.0
5.5
5.0
4.5
1
2
t−Test: p−value = 0.52

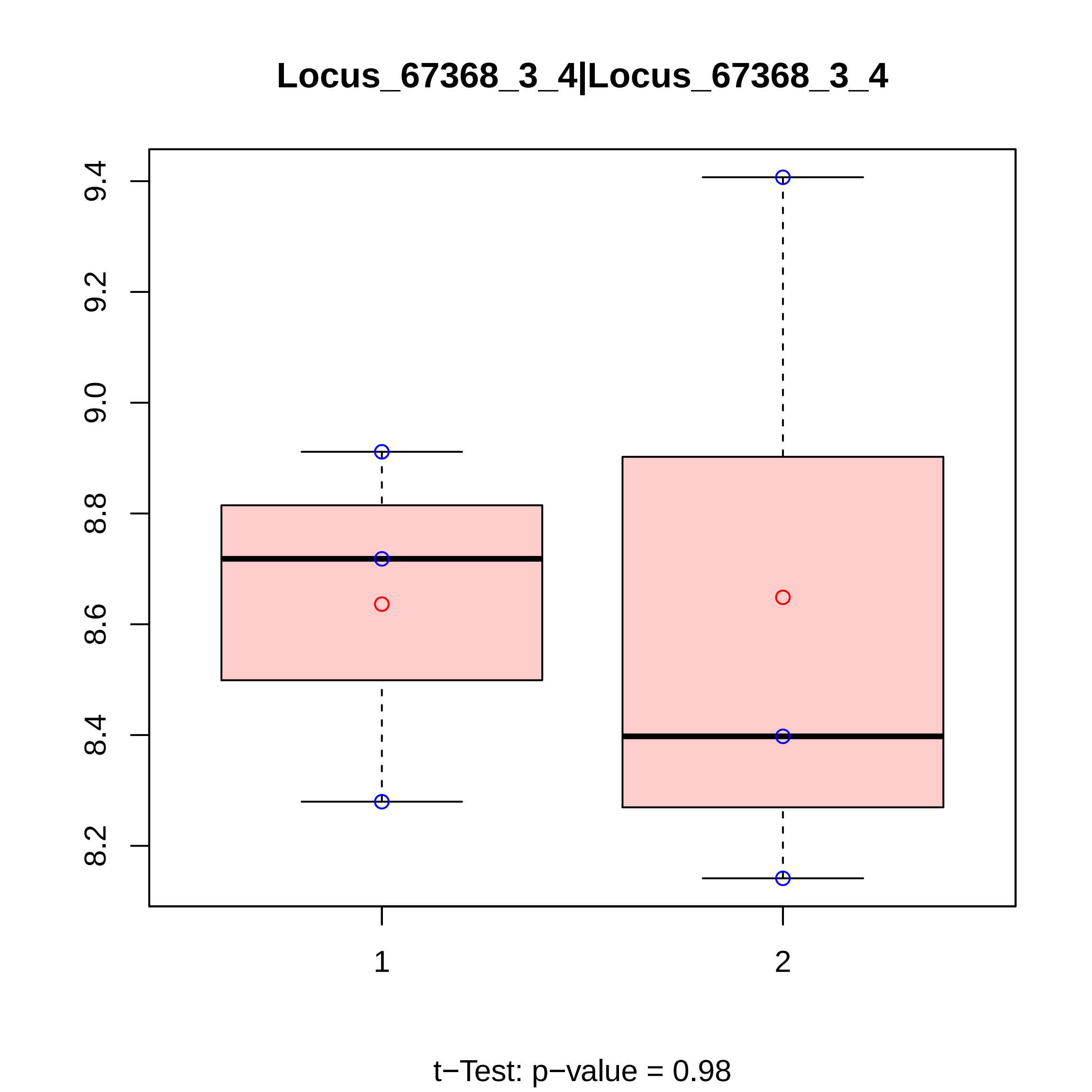

Locus_67368_3_4|Locus_67368_3_4
9.4
9.2
9.0
8.8
8.6
8.4
8.2
1
2
t−Test: p−value = 0.98

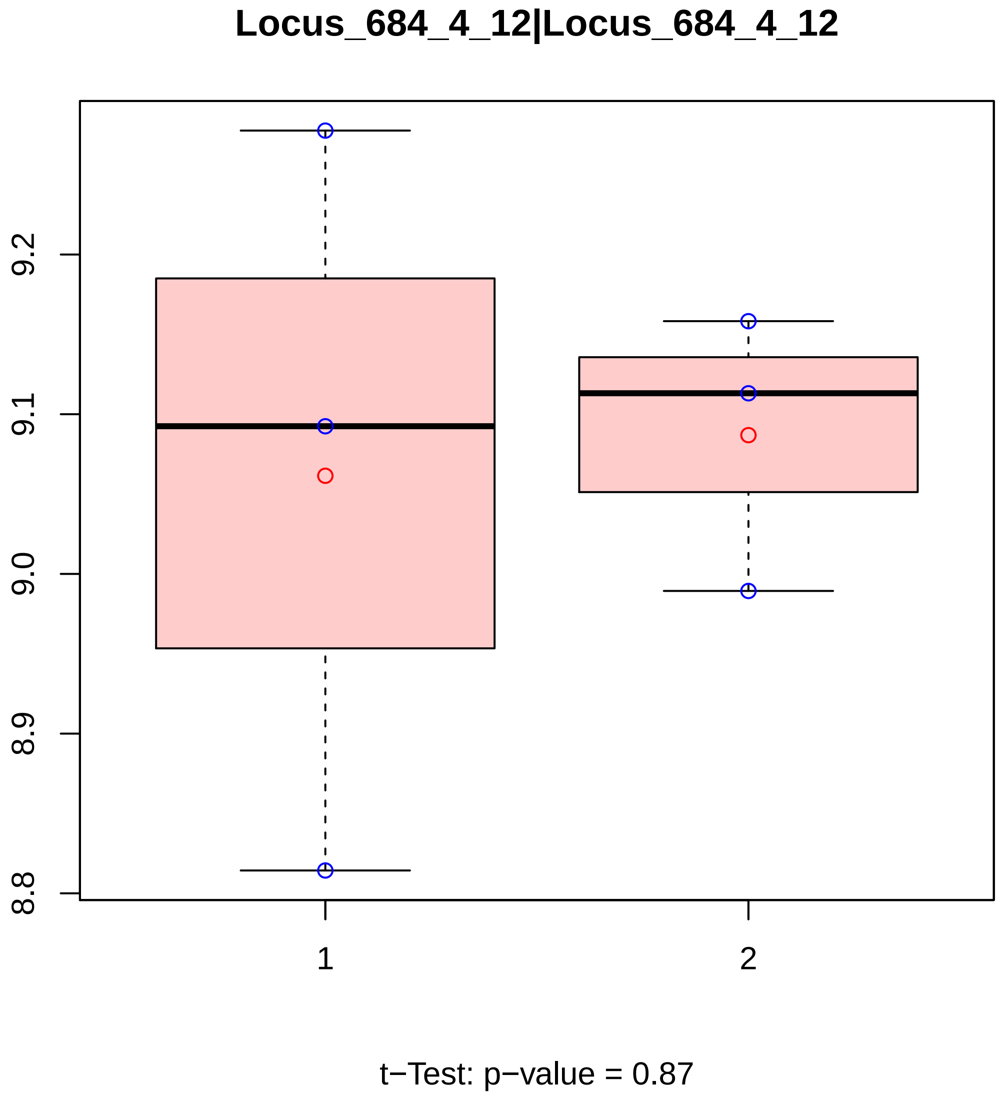

Locus_684_4_12|Locus_684_4_12
9.2
9.1
9.0
8.9
8.8
1
2
t−Test: p−value = 0.87

# Locus_684_8_12|Locus_684_8_12

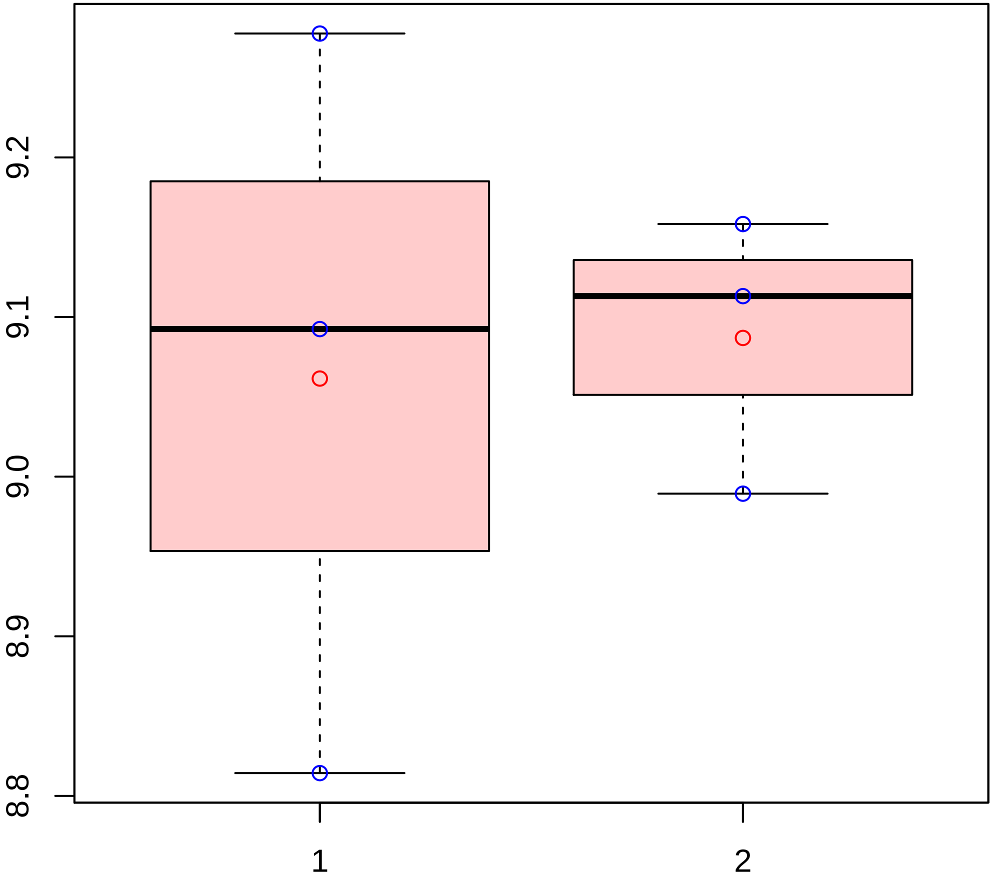


t−Test: p−value = 0.87

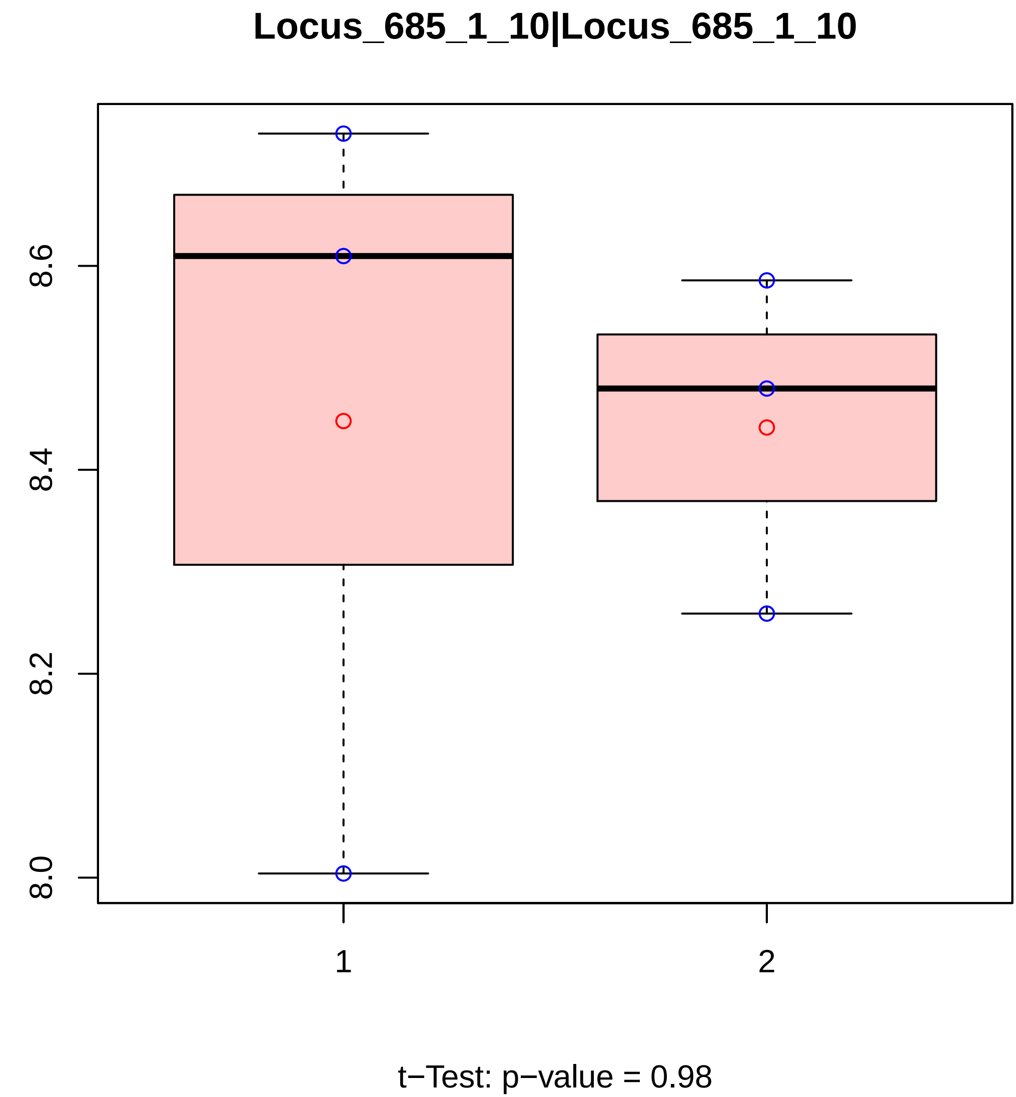

Locus_685_1_10|Locus_685_1_10
8.0
8.2
8.4
8.6
1
2
t−Test: p−value = 0.98

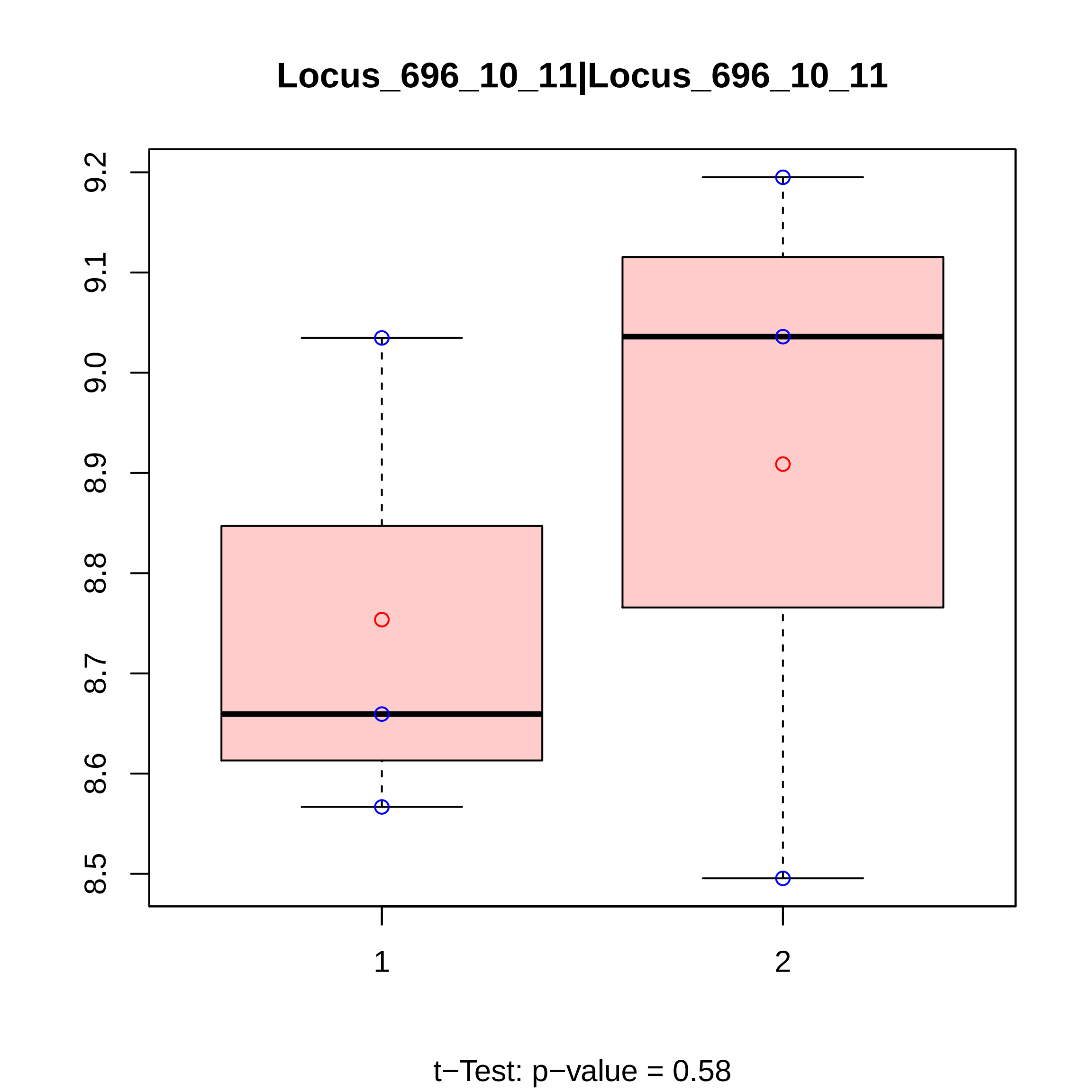
Locus_696_10_11|Locus_696_10_11
9.2
9.1
9.0
8.9
8.8
8.7
8.6
8.5
1
2
t−Test: p−value = 0.58

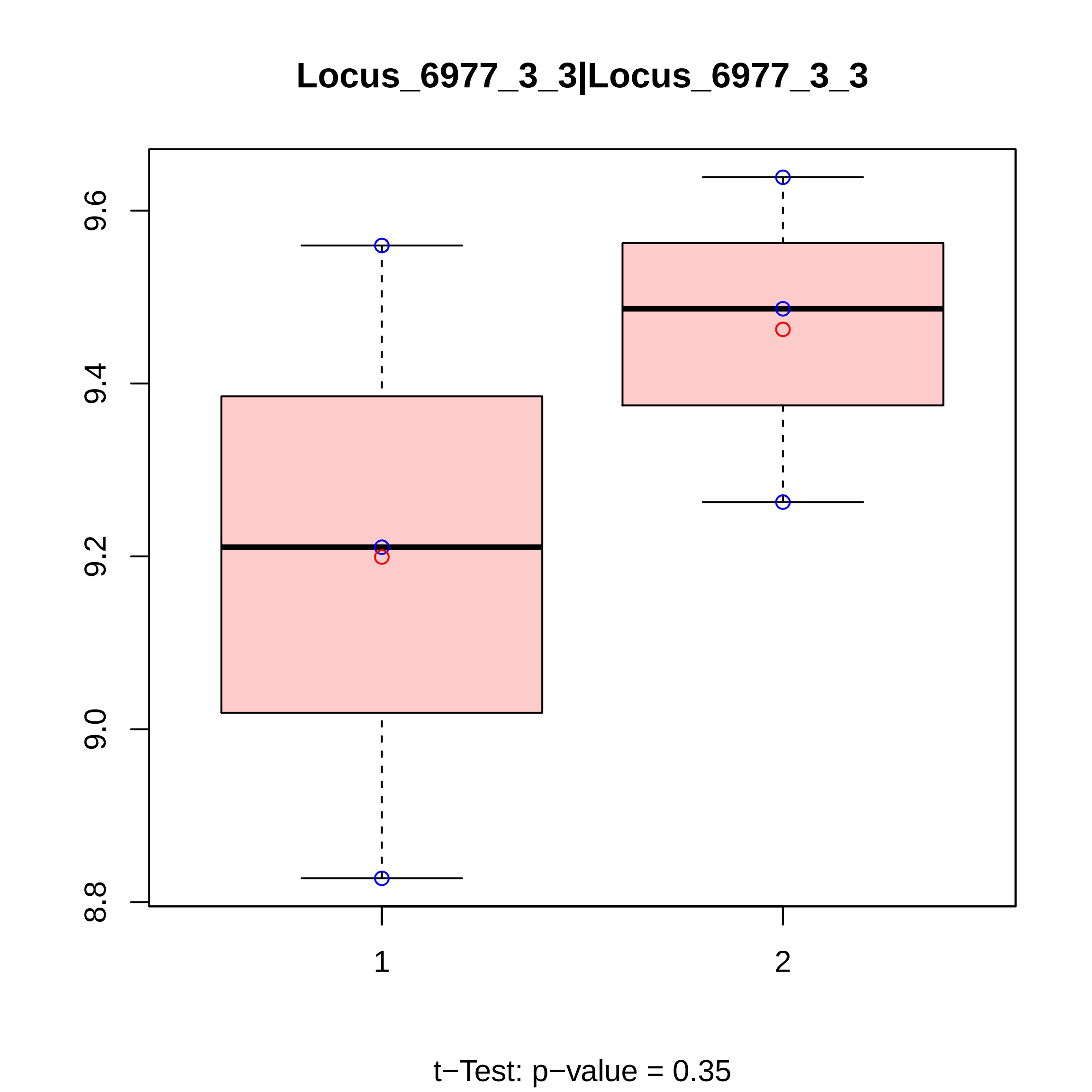
Locus_6977_3_3|Locus_6977_3_3
9.6
9.4
9.2
9.0
8.8
1
2
t−Test: p−value = 0.35

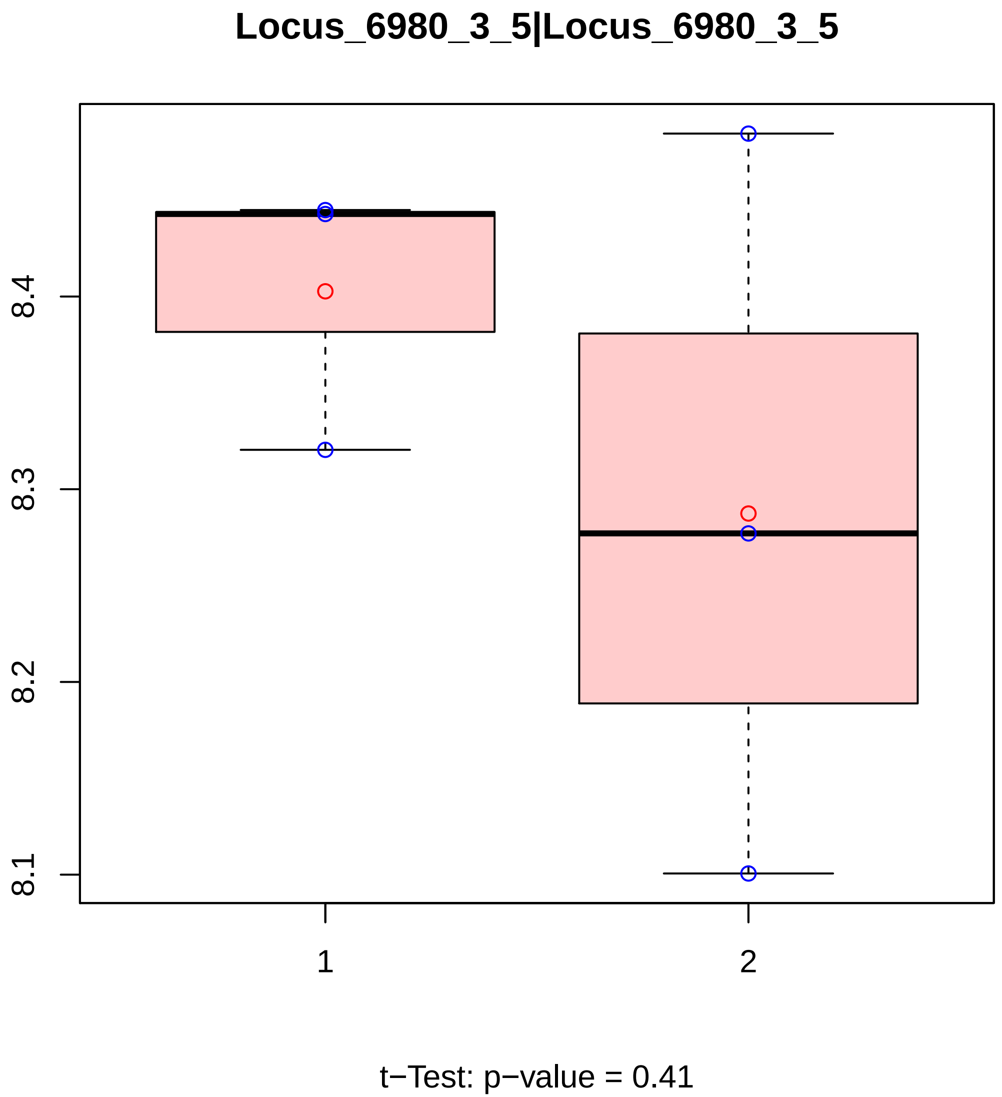
Locus_6980_3_5|Locus_6980_3_5
8.1
8.2
8.3
8.4
1
2
t−Test: p−value = 0.41

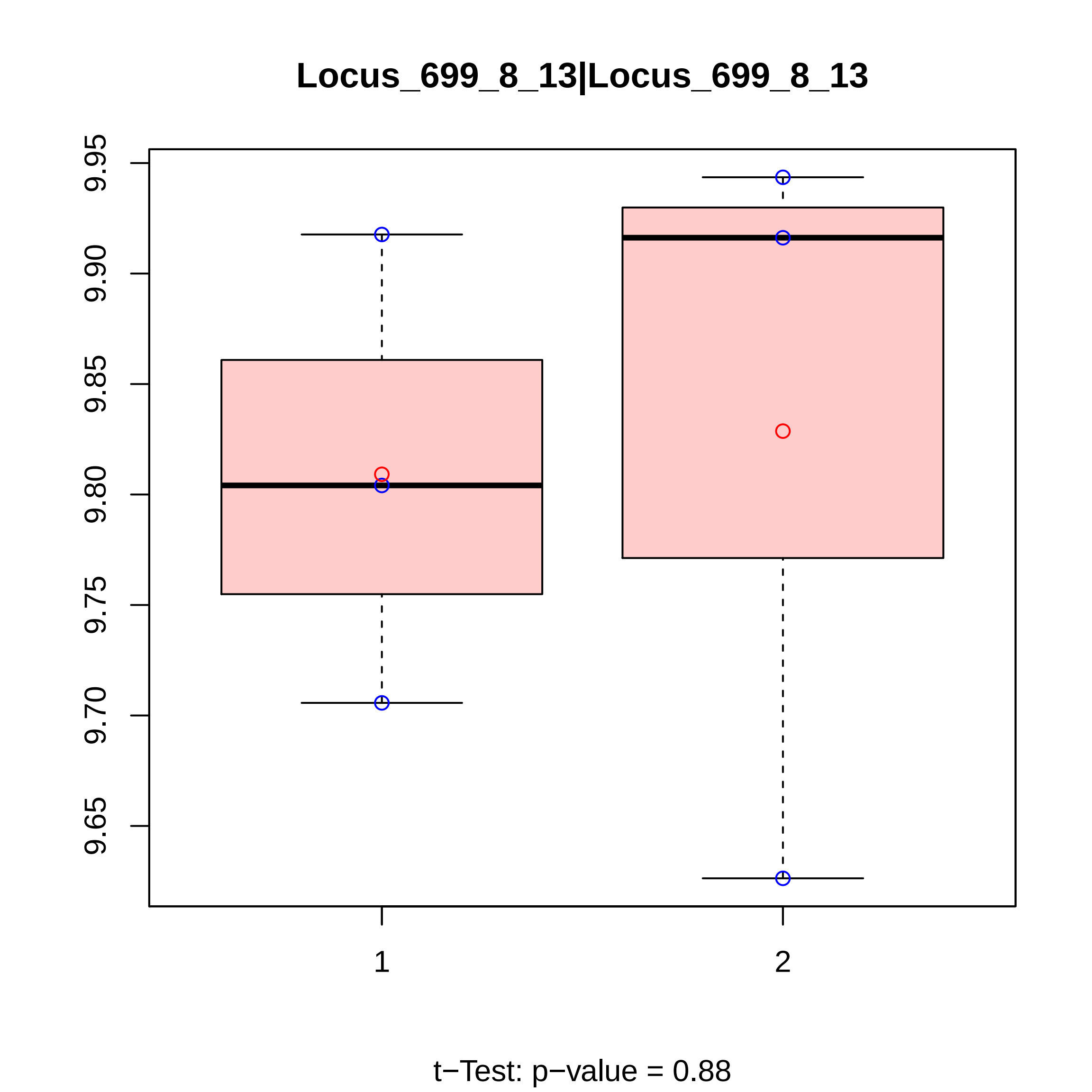
Locus_699_8_13|Locus_699_8_13
9.95
9.90
9.85
9.80
9.75
9.70
9.65
1
2
t−Test: p−value = 0.88

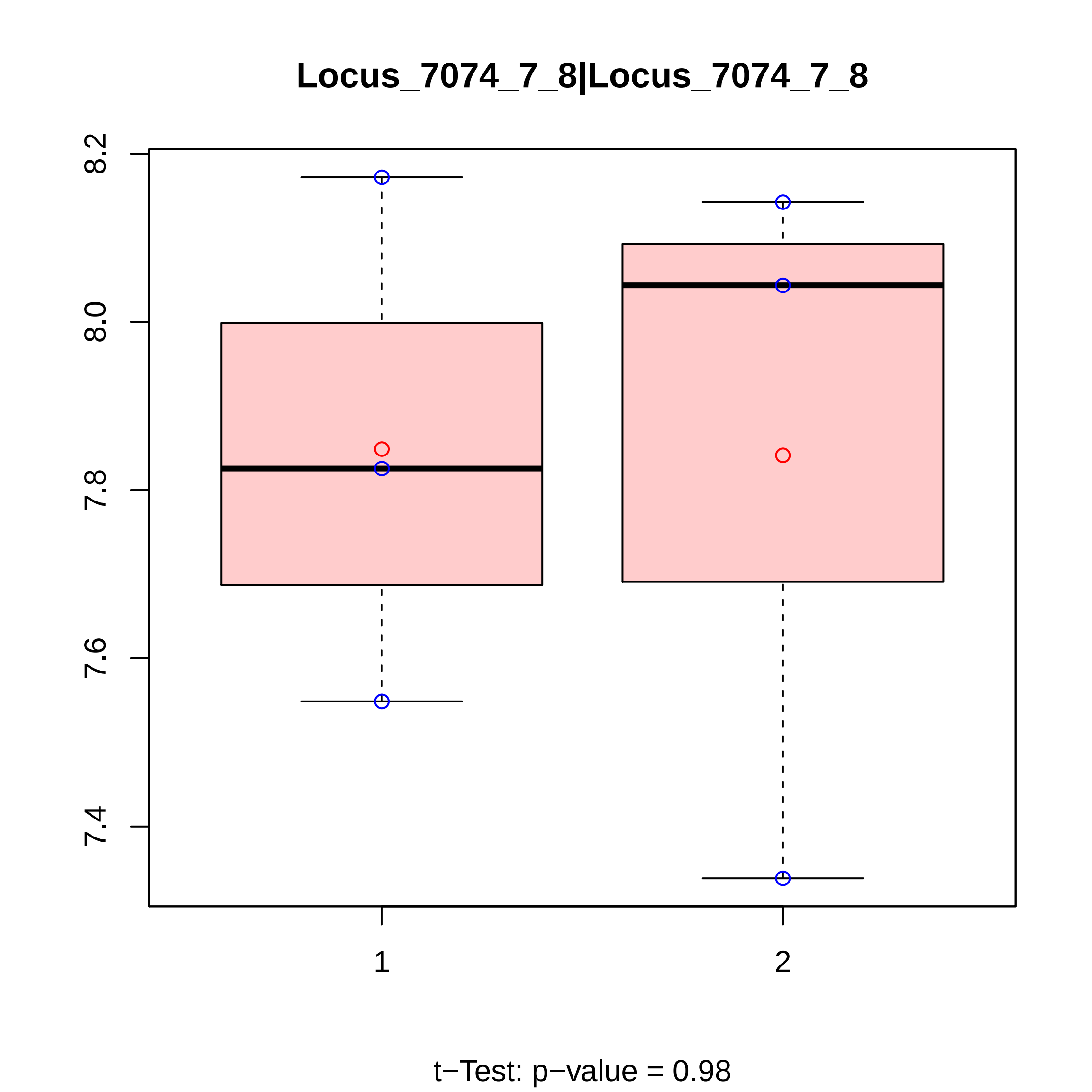

Locus_7074_7_8|Locus_7074_7_8
8.2
8.0
7.8
7.6
7.4
1
2
t−Test: p−value = 0.98

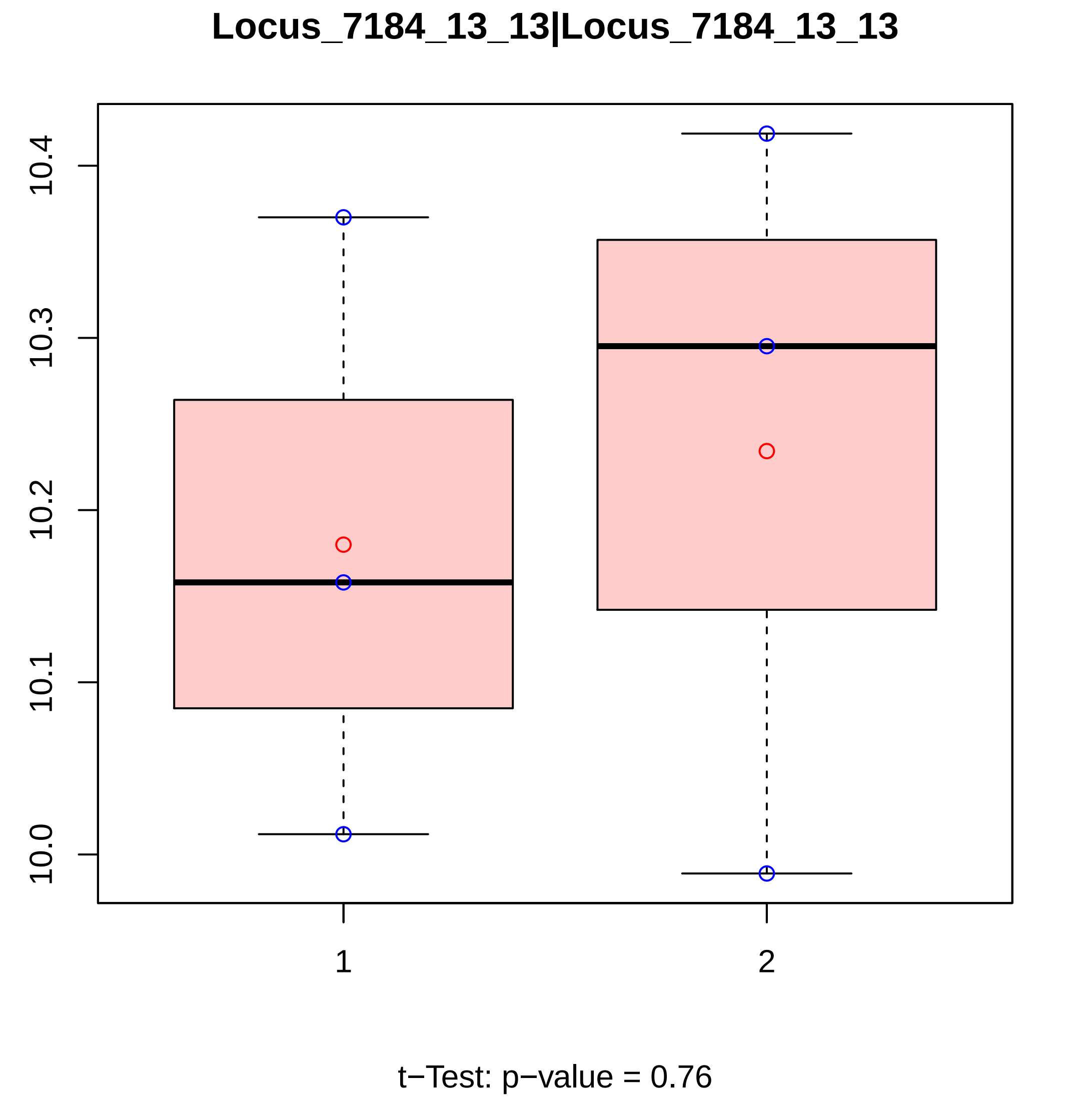

Locus_7184_13_13|Locus_7184_13_13
10.0
10.1
10.2
10.3
10.4
1
2
t−Test: p−value = 0.76

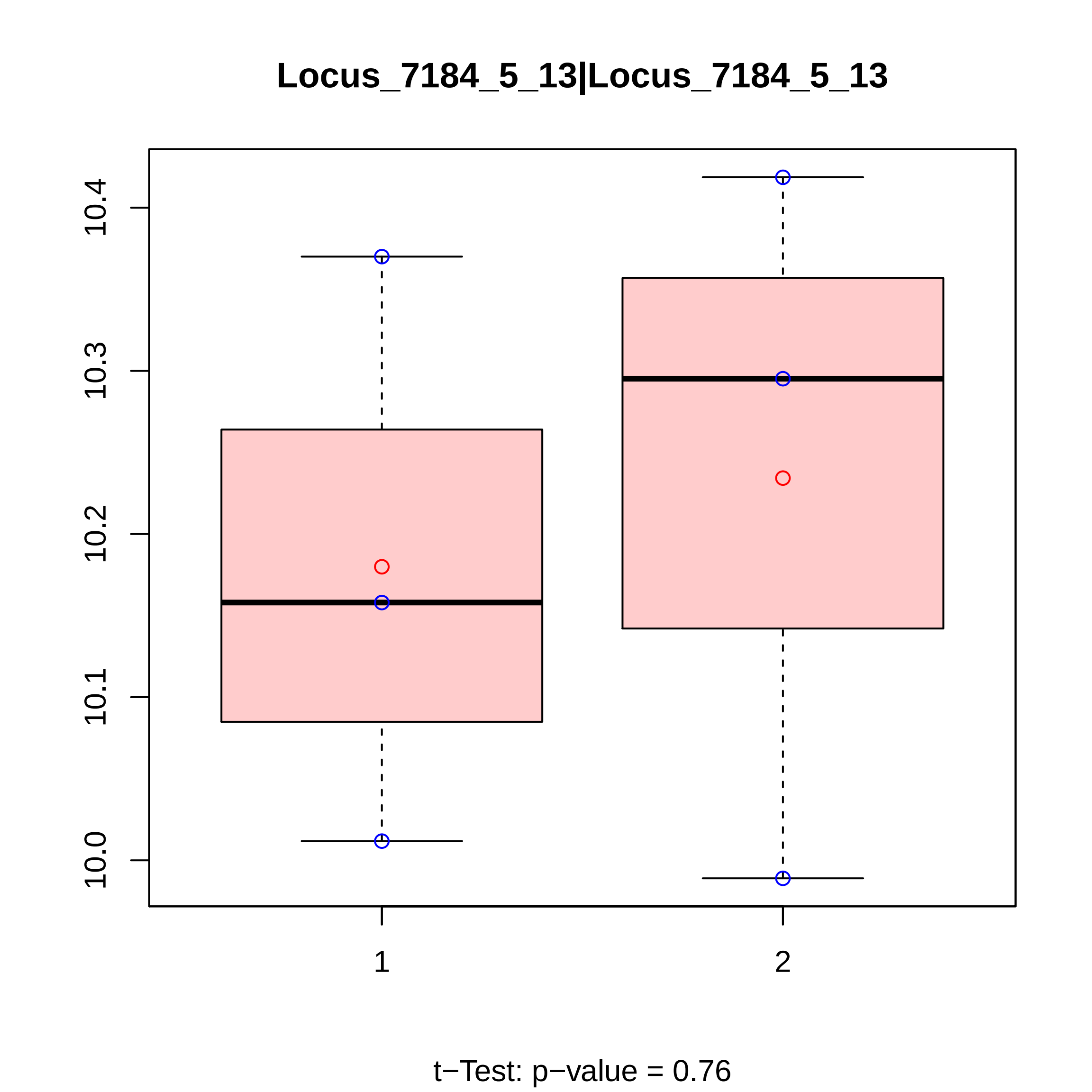

Locus_7184_5_13|Locus_7184_5_13
10.0
10.1
10.2
10.3
10.4
1
2
t−Test: p−value = 0.76

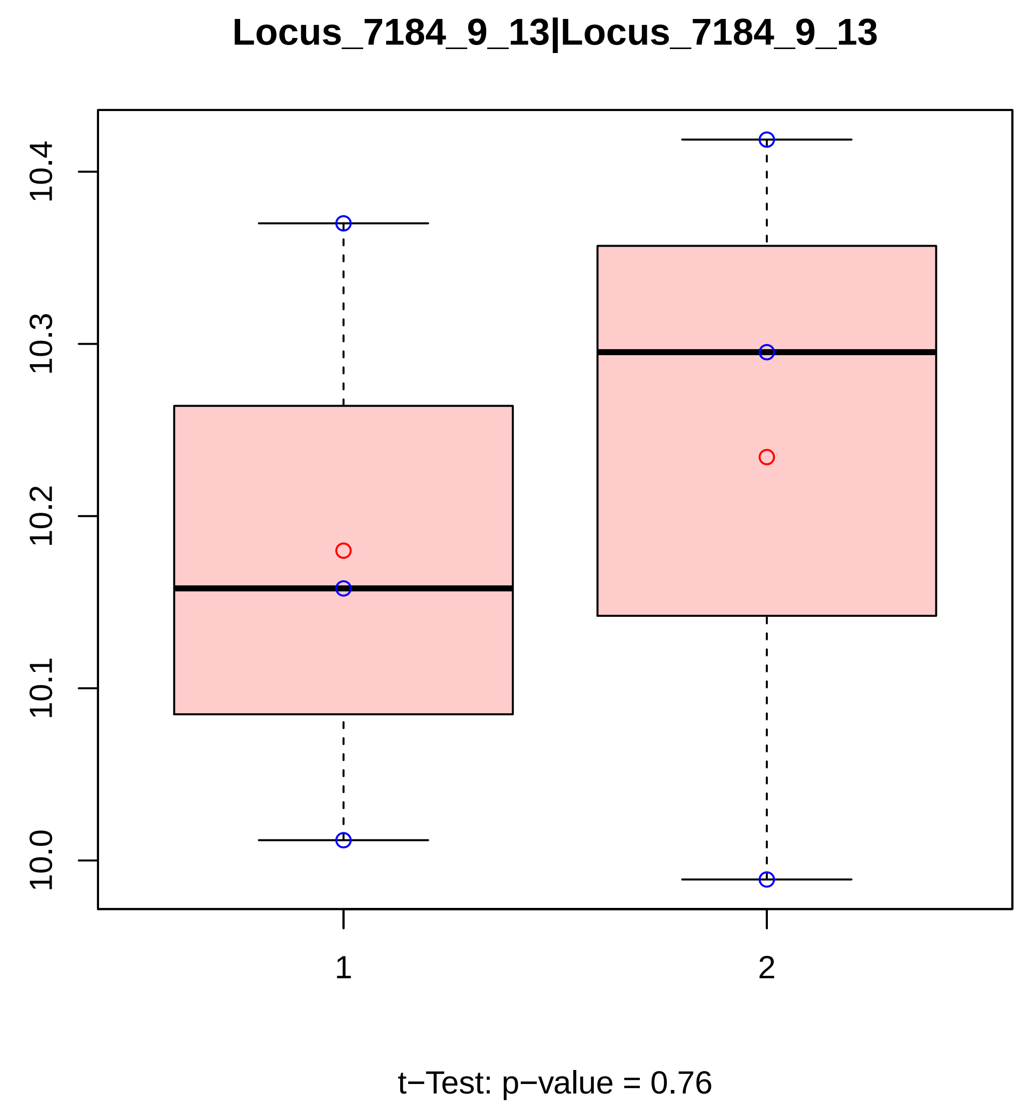
Locus_7184_9_13|Locus_7184_9_13
10.0
10.1
10.2
10.3
10.4
1
2
t−Test: p−value = 0.76

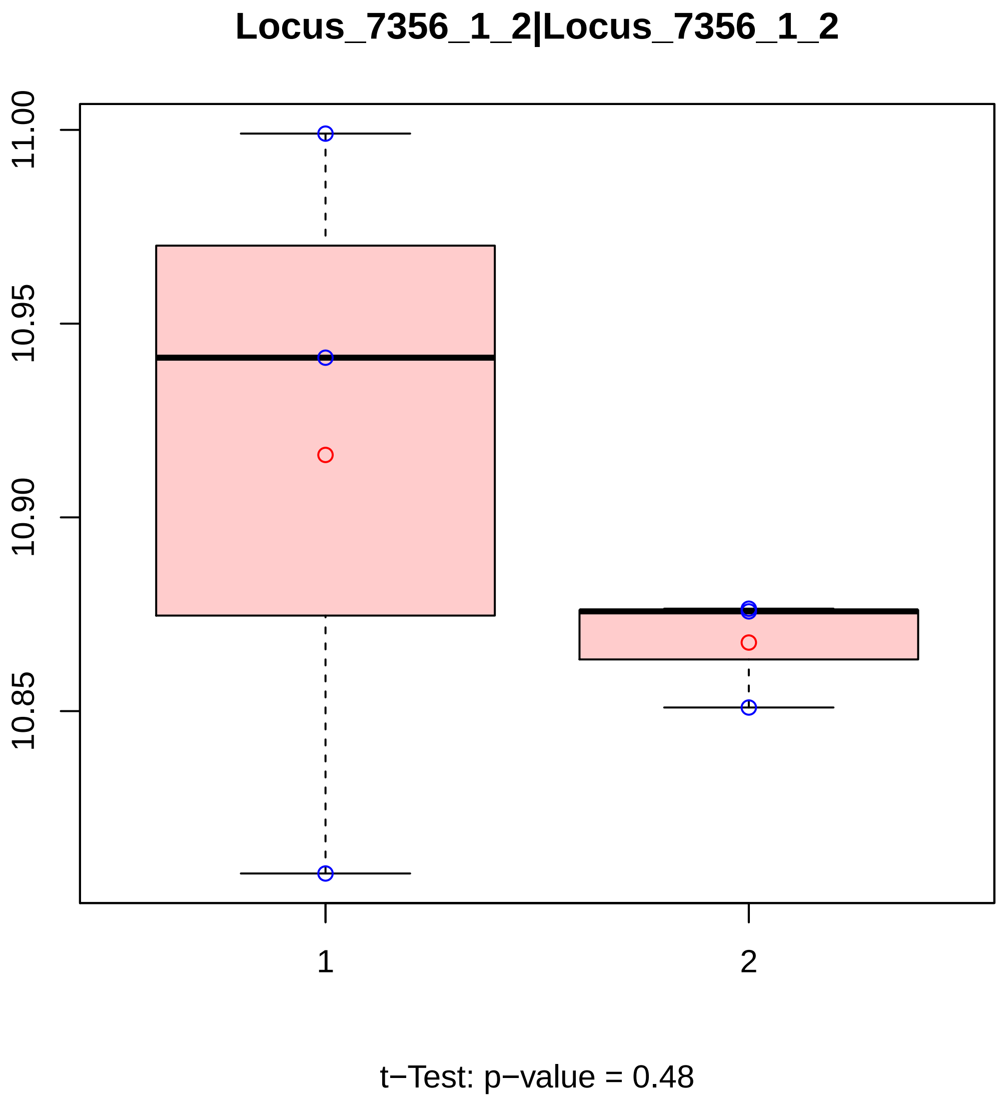
Locus_7356_1_2|Locus_7356_1_2
11.00
10.95
10.90
10.85
1
2
t−Test: p−value = 0.48

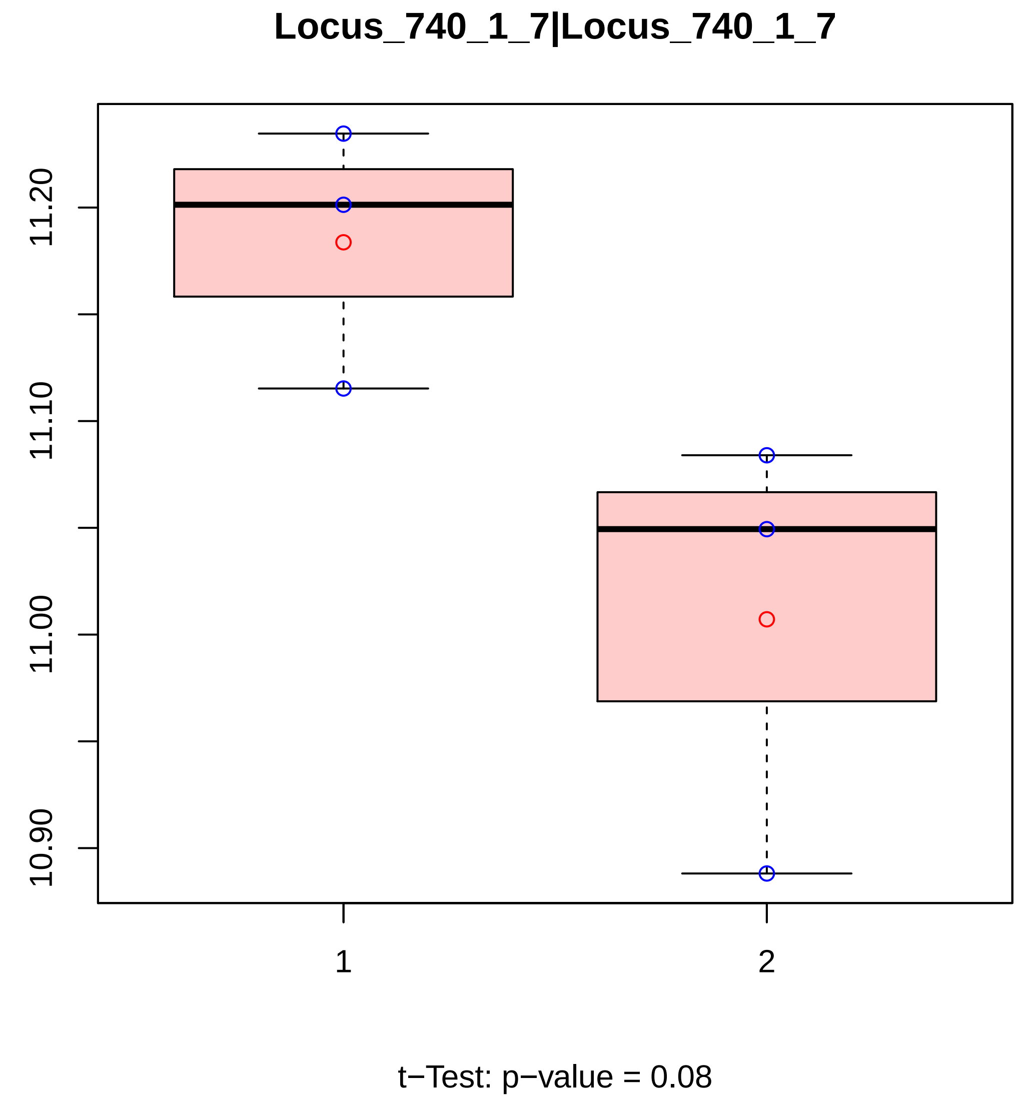

Locus_740_1_7|Locus_740_1_7
11.20
11.10
11.00
10.90
1
2
t−Test: p−value = 0.08

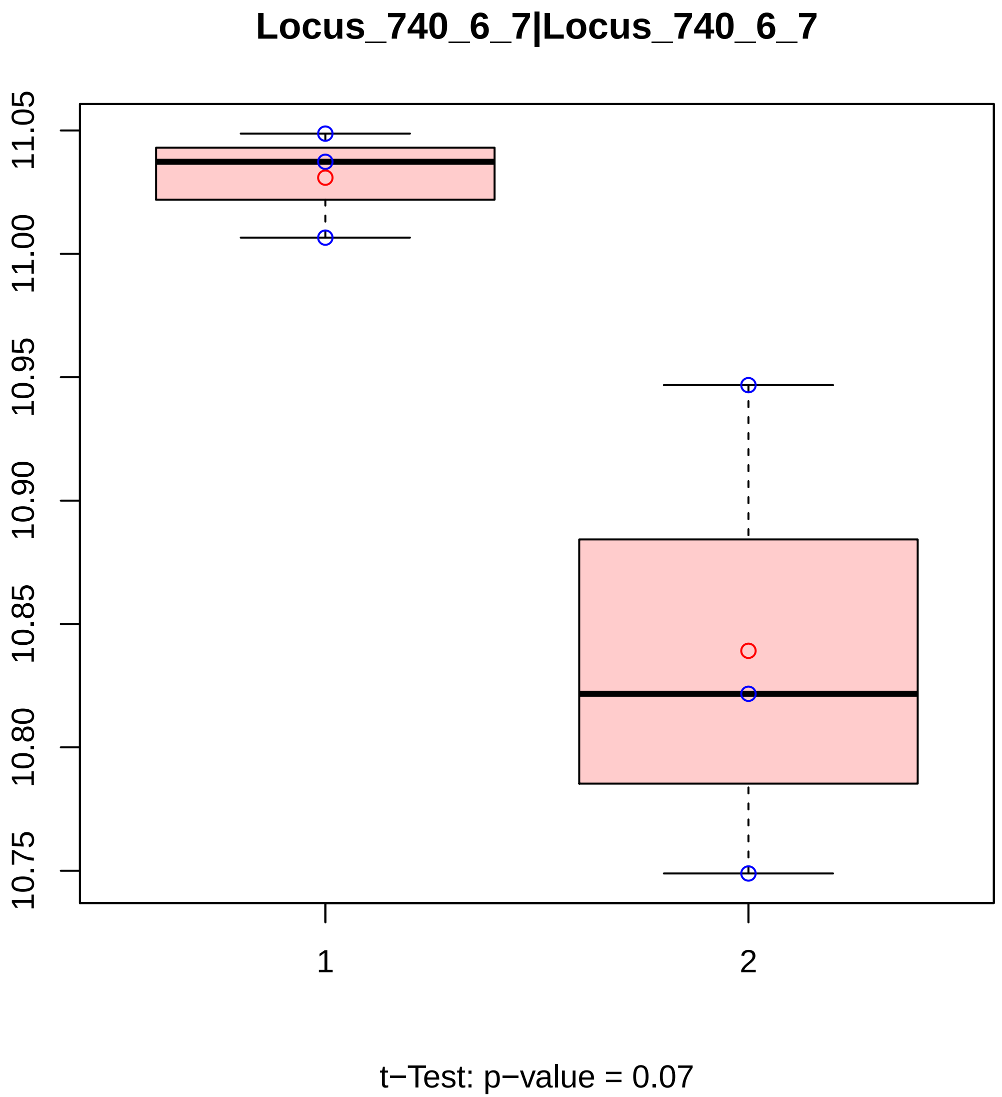
Locus_740_6_7|Locus_740_6_7
11.05
11.00
10.95
10.90
10.85
10.80
10.75
1
2
t−Test: p−value = 0.07

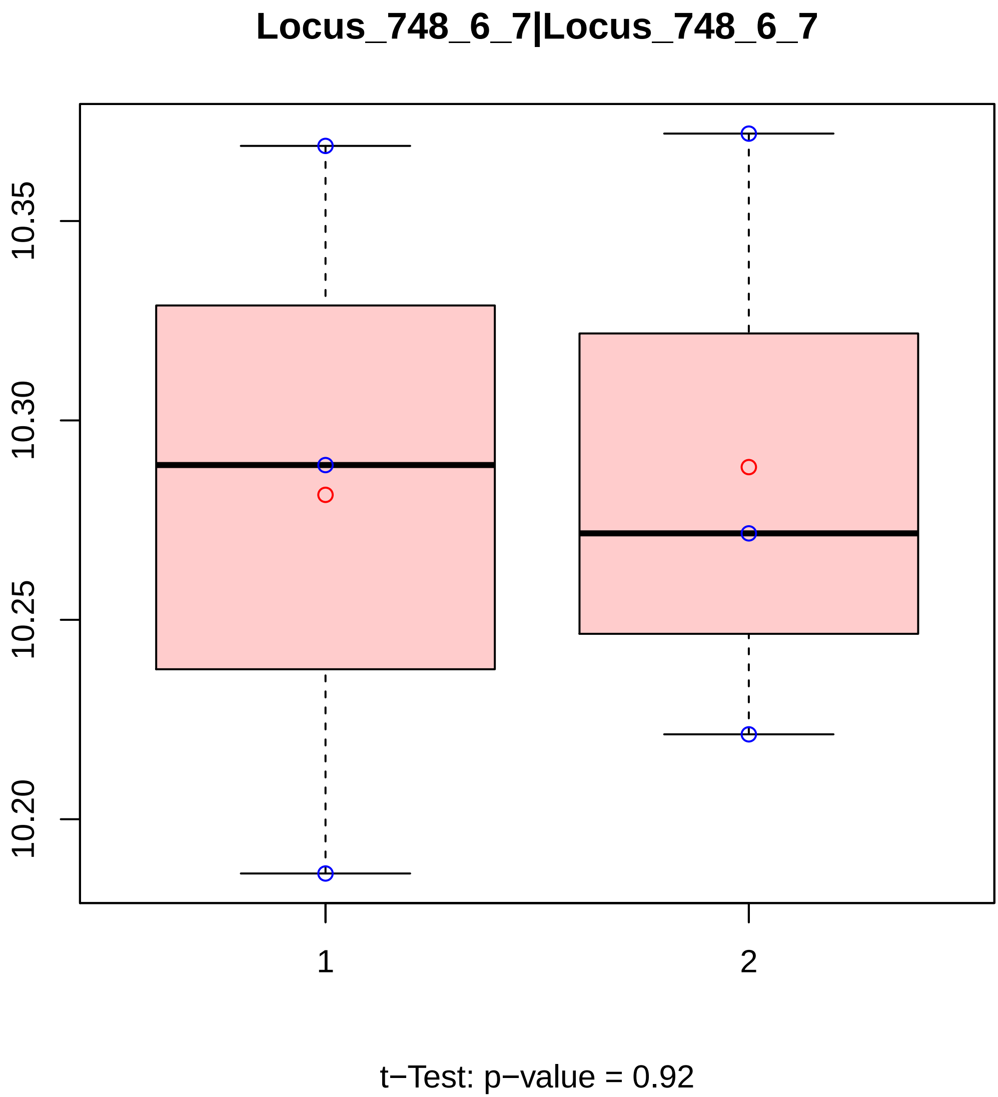
Locus_748_6_7|Locus_748_6_7
10.35
10.30
10.25
10.20
1
2
t−Test: p−value = 0.92

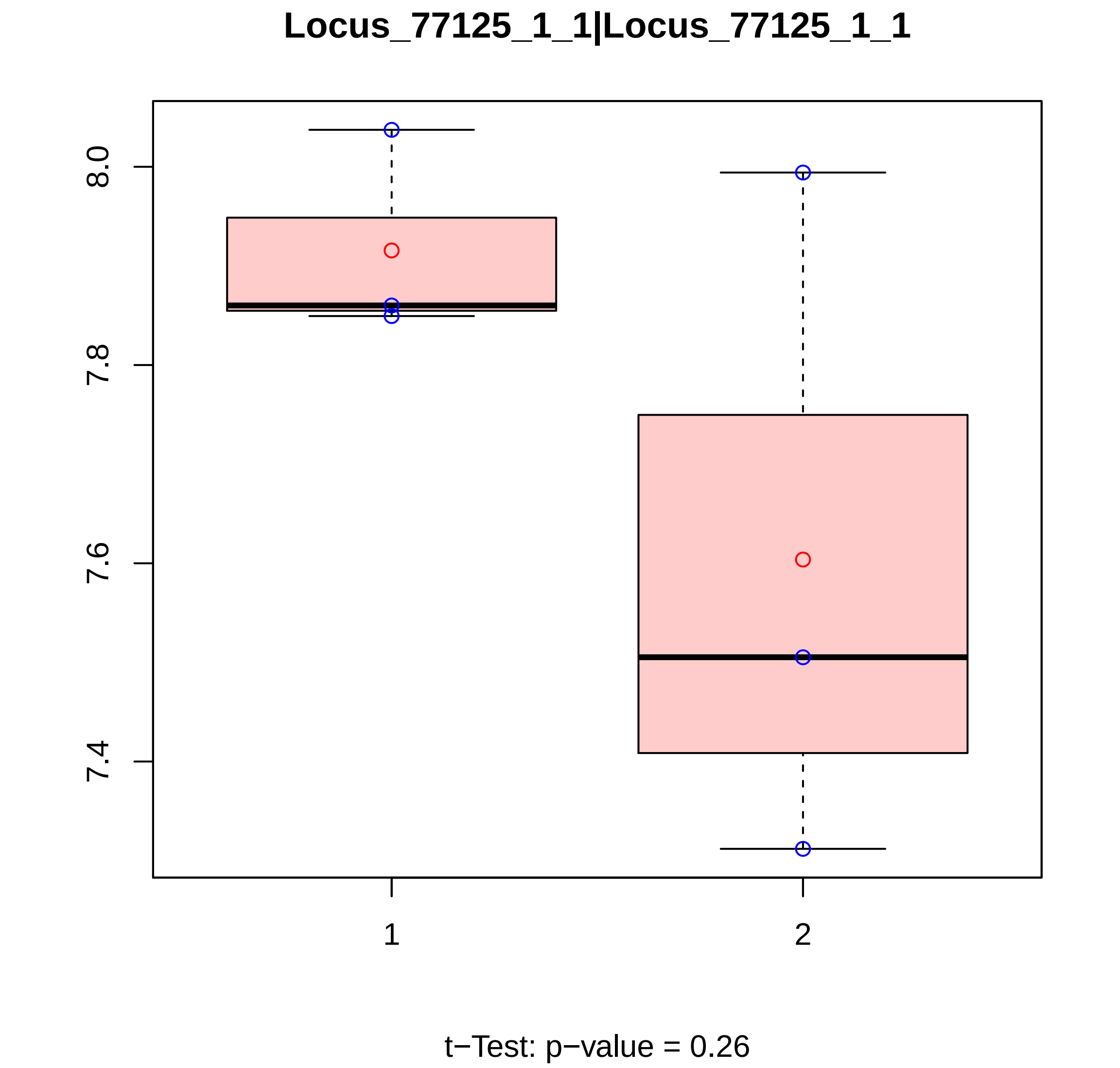

Locus_77125_1_1|Locus_77125_1_1
8.0
7.8
7.6
7.4
1
2
t−Test: p−value = 0.26

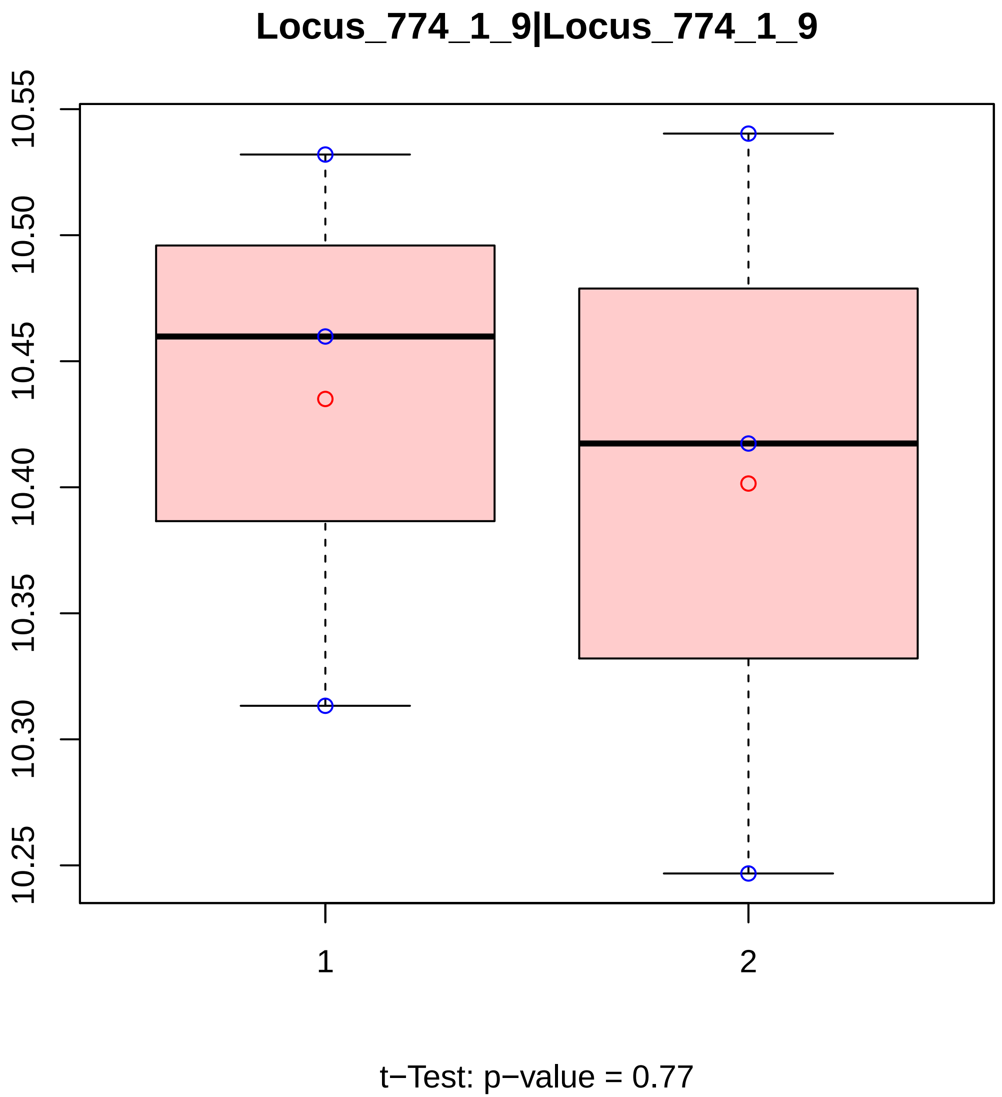

Locus_774_1_9|Locus_774_1_9
10.55
10.50
10.45
10.40
10.35
10.30
10.25
1
2
t−Test: p−value = 0.77

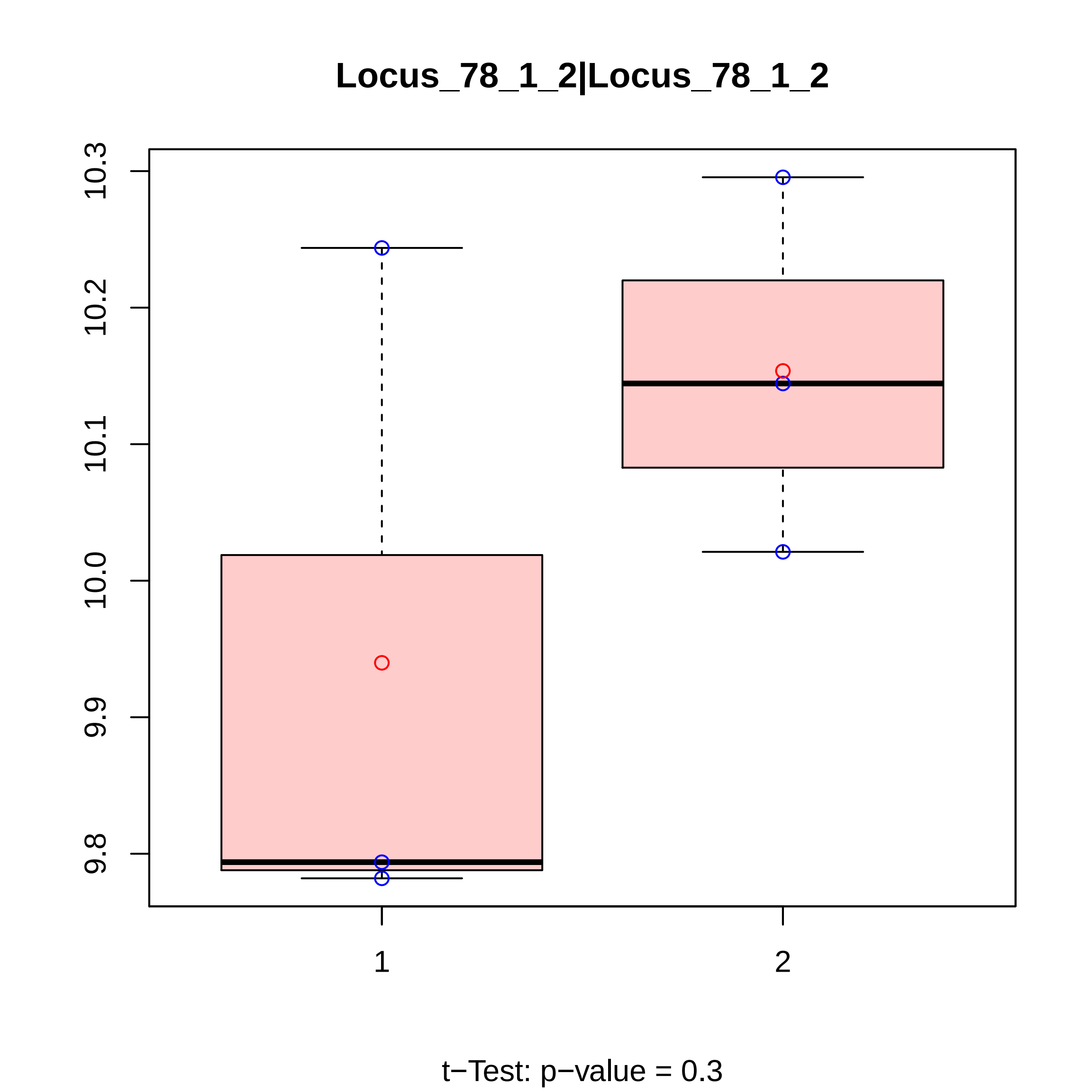

Locus_78_1_2|Locus_78_1_2
10.3
10.2
10.1
10.0
9.9
9.8
1
2
t−Test: p−value = 0.3

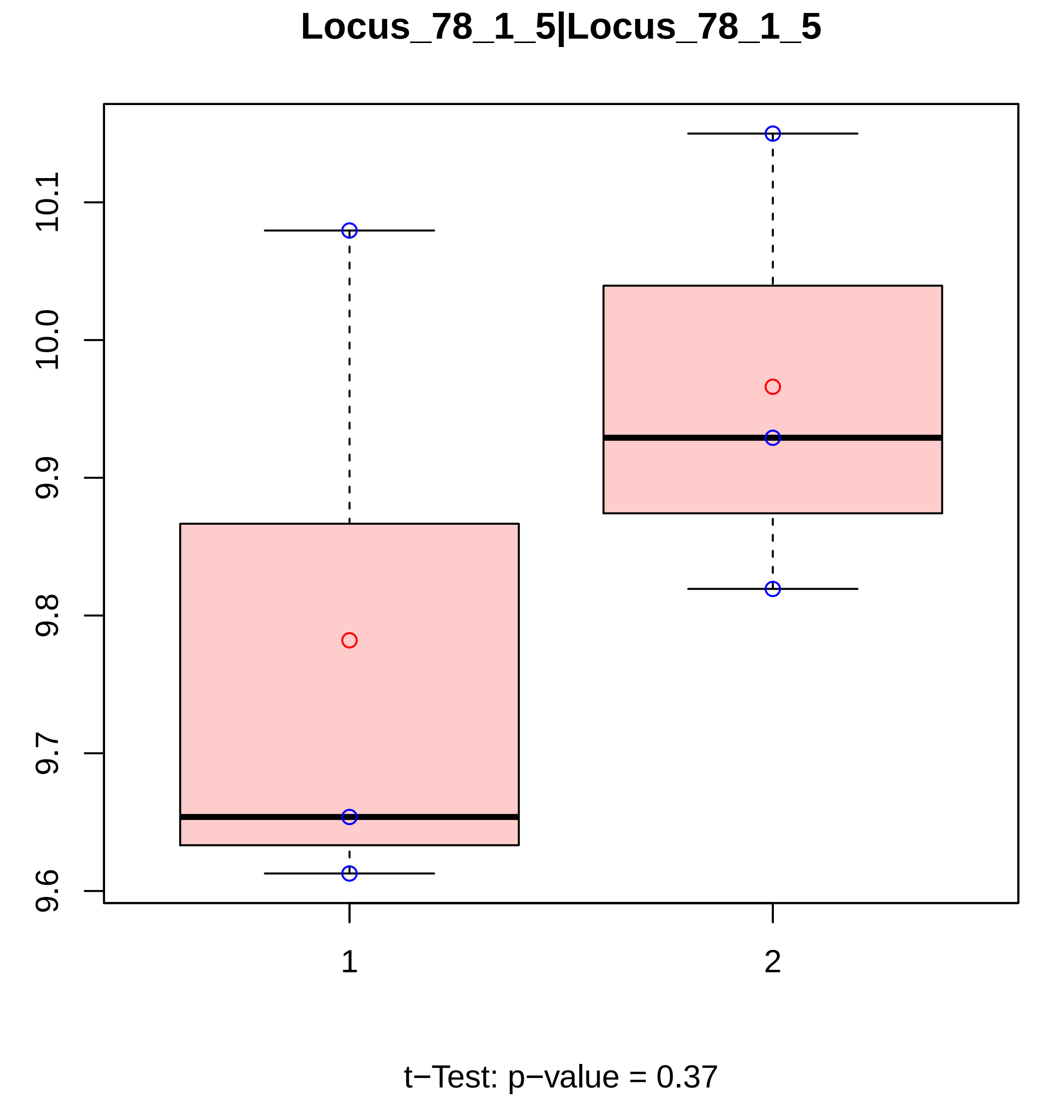
Locus_78_1_5|Locus_78_1_5
10.1
10.0
9.9
9.8
9.7
9.6
1
2
t−Test: p−value = 0.37

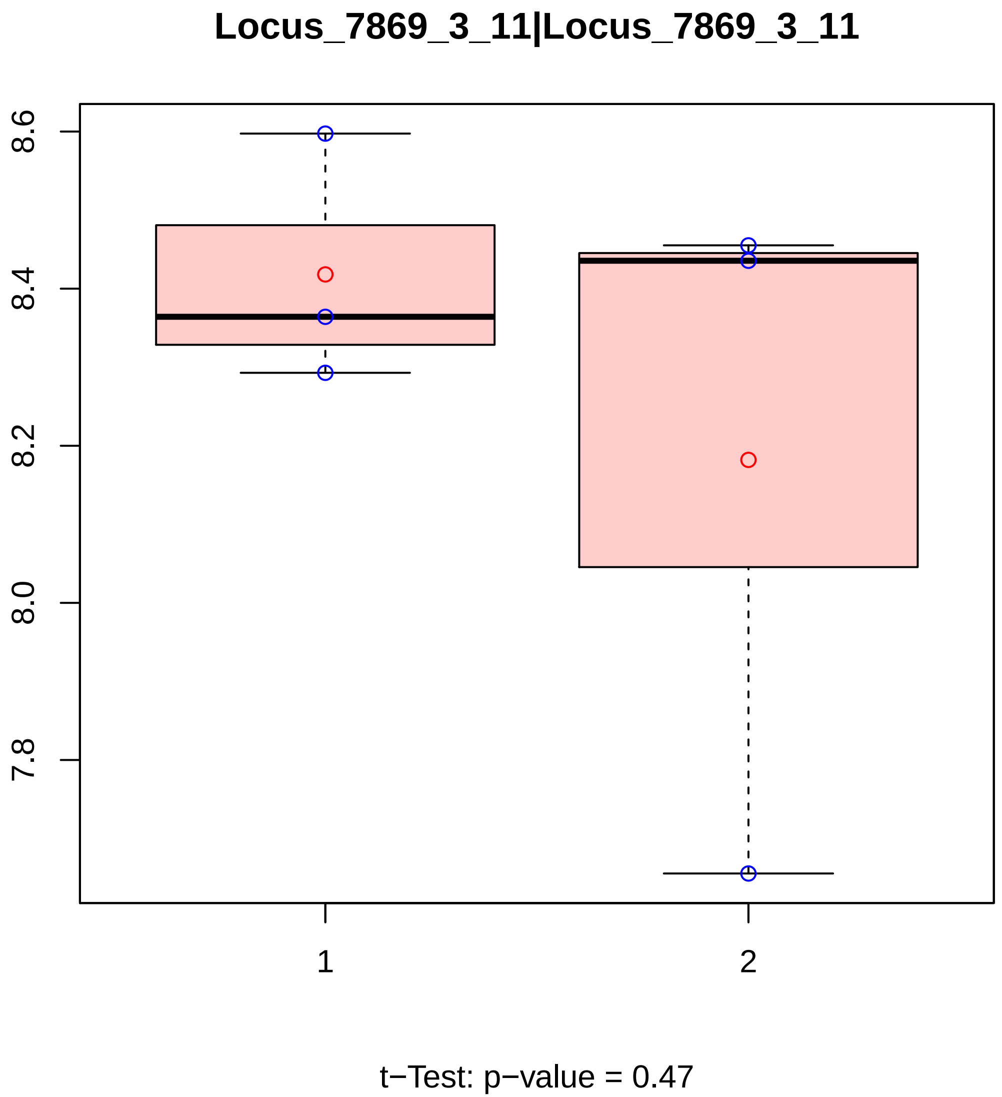

Locus_7869_3_11|Locus_7869_3_11
8.6
8.4
8.2
8.0
7.8
1
2
t−Test: p−value = 0.47

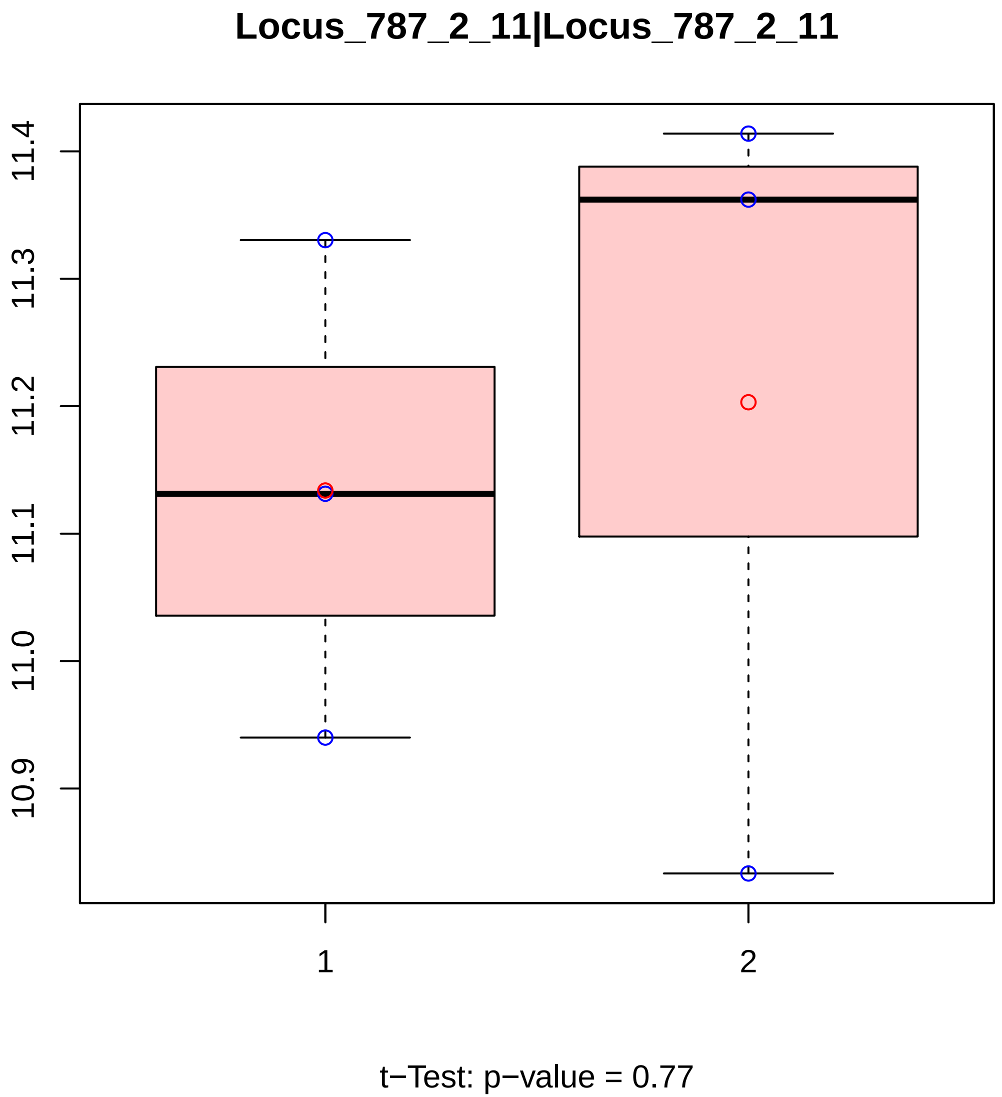
Locus_787_2_11|Locus_787_2_11
10.9
11.0
11.1
11.2
11.3
11.4
1
2
t−Test: p−value = 0.77

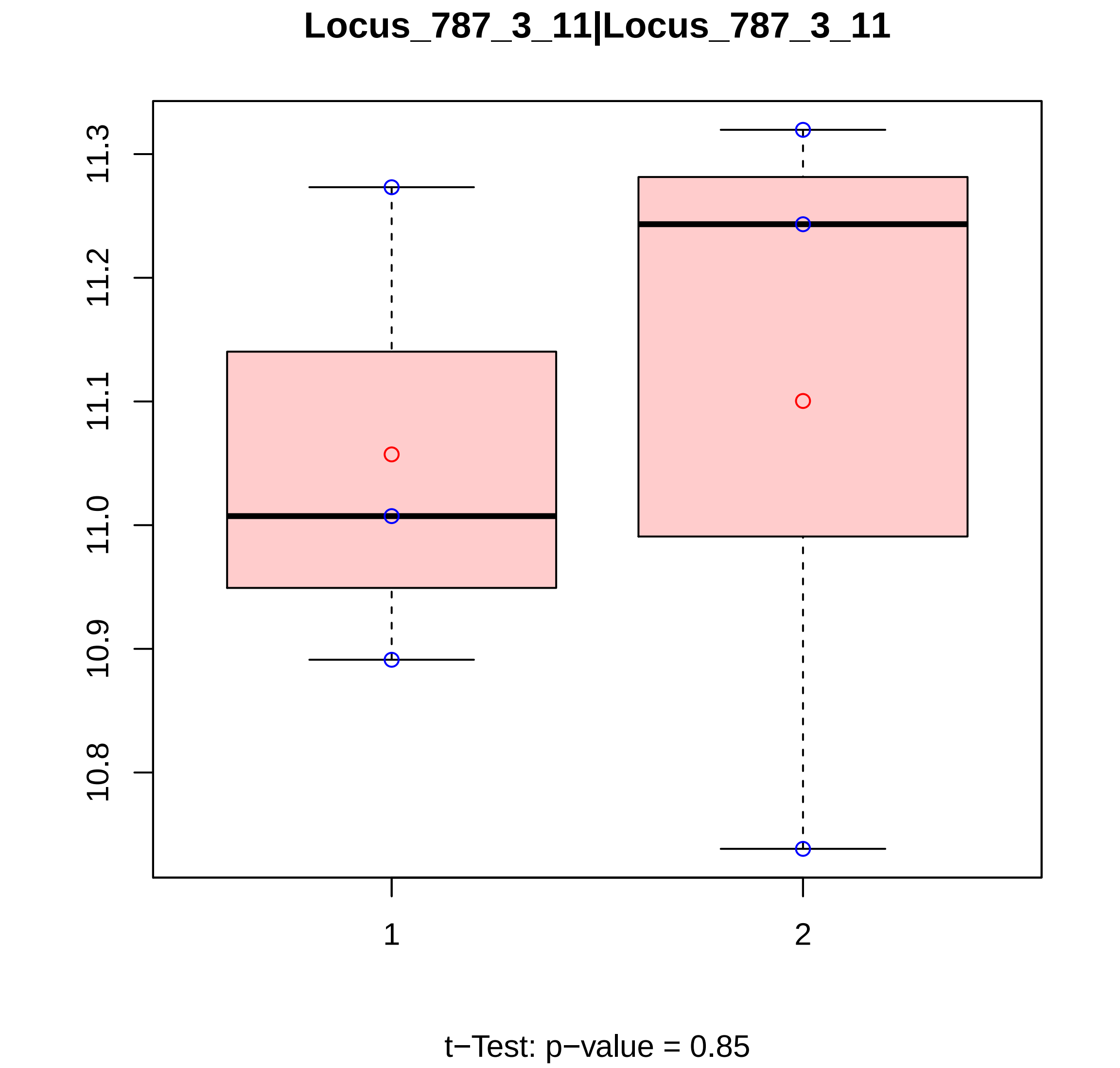

Locus_787_3_11|Locus_787_3_11
11.3
11.2
11.1
11.0
10.9
10.8
1
2
t−Test: p−value = 0.85

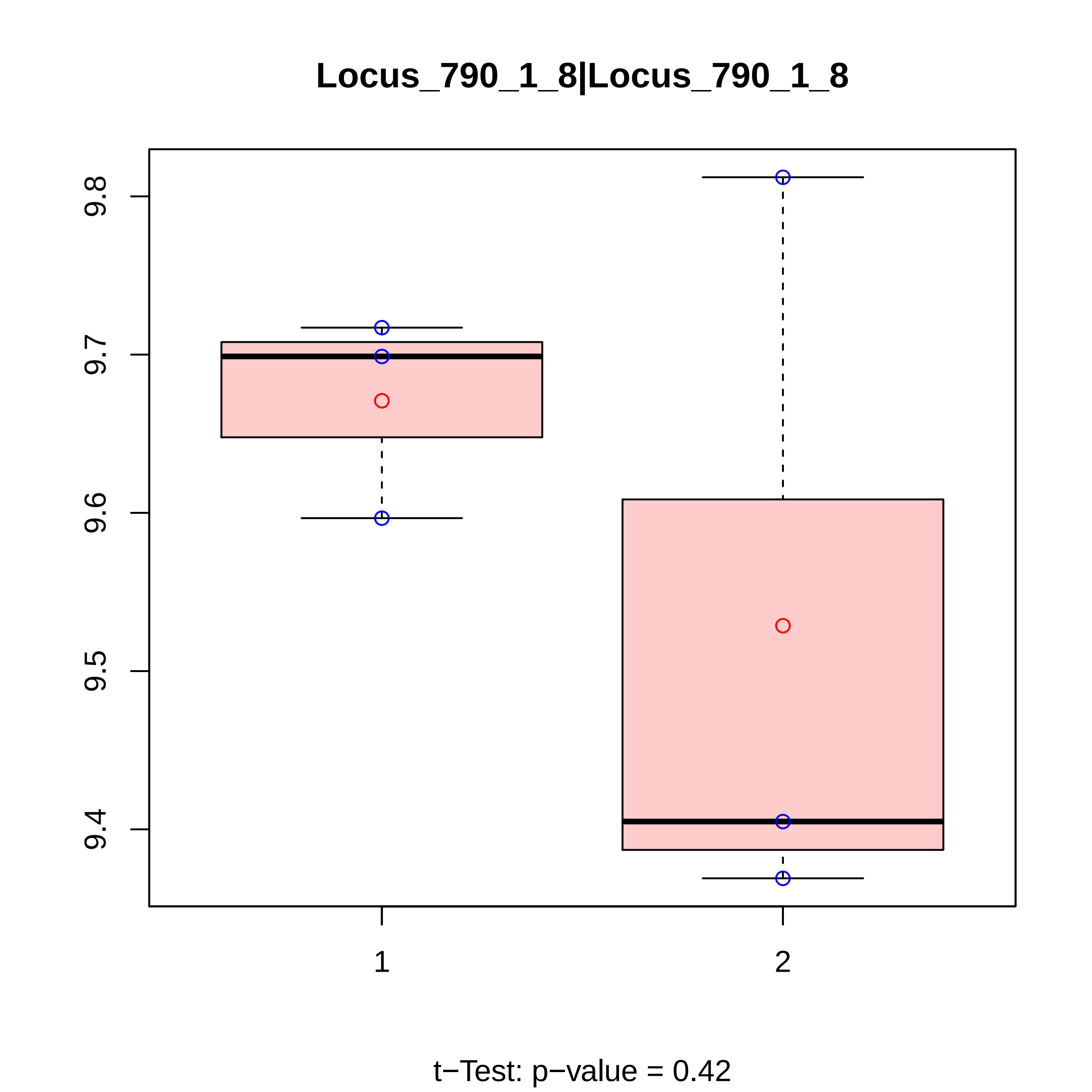

Locus_790_1_8|Locus_790_1_8
9.4
9.5
9.6
9.7
9.8
1
2
t−Test: p−value = 0.42

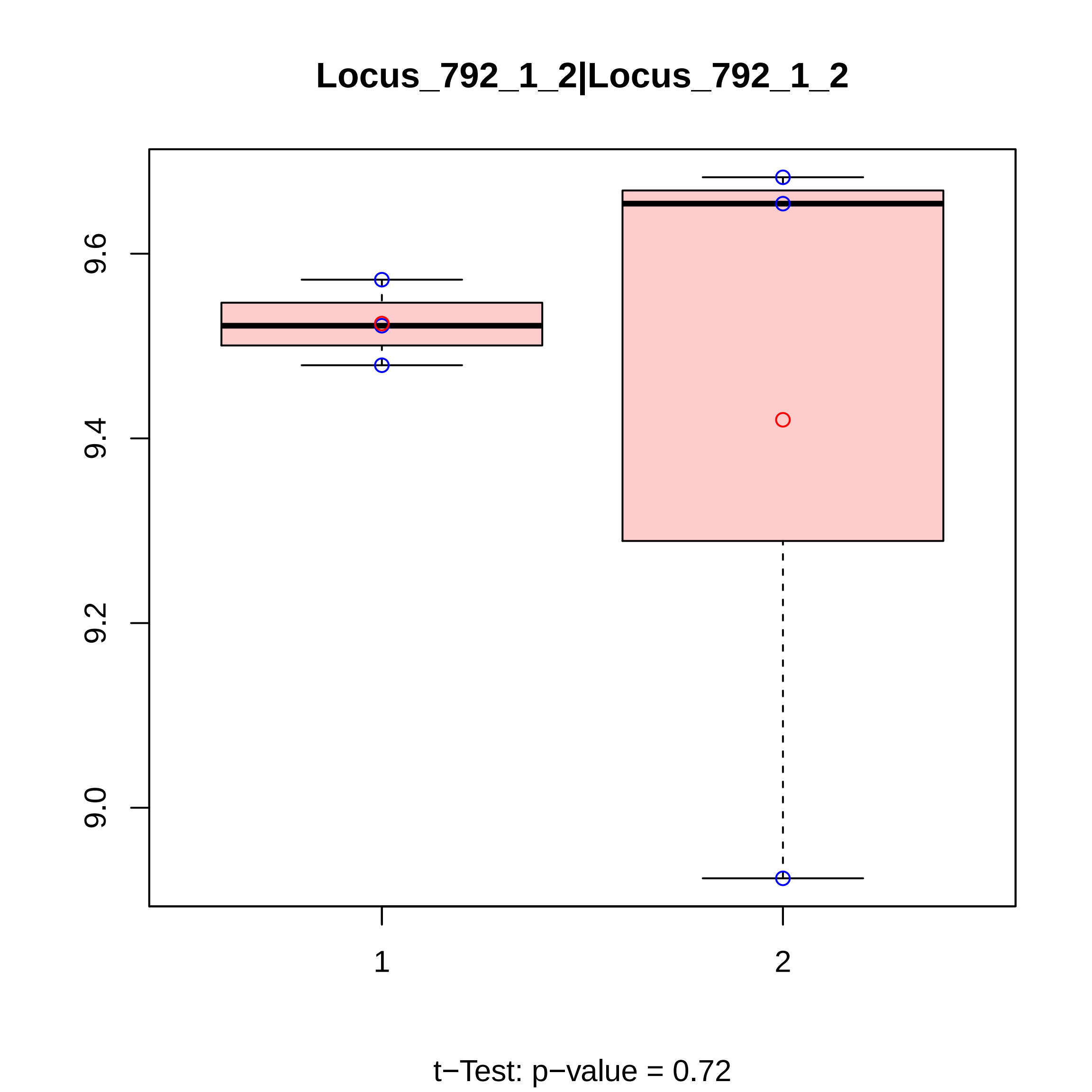
Locus_792_1_2|Locus_792_1_2
9.0
9.2
9.4
9.6
1
2
t−Test: p−value = 0.72

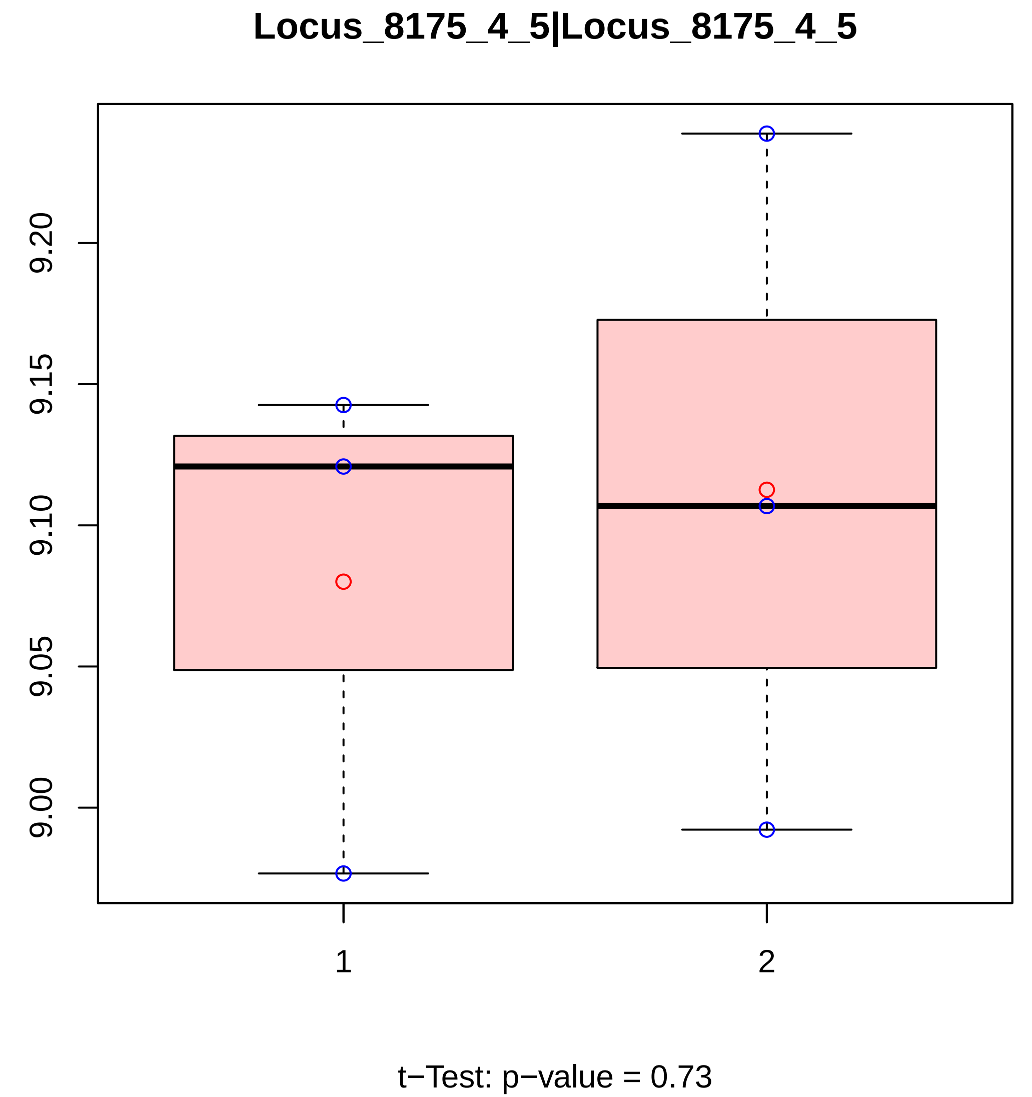
Locus_8175_4_5|Locus_8175_4_5
9.00
9.05
9.10
9.15
9.20
1
2
t−Test: p−value = 0.73

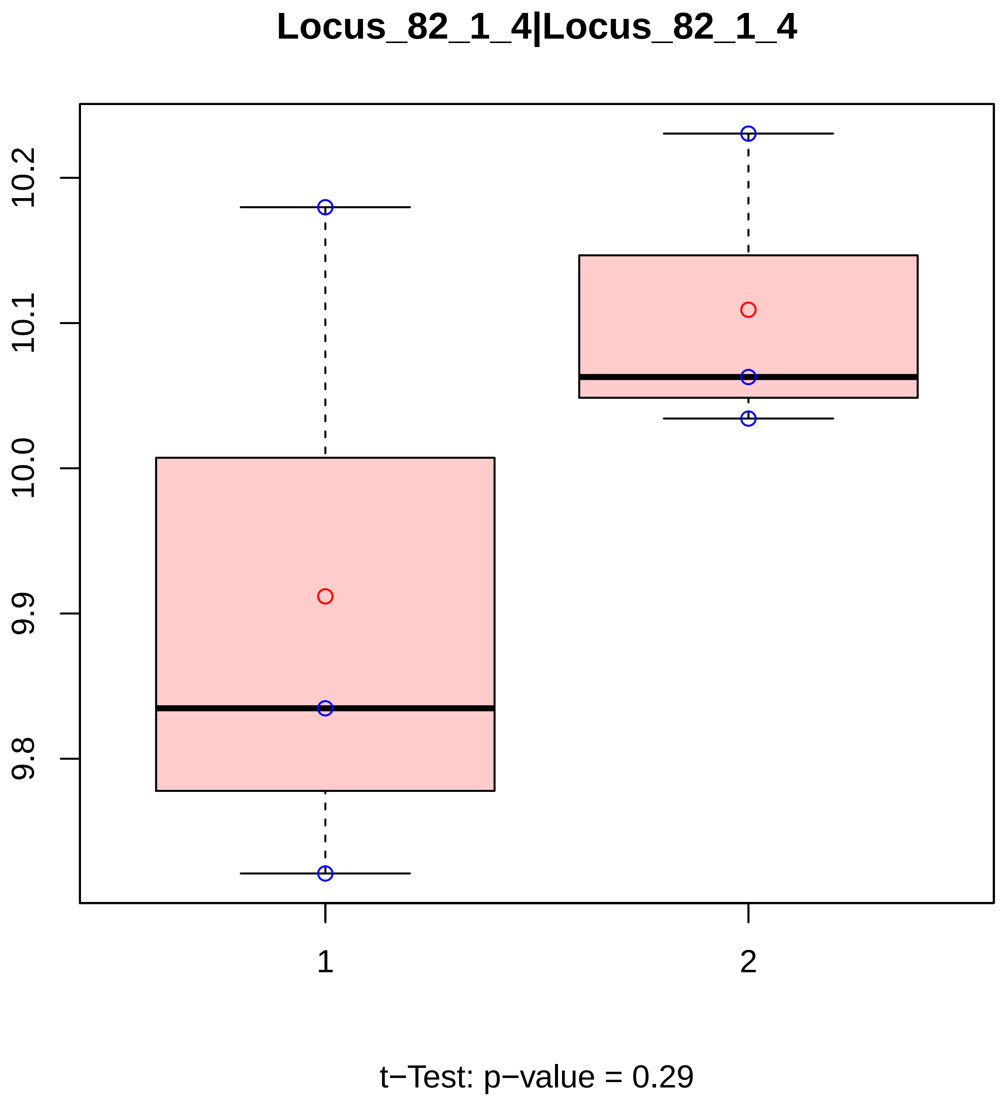

Locus_82_1_4|Locus_82_1_4
10.2
10.1
10.0
9.9
9.8
1
2
t−Test: p−value = 0.29

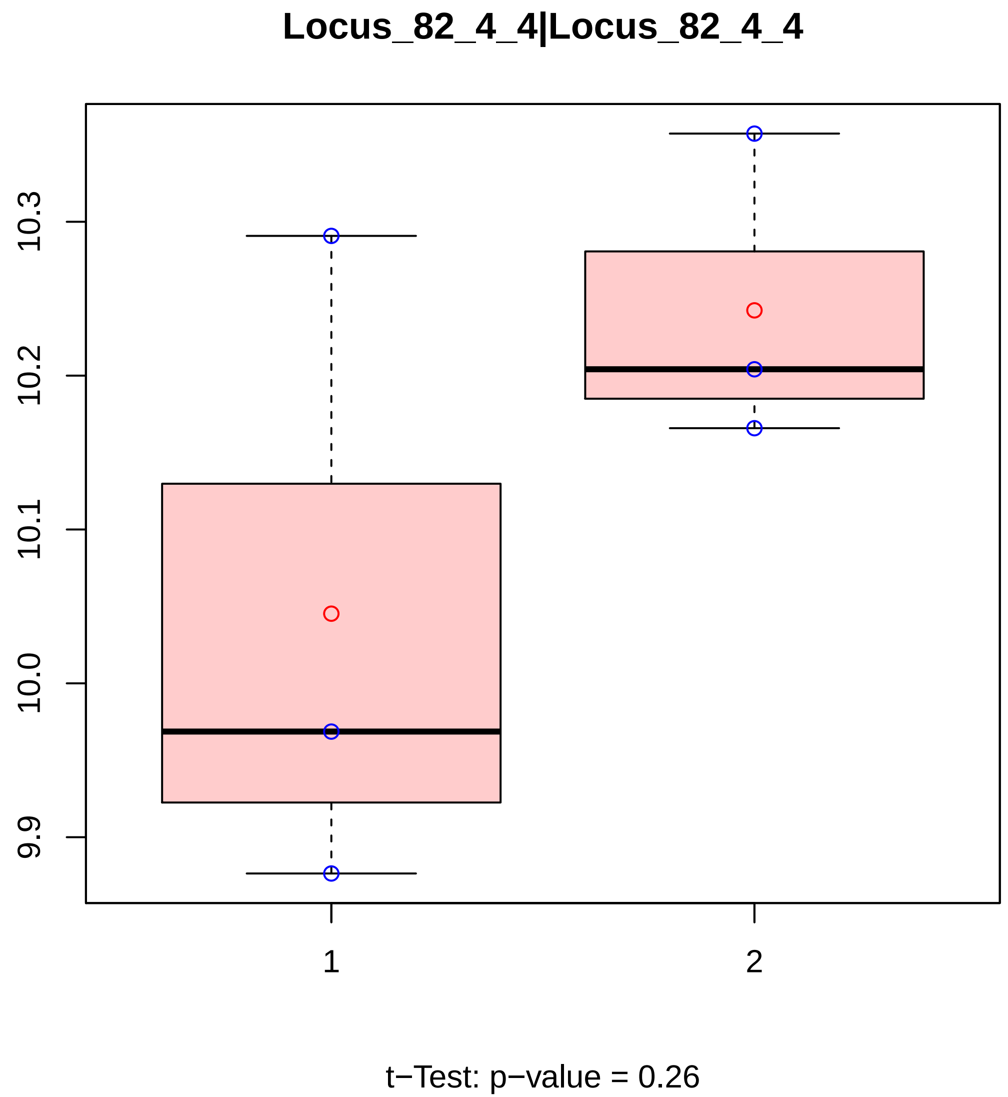

Locus_82_4_4|Locus_82_4_4
10.3
10.2
10.1
10.0
9.9
1
2
t−Test: p−value = 0.26

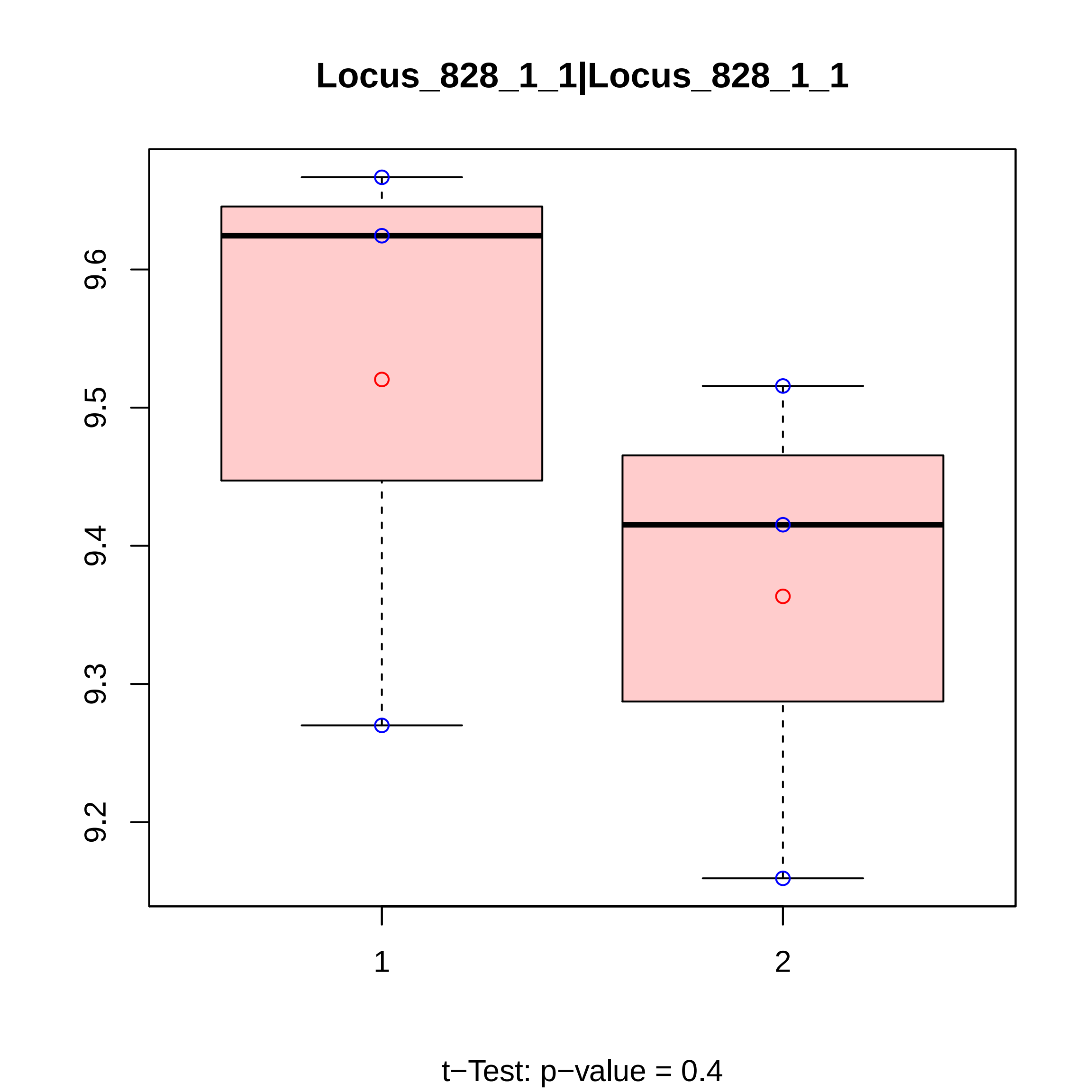
Locus_828_1_1|Locus_828_1_1
9.2
9.3
9.4
9.5
9.6
1
2
t−Test: p−value = 0.4

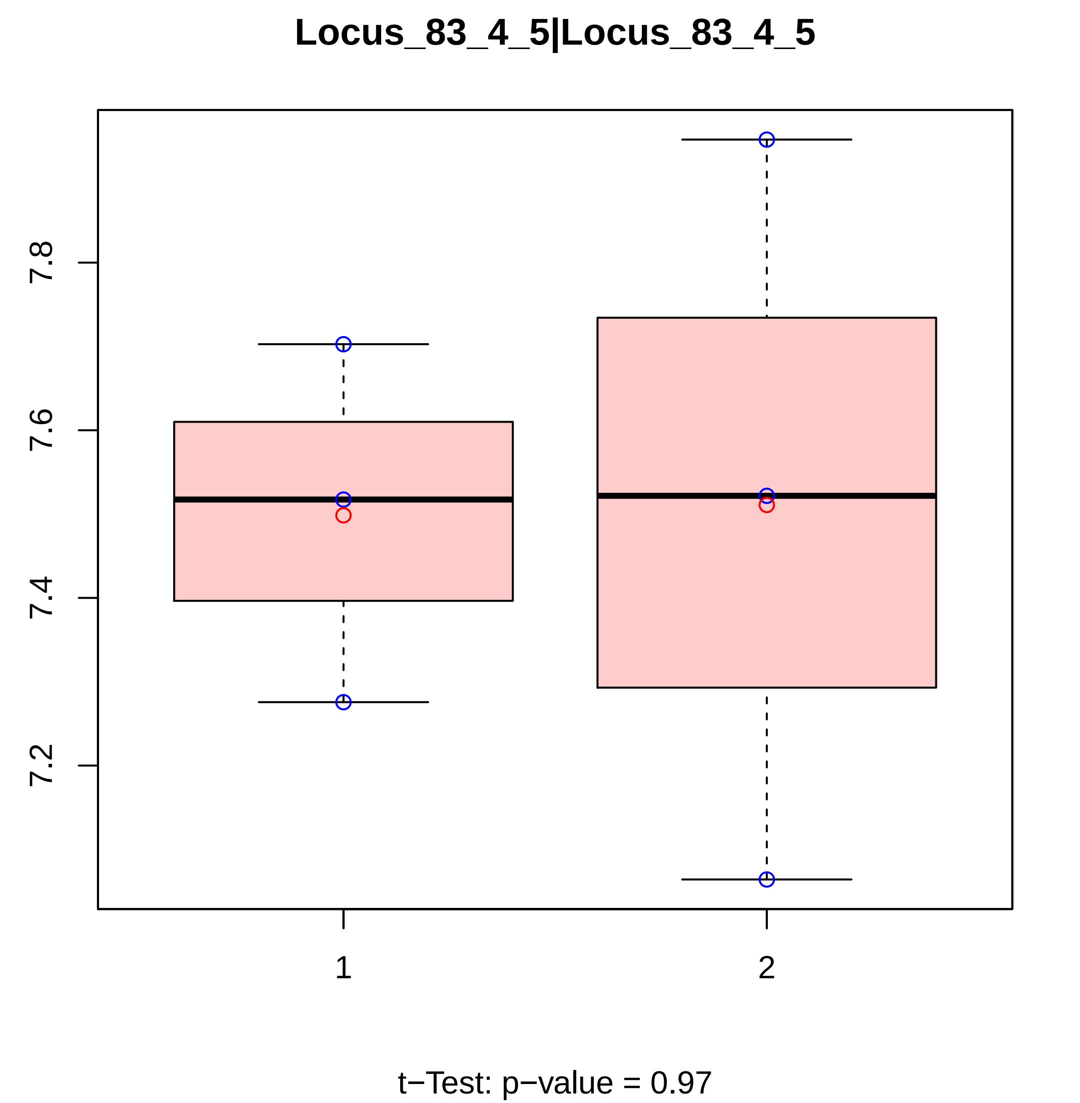

Locus_83_4_5|Locus_83_4_5
7.2
7.4
7.6
7.8
1
2
t−Test: p−value = 0.97

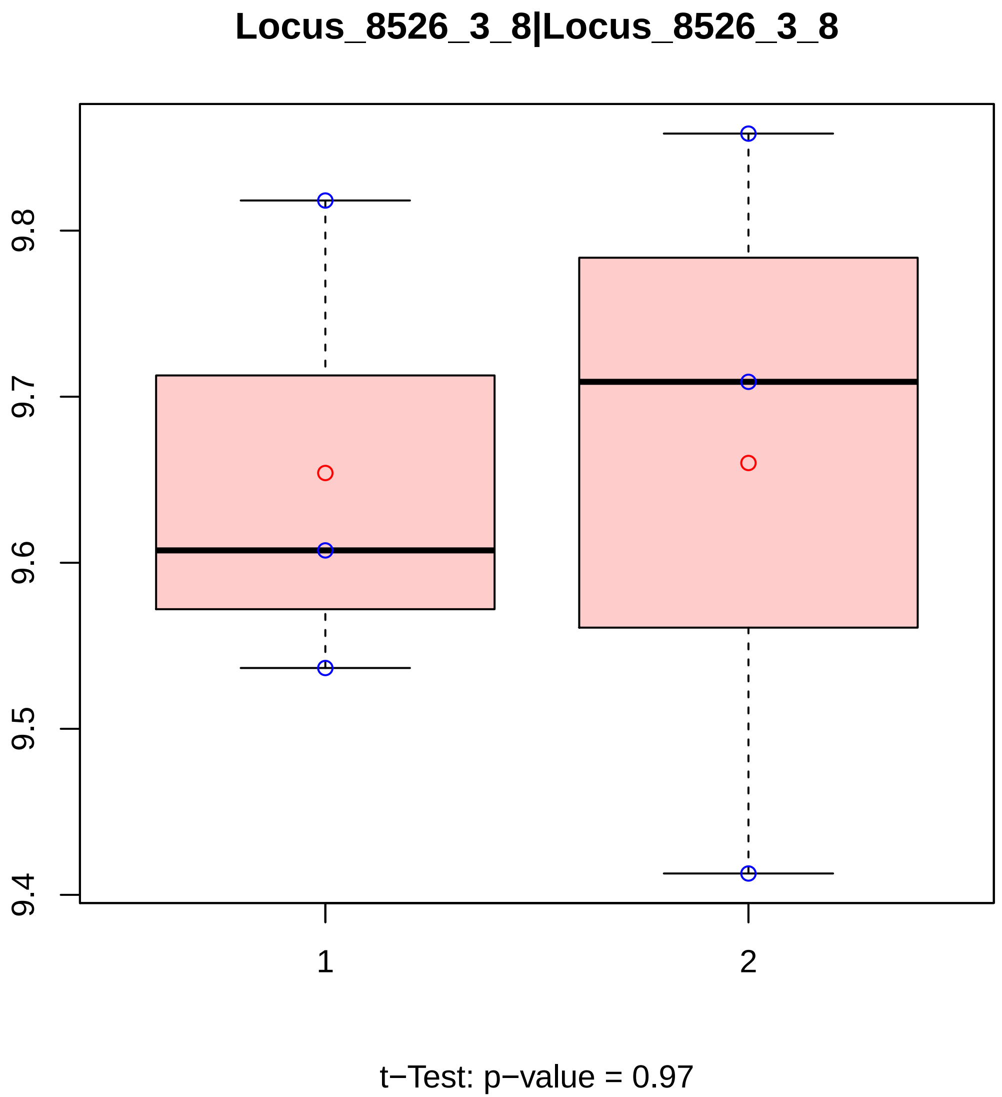
Locus_8526_3_8|Locus_8526_3_8
9.4
9.5
9.6
9.7
9.8
1
2
t−Test: p−value = 0.97

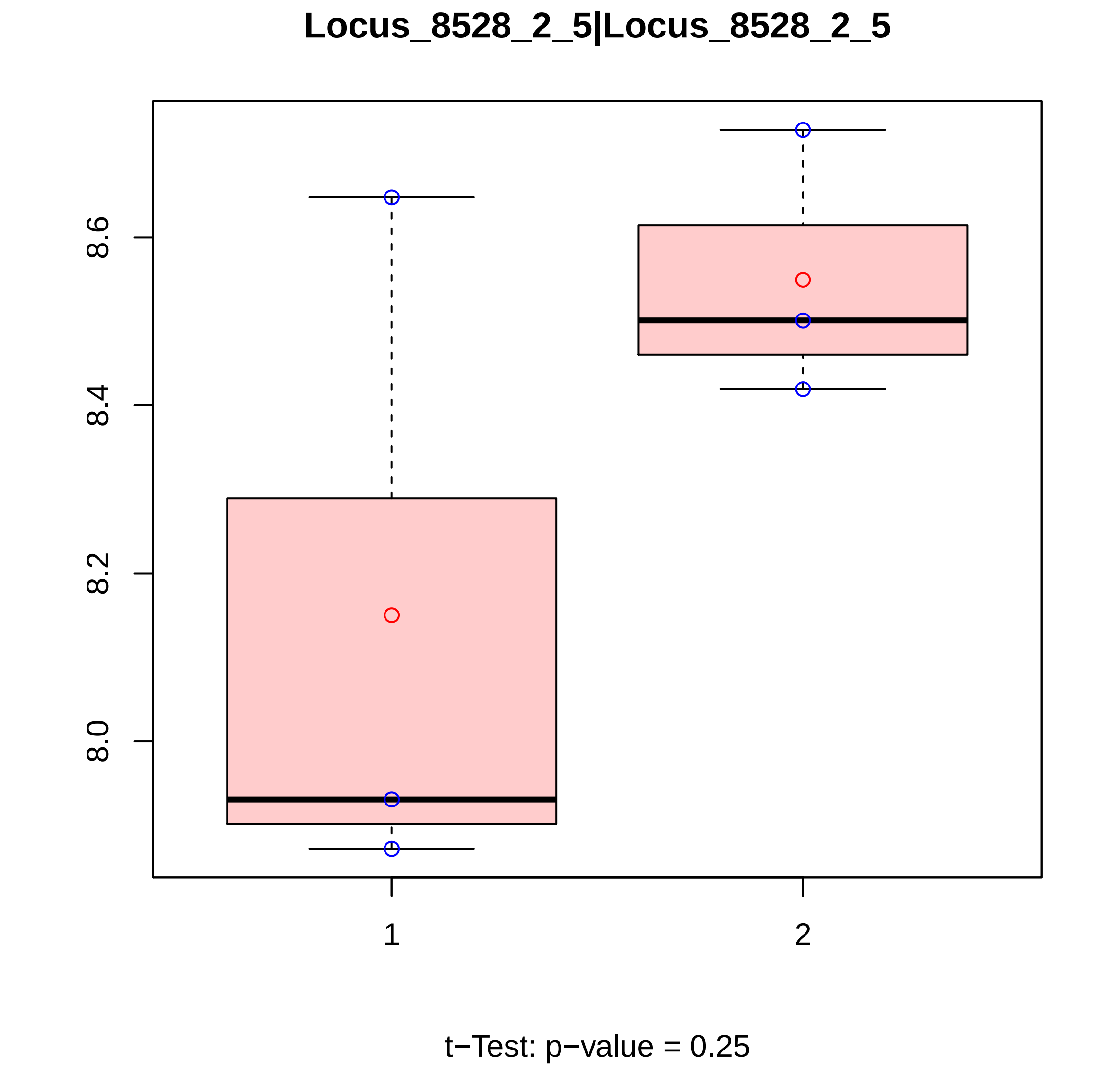
Locus_8528_2_5|Locus_8528_2_5
8.0
8.2
8.4
8.6
1
2
t−Test: p−value = 0.25

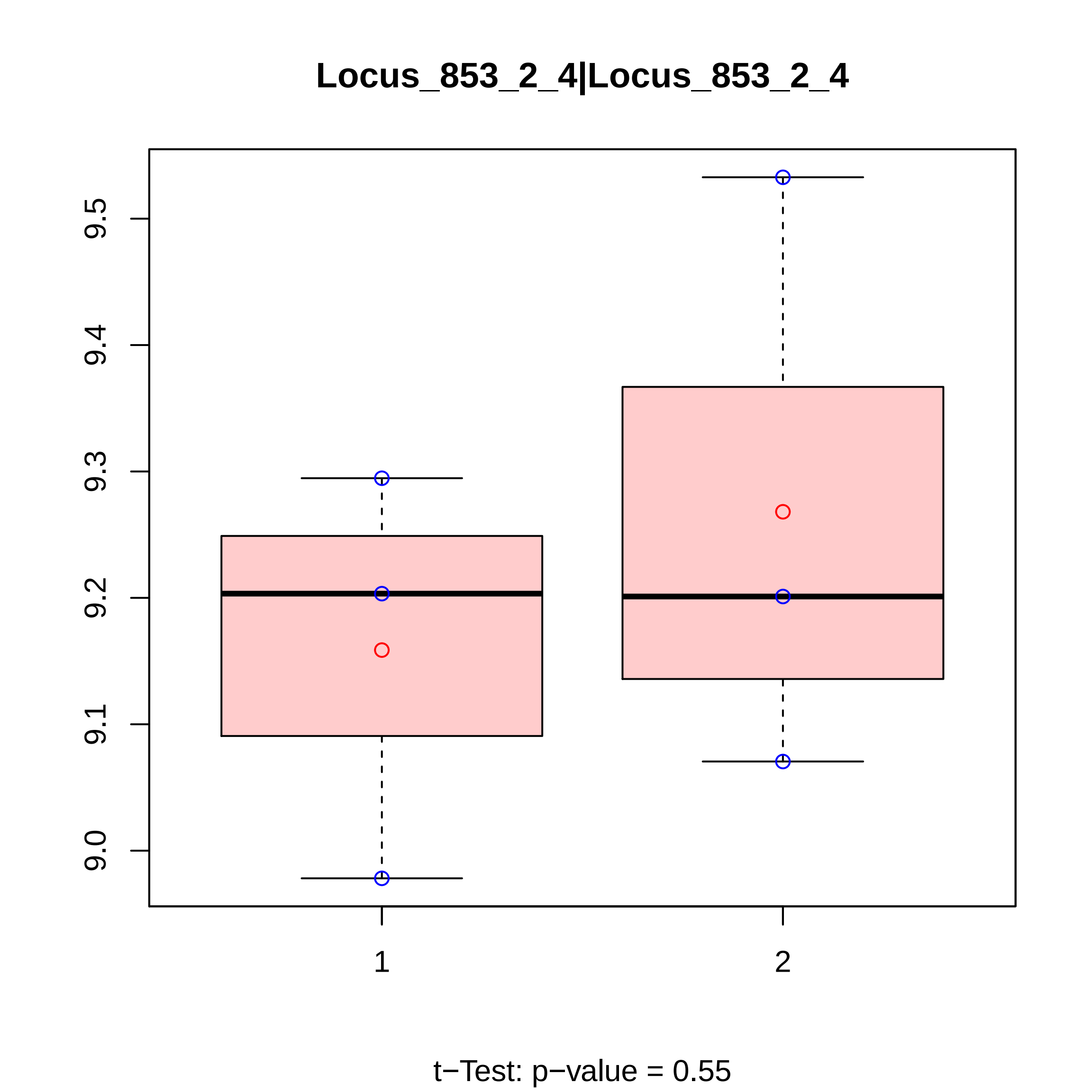
Locus_853_2_4|Locus_853_2_4
9.0
9.1
9.2
9.3
9.4
9.5
1
2
t−Test: p−value = 0.55

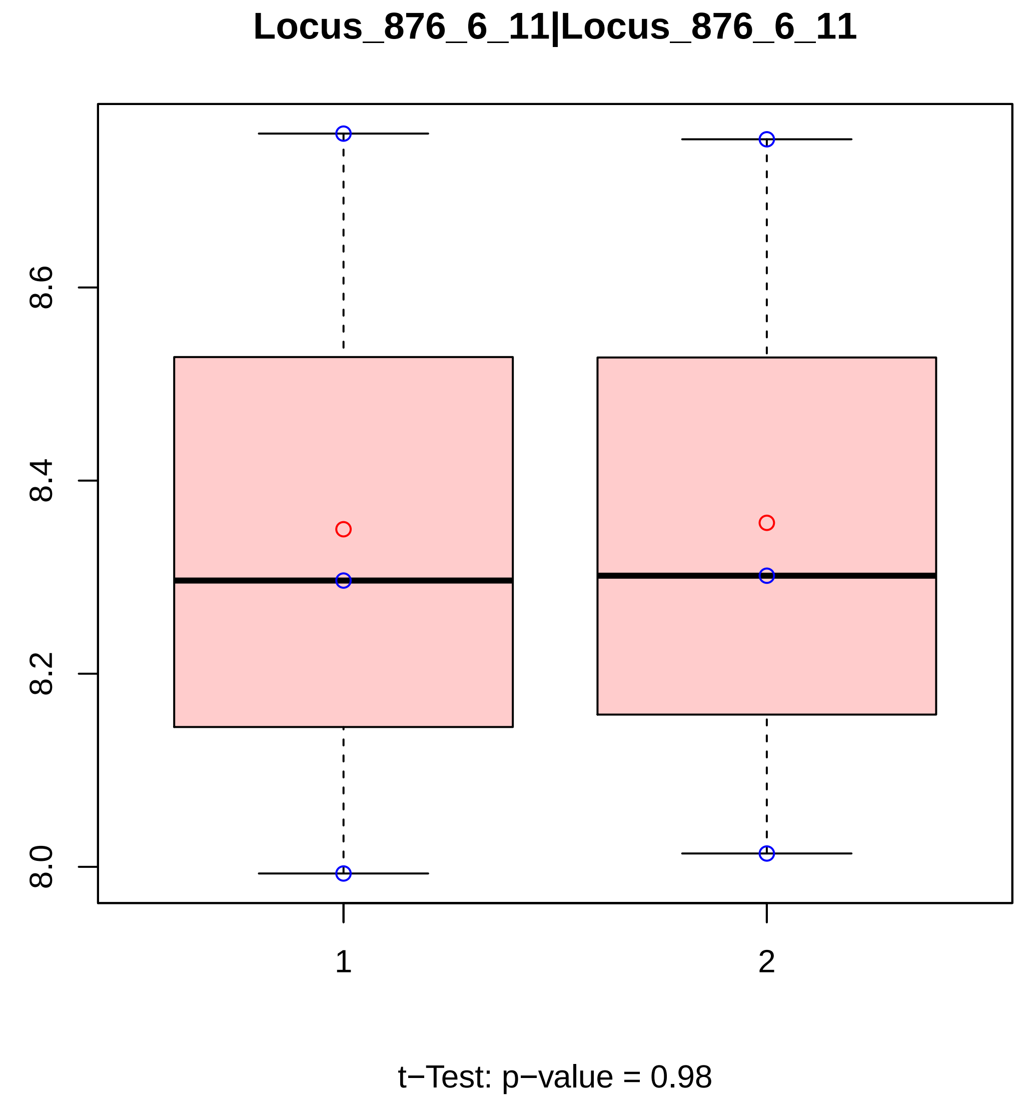
Locus_876_6_11|Locus_876_6_11
8.0
8.2
8.4
8.6
1
2
t−Test: p−value = 0.98

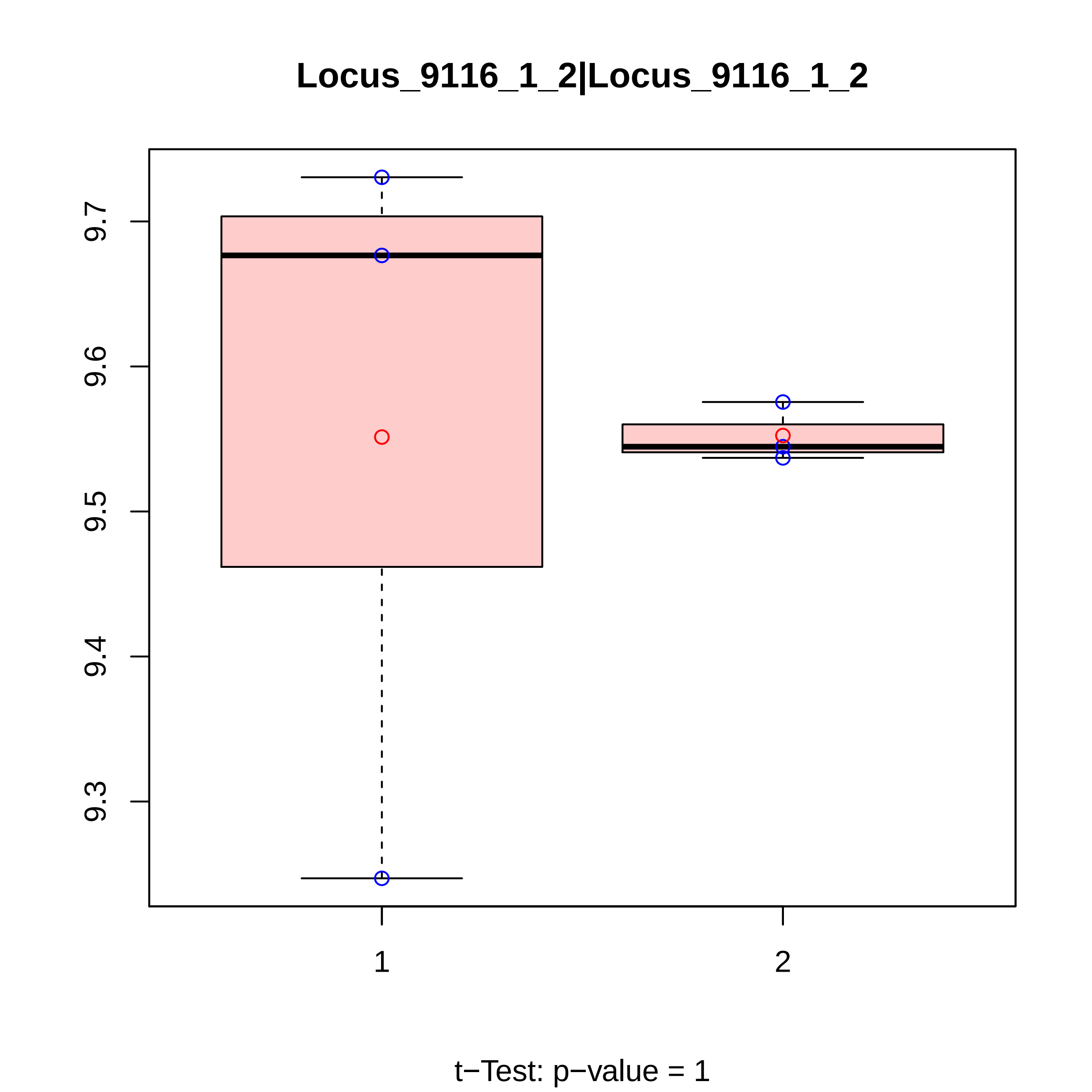
Locus_9116_1_2|Locus_9116_1_2
9.3
9.4
9.5
9.6
9.7
1
2
t−Test: p−value = 1

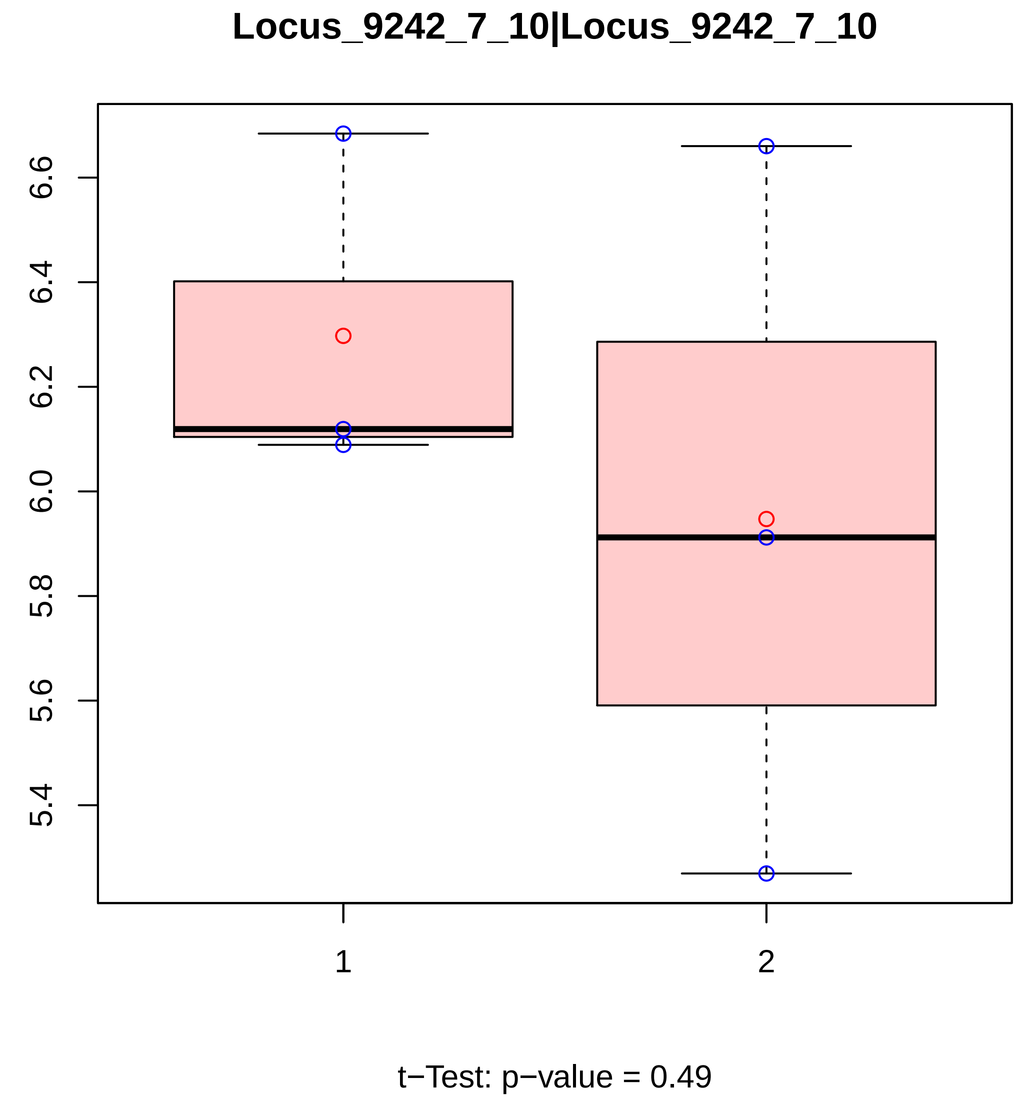

Locus_9242_7_10|Locus_9242_7_10
6.6
6.4
6.2
6.0
5.8
5.6
5.4
1
2
t−Test: p−value = 0.49

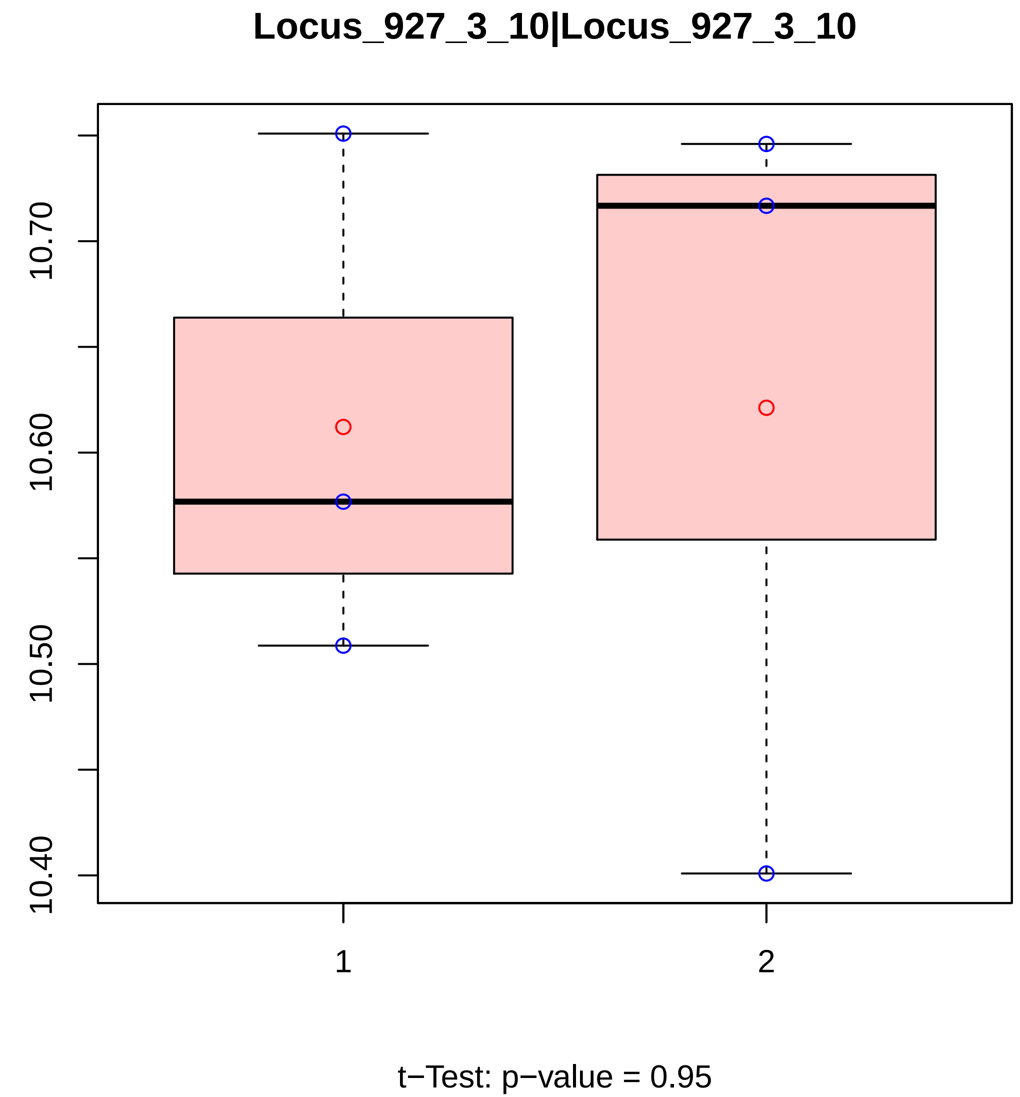

Locus_927_3_10|Locus_927_3_10
10.40
10.50
10.60
10.70
1
2
t−Test: p−value = 0.95

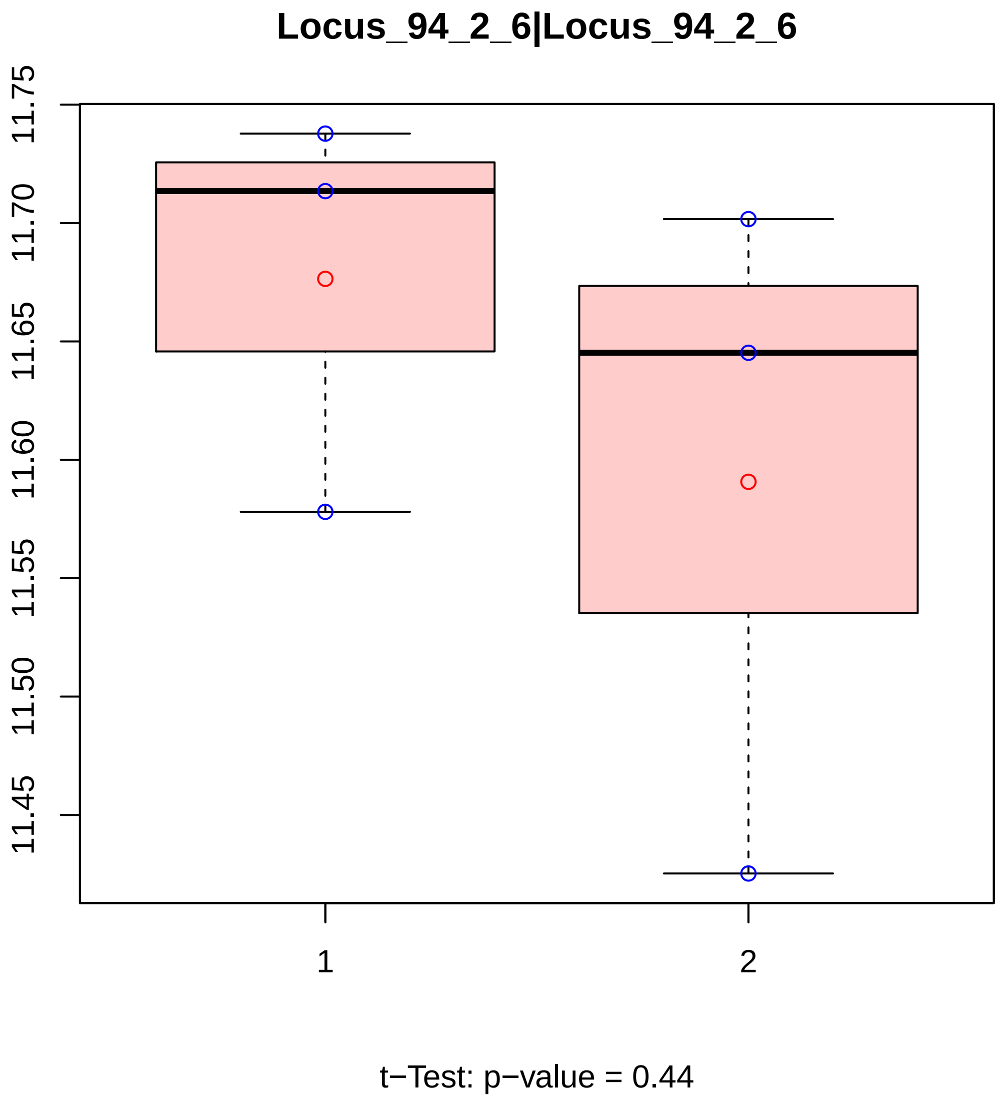

Locus_94_2_6|Locus_94_2_6
11.45
11.50
11.55
11.60
11.65
11.70
11.75
1
2
t−Test: p−value = 0.44

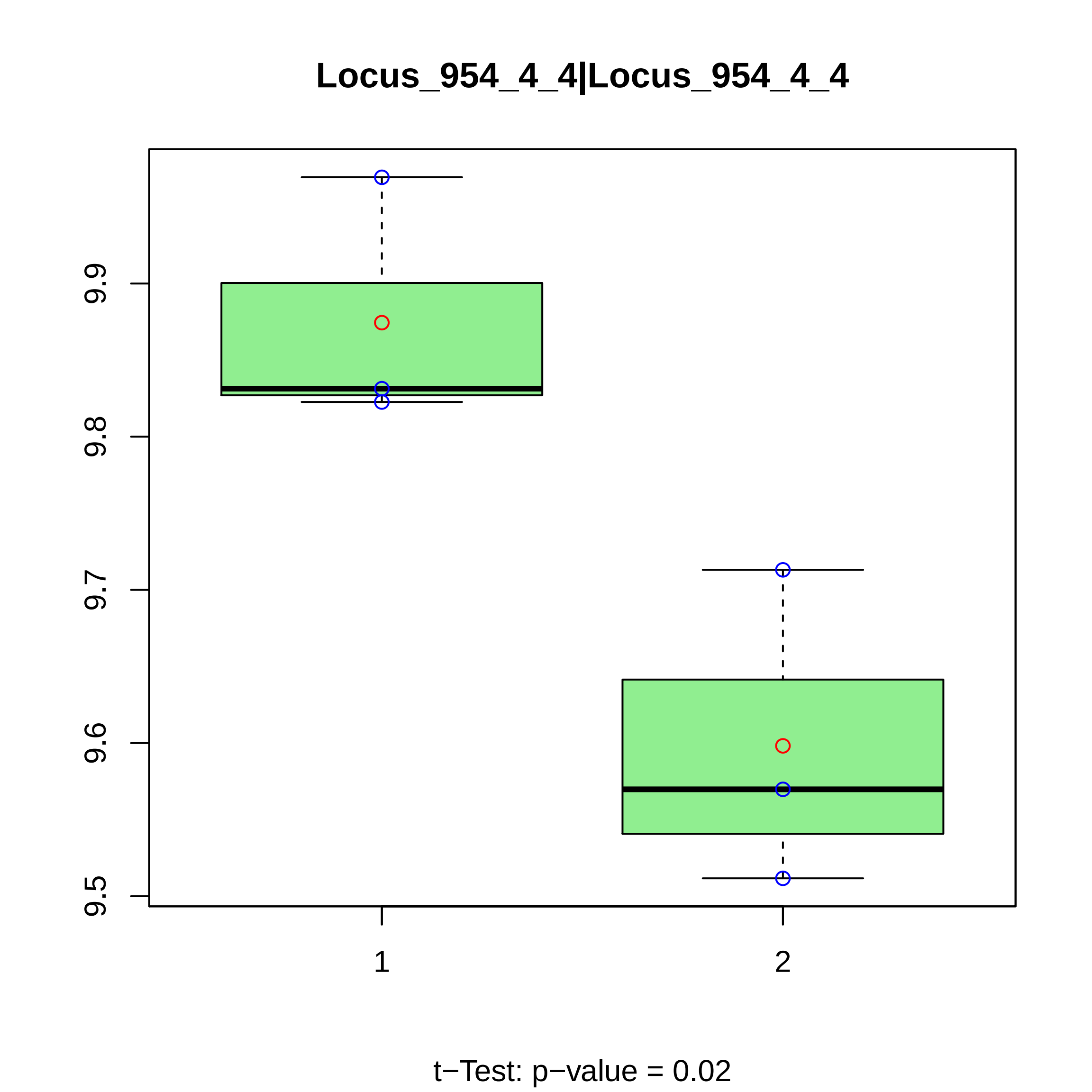

Locus_954_4_4|Locus_954_4_4
9.5
9.6
9.7
9.8
9.9
1
2
t−Test: p−value = 0.02

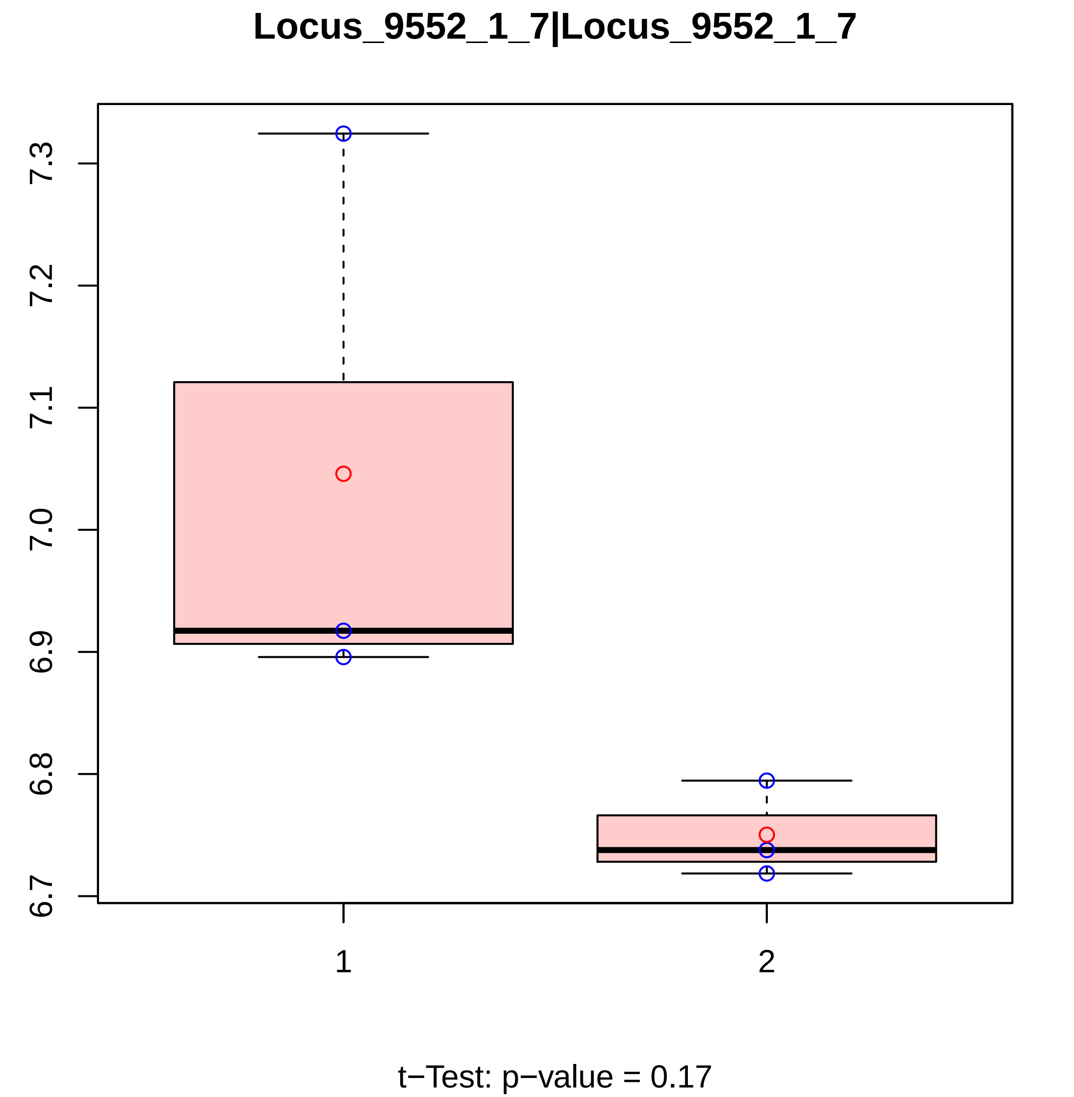

Locus_9552_1_7|Locus_9552_1_7
7.3
7.2
7.1
7.0
6.9
6.8
6.7
1
2
t−Test: p−value = 0.17

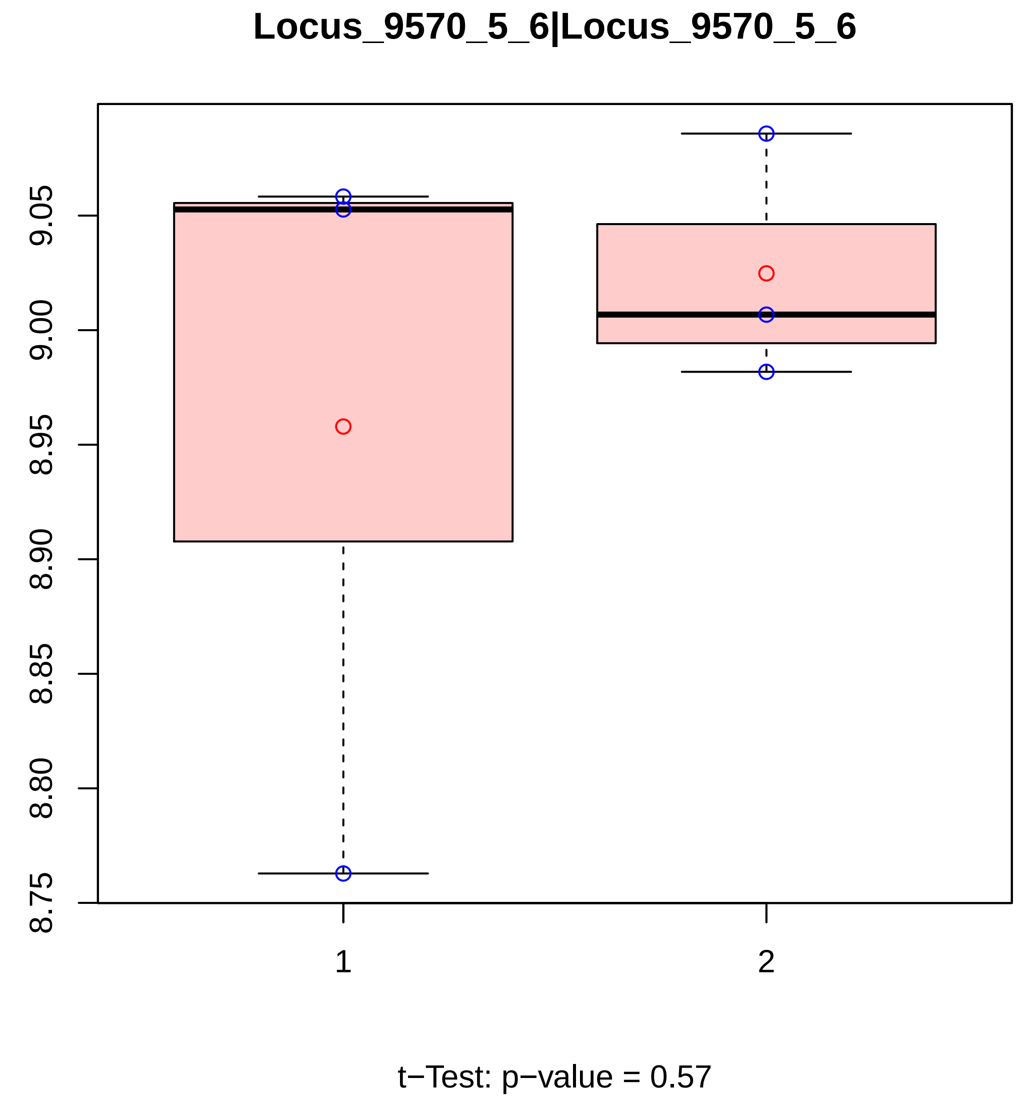

Locus_9570_5_6|Locus_9570_5_6
9.05
9.00
8.95
8.90
8.85
8.80
8.75
1
2
t−Test: p−value = 0.57

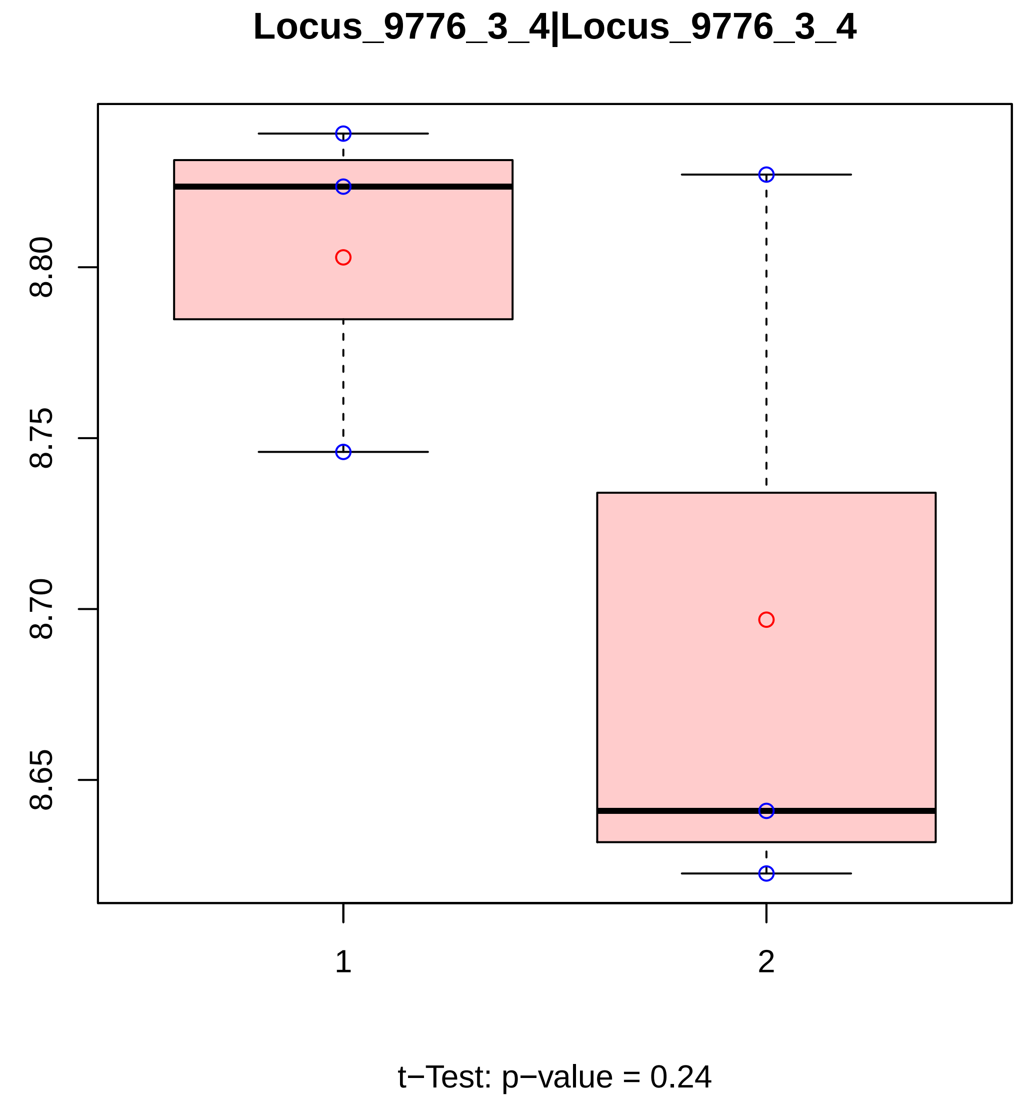

Locus_9776_3_4|Locus_9776_3_4
8.65
8.70
8.75
8.80
1
2
t−Test: p−value = 0.24

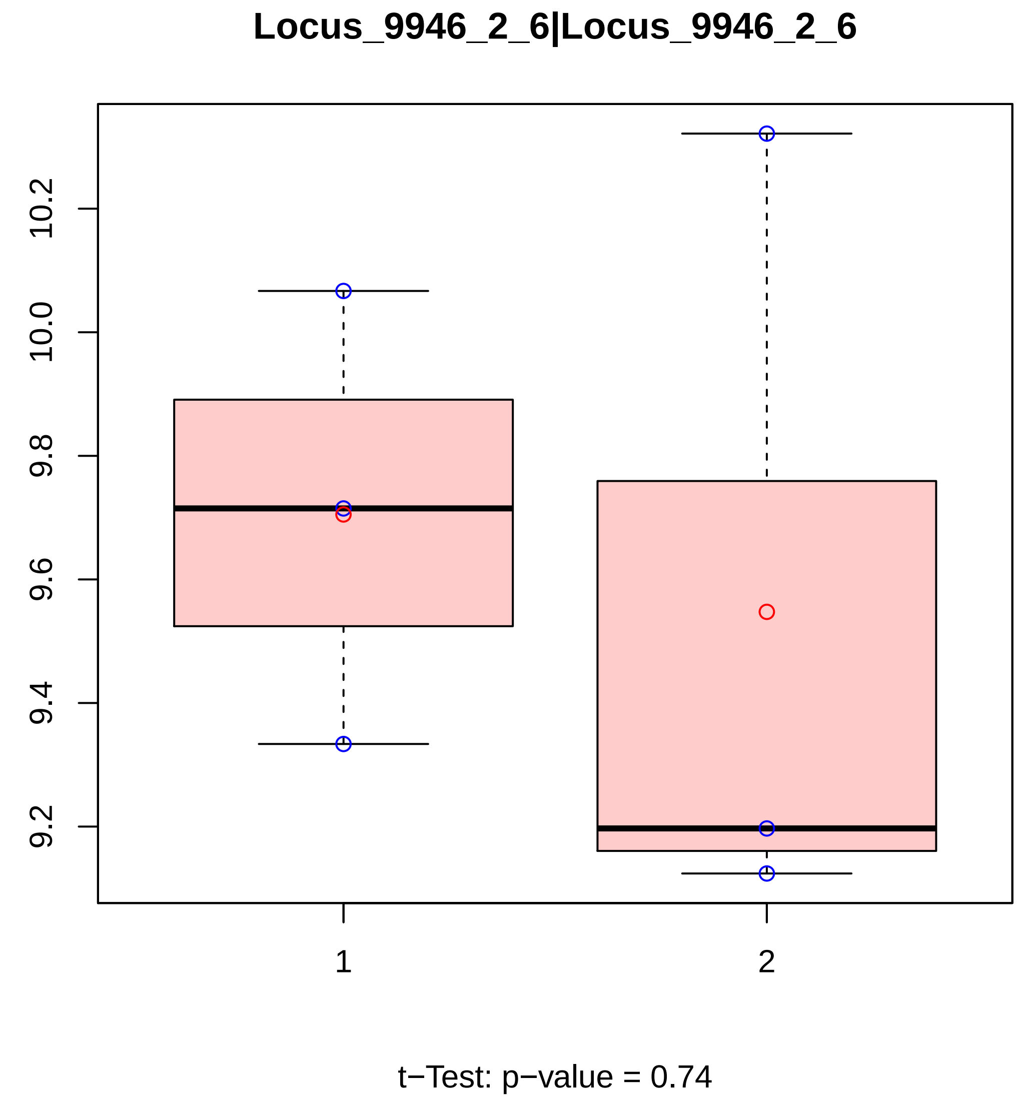
Locus_9946_2_6|Locus_9946_2_6
10.2
10.0
9.8
9.6
9.4
9.2
1
2
t−Test: p−value = 0.74

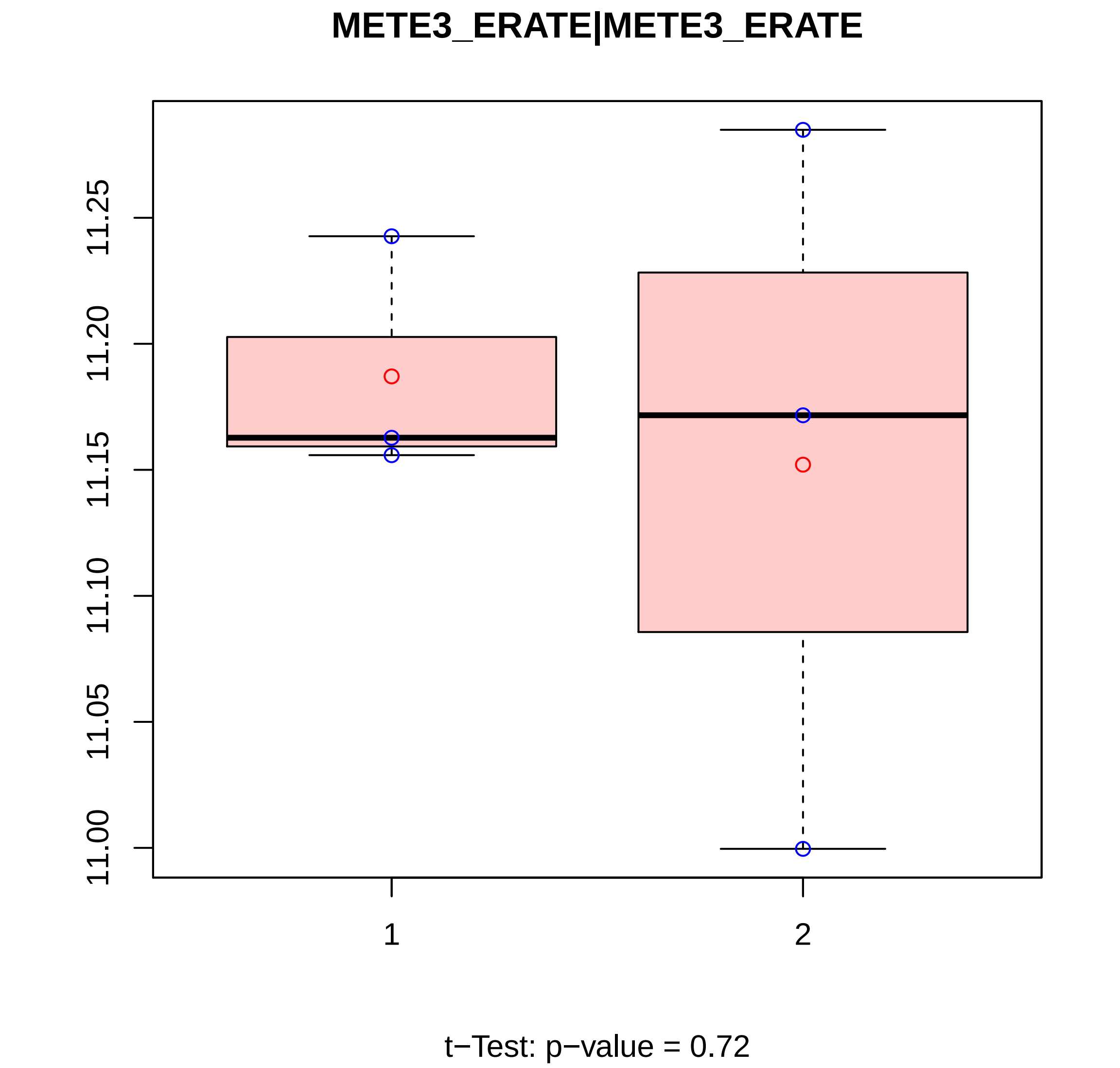
METE3_ERATE|METE3_ERATE
11.00
11.05
11.10
11.15
11.20
11.25
1
2
t−Test: p−value = 0.72

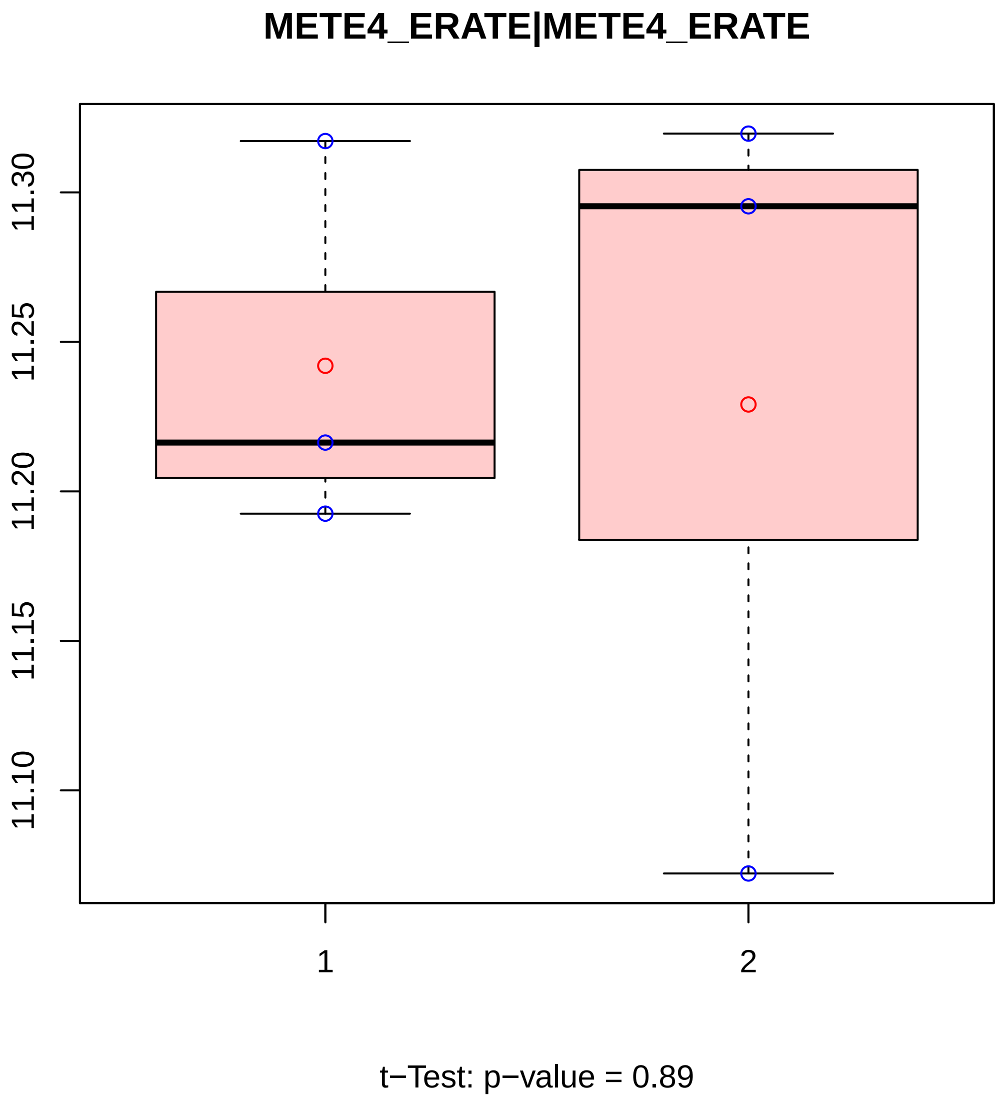
METE4_ERATE|METE4_ERATE
11.30
11.25
11.20
11.15
11.10
1
2
t−Test: p−value = 0.89

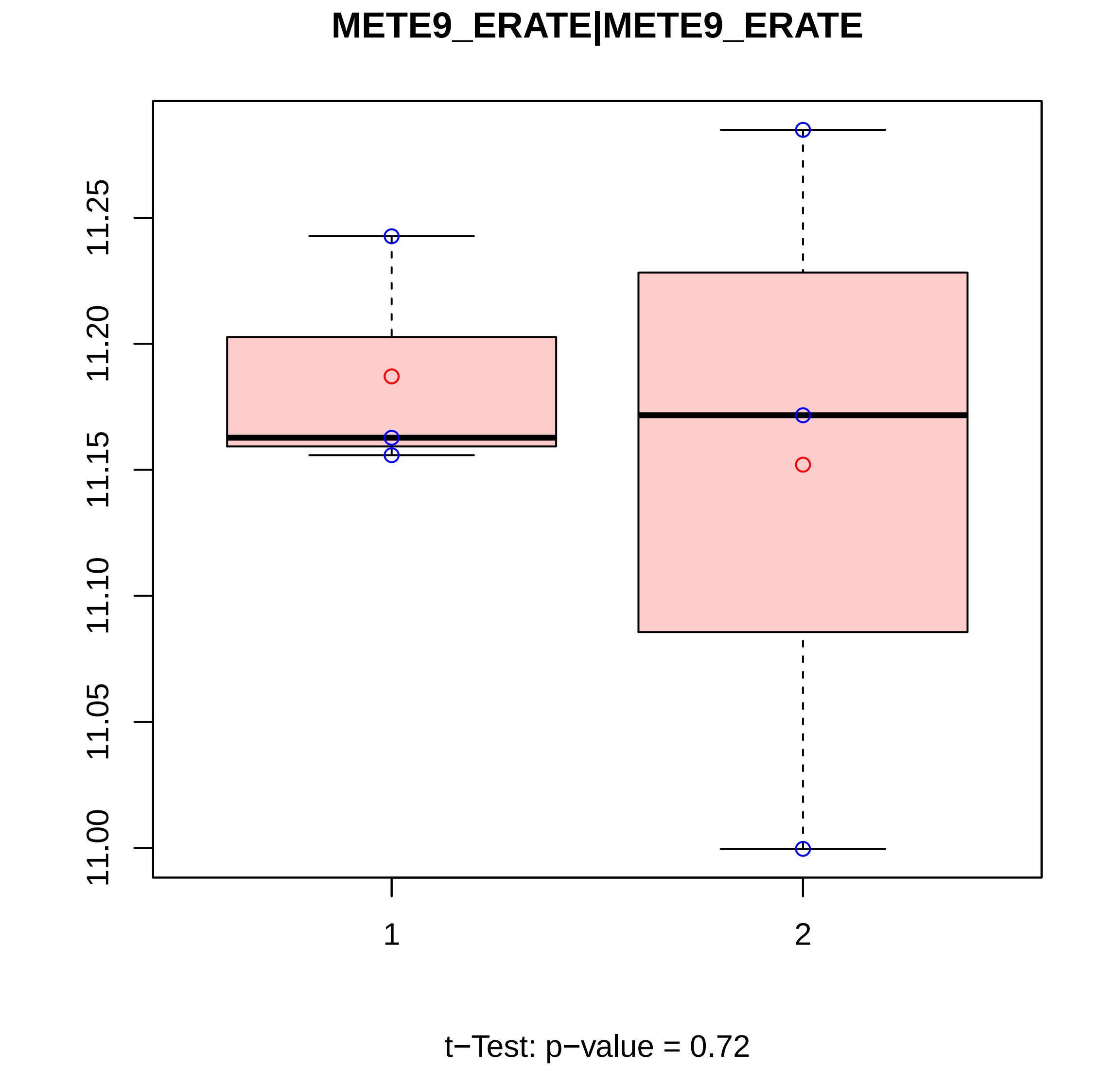
METE9_ERATE|METE9_ERATE
11.00
11.05
11.10
11.15
11.20
11.25
1
2
t−Test: p−value = 0.72

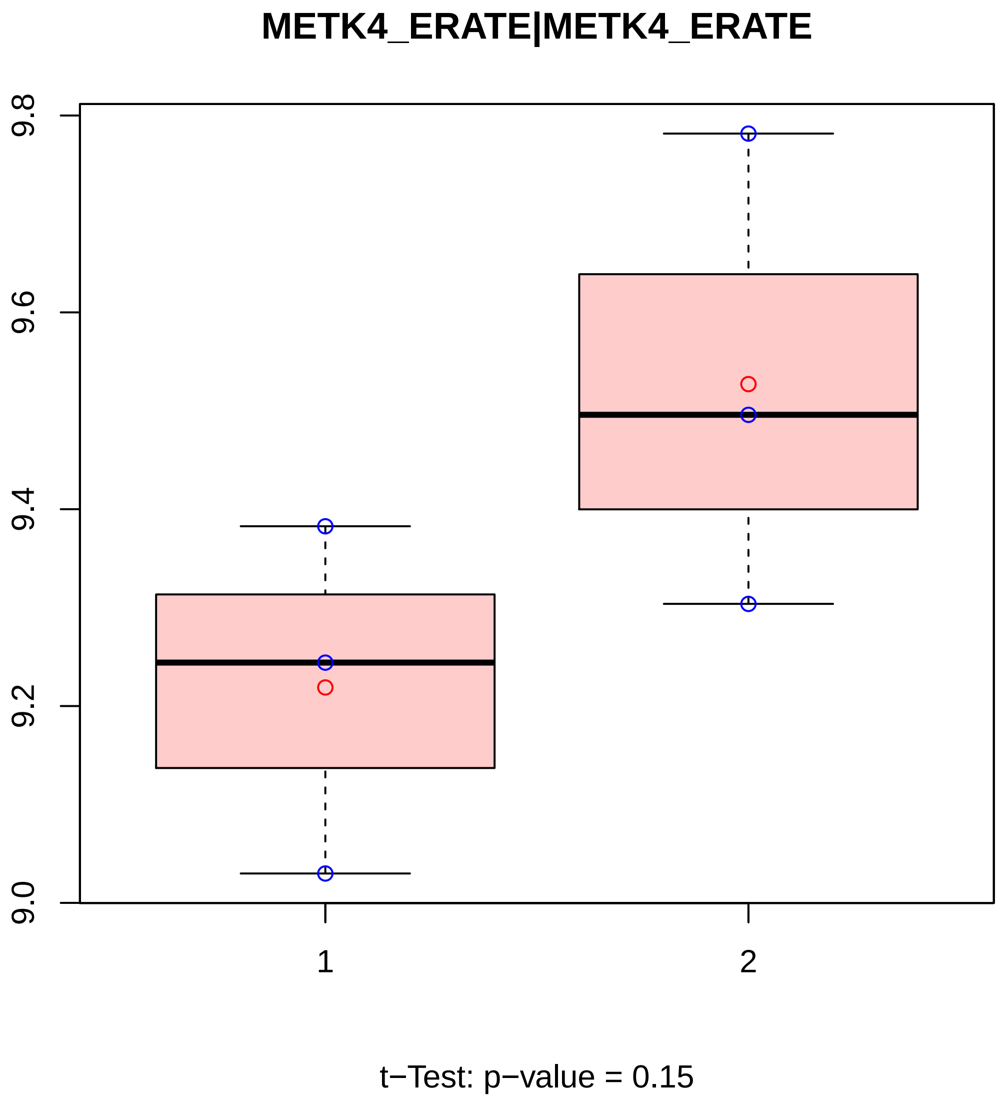
METK4_ERATE|METK4_ERATE
9.0
9.2
9.4
9.6
9.8
1
2
t−Test: p−value = 0.15

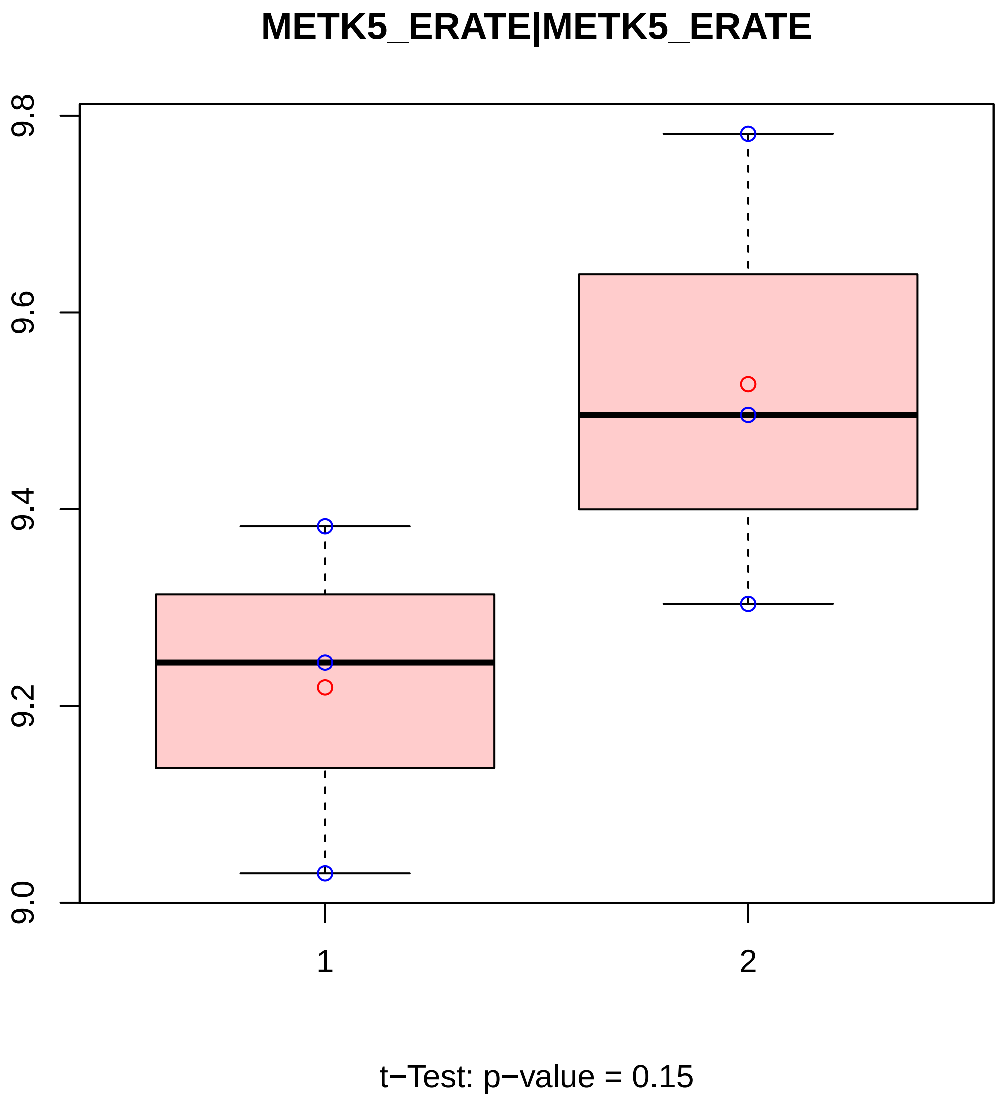
METK5_ERATE|METK5_ERATE
9.0
9.2
9.4
9.6
9.8
1
2
t−Test: p−value = 0.15

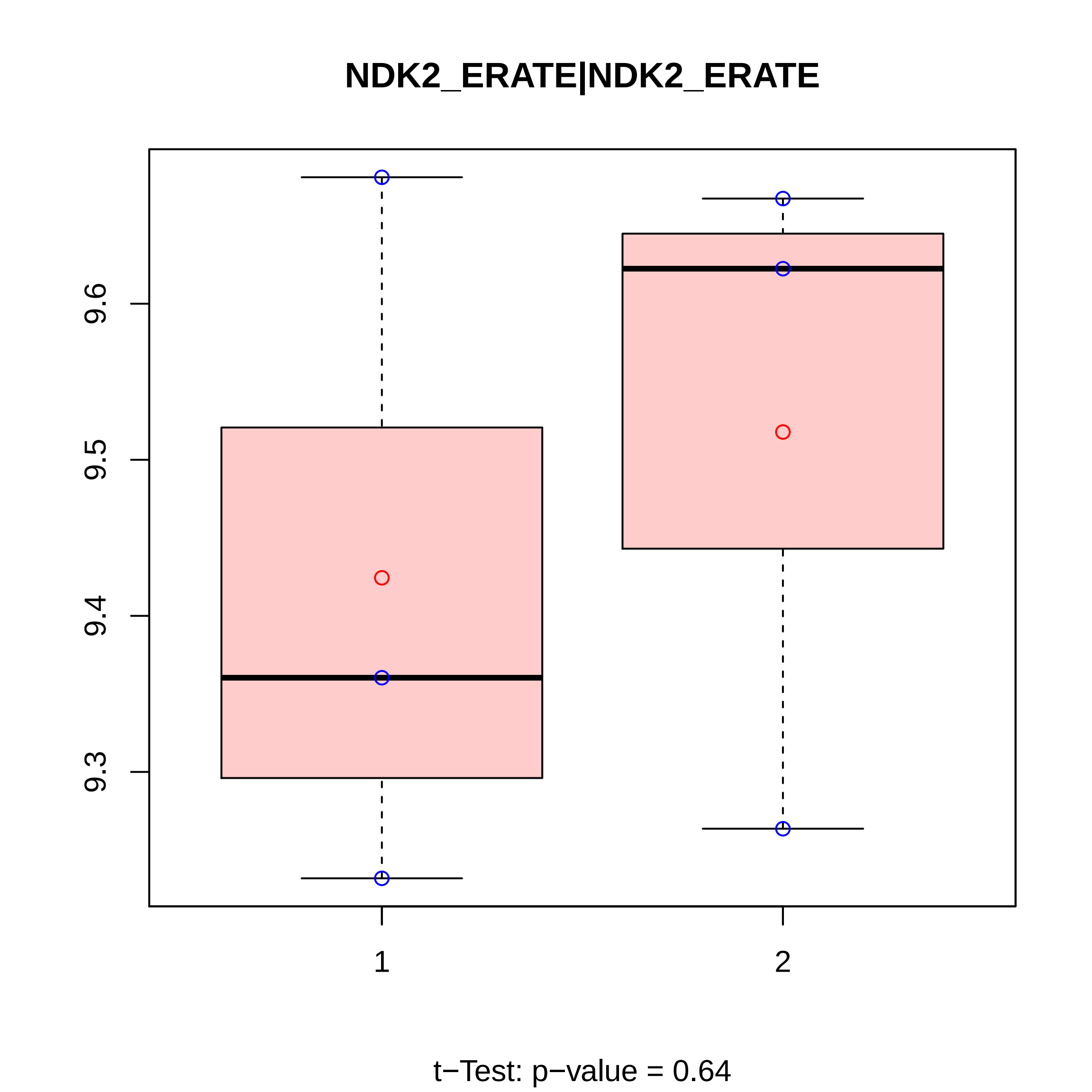
NDK2_ERATE|NDK2_ERATE
9.3
9.4
9.5
9.6
1
2
t−Test: p−value = 0.64

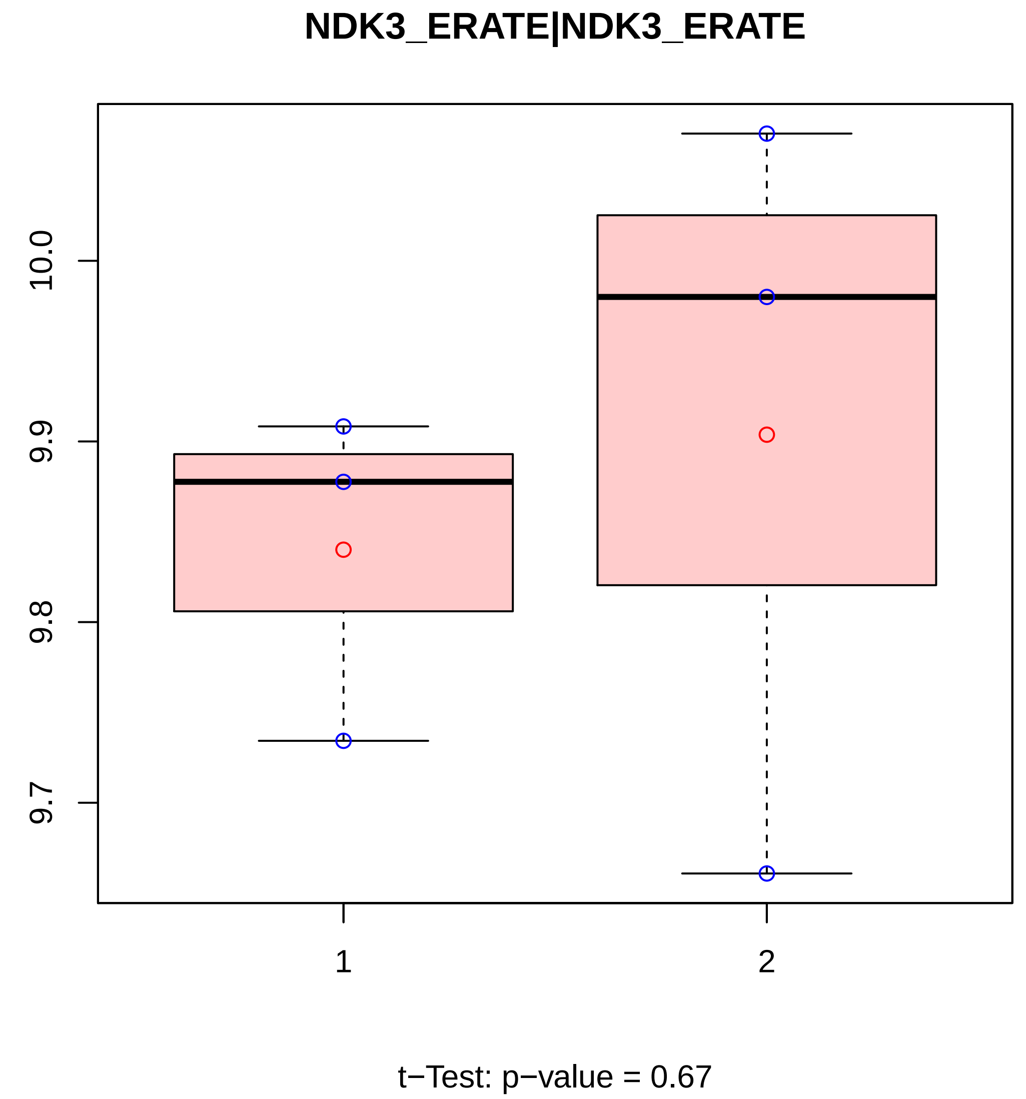

NDK3_ERATE|NDK3_ERATE
10.0
9.9
9.8
9.7
1
2
t−Test: p−value = 0.67

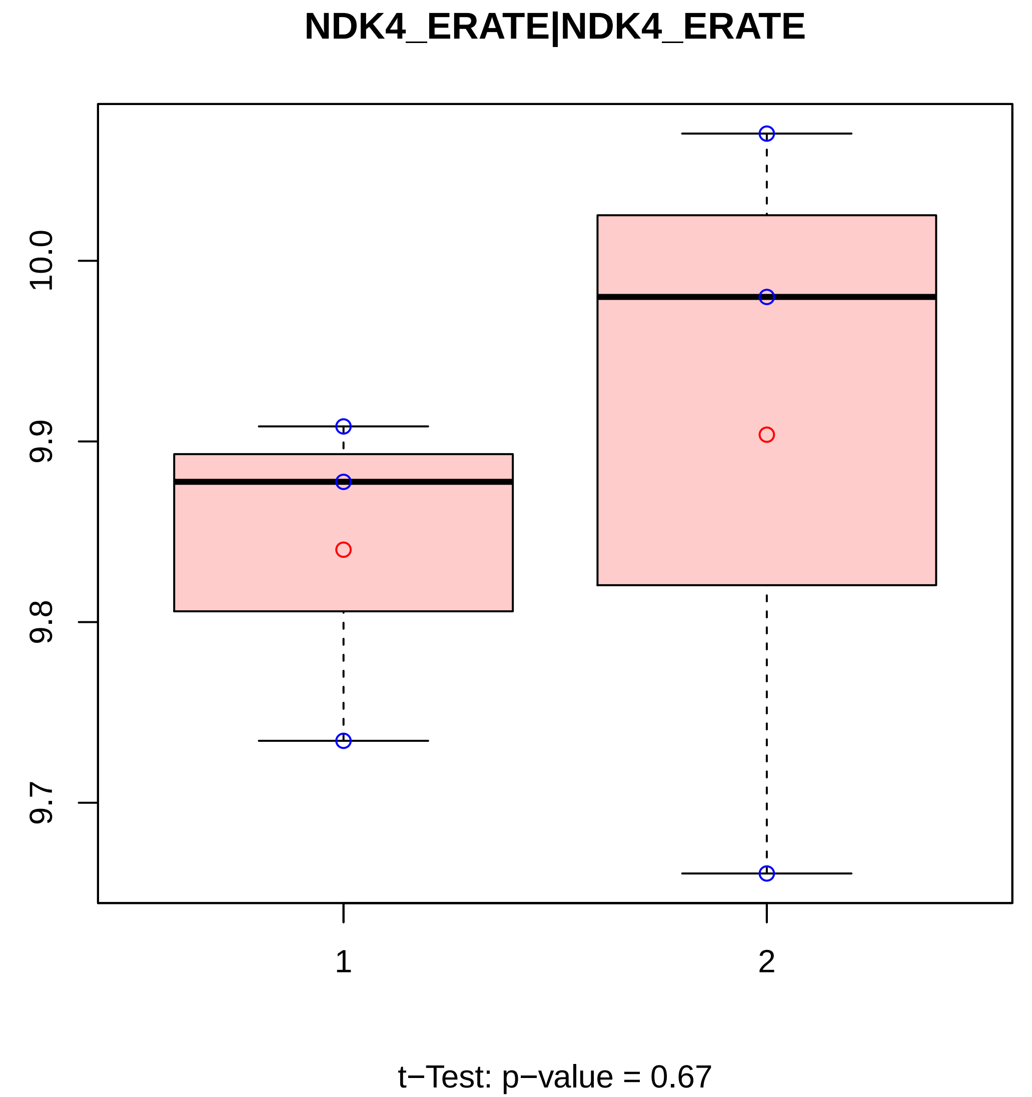

NDK4_ERATE|NDK4_ERATE
10.0
9.9
9.8
9.7
1
2
t−Test: p−value = 0.67

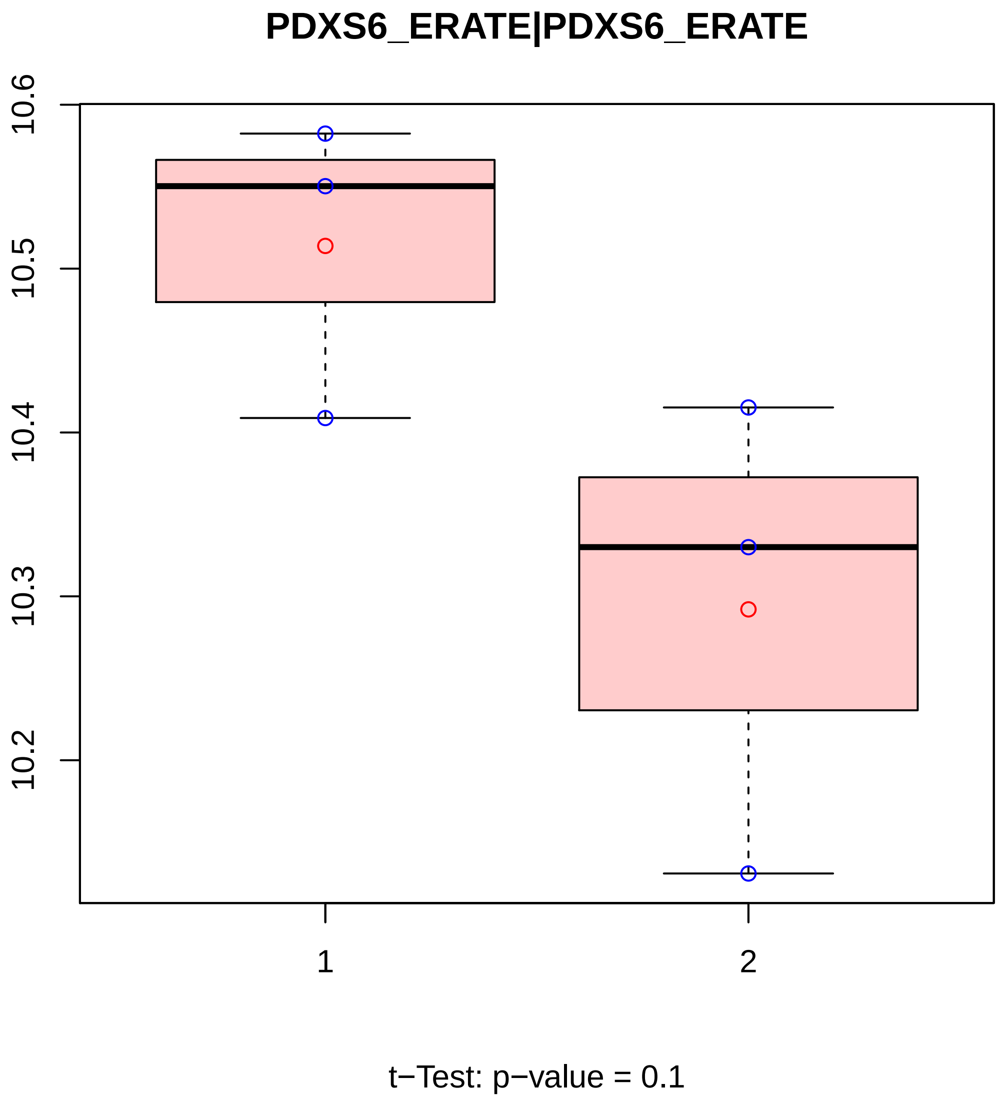

PDXS6_ERATE|PDXS6_ERATE
10.2
10.3
10.4
10.5
10.6
1
2
t−Test: p−value = 0.1

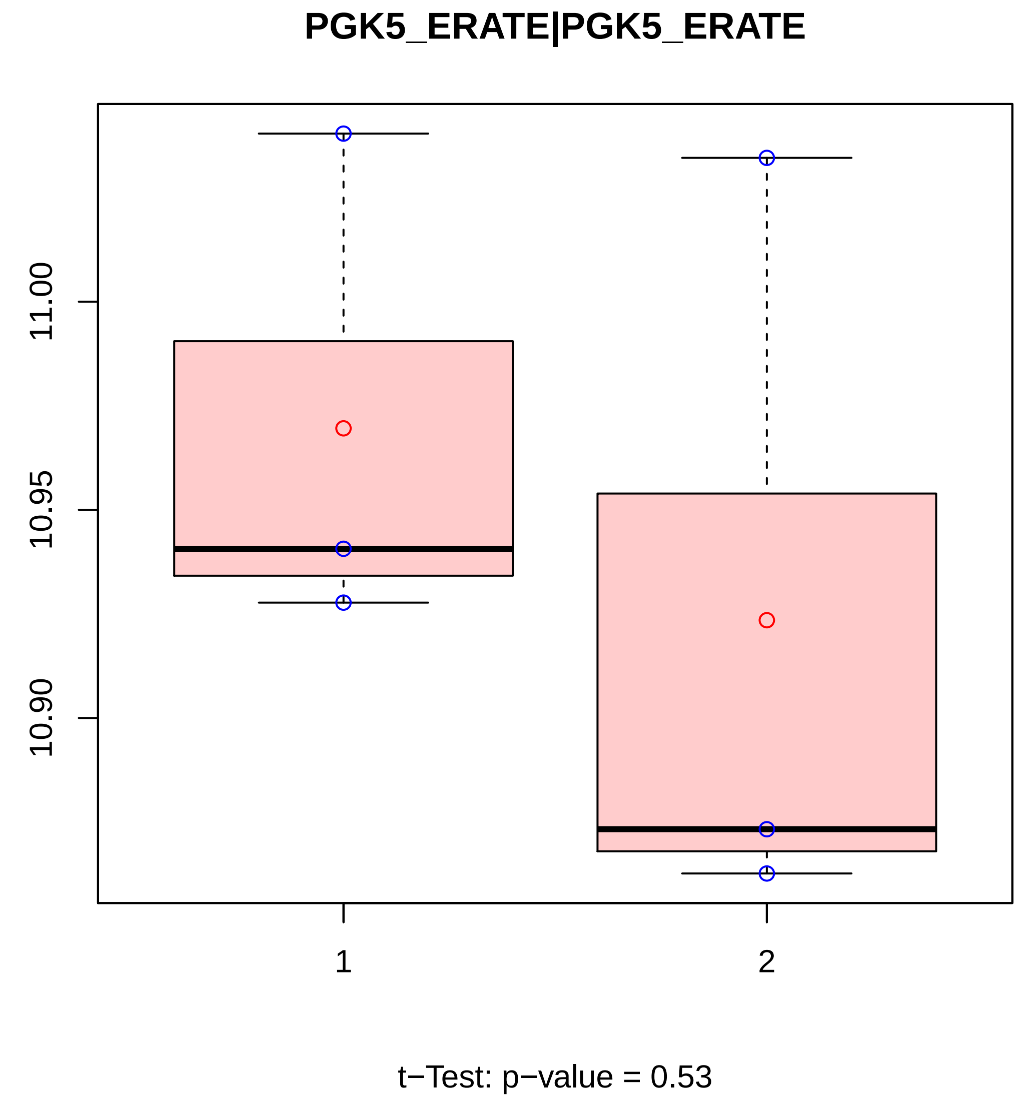
PGK5_ERATE|PGK5_ERATE
11.00
10.95
10.90
1
2
t−Test: p−value = 0.53

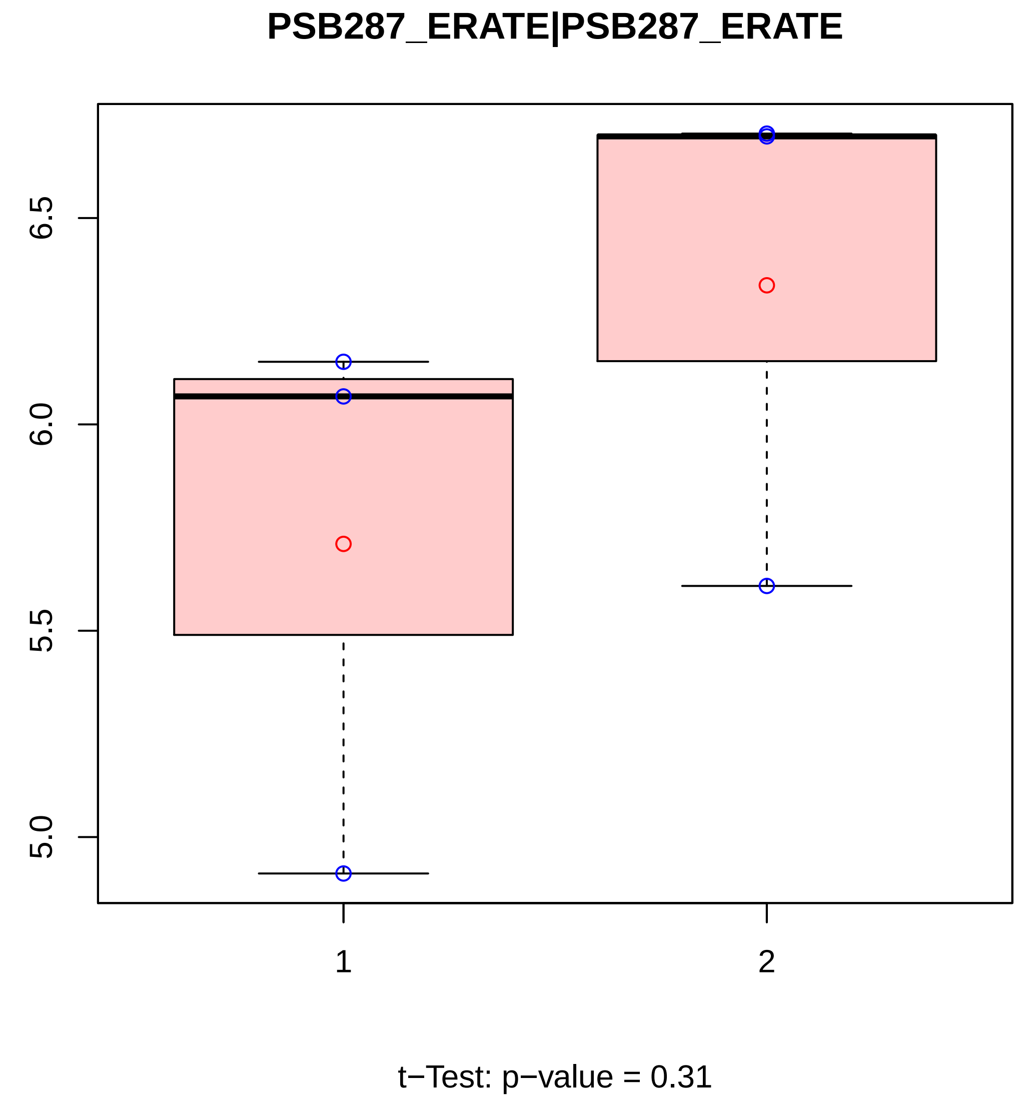

PSB287_ERATE|PSB287_ERATE
6.5
6.0
5.5
5.0
1
2
t−Test: p−value = 0.31

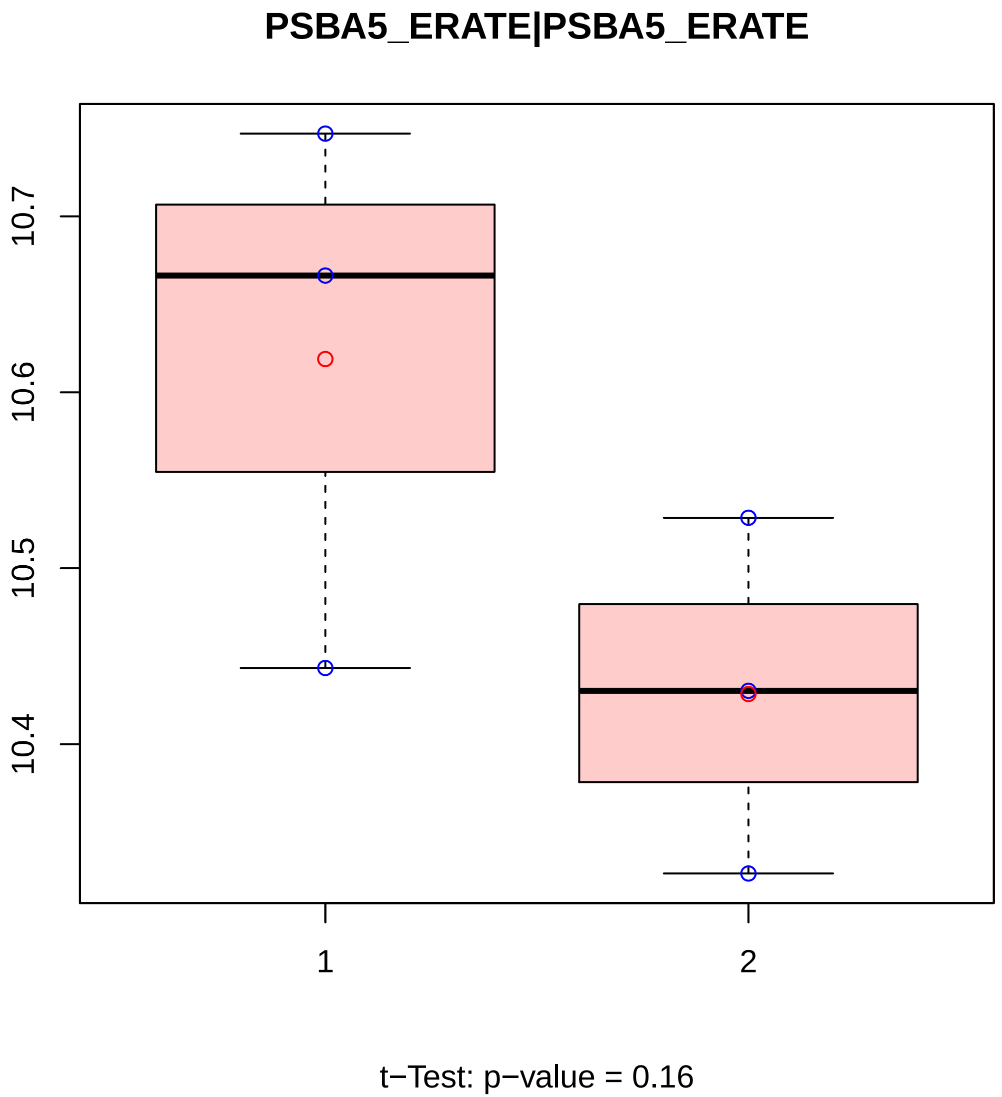
PSBA5_ERATE|PSBA5_ERATE
10.4
10.5
10.6
10.7
1
2
t−Test: p−value = 0.16

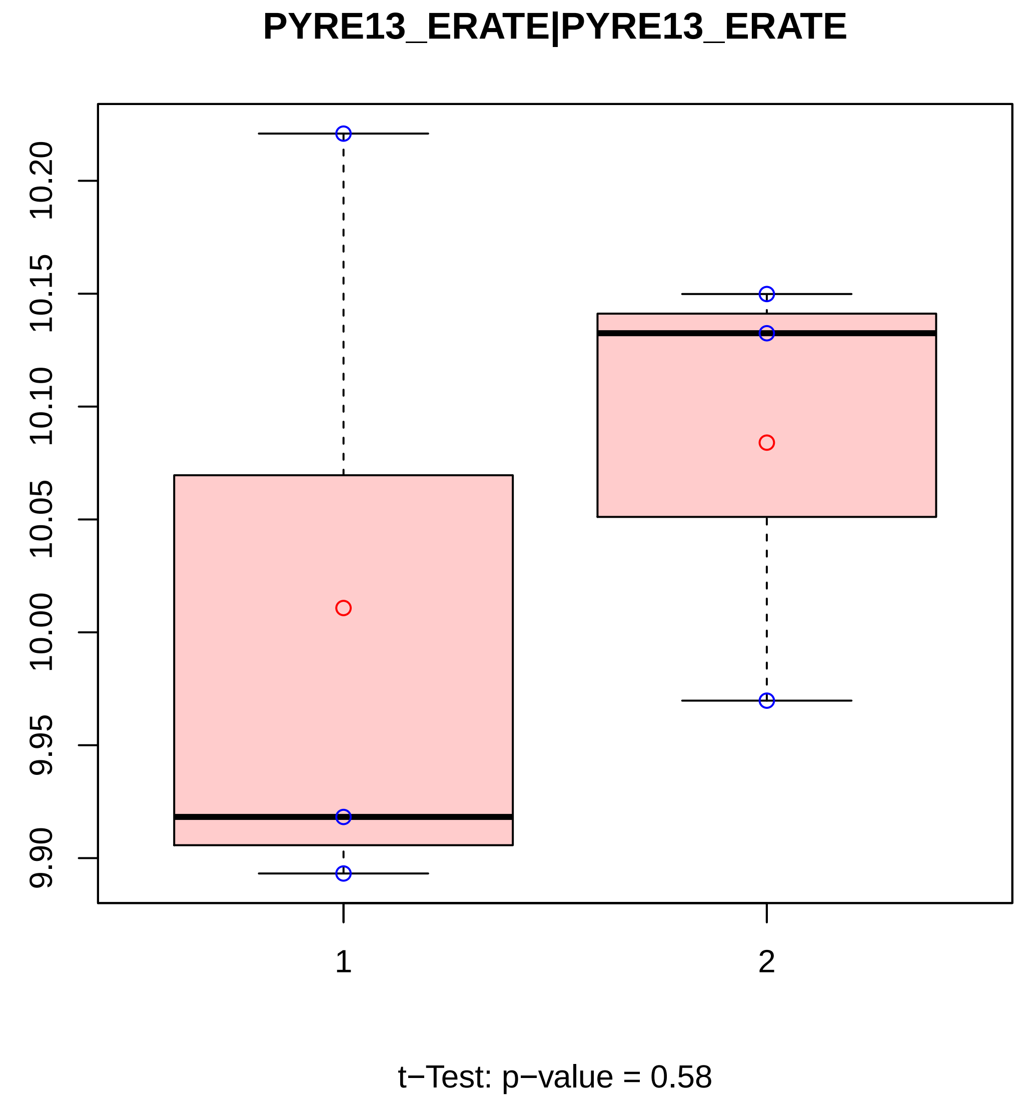
PYRE13_ERATE|PYRE13_ERATE
10.20
10.15
10.10
10.05
10.00
9.95
9.90
1
2
t−Test: p−value = 0.58

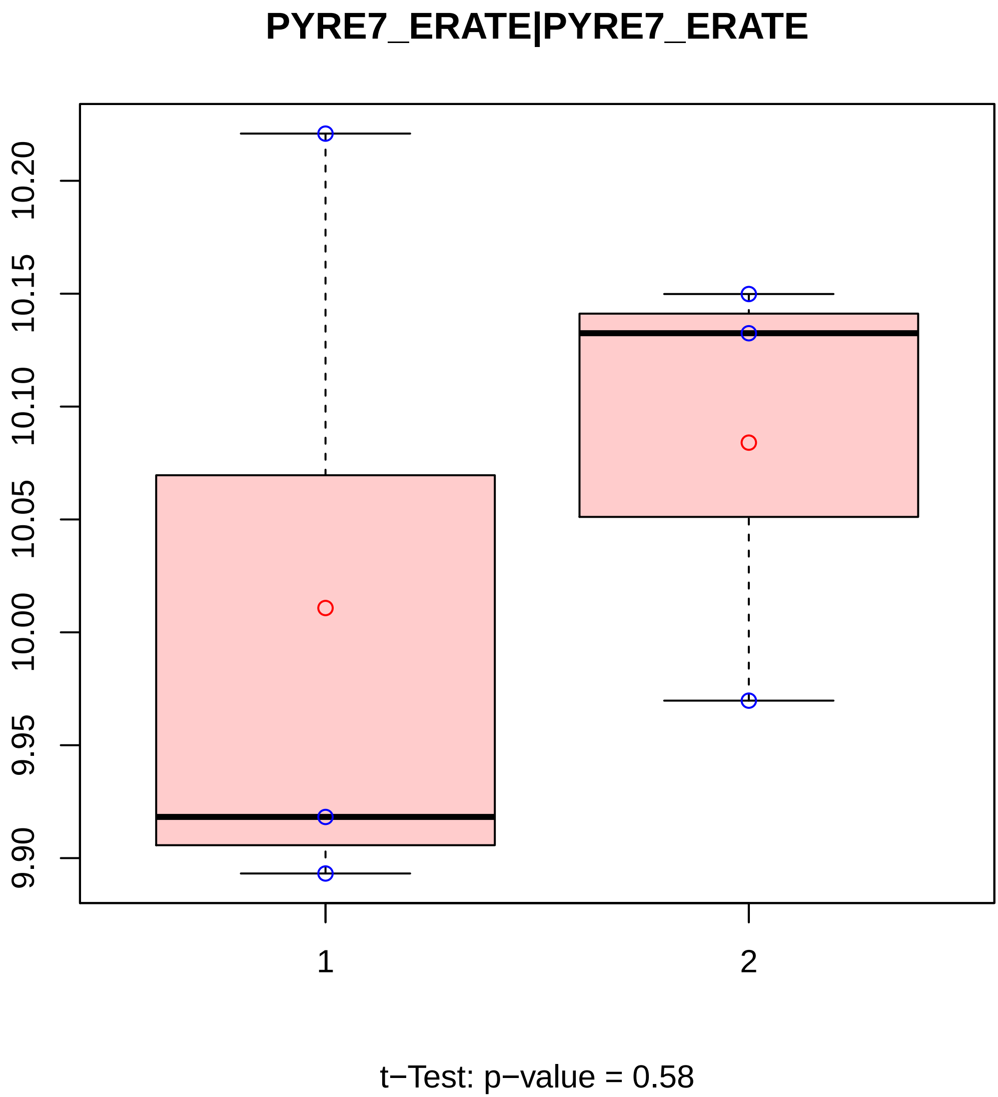
PYRE7_ERATE|PYRE7_ERATE
10.20
10.15
10.10
10.05
10.00
9.95
9.90
1
2
t−Test: p−value = 0.58

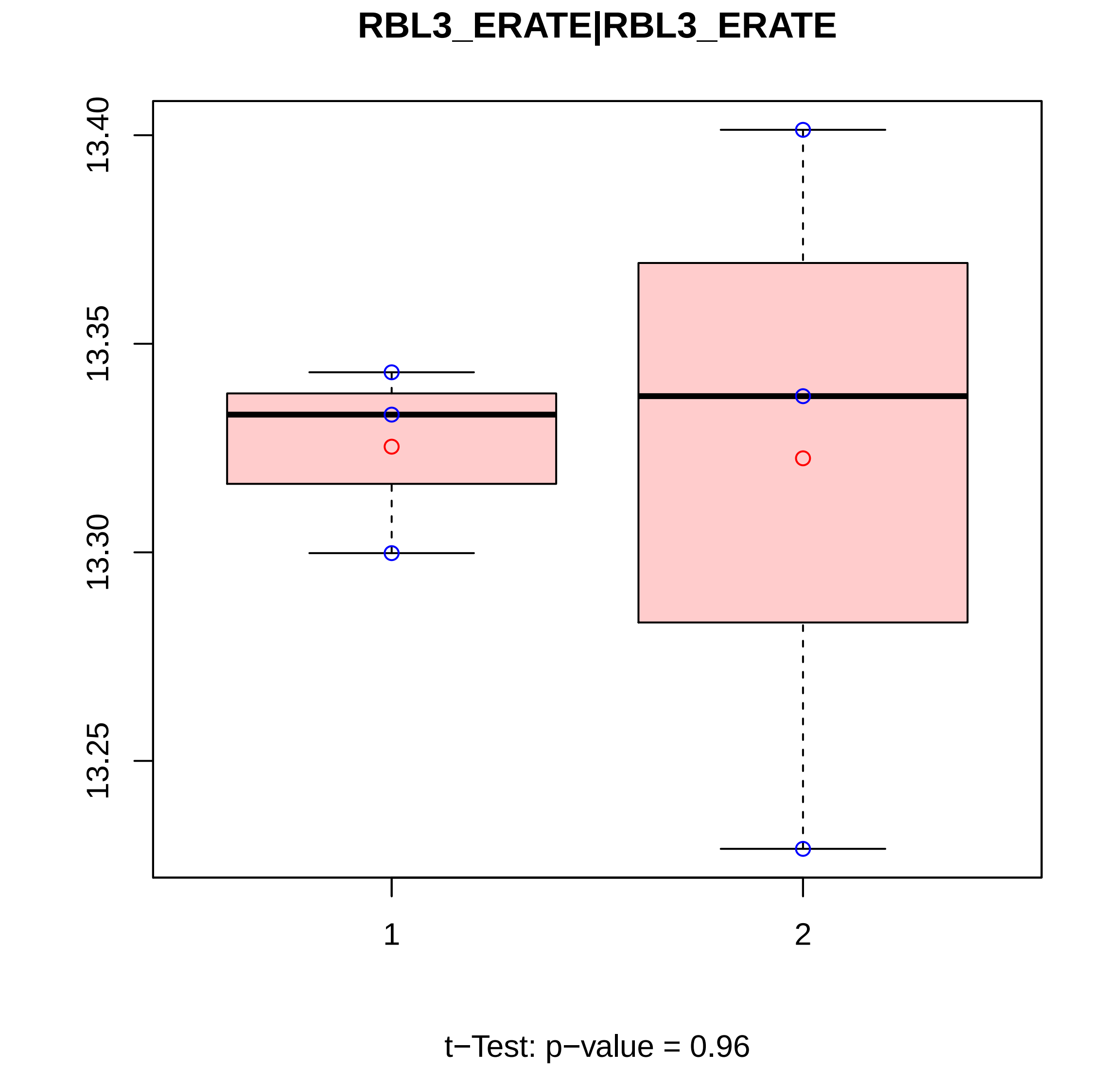

RBL3_ERATE|RBL3_ERATE
13.40
13.35
13.30
13.25
1
2
t−Test: p−value = 0.96

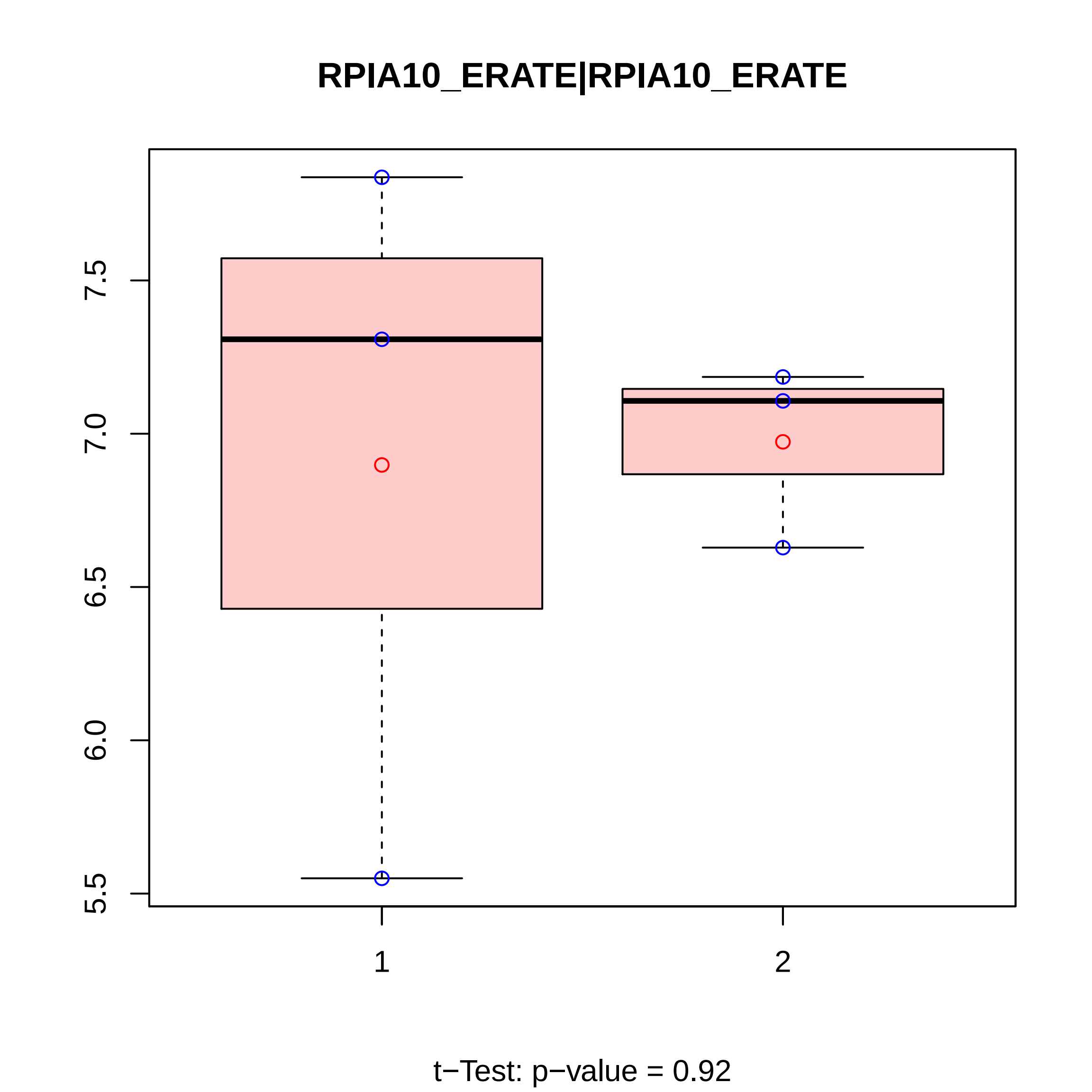
RPIA10_ERATE|RPIA10_ERATE
7.5
7.0
6.5
6.0
5.5
1
2
t−Test: p−value = 0.92

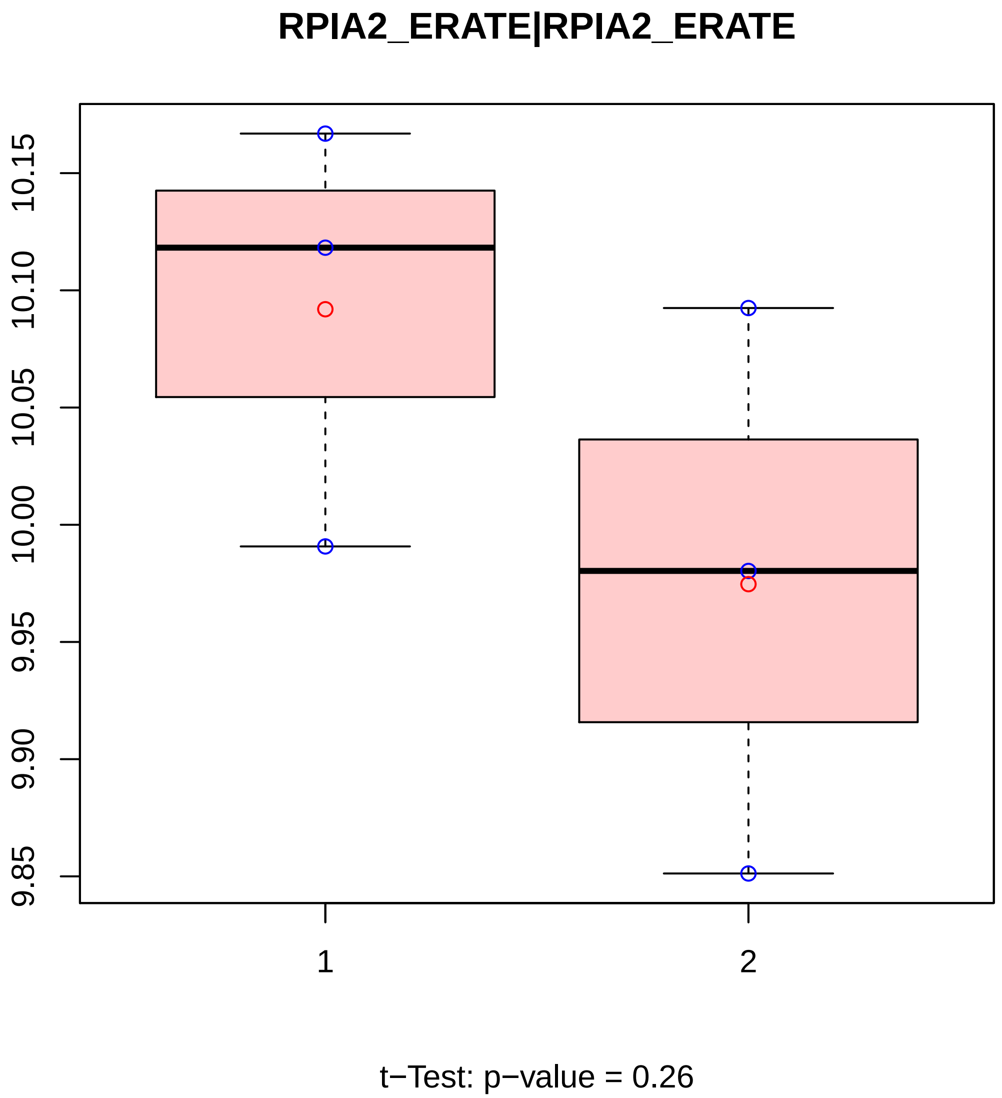
RPIA2_ERATE|RPIA2_ERATE
10.15
10.10
10.05
10.00
9.95
9.90
9.85
1
2
t−Test: p−value = 0.26

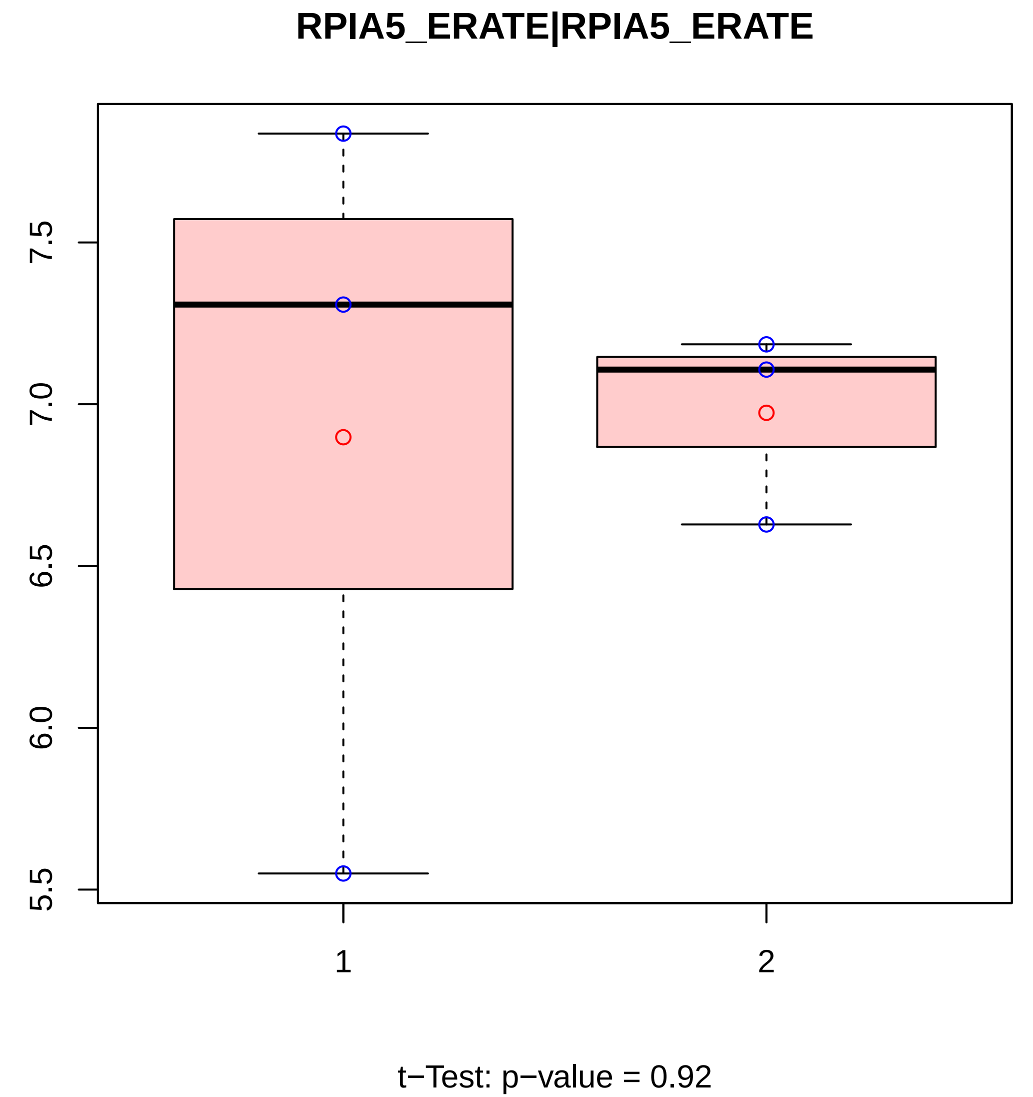
RPIA5_ERATE|RPIA5_ERATE
7.5
7.0
6.5
6.0
5.5
1
2
t−Test: p−value = 0.92

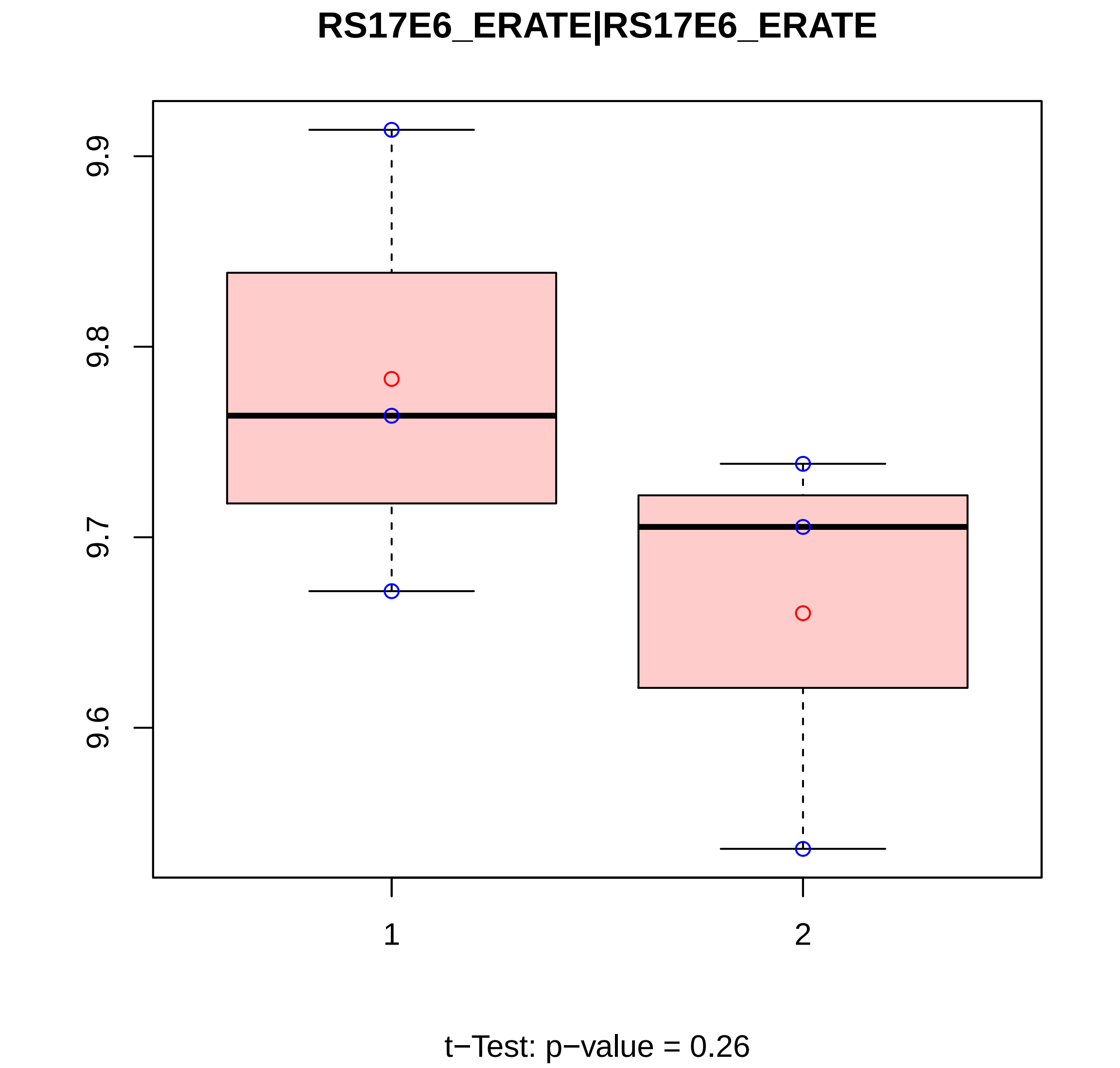
RS17E6_ERATE|RS17E6_ERATE
9.6
9.7
9.8
9.9
1
2
t−Test: p−value = 0.26

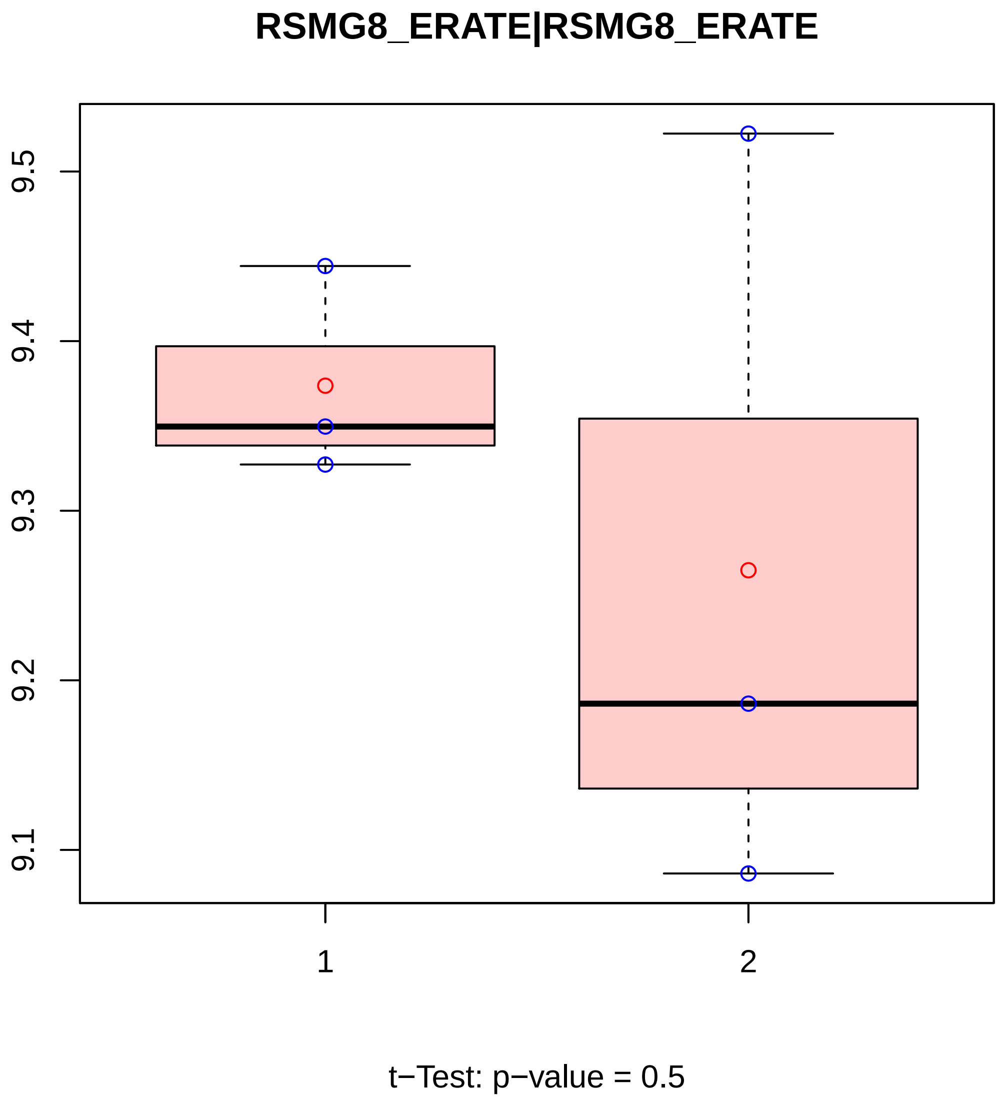
RSMG8_ERATE|RSMG8_ERATE
9.1
9.2
9.3
9.4
9.5
1
2
t−Test: p−value = 0.5

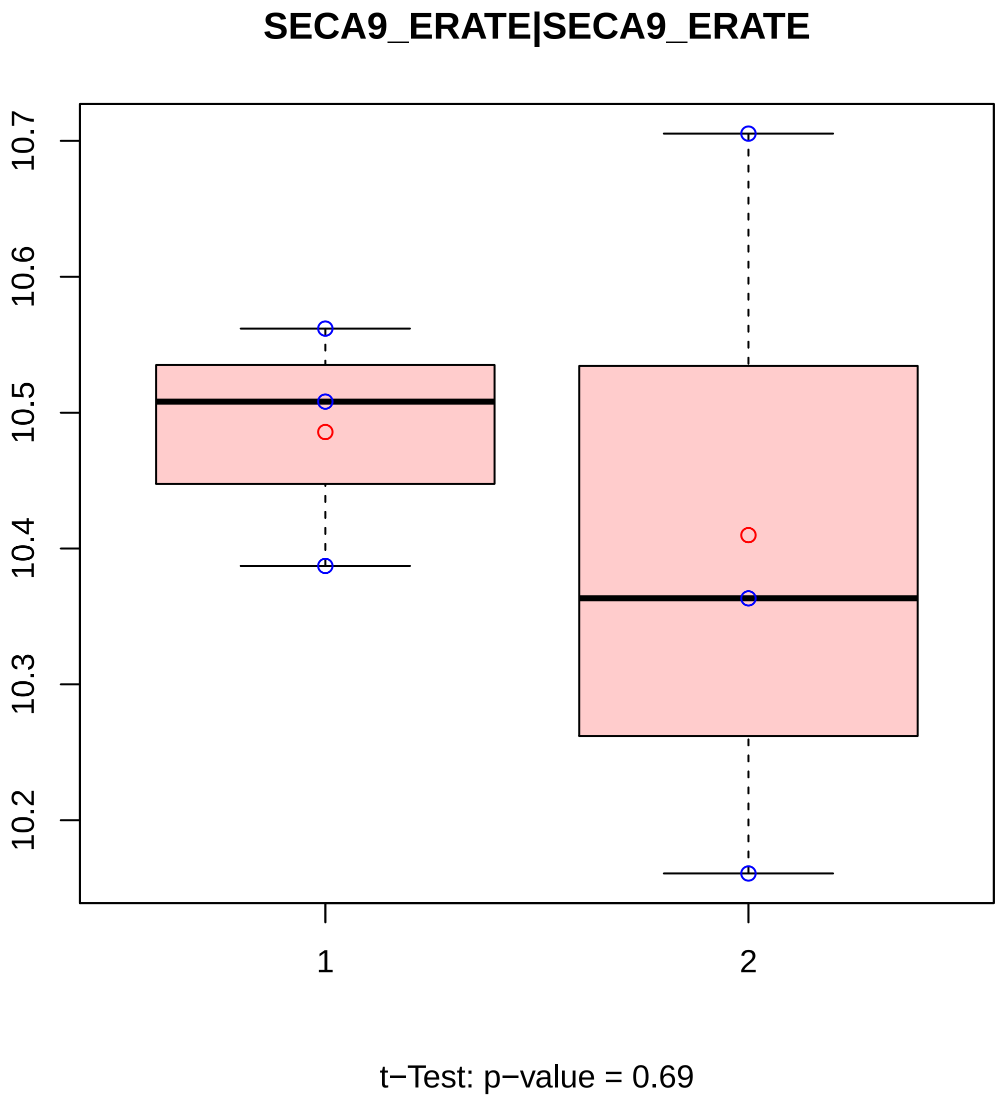

SECA9_ERATE|SECA9_ERATE
10.7
10.6
10.5
10.4
10.3
10.2
1
2
t−Test: p−value = 0.69

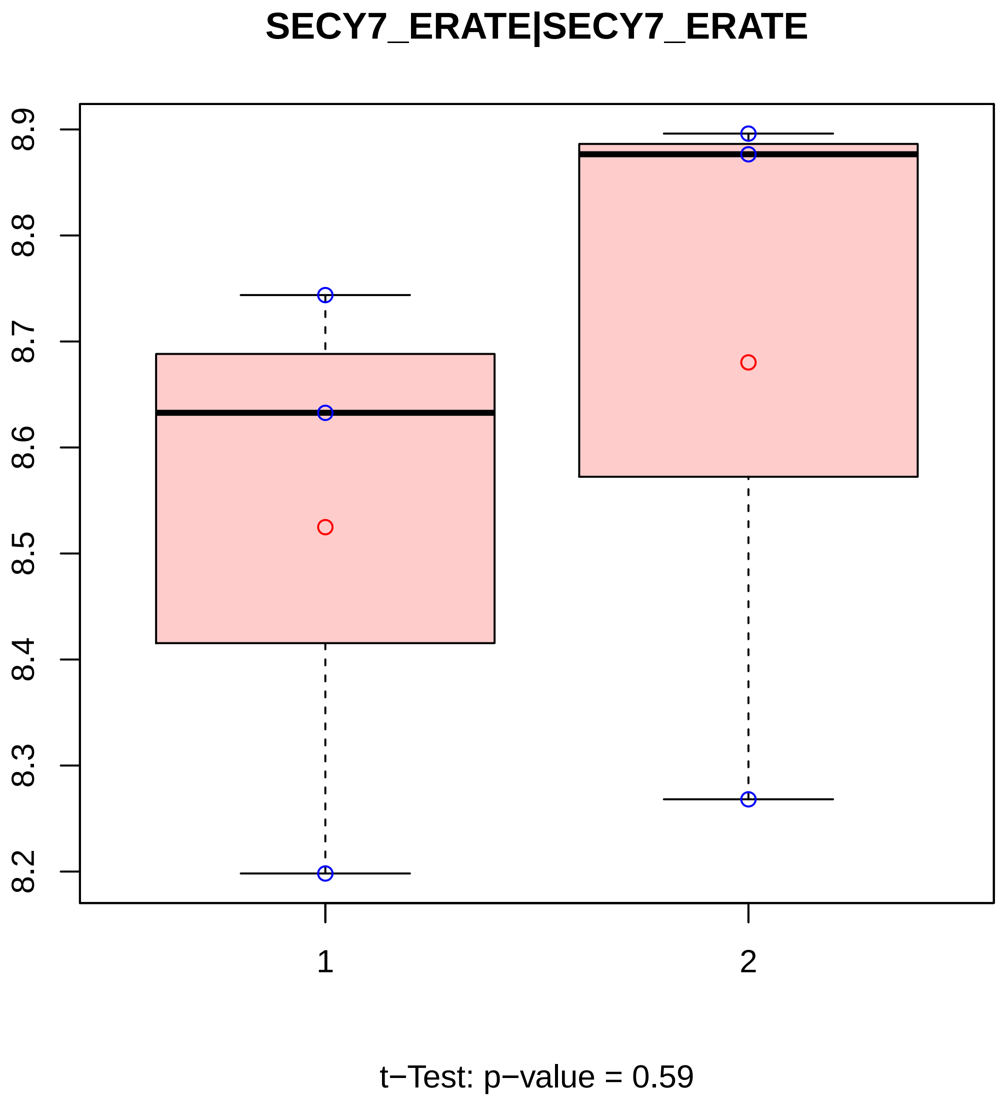

SECY7_ERATE|SECY7_ERATE
8.2
8.3
8.4
8.5
8.6
8.7
8.8
8.9
1
2
t−Test: p−value = 0.59

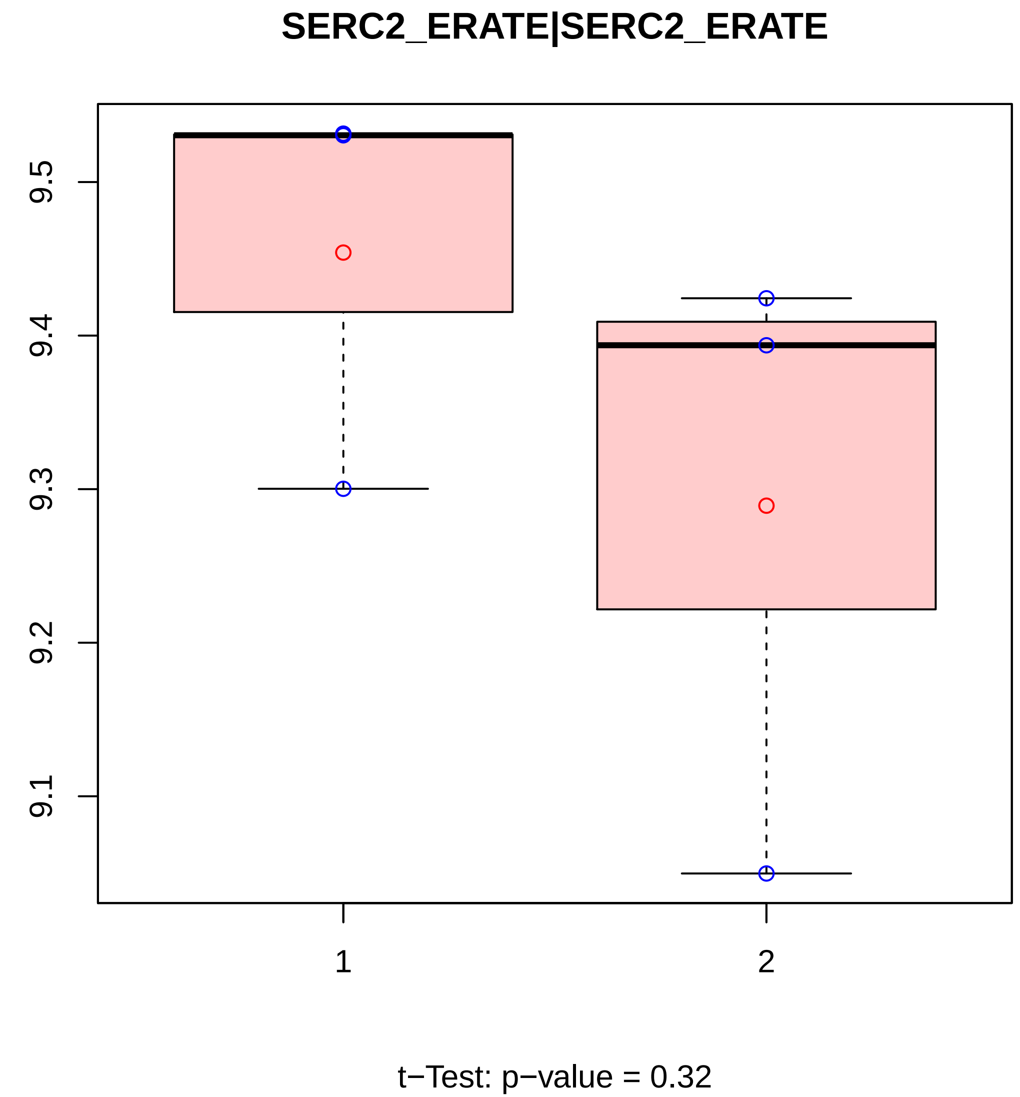
SERC2_ERATE|SERC2_ERATE
9.5
9.4
9.3
9.2
9.1
1
2
t−Test: p−value = 0.32

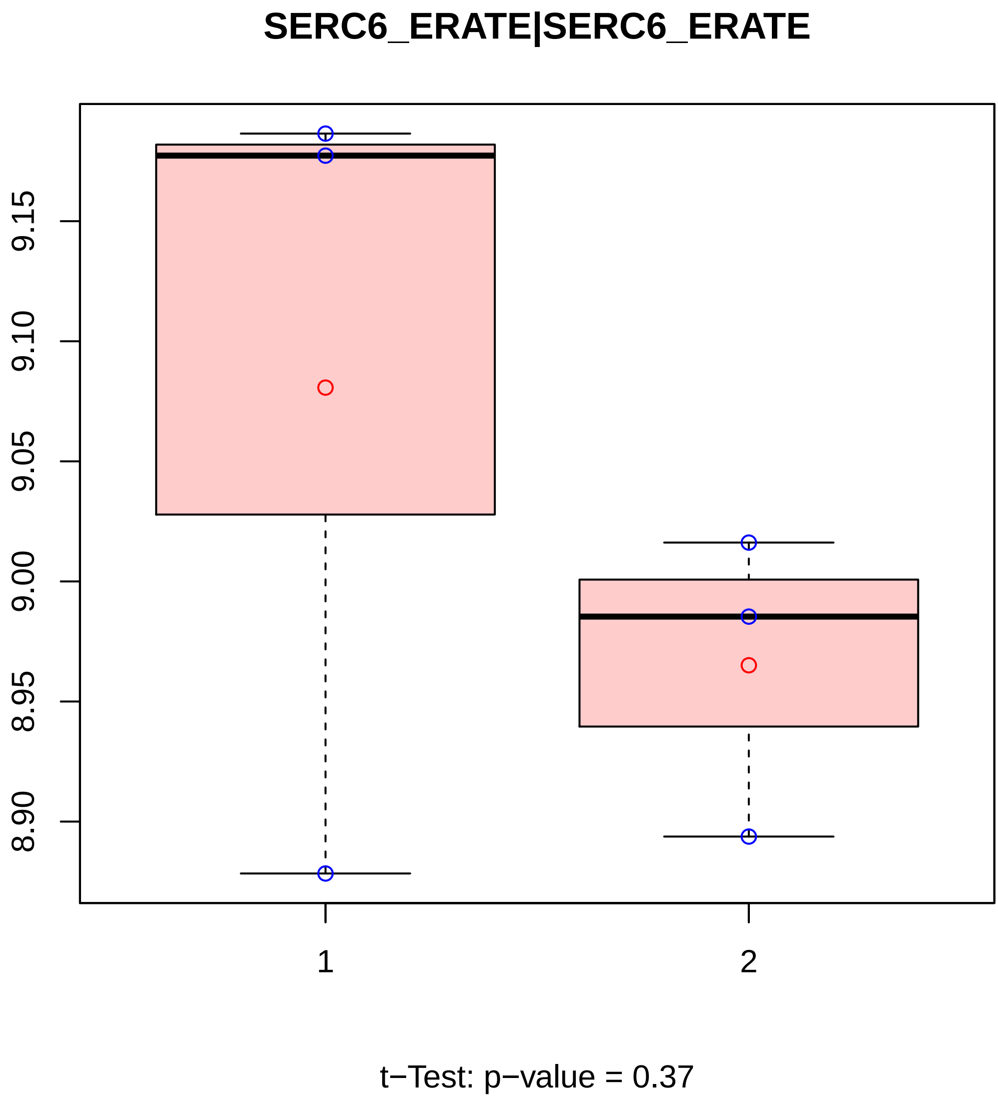

SERC6_ERATE|SERC6_ERATE
9.15
9.10
9.05
9.00
8.95
8.90
1
2
t−Test: p−value = 0.37

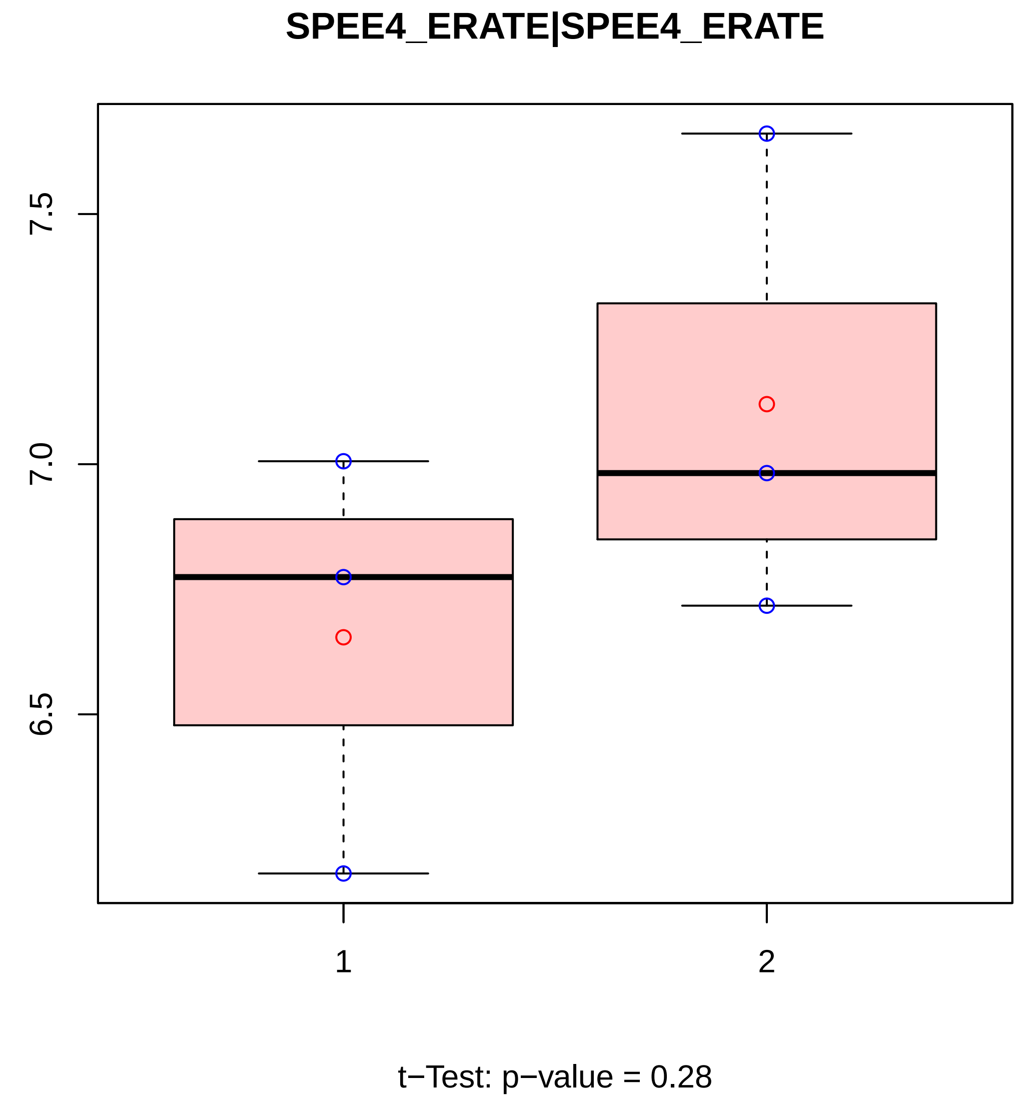

SPEE4_ERATE|SPEE4_ERATE
7.5
7.0
6.5
1
2
t−Test: p−value = 0.28

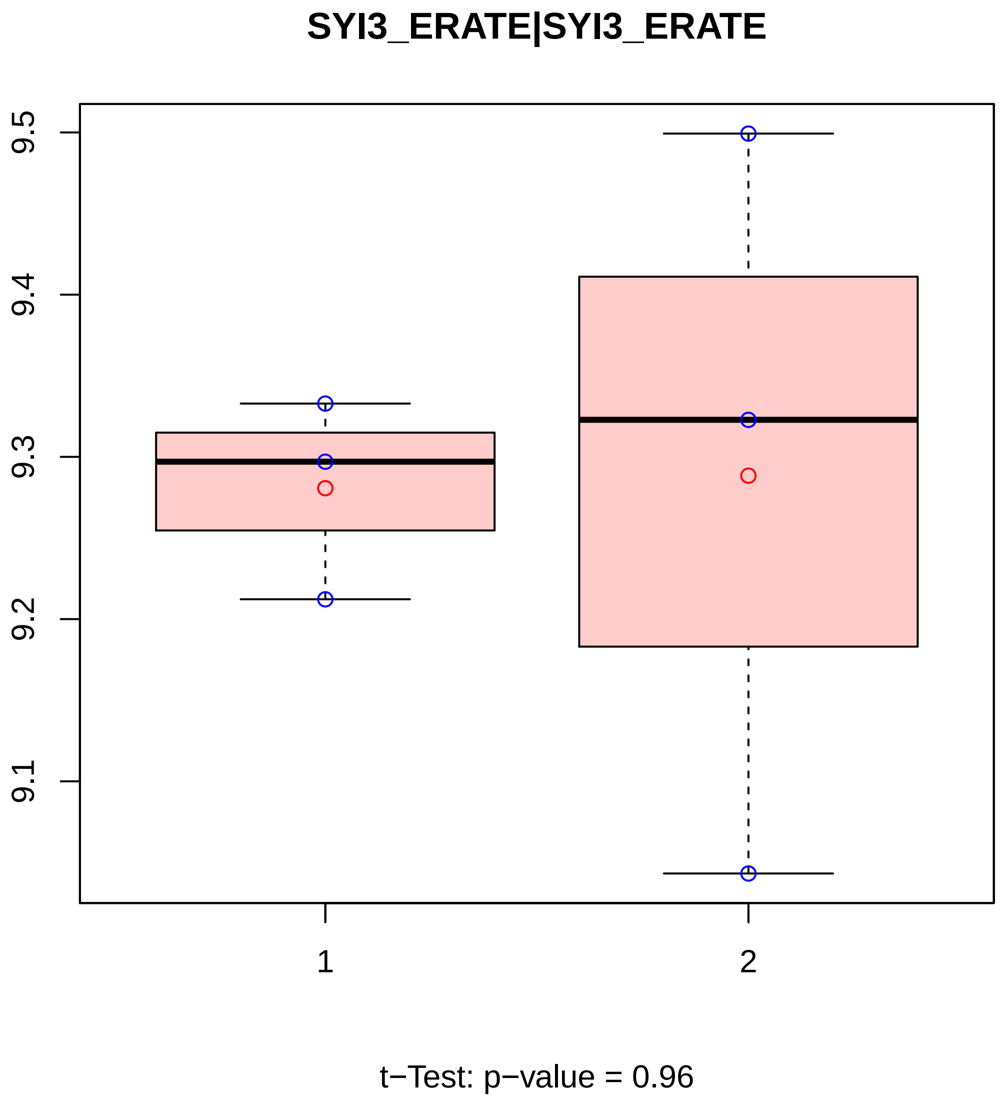

SYI3_ERATE|SYI3_ERATE
9.5
9.4
9.3
9.2
9.1
1
2
t−Test: p−value = 0.96

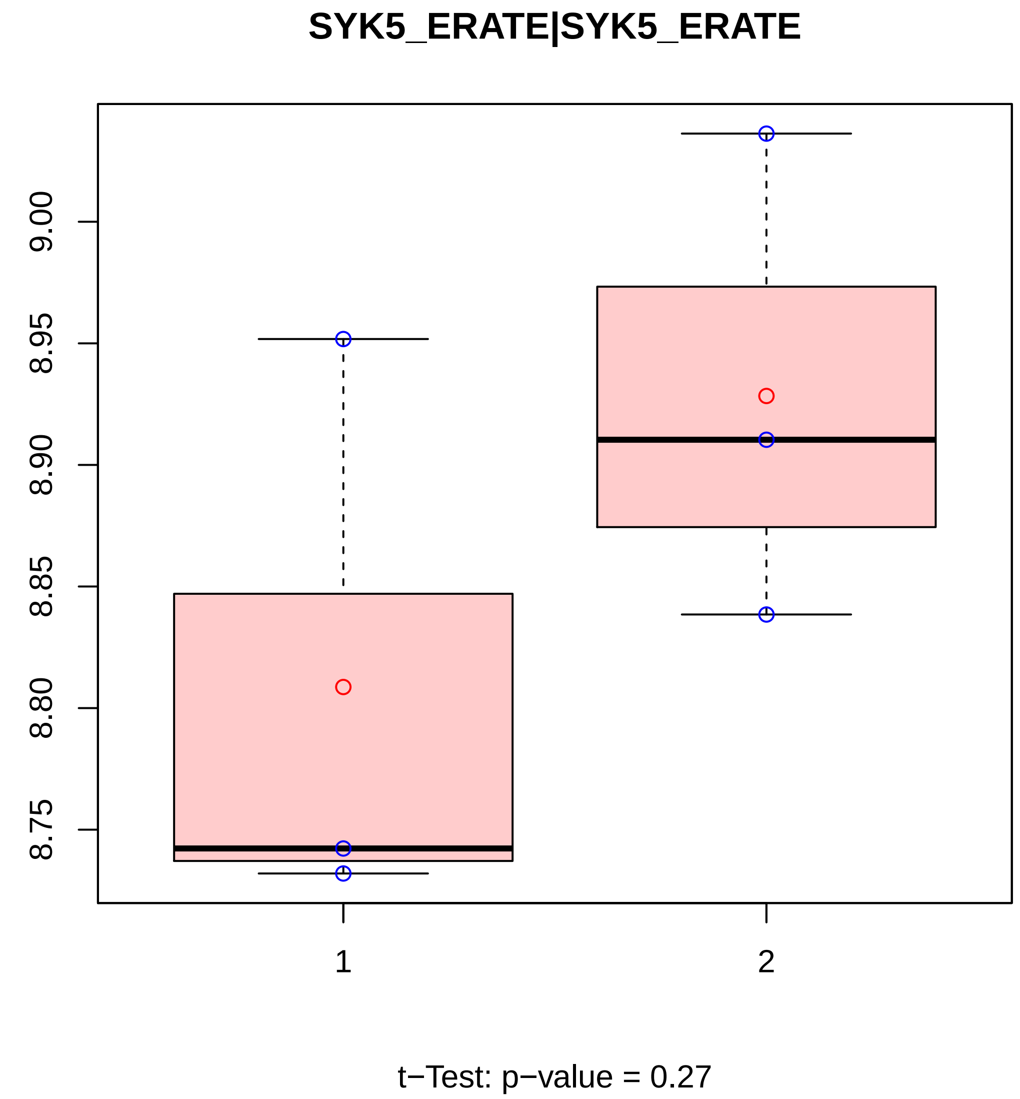

SYK5_ERATE|SYK5_ERATE
9.00
8.95
8.90
8.85
8.80
8.75
1
2
t−Test: p−value = 0.27

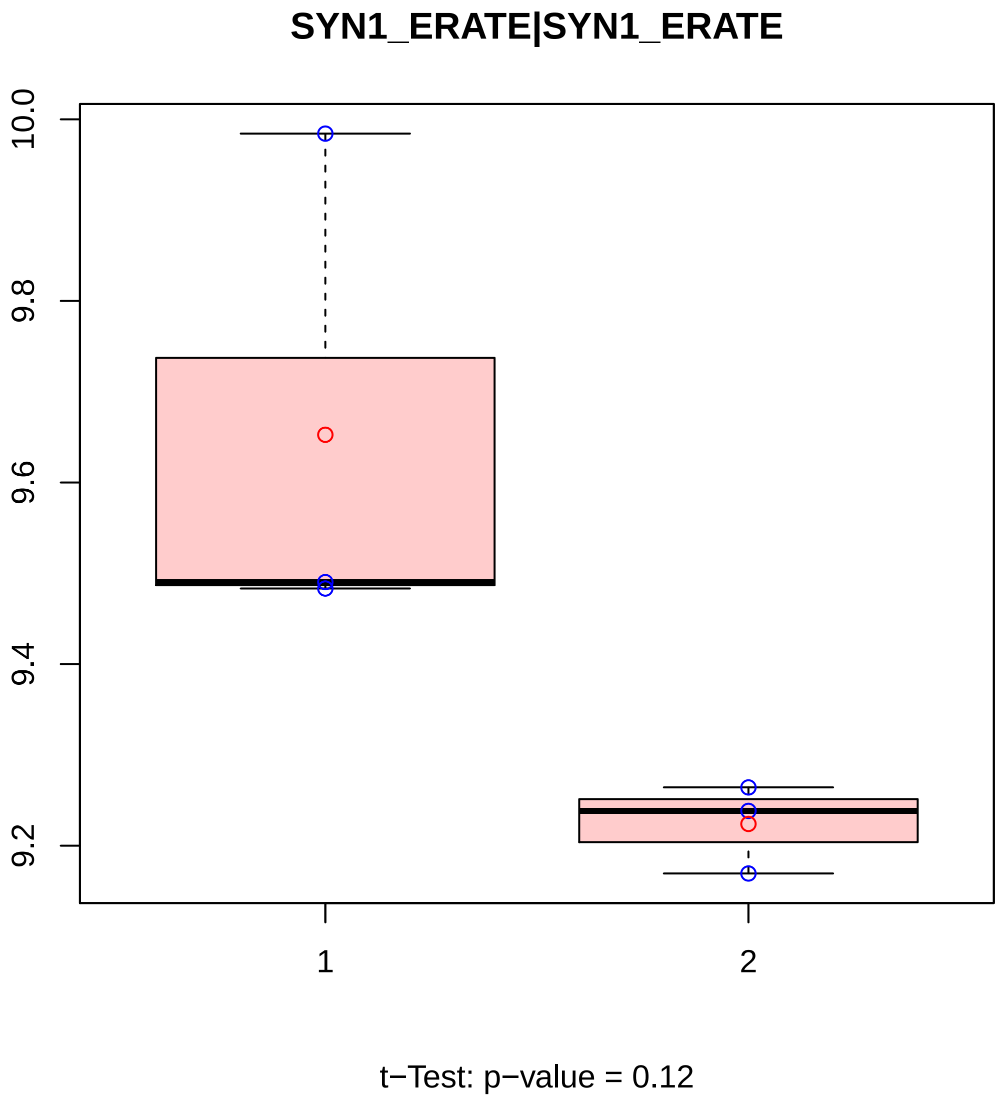
SYN1_ERATE|SYN1_ERATE
10.0
9.8
9.6
9.4
9.2
1
2
t−Test: p−value = 0.12

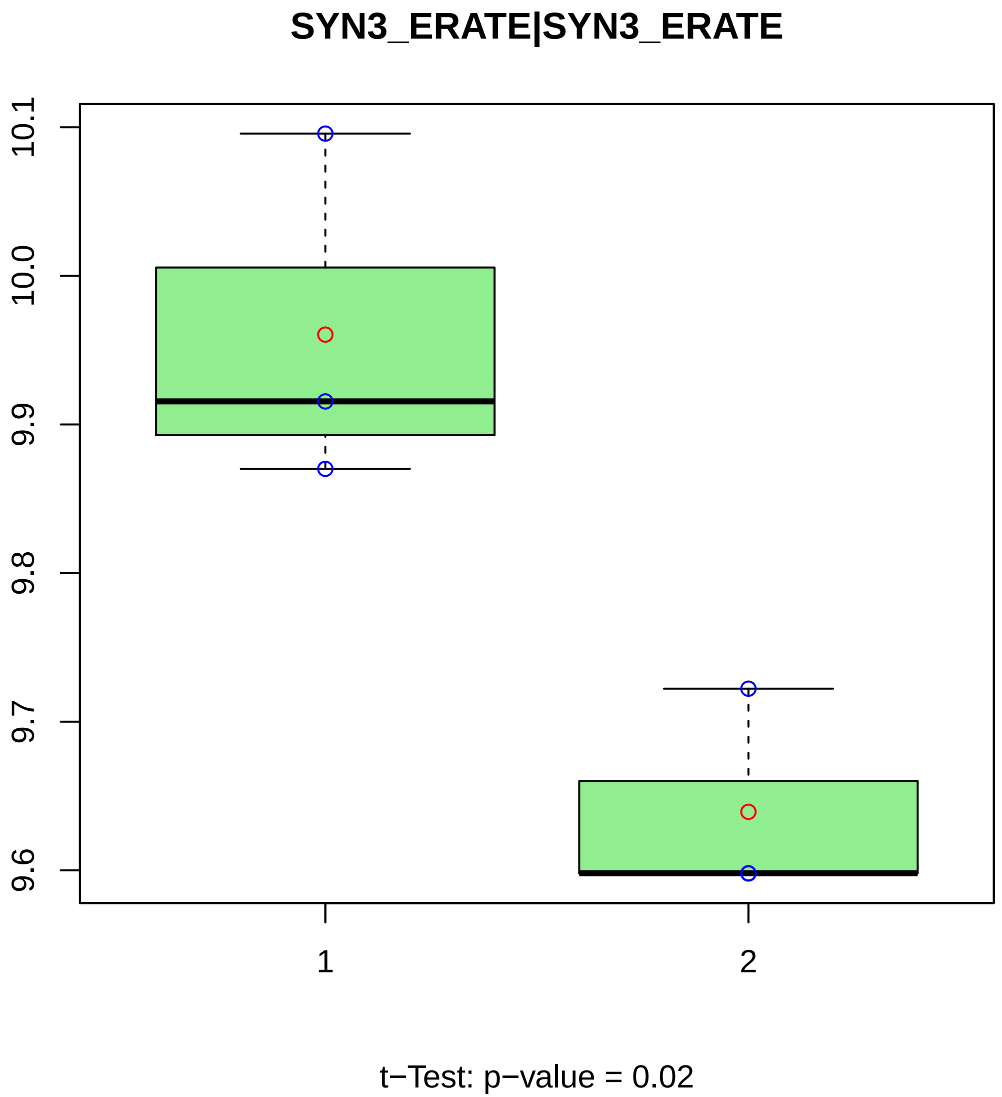

SYN3_ERATE|SYN3_ERATE
10.1
10.0
9.9
9.8
9.7
9.6
1
2
t−Test: p−value = 0.02

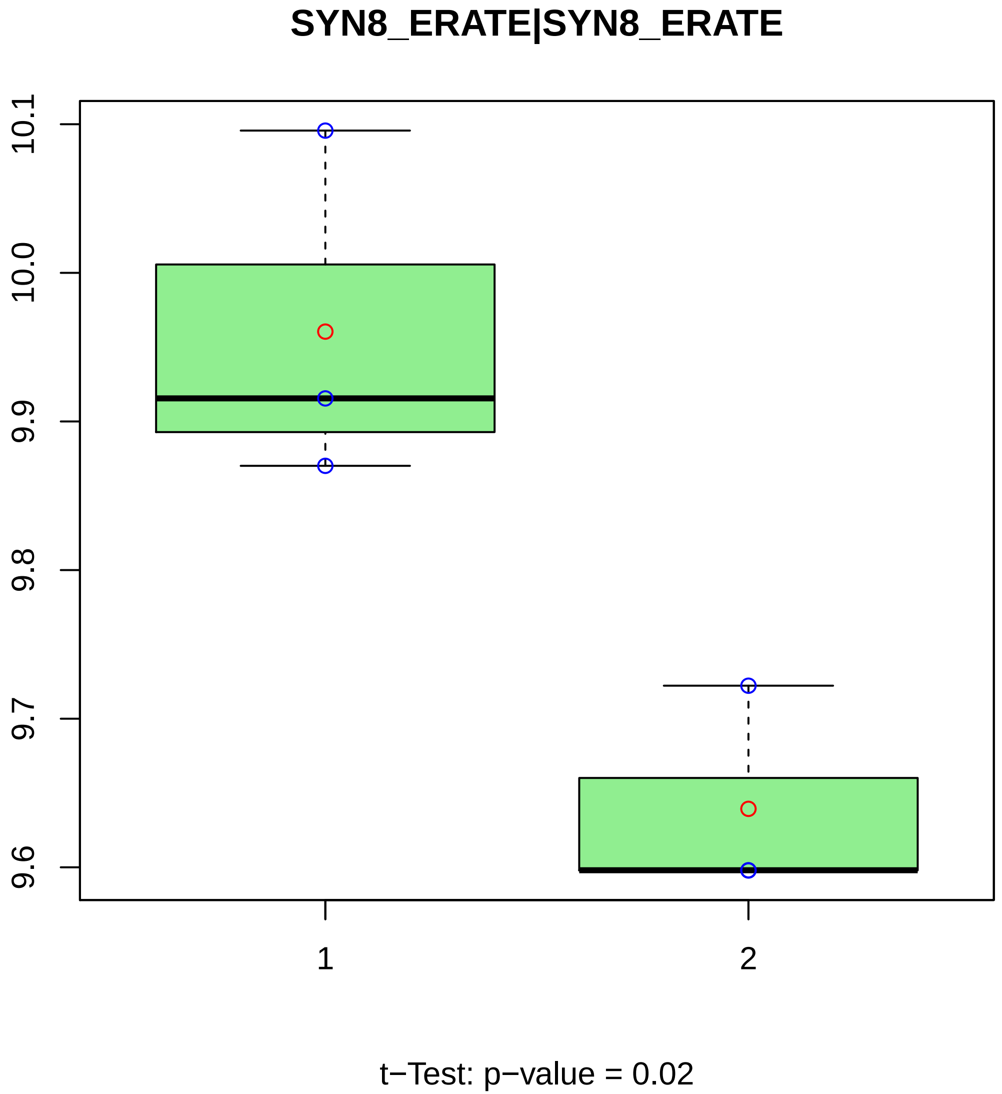

SYN8_ERATE|SYN8_ERATE
10.1
10.0
9.9
9.8
9.7
9.6
1
2
t−Test: p−value = 0.02

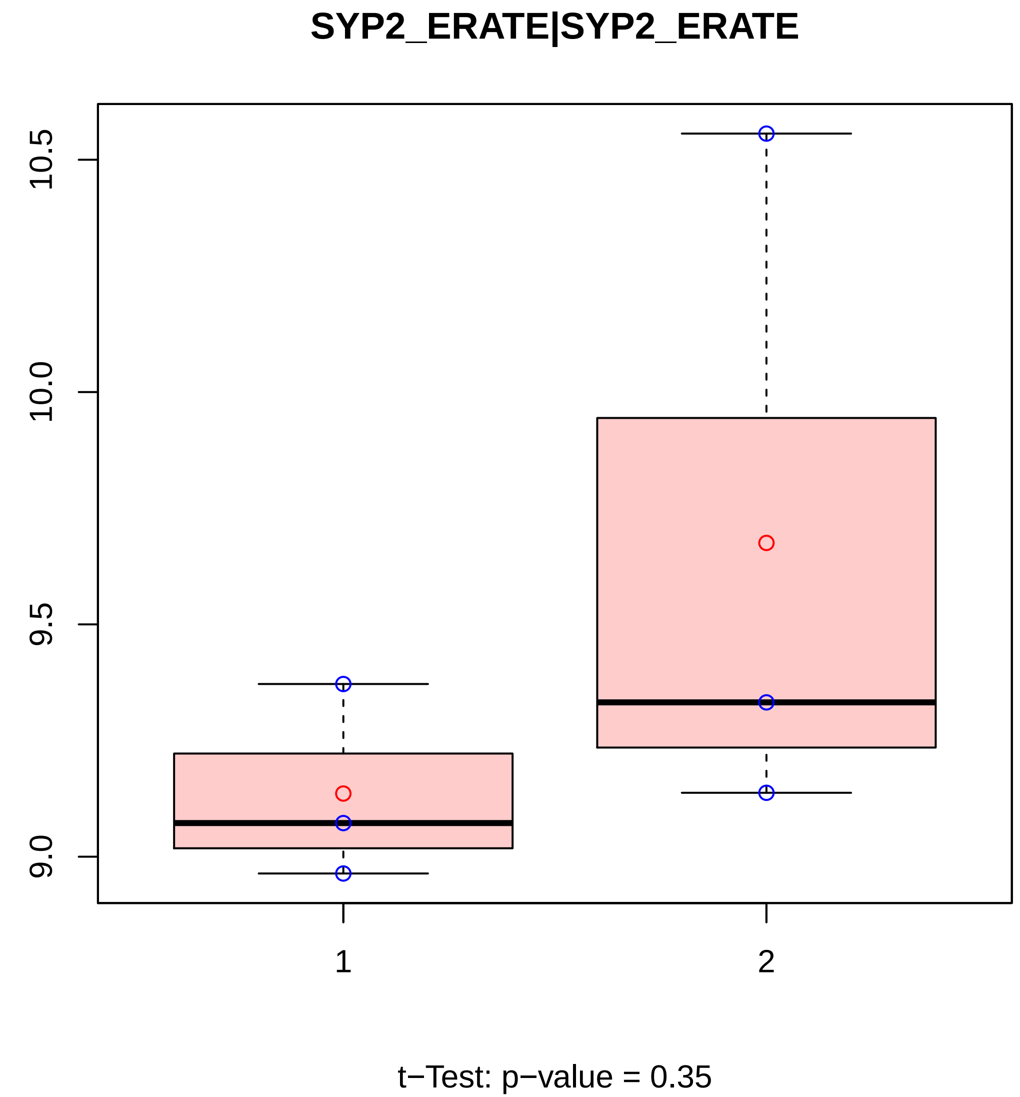
SYP2_ERATE|SYP2_ERATE
10.5
10.0
9.5
9.0
1
2
t−Test: p−value = 0.35

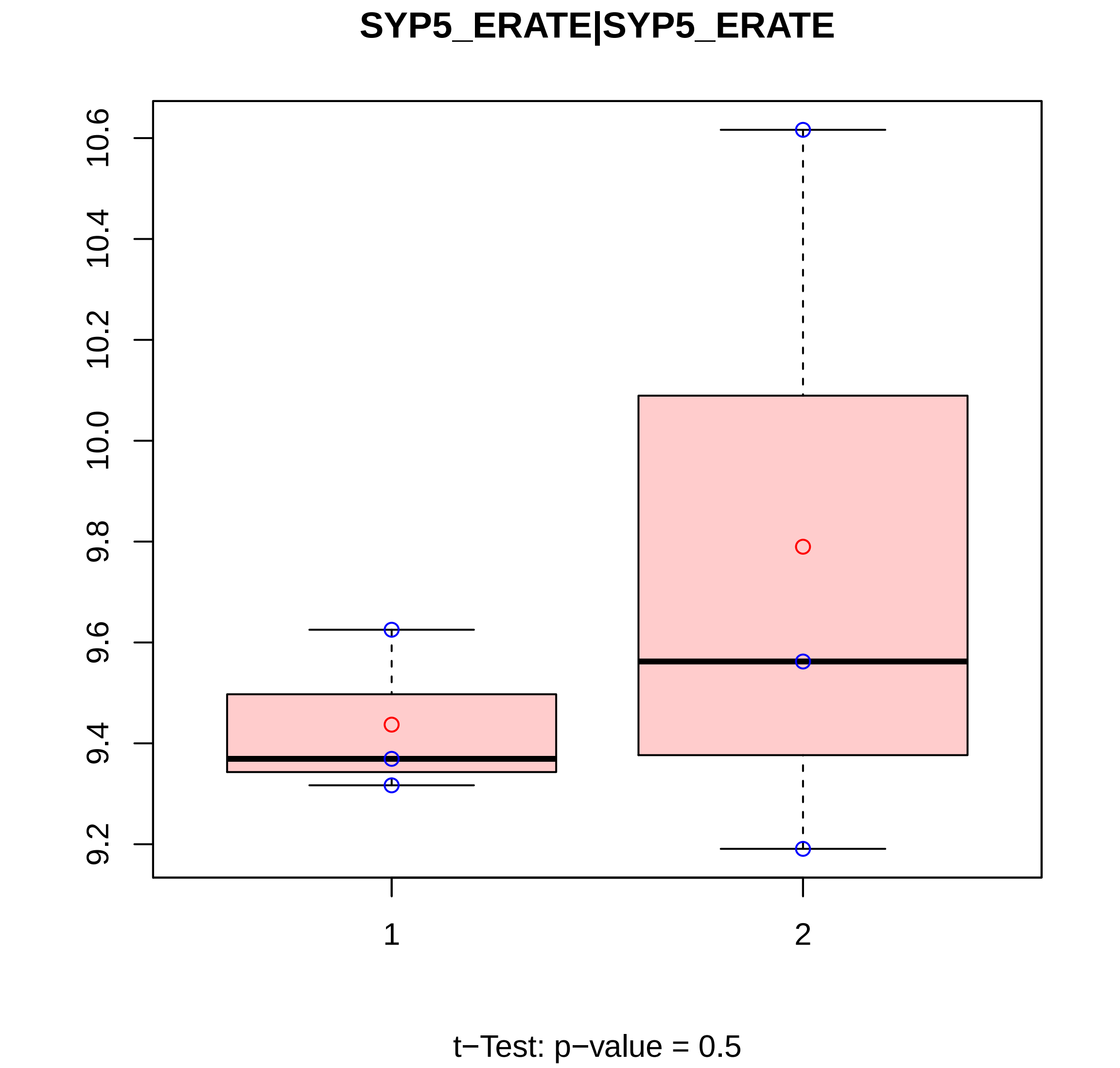
SYP5_ERATE|SYP5_ERATE
9.2
9.4
9.6
9.8
10.0
10.2
10.4
10.6
1
2
t−Test: p−value = 0.5

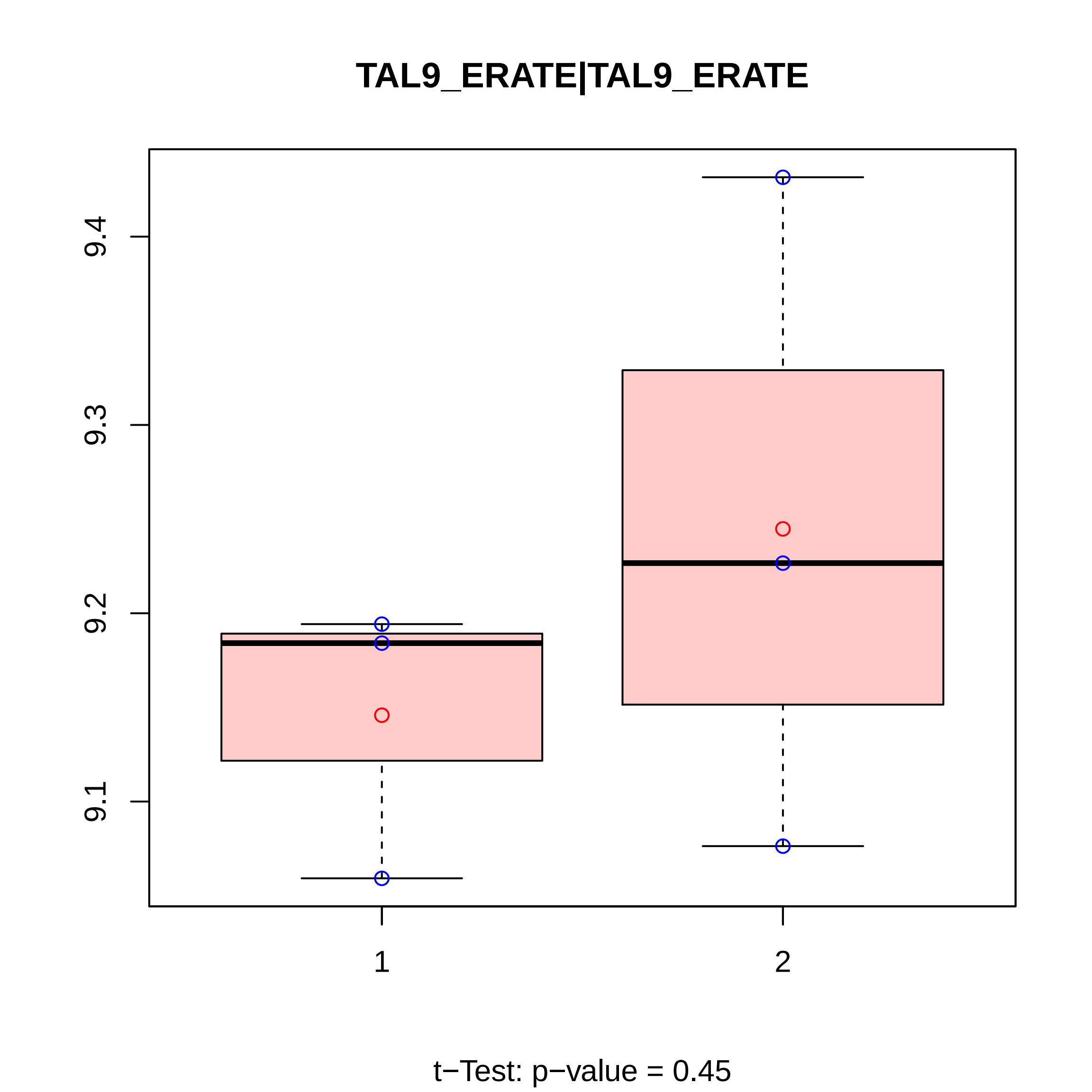

TAL9_ERATE|TAL9_ERATE
9.1
9.2
9.3
9.4
1
2
t−Test: p−value = 0.45

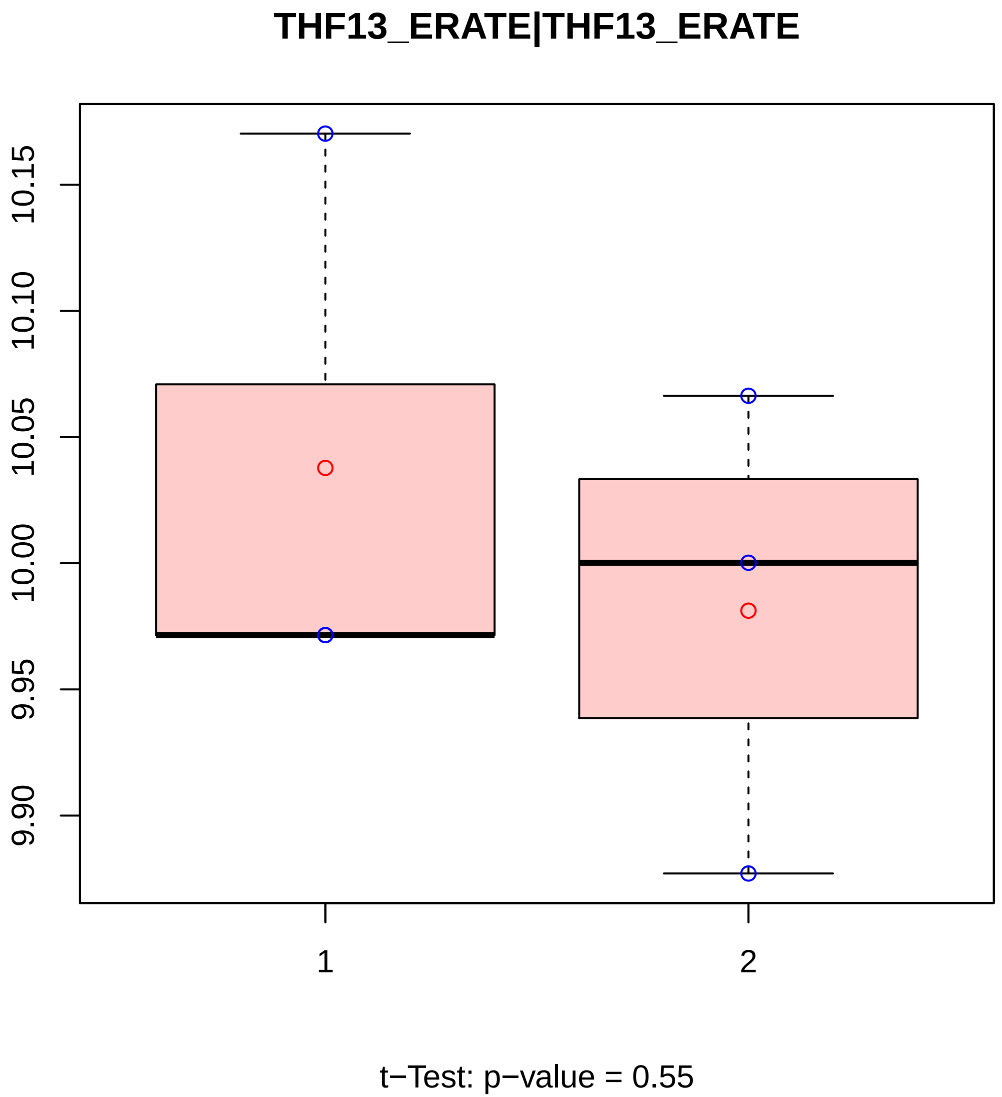
THF13_ERATE|THF13_ERATE
10.15
10.10
10.05
10.00
9.95
9.90
1
2
t−Test: p−value = 0.55

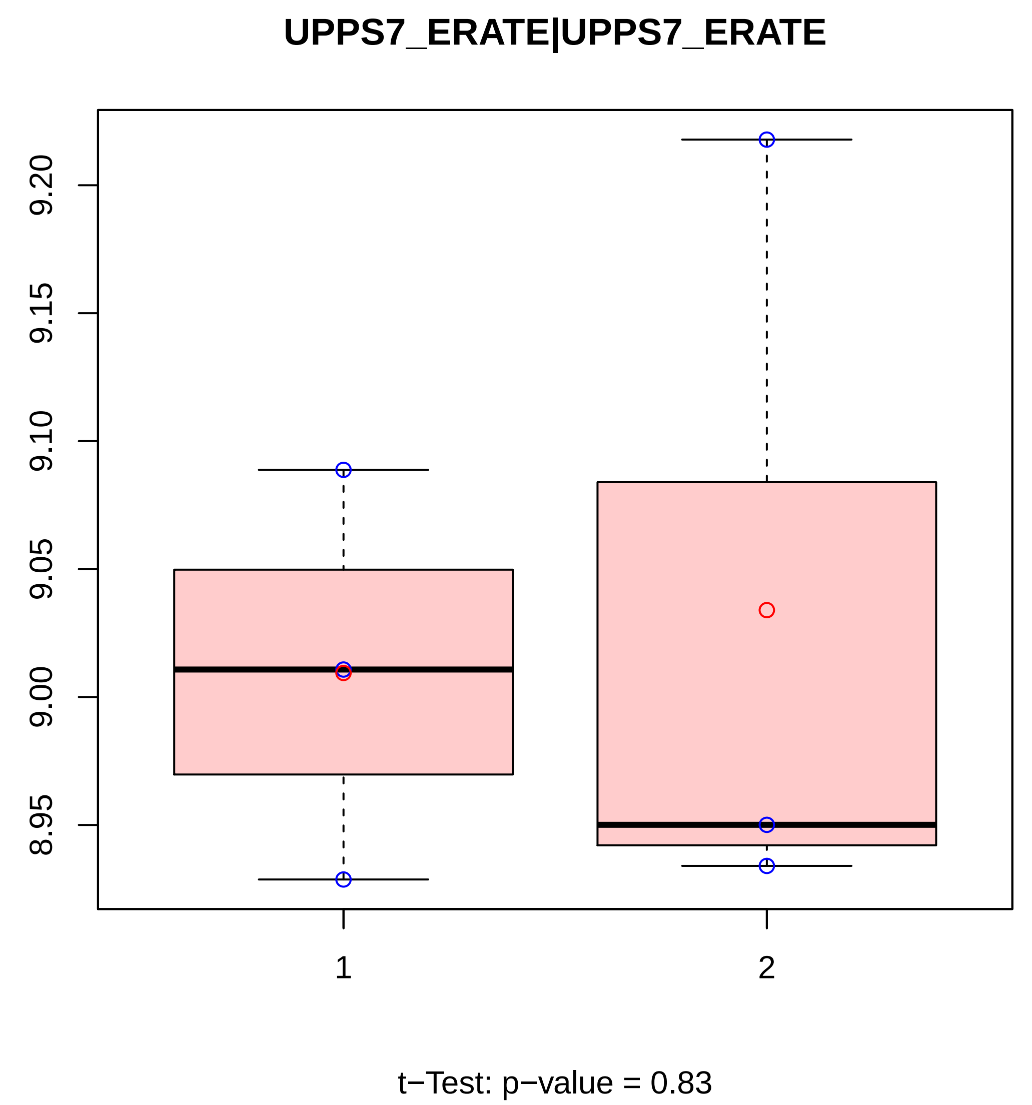
UPPS7_ERATE|UPPS7_ERATE
9.20
9.15
9.10
9.05
9.00
8.95
1
2
t−Test: p−value = 0.83

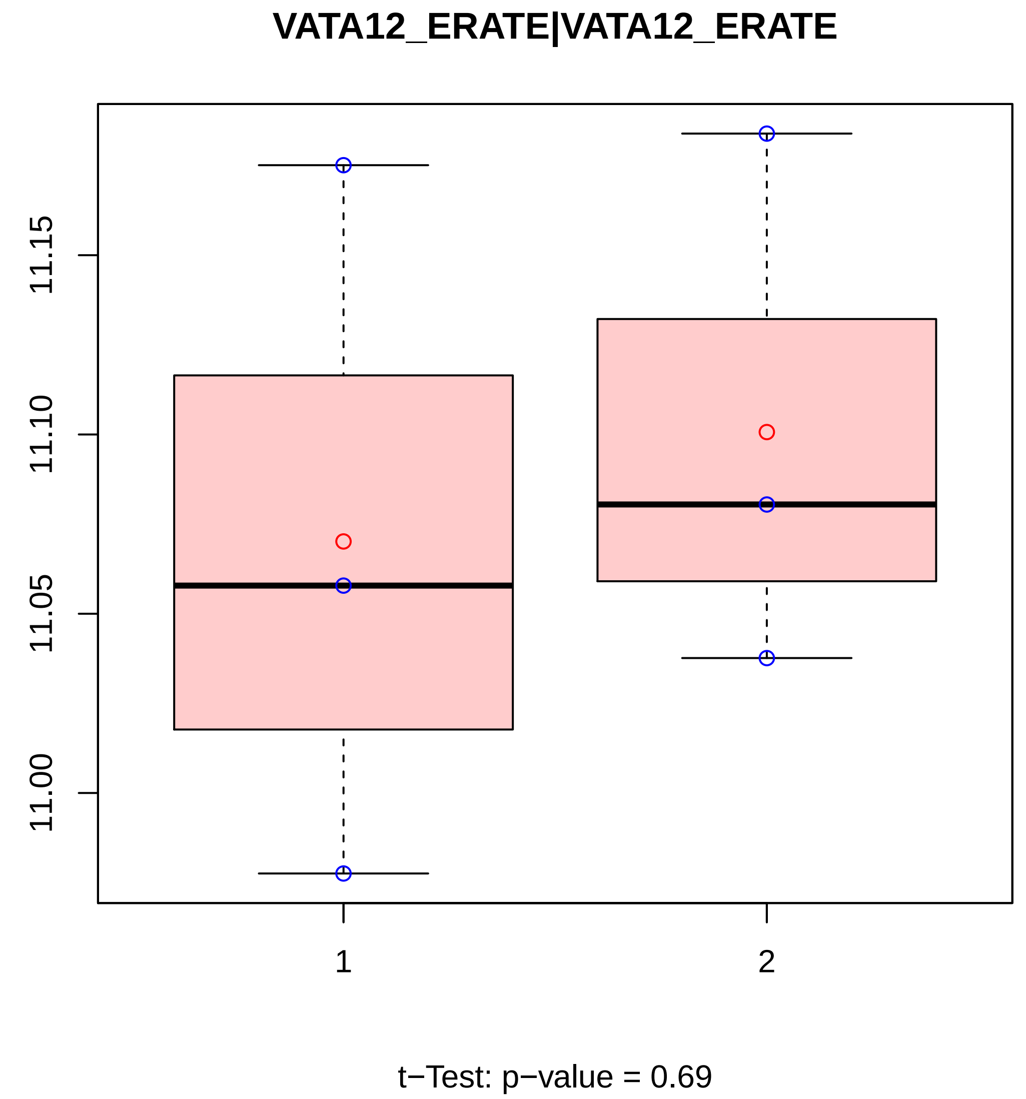
VATA12_ERATE|VATA12_ERATE
11.00
11.05
11.10
11.15
1
2
t−Test: p−value = 0.69

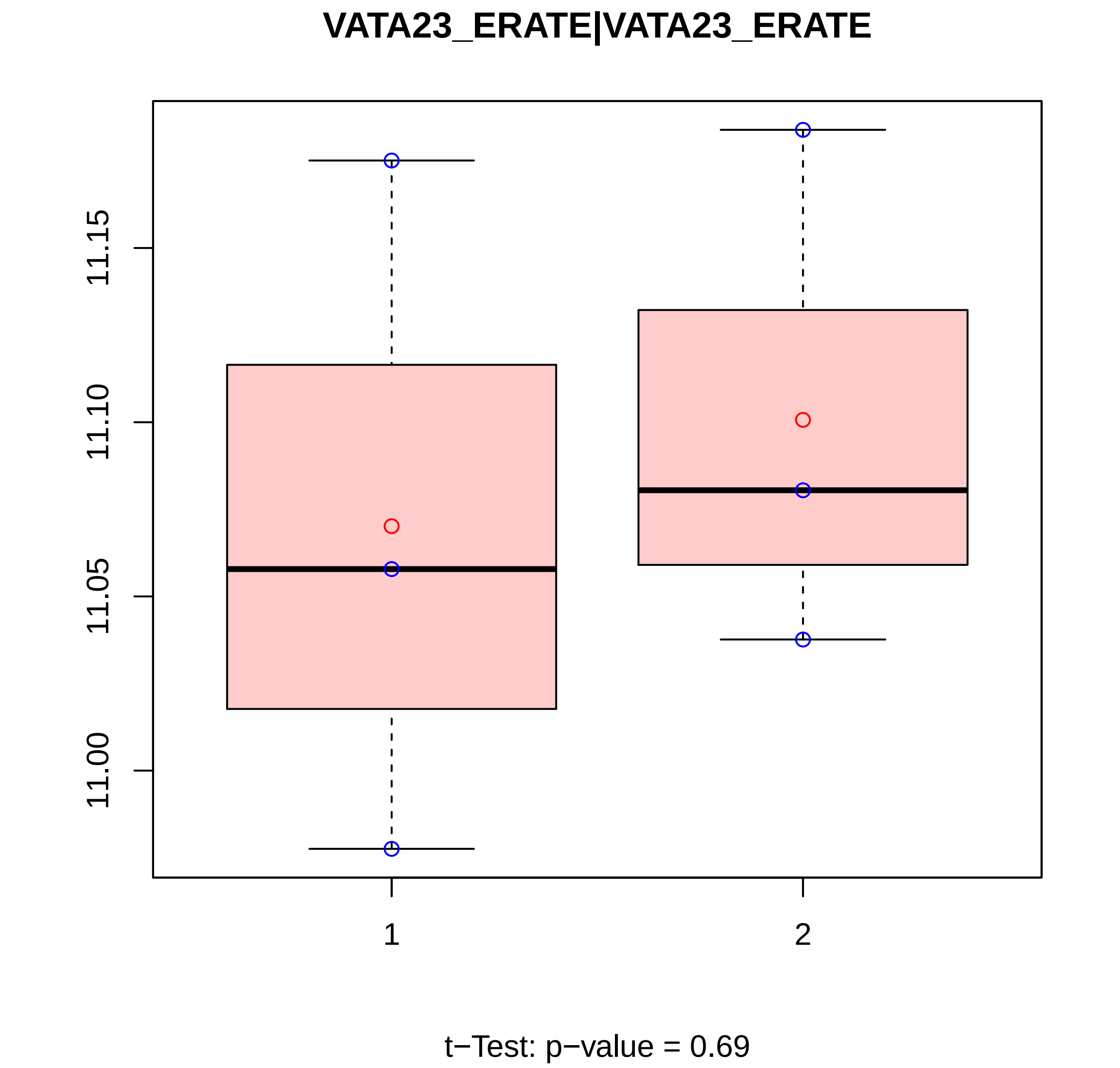
VATA23_ERATE|VATA23_ERATE
11.00
11.05
11.10
11.15
1
2
t−Test: p−value = 0.69

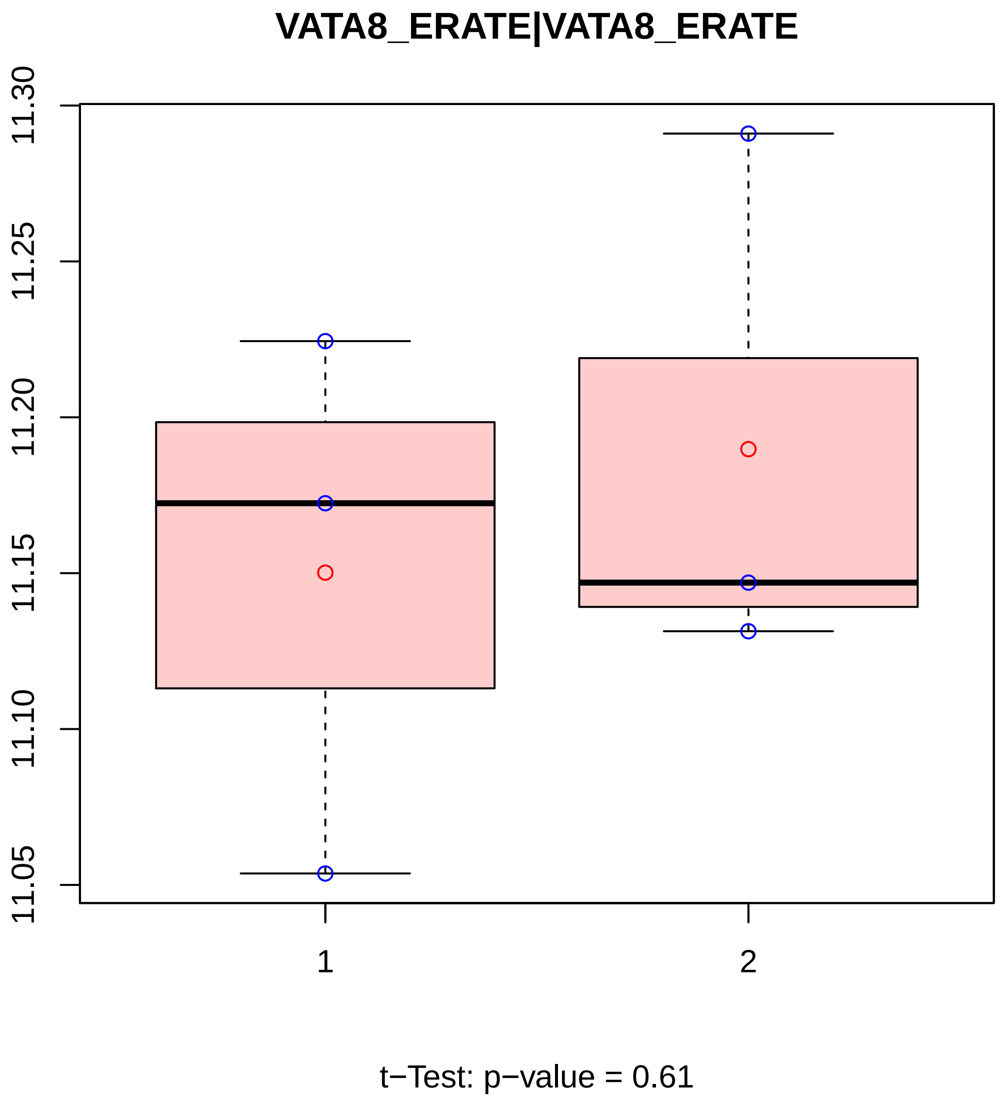
VATA8_ERATE|VATA8_ERATE
11.05
11.10
11.15
11.20
11.25
11.30
1
2
t−Test: p−value = 0.61

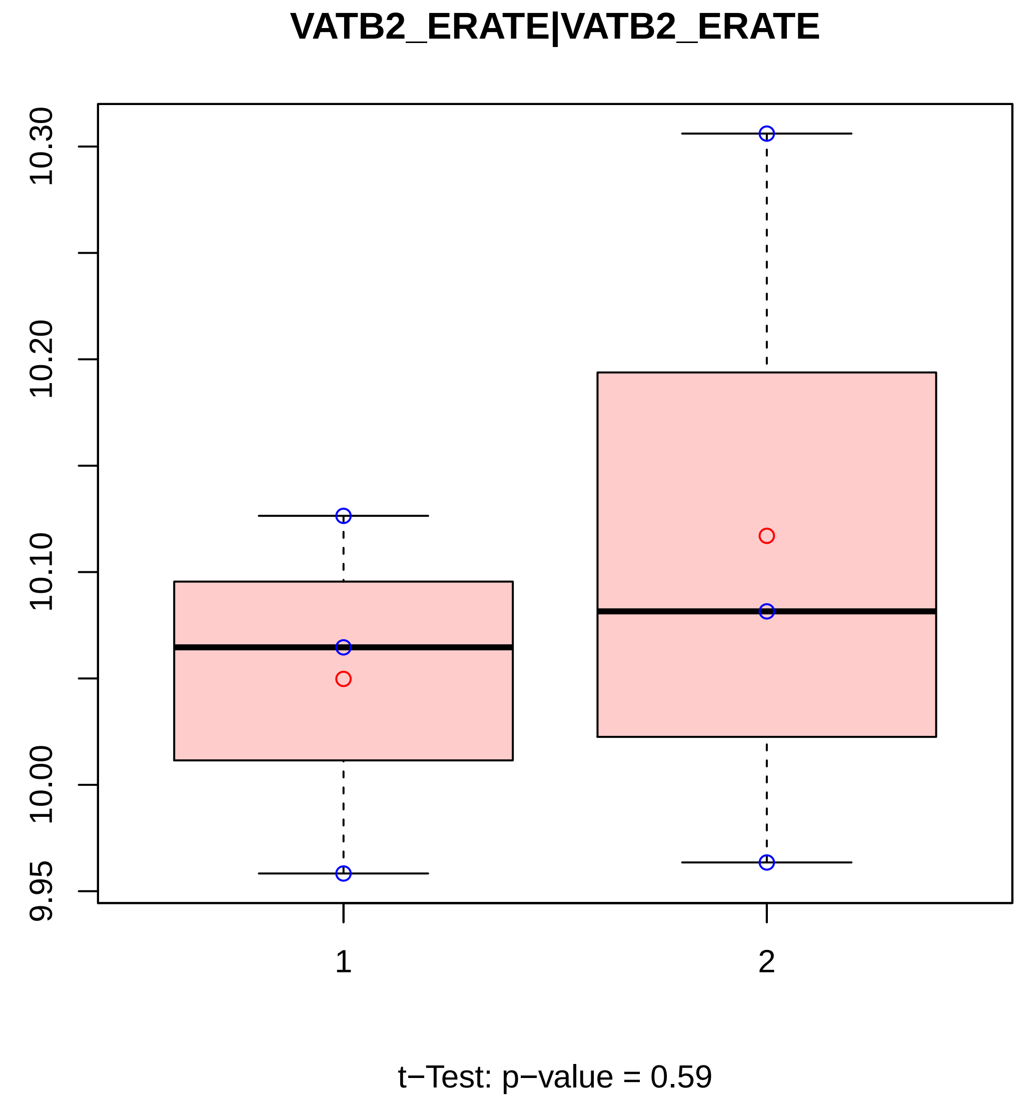
VATB2_ERATE|VATB2_ERATE
10.30
10.20
10.10
10.00
9.95
1
2
t−Test: p−value = 0.59

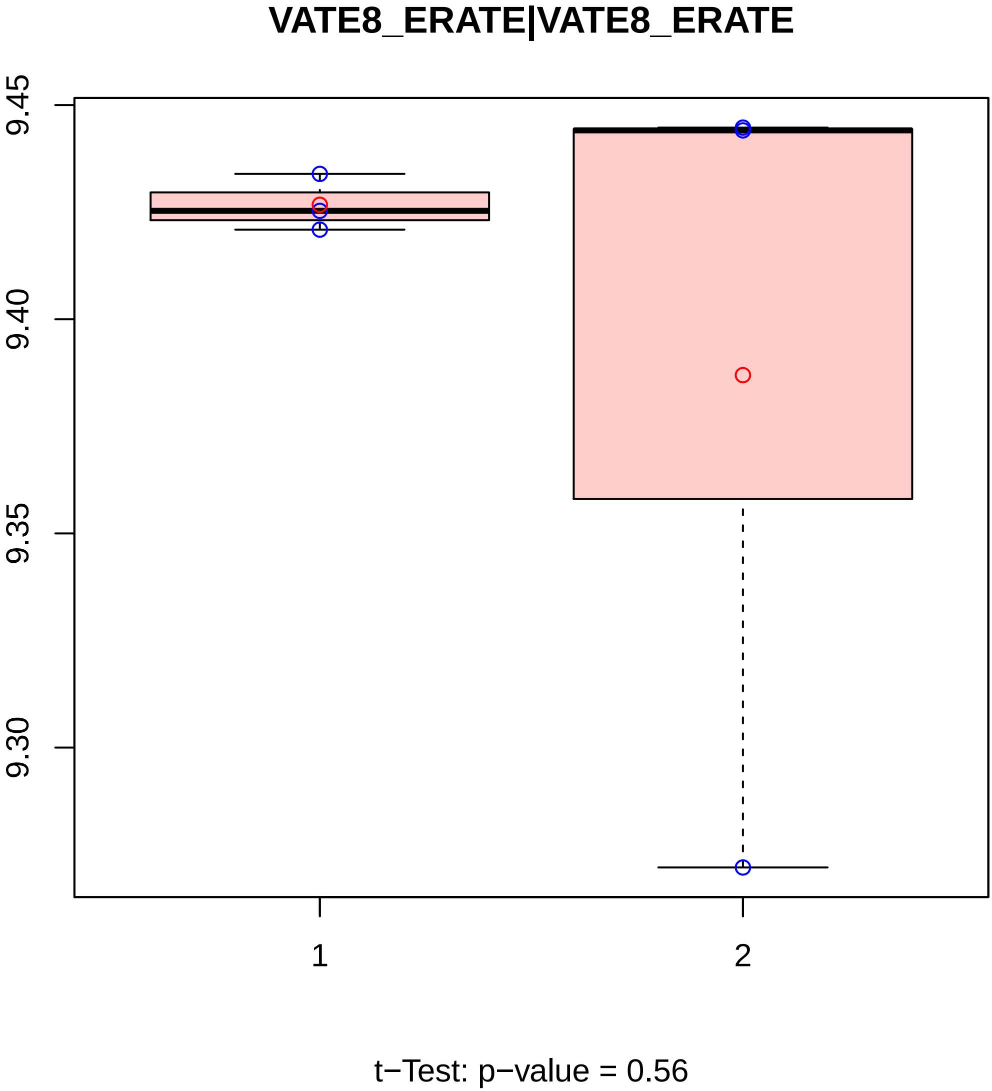

VATE8_ERATE|VATE8_ERATE
9.45
9.40
9.35
9.30
1
2
t−Test: p−value = 0.56
