# Supplementary material for: A Proteomic Approach to Investigate the Drought Response in the Orphan Crop Eragrostis tef
Source: Proteomes. 2017 Nov 15;5(4):32. doi: 10.3390/proteomes5040032 (PMC5748567; doi:10.3390/proteomes5040032)
Supplement: Supplementary file 1 [file S3.2 TEU two group analysis (box-plots).pdf › proteomes-208511 supplementary final/S3.2 TEU two group analysis (box-plots).pdf]

# ACSF1_ERATE|ACSF1_ERATE

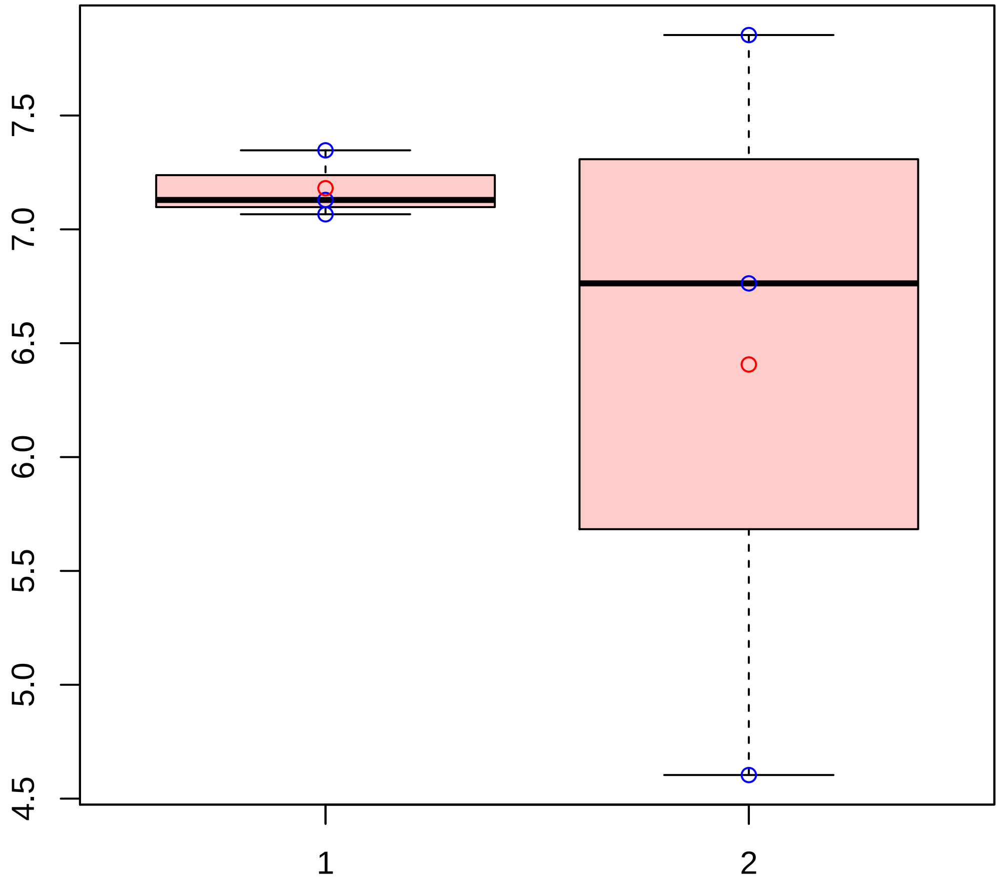


t−Test: p−value = 0.5

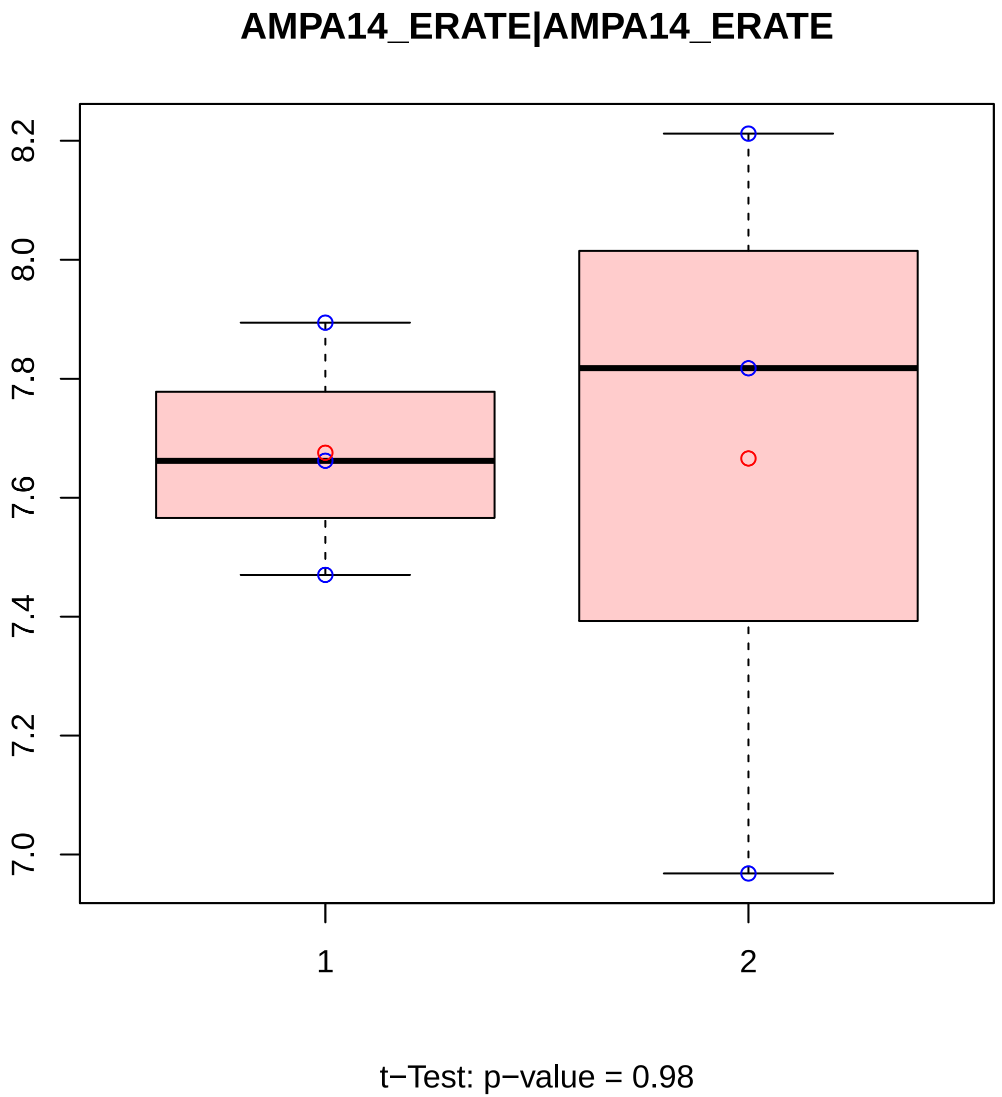

AMPA14_ERATE|AMPA14_ERATE
8.2
8.0
7.8
7.6
7.4
7.2
7.0
1
2
t−Test: p−value = 0.98

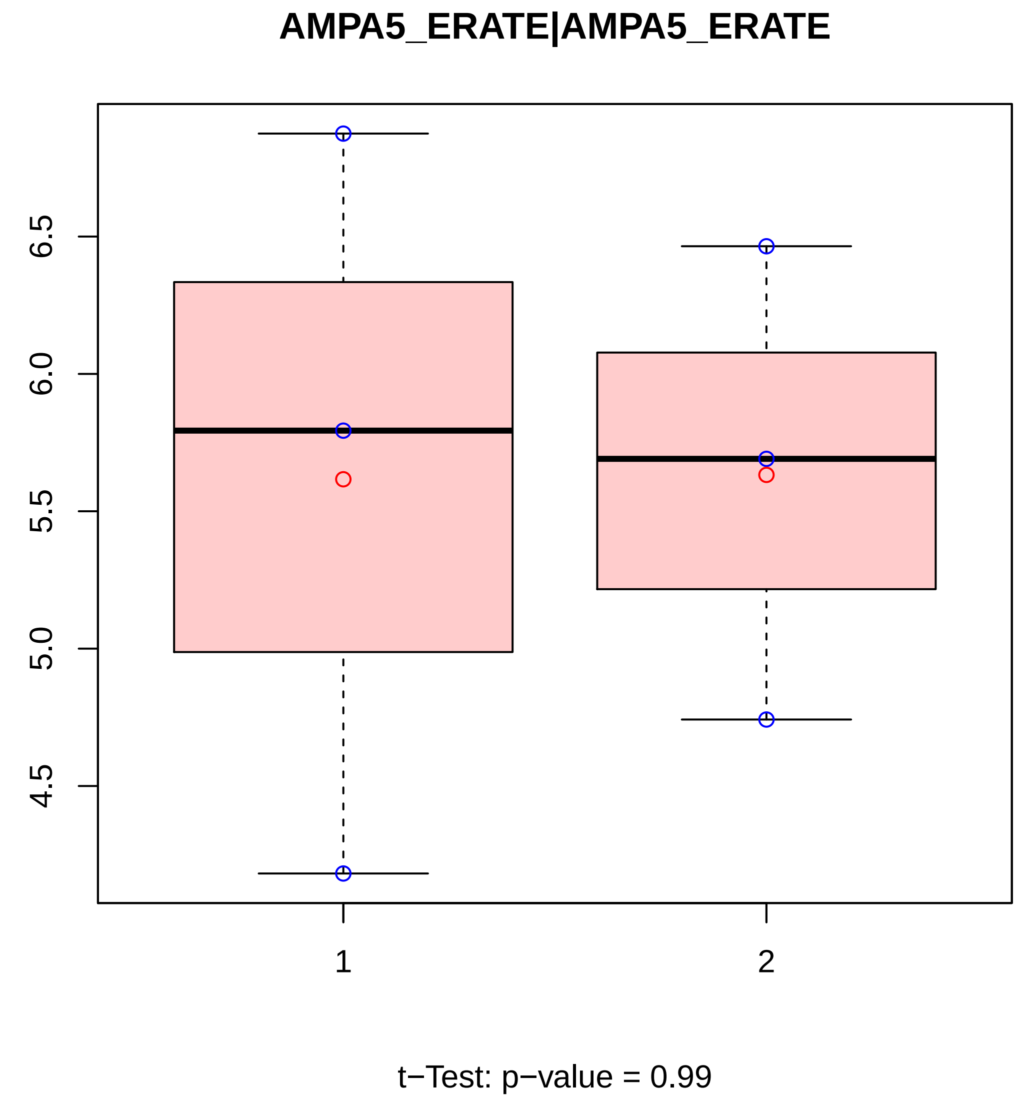
AMPA5_ERATE|AMPA5_ERATE
6.5
6.0
5.5
5.0
4.5
1
2
t−Test: p−value = 0.99

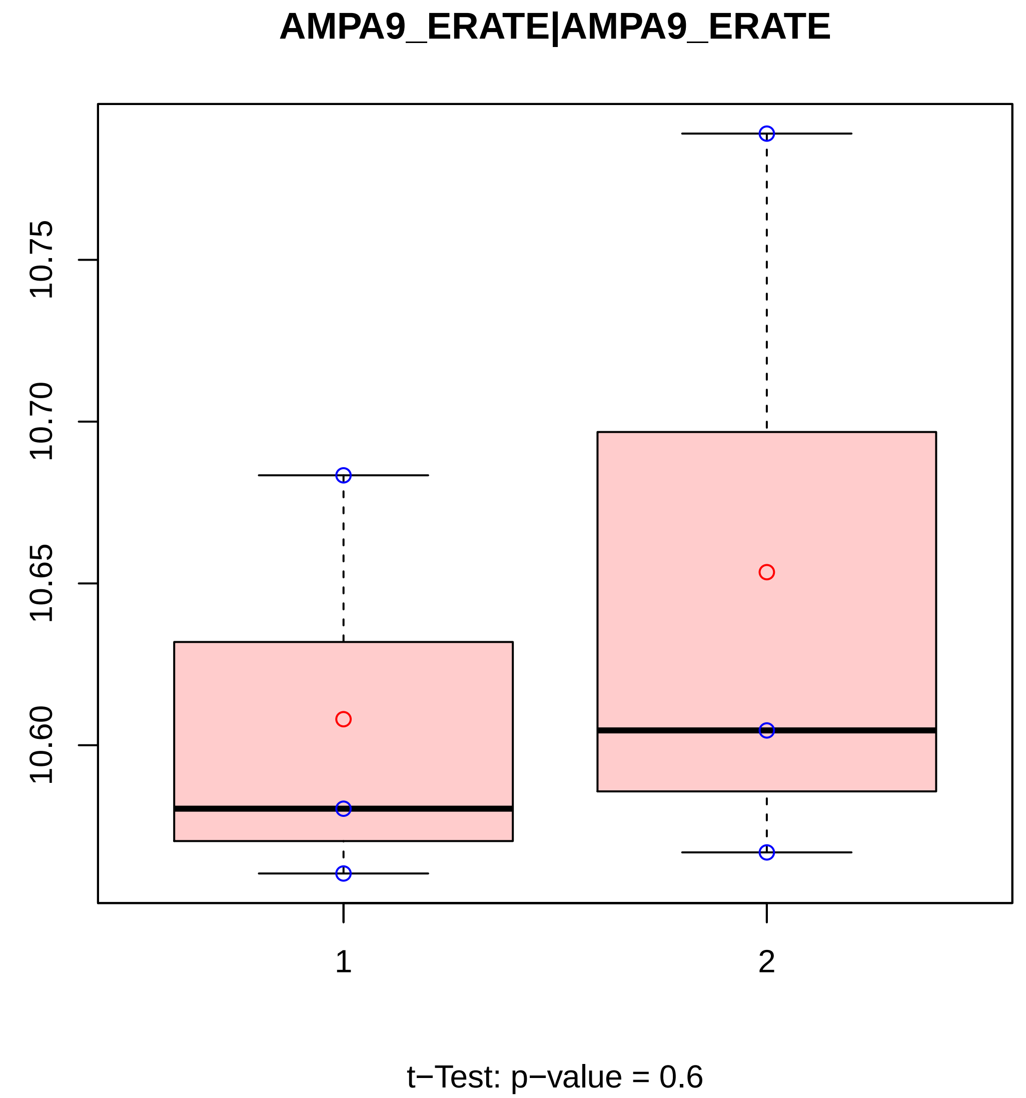
AMPA9_ERATE|AMPA9_ERATE
10.60
10.65
10.70
10.75
1
2
t−Test: p−value = 0.6

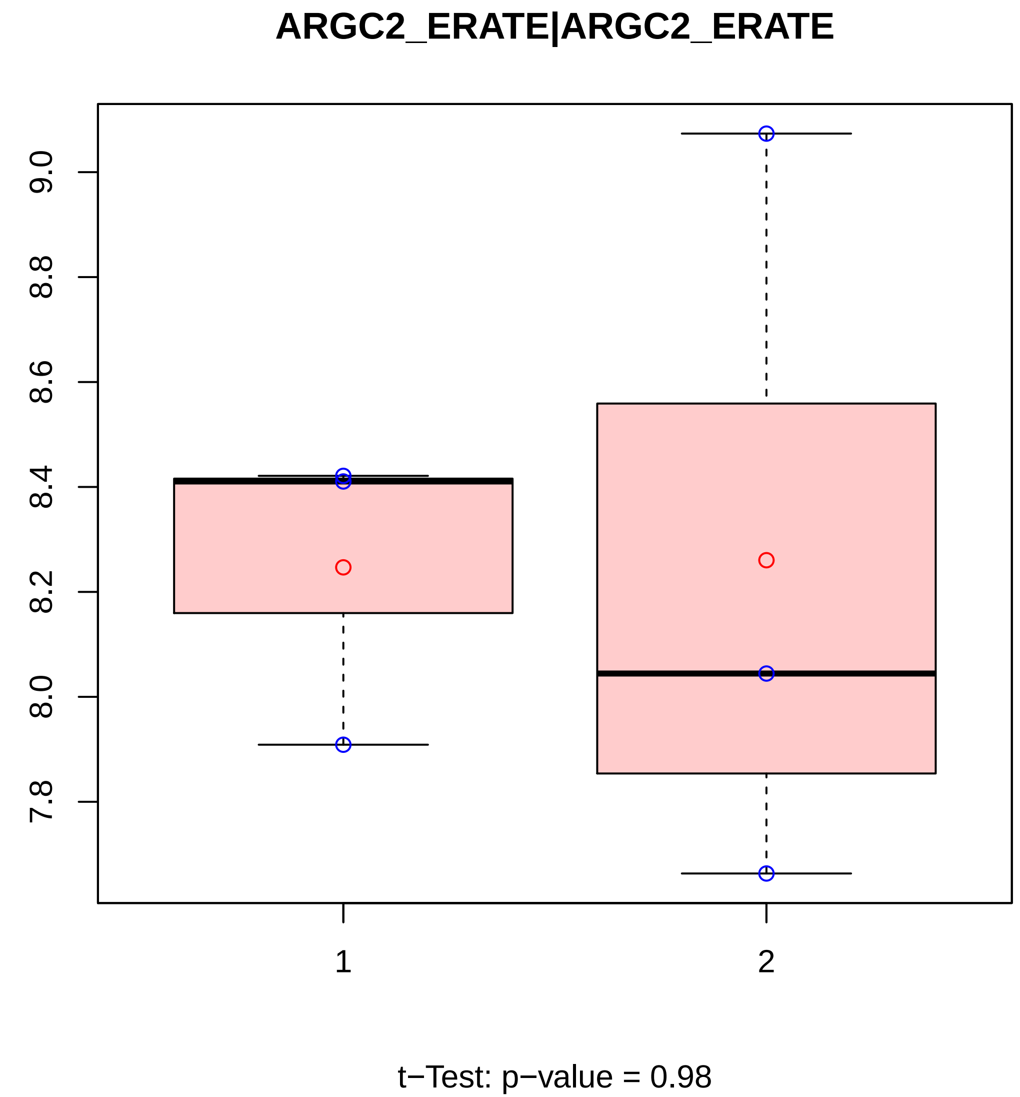

ARGC2_ERATE|ARGC2_ERATE
9.0
8.8
8.6
8.4
8.2
8.0
7.8
1
2
t−Test: p−value = 0.98

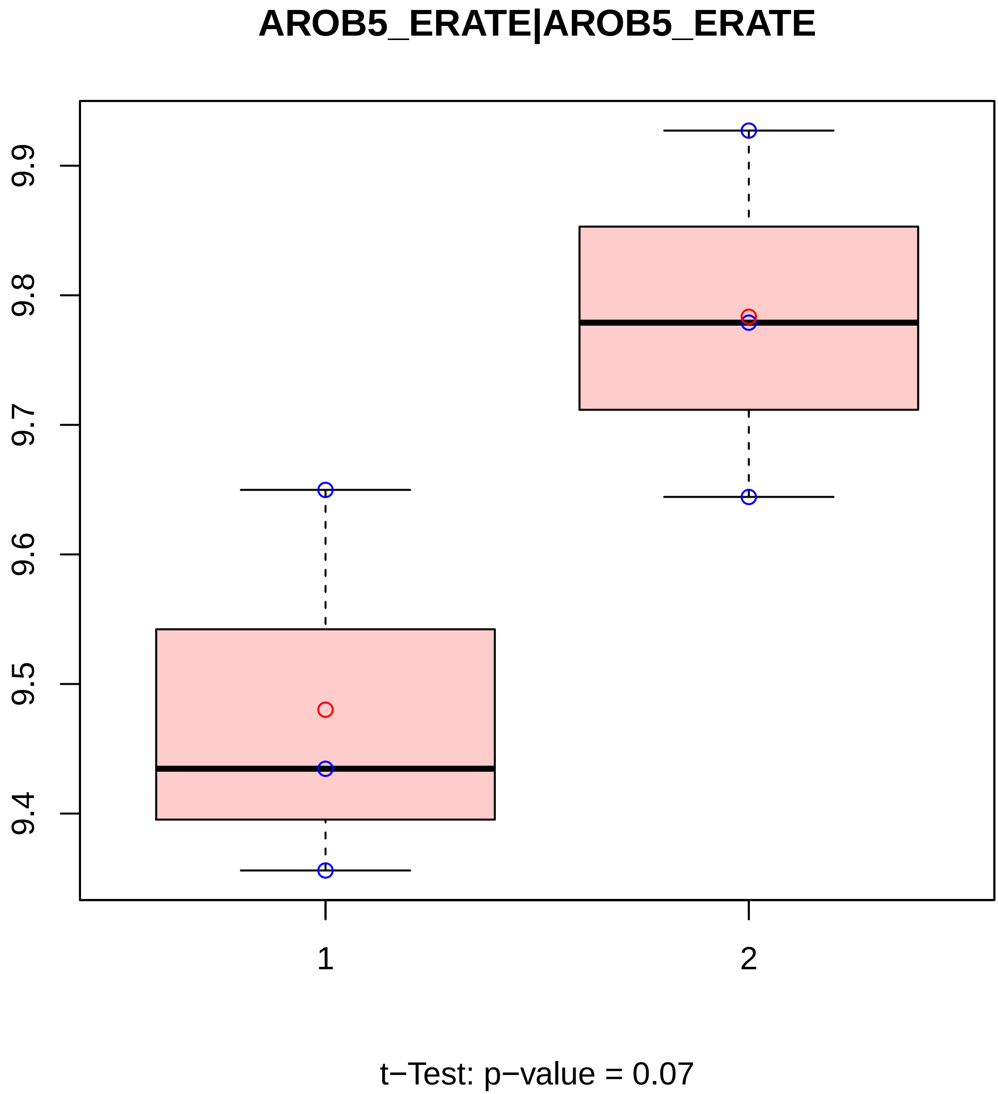

AROB5_ERATE|AROB5_ERATE
9.4
9.5
9.6
9.7
9.8
9.9
1
2
t−Test: p−value = 0.07

# ATPA6_ERATE|ATPA6_ERATE

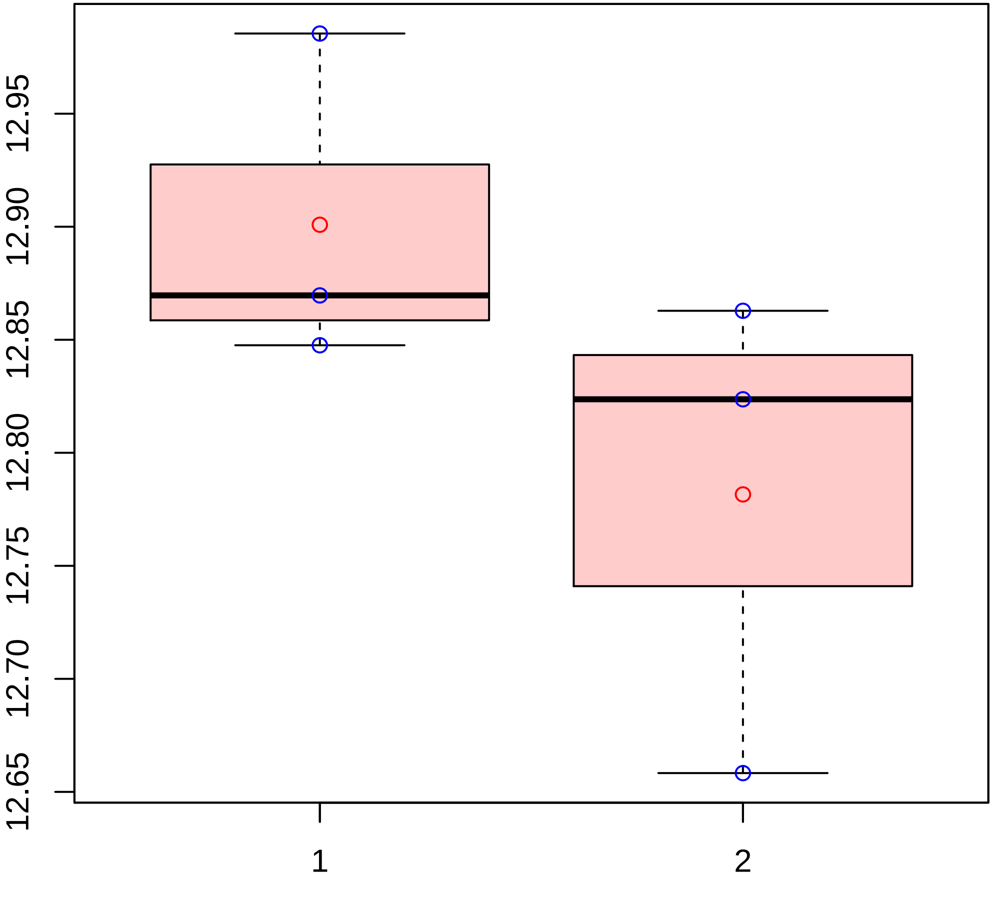


t−Test: p−value = 0.2

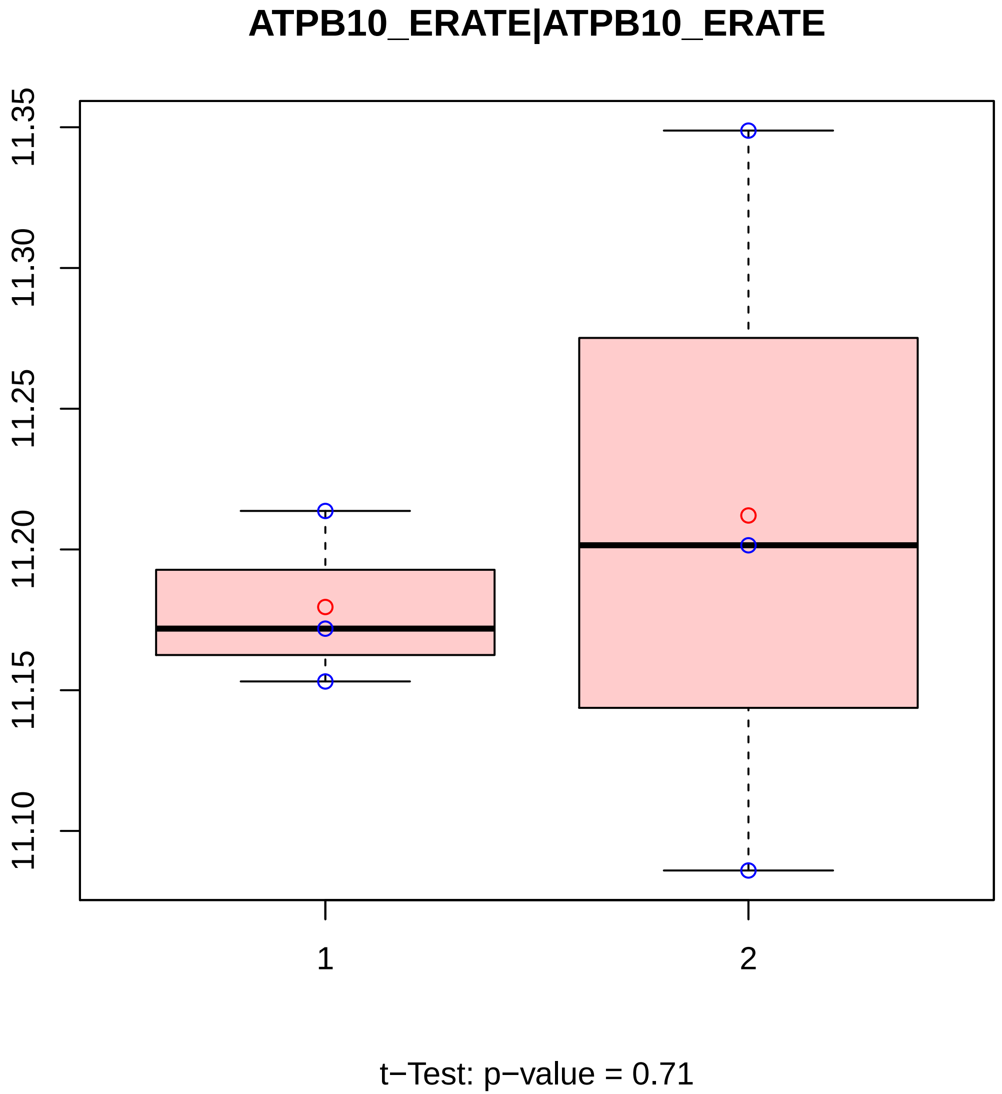

ATPB10_ERATE|ATPB10_ERATE
11.10
11.15
11.20
11.25
11.30
11.35
1
2
t−Test: p−value = 0.71

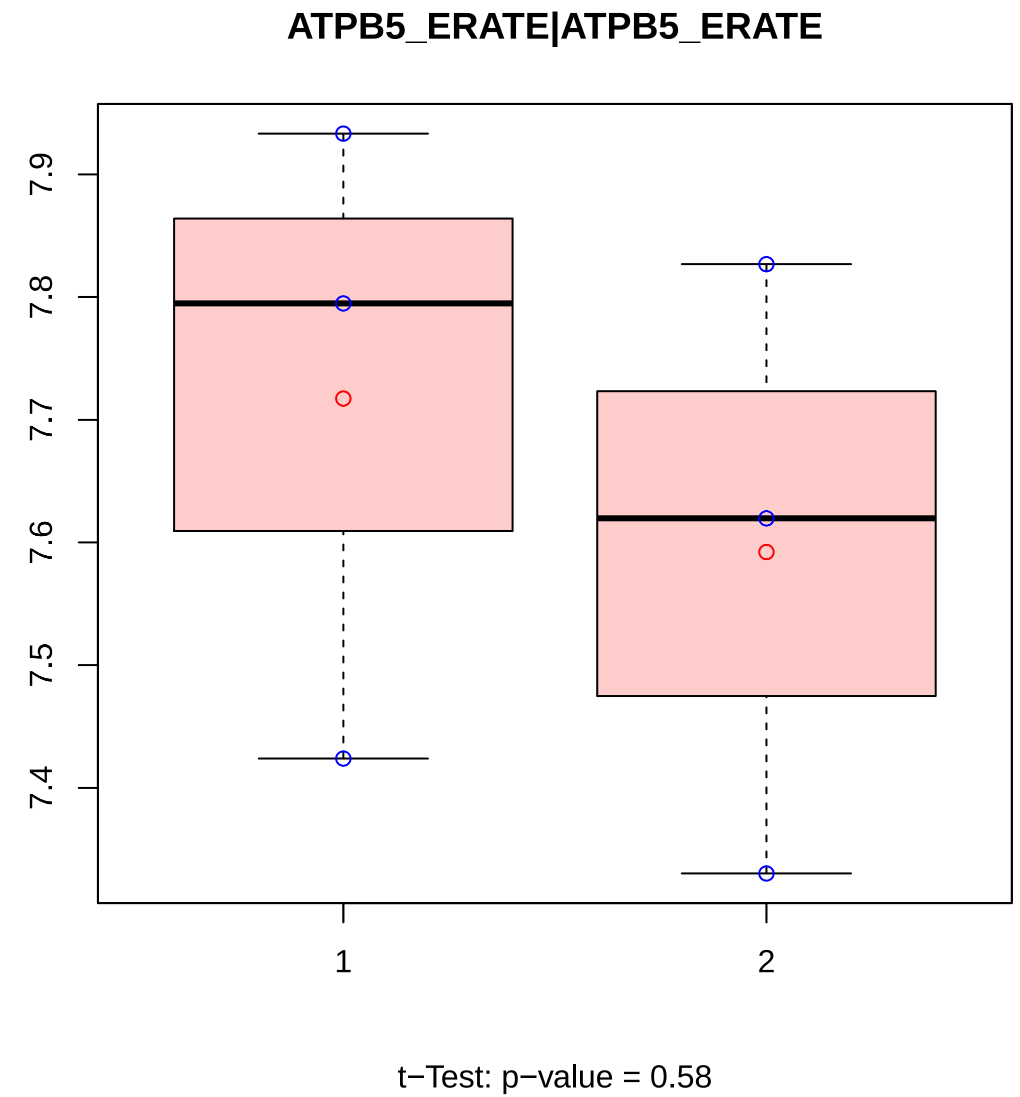
ATPB5_ERATE|ATPB5_ERATE
7.4
7.5
7.6
7.7
7.8
7.9
1
2
t−Test: p−value = 0.58

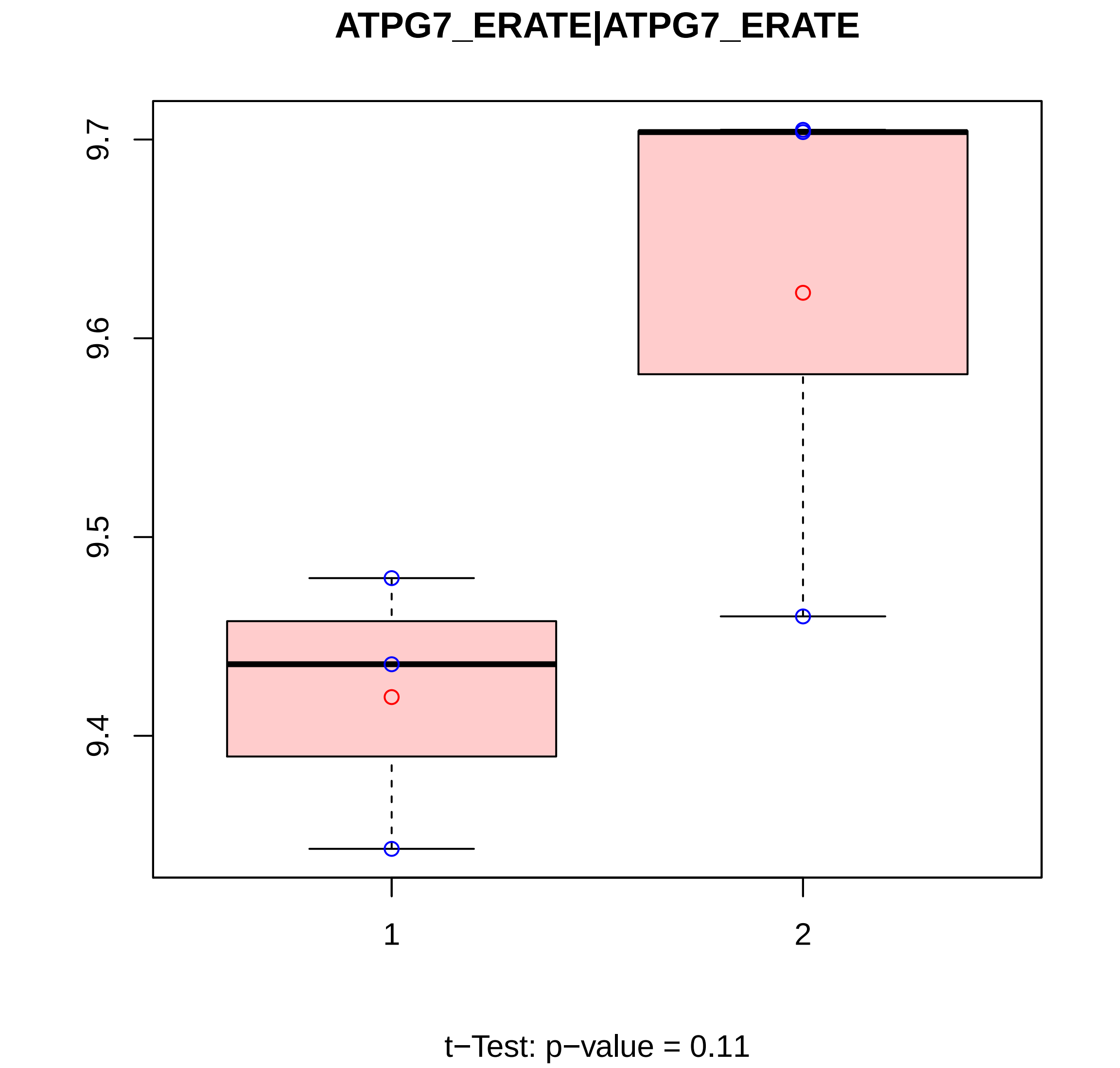
ATPG7_ERATE|ATPG7_ERATE
9.4
9.5
9.6
9.7
1
2
t−Test: p−value = 0.11

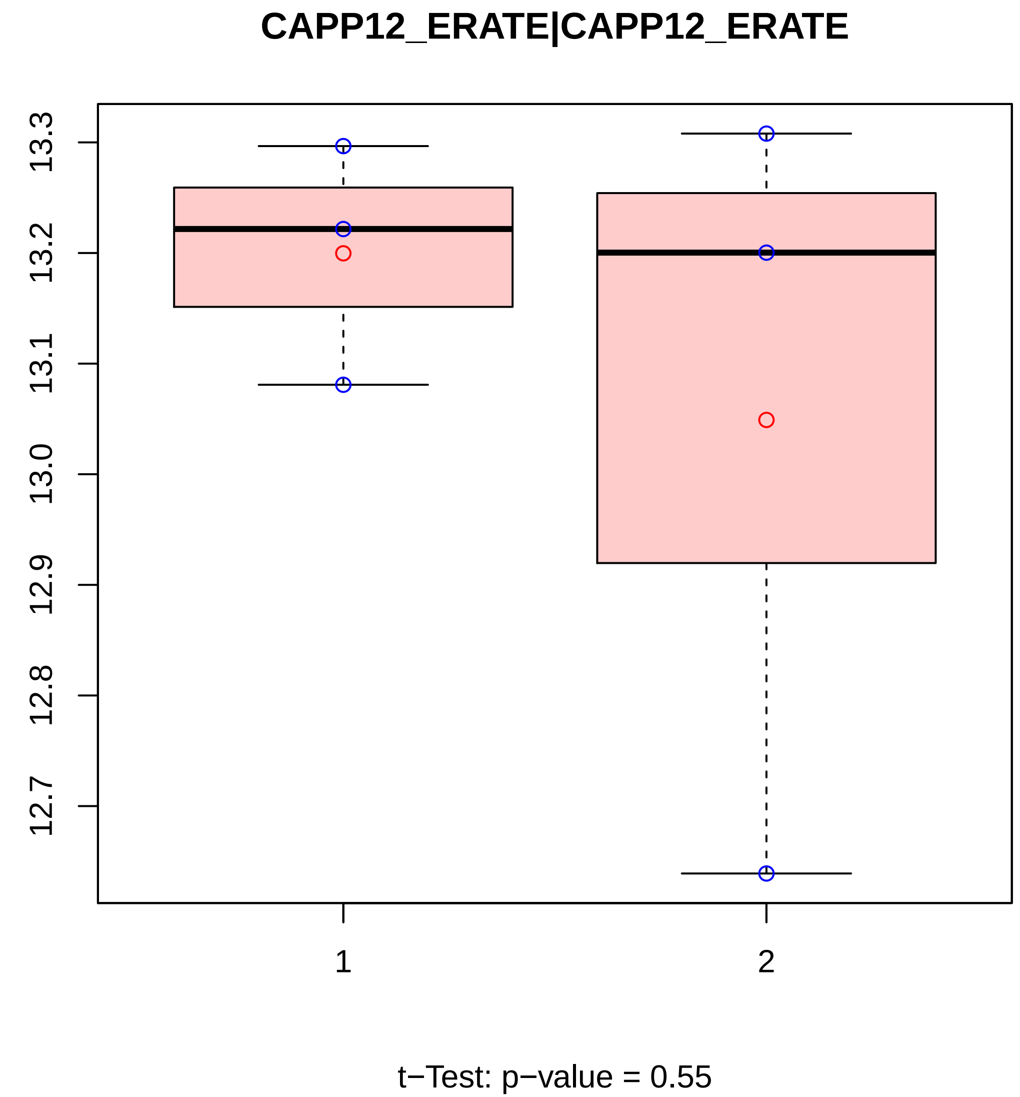
CAPP12_ERATE|CAPP12_ERATE
13.3
13.2
13.1
13.0
12.9
12.8
12.7
1
2
t−Test: p−value = 0.55

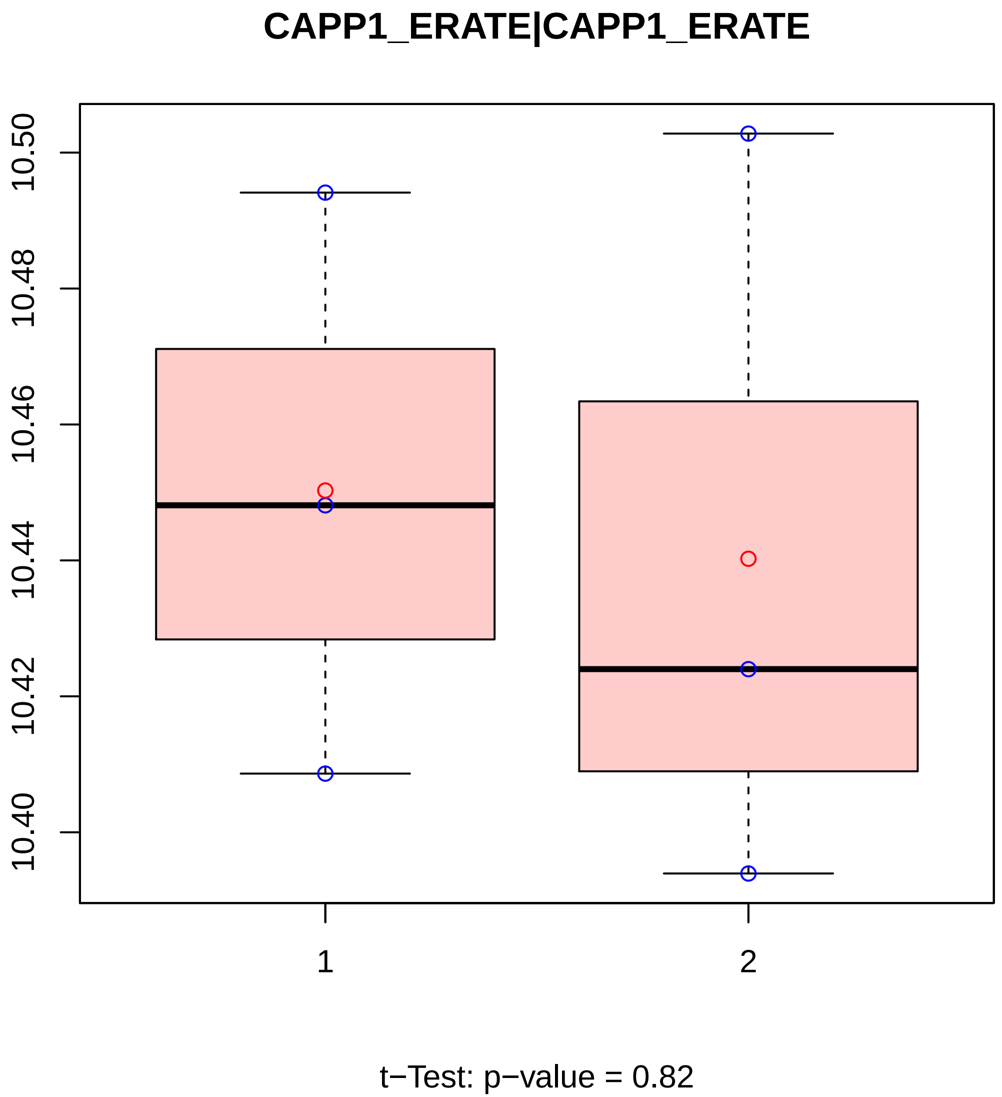

CAPP1_ERATE|CAPP1_ERATE
10.40
10.42
10.44
10.46
10.48
10.50
1
2
t−Test: p−value = 0.82

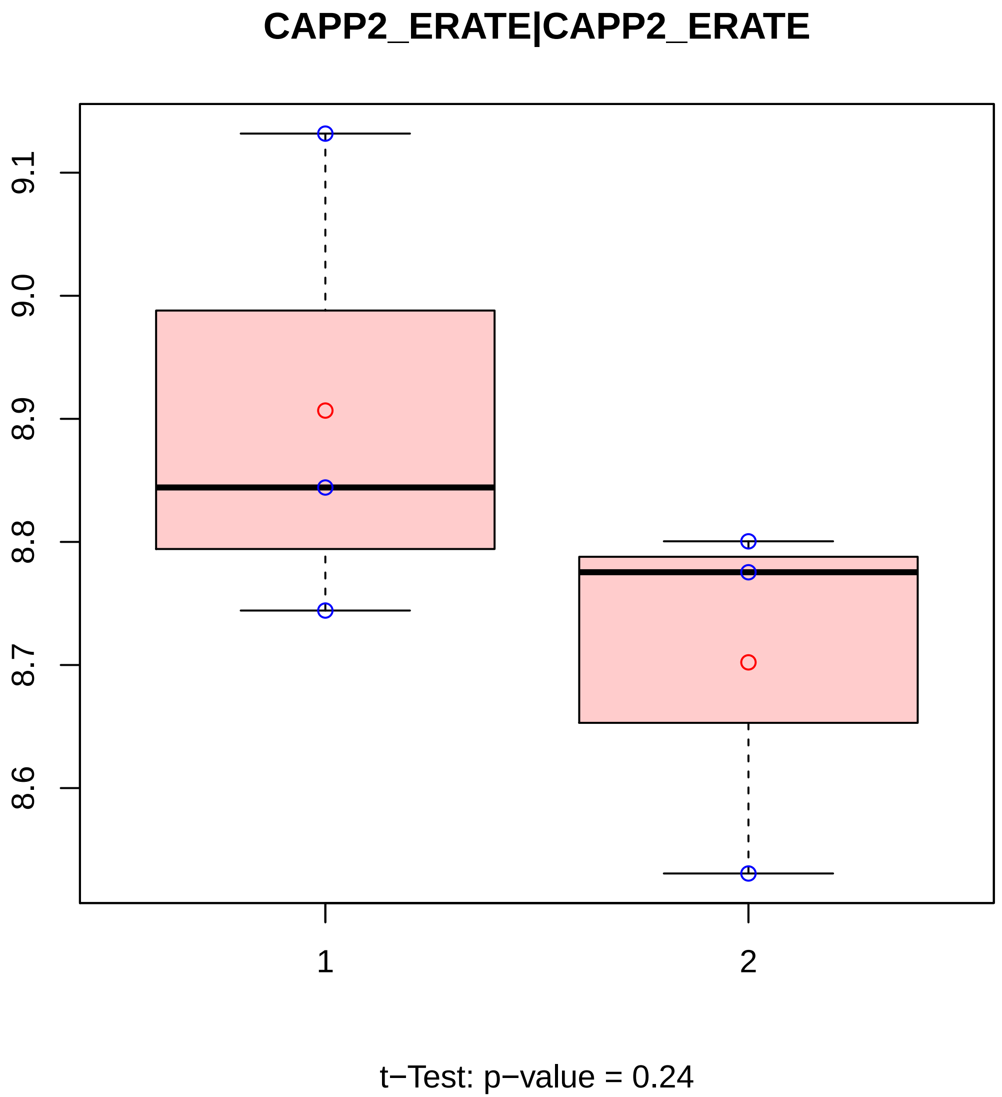
CAPP2_ERATE|CAPP2_ERATE
9.1
9.0
8.9
8.8
8.7
8.6
1
2
t−Test: p−value = 0.24

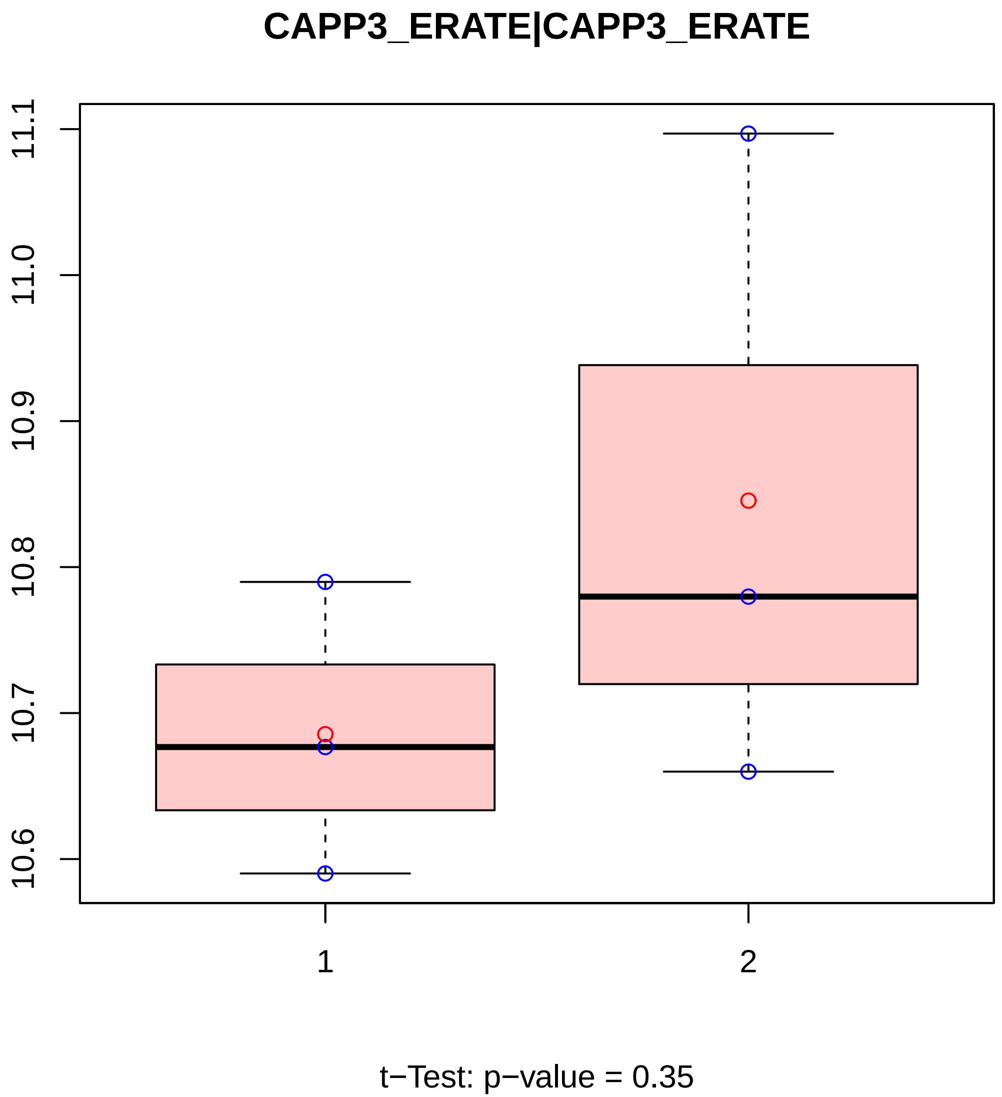

CAPP3_ERATE|CAPP3_ERATE
10.6
10.7
10.8
10.9
11.0
11.1
1
2
t−Test: p−value = 0.35

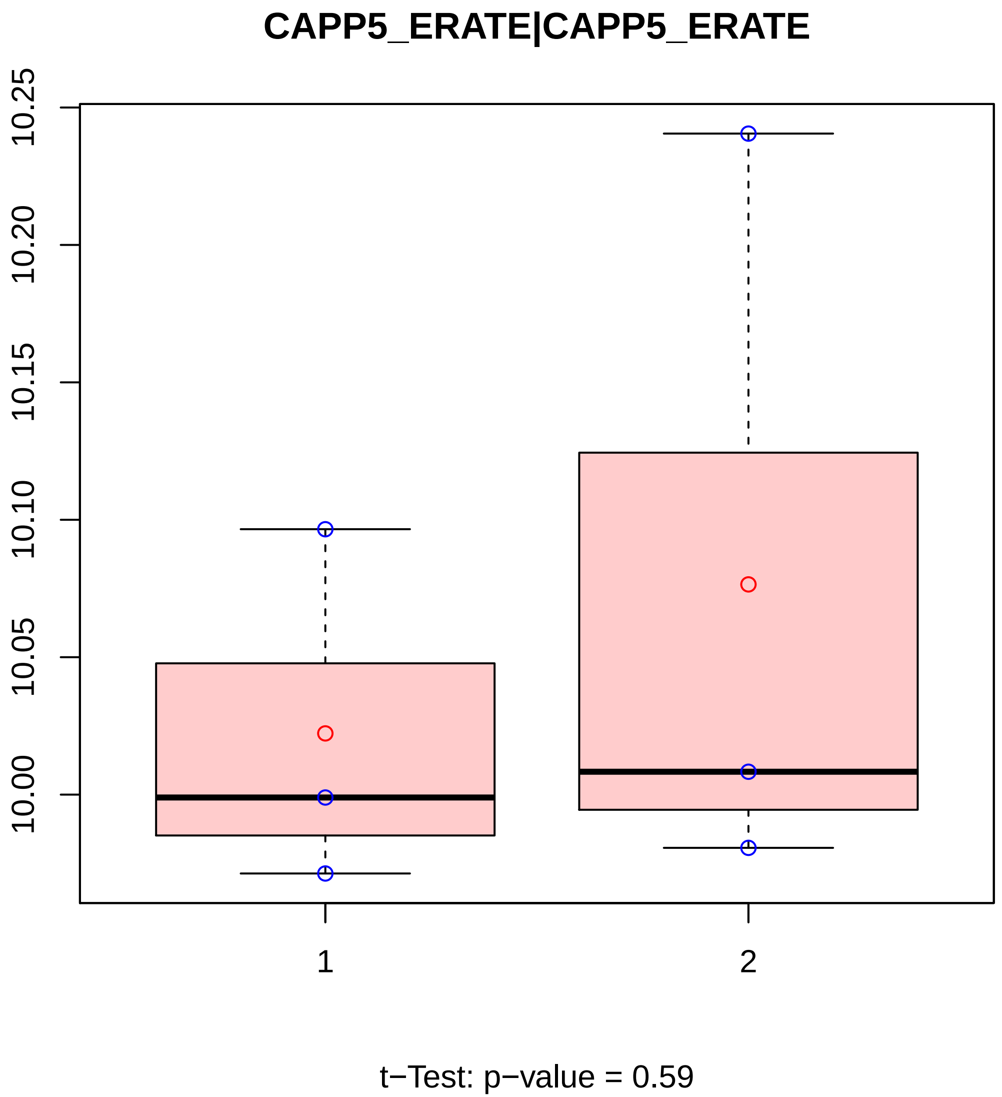
CAPP5_ERATE|CAPP5_ERATE
10.00
10.05
10.10
10.15
10.20
10.25
1
2
t−Test: p−value = 0.59

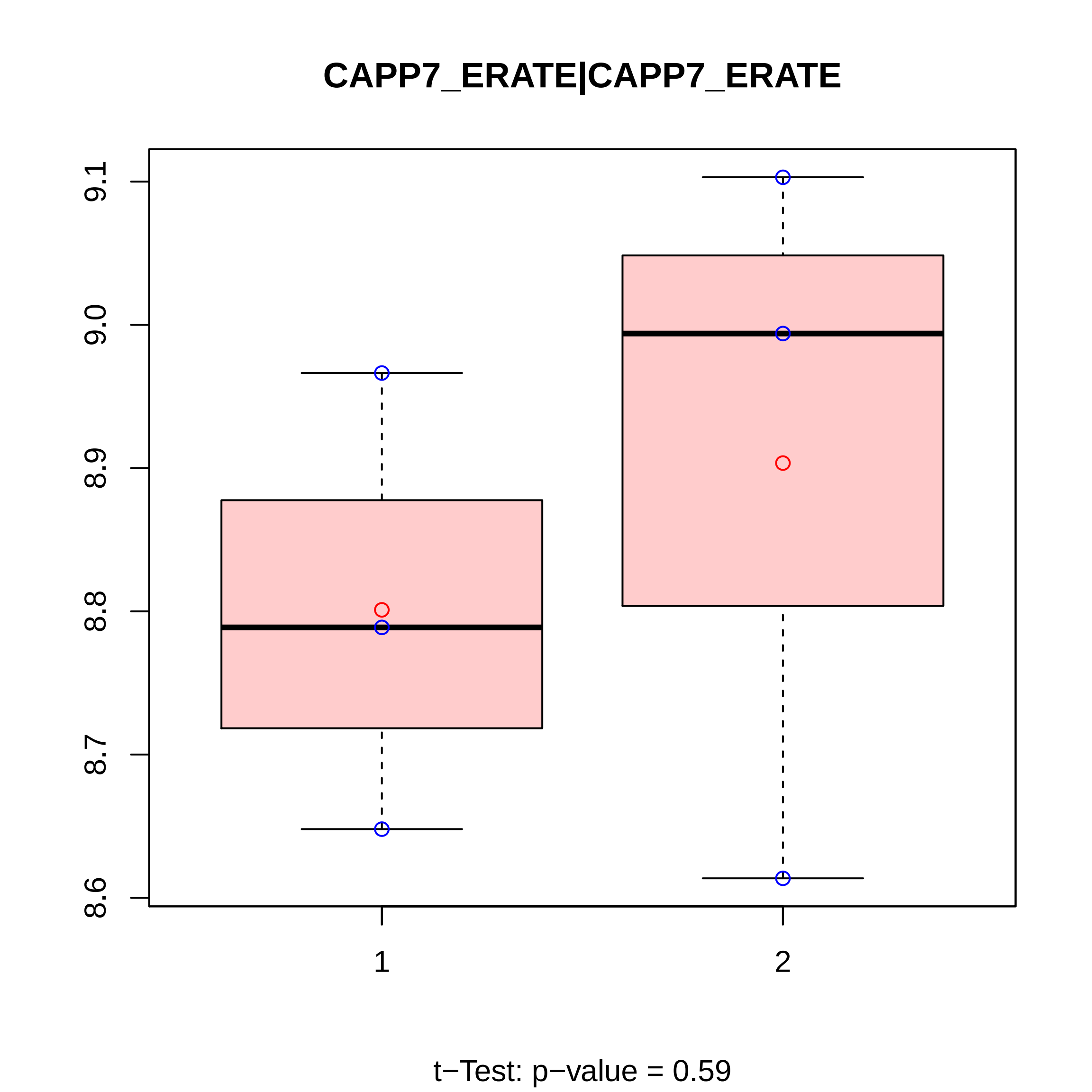
CAPP7_ERATE|CAPP7_ERATE
9.1
9.0
8.9
8.8
8.7
8.6
1
2
t−Test: p−value = 0.59

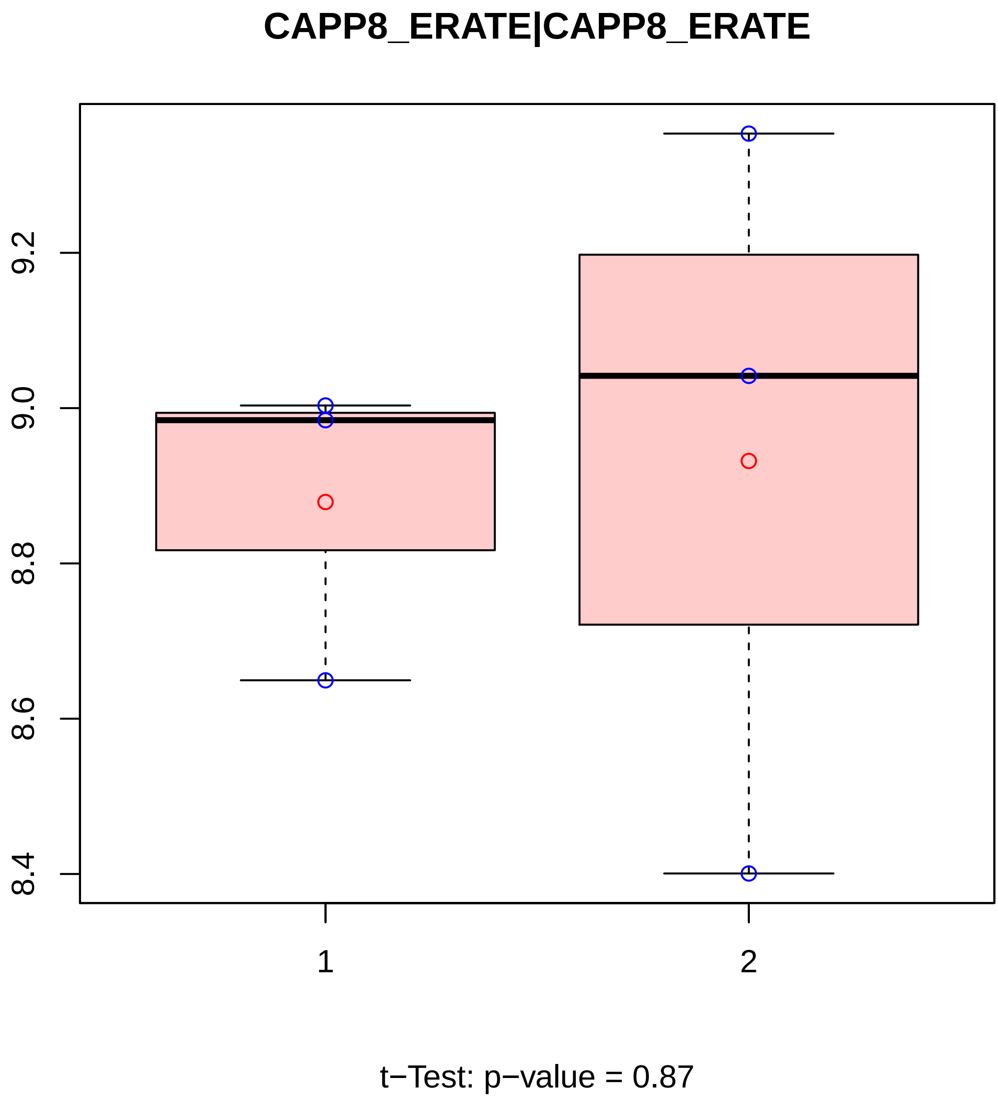

CAPP8_ERATE|CAPP8_ERATE
9.2
9.0
8.8
8.6
8.4
1
2
t−Test: p−value = 0.87

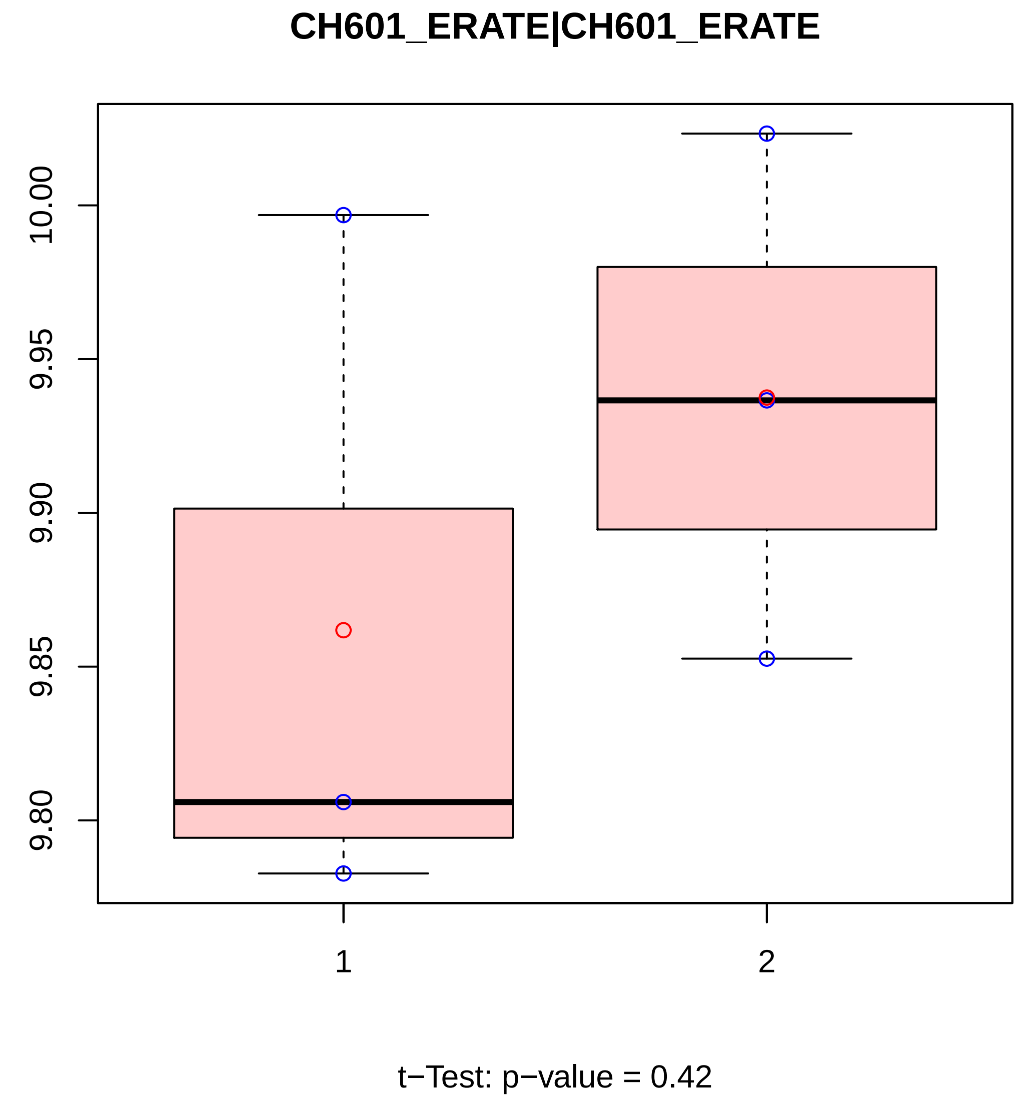
CH601_ERATE|CH601_ERATE
10.00
9.95
9.90
9.85
9.80
1
2
t−Test: p−value = 0.42

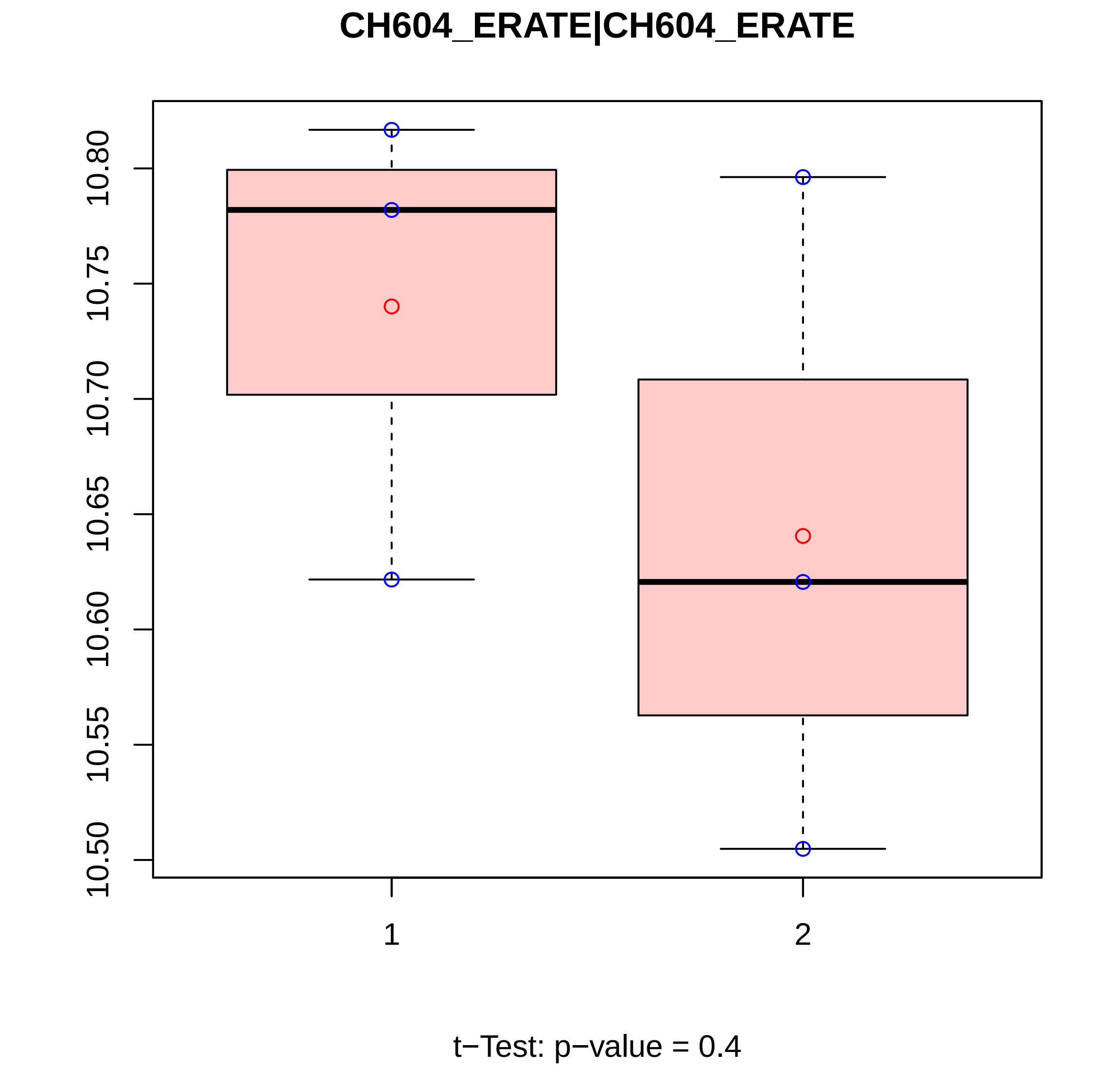
CH604_ERATE|CH604_ERATE
10.50
10.55
10.60
10.65
10.70
10.75
10.80
1
2
t−Test: p−value = 0.4

# CL10027Contig2|CL10027Contig2

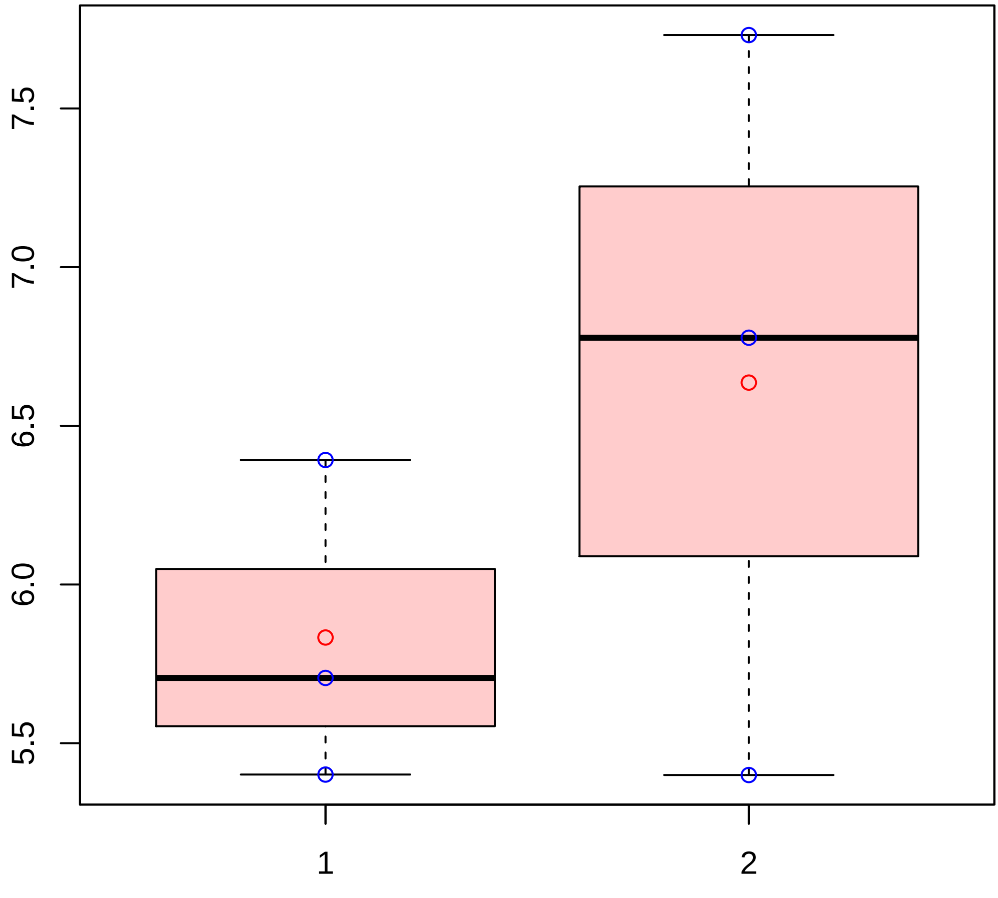


t−Test: p−value = 0.36

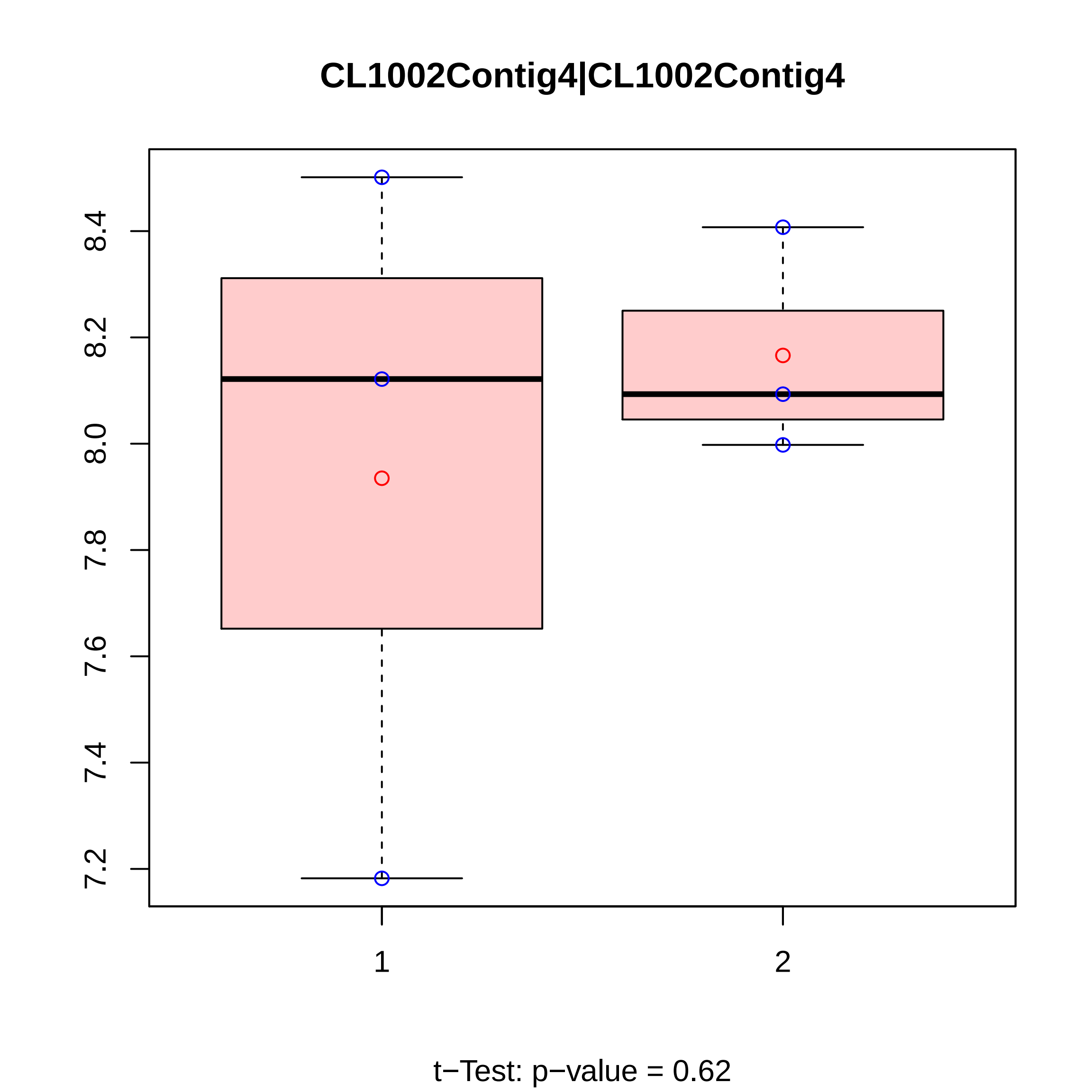

CL1002Contig4|CL1002Contig4
8.4
8.2
8.0
7.8
7.6
7.4
7.2
1
2
t−Test: p−value = 0.62

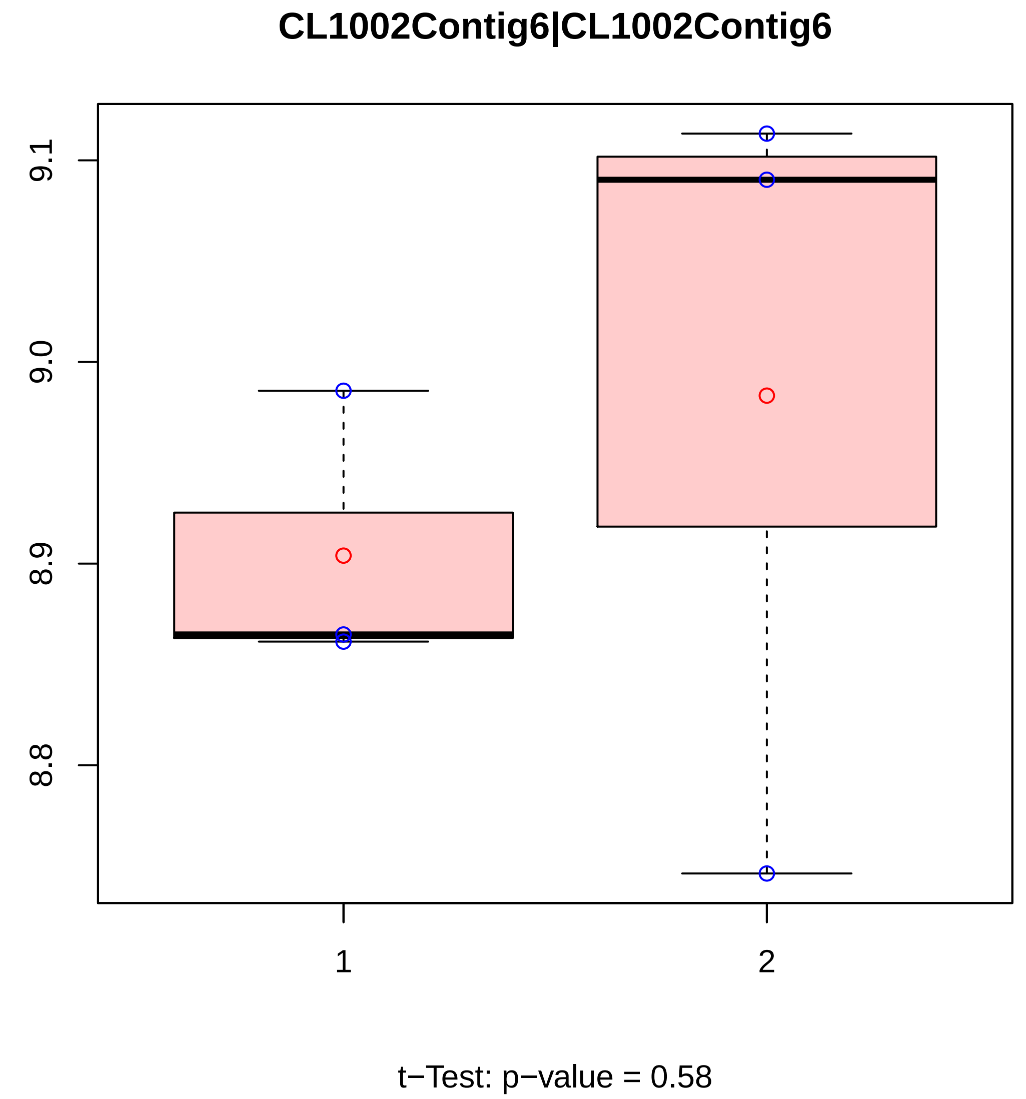
CL1002Contig6|CL1002Contig6
9.1
9.0
8.9
8.8
1
2
t−Test: p−value = 0.58

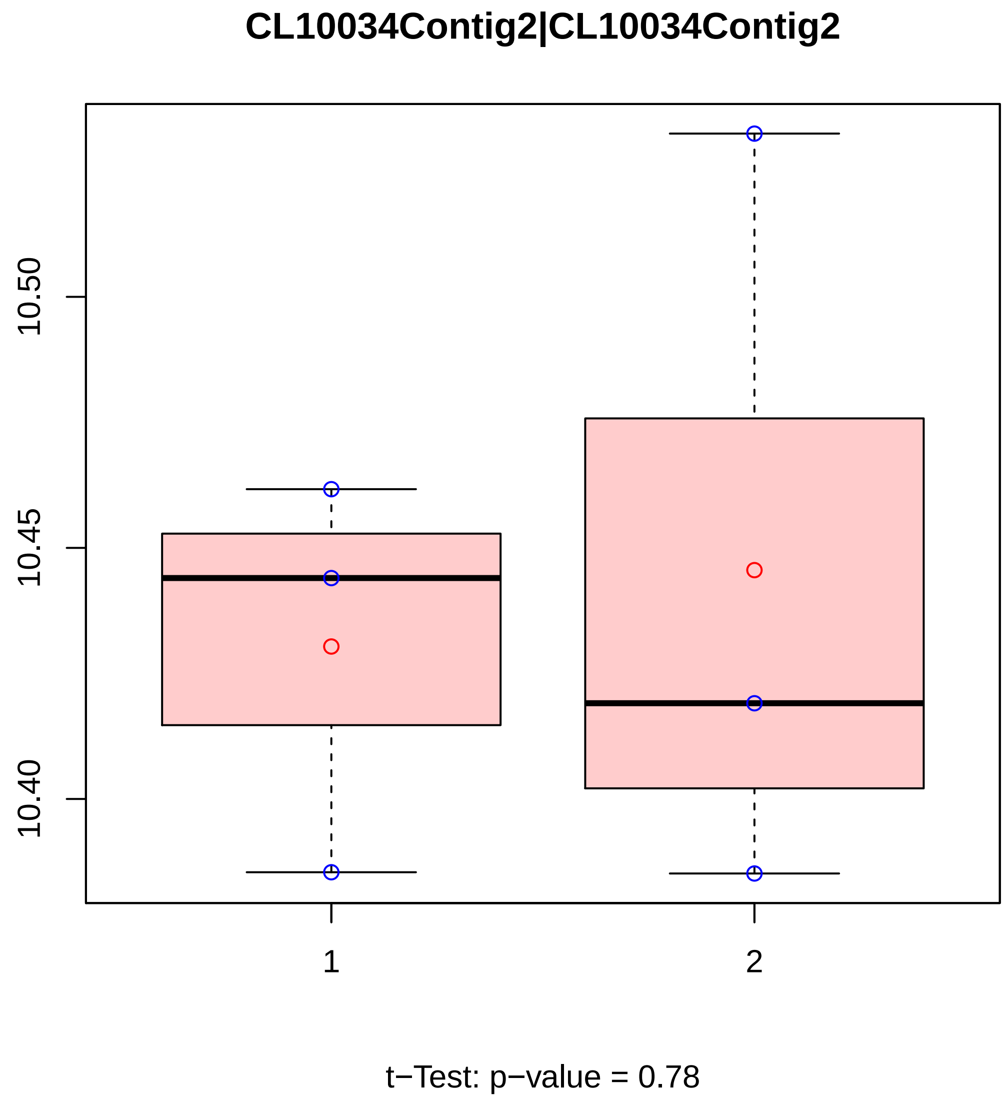
CL10034Contig2|CL10034Contig2
10.40
10.45
10.50
1
2
t−Test: p−value = 0.78

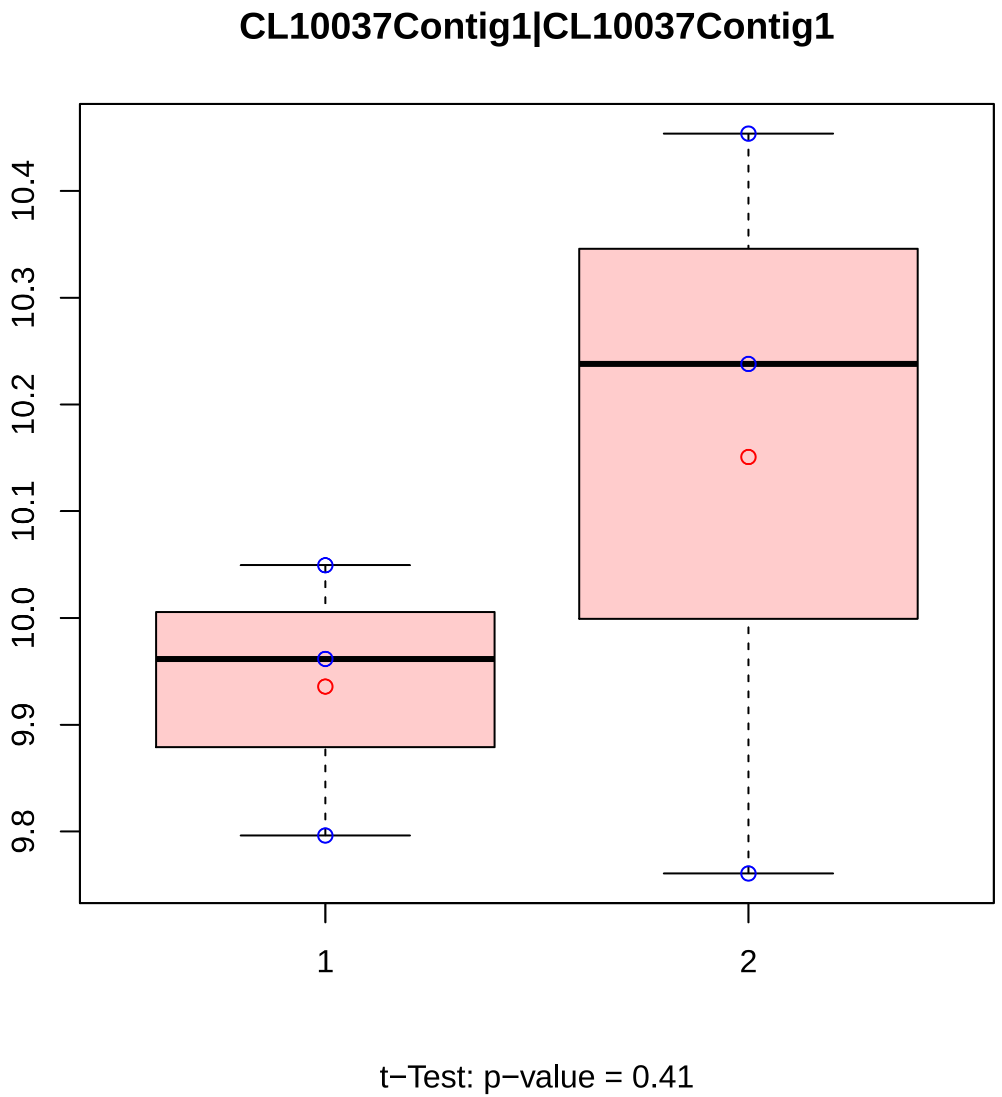
CL10037Contig1|CL10037Contig1
10.4
10.3
10.2
10.1
10.0
9.9
9.8
1
2
t−Test: p−value = 0.41

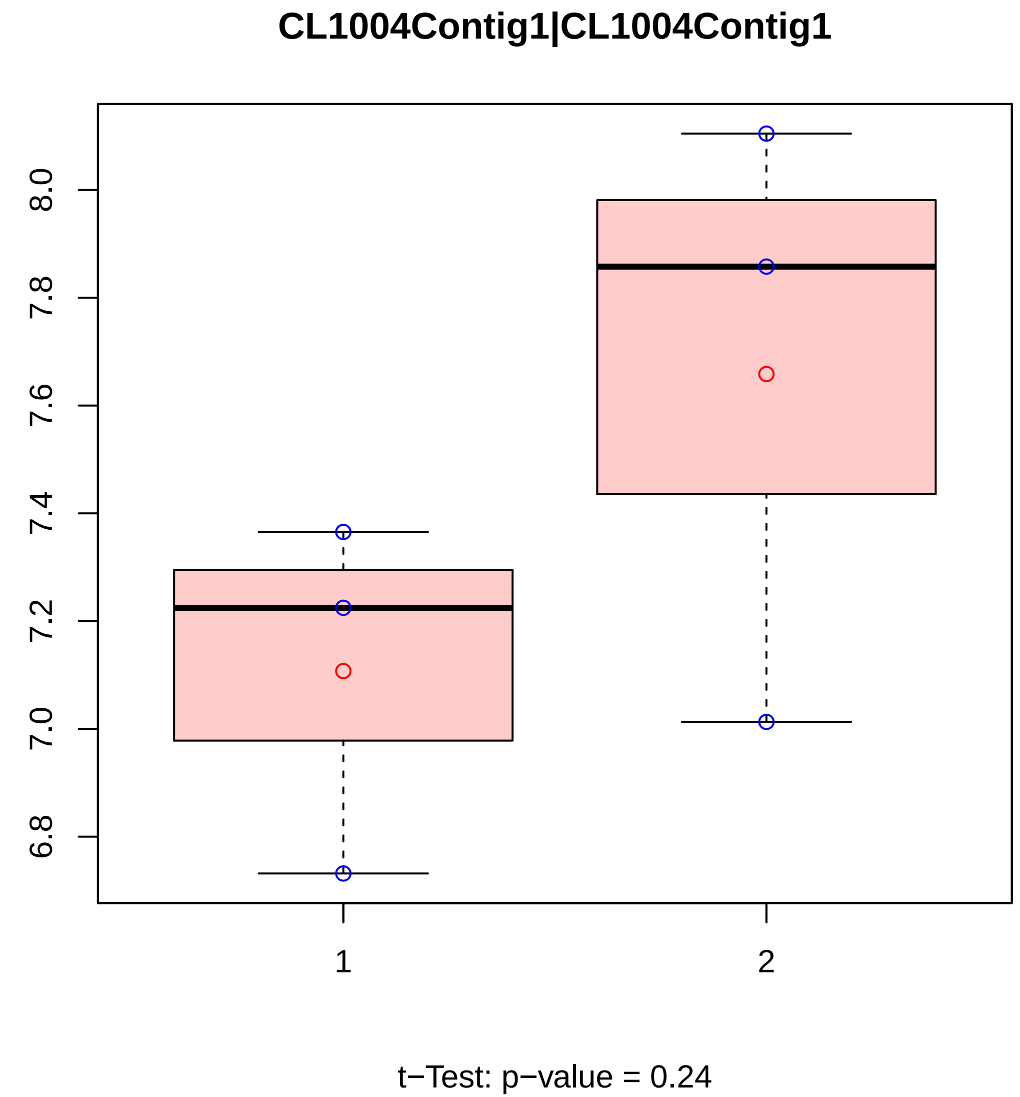
CL1004Contig1|CL1004Contig1
8.0
7.8
7.6
7.4
7.2
7.0
6.8
1
2
t−Test: p−value = 0.24

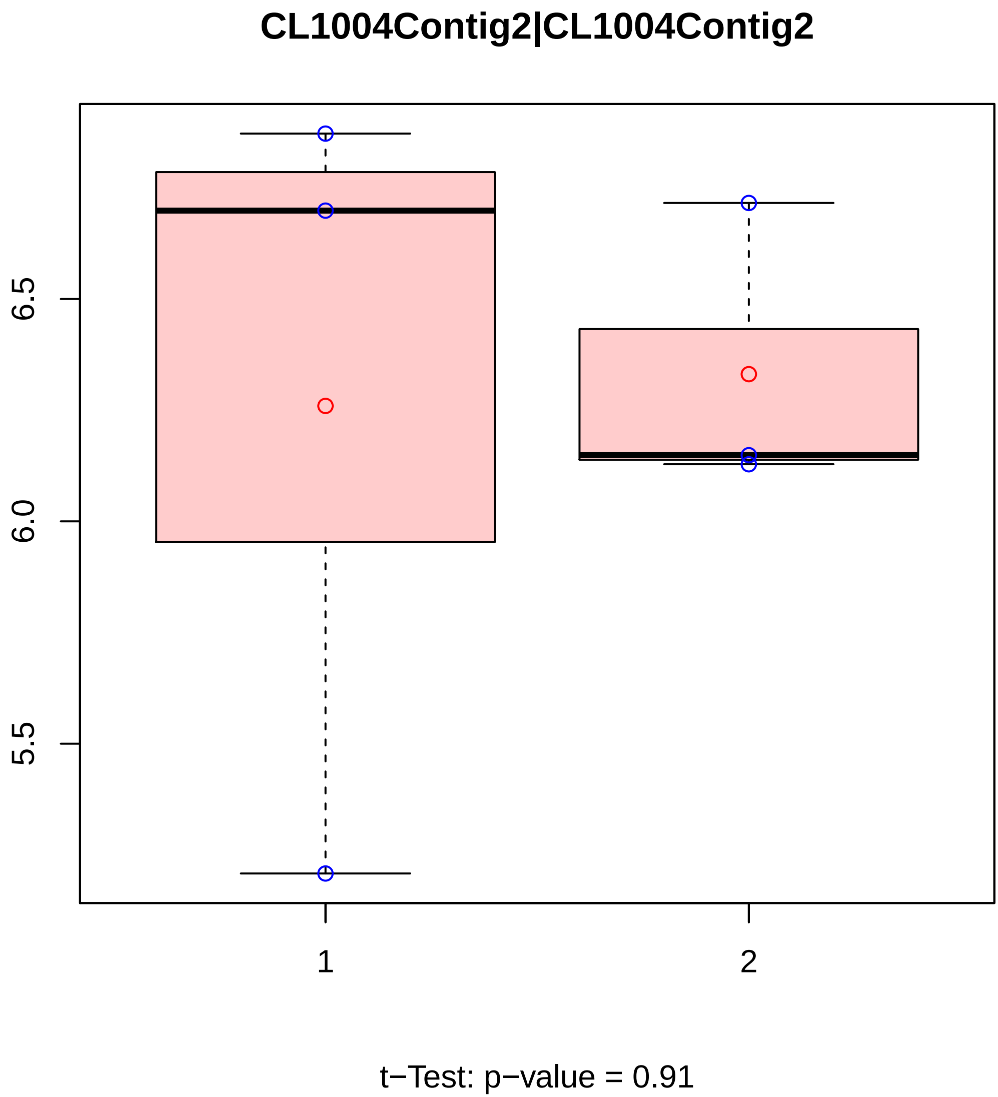
CL1004Contig2|CL1004Contig2
6.5
6.0
5.5
1
2
t−Test: p−value = 0.91

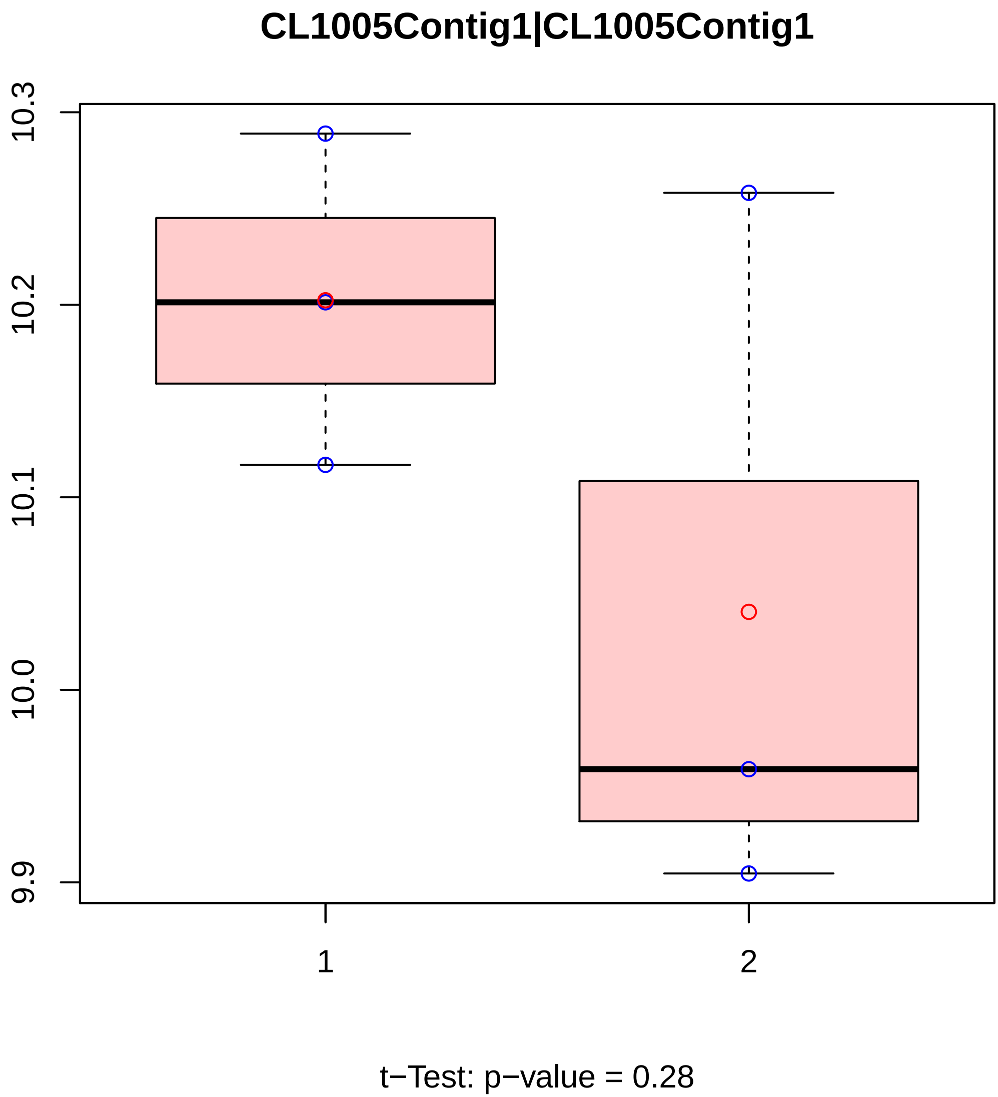

CL1005Contig1|CL1005Contig1
10.3
10.2
10.1
10.0
9.9
1
2
t−Test: p−value = 0.28

## CL10079Contig1|CL10079Contig1

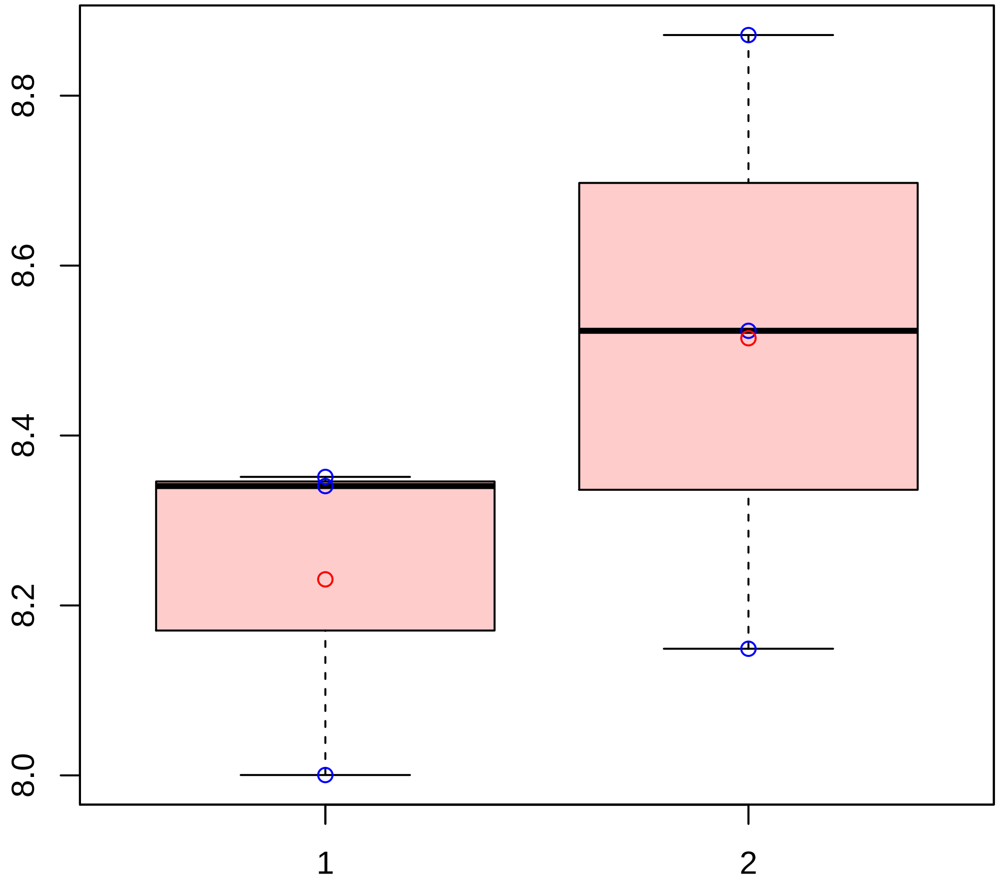


t−Test: p−value = 0.32

**CL100Contig17|CL100Contig17**

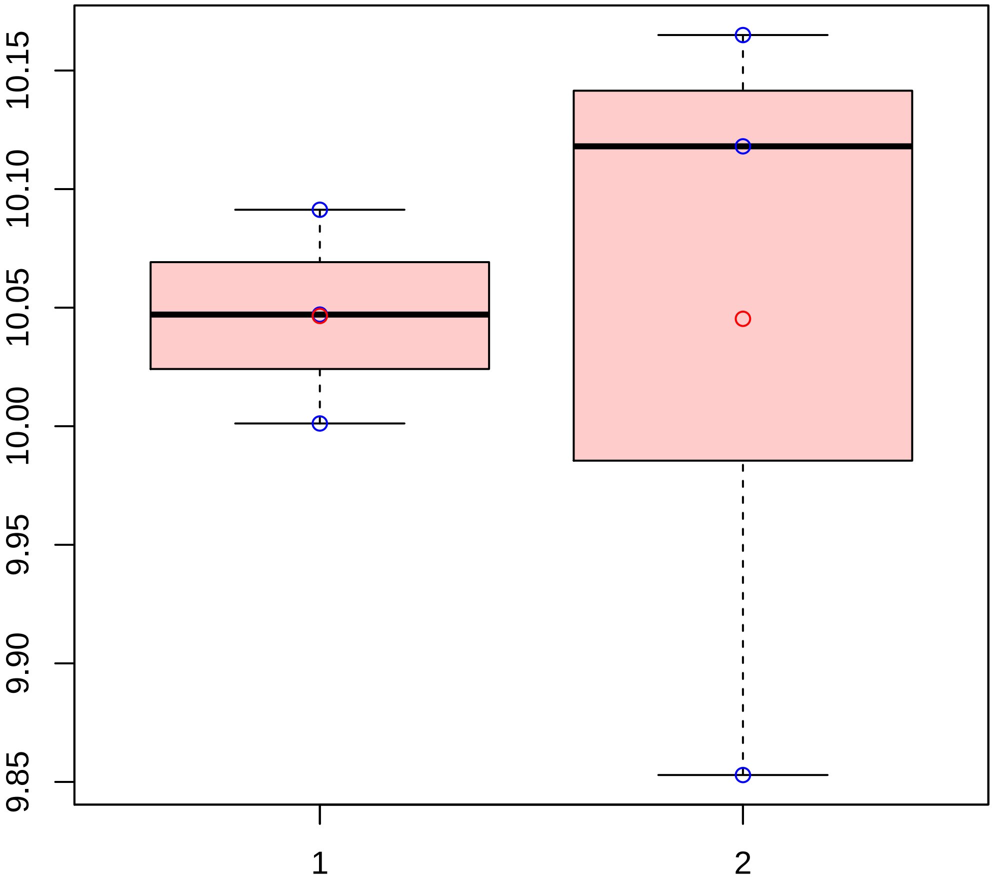


t−Test: p−value = 0.99

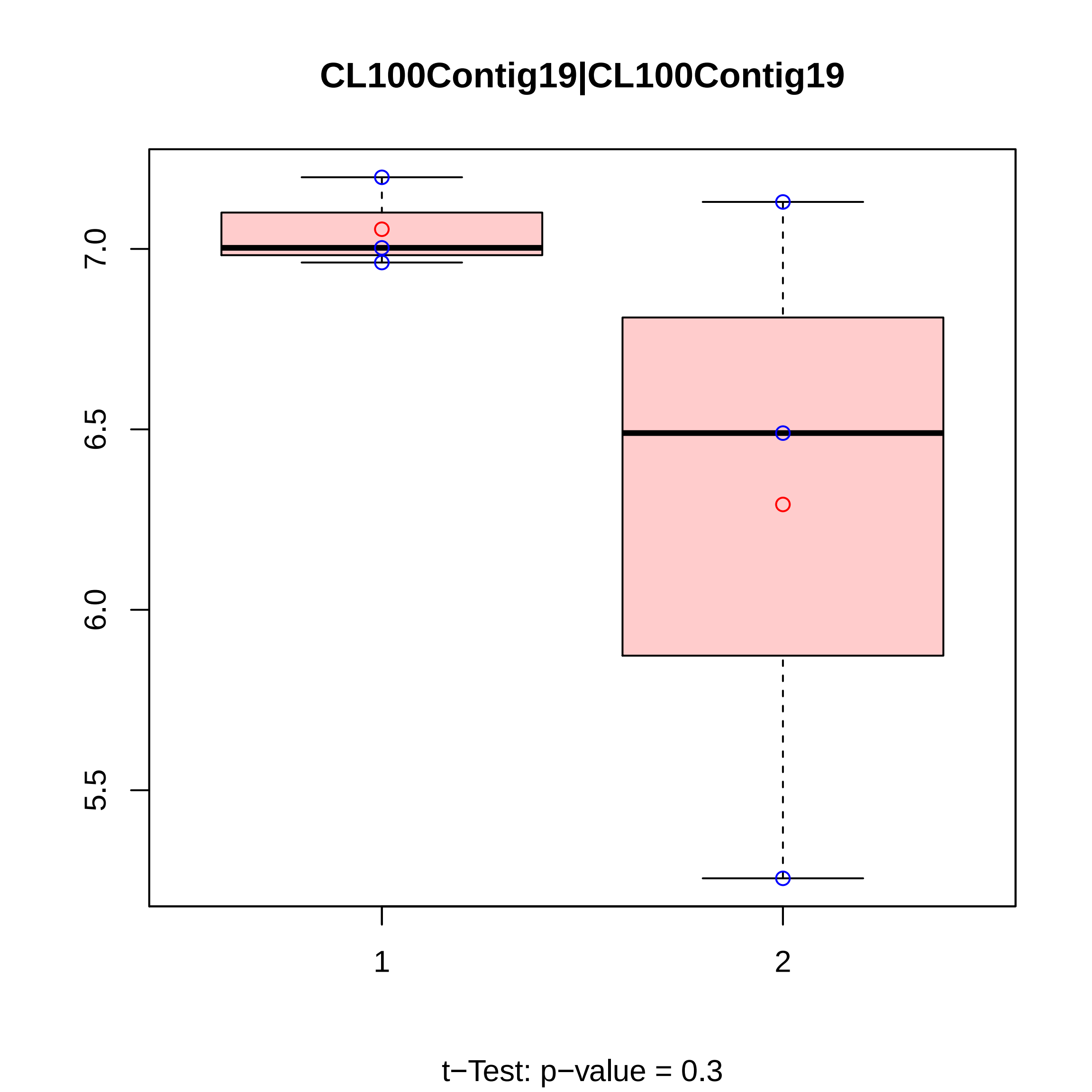
CL100Contig19|CL100Contig19
7.0
6.5
6.0
5.5
1
2
t−Test: p−value = 0.3

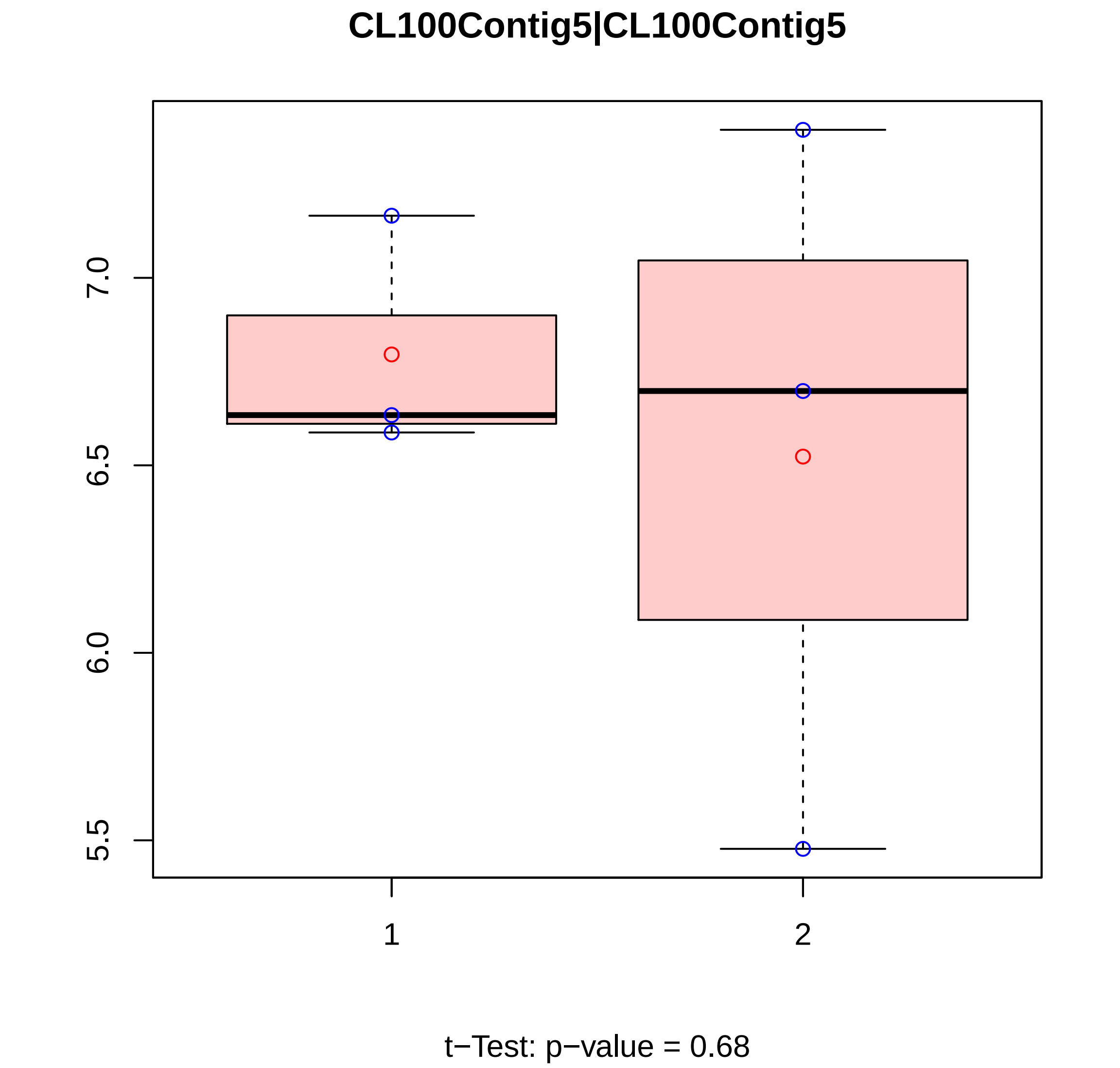
CL100Contig5|CL100Contig5
7.0
6.5
6.0
5.5
1
2
t−Test: p−value = 0.68

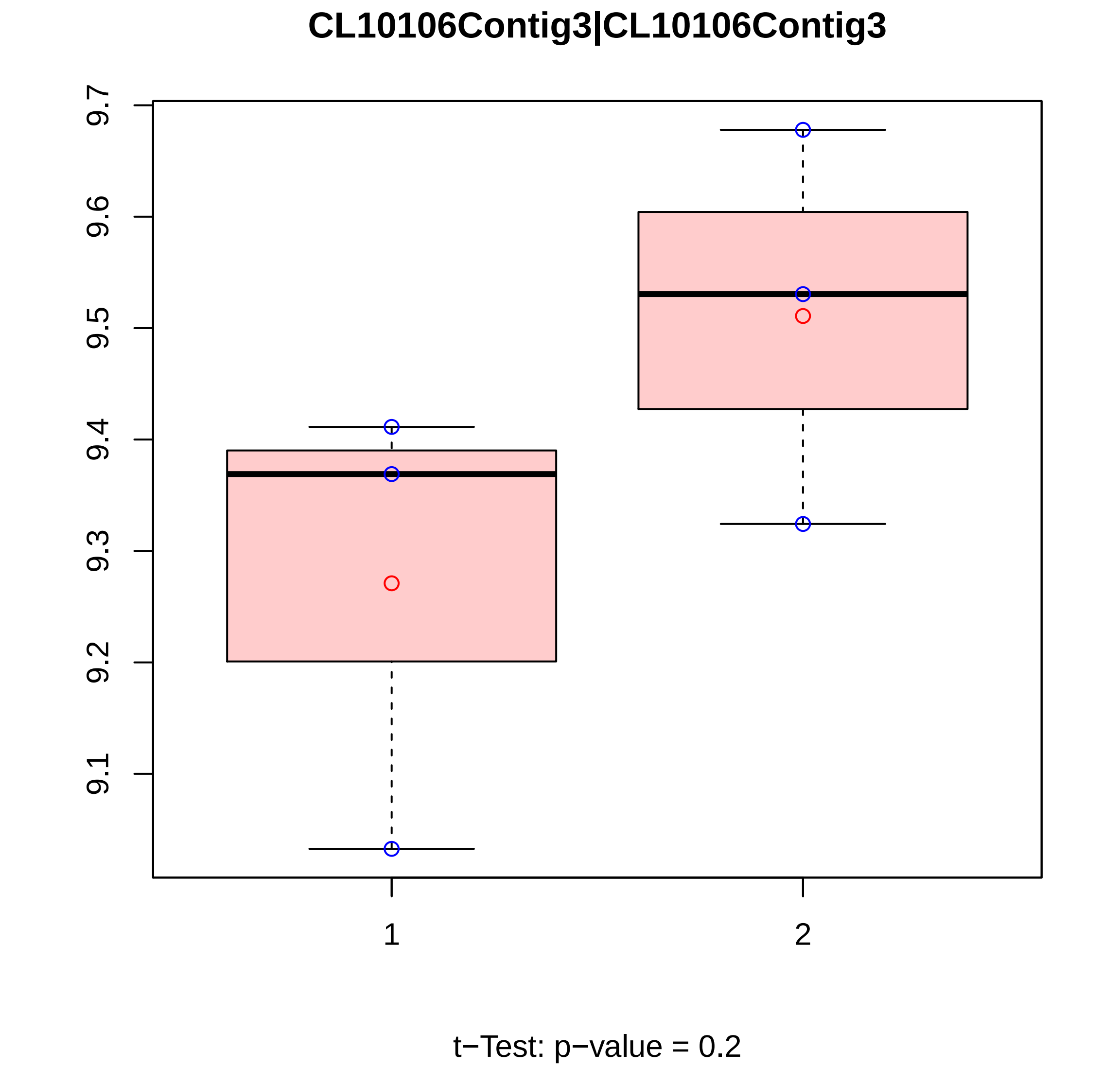
CL10106Contig3|CL10106Contig3
9.1
9.2
9.3
9.4
9.5
9.6
9.7
1
2
t−Test: p−value = 0.2

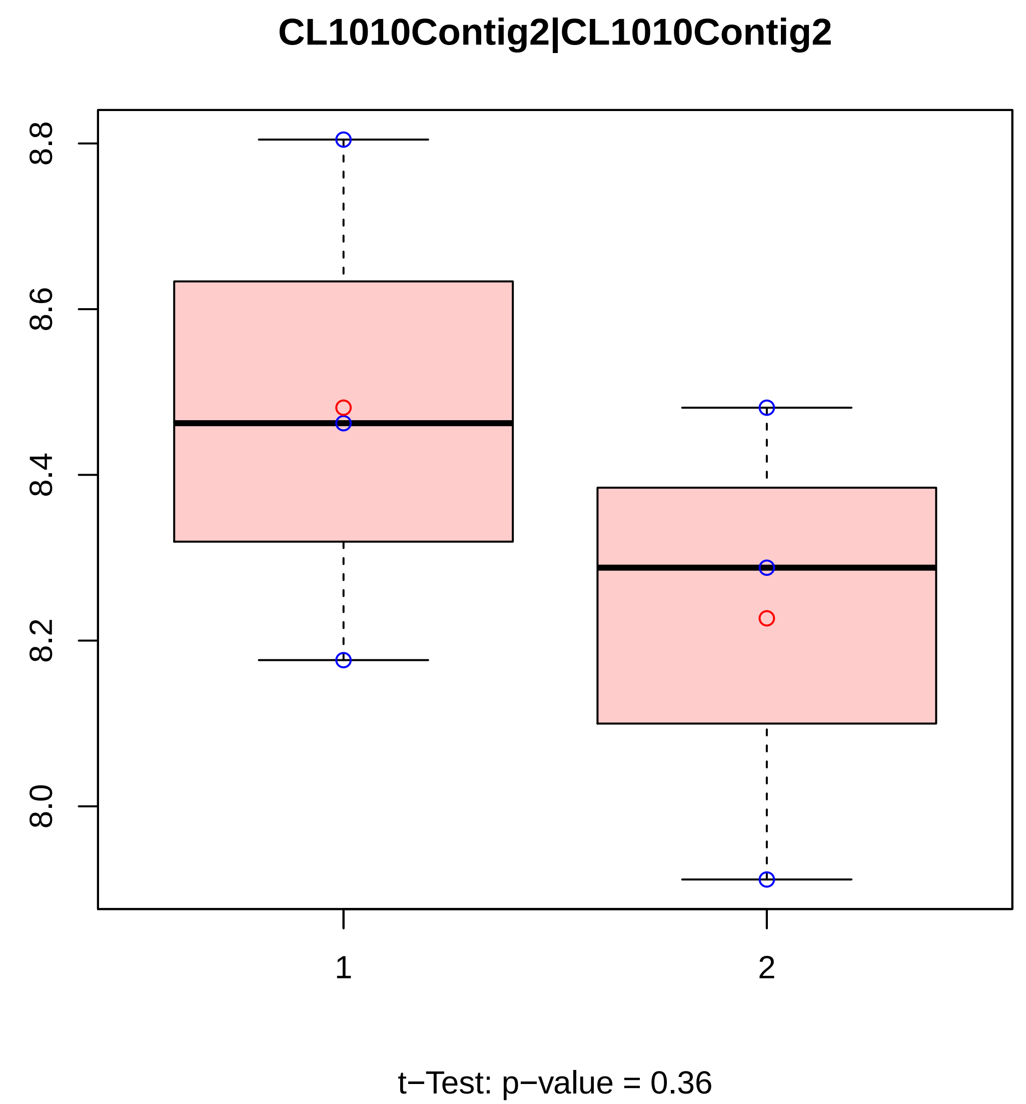
CL1010Contig2|CL1010Contig2
8.0
8.2
8.4
8.6
8.8
1
2
t−Test: p−value = 0.36

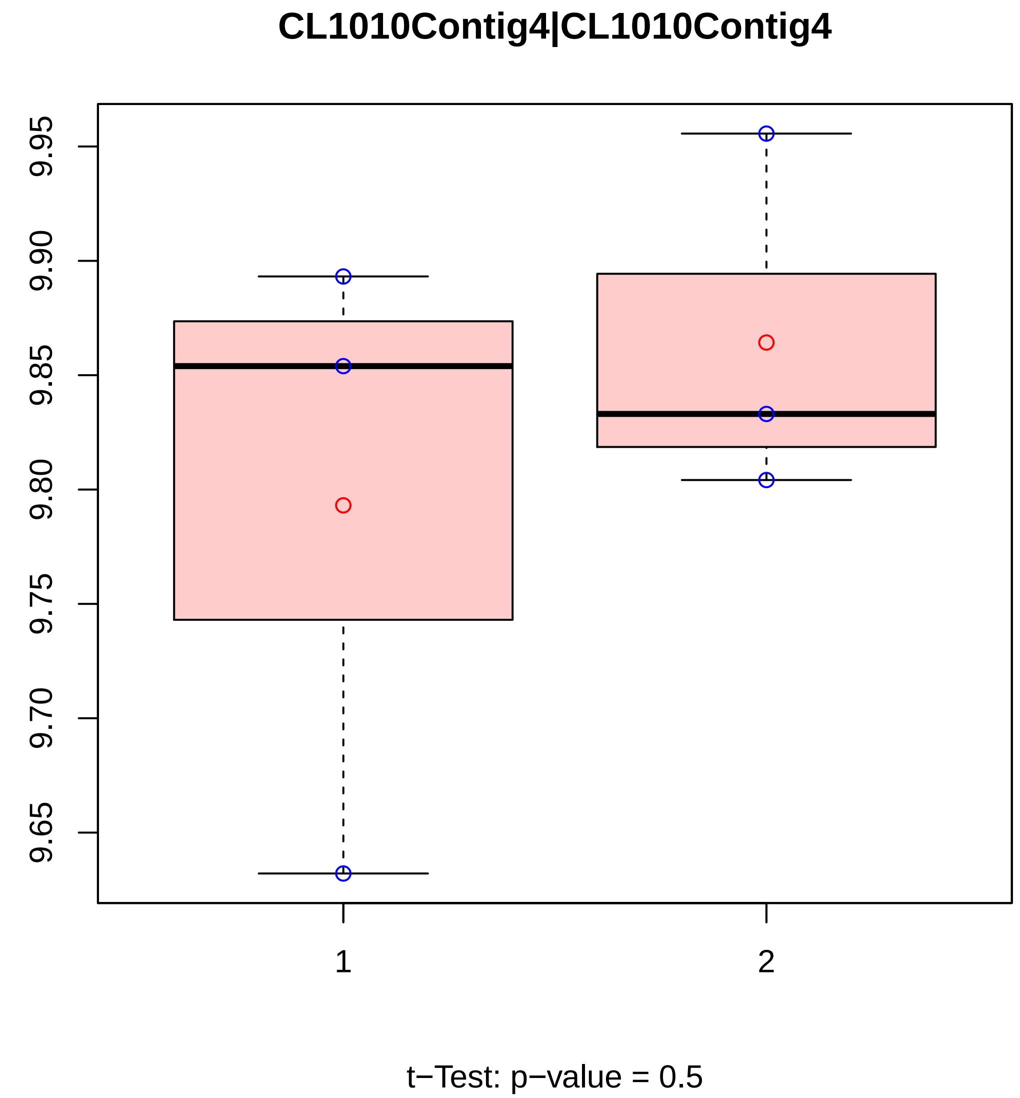
CL1010Contig4|CL1010Contig4
9.65
9.70
9.75
9.80
9.85
9.90
9.95
1
2
t−Test: p−value = 0.5

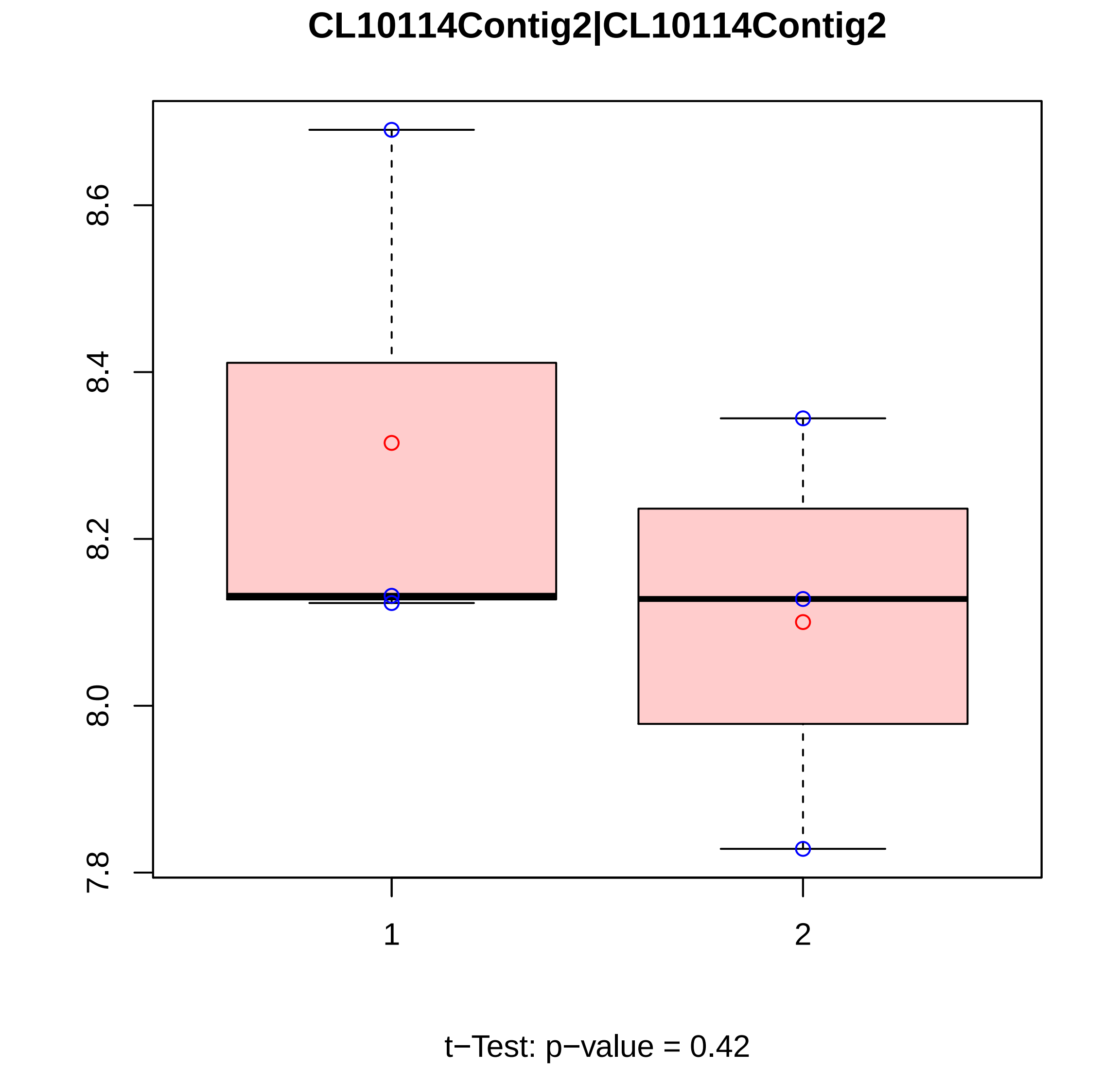
CL10114Contig2|CL10114Contig2
8.6
8.4
8.2
8.0
7.8
1
2
t−Test: p−value = 0.42

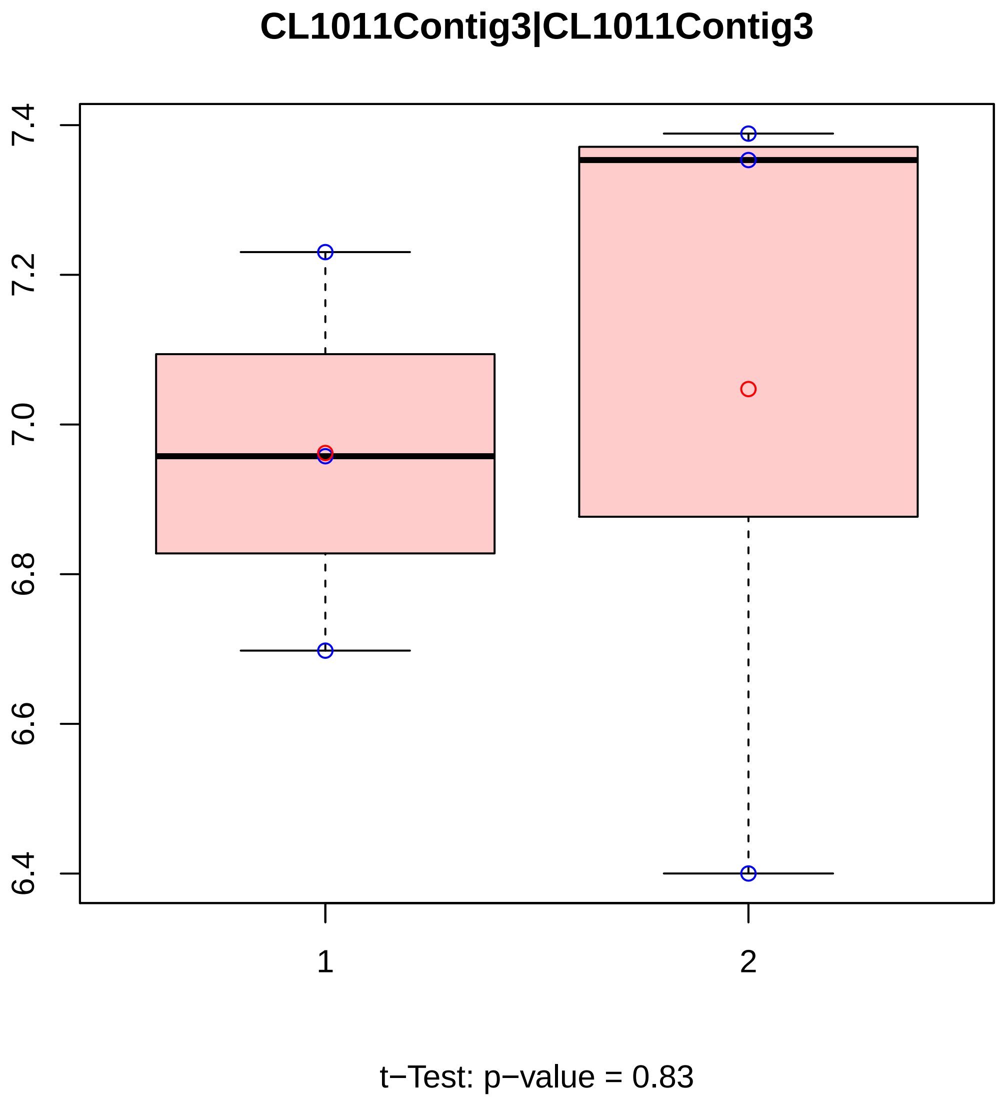
CL1011Contig3|CL1011Contig3
7.4
7.2
7.0
6.8
6.6
6.4
1
2
t−Test: p−value = 0.83

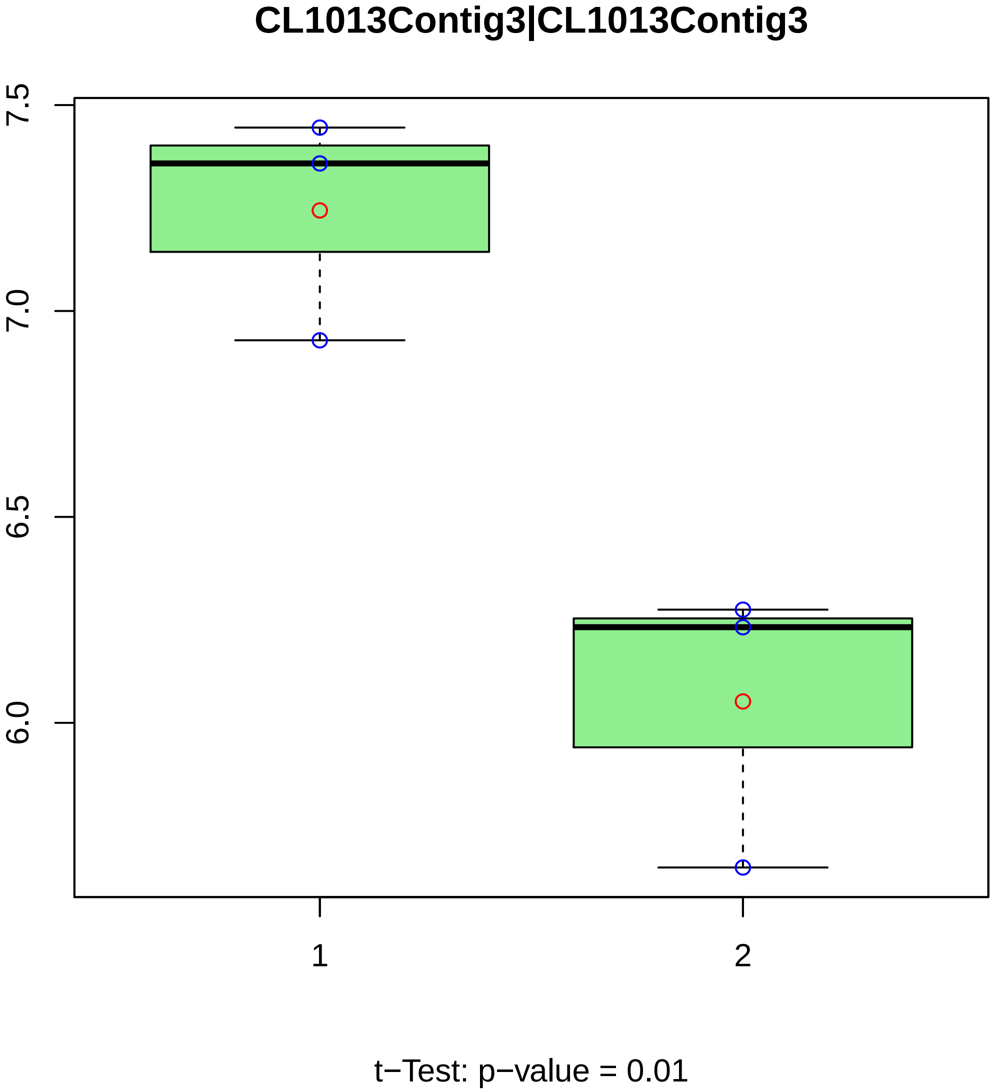

CL1013Contig3|CL1013Contig3
7.5
7.0
6.5
6.0
1
2
t−Test: p−value = 0.01

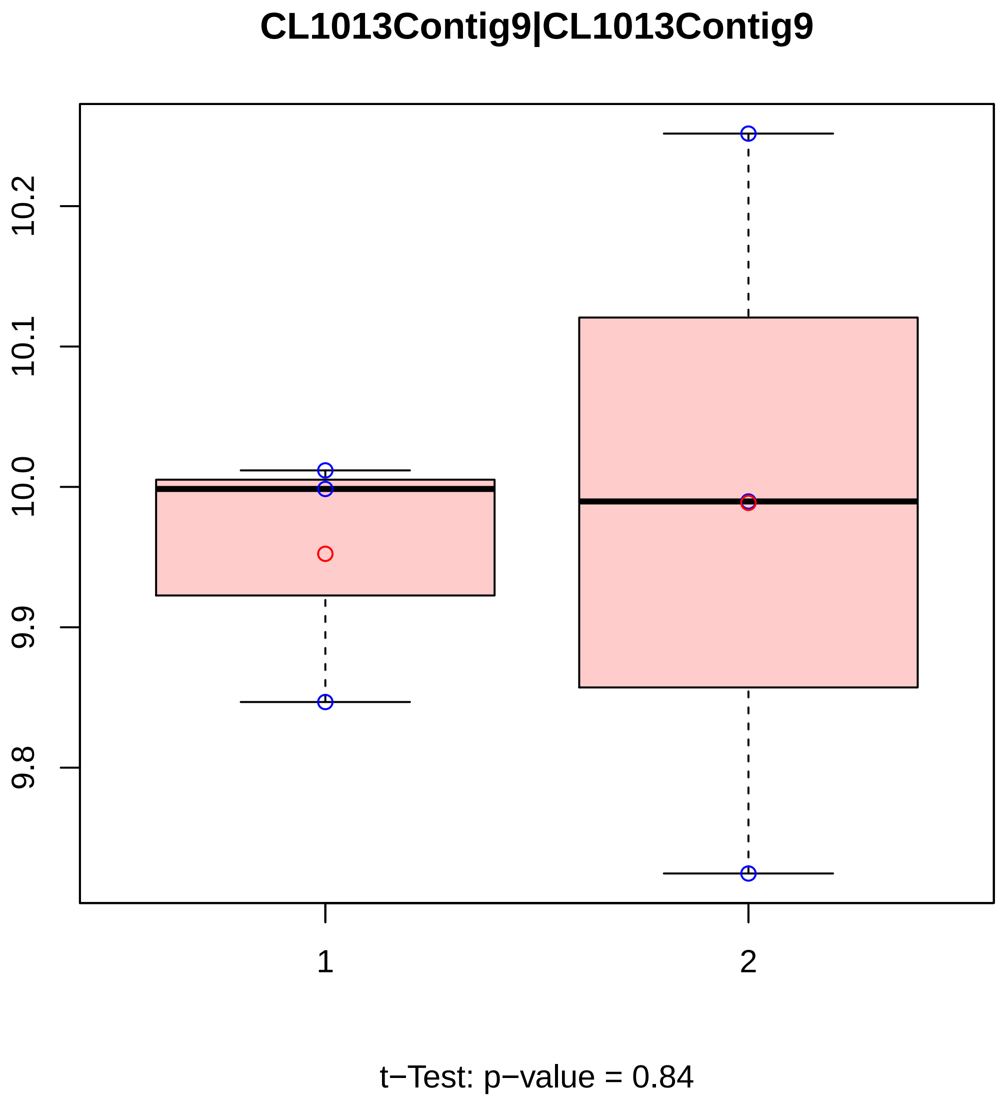
CL1013Contig9|CL1013Contig9
10.2
10.1
10.0
9.9
9.8
1
2
t−Test: p−value = 0.84

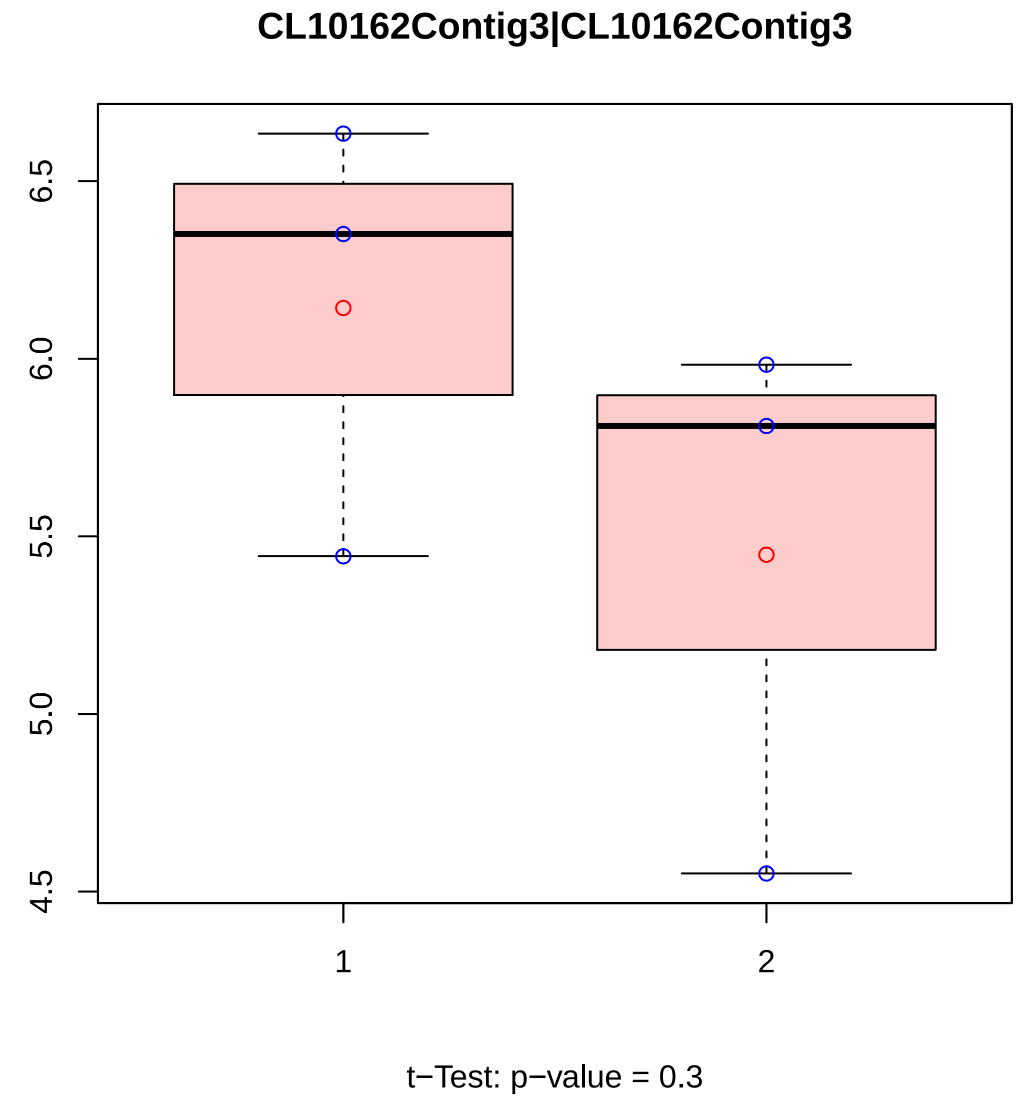
CL10162Contig3|CL10162Contig3
6.5
6.0
5.5
5.0
4.5
1
2
t−Test: p−value = 0.3

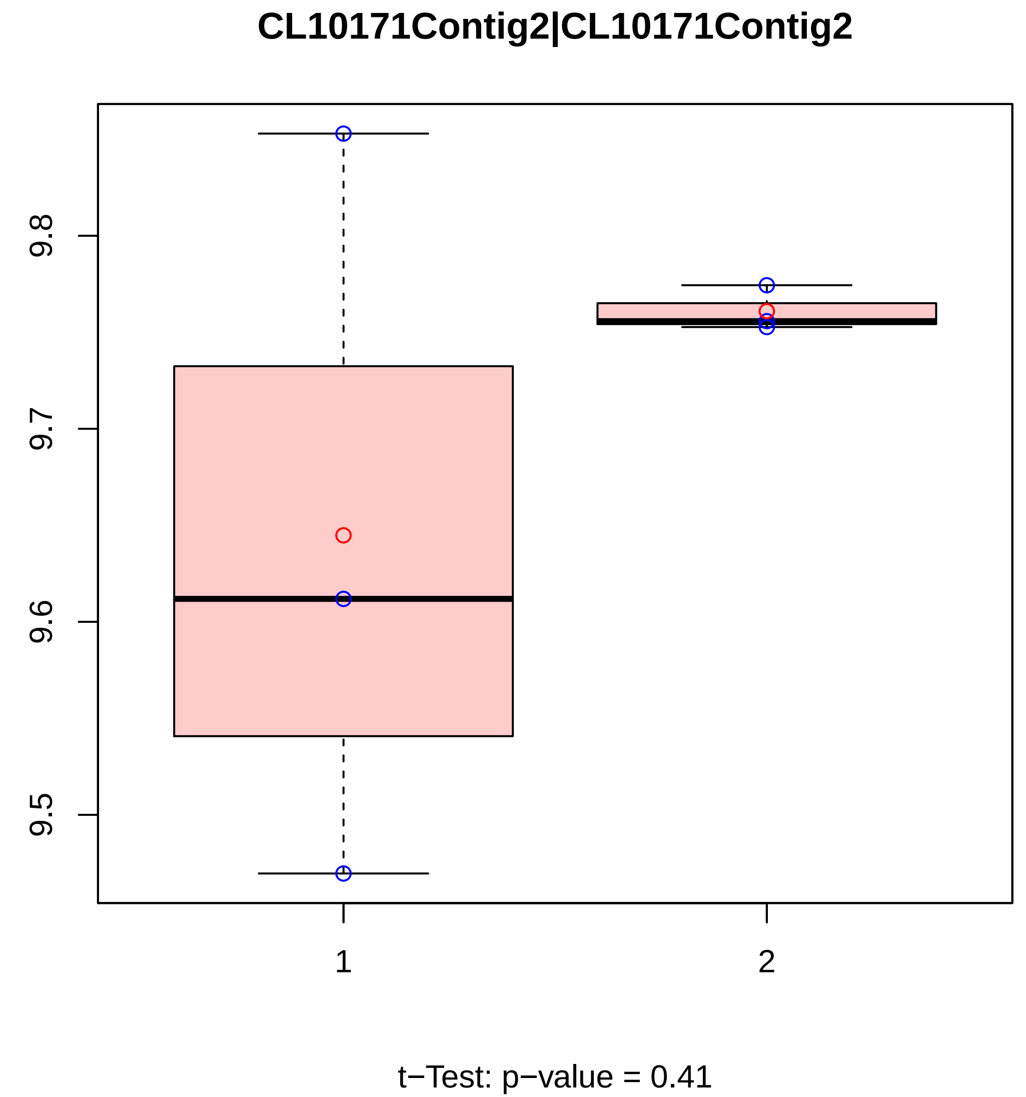
CL10171Contig2|CL10171Contig2
9.5
9.6
9.7
9.8
1
2
t−Test: p−value = 0.41

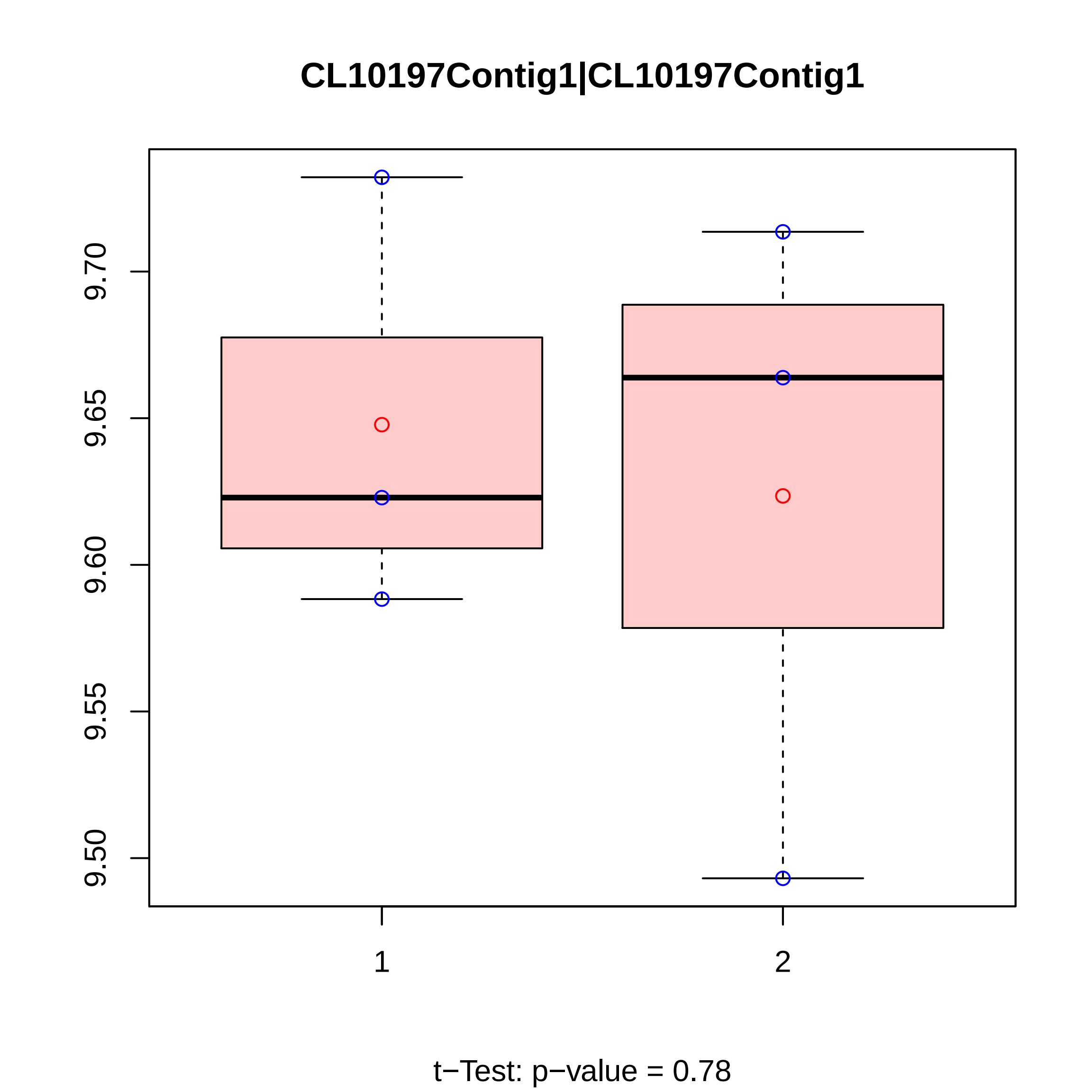

CL10197Contig1|CL10197Contig1
9.50
9.55
9.60
9.65
9.70
1
2
t−Test: p−value = 0.78

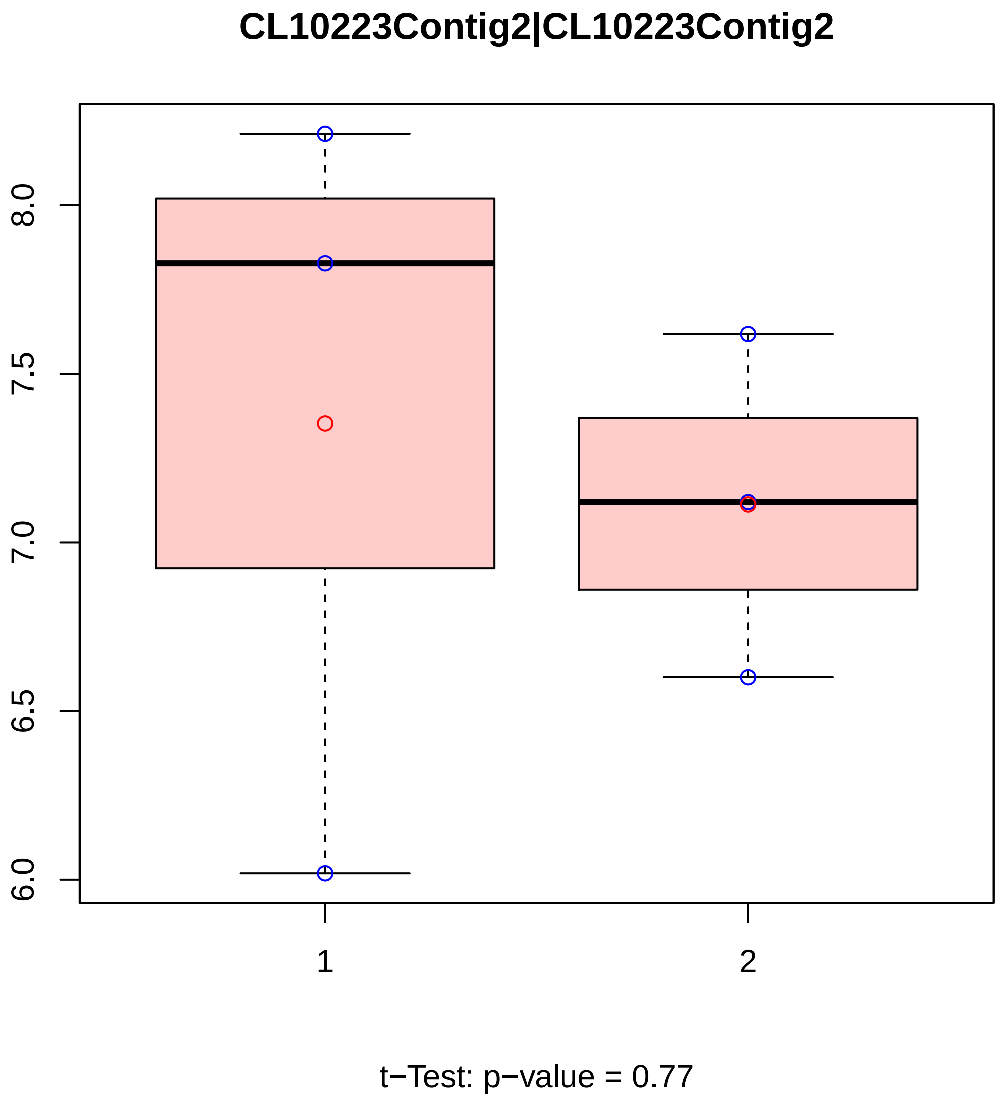
CL10223Contig2|CL10223Contig2
6.0
6.5
7.0
7.5
8.0
1
2
t−Test: p−value = 0.77

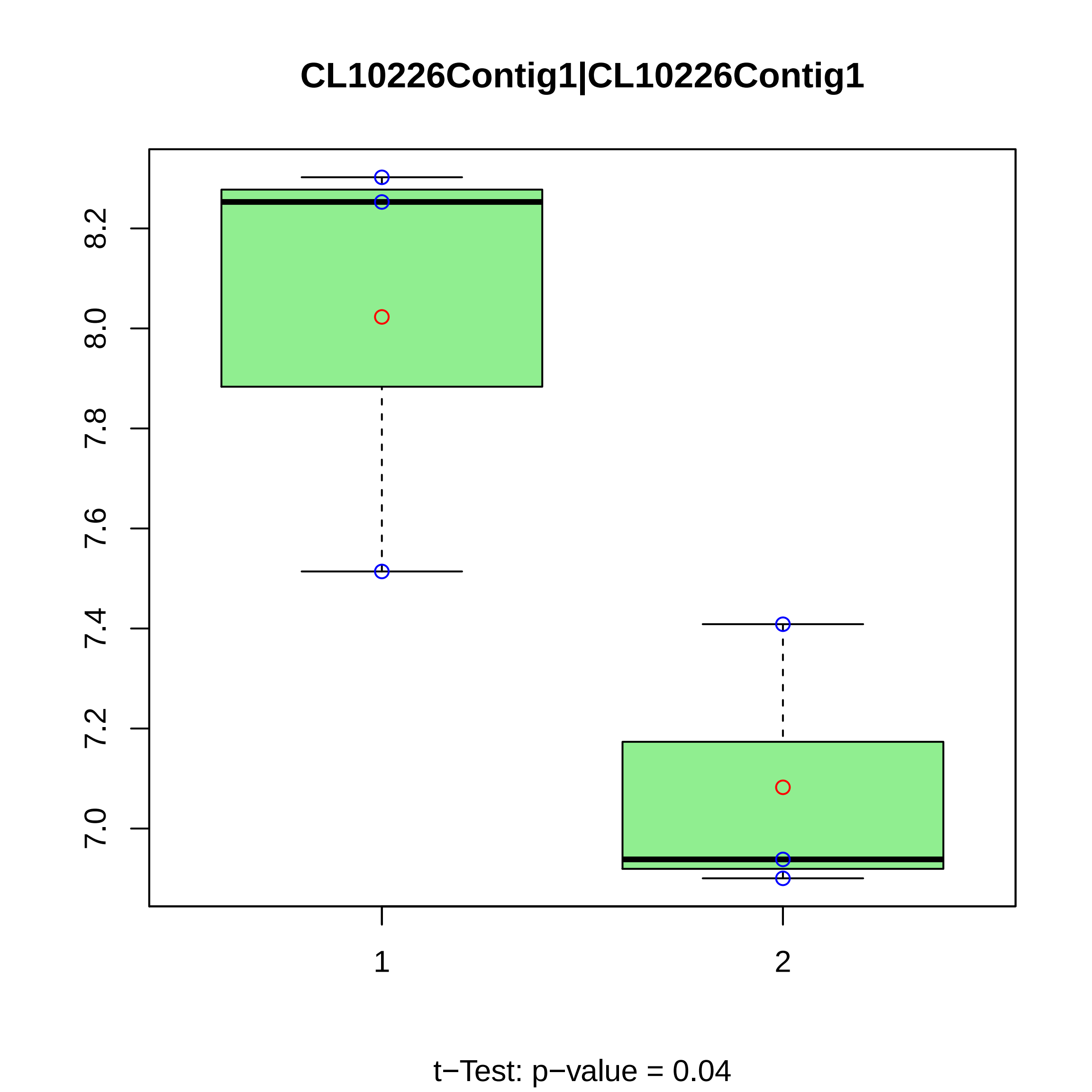
CL10226Contig1|CL10226Contig1
8.2
8.0
7.8
7.6
7.4
7.2
7.0
1
2
t−Test: p−value = 0.04

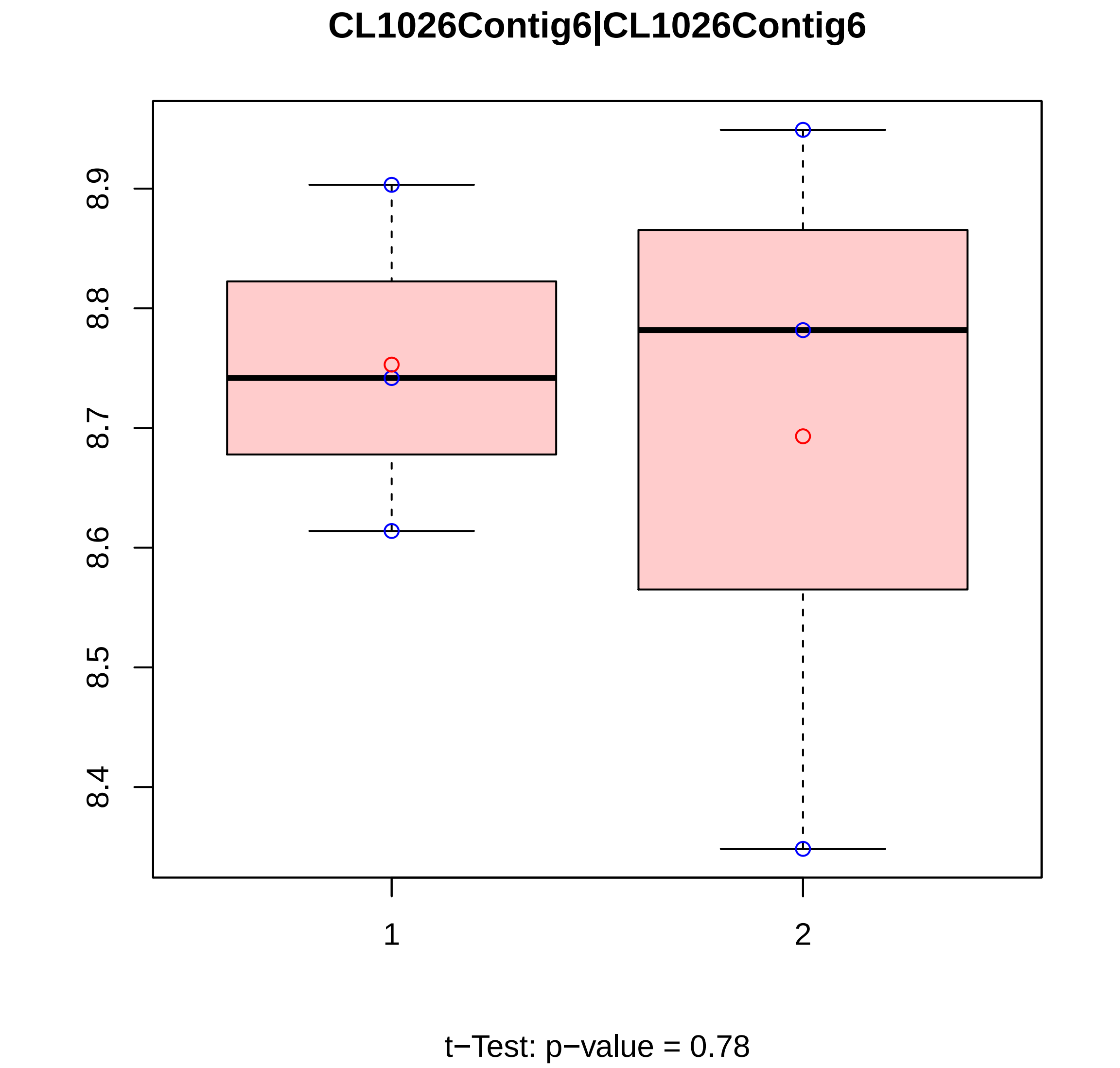
CL1026Contig6|CL1026Contig6
8.4
8.5
8.6
8.7
8.8
8.9
1
2
t−Test: p−value = 0.78

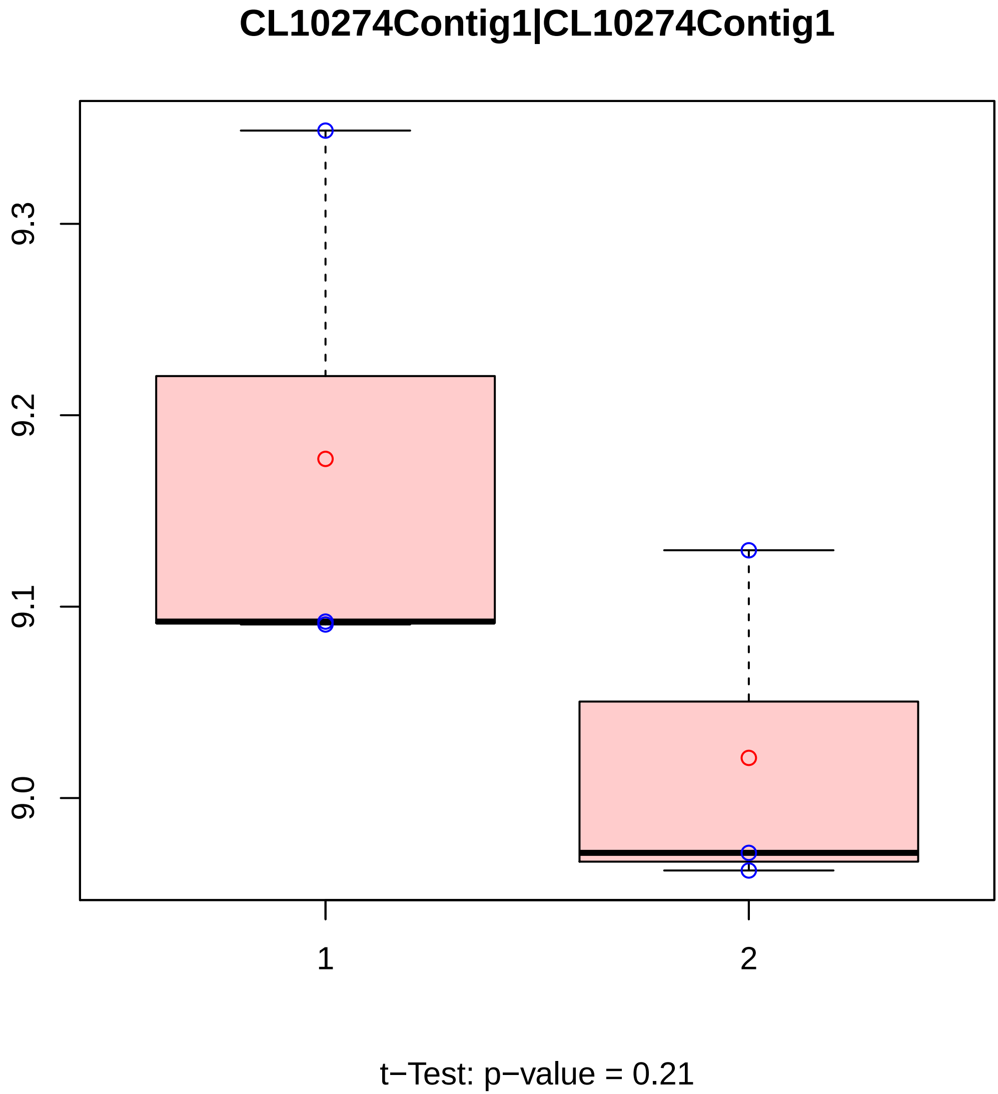
CL10274Contig1|CL10274Contig1
9.3
9.2
9.1
9.0
1
2
t−Test: p−value = 0.21

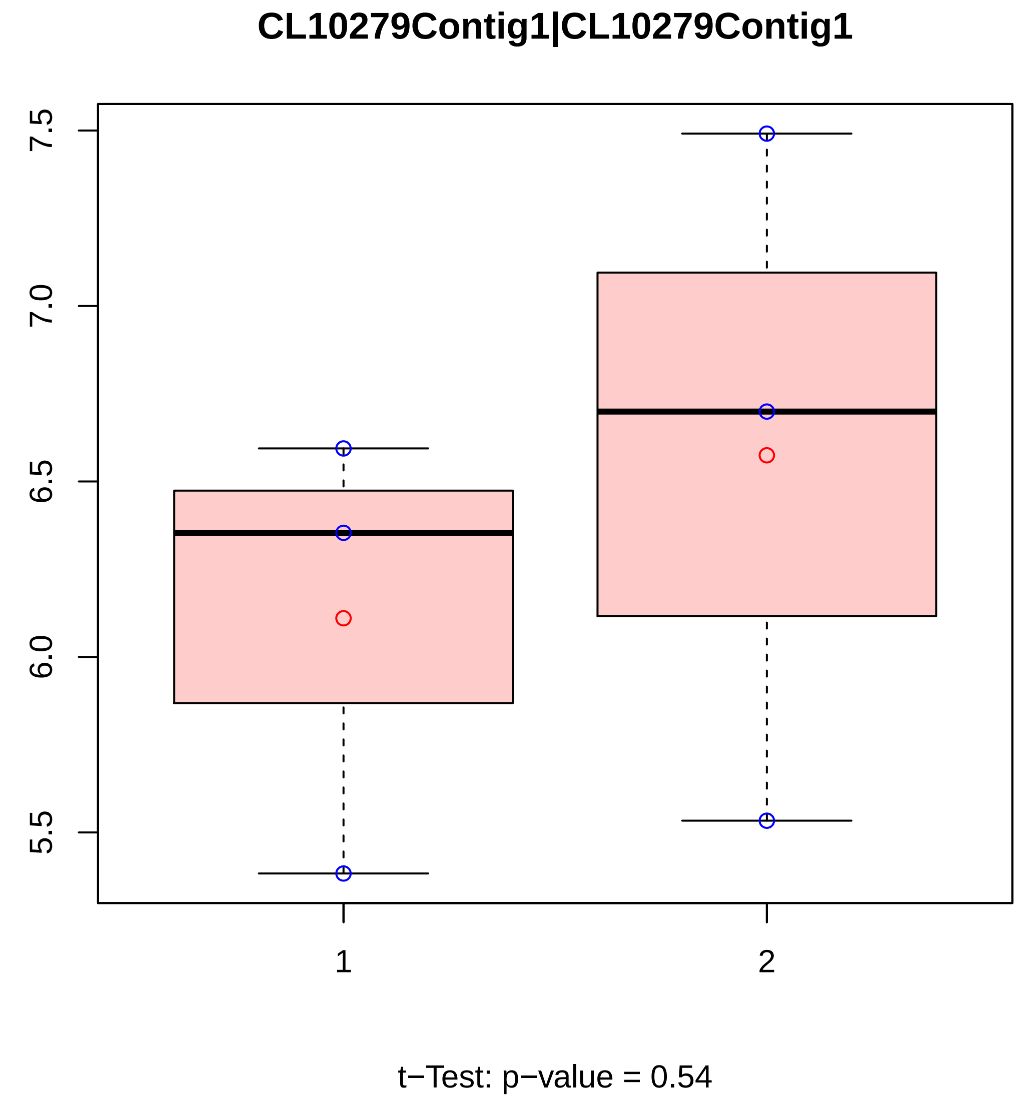
CL10279Contig1|CL10279Contig1
7.5
7.0
6.5
6.0
5.5
1
2
t−Test: p−value = 0.54

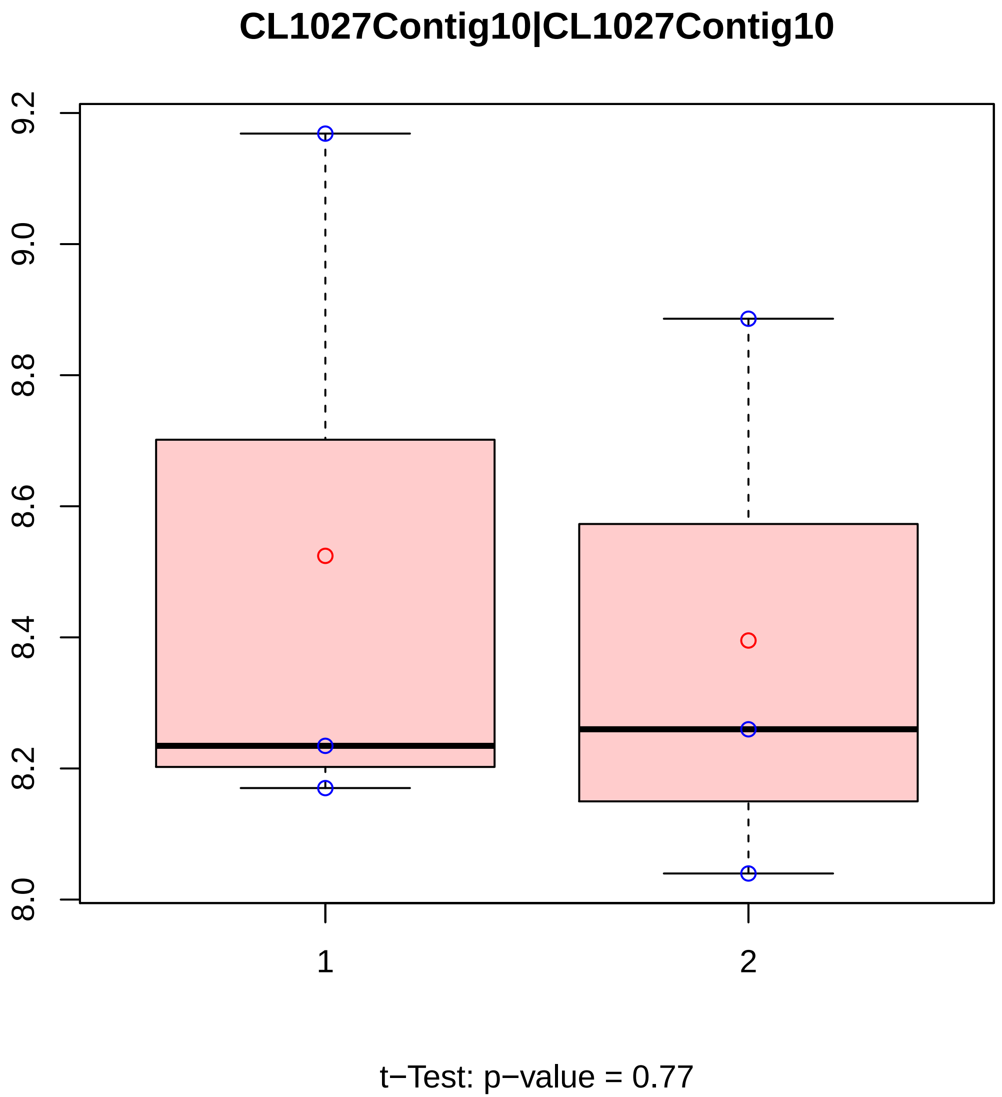
CL1027Contig10|CL1027Contig10
9.2
9.0
8.8
8.6
8.4
8.2
8.0
1
2
t−Test: p−value = 0.77

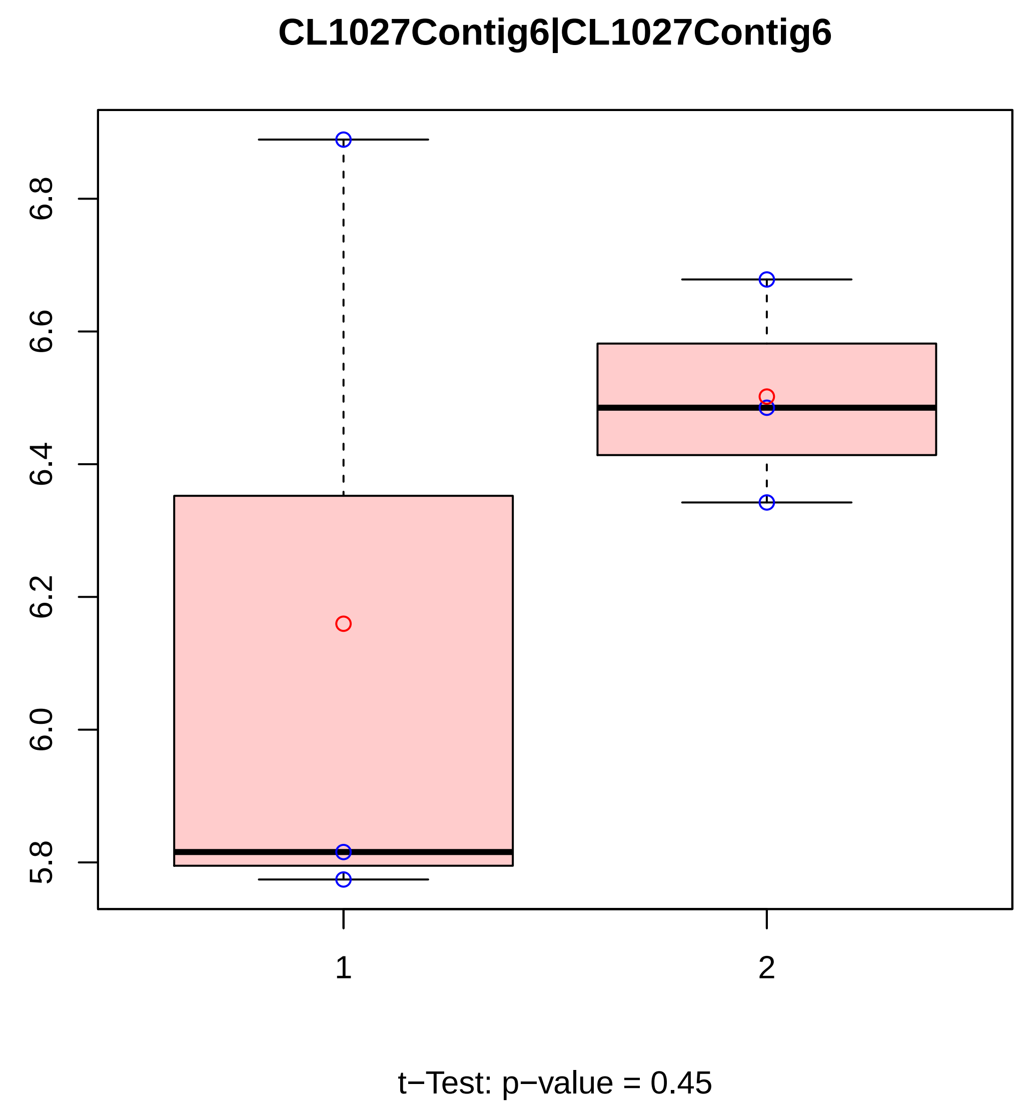
CL1027Contig6|CL1027Contig6
6.8
6.6
6.4
6.2
6.0
5.8
1
2
t−Test: p−value = 0.45

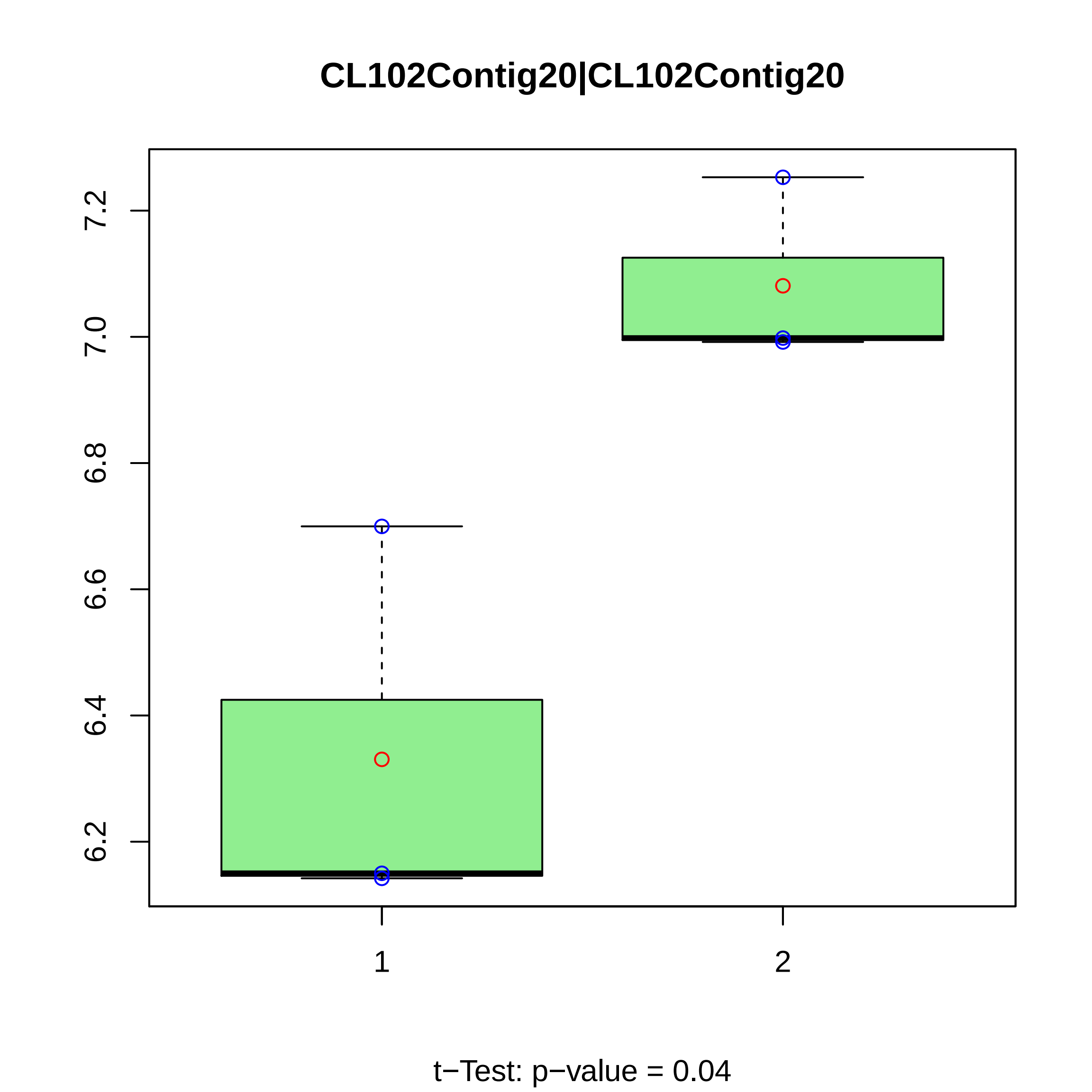
CL102Contig20|CL102Contig20
7.2
7.0
6.8
6.6
6.4
6.2
1
2
t−Test: p−value = 0.04

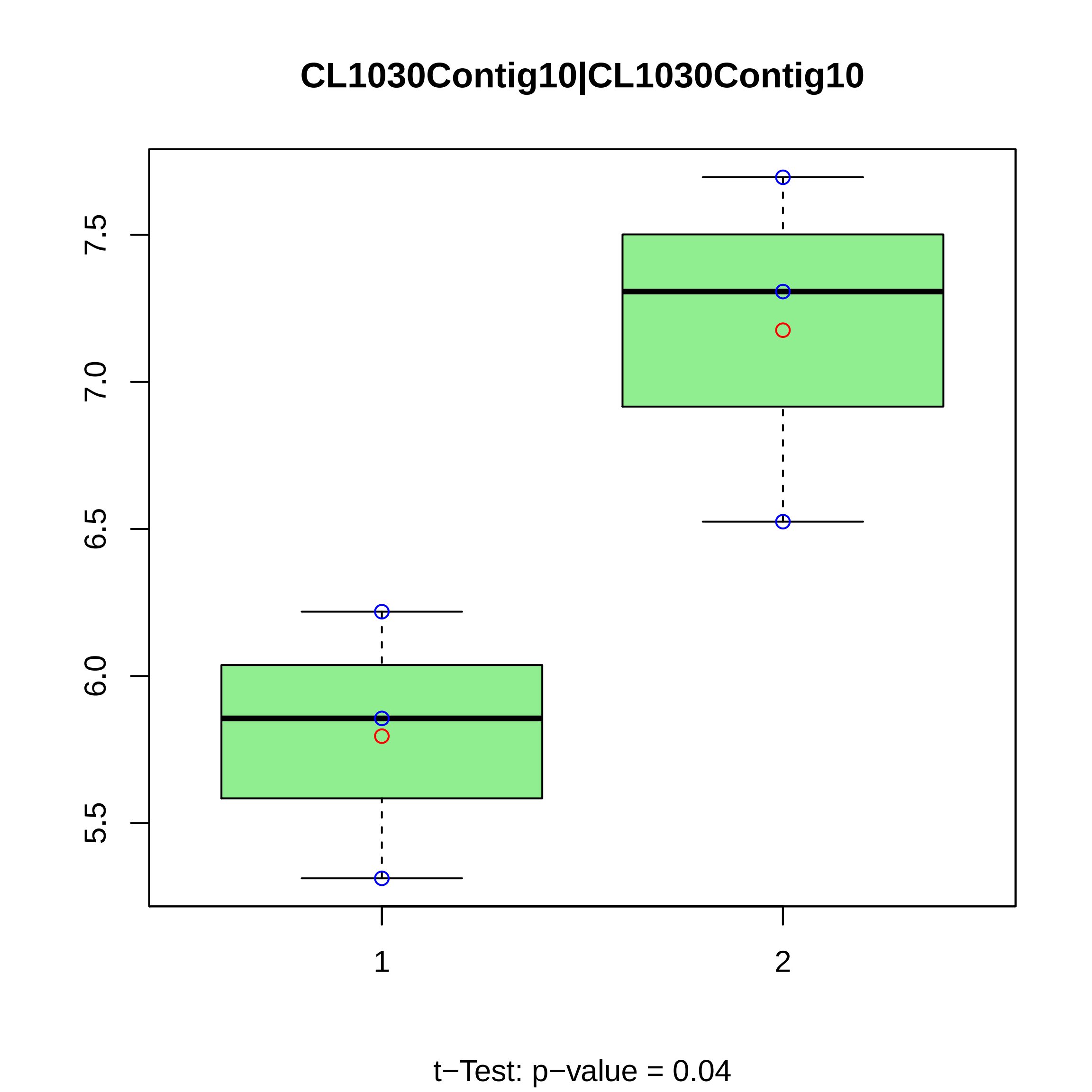
CL1030Contig10|CL1030Contig10
7.5
7.0
6.5
6.0
5.5
1
2
t−Test: p−value = 0.04

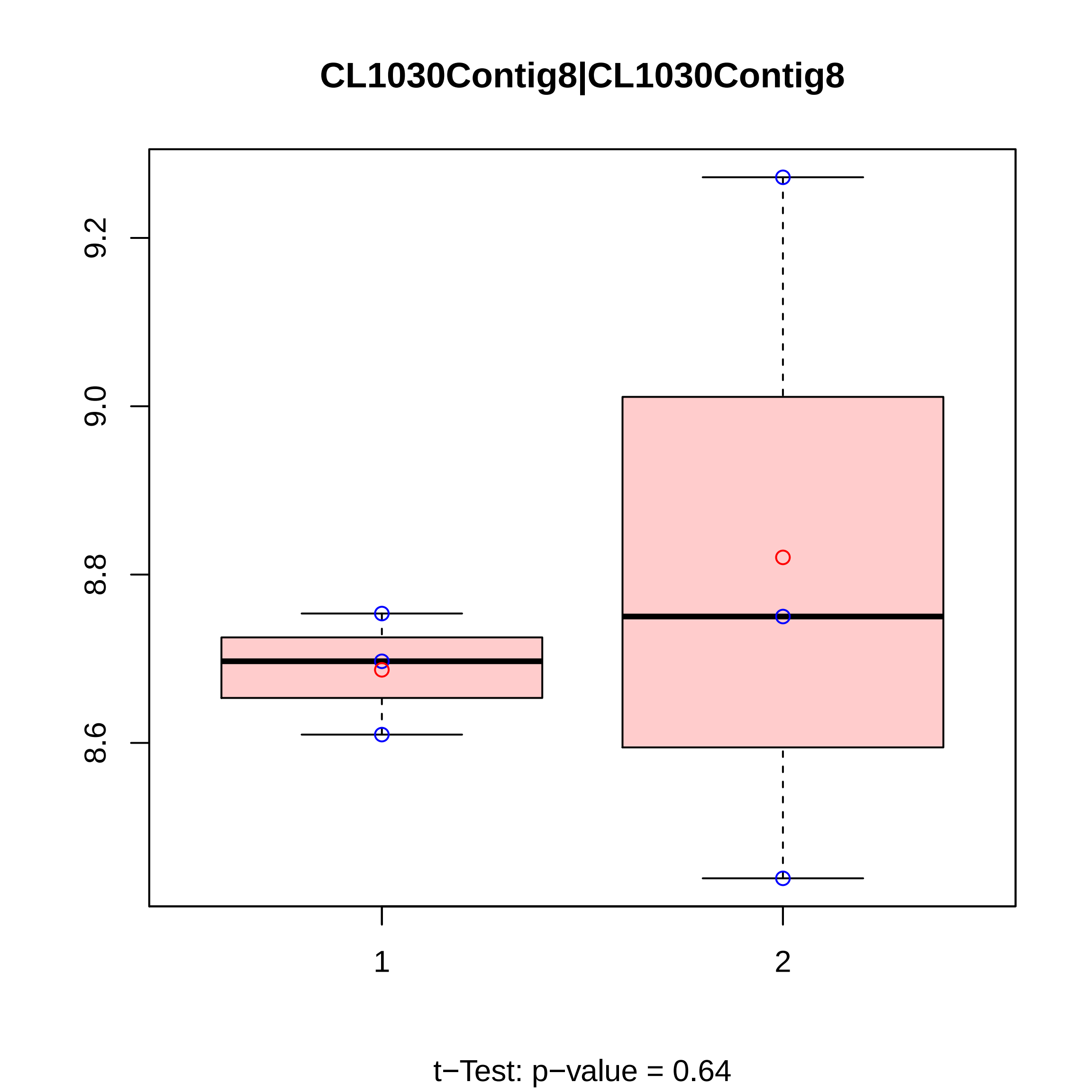
CL1030Contig8|CL1030Contig8
9.2
9.0
8.8
8.6
1
2
t−Test: p−value = 0.64

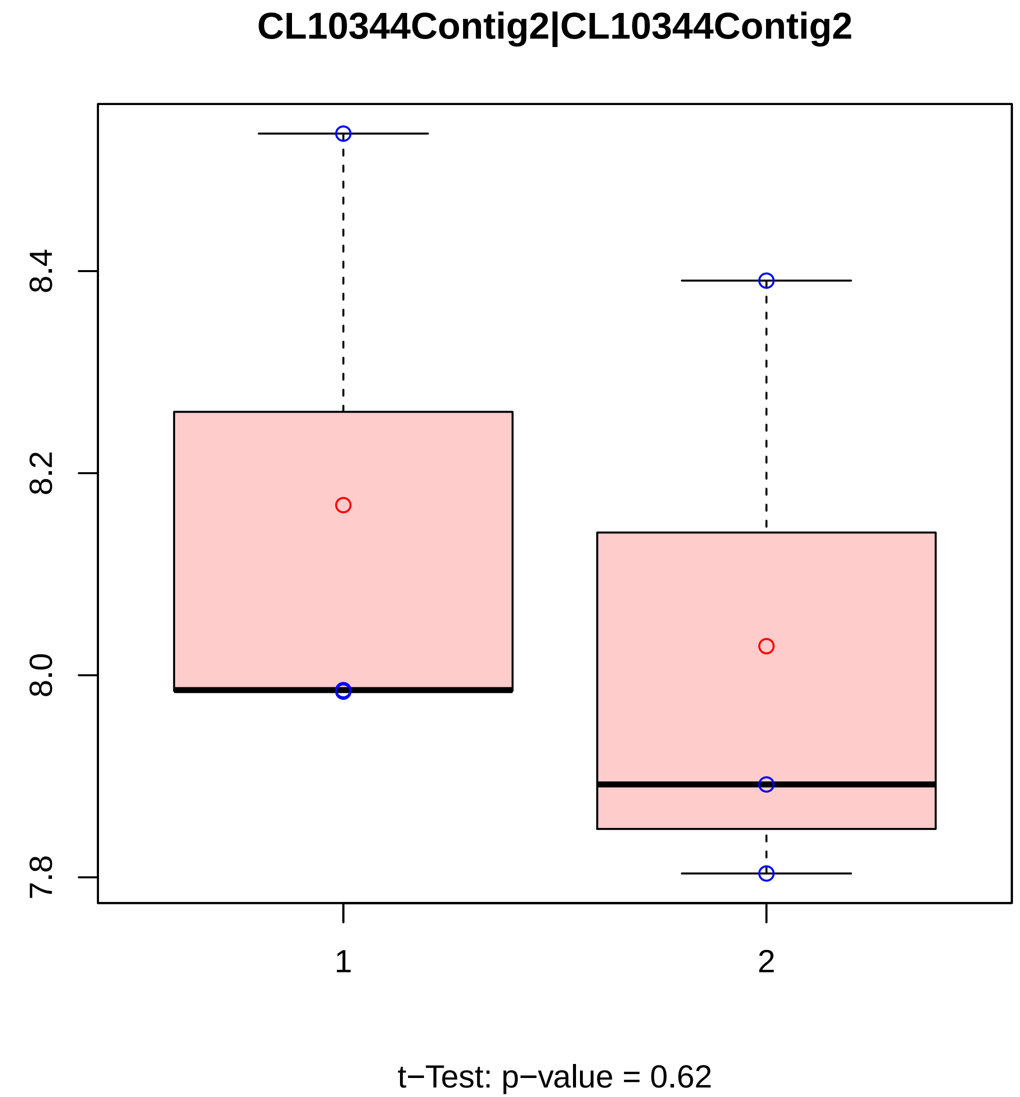
CL10344Contig2|CL10344Contig2
8.4
8.2
8.0
7.8
1
2
t−Test: p−value = 0.62

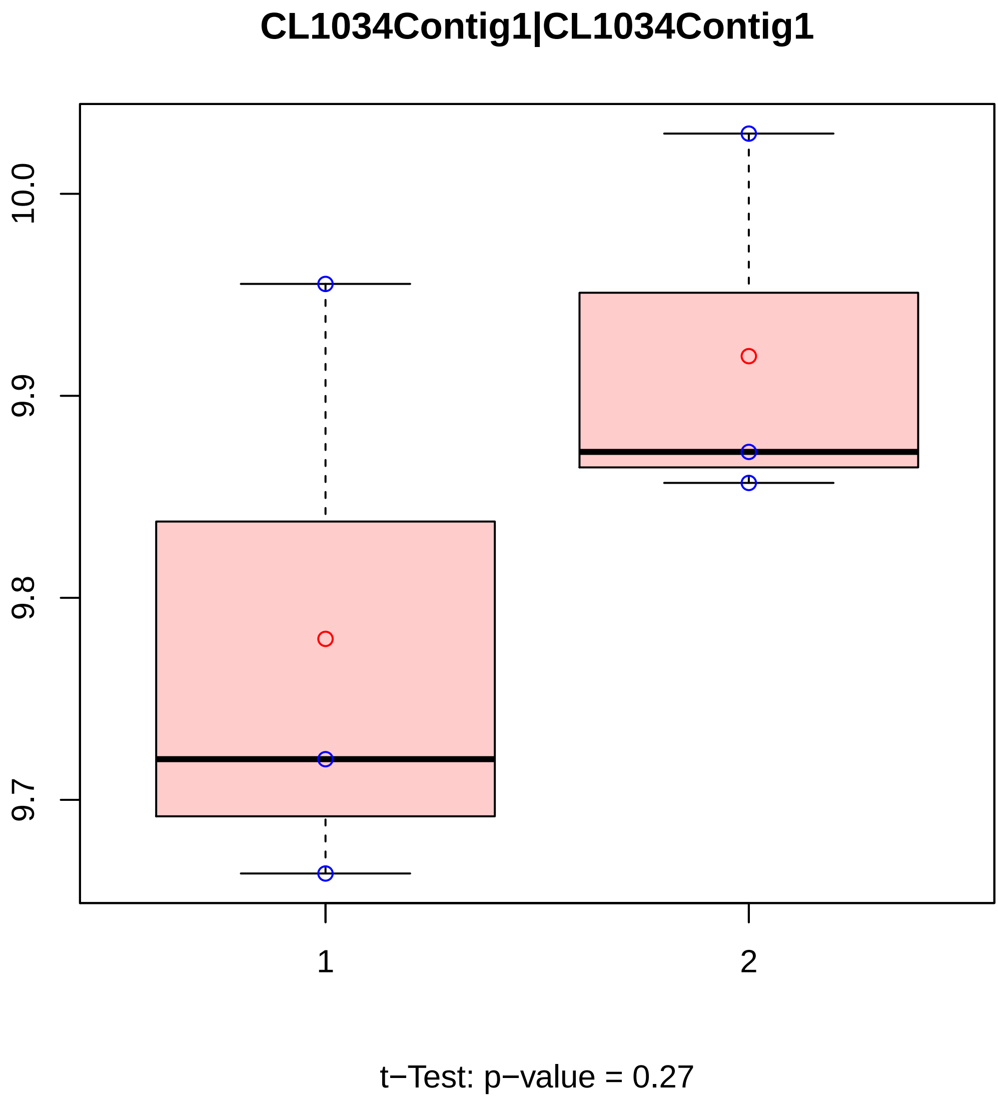
CL1034Contig1|CL1034Contig1
10.0
9.9
9.8
9.7
1
2
t−Test: p−value = 0.27

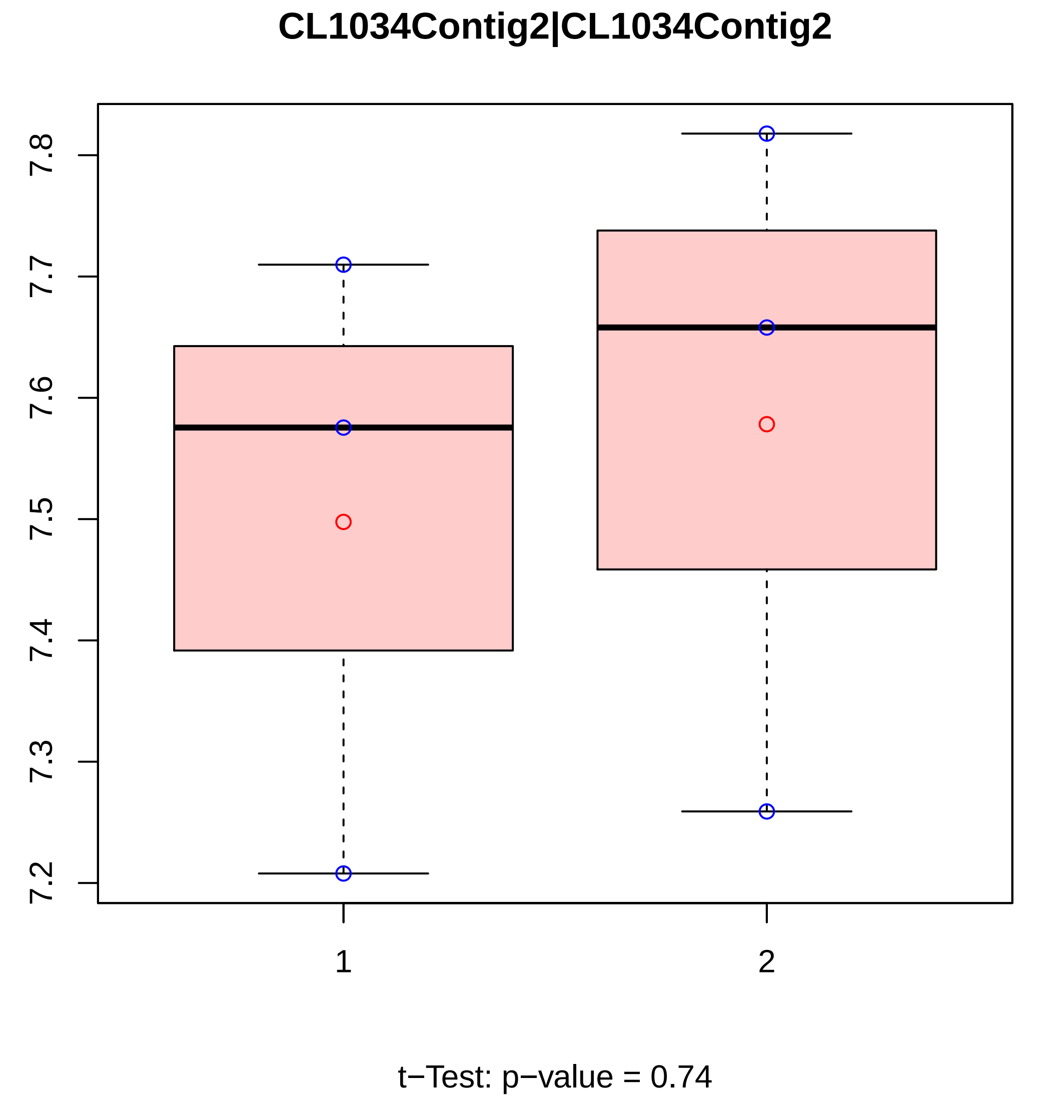

CL1034Contig2|CL1034Contig2
7.2
7.3
7.4
7.5
7.6
7.7
7.8
1
2
t−Test: p−value = 0.74

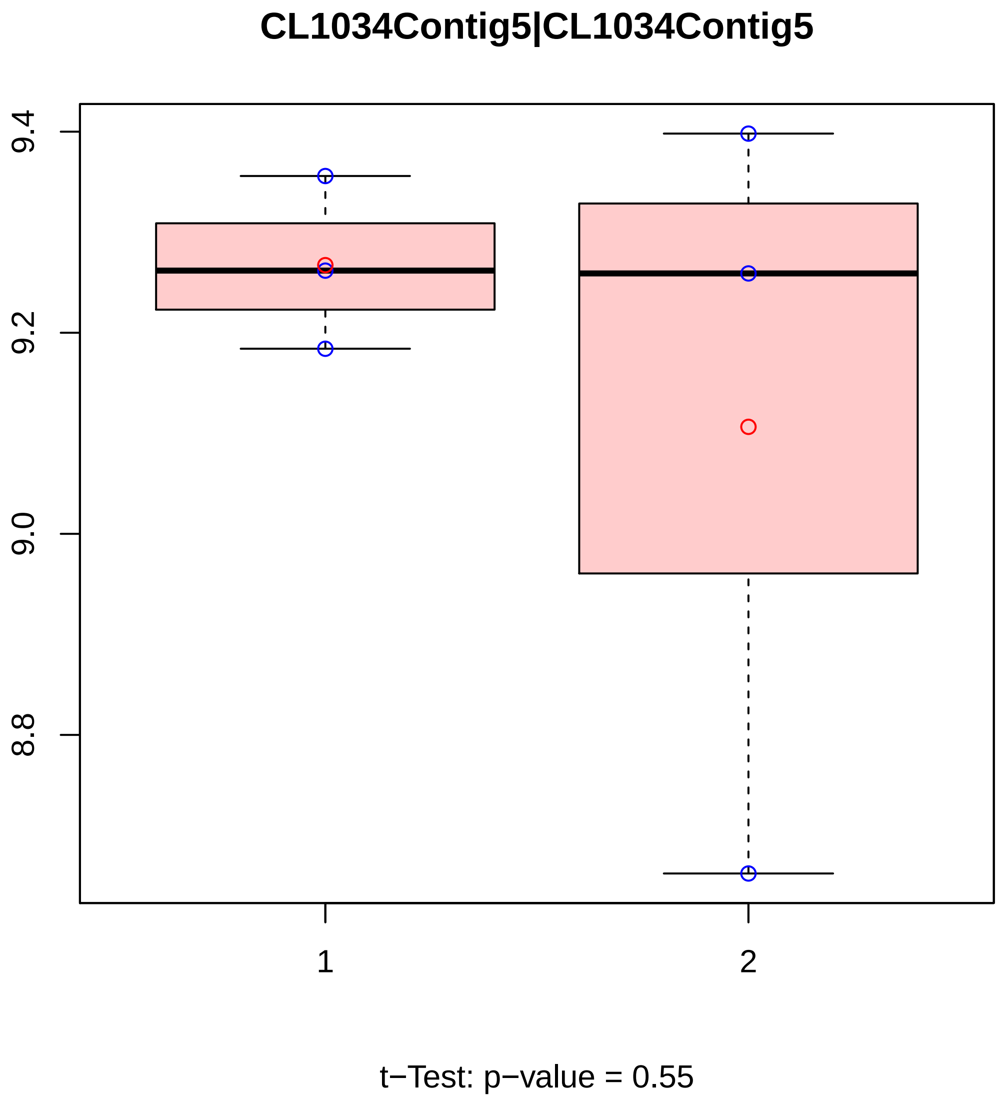
CL1034Contig5|CL1034Contig5
9.4
9.2
9.0
8.8
1
2
t−Test: p−value = 0.55

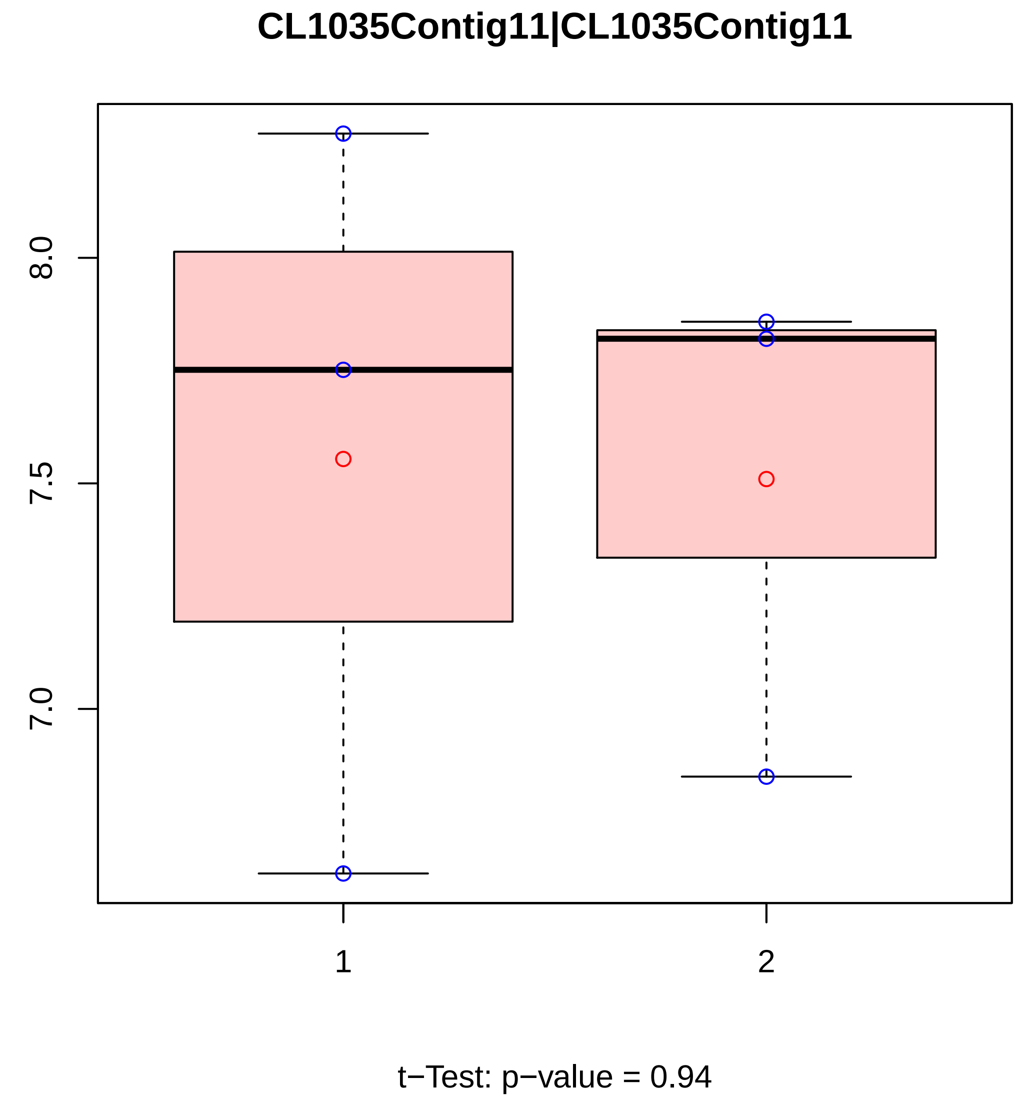

CL1035Contig11|CL1035Contig11
8.0
7.5
7.0
1
2
t−Test: p−value = 0.94

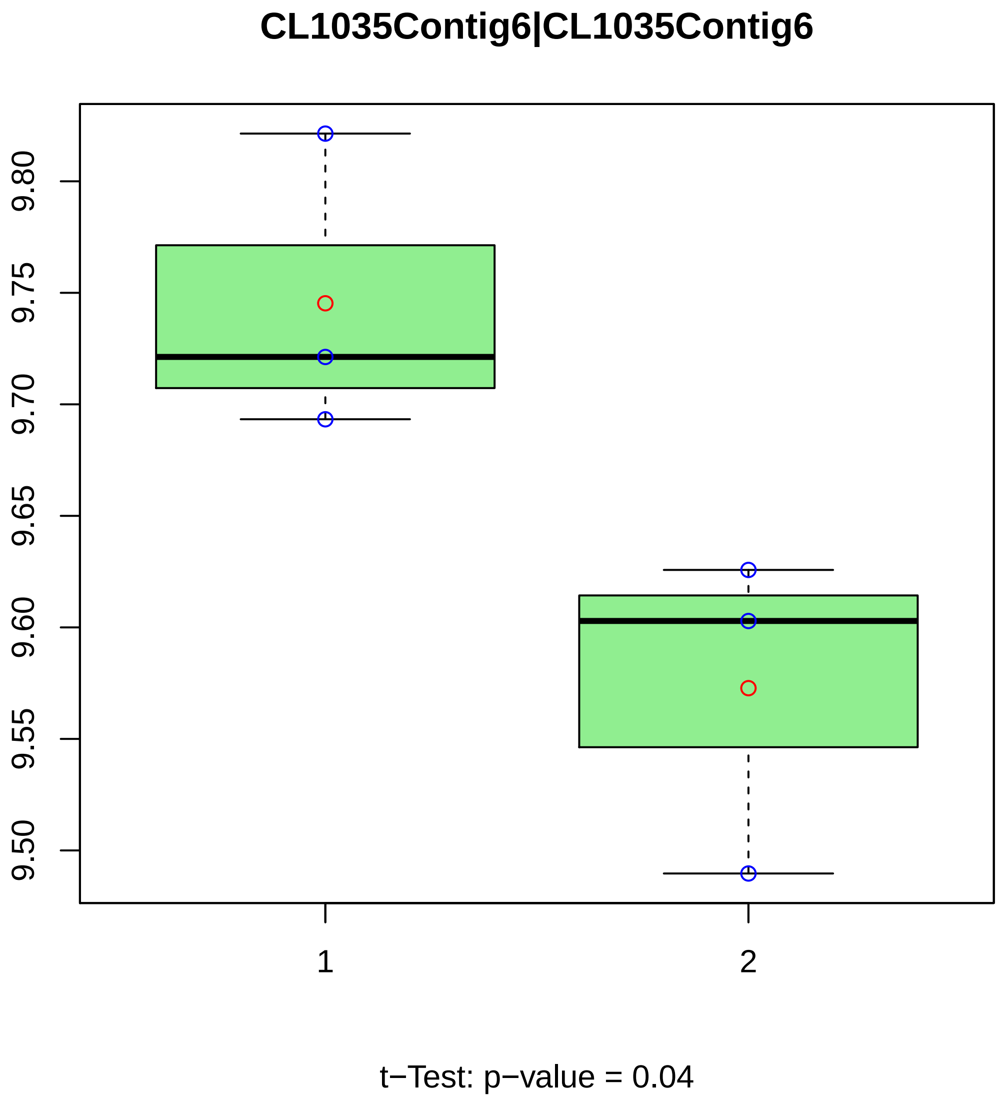

CL1035Contig6|CL1035Contig6
9.50
9.55
9.60
9.65
9.70
9.75
9.80
1
2
t−Test: p−value = 0.04

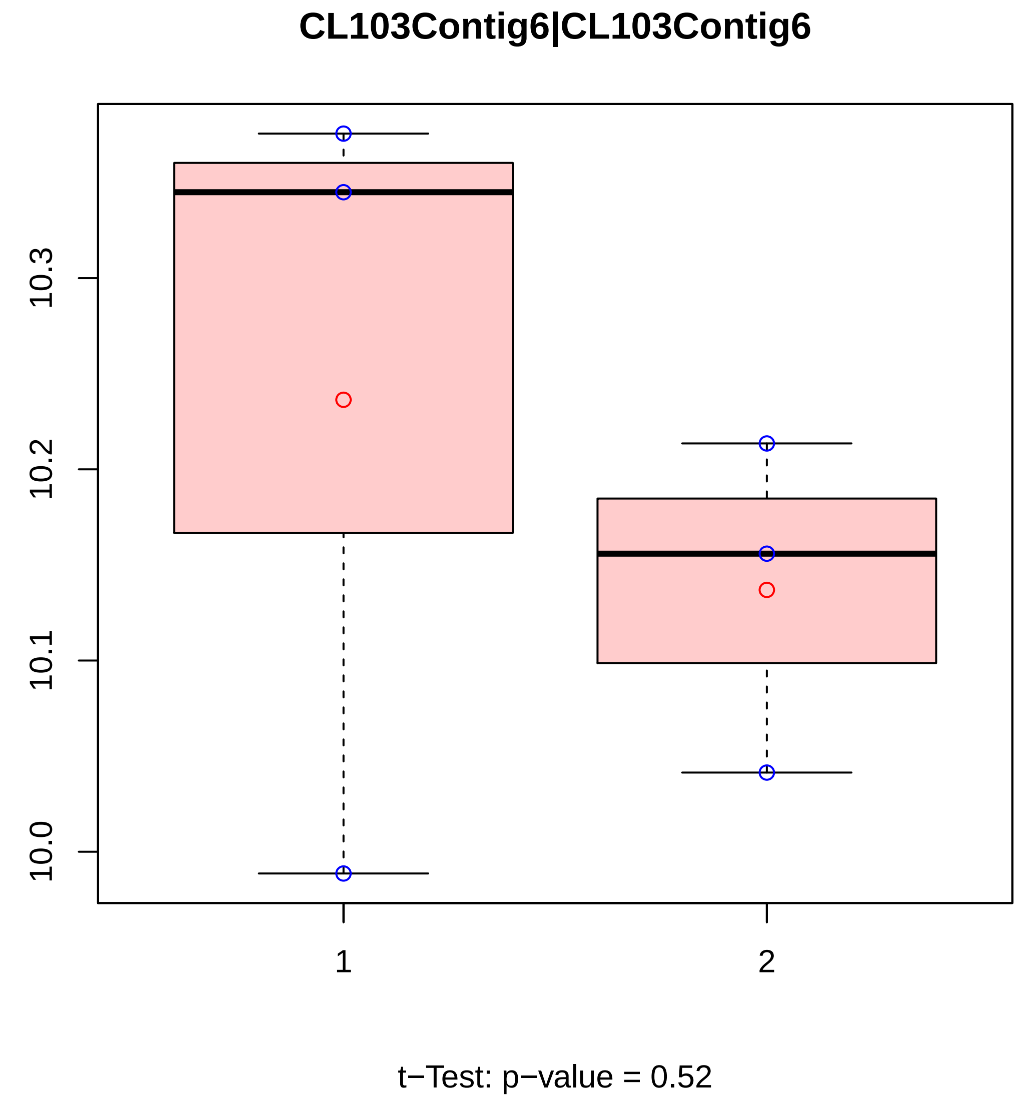
CL103Contig6|CL103Contig6
10.0
10.1
10.2
10.3
1
2
t−Test: p−value = 0.52

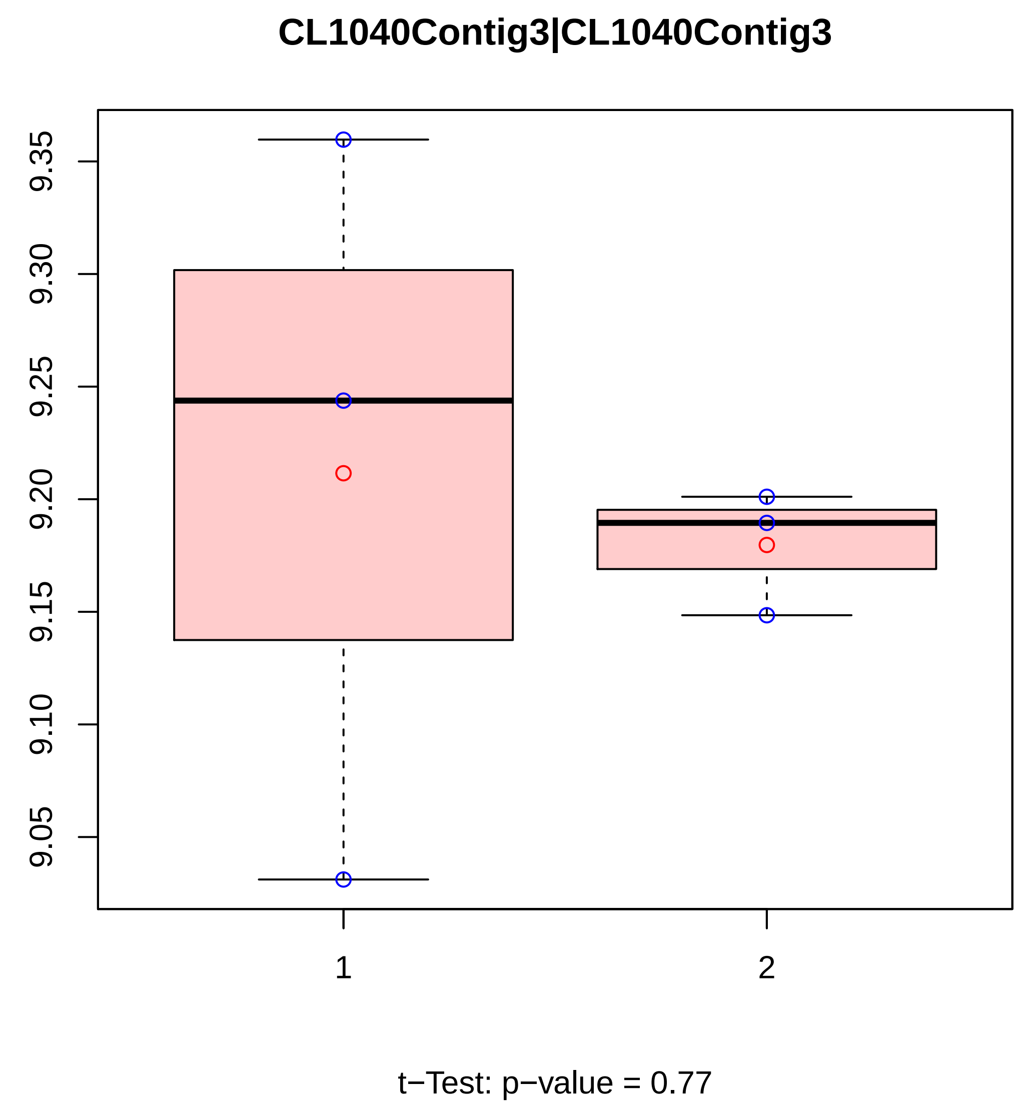
CL1040Contig3|CL1040Contig3
9.05
9.10
9.15
9.20
9.25
9.30
9.35
1
2
t−Test: p−value = 0.77

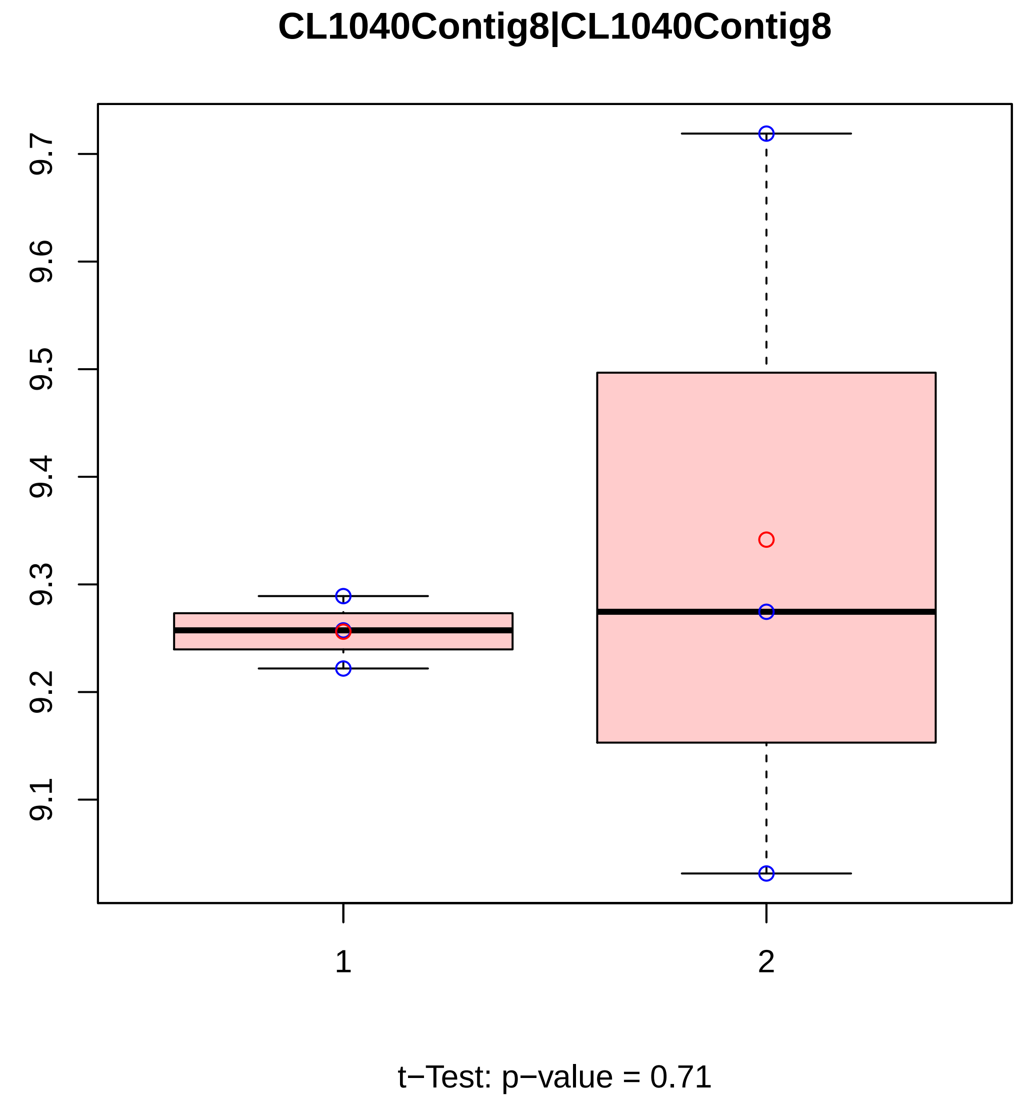
CL1040Contig8|CL1040Contig8
9.1
9.2
9.3
9.4
9.5
9.6
9.7
1
2
t−Test: p−value = 0.71

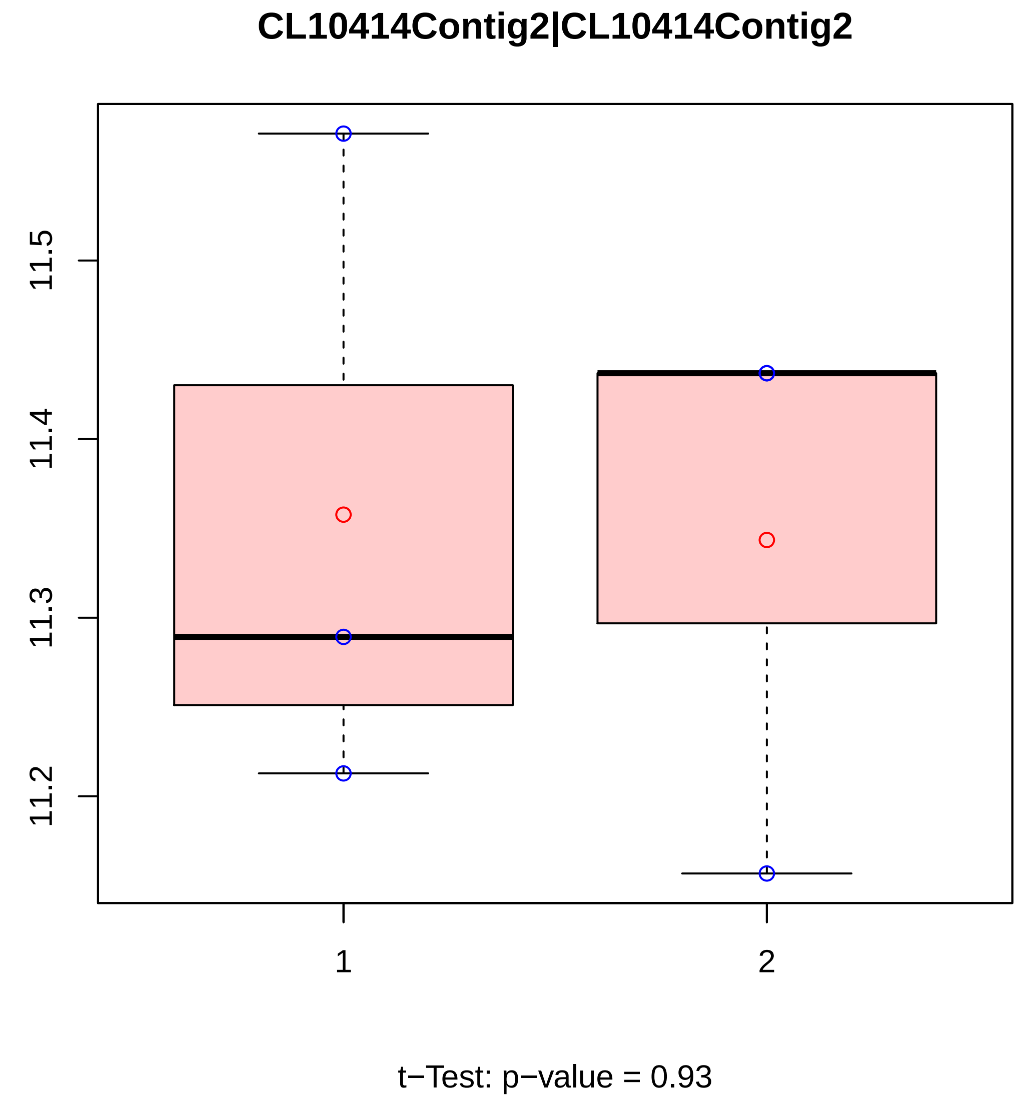
CL10414Contig2|CL10414Contig2
11.2
11.3
11.4
11.5
1
2
t−Test: p−value = 0.93

# CL1042Contig2|CL1042Contig2

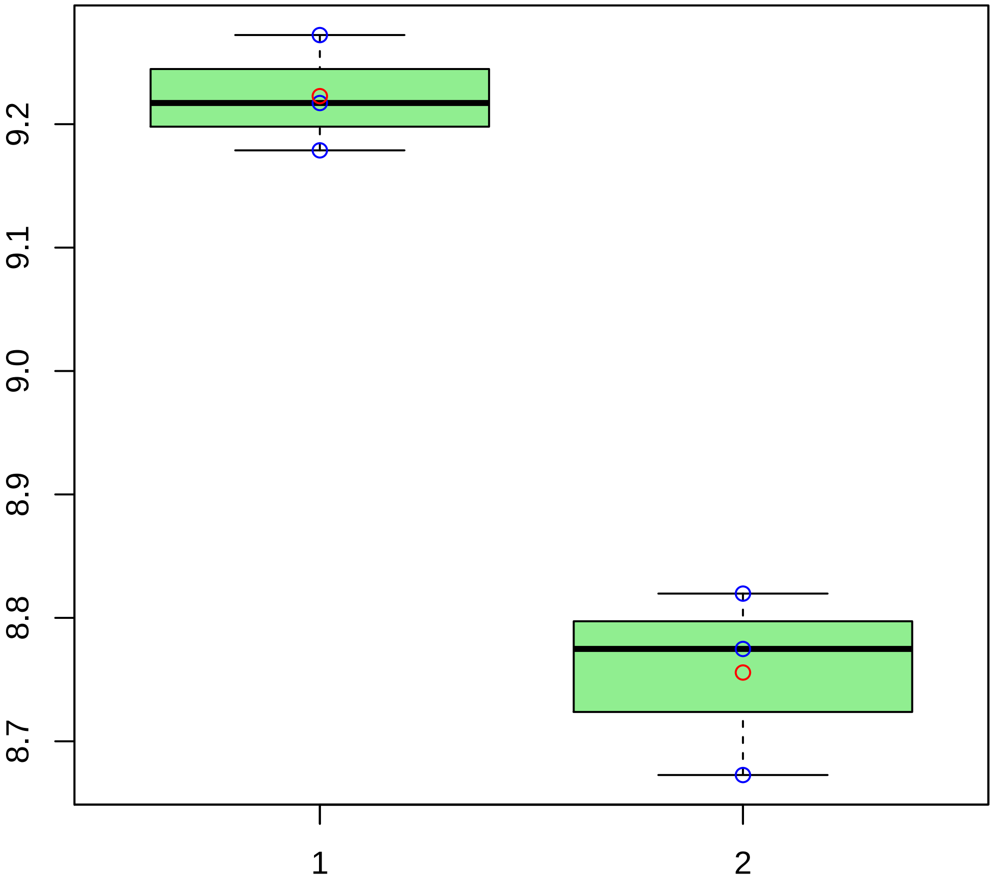


t−Test: p−value = 0

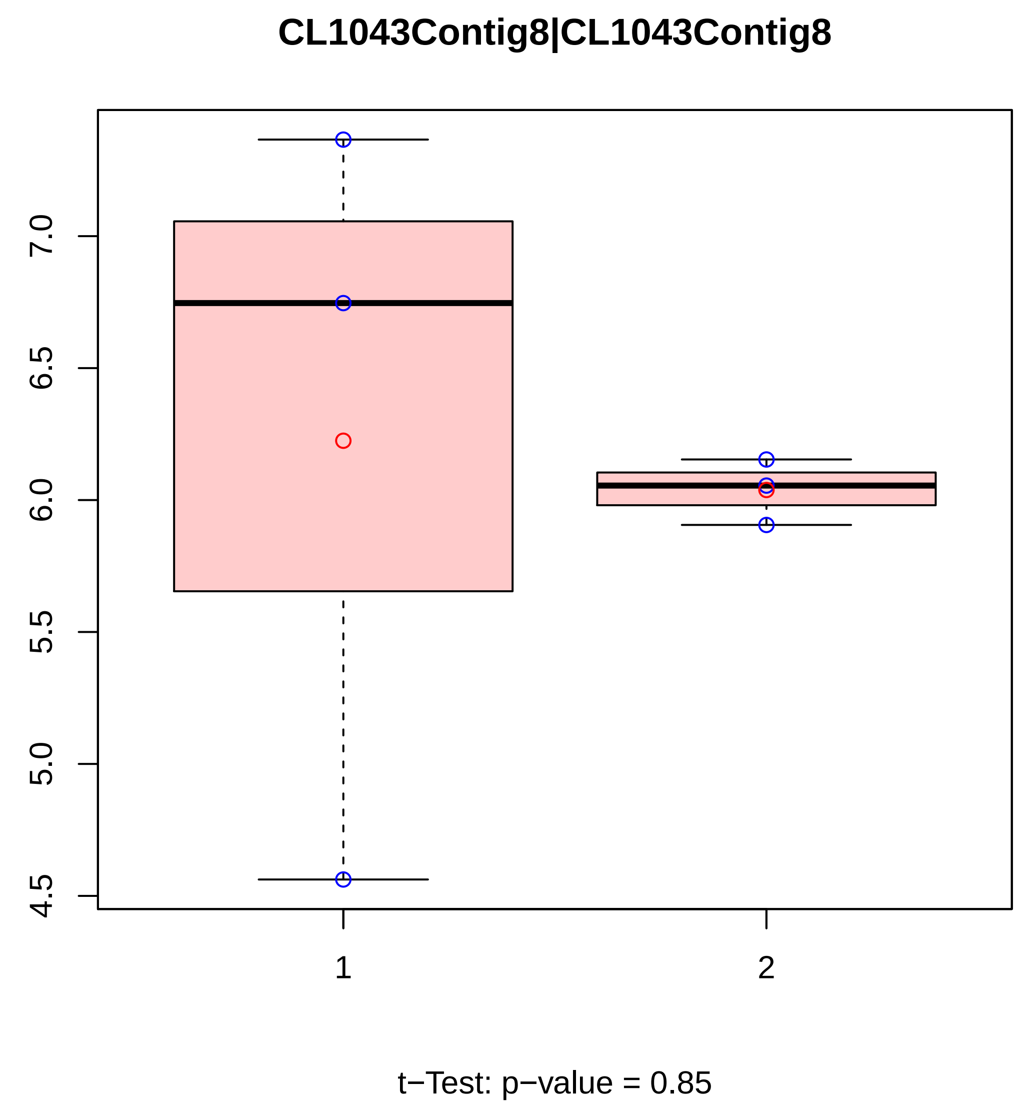

CL1043Contig8|CL1043Contig8
4.5
5.0
5.5
6.0
6.5
7.0
1
2
t−Test: p−value = 0.85

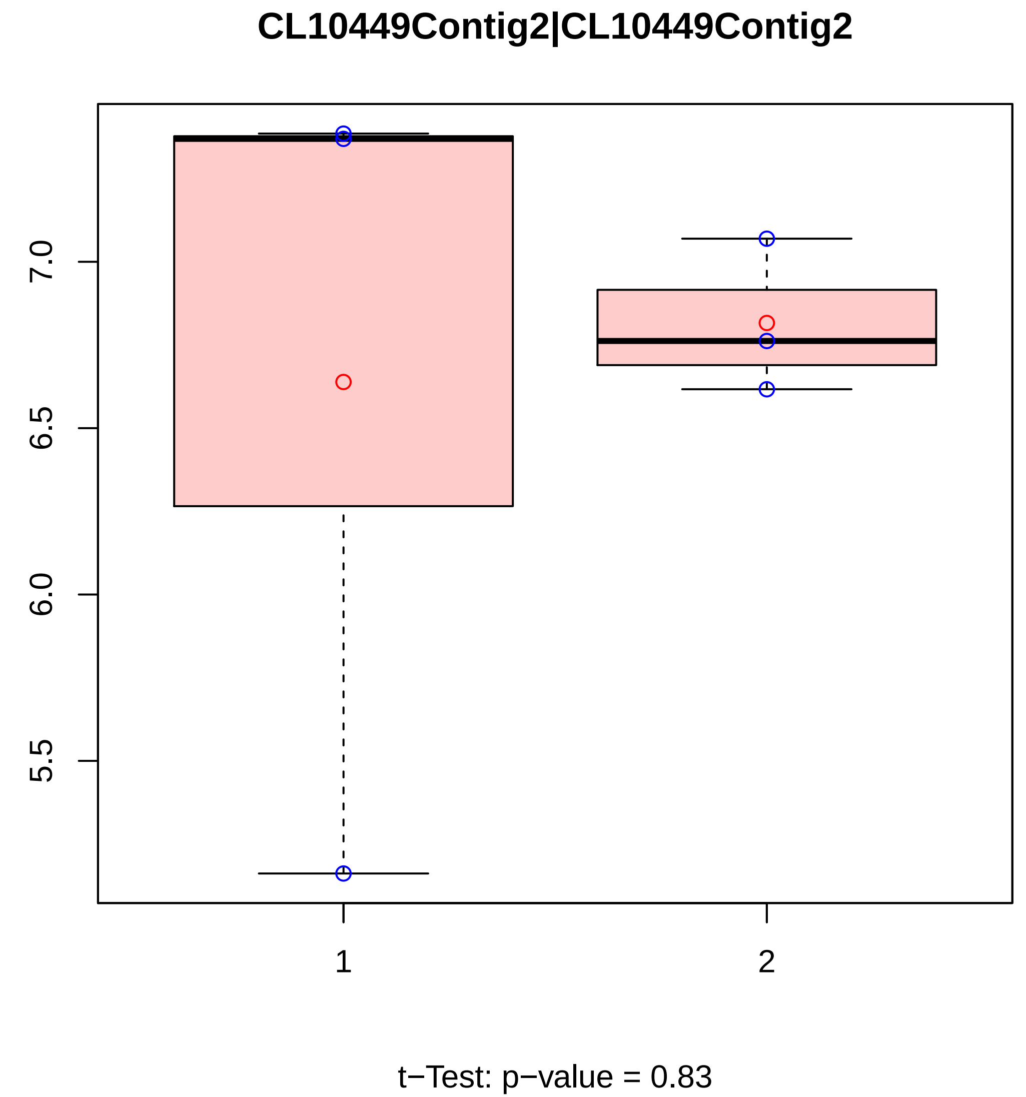
CL10449Contig2|CL10449Contig2
7.0
6.5
6.0
5.5
1
2
t−Test: p−value = 0.83

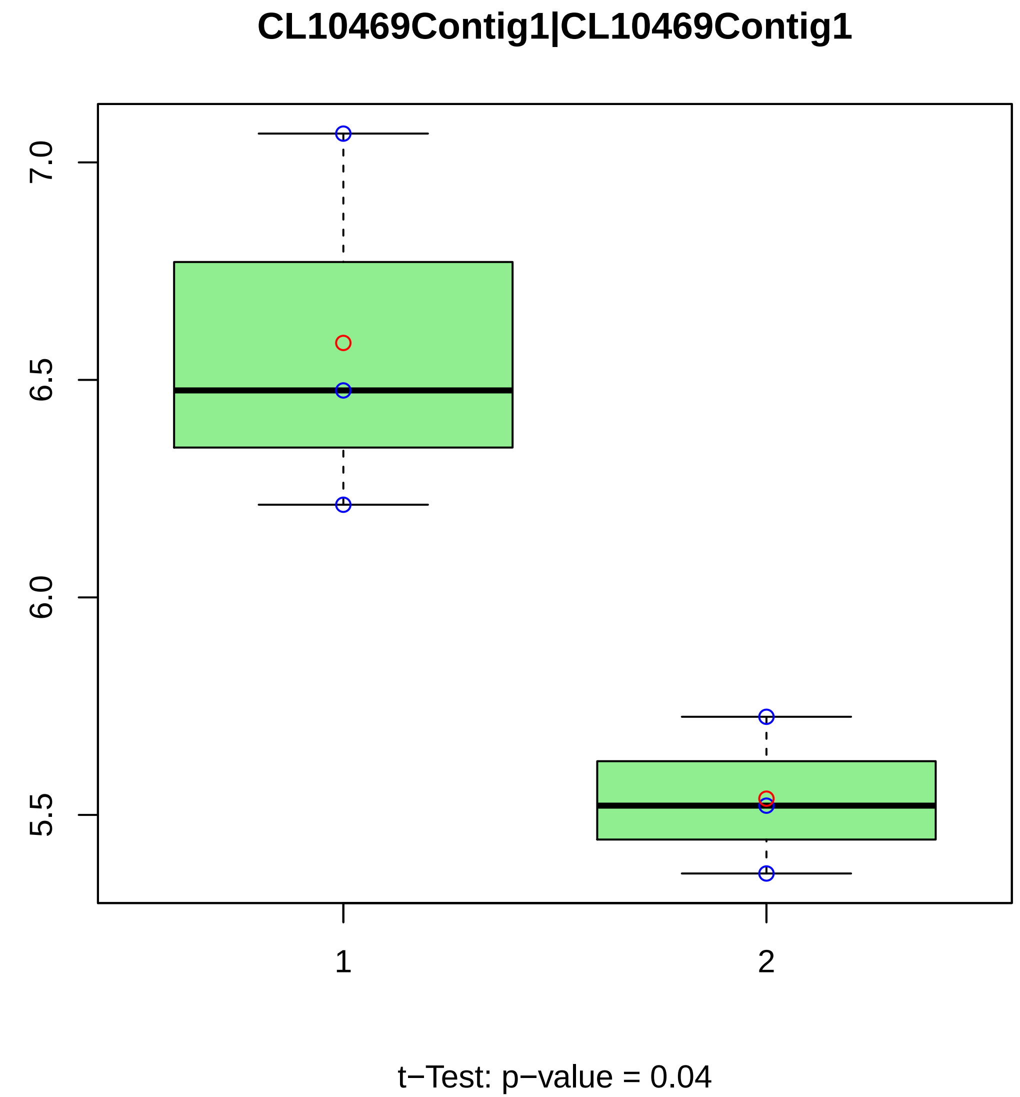
CL10469Contig1|CL10469Contig1
7.0
6.5
6.0
5.5
1
2
t−Test: p−value = 0.04

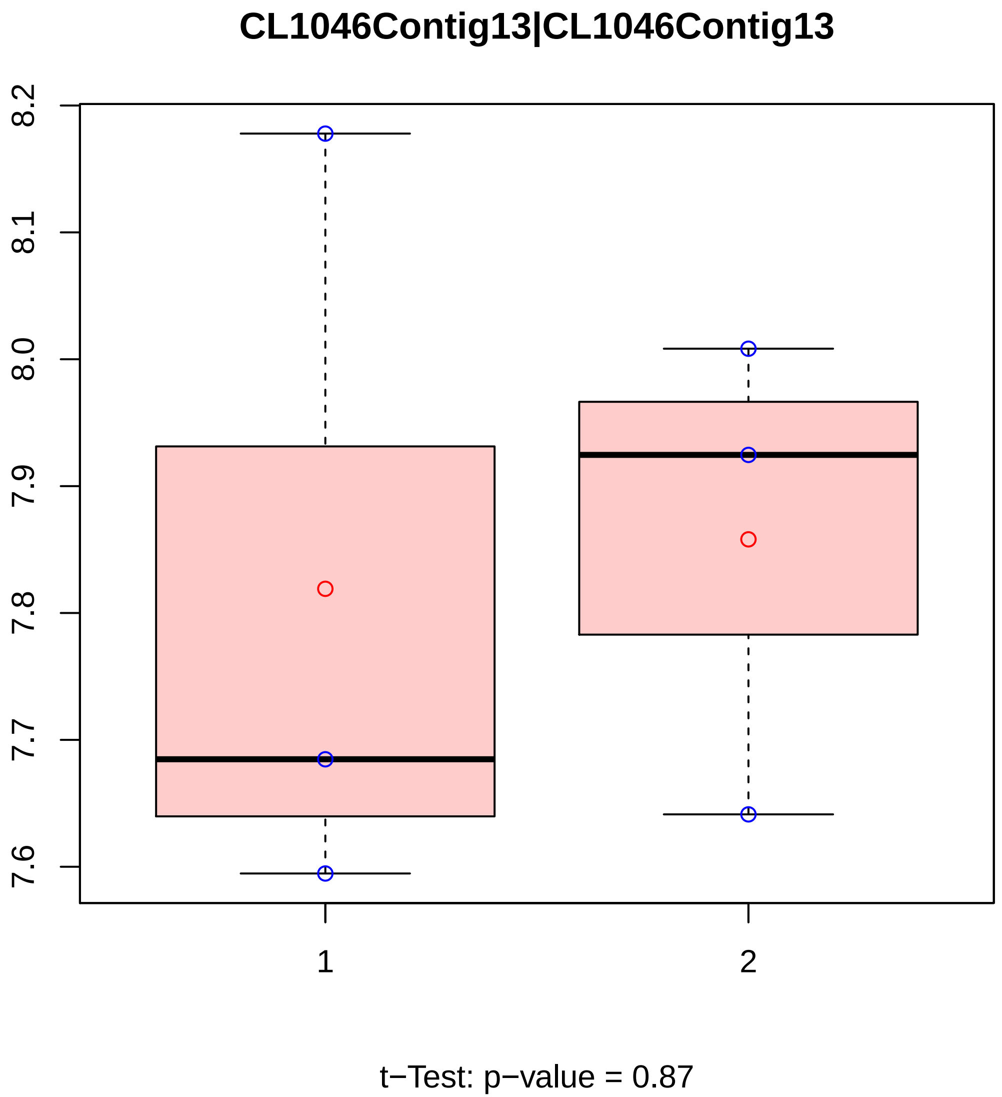
CL1046Contig13|CL1046Contig13
8.2
8.1
8.0
7.9
7.8
7.7
7.6
1
2
t−Test: p−value = 0.87

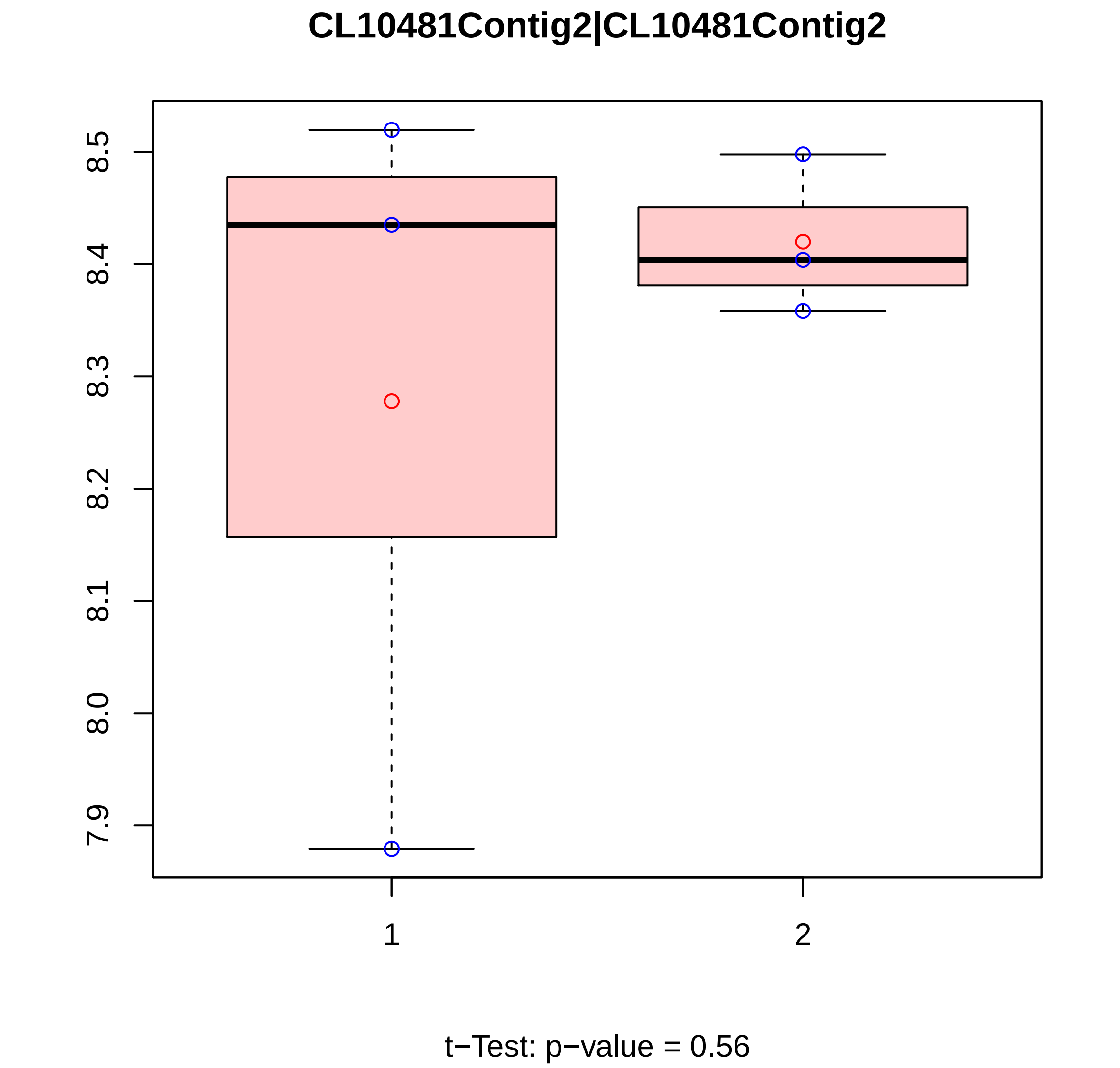
CL10481Contig2|CL10481Contig2
8.5
8.4
8.3
8.2
8.1
8.0
7.9
1
2
t−Test: p−value = 0.56

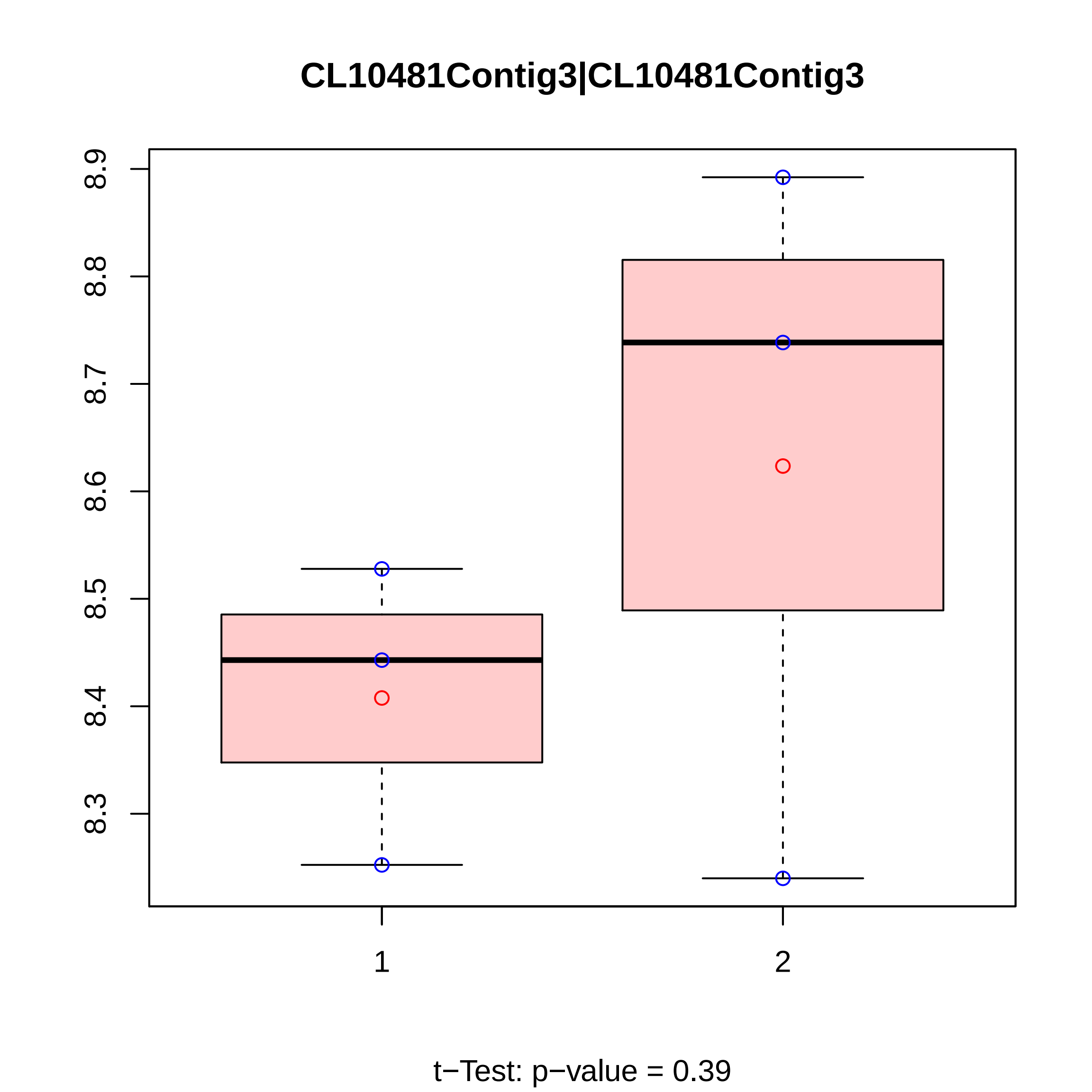
CL10481Contig3|CL10481Contig3
8.3
8.4
8.5
8.6
8.7
8.8
8.9
1
2
t−Test: p−value = 0.39

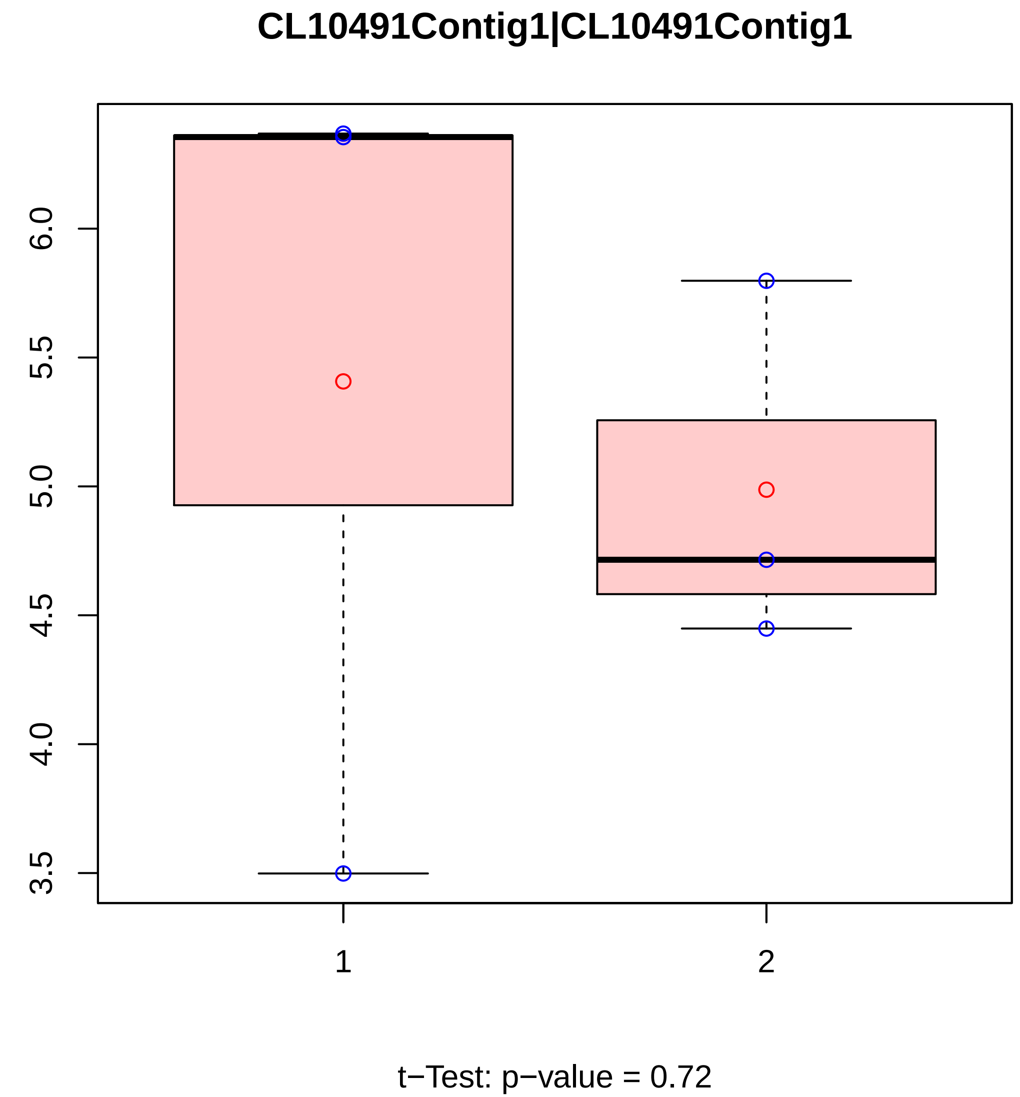
CL10491Contig1|CL10491Contig1
6.0
5.5
5.0
4.5
4.0
3.5
1
2
t−Test: p−value = 0.72

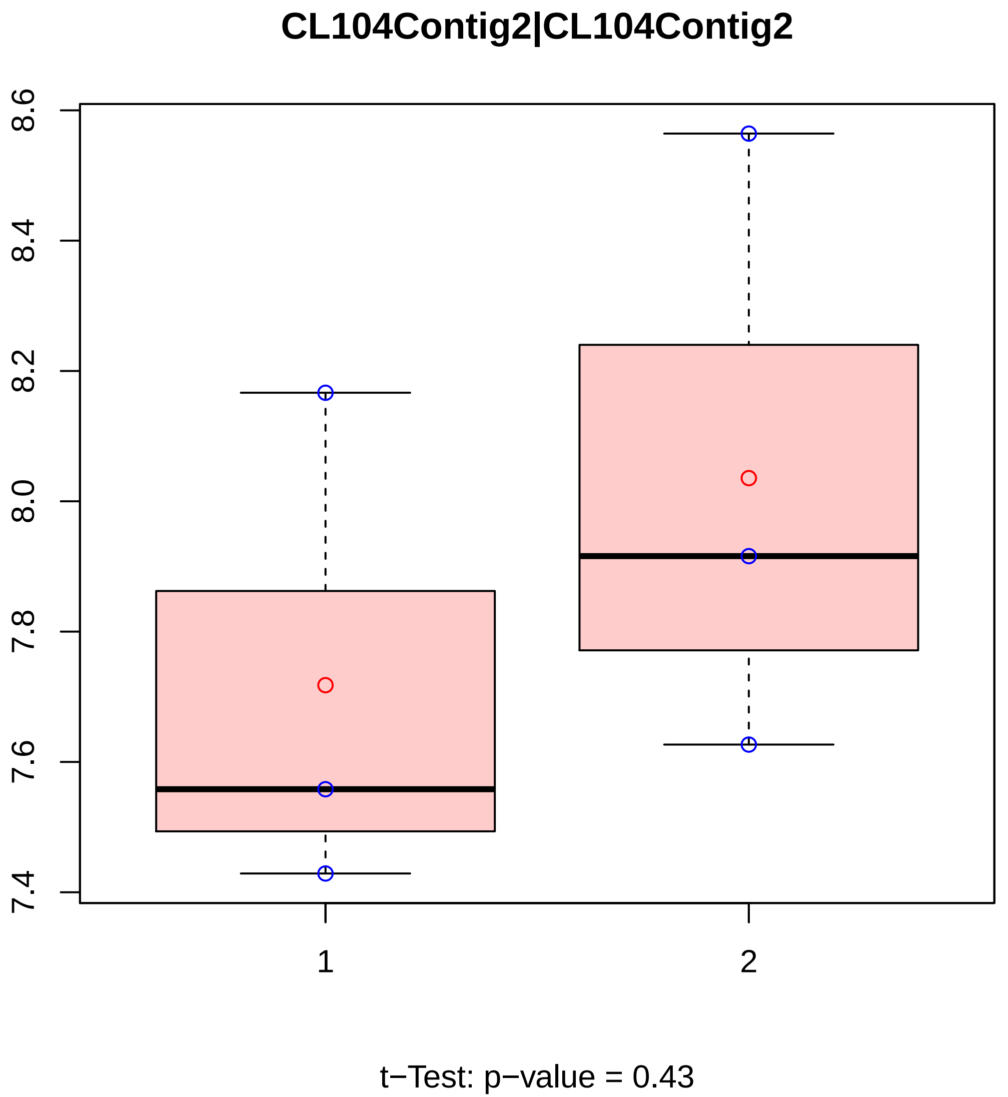

CL104Contig2|CL104Contig2
8.6
8.4
8.2
8.0
7.8
7.6
7.4
1
2
t−Test: p−value = 0.43

# CL10519Contig1|CL10519Contig1

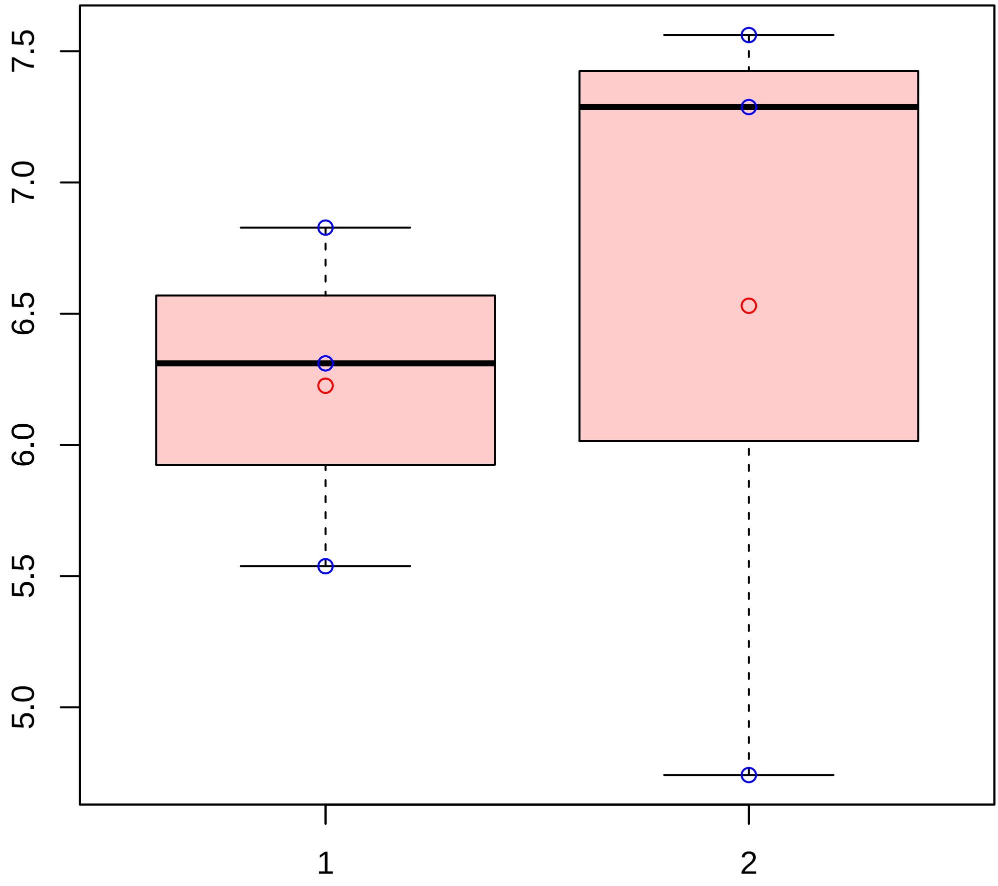


t−Test: p−value = 0.78

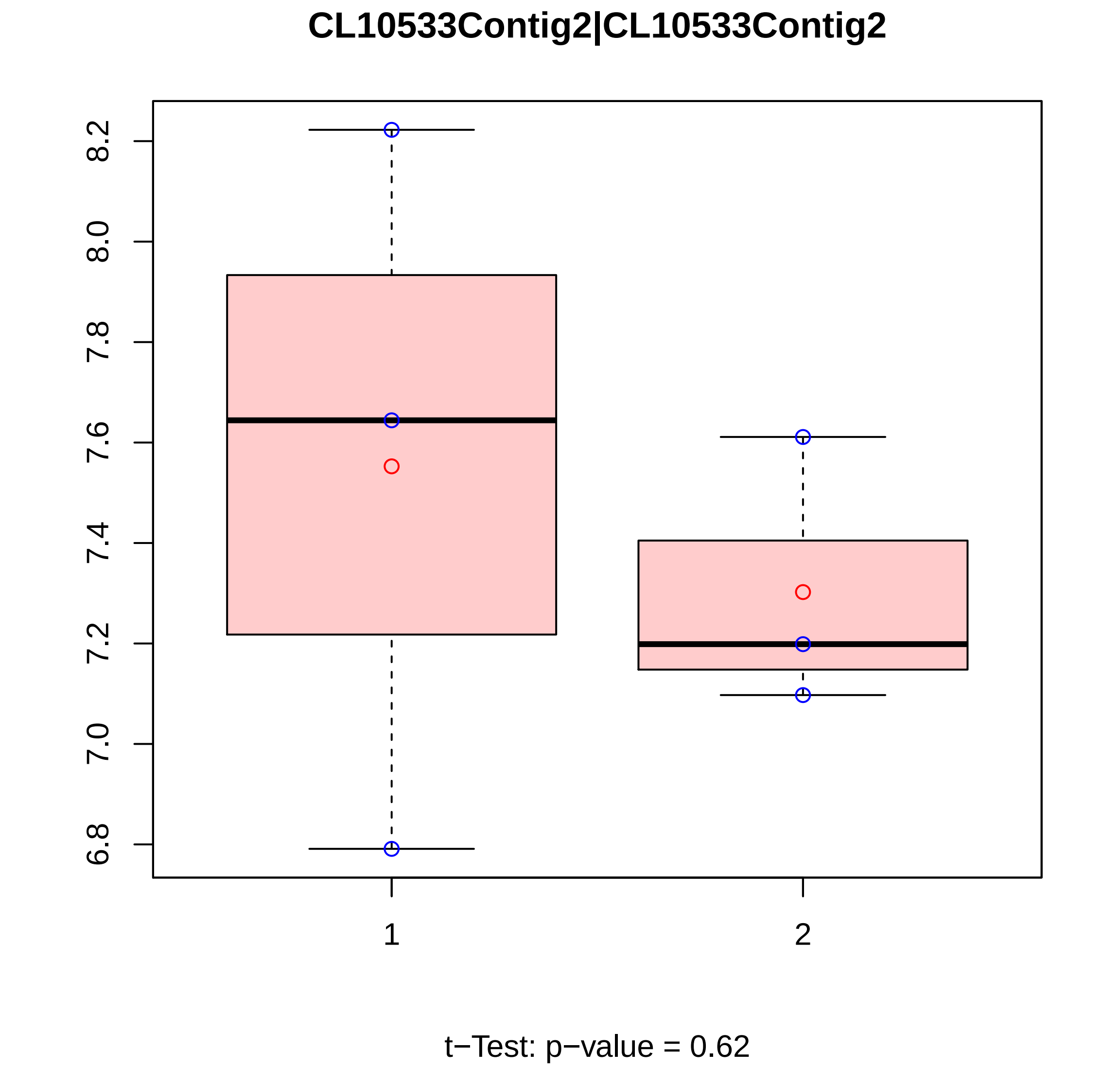
CL10533Contig2|CL10533Contig2
8.2
8.0
7.8
7.6
7.4
7.2
7.0
6.8
1
2
t−Test: p−value = 0.62

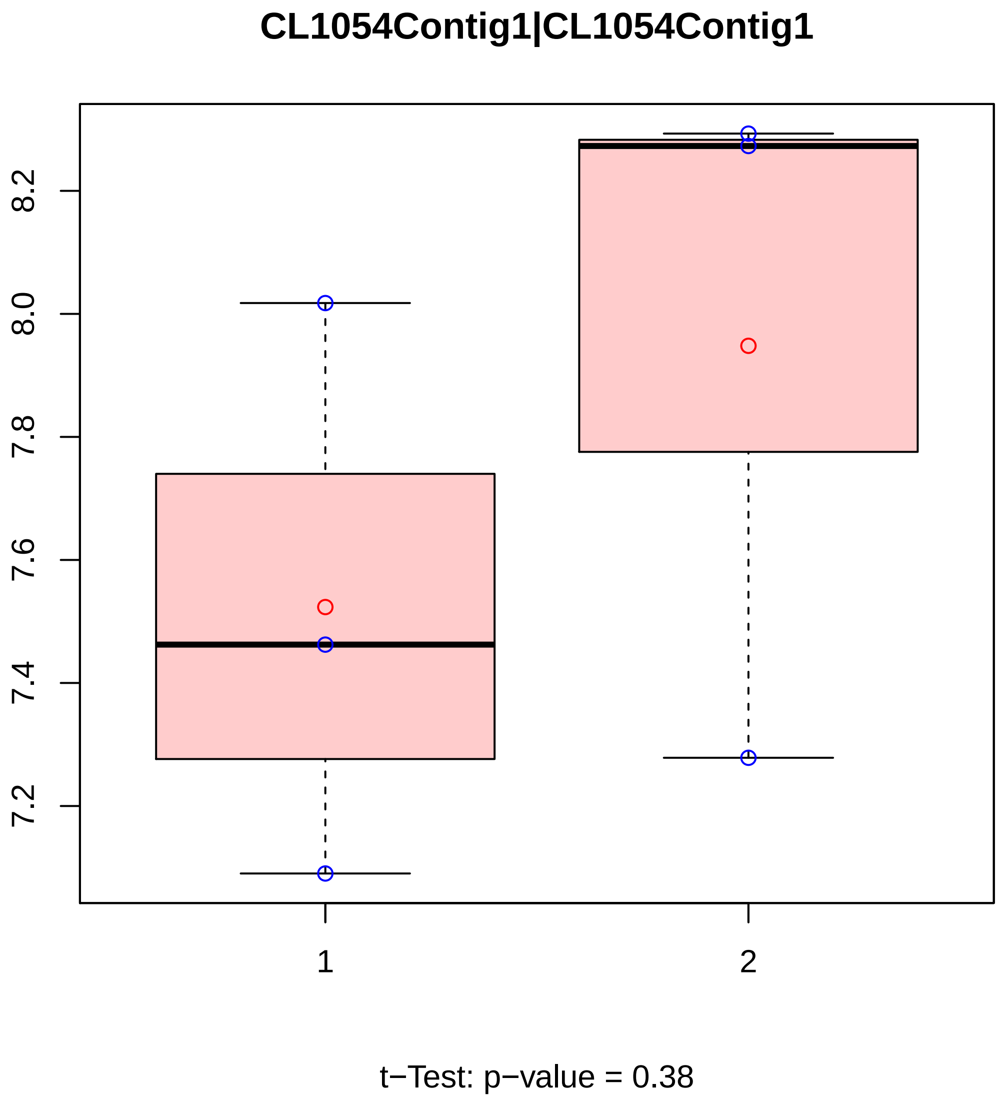
CL1054Contig1|CL1054Contig1
8.2
8.0
7.8
7.6
7.4
7.2
1
2
t−Test: p−value = 0.38

# CL1056Contig5|CL1056Contig5

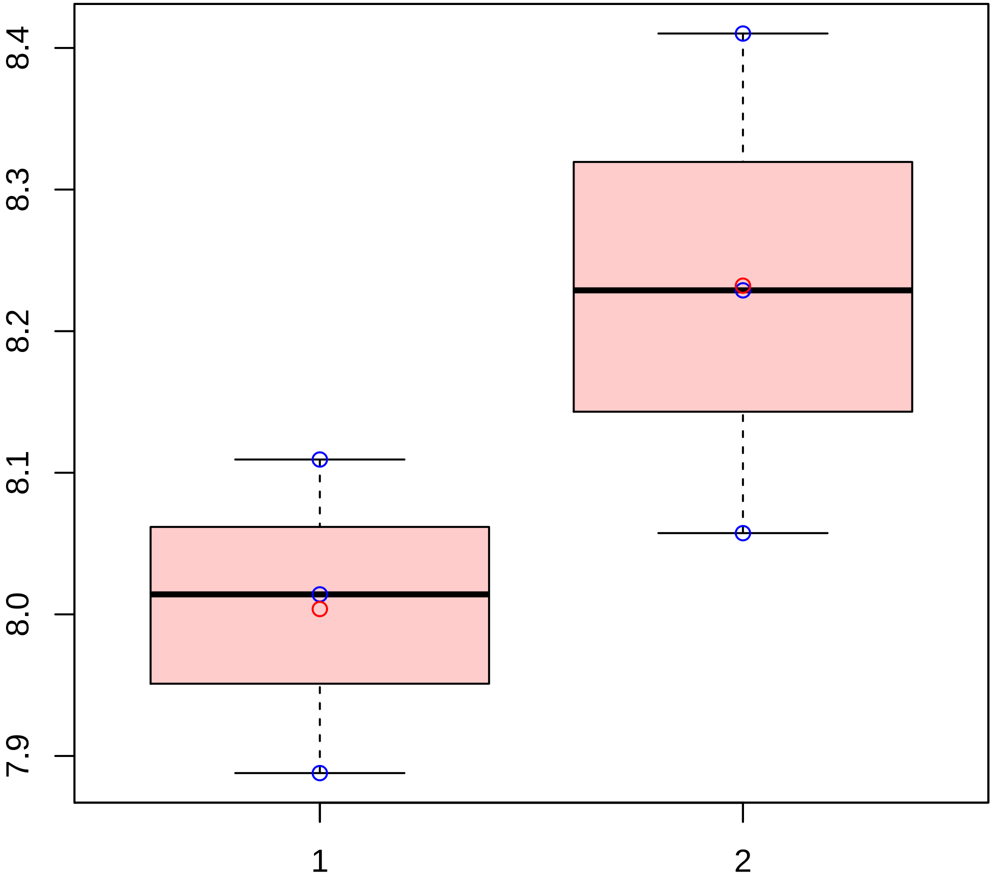


t−Test: p−value = 0.14

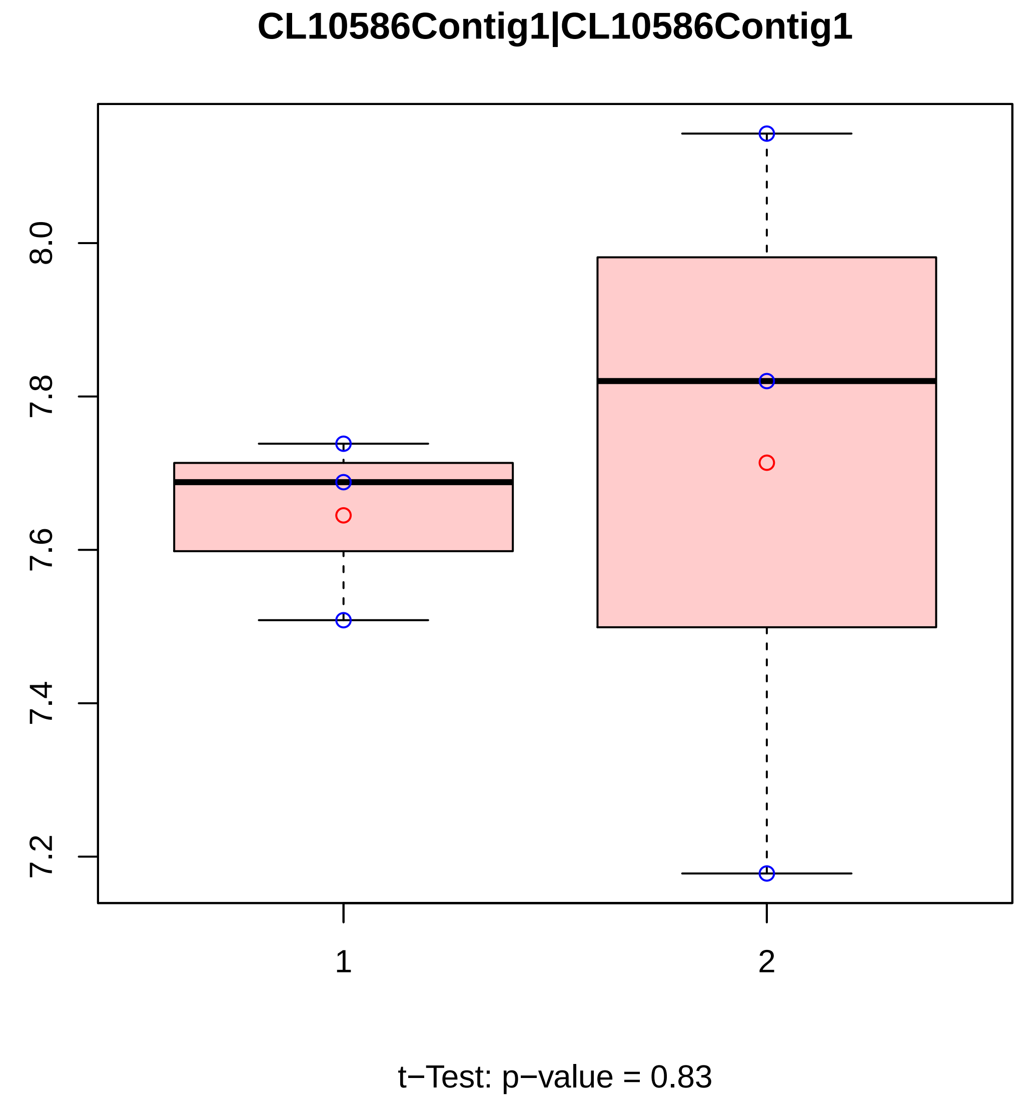
CL10586Contig1|CL10586Contig1
8.0
7.8
7.6
7.4
7.2
1
2
t−Test: p−value = 0.83

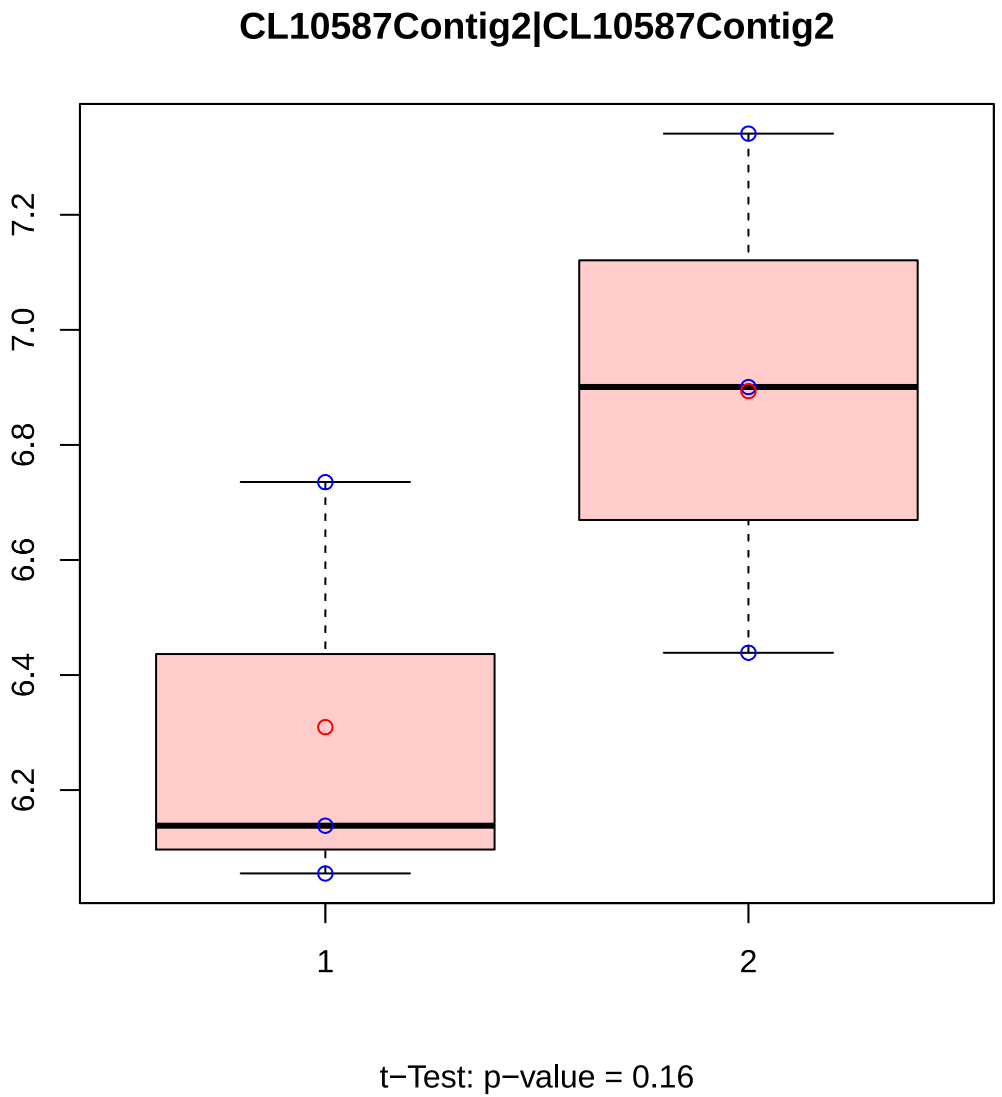

CL10587Contig2|CL10587Contig2
6.2
6.4
6.6
6.8
7.0
7.2
1
2
t−Test: p−value = 0.16

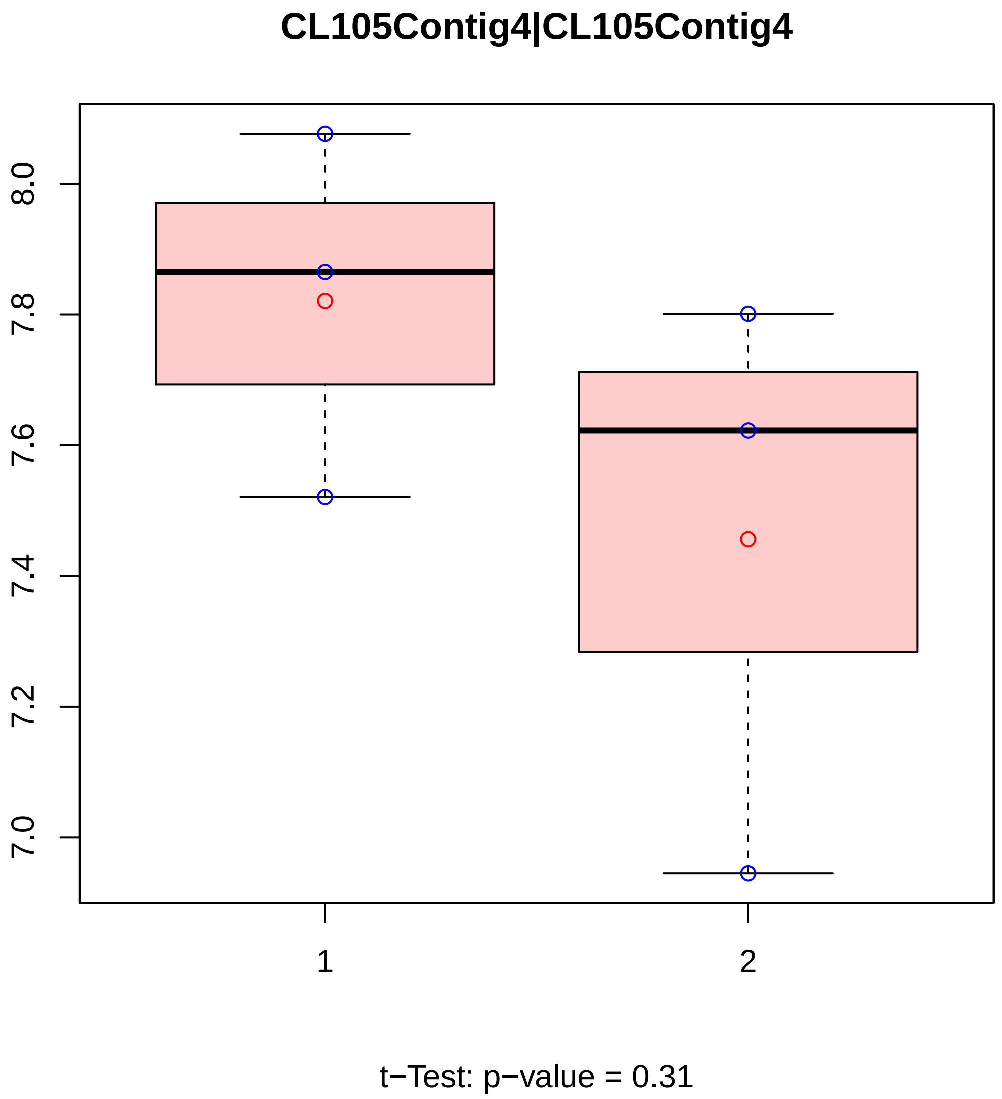

CL105Contig4|CL105Contig4
8.0
7.8
7.6
7.4
7.2
7.0
1
2
t−Test: p−value = 0.31

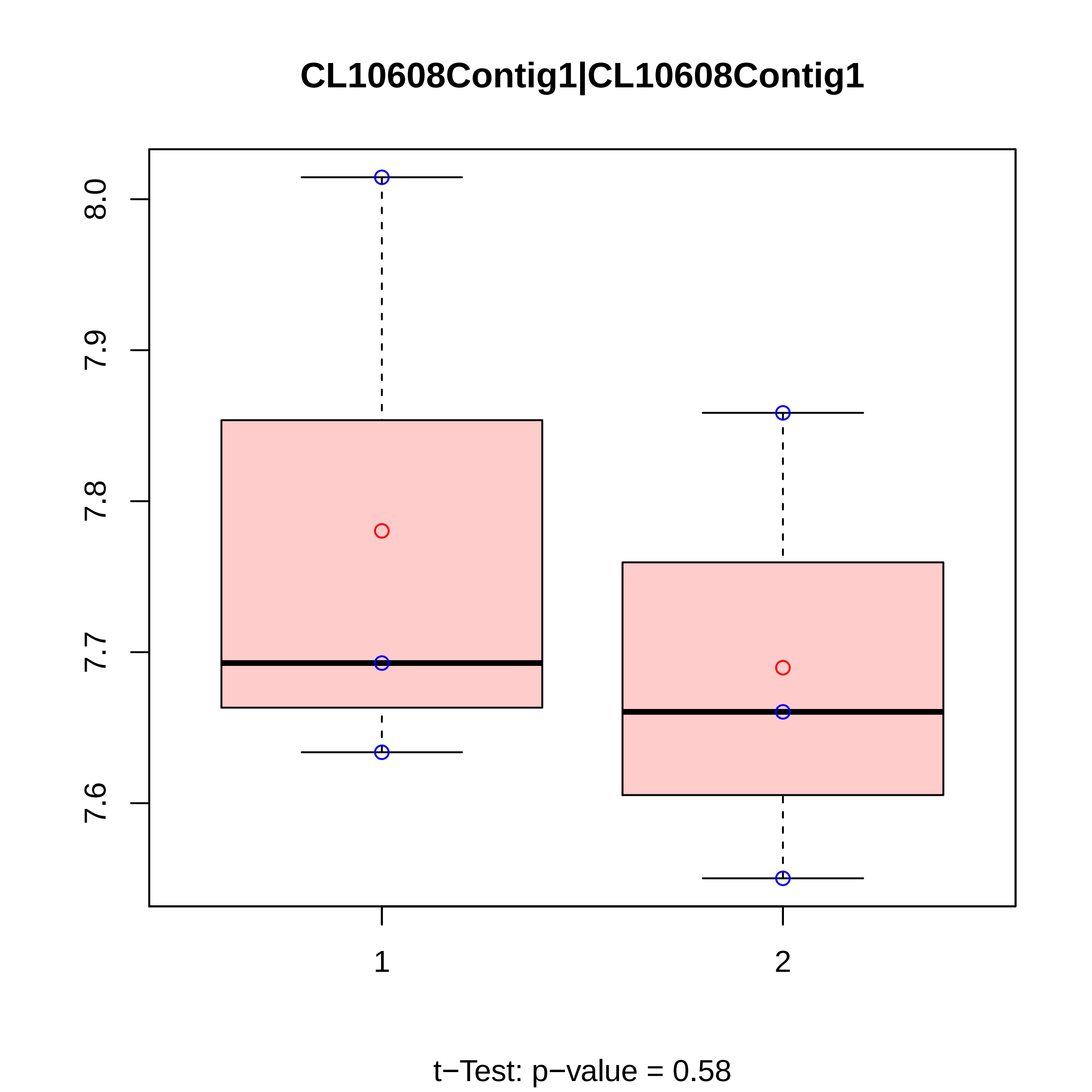
CL10608Contig1|CL10608Contig1
8.0
7.9
7.8
7.7
7.6
1
2
t−Test: p−value = 0.58

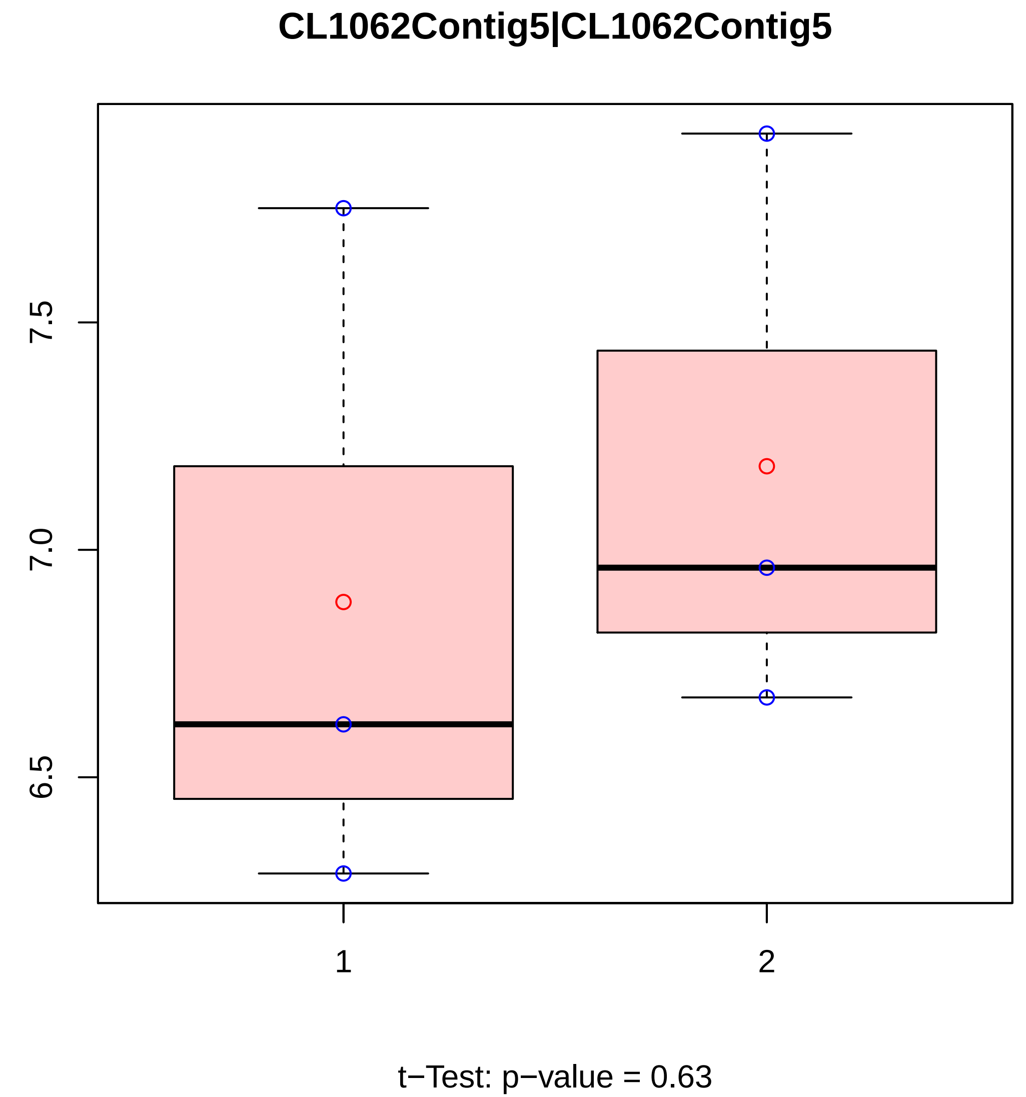
CL1062Contig5|CL1062Contig5
7.5
7.0
6.5
1
2
t−Test: p−value = 0.63

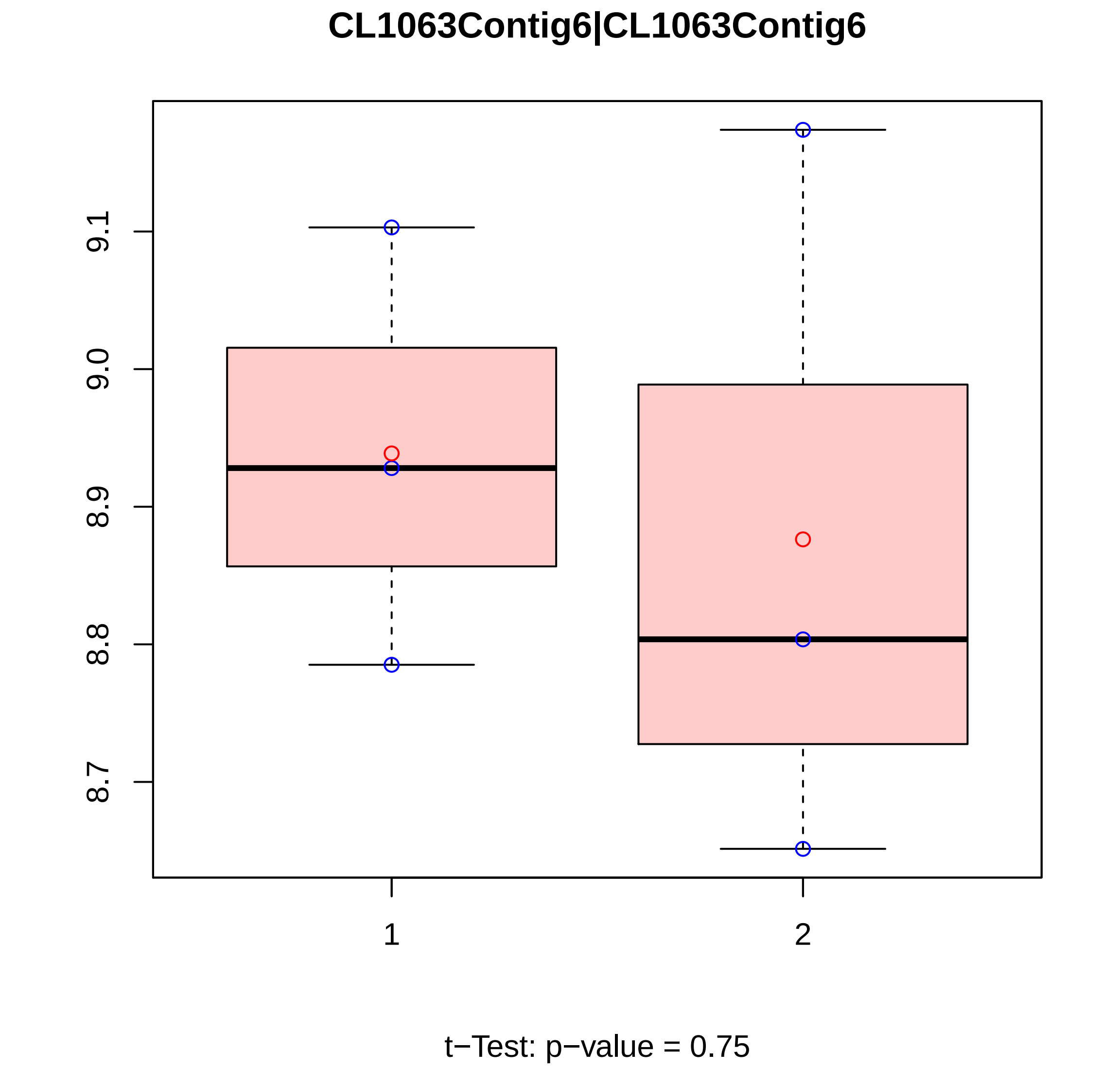
CL1063Contig6|CL1063Contig6
9.1
9.0
8.9
8.8
8.7
1
2
t−Test: p−value = 0.75

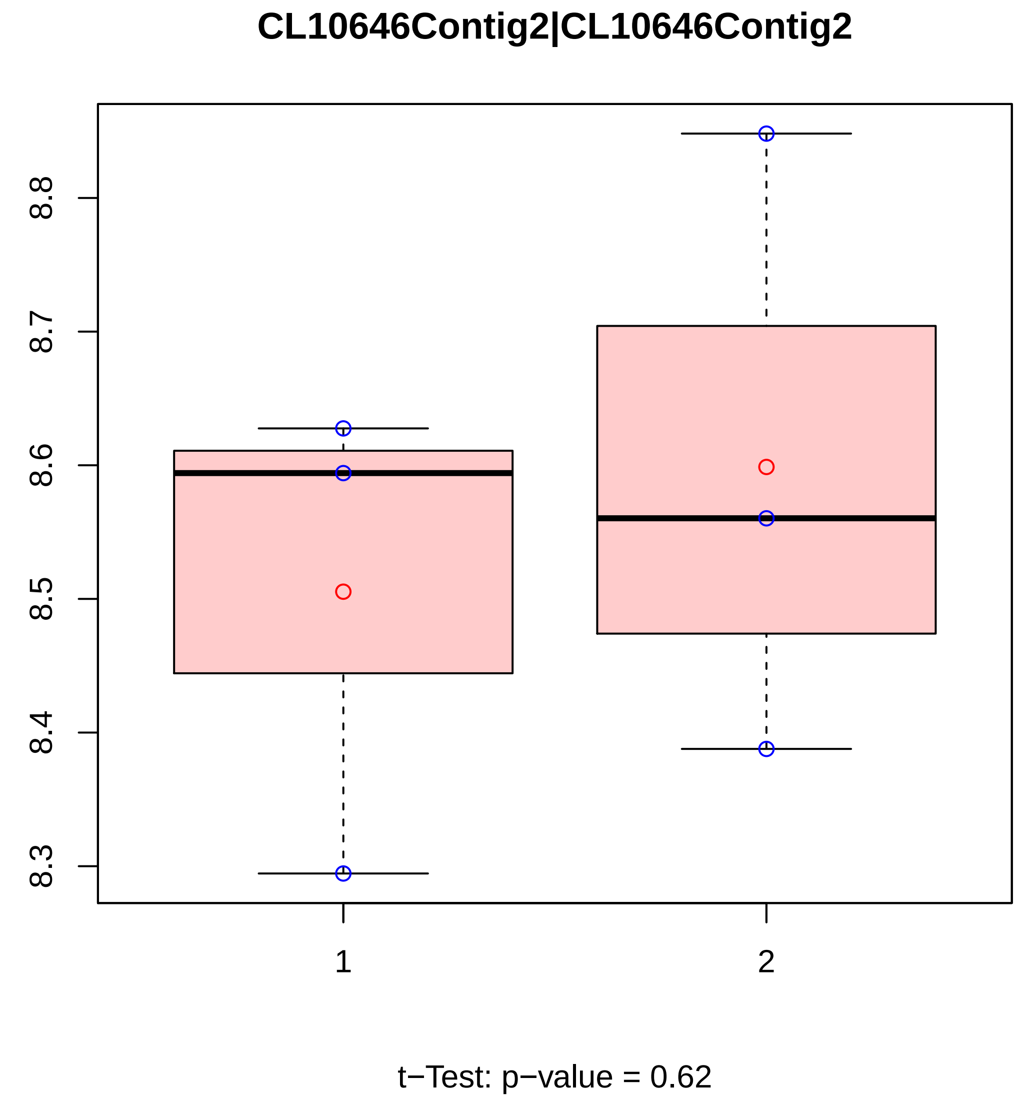
CL10646Contig2|CL10646Contig2
8.3
8.4
8.5
8.6
8.7
8.8
1
2
t−Test: p−value = 0.62

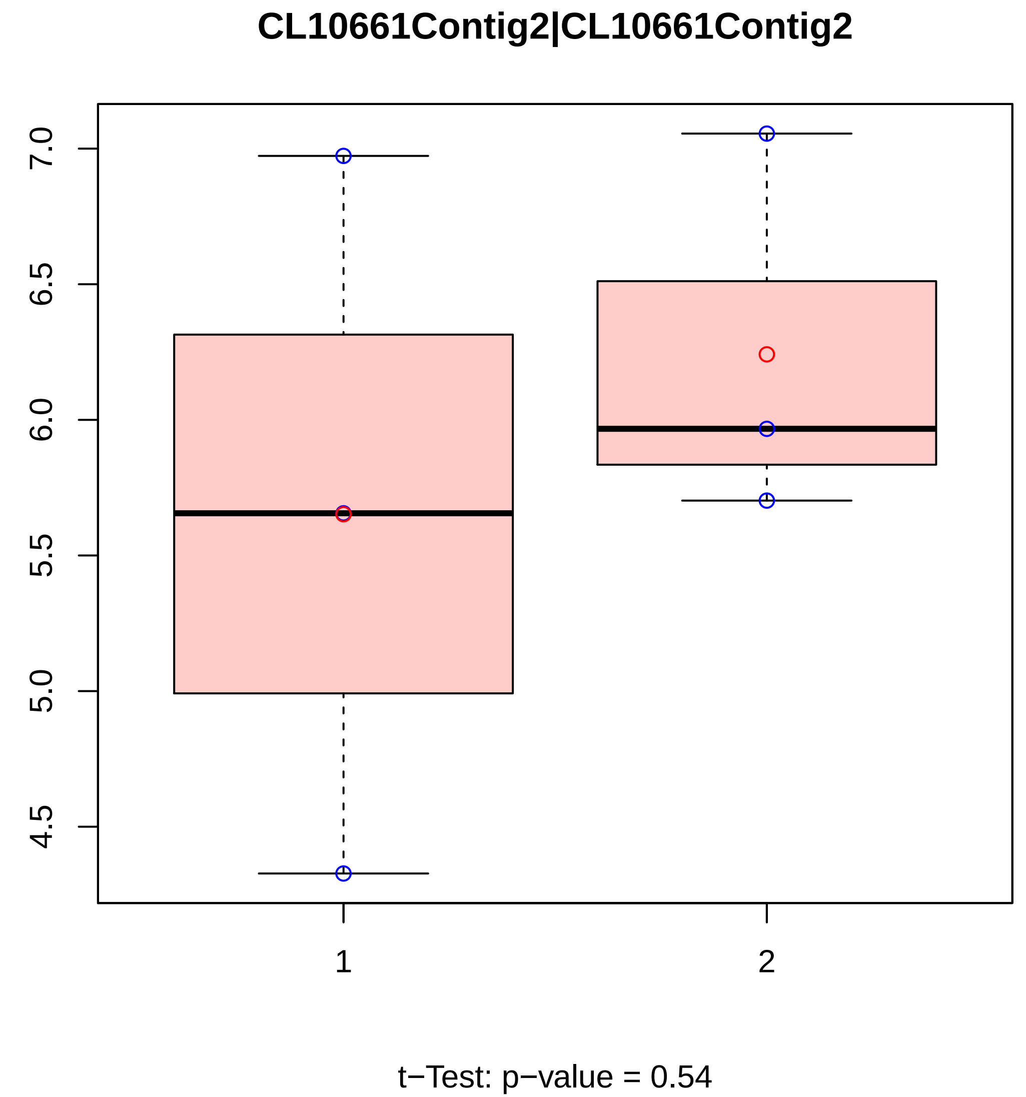
CL10661Contig2|CL10661Contig2
7.0
6.5
6.0
5.5
5.0
4.5
1
2
t−Test: p−value = 0.54

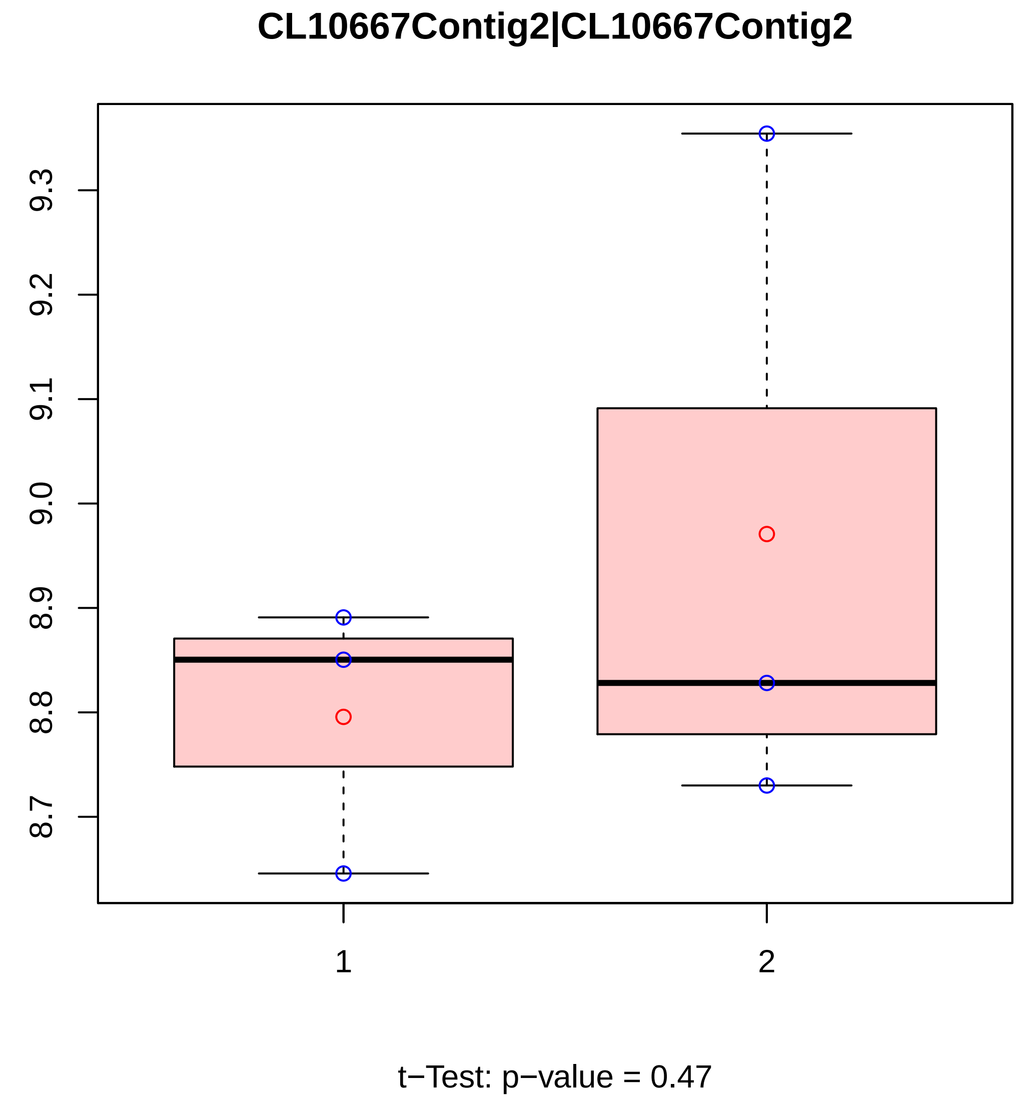
CL10667Contig2|CL10667Contig2
9.3
9.2
9.1
9.0
8.9
8.8
8.7
1
2
t−Test: p−value = 0.47

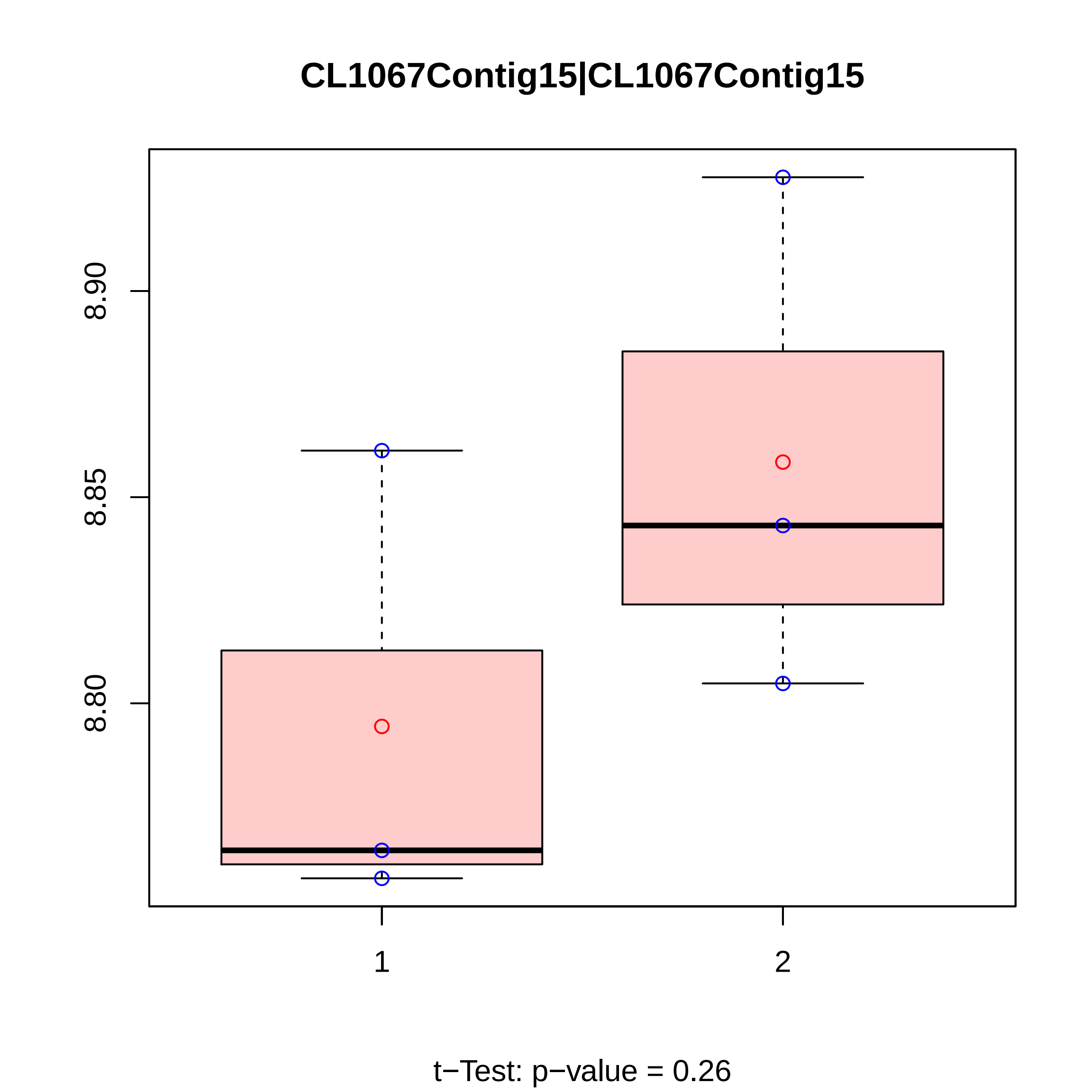
CL1067Contig15|CL1067Contig15
8.90
8.85
8.80
1
2
t−Test: p−value = 0.26

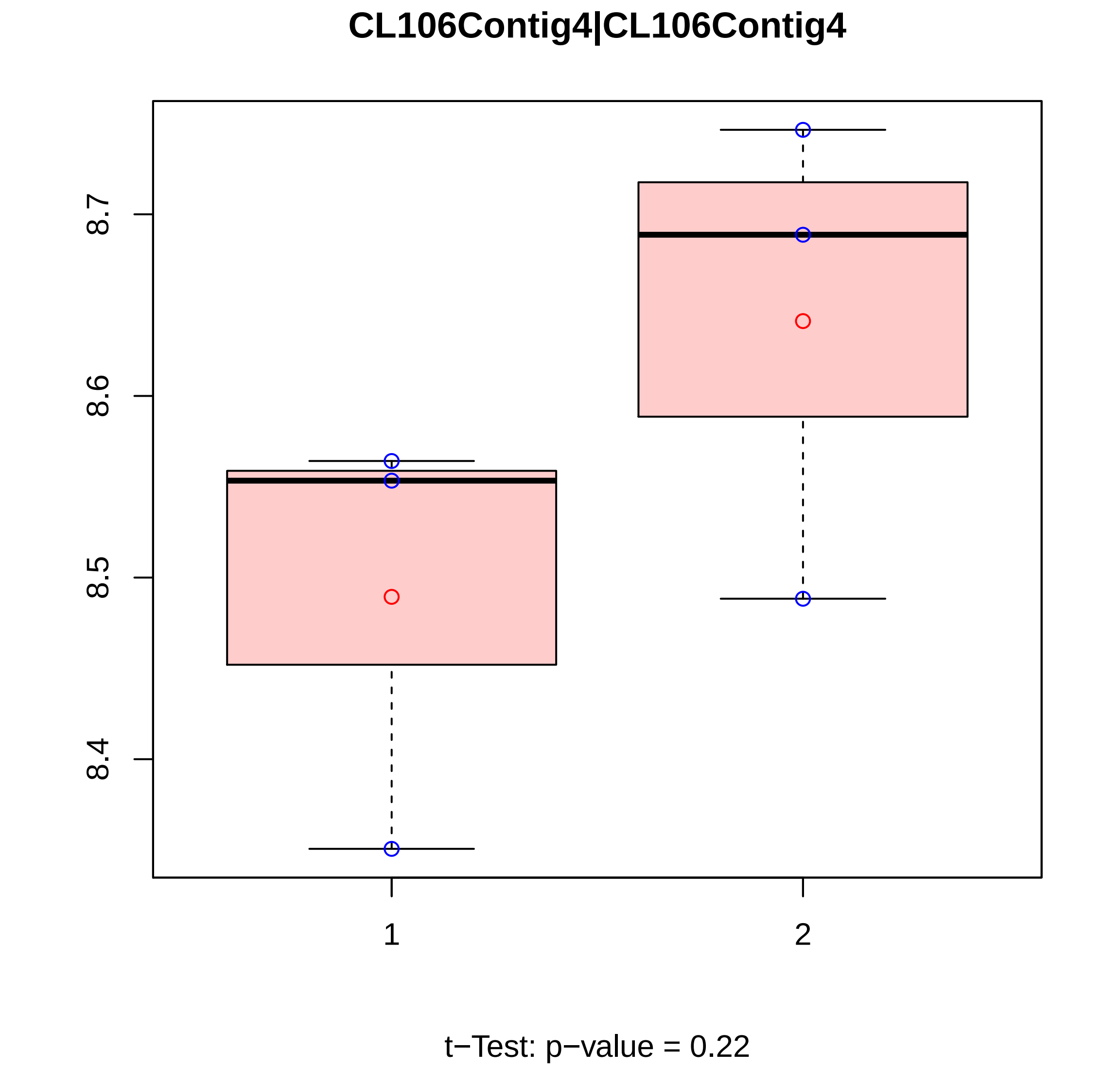

CL106Contig4|CL106Contig4
8.4
8.5
8.6
8.7
1
2
t−Test: p−value = 0.22

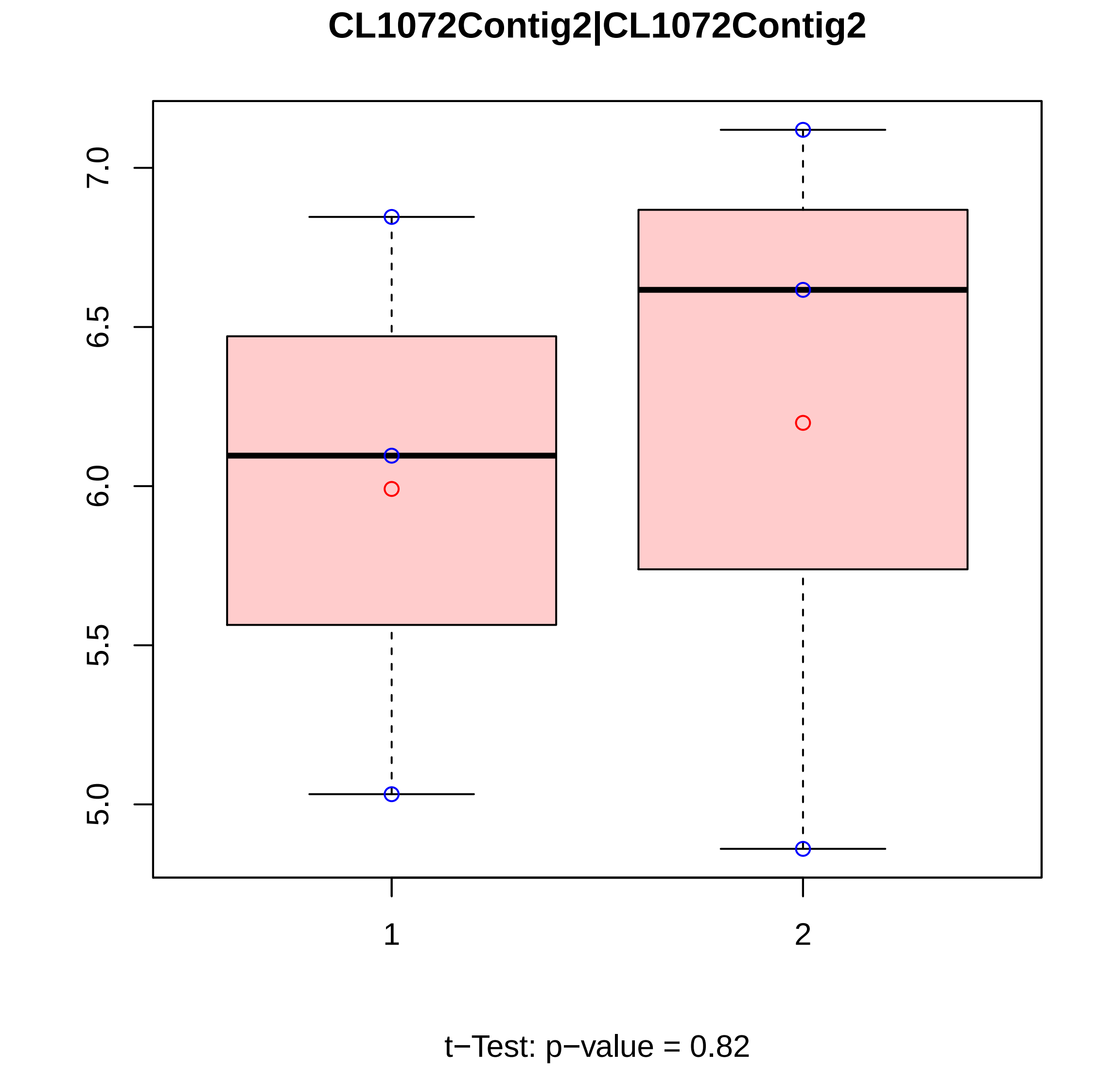
CL1072Contig2|CL1072Contig2
7.0
6.5
6.0
5.5
5.0
1
2
t−Test: p−value = 0.82

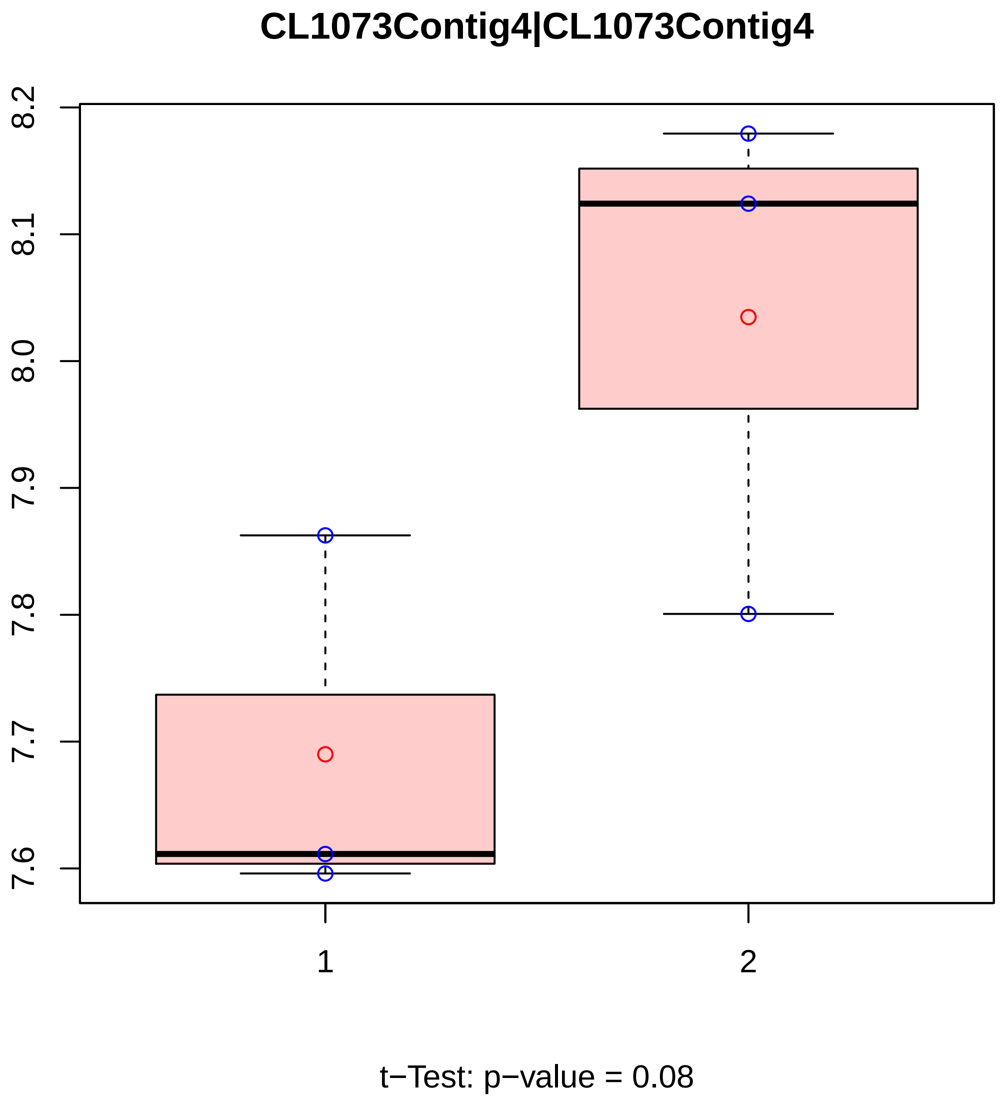
CL1073Contig4|CL1073Contig4
8.2
8.1
8.0
7.9
7.8
7.7
7.6
1
2
t−Test: p−value = 0.08

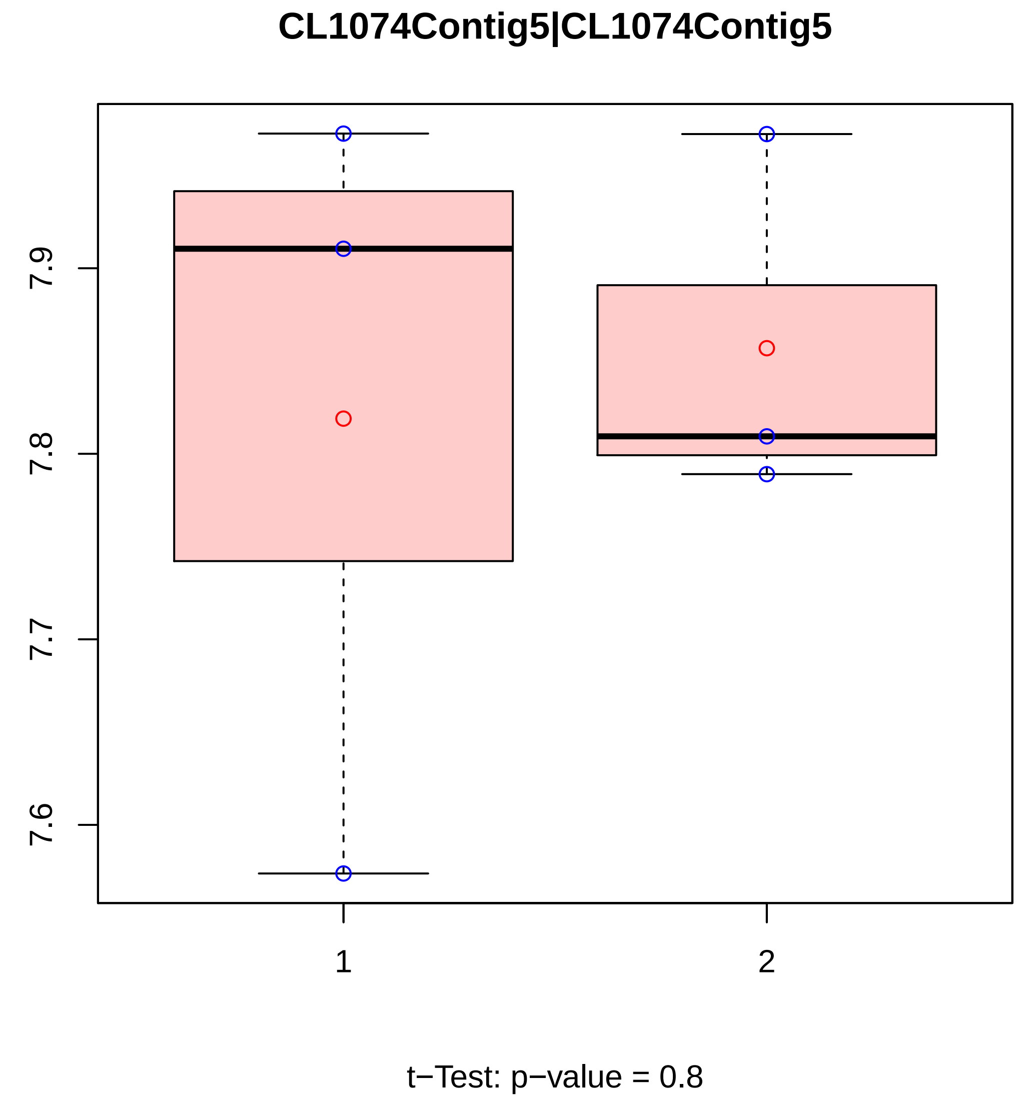
CL1074Contig5|CL1074Contig5
7.6
7.7
7.8
7.9
1
2
t−Test: p−value = 0.8

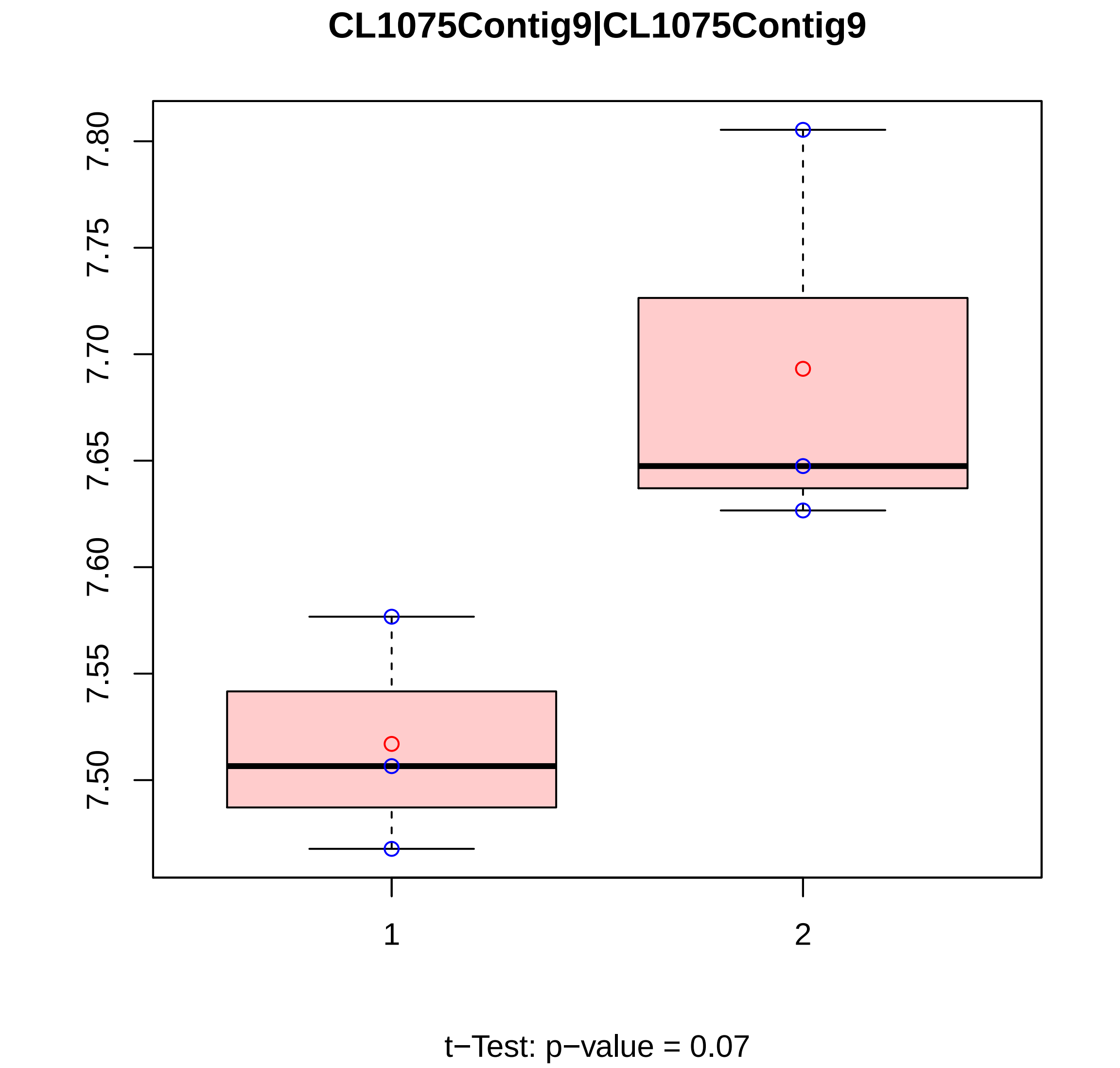
CL1075Contig9|CL1075Contig9
7.50
7.55
7.60
7.65
7.70
7.75
7.80
1
2
t−Test: p−value = 0.07

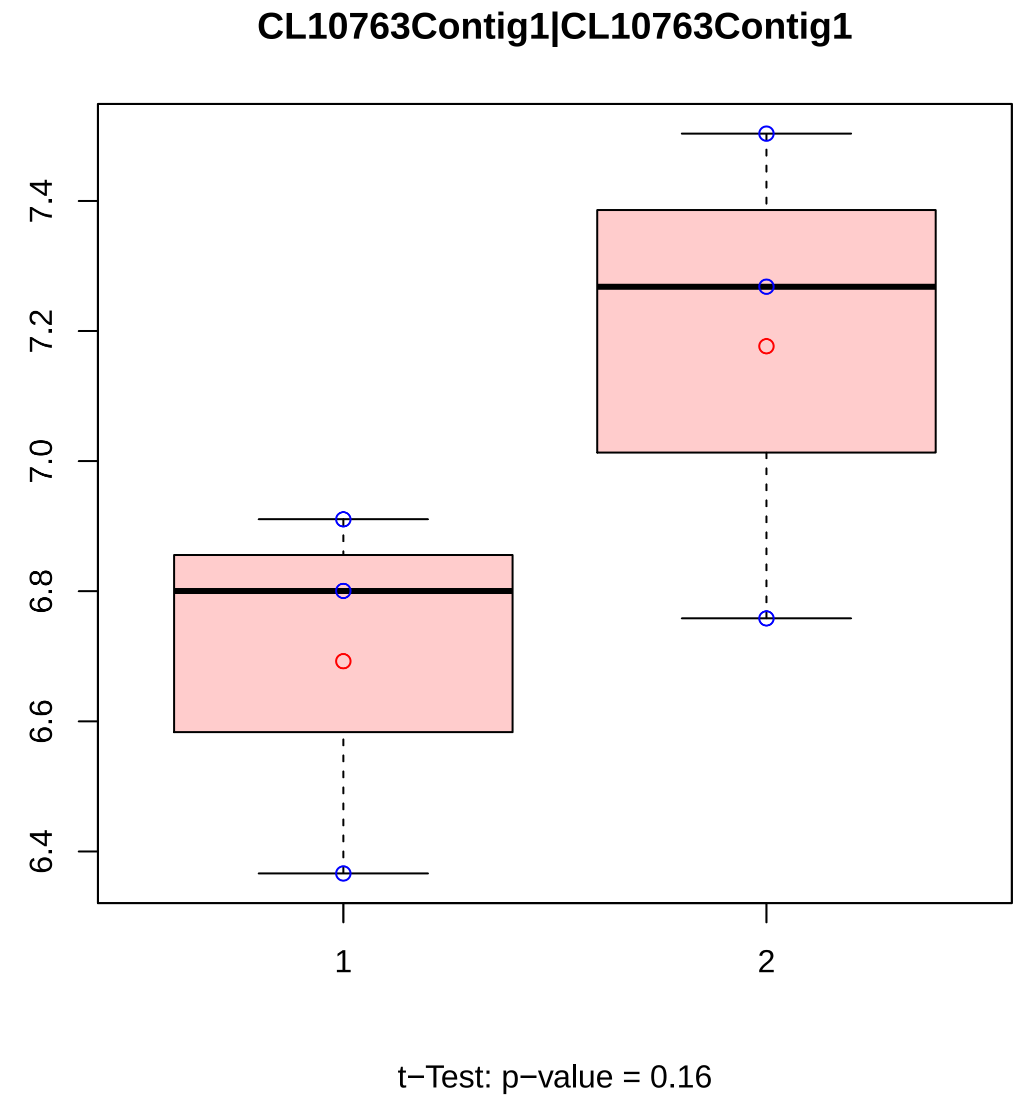
CL10763Contig1|CL10763Contig1
7.4
7.2
7.0
6.8
6.6
6.4
1
2
t−Test: p−value = 0.16

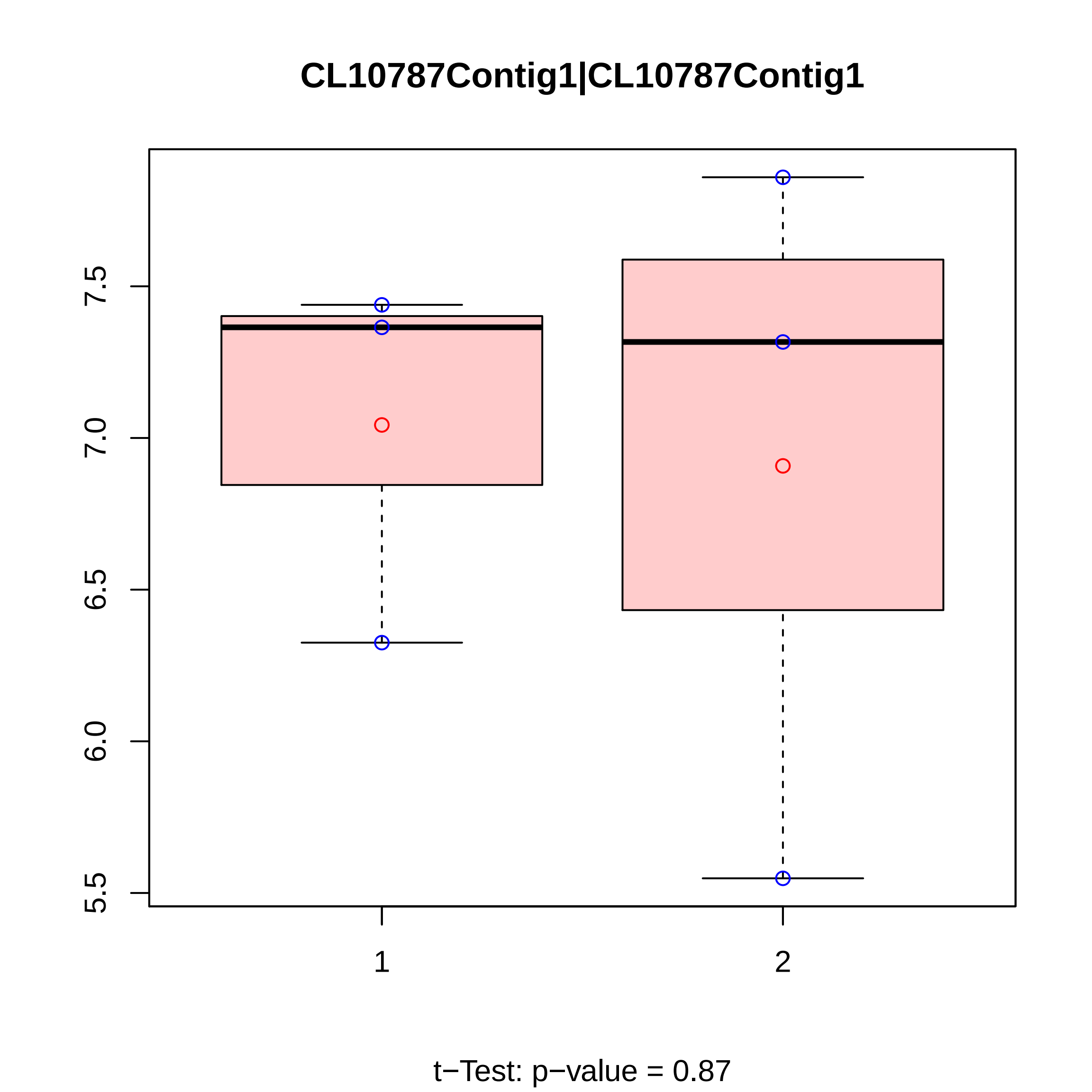
CL10787Contig1|CL10787Contig1
7.5
7.0
6.5
6.0
5.5
1
2
t−Test: p−value = 0.87

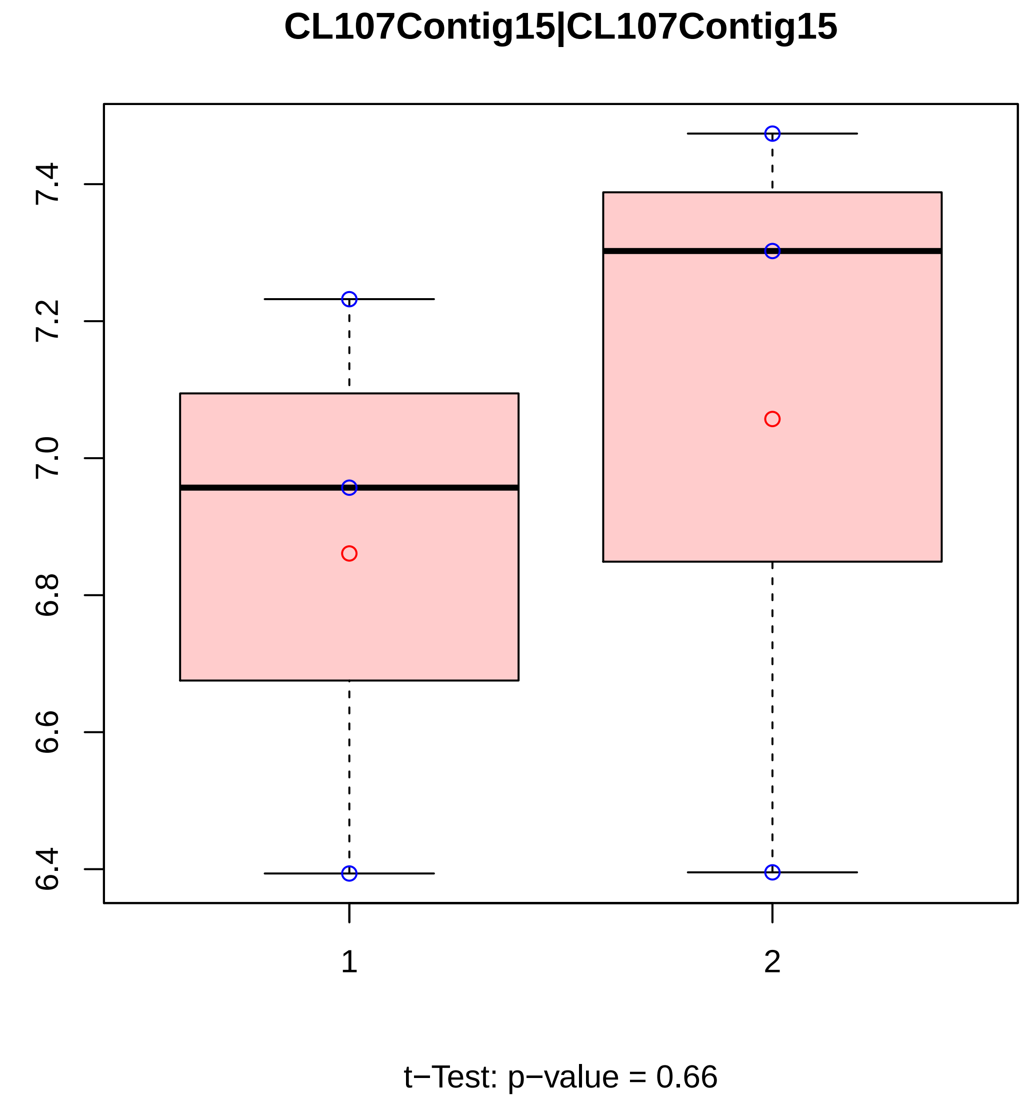
CL107Contig15|CL107Contig15
7.4
7.2
7.0
6.8
6.6
6.4
1
2
t−Test: p−value = 0.66

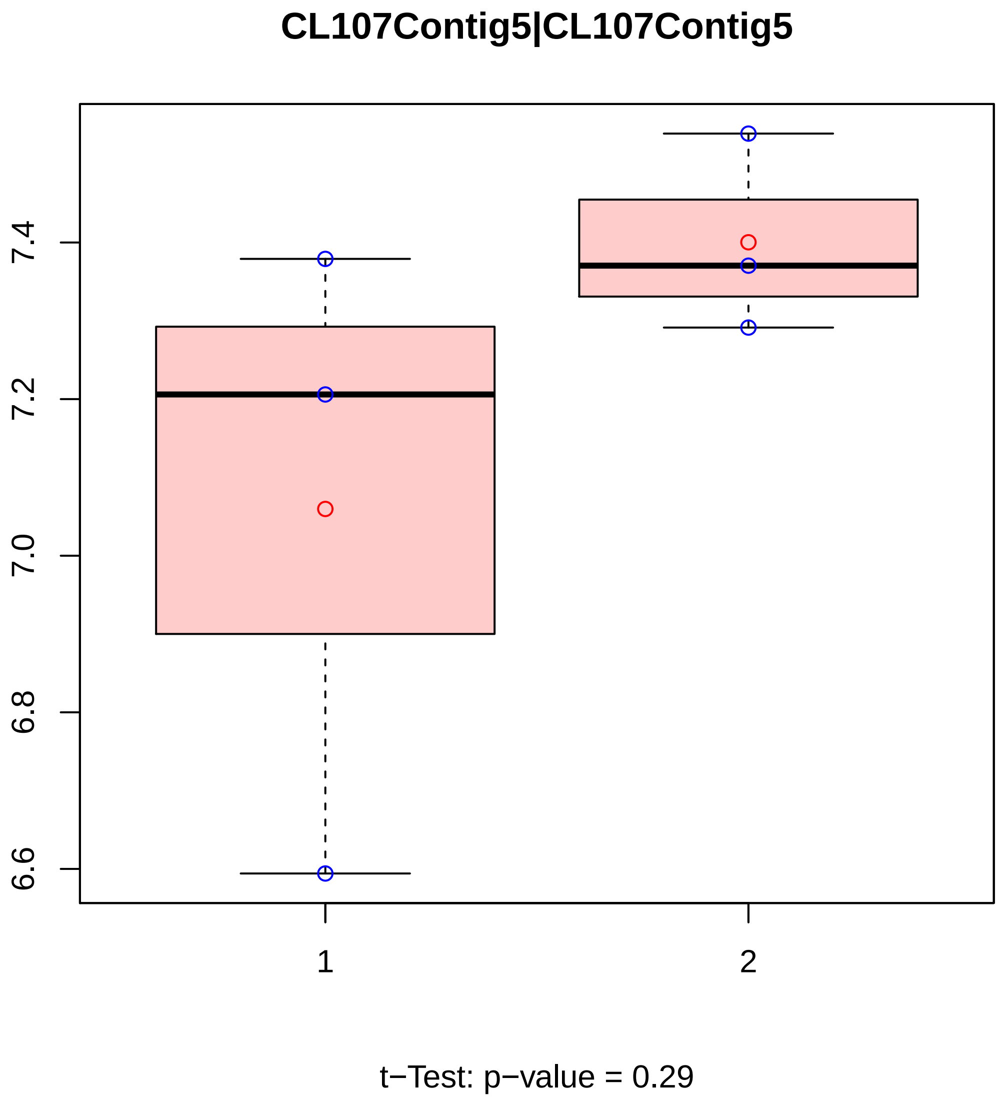

CL107Contig5|CL107Contig5
7.4
7.2
7.0
6.8
6.6
1
2
t−Test: p−value = 0.29

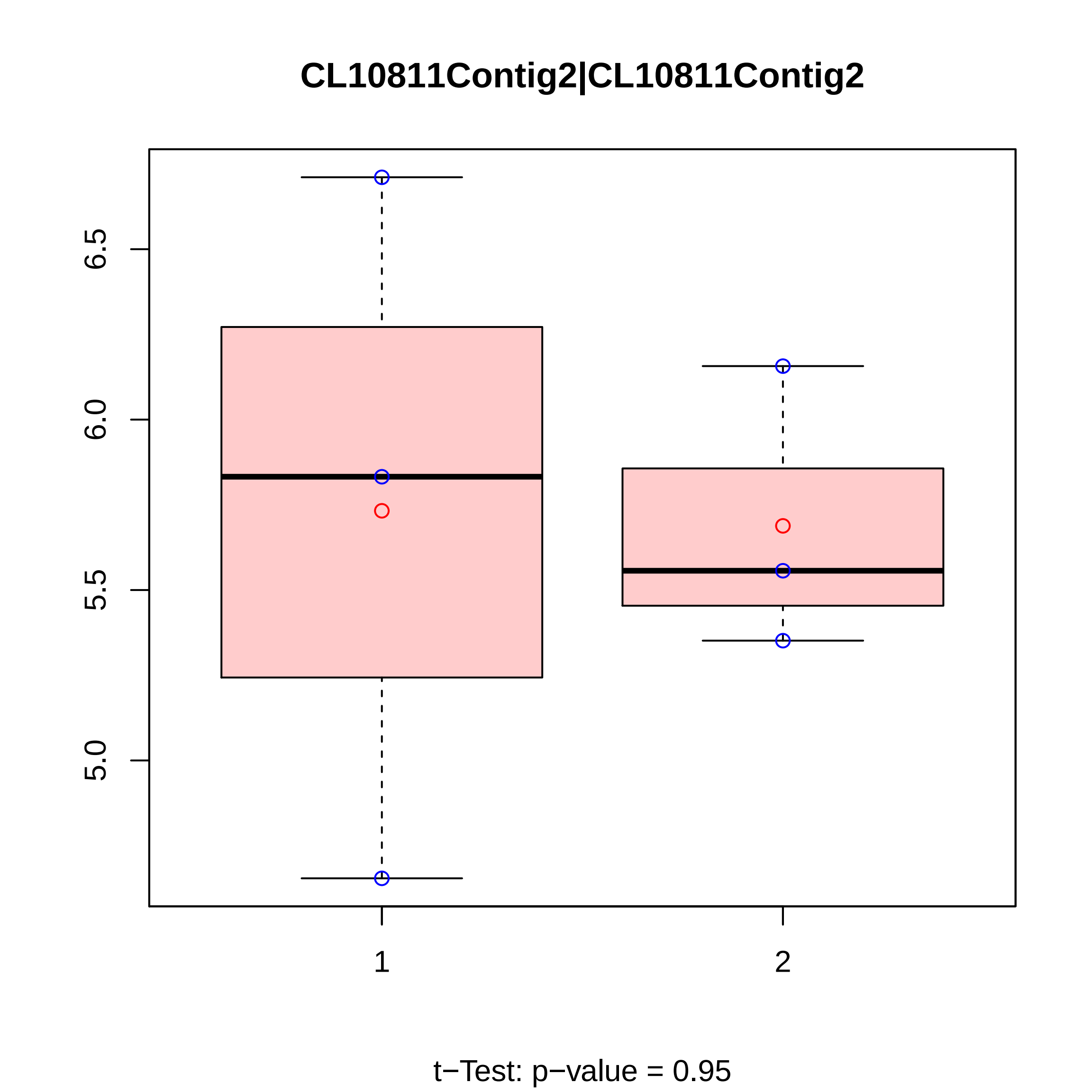

CL10811Contig2|CL10811Contig2
6.5
6.0
5.5
5.0
1
2
t−Test: p−value = 0.95

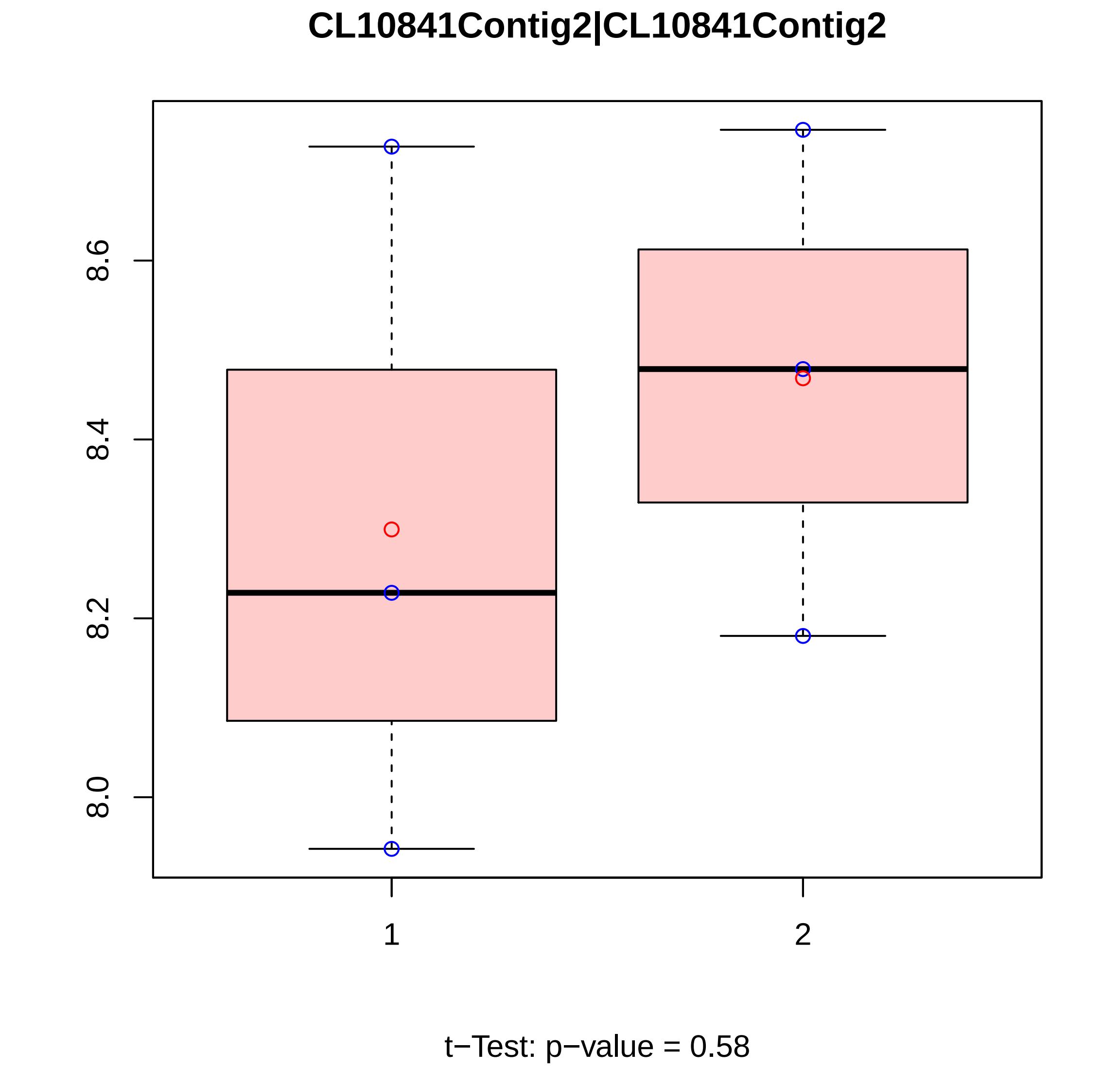
CL10841Contig2|CL10841Contig2
8.0
8.2
8.4
8.6
1
2
t−Test: p−value = 0.58

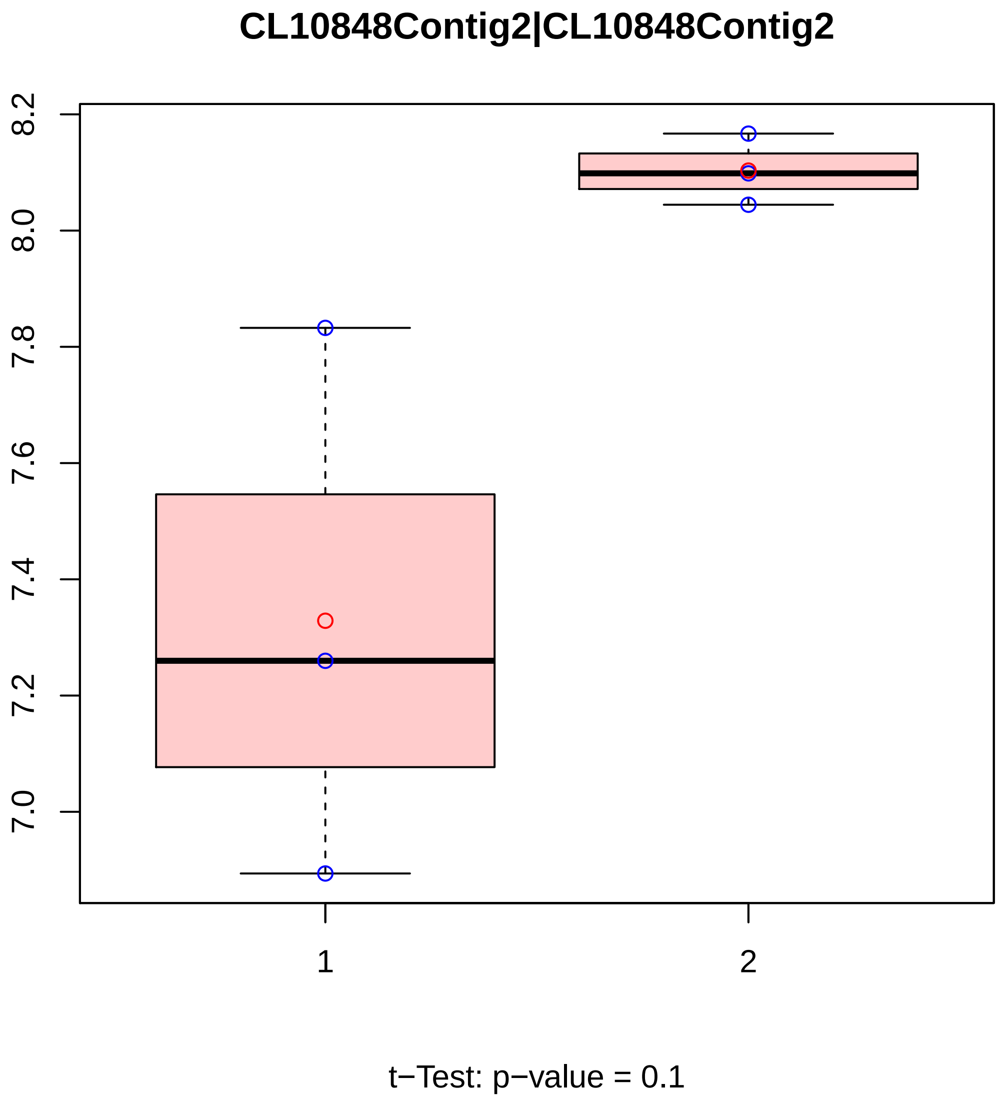
CL10848Contig2|CL10848Contig2
8.2
8.0
7.8
7.6
7.4
7.2
7.0
1
2
t−Test: p−value = 0.1

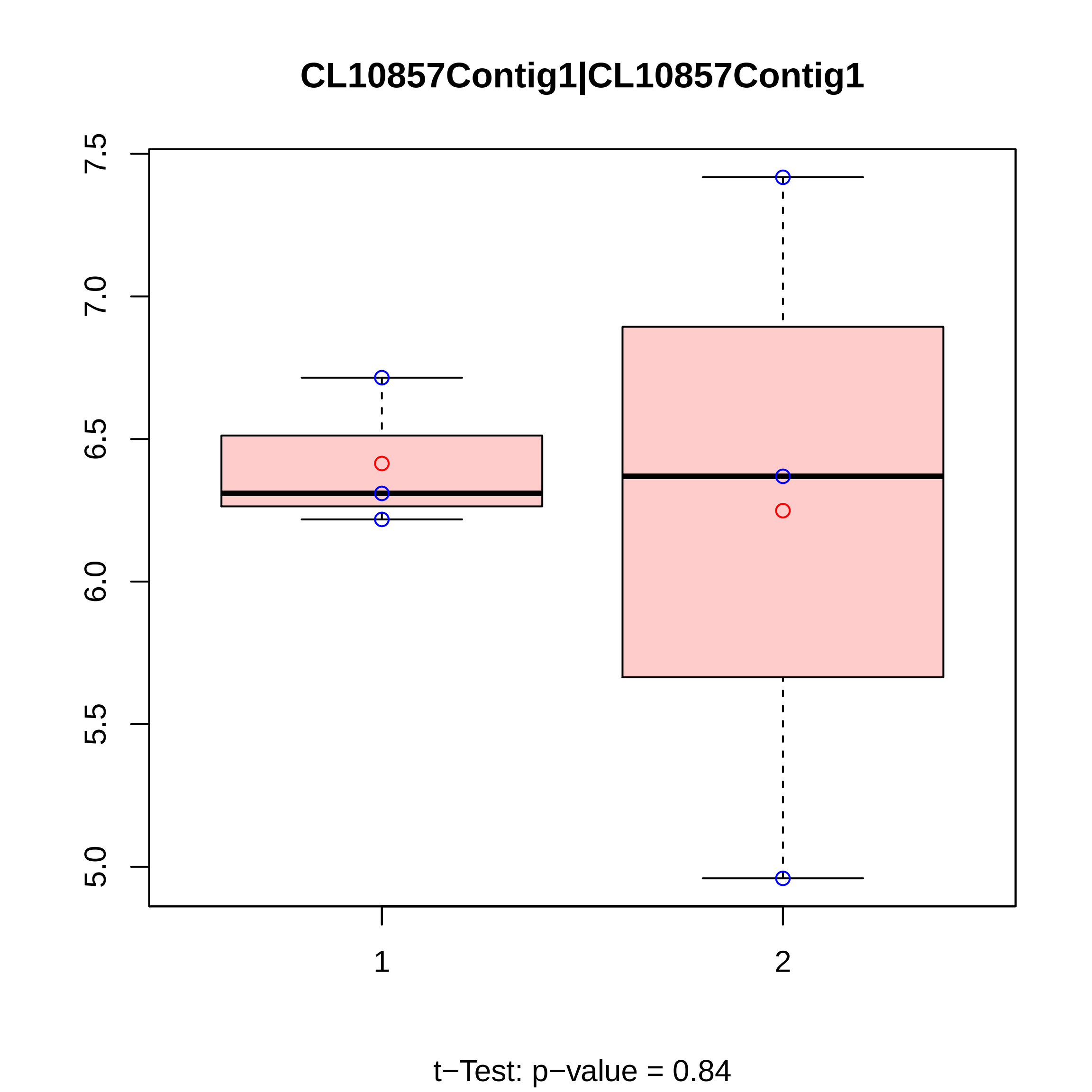
CL10857Contig1|CL10857Contig1
7.5
7.0
6.5
6.0
5.5
5.0
1
2
t−Test: p−value = 0.84

**CL1085Contig2|CL1085Contig2**

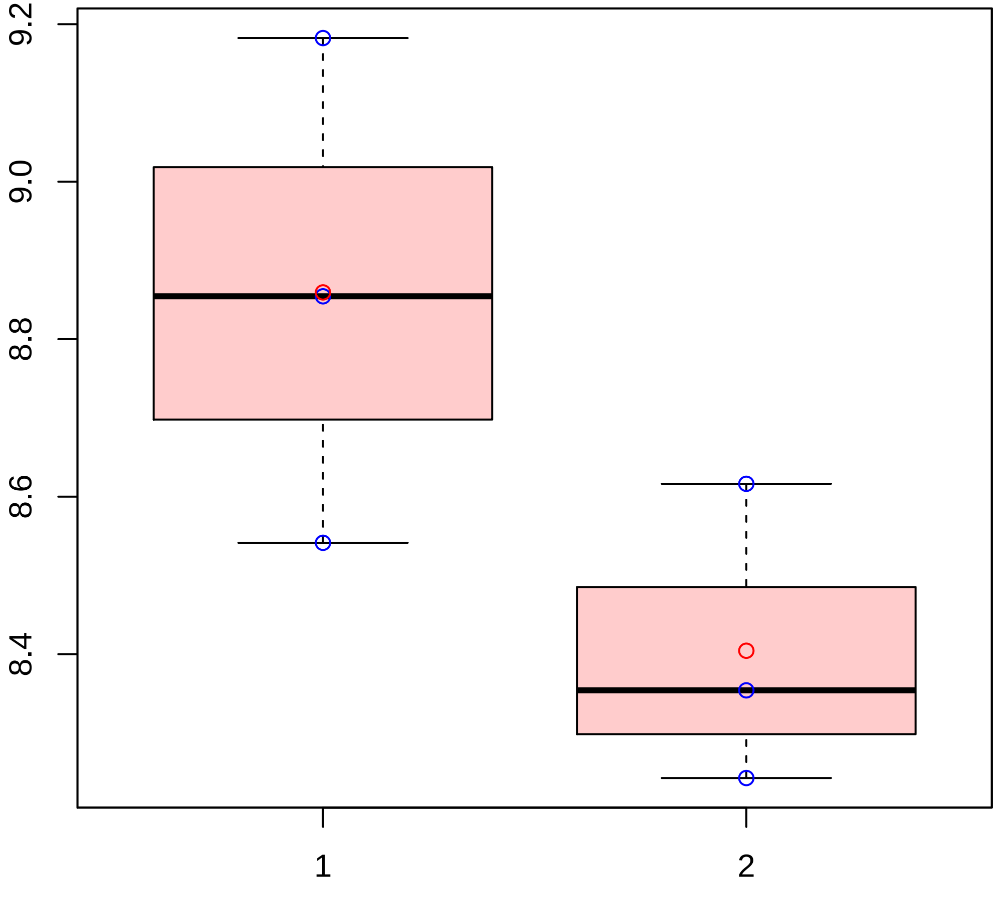


t−Test: p−value = 0.12

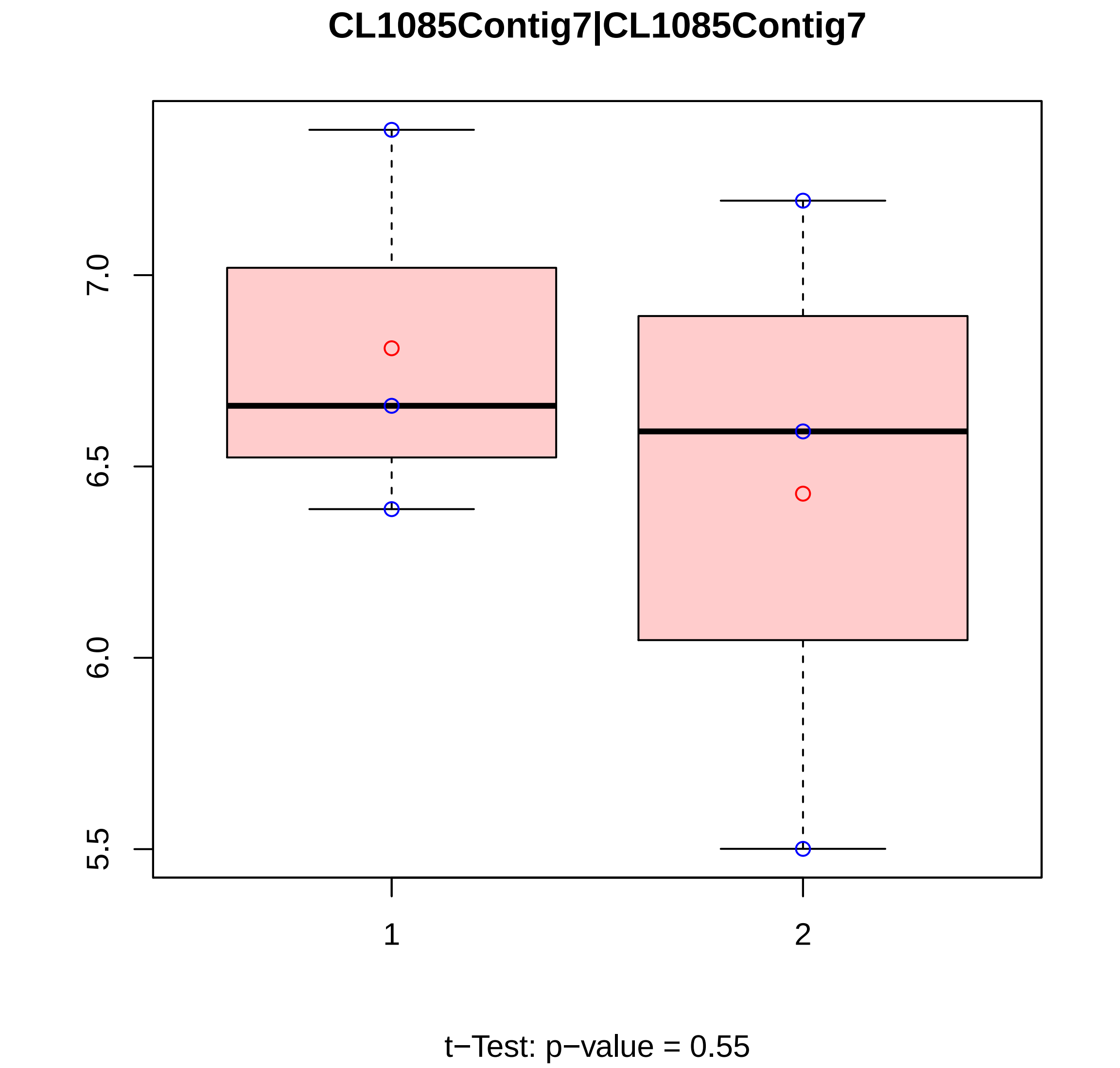
CL1085Contig7|CL1085Contig7
7.0
6.5
6.0
5.5
1
2
t−Test: p−value = 0.55

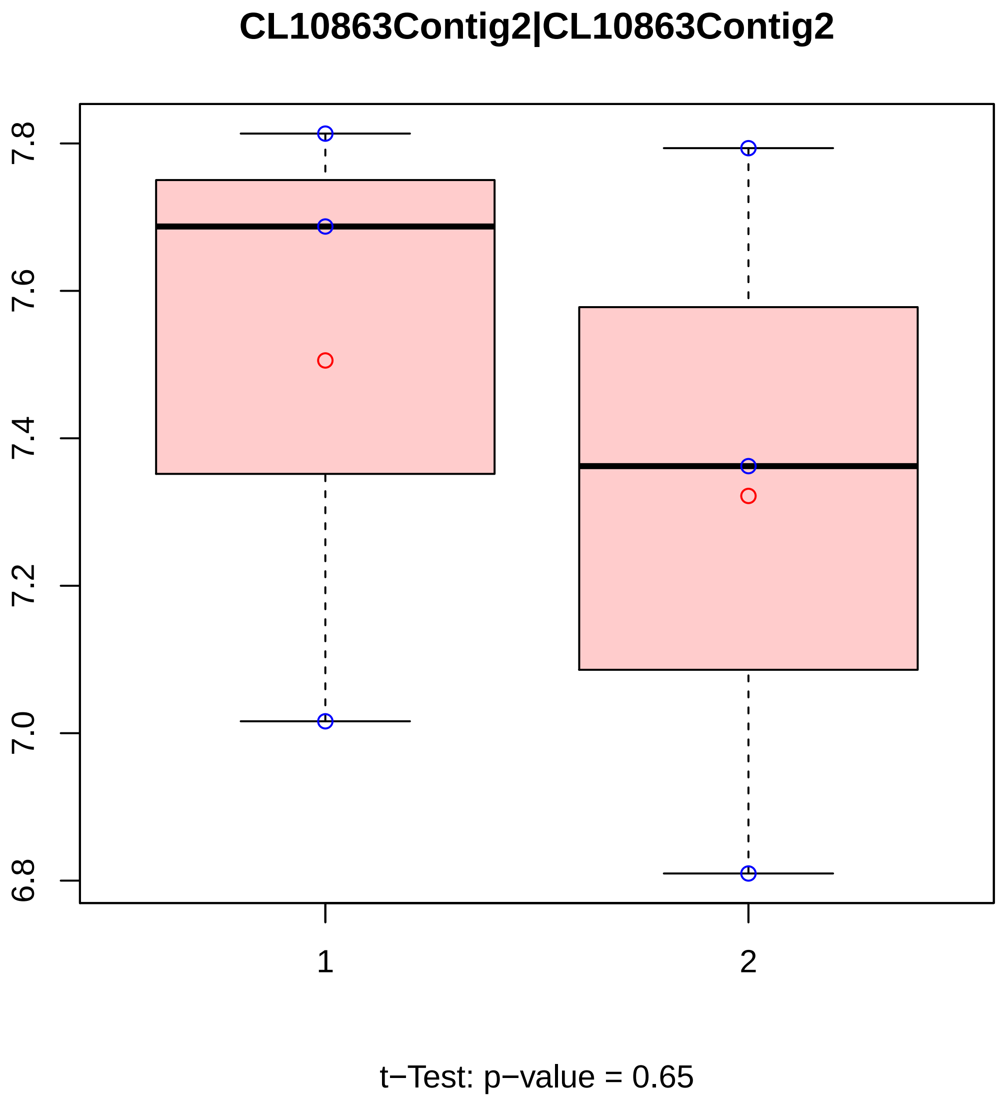
CL10863Contig2|CL10863Contig2
7.8
7.6
7.4
7.2
7.0
6.8
1
2
t−Test: p−value = 0.65

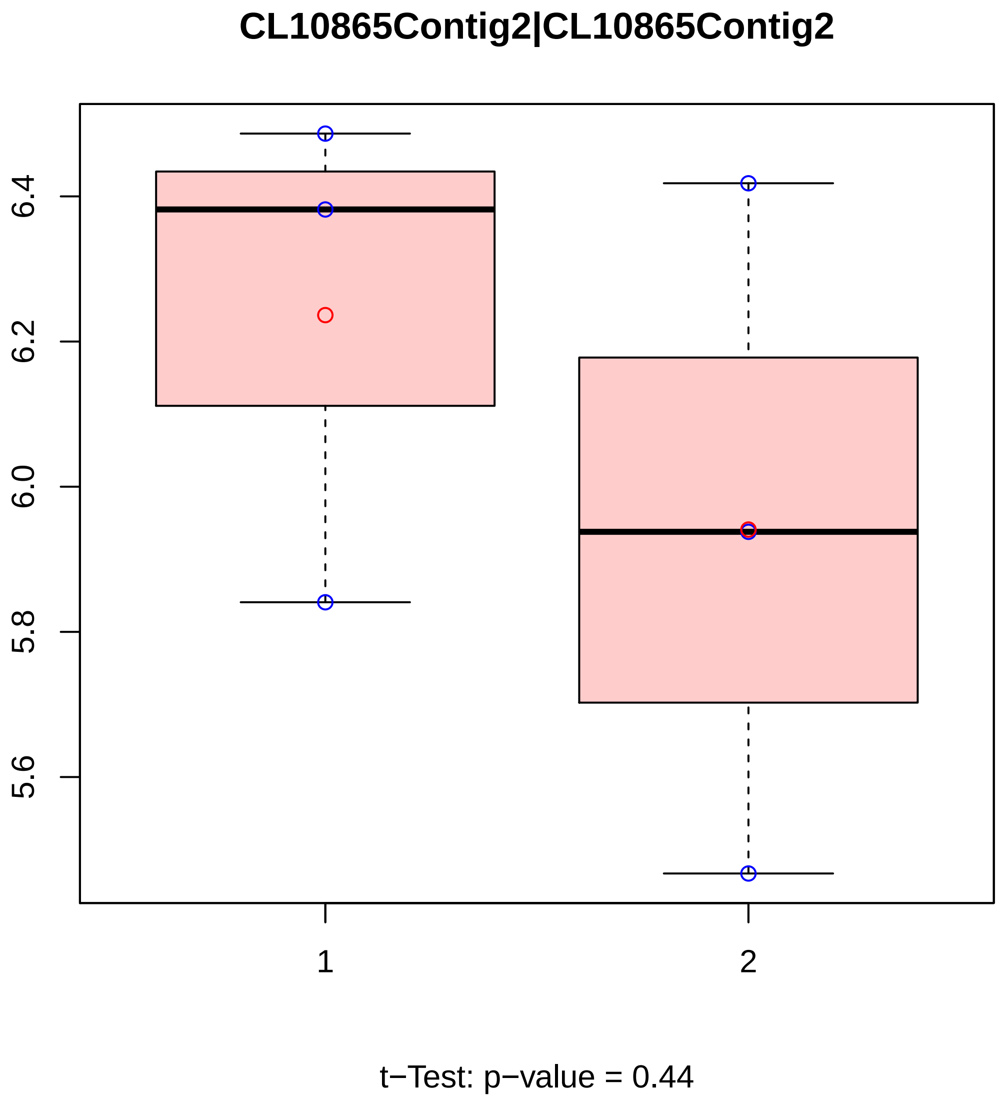

CL10865Contig2|CL10865Contig2
6.4
6.2
6.0
5.8
5.6
1
2
t−Test: p−value = 0.44

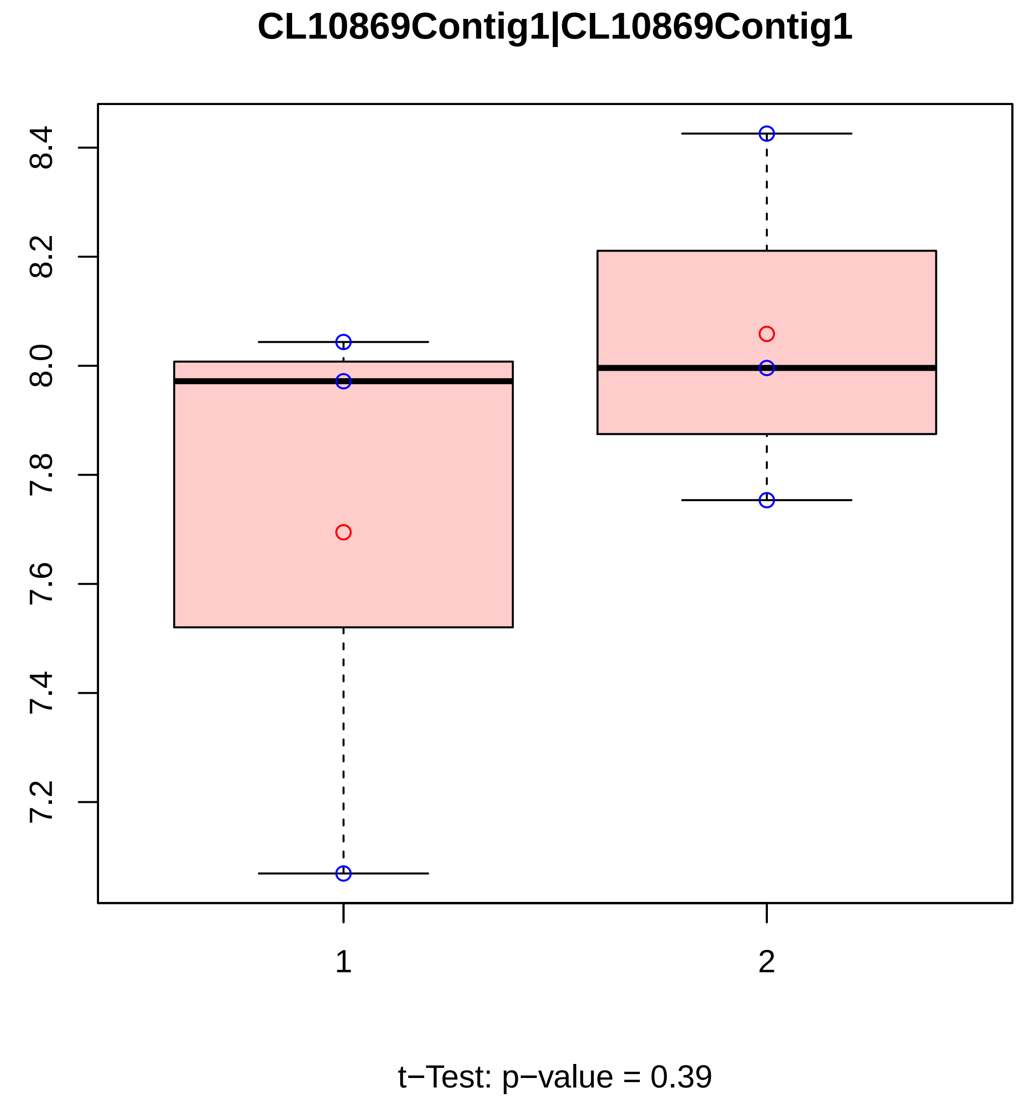
CL10869Contig1|CL10869Contig1
8.4
8.2
8.0
7.8
7.6
7.4
7.2
1
2
t−Test: p−value = 0.39

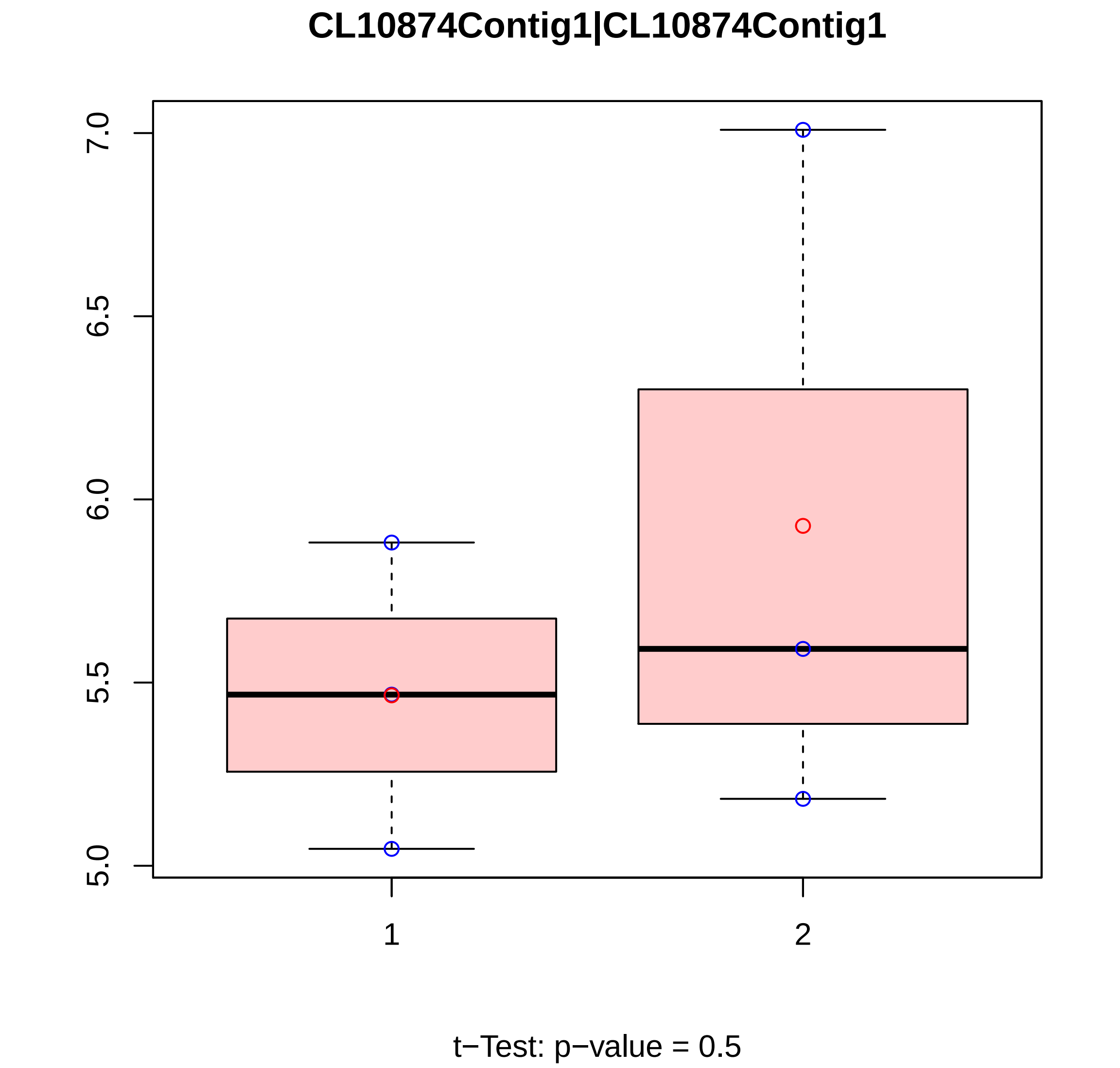
CL10874Contig1|CL10874Contig1
7.0
6.5
6.0
5.5
5.0
1
2
t−Test: p−value = 0.5

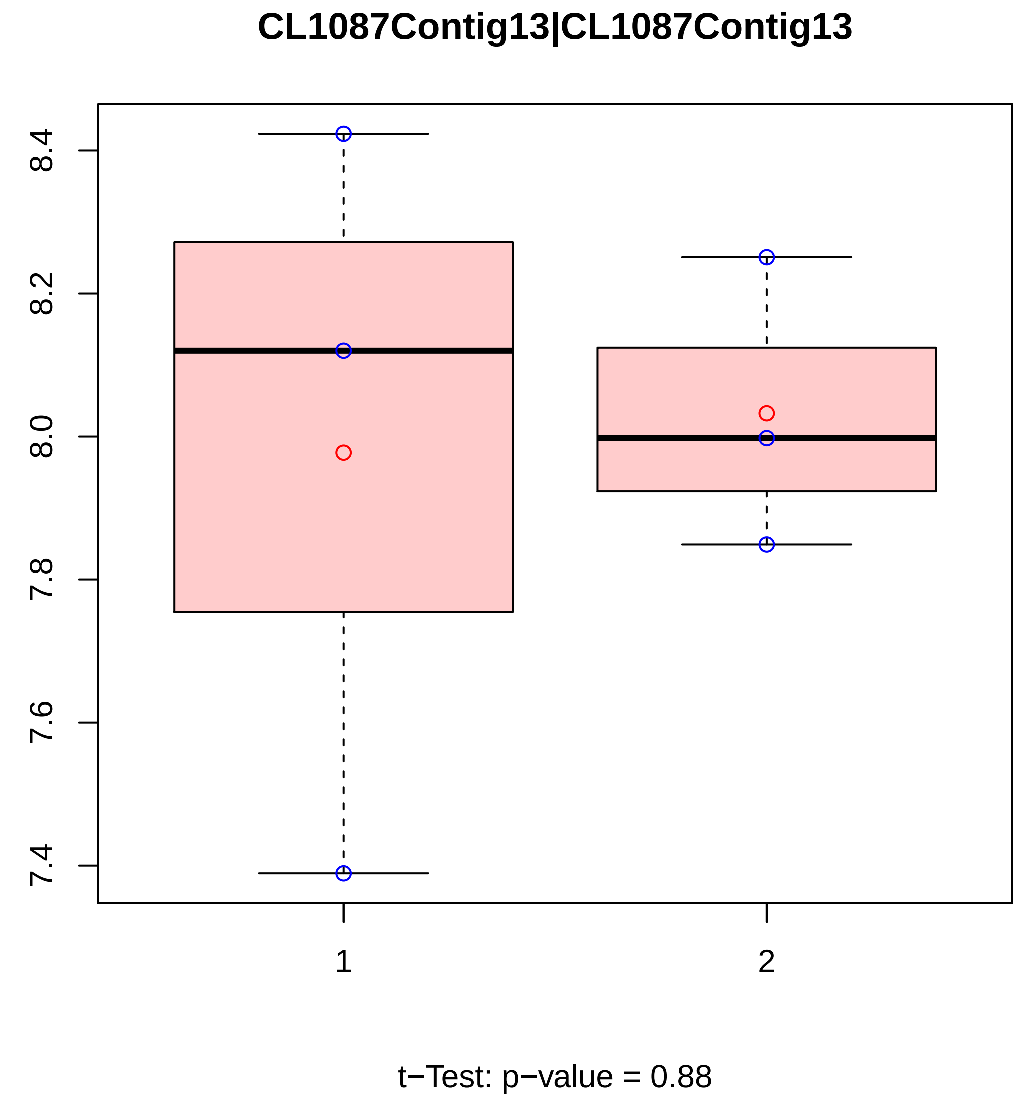
CL1087Contig13|CL1087Contig13
8.4
8.2
8.0
7.8
7.6
7.4
1
2
t−Test: p−value = 0.88

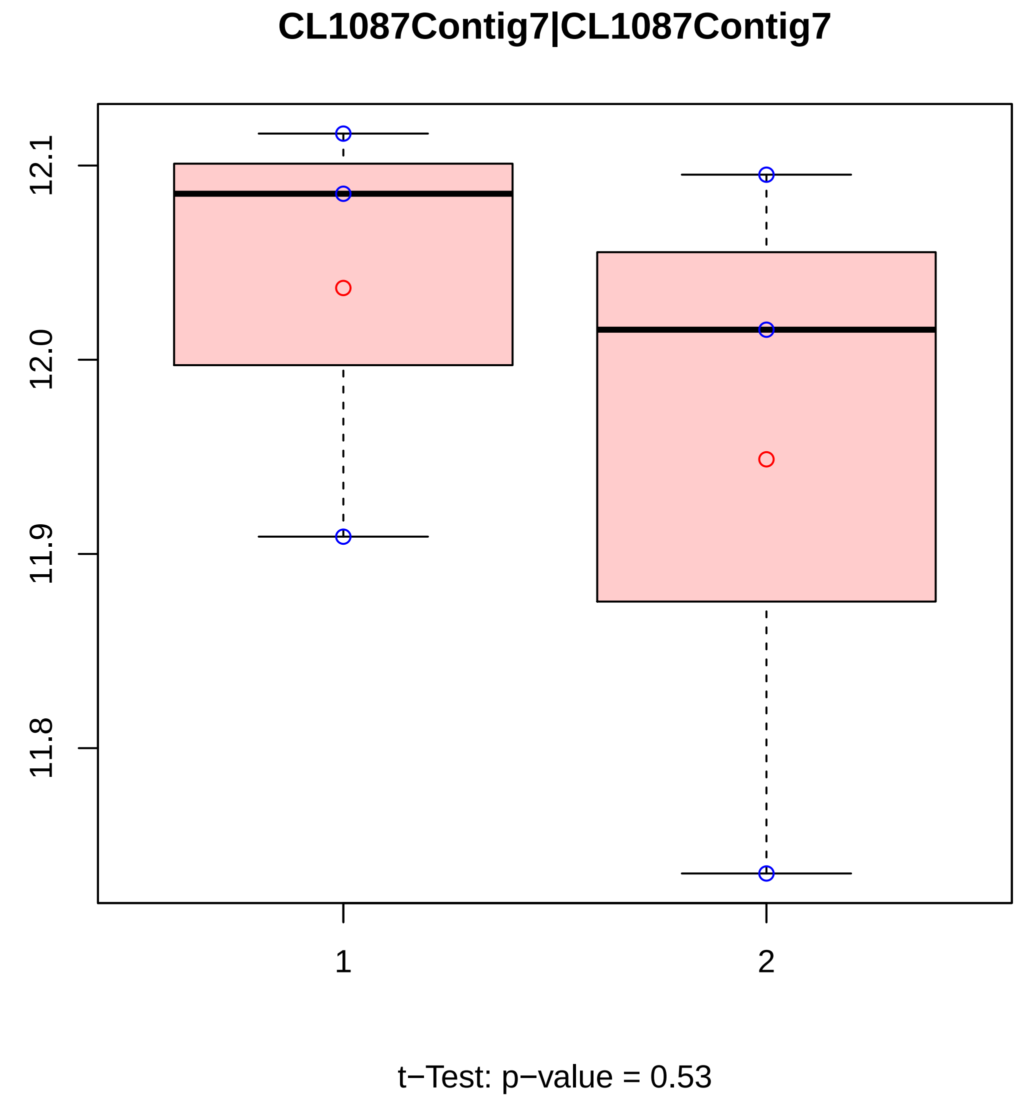
CL1087Contig7|CL1087Contig7
12.1
12.0
11.9
11.8
1
2
t−Test: p−value = 0.53

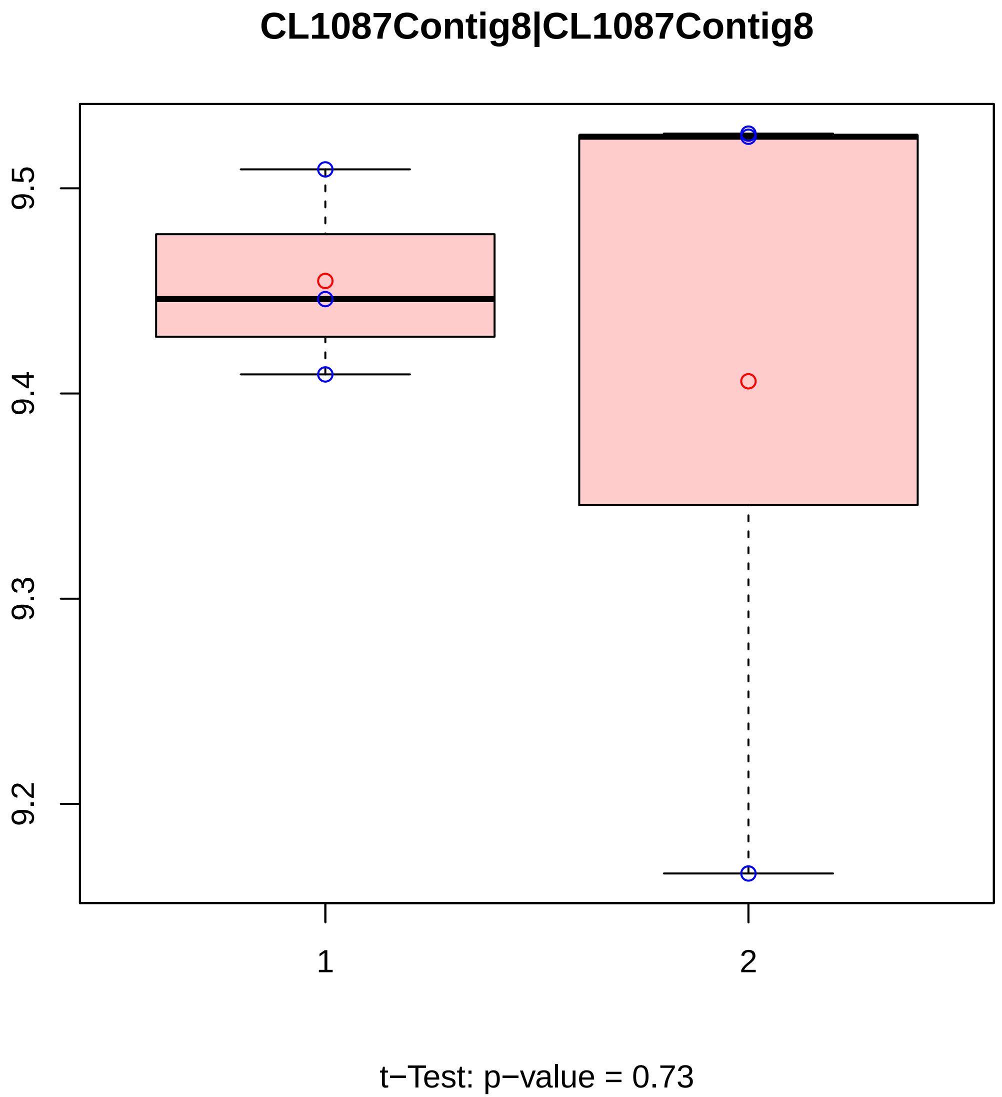
CL1087Contig8|CL1087Contig8
9.2
9.3
9.4
9.5
1
2
t−Test: p−value = 0.73

## CL10896Contig1|CL10896Contig1

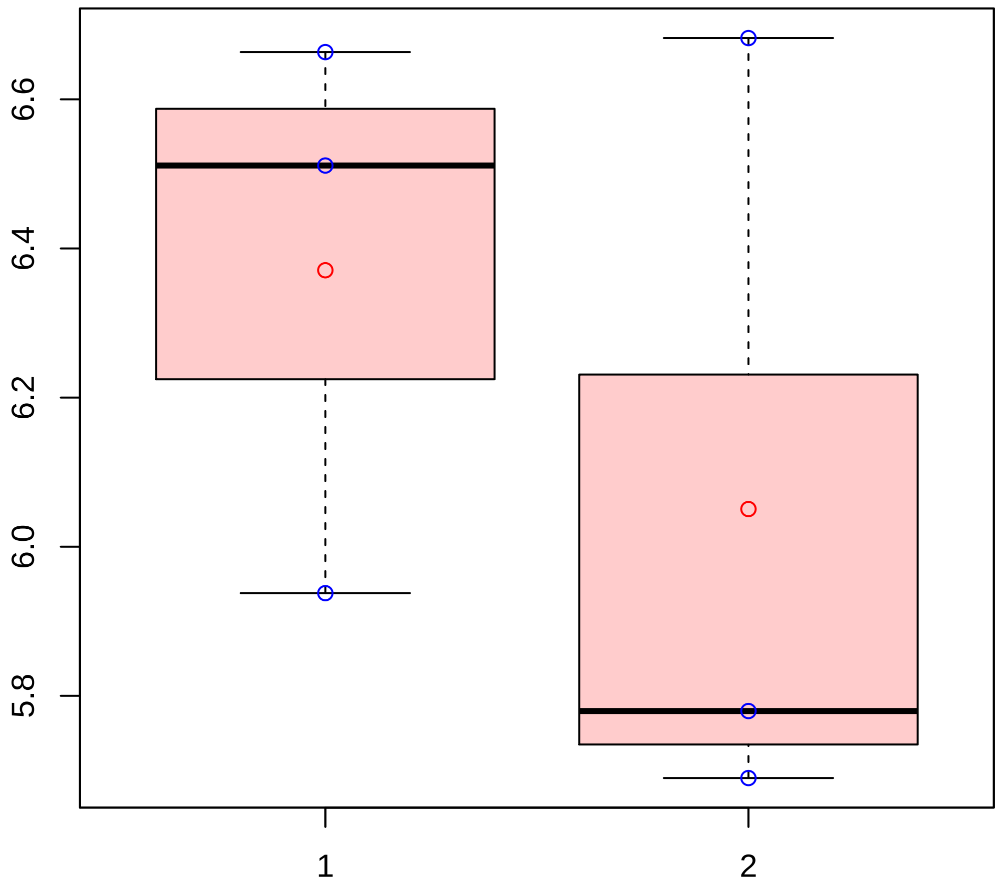


t−Test: p−value = 0.46

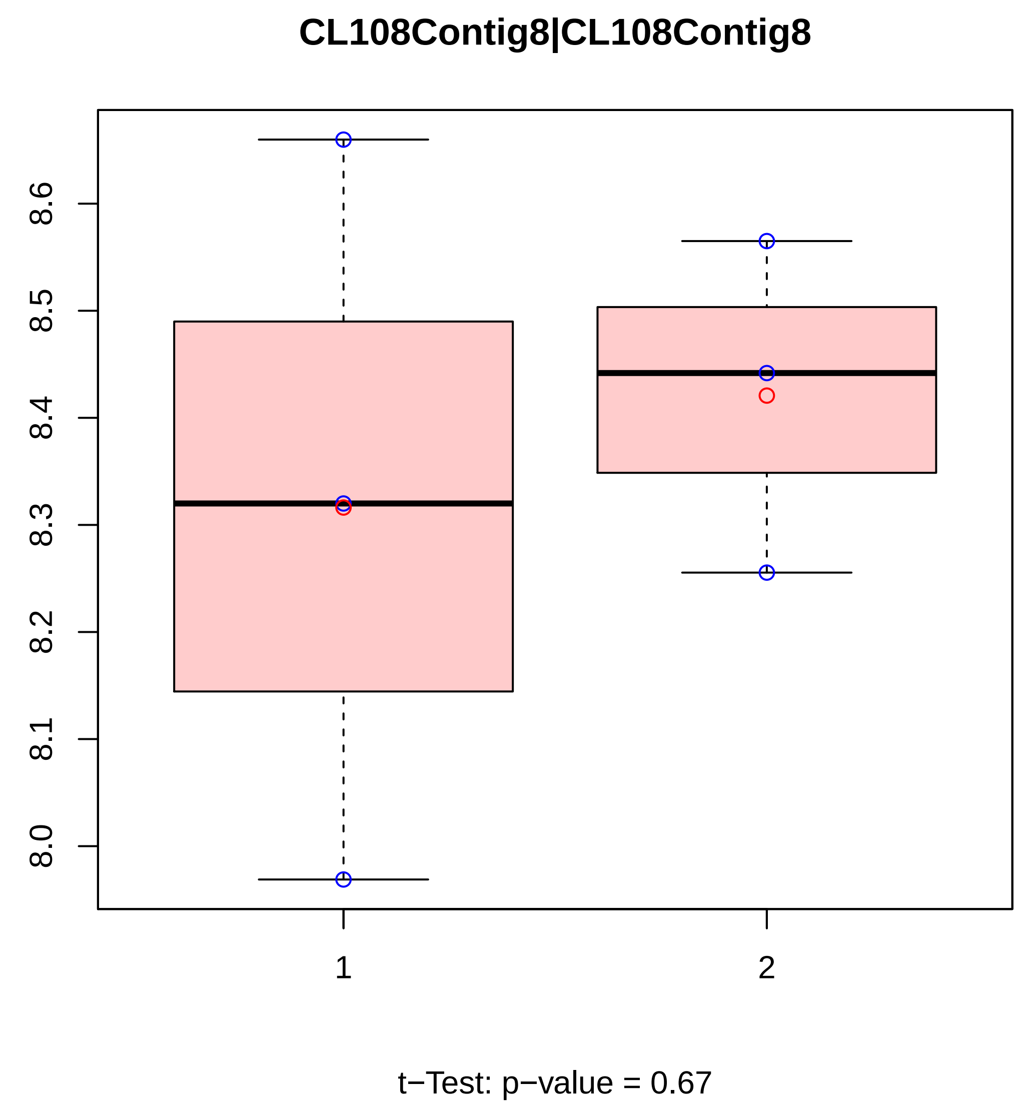
CL108Contig8|CL108Contig8
8.0
8.1
8.2
8.3
8.4
8.5
8.6
1
2
t−Test: p−value = 0.67

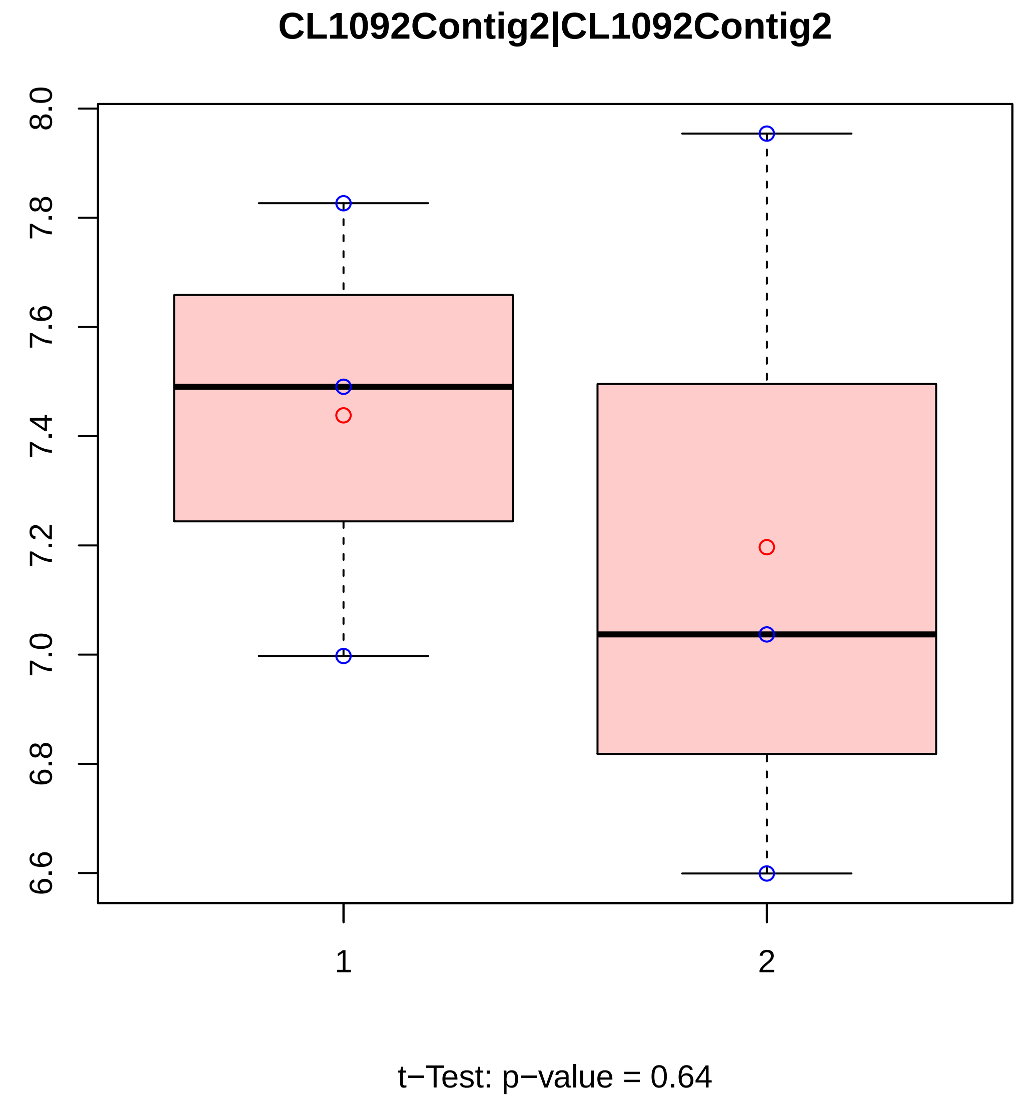
CL1092Contig2|CL1092Contig2
8.0
7.8
7.6
7.4
7.2
7.0
6.8
6.6
1
2
t−Test: p−value = 0.64

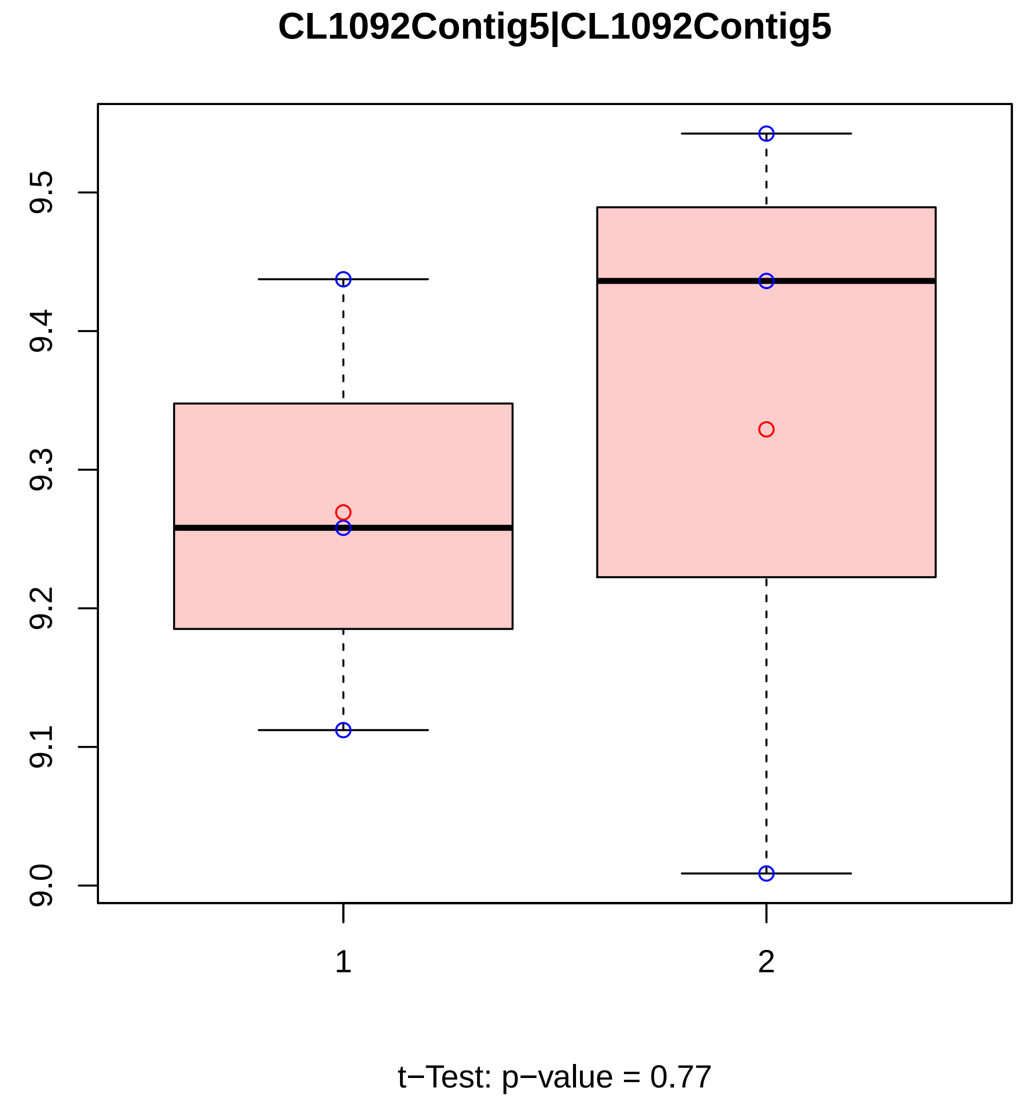
CL1092Contig5|CL1092Contig5
9.0
9.1
9.2
9.3
9.4
9.5
1
2
t−Test: p−value = 0.77

## CL10931Contig2|CL10931Contig2

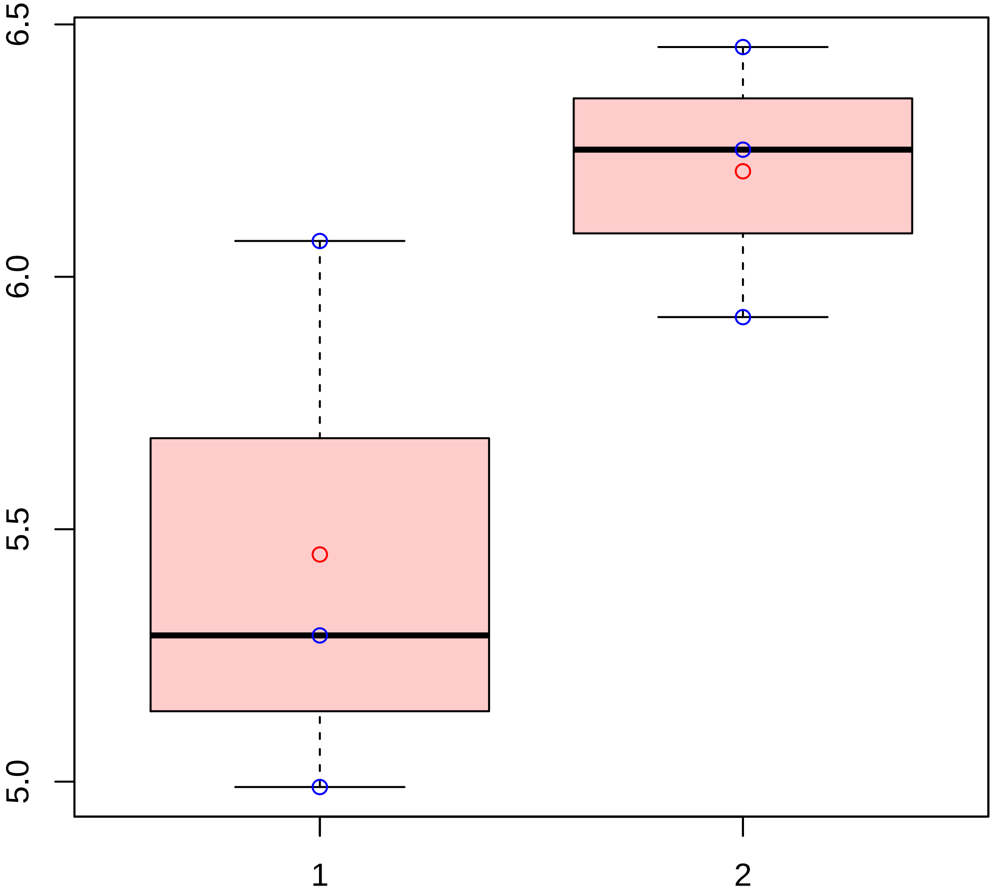


t−Test: p−value = 0.13

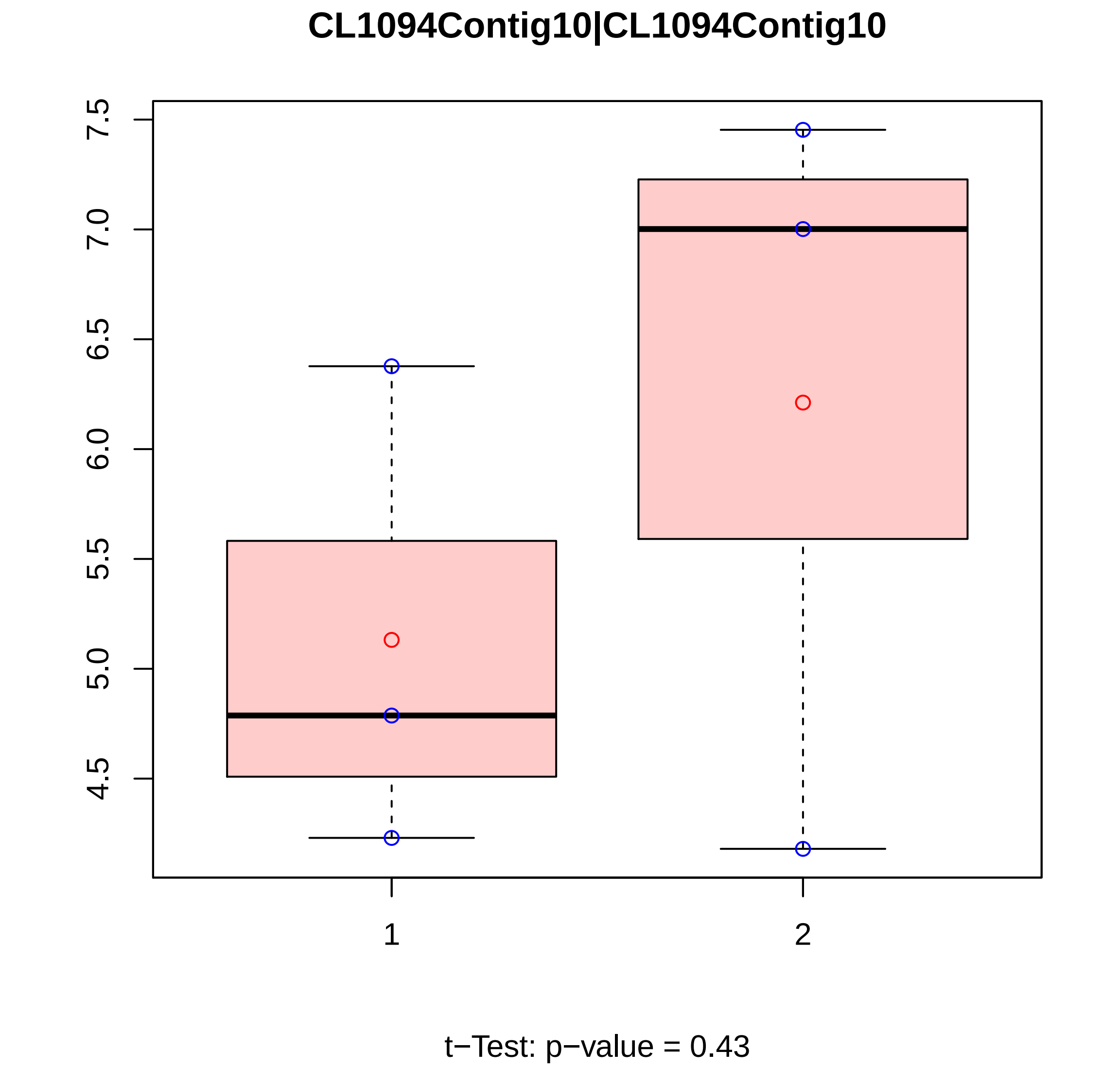
CL1094Contig10|CL1094Contig10
7.5
7.0
6.5
6.0
5.5
5.0
4.5
1
2
t−Test: p−value = 0.43

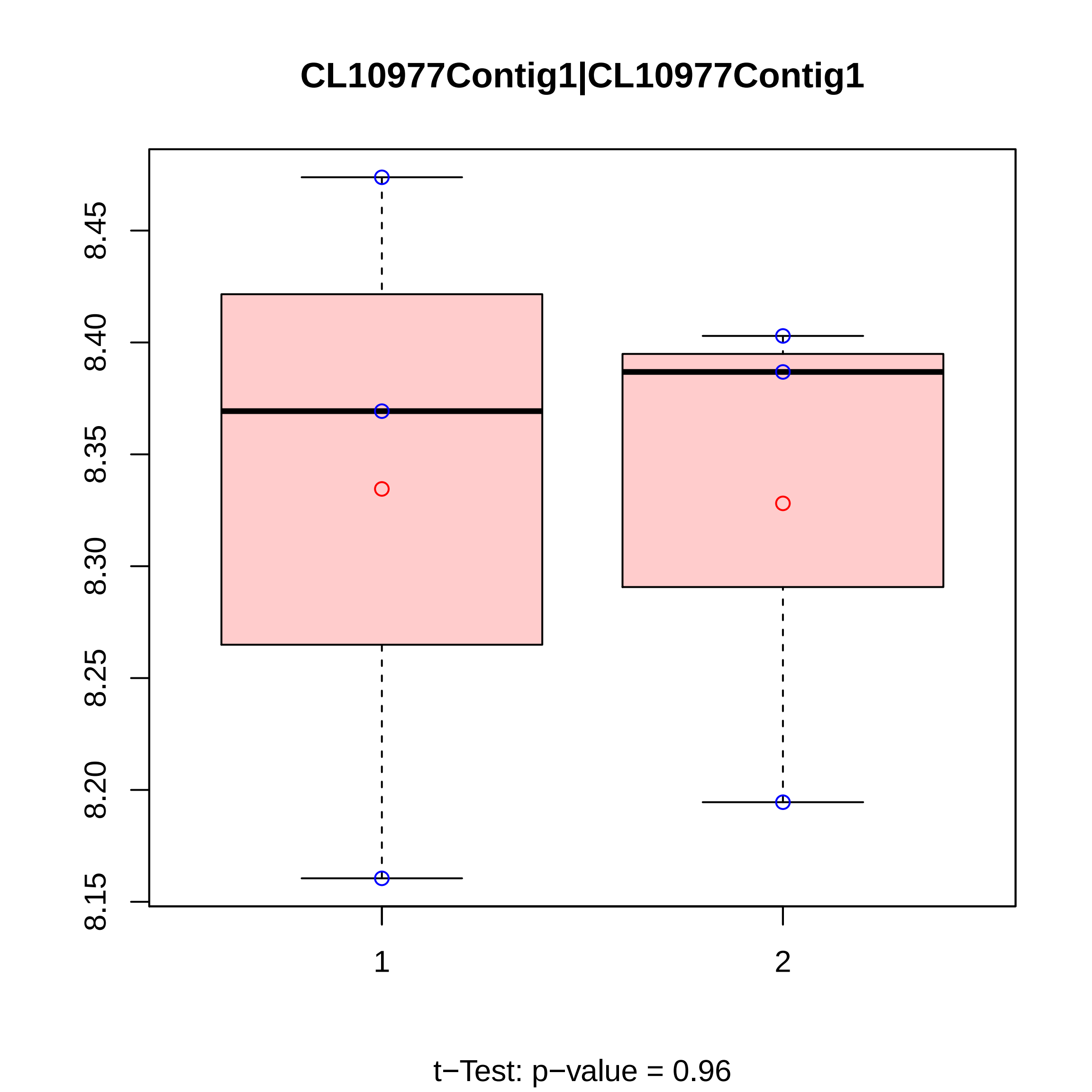
CL10977Contig1|CL10977Contig1
8.15
8.20
8.25
8.30
8.35
8.40
8.45
1
2
t−Test: p−value = 0.96

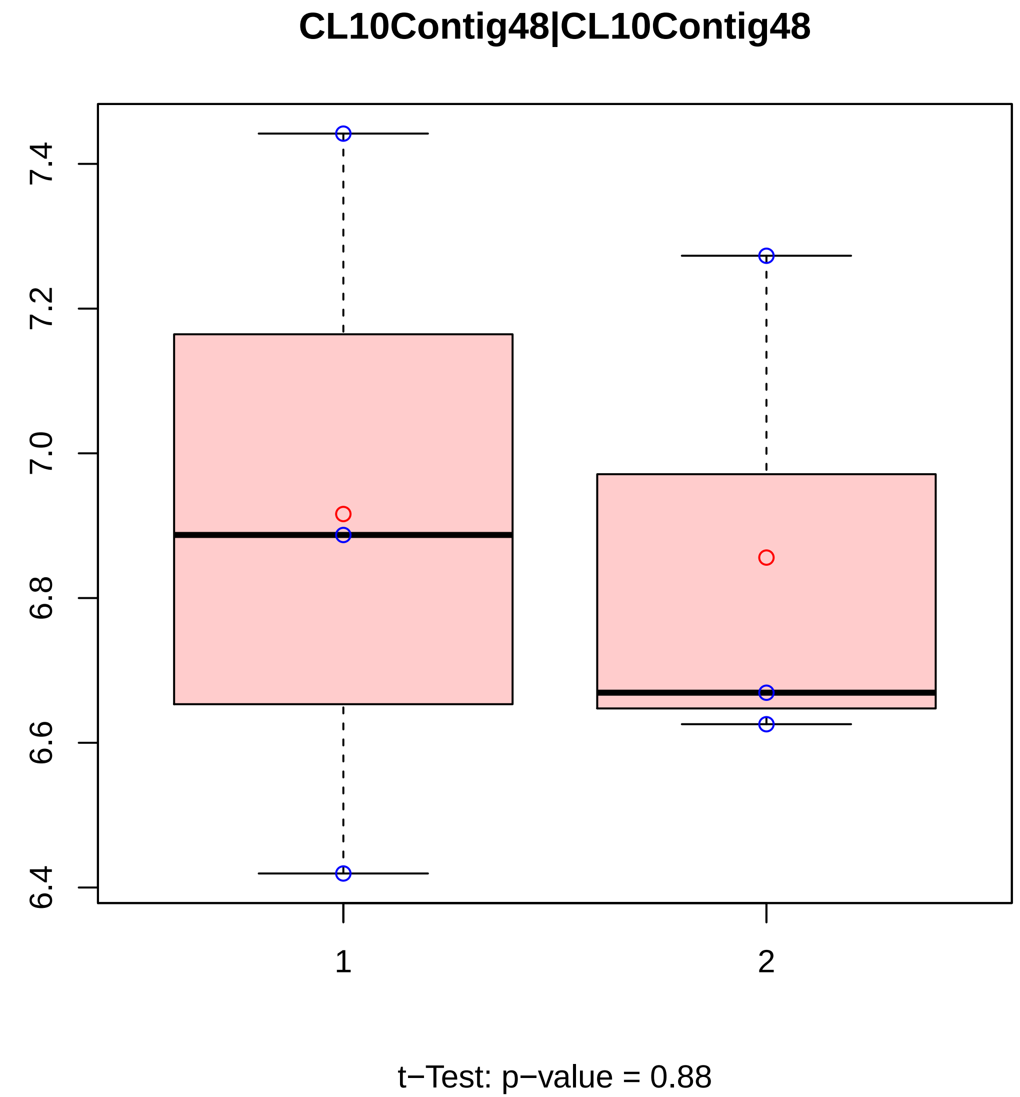
CL10Contig48|CL10Contig48
7.4
7.2
7.0
6.8
6.6
6.4
1
2
t−Test: p−value = 0.88

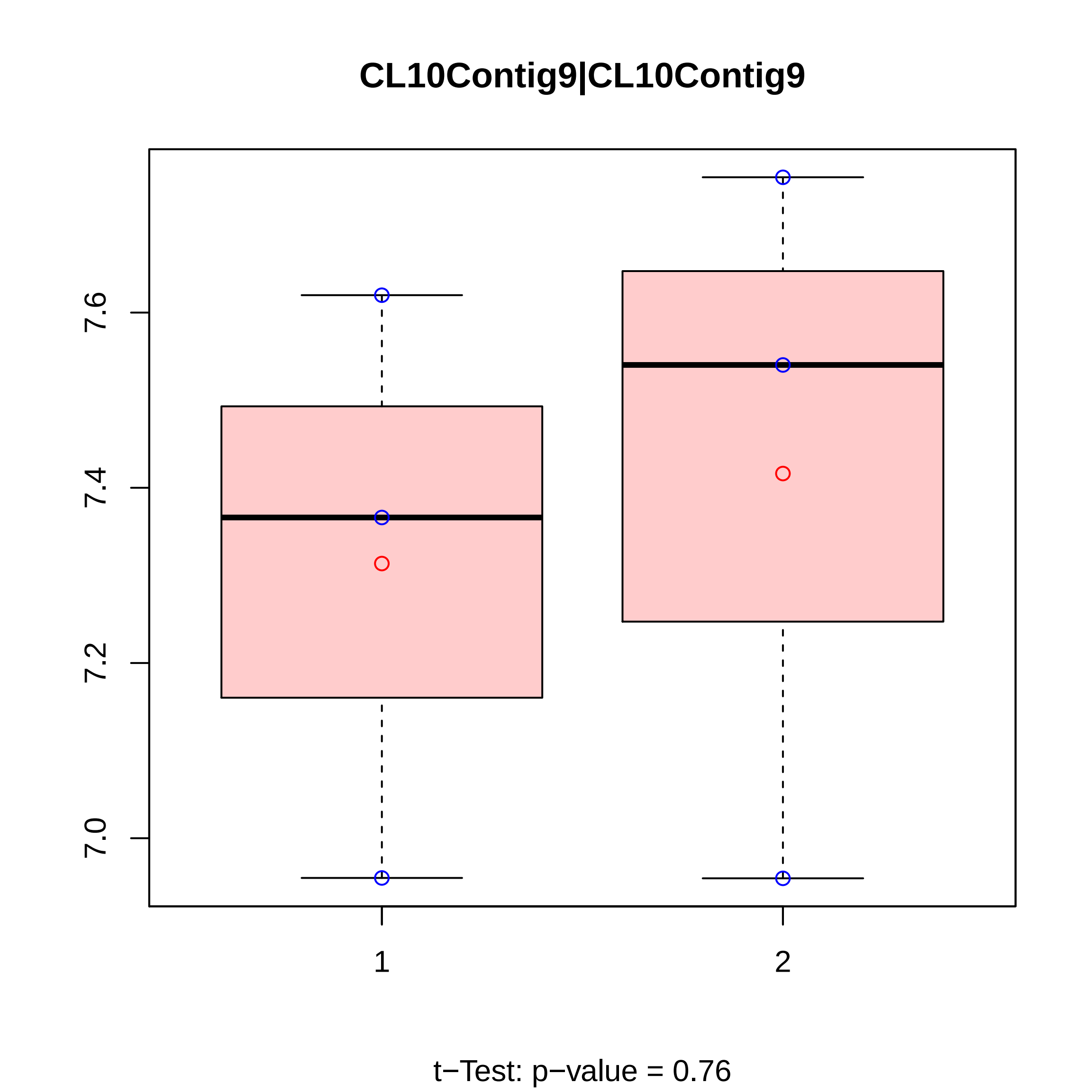
CL10Contig9|CL10Contig9
7.0
7.2
7.4
7.6
1
2
t−Test: p−value = 0.76

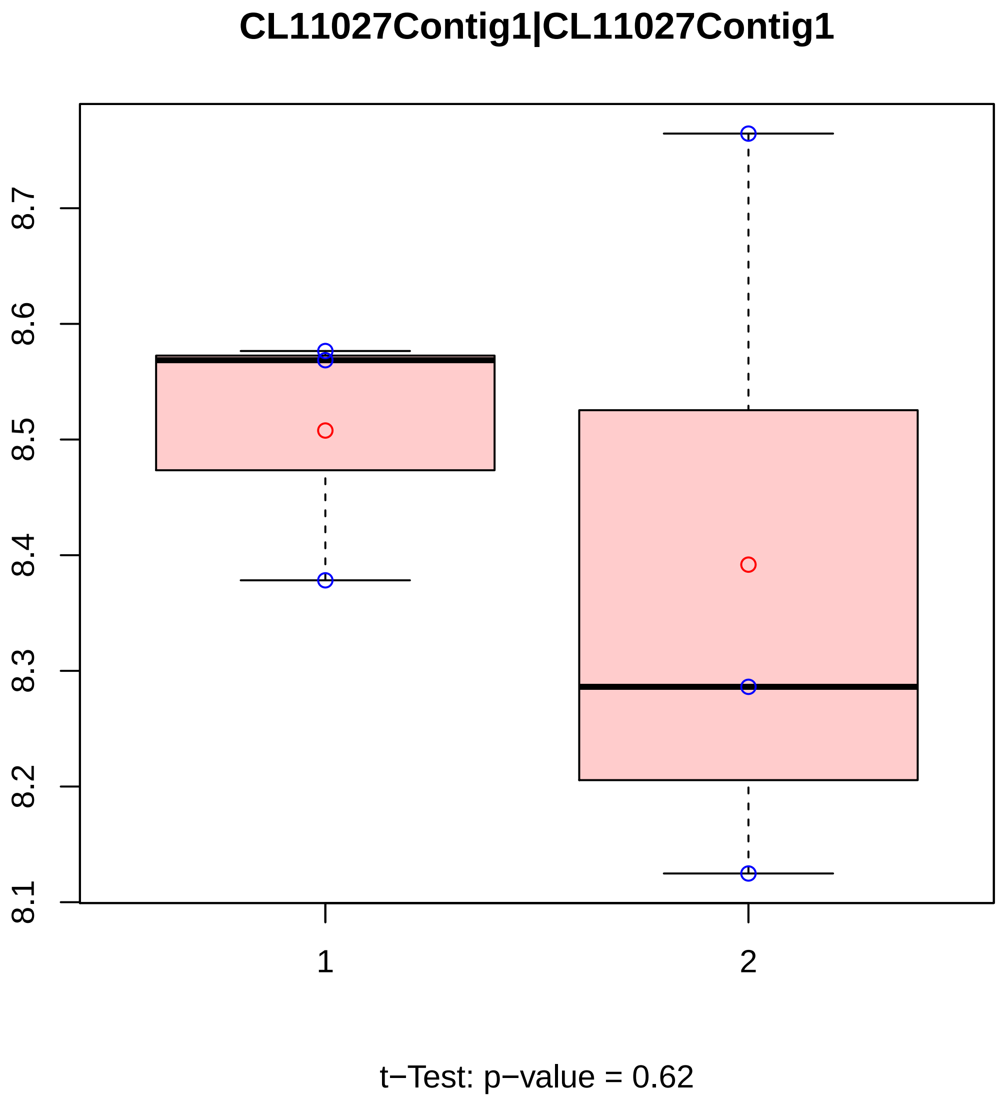
CL11027Contig1|CL11027Contig1
8.1
8.2
8.3
8.4
8.5
8.6
8.7
1
2
t−Test: p−value = 0.62

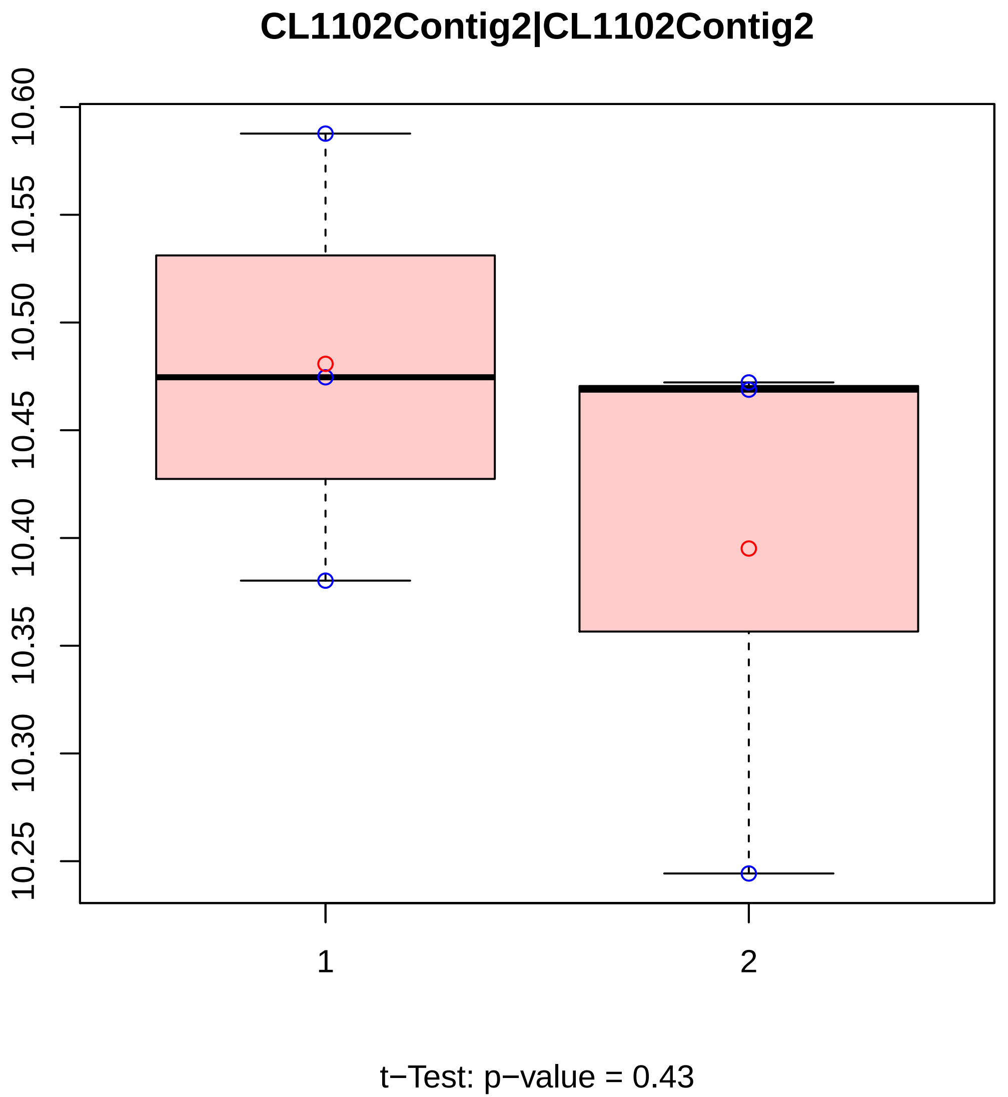
CL1102Contig2|CL1102Contig2
10.60
10.55
10.50
10.45
10.40
10.35
10.30
10.25
1
2
t−Test: p−value = 0.43

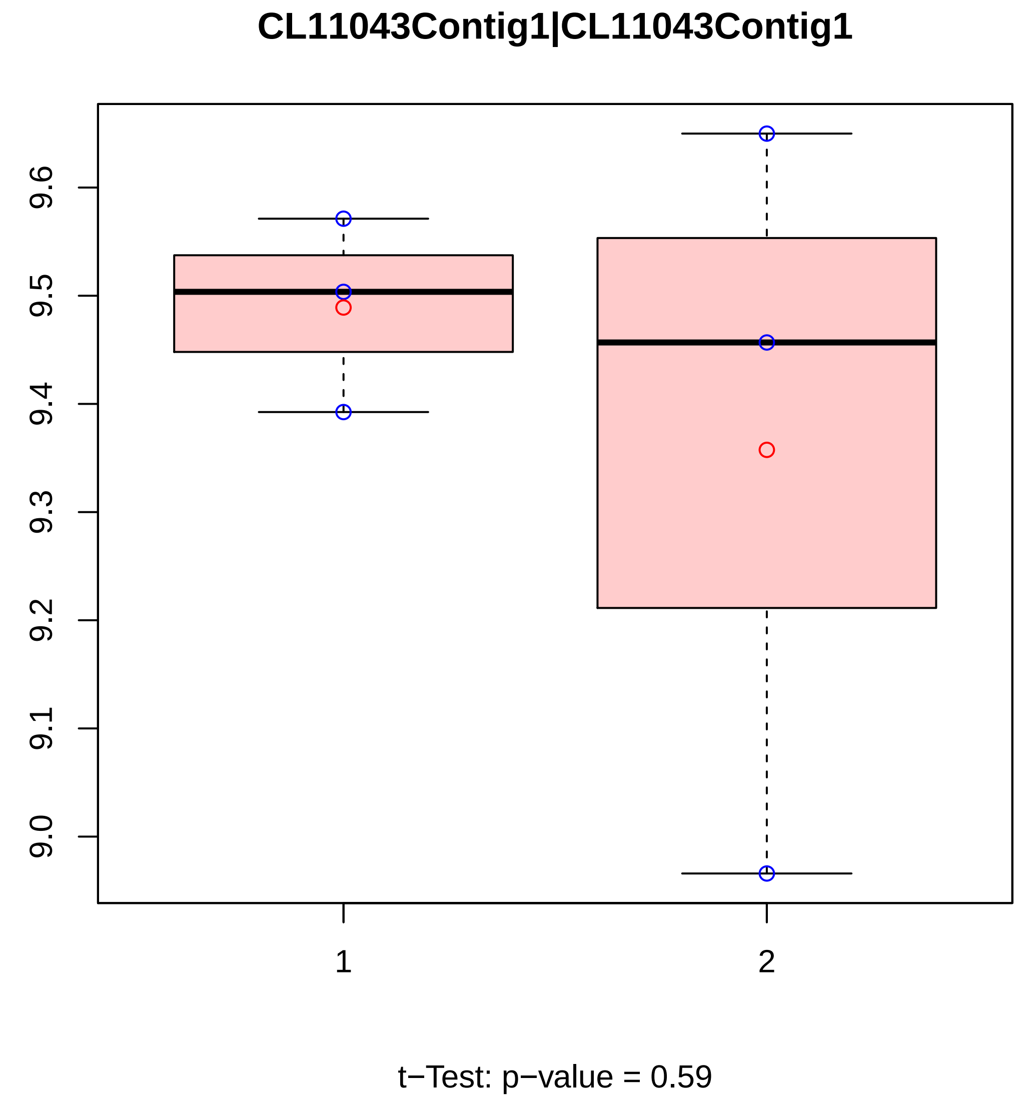
CL11043Contig1|CL11043Contig1
9.0
9.1
9.2
9.3
9.4
9.5
9.6
1
2
t−Test: p−value = 0.59

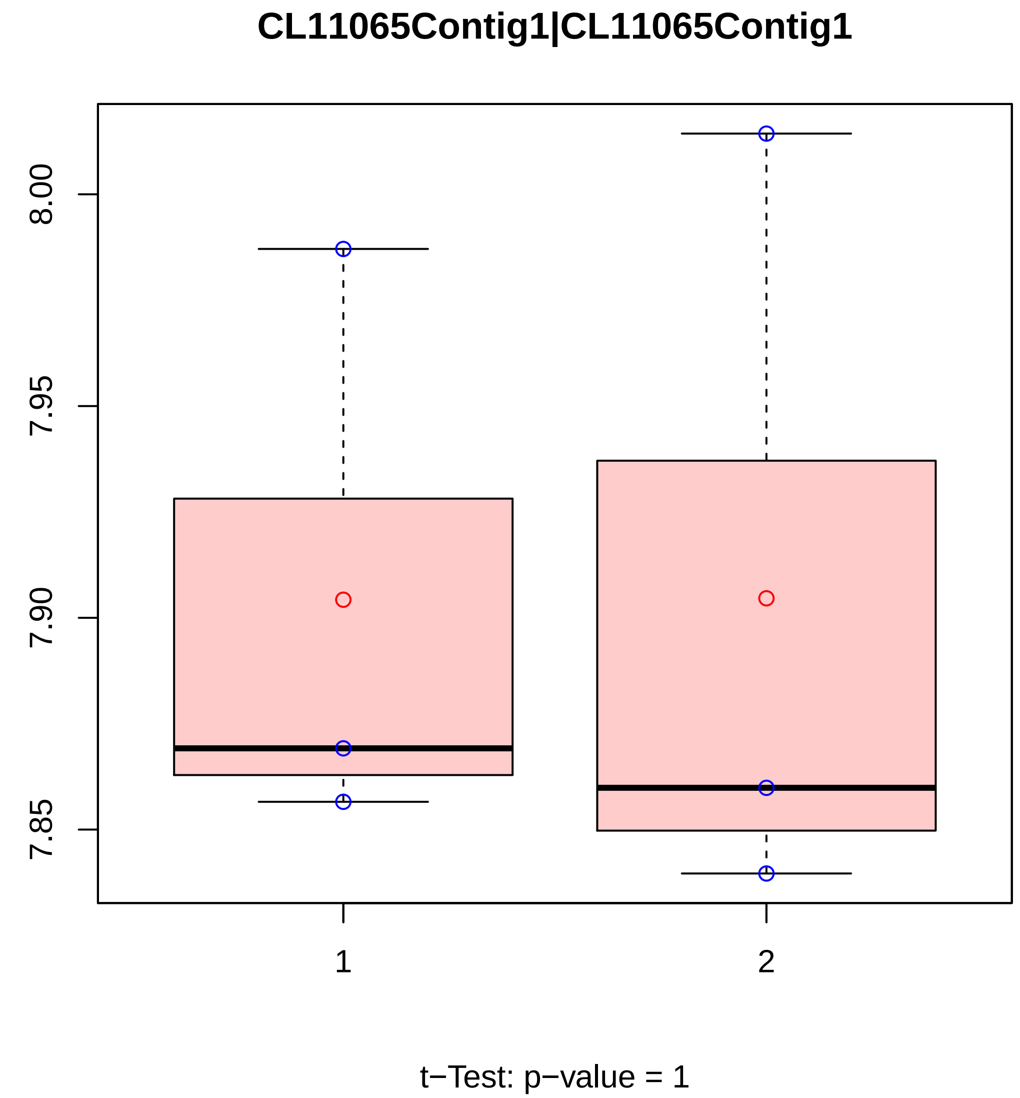
CL11065Contig1|CL11065Contig1
8.00
7.95
7.90
7.85
1
2
t−Test: p−value = 1

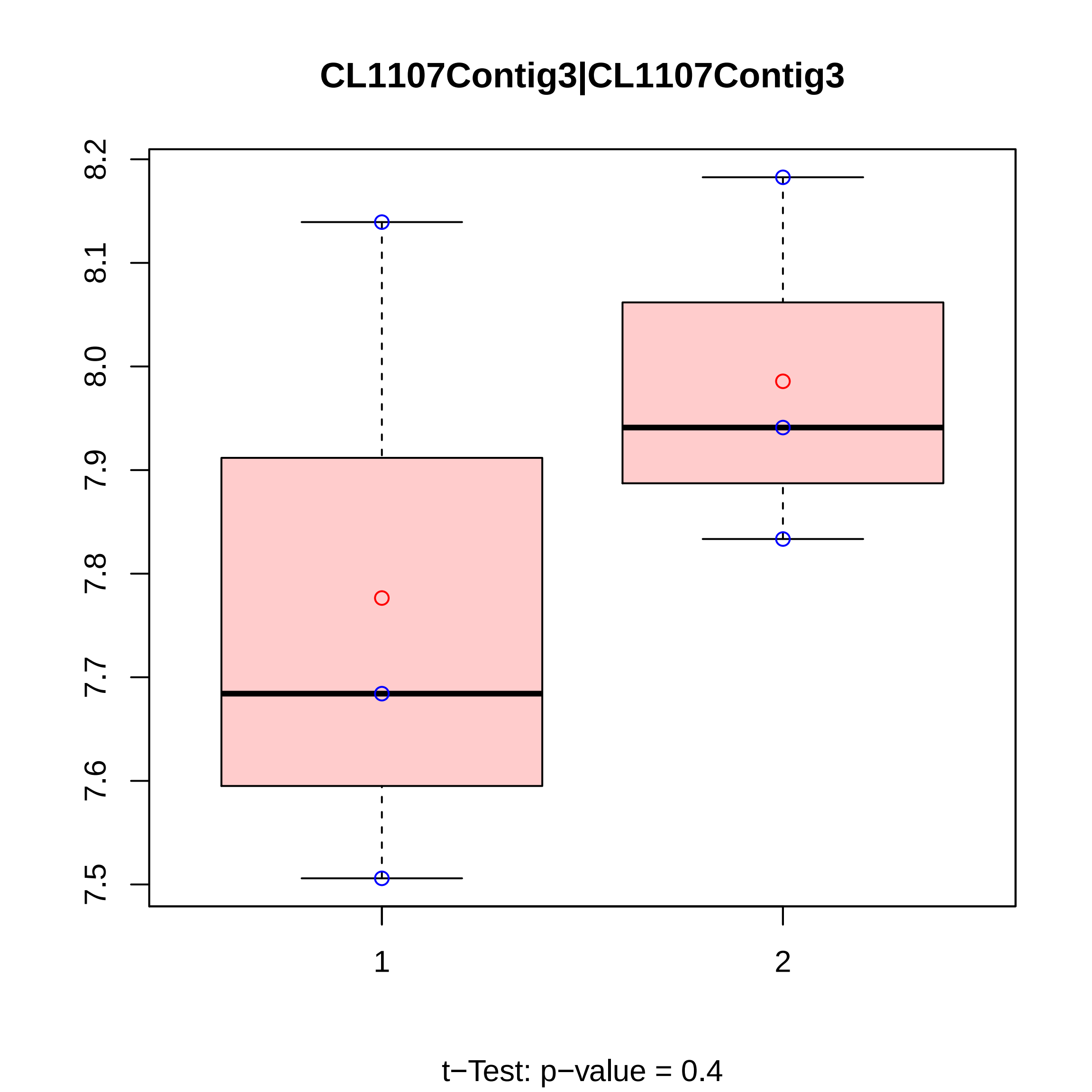
CL1107Contig3|CL1107Contig3
8.2
8.1
8.0
7.9
7.8
7.7
7.6
7.5
1
2
t−Test: p−value = 0.4

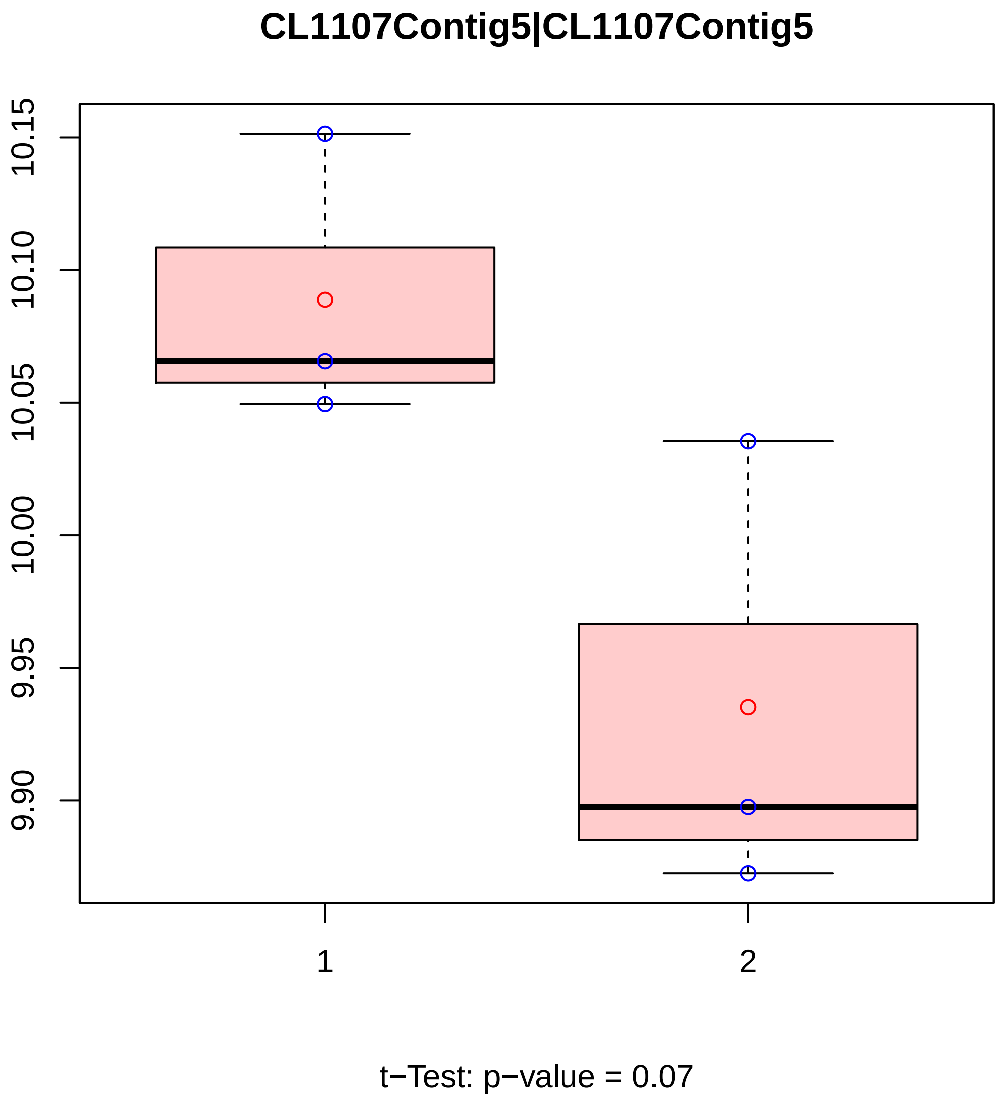
CL1107Contig5|CL1107Contig5
10.15
10.10
10.05
10.00
9.95
9.90
1
2
t−Test: p−value = 0.07

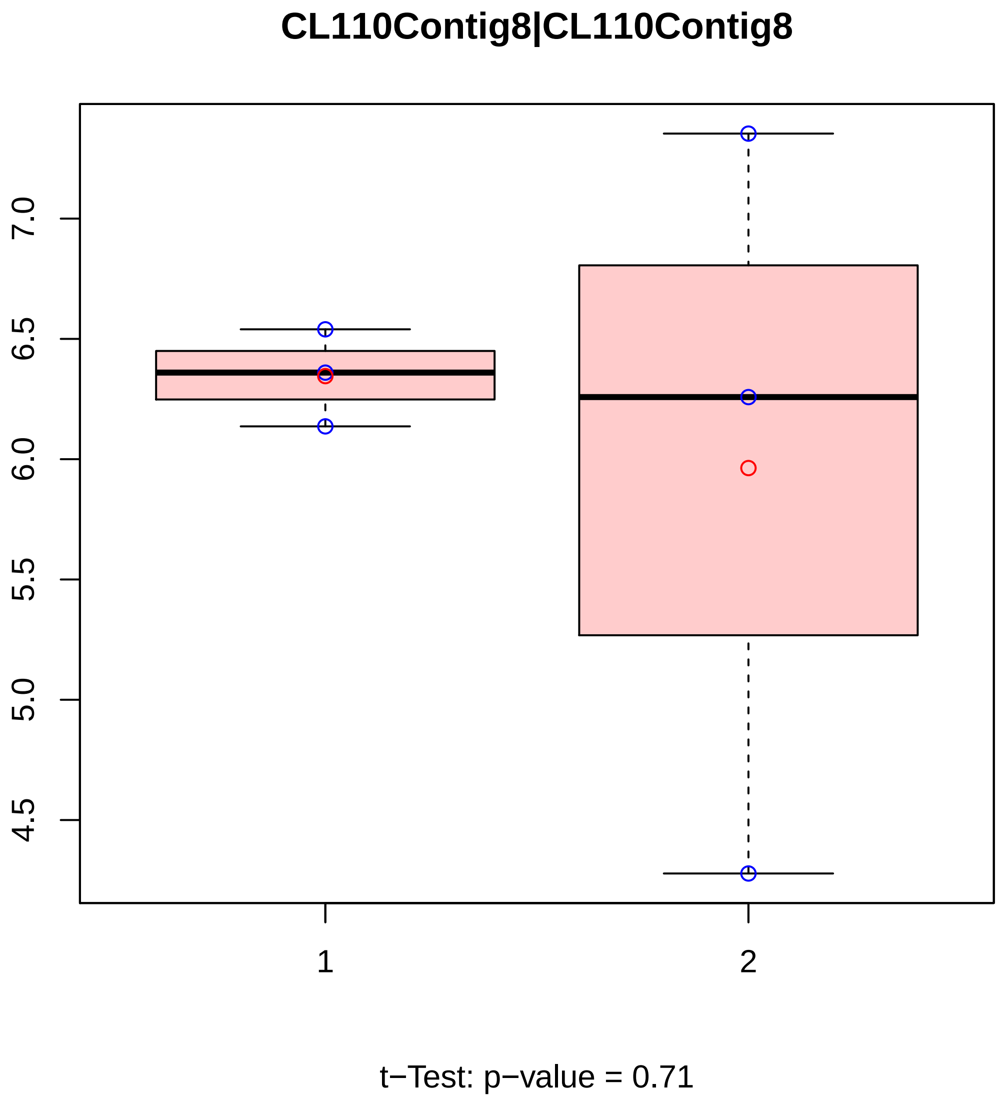

CL110Contig8|CL110Contig8
7.0
6.5
6.0
5.5
5.0
4.5
1
2
t−Test: p−value = 0.71

# CL11137Contig2|CL11137Contig2

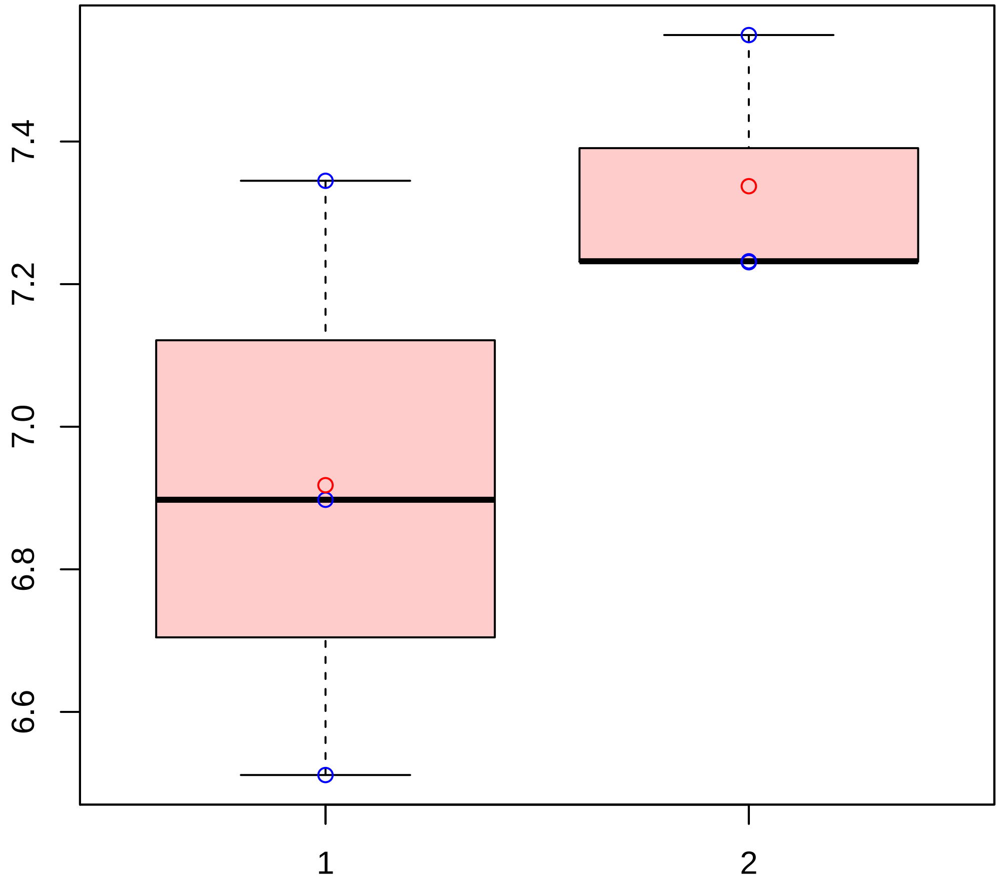


t−Test: p−value = 0.22

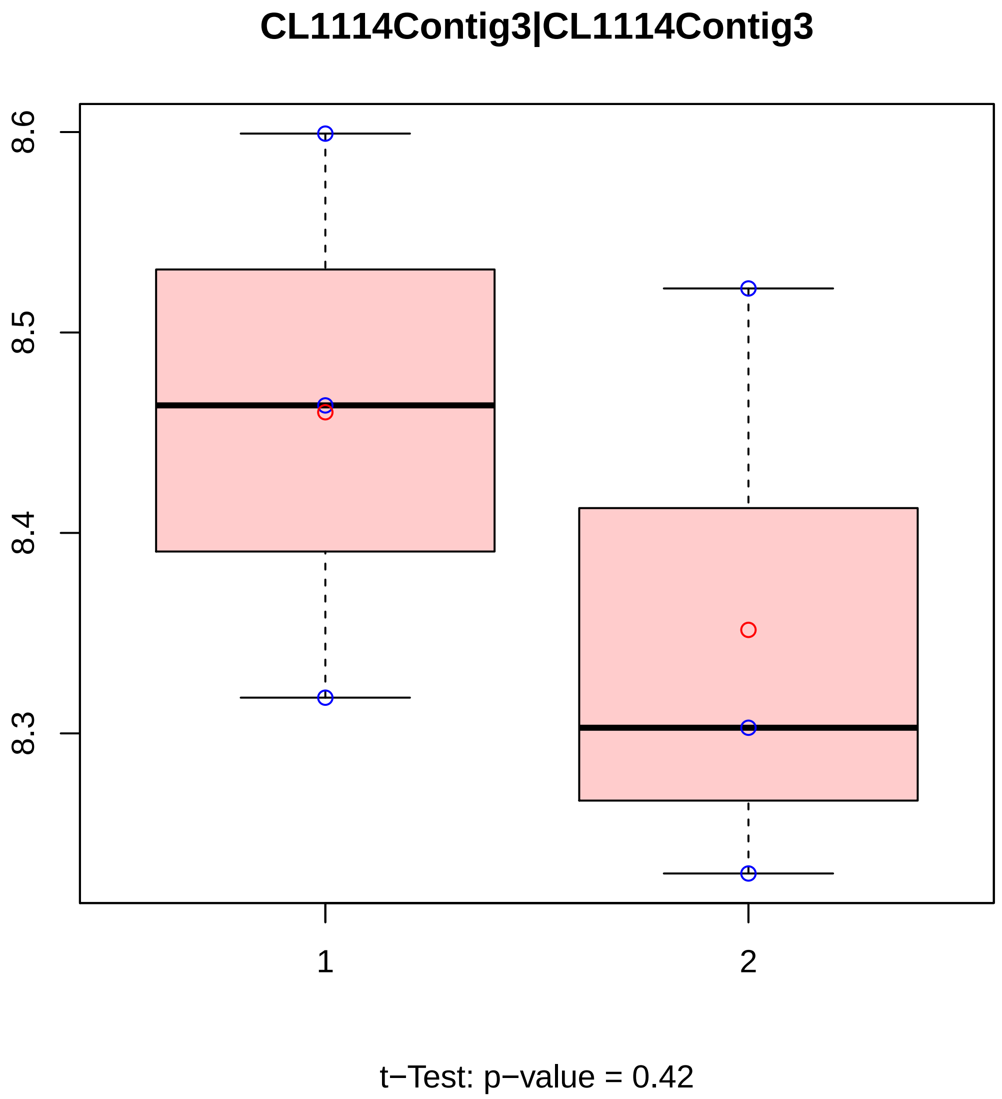
CL1114Contig3|CL1114Contig3
8.6
8.5
8.4
8.3
1
2
t−Test: p−value = 0.42

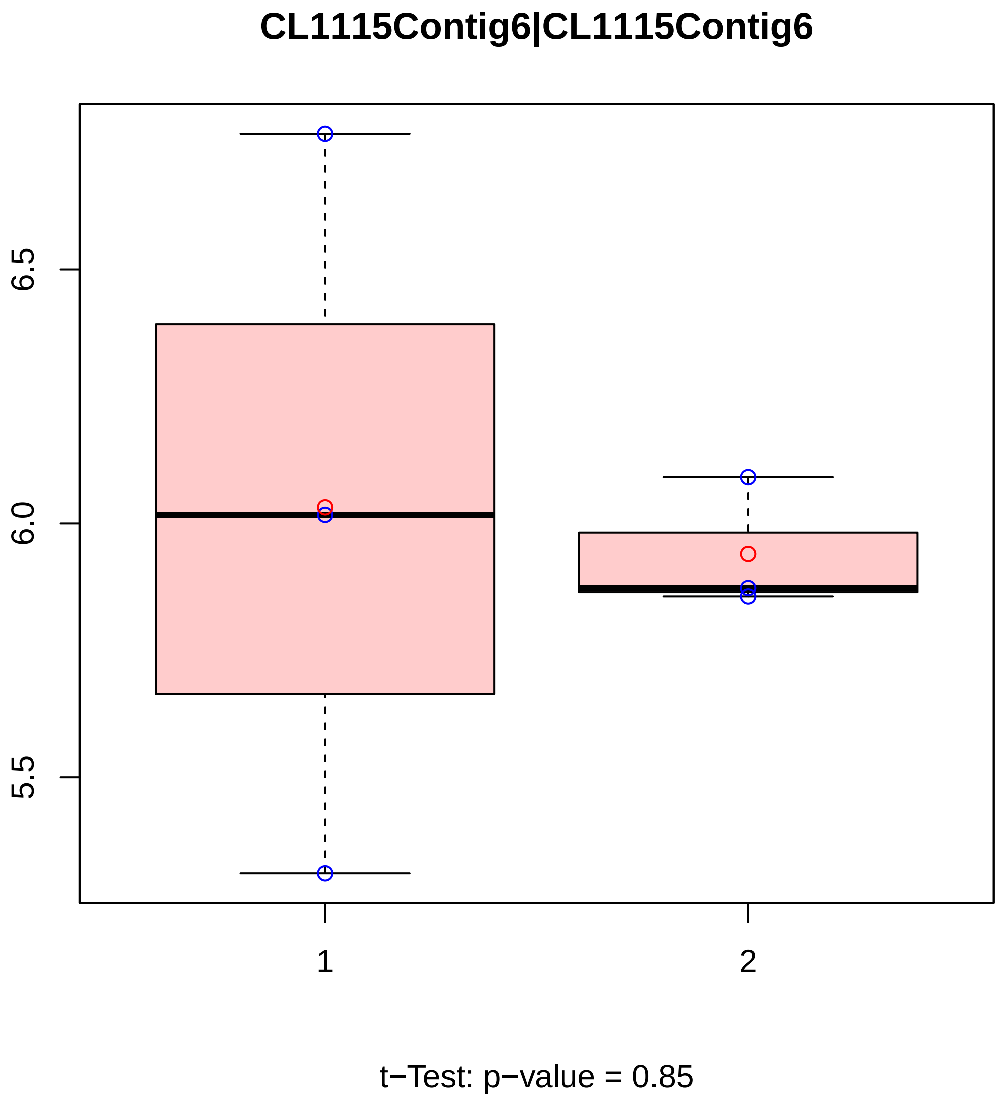
CL1115Contig6|CL1115Contig6
6.5
6.0
5.5
1
2
t−Test: p−value = 0.85

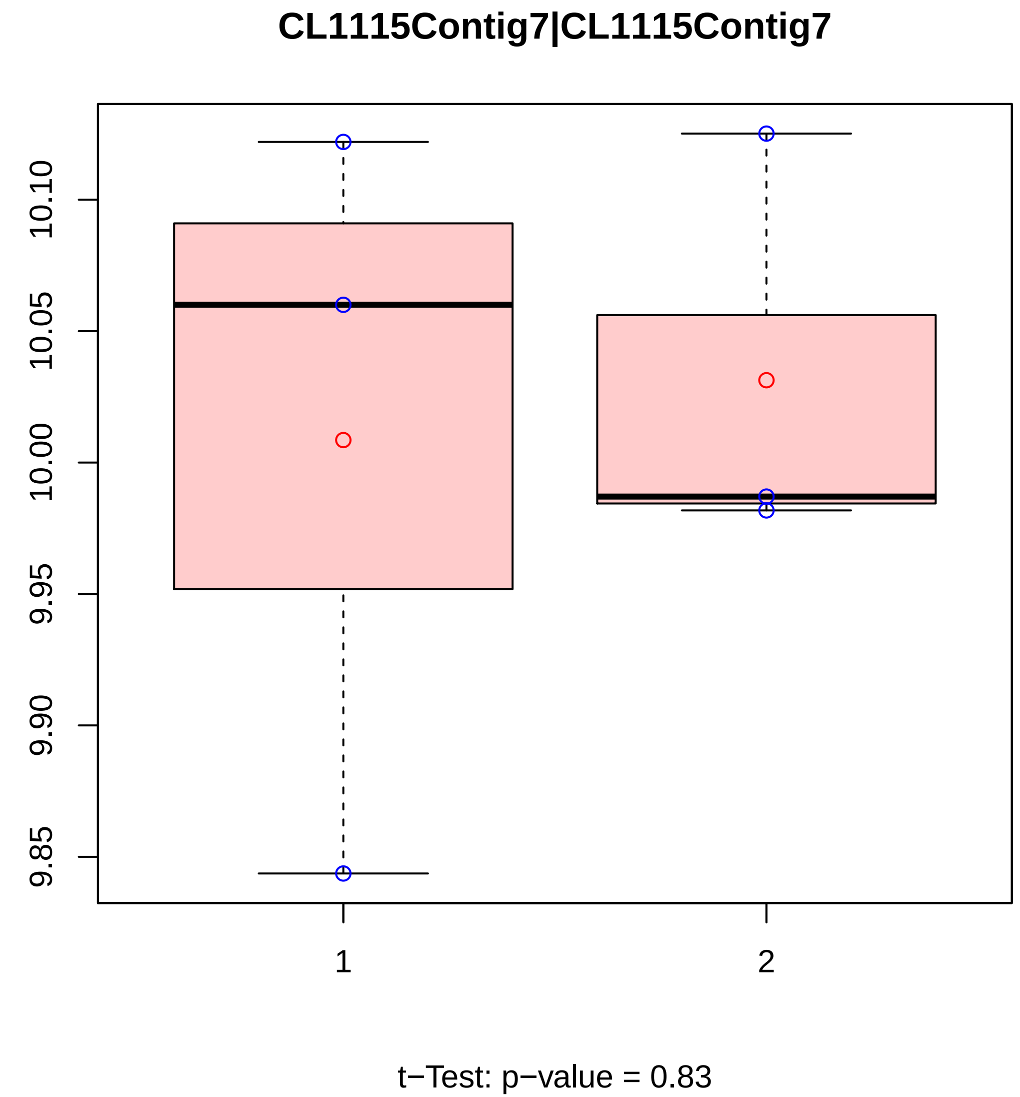
CL1115Contig7|CL1115Contig7
10.10
10.05
10.00
9.95
9.90
9.85
1
2
t−Test: p−value = 0.83

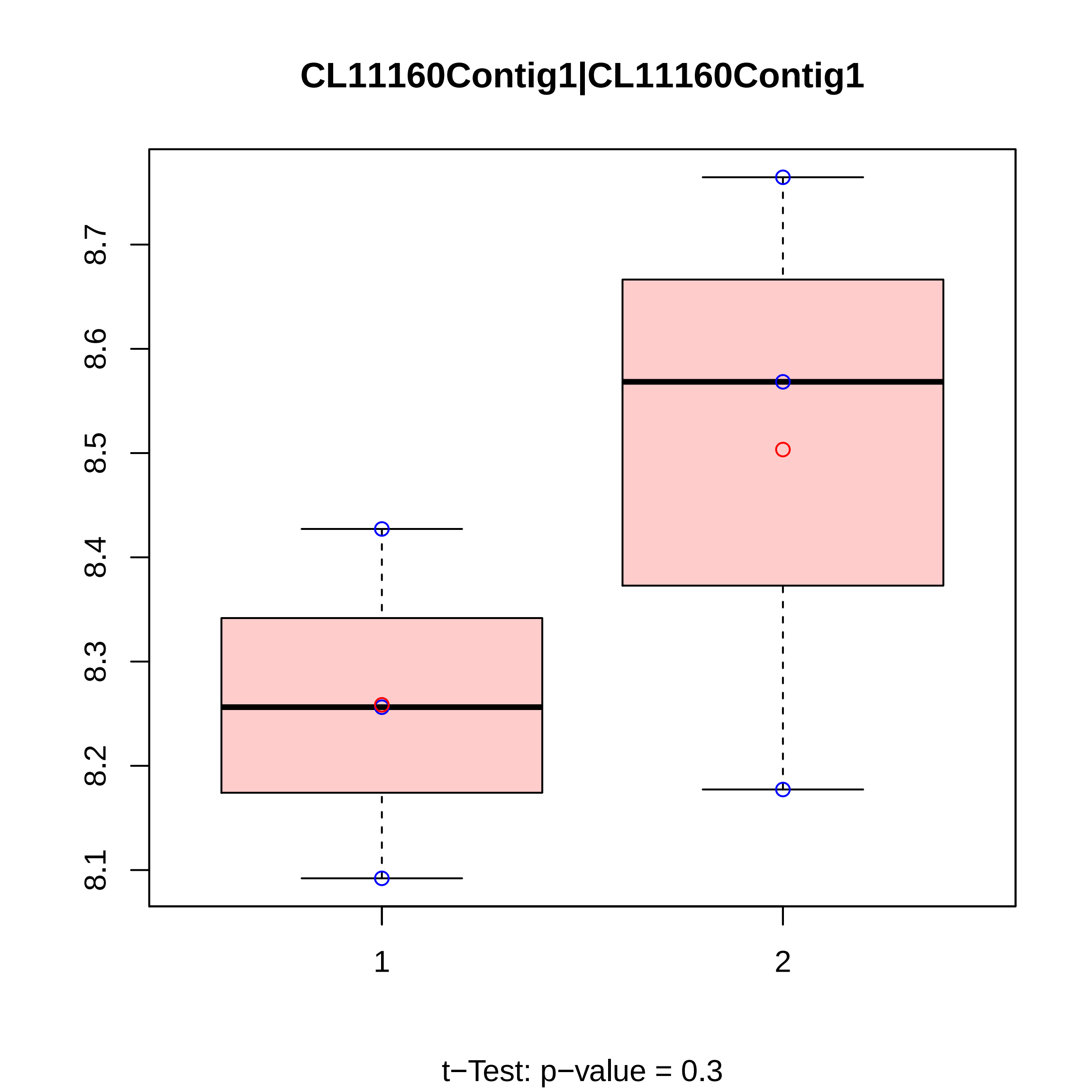

CL11160Contig1|CL11160Contig1
8.1
8.2
8.3
8.4
8.5
8.6
8.7
1
2
t−Test: p−value = 0.3

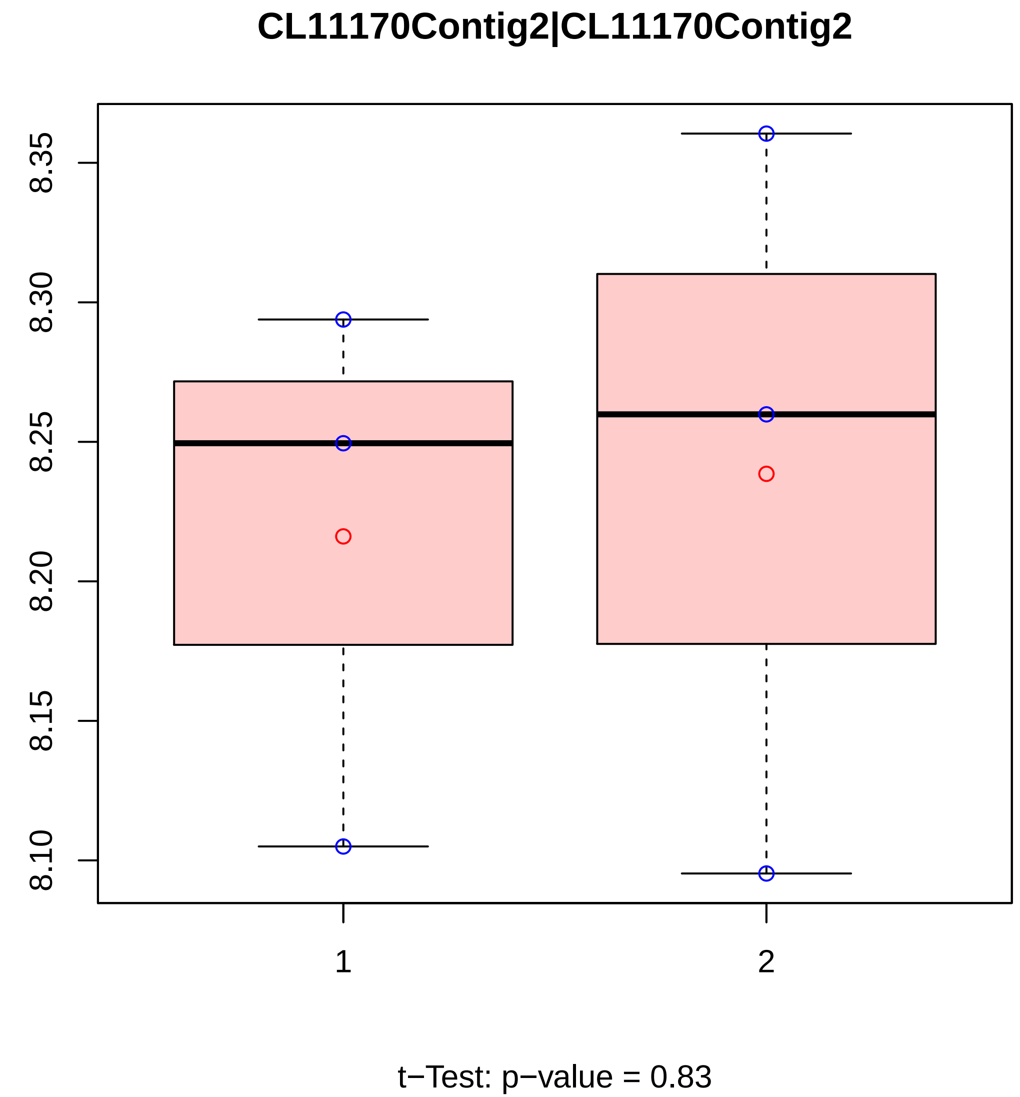
CL11170Contig2|CL11170Contig2
8.10
8.15
8.20
8.25
8.30
8.35
1
2
t−Test: p−value = 0.83

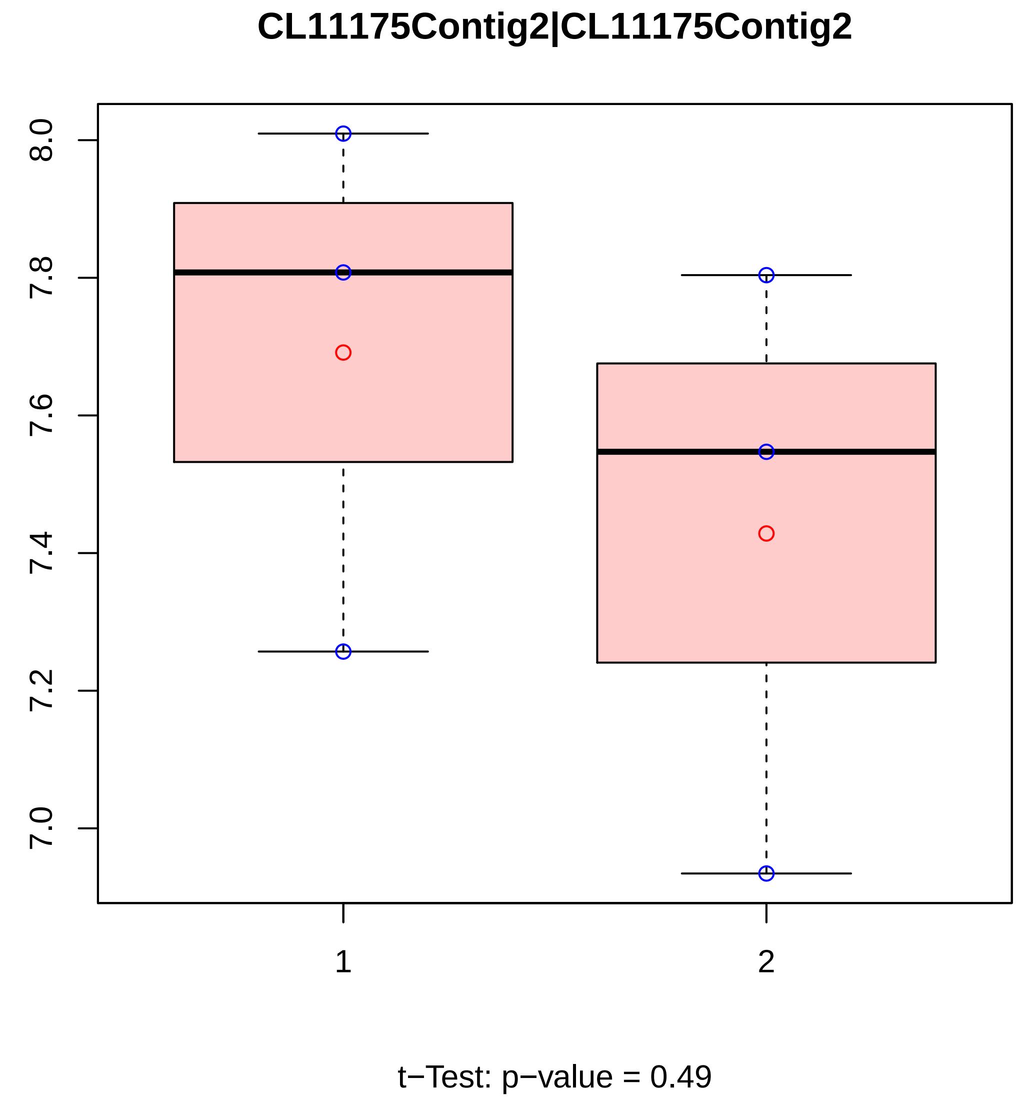

CL11175Contig2|CL11175Contig2
8.0
7.8
7.6
7.4
7.2
7.0
1
2
t−Test: p−value = 0.49

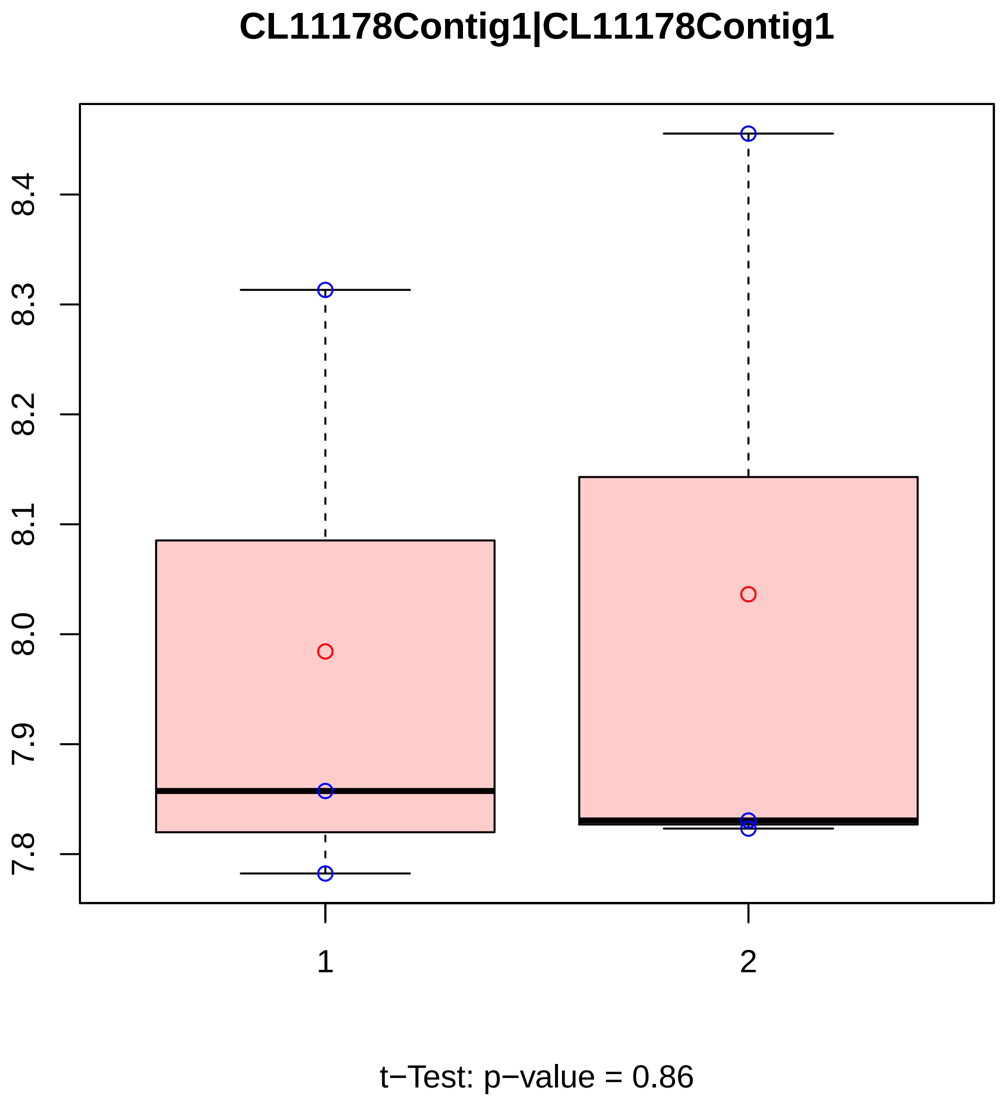
CL11178Contig1|CL11178Contig1
8.4
8.3
8.2
8.1
8.0
7.9
7.8
1
2
t−Test: p−value = 0.86

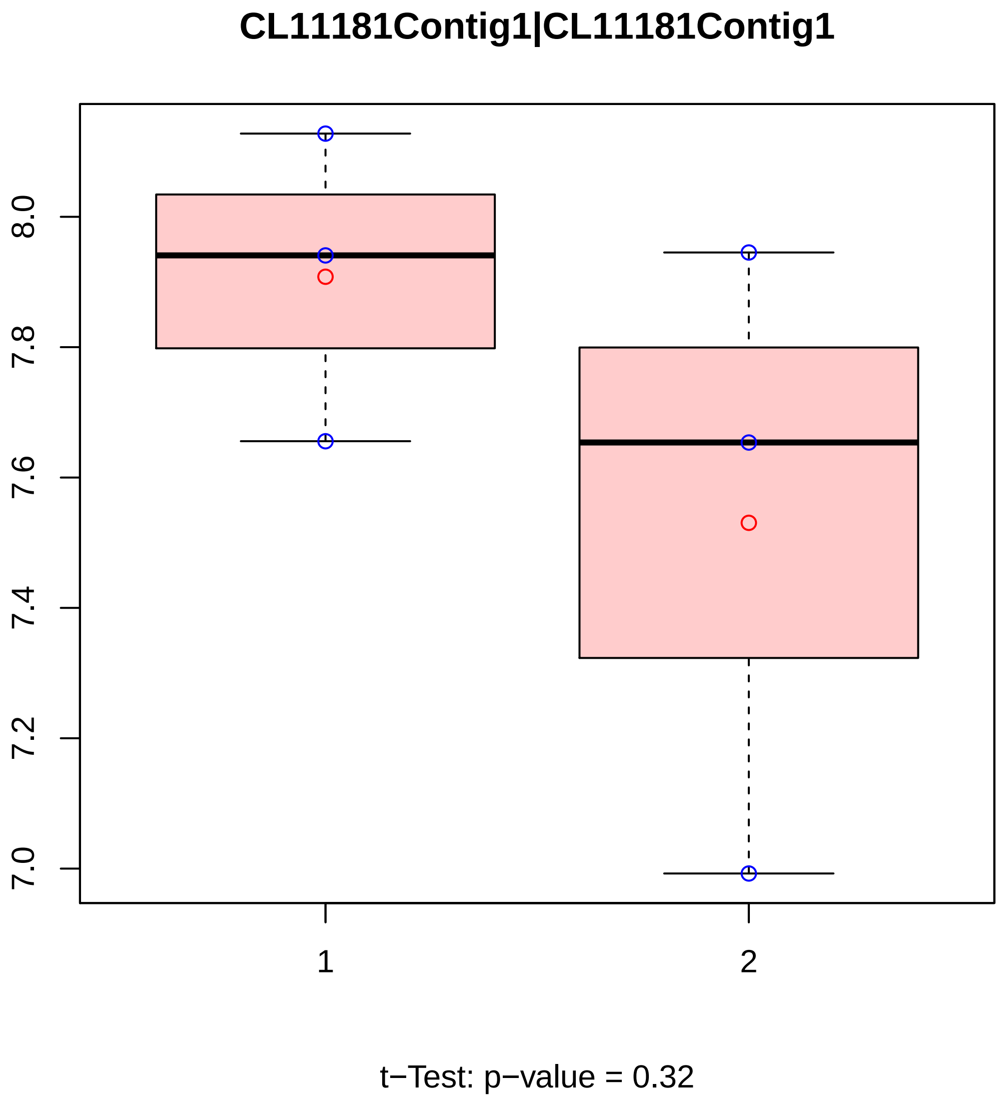
CL11181Contig1|CL11181Contig1
8.0
7.8
7.6
7.4
7.2
7.0
1
2
t−Test: p−value = 0.32

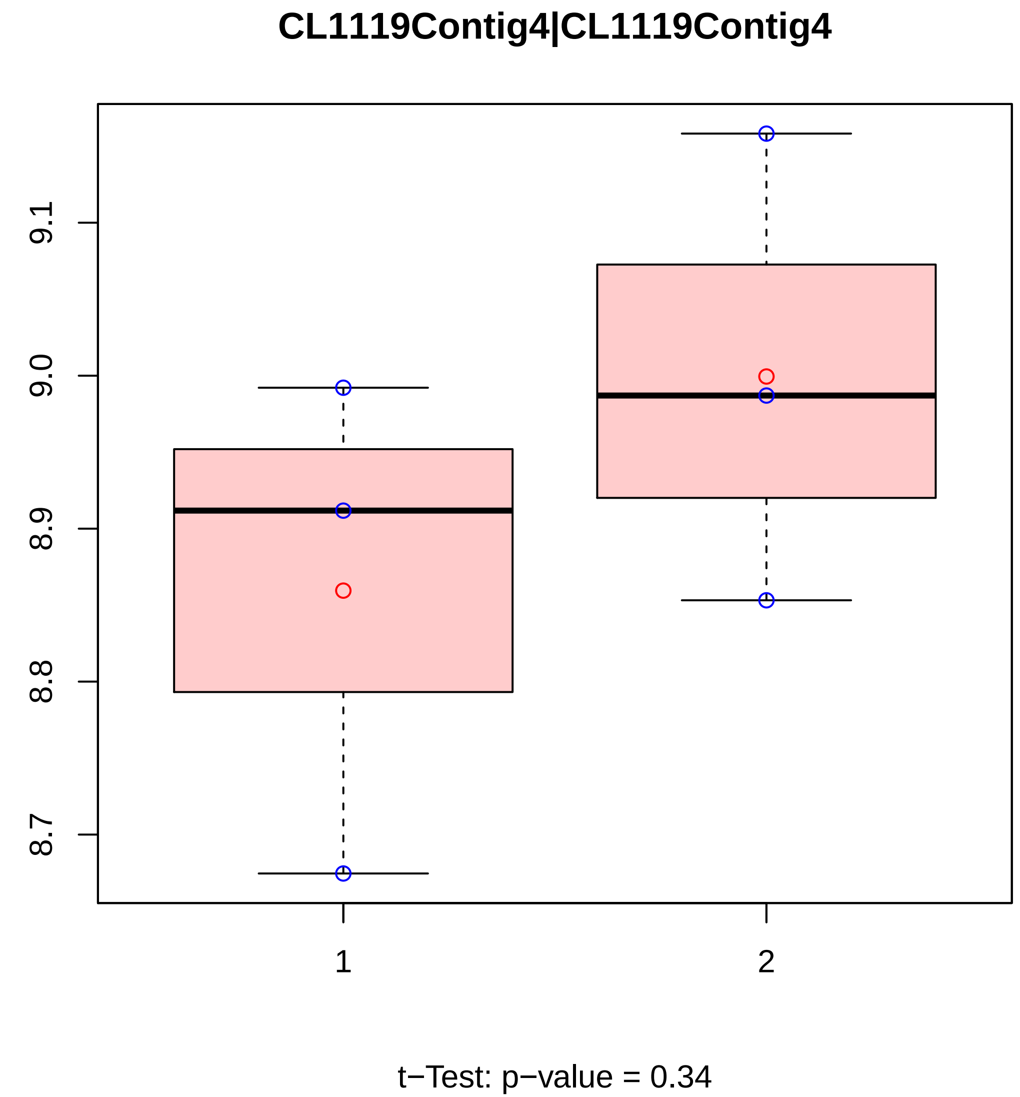
CL1119Contig4|CL1119Contig4
9.1
9.0
8.9
8.8
8.7
1
2
t−Test: p−value = 0.34

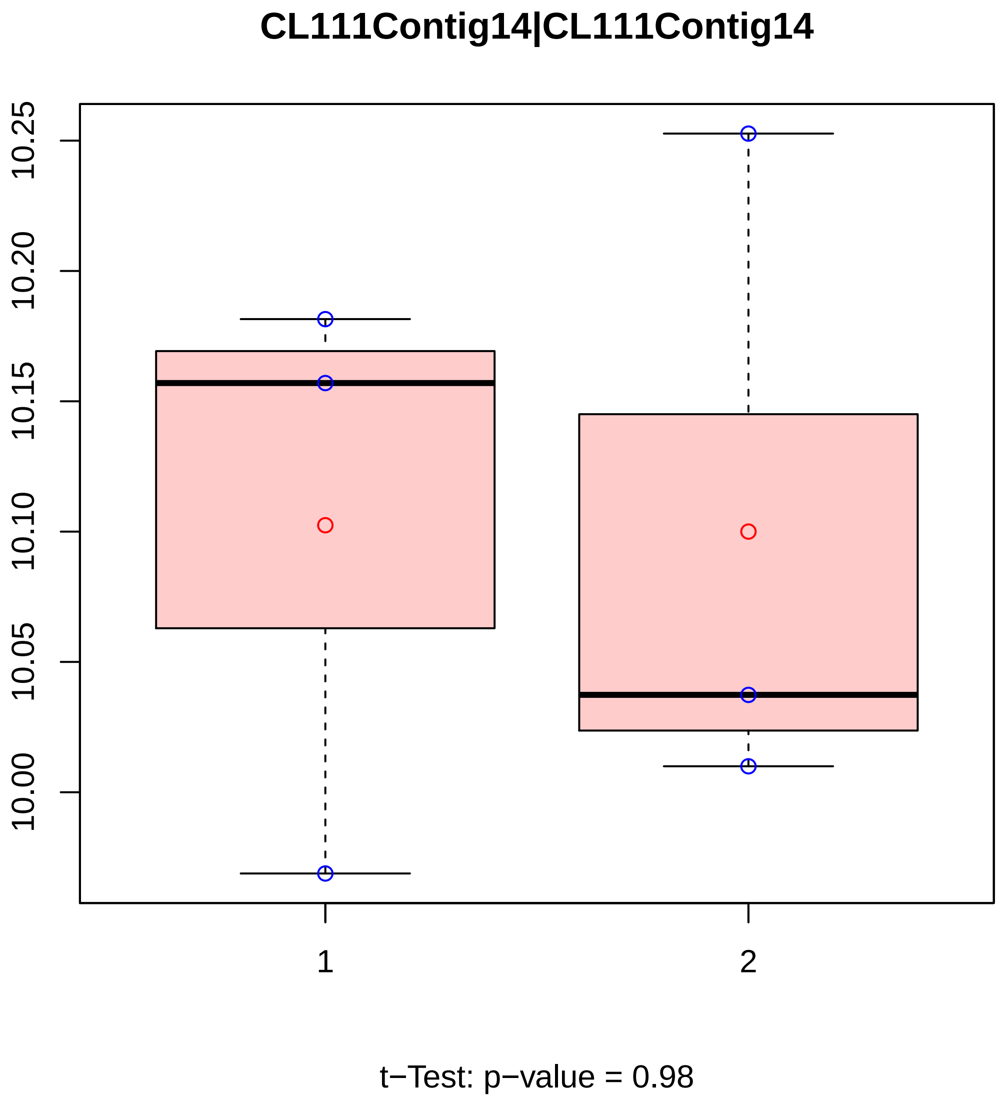
CL111Contig14|CL111Contig14
10.25
10.20
10.15
10.10
10.05
10.00
1
2
t−Test: p−value = 0.98

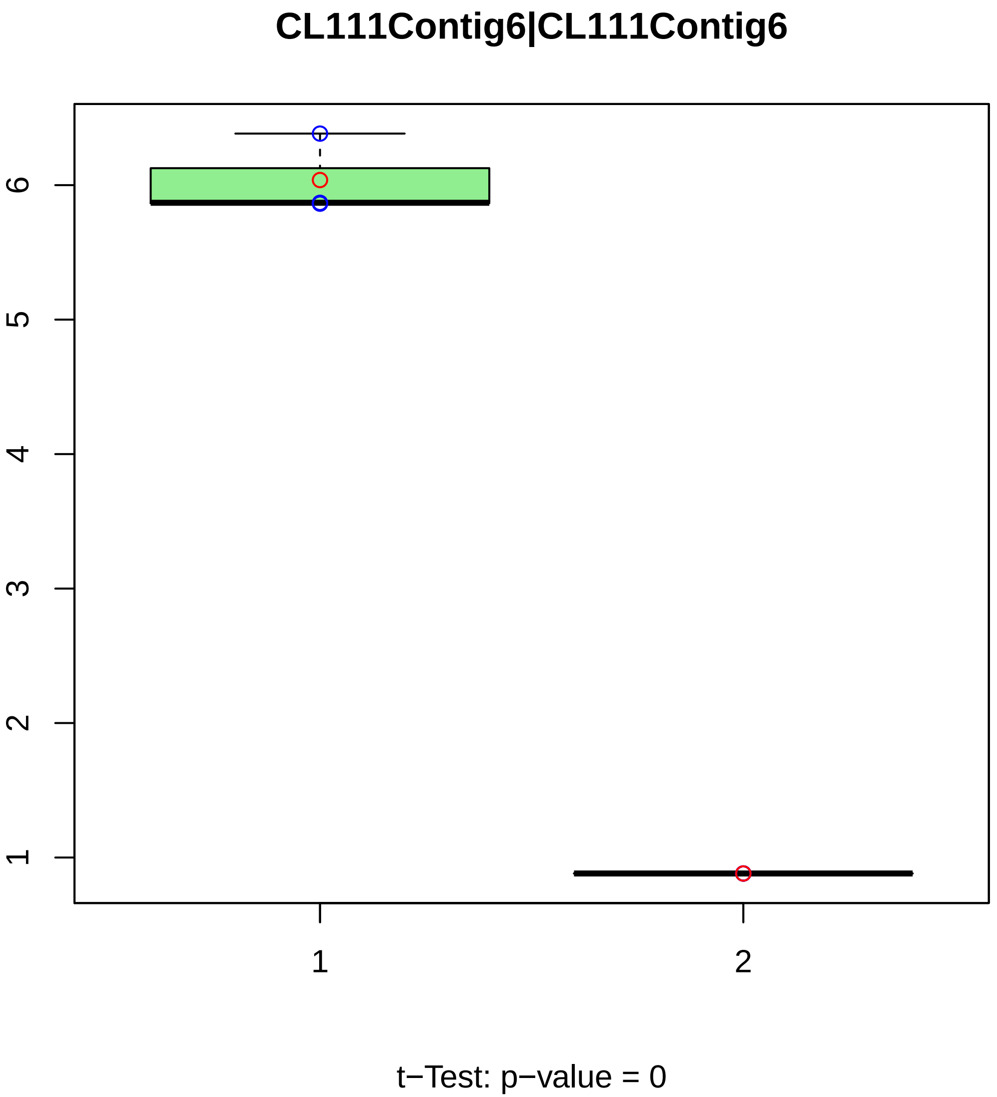
CL111Contig6|CL111Contig6
1
2
3
4
5
6
1
2
t−Test: p−value = 0

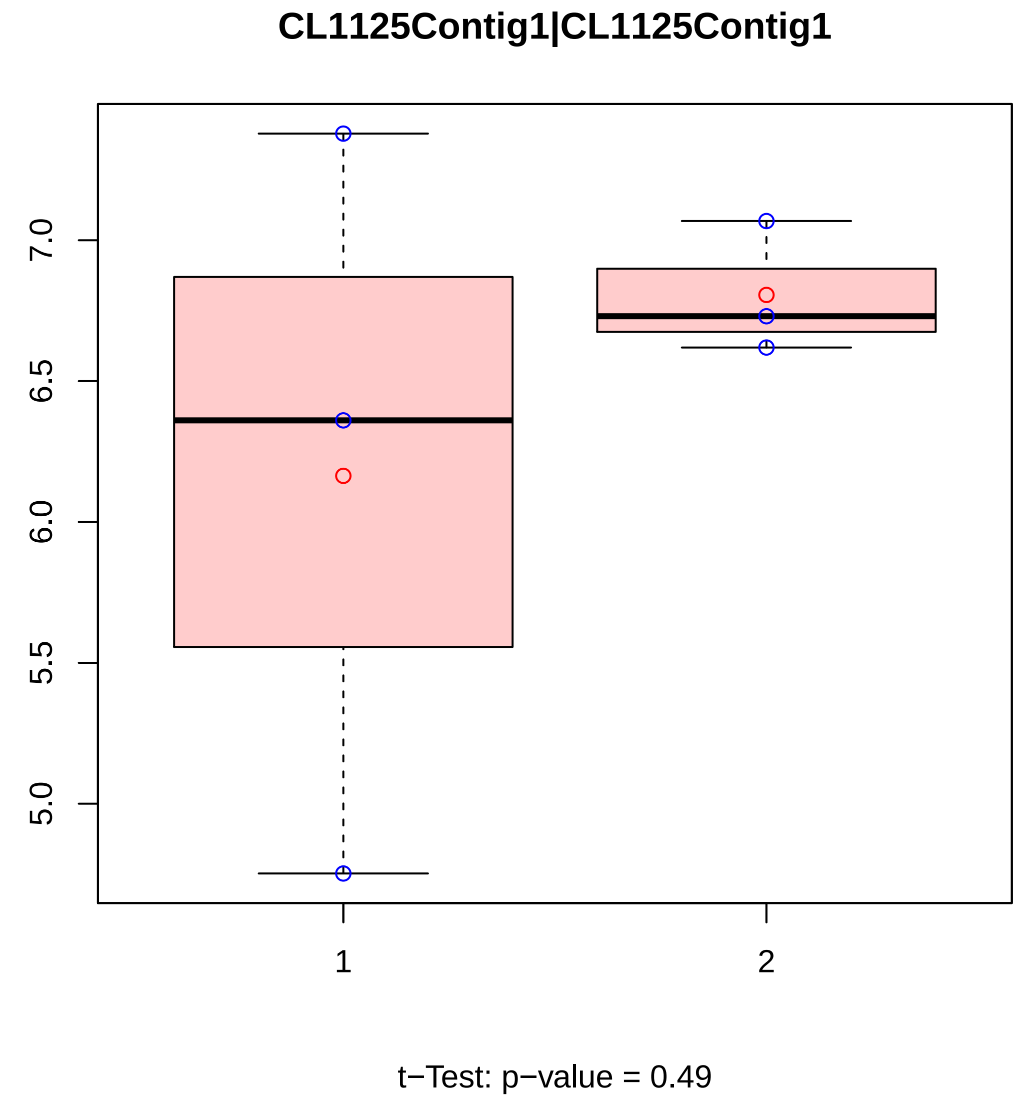
CL1125Contig1|CL1125Contig1
7.0
6.5
6.0
5.5
5.0
1
2
t−Test: p−value = 0.49

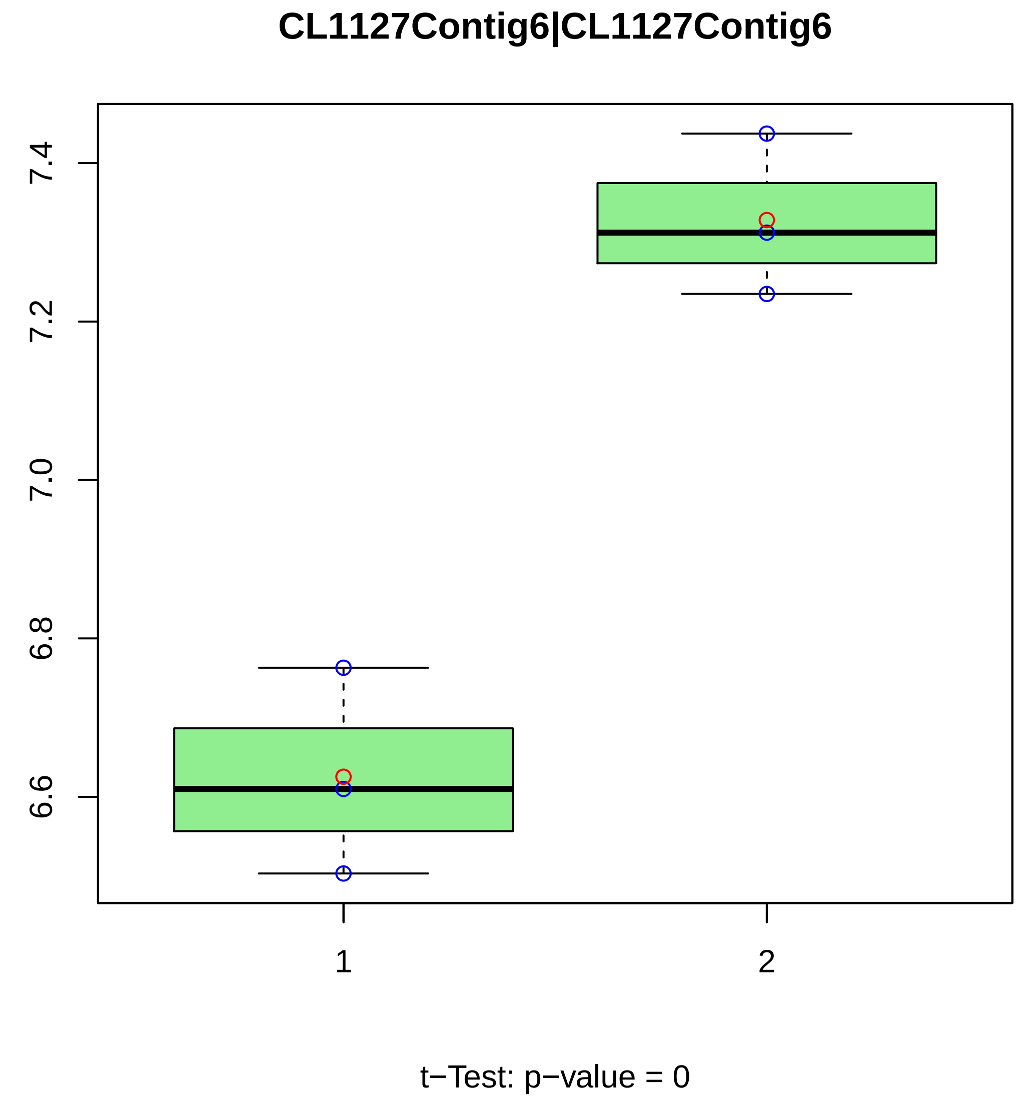
CL1127Contig6|CL1127Contig6
7.4
7.2
7.0
6.8
6.6
1
2
t−Test: p−value = 0

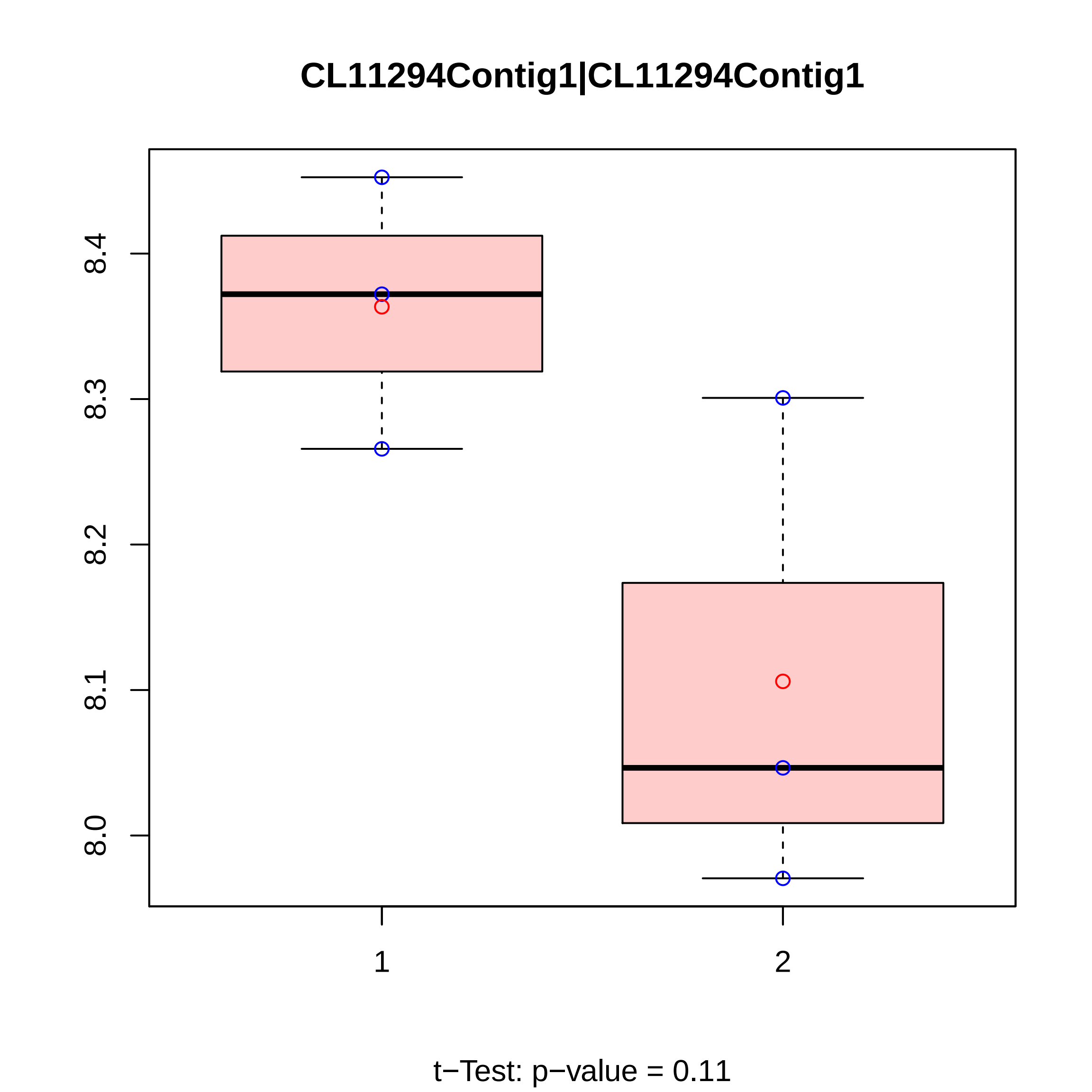
CL11294Contig1|CL11294Contig1
8.0
8.1
8.2
8.3
8.4
1
2
t−Test: p−value = 0.11

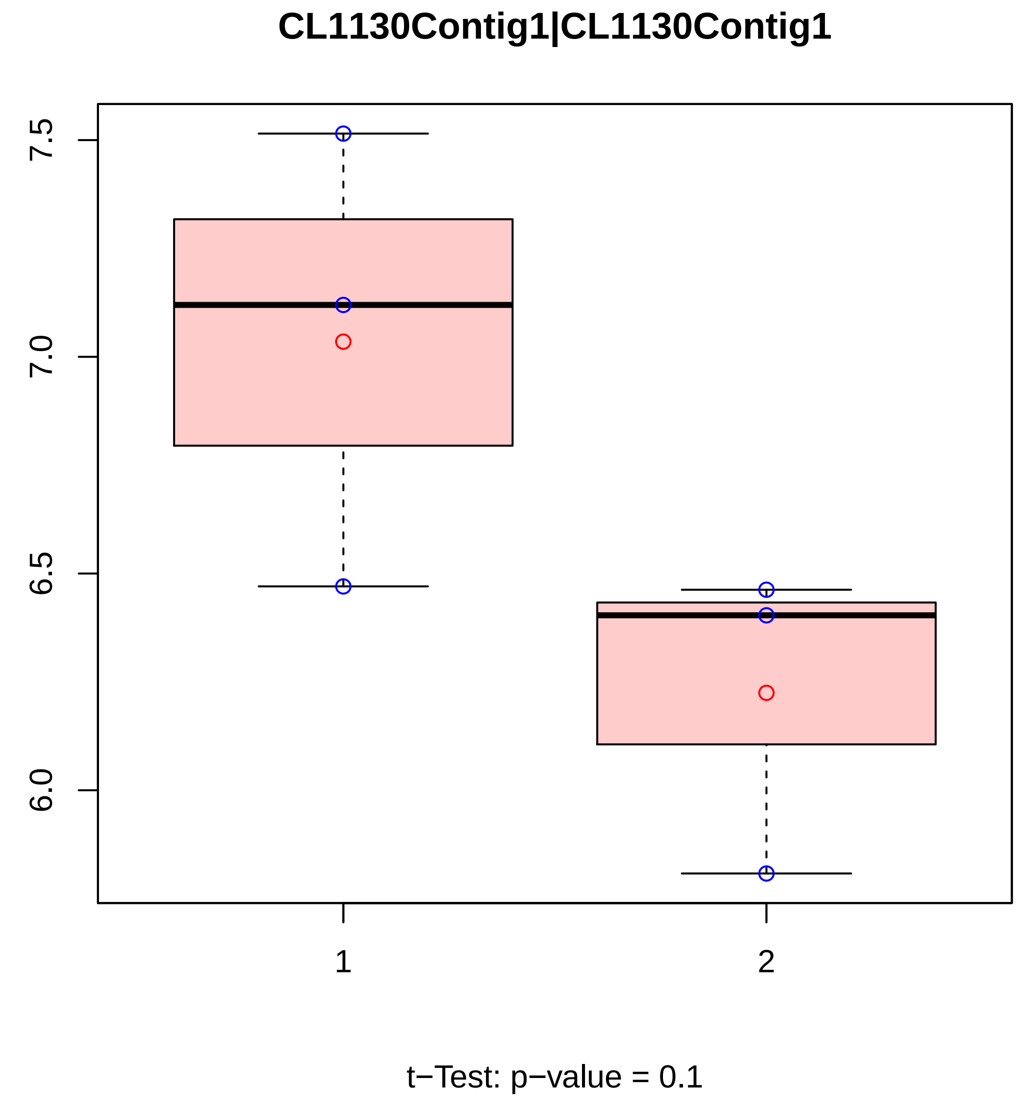
CL1130Contig1|CL1130Contig1
7.5
7.0
6.5
6.0
1
2
t−Test: p−value = 0.1

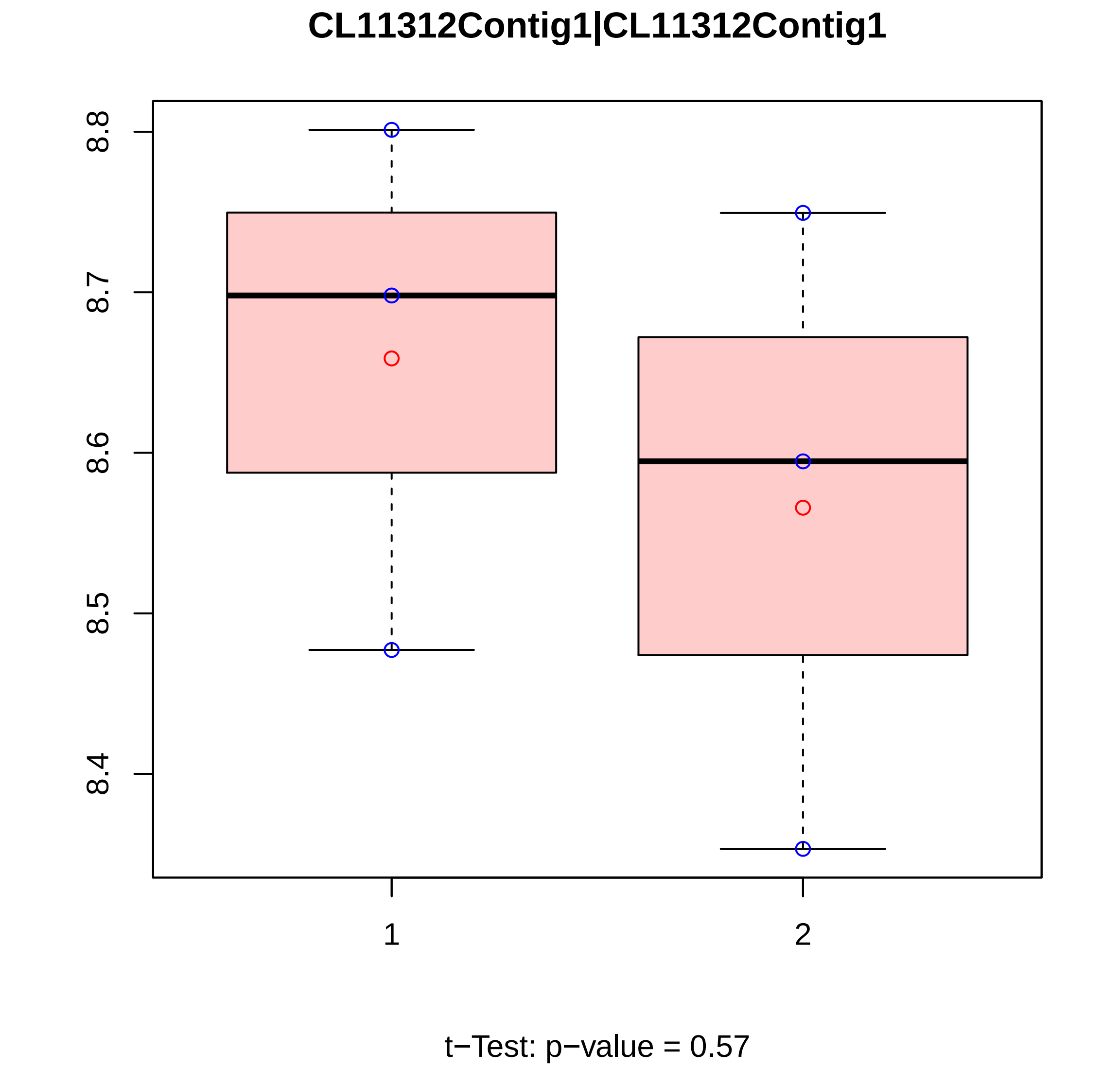
CL11312Contig1|CL11312Contig1
8.4
8.5
8.6
8.7
8.8
1
2
t−Test: p−value = 0.57

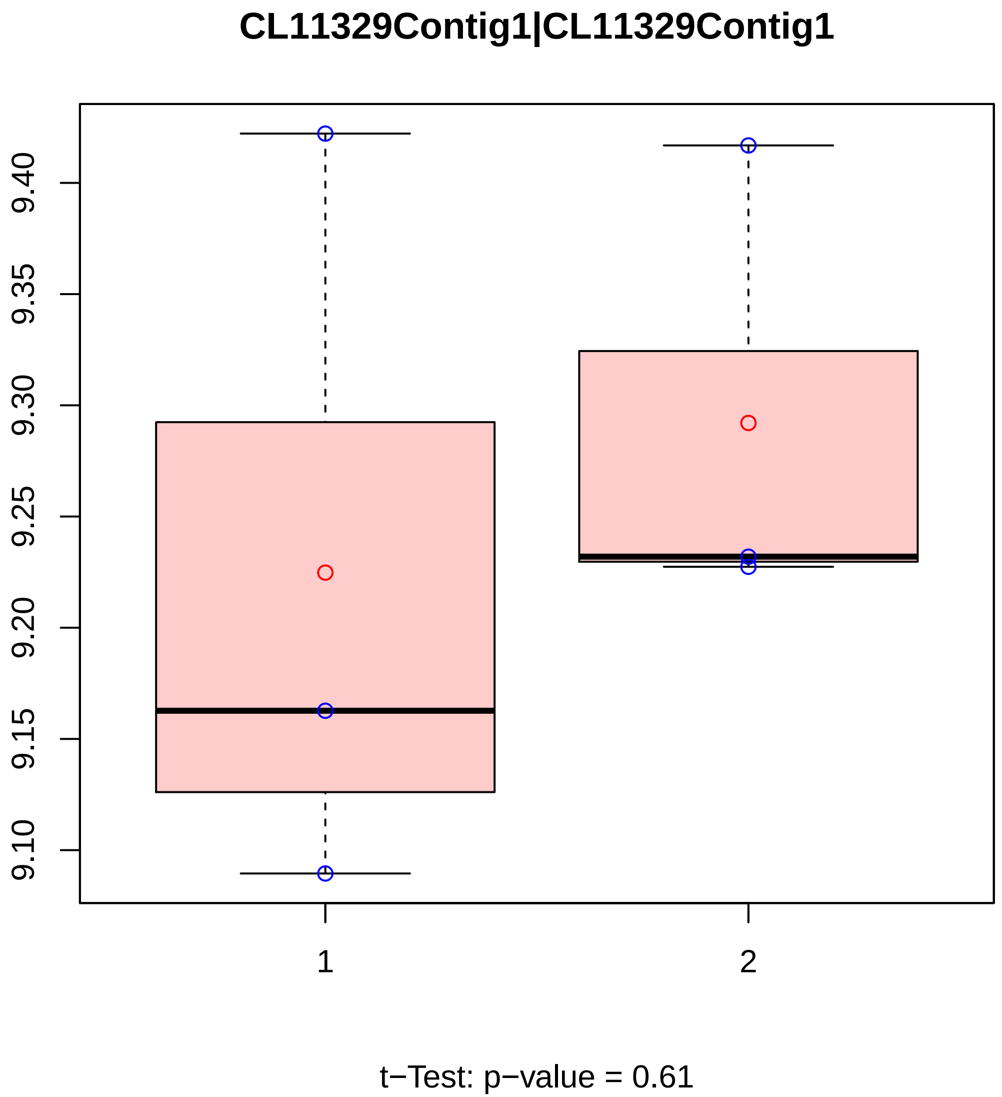
CL11329Contig1|CL11329Contig1
9.10
9.15
9.20
9.25
9.30
9.35
9.40
1
2
t−Test: p−value = 0.61

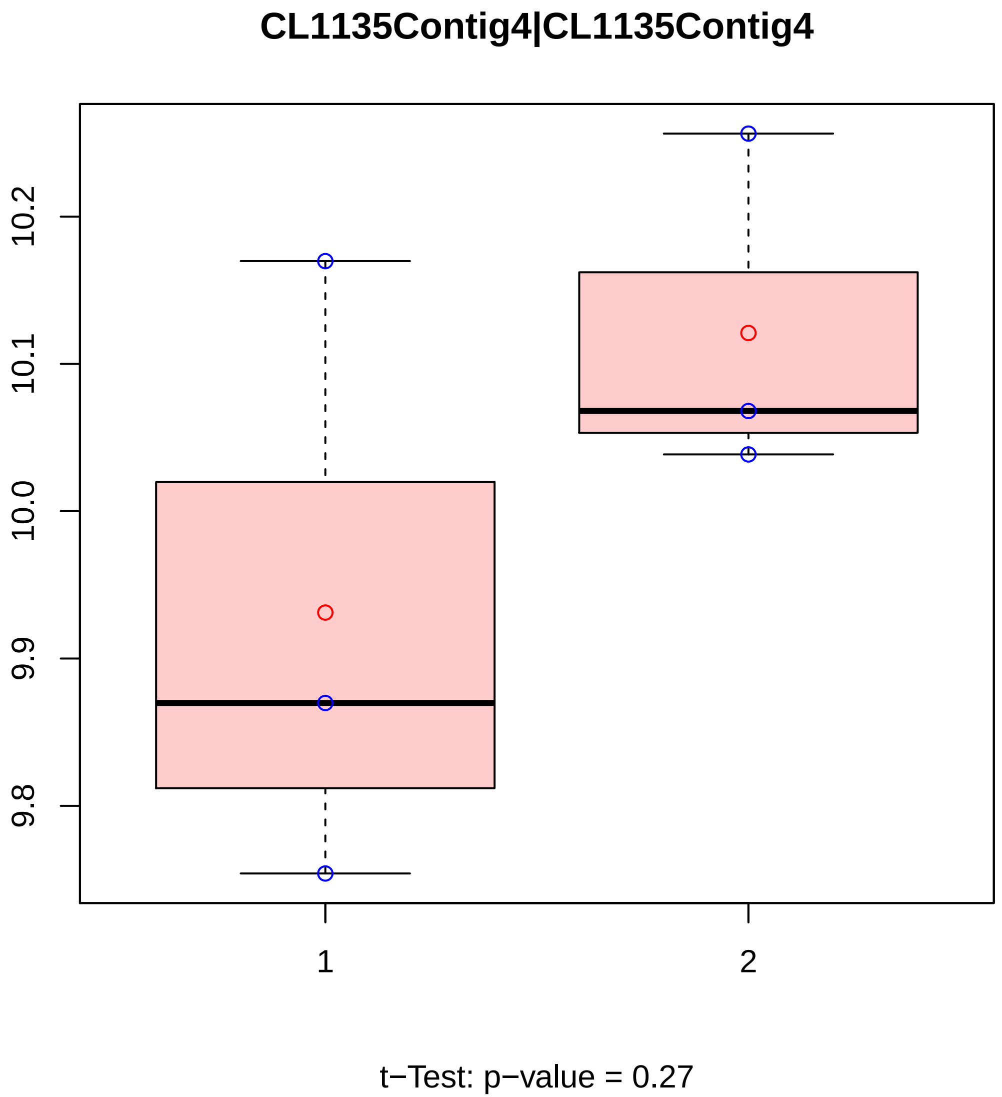
CL1135Contig4|CL1135Contig4
10.2
10.1
10.0
9.9
9.8
1
2
t−Test: p−value = 0.27

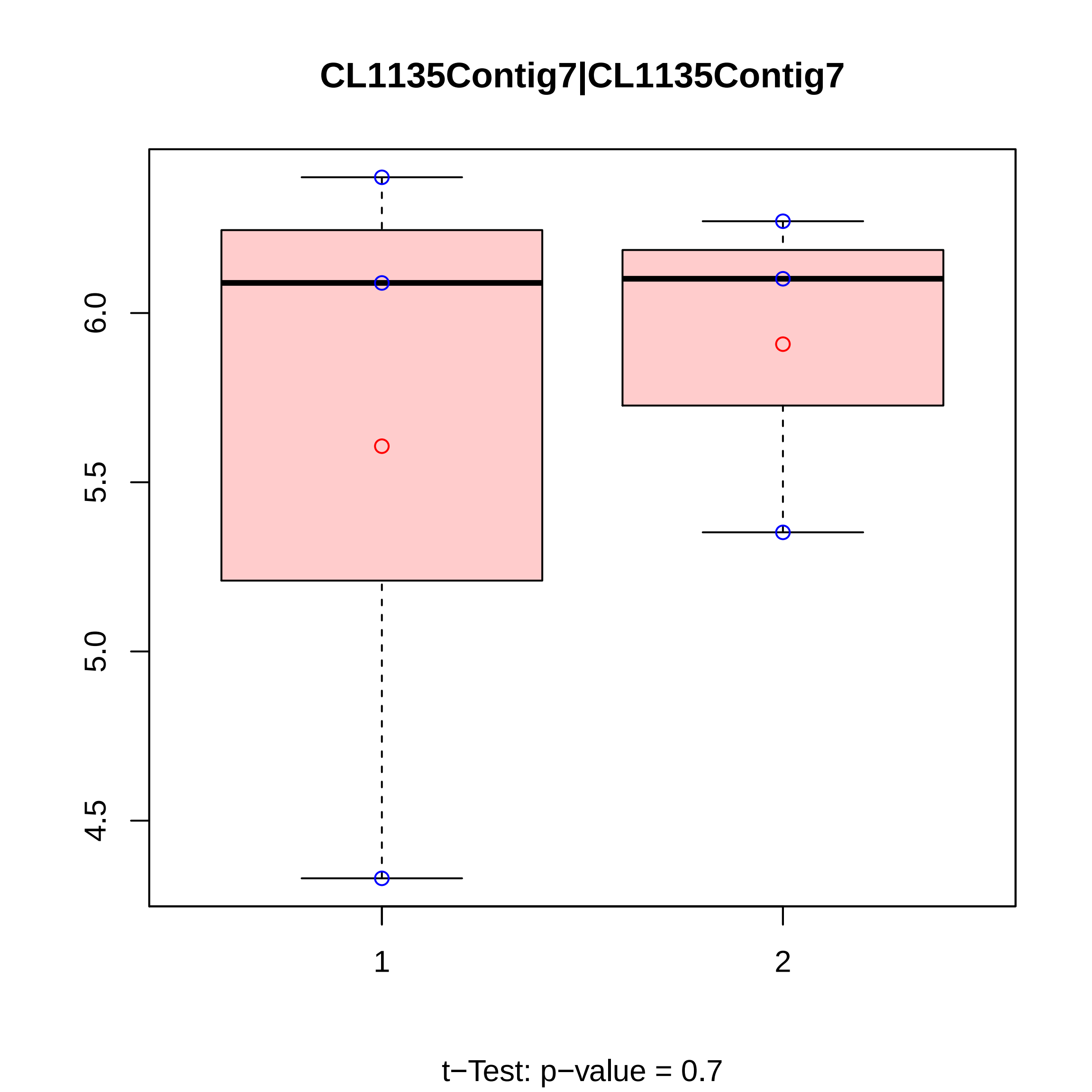
CL1135Contig7|CL1135Contig7
6.0
5.5
5.0
4.5
1
2
t−Test: p−value = 0.7

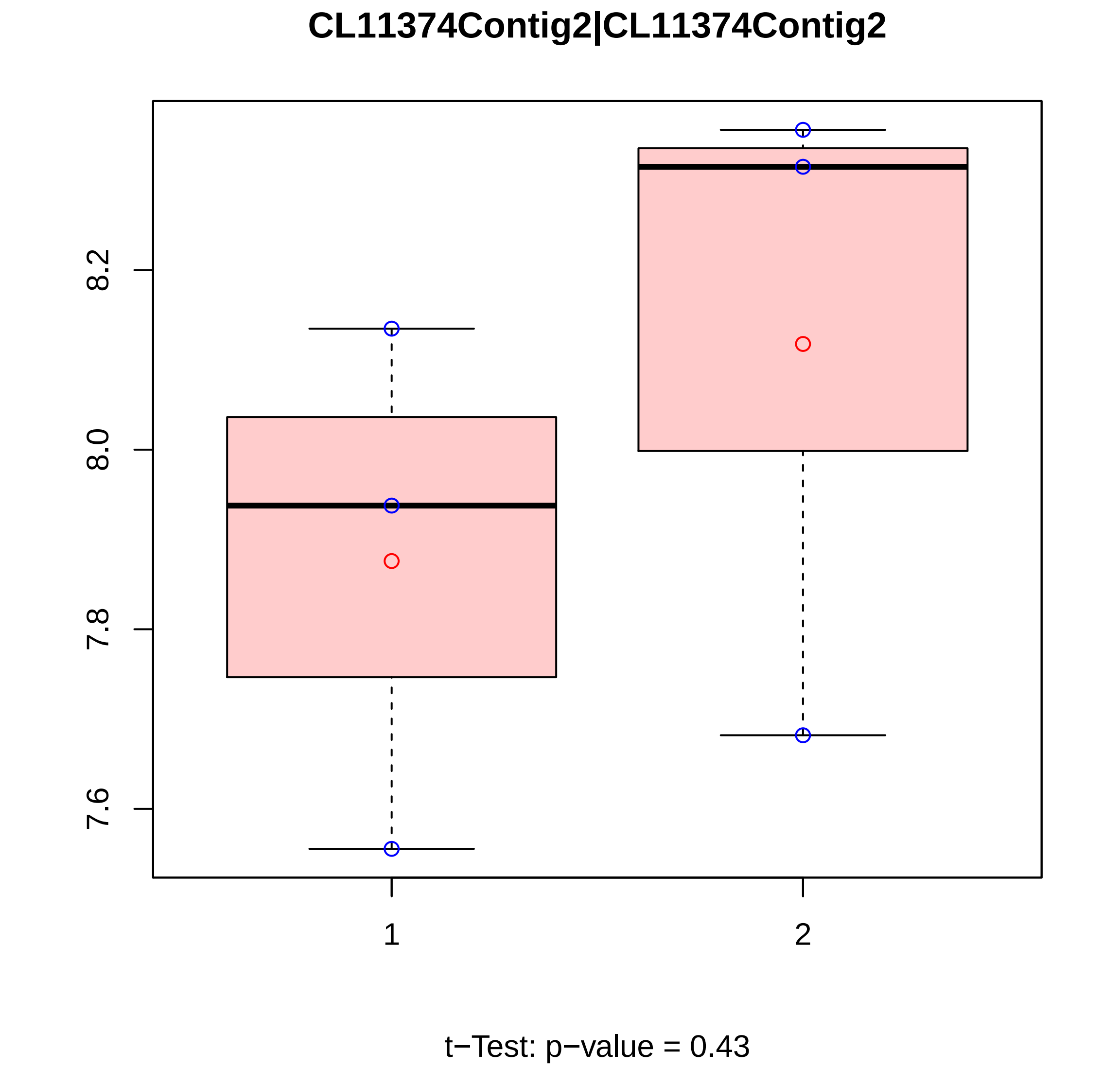

CL11374Contig2|CL11374Contig2
8.2
8.0
7.8
7.6
1
2
t−Test: p−value = 0.43

# CL11382Contig2|CL11382Contig2

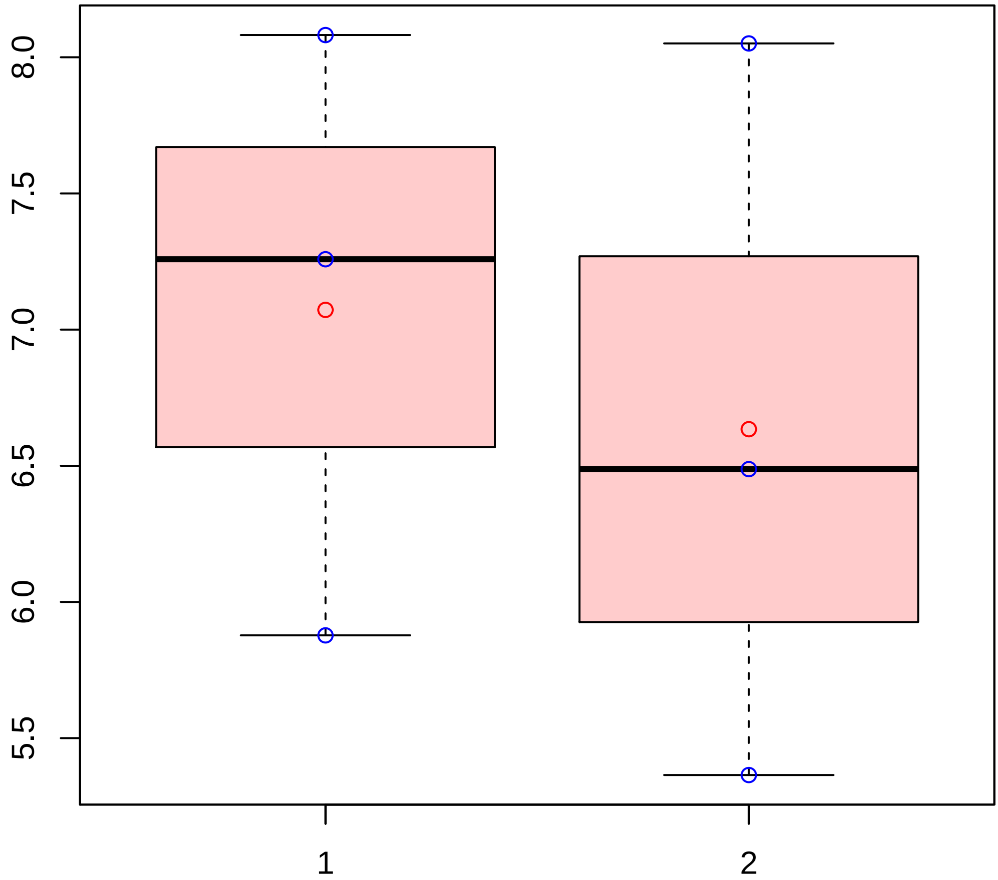


t−Test: p−value = 0.69

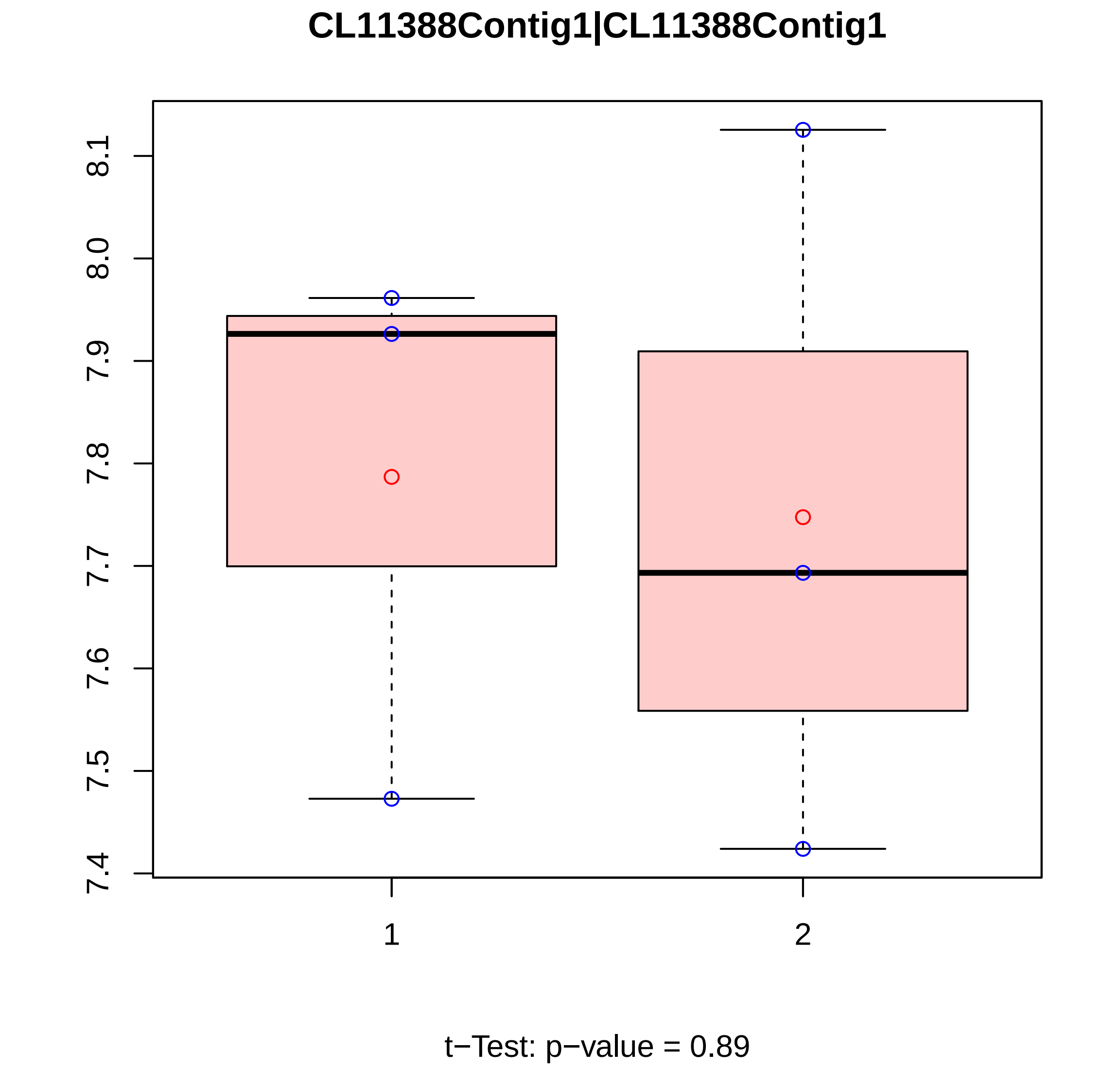
CL11388Contig1|CL11388Contig1
8.1
8.0
7.9
7.8
7.7
7.6
7.5
7.4
1
2
t−Test: p−value = 0.89

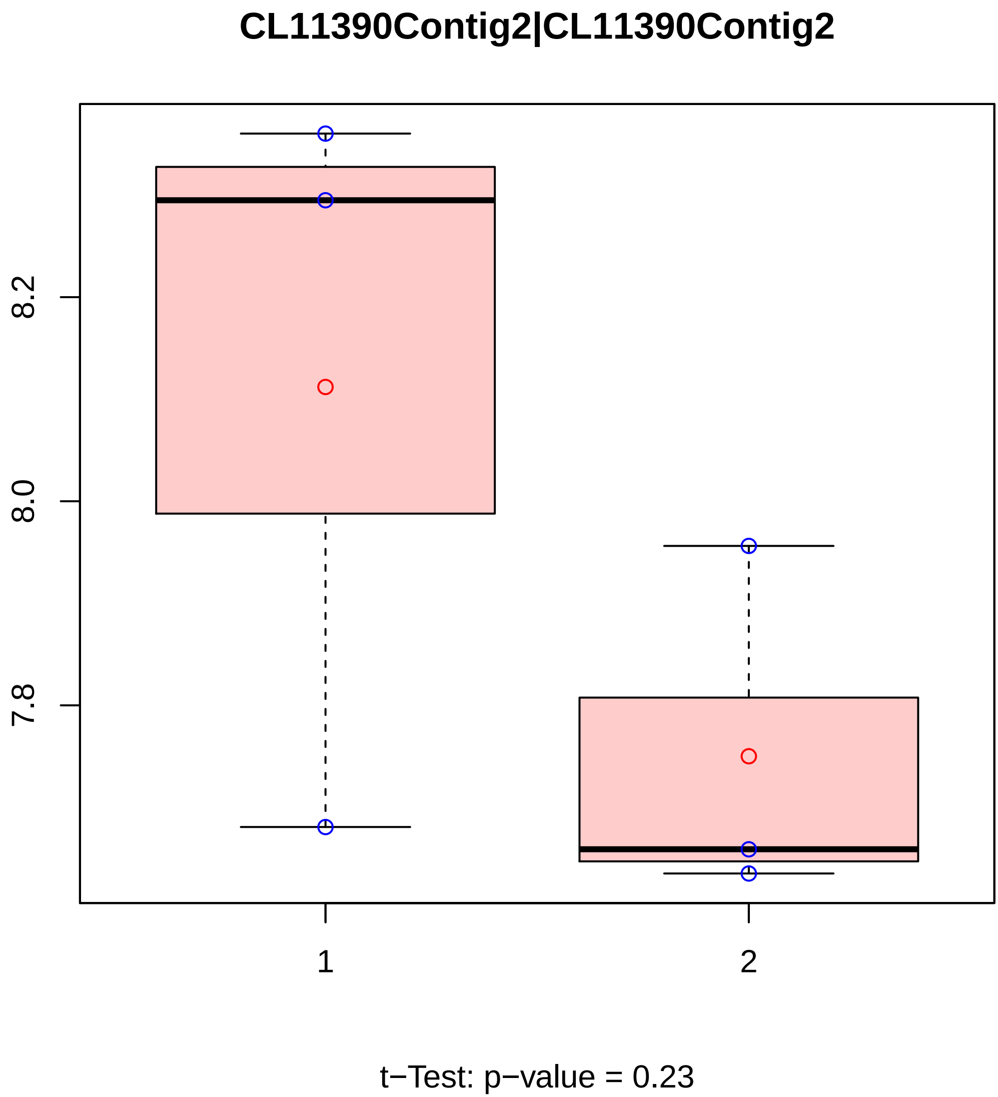

CL11390Contig2|CL11390Contig2
8.2
8.0
7.8
1
2
t−Test: p−value = 0.23

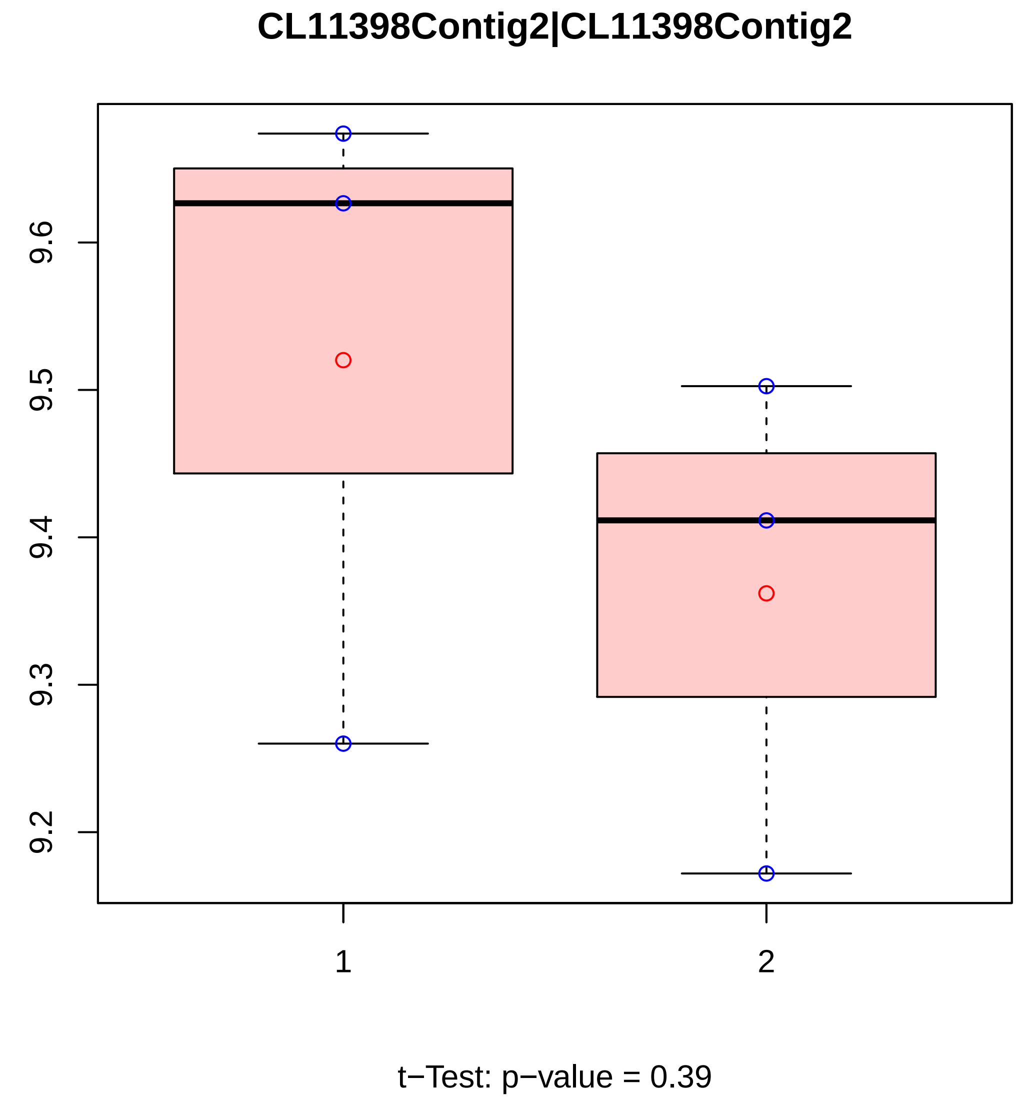
CL11398Contig2|CL11398Contig2
9.2
9.3
9.4
9.5
9.6
1
2
t−Test: p−value = 0.39

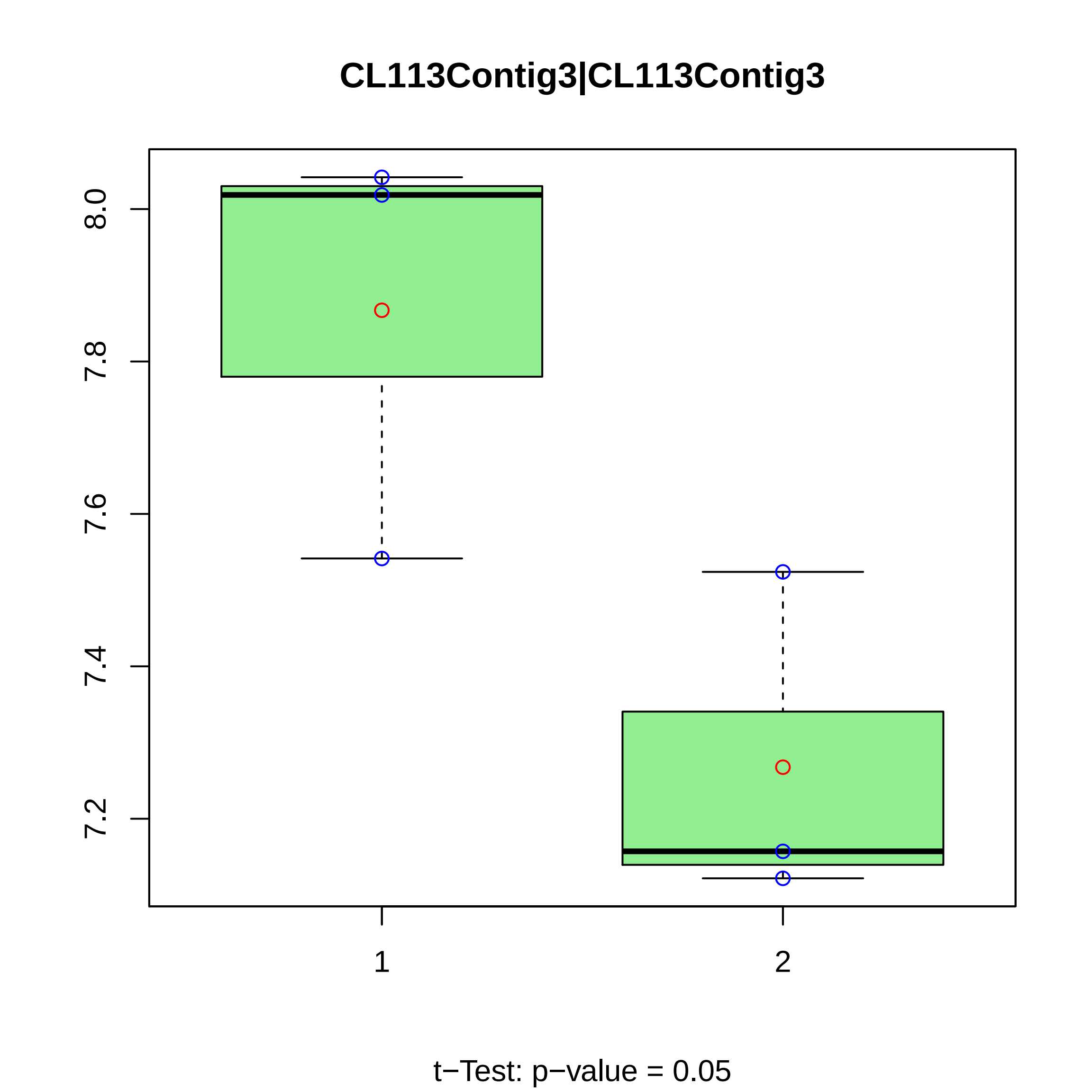
CL113Contig3|CL113Contig3
8.0
7.8
7.6
7.4
7.2
1
2
t−Test: p−value = 0.05

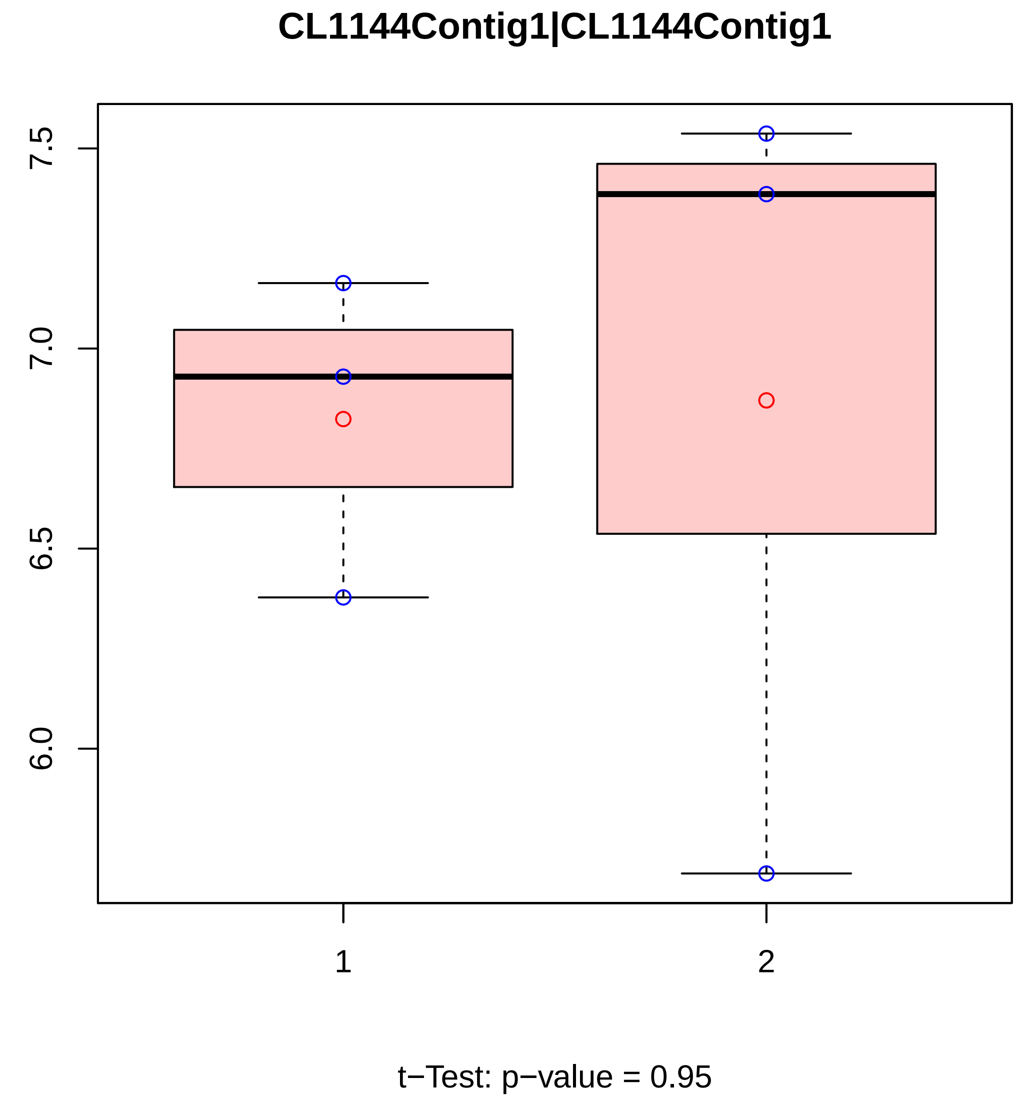
CL1144Contig1|CL1144Contig1
7.5
7.0
6.5
6.0
1
2
t−Test: p−value = 0.95

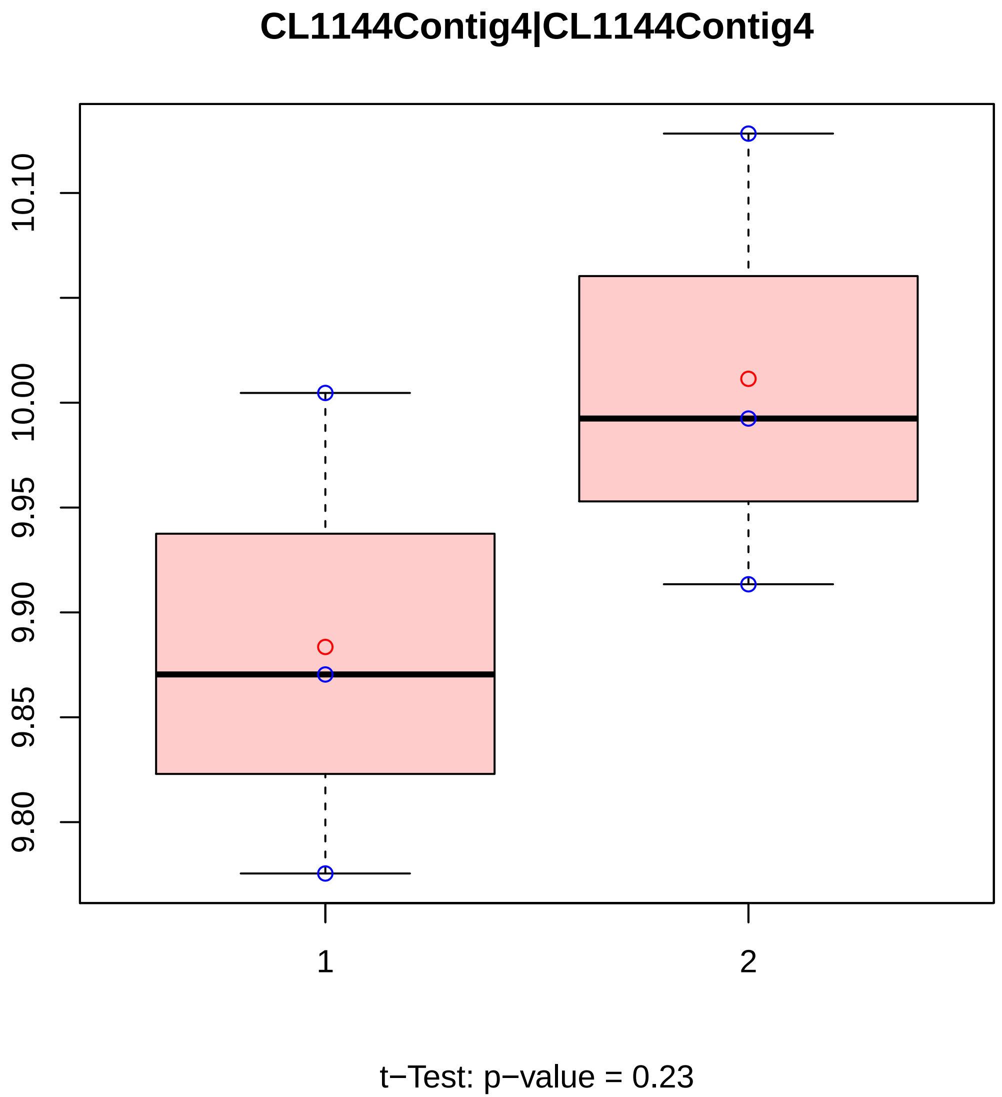
CL1144Contig4|CL1144Contig4
10.10
10.00
9.95
9.90
9.85
9.80
1
2
t−Test: p−value = 0.23

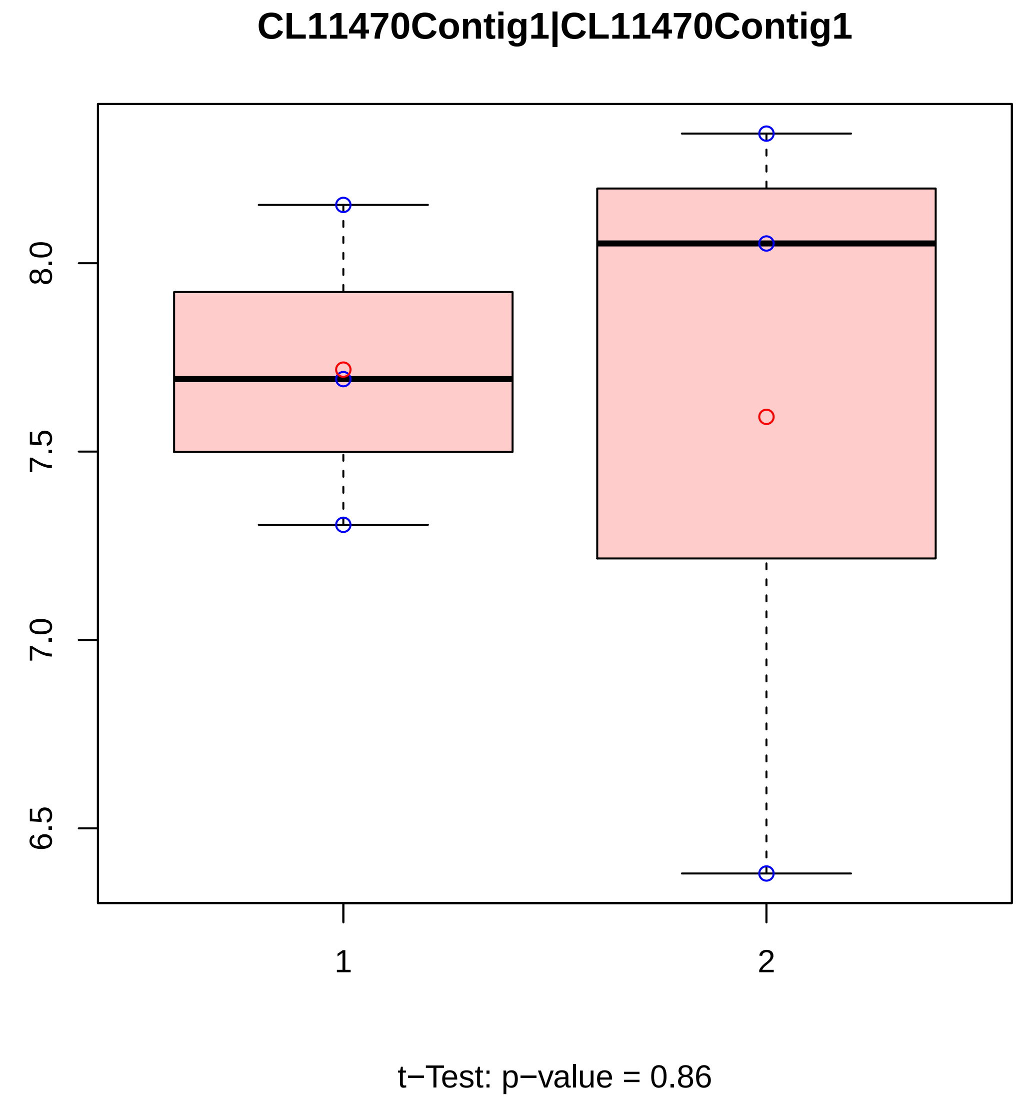
CL11470Contig1|CL11470Contig1
8.0
7.5
7.0
6.5
1
2
t−Test: p−value = 0.86

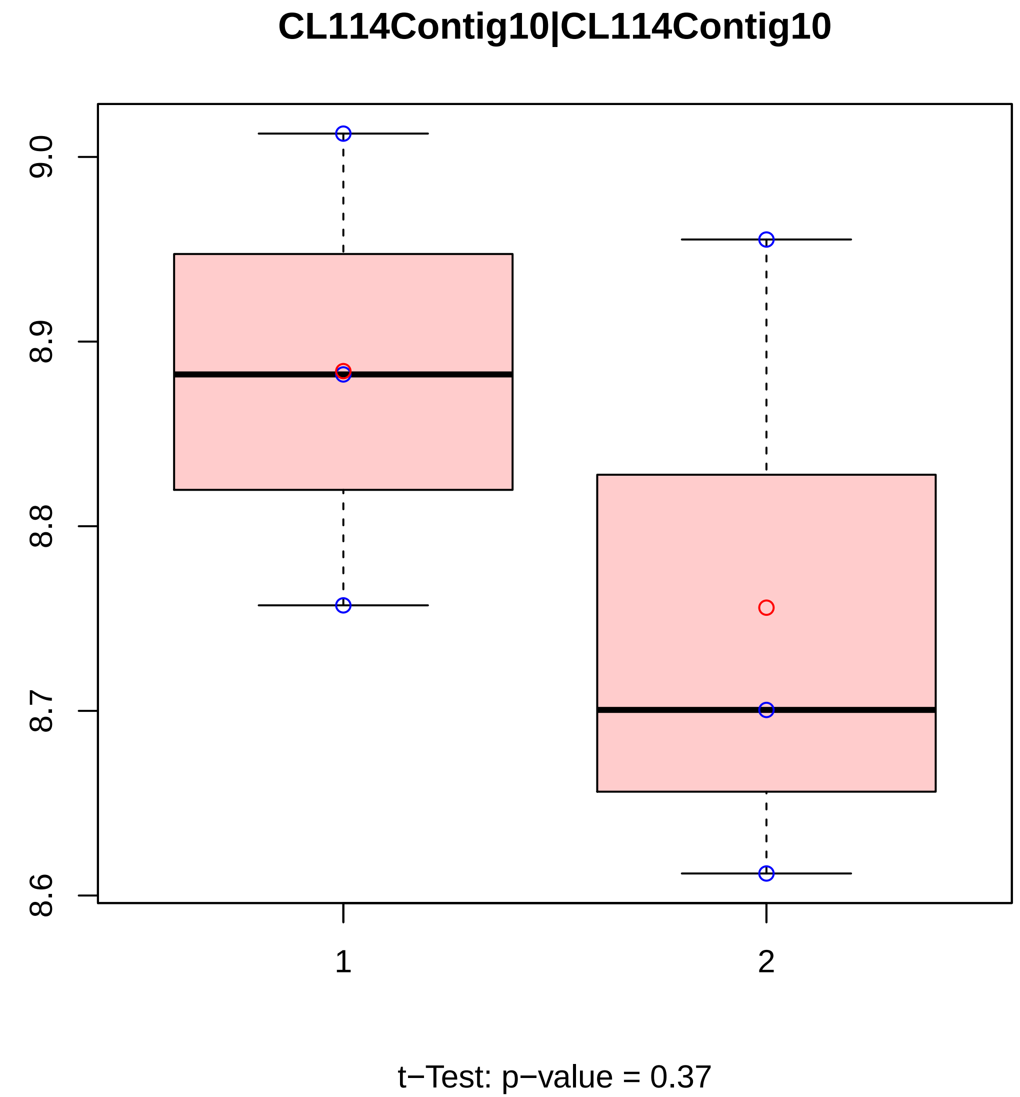
CL114Contig10|CL114Contig10
9.0
8.9
8.8
8.7
8.6
1
2
t−Test: p−value = 0.37

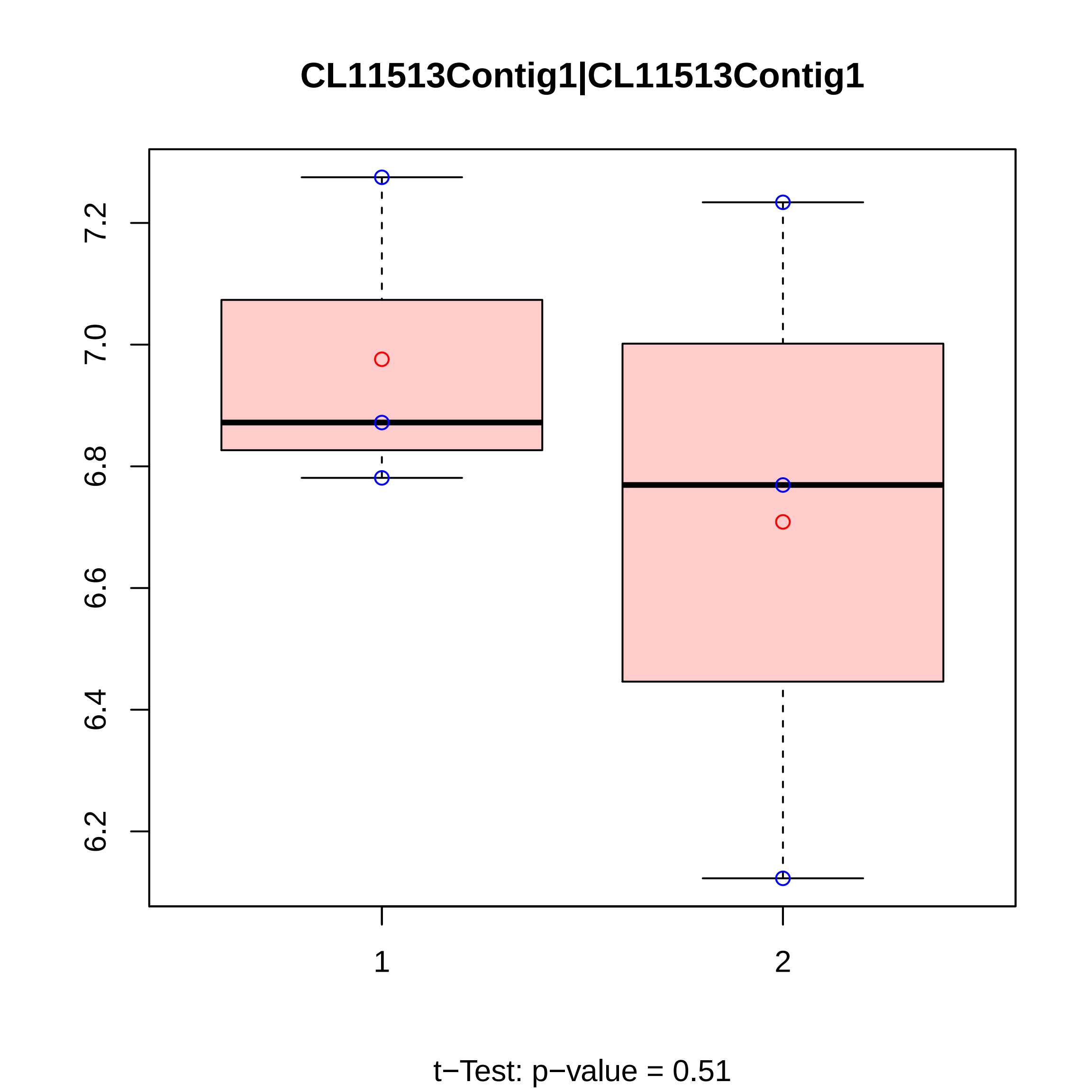
CL11513Contig1|CL11513Contig1
7.2
7.0
6.8
6.6
6.4
6.2
1
2
t−Test: p−value = 0.51

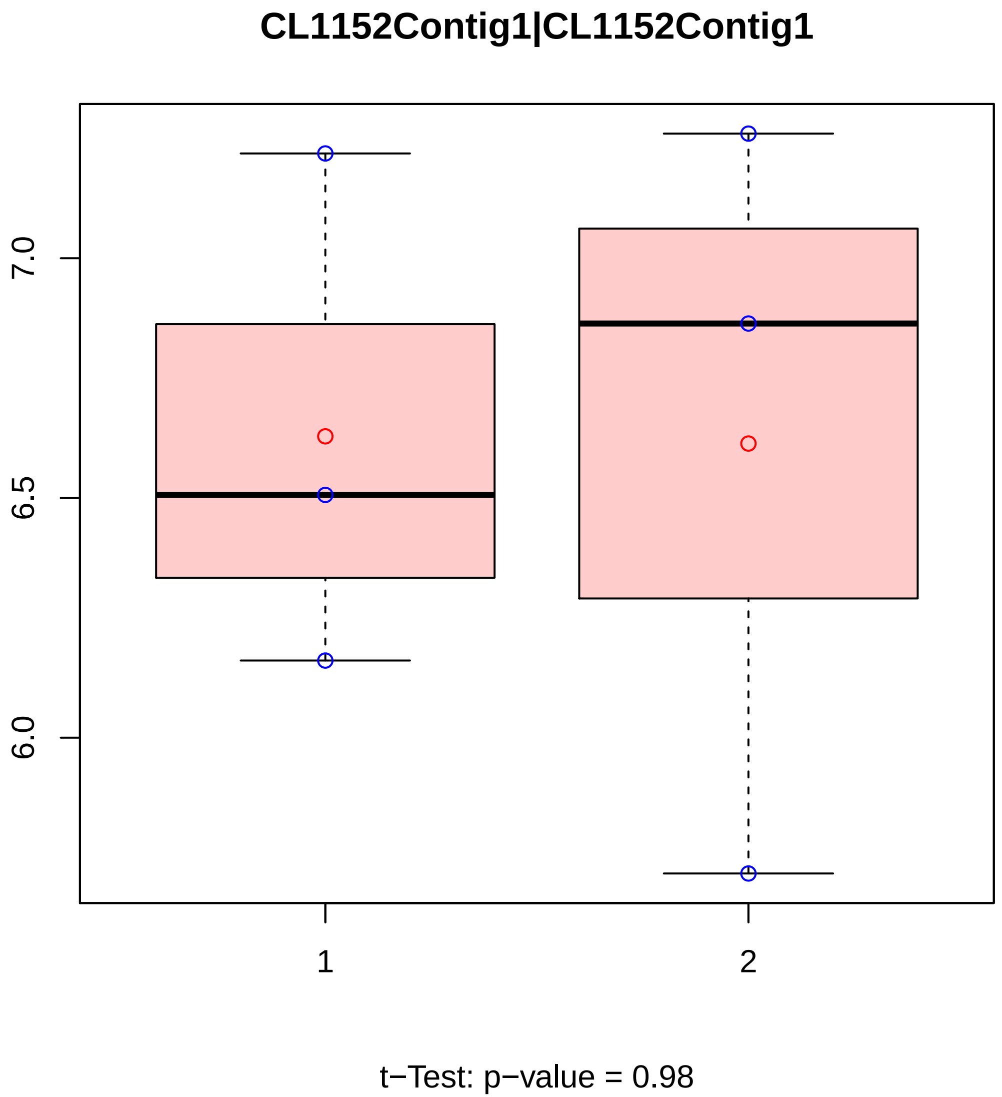
CL1152Contig1|CL1152Contig1
7.0
6.5
6.0
1
2
t−Test: p−value = 0.98

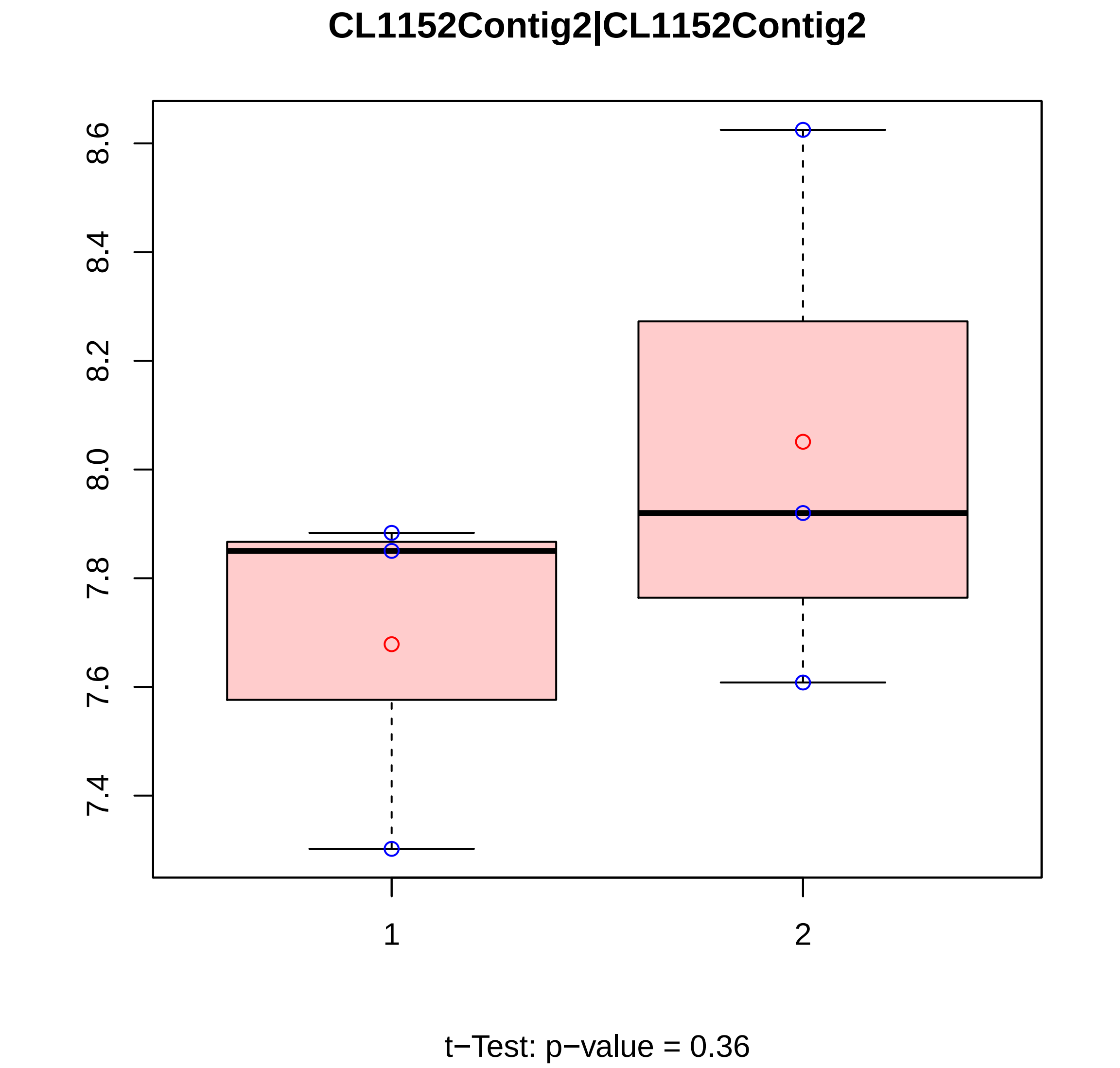
CL1152Contig2|CL1152Contig2
8.6
8.4
8.2
8.0
7.8
7.6
7.4
1
2
t−Test: p−value = 0.36

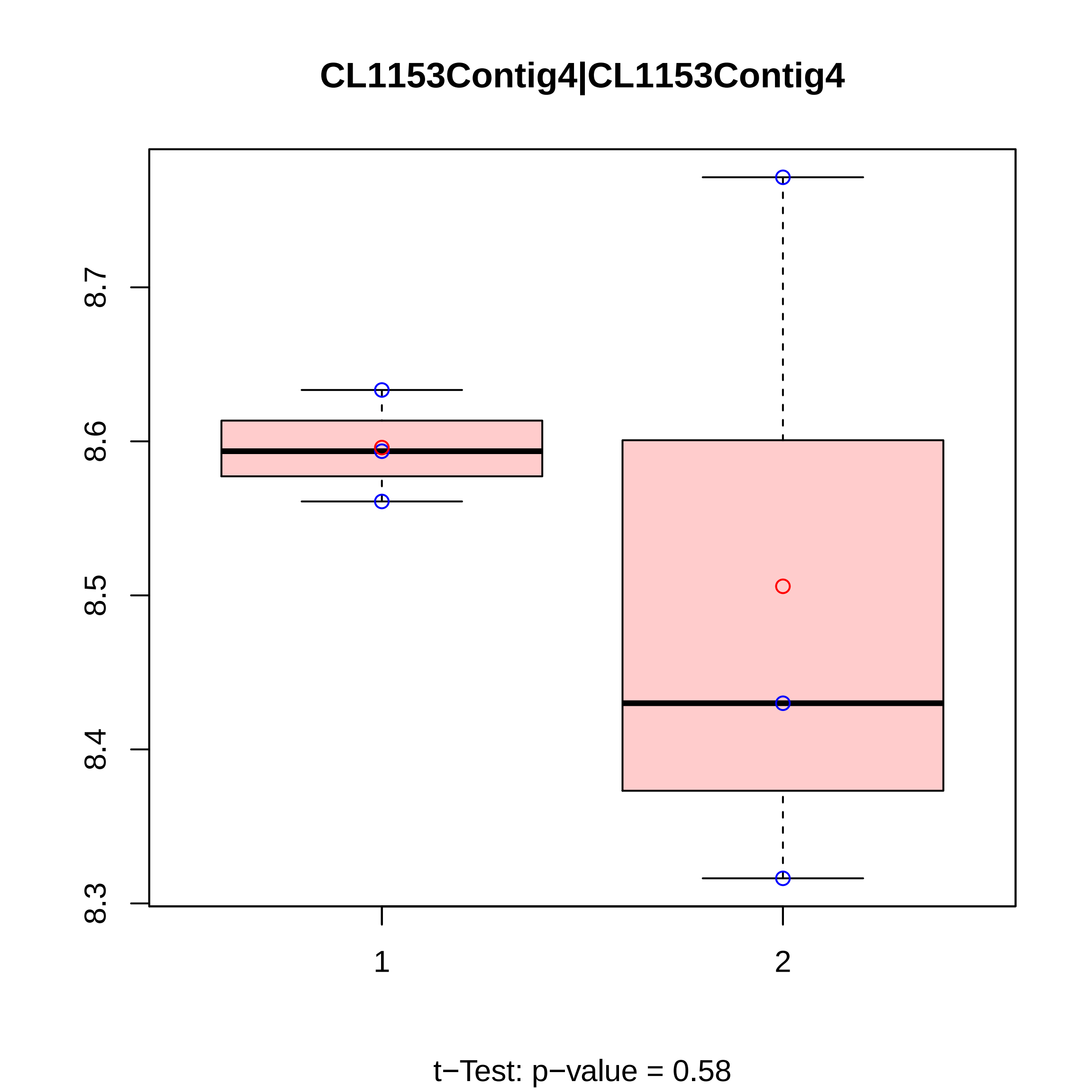
CL1153Contig4|CL1153Contig4
8.3
8.4
8.5
8.6
8.7
1
2
t−Test: p−value = 0.58

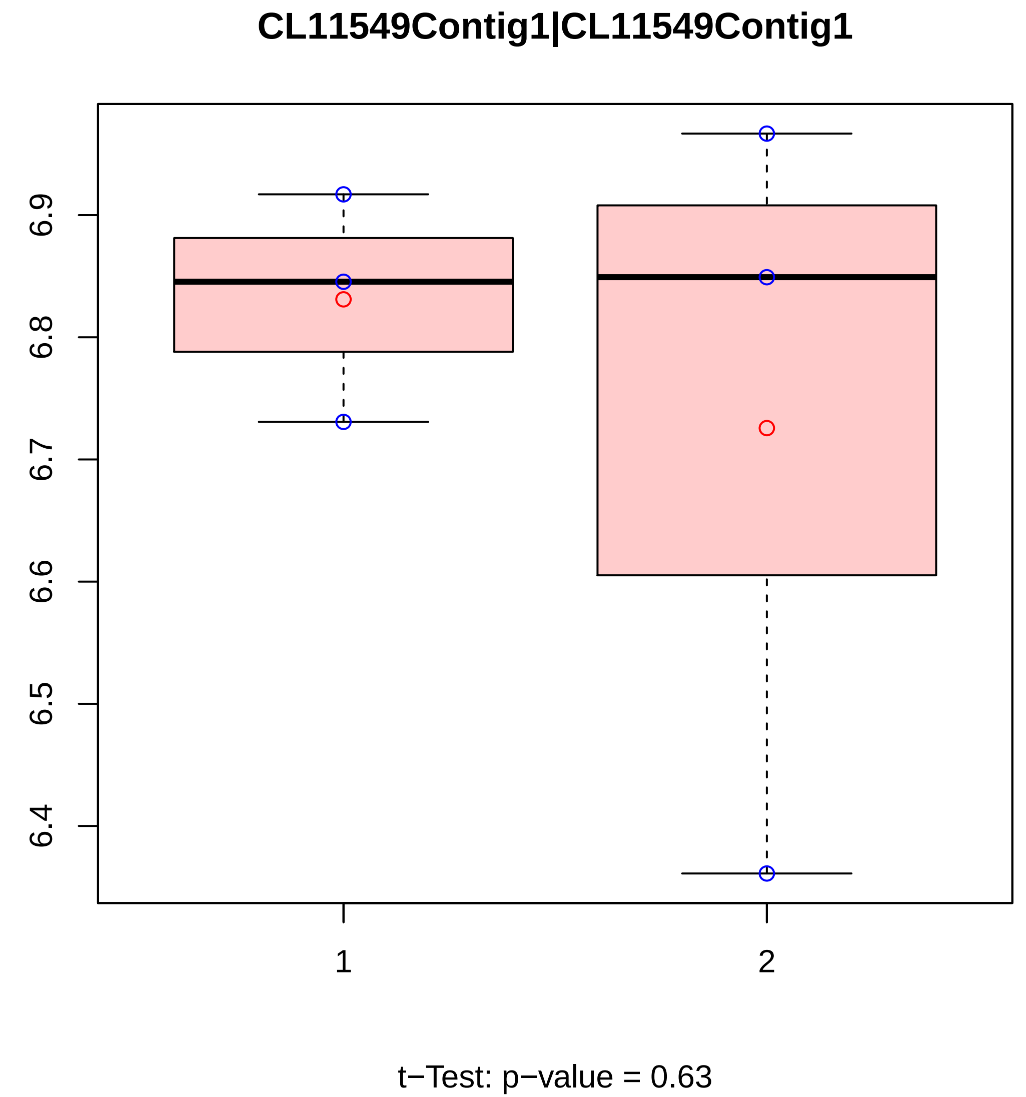
CL11549Contig1|CL11549Contig1
6.4
6.5
6.6
6.7
6.8
6.9
1
2
t−Test: p−value = 0.63

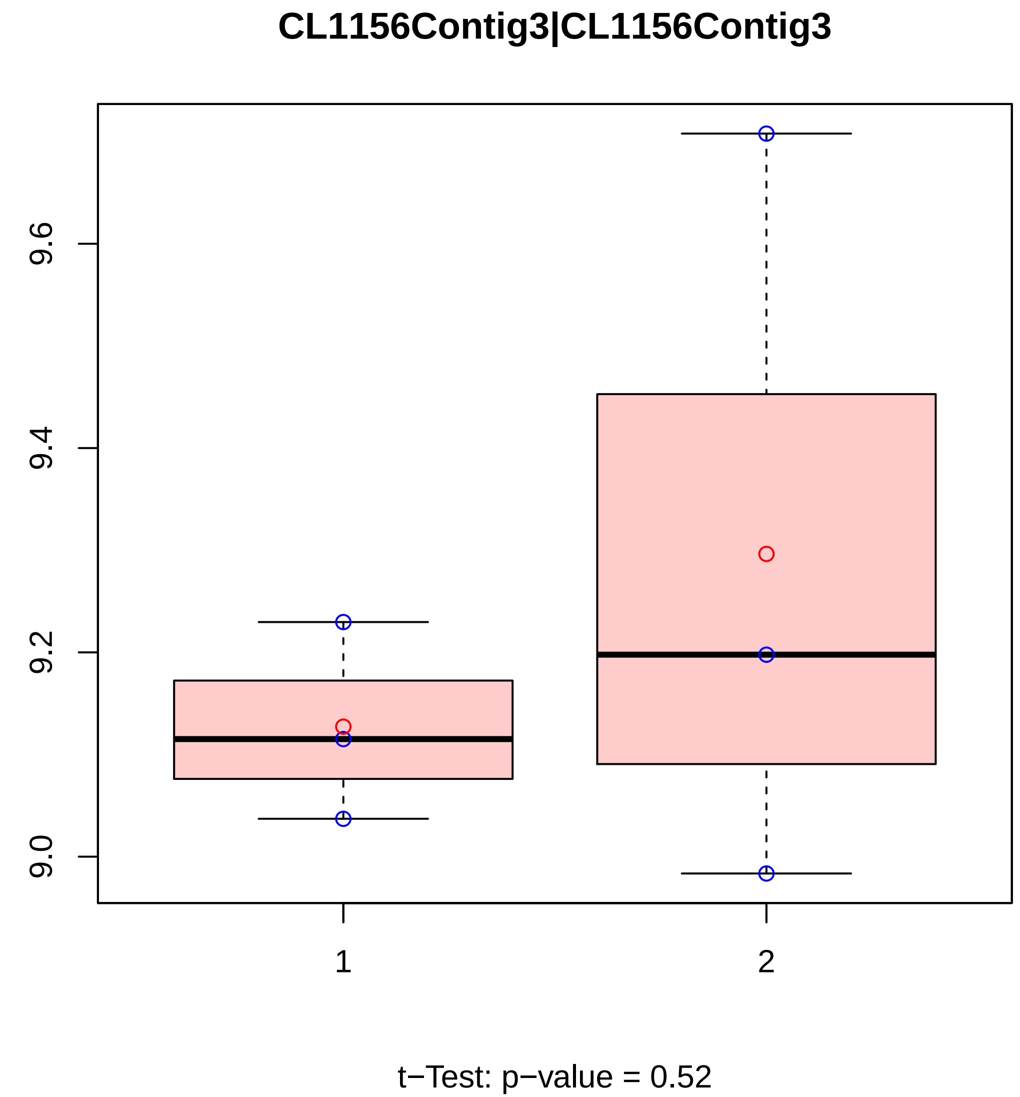
CL1156Contig3|CL1156Contig3
9.0
9.2
9.4
9.6
1
2
t−Test: p−value = 0.52

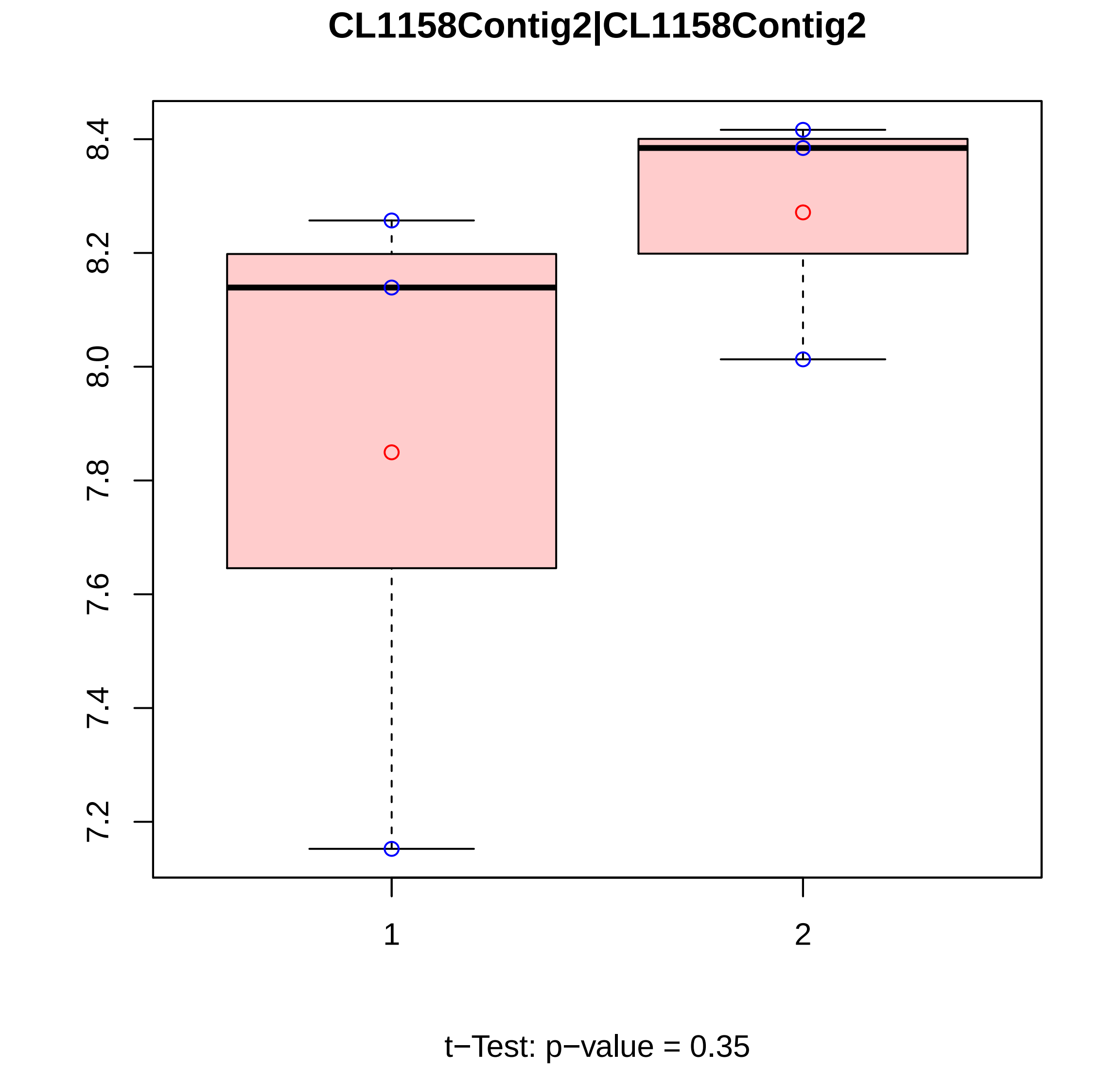
CL1158Contig2|CL1158Contig2
8.4
8.2
8.0
7.8
7.6
7.4
7.2
1
2
t−Test: p−value = 0.35

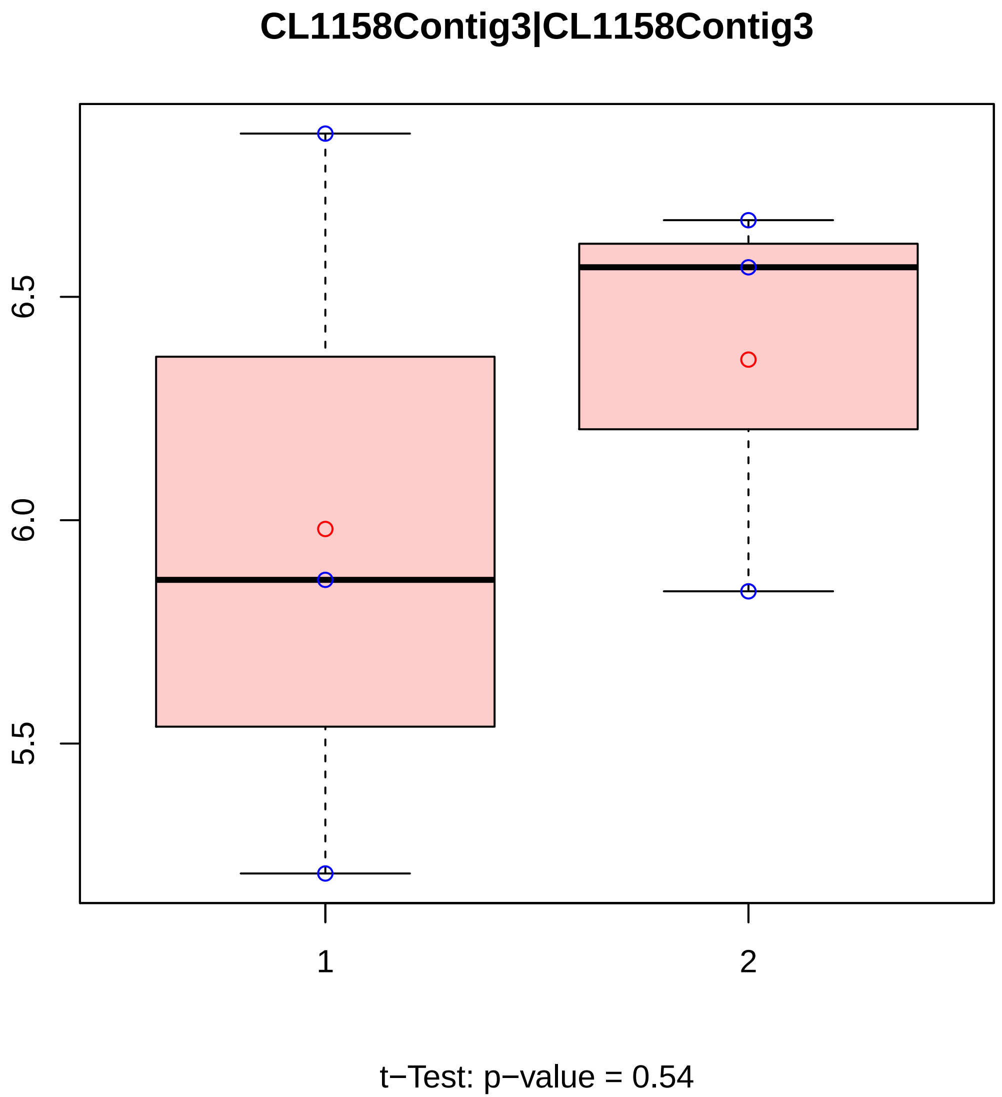

CL1158Contig3|CL1158Contig3
6.5
6.0
5.5
1
2
t−Test: p−value = 0.54

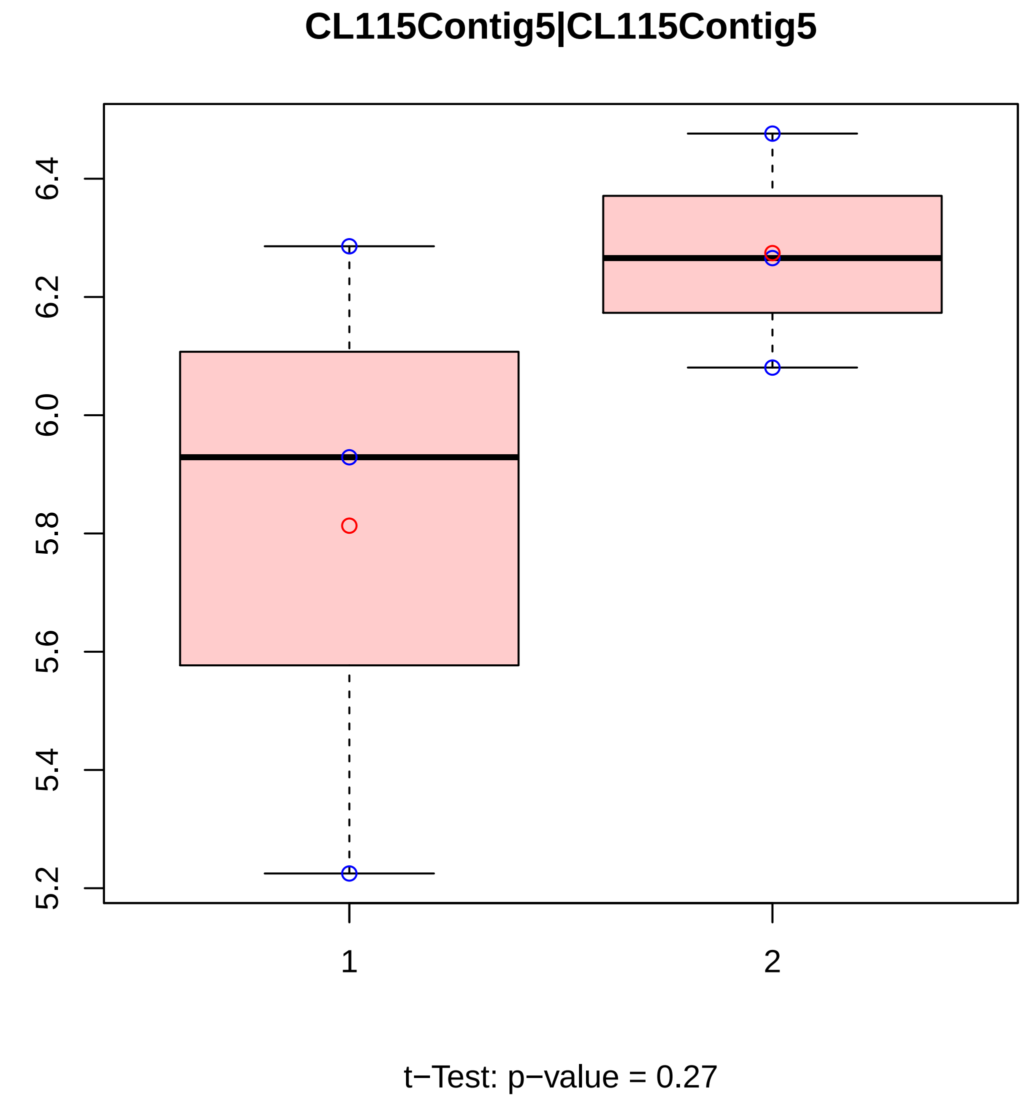
CL115Contig5|CL115Contig5
6.4
6.2
6.0
5.8
5.6
5.4
5.2
1
2
t−Test: p−value = 0.27

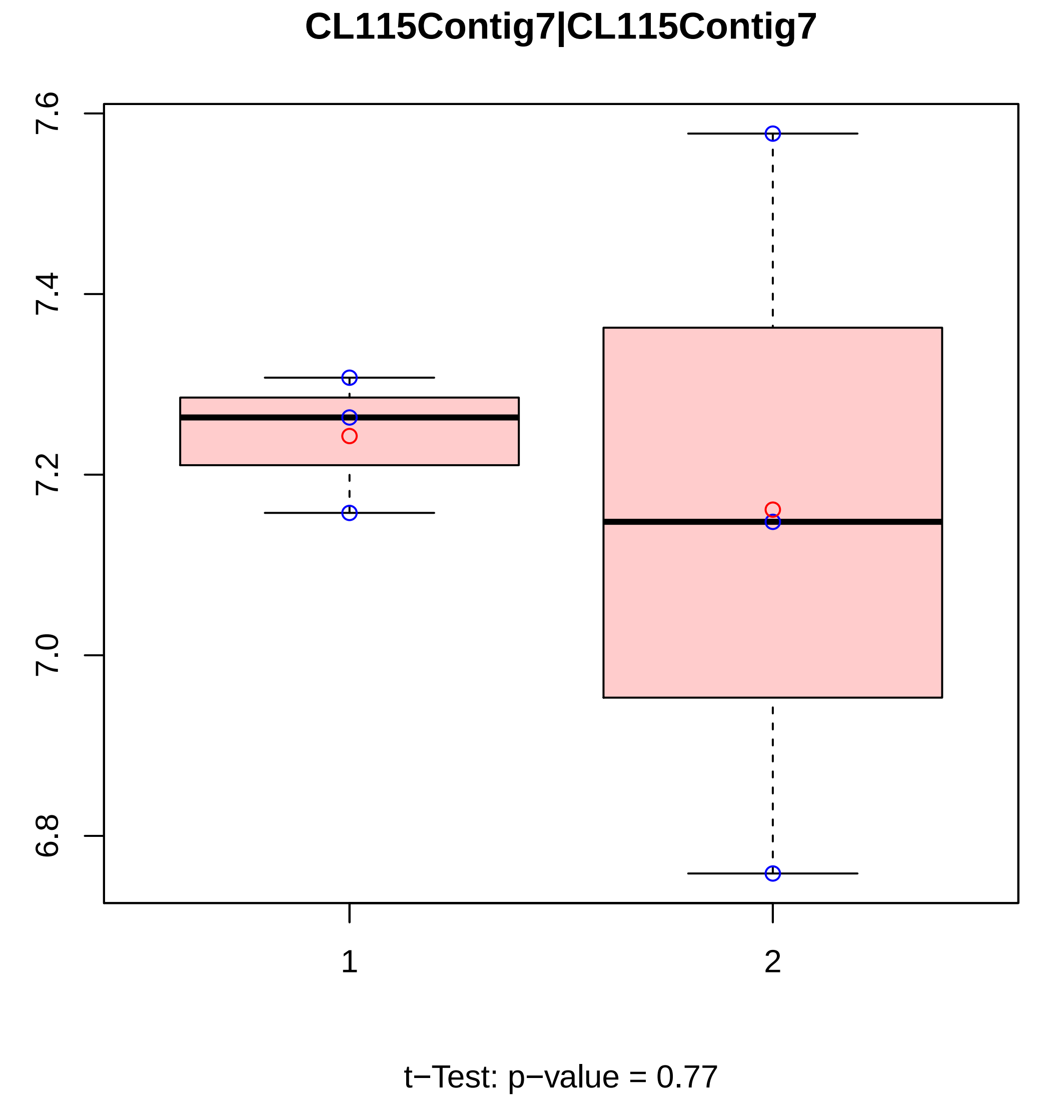
CL115Contig7|CL115Contig7
7.6
7.4
7.2
7.0
6.8
1
2
t−Test: p−value = 0.77

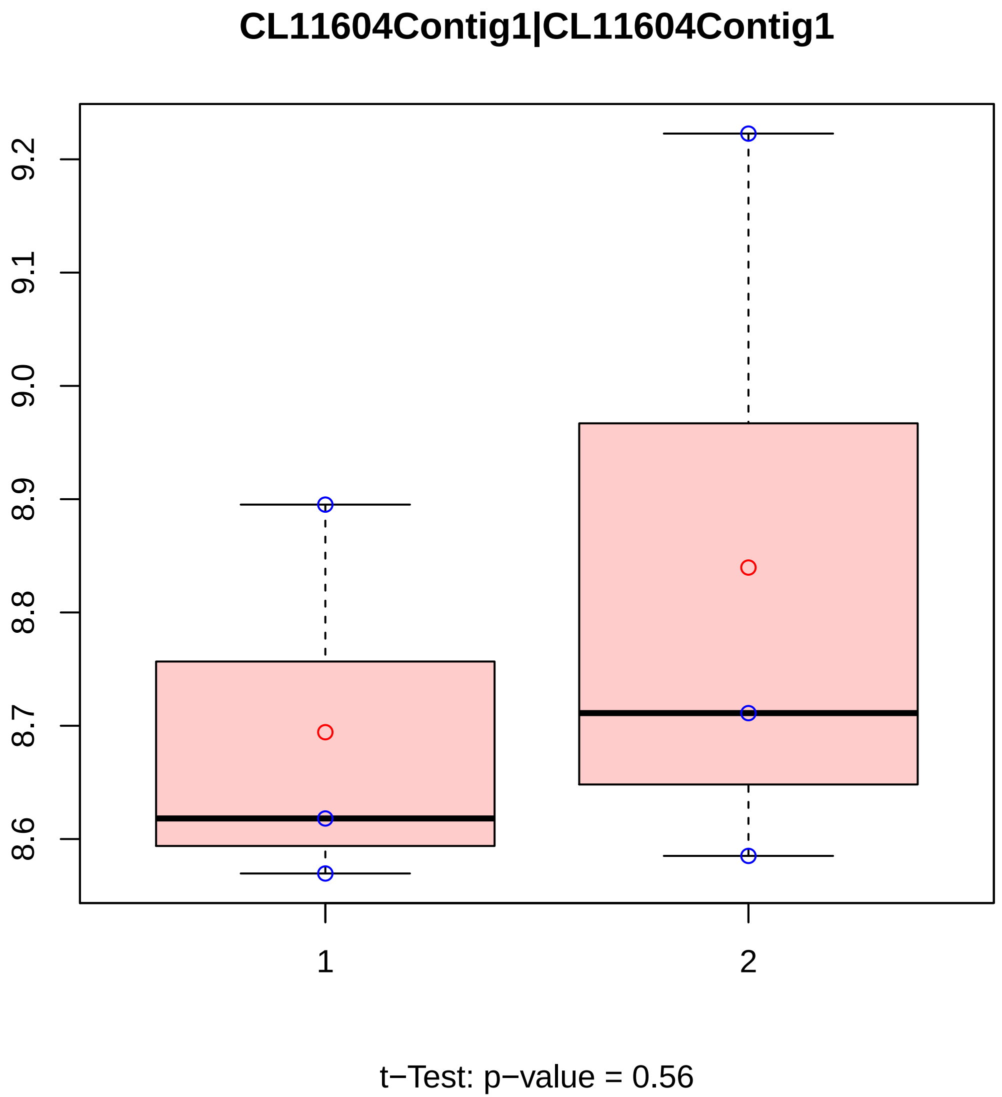
CL11604Contig1|CL11604Contig1
9.2
9.1
9.0
8.9
8.8
8.7
8.6
1
2
t−Test: p−value = 0.56

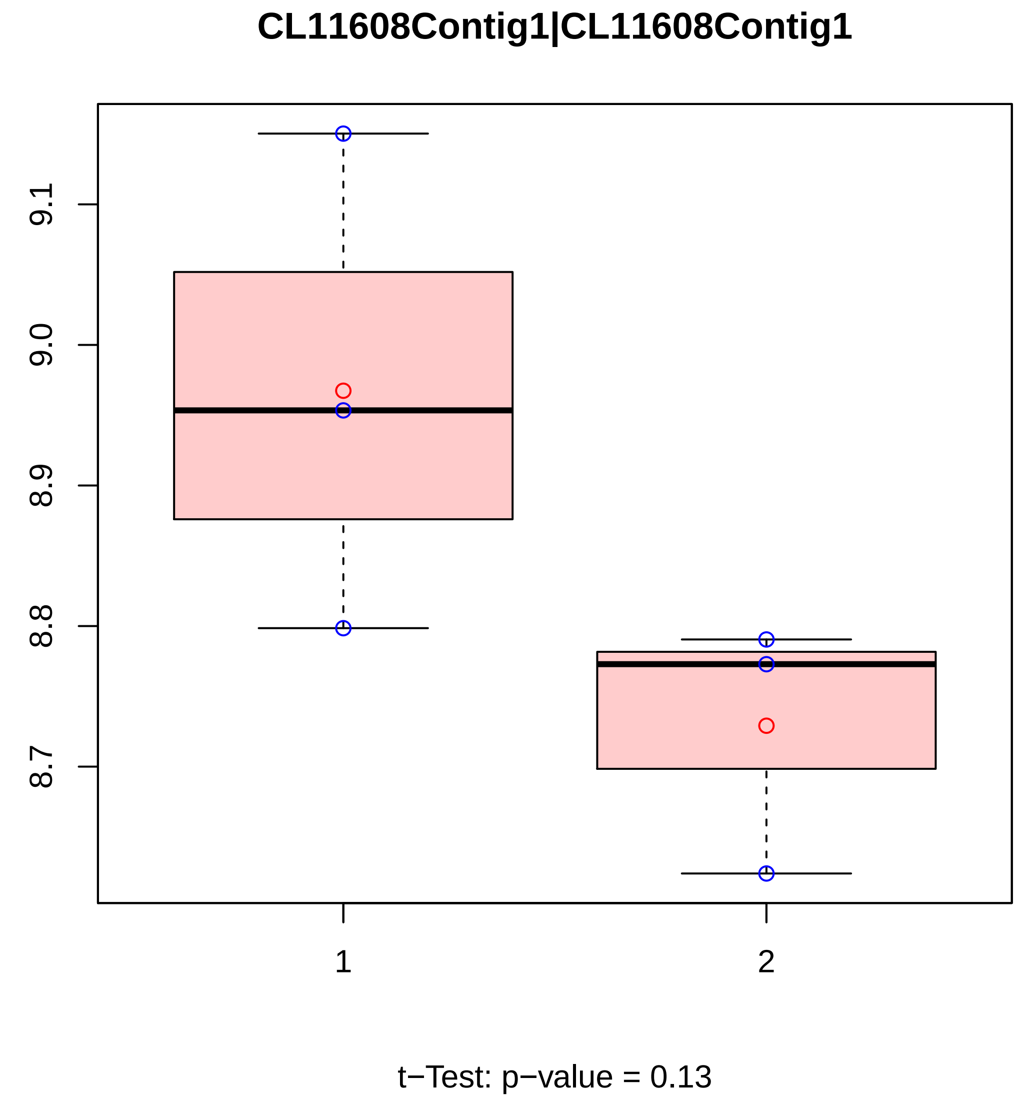
CL11608Contig1|CL11608Contig1
9.1
9.0
8.9
8.8
8.7
1
2
t−Test: p−value = 0.13

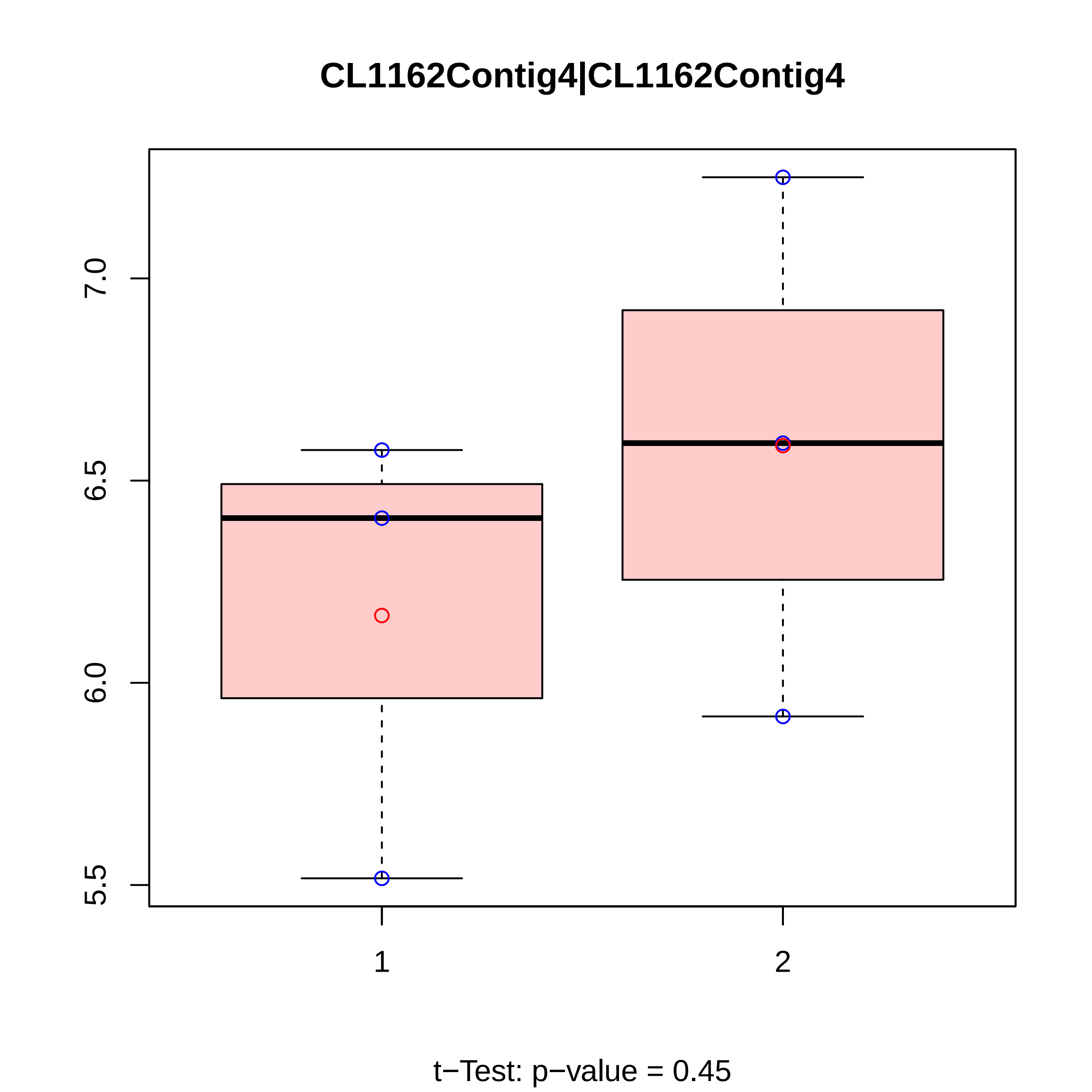
CL1162Contig4|CL1162Contig4
7.0
6.5
6.0
5.5
1
2
t−Test: p−value = 0.45

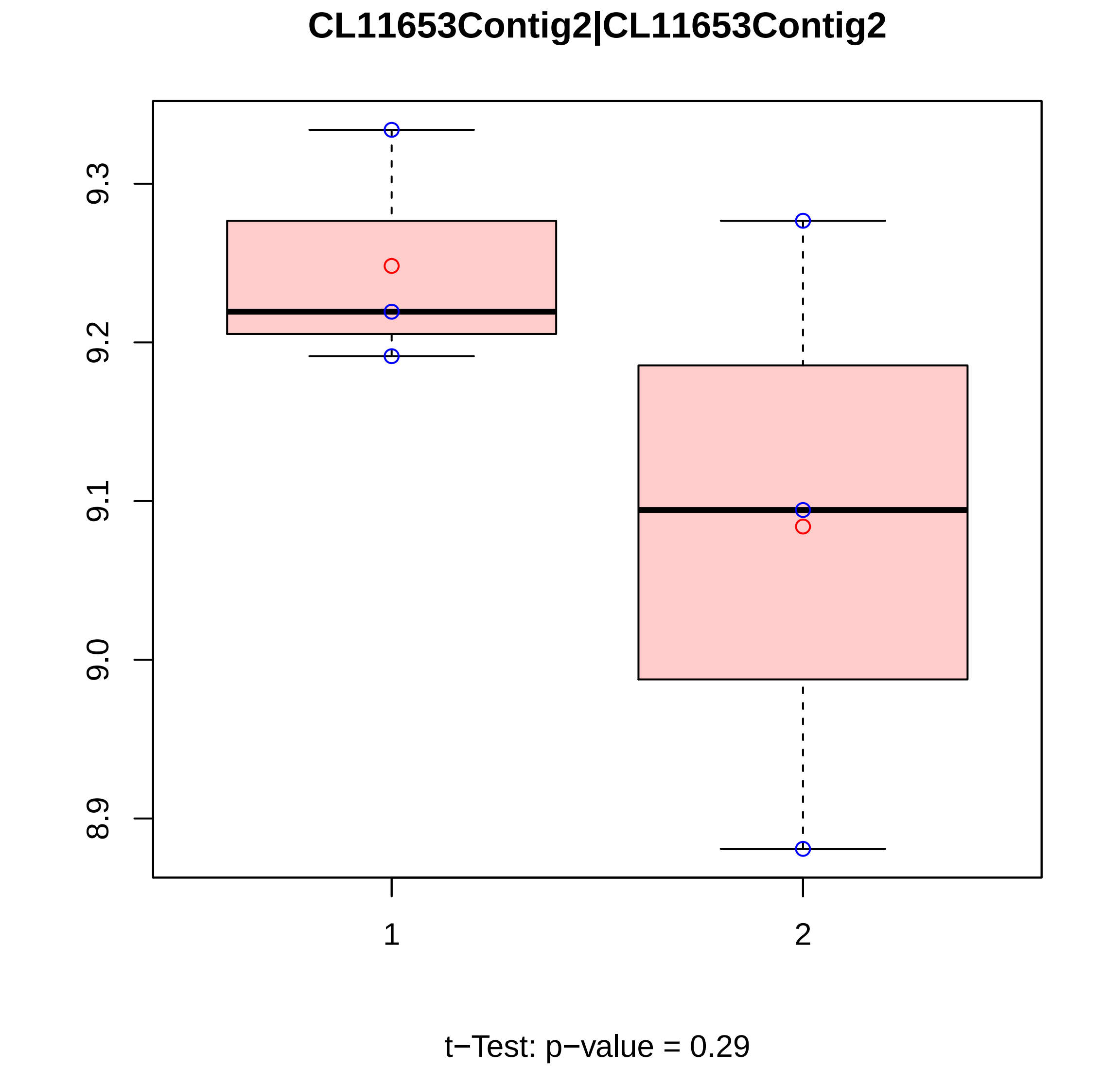
CL11653Contig2|CL11653Contig2
9.3
9.2
9.1
9.0
8.9
1
2
t−Test: p−value = 0.29

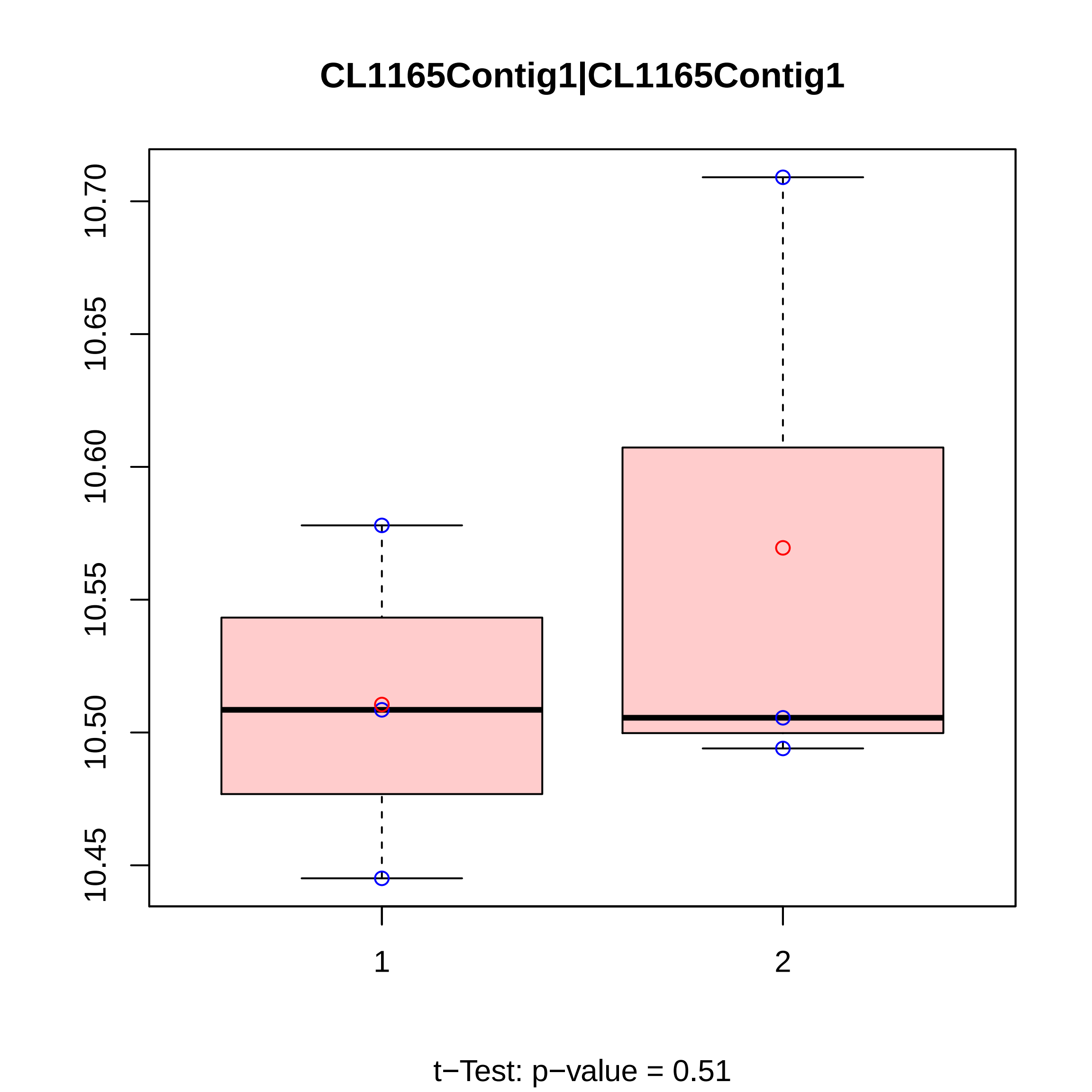
CL1165Contig1|CL1165Contig1
10.45
10.50
10.55
10.60
10.65
10.70
1
2
t−Test: p−value = 0.51

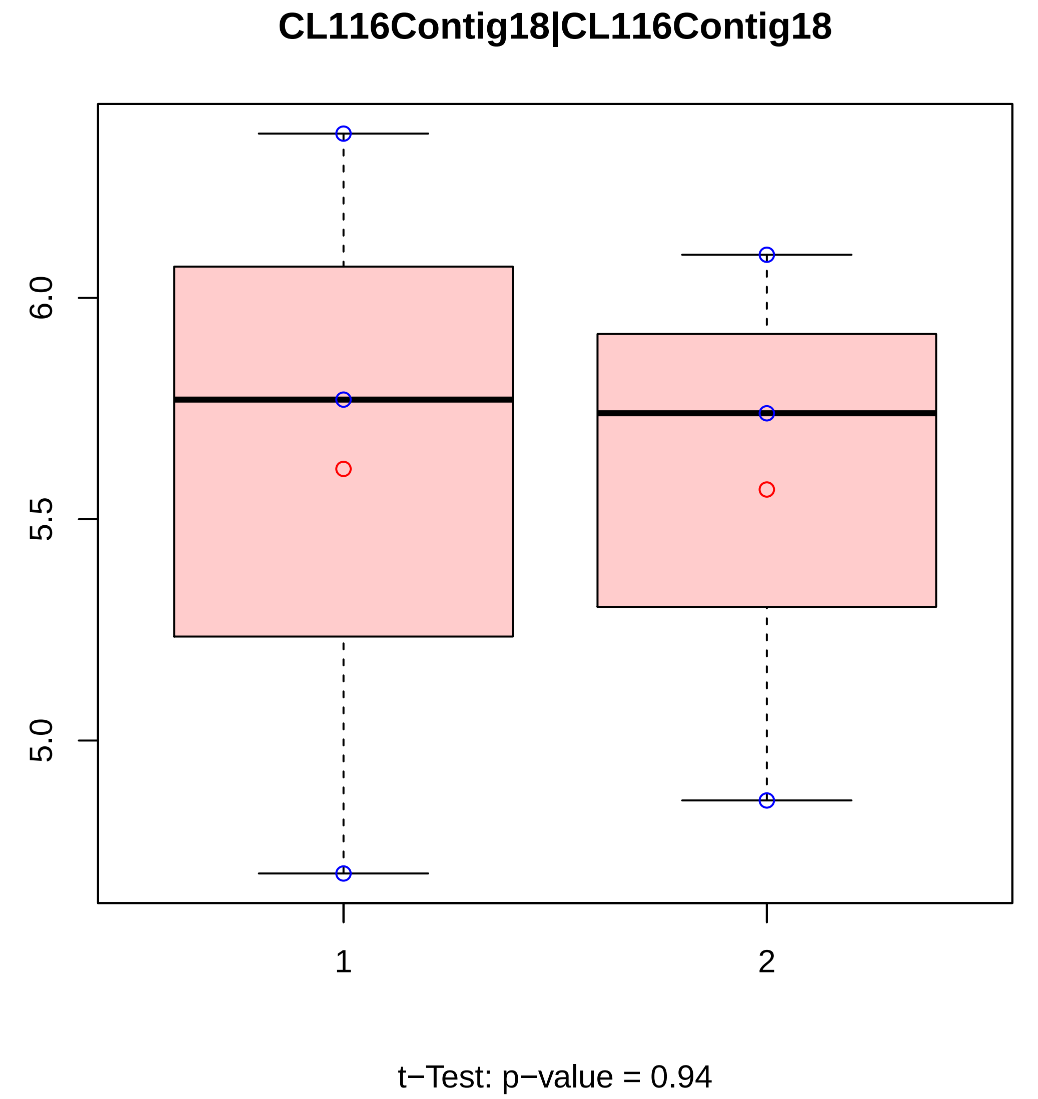

CL116Contig18|CL116Contig18
6.0
5.5
5.0
1
2
t−Test: p−value = 0.94

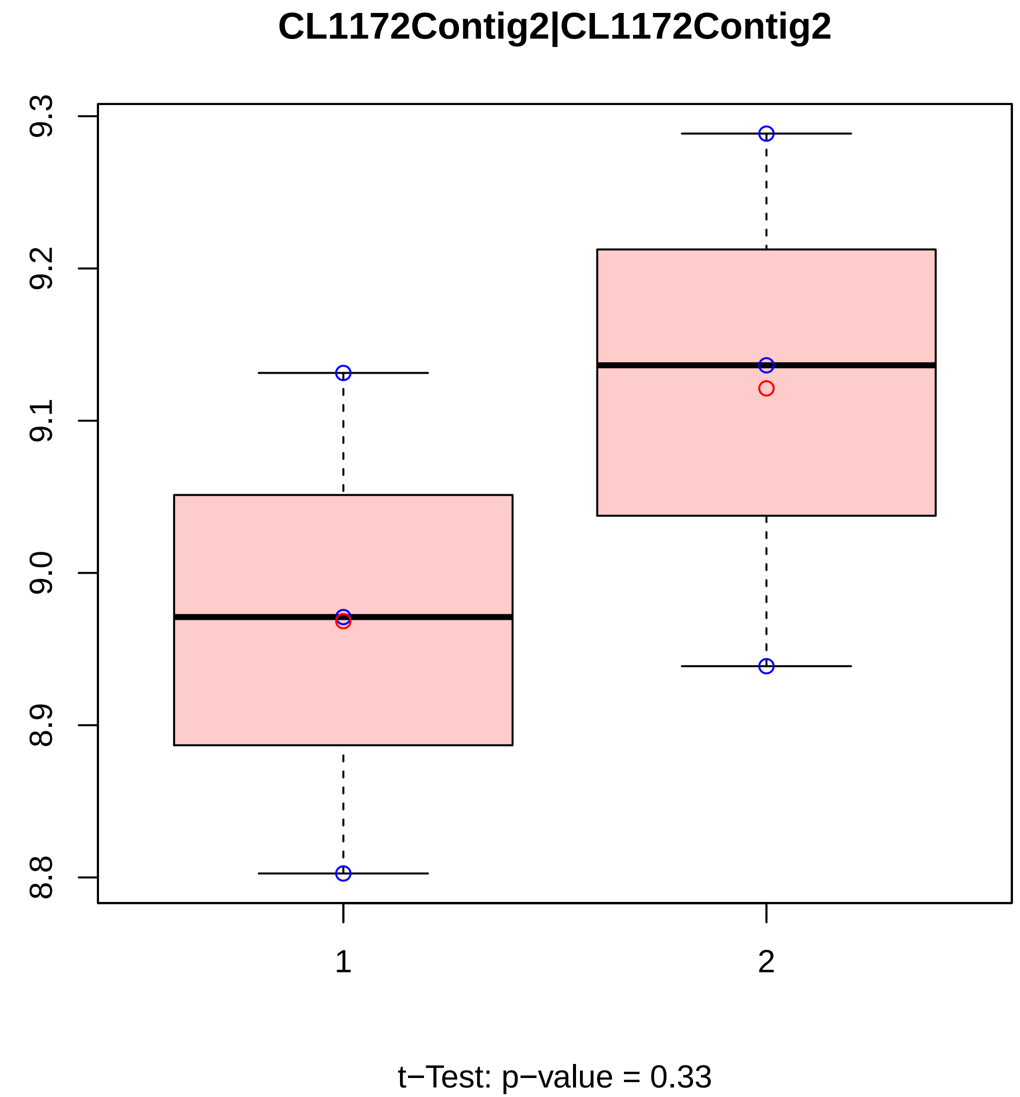

CL1172Contig2|CL1172Contig2
9.3
9.2
9.1
9.0
8.9
8.8
1
2
t−Test: p−value = 0.33

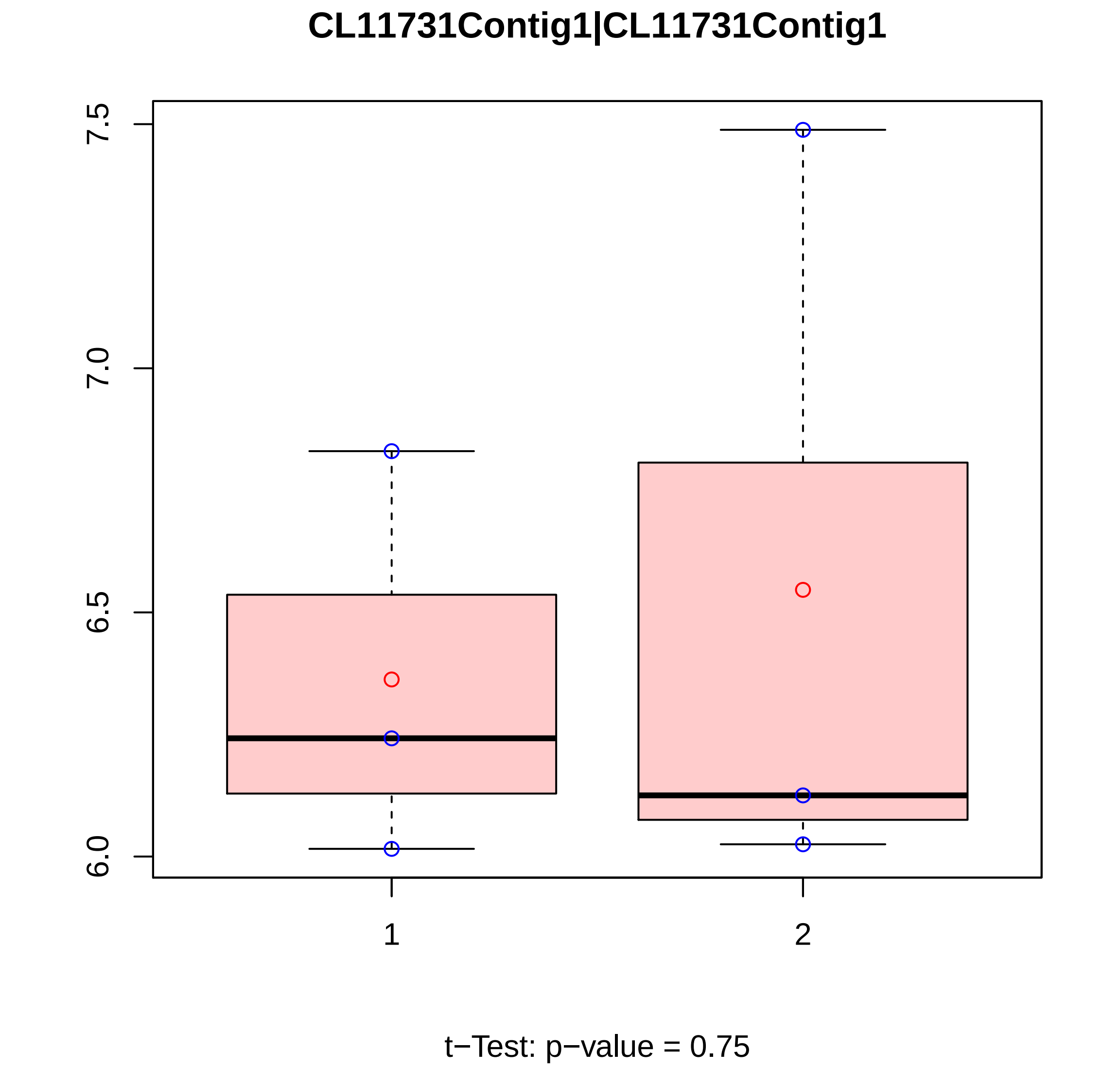

CL11731Contig1|CL11731Contig1
7.5
7.0
6.5
6.0
1
2
t−Test: p−value = 0.75

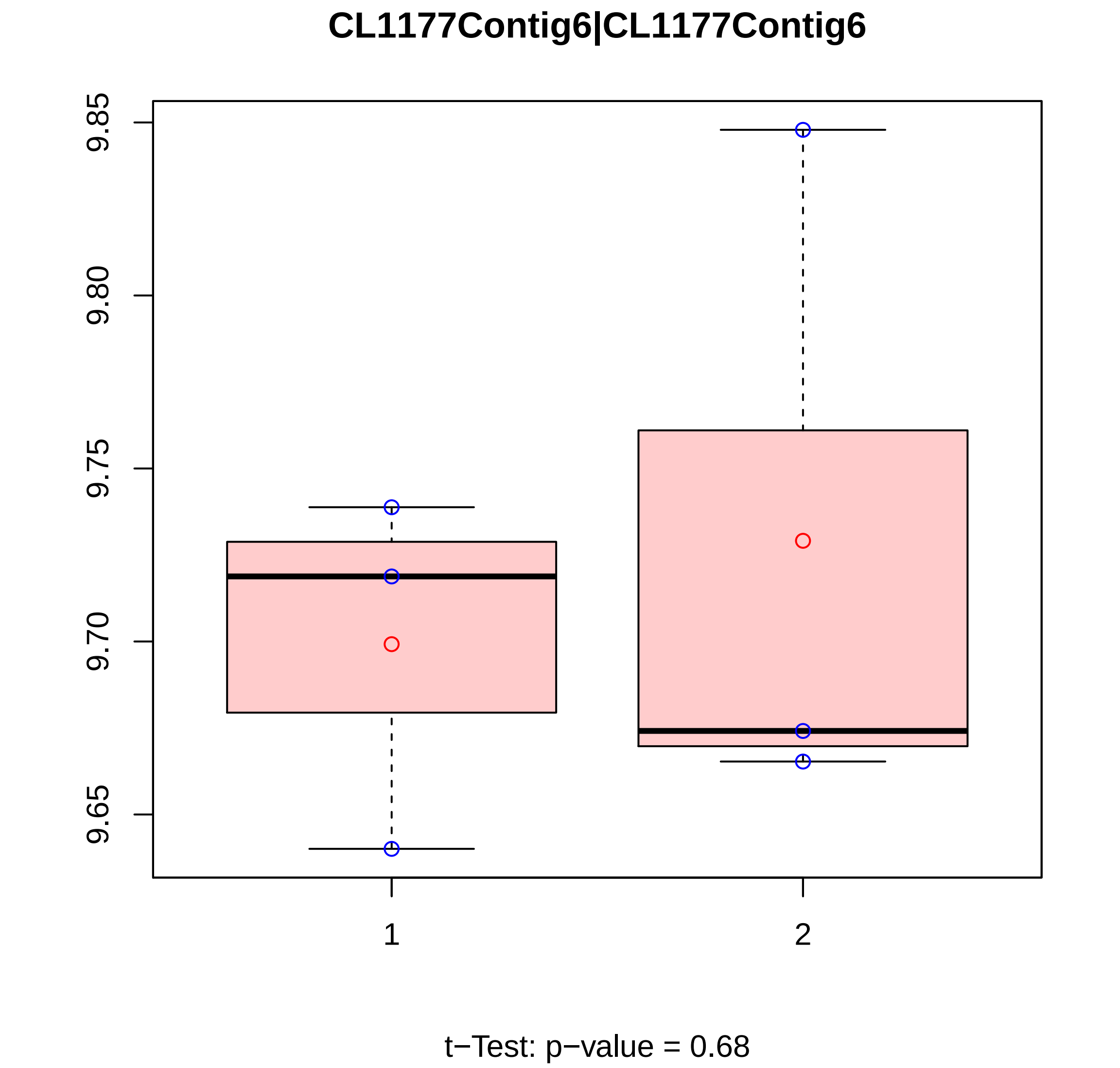

CL1177Contig6|CL1177Contig6
9.65
9.70
9.75
9.80
9.85
1
2
t−Test: p−value = 0.68

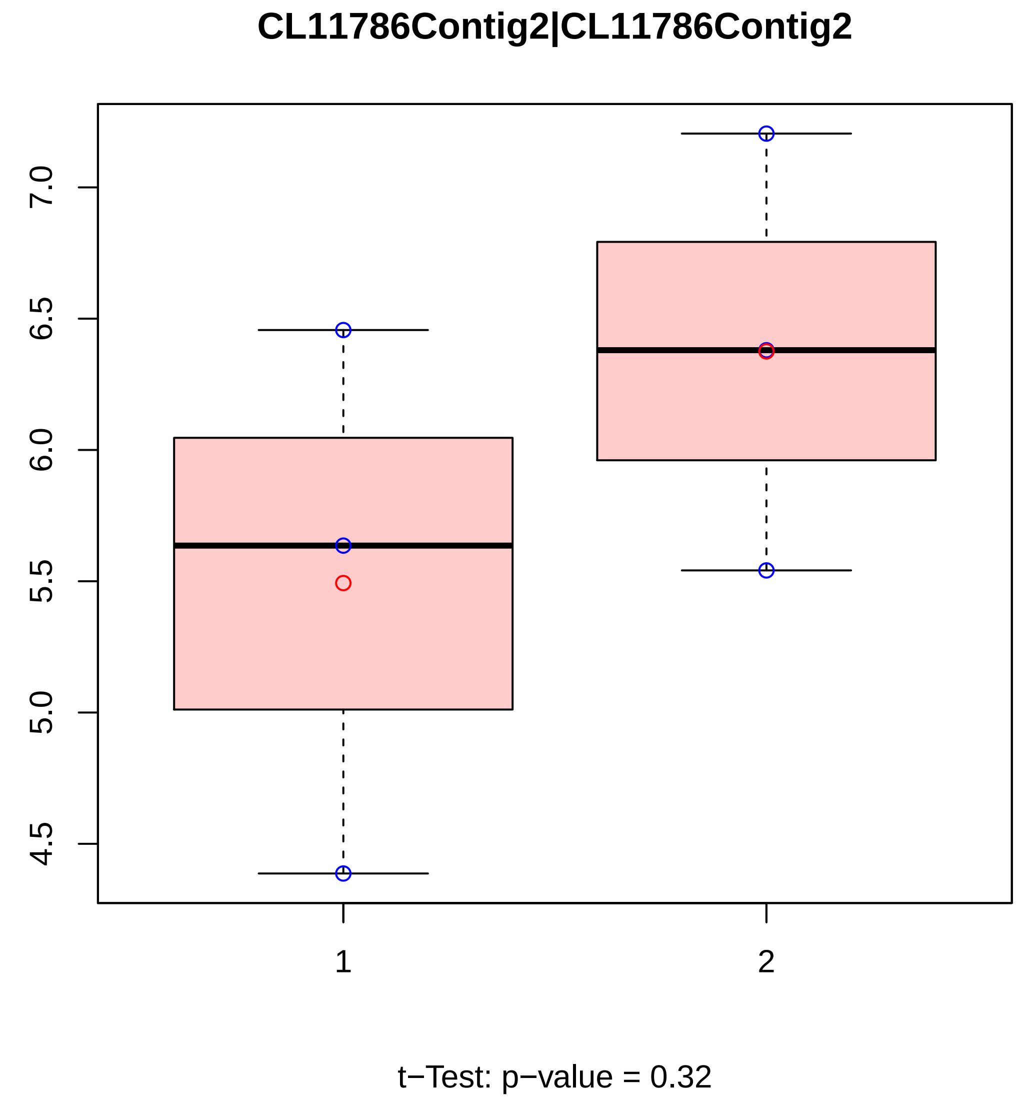
CL11786Contig2|CL11786Contig2
7.0
6.5
6.0
5.5
5.0
4.5
1
2
t−Test: p−value = 0.32

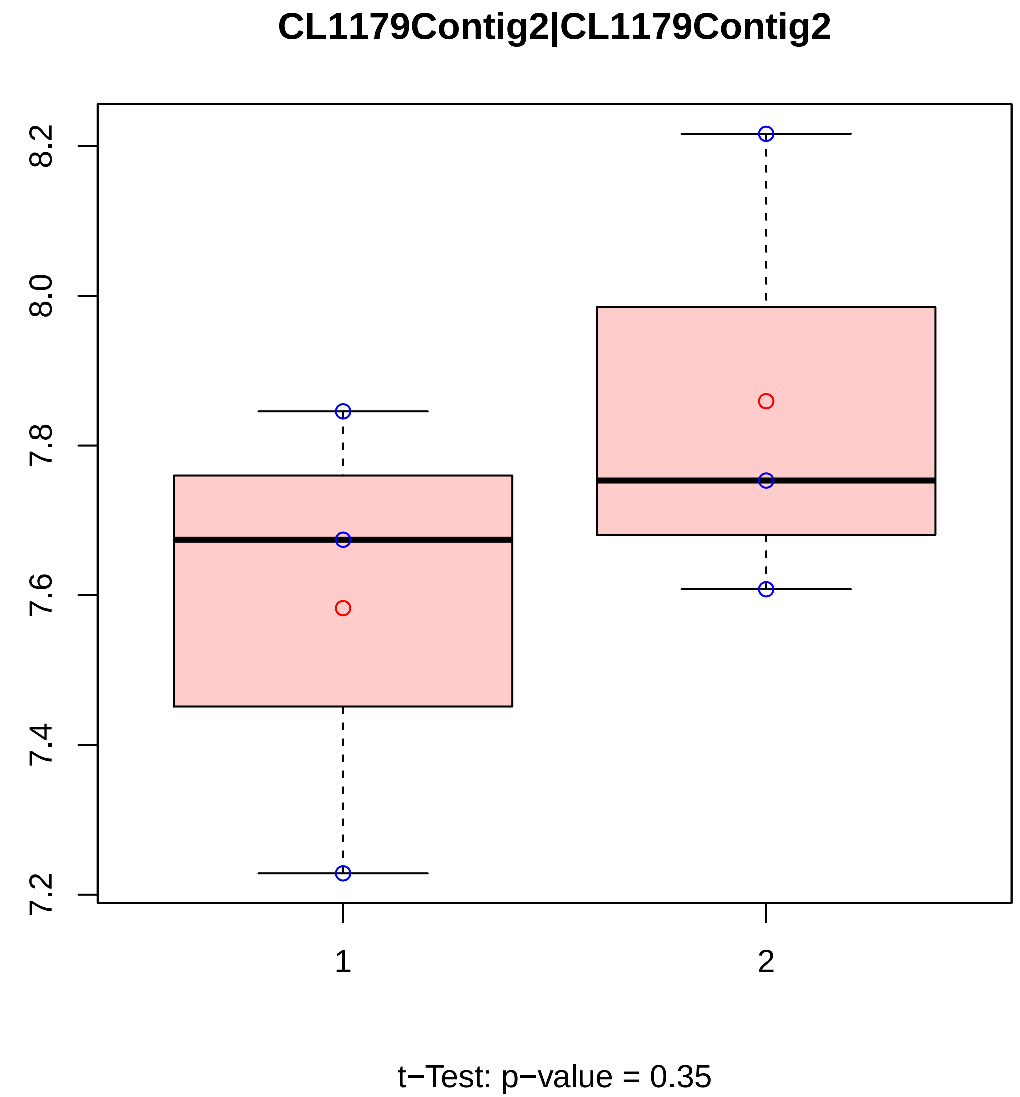
CL1179Contig2|CL1179Contig2
8.2
8.0
7.8
7.6
7.4
7.2
1
2
t−Test: p−value = 0.35

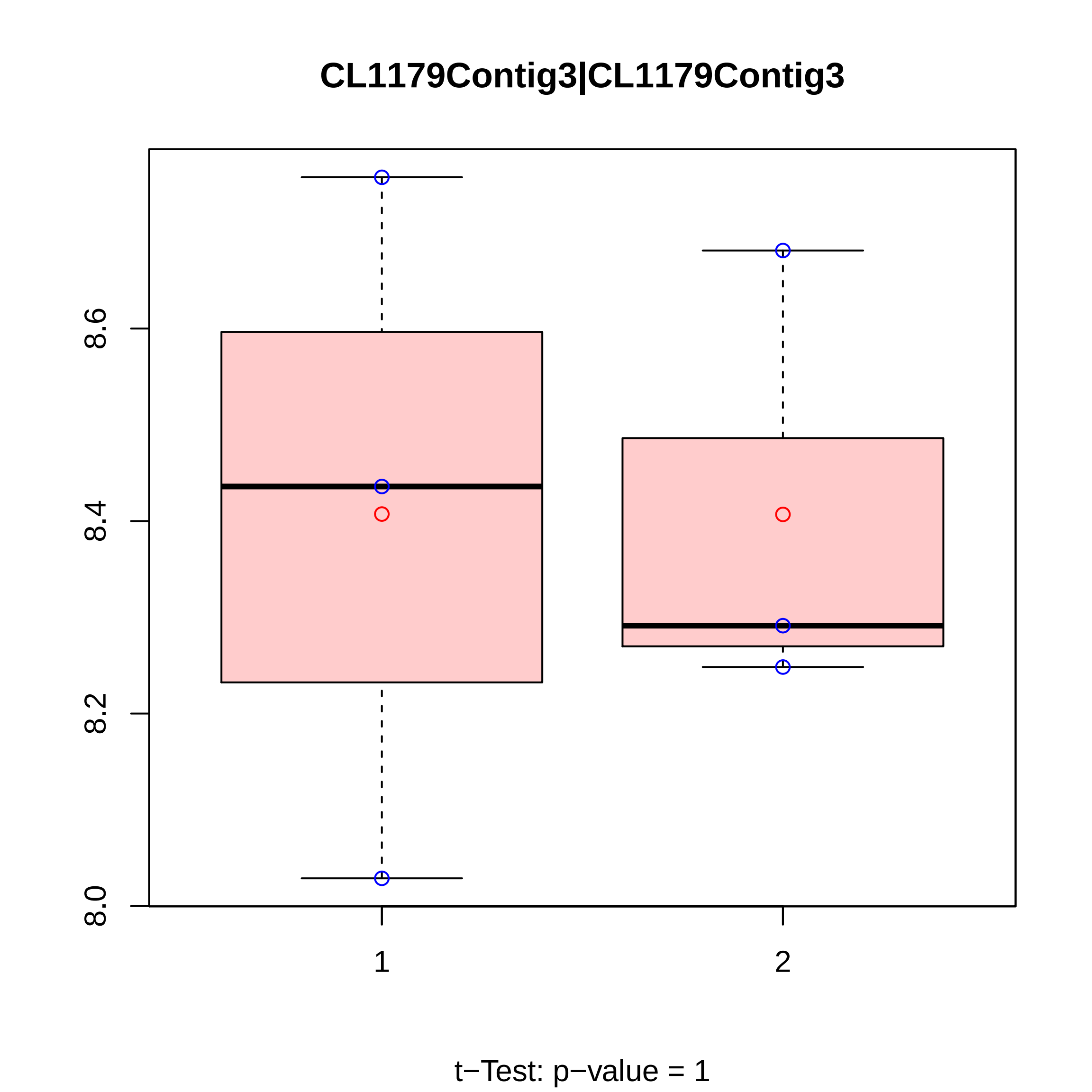
CL1179Contig3|CL1179Contig3
8.0
8.2
8.4
8.6
1
2
t−Test: p−value = 1

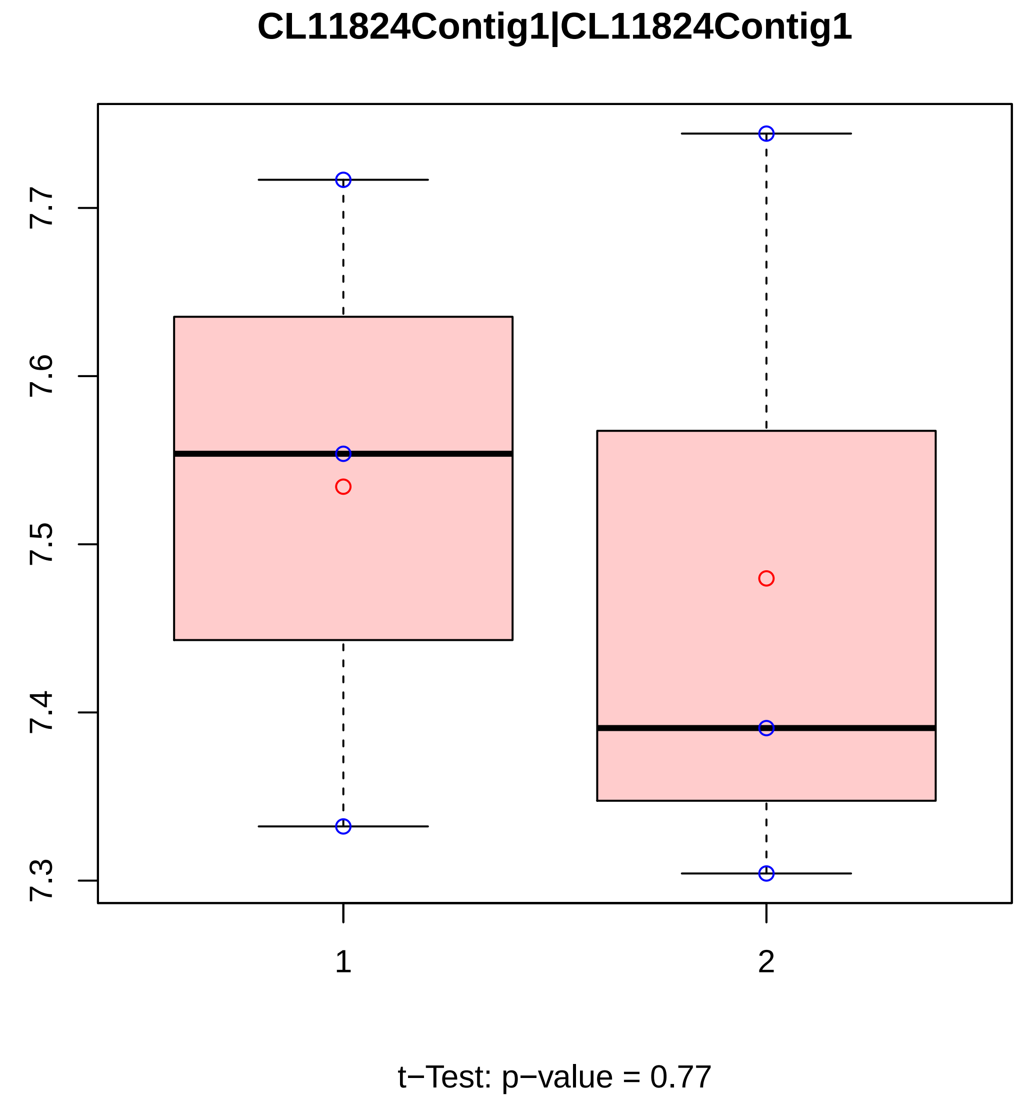
CL11824Contig1|CL11824Contig1
7.3
7.4
7.5
7.6
7.7
1
2
t−Test: p−value = 0.77

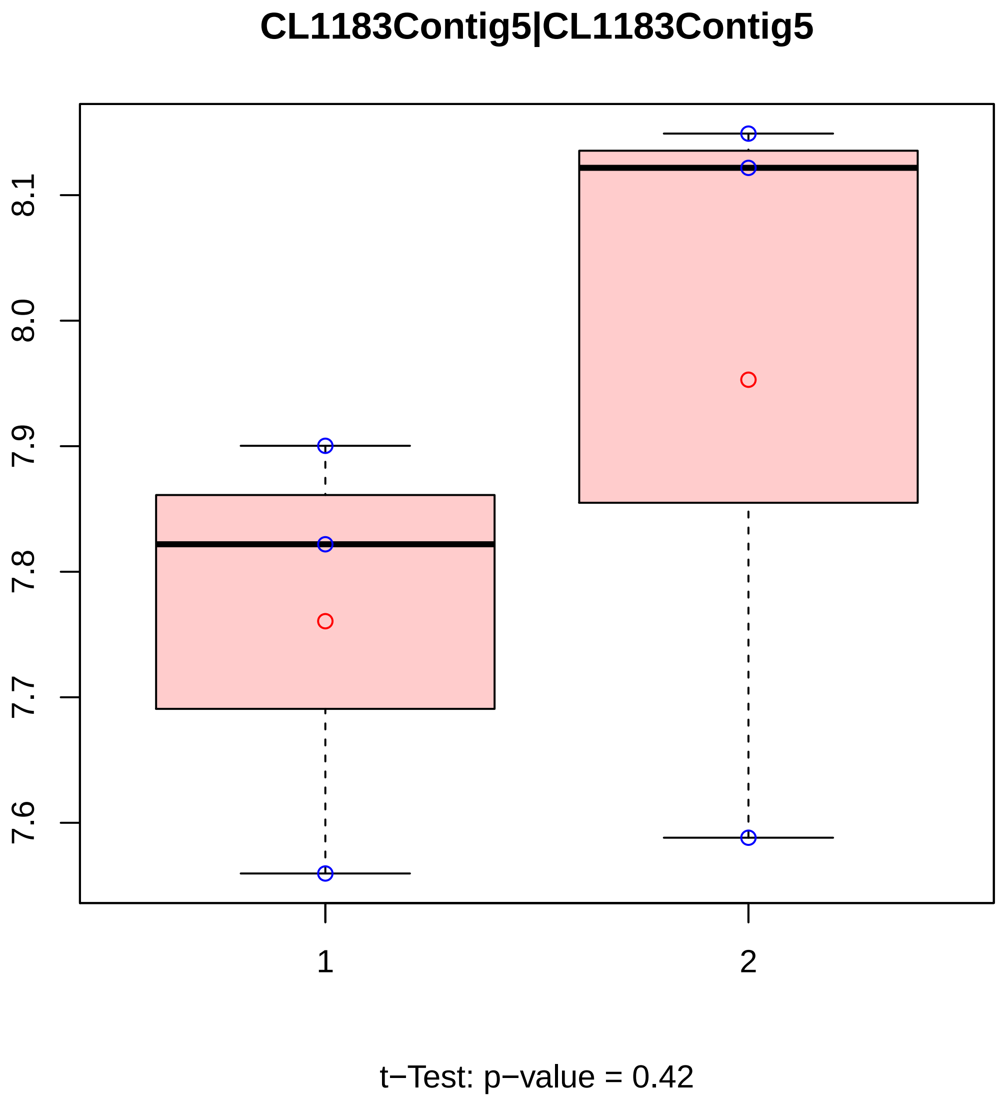

CL1183Contig5|CL1183Contig5
8.1
8.0
7.9
7.8
7.7
7.6
1
2
t−Test: p−value = 0.42

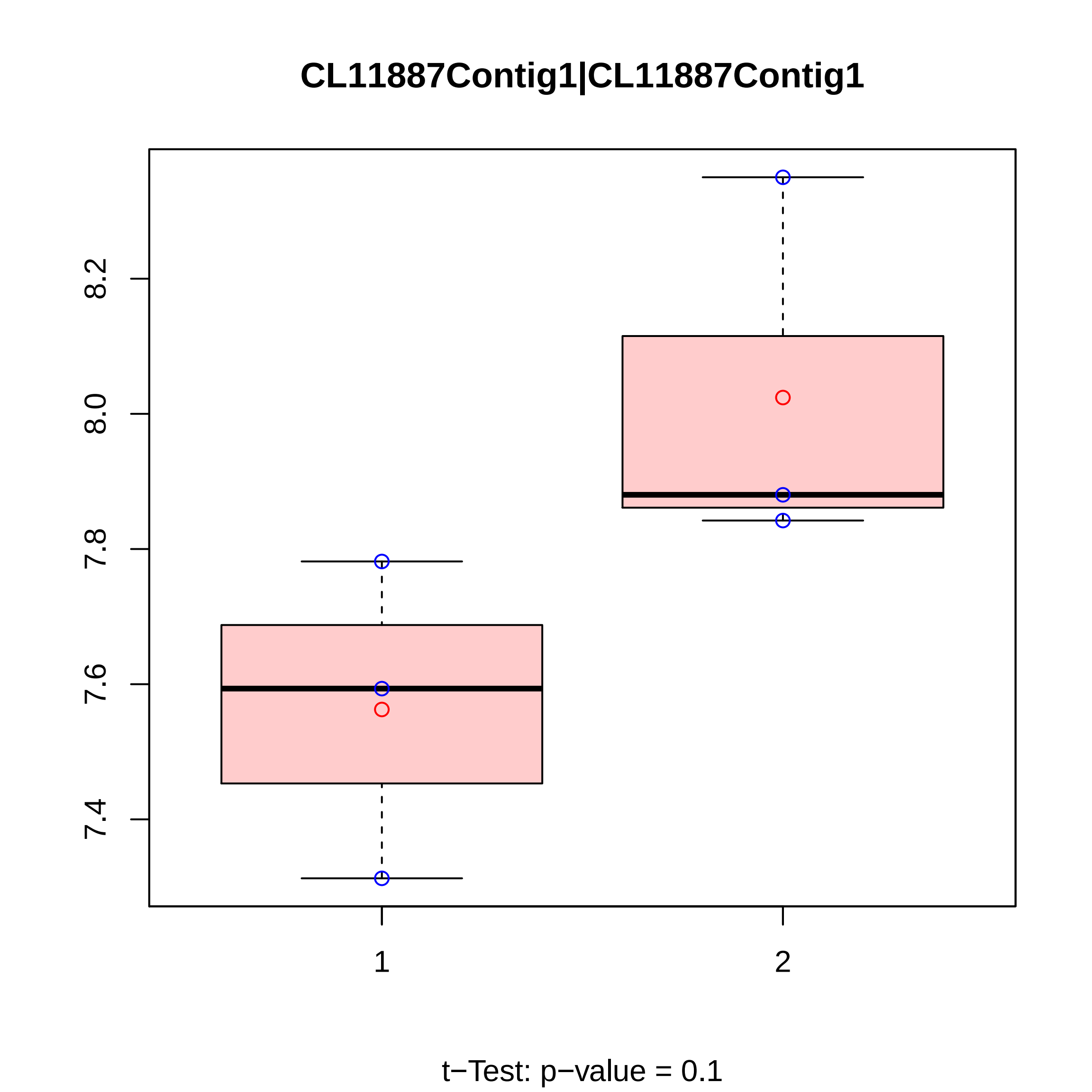
CL11887Contig1|CL11887Contig1
8.2
8.0
7.8
7.6
7.4
1
2
t−Test: p−value = 0.1

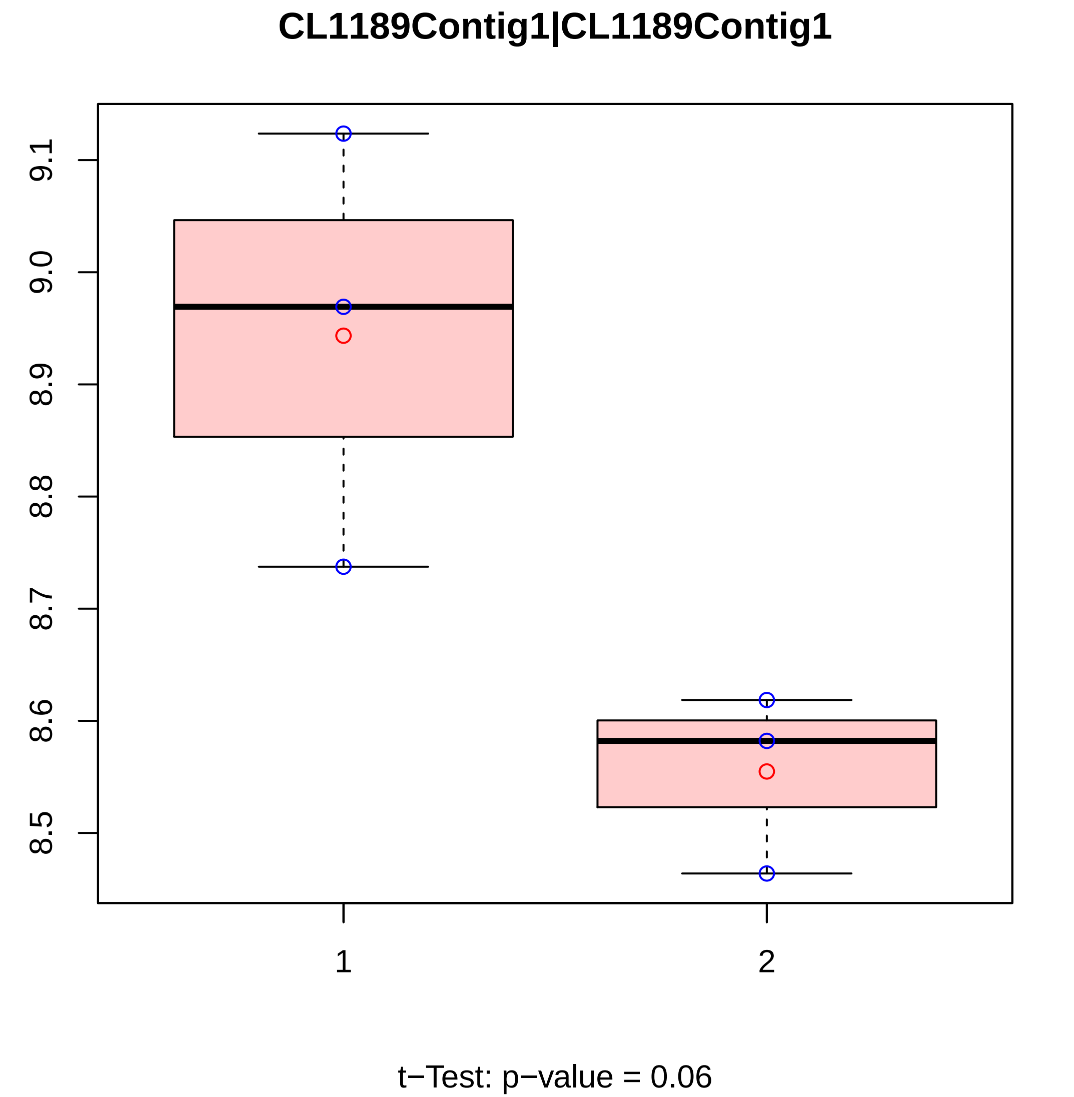
CL1189Contig1|CL1189Contig1
9.1
9.0
8.9
8.8
8.7
8.6
8.5
1
2
t−Test: p−value = 0.06

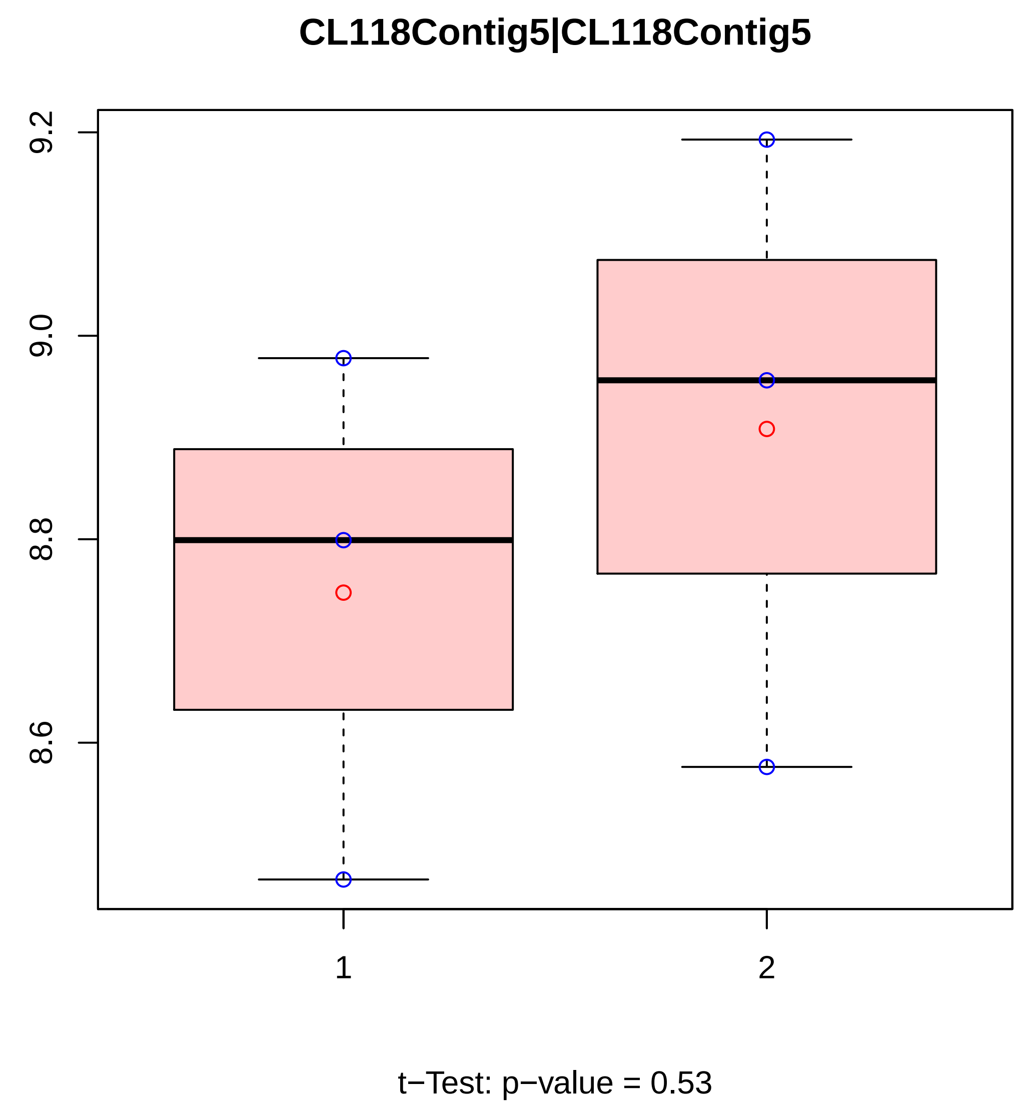
CL118Contig5|CL118Contig5
9.2
9.0
8.8
8.6
1
2
t−Test: p−value = 0.53

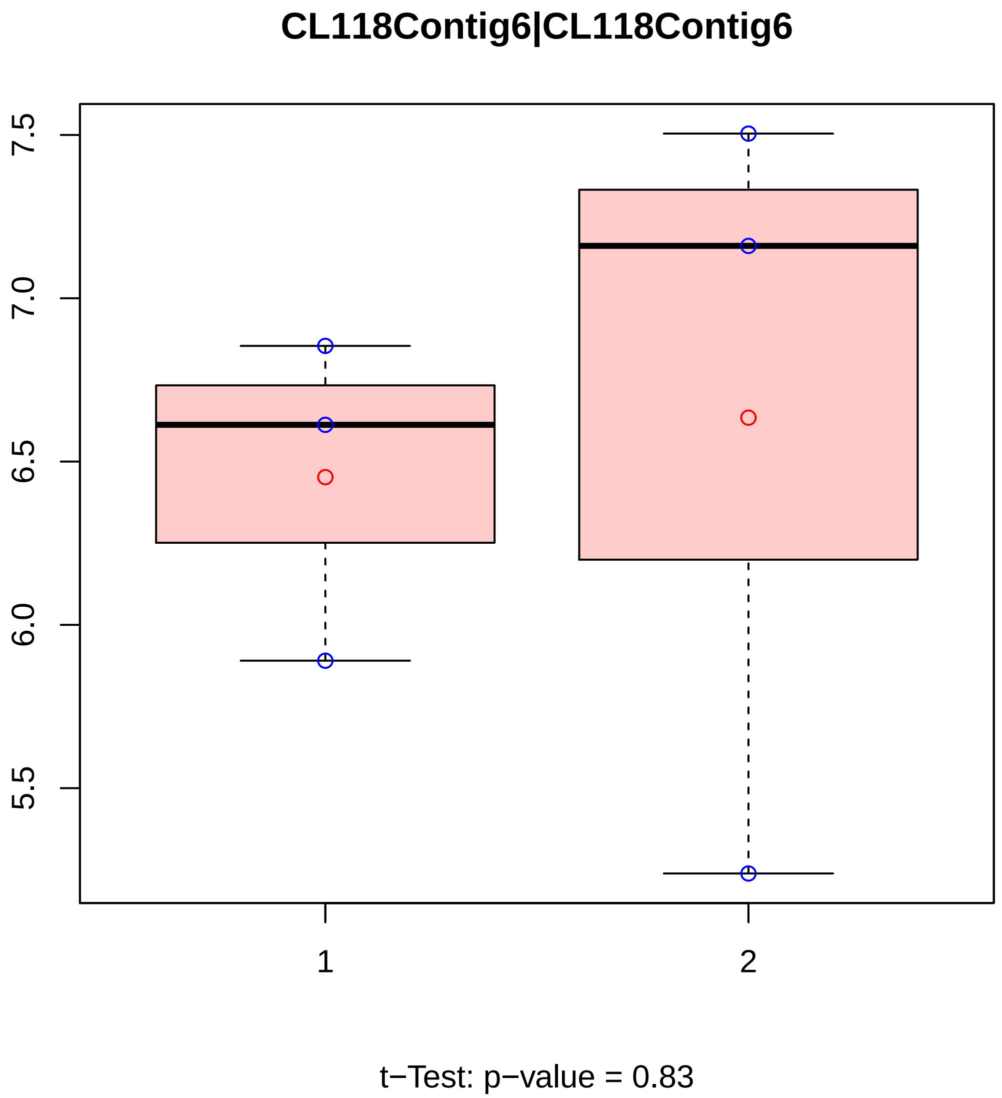
CL118Contig6|CL118Contig6
7.5
7.0
6.5
6.0
5.5
1
2
t−Test: p−value = 0.83

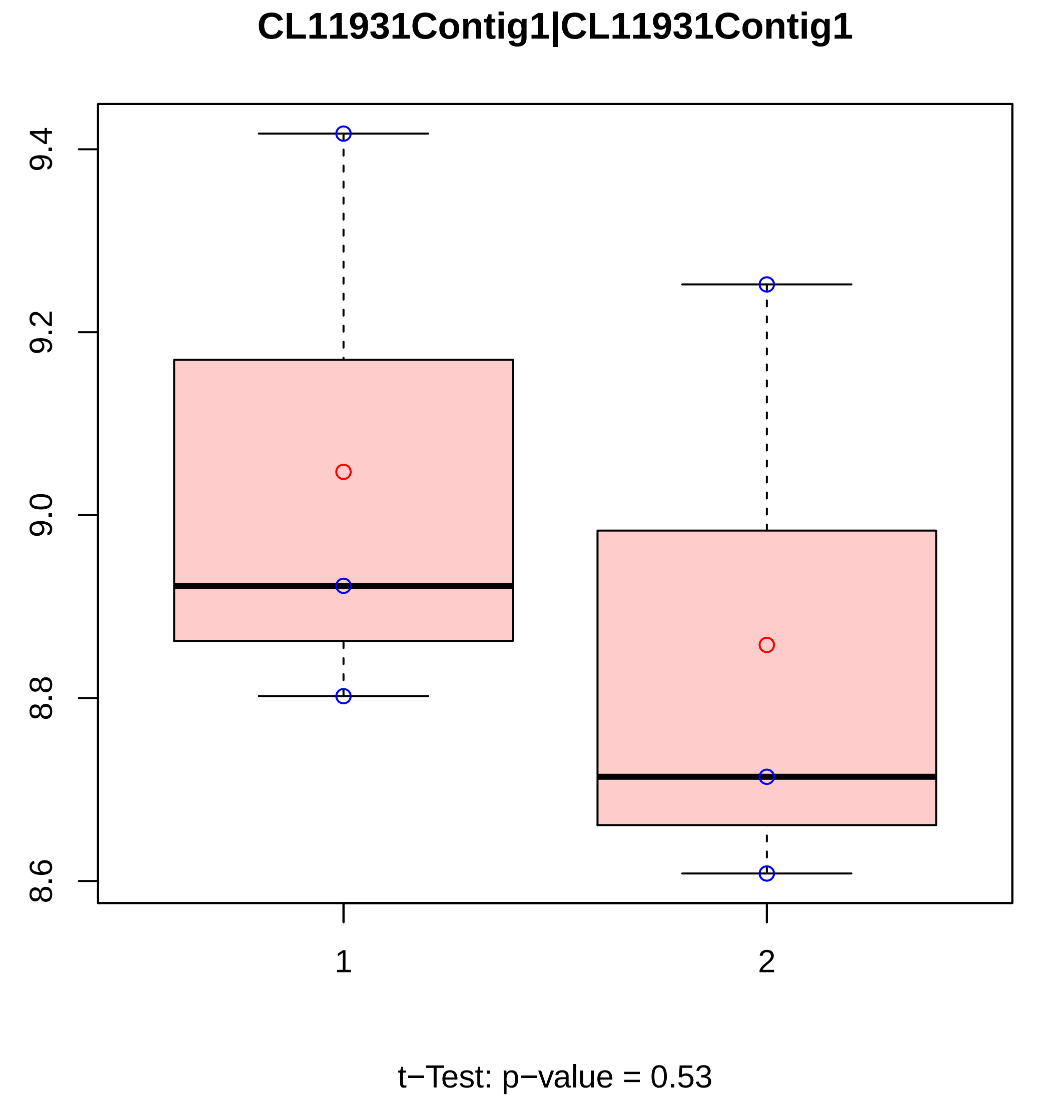
CL11931Contig1|CL11931Contig1
9.4
9.2
9.0
8.8
8.6
1
2
t−Test: p−value = 0.53

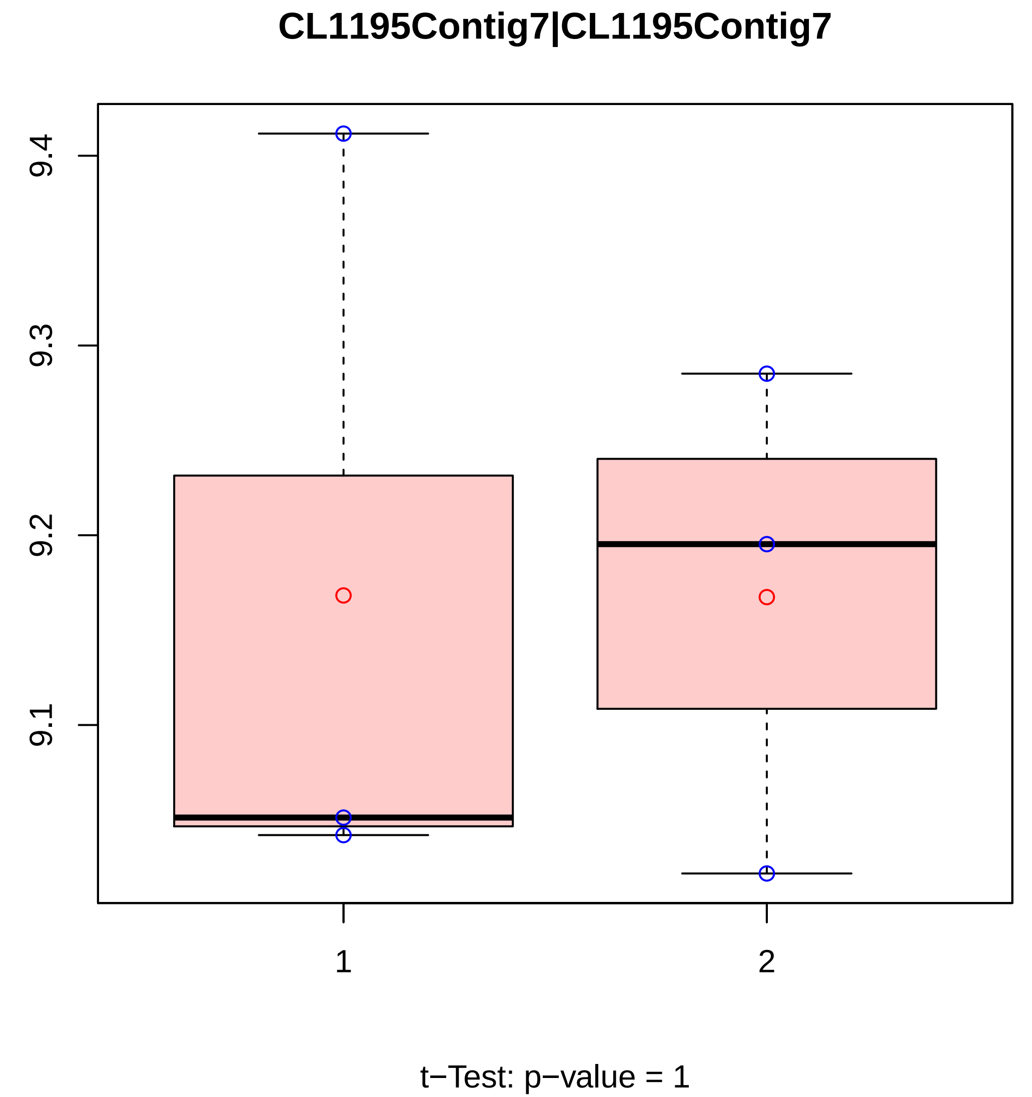
CL1195Contig7|CL1195Contig7
9.1
9.2
9.3
9.4
1
2
t−Test: p−value = 1

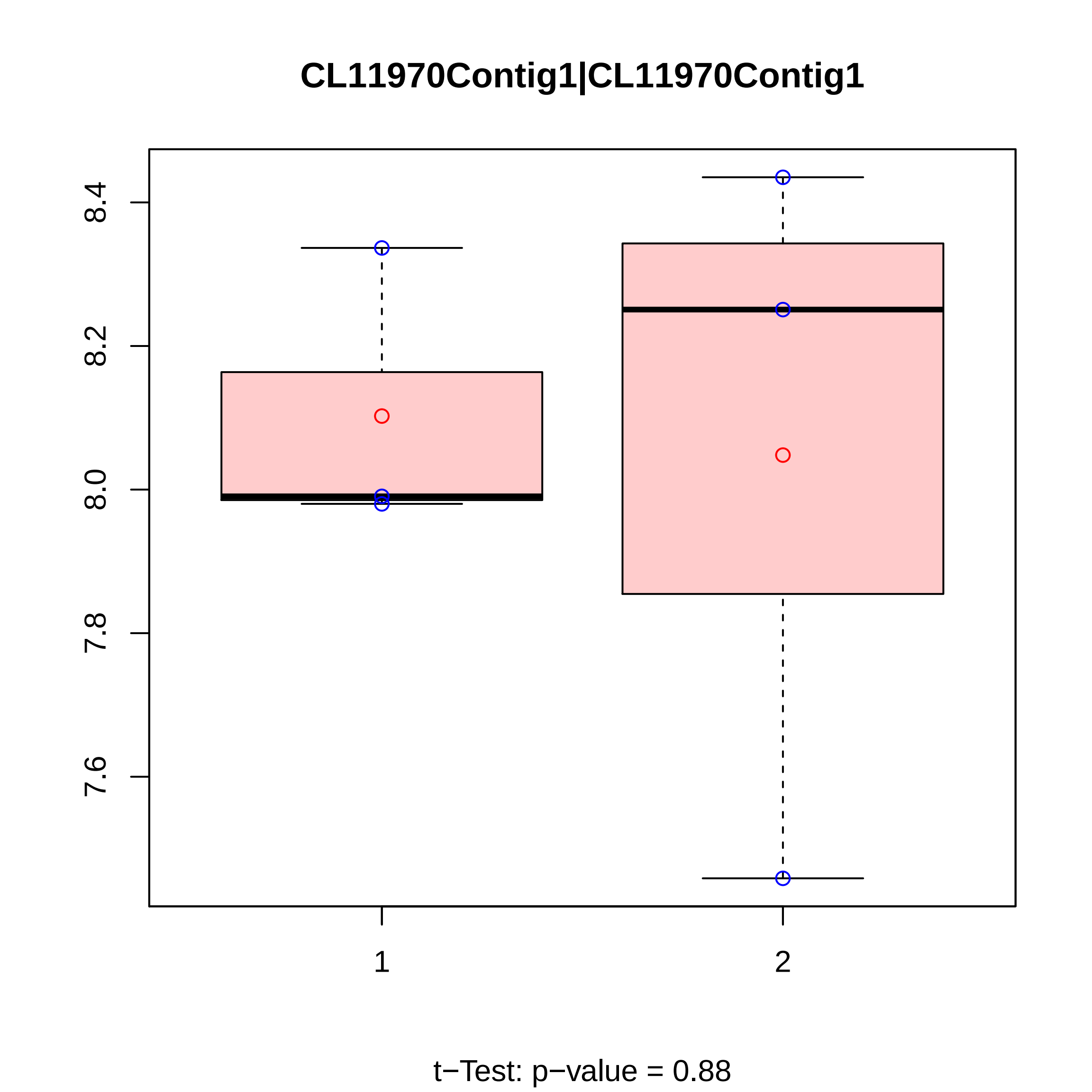
CL11970Contig1|CL11970Contig1
8.4
8.2
8.0
7.8
7.6
1
2
t−Test: p−value = 0.88

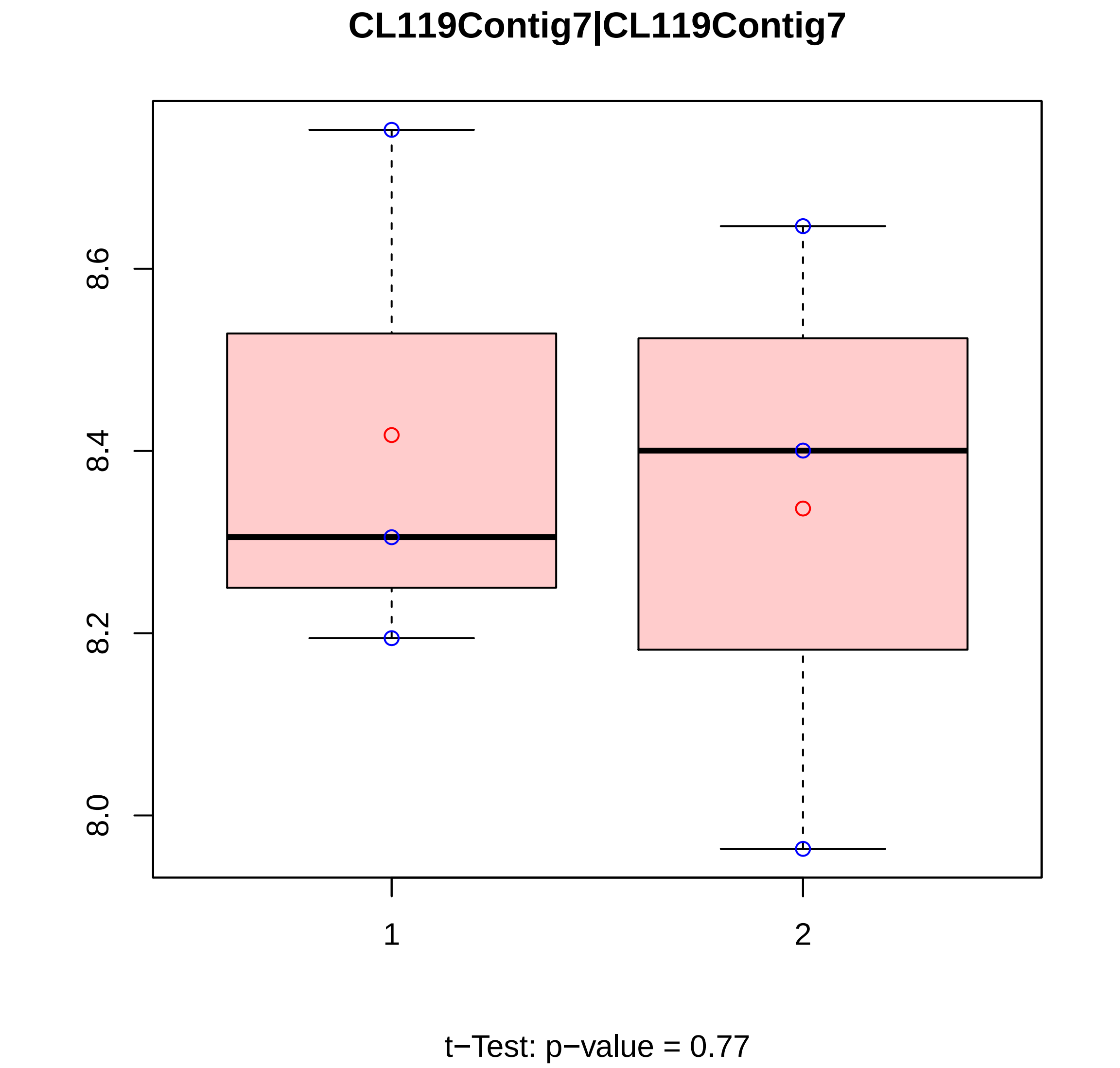
CL119Contig7|CL119Contig7
8.0
8.2
8.4
8.6
1
2
t−Test: p−value = 0.77

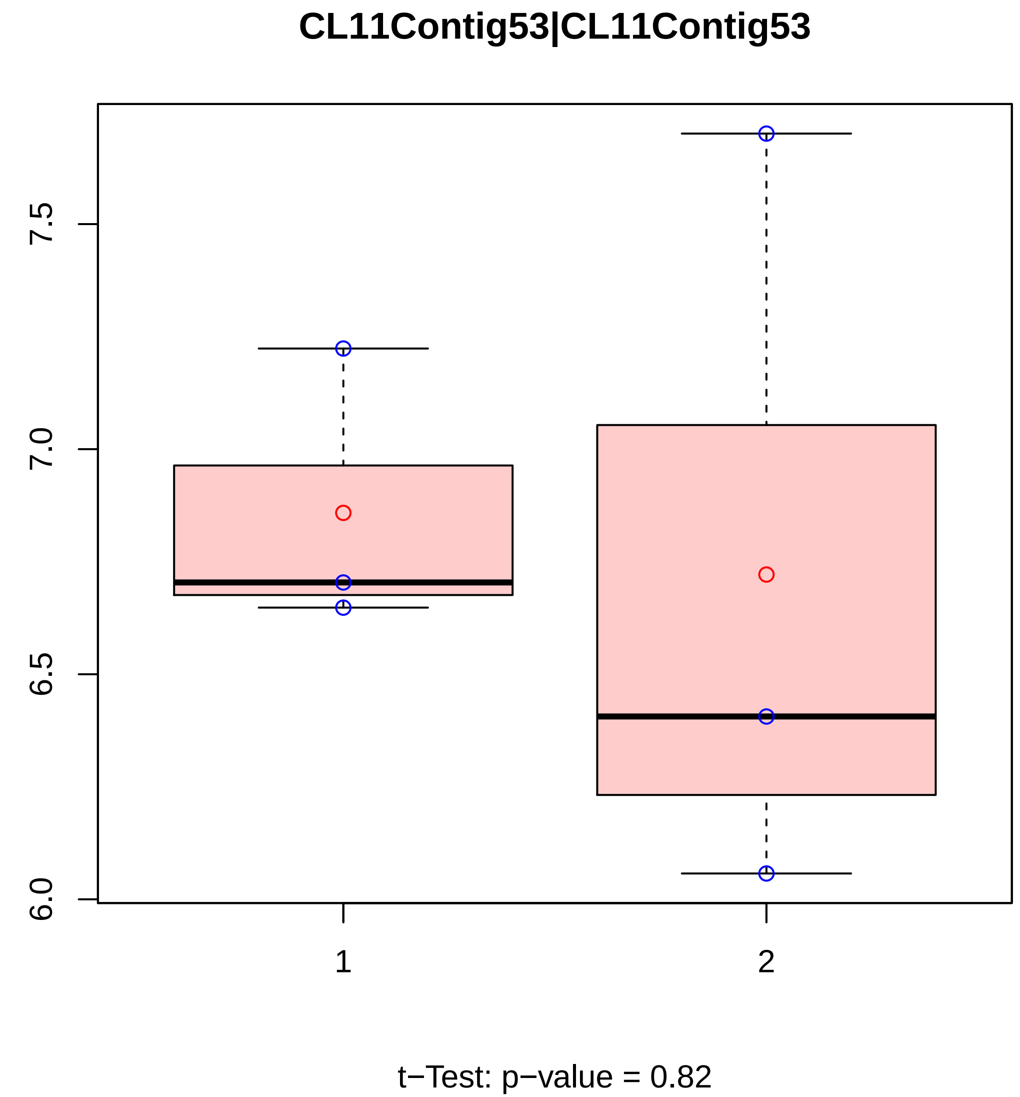
CL11Contig53|CL11Contig53
7.5
7.0
6.5
6.0
1
2
t−Test: p−value = 0.82

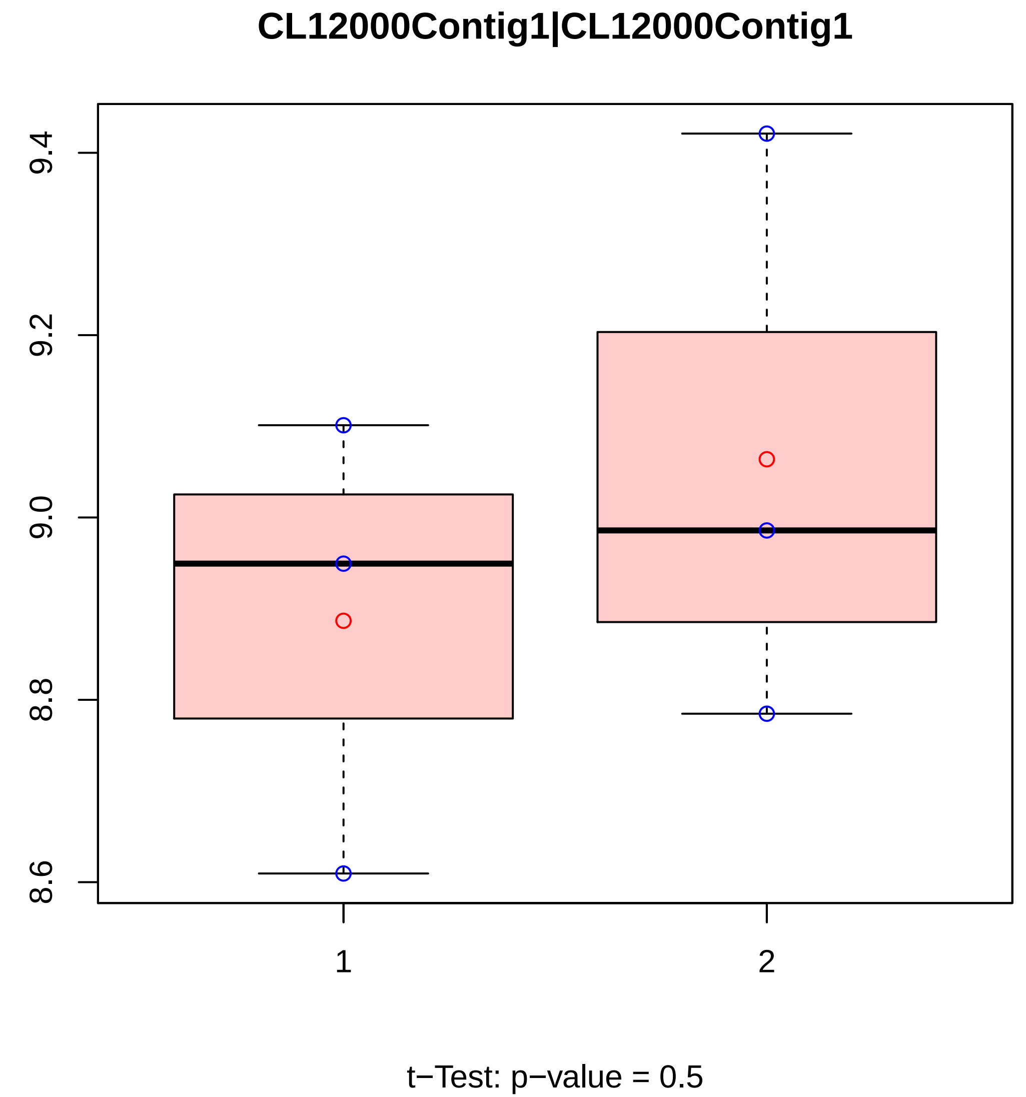
CL12000Contig1|CL12000Contig1
9.4
9.2
9.0
8.8
8.6
1
2
t−Test: p−value = 0.5

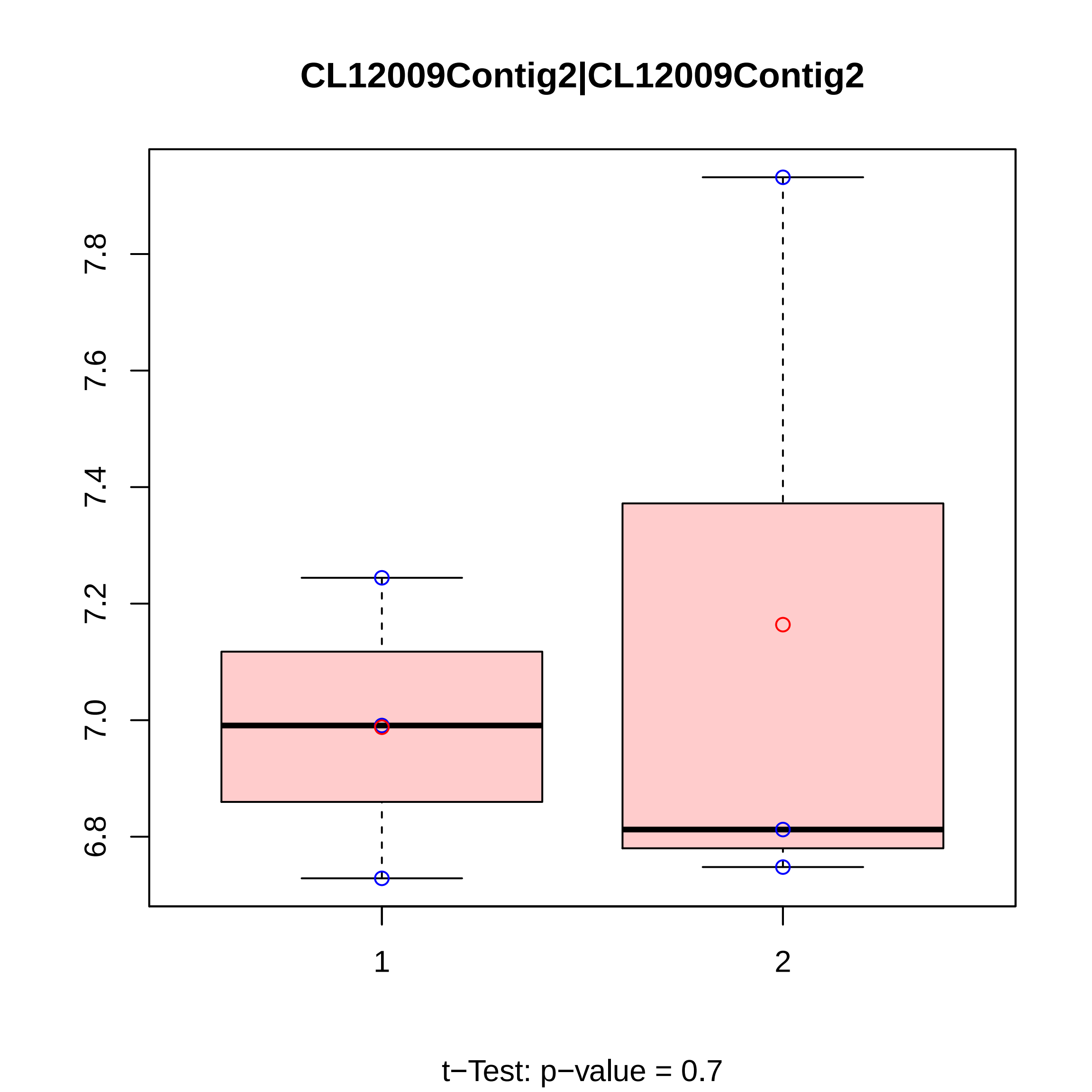
CL12009Contig2|CL12009Contig2
7.8
7.6
7.4
7.2
7.0
6.8
1
2
t−Test: p−value = 0.7

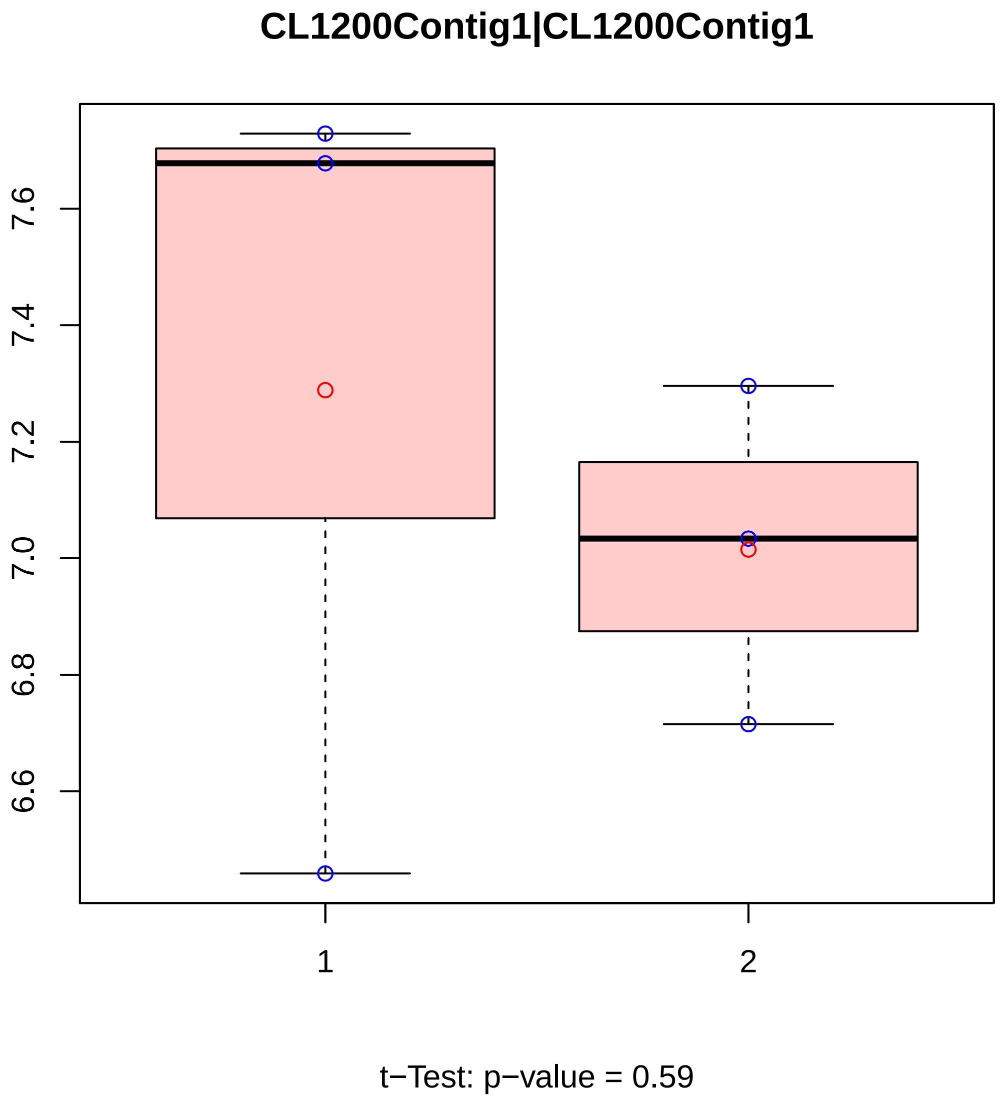
CL1200Contig1|CL1200Contig1
7.6
7.4
7.2
7.0
6.8
6.6
1
2
t−Test: p−value = 0.59

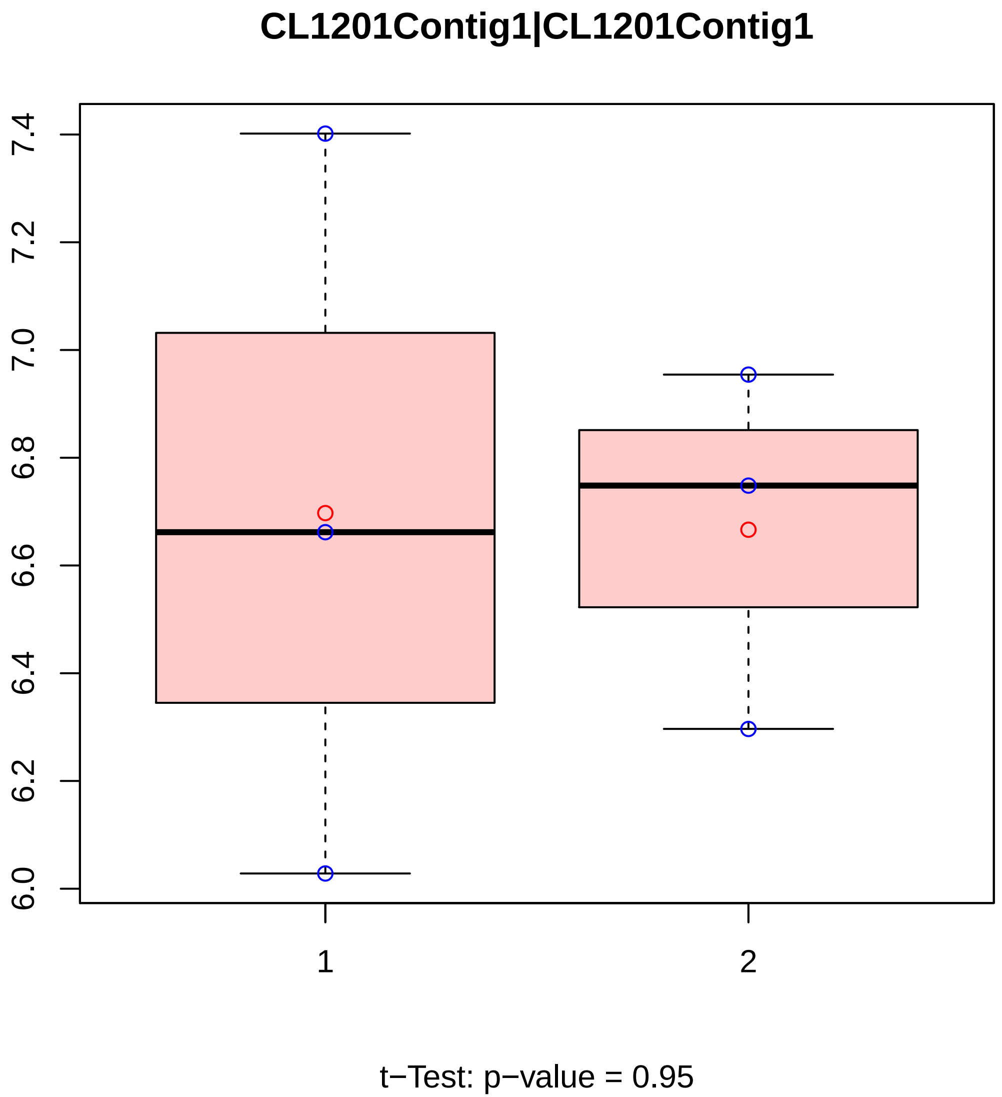
CL1201Contig1|CL1201Contig1
7.4
7.2
7.0
6.8
6.6
6.4
6.2
6.0
1
2
t−Test: p−value = 0.95

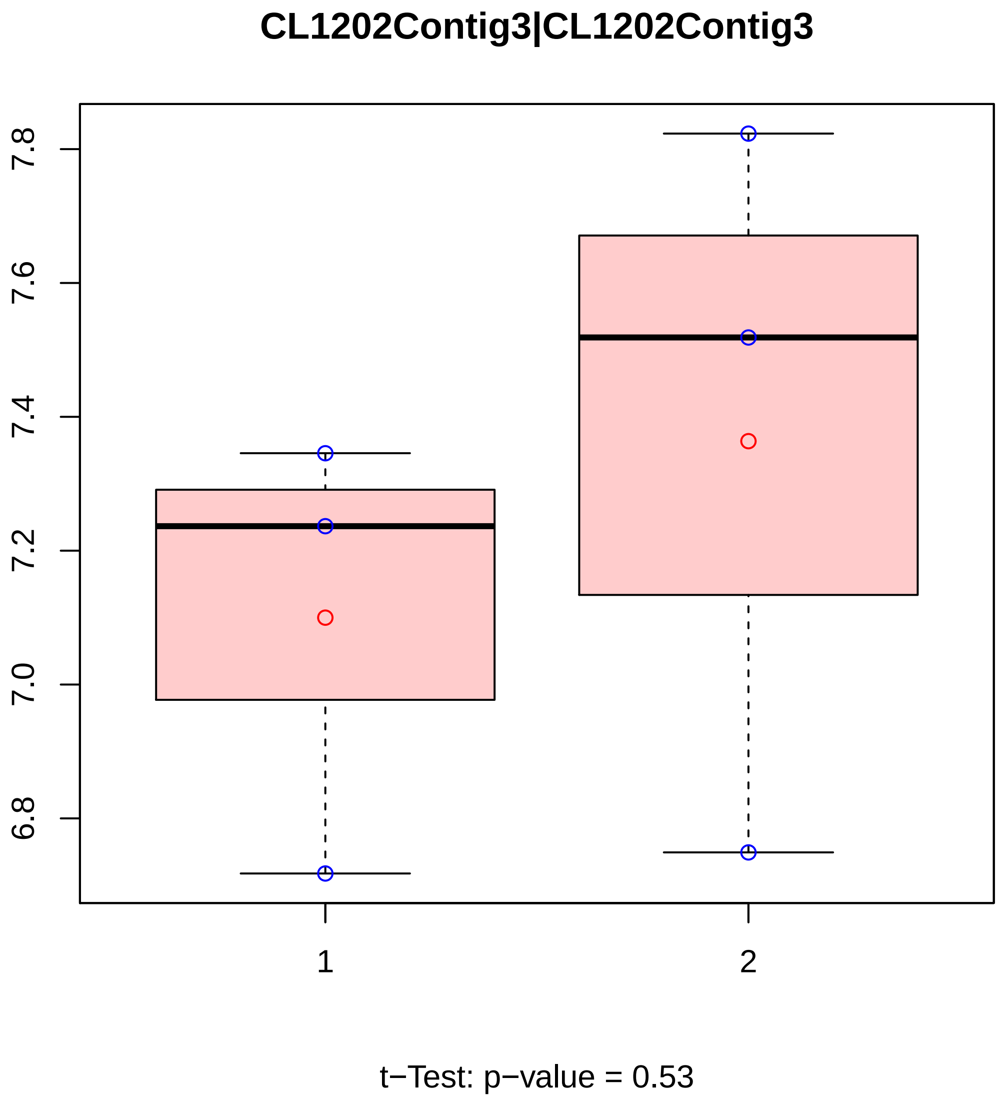

CL1202Contig3|CL1202Contig3
7.8
7.6
7.4
7.2
7.0
6.8
1
2
t−Test: p−value = 0.53

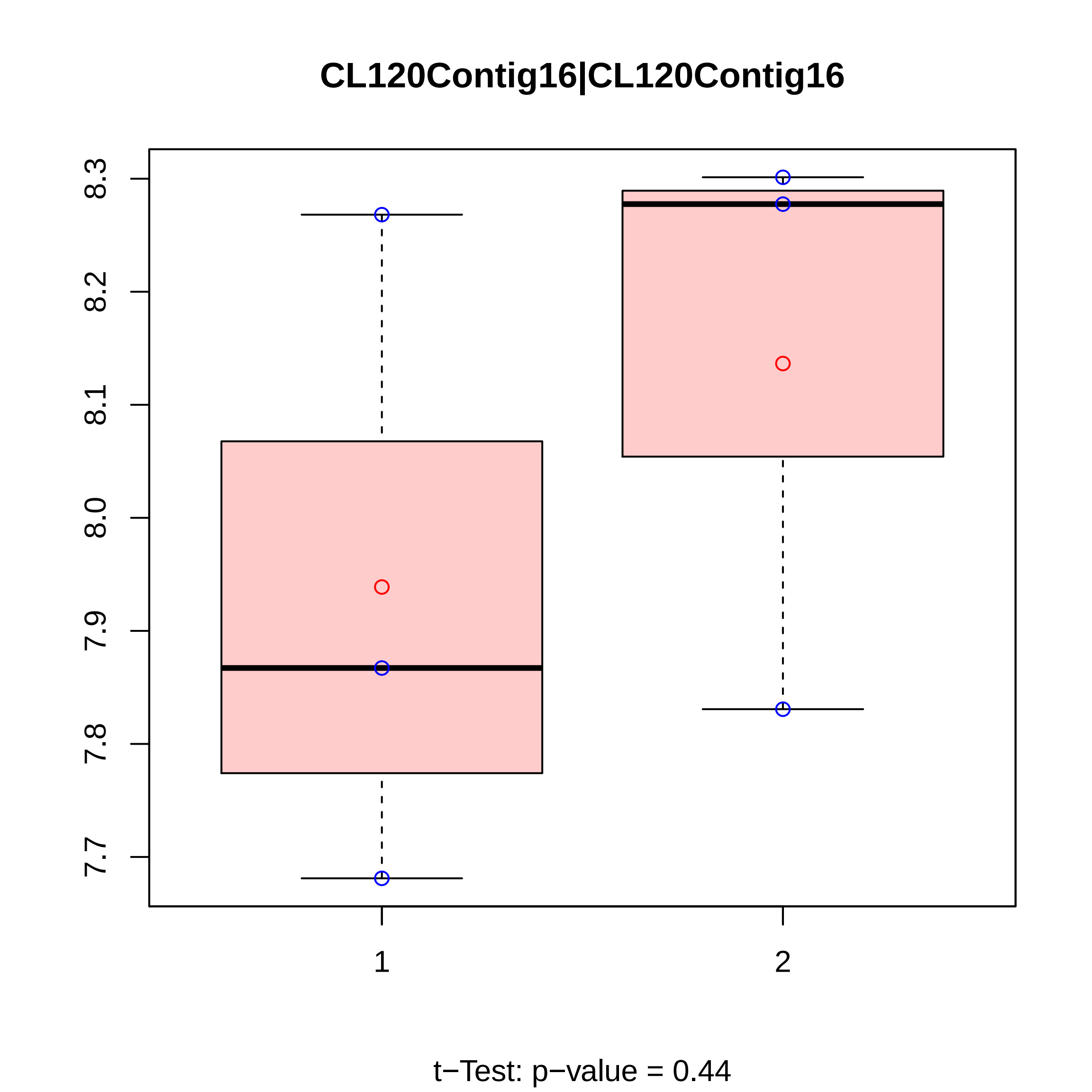
CL120Contig16|CL120Contig16
8.3
8.2
8.1
8.0
7.9
7.8
7.7
1
2
t−Test: p−value = 0.44

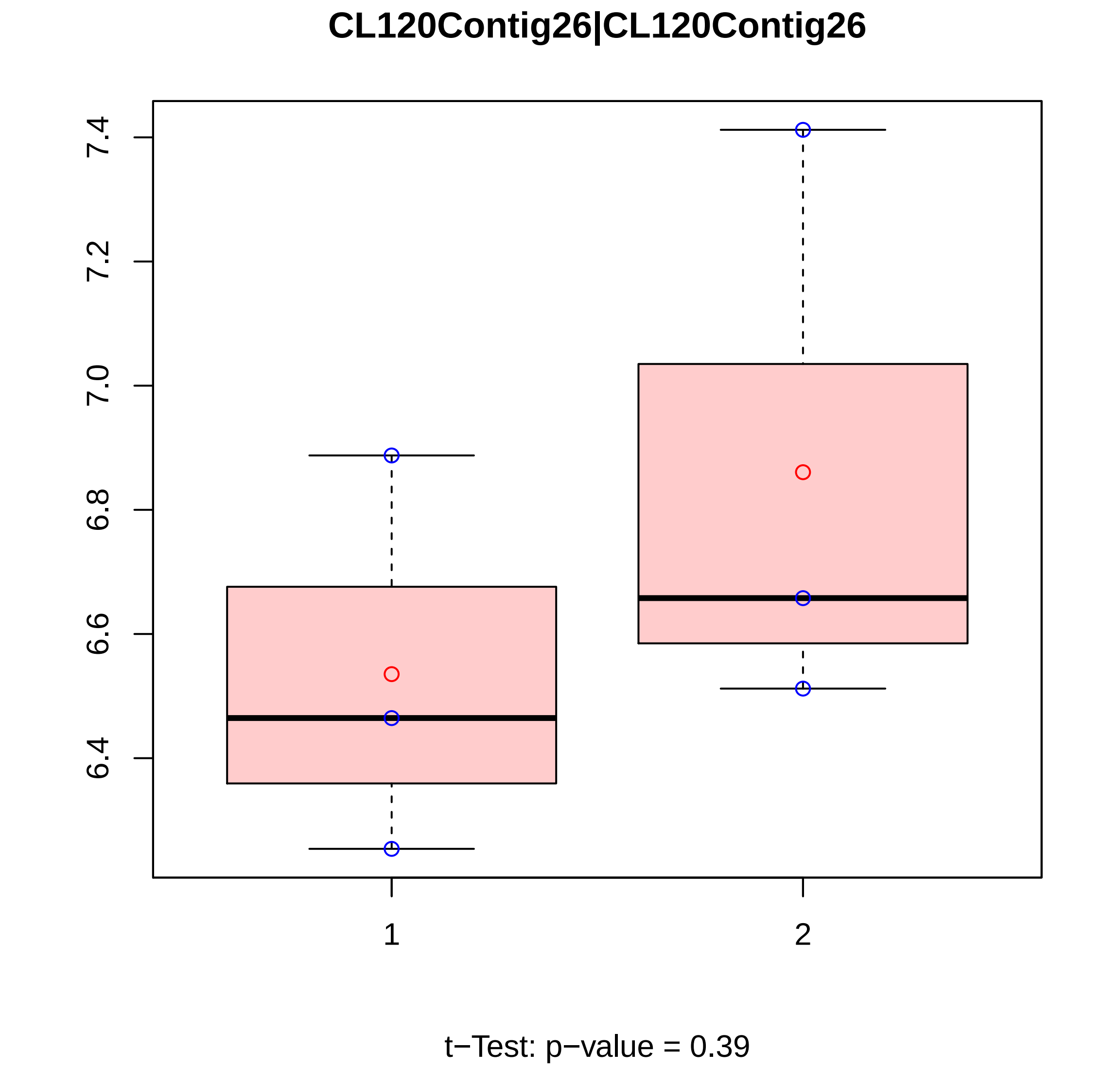
CL120Contig26|CL120Contig26
7.4
7.2
7.0
6.8
6.6
6.4
1
2
t−Test: p−value = 0.39

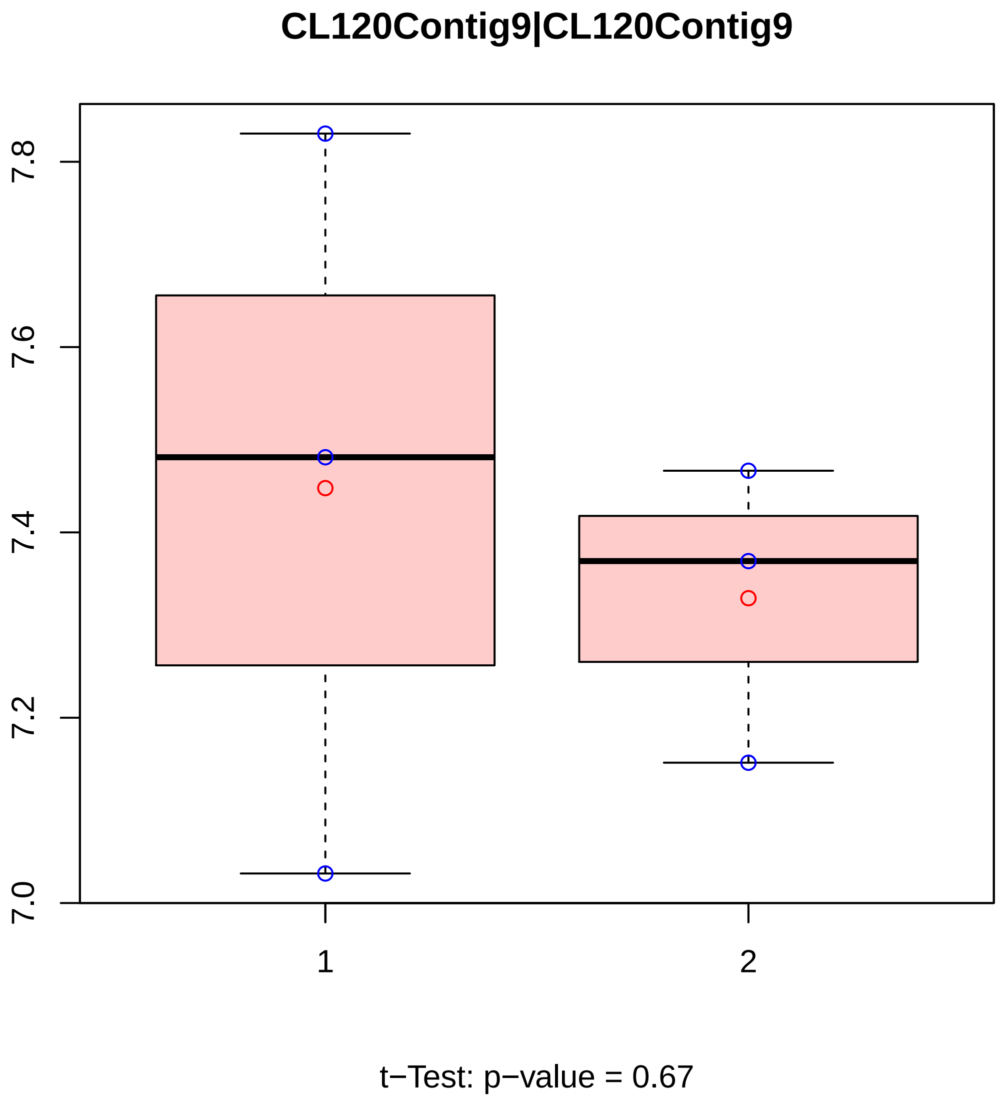
CL120Contig9|CL120Contig9
7.0
7.2
7.4
7.6
7.8
1
2
t−Test: p−value = 0.67

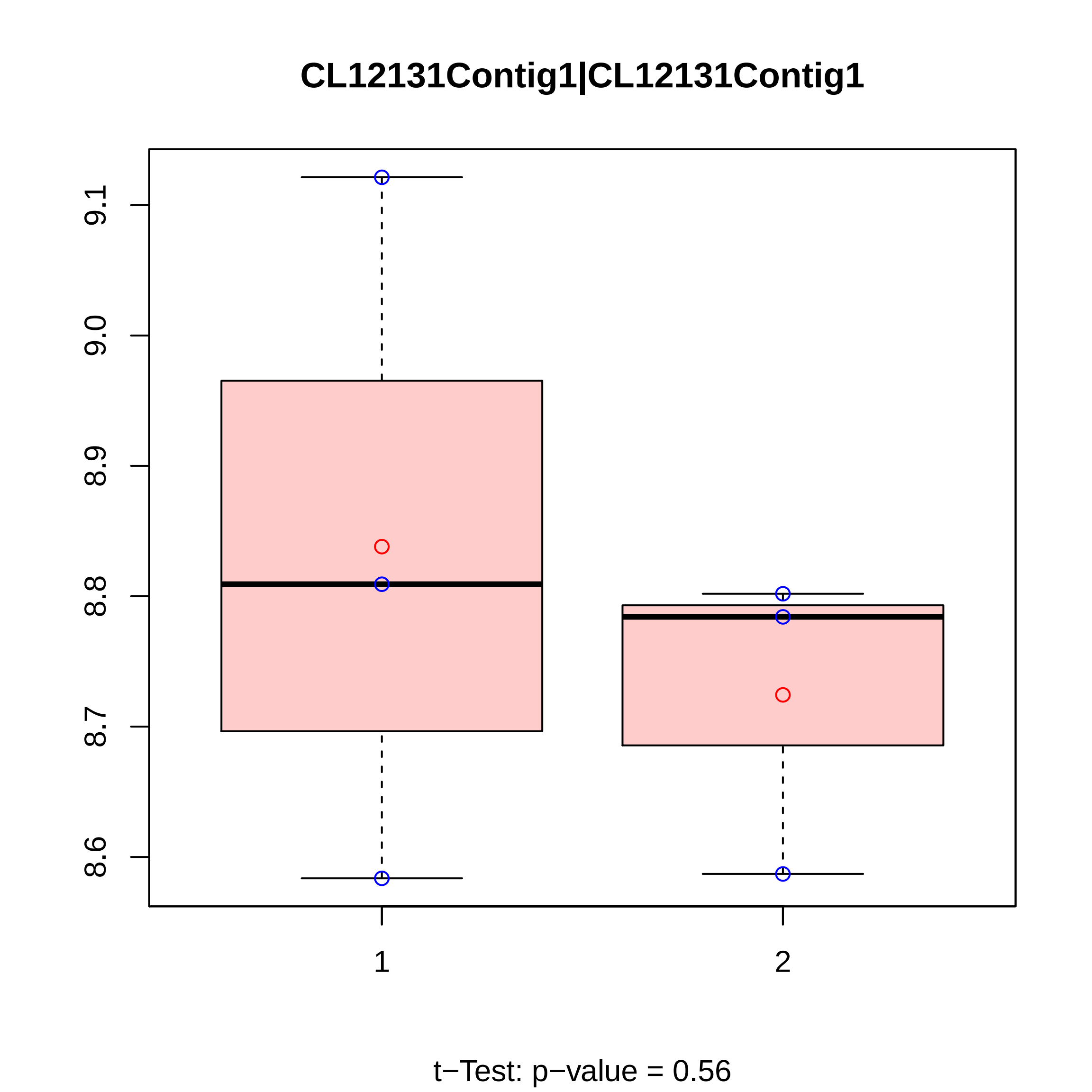

CL12131Contig1|CL12131Contig1
9.1
9.0
8.9
8.8
8.7
8.6
1
2
t−Test: p−value = 0.56

## CL1215Contig1|CL1215Contig1

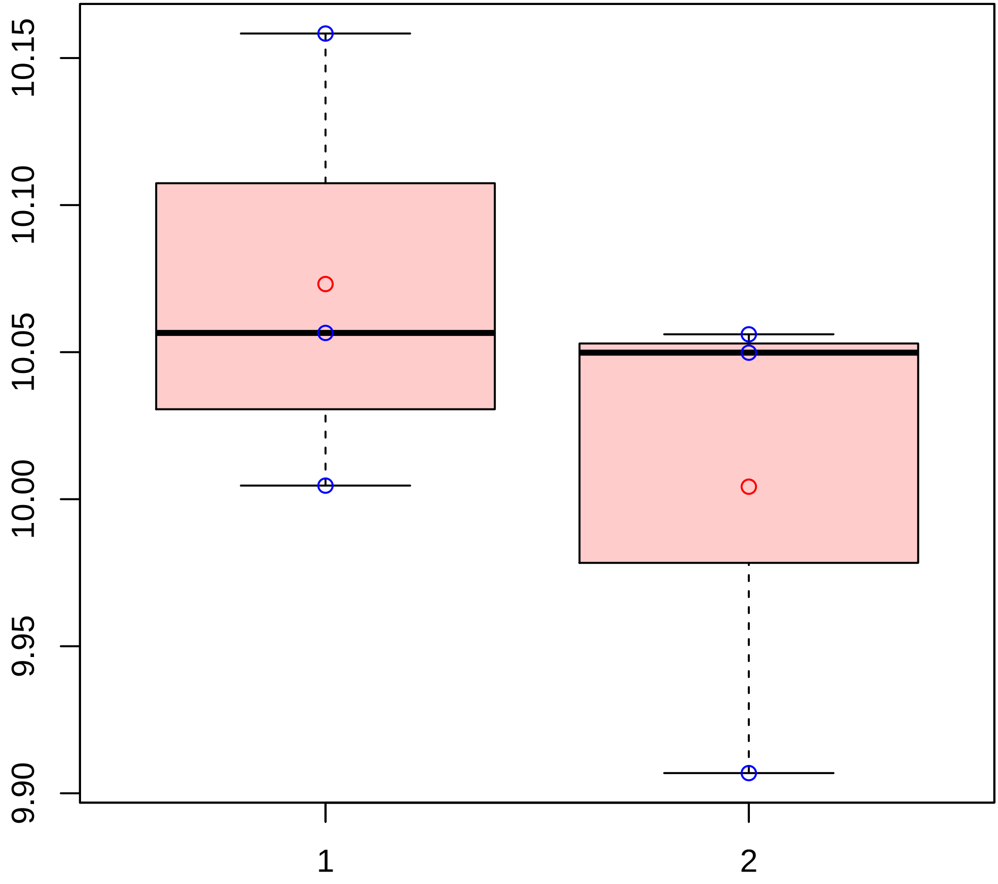


t−Test: p−value = 0.36

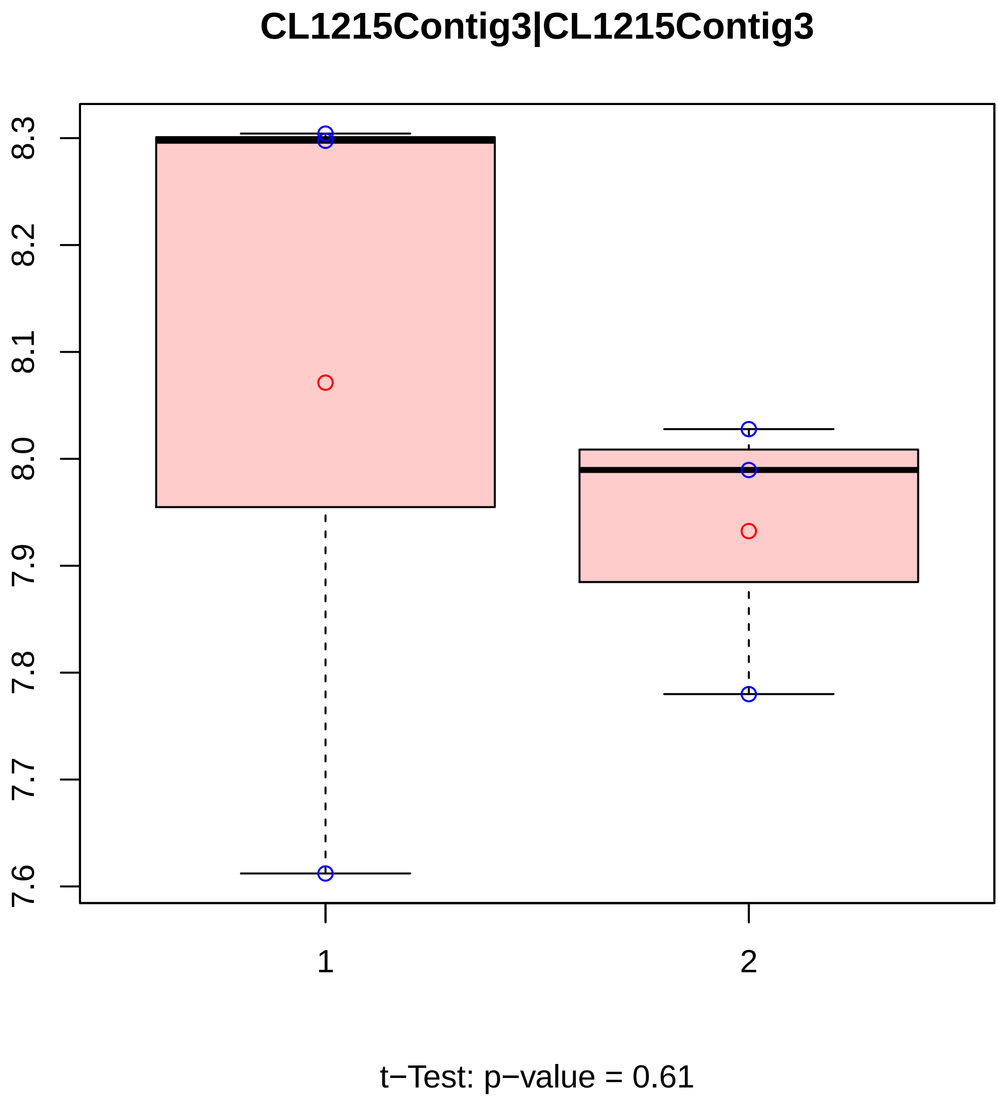

CL1215Contig3|CL1215Contig3
8.3
8.2
8.1
8.0
7.9
7.8
7.7
7.6
1
2
t−Test: p−value = 0.61

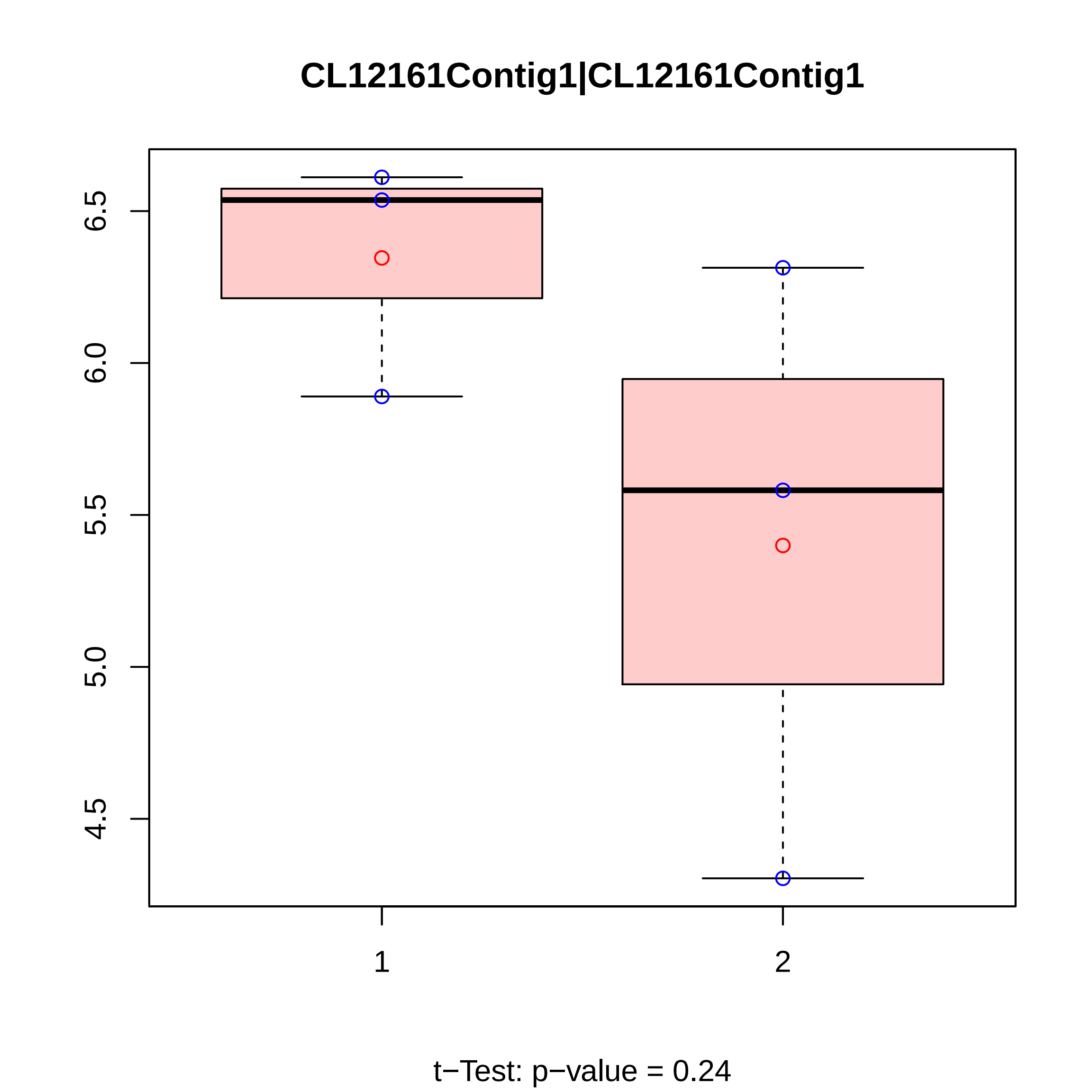
CL12161Contig1|CL12161Contig1
6.5
6.0
5.5
5.0
4.5
1
2
t−Test: p−value = 0.24

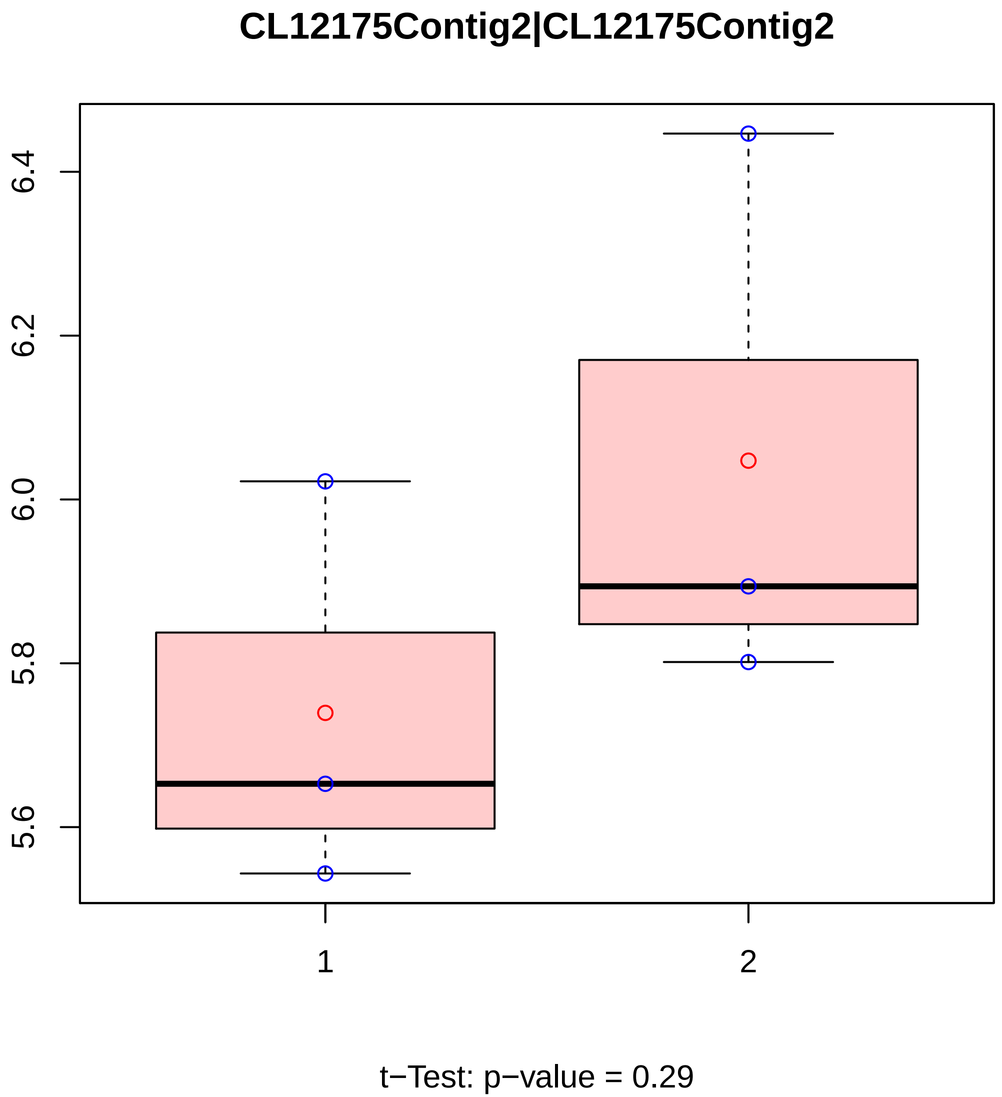
CL12175Contig2|CL12175Contig2
6.4
6.2
6.0
5.8
5.6
1
2
t−Test: p−value = 0.29

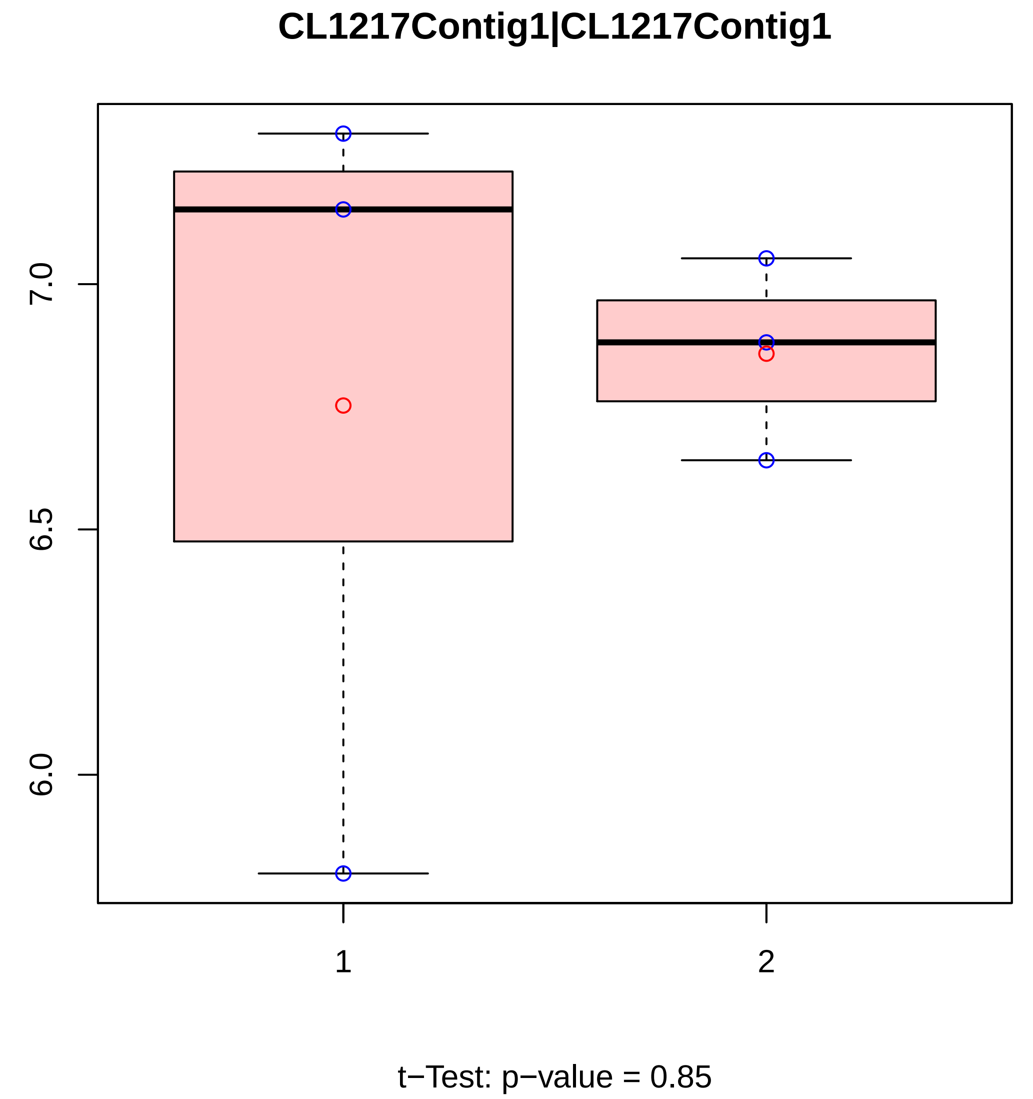
CL1217Contig1|CL1217Contig1
7.0
6.5
6.0
1
2
t−Test: p−value = 0.85

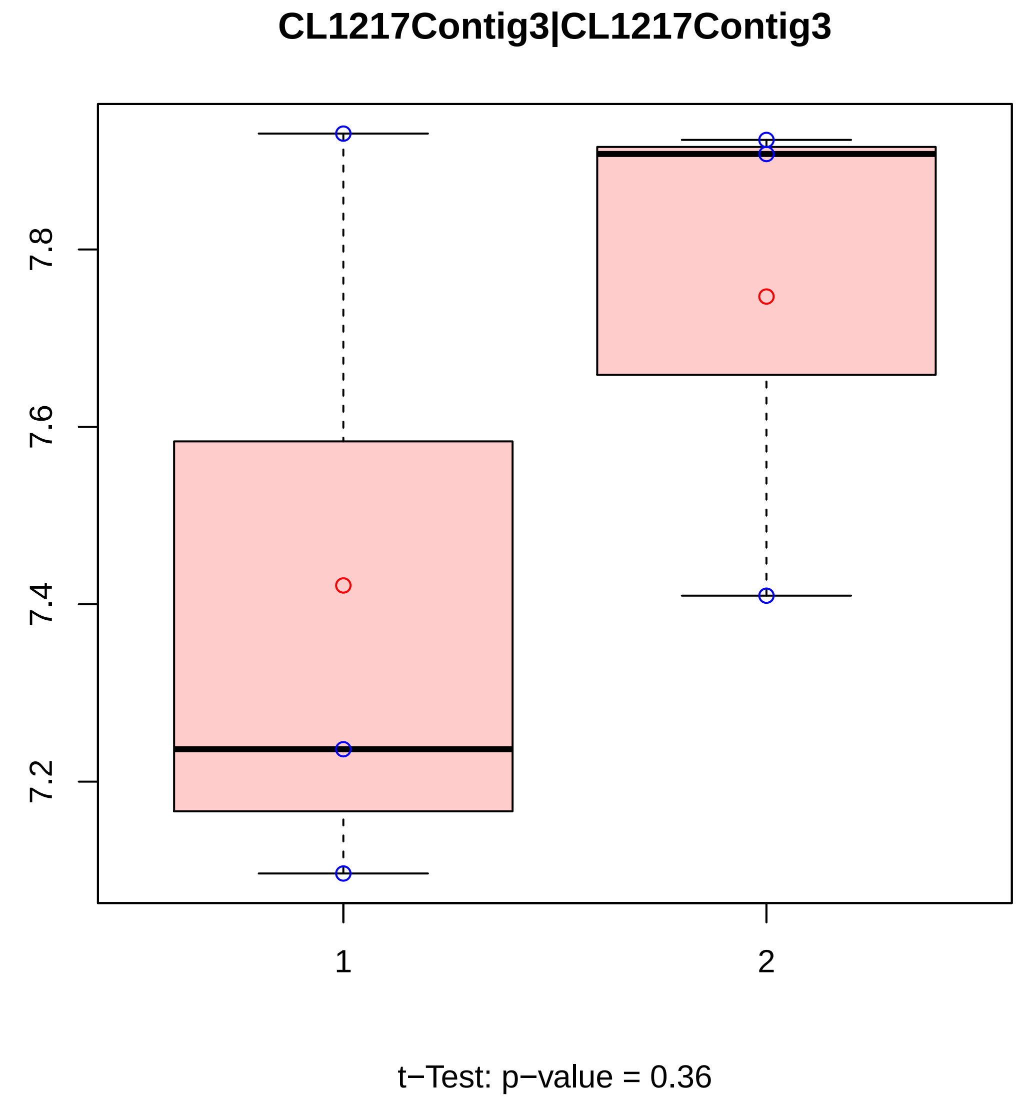
CL1217Contig3|CL1217Contig3
7.2
7.4
7.6
7.8
1
2
t−Test: p−value = 0.36

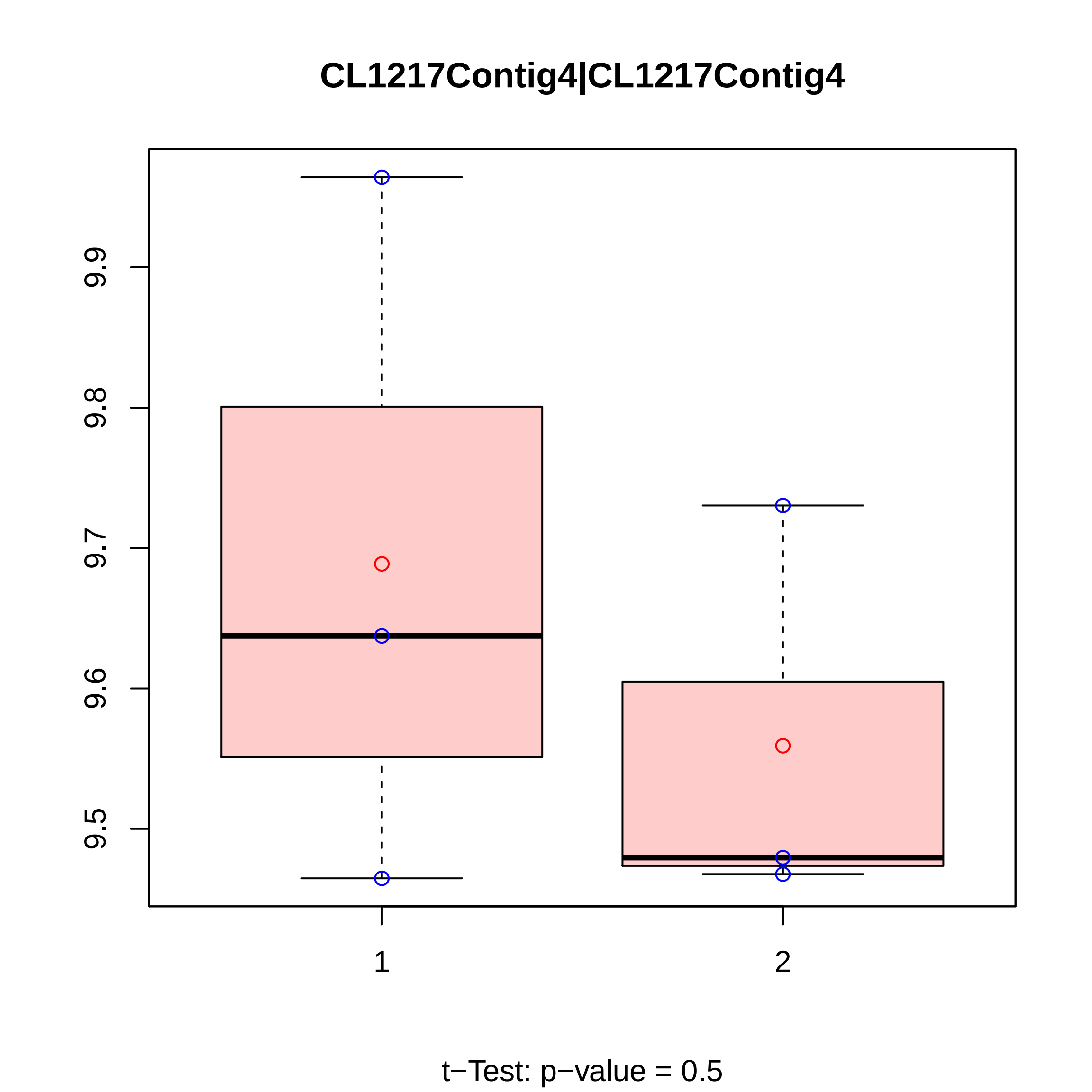
CL1217Contig4|CL1217Contig4
9.5
9.6
9.7
9.8
9.9
1
2
t−Test: p−value = 0.5

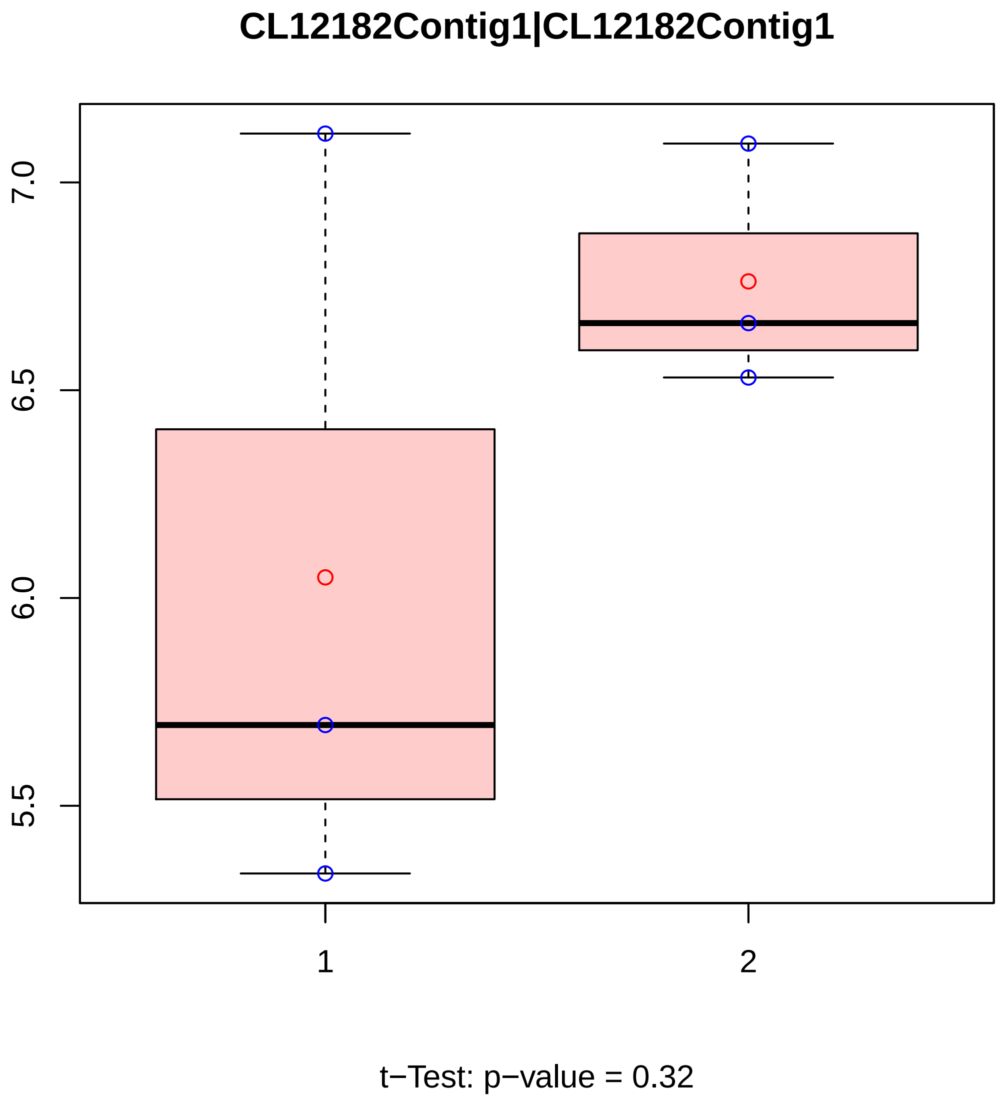
CL12182Contig1|CL12182Contig1
7.0
6.5
6.0
5.5
1
2
t−Test: p−value = 0.32

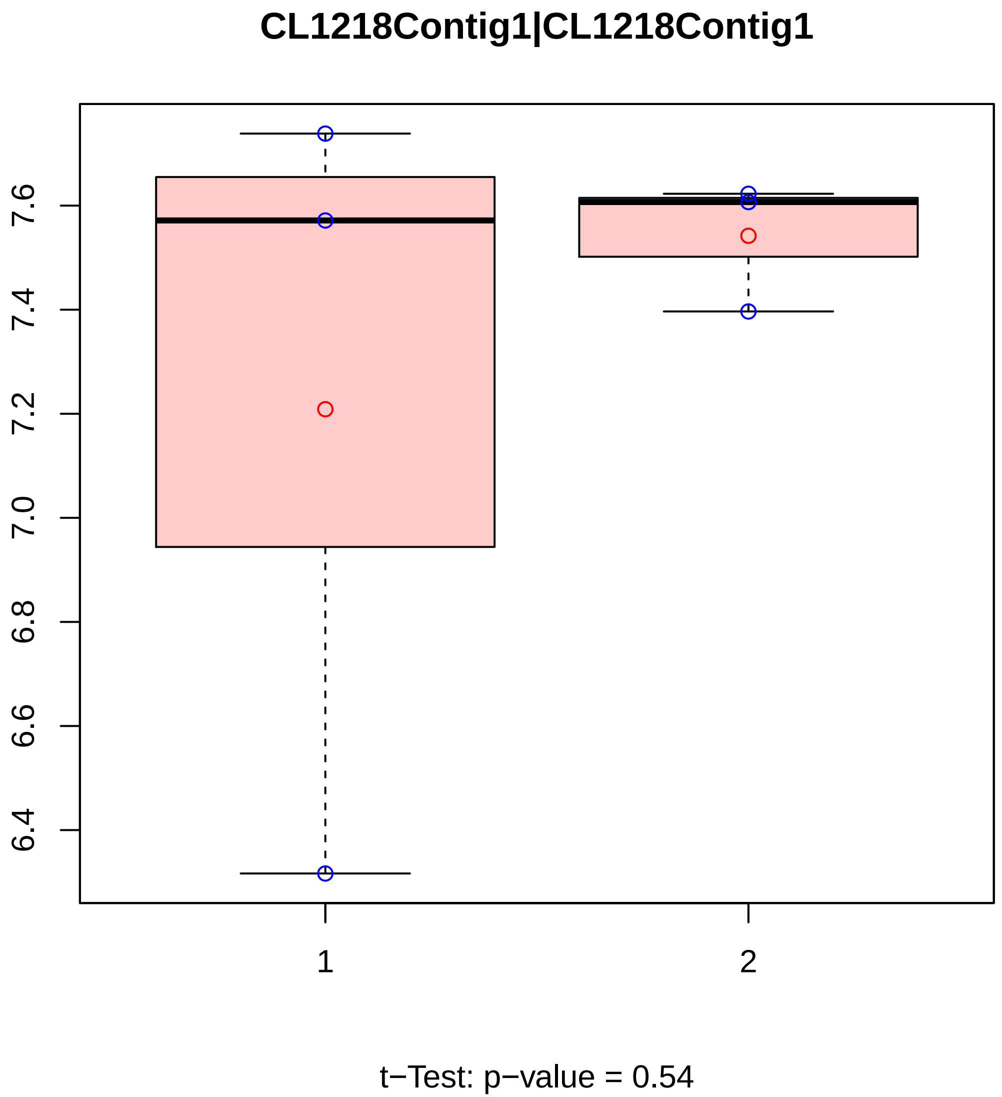

CL1218Contig1|CL1218Contig1
7.6
7.4
7.2
7.0
6.8
6.6
6.4
1
2
t−Test: p−value = 0.54

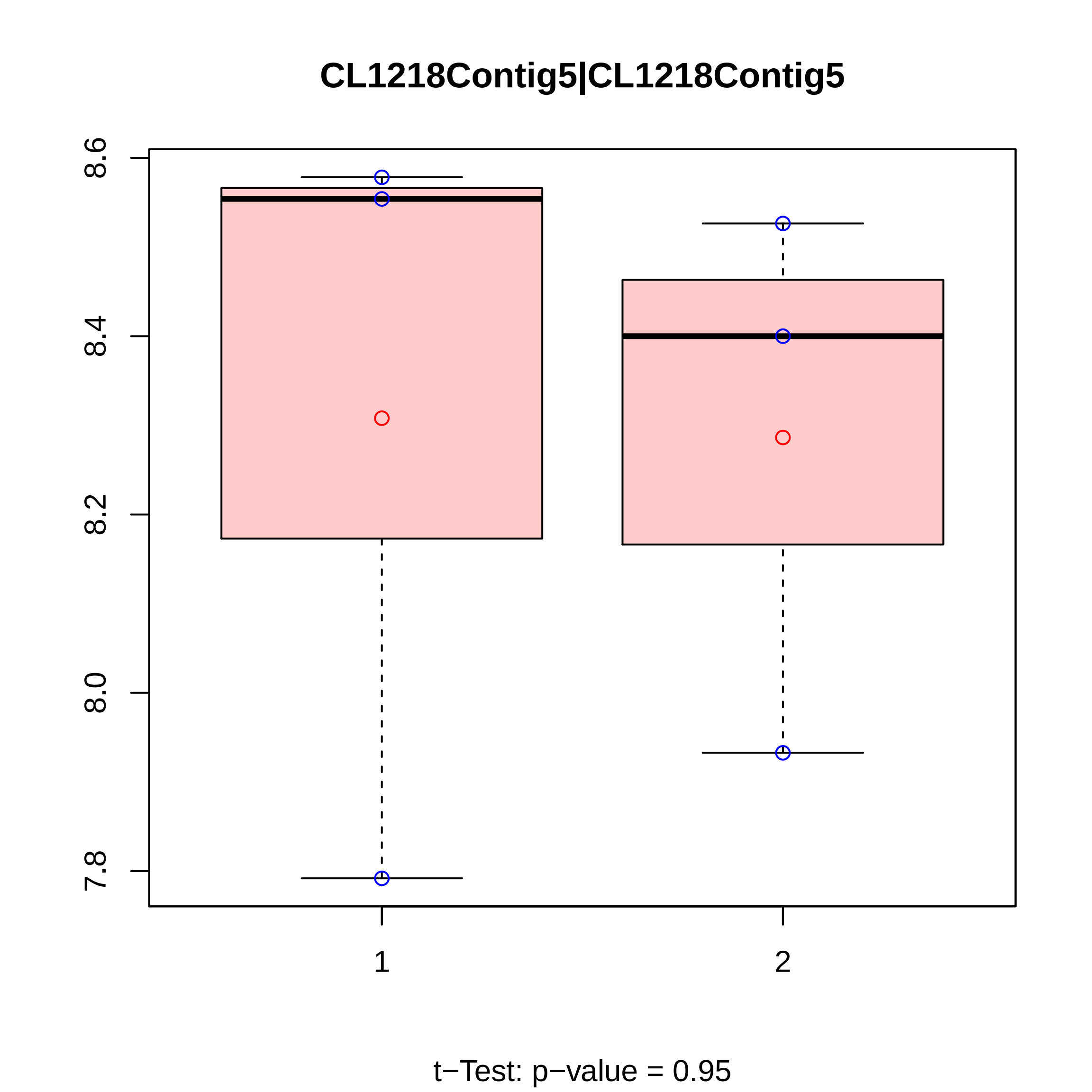
CL1218Contig5|CL1218Contig5
8.6
8.4
8.2
8.0
7.8
1
2
t−Test: p−value = 0.95

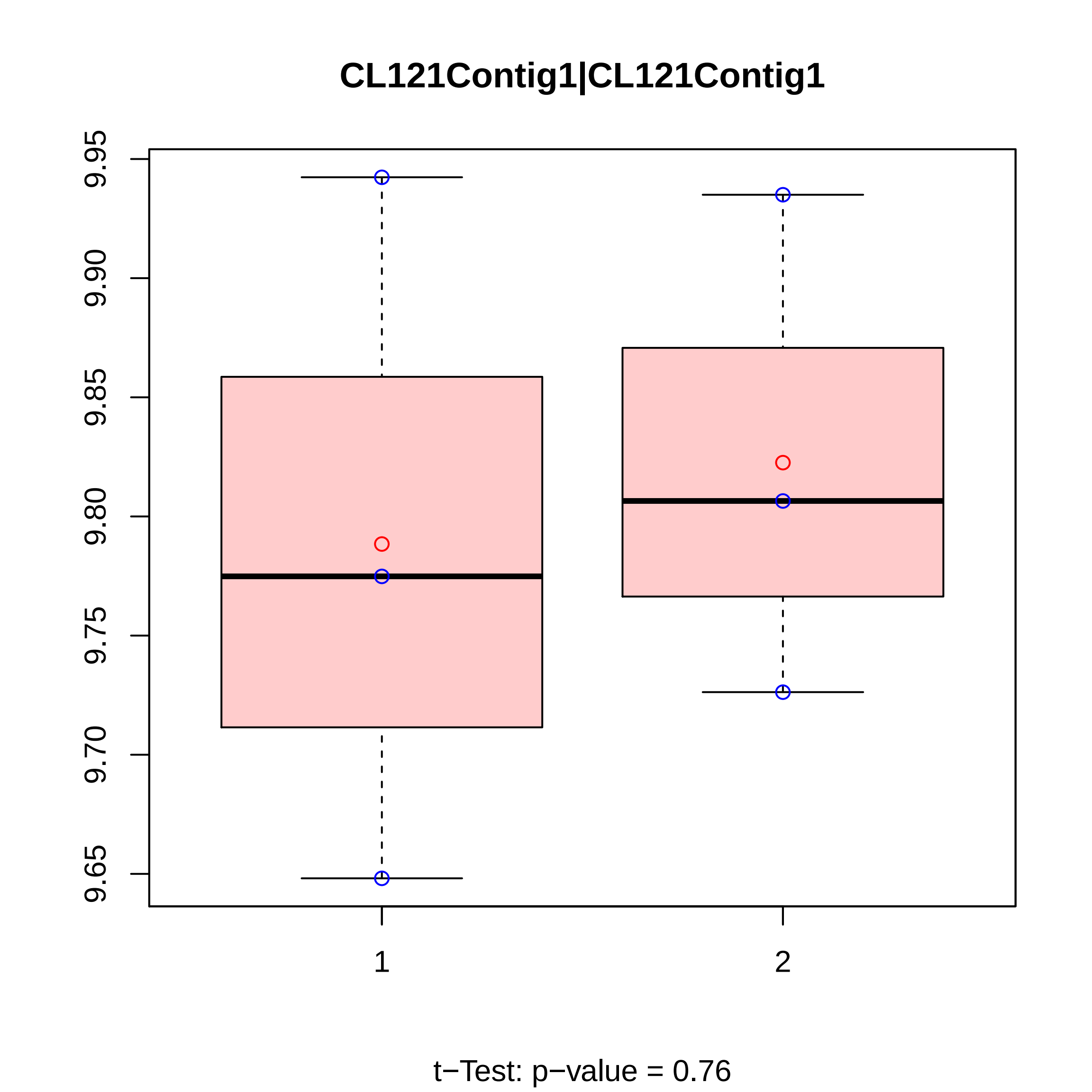

CL121Contig1|CL121Contig1
9.65
9.70
9.75
9.80
9.85
9.90
9.95
1
2
t−Test: p−value = 0.76

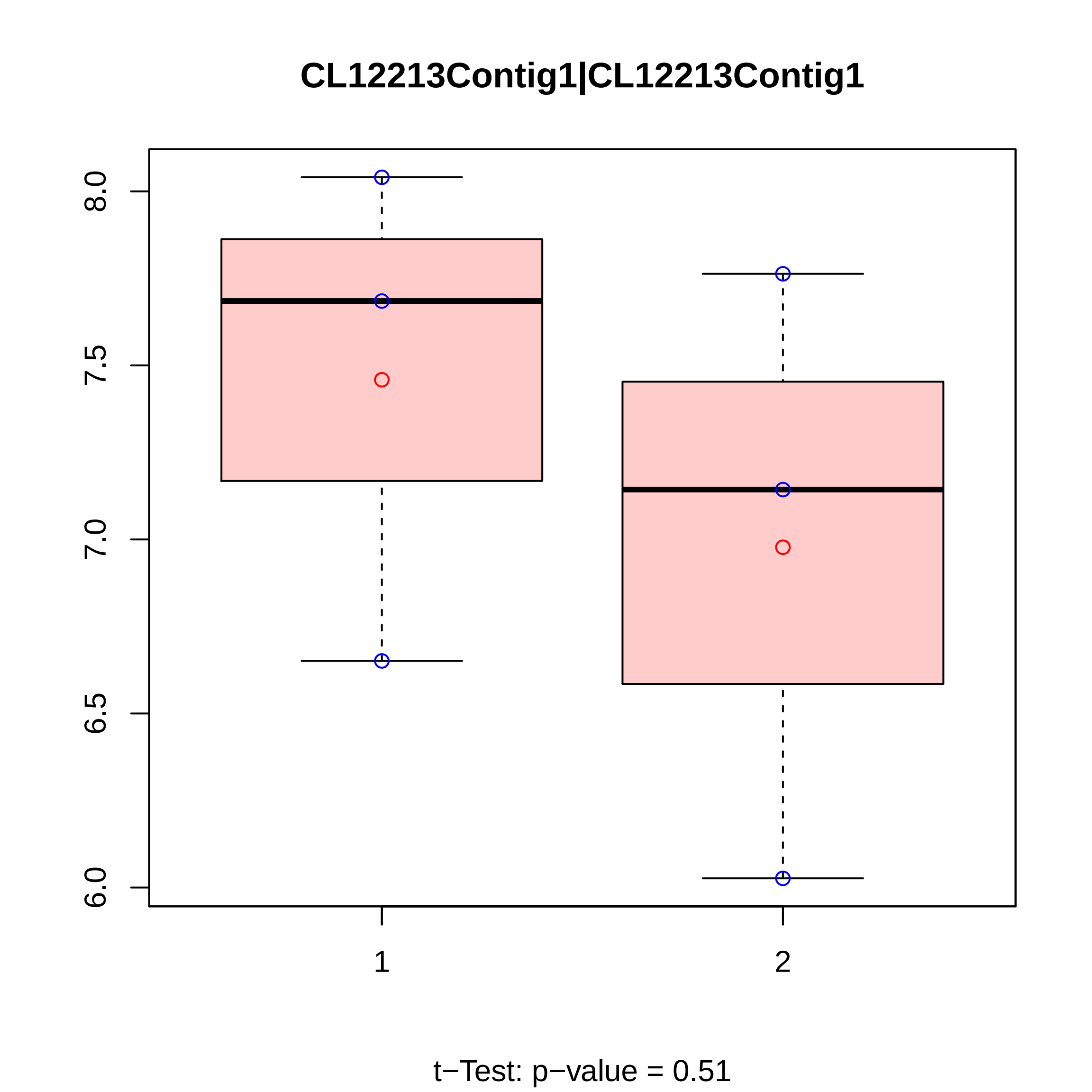
CL12213Contig1|CL12213Contig1
8.0
7.5
7.0
6.5
6.0
1
2
t−Test: p−value = 0.51

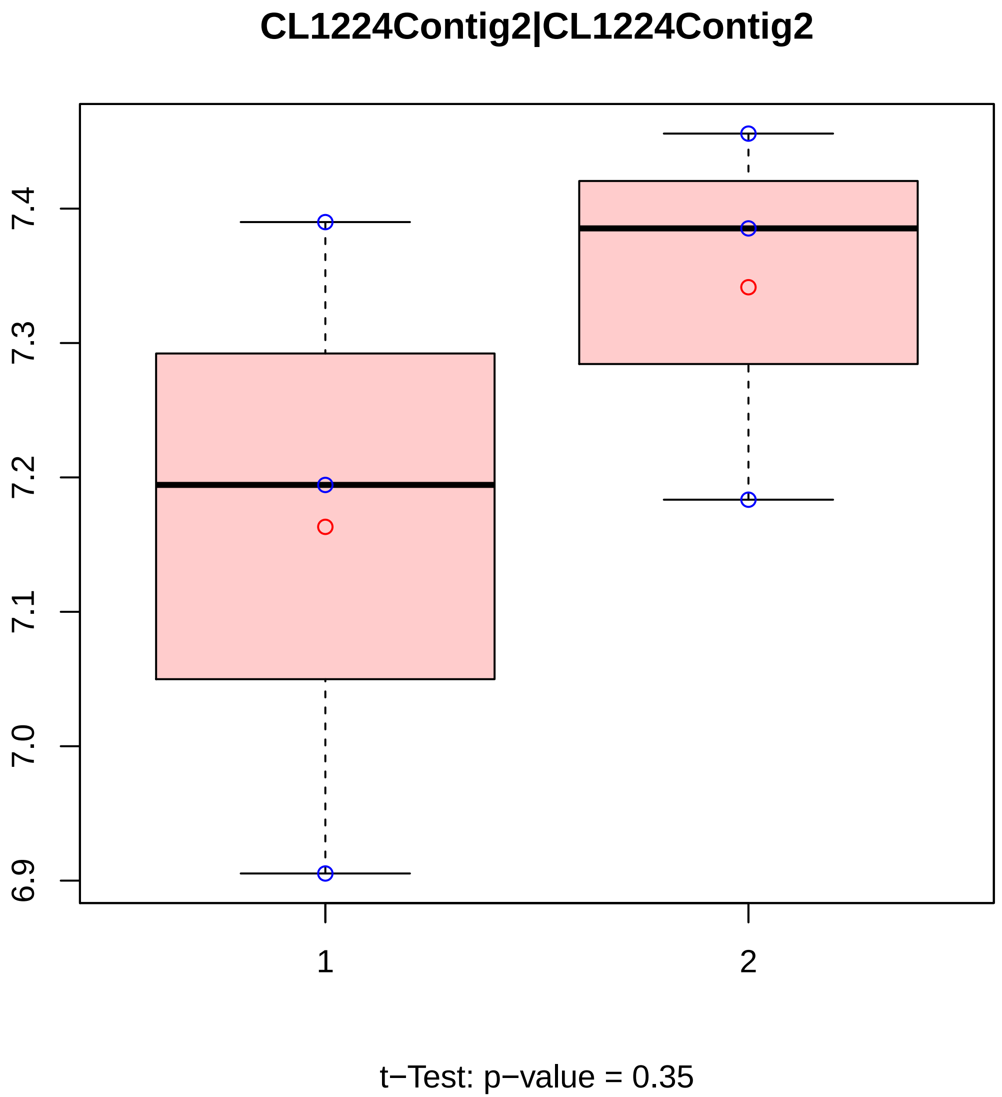
CL1224Contig2|CL1224Contig2
7.4
7.3
7.2
7.1
7.0
6.9
1
2
t−Test: p−value = 0.35

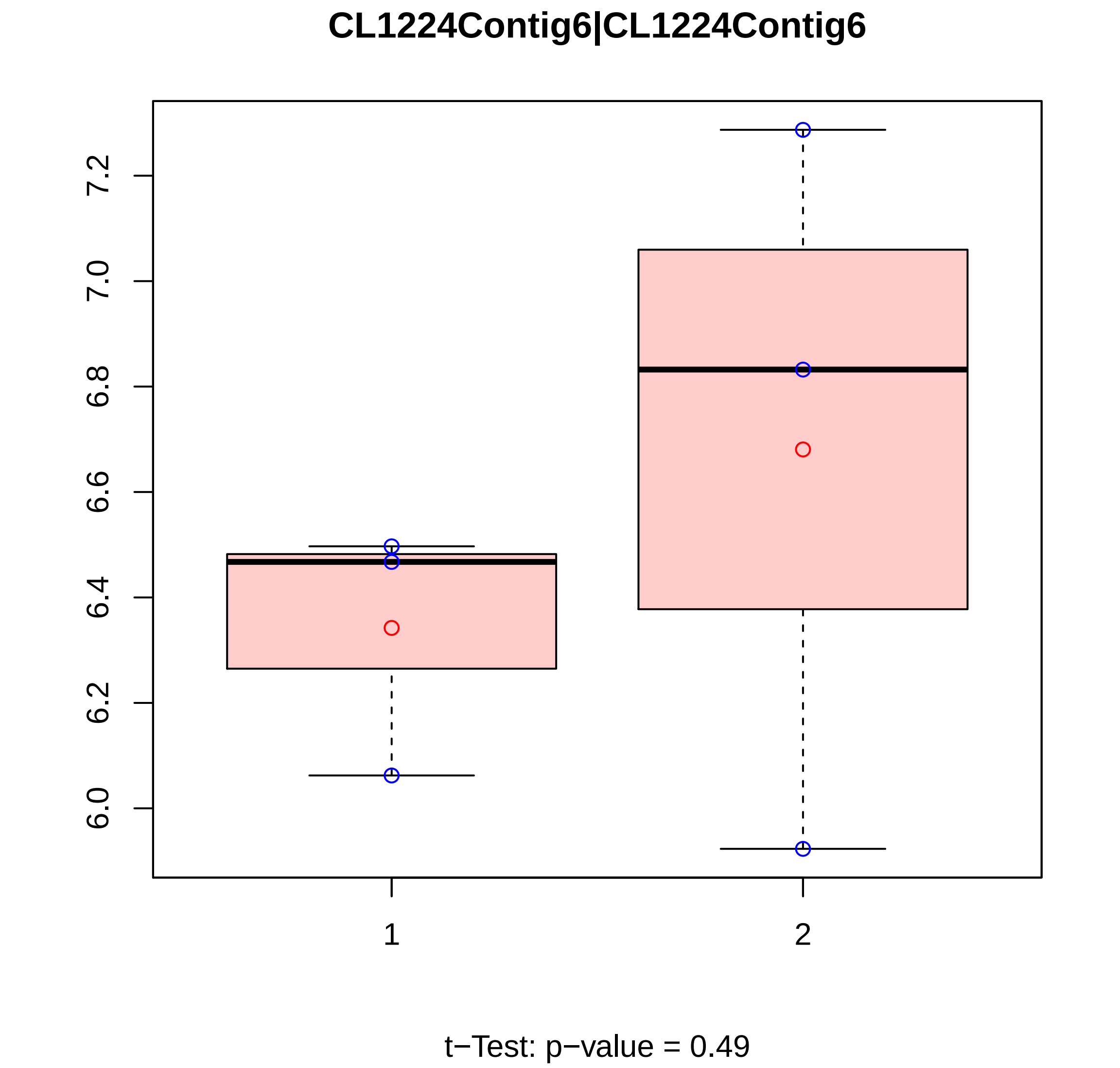
CL1224Contig6|CL1224Contig6
7.2
7.0
6.8
6.6
6.4
6.2
6.0
1
2
t−Test: p−value = 0.49

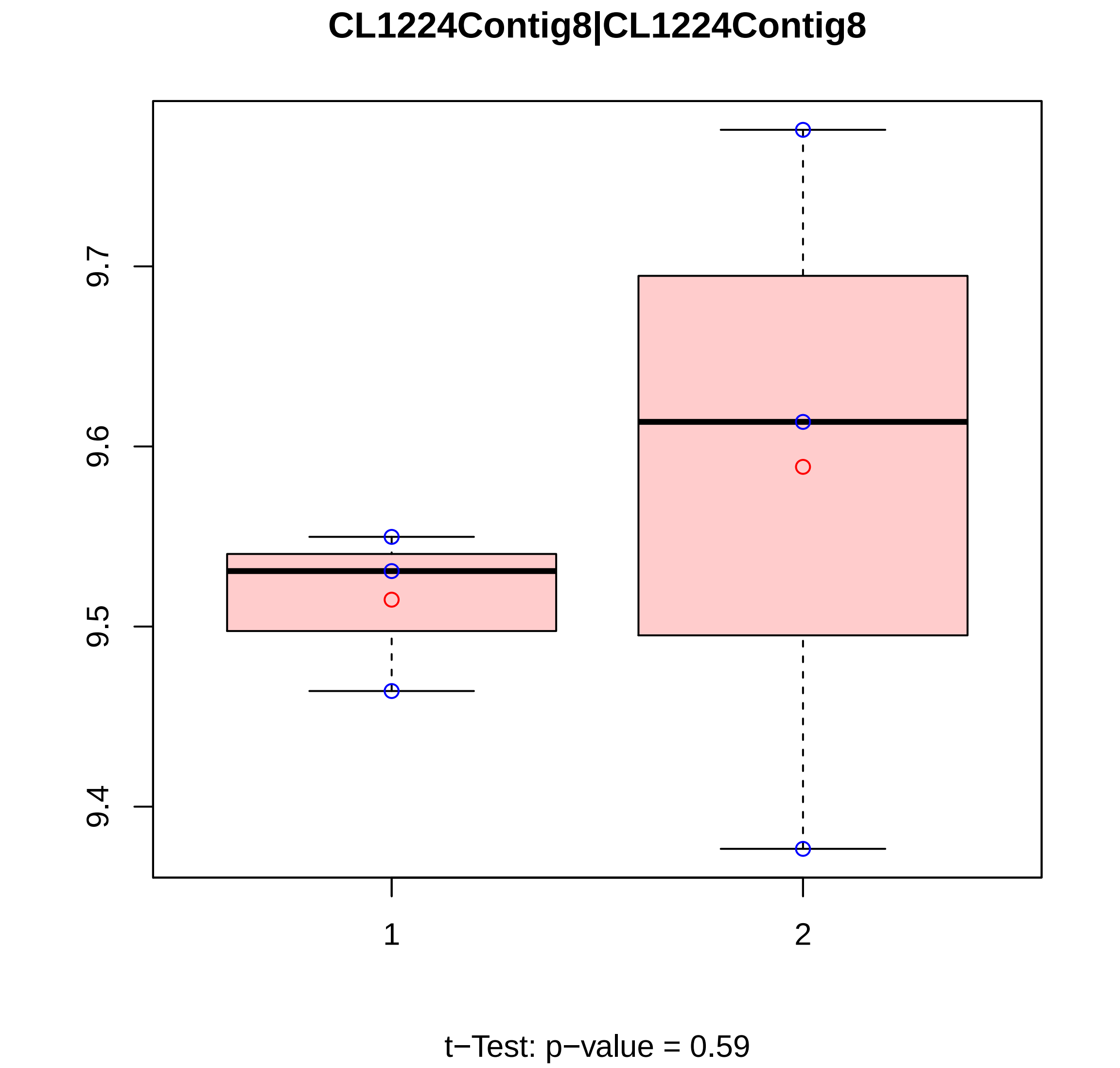
CL1224Contig8|CL1224Contig8
9.4
9.5
9.6
9.7
1
2
t−Test: p−value = 0.59

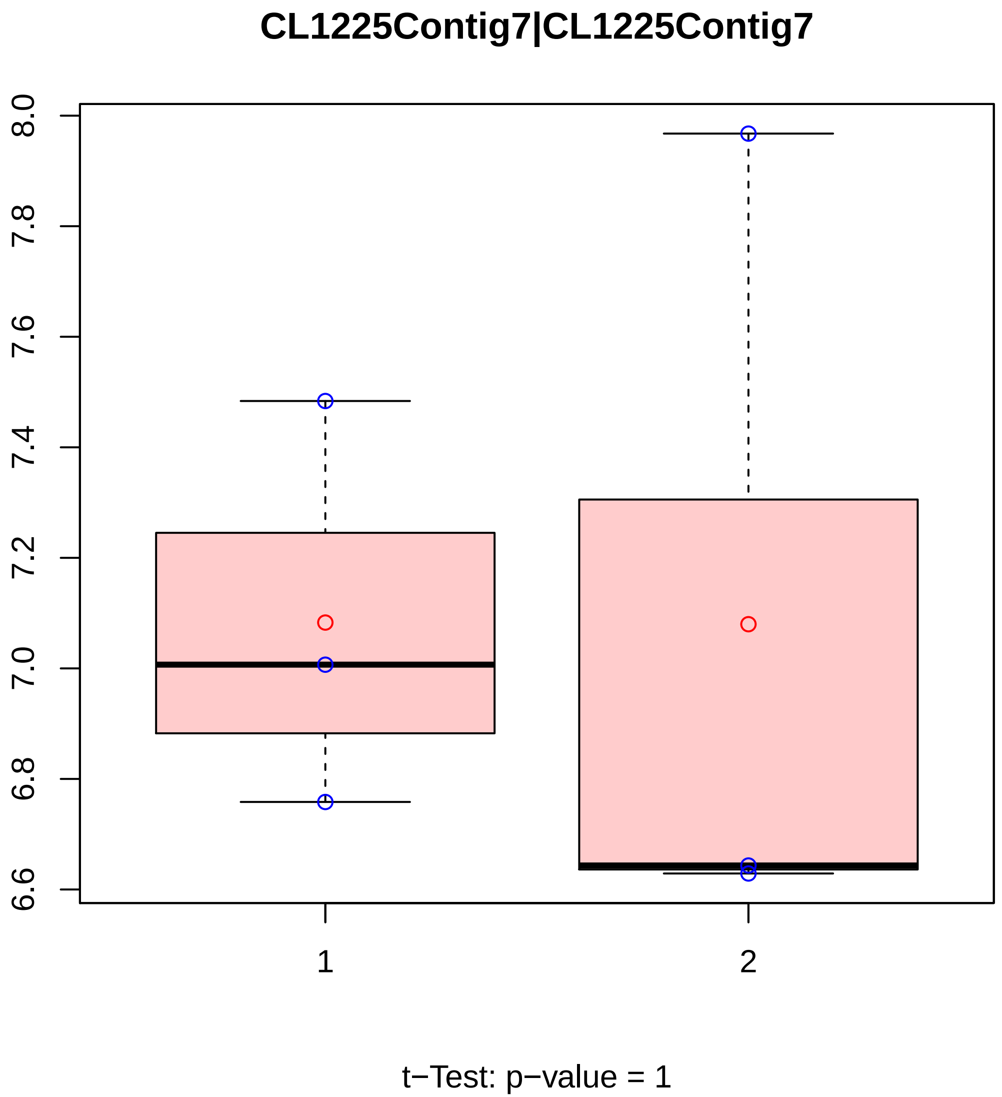
CL1225Contig7|CL1225Contig7
8.0
7.8
7.6
7.4
7.2
7.0
6.8
6.6
1
2
t−Test: p−value = 1

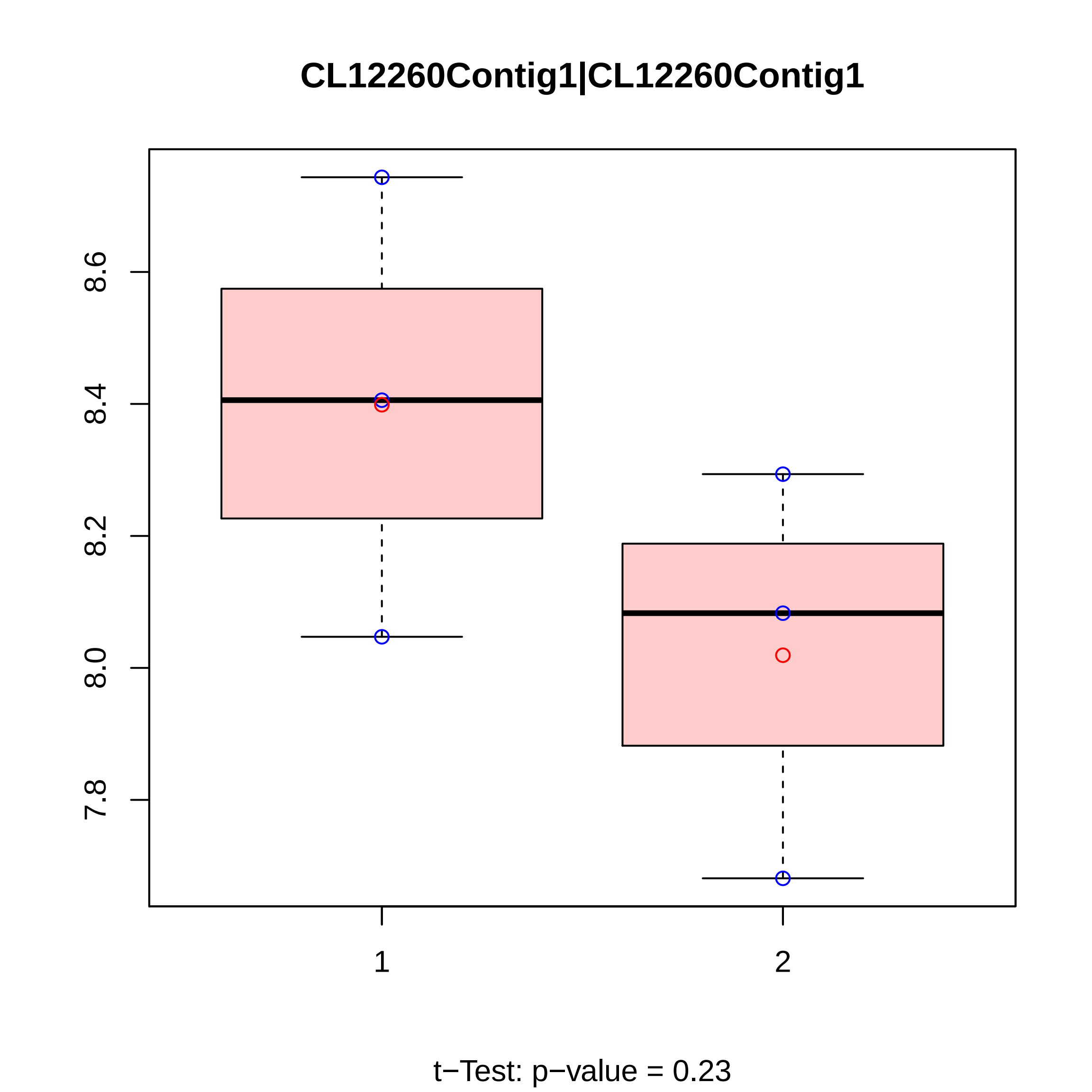

CL12260Contig1|CL12260Contig1
8.6
8.4
8.2
8.0
7.8
1
2
t−Test: p−value = 0.23

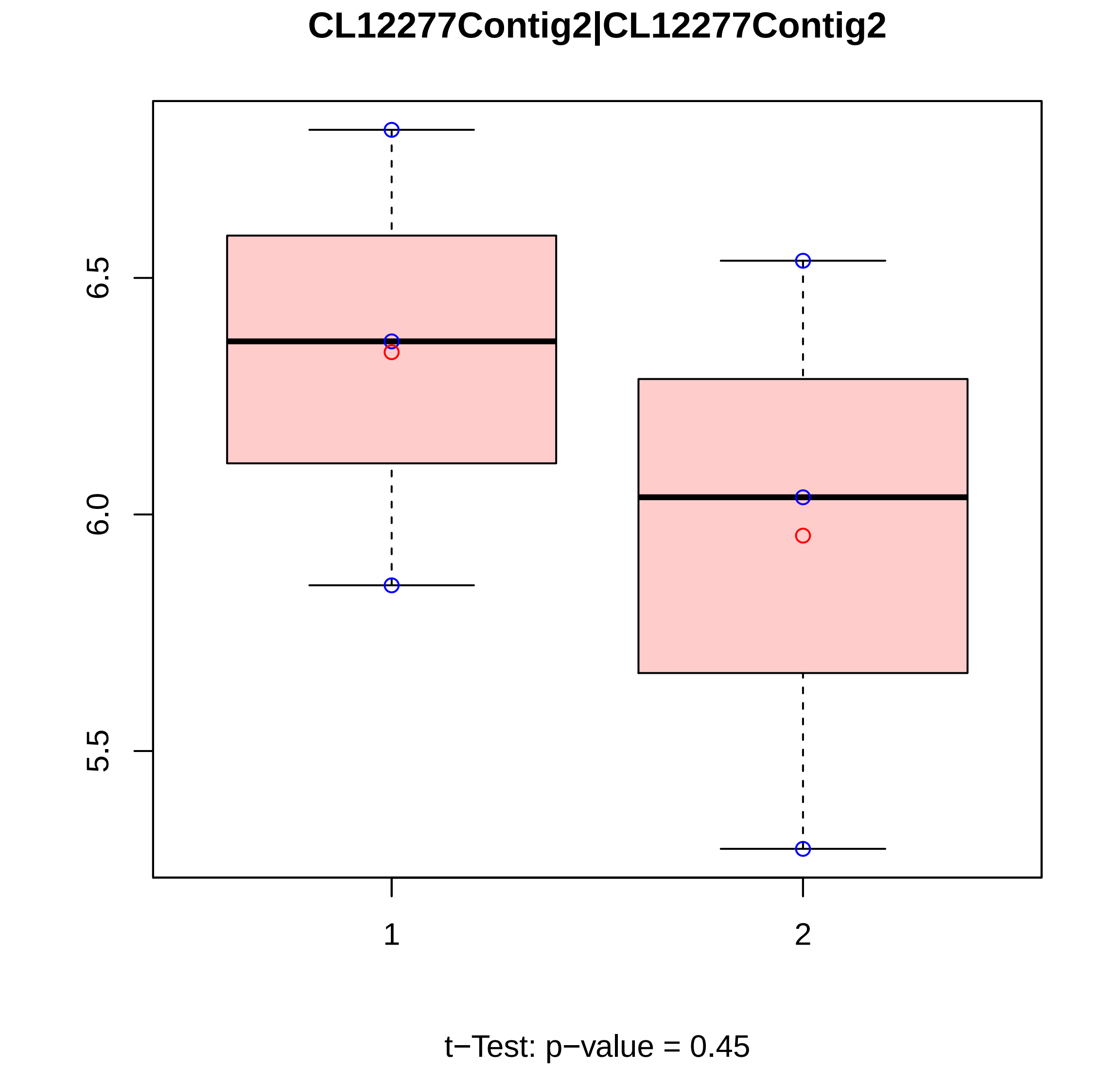
CL12277Contig2|CL12277Contig2
6.5
6.0
5.5
1
2
t−Test: p−value = 0.45

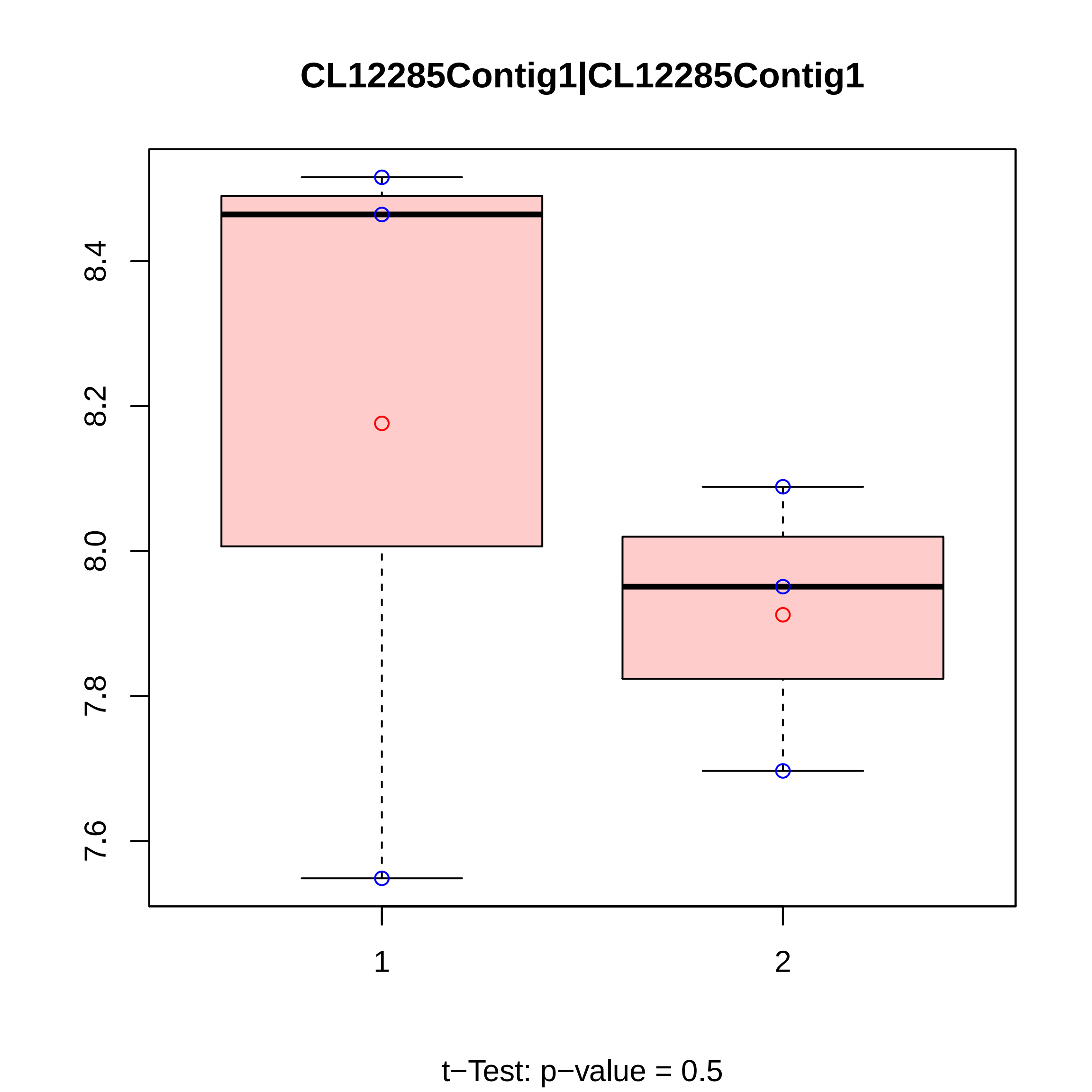
CL12285Contig1|CL12285Contig1
8.4
8.2
8.0
7.8
7.6
1
2
t−Test: p−value = 0.5

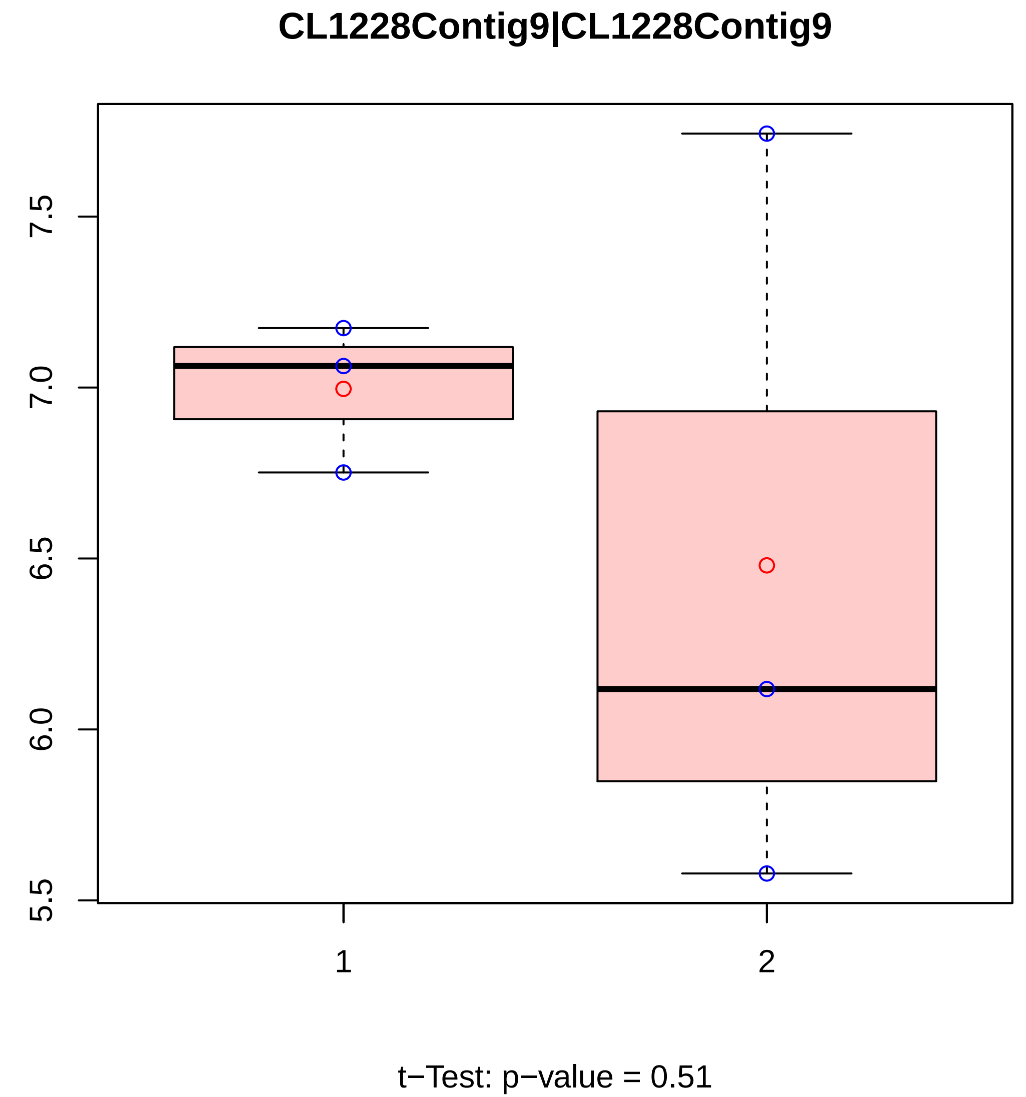
CL1228Contig9|CL1228Contig9
7.5
7.0
6.5
6.0
5.5
1
2
t−Test: p−value = 0.51

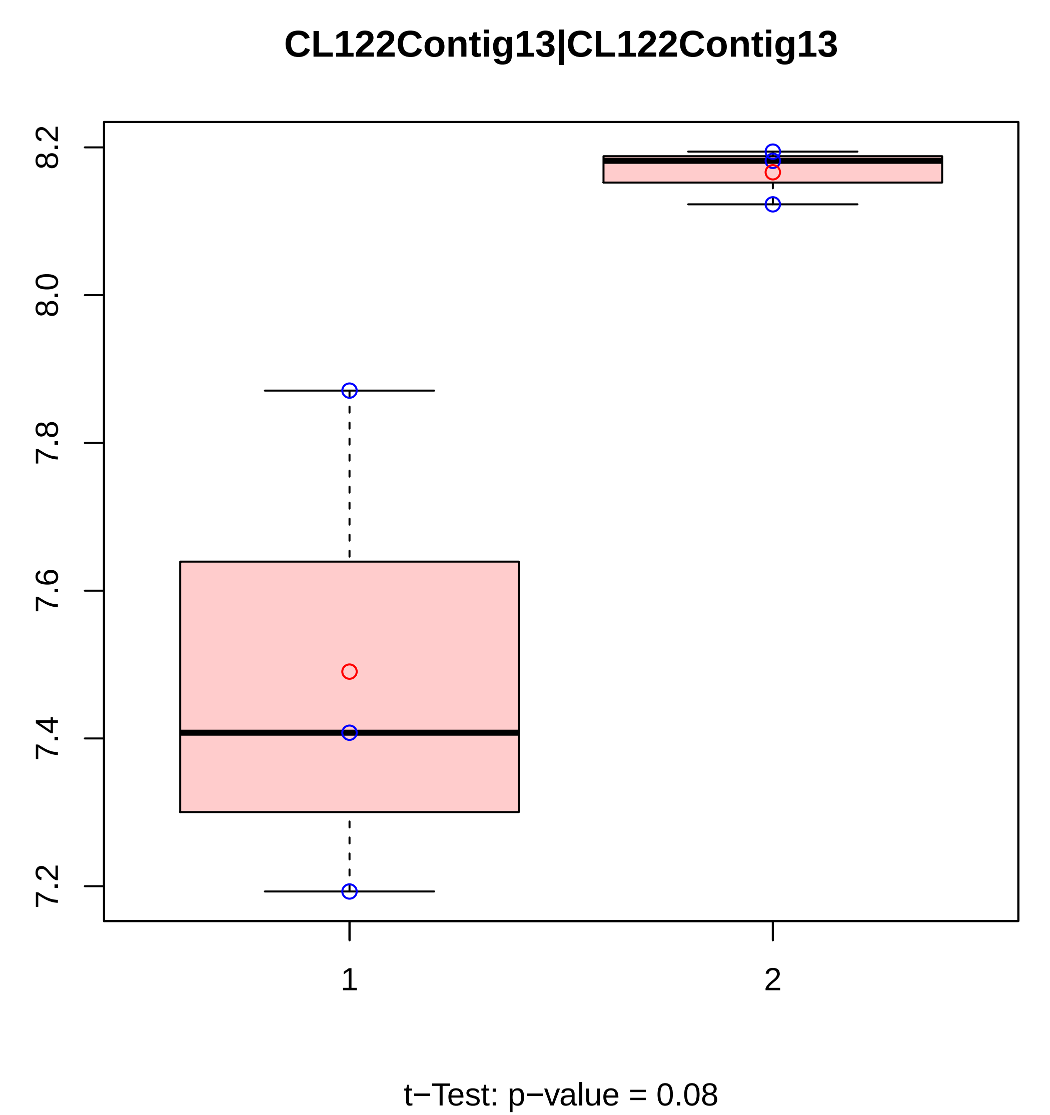
CL122Contig13|CL122Contig13
8.2
8.0
7.8
7.6
7.4
7.2
1
2
t−Test: p−value = 0.08

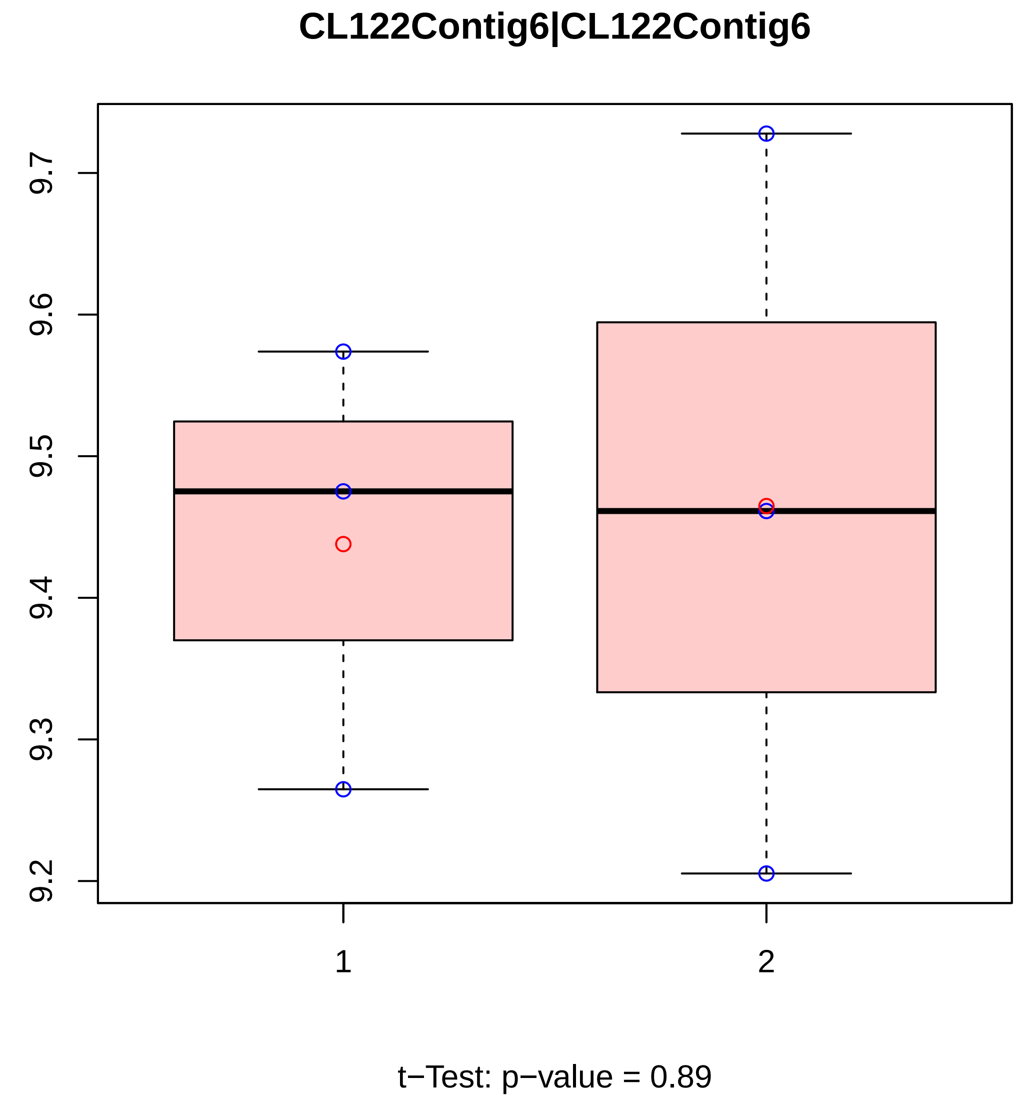
CL122Contig6|CL122Contig6
9.2
9.3
9.4
9.5
9.6
9.7
1
2
t−Test: p−value = 0.89

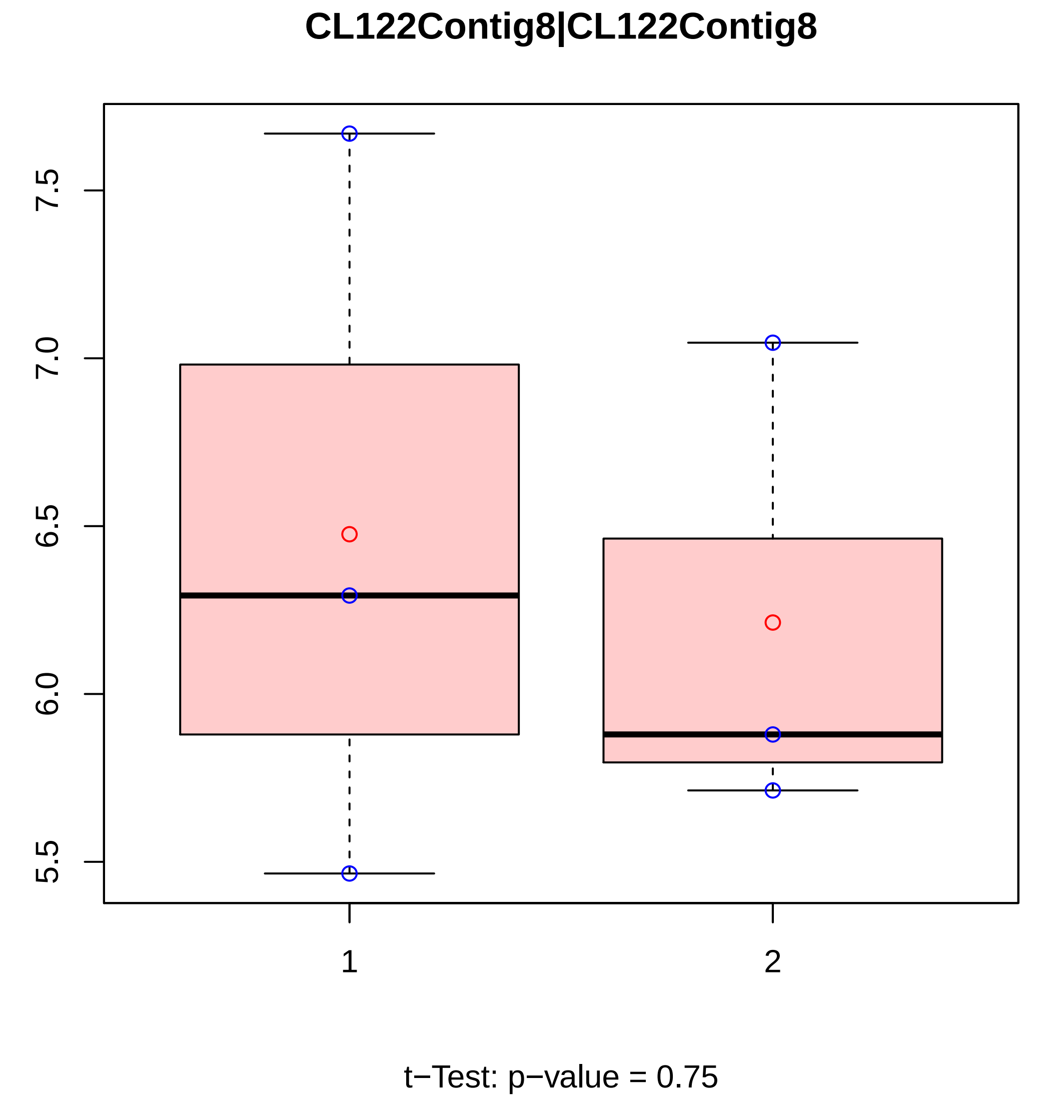
CL122Contig8|CL122Contig8
7.5
7.0
6.5
6.0
5.5
1
2
t−Test: p−value = 0.75

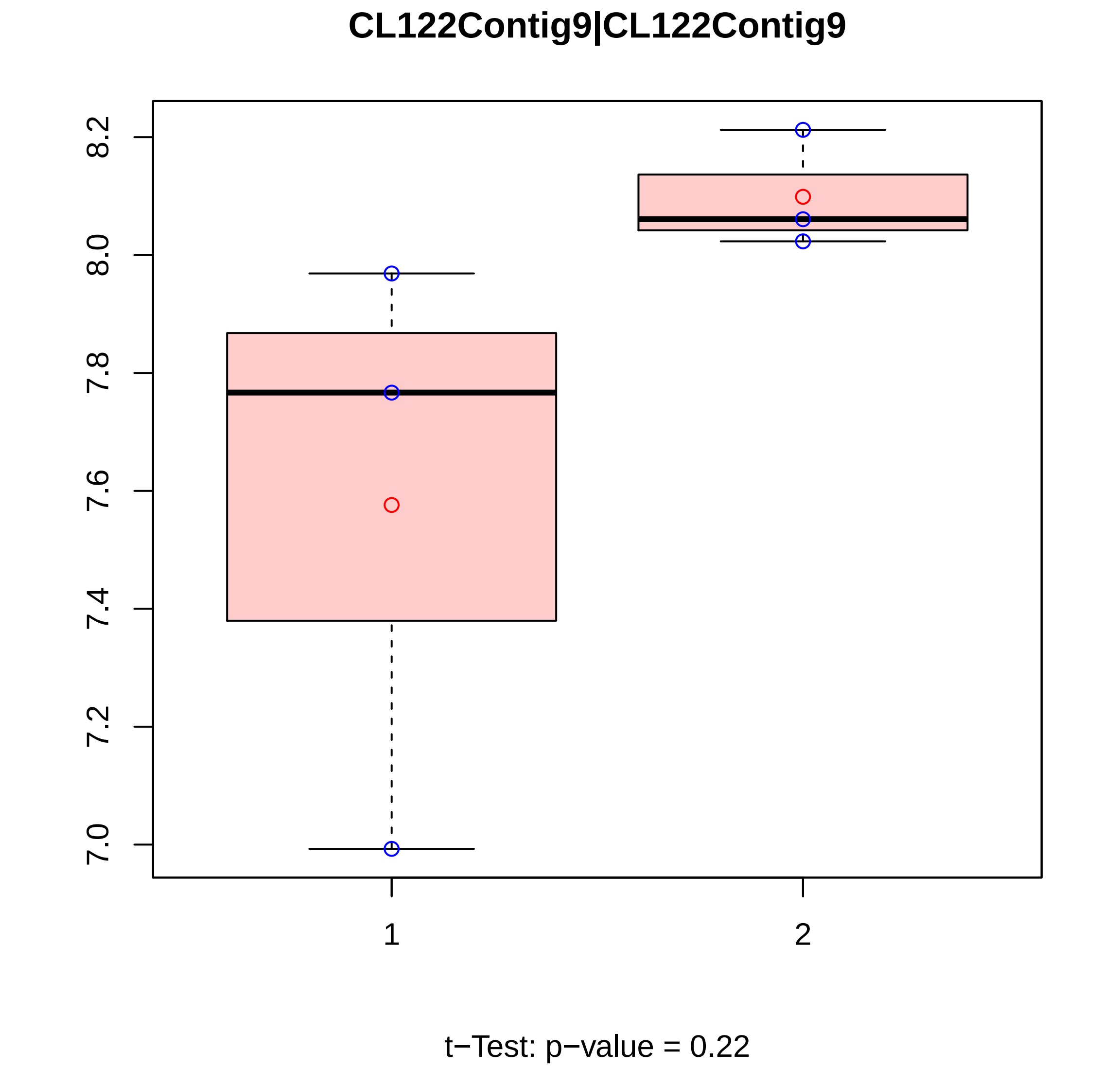
CL122Contig9|CL122Contig9
8.2
8.0
7.8
7.6
7.4
7.2
7.0
1
2
t−Test: p−value = 0.22

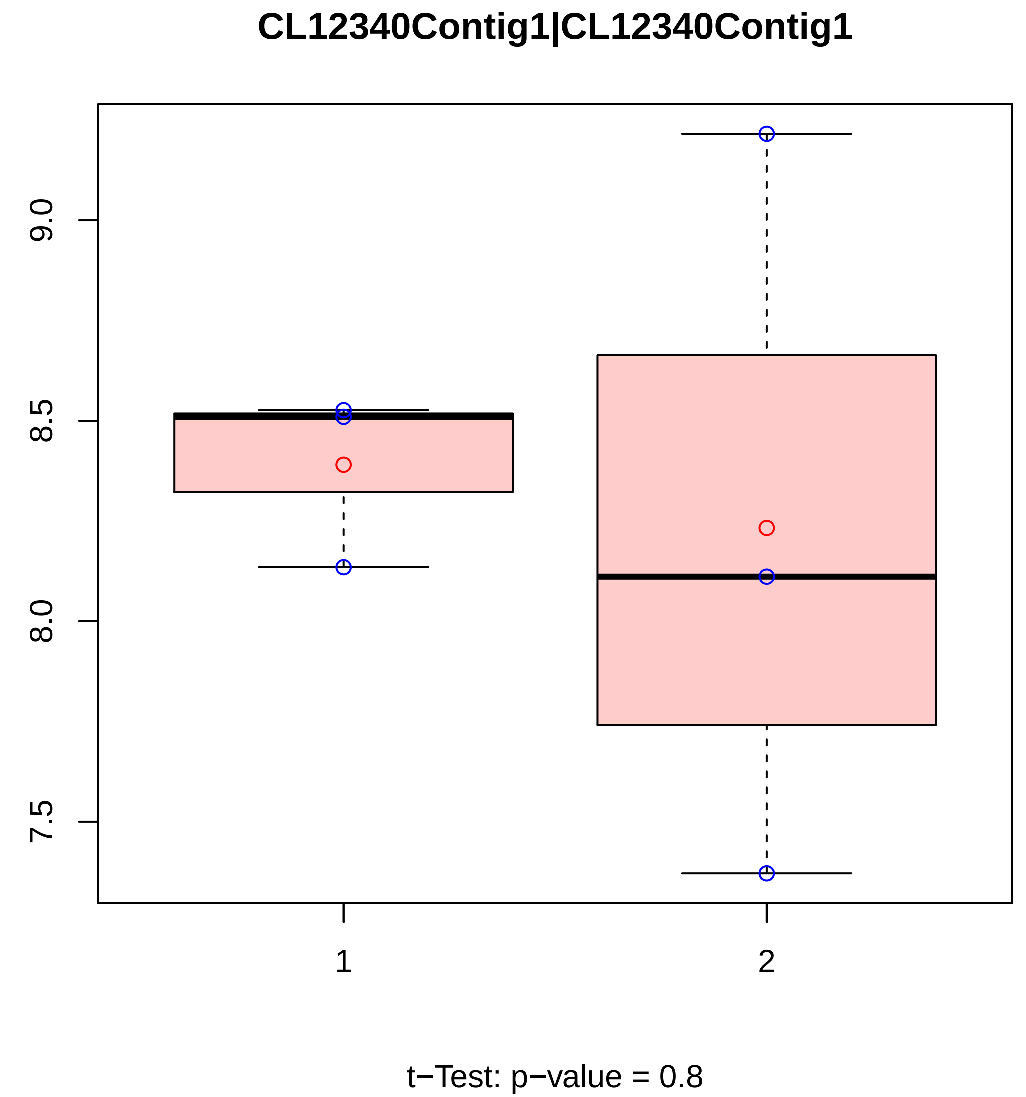
CL12340Contig1|CL12340Contig1
9.0
8.5
8.0
7.5
1
2
t−Test: p−value = 0.8

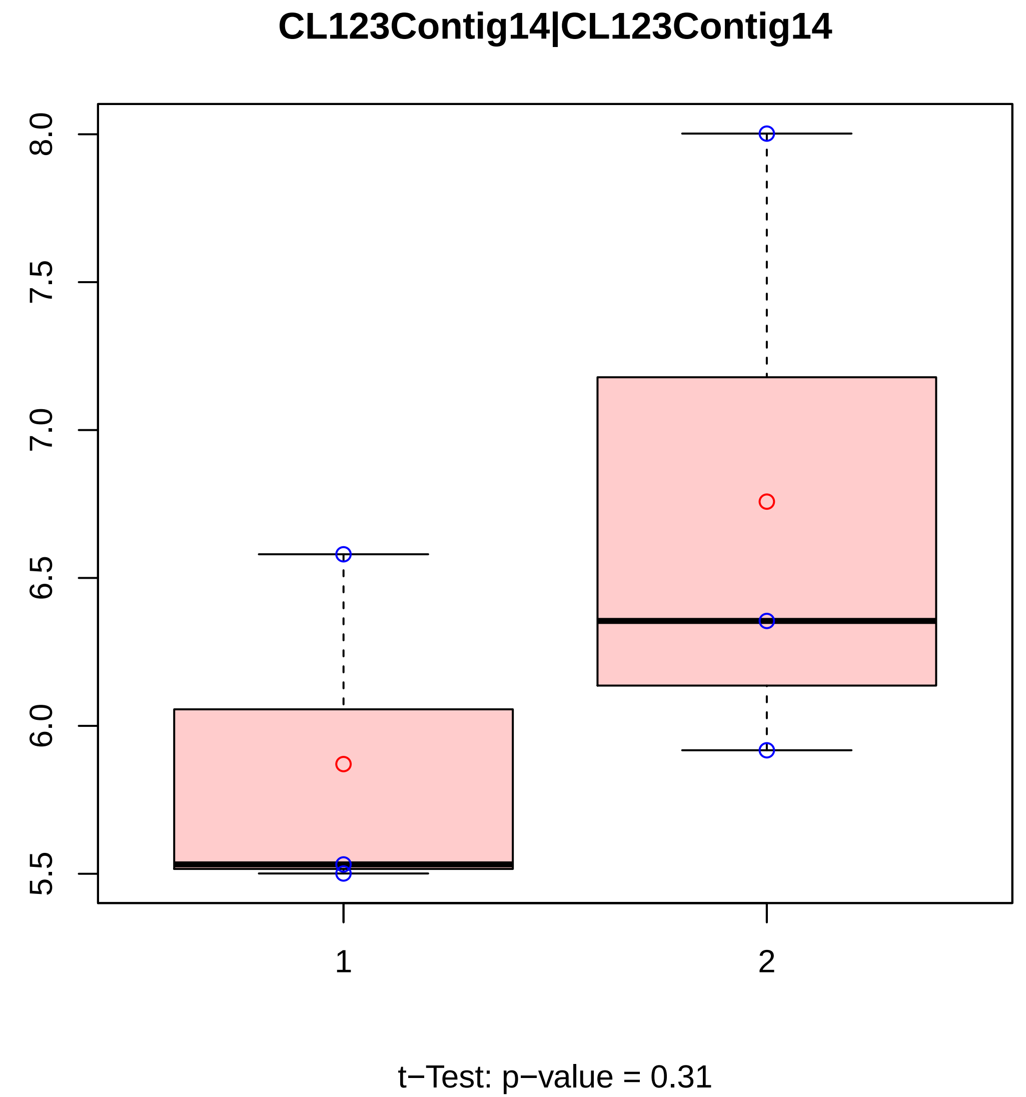
CL123Contig14|CL123Contig14
8.0
7.5
7.0
6.5
6.0
5.5
1
2
t−Test: p−value = 0.31

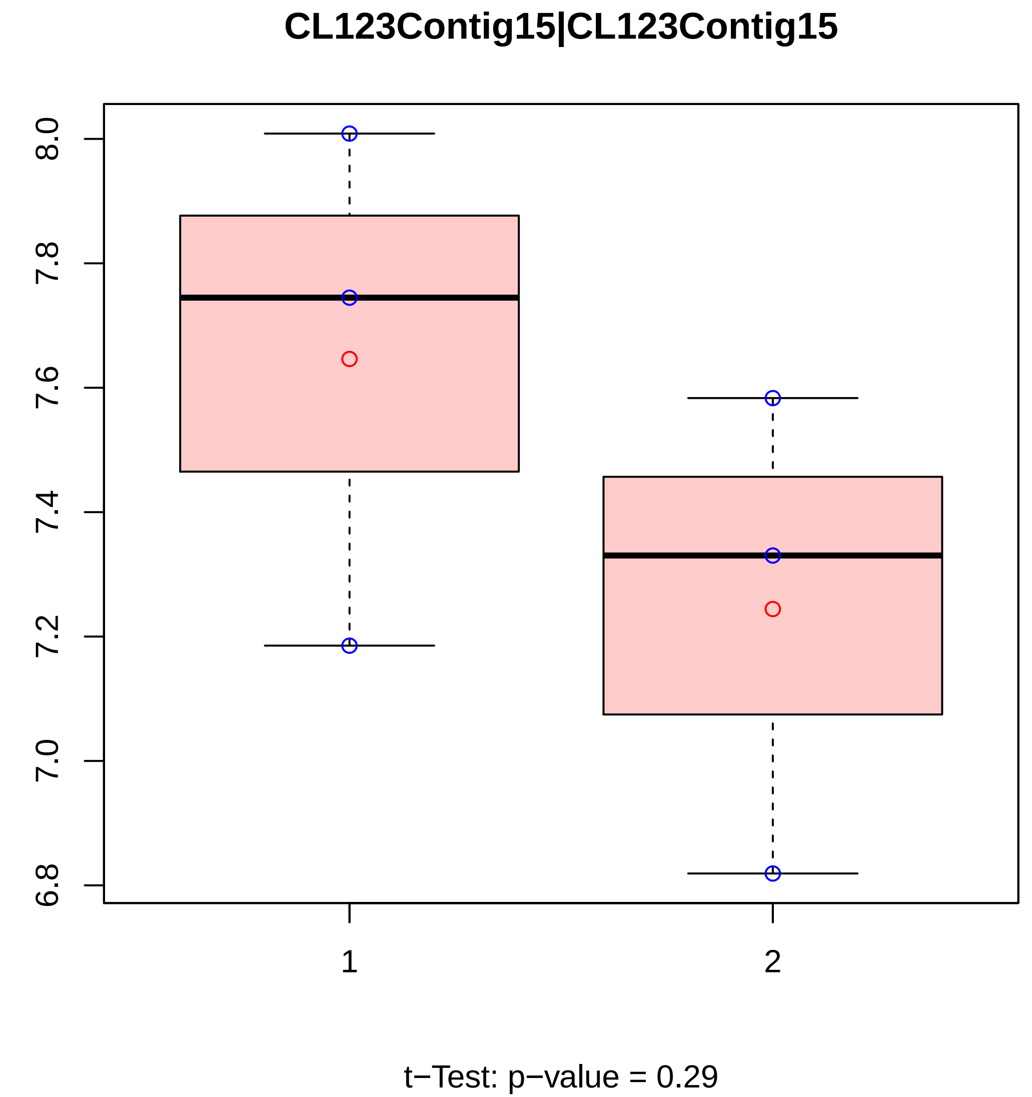
CL123Contig15|CL123Contig15
8.0
7.8
7.6
7.4
7.2
7.0
6.8
1
2
t−Test: p−value = 0.29

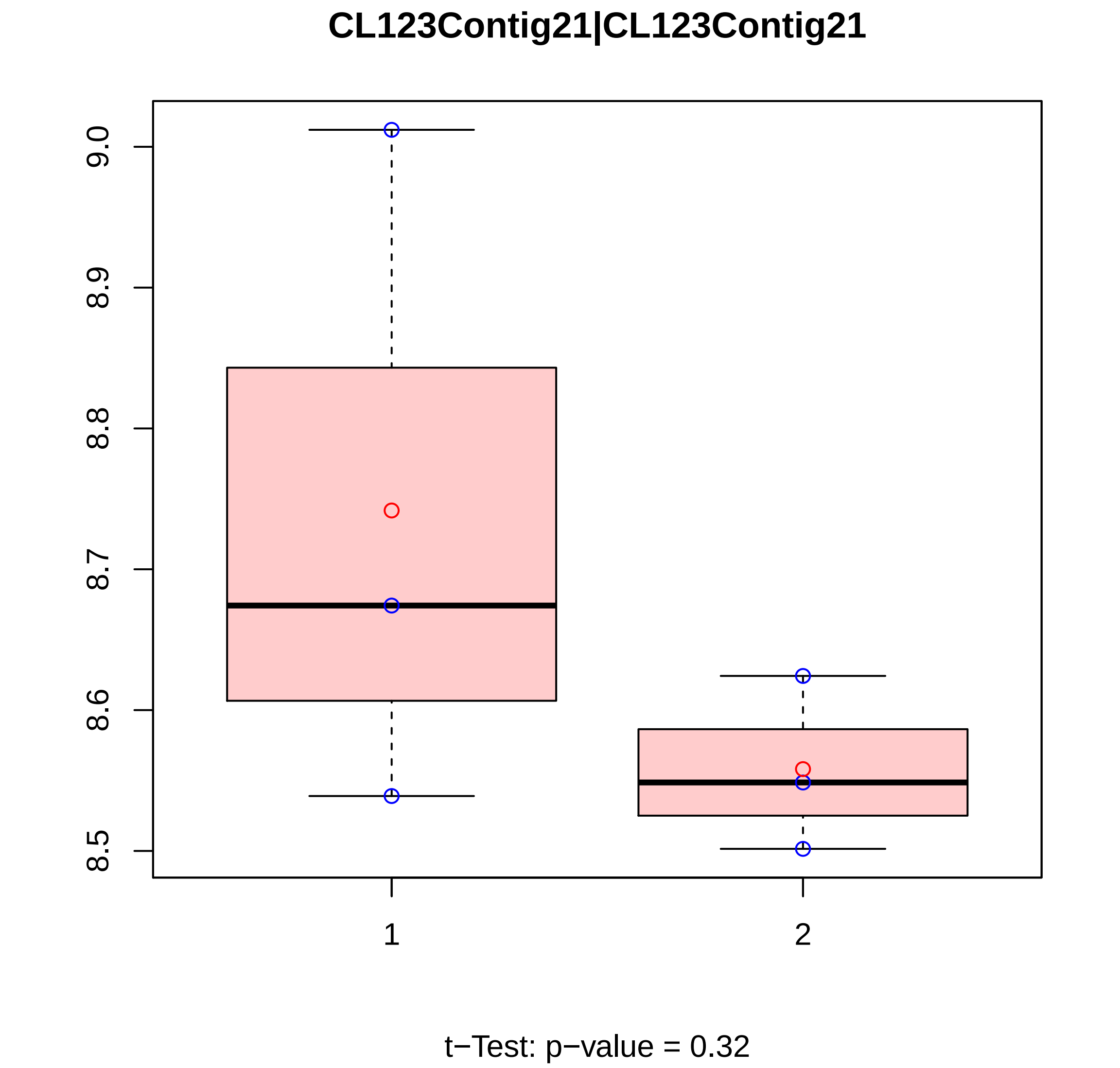
CL123Contig21|CL123Contig21
9.0
8.9
8.8
8.7
8.6
8.5
1
2
t−Test: p−value = 0.32

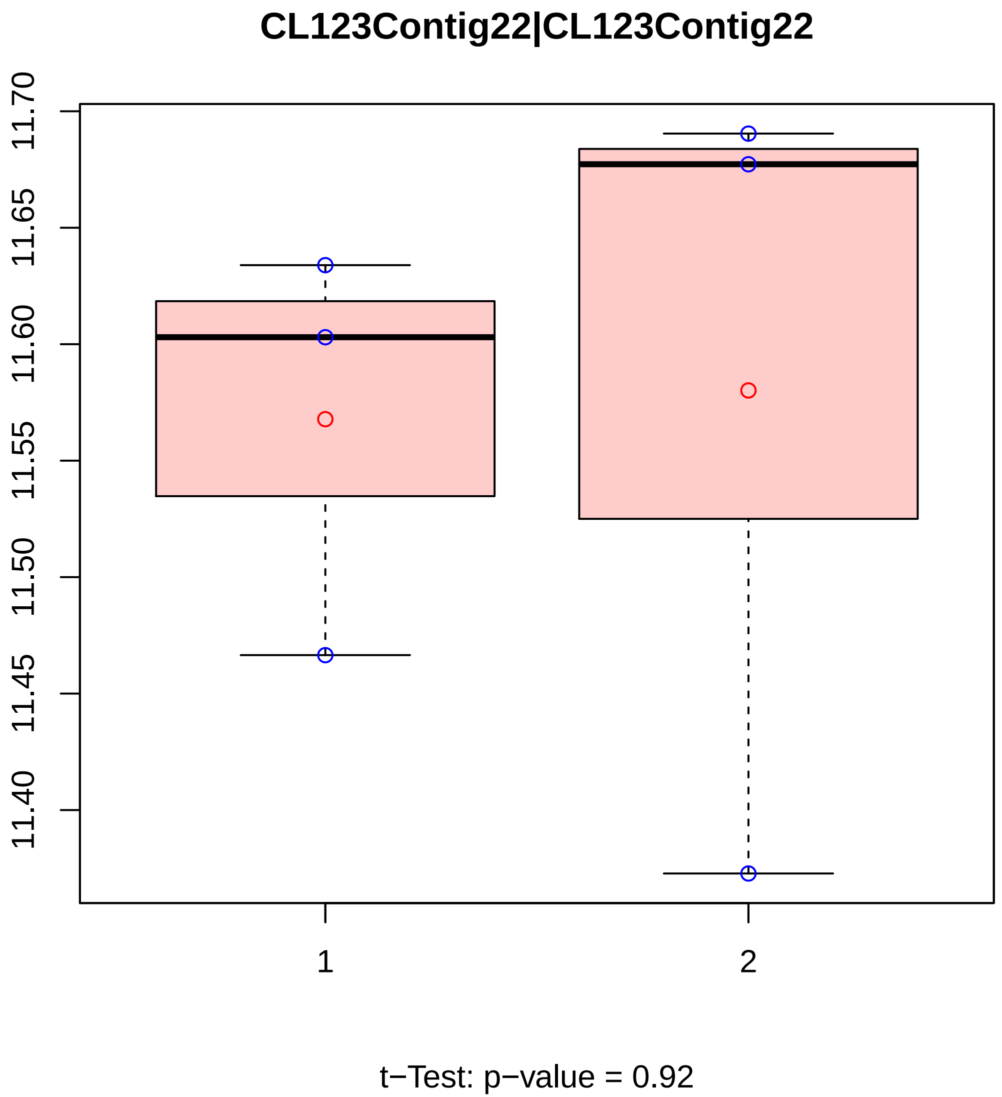
CL123Contig22|CL123Contig22
11.70
11.65
11.60
11.55
11.50
11.45
11.40
1
2
t−Test: p−value = 0.92

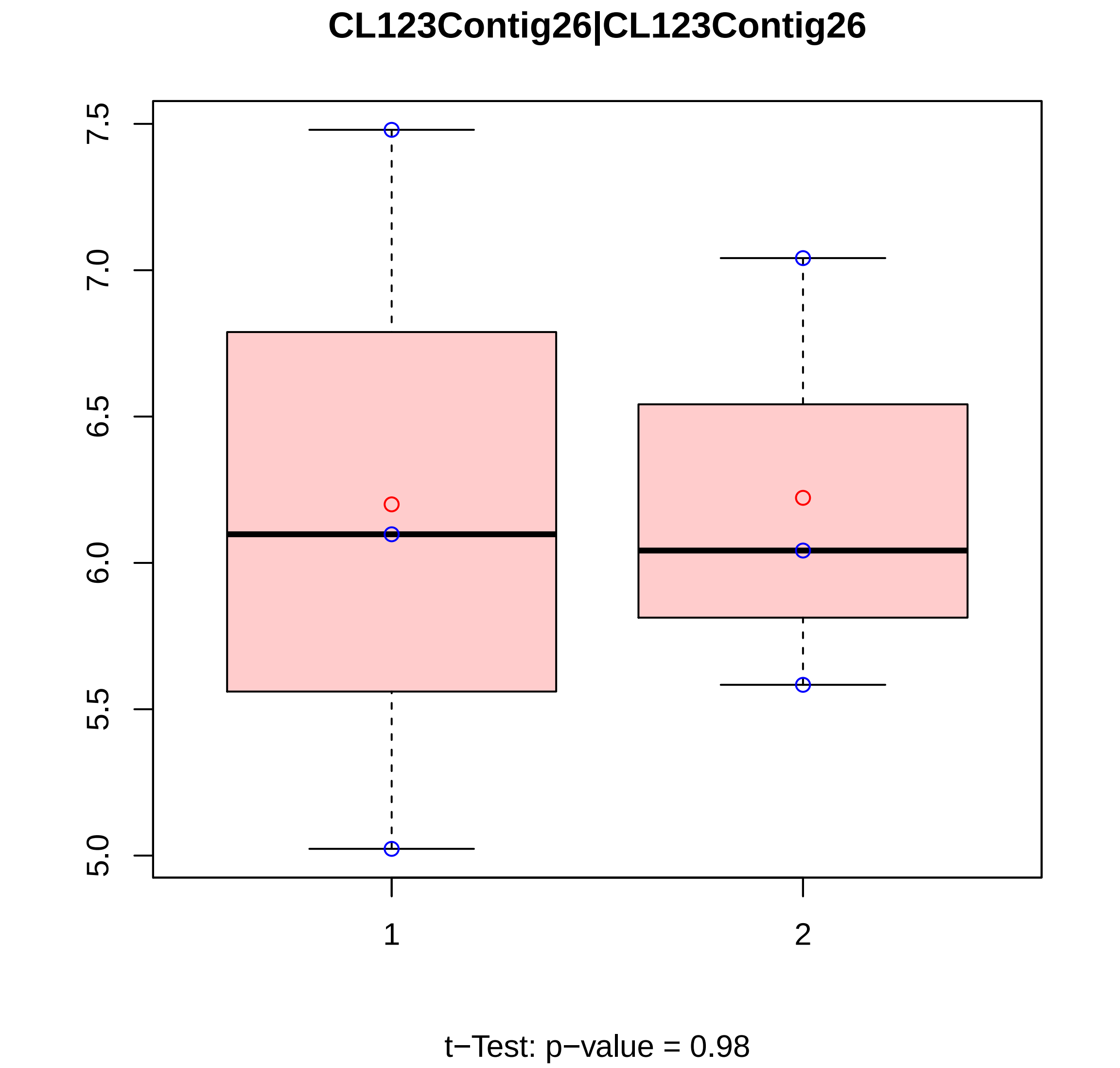
CL123Contig26|CL123Contig26
7.5
7.0
6.5
6.0
5.5
5.0
1
2
t−Test: p−value = 0.98

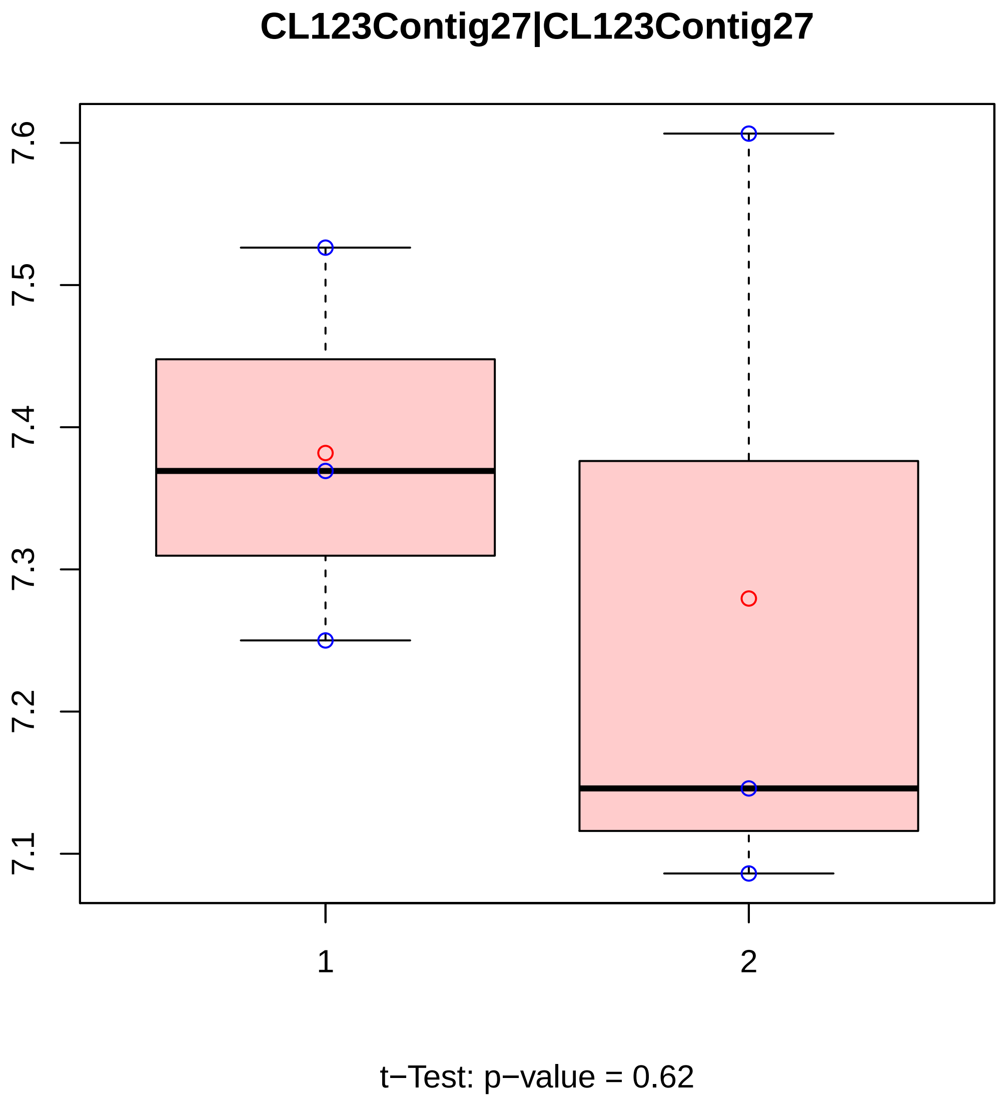
CL123Contig27|CL123Contig27
7.6
7.5
7.4
7.3
7.2
7.1
1
2
t−Test: p−value = 0.62

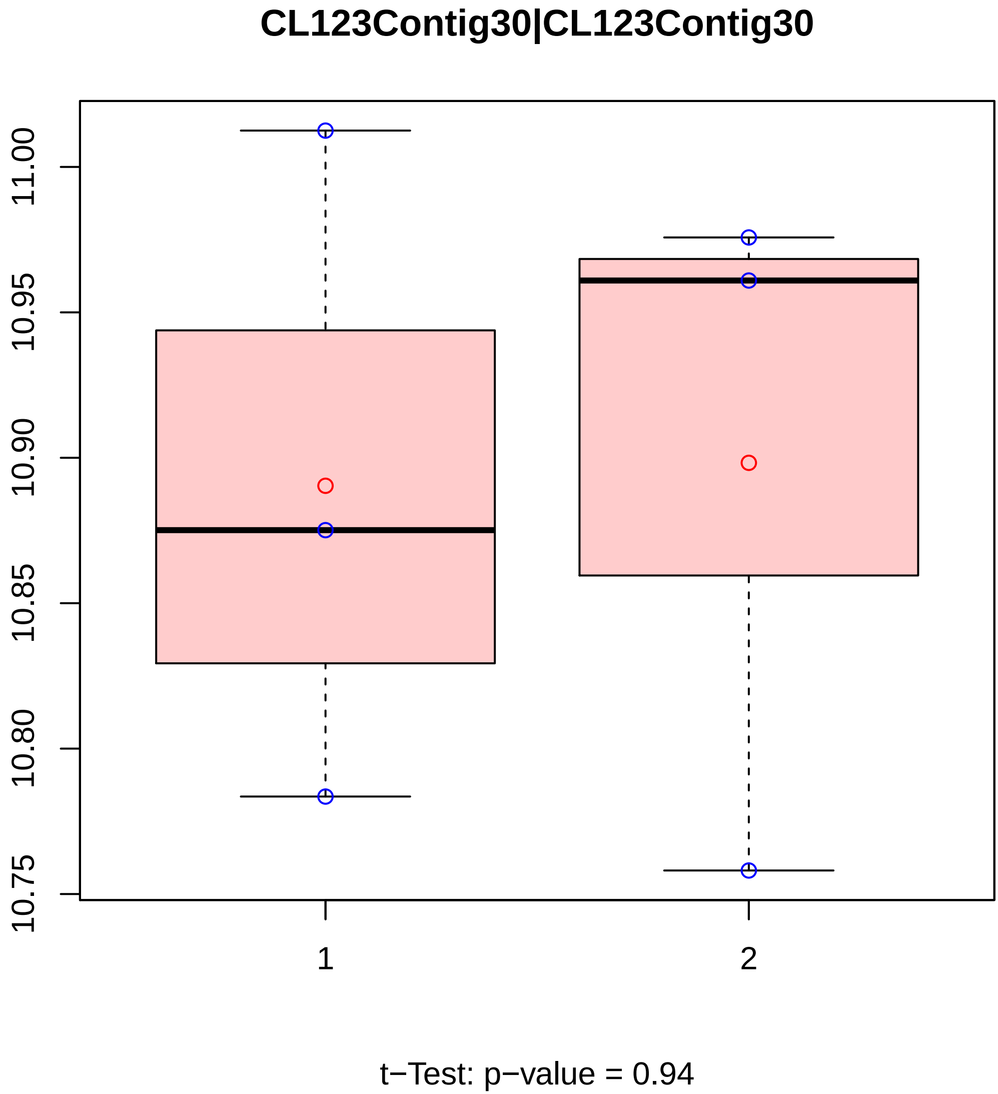
CL123Contig30|CL123Contig30
11.00
10.95
10.90
10.85
10.80
10.75
1
2
t−Test: p−value = 0.94

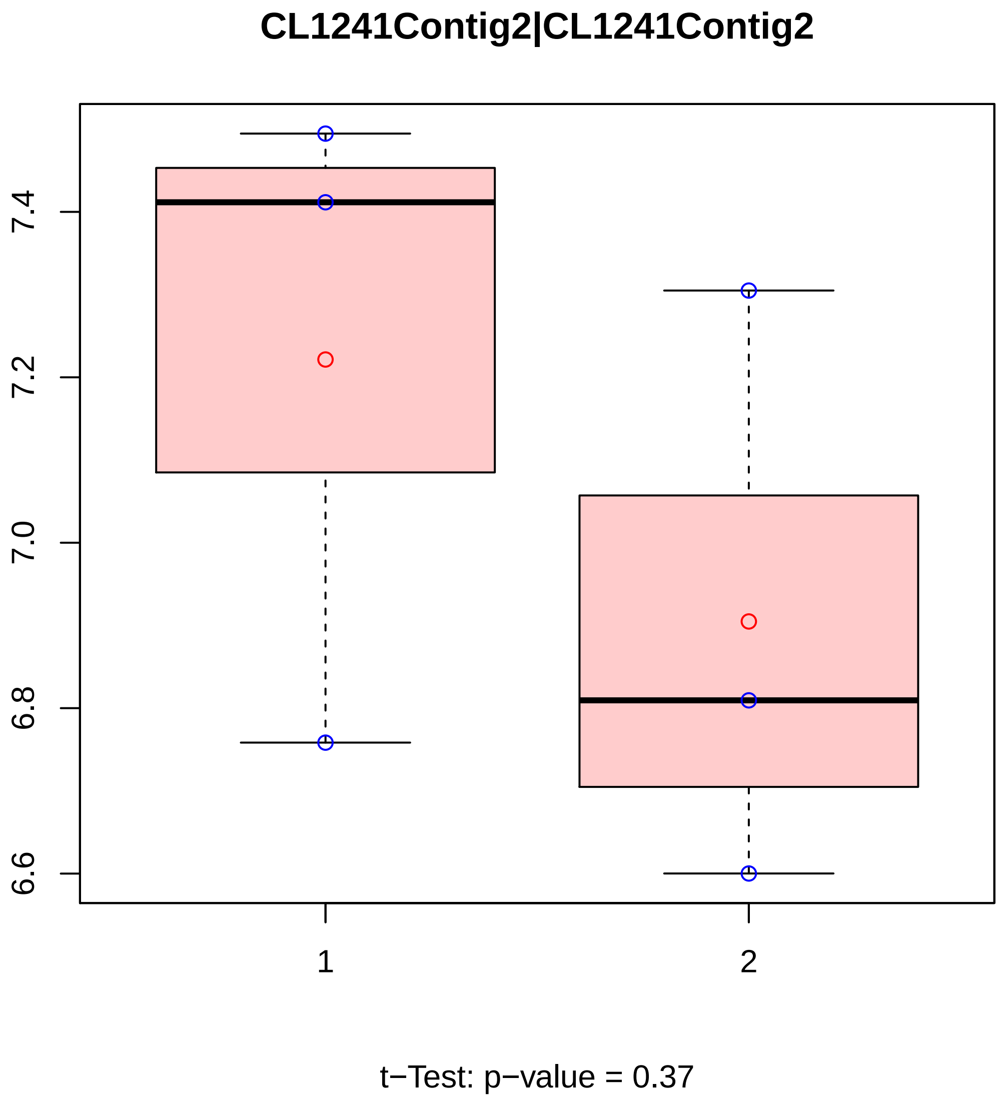
CL1241Contig2|CL1241Contig2
7.4
7.2
7.0
6.8
6.6
1
2
t−Test: p−value = 0.37

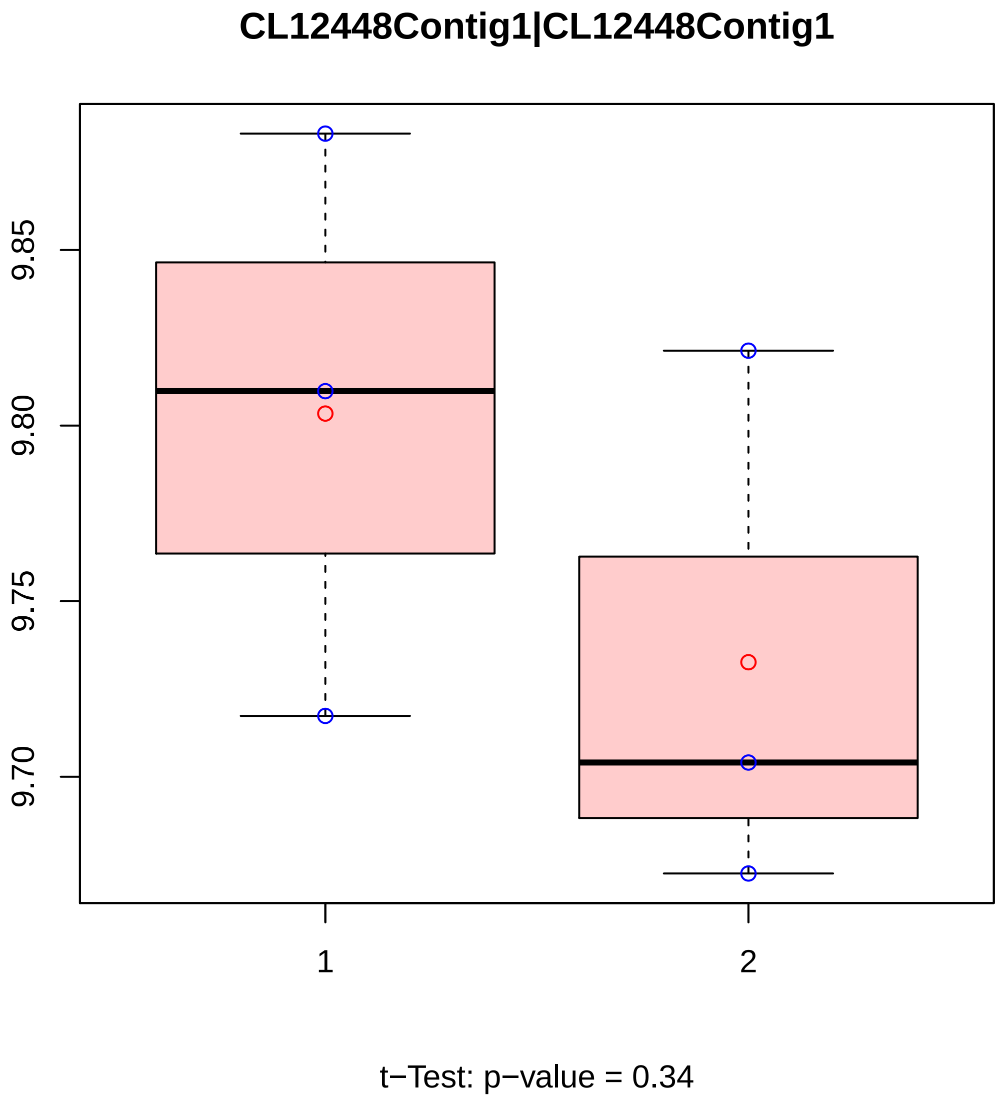
CL12448Contig1|CL12448Contig1
9.70
9.75
9.80
9.85
1
2
t−Test: p−value = 0.34

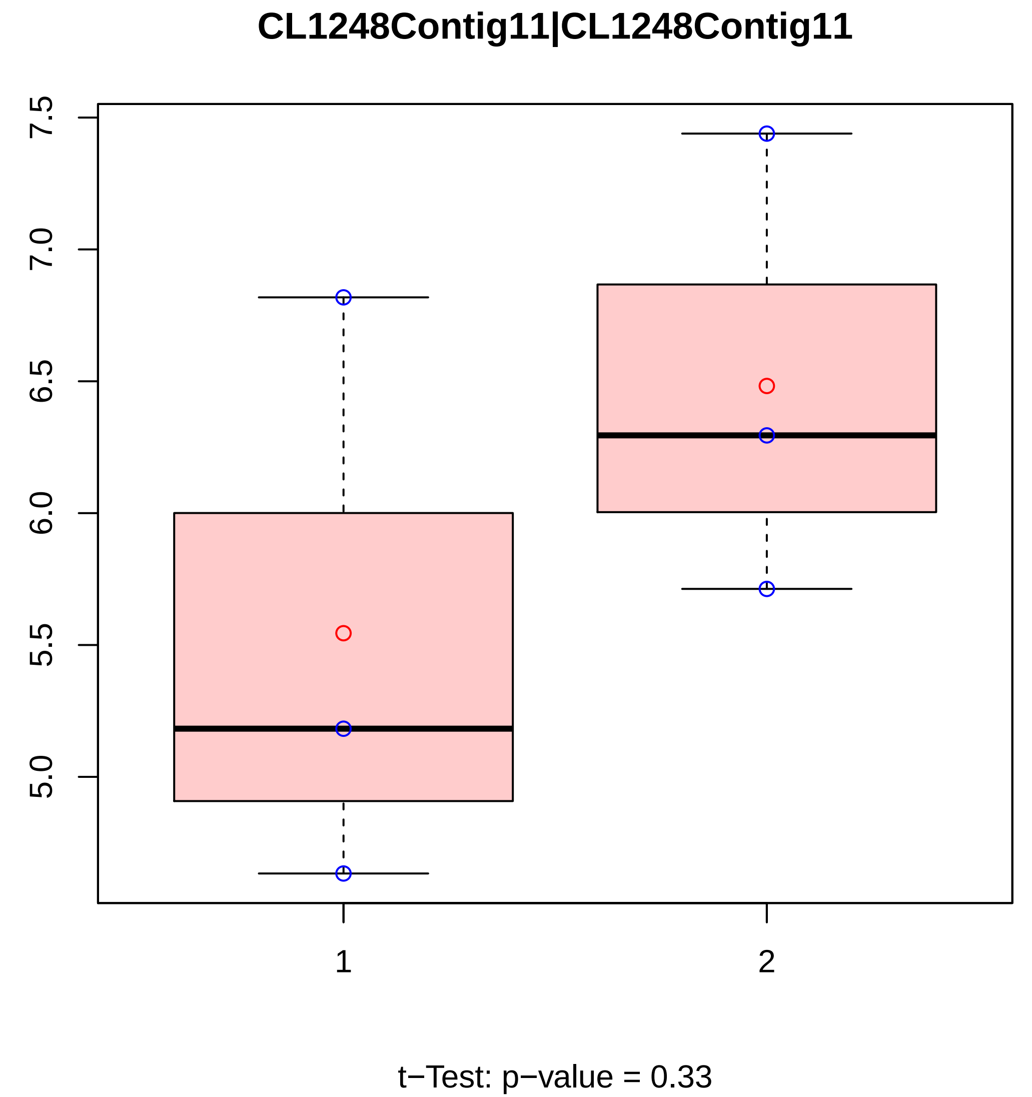
CL1248Contig11|CL1248Contig11
7.5
7.0
6.5
6.0
5.5
5.0
1
2
t−Test: p−value = 0.33

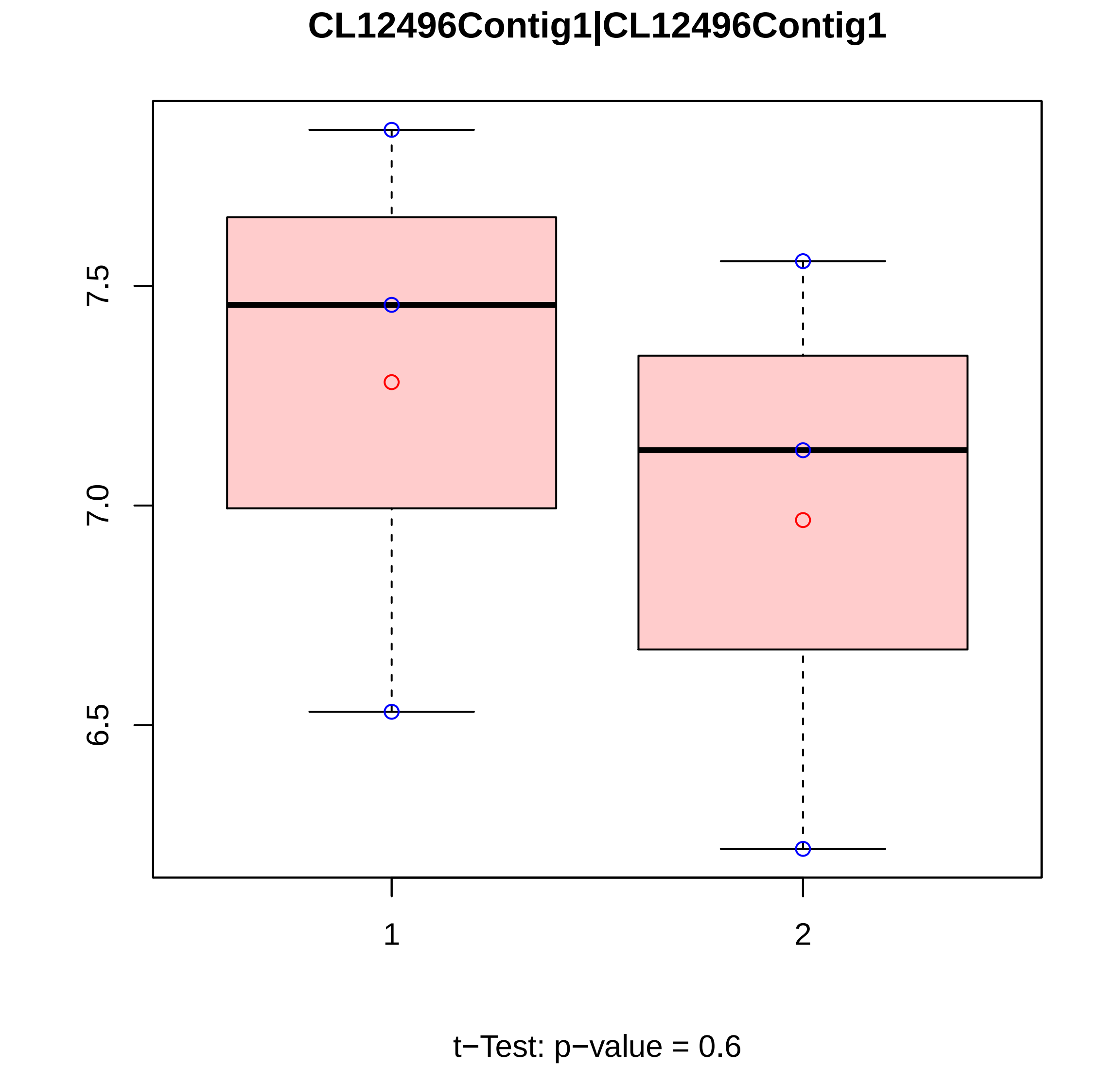

CL12496Contig1|CL12496Contig1
7.5
7.0
6.5
1
2
t−Test: p−value = 0.6

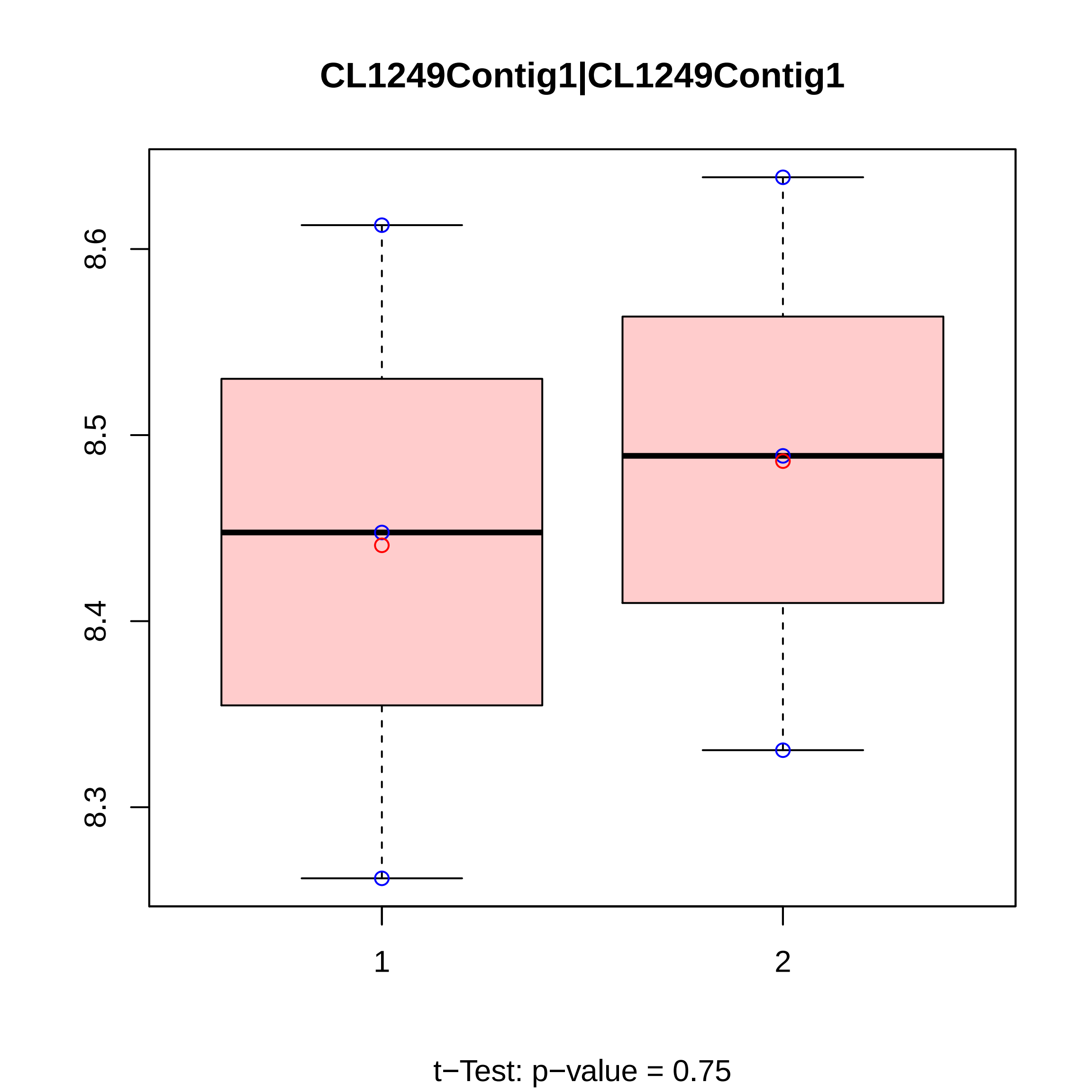
CL1249Contig1|CL1249Contig1
8.3
8.4
8.5
8.6
1
2
t−Test: p−value = 0.75

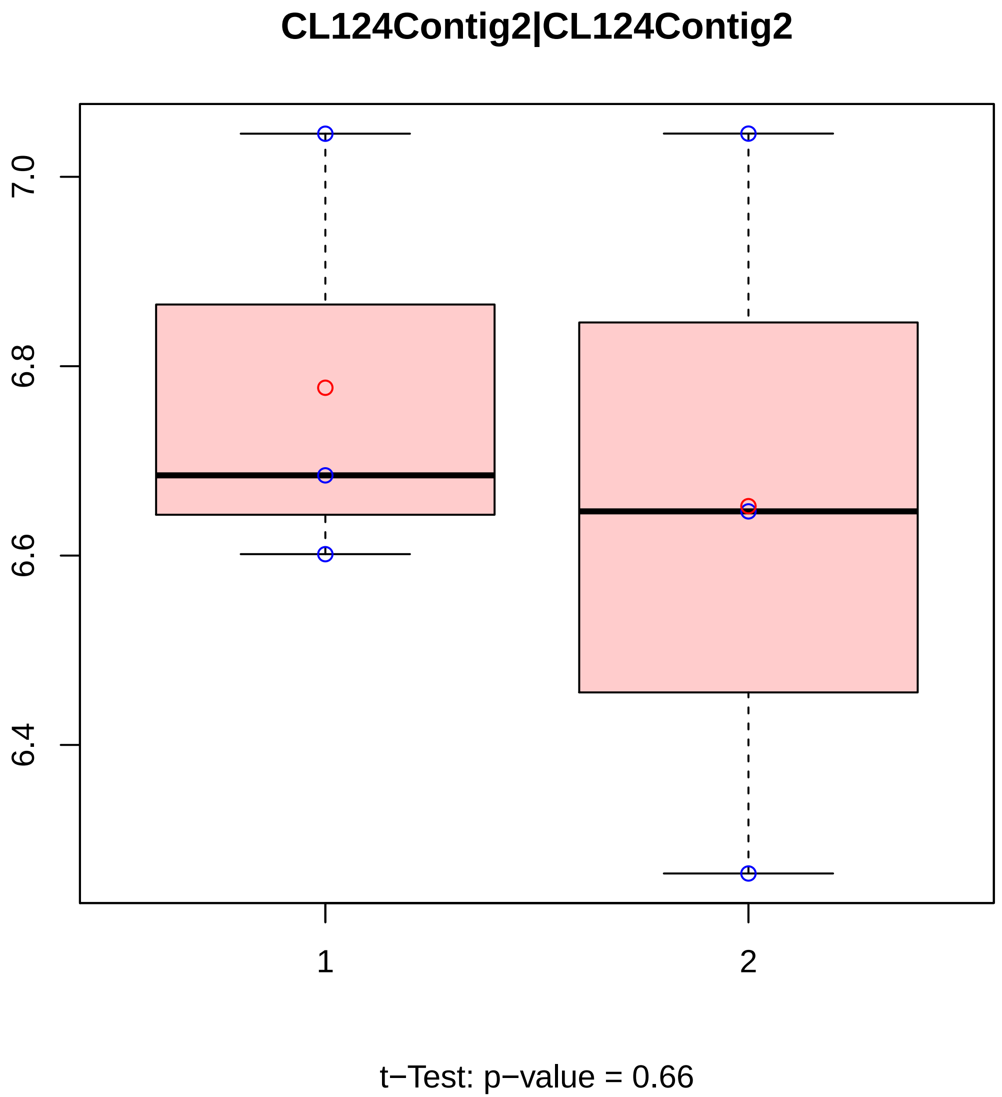
CL124Contig2|CL124Contig2
7.0
6.8
6.6
6.4
1
2
t−Test: p−value = 0.66

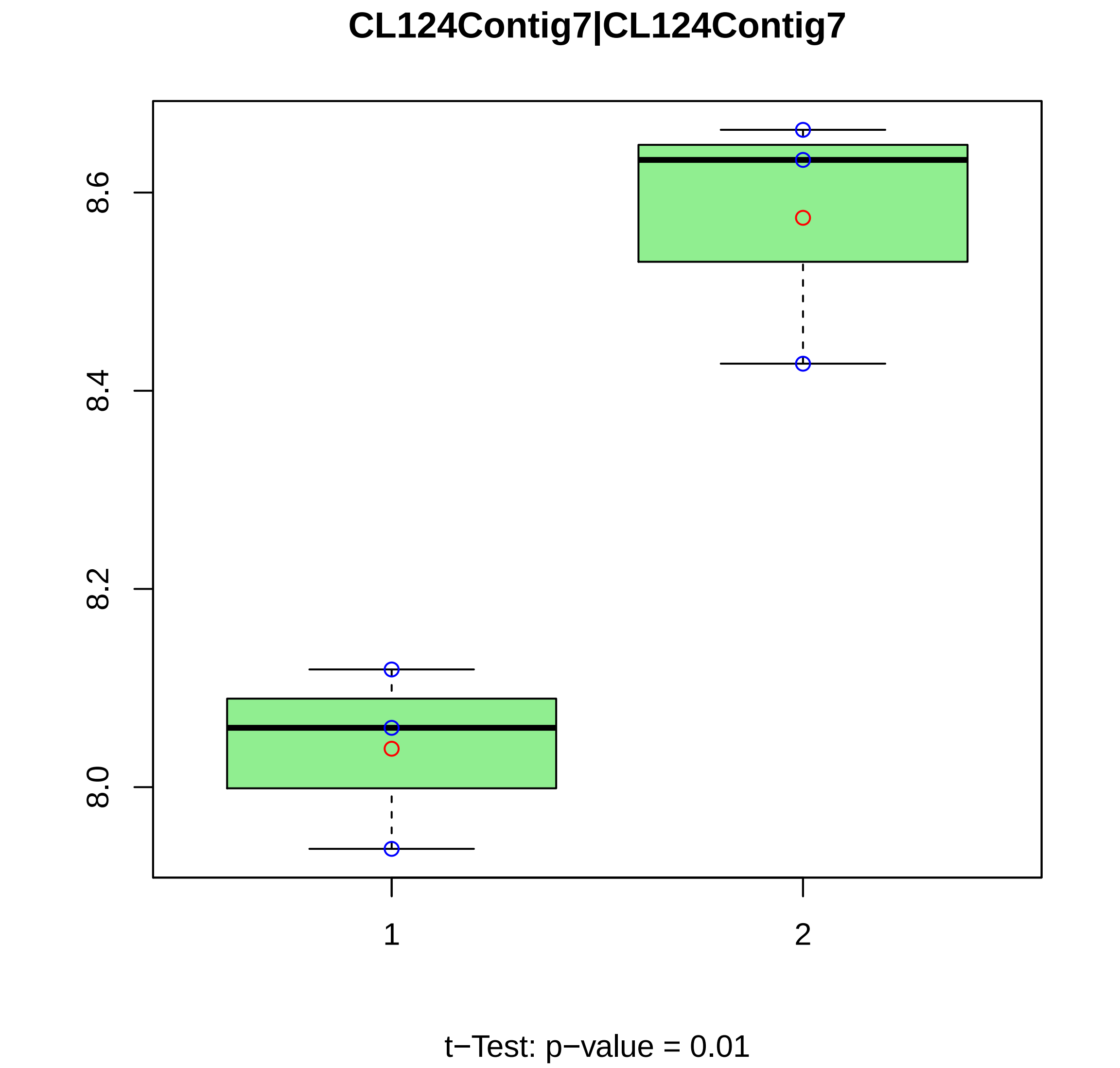
CL124Contig7|CL124Contig7
8.0
8.2
8.4
8.6
1
2
t−Test: p−value = 0.01

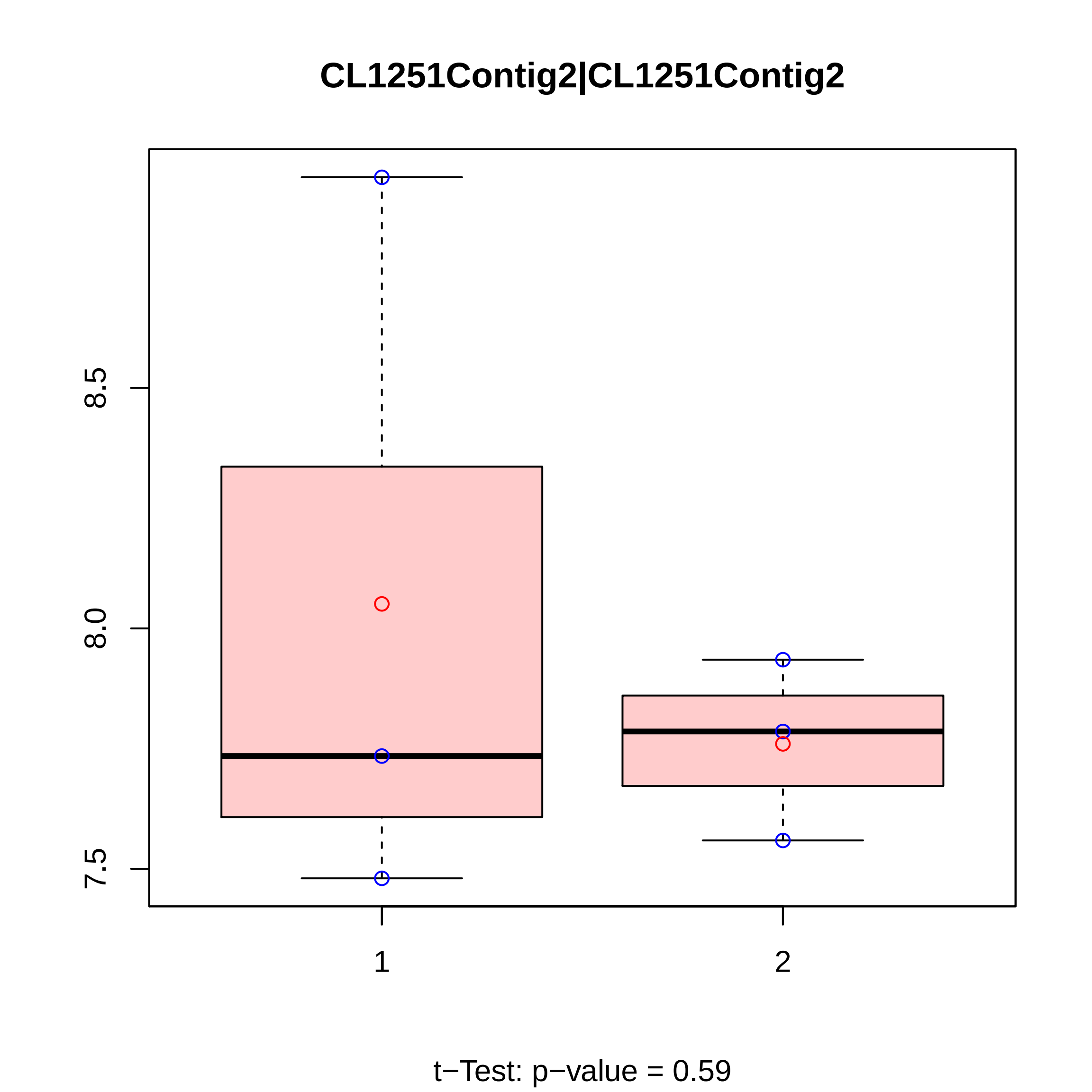
CL1251Contig2|CL1251Contig2
8.5
8.0
7.5
1
2
t−Test: p−value = 0.59

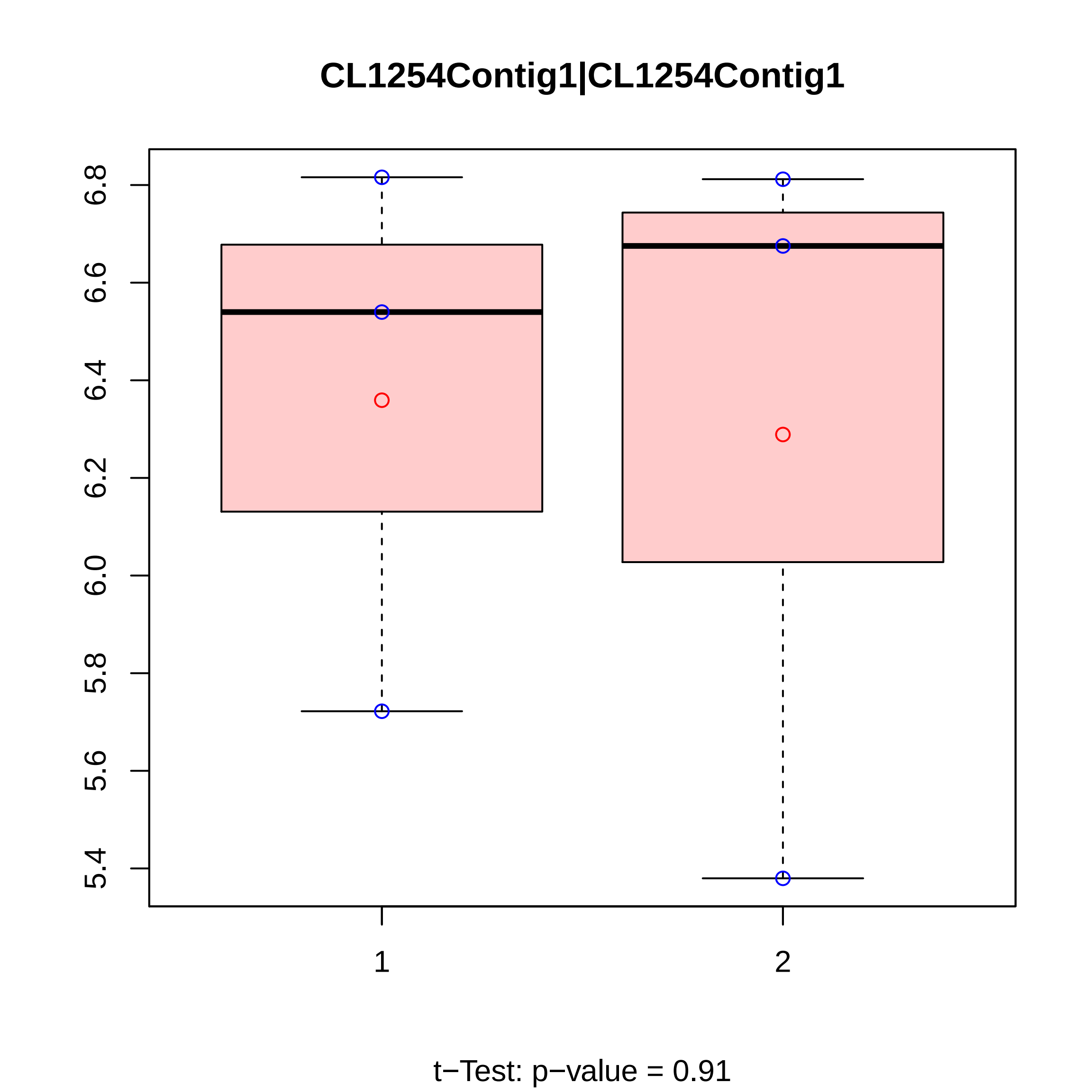
CL1254Contig1|CL1254Contig1
6.8
6.6
6.4
6.2
6.0
5.8
5.6
5.4
1
2
t−Test: p−value = 0.91

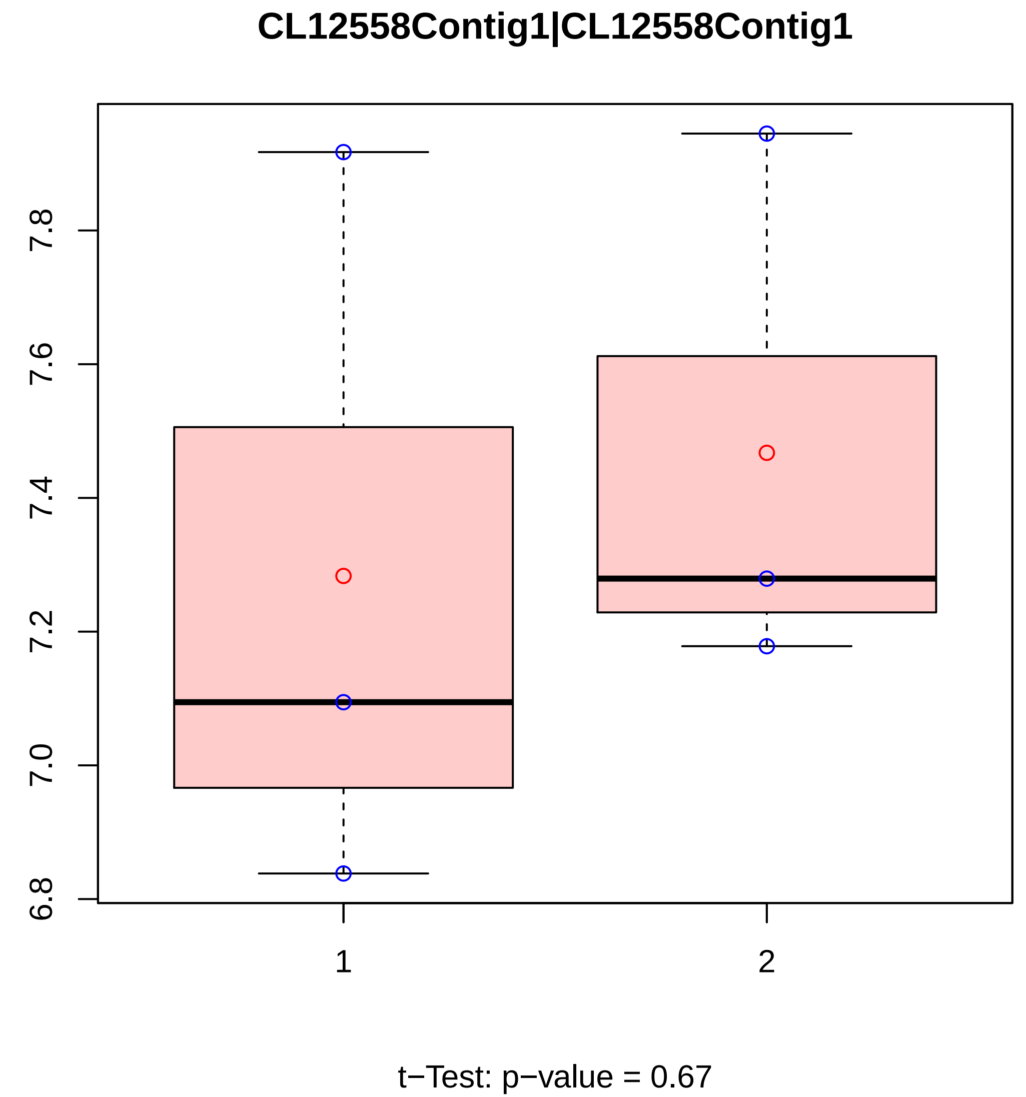

CL12558Contig1|CL12558Contig1
6.8
7.0
7.2
7.4
7.6
7.8
1
2
t−Test: p−value = 0.67

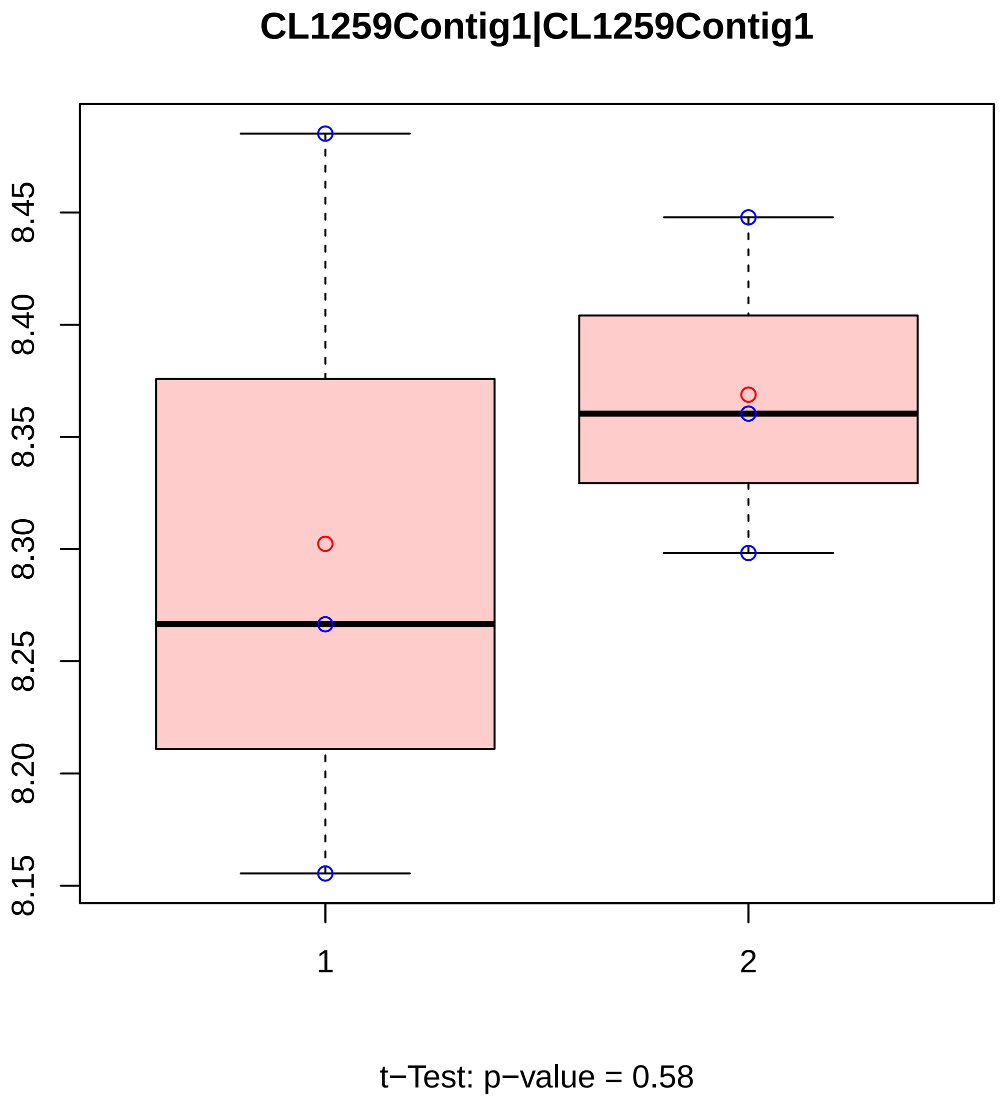
CL1259Contig1|CL1259Contig1
8.15
8.20
8.25
8.30
8.35
8.40
8.45
1
2
t−Test: p−value = 0.58

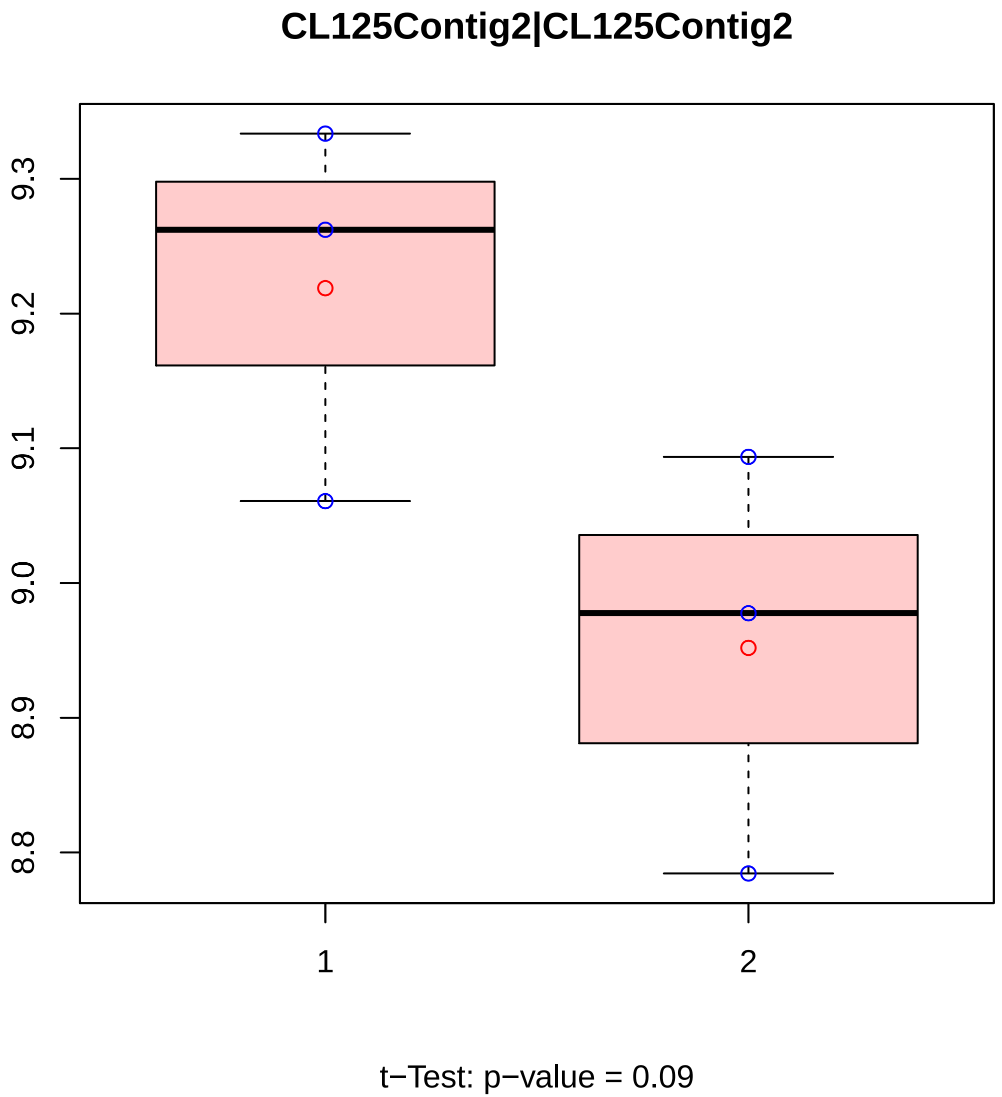

CL125Contig2|CL125Contig2
9.3
9.2
9.1
9.0
8.9
8.8
1
2
t−Test: p−value = 0.09

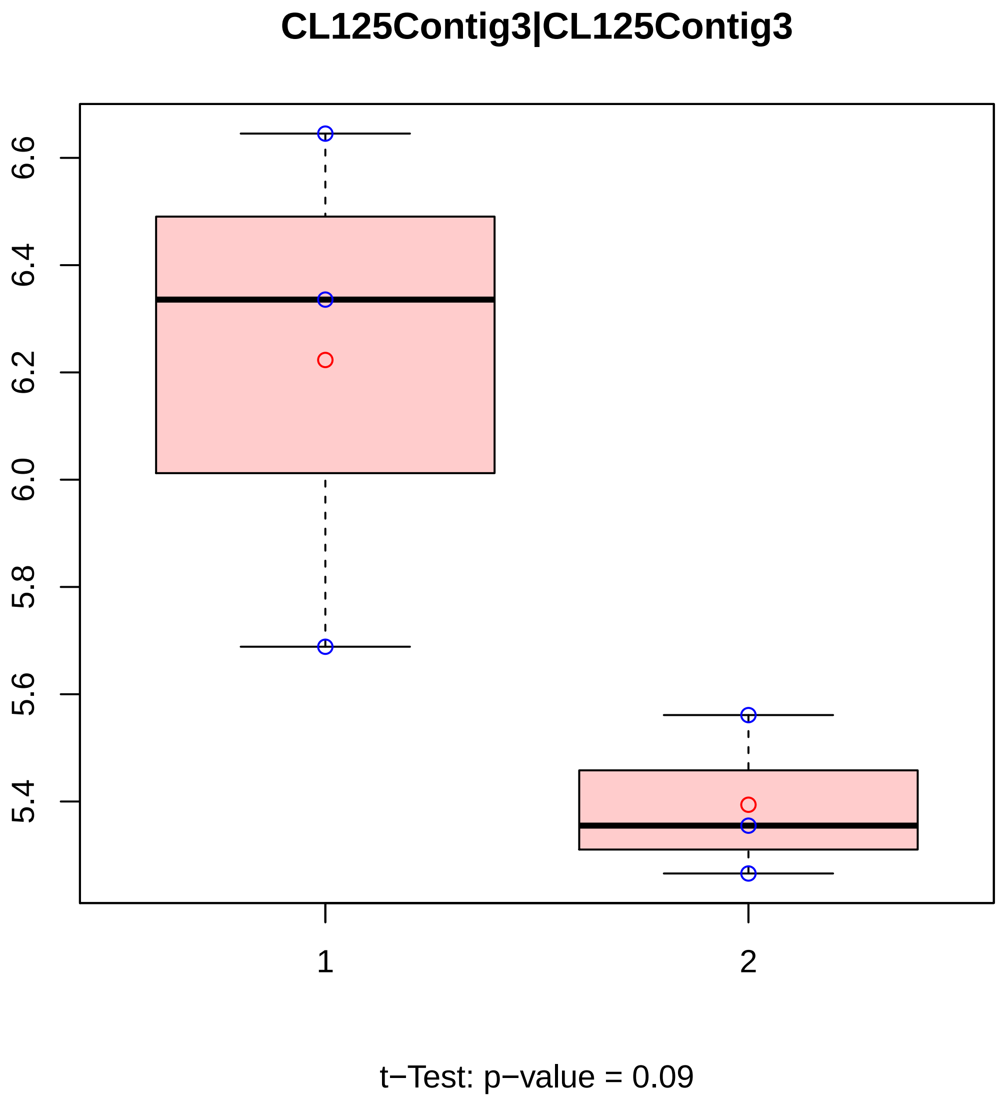
CL125Contig3|CL125Contig3
6.6
6.4
6.2
6.0
5.8
5.6
5.4
1
2
t−Test: p−value = 0.09

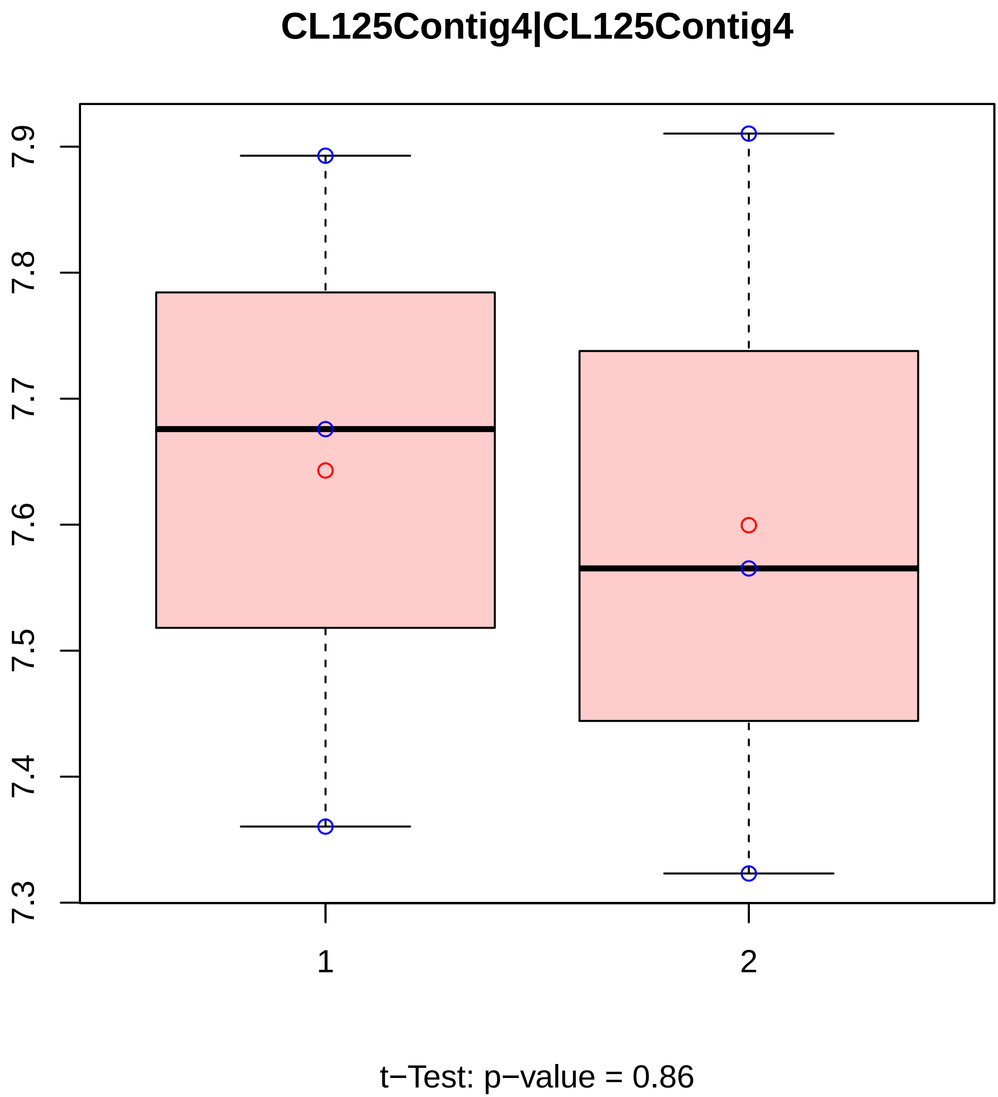

CL125Contig4|CL125Contig4
7.3
7.4
7.5
7.6
7.7
7.8
7.9
1
2
t−Test: p−value = 0.86

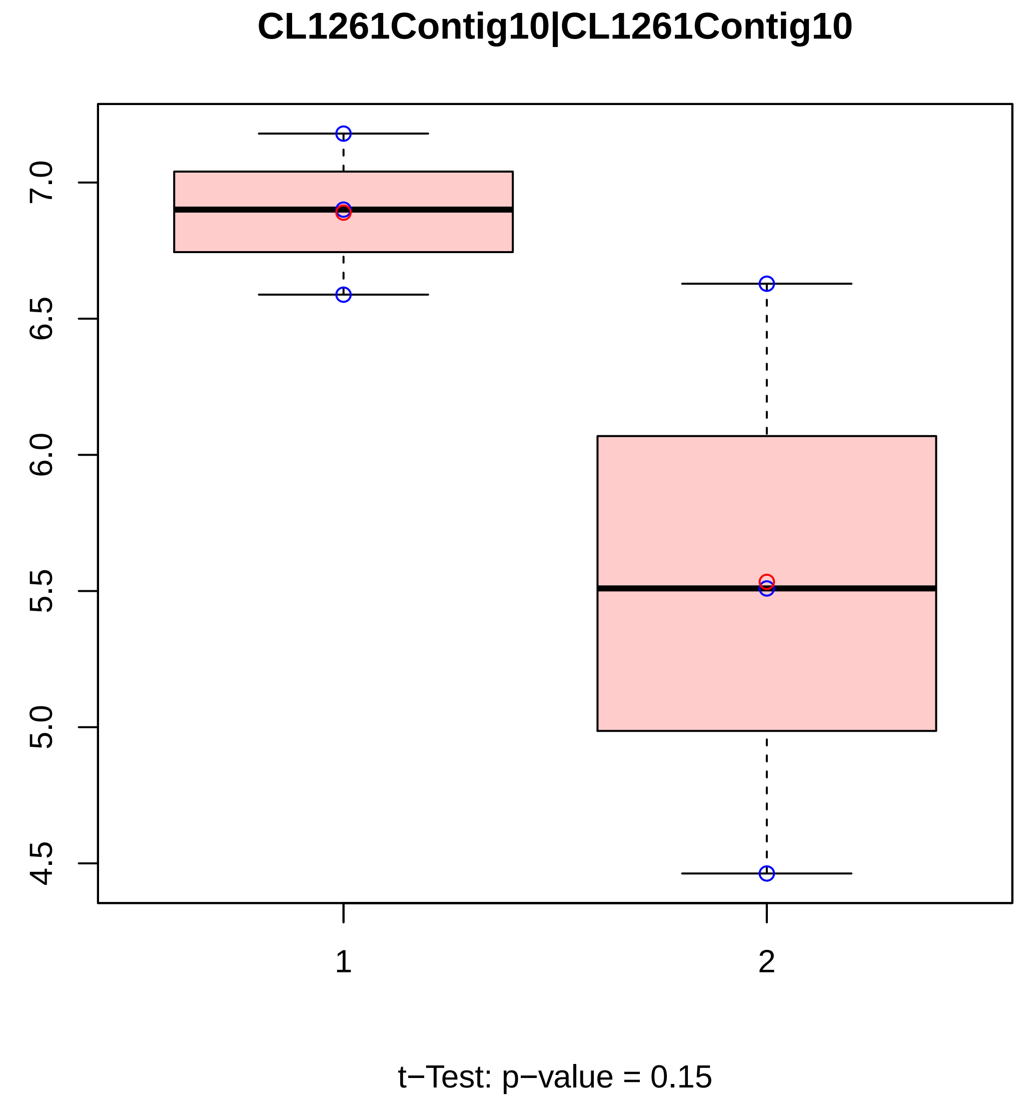
CL1261Contig10|CL1261Contig10
7.0
6.5
6.0
5.5
5.0
4.5
1
2
t−Test: p−value = 0.15

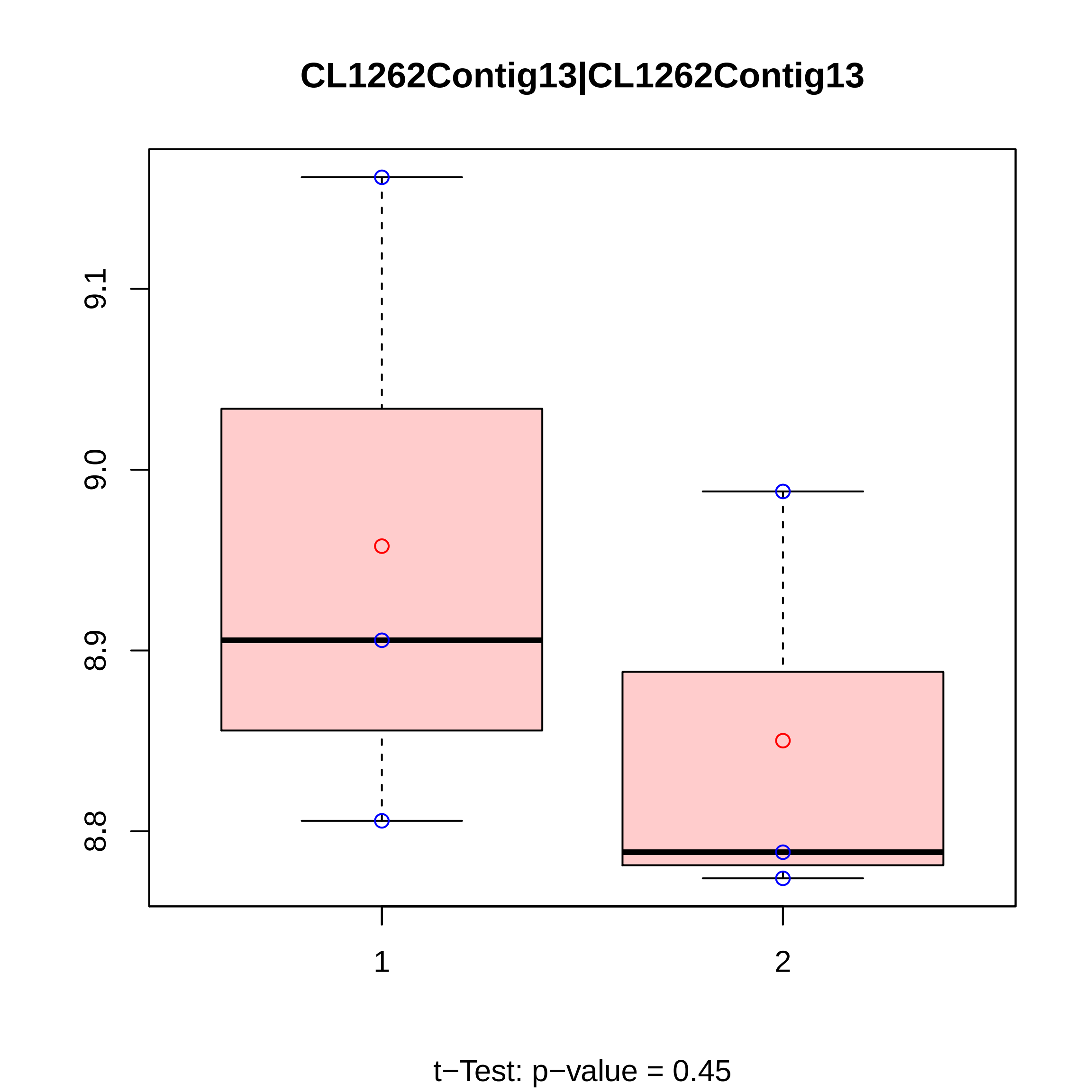
CL1262Contig13|CL1262Contig13
9.1
9.0
8.9
8.8
1
2
t−Test: p−value = 0.45

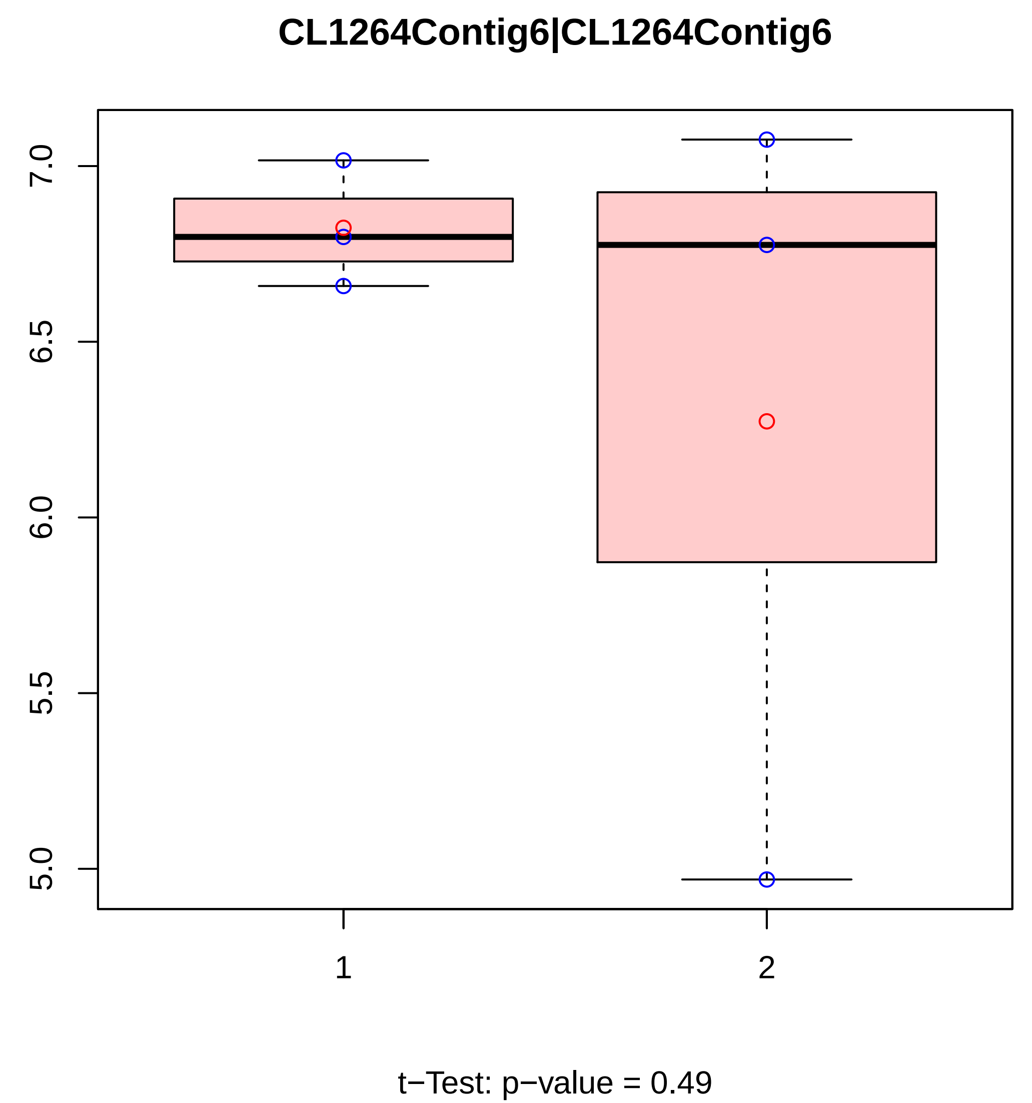
CL1264Contig6|CL1264Contig6
7.0
6.5
6.0
5.5
5.0
1
2
t−Test: p−value = 0.49

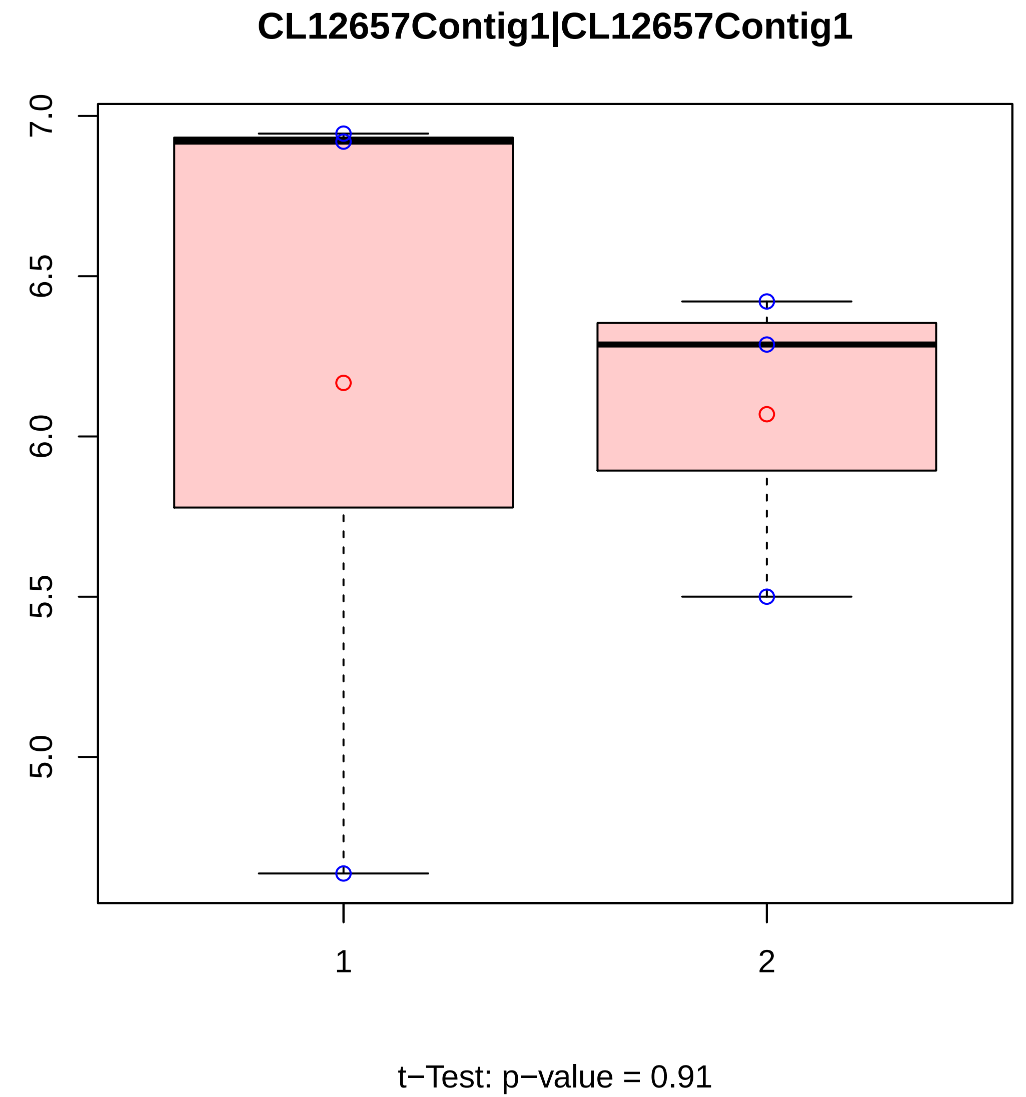

CL12657Contig1|CL12657Contig1
7.0
6.5
6.0
5.5
5.0
1
2
t−Test: p−value = 0.91

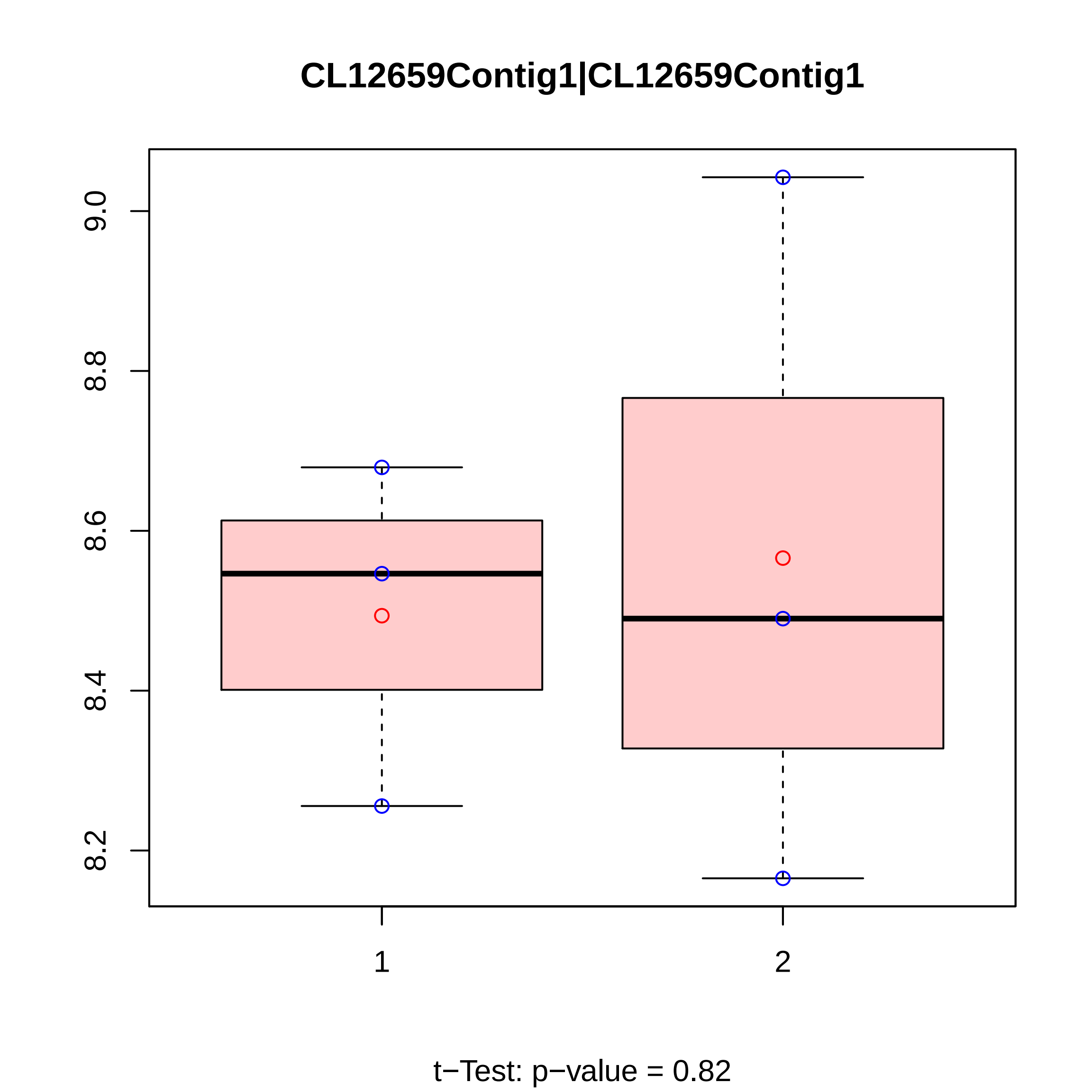
CL12659Contig1|CL12659Contig1
9.0
8.8
8.6
8.4
8.2
1
2
t−Test: p−value = 0.82

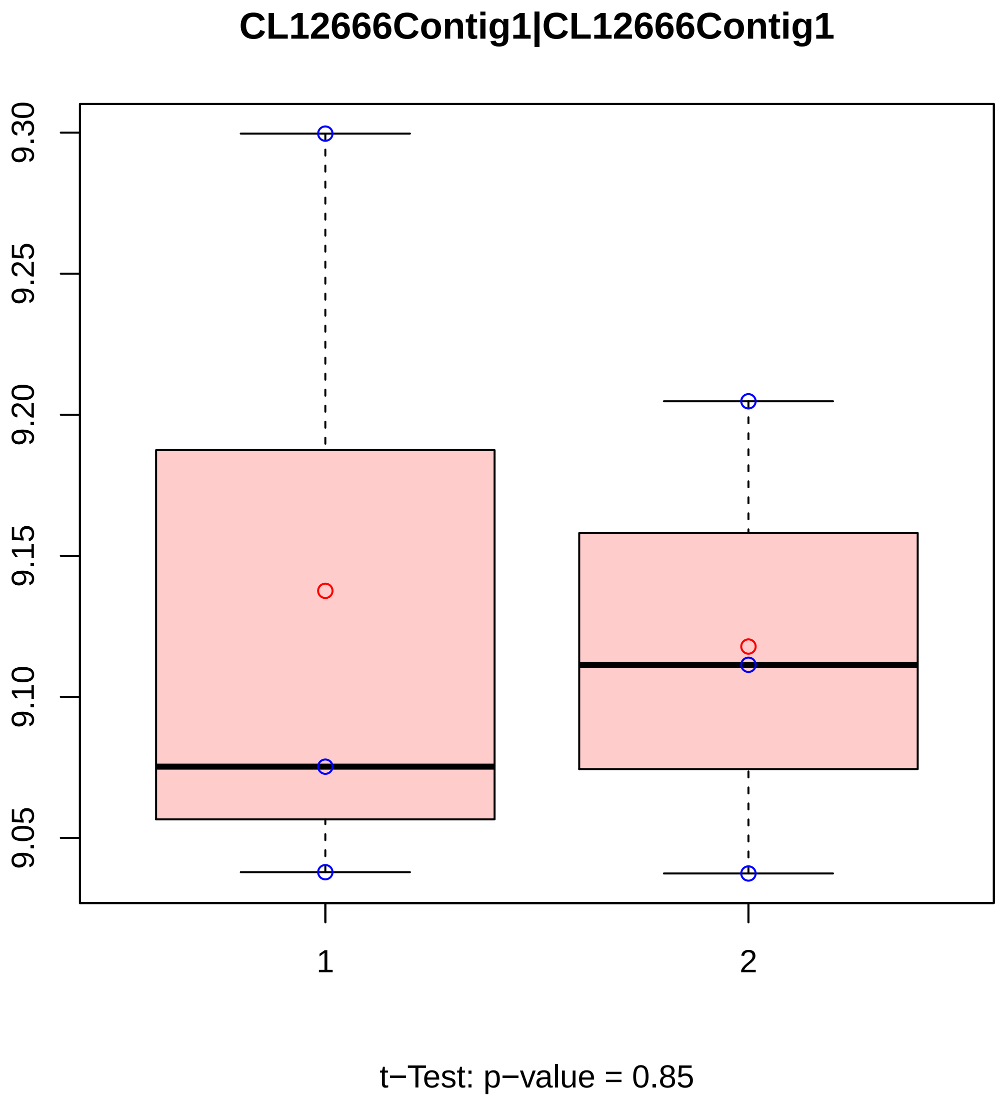
CL12666Contig1|CL12666Contig1
9.30
9.25
9.20
9.15
9.10
9.05
1
2
t−Test: p−value = 0.85

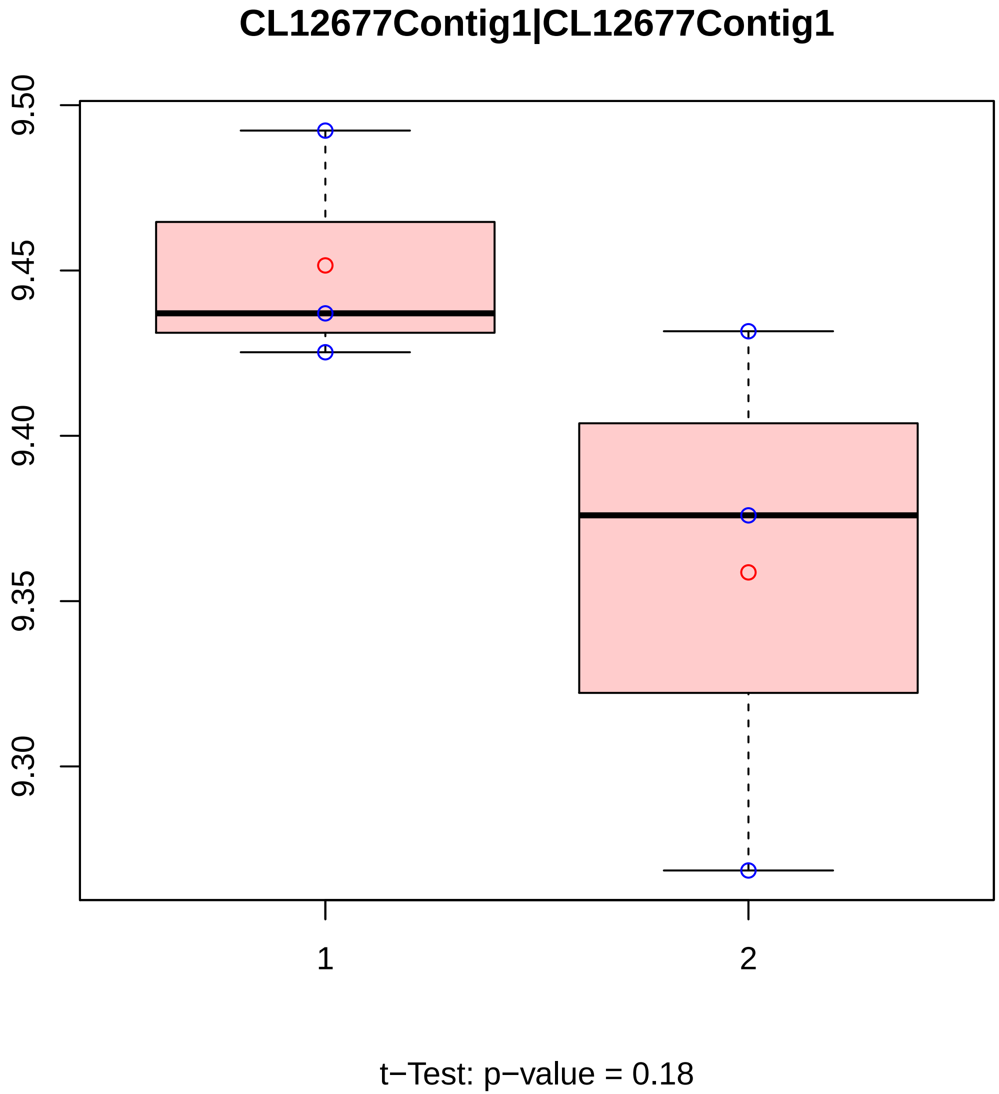

CL12677Contig1|CL12677Contig1
9.50
9.45
9.40
9.35
9.30
1
2
t−Test: p−value = 0.18

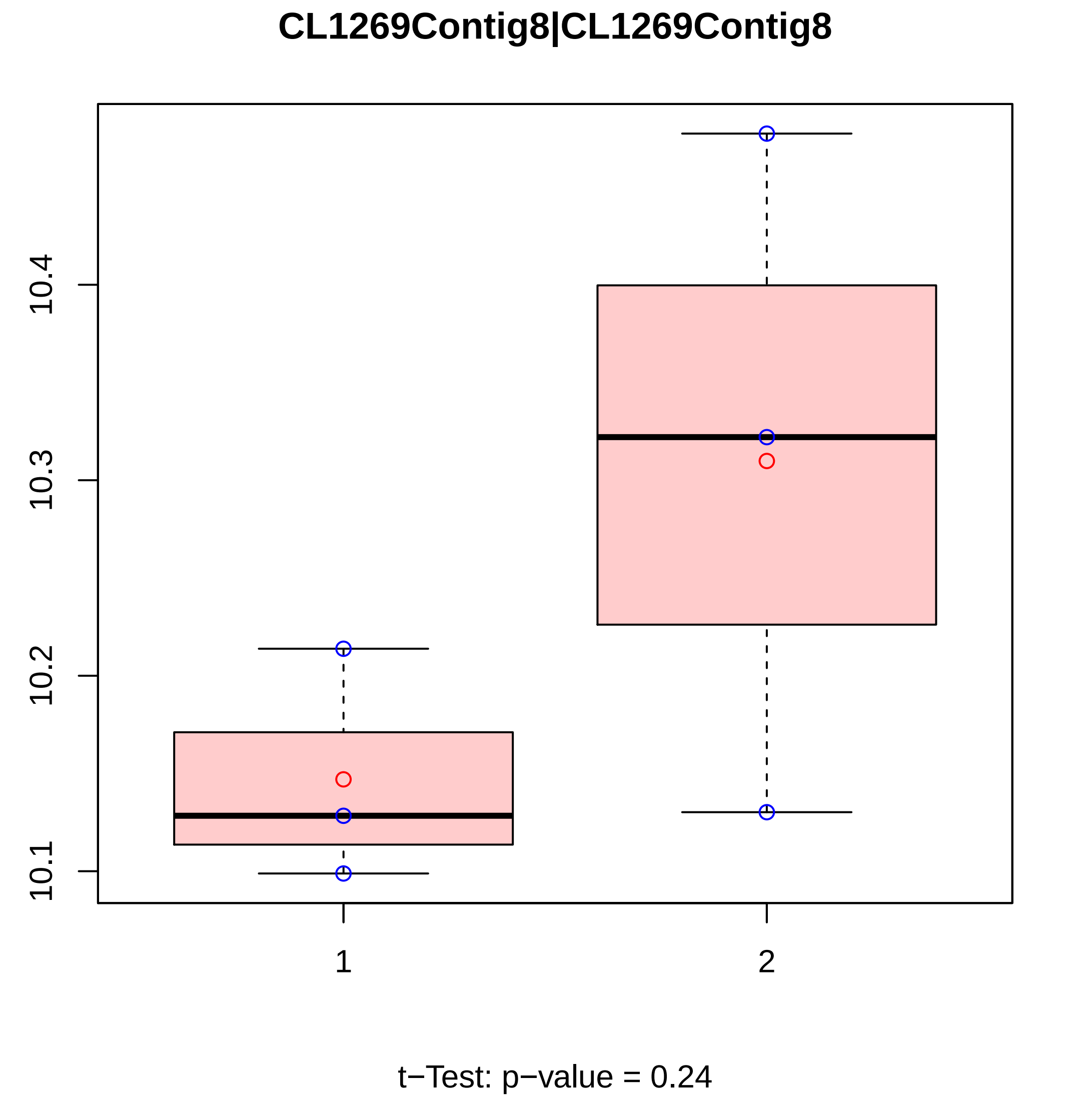
CL1269Contig8|CL1269Contig8
10.1
10.2
10.3
10.4
1
2
t−Test: p−value = 0.24

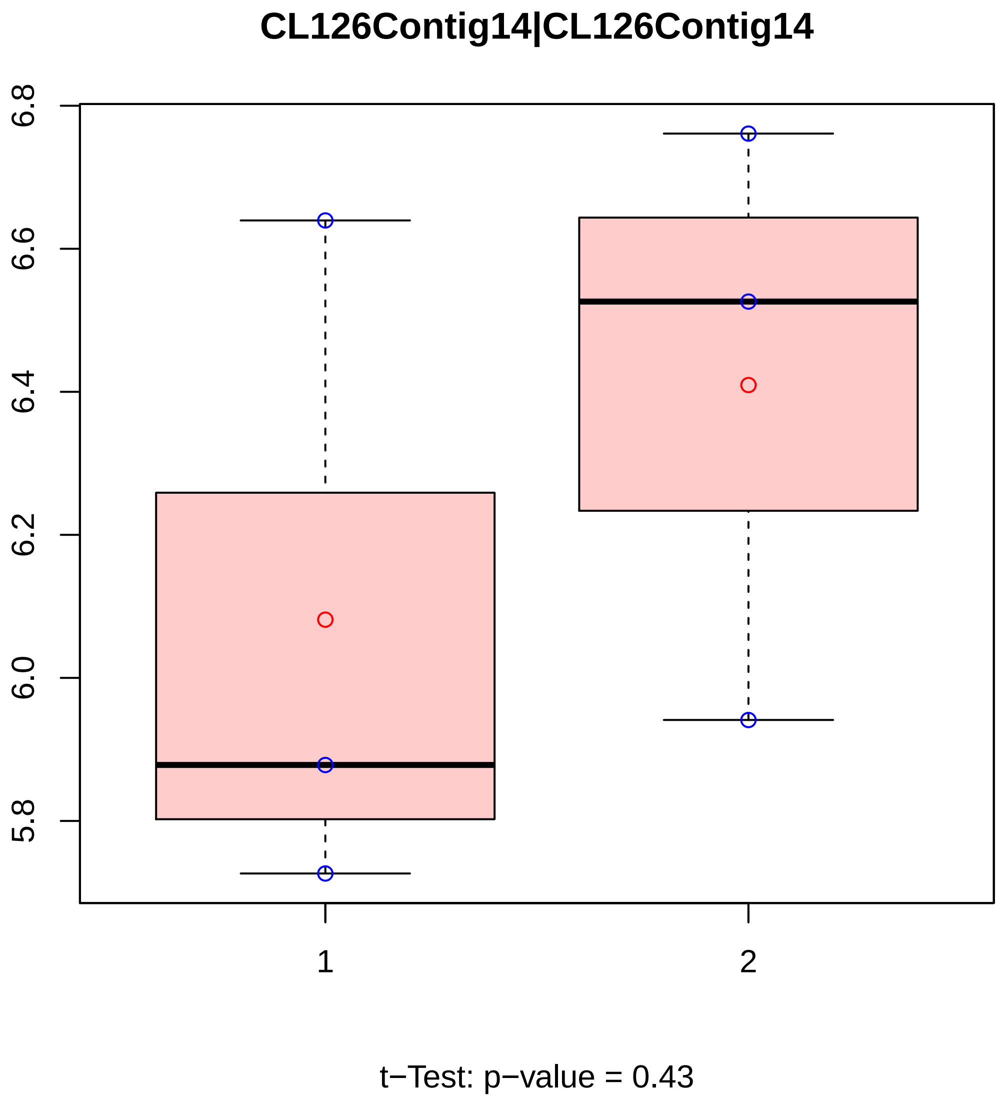

CL126Contig14|CL126Contig14
6.8
6.6
6.4
6.2
6.0
5.8
1
2
t−Test: p−value = 0.43

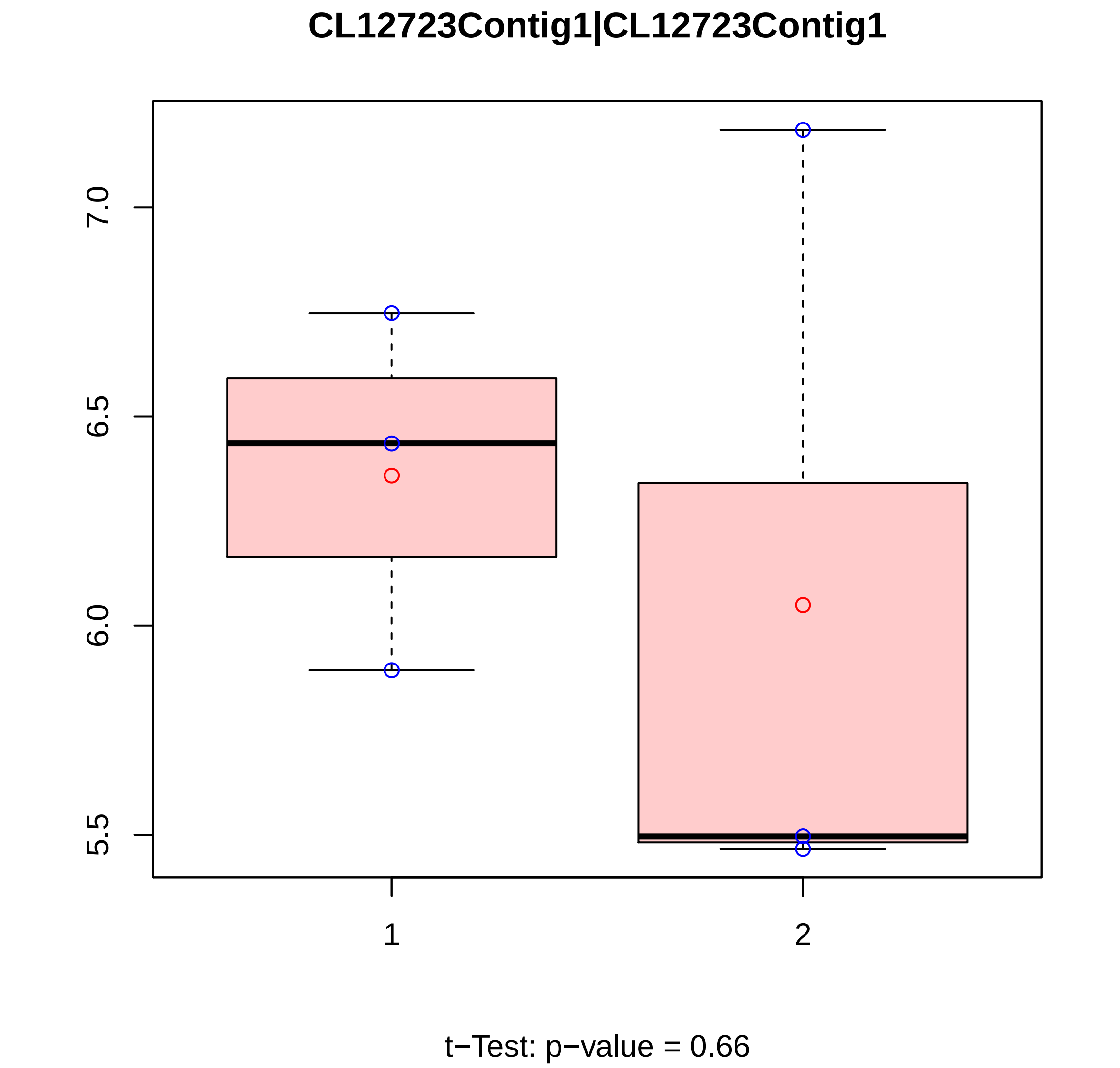

CL12723Contig1|CL12723Contig1
7.0
6.5
6.0
5.5
1
2
t−Test: p−value = 0.66

# CL12771Contig1|CL12771Contig1

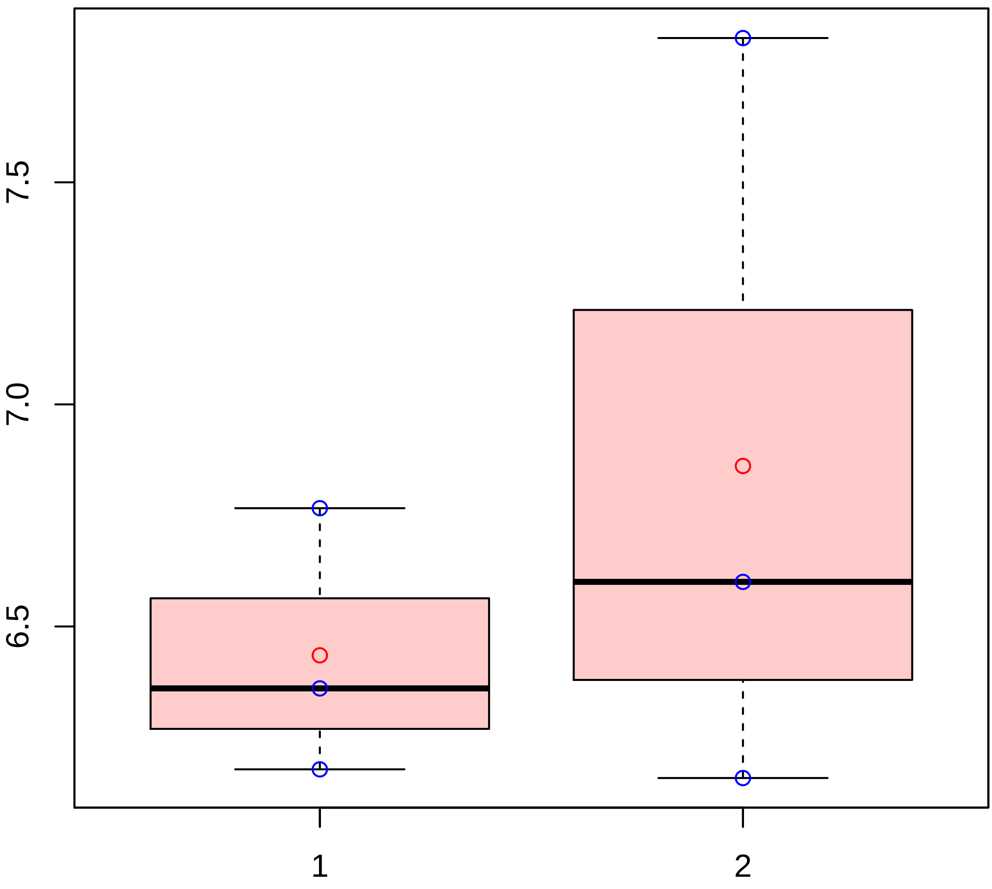


t−Test: p−value = 0.49

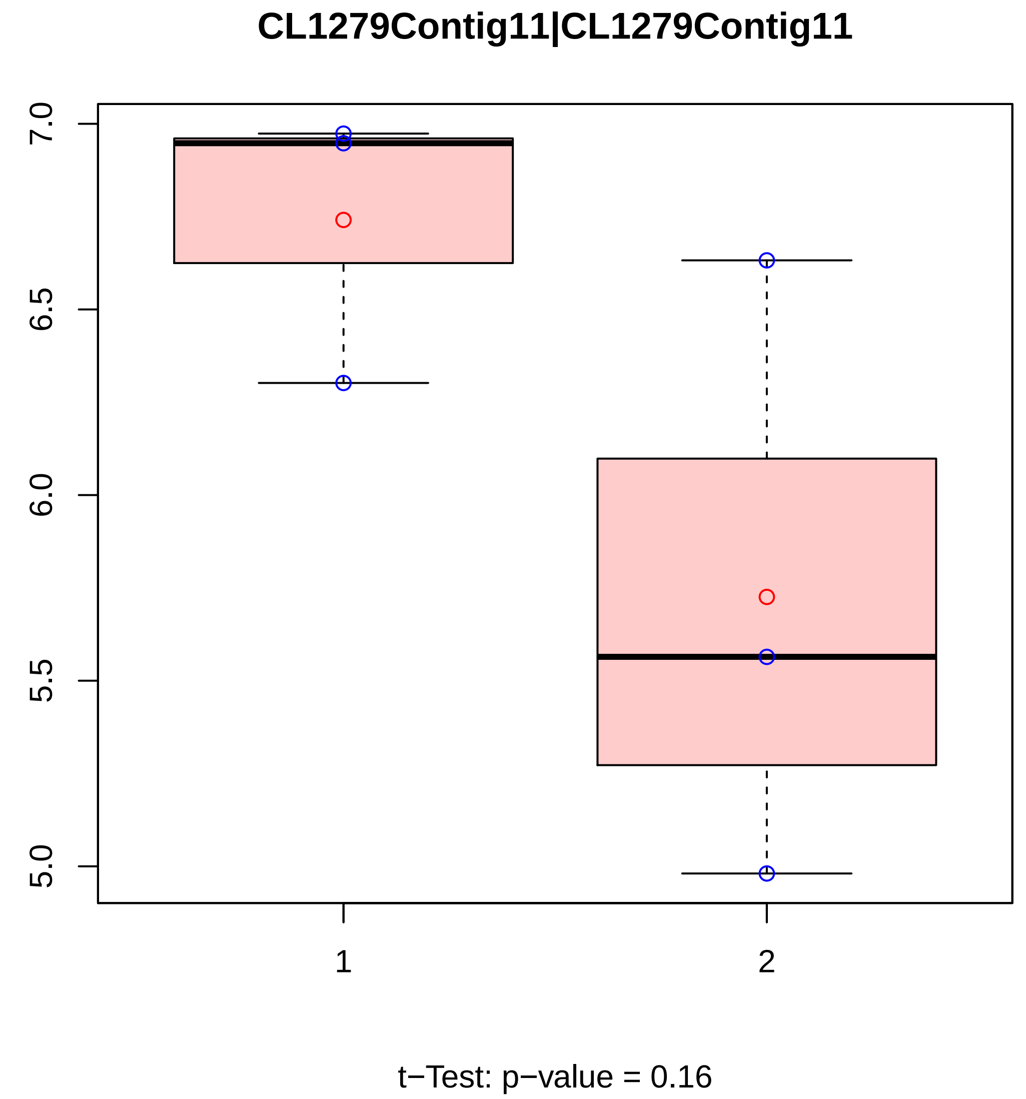
CL1279Contig11|CL1279Contig11
7.0
6.5
6.0
5.5
5.0
1
2
t−Test: p−value = 0.16

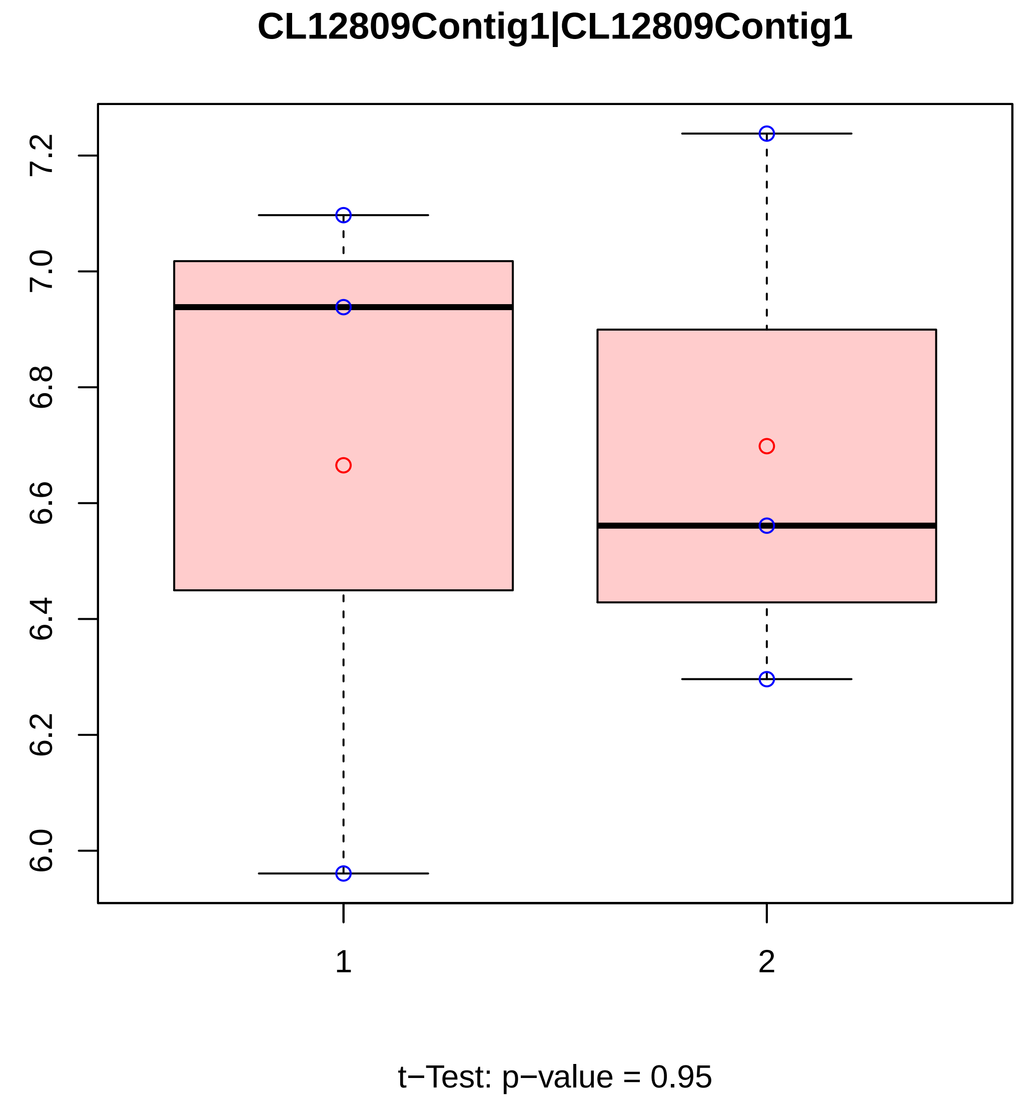
CL12809Contig1|CL12809Contig1
7.2
7.0
6.8
6.6
6.4
6.2
6.0
1
2
t−Test: p−value = 0.95

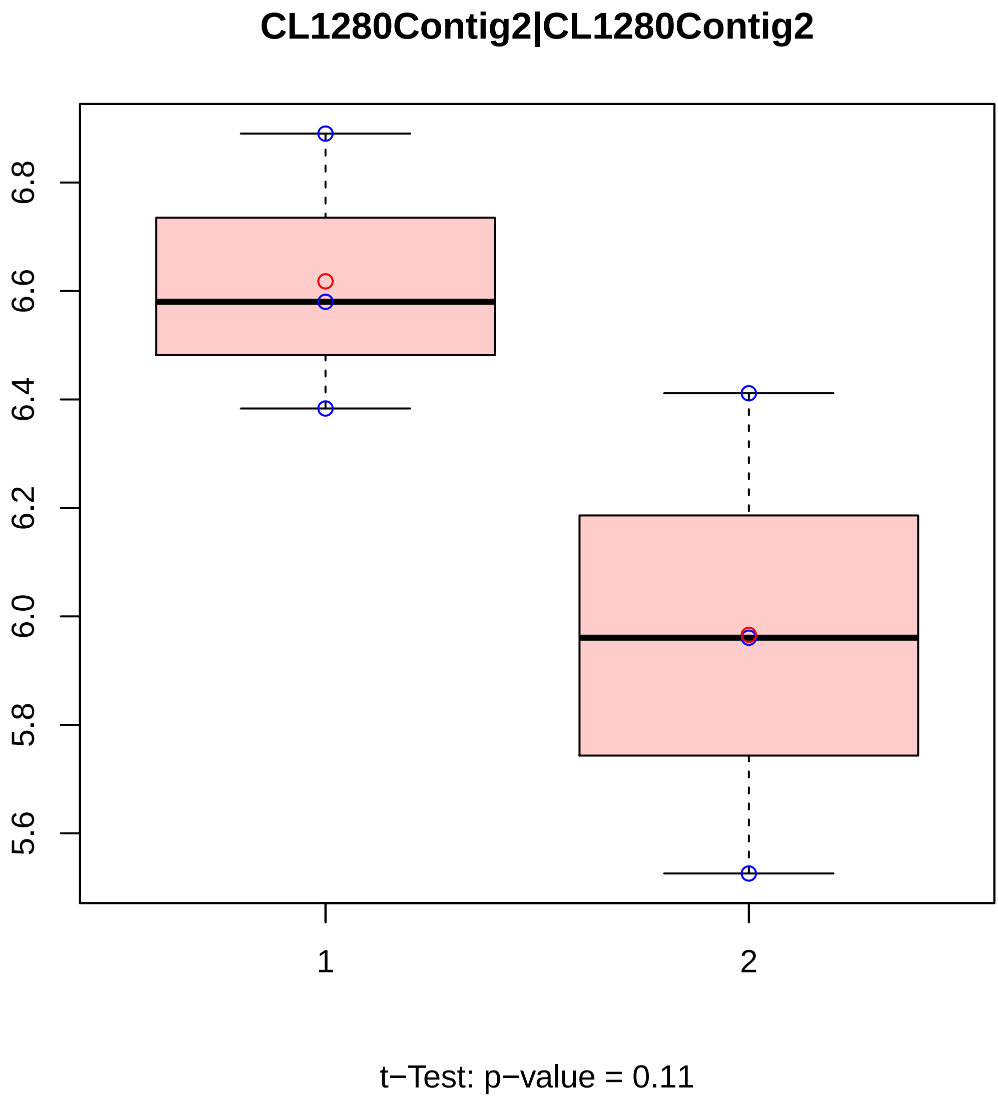
CL1280Contig2|CL1280Contig2
6.8
6.6
6.4
6.2
6.0
5.8
5.6
1
2
t−Test: p−value = 0.11

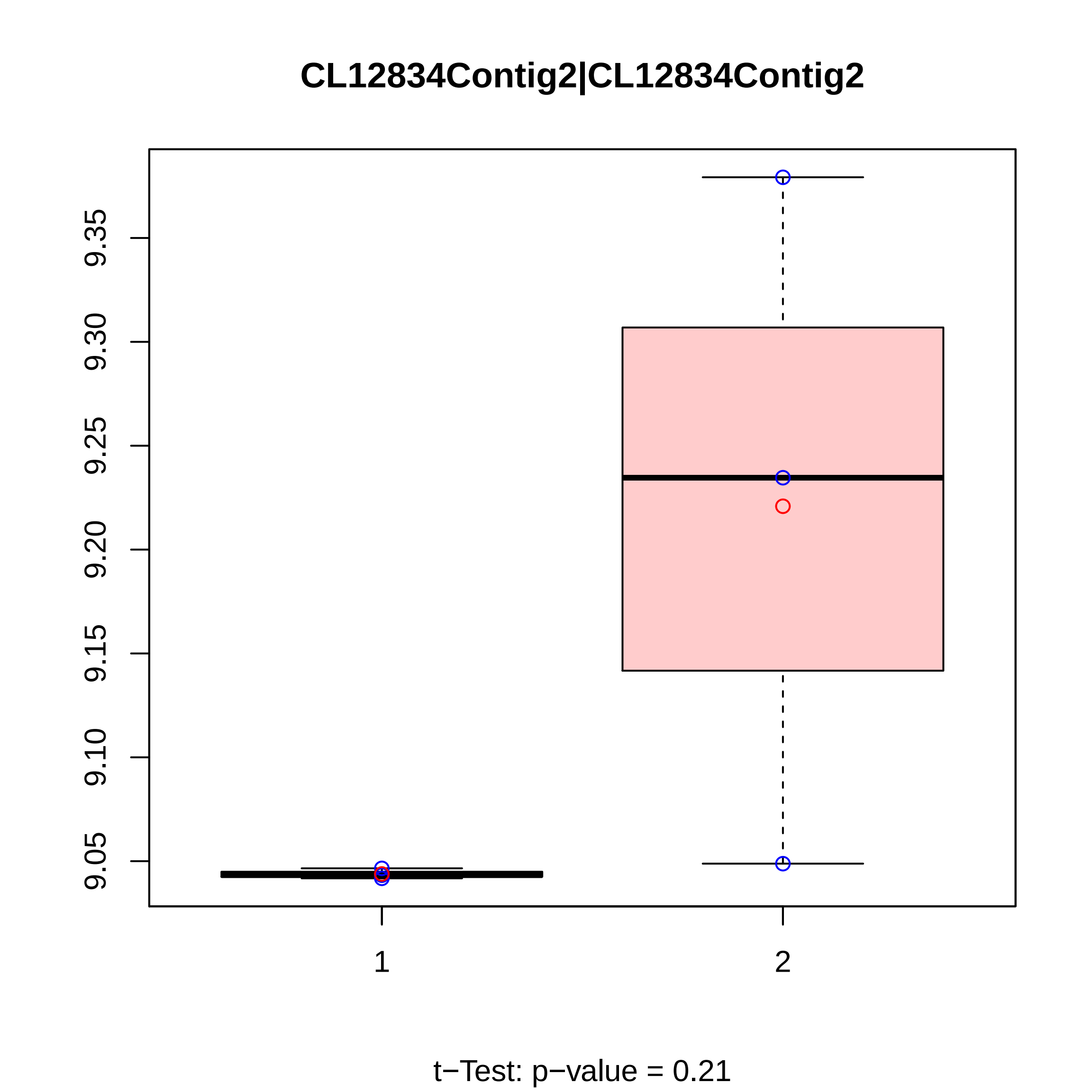
CL12834Contig2|CL12834Contig2
9.05
9.10
9.15
9.20
9.25
9.30
9.35
1
2
t−Test: p−value = 0.21

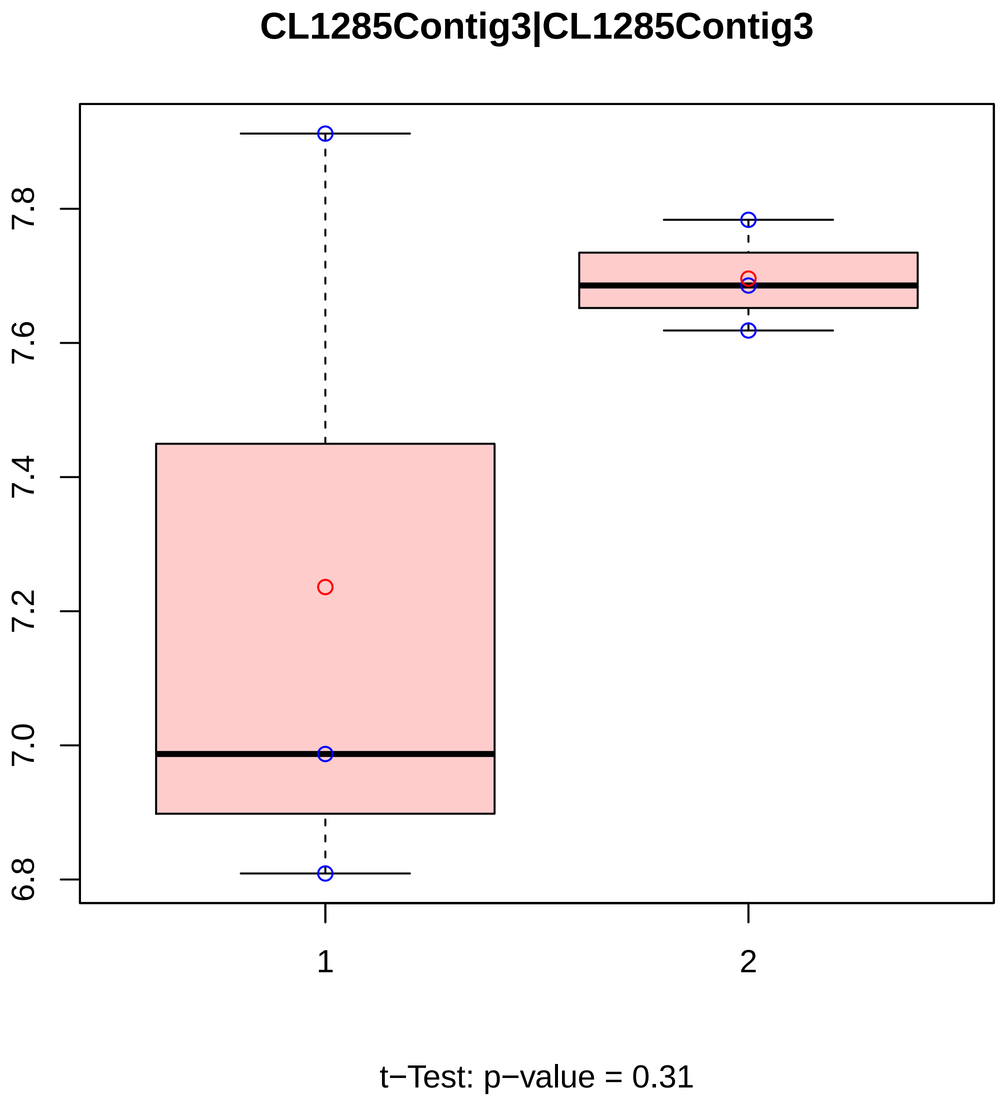
CL1285Contig3|CL1285Contig3
6.8
7.0
7.2
7.4
7.6
7.8
1
2
t−Test: p−value = 0.31

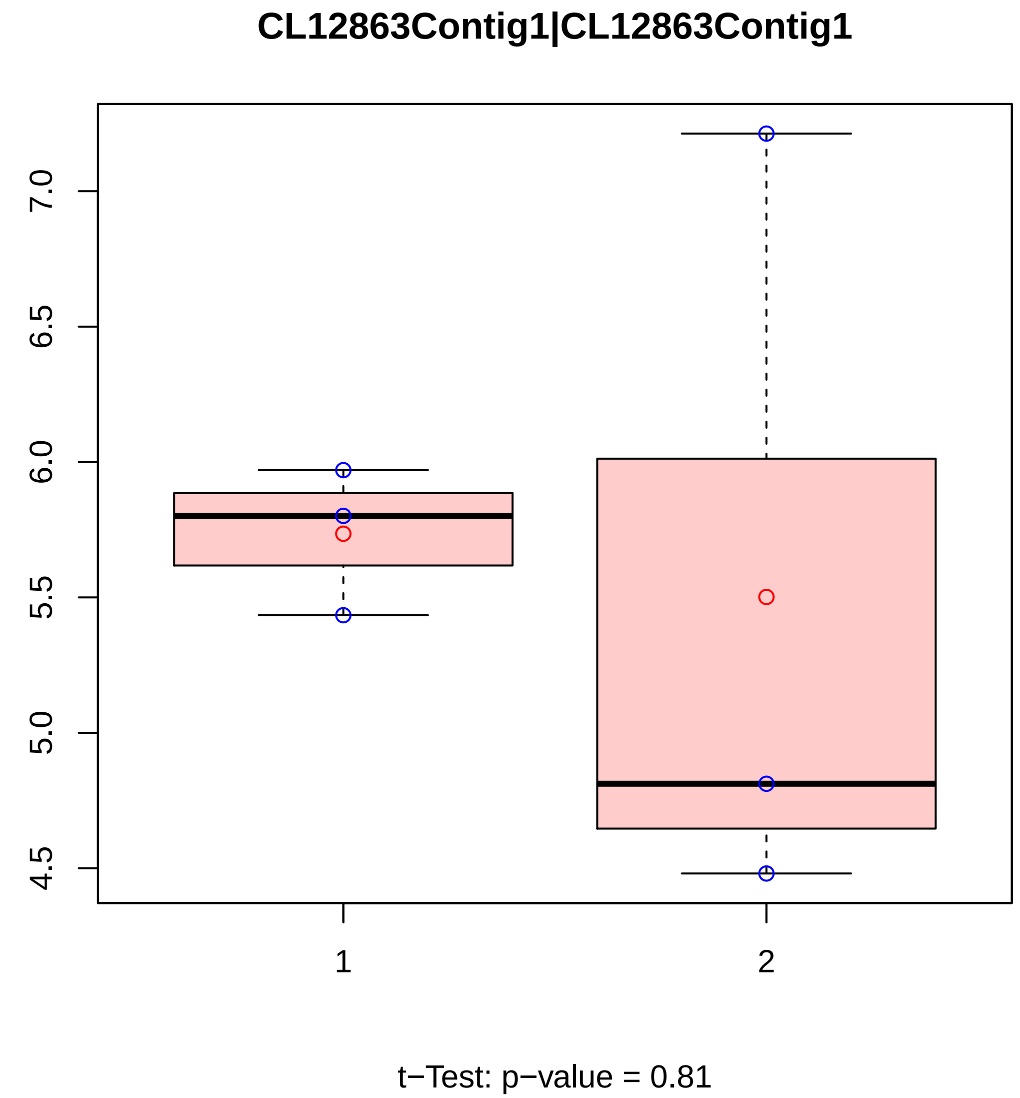
CL12863Contig1|CL12863Contig1
7.0
6.5
6.0
5.5
5.0
4.5
1
2
t−Test: p−value = 0.81

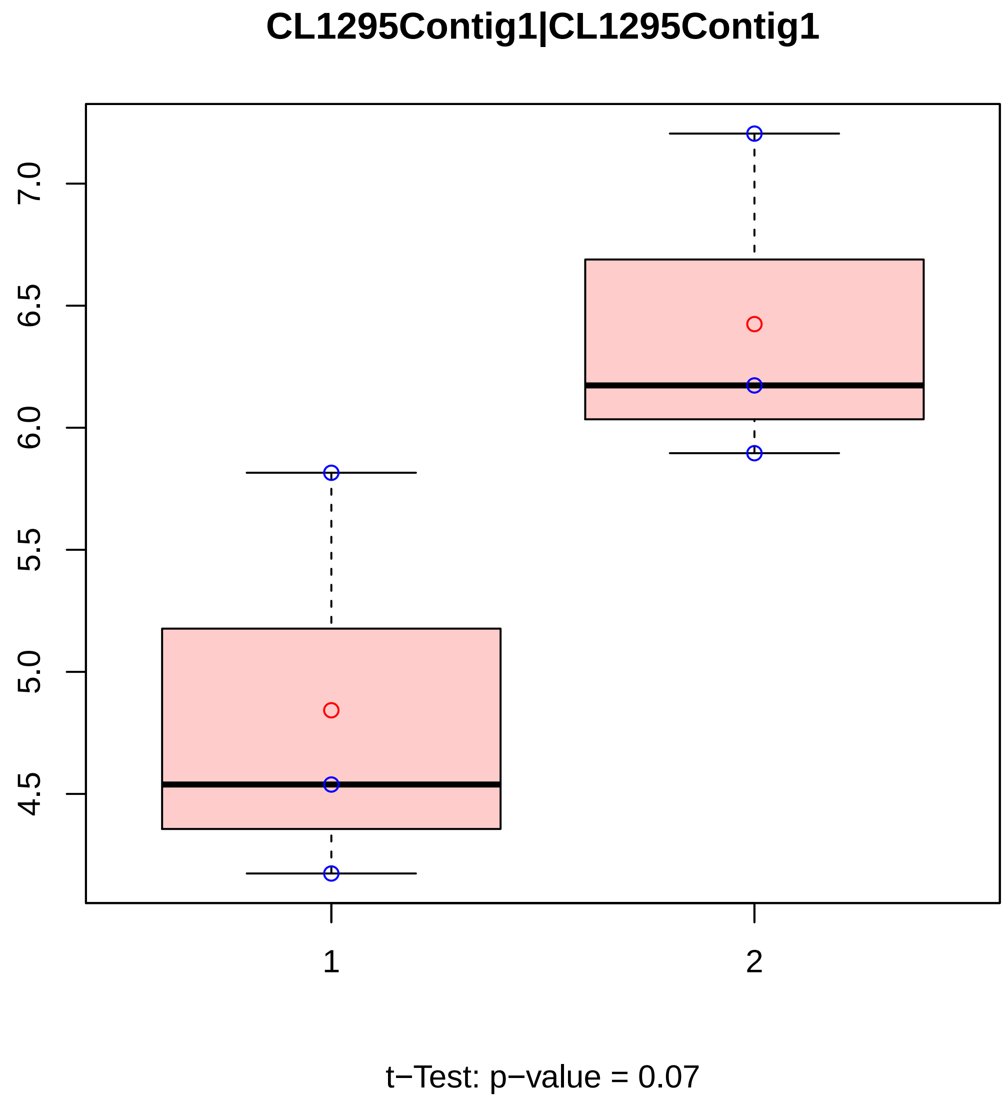

CL1295Contig1|CL1295Contig1
7.0
6.5
6.0
5.5
5.0
4.5
1
2
t−Test: p−value = 0.07

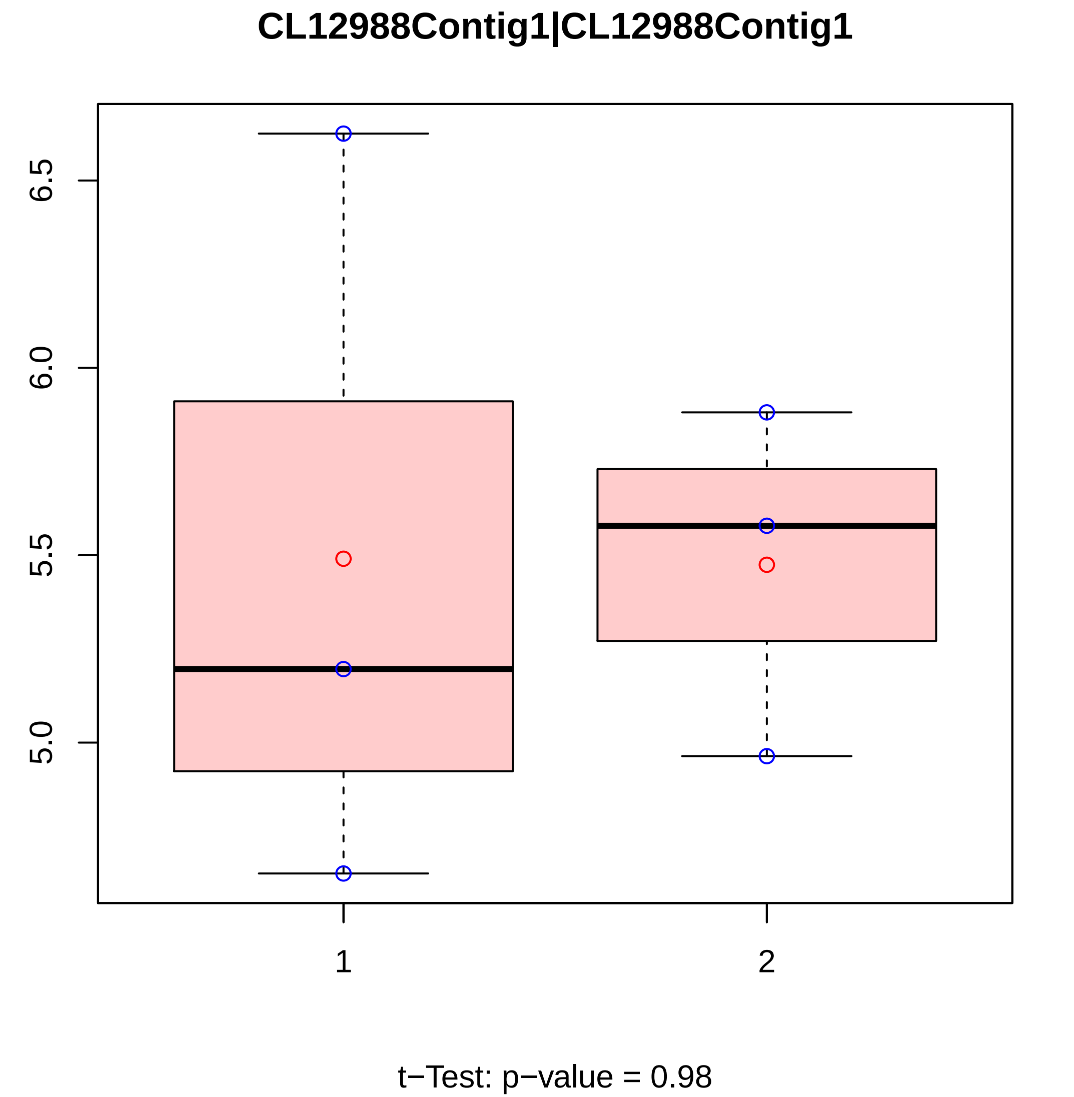
CL12988Contig1|CL12988Contig1
6.5
6.0
5.5
5.0
1
2
t−Test: p−value = 0.98

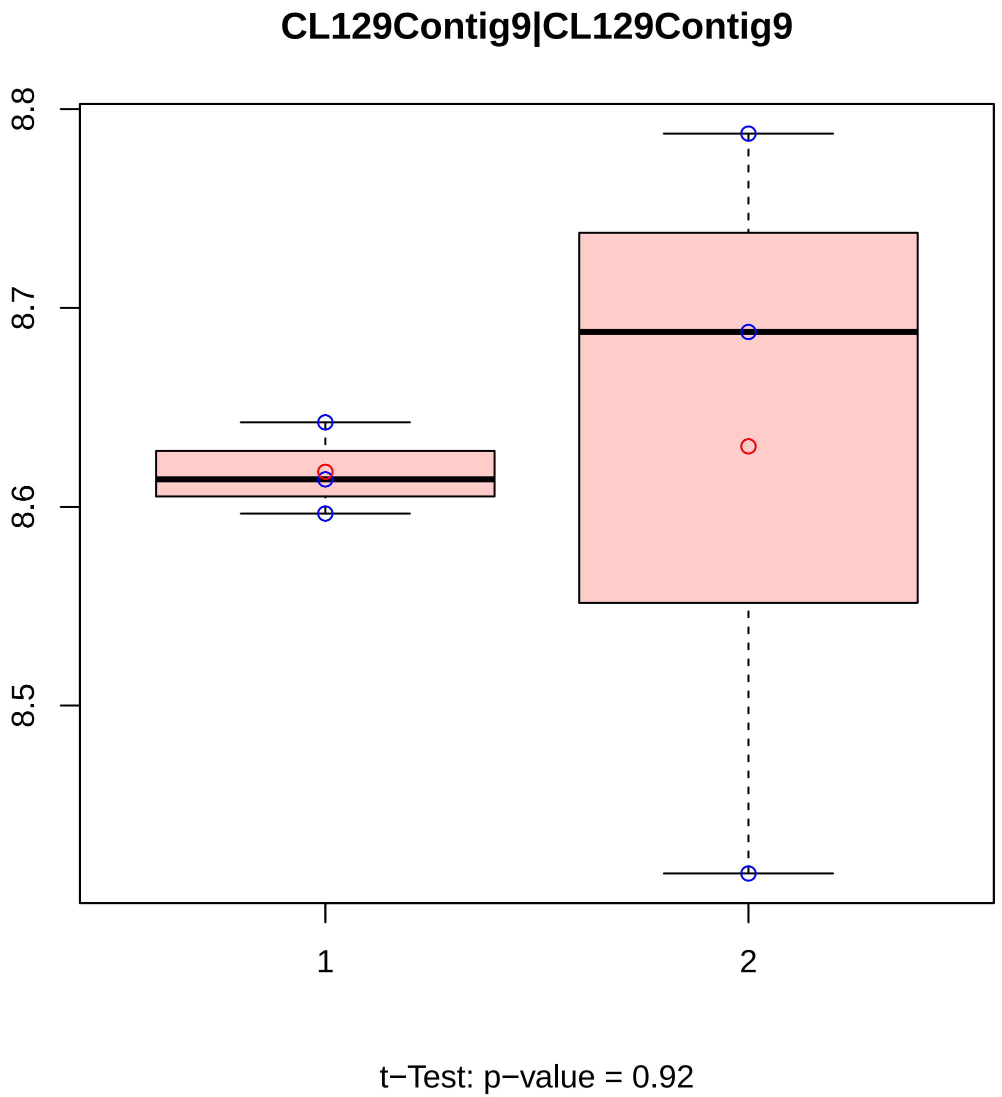
CL129Contig9|CL129Contig9
8.8
8.7
8.6
8.5
1
2
t−Test: p−value = 0.92

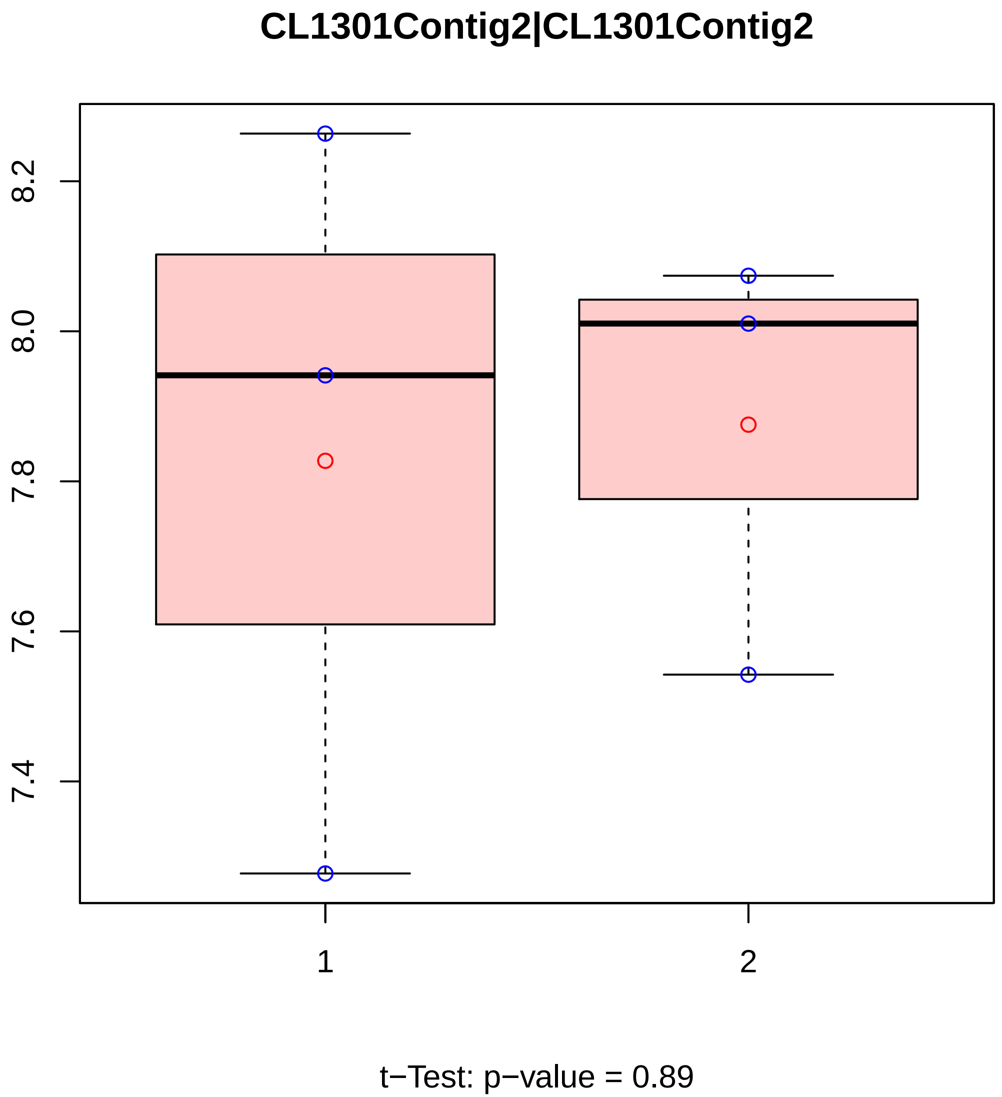
CL1301Contig2|CL1301Contig2
8.2
8.0
7.8
7.6
7.4
1
2
t−Test: p−value = 0.89

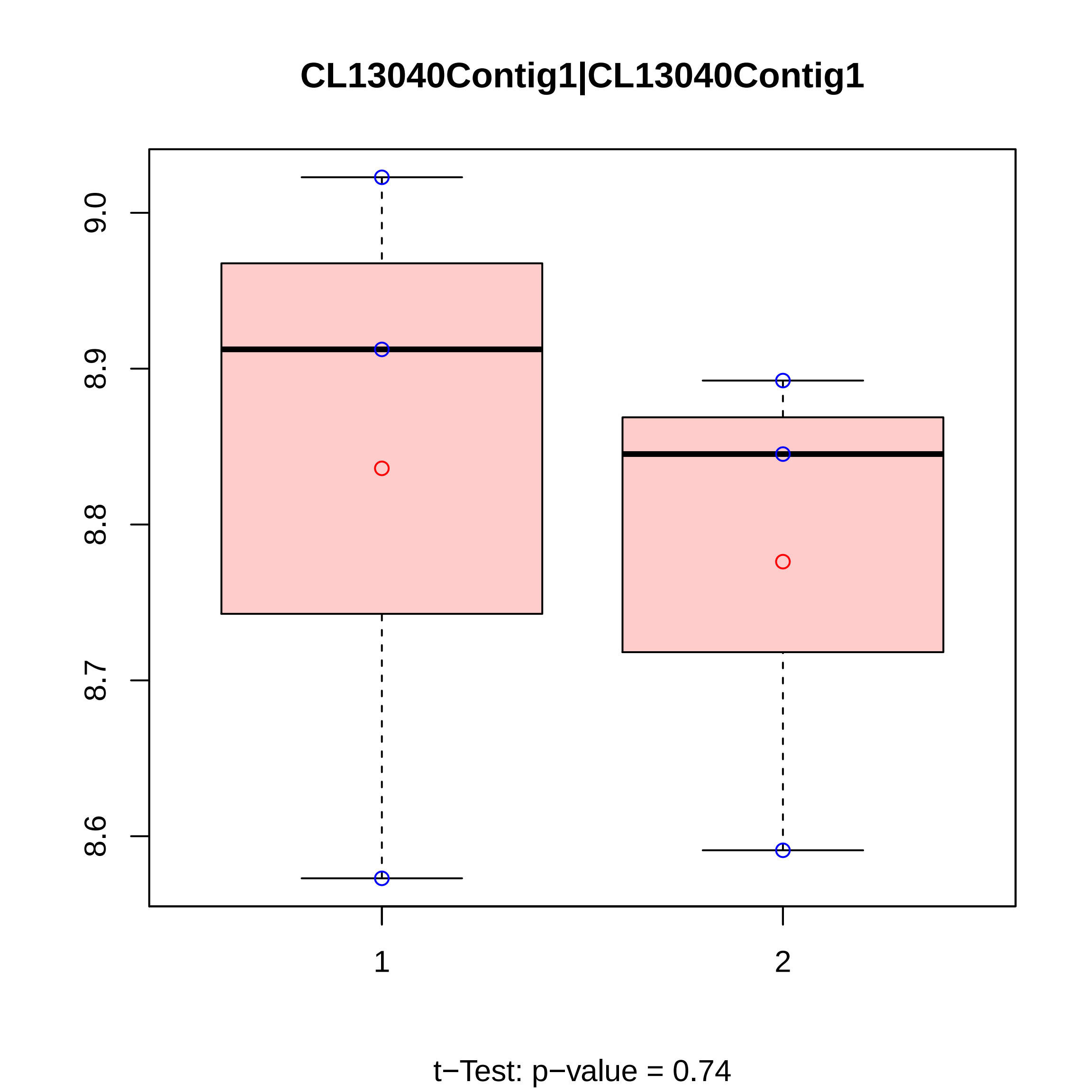
CL13040Contig1|CL13040Contig1
9.0
8.9
8.8
8.7
8.6
1
2
t−Test: p−value = 0.74

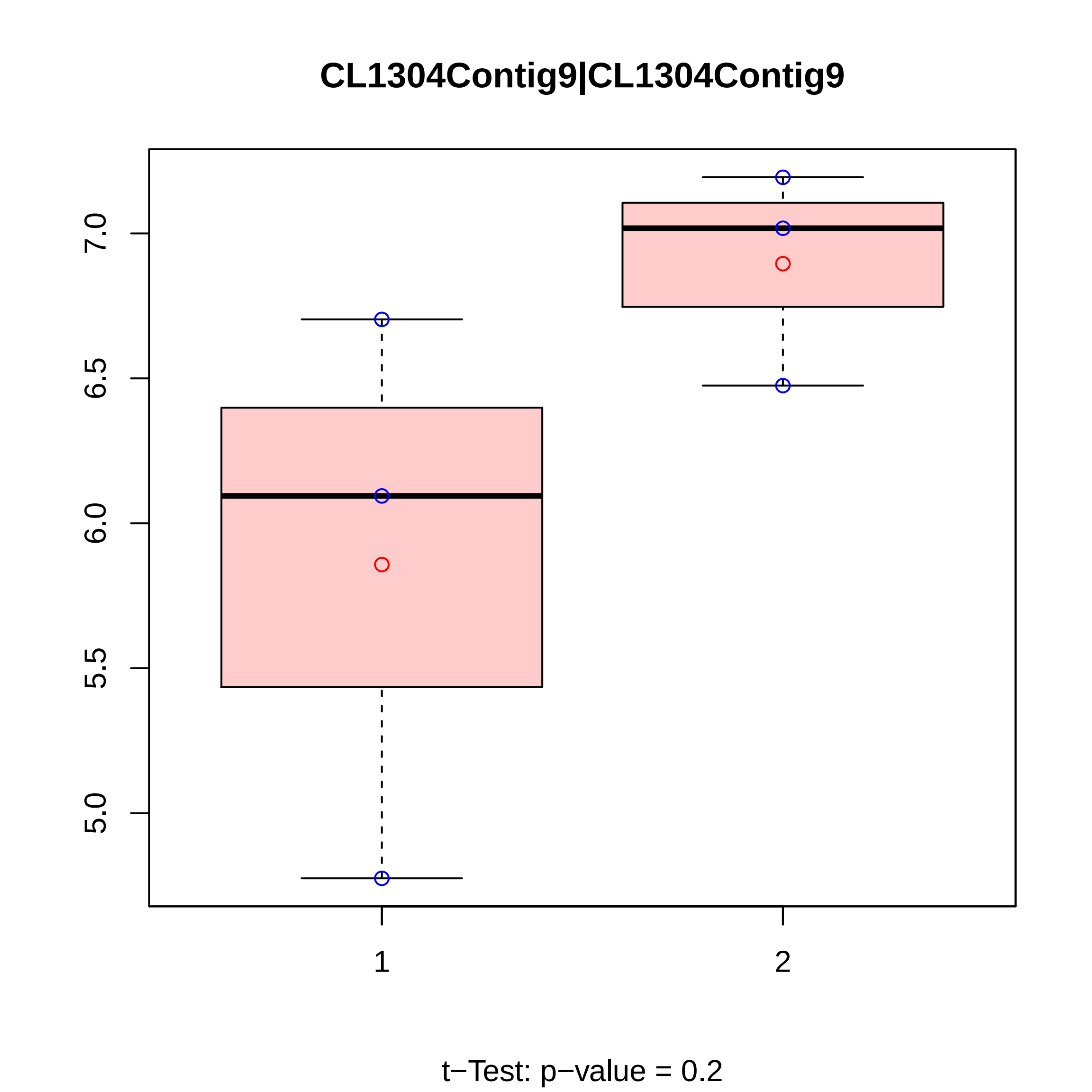
CL1304Contig9|CL1304Contig9
7.0
6.5
6.0
5.5
5.0
1
2
t−Test: p−value = 0.2

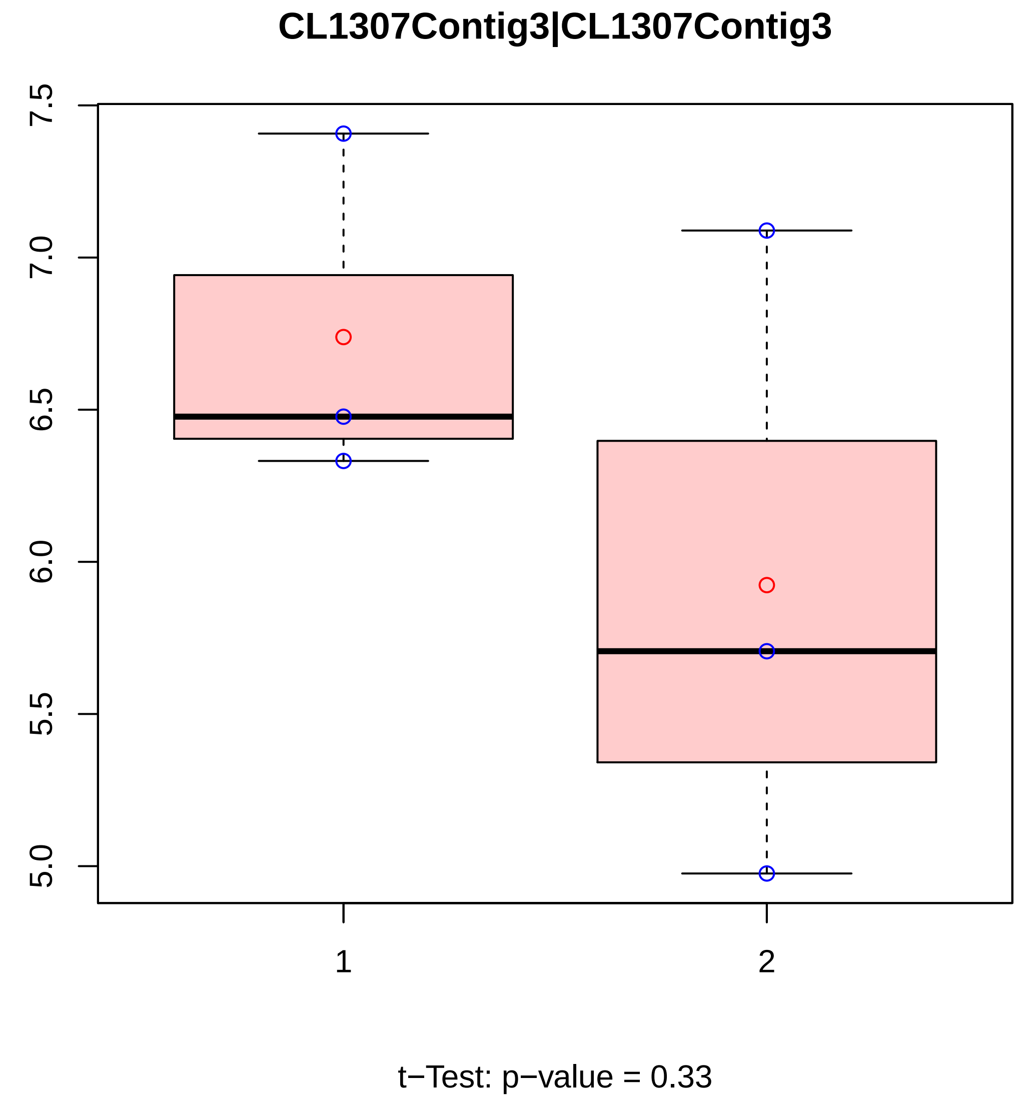

CL1307Contig3|CL1307Contig3
7.5
7.0
6.5
6.0
5.5
5.0
1
2
t−Test: p−value = 0.33

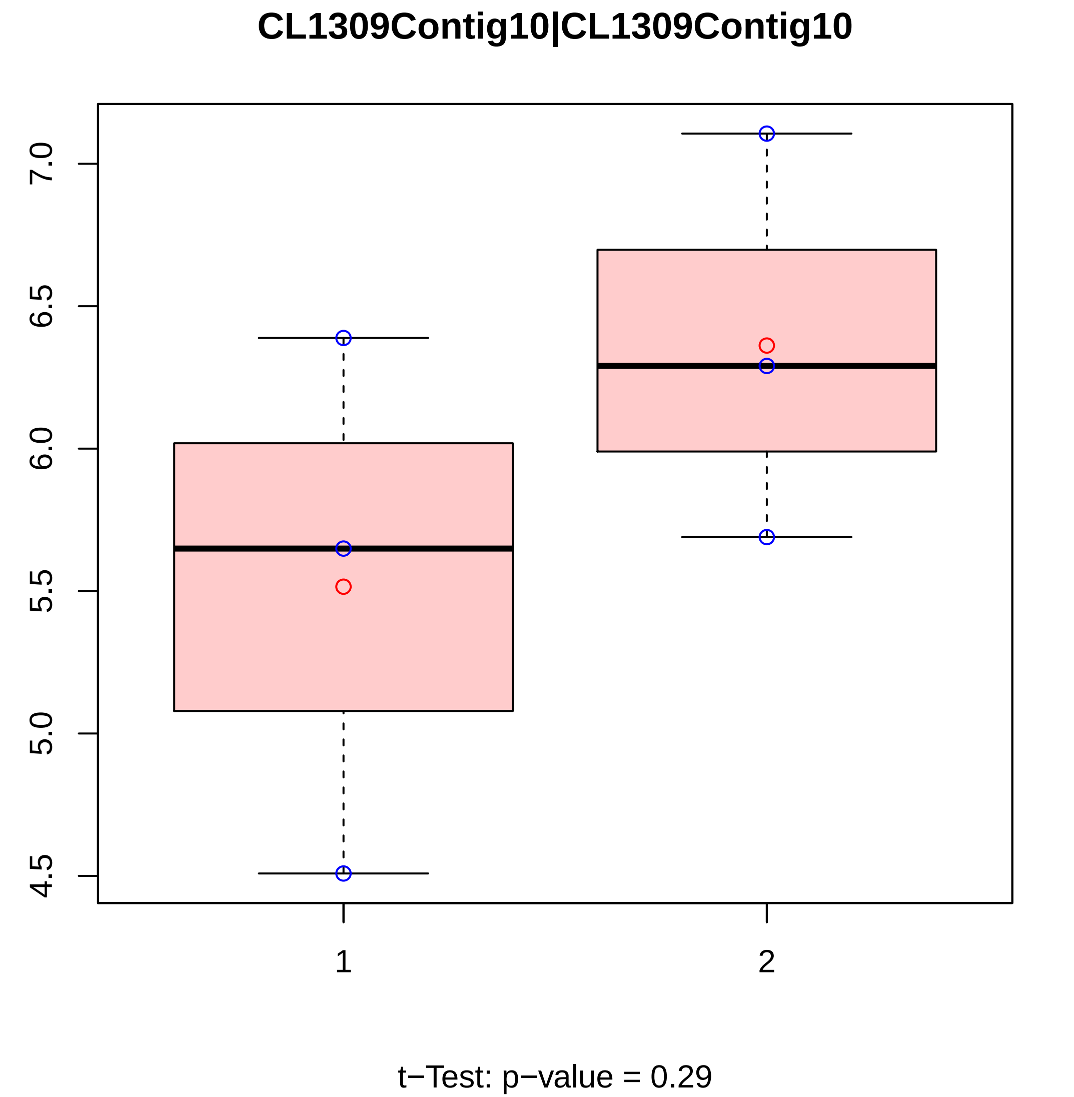
CL1309Contig10|CL1309Contig10
7.0
6.5
6.0
5.5
5.0
4.5
1
2
t−Test: p−value = 0.29

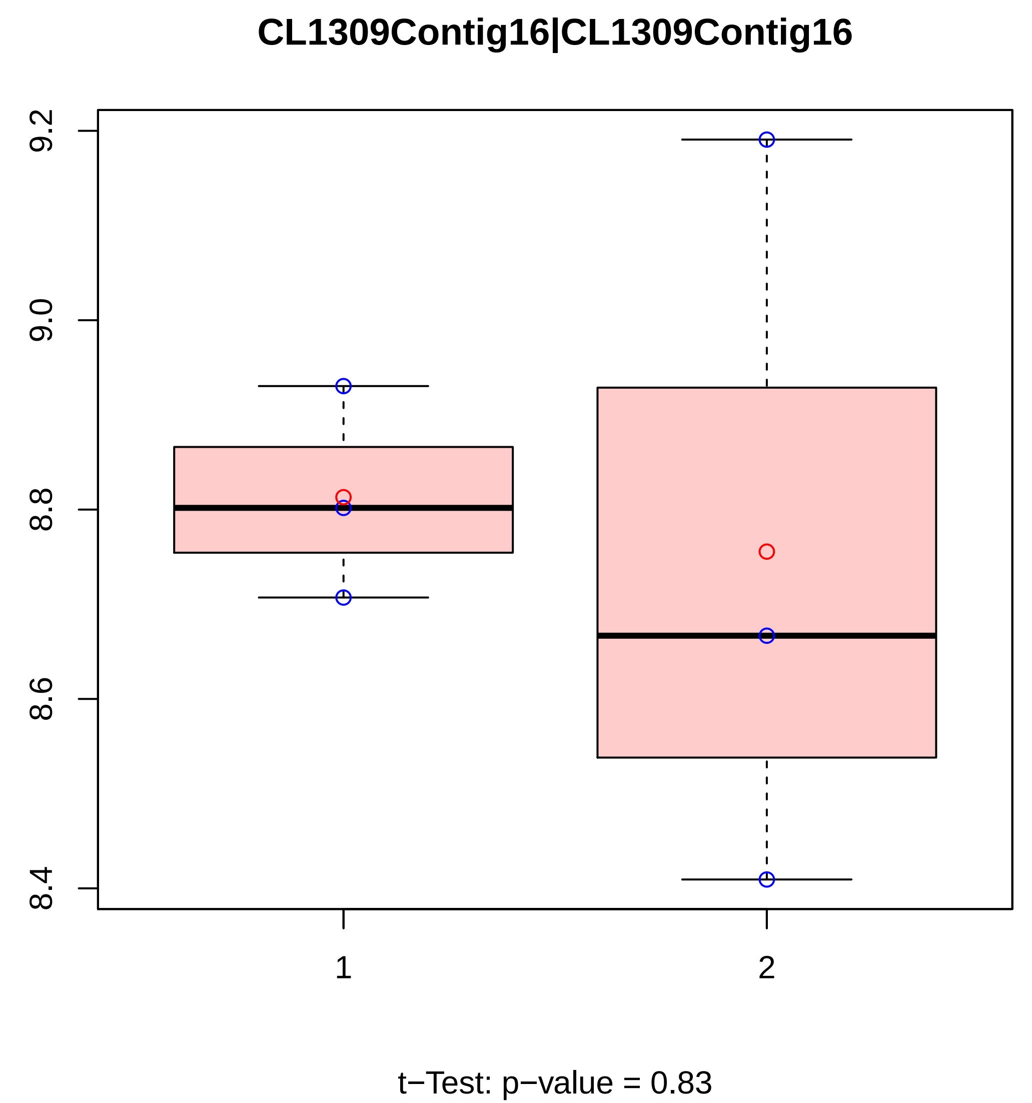
CL1309Contig16|CL1309Contig16
9.2
9.0
8.8
8.6
8.4
1
2
t−Test: p−value = 0.83

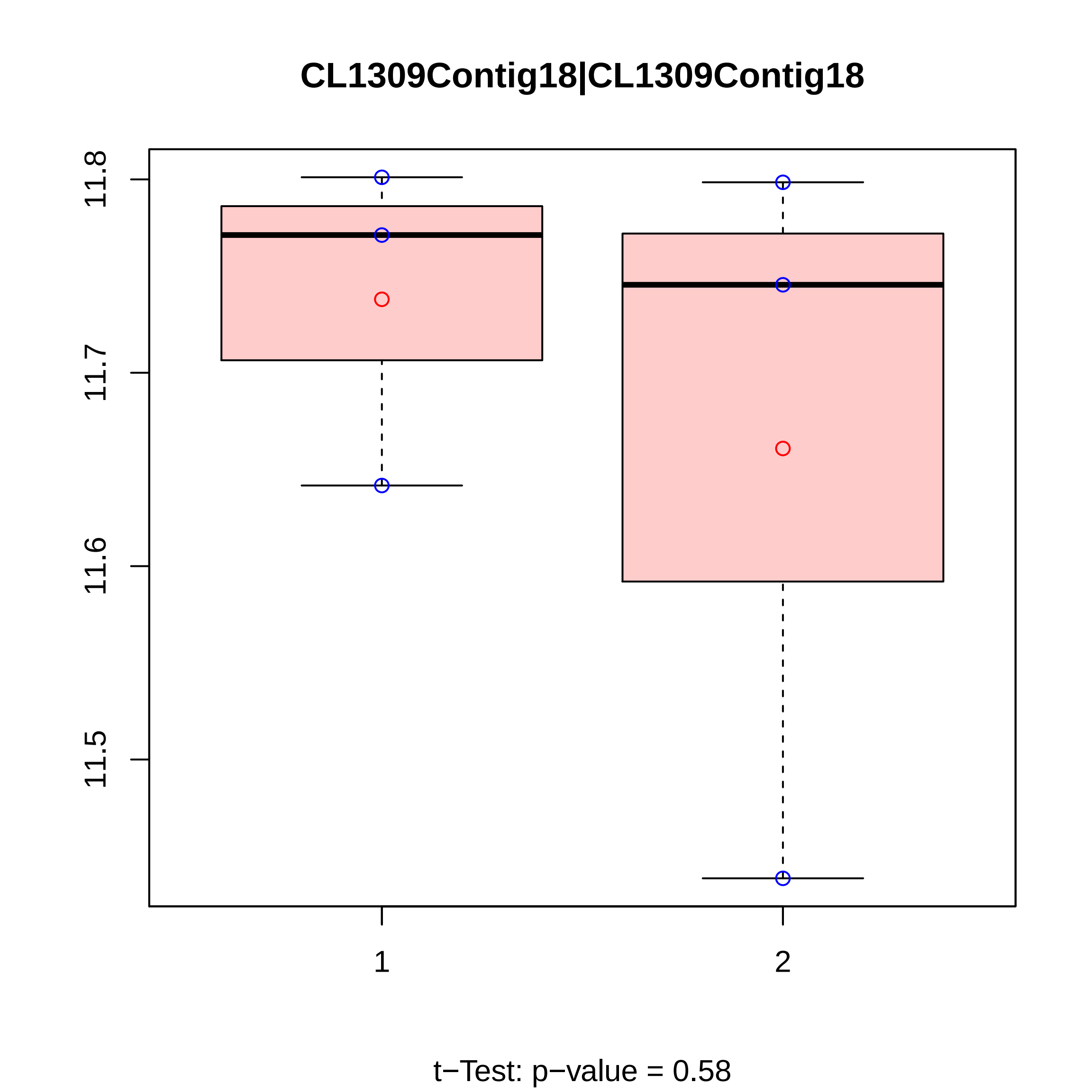
CL1309Contig18|CL1309Contig18
11.8
11.7
11.6
11.5
1
2
t−Test: p−value = 0.58

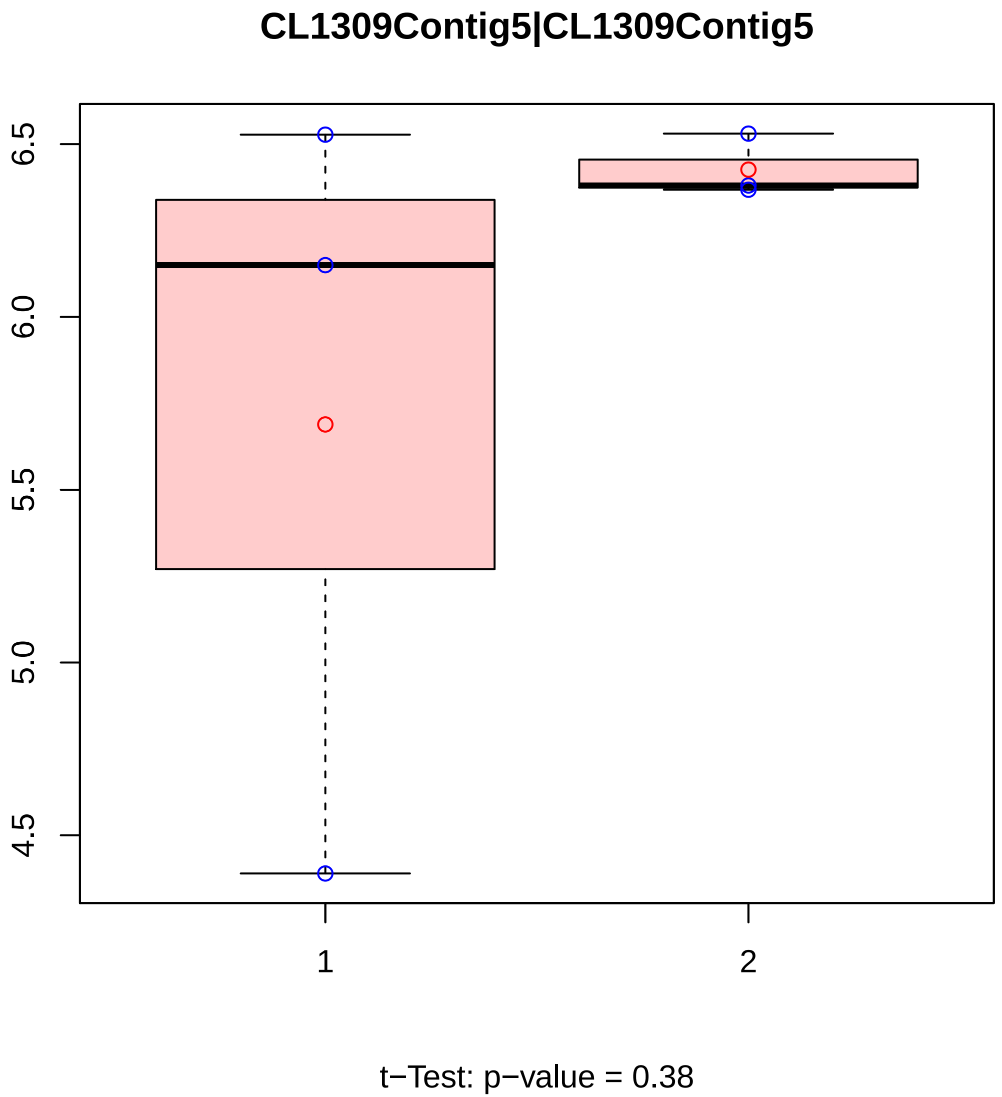
CL1309Contig5|CL1309Contig5
6.5
6.0
5.5
5.0
4.5
1
2
t−Test: p−value = 0.38

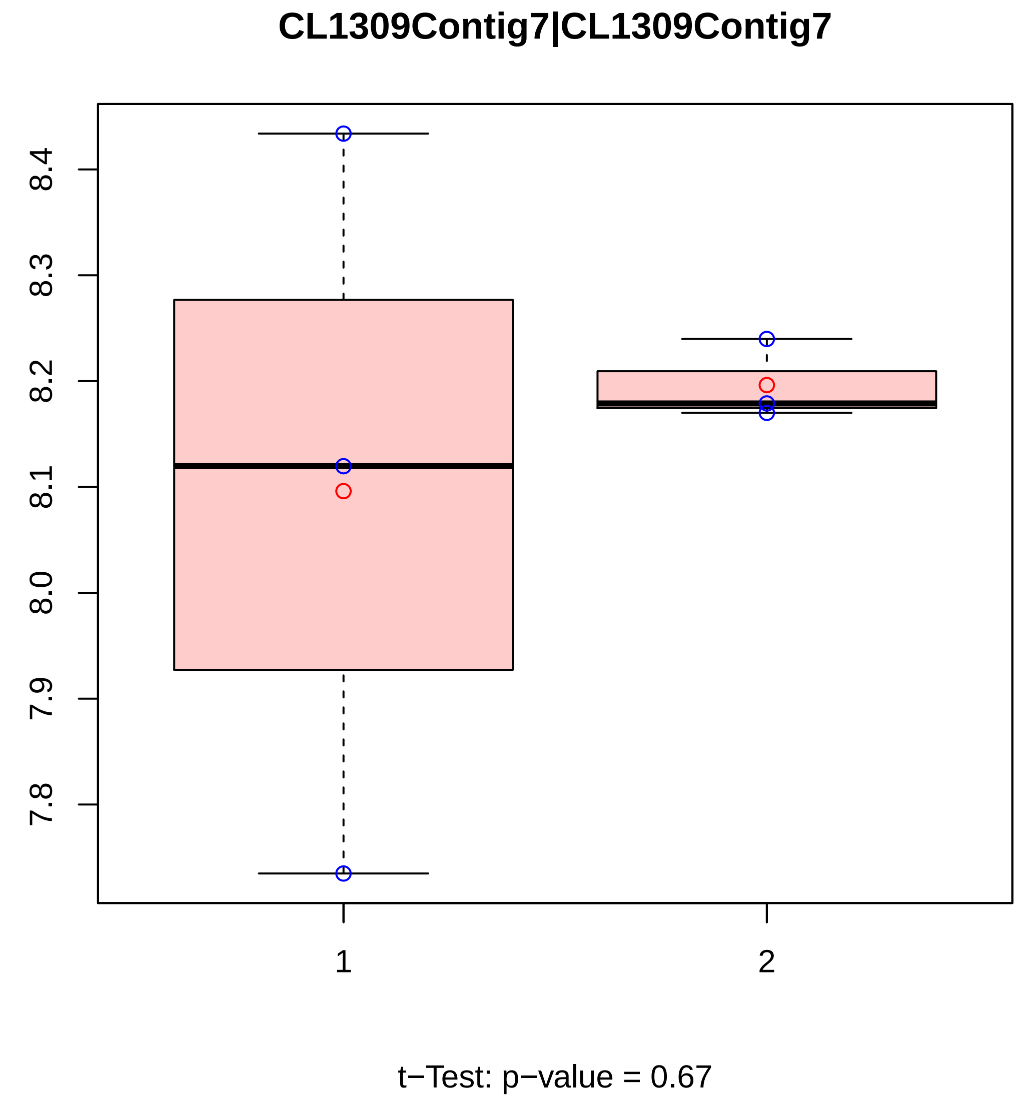
CL1309Contig7|CL1309Contig7
8.4
8.3
8.2
8.1
8.0
7.9
7.8
1
2
t−Test: p−value = 0.67

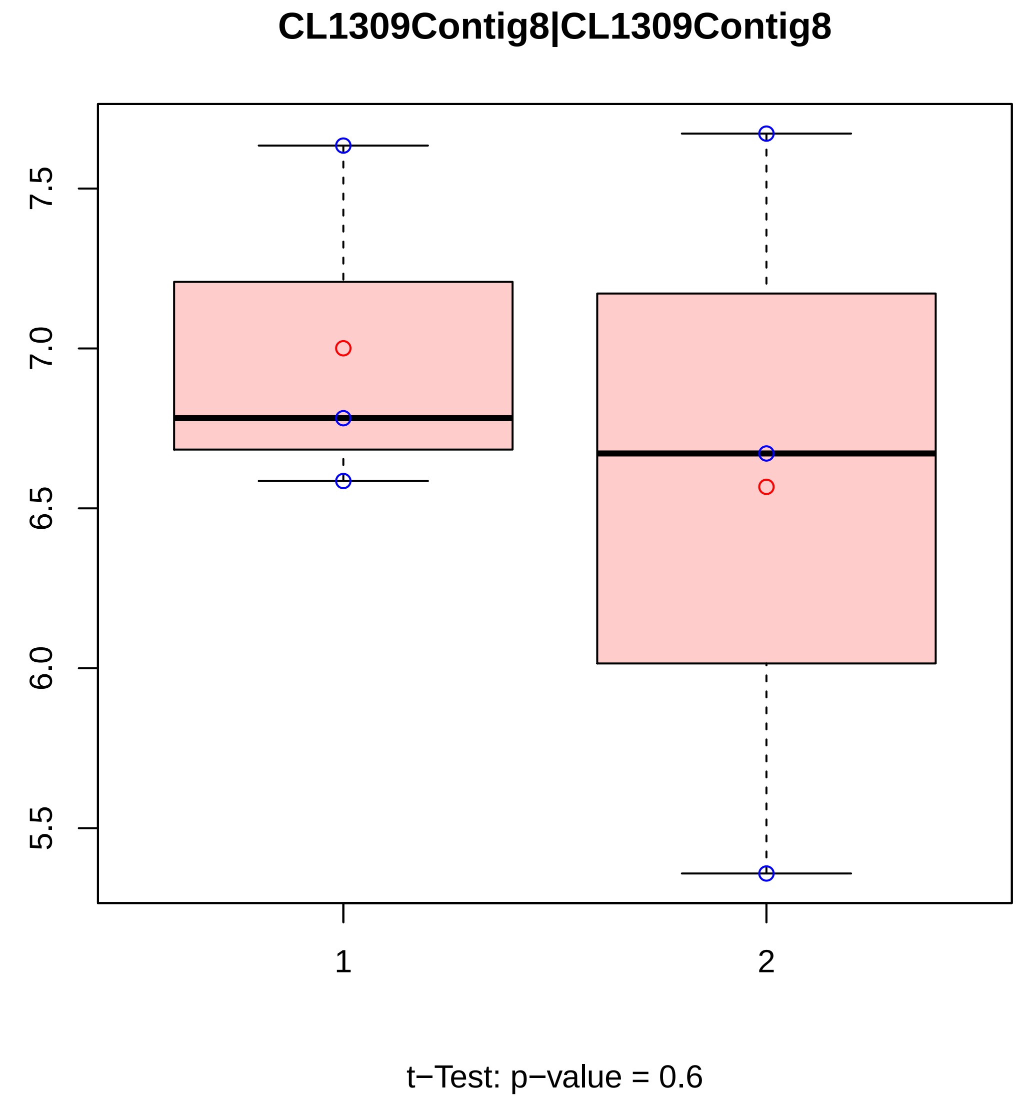
CL1309Contig8|CL1309Contig8
7.5
7.0
6.5
6.0
5.5
1
2
t−Test: p−value = 0.6

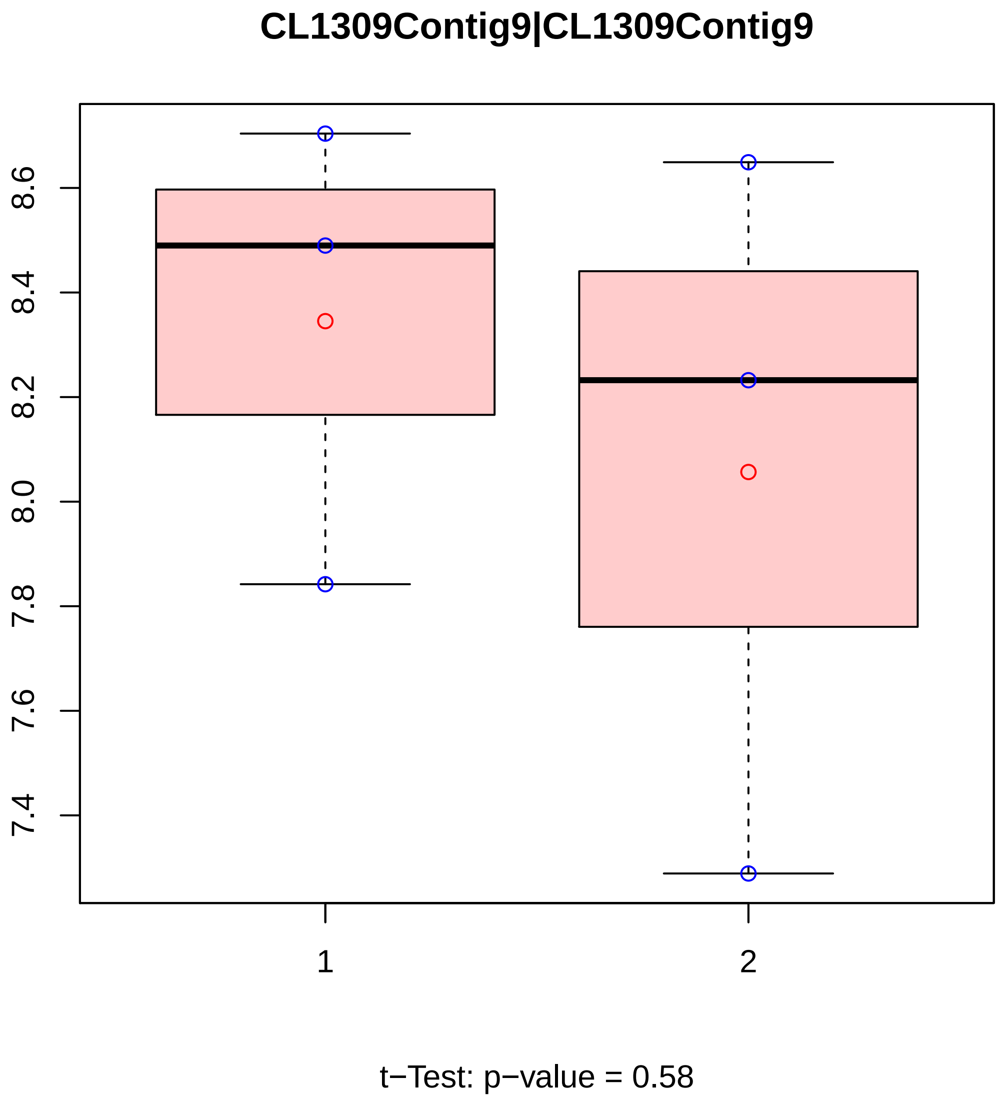
CL1309Contig9|CL1309Contig9
8.6
8.4
8.2
8.0
7.8
7.6
7.4
1
2
t−Test: p−value = 0.58

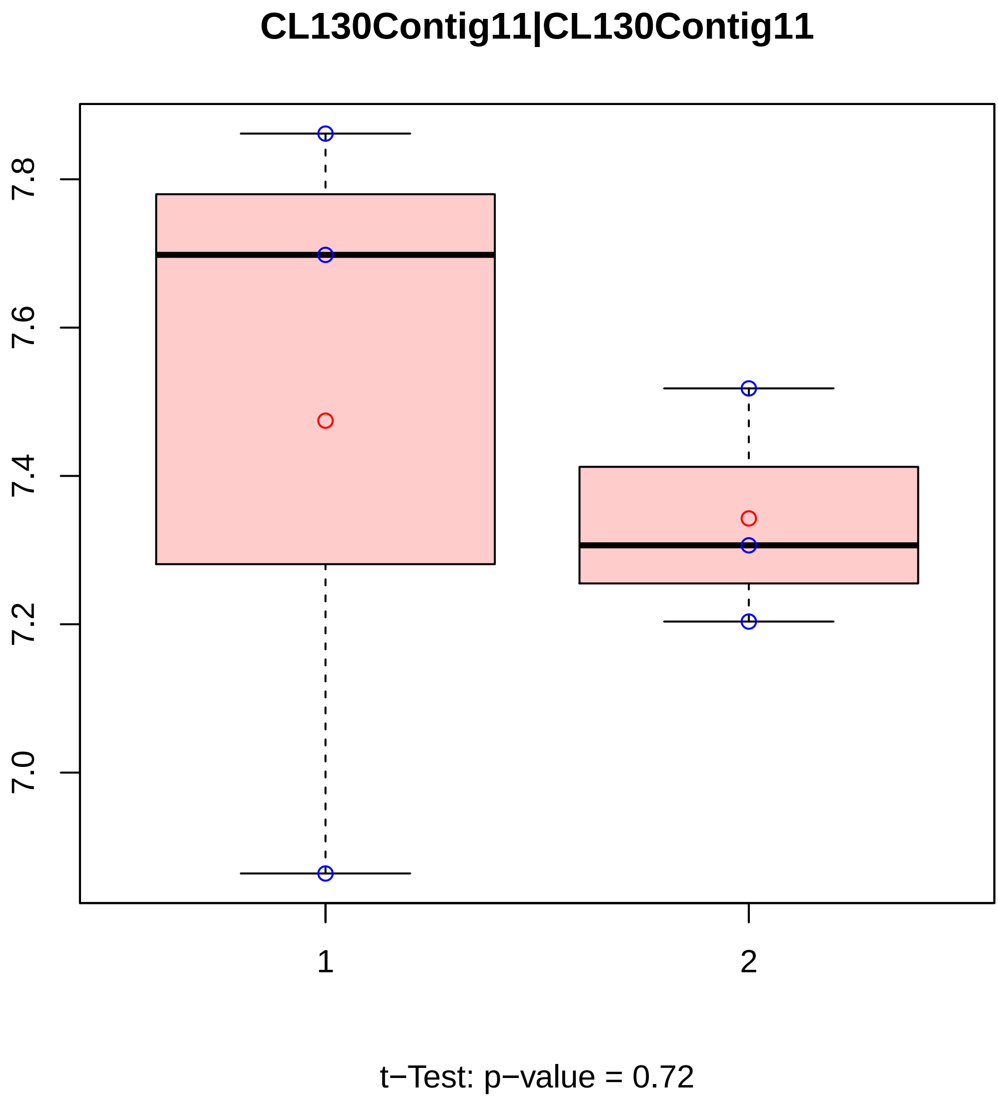
CL130Contig11|CL130Contig11
7.0
7.2
7.4
7.6
7.8
1
2
t−Test: p−value = 0.72

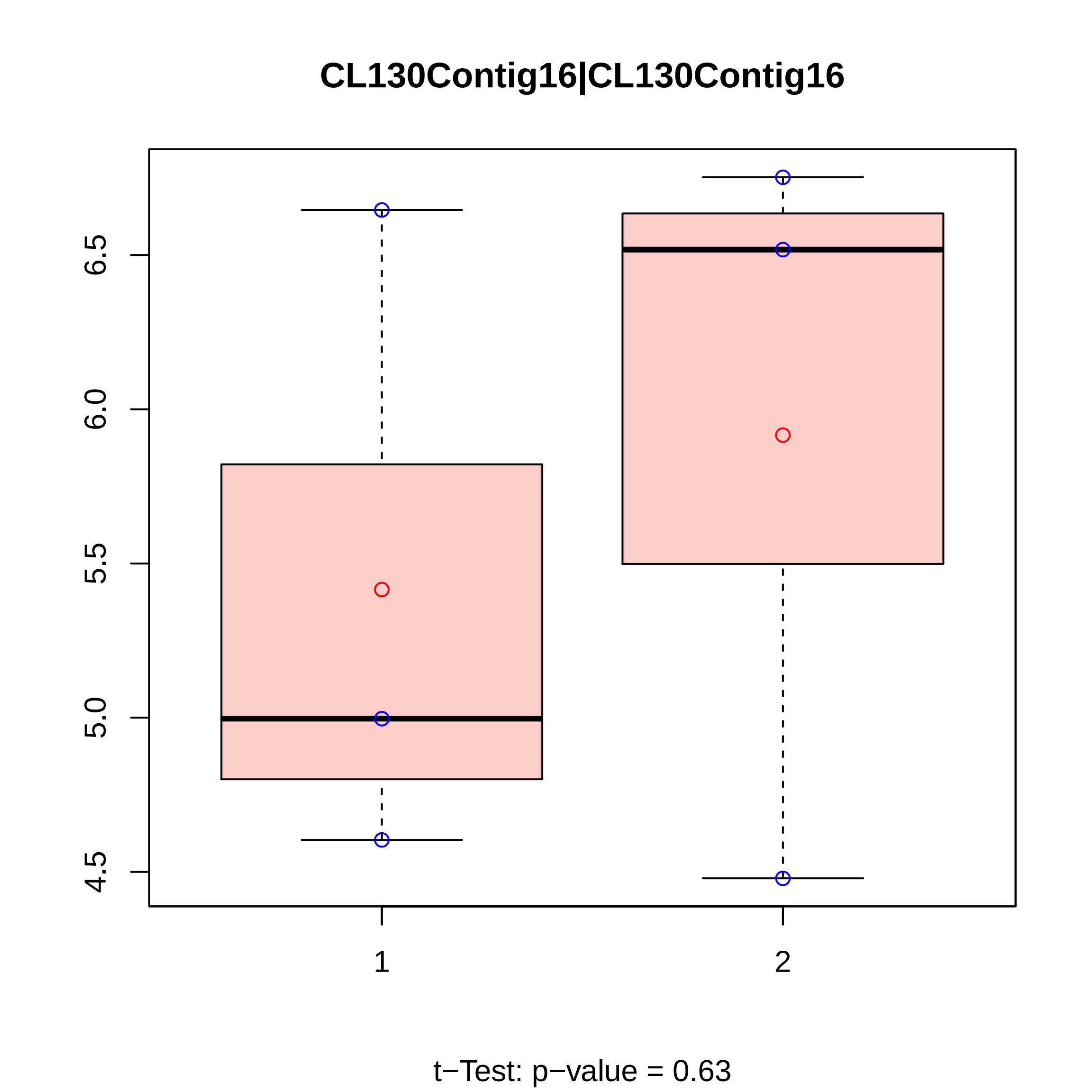
CL130Contig16|CL130Contig16
6.5
6.0
5.5
5.0
4.5
1
2
t−Test: p−value = 0.63

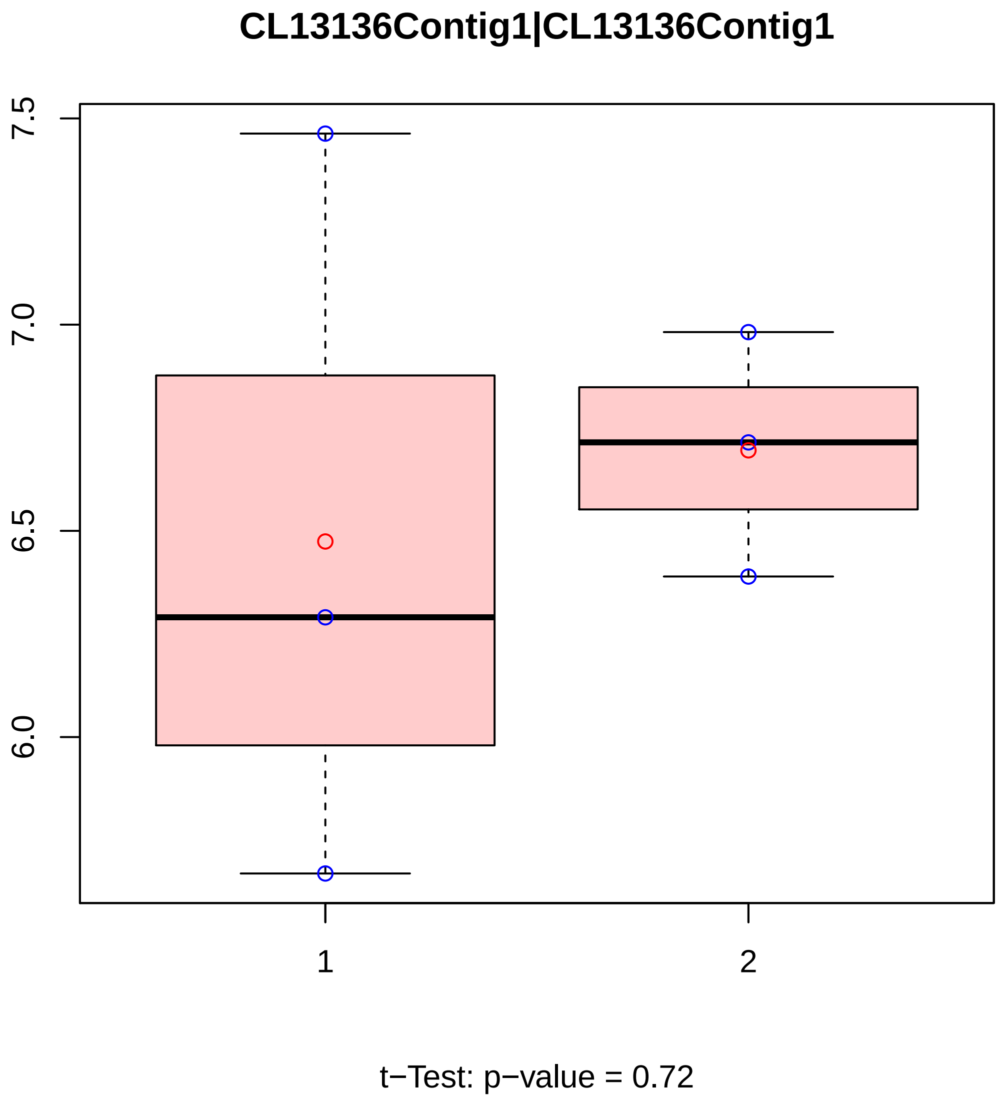
CL13136Contig1|CL13136Contig1
7.5
7.0
6.5
6.0
1
2
t−Test: p−value = 0.72

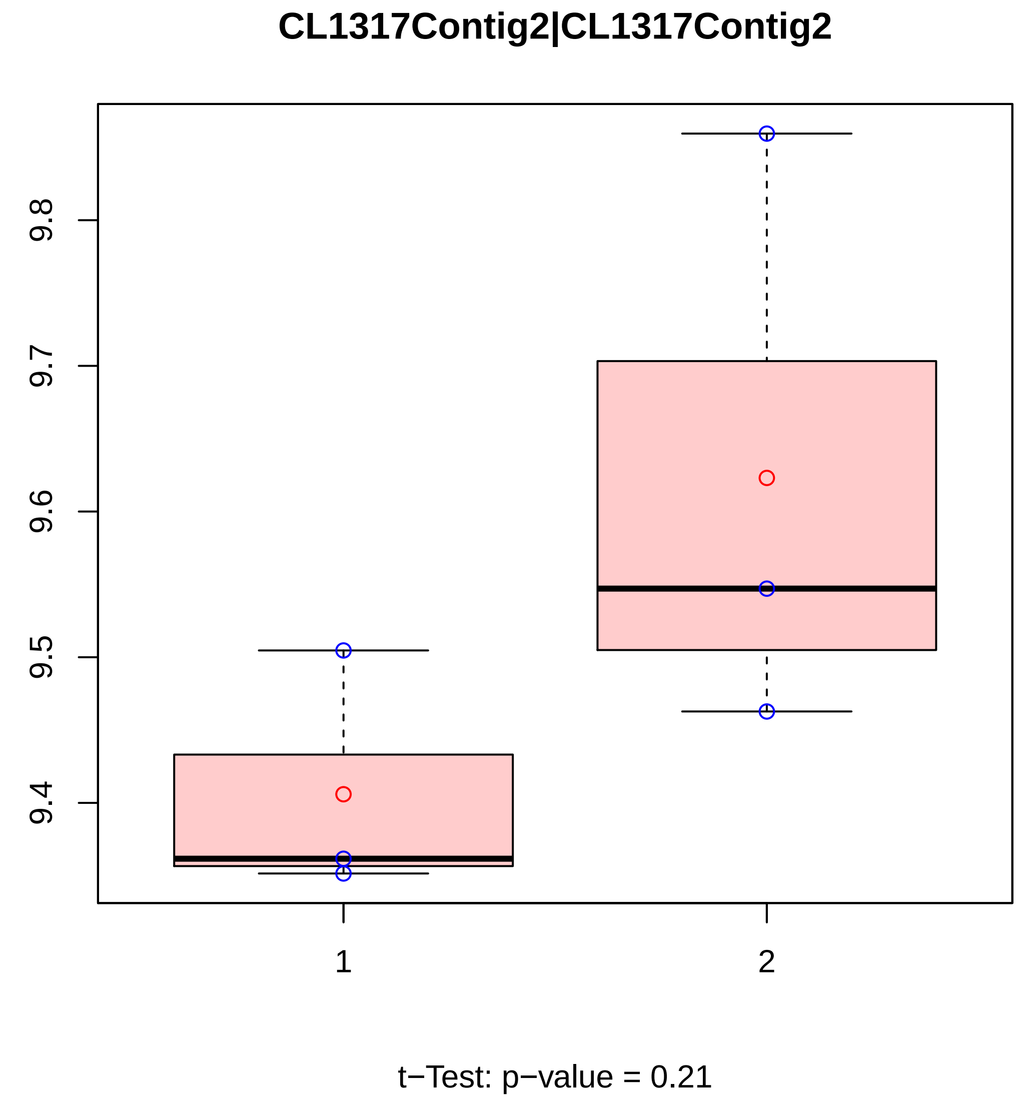
CL1317Contig2|CL1317Contig2
9.4
9.5
9.6
9.7
9.8
1
2
t−Test: p−value = 0.21

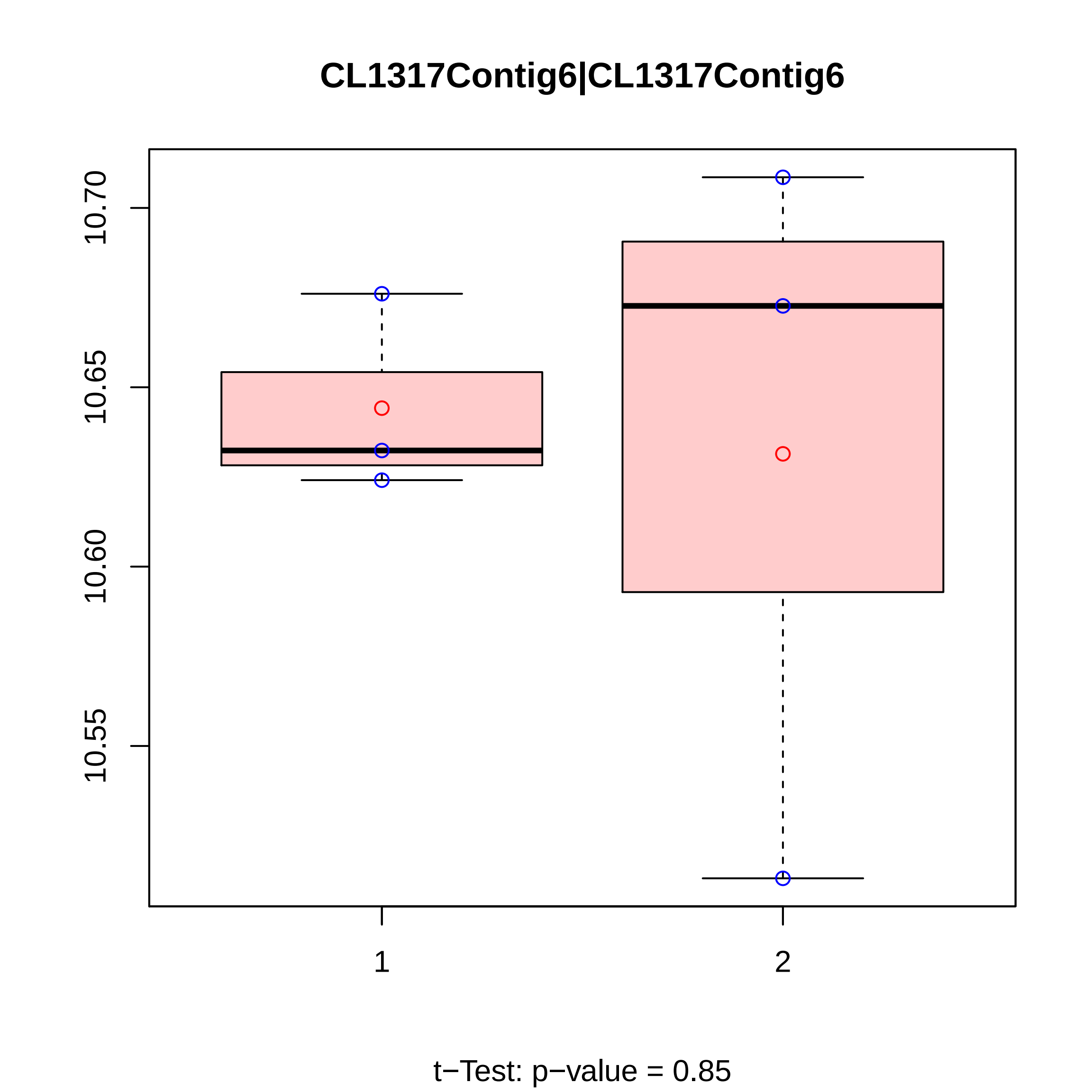
CL1317Contig6|CL1317Contig6
10.70
10.65
10.60
10.55
1
2
t−Test: p−value = 0.85

# CL1317Contig7|CL1317Contig7

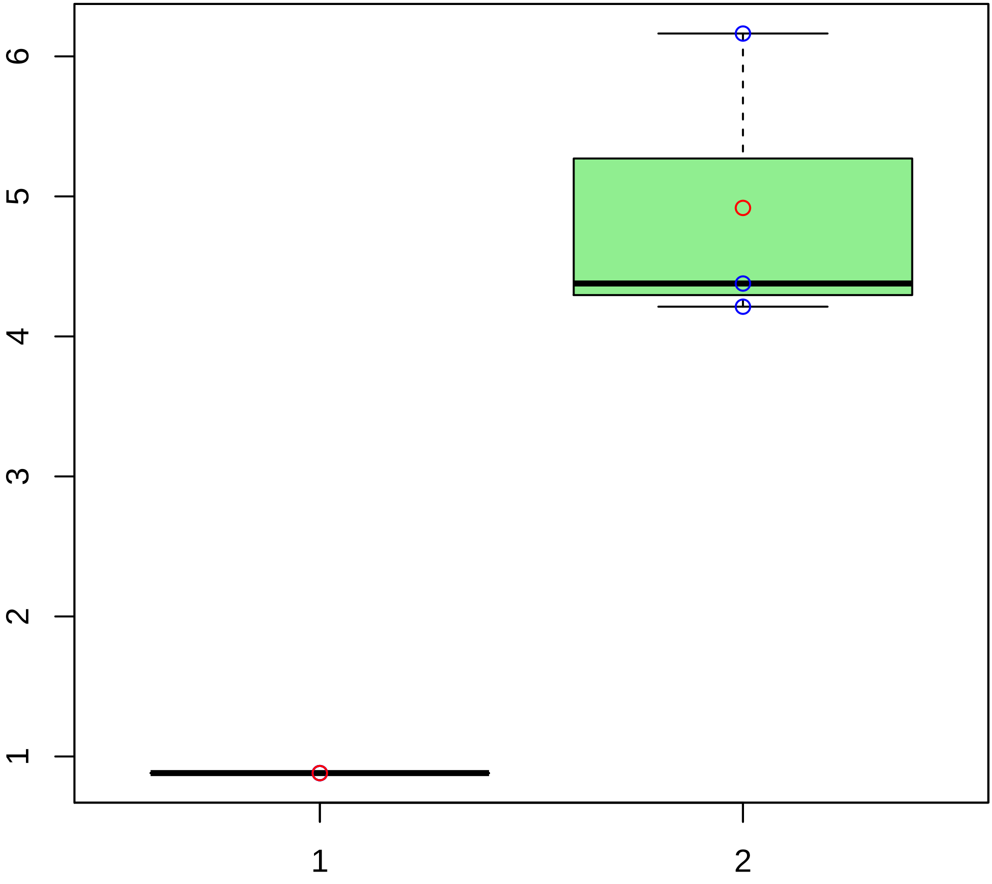


t−Test: p−value = 0.02

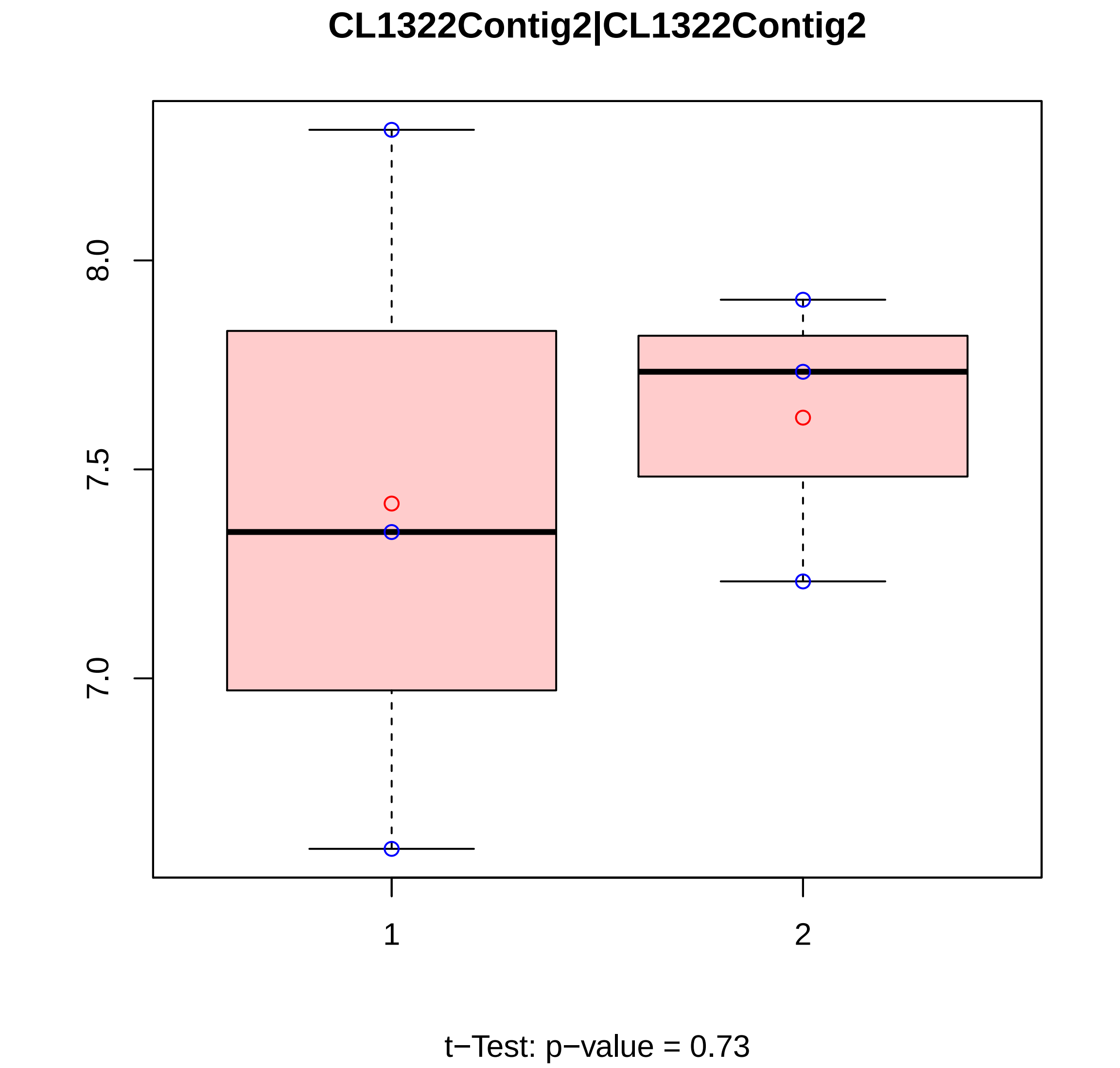
CL1322Contig2|CL1322Contig2
8.0
7.5
7.0
1
2
t−Test: p−value = 0.73

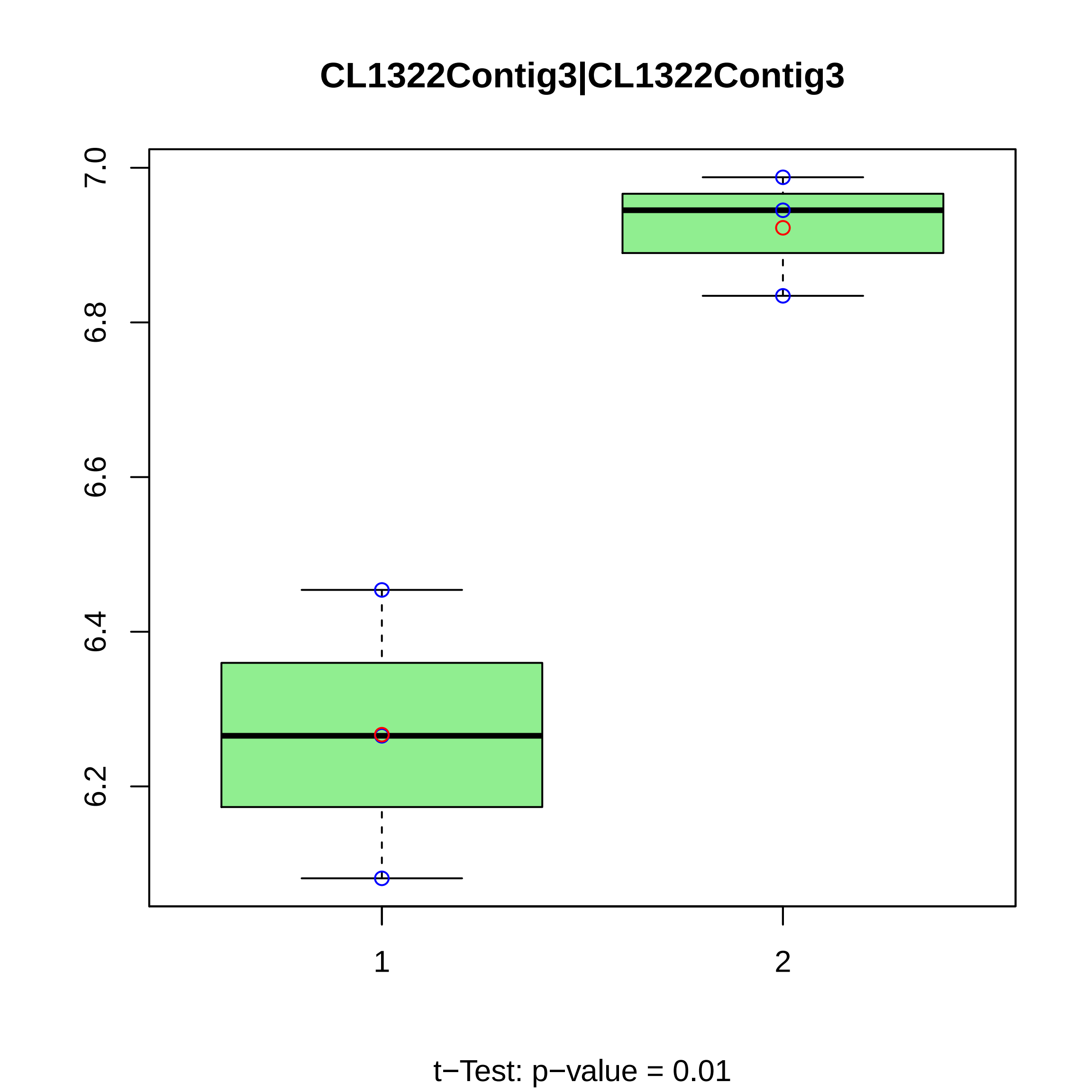
CL1322Contig3|CL1322Contig3
7.0
6.8
6.6
6.4
6.2
1
2
t−Test: p−value = 0.01

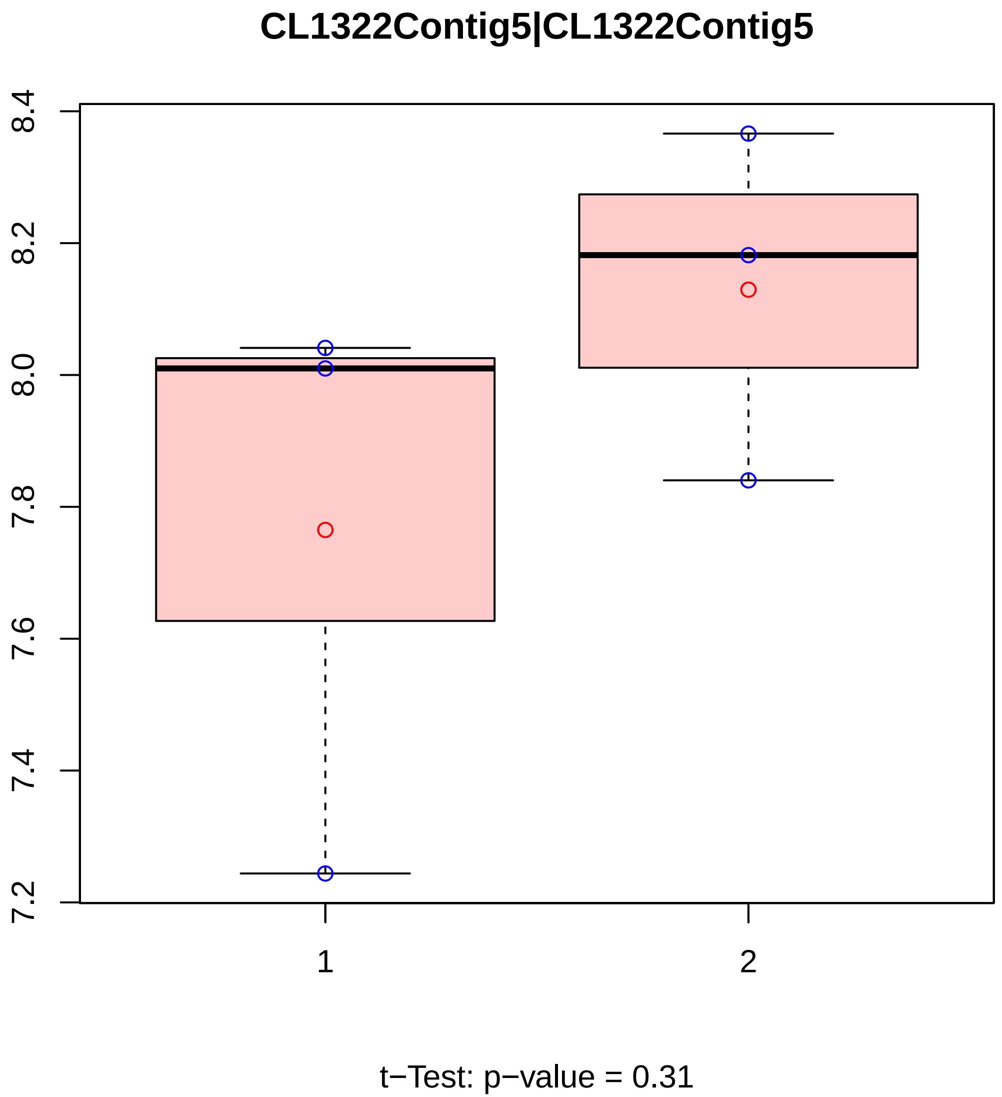
CL1322Contig5|CL1322Contig5
8.4
8.2
8.0
7.8
7.6
7.4
7.2
1
2
t−Test: p−value = 0.31

# CL13244Contig1|CL13244Contig1

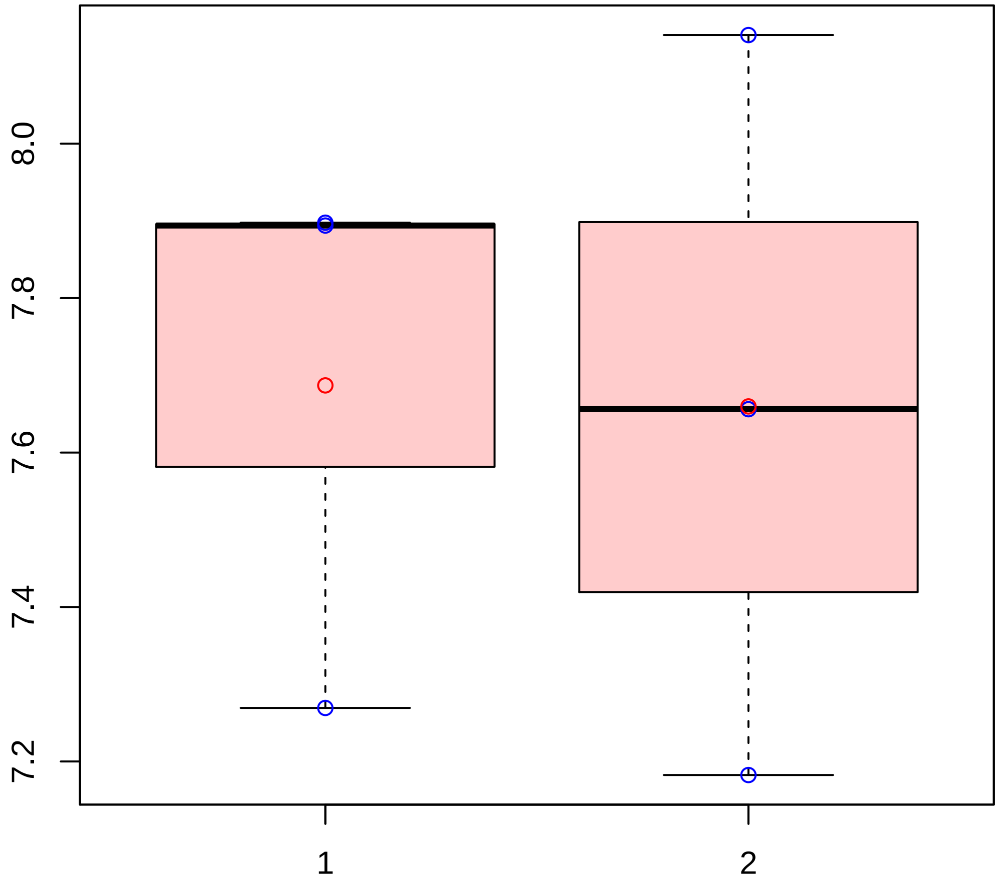


t−Test: p−value = 0.94

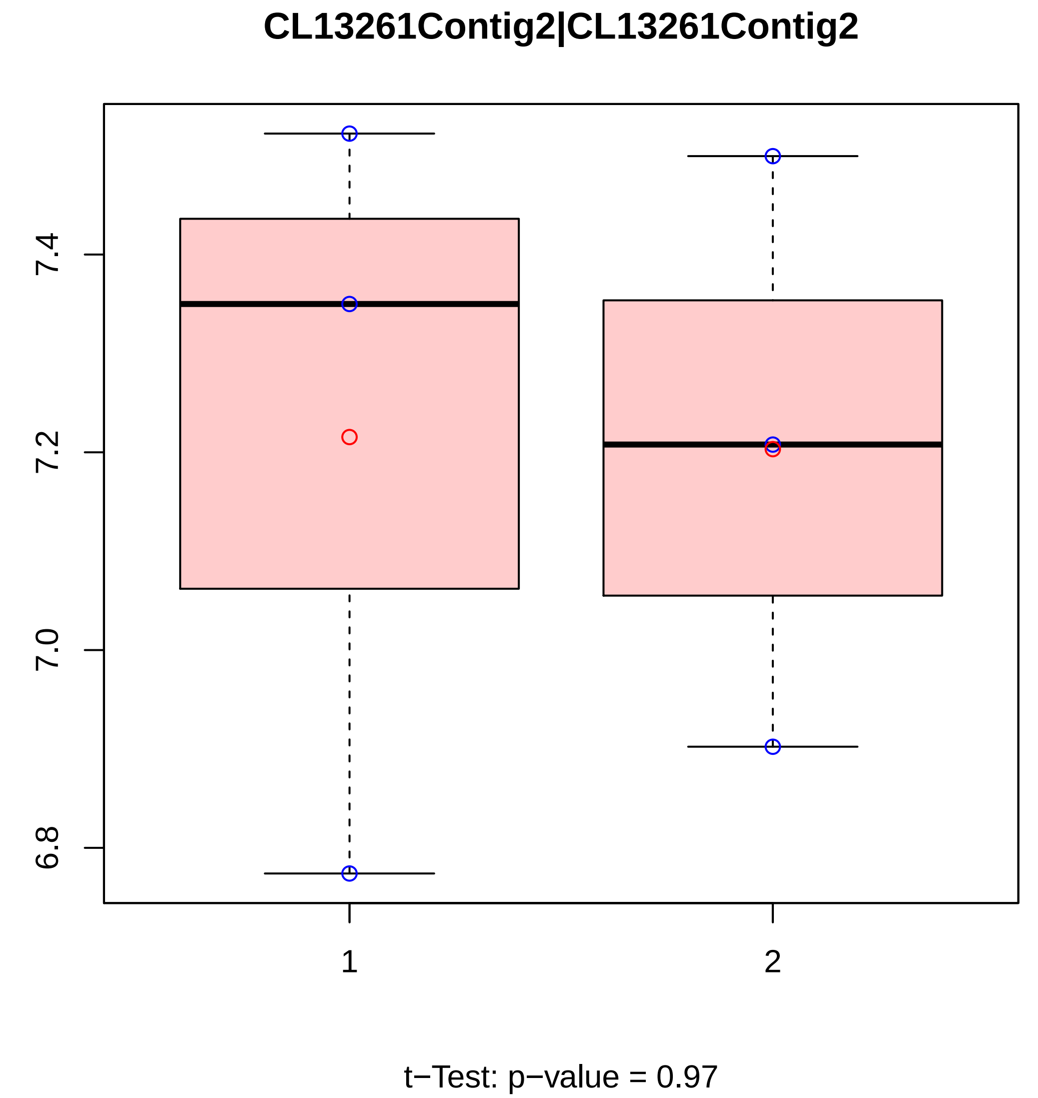
CL13261Contig2|CL13261Contig2
7.4
7.2
7.0
6.8
1
2
t−Test: p−value = 0.97

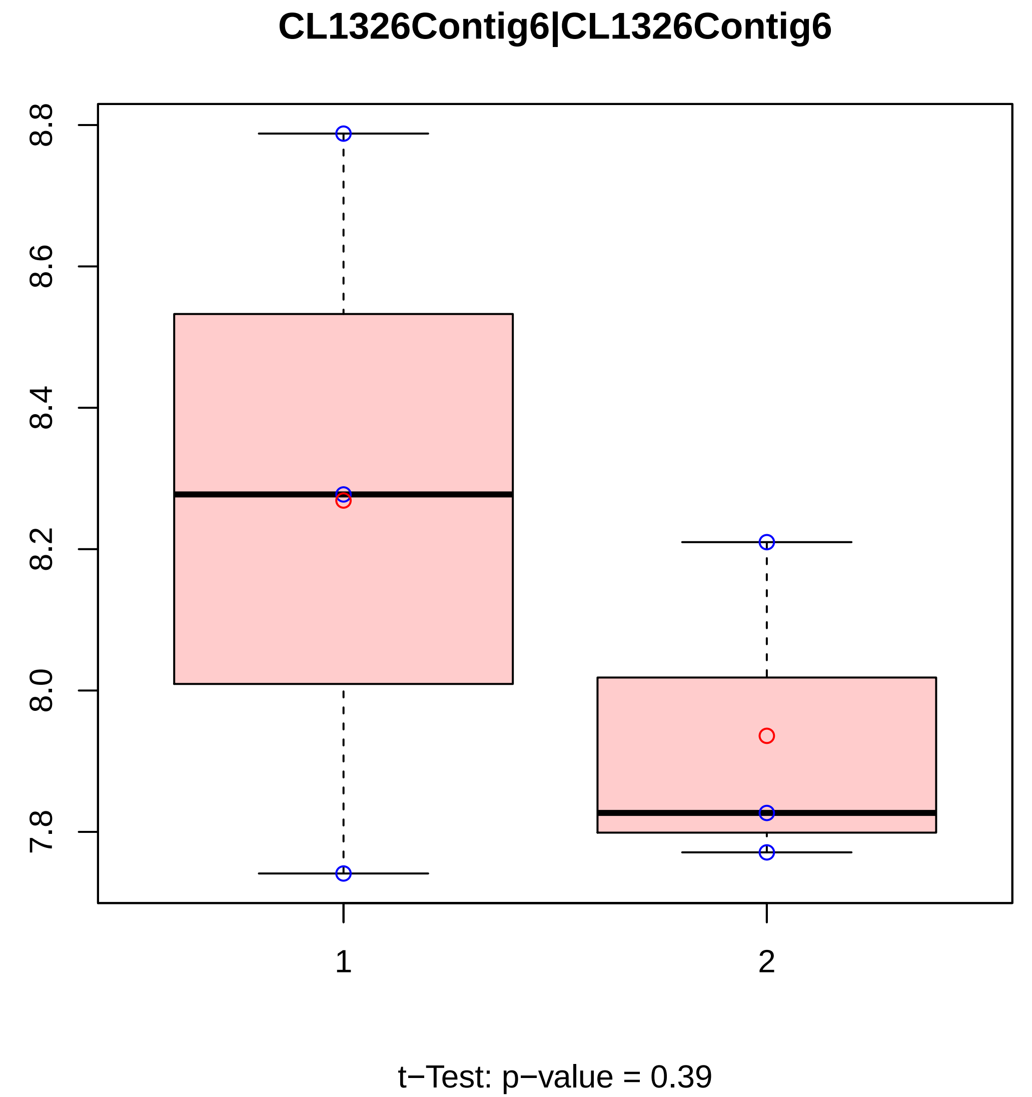
CL1326Contig6|CL1326Contig6
8.8
8.6
8.4
8.2
8.0
7.8
1
2
t−Test: p−value = 0.39

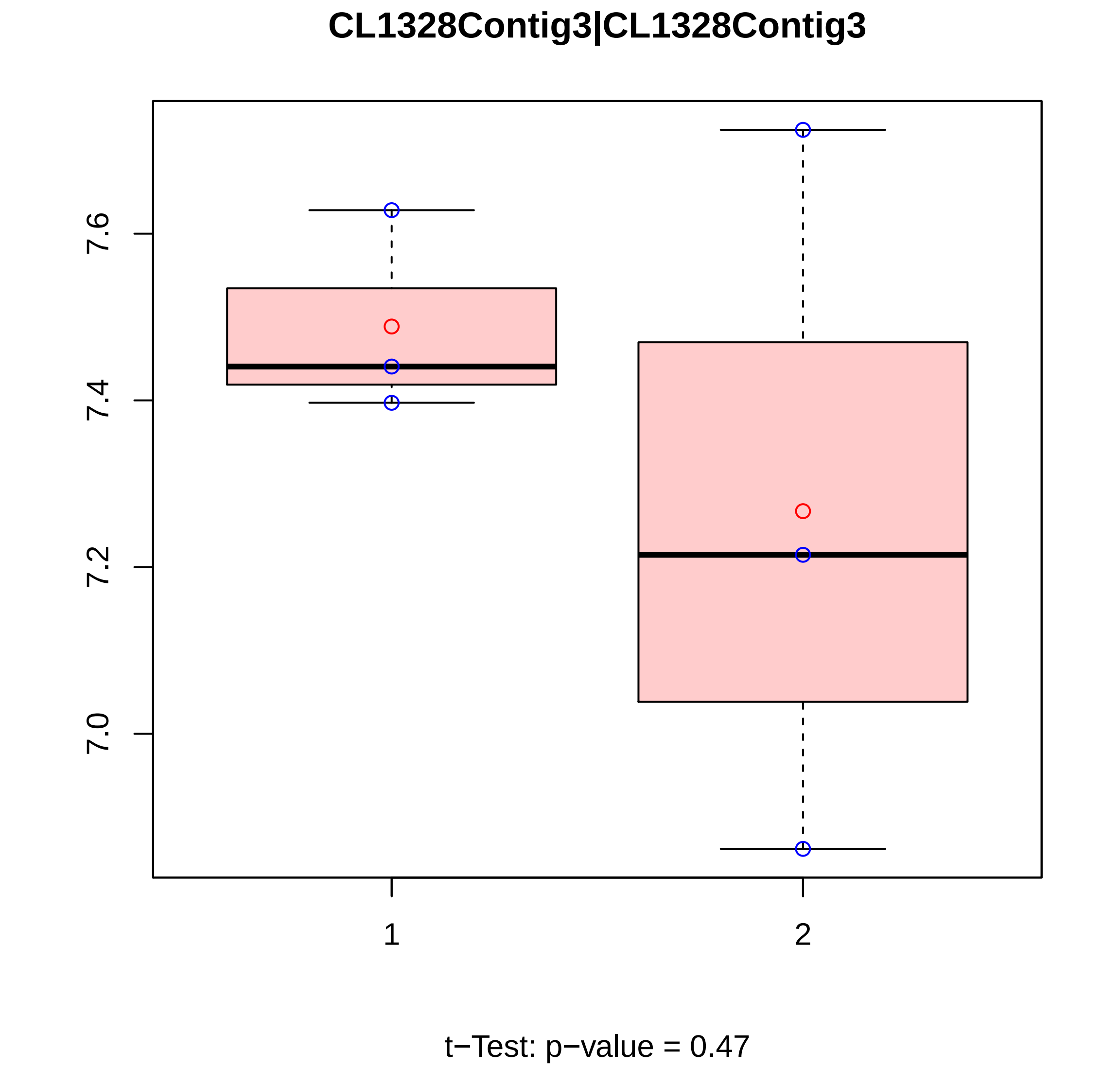

CL1328Contig3|CL1328Contig3
7.0
7.2
7.4
7.6
1
2
t−Test: p−value = 0.47

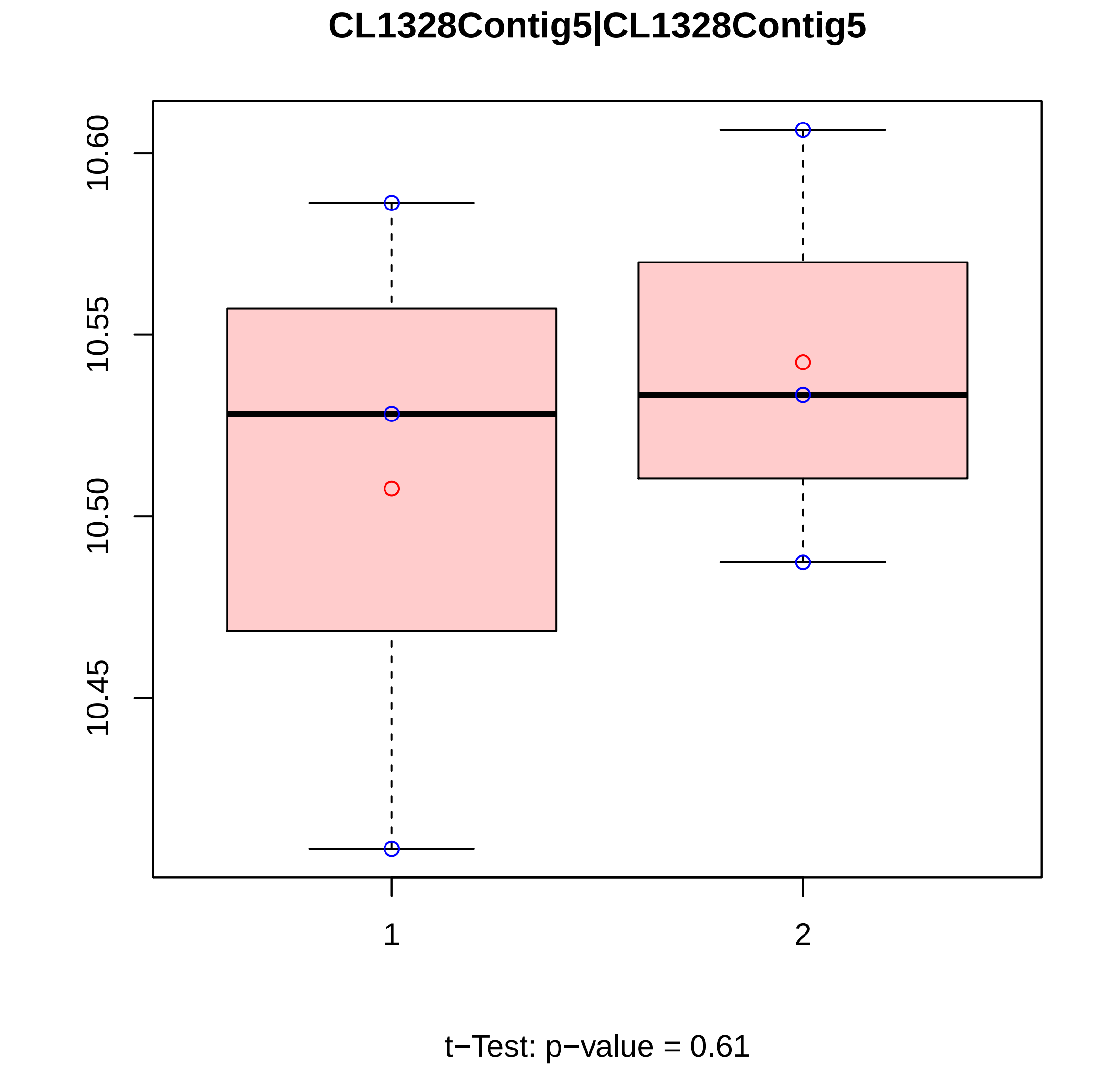
CL1328Contig5|CL1328Contig5
10.60
10.55
10.50
10.45
1
2
t−Test: p−value = 0.61

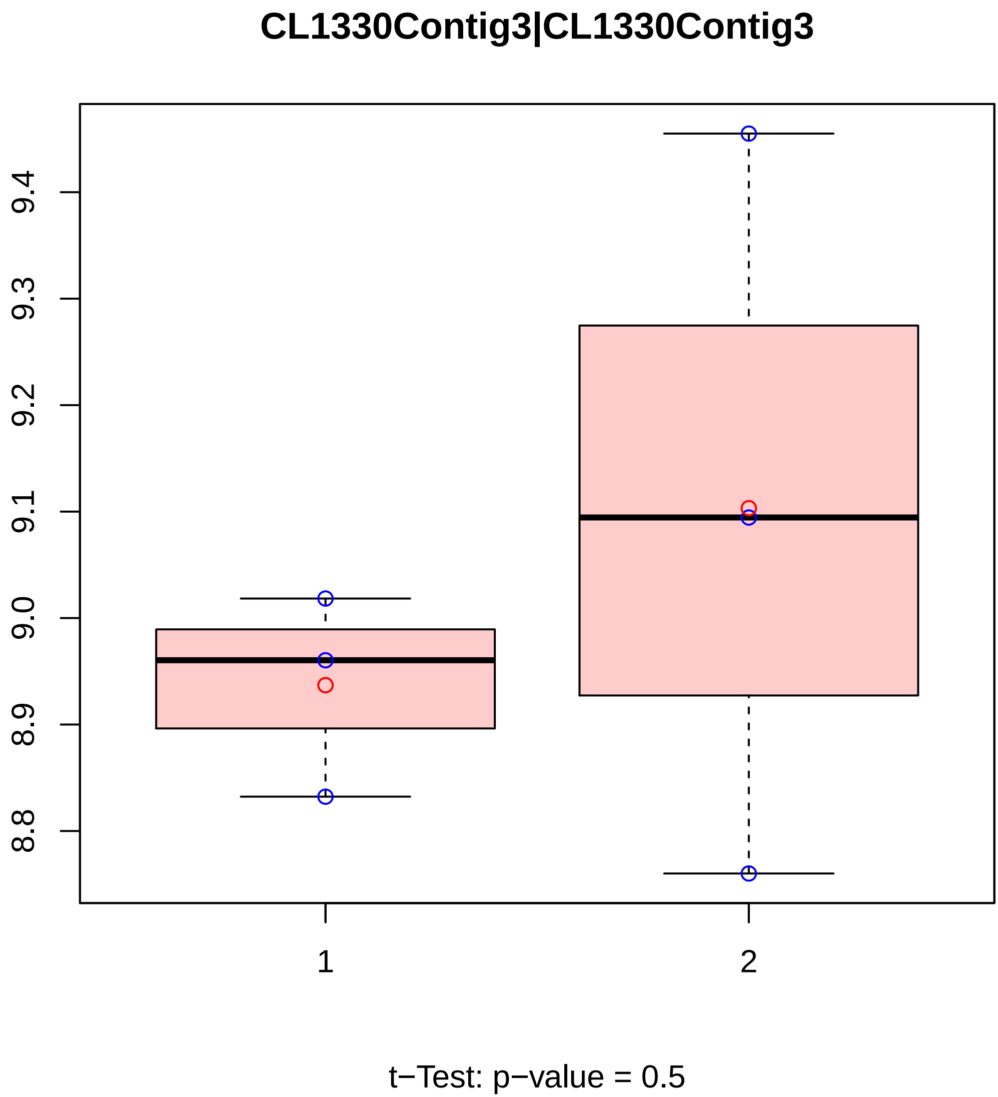
CL1330Contig3|CL1330Contig3
9.4
9.3
9.2
9.1
9.0
8.9
8.8
1
2
t−Test: p−value = 0.5

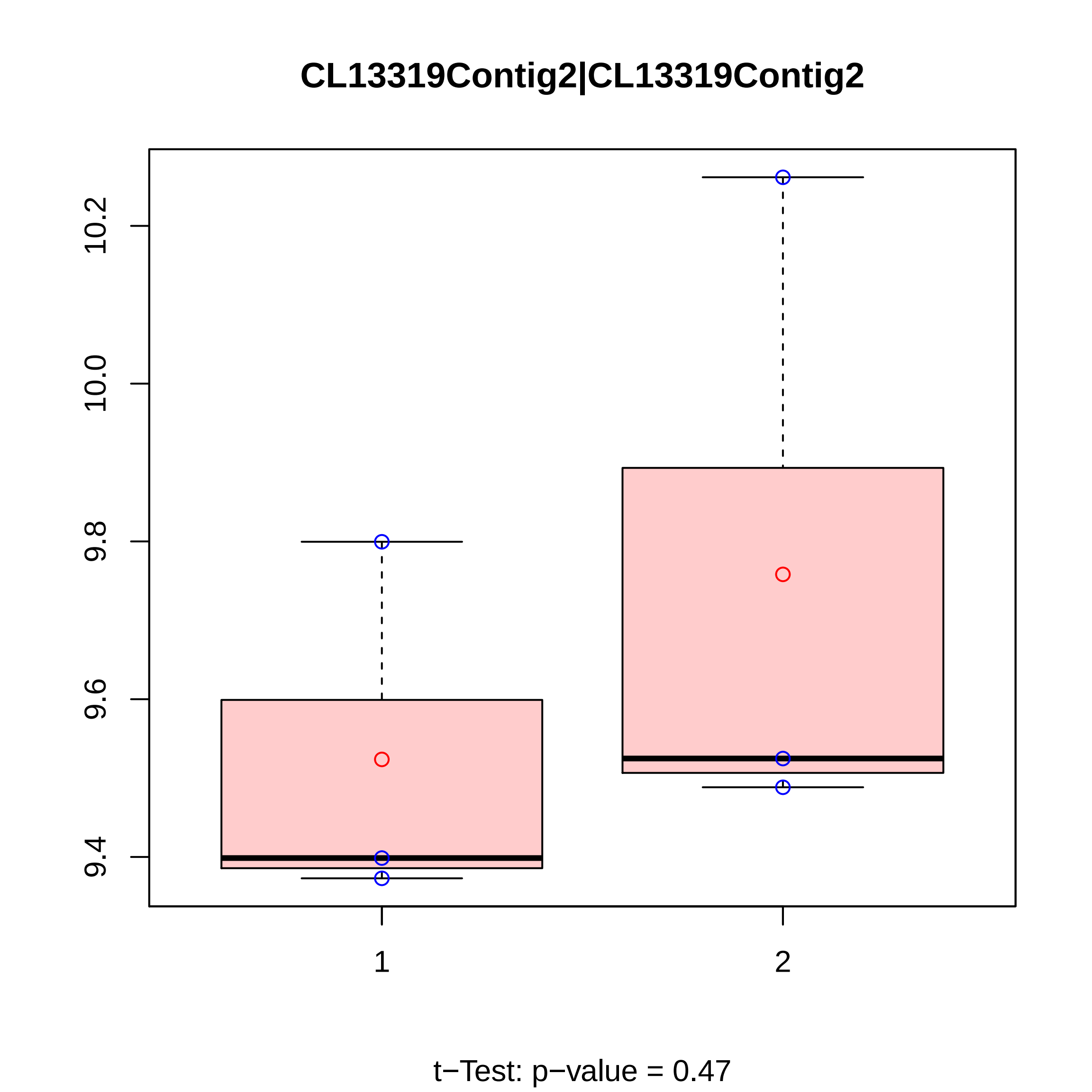

CL13319Contig2|CL13319Contig2
10.2
10.0
9.8
9.6
9.4
1
2
t−Test: p−value = 0.47

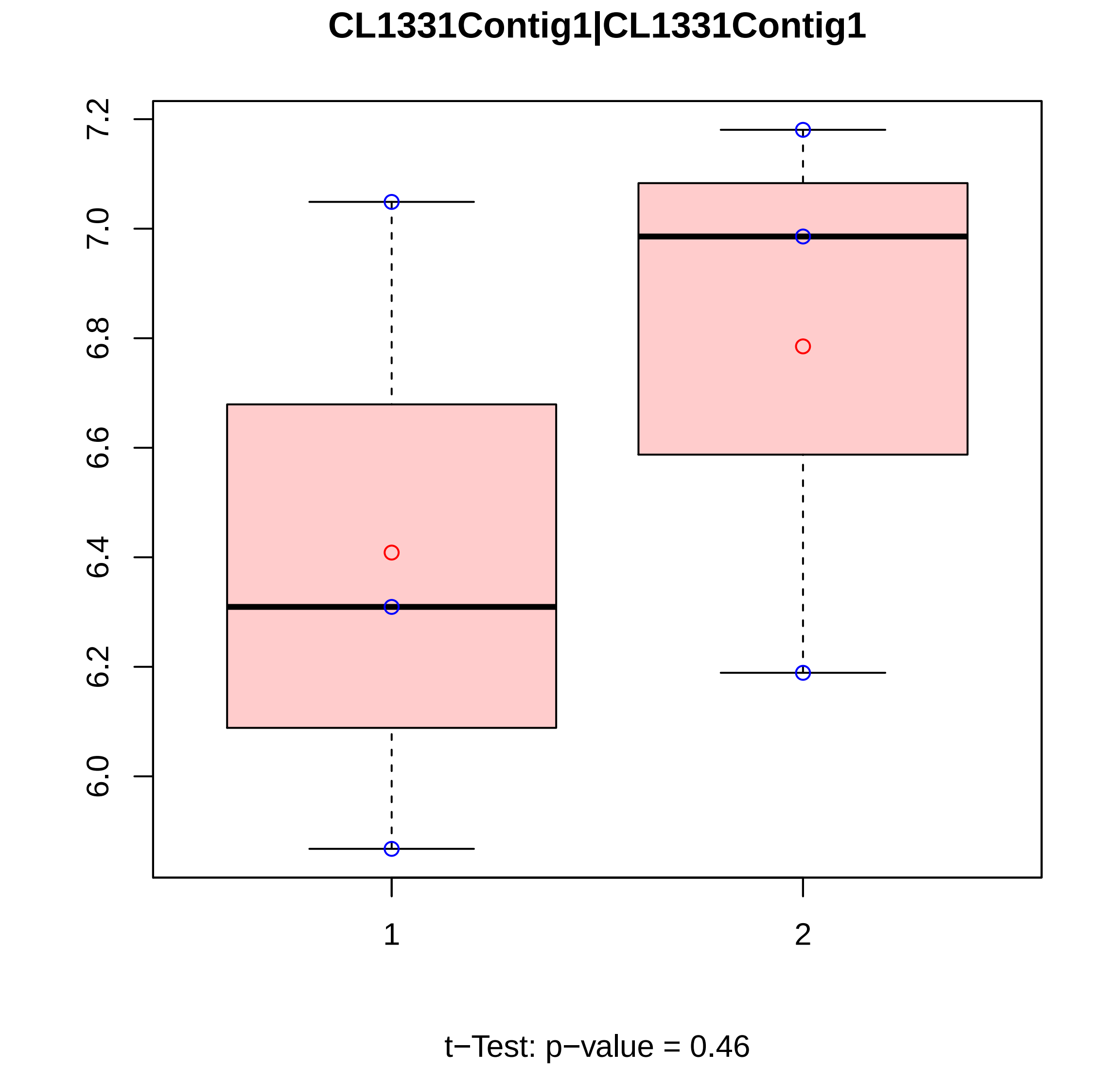
CL1331Contig1|CL1331Contig1
7.2
7.0
6.8
6.6
6.4
6.2
6.0
1
2
t−Test: p−value = 0.46

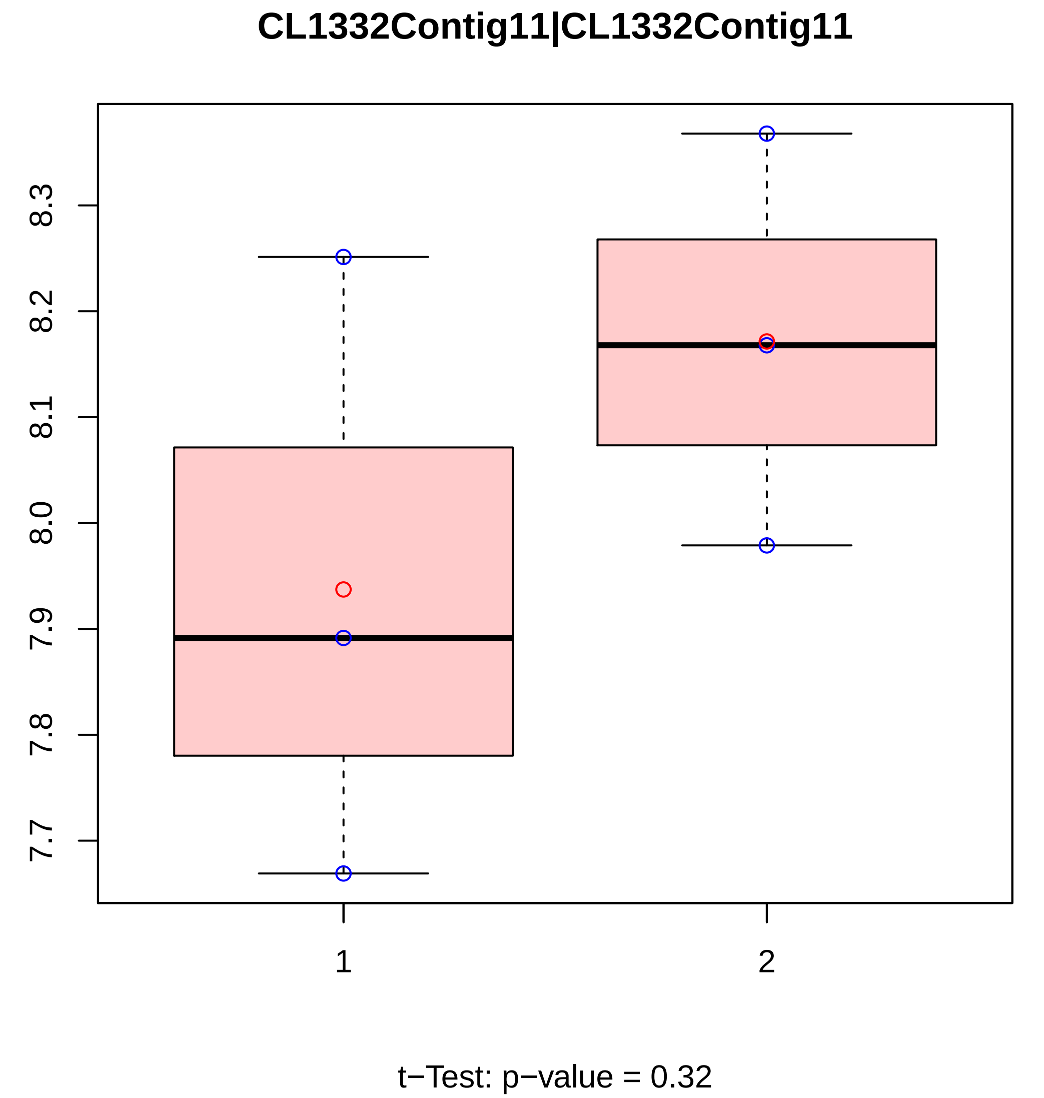
CL1332Contig11|CL1332Contig11
8.3
8.2
8.1
8.0
7.9
7.8
7.7
1
2
t−Test: p−value = 0.32

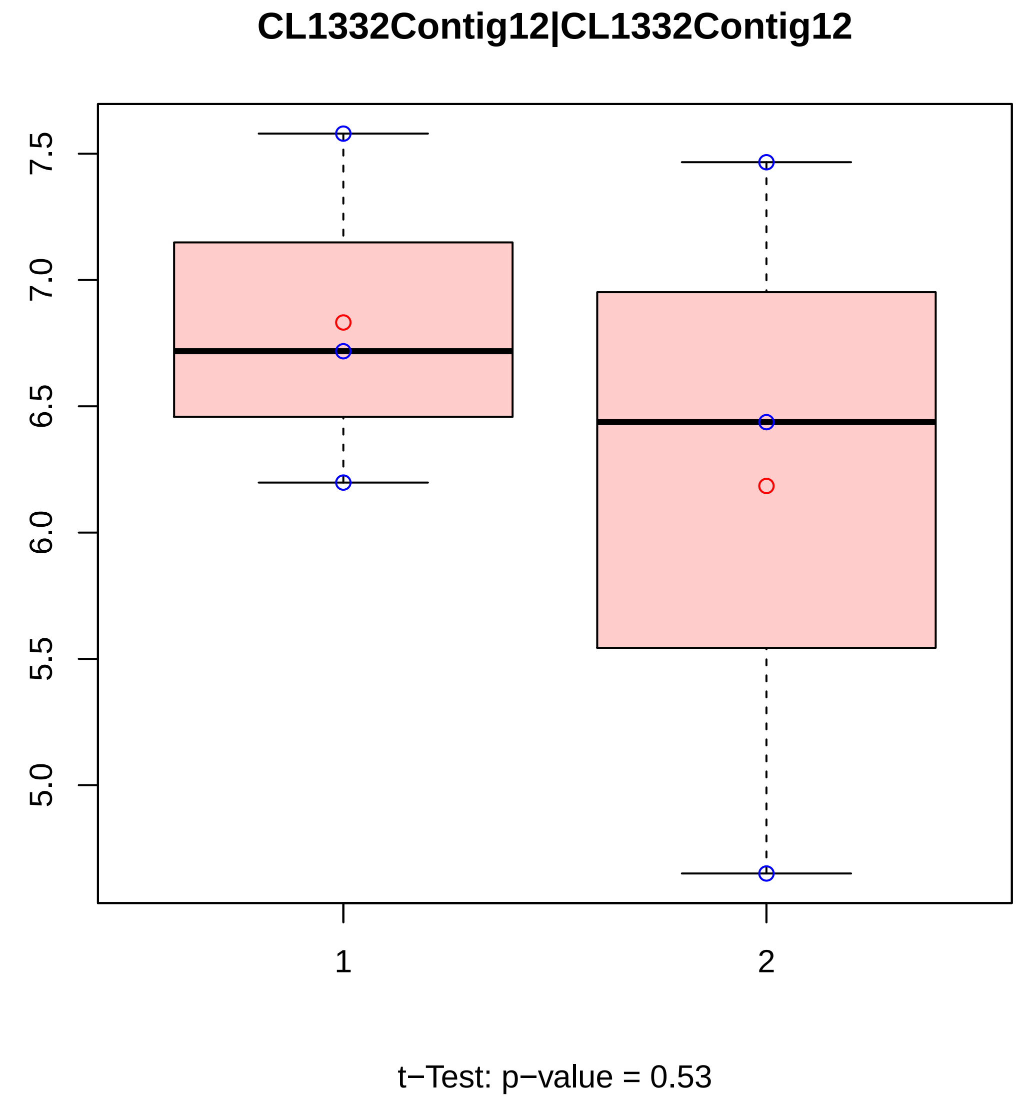

CL1332Contig12|CL1332Contig12
7.5
7.0
6.5
6.0
5.5
5.0
1
2
t−Test: p−value = 0.53

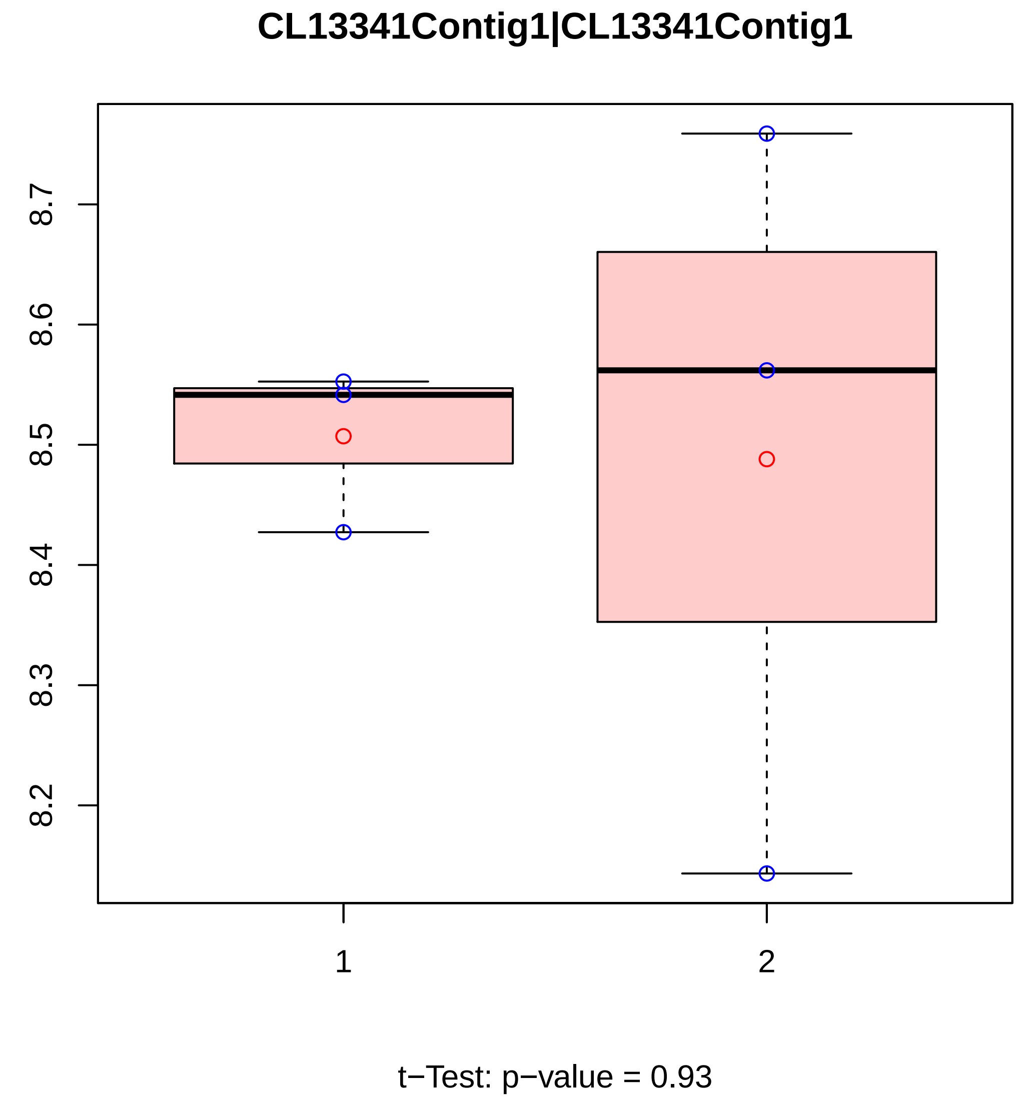
CL13341Contig1|CL13341Contig1
8.2
8.3
8.4
8.5
8.6
8.7
1
2
t−Test: p−value = 0.93

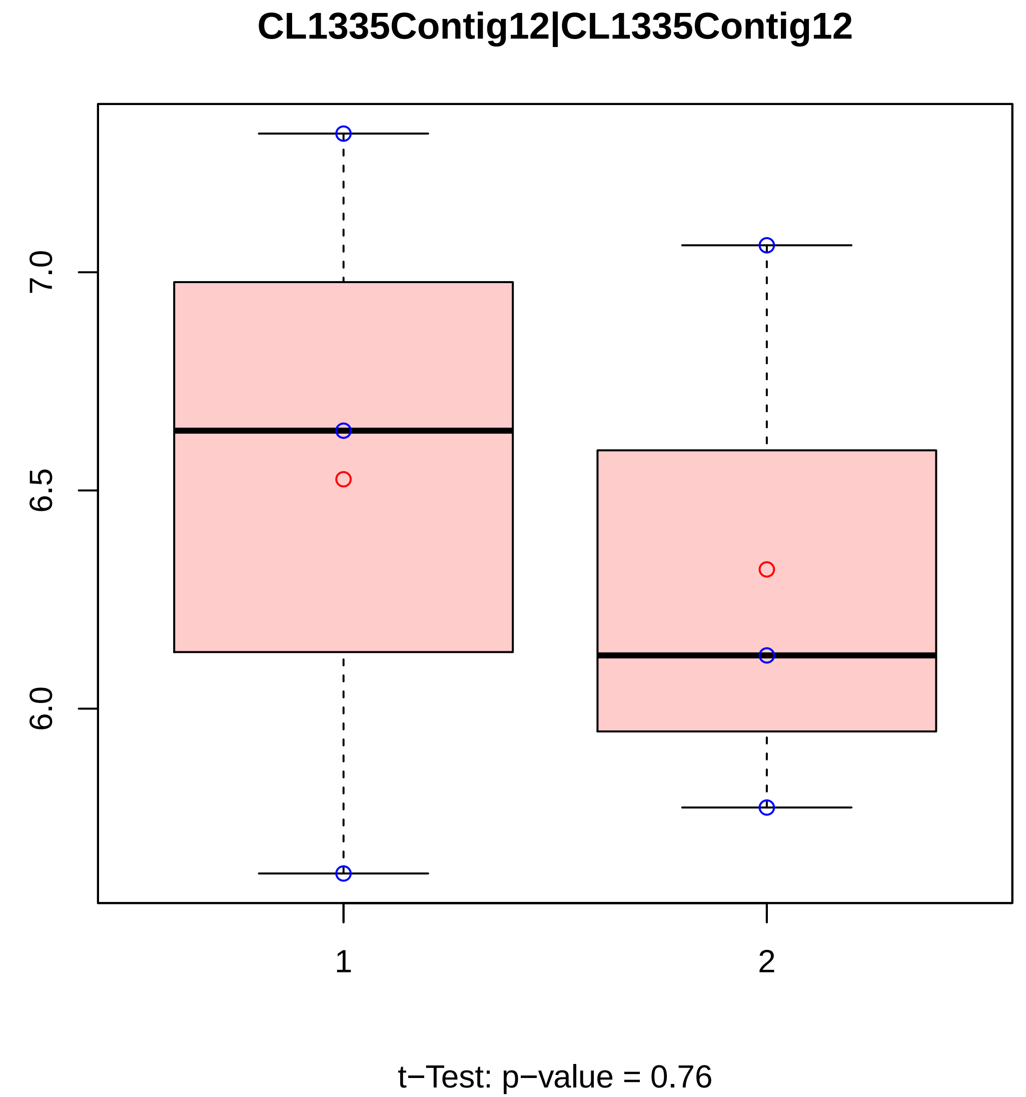
CL1335Contig12|CL1335Contig12
7.0
6.5
6.0
1
2
t−Test: p−value = 0.76

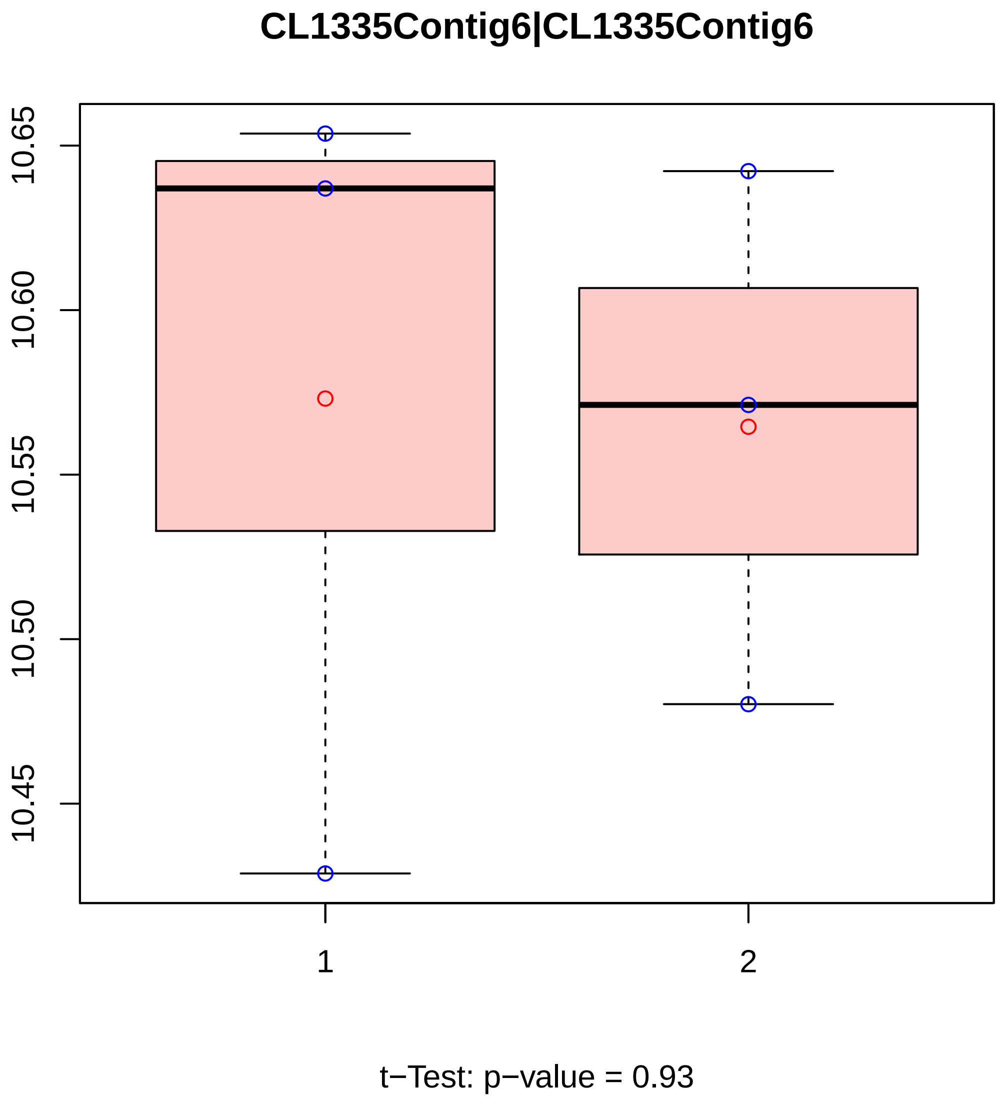
CL1335Contig6|CL1335Contig6
10.65
10.60
10.55
10.50
10.45
1
2
t−Test: p−value = 0.93

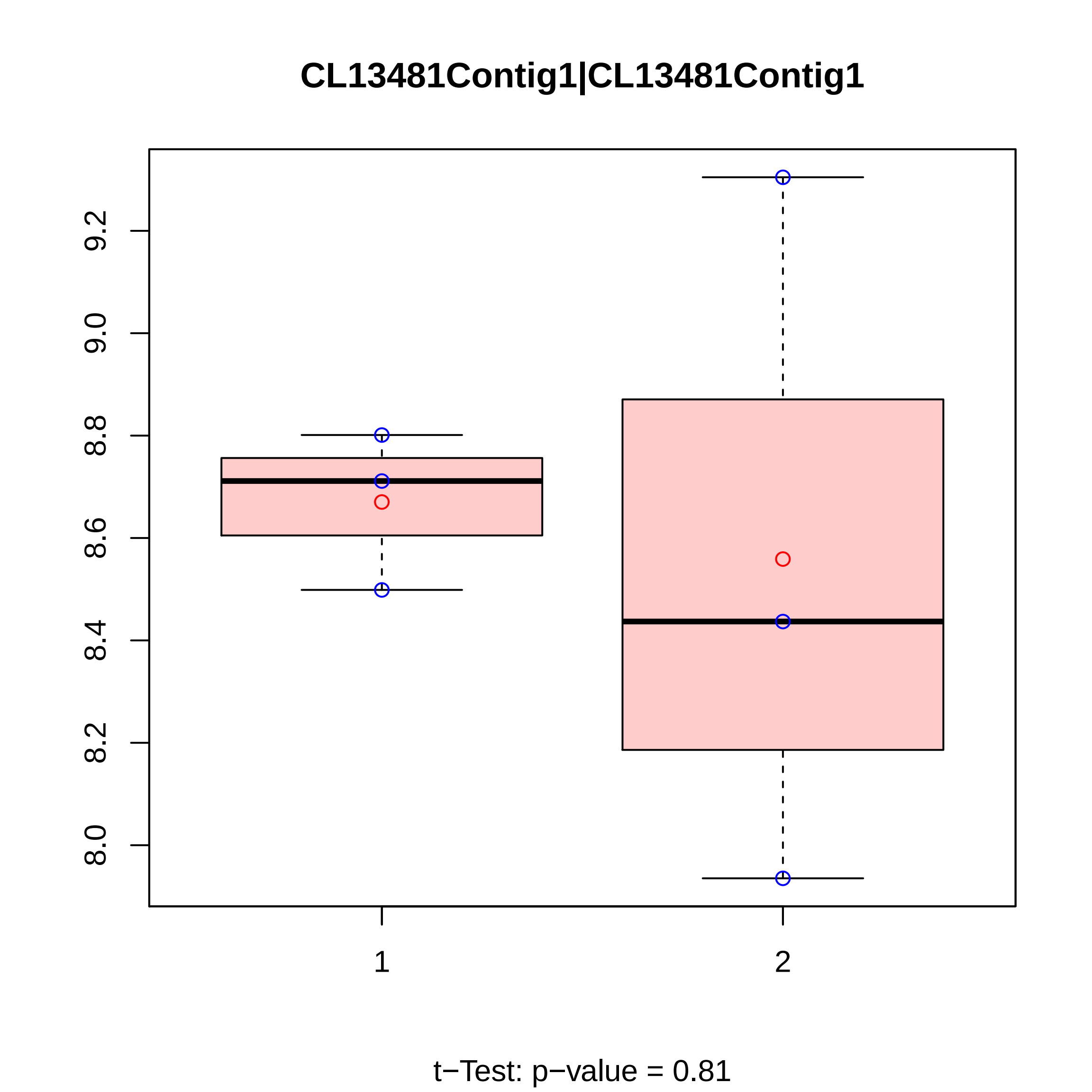
CL13481Contig1|CL13481Contig1
8.0
8.2
8.4
8.6
8.8
9.0
9.2
1
2
t−Test: p−value = 0.81

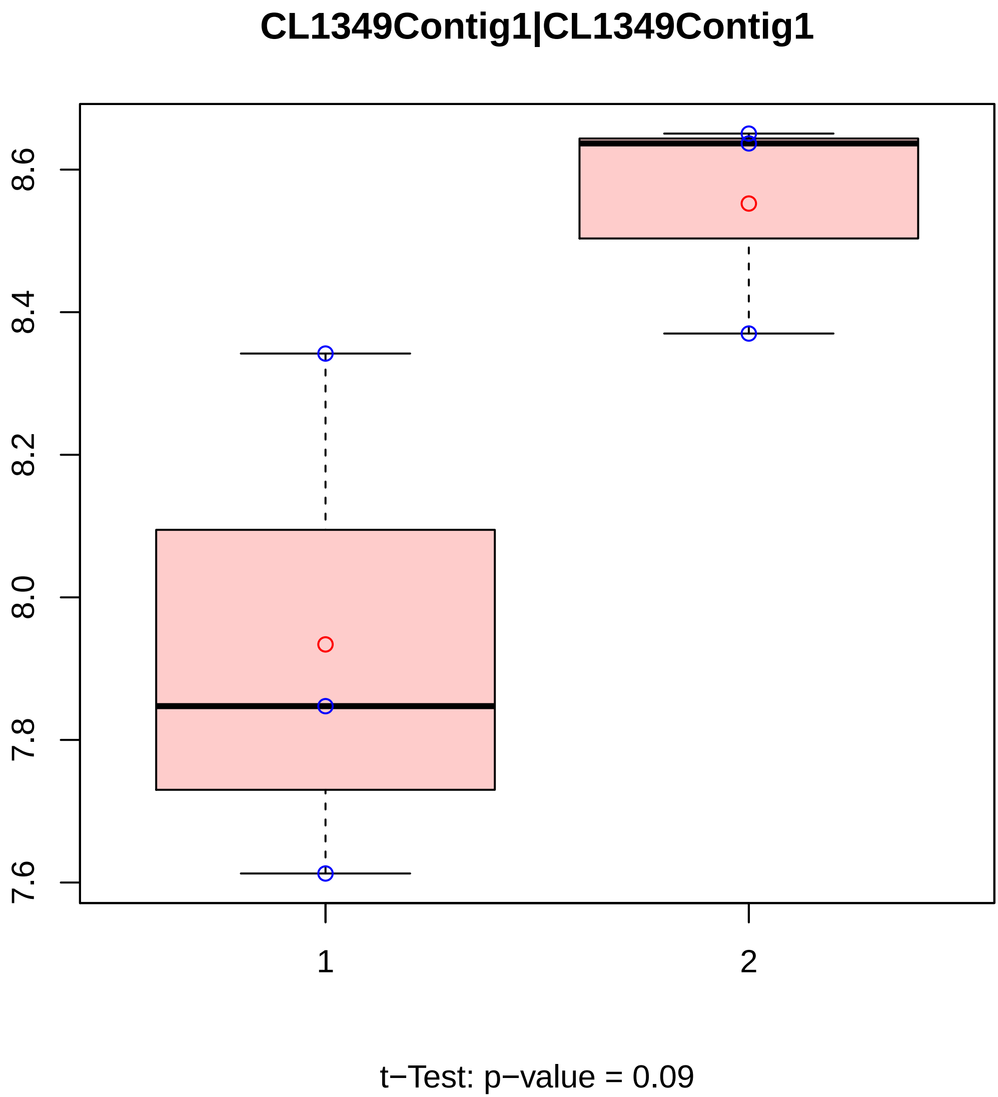

CL1349Contig1|CL1349Contig1
8.6
8.4
8.2
8.0
7.8
7.6
1
2
t−Test: p−value = 0.09

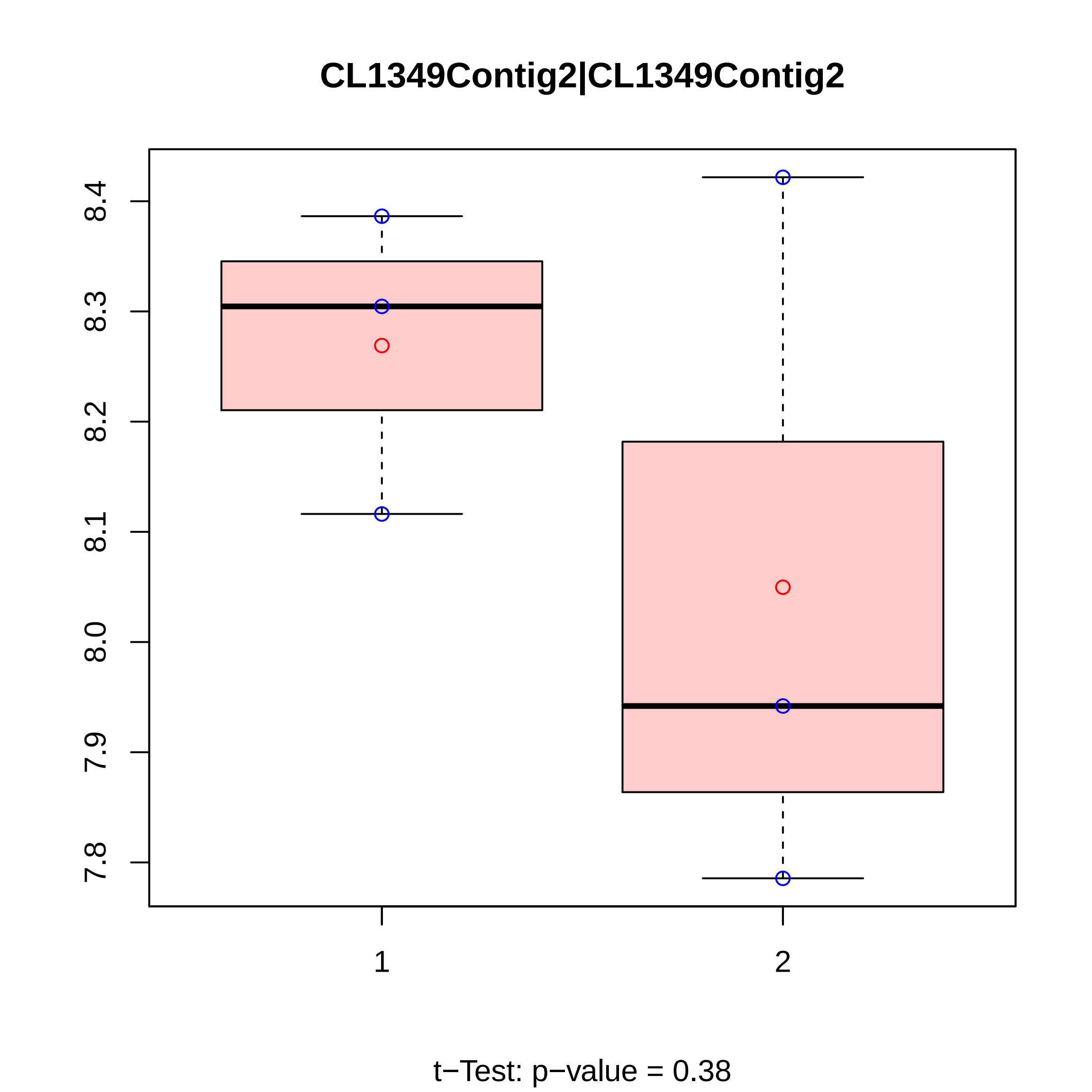
CL1349Contig2|CL1349Contig2
8.4
8.3
8.2
8.1
8.0
7.9
7.8
1
2
t−Test: p−value = 0.38

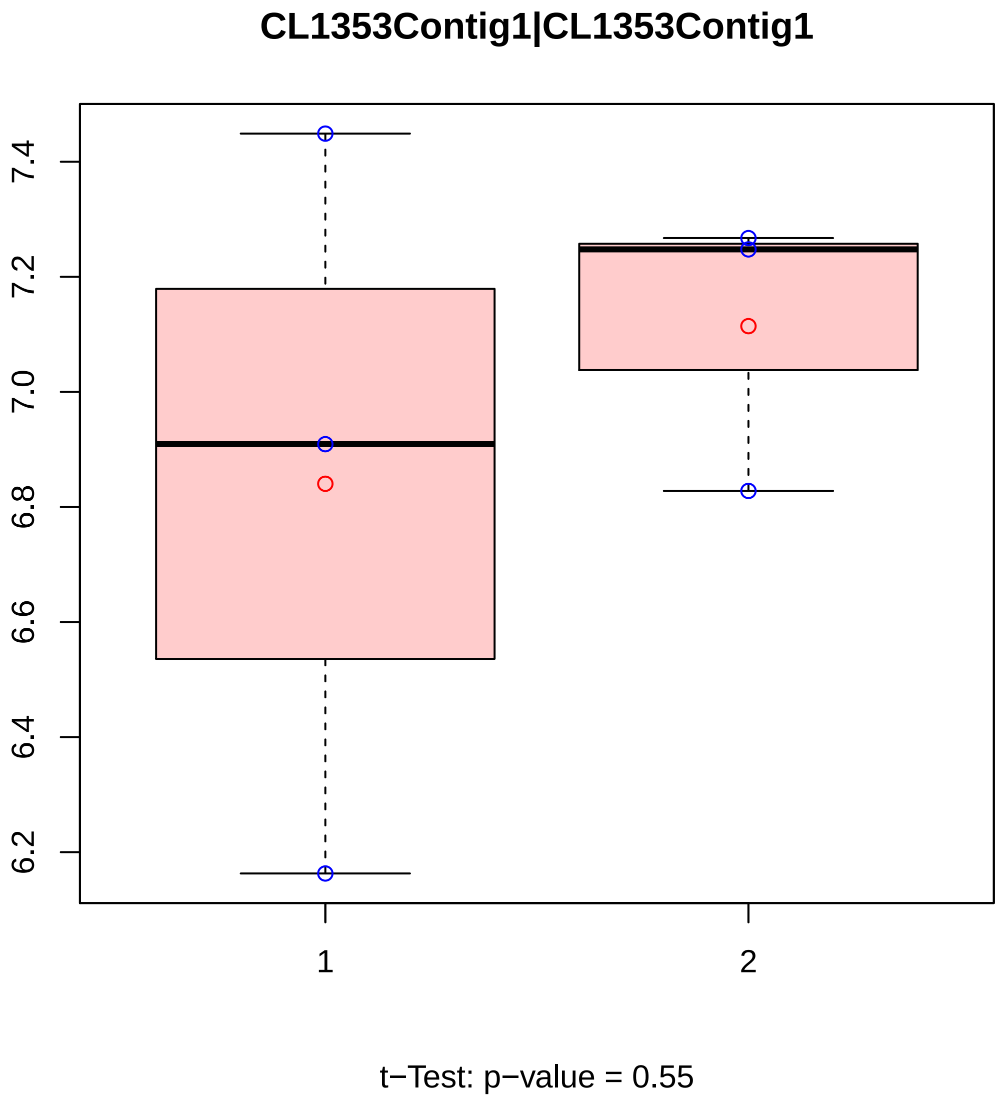
CL1353Contig1|CL1353Contig1
7.4
7.2
7.0
6.8
6.6
6.4
6.2
1
2
t−Test: p−value = 0.55

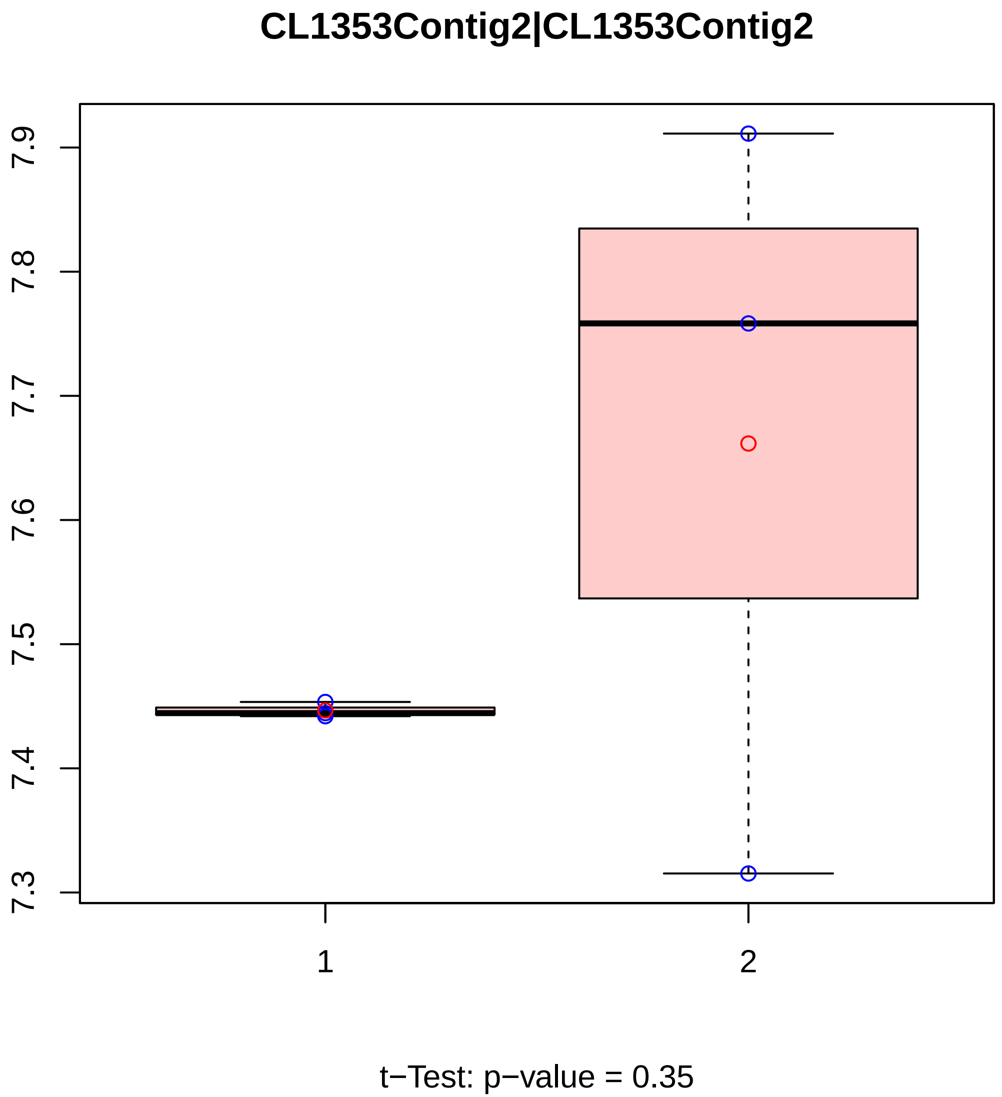
CL1353Contig2|CL1353Contig2
7.3
7.4
7.5
7.6
7.7
7.8
7.9
1
2
t−Test: p−value = 0.35

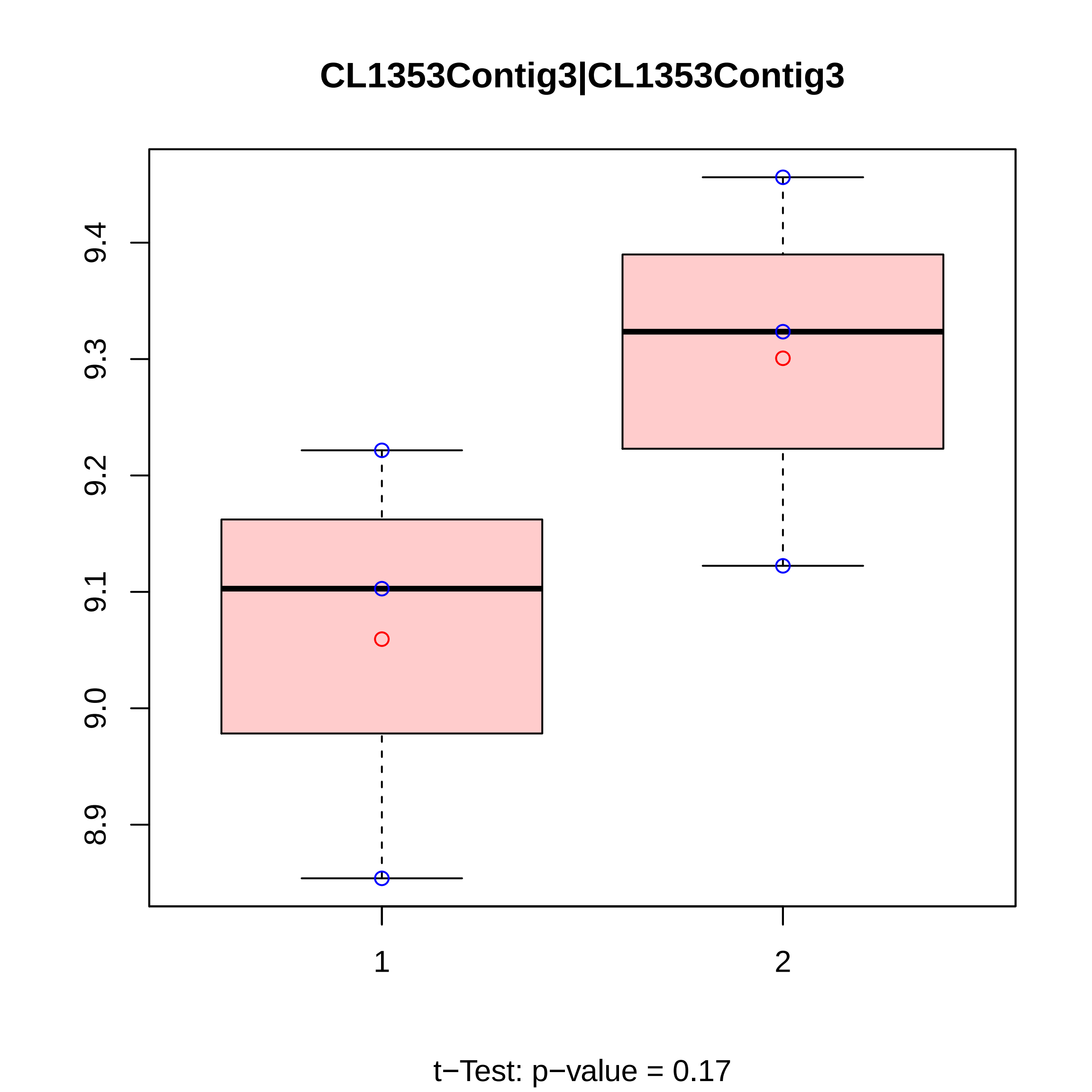
CL1353Contig3|CL1353Contig3
9.4
9.3
9.2
9.1
9.0
8.9
1
2
t−Test: p−value = 0.17

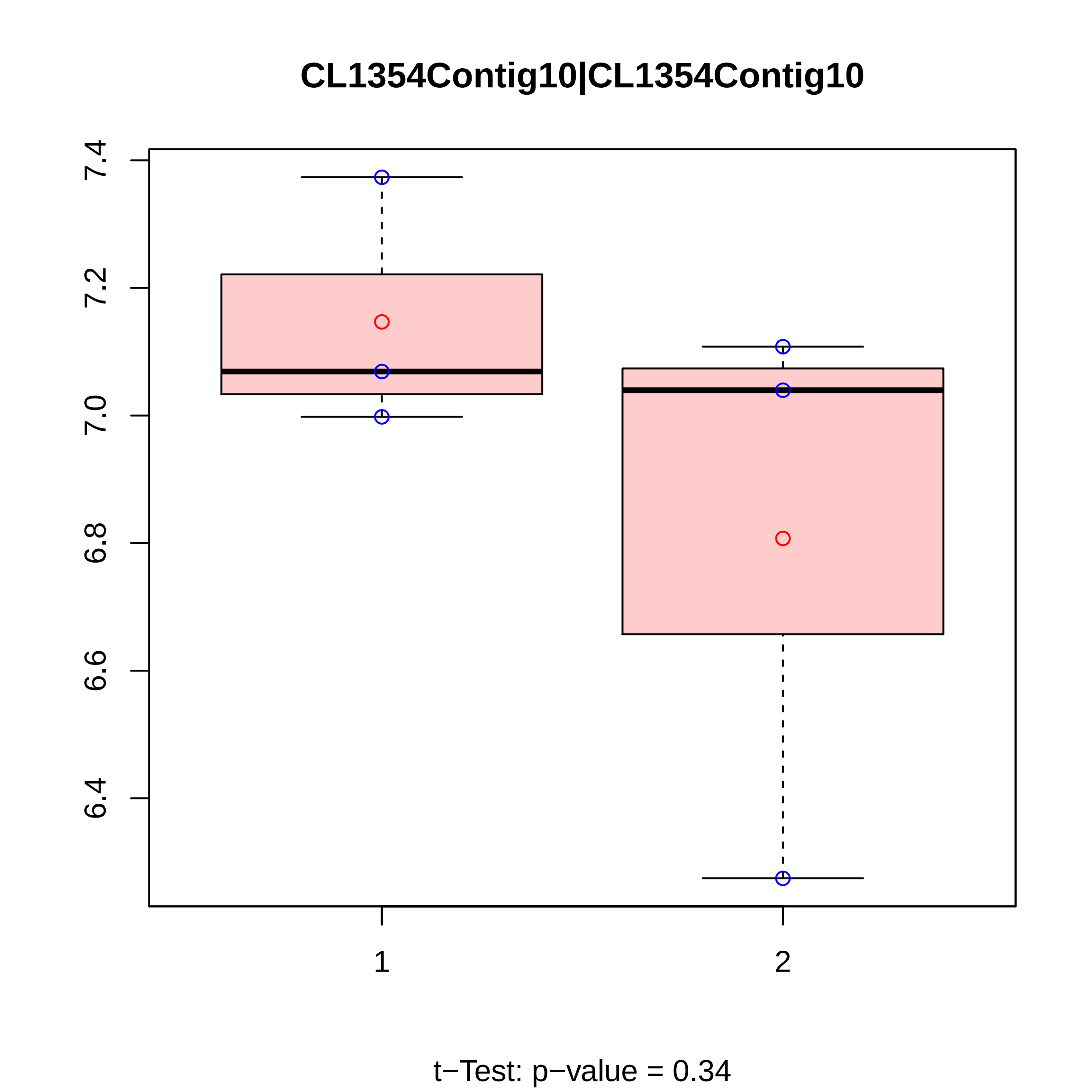

CL1354Contig10|CL1354Contig10
7.4
7.2
7.0
6.8
6.6
6.4
1
2
t−Test: p−value = 0.34

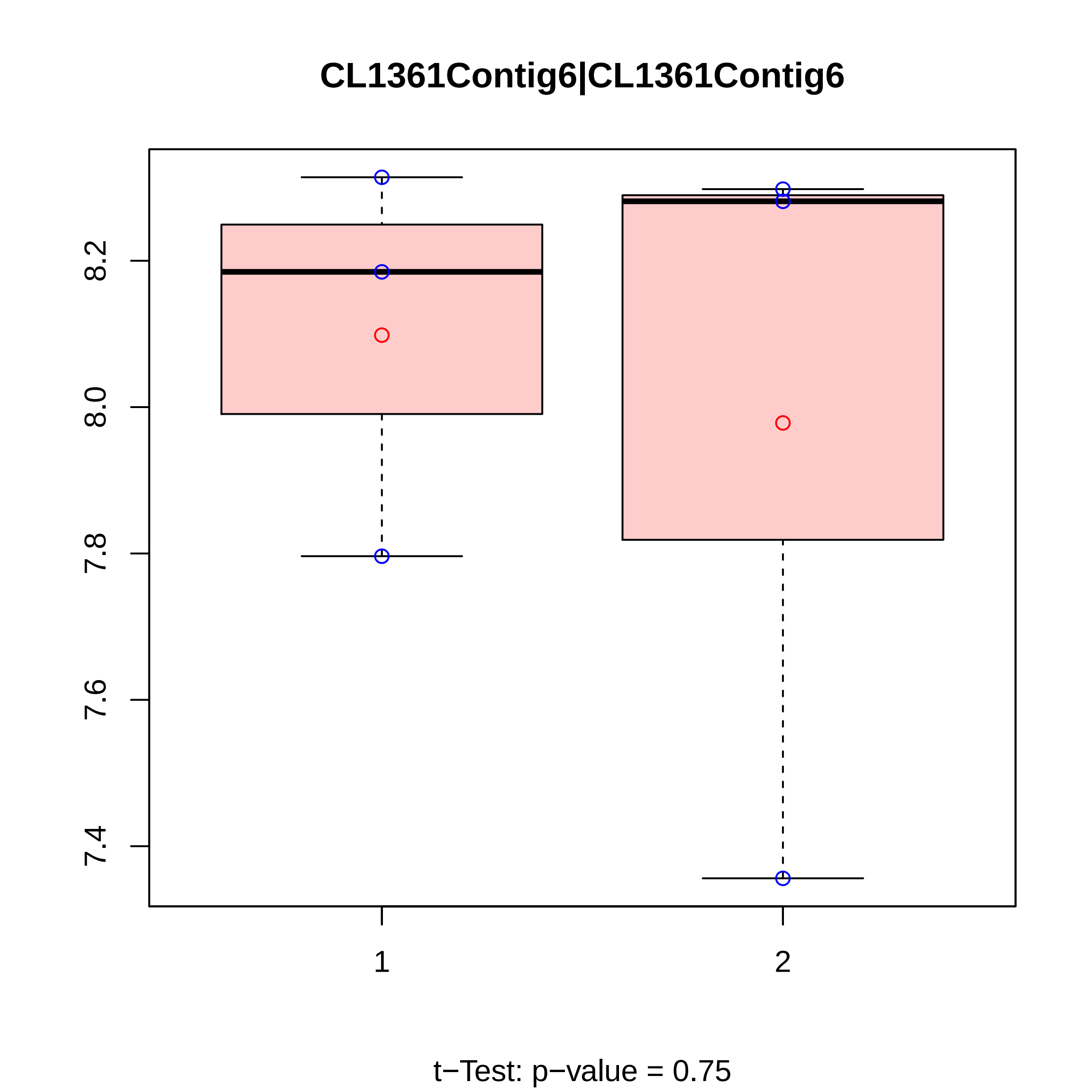
CL1361Contig6|CL1361Contig6
8.2
8.0
7.8
7.6
7.4
1
2
t−Test: p−value = 0.75

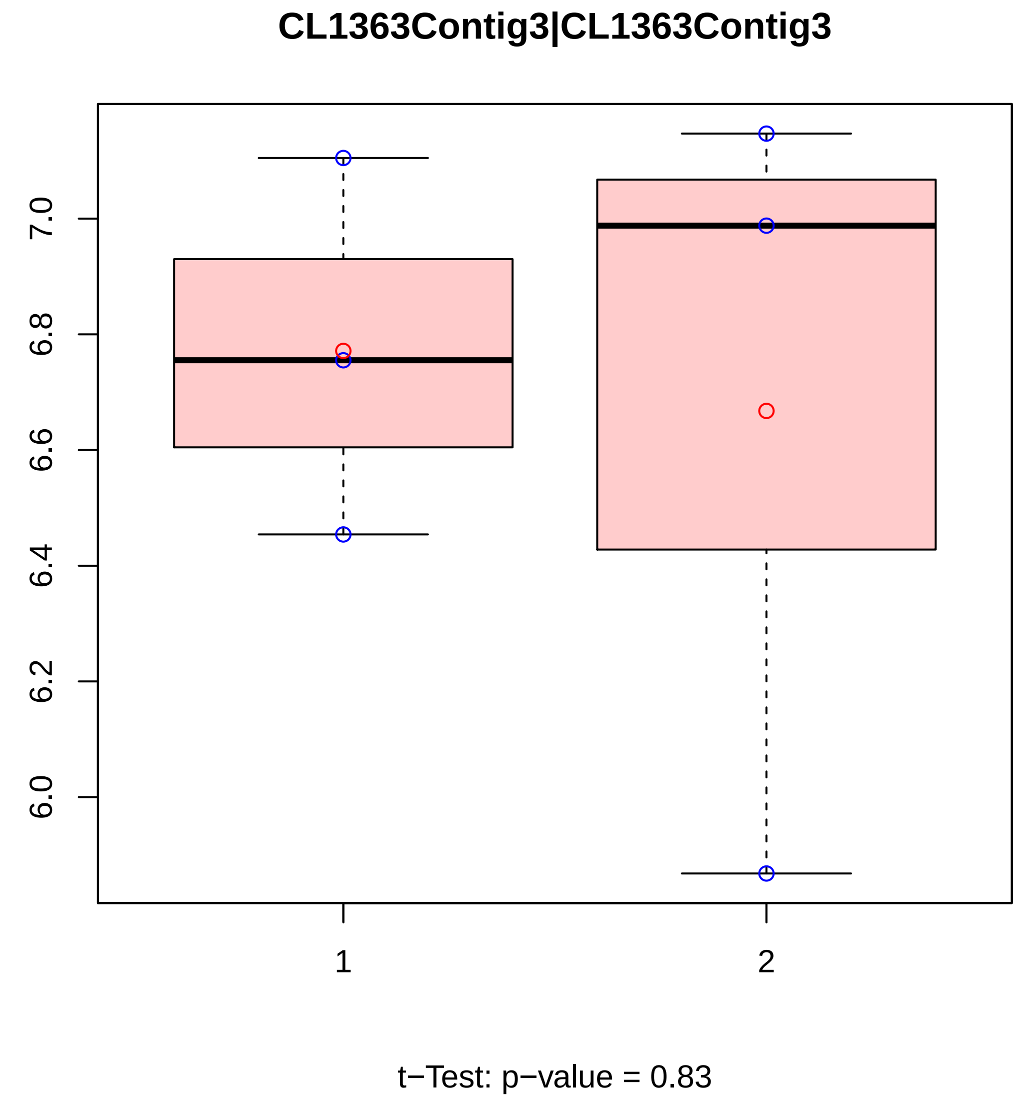

CL1363Contig3|CL1363Contig3
7.0
6.8
6.6
6.4
6.2
6.0
1
2
t−Test: p−value = 0.83

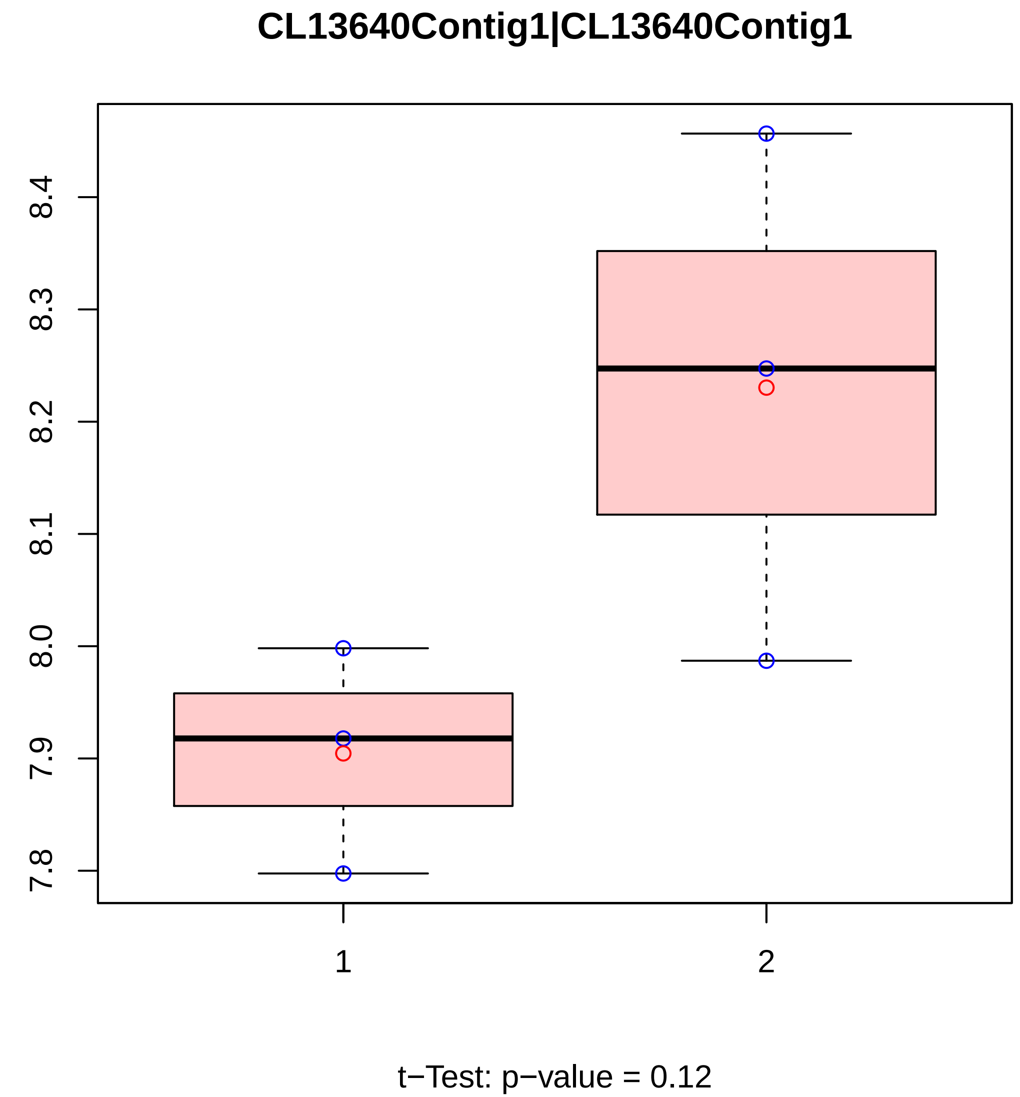
CL13640Contig1|CL13640Contig1
8.4
8.3
8.2
8.1
8.0
7.9
7.8
1
2
t−Test: p−value = 0.12

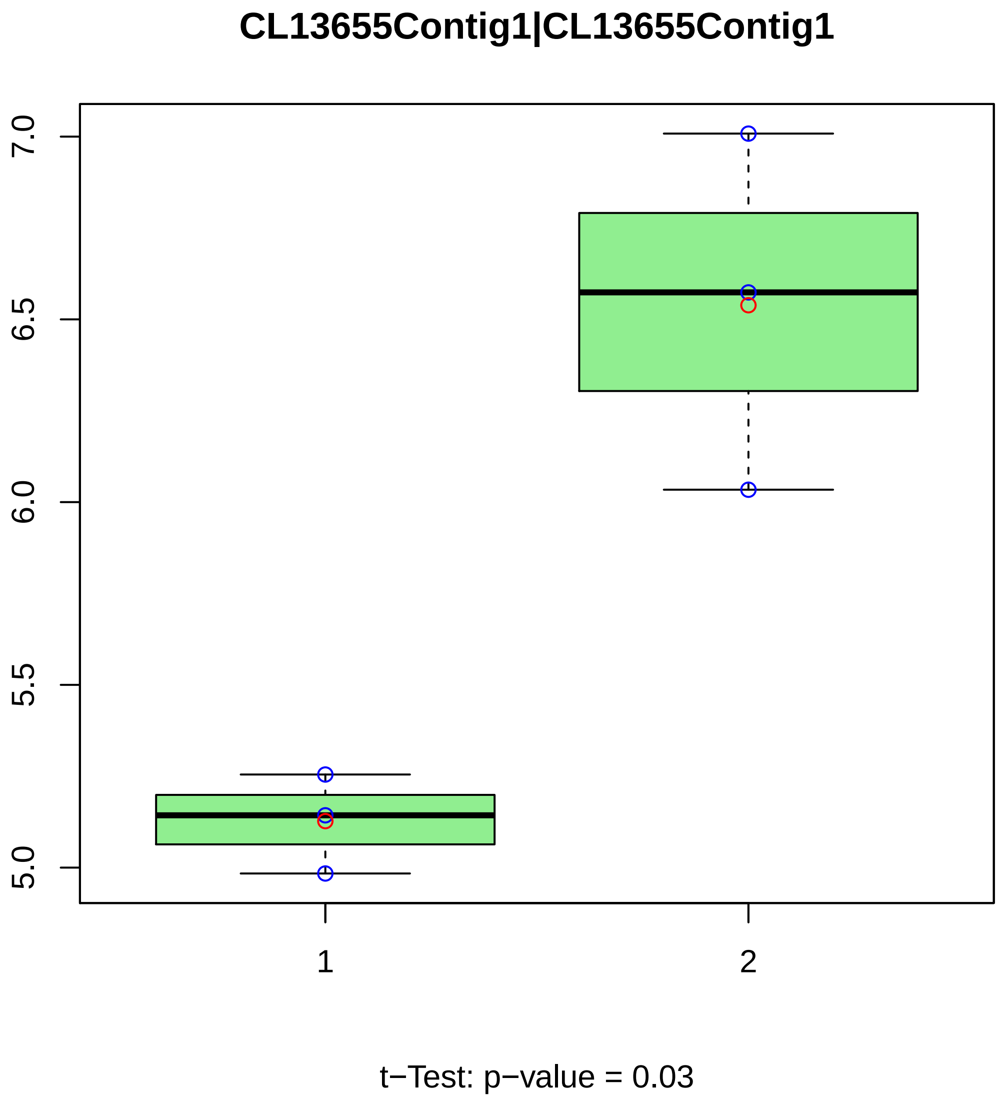
CL13655Contig1|CL13655Contig1
7.0
6.5
6.0
5.5
5.0
1
2
t−Test: p−value = 0.03

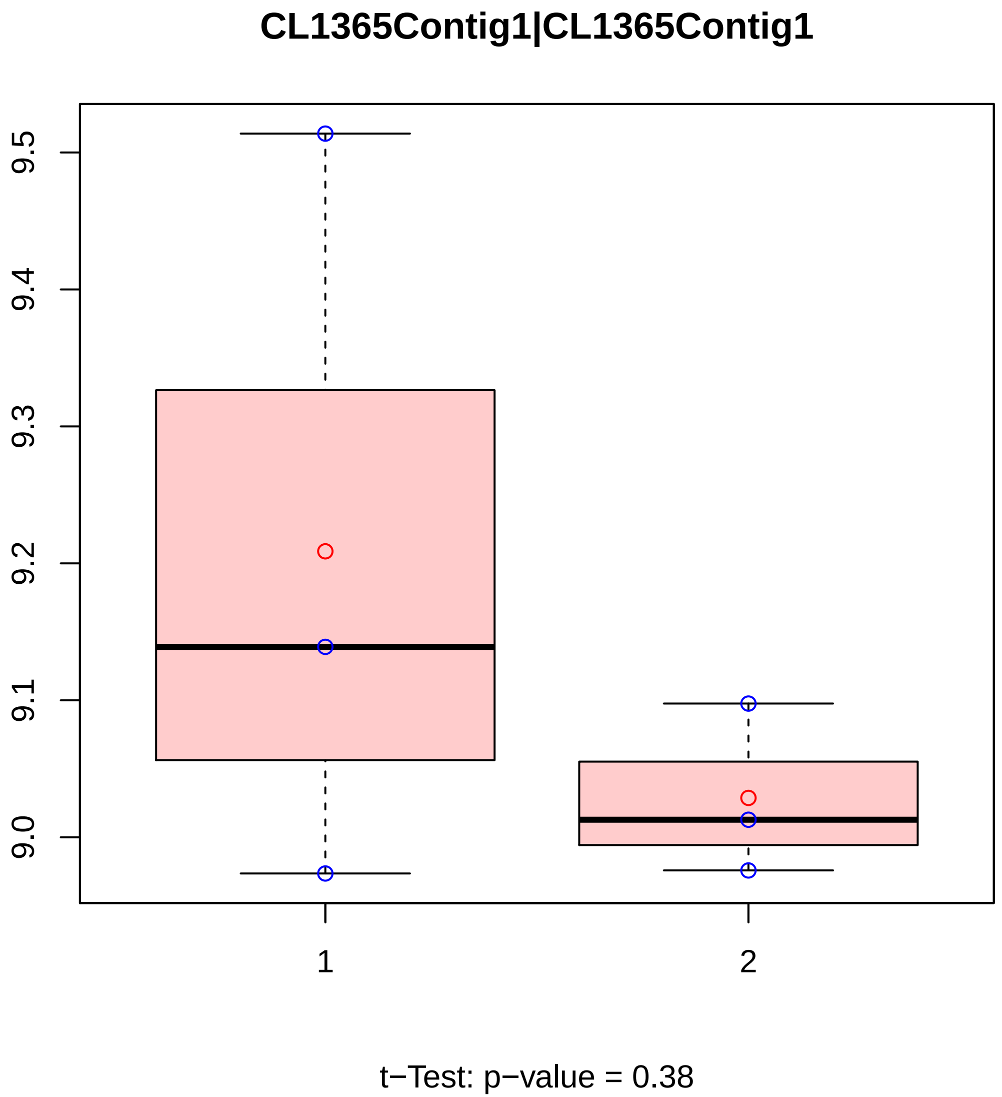
CL1365Contig1|CL1365Contig1
9.0
9.1
9.2
9.3
9.4
9.5
1
2
t−Test: p−value = 0.38

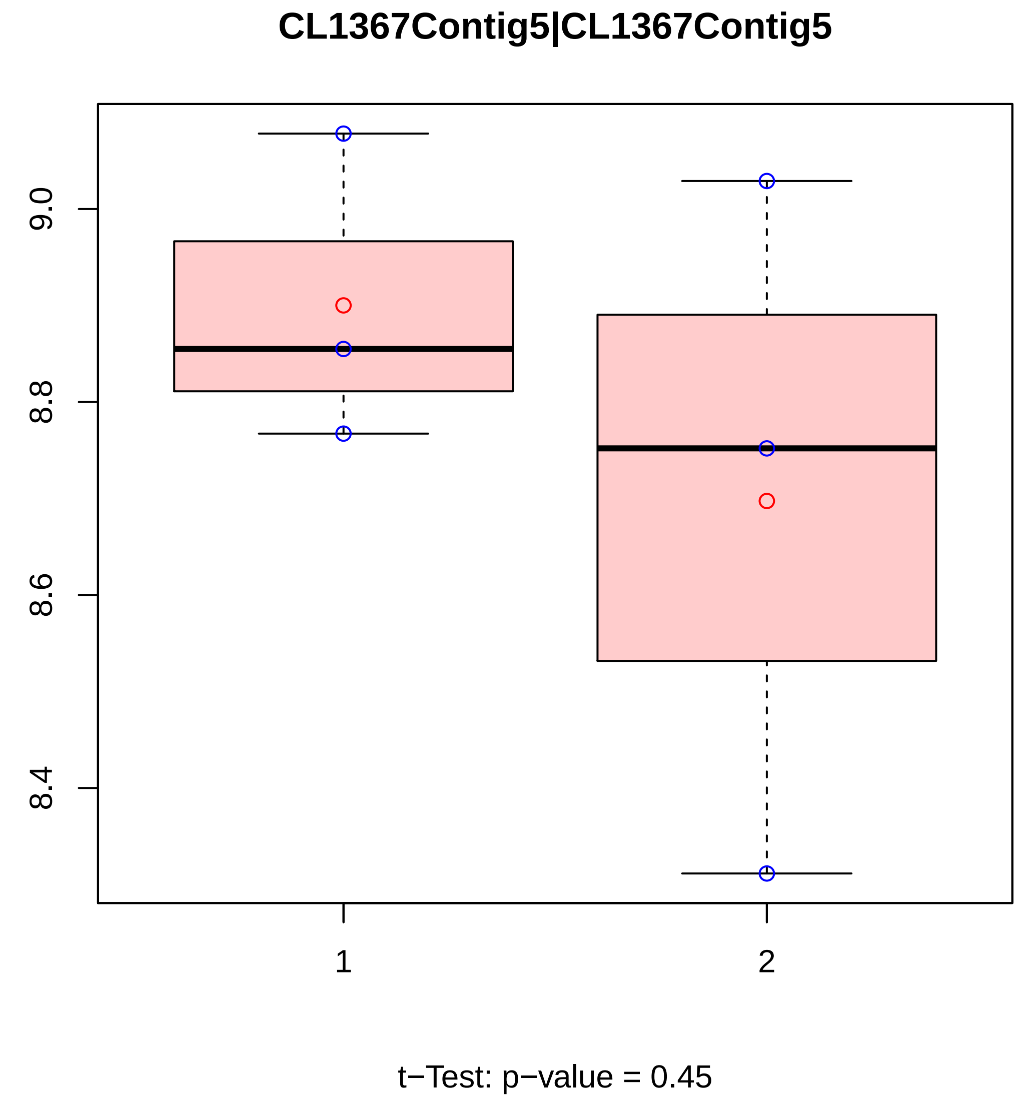
CL1367Contig5|CL1367Contig5
9.0
8.8
8.6
8.4
1
2
t−Test: p−value = 0.45

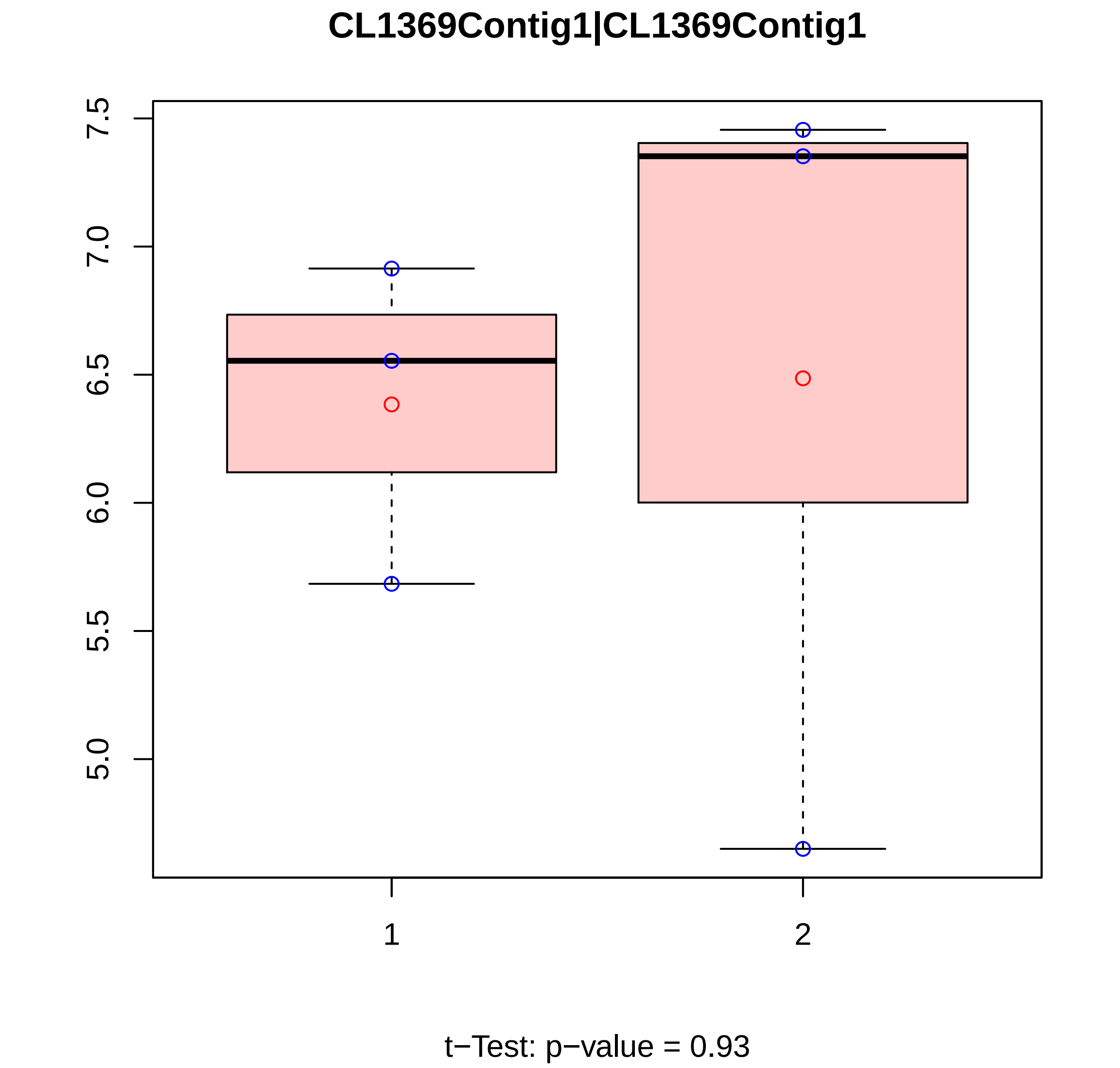
CL1369Contig1|CL1369Contig1
7.5
7.0
6.5
6.0
5.5
5.0
1
2
t−Test: p−value = 0.93

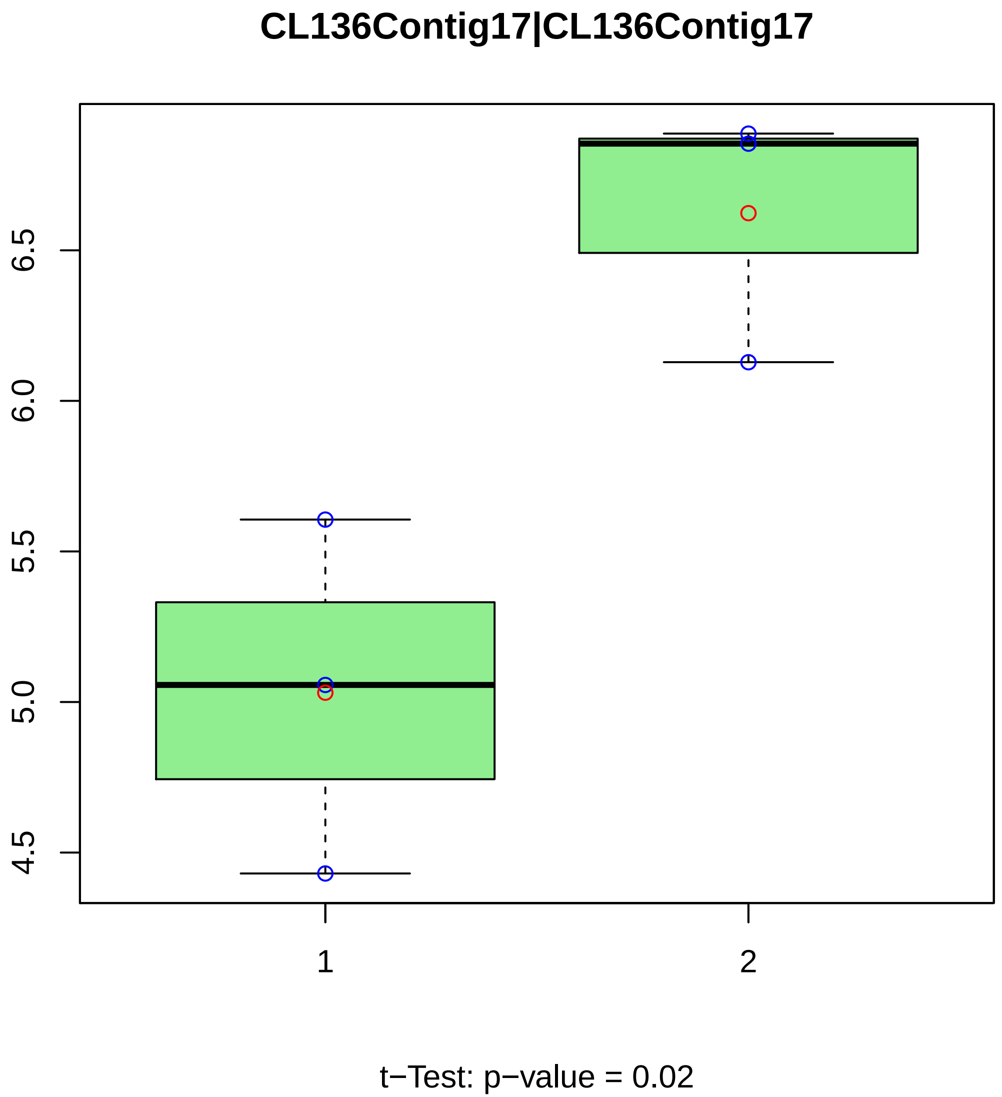
CL136Contig17|CL136Contig17
6.5
6.0
5.5
5.0
4.5
1
2
t−Test: p−value = 0.02

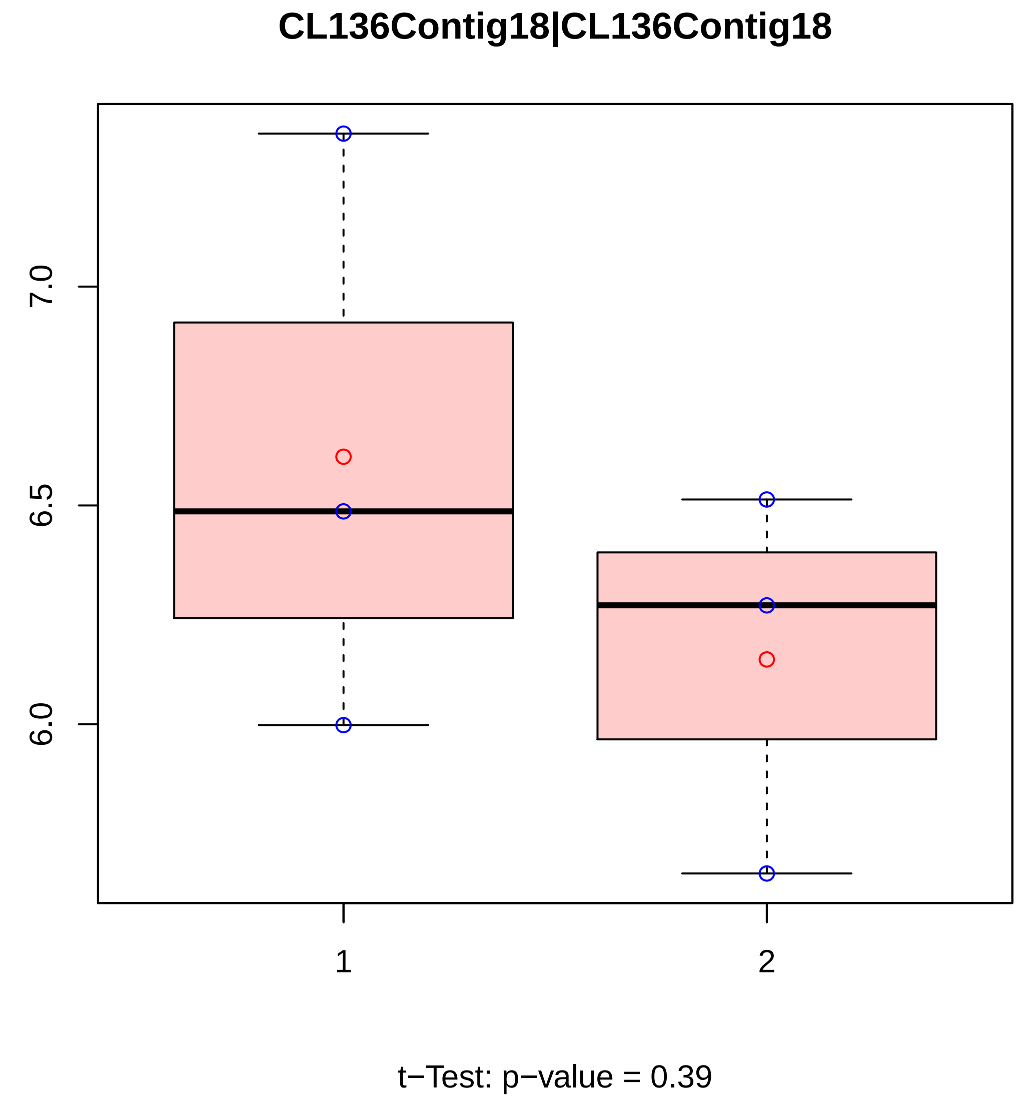
CL136Contig18|CL136Contig18
7.0
6.5
6.0
1
2
t−Test: p−value = 0.39

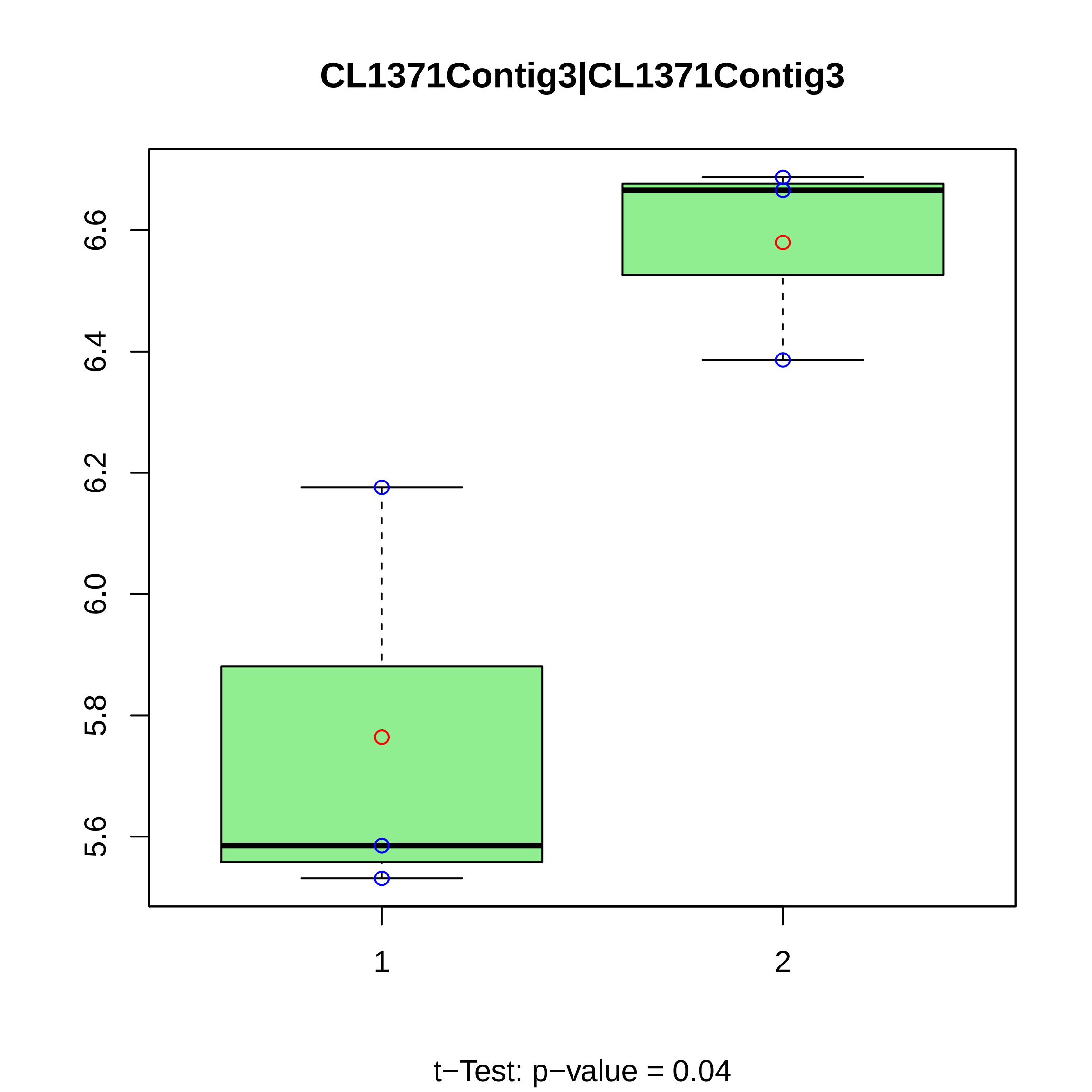
CL1371Contig3|CL1371Contig3
5.6
5.8
6.0
6.2
6.4
6.6
1
2
t−Test: p−value = 0.04

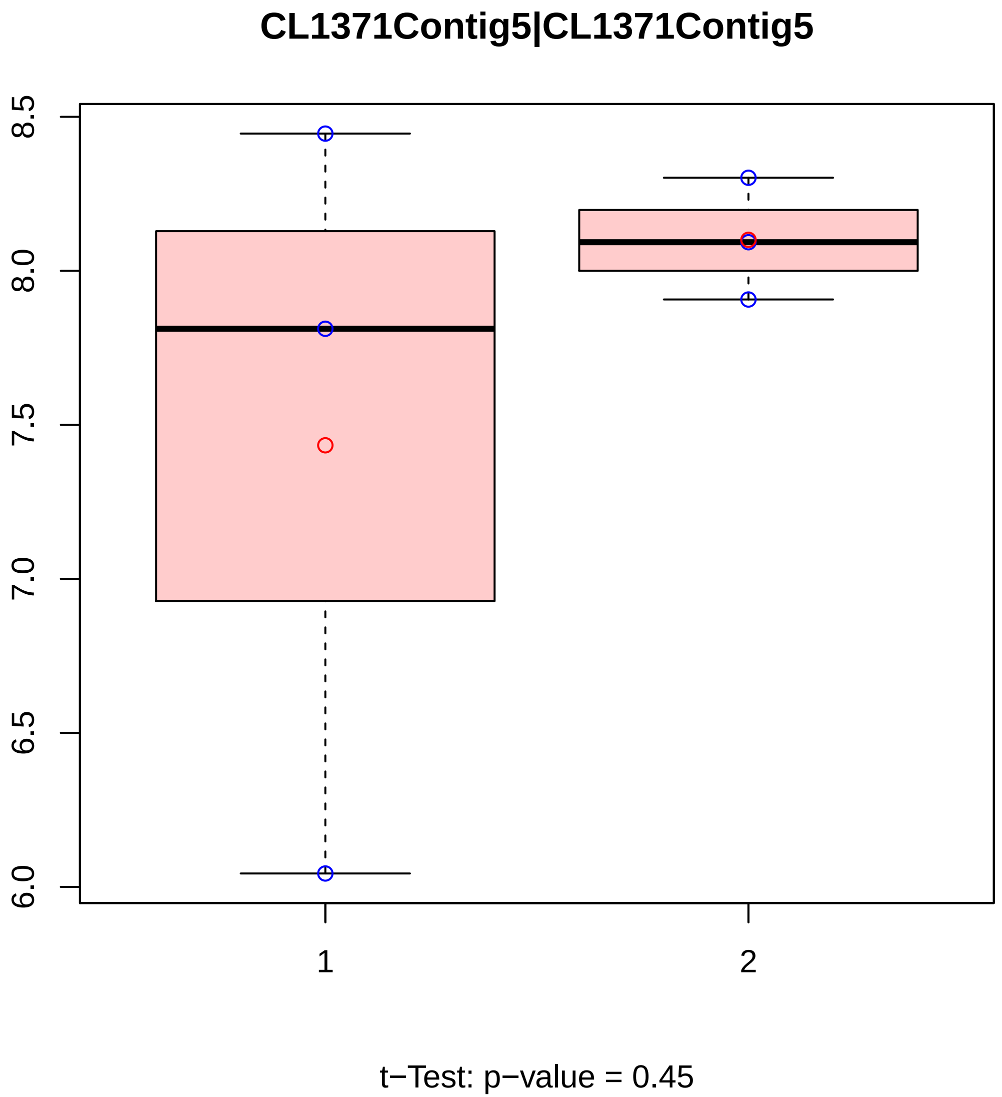
CL1371Contig5|CL1371Contig5
8.5
8.0
7.5
7.0
6.5
6.0
1
2
t−Test: p−value = 0.45

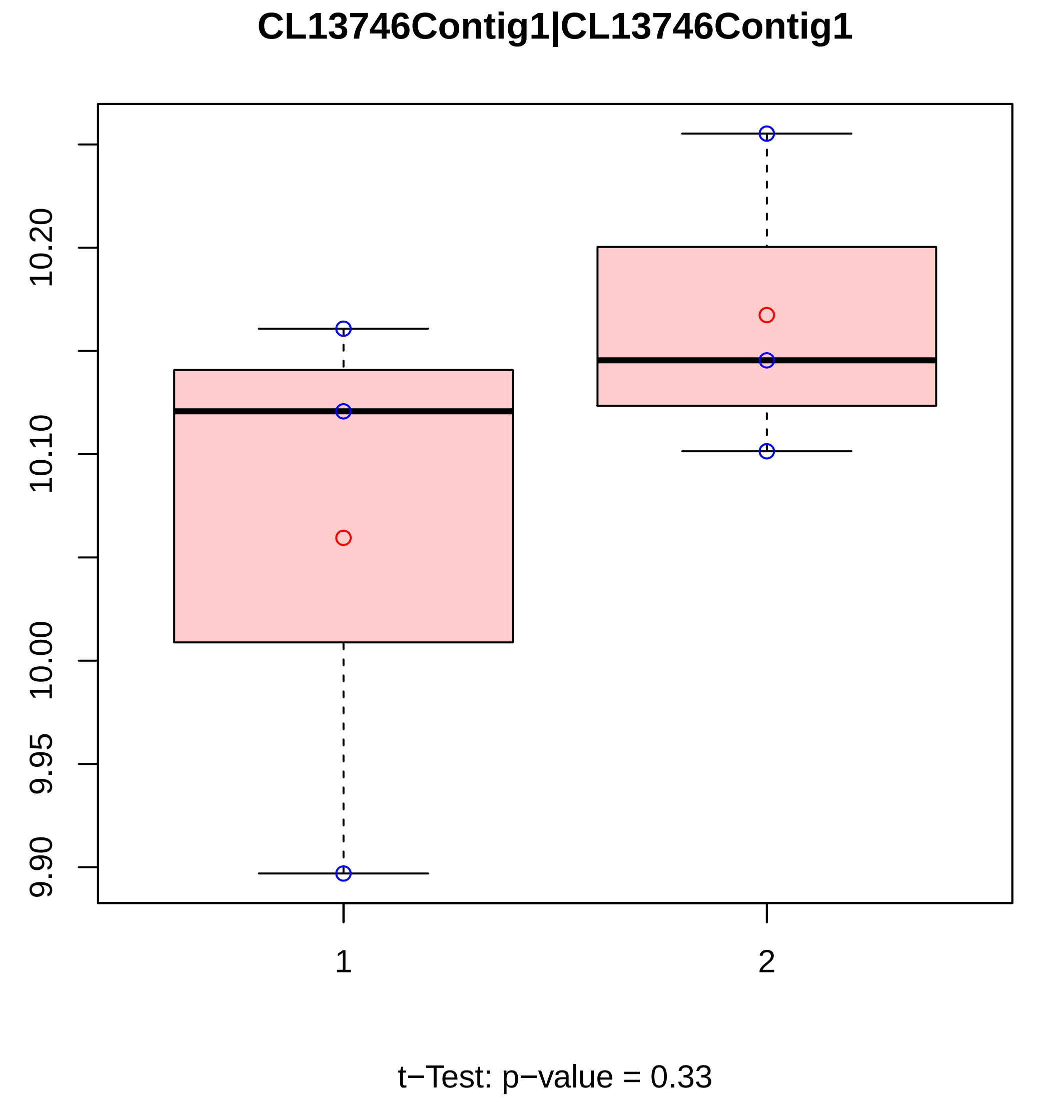
CL13746Contig1|CL13746Contig1
10.20
10.10
10.00
9.95
9.90
1
2
t−Test: p−value = 0.33

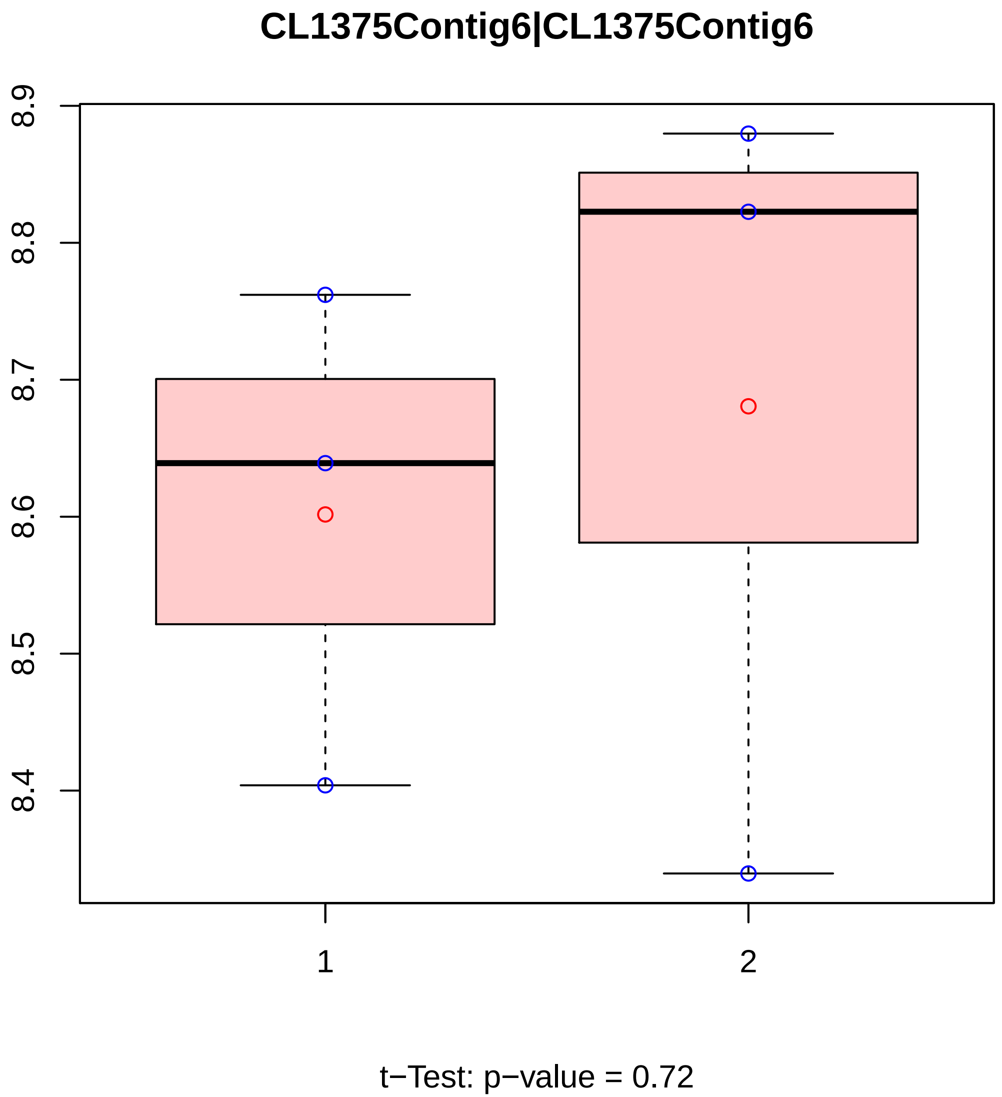
CL1375Contig6|CL1375Contig6
8.4
8.5
8.6
8.7
8.8
8.9
1
2
t−Test: p−value = 0.72

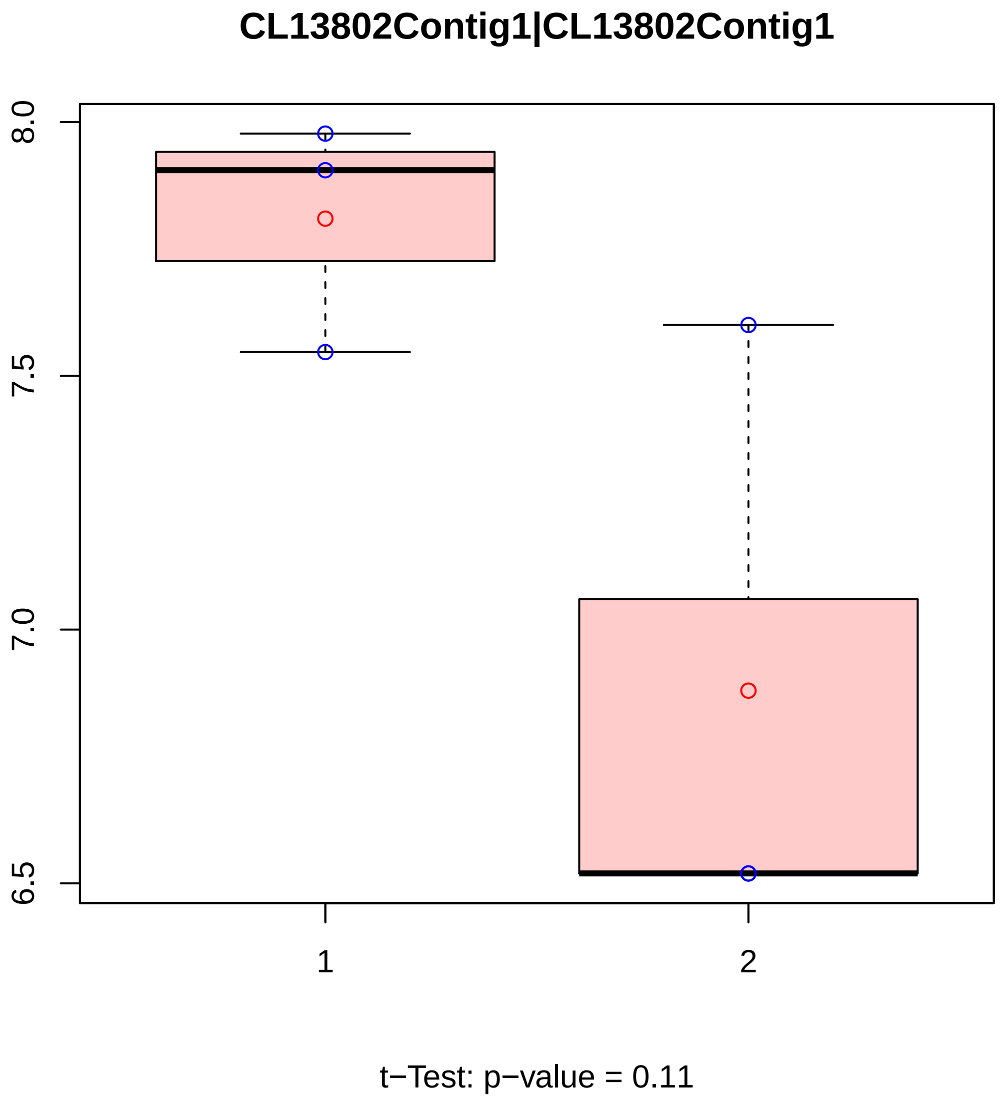
CL13802Contig1|CL13802Contig1
8.0
7.5
7.0
6.5
1
2
t−Test: p−value = 0.11

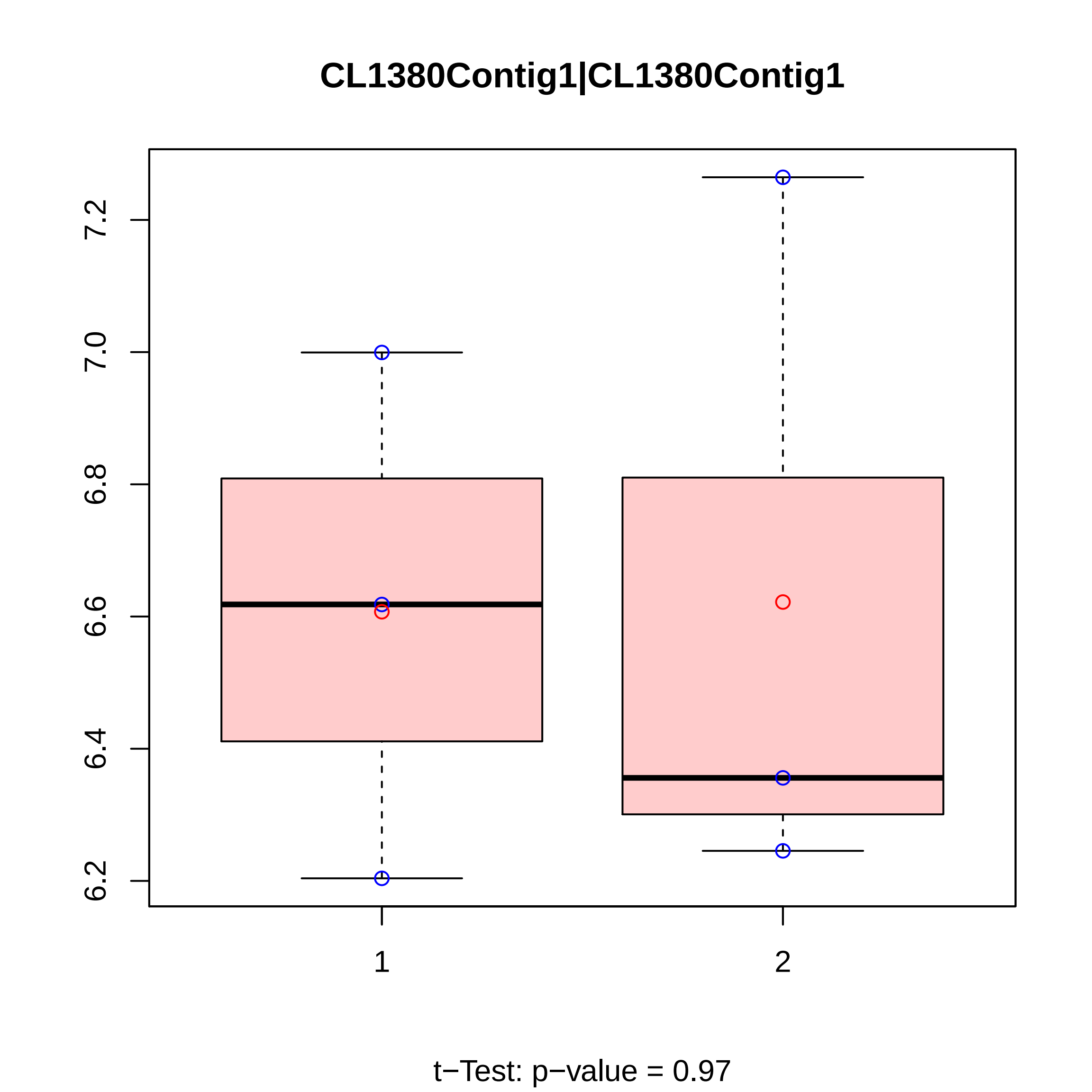

CL1380Contig1|CL1380Contig1
7.2
7.0
6.8
6.6
6.4
6.2
1
2
t−Test: p−value = 0.97

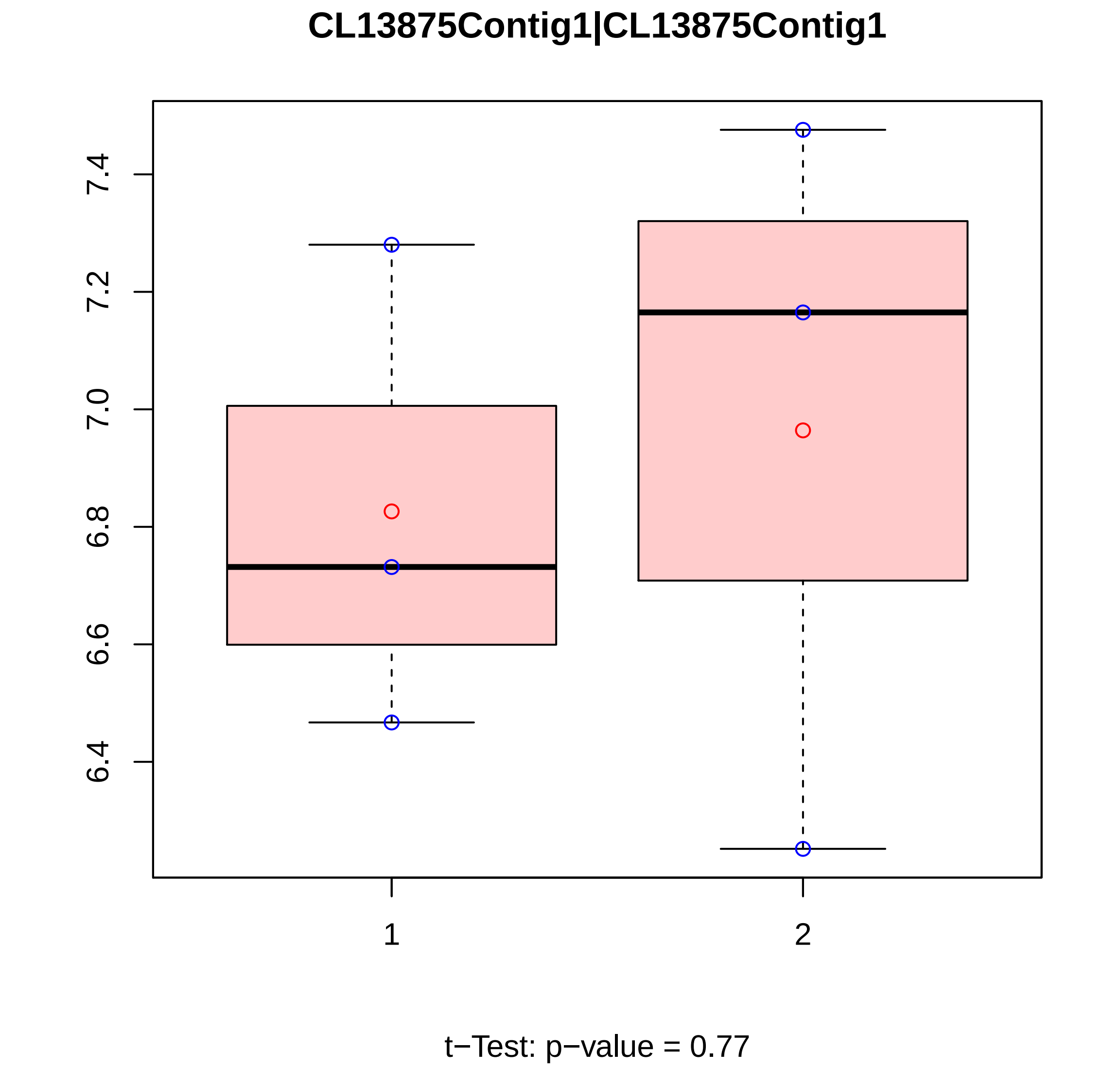
CL13875Contig1|CL13875Contig1
7.4
7.2
7.0
6.8
6.6
6.4
1
2
t−Test: p−value = 0.77

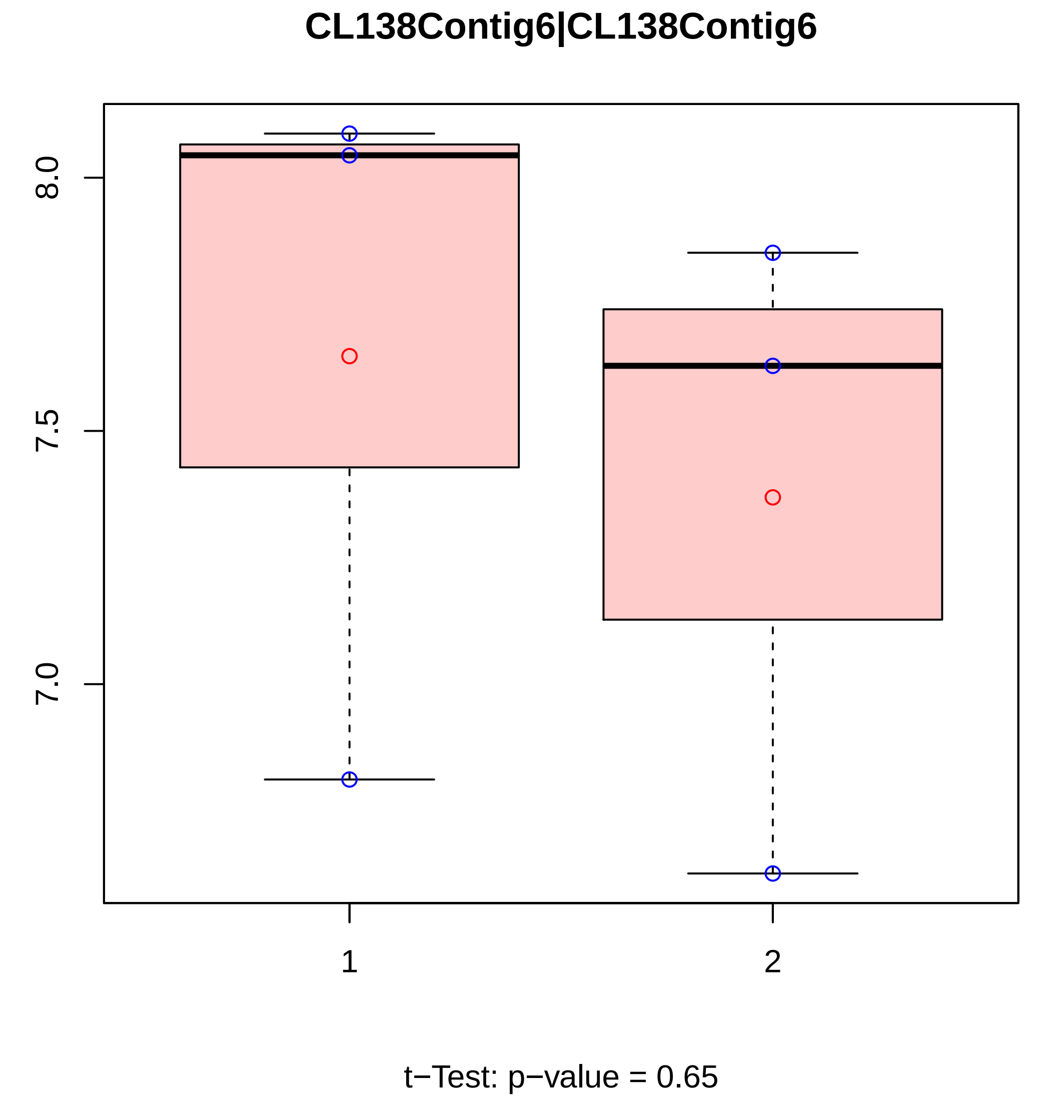
CL138Contig6|CL138Contig6
8.0
7.5
7.0
1
2
t−Test: p−value = 0.65

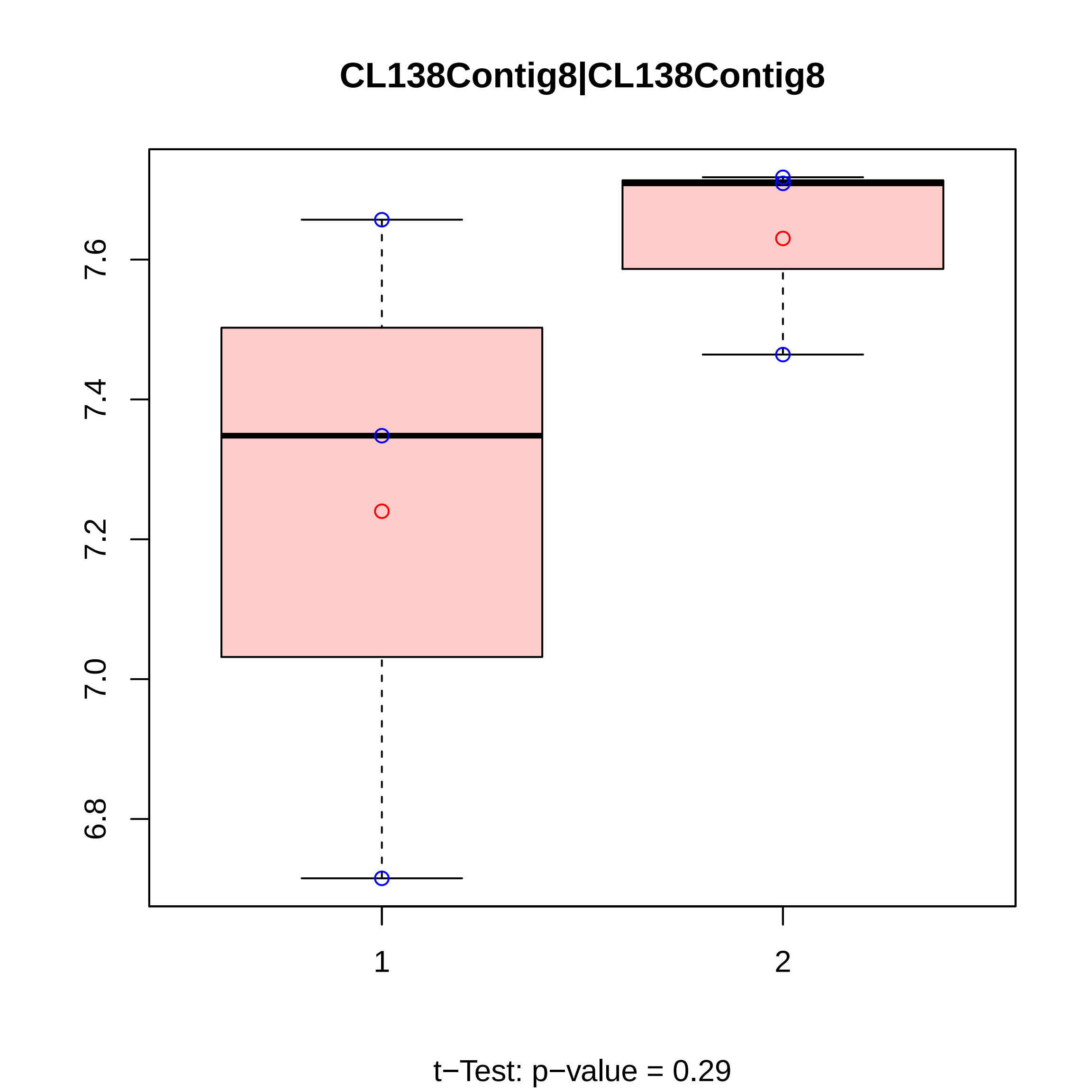
CL138Contig8|CL138Contig8
7.6
7.4
7.2
7.0
6.8
1
2
t−Test: p−value = 0.29

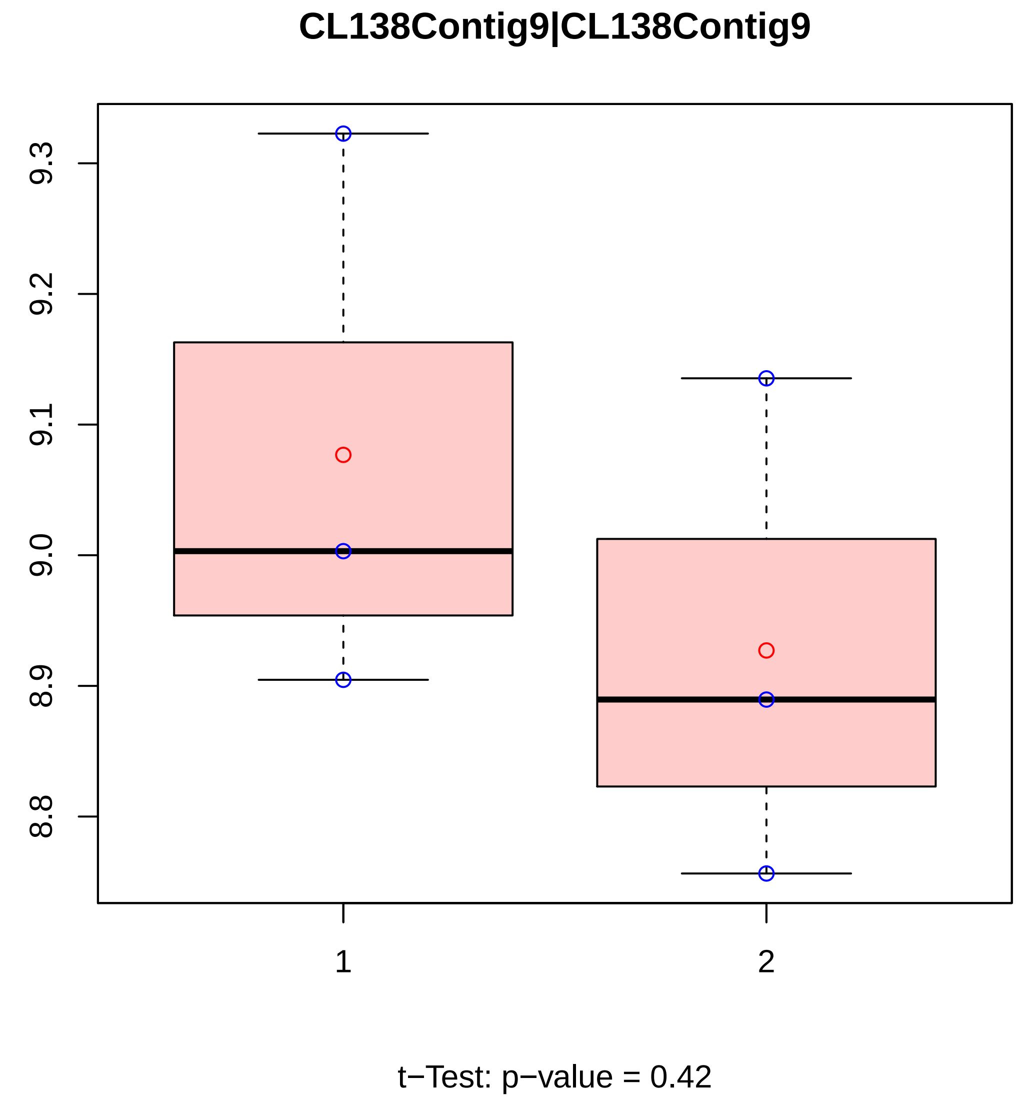
CL138Contig9|CL138Contig9
9.3
9.2
9.1
9.0
8.9
8.8
1
2
t−Test: p−value = 0.42

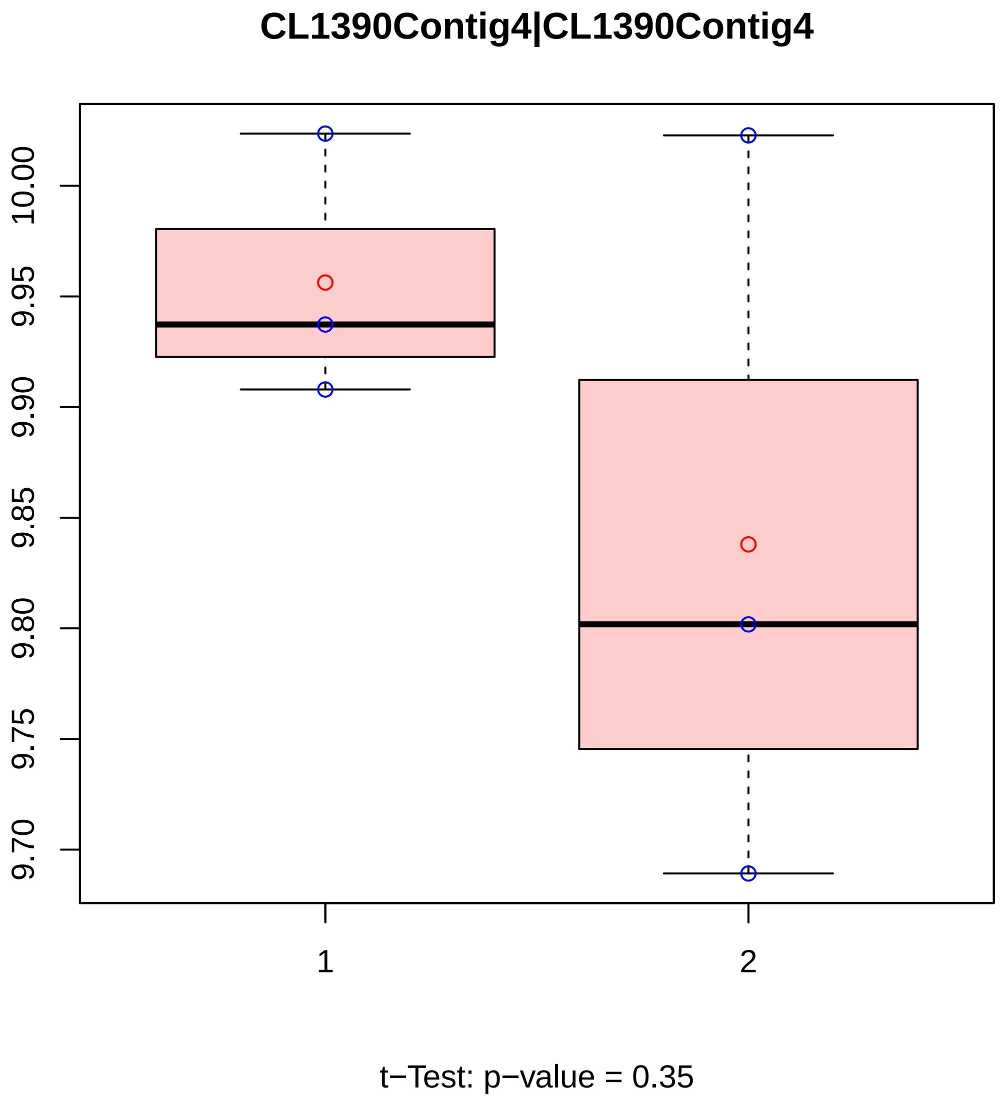
CL1390Contig4|CL1390Contig4
10.00
9.95
9.90
9.85
9.80
9.75
9.70
1
2
t−Test: p−value = 0.35

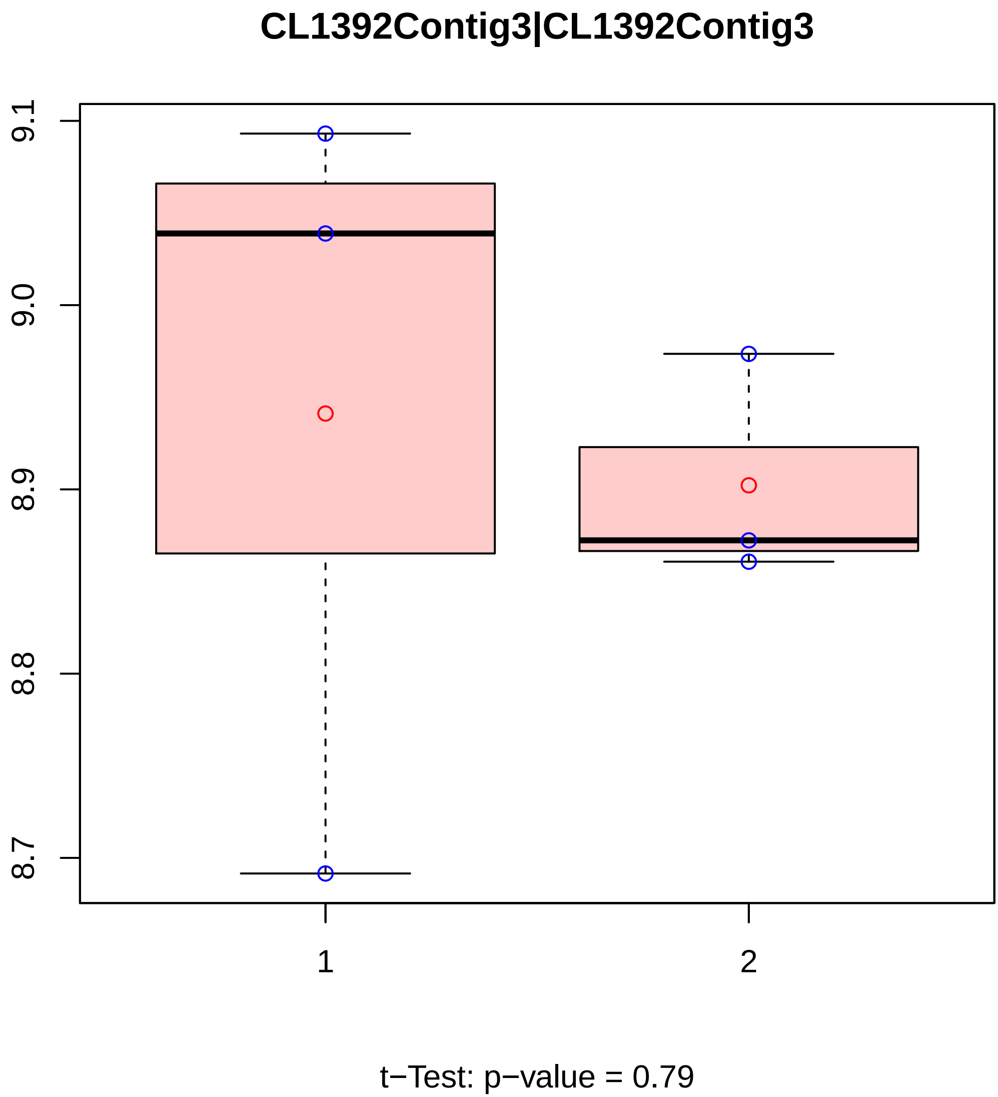

CL1392Contig3|CL1392Contig3
9.1
9.0
8.9
8.8
8.7
1
2
t−Test: p−value = 0.79

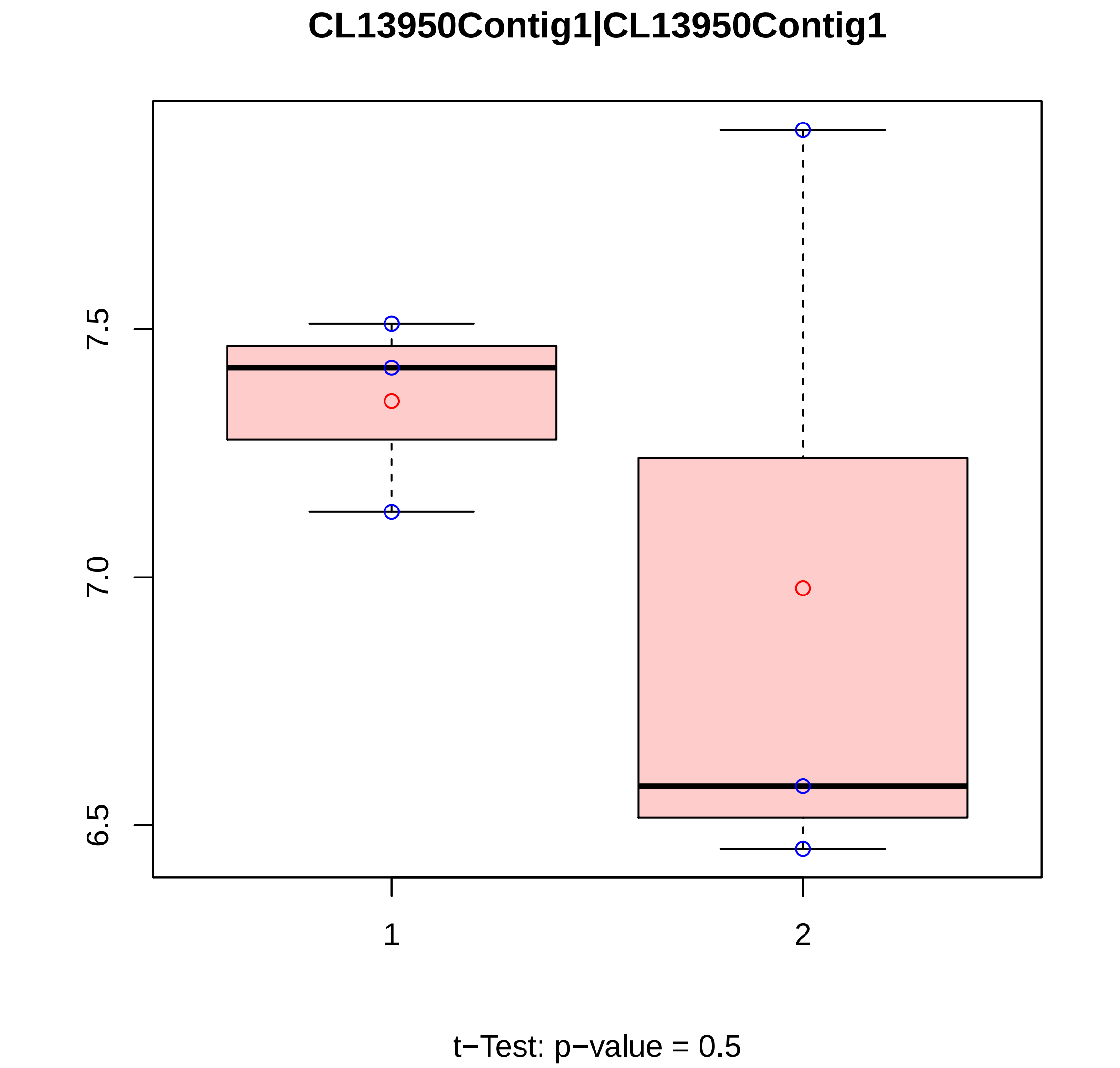

CL13950Contig1|CL13950Contig1
7.5
7.0
6.5
1
2
t−Test: p−value = 0.5

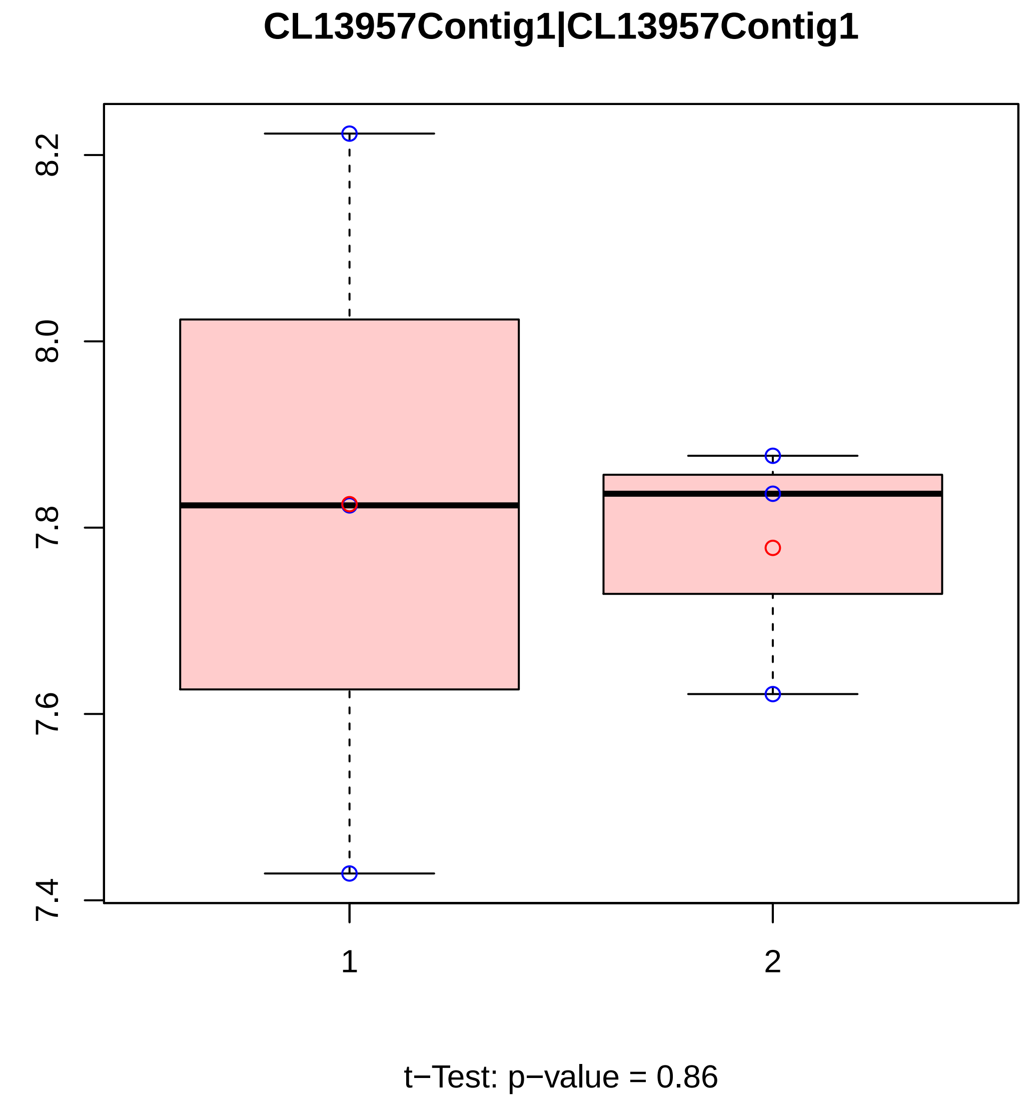
CL13957Contig1|CL13957Contig1
8.2
8.0
7.8
7.6
7.4
1
2
t−Test: p−value = 0.86

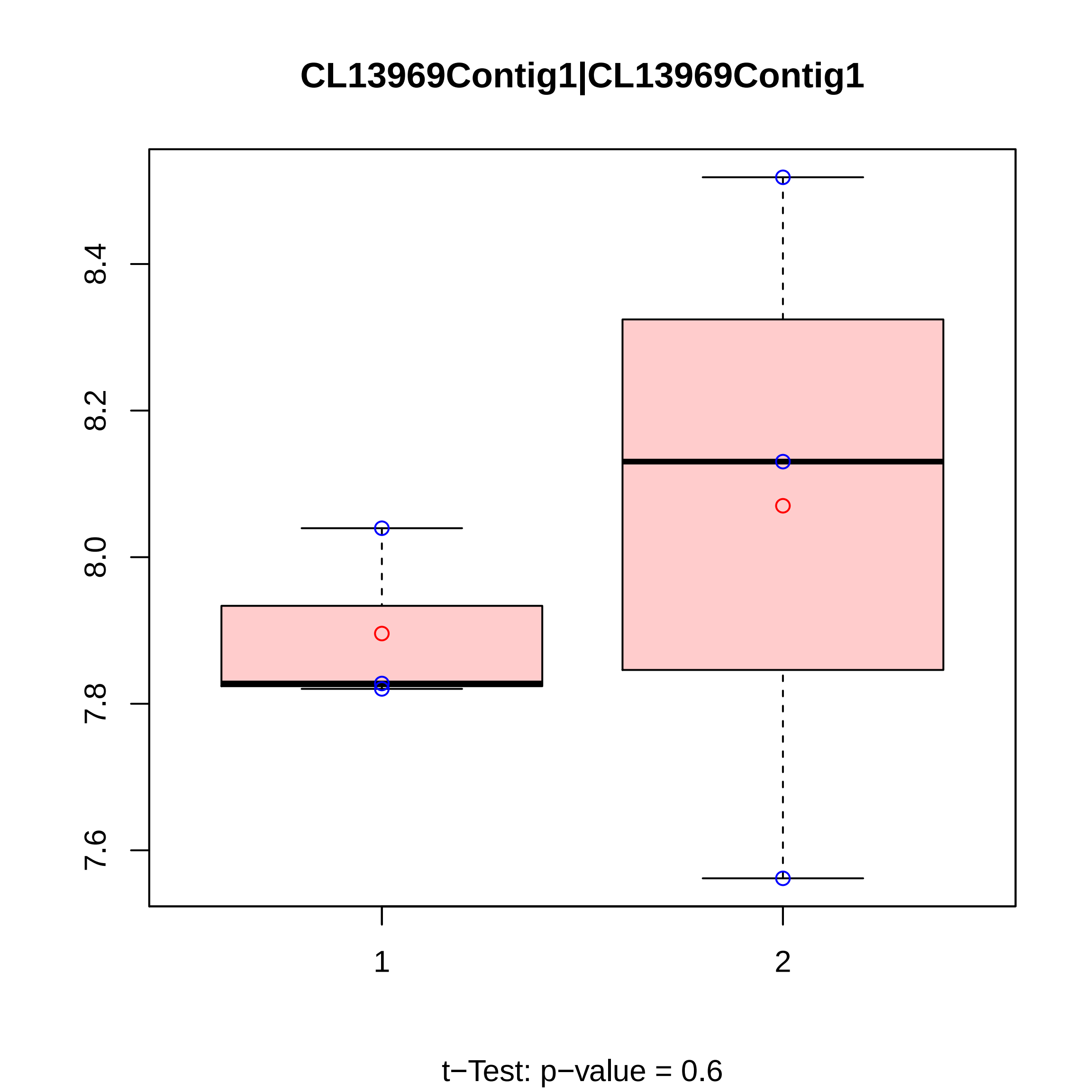
CL13969Contig1|CL13969Contig1
8.4
8.2
8.0
7.8
7.6
1
2
t−Test: p−value = 0.6

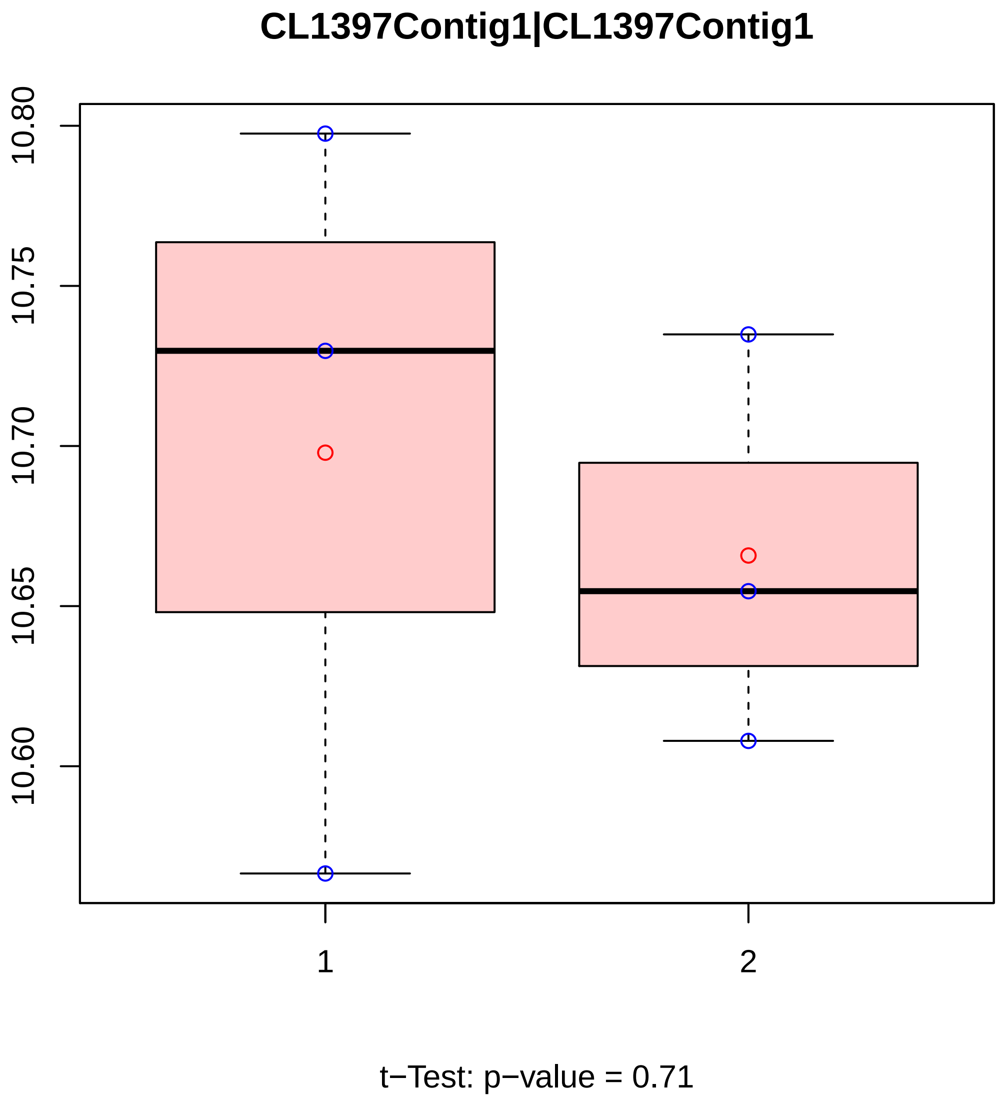
CL1397Contig1|CL1397Contig1
10.80
10.75
10.70
10.65
10.60
1
2
t−Test: p−value = 0.71

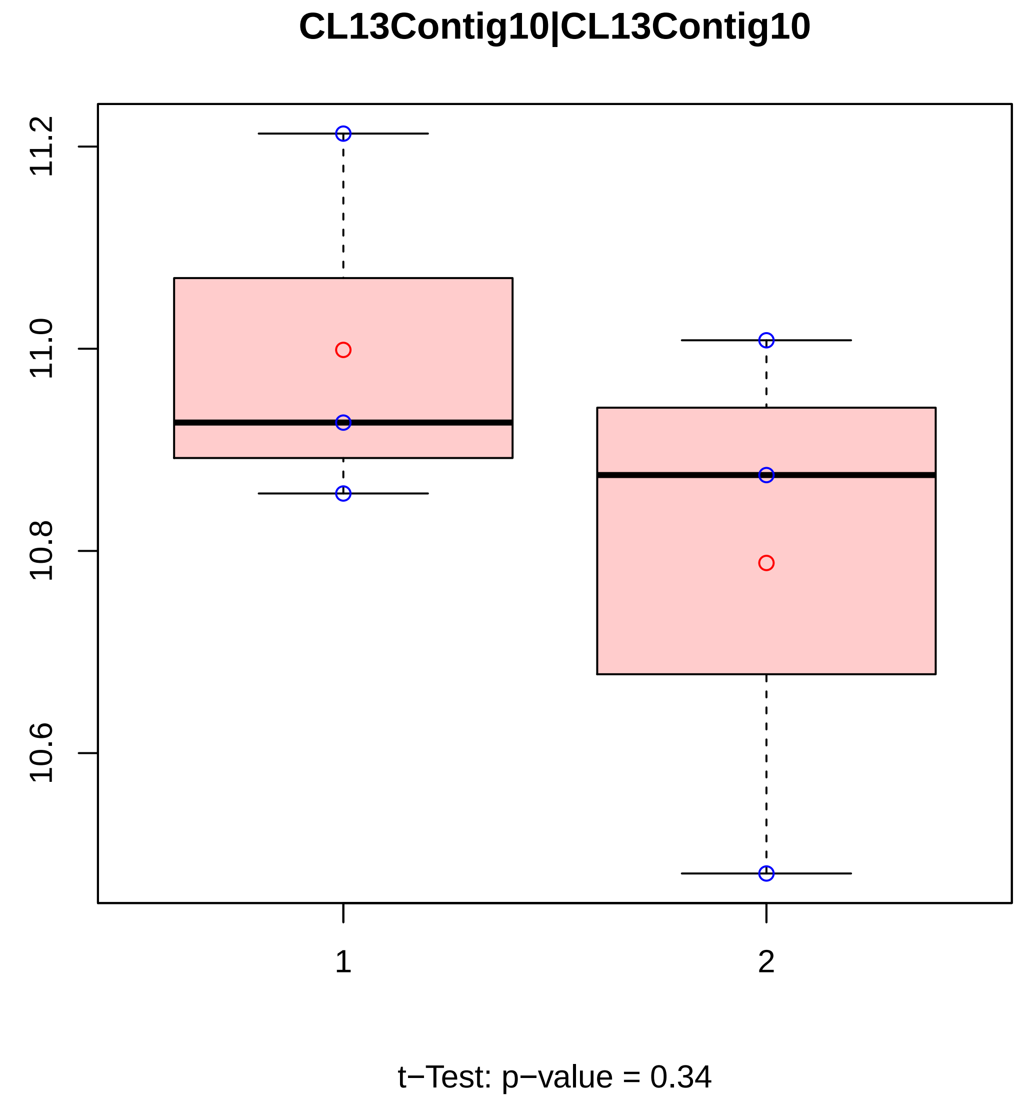
CL13Contig10|CL13Contig10
11.2
11.0
10.8
10.6
1
2
t−Test: p−value = 0.34

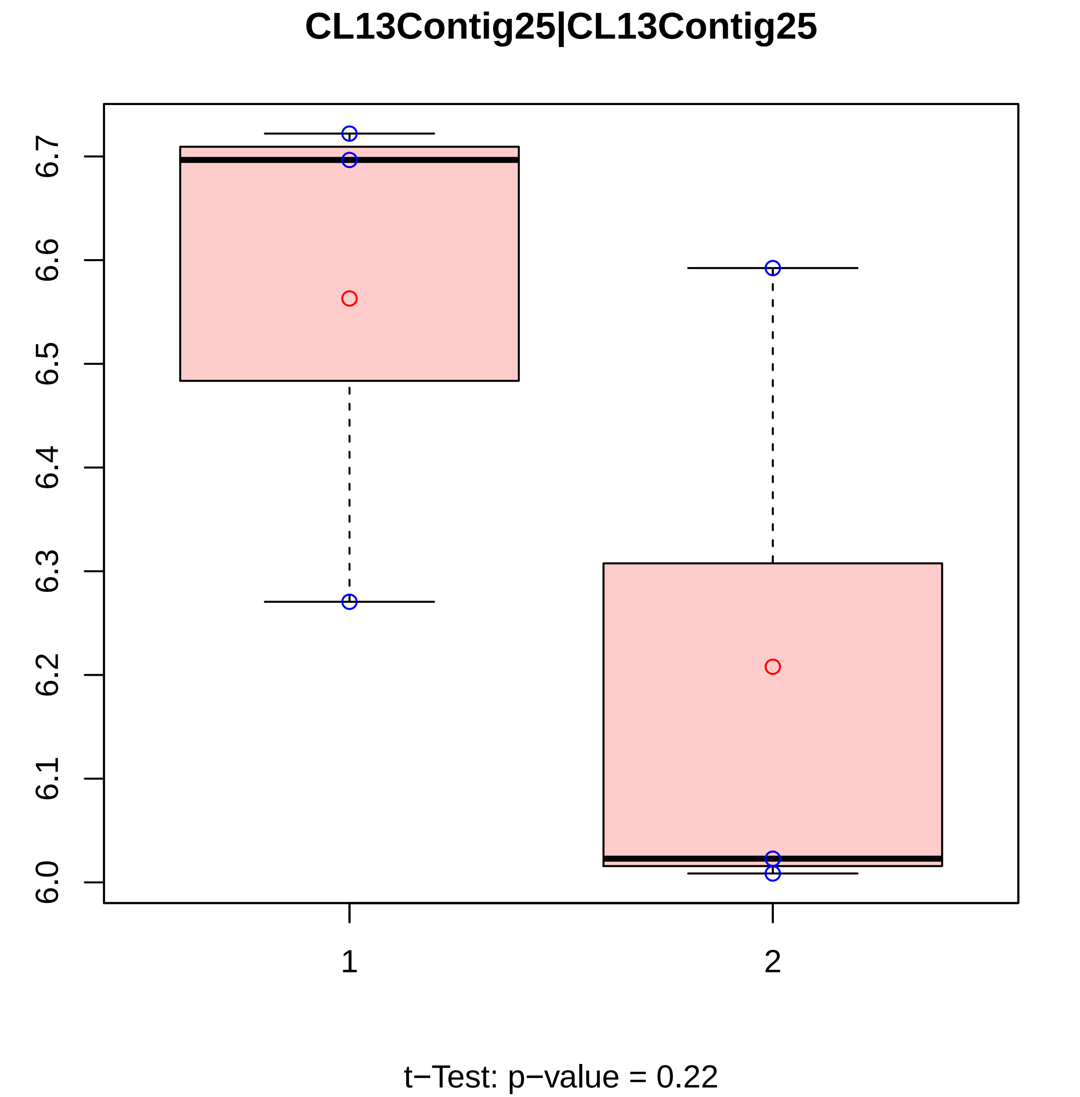
CL13Contig25|CL13Contig25
6.0
6.1
6.2
6.3
6.4
6.5
6.6
6.7
1
2
t−Test: p−value = 0.22

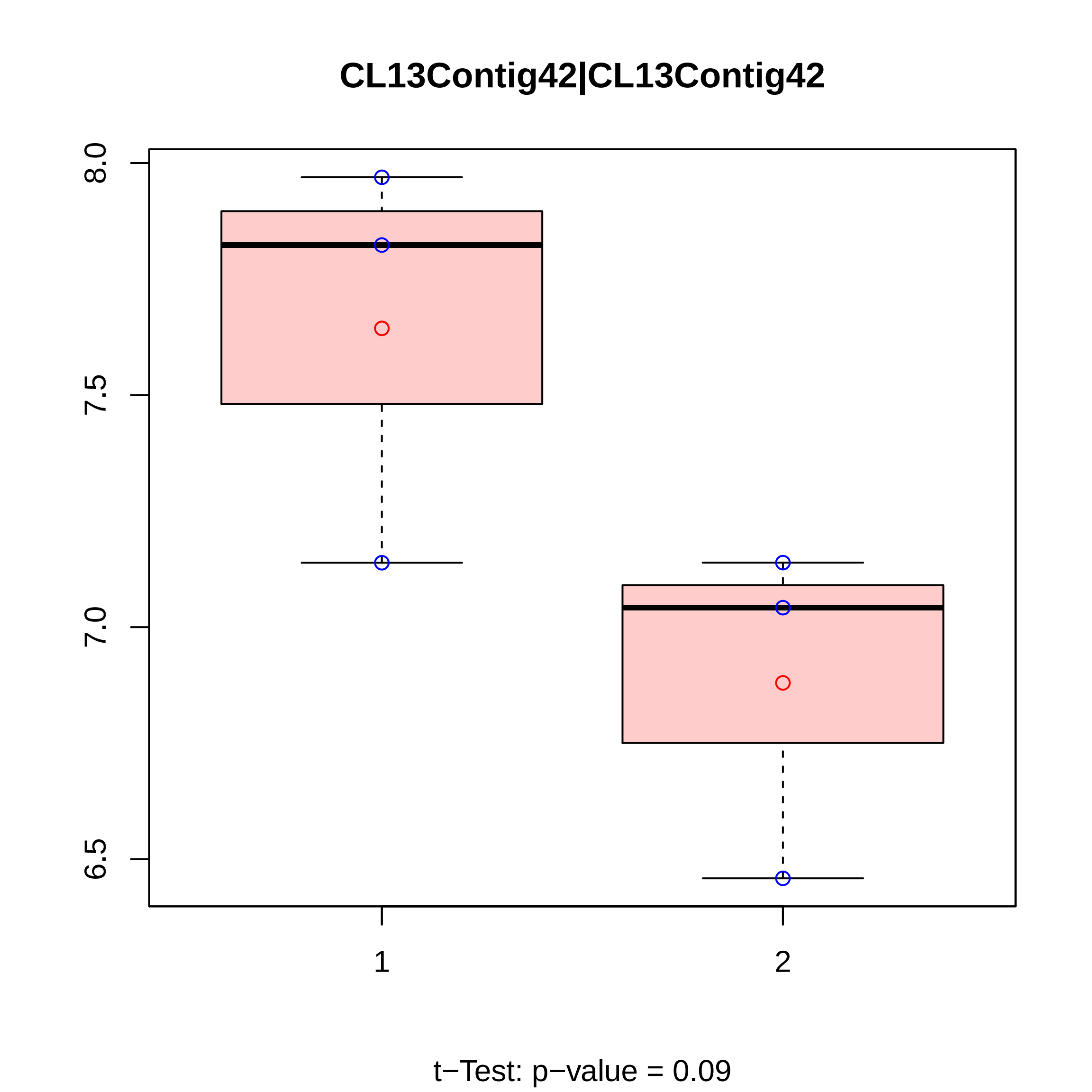
CL13Contig42|CL13Contig42
8.0
7.5
7.0
6.5
1
2
t−Test: p−value = 0.09

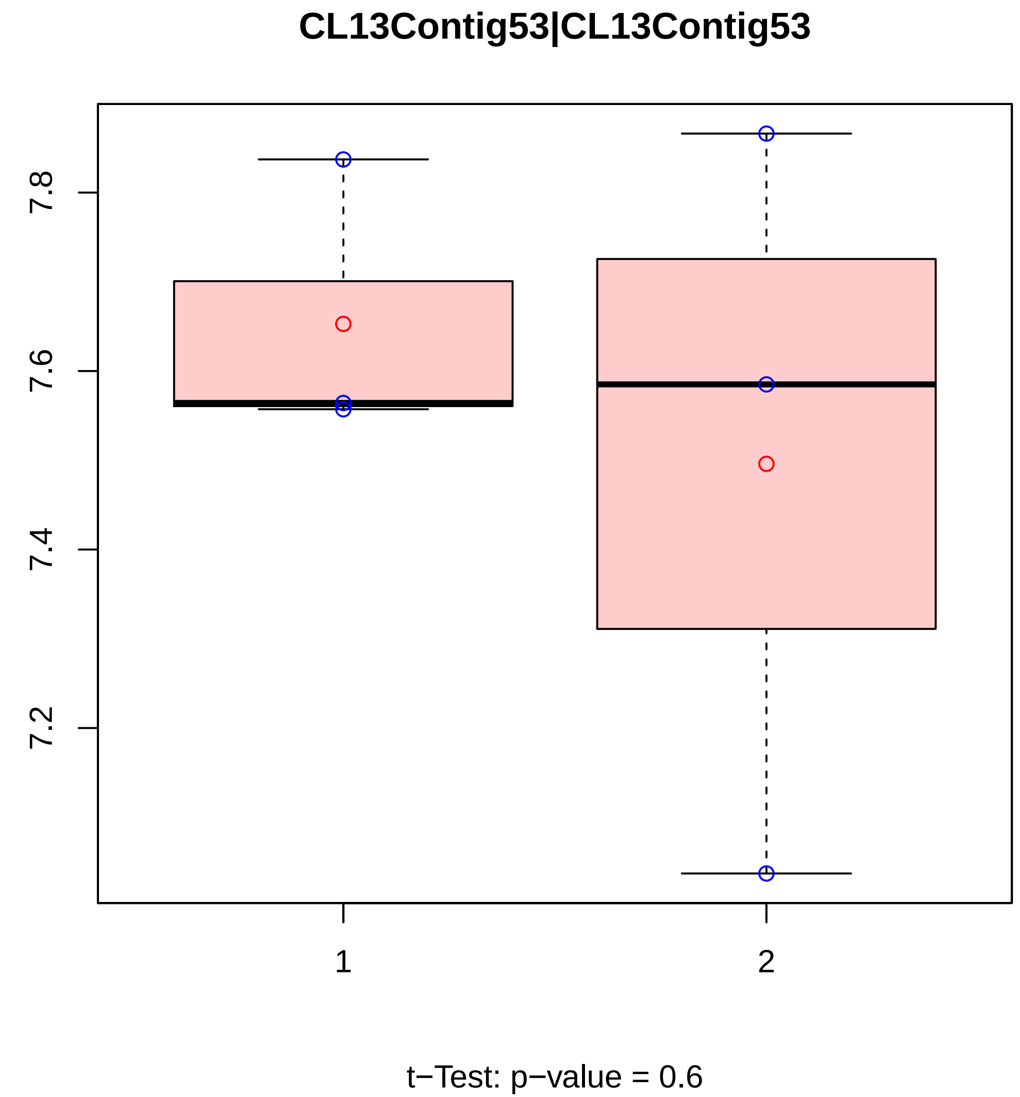
CL13Contig53|CL13Contig53
7.2
7.4
7.6
7.8
1
2
t−Test: p−value = 0.6

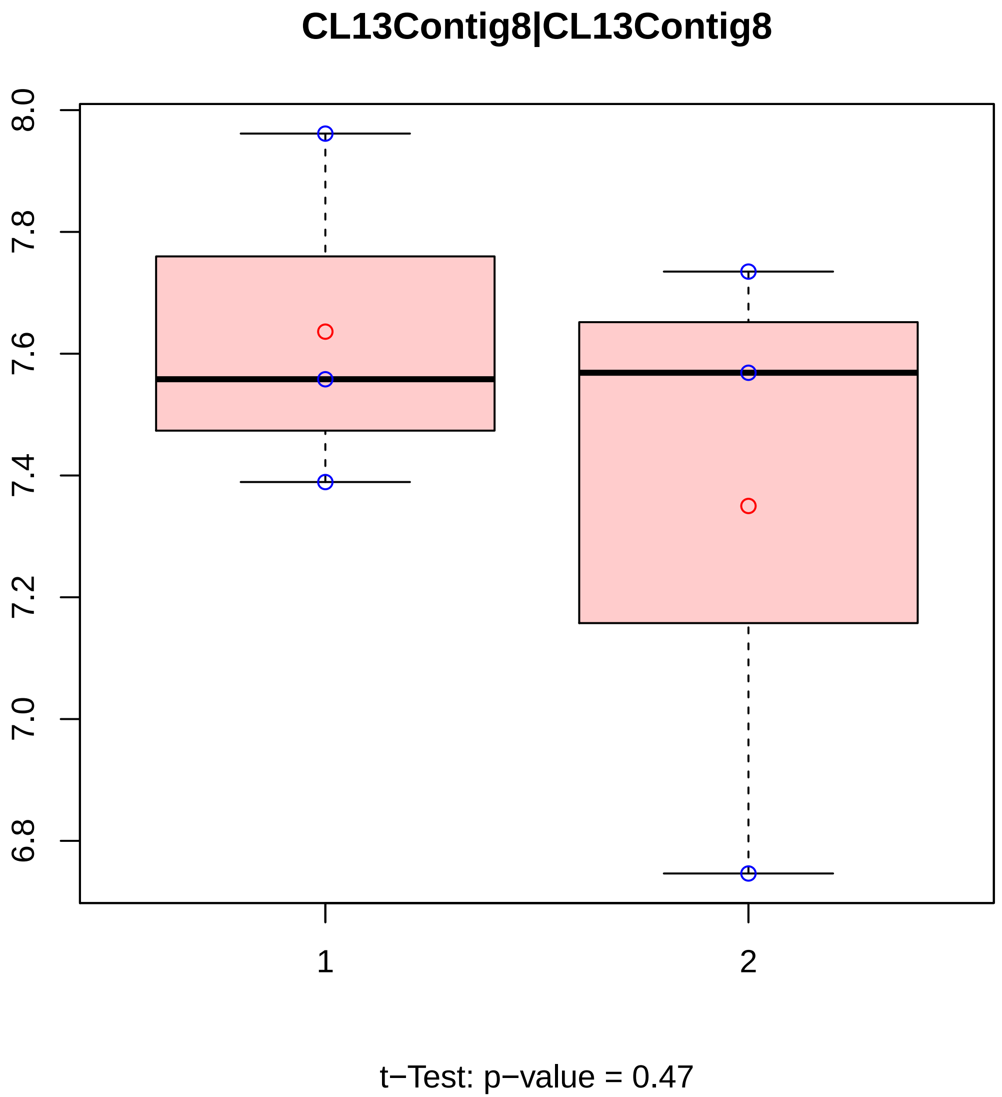
CL13Contig8|CL13Contig8
8.0
7.8
7.6
7.4
7.2
7.0
6.8
1
2
t−Test: p−value = 0.47

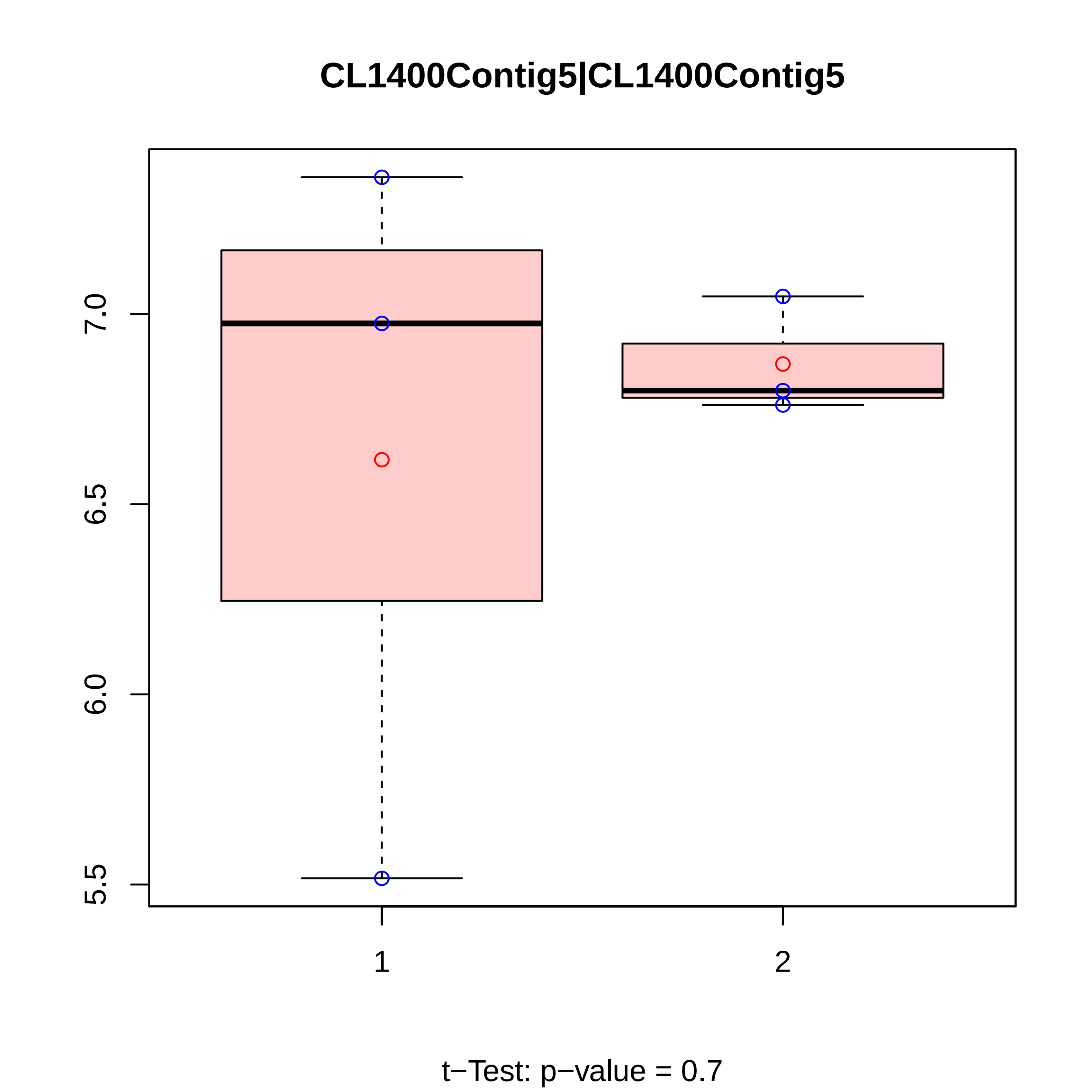
CL1400Contig5|CL1400Contig5
7.0
6.5
6.0
5.5
1
2
t−Test: p−value = 0.7

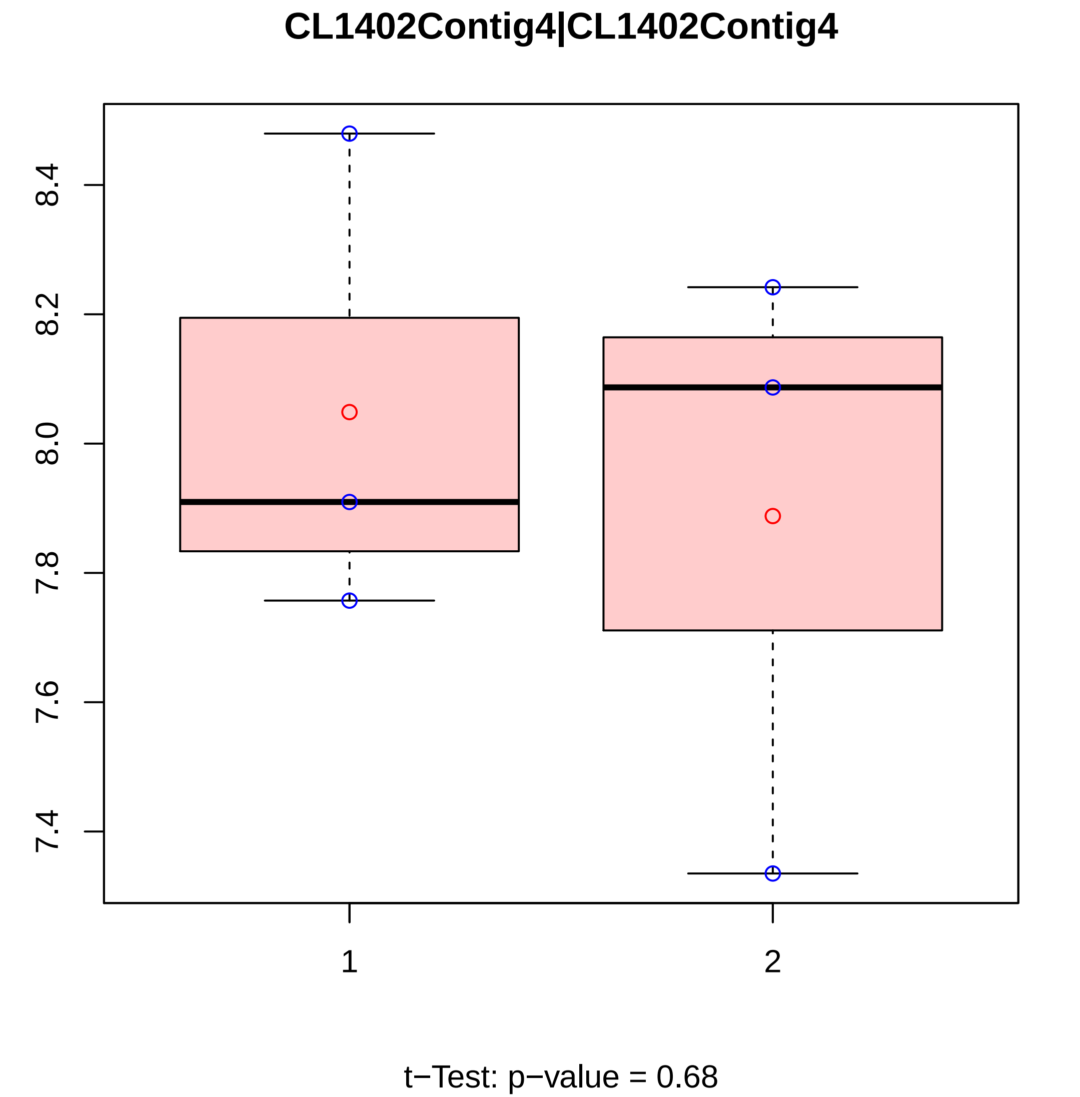
CL1402Contig4|CL1402Contig4
8.4
8.2
8.0
7.8
7.6
7.4
1
2
t−Test: p−value = 0.68

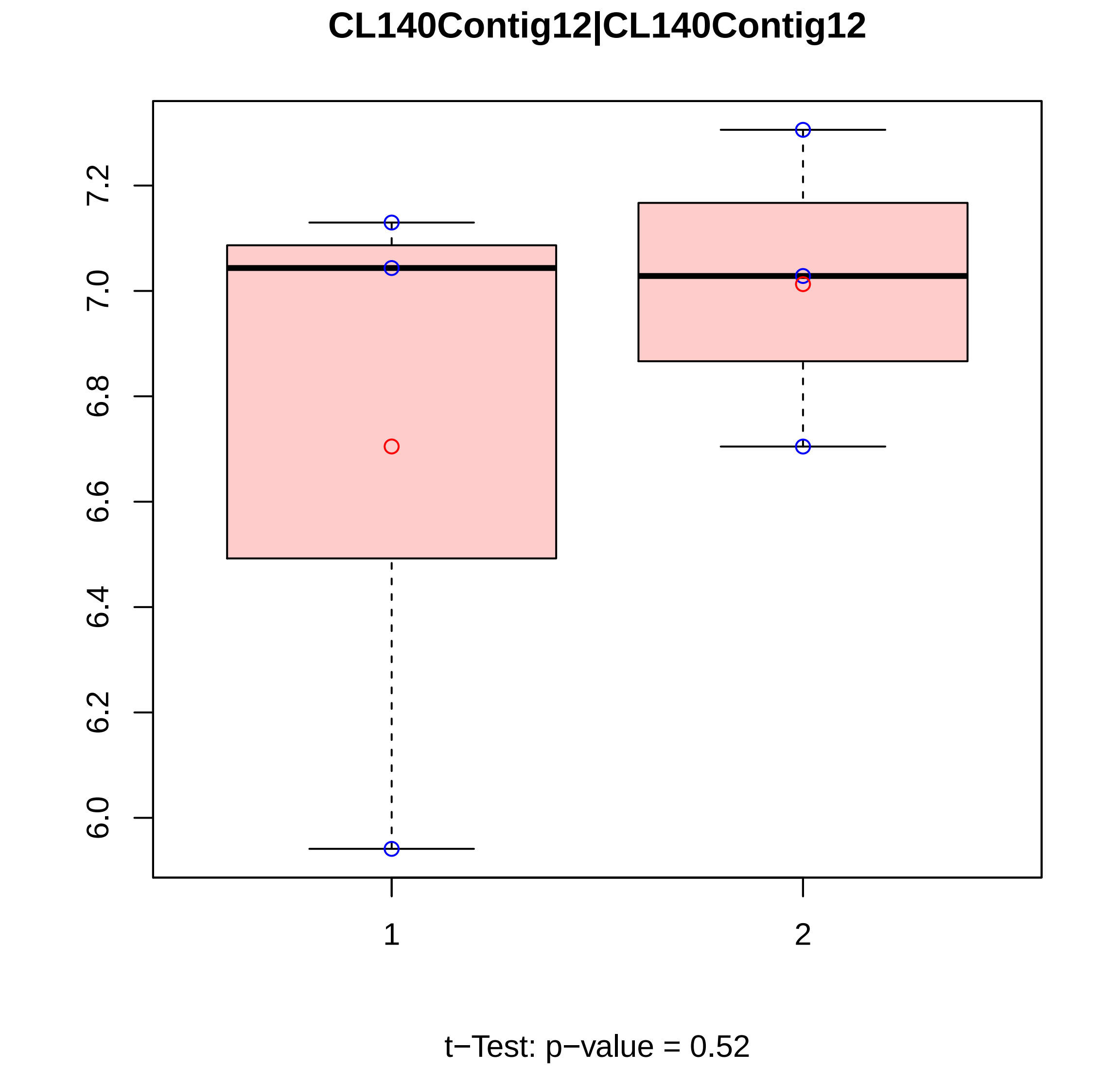
CL140Contig12|CL140Contig12
6.0
6.2
6.4
6.6
6.8
7.0
7.2
1
2
t−Test: p−value = 0.52

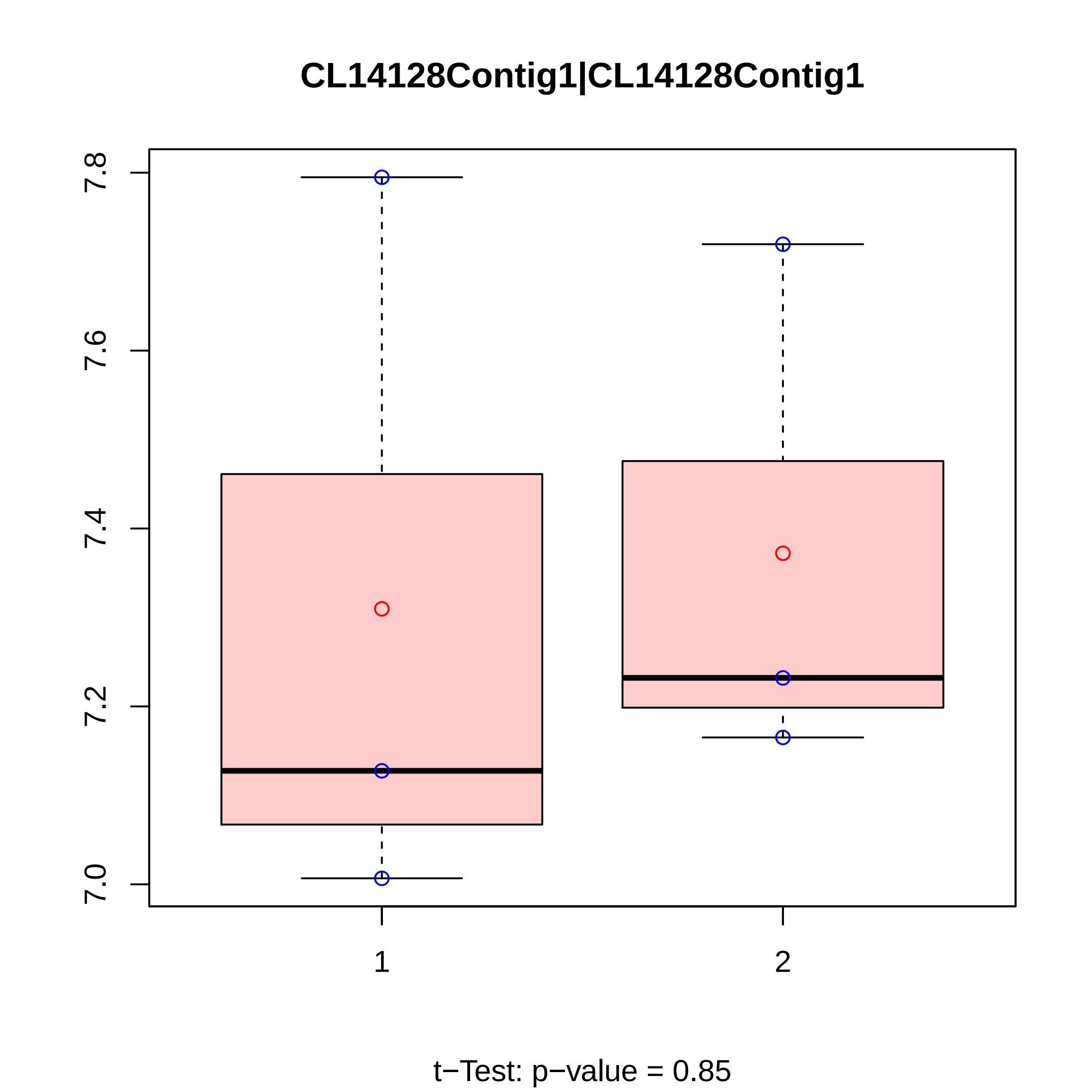
CL14128Contig1|CL14128Contig1
7.0
7.2
7.4
7.6
7.8
1
2
t−Test: p−value = 0.85

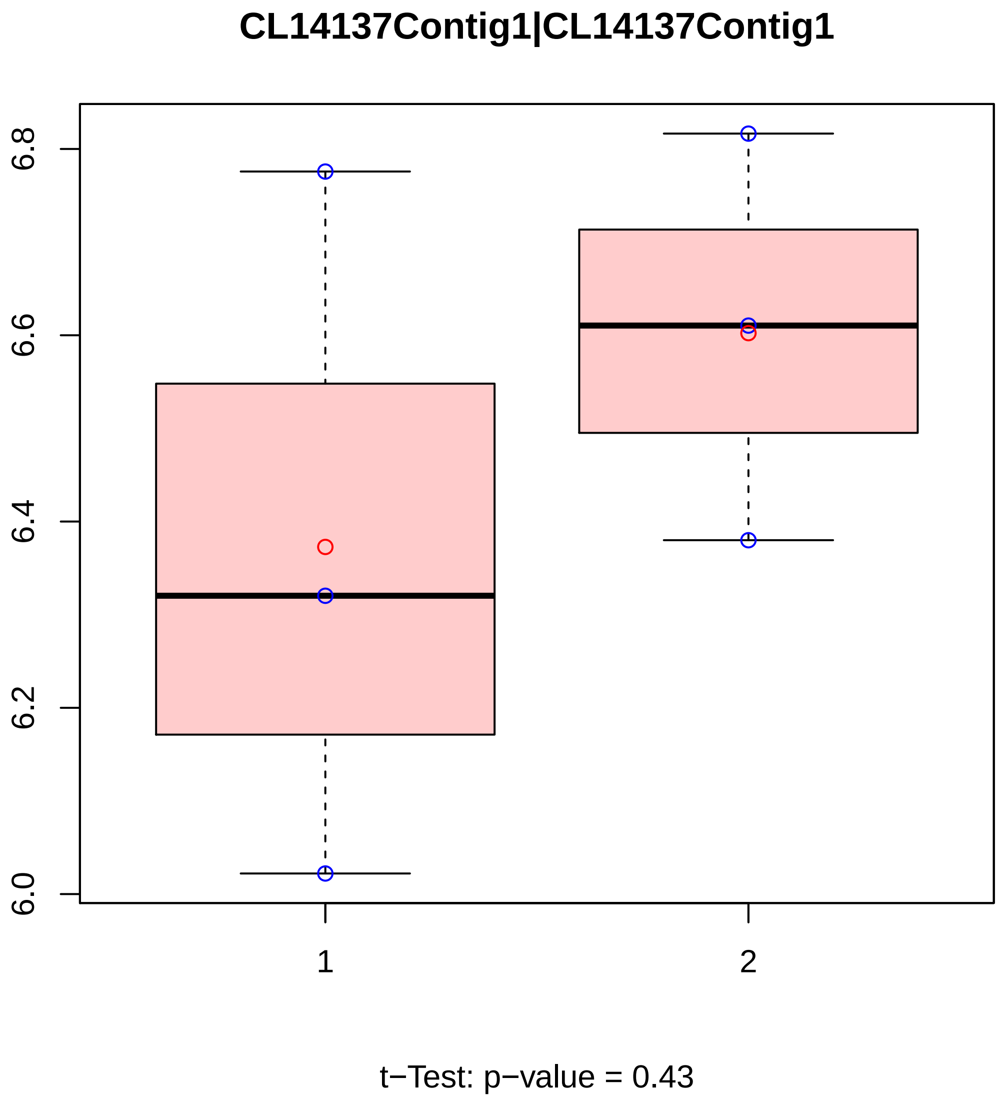

CL14137Contig1|CL14137Contig1
6.0
6.2
6.4
6.6
6.8
1
2
t−Test: p−value = 0.43

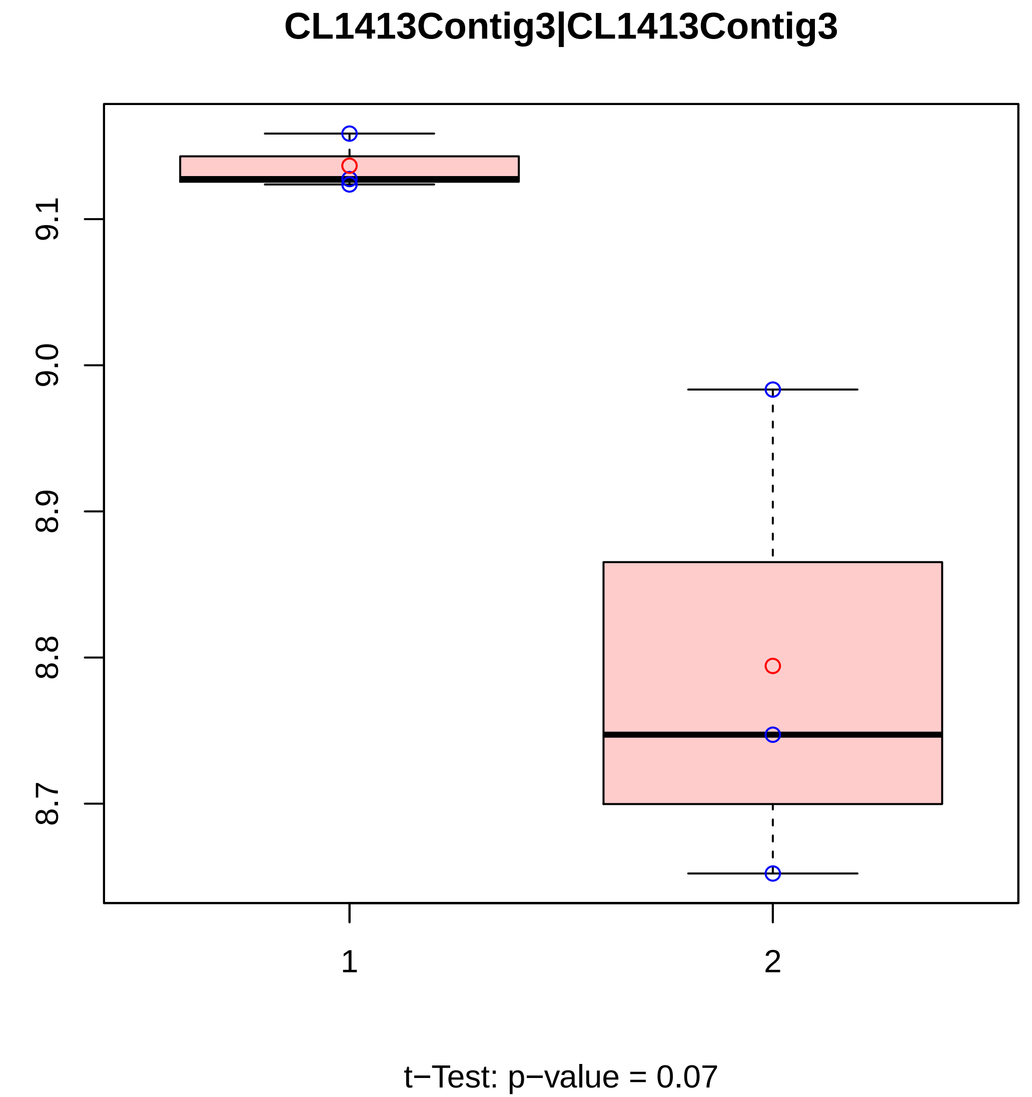
CL1413Contig3|CL1413Contig3
9.1
9.0
8.9
8.8
8.7
1
2
t−Test: p−value = 0.07

# CL1414Contig3|CL1414Contig3

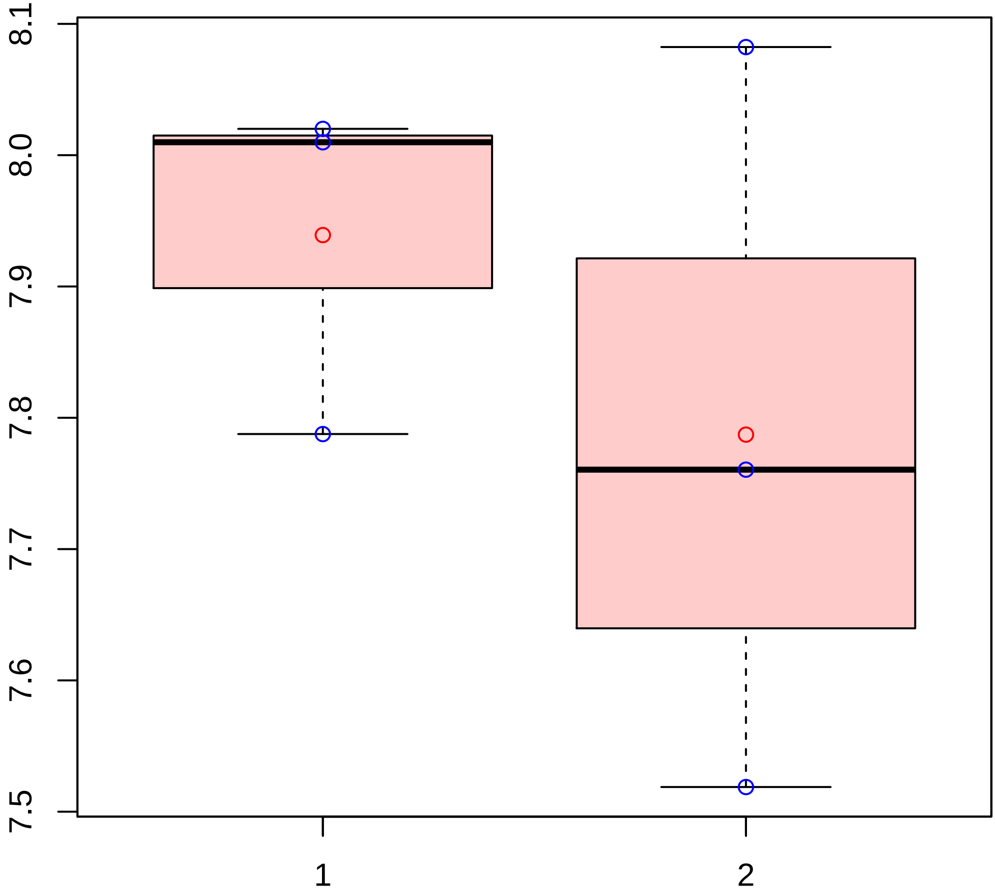


t−Test: p−value = 0.46

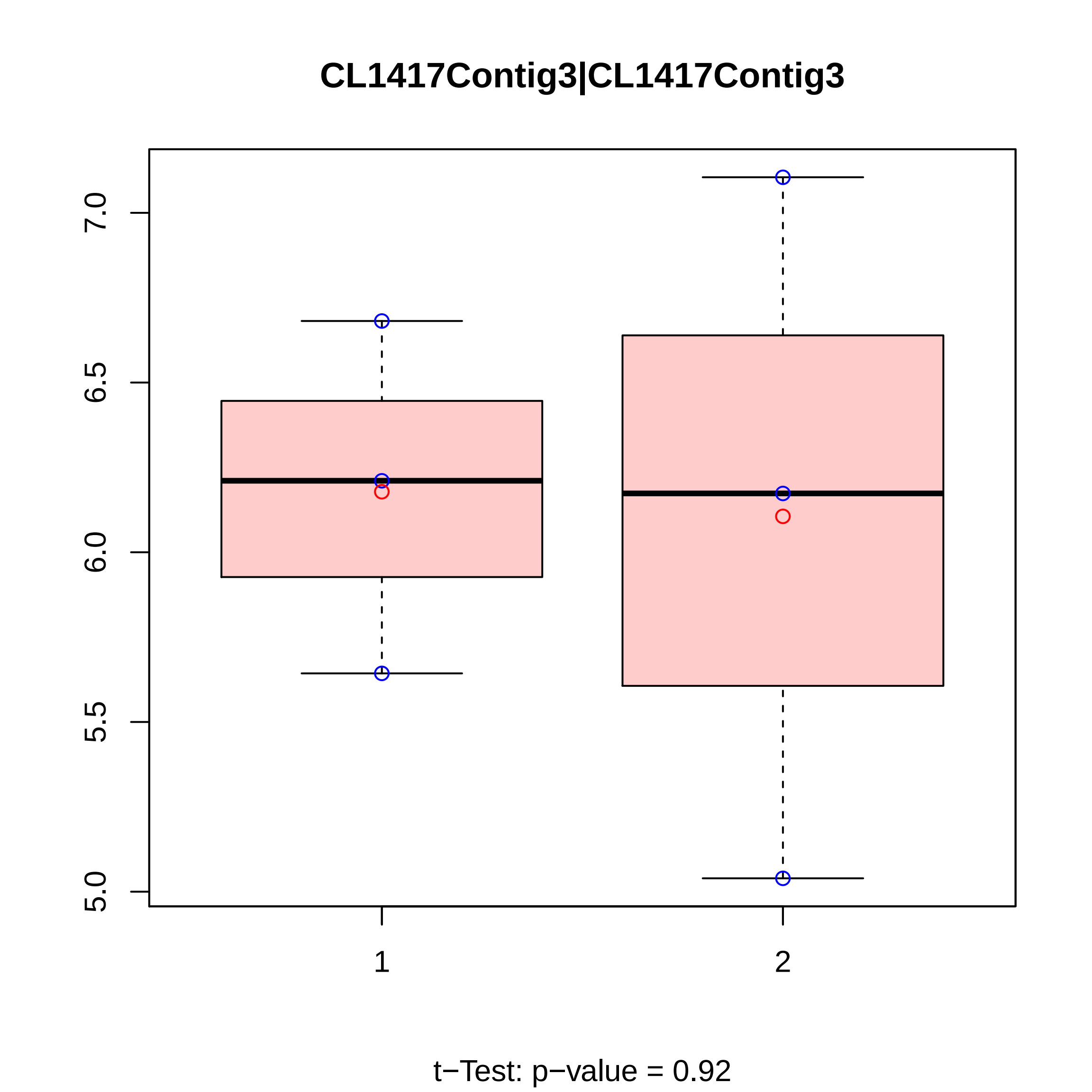
CL1417Contig3|CL1417Contig3
7.0
6.5
6.0
5.5
5.0
1
2
t−Test: p−value = 0.92

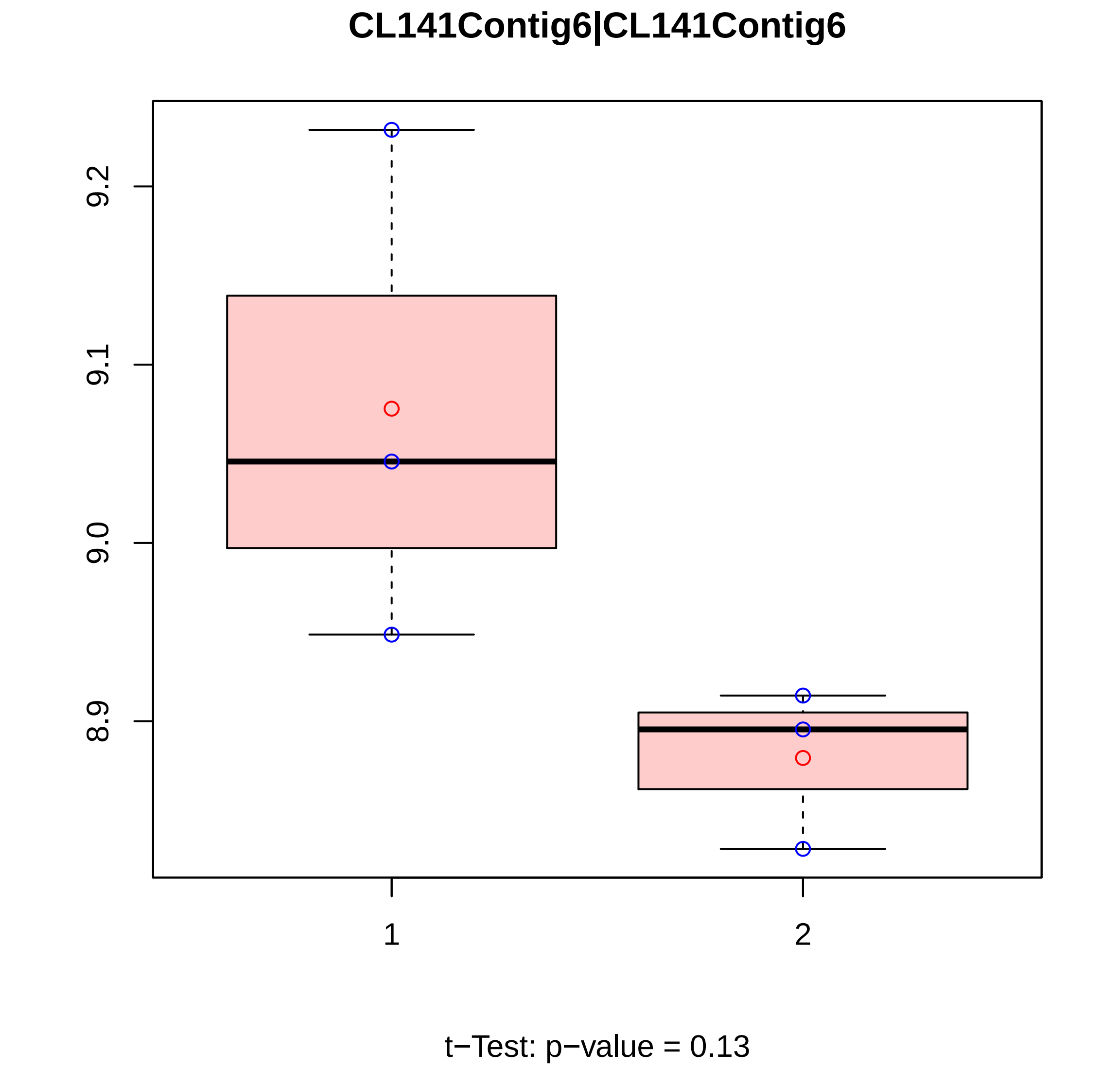
CL141Contig6|CL141Contig6
9.2
9.1
9.0
8.9
1
2
t−Test: p−value = 0.13

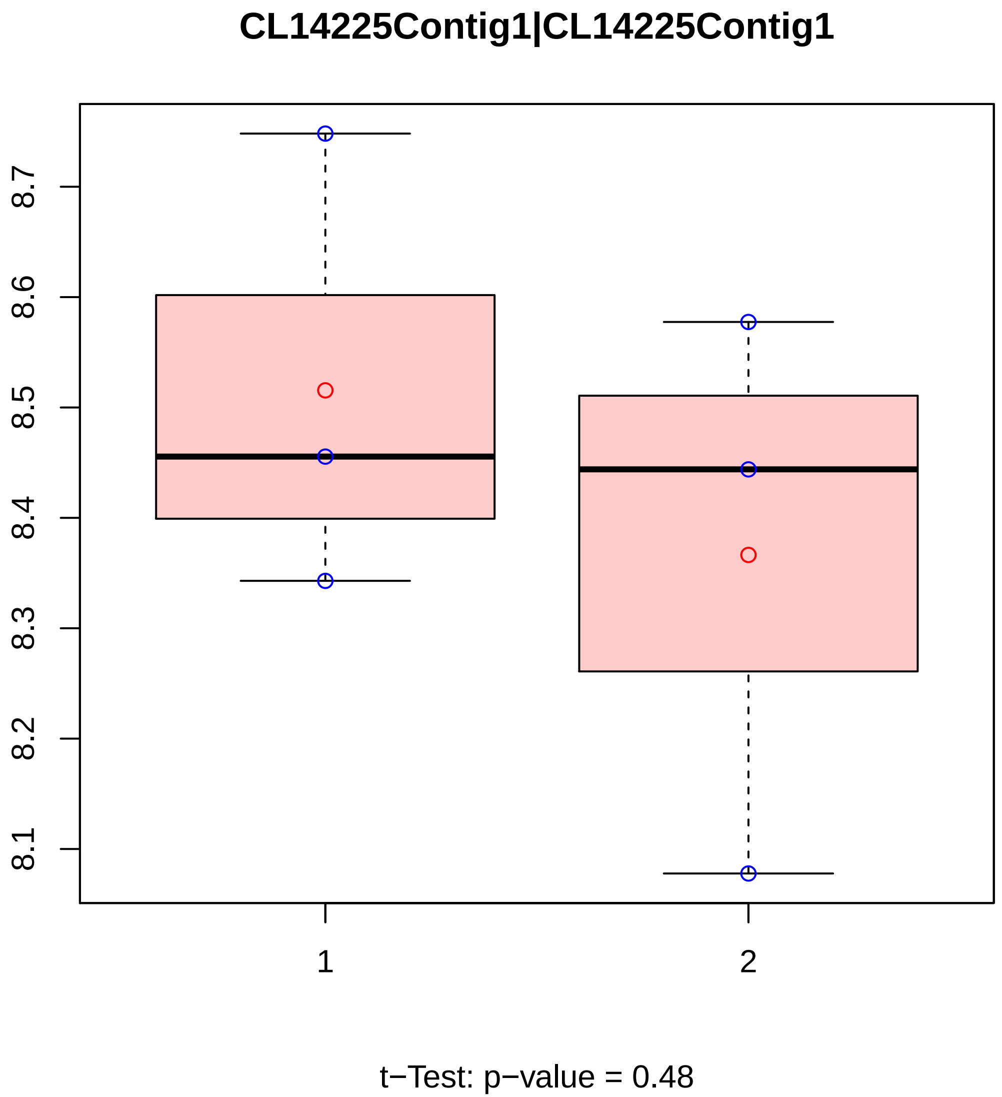
CL14225Contig1|CL14225Contig1
8.1
8.2
8.3
8.4
8.5
8.6
8.7
1
2
t−Test: p−value = 0.48

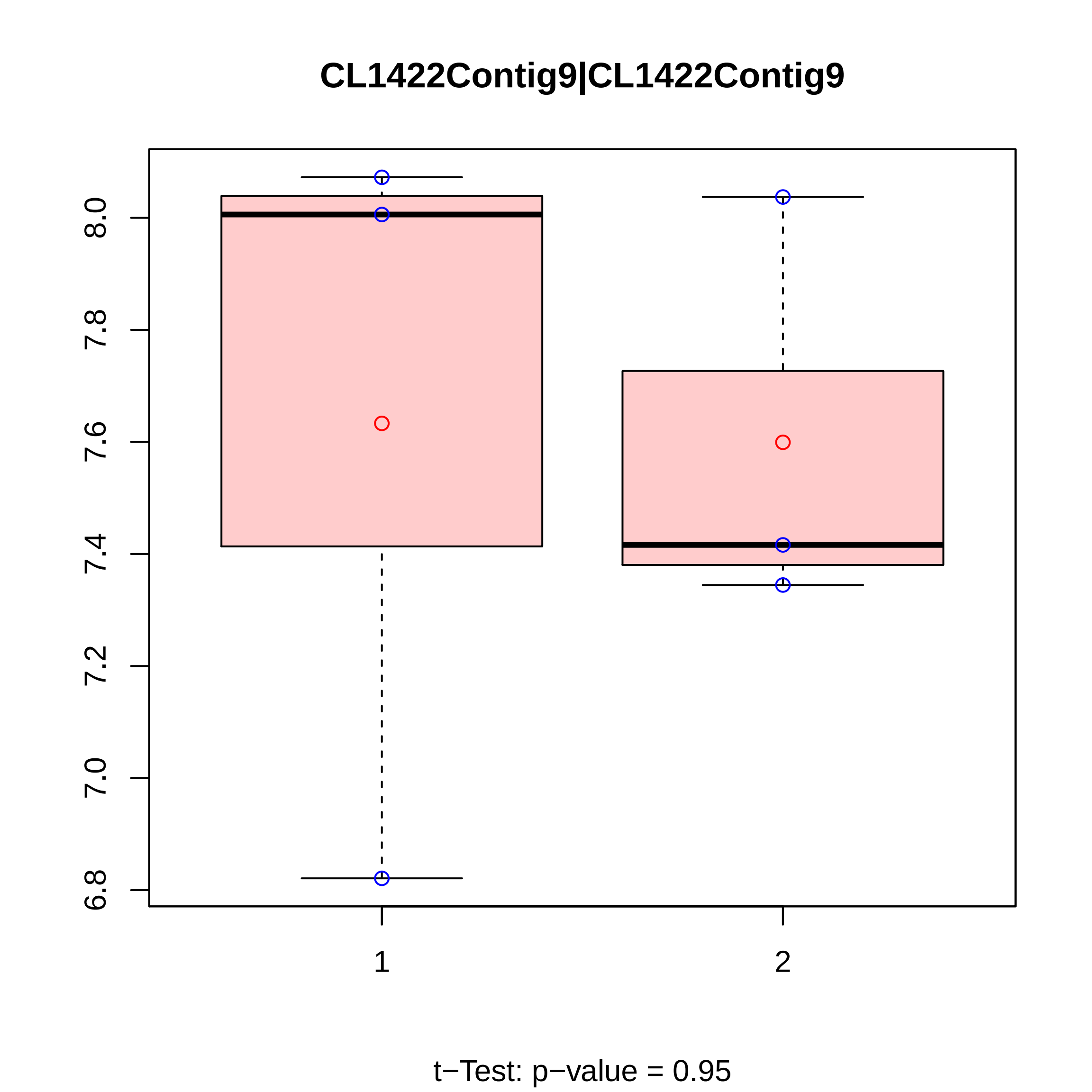
CL1422Contig9|CL1422Contig9
8.0
7.8
7.6
7.4
7.2
7.0
6.8
1
2
t−Test: p−value = 0.95

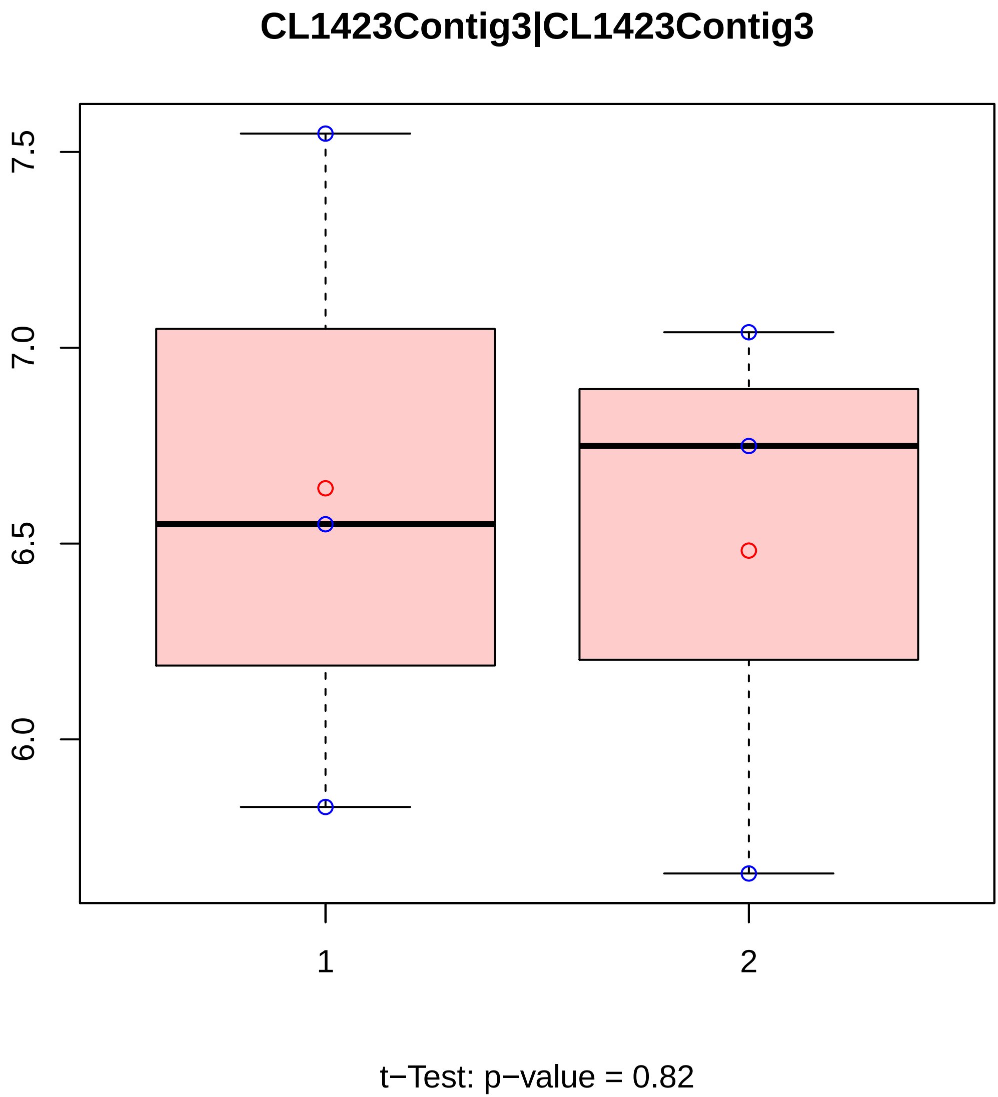
CL1423Contig3|CL1423Contig3
7.5
7.0
6.5
6.0
1
2
t−Test: p−value = 0.82

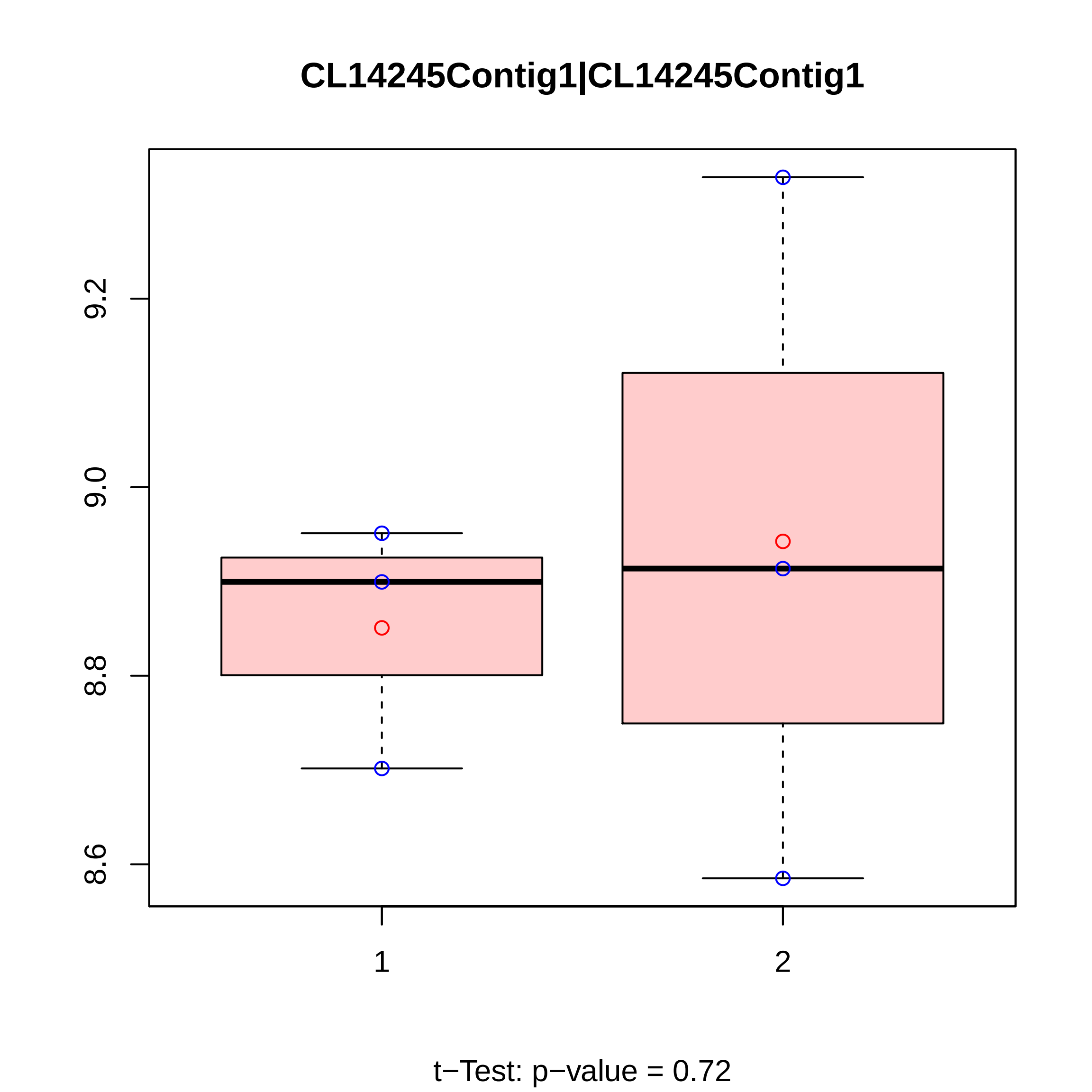
CL14245Contig1|CL14245Contig1
9.2
9.0
8.8
8.6
1
2
t−Test: p−value = 0.72

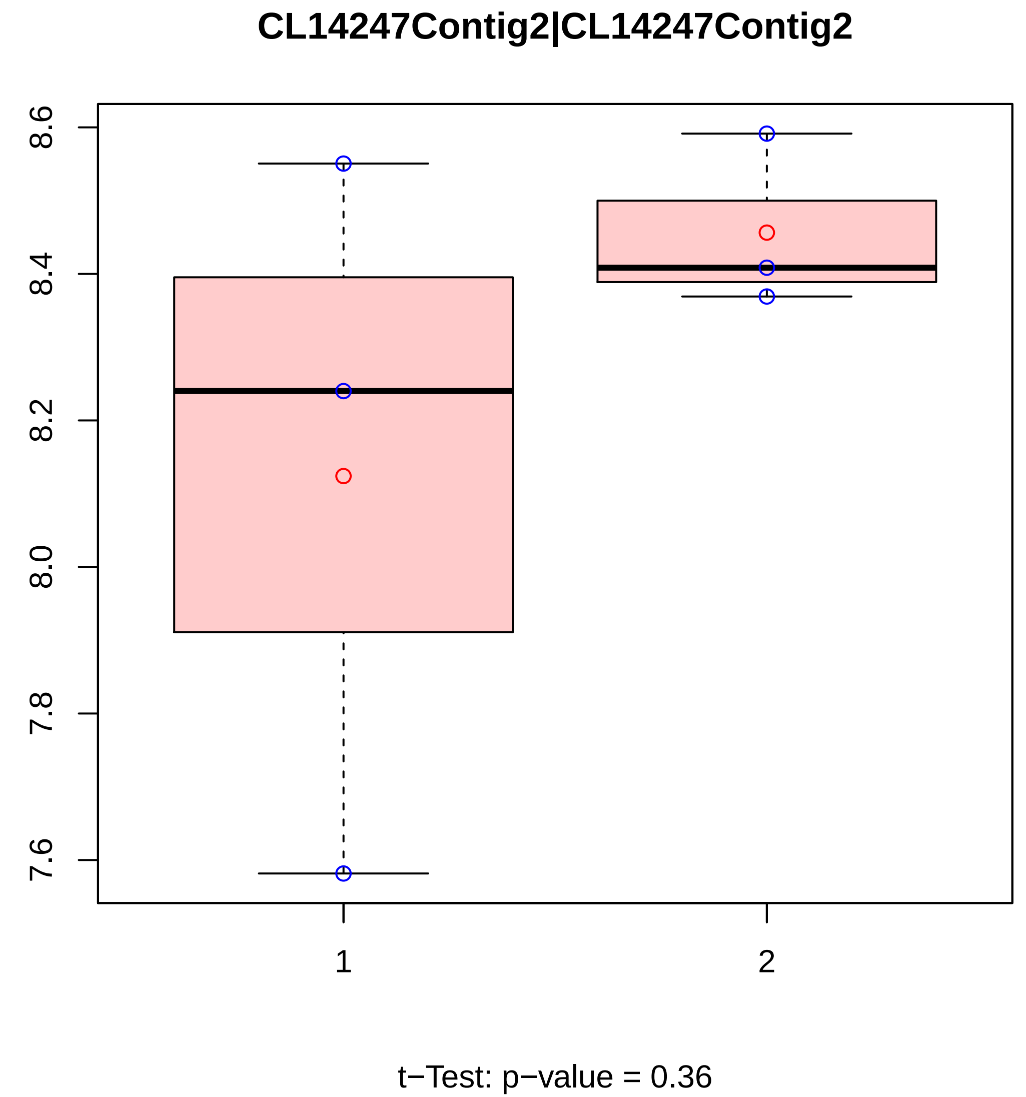
CL14247Contig2|CL14247Contig2
8.6
8.4
8.2
8.0
7.8
7.6
1
2
t−Test: p−value = 0.36

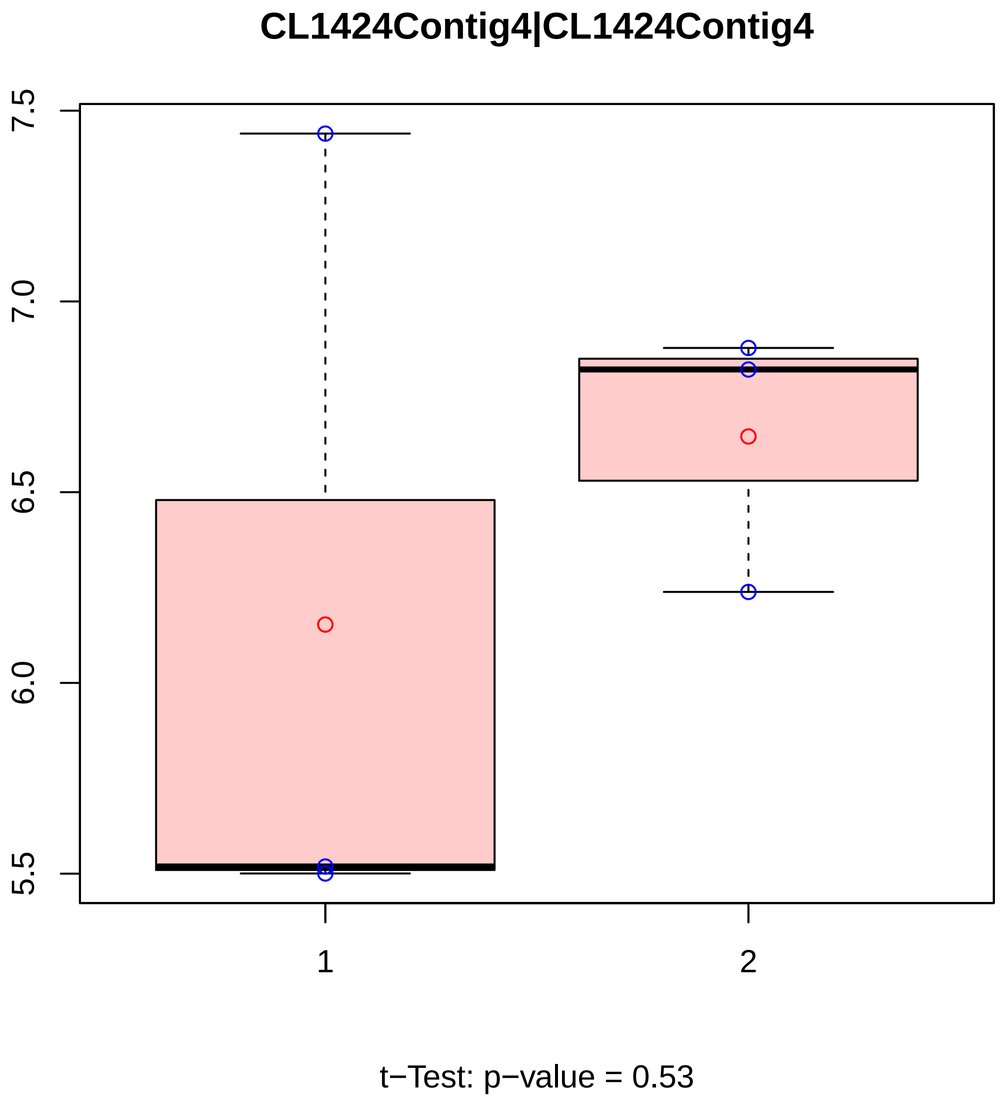
CL1424Contig4|CL1424Contig4
7.5
7.0
6.5
6.0
5.5
1
2
t−Test: p−value = 0.53

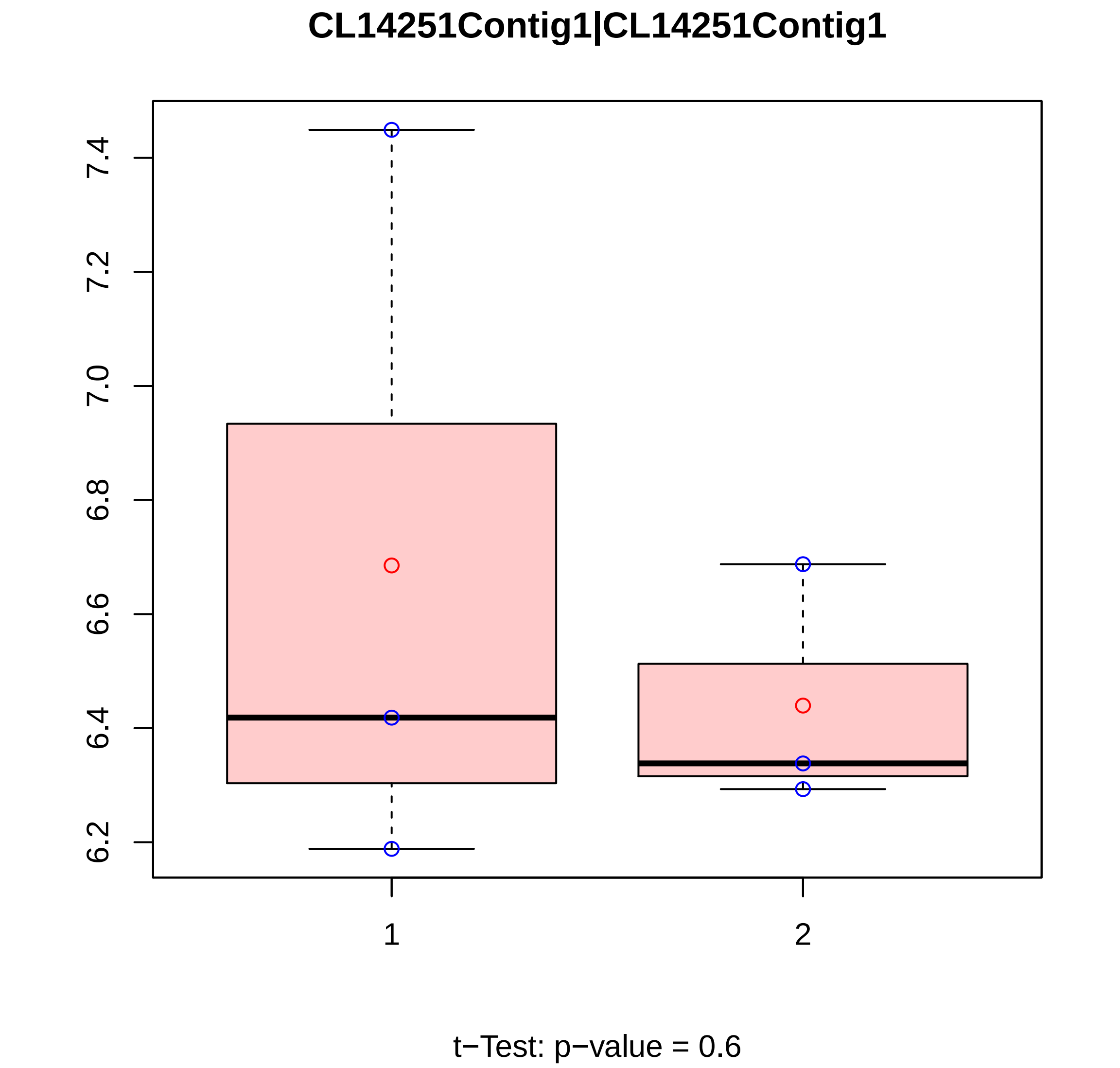
CL14251Contig1|CL14251Contig1
7.4
7.2
7.0
6.8
6.6
6.4
6.2
1
2
t−Test: p−value = 0.6

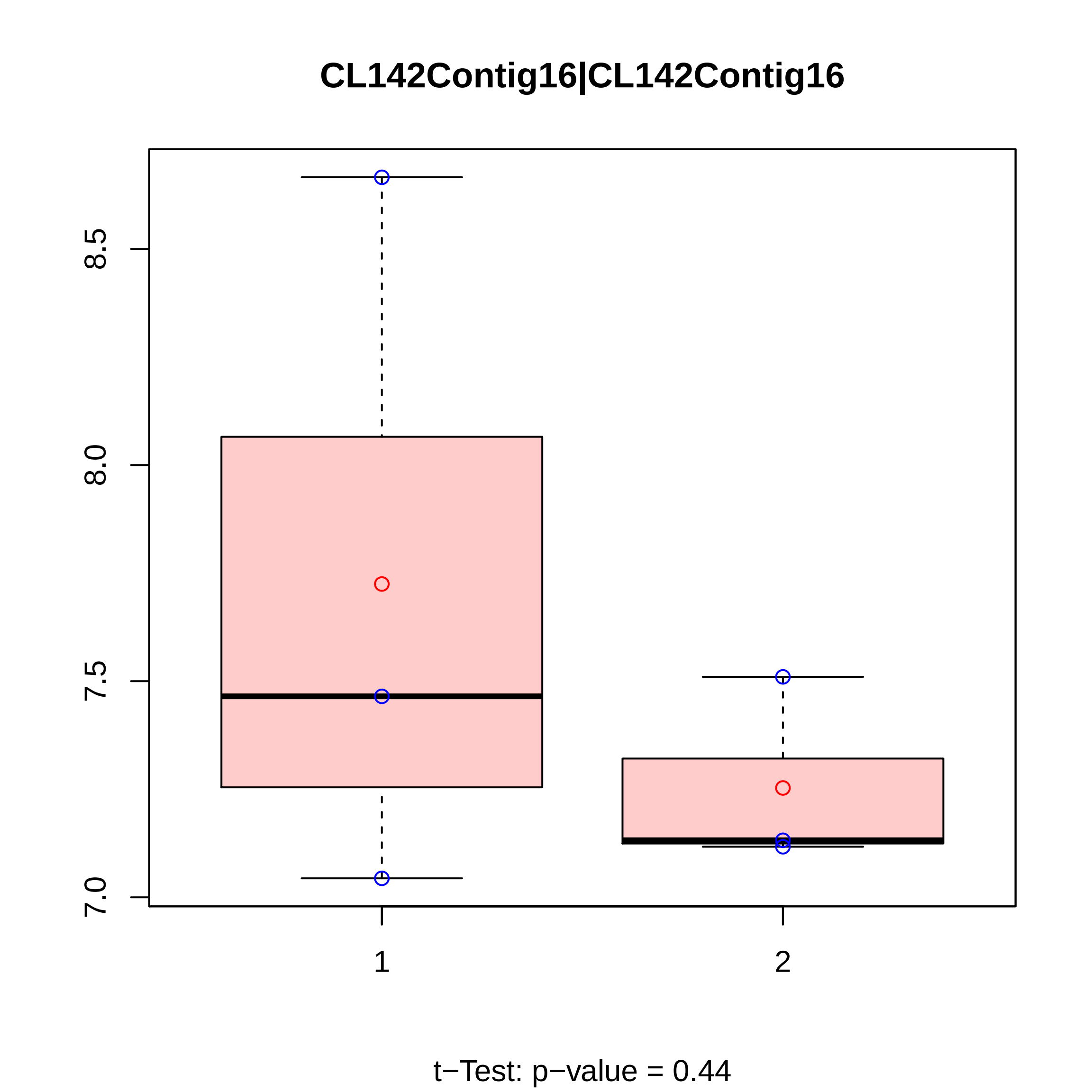
CL142Contig16|CL142Contig16
8.5
8.0
7.5
7.0
1
2
t−Test: p−value = 0.44

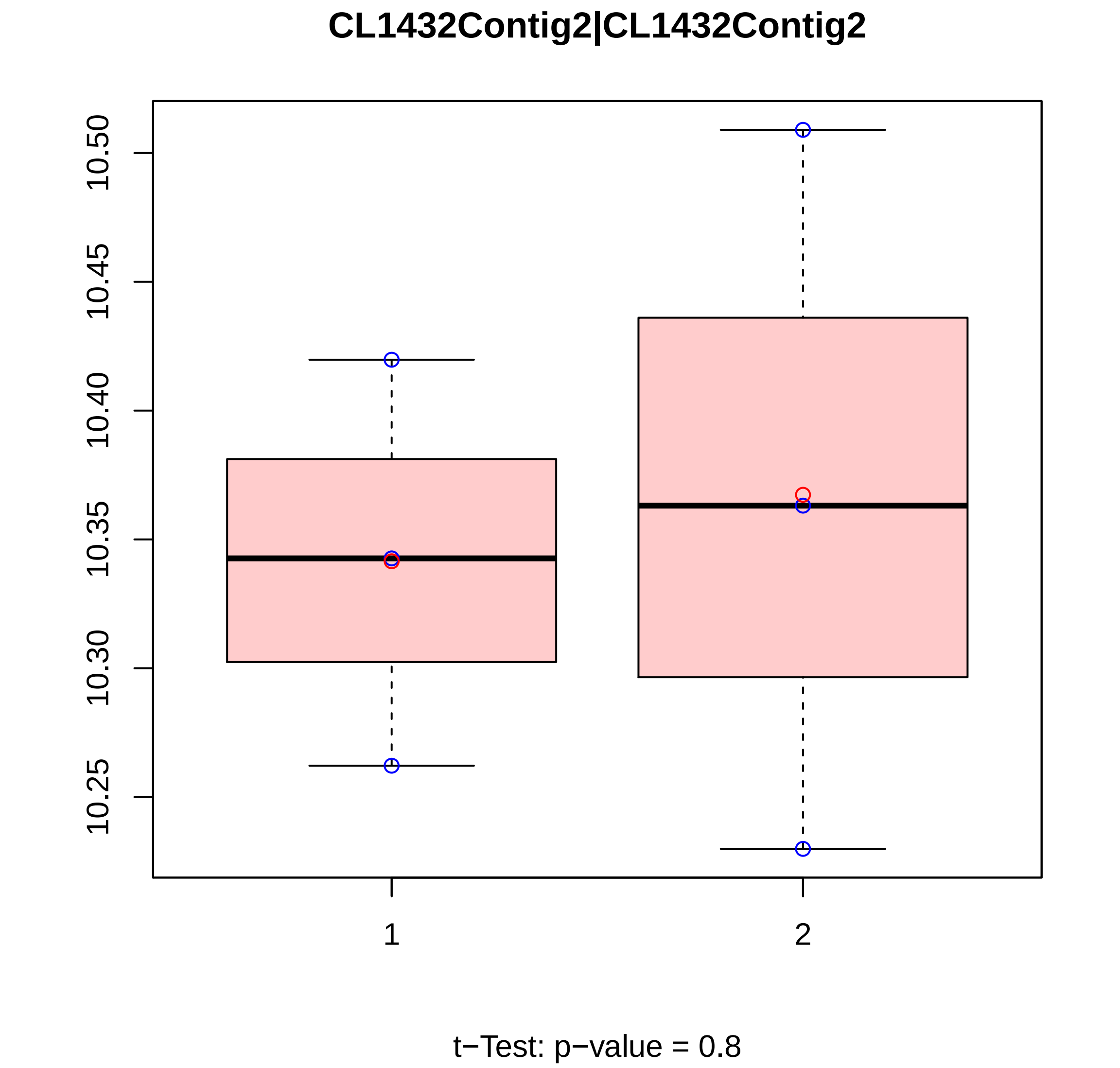

CL1432Contig2|CL1432Contig2
10.25
10.30
10.35
10.40
10.45
10.50
1
2
t−Test: p−value = 0.8

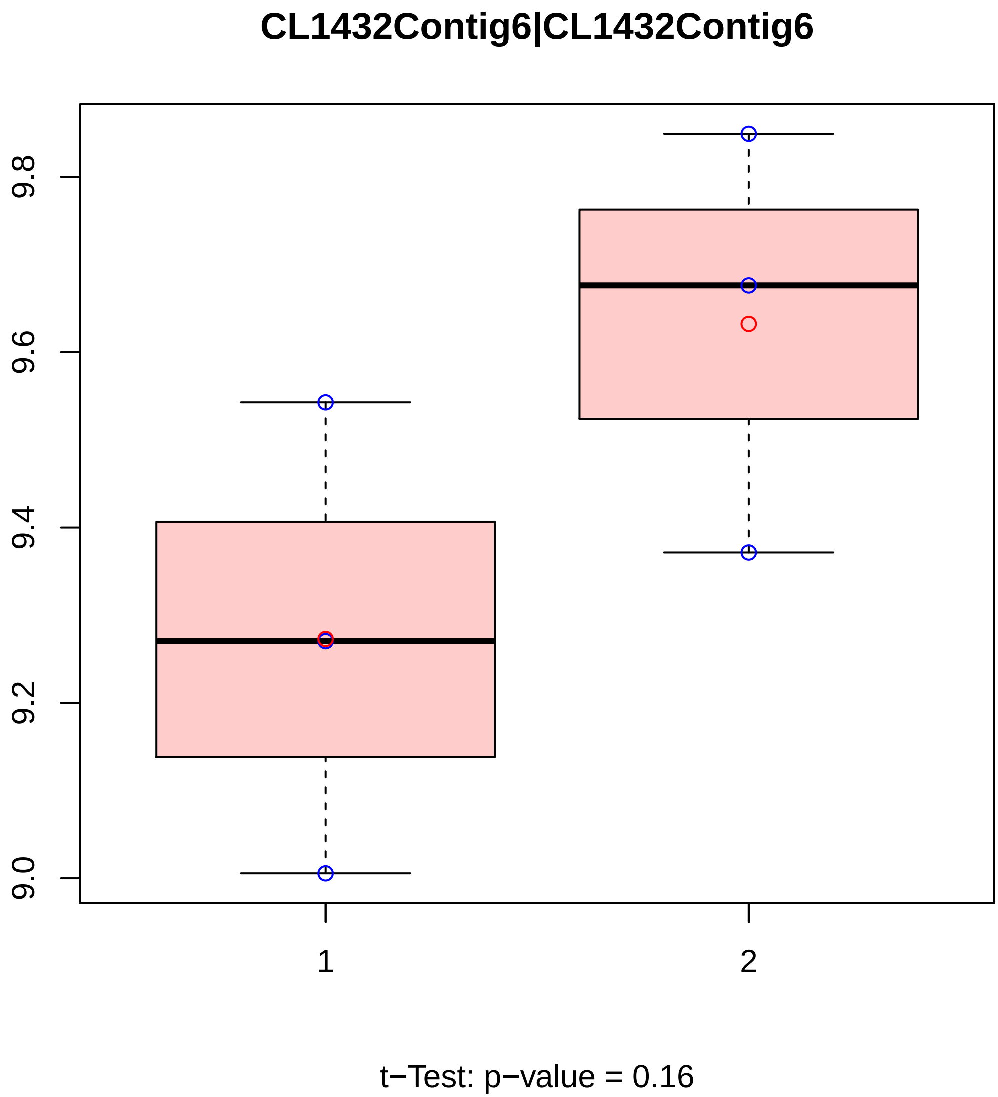
CL1432Contig6|CL1432Contig6
9.0
9.2
9.4
9.6
9.8
1
2
t−Test: p−value = 0.16

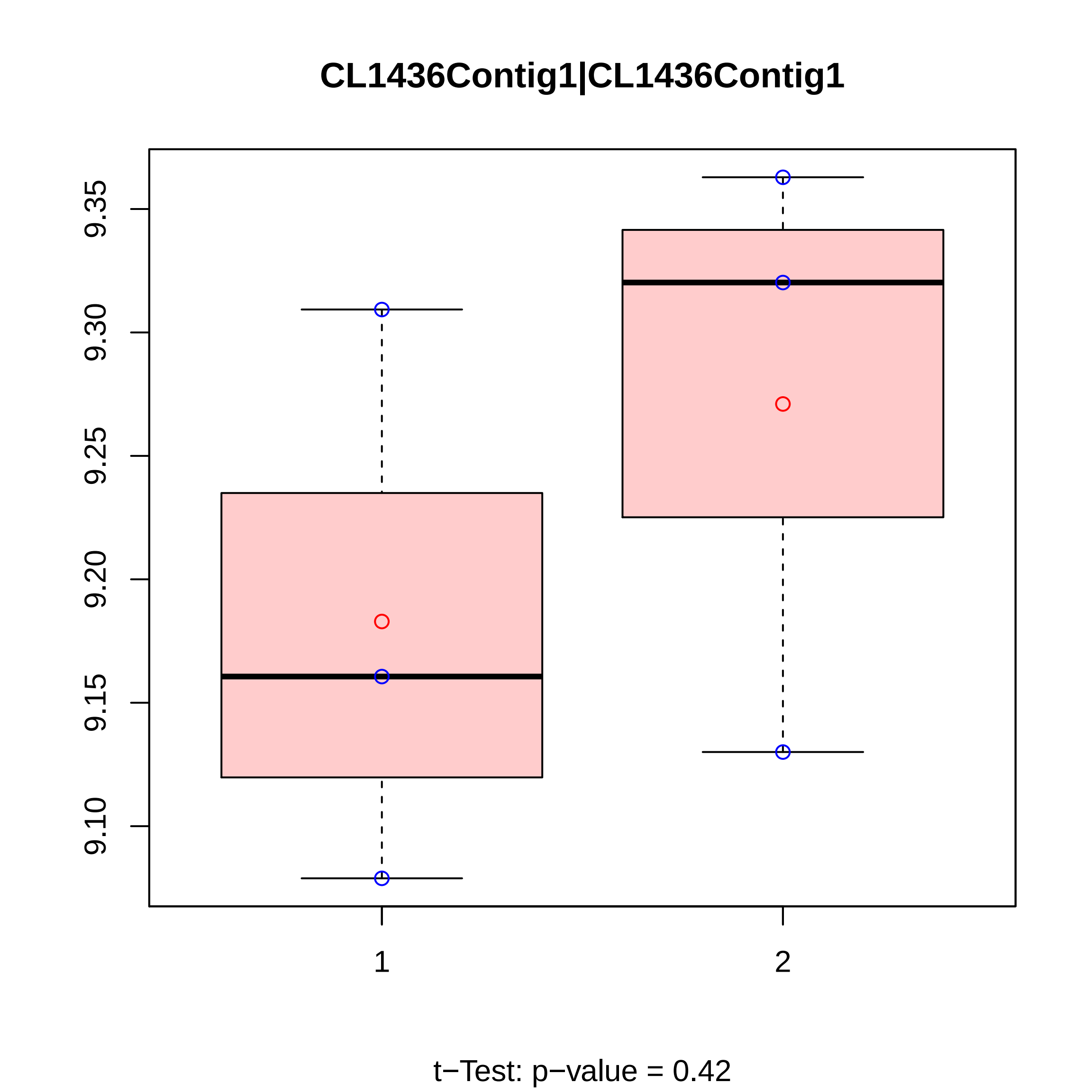
CL1436Contig1|CL1436Contig1
9.10
9.15
9.20
9.25
9.30
9.35
1
2
t−Test: p−value = 0.42

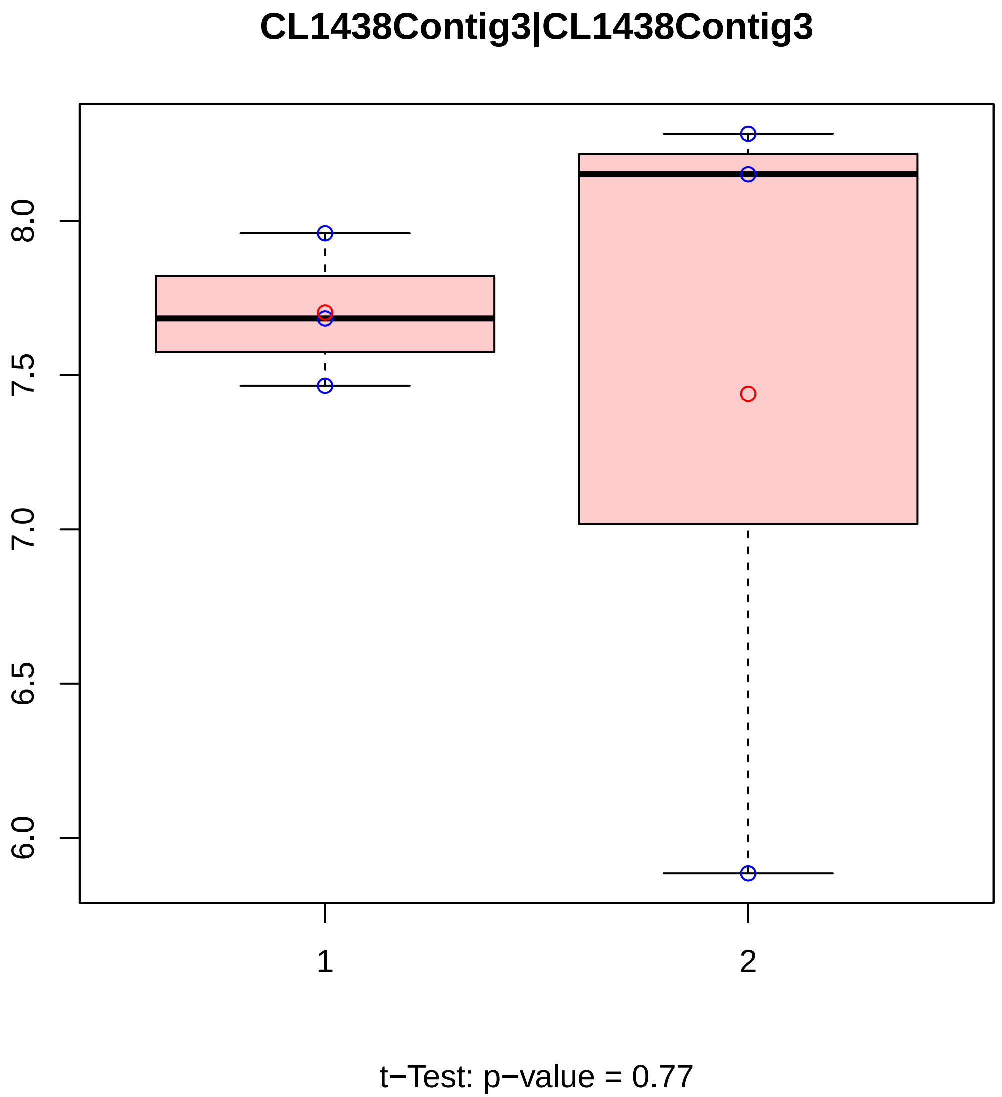
CL1438Contig3|CL1438Contig3
8.0
7.5
7.0
6.5
6.0
1
2
t−Test: p−value = 0.77

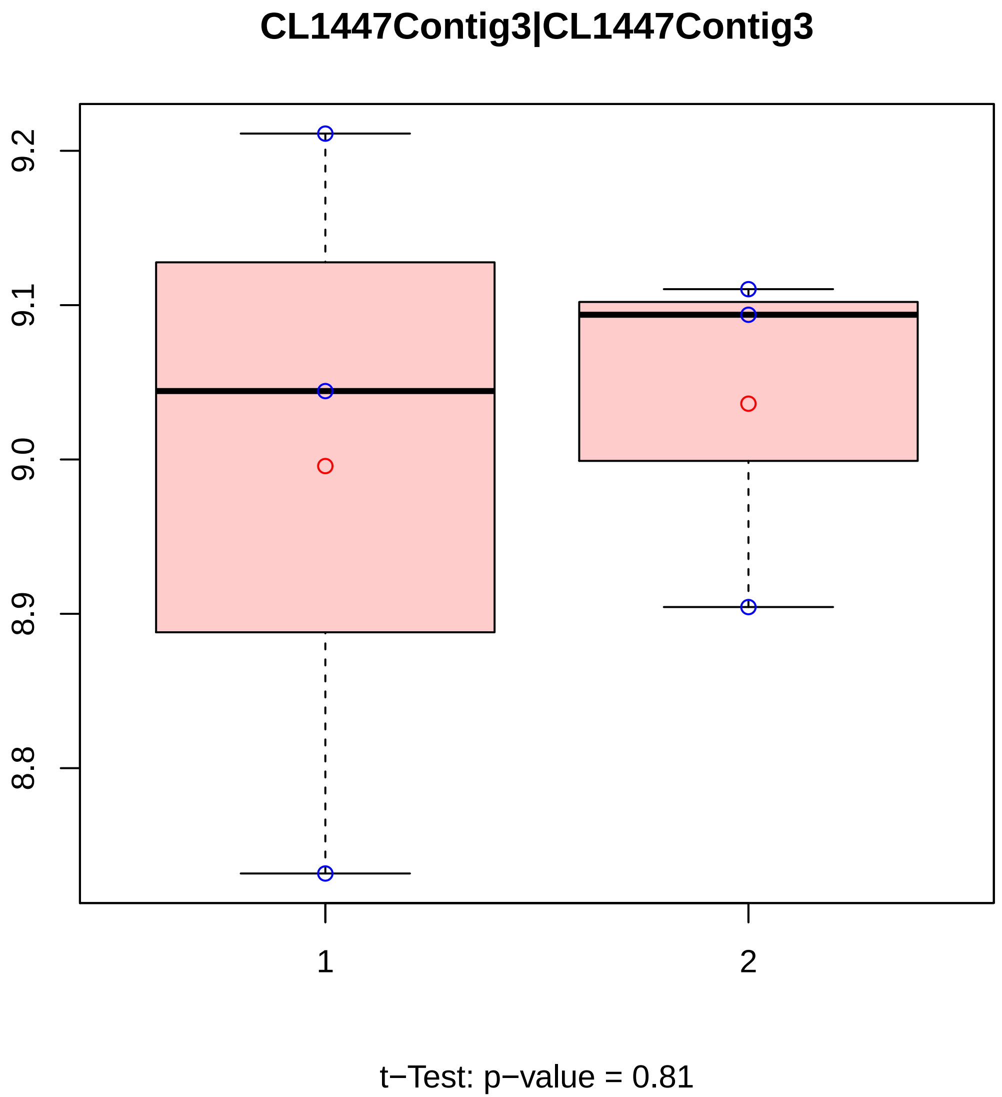

CL1447Contig3|CL1447Contig3
9.2
9.1
9.0
8.9
8.8
1
2
t−Test: p−value = 0.81

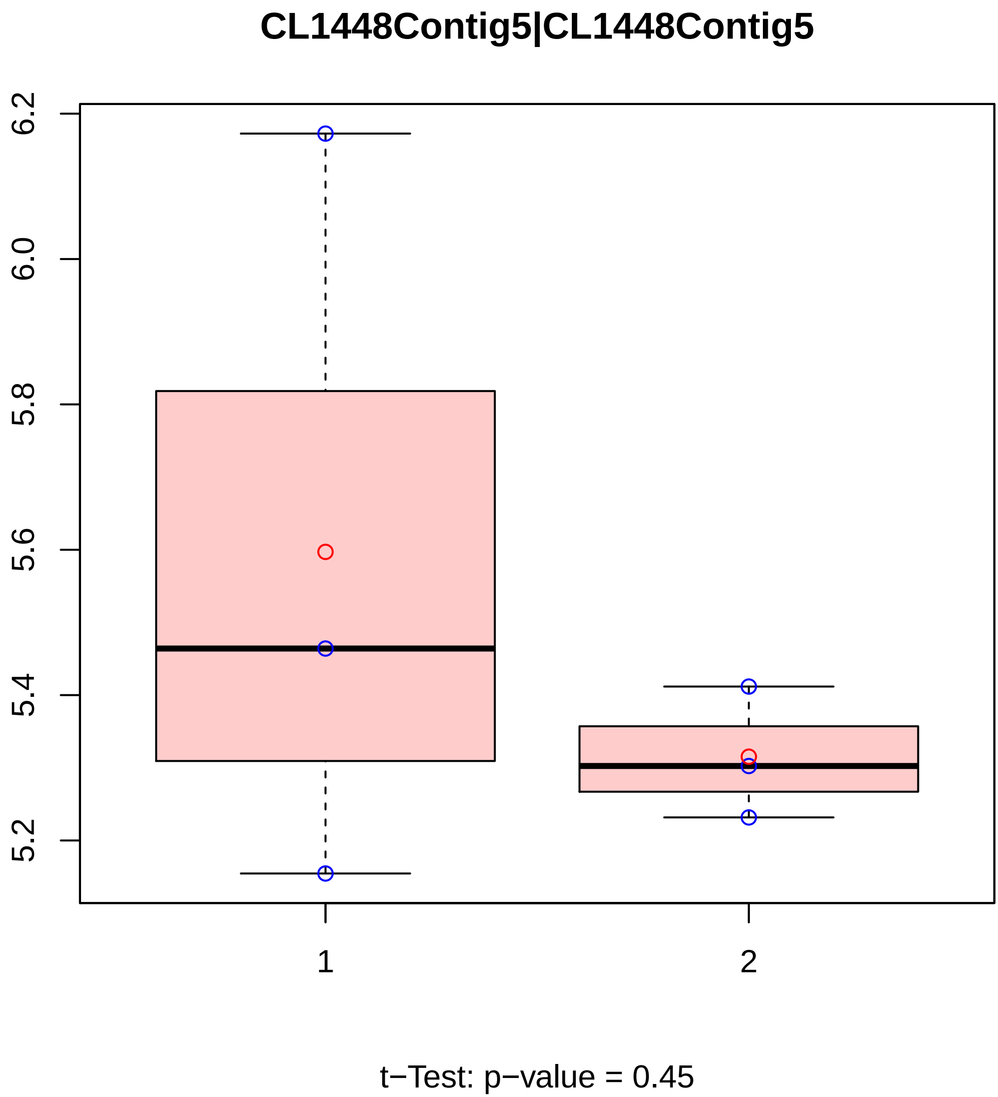
CL1448Contig5|CL1448Contig5
6.2
6.0
5.8
5.6
5.4
5.2
1
2
t−Test: p−value = 0.45

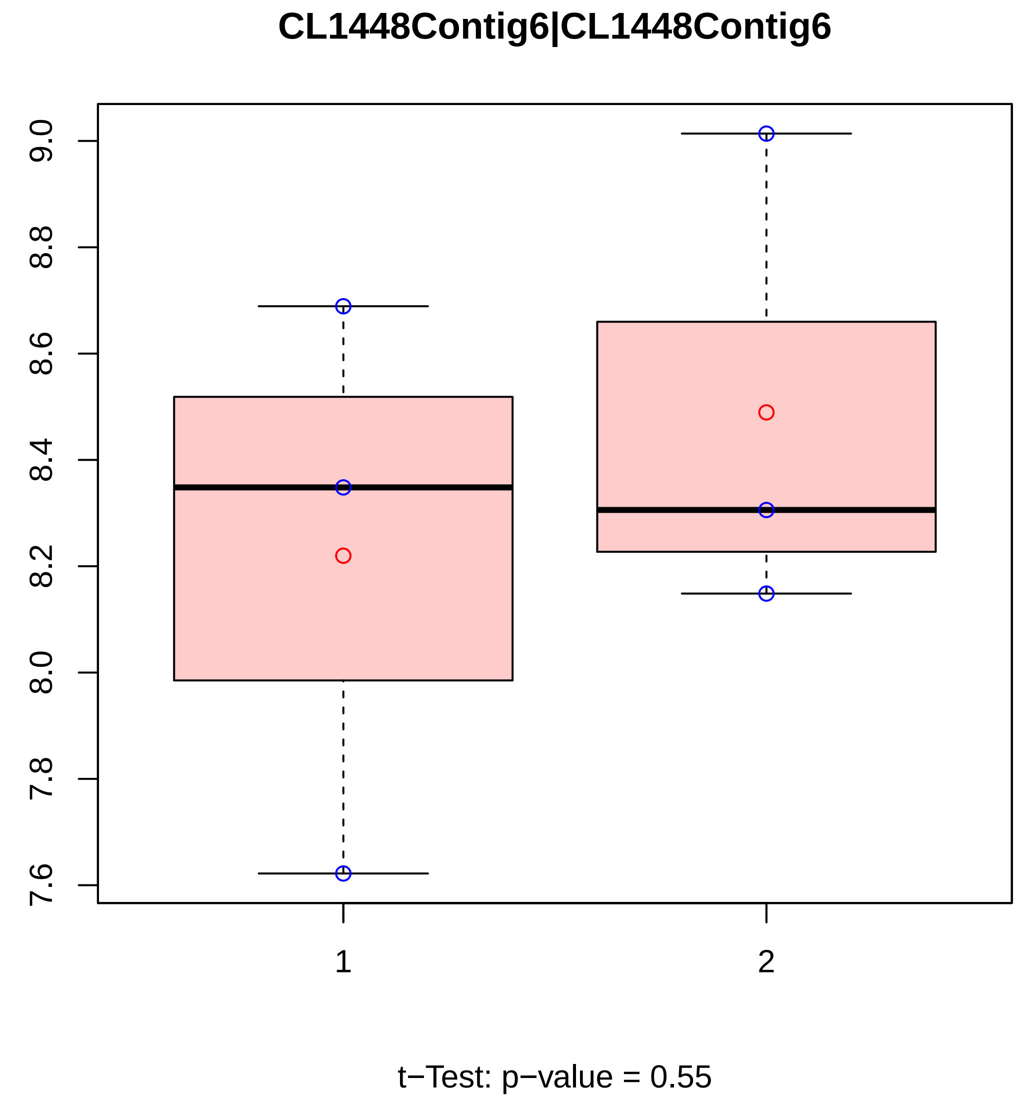
CL1448Contig6|CL1448Contig6
9.0
8.8
8.6
8.4
8.2
8.0
7.8
7.6
1
2
t−Test: p−value = 0.55

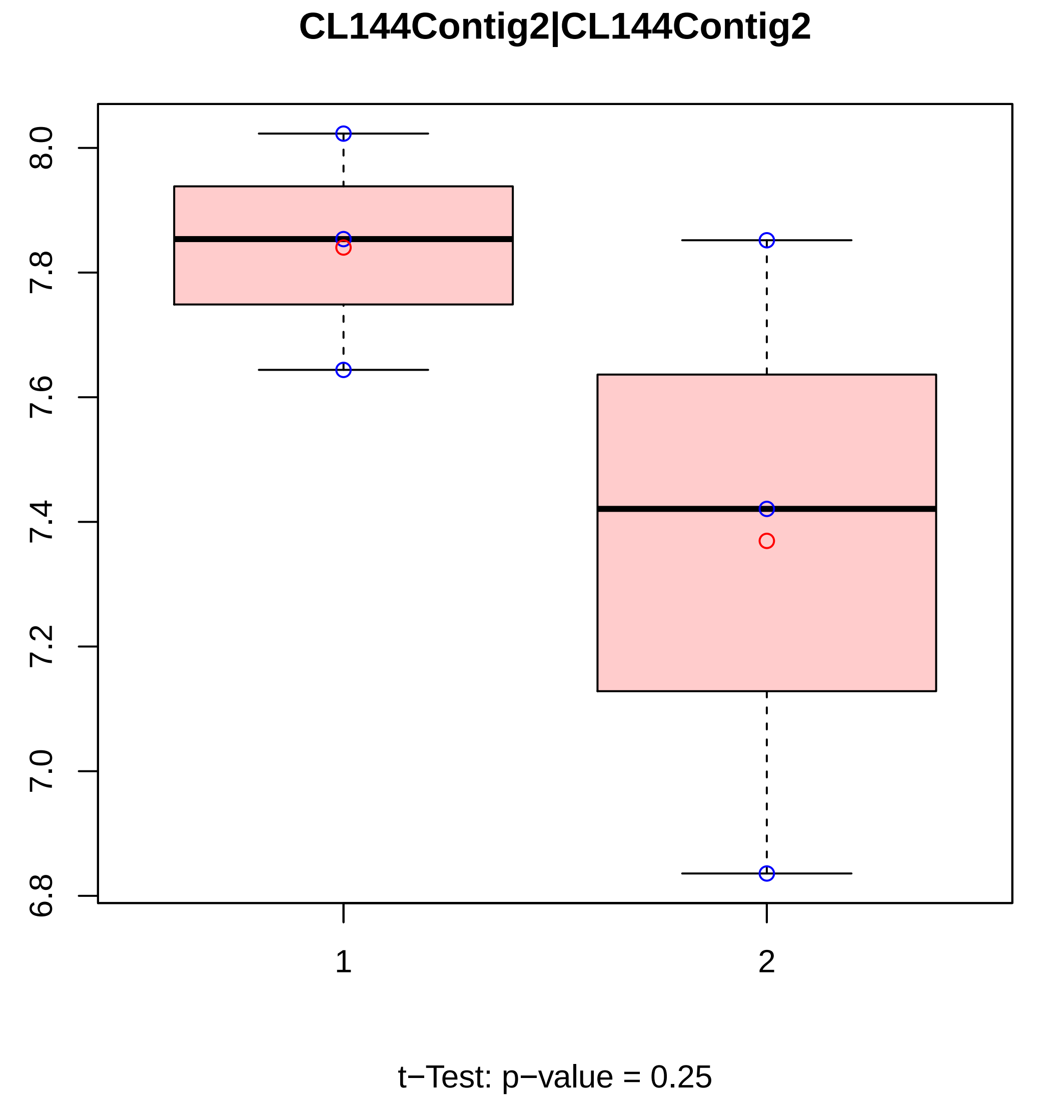
CL144Contig2|CL144Contig2
8.0
7.8
7.6
7.4
7.2
7.0
6.8
1
2
t−Test: p−value = 0.25

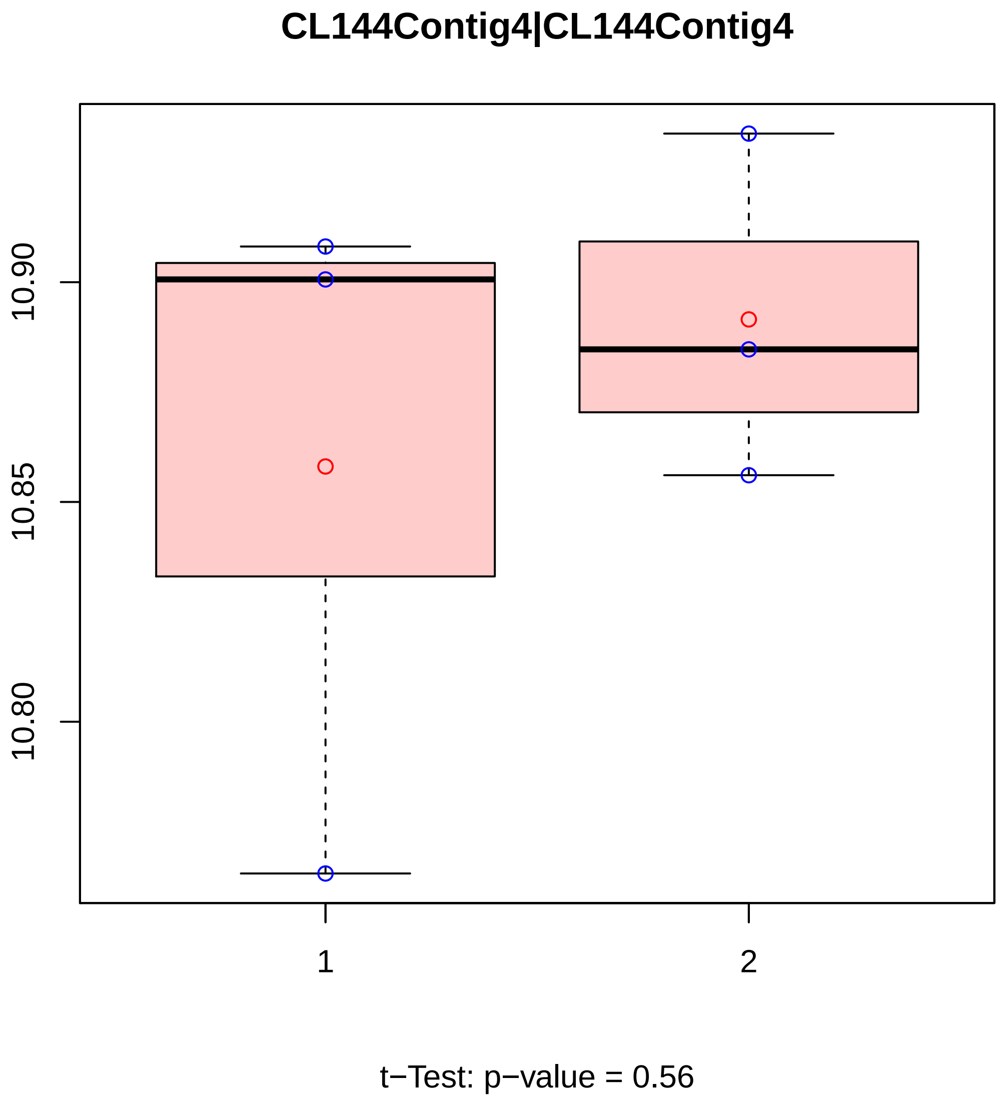

CL144Contig4|CL144Contig4
10.80
10.85
10.90
1
2
t−Test: p−value = 0.56

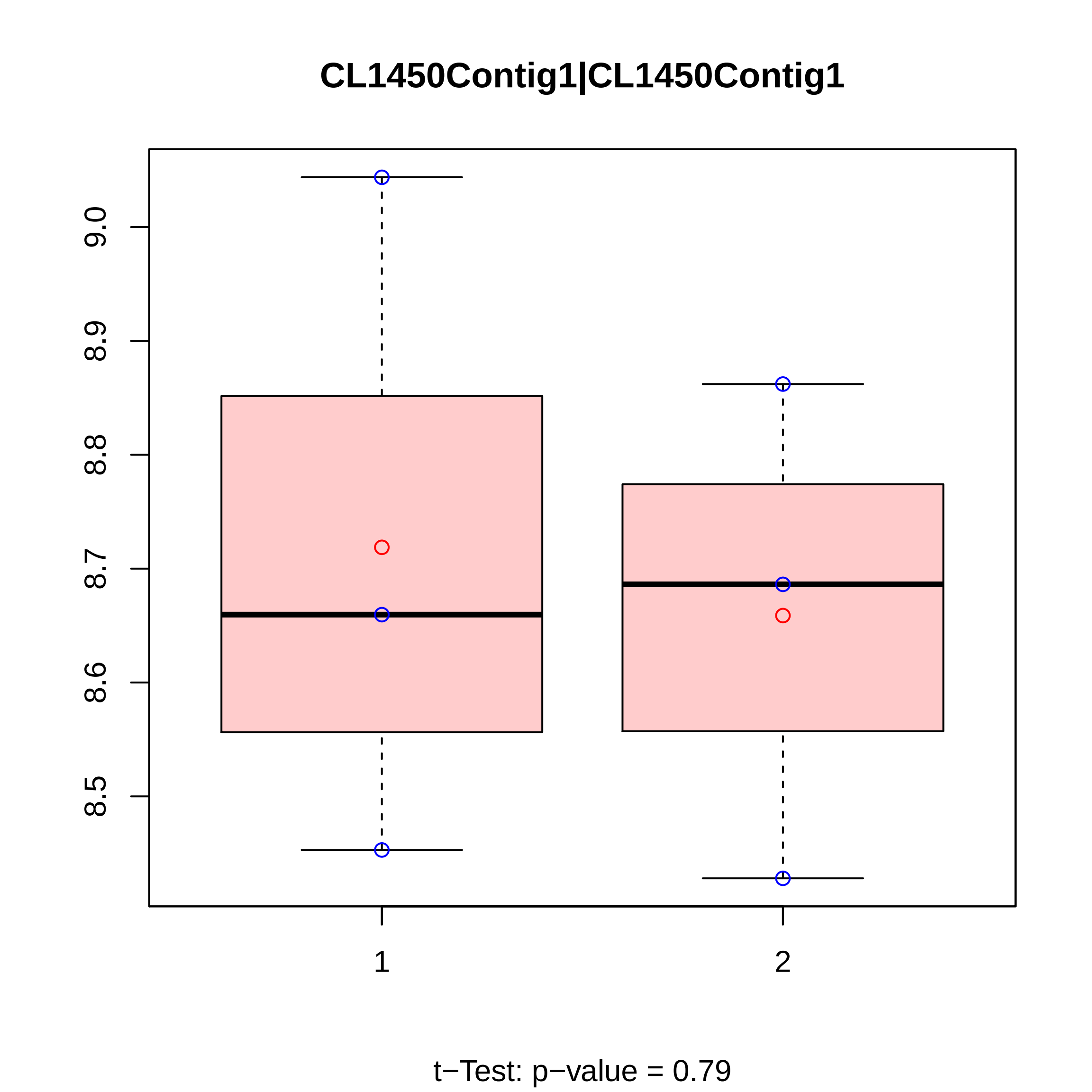

CL1450Contig1|CL1450Contig1
9.0
8.9
8.8
8.7
8.6
8.5
1
2
t−Test: p−value = 0.79

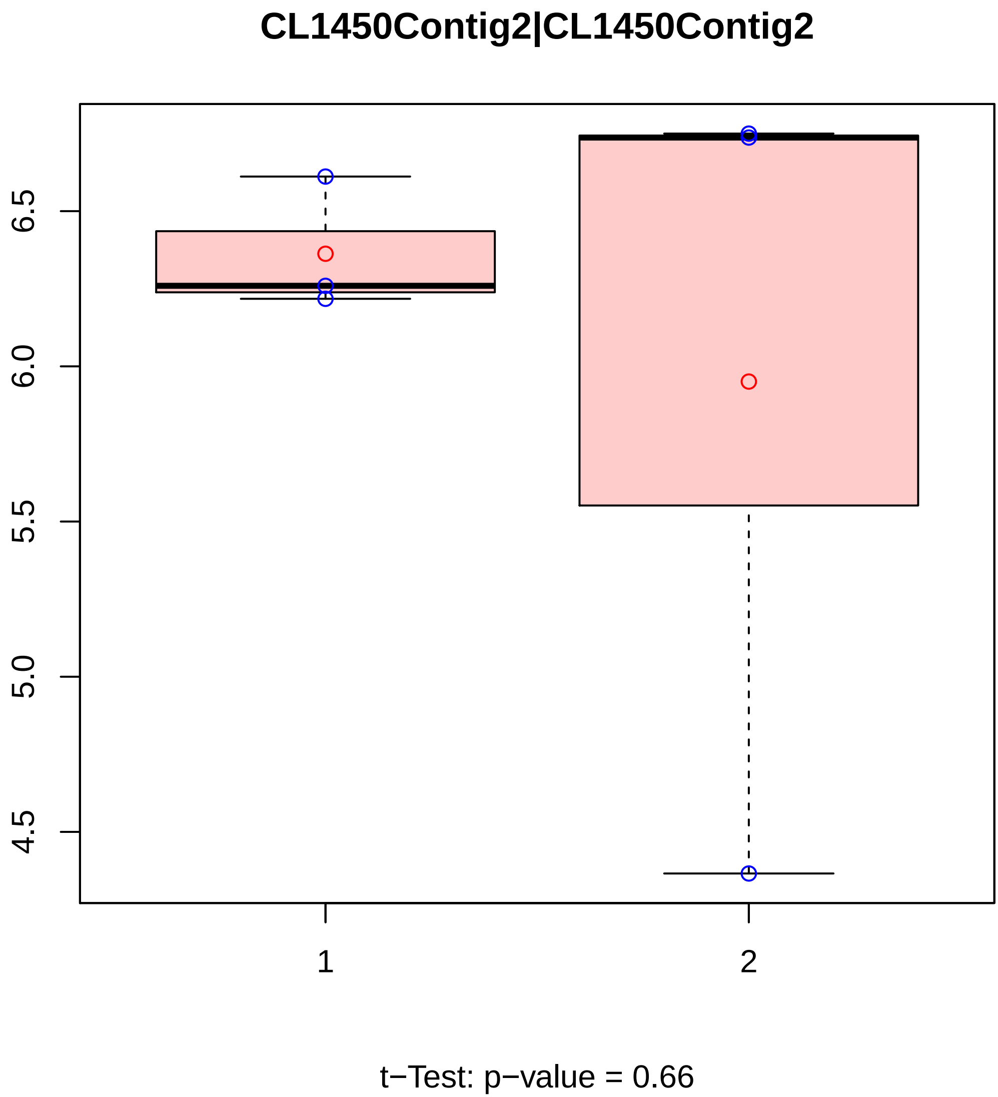

CL1450Contig2|CL1450Contig2
4.5
5.0
5.5
6.0
6.5
1
2
t−Test: p−value = 0.66

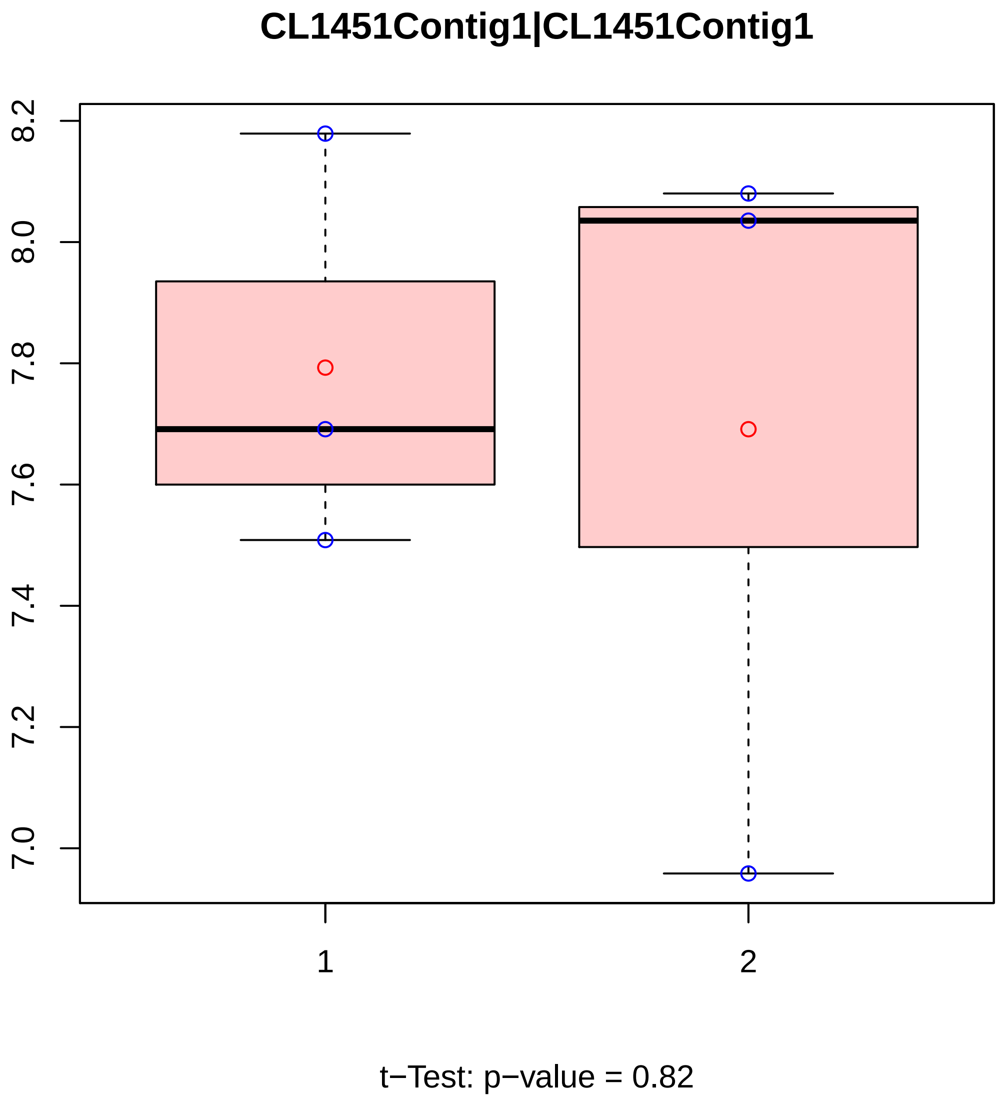
CL1451Contig1|CL1451Contig1
8.2
8.0
7.8
7.6
7.4
7.2
7.0
1
2
t−Test: p−value = 0.82

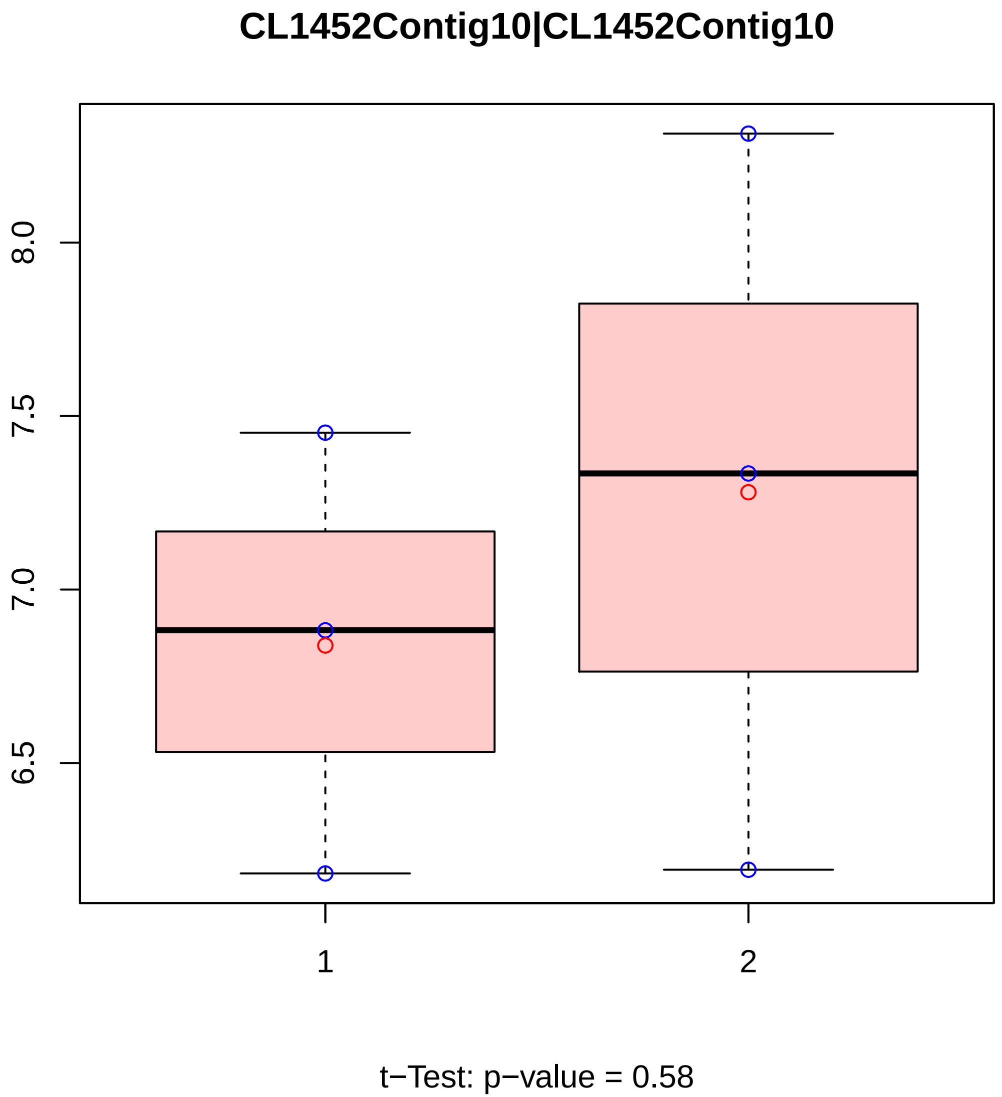
CL1452Contig10|CL1452Contig10
6.5
7.0
7.5
8.0
1
2
t−Test: p−value = 0.58

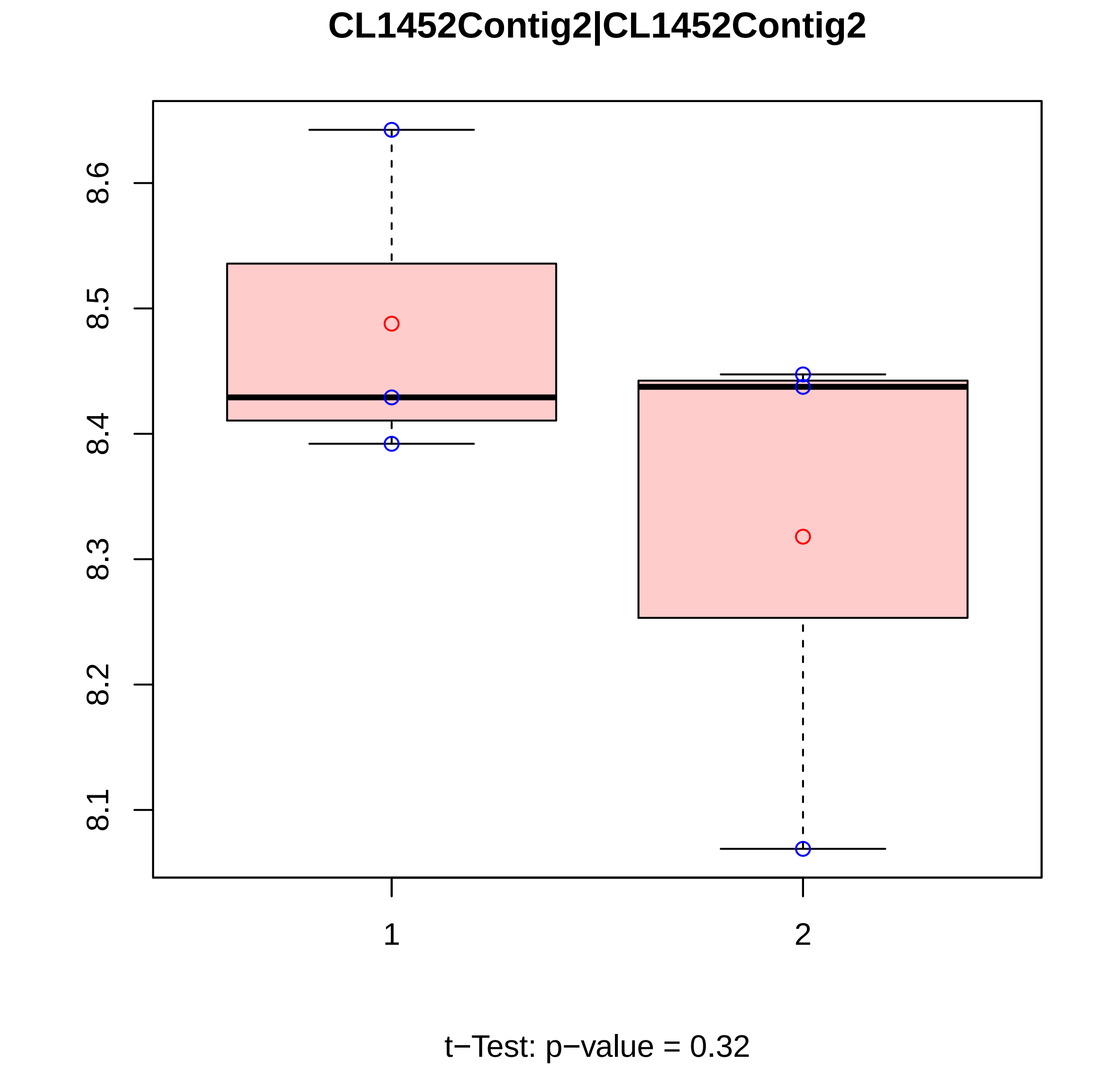
CL1452Contig2|CL1452Contig2
8.1
8.2
8.3
8.4
8.5
8.6
1
2
t−Test: p−value = 0.32

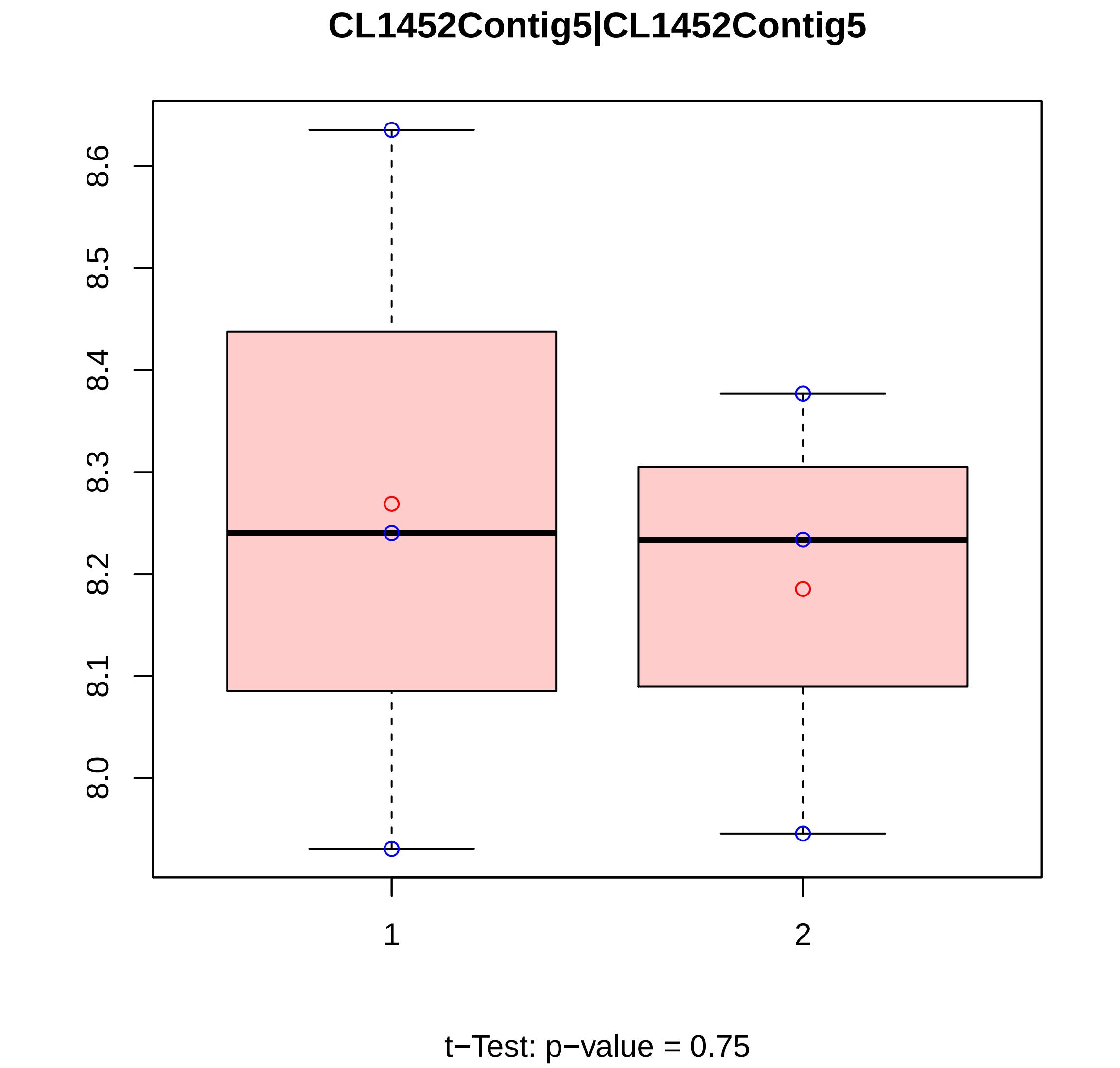
CL1452Contig5|CL1452Contig5
8.0
8.1
8.2
8.3
8.4
8.5
8.6
1
2
t−Test: p−value = 0.75

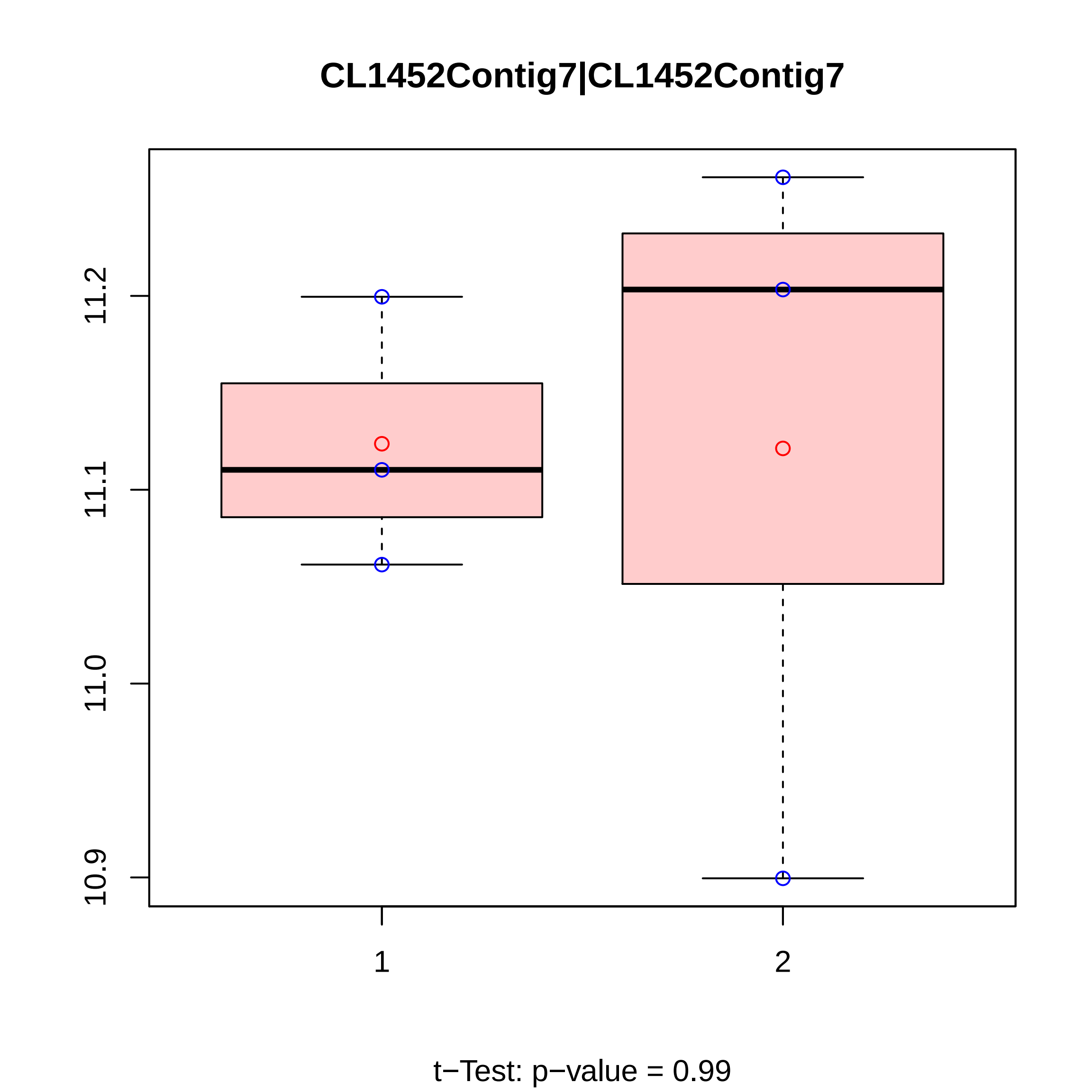
CL1452Contig7|CL1452Contig7
11.2
11.1
11.0
10.9
1
2
t−Test: p−value = 0.99

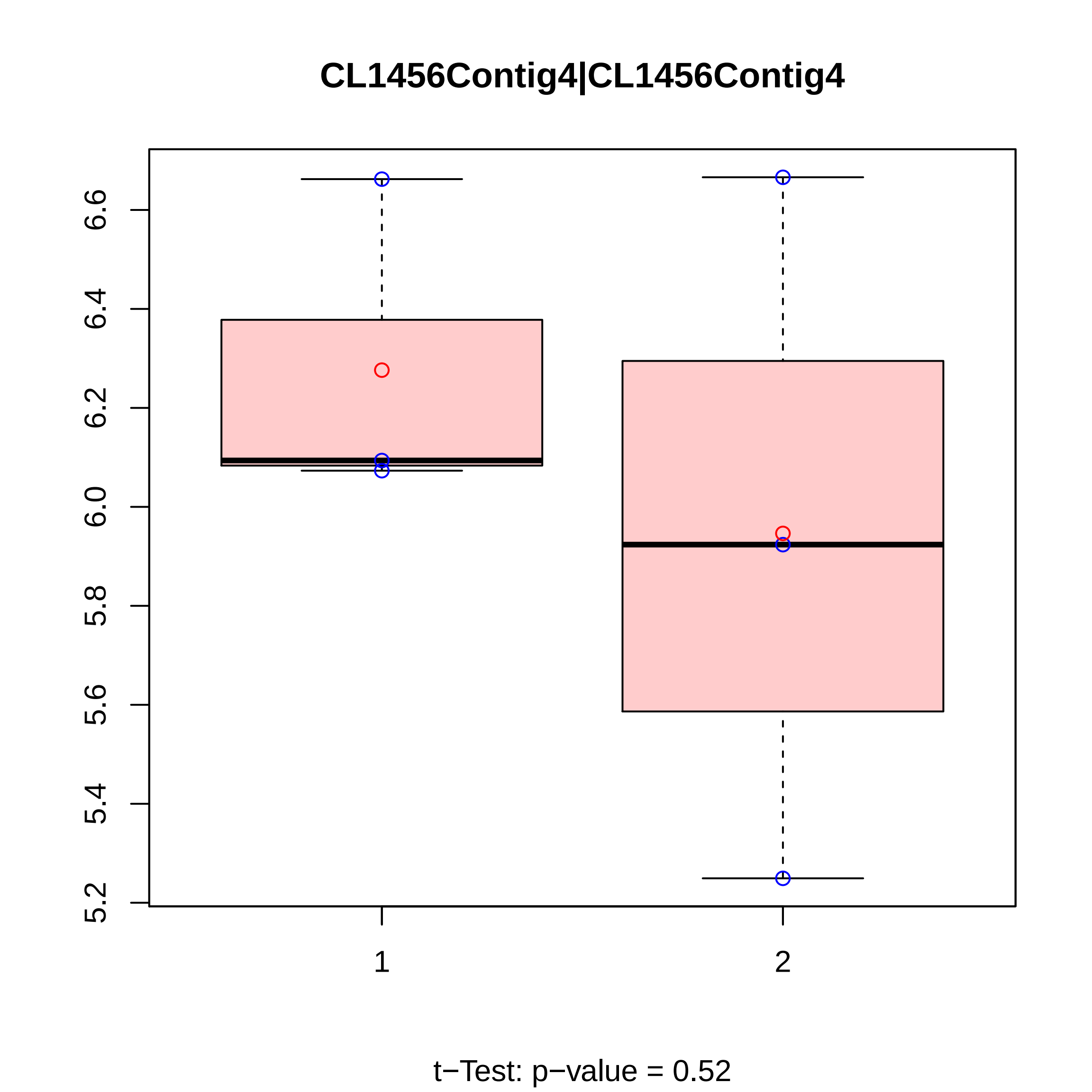

CL1456Contig4|CL1456Contig4
6.6
6.4
6.2
6.0
5.8
5.6
5.4
5.2
1
2
t−Test: p−value = 0.52

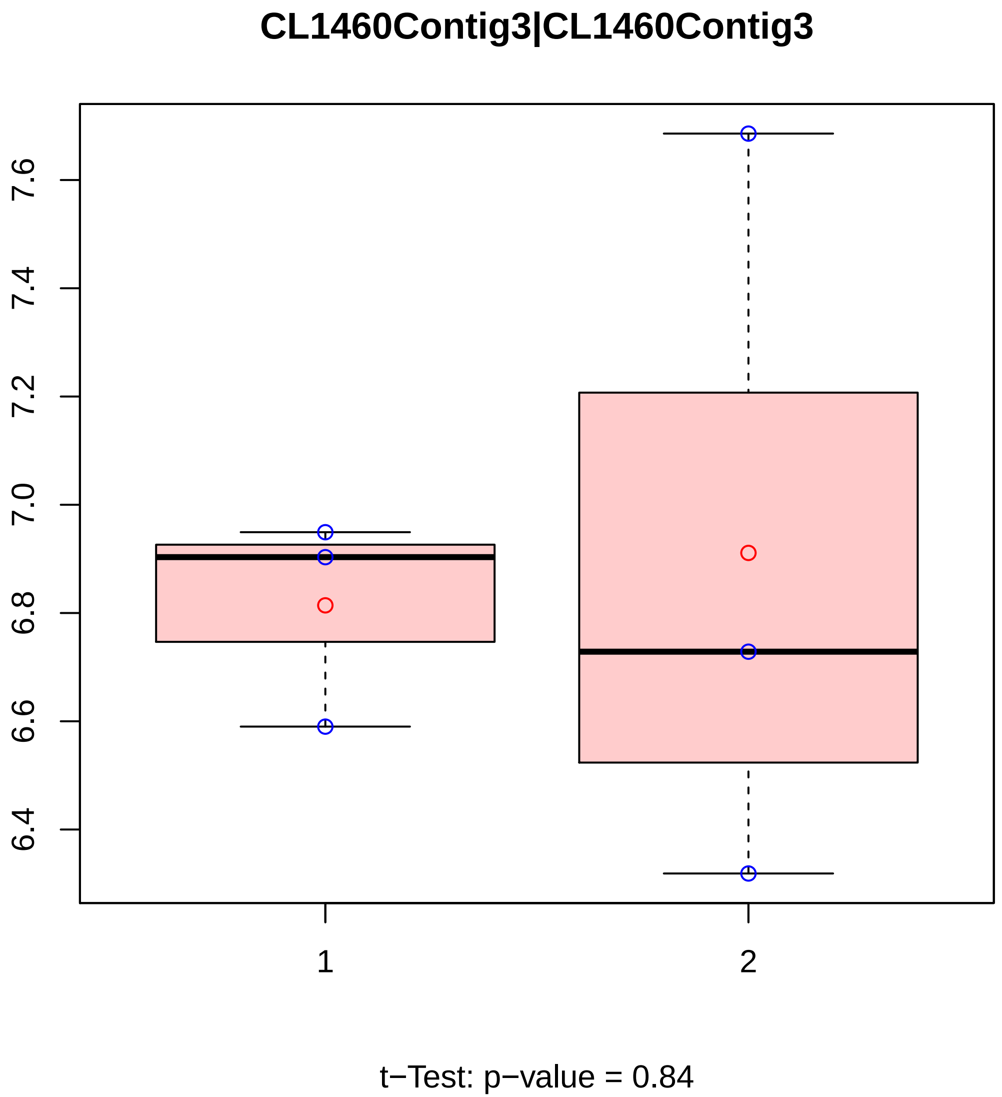
CL1460Contig3|CL1460Contig3
7.6
7.4
7.2
7.0
6.8
6.6
6.4
1
2
t−Test: p−value = 0.84

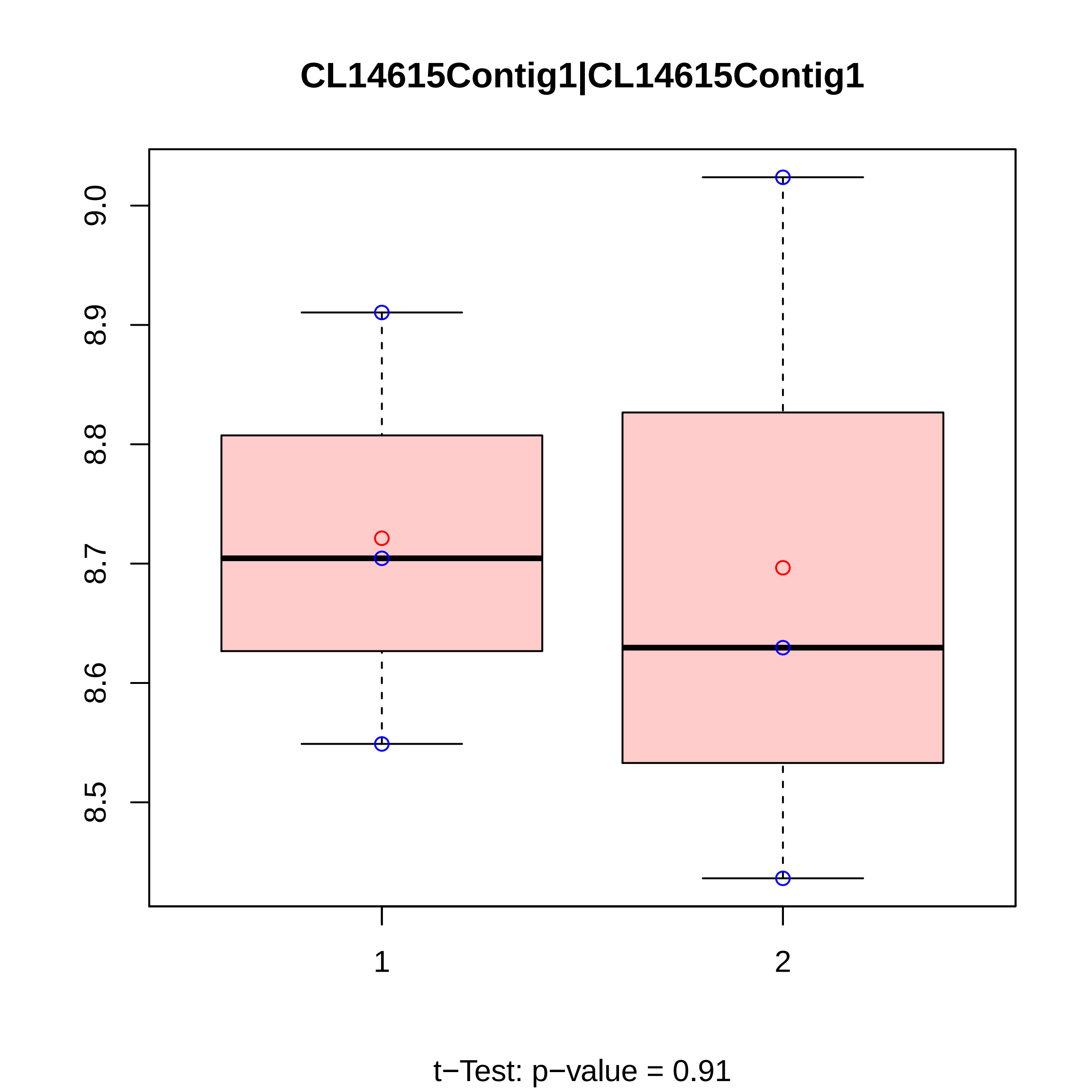
CL14615Contig1|CL14615Contig1
9.0
8.9
8.8
8.7
8.6
8.5
1
2
t−Test: p−value = 0.91

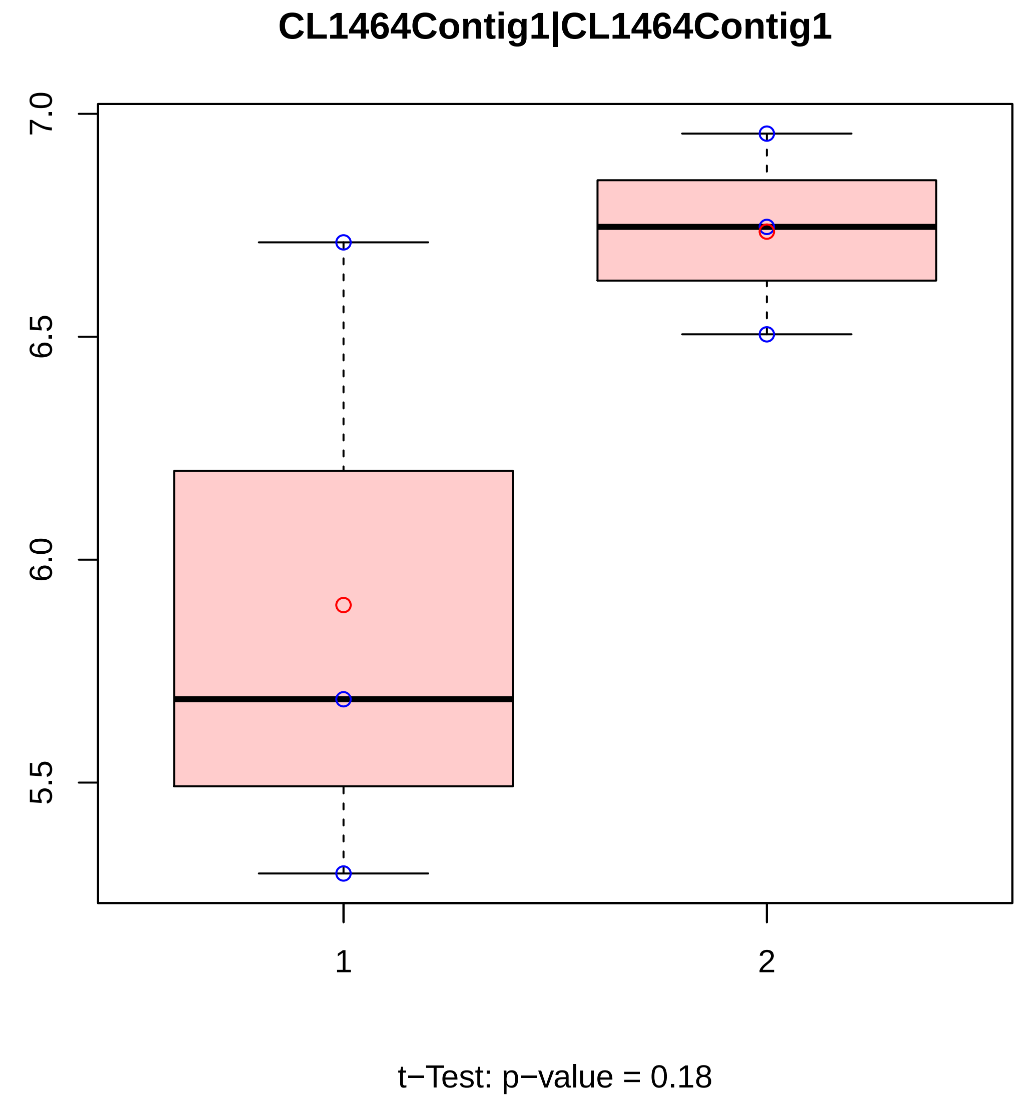
CL1464Contig1|CL1464Contig1
7.0
6.5
6.0
5.5
1
2
t−Test: p−value = 0.18

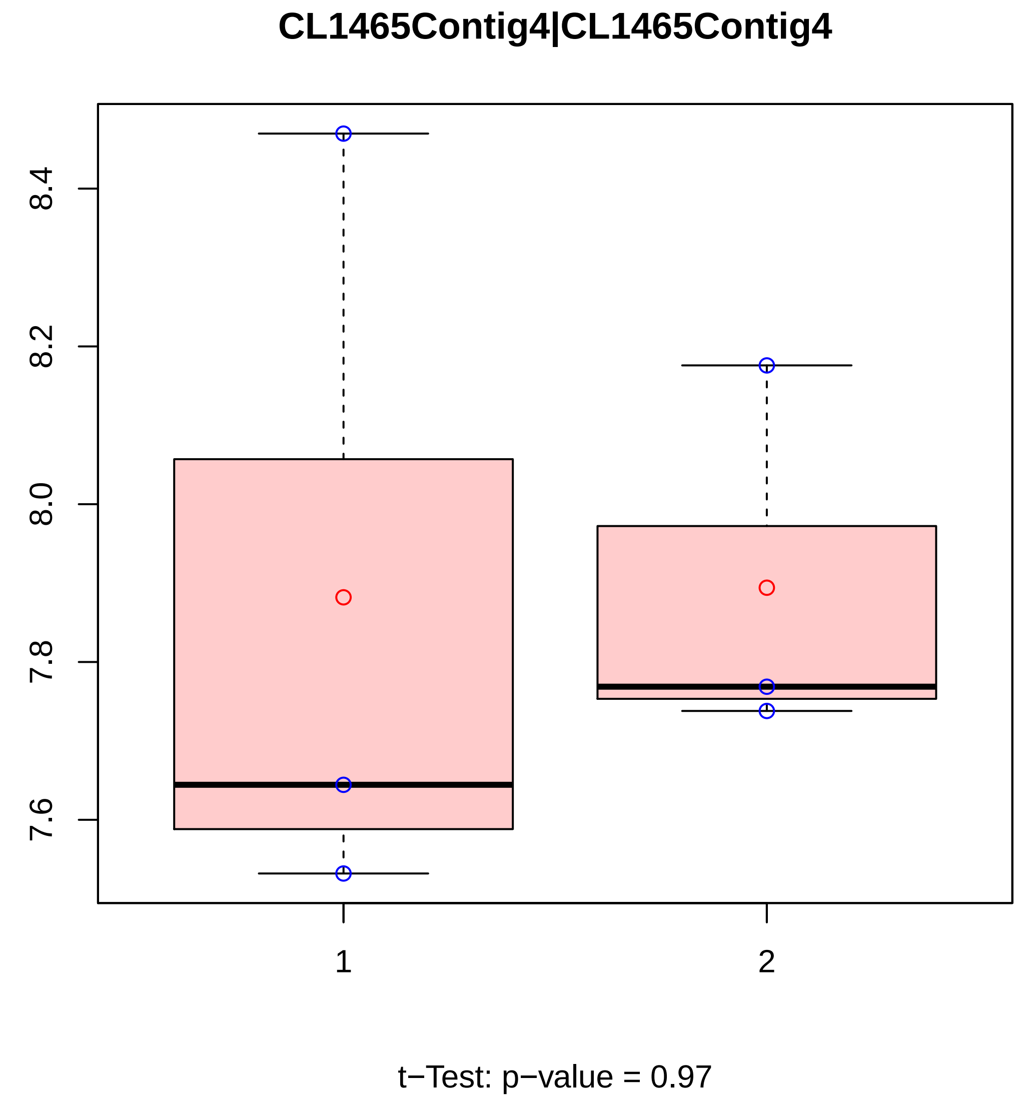

CL1465Contig4|CL1465Contig4
8.4
8.2
8.0
7.8
7.6
1
2
t−Test: p−value = 0.97

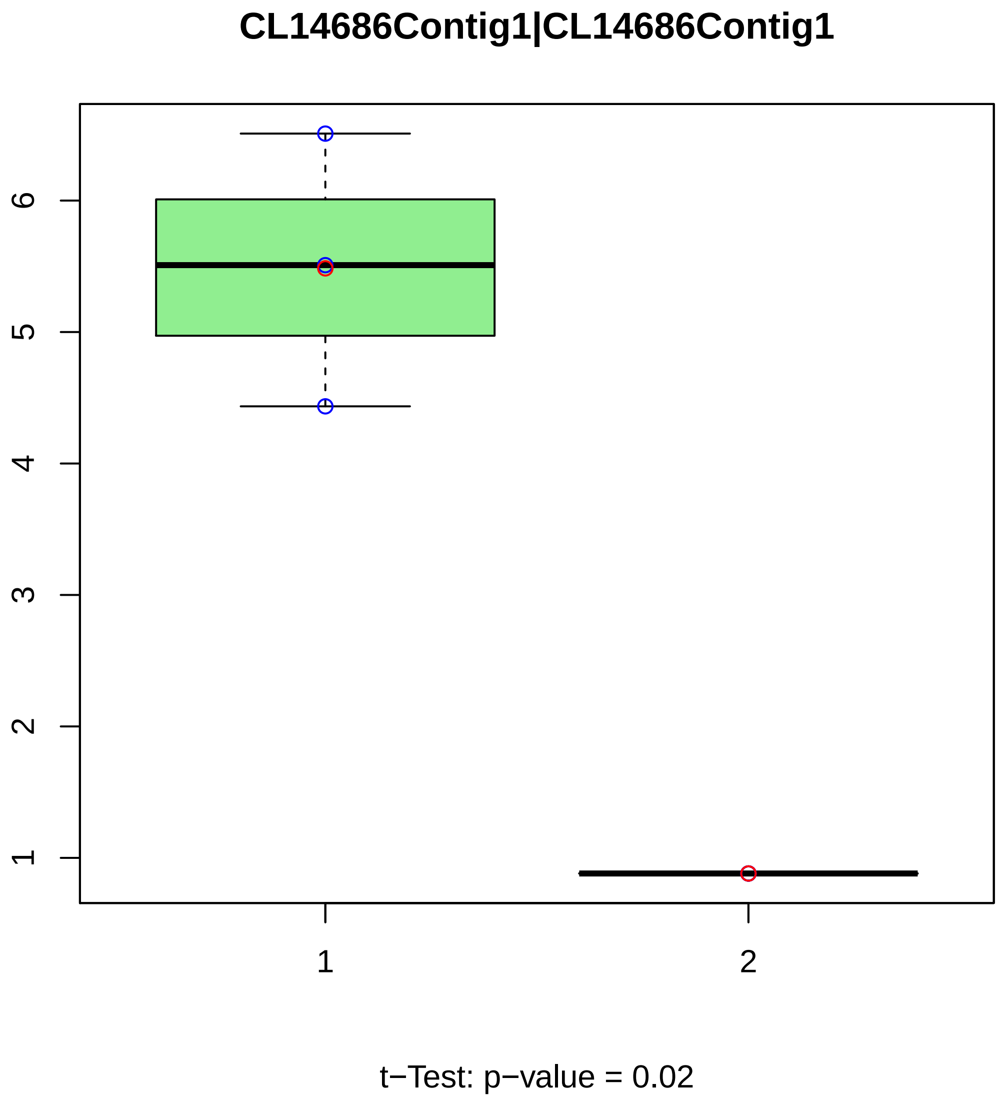

CL14686Contig1|CL14686Contig1
1
2
3
4
5
6
1
2
t−Test: p−value = 0.02

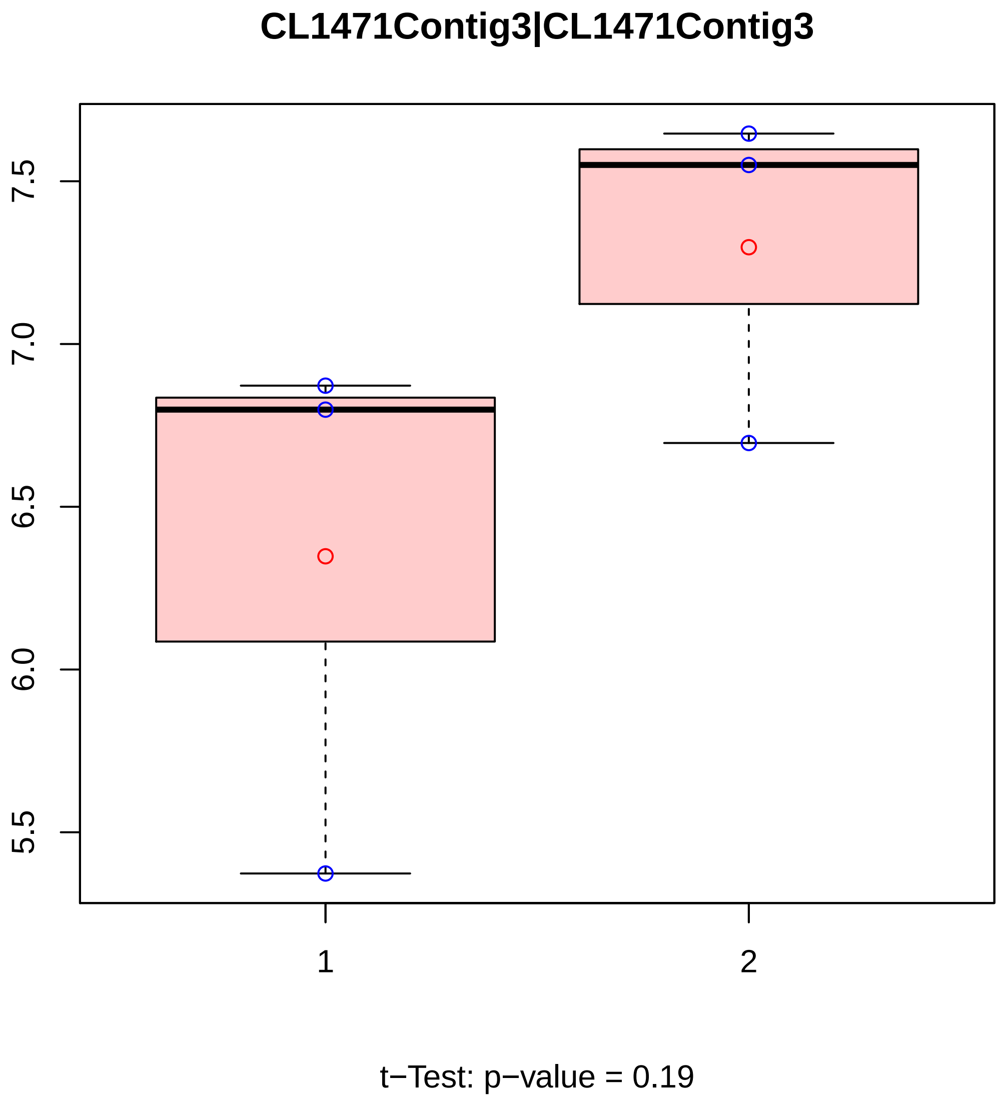

CL1471Contig3|CL1471Contig3
7.5
7.0
6.5
6.0
5.5
1
2
t−Test: p−value = 0.19

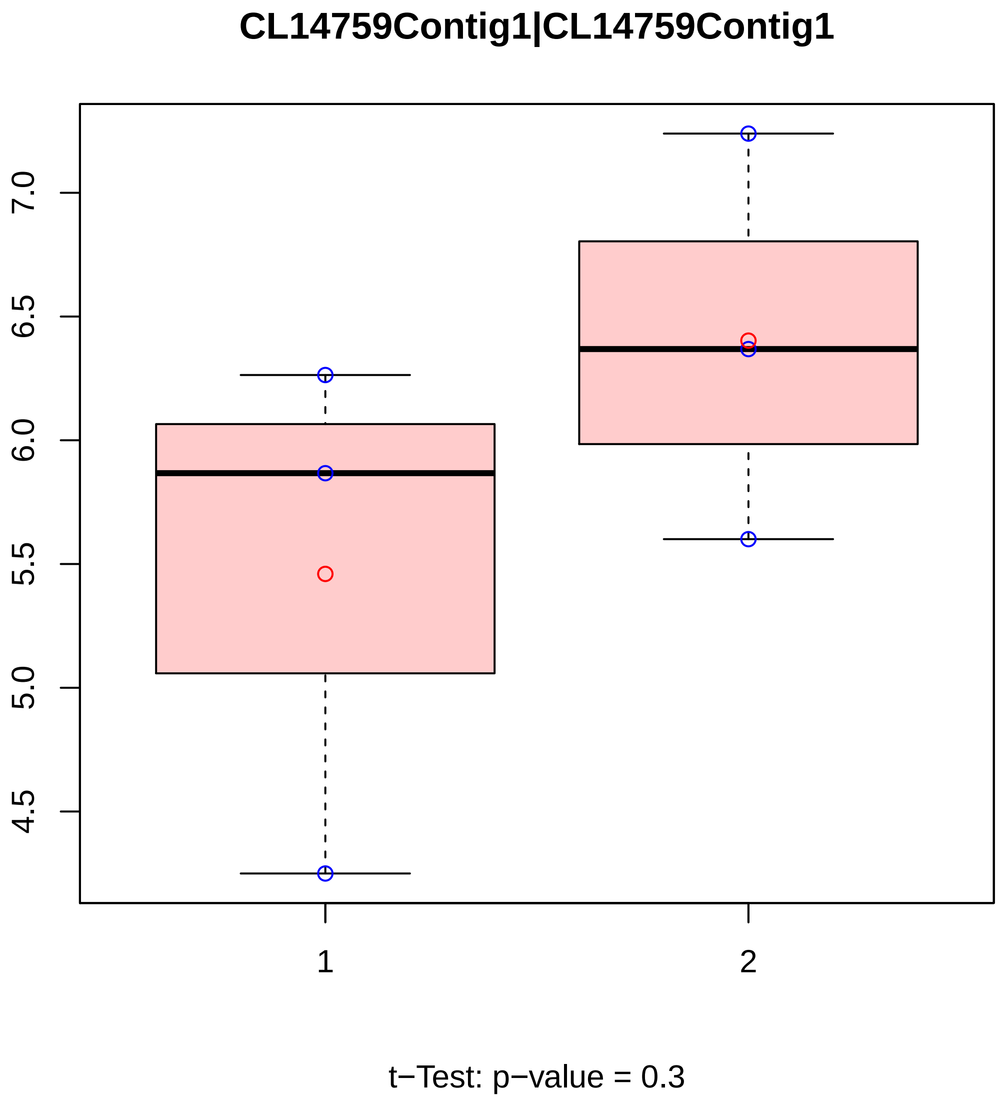
CL14759Contig1|CL14759Contig1
7.0
6.5
6.0
5.5
5.0
4.5
1
2
t−Test: p−value = 0.3

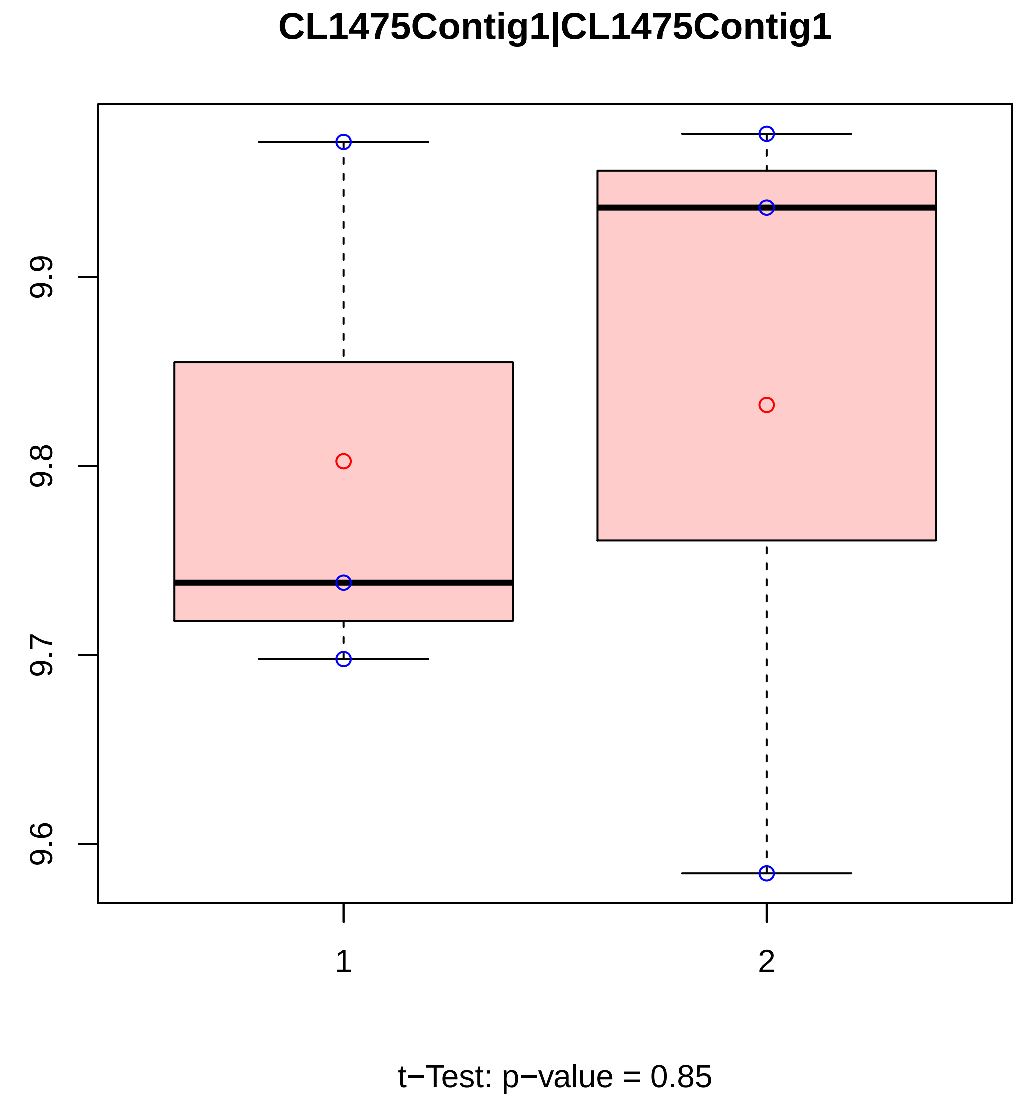
CL1475Contig1|CL1475Contig1
9.6
9.7
9.8
9.9
1
2
t−Test: p−value = 0.85

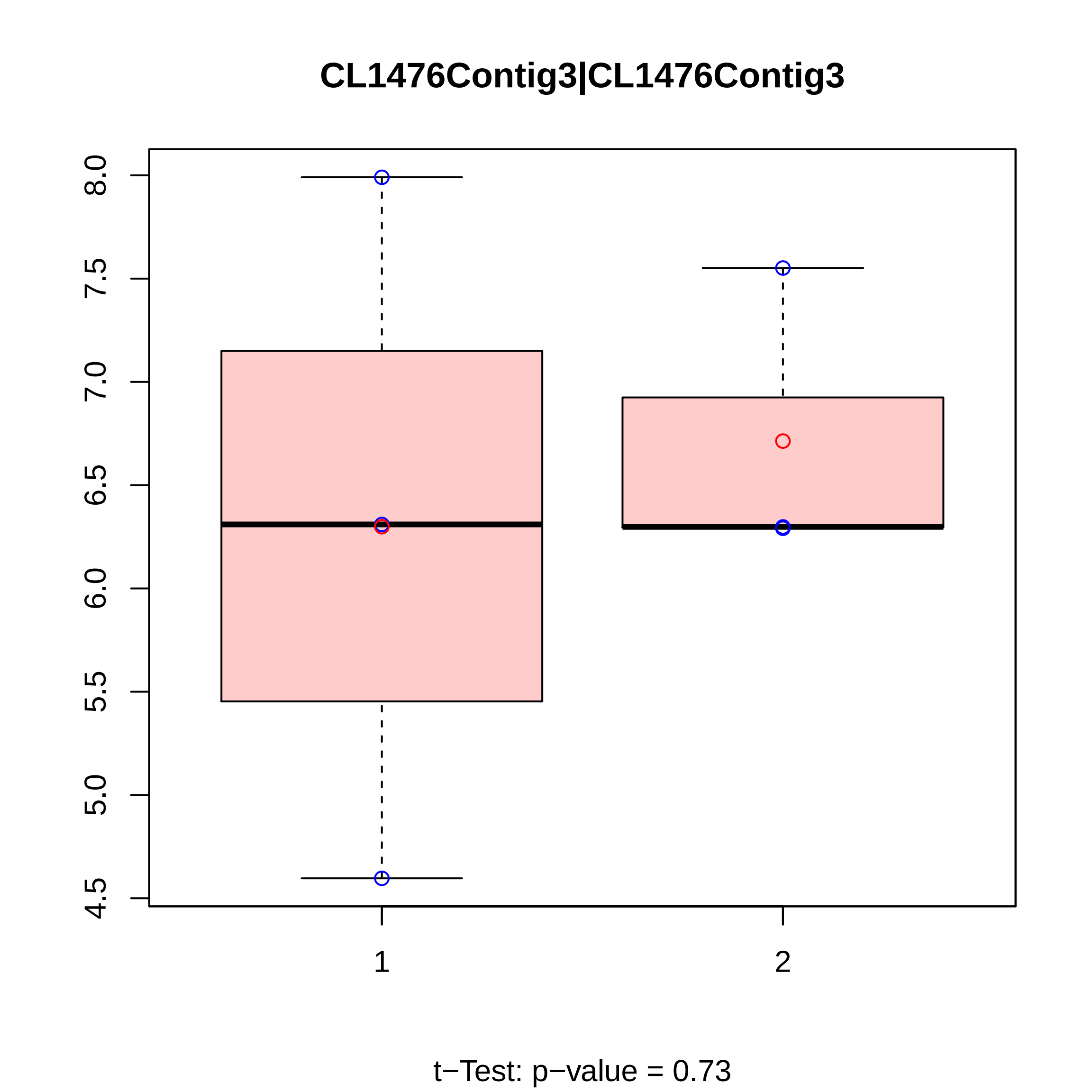
CL1476Contig3|CL1476Contig3
8.0
7.5
7.0
6.5
6.0
5.5
5.0
4.5
1
2
t−Test: p−value = 0.73

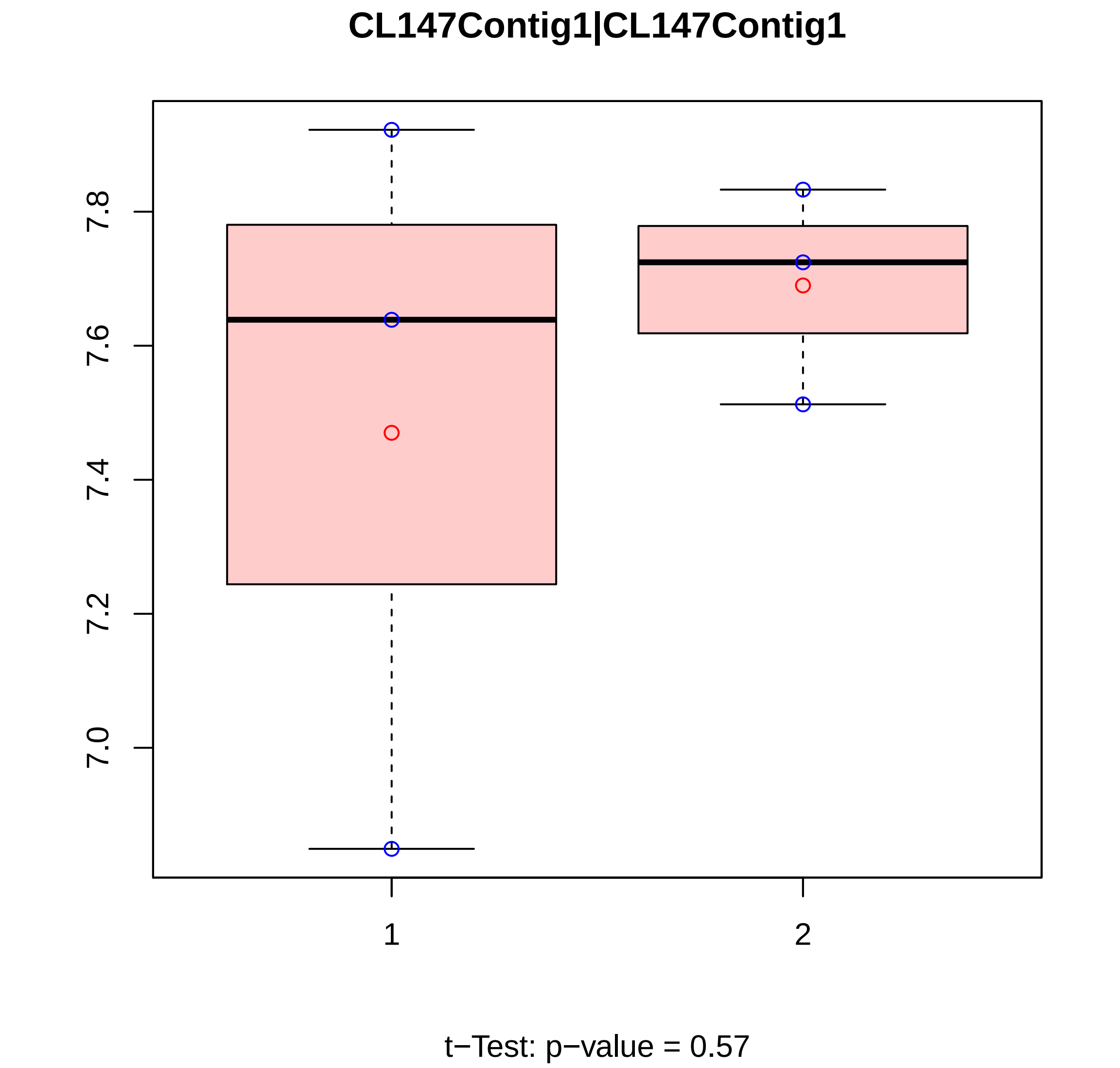
CL147Contig1|CL147Contig1
7.0
7.2
7.4
7.6
7.8
1
2
t−Test: p−value = 0.57

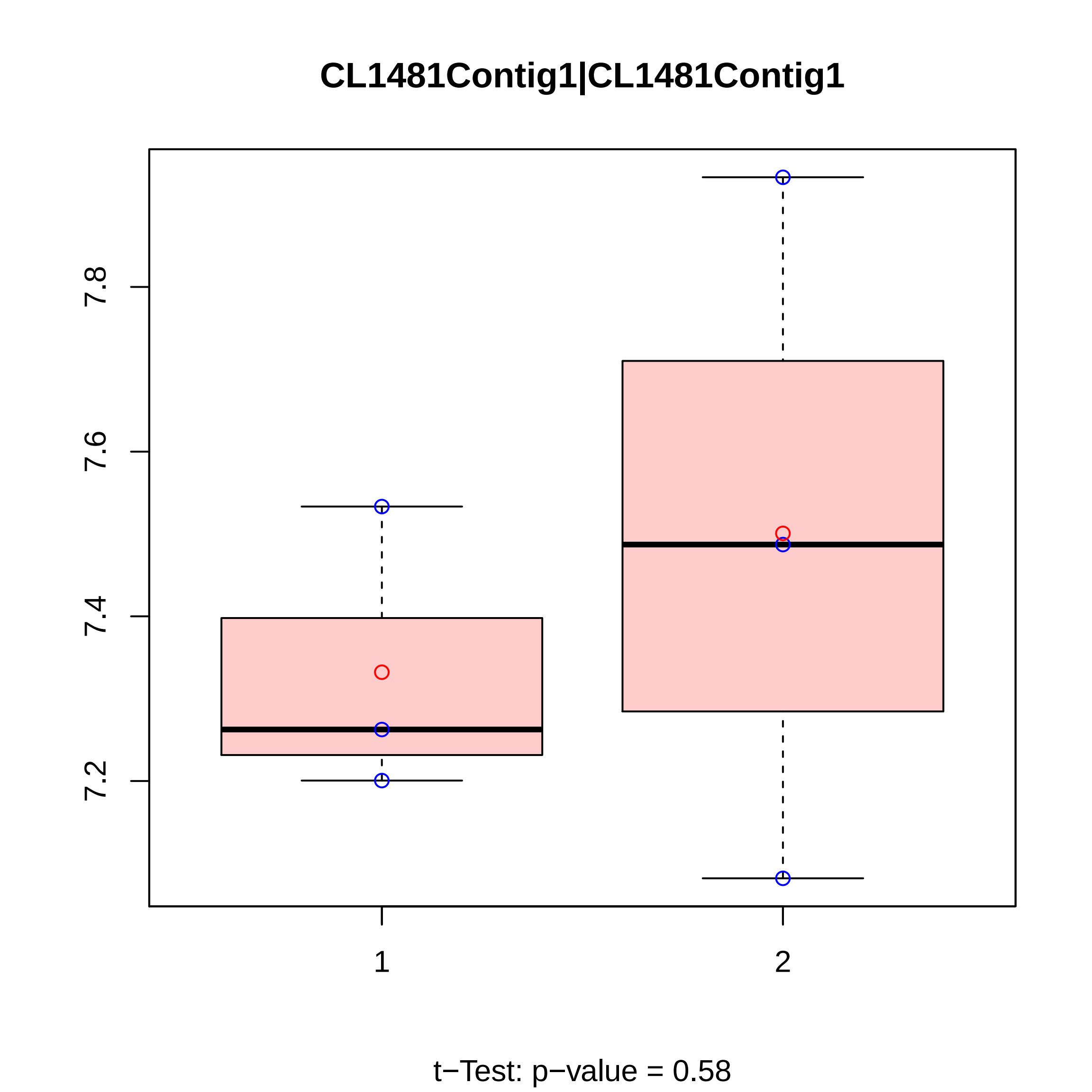
CL1481Contig1|CL1481Contig1
7.2
7.4
7.6
7.8
1
2
t−Test: p−value = 0.58

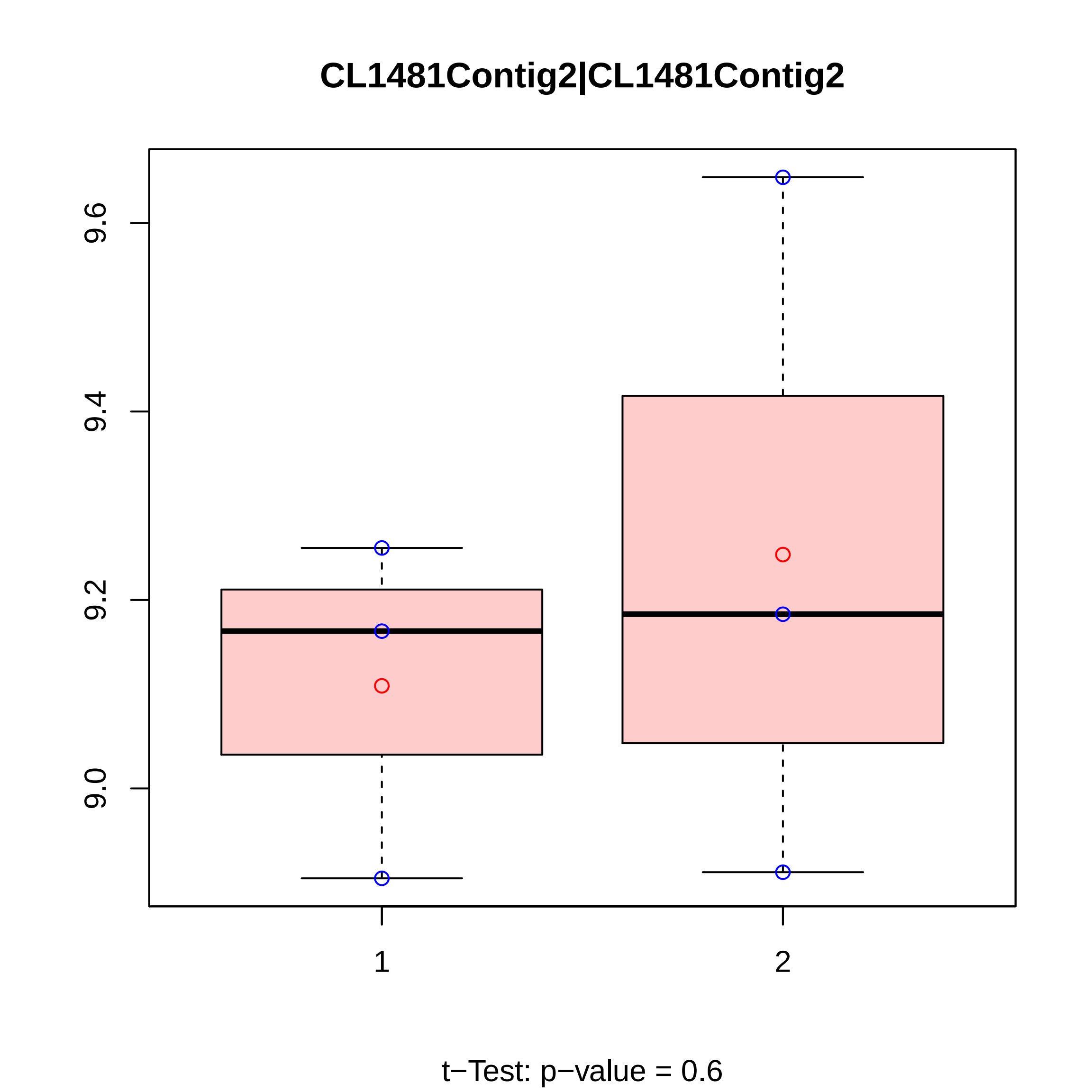
CL1481Contig2|CL1481Contig2
9.0
9.2
9.4
9.6
1
2
t−Test: p−value = 0.6

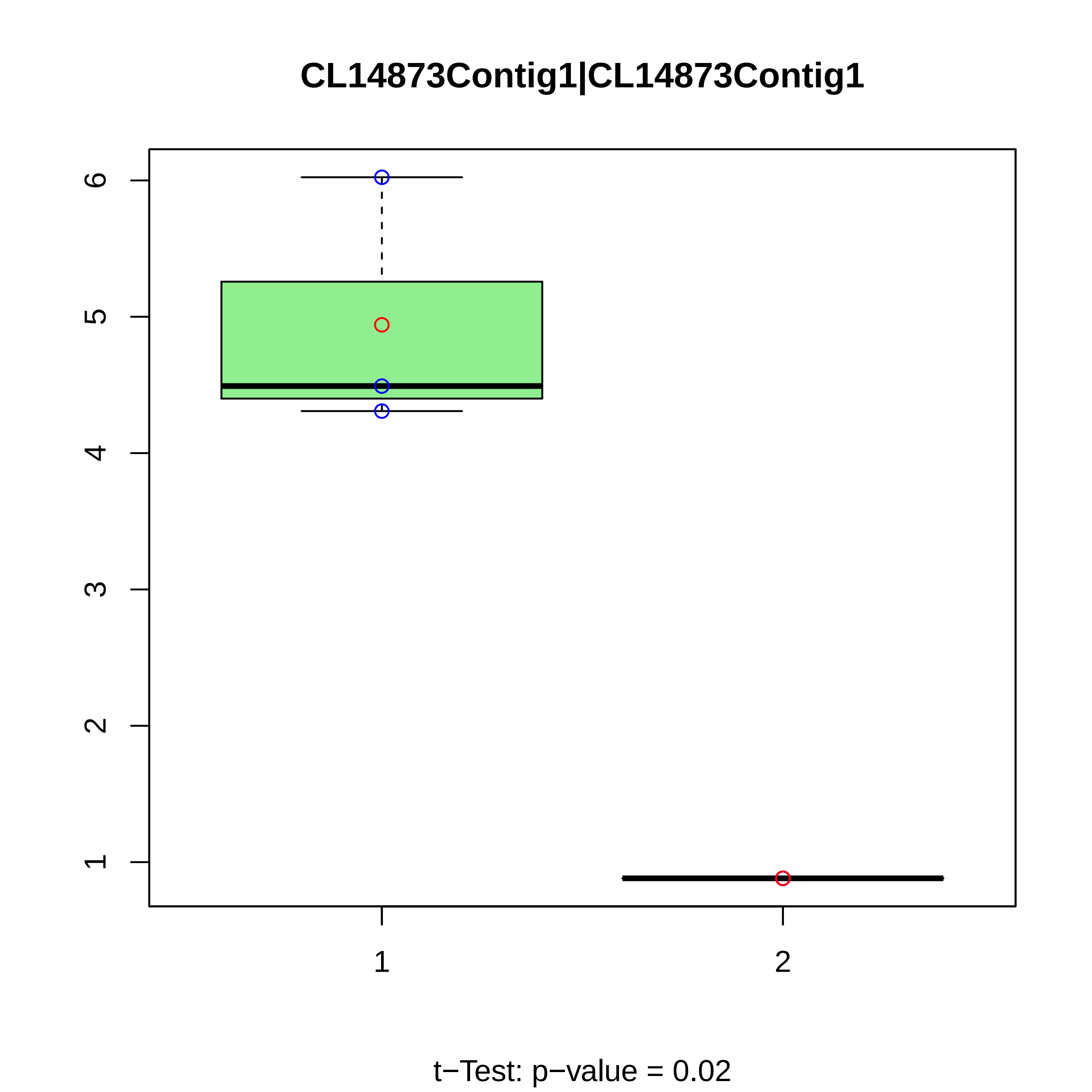

CL14873Contig1|CL14873Contig1
1
2
3
4
5
6
1
2
t−Test: p−value = 0.02

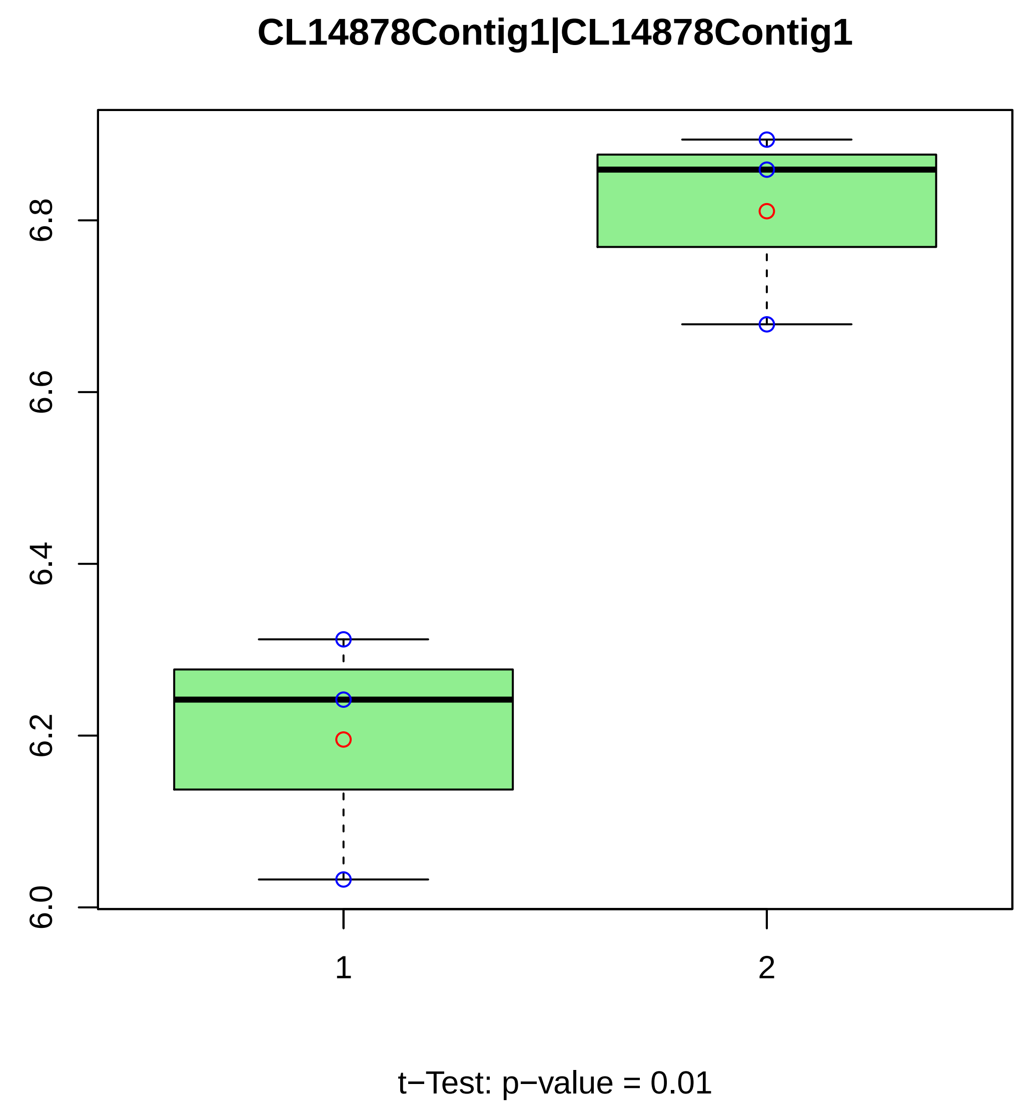
CL14878Contig1|CL14878Contig1
6.0
6.2
6.4
6.6
6.8
1
2
t−Test: p−value = 0.01

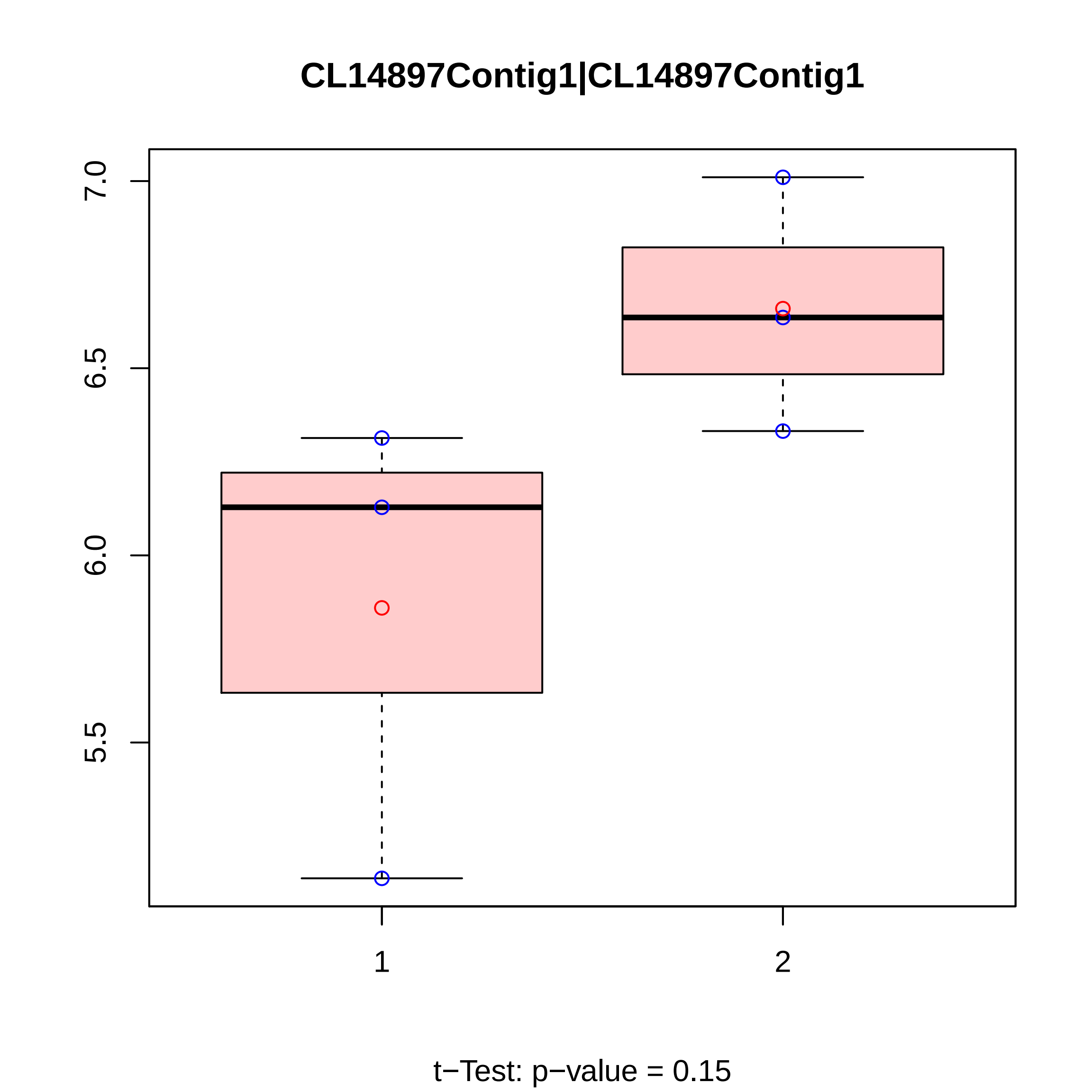
CL14897Contig1|CL14897Contig1
7.0
6.5
6.0
5.5
1
2
t−Test: p−value = 0.15

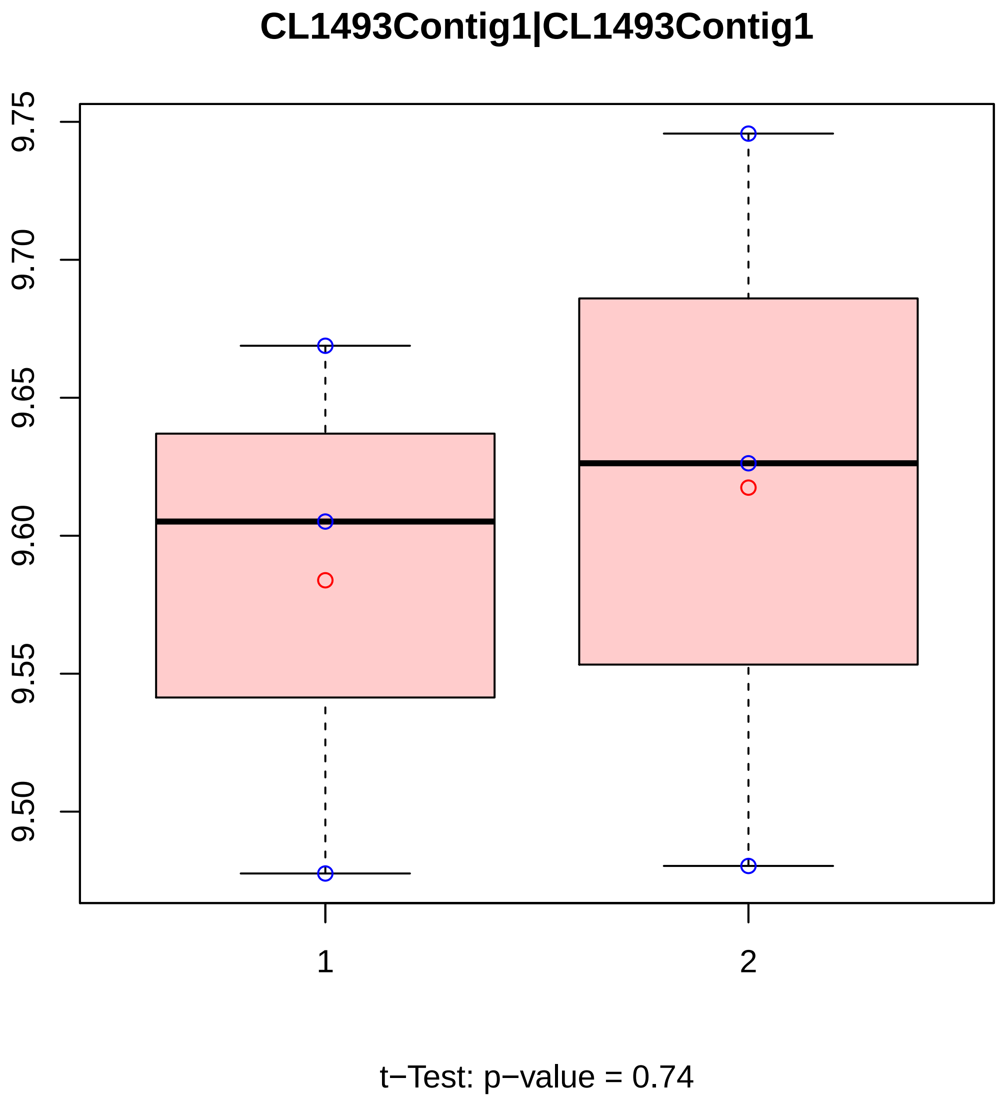
CL1493Contig1|CL1493Contig1
9.50
9.55
9.60
9.65
9.70
9.75
1
2
t−Test: p−value = 0.74

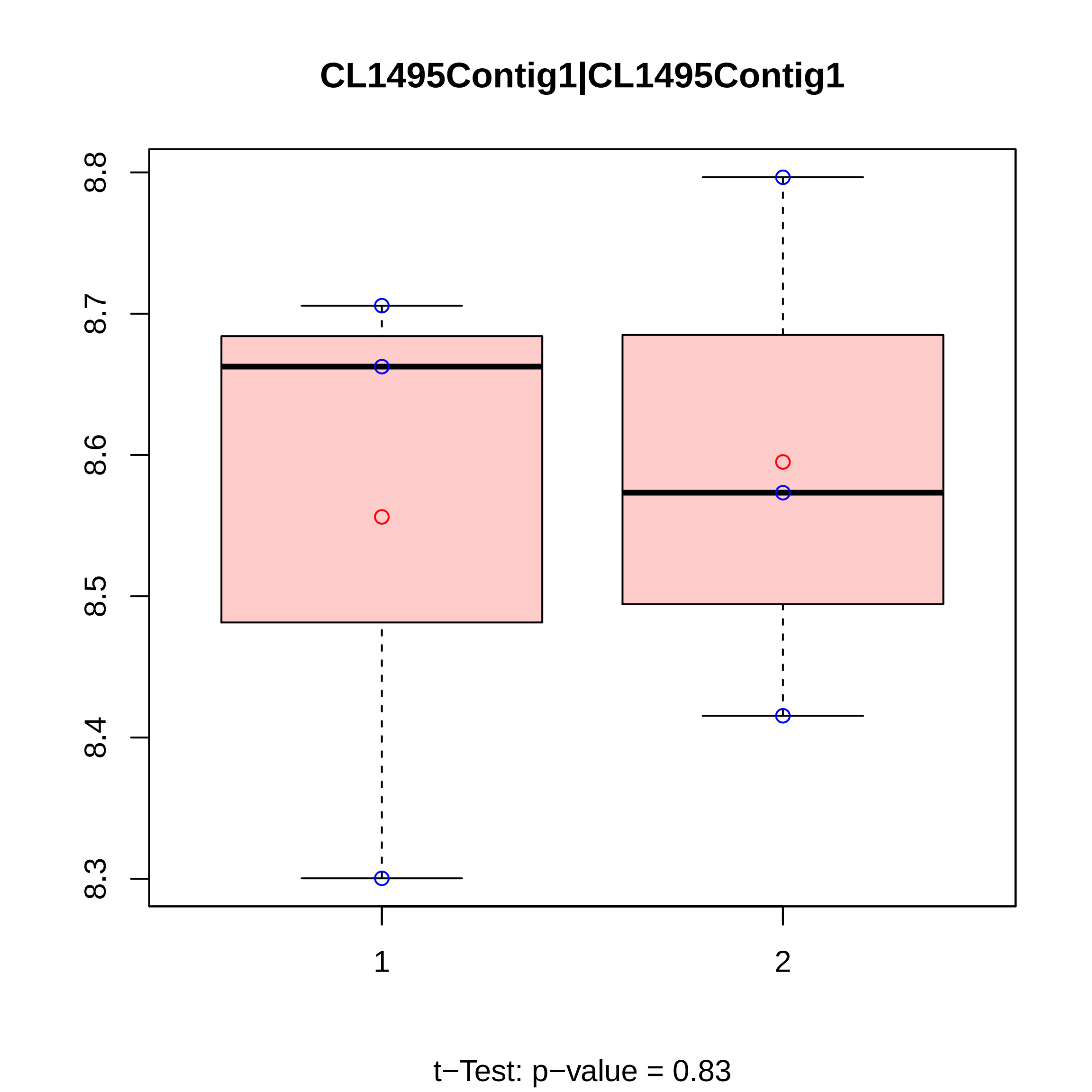
CL1495Contig1|CL1495Contig1
8.3
8.4
8.5
8.6
8.7
8.8
1
2
t−Test: p−value = 0.83

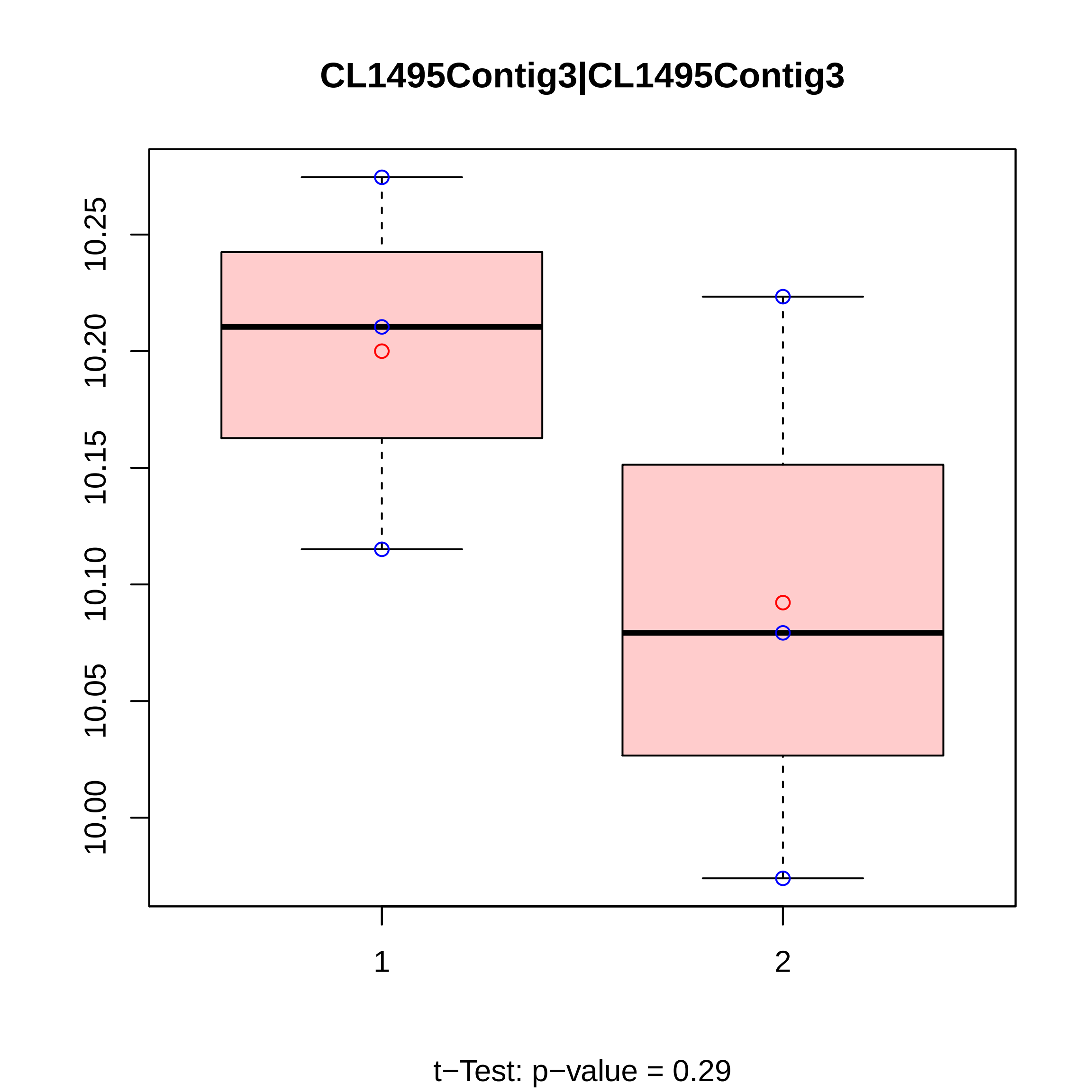
CL1495Contig3|CL1495Contig3
10.00
10.05
10.10
10.15
10.20
10.25
1
2
t−Test: p−value = 0.29

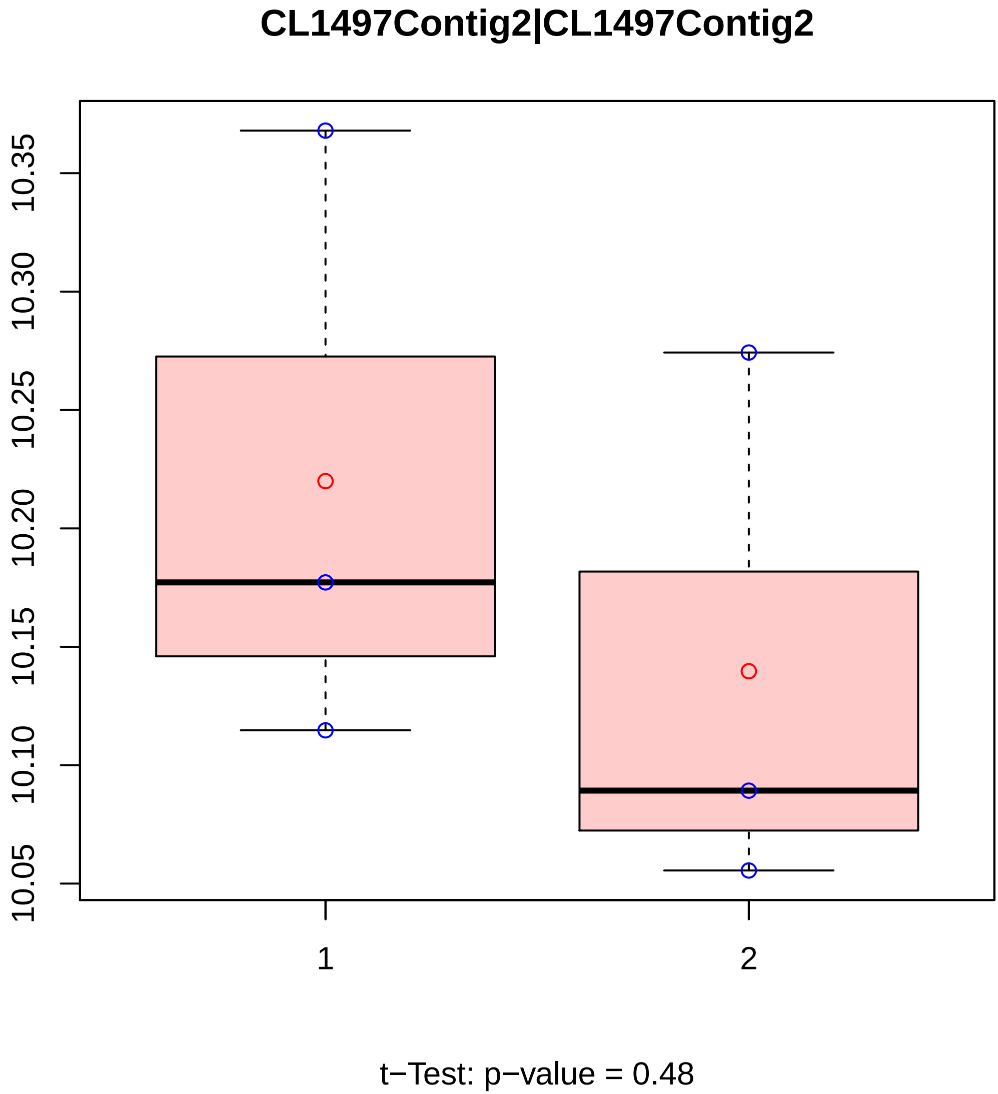
CL1497Contig2|CL1497Contig2
10.05
10.10
10.15
10.20
10.25
10.30
10.35
1
2
t−Test: p−value = 0.48

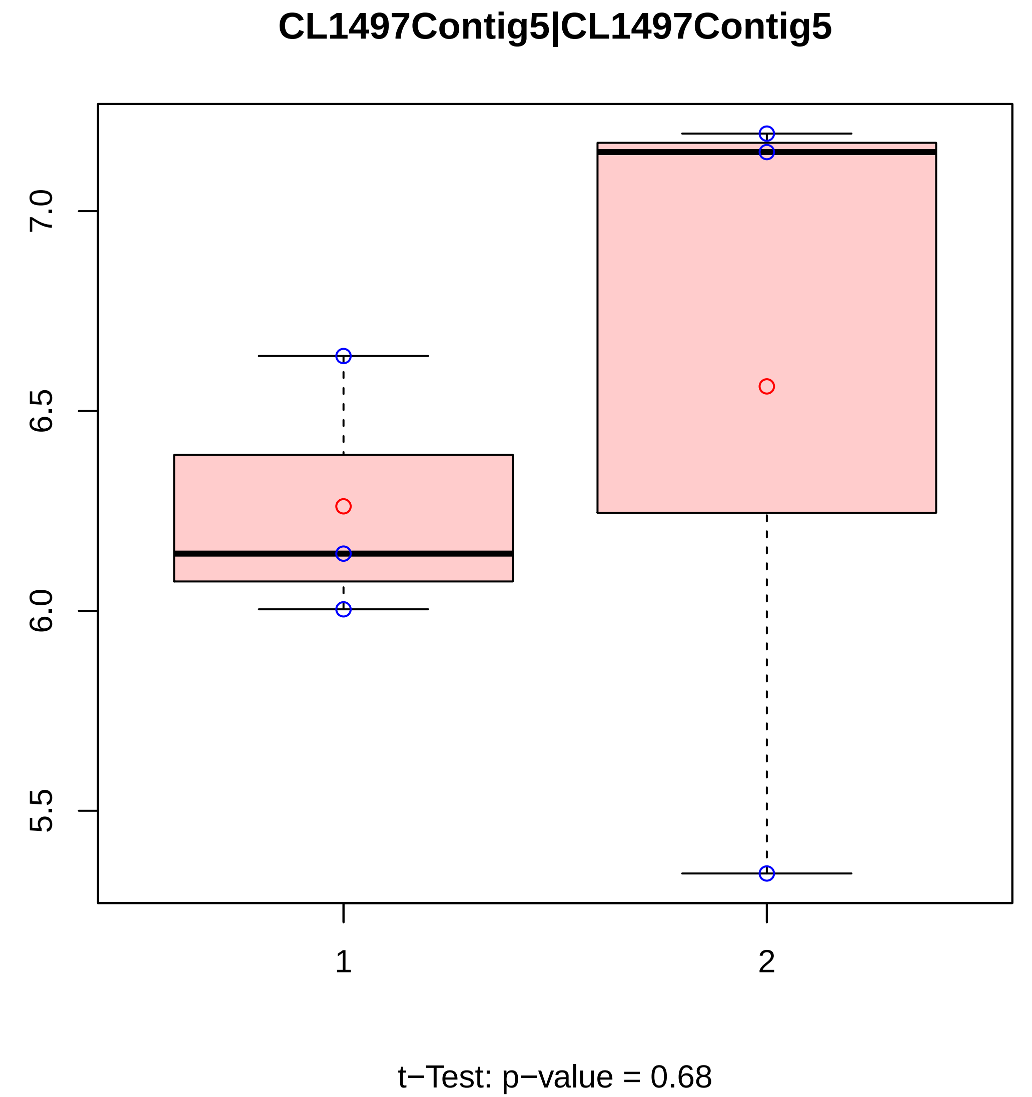
CL1497Contig5|CL1497Contig5
7.0
6.5
6.0
5.5
1
2
t−Test: p−value = 0.68

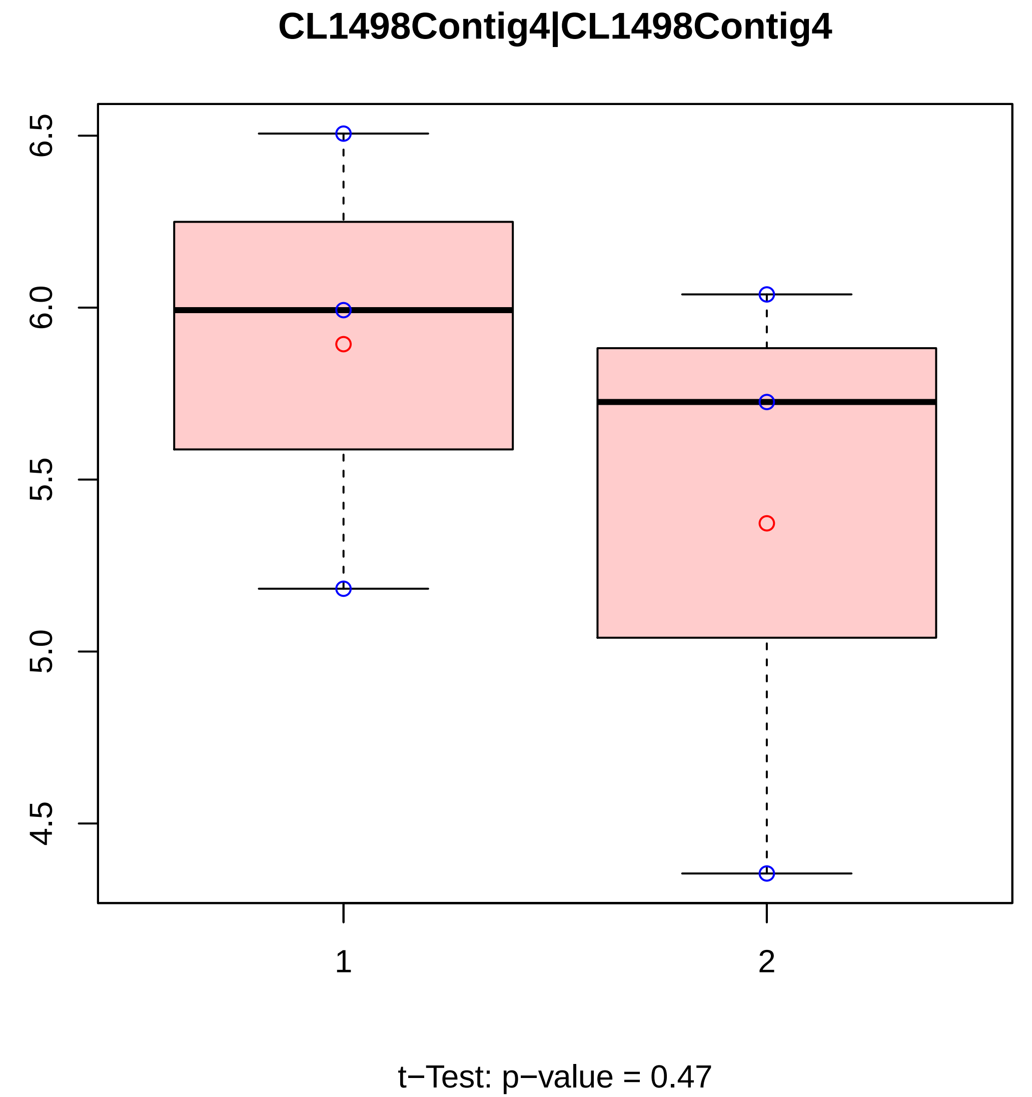
CL1498Contig4|CL1498Contig4
6.5
6.0
5.5
5.0
4.5
1
2
t−Test: p−value = 0.47

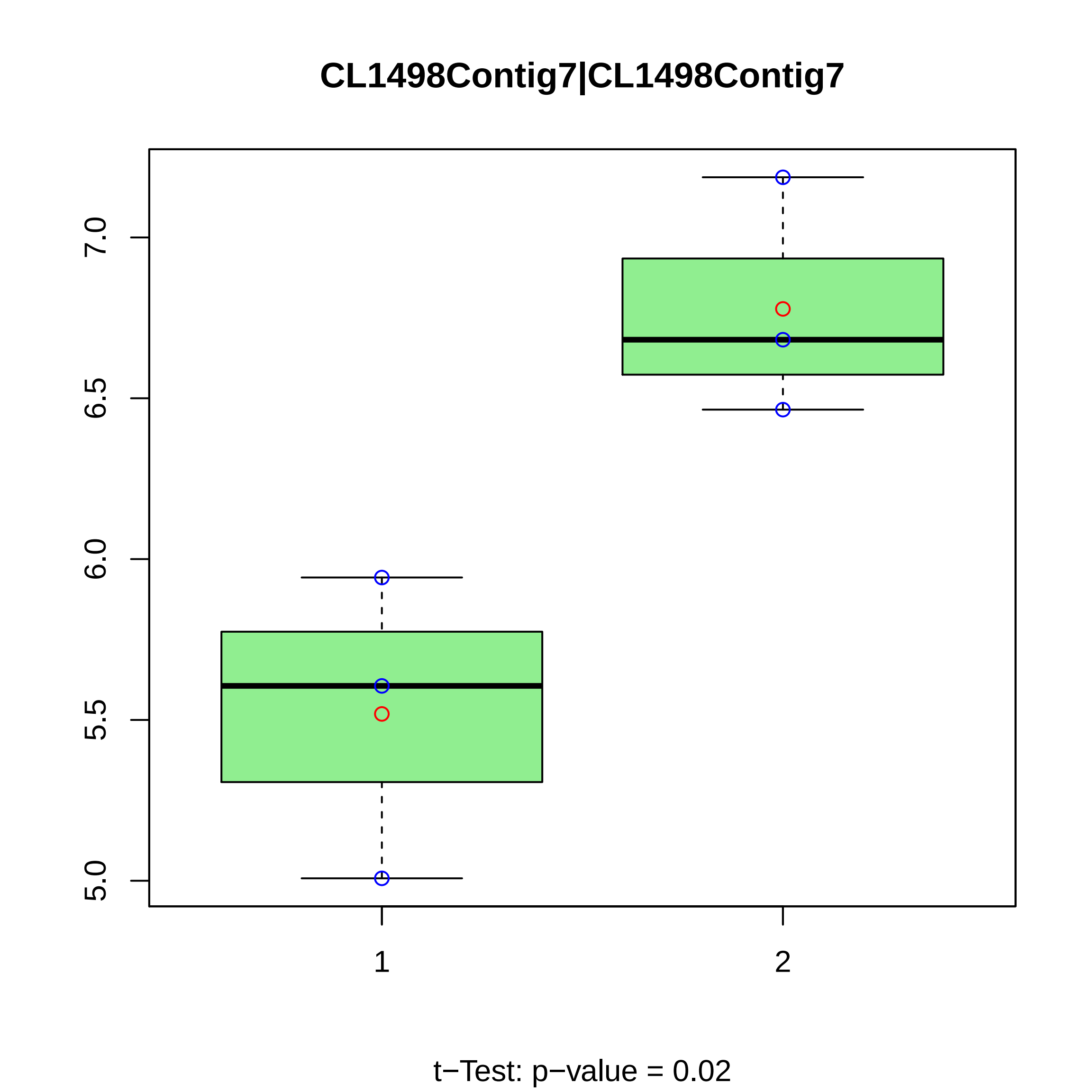
CL1498Contig7|CL1498Contig7
7.0
6.5
6.0
5.5
5.0
1
2
t−Test: p−value = 0.02

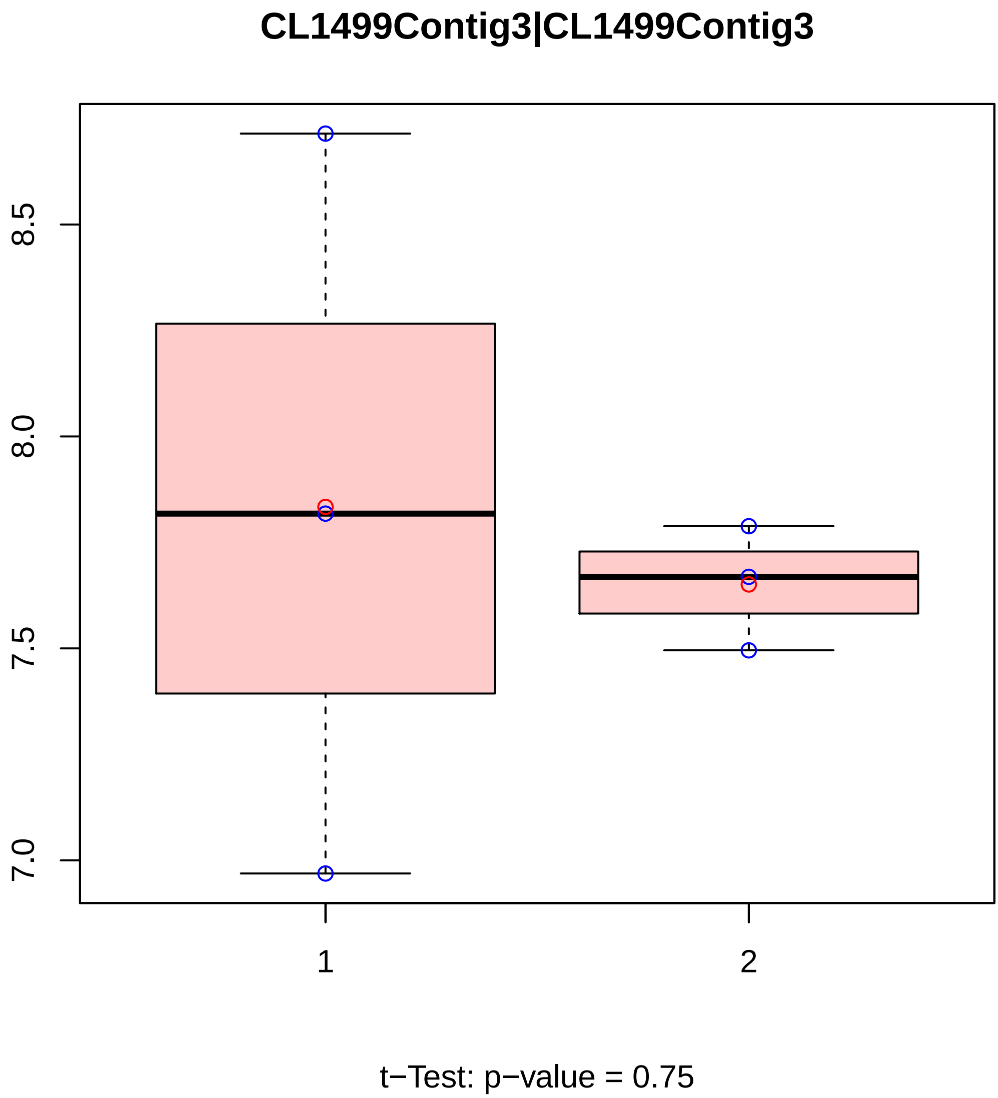

CL1499Contig3|CL1499Contig3
8.5
8.0
7.5
7.0
1
2
t−Test: p−value = 0.75

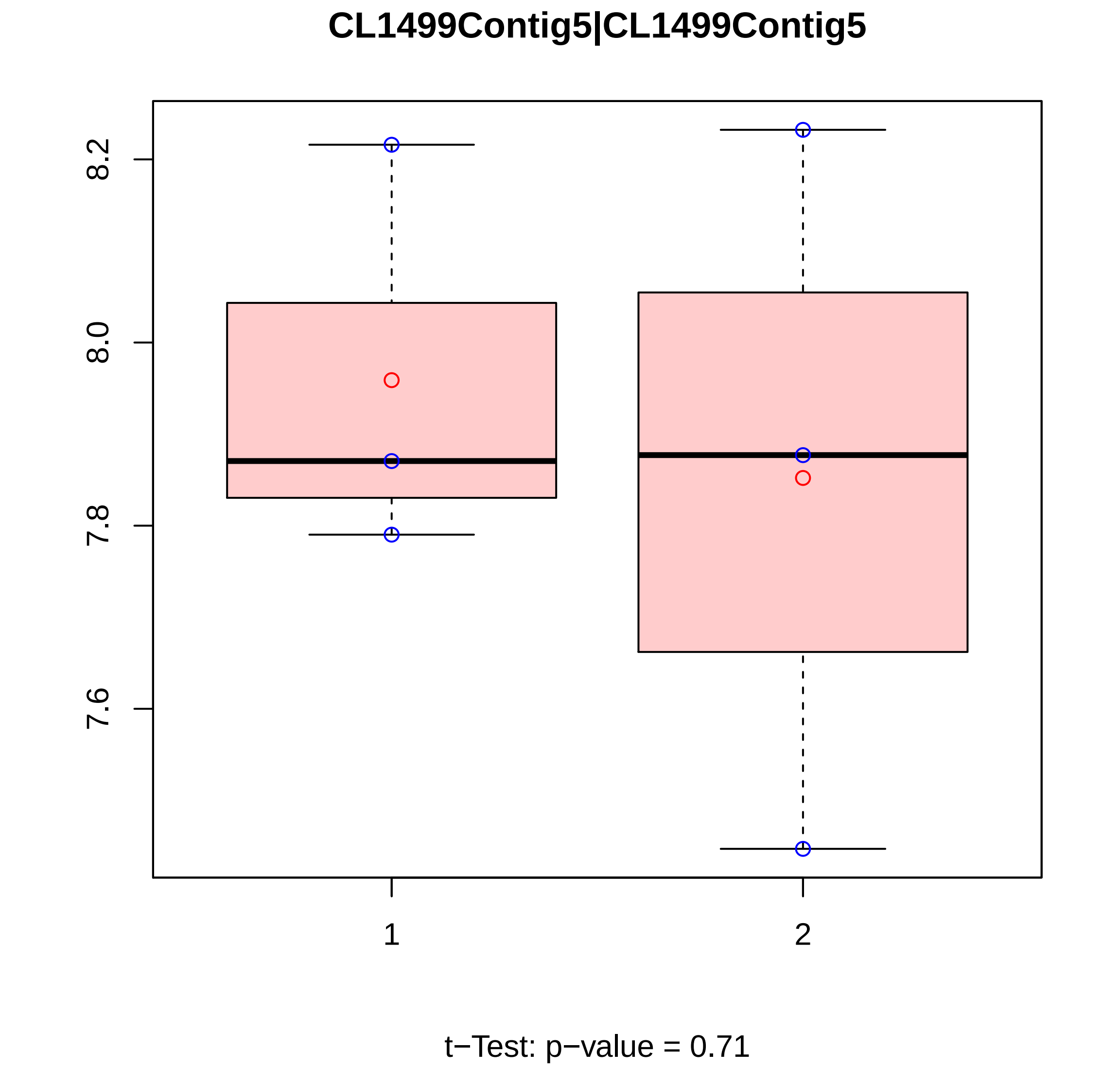
CL1499Contig5|CL1499Contig5
8.2
8.0
7.8
7.6
1
2
t−Test: p−value = 0.71

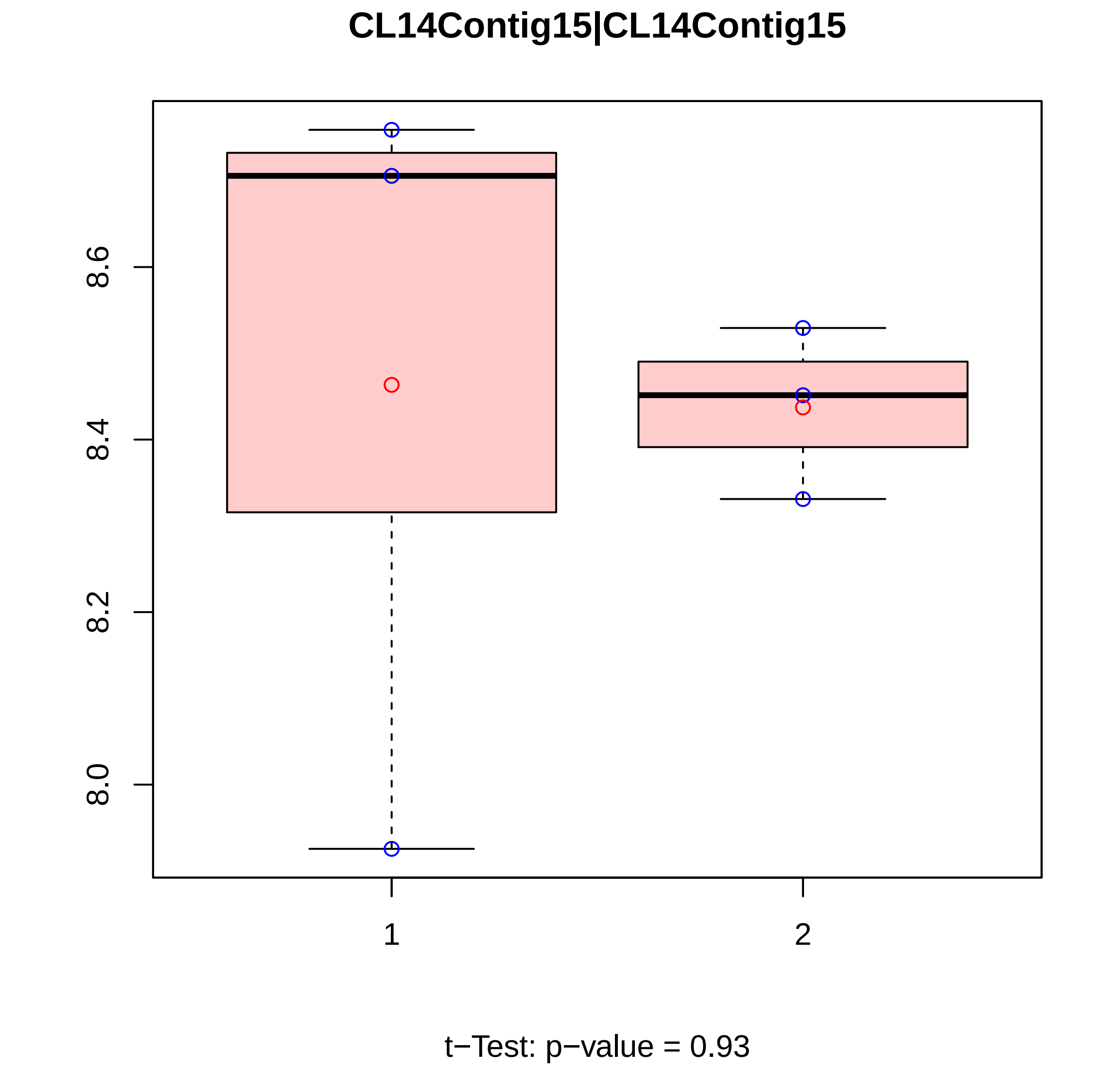

CL14Contig15|CL14Contig15
8.0
8.2
8.4
8.6
1
2
t−Test: p−value = 0.93

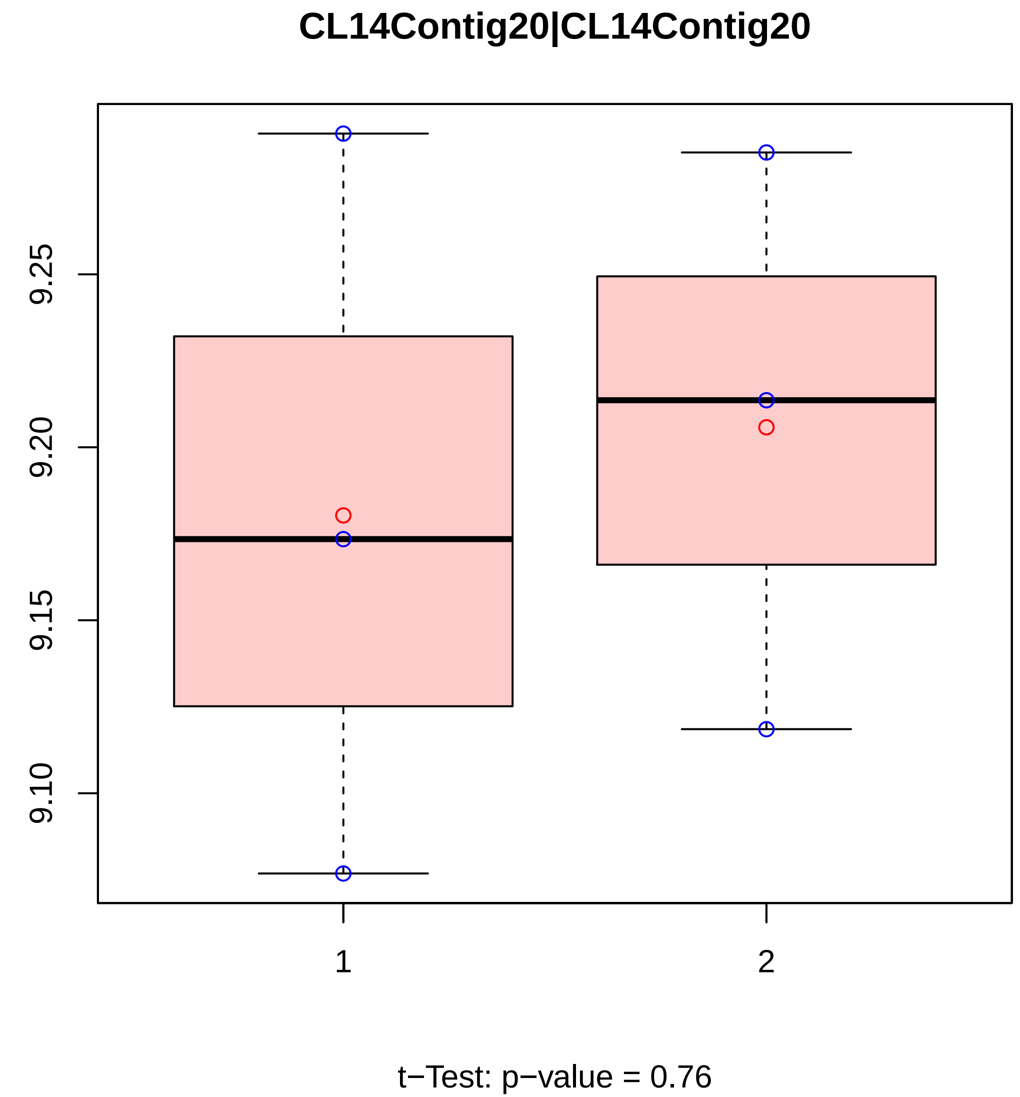
CL14Contig20|CL14Contig20
9.10
9.15
9.20
9.25
1
2
t−Test: p−value = 0.76

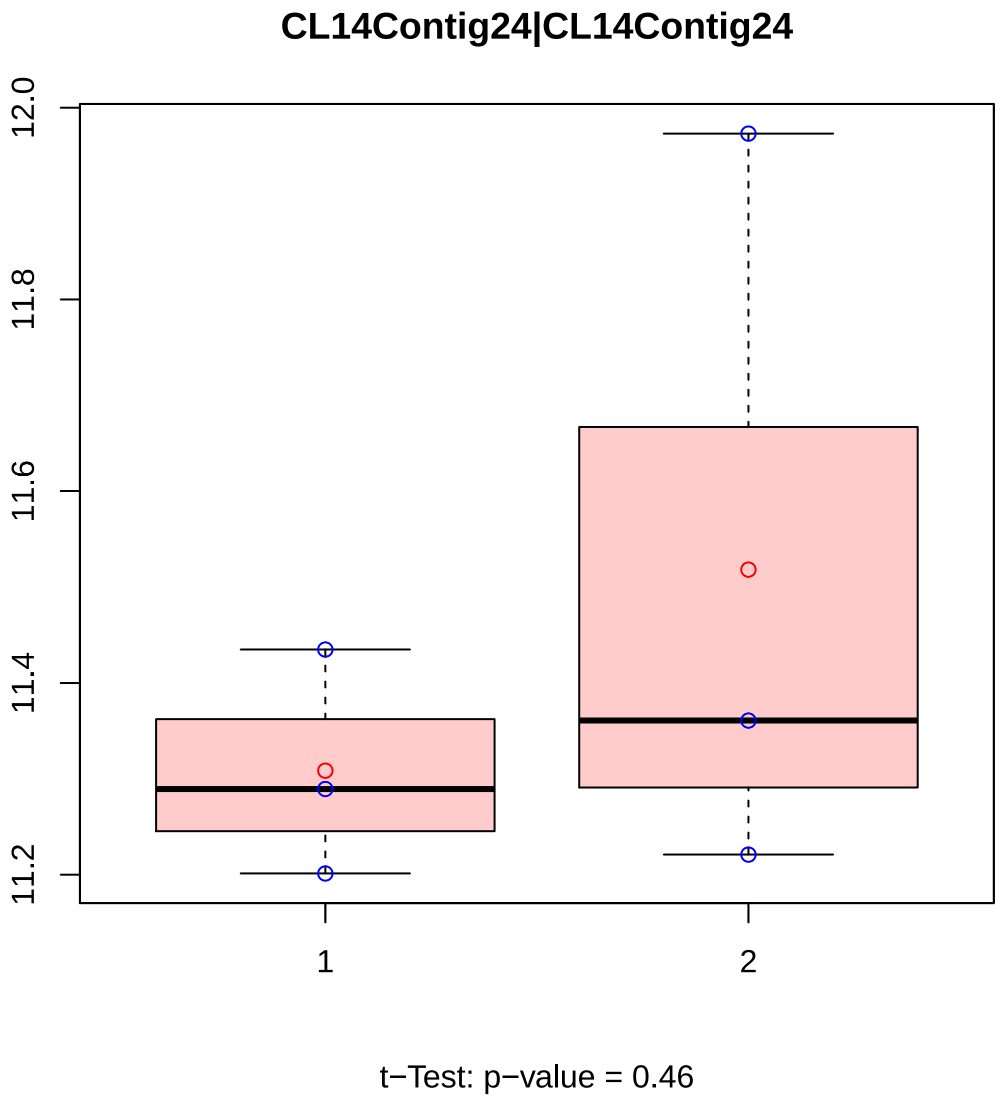

CL14Contig24|CL14Contig24
12.0
11.8
11.6
11.4
11.2
1
2
t−Test: p−value = 0.46

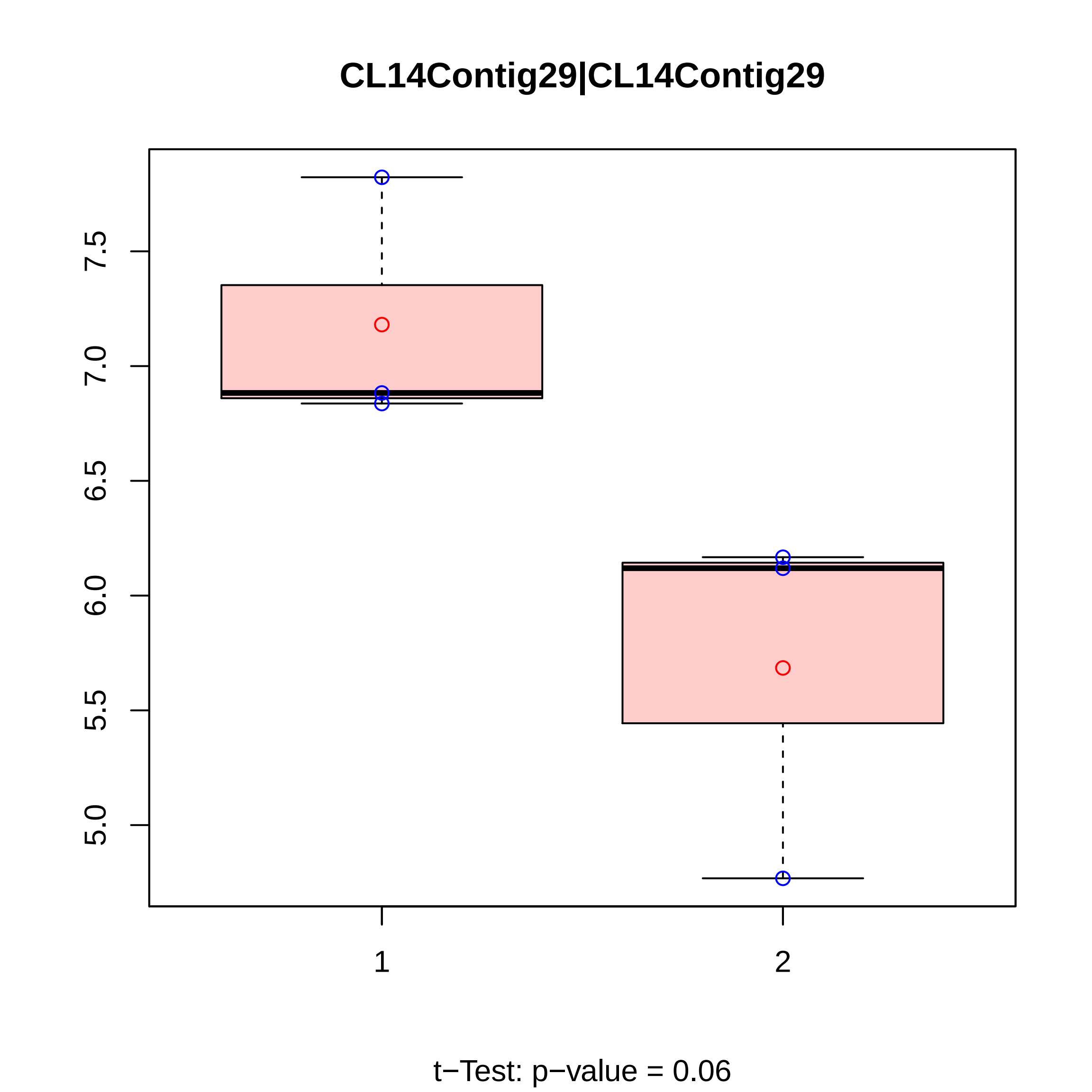
CL14Contig29|CL14Contig29
7.5
7.0
6.5
6.0
5.5
5.0
1
2
t−Test: p−value = 0.06

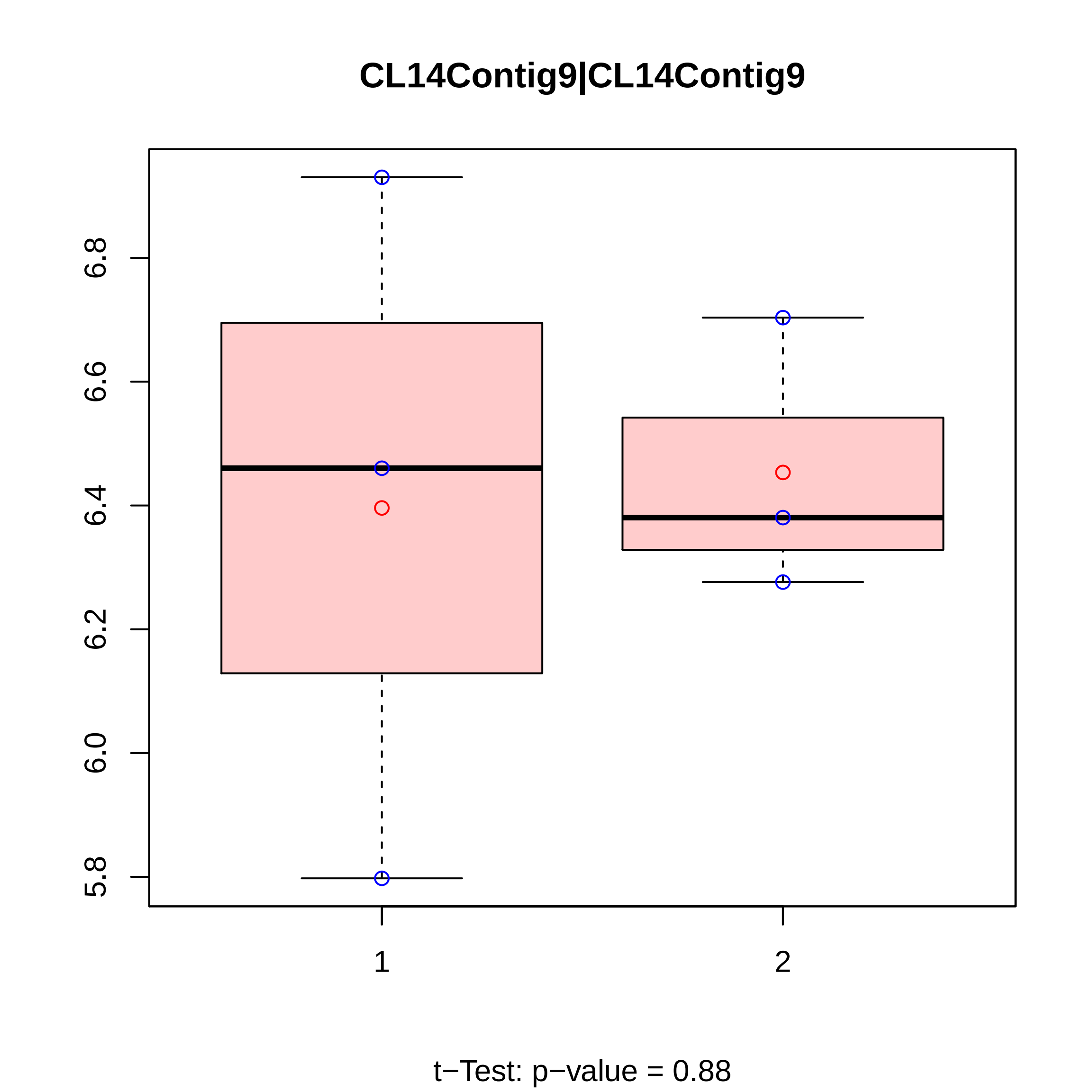

CL14Contig9|CL14Contig9
5.8
6.0
6.2
6.4
6.6
6.8
1
2
t−Test: p−value = 0.88

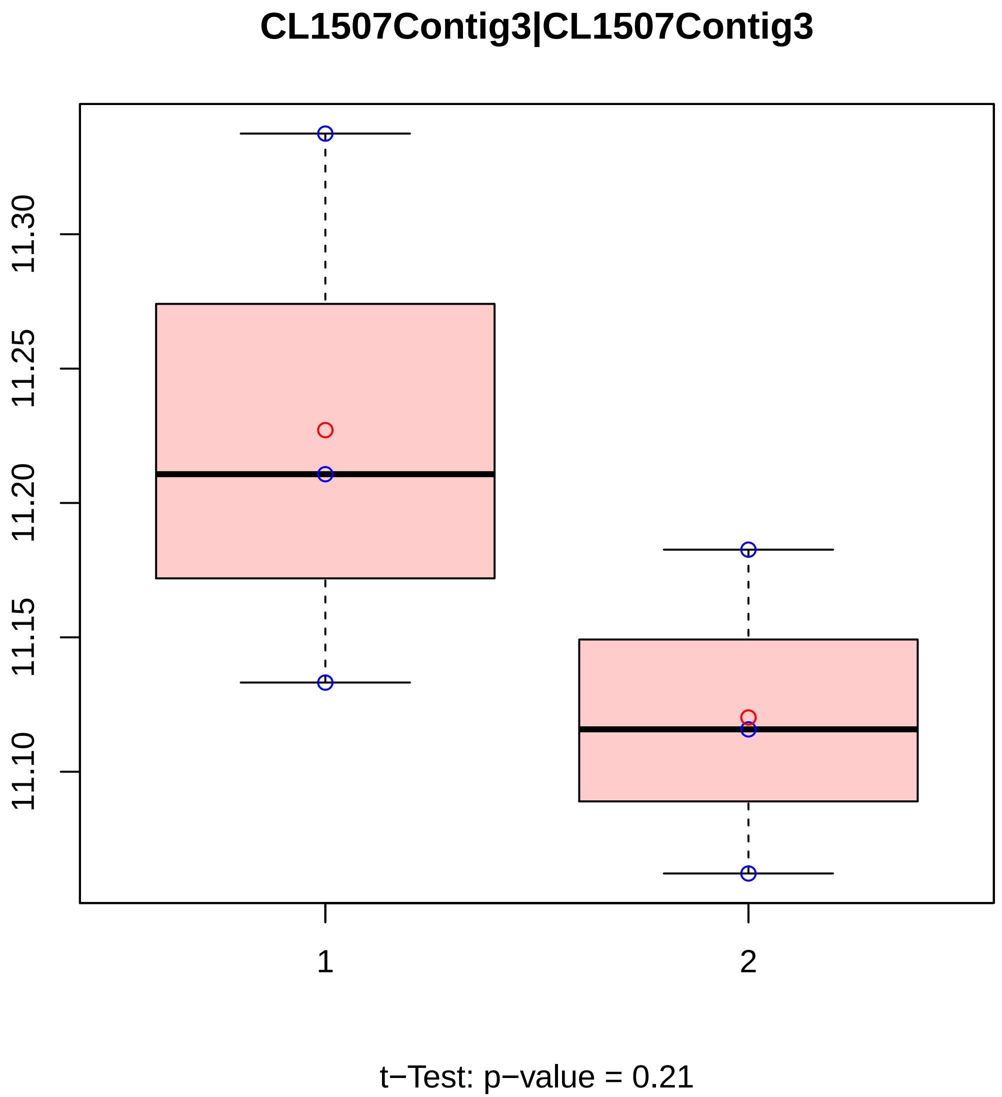

CL1507Contig3|CL1507Contig3
11.10
11.15
11.20
11.25
11.30
1
2
t−Test: p−value = 0.21

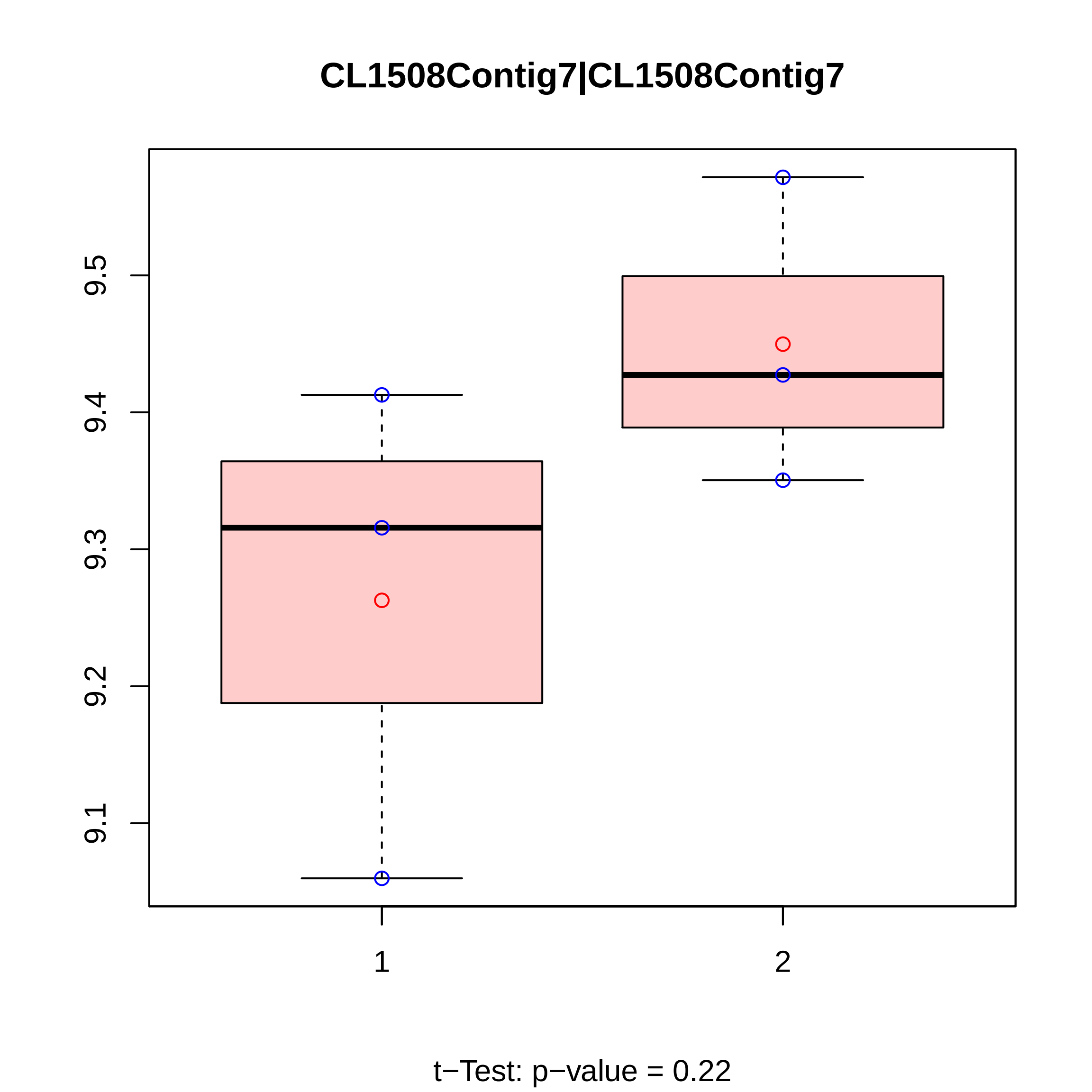
CL1508Contig7|CL1508Contig7
9.1
9.2
9.3
9.4
9.5
1
2
t−Test: p−value = 0.22

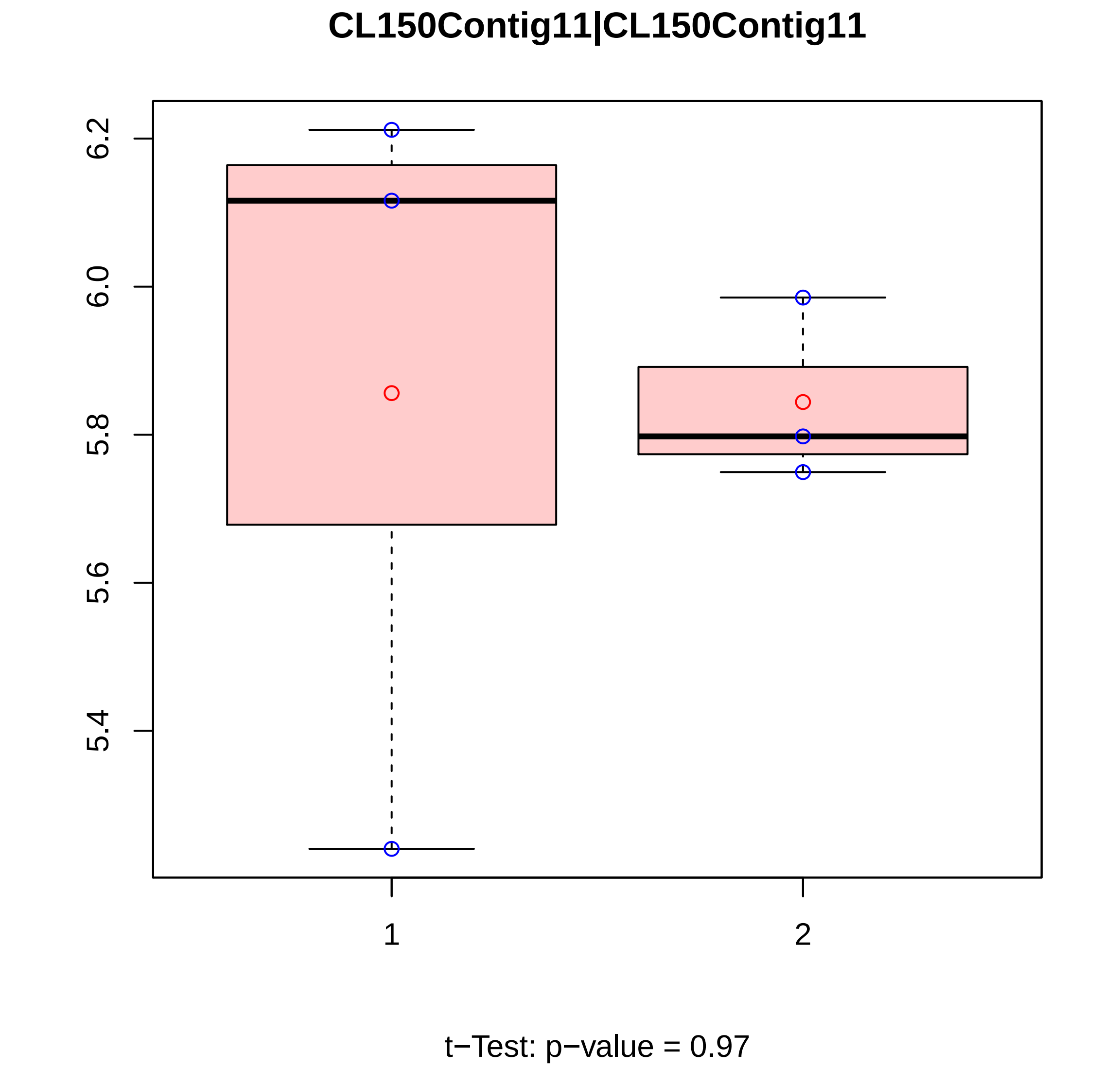
CL150Contig11|CL150Contig11
6.2
6.0
5.8
5.6
5.4
1
2
t−Test: p−value = 0.97

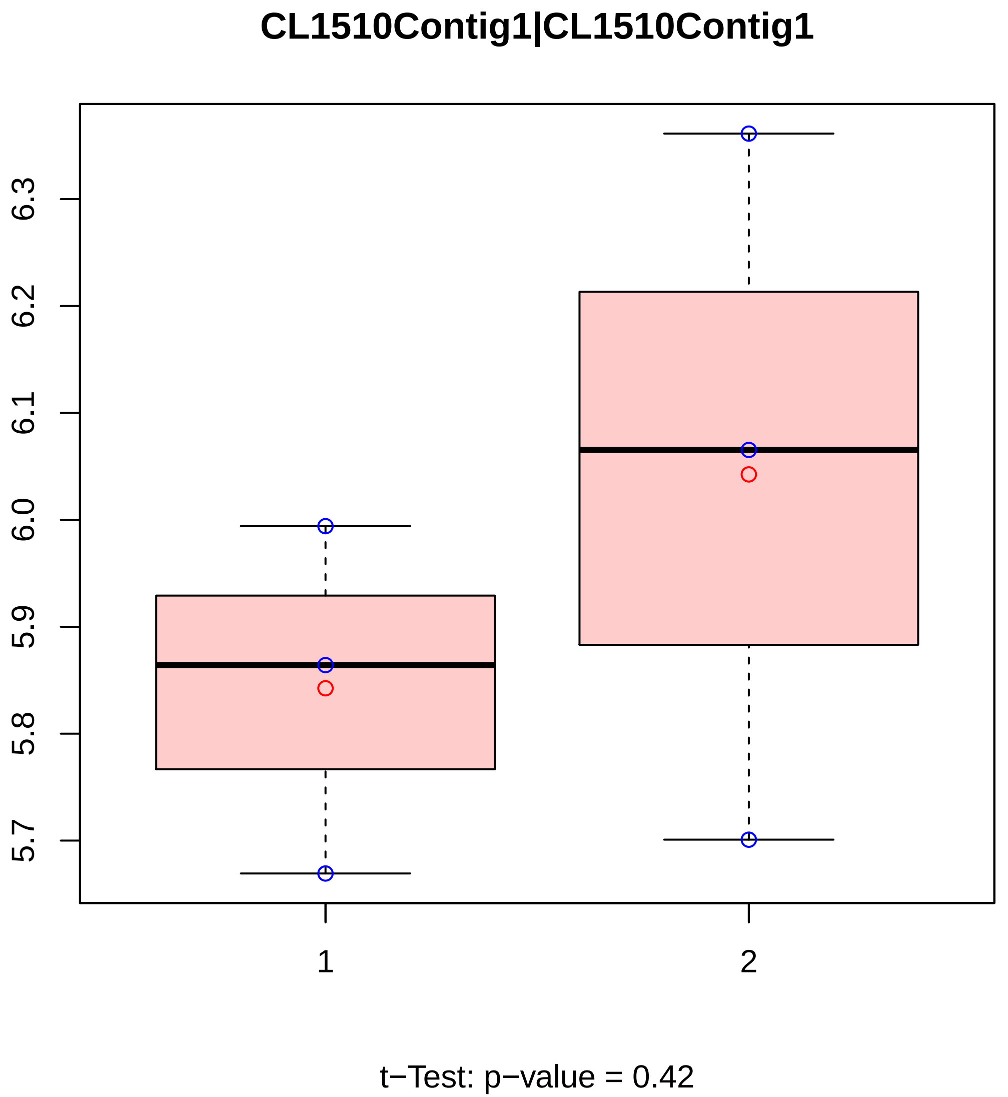
CL1510Contig1|CL1510Contig1
6.3
6.2
6.1
6.0
5.9
5.8
5.7
1
2
t−Test: p−value = 0.42

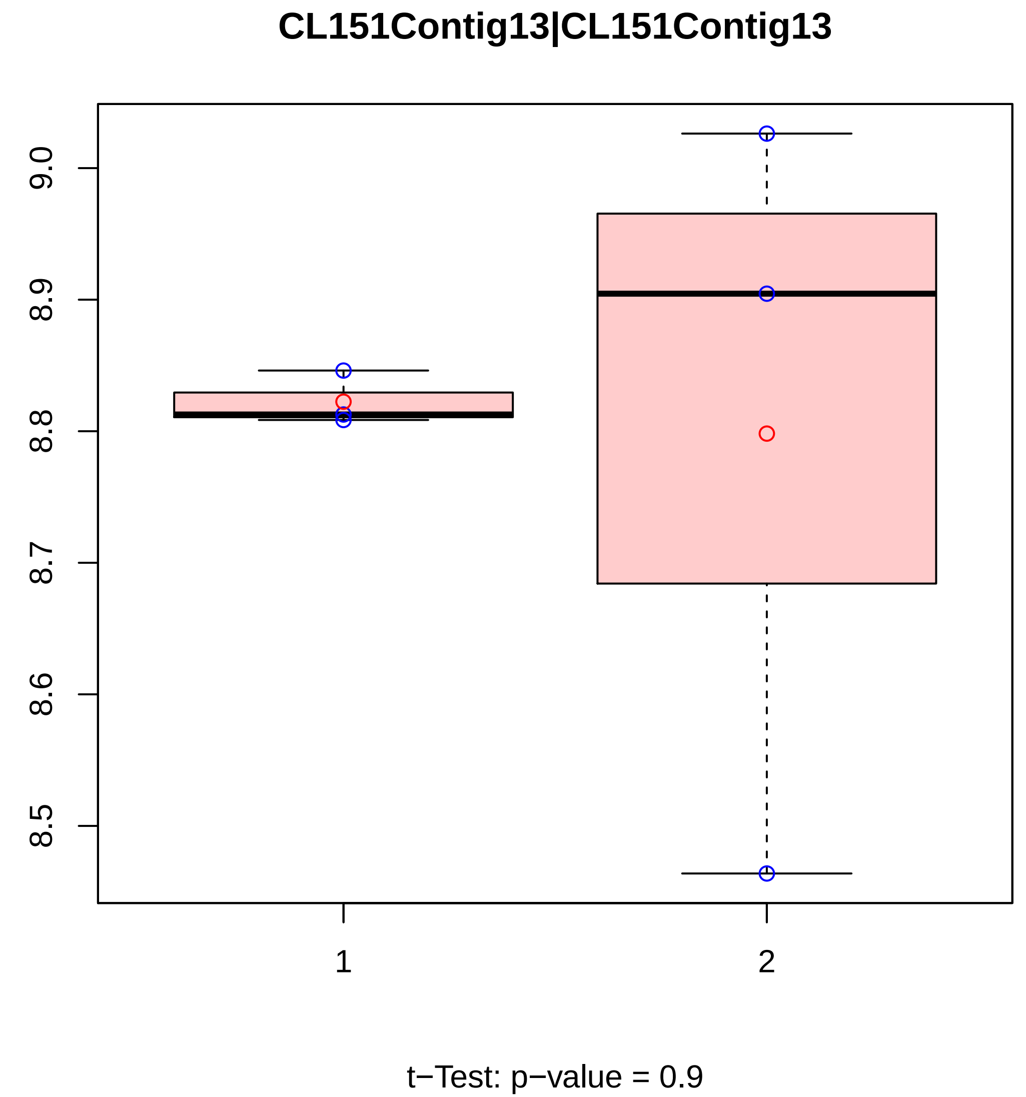
CL151Contig13|CL151Contig13
9.0
8.9
8.8
8.7
8.6
8.5
1
2
t−Test: p−value = 0.9

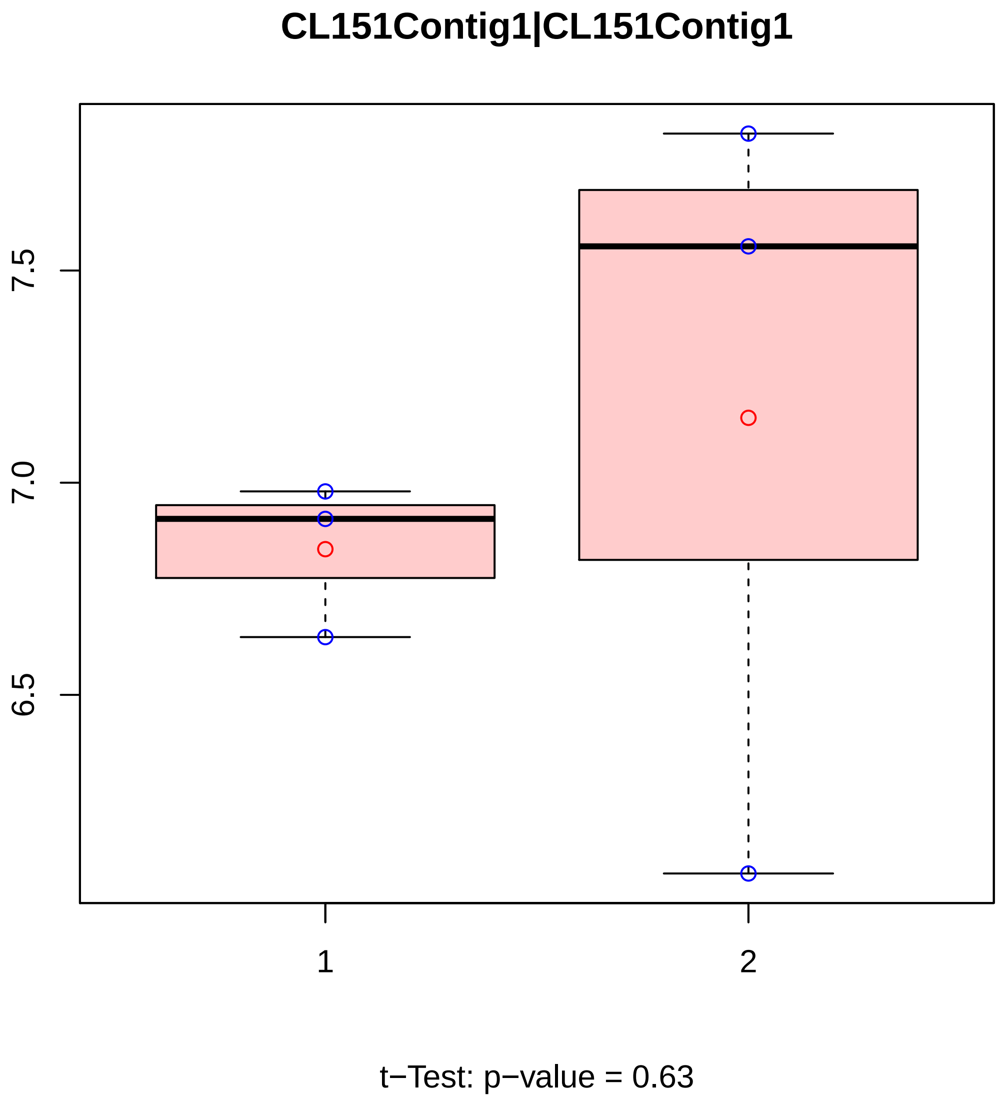
CL151Contig1|CL151Contig1
7.5
7.0
6.5
1
2
t−Test: p−value = 0.63

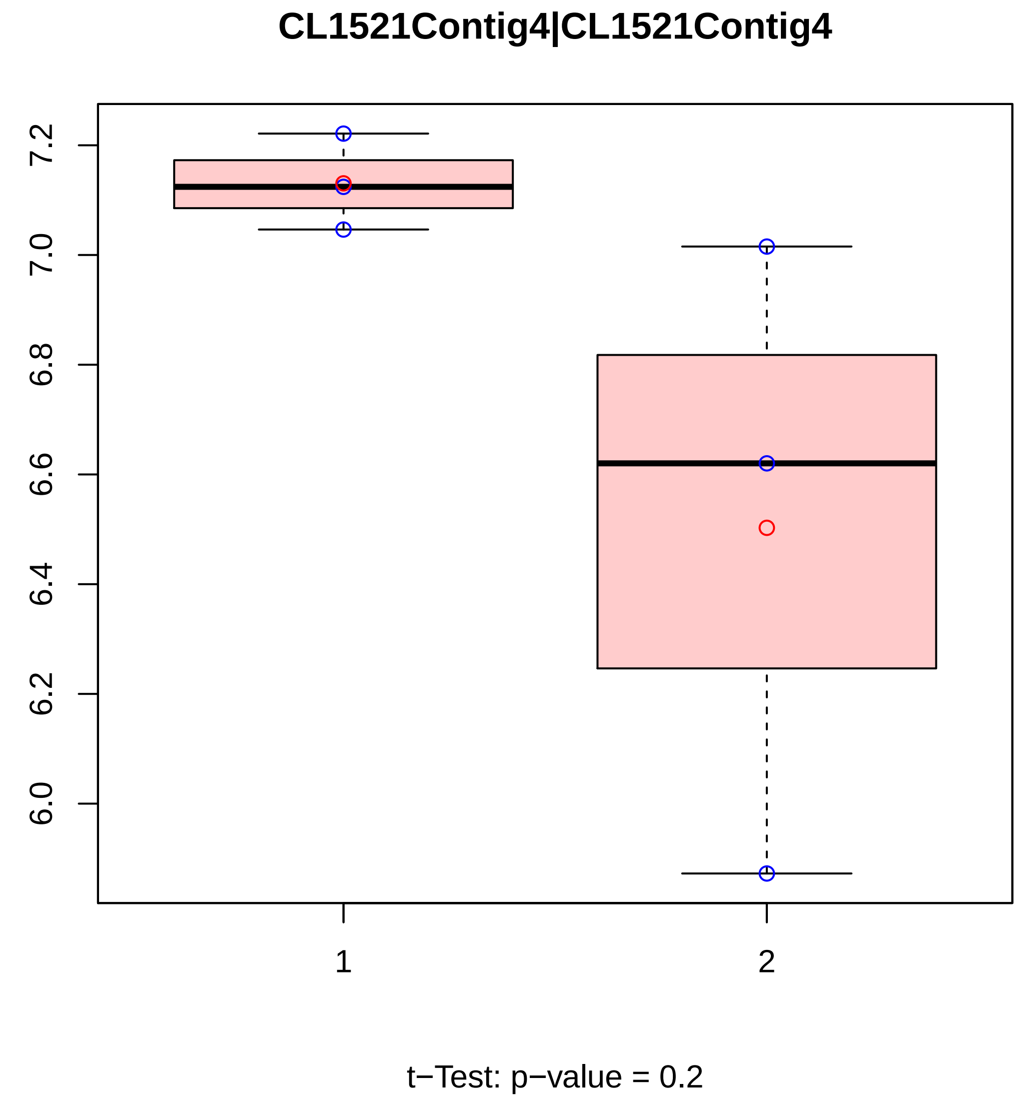
CL1521Contig4|CL1521Contig4
7.2
7.0
6.8
6.6
6.4
6.2
6.0
1
2
t−Test: p−value = 0.2

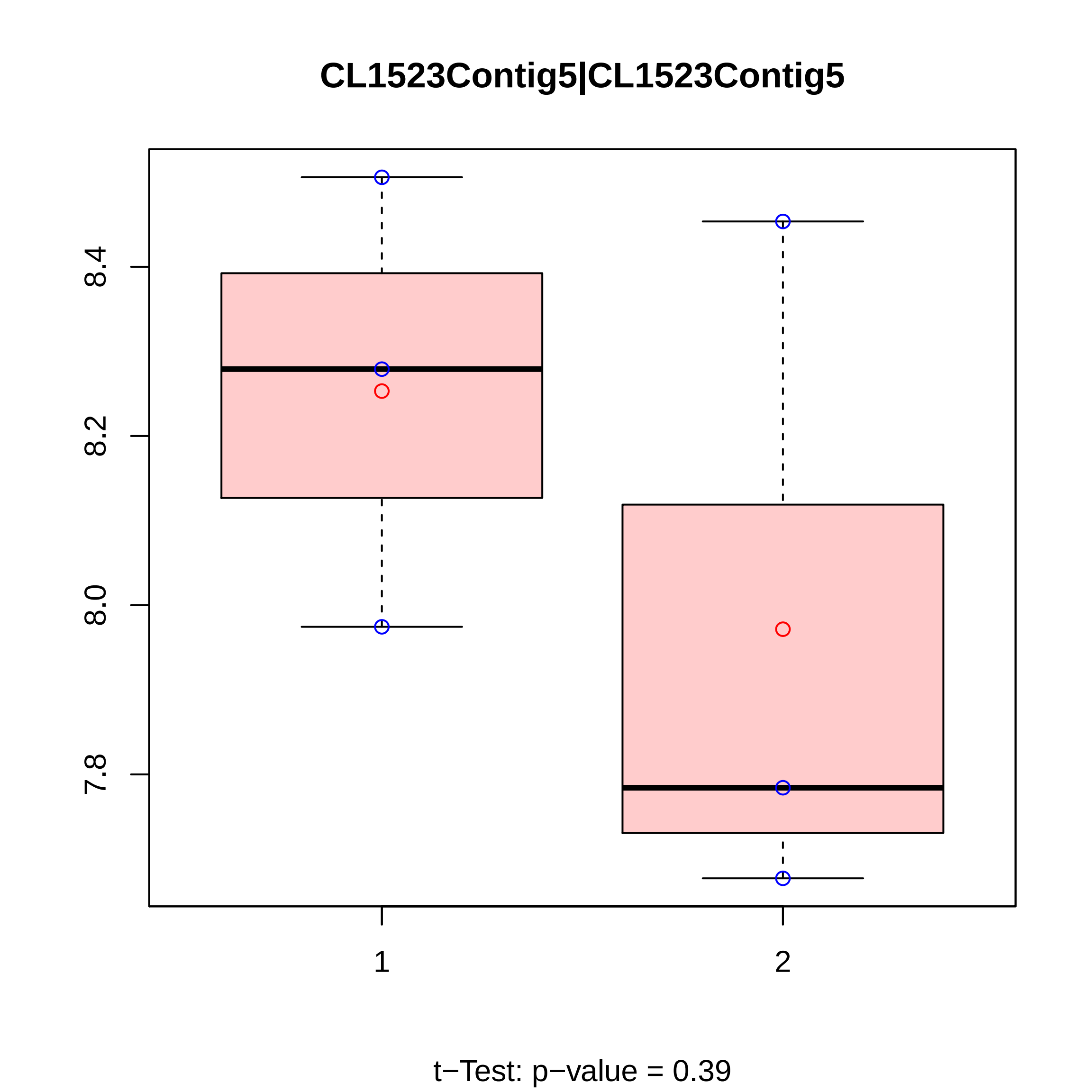
CL1523Contig5|CL1523Contig5
8.4
8.2
8.0
7.8
1
2
t−Test: p−value = 0.39

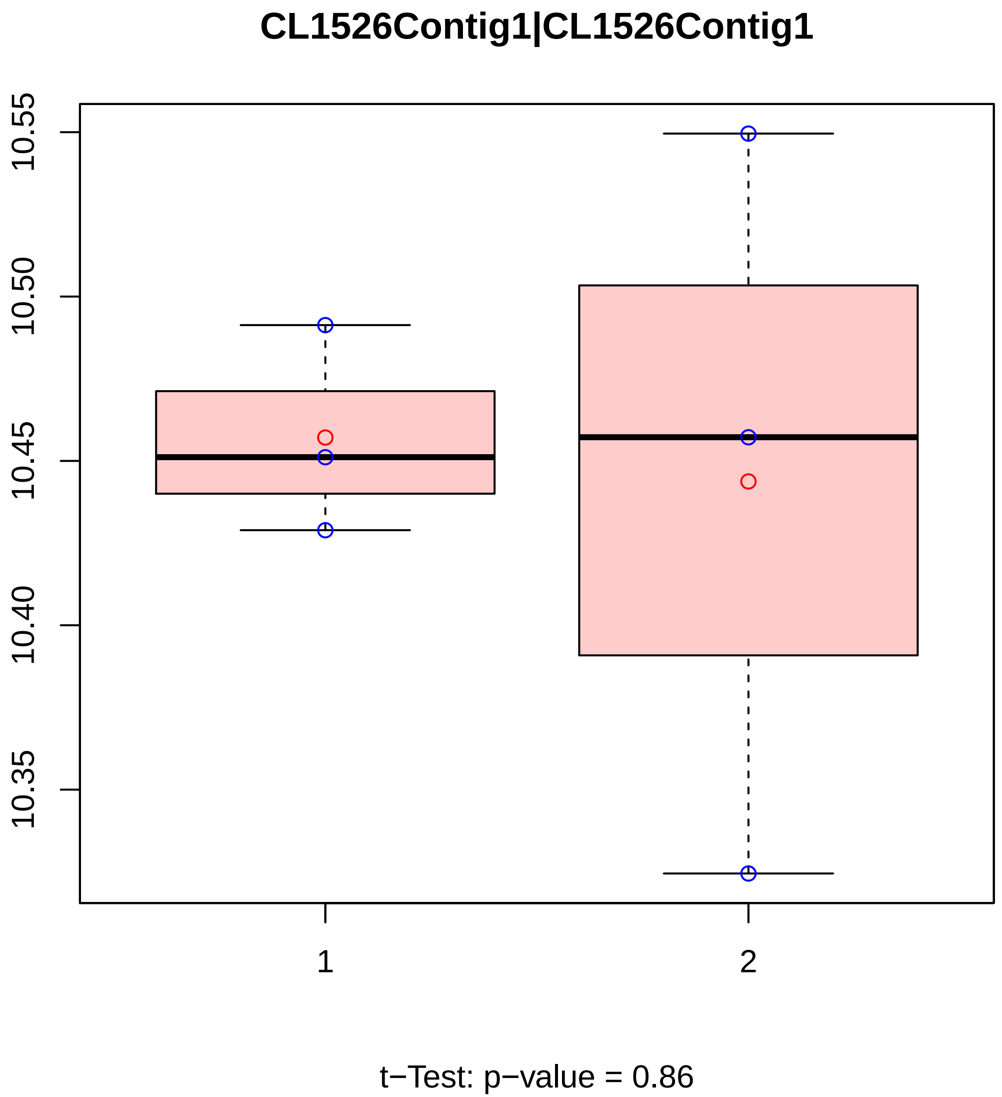

CL1526Contig1|CL1526Contig1
10.35
10.40
10.45
10.50
10.55
1
2
t−Test: p−value = 0.86

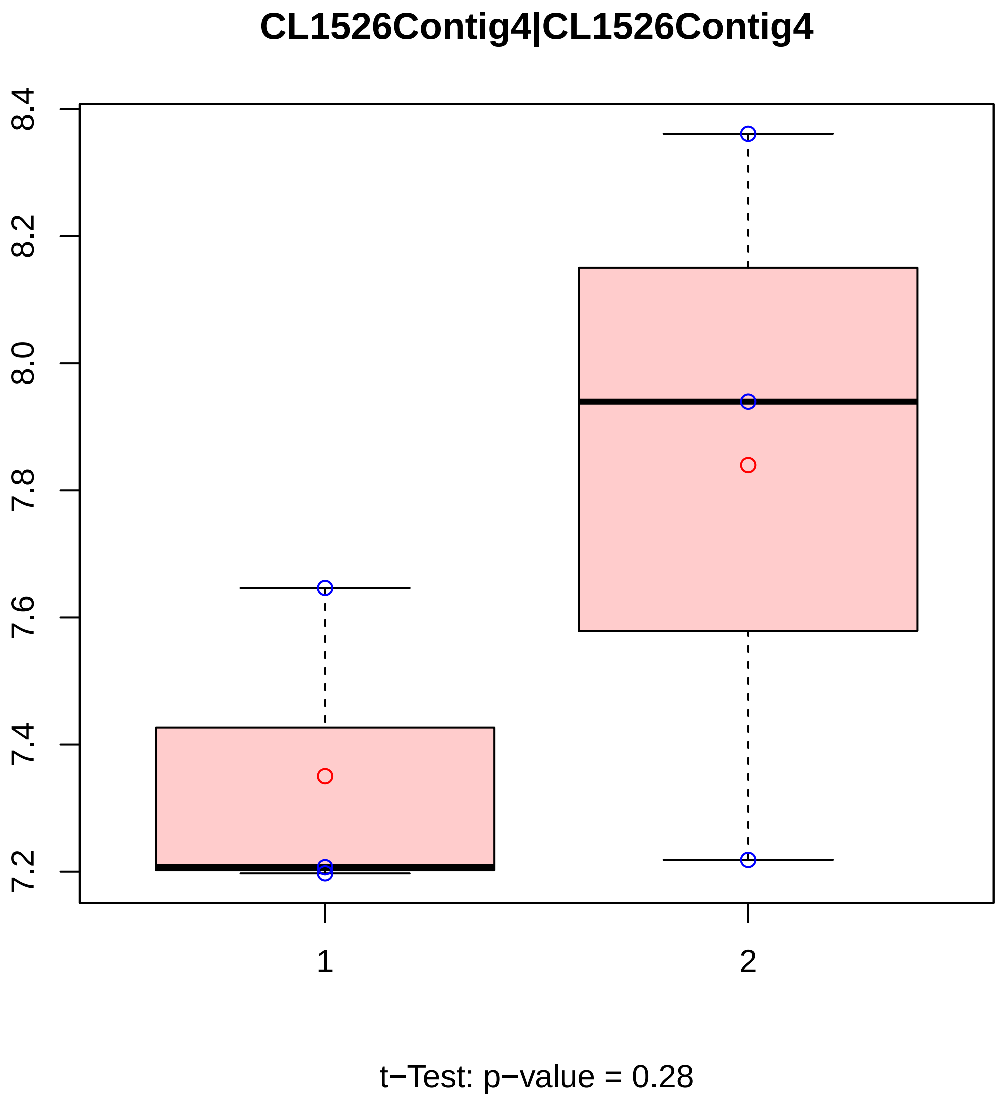
CL1526Contig4|CL1526Contig4
8.4
8.2
8.0
7.8
7.6
7.4
7.2
1
2
t−Test: p−value = 0.28

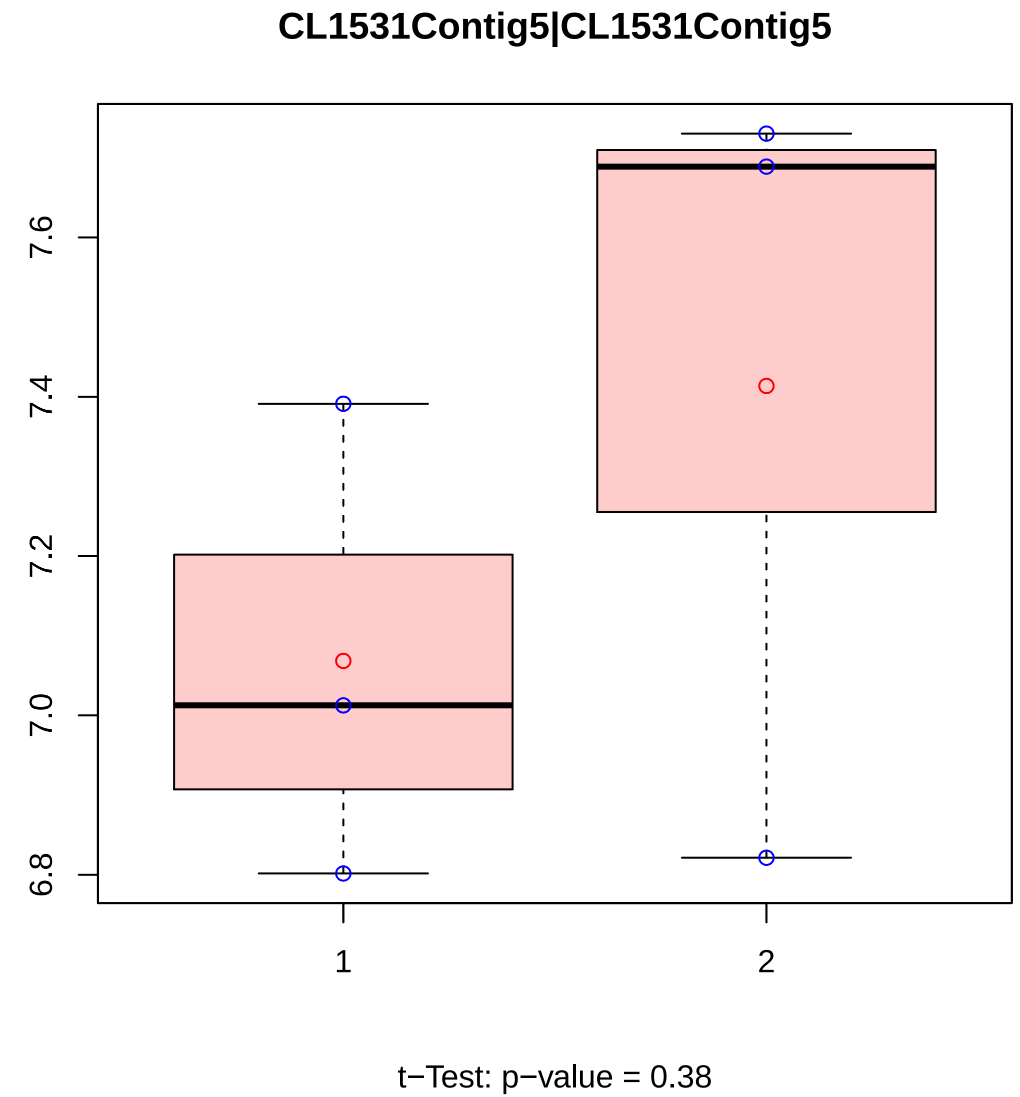
CL1531Contig5|CL1531Contig5
7.6
7.4
7.2
7.0
6.8
1
2
t−Test: p−value = 0.38

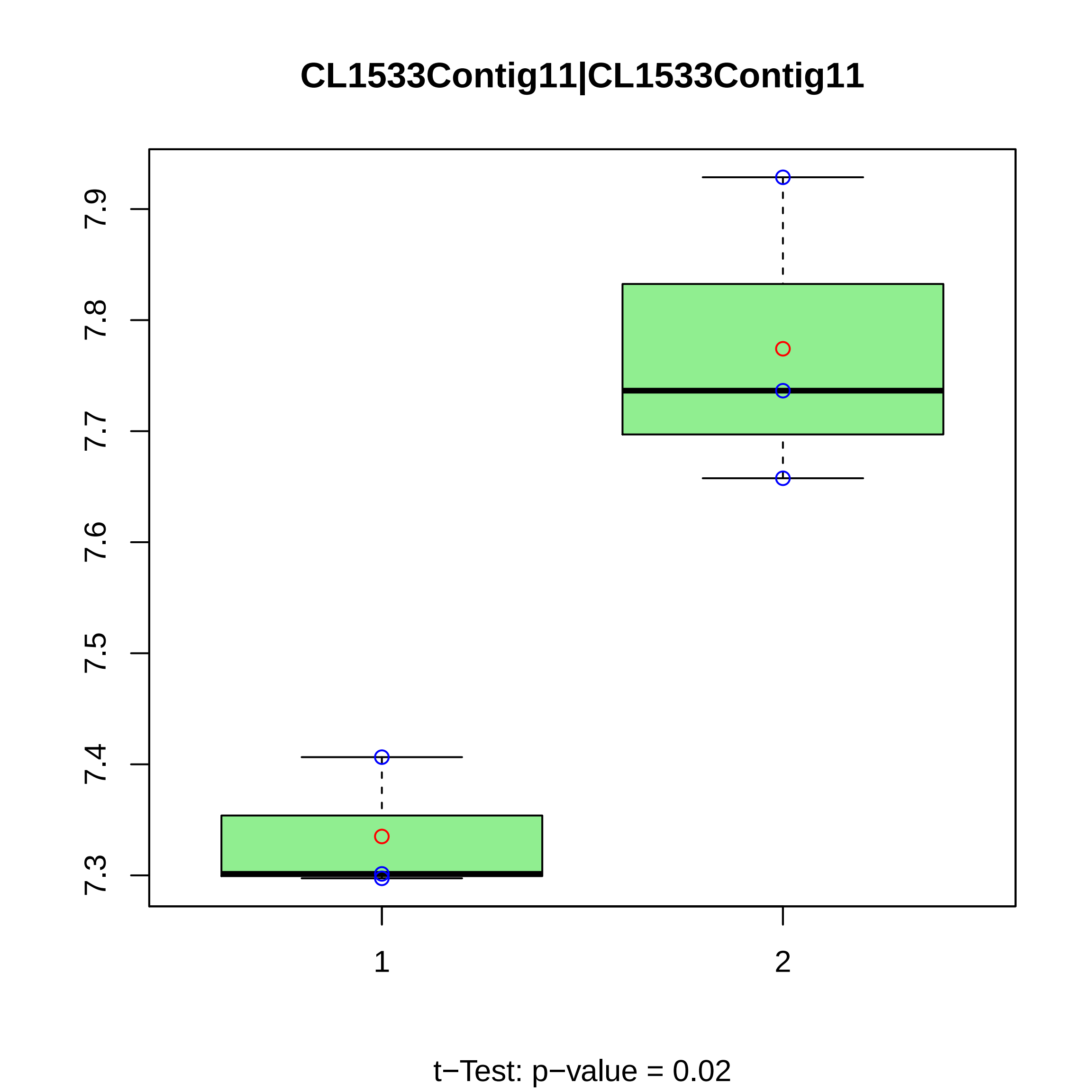
CL1533Contig11|CL1533Contig11
7.9
7.8
7.7
7.6
7.5
7.4
7.3
1
2
t−Test: p−value = 0.02

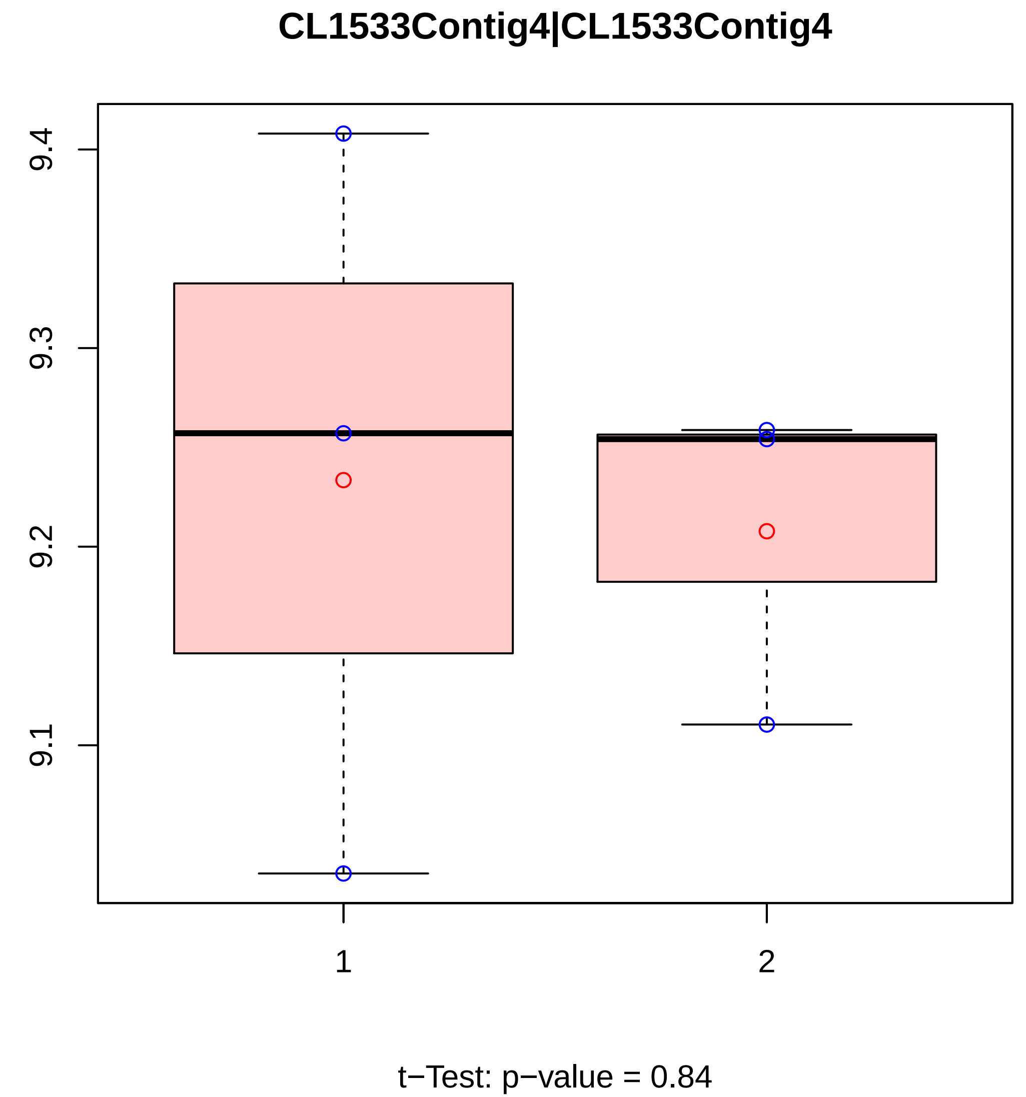
CL1533Contig4|CL1533Contig4
9.1
9.2
9.3
9.4
1
2
t−Test: p−value = 0.84

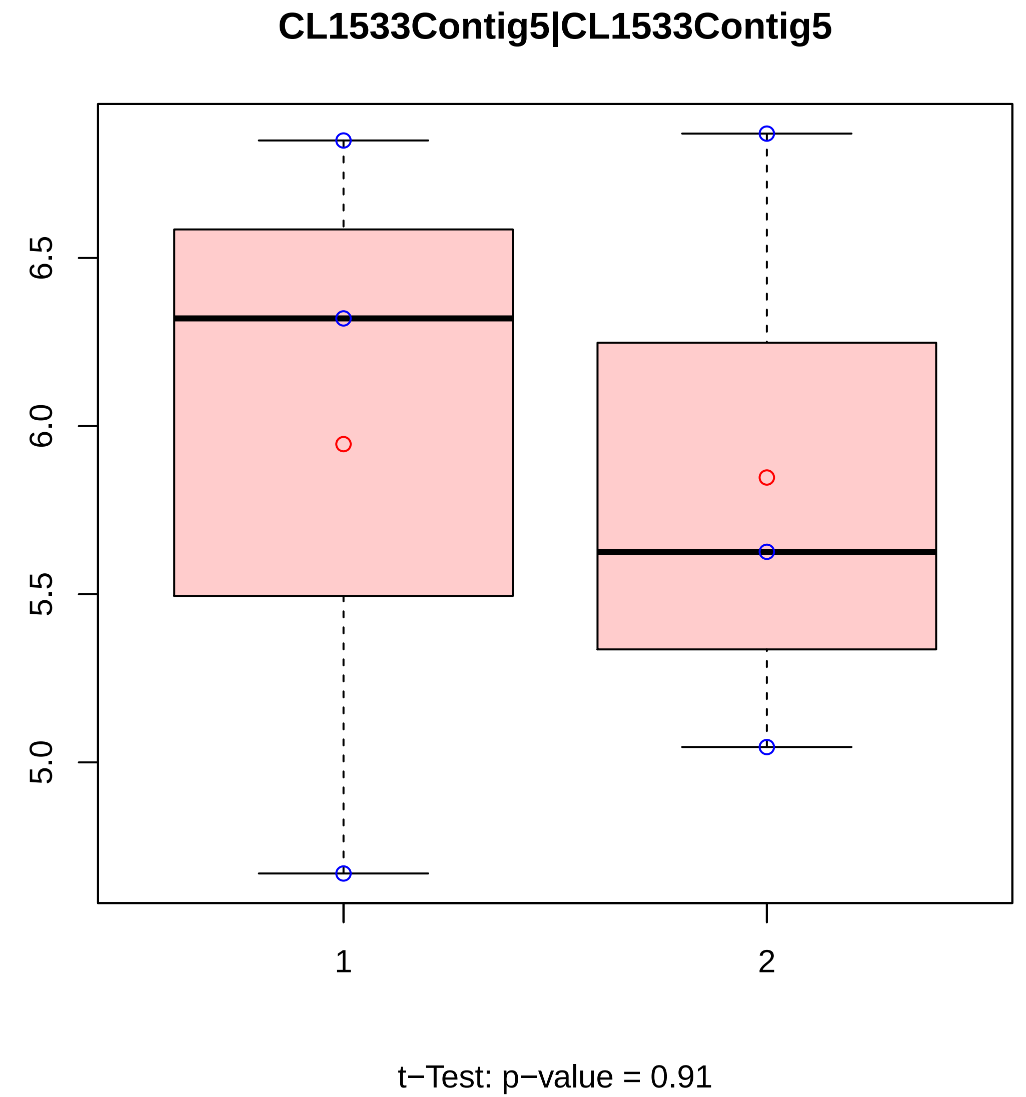
CL1533Contig5|CL1533Contig5
6.5
6.0
5.5
5.0
1
2
t−Test: p−value = 0.91

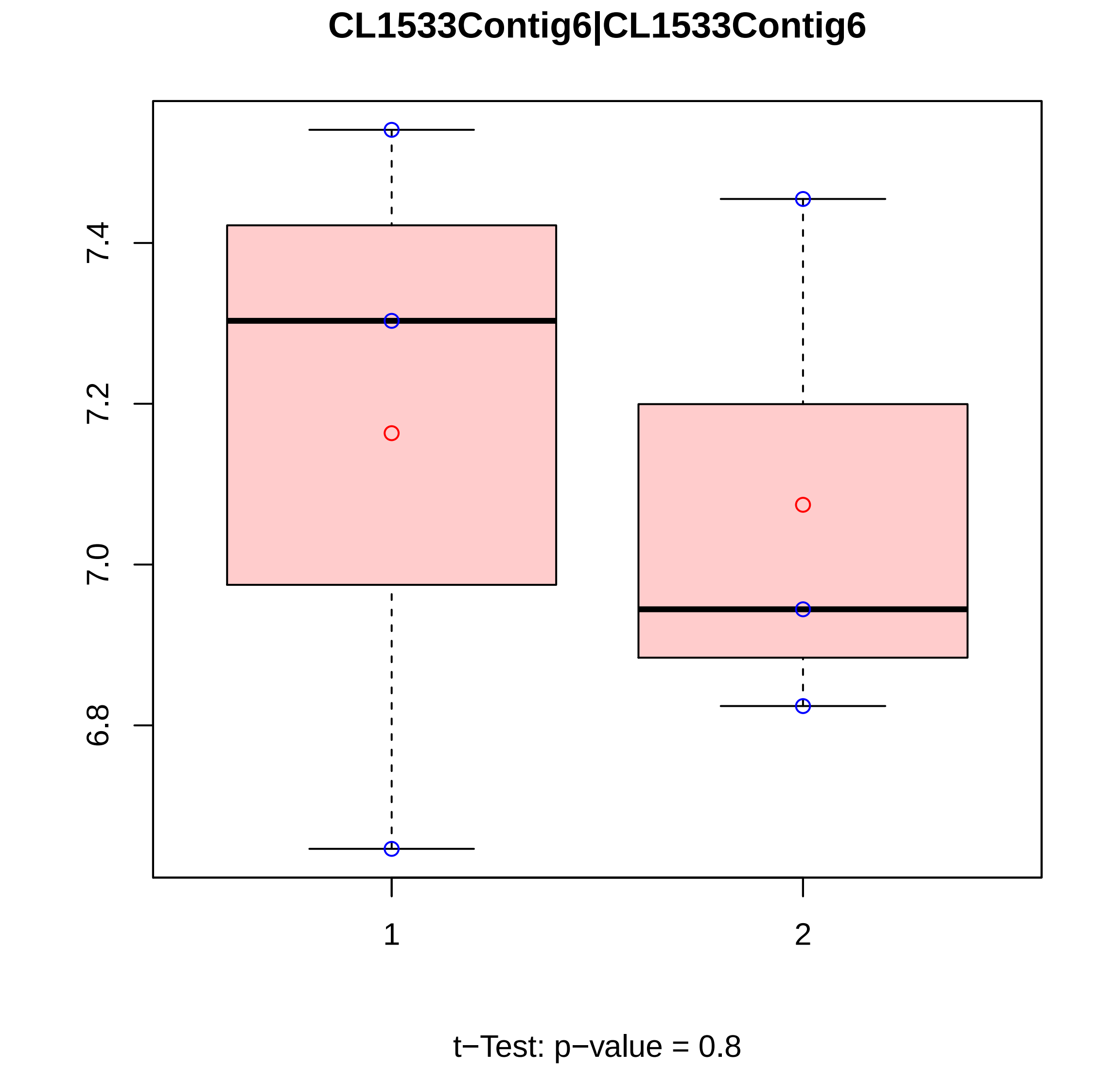
CL1533Contig6|CL1533Contig6
7.4
7.2
7.0
6.8
1
2
t−Test: p−value = 0.8

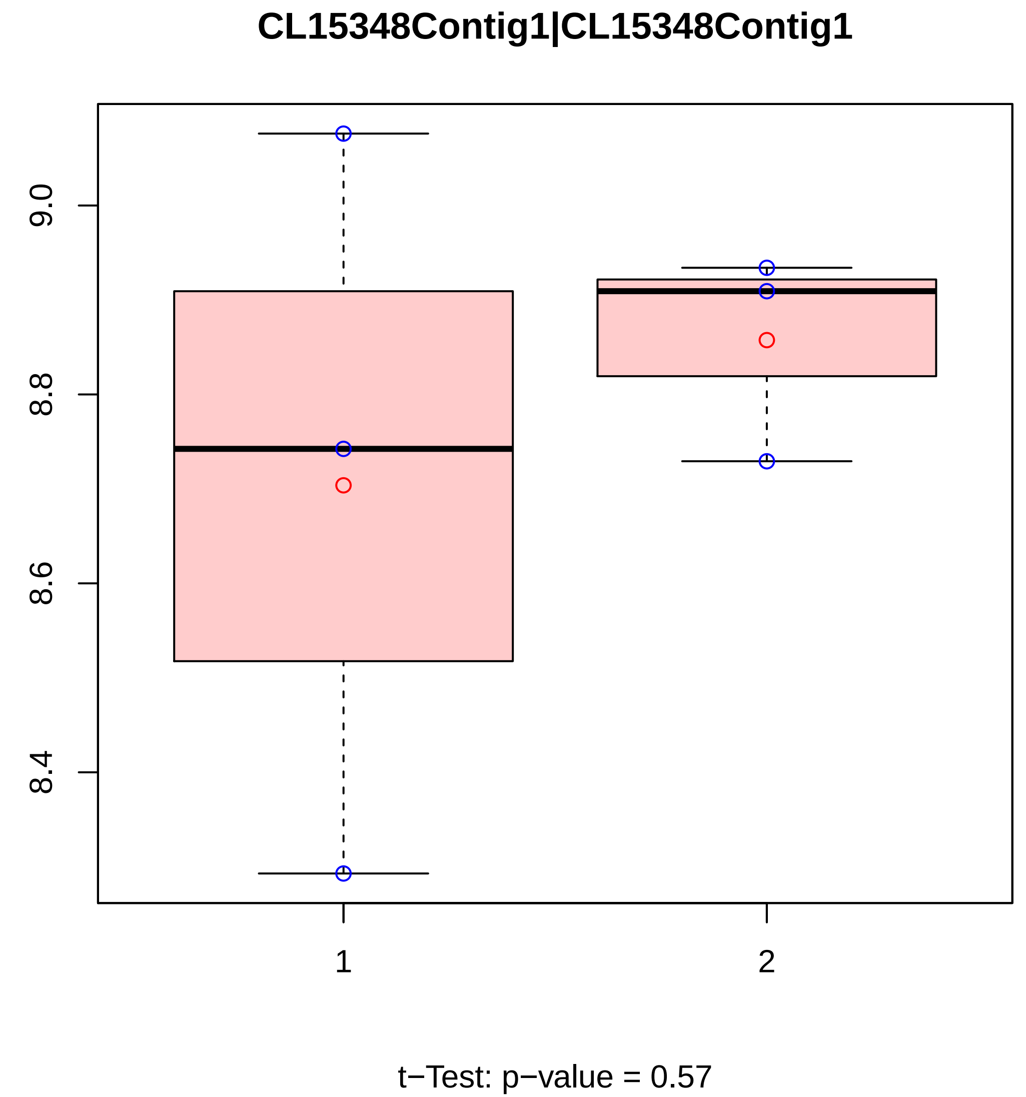
CL15348Contig1|CL15348Contig1
9.0
8.8
8.6
8.4
1
2
t−Test: p−value = 0.57

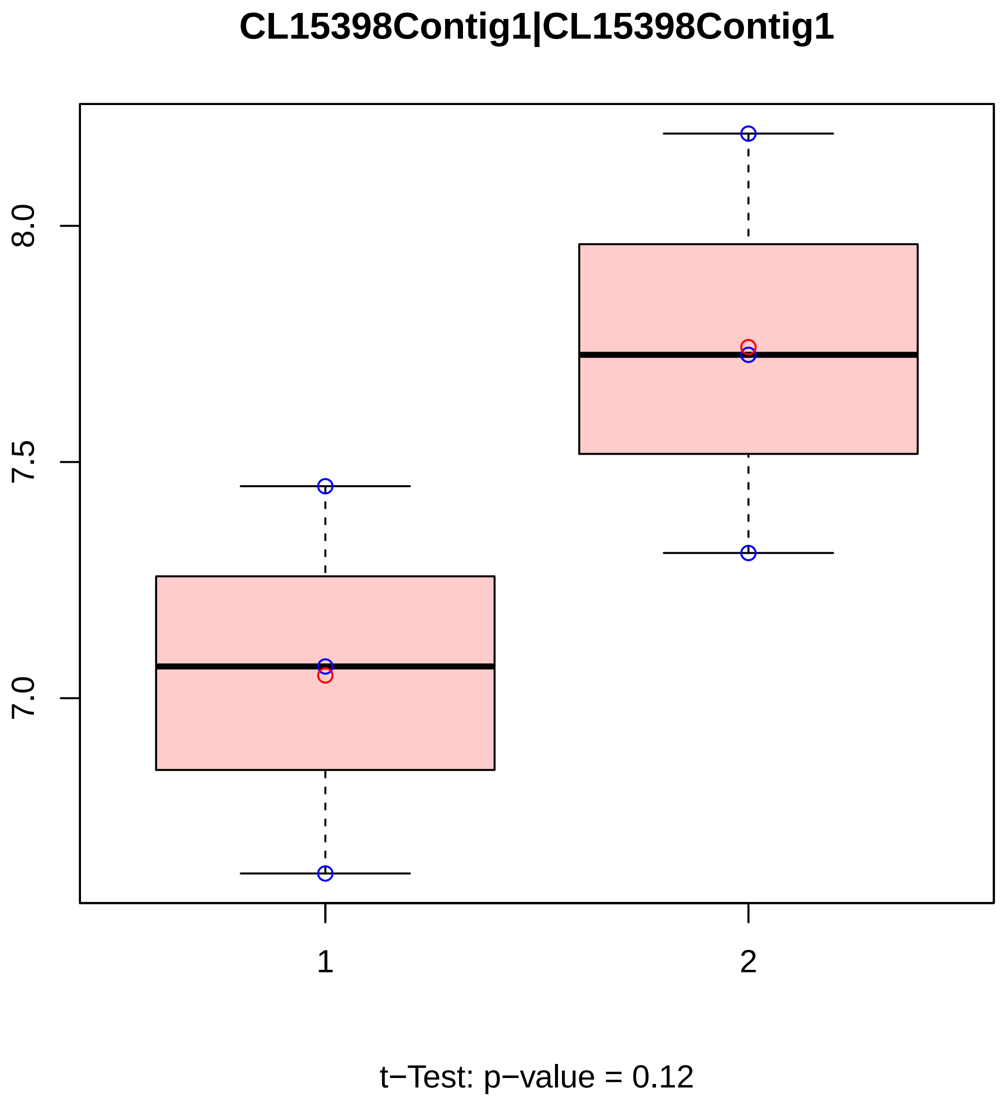
CL15398Contig1|CL15398Contig1
8.0
7.5
7.0
1
2
t−Test: p−value = 0.12

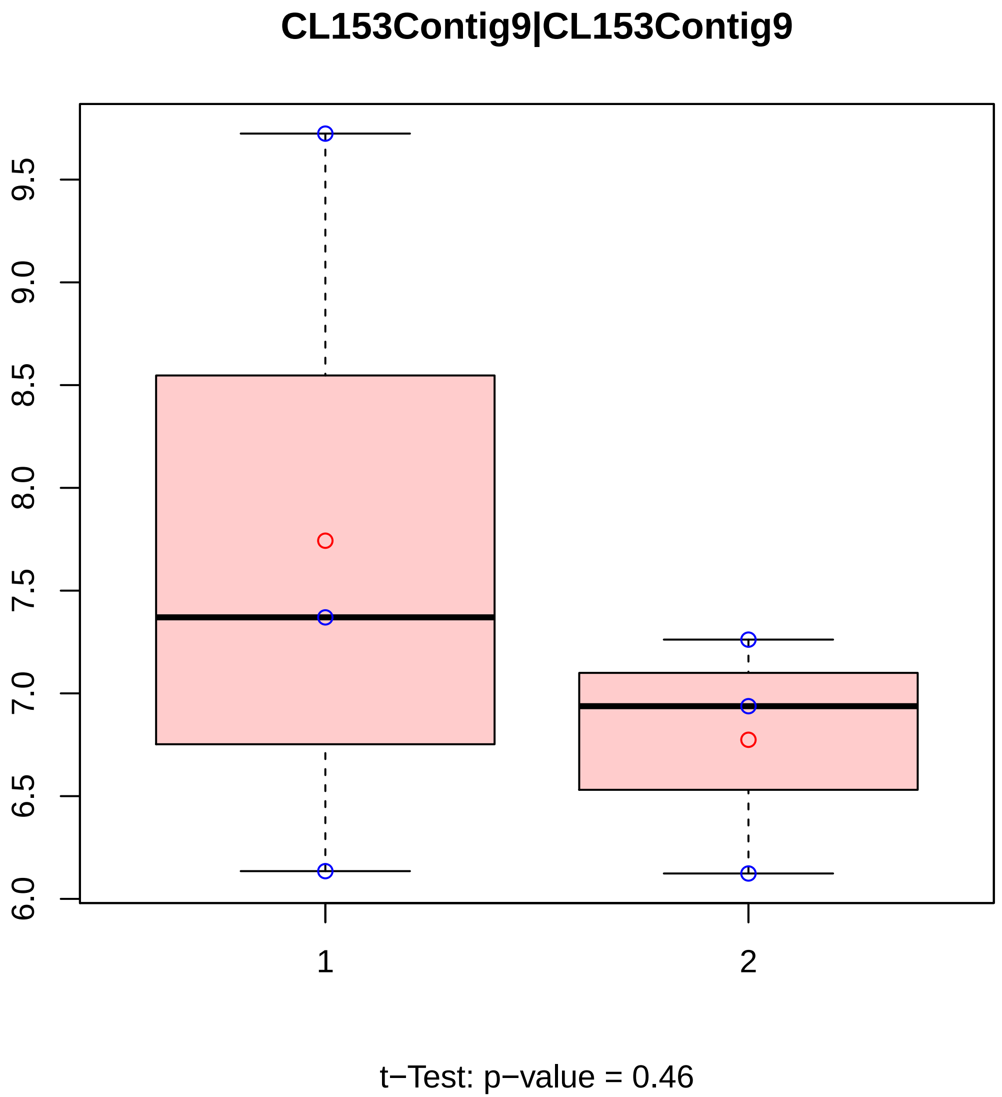
CL153Contig9|CL153Contig9
9.5
9.0
8.5
8.0
7.5
7.0
6.5
6.0
1
2
t−Test: p−value = 0.46

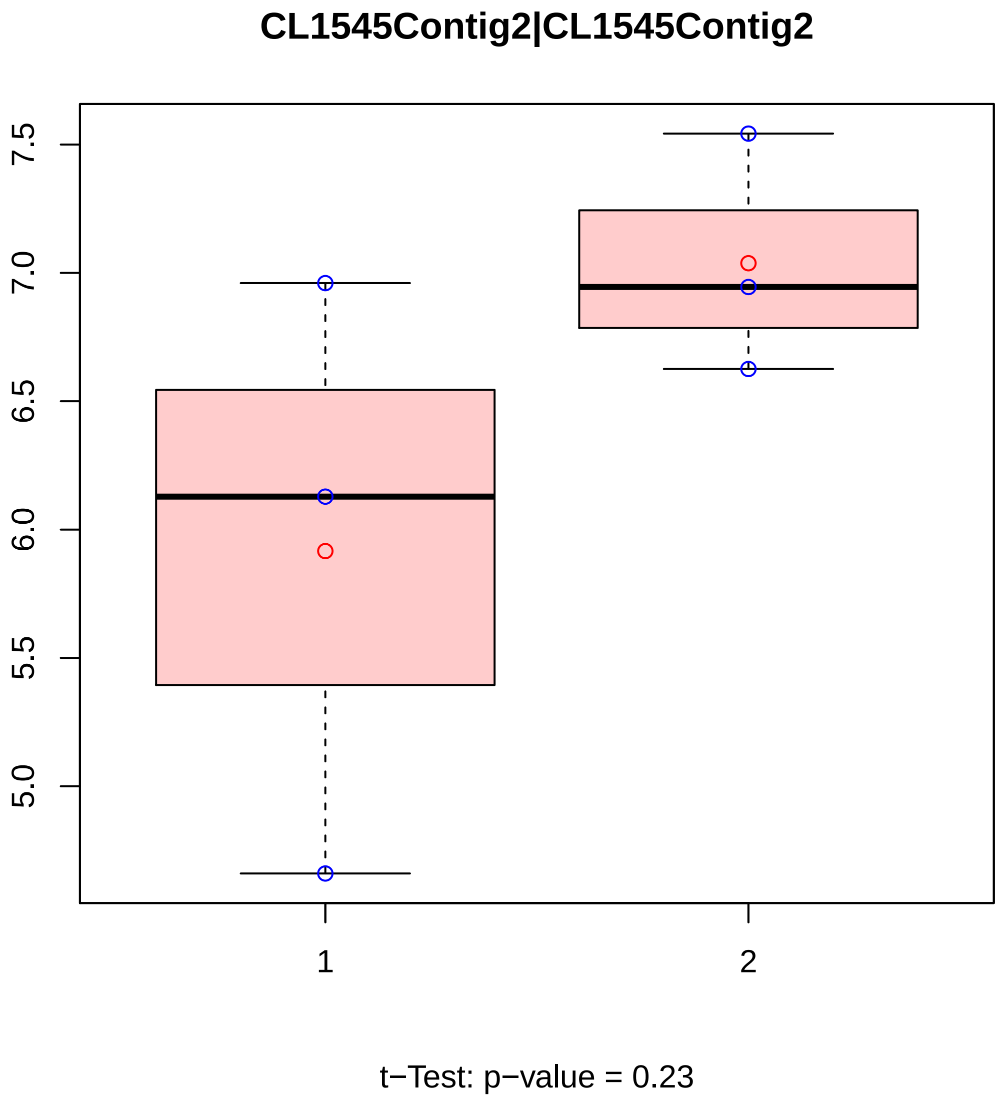
CL1545Contig2|CL1545Contig2
7.5
7.0
6.5
6.0
5.5
5.0
1
2
t−Test: p−value = 0.23

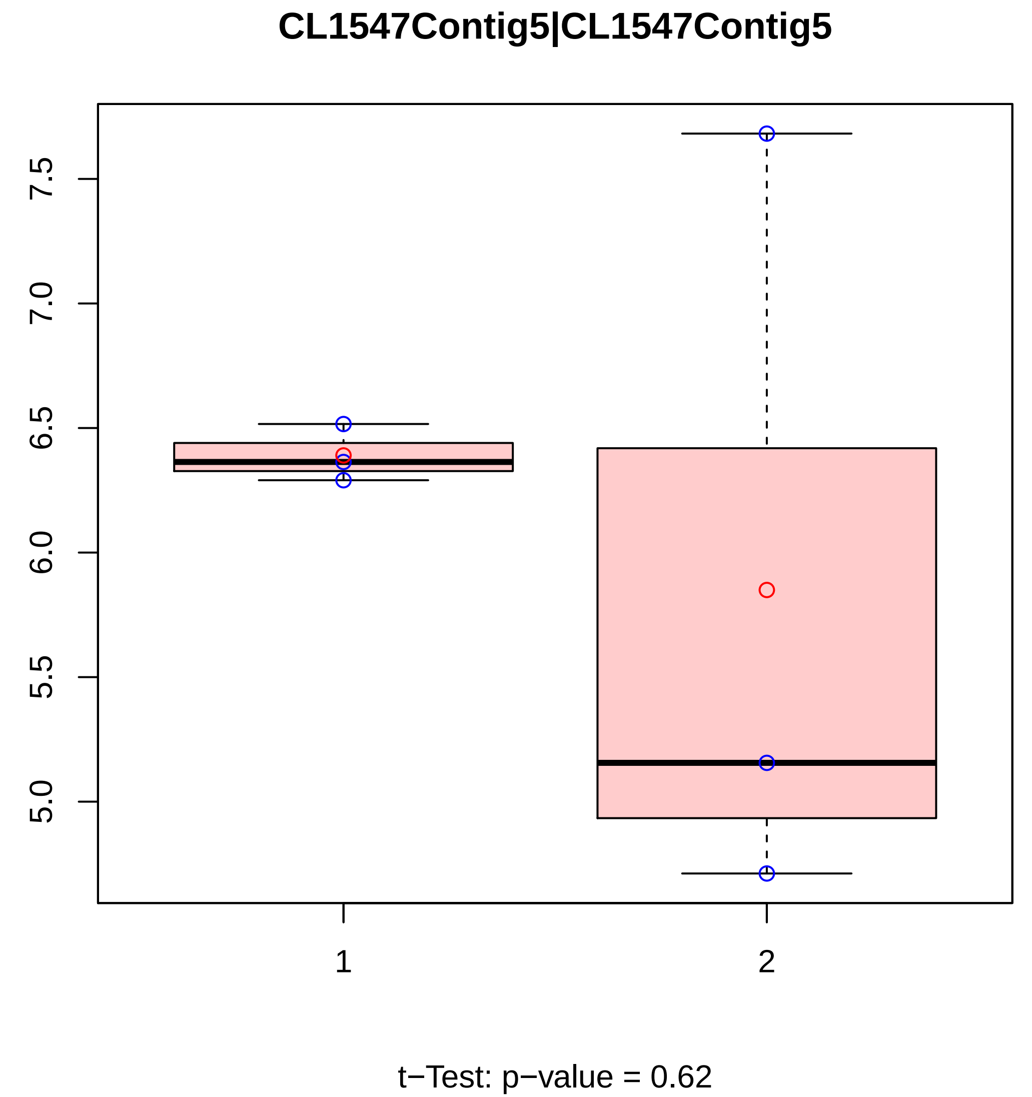
CL1547Contig5|CL1547Contig5
7.5
7.0
6.5
6.0
5.5
5.0
1
2
t−Test: p−value = 0.62

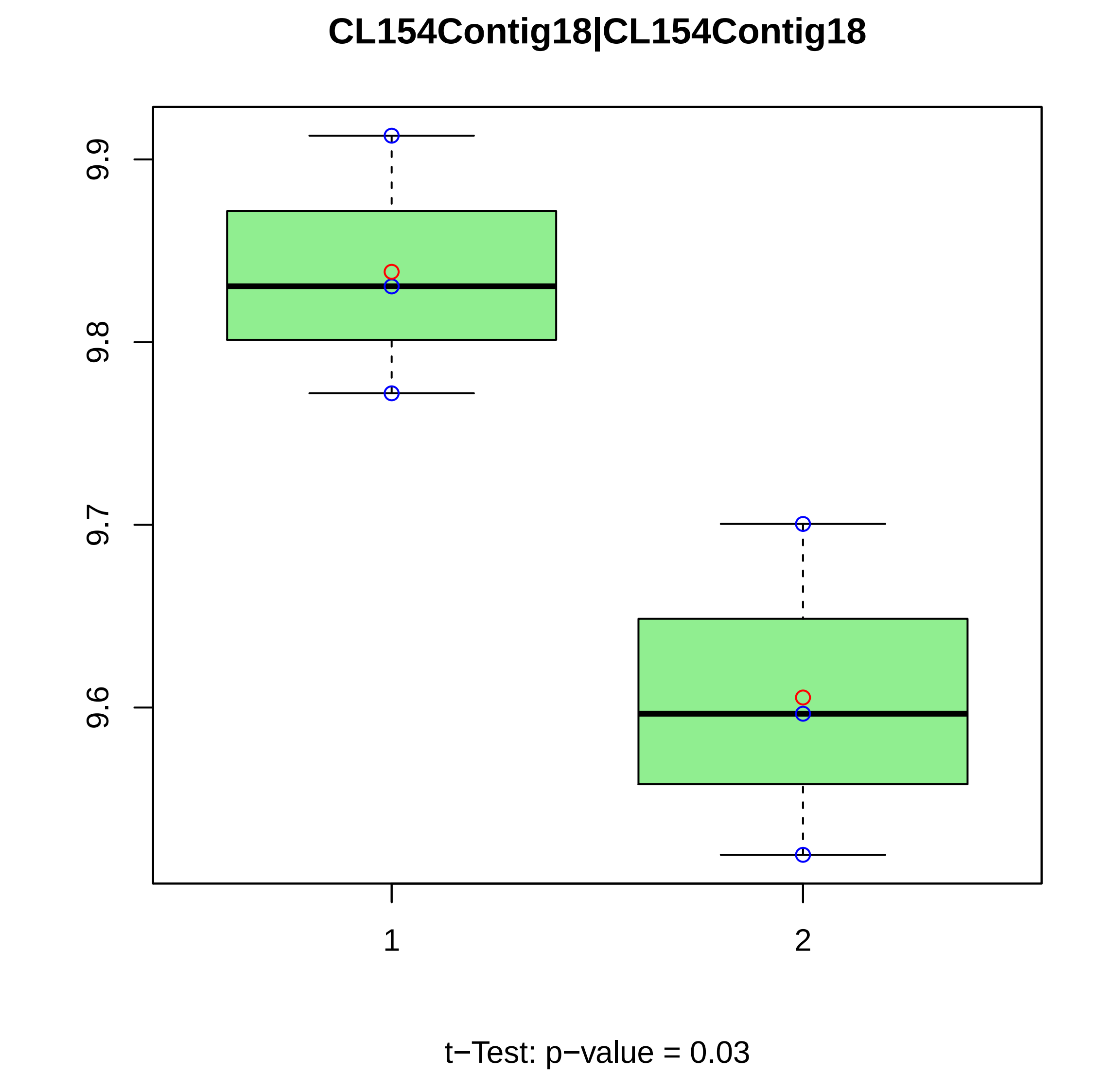
CL154Contig18|CL154Contig18
9.9
9.8
9.7
9.6
1
2
t−Test: p−value = 0.03

# CL1551Contig4|CL1551Contig4

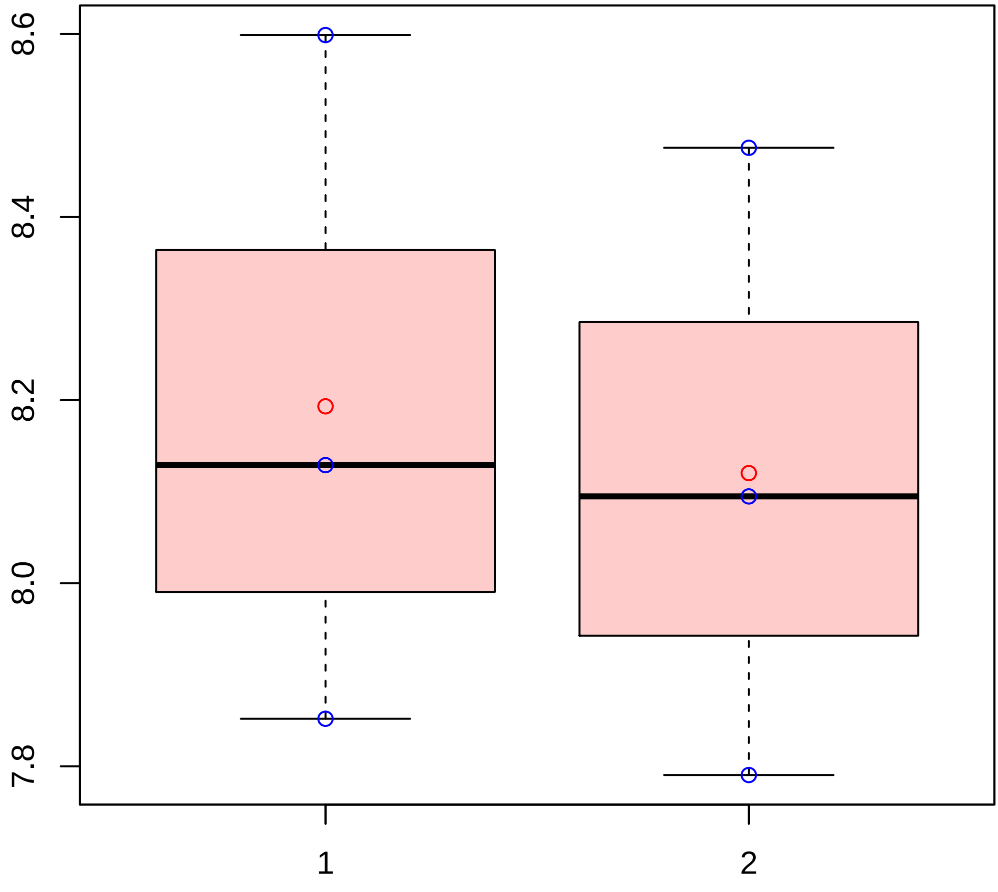


t−Test: p−value = 0.82

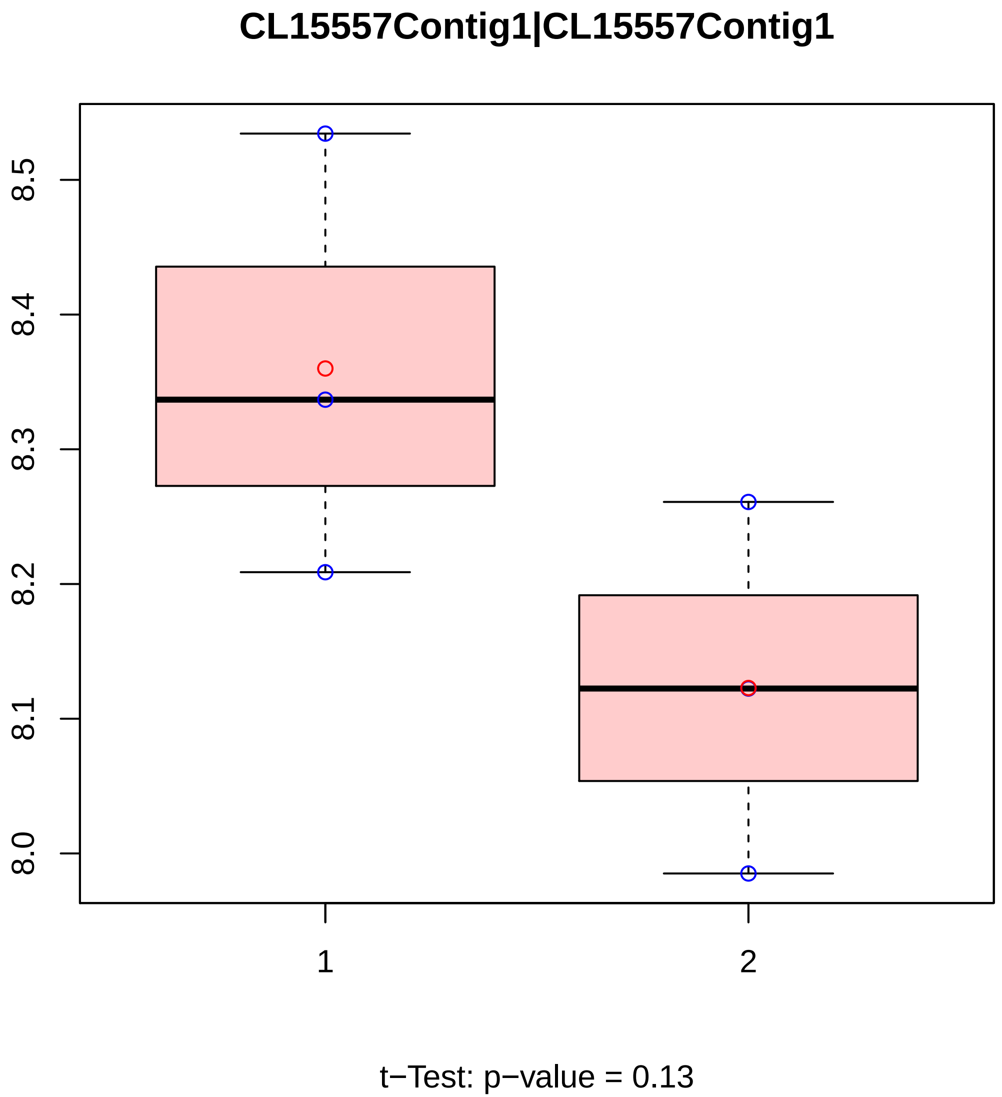
CL15557Contig1|CL15557Contig1
8.0
8.1
8.2
8.3
8.4
8.5
1
2
t−Test: p−value = 0.13

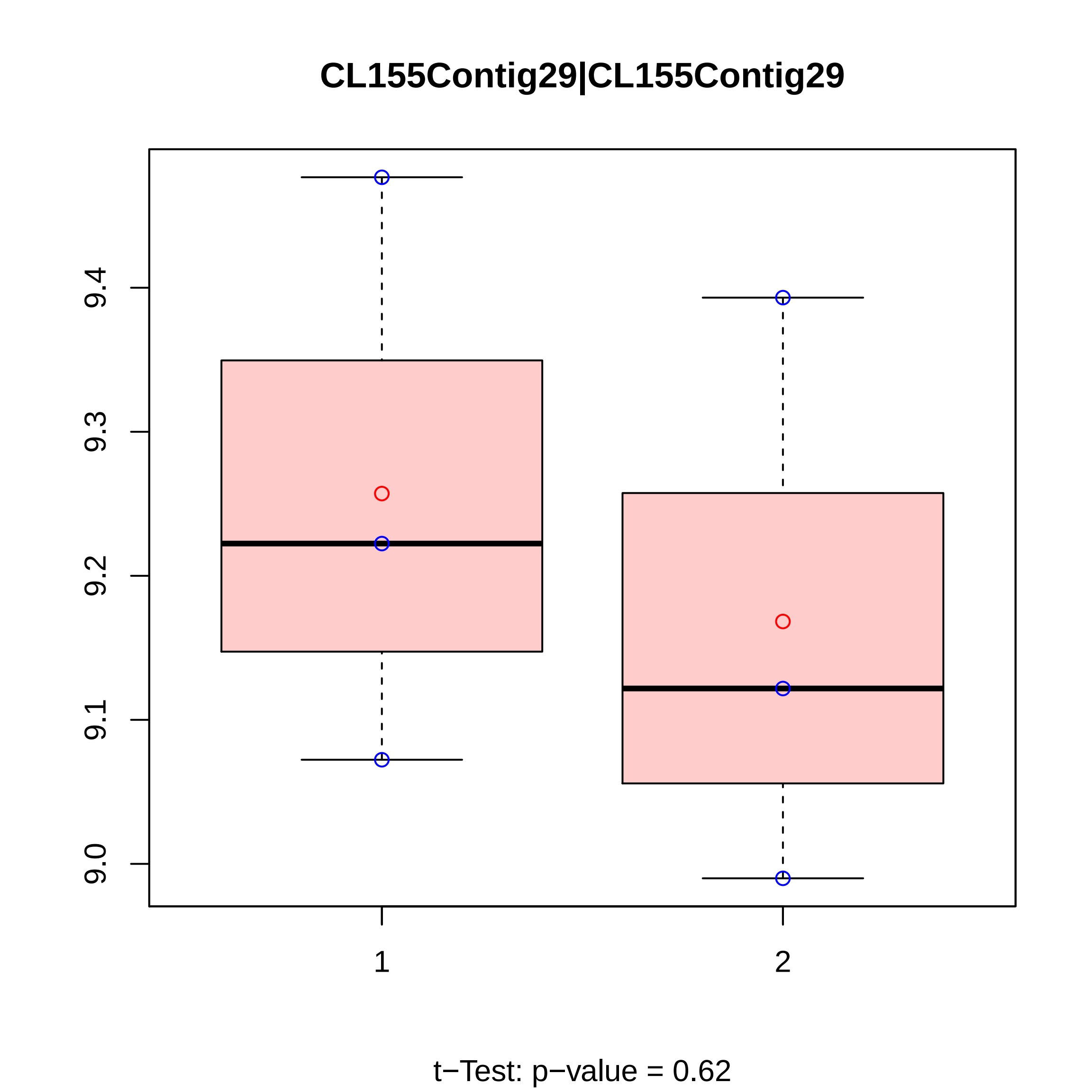

CL155Contig29|CL155Contig29
9.0
9.1
9.2
9.3
9.4
1
2
t−Test: p−value = 0.62

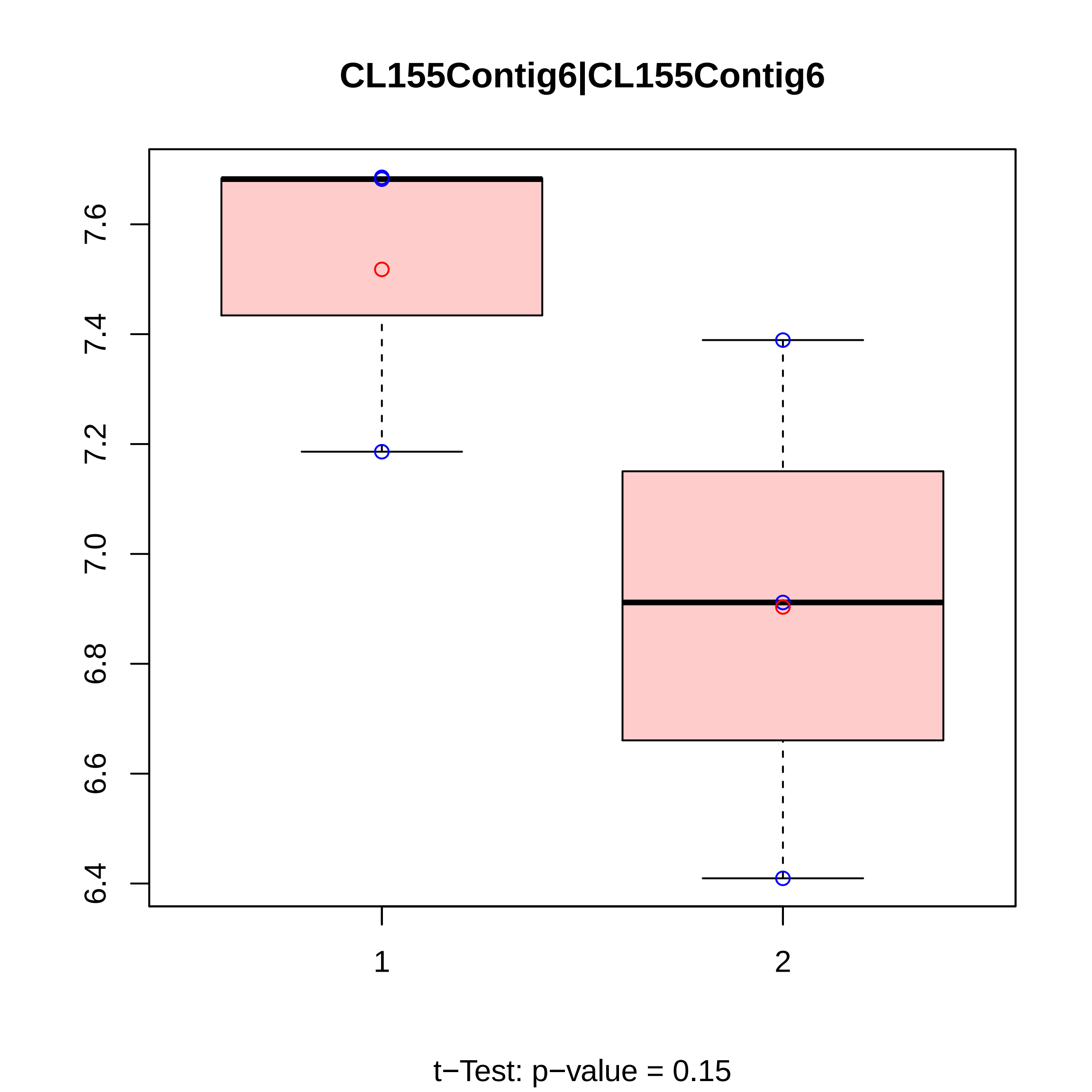
CL155Contig6|CL155Contig6
7.6
7.4
7.2
7.0
6.8
6.6
6.4
1
2
t−Test: p−value = 0.15

# CL1567Contig1|CL1567Contig1

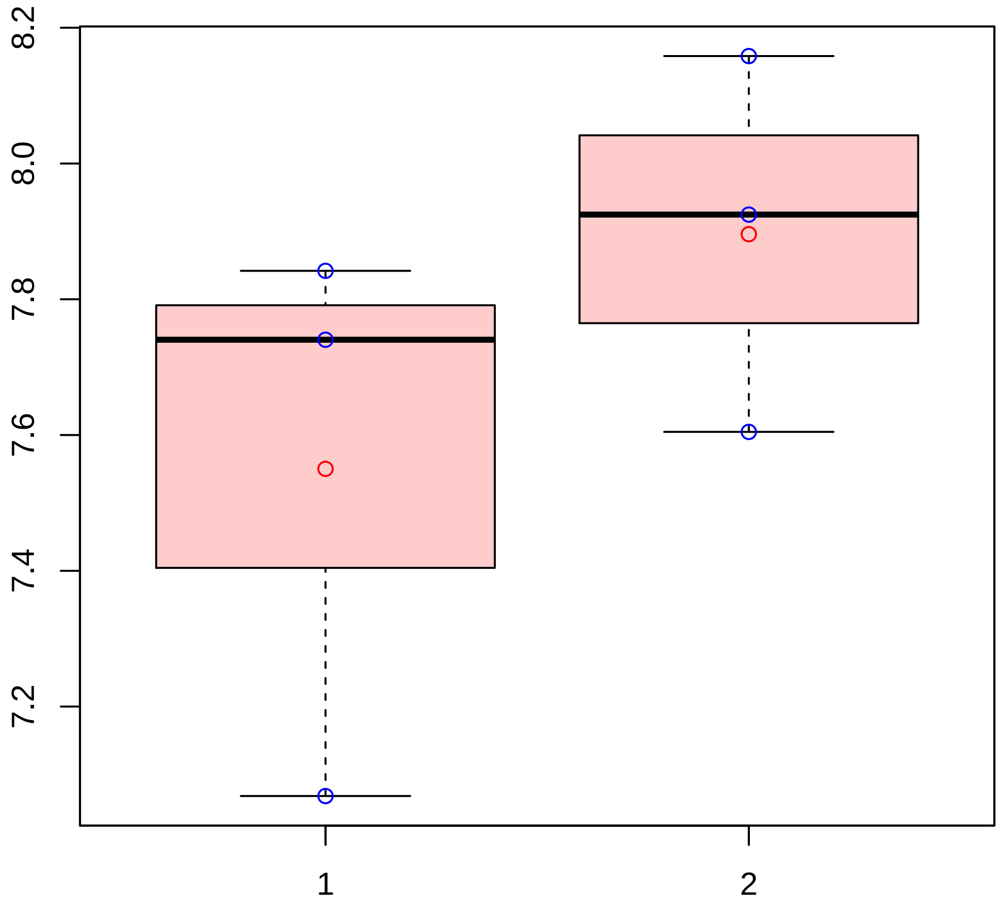


t−Test: p−value = 0.31

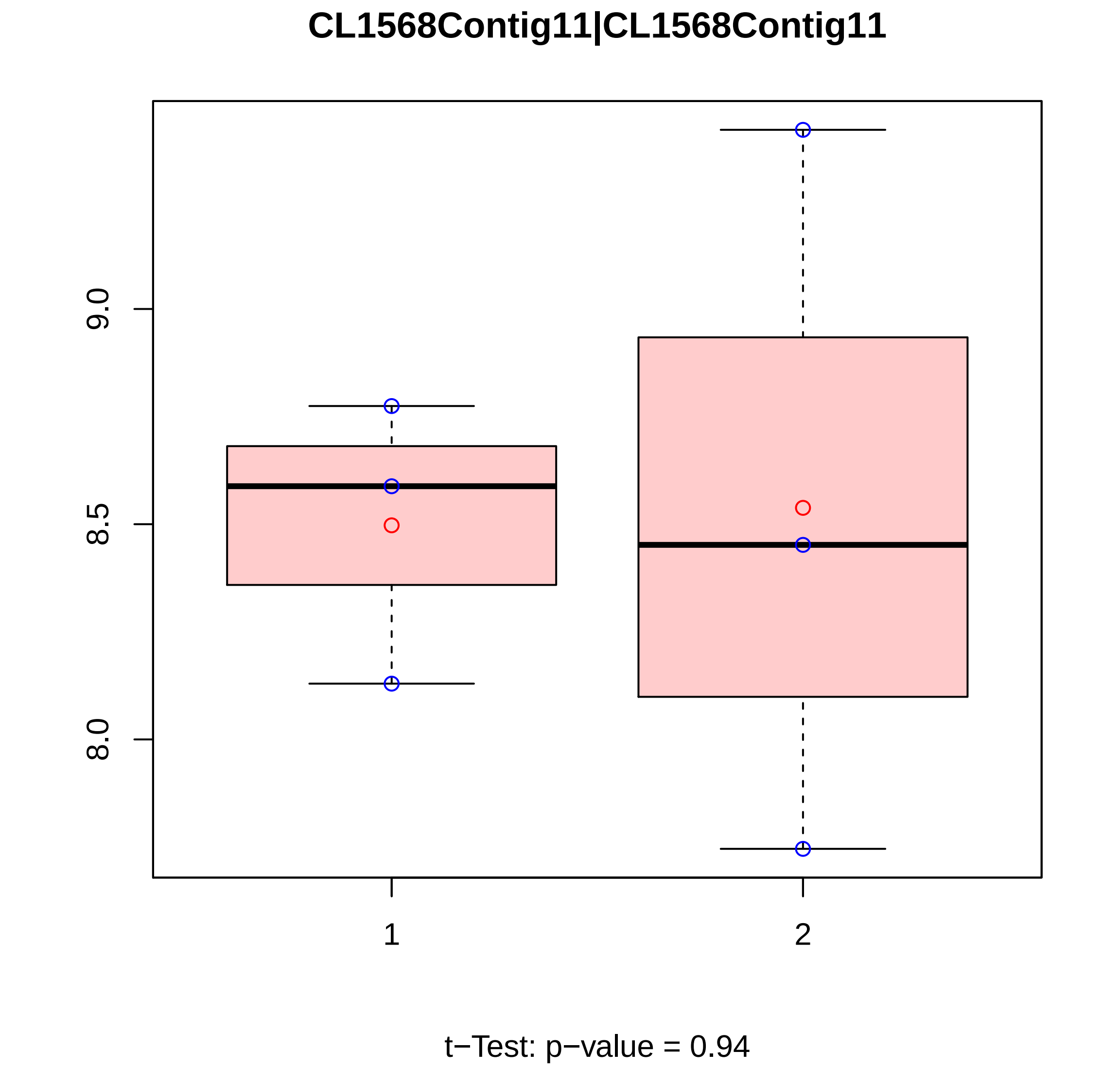
CL1568Contig11|CL1568Contig11
9.0
8.5
8.0
1
2
t−Test: p−value = 0.94

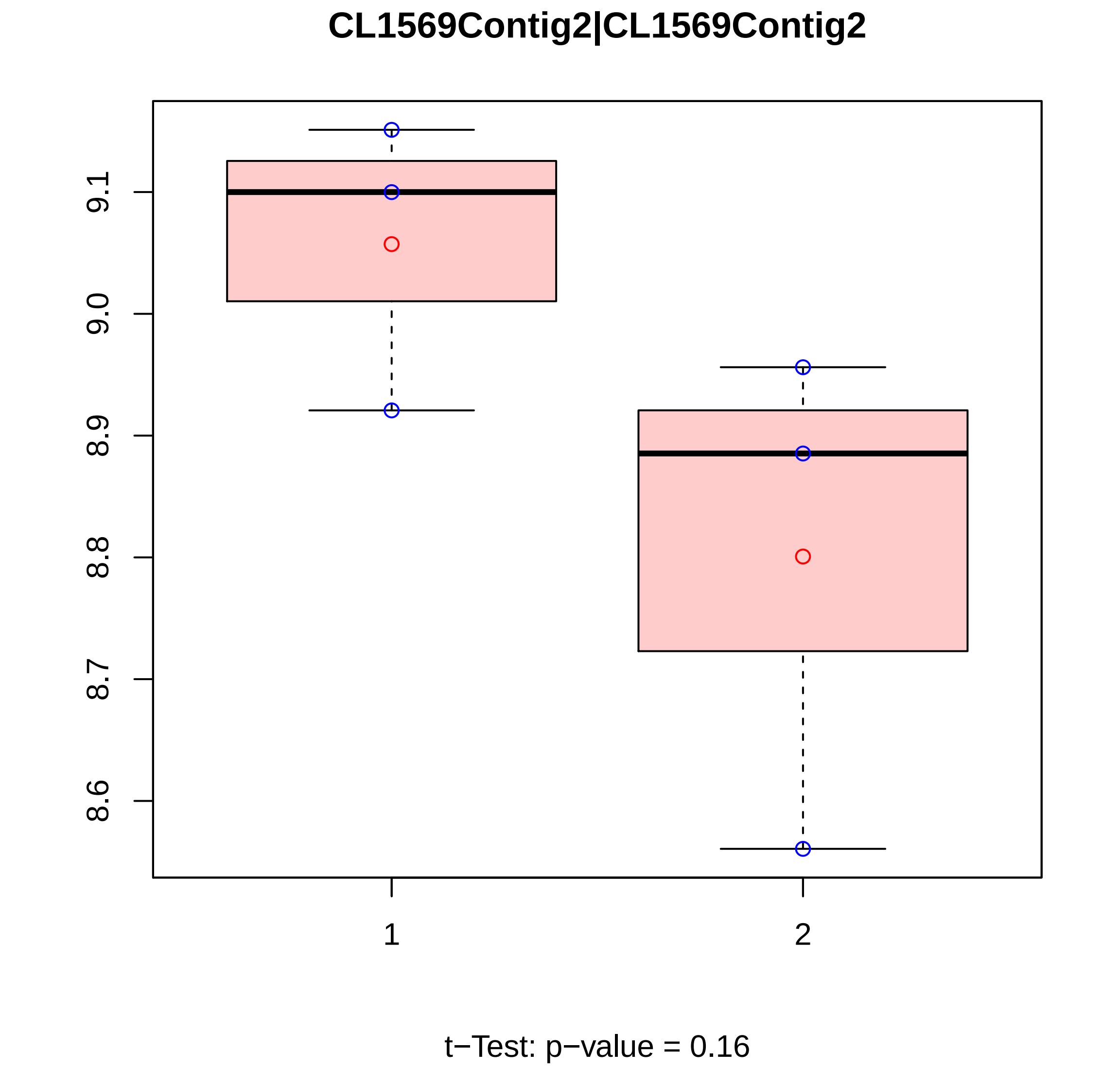
CL1569Contig2|CL1569Contig2
9.1
9.0
8.9
8.8
8.7
8.6
1
2
t−Test: p−value = 0.16

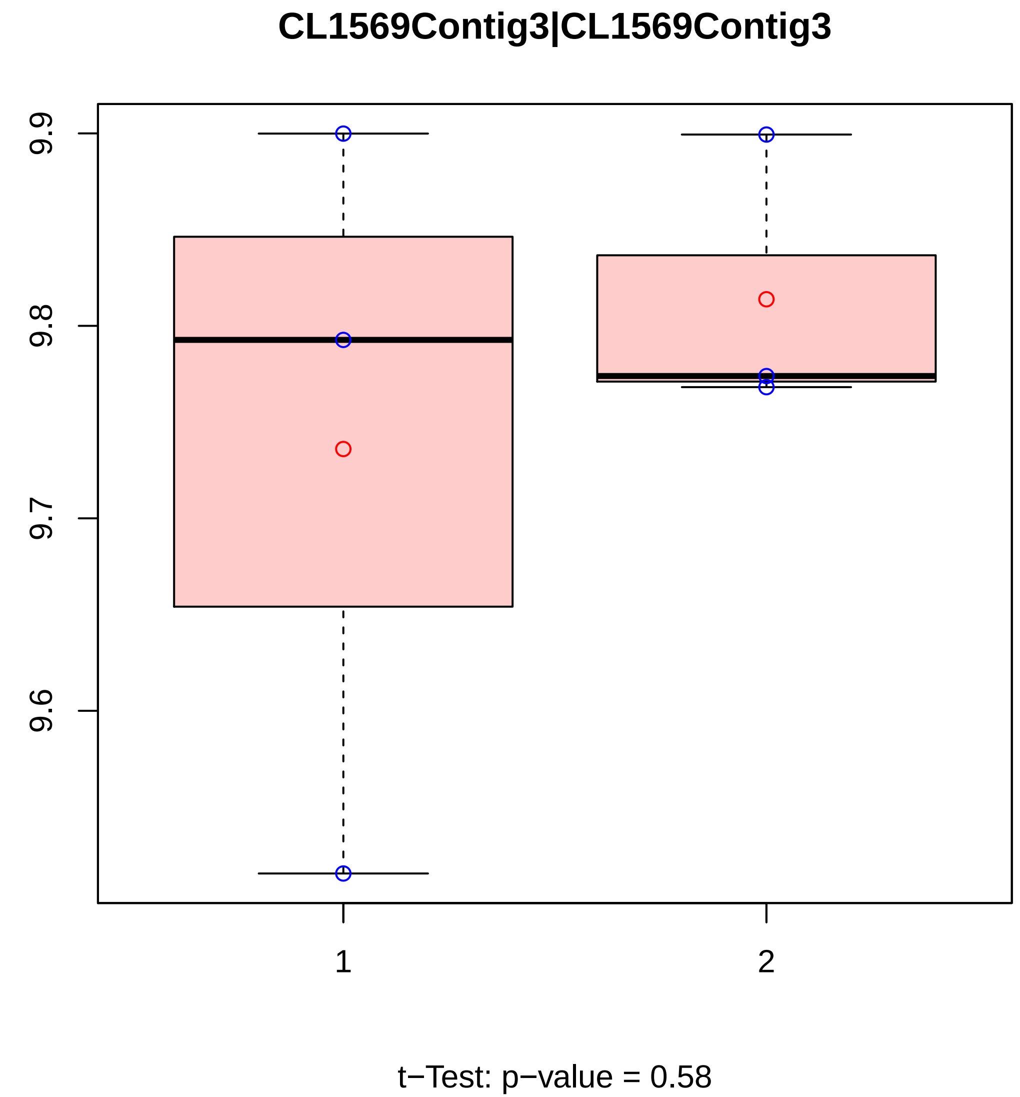
CL1569Contig3|CL1569Contig3
9.9
9.8
9.7
9.6
1
2
t−Test: p−value = 0.58

# CL1571Contig5|CL1571Contig5

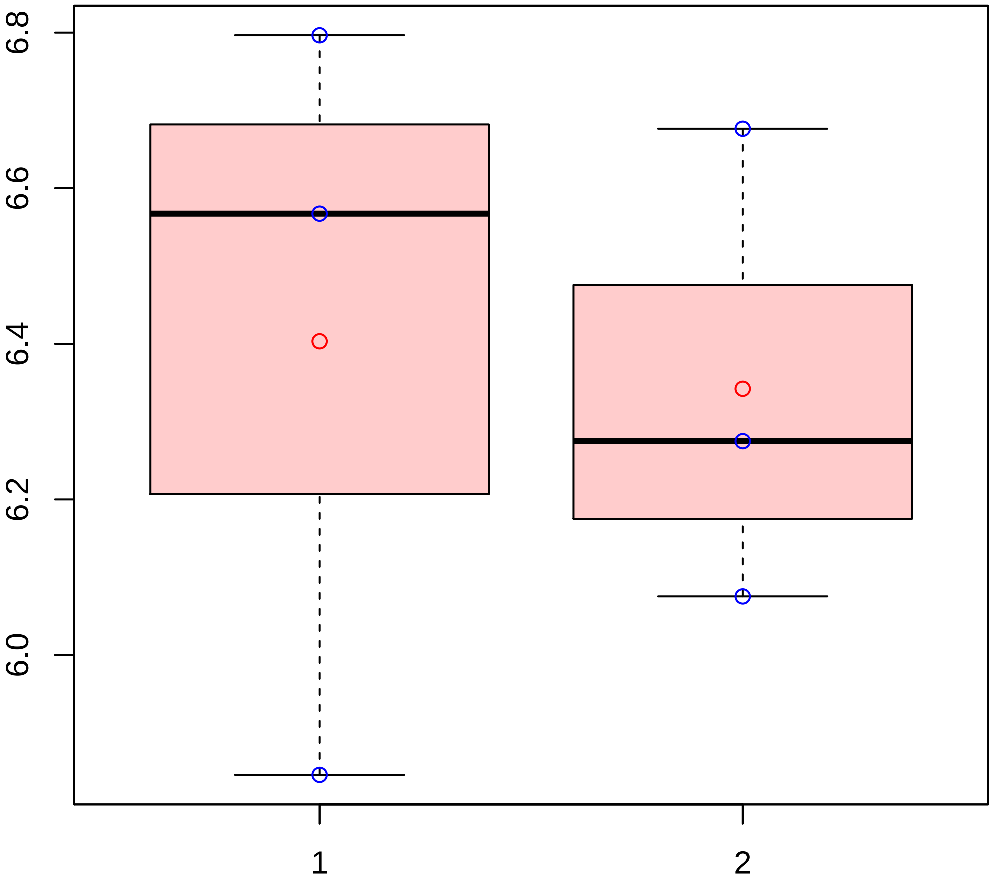


t−Test: p−value = 0.87

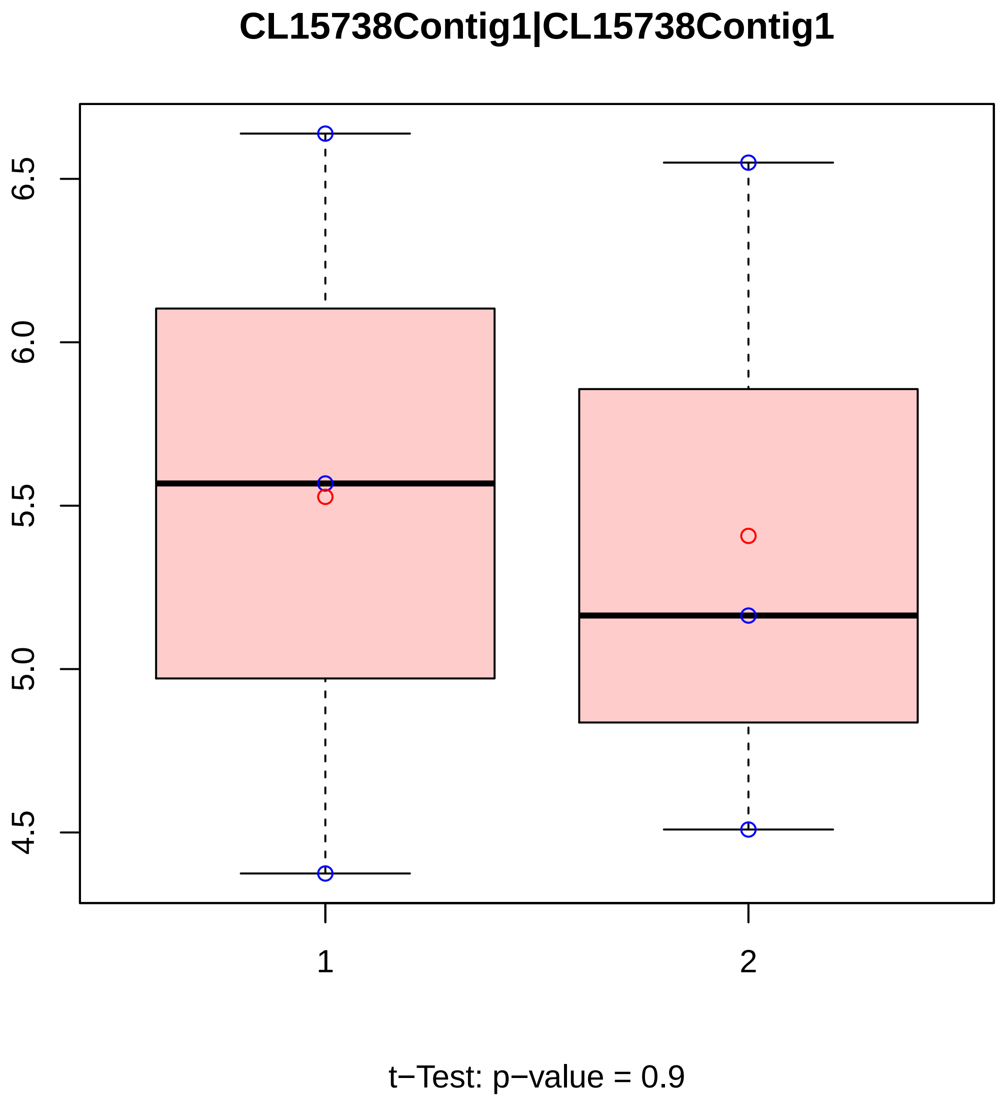

CL15738Contig1|CL15738Contig1
6.5
6.0
5.5
5.0
4.5
1
2
t−Test: p−value = 0.9

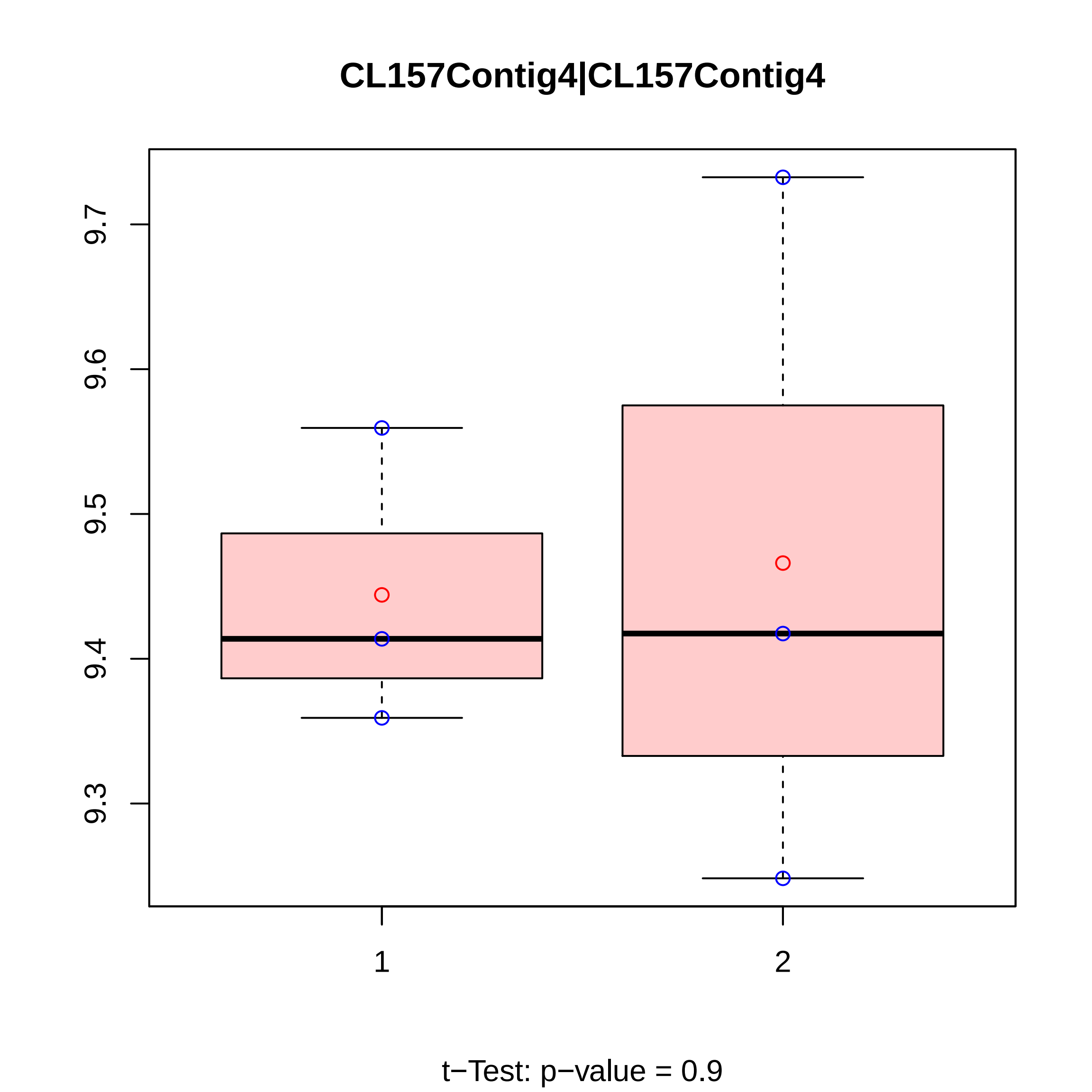
CL157Contig4|CL157Contig4
9.3
9.4
9.5
9.6
9.7
1
2
t−Test: p−value = 0.9

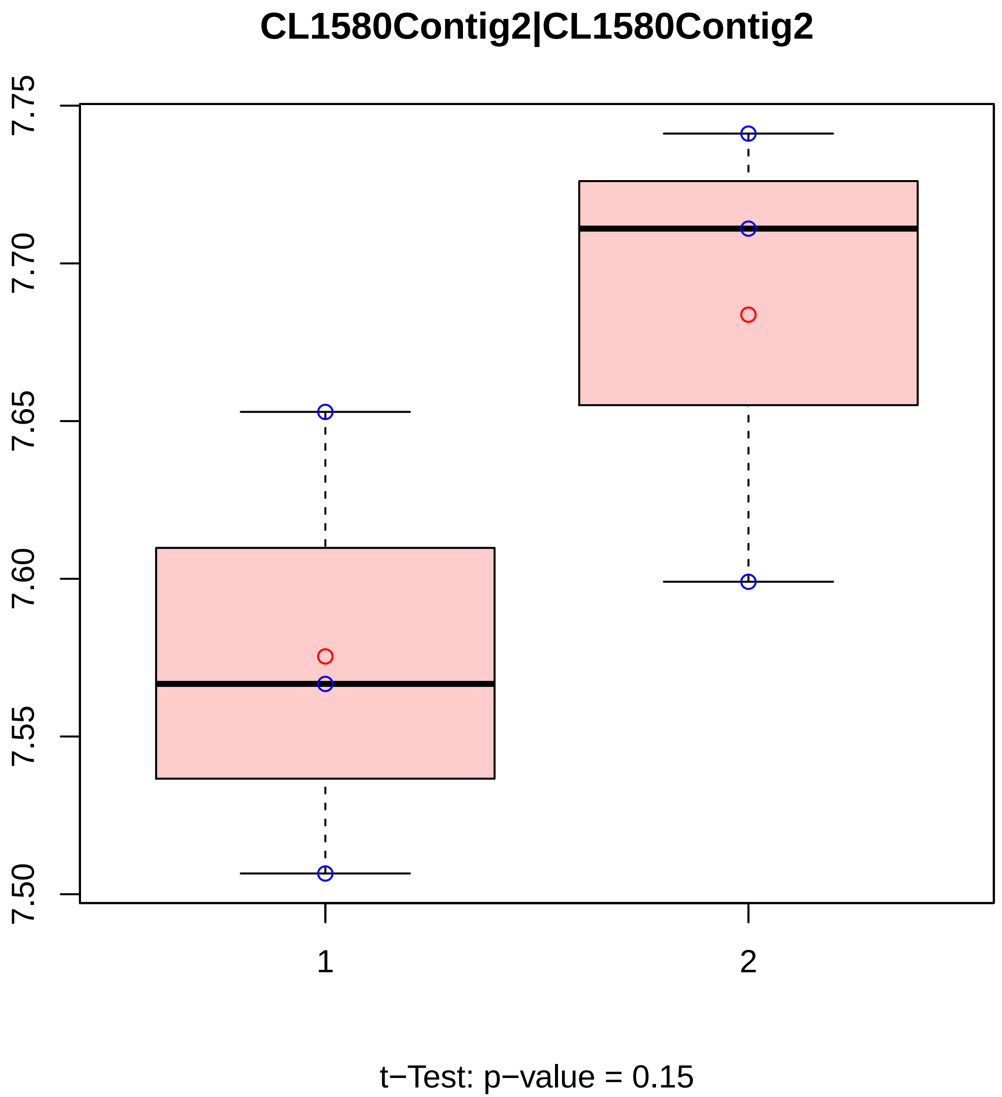

CL1580Contig2|CL1580Contig2
7.50
7.55
7.60
7.65
7.70
7.75
1
2
t−Test: p−value = 0.15

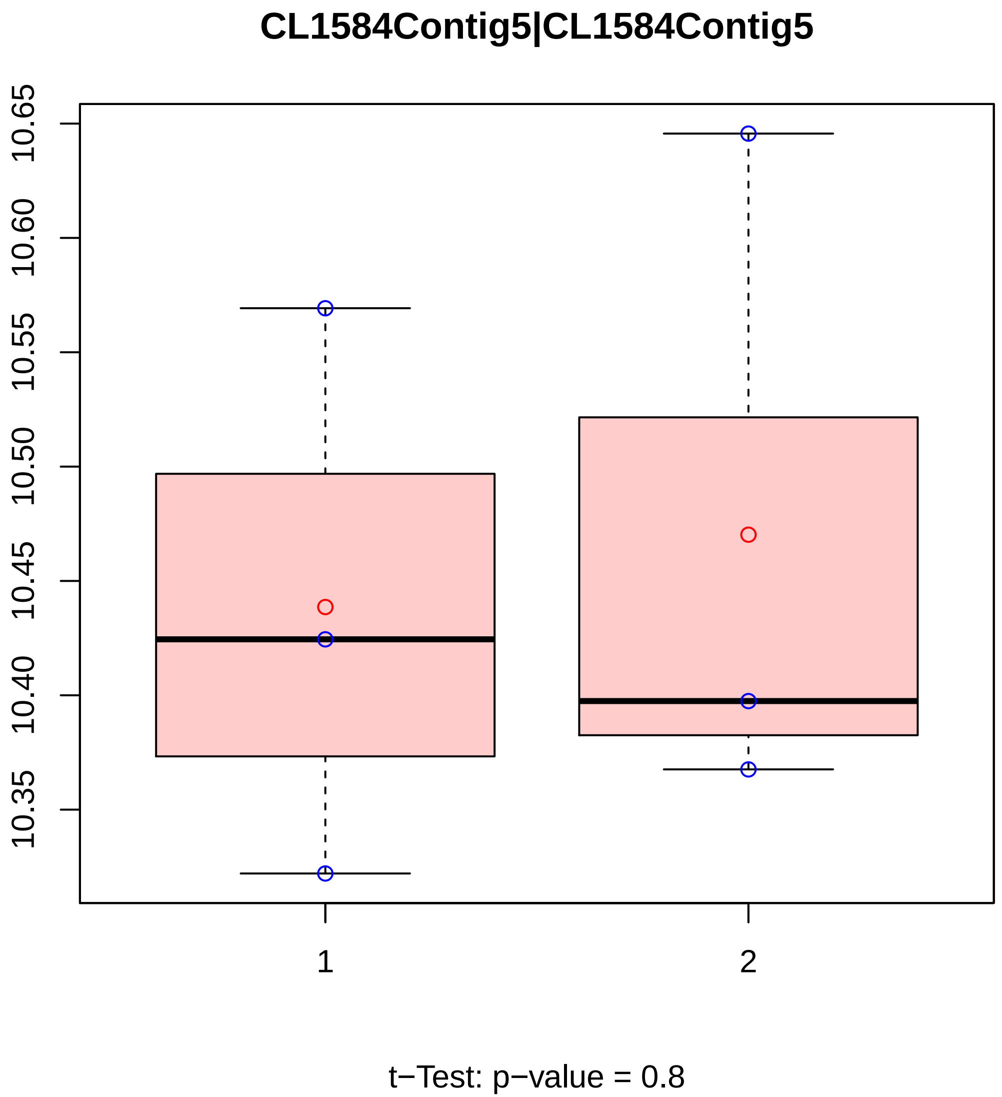
CL1584Contig5|CL1584Contig5
10.65
10.60
10.55
10.50
10.45
10.40
10.35
1
2
t−Test: p−value = 0.8

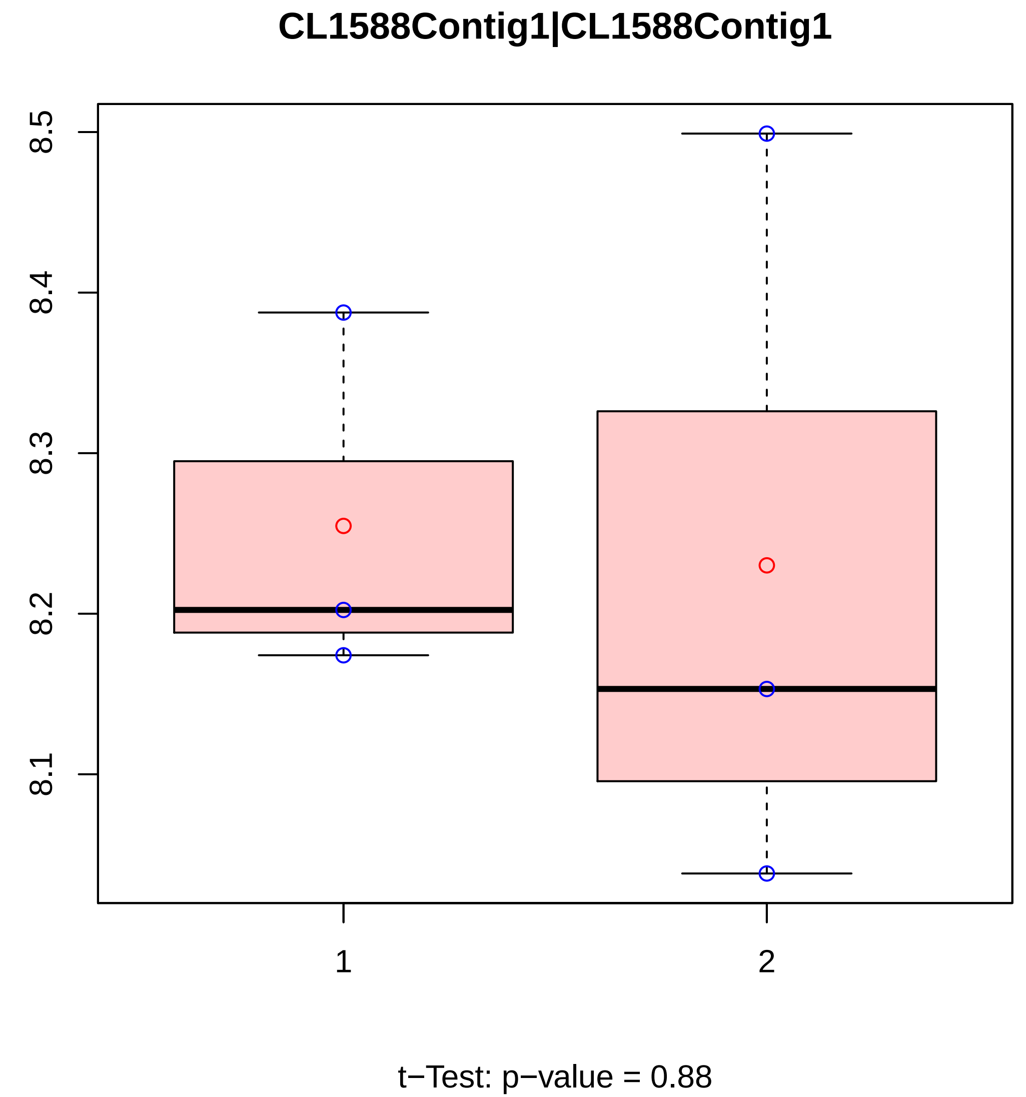
CL1588Contig1|CL1588Contig1
8.1
8.2
8.3
8.4
8.5
1
2
t−Test: p−value = 0.88

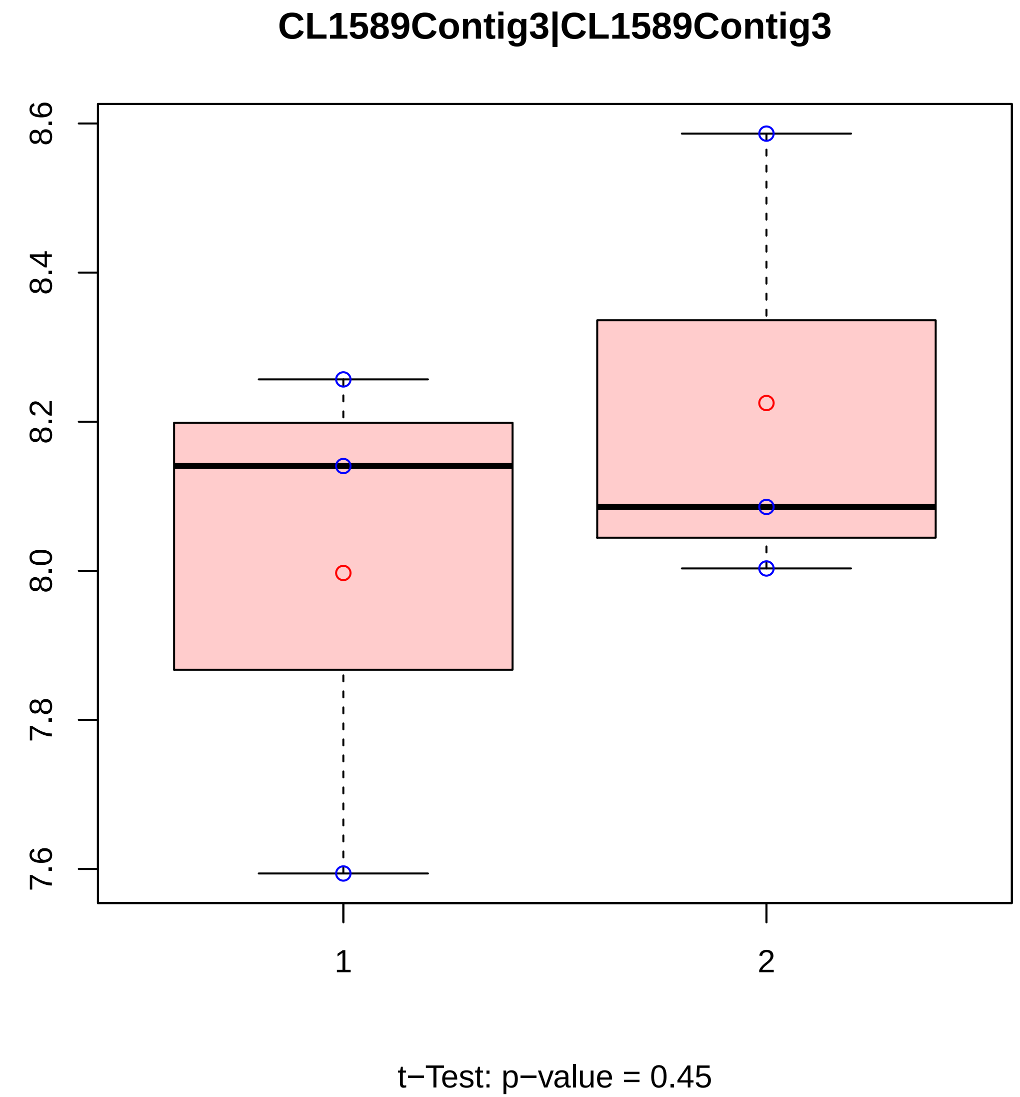
CL1589Contig3|CL1589Contig3
8.6
8.4
8.2
8.0
7.8
7.6
1
2
t−Test: p−value = 0.45

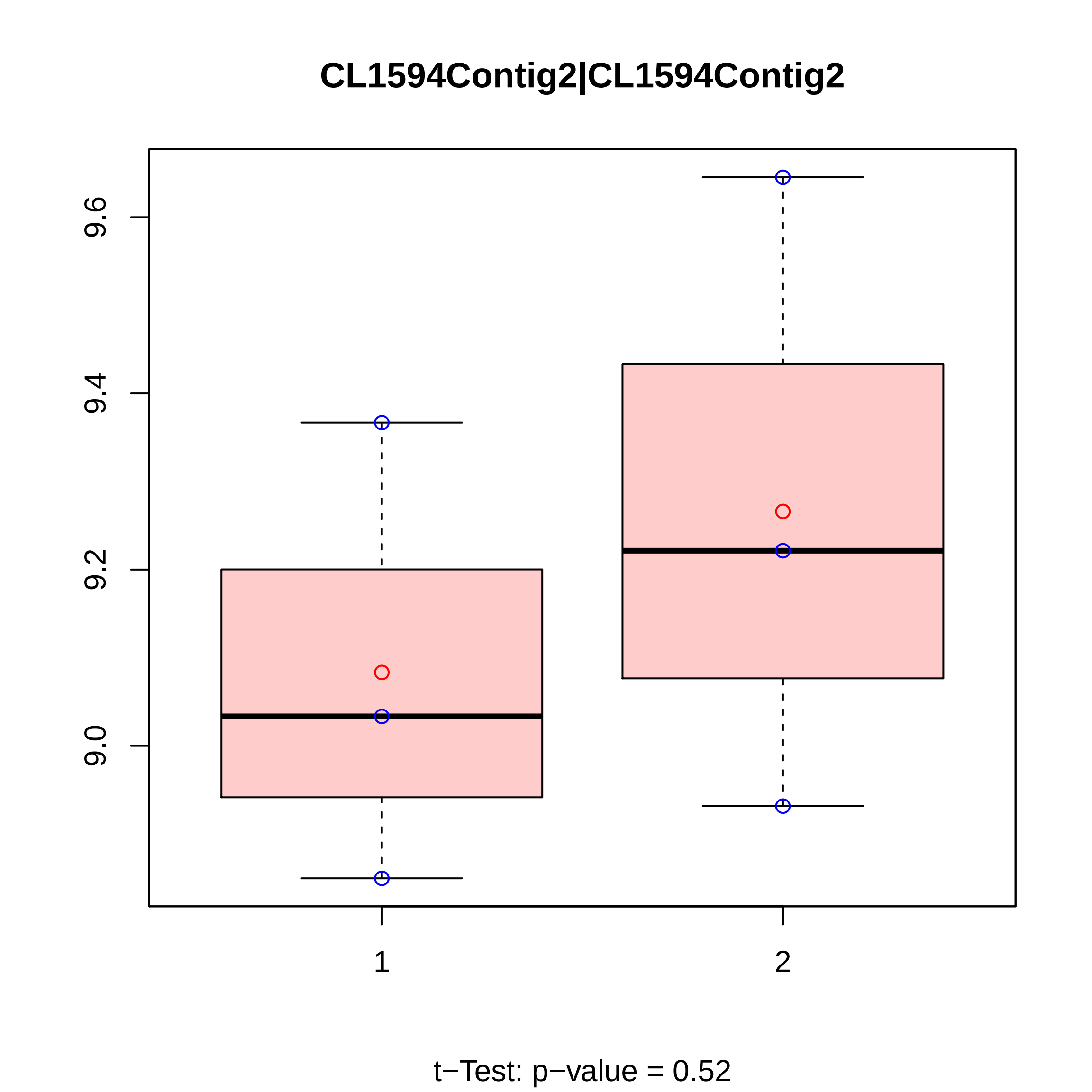
CL1594Contig2|CL1594Contig2
9.0
9.2
9.4
9.6
1
2
t−Test: p−value = 0.52

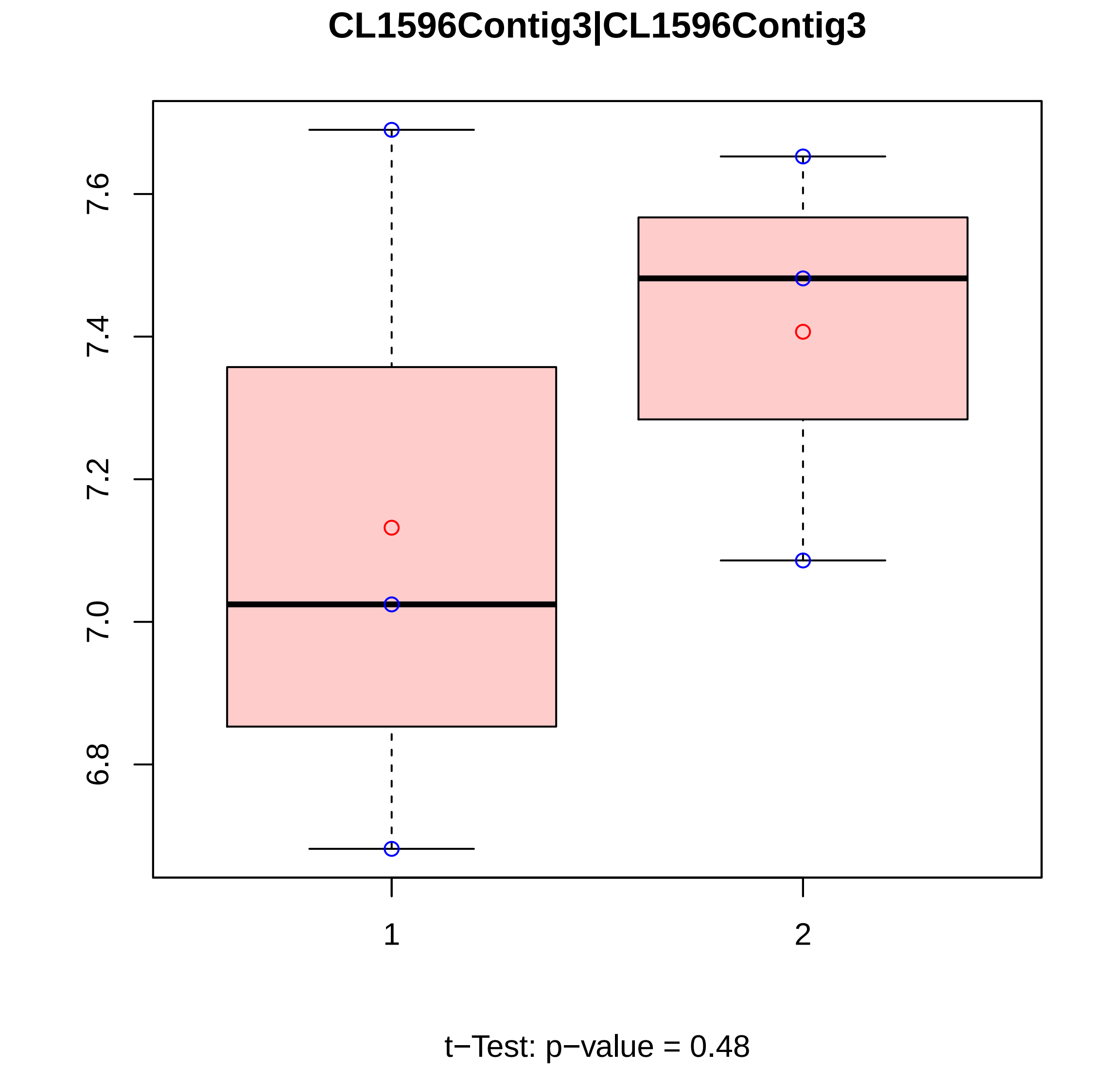
CL1596Contig3|CL1596Contig3
7.6
7.4
7.2
7.0
6.8
1
2
t−Test: p−value = 0.48

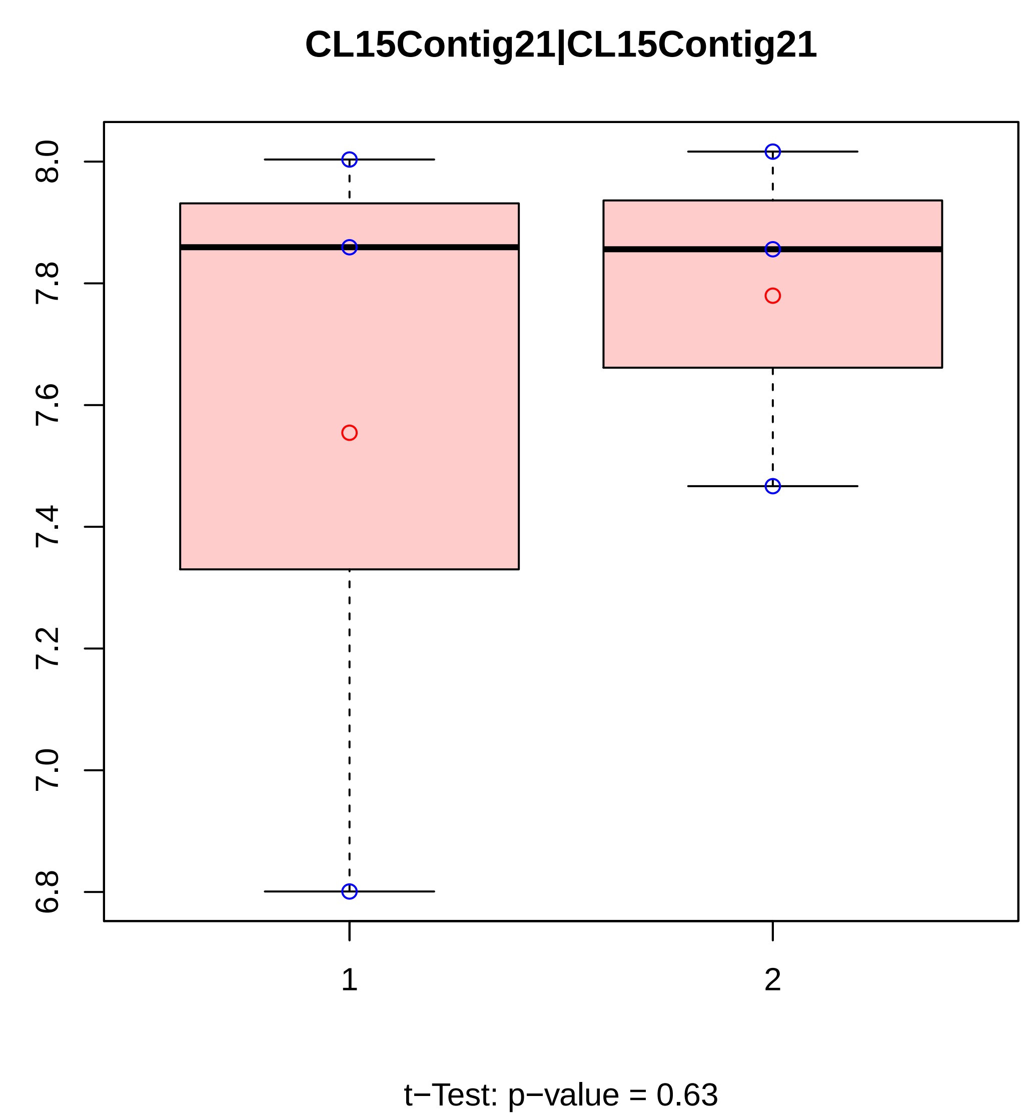
CL15Contig21|CL15Contig21
8.0
7.8
7.6
7.4
7.2
7.0
6.8
1
2
t−Test: p−value = 0.63

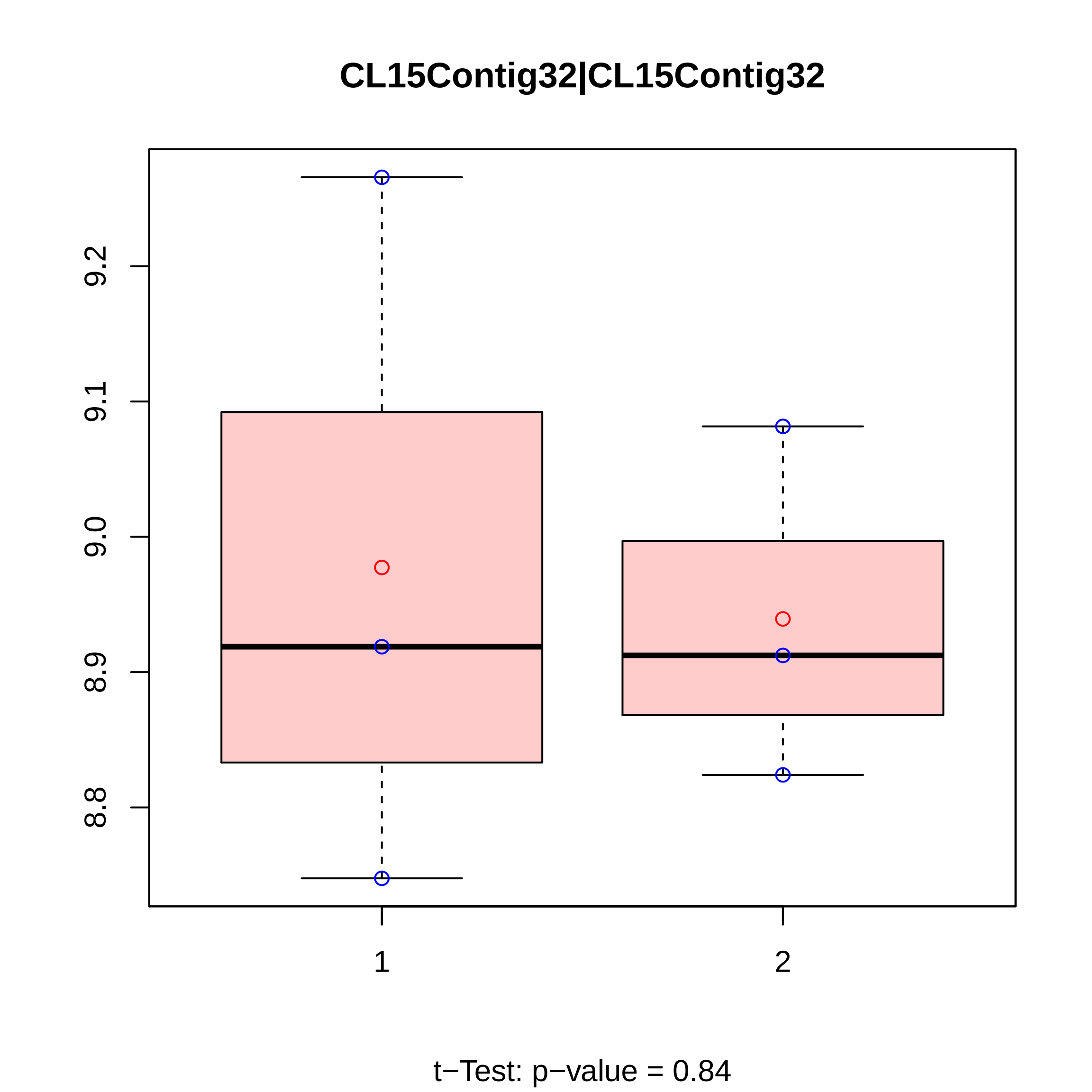

CL15Contig32|CL15Contig32
9.2
9.1
9.0
8.9
8.8
1
2
t−Test: p−value = 0.84

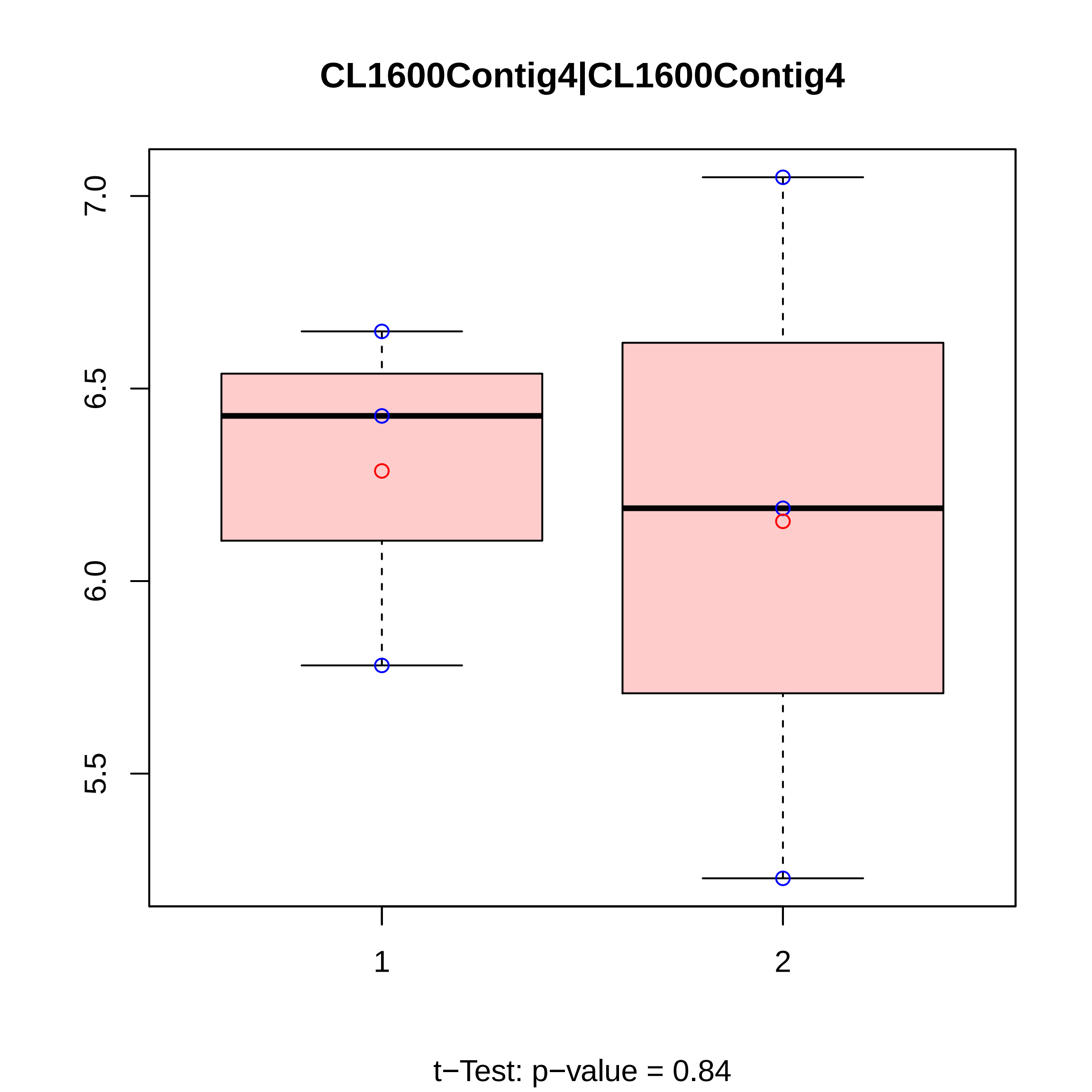
CL1600Contig4|CL1600Contig4
7.0
6.5
6.0
5.5
1
2
t−Test: p−value = 0.84

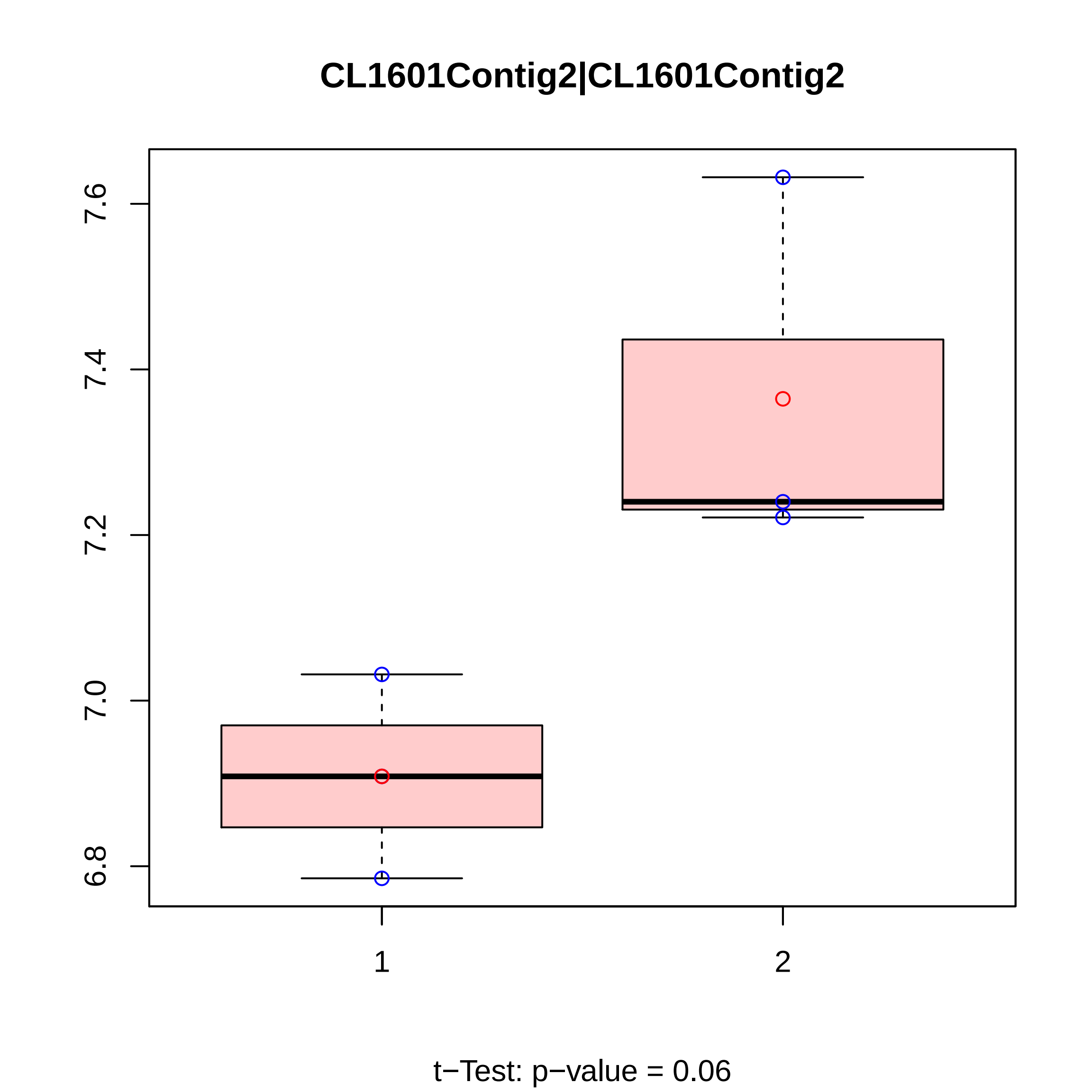
CL1601Contig2|CL1601Contig2
7.6
7.4
7.2
7.0
6.8
1
2
t−Test: p−value = 0.06

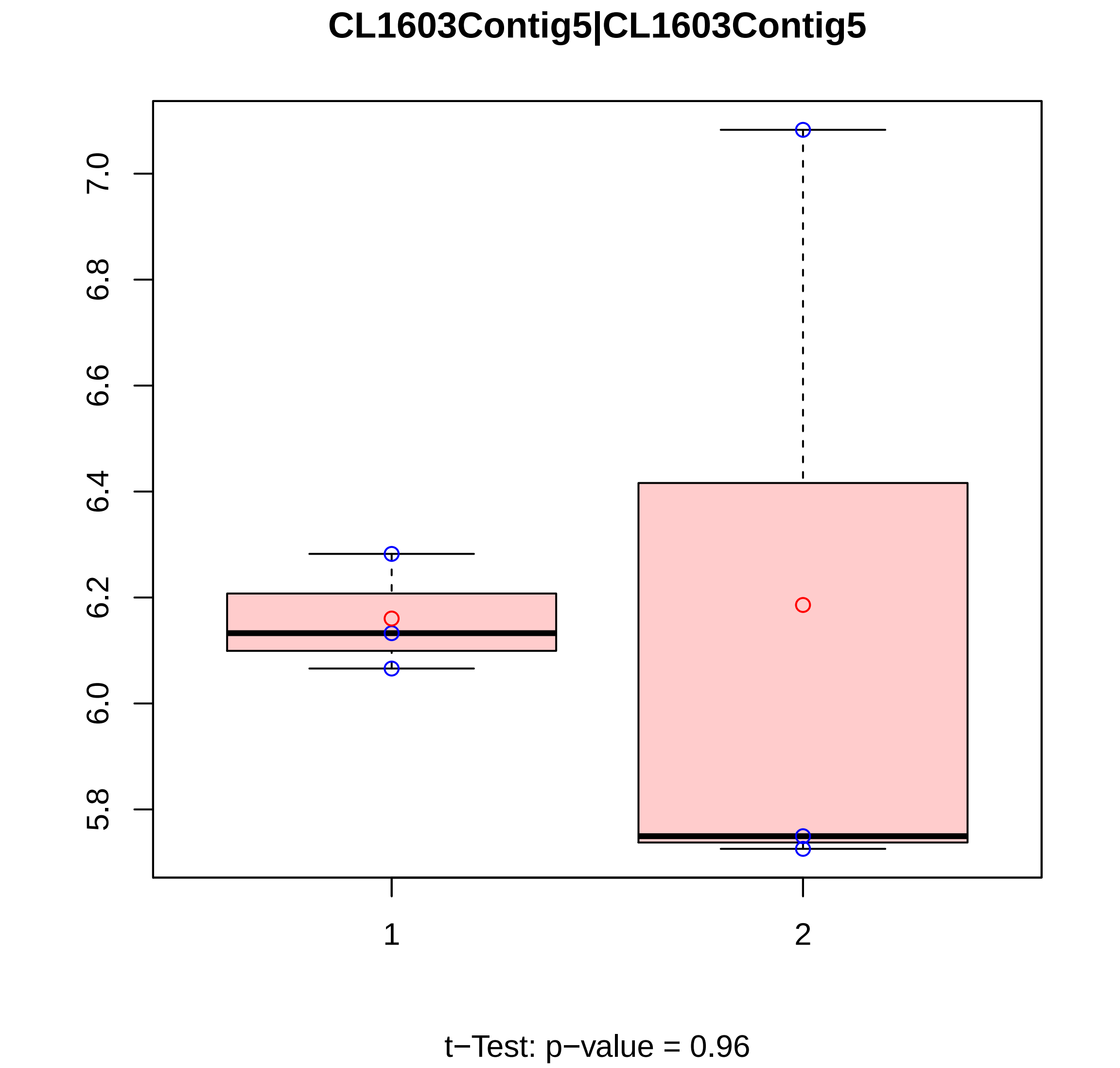

CL1603Contig5|CL1603Contig5
7.0
6.8
6.6
6.4
6.2
6.0
5.8
1
2
t−Test: p−value = 0.96

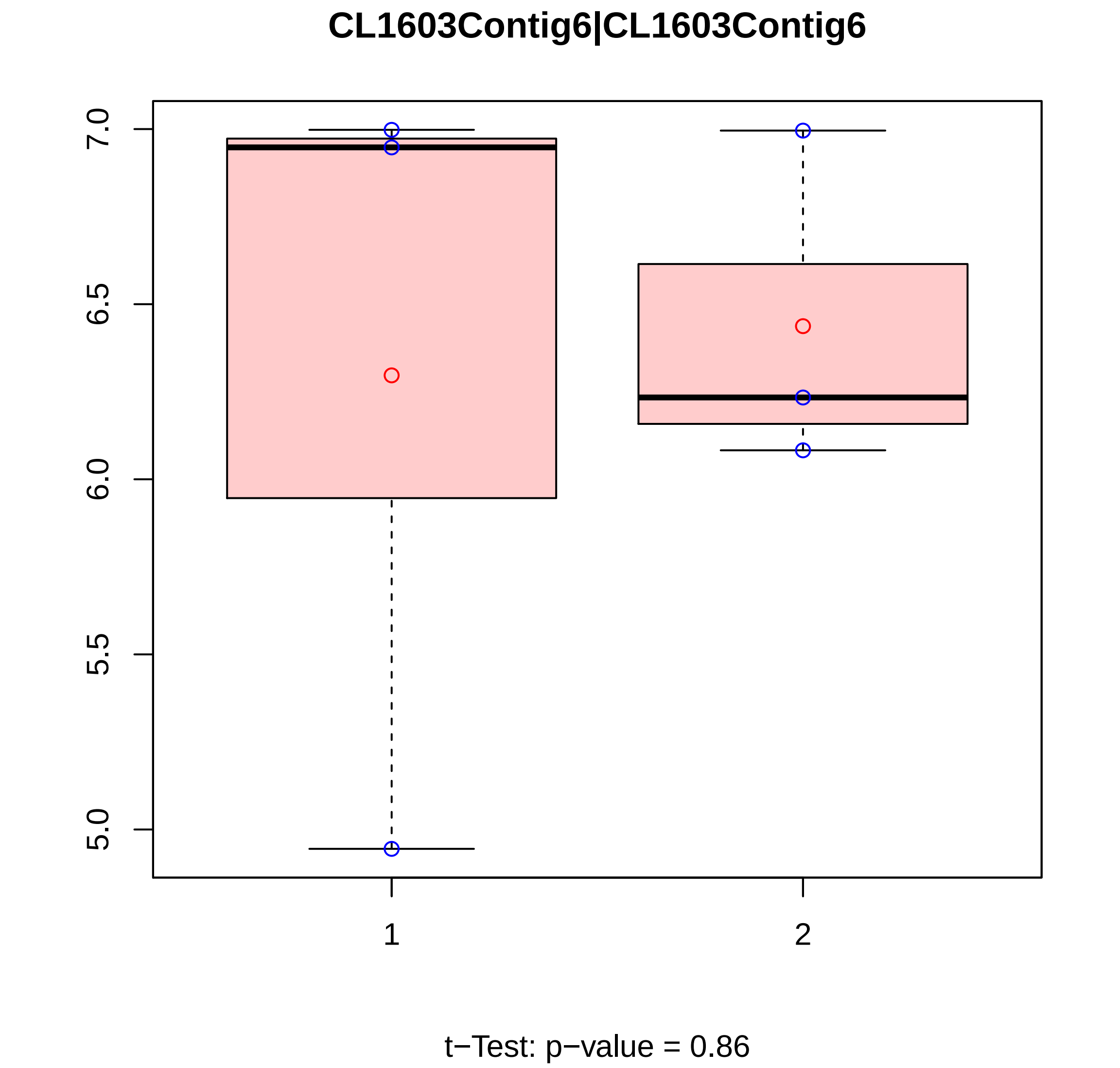
CL1603Contig6|CL1603Contig6
7.0
6.5
6.0
5.5
5.0
1
2
t−Test: p−value = 0.86

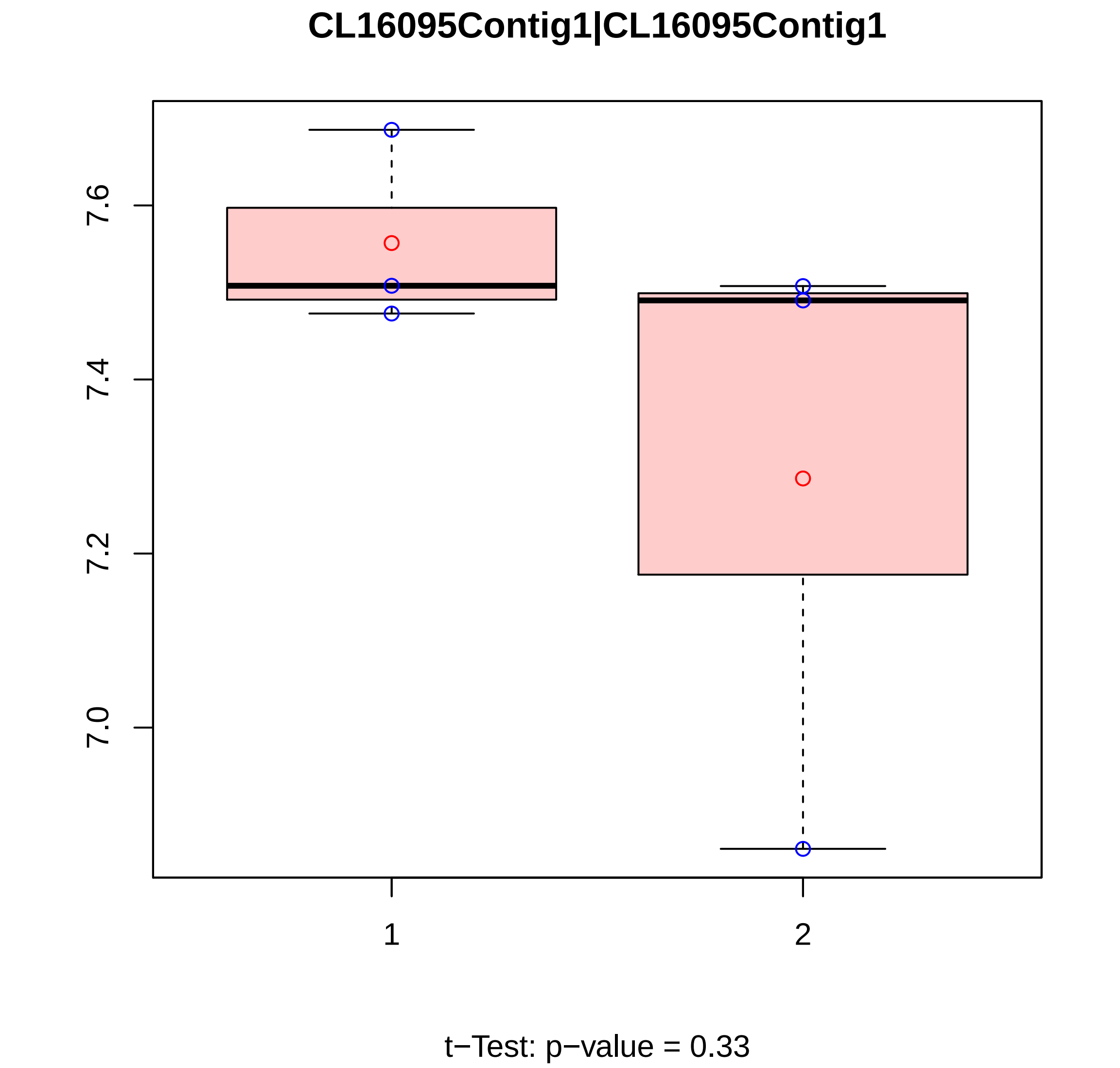
CL16095Contig1|CL16095Contig1
7.0
7.2
7.4
7.6
1
2
t−Test: p−value = 0.33

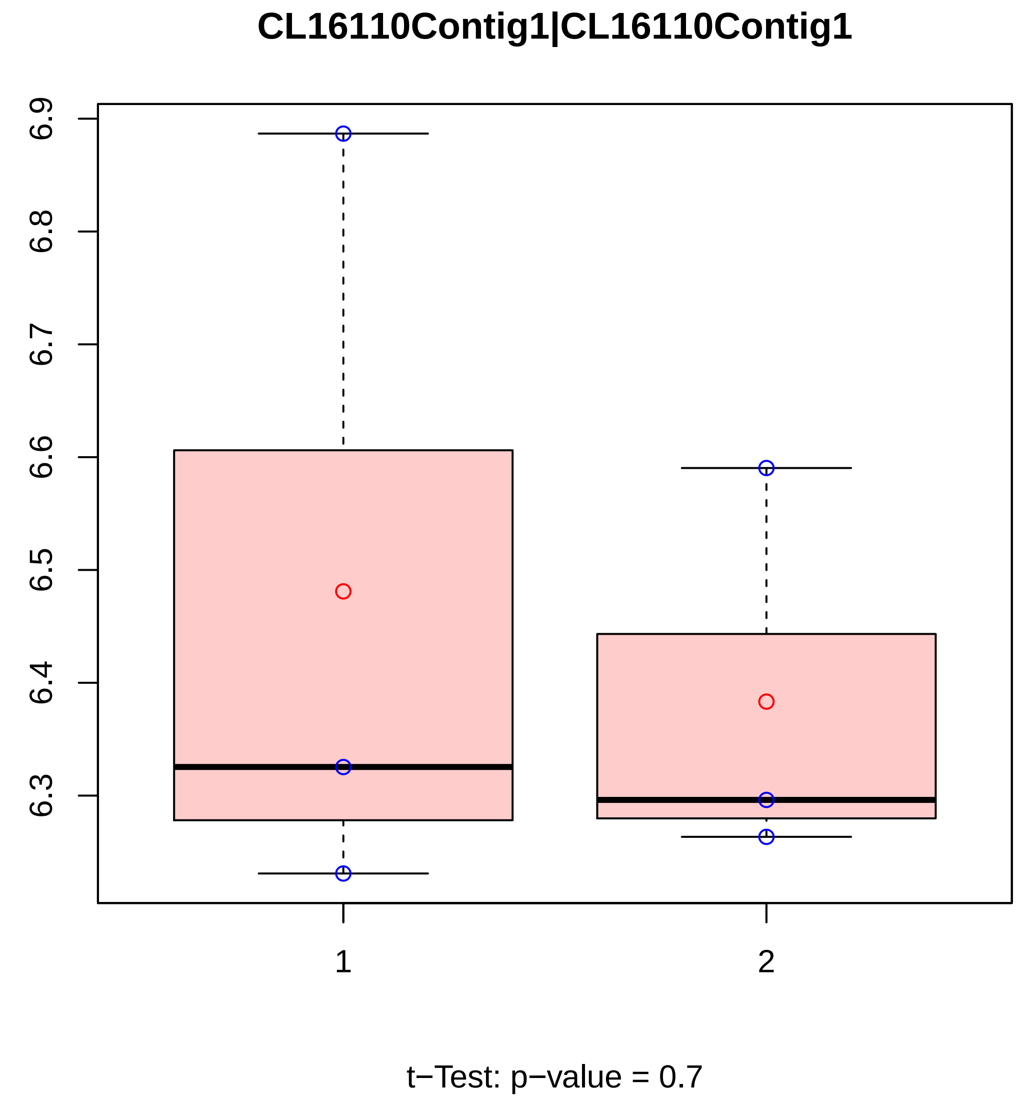
CL16110Contig1|CL16110Contig1
6.3
6.4
6.5
6.6
6.7
6.8
6.9
1
2
t−Test: p−value = 0.7

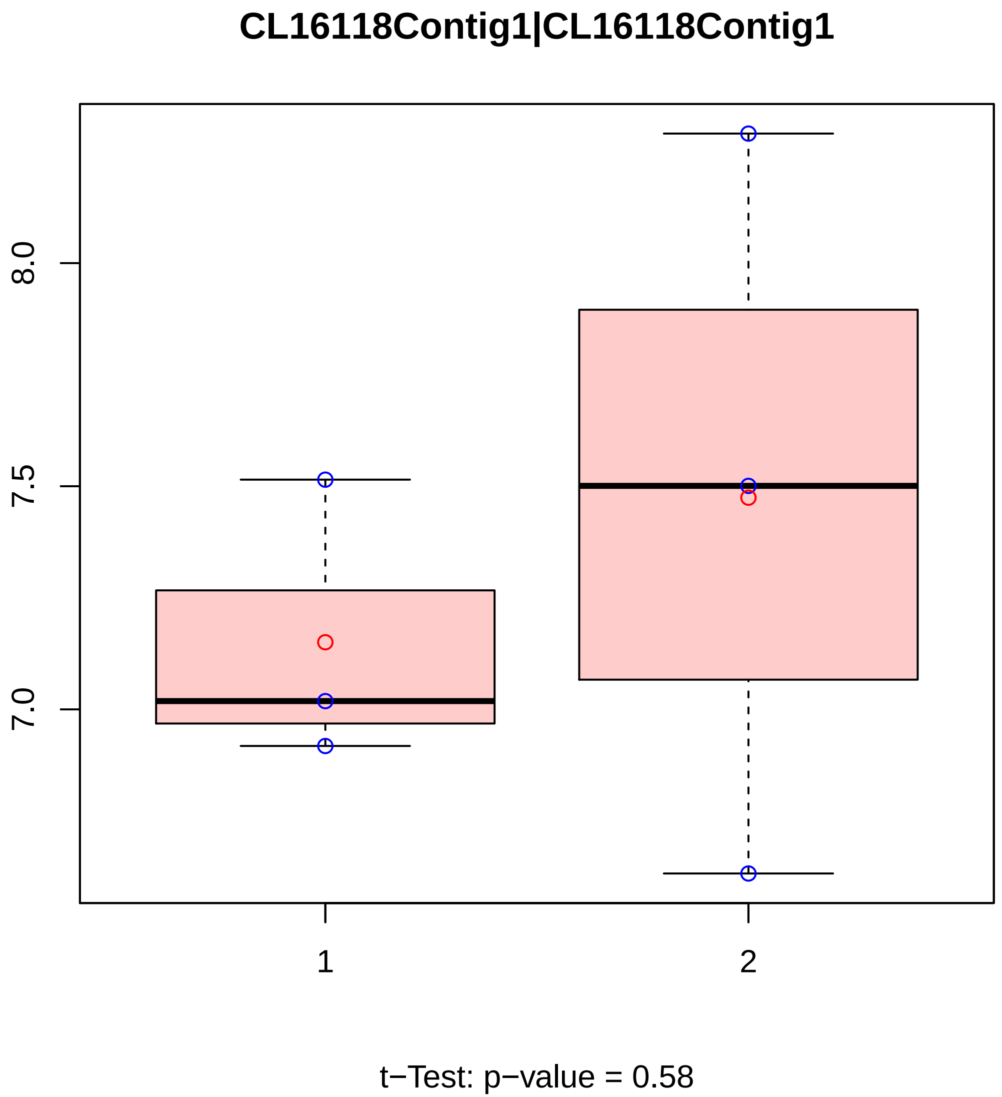
CL16118Contig1|CL16118Contig1
8.0
7.5
7.0
1
2
t−Test: p−value = 0.58

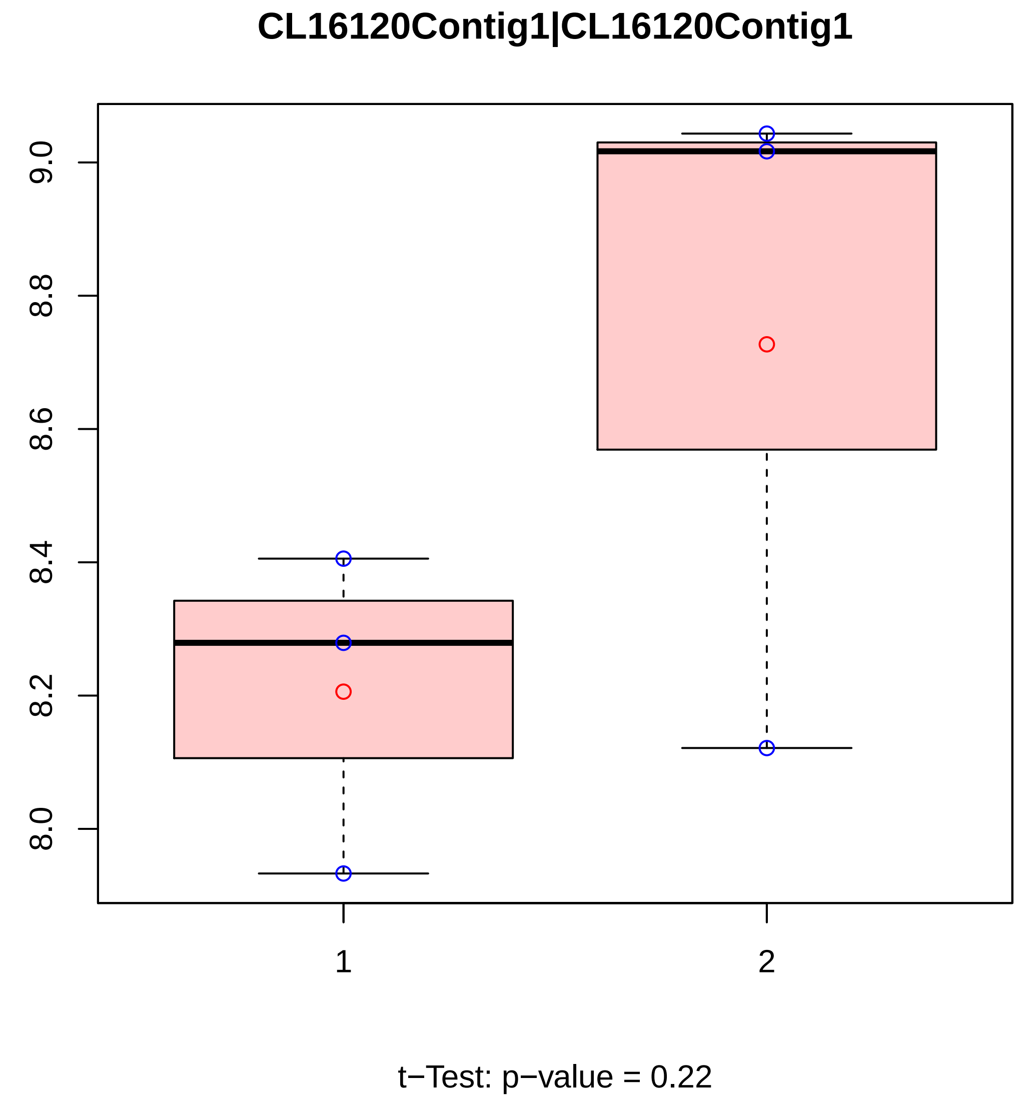
CL16120Contig1|CL16120Contig1
9.0
8.8
8.6
8.4
8.2
8.0
1
2
t−Test: p−value = 0.22

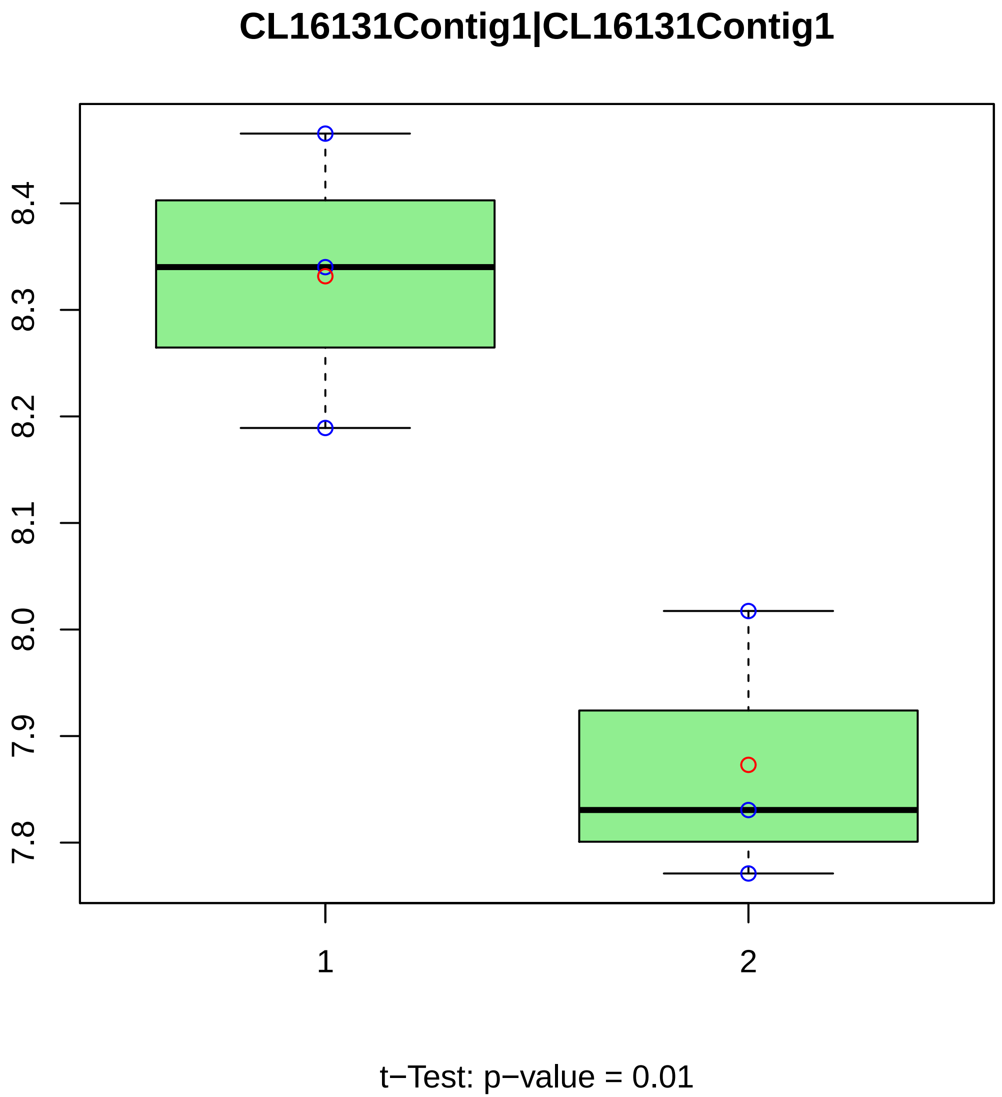
CL16131Contig1|CL16131Contig1
8.4
8.3
8.2
8.1
8.0
7.9
7.8
1
2
t−Test: p−value = 0.01

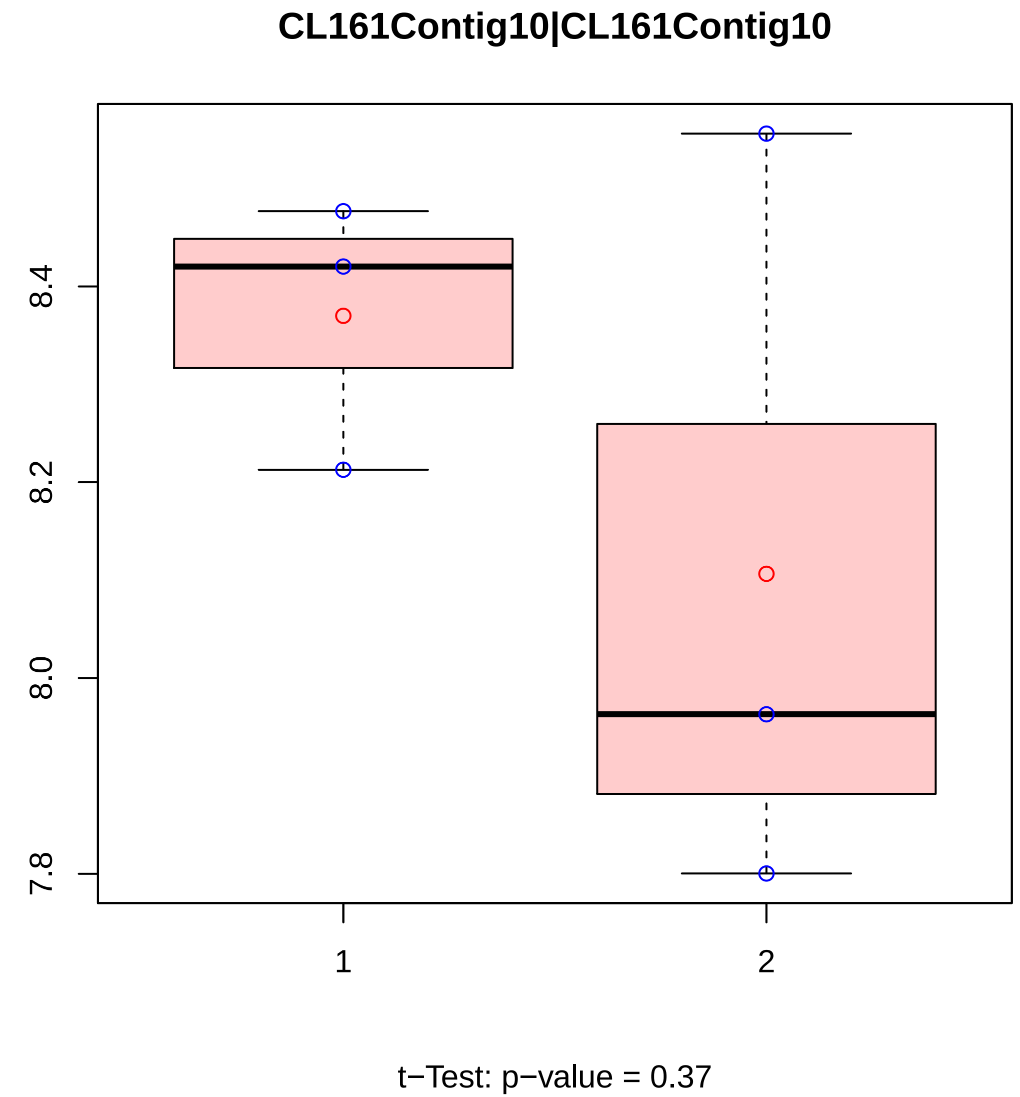
CL161Contig10|CL161Contig10
8.4
8.2
8.0
7.8
1
2
t−Test: p−value = 0.37

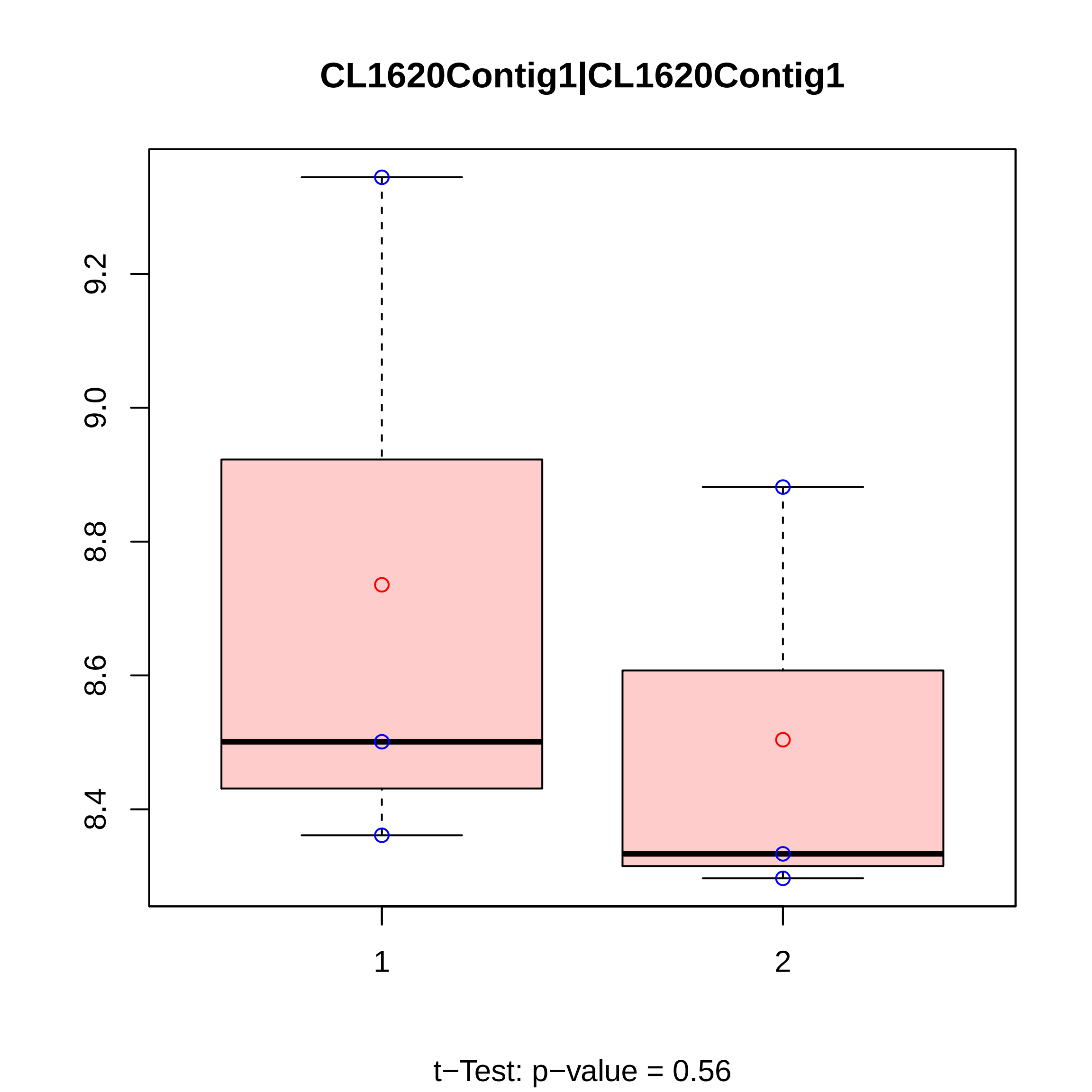
CL1620Contig1|CL1620Contig1
9.2
9.0
8.8
8.6
8.4
1
2
t−Test: p−value = 0.56

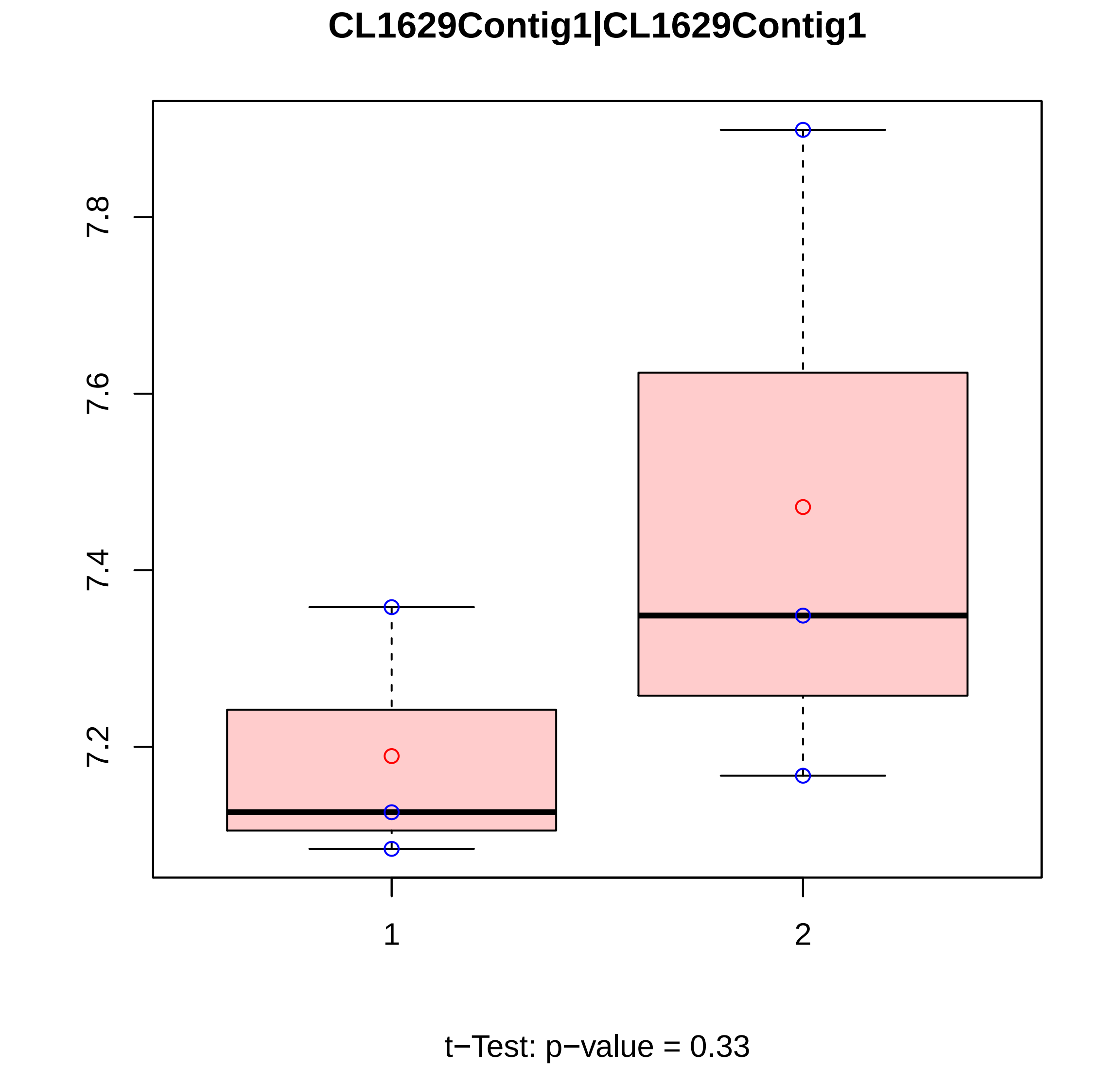
CL1629Contig1|CL1629Contig1
7.2
7.4
7.6
7.8
1
2
t−Test: p−value = 0.33

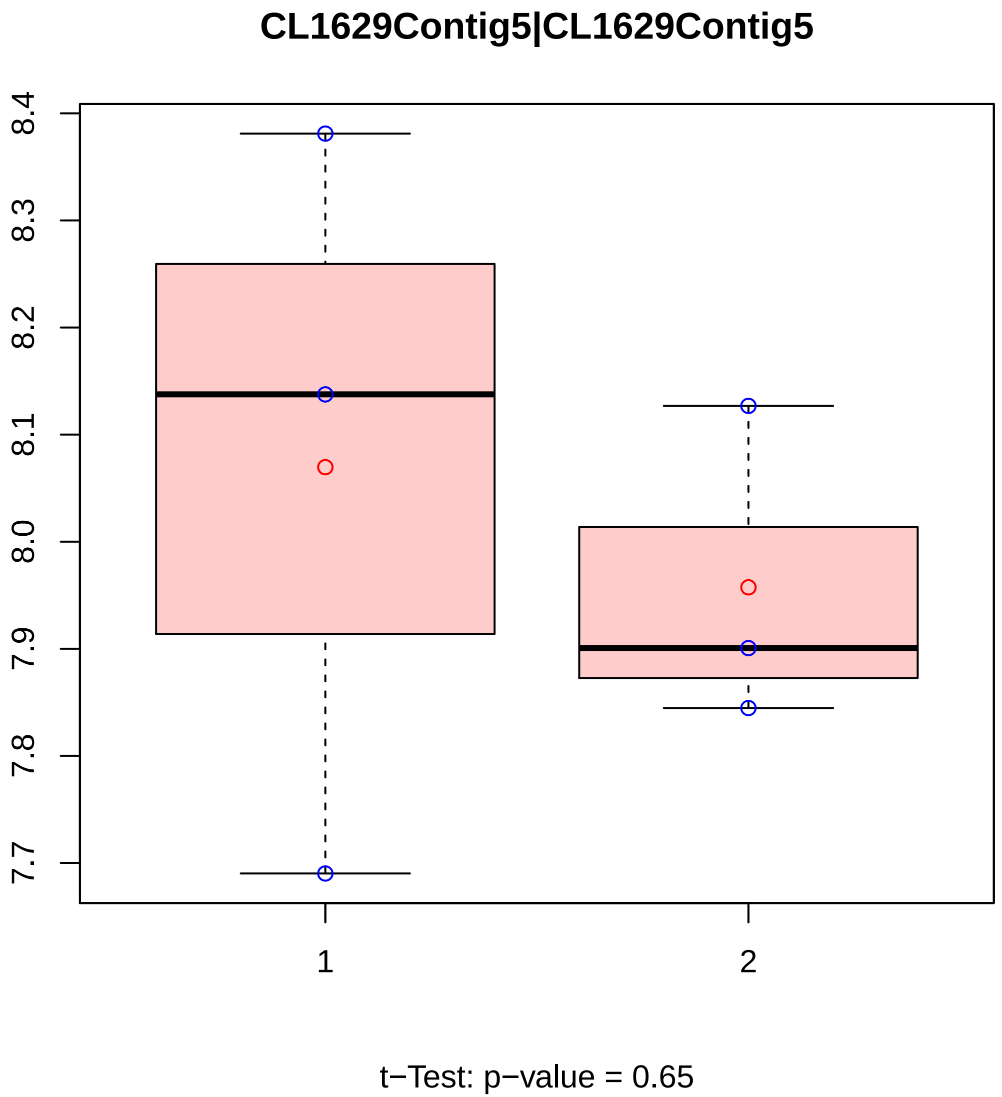
CL1629Contig5|CL1629Contig5
8.4
8.3
8.2
8.1
8.0
7.9
7.8
7.7
1
2
t−Test: p−value = 0.65

# CL1629Contig7|CL1629Contig7

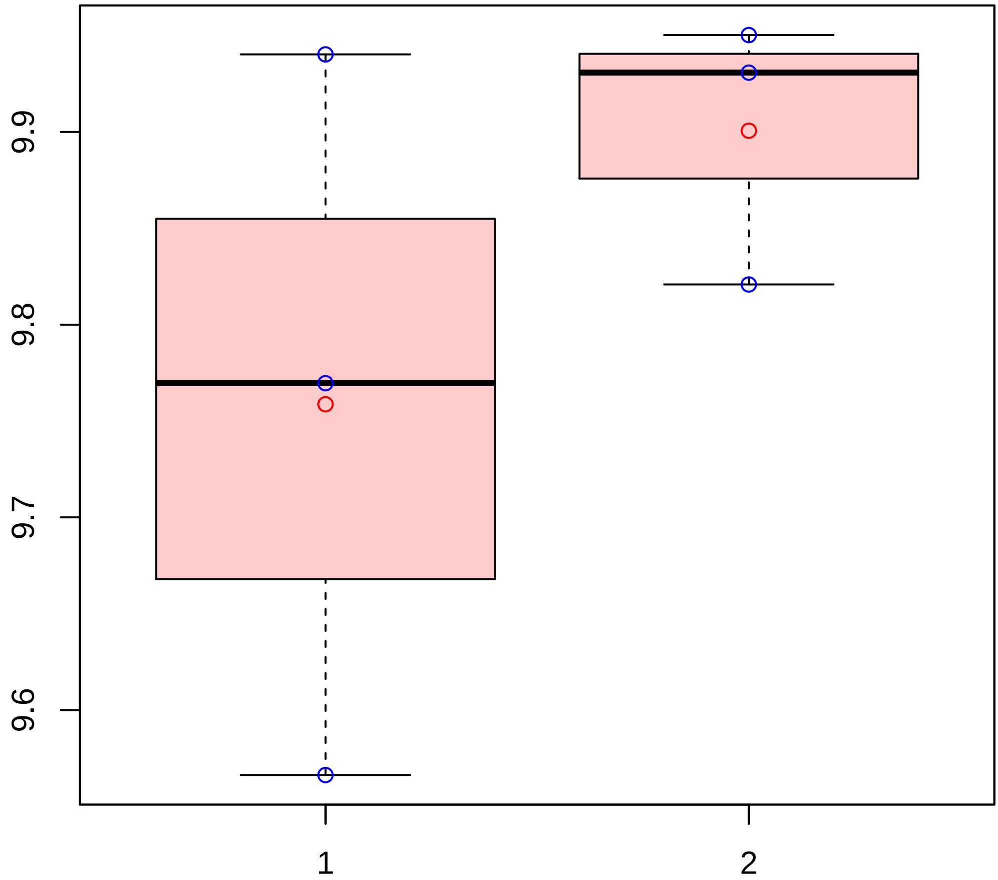


t−Test: p−value = 0.32

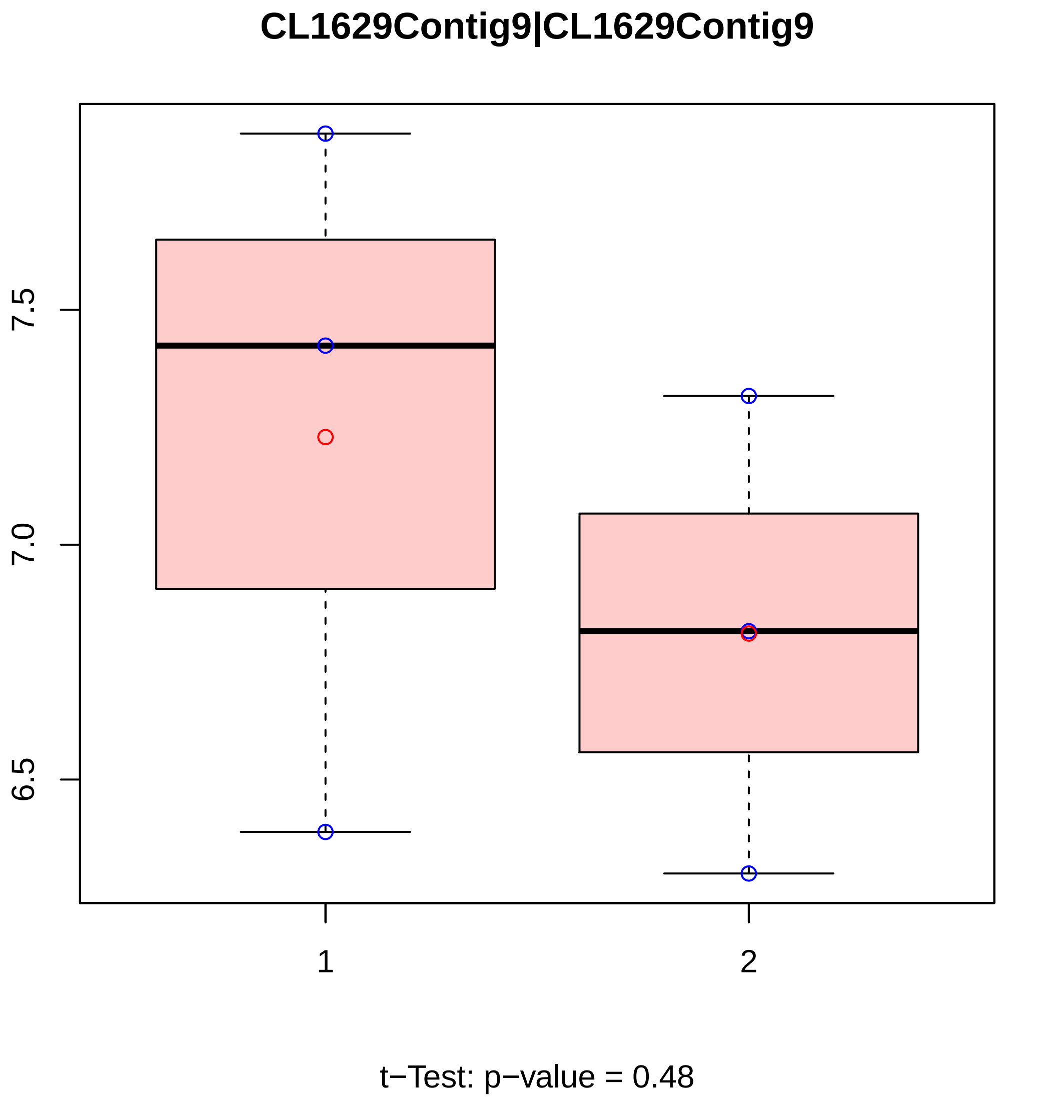

CL1629Contig9|CL1629Contig9
7.5
7.0
6.5
1
2
t−Test: p−value = 0.48

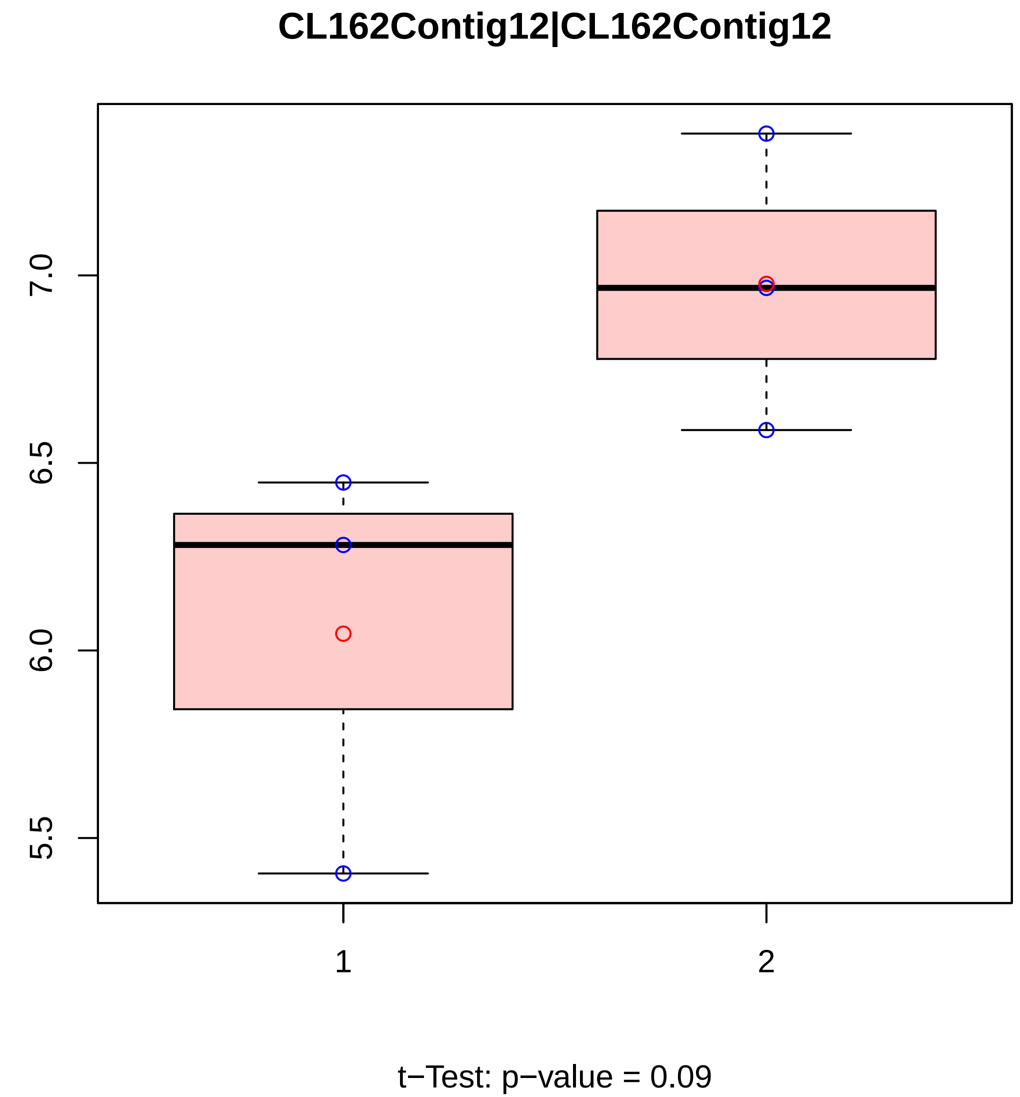
CL162Contig12|CL162Contig12
7.0
6.5
6.0
5.5
1
2
t−Test: p−value = 0.09

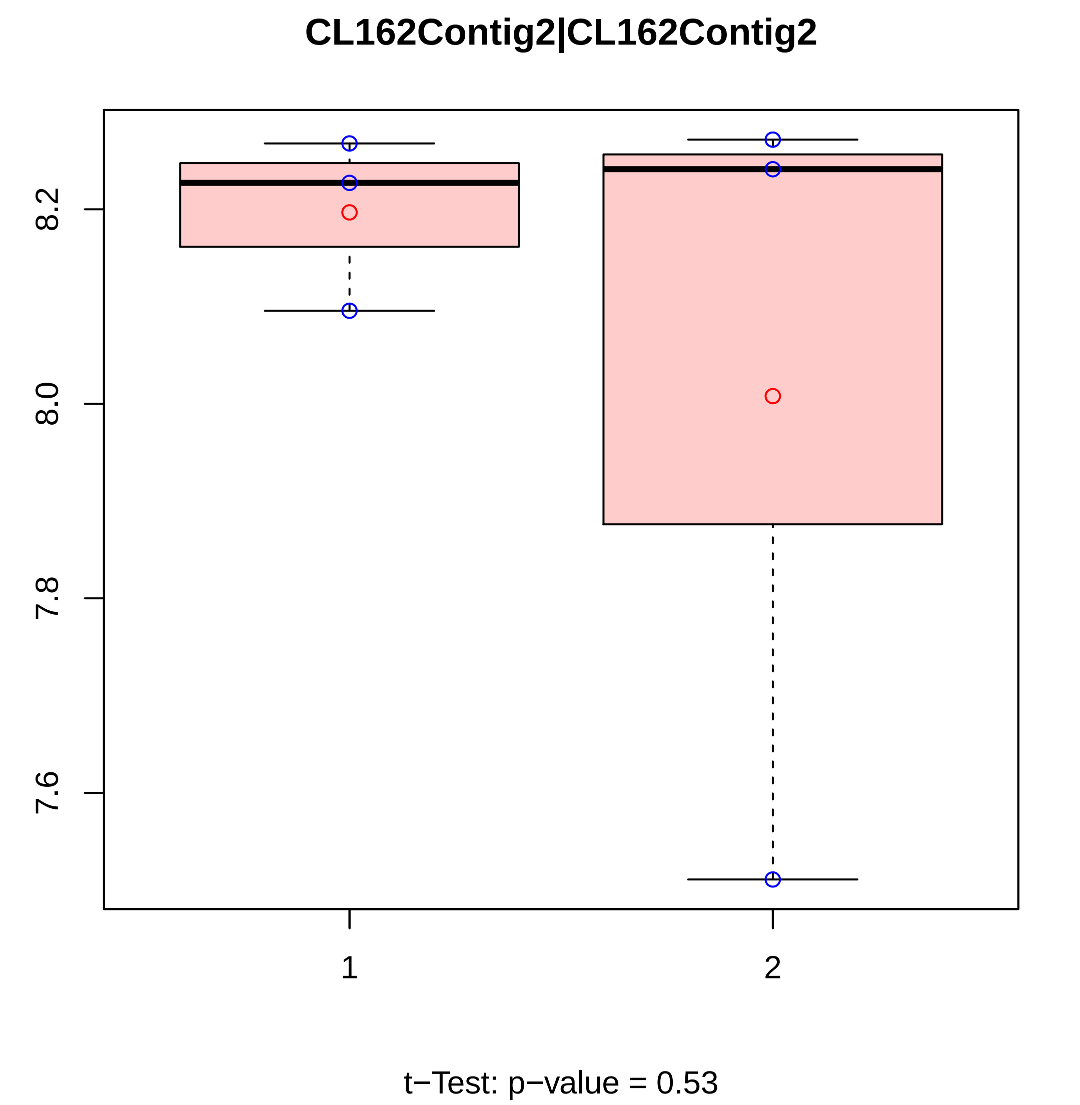
CL162Contig2|CL162Contig2
8.2
8.0
7.8
7.6
1
2
t−Test: p−value = 0.53

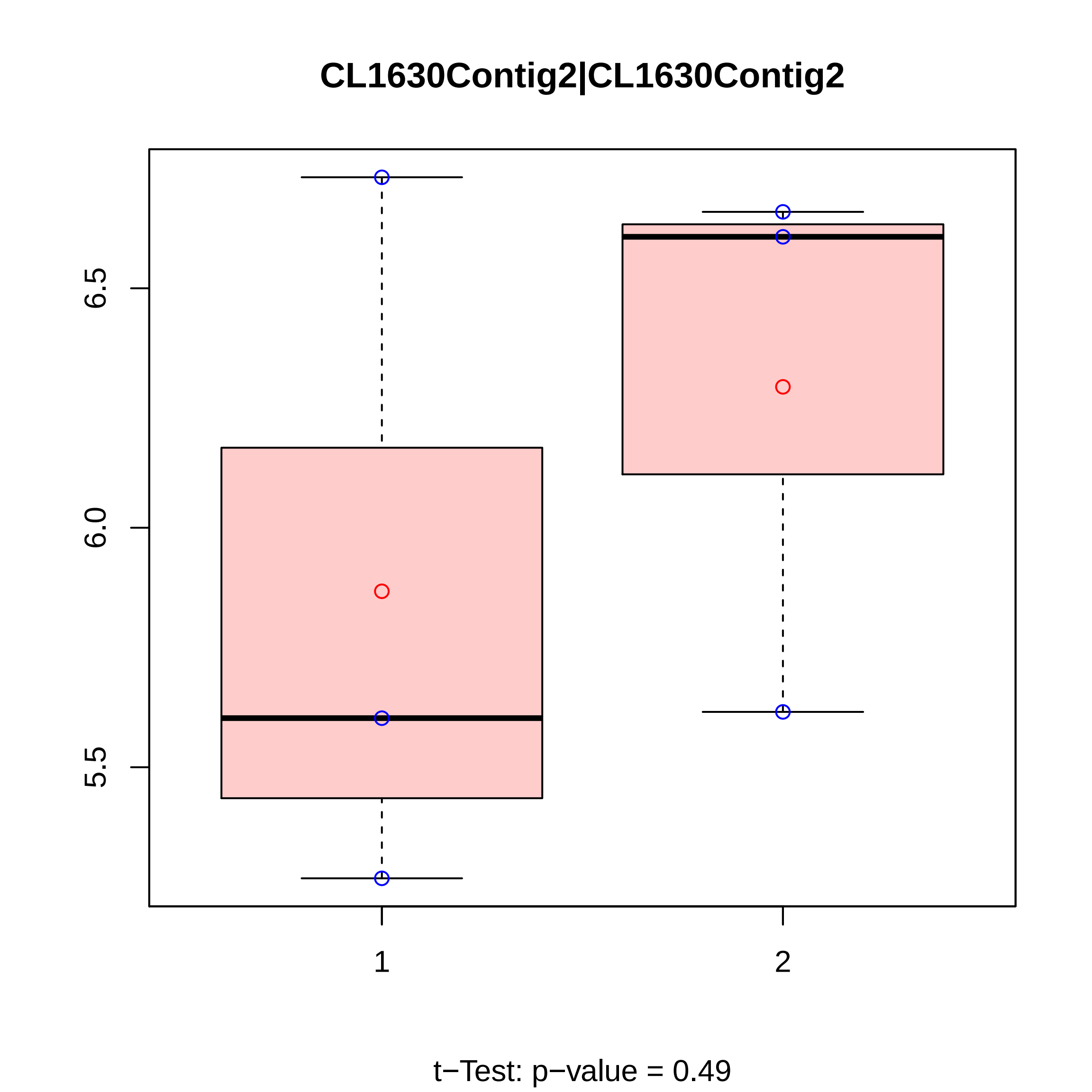
CL1630Contig2|CL1630Contig2
6.5
6.0
5.5
1
2
t−Test: p−value = 0.49

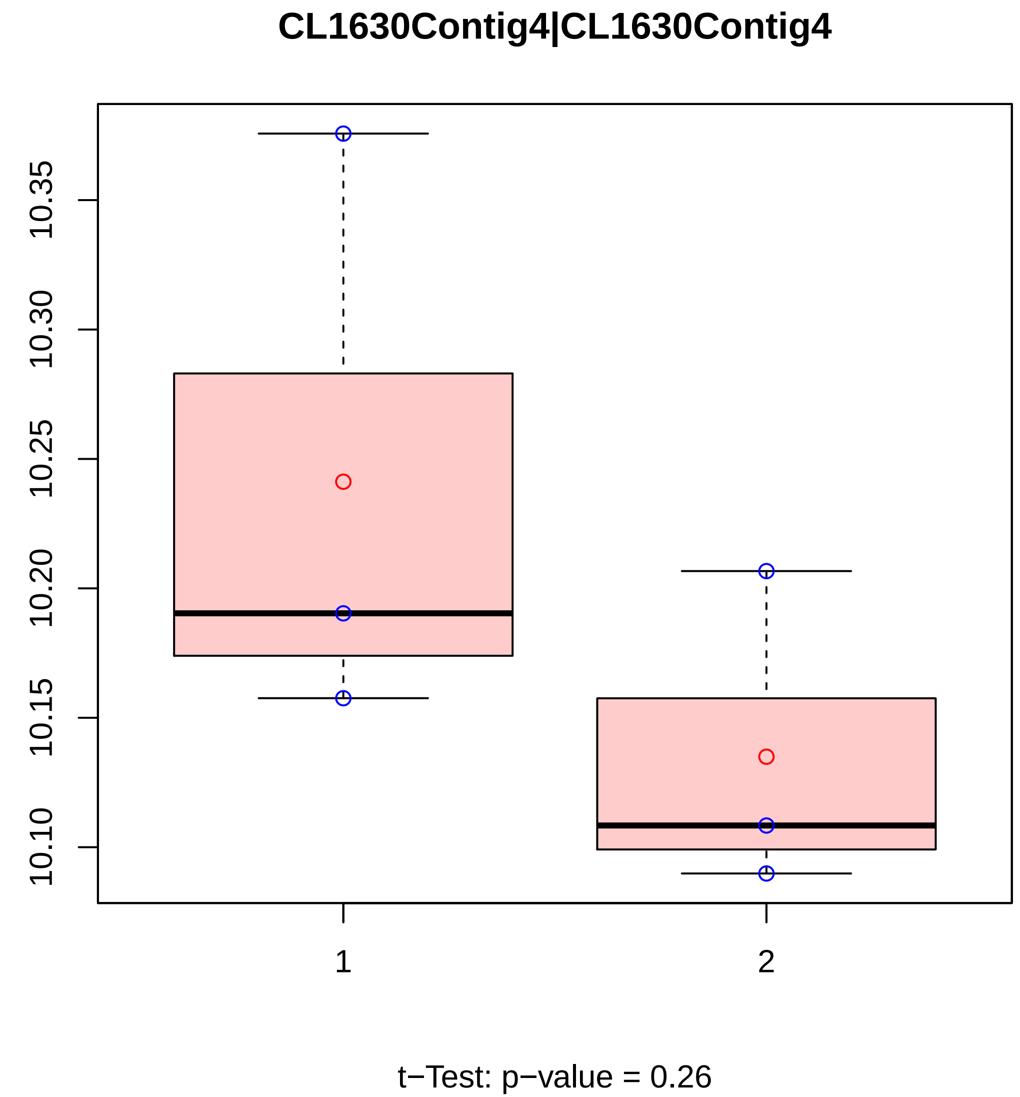
CL1630Contig4|CL1630Contig4
10.10
10.15
10.20
10.25
10.30
10.35
1
2
t−Test: p−value = 0.26

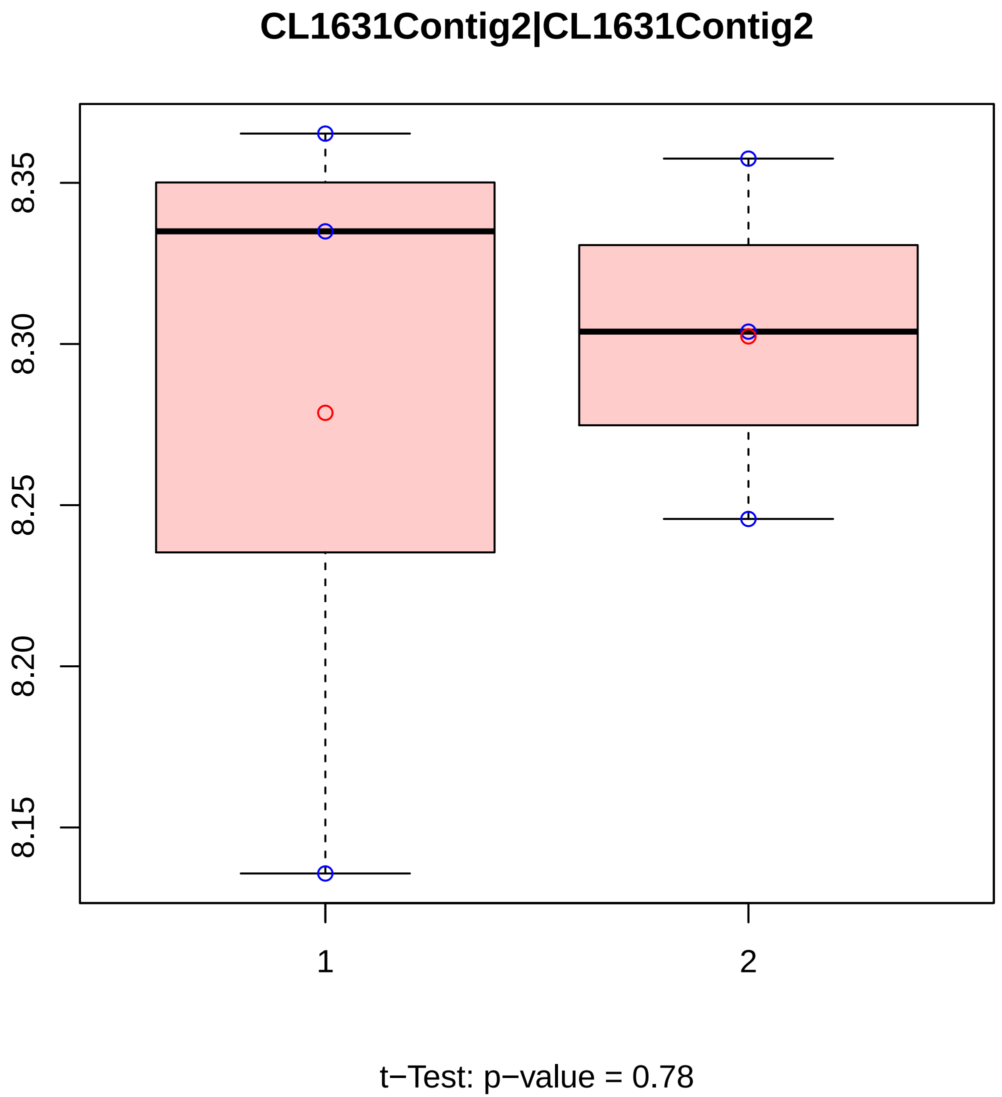
CL1631Contig2|CL1631Contig2
8.35
8.30
8.25
8.20
8.15
1
2
t−Test: p−value = 0.78

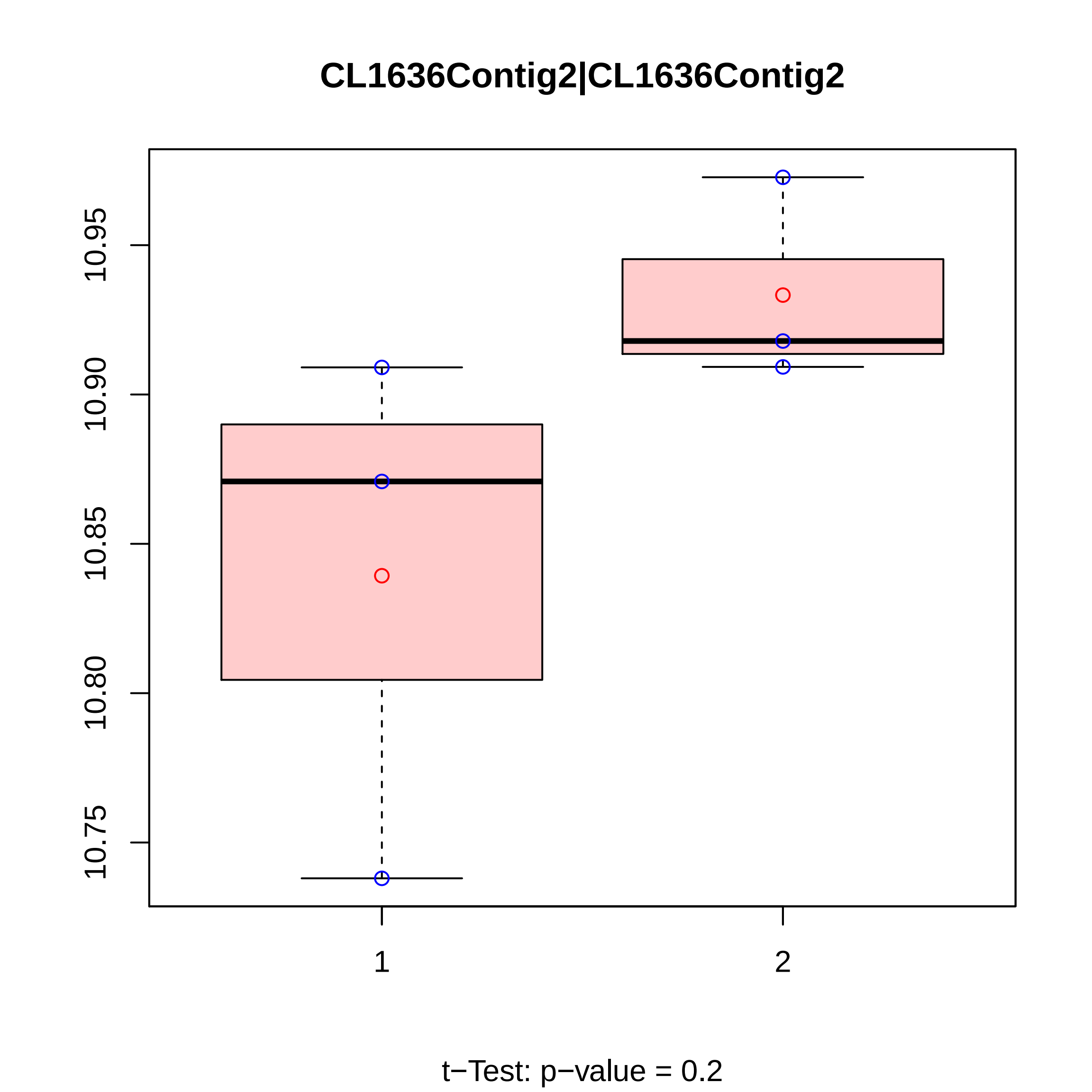
CL1636Contig2|CL1636Contig2
10.75
10.80
10.85
10.90
10.95
1
2
t−Test: p−value = 0.2

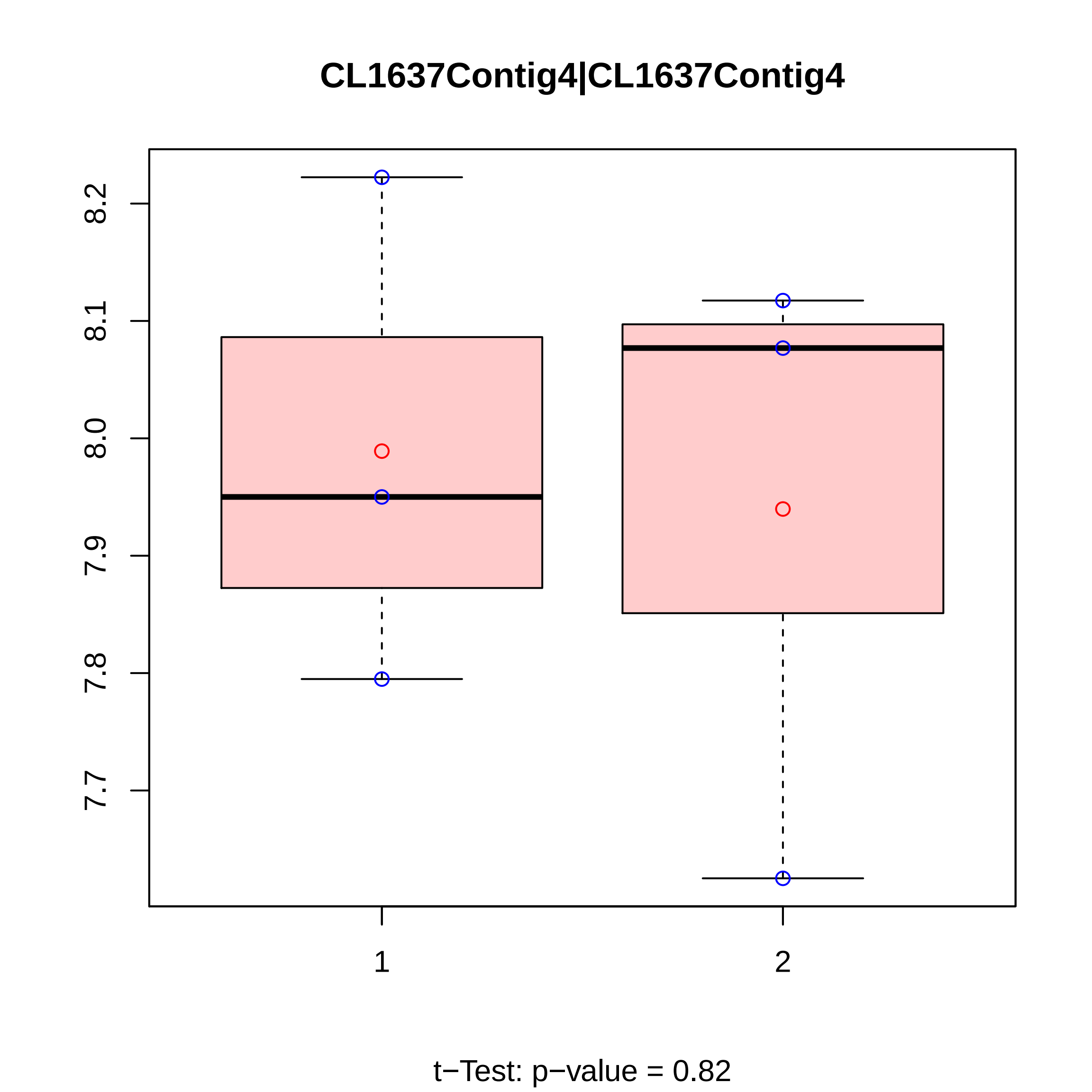

CL1637Contig4|CL1637Contig4
8.2
8.1
8.0
7.9
7.8
7.7
1
2
t−Test: p−value = 0.82

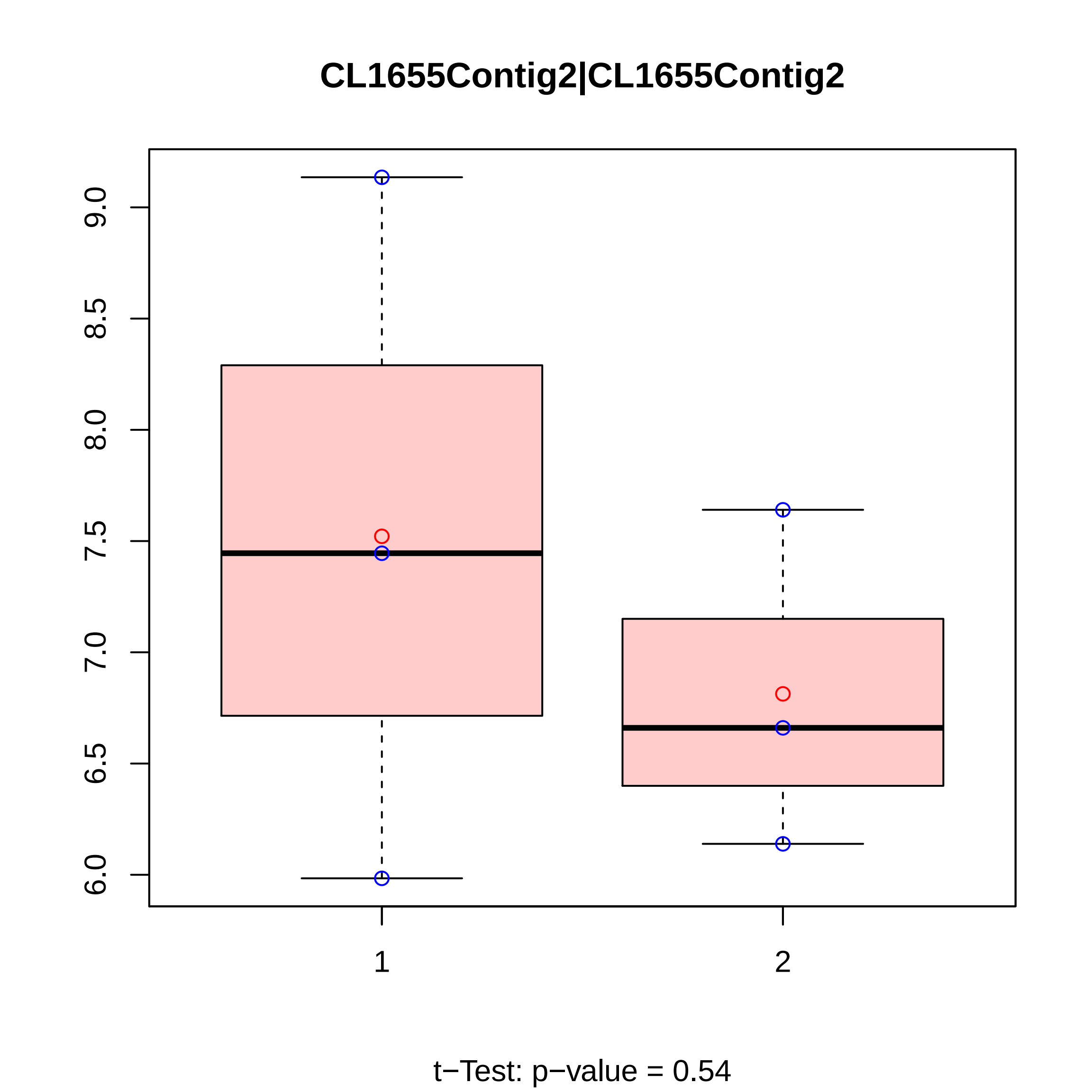

CL1655Contig2|CL1655Contig2
9.0
8.5
8.0
7.5
7.0
6.5
6.0
1
2
t−Test: p−value = 0.54

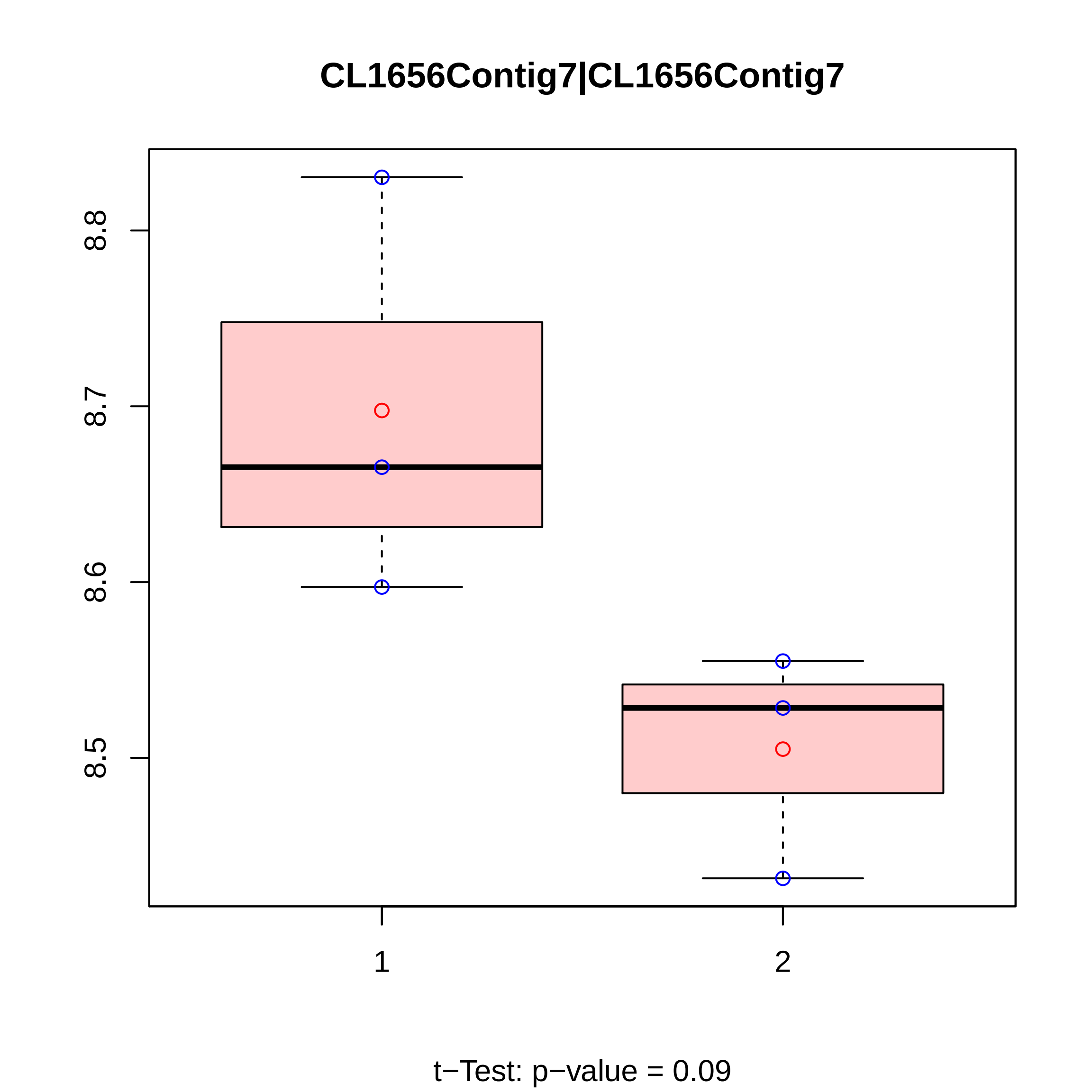
CL1656Contig7|CL1656Contig7
8.8
8.7
8.6
8.5
1
2
t−Test: p−value = 0.09

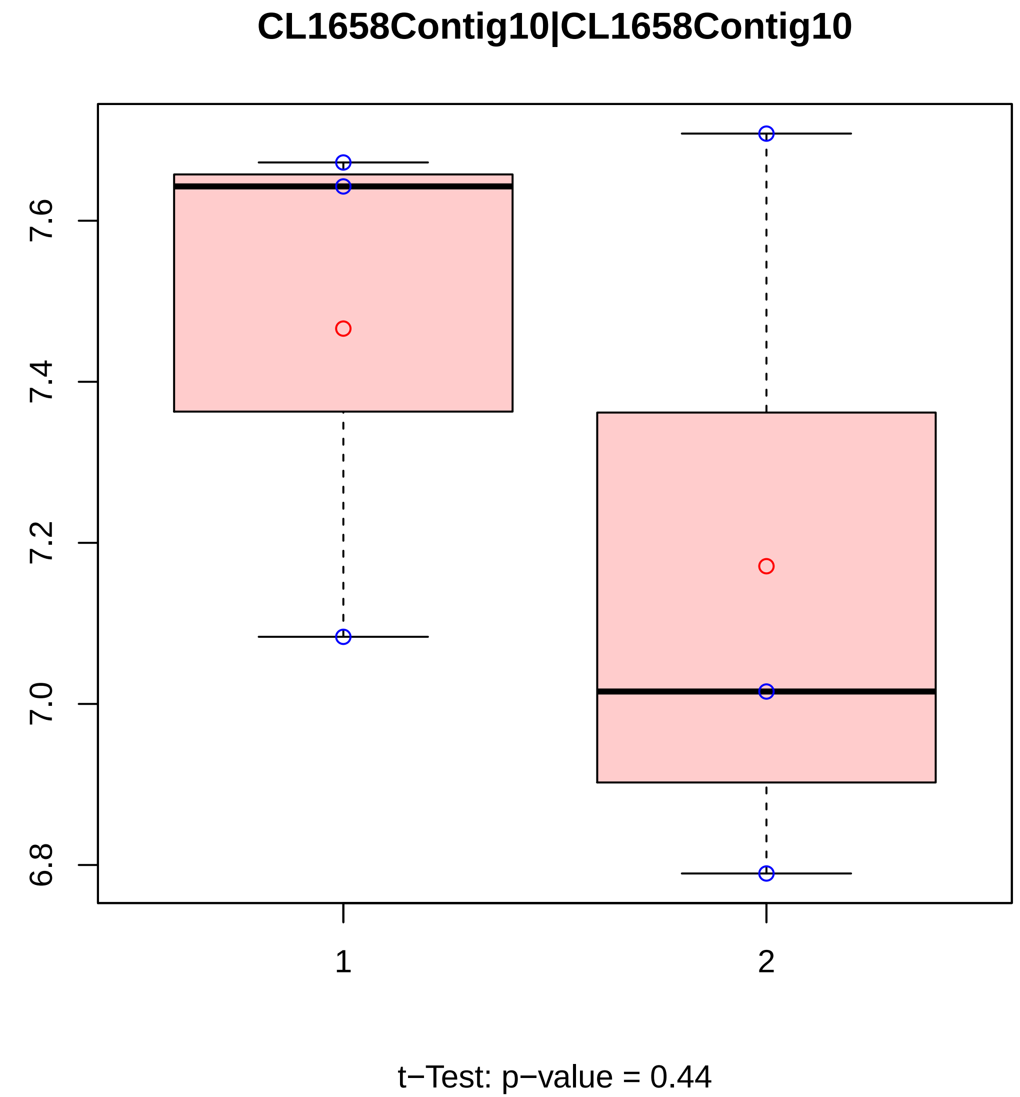

CL1658Contig10|CL1658Contig10
7.6
7.4
7.2
7.0
6.8
1
2
t−Test: p−value = 0.44

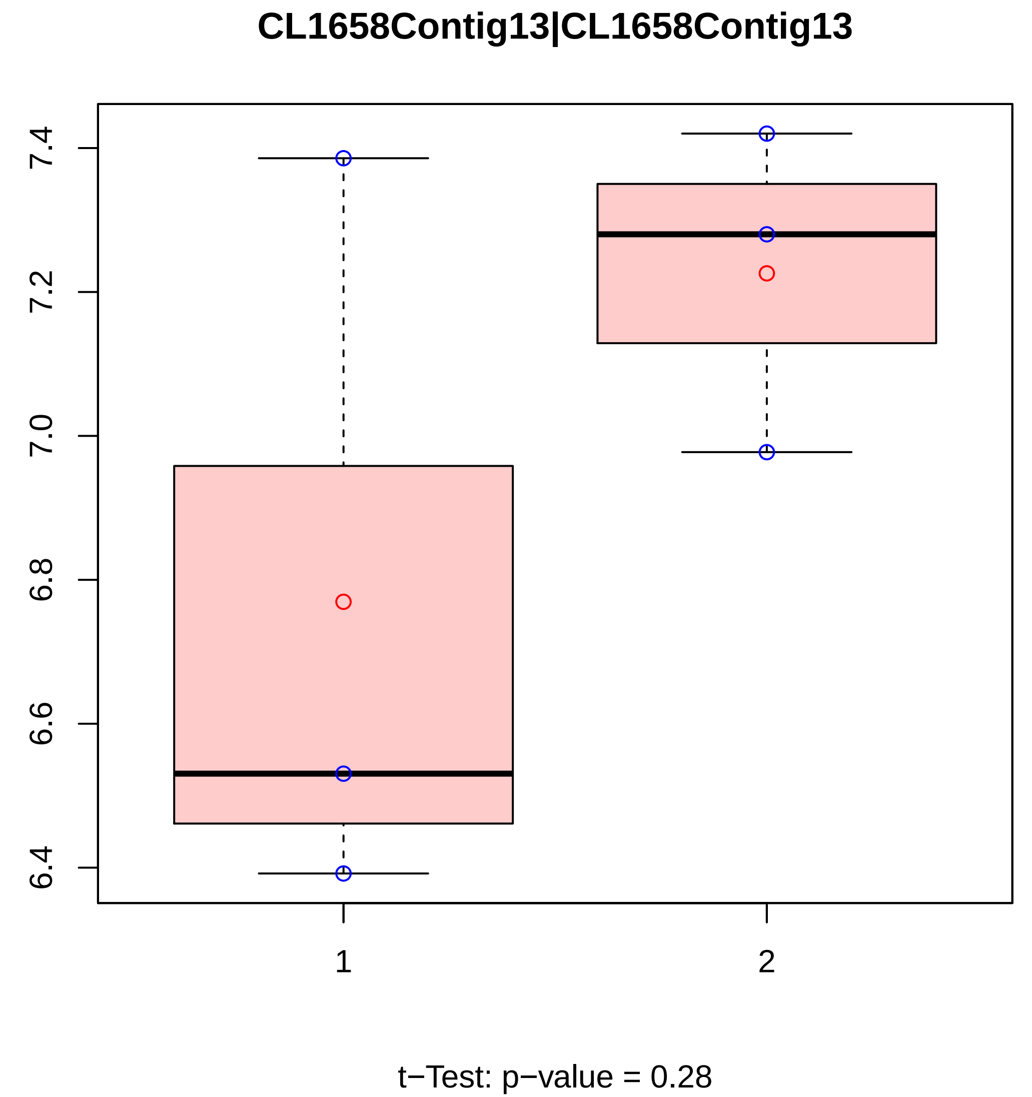
CL1658Contig13|CL1658Contig13
7.4
7.2
7.0
6.8
6.6
6.4
1
2
t−Test: p−value = 0.28

## CL1658Contig8|CL1658Contig8

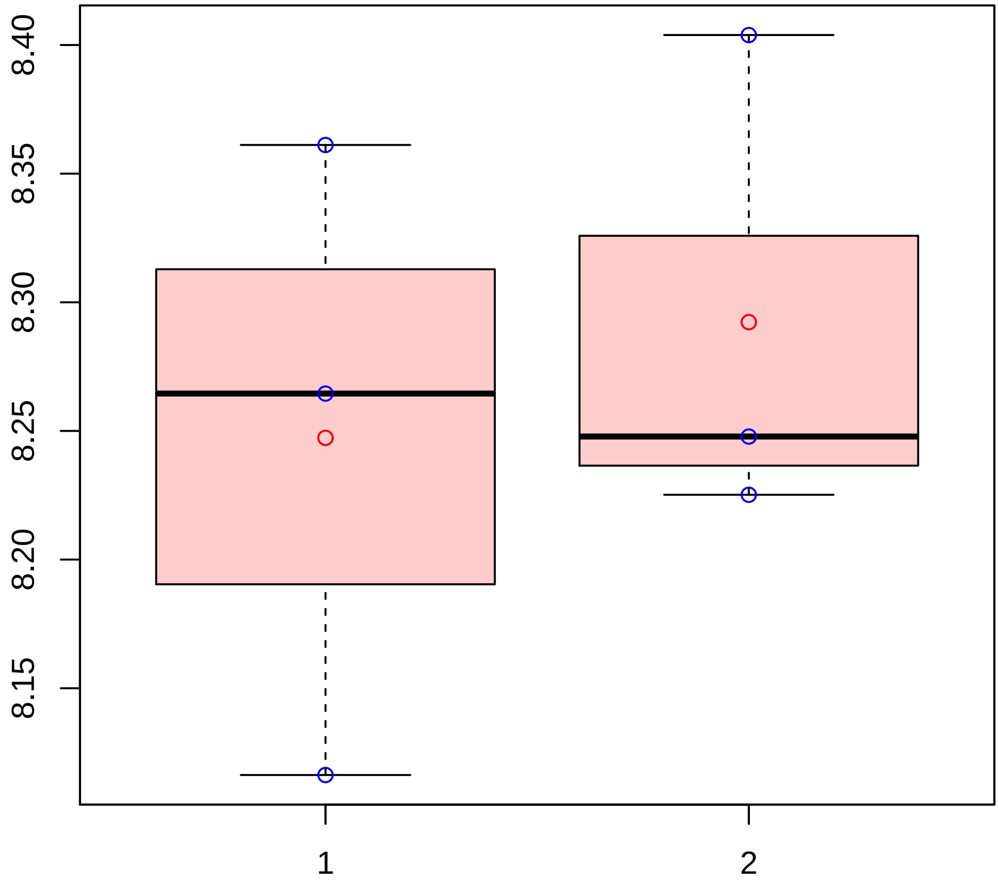


t−Test: p−value = 0.65

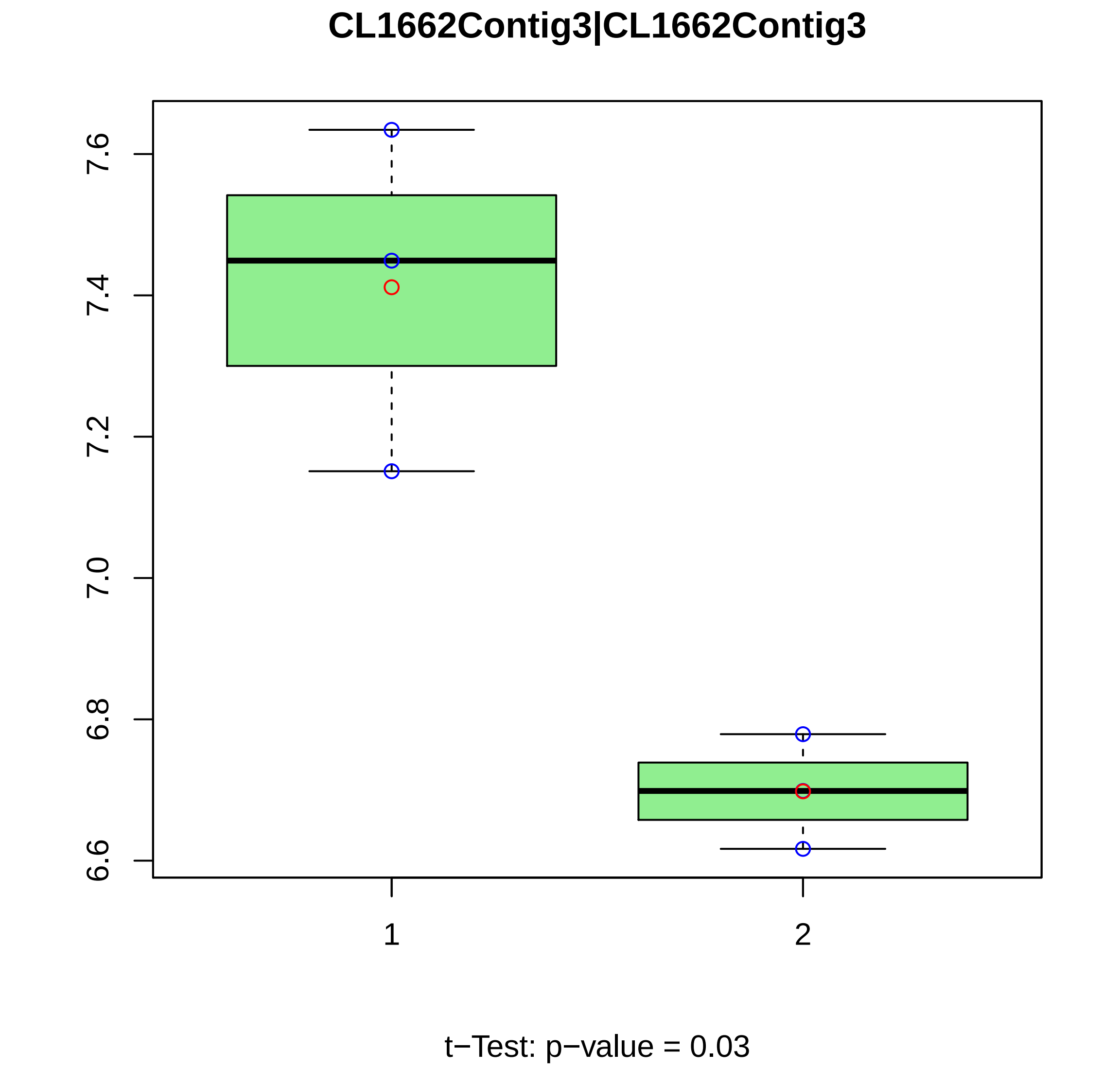
CL1662Contig3|CL1662Contig3
7.6
7.4
7.2
7.0
6.8
6.6
1
2
t−Test: p−value = 0.03

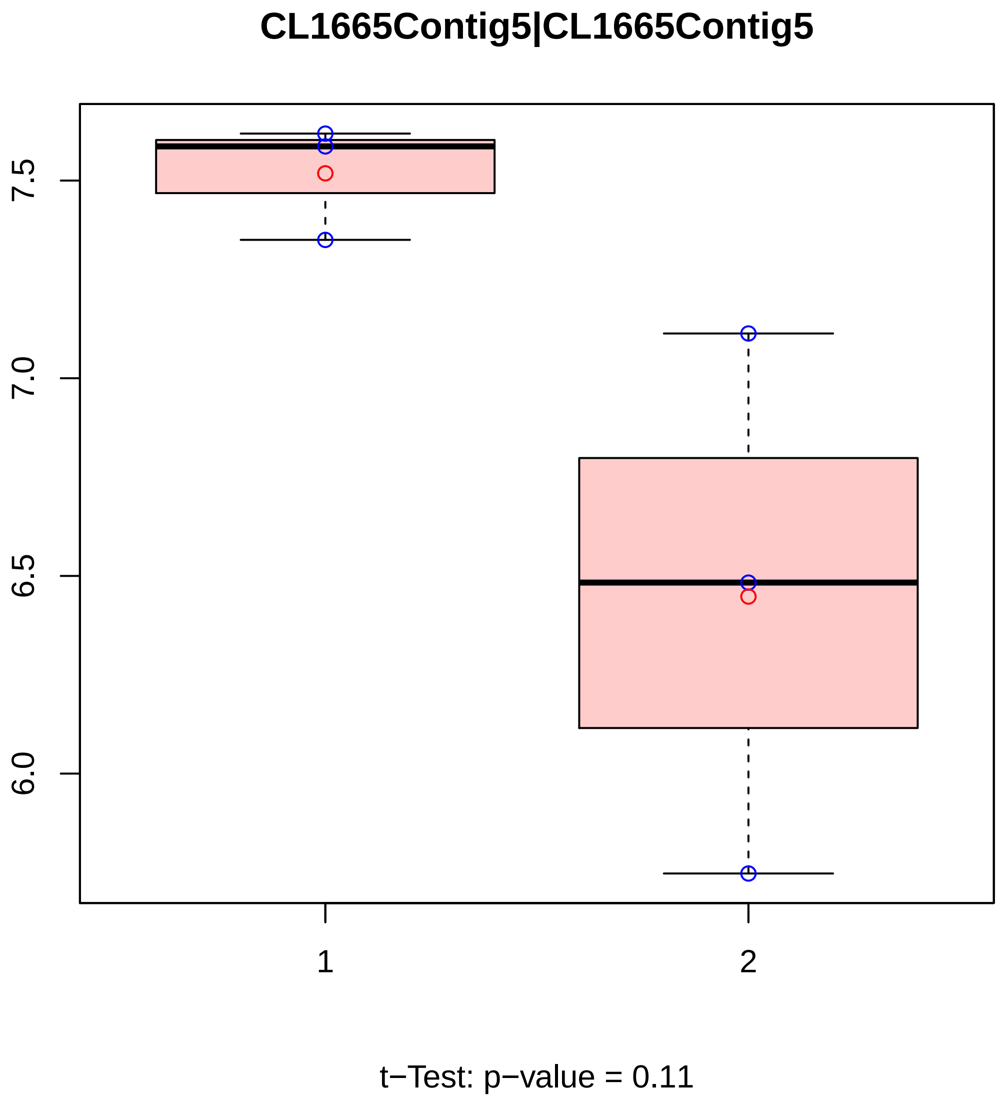
CL1665Contig5|CL1665Contig5
7.5
7.0
6.5
6.0
1
2
t−Test: p−value = 0.11

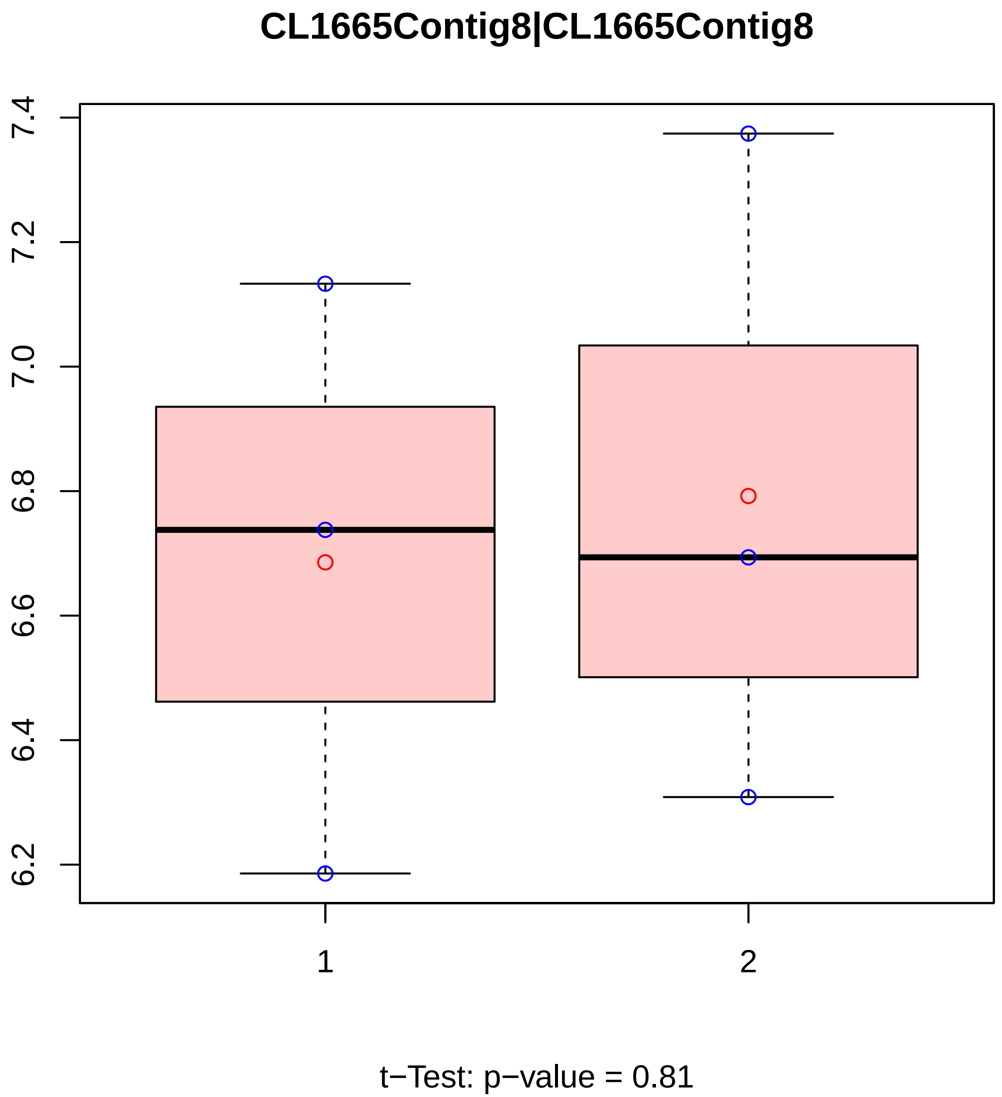

CL1665Contig8|CL1665Contig8
7.4
7.2
7.0
6.8
6.6
6.4
6.2
1
2
t−Test: p−value = 0.81

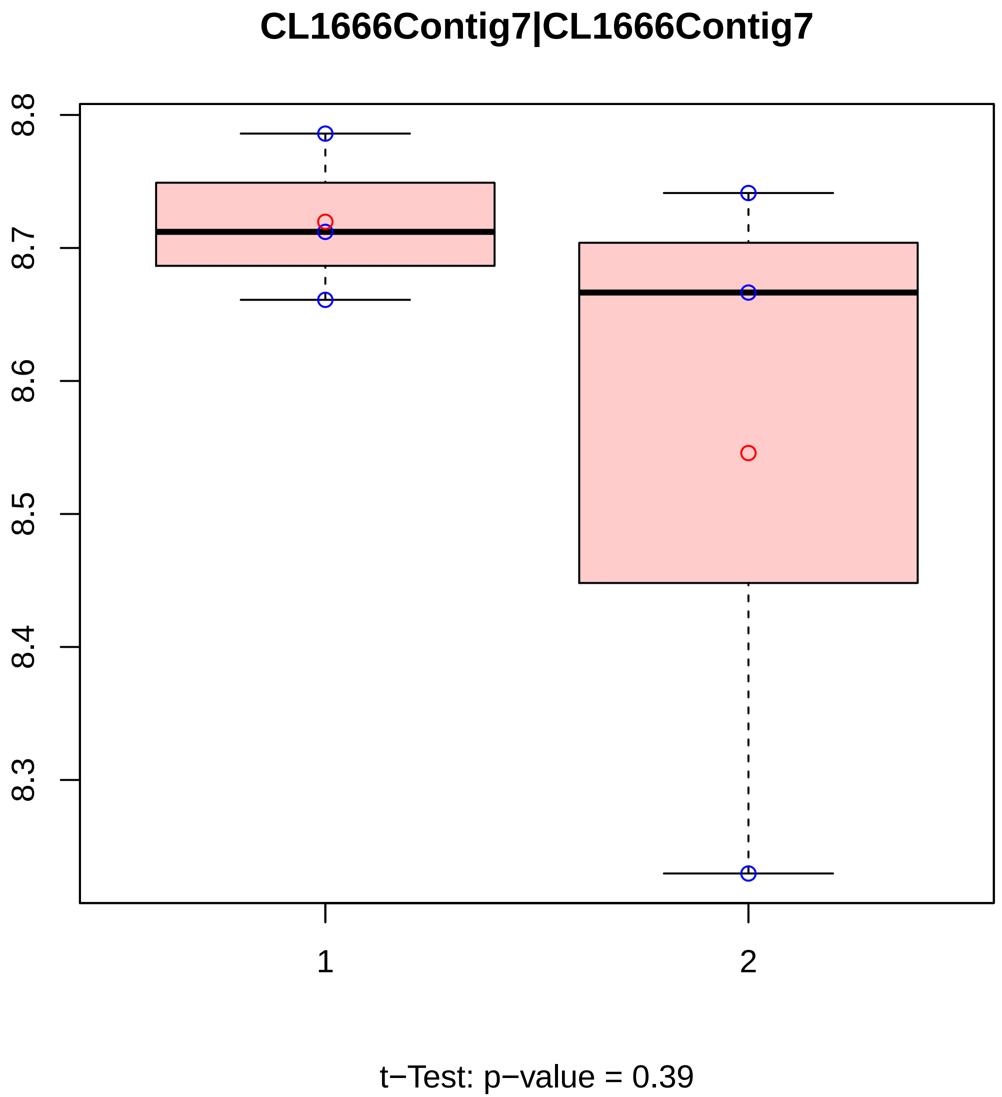
CL1666Contig7|CL1666Contig7
8.8
8.7
8.6
8.5
8.4
8.3
1
2
t−Test: p−value = 0.39

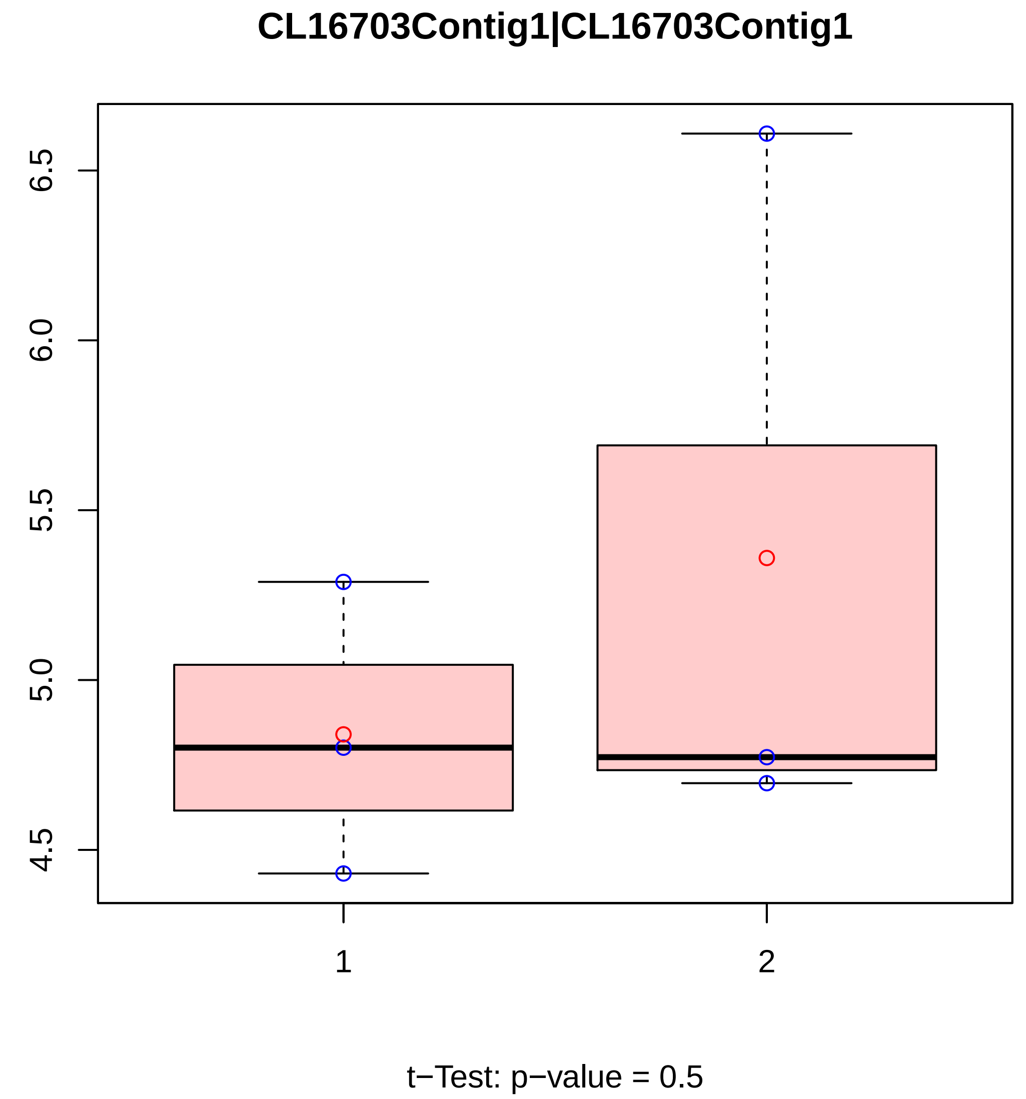
CL16703Contig1|CL16703Contig1
6.5
6.0
5.5
5.0
4.5
1
2
t−Test: p−value = 0.5

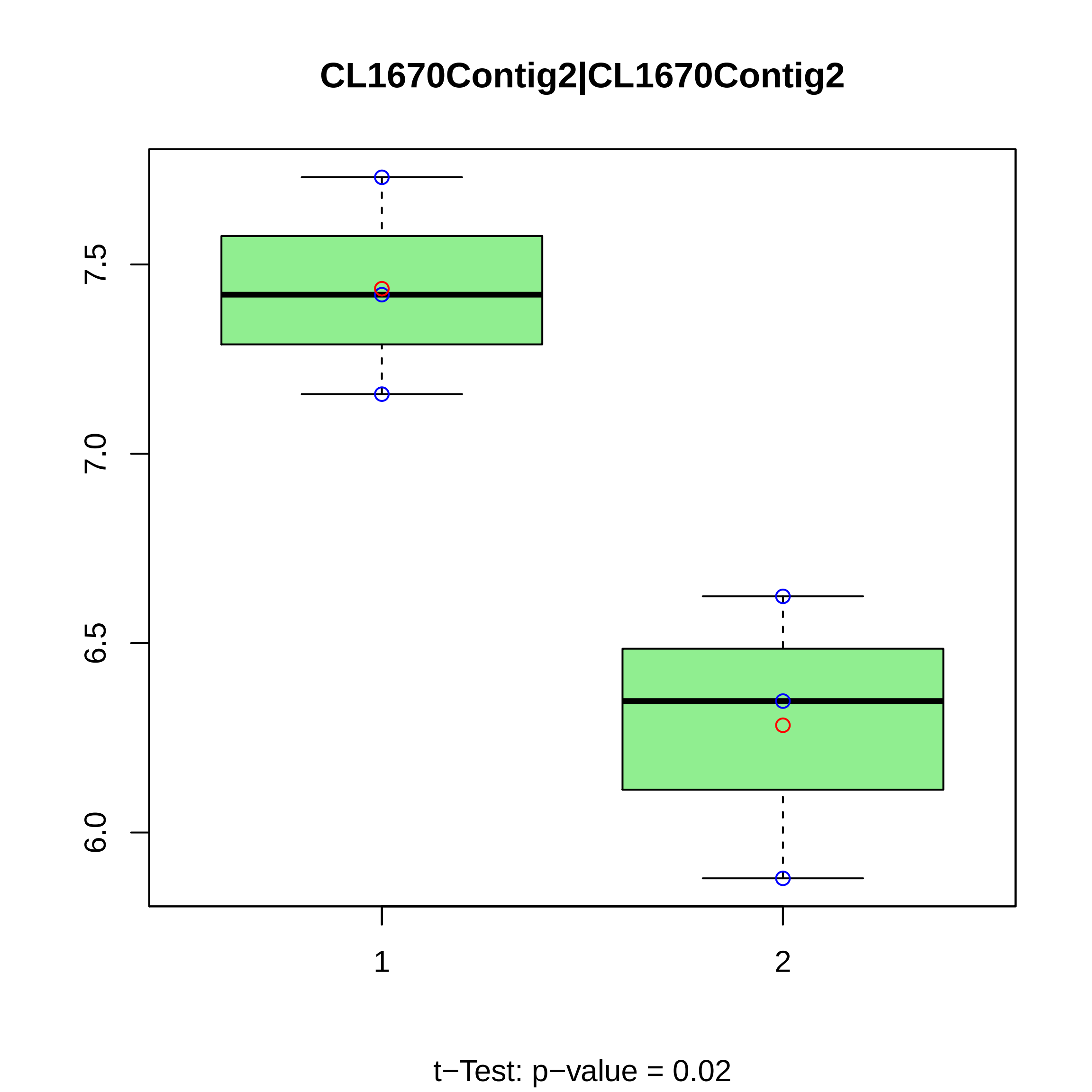

CL1670Contig2|CL1670Contig2
7.5
7.0
6.5
6.0
1
2
t−Test: p−value = 0.02

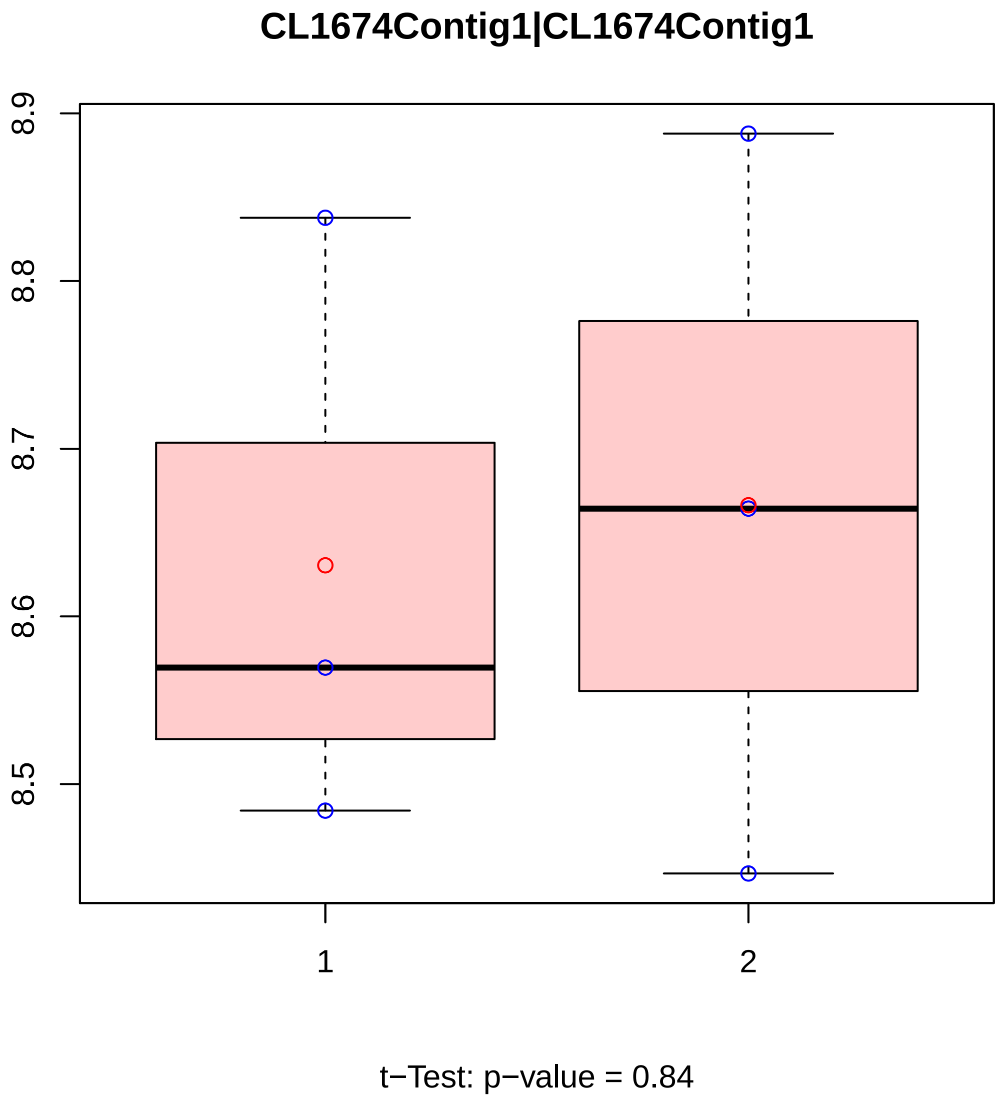

CL1674Contig1|CL1674Contig1
8.5
8.6
8.7
8.8
8.9
1
2
t−Test: p−value = 0.84

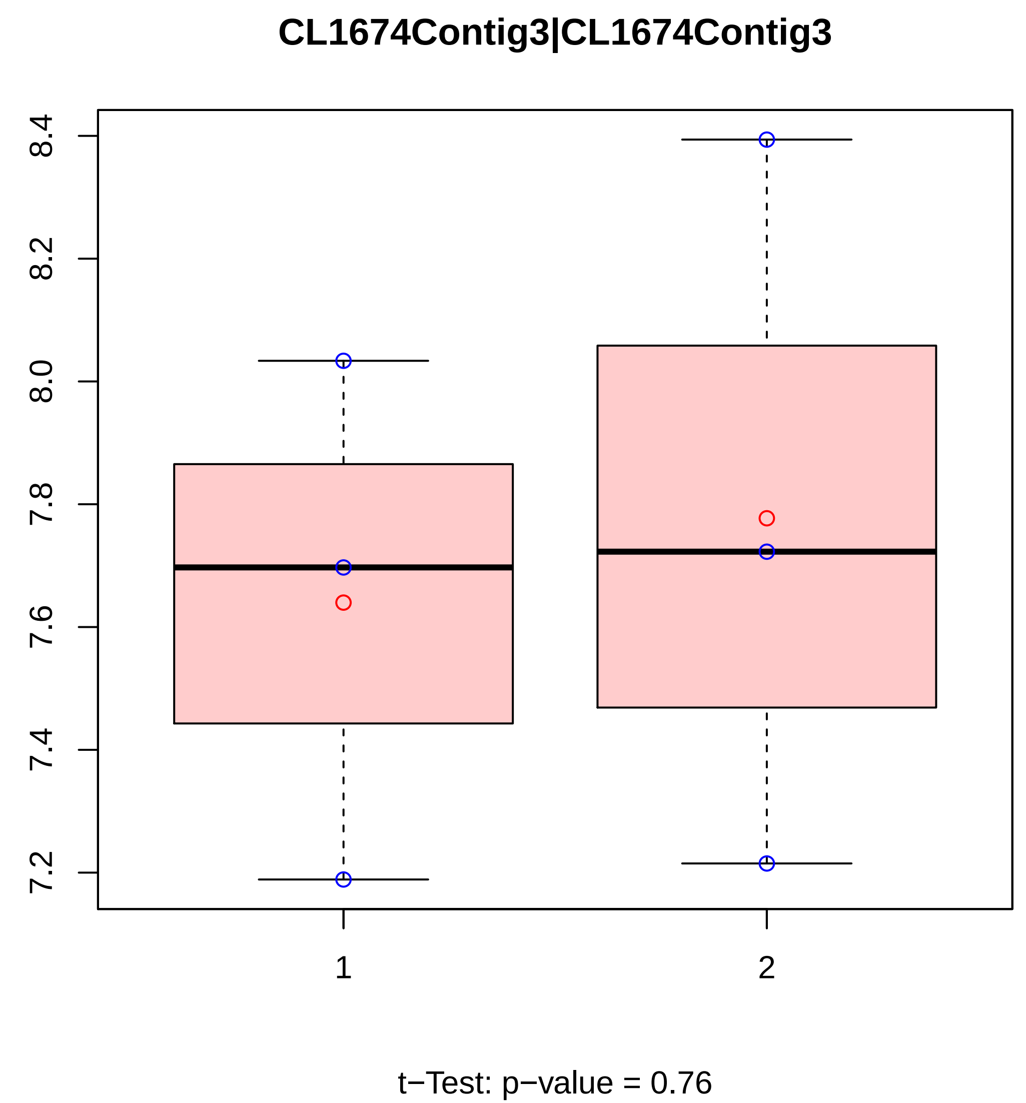
CL1674Contig3|CL1674Contig3
8.4
8.2
8.0
7.8
7.6
7.4
7.2
1
2
t−Test: p−value = 0.76

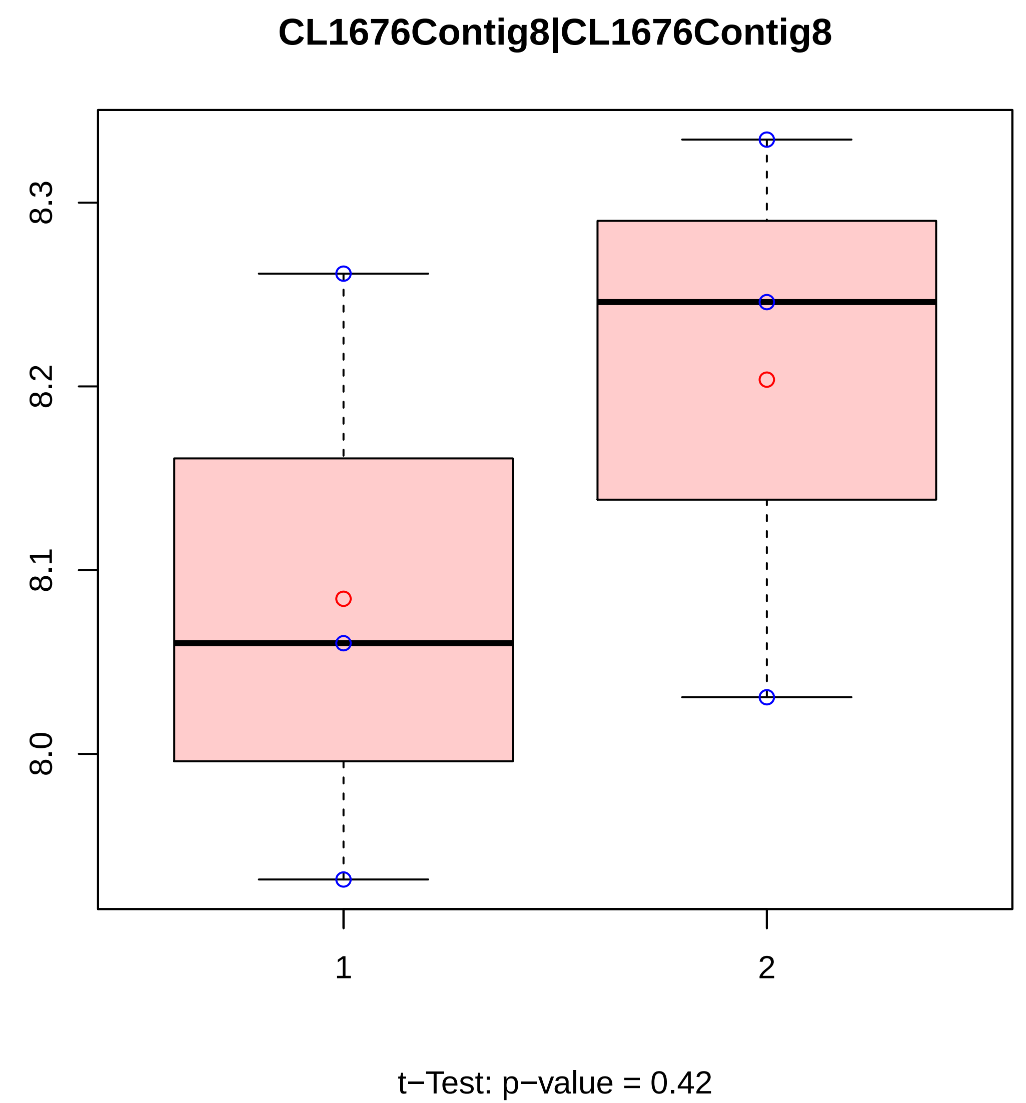
CL1676Contig8|CL1676Contig8
8.0
8.1
8.2
8.3
1
2
t−Test: p−value = 0.42

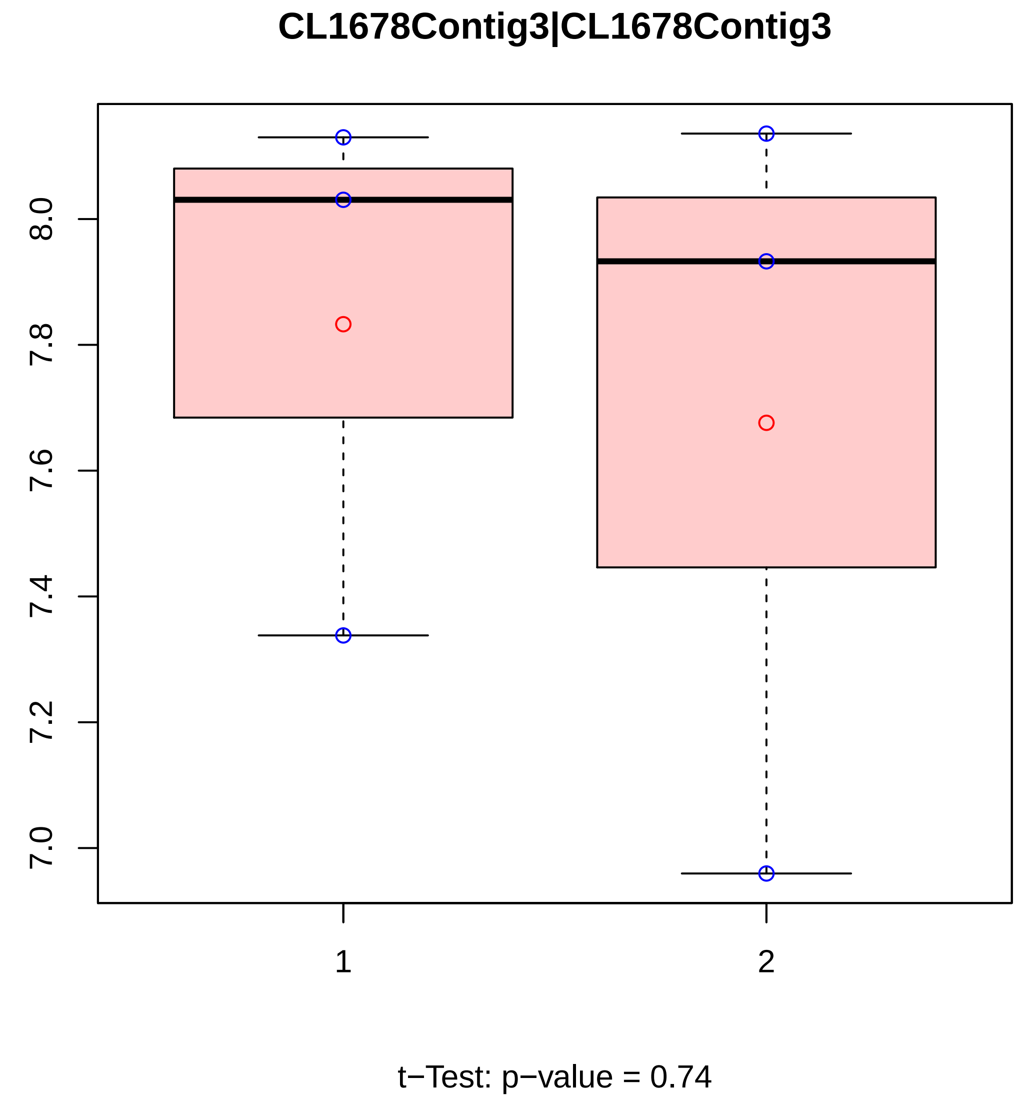
CL1678Contig3|CL1678Contig3
8.0
7.8
7.6
7.4
7.2
7.0
1
2
t−Test: p−value = 0.74

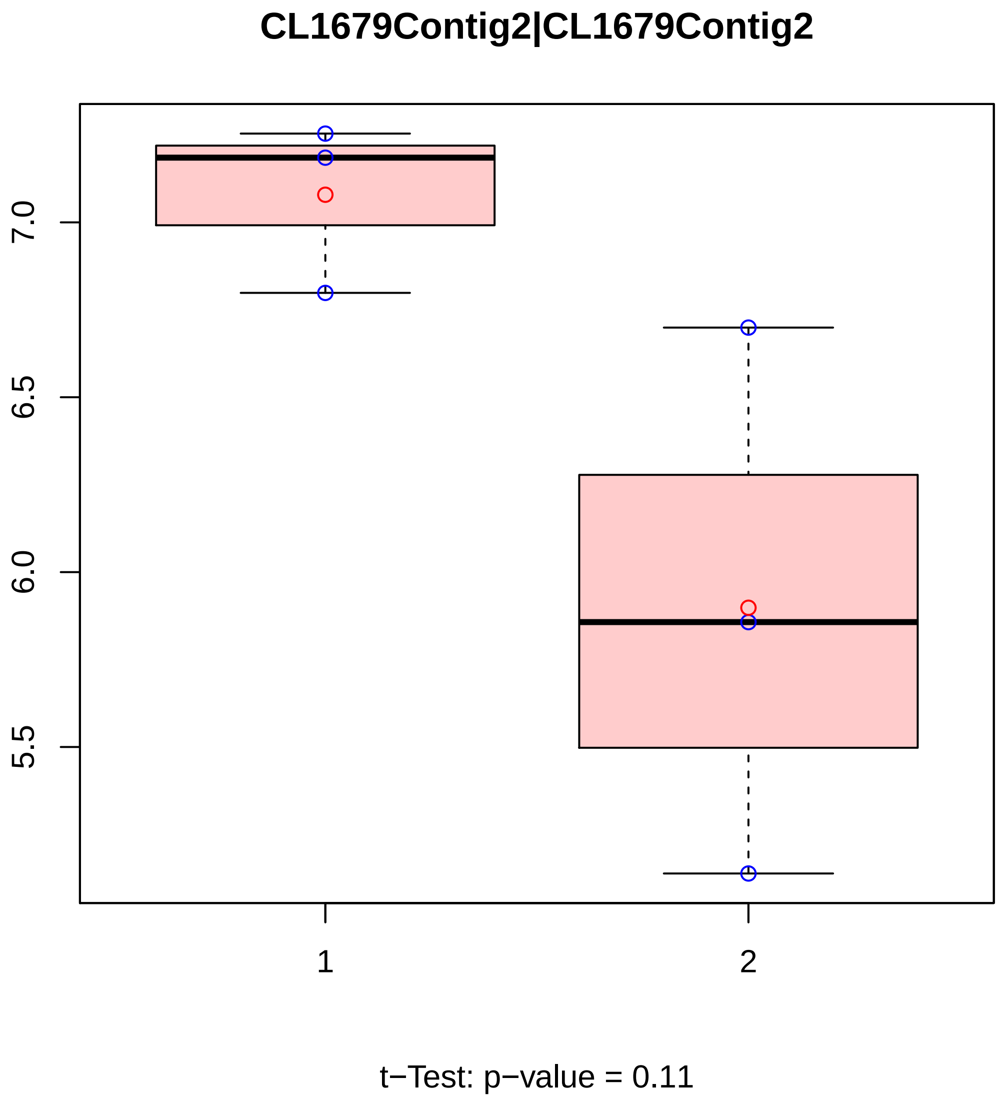

CL1679Contig2|CL1679Contig2
7.0
6.5
6.0
5.5
1
2
t−Test: p−value = 0.11

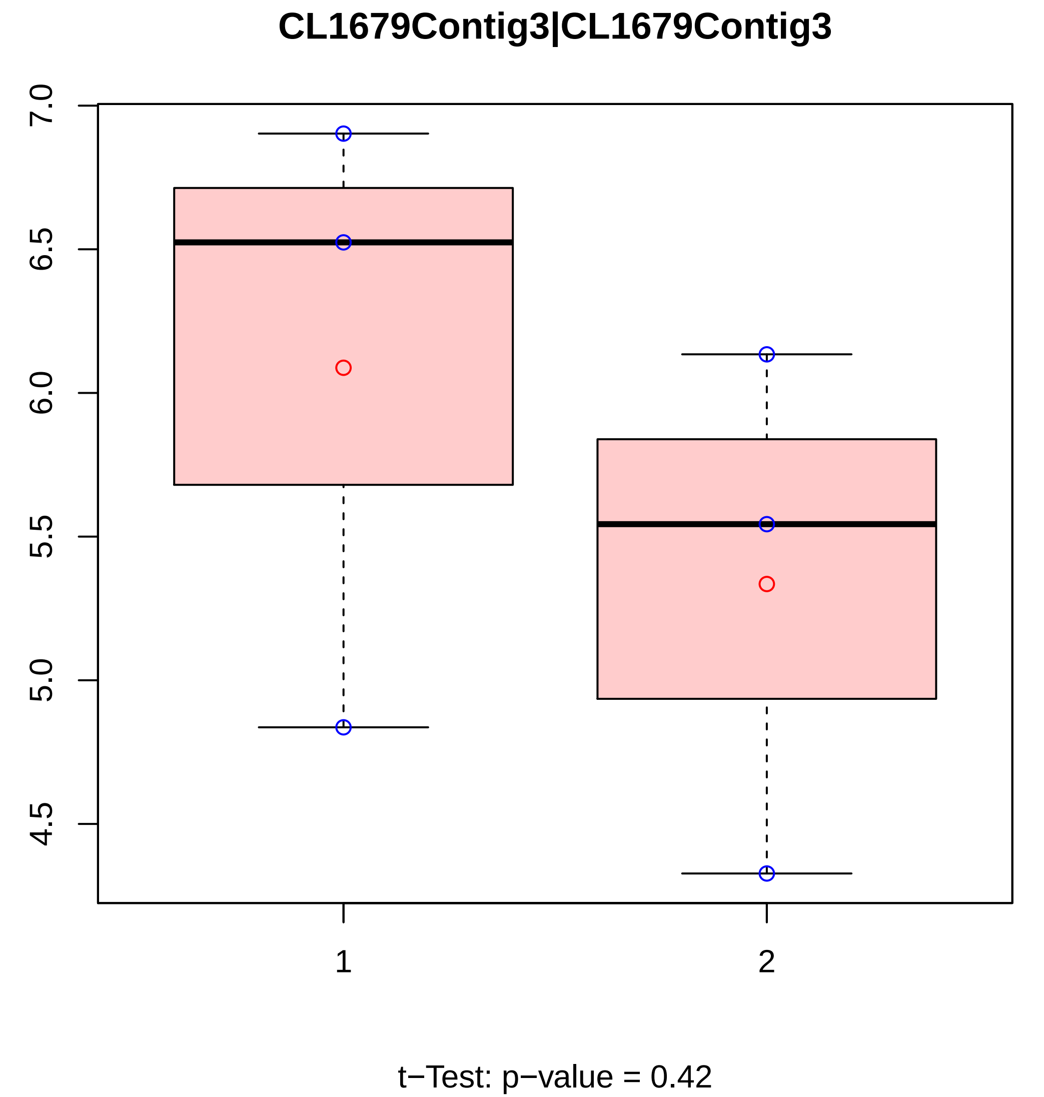
CL1679Contig3|CL1679Contig3
7.0
6.5
6.0
5.5
5.0
4.5
1
2
t−Test: p−value = 0.42

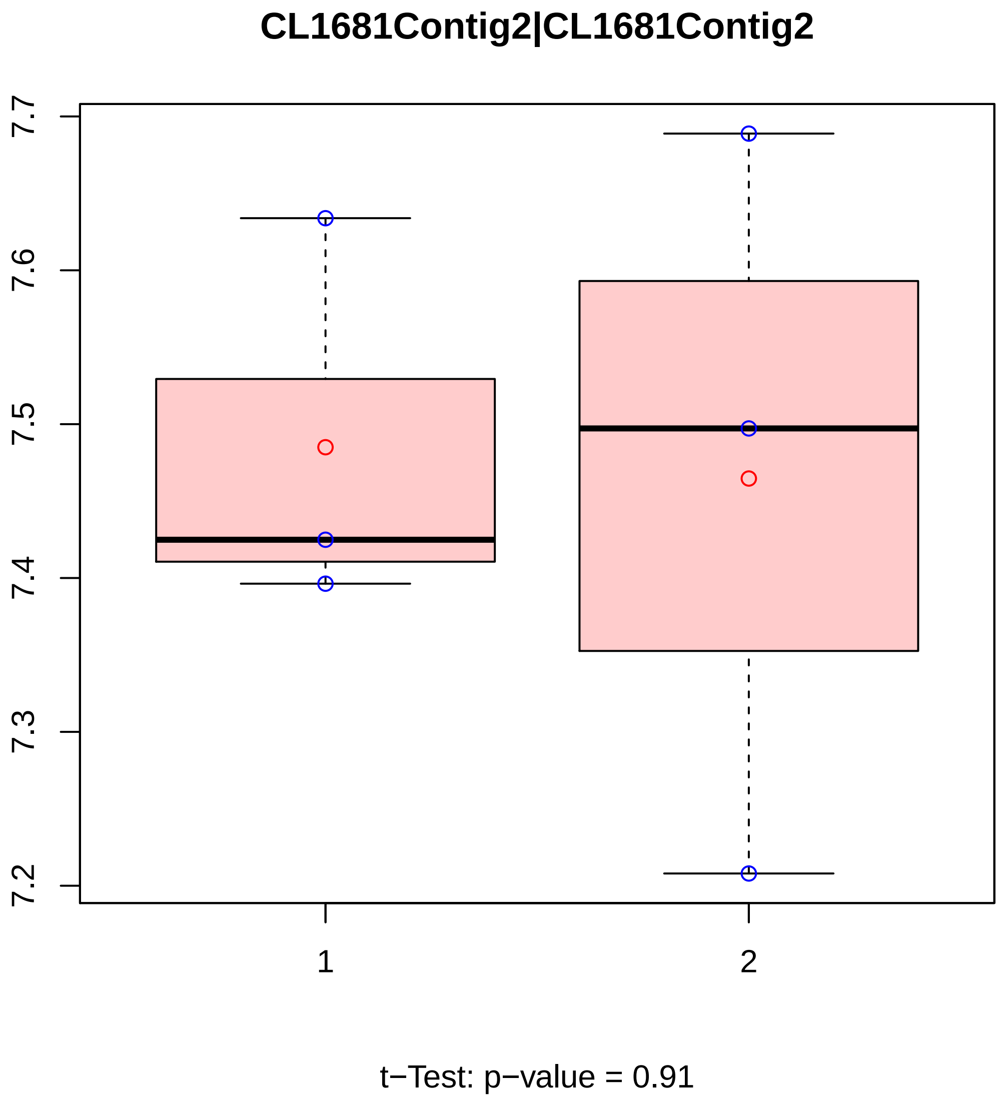
CL1681Contig2|CL1681Contig2
7.2
7.3
7.4
7.5
7.6
7.7
1
2
t−Test: p−value = 0.91

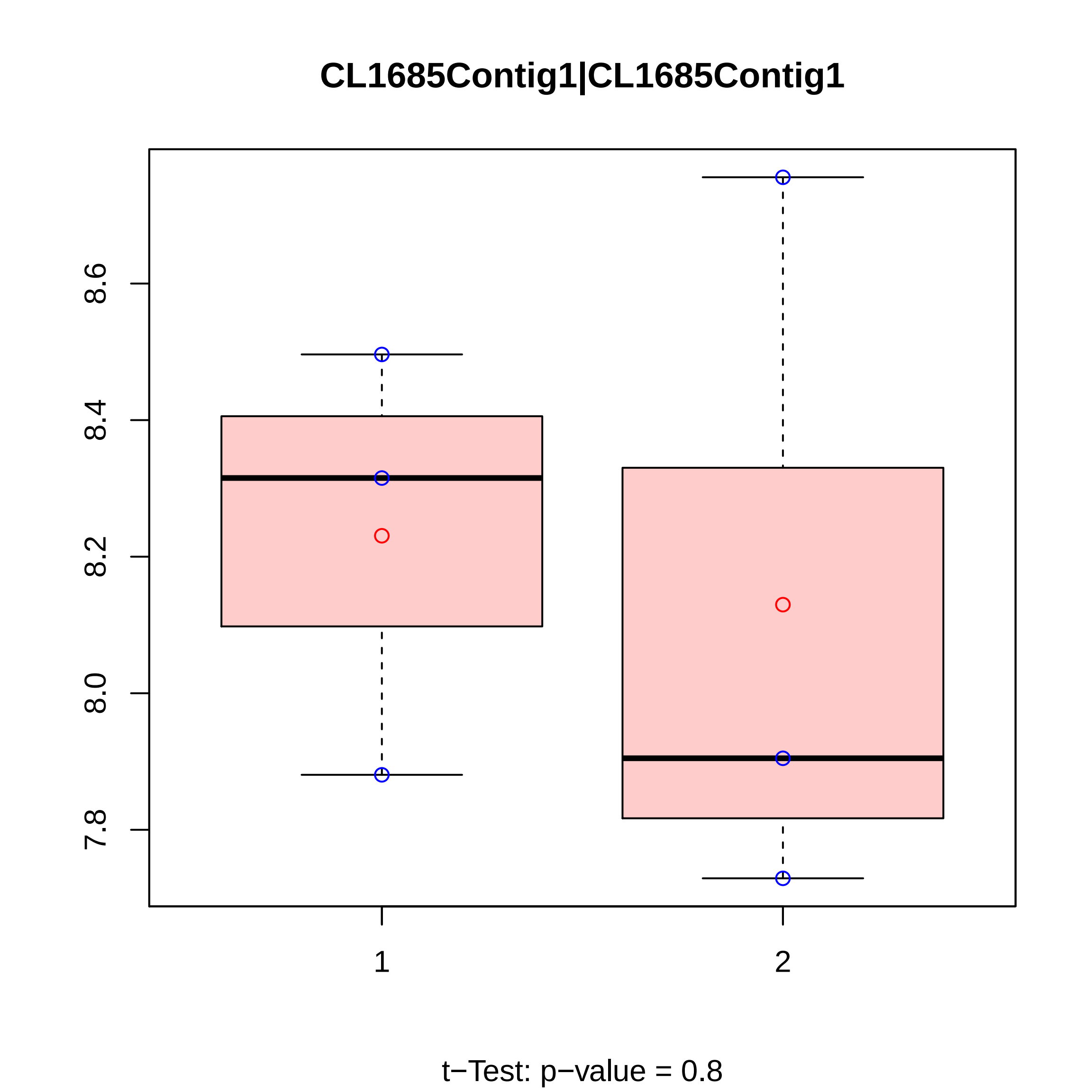
CL1685Contig1|CL1685Contig1
8.6
8.4
8.2
8.0
7.8
1
2
t−Test: p−value = 0.8

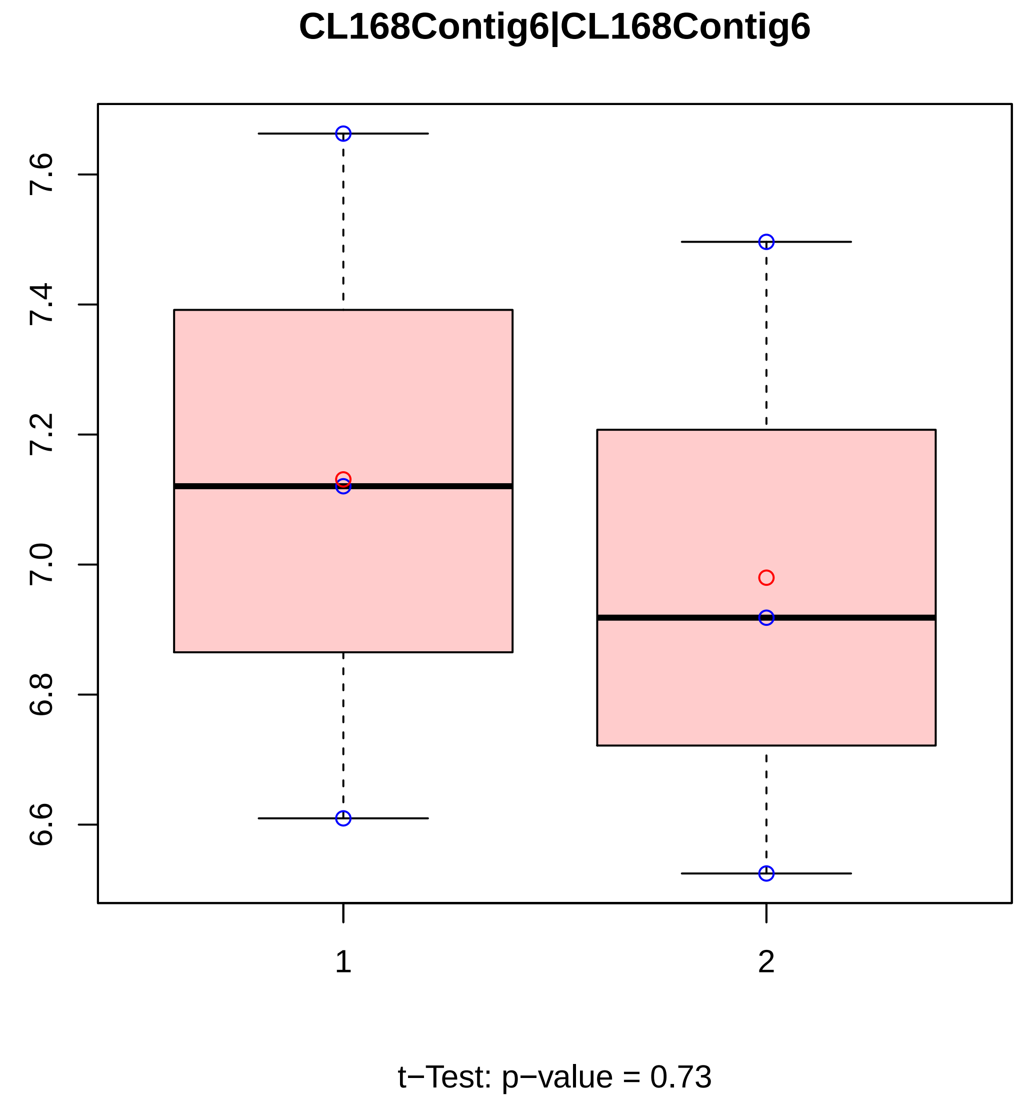
CL168Contig6|CL168Contig6
7.6
7.4
7.2
7.0
6.8
6.6
1
2
t−Test: p−value = 0.73

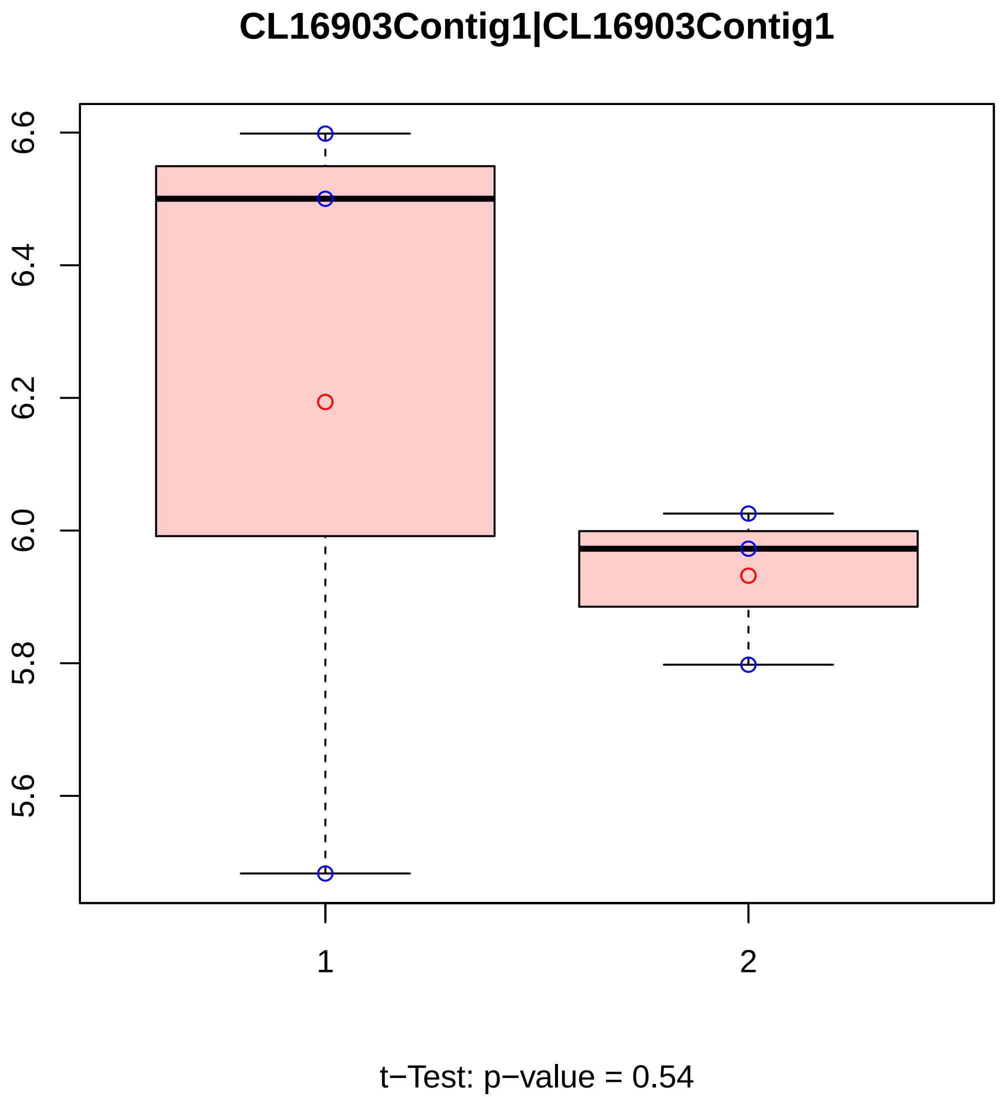
CL16903Contig1|CL16903Contig1
6.6
6.4
6.2
6.0
5.8
5.6
1
2
t−Test: p−value = 0.54

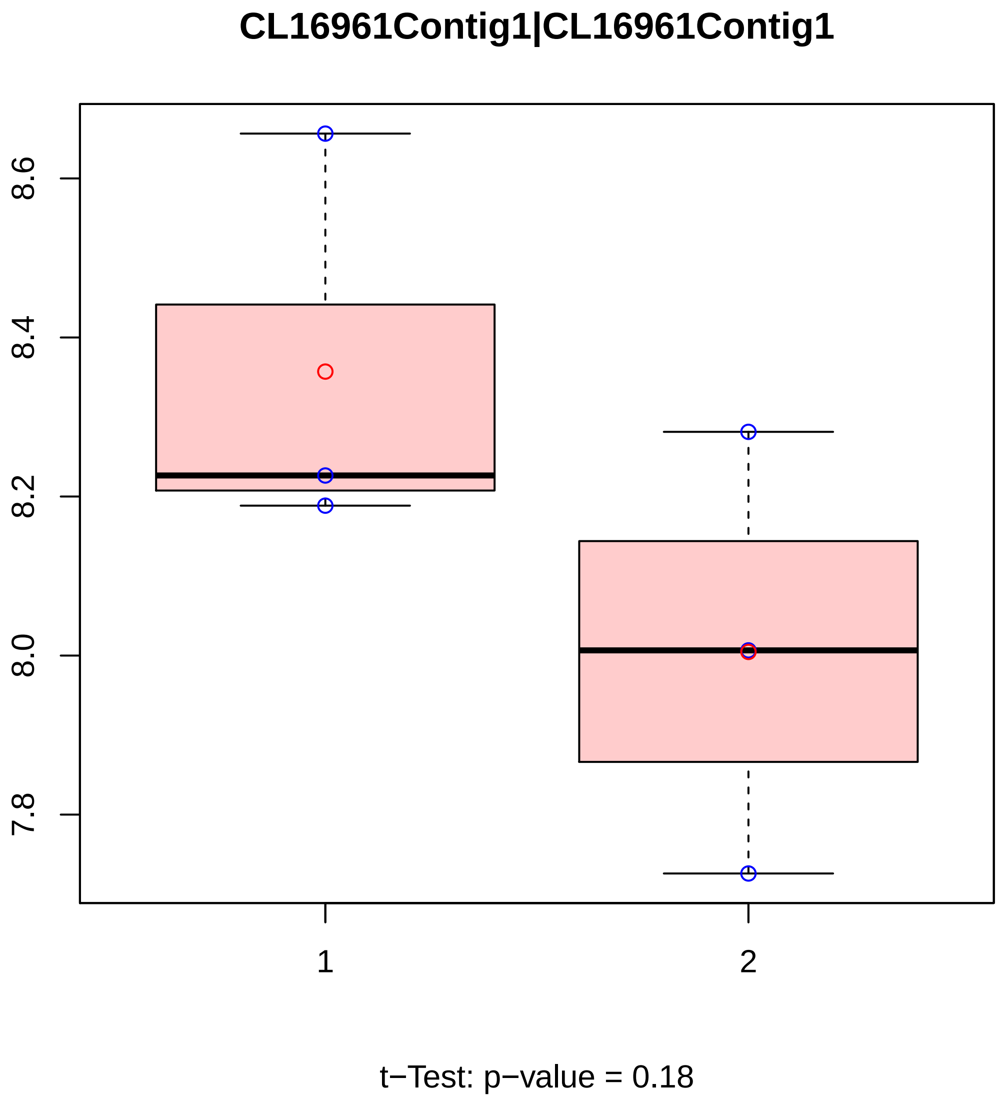
CL16961Contig1|CL16961Contig1
8.6
8.4
8.2
8.0
7.8
1
2
t−Test: p−value = 0.18

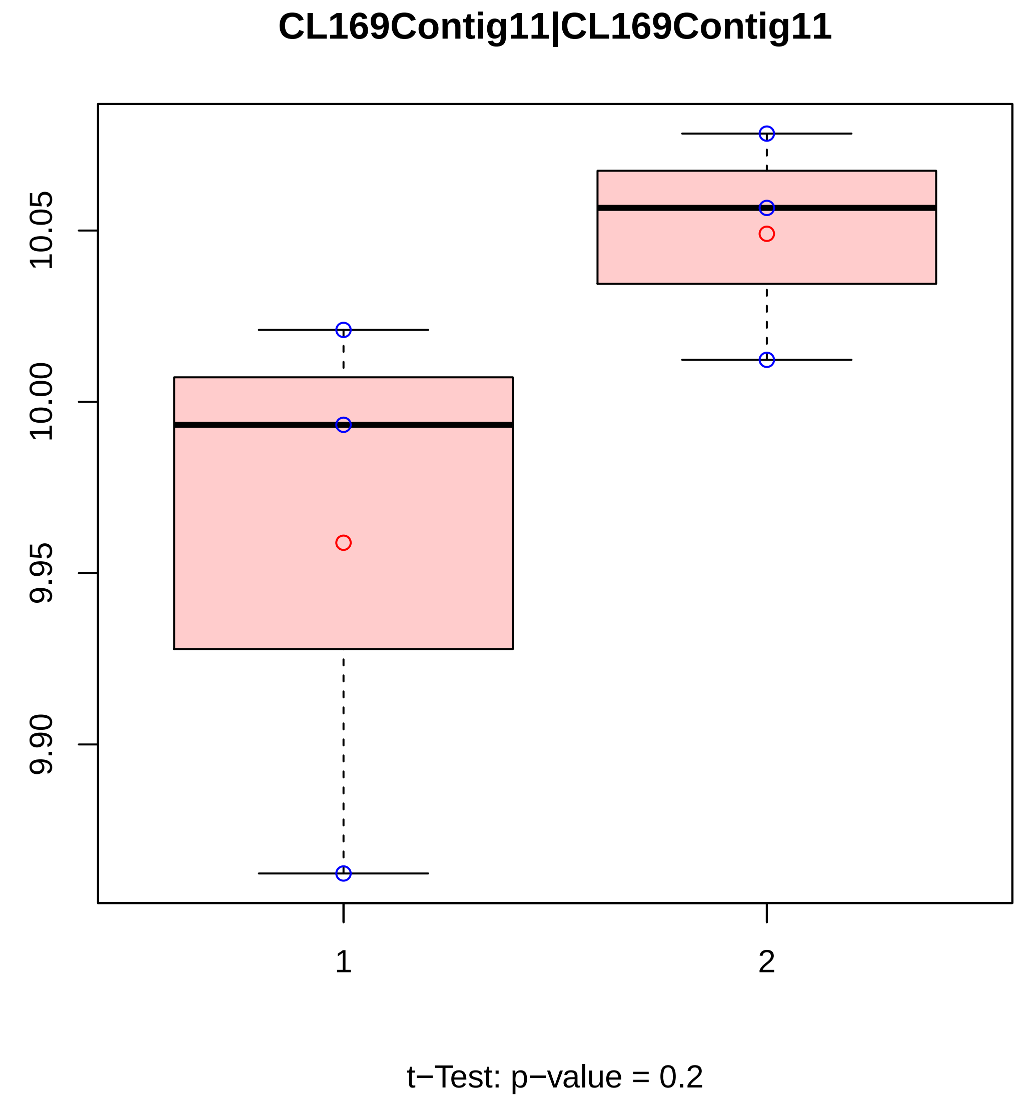
CL169Contig11|CL169Contig11
10.05
10.00
9.95
9.90
1
2
t−Test: p−value = 0.2

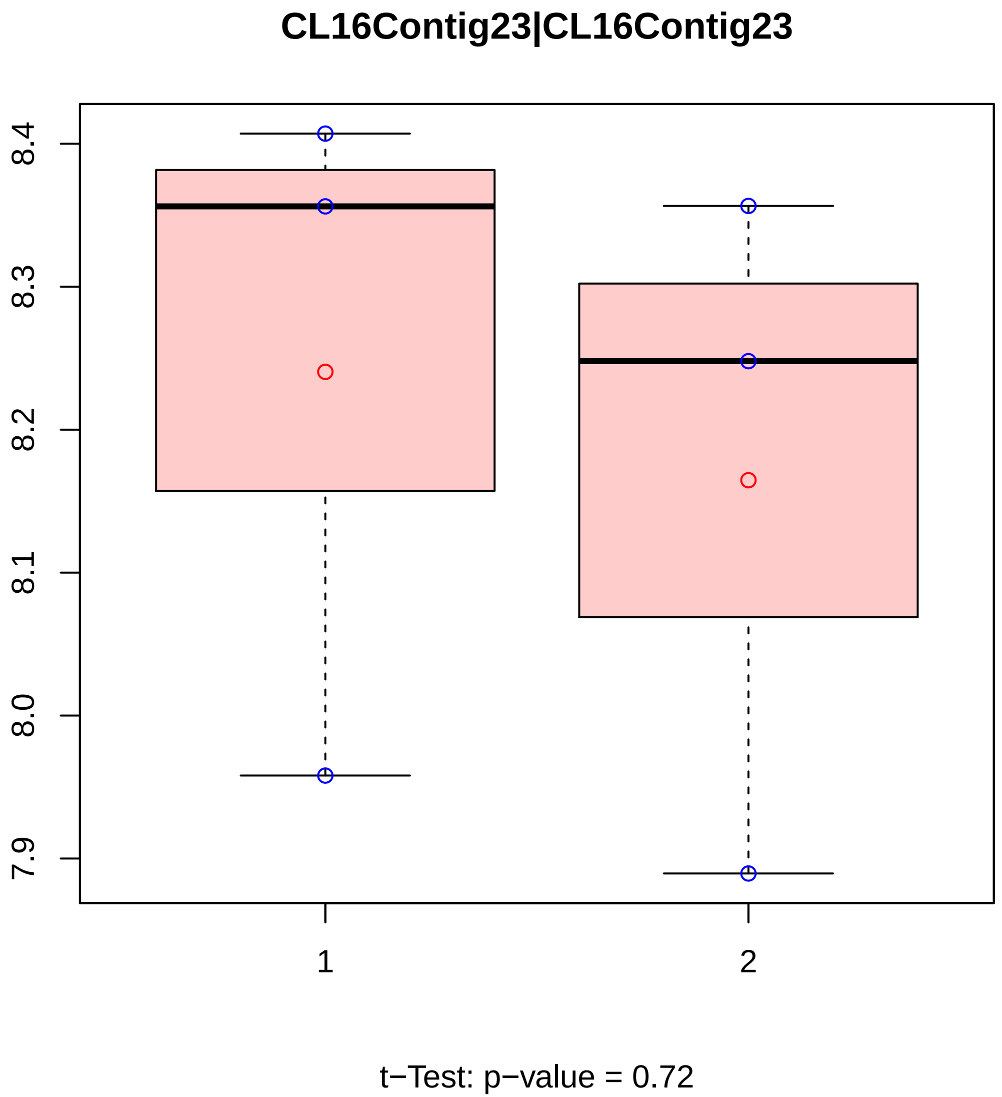

CL16Contig23|CL16Contig23
8.4
8.3
8.2
8.1
8.0
7.9
1
2
t−Test: p−value = 0.72

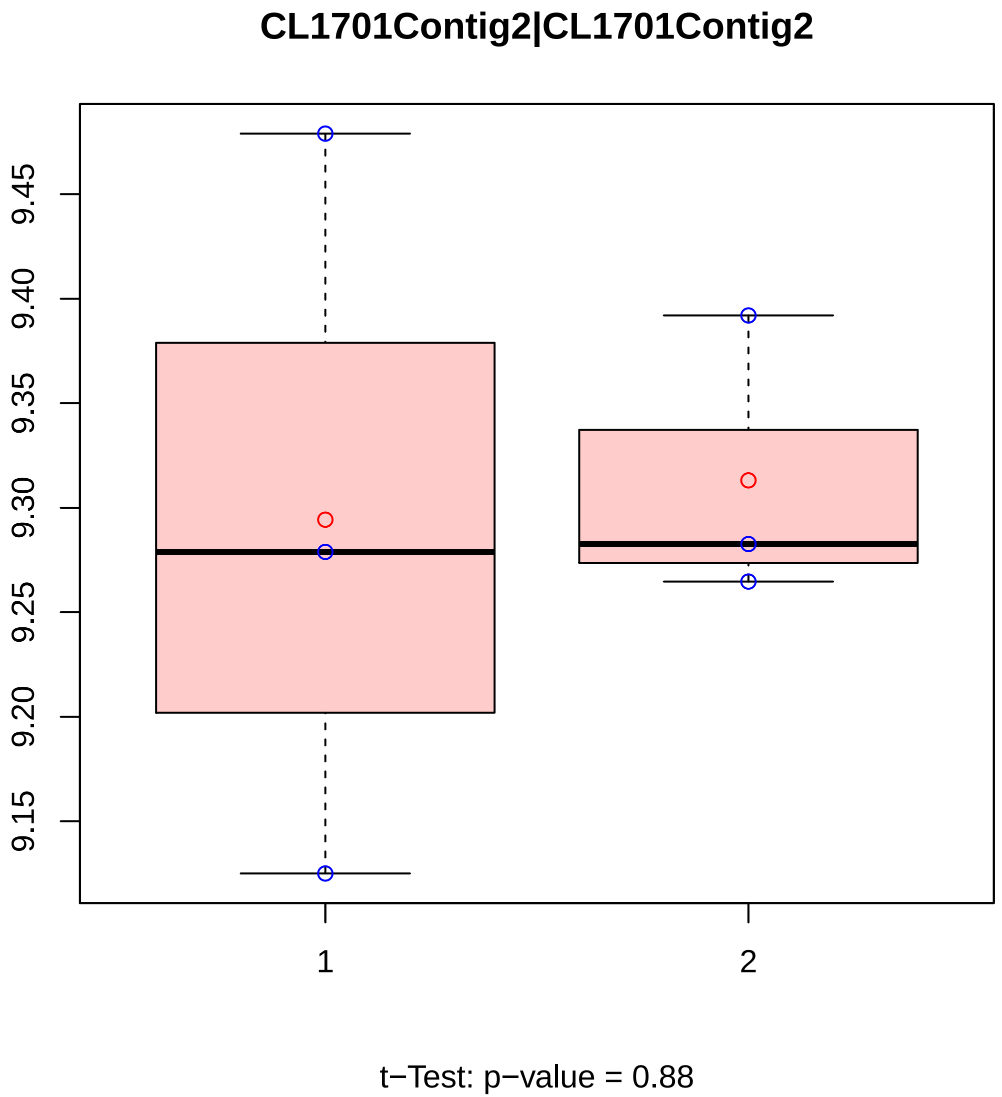
CL1701Contig2|CL1701Contig2
9.15
9.20
9.25
9.30
9.35
9.40
9.45
1
2
t−Test: p−value = 0.88

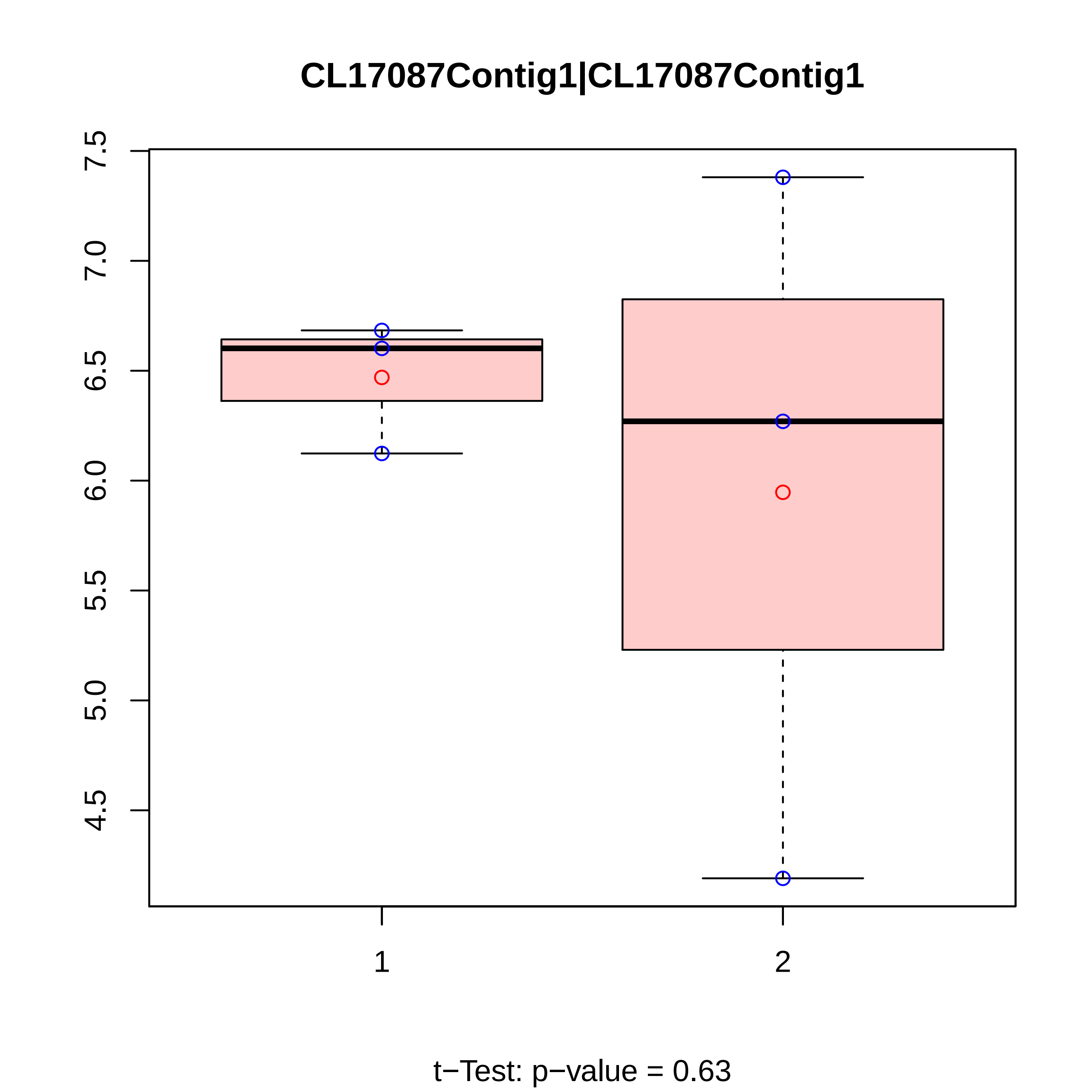
CL17087Contig1|CL17087Contig1
7.5
7.0
6.5
6.0
5.5
5.0
4.5
1
2
t−Test: p−value = 0.63

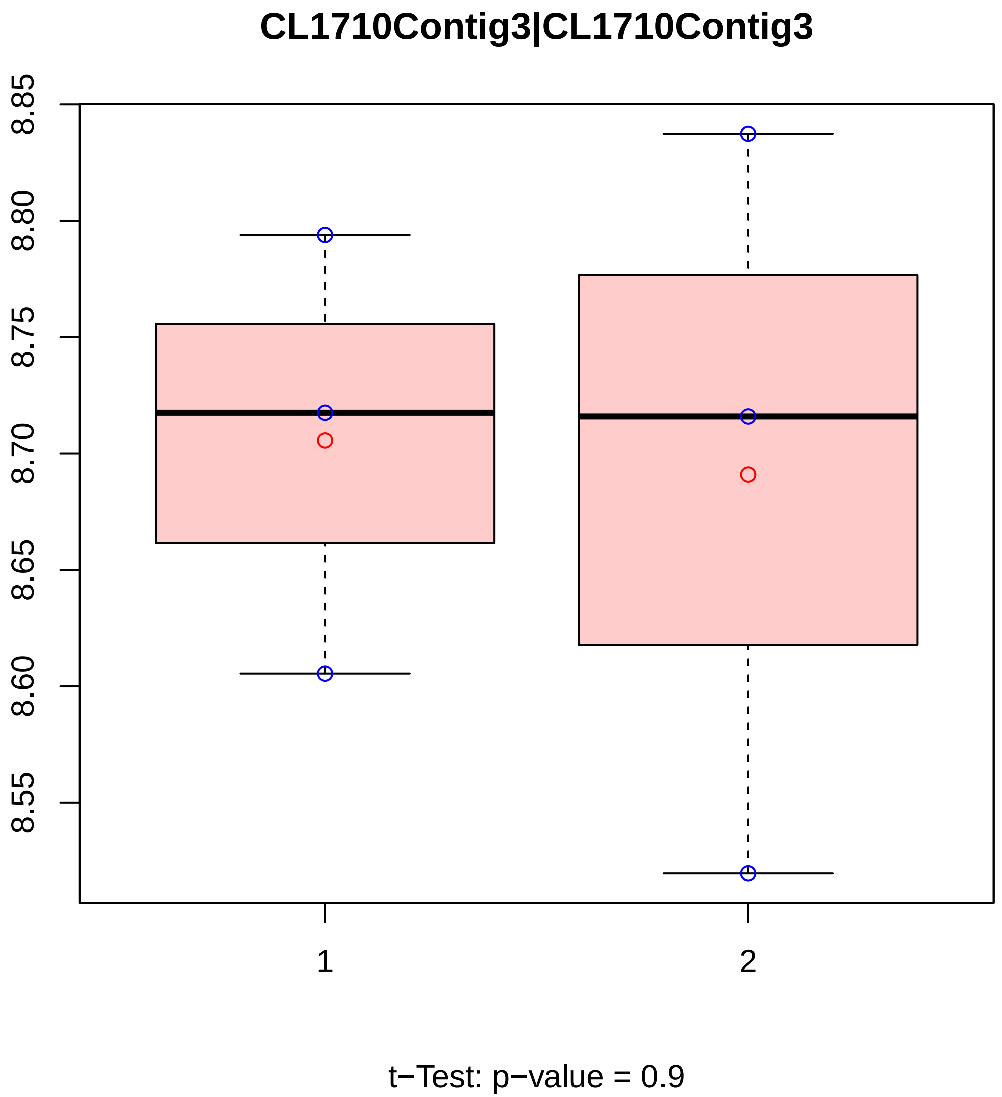
CL1710Contig3|CL1710Contig3
8.85
8.80
8.75
8.70
8.65
8.60
8.55
1
2
t−Test: p−value = 0.9

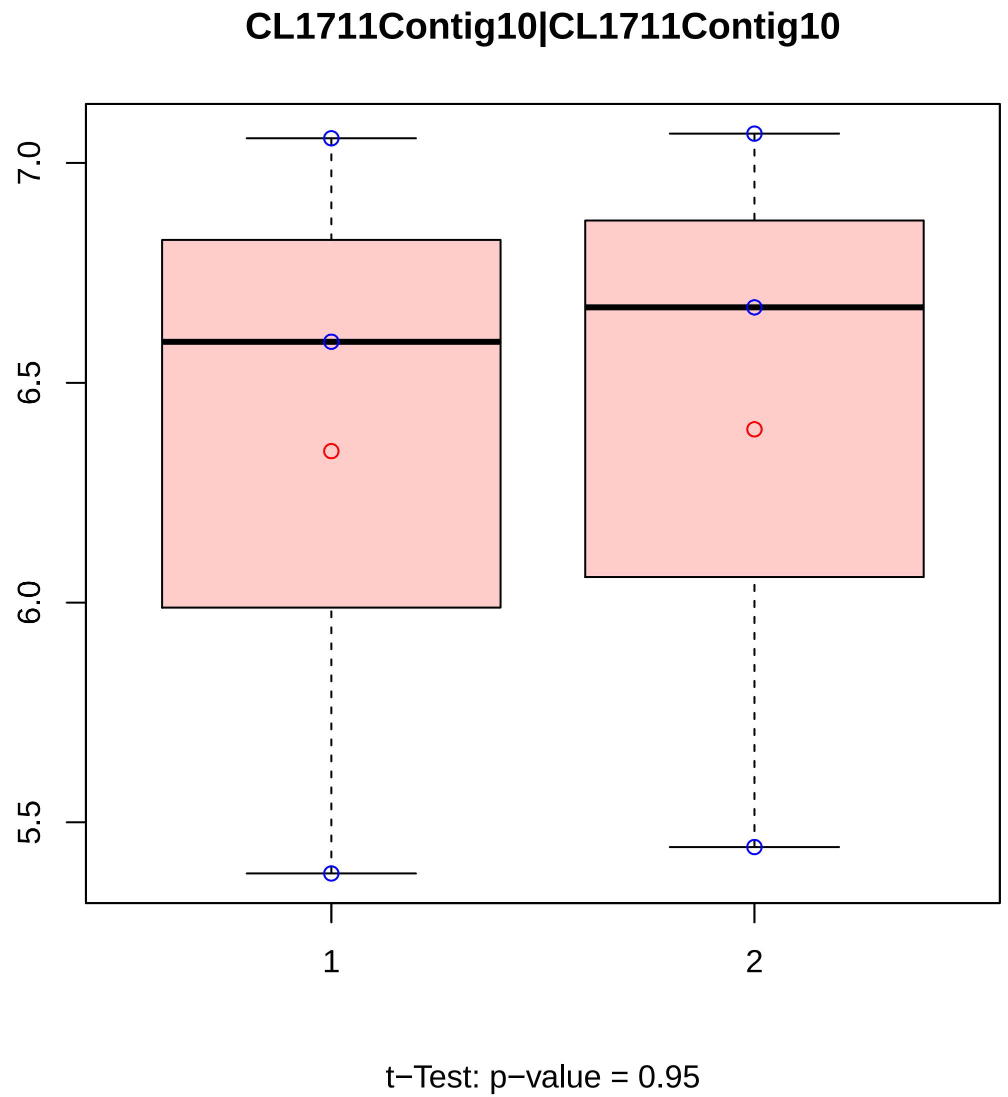
CL1711Contig10|CL1711Contig10
7.0
6.5
6.0
5.5
1
2
t−Test: p−value = 0.95

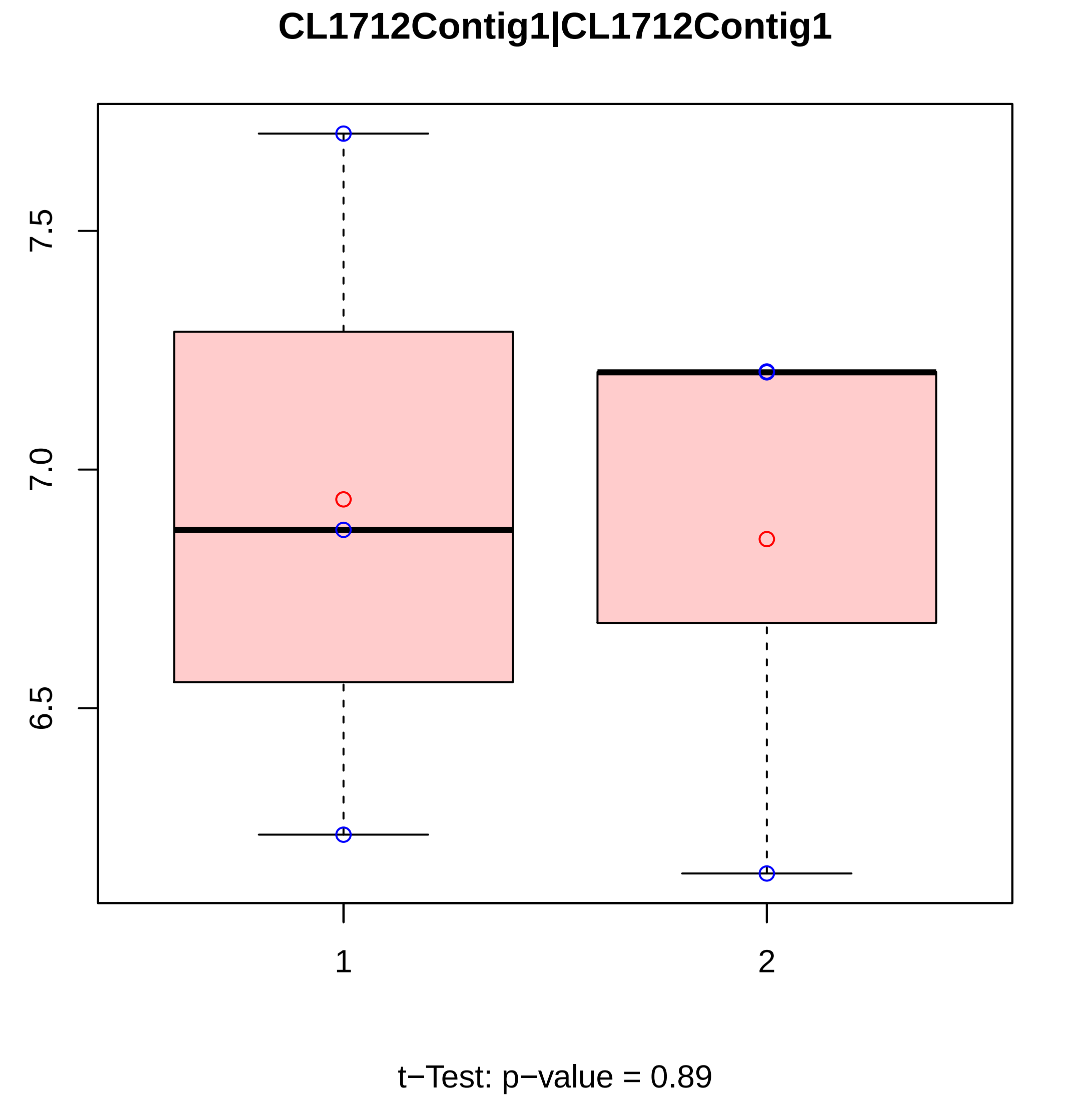
CL1712Contig1|CL1712Contig1
7.5
7.0
6.5
1
2
t−Test: p−value = 0.89

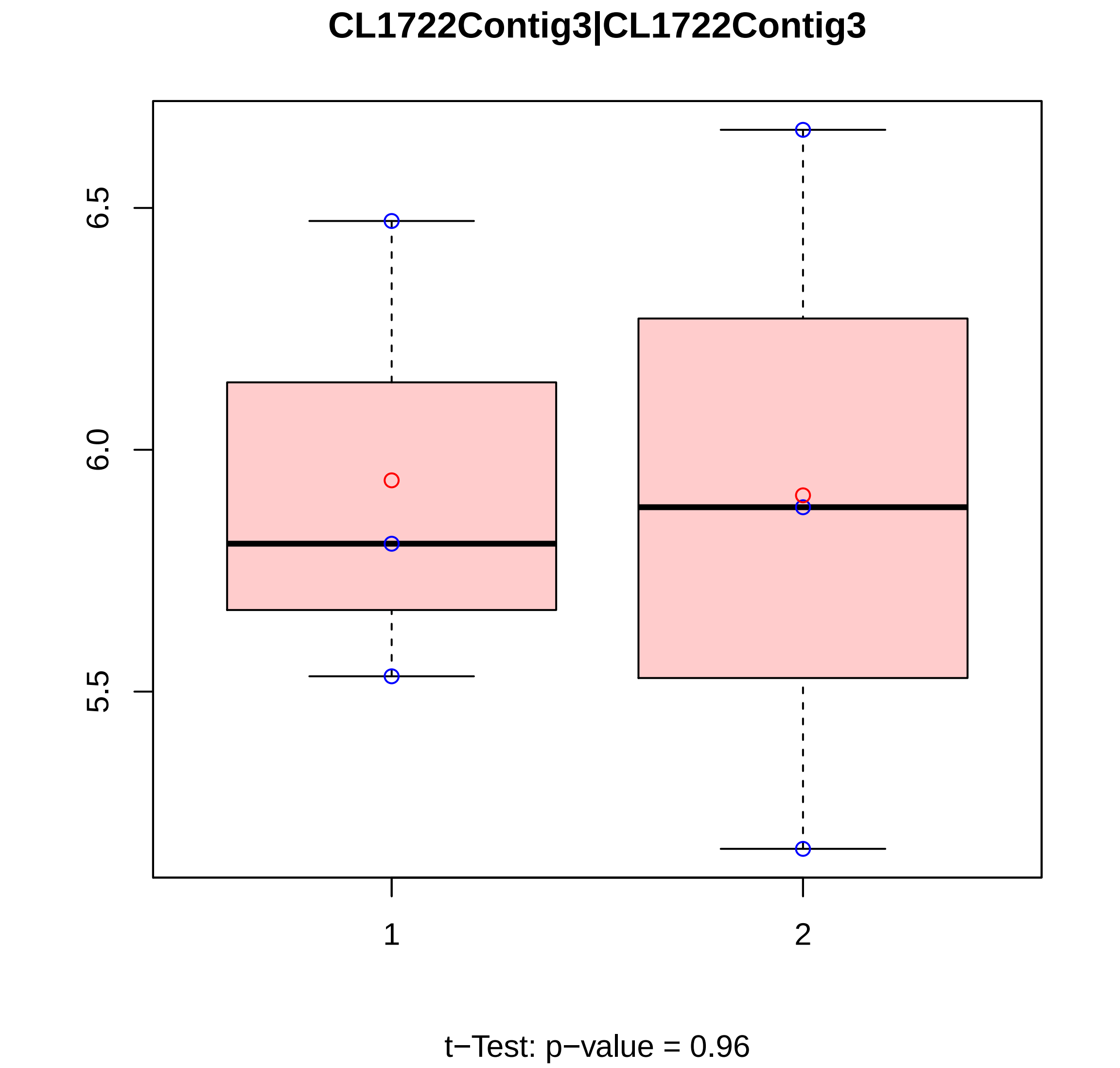
CL1722Contig3|CL1722Contig3
6.5
6.0
5.5
1
2
t−Test: p−value = 0.96

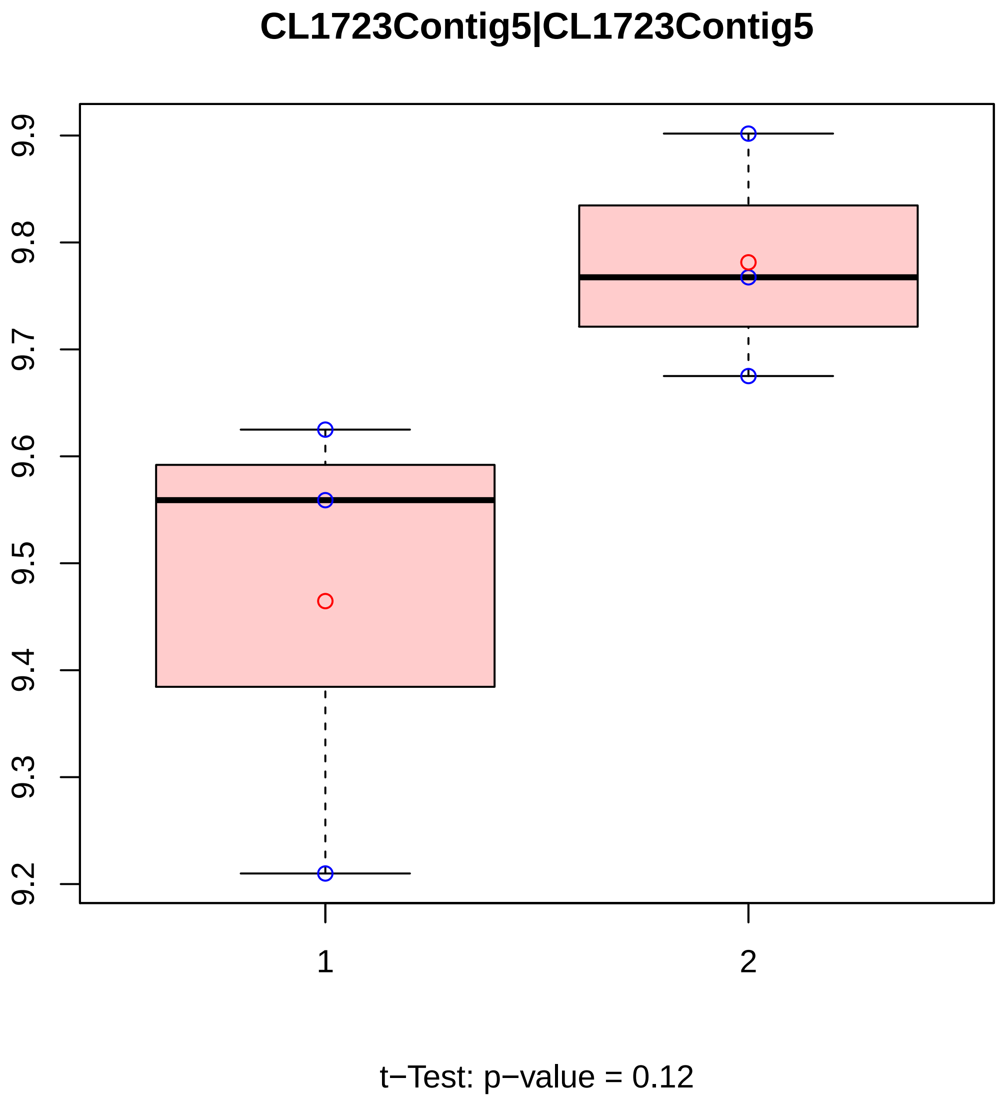
CL1723Contig5|CL1723Contig5
9.2
9.3
9.4
9.5
9.6
9.7
9.8
9.9
1
2
t−Test: p−value = 0.12

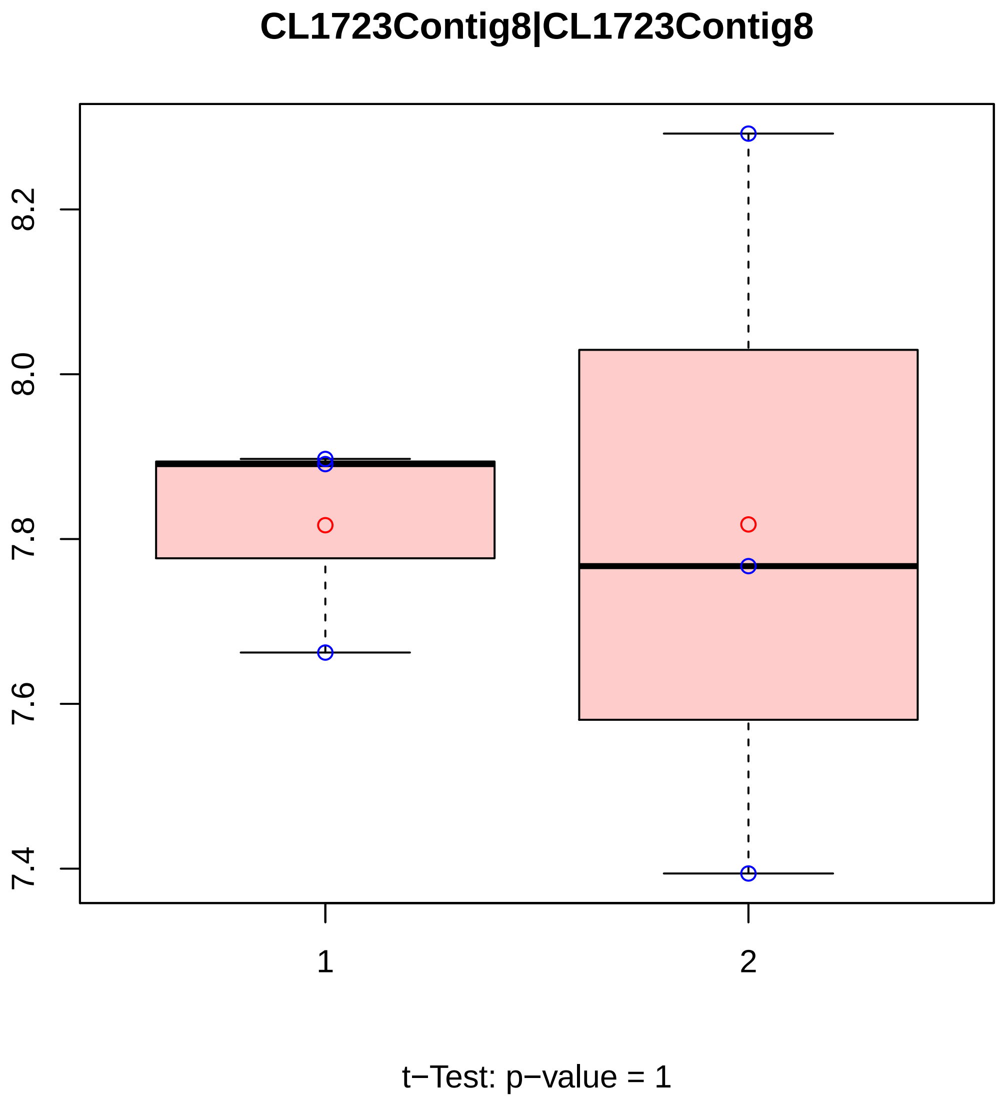
CL1723Contig8|CL1723Contig8
7.4
7.6
7.8
8.0
8.2
1
2
t−Test: p−value = 1

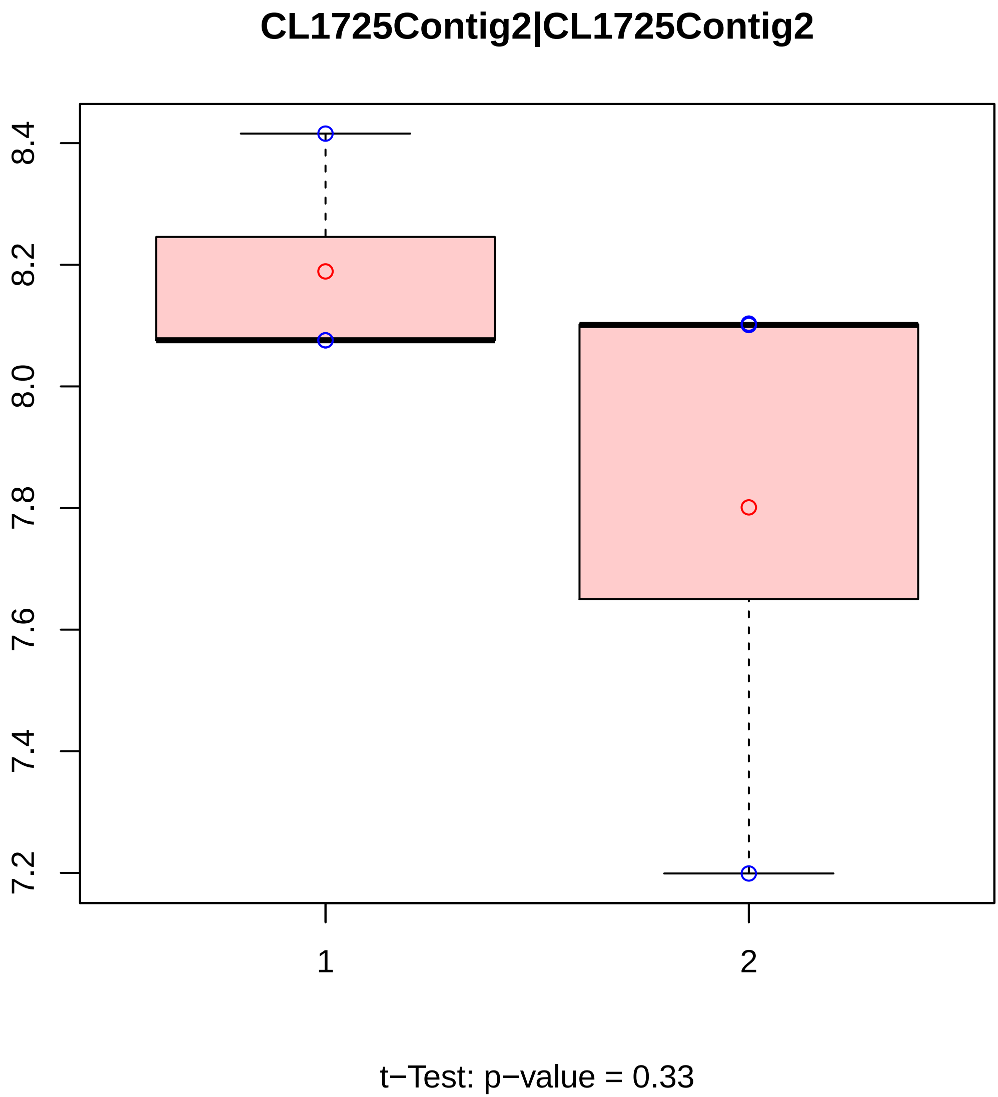
CL1725Contig2|CL1725Contig2
8.4
8.2
8.0
7.8
7.6
7.4
7.2
1
2
t−Test: p−value = 0.33

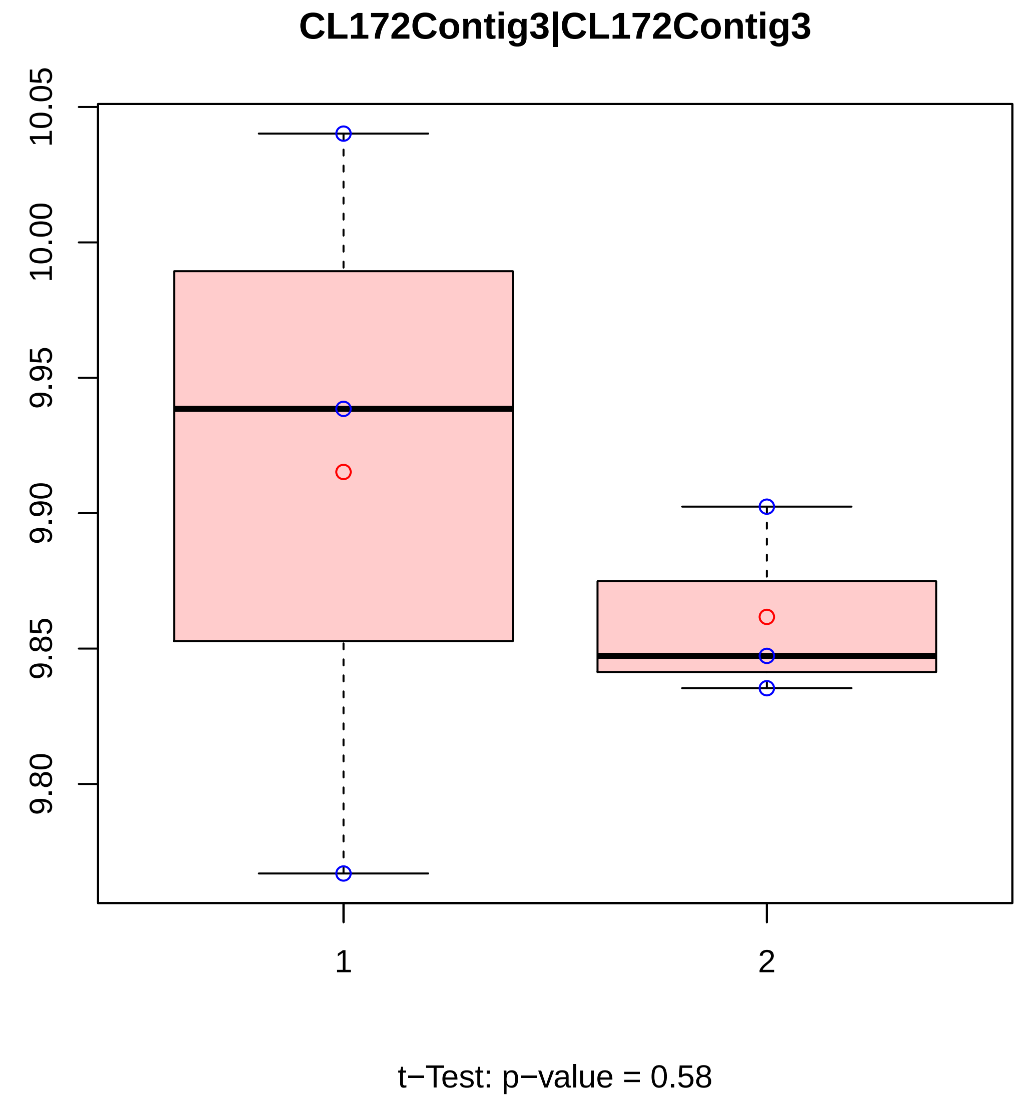

CL172Contig3|CL172Contig3
10.05
10.00
9.95
9.90
9.85
9.80
1
2
t−Test: p−value = 0.58

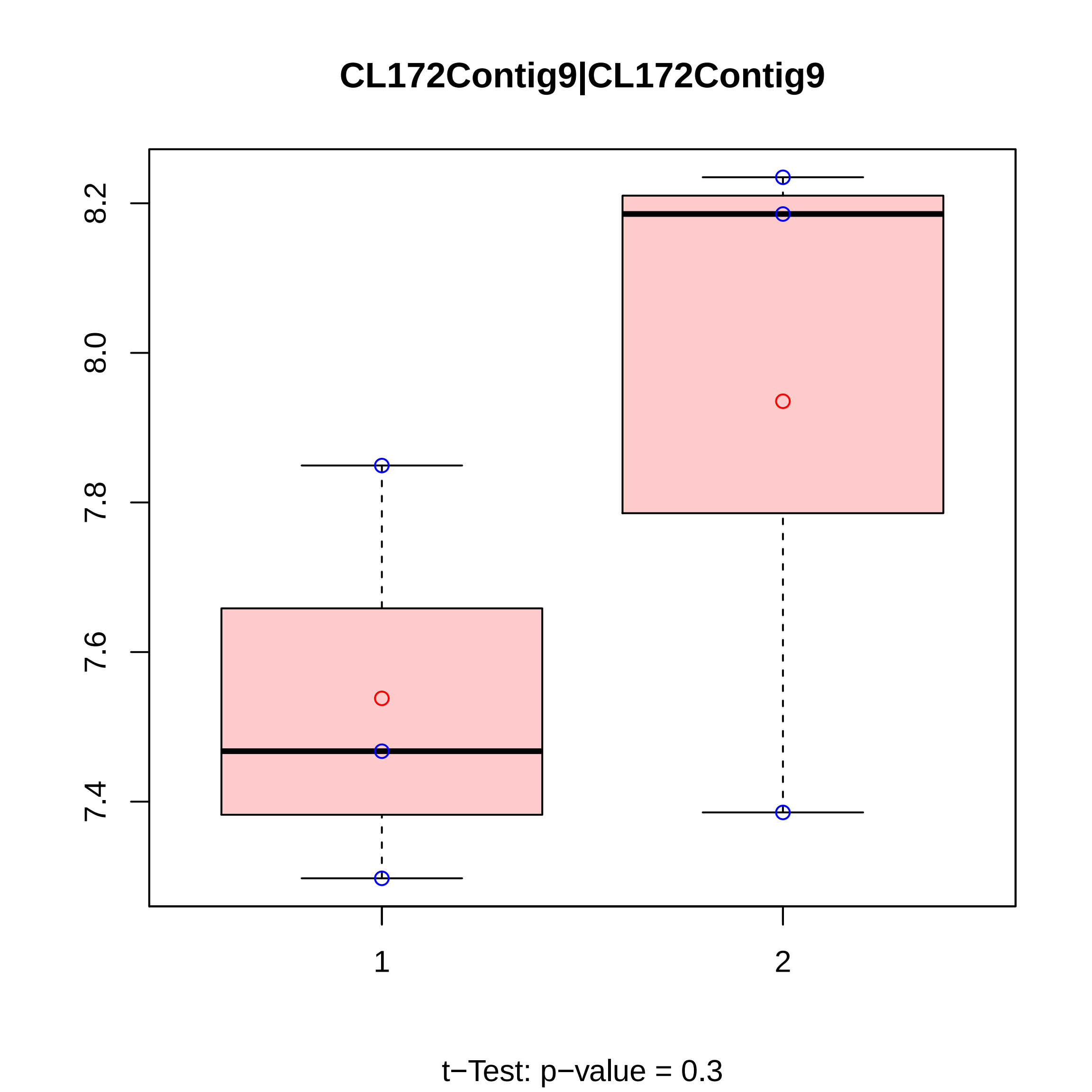
CL172Contig9|CL172Contig9
8.2
8.0
7.8
7.6
7.4
1
2
t−Test: p−value = 0.3

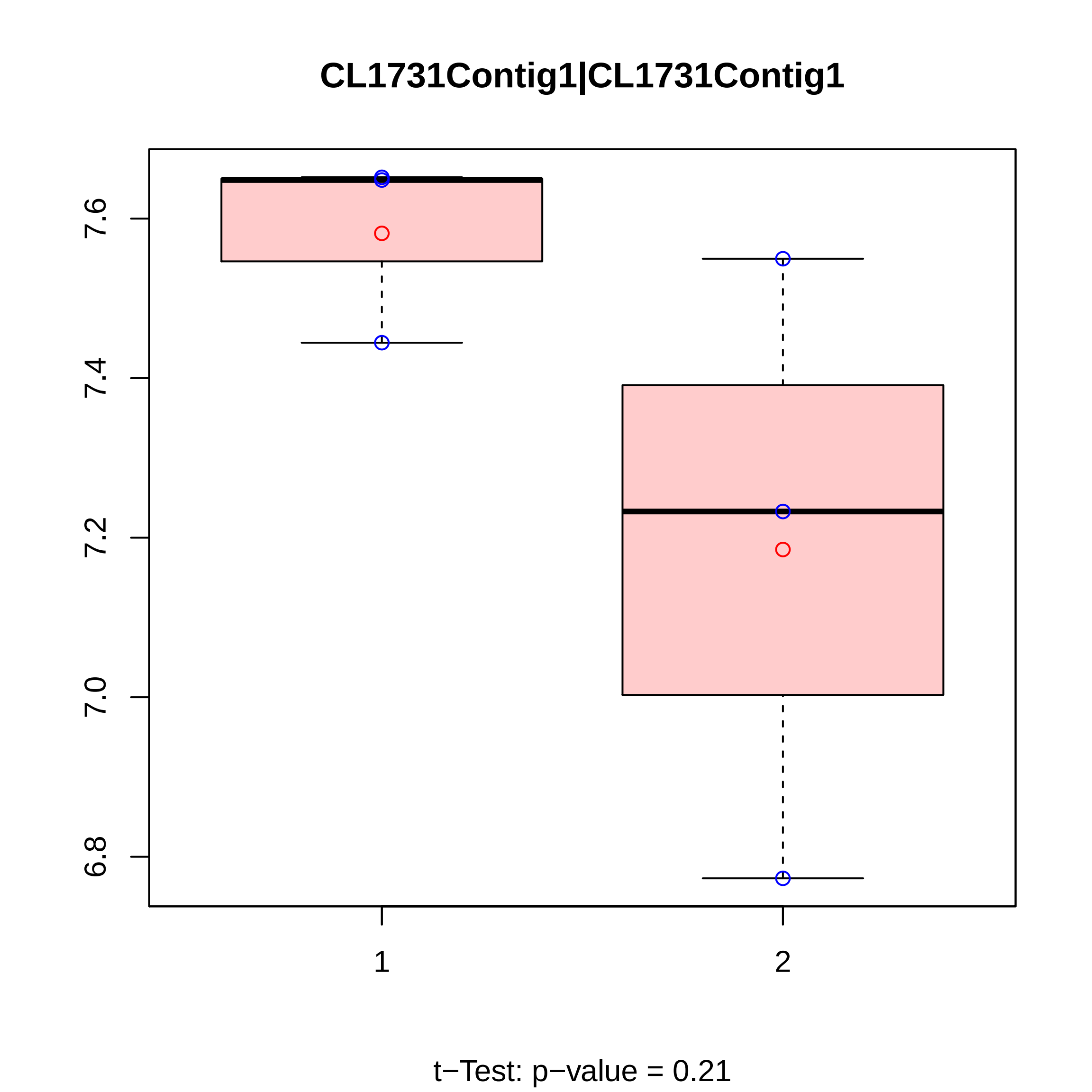
CL1731Contig1|CL1731Contig1
7.6
7.4
7.2
7.0
6.8
1
2
t−Test: p−value = 0.21

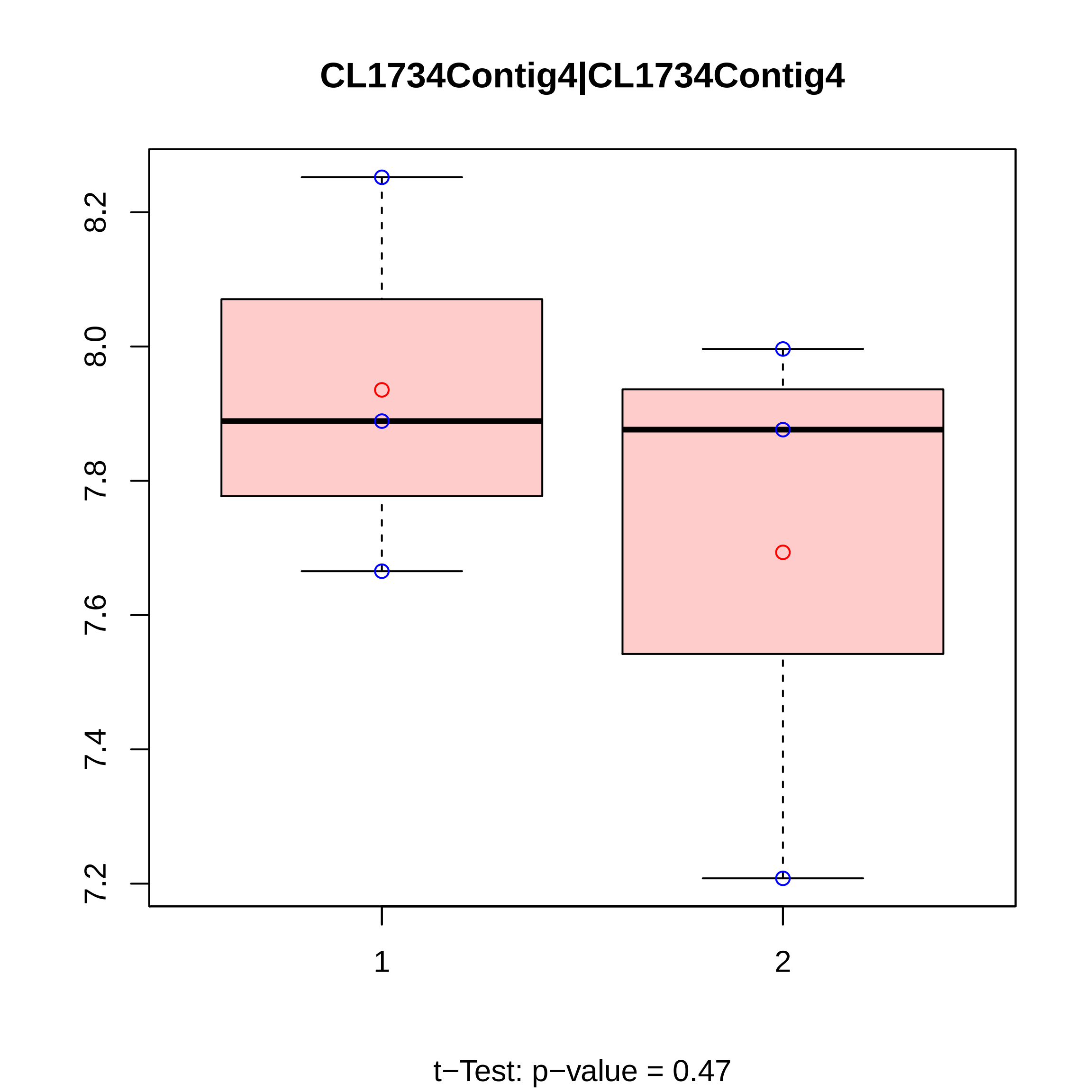
CL1734Contig4|CL1734Contig4
8.2
8.0
7.8
7.6
7.4
7.2
1
2
t−Test: p−value = 0.47

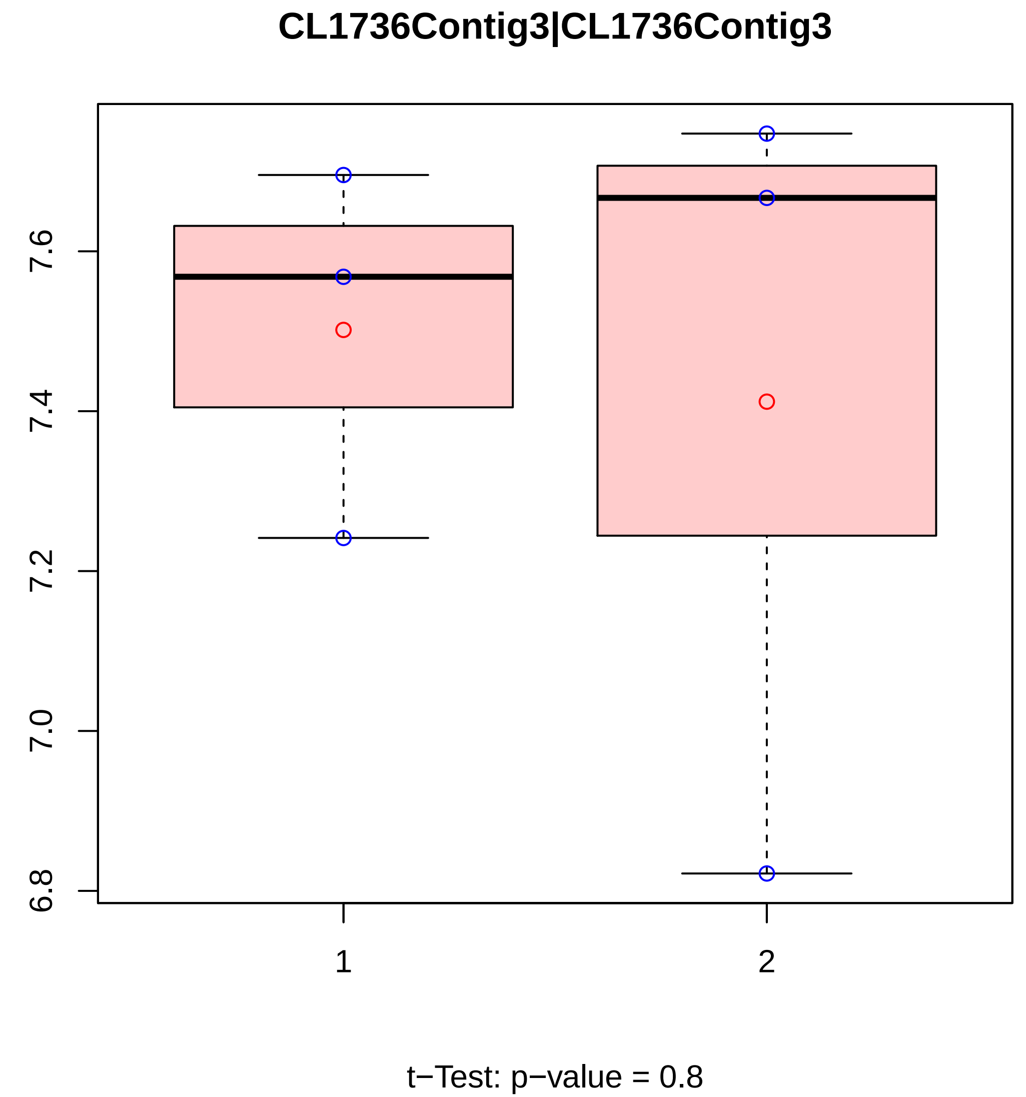
CL1736Contig3|CL1736Contig3
7.6
7.4
7.2
7.0
6.8
1
2
t−Test: p−value = 0.8

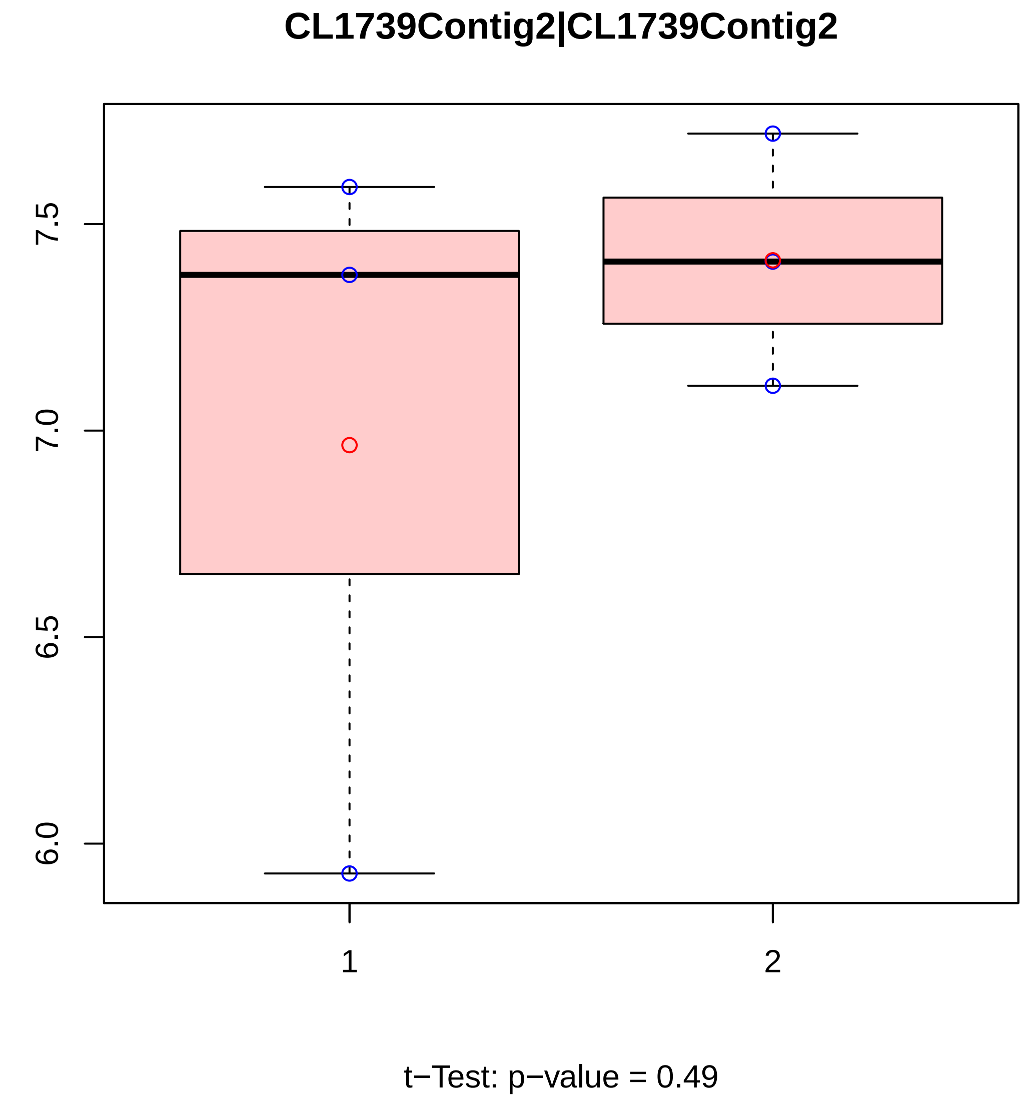
CL1739Contig2|CL1739Contig2
7.5
7.0
6.5
6.0
1
2
t−Test: p−value = 0.49

# CL1742Contig1|CL1742Contig1

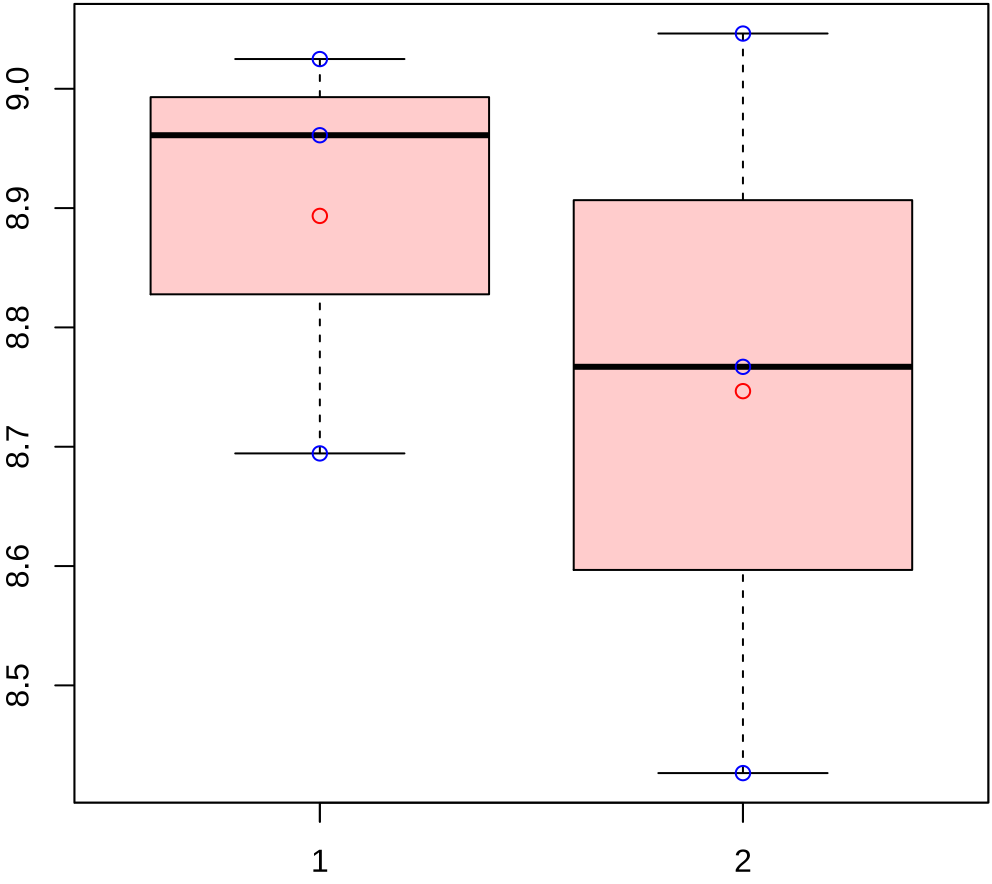


t−Test: p−value = 0.52

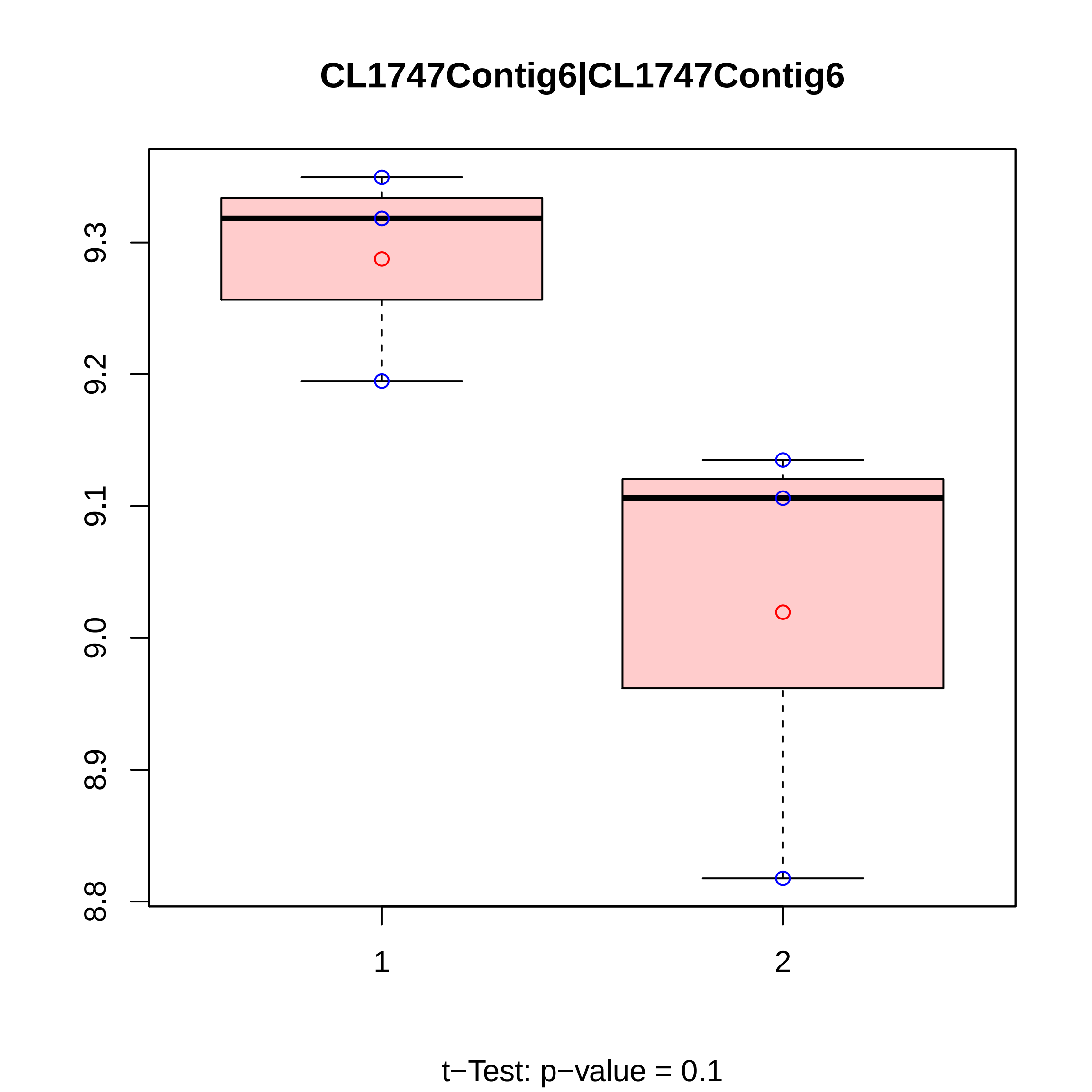

CL1747Contig6|CL1747Contig6
9.3
9.2
9.1
9.0
8.9
8.8
1
2
t−Test: p−value = 0.1

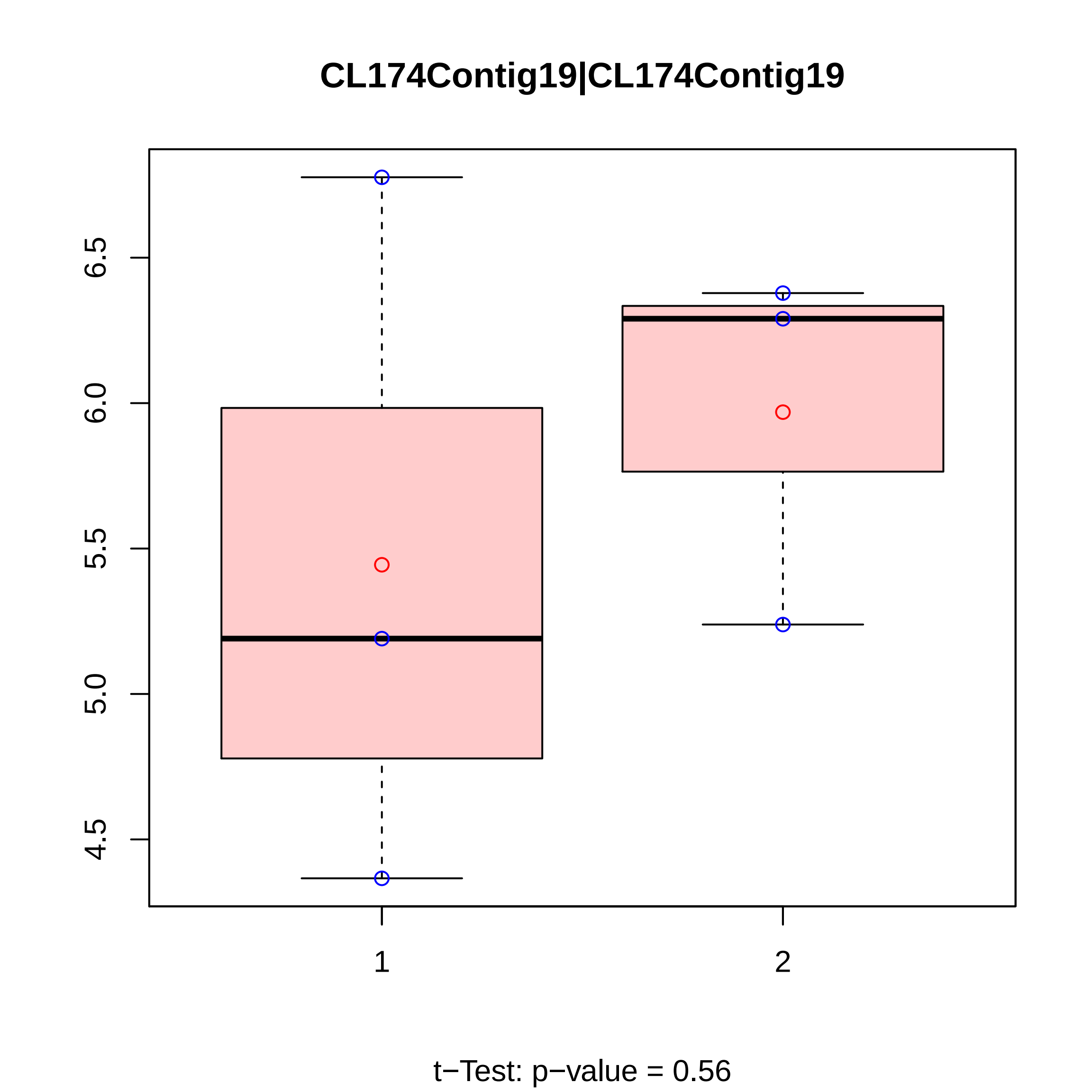
CL174Contig19|CL174Contig19
6.5
6.0
5.5
5.0
4.5
1
2
t−Test: p−value = 0.56

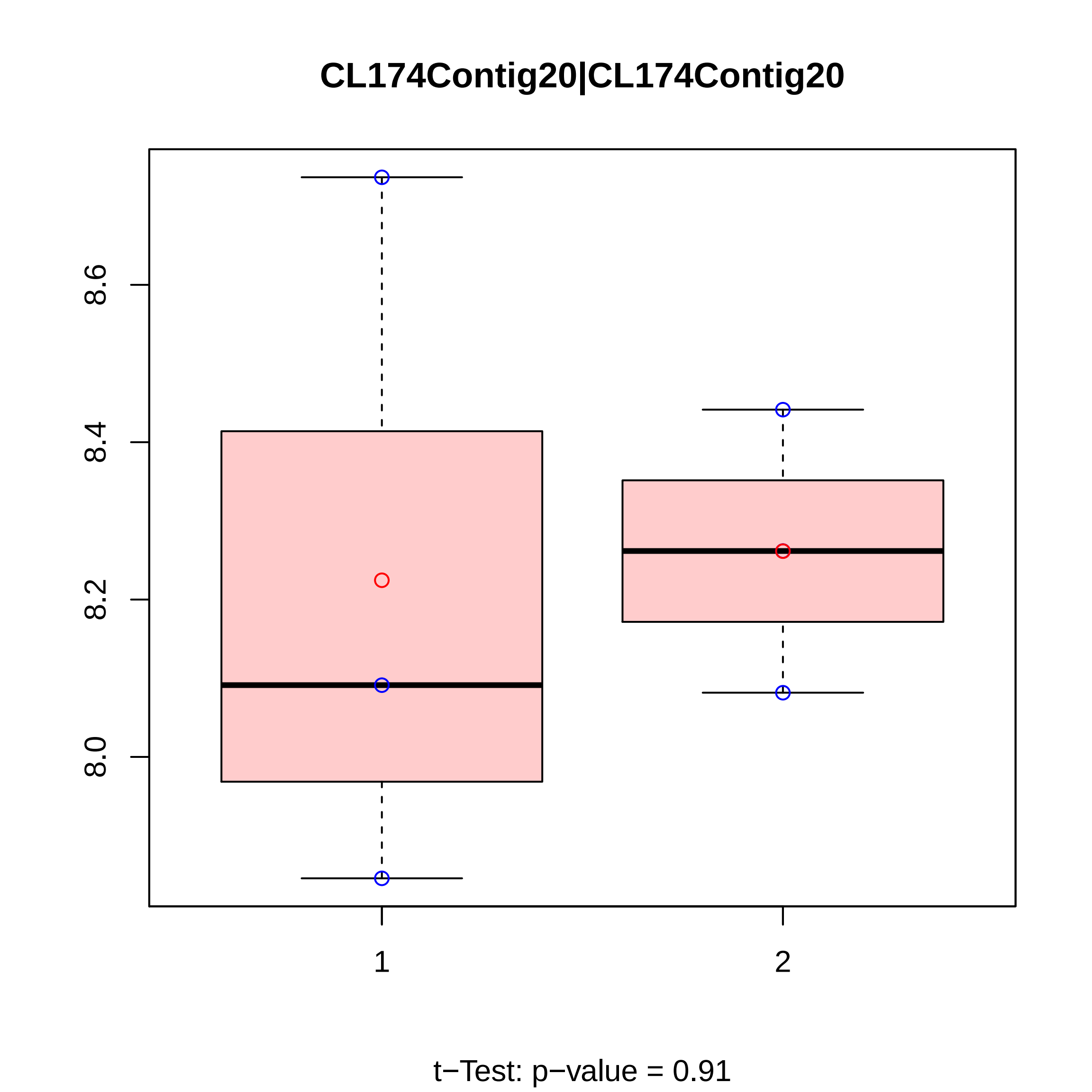
CL174Contig20|CL174Contig20
8.0
8.2
8.4
8.6
1
2
t−Test: p−value = 0.91

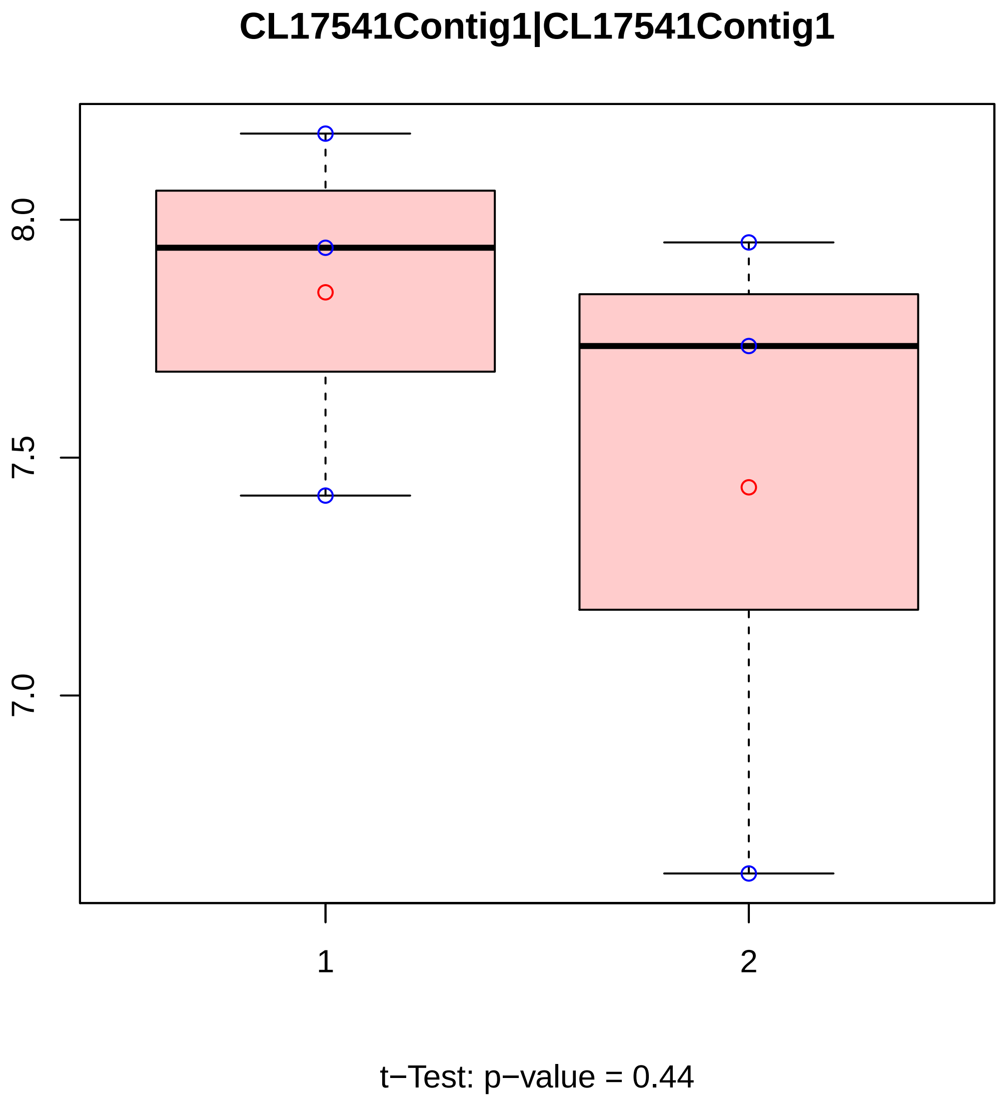

CL17541Contig1|CL17541Contig1
8.0
7.5
7.0
1
2
t−Test: p−value = 0.44

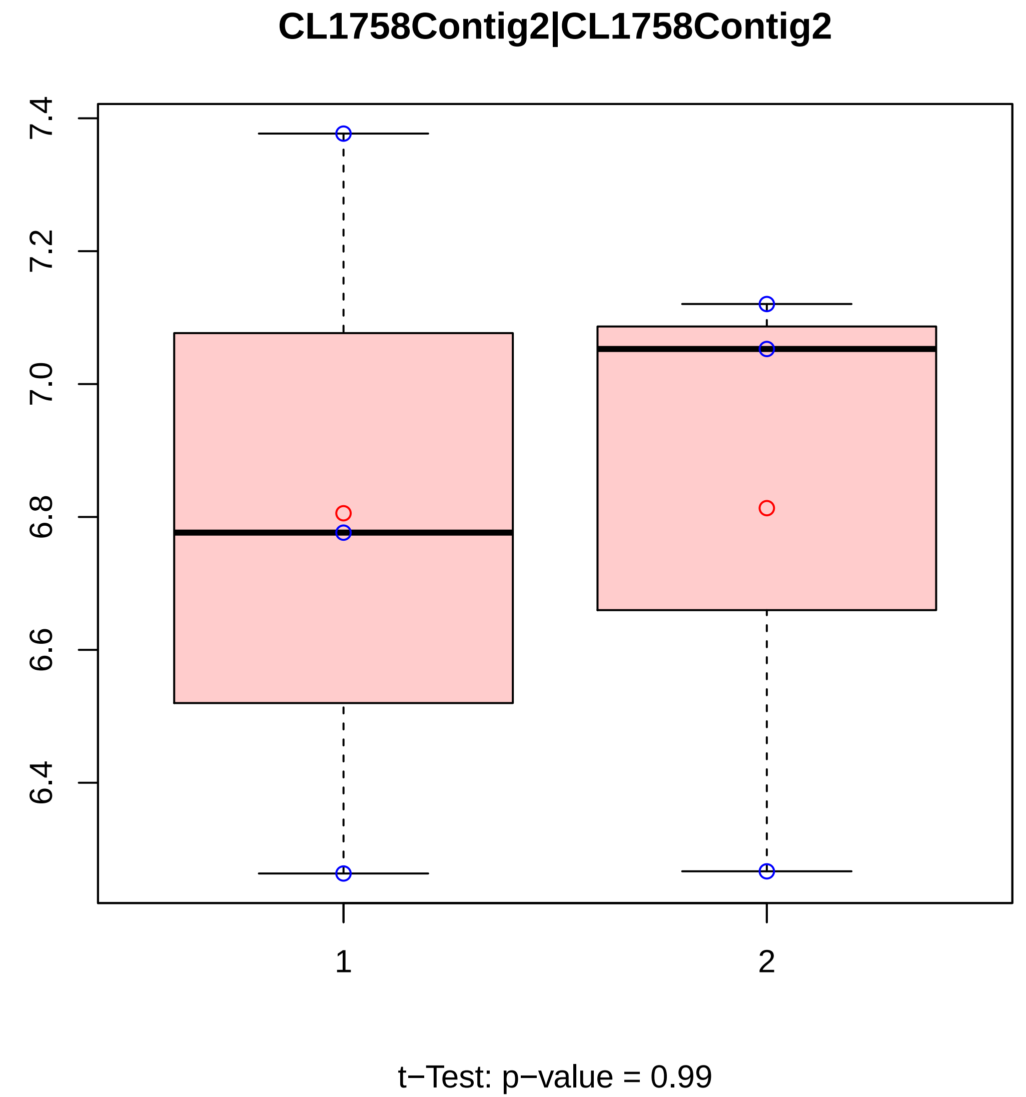
CL1758Contig2|CL1758Contig2
7.4
7.2
7.0
6.8
6.6
6.4
1
2
t−Test: p−value = 0.99

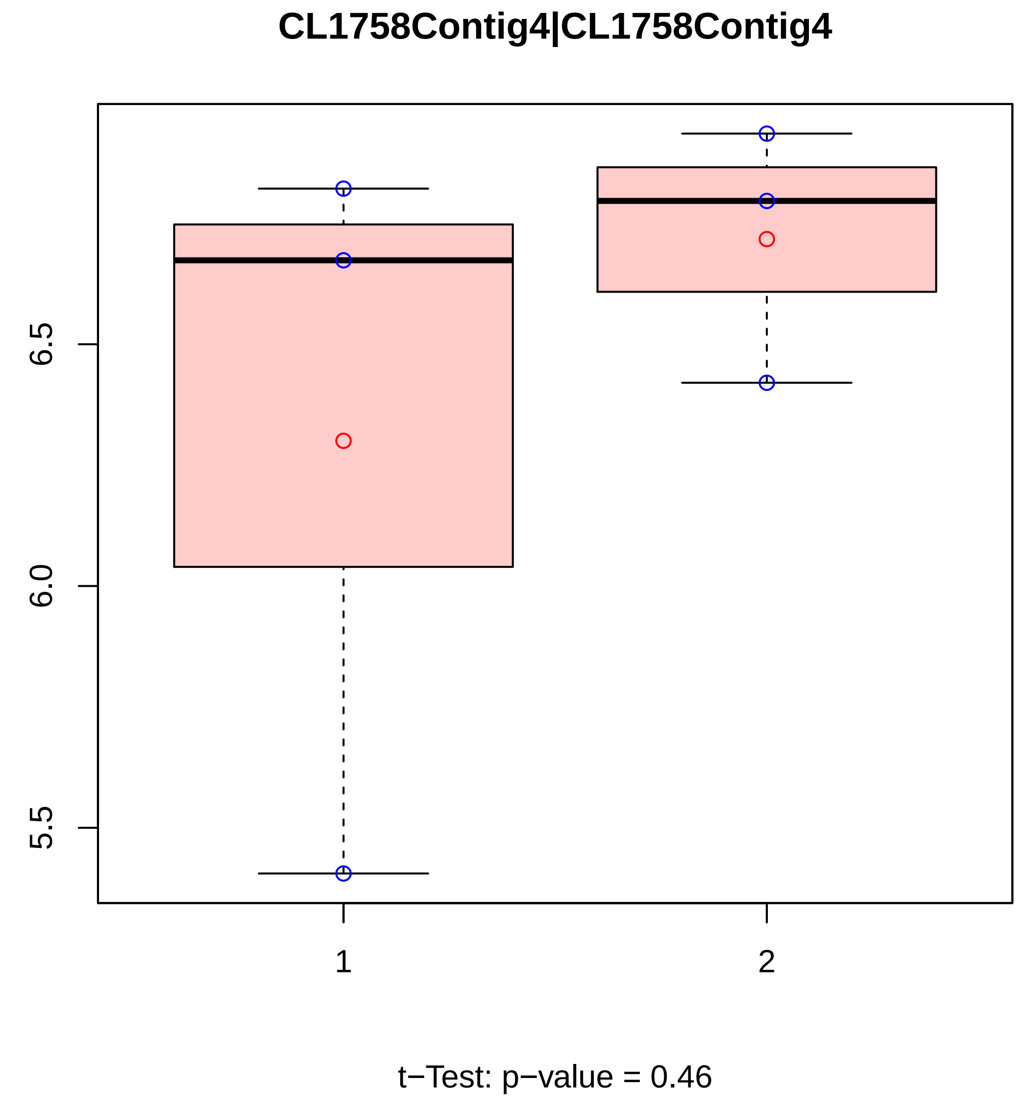
CL1758Contig4|CL1758Contig4
6.5
6.0
5.5
1
2
t−Test: p−value = 0.46

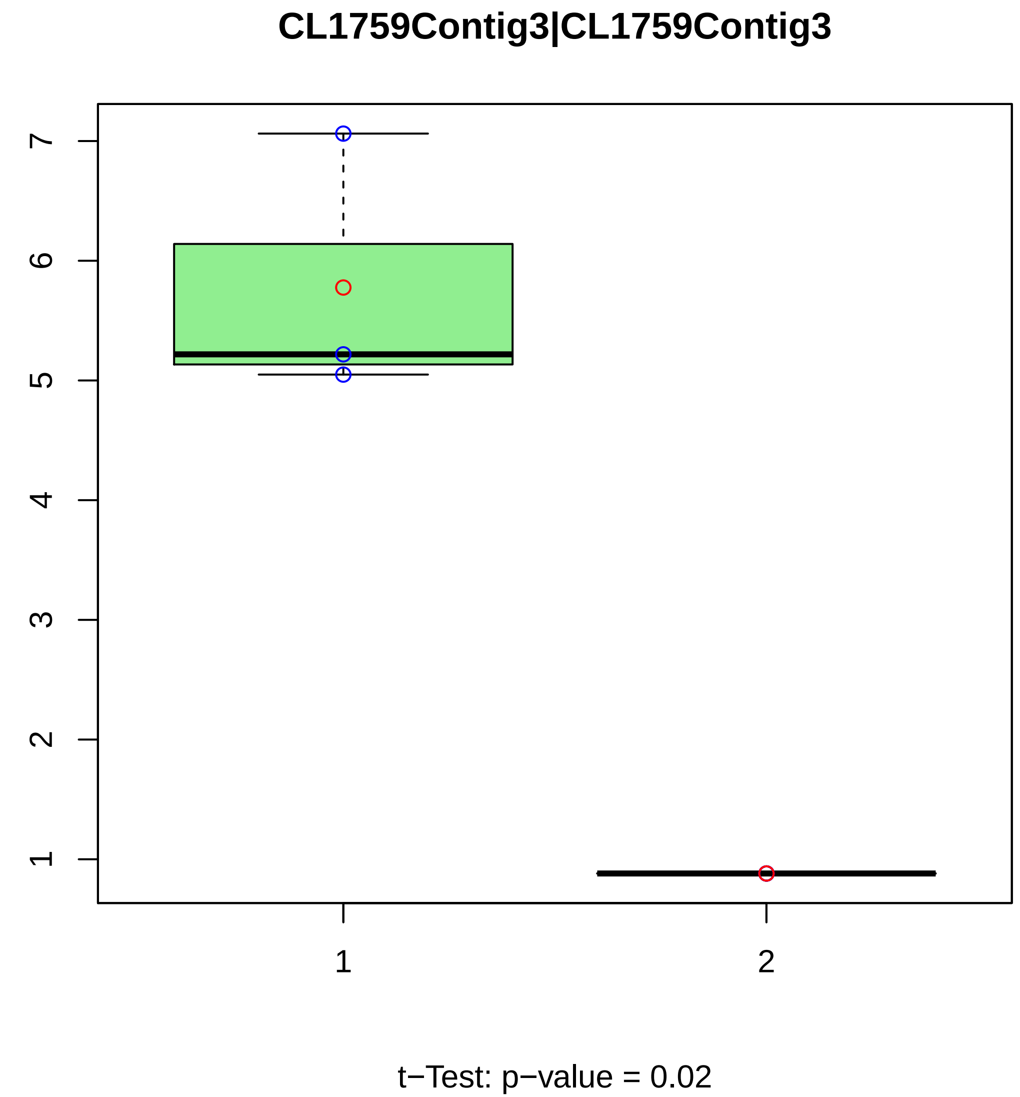
CL1759Contig3|CL1759Contig3
1
2
3
4
5
6
7
1
2
t−Test: p−value = 0.02

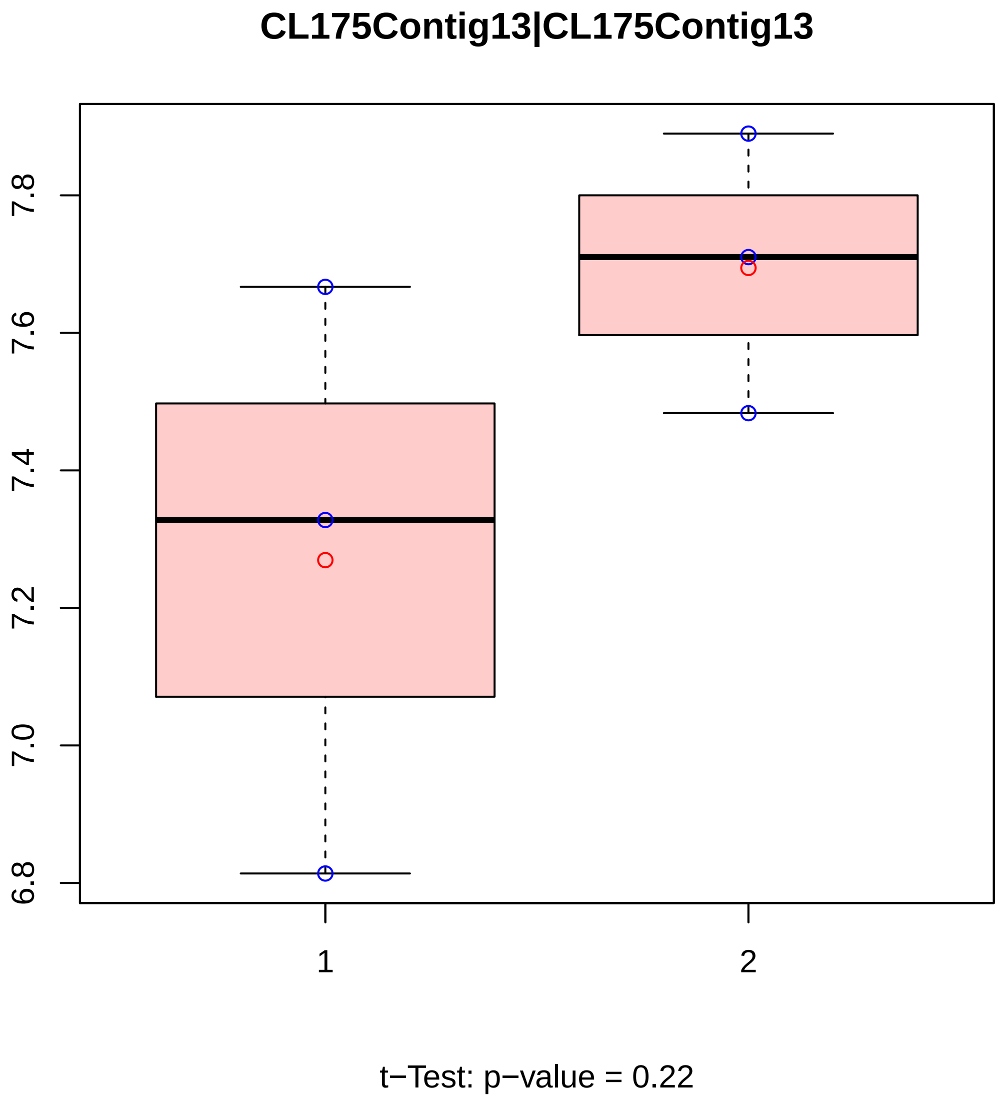
CL175Contig13|CL175Contig13
7.8
7.6
7.4
7.2
7.0
6.8
1
2
t−Test: p−value = 0.22

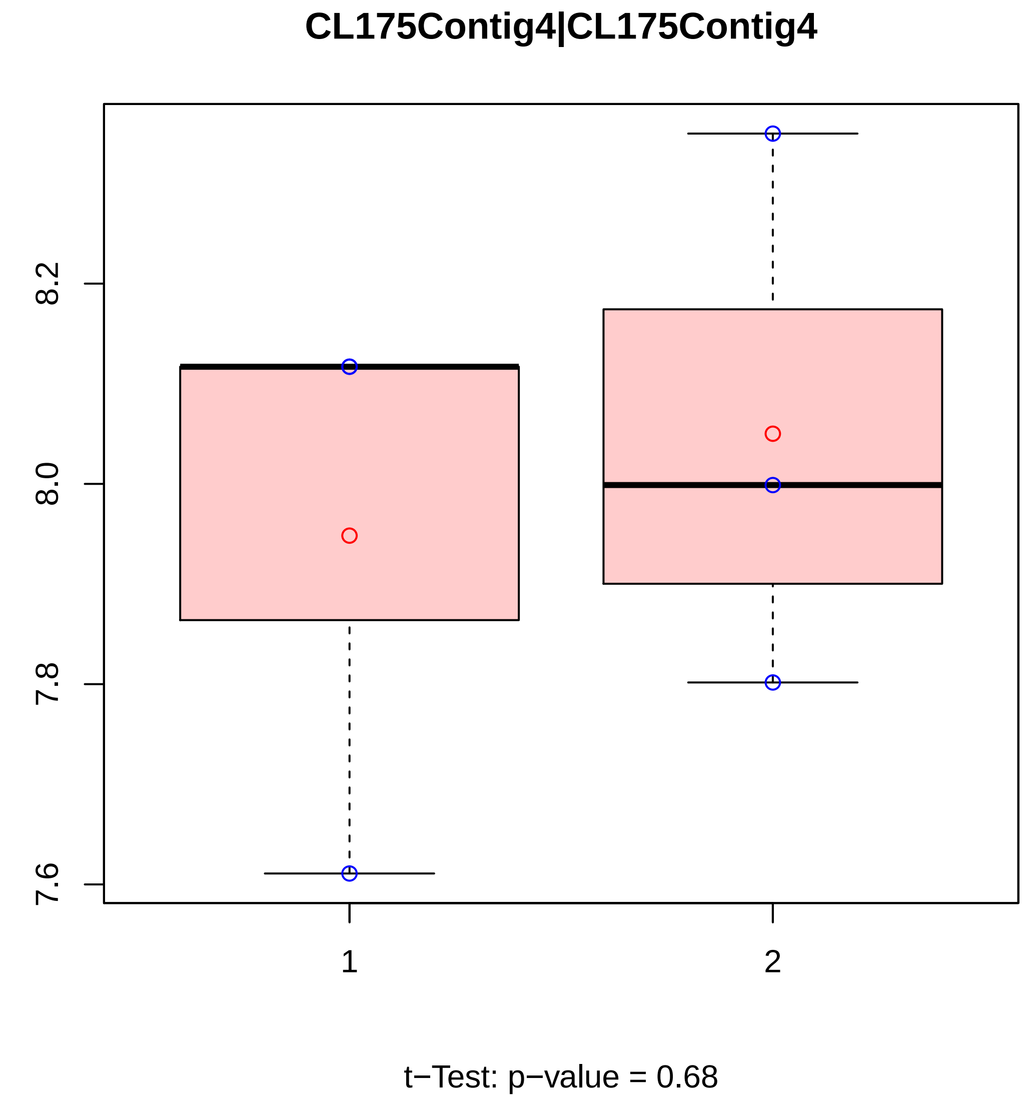
CL175Contig4|CL175Contig4
8.2
8.0
7.8
7.6
1
2
t−Test: p−value = 0.68

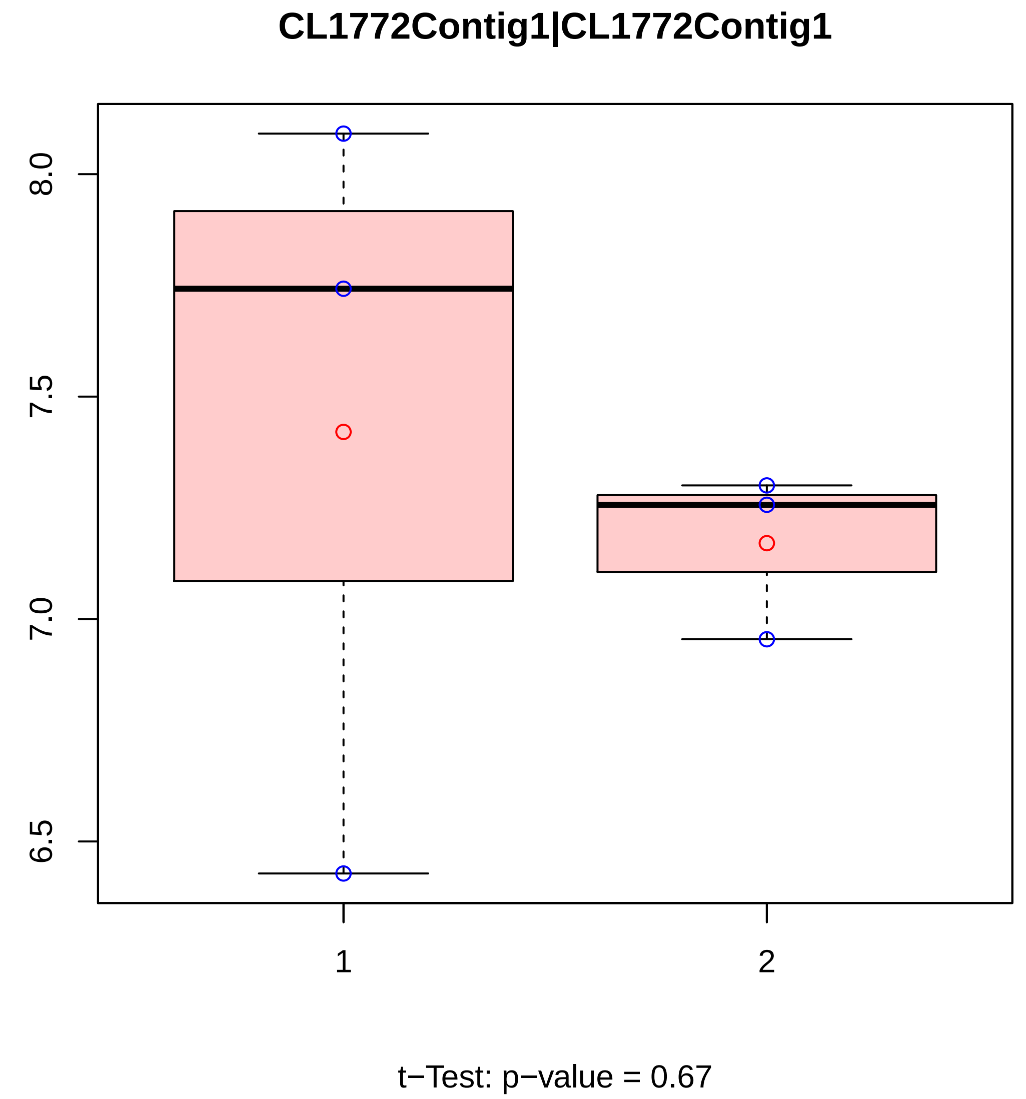
CL1772Contig1|CL1772Contig1
8.0
7.5
7.0
6.5
1
2
t−Test: p−value = 0.67

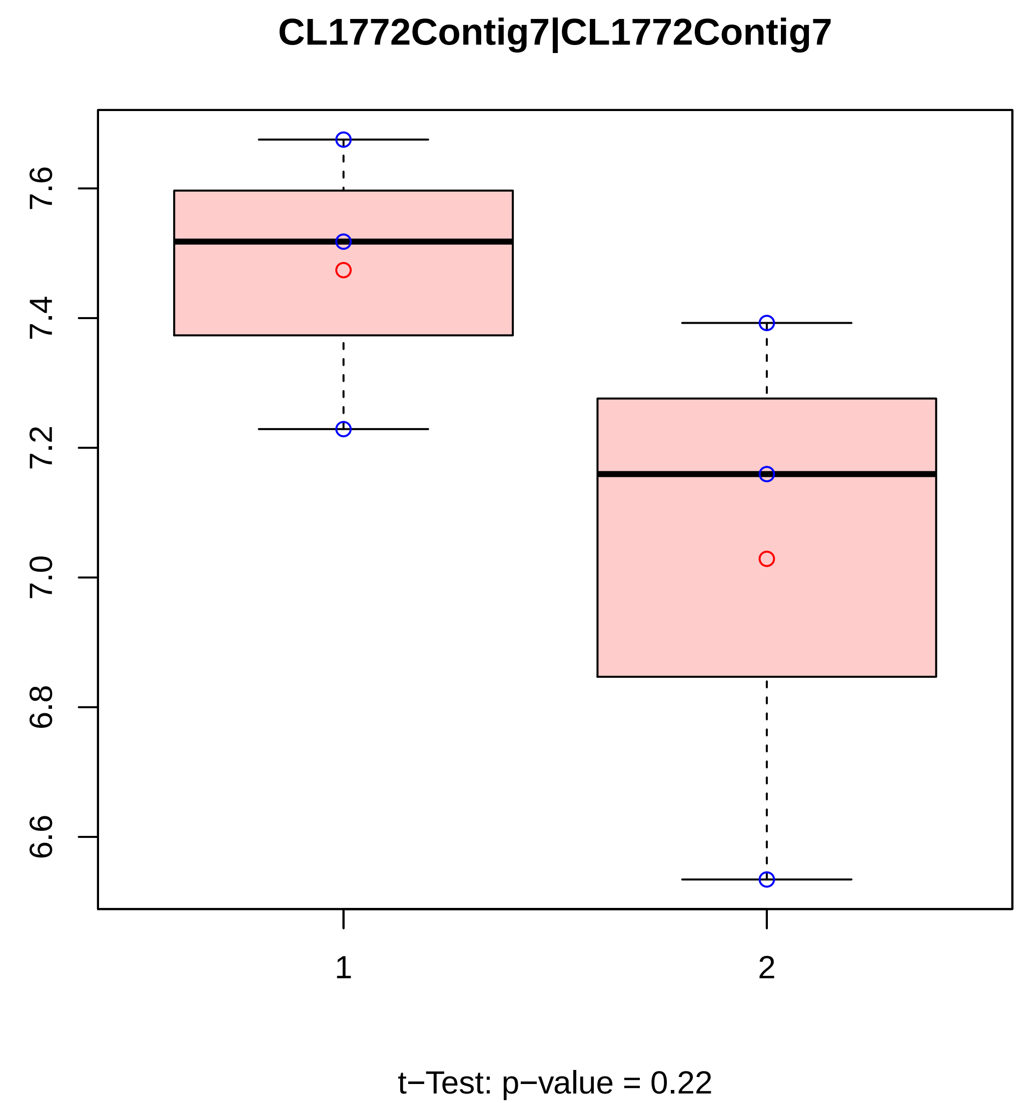

CL1772Contig7|CL1772Contig7
7.6
7.4
7.2
7.0
6.8
6.6
1
2
t−Test: p−value = 0.22

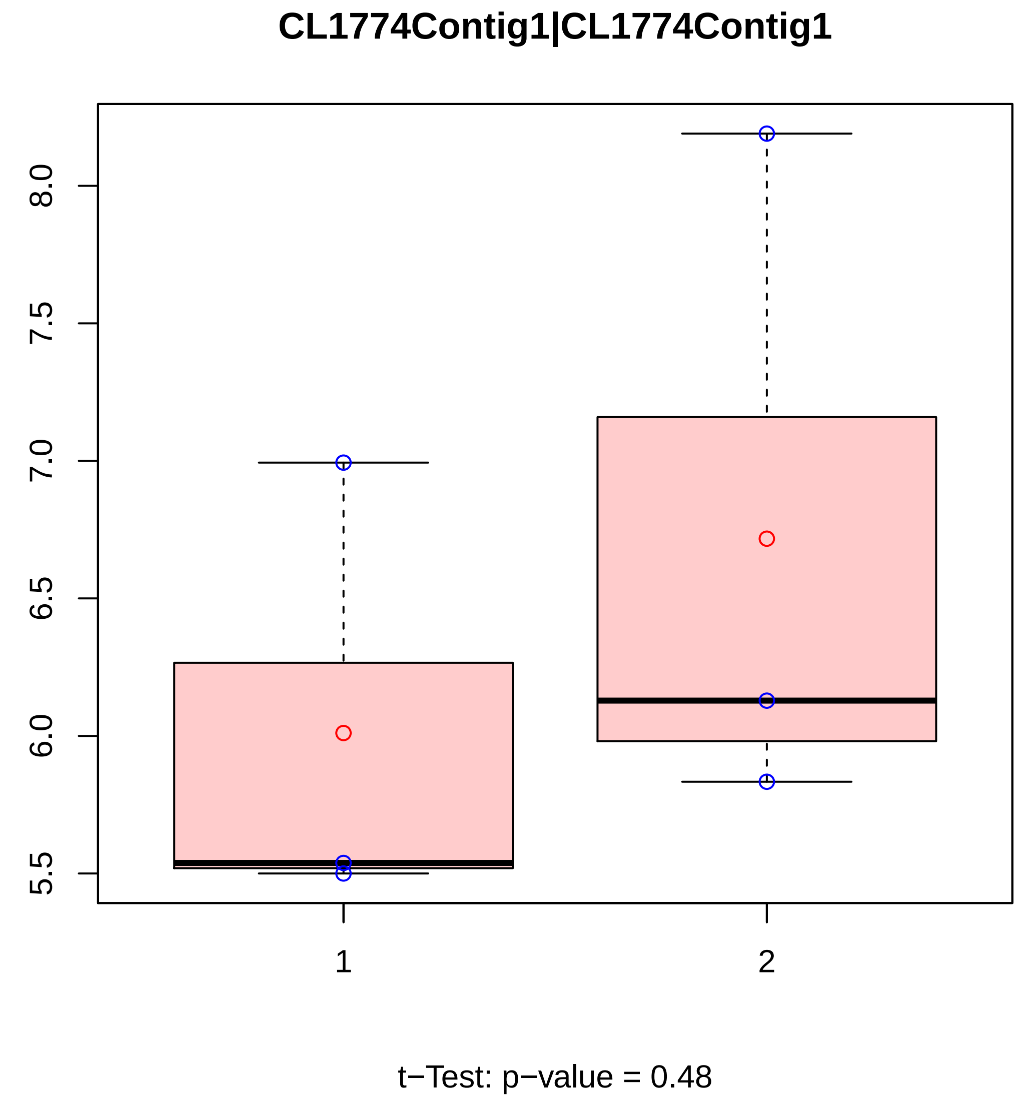
CL1774Contig1|CL1774Contig1
8.0
7.5
7.0
6.5
6.0
5.5
1
2
t−Test: p−value = 0.48

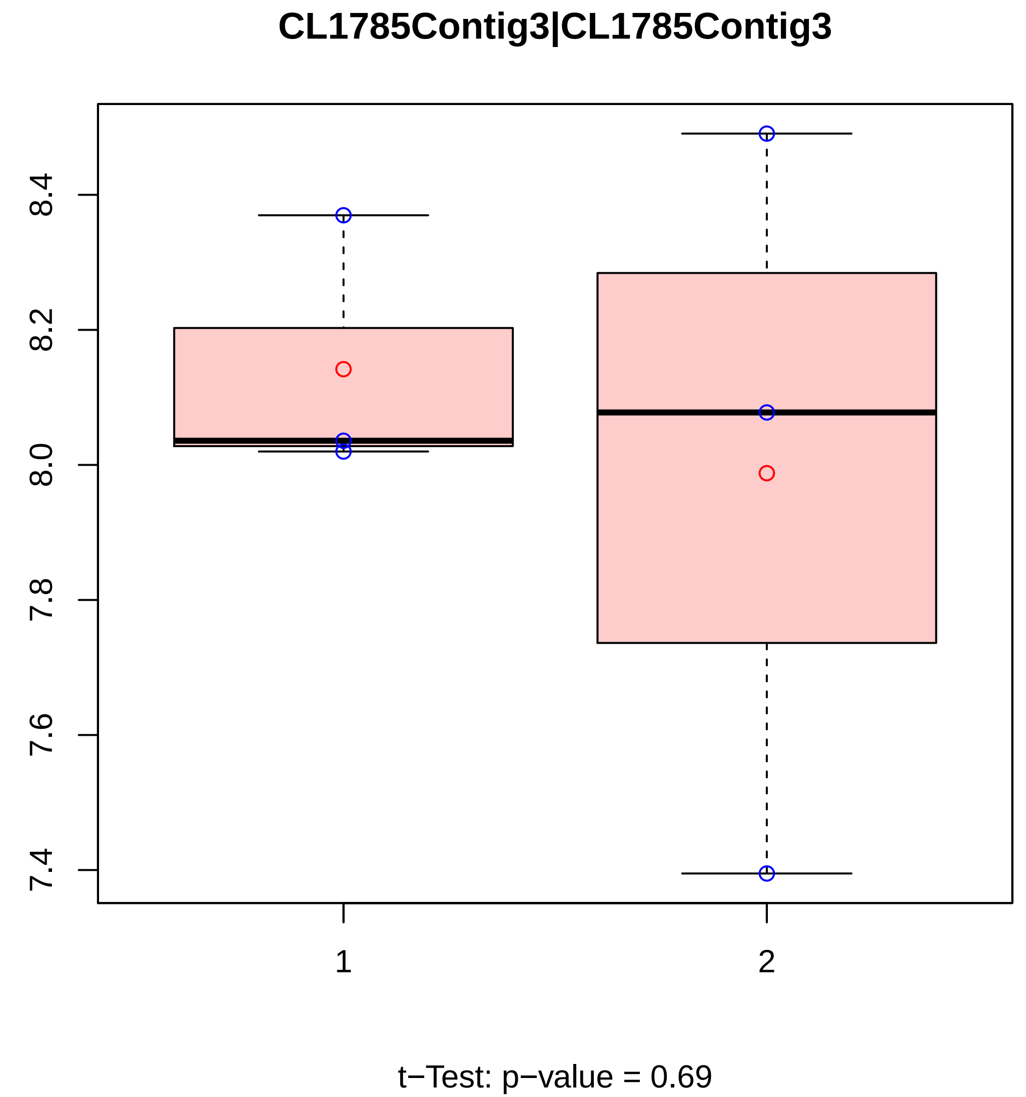
CL1785Contig3|CL1785Contig3
8.4
8.2
8.0
7.8
7.6
7.4
1
2
t−Test: p−value = 0.69

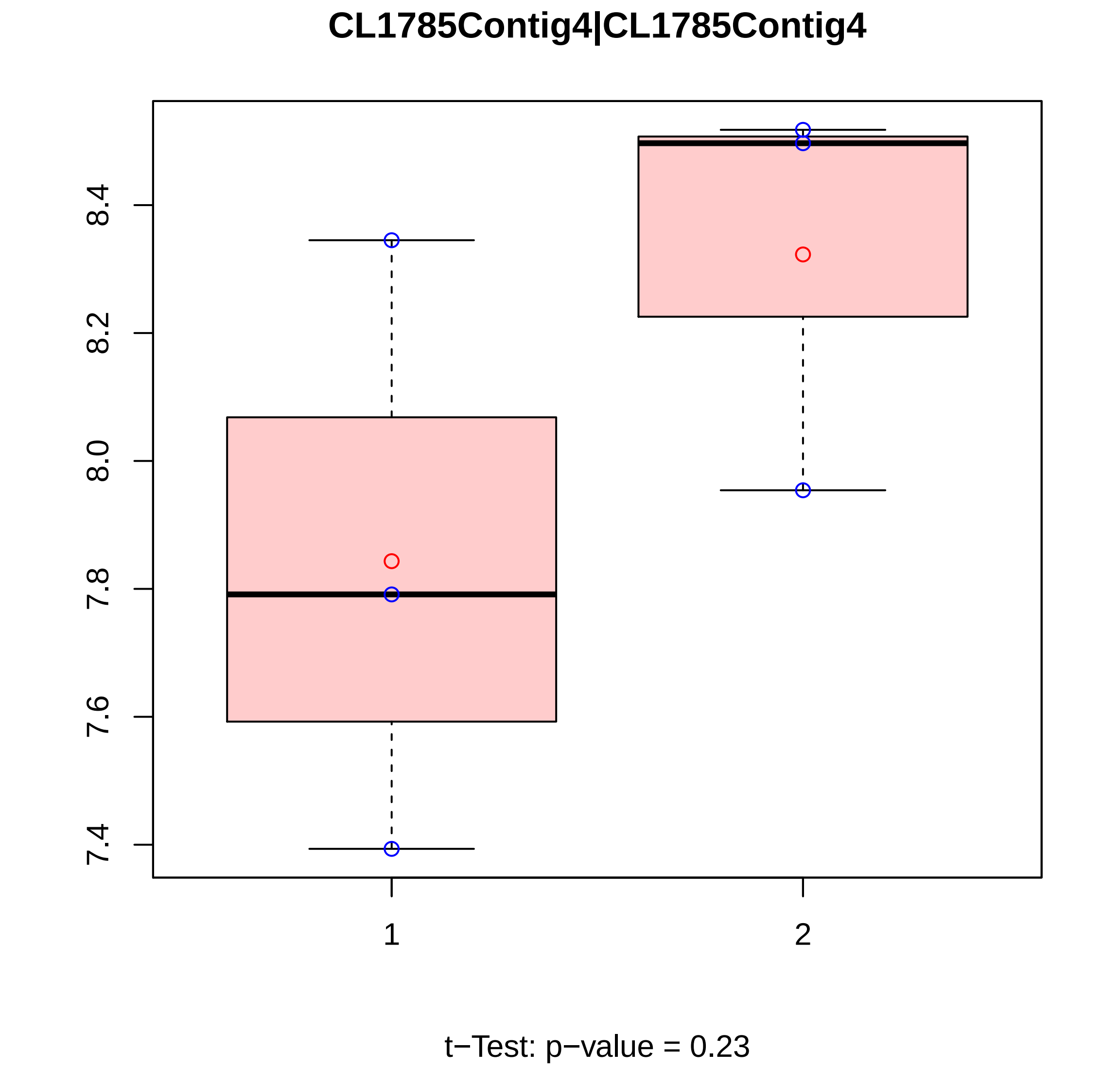
CL1785Contig4|CL1785Contig4
8.4
8.2
8.0
7.8
7.6
7.4
1
2
t−Test: p−value = 0.23

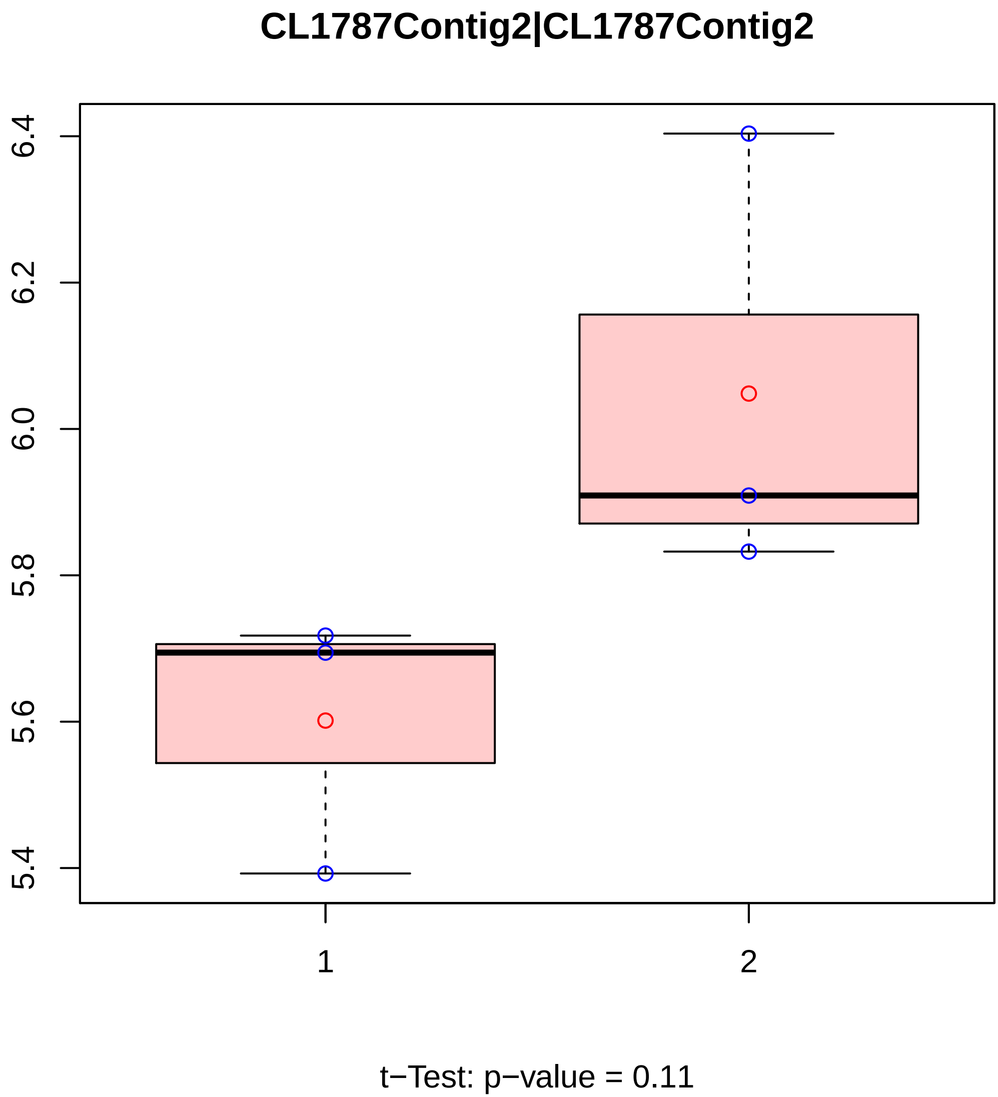
CL1787Contig2|CL1787Contig2
6.4
6.2
6.0
5.8
5.6
5.4
1
2
t−Test: p−value = 0.11

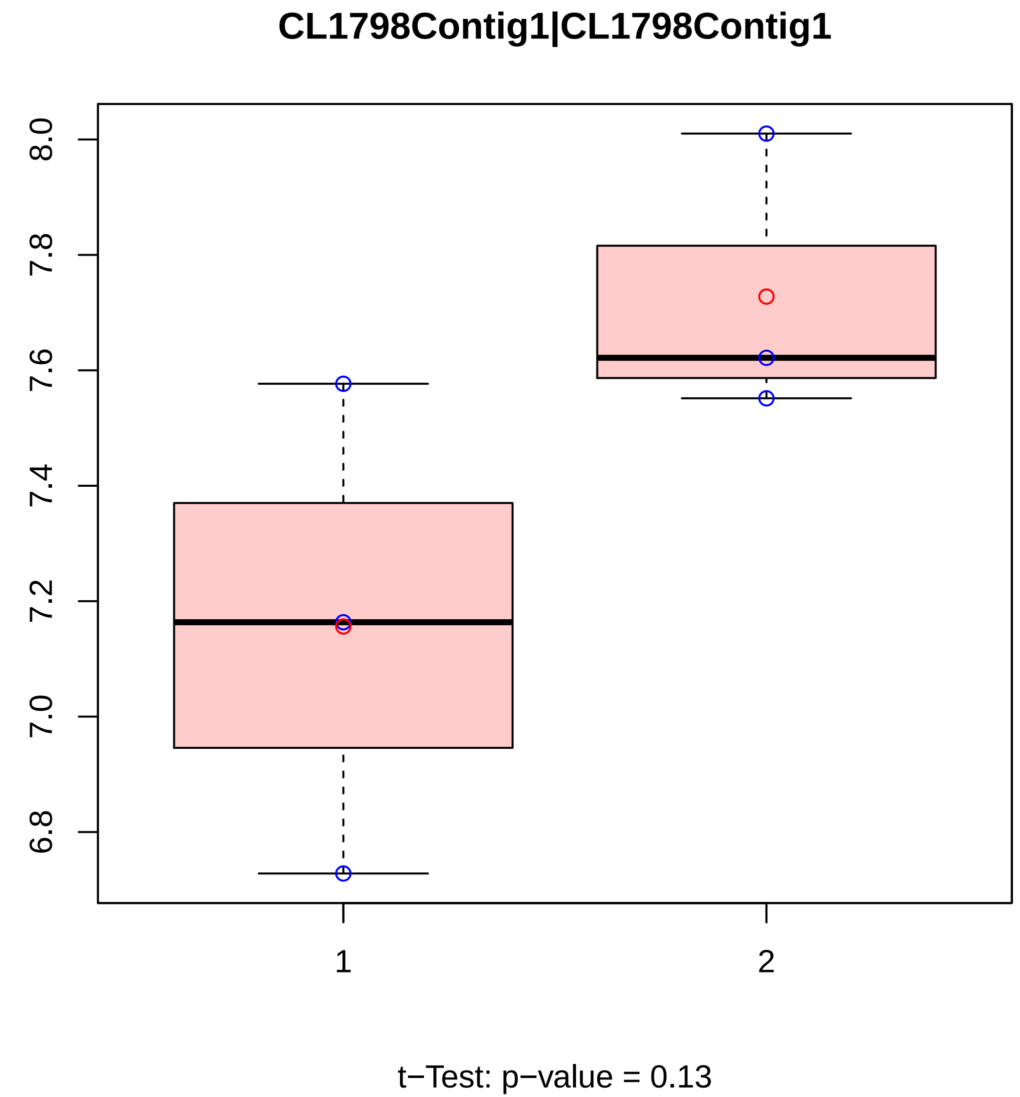
CL1798Contig1|CL1798Contig1
8.0
7.8
7.6
7.4
7.2
7.0
6.8
1
2
t−Test: p−value = 0.13

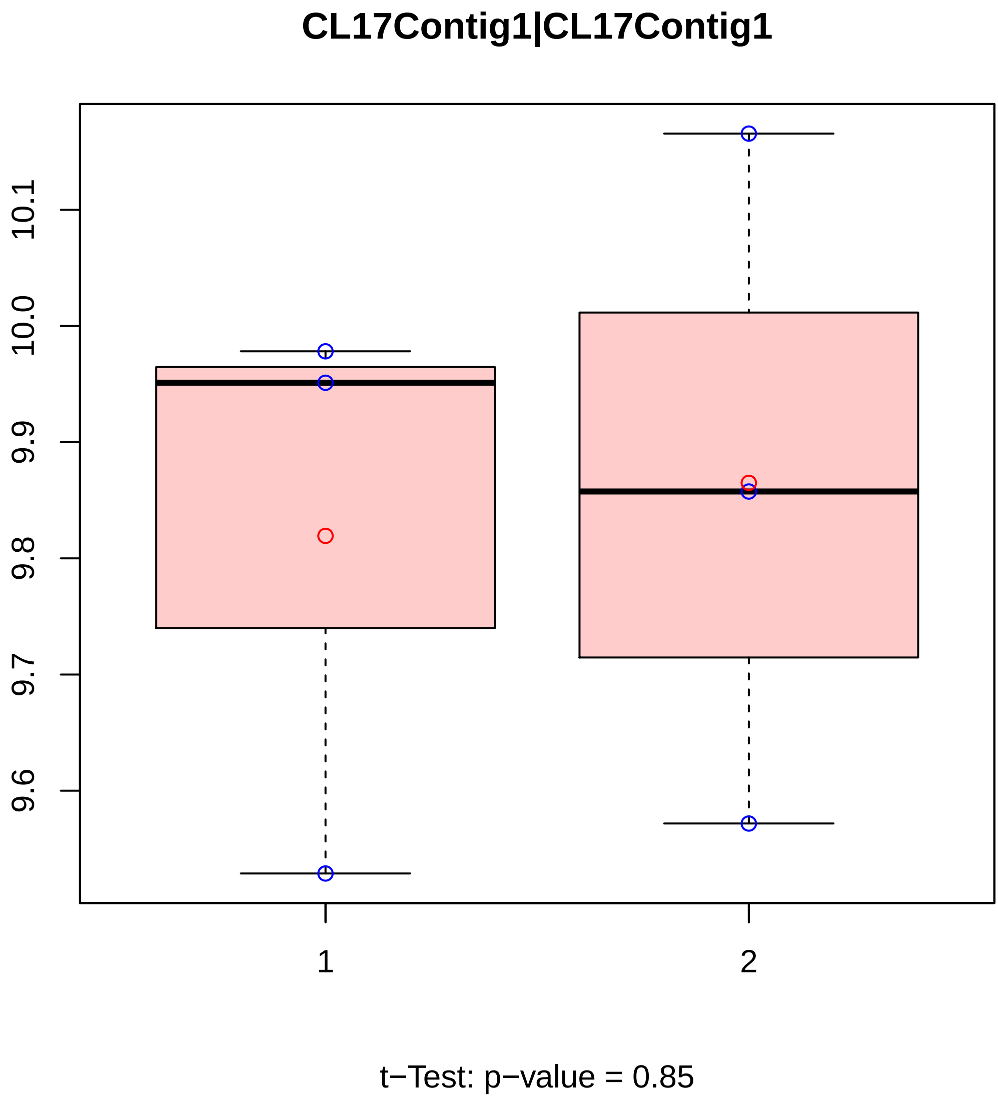
CL17Contig1|CL17Contig1
10.1
10.0
9.9
9.8
9.7
9.6
1
2
t−Test: p−value = 0.85

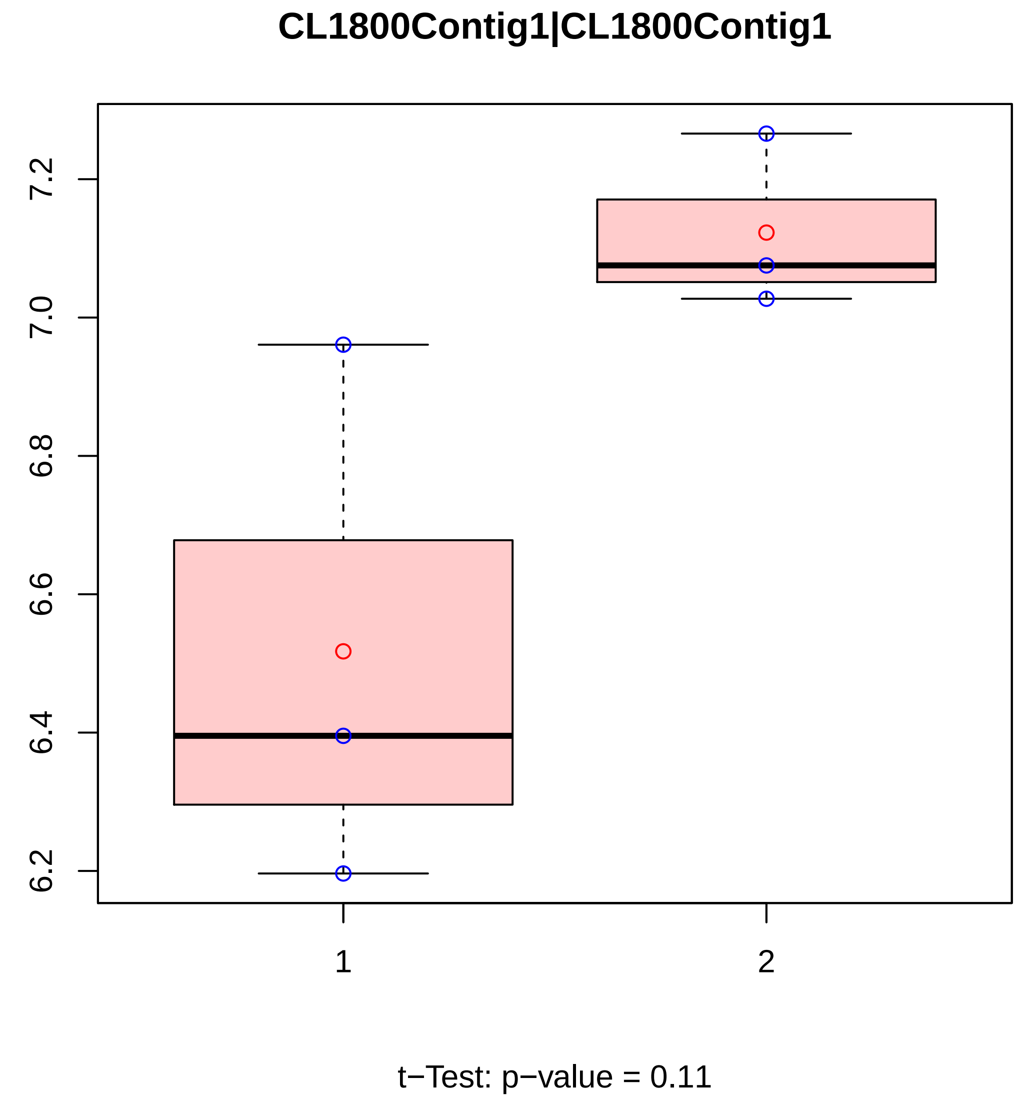
CL1800Contig1|CL1800Contig1
7.2
7.0
6.8
6.6
6.4
6.2
1
2
t−Test: p−value = 0.11

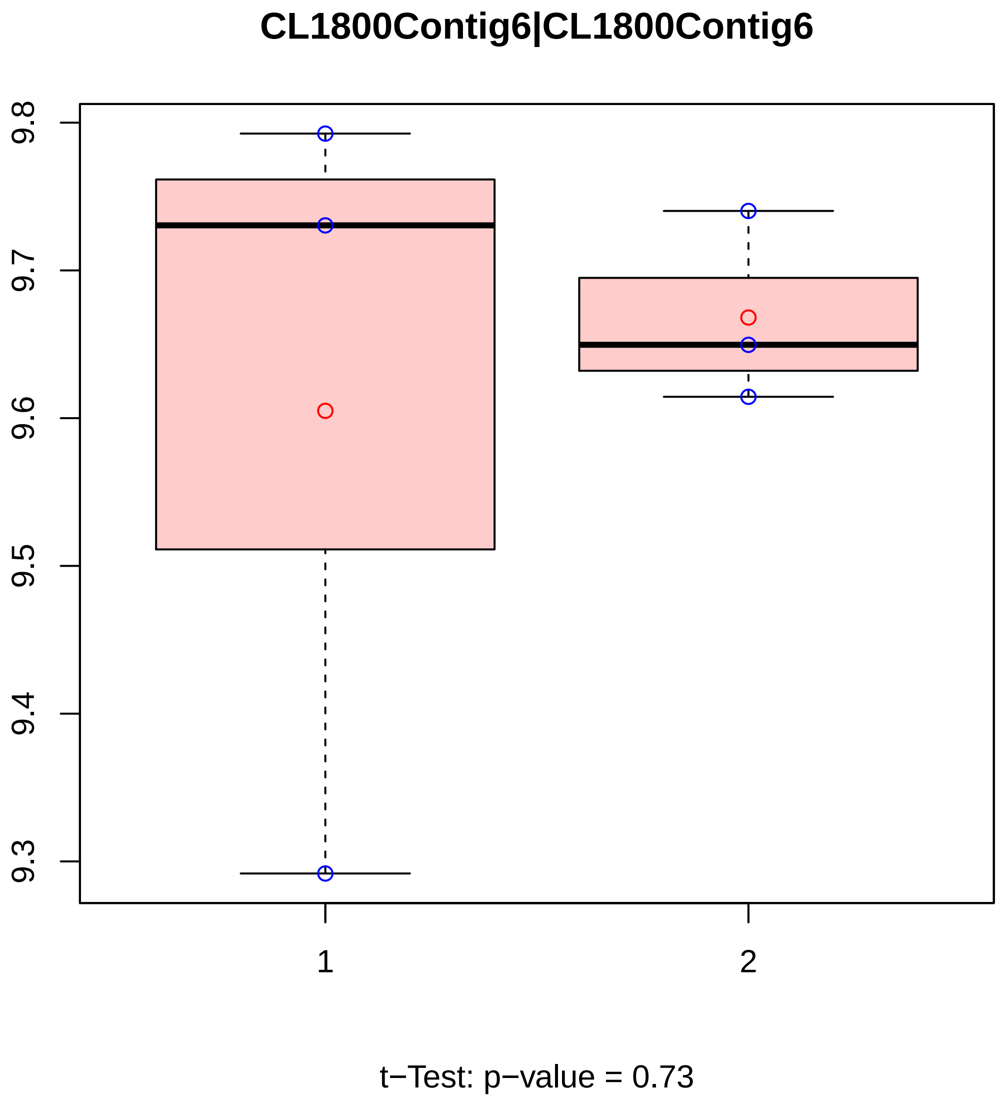

CL1800Contig6|CL1800Contig6
9.3
9.4
9.5
9.6
9.7
9.8
1
2
t−Test: p−value = 0.73

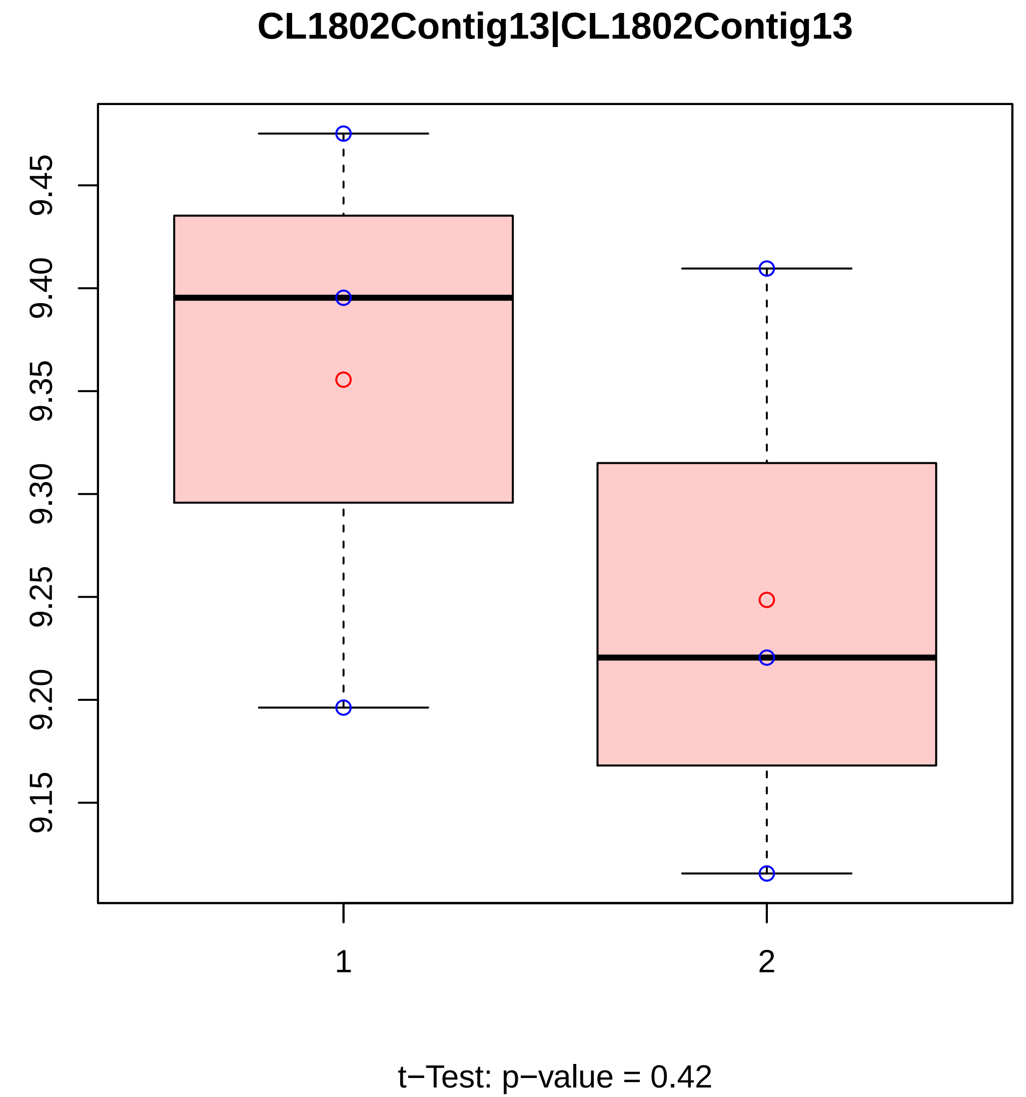

CL1802Contig13|CL1802Contig13
9.15
9.20
9.25
9.30
9.35
9.40
9.45
1
2
t−Test: p−value = 0.42

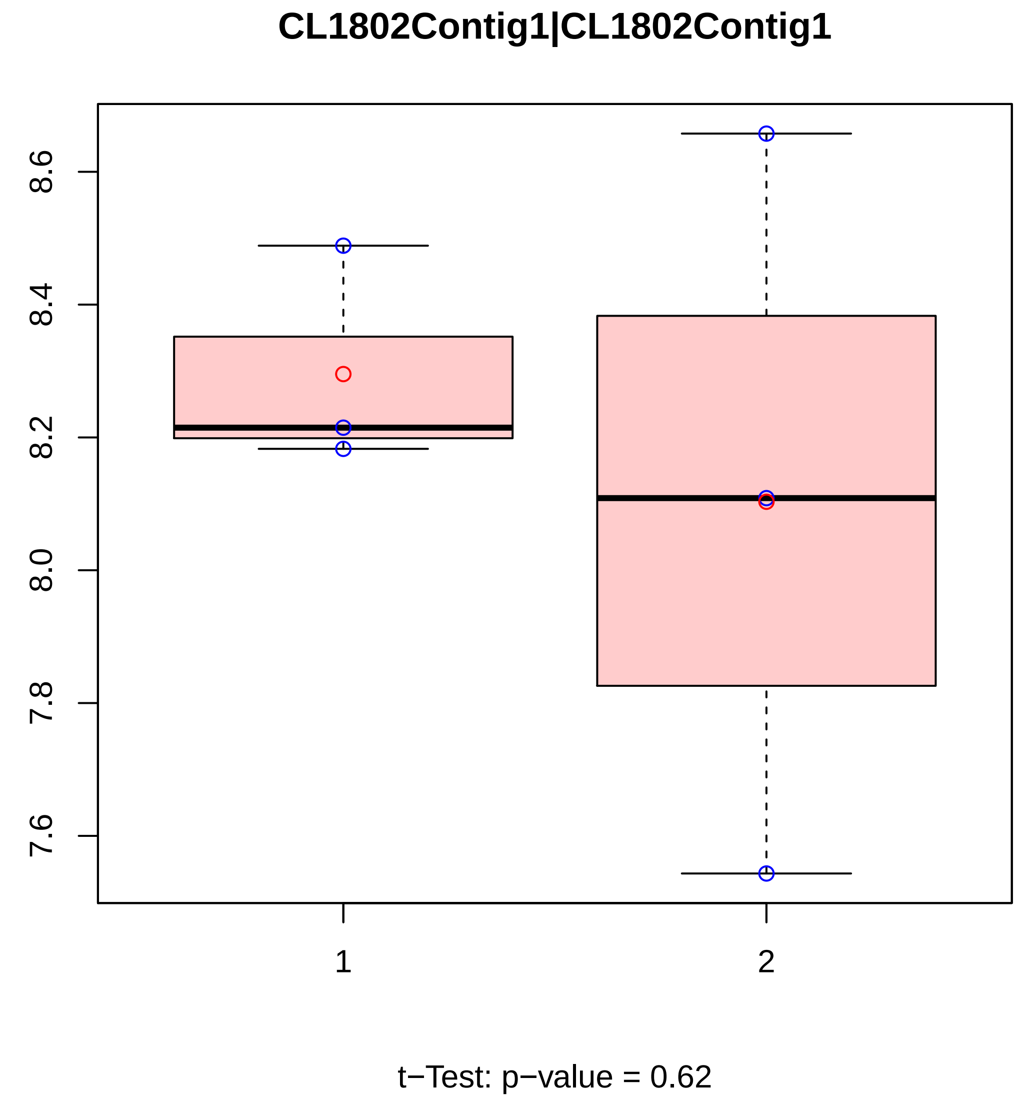
CL1802Contig1|CL1802Contig1
8.6
8.4
8.2
8.0
7.8
7.6
1
2
t−Test: p−value = 0.62

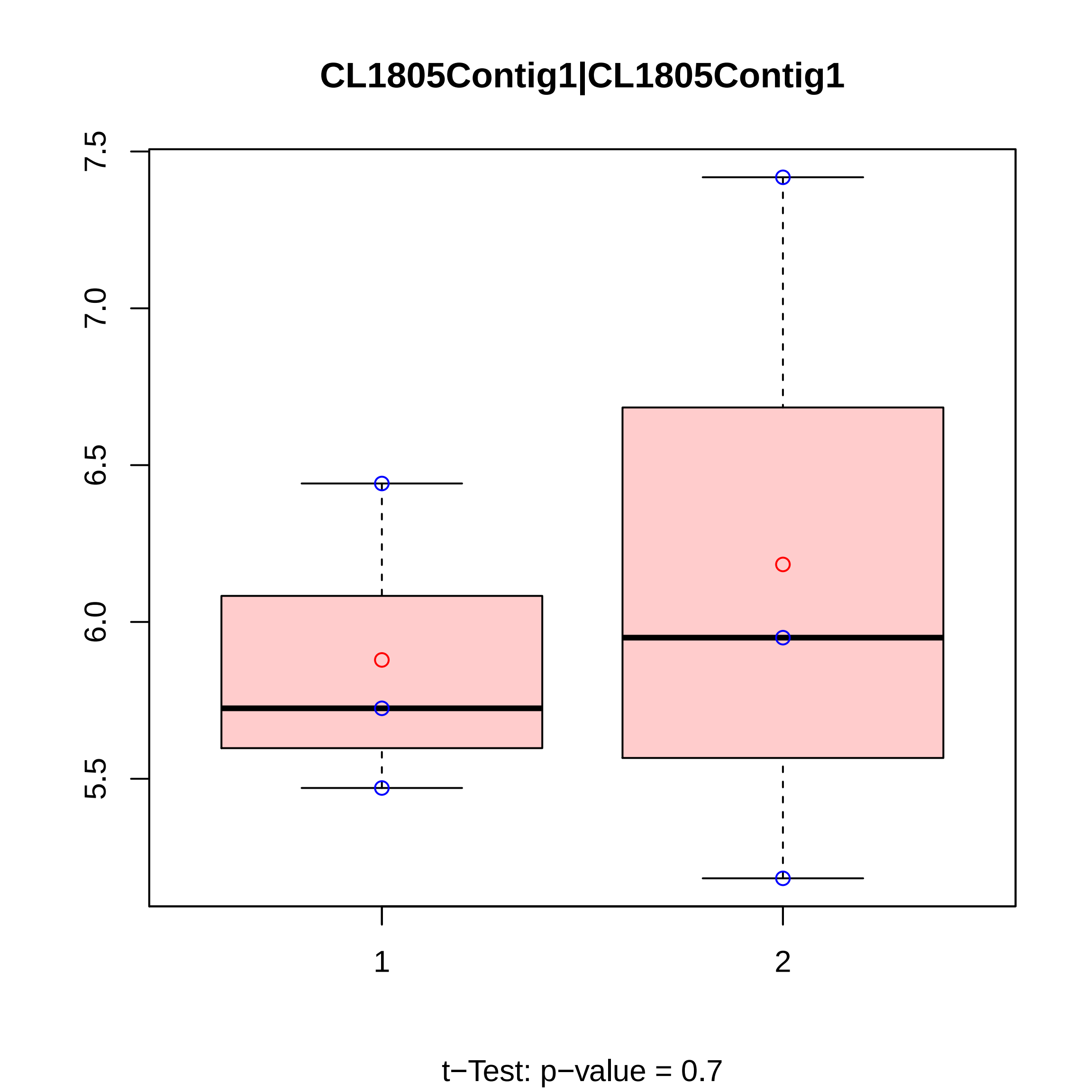
CL1805Contig1|CL1805Contig1
7.5
7.0
6.5
6.0
5.5
1
2
t−Test: p−value = 0.7

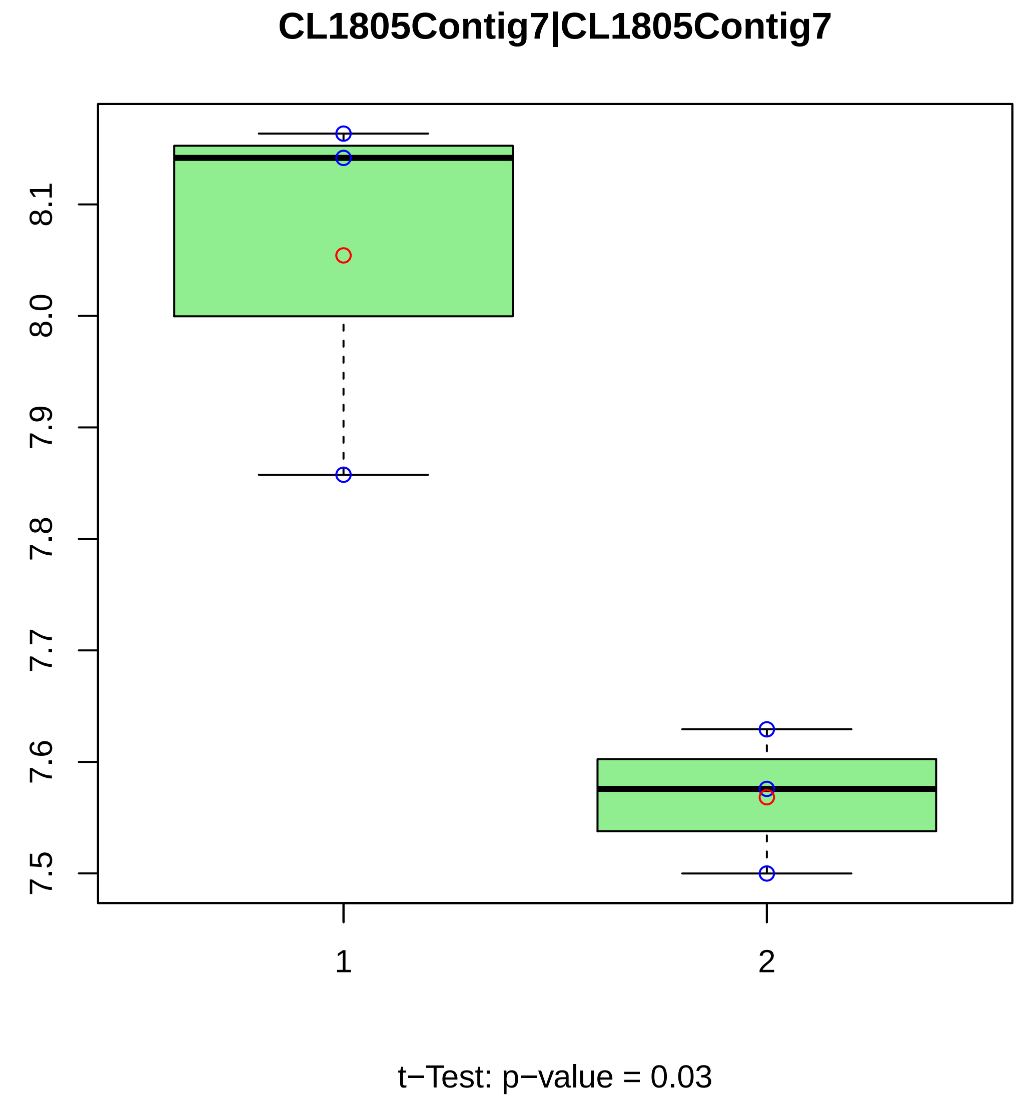
CL1805Contig7|CL1805Contig7
8.1
8.0
7.9
7.8
7.7
7.6
7.5
1
2
t−Test: p−value = 0.03

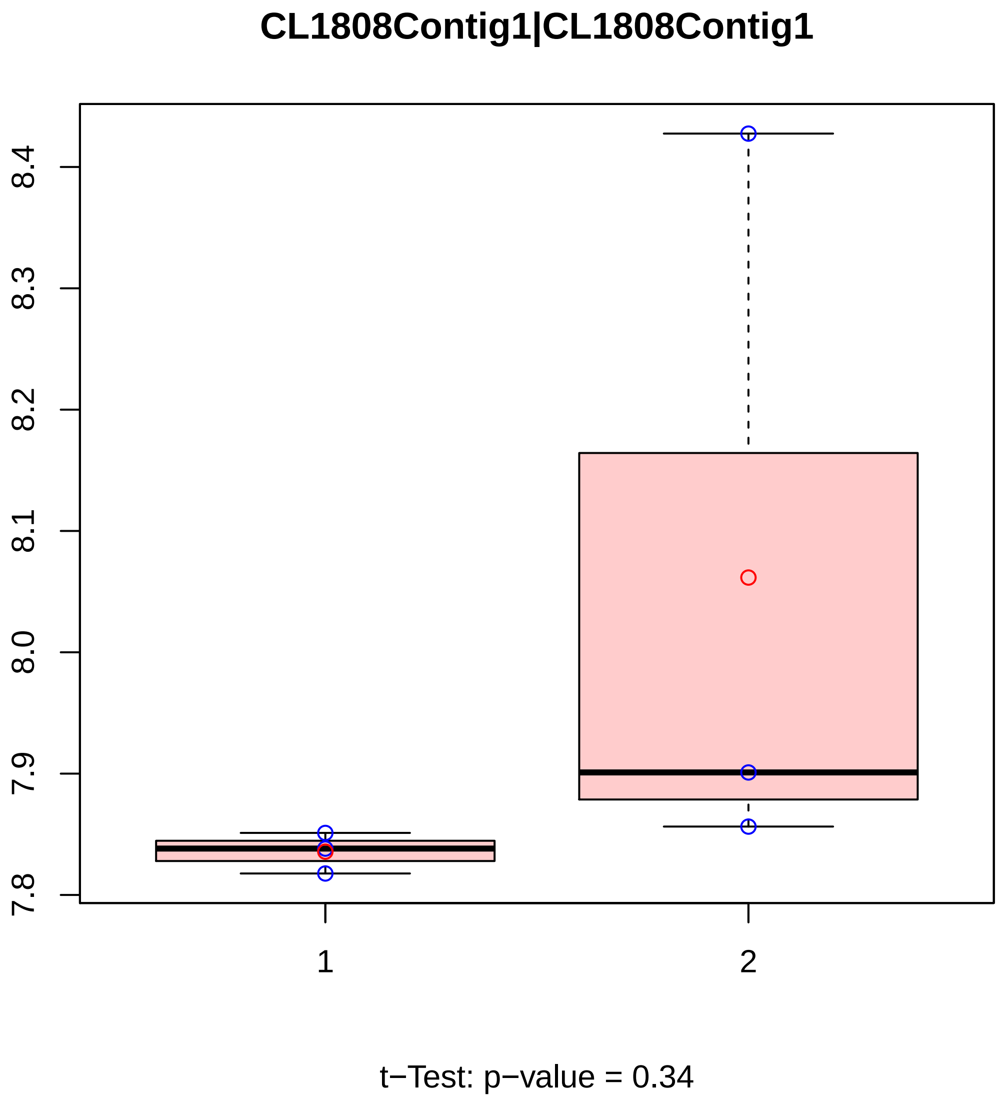
CL1808Contig1|CL1808Contig1
8.4
8.3
8.2
8.1
8.0
7.9
7.8
1
2
t−Test: p−value = 0.34

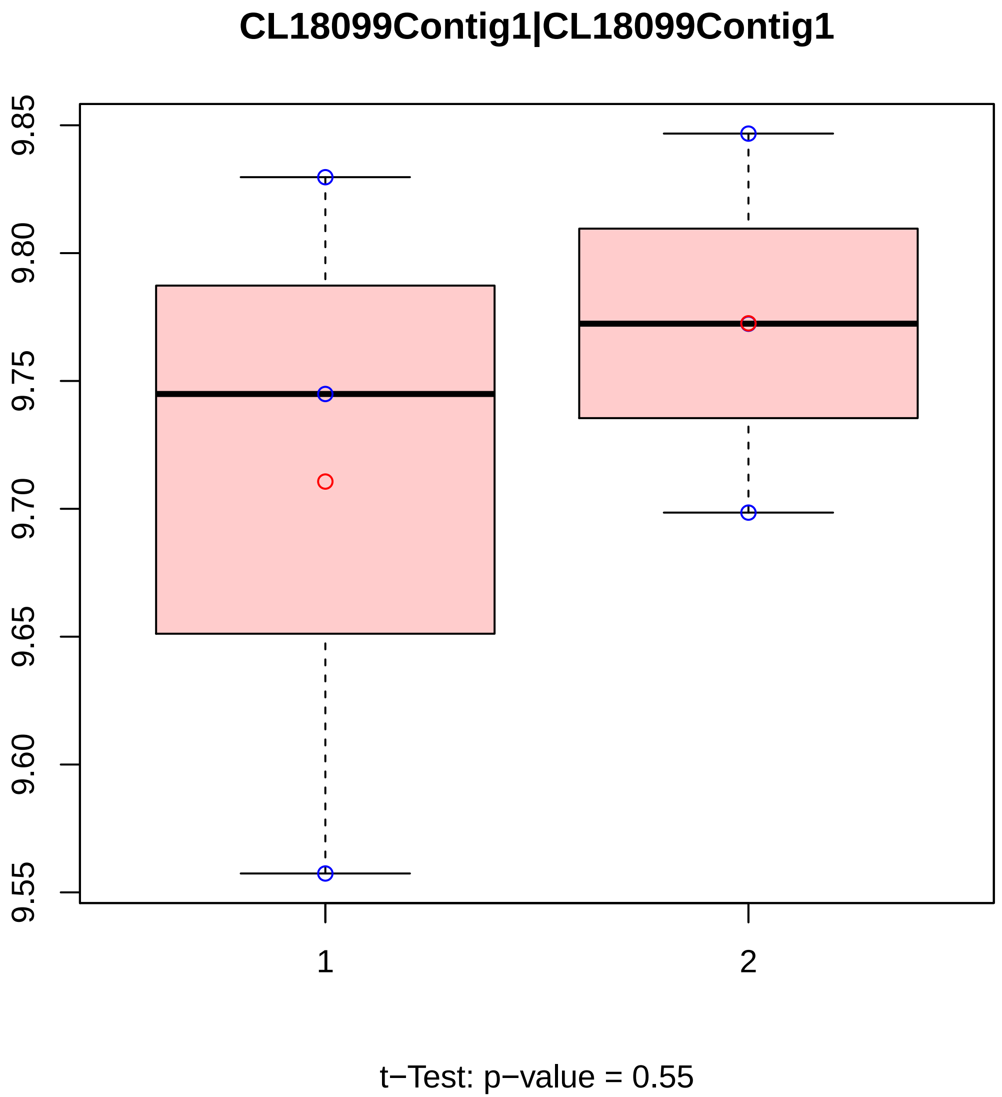
CL18099Contig1|CL18099Contig1
9.55
9.60
9.65
9.70
9.75
9.80
9.85
1
2
t−Test: p−value = 0.55

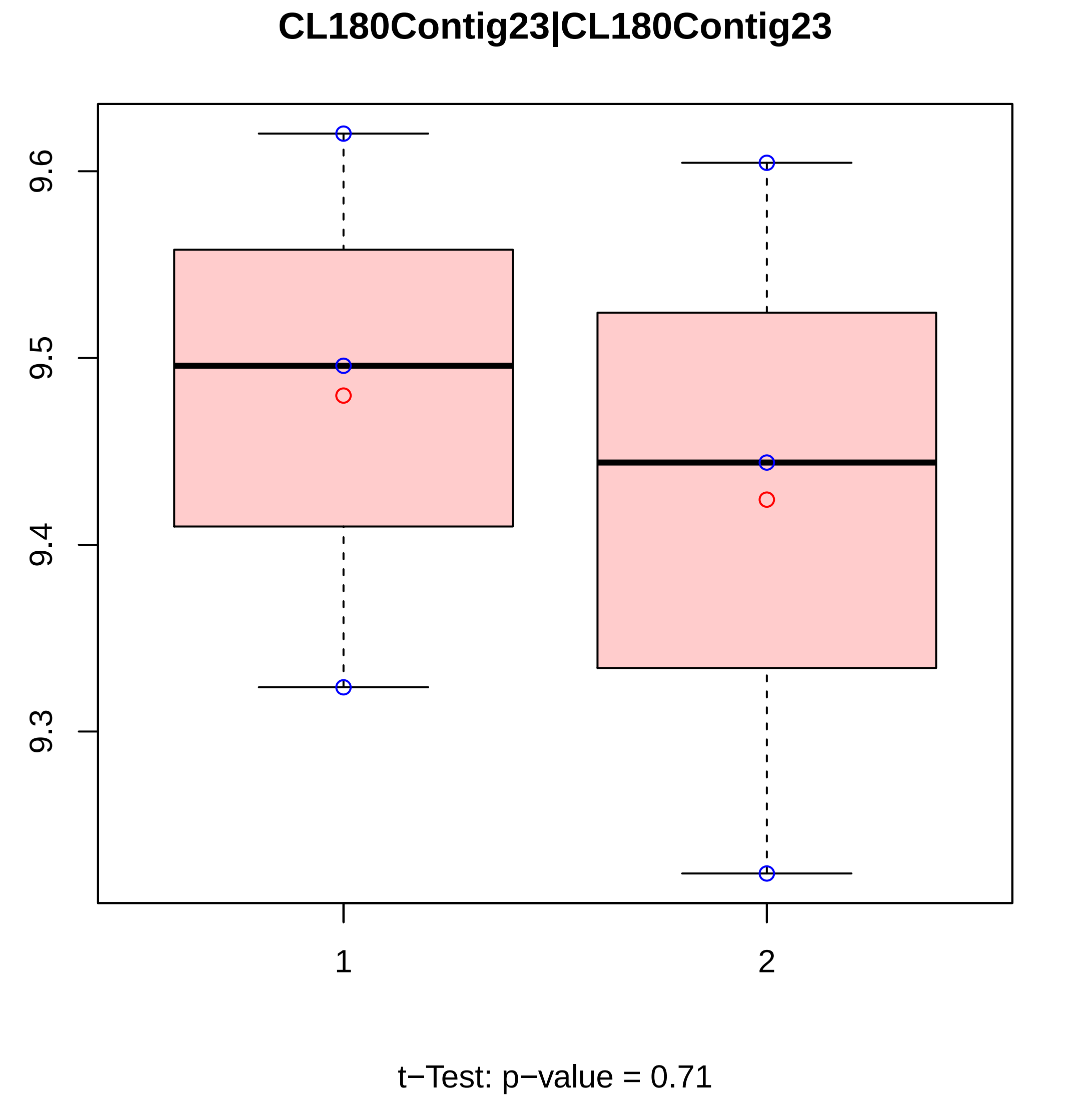
CL180Contig23|CL180Contig23
9.3
9.4
9.5
9.6
1
2
t−Test: p−value = 0.71

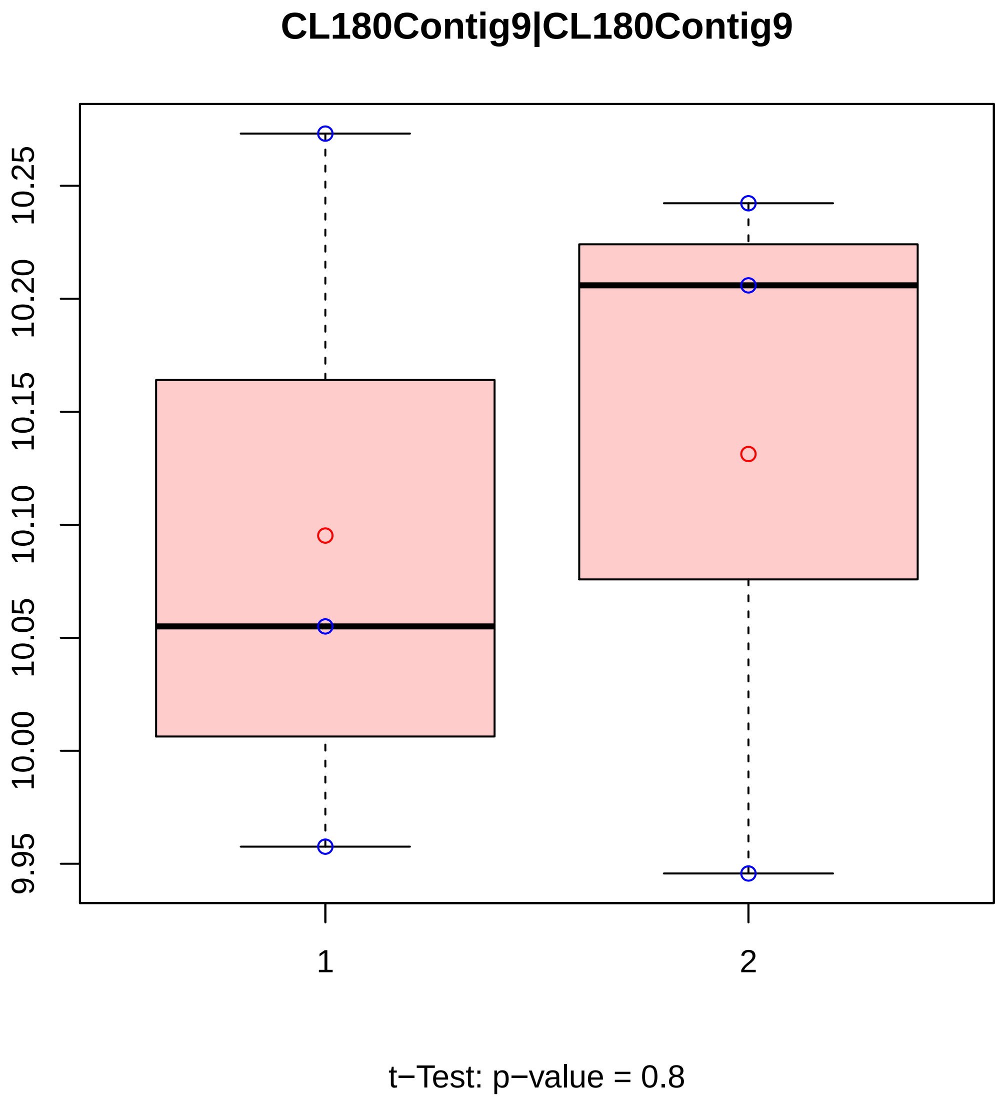
CL180Contig9|CL180Contig9
10.25
10.20
10.15
10.10
10.05
10.00
9.95
1
2
t−Test: p−value = 0.8

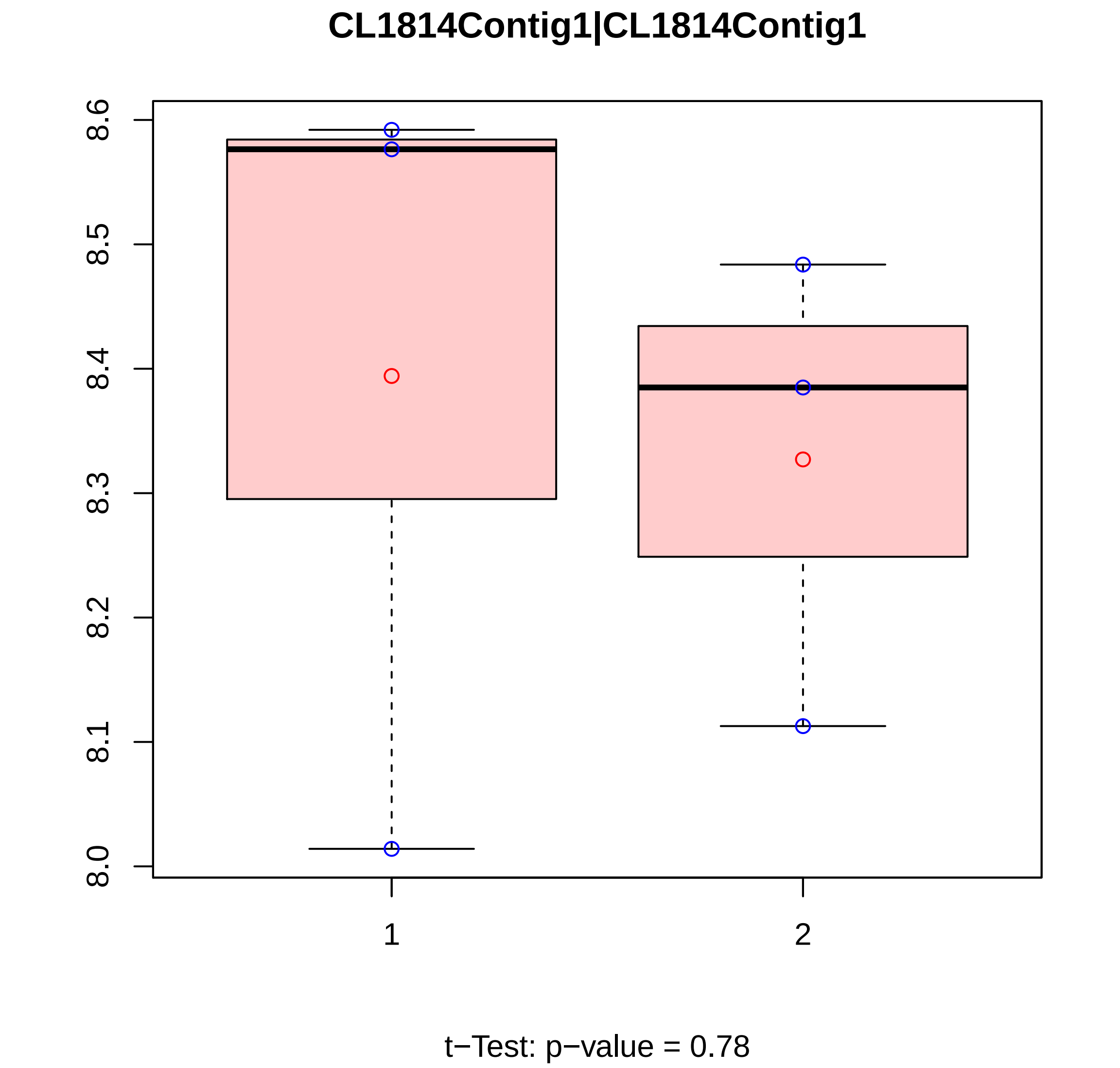
CL1814Contig1|CL1814Contig1
8.0
8.1
8.2
8.3
8.4
8.5
8.6
1
2
t−Test: p−value = 0.78

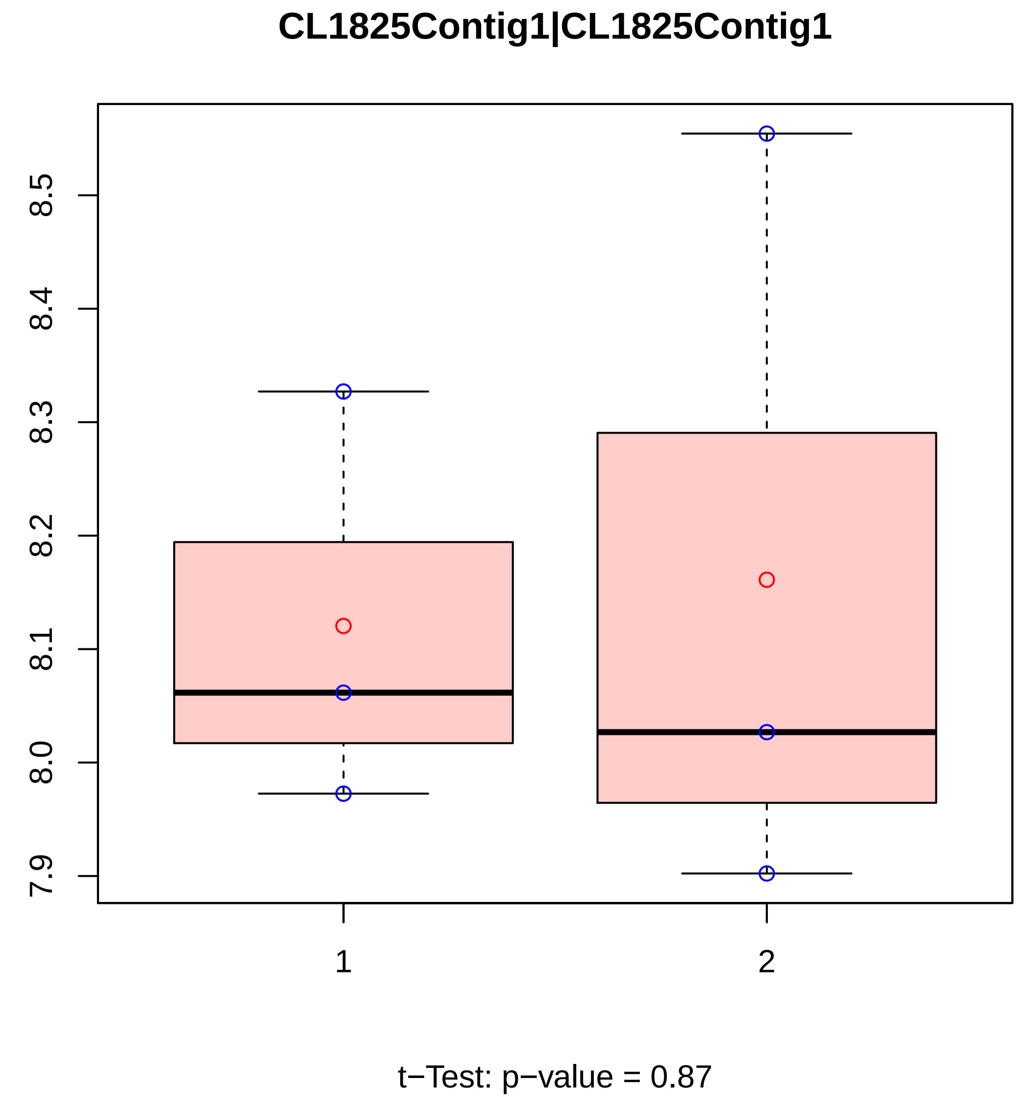
CL1825Contig1|CL1825Contig1
8.5
8.4
8.3
8.2
8.1
8.0
7.9
1
2
t−Test: p−value = 0.87

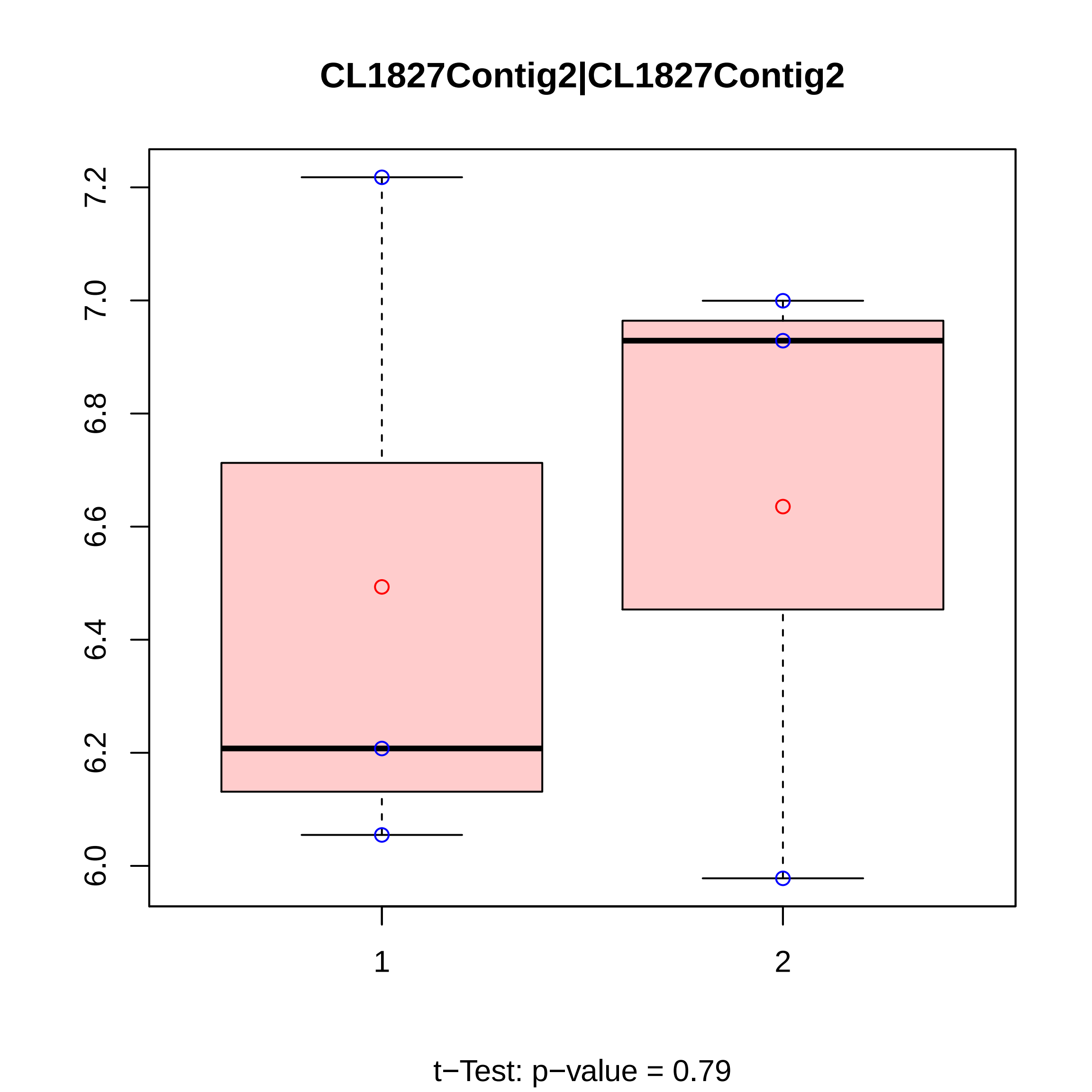
CL1827Contig2|CL1827Contig2
7.2
7.0
6.8
6.6
6.4
6.2
6.0
1
2
t−Test: p−value = 0.79

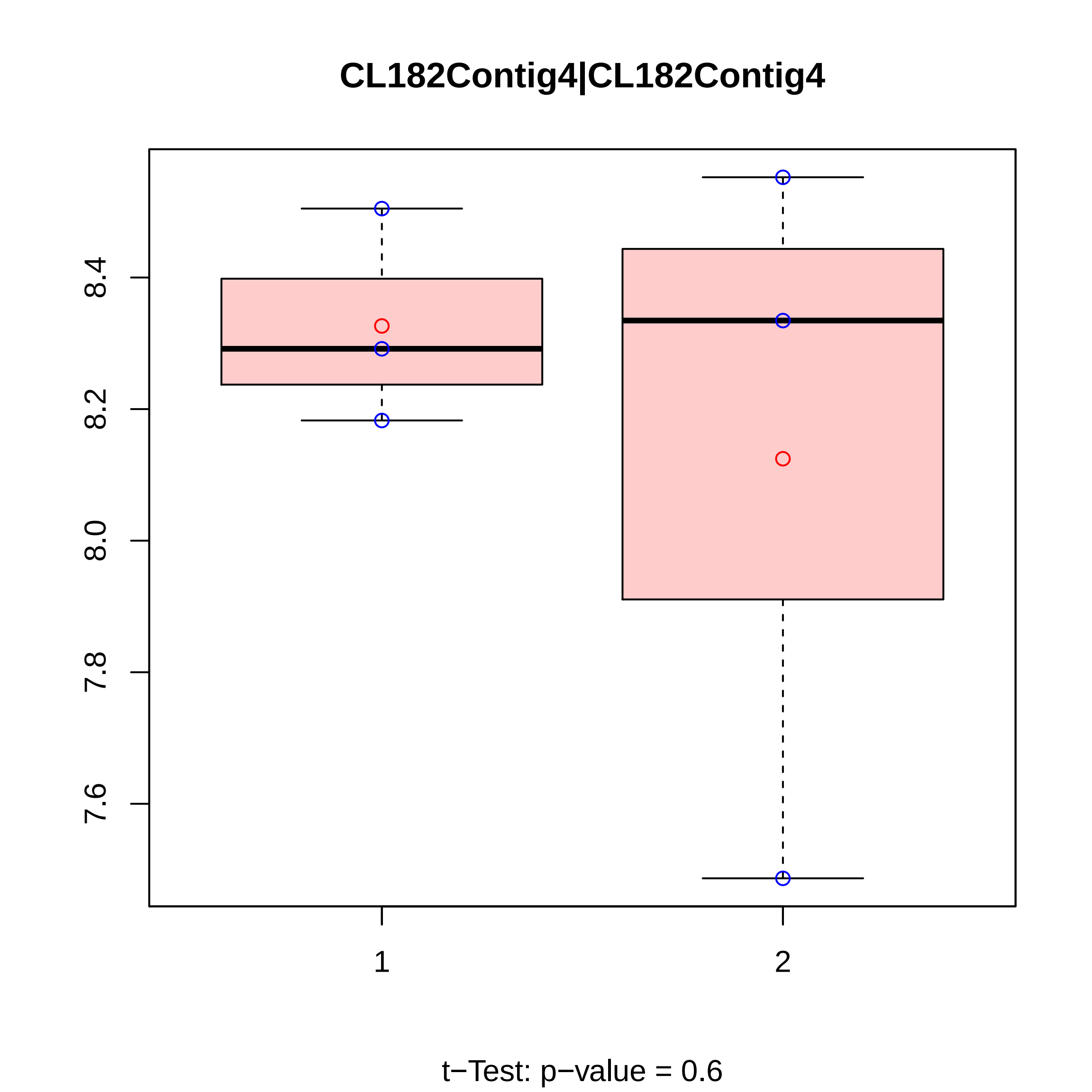
CL182Contig4|CL182Contig4
8.4
8.2
8.0
7.8
7.6
1
2
t−Test: p−value = 0.6

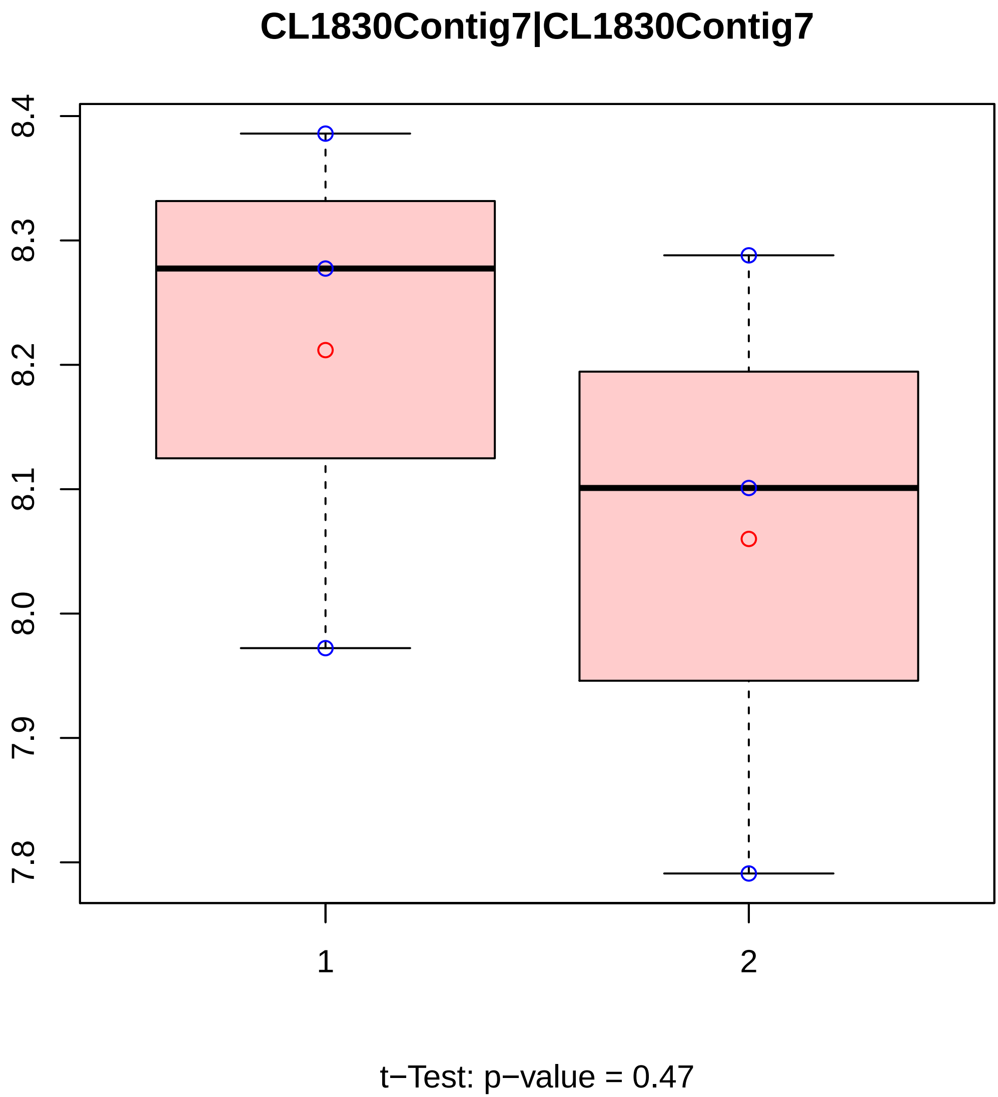
CL1830Contig7|CL1830Contig7
8.4
8.3
8.2
8.1
8.0
7.9
7.8
1
2
t−Test: p−value = 0.47

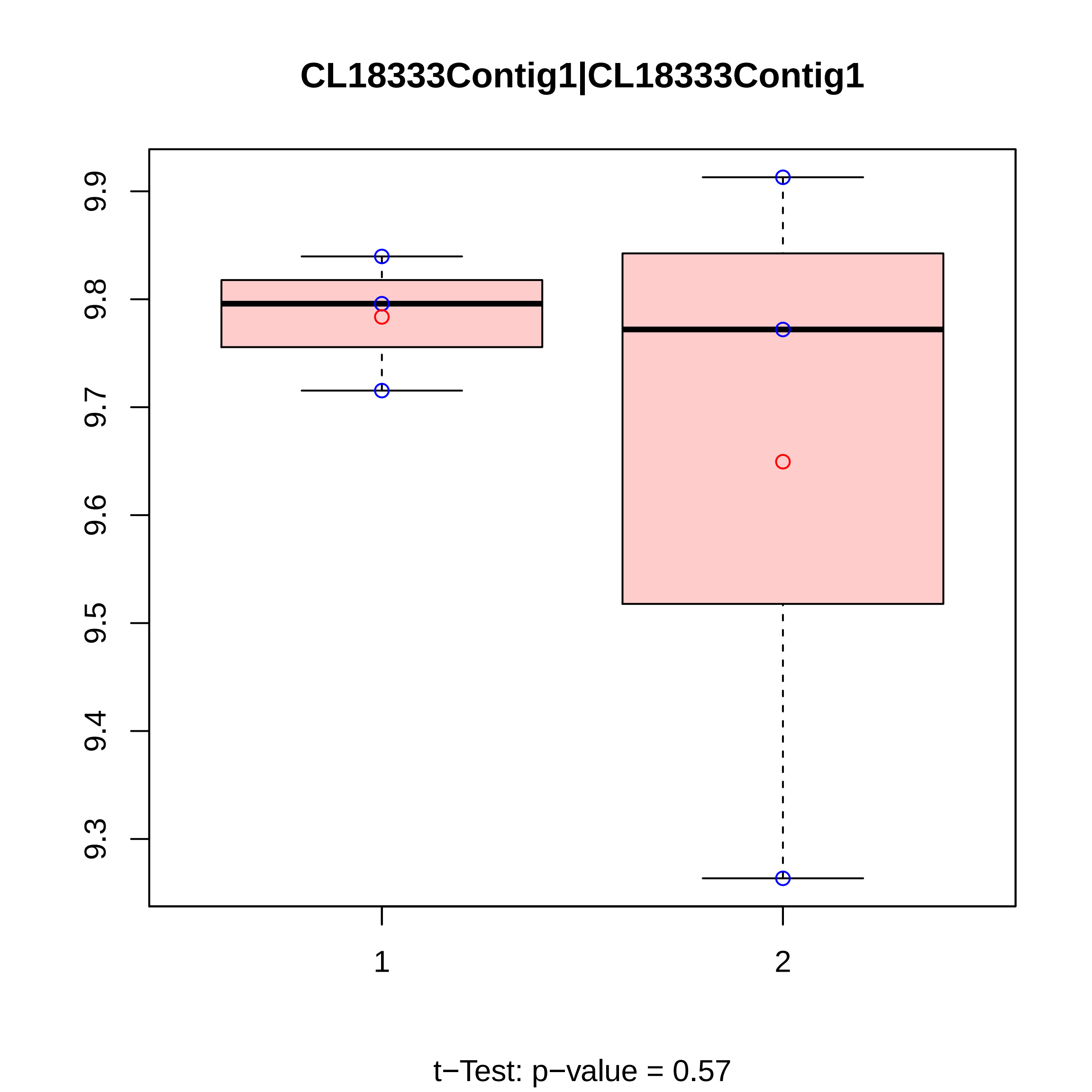

CL18333Contig1|CL18333Contig1
9.3
9.4
9.5
9.6
9.7
9.8
9.9
1
2
t−Test: p−value = 0.57

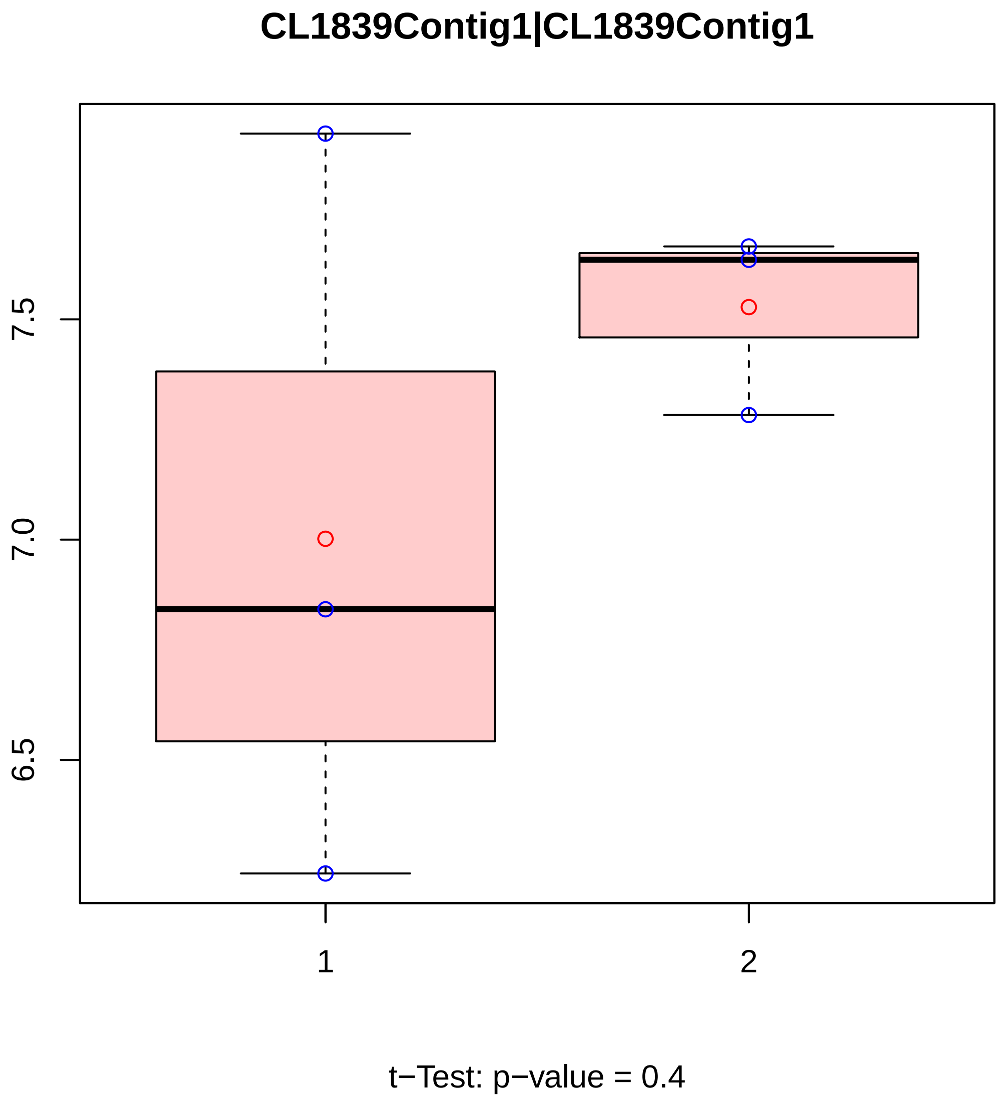
CL1839Contig1|CL1839Contig1
7.5
7.0
6.5
1
2
t−Test: p−value = 0.4

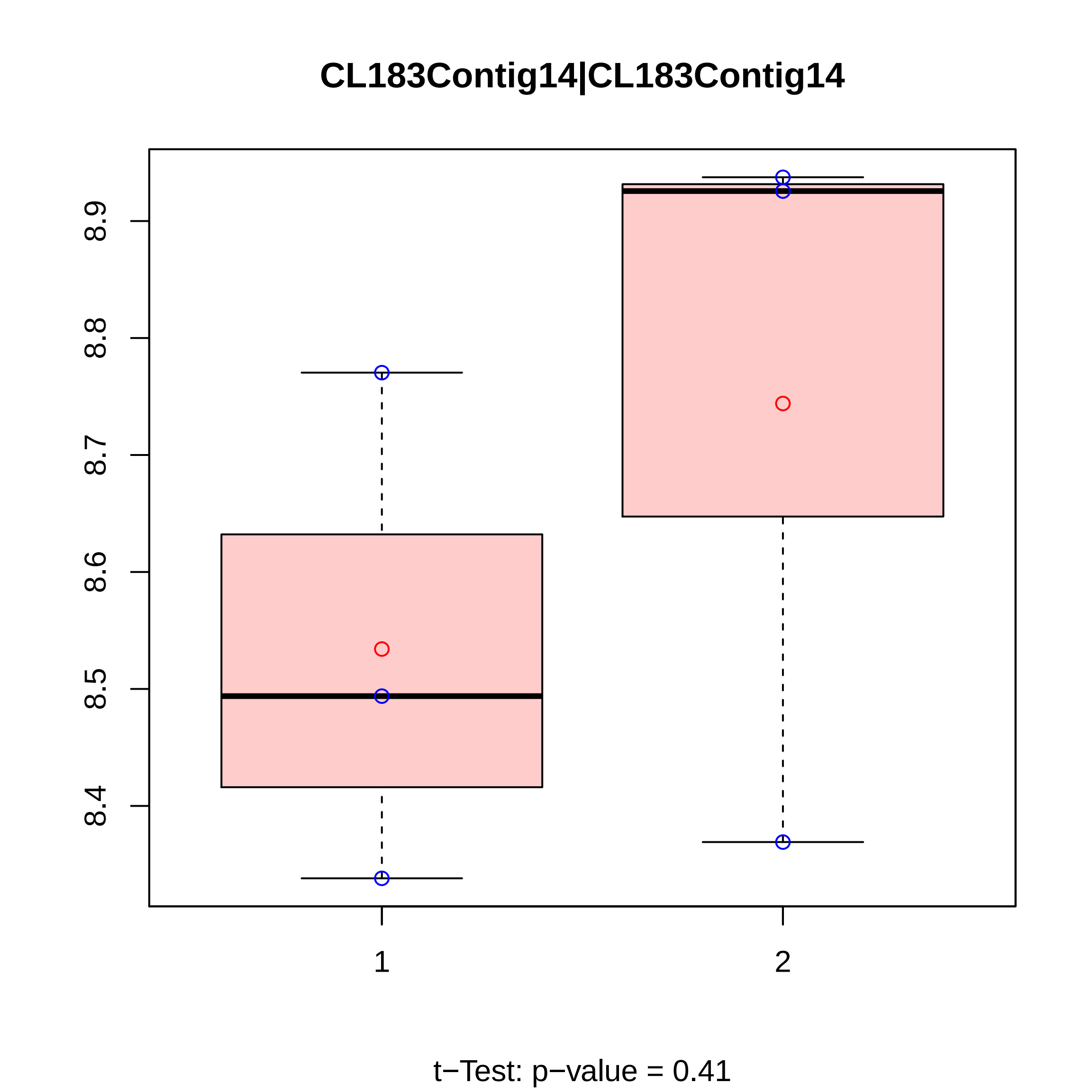
CL183Contig14|CL183Contig14
8.4
8.5
8.6
8.7
8.8
8.9
1
2
t−Test: p−value = 0.41

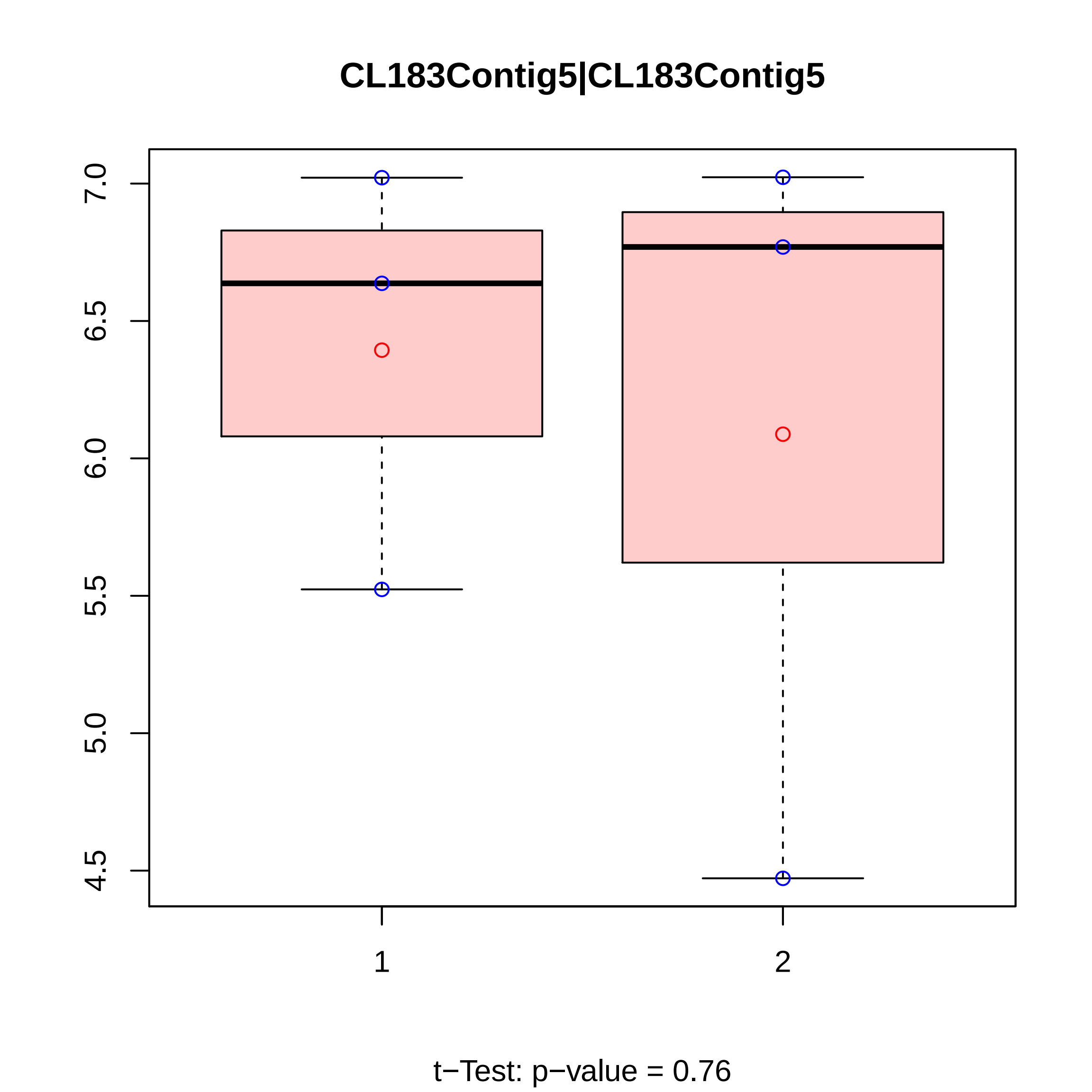
CL183Contig5|CL183Contig5
7.0
6.5
6.0
5.5
5.0
4.5
1
2
t−Test: p−value = 0.76

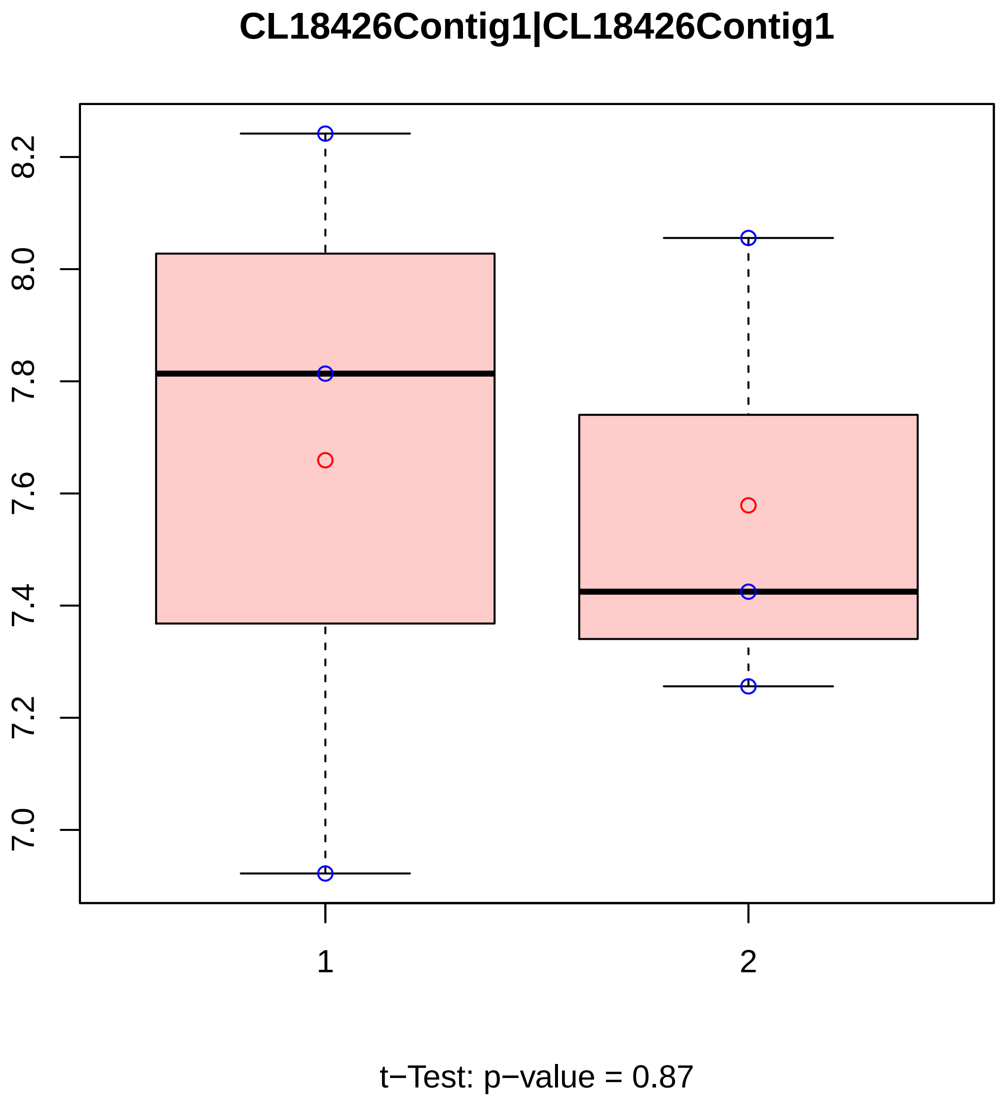
CL18426Contig1|CL18426Contig1
8.2
8.0
7.8
7.6
7.4
7.2
7.0
1
2
t−Test: p−value = 0.87

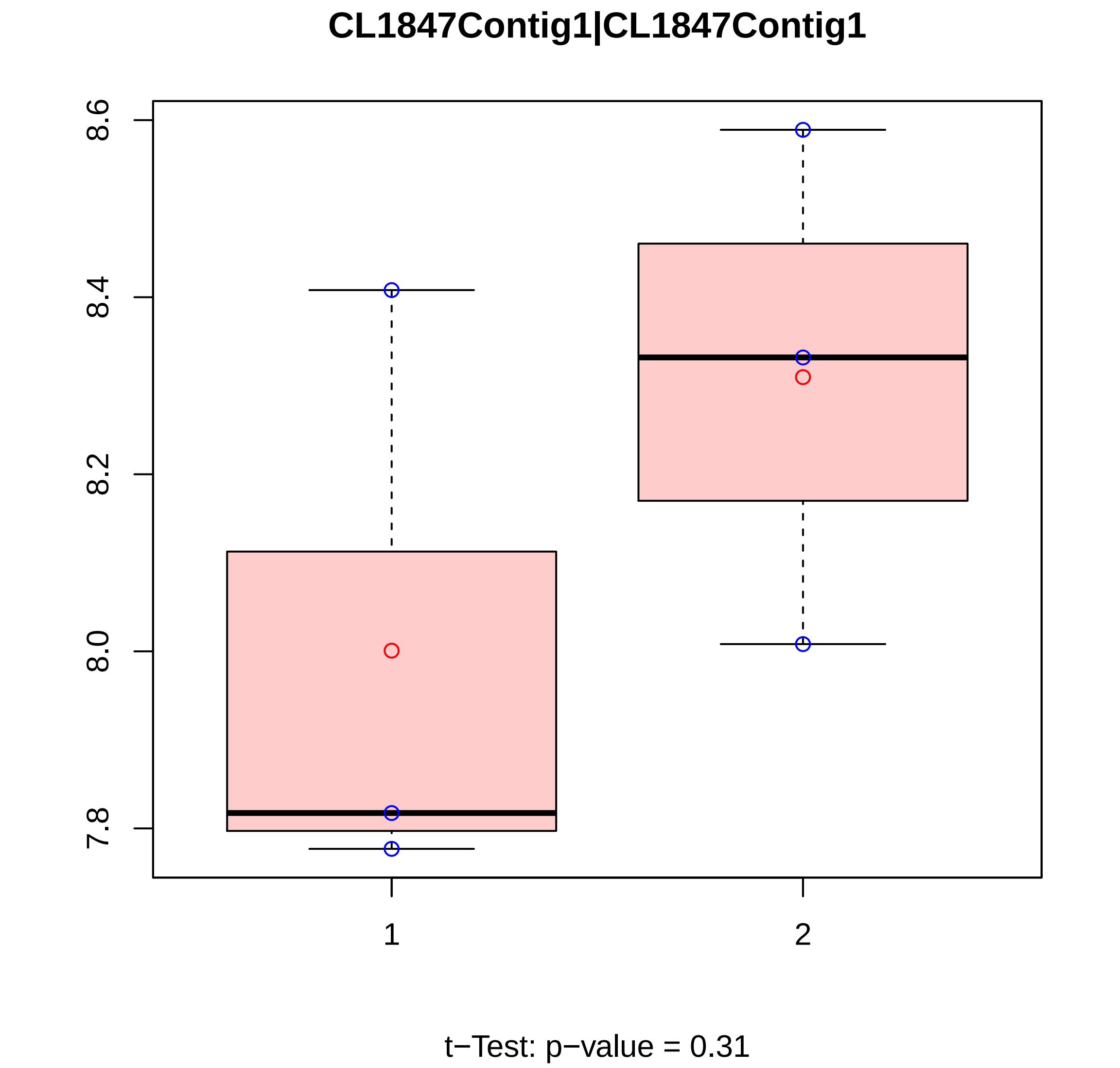

CL1847Contig1|CL1847Contig1
8.6
8.4
8.2
8.0
7.8
1
2
t−Test: p−value = 0.31

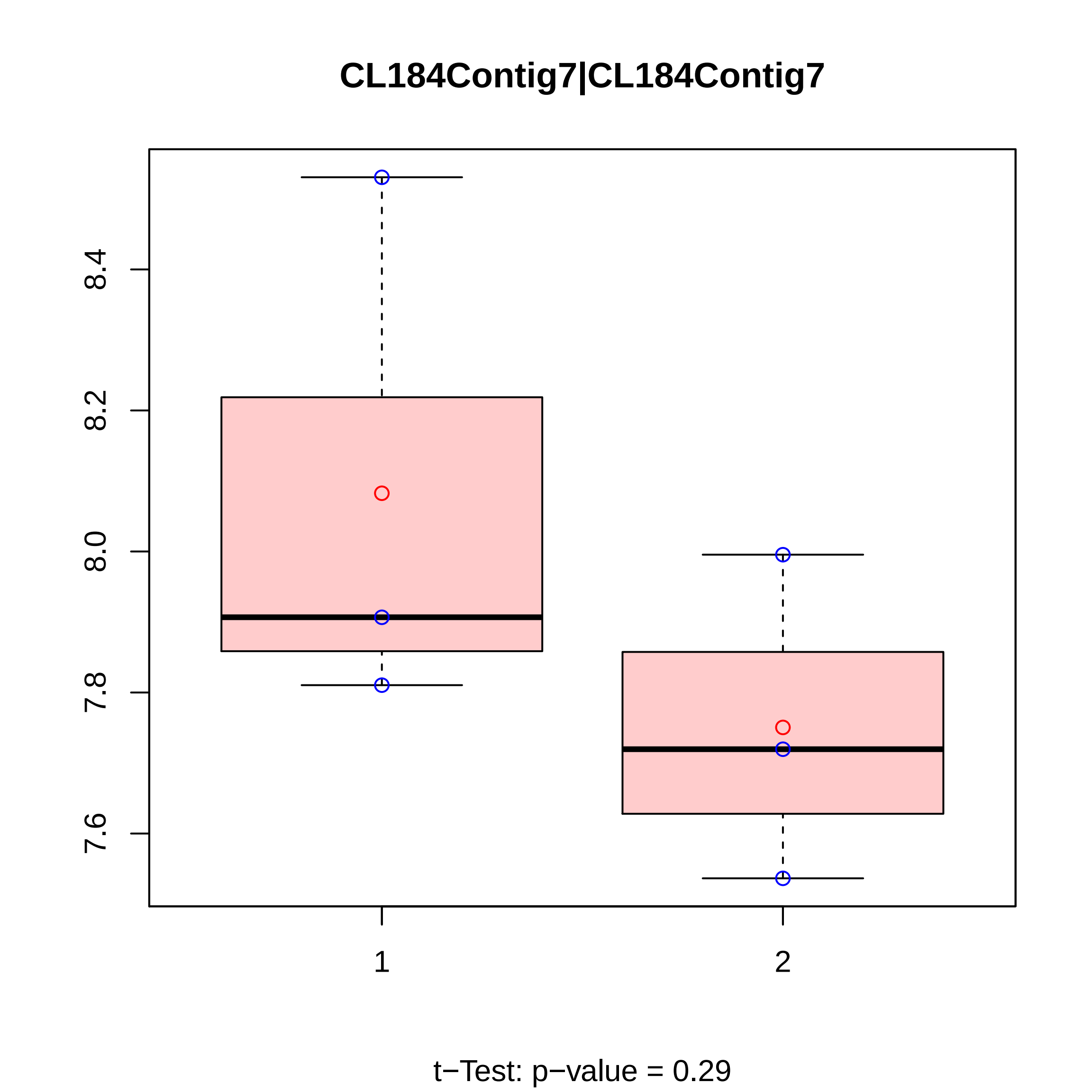
CL184Contig7|CL184Contig7
8.4
8.2
8.0
7.8
7.6
1
2
t−Test: p−value = 0.29

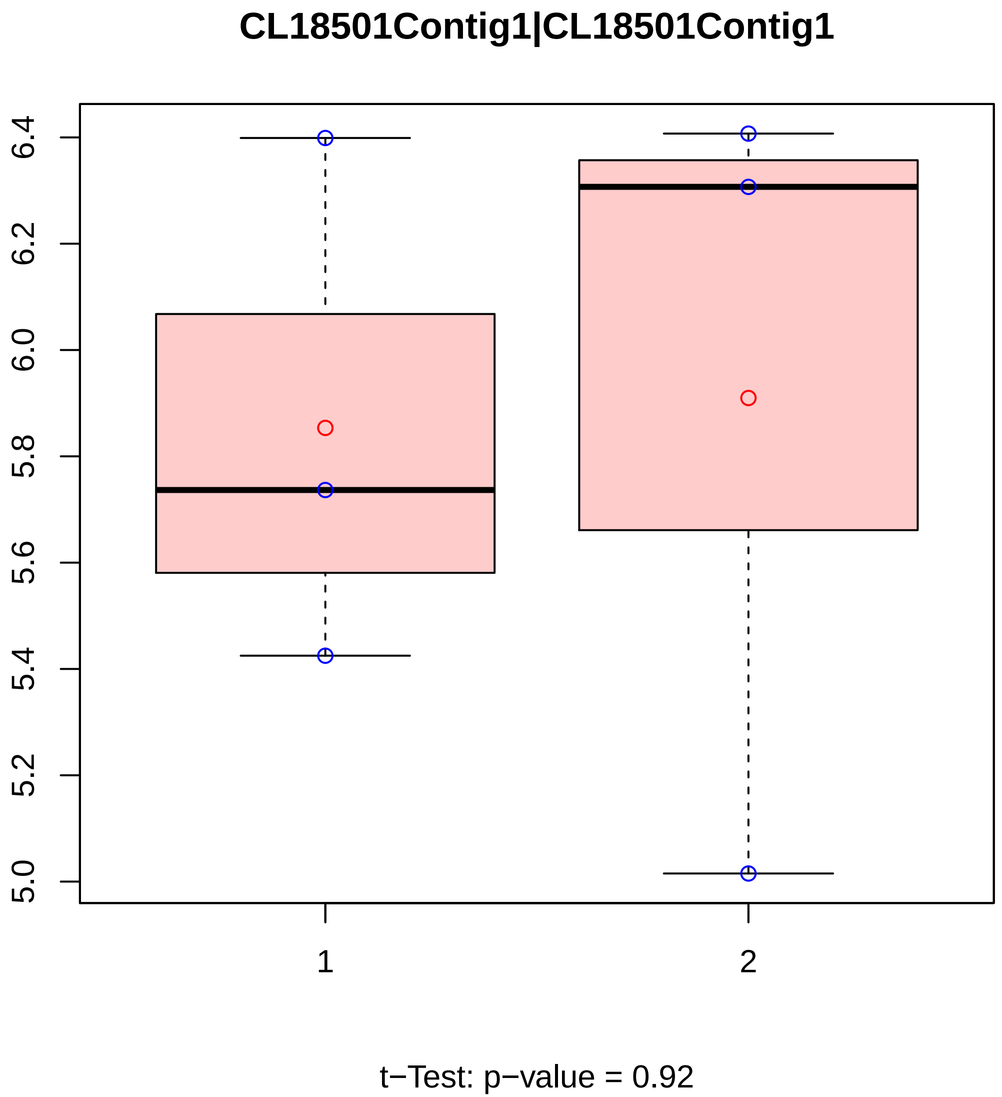

CL18501Contig1|CL18501Contig1
6.4
6.2
6.0
5.8
5.6
5.4
5.2
5.0
1
2
t−Test: p−value = 0.92

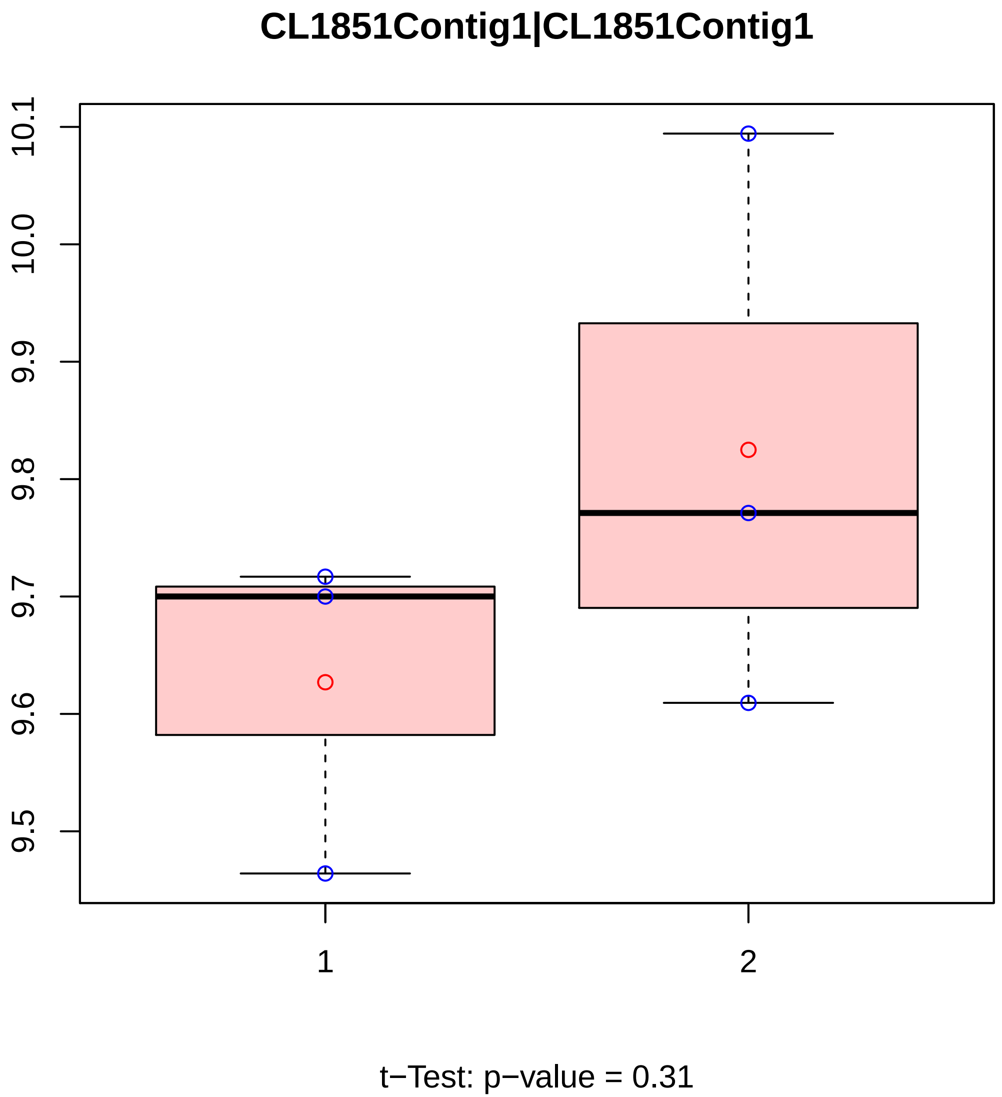
CL1851Contig1|CL1851Contig1
10.1
10.0
9.9
9.8
9.7
9.6
9.5
1
2
t−Test: p−value = 0.31

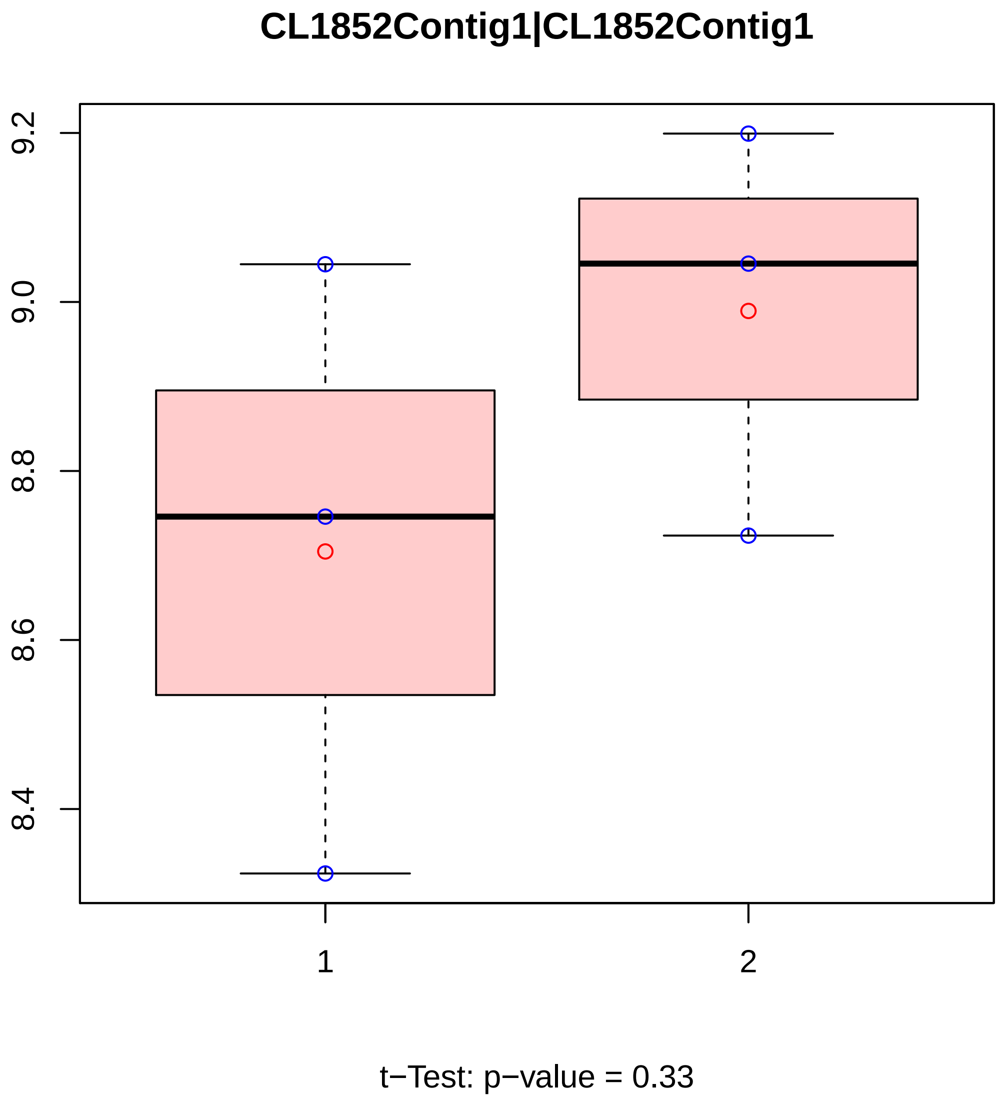
CL1852Contig1|CL1852Contig1
9.2
9.0
8.8
8.6
8.4
1
2
t−Test: p−value = 0.33

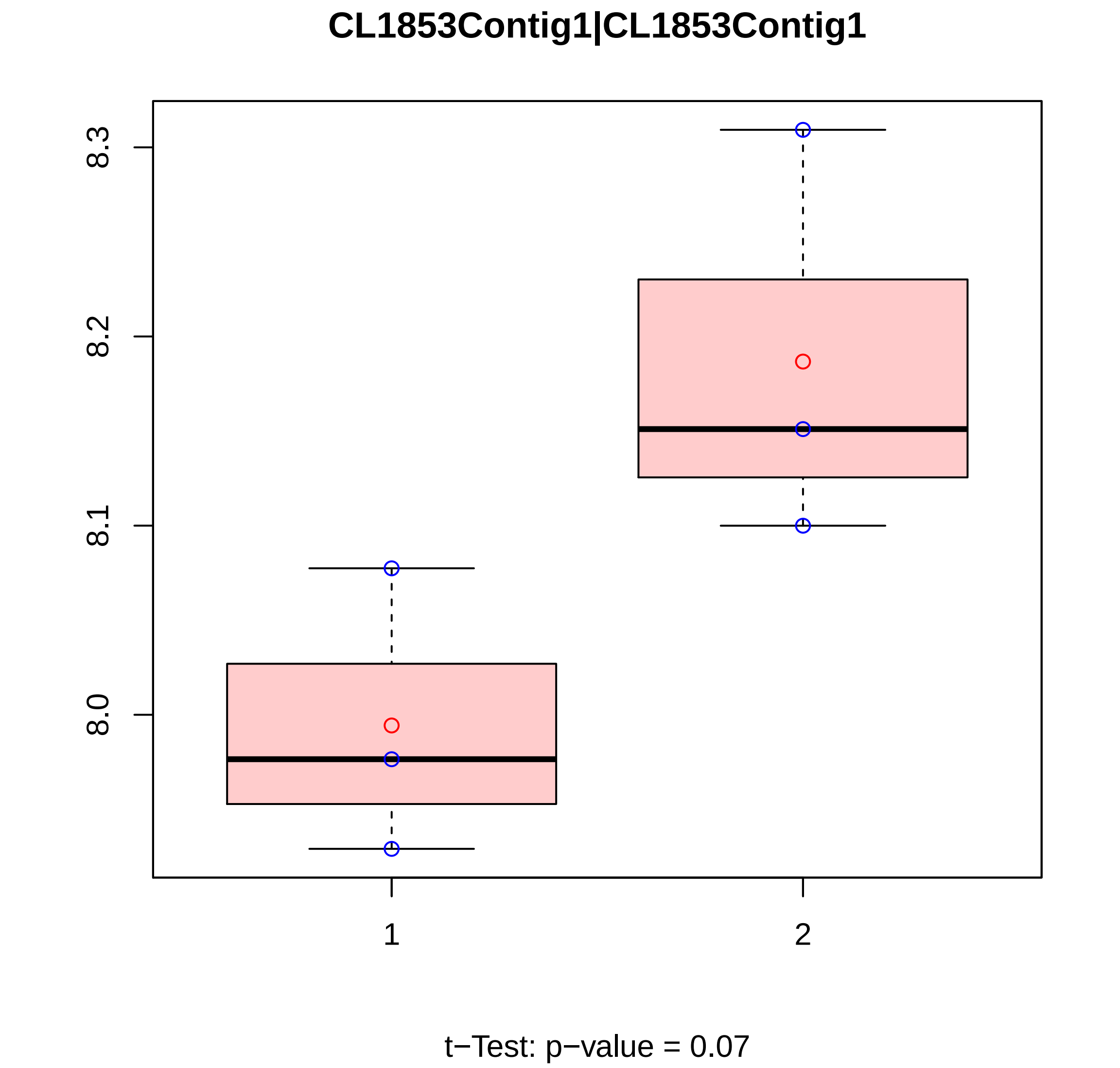
CL1853Contig1|CL1853Contig1
8.0
8.1
8.2
8.3
1
2
t−Test: p−value = 0.07

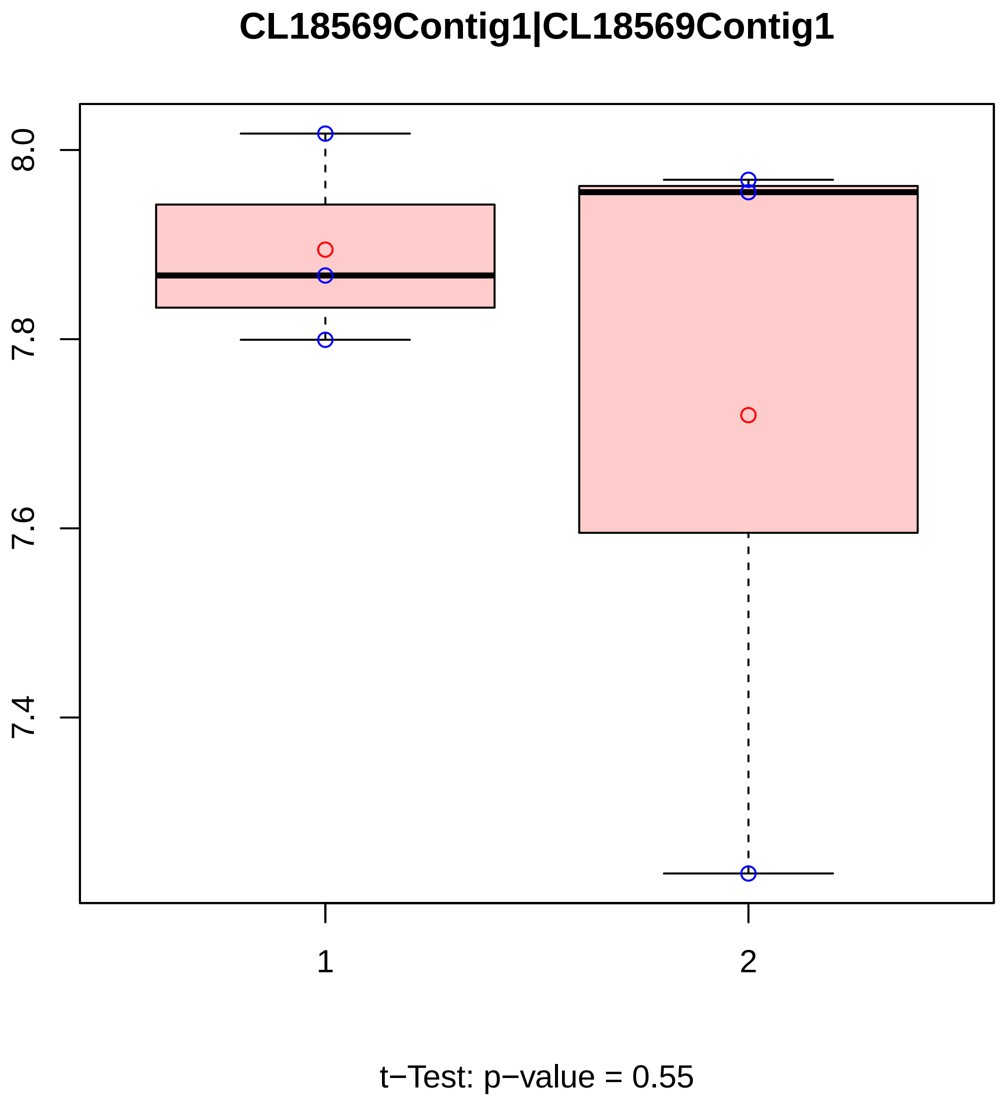
CL18569Contig1|CL18569Contig1
8.0
7.8
7.6
7.4
1
2
t−Test: p−value = 0.55

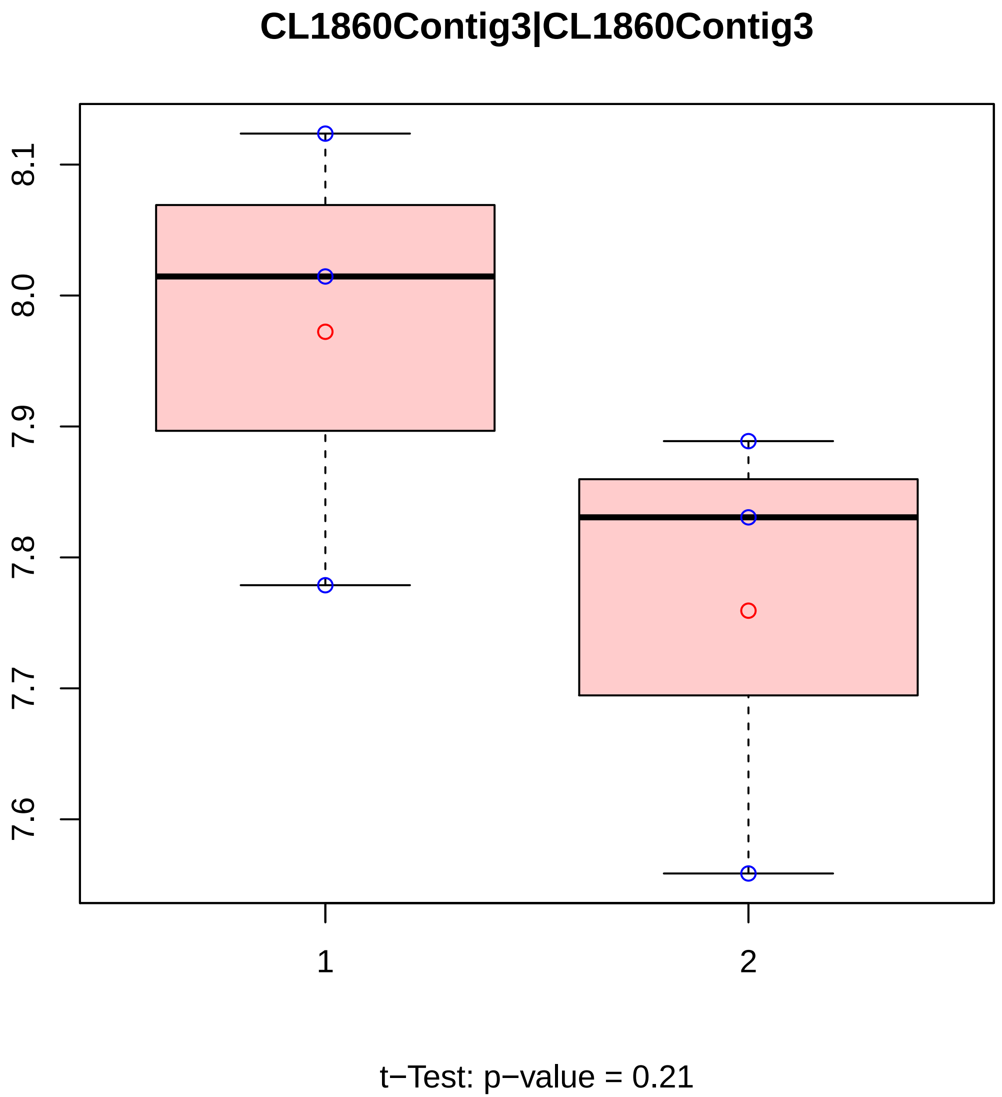
CL1860Contig3|CL1860Contig3
8.1
8.0
7.9
7.8
7.7
7.6
1
2
t−Test: p−value = 0.21

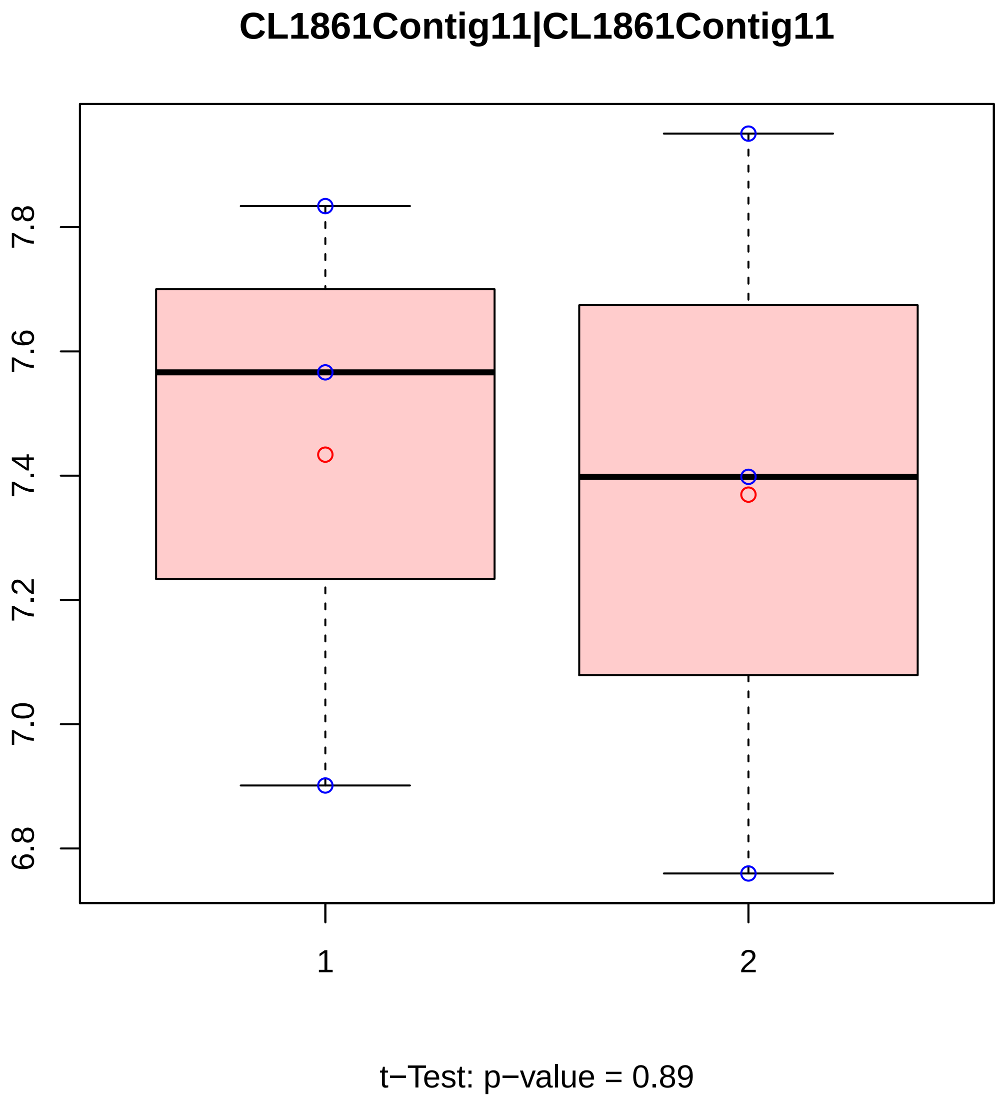
CL1861Contig11|CL1861Contig11
7.8
7.6
7.4
7.2
7.0
6.8
1
2
t−Test: p−value = 0.89

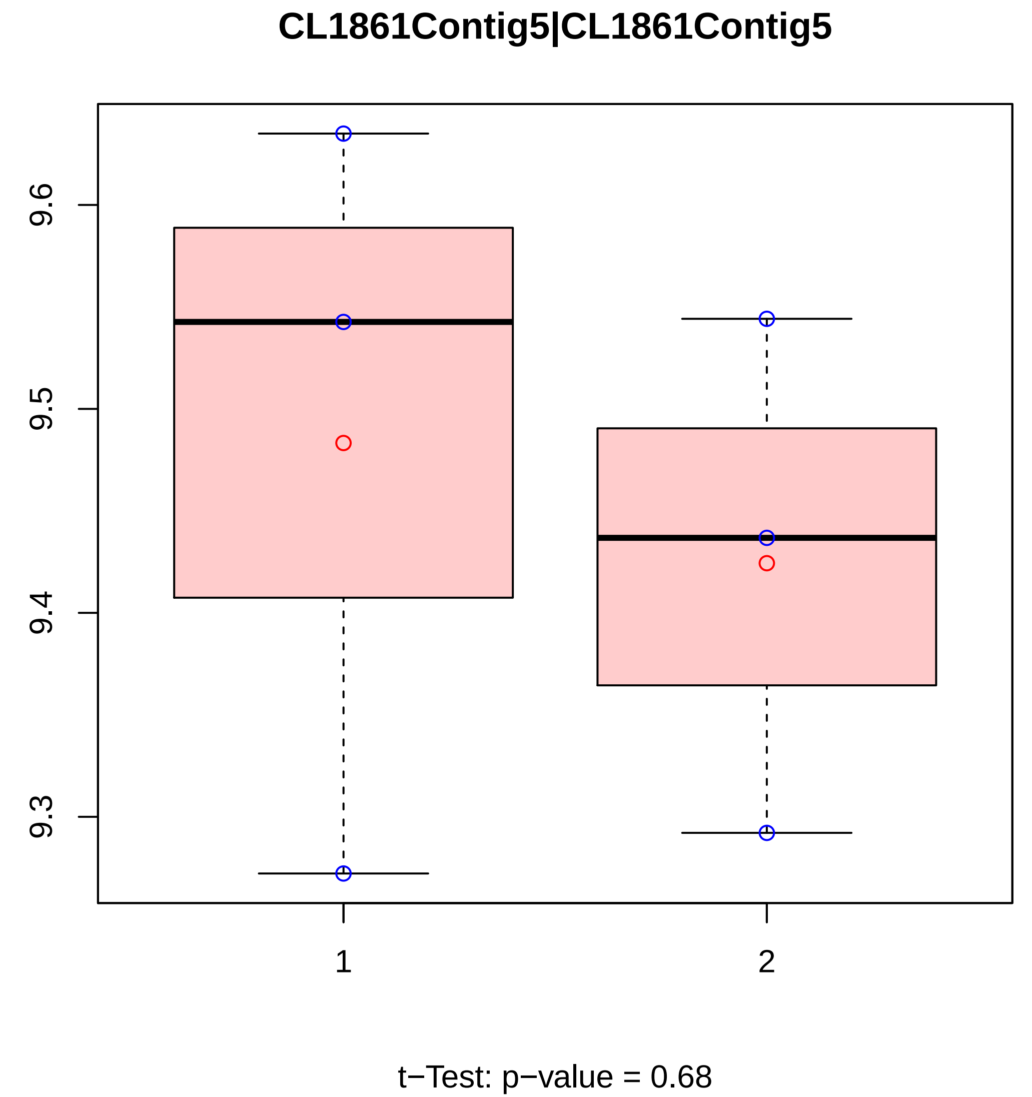
CL1861Contig5|CL1861Contig5
9.3
9.4
9.5
9.6
1
2
t−Test: p−value = 0.68

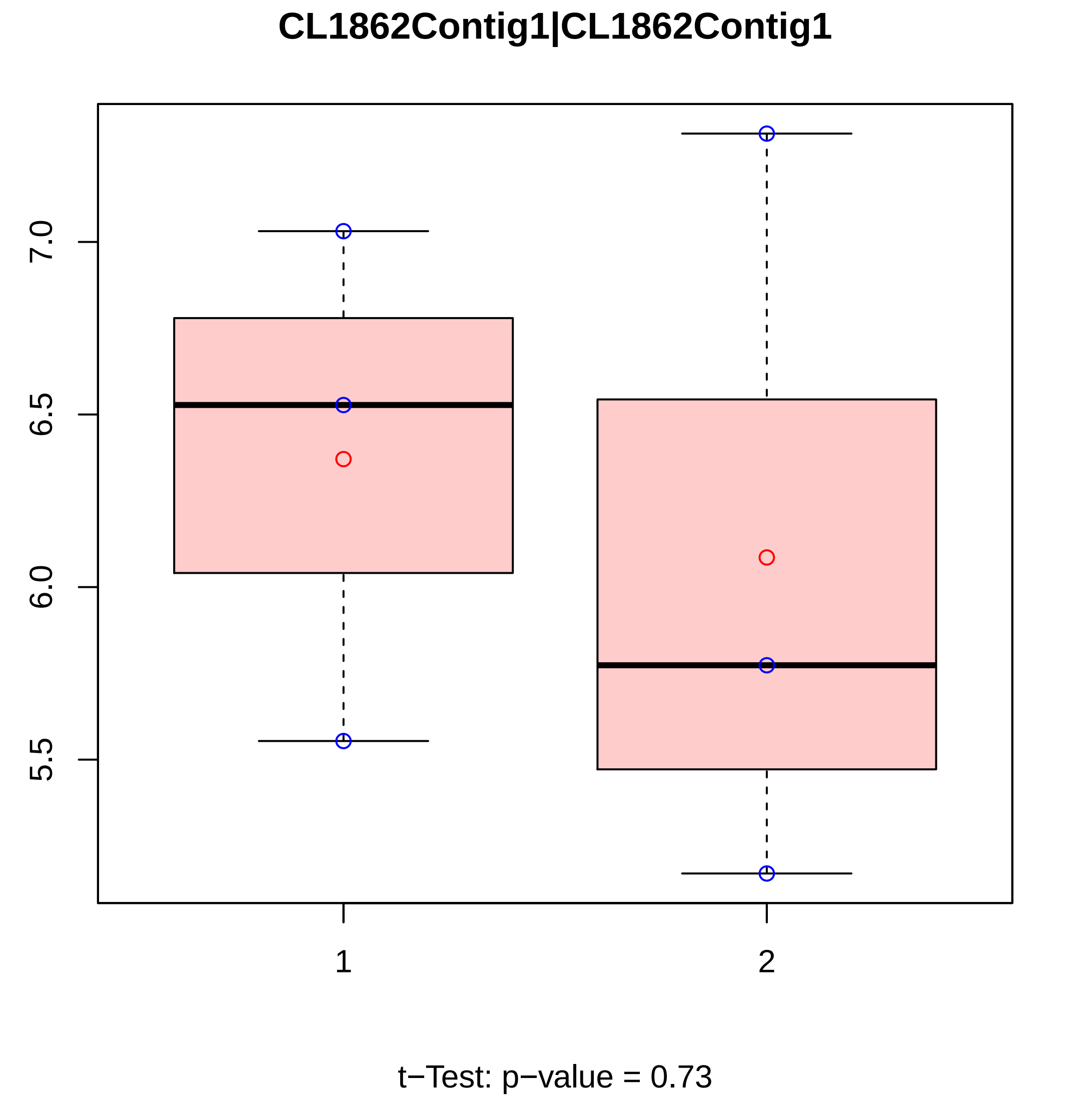
CL1862Contig1|CL1862Contig1
7.0
6.5
6.0
5.5
1
2
t−Test: p−value = 0.73

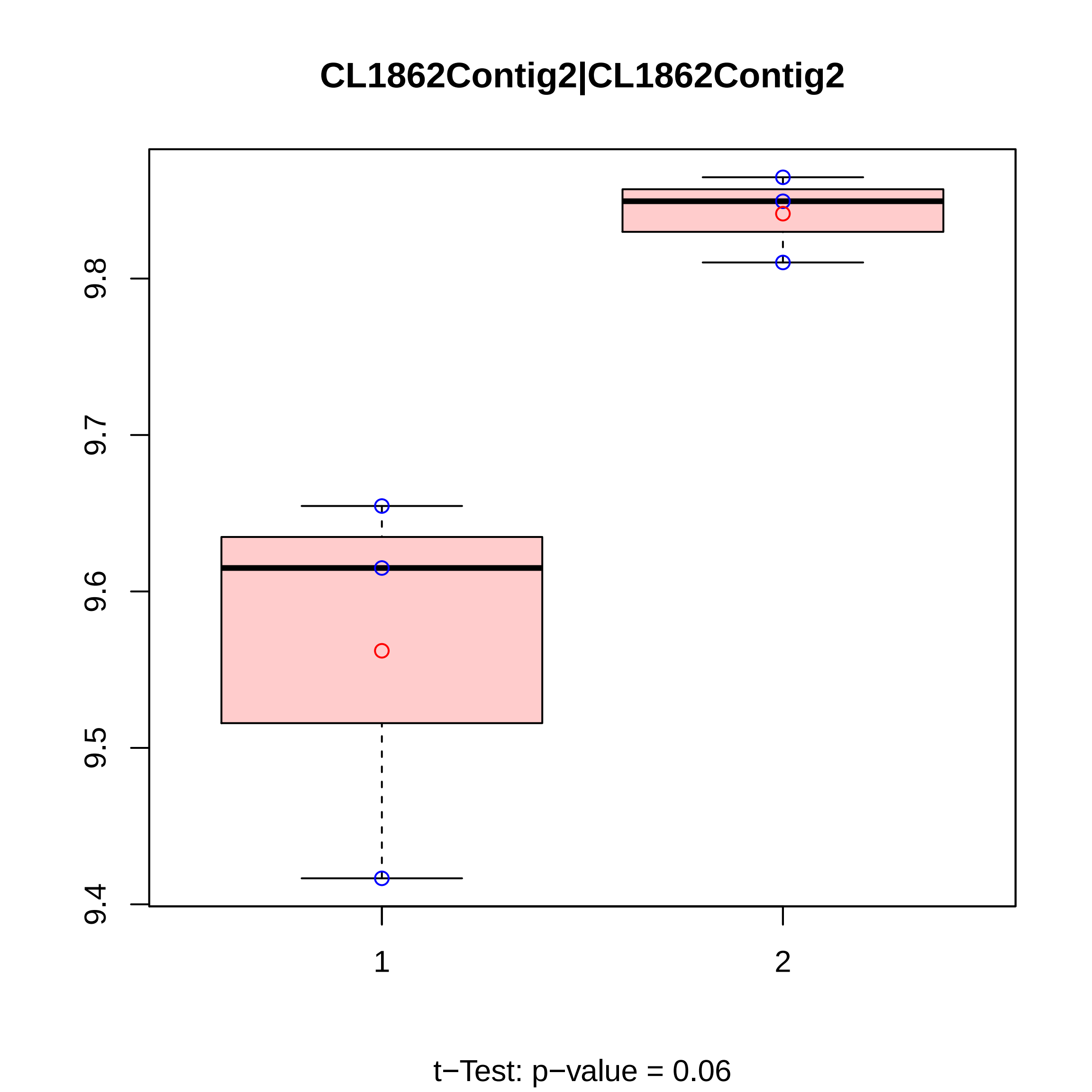

CL1862Contig2|CL1862Contig2
9.4
9.5
9.6
9.7
9.8
1
2
t−Test: p−value = 0.06

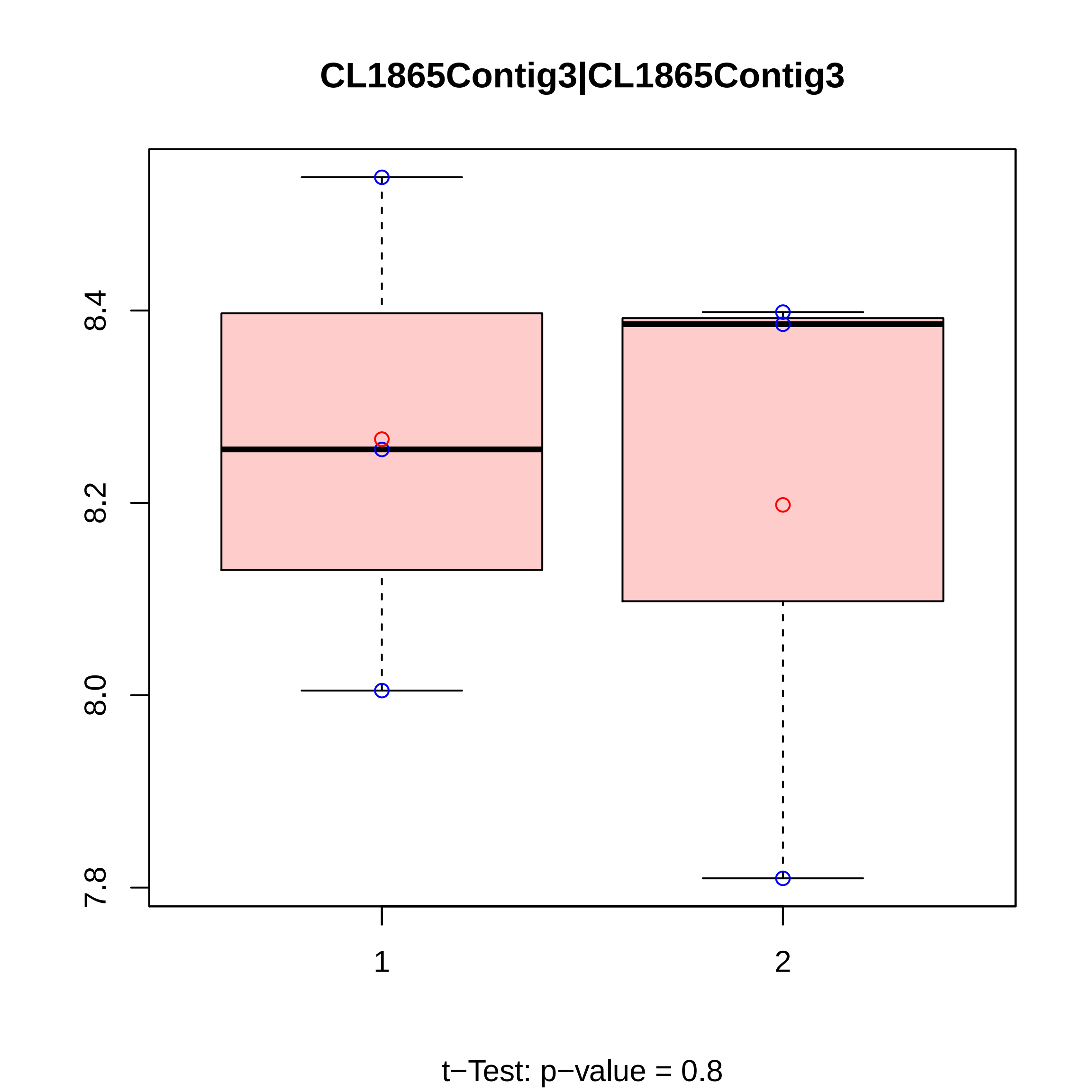

CL1865Contig3|CL1865Contig3
8.4
8.2
8.0
7.8
1
2
t−Test: p−value = 0.8

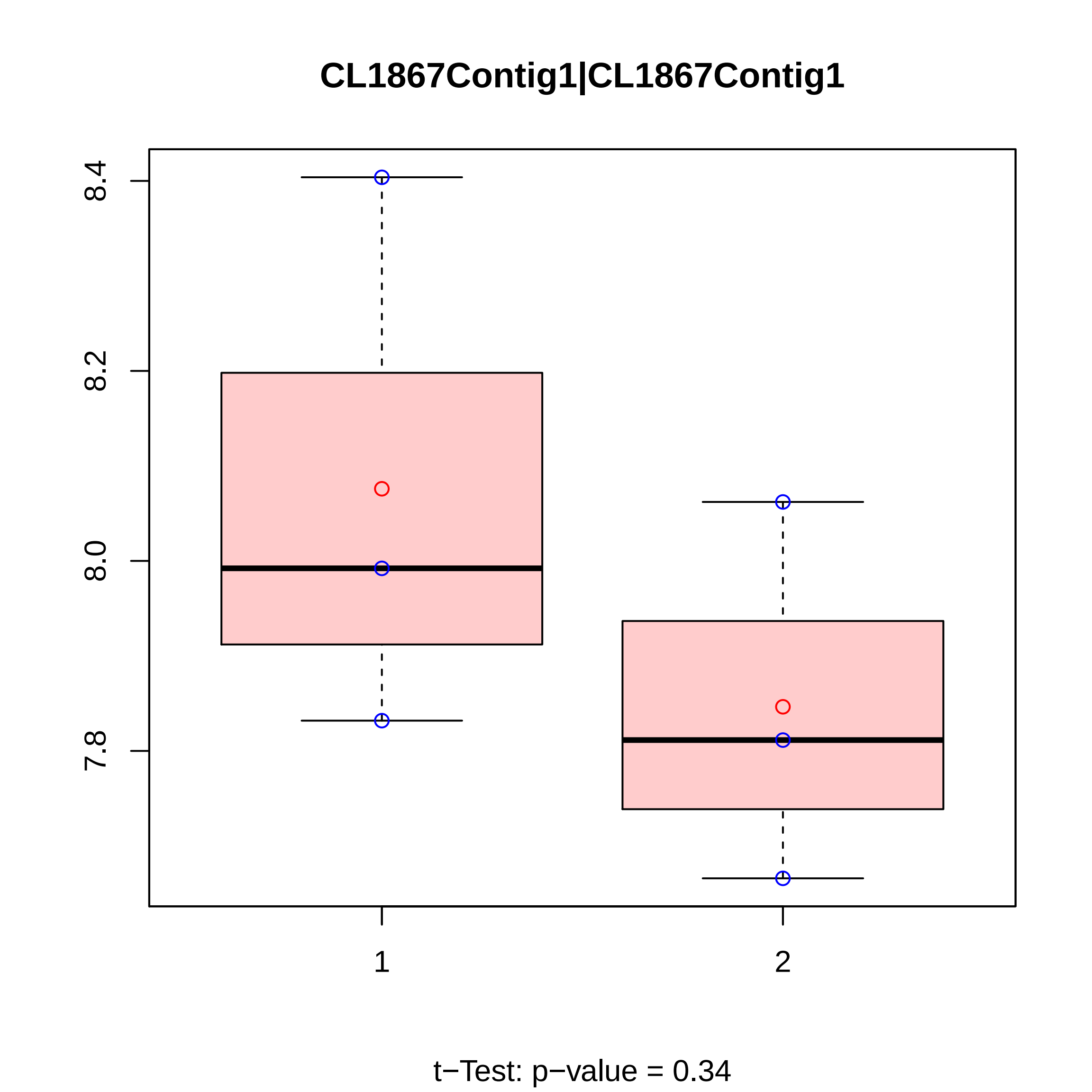
CL1867Contig1|CL1867Contig1
8.4
8.2
8.0
7.8
1
2
t−Test: p−value = 0.34

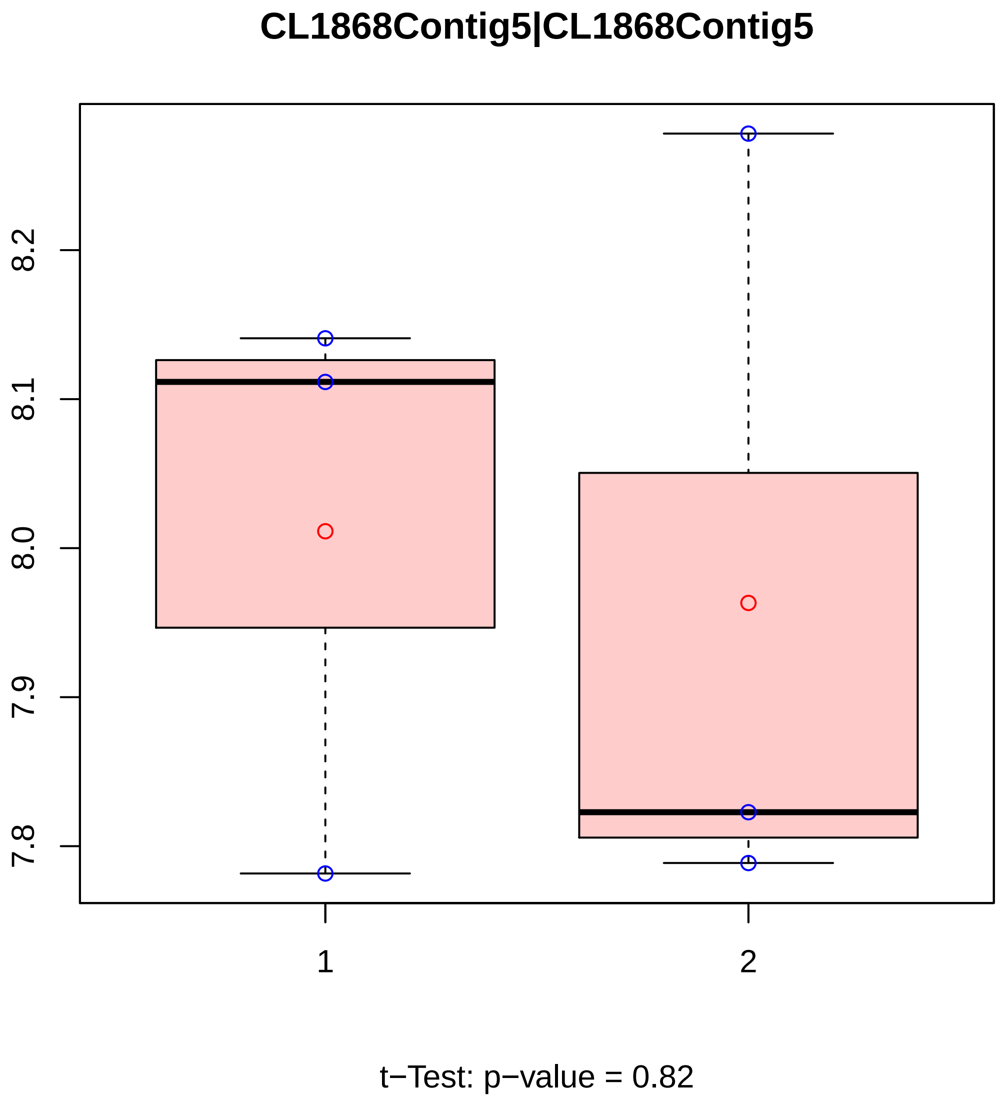

CL1868Contig5|CL1868Contig5
8.2
8.1
8.0
7.9
7.8
1
2
t−Test: p−value = 0.82

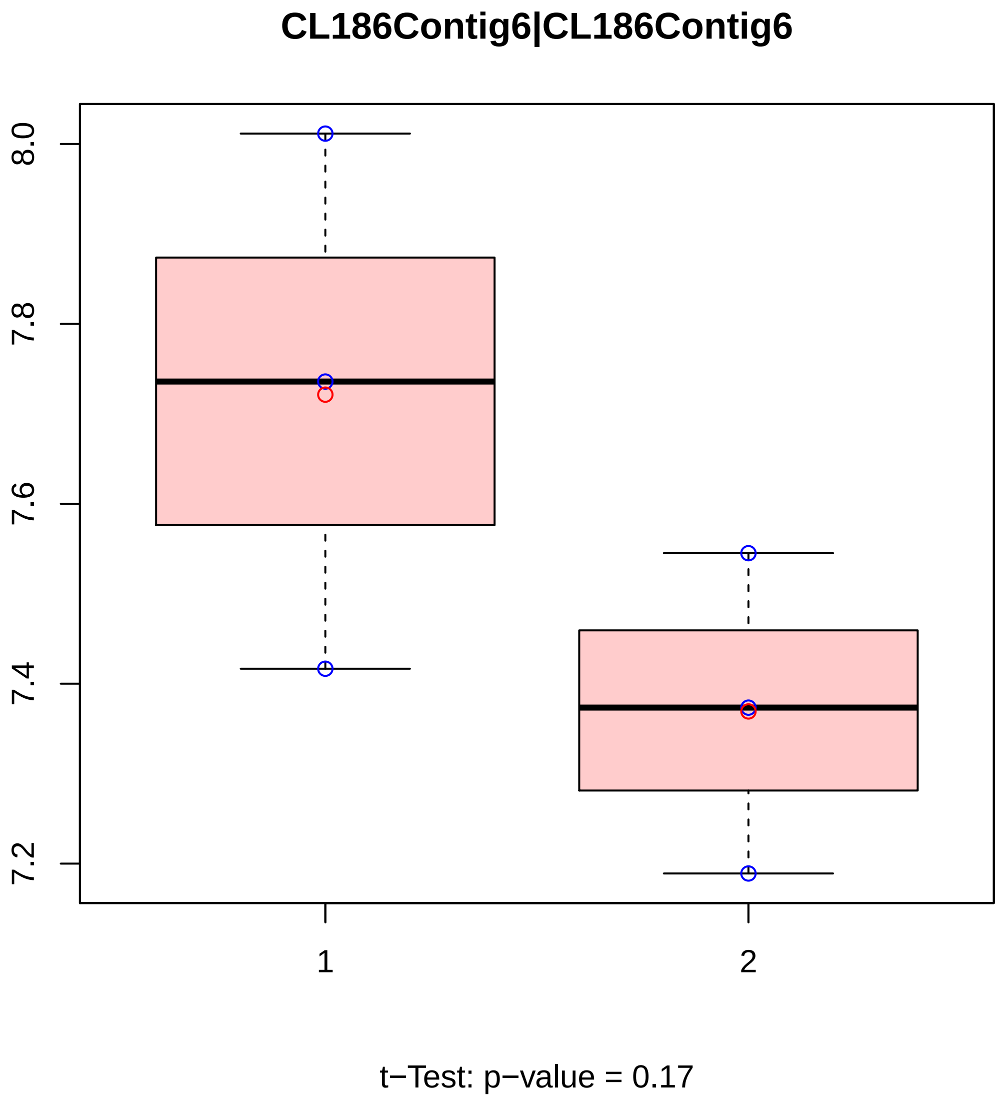
CL186Contig6|CL186Contig6
8.0
7.8
7.6
7.4
7.2
1
2
t−Test: p−value = 0.17

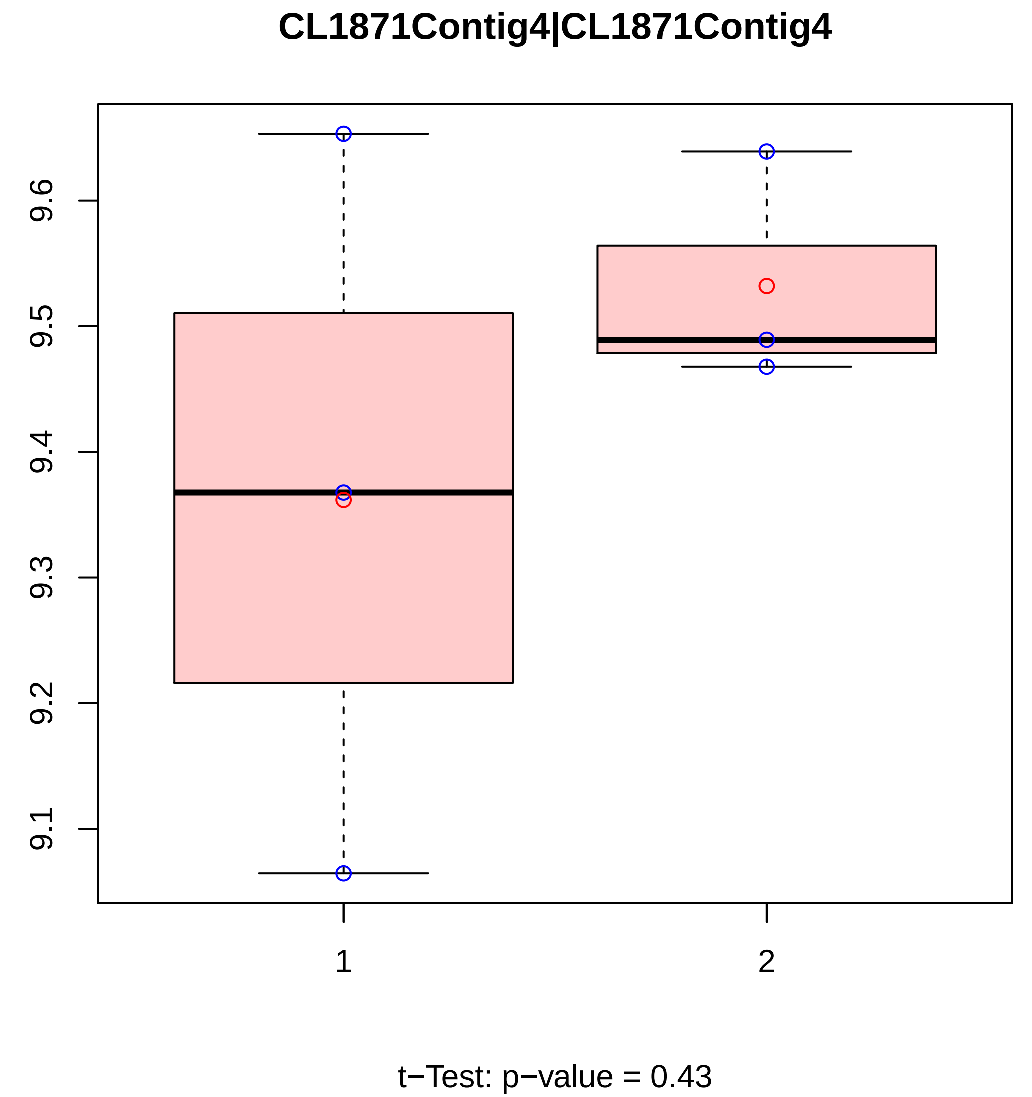
CL1871Contig4|CL1871Contig4
9.1
9.2
9.3
9.4
9.5
9.6
1
2
t−Test: p−value = 0.43

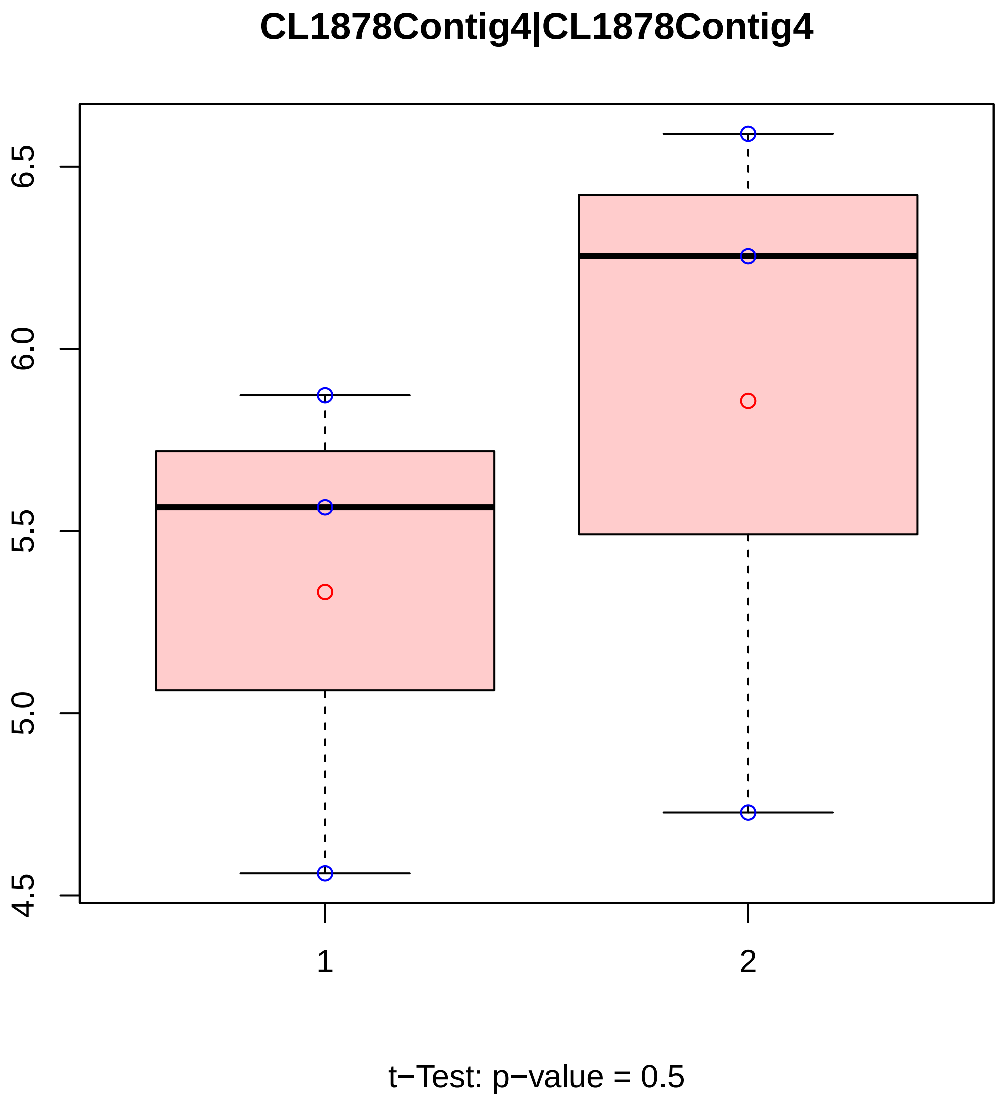

CL1878Contig4|CL1878Contig4
6.5
6.0
5.5
5.0
4.5
1
2
t−Test: p−value = 0.5

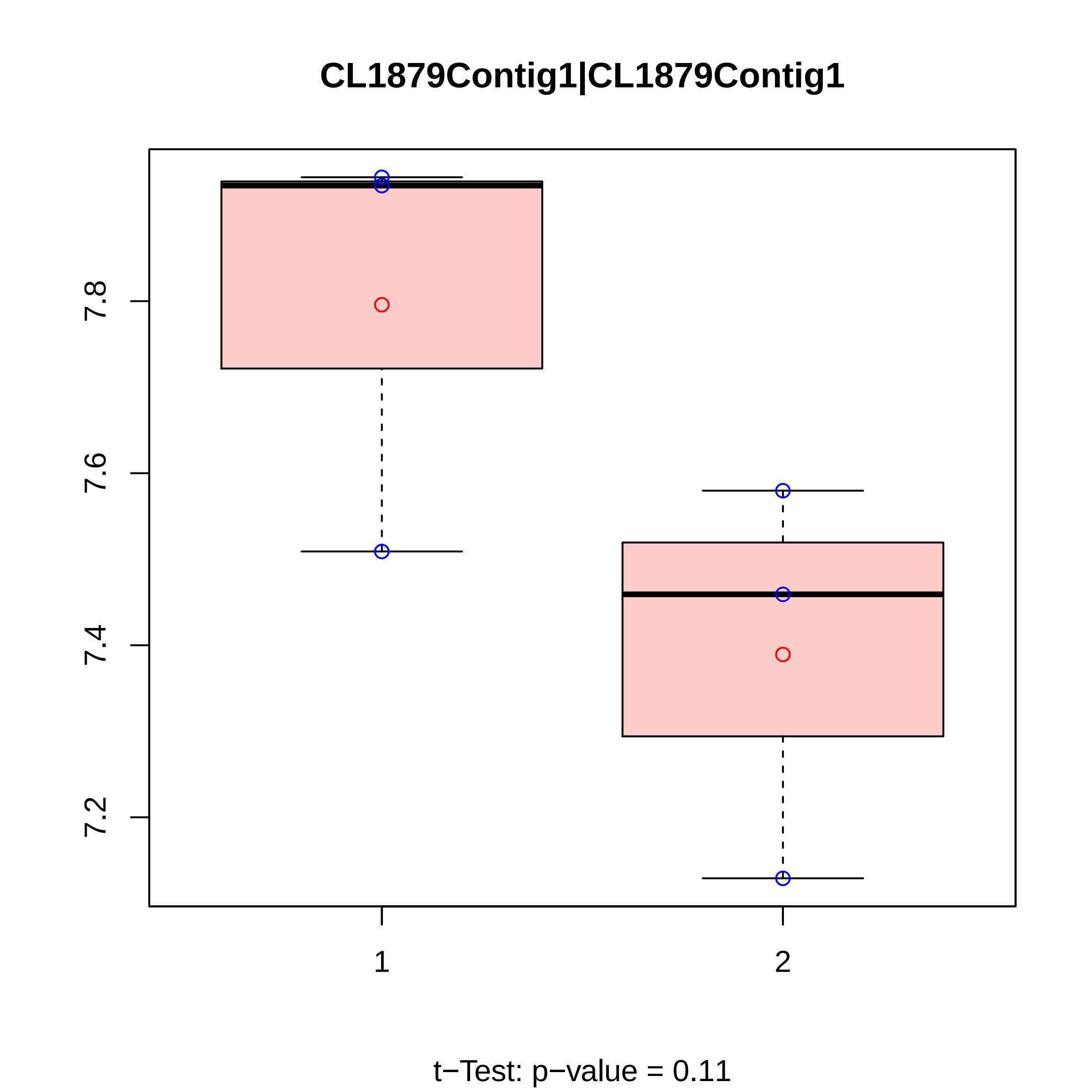

CL1879Contig1|CL1879Contig1
7.2
7.4
7.6
7.8
1
2
t−Test: p−value = 0.11

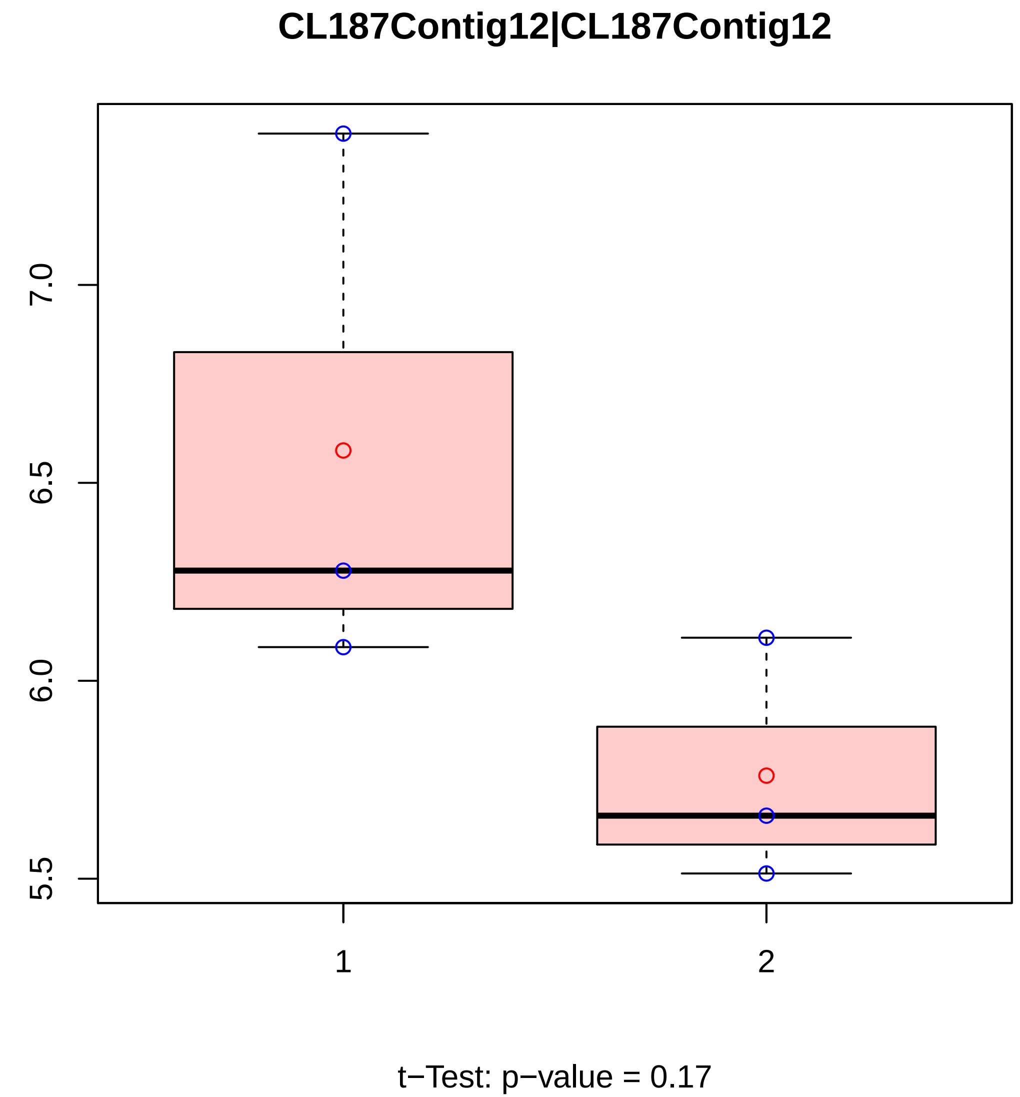
CL187Contig12|CL187Contig12
7.0
6.5
6.0
5.5
1
2
t−Test: p−value = 0.17

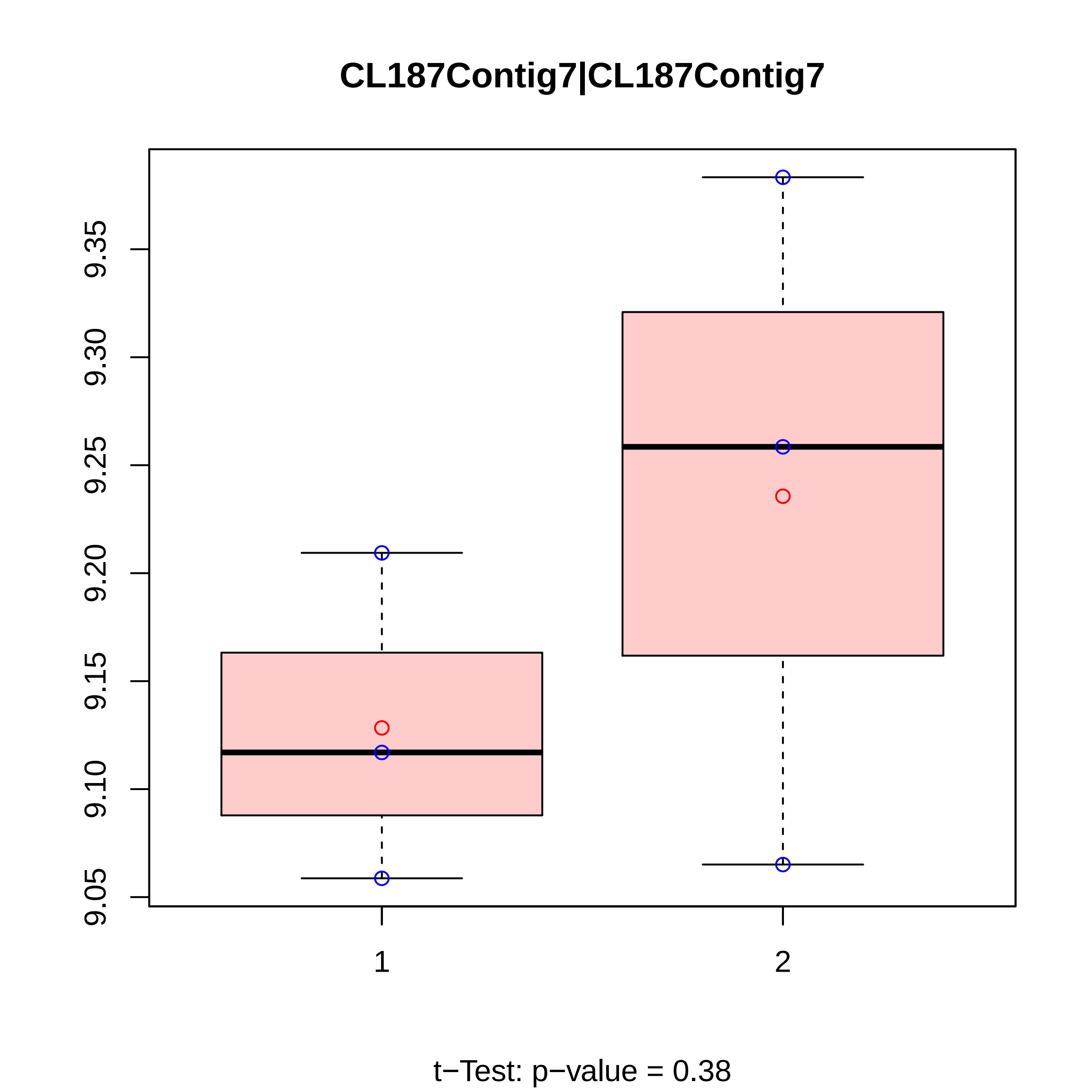

CL187Contig7|CL187Contig7
9.05
9.10
9.15
9.20
9.25
9.30
9.35
1
2
t−Test: p−value = 0.38

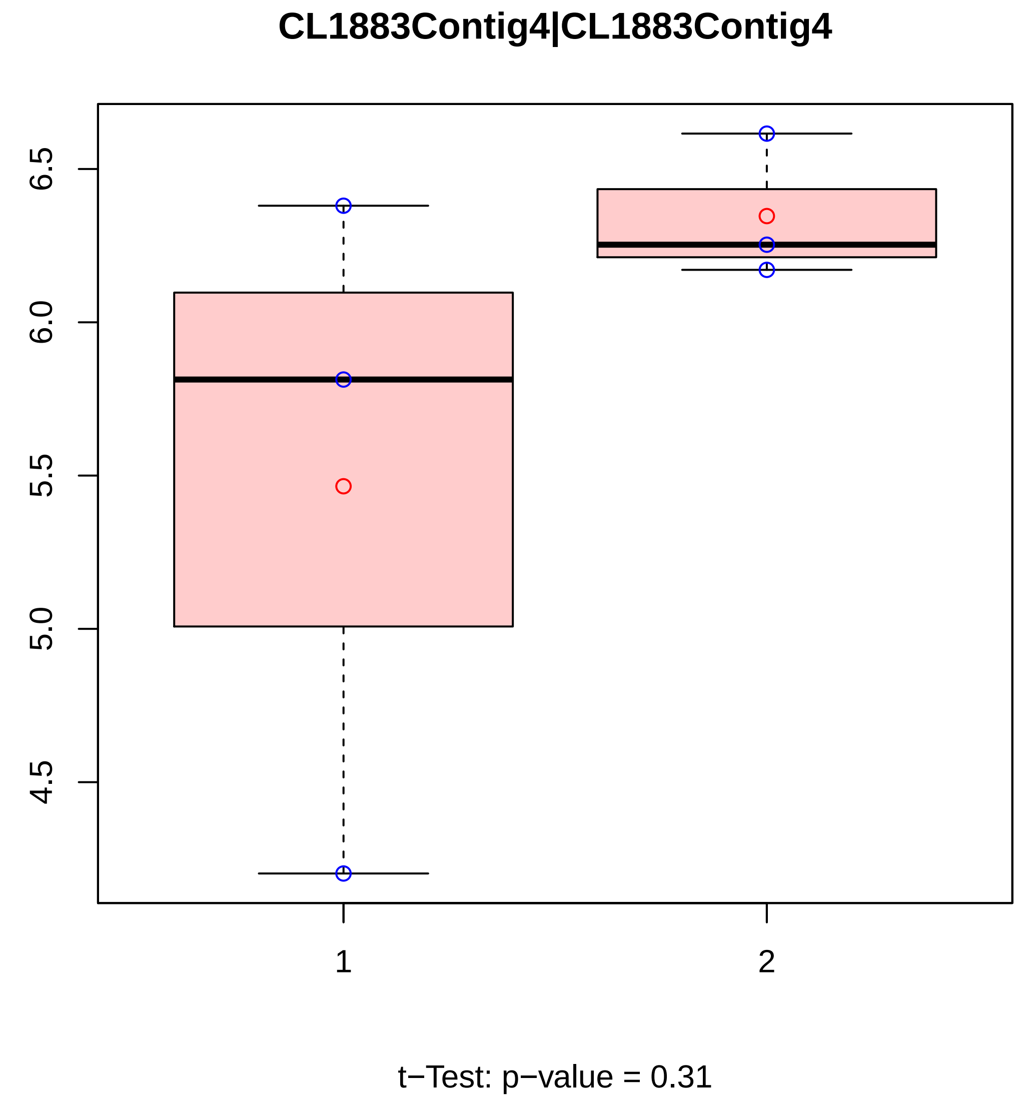
CL1883Contig4|CL1883Contig4
6.5
6.0
5.5
5.0
4.5
1
2
t−Test: p−value = 0.31

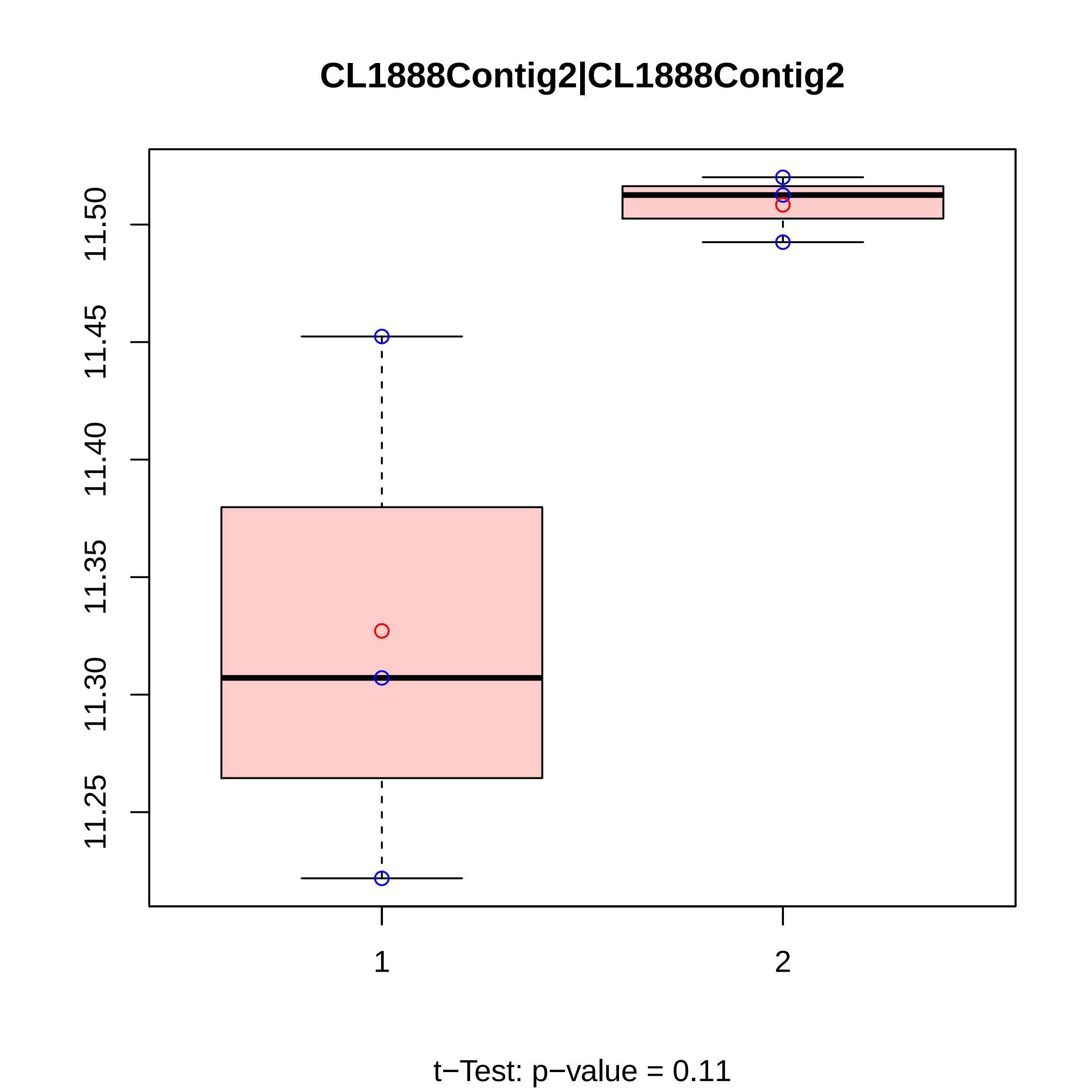

CL1888Contig2|CL1888Contig2
11.25
11.30
11.35
11.40
11.45
11.50
1
2
t−Test: p−value = 0.11

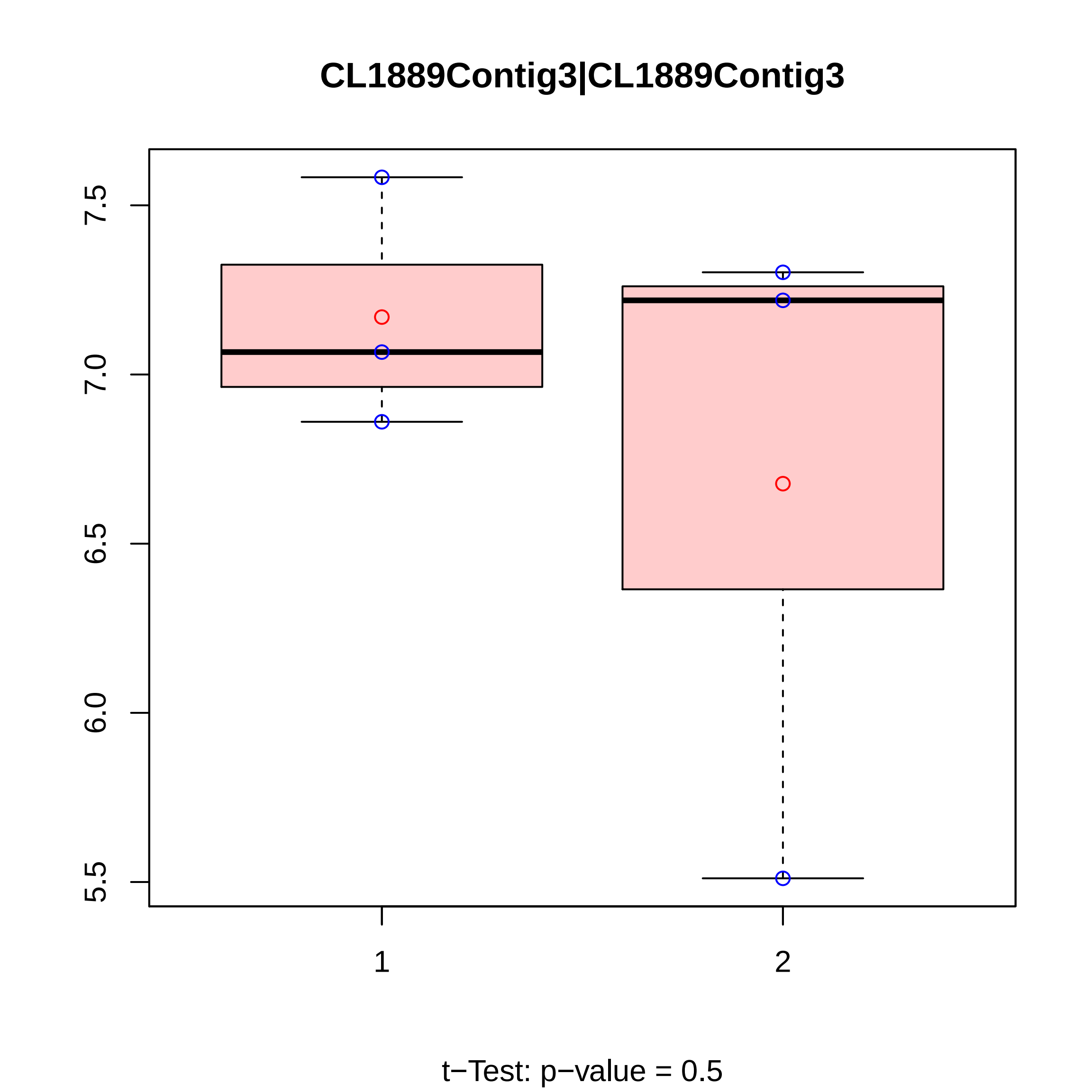
CL1889Contig3|CL1889Contig3
7.5
7.0
6.5
6.0
5.5
1
2
t−Test: p−value = 0.5

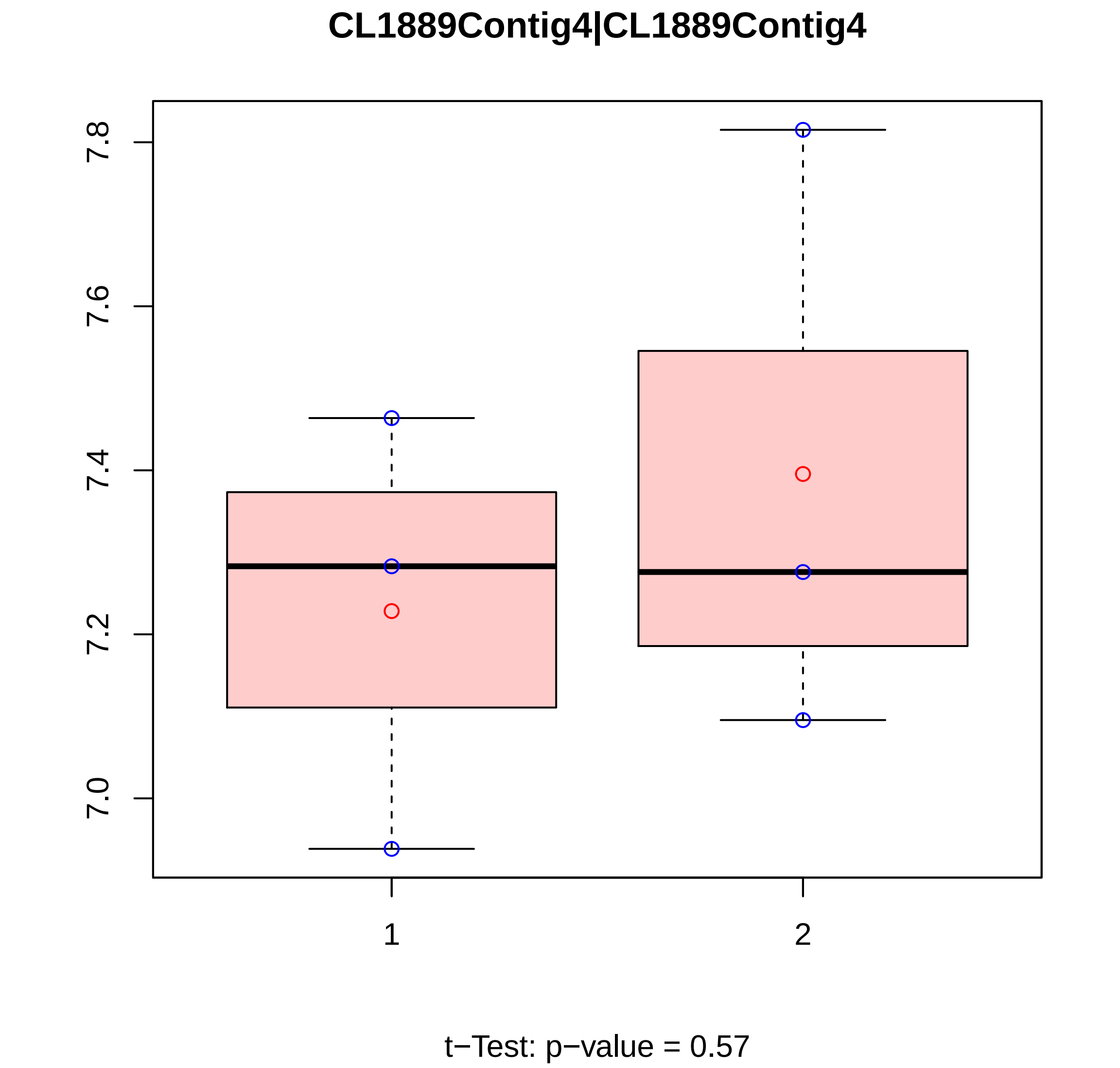
CL1889Contig4|CL1889Contig4
7.0
7.2
7.4
7.6
7.8
1
2
t−Test: p−value = 0.57

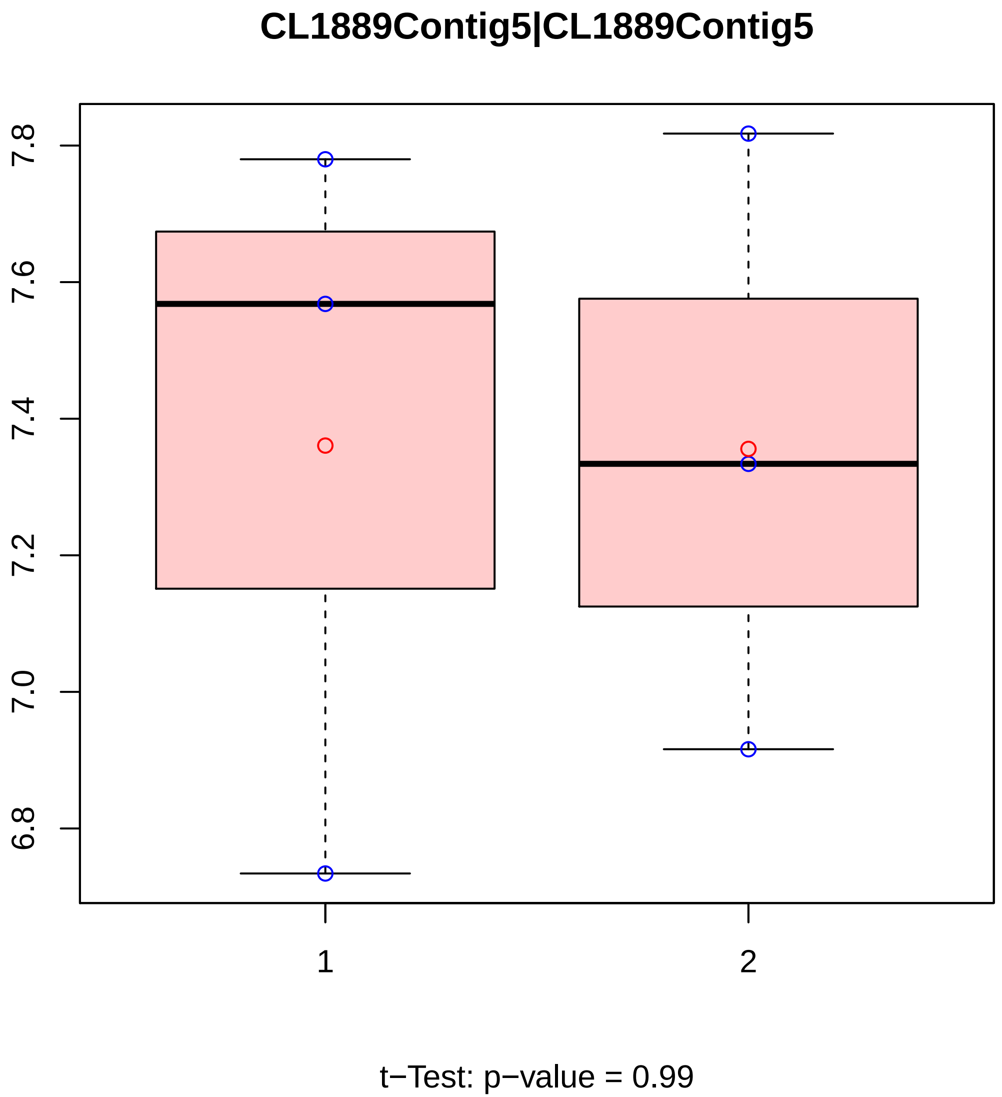
CL1889Contig5|CL1889Contig5
7.8
7.6
7.4
7.2
7.0
6.8
1
2
t−Test: p−value = 0.99

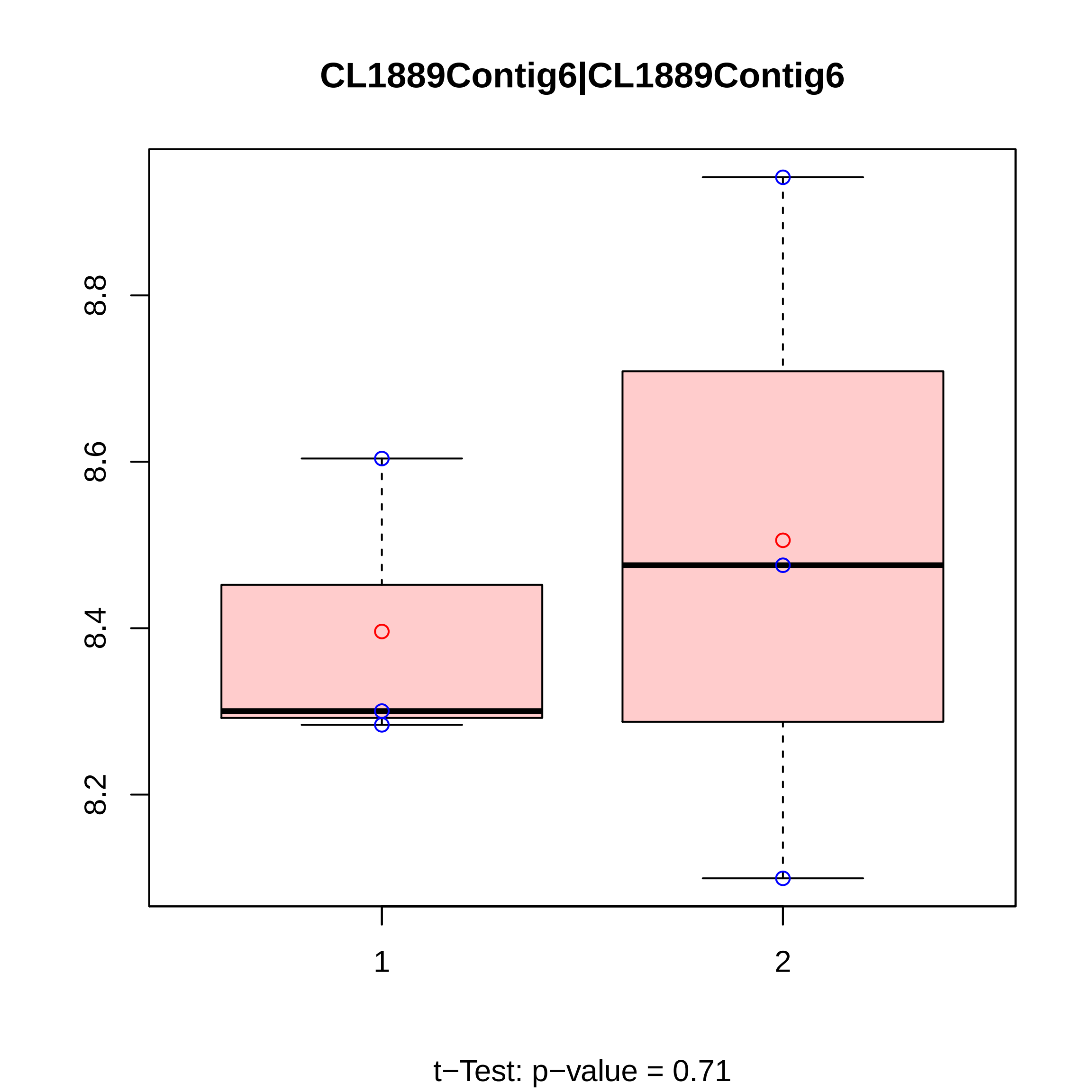
CL1889Contig6|CL1889Contig6
8.2
8.4
8.6
8.8
1
2
t−Test: p−value = 0.71

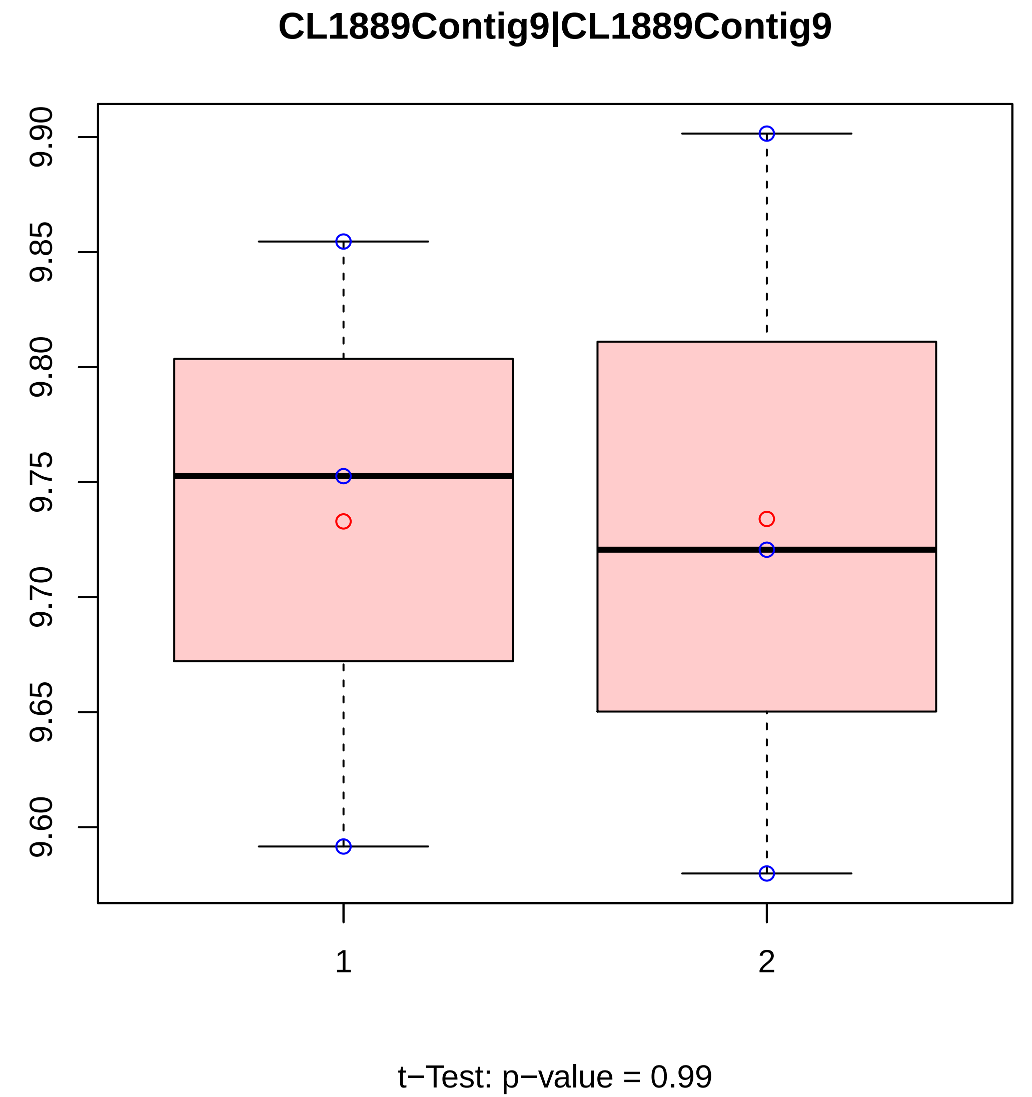
CL1889Contig9|CL1889Contig9
9.60
9.65
9.70
9.75
9.80
9.85
9.90
1
2
t−Test: p−value = 0.99

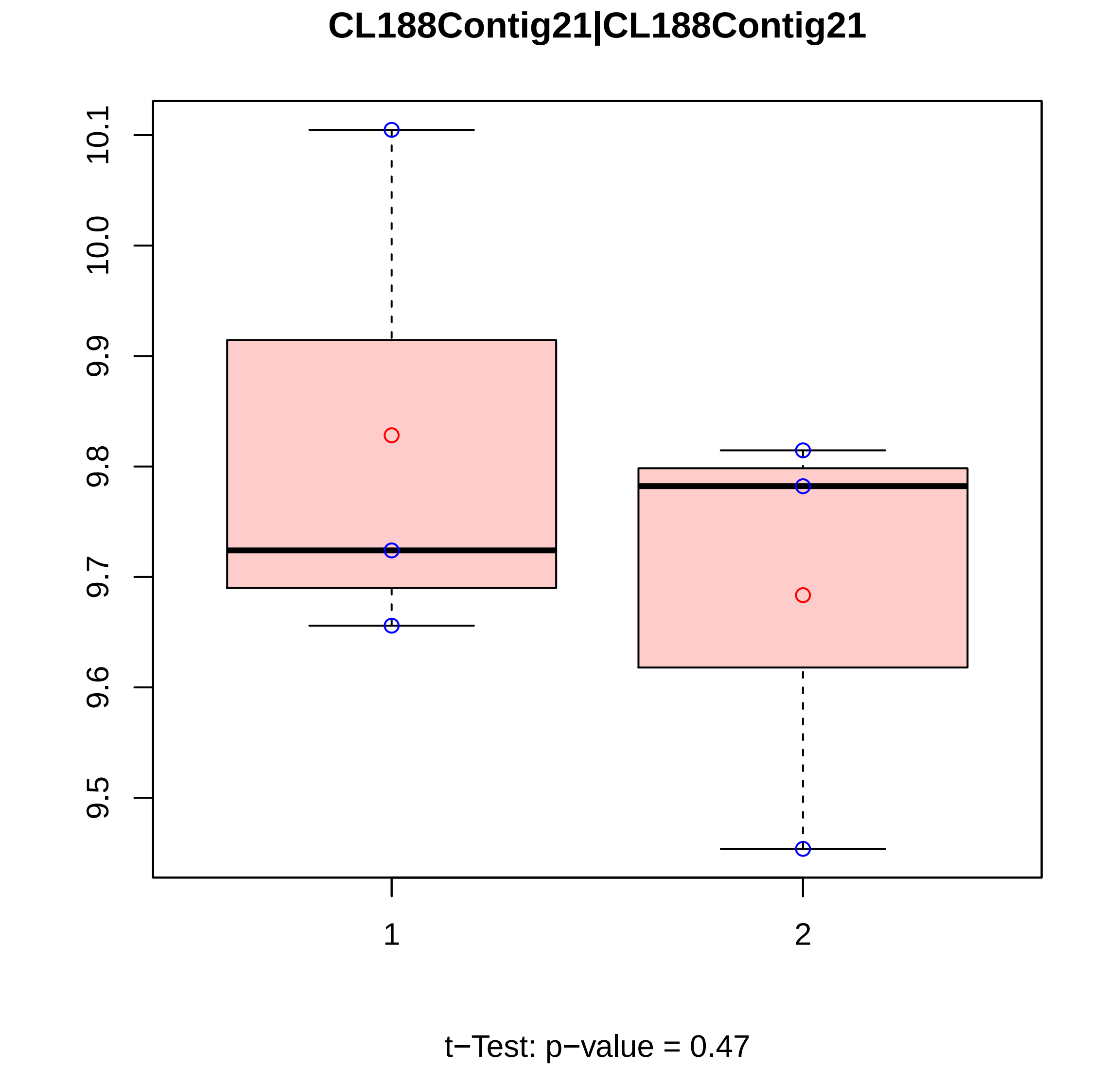
CL188Contig21|CL188Contig21
10.1
10.0
9.9
9.8
9.7
9.6
9.5
1
2
t−Test: p−value = 0.47

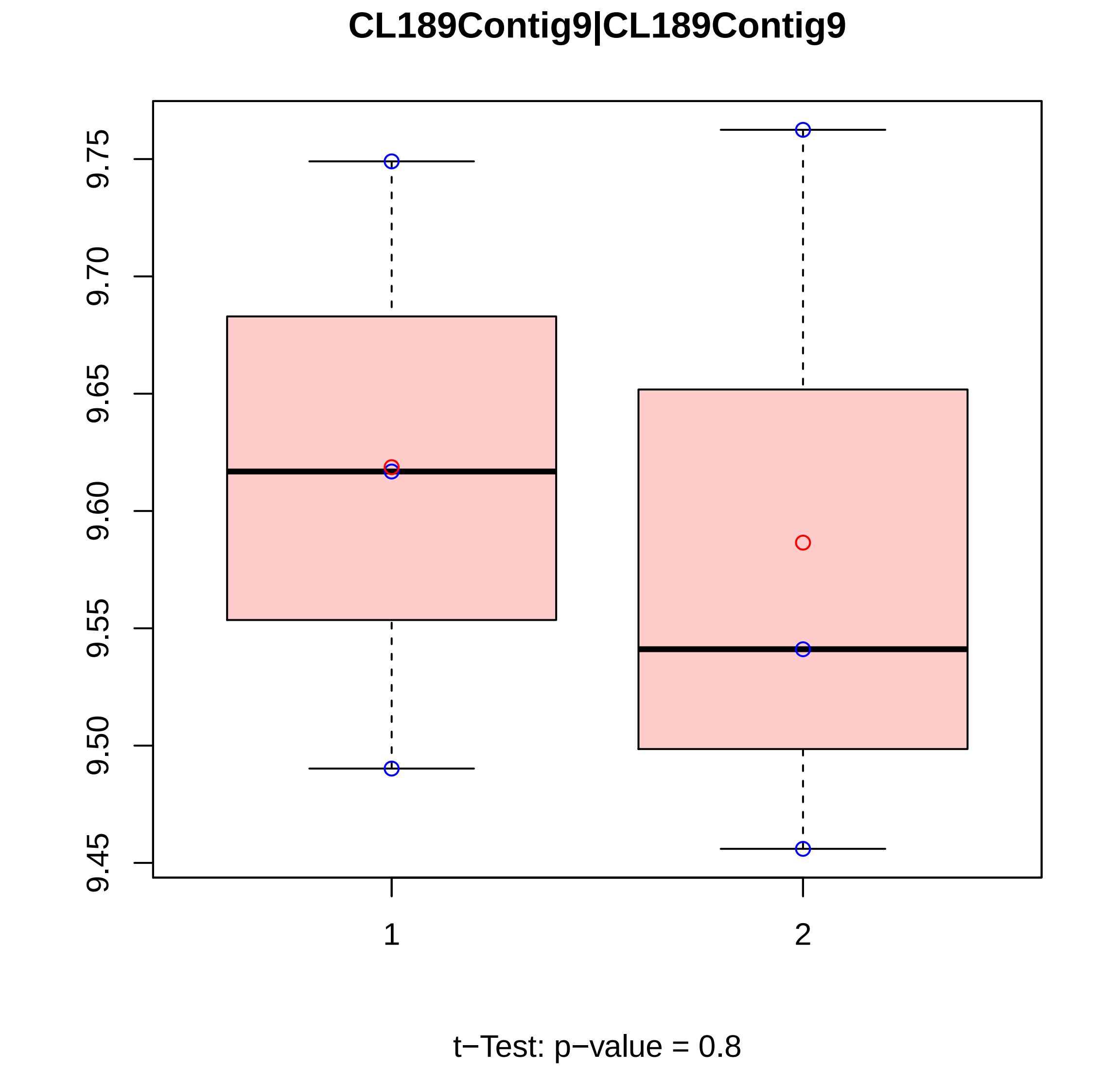
CL189Contig9|CL189Contig9
9.45
9.50
9.55
9.60
9.65
9.70
9.75
1
2
t−Test: p−value = 0.8

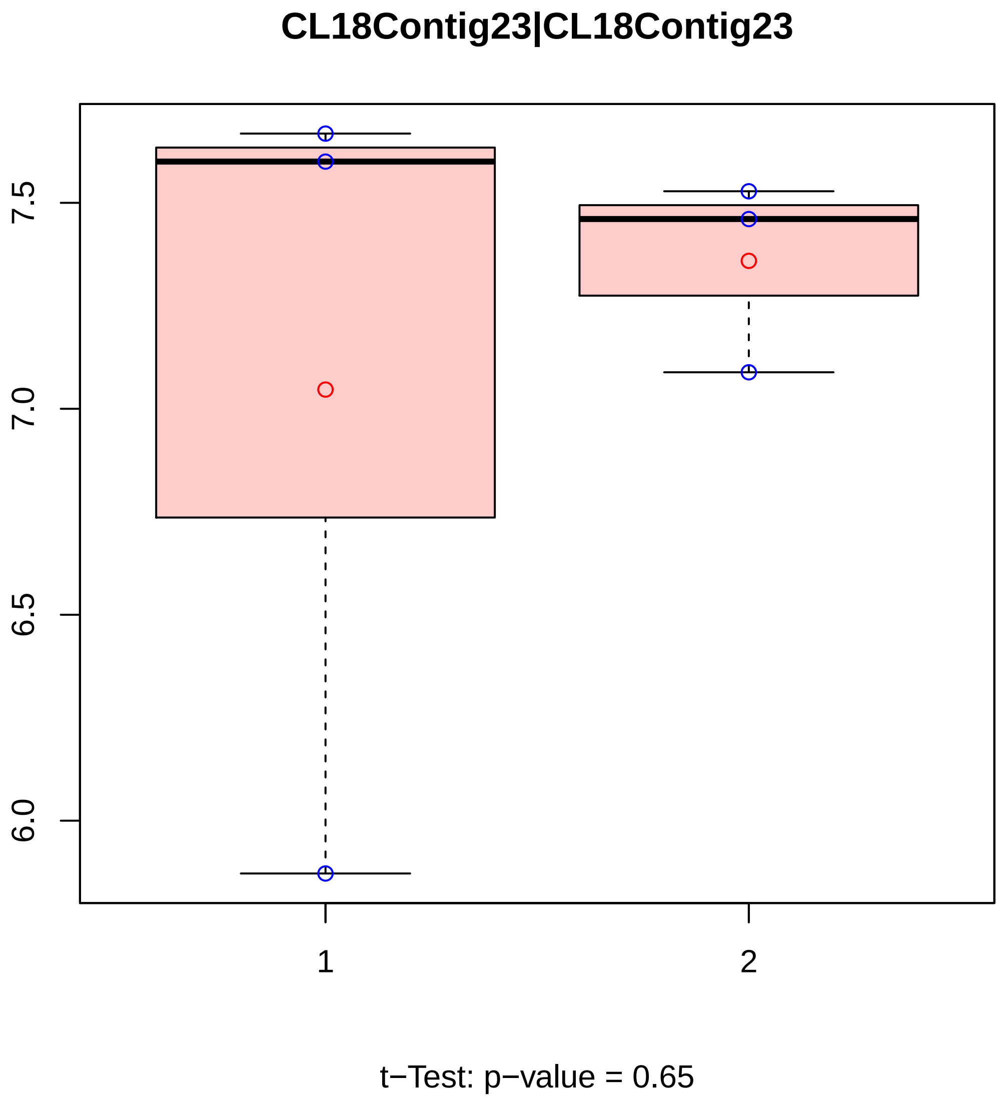
CL18Contig23|CL18Contig23
7.5
7.0
6.5
6.0
1
2
t−Test: p−value = 0.65

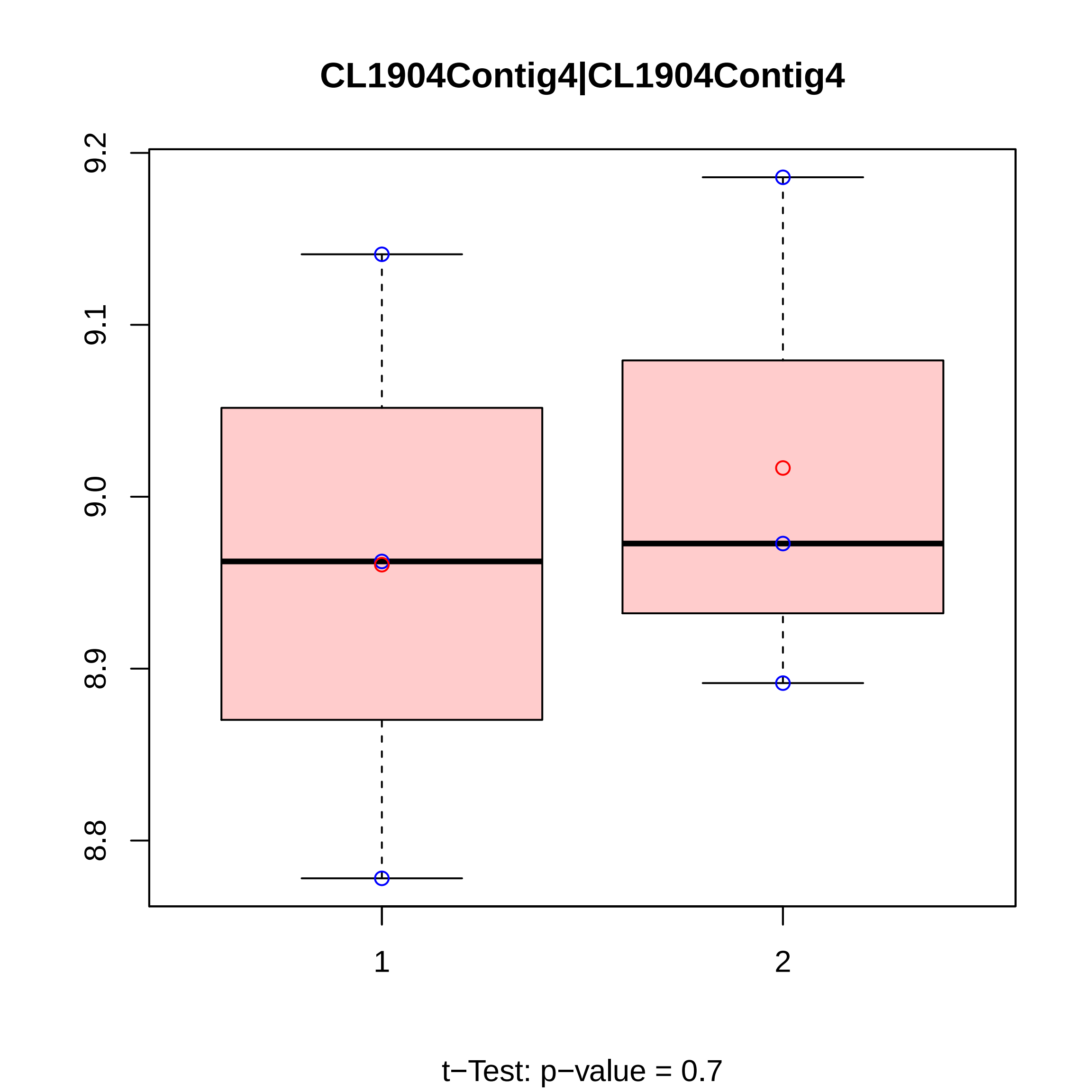
CL1904Contig4|CL1904Contig4
9.2
9.1
9.0
8.9
8.8
1
2
t−Test: p−value = 0.7

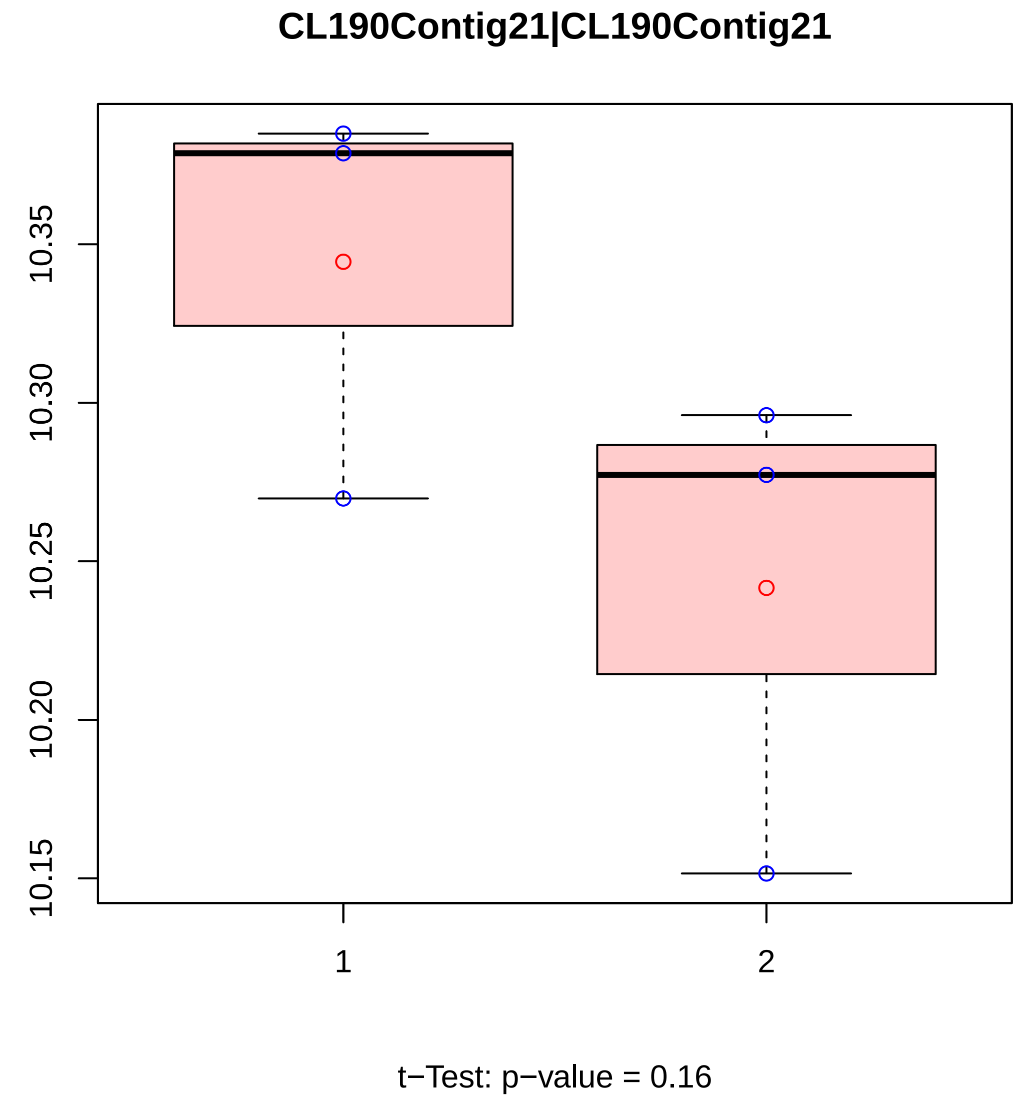
CL190Contig21|CL190Contig21
10.15
10.20
10.25
10.30
10.35
1
2
t−Test: p−value = 0.16

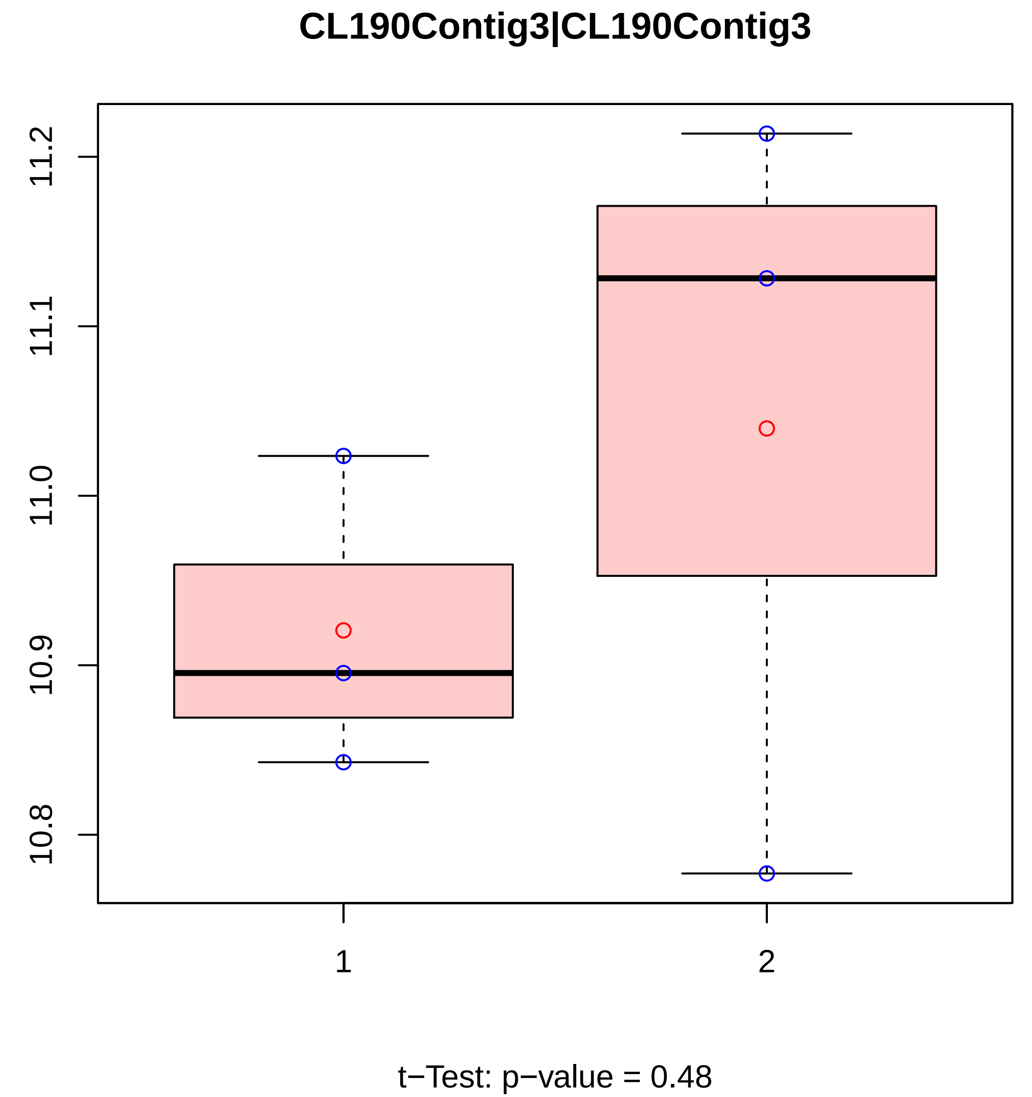
CL190Contig3|CL190Contig3
11.2
11.1
11.0
10.9
10.8
1
2
t−Test: p−value = 0.48

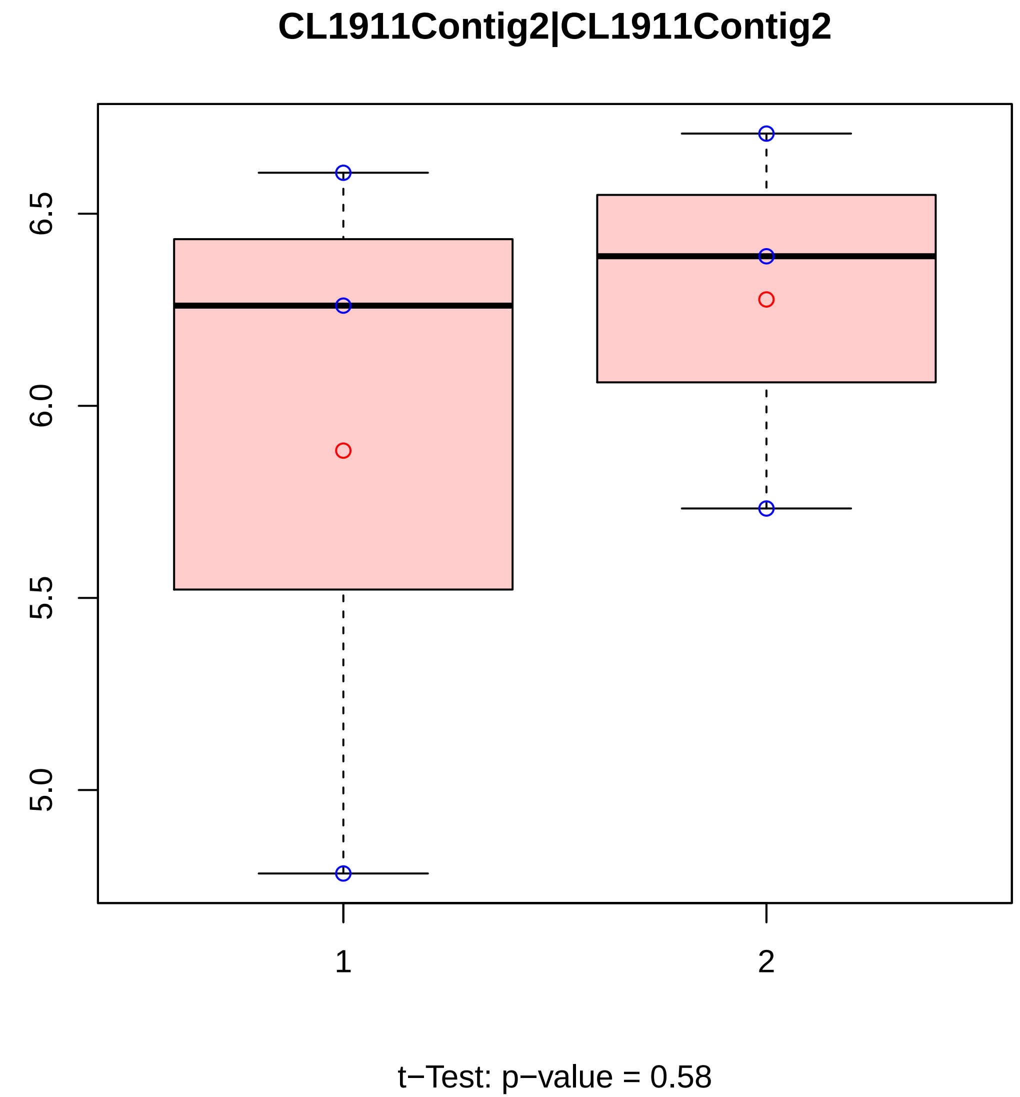
CL1911Contig2|CL1911Contig2
6.5
6.0
5.5
5.0
1
2
t−Test: p−value = 0.58

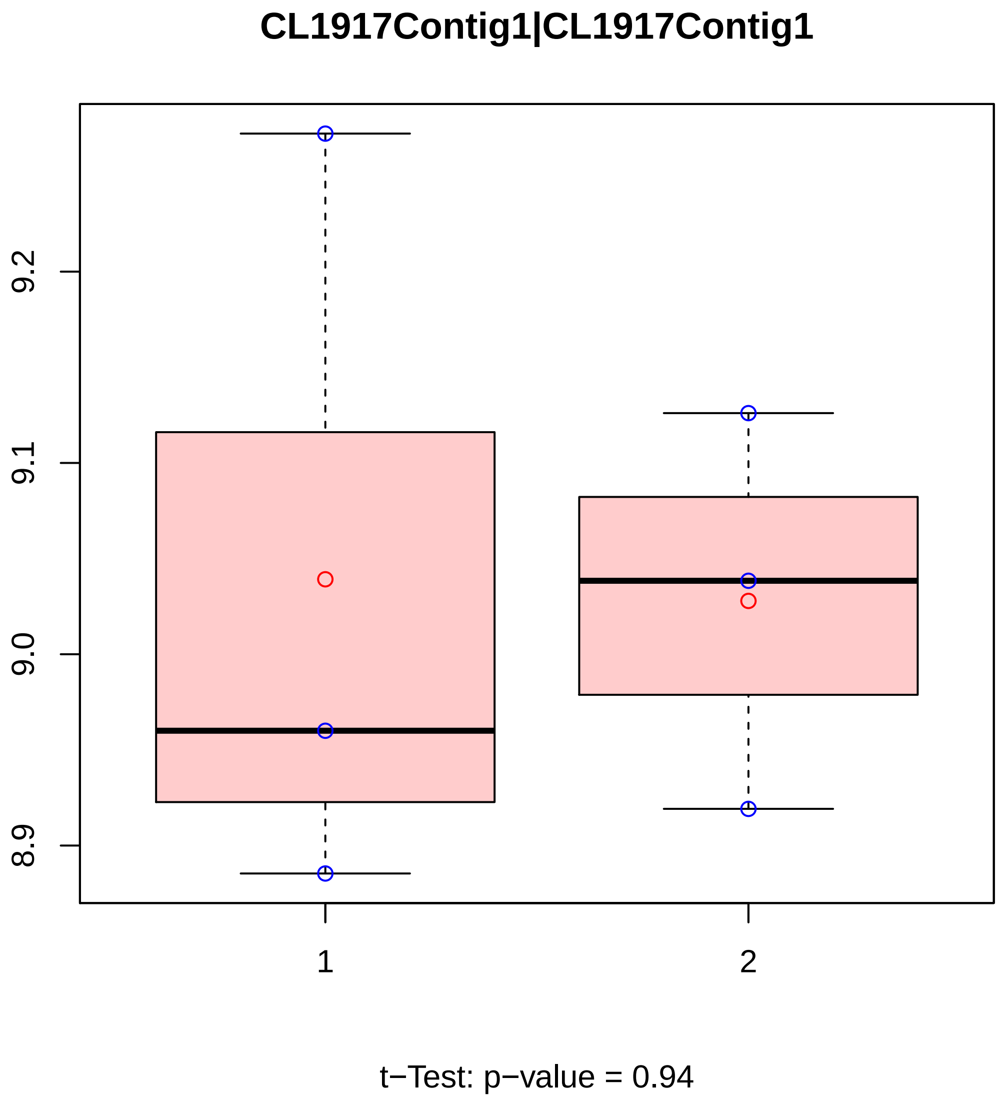
CL1917Contig1|CL1917Contig1
9.2
9.1
9.0
8.9
1
2
t−Test: p−value = 0.94

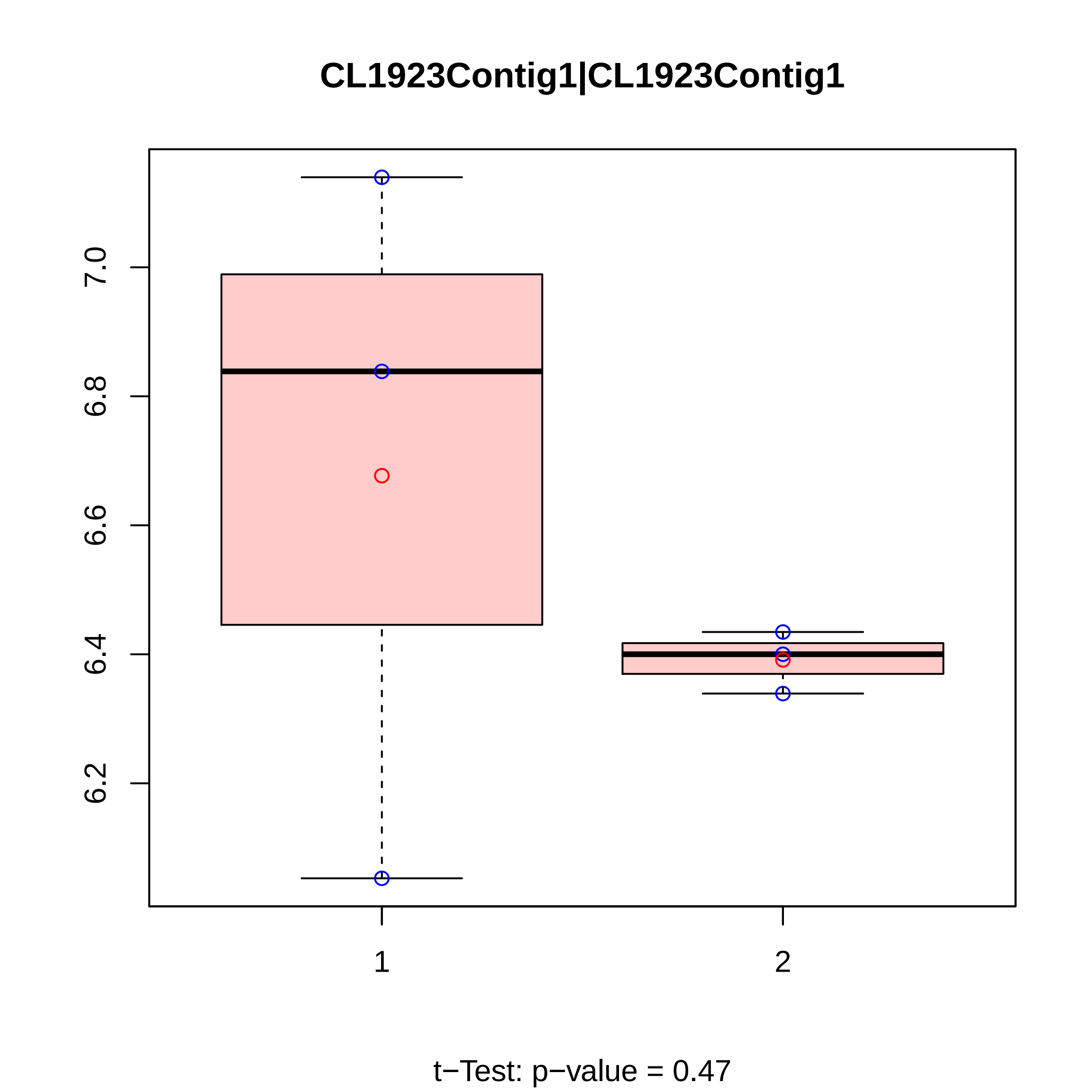
CL1923Contig1|CL1923Contig1
7.0
6.8
6.6
6.4
6.2
1
2
t−Test: p−value = 0.47

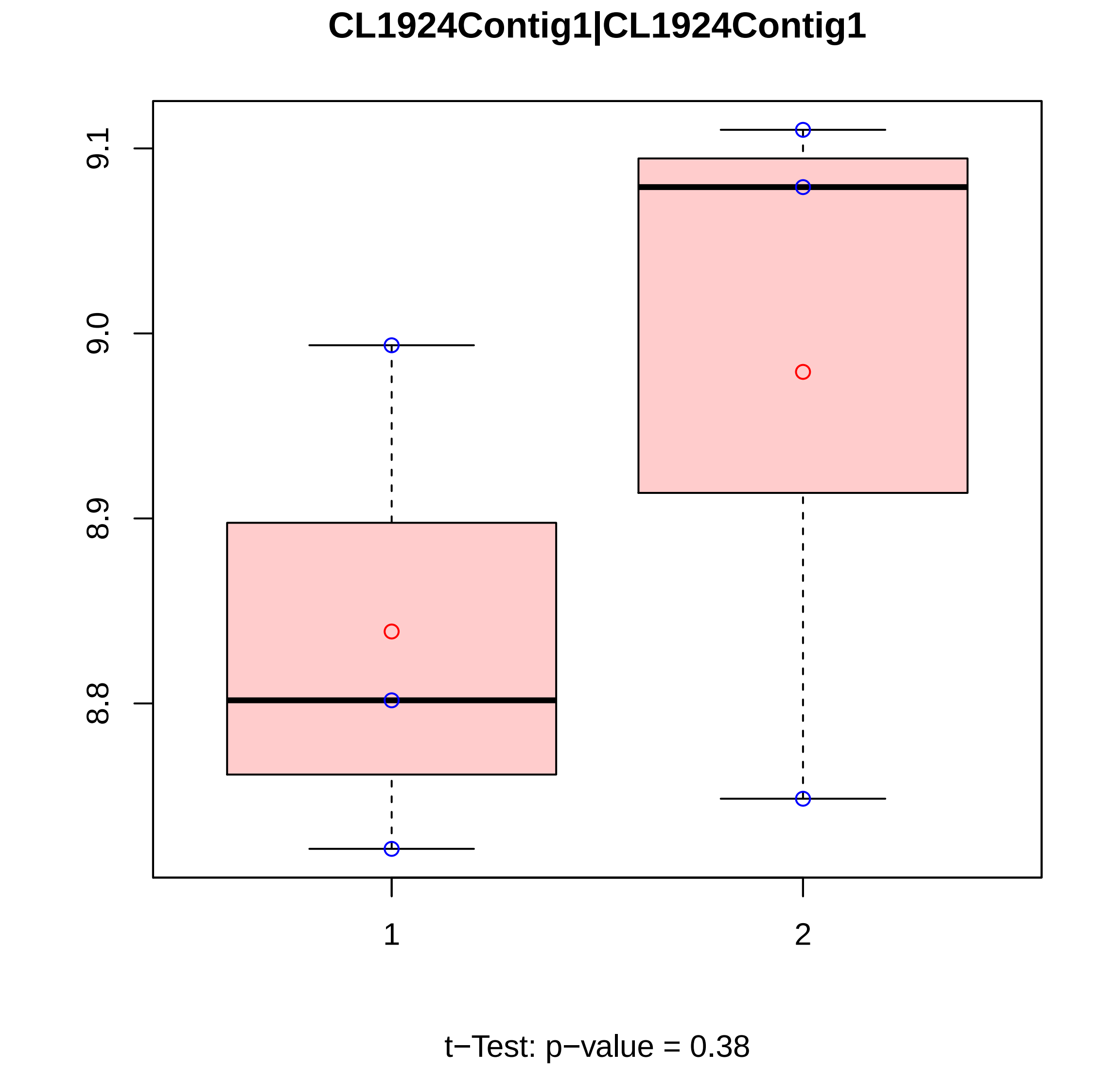
CL1924Contig1|CL1924Contig1
9.1
9.0
8.9
8.8
1
2
t−Test: p−value = 0.38

# CL1937Contig7|CL1937Contig7

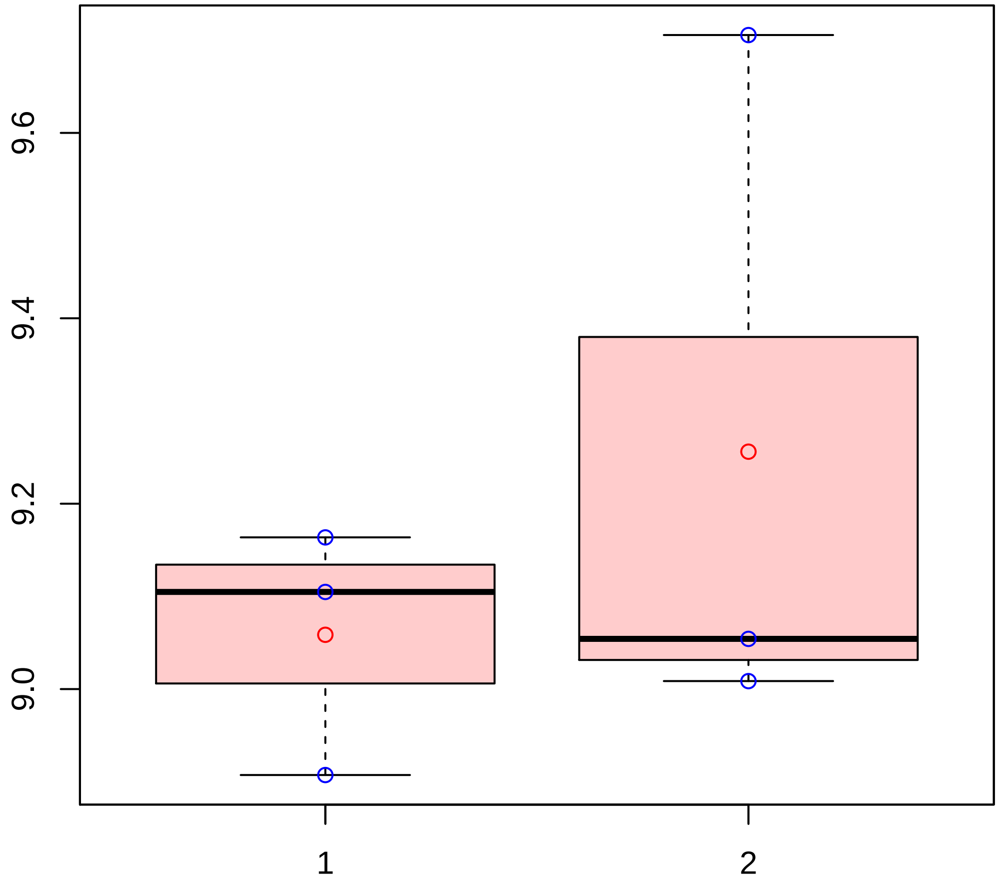


t−Test: p−value = 0.48

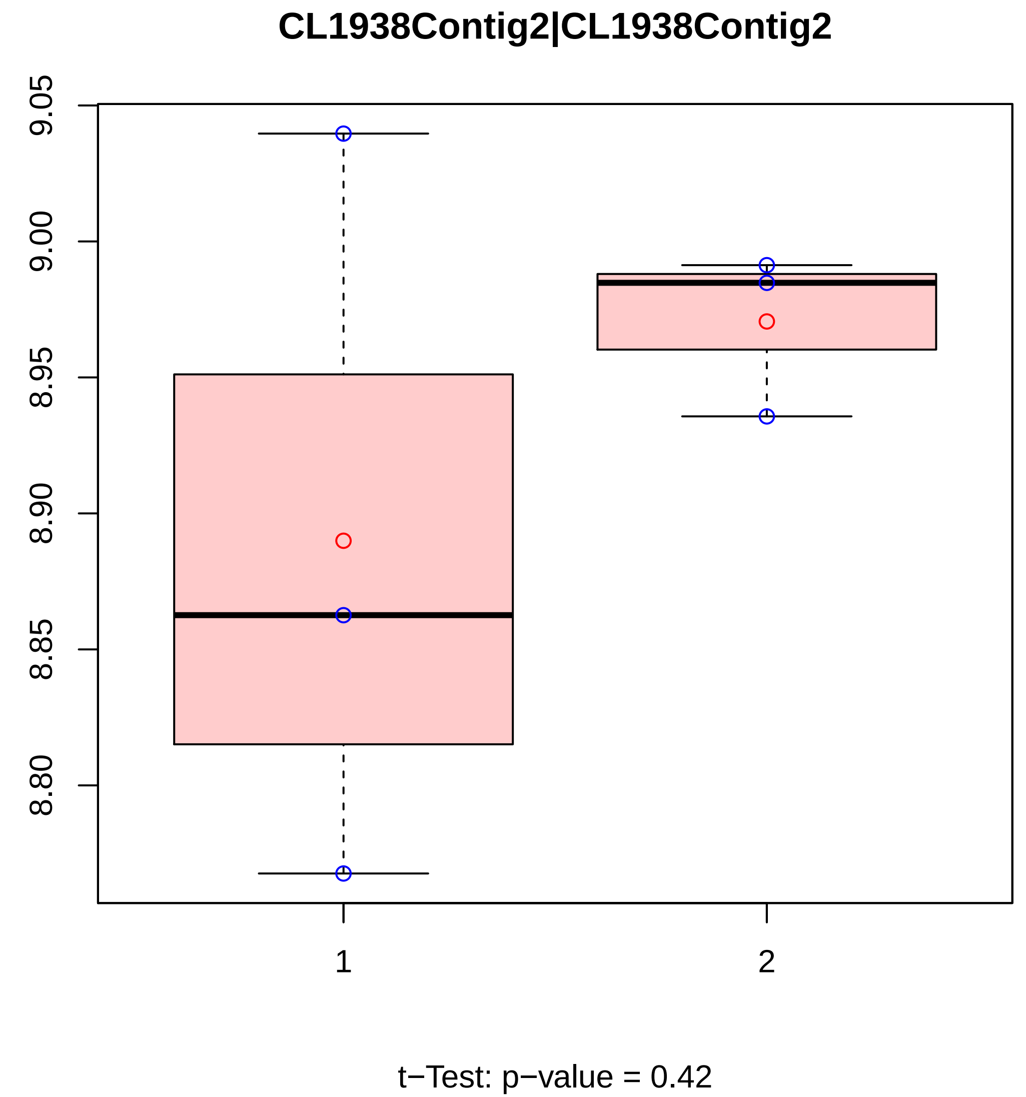
CL1938Contig2|CL1938Contig2
9.05
9.00
8.95
8.90
8.85
8.80
1
2
t−Test: p−value = 0.42

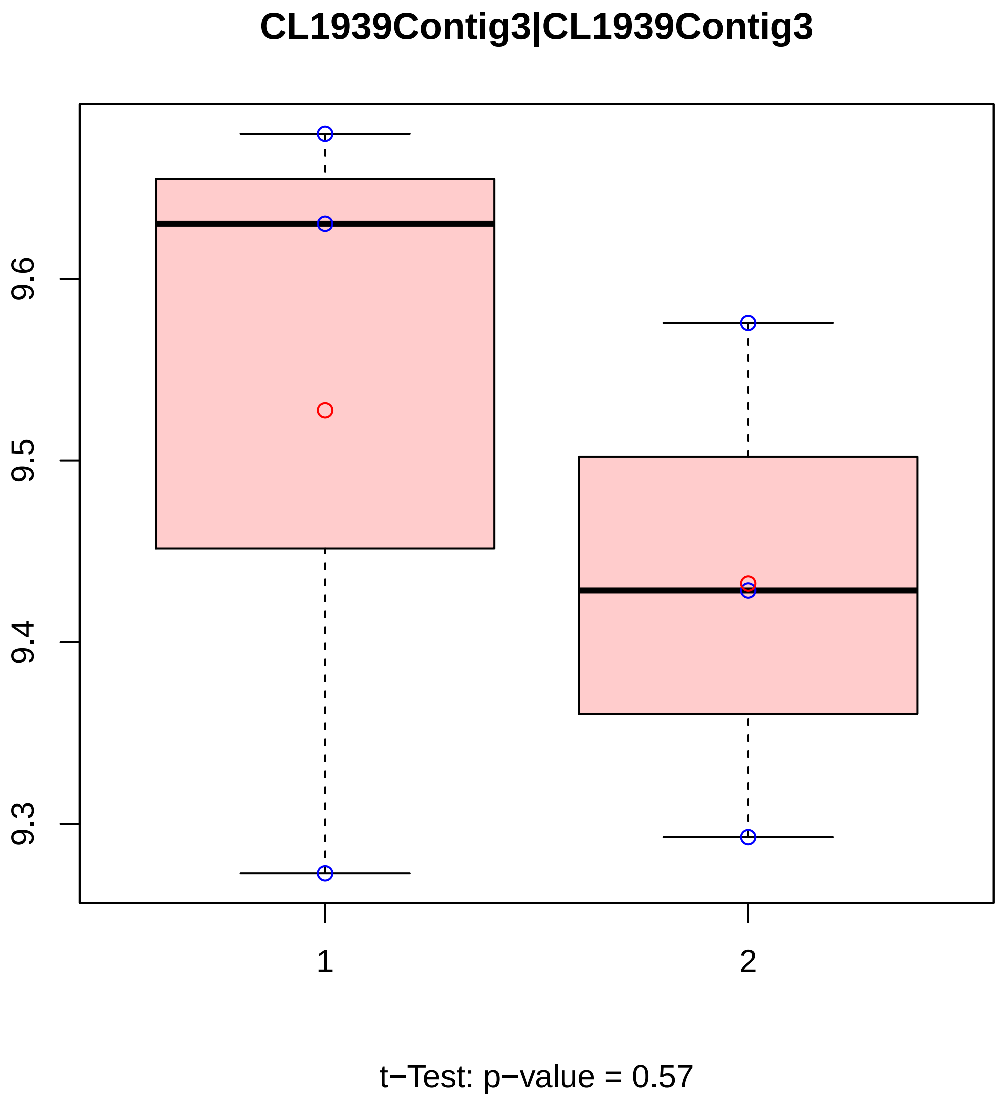
CL1939Contig3|CL1939Contig3
9.3
9.4
9.5
9.6
1
2
t−Test: p−value = 0.57

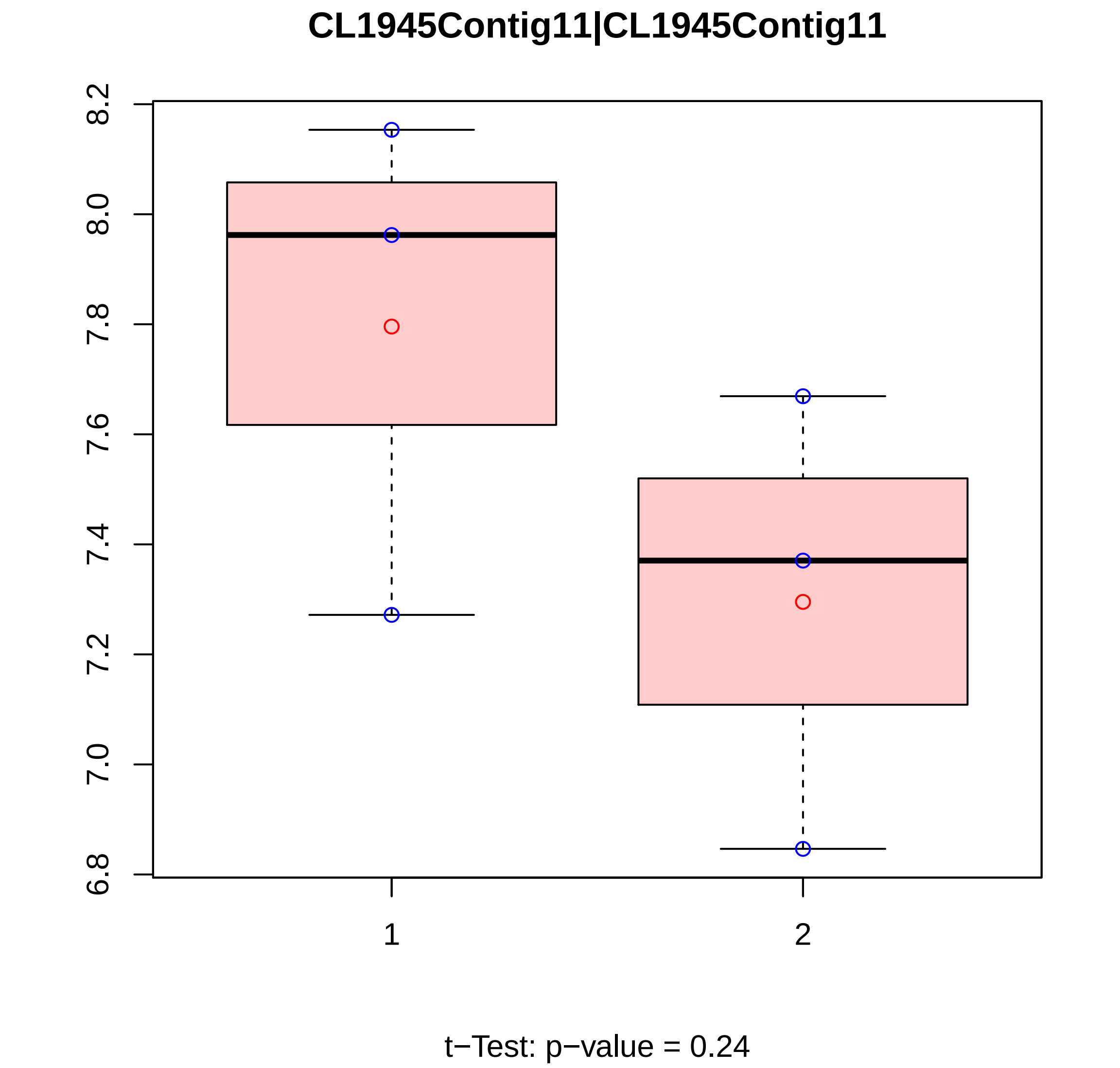
CL1945Contig11|CL1945Contig11
8.2
8.0
7.8
7.6
7.4
7.2
7.0
6.8
1
2
t−Test: p−value = 0.24

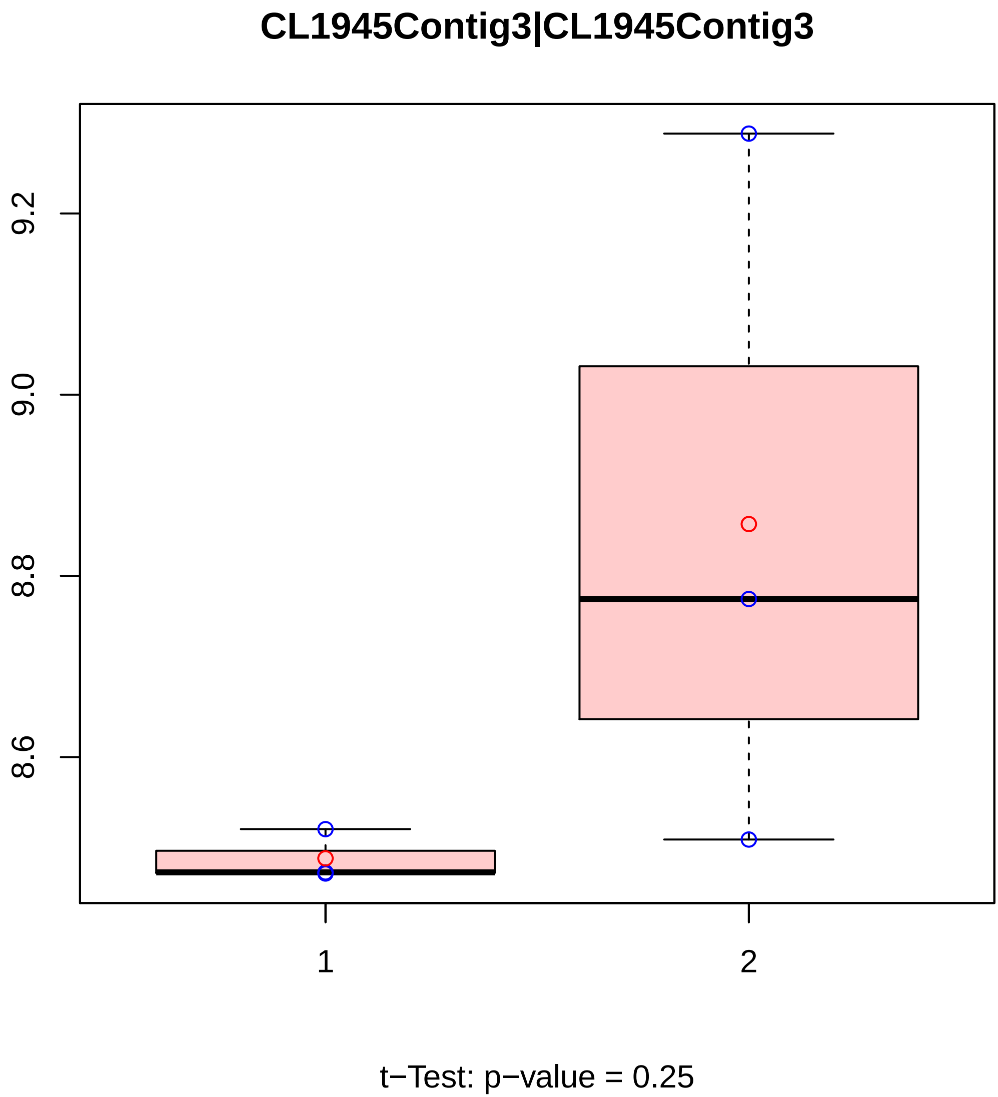
CL1945Contig3|CL1945Contig3
9.2
9.0
8.8
8.6
1
2
t−Test: p−value = 0.25

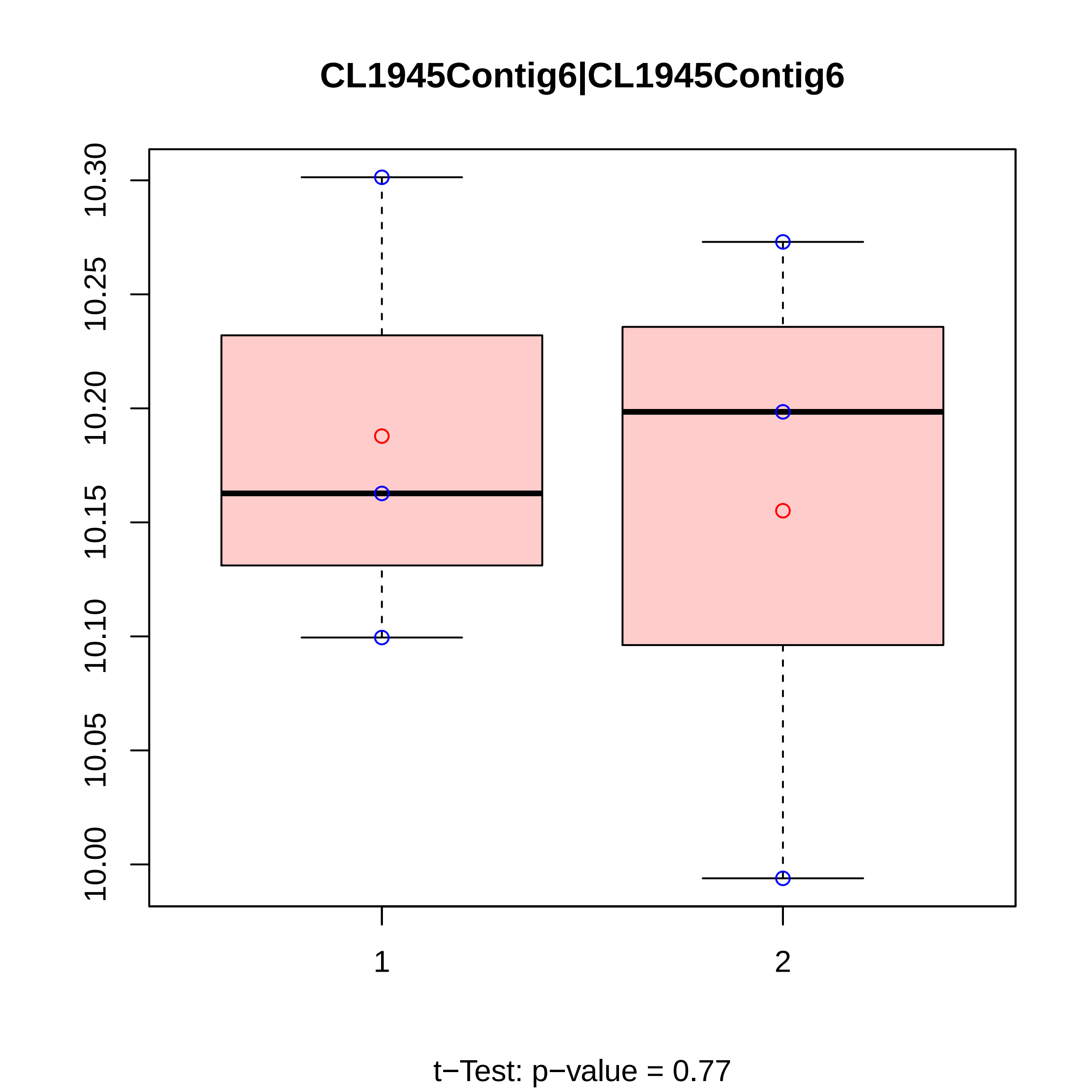

CL1945Contig6|CL1945Contig6
10.00
10.05
10.10
10.15
10.20
10.25
10.30
1
2
t−Test: p−value = 0.77

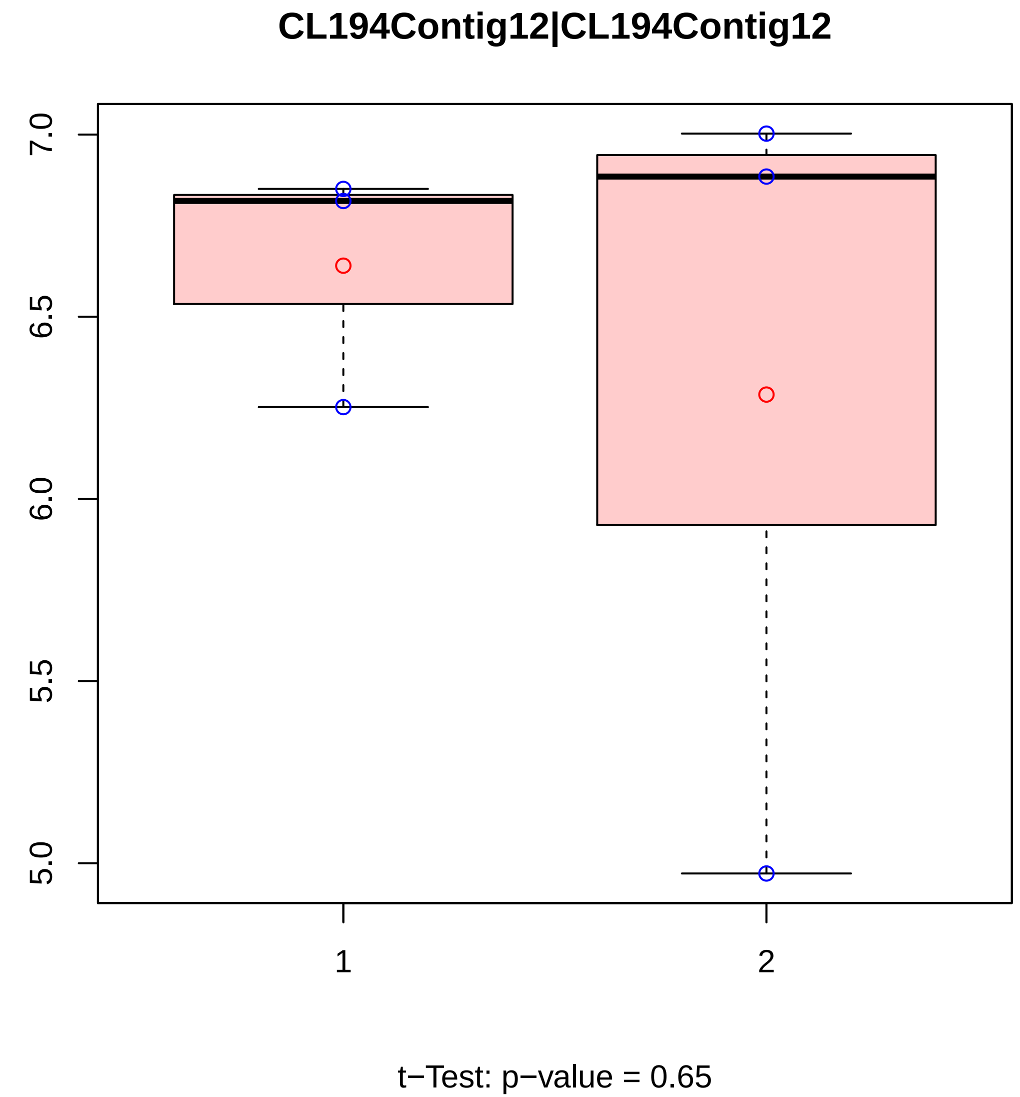
CL194Contig12|CL194Contig12
7.0
6.5
6.0
5.5
5.0
1
2
t−Test: p−value = 0.65

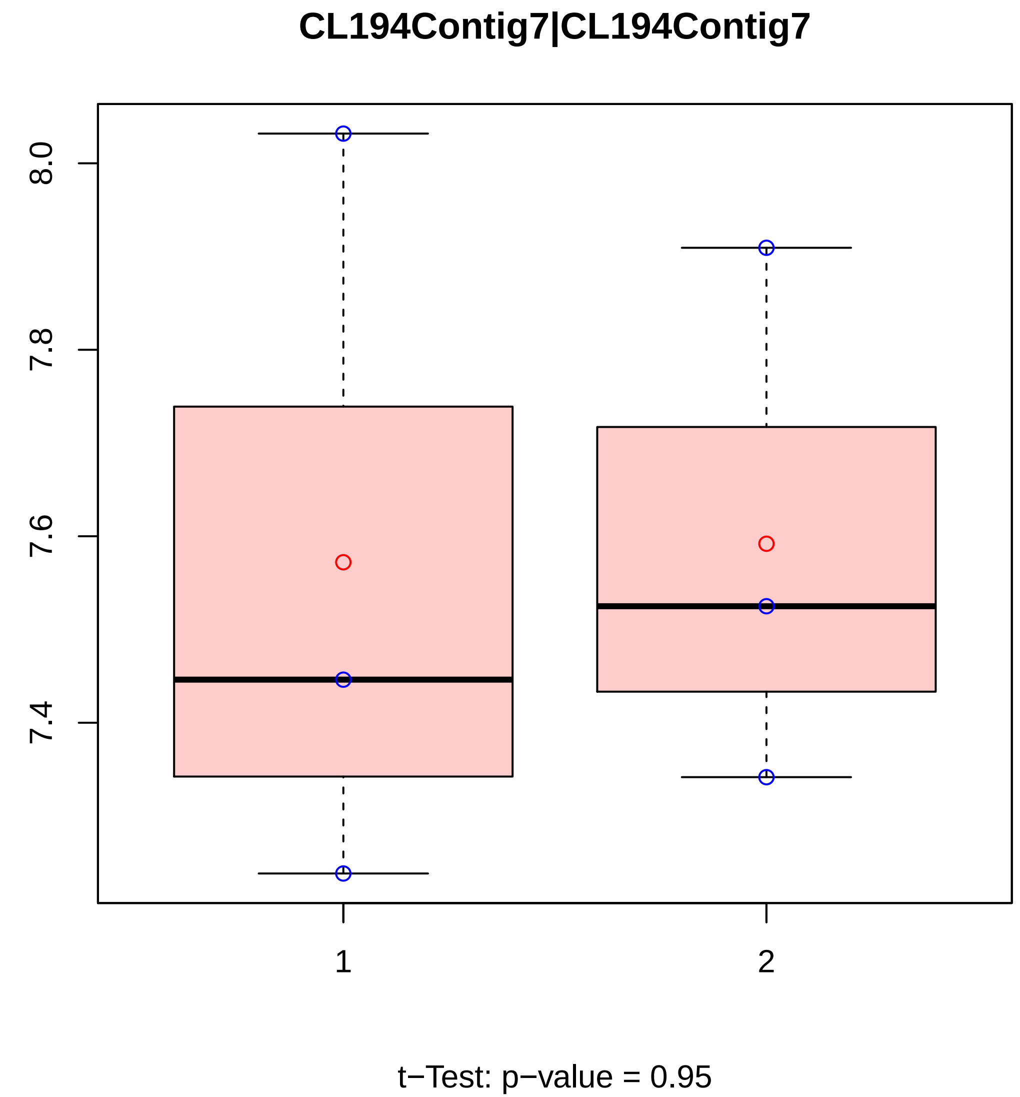

CL194Contig7|CL194Contig7
8.0
7.8
7.6
7.4
1
2
t−Test: p−value = 0.95

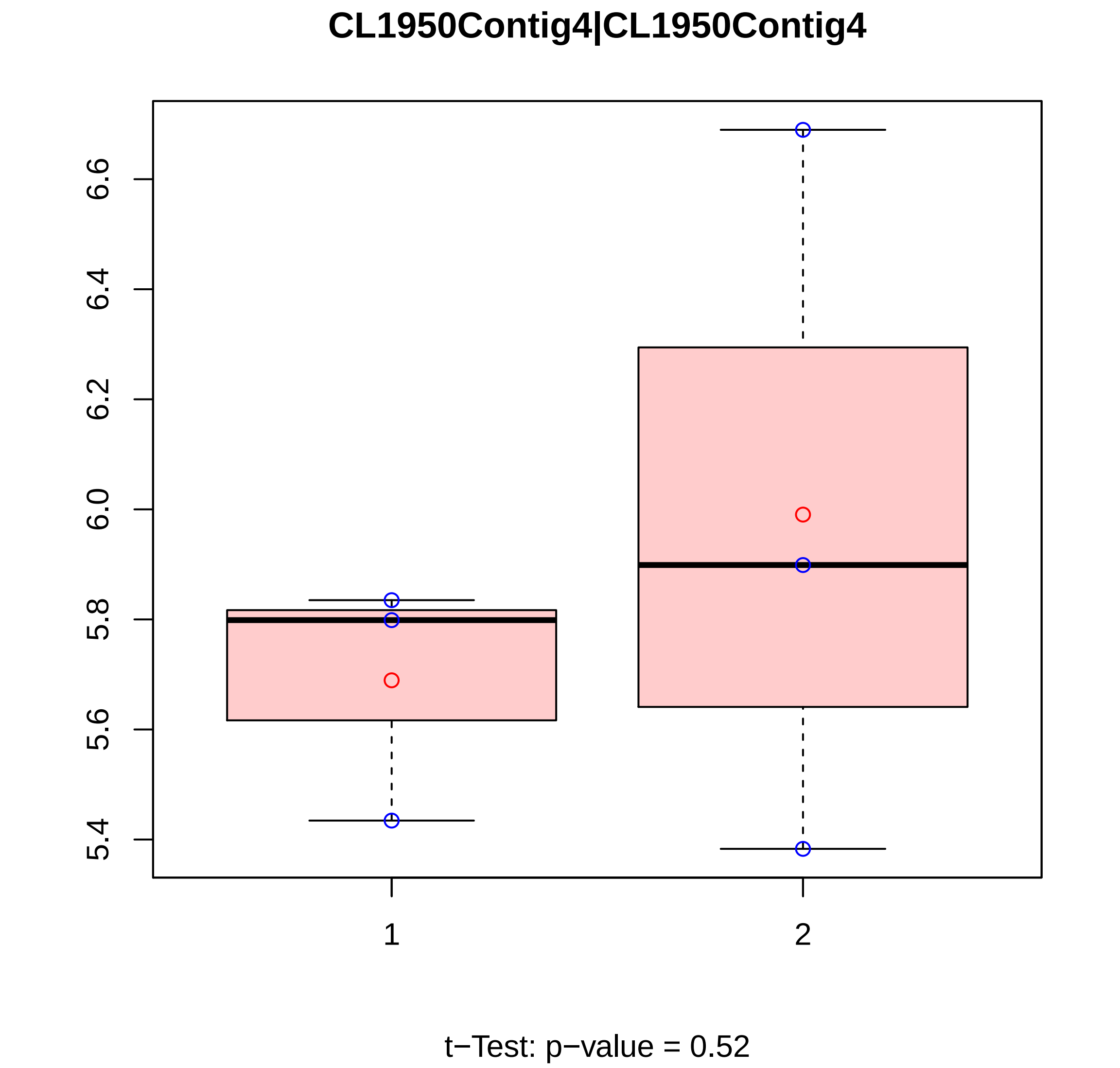
CL1950Contig4|CL1950Contig4
6.6
6.4
6.2
6.0
5.8
5.6
5.4
1
2
t−Test: p−value = 0.52

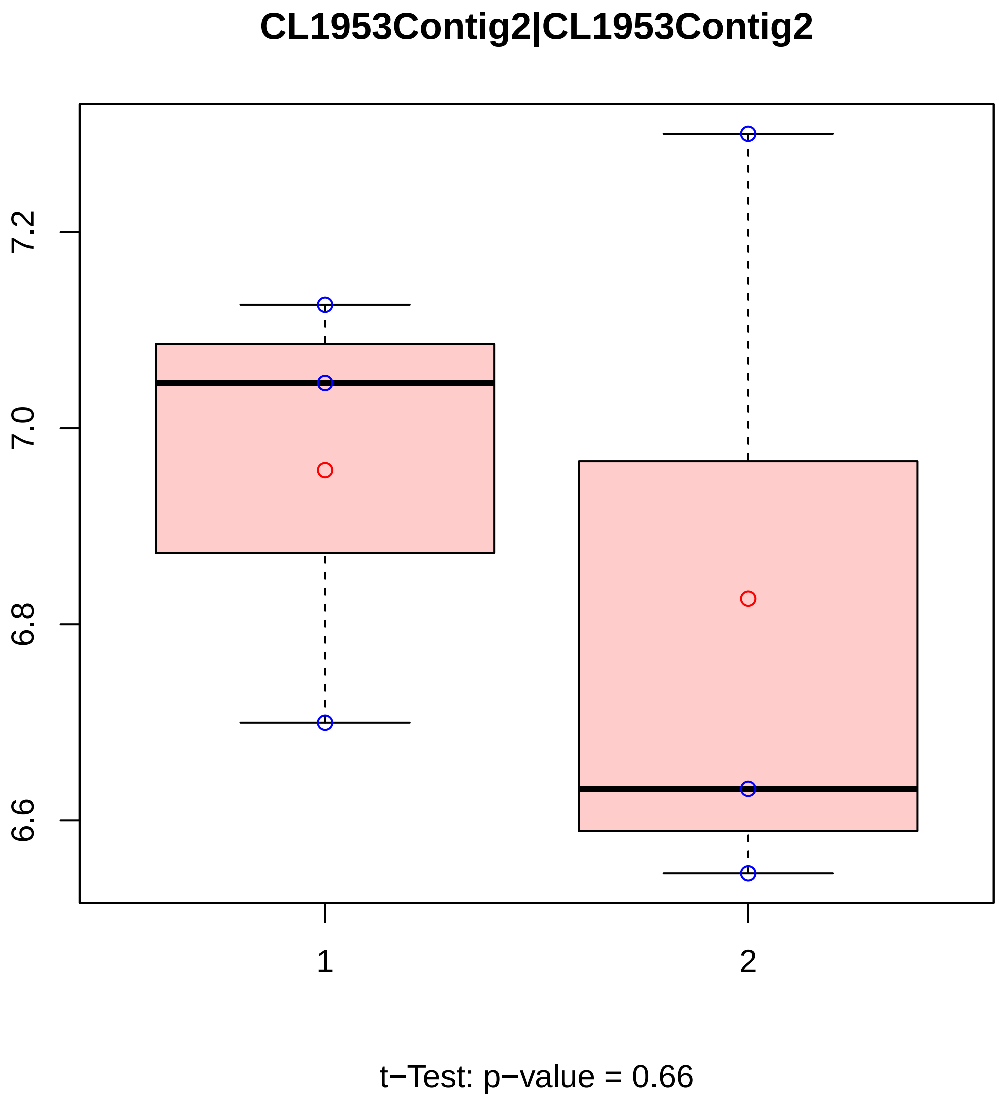

CL1953Contig2|CL1953Contig2
7.2
7.0
6.8
6.6
1
2
t−Test: p−value = 0.66

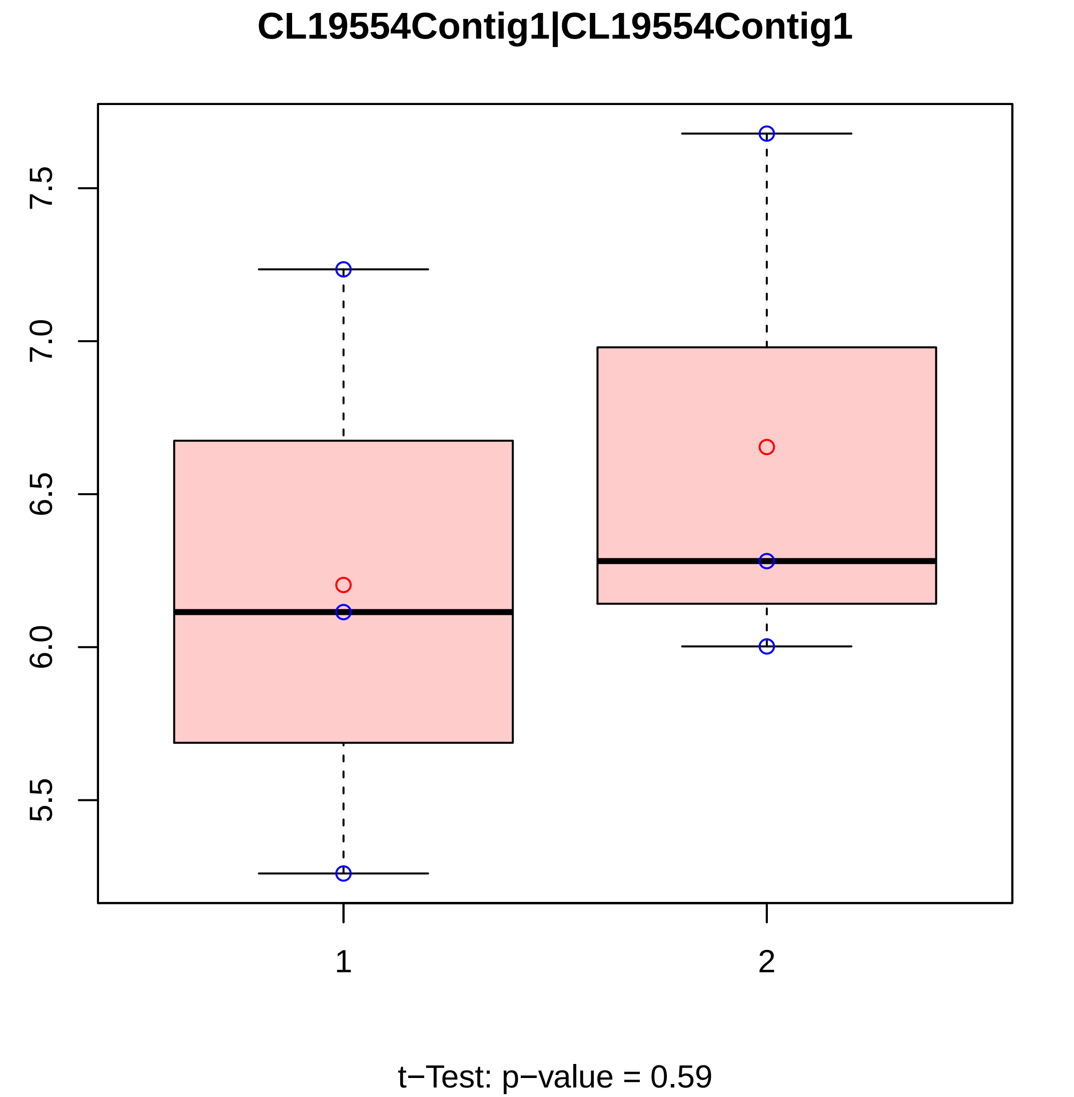
CL19554Contig1|CL19554Contig1
7.5
7.0
6.5
6.0
5.5
1
2
t−Test: p−value = 0.59

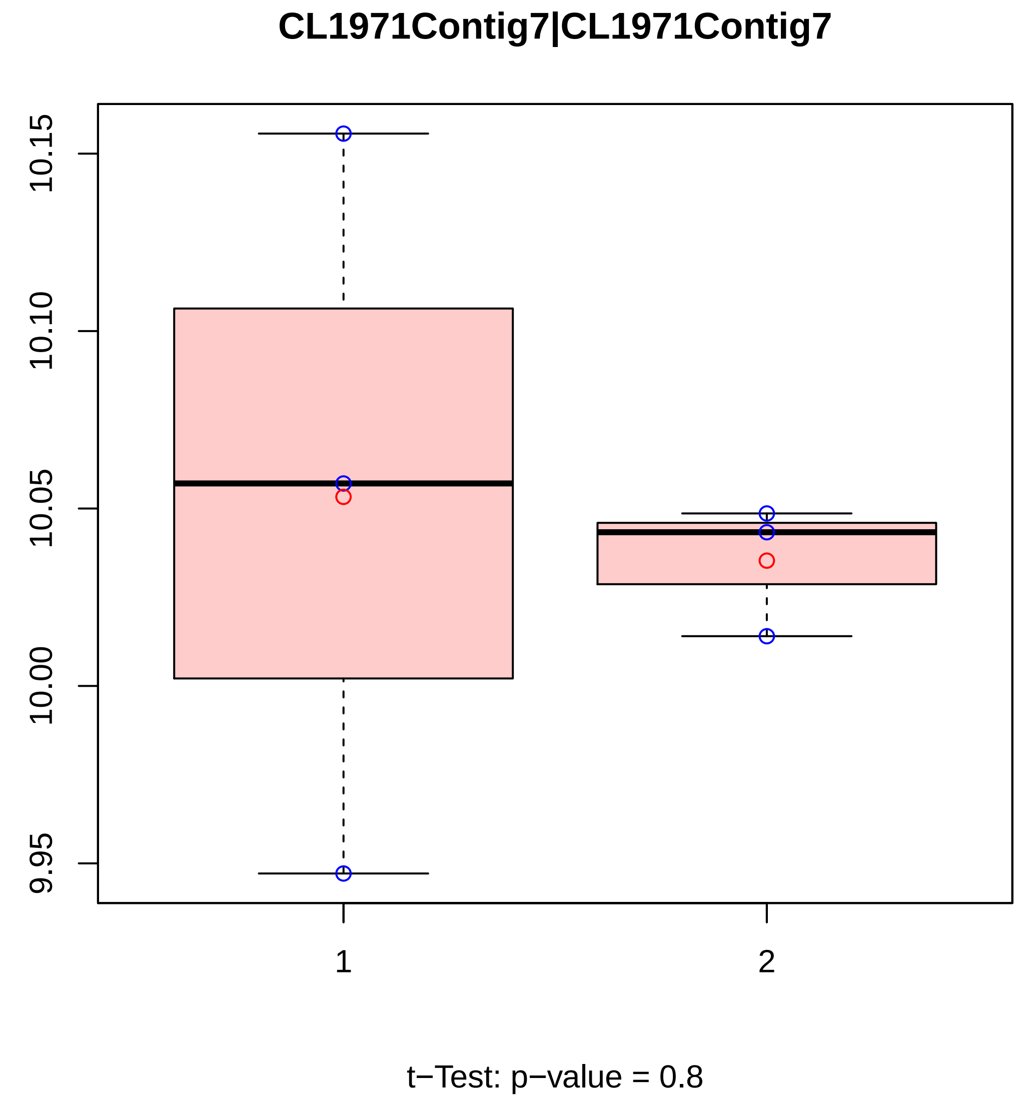
CL1971Contig7|CL1971Contig7
10.15
10.10
10.05
10.00
9.95
1
2
t−Test: p−value = 0.8

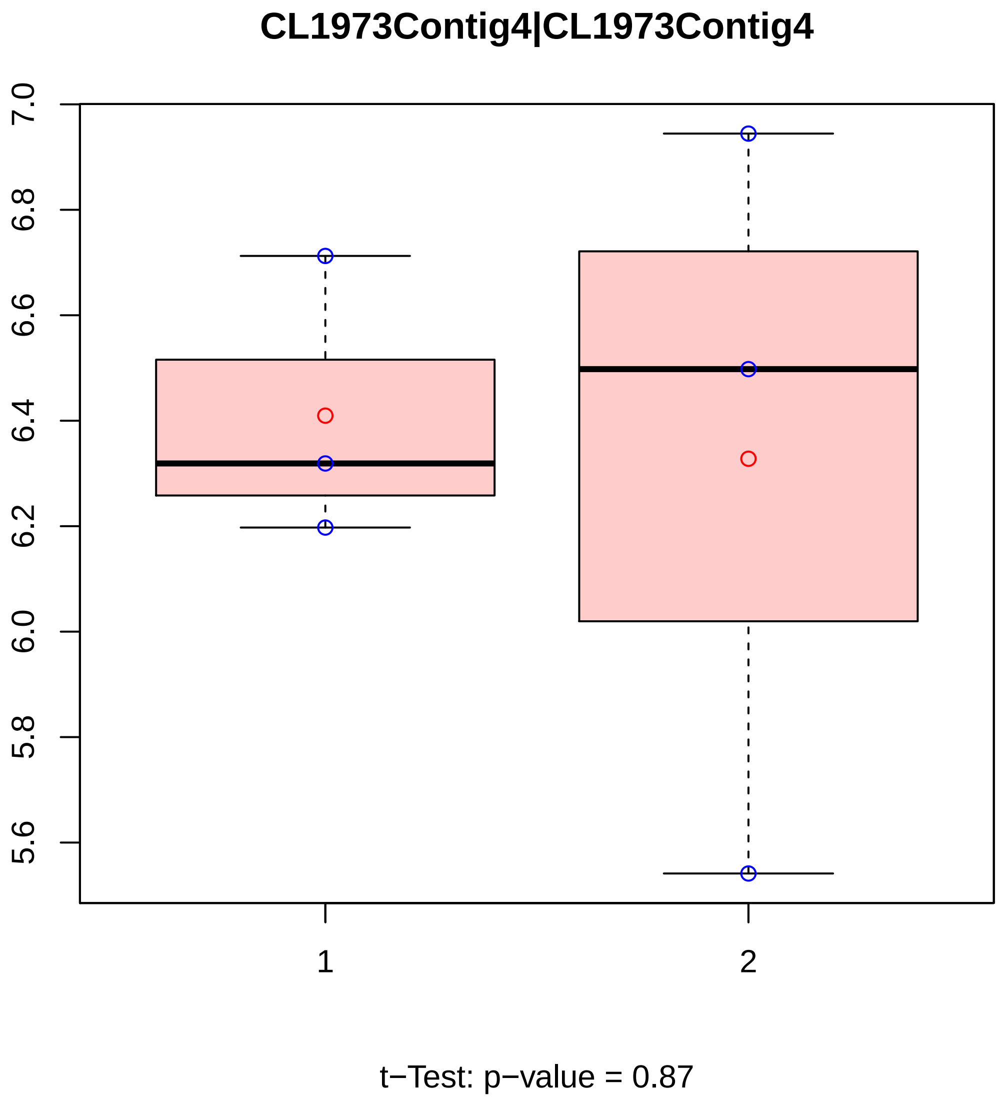
CL1973Contig4|CL1973Contig4
7.0
6.8
6.6
6.4
6.2
6.0
5.8
5.6
1
2
t−Test: p−value = 0.87

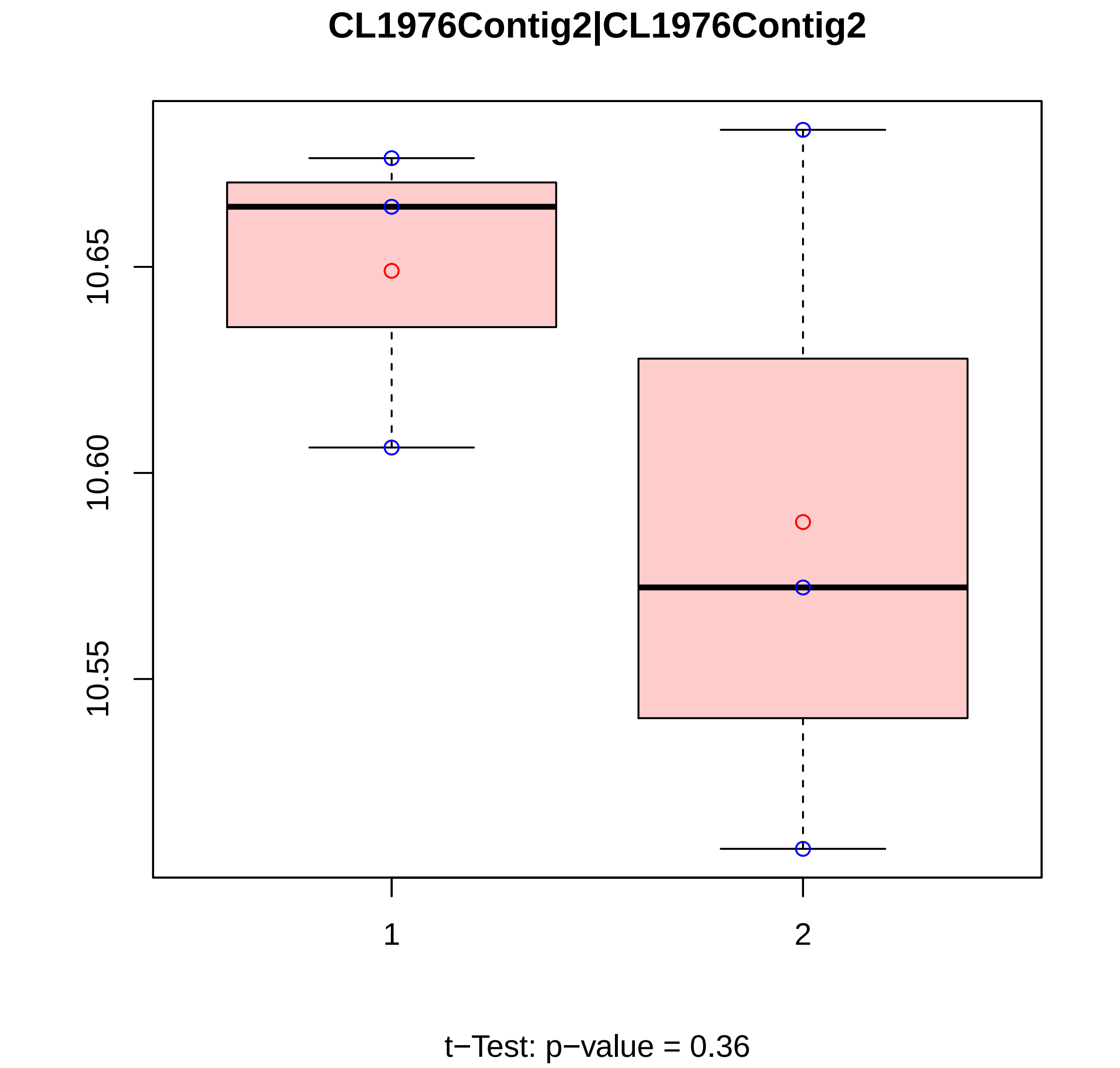
CL1976Contig2|CL1976Contig2
10.55
10.60
10.65
1
2
t−Test: p−value = 0.36

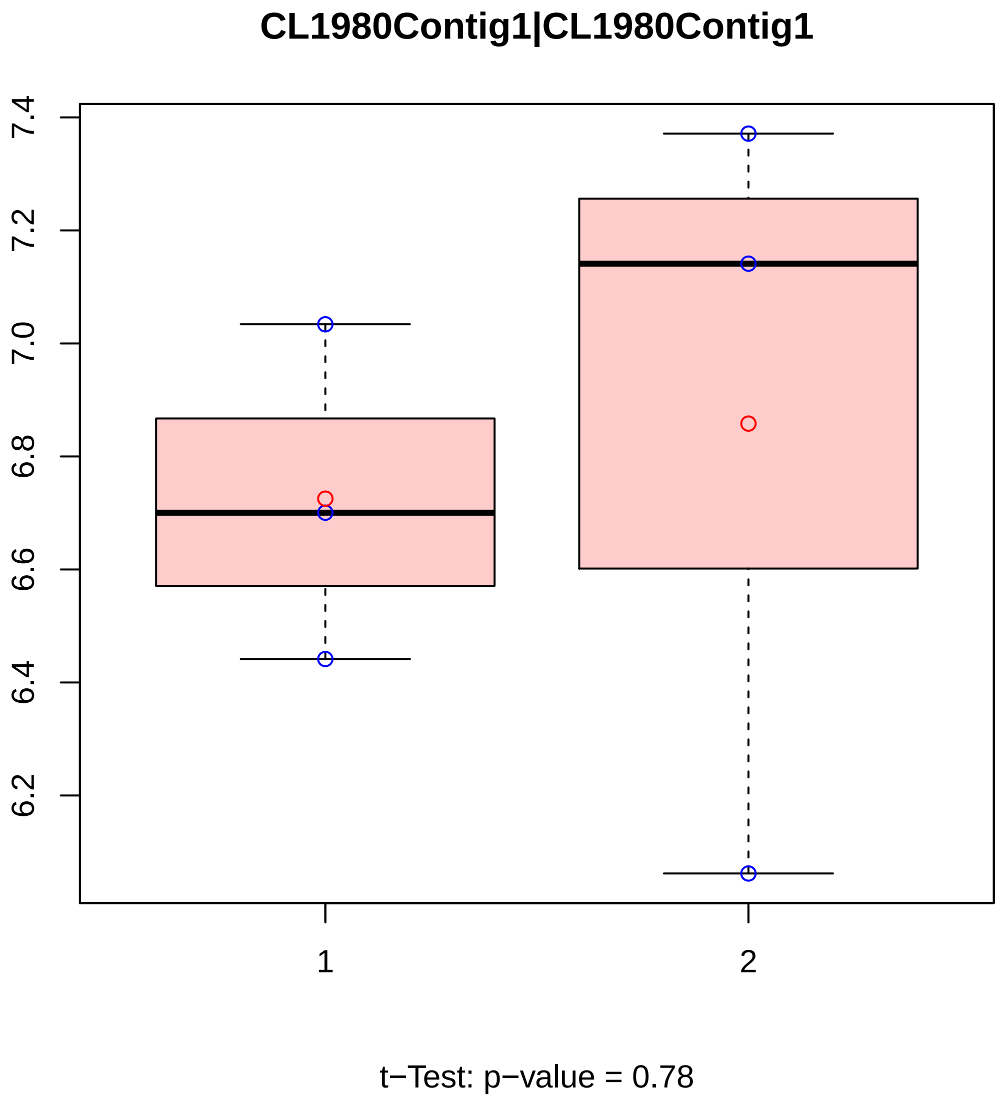
CL1980Contig1|CL1980Contig1
7.4
7.2
7.0
6.8
6.6
6.4
6.2
1
2
t−Test: p−value = 0.78

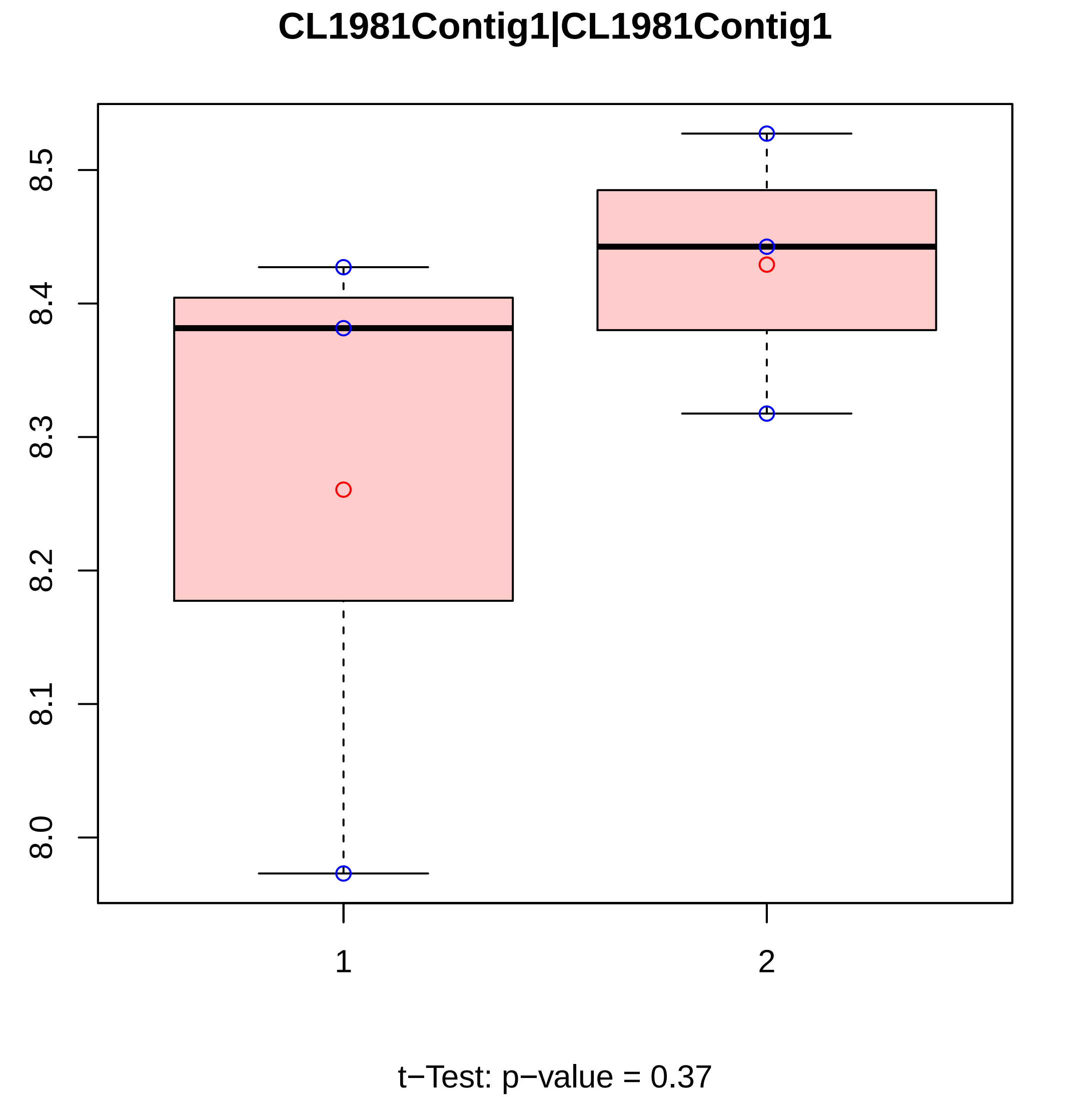
CL1981Contig1|CL1981Contig1
8.0
8.1
8.2
8.3
8.4
8.5
1
2
t−Test: p−value = 0.37

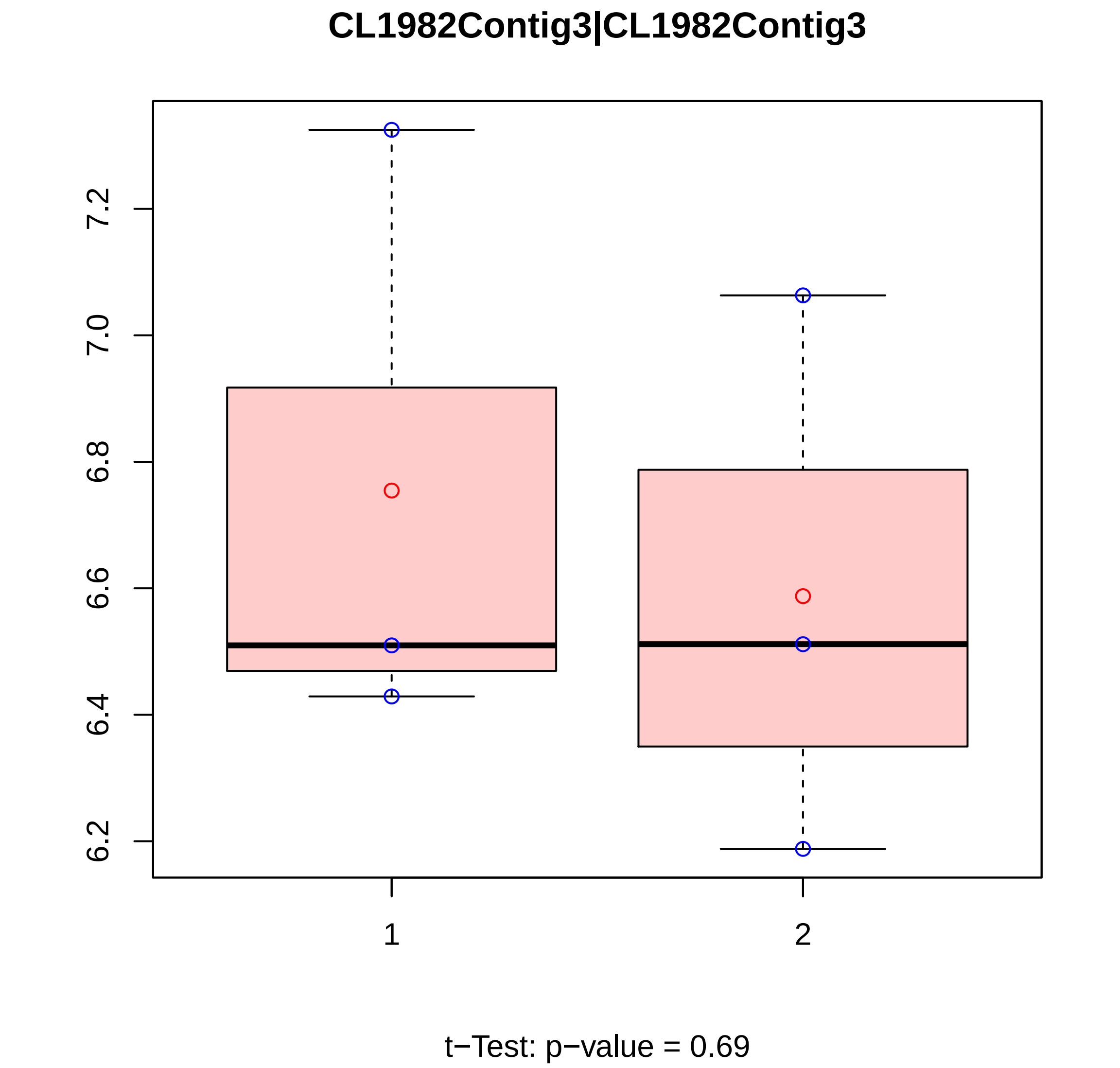

CL1982Contig3|CL1982Contig3
7.2
7.0
6.8
6.6
6.4
6.2
1
2
t−Test: p−value = 0.69

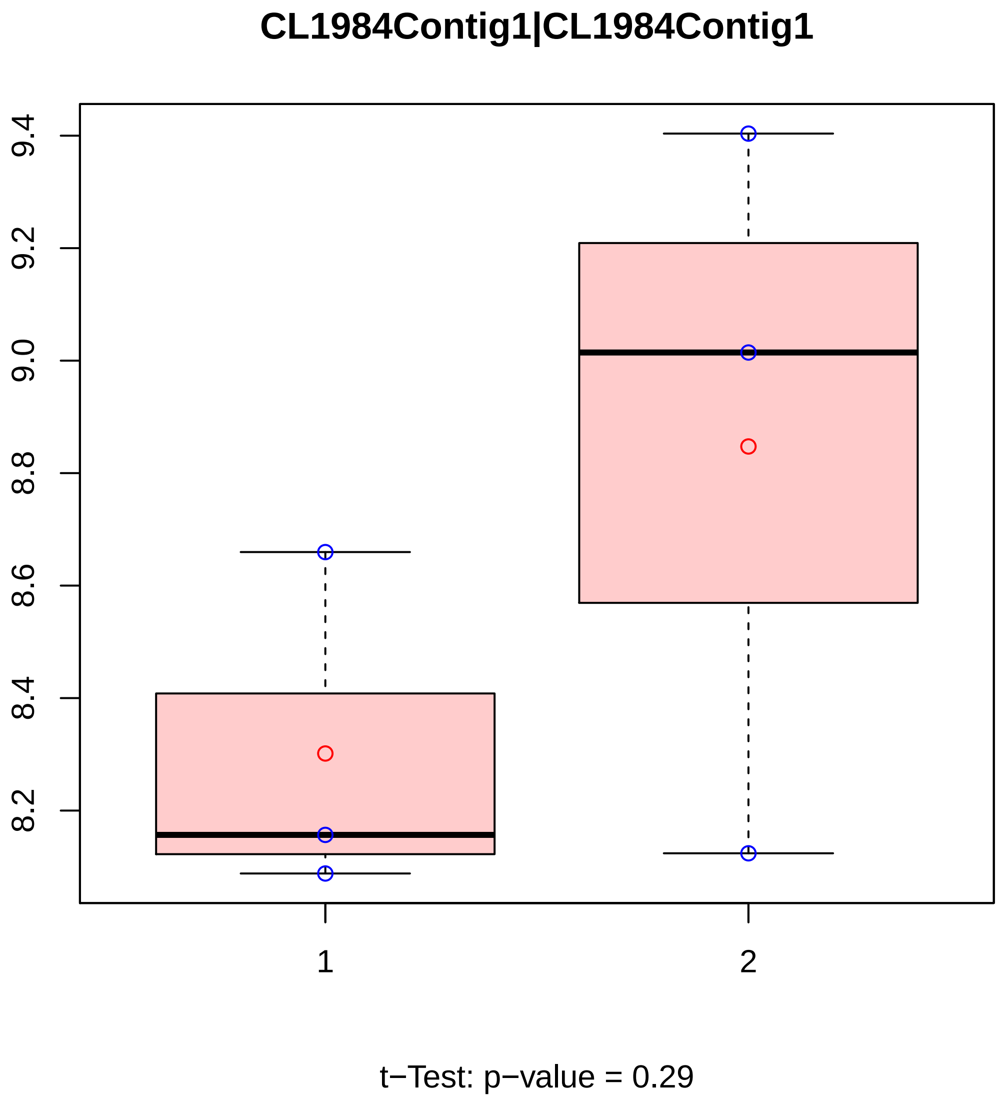
CL1984Contig1|CL1984Contig1
9.4
9.2
9.0
8.8
8.6
8.4
8.2
1
2
t−Test: p−value = 0.29

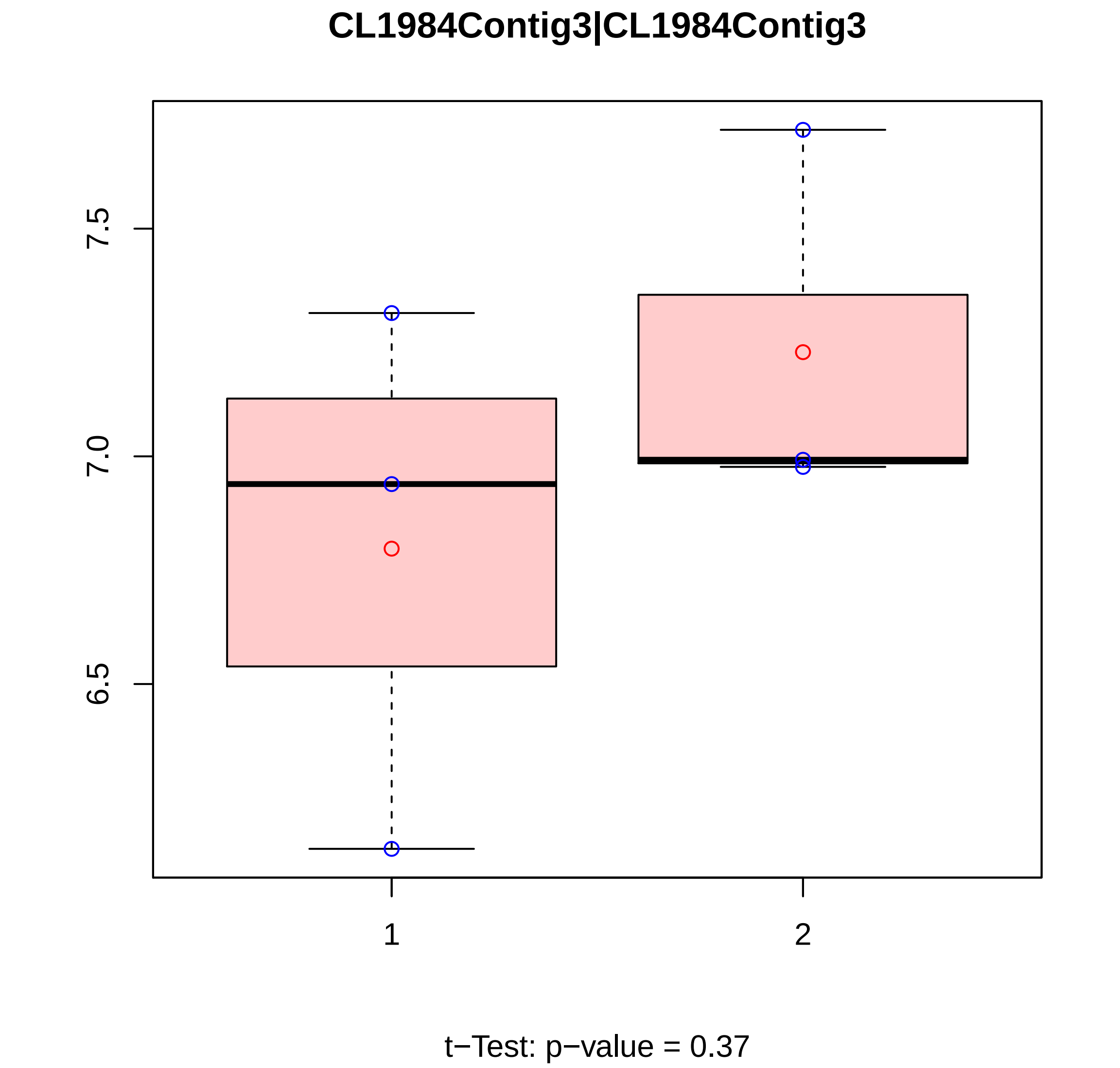
CL1984Contig3|CL1984Contig3
7.5
7.0
6.5
1
2
t−Test: p−value = 0.37

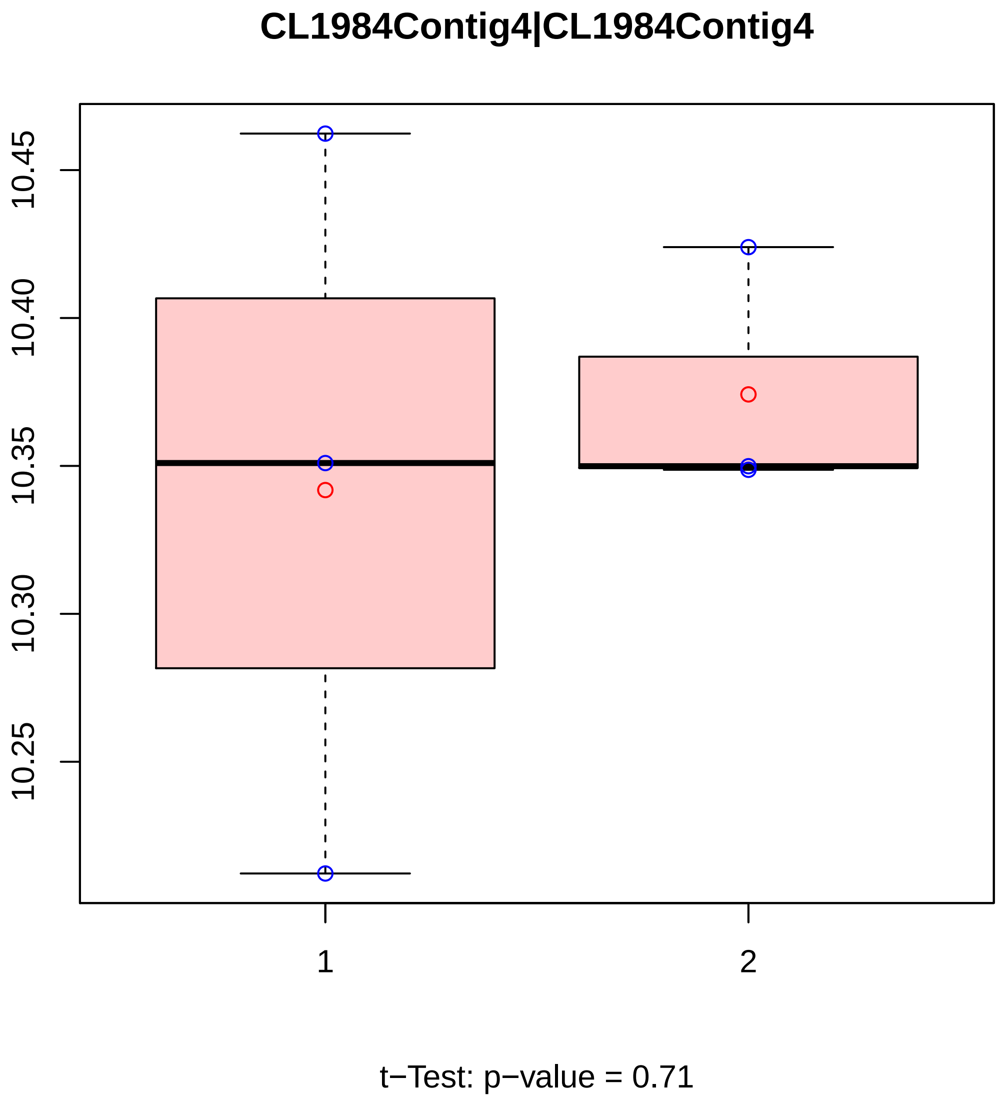
CL1984Contig4|CL1984Contig4
10.45
10.40
10.35
10.30
10.25
1
2
t−Test: p−value = 0.71

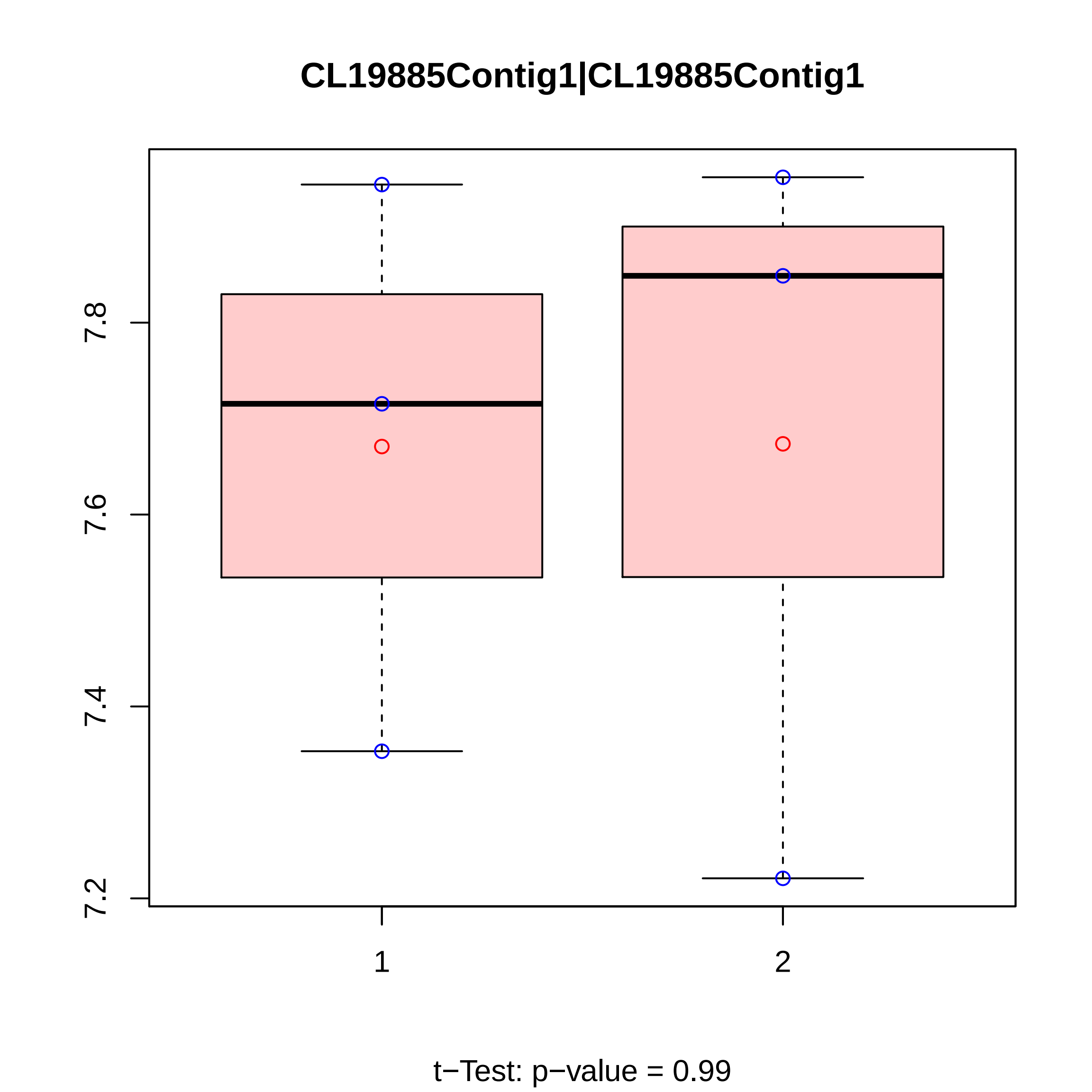
CL19885Contig1|CL19885Contig1
7.2
7.4
7.6
7.8
1
2
t−Test: p−value = 0.99

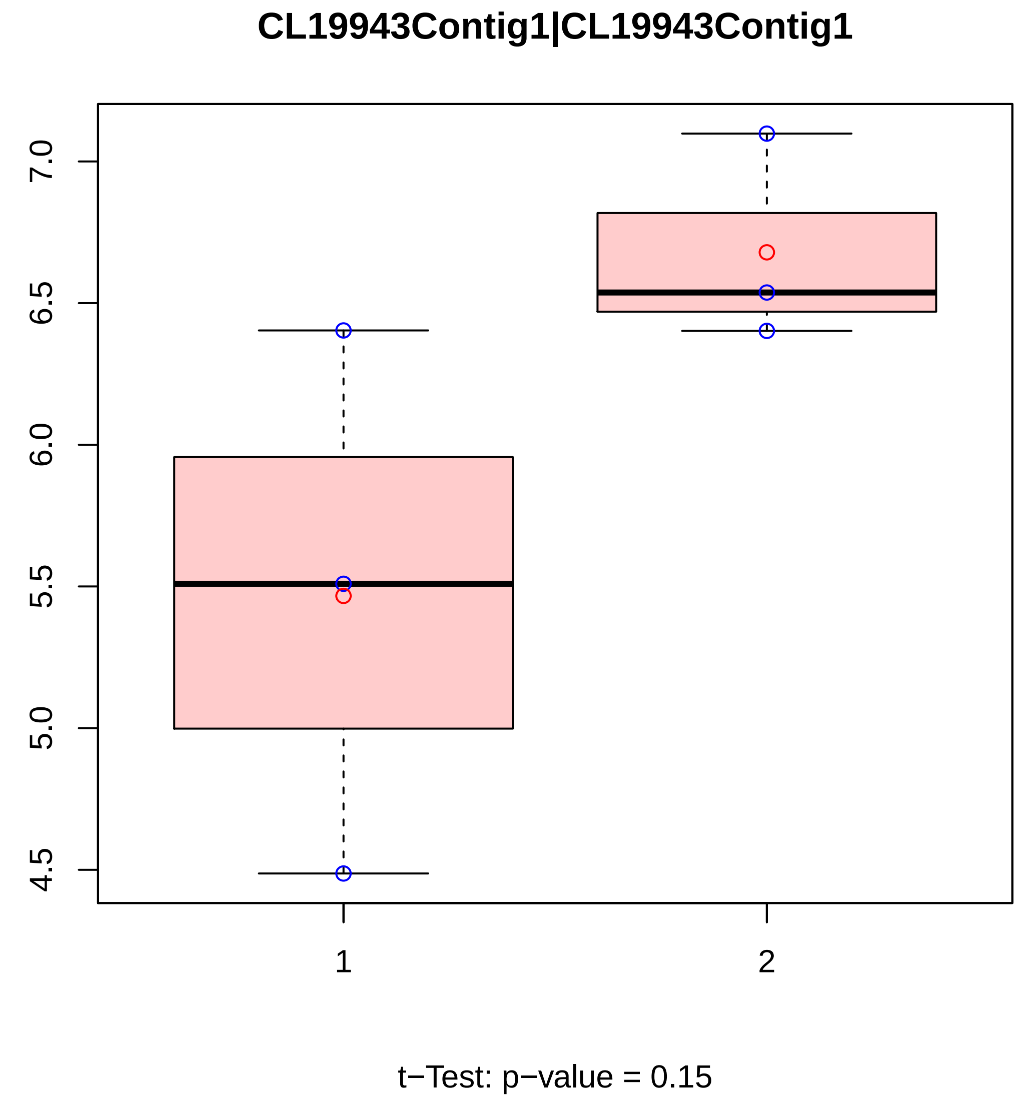
CL19943Contig1|CL19943Contig1
7.0
6.5
6.0
5.5
5.0
4.5
1
2
t−Test: p−value = 0.15

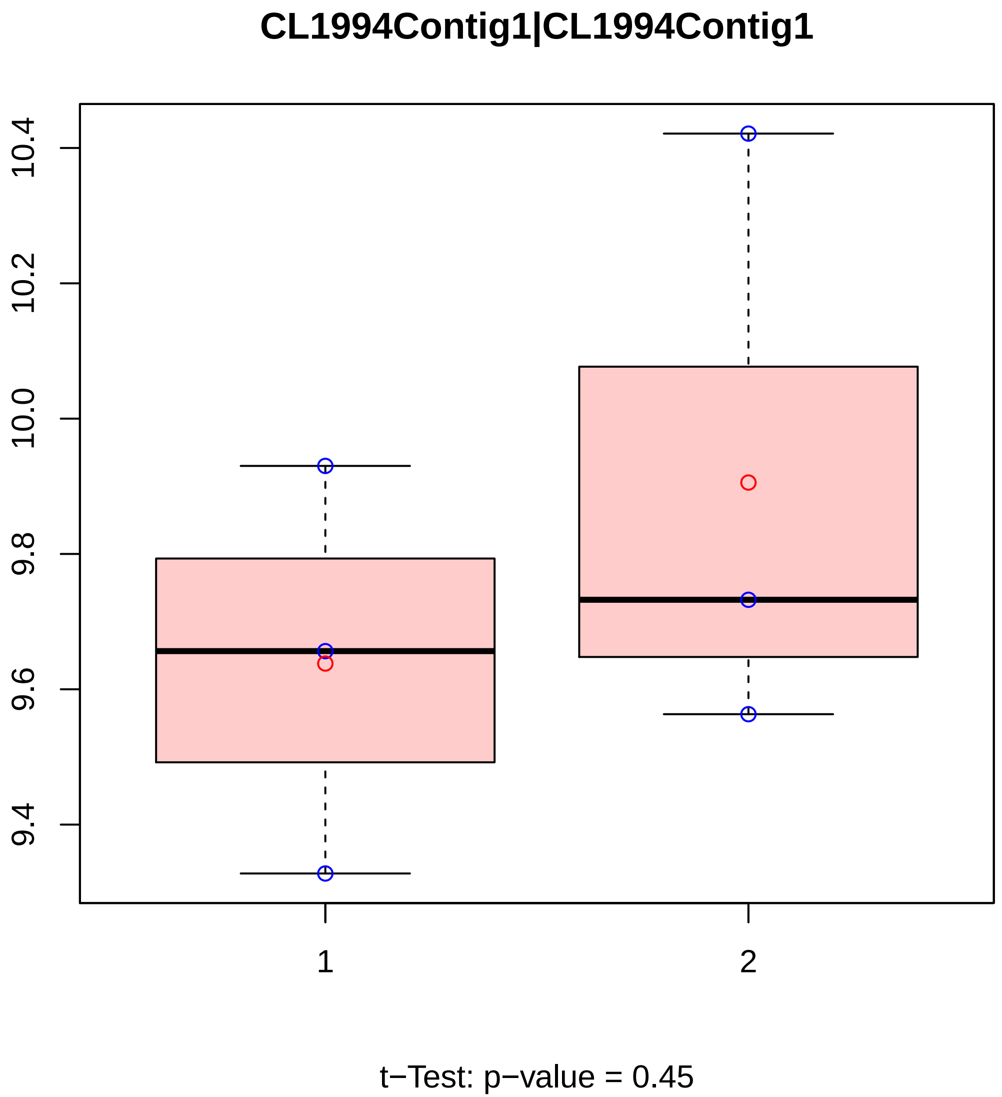
CL1994Contig1|CL1994Contig1
10.4
10.2
10.0
9.8
9.6
9.4
1
2
t−Test: p−value = 0.45

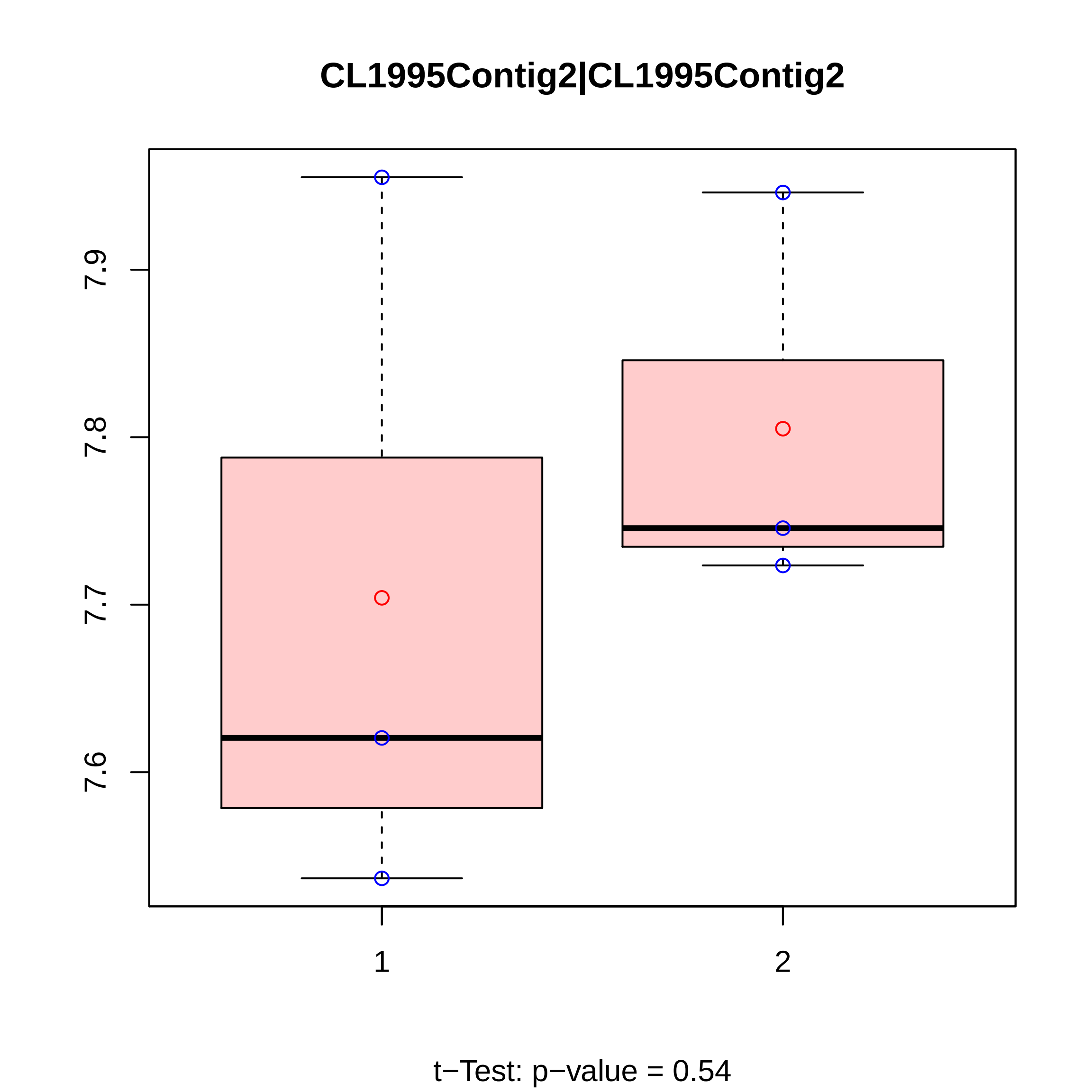

CL1995Contig2|CL1995Contig2
7.6
7.7
7.8
7.9
1
2
t−Test: p−value = 0.54

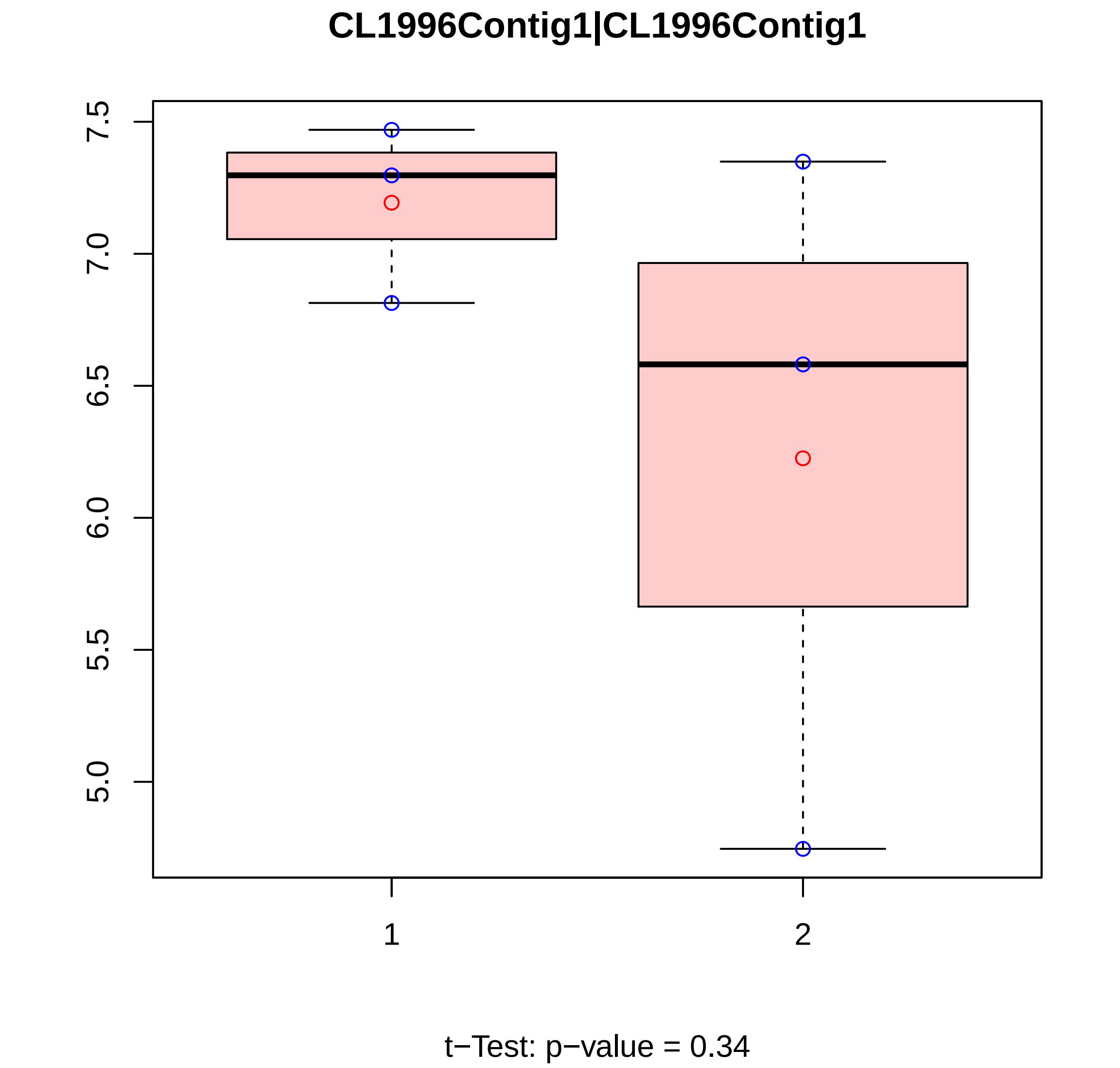
CL1996Contig1|CL1996Contig1
7.5
7.0
6.5
6.0
5.5
5.0
1
2
t−Test: p−value = 0.34

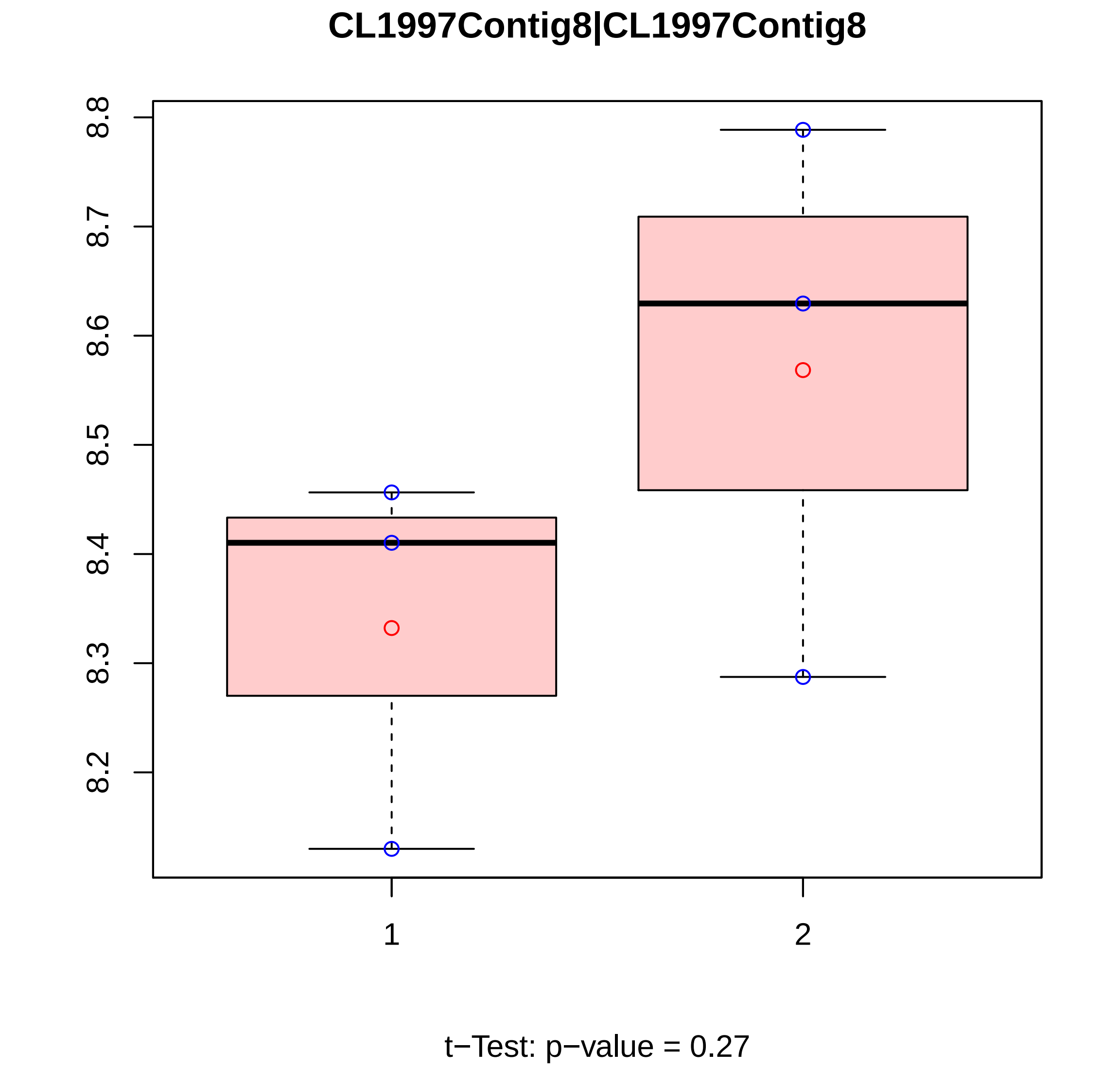

CL1997Contig8|CL1997Contig8
8.8
8.7
8.6
8.5
8.4
8.3
8.2
1
2
t−Test: p−value = 0.27

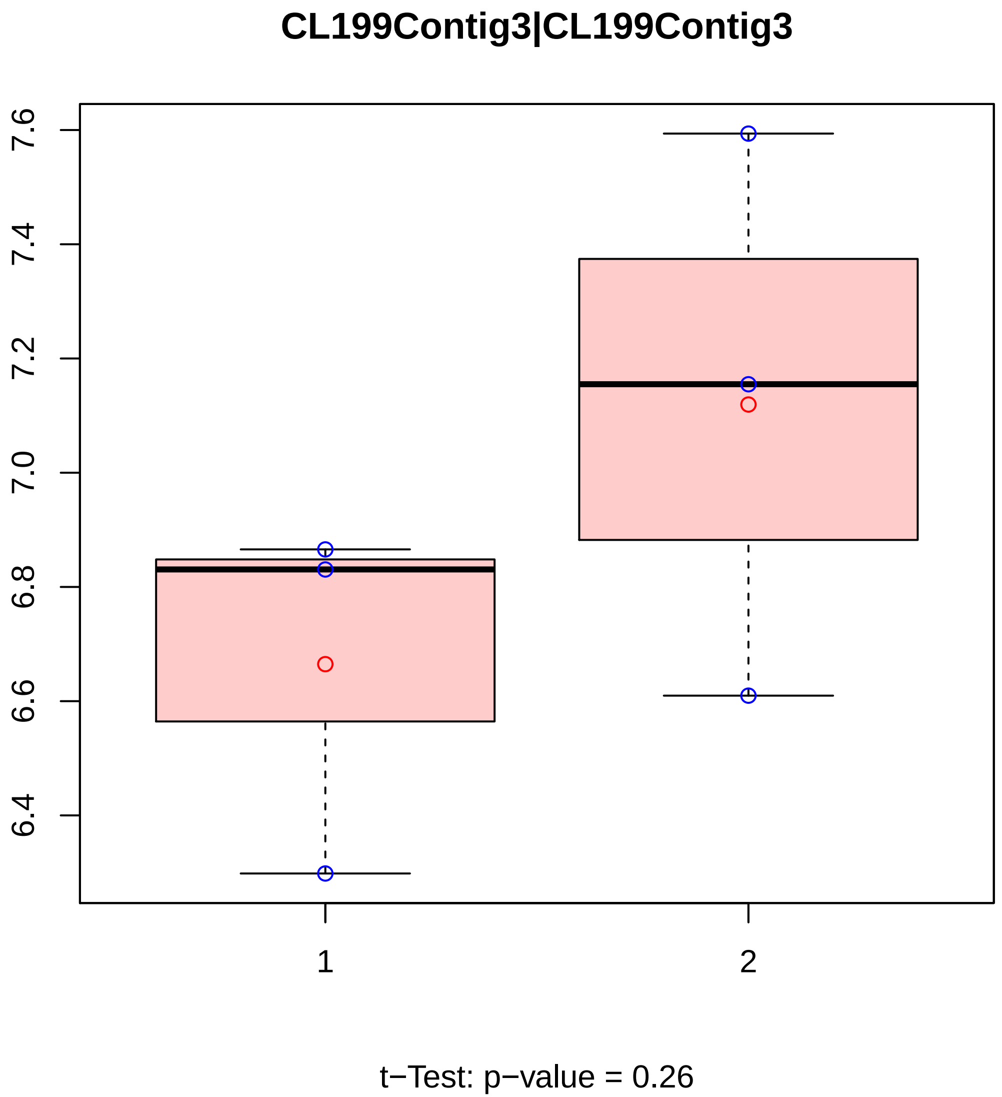
CL199Contig3|CL199Contig3
7.6
7.4
7.2
7.0
6.8
6.6
6.4
1
2
t−Test: p−value = 0.26

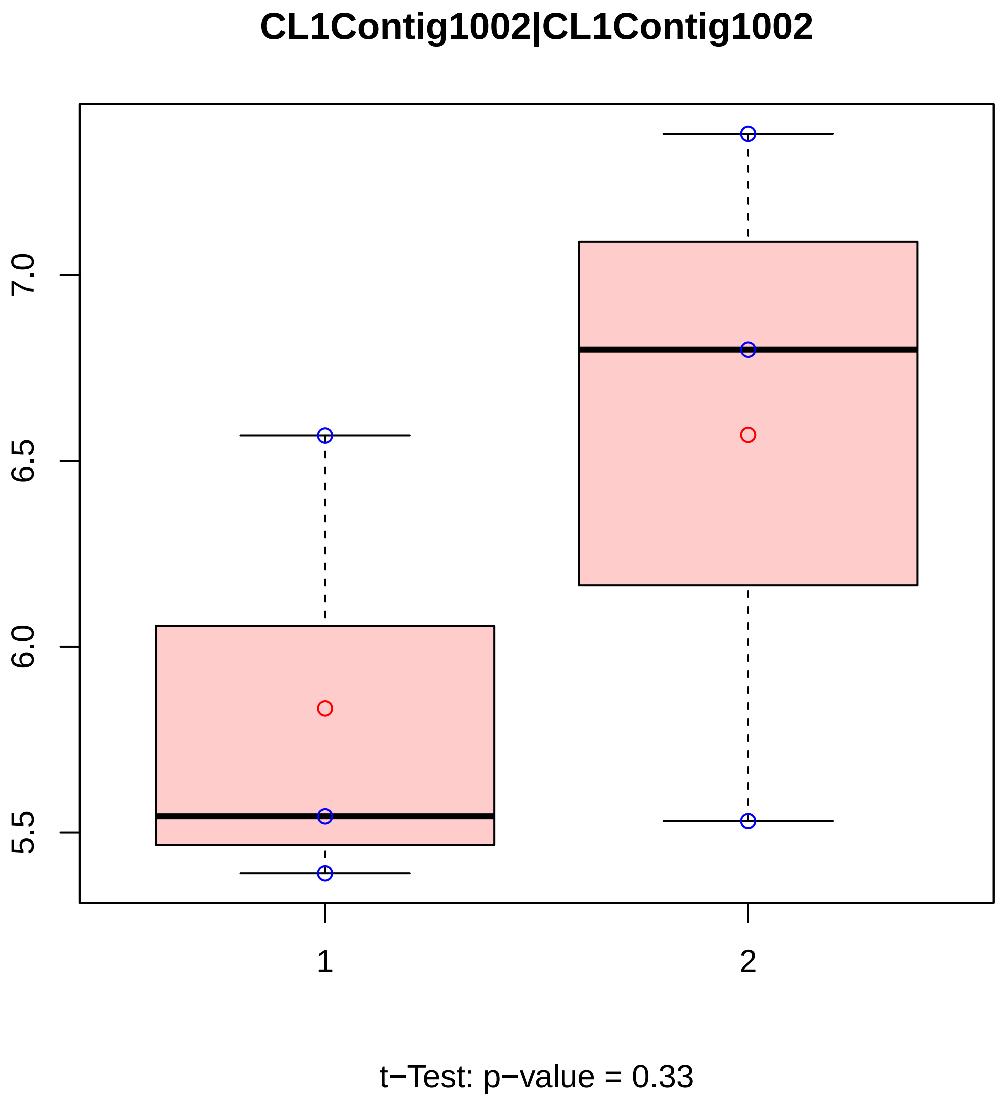
CL1Contig1002|CL1Contig1002
7.0
6.5
6.0
5.5
1
2
t−Test: p−value = 0.33

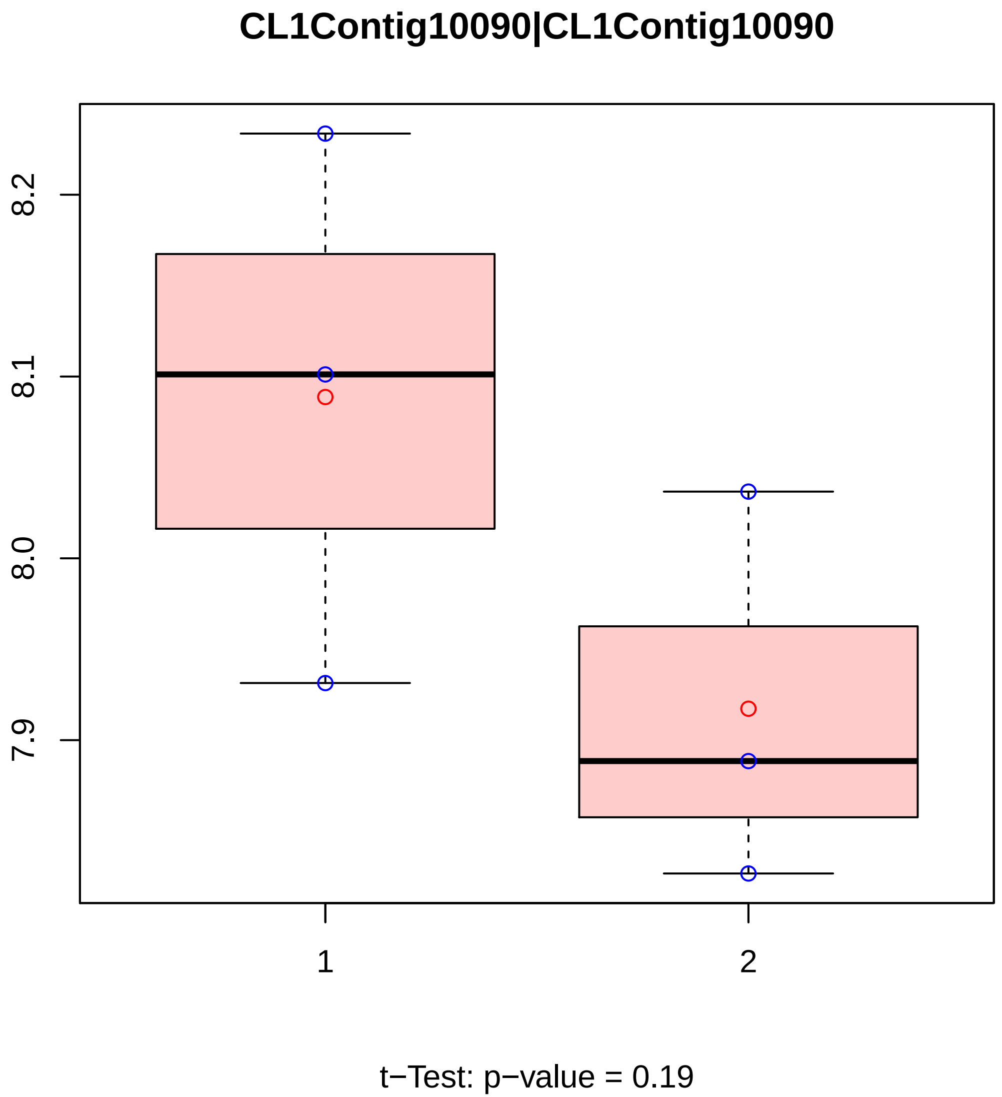
CL1Contig10090|CL1Contig10090
8.2
8.1
8.0
7.9
1
2
t−Test: p−value = 0.19

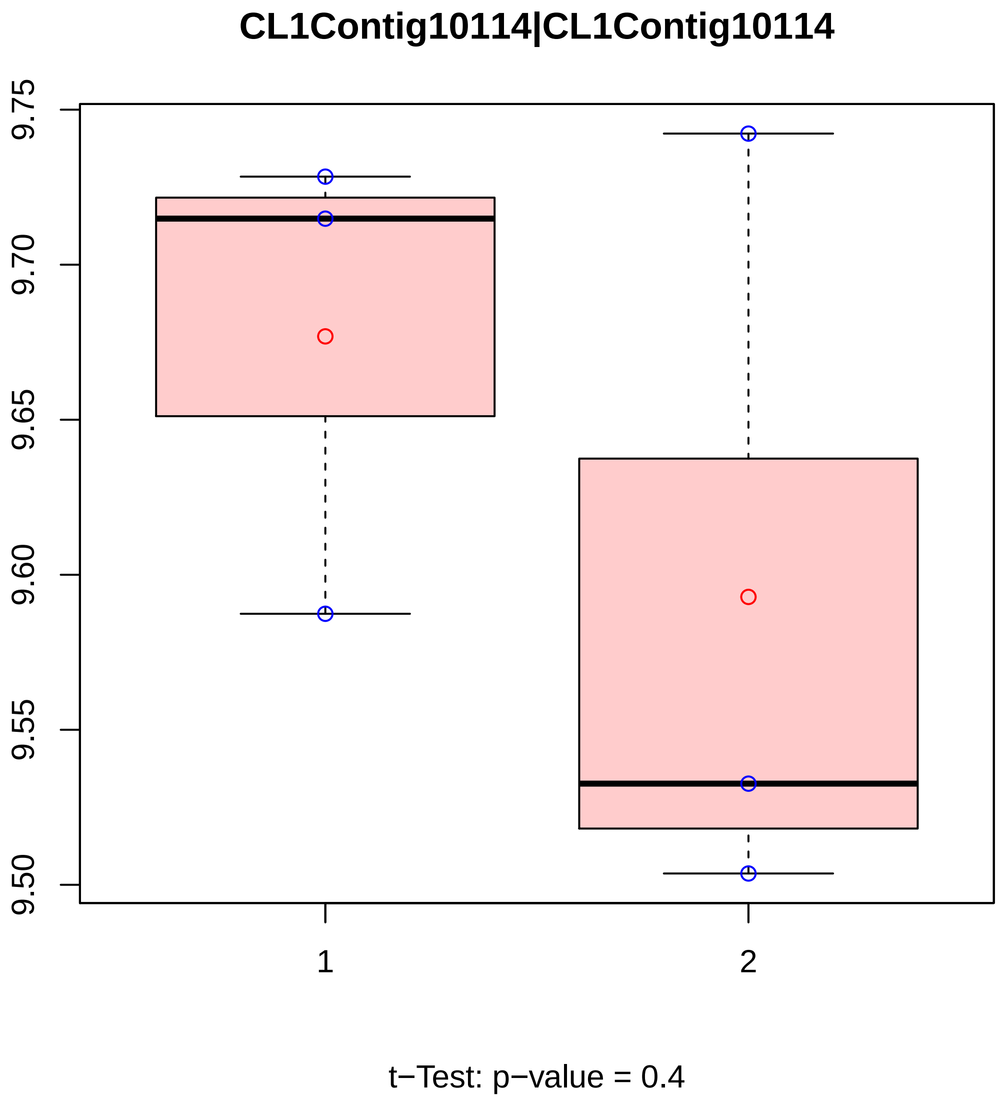
CL1Contig10114|CL1Contig10114
9.50
9.55
9.60
9.65
9.70
9.75
1
2
t−Test: p−value = 0.4

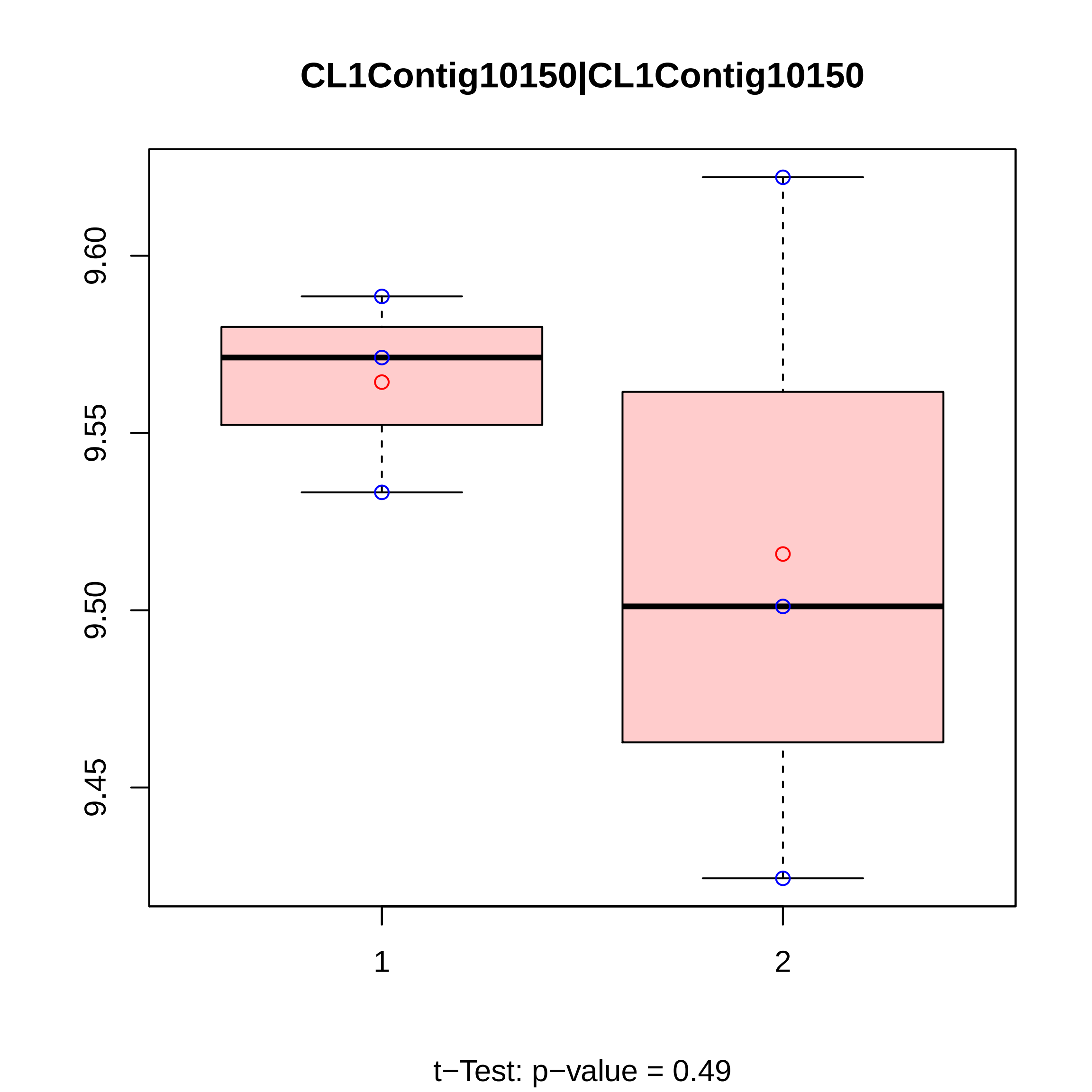
CL1Contig10150|CL1Contig10150
9.60
9.55
9.50
9.45
1
2
t−Test: p−value = 0.49

# CL1Contig10194|CL1Contig10194

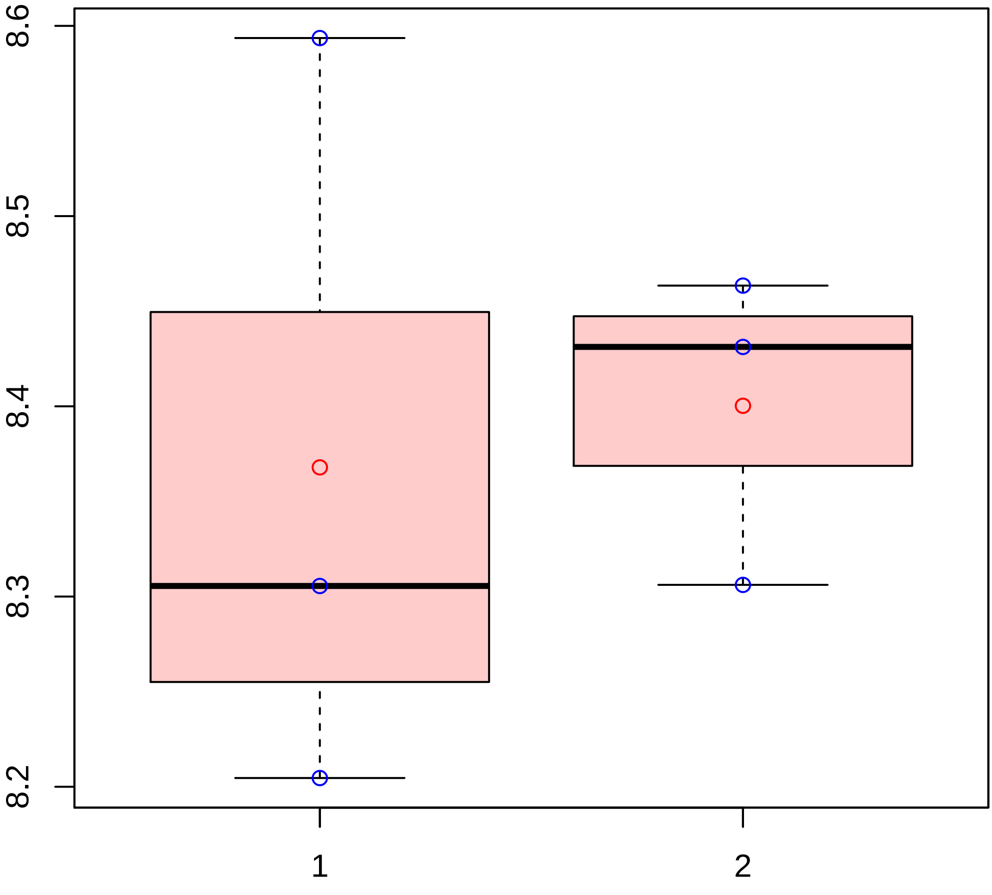


t−Test: p−value = 0.82

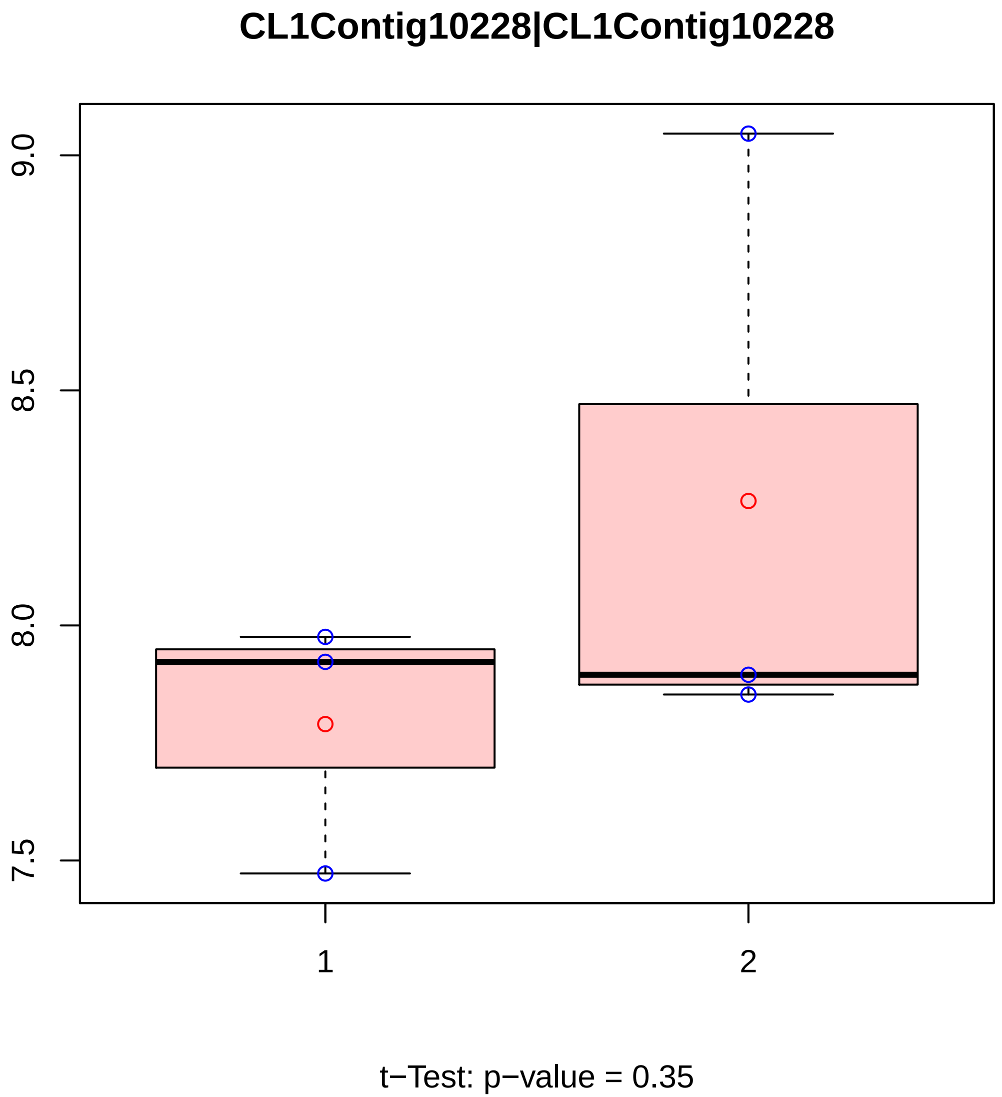
CL1Contig10228|CL1Contig10228
9.0
8.5
8.0
7.5
1
2
t−Test: p−value = 0.35

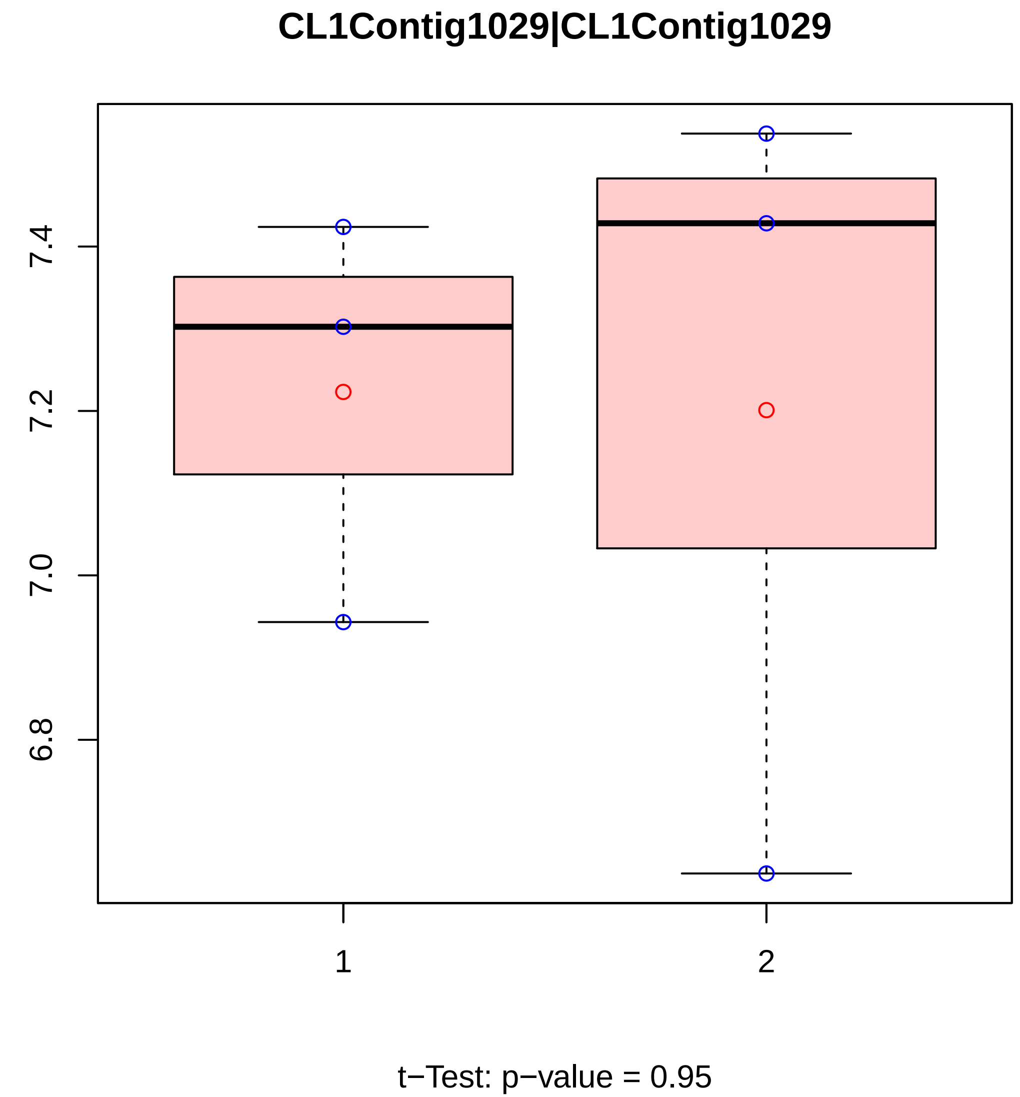
CL1Contig1029|CL1Contig1029
6.8
7.0
7.2
7.4
1
2
t−Test: p−value = 0.95

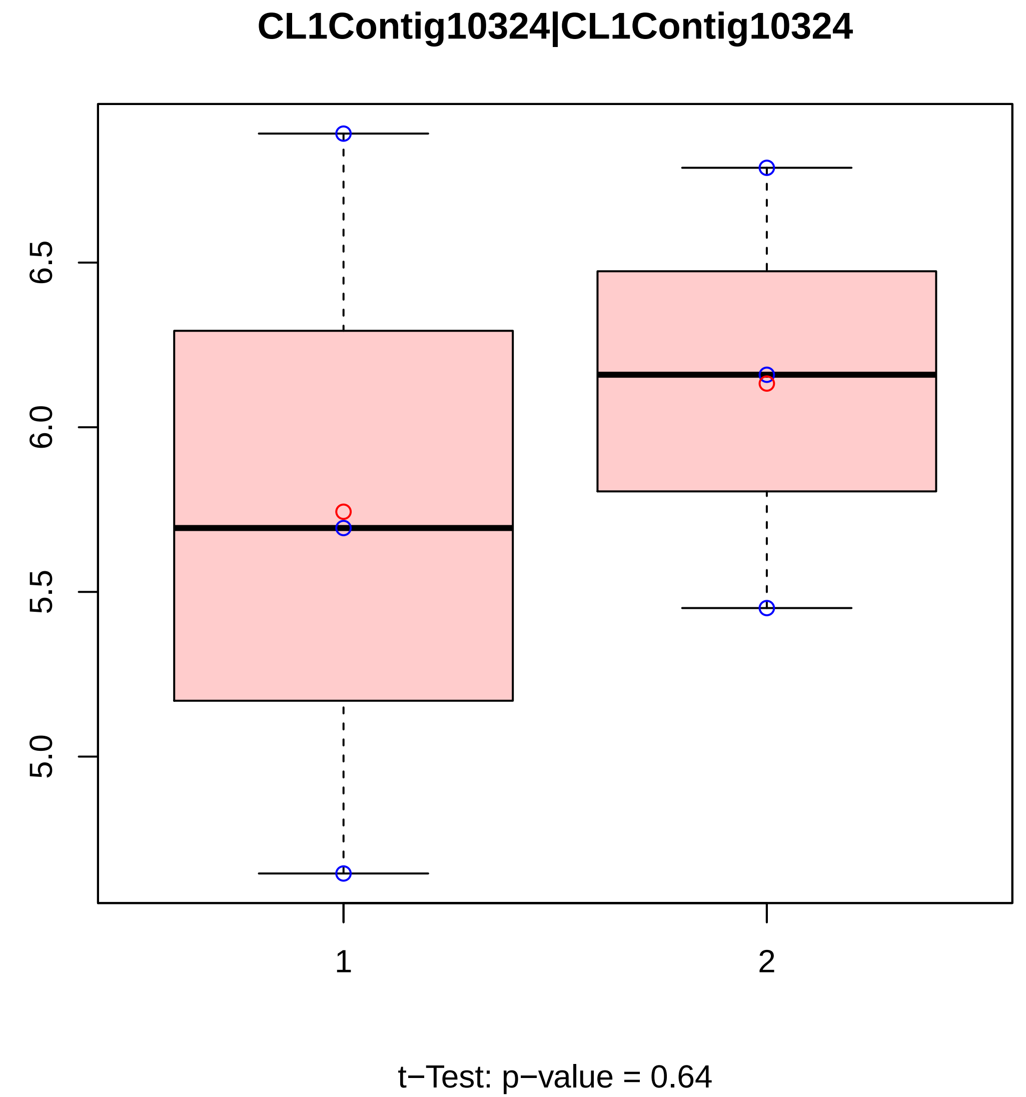
CL1Contig10324|CL1Contig10324
6.5
6.0
5.5
5.0
1
2
t−Test: p−value = 0.64

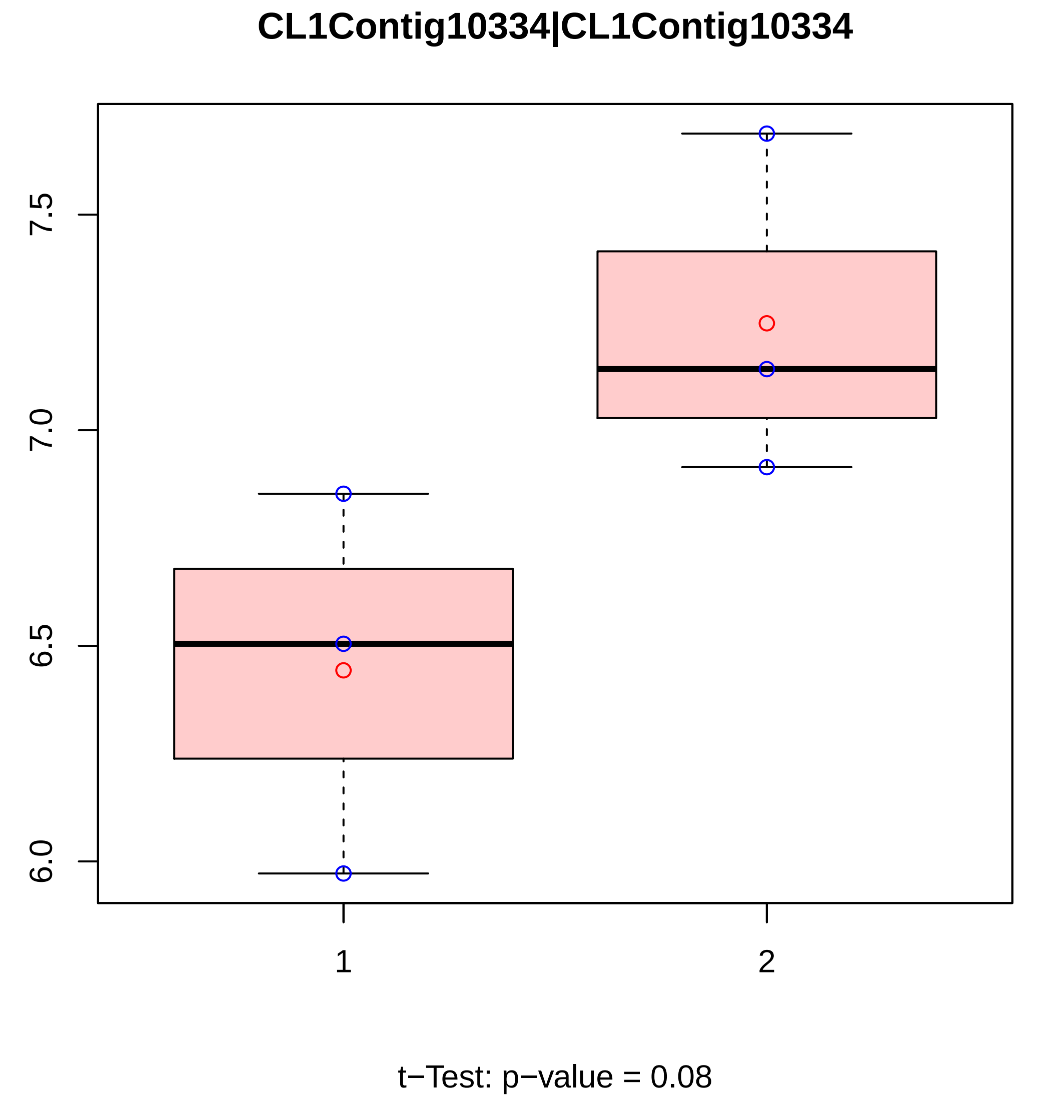
CL1Contig10334|CL1Contig10334
7.5
7.0
6.5
6.0
1
2
t−Test: p−value = 0.08

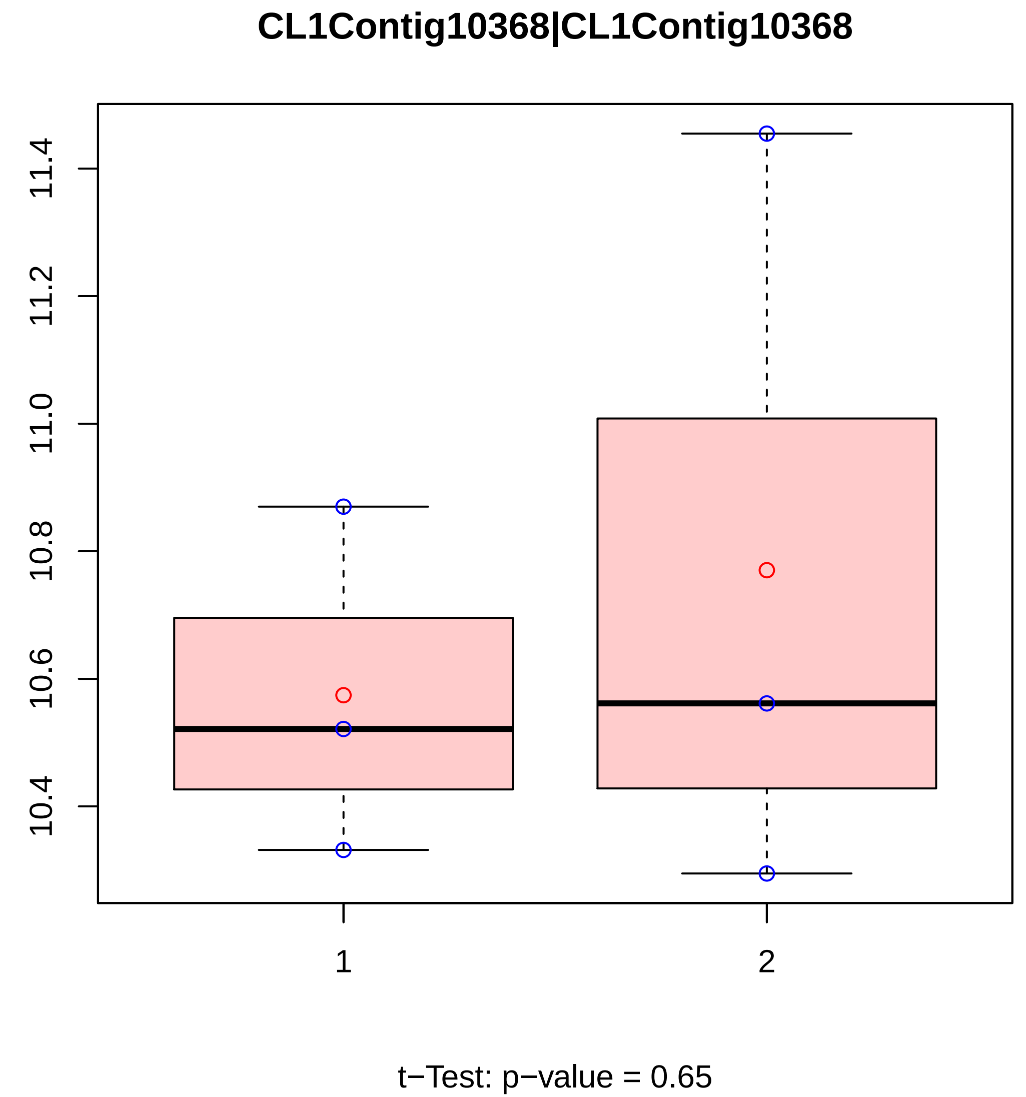
CL1Contig10368|CL1Contig10368
11.4
11.2
11.0
10.8
10.6
10.4
1
2
t−Test: p−value = 0.65

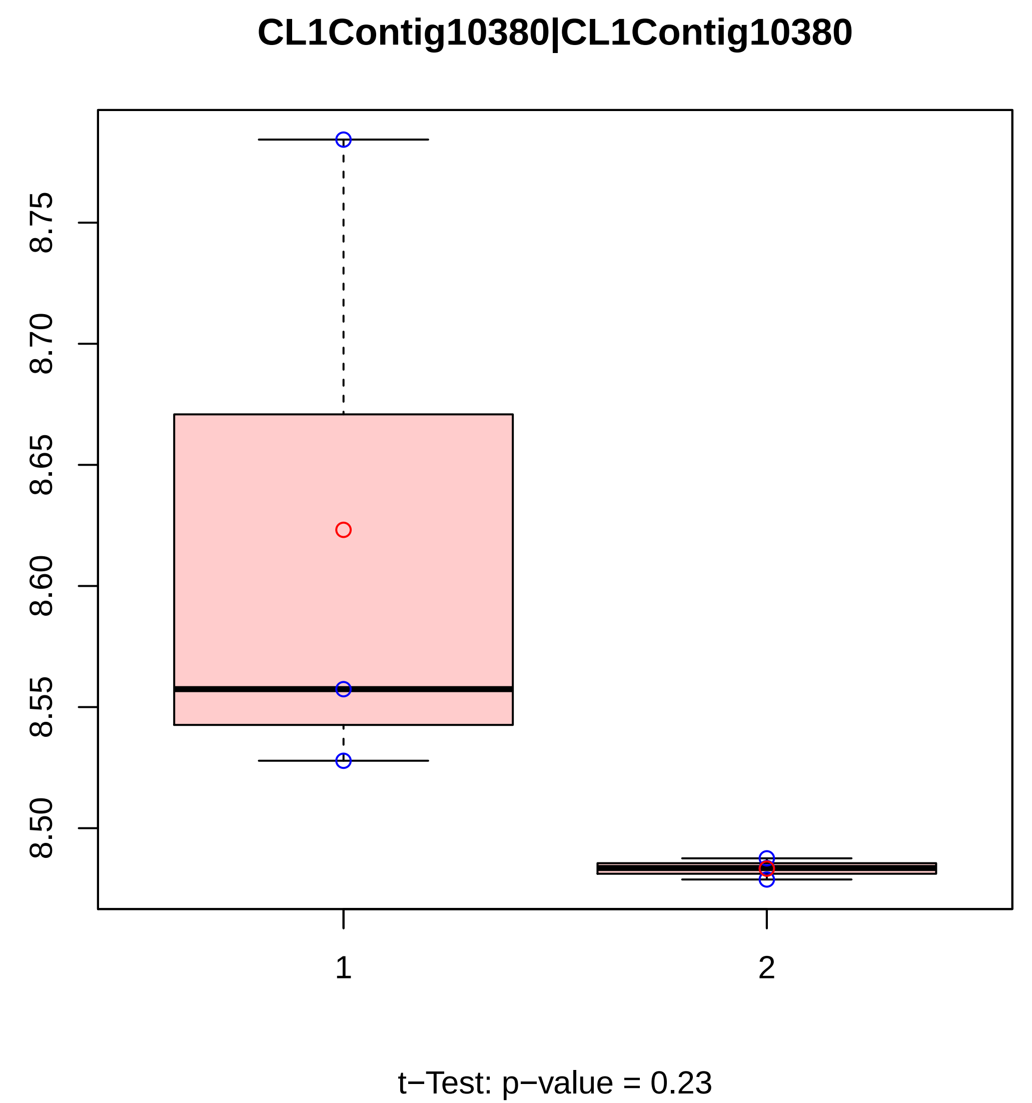
CL1Contig10380|CL1Contig10380
8.50
8.55
8.60
8.65
8.70
8.75
1
2
t−Test: p−value = 0.23

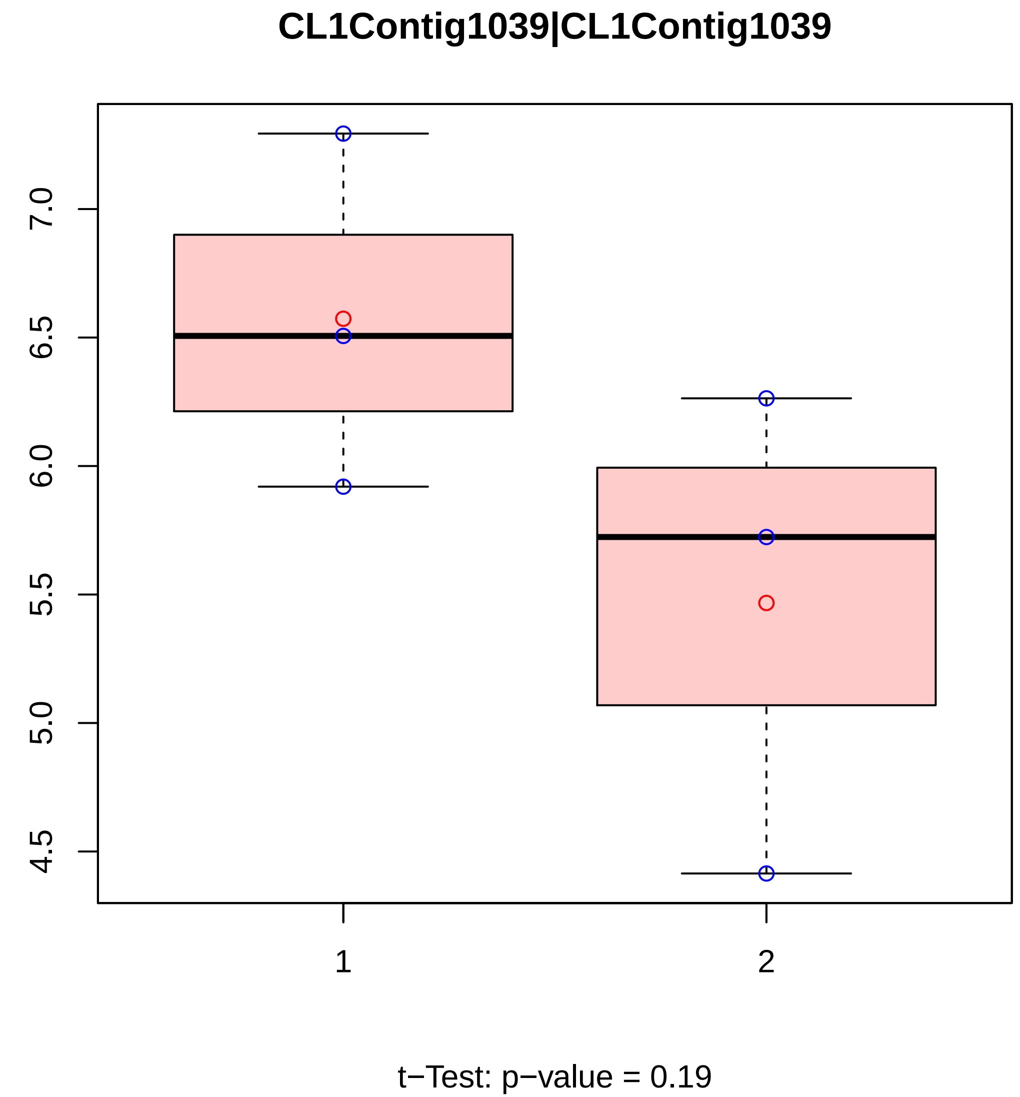
CL1Contig1039|CL1Contig1039
7.0
6.5
6.0
5.5
5.0
4.5
1
2
t−Test: p−value = 0.19

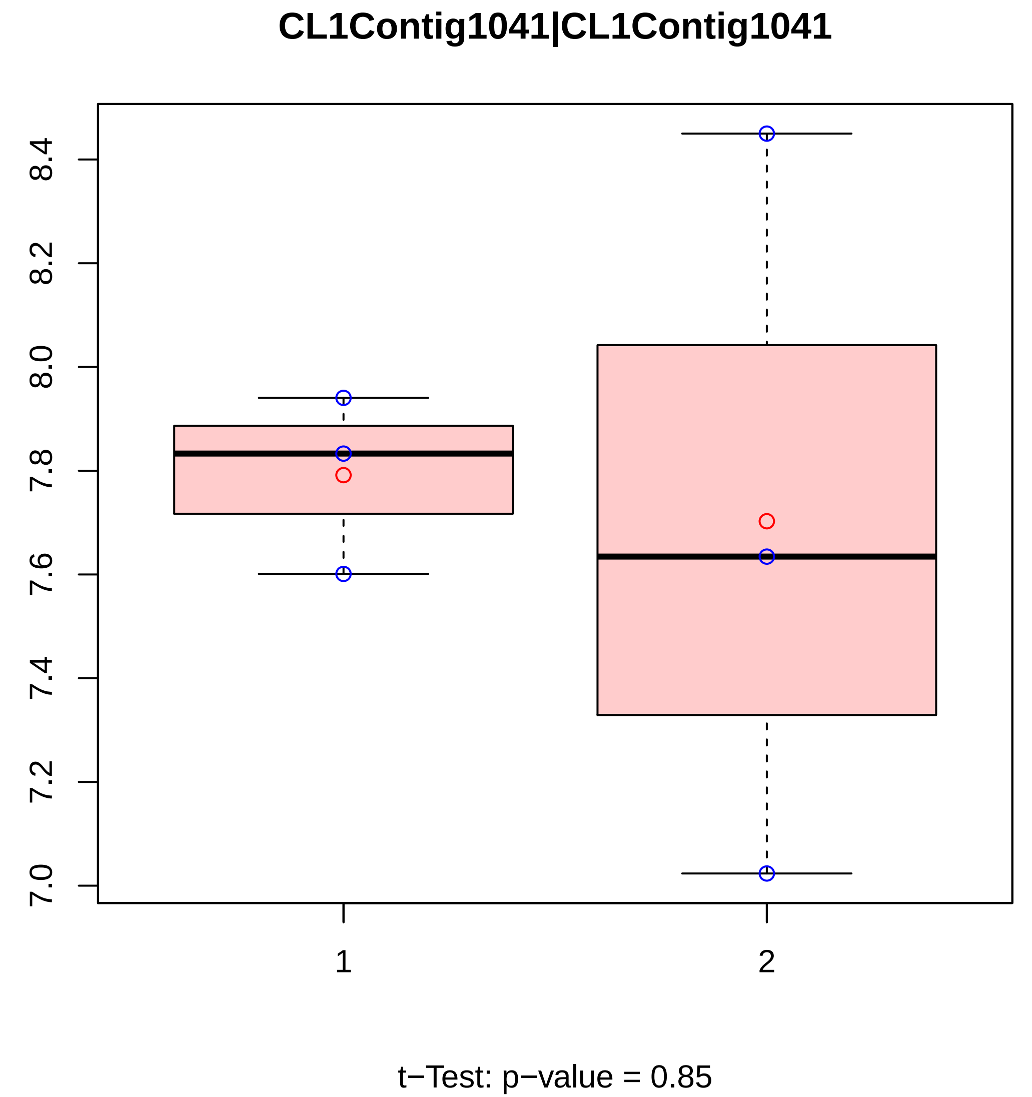
CL1Contig1041|CL1Contig1041
7.0
7.2
7.4
7.6
7.8
8.0
8.2
8.4
1
2
t−Test: p−value = 0.85

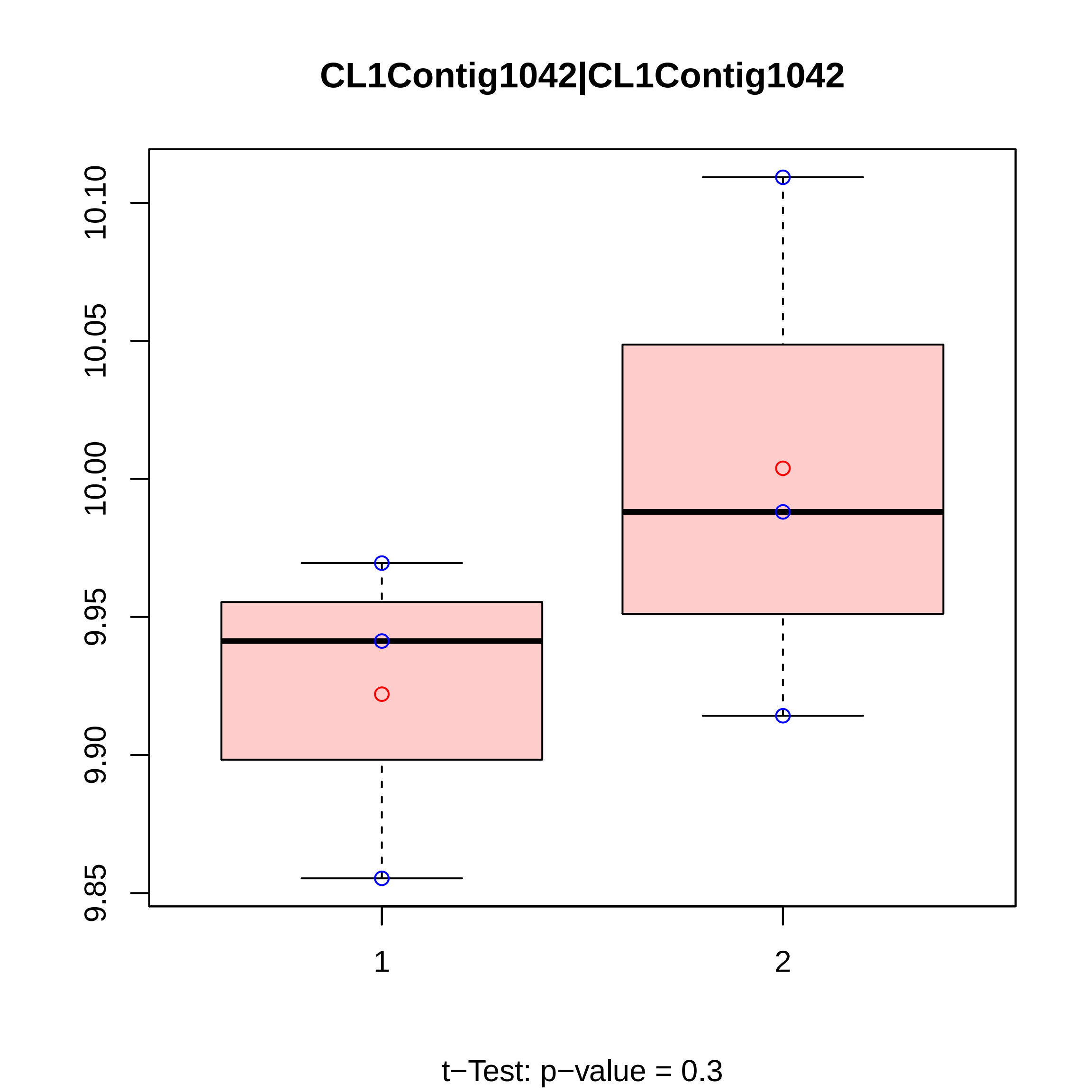
CL1Contig1042|CL1Contig1042
10.10
10.05
10.00
9.95
9.90
9.85
1
2
t−Test: p−value = 0.3

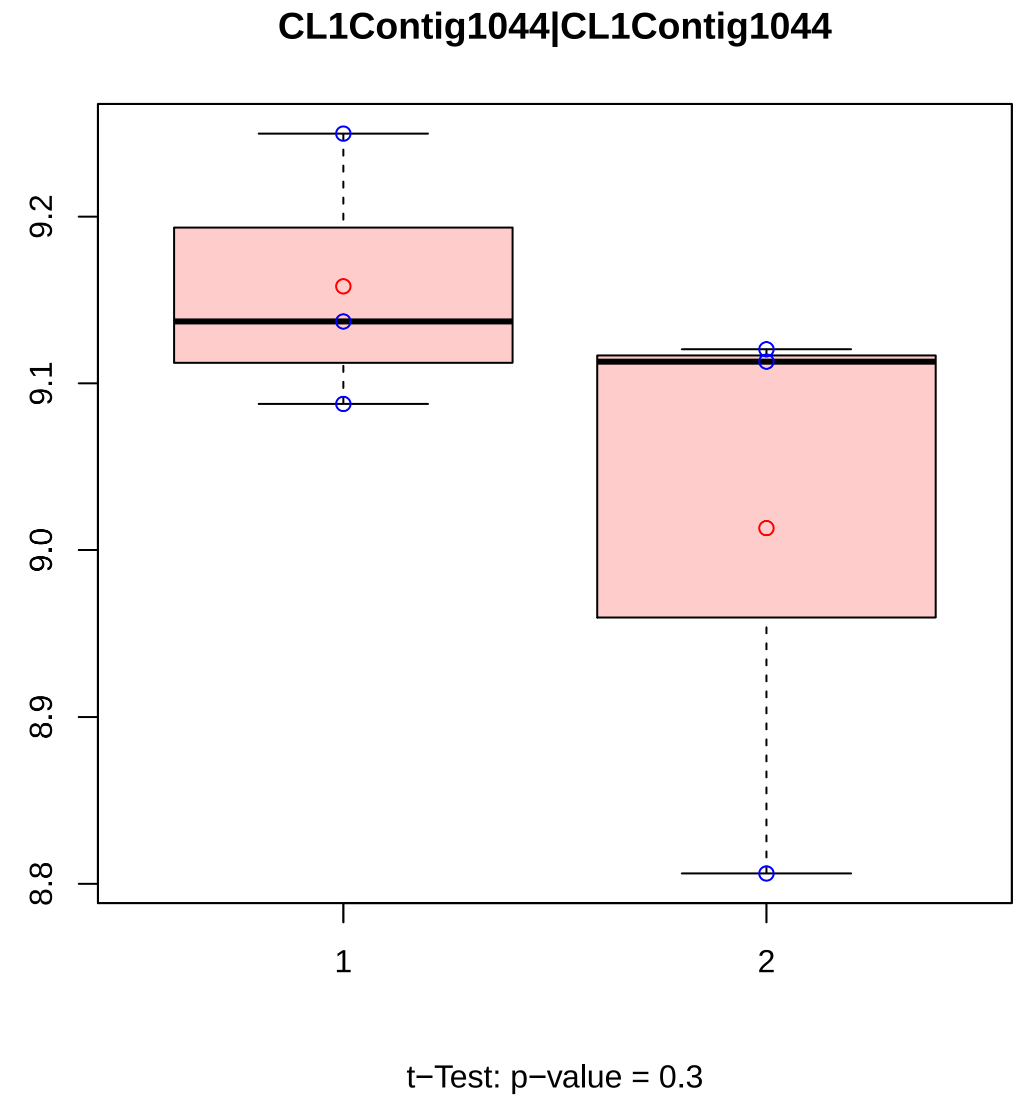

CL1Contig1044|CL1Contig1044
9.2
9.1
9.0
8.9
8.8
1
2
t−Test: p−value = 0.3

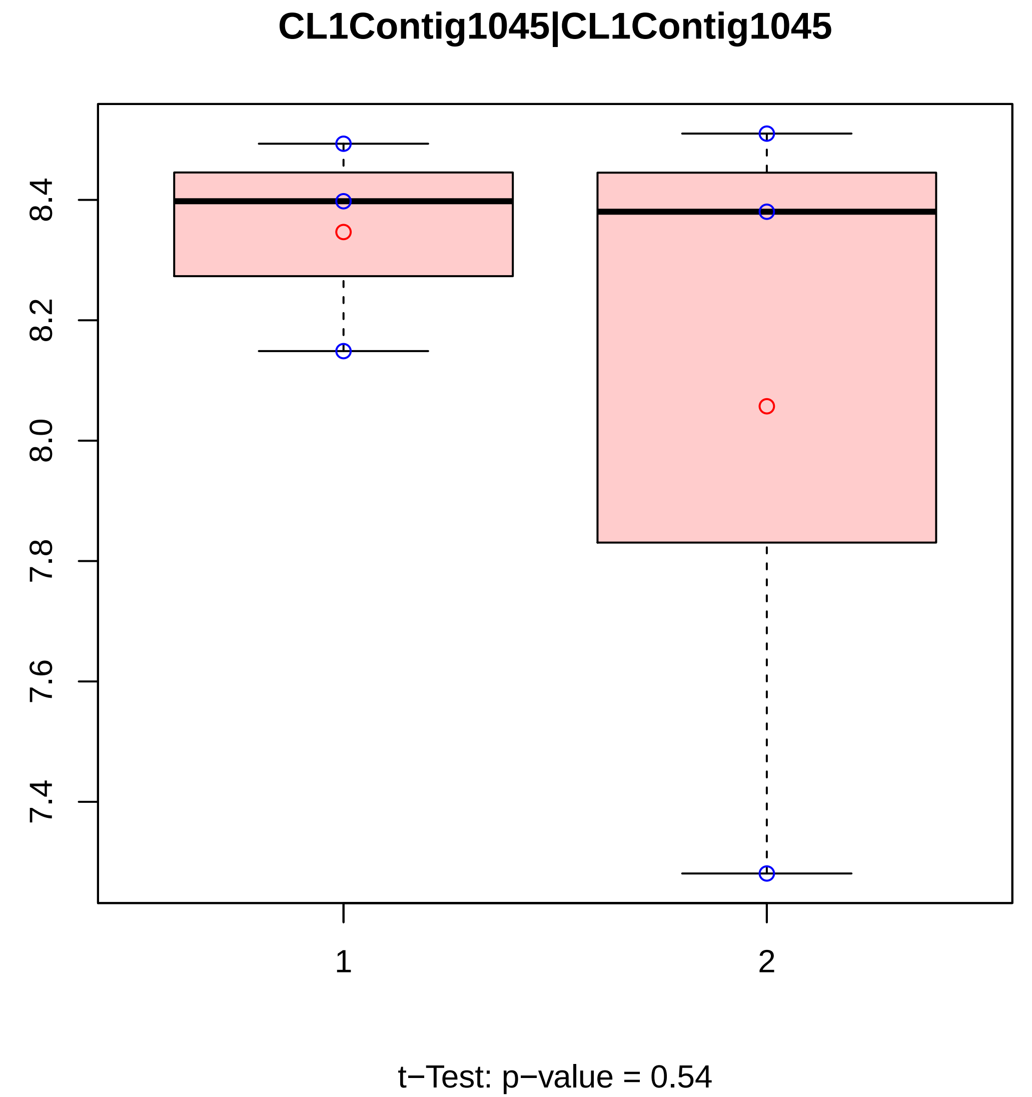
CL1Contig1045|CL1Contig1045
8.4
8.2
8.0
7.8
7.6
7.4
1
2
t−Test: p−value = 0.54

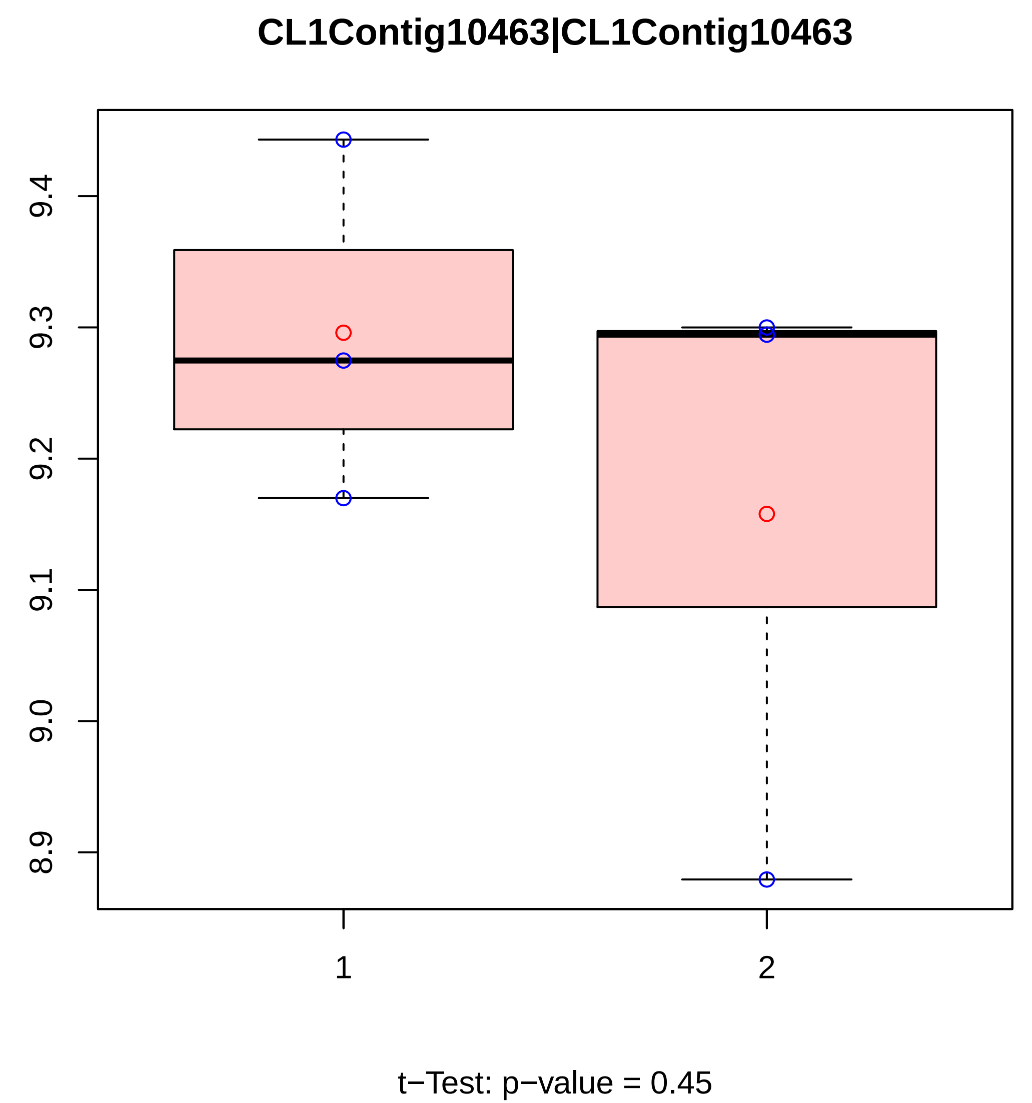
CL1Contig10463|CL1Contig10463
9.4
9.3
9.2
9.1
9.0
8.9
1
2
t−Test: p−value = 0.45

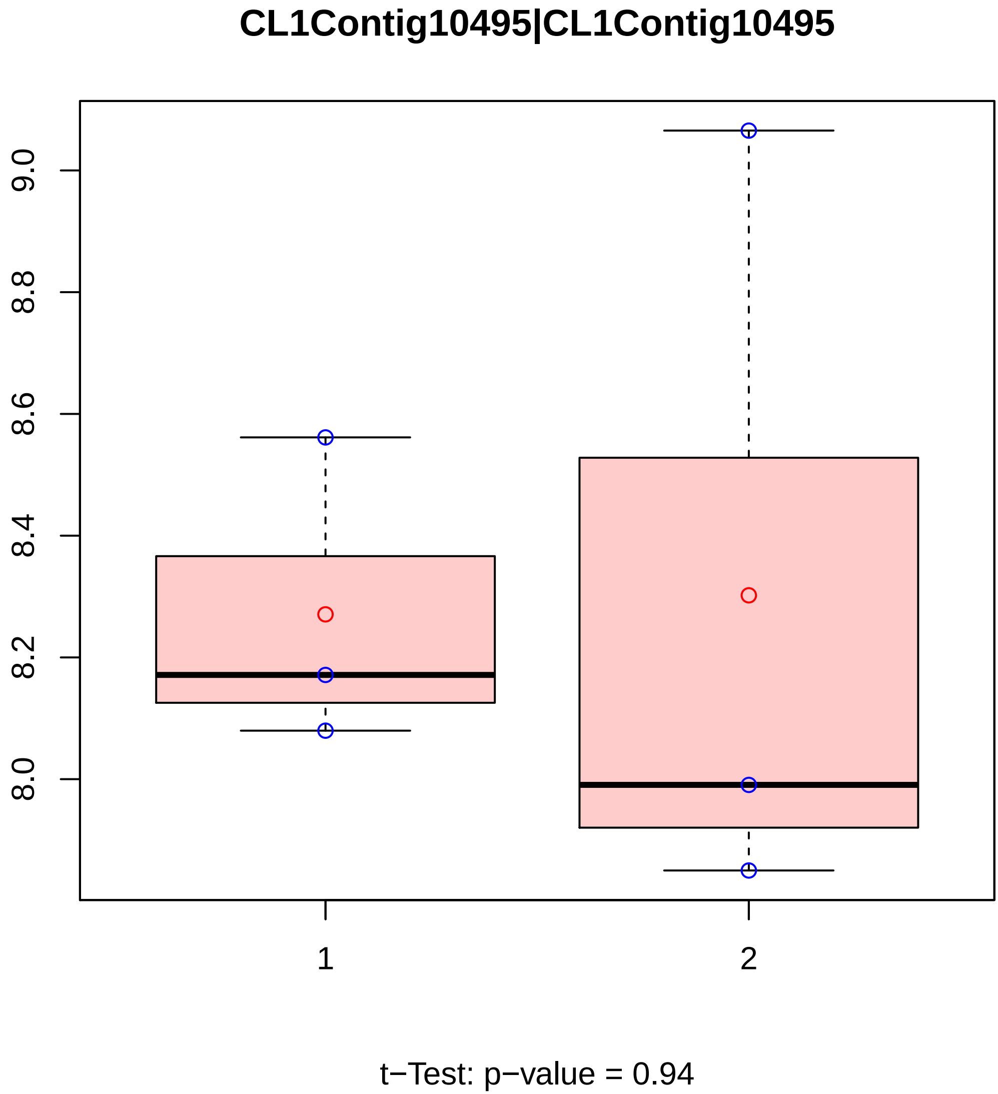

CL1Contig10495|CL1Contig10495
9.0
8.8
8.6
8.4
8.2
8.0
1
2
t−Test: p−value = 0.94

## CL1Contig10521|CL1Contig10521

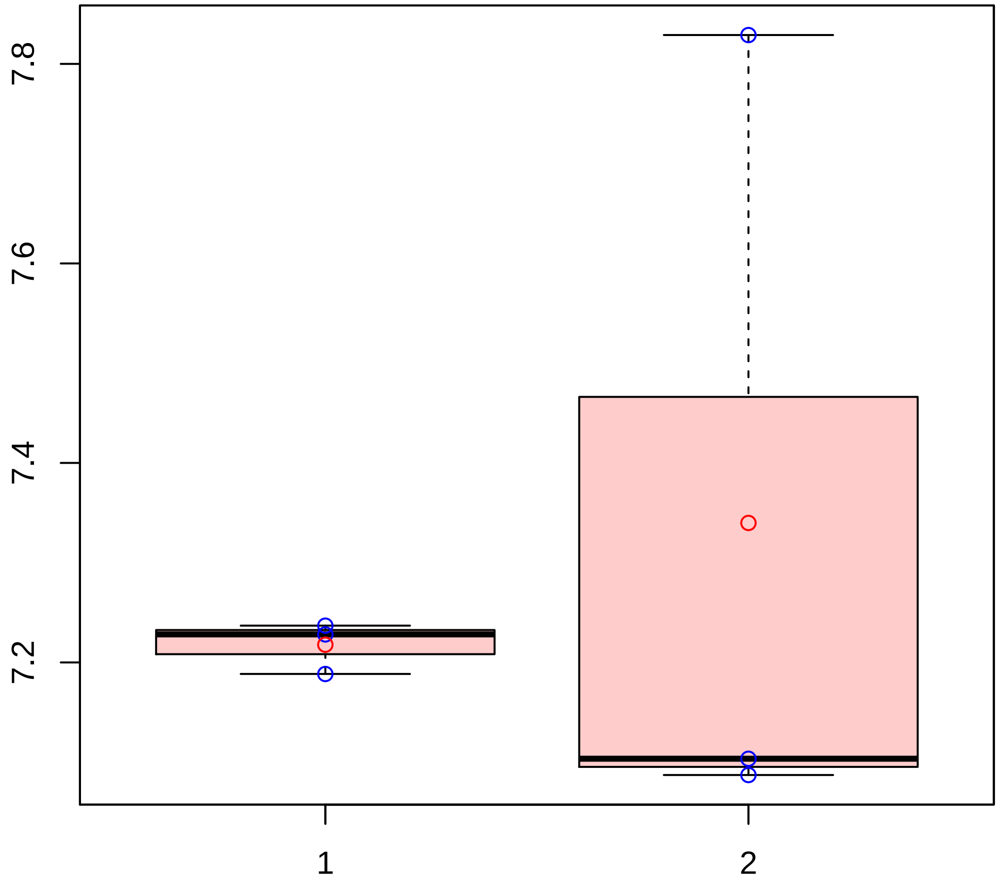


t−Test: p−value = 0.67

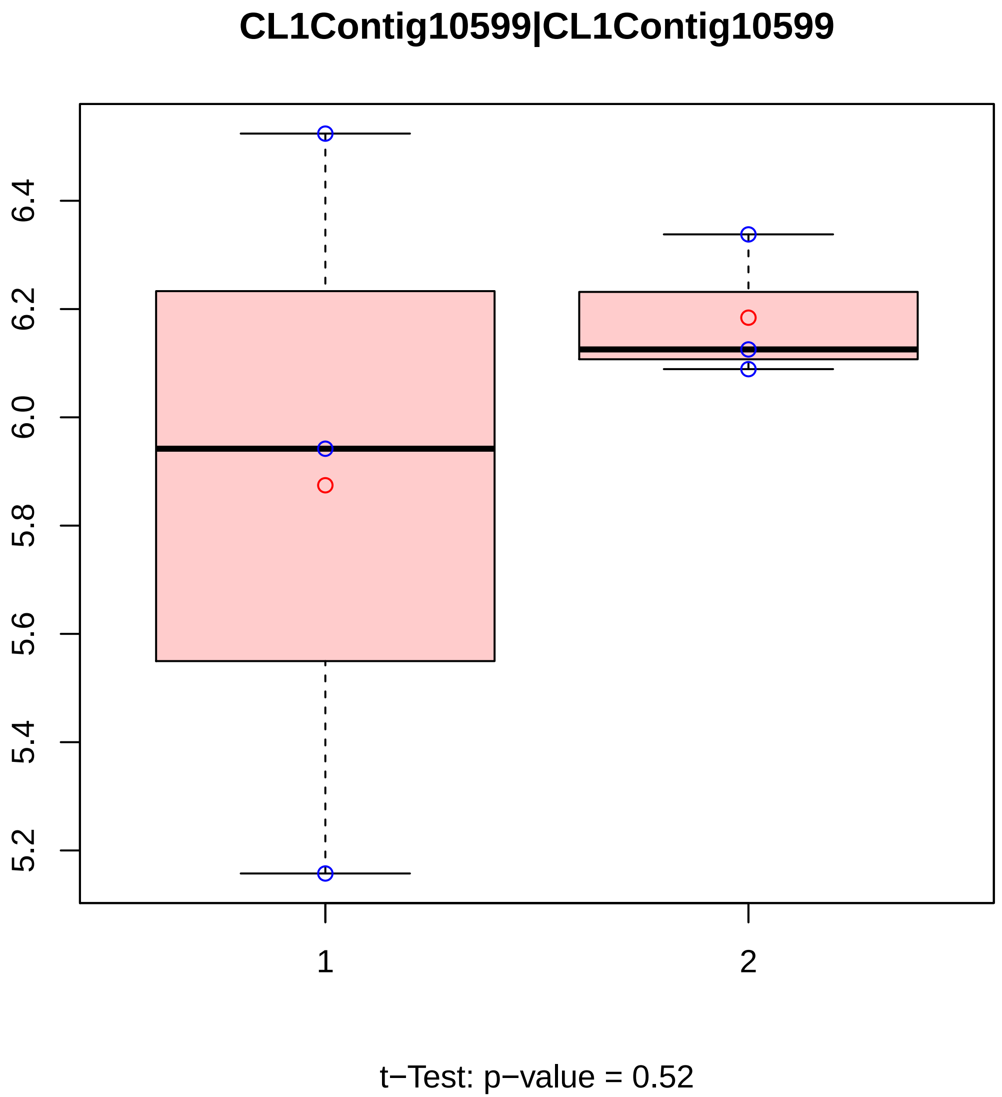
CL1Contig10599|CL1Contig10599
6.4
6.2
6.0
5.8
5.6
5.4
5.2
1
2
t−Test: p−value = 0.52

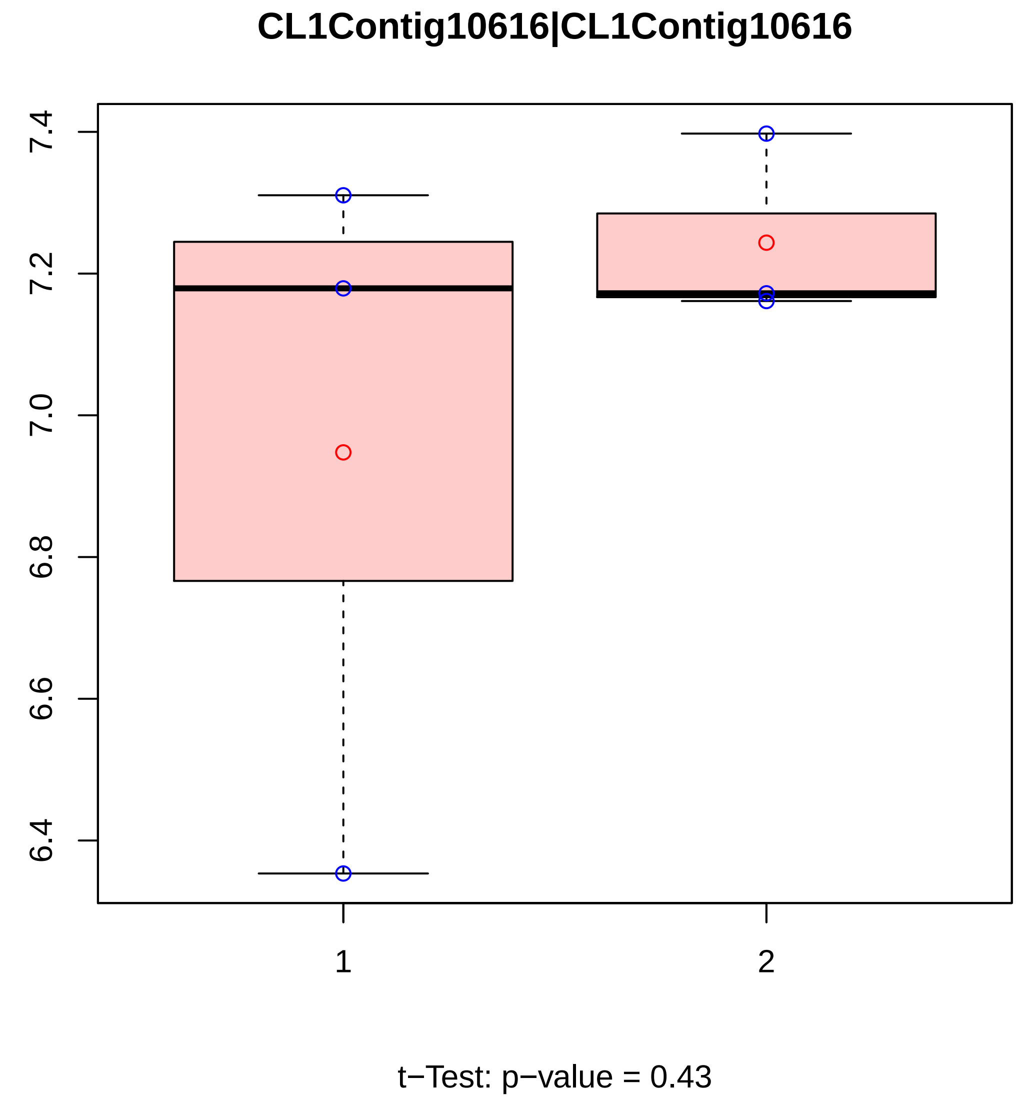
CL1Contig10616|CL1Contig10616
7.4
7.2
7.0
6.8
6.6
6.4
1
2
t−Test: p−value = 0.43

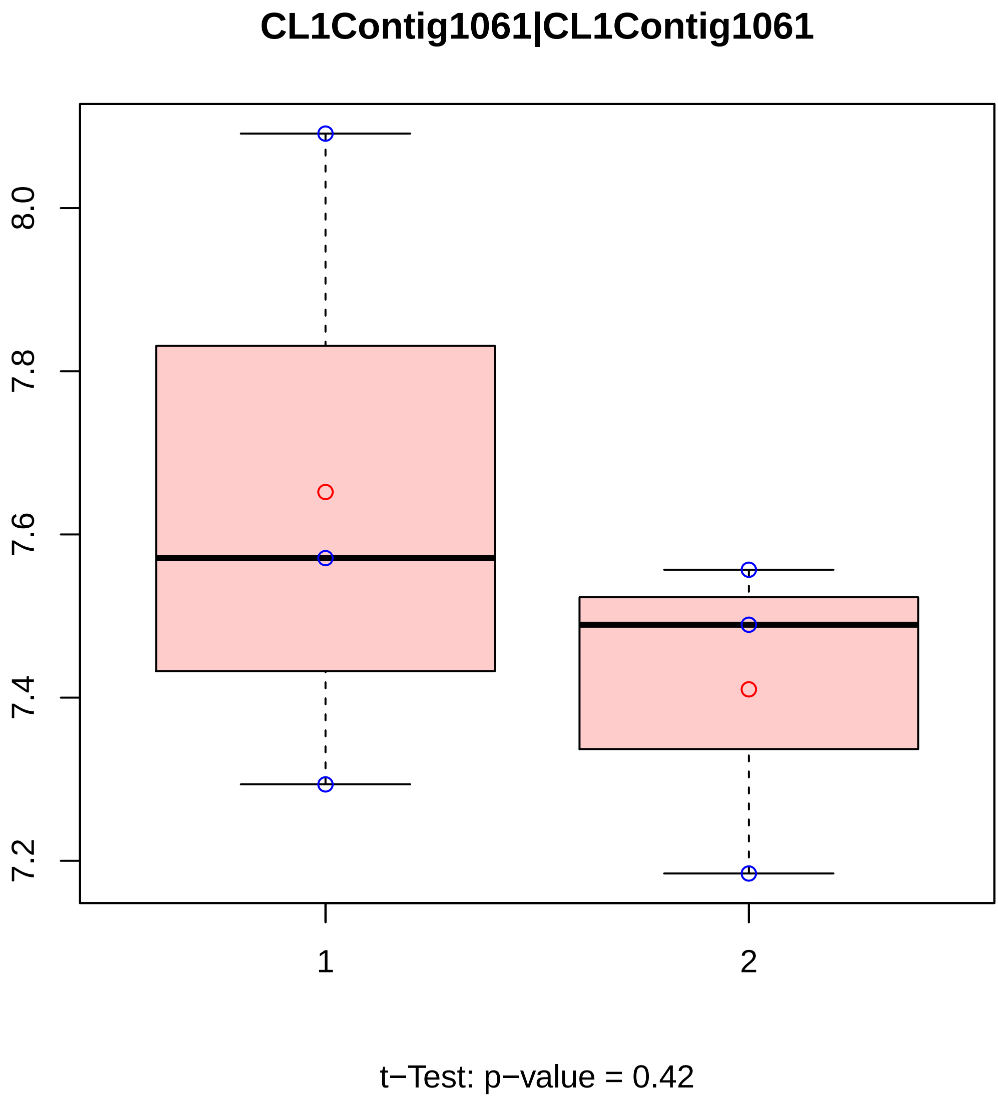
CL1Contig1061|CL1Contig1061
8.0
7.8
7.6
7.4
7.2
1
2
t−Test: p−value = 0.42

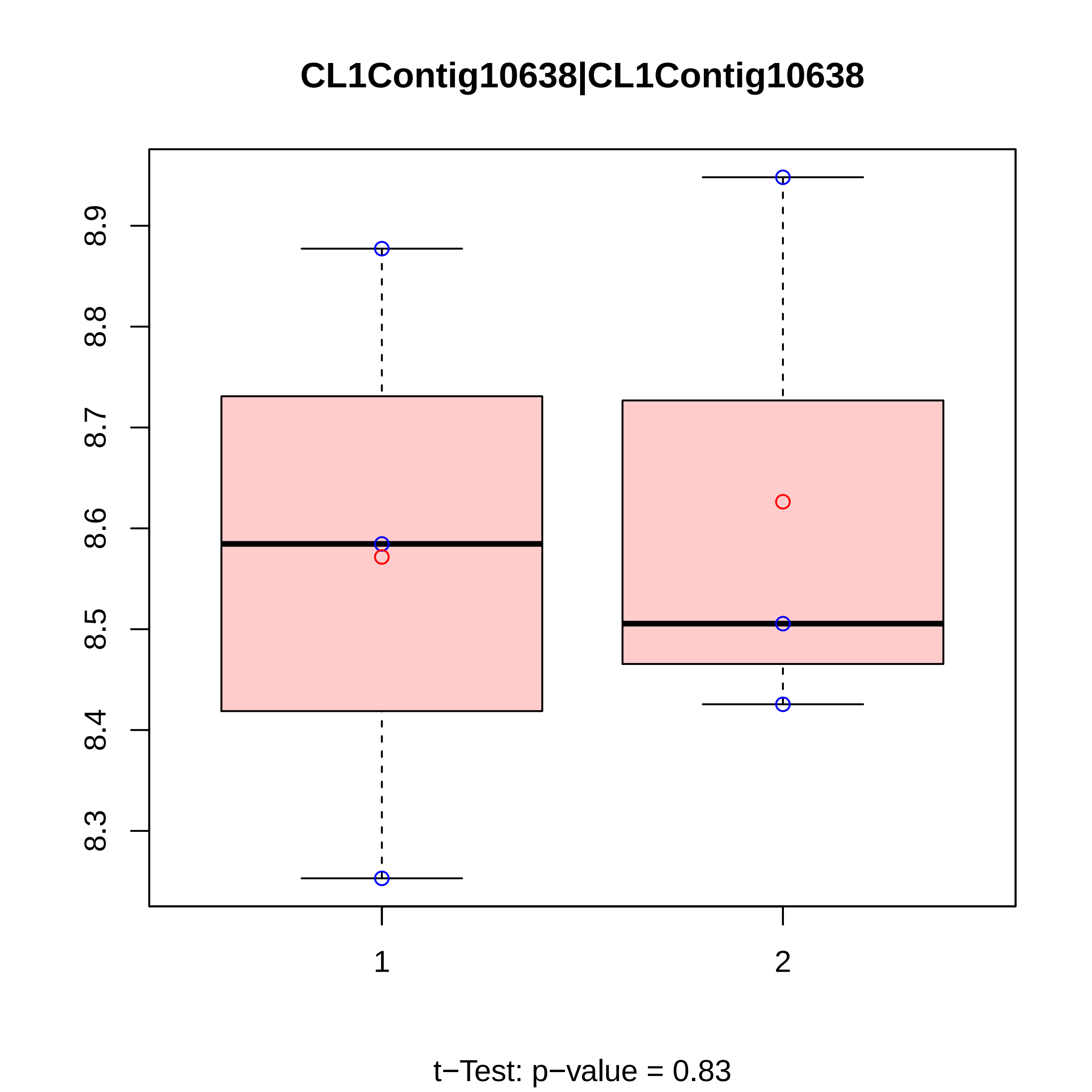
CL1Contig10638|CL1Contig10638
8.3
8.4
8.5
8.6
8.7
8.8
8.9
1
2
t−Test: p−value = 0.83

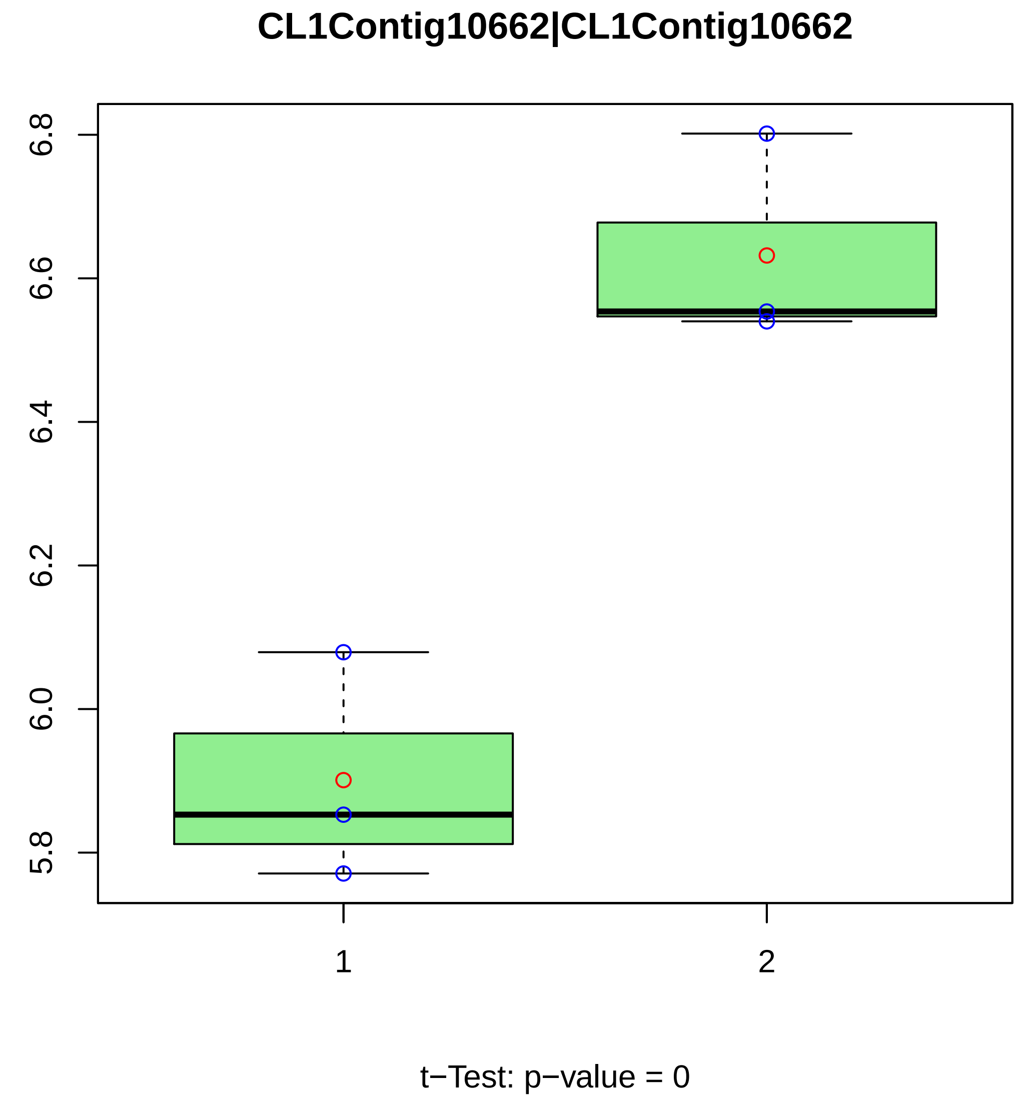

CL1Contig10662|CL1Contig10662
6.8
6.6
6.4
6.2
6.0
5.8
1
2
t−Test: p−value = 0

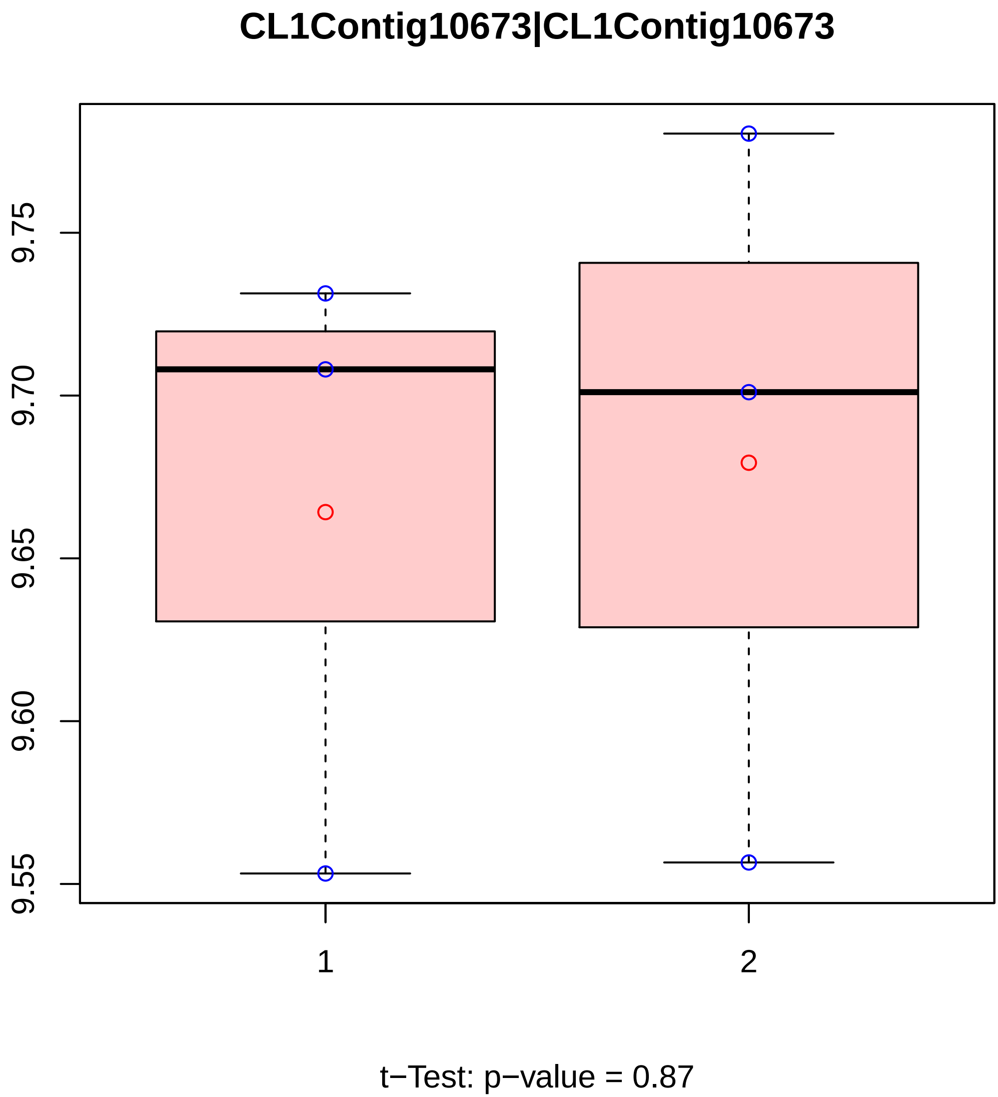
CL1Contig10673|CL1Contig10673
9.55
9.60
9.65
9.70
9.75
1
2
t−Test: p−value = 0.87

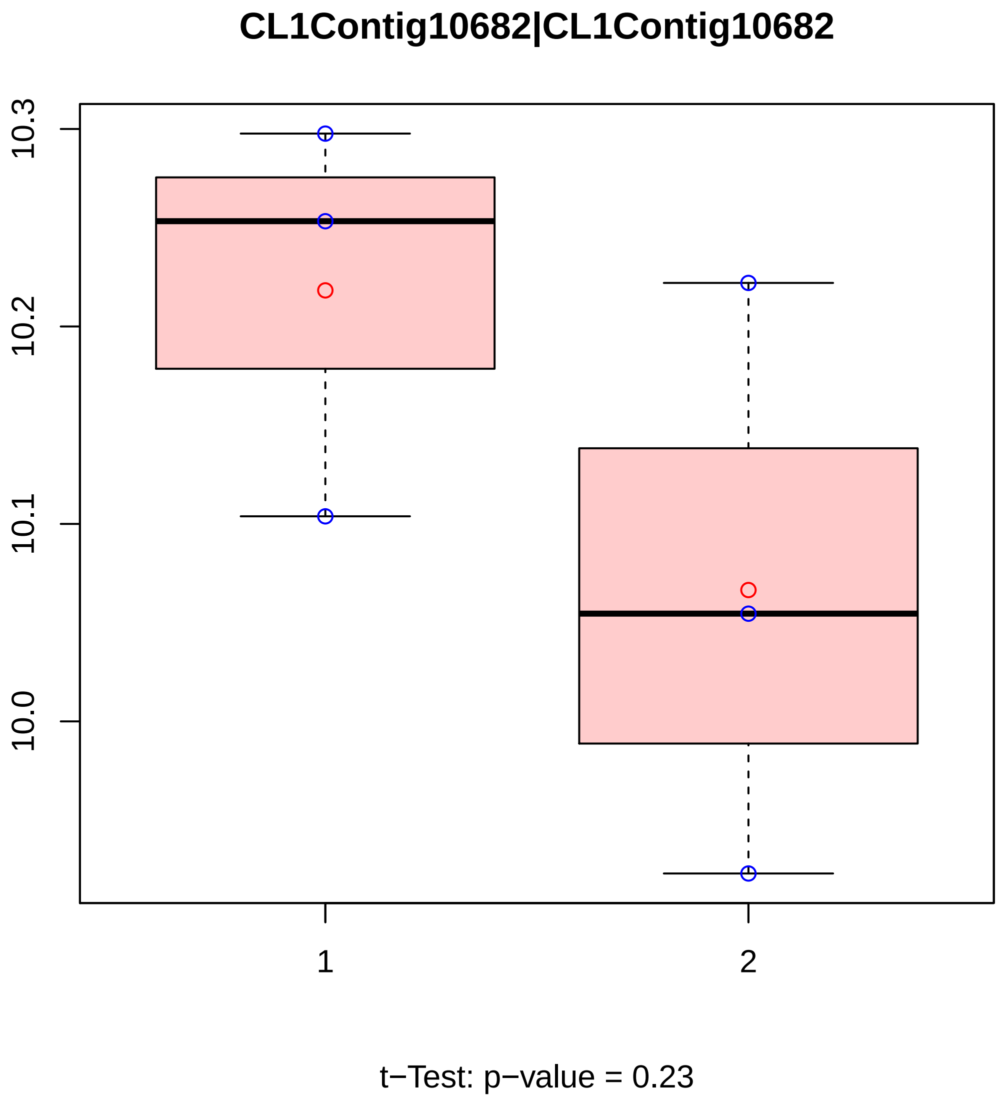
CL1Contig10682|CL1Contig10682
10.3
10.2
10.1
10.0
1
2
t−Test: p−value = 0.23

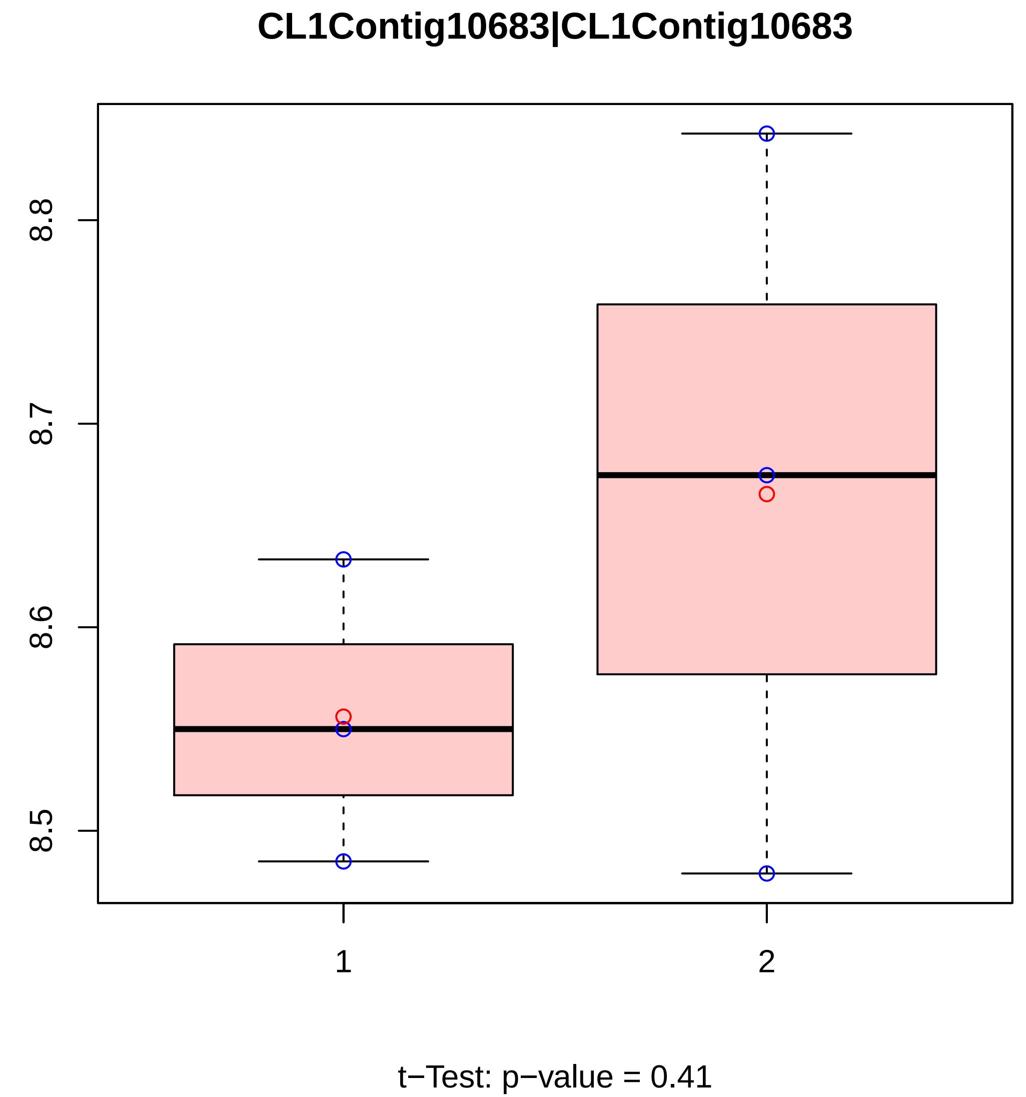

CL1Contig10683|CL1Contig10683
8.5
8.6
8.7
8.8
1
2
t−Test: p−value = 0.41

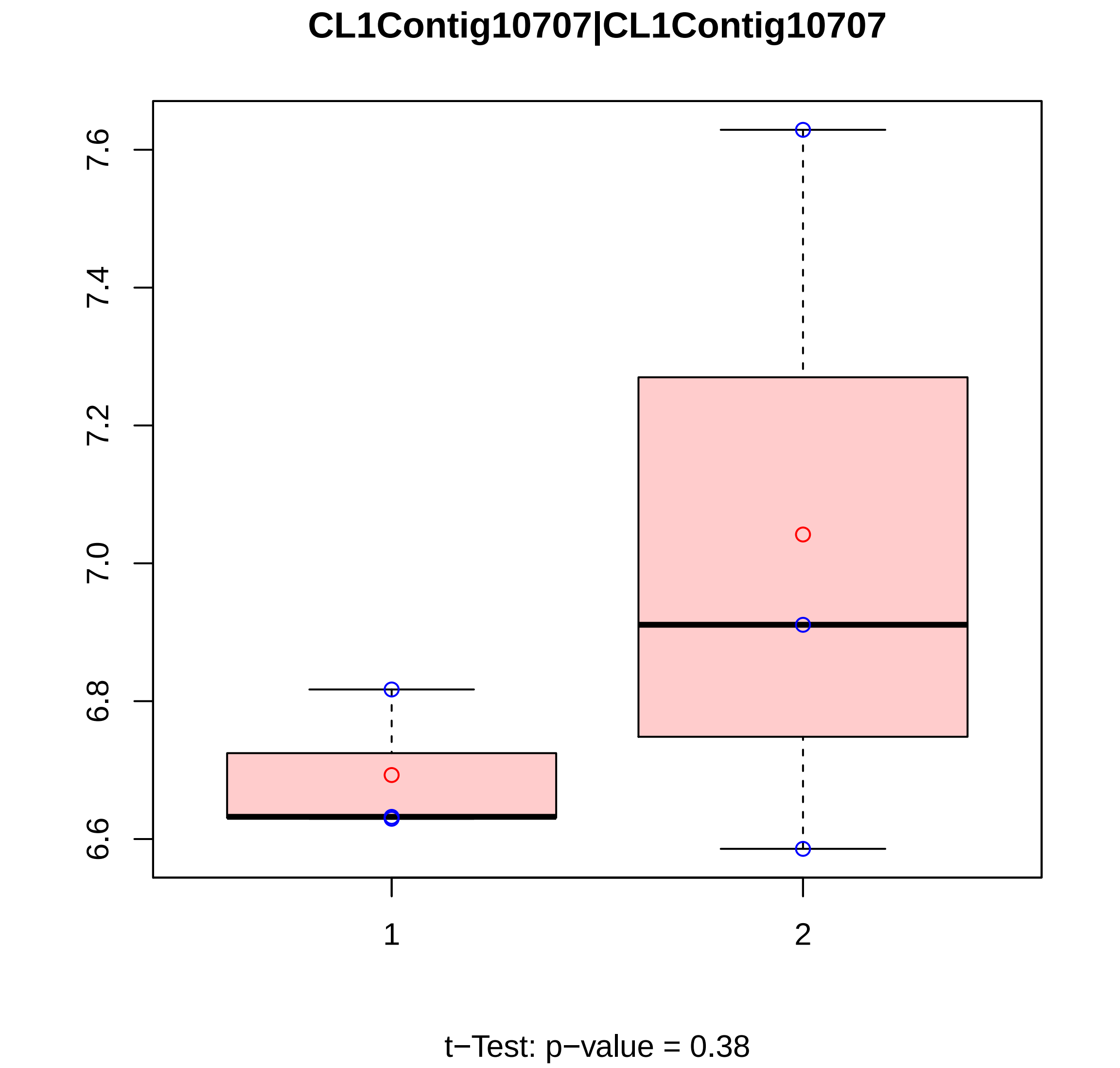
CL1Contig10707|CL1Contig10707
7.6
7.4
7.2
7.0
6.8
6.6
1
2
t−Test: p−value = 0.38

**CL1Contig10729|CL1Contig10729**

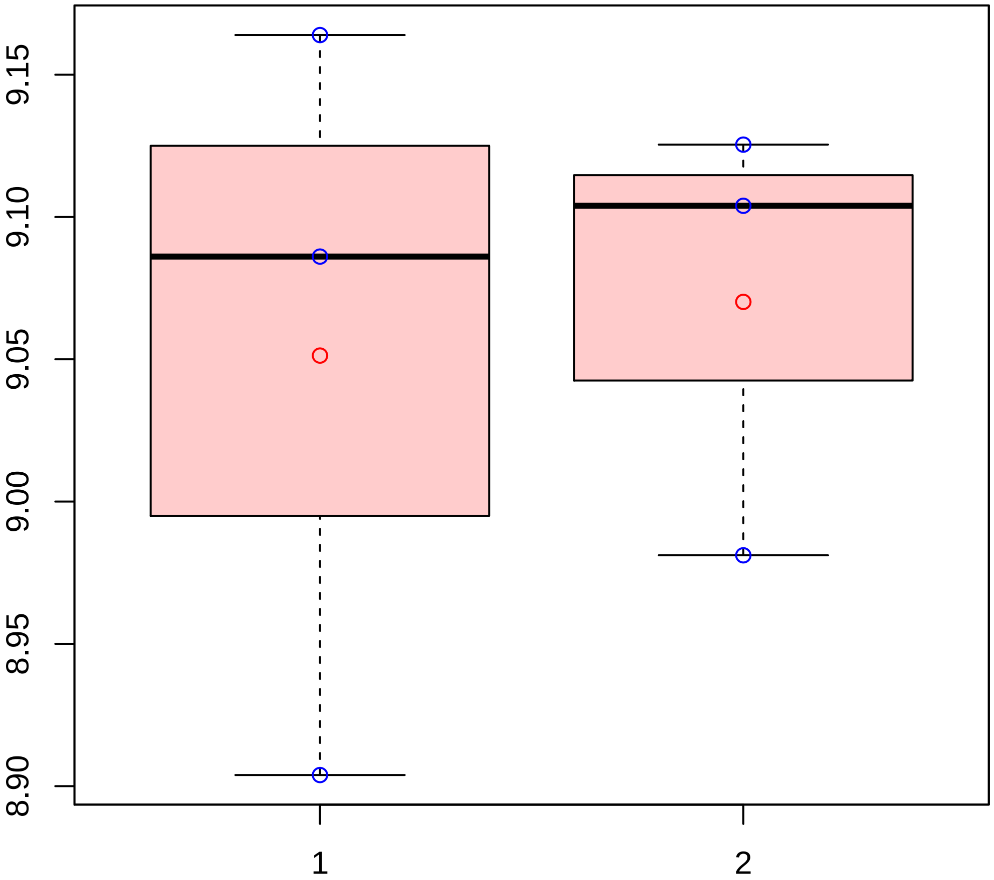


t−Test: p−value = 0.85

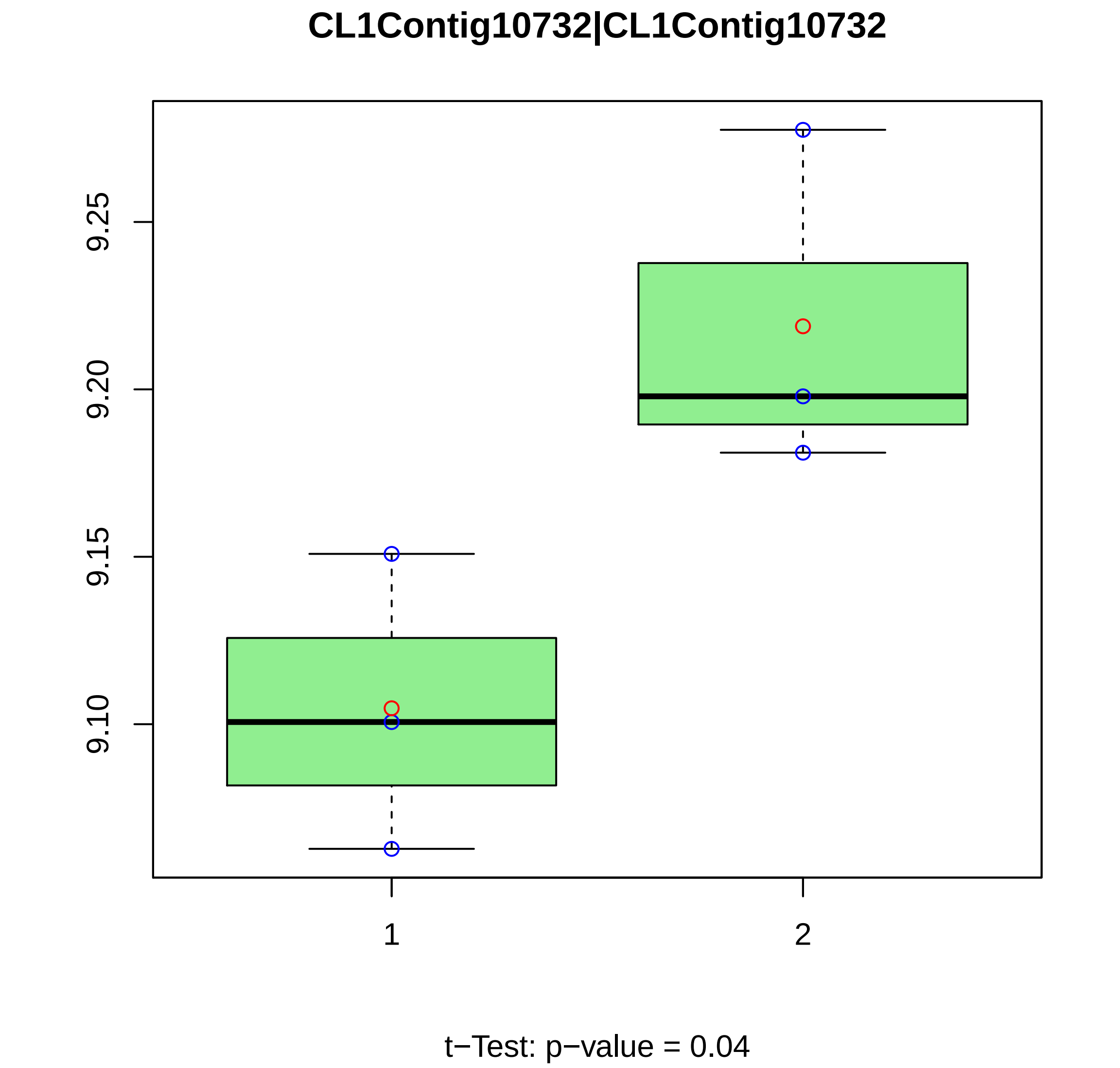
CL1Contig10732|CL1Contig10732
9.25
9.20
9.15
9.10
1
2
t−Test: p−value = 0.04

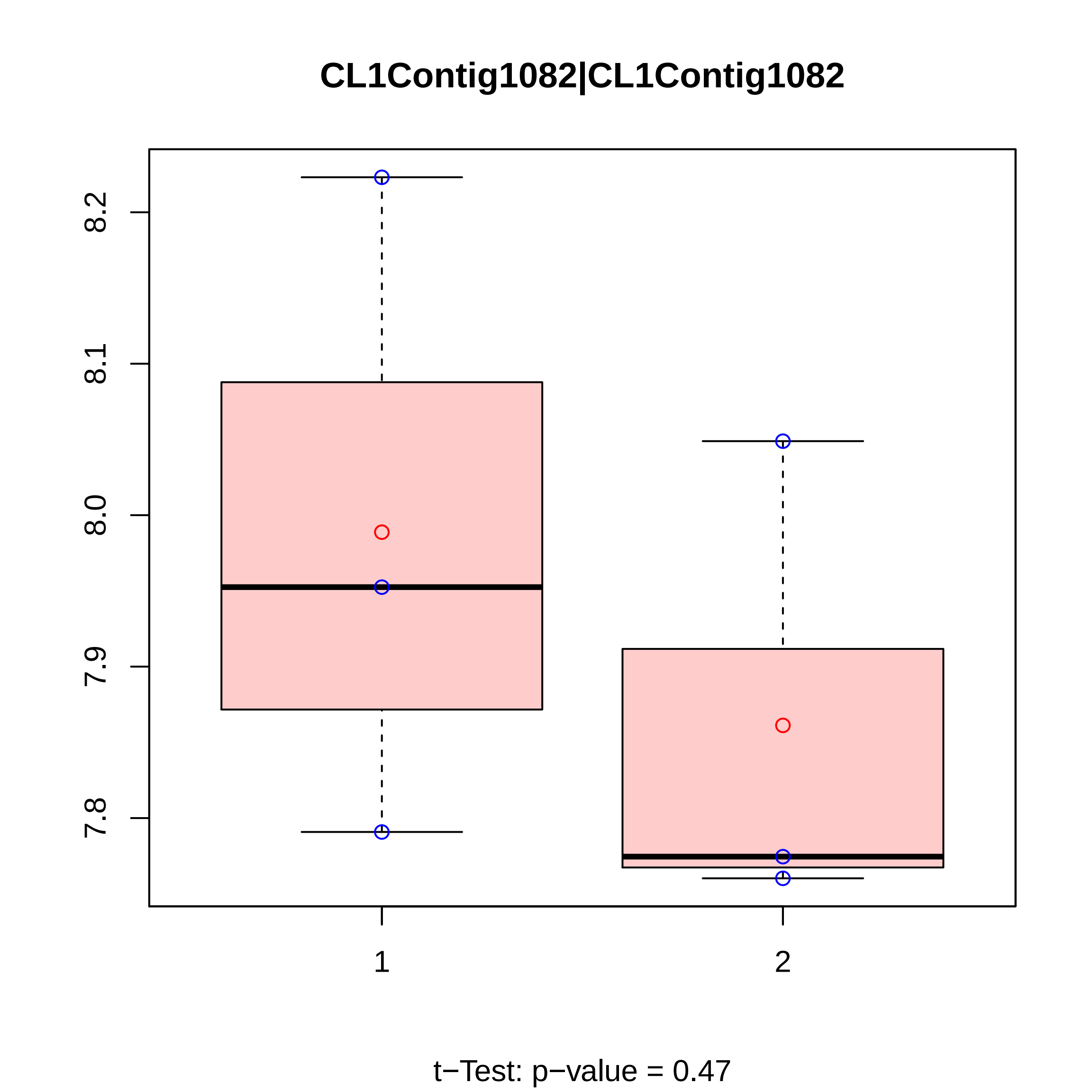
CL1Contig1082|CL1Contig1082
8.2
8.1
8.0
7.9
7.8
1
2
t−Test: p−value = 0.47

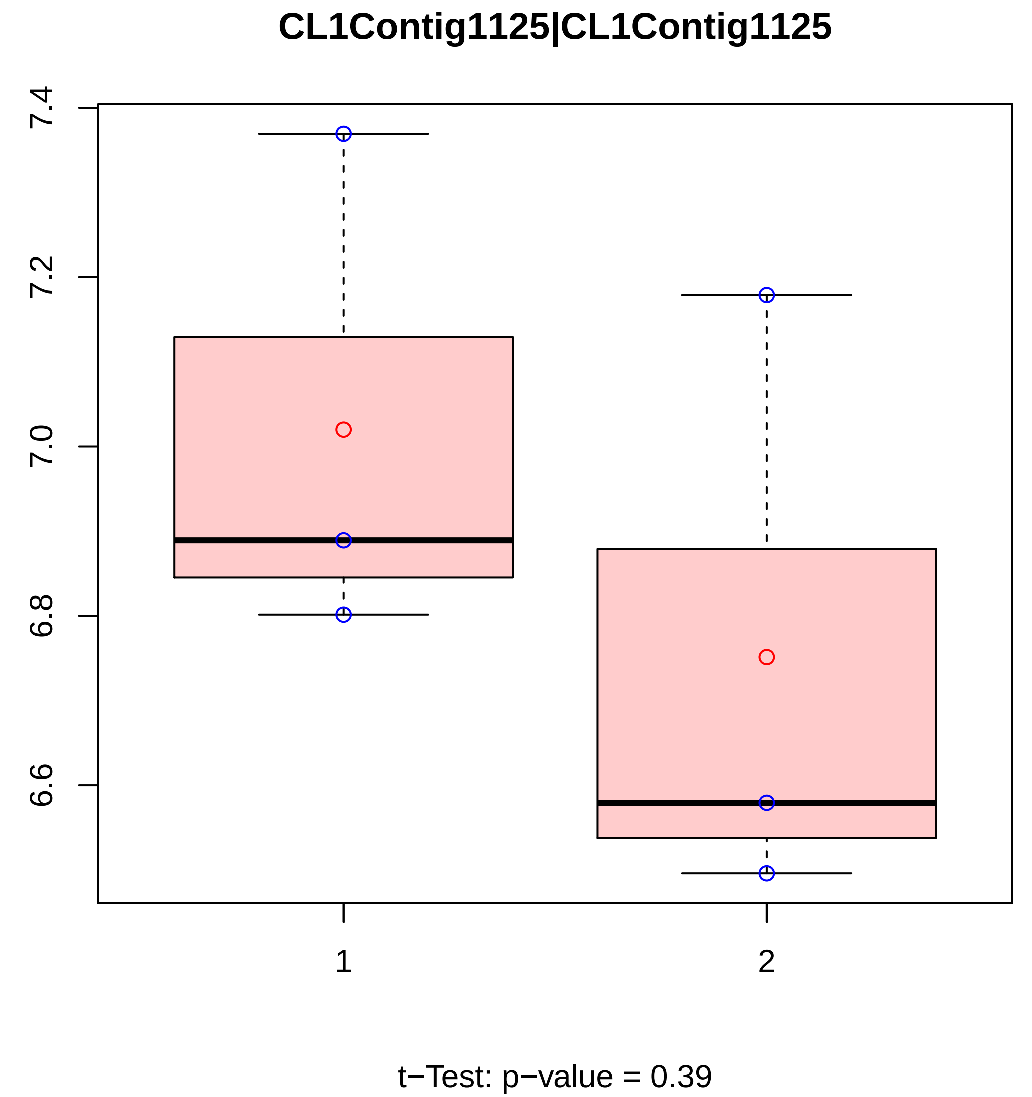
CL1Contig1125|CL1Contig1125
7.4
7.2
7.0
6.8
6.6
1
2
t−Test: p−value = 0.39

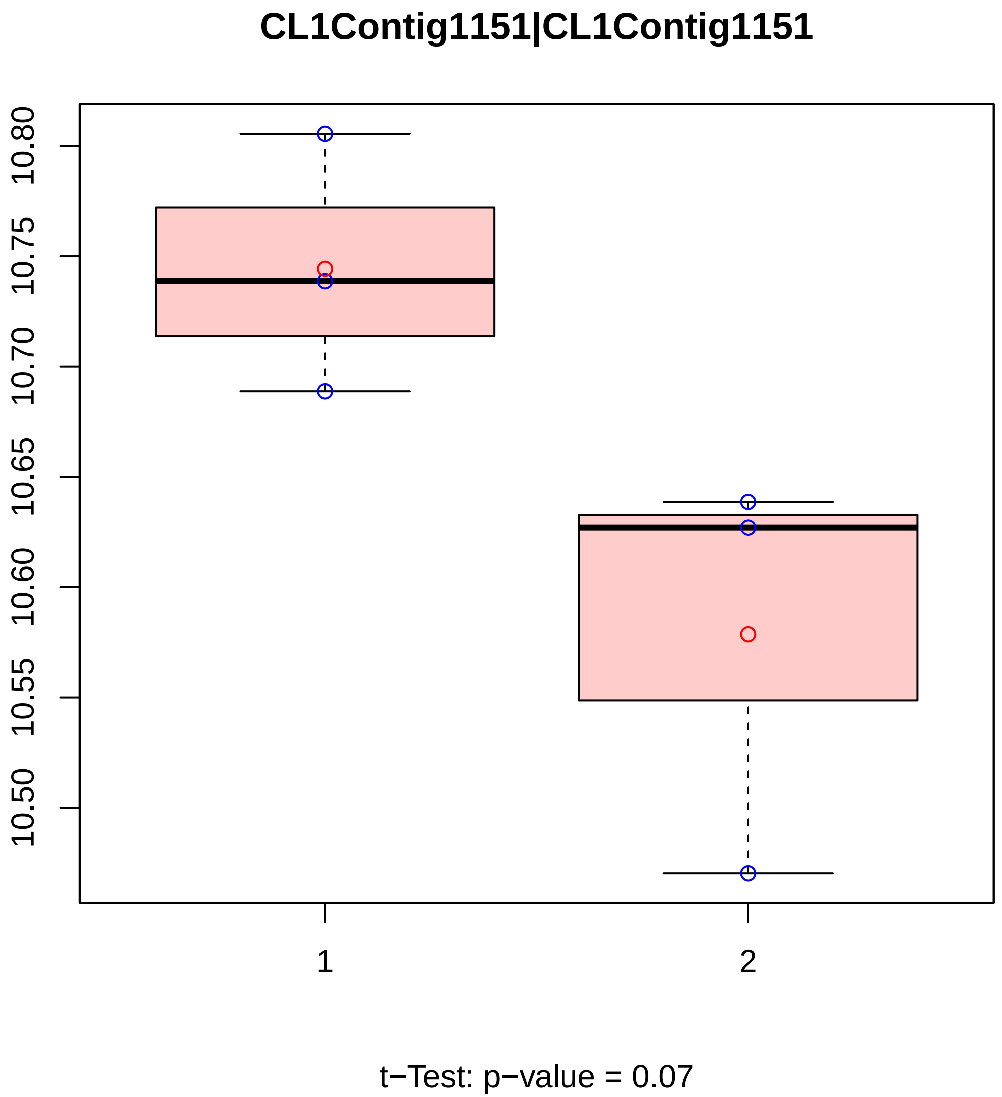
CL1Contig1151|CL1Contig1151
10.50
10.55
10.60
10.65
10.70
10.75
10.80
1
2
t−Test: p−value = 0.07

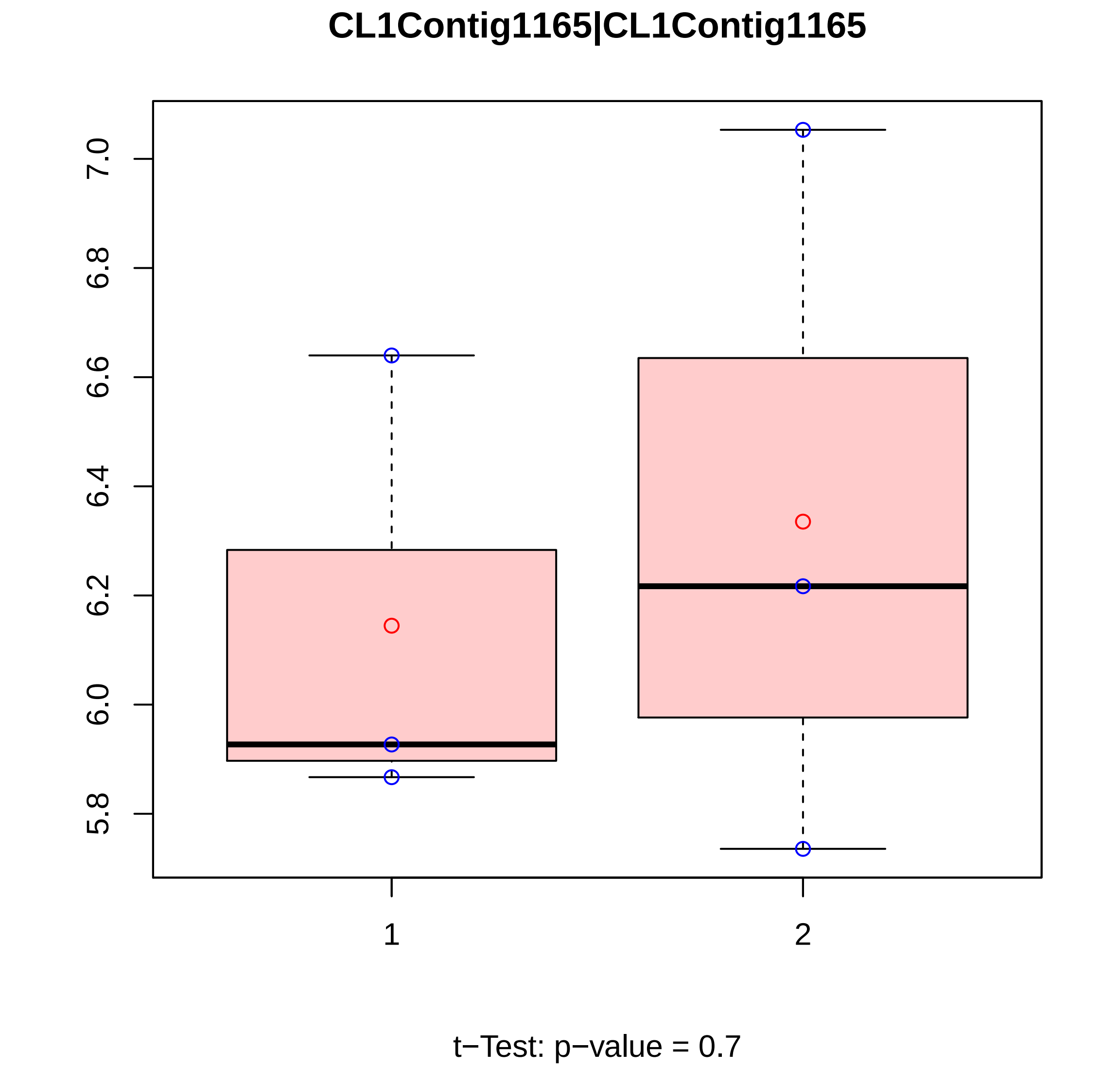
CL1Contig1165|CL1Contig1165
7.0
6.8
6.6
6.4
6.2
6.0
5.8
1
2
t−Test: p−value = 0.7

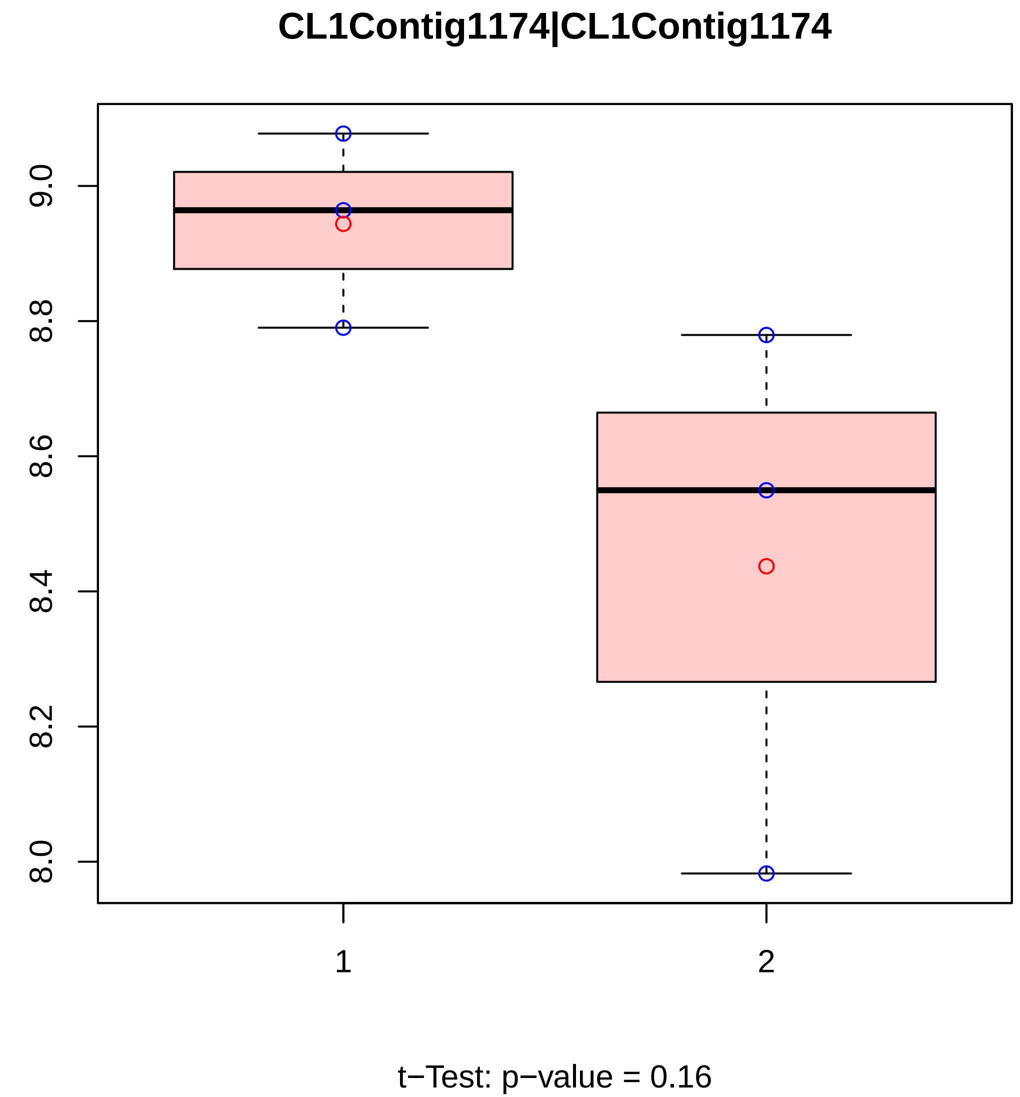
CL1Contig1174|CL1Contig1174
9.0
8.8
8.6
8.4
8.2
8.0
1
2
t−Test: p−value = 0.16

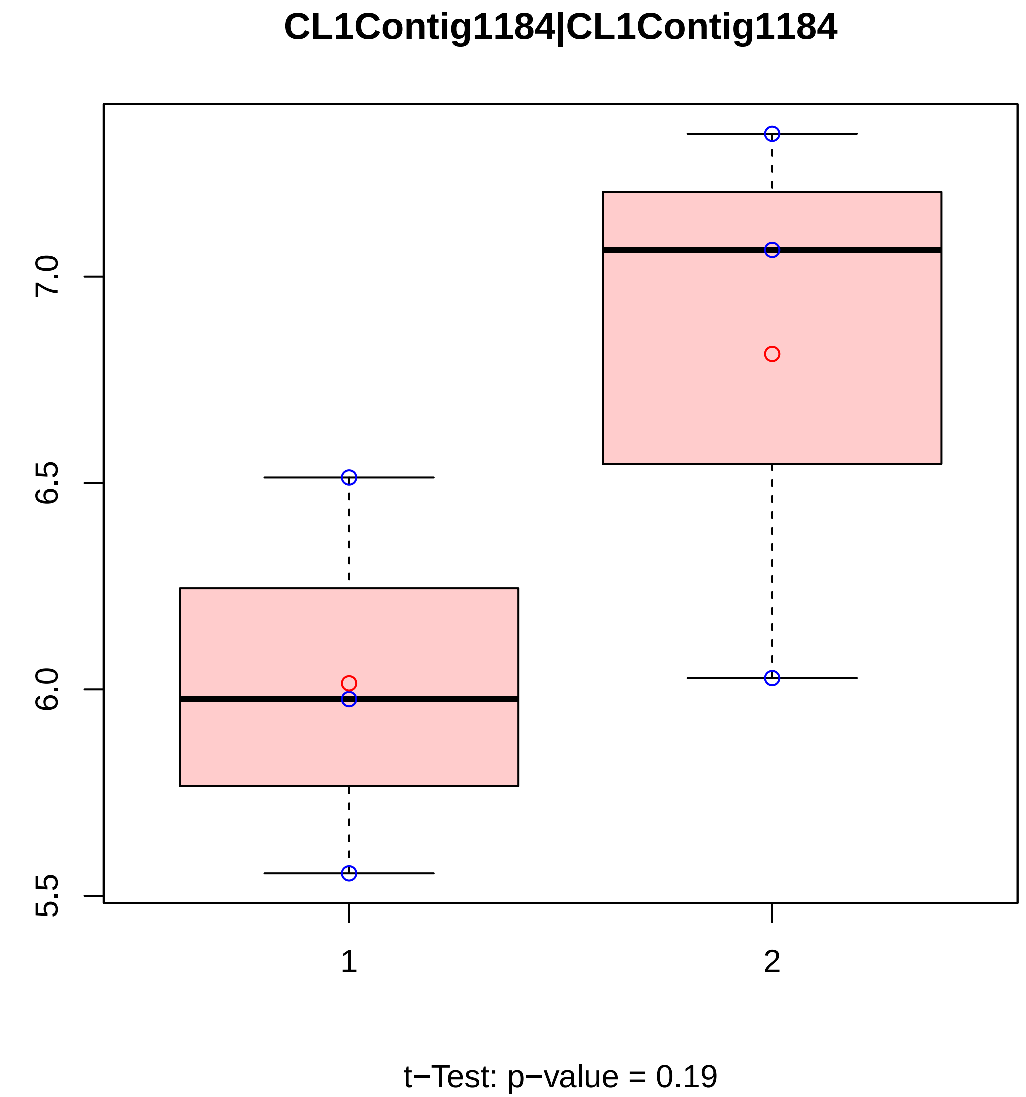

CL1Contig1184|CL1Contig1184
7.0
6.5
6.0
5.5
1
2
t−Test: p−value = 0.19

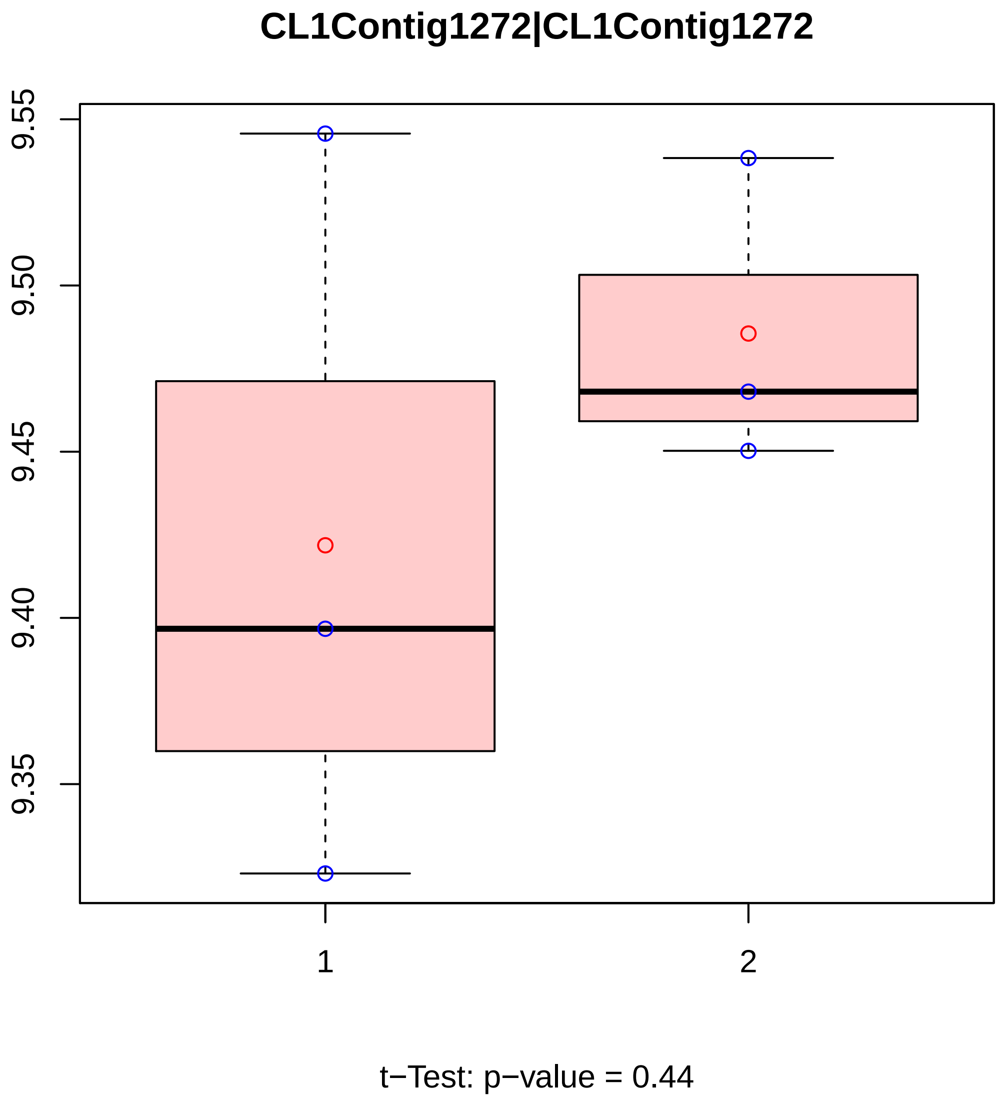

CL1Contig1272|CL1Contig1272
9.35
9.40
9.45
9.50
9.55
1
2
t−Test: p−value = 0.44

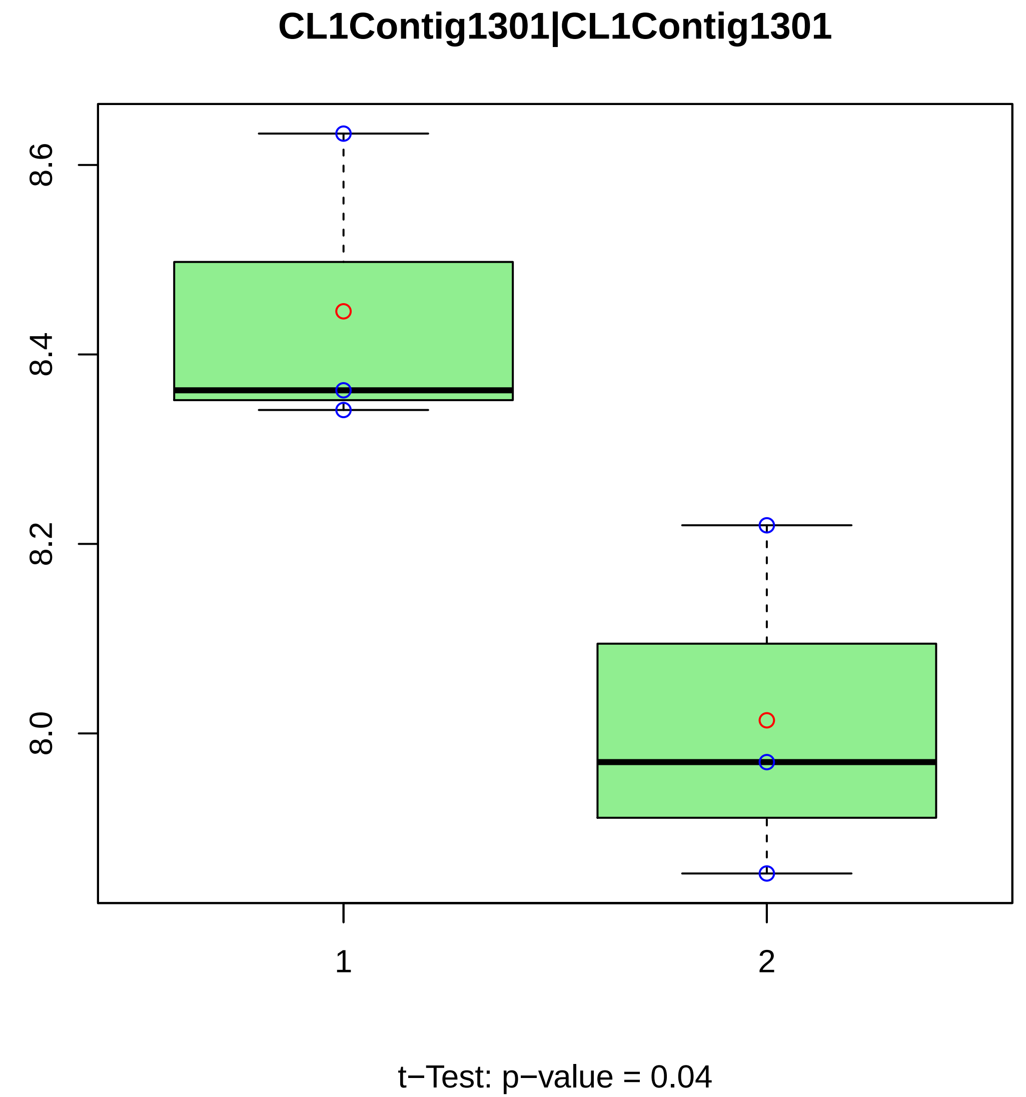
CL1Contig1301|CL1Contig1301
8.0
8.2
8.4
8.6
1
2
t−Test: p−value = 0.04

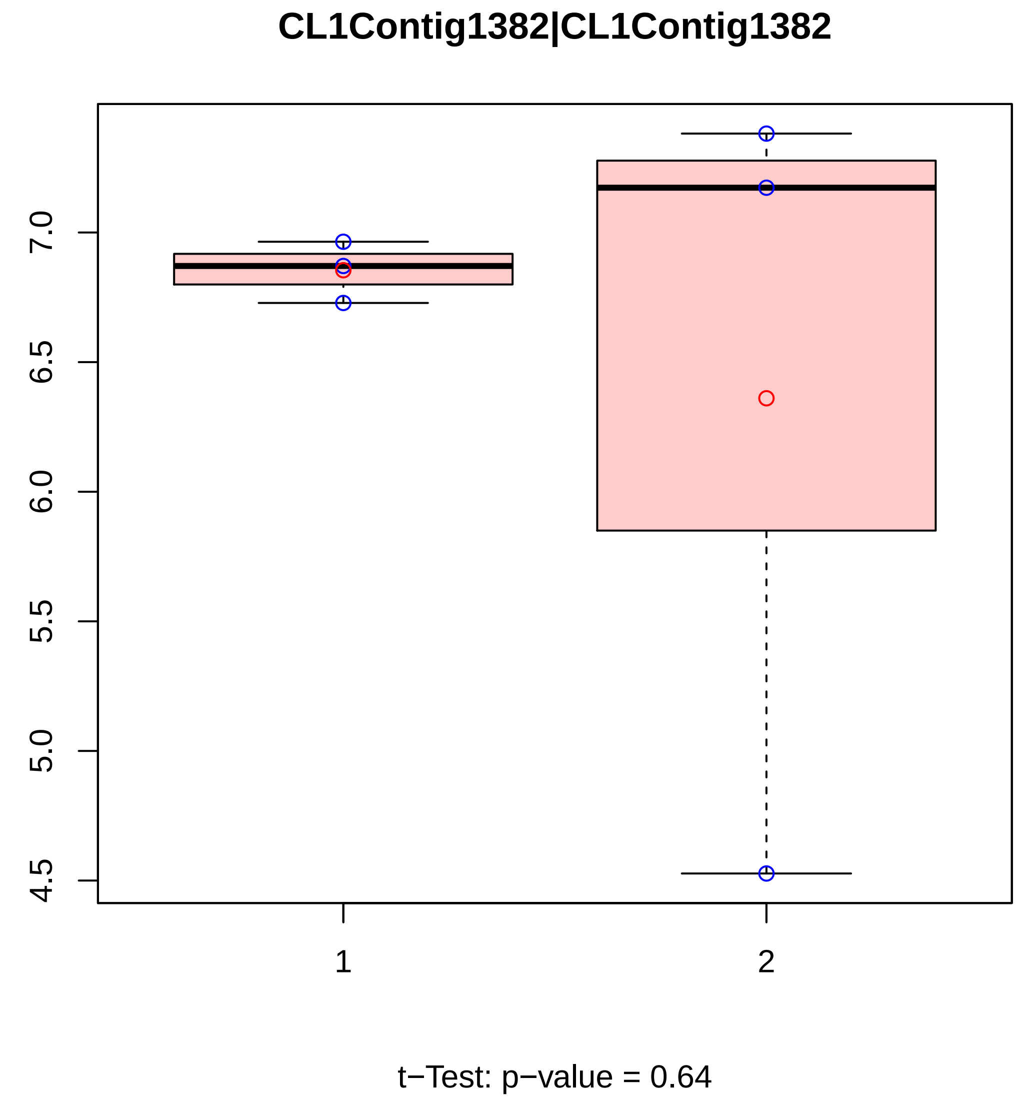
CL1Contig1382|CL1Contig1382
7.0
6.5
6.0
5.5
5.0
4.5
1
2
t−Test: p−value = 0.64

# CL1Contig1458|CL1Contig1458

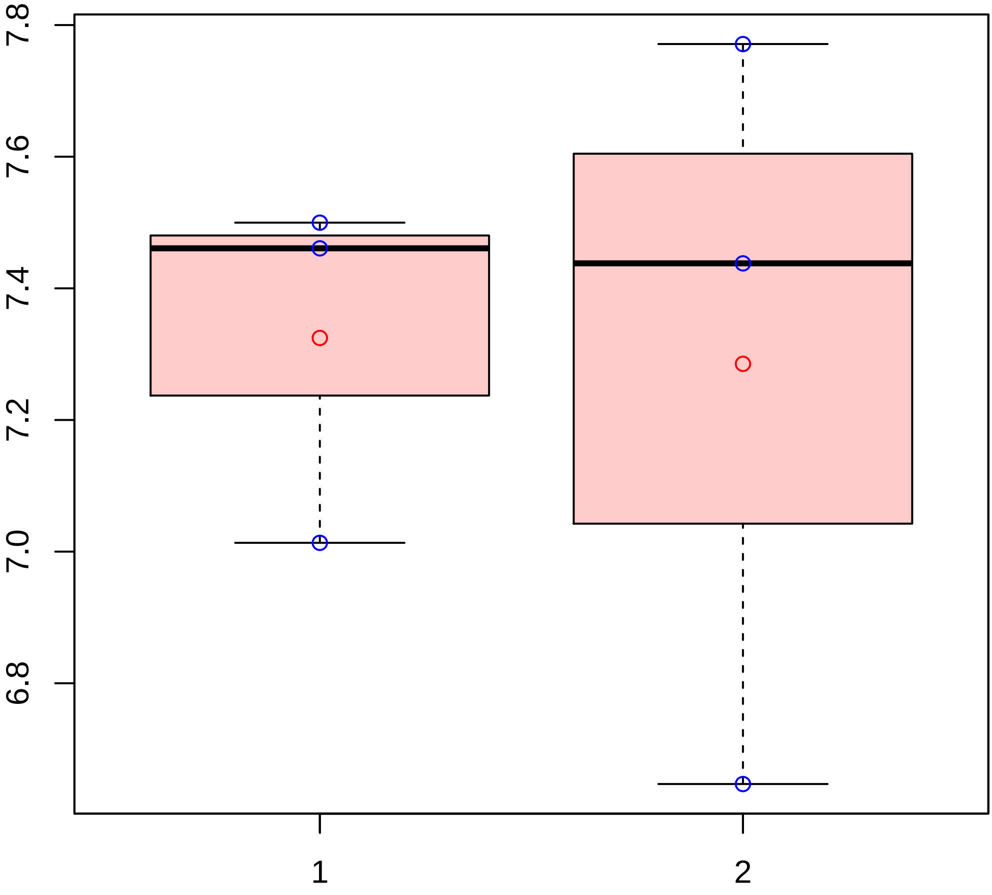


t−Test: p−value = 0.92

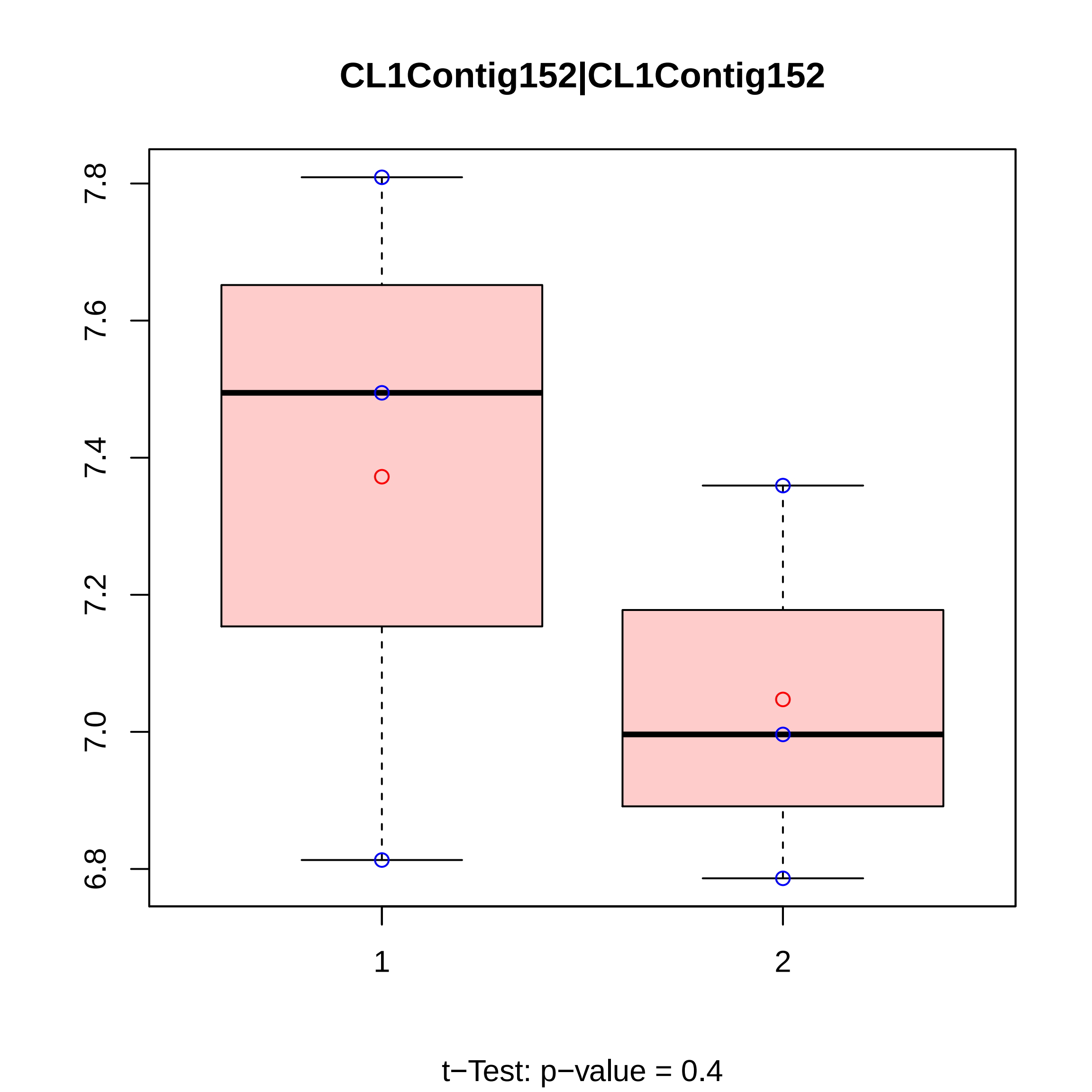

CL1Contig152|CL1Contig152
7.8
7.6
7.4
7.2
7.0
6.8
1
2
t−Test: p−value = 0.4

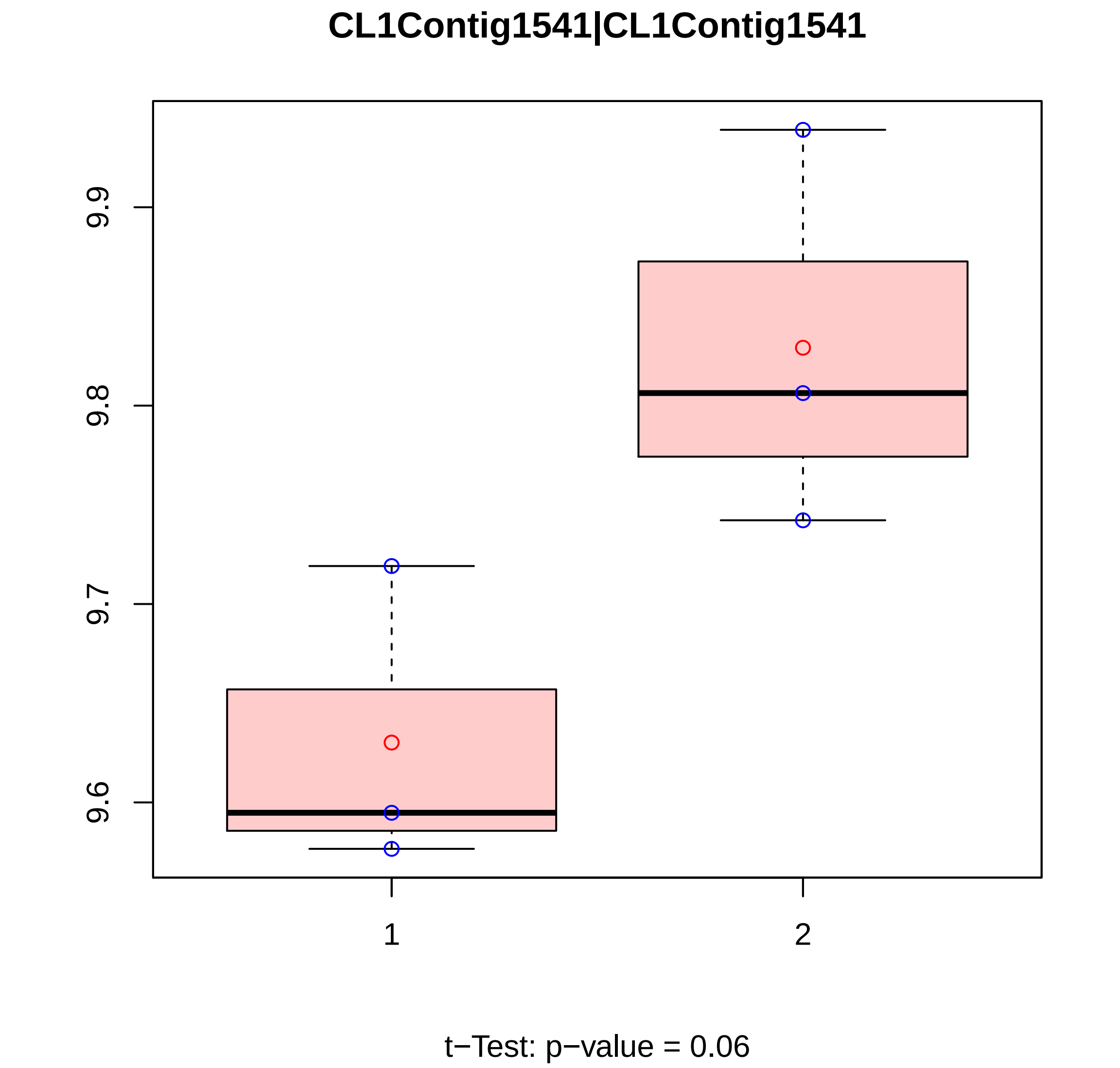
CL1Contig1541|CL1Contig1541
9.6
9.7
9.8
9.9
1
2
t−Test: p−value = 0.06

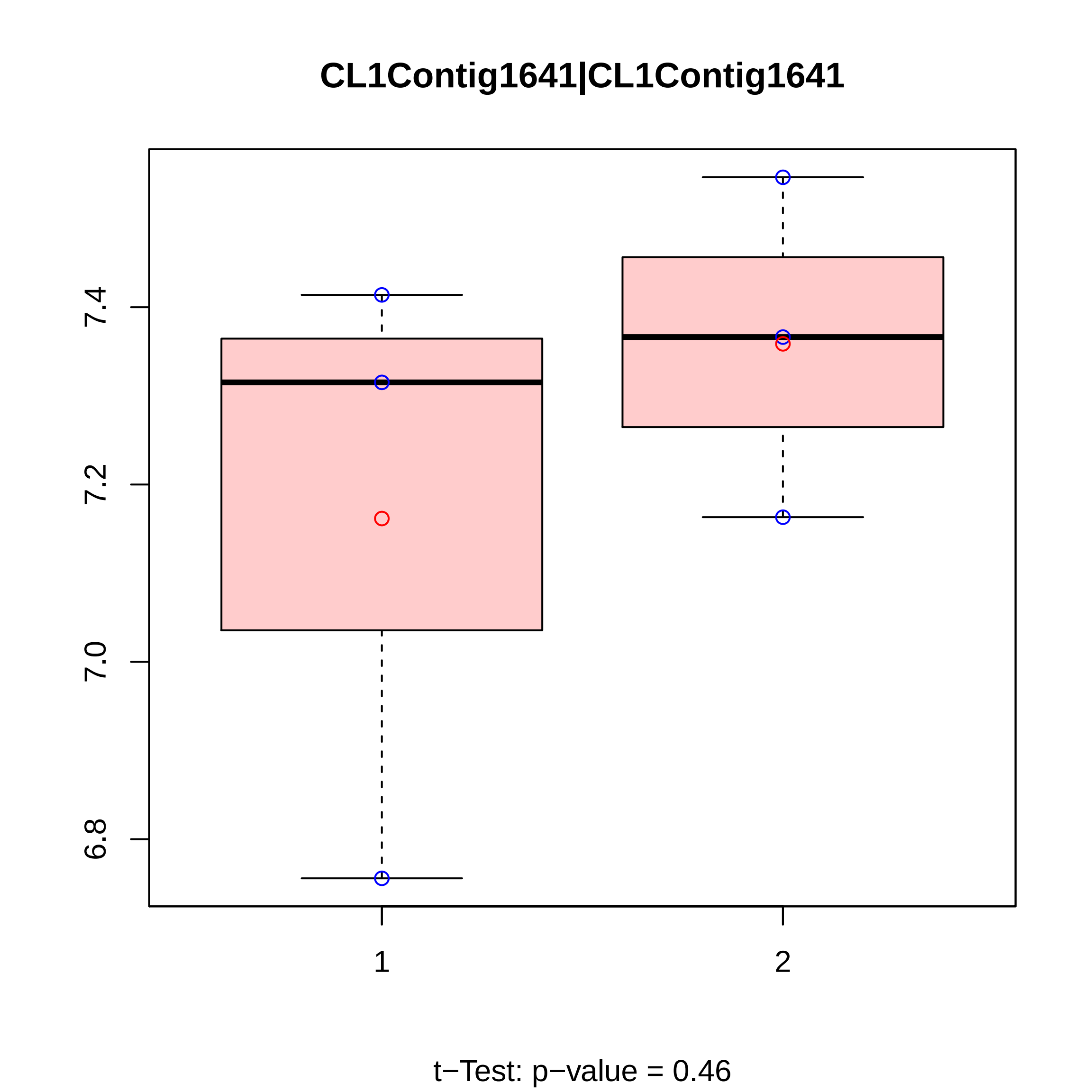
CL1Contig1641|CL1Contig1641
6.8
7.0
7.2
7.4
1
2
t−Test: p−value = 0.46

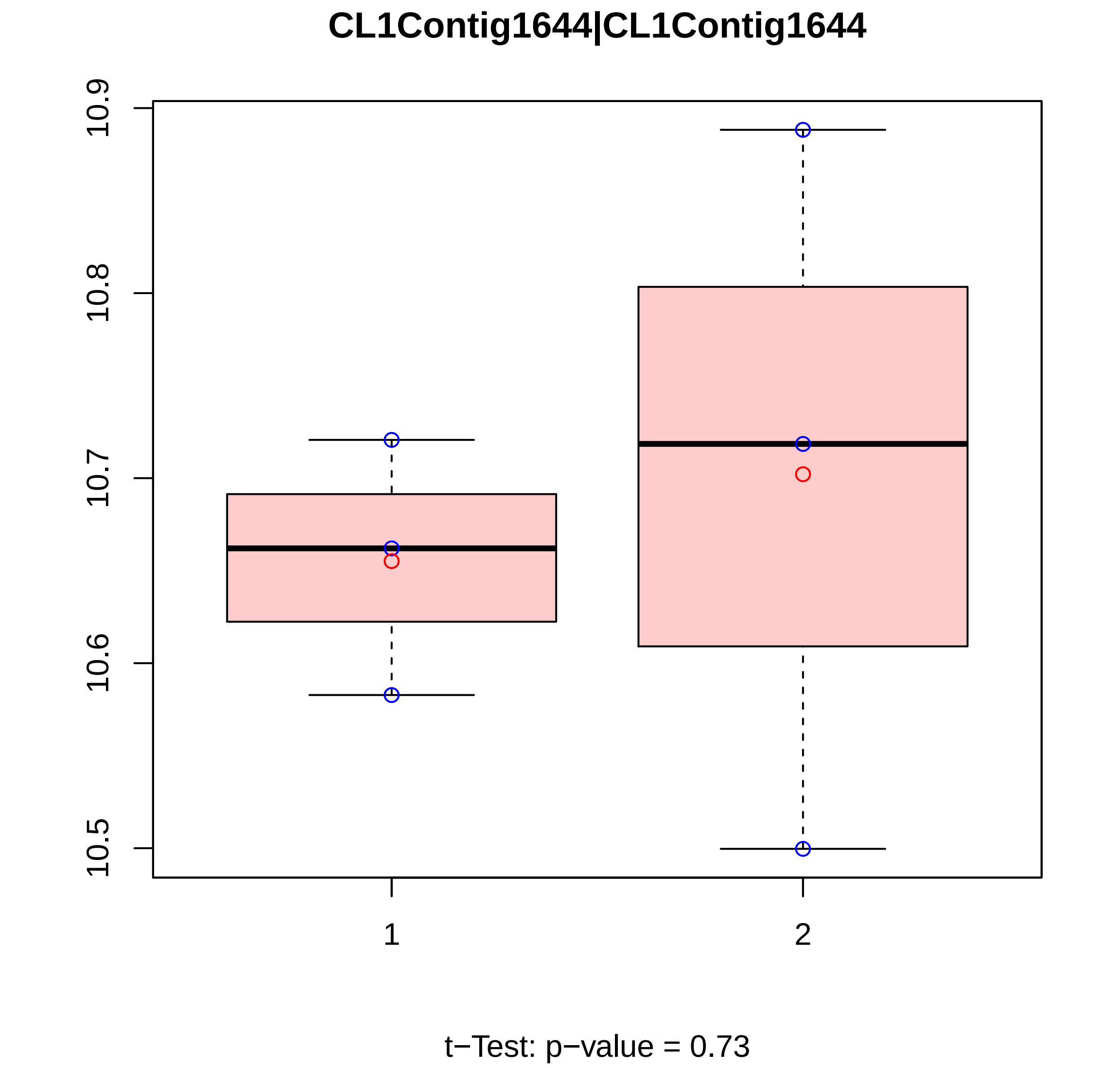
CL1Contig1644|CL1Contig1644
10.9
10.8
10.7
10.6
10.5
1
2
t−Test: p−value = 0.73

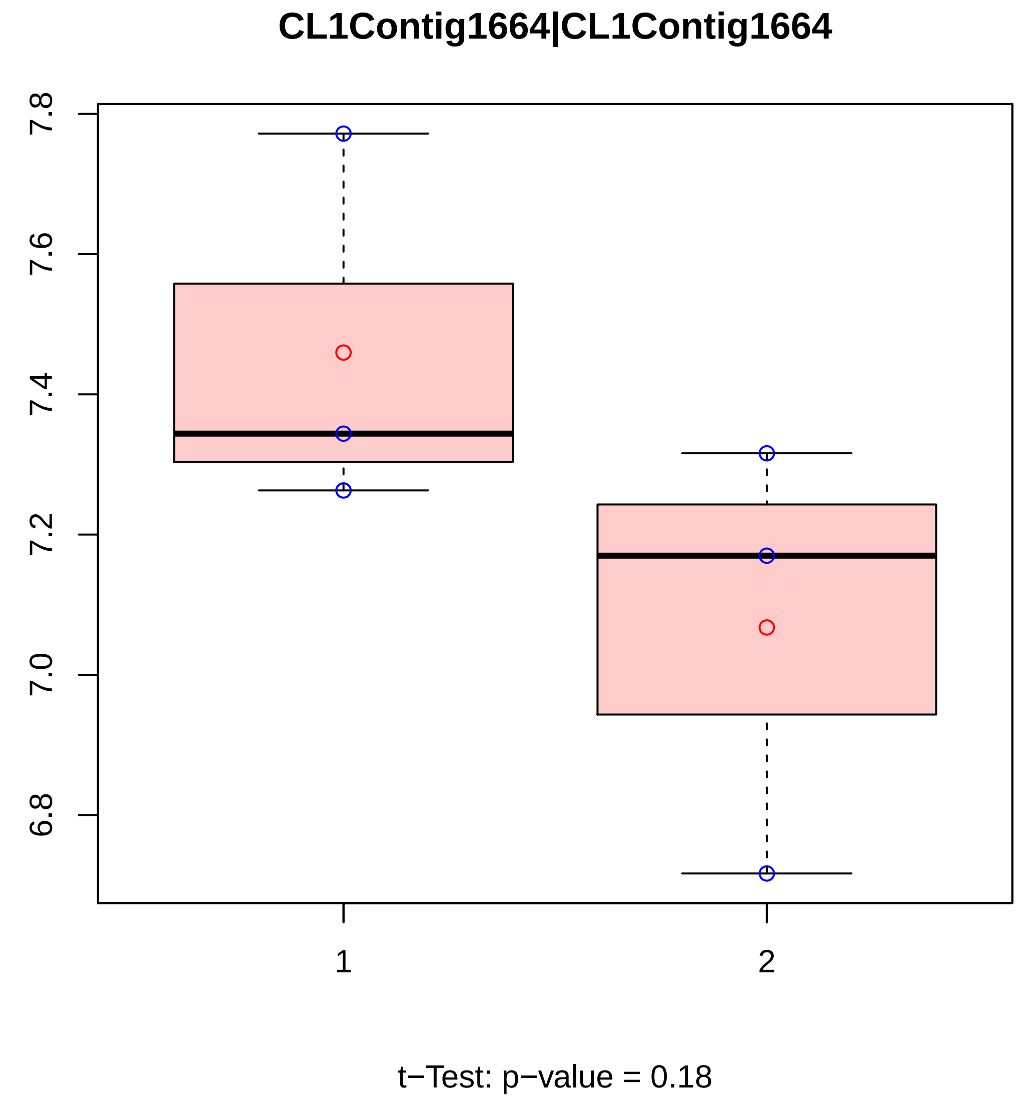

CL1Contig1664|CL1Contig1664
7.8
7.6
7.4
7.2
7.0
6.8
1
2
t−Test: p−value = 0.18

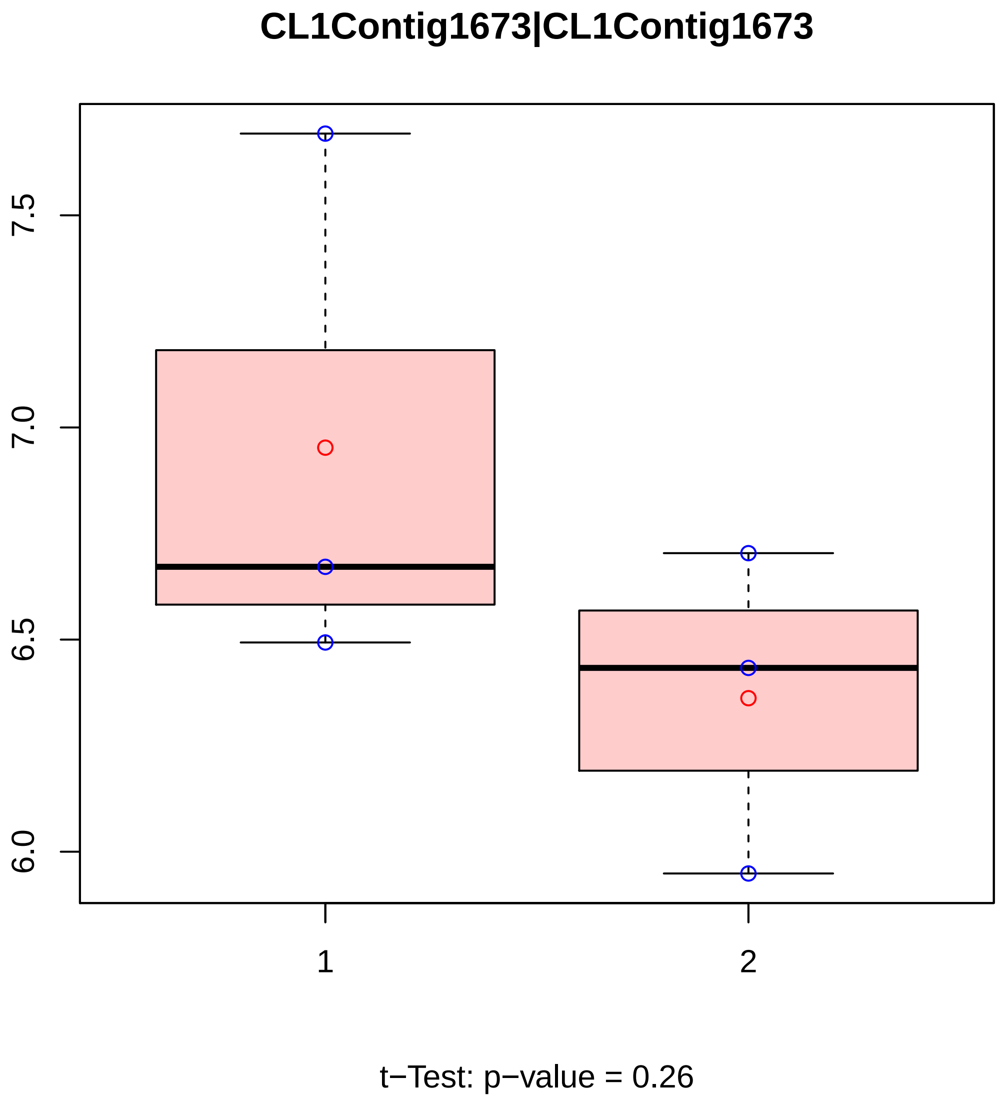
CL1Contig1673|CL1Contig1673
7.5
7.0
6.5
6.0
1
2
t−Test: p−value = 0.26

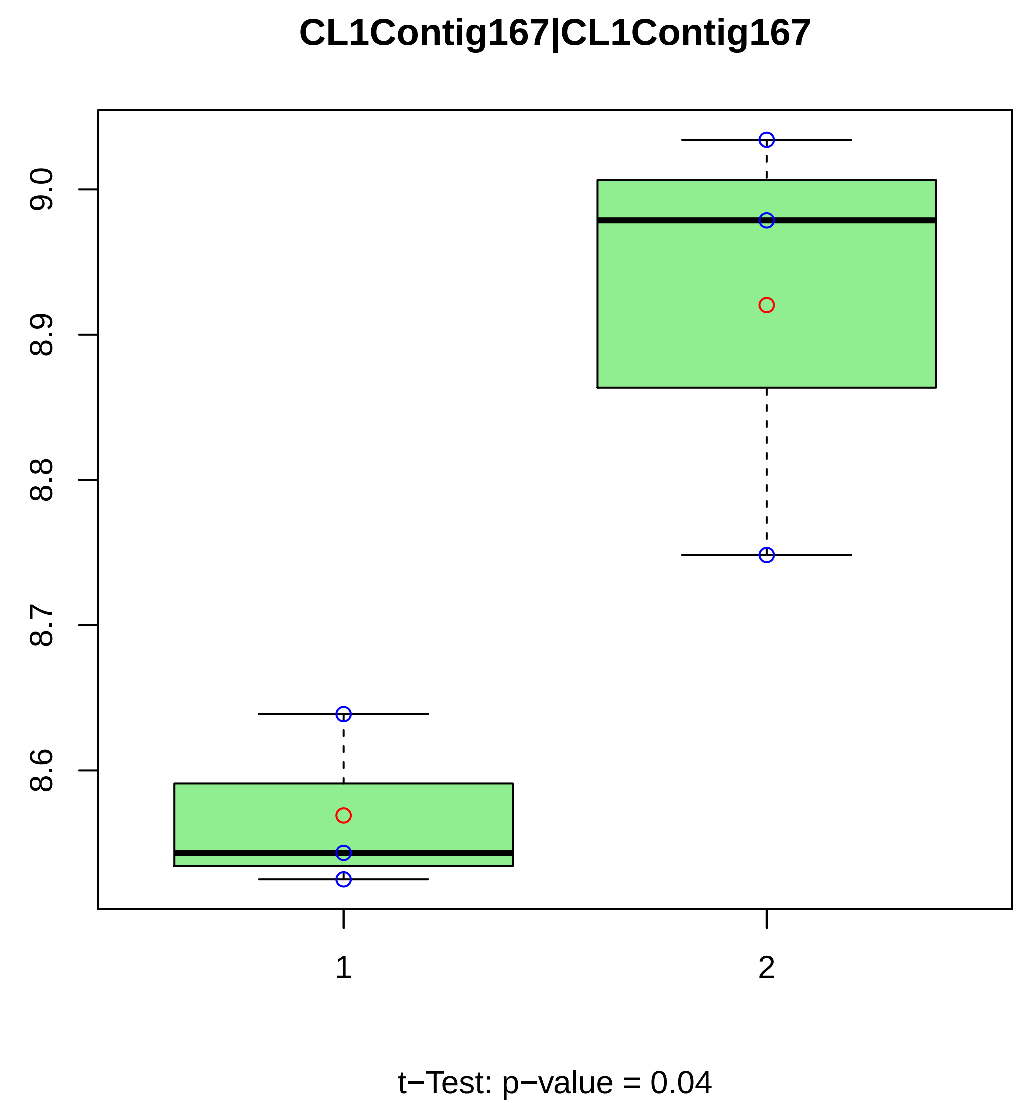
CL1Contig167|CL1Contig167
9.0
8.9
8.8
8.7
8.6
1
2
t−Test: p−value = 0.04

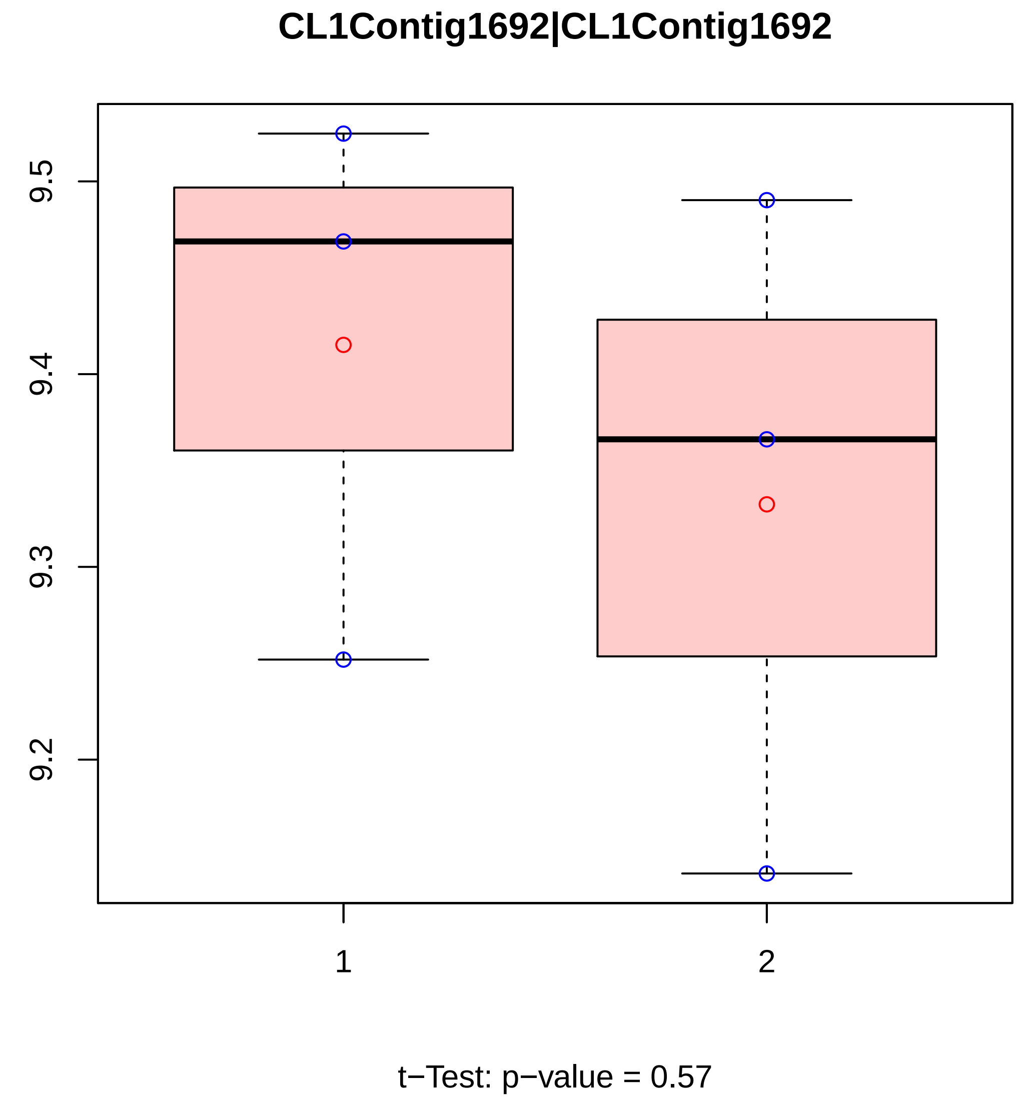
CL1Contig1692|CL1Contig1692
9.5
9.4
9.3
9.2
1
2
t−Test: p−value = 0.57

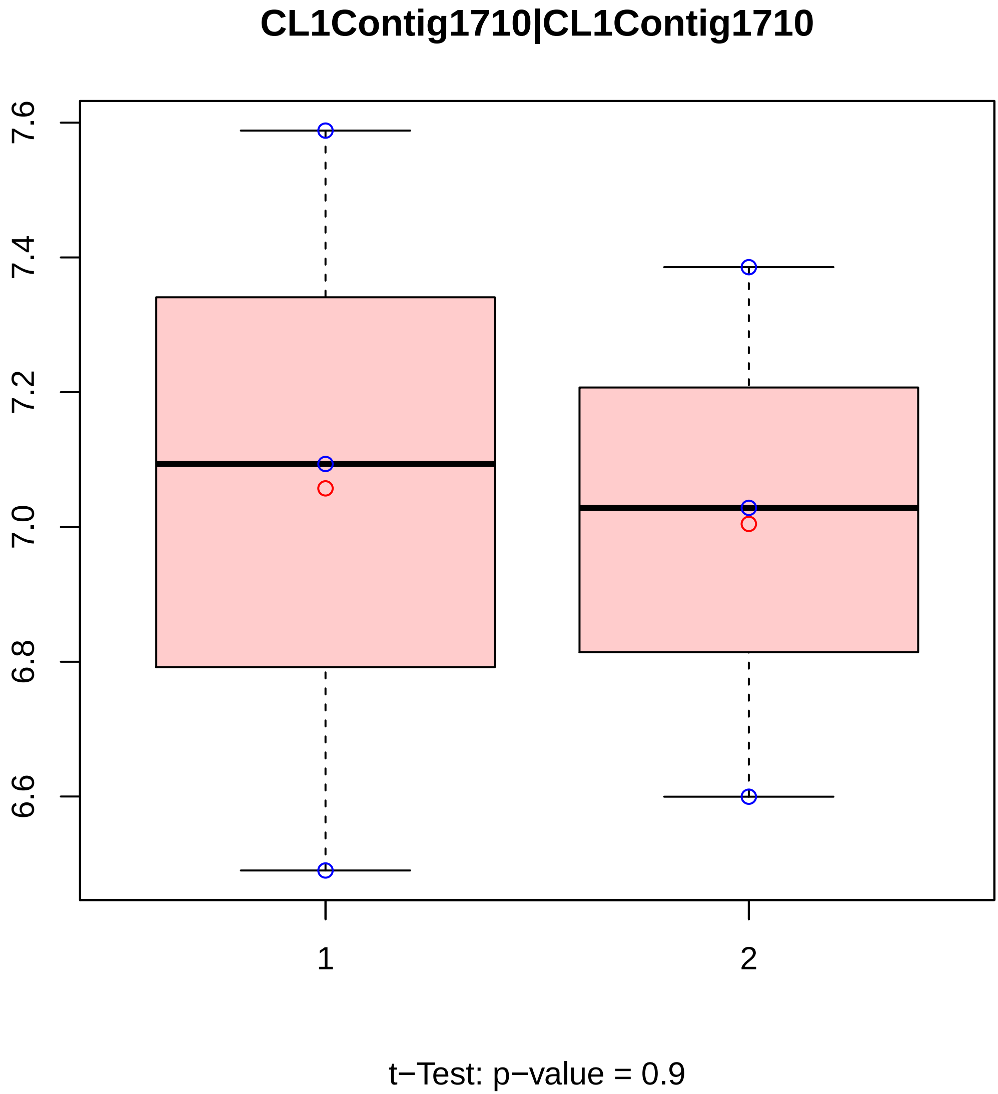
CL1Contig1710|CL1Contig1710
7.6
7.4
7.2
7.0
6.8
6.6
1
2
t−Test: p−value = 0.9

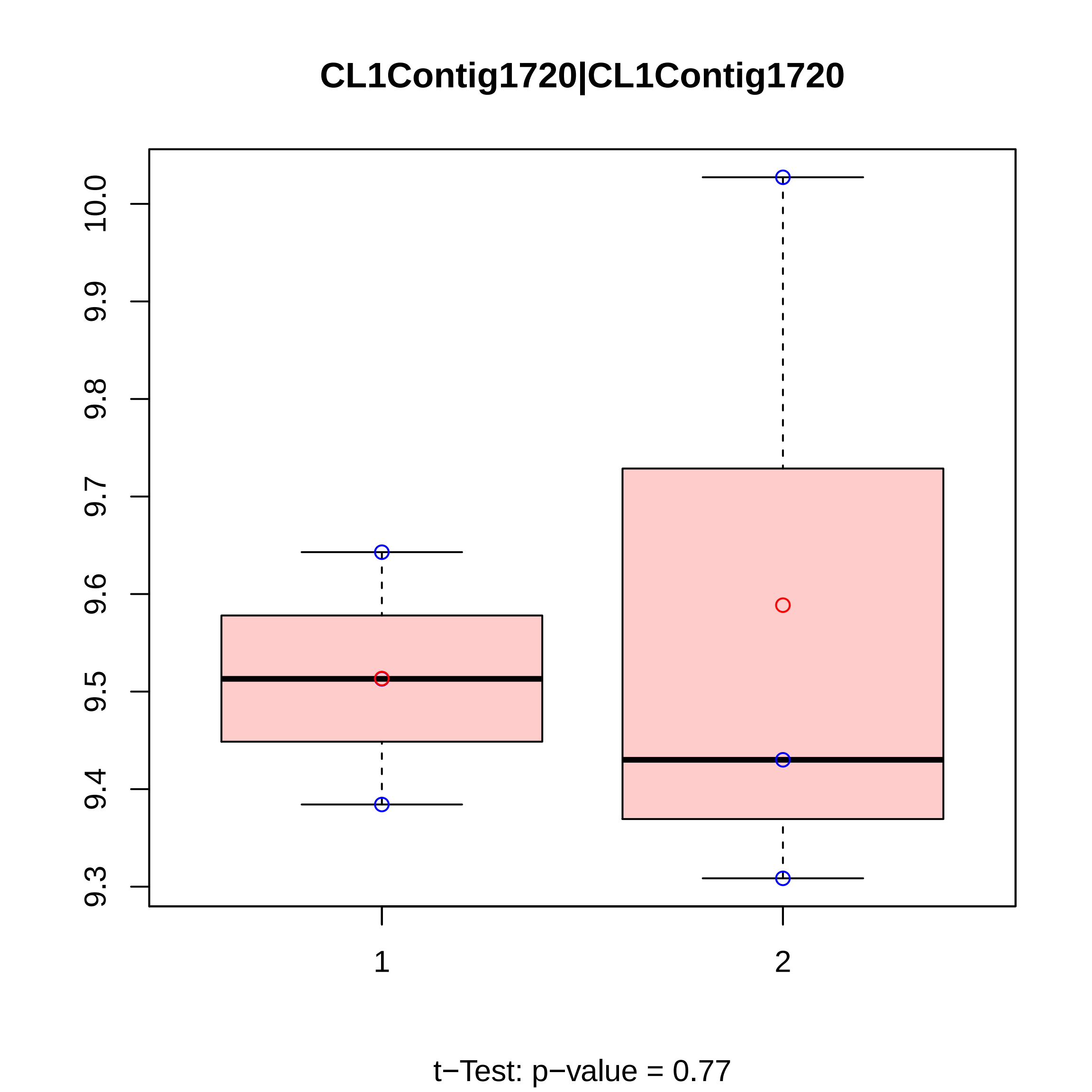

CL1Contig1720|CL1Contig1720
10.0
9.9
9.8
9.7
9.6
9.5
9.4
9.3
1
2
t−Test: p−value = 0.77

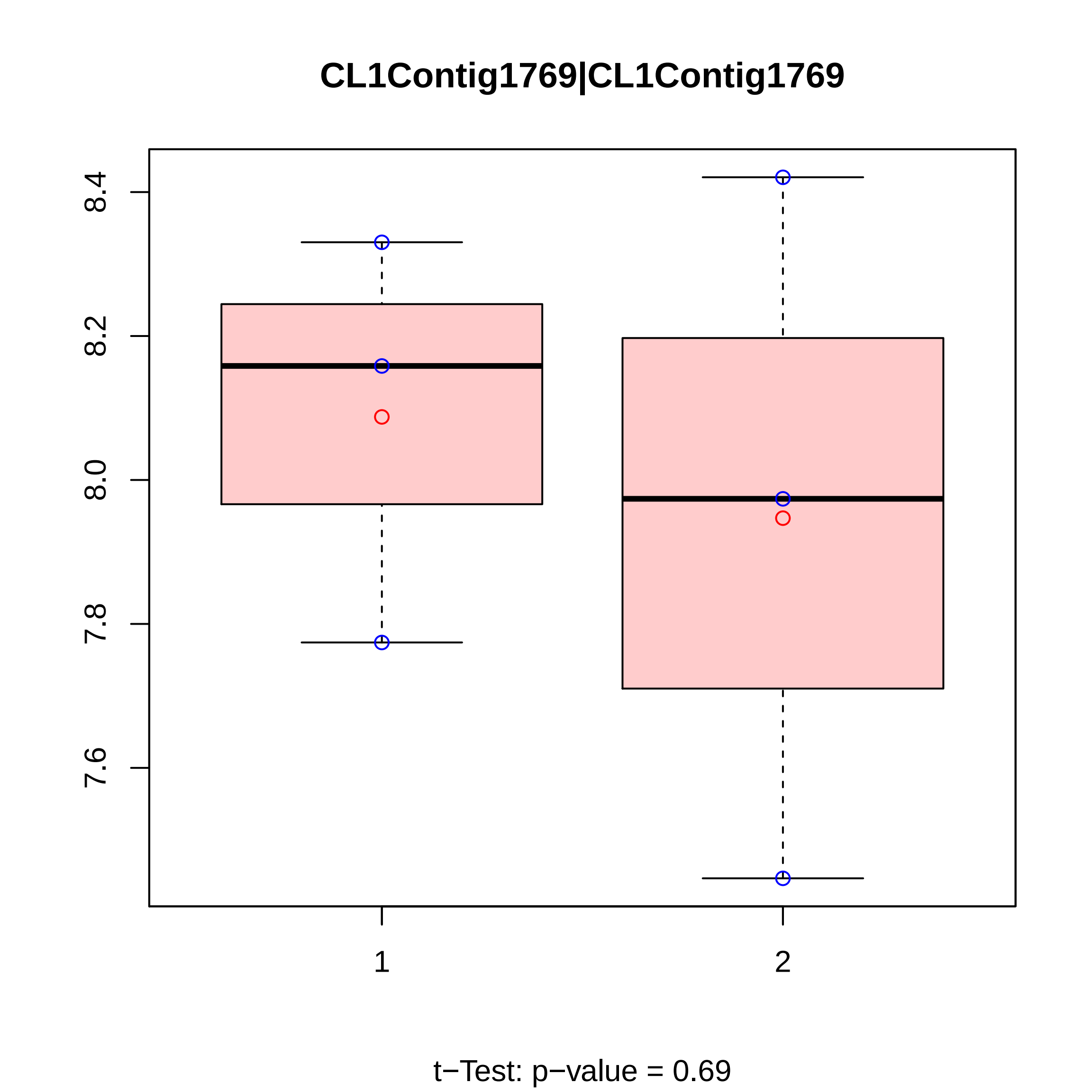
CL1Contig1769|CL1Contig1769
8.4
8.2
8.0
7.8
7.6
1
2
t−Test: p−value = 0.69

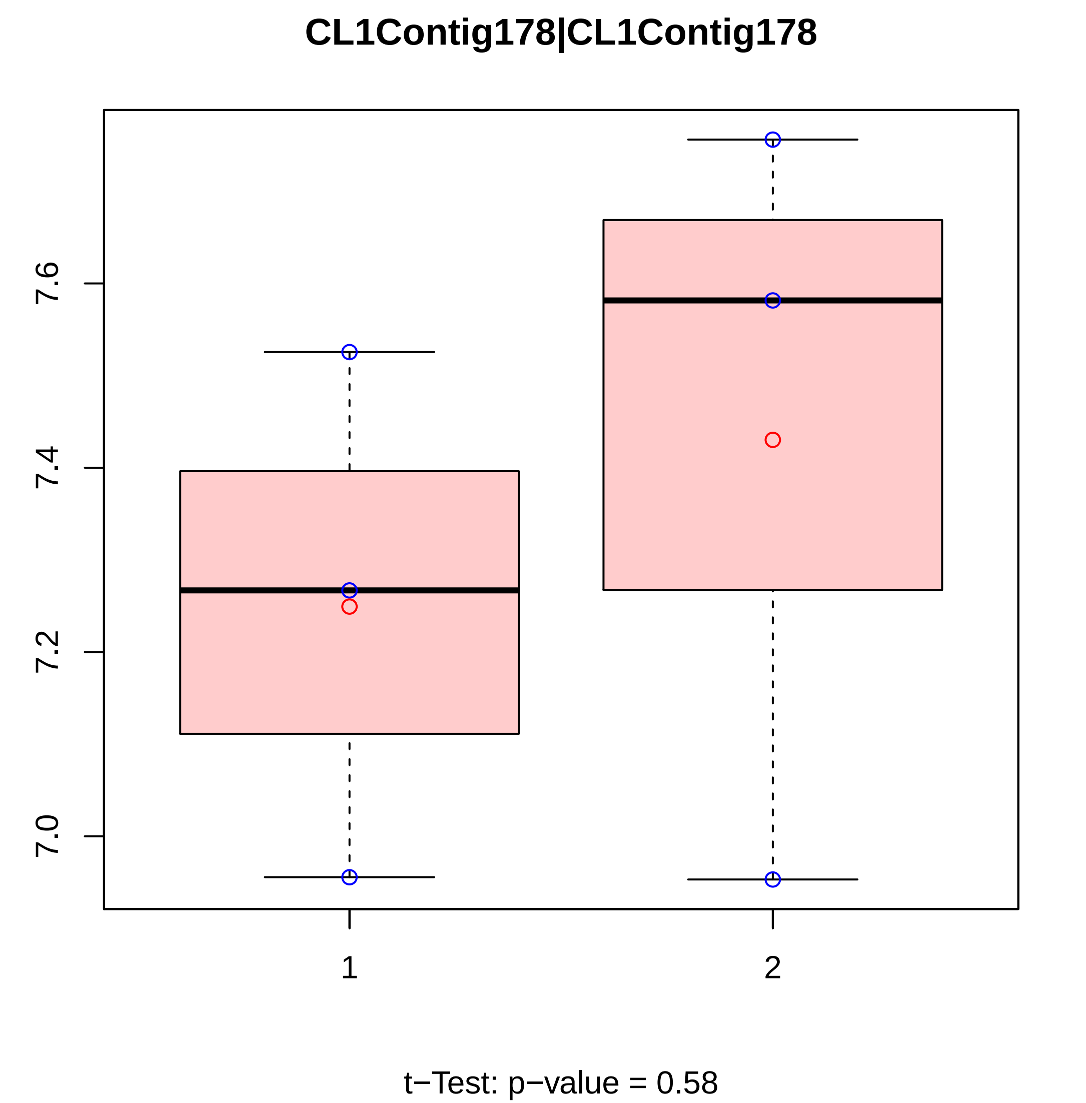
CL1Contig178|CL1Contig178
7.0
7.2
7.4
7.6
1
2
t−Test: p−value = 0.58

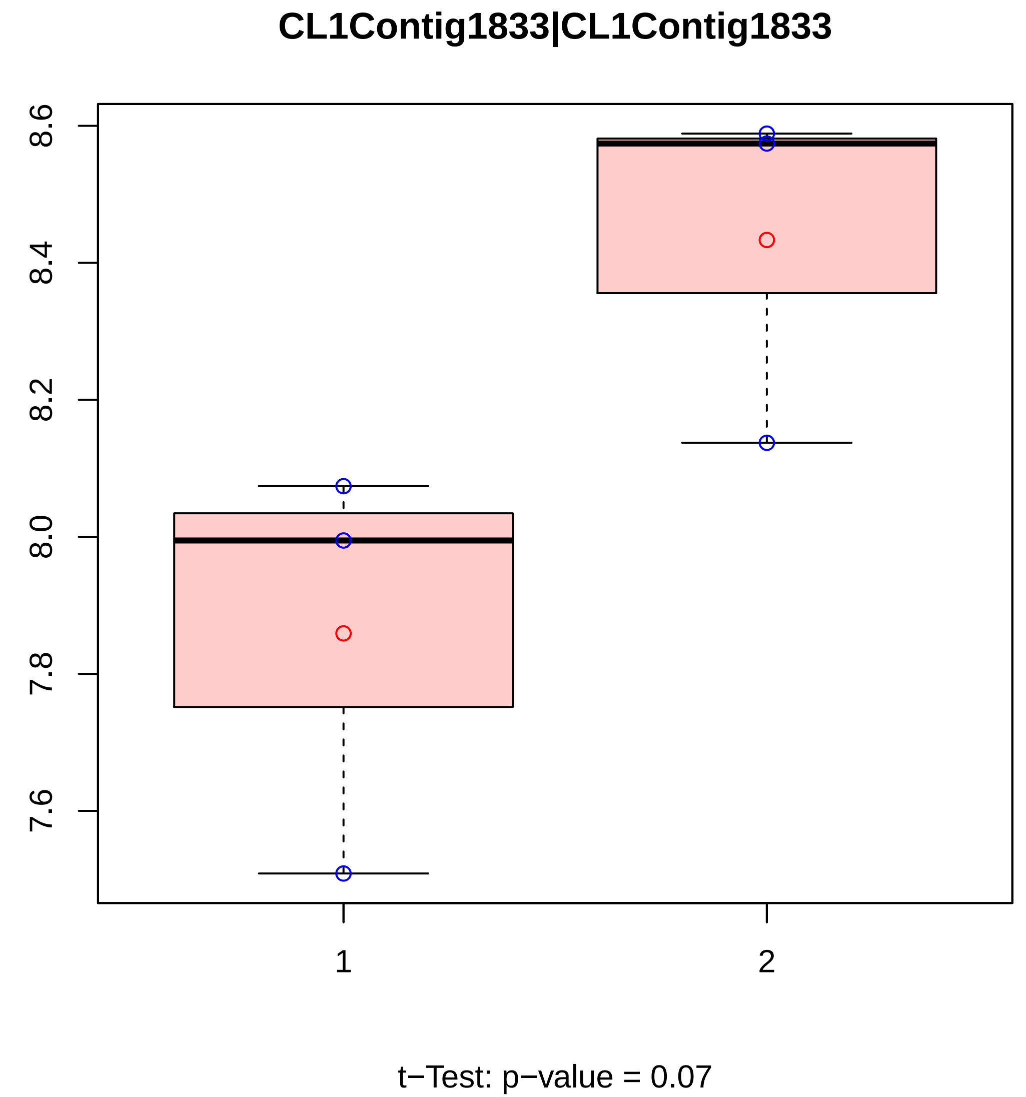

CL1Contig1833|CL1Contig1833
8.6
8.4
8.2
8.0
7.8
7.6
1
2
t−Test: p−value = 0.07

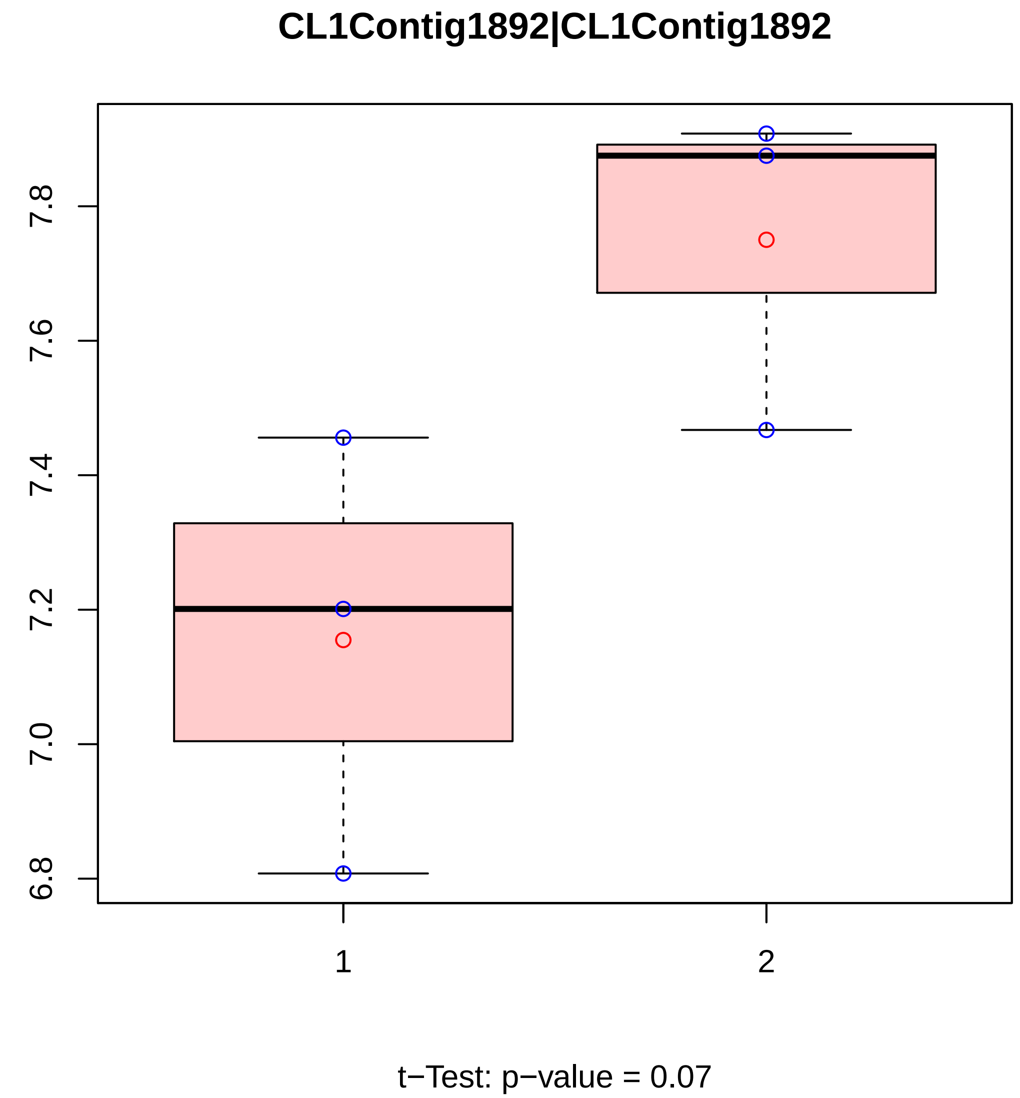
CL1Contig1892|CL1Contig1892
6.8
7.0
7.2
7.4
7.6
7.8
1
2
t−Test: p−value = 0.07

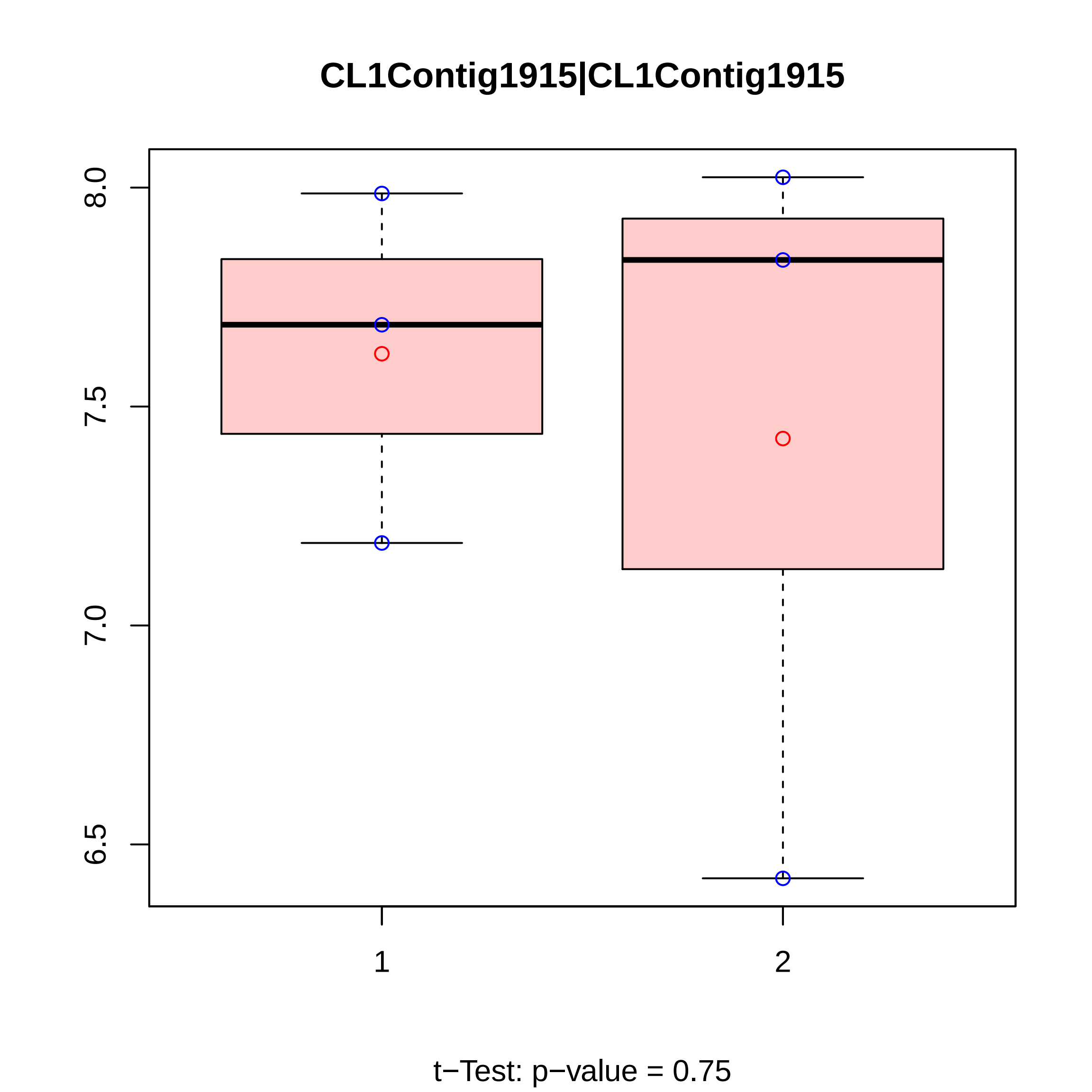
CL1Contig1915|CL1Contig1915
8.0
7.5
7.0
6.5
1
2
t−Test: p−value = 0.75

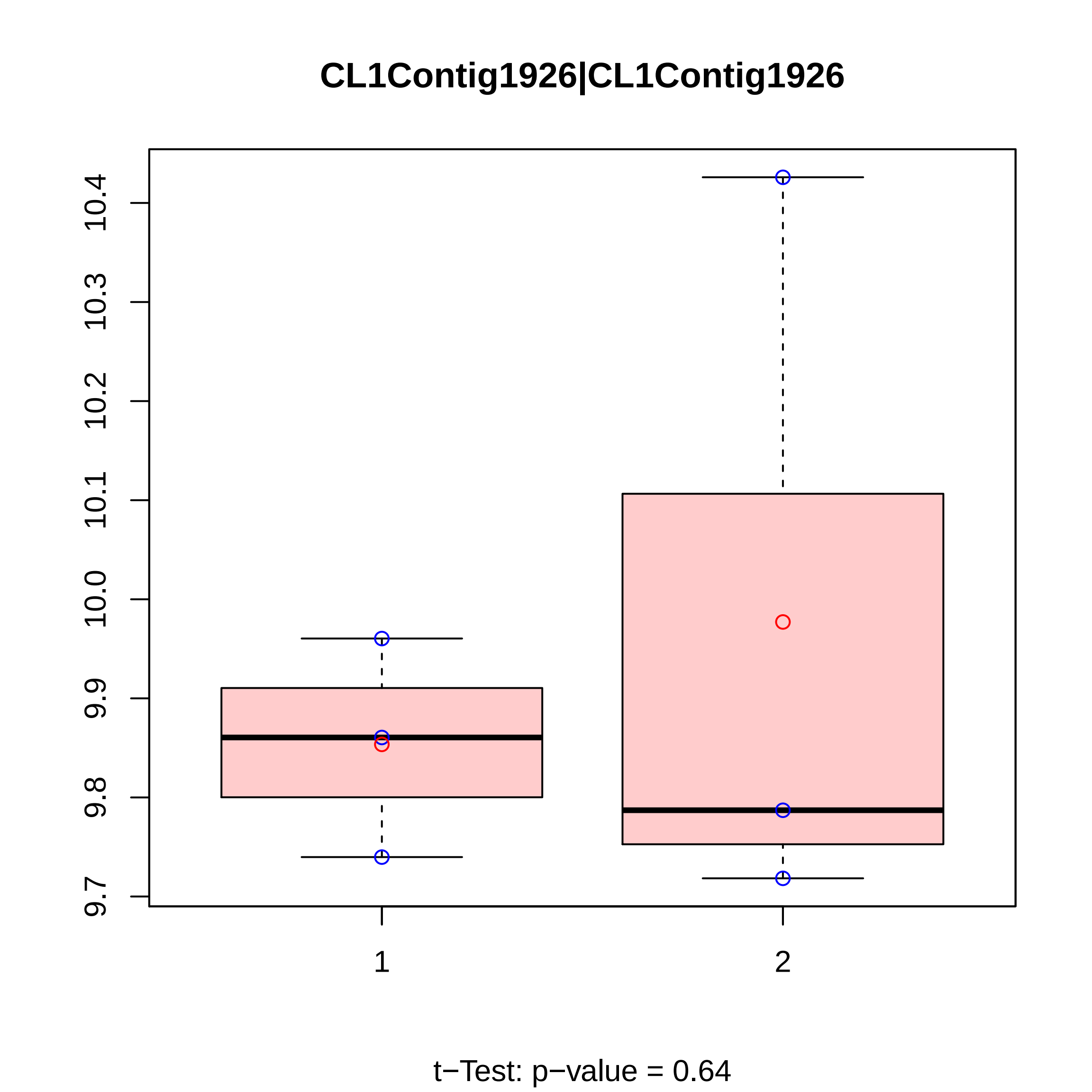
CL1Contig1926|CL1Contig1926
10.4
10.3
10.2
10.1
10.0
9.9
9.8
9.7
1
2
t−Test: p−value = 0.64

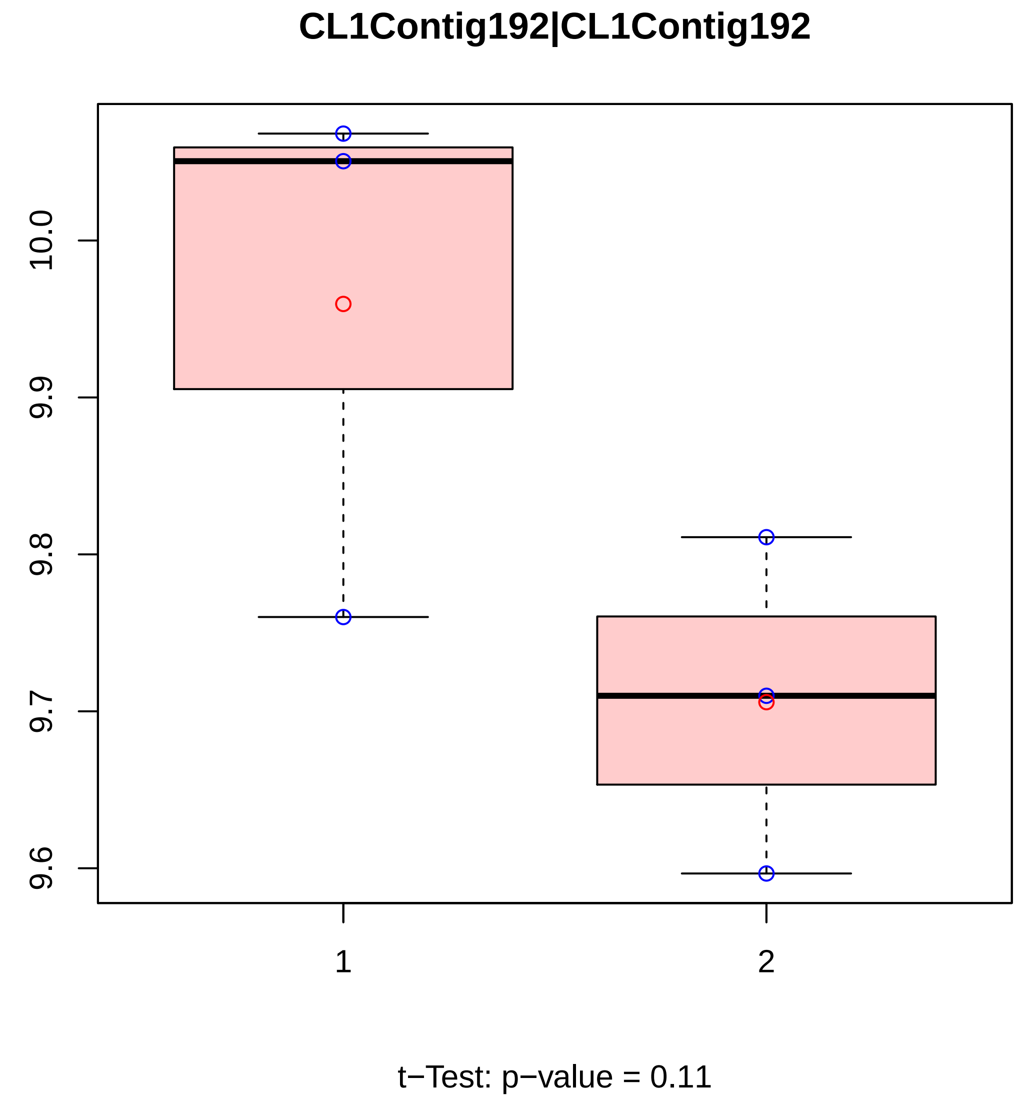

CL1Contig192|CL1Contig192
10.0
9.9
9.8
9.7
9.6
1
2
t−Test: p−value = 0.11

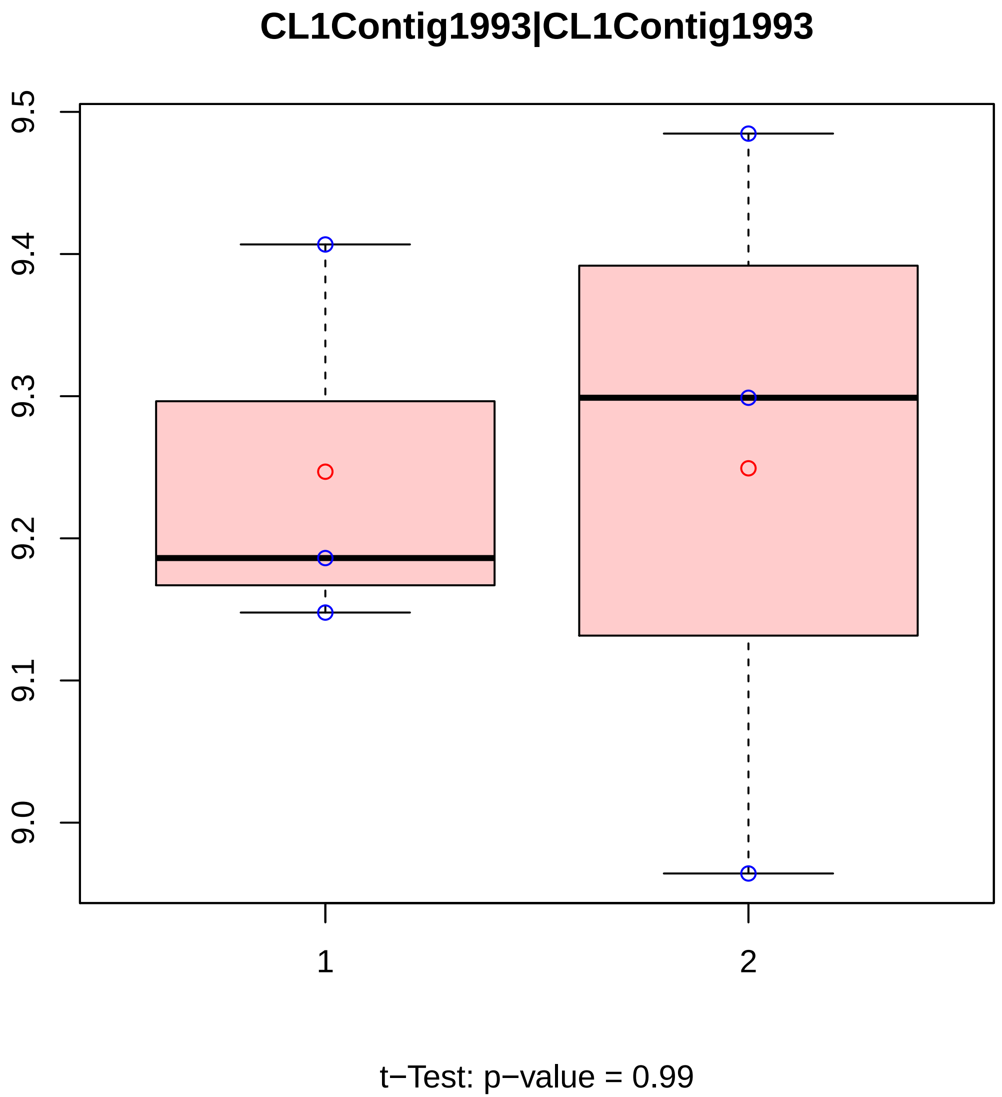
CL1Contig1993|CL1Contig1993
9.0
9.1
9.2
9.3
9.4
9.5
1
2
t−Test: p−value = 0.99

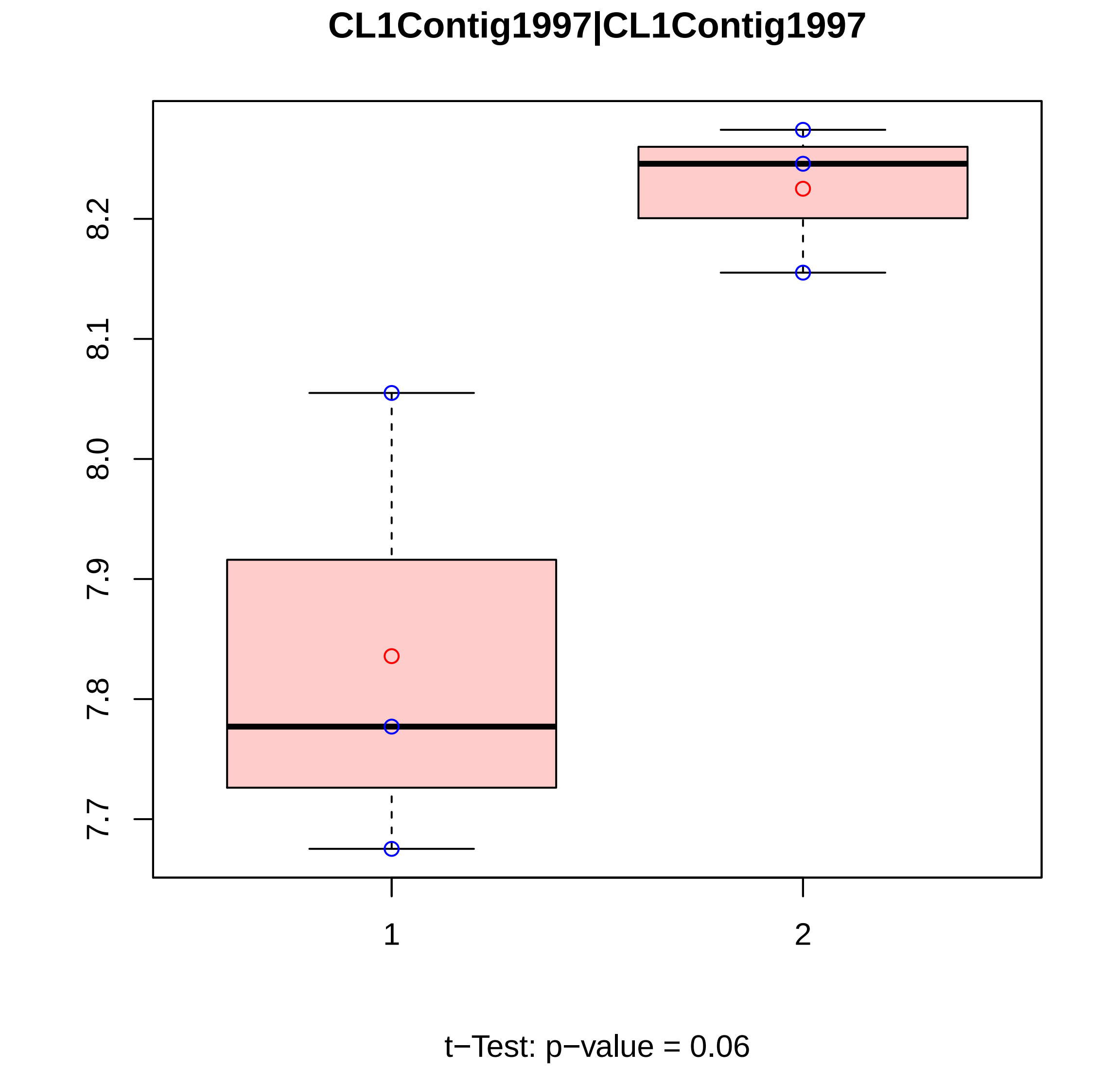
CL1Contig1997|CL1Contig1997
8.2
8.1
8.0
7.9
7.8
7.7
1
2
t−Test: p−value = 0.06

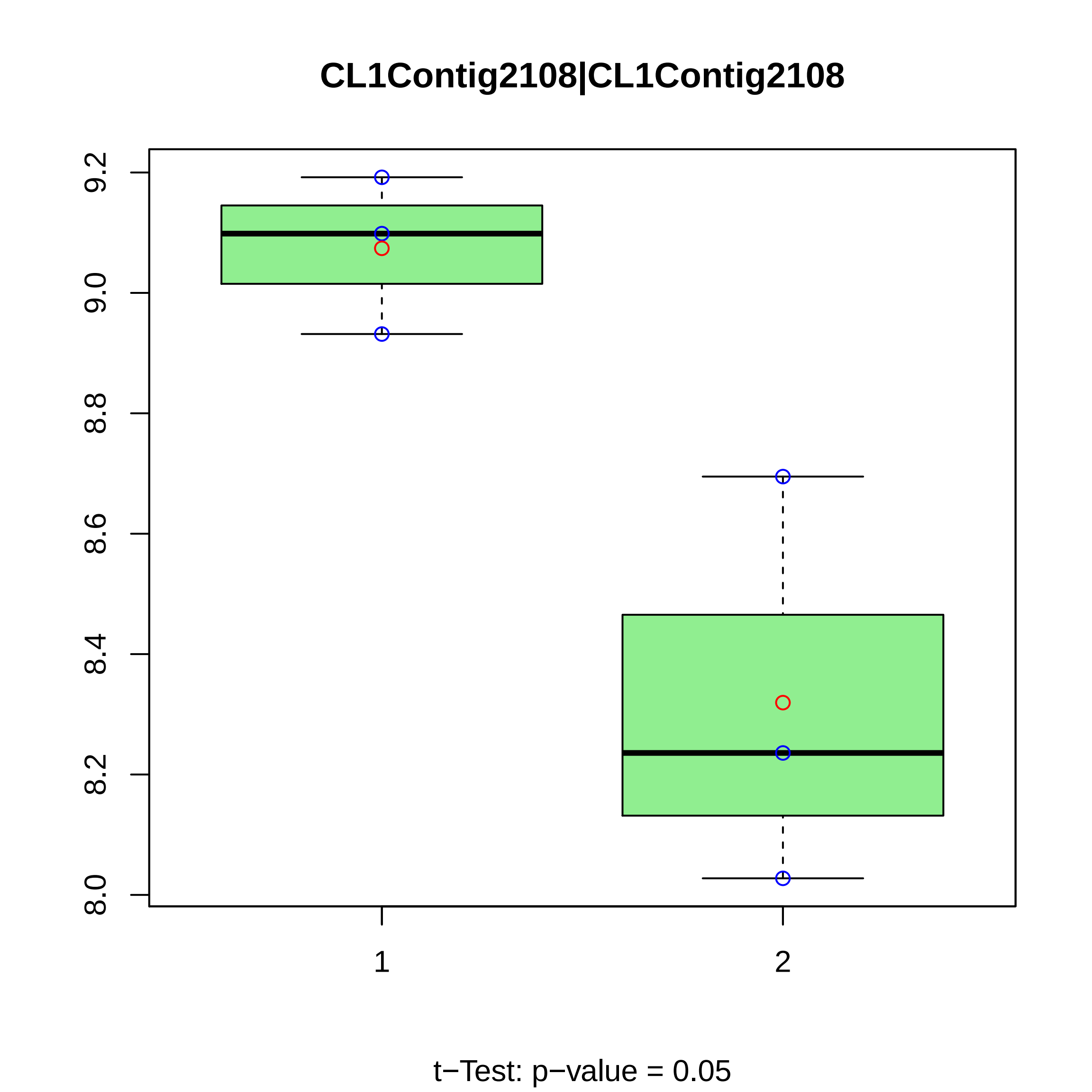

CL1Contig2108|CL1Contig2108
9.2
9.0
8.8
8.6
8.4
8.2
8.0
1
2
t−Test: p−value = 0.05

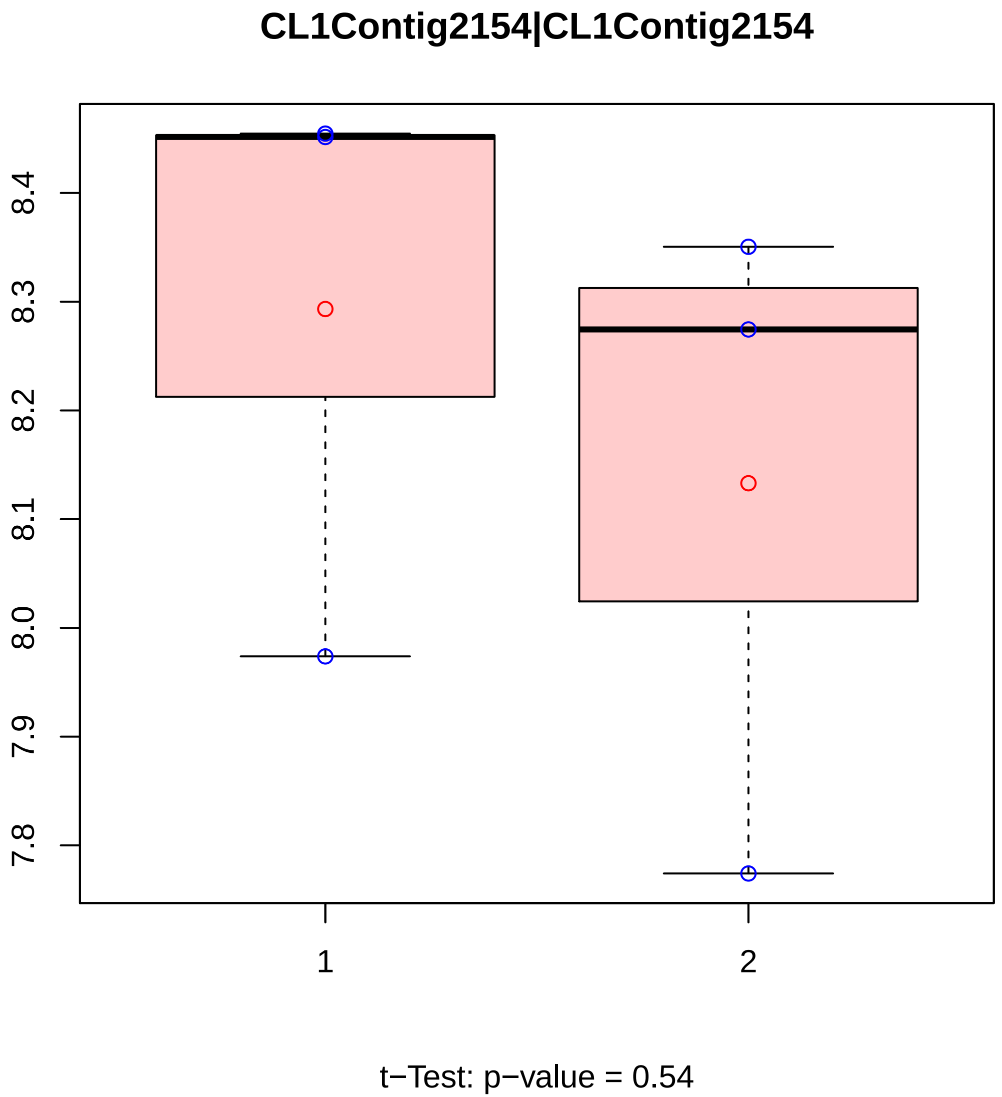
CL1Contig2154|CL1Contig2154
8.4
8.3
8.2
8.1
8.0
7.9
7.8
1
2
t−Test: p−value = 0.54

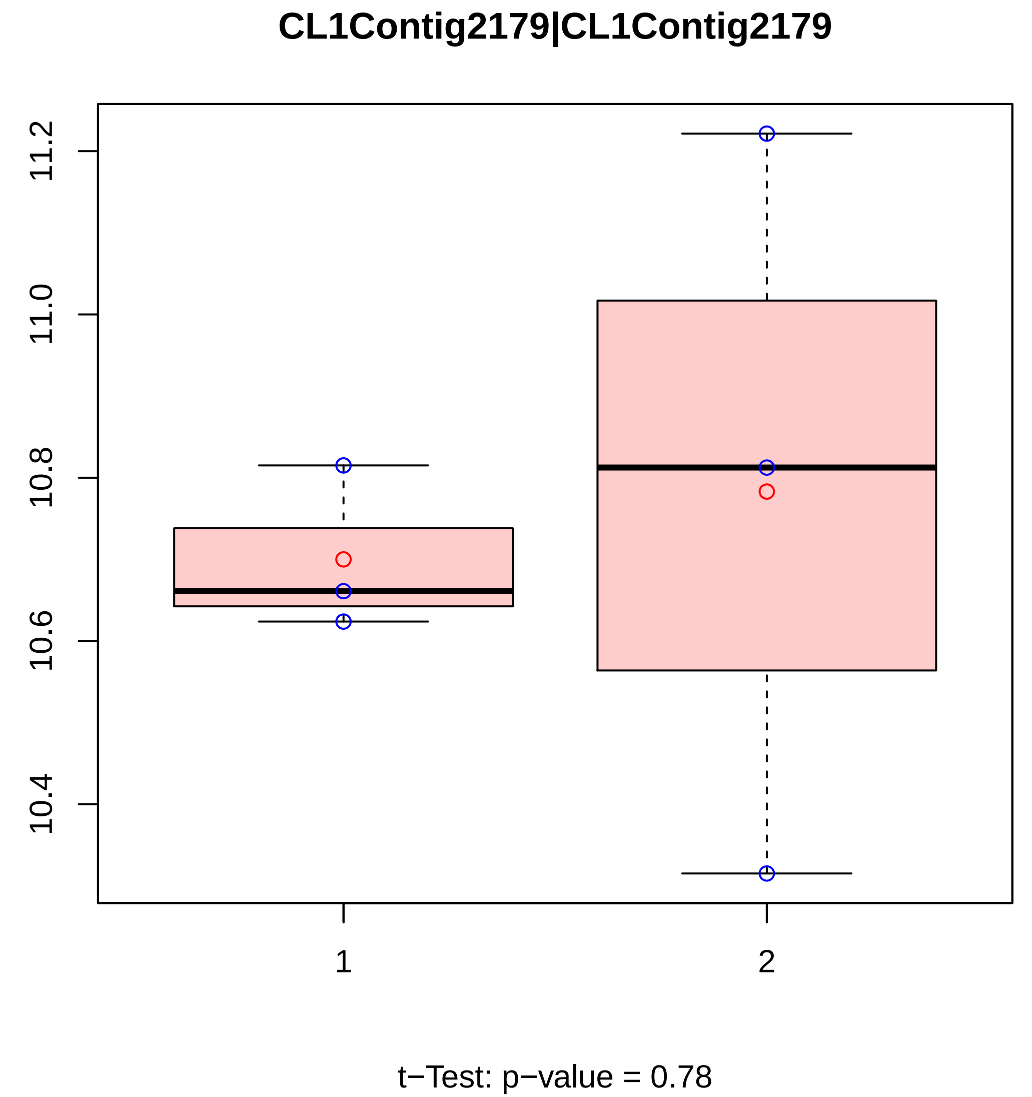
CL1Contig2179|CL1Contig2179
11.2
11.0
10.8
10.6
10.4
1
2
t−Test: p−value = 0.78

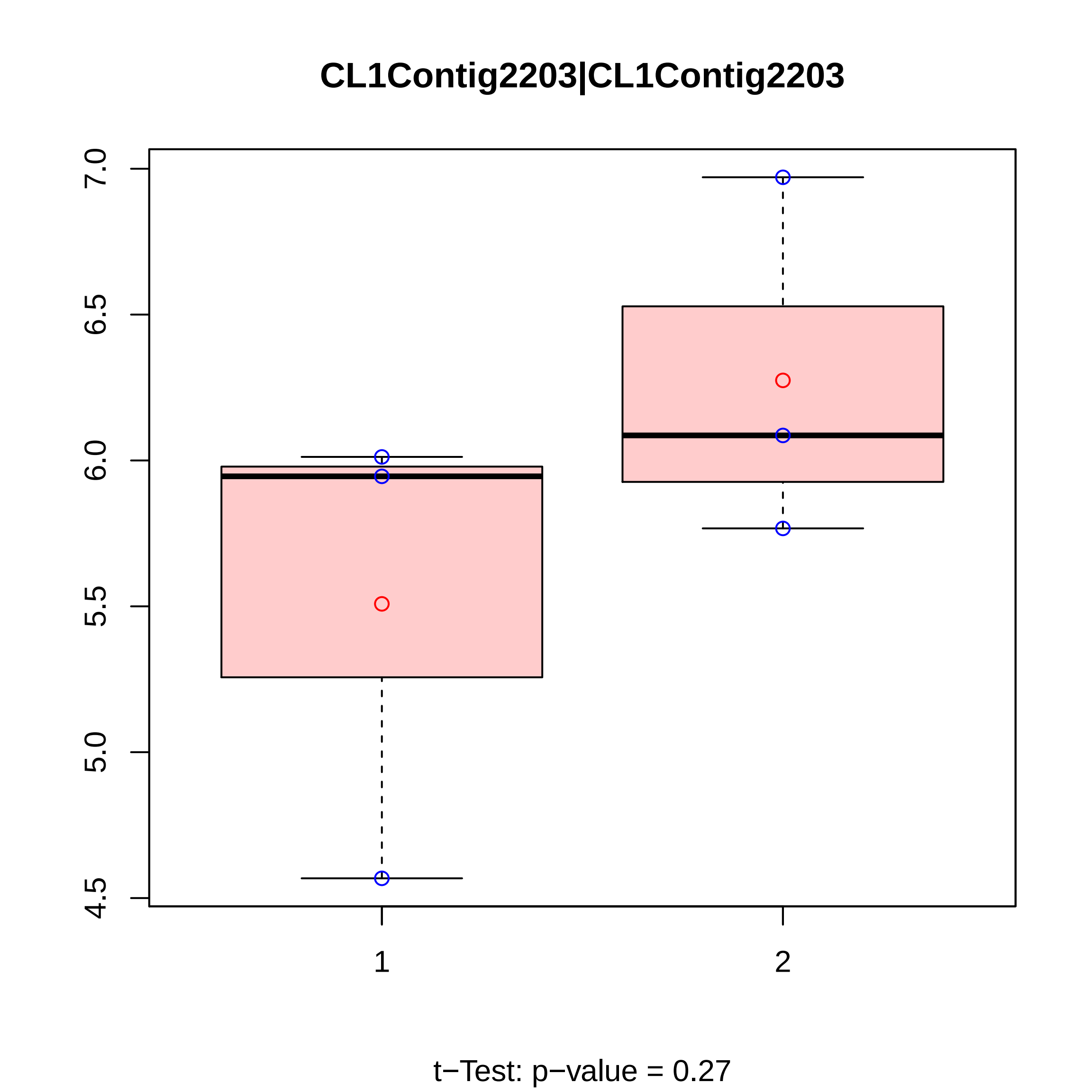
CL1Contig2203|CL1Contig2203
7.0
6.5
6.0
5.5
5.0
4.5
1
2
t−Test: p−value = 0.27

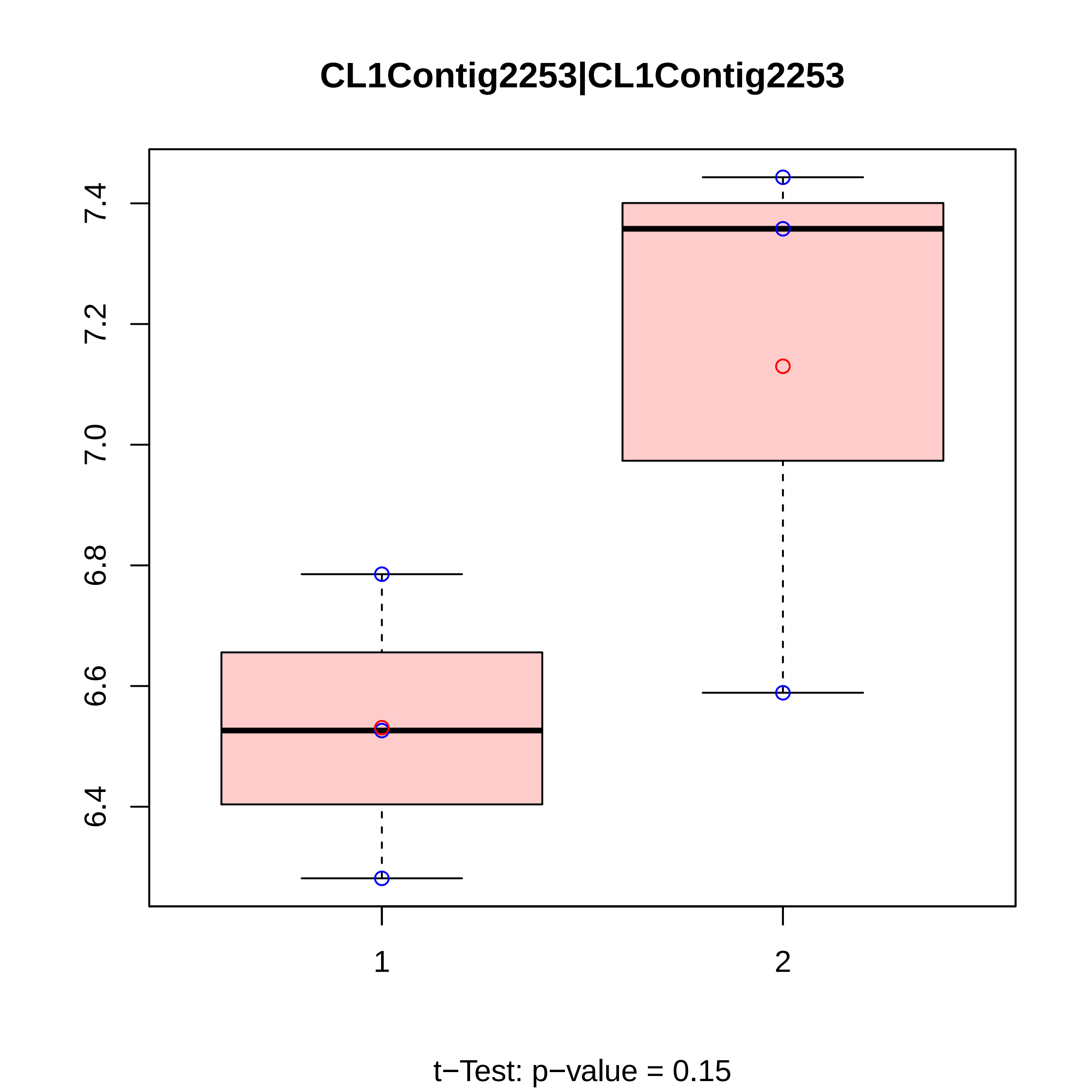

CL1Contig2253|CL1Contig2253
7.4
7.2
7.0
6.8
6.6
6.4
1
2
t−Test: p−value = 0.15

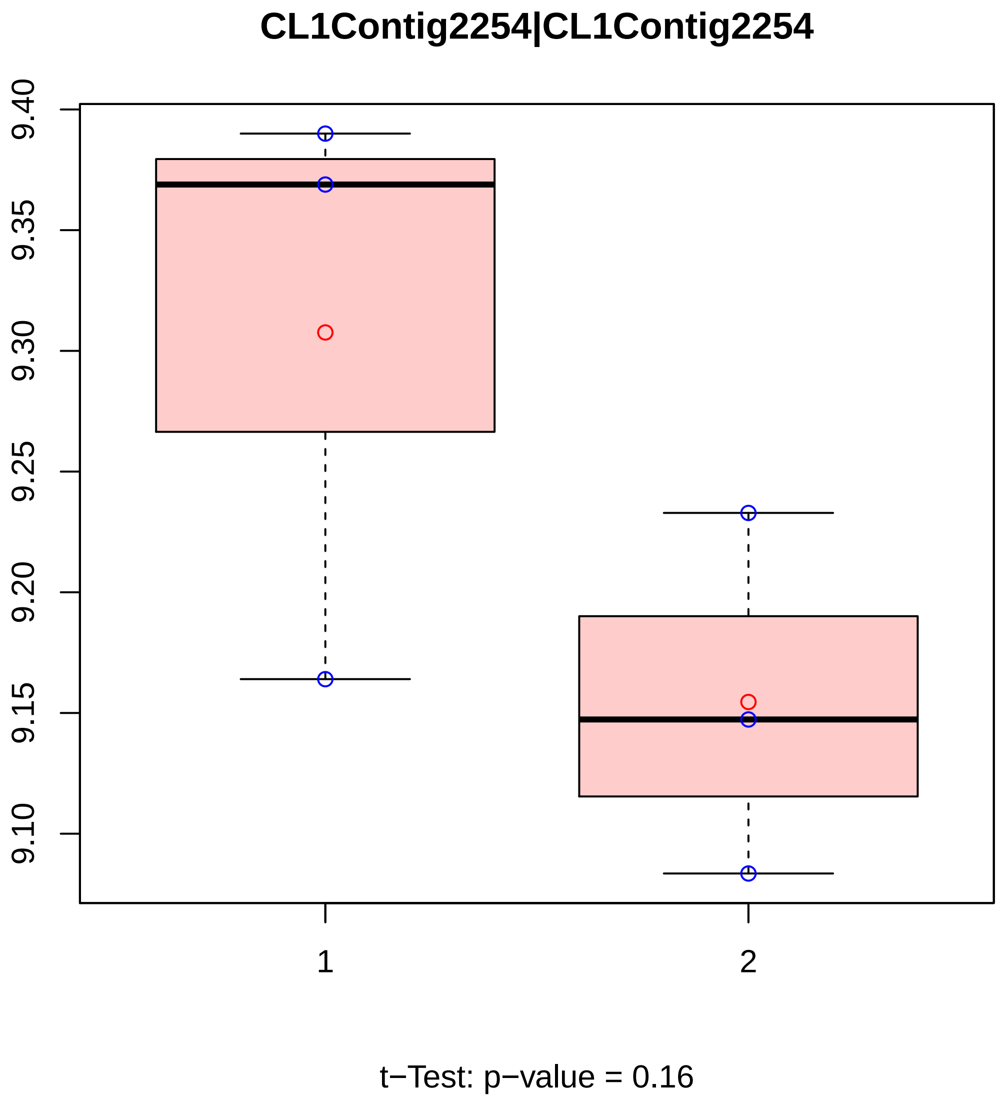
CL1Contig2254|CL1Contig2254
9.40
9.35
9.30
9.25
9.20
9.15
9.10
1
2
t−Test: p−value = 0.16

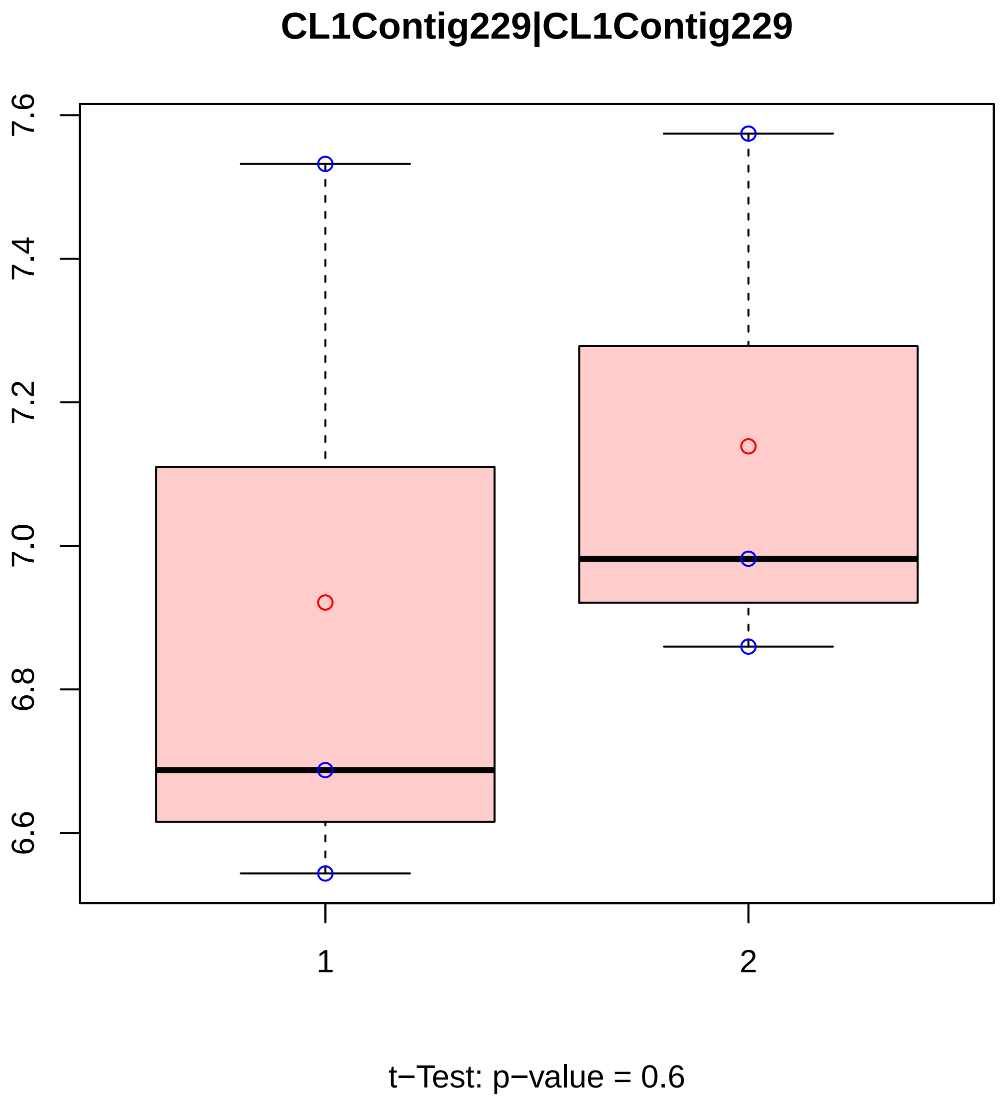

CL1Contig229|CL1Contig229
7.6
7.4
7.2
7.0
6.8
6.6
1
2
t−Test: p−value = 0.6

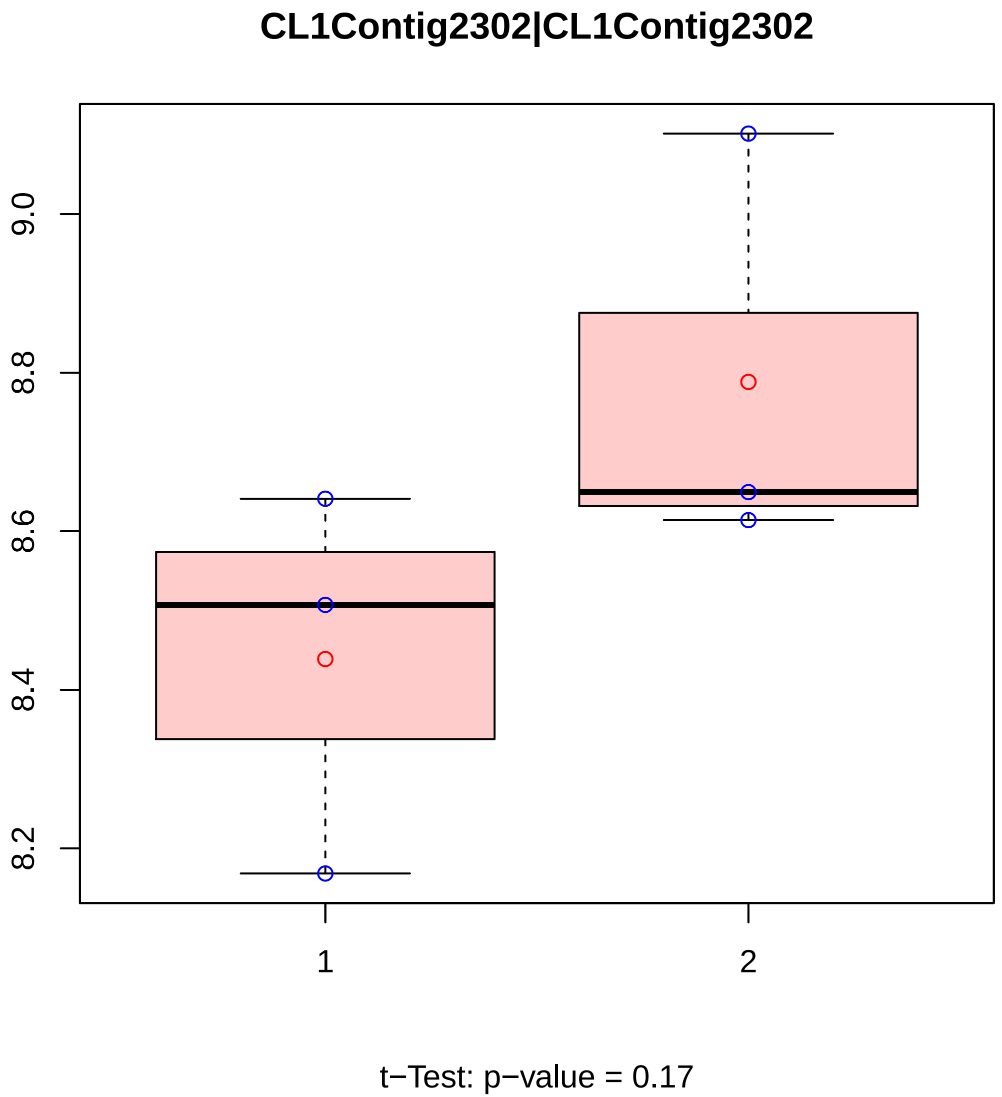
CL1Contig2302|CL1Contig2302
9.0
8.8
8.6
8.4
8.2
1
2
t−Test: p−value = 0.17

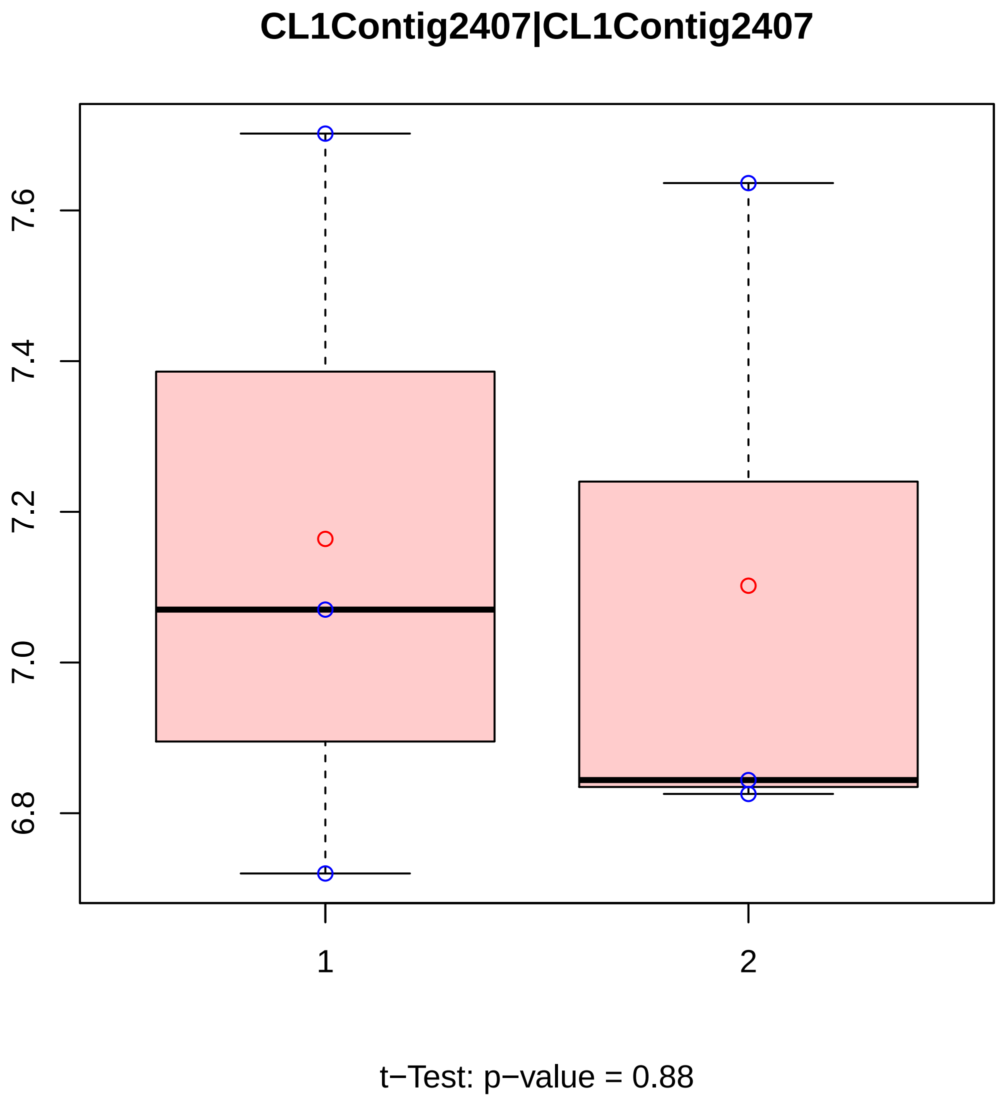

CL1Contig2407|CL1Contig2407
7.6
7.4
7.2
7.0
6.8
1
2
t−Test: p−value = 0.88

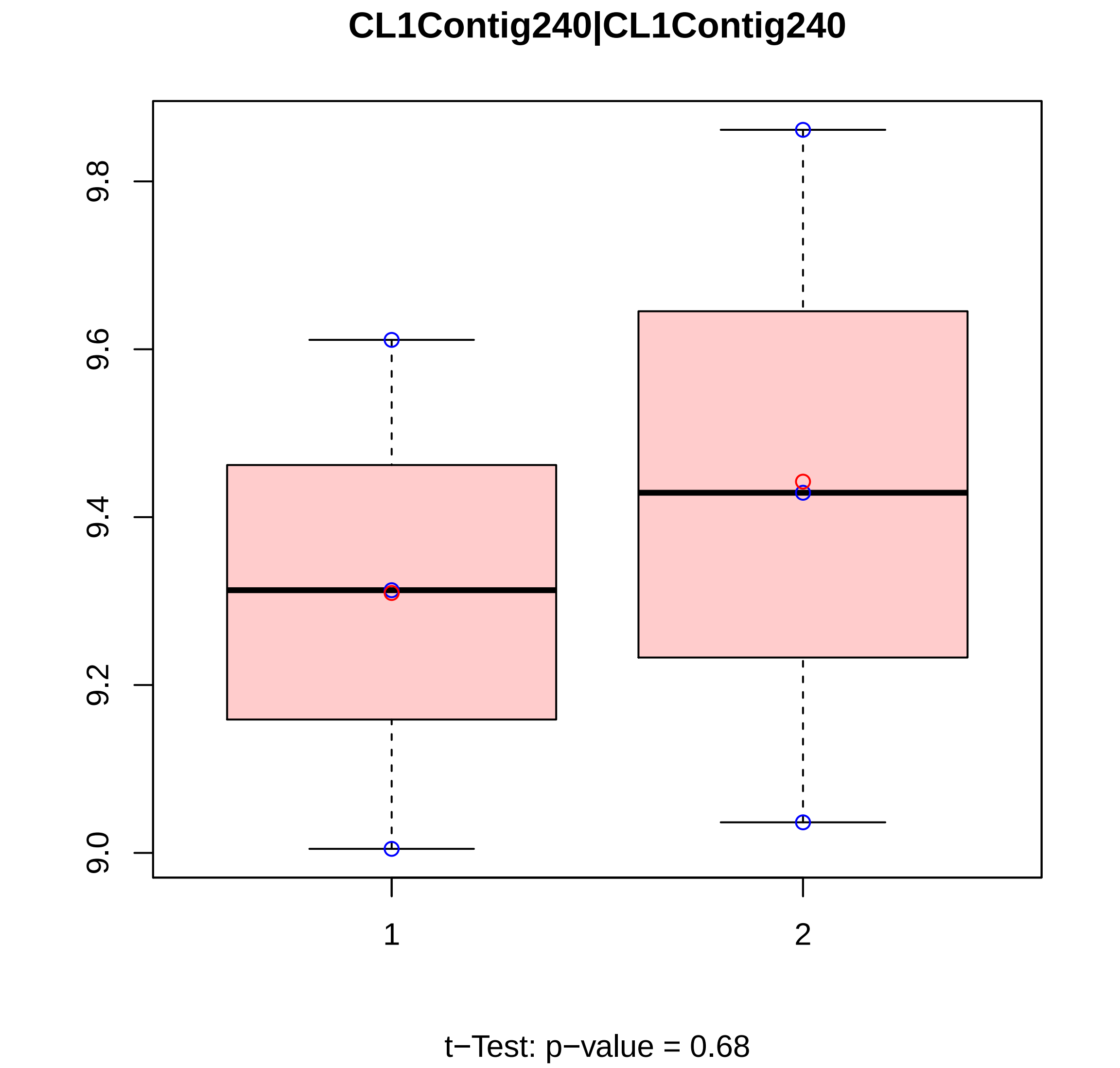
CL1Contig240|CL1Contig240
9.0
9.2
9.4
9.6
9.8
1
2
t−Test: p−value = 0.68

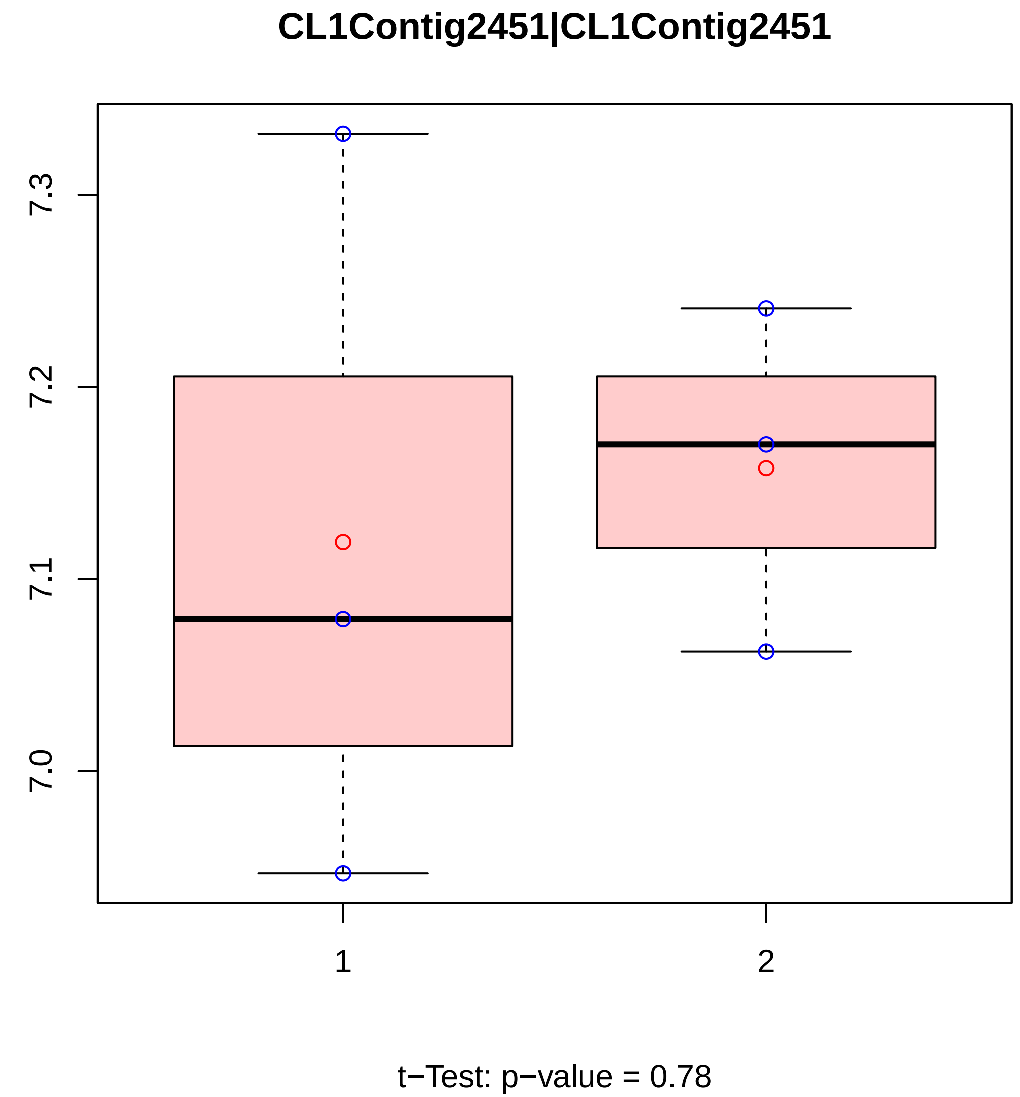
CL1Contig2451|CL1Contig2451
7.0
7.1
7.2
7.3
1
2
t−Test: p−value = 0.78

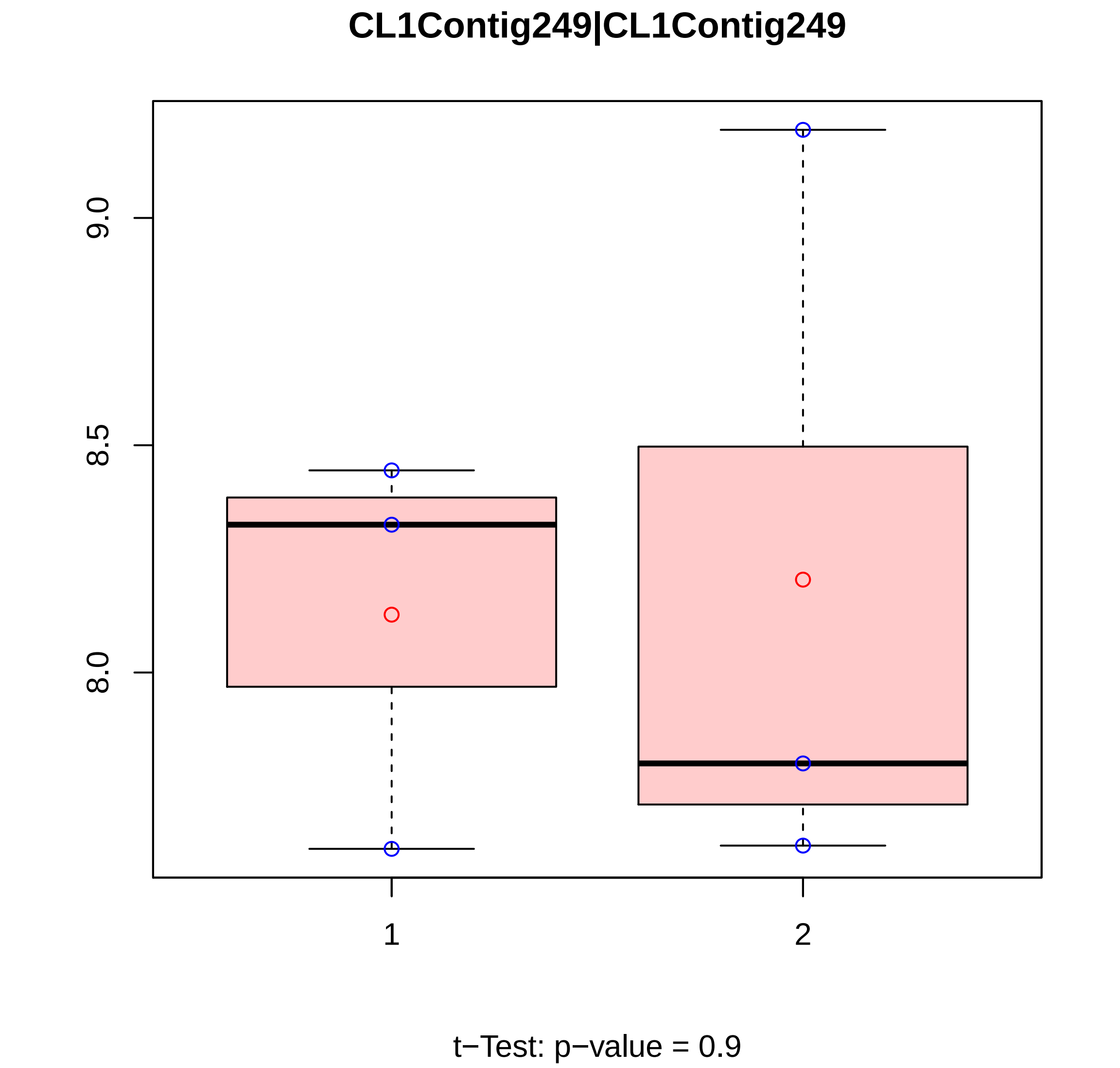
CL1Contig249|CL1Contig249
9.0
8.5
8.0
1
2
t−Test: p−value = 0.9

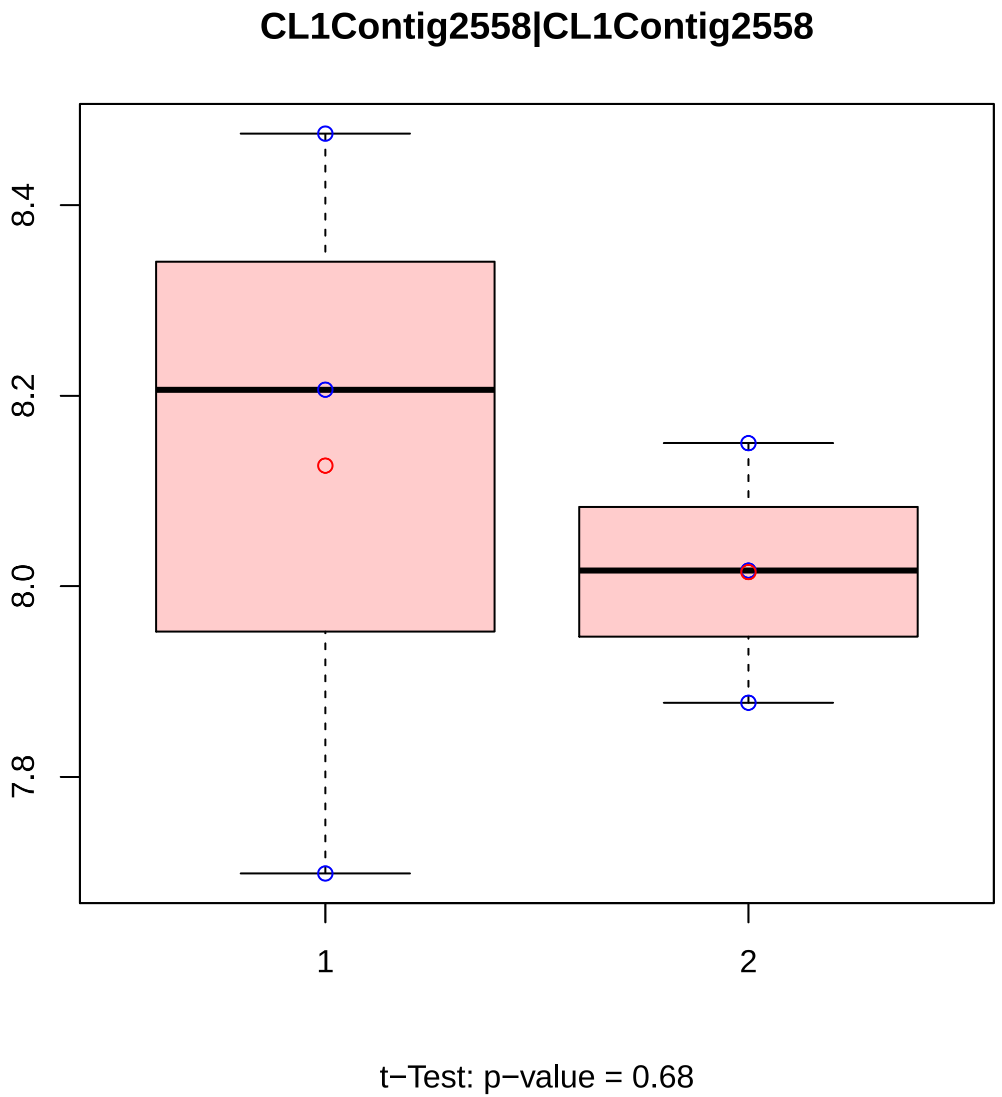
CL1Contig2558|CL1Contig2558
8.4
8.2
8.0
7.8
1
2
t−Test: p−value = 0.68

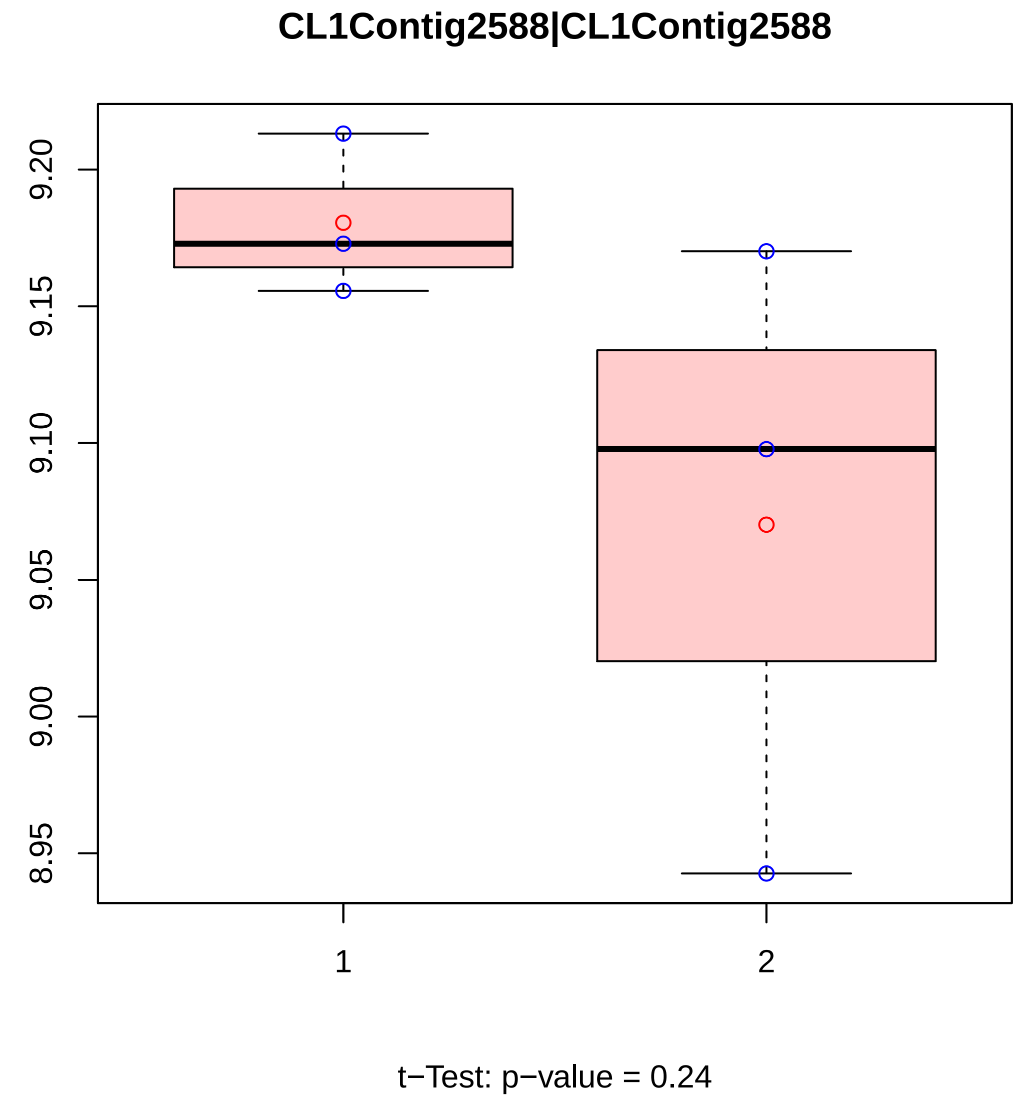
CL1Contig2588|CL1Contig2588
9.20
9.15
9.10
9.05
9.00
8.95
1
2
t−Test: p−value = 0.24

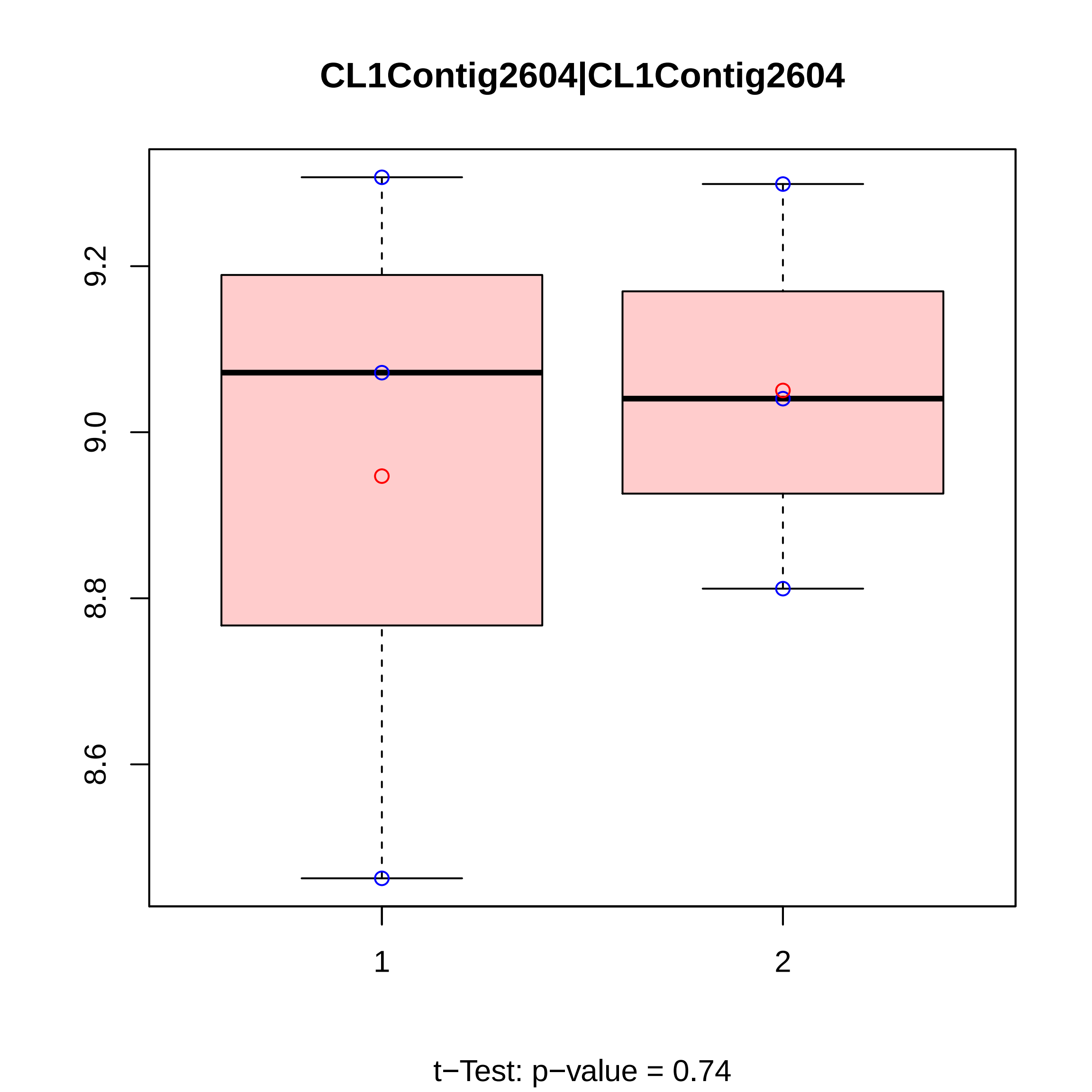

CL1Contig2604|CL1Contig2604
9.2
9.0
8.8
8.6
1
2
t−Test: p−value = 0.74

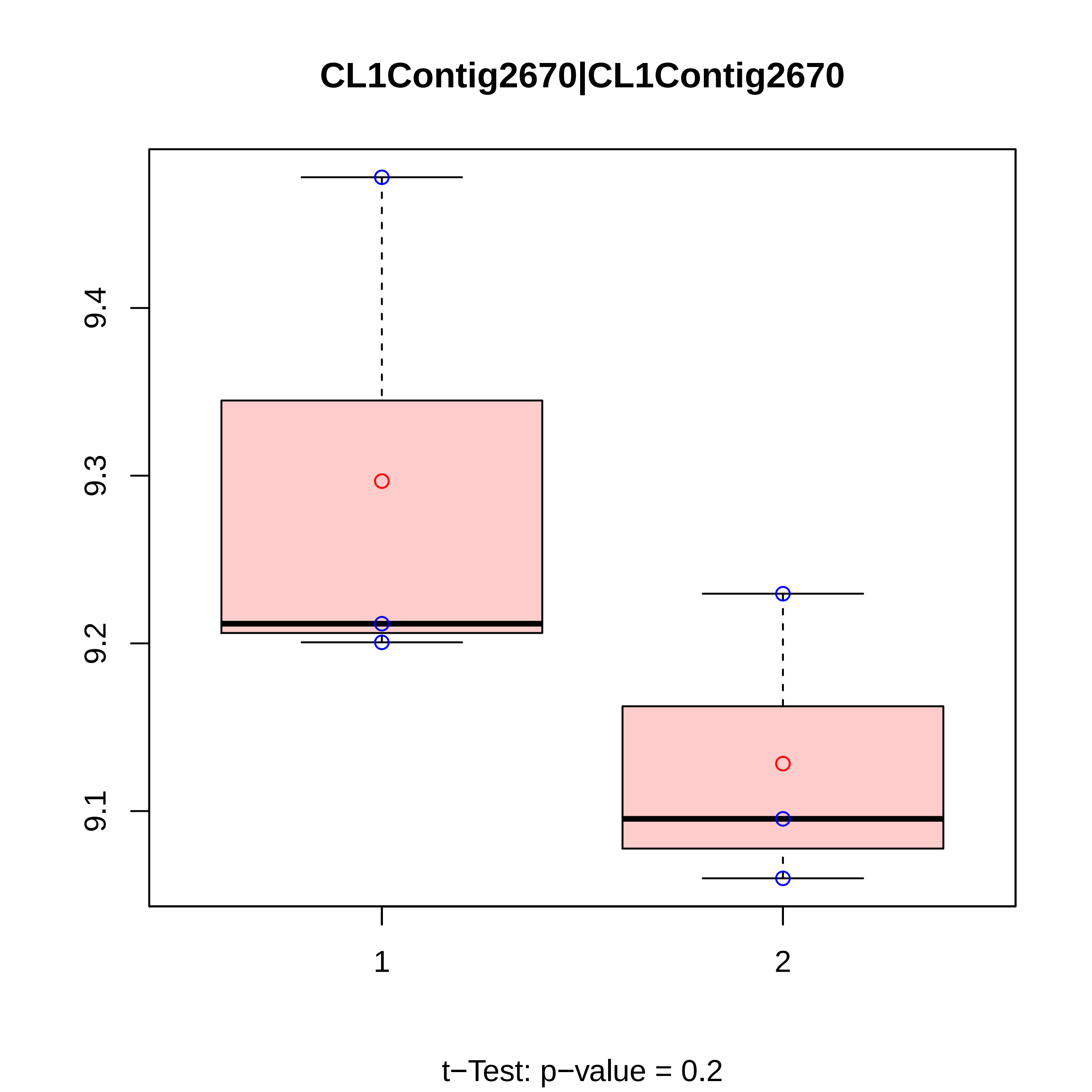

CL1Contig2670|CL1Contig2670
9.1
9.2
9.3
9.4
1
2
t−Test: p−value = 0.2

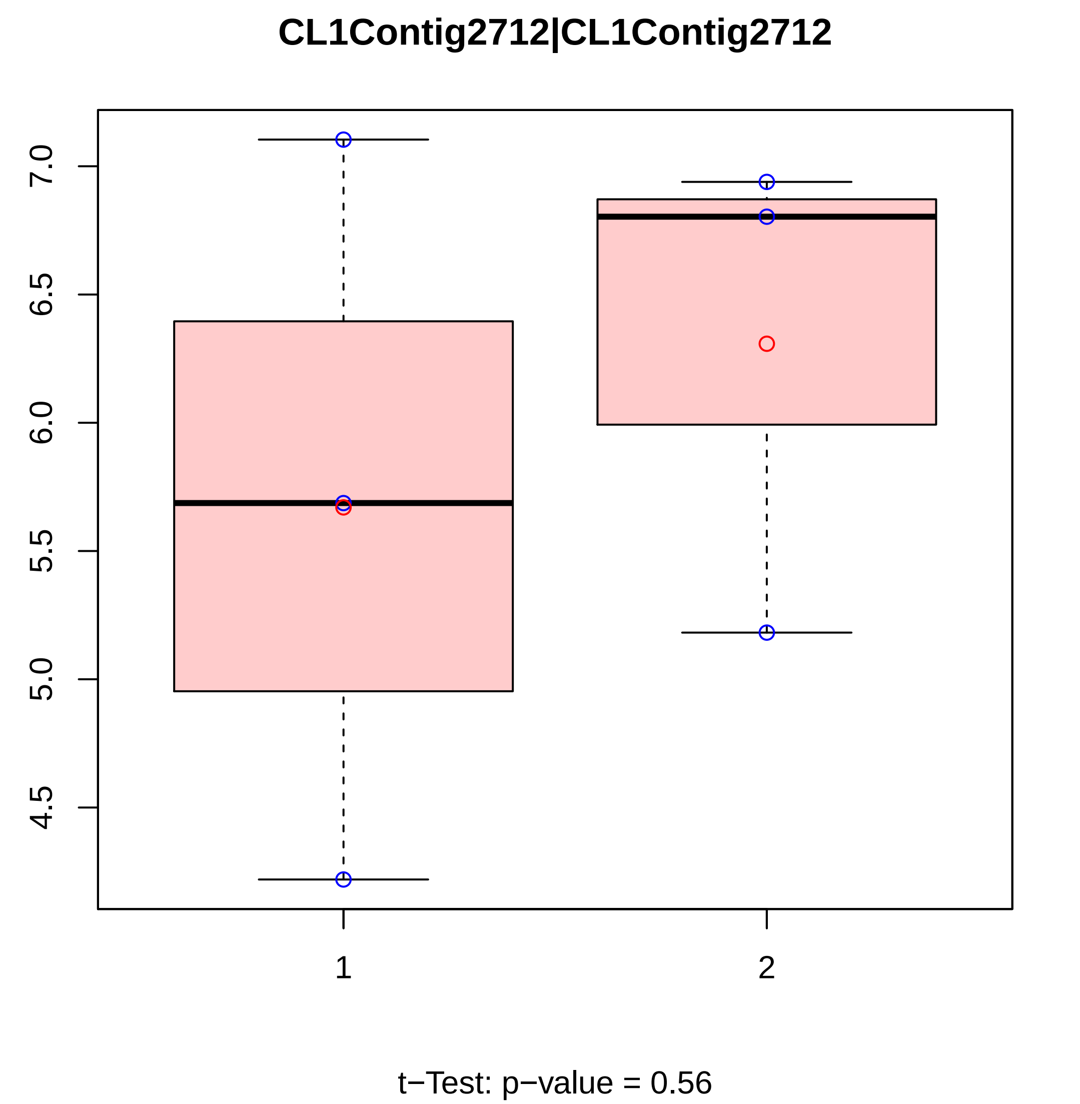
CL1Contig2712|CL1Contig2712
7.0
6.5
6.0
5.5
5.0
4.5
1
2
t−Test: p−value = 0.56

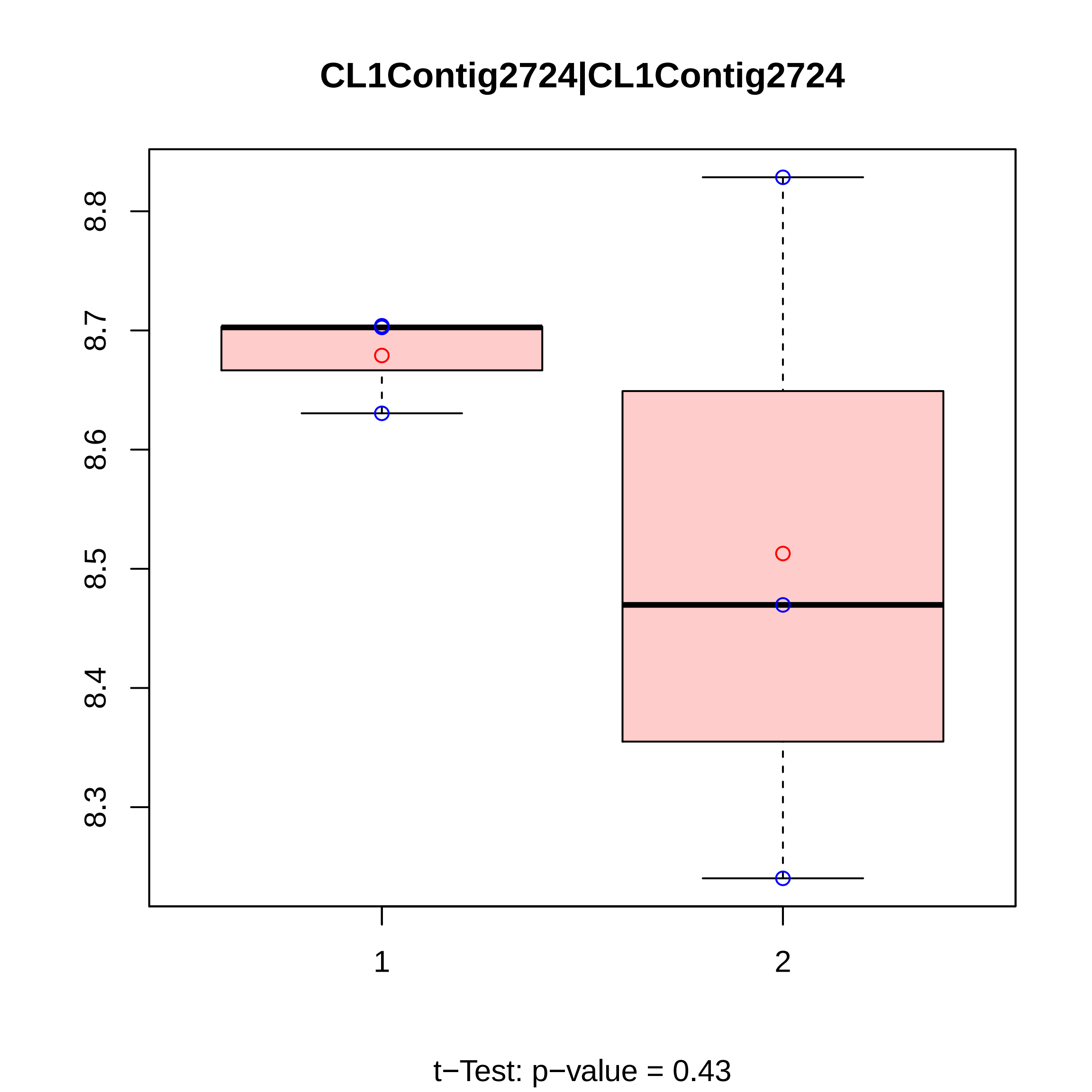

CL1Contig2724|CL1Contig2724
8.3
8.4
8.5
8.6
8.7
8.8
1
2
t−Test: p−value = 0.43

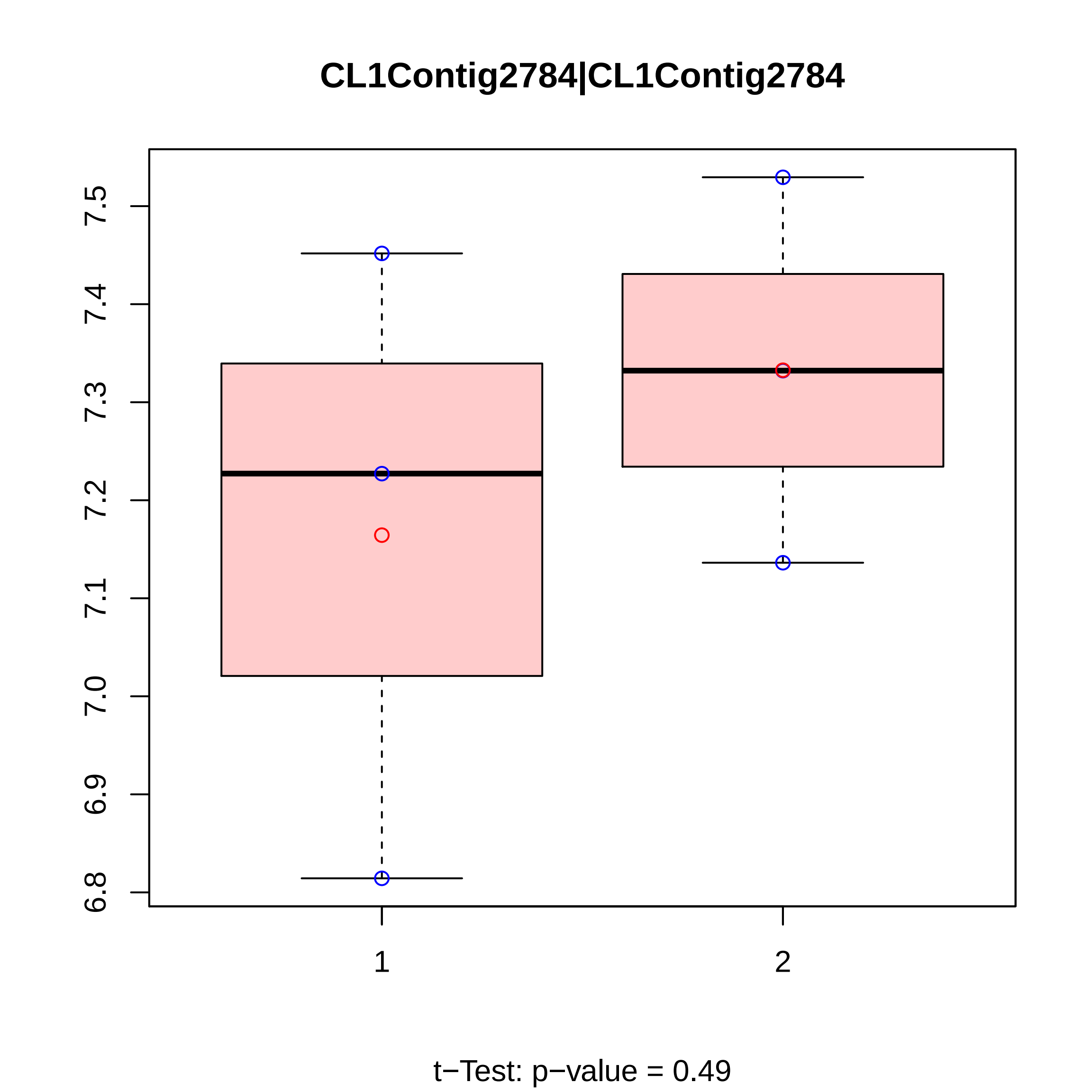

CL1Contig2784|CL1Contig2784
7.5
7.4
7.3
7.2
7.1
7.0
6.9
6.8
1
2
t−Test: p−value = 0.49

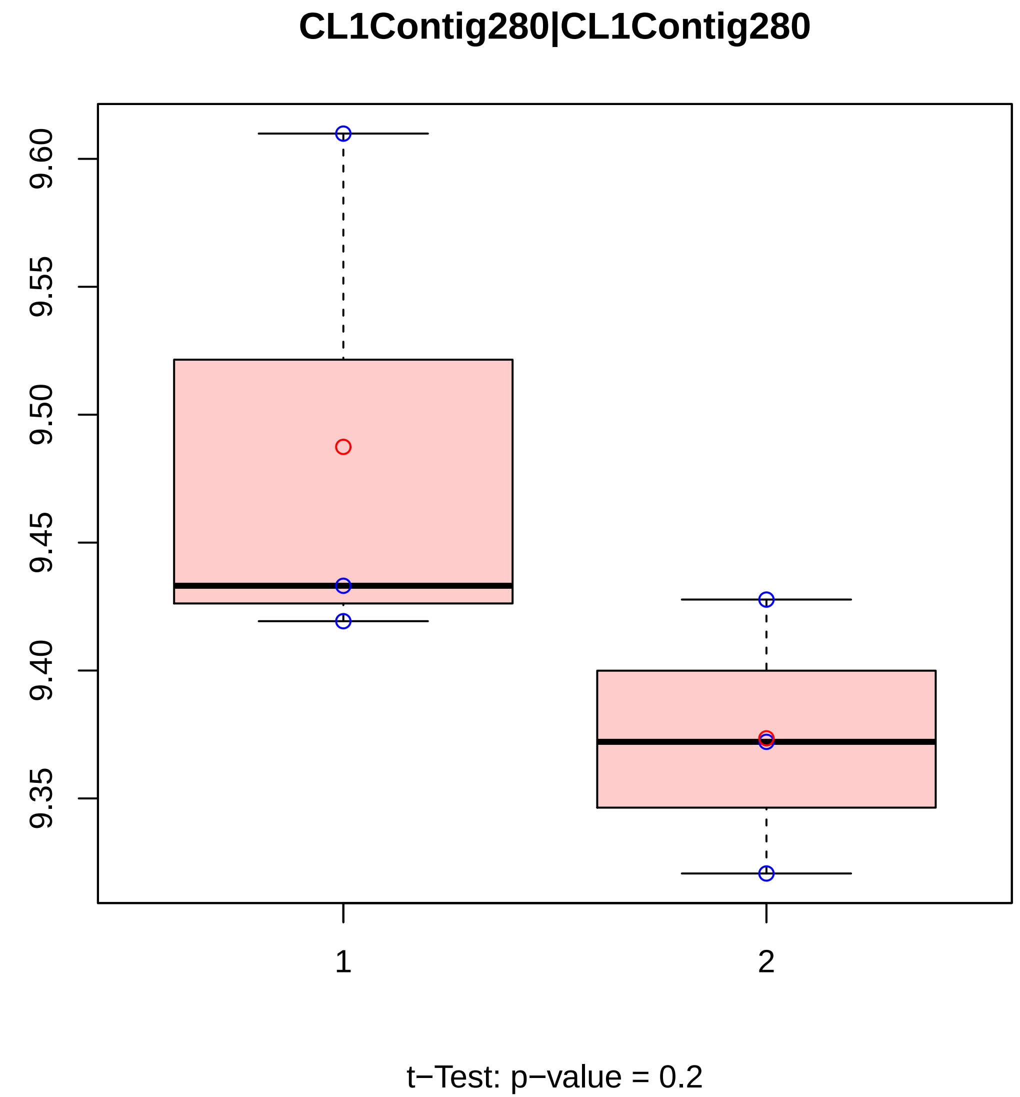

CL1Contig280|CL1Contig280
9.35
9.40
9.45
9.50
9.55
9.60
1
2
t−Test: p−value = 0.2

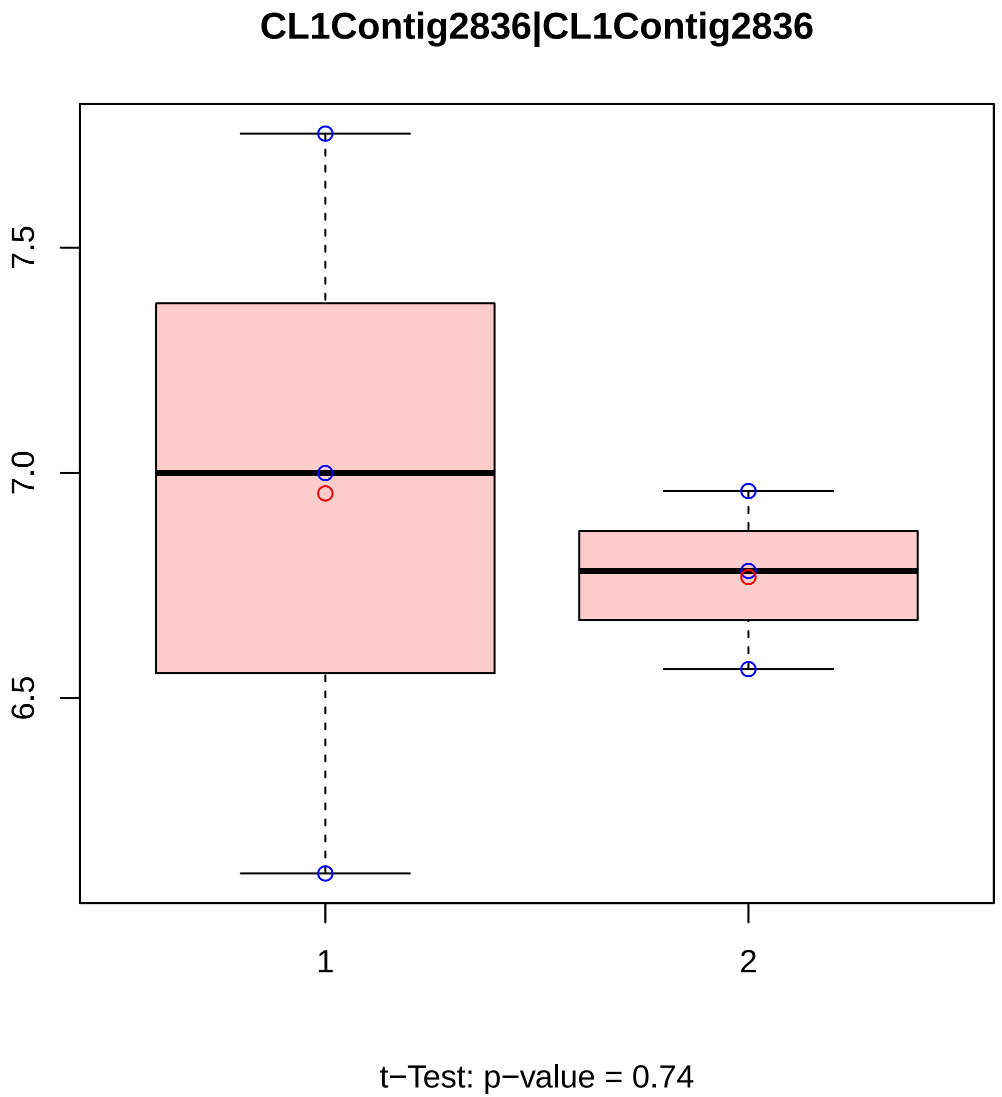
CL1Contig2836|CL1Contig2836
7.5
7.0
6.5
1
2
t−Test: p−value = 0.74

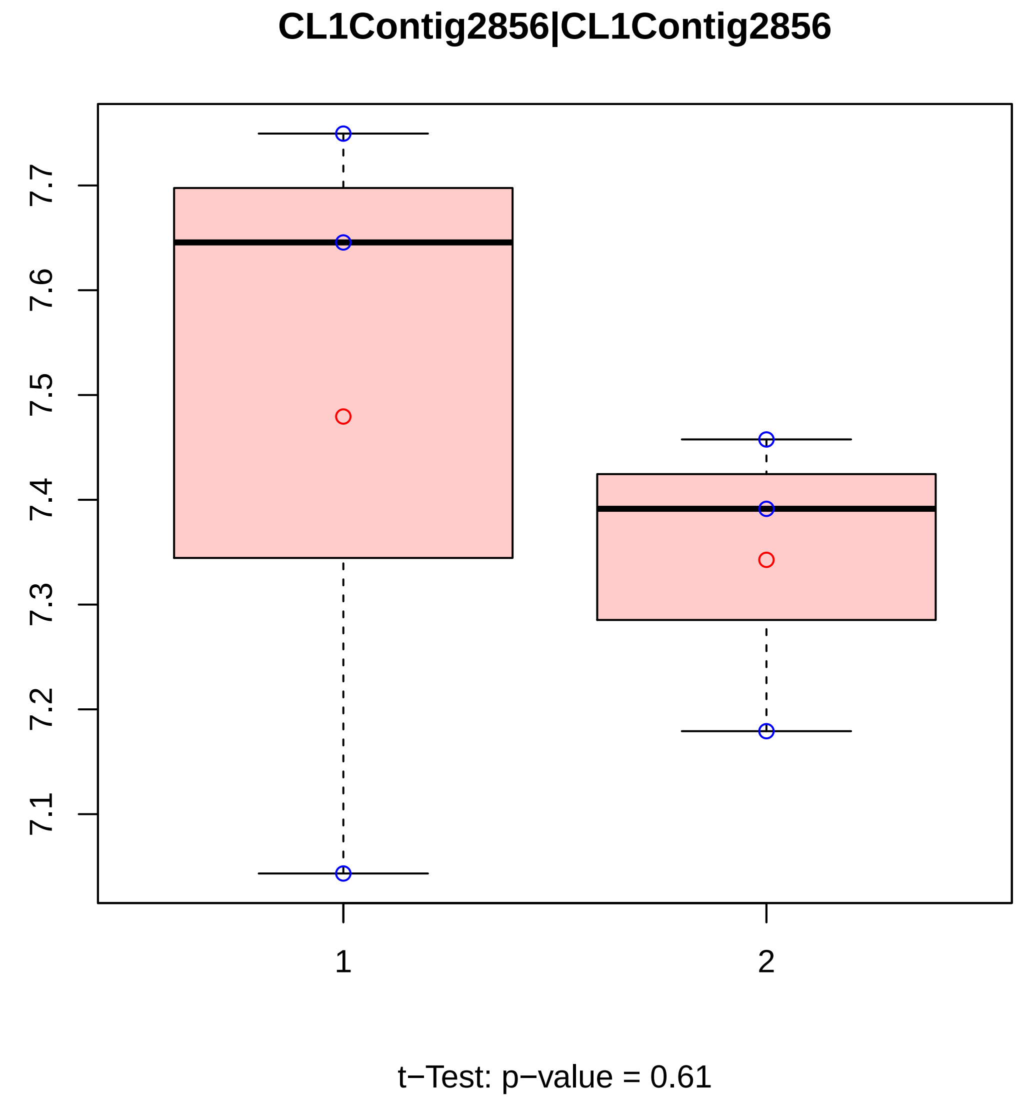
CL1Contig2856|CL1Contig2856
7.1
7.2
7.3
7.4
7.5
7.6
7.7
1
2
t−Test: p−value = 0.61

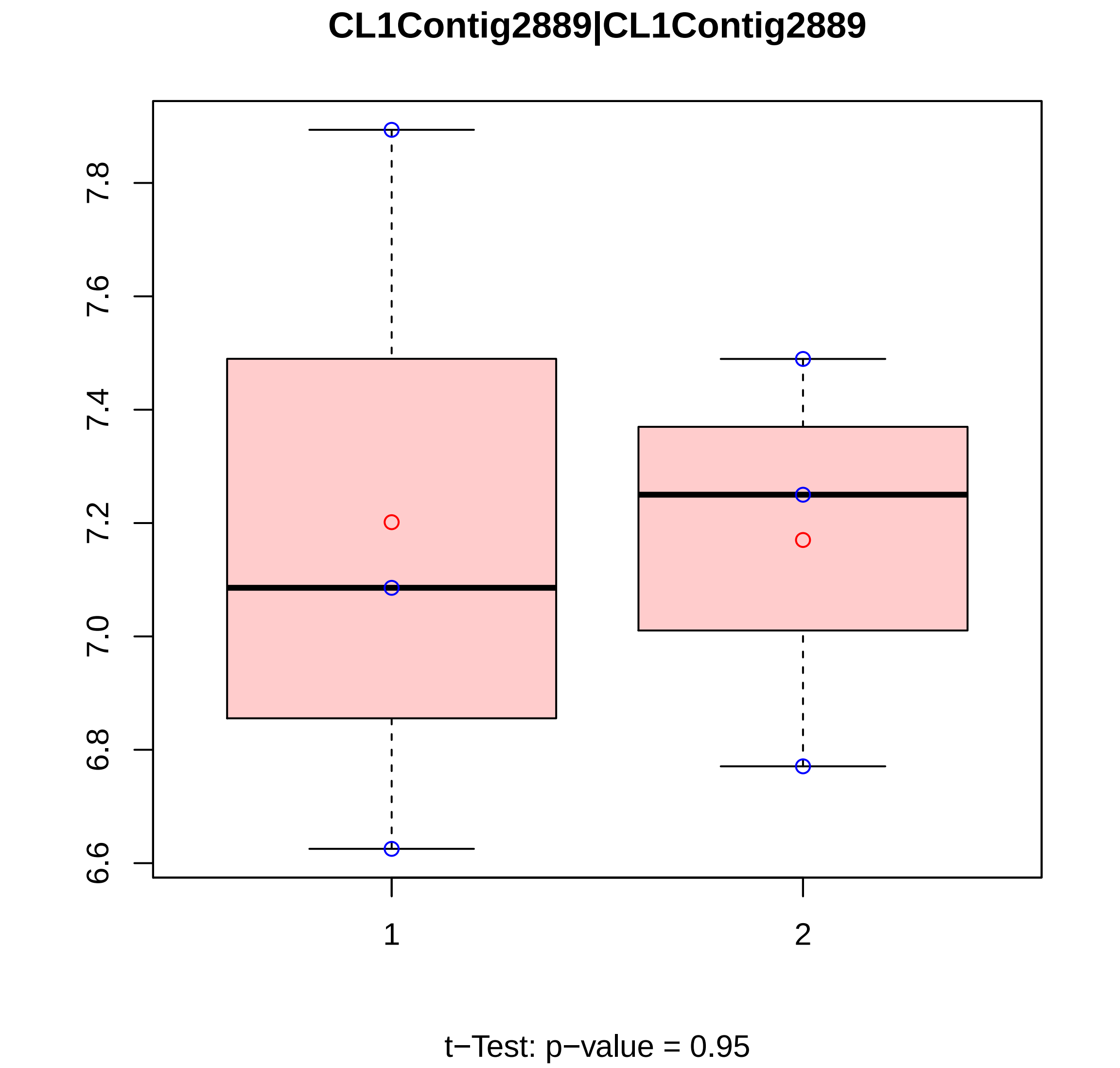

CL1Contig2889|CL1Contig2889
6.6
6.8
7.0
7.2
7.4
7.6
7.8
1
2
t−Test: p−value = 0.95

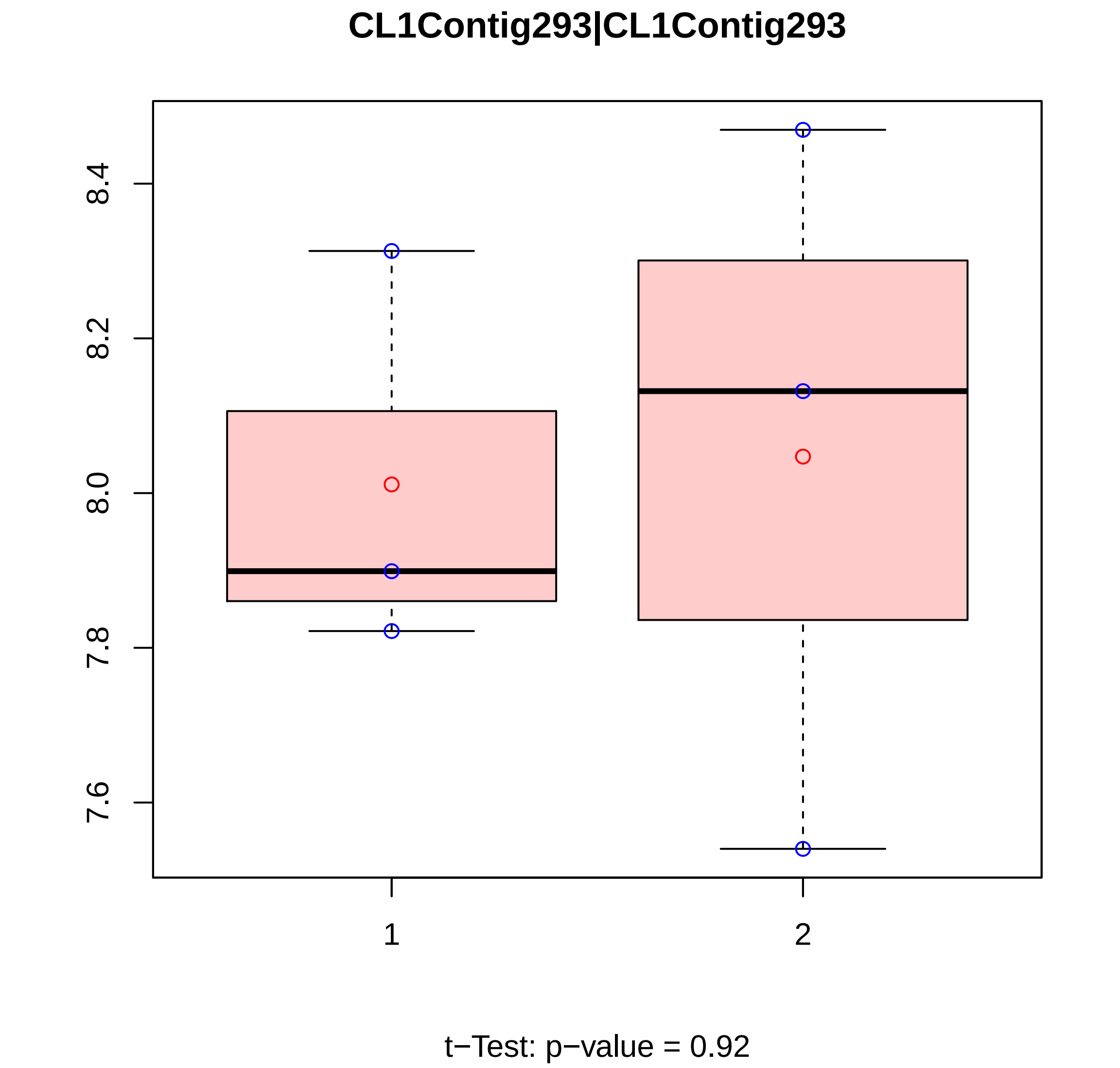
CL1Contig293|CL1Contig293
8.4
8.2
8.0
7.8
7.6
1
2
t−Test: p−value = 0.92

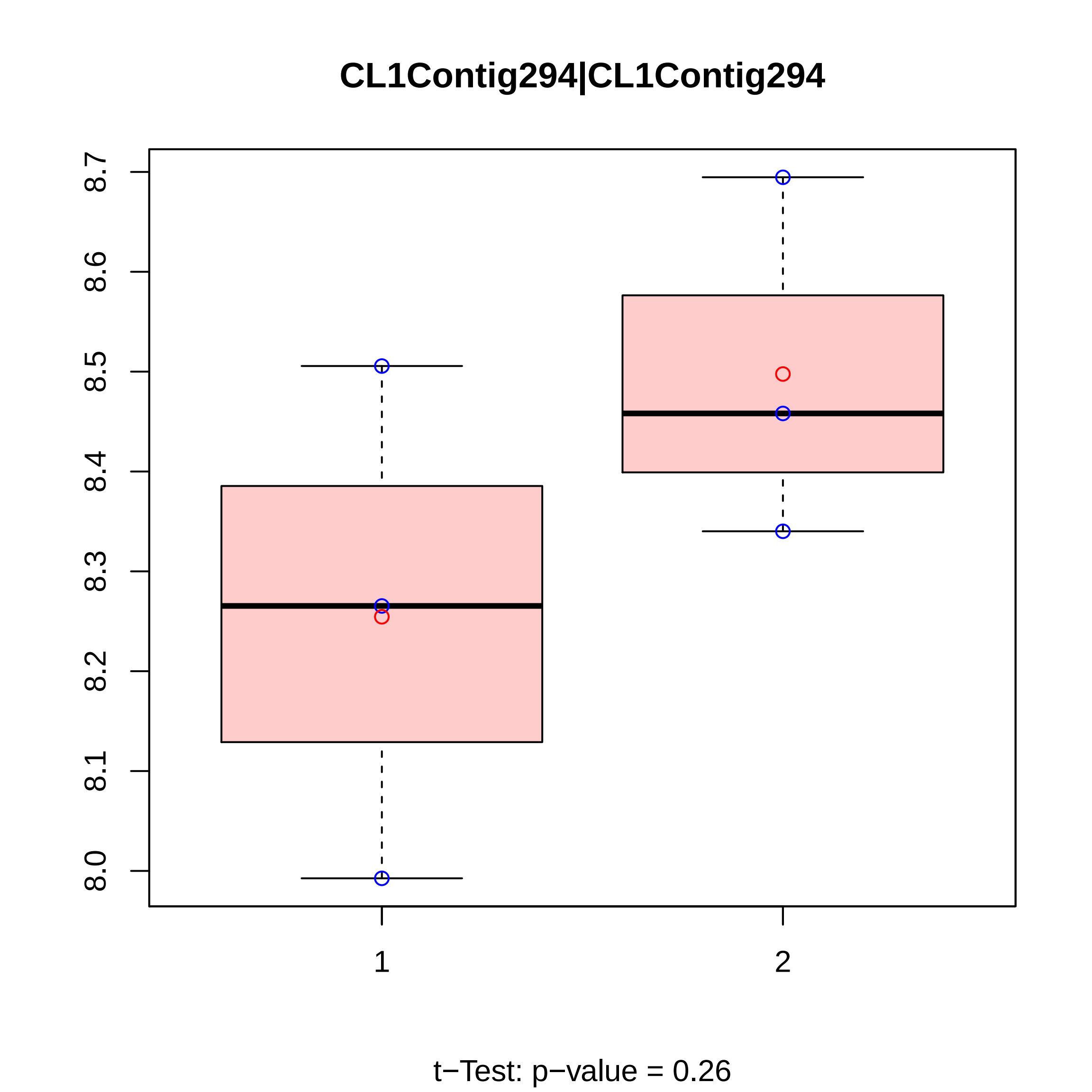
CL1Contig294|CL1Contig294
8.0
8.1
8.2
8.3
8.4
8.5
8.6
8.7
1
2
t−Test: p−value = 0.26

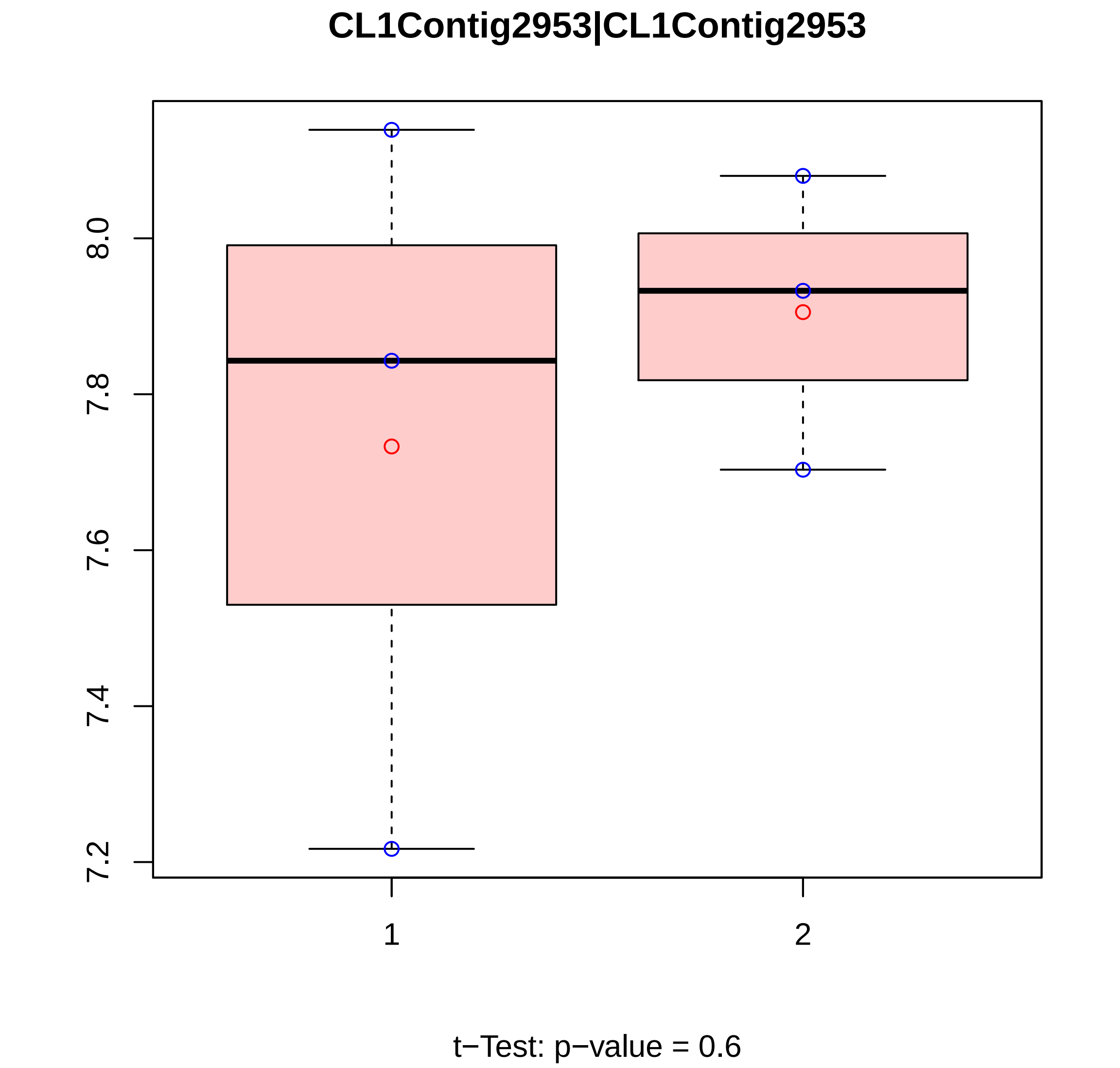
CL1Contig2953|CL1Contig2953
7.2
7.4
7.6
7.8
8.0
1
2
t−Test: p−value = 0.6

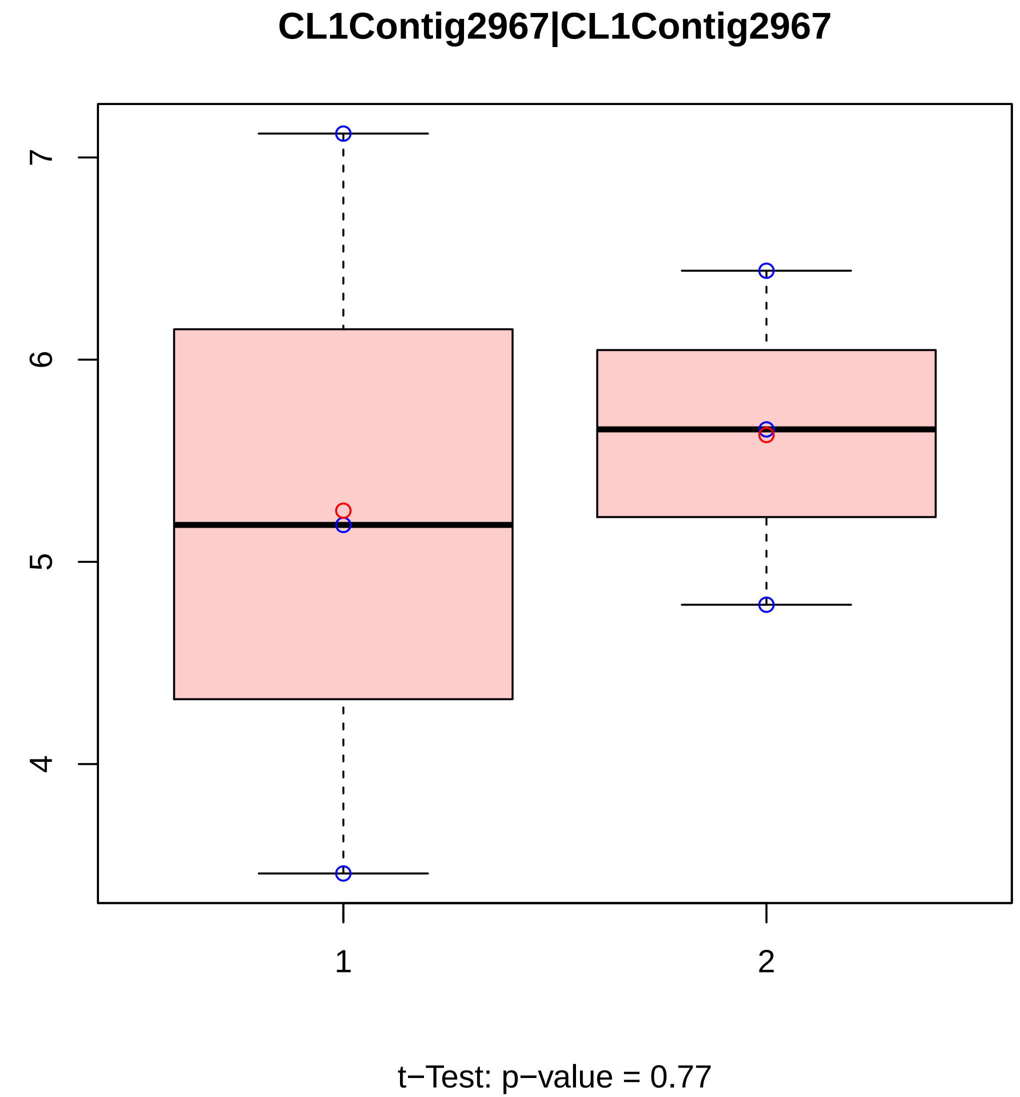
CL1Contig2967|CL1Contig2967
7
6
5
4
1
2
t−Test: p−value = 0.77

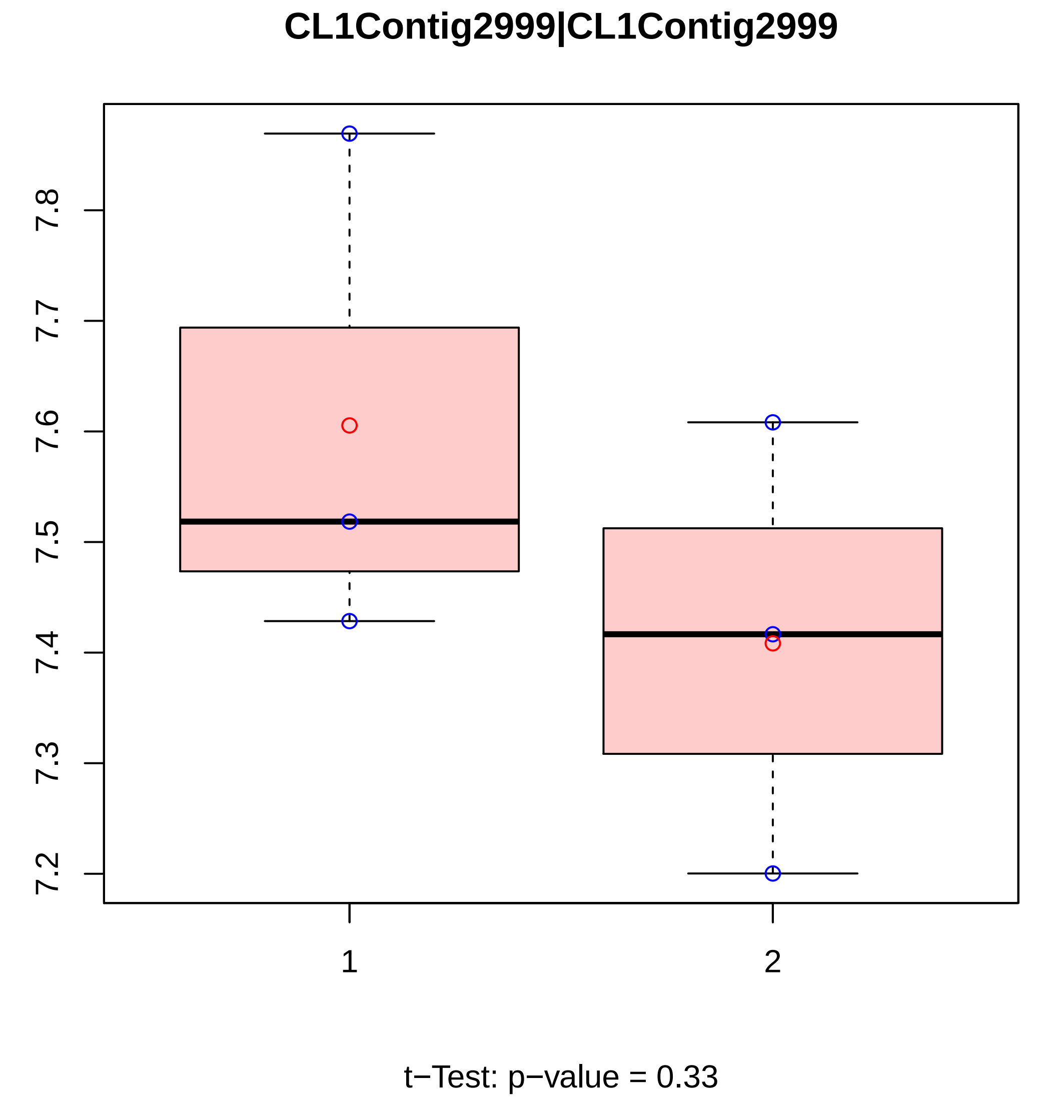
CL1Contig2999|CL1Contig2999
7.2
7.3
7.4
7.5
7.6
7.7
7.8
1
2
t−Test: p−value = 0.33

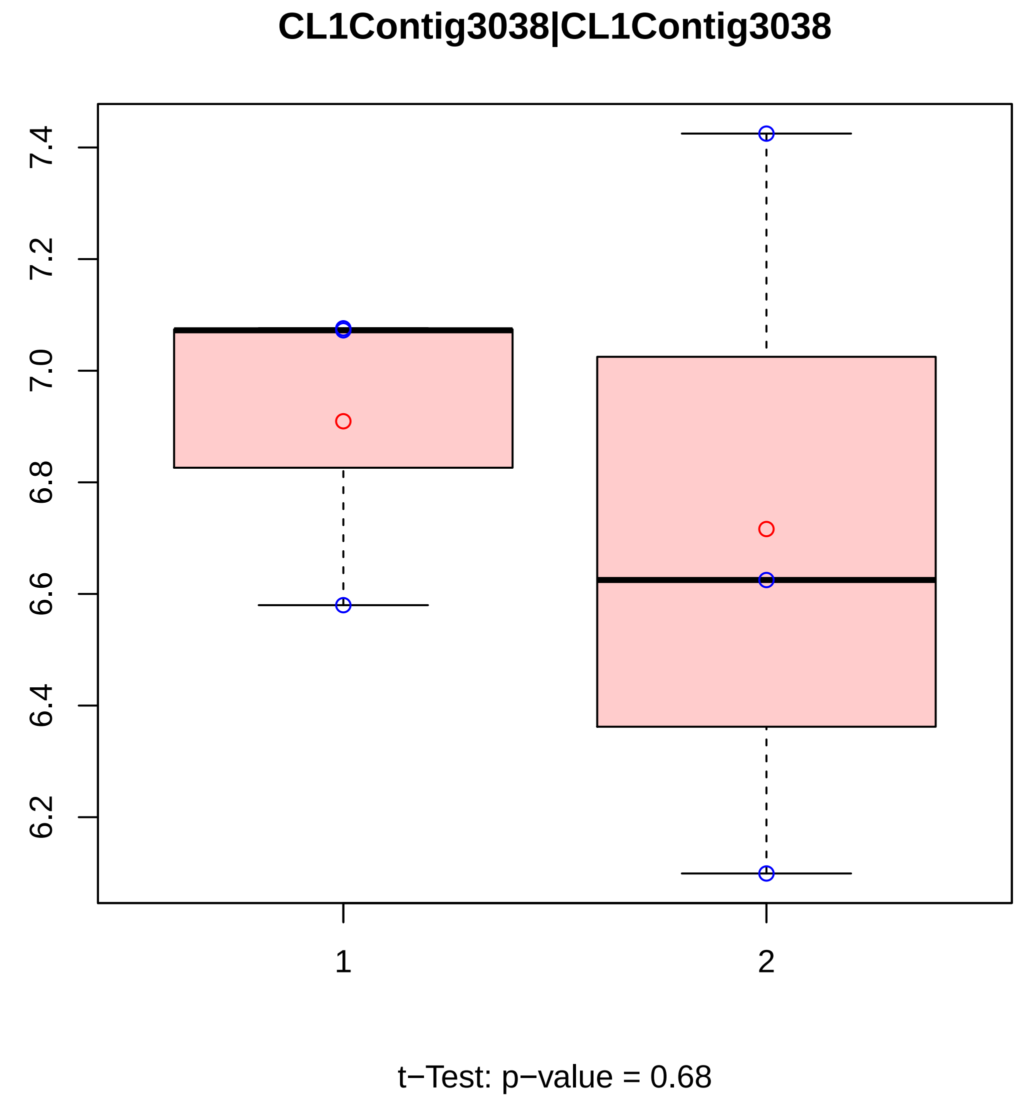

CL1Contig3038|CL1Contig3038
7.4
7.2
7.0
6.8
6.6
6.4
6.2
1
2
t−Test: p−value = 0.68

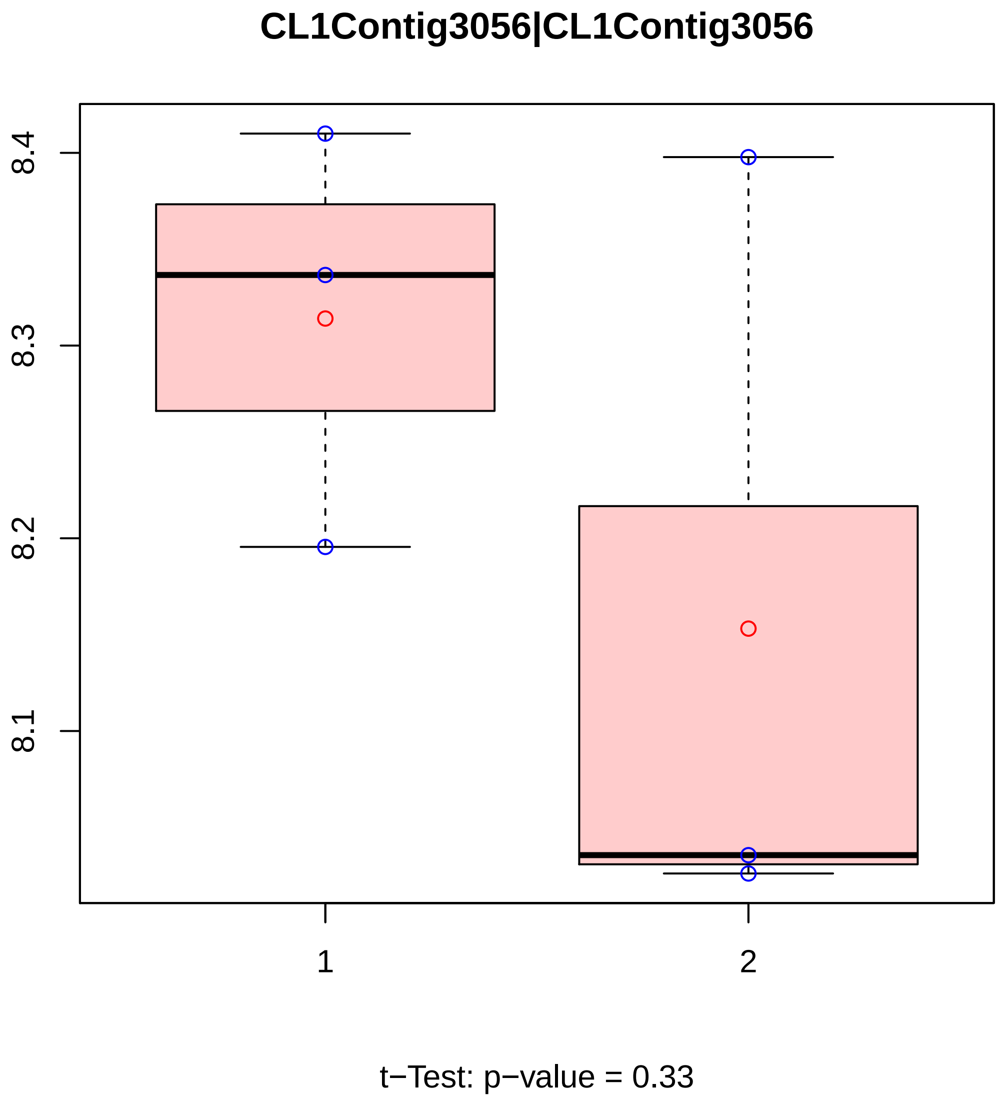
CL1Contig3056|CL1Contig3056
8.4
8.3
8.2
8.1
1
2
t−Test: p−value = 0.33

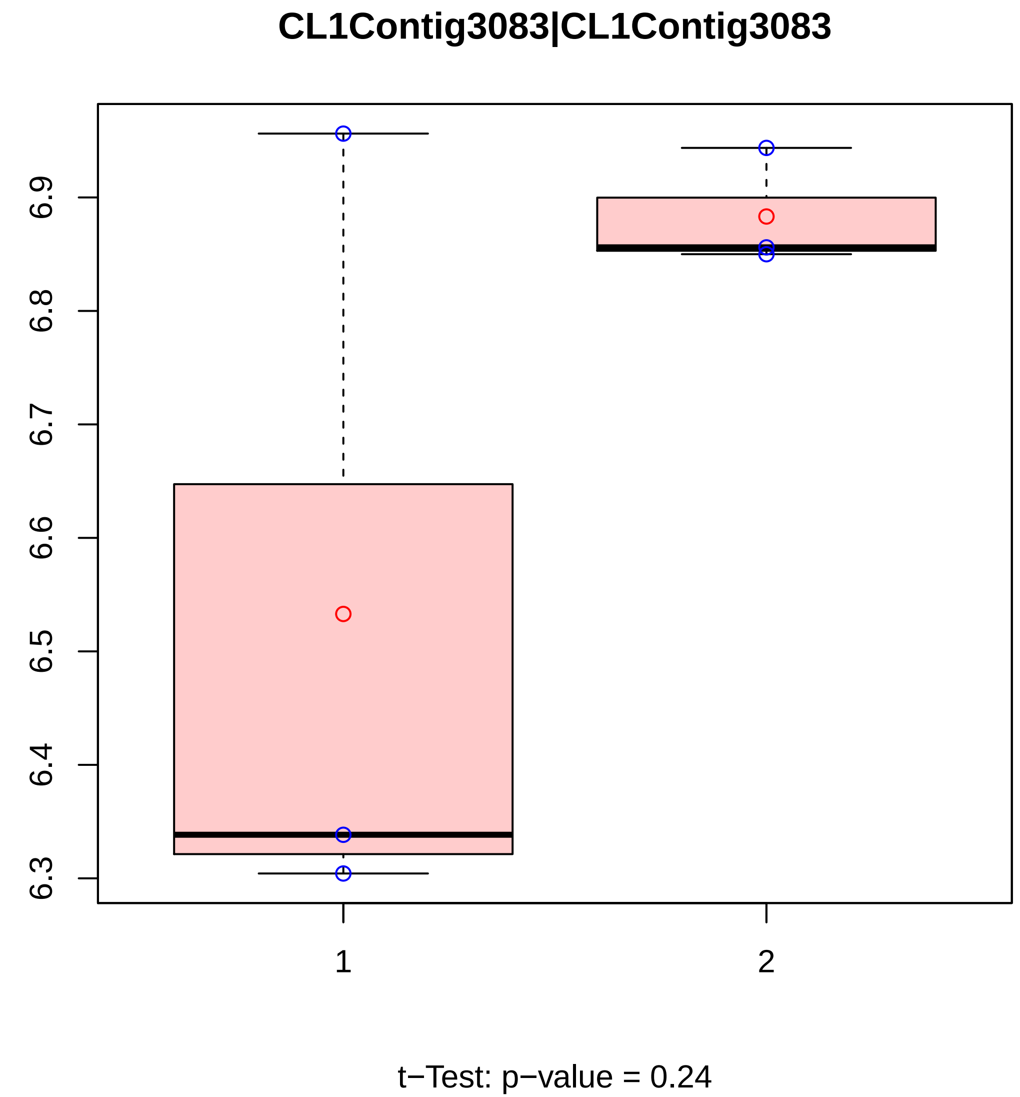
CL1Contig3083|CL1Contig3083
6.3
6.4
6.5
6.6
6.7
6.8
6.9
1
2
t−Test: p−value = 0.24

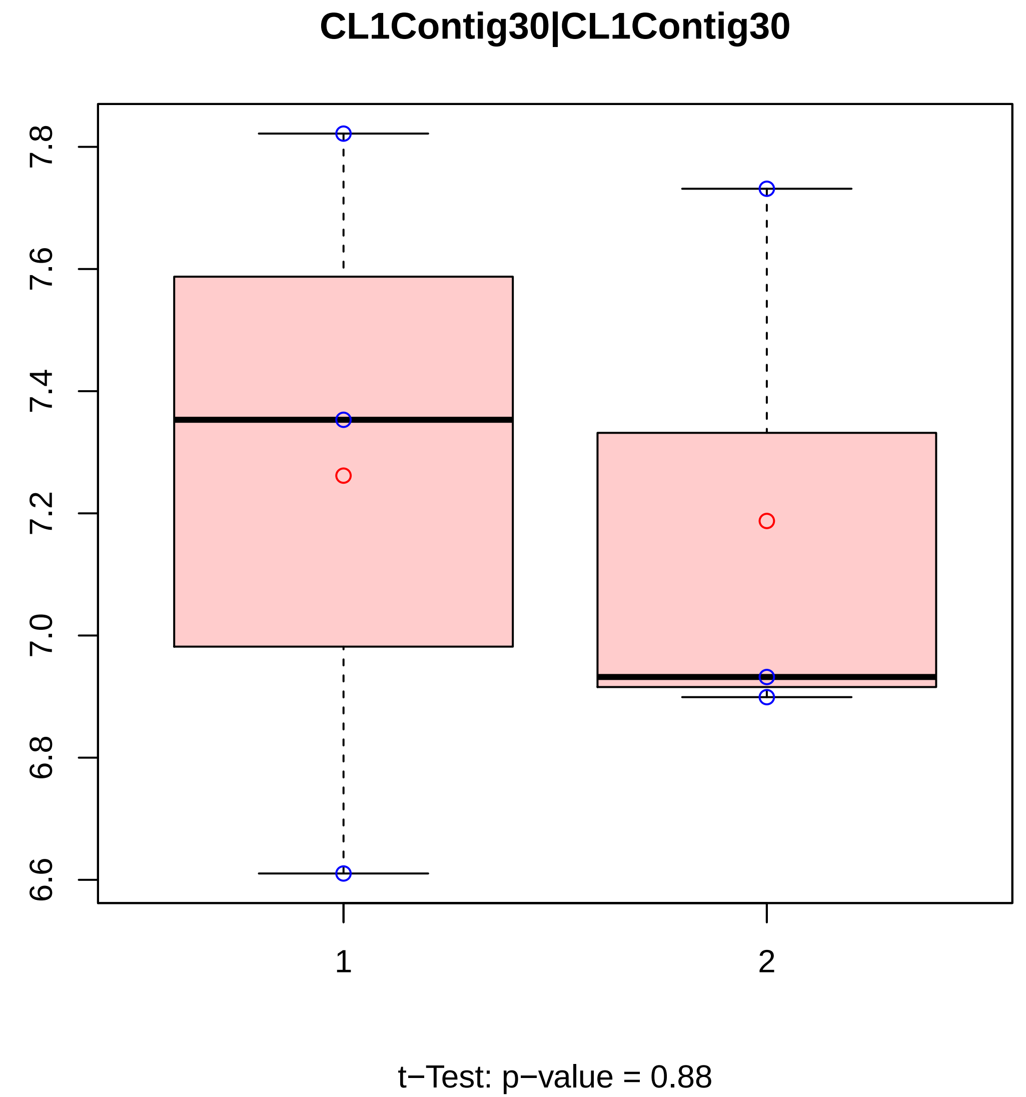

CL1Contig30|CL1Contig30
7.8
7.6
7.4
7.2
7.0
6.8
6.6
1
2
t−Test: p−value = 0.88

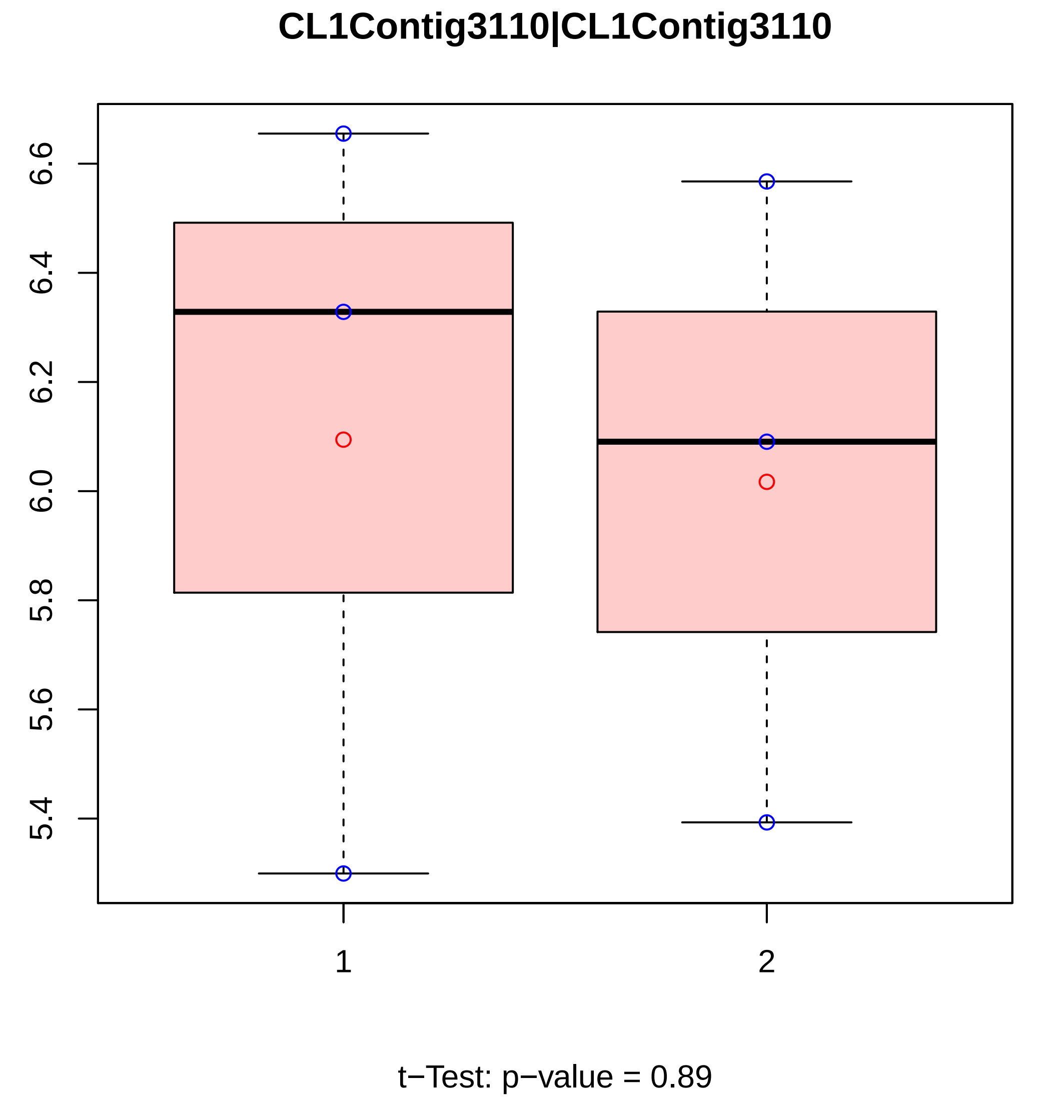
CL1Contig3110|CL1Contig3110
6.6
6.4
6.2
6.0
5.8
5.6
5.4
1
2
t−Test: p−value = 0.89

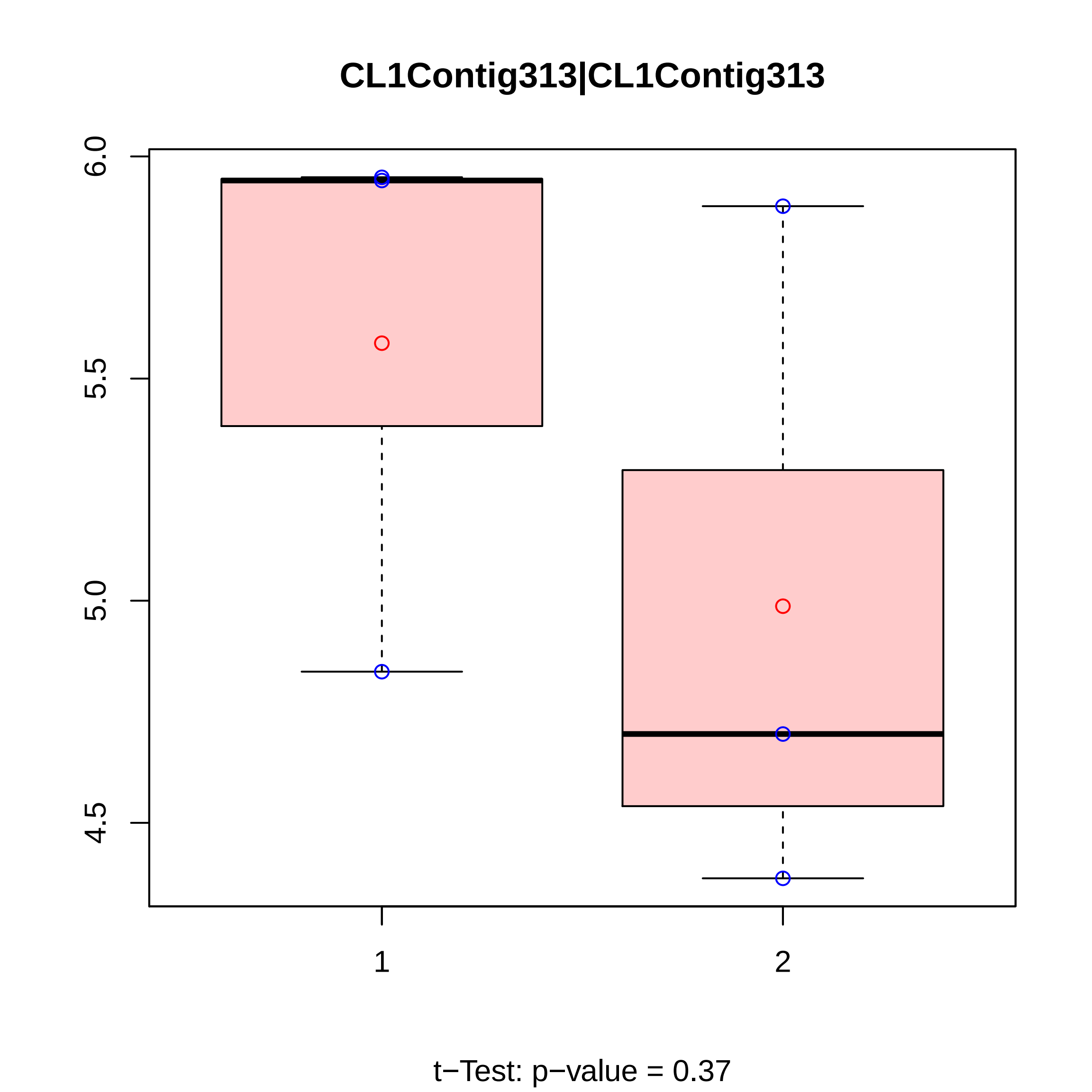
CL1Contig313|CL1Contig313
6.0
5.5
5.0
4.5
1
2
t−Test: p−value = 0.37

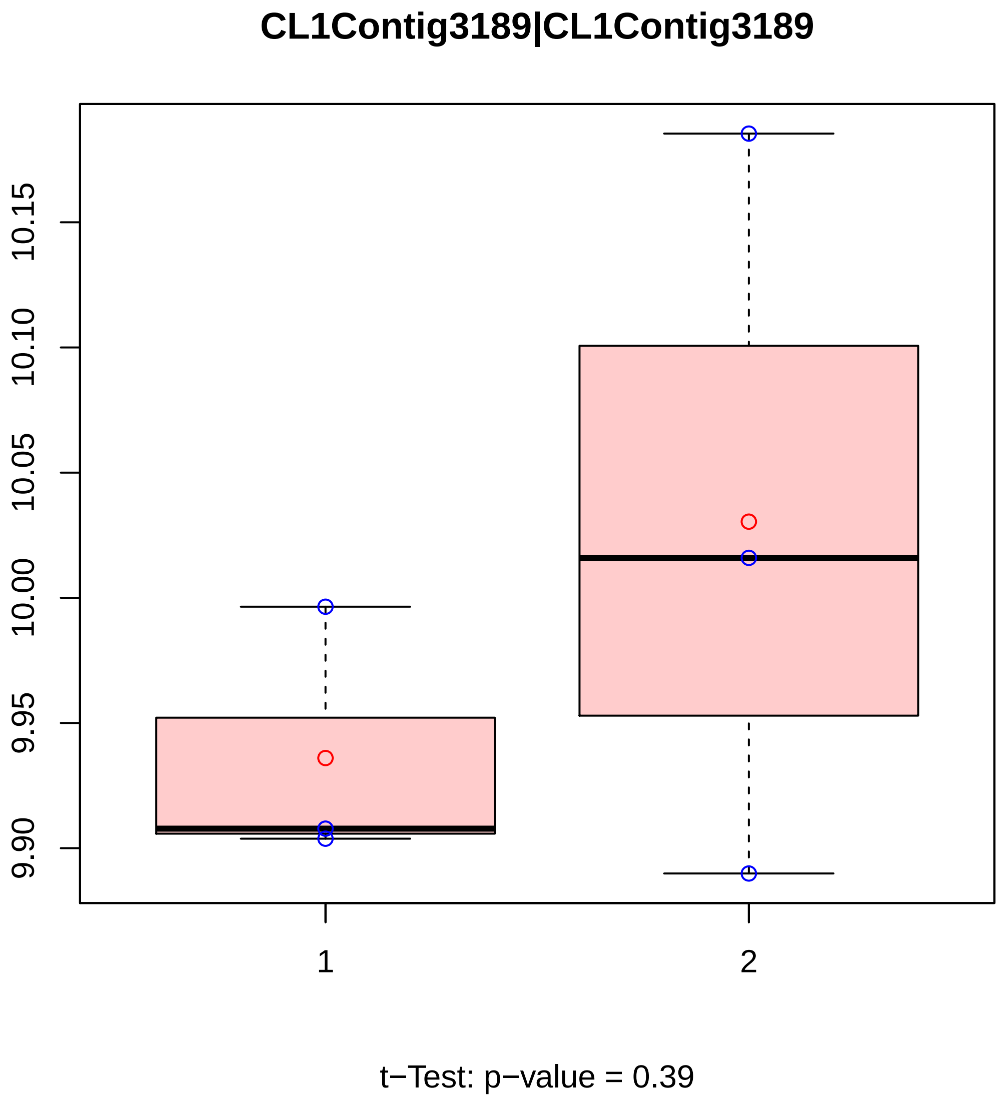
CL1Contig3189|CL1Contig3189
10.15
10.10
10.05
10.00
9.95
9.90
1
2
t−Test: p−value = 0.39

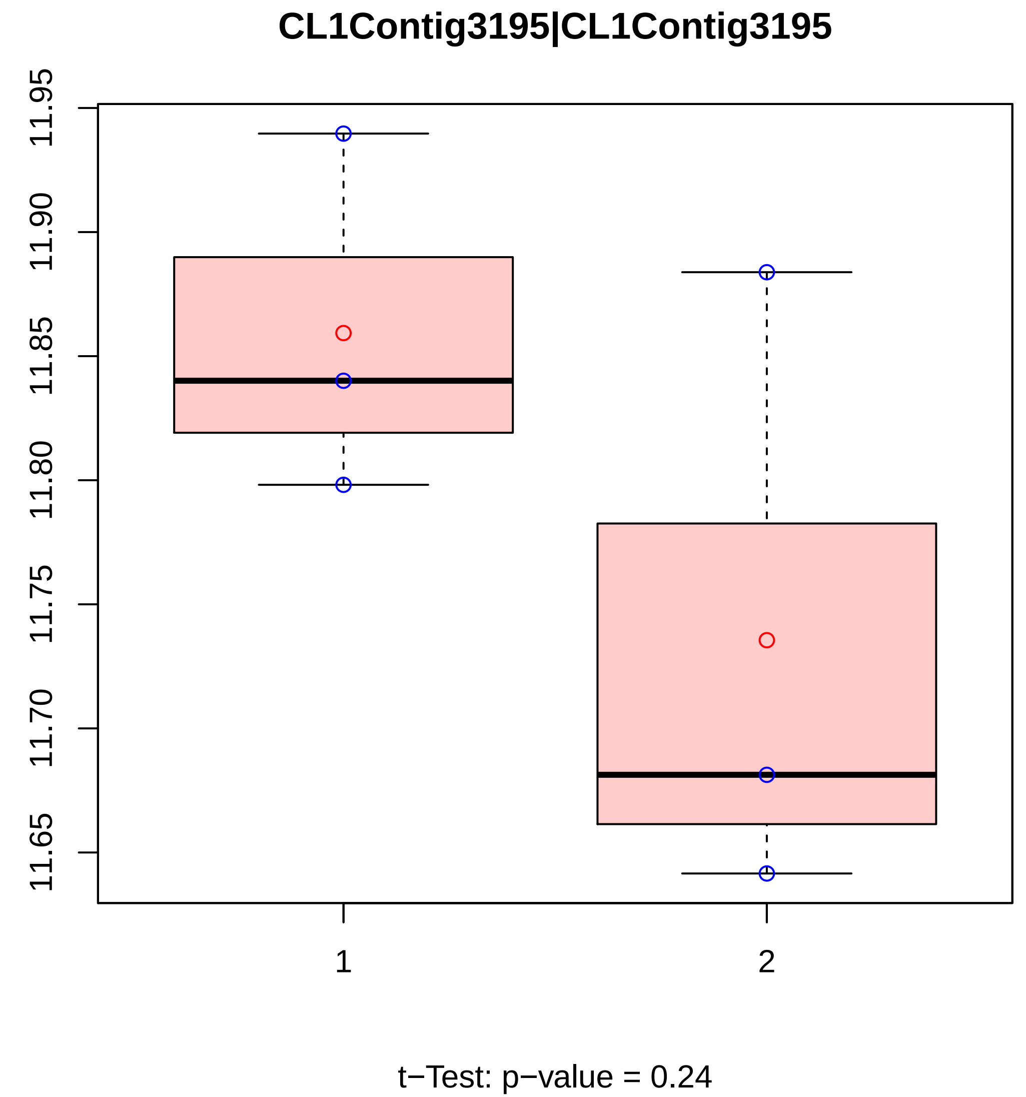

CL1Contig3195|CL1Contig3195
11.95
11.90
11.85
11.80
11.75
11.70
11.65
1
2
t−Test: p−value = 0.24

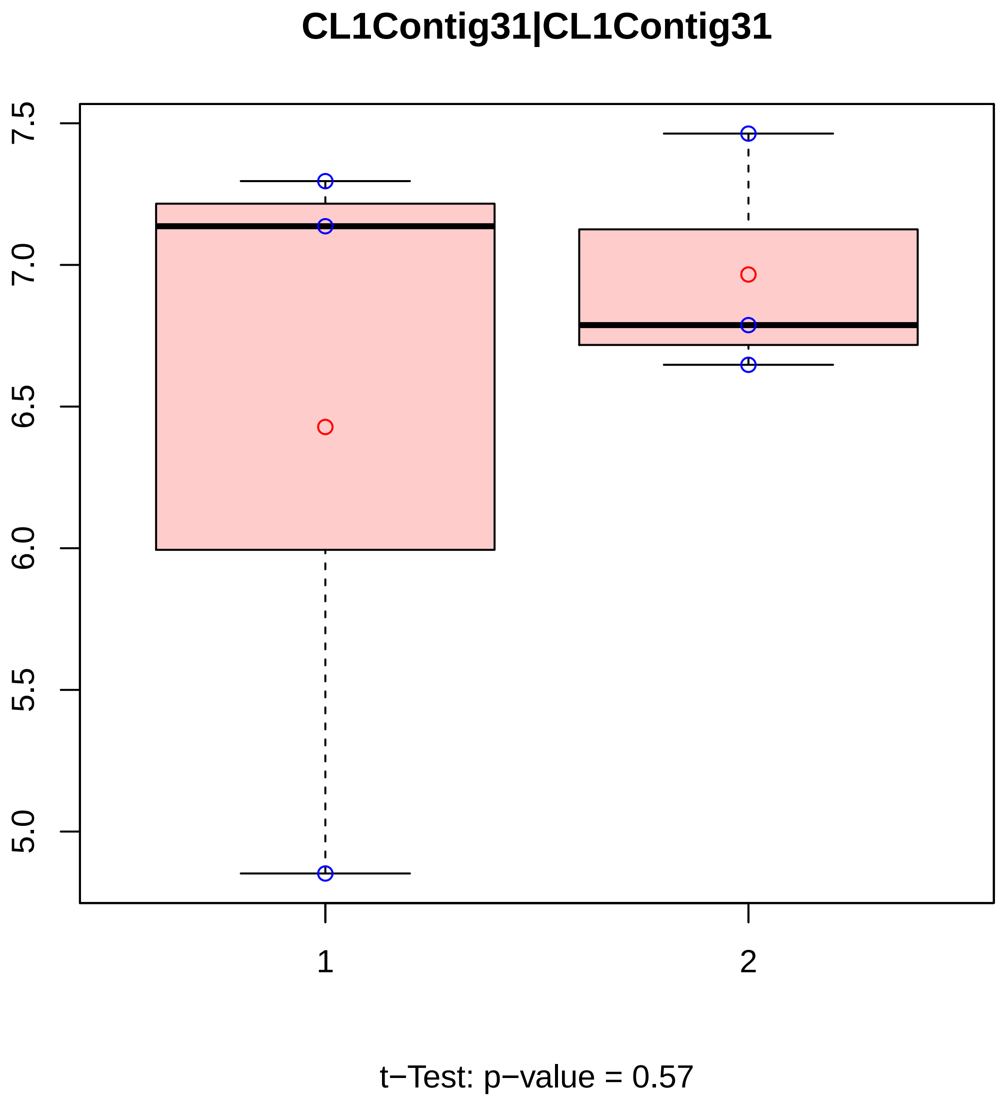

CL1Contig31|CL1Contig31
7.5
7.0
6.5
6.0
5.5
5.0
1
2
t−Test: p−value = 0.57

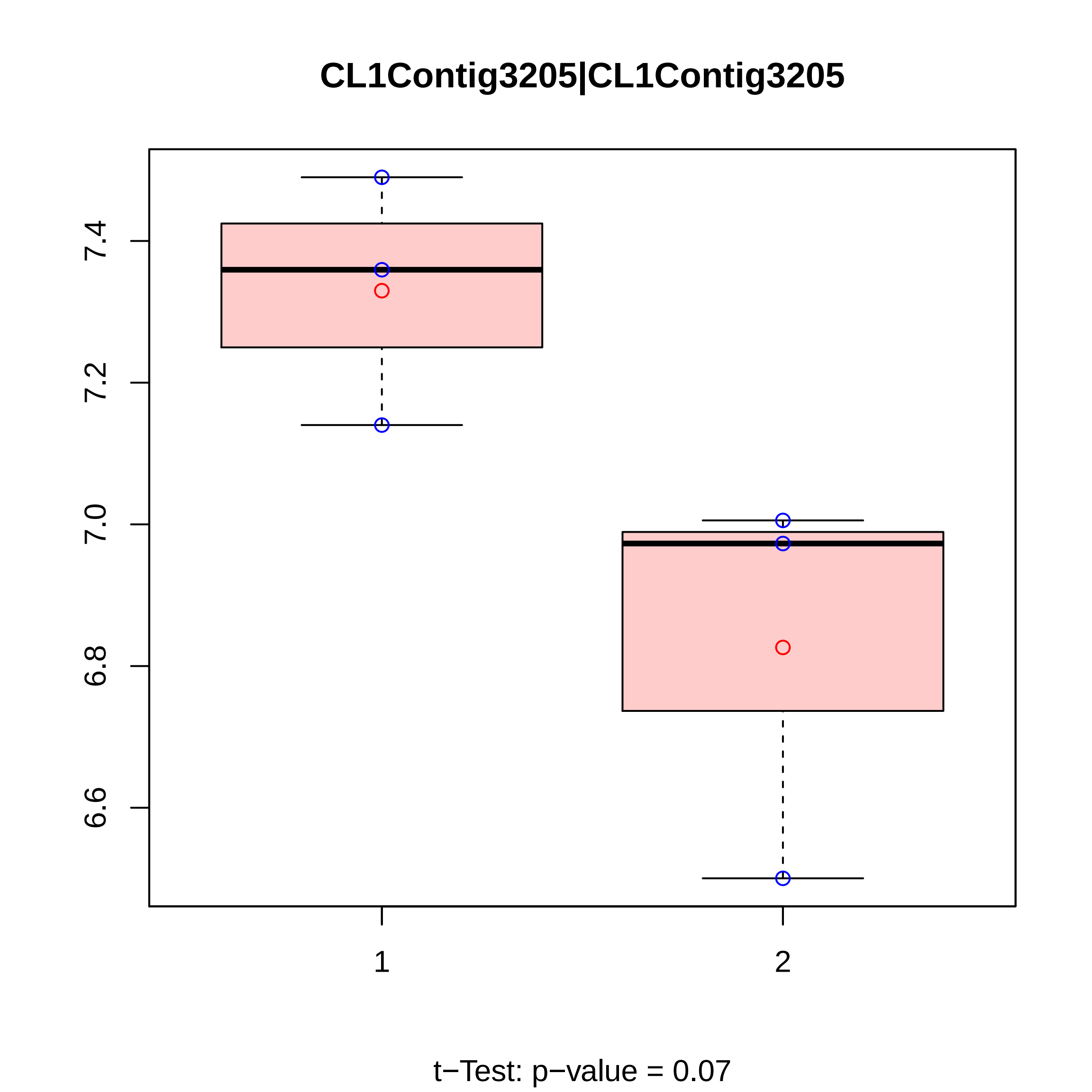

CL1Contig3205|CL1Contig3205
7.4
7.2
7.0
6.8
6.6
1
2
t−Test: p−value = 0.07

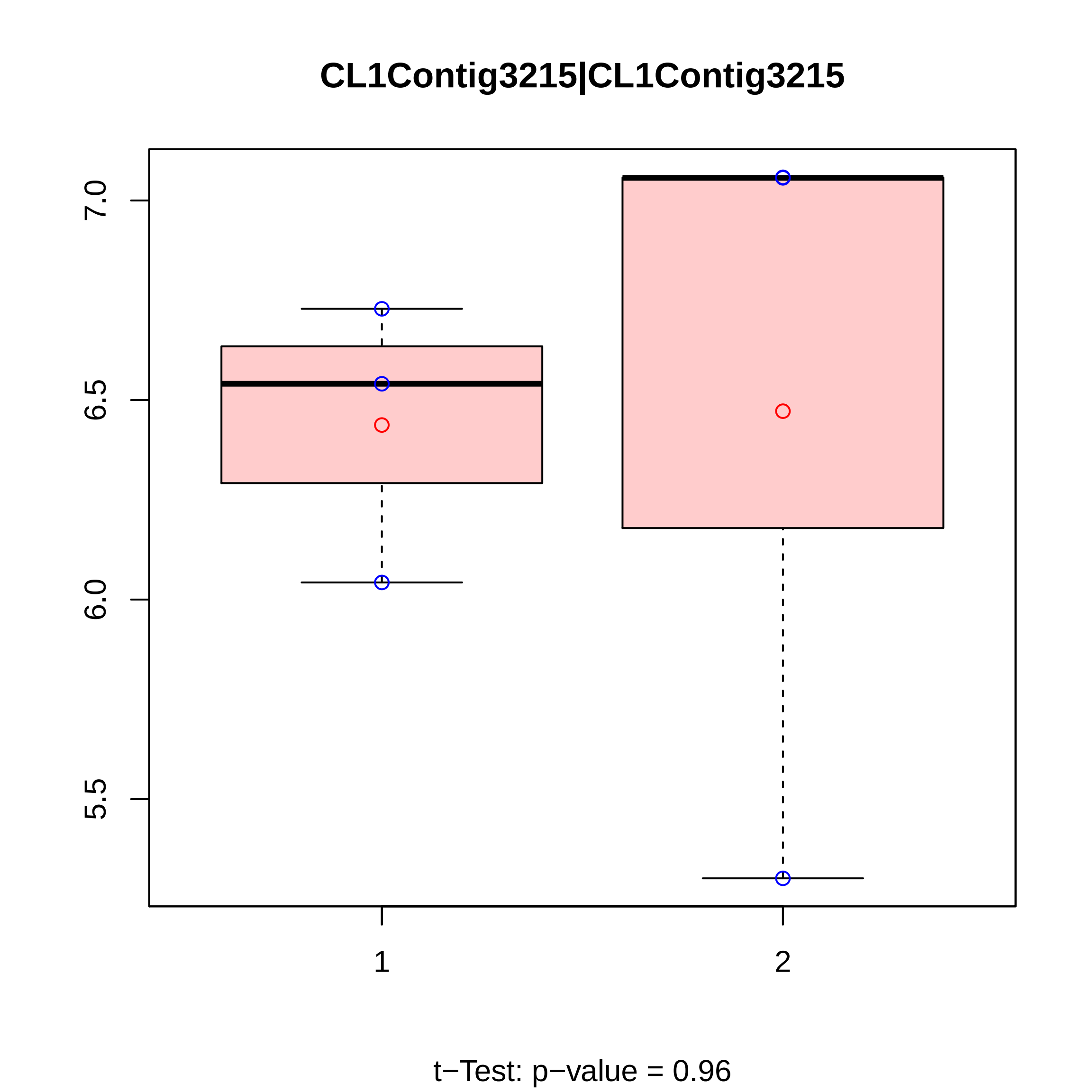
CL1Contig3215|CL1Contig3215
7.0
6.5
6.0
5.5
1
2
t−Test: p−value = 0.96

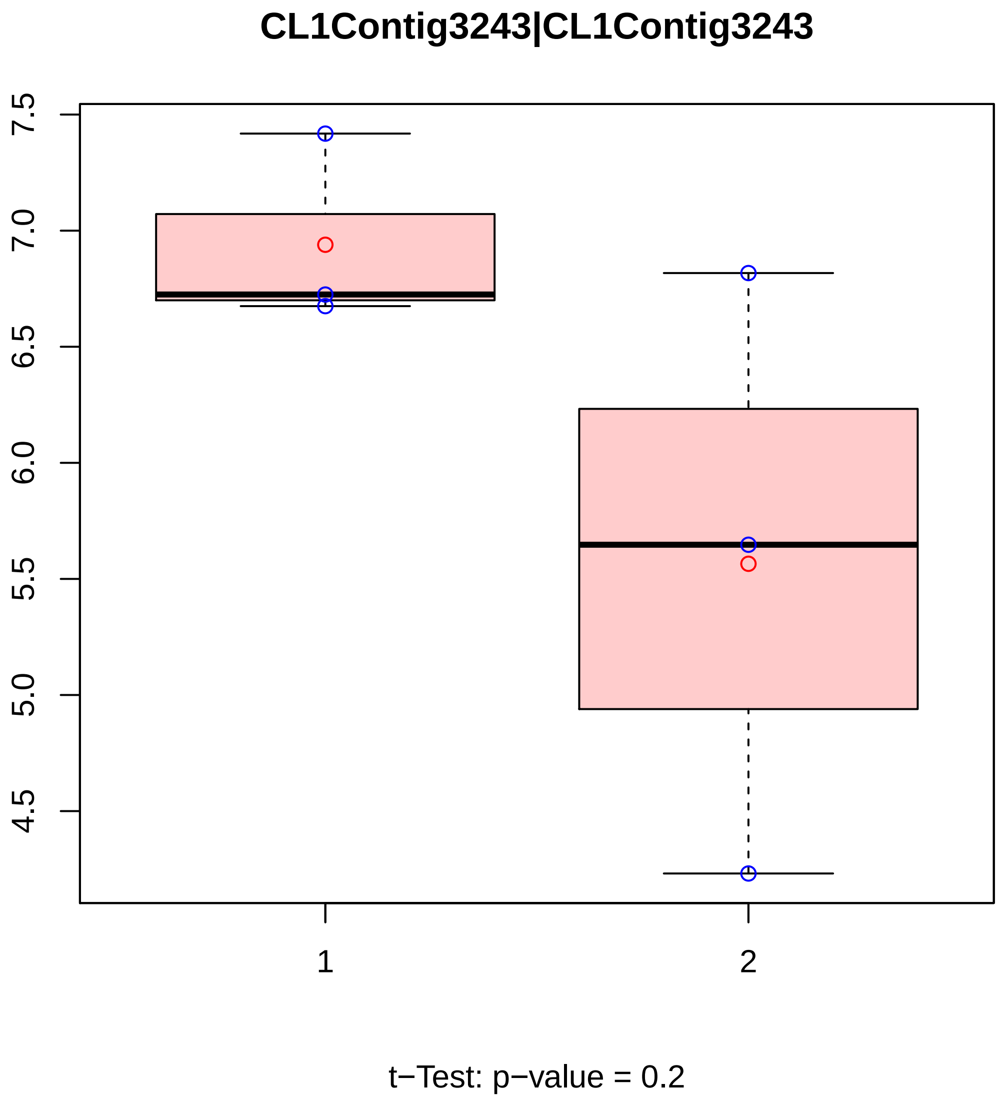
CL1Contig3243|CL1Contig3243
7.5
7.0
6.5
6.0
5.5
5.0
4.5
1
2
t−Test: p−value = 0.2

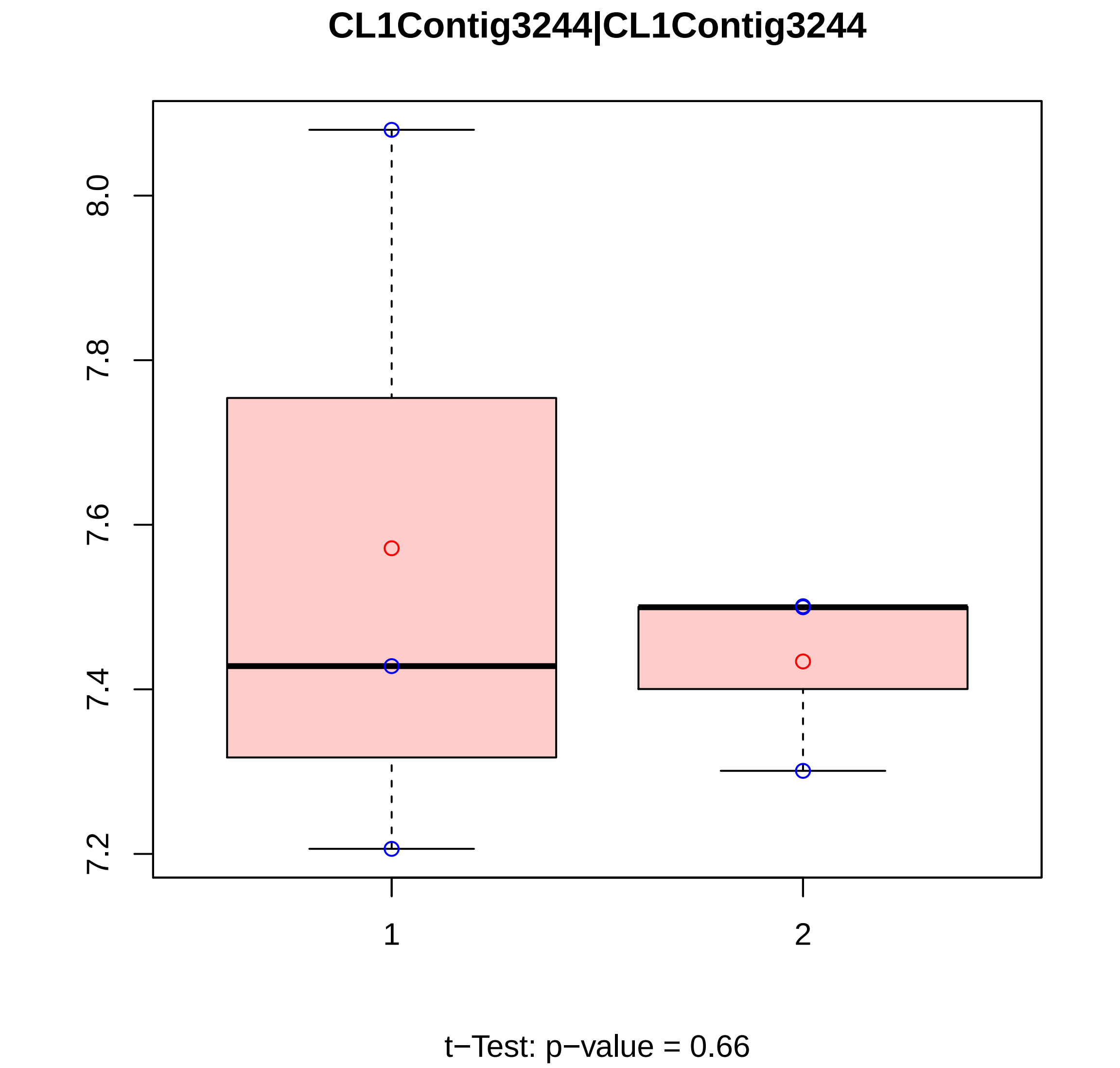
CL1Contig3244|CL1Contig3244
7.2
7.4
7.6
7.8
8.0
1
2
t−Test: p−value = 0.66

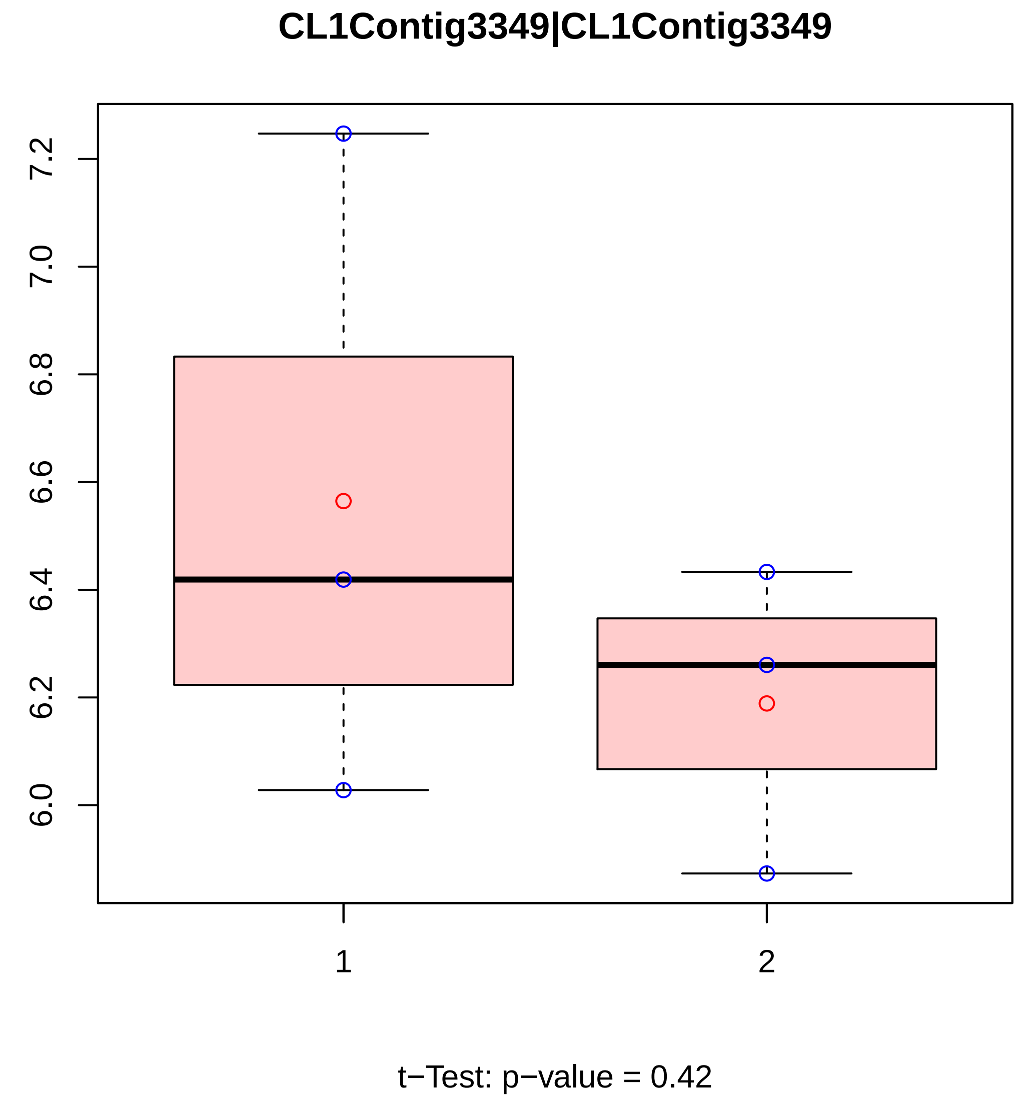
CL1Contig3349|CL1Contig3349
7.2
7.0
6.8
6.6
6.4
6.2
6.0
1
2
t−Test: p−value = 0.42

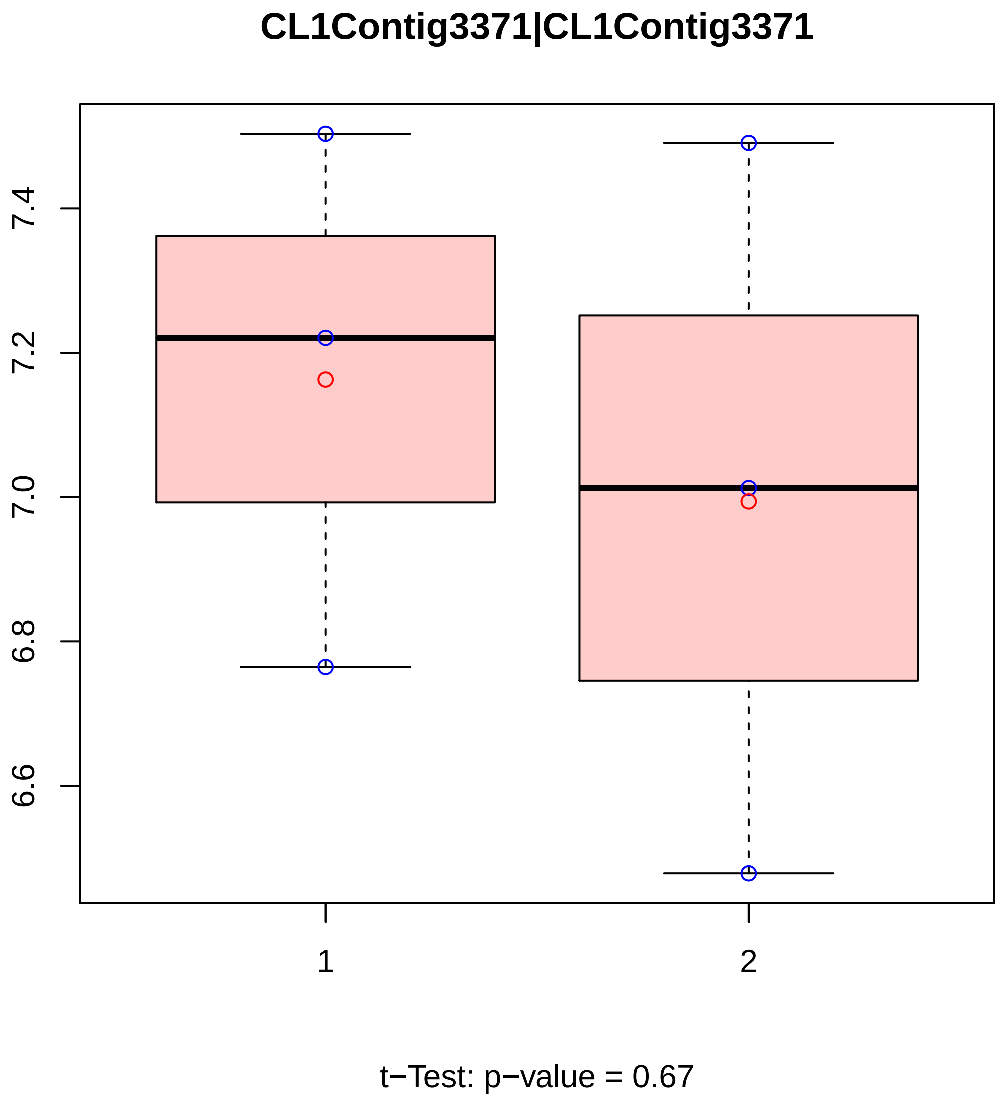

CL1Contig3371|CL1Contig3371
7.4
7.2
7.0
6.8
6.6
1
2
t−Test: p−value = 0.67

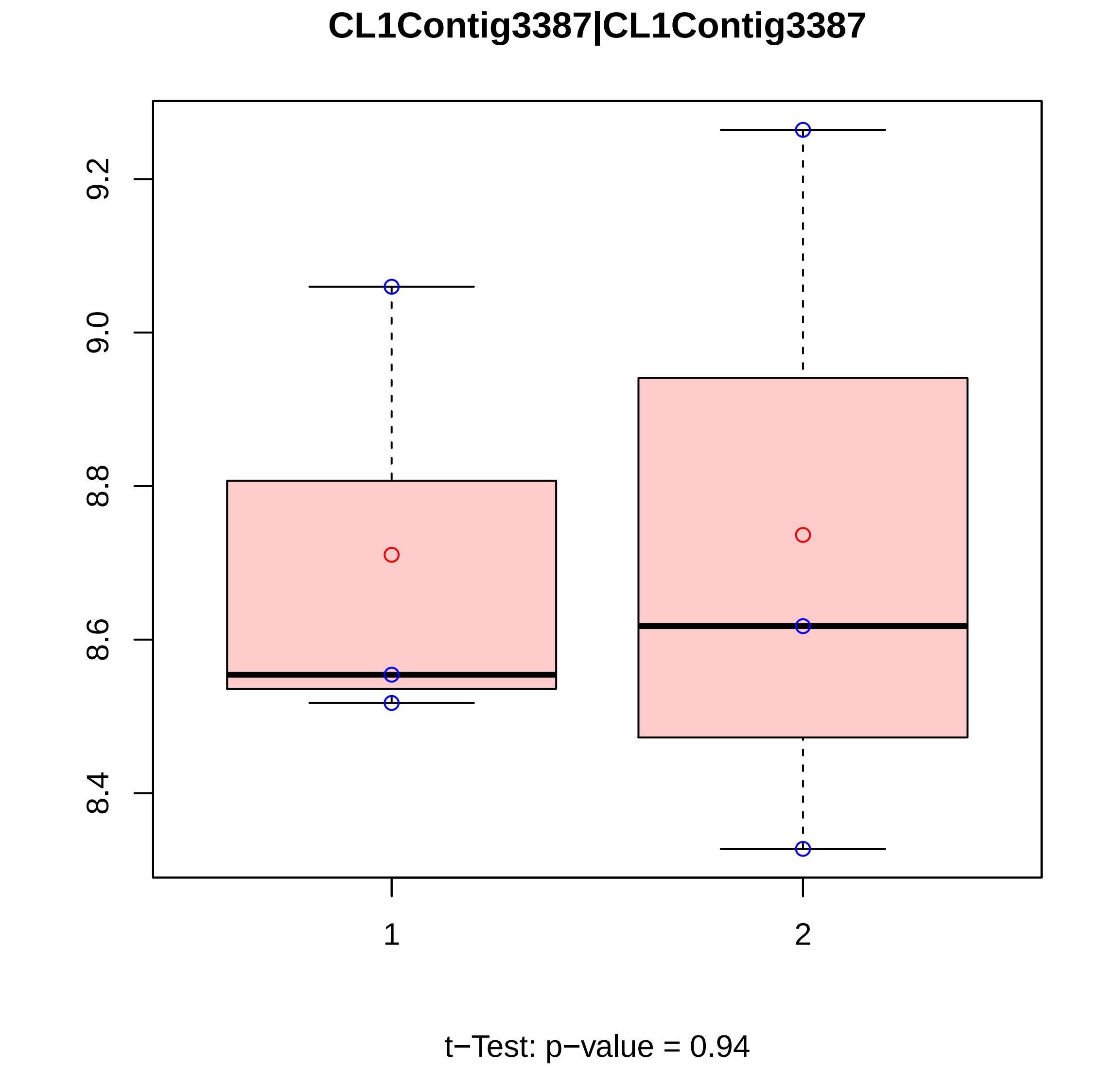
CL1Contig3387|CL1Contig3387
9.2
9.0
8.8
8.6
8.4
1
2
t−Test: p−value = 0.94

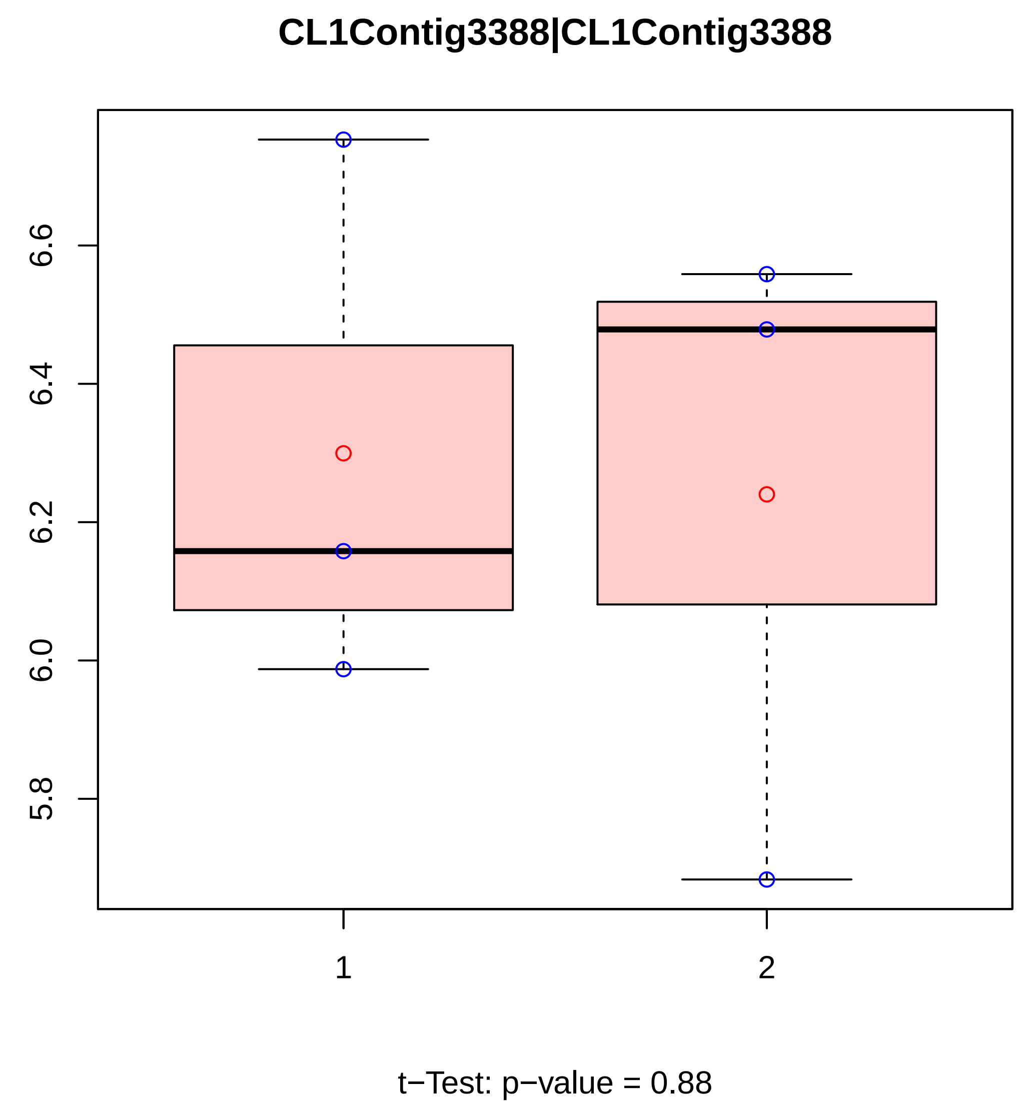
CL1Contig3388|CL1Contig3388
6.6
6.4
6.2
6.0
5.8
1
2
t−Test: p−value = 0.88

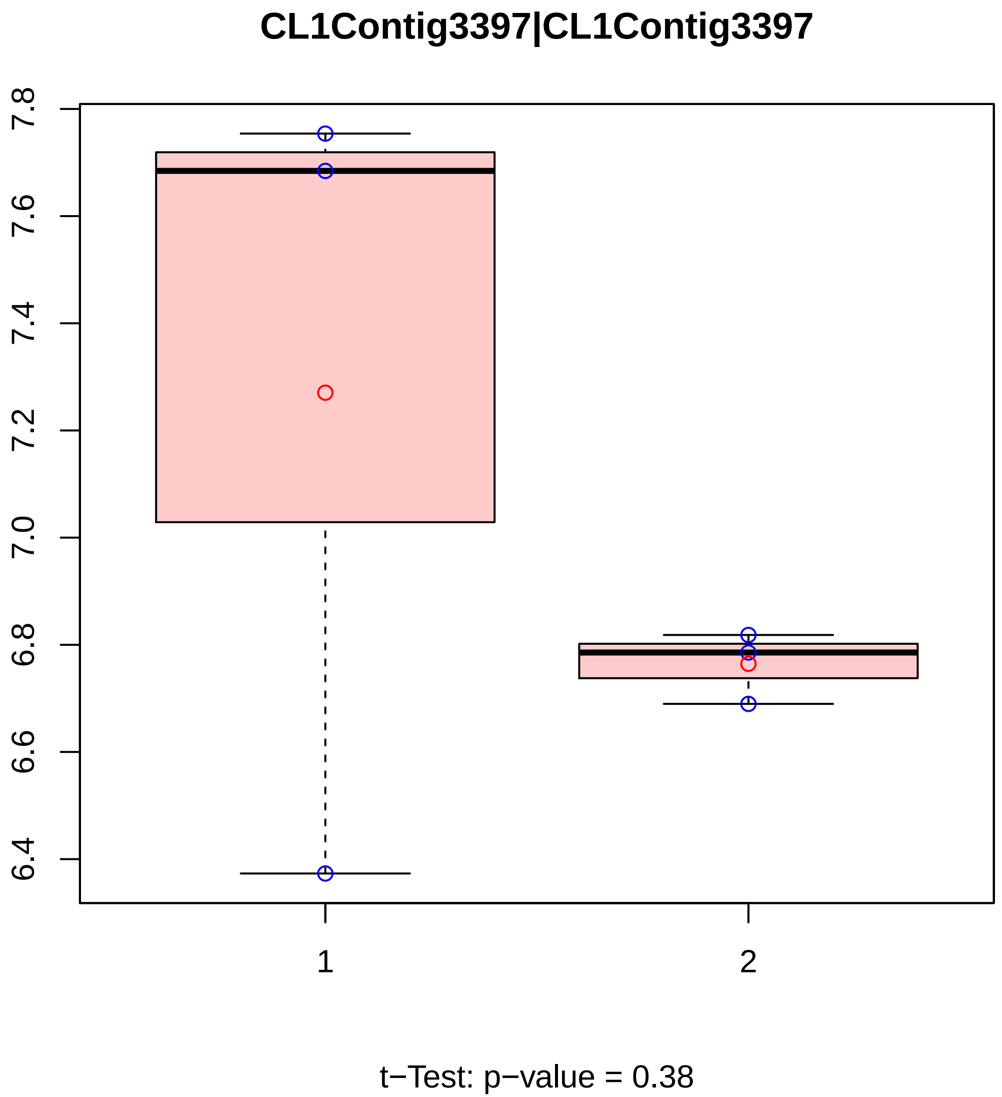
CL1Contig3397|CL1Contig3397
7.8
7.6
7.4
7.2
7.0
6.8
6.6
6.4
1
2
t−Test: p−value = 0.38

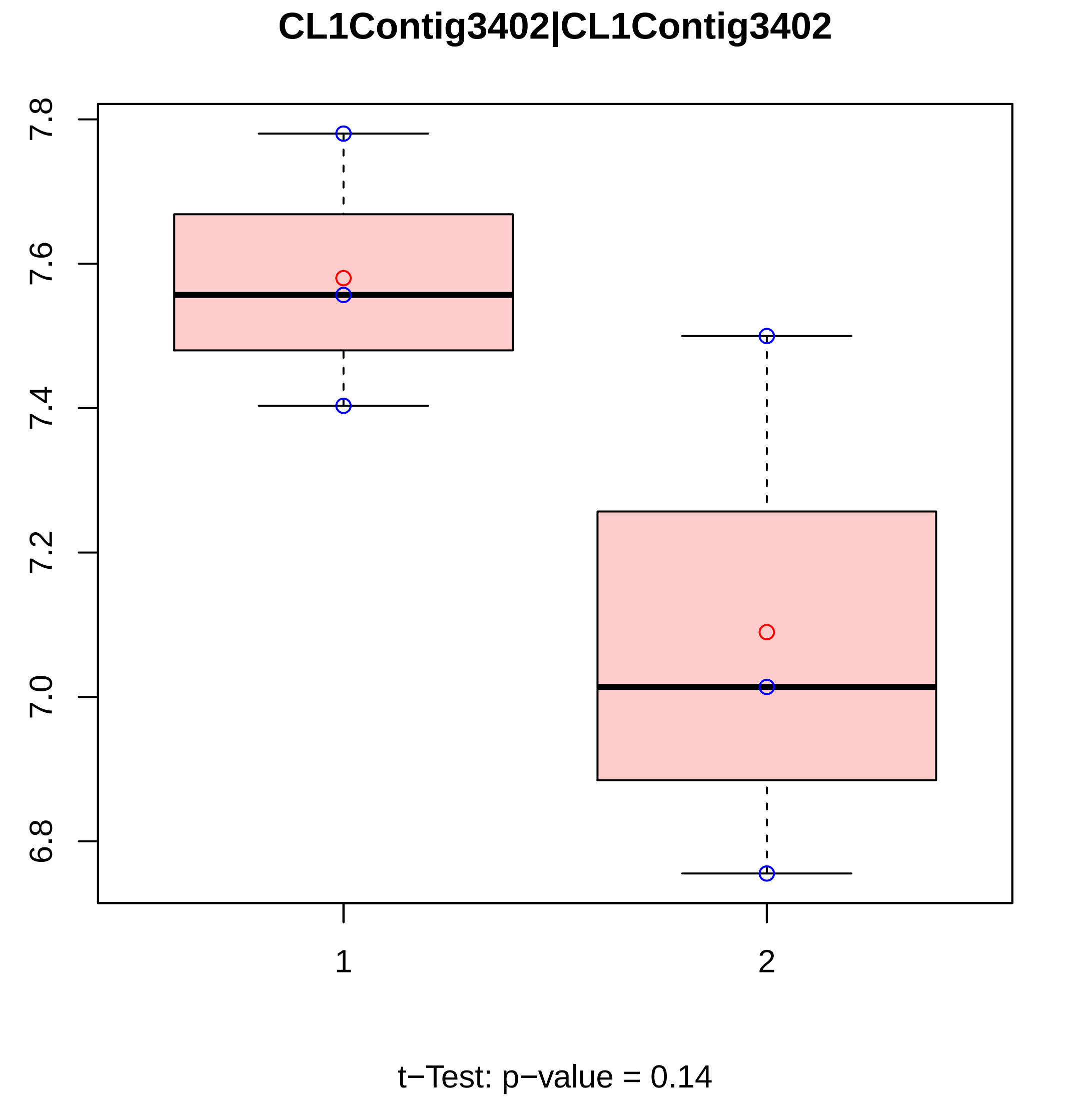
CL1Contig3402|CL1Contig3402
7.8
7.6
7.4
7.2
7.0
6.8
1
2
t−Test: p−value = 0.14

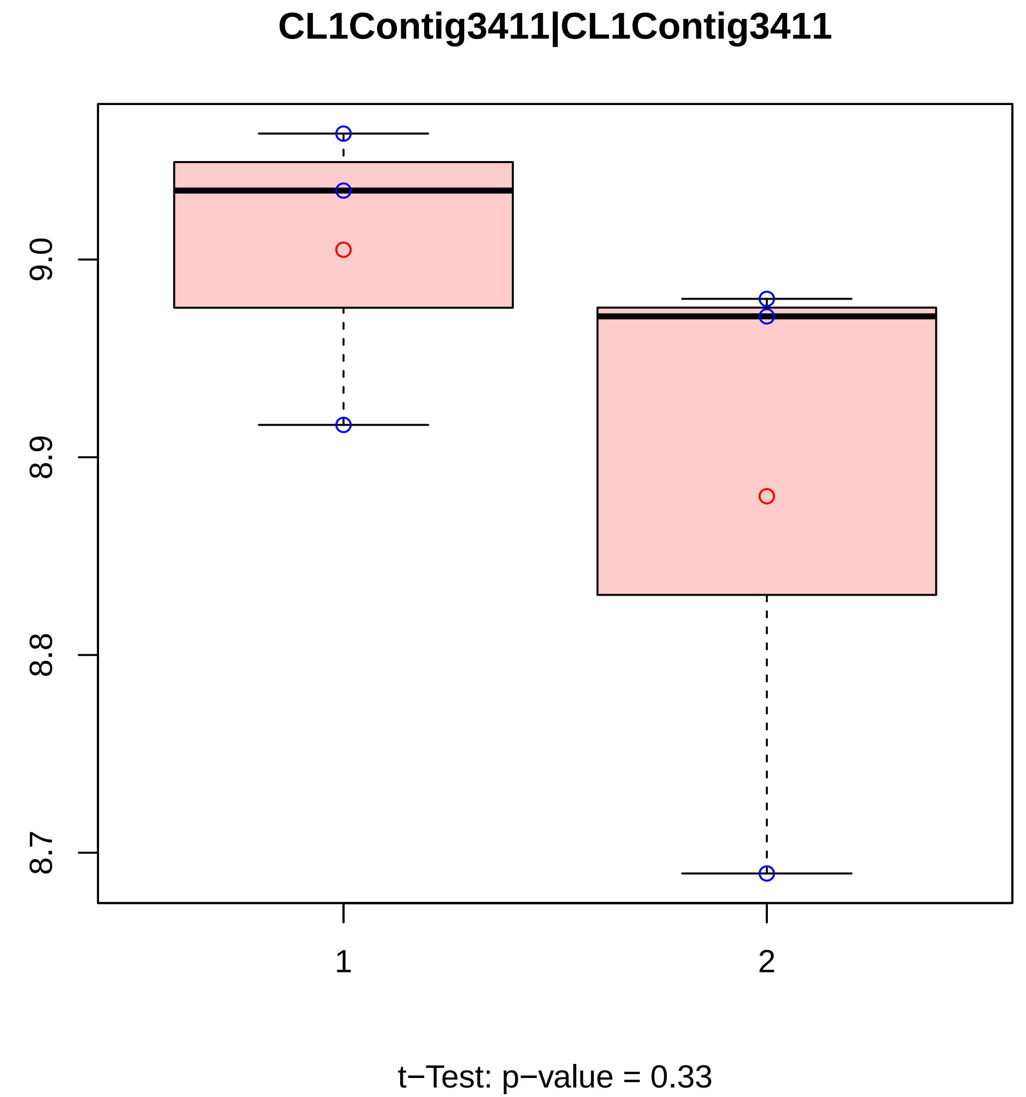

CL1Contig3411|CL1Contig3411
9.0
8.9
8.8
8.7
1
2
t−Test: p−value = 0.33

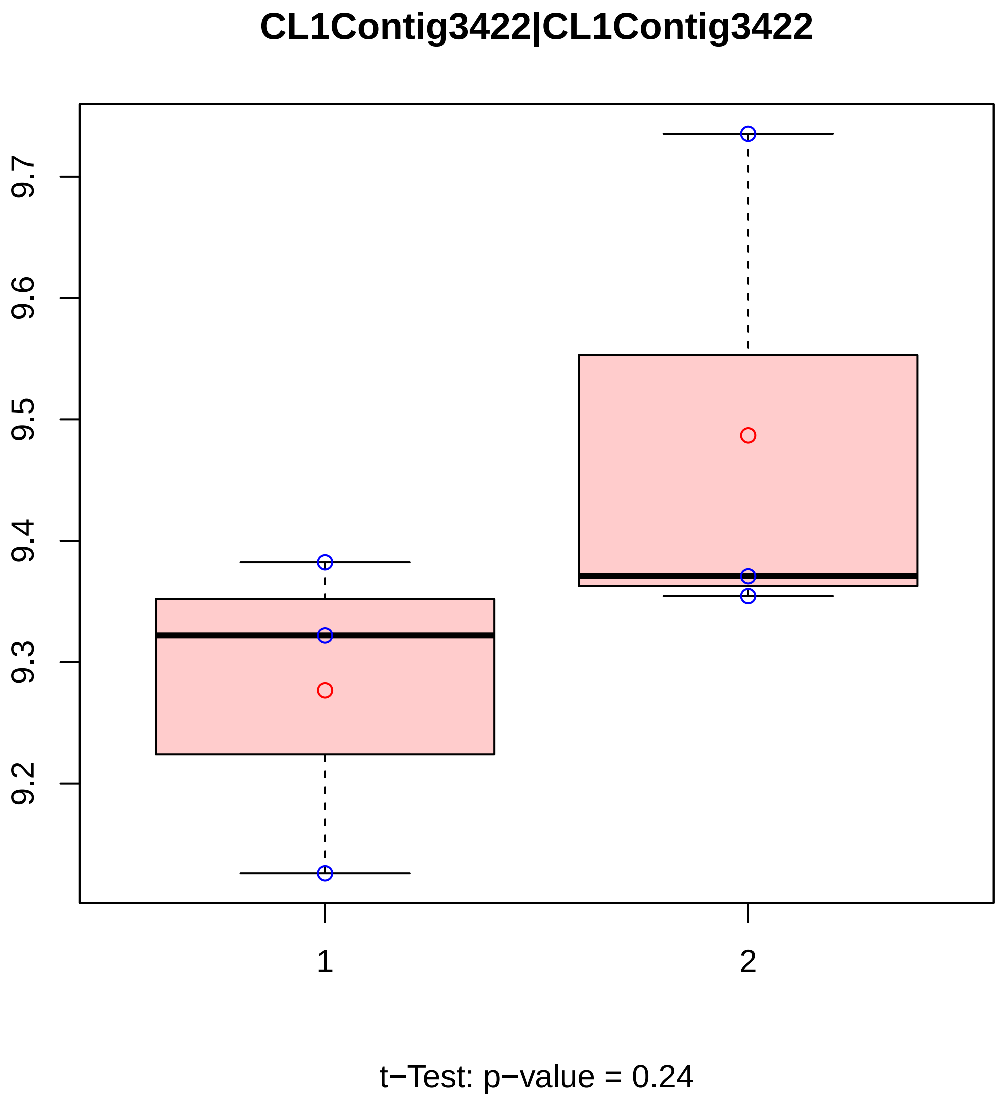
CL1Contig3422|CL1Contig3422
9.2
9.3
9.4
9.5
9.6
9.7
1
2
t−Test: p−value = 0.24

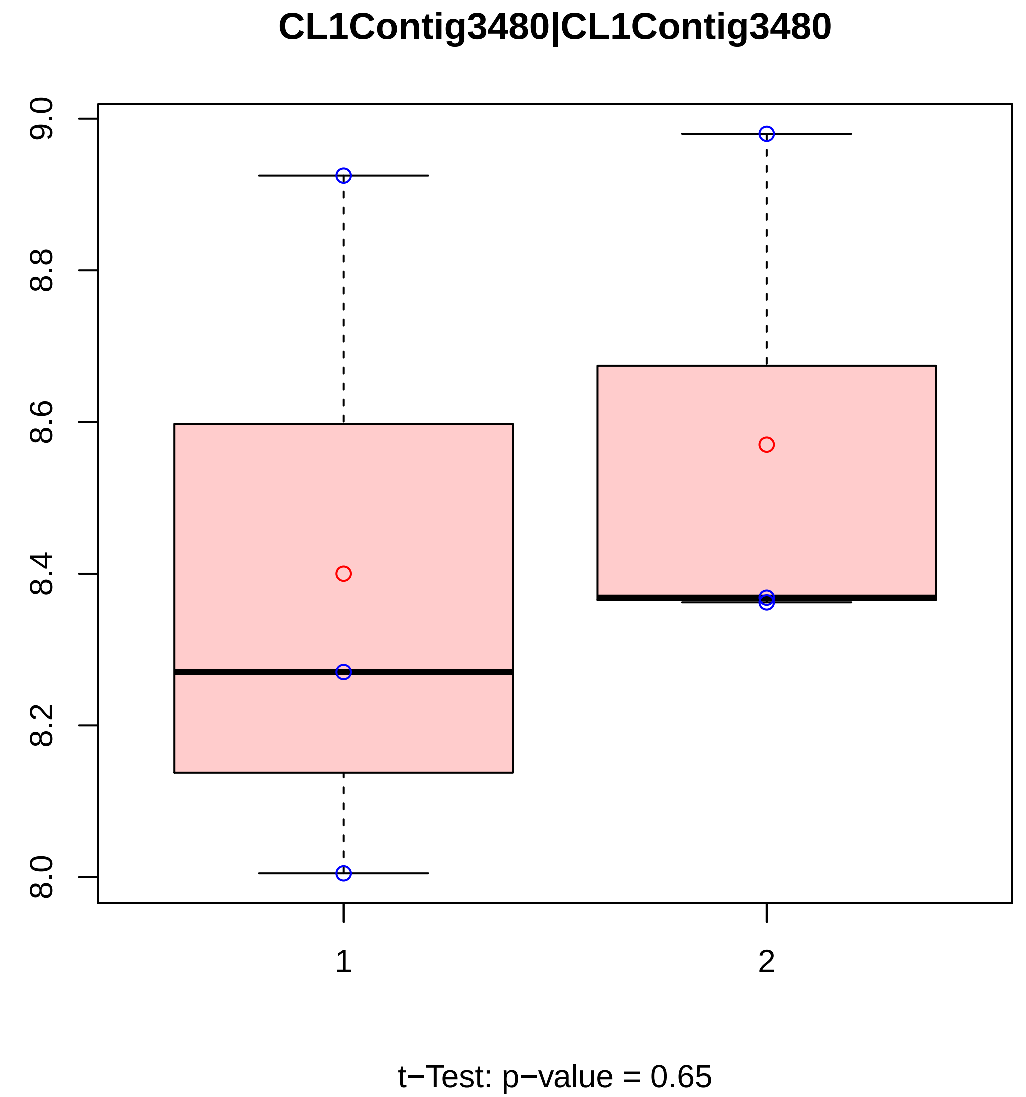
CL1Contig3480|CL1Contig3480
9.0
8.8
8.6
8.4
8.2
8.0
1
2
t−Test: p−value = 0.65

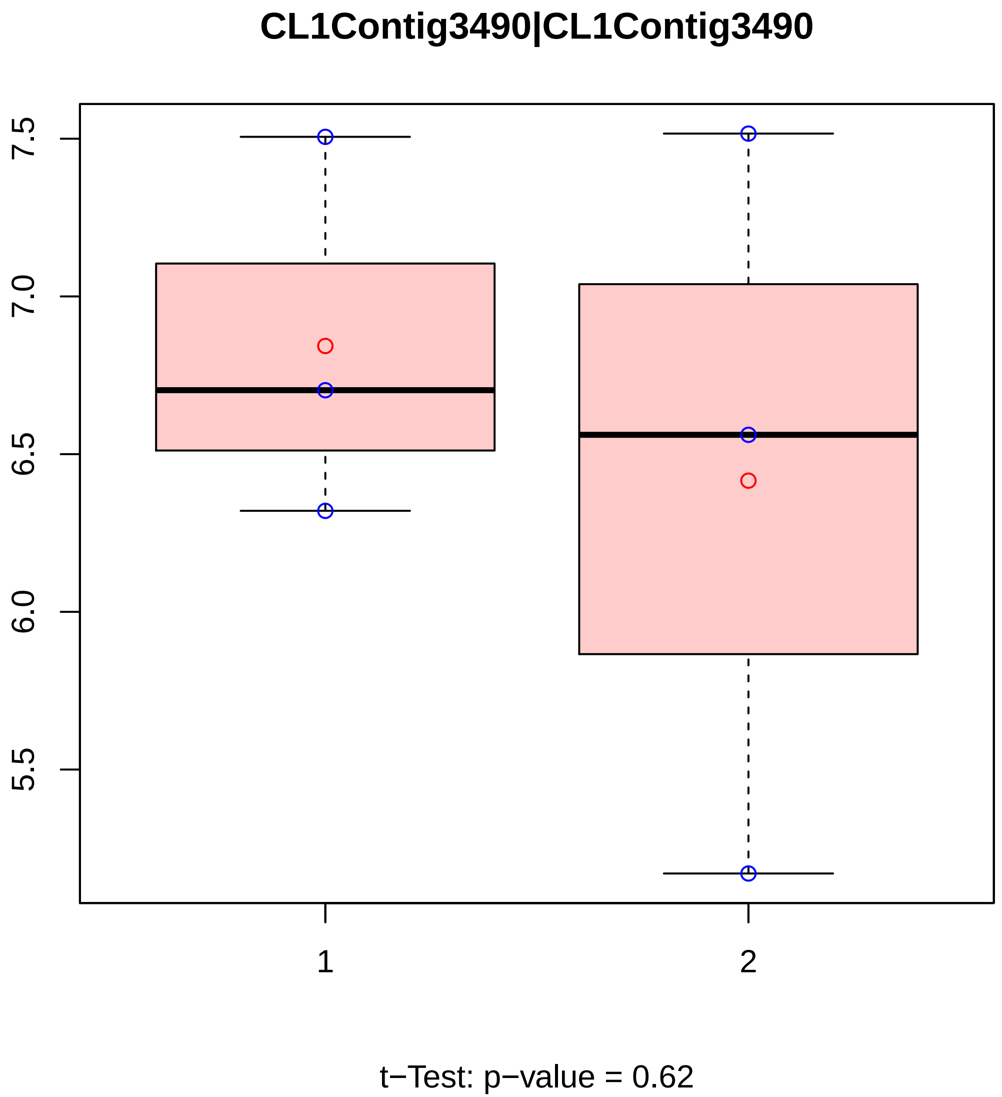
CL1Contig3490|CL1Contig3490
7.5
7.0
6.5
6.0
5.5
1
2
t−Test: p−value = 0.62

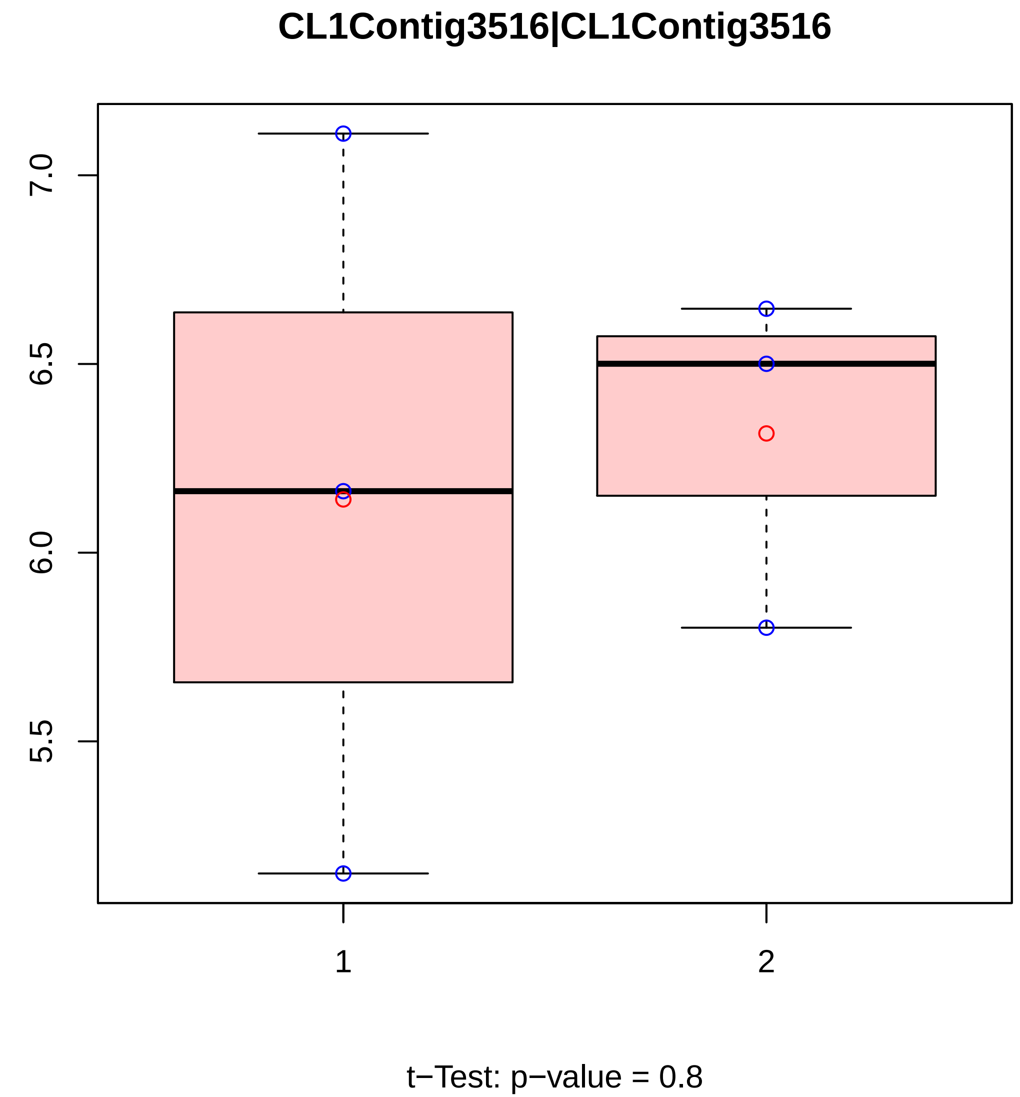
CL1Contig3516|CL1Contig3516
7.0
6.5
6.0
5.5
1
2
t−Test: p−value = 0.8

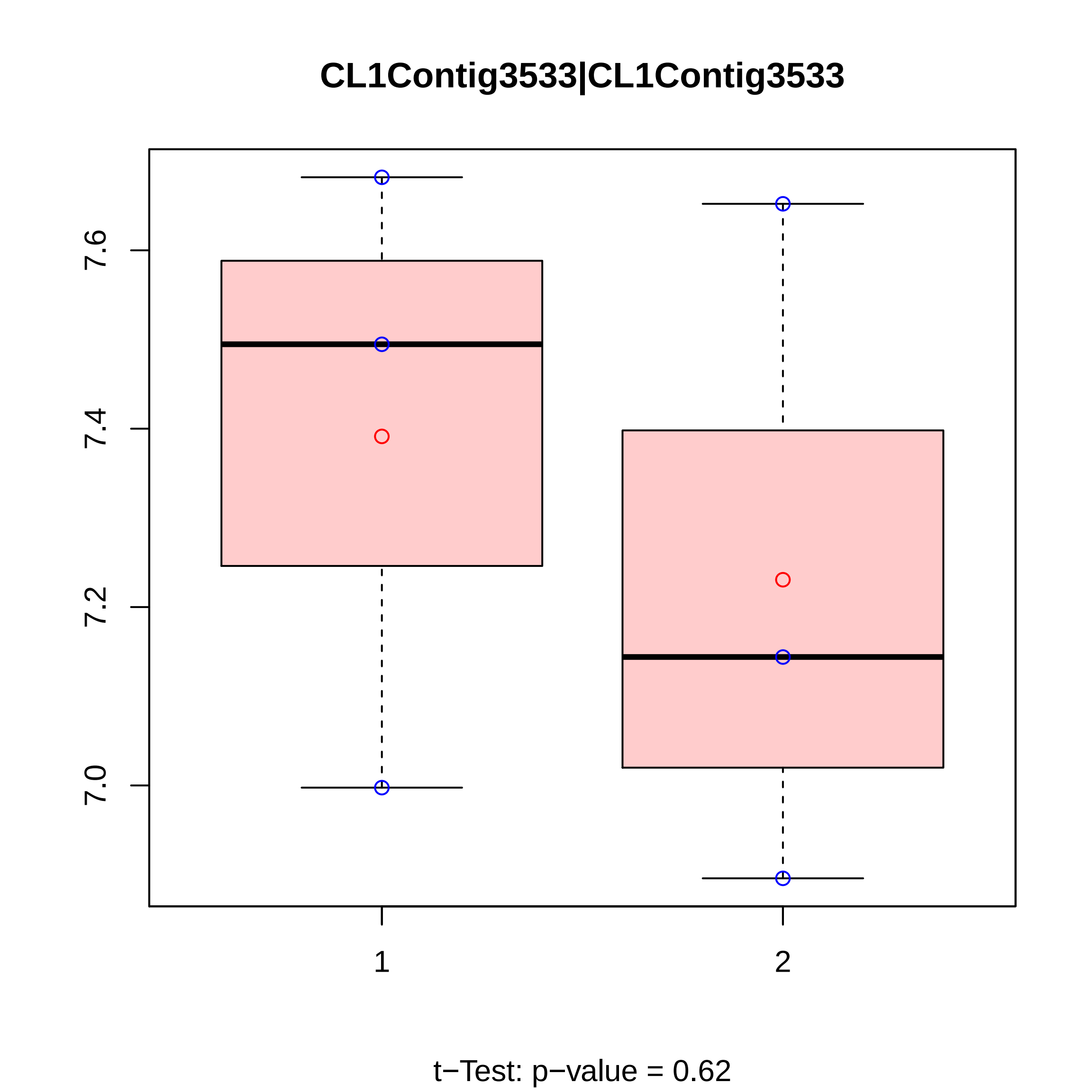

CL1Contig3533|CL1Contig3533
7.0
7.2
7.4
7.6
1
2
t−Test: p−value = 0.62

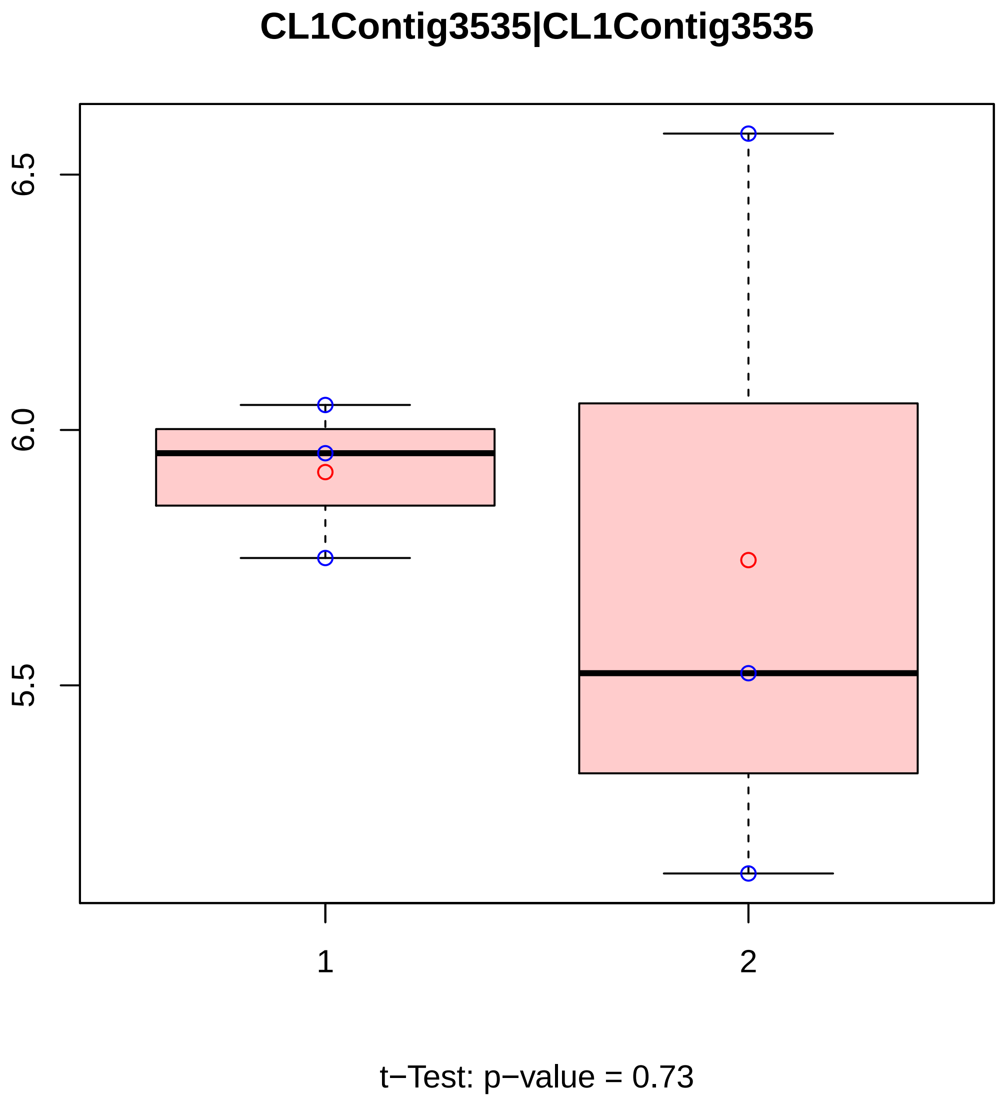

CL1Contig3535|CL1Contig3535
6.5
6.0
5.5
1
2
t−Test: p−value = 0.73

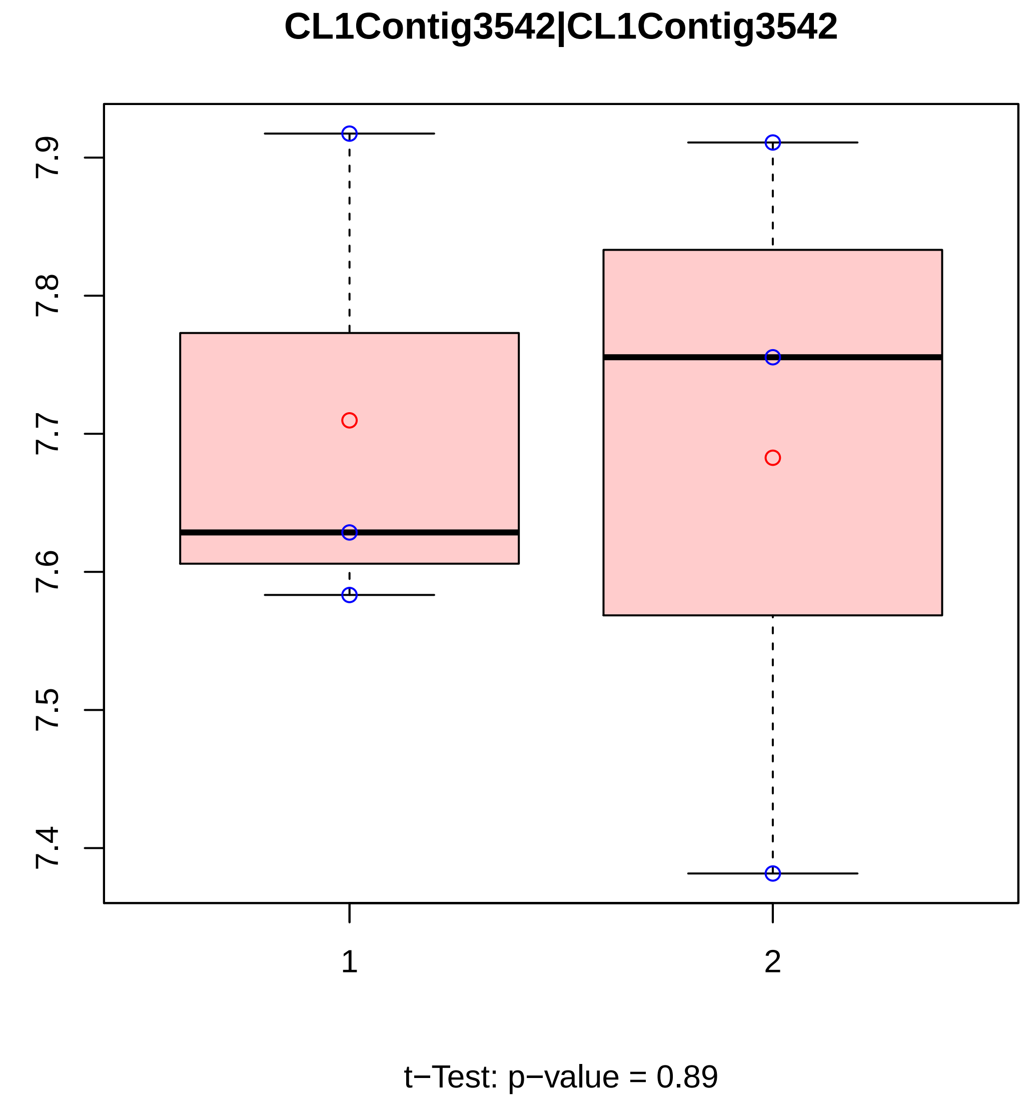
CL1Contig3542|CL1Contig3542
7.9
7.8
7.7
7.6
7.5
7.4
1
2
t−Test: p−value = 0.89

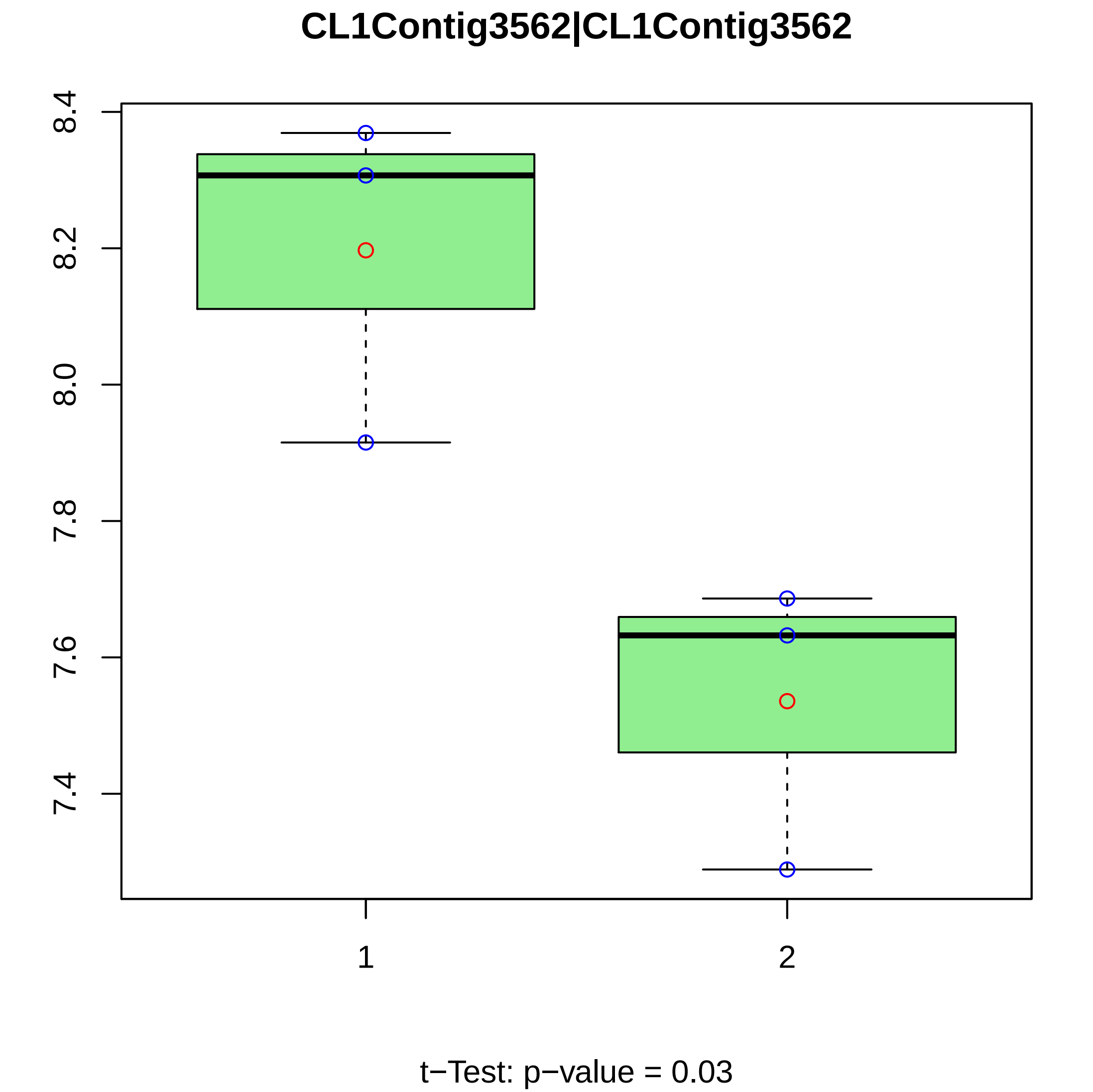
CL1Contig3562|CL1Contig3562
8.4
8.2
8.0
7.8
7.6
7.4
1
2
t−Test: p−value = 0.03

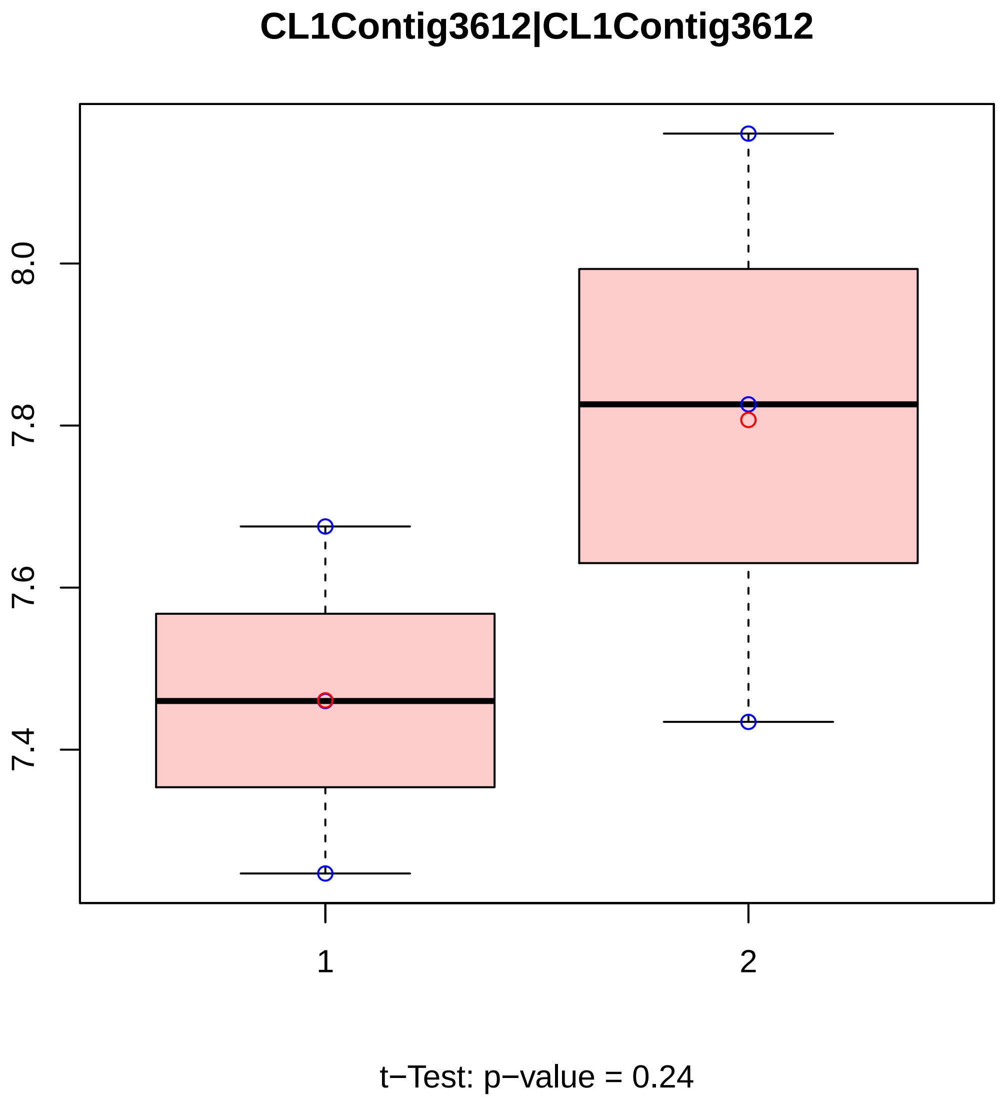
CL1Contig3612|CL1Contig3612
8.0
7.8
7.6
7.4
1
2
t−Test: p−value = 0.24

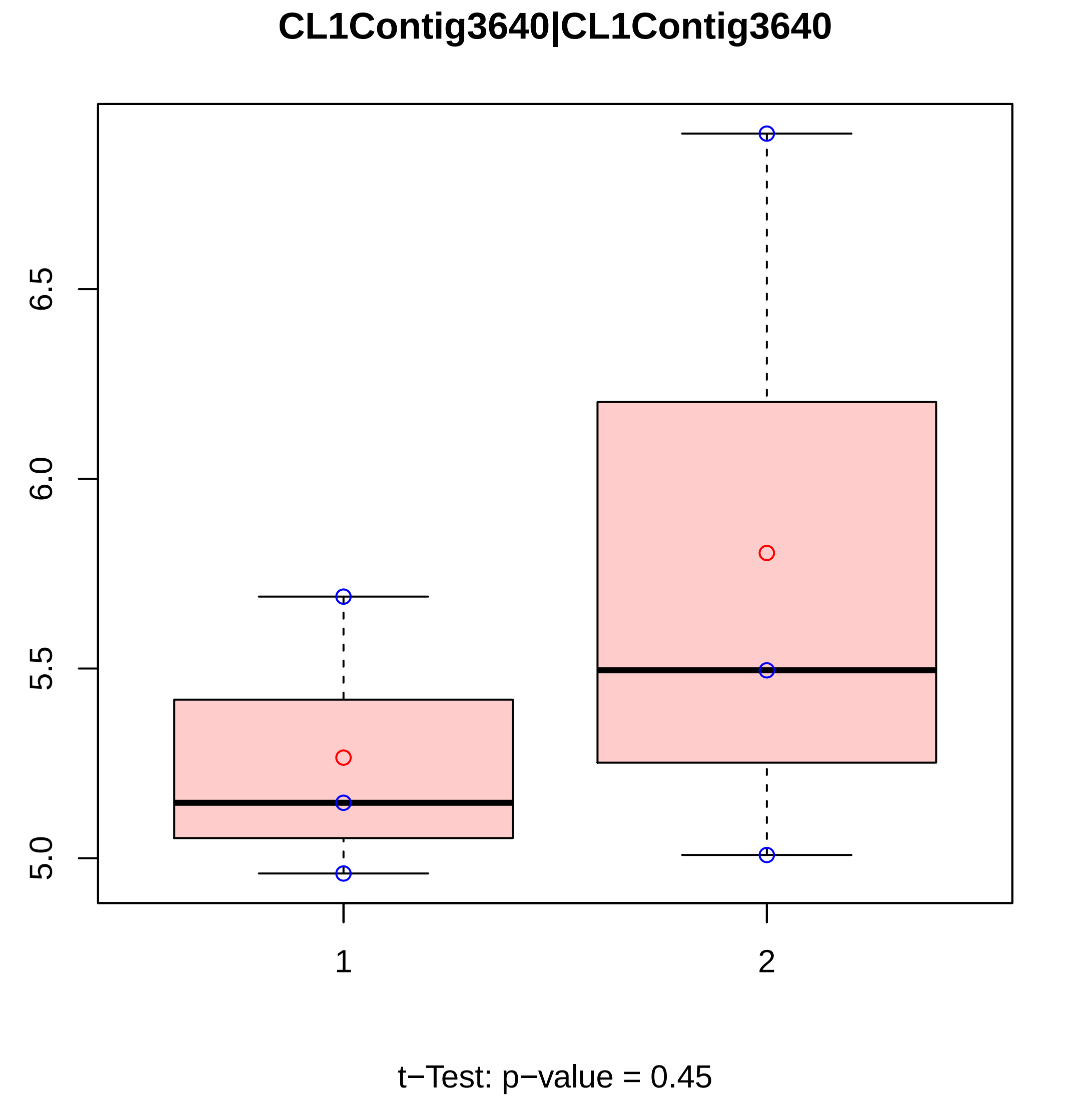
CL1Contig3640|CL1Contig3640
5.0
5.5
6.0
6.5
1
2
t−Test: p−value = 0.45

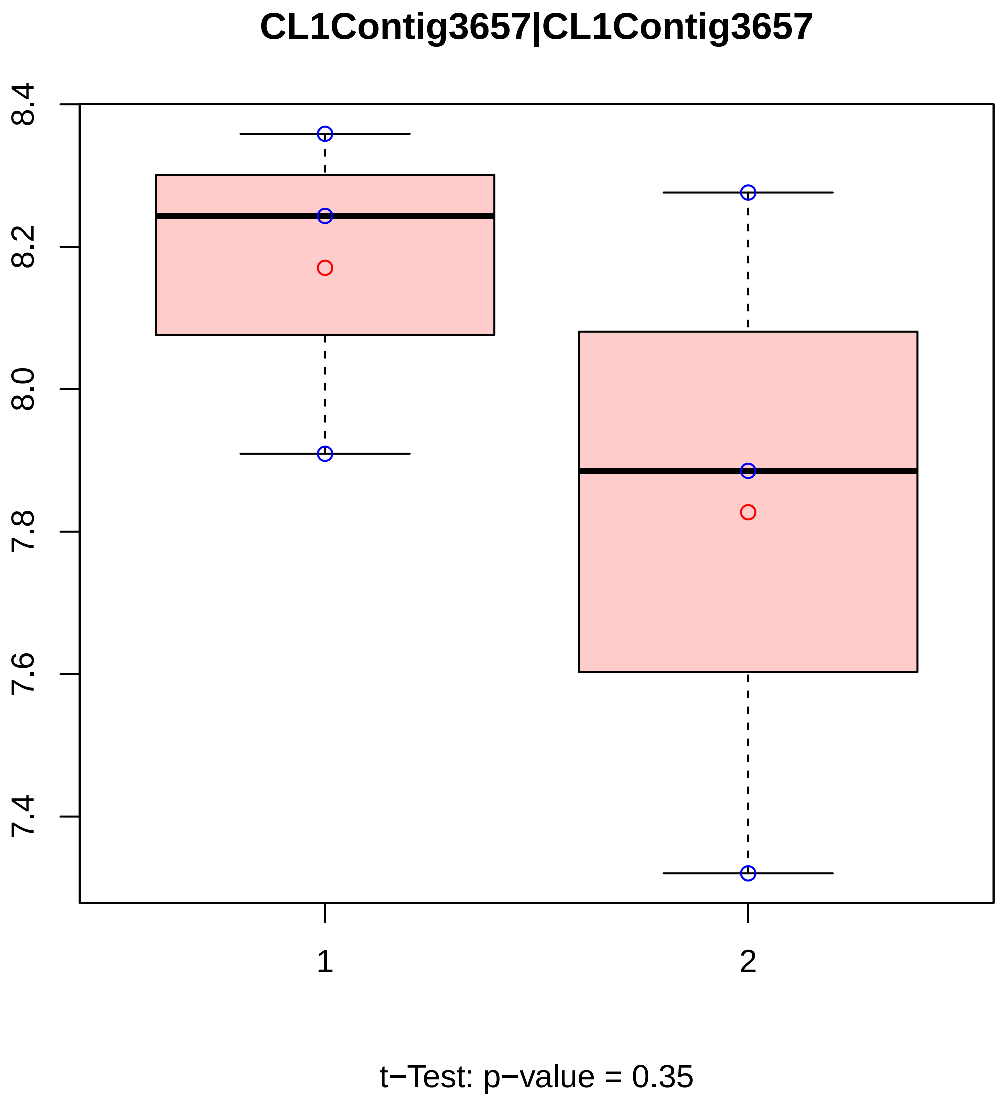
CL1Contig3657|CL1Contig3657
8.4
8.2
8.0
7.8
7.6
7.4
1
2
t−Test: p−value = 0.35

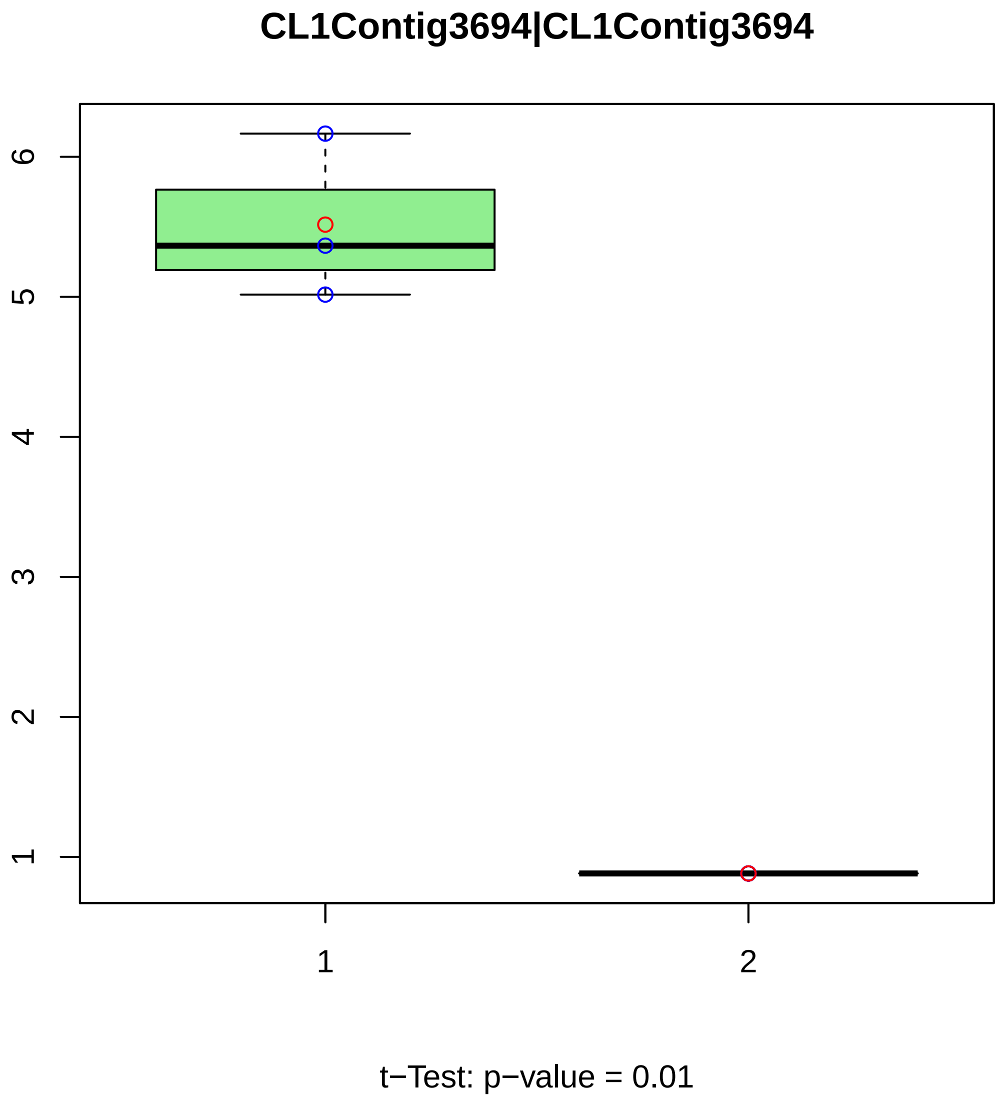

CL1Contig3694|CL1Contig3694
6
5
4
3
2
1
1
2
t−Test: p−value = 0.01

# CL1Contig36|CL1Contig36

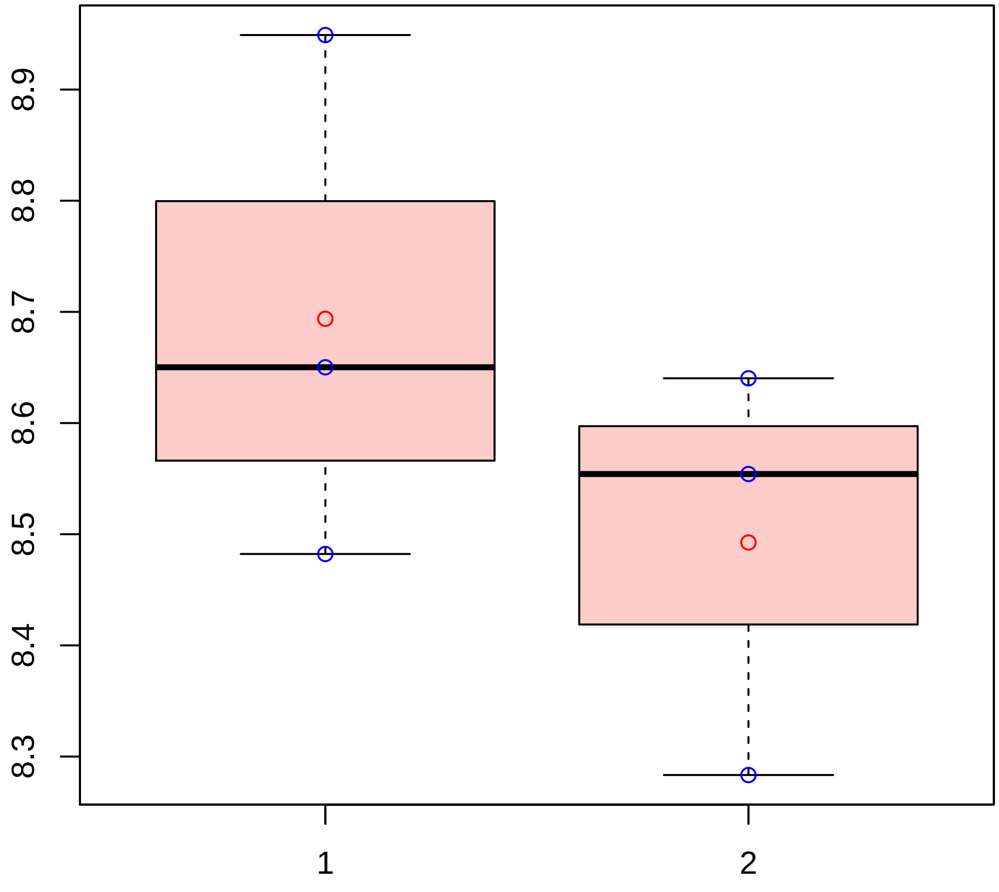


t−Test: p−value = 0.31

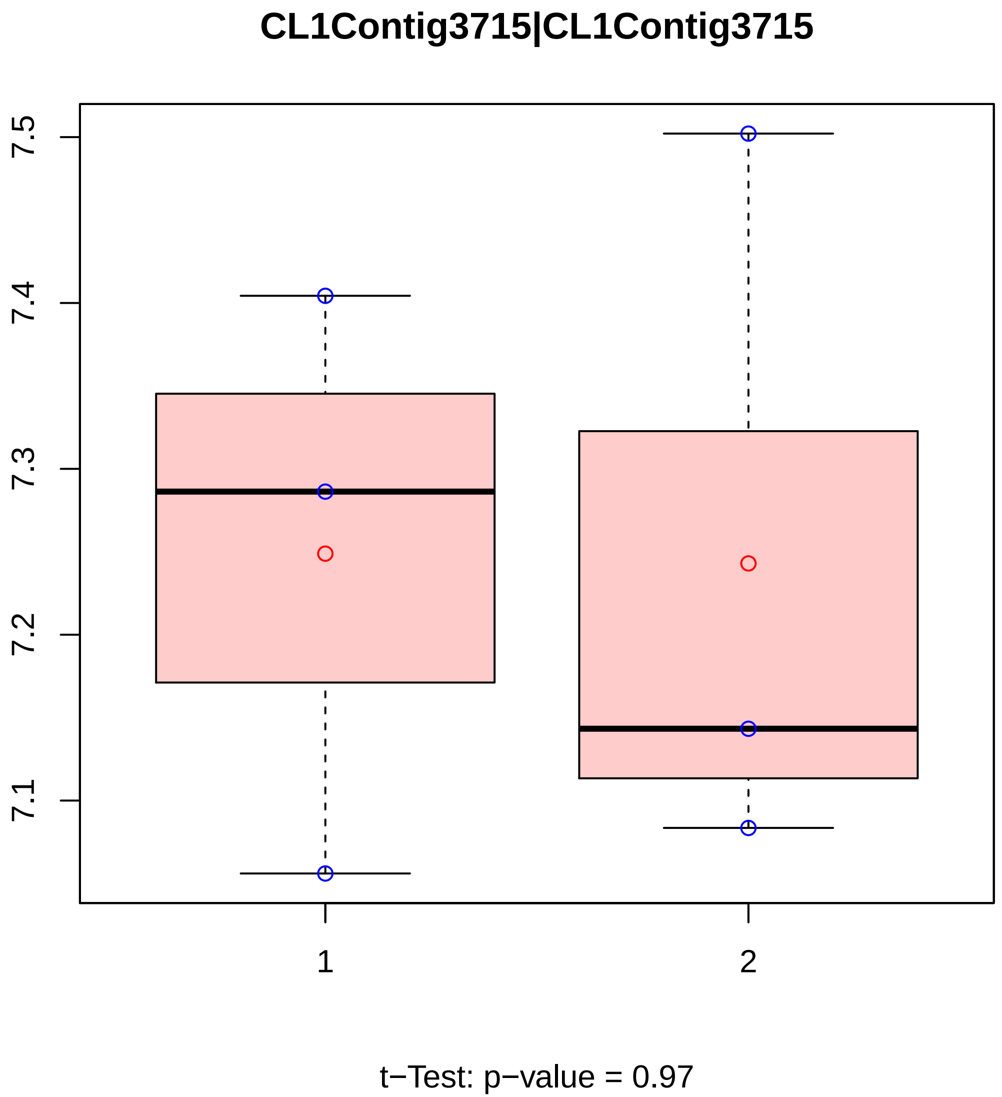
CL1Contig3715|CL1Contig3715
7.5
7.4
7.3
7.2
7.1
1
2
t−Test: p−value = 0.97

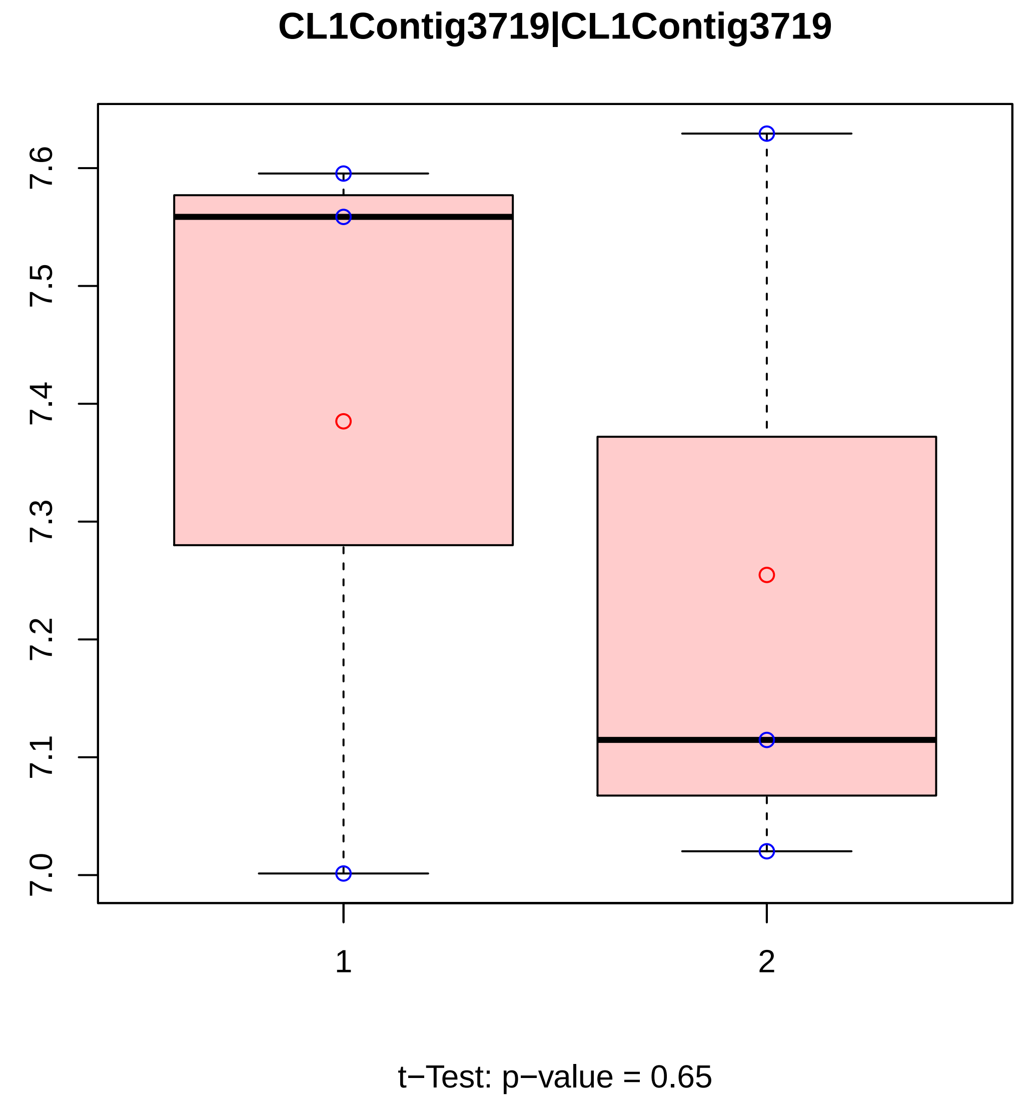
CL1Contig3719|CL1Contig3719
7.0
7.1
7.2
7.3
7.4
7.5
7.6
1
2
t−Test: p−value = 0.65

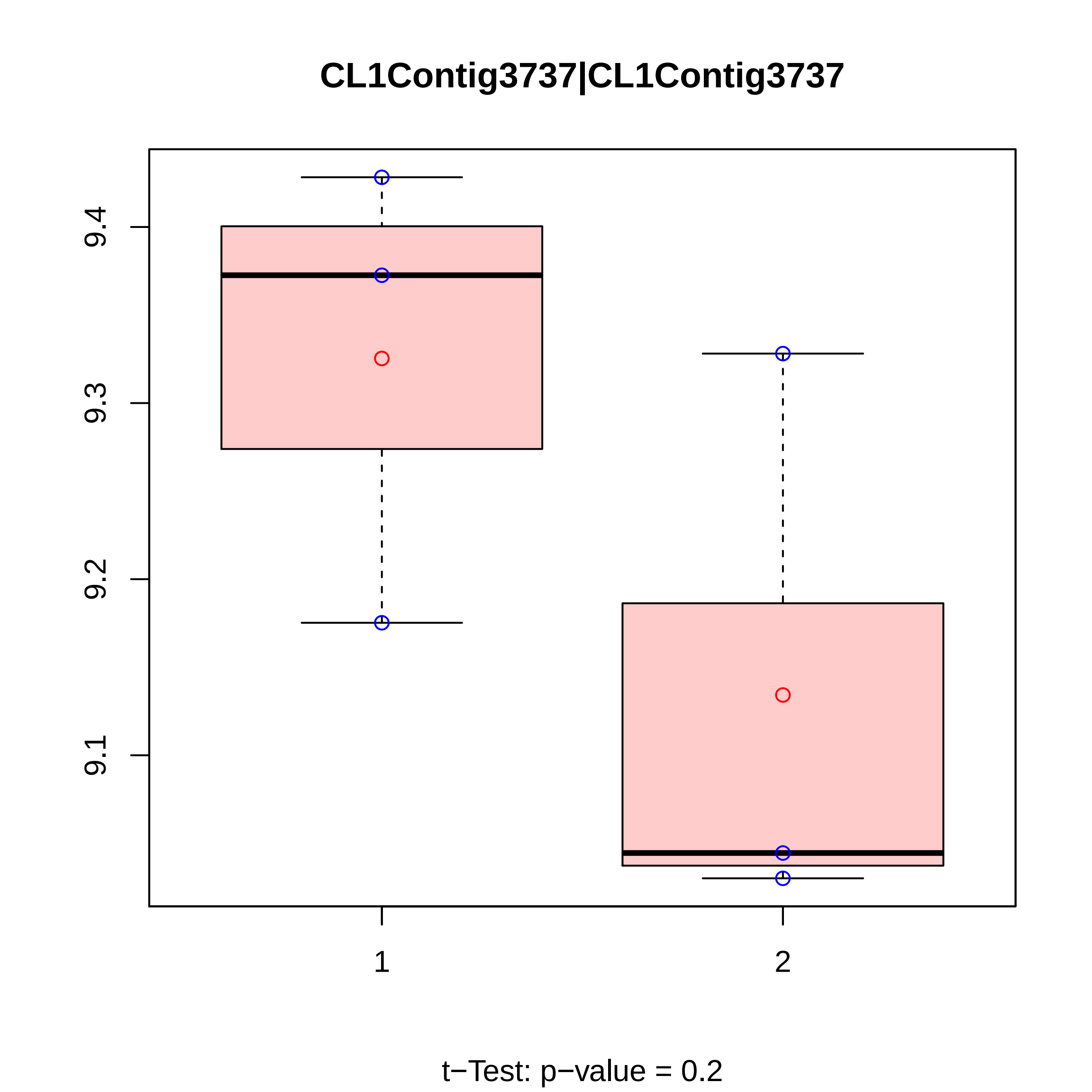
CL1Contig3737|CL1Contig3737
9.4
9.3
9.2
9.1
1
2
t−Test: p−value = 0.2

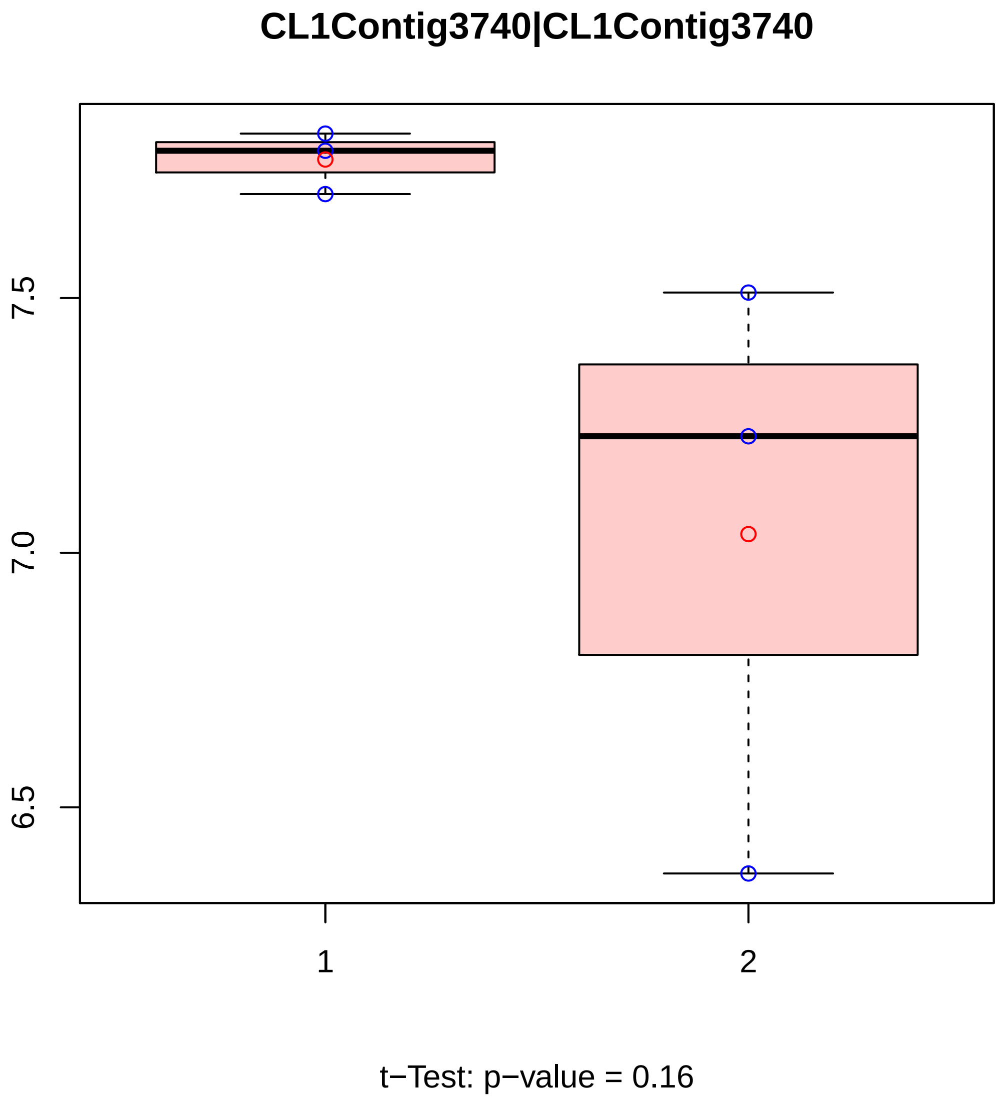
CL1Contig3740|CL1Contig3740
7.5
7.0
6.5
1
2
t−Test: p−value = 0.16

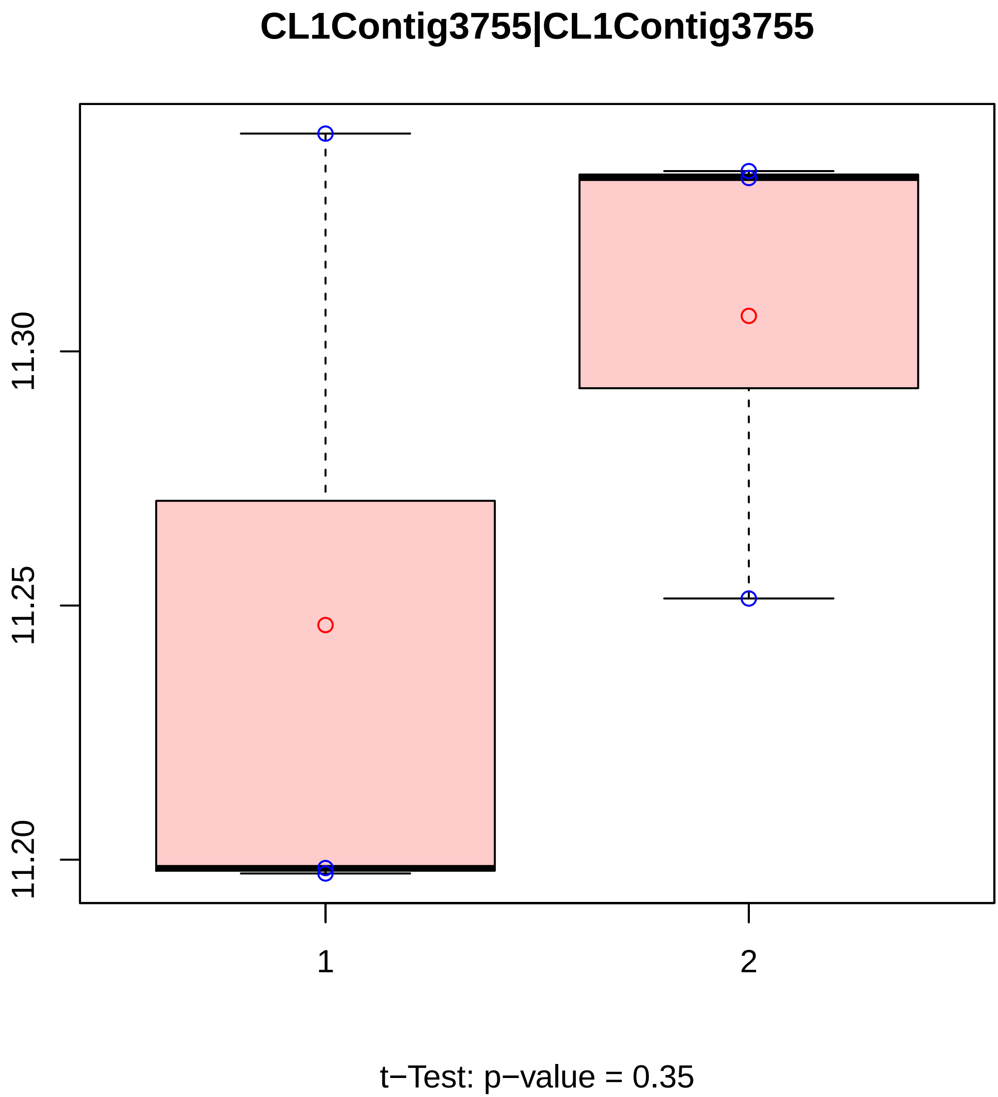
CL1Contig3755|CL1Contig3755
11.20
11.25
11.30
1
2
t−Test: p−value = 0.35

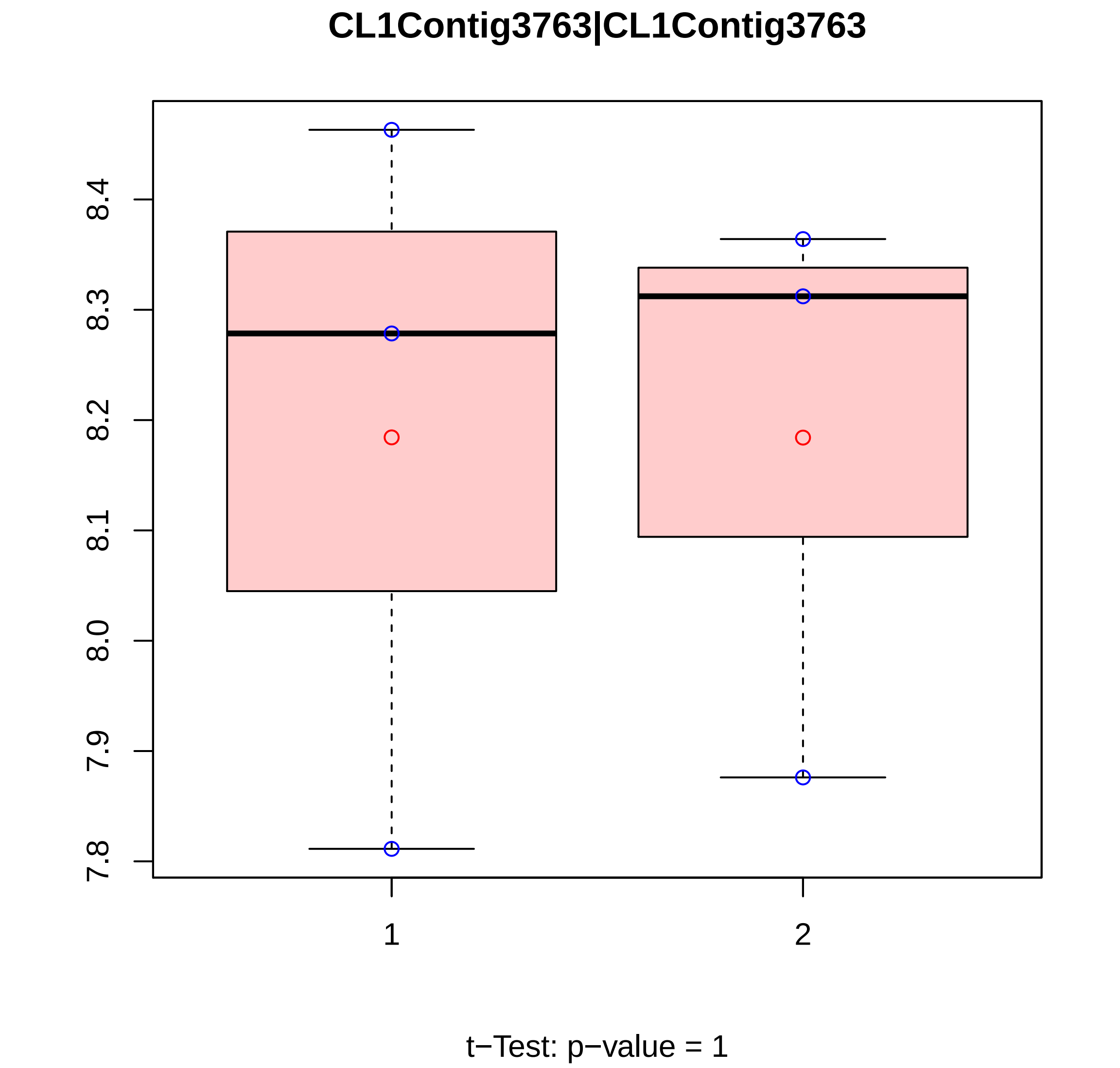
CL1Contig3763|CL1Contig3763
7.8
7.9
8.0
8.1
8.2
8.3
8.4
1
2
t−Test: p−value = 1

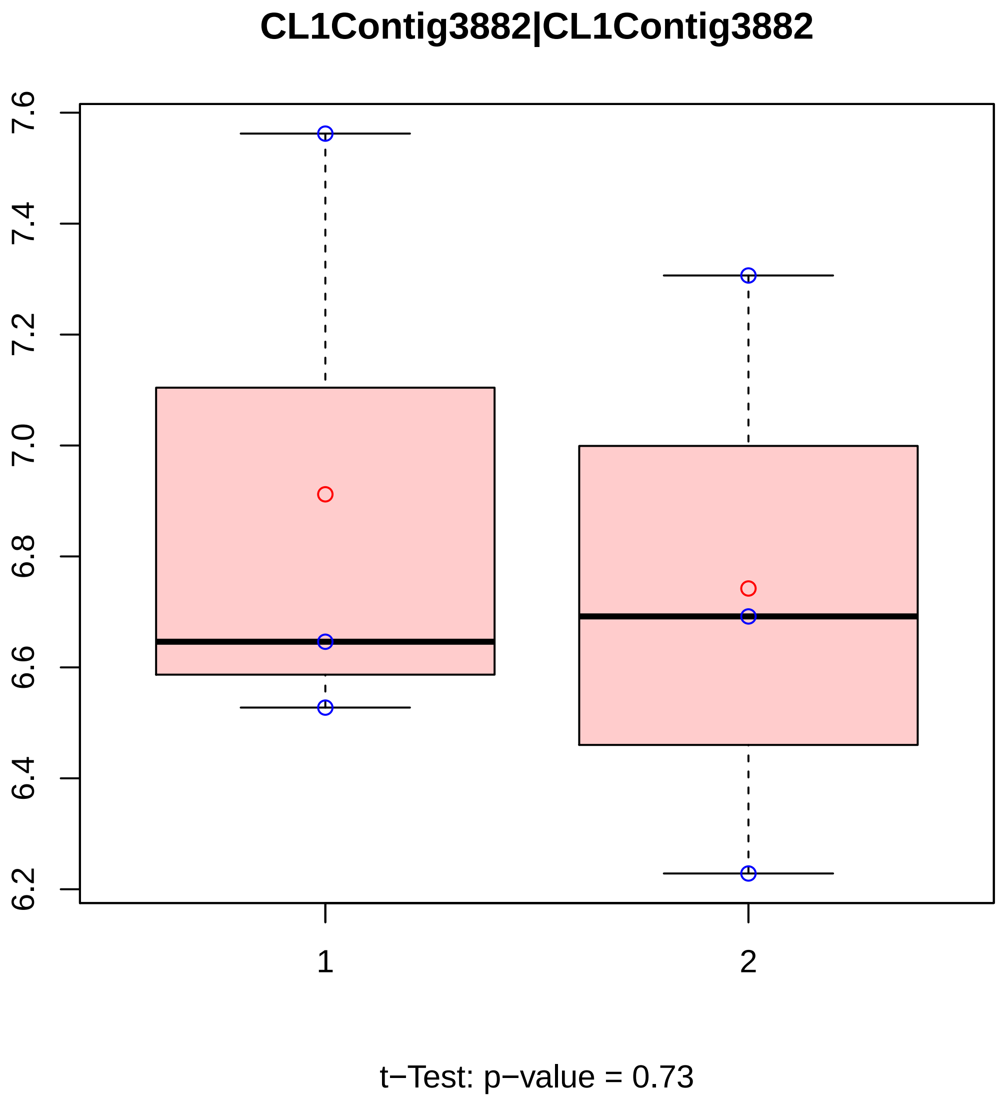
CL1Contig3882|CL1Contig3882
7.6
7.4
7.2
7.0
6.8
6.6
6.4
6.2
1
2
t−Test: p−value = 0.73

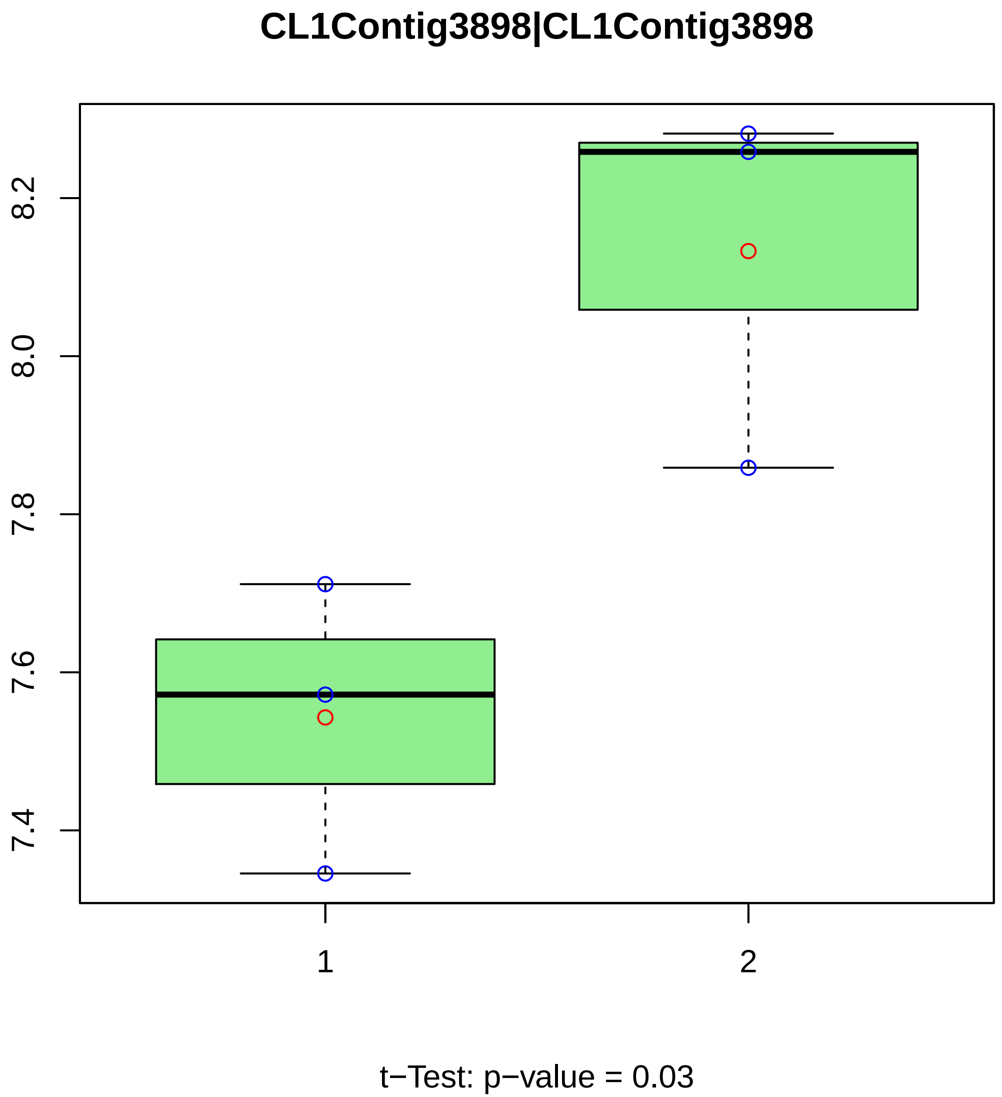

CL1Contig3898|CL1Contig3898
8.2
8.0
7.8
7.6
7.4
1
2
t−Test: p−value = 0.03

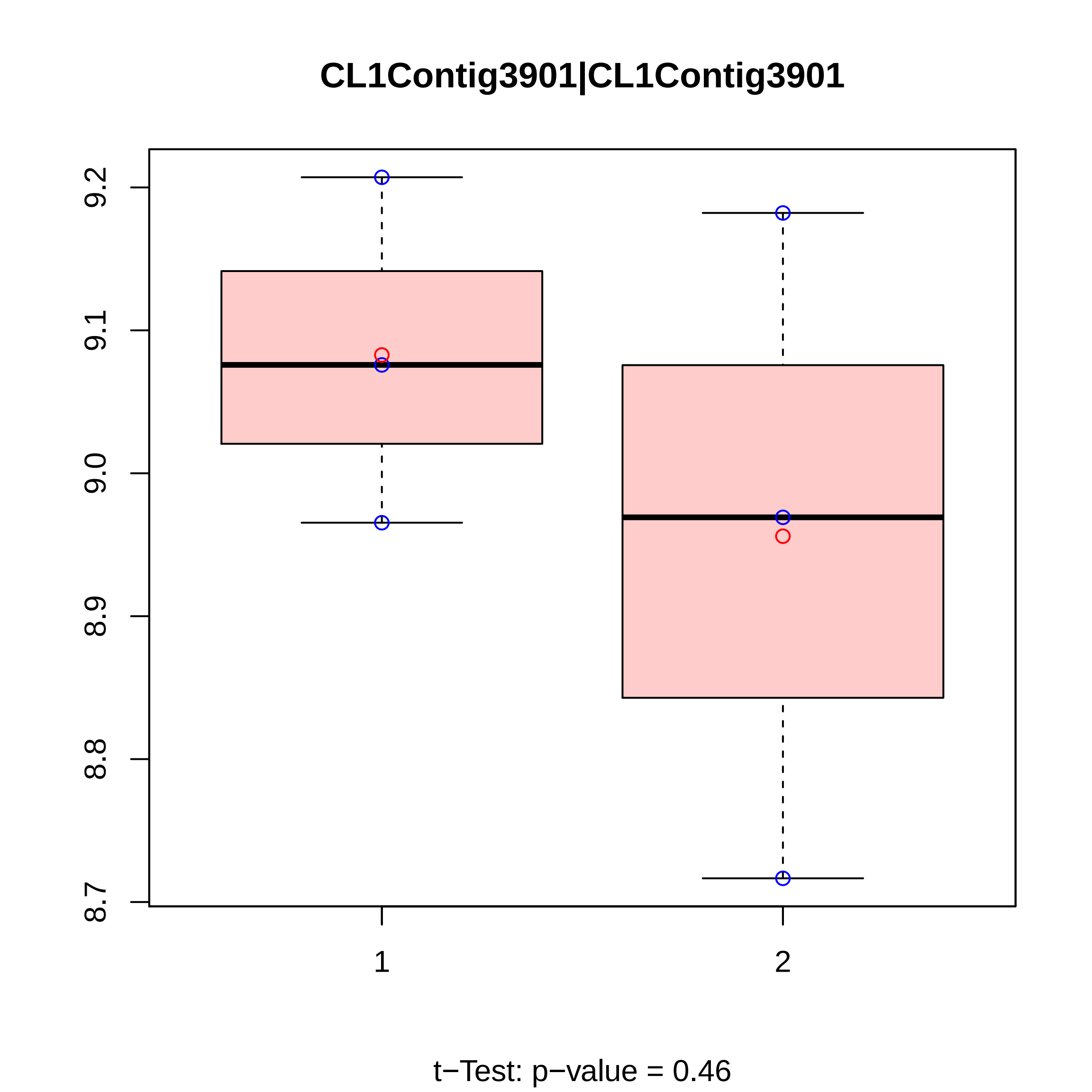
CL1Contig3901|CL1Contig3901
9.2
9.1
9.0
8.9
8.8
8.7
1
2
t−Test: p−value = 0.46

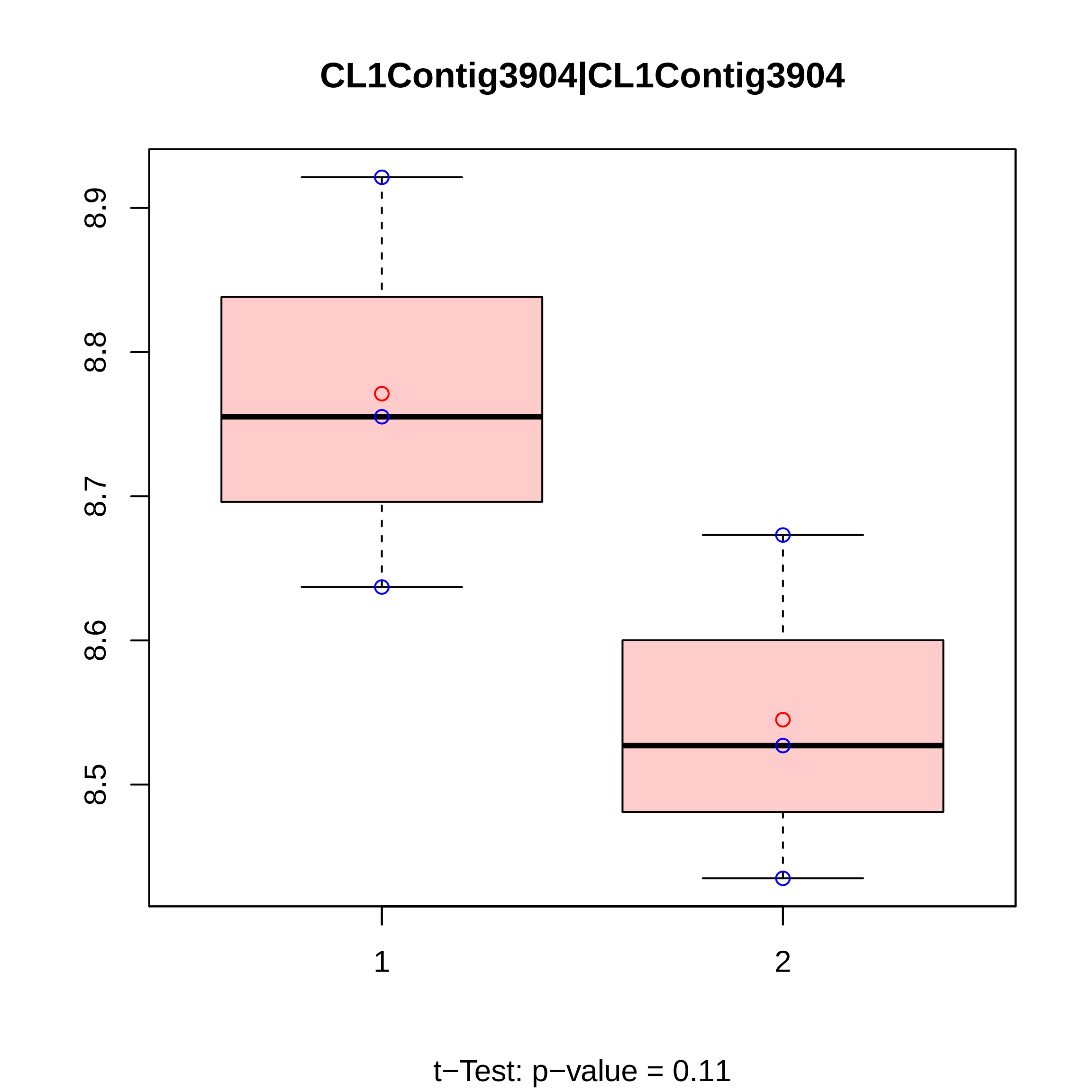
CL1Contig3904|CL1Contig3904
8.5
8.6
8.7
8.8
8.9
1
2
t−Test: p−value = 0.11

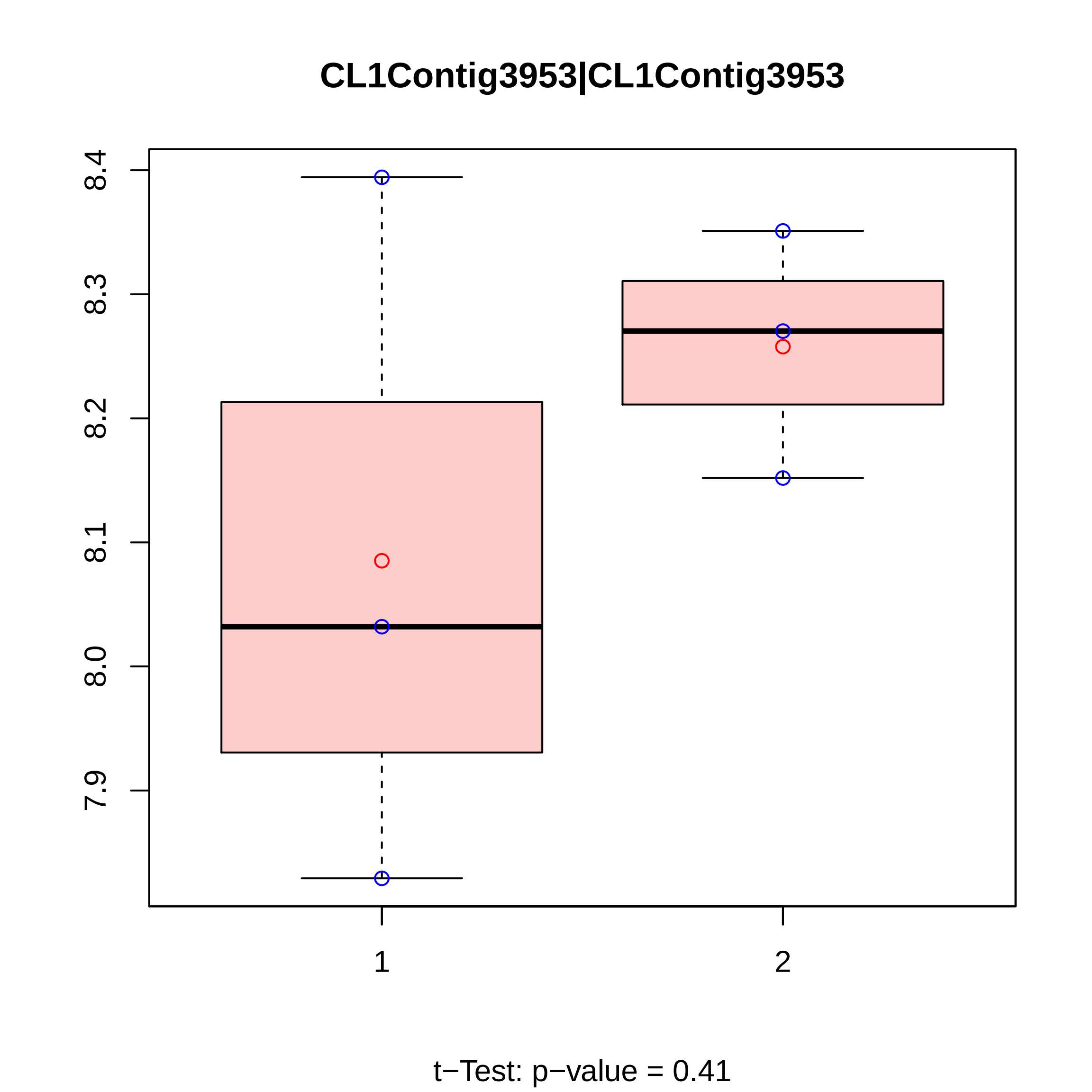
CL1Contig3953|CL1Contig3953
8.4
8.3
8.2
8.1
8.0
7.9
1
2
t−Test: p−value = 0.41

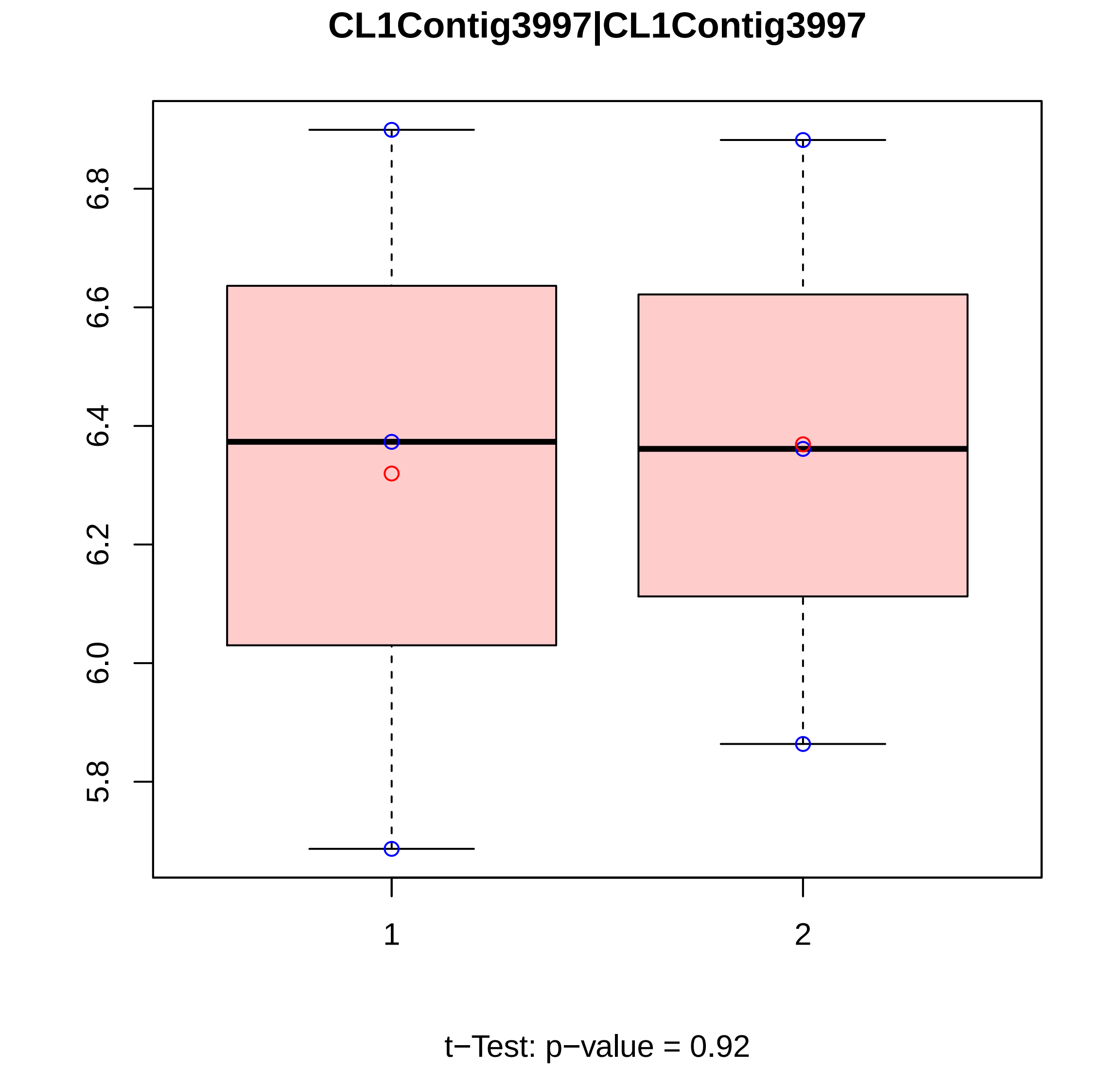
CL1Contig3997|CL1Contig3997
6.8
6.6
6.4
6.2
6.0
5.8
1
2
t−Test: p−value = 0.92

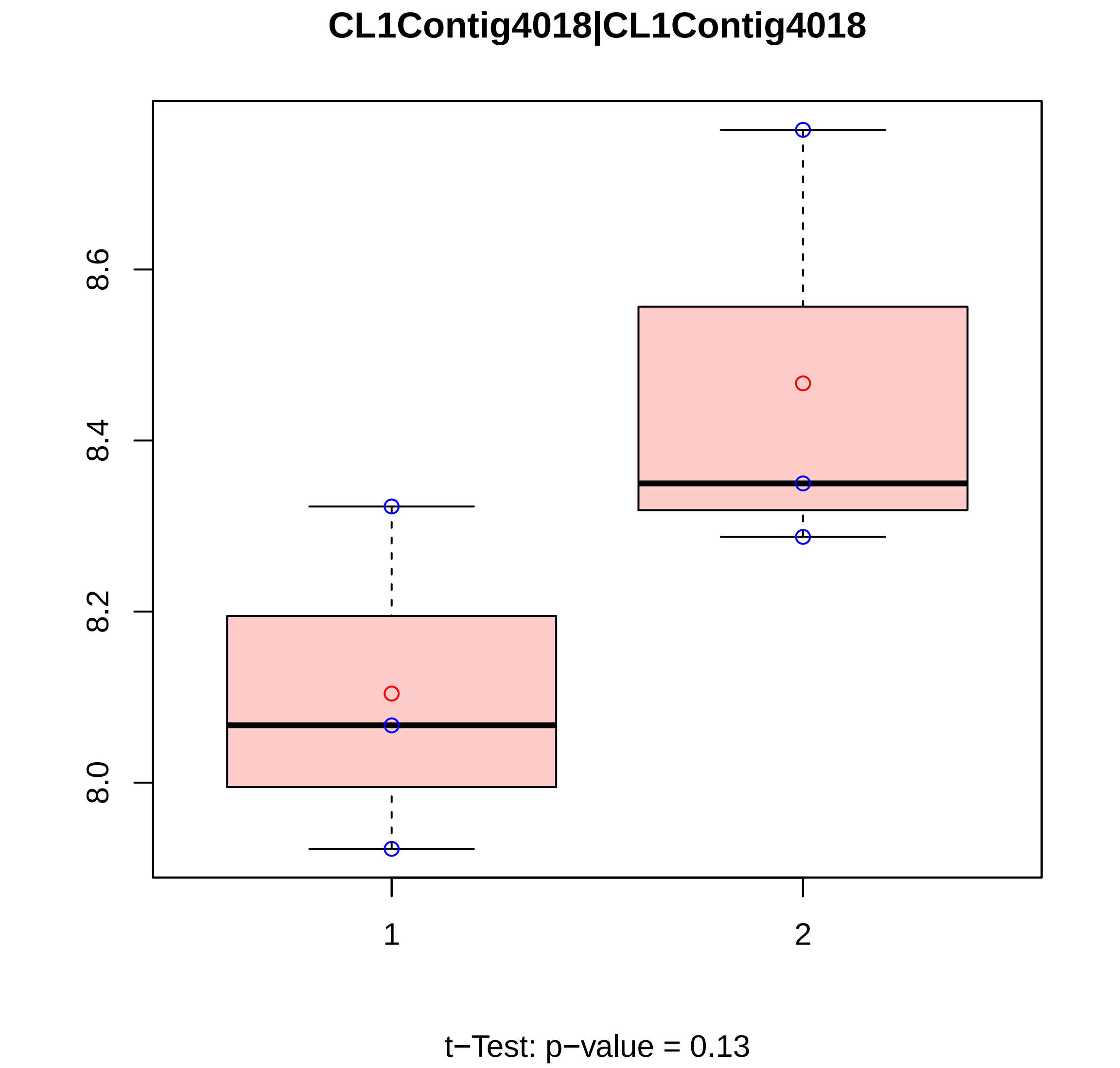
CL1Contig4018|CL1Contig4018
8.0
8.2
8.4
8.6
1
2
t−Test: p−value = 0.13

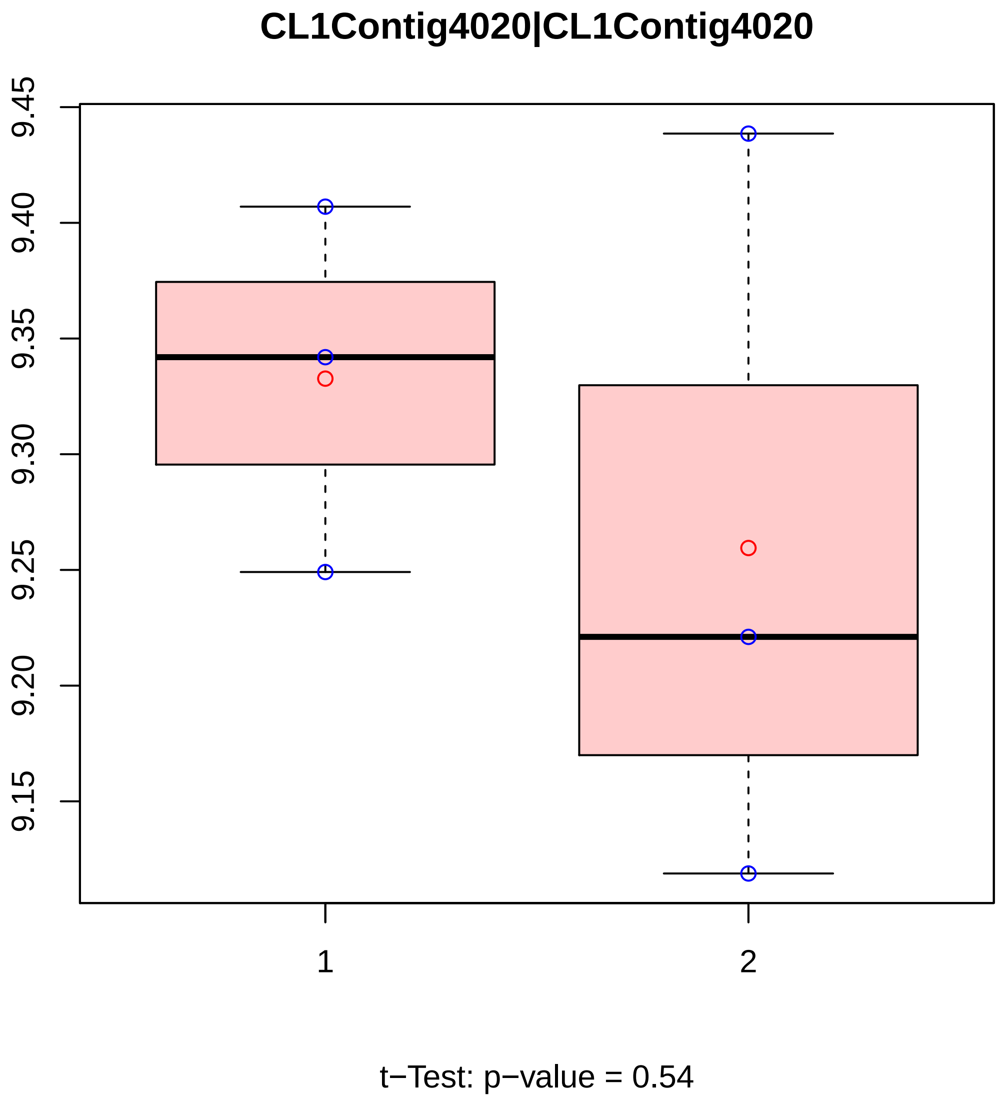
CL1Contig4020|CL1Contig4020
9.45
9.40
9.35
9.30
9.25
9.20
9.15
1
2
t−Test: p−value = 0.54

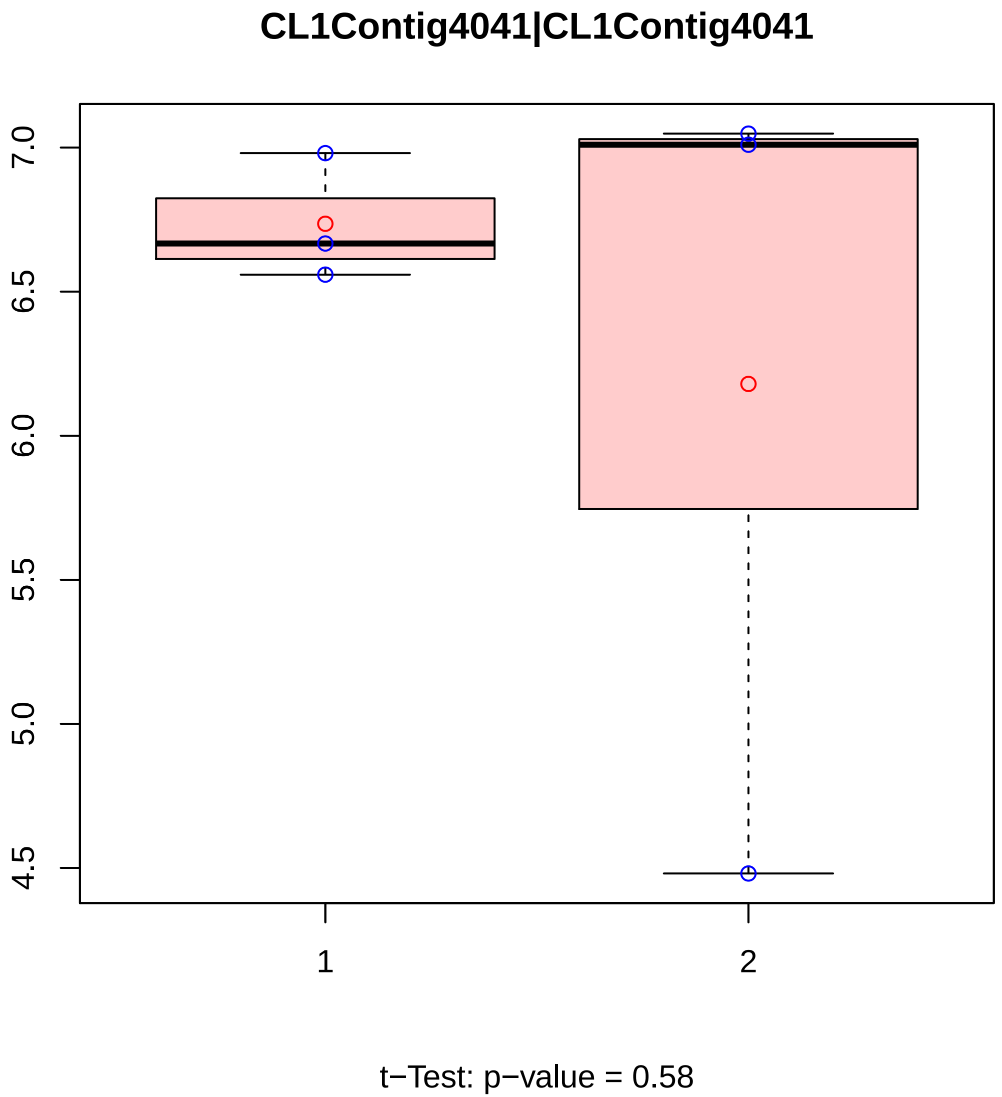
CL1Contig4041|CL1Contig4041
7.0
6.5
6.0
5.5
5.0
4.5
1
2
t−Test: p−value = 0.58

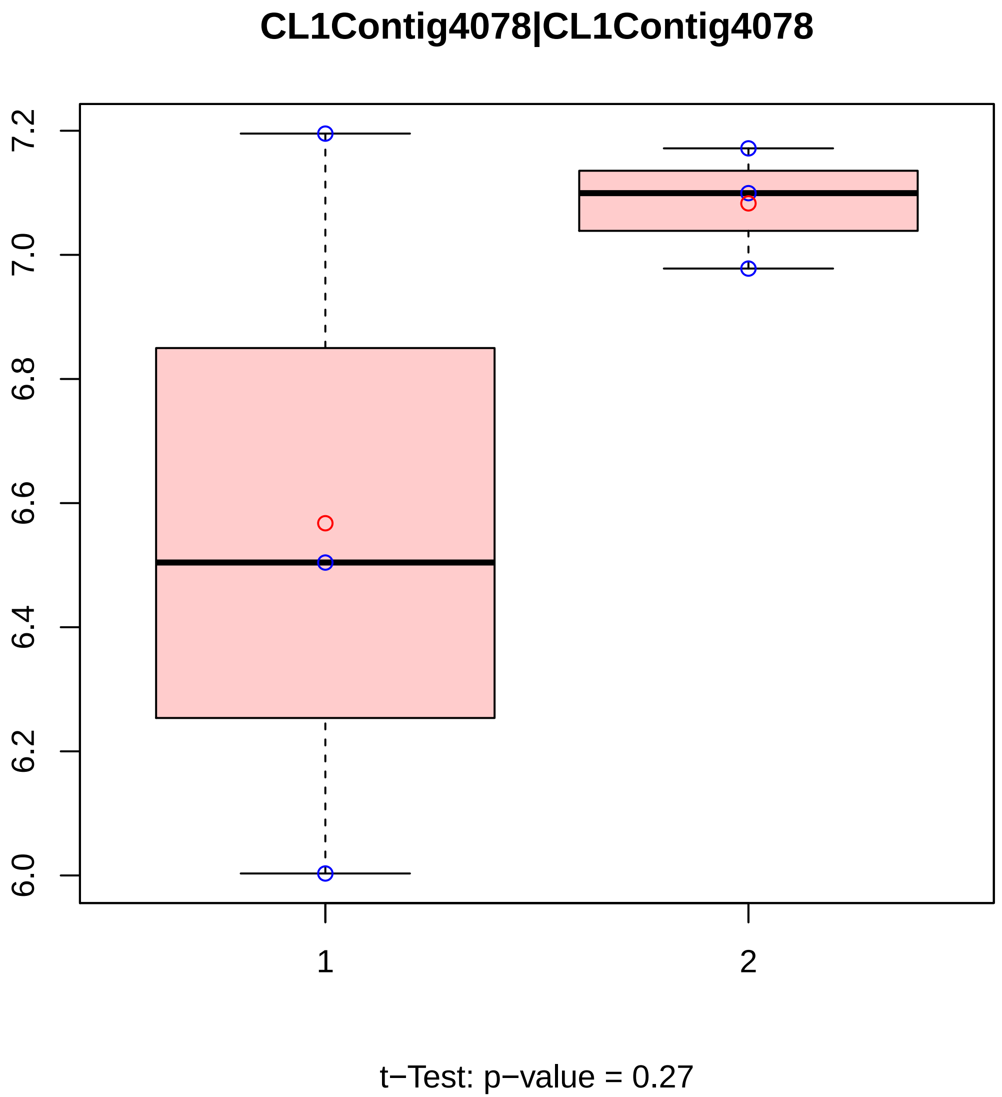

CL1Contig4078|CL1Contig4078
7.2
7.0
6.8
6.6
6.4
6.2
6.0
1
2
t−Test: p−value = 0.27

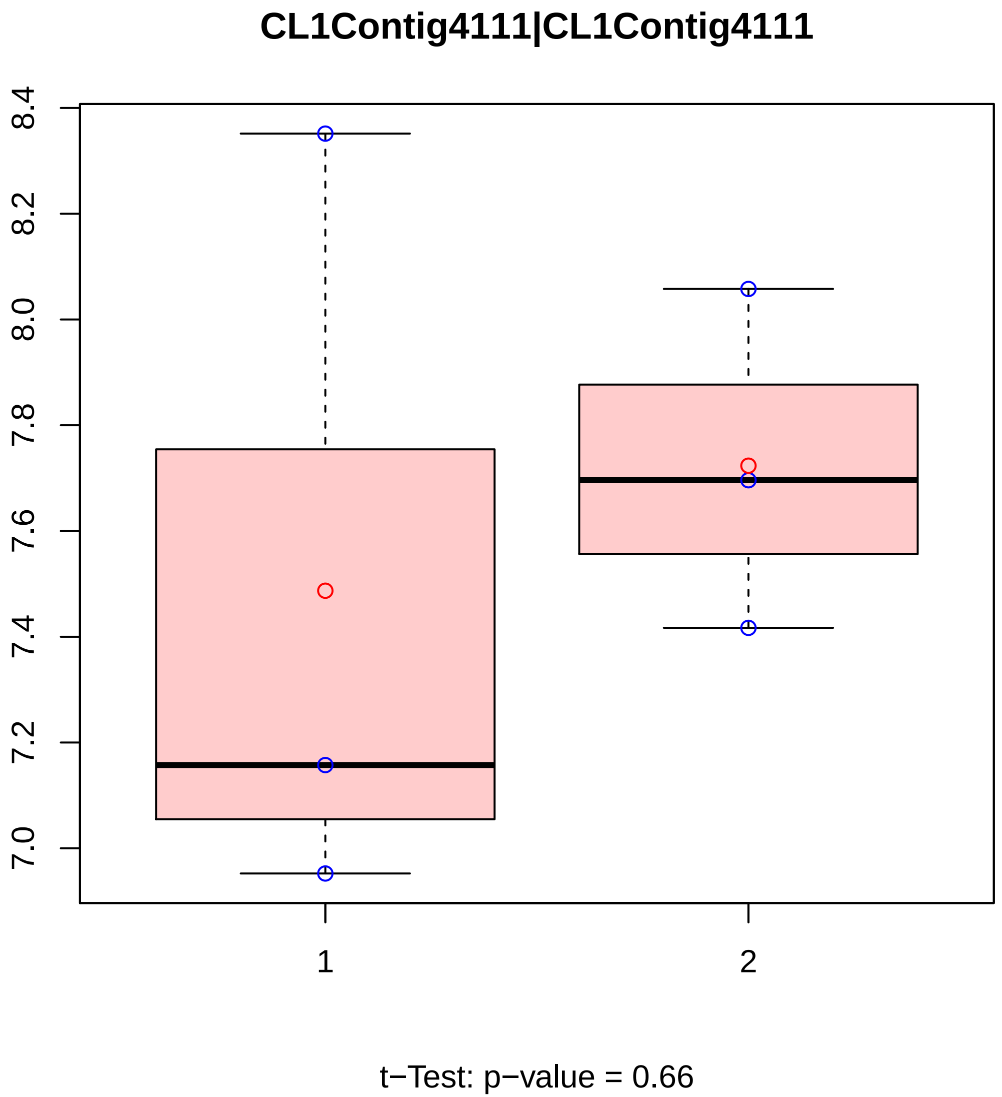

CL1Contig4111|CL1Contig4111
8.4
8.2
8.0
7.8
7.6
7.4
7.2
7.0
1
2
t−Test: p−value = 0.66

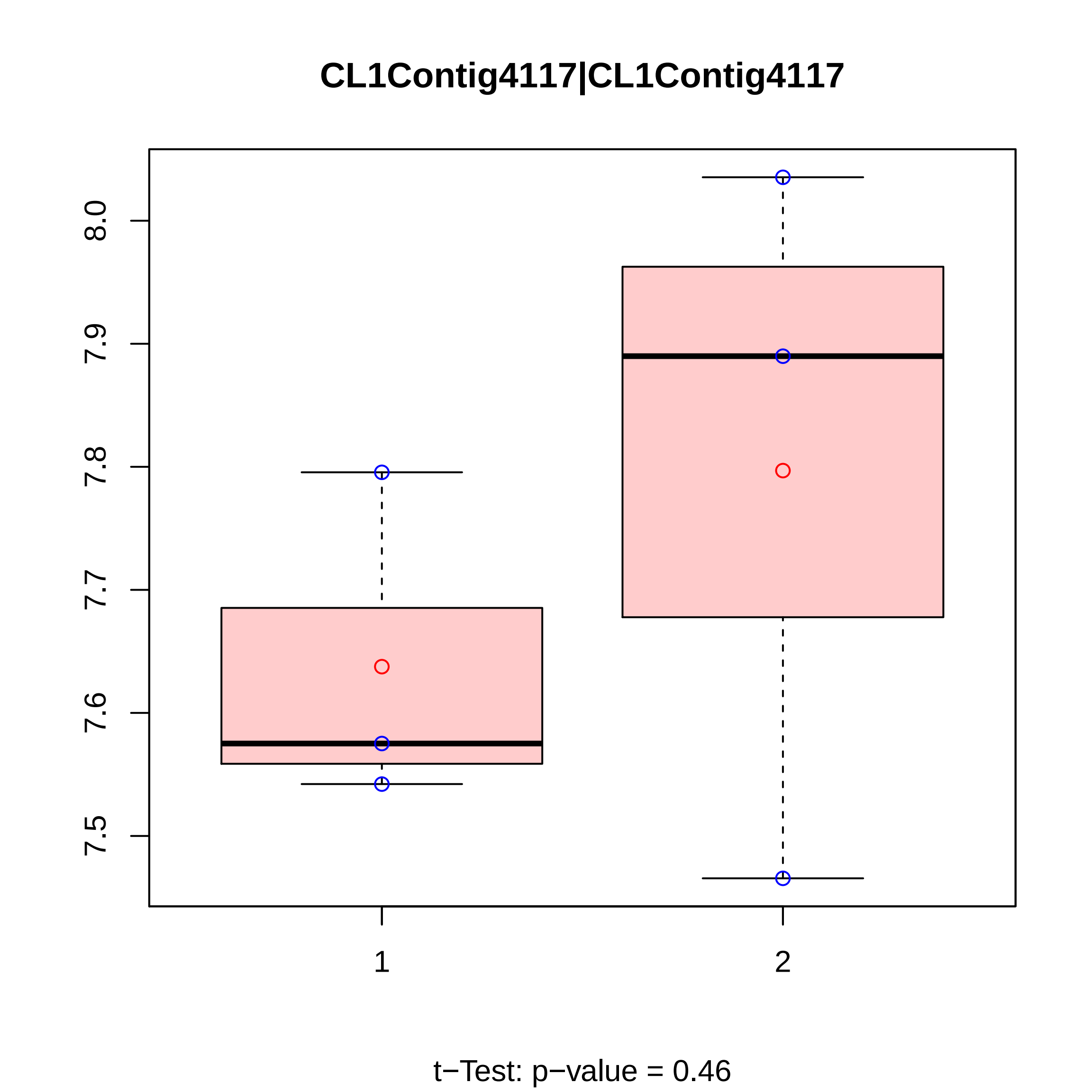
CL1Contig4117|CL1Contig4117
8.0
7.9
7.8
7.7
7.6
7.5
1
2
t−Test: p−value = 0.46

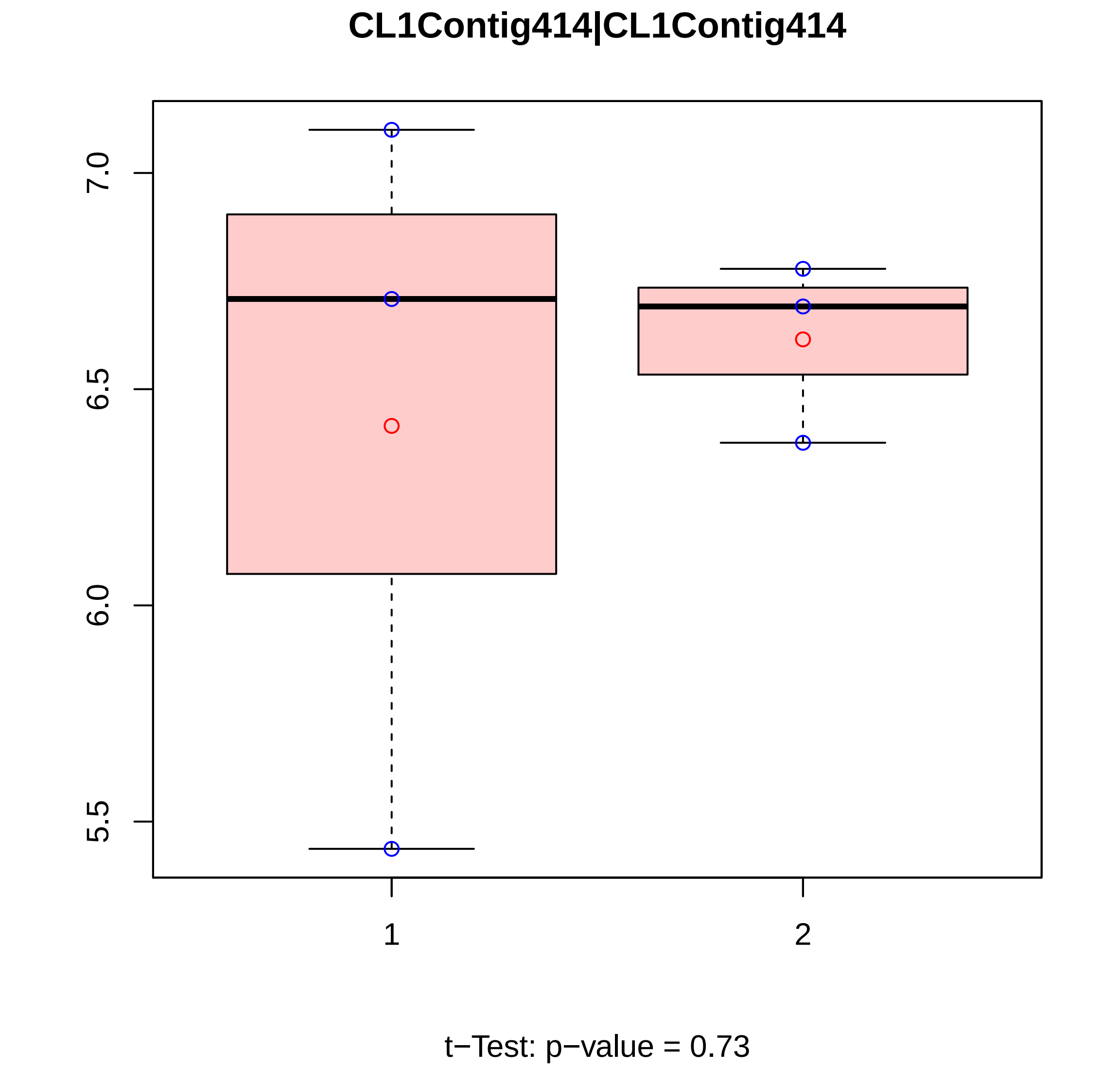
CL1Contig414|CL1Contig414
7.0
6.5
6.0
5.5
1
2
t−Test: p−value = 0.73

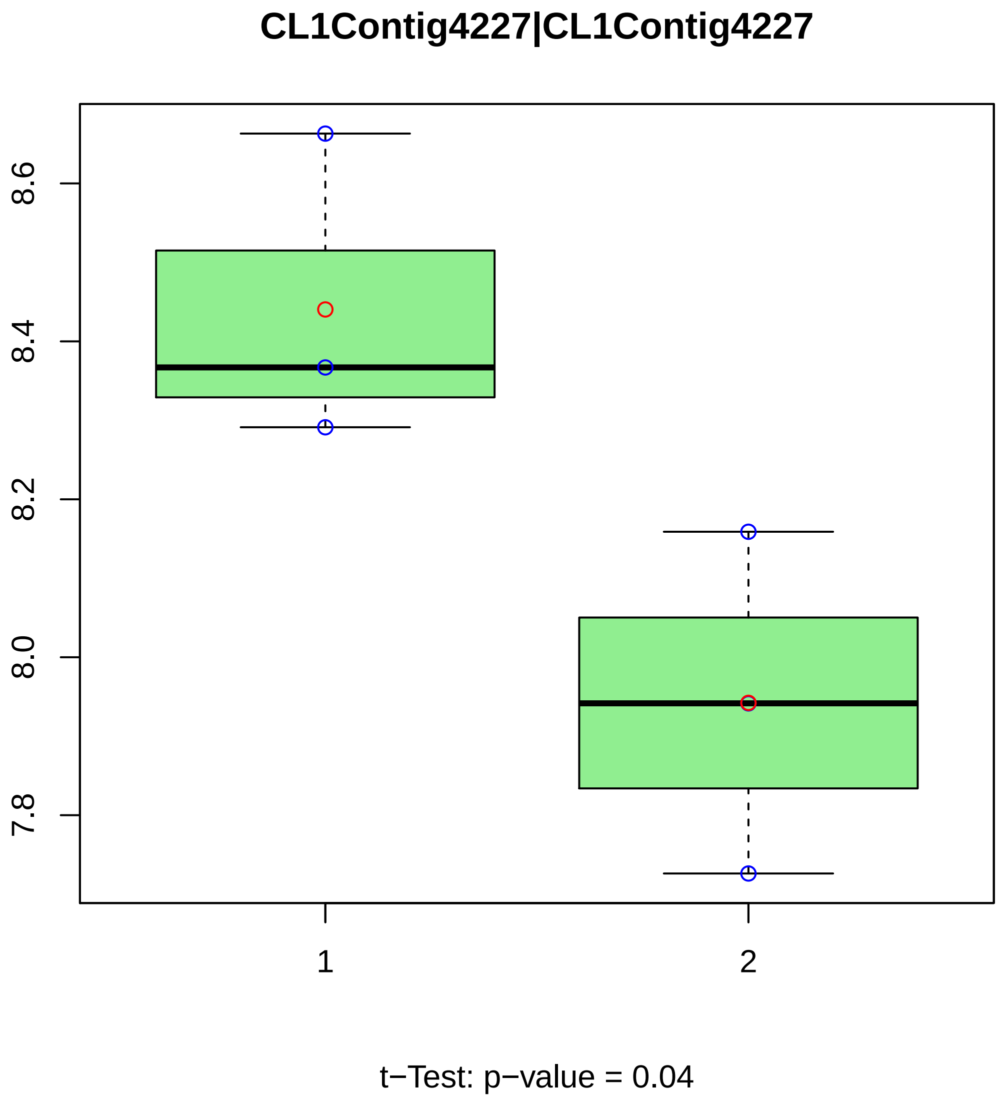

CL1Contig4227|CL1Contig4227
8.6
8.4
8.2
8.0
7.8
1
2
t−Test: p−value = 0.04

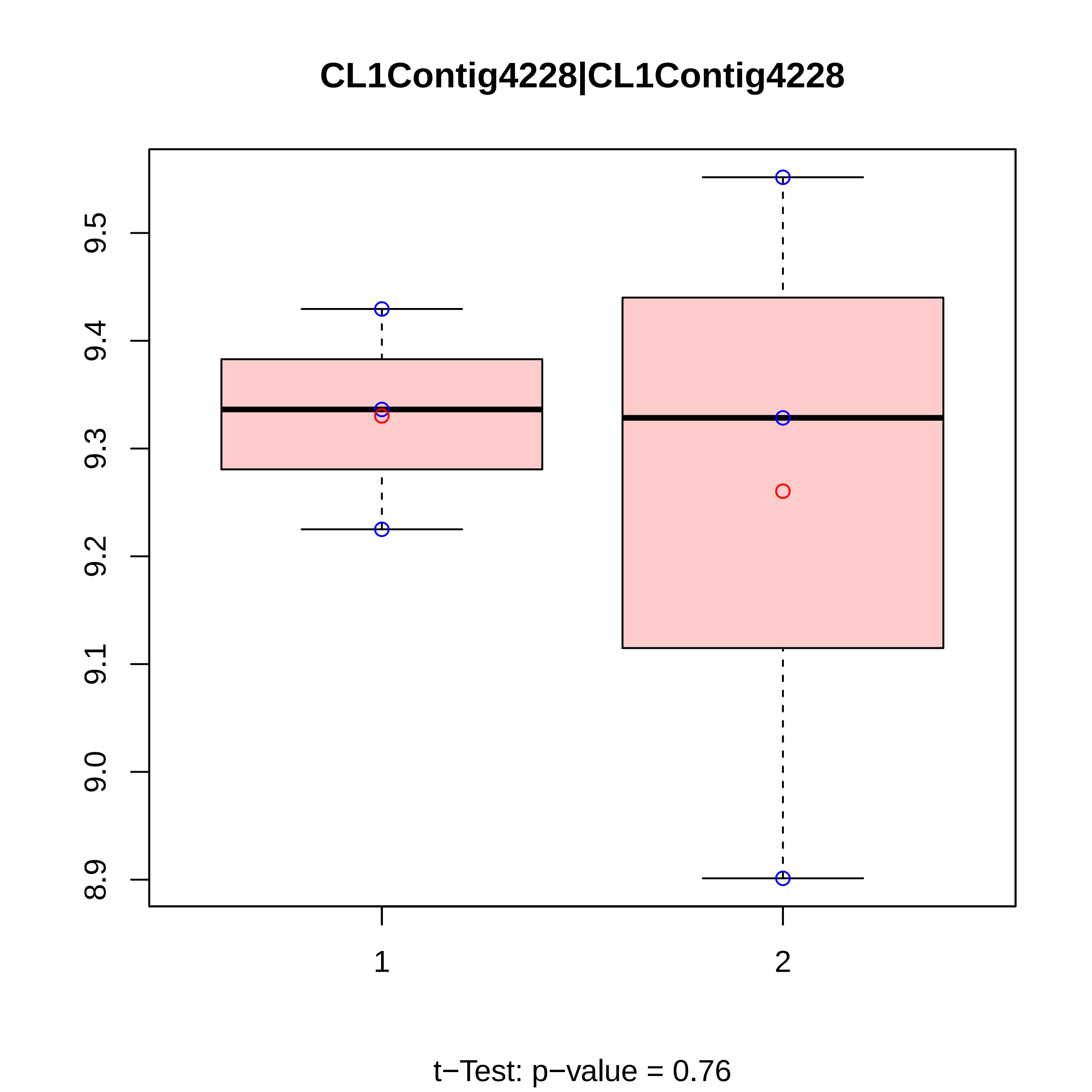
CL1Contig4228|CL1Contig4228
9.5
9.4
9.3
9.2
9.1
9.0
8.9
1
2
t−Test: p−value = 0.76

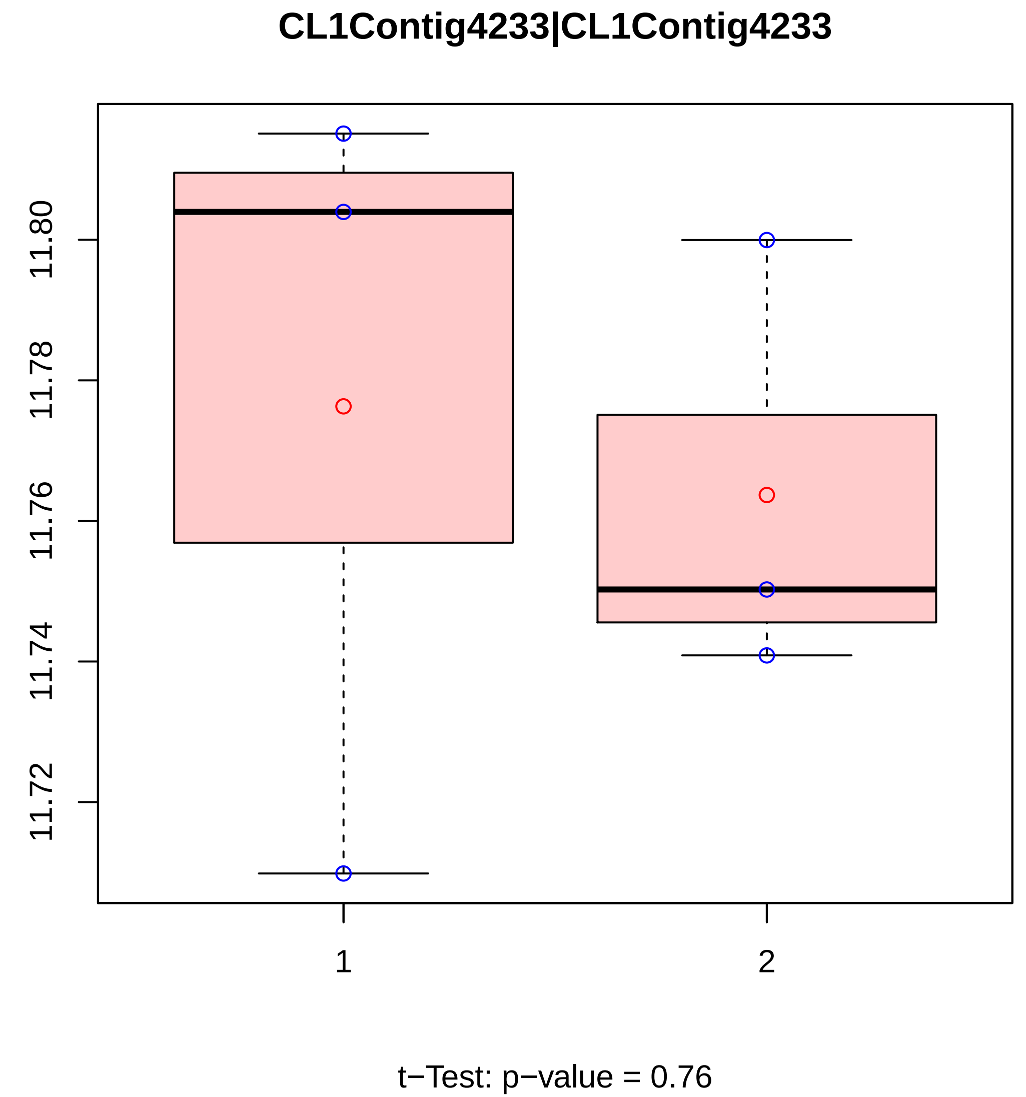
CL1Contig4233|CL1Contig4233
11.72
11.74
11.76
11.78
11.80
1
2
t−Test: p−value = 0.76

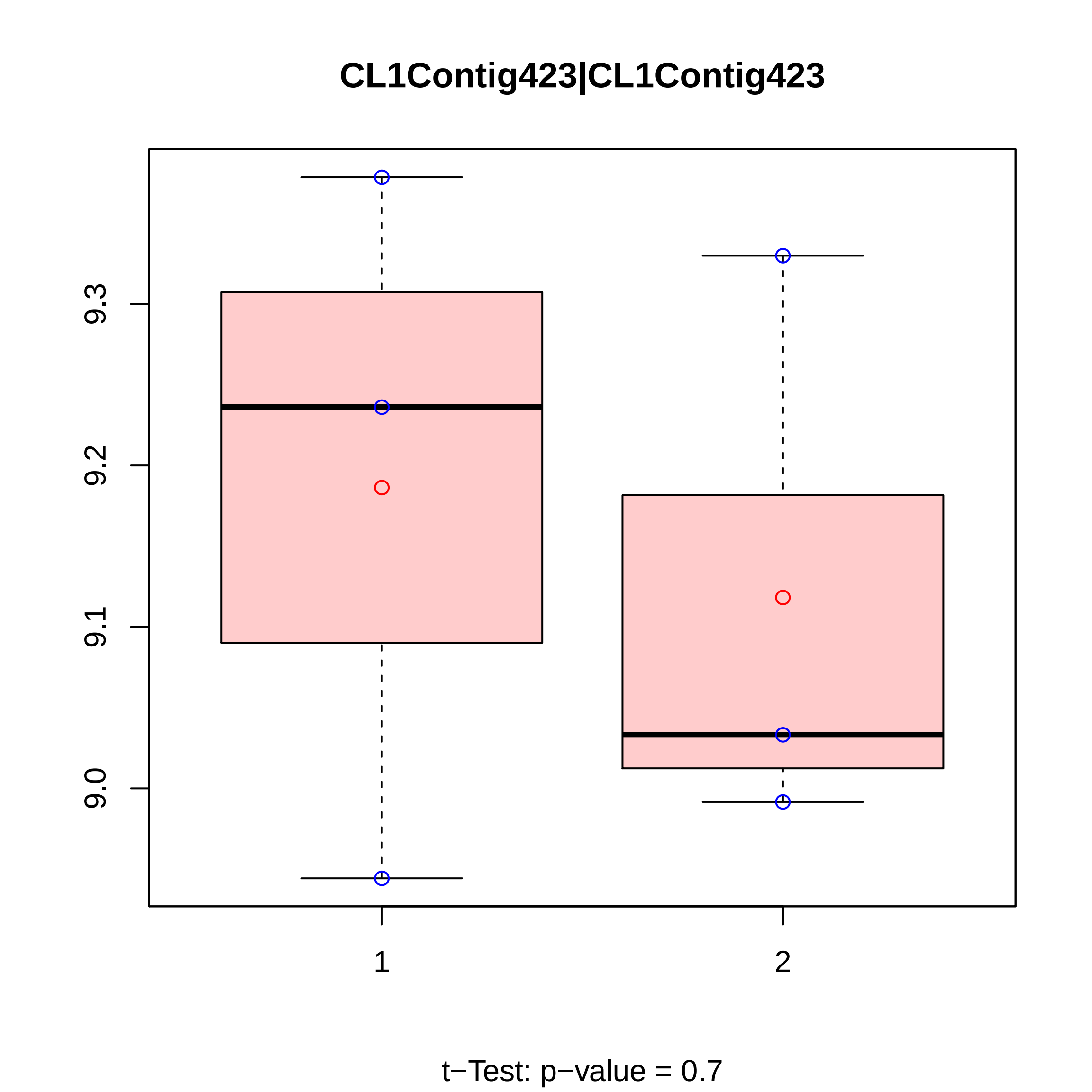

CL1Contig423|CL1Contig423
9.0
9.1
9.2
9.3
1
2
t−Test: p−value = 0.7

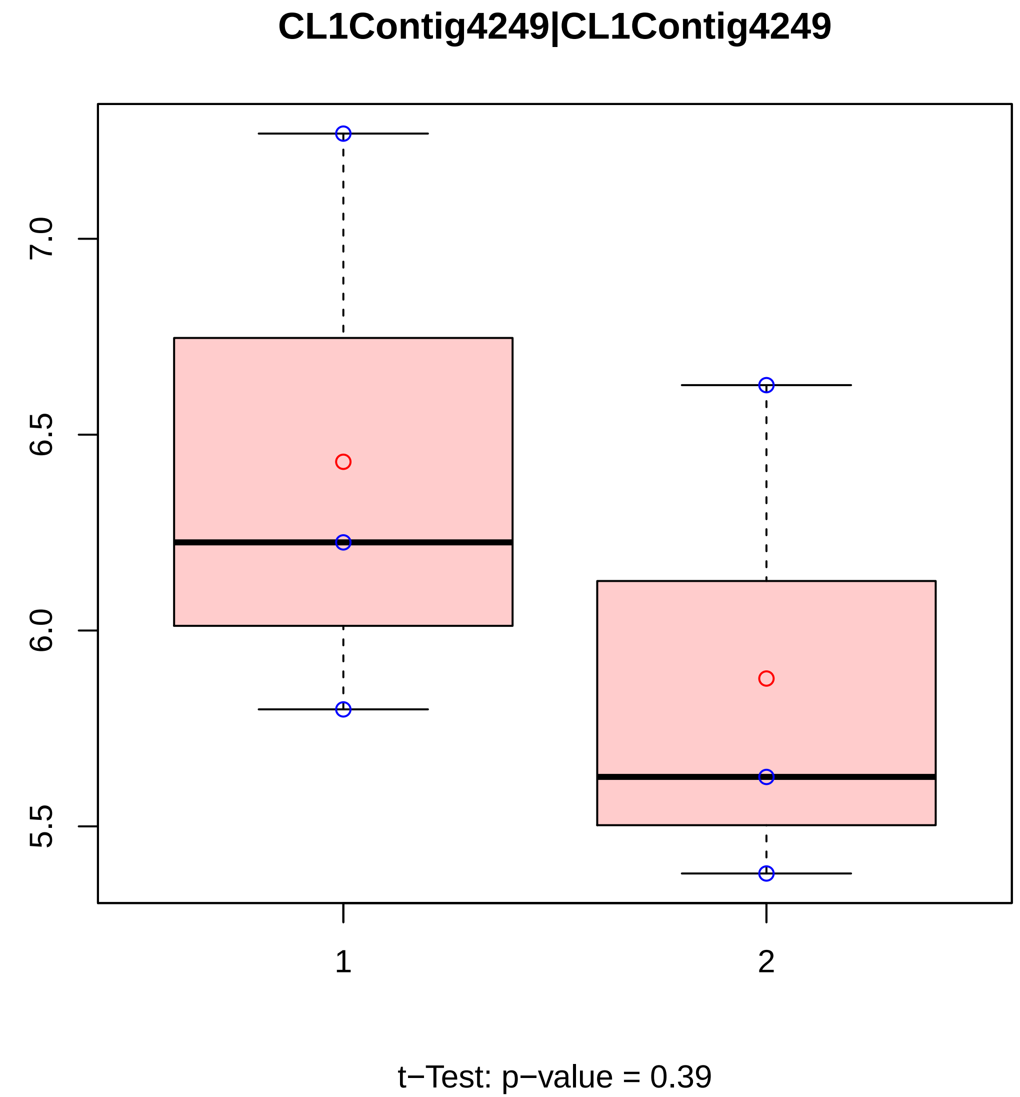
CL1Contig4249|CL1Contig4249
7.0
6.5
6.0
5.5
1
2
t−Test: p−value = 0.39

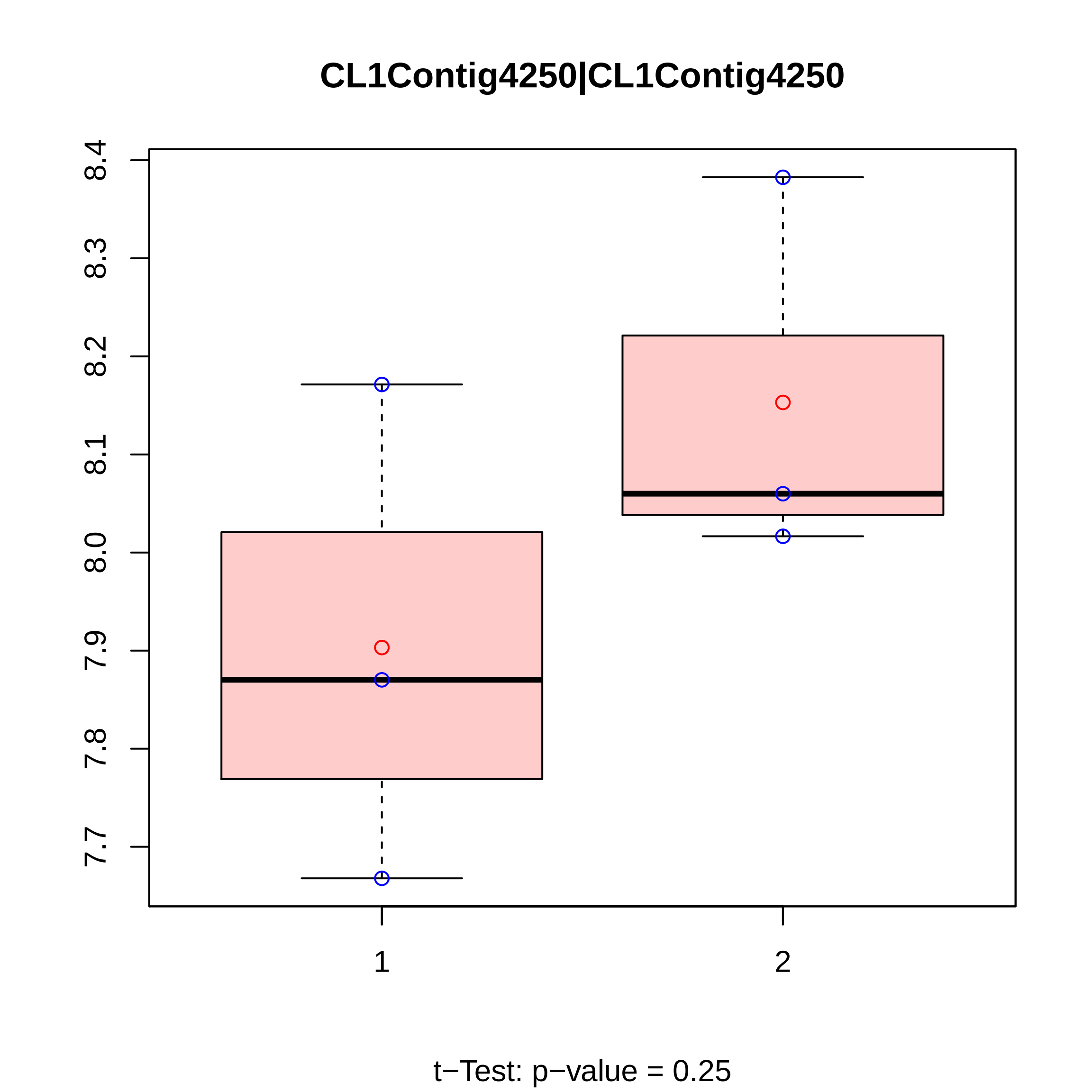
CL1Contig4250|CL1Contig4250
8.4
8.3
8.2
8.1
8.0
7.9
7.8
7.7
1
2
t−Test: p−value = 0.25

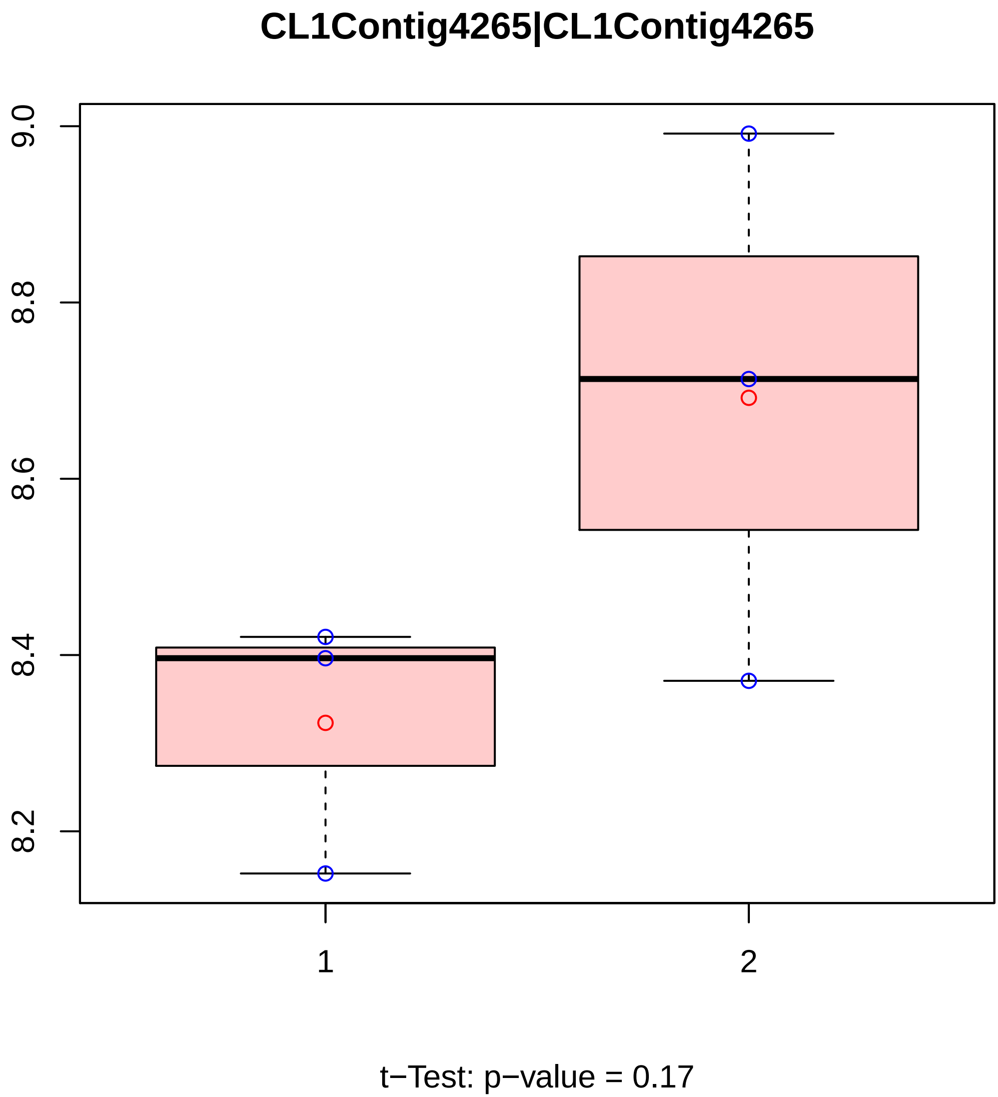

CL1Contig4265|CL1Contig4265
9.0
8.8
8.6
8.4
8.2
1
2
t−Test: p−value = 0.17

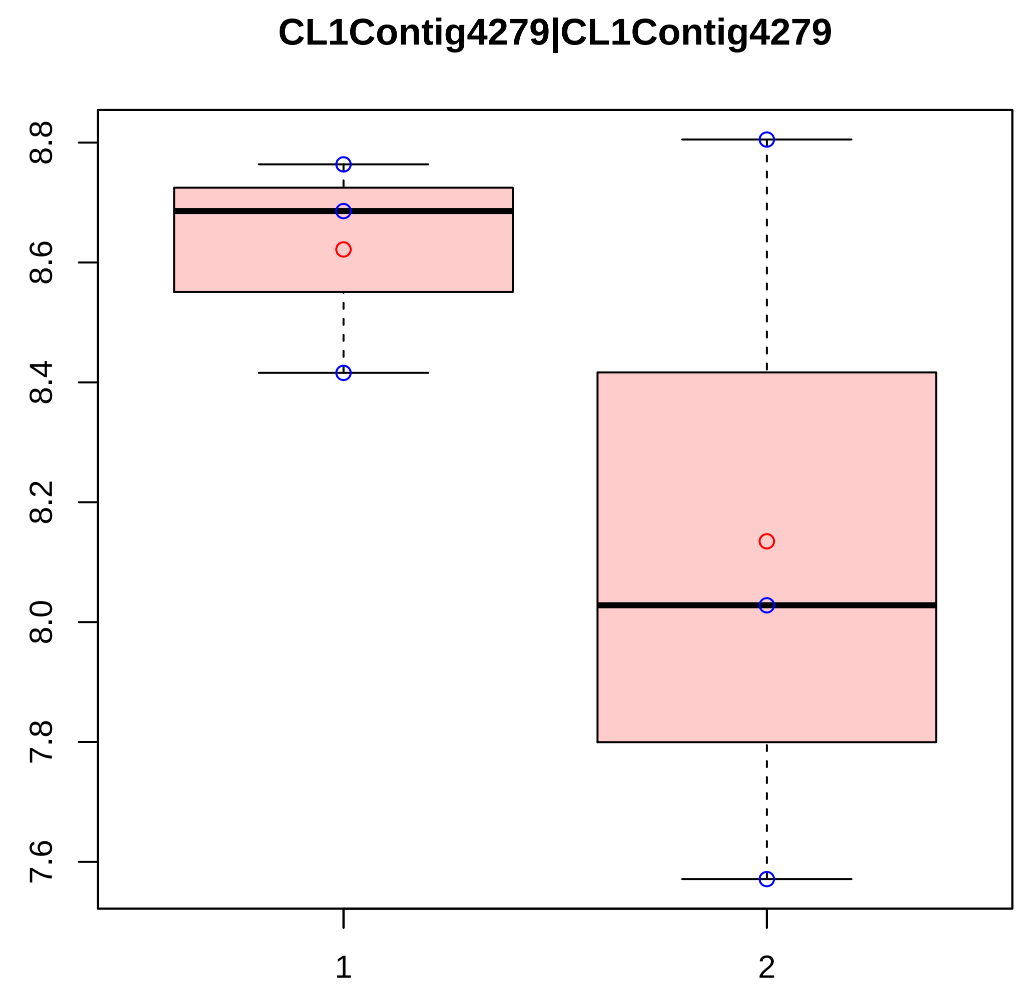


t−Test: p−value = 0.31

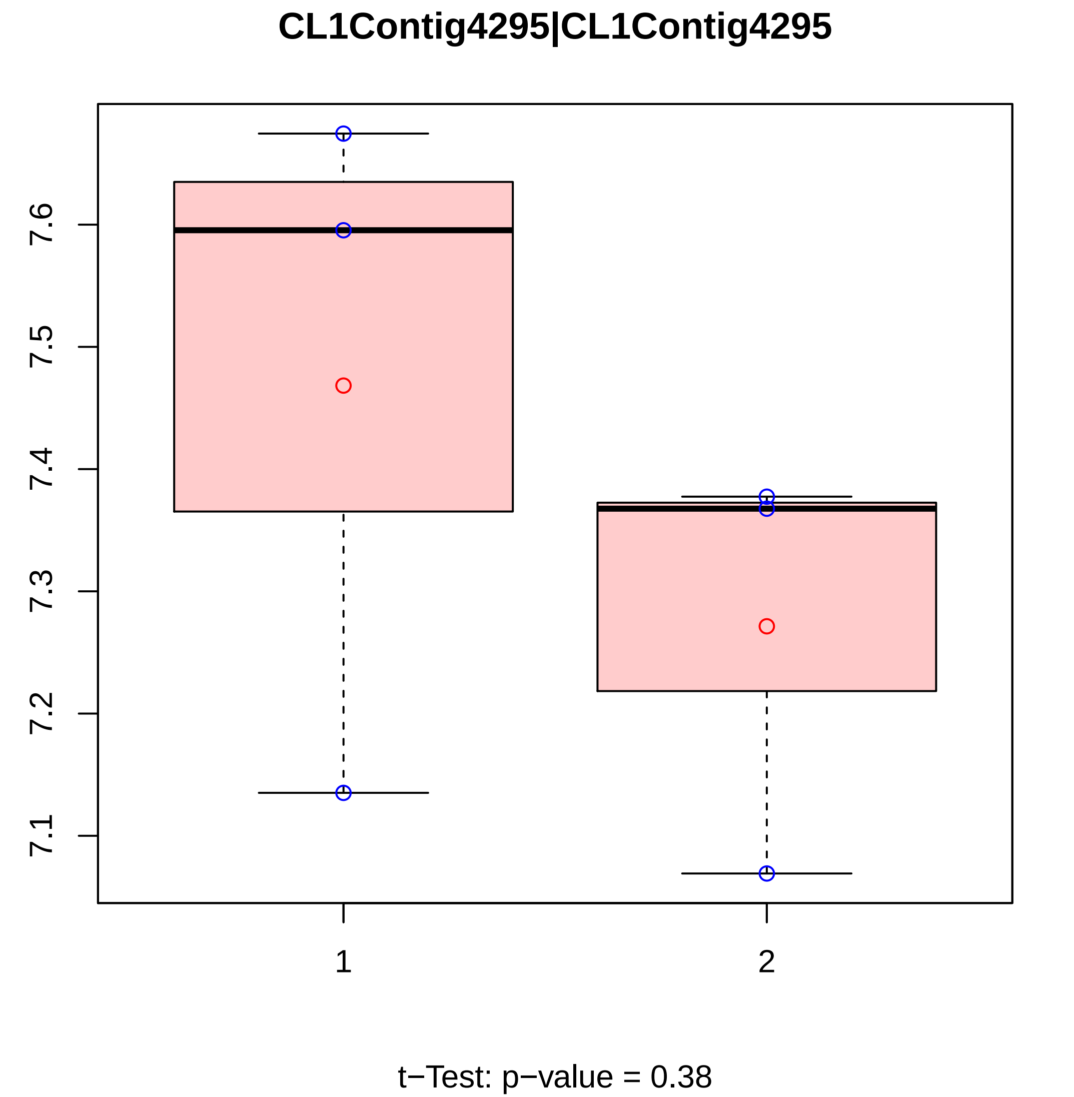
CL1Contig4295|CL1Contig4295
7.1
7.2
7.3
7.4
7.5
7.6
1
2
t−Test: p−value = 0.38

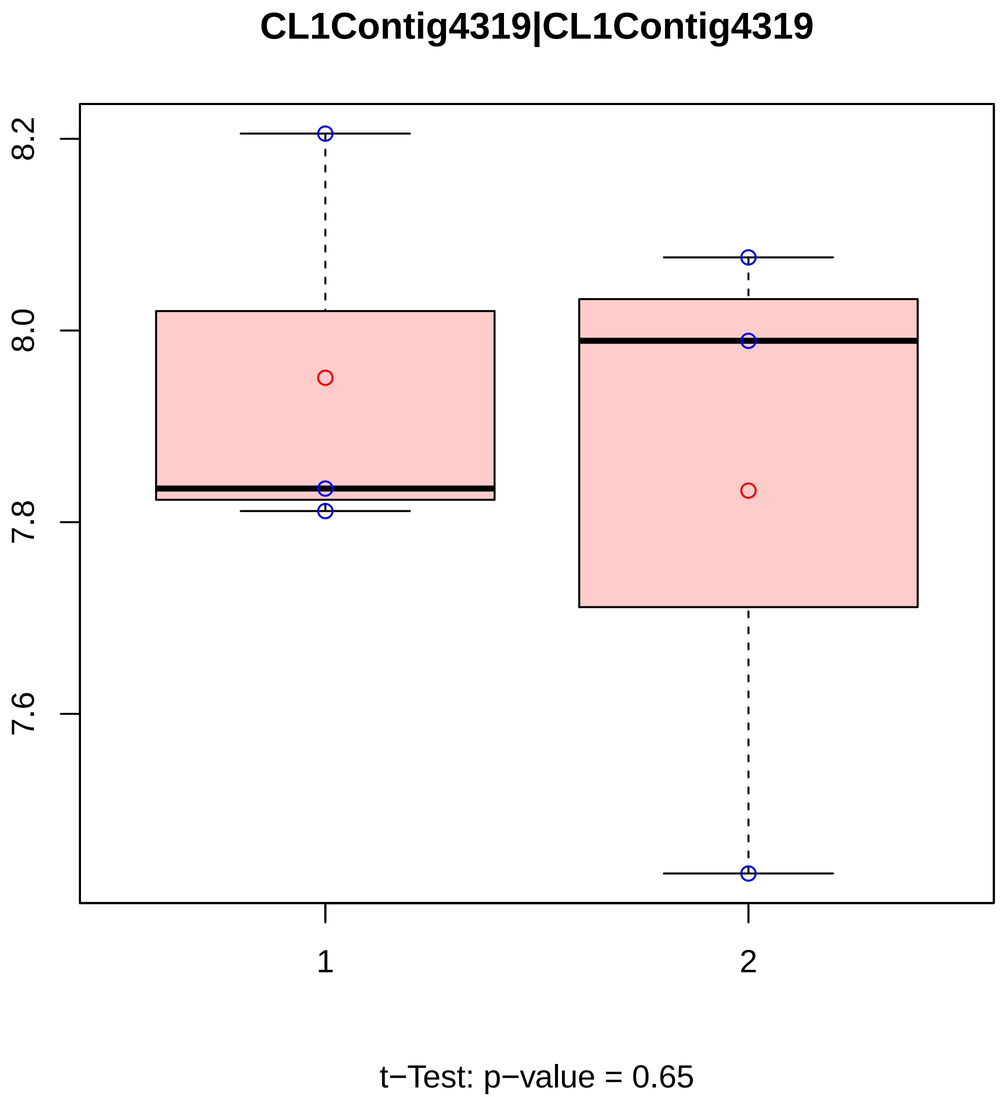
CL1Contig4319|CL1Contig4319
8.2
8.0
7.8
7.6
1
2
t−Test: p−value = 0.65

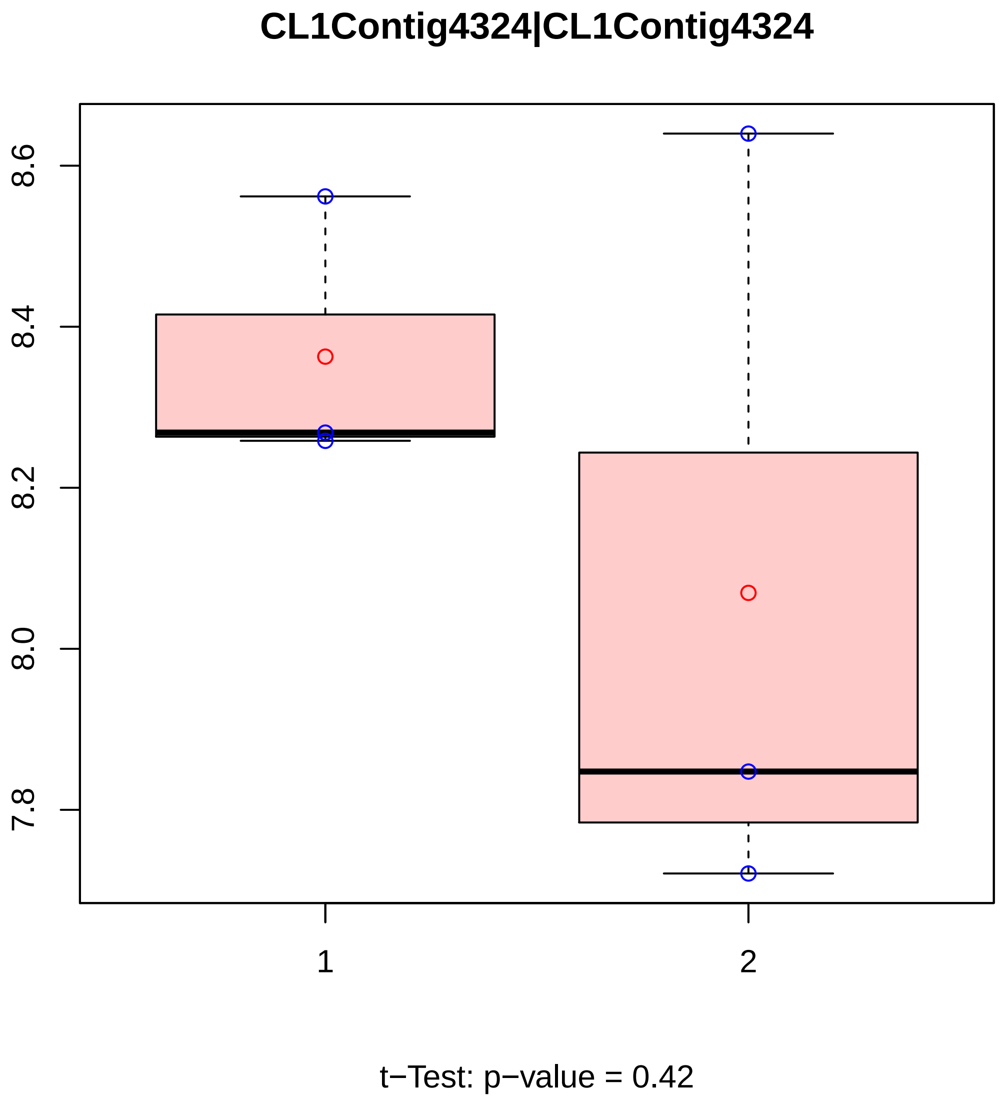
CL1Contig4324|CL1Contig4324
8.6
8.4
8.2
8.0
7.8
1
2
t−Test: p−value = 0.42

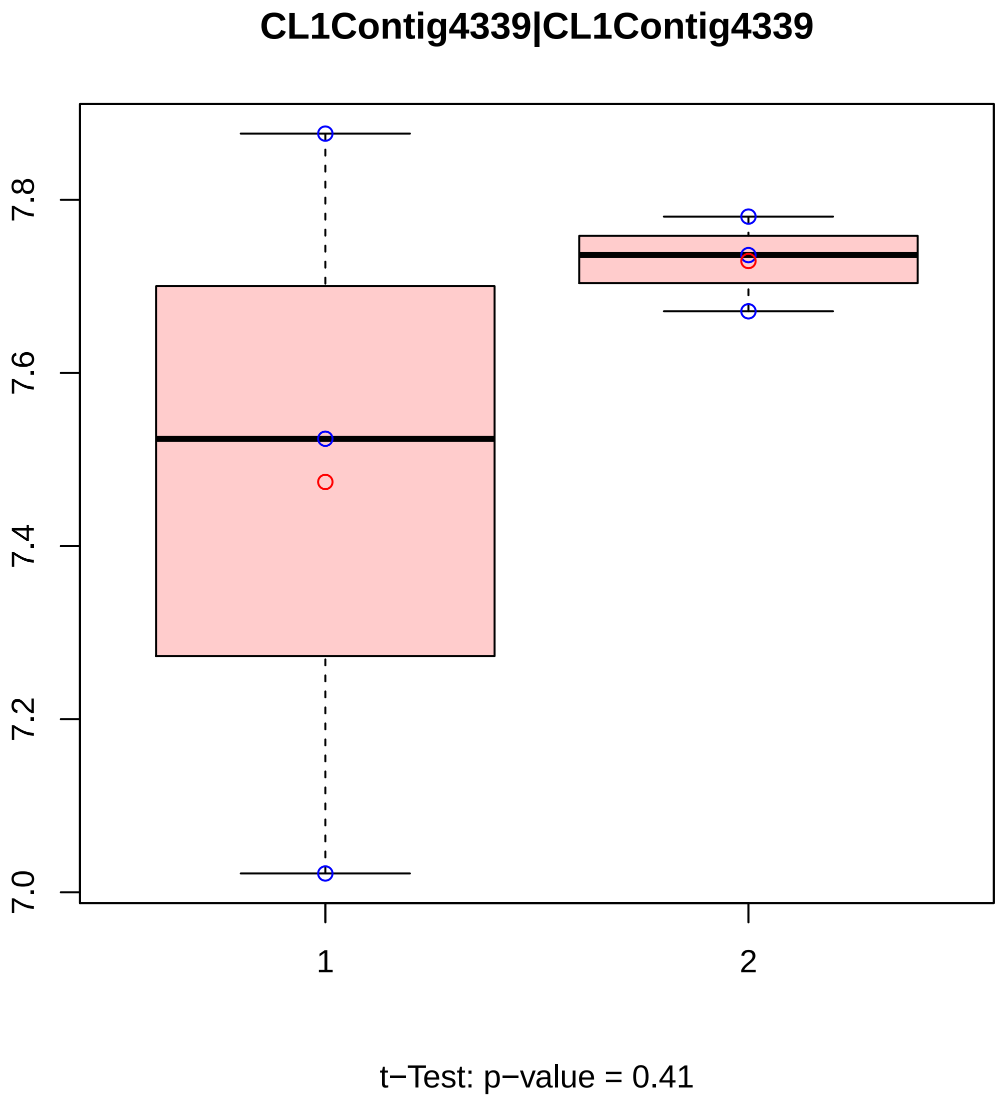
CL1Contig4339|CL1Contig4339
7.0
7.2
7.4
7.6
7.8
1
2
t−Test: p−value = 0.41

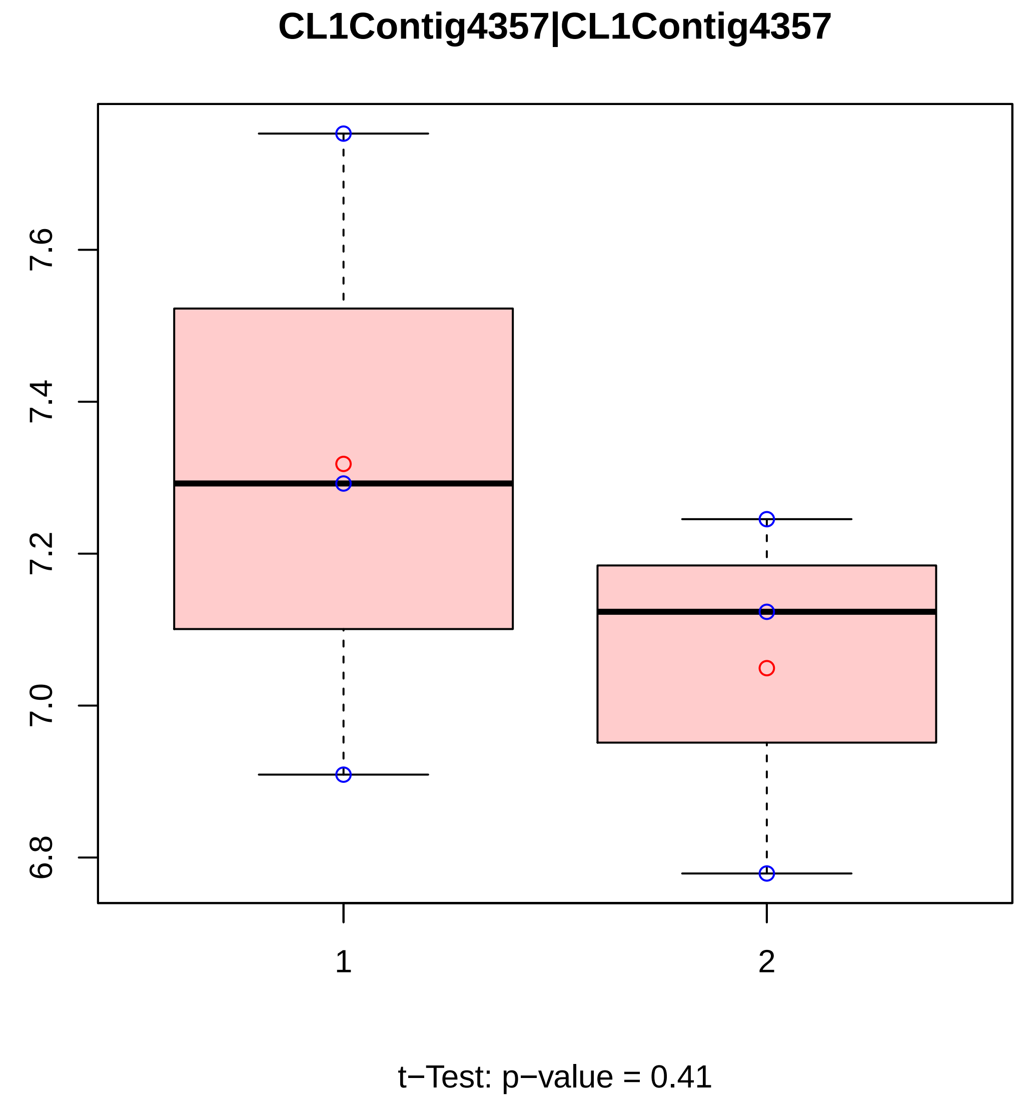
CL1Contig4357|CL1Contig4357
7.6
7.4
7.2
7.0
6.8
1
2
t−Test: p−value = 0.41

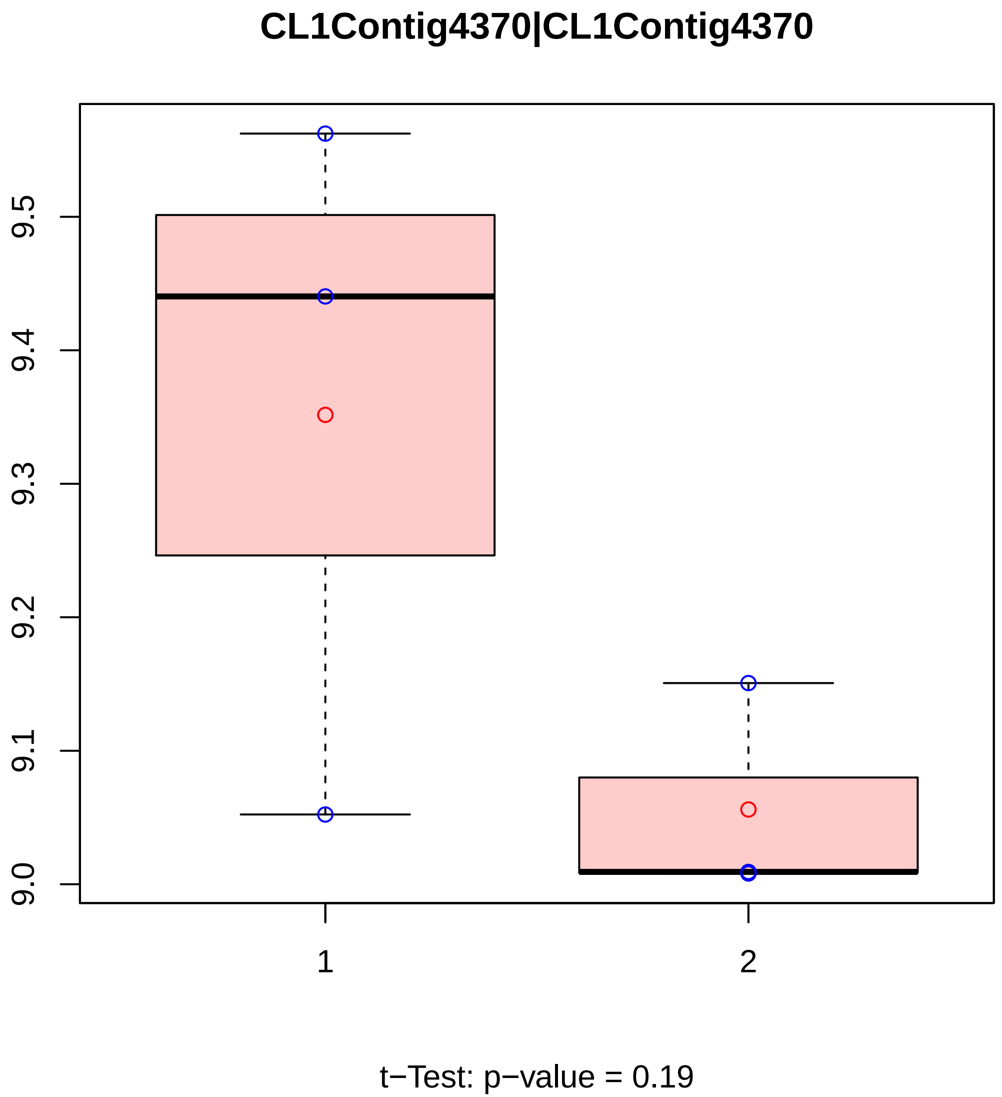

CL1Contig4370|CL1Contig4370
9.0
9.1
9.2
9.3
9.4
9.5
1
2
t−Test: p−value = 0.19

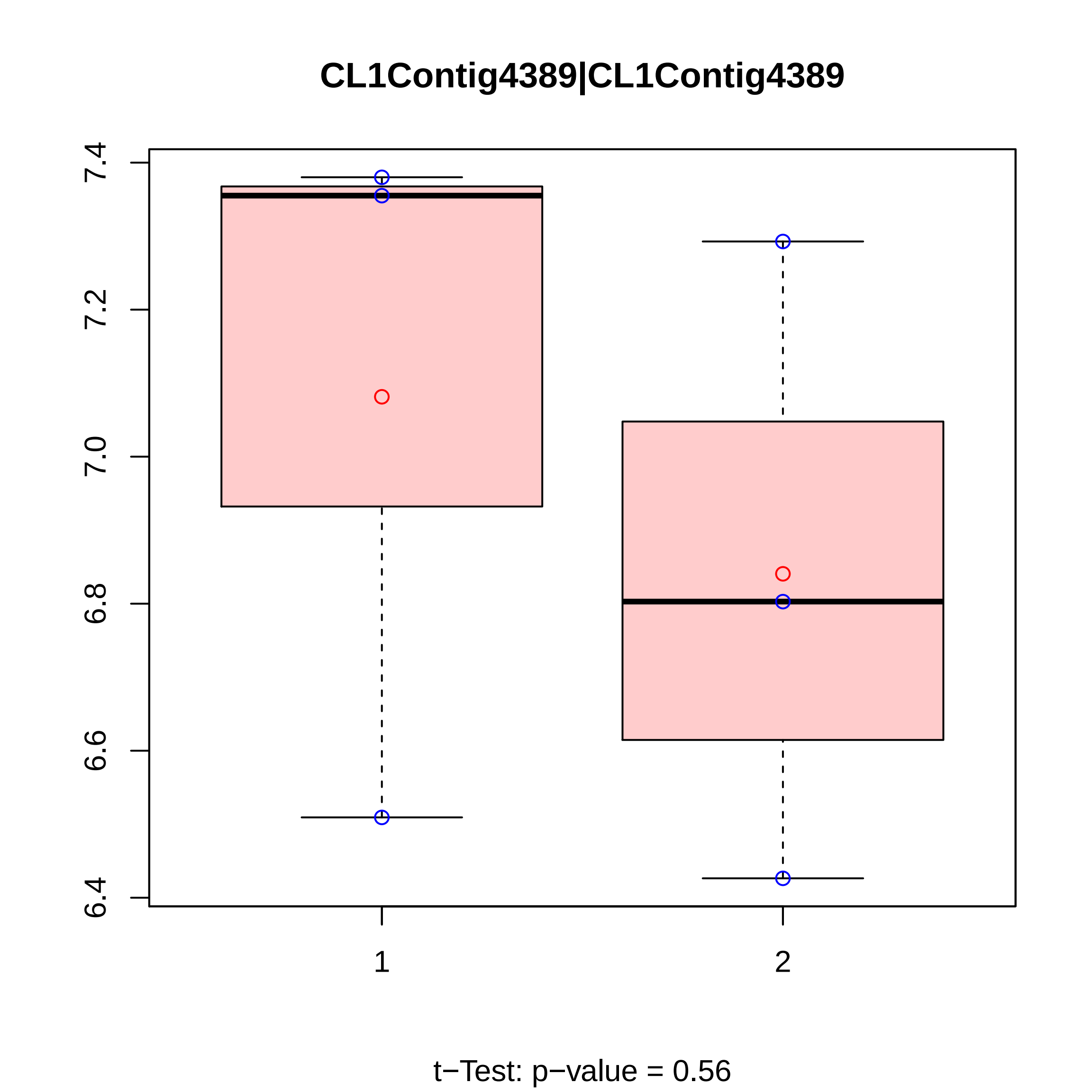

CL1Contig4389|CL1Contig4389
7.4
7.2
7.0
6.8
6.6
6.4
1
2
t−Test: p−value = 0.56

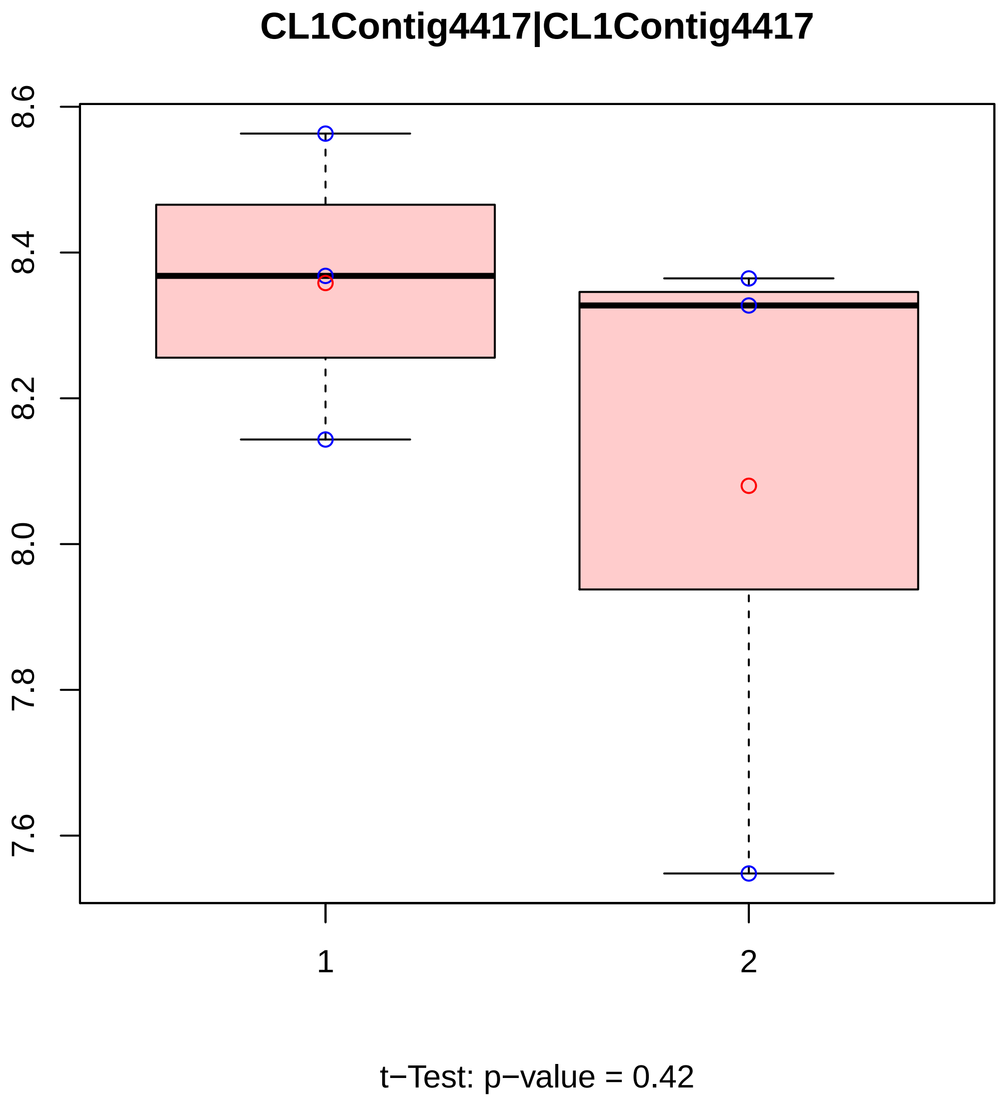

CL1Contig4417|CL1Contig4417
8.6
8.4
8.2
8.0
7.8
7.6
1
2
t−Test: p−value = 0.42

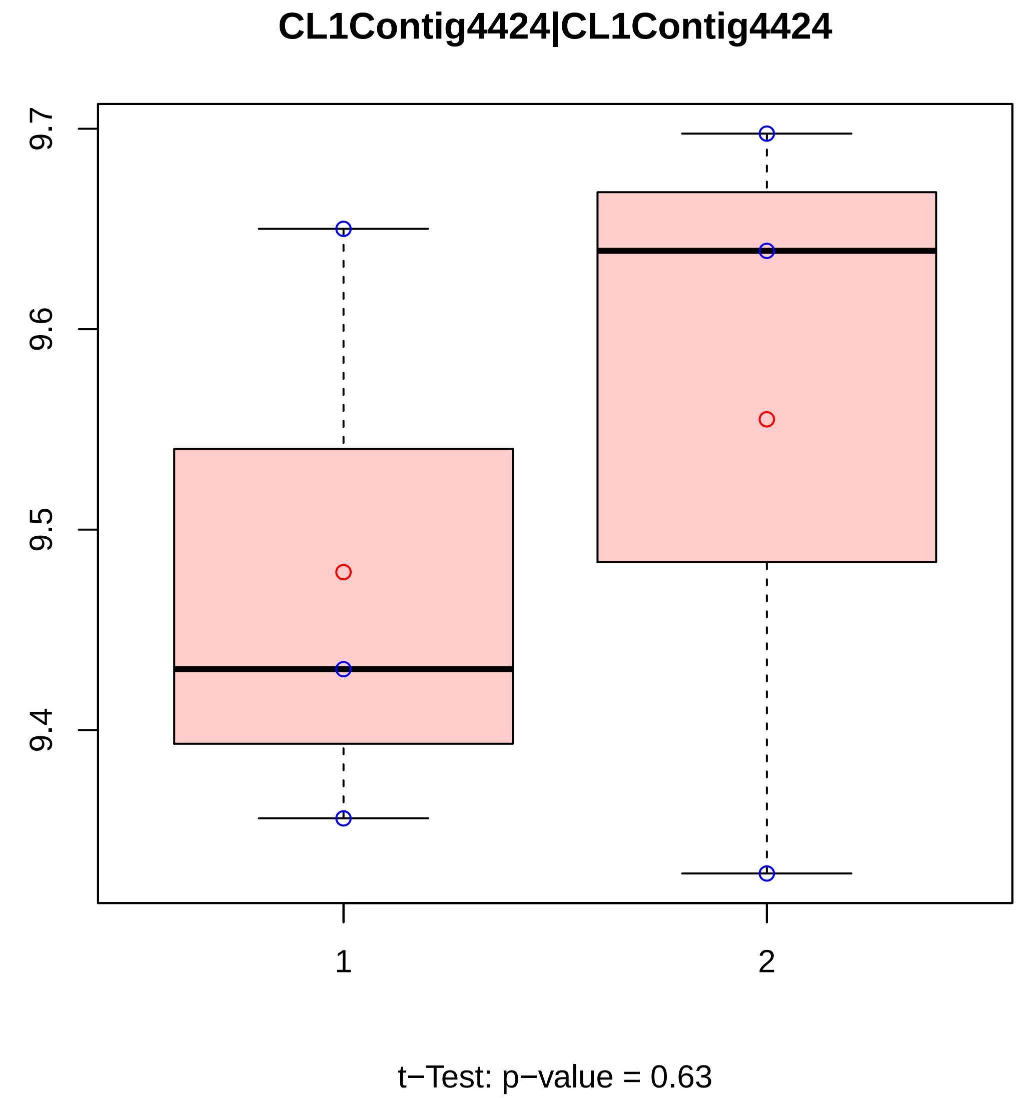

CL1Contig4424|CL1Contig4424
9.4
9.5
9.6
9.7
1
2
t−Test: p−value = 0.63

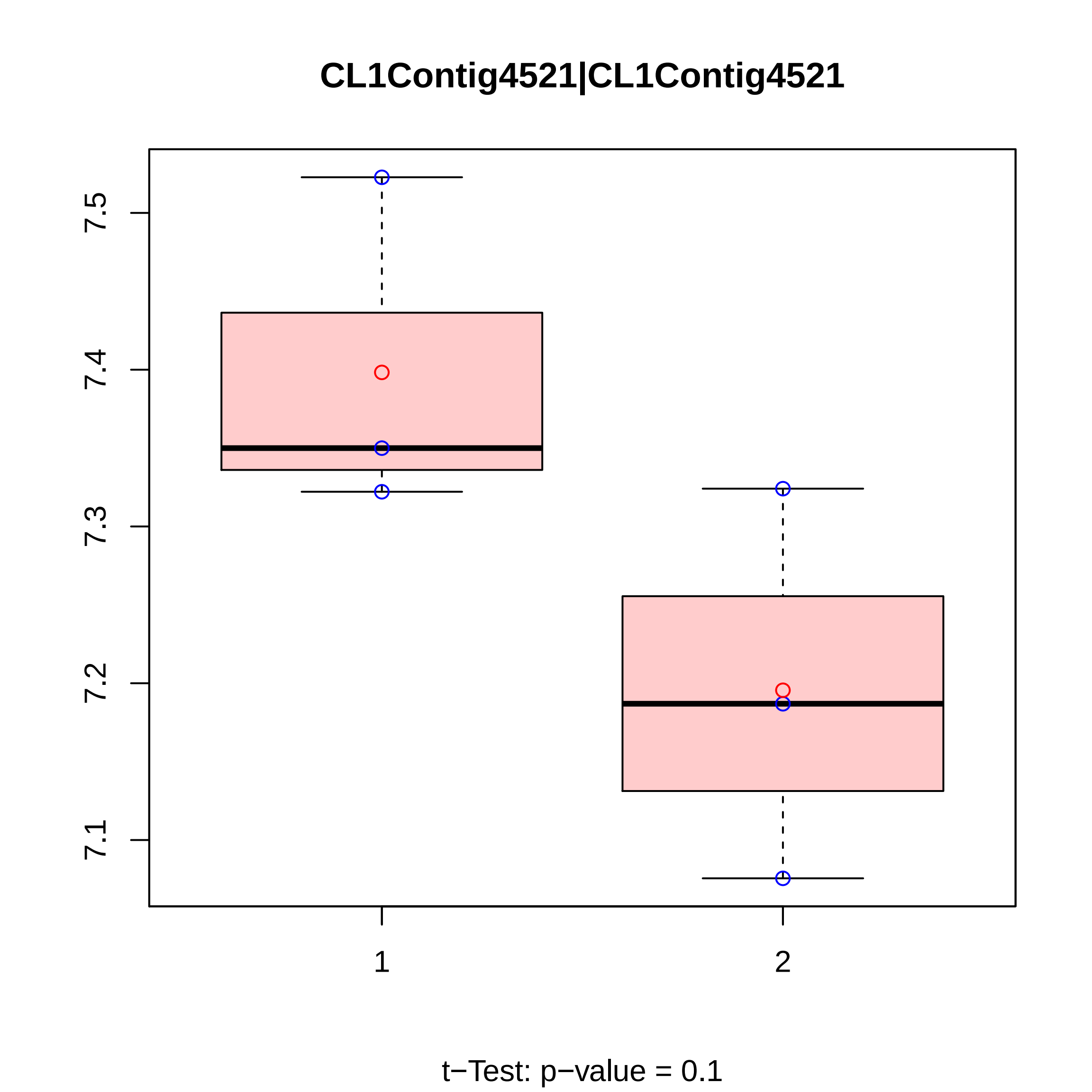
CL1Contig4521|CL1Contig4521
7.1
7.2
7.3
7.4
7.5
1
2
t−Test: p−value = 0.1

# CL1Contig4551|CL1Contig4551

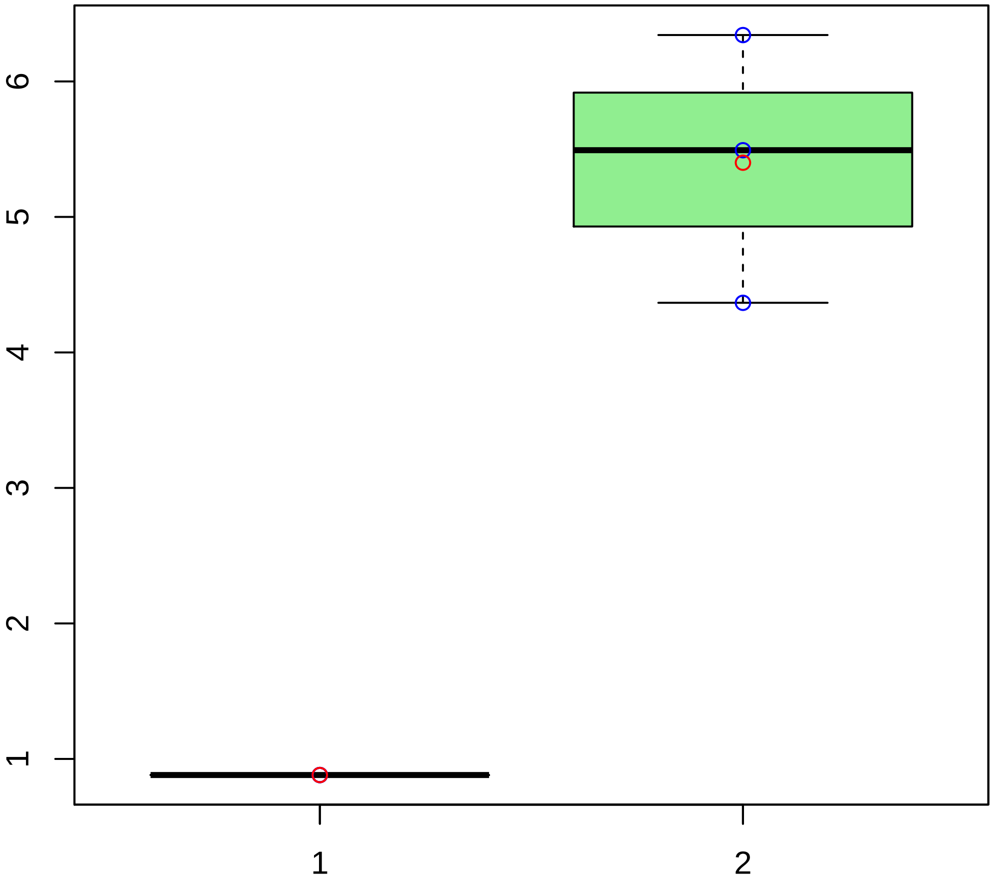


t−Test: p−value = 0.02

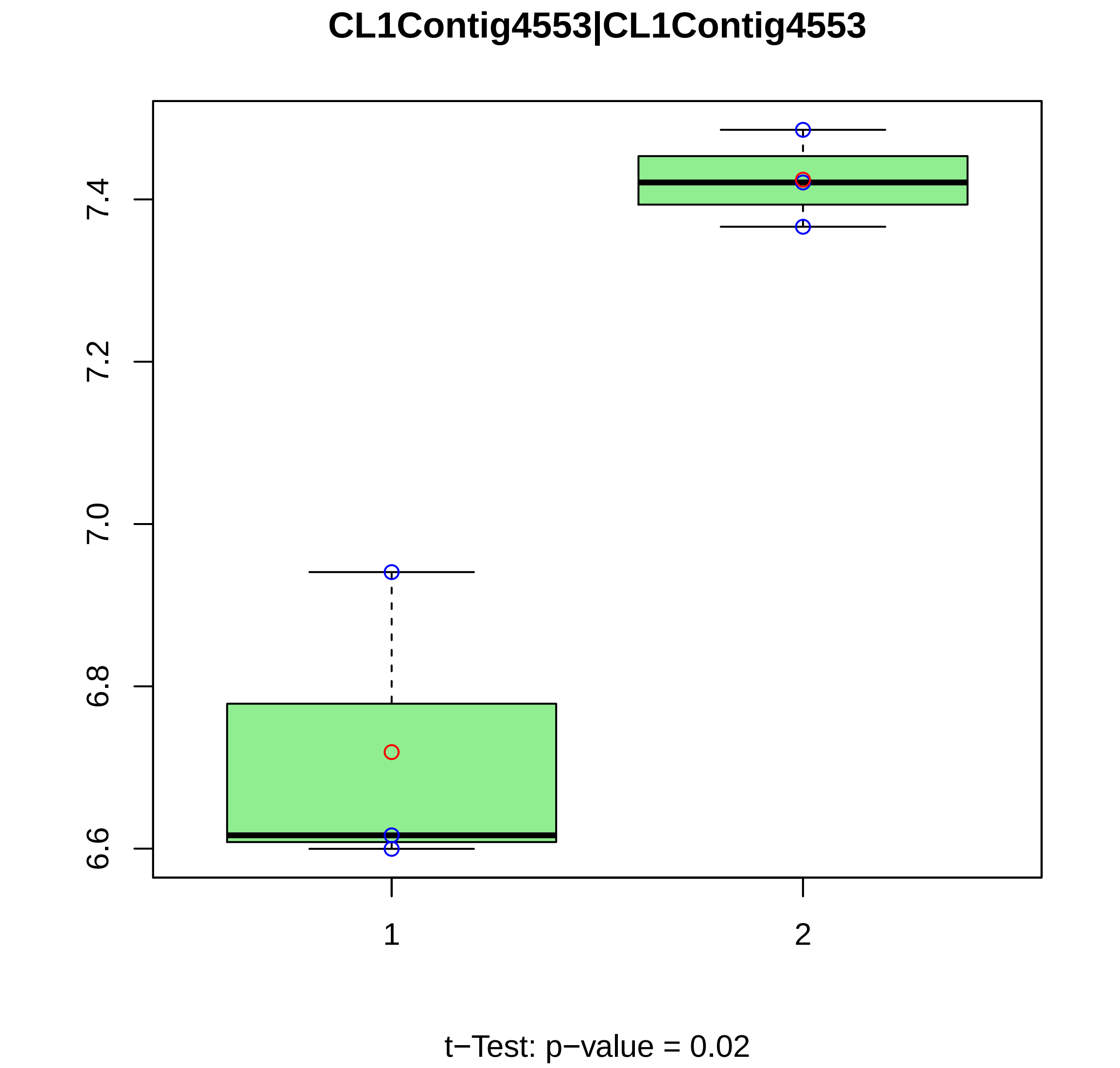
CL1Contig4553|CL1Contig4553
7.4
7.2
7.0
6.8
6.6
1
2
t−Test: p−value = 0.02

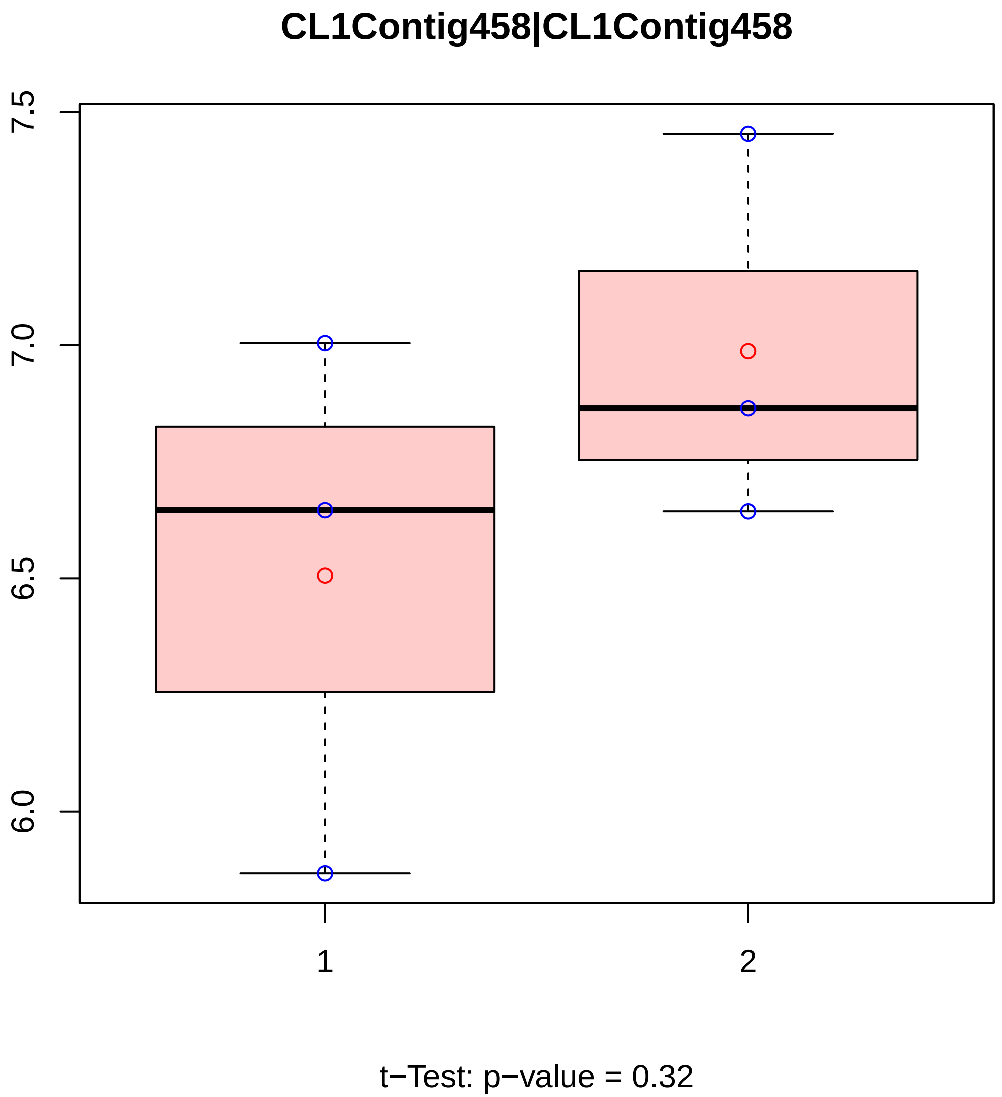
CL1Contig458|CL1Contig458
7.5
7.0
6.5
6.0
1
2
t−Test: p−value = 0.32

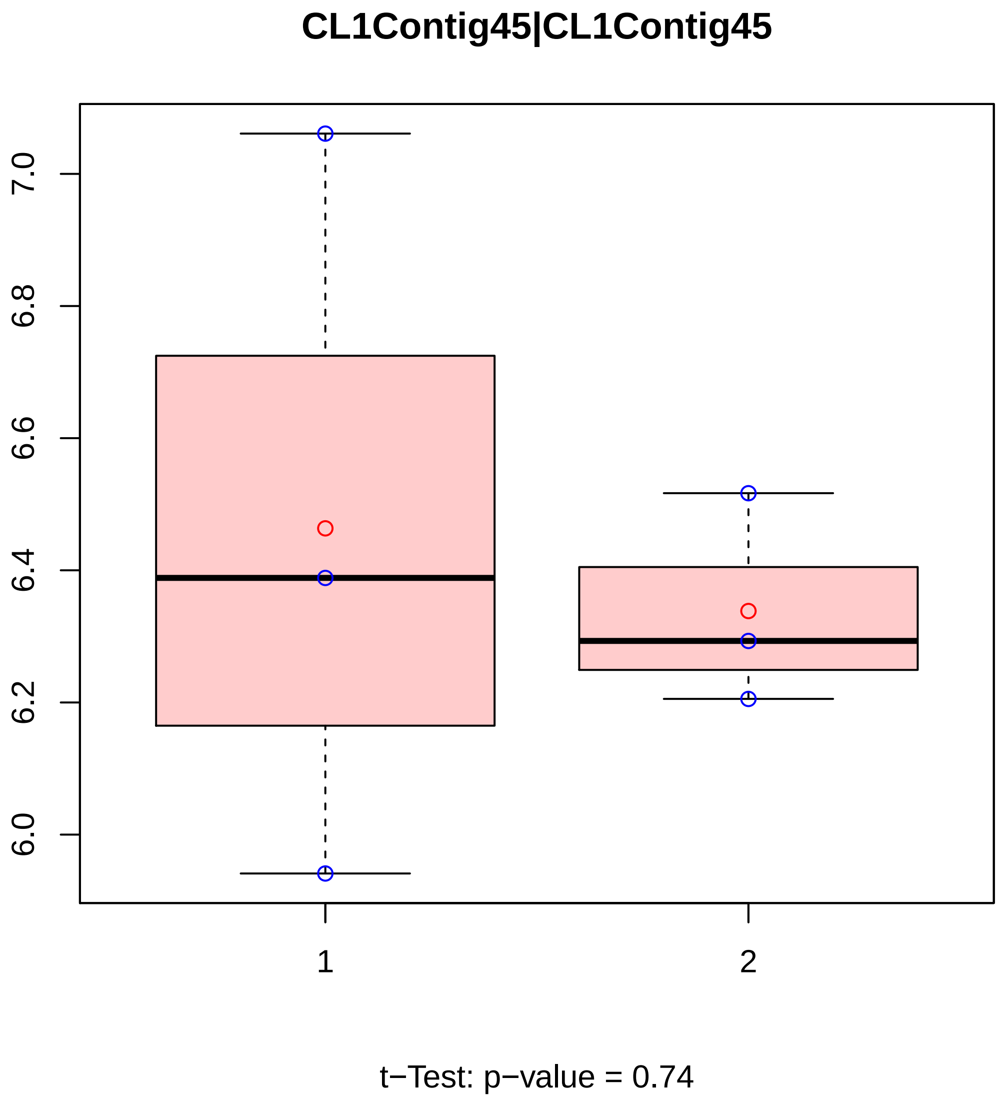
CL1Contig45|CL1Contig45
6.0
6.2
6.4
6.6
6.8
7.0
1
2
t−Test: p−value = 0.74

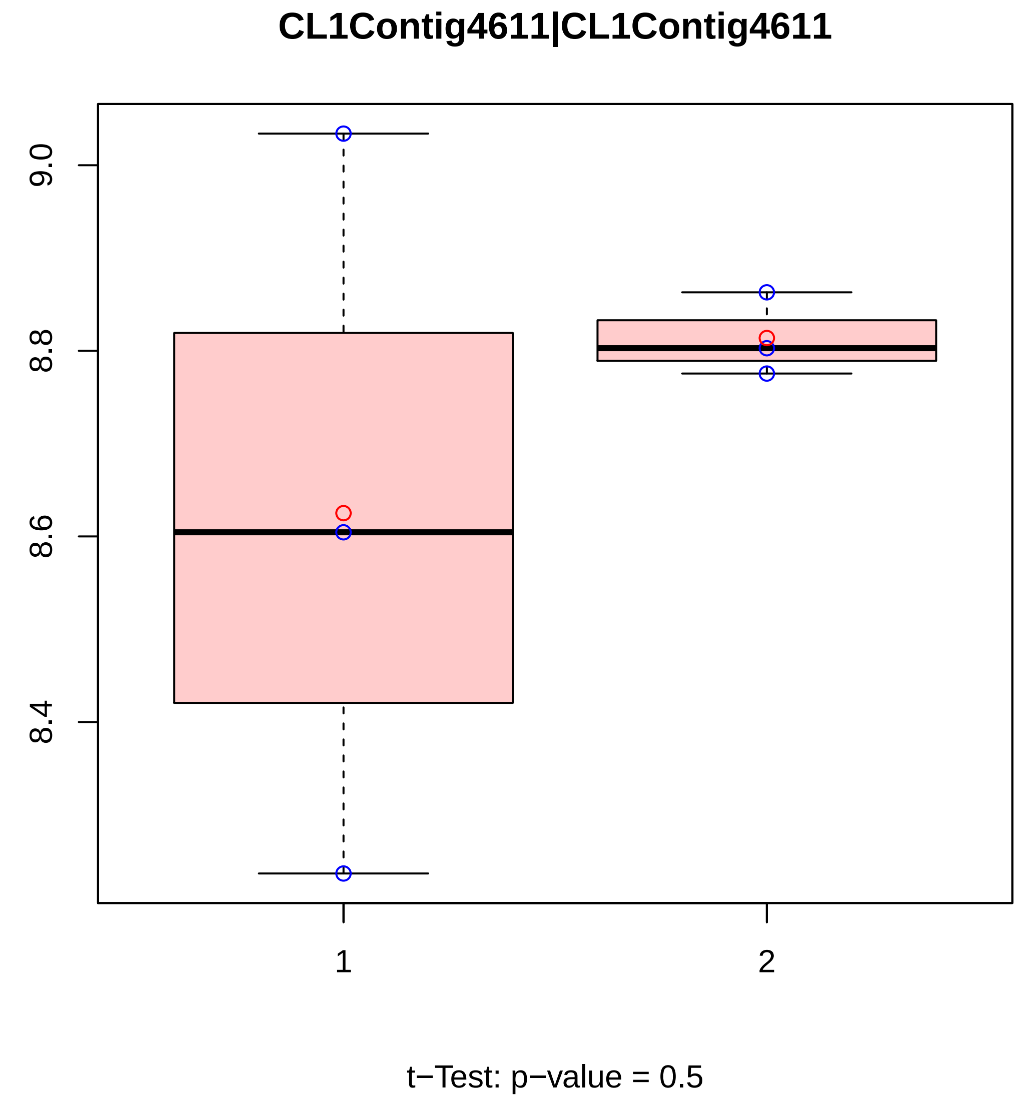
CL1Contig4611|CL1Contig4611
9.0
8.8
8.6
8.4
1
2
t−Test: p−value = 0.5

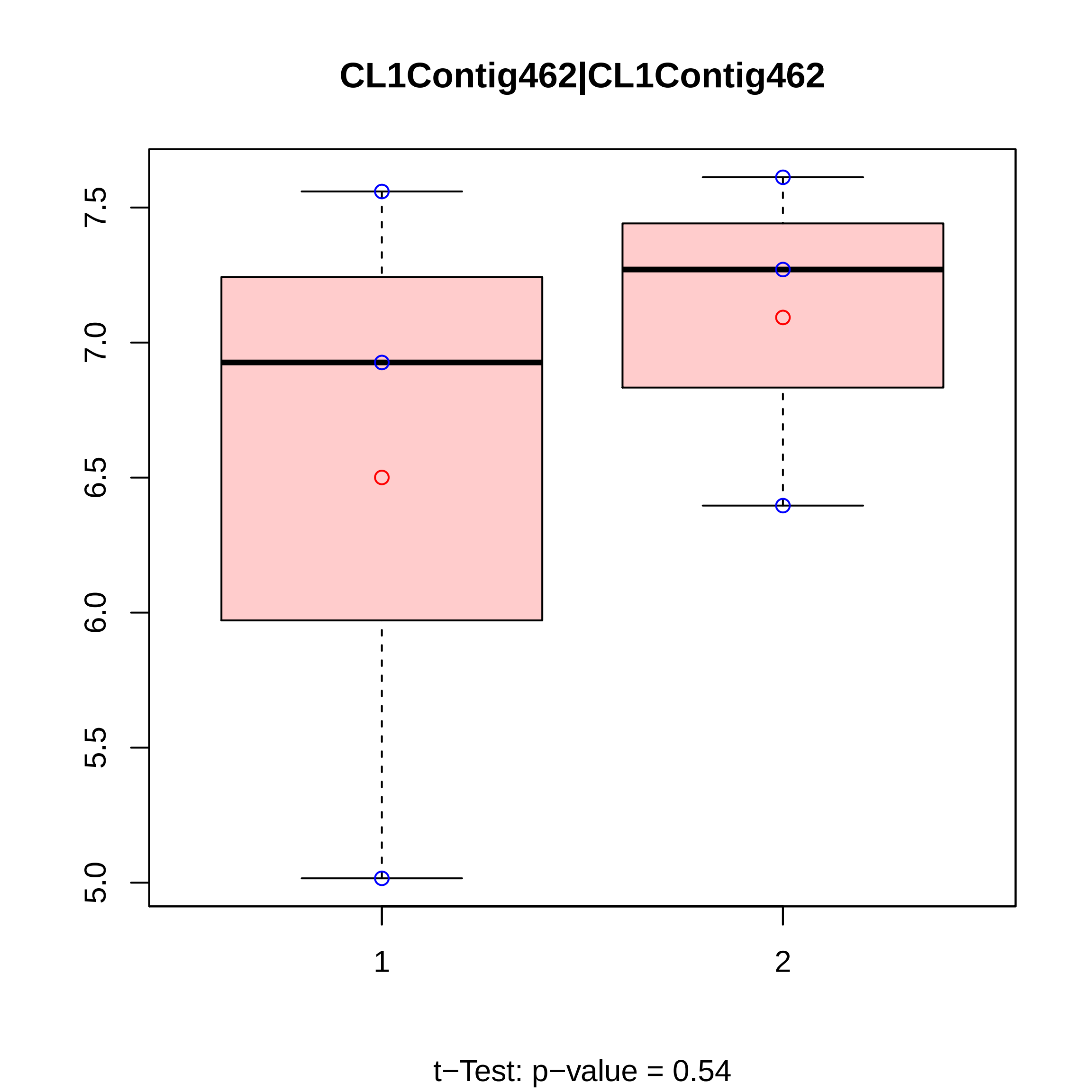
CL1Contig462|CL1Contig462
7.5
7.0
6.5
6.0
5.5
5.0
1
2
t−Test: p−value = 0.54

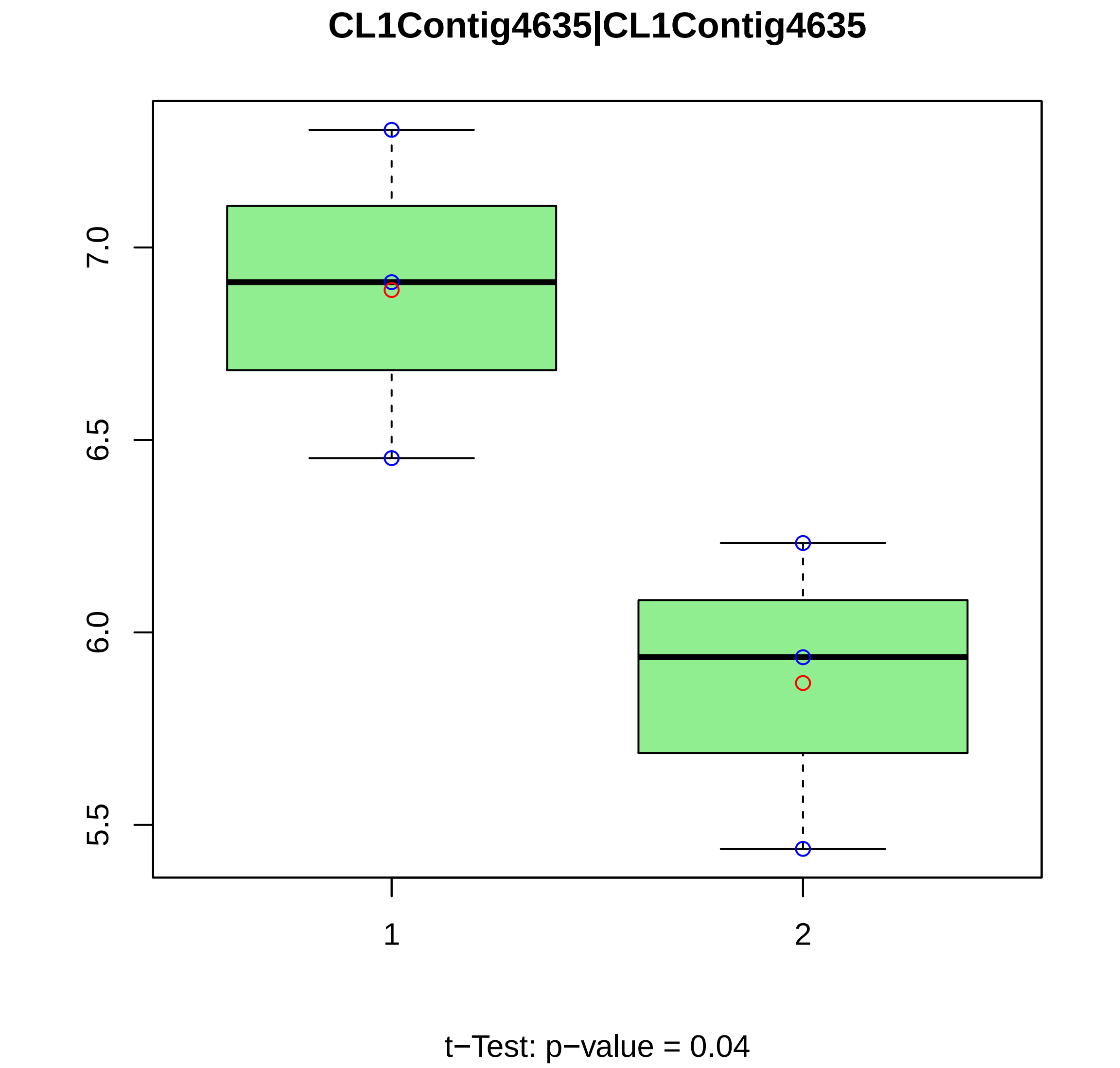
CL1Contig4635|CL1Contig4635
7.0
6.5
6.0
5.5
1
2
t−Test: p−value = 0.04

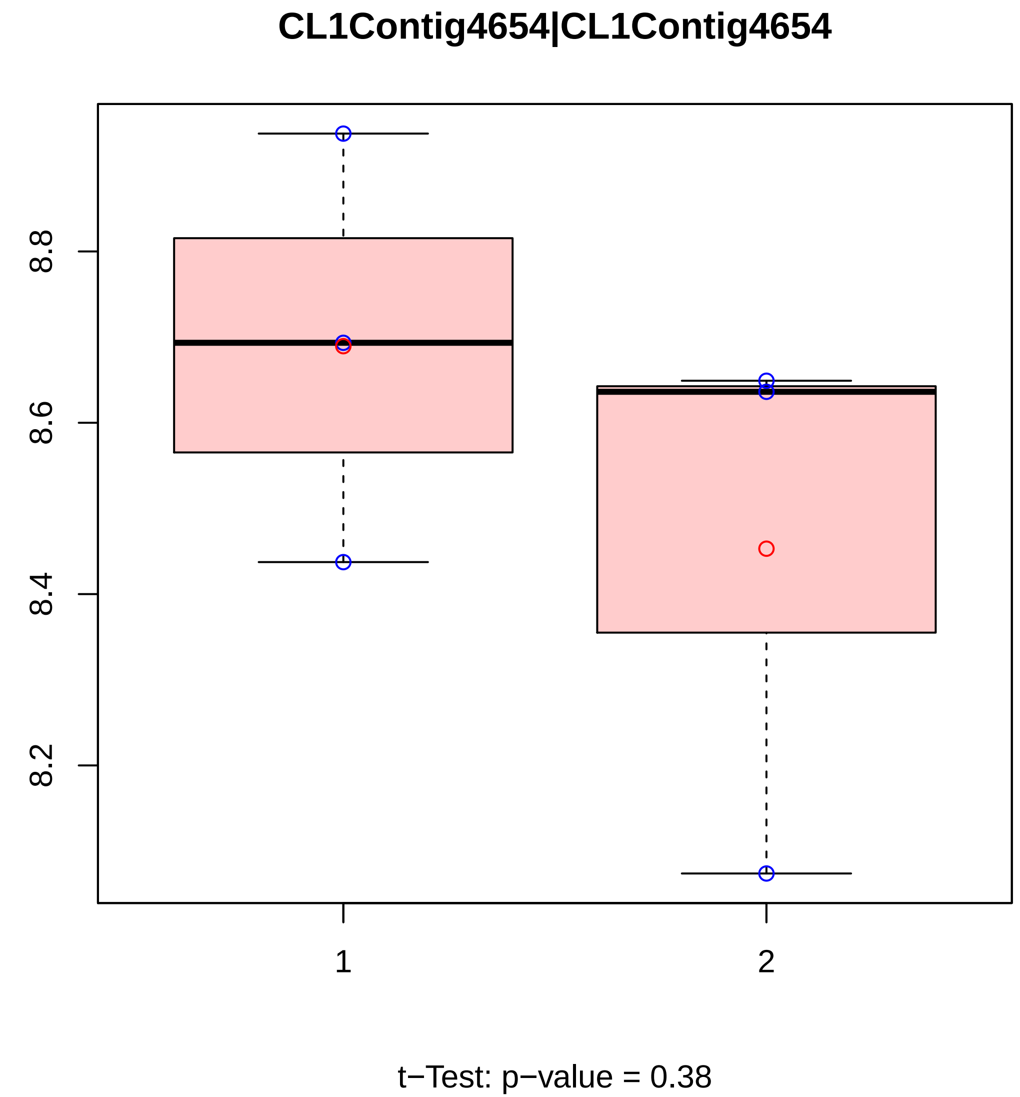
CL1Contig4654|CL1Contig4654
8.2
8.4
8.6
8.8
1
2
t−Test: p−value = 0.38

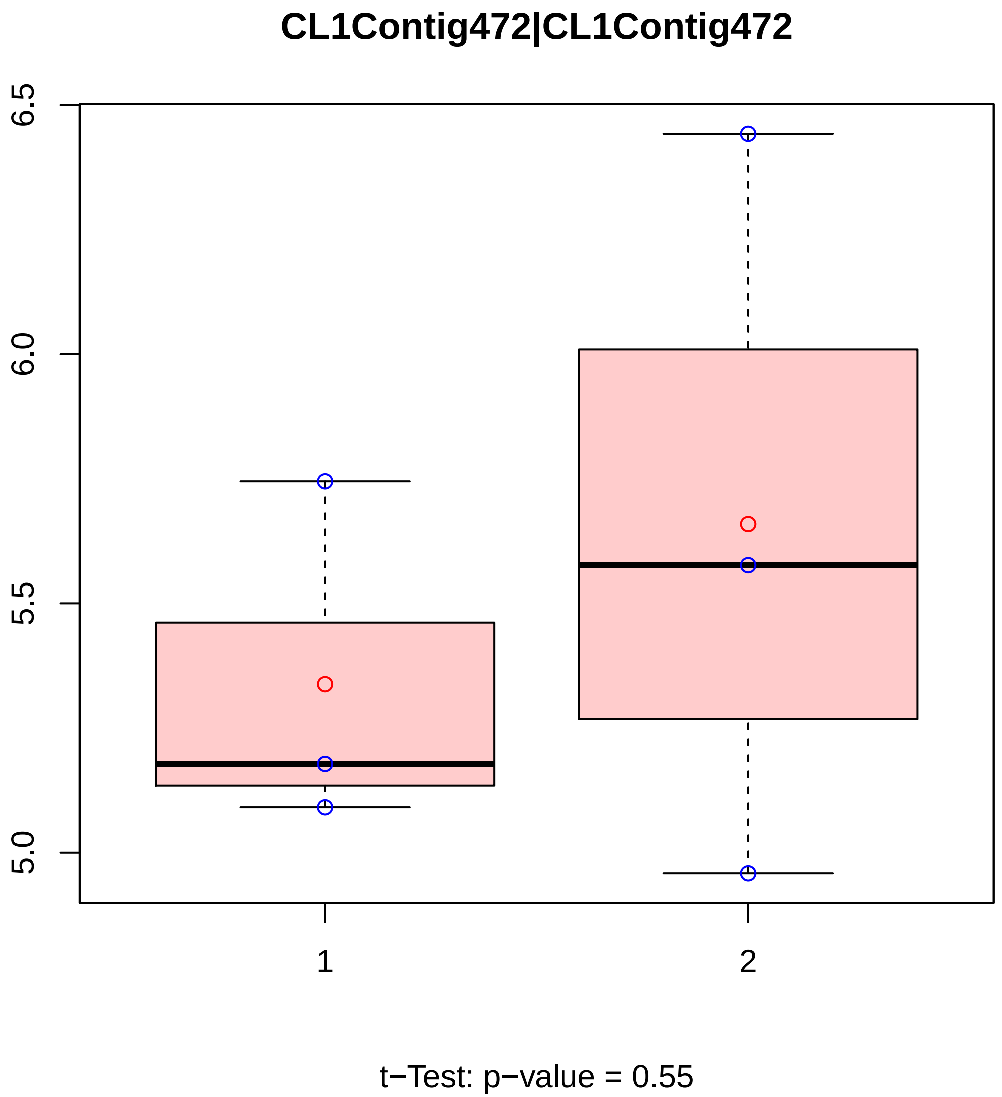
CL1Contig472|CL1Contig472
6.5
6.0
5.5
5.0
1
2
t−Test: p−value = 0.55

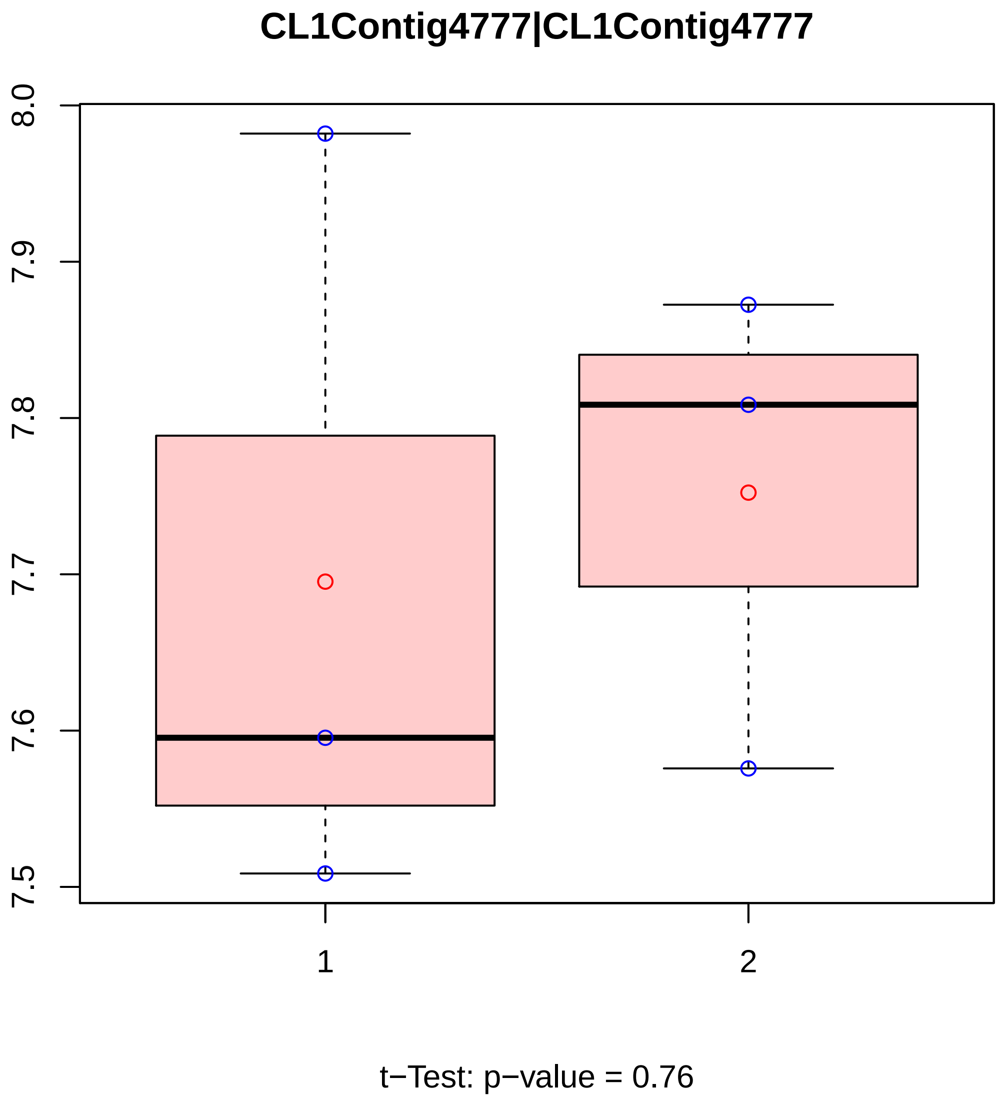
CL1Contig4777|CL1Contig4777
8.0
7.9
7.8
7.7
7.6
7.5
1
2
t−Test: p−value = 0.76

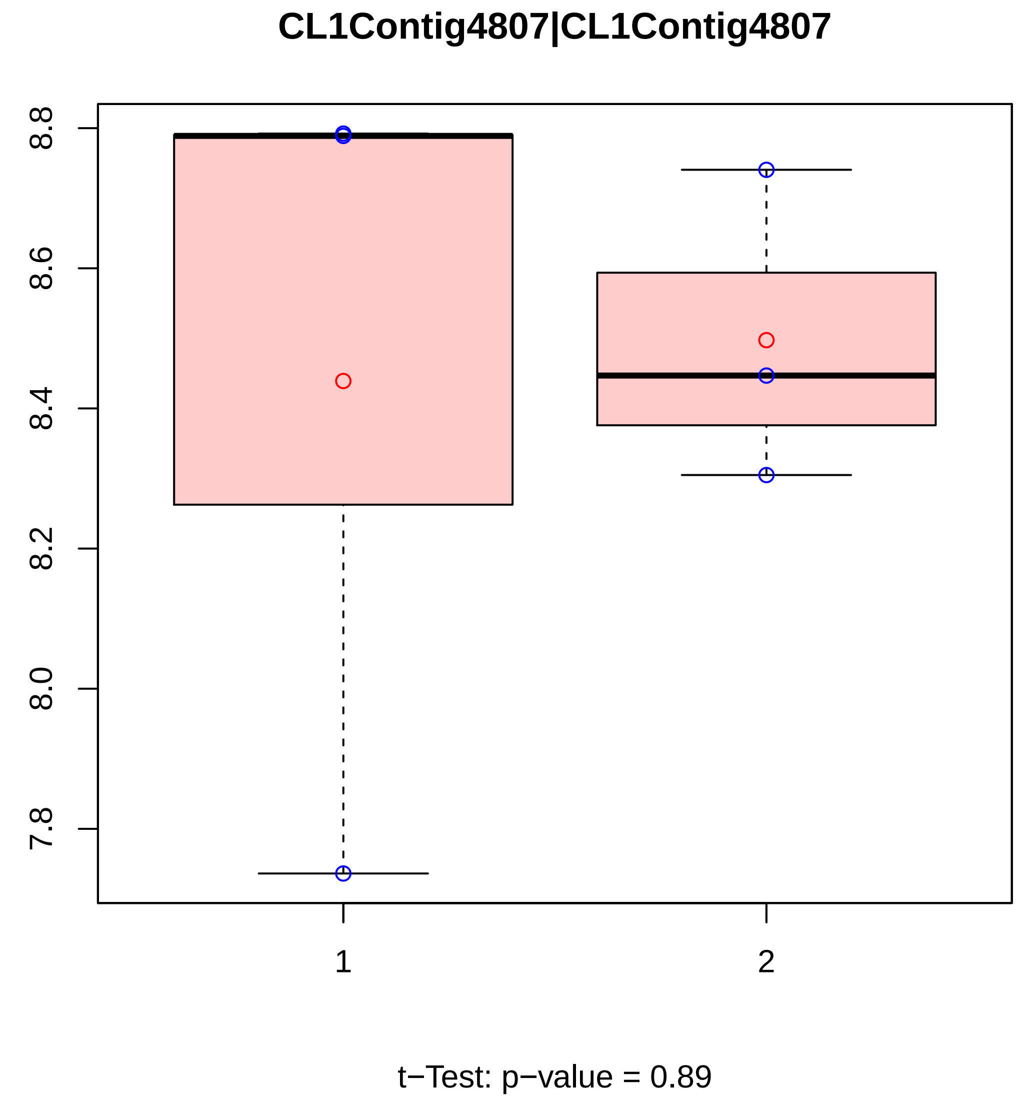
CL1Contig4807|CL1Contig4807
8.8
8.6
8.4
8.2
8.0
7.8
1
2
t−Test: p−value = 0.89

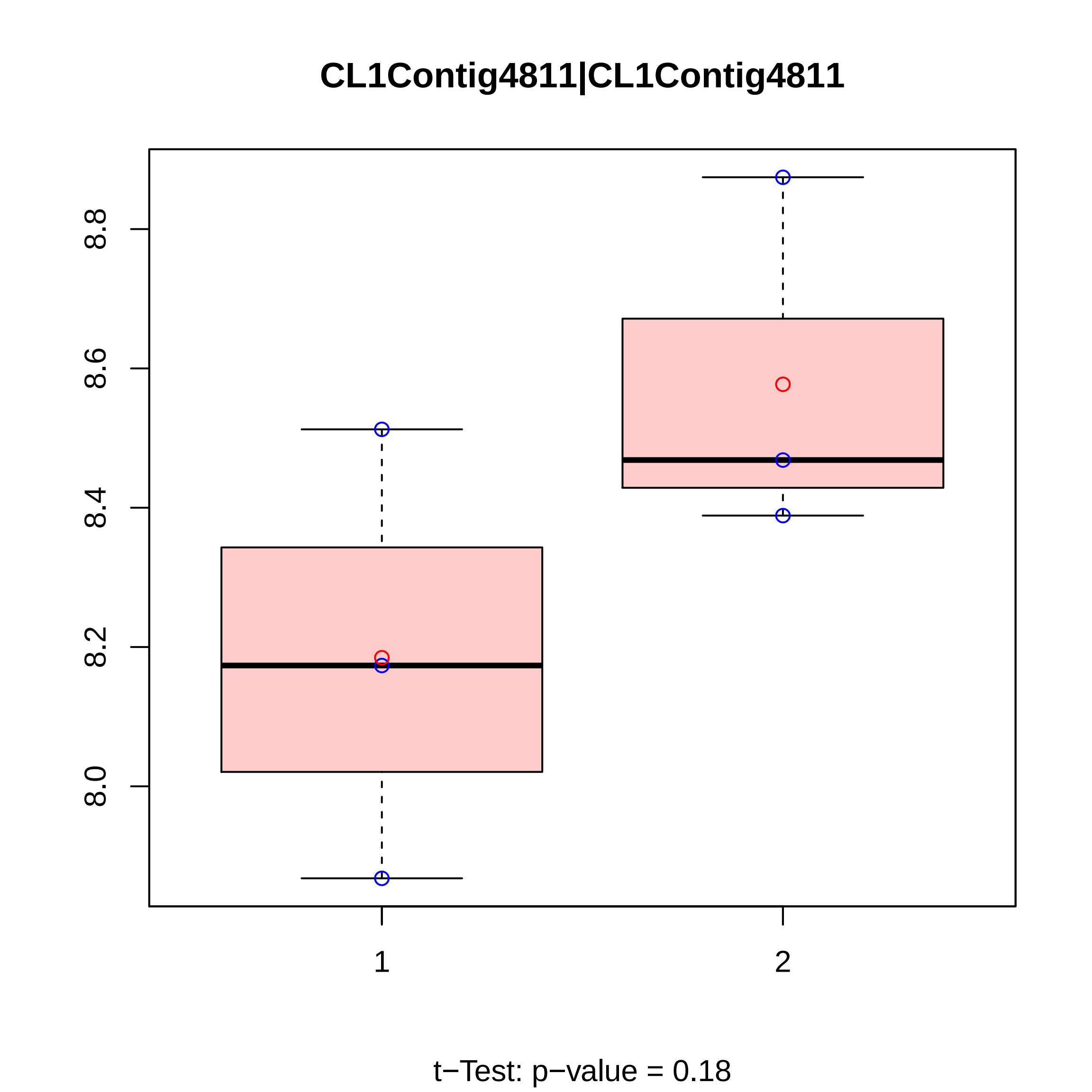
CL1Contig4811|CL1Contig4811
8.0
8.2
8.4
8.6
8.8
1
2
t−Test: p−value = 0.18

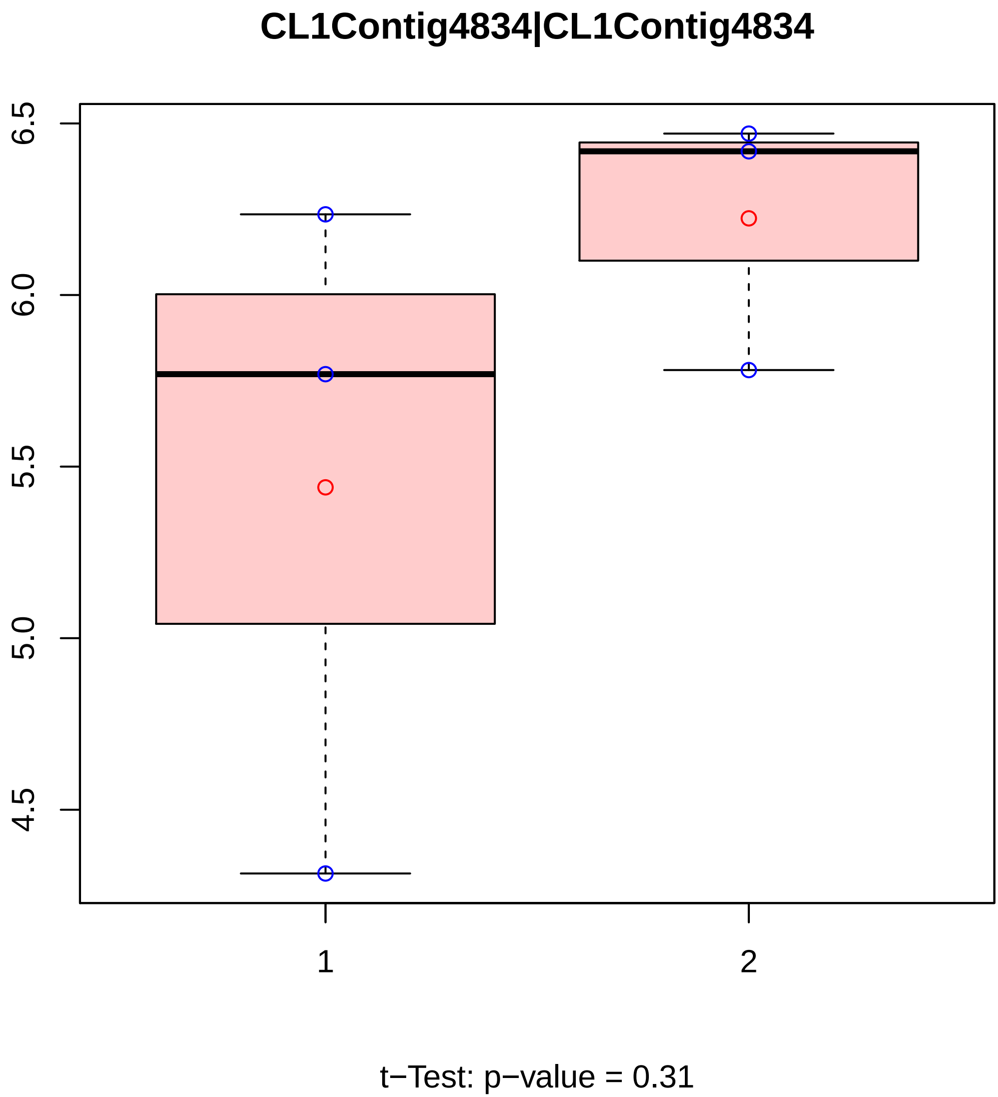
CL1Contig4834|CL1Contig4834
6.5
6.0
5.5
5.0
4.5
1
2
t−Test: p−value = 0.31

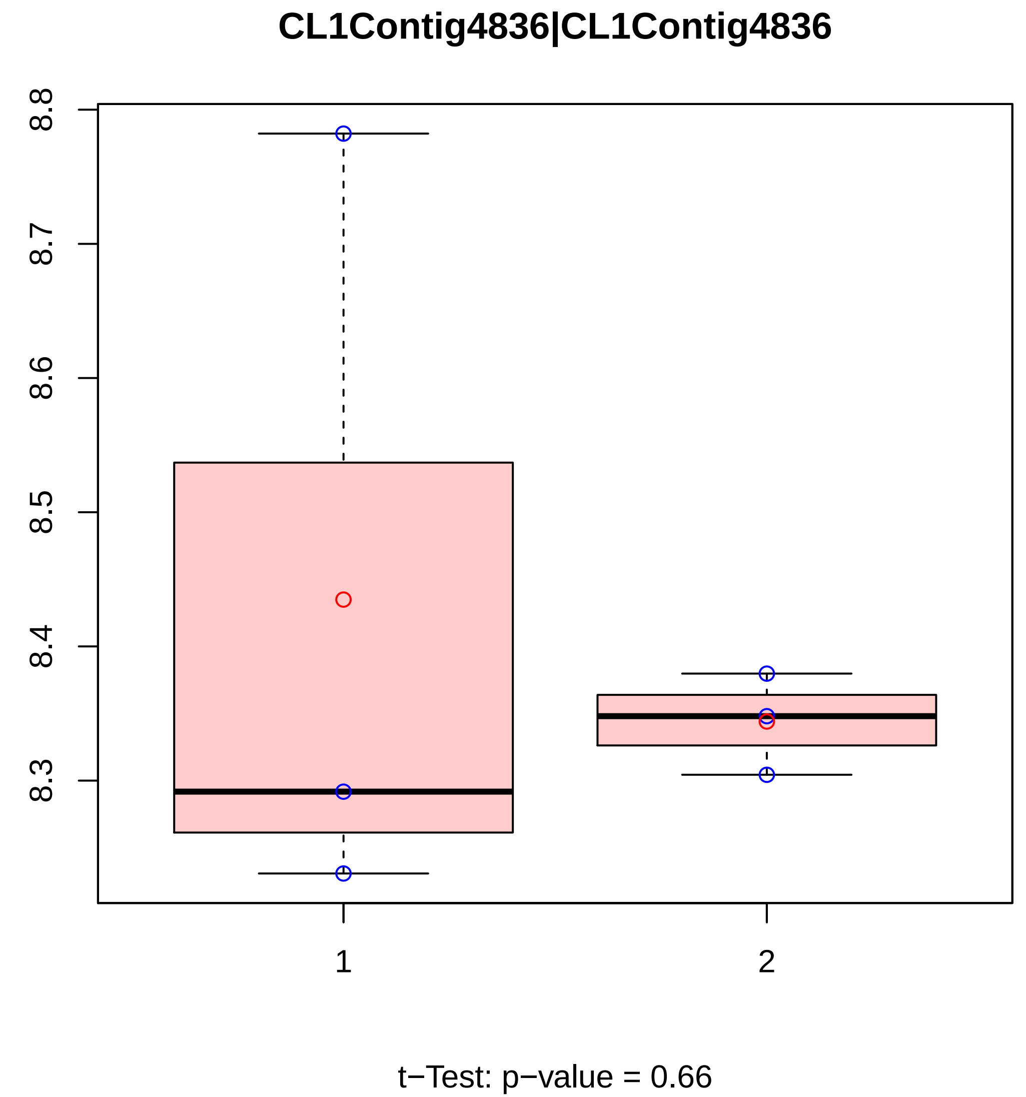
CL1Contig4836|CL1Contig4836
8.8
8.7
8.6
8.5
8.4
8.3
1
2
t−Test: p−value = 0.66

## CL1Contig4859|CL1Contig4859

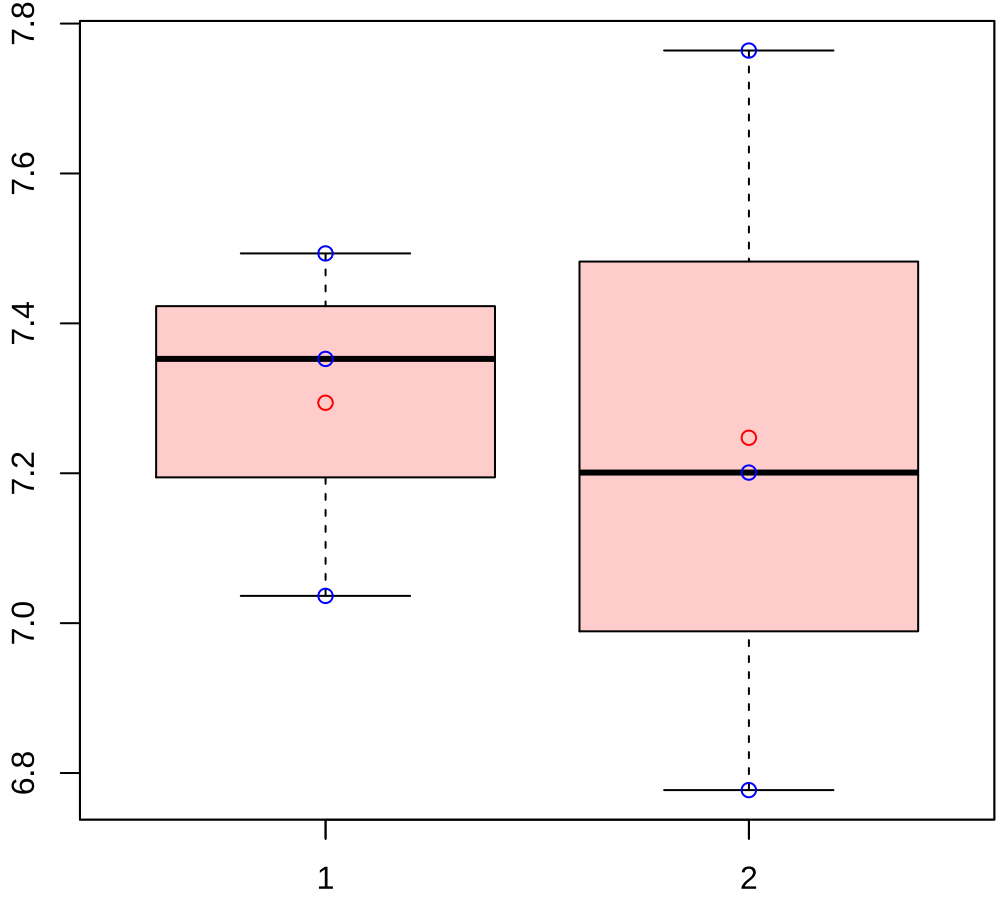


t−Test: p−value = 0.89

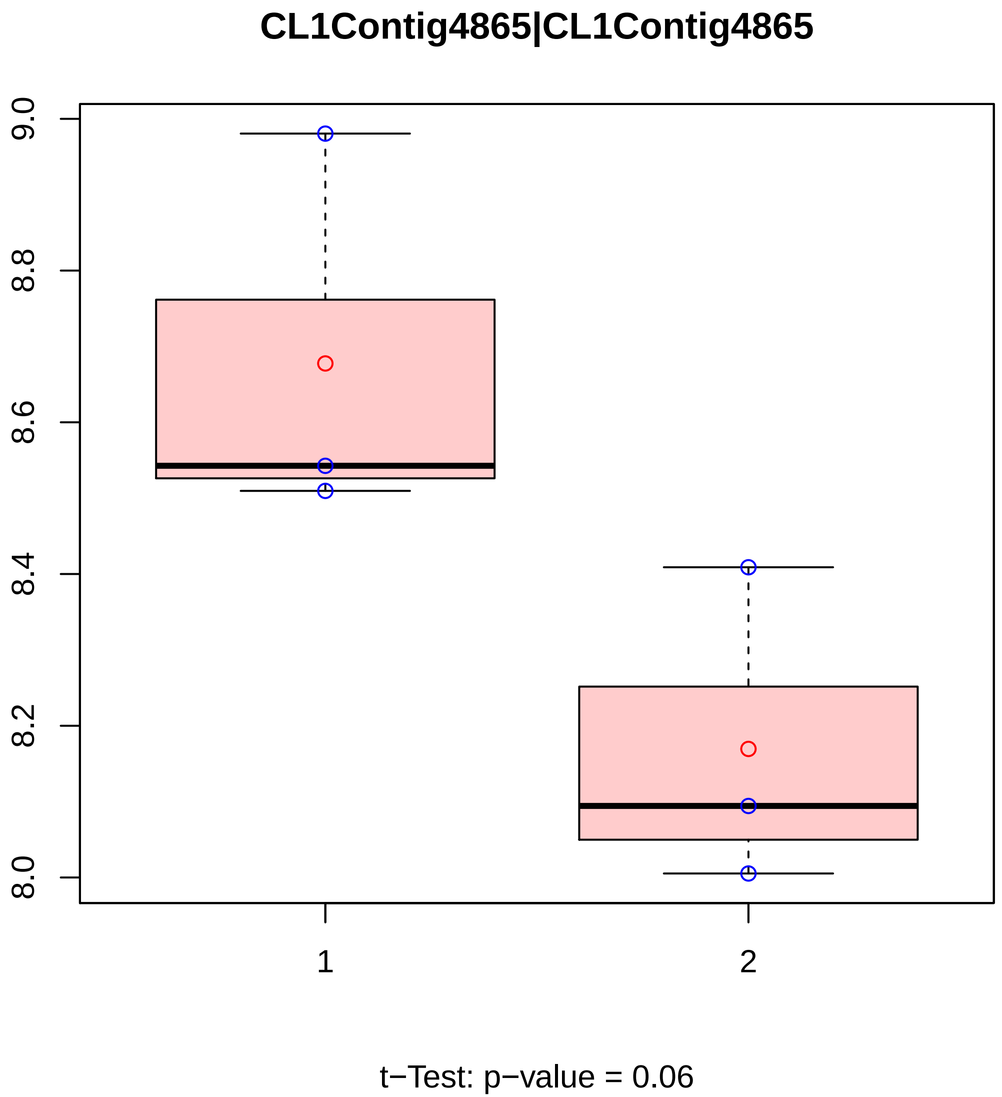
CL1Contig4865|CL1Contig4865
9.0
8.8
8.6
8.4
8.2
8.0
1
2
t−Test: p−value = 0.06

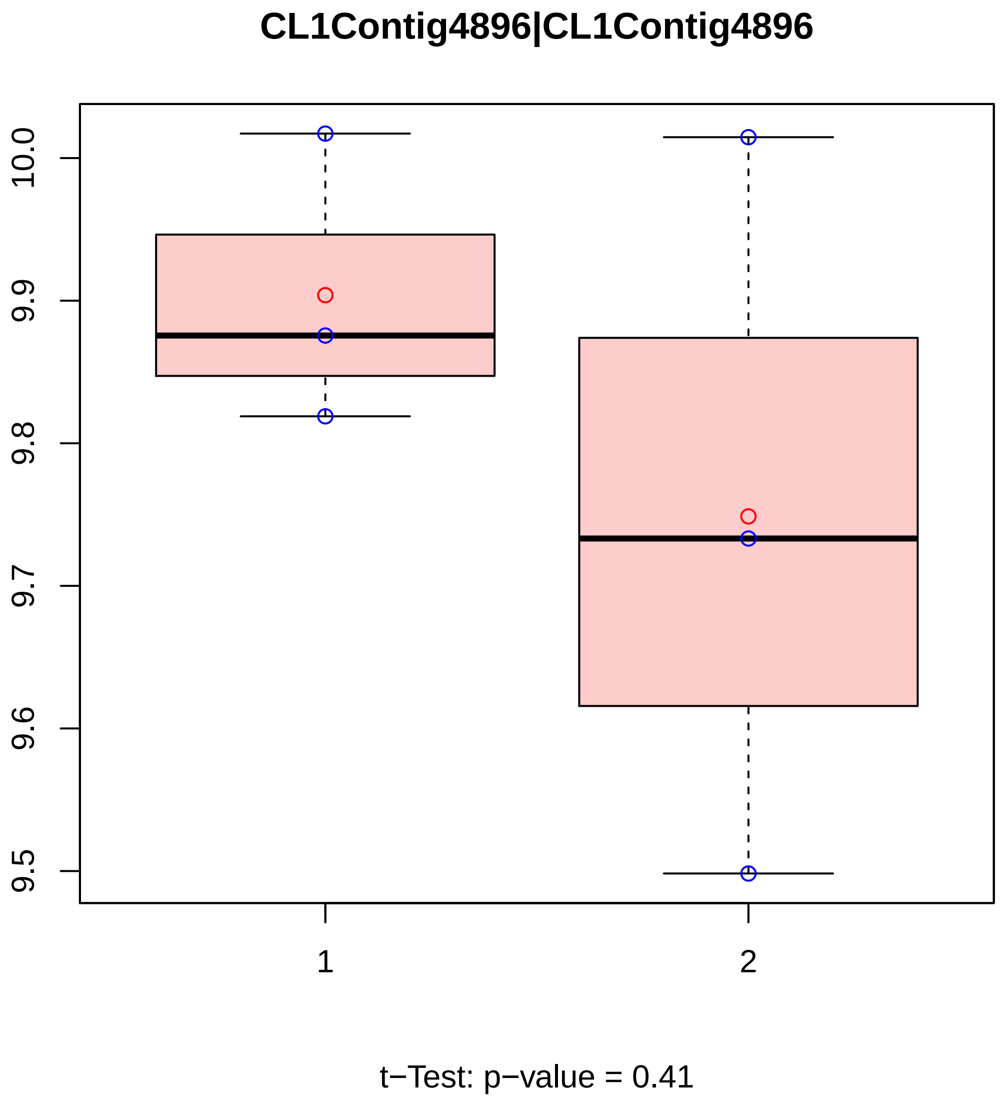

CL1Contig4896|CL1Contig4896
10.0
9.9
9.8
9.7
9.6
9.5
1
2
t−Test: p−value = 0.41

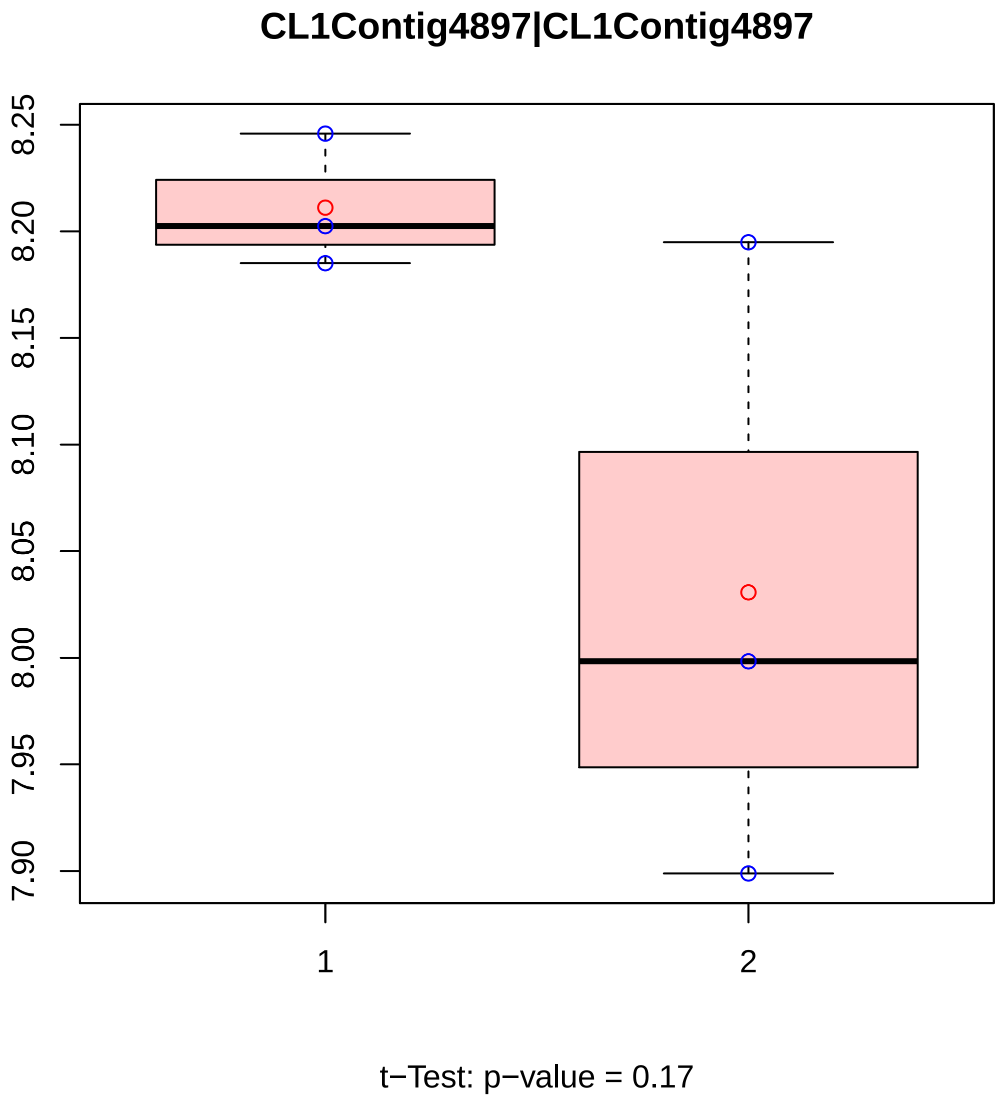
CL1Contig4897|CL1Contig4897
8.25
8.20
8.15
8.10
8.05
8.00
7.95
7.90
1
2
t−Test: p−value = 0.17

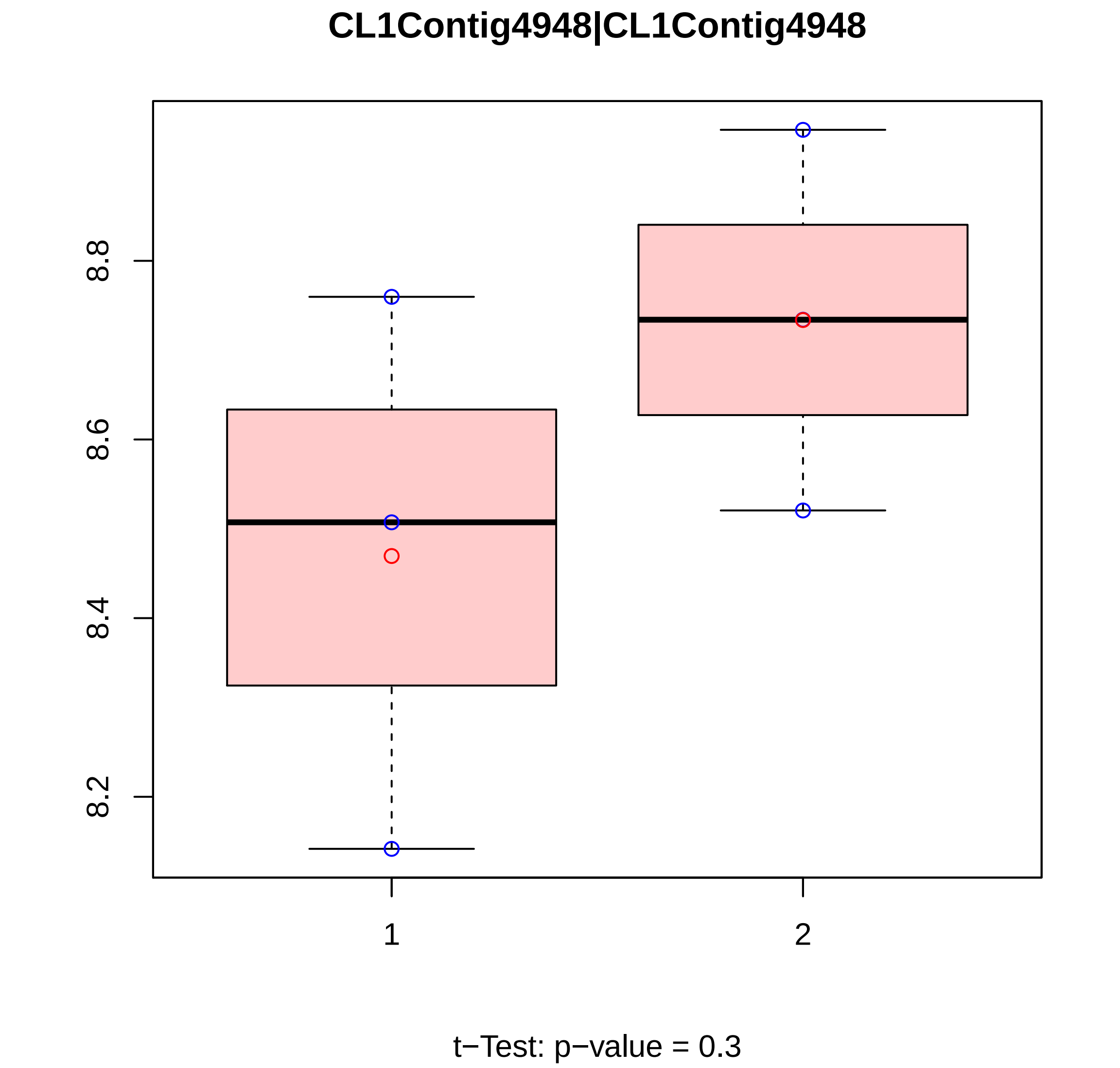
CL1Contig4948|CL1Contig4948
8.2
8.4
8.6
8.8
1
2
t−Test: p−value = 0.3

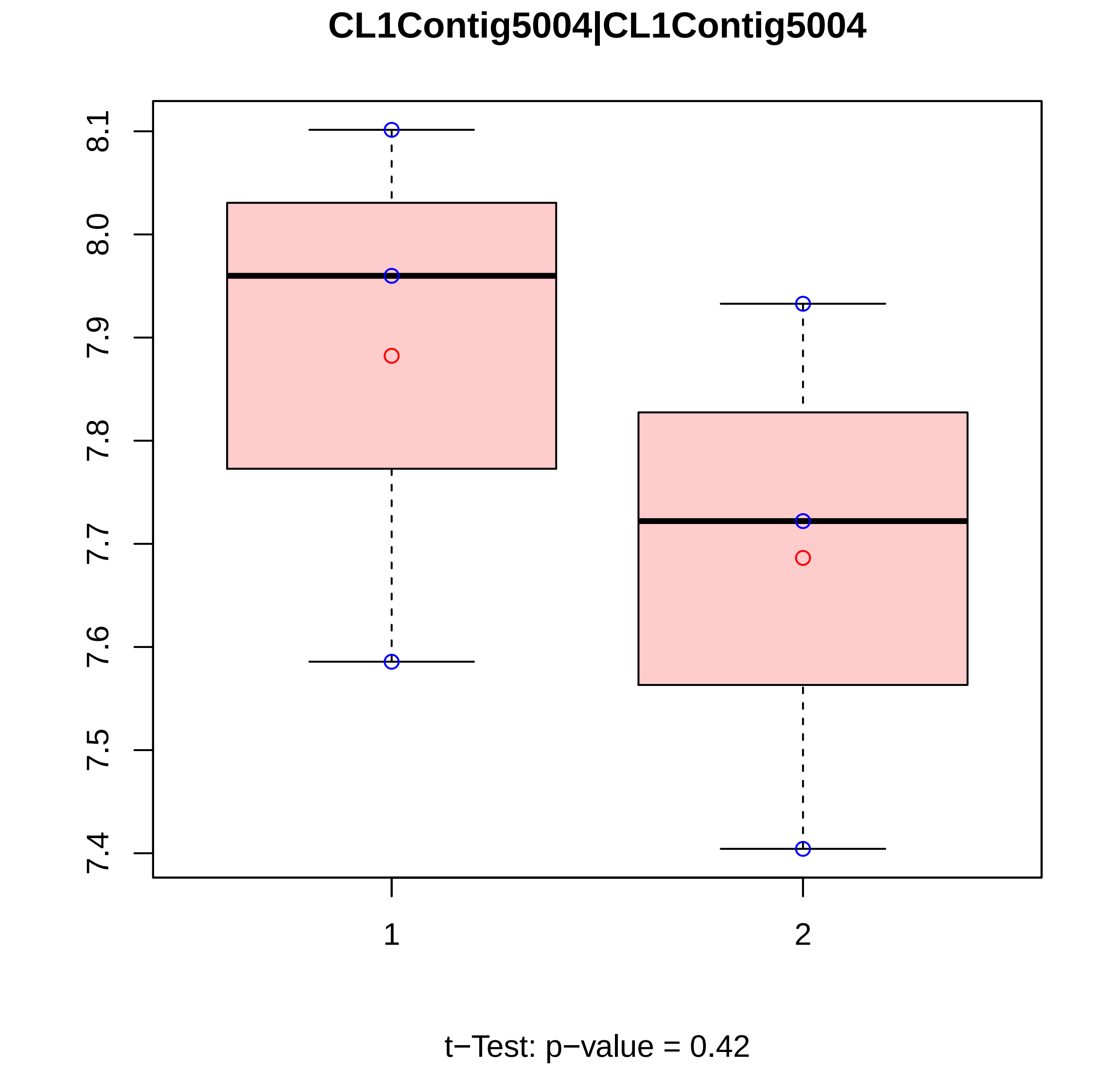
CL1Contig5004|CL1Contig5004
8.1
8.0
7.9
7.8
7.7
7.6
7.5
7.4
1
2
t−Test: p−value = 0.42

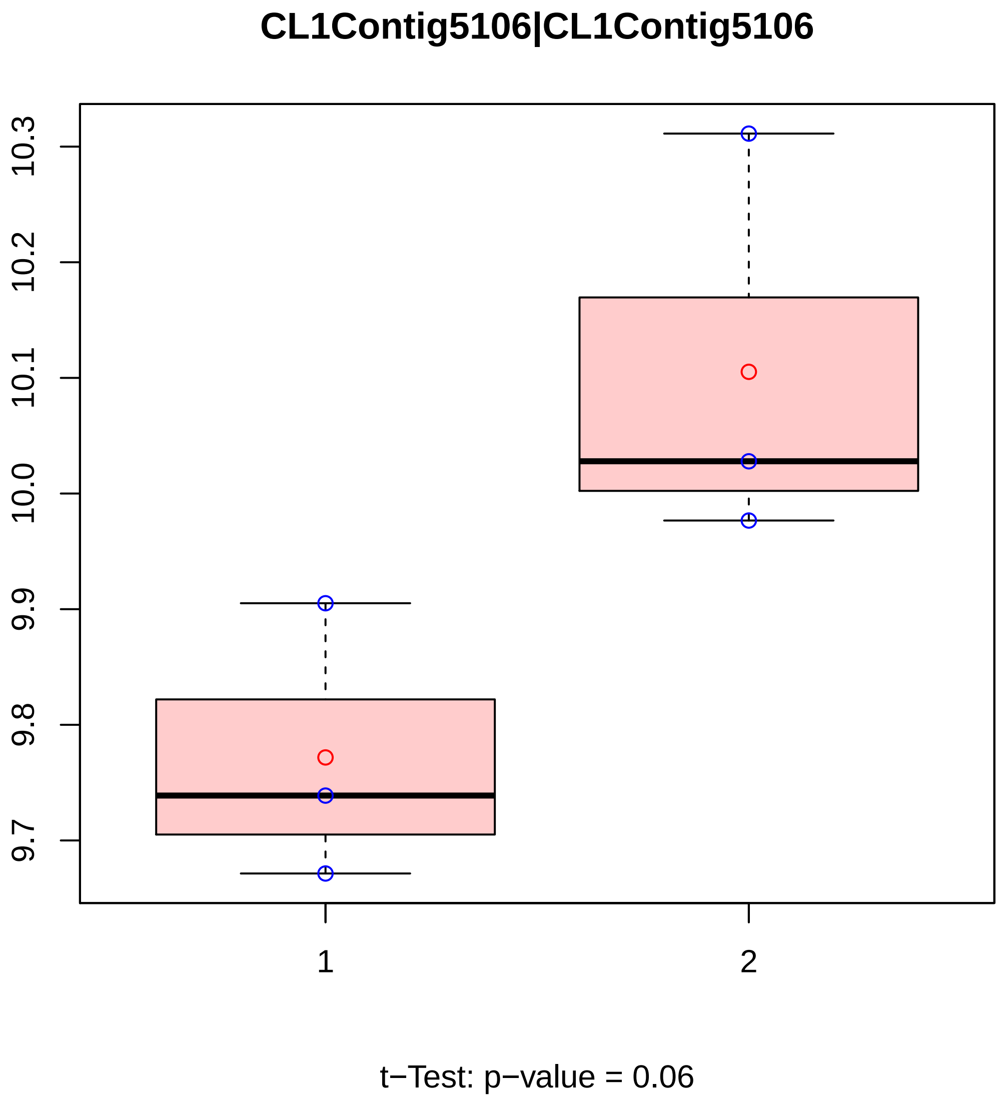
CL1Contig5106|CL1Contig5106
10.3
10.2
10.1
10.0
9.9
9.8
9.7
1
2
t−Test: p−value = 0.06

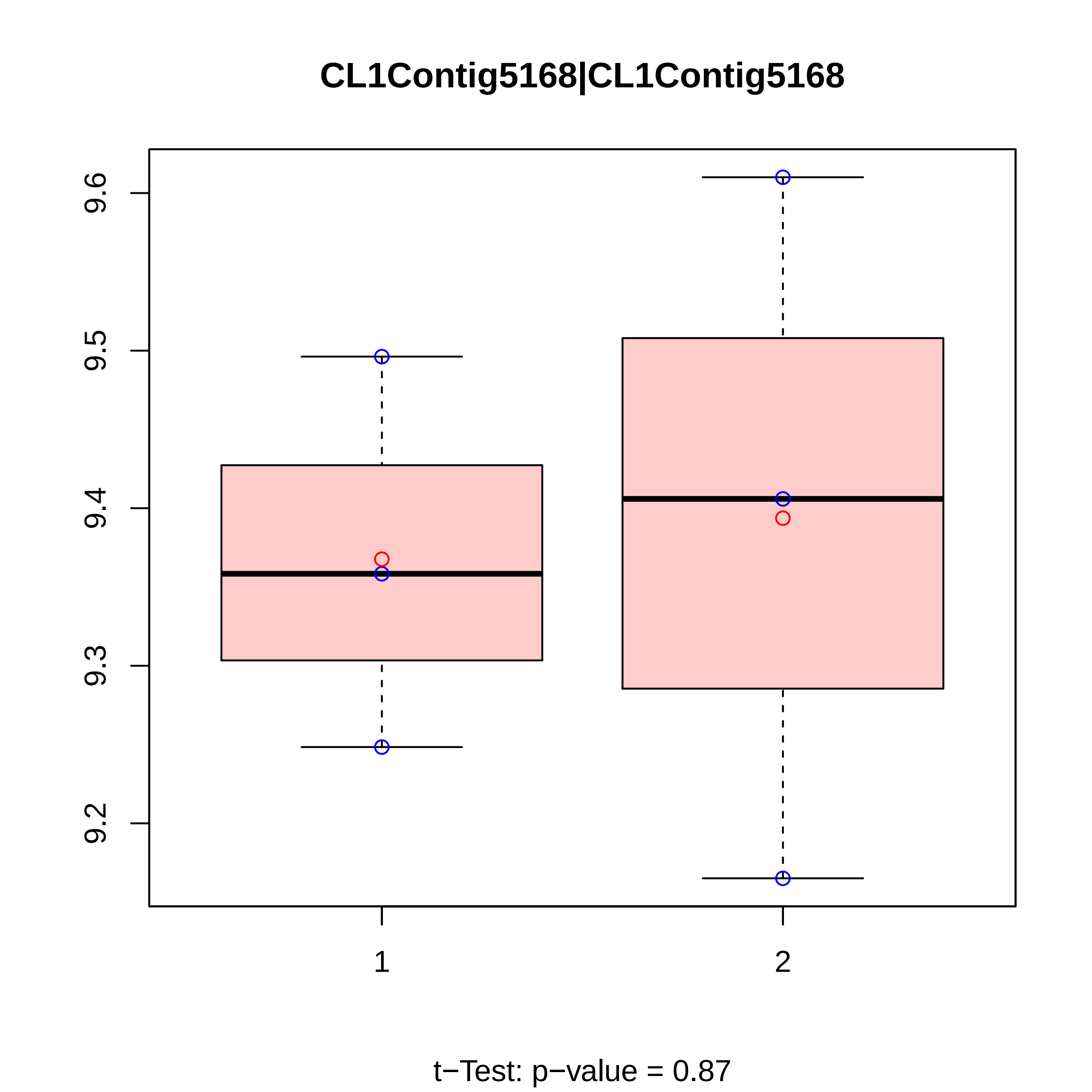
CL1Contig5168|CL1Contig5168
9.2
9.3
9.4
9.5
9.6
1
2
t−Test: p−value = 0.87

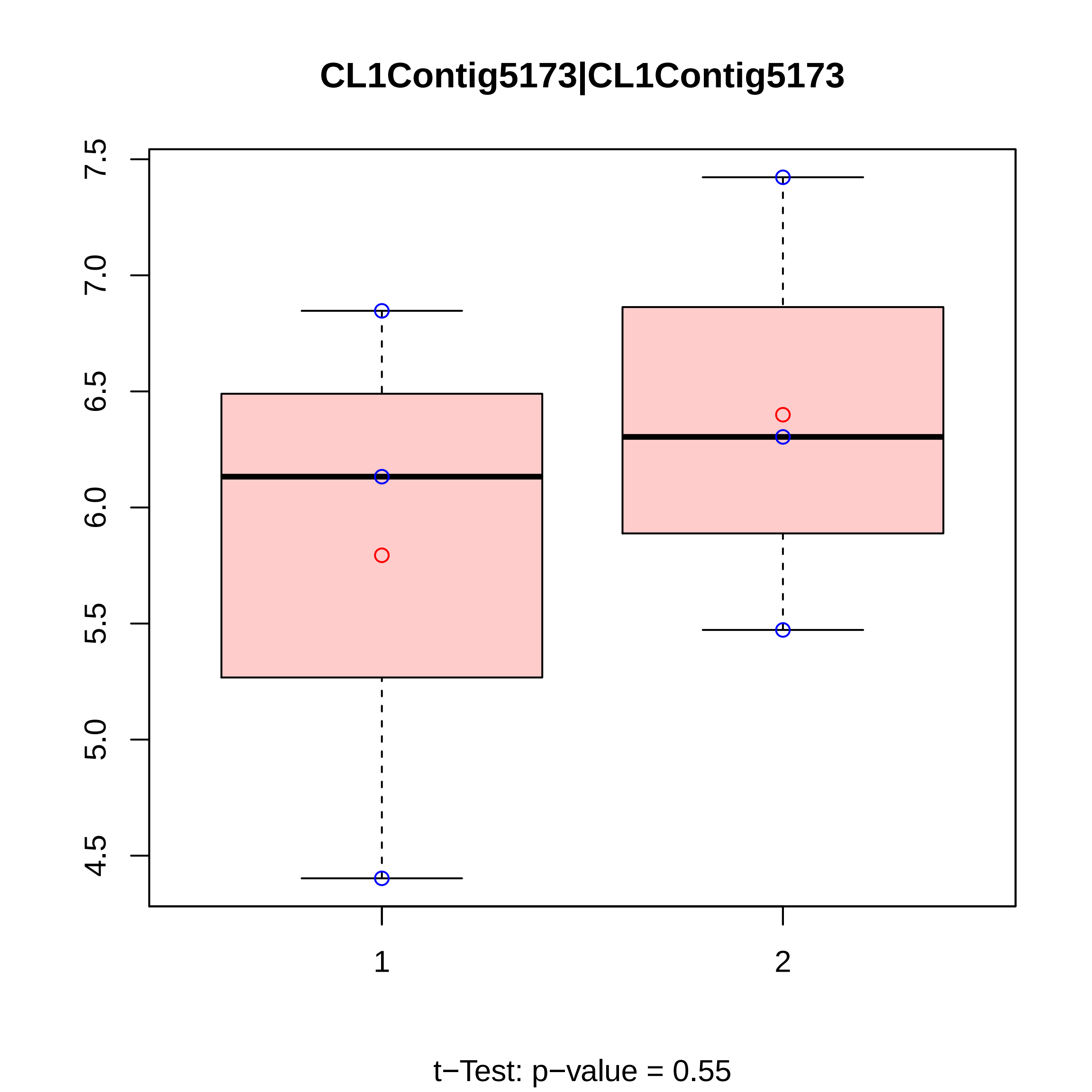
CL1Contig5173|CL1Contig5173
7.5
7.0
6.5
6.0
5.5
5.0
4.5
1
2
t−Test: p−value = 0.55

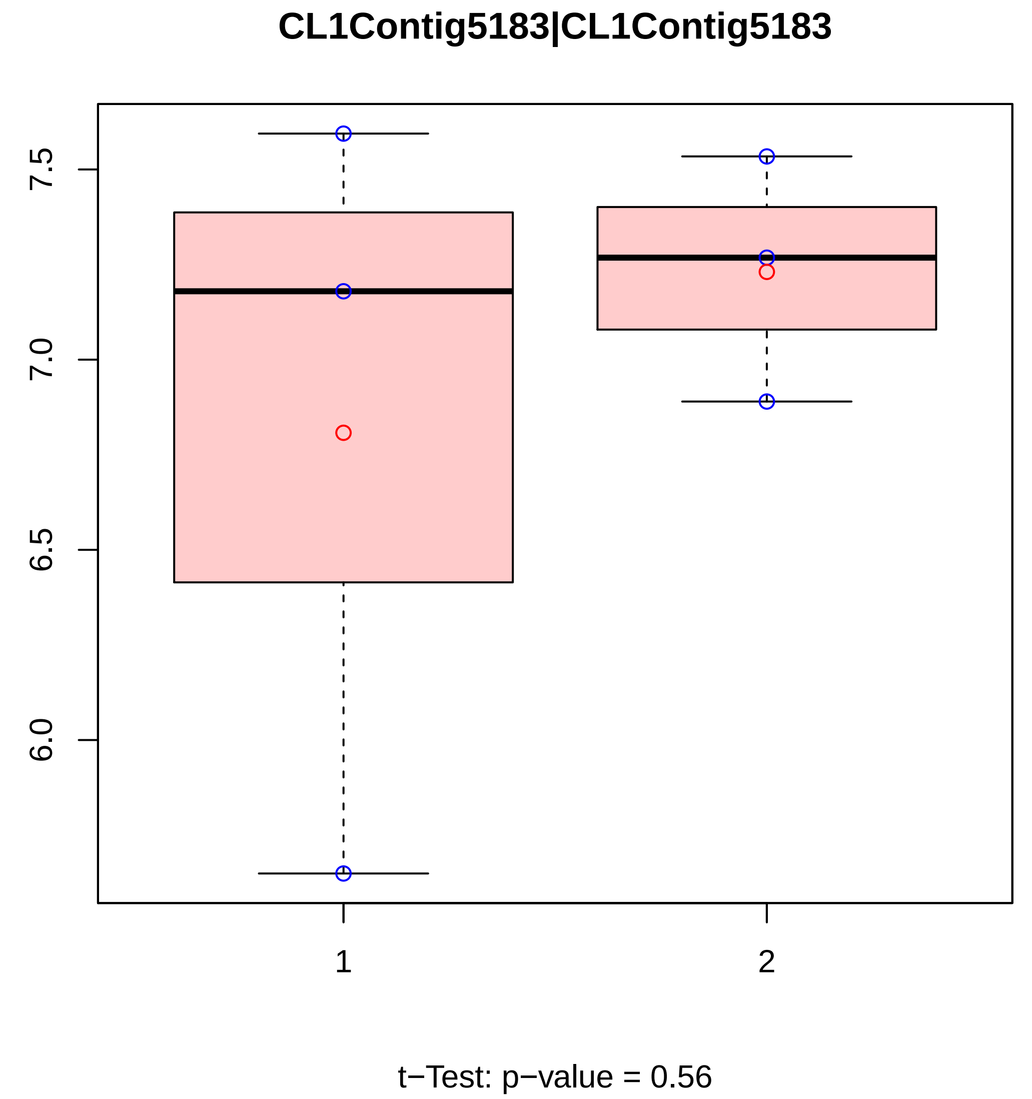
CL1Contig5183|CL1Contig5183
7.5
7.0
6.5
6.0
1
2
t−Test: p−value = 0.56

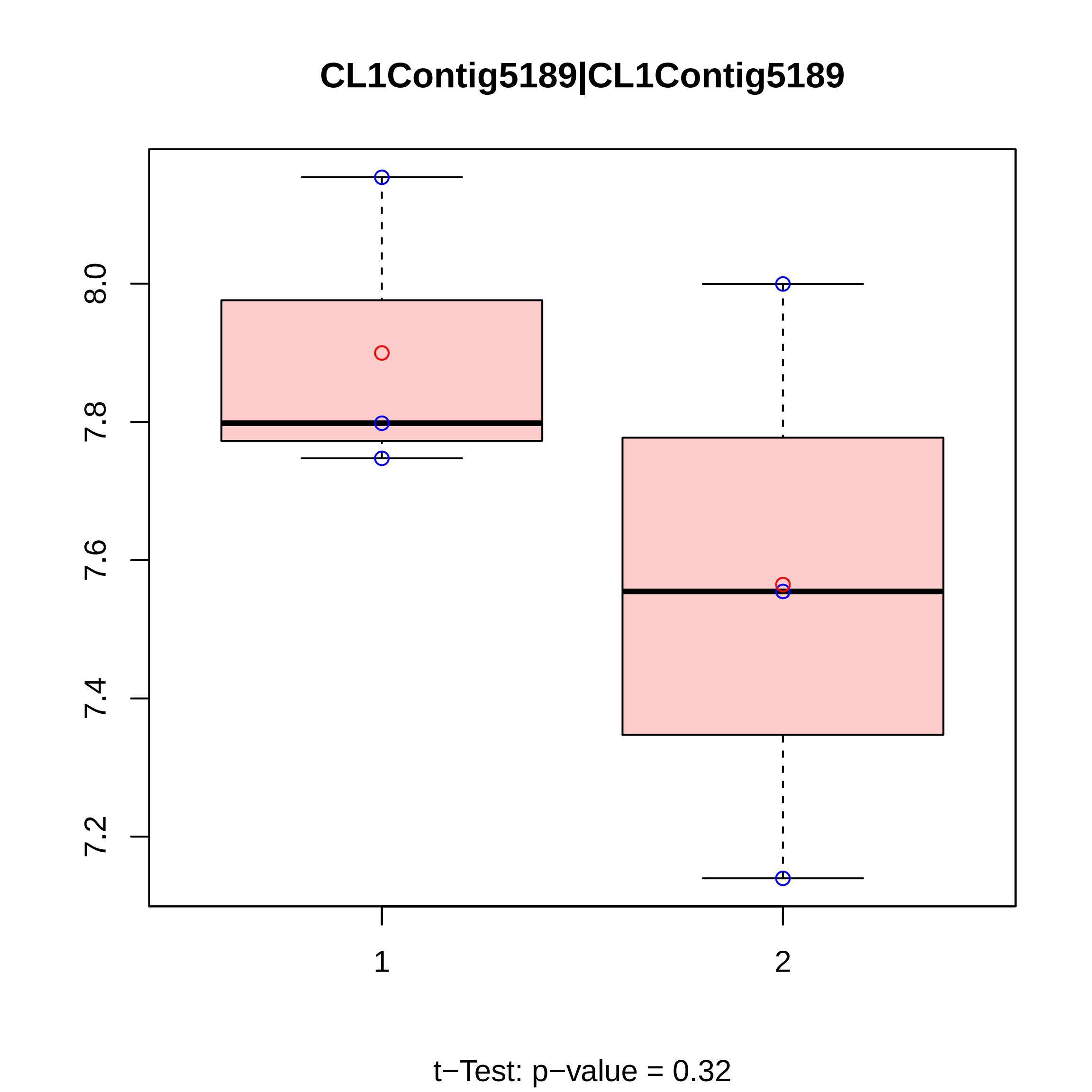
CL1Contig5189|CL1Contig5189
8.0
7.8
7.6
7.4
7.2
1
2
t−Test: p−value = 0.32

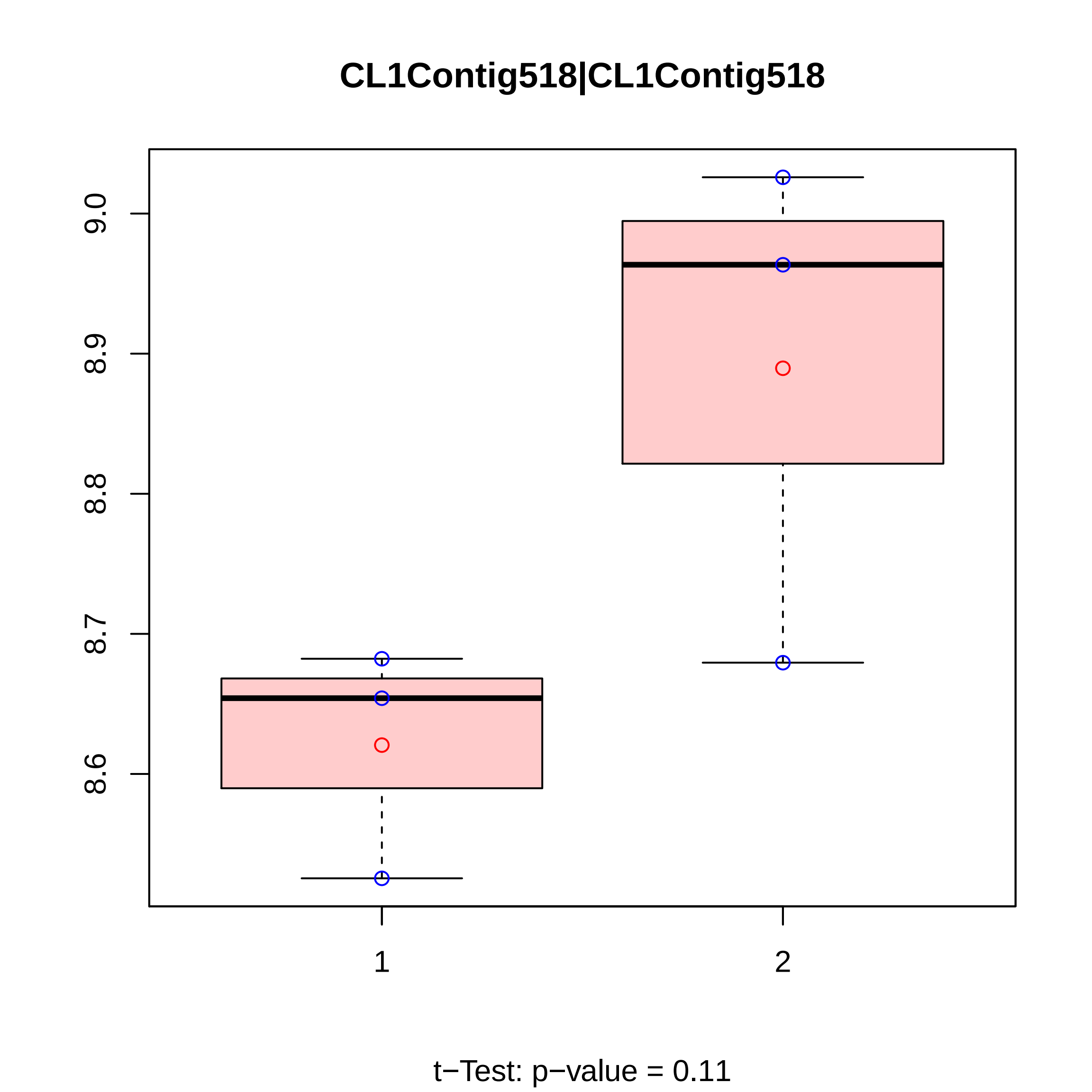
CL1Contig518|CL1Contig518
9.0
8.9
8.8
8.7
8.6
1
2
t−Test: p−value = 0.11

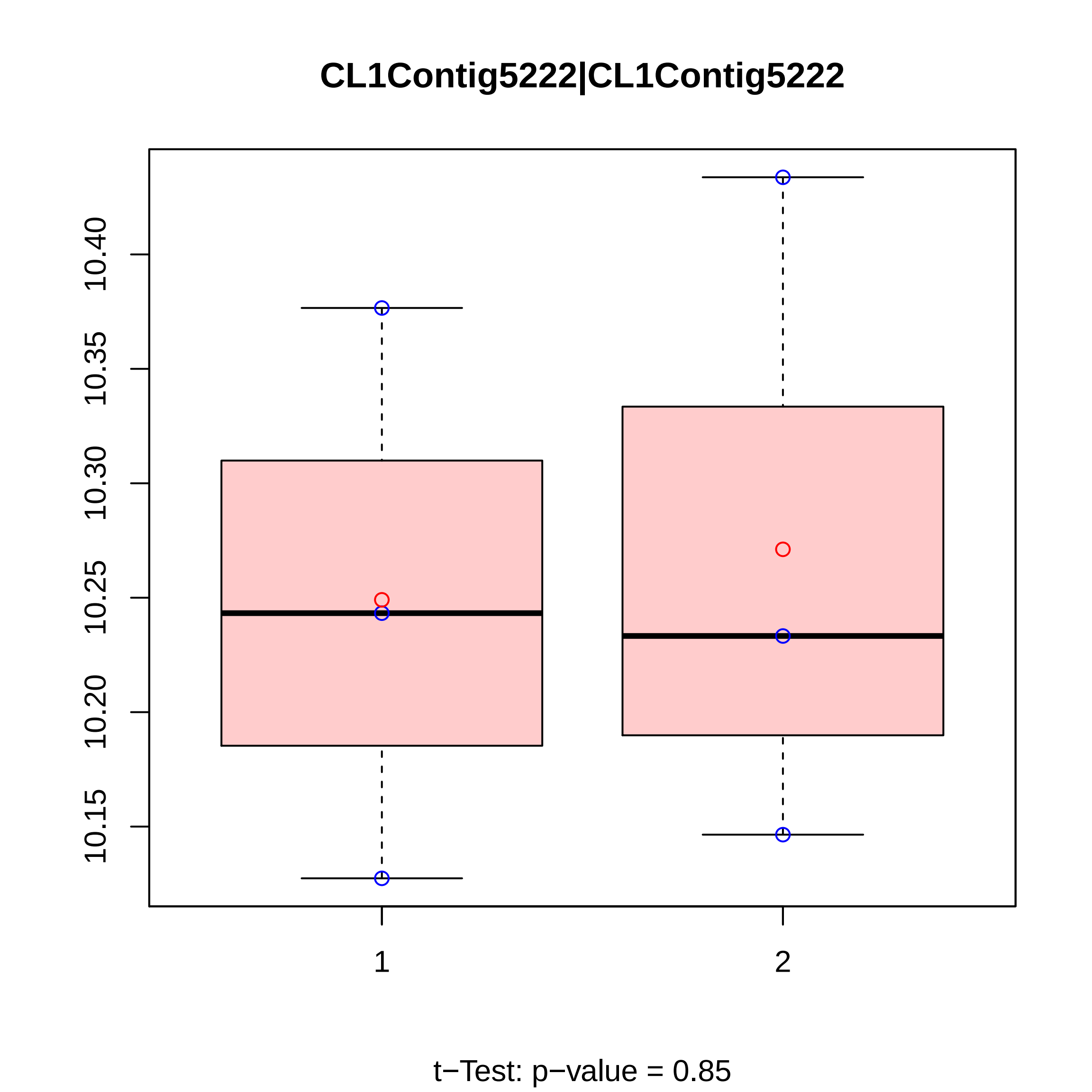
CL1Contig5222|CL1Contig5222
10.15
10.20
10.25
10.30
10.35
10.40
1
2
t−Test: p−value = 0.85

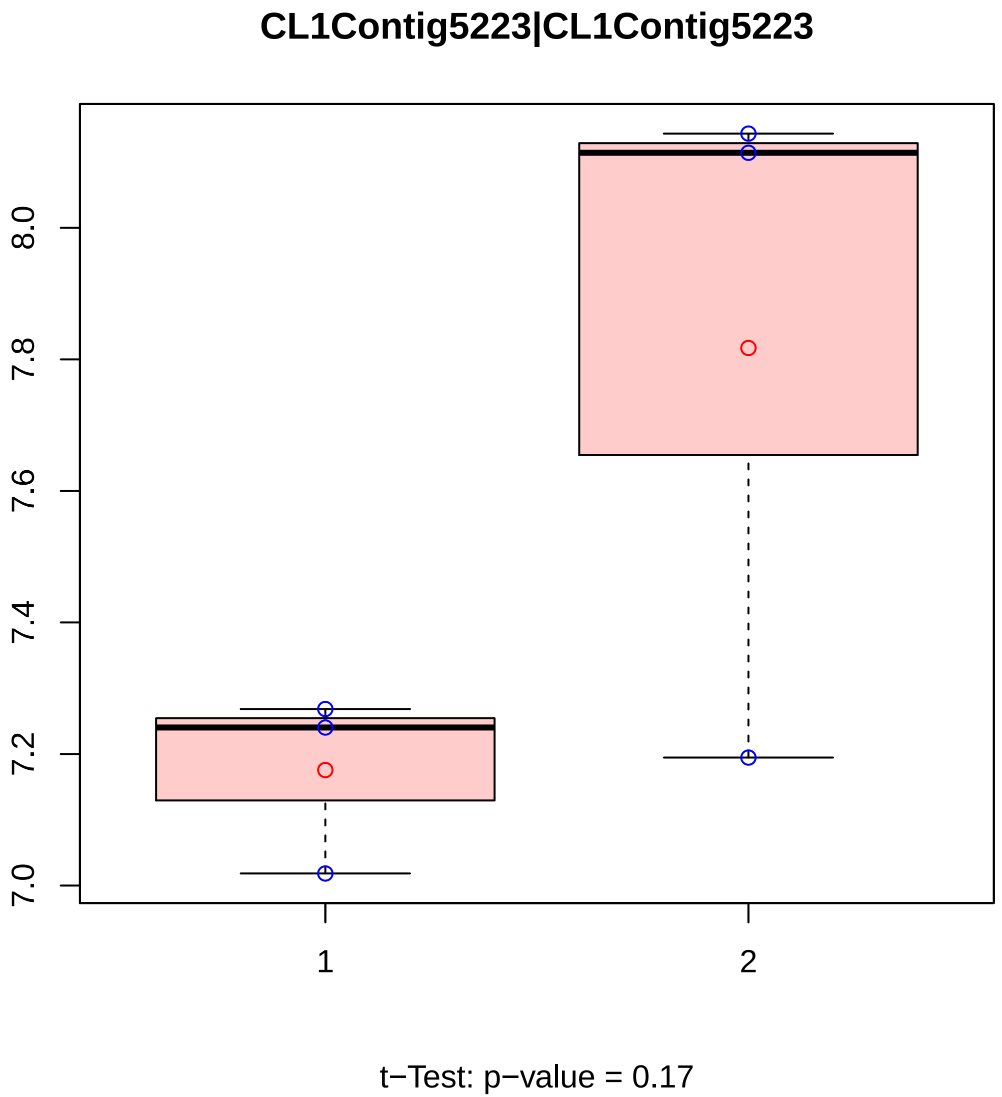
CL1Contig5223|CL1Contig5223
7.0
7.2
7.4
7.6
7.8
8.0
1
2
t−Test: p−value = 0.17

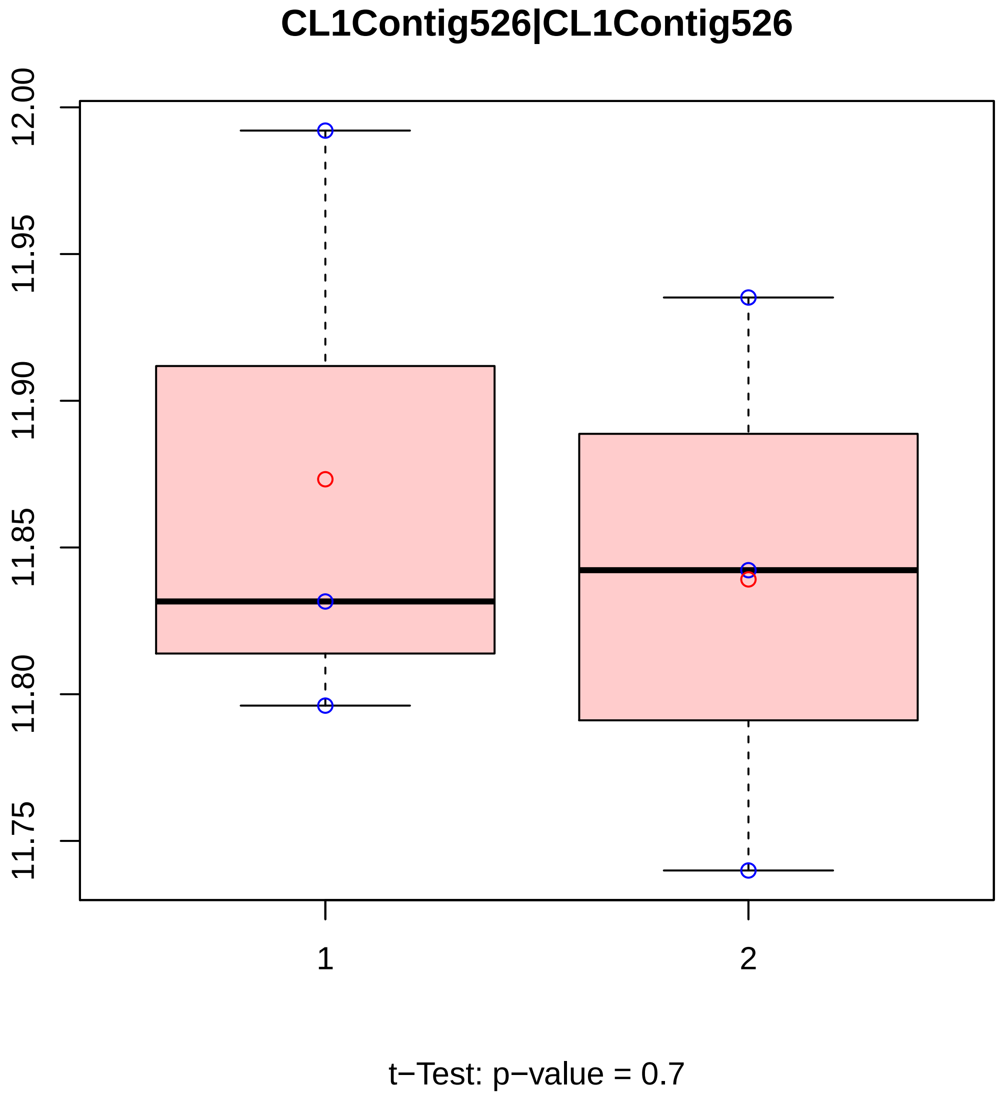
CL1Contig526|CL1Contig526
12.00
11.95
11.90
11.85
11.80
11.75
1
2
t−Test: p−value = 0.7

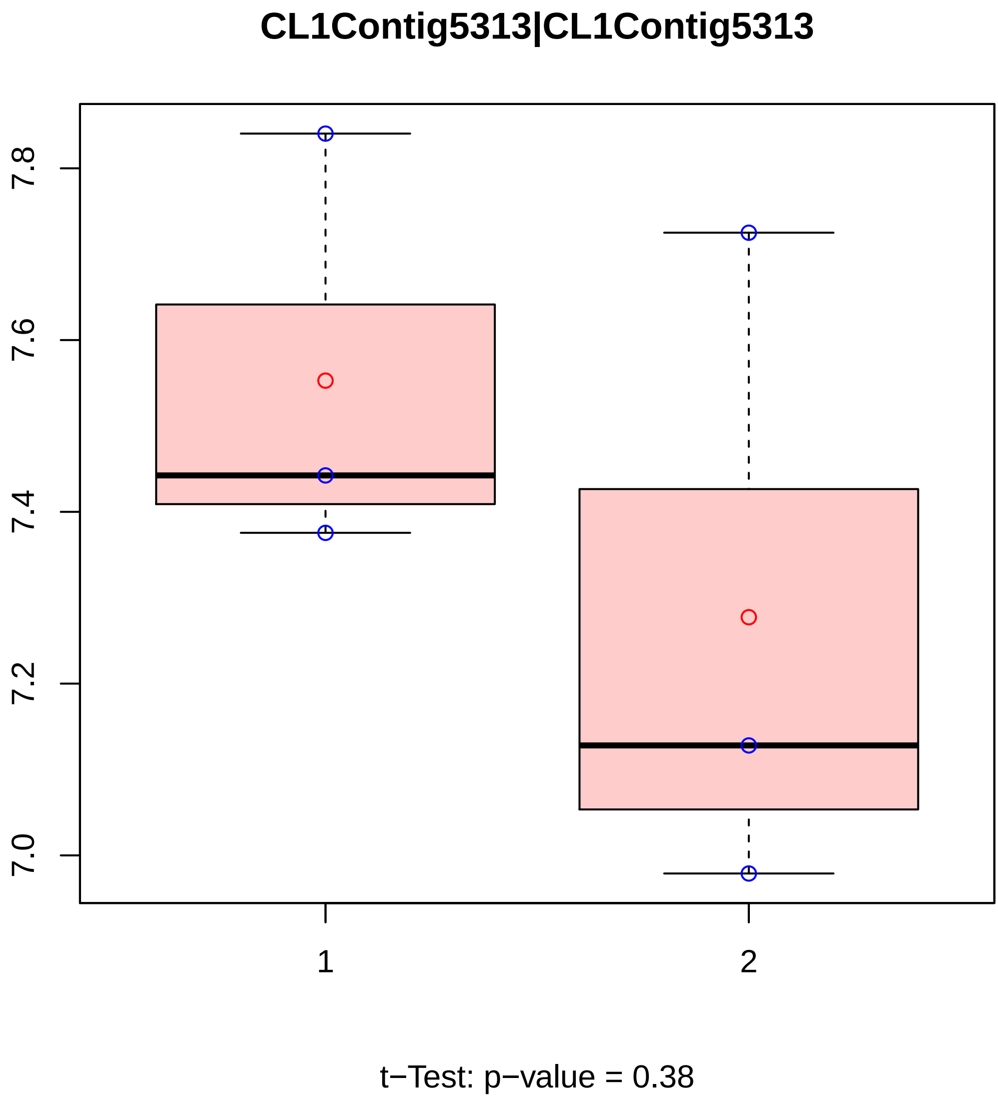

CL1Contig5313|CL1Contig5313
7.0
7.2
7.4
7.6
7.8
1
2
t−Test: p−value = 0.38

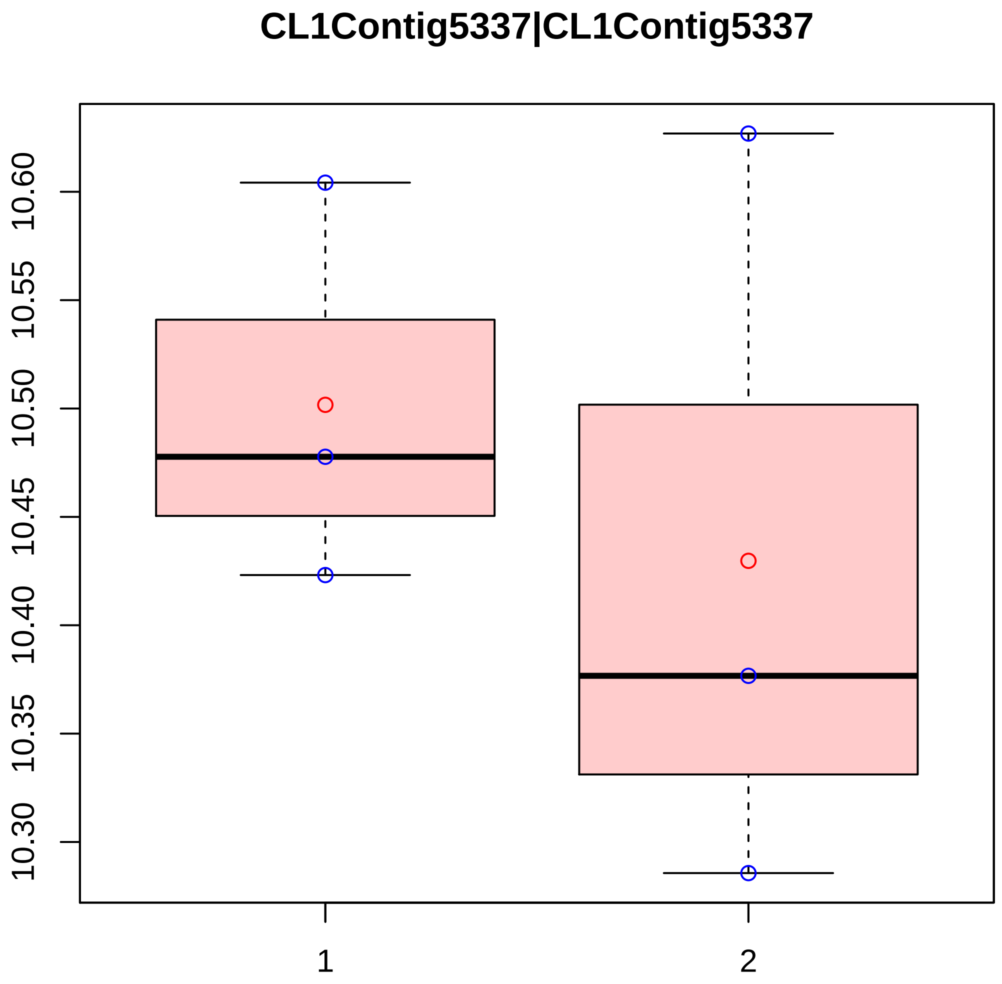


t−Test: p−value = 0.58

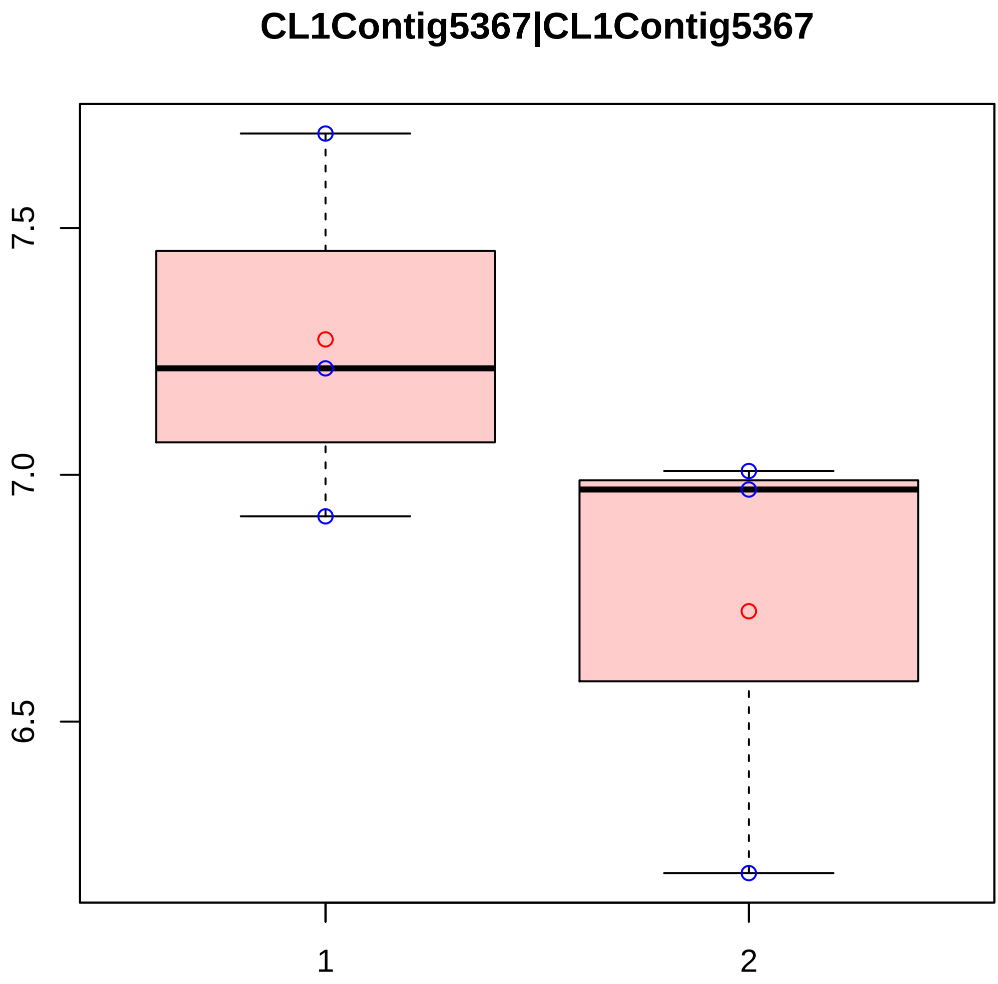


t−Test: p−value = 0.19

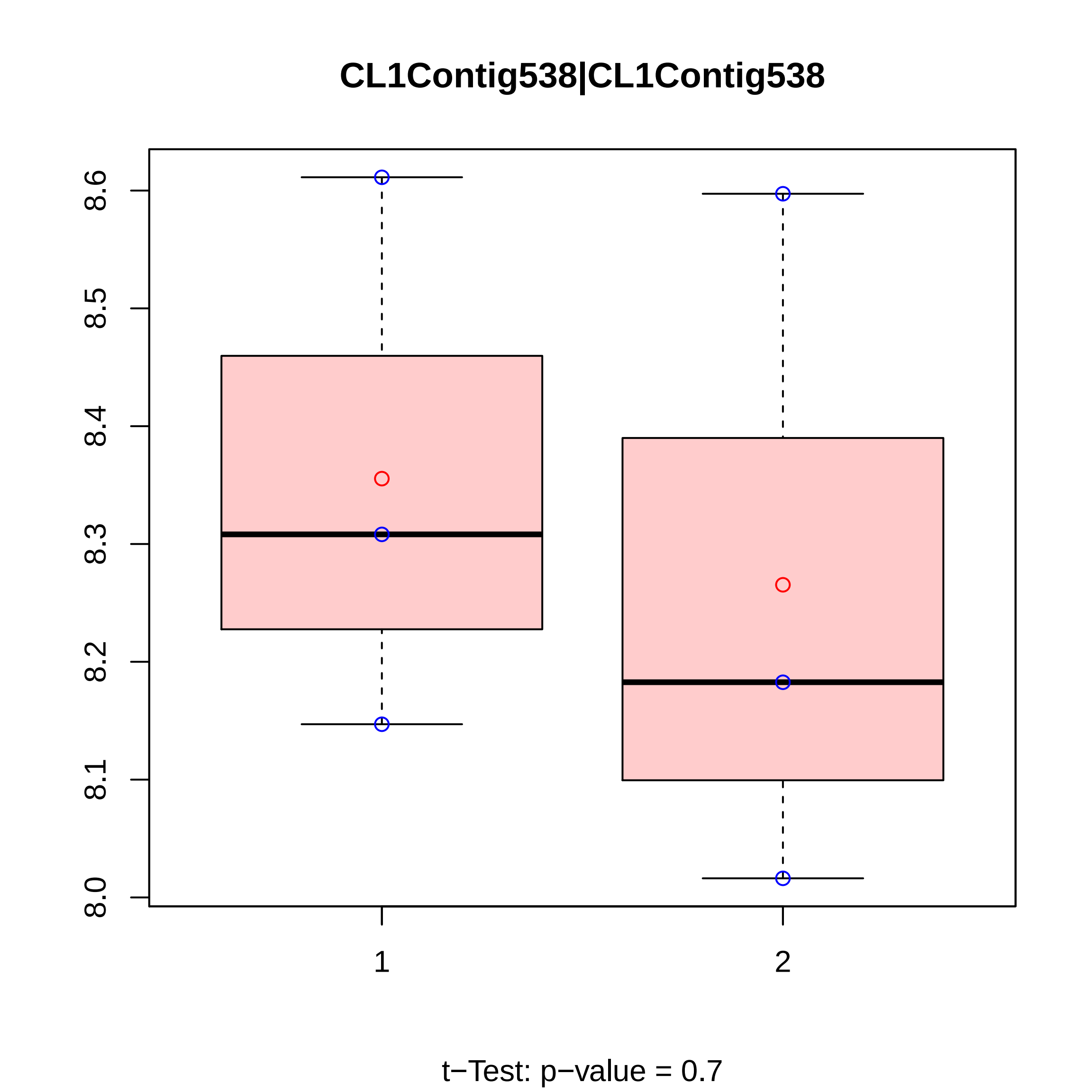

CL1Contig538|CL1Contig538
8.0
8.1
8.2
8.3
8.4
8.5
8.6
1
2
t−Test: p−value = 0.7

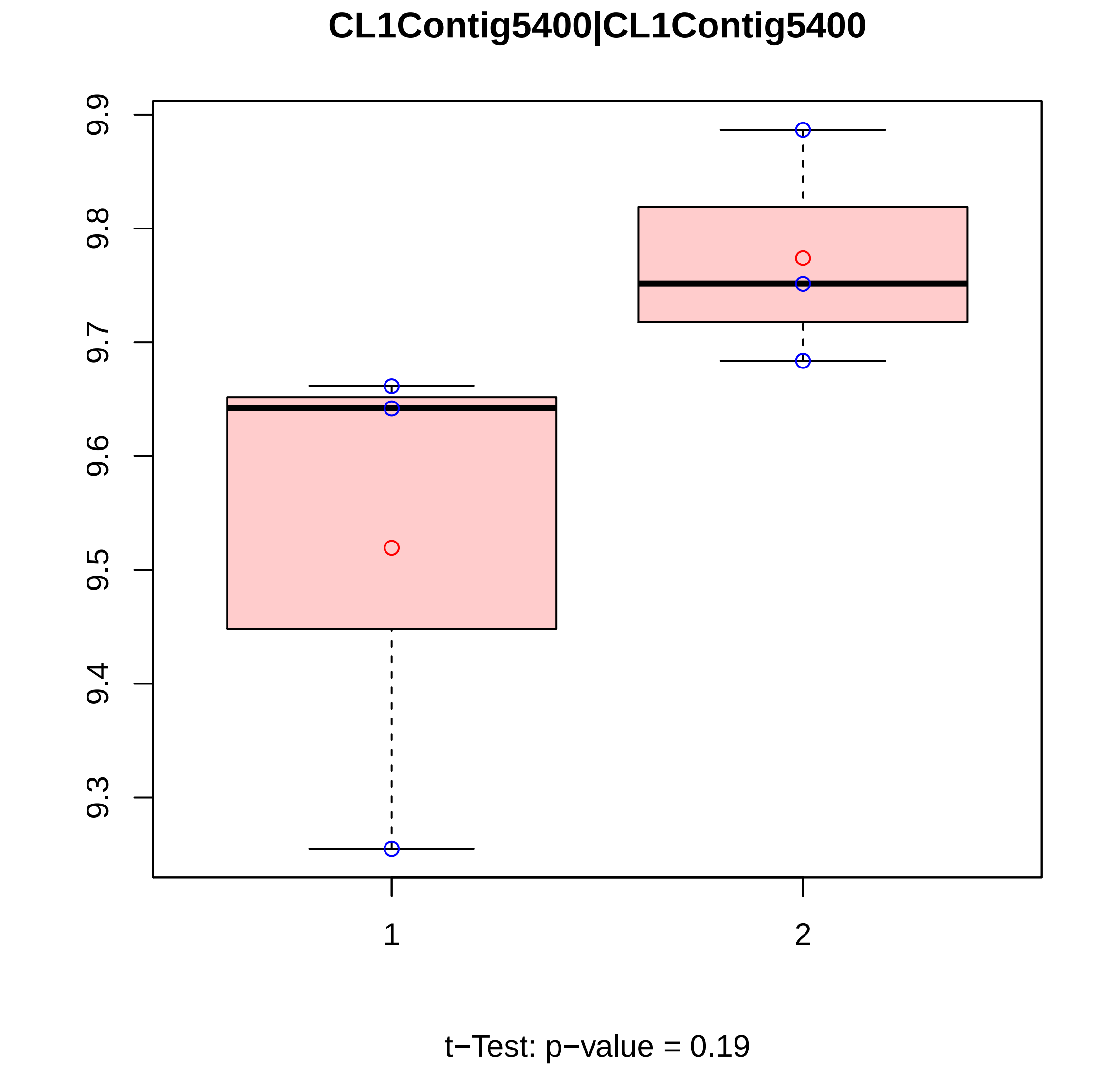
CL1Contig5400|CL1Contig5400
9.3
9.4
9.5
9.6
9.7
9.8
9.9
1
2
t−Test: p−value = 0.19

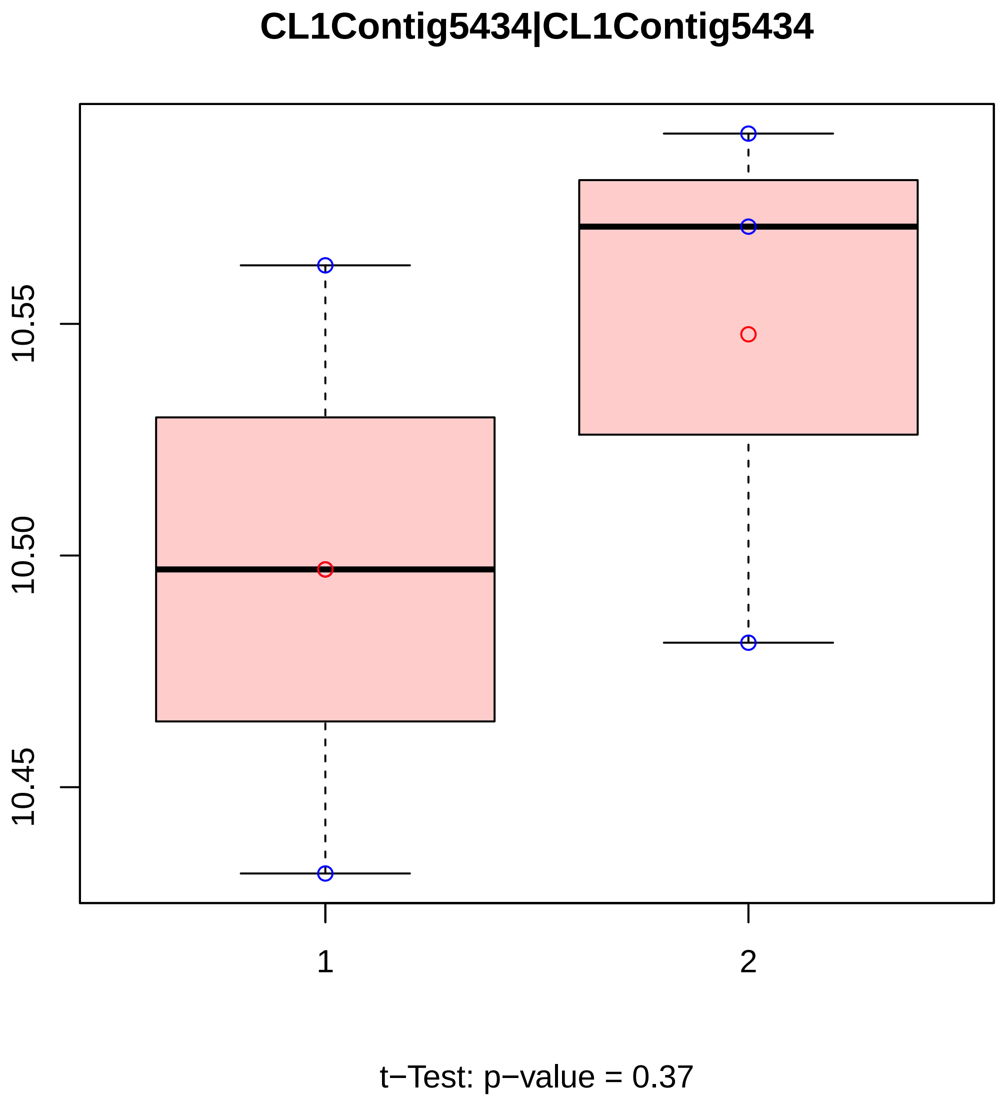
CL1Contig5434|CL1Contig5434
10.45
10.50
10.55
1
2
t−Test: p−value = 0.37

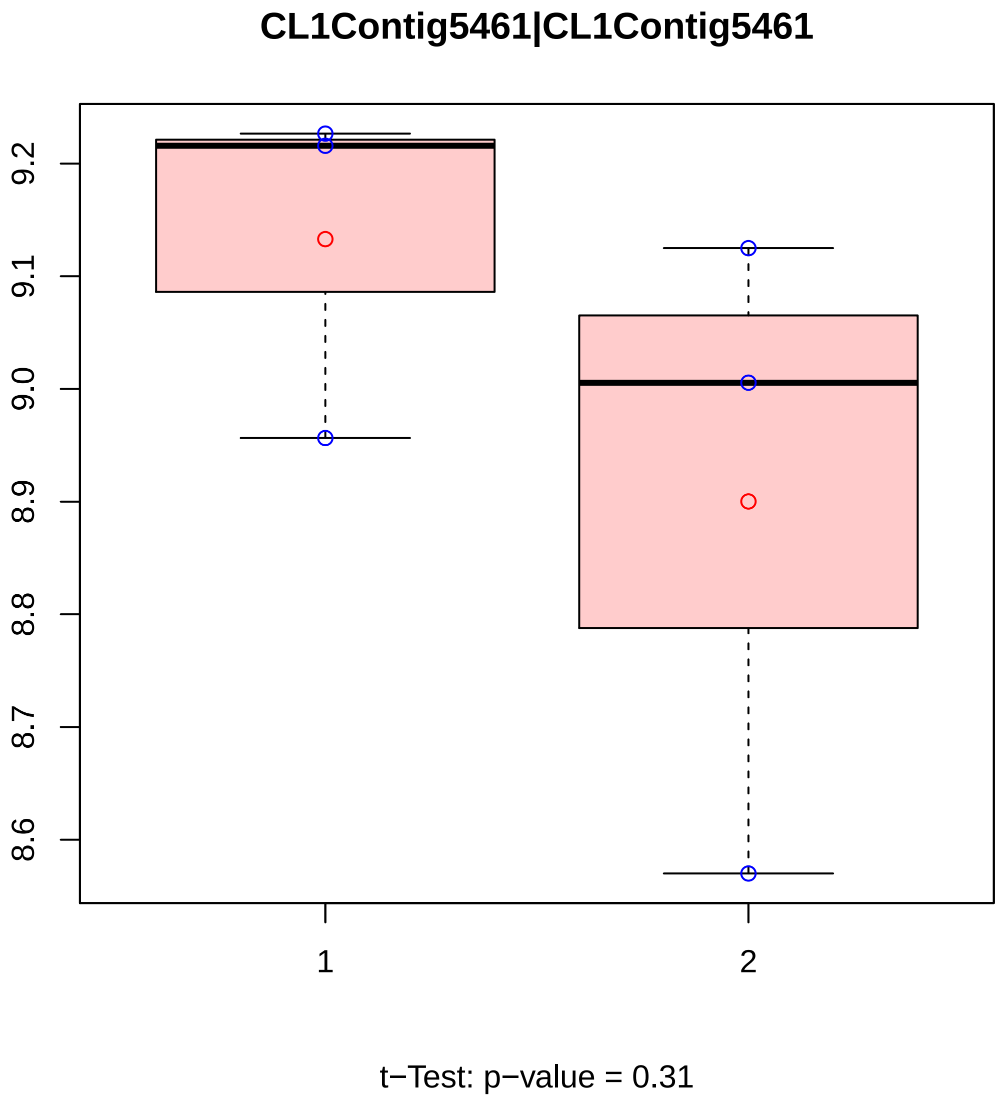
CL1Contig5461|CL1Contig5461
9.2
9.1
9.0
8.9
8.8
8.7
8.6
1
2
t−Test: p−value = 0.31

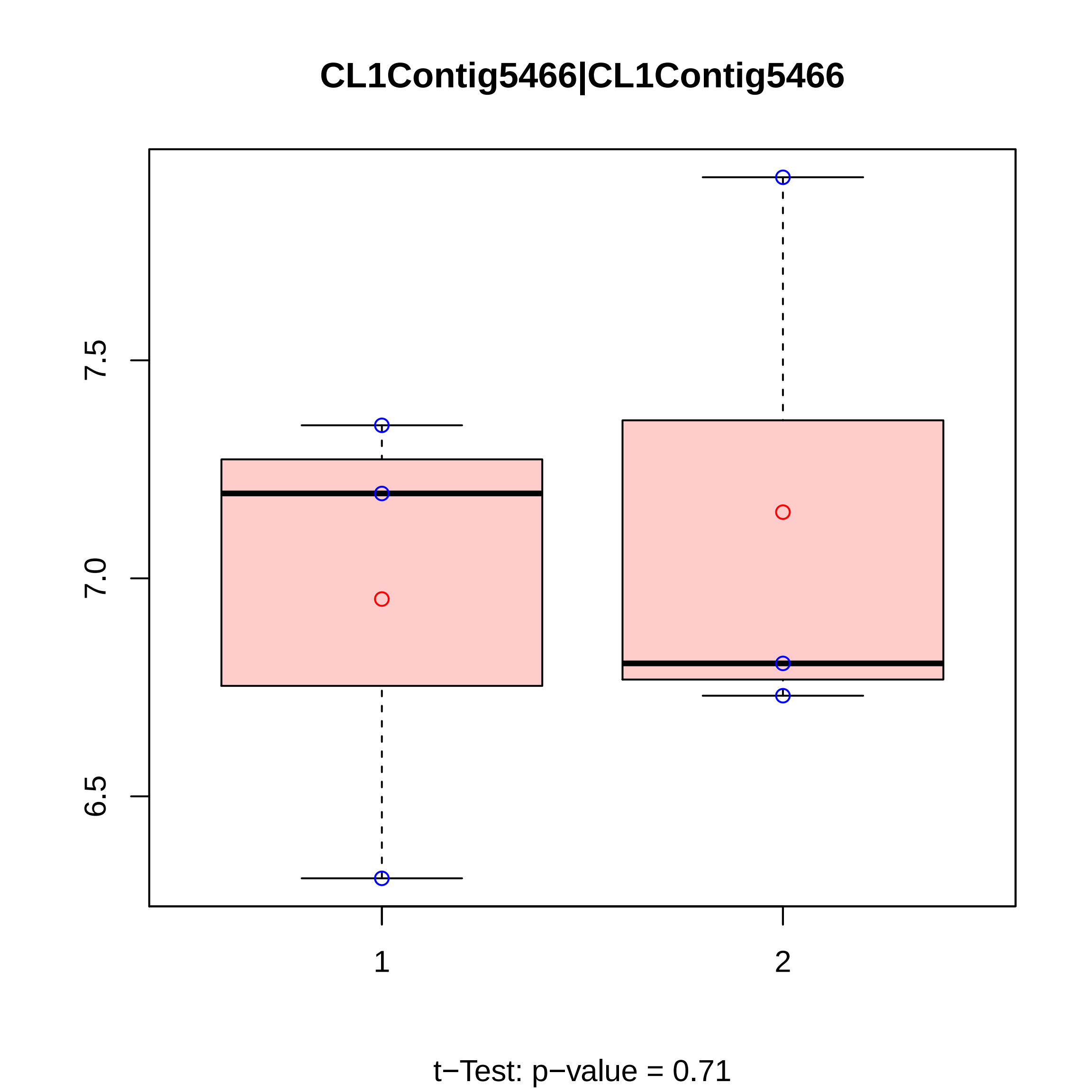
CL1Contig5466|CL1Contig5466
7.5
7.0
6.5
1
2
t−Test: p−value = 0.71

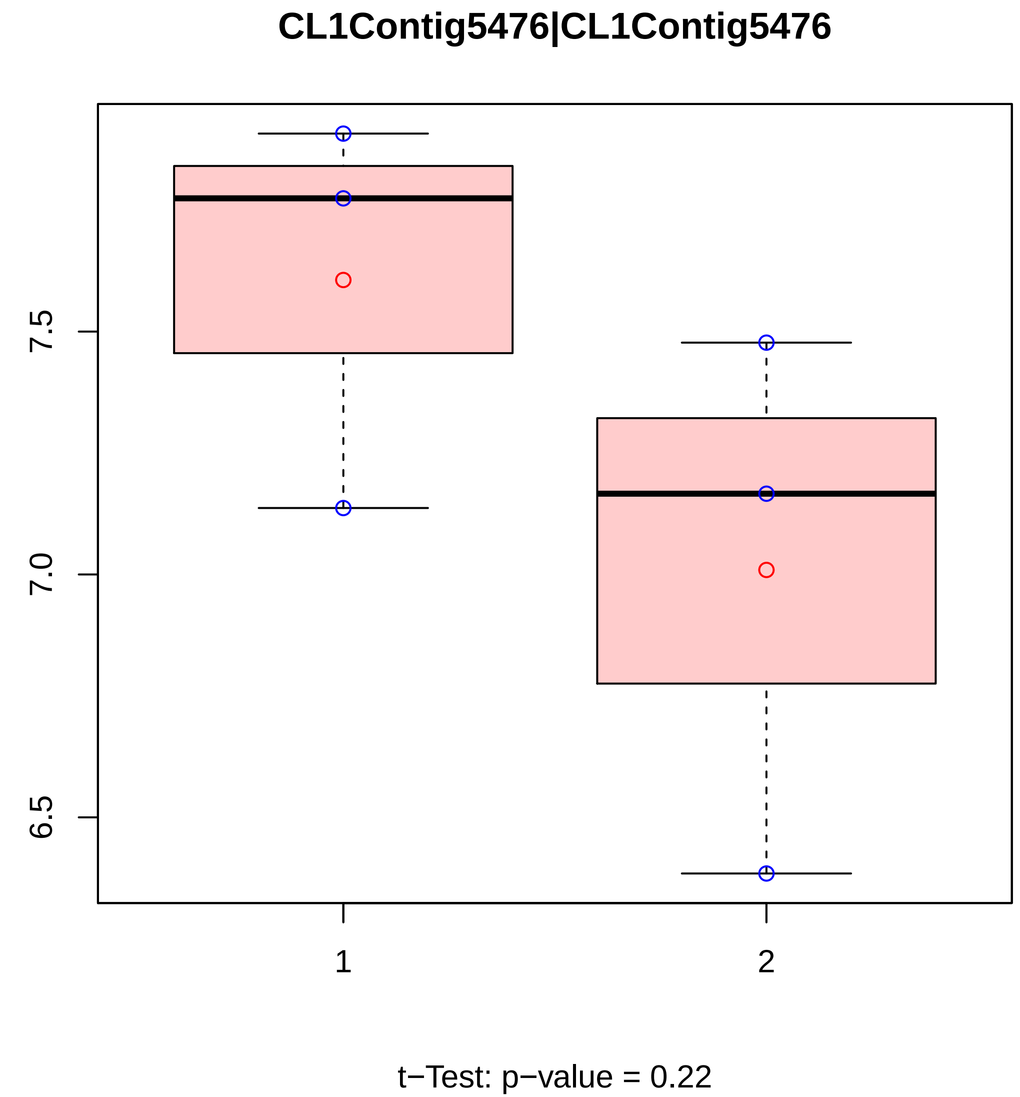
CL1Contig5476|CL1Contig5476
7.5
7.0
6.5
1
2
t−Test: p−value = 0.22

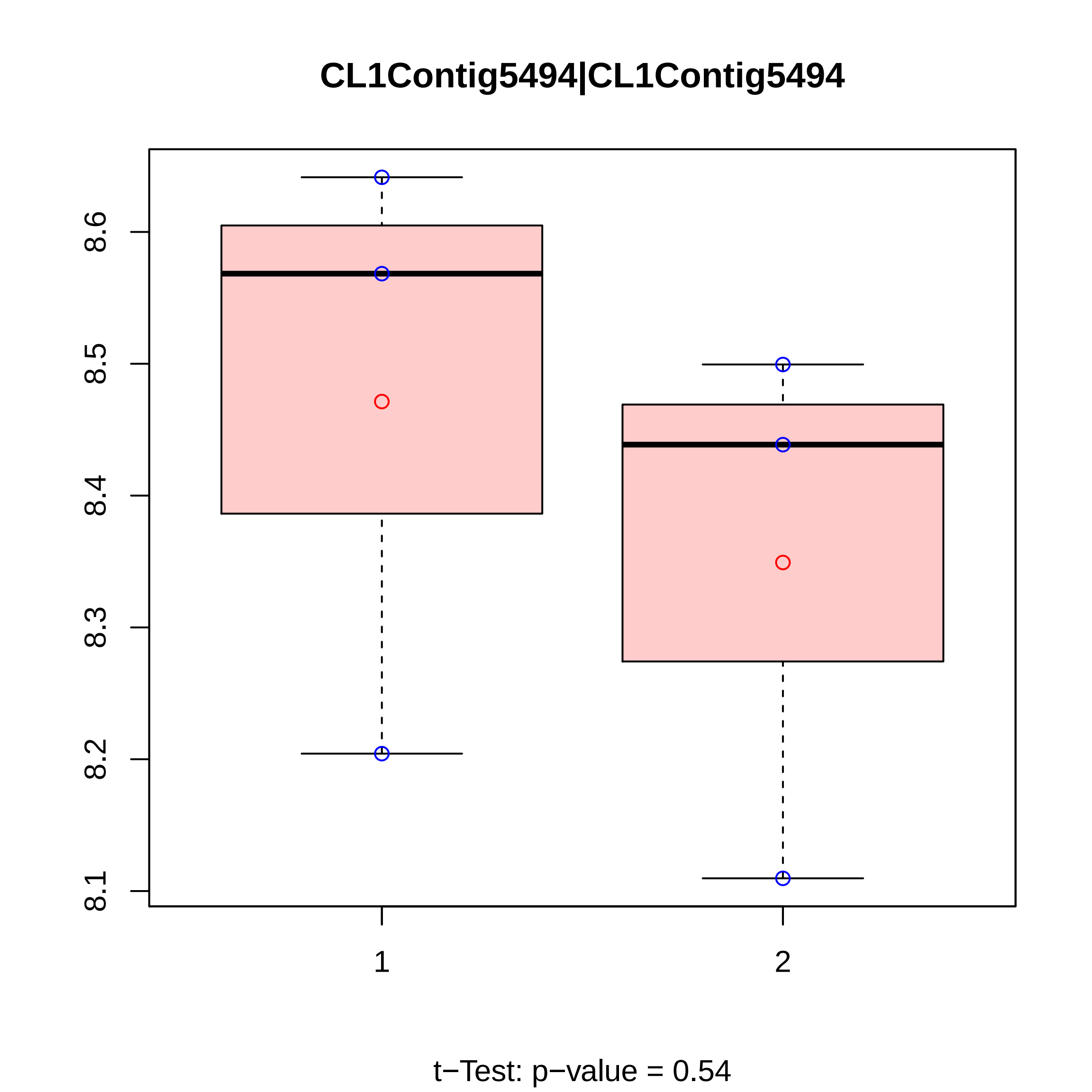

CL1Contig5494|CL1Contig5494
8.1
8.2
8.3
8.4
8.5
8.6
1
2
t−Test: p−value = 0.54

**CL1Contig5500|CL1Contig5500**

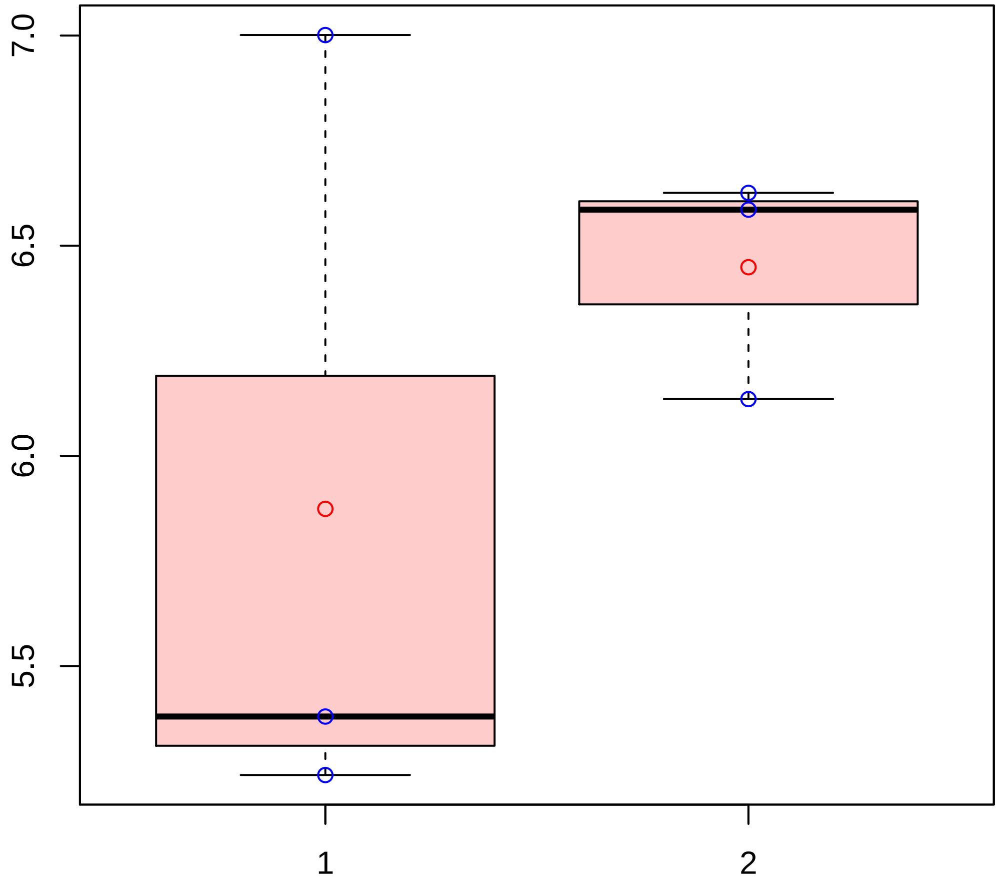


t−Test: p−value = 0.42

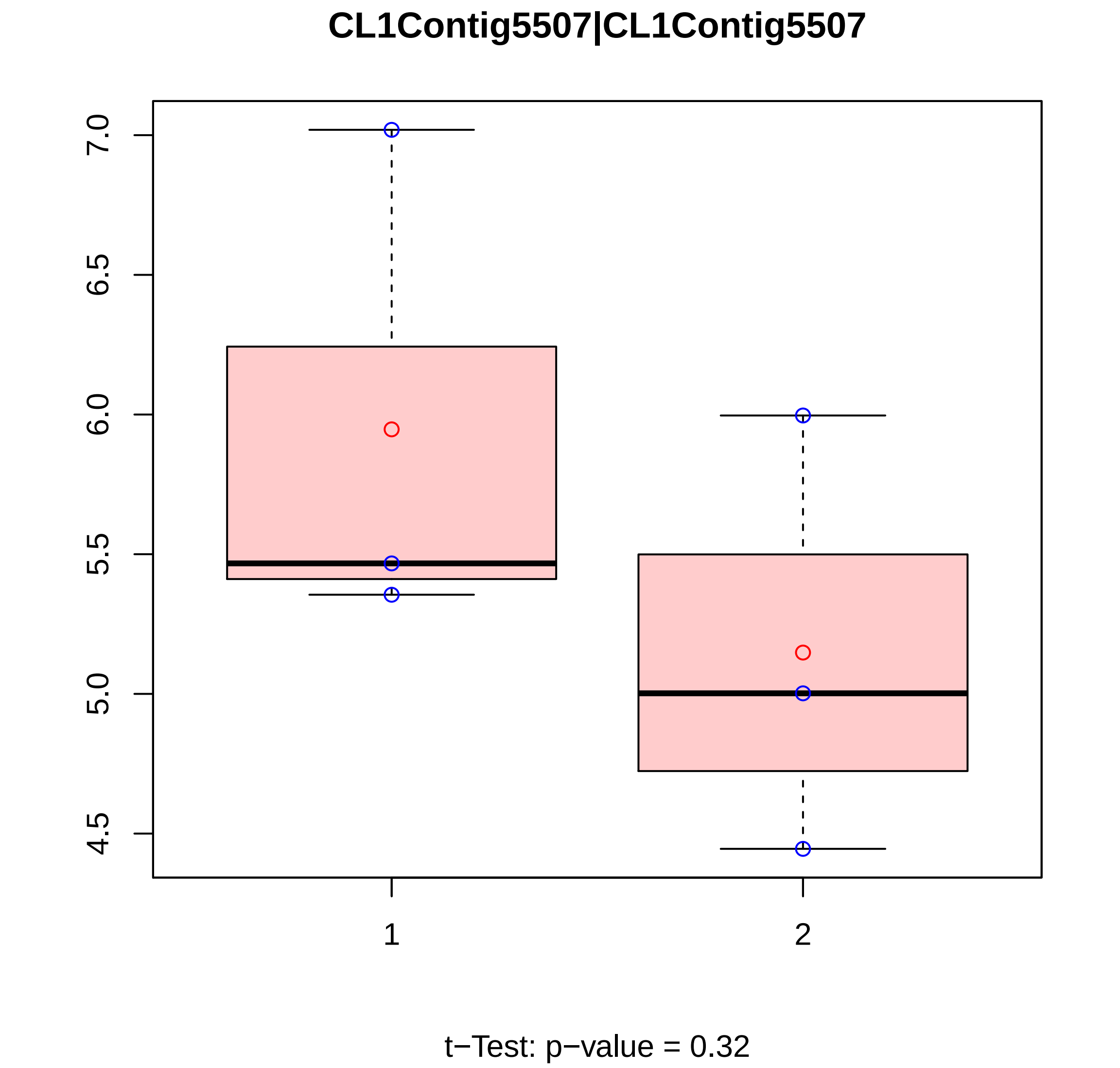
CL1Contig5507|CL1Contig5507
7.0
6.5
6.0
5.5
5.0
4.5
1
2
t−Test: p−value = 0.32

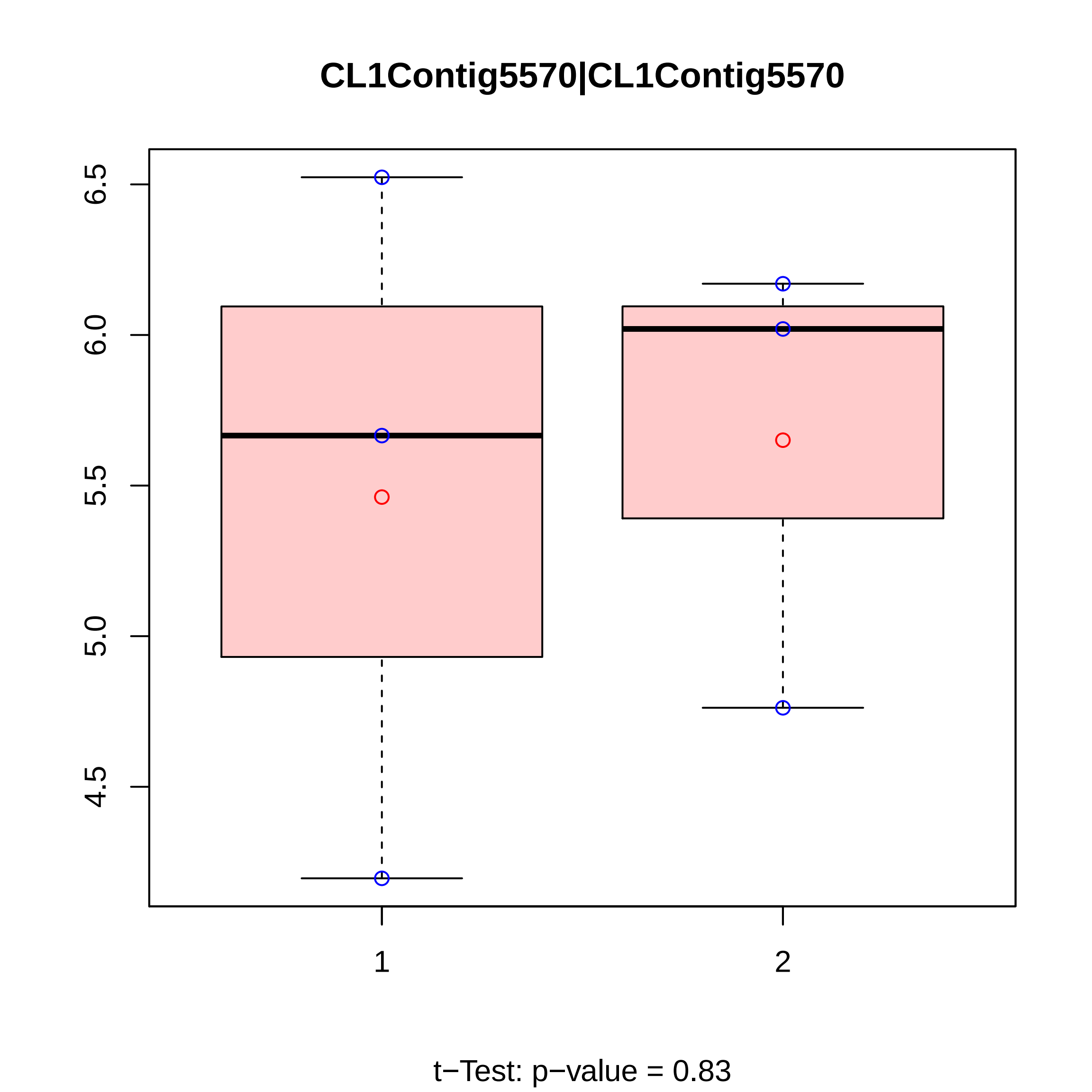
CL1Contig5570|CL1Contig5570
6.5
6.0
5.5
5.0
4.5
1
2
t−Test: p−value = 0.83

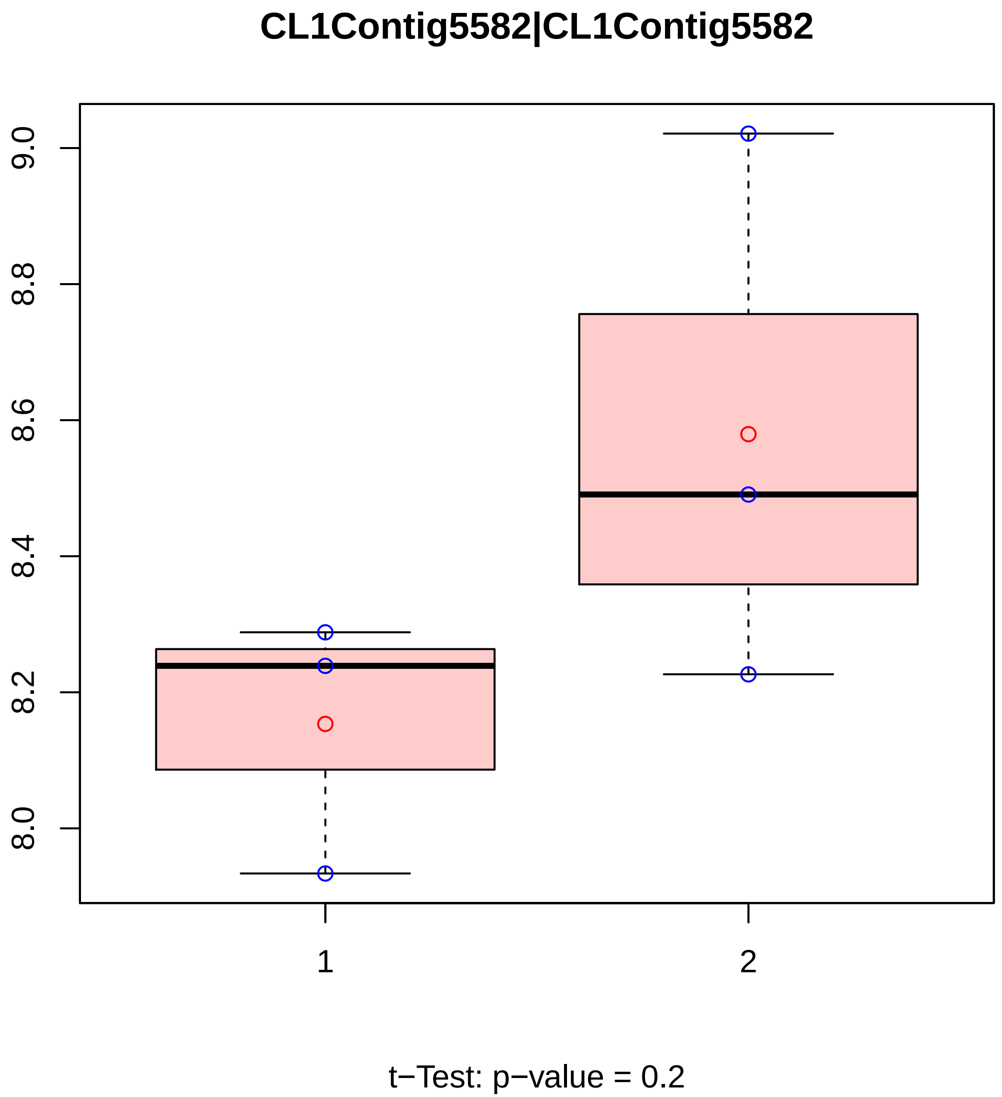

CL1Contig5582|CL1Contig5582
9.0
8.8
8.6
8.4
8.2
8.0
1
2
t−Test: p−value = 0.2

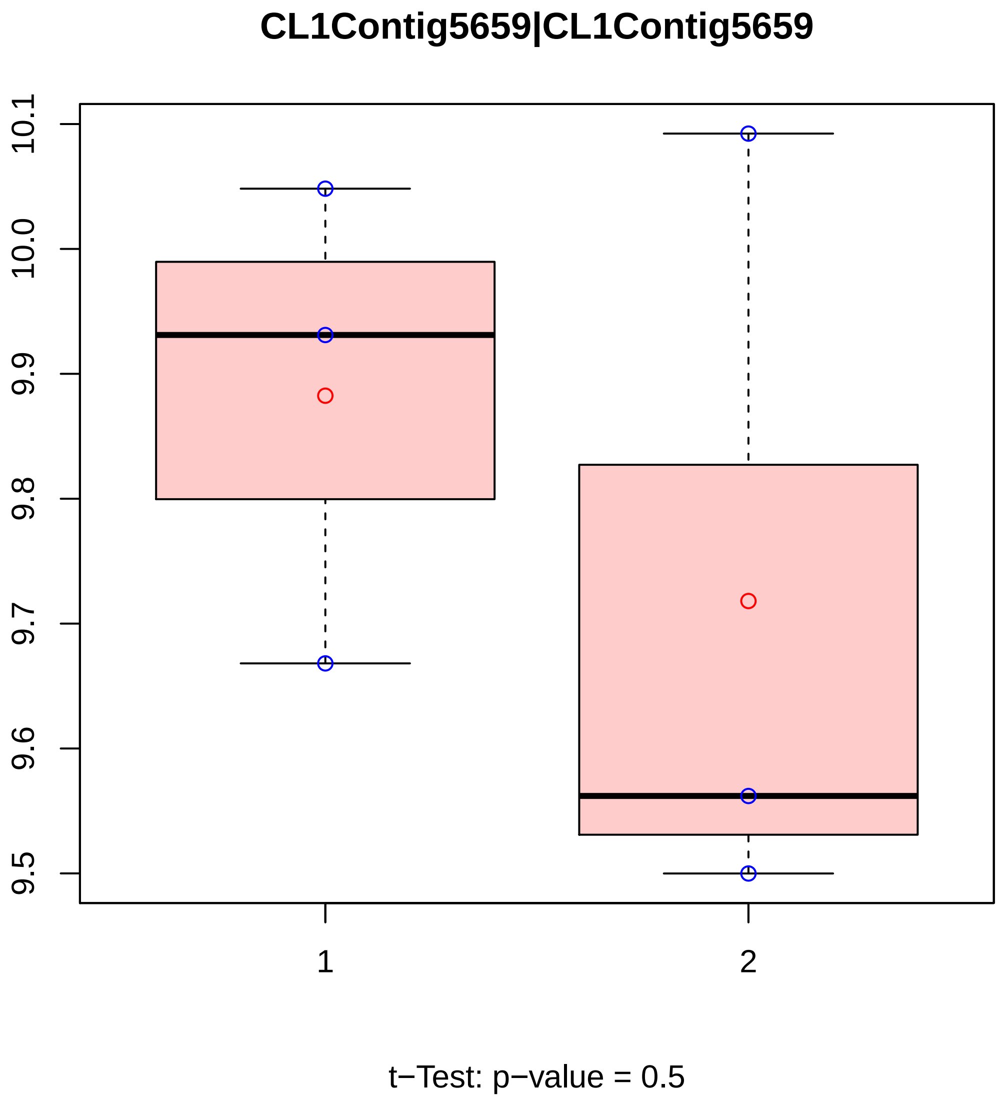
CL1Contig5659|CL1Contig5659
10.1
10.0
9.9
9.8
9.7
9.6
9.5
1
2
t−Test: p−value = 0.5

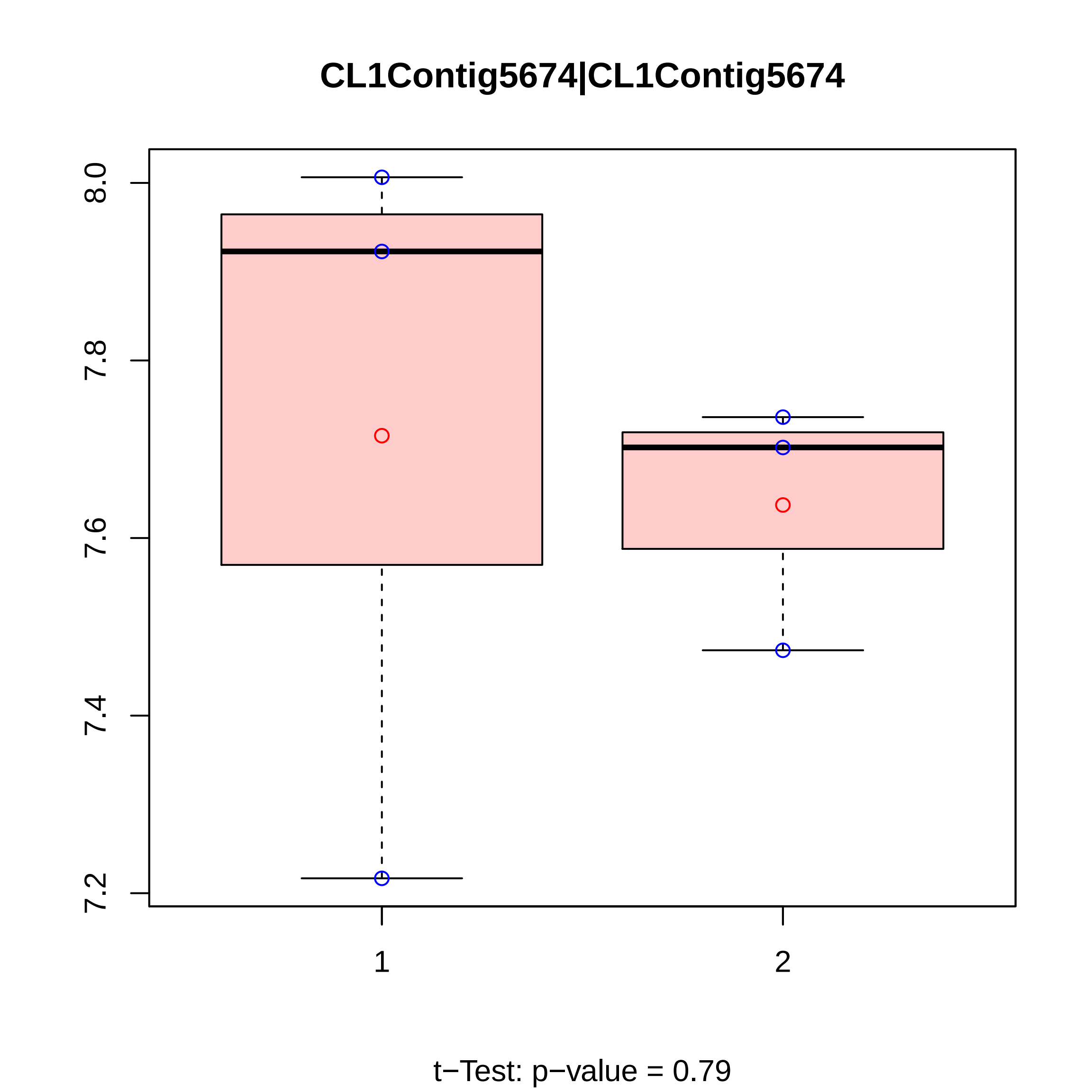
CL1Contig5674|CL1Contig5674
7.2
7.4
7.6
7.8
8.0
1
2
t−Test: p−value = 0.79

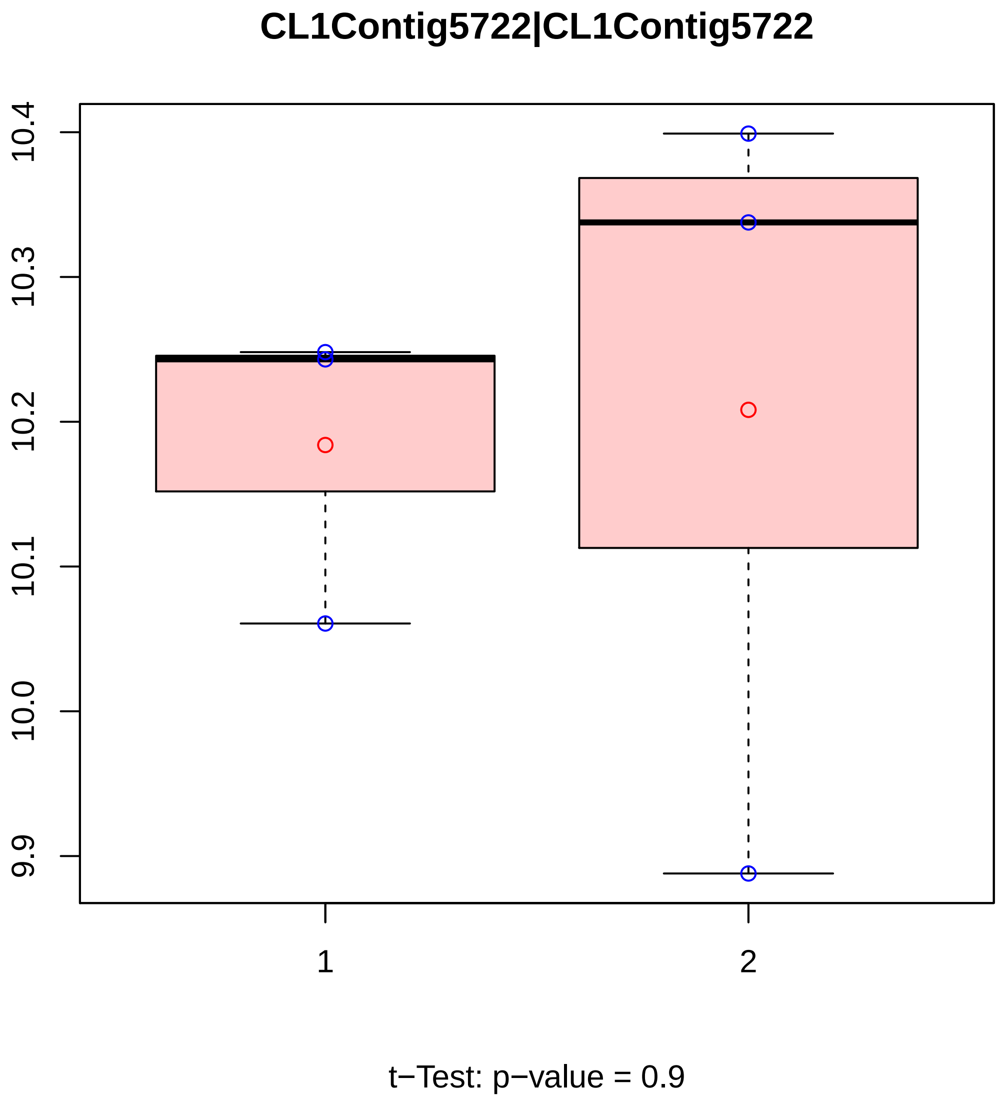
CL1Contig5722|CL1Contig5722
10.4
10.3
10.2
10.1
10.0
9.9
1
2
t−Test: p−value = 0.9

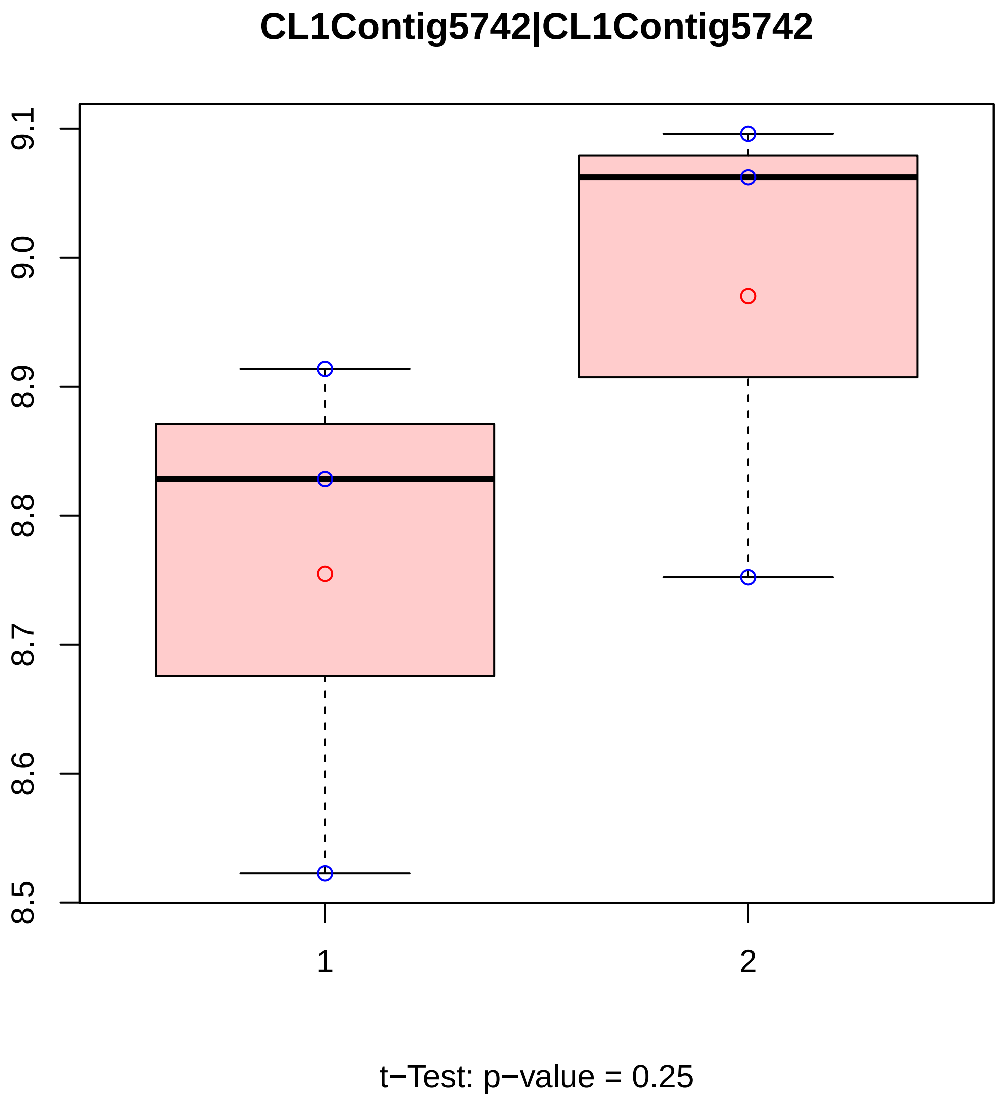
CL1Contig5742|CL1Contig5742
9.1
9.0
8.9
8.8
8.7
8.6
8.5
1
2
t−Test: p−value = 0.25

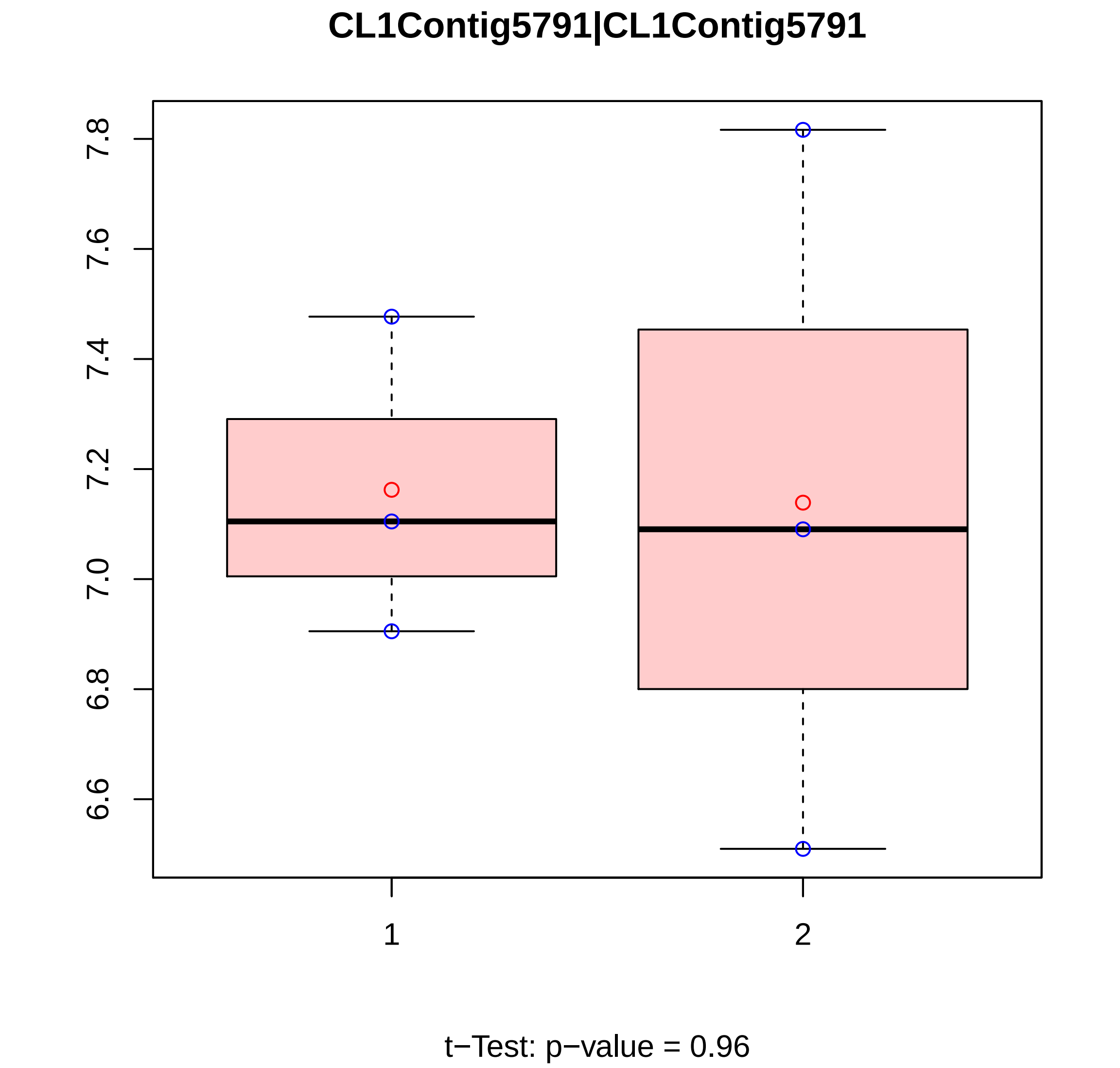
CL1Contig5791|CL1Contig5791
7.8
7.6
7.4
7.2
7.0
6.8
6.6
1
2
t−Test: p−value = 0.96

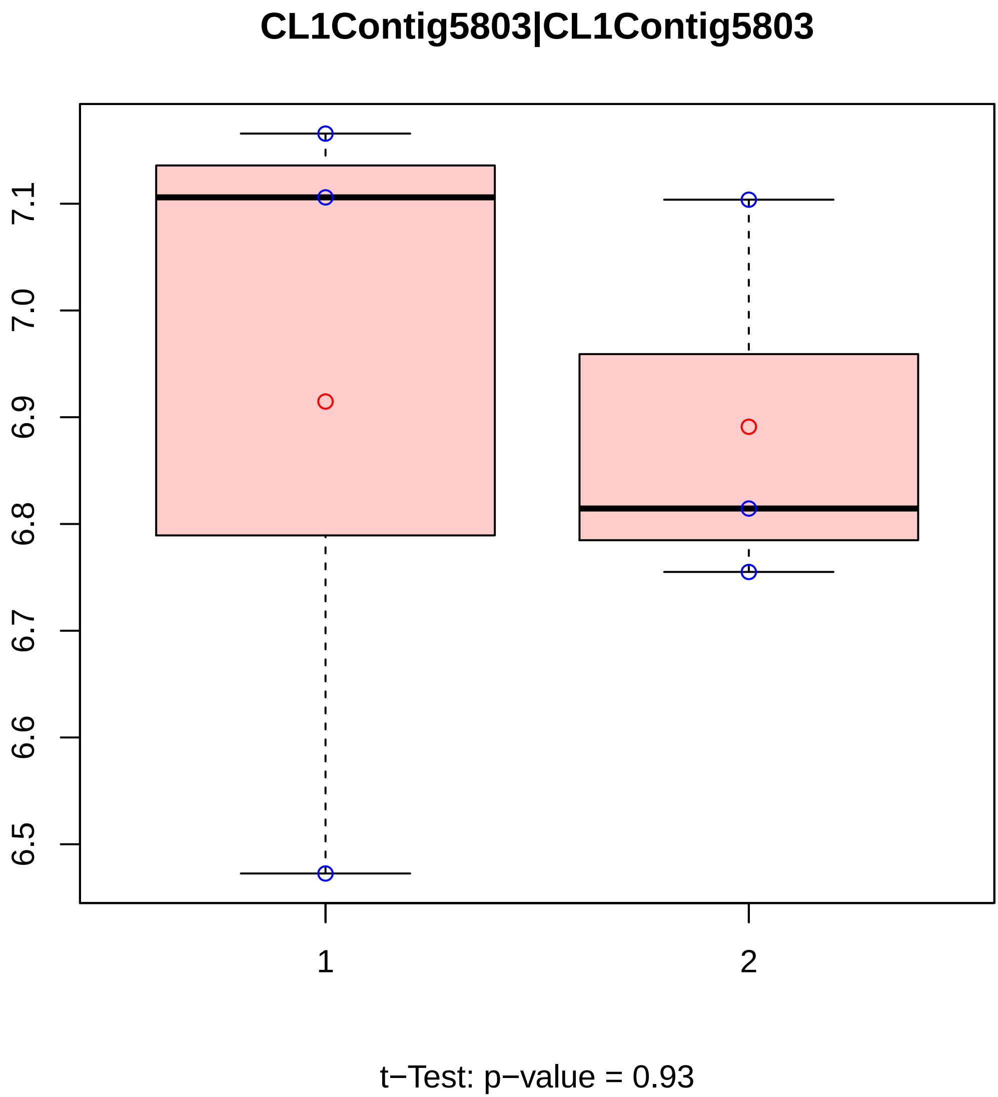
CL1Contig5803|CL1Contig5803
7.1
7.0
6.9
6.8
6.7
6.6
6.5
1
2
t−Test: p−value = 0.93

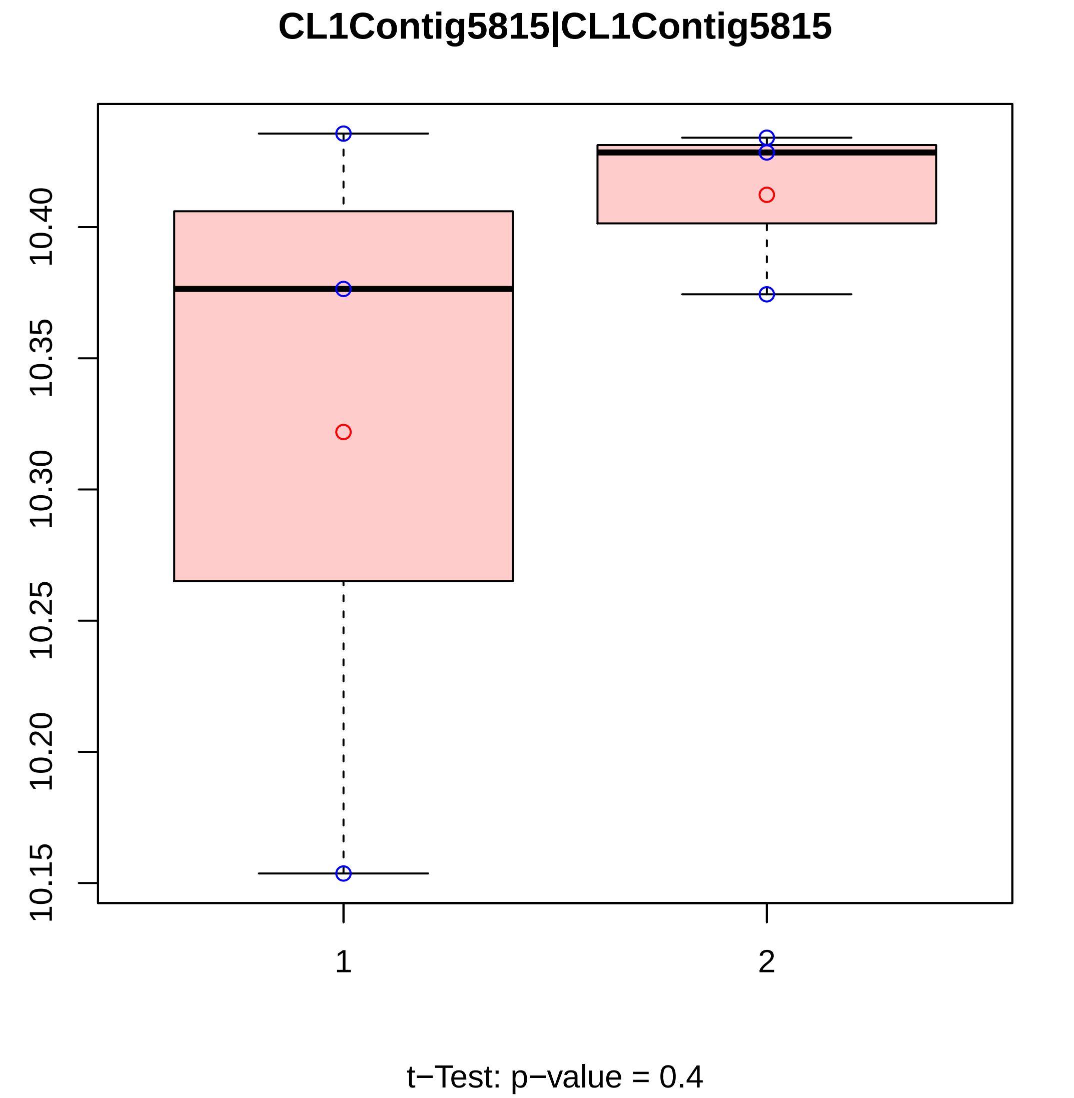
CL1Contig5815|CL1Contig5815
10.15
10.20
10.25
10.30
10.35
10.40
1
2
t−Test: p−value = 0.4

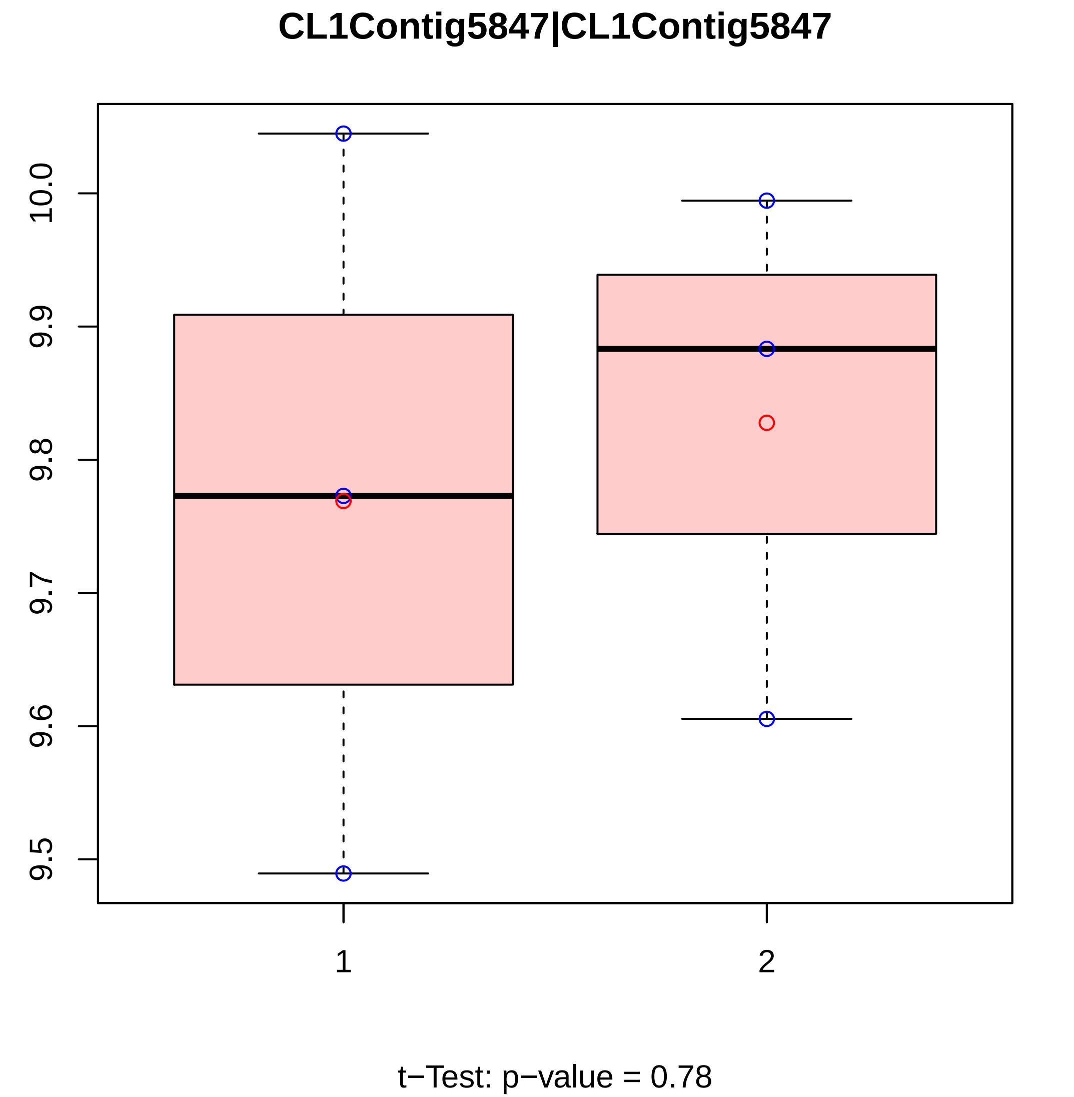
CL1Contig5847|CL1Contig5847
10.0
9.9
9.8
9.7
9.6
9.5
1
2
t−Test: p−value = 0.78

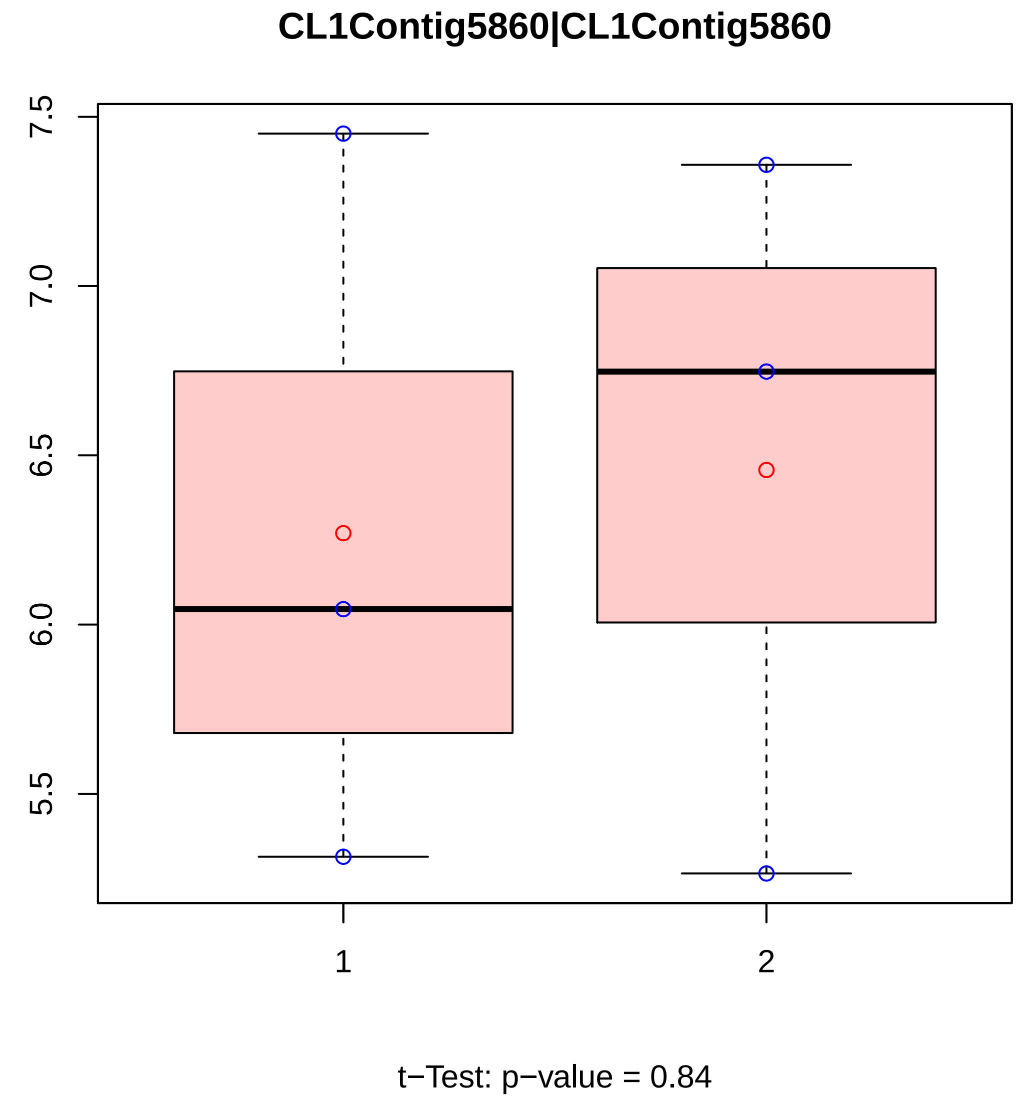
CL1Contig5860|CL1Contig5860
7.5
7.0
6.5
6.0
5.5
1
2
t−Test: p−value = 0.84

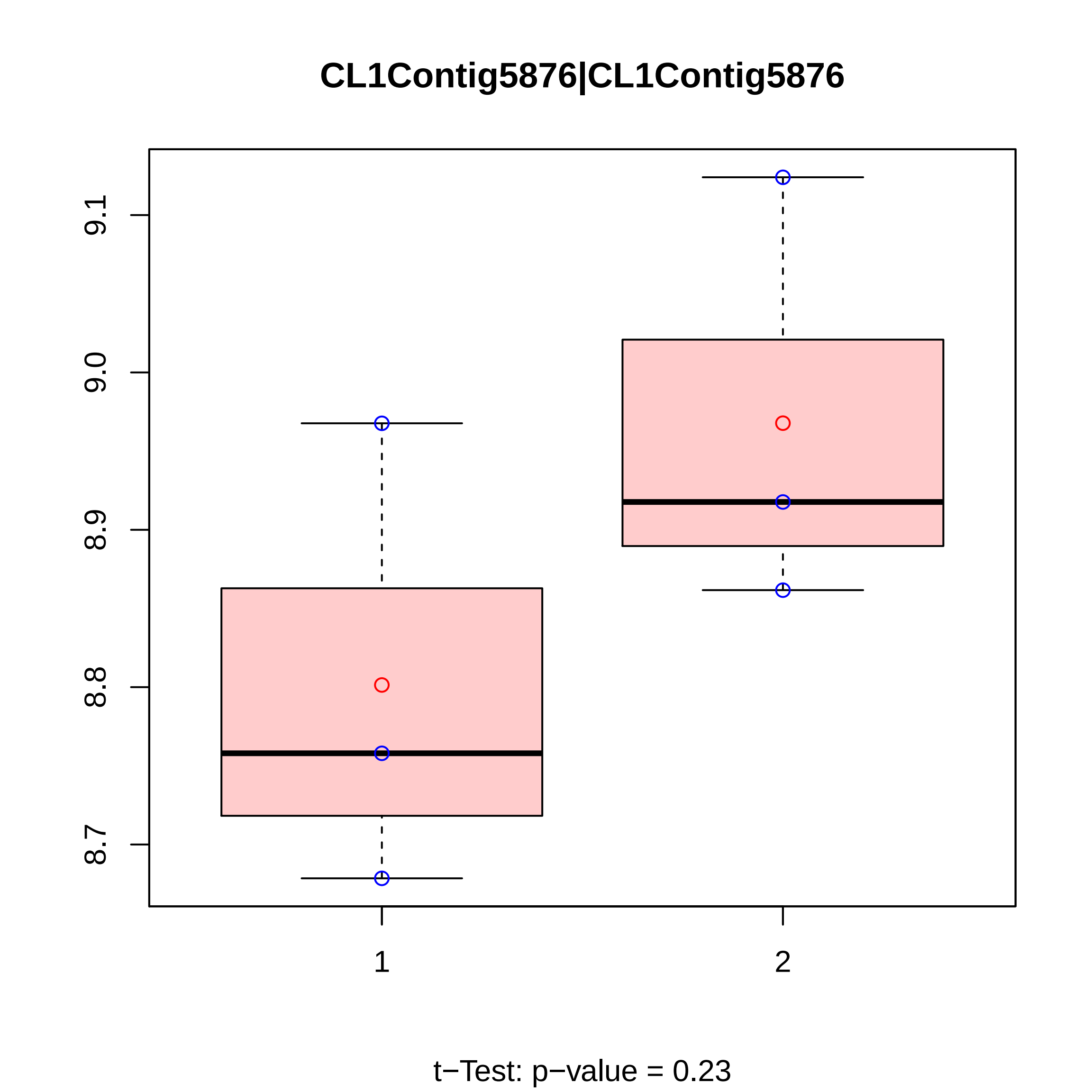
CL1Contig5876|CL1Contig5876
9.1
9.0
8.9
8.8
8.7
1
2
t−Test: p−value = 0.23

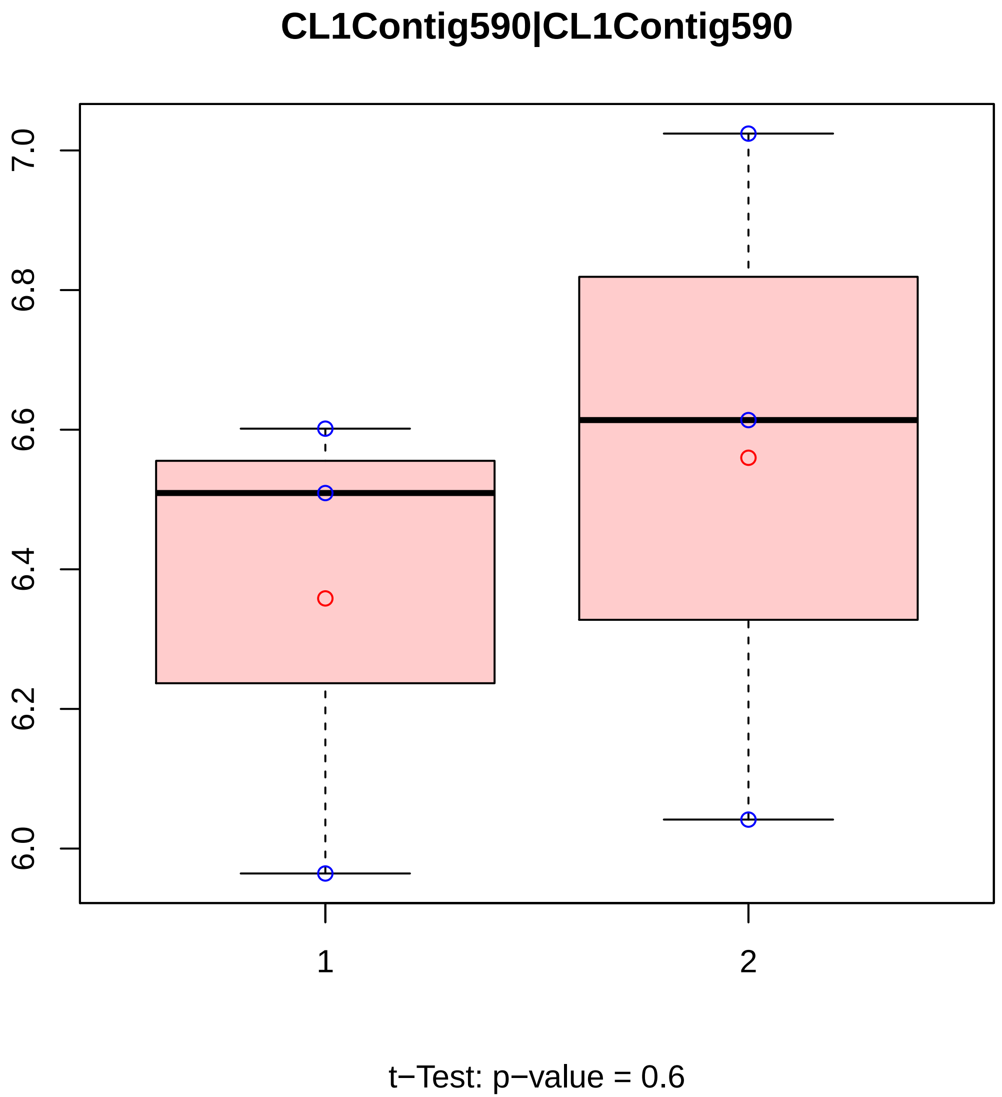
CL1Contig590|CL1Contig590
7.0
6.8
6.6
6.4
6.2
6.0
1
2
t−Test: p−value = 0.6

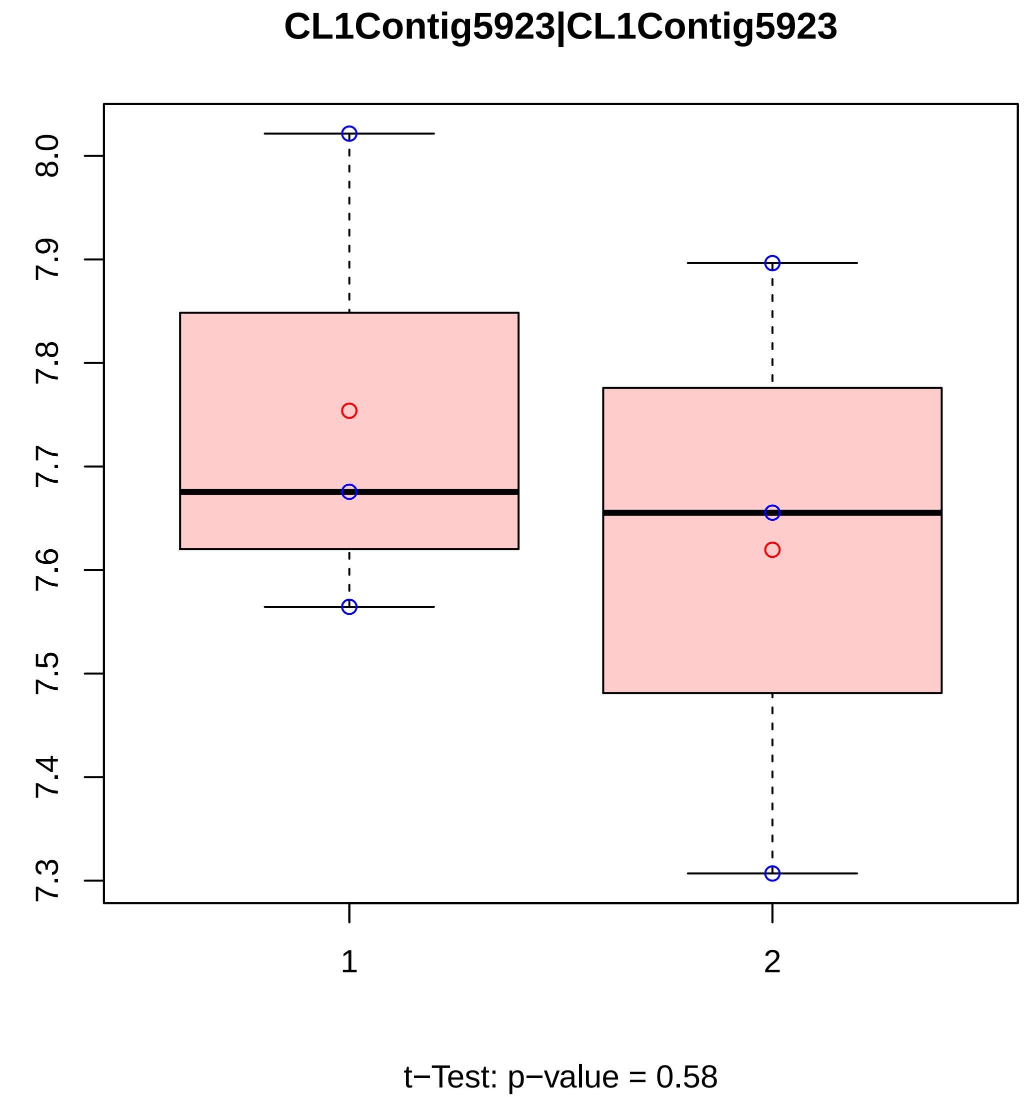
CL1Contig5923|CL1Contig5923
8.0
7.9
7.8
7.7
7.6
7.5
7.4
7.3
1
2
t−Test: p−value = 0.58

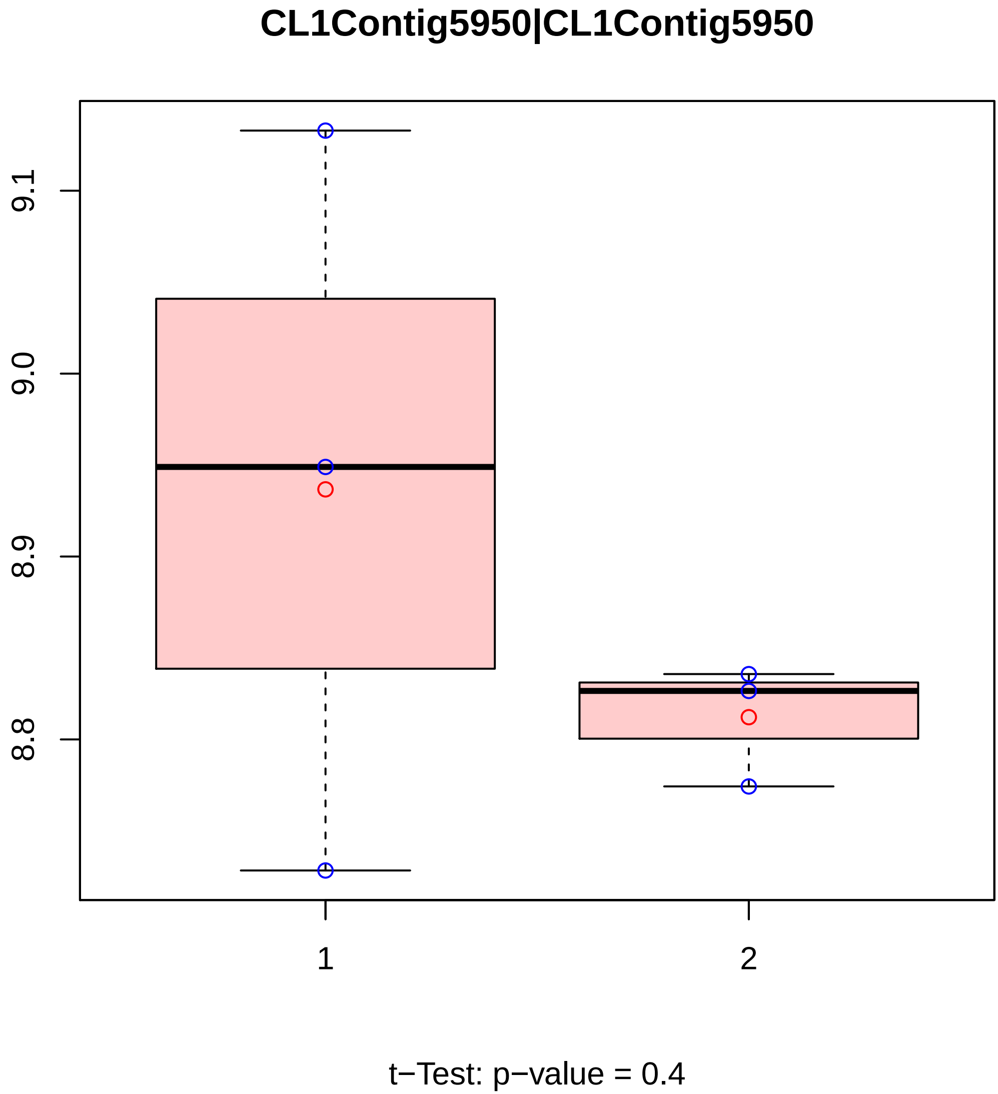
CL1Contig5950|CL1Contig5950
9.1
9.0
8.9
8.8
1
2
t−Test: p−value = 0.4

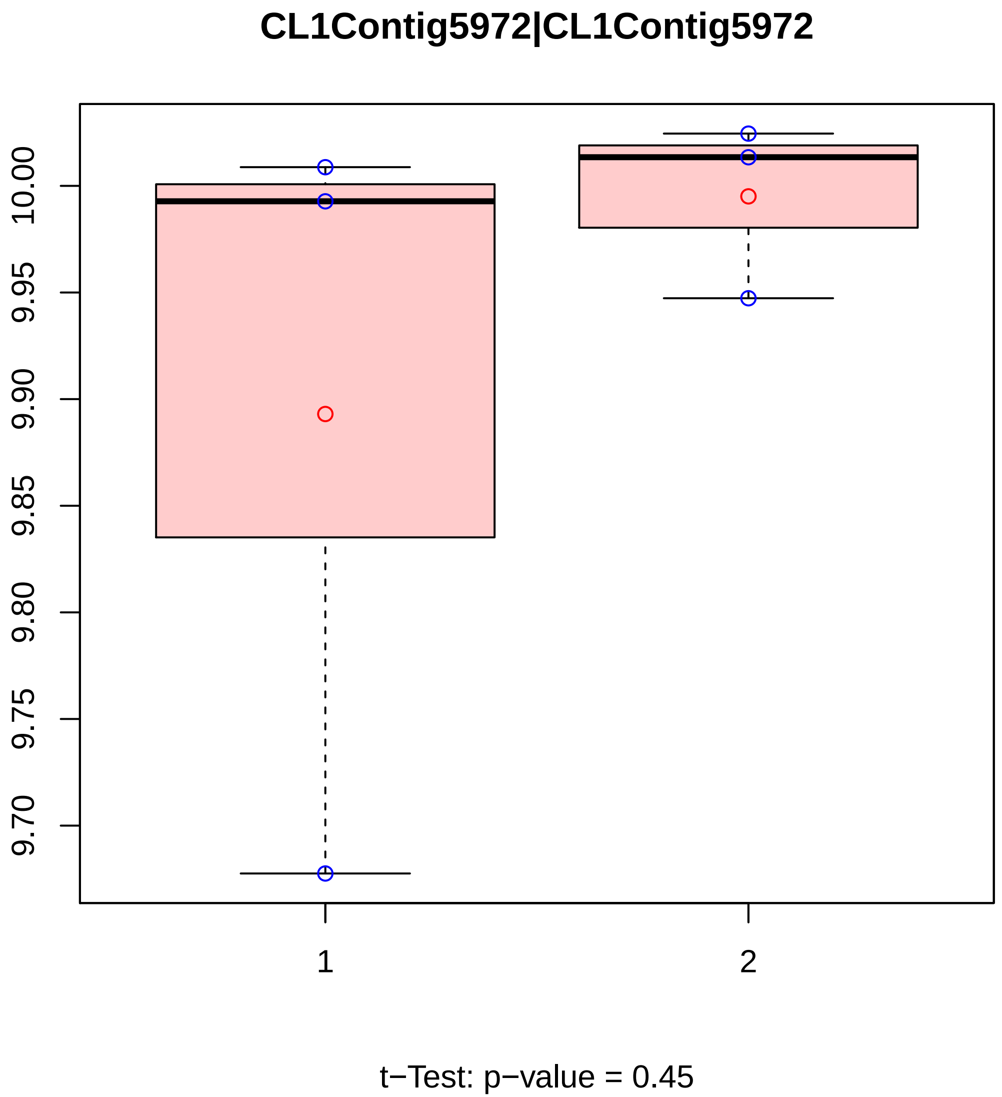

CL1Contig5972|CL1Contig5972
10.00
9.95
9.90
9.85
9.80
9.75
9.70
1
2
t−Test: p−value = 0.45

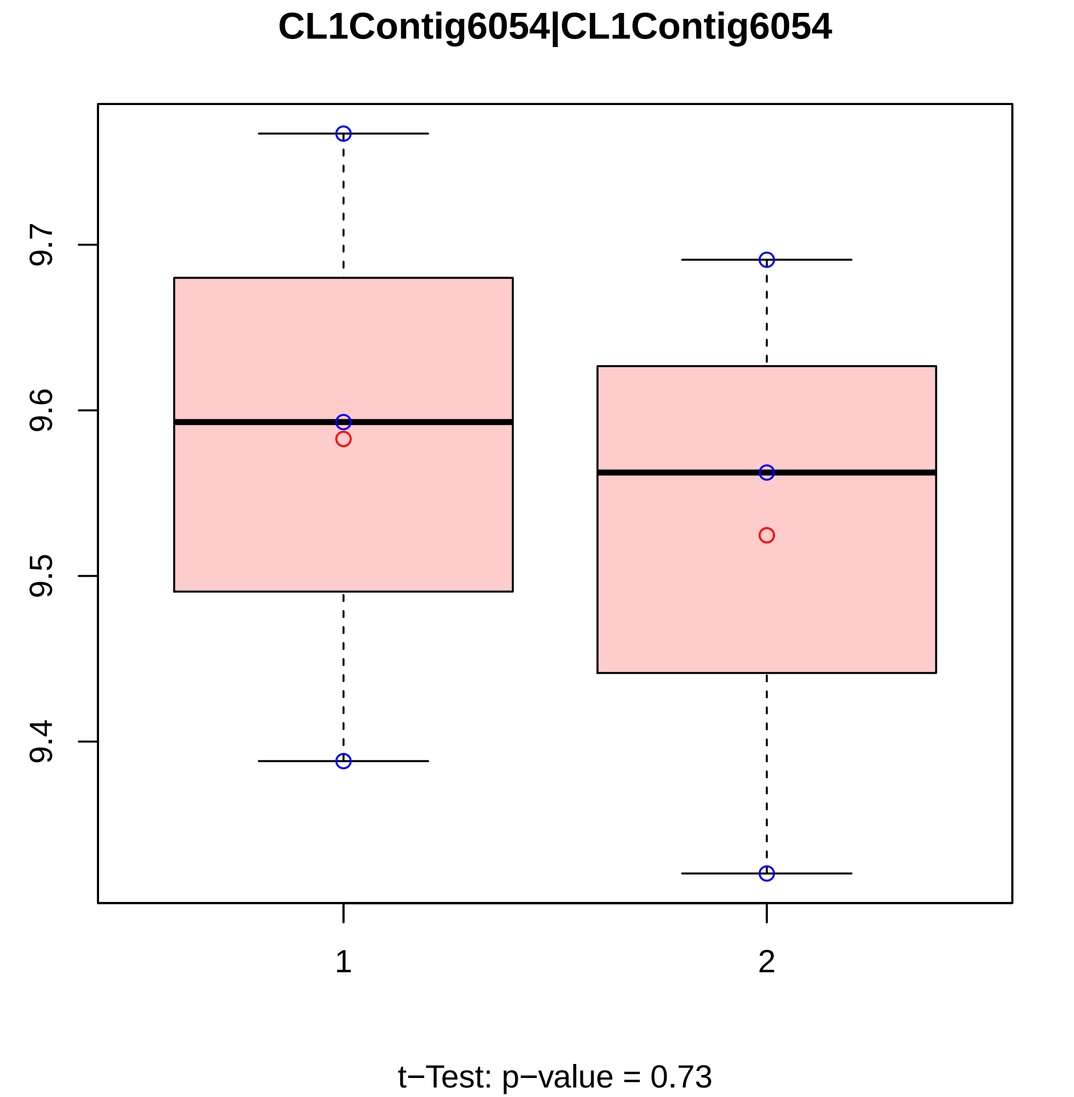
CL1Contig6054|CL1Contig6054
9.4
9.5
9.6
9.7
1
2
t−Test: p−value = 0.73

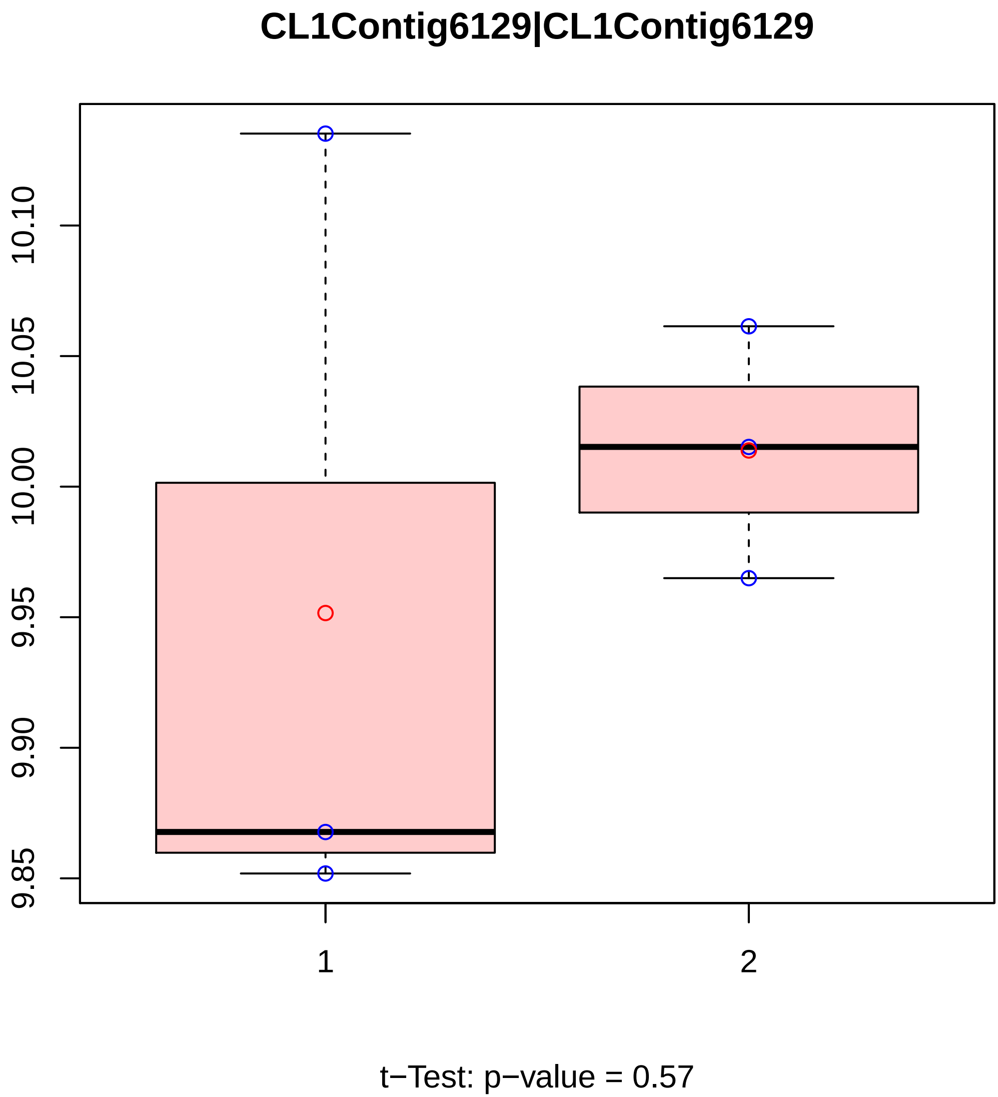

CL1Contig6129|CL1Contig6129
10.10
10.05
10.00
9.95
9.90
9.85
1
2
t−Test: p−value = 0.57

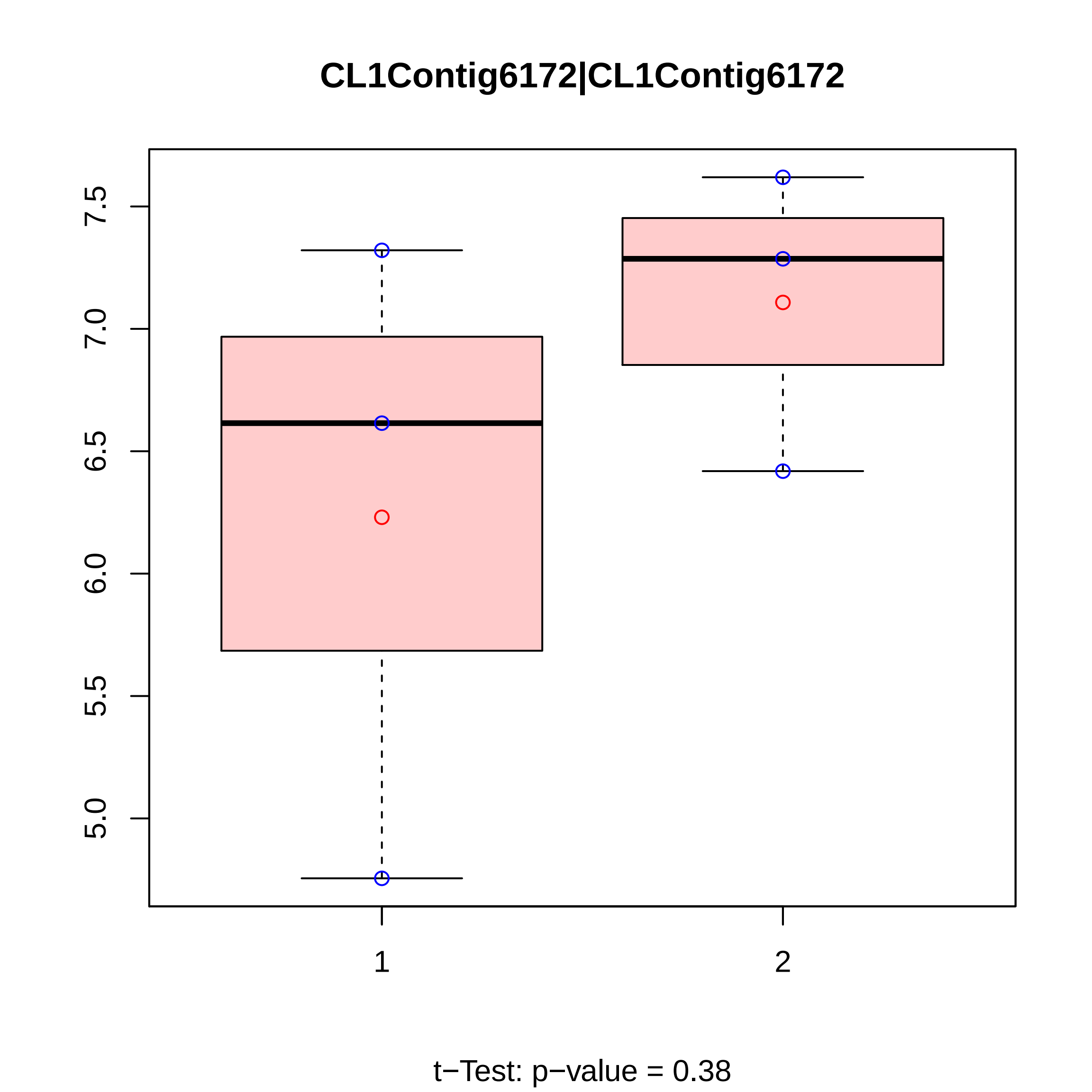
CL1Contig6172|CL1Contig6172
7.5
7.0
6.5
6.0
5.5
5.0
1
2
t−Test: p−value = 0.38

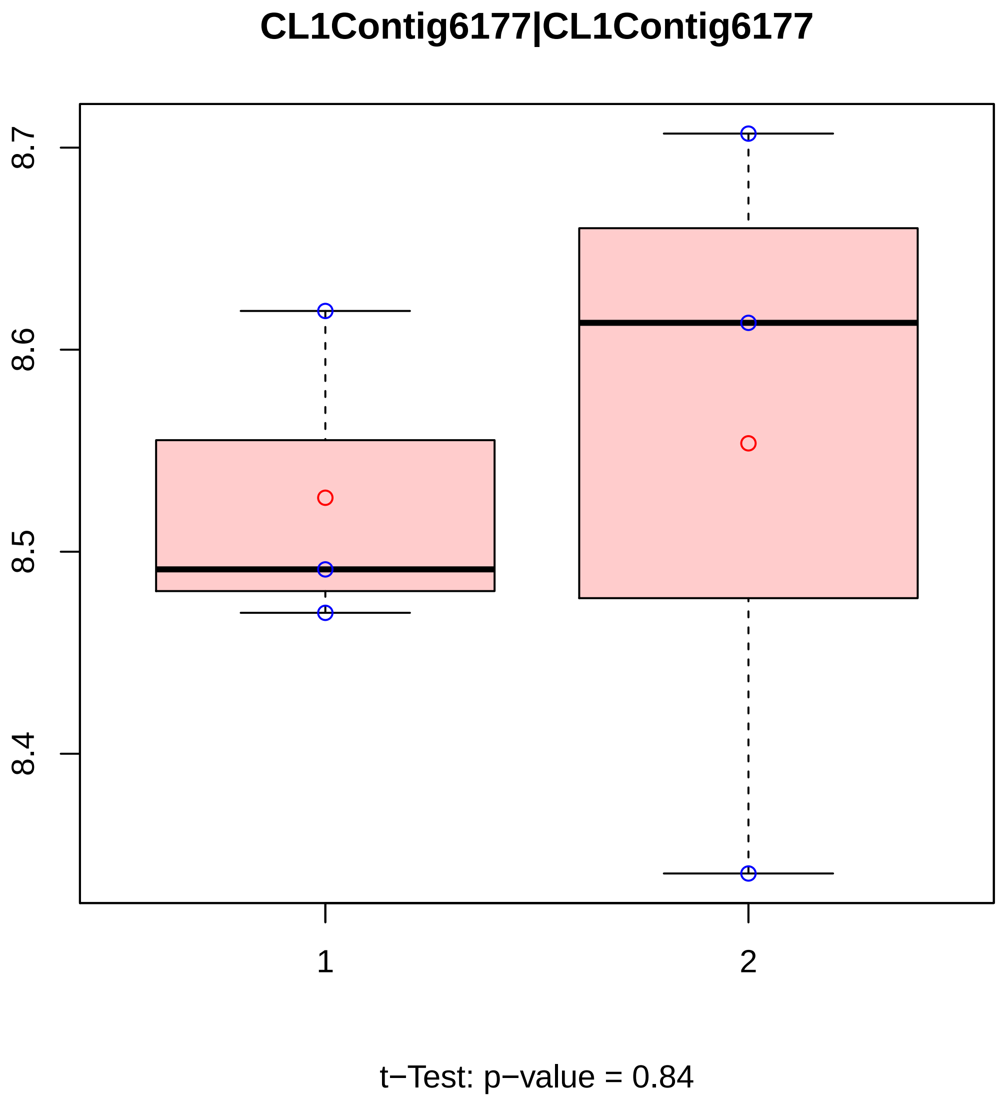

CL1Contig6177|CL1Contig6177
8.7
8.6
8.5
8.4
1
2
t−Test: p−value = 0.84

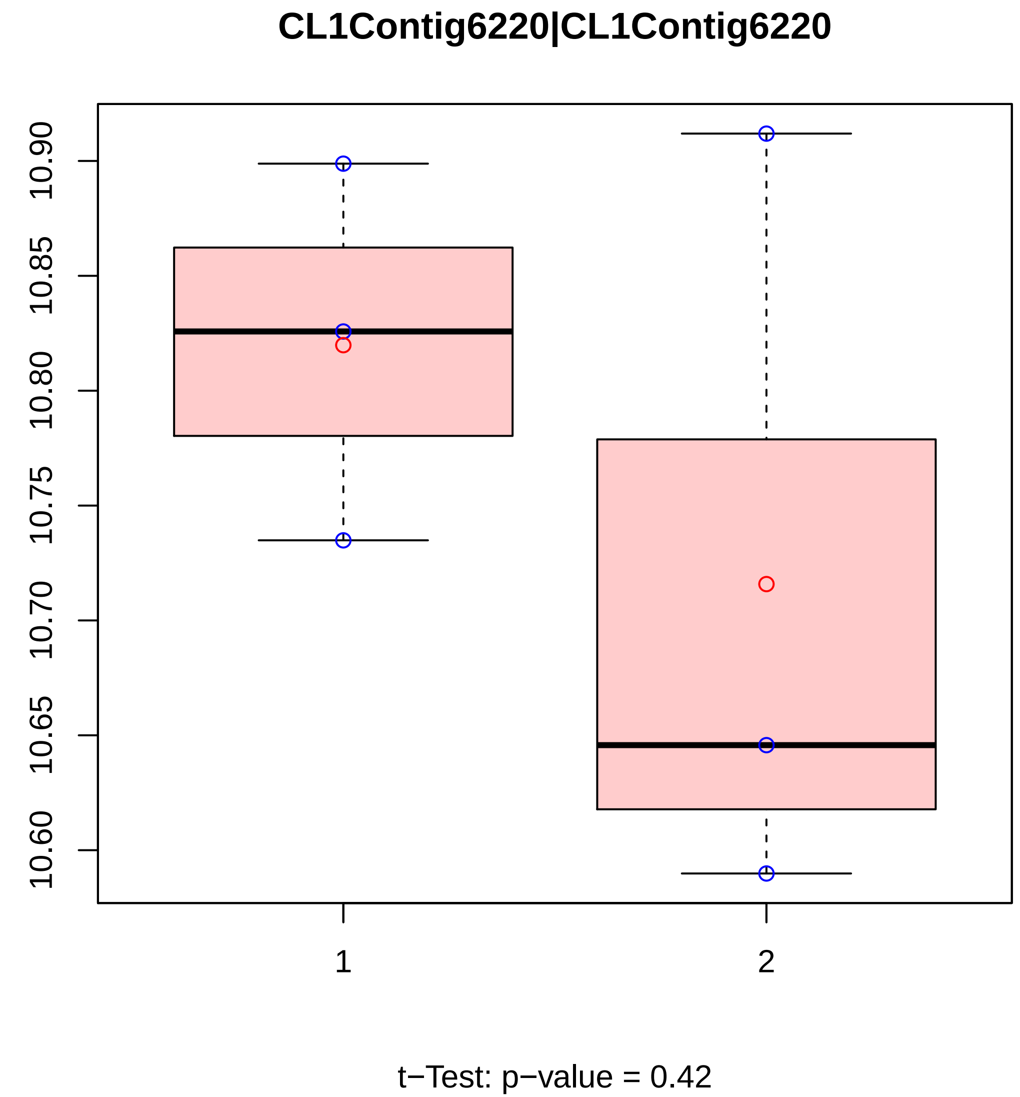

CL1Contig6220|CL1Contig6220
10.60
10.65
10.70
10.75
10.80
10.85
10.90
1
2
t−Test: p−value = 0.42

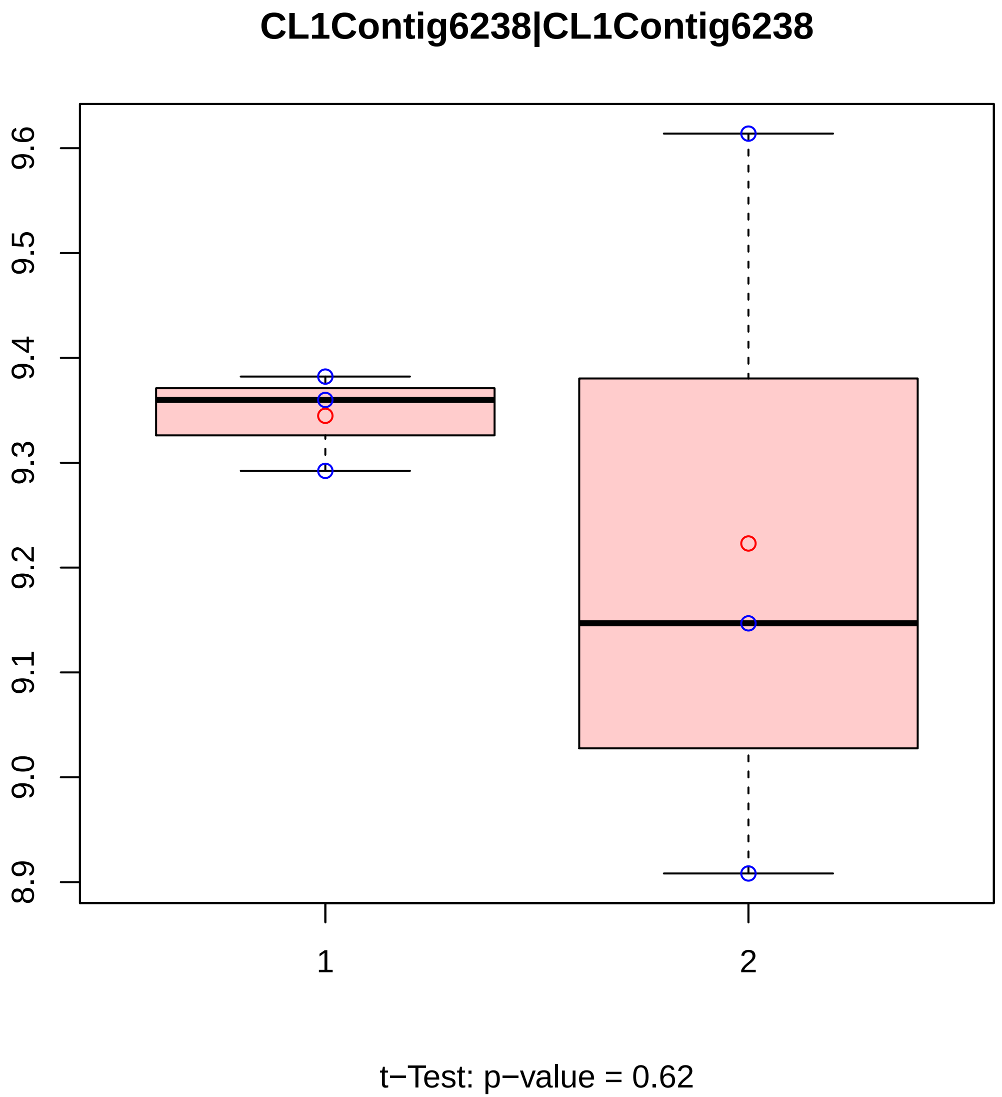
CL1Contig6238|CL1Contig6238
9.6
9.5
9.4
9.3
9.2
9.1
9.0
8.9
1
2
t−Test: p−value = 0.62

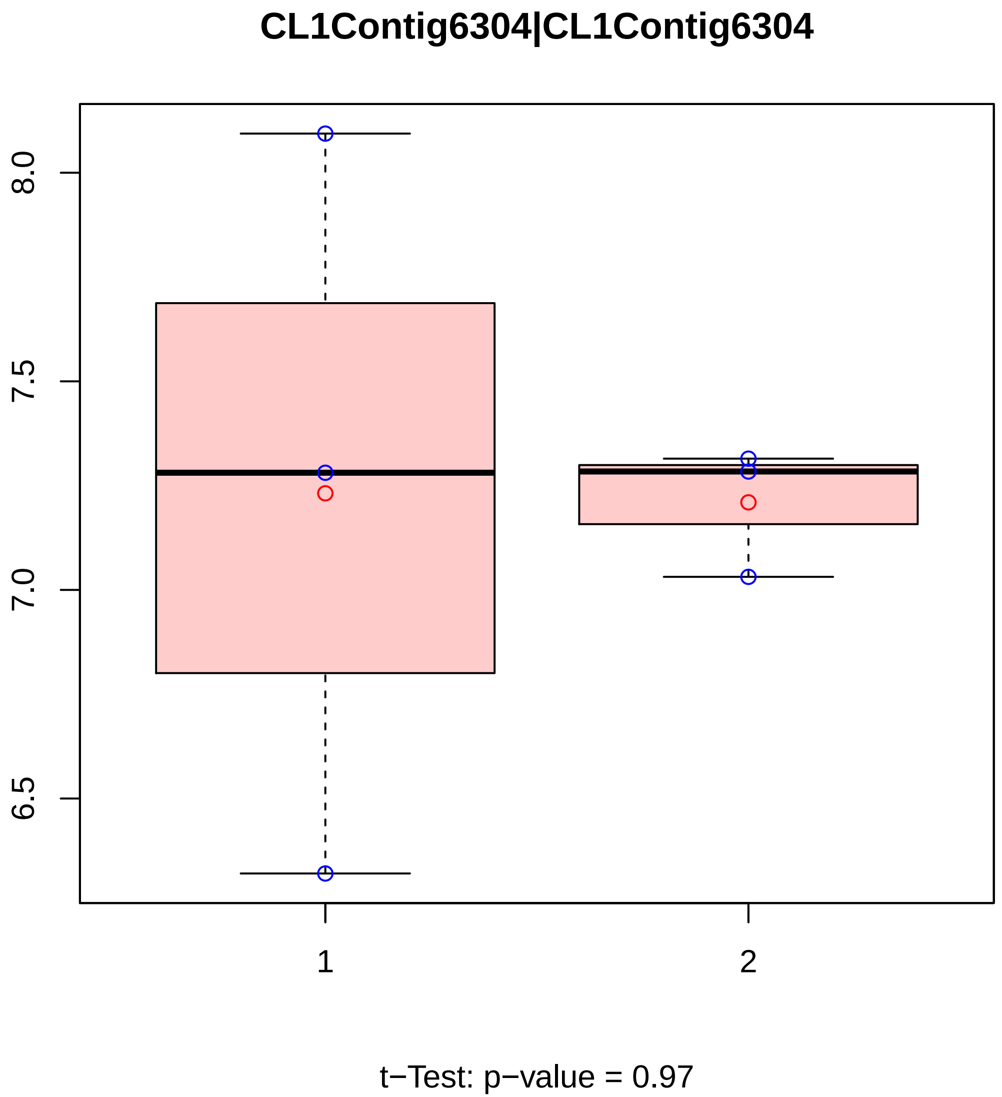
CL1Contig6304|CL1Contig6304
8.0
7.5
7.0
6.5
1
2
t−Test: p−value = 0.97

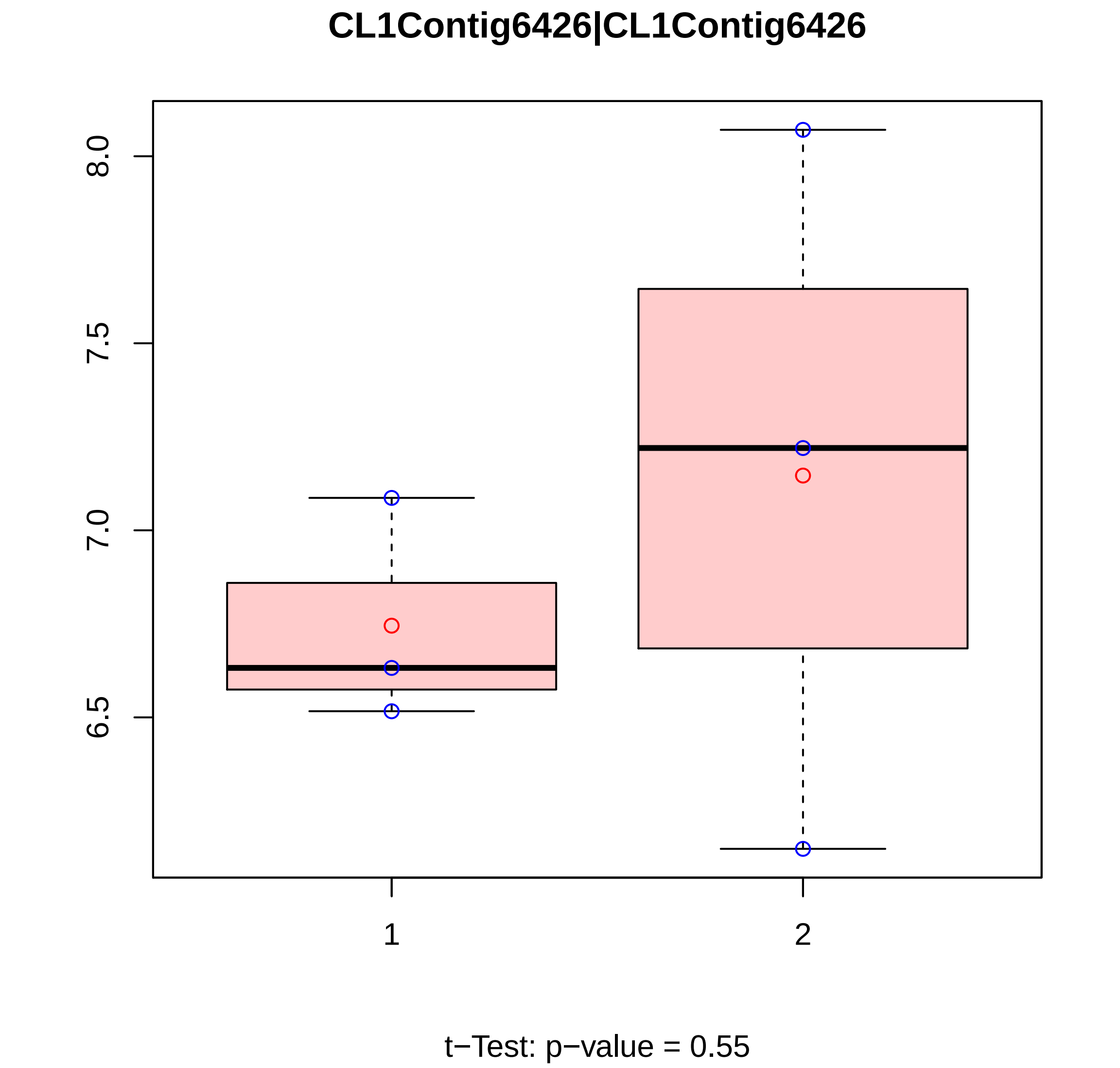
CL1Contig6426|CL1Contig6426
8.0
7.5
7.0
6.5
1
2
t−Test: p−value = 0.55

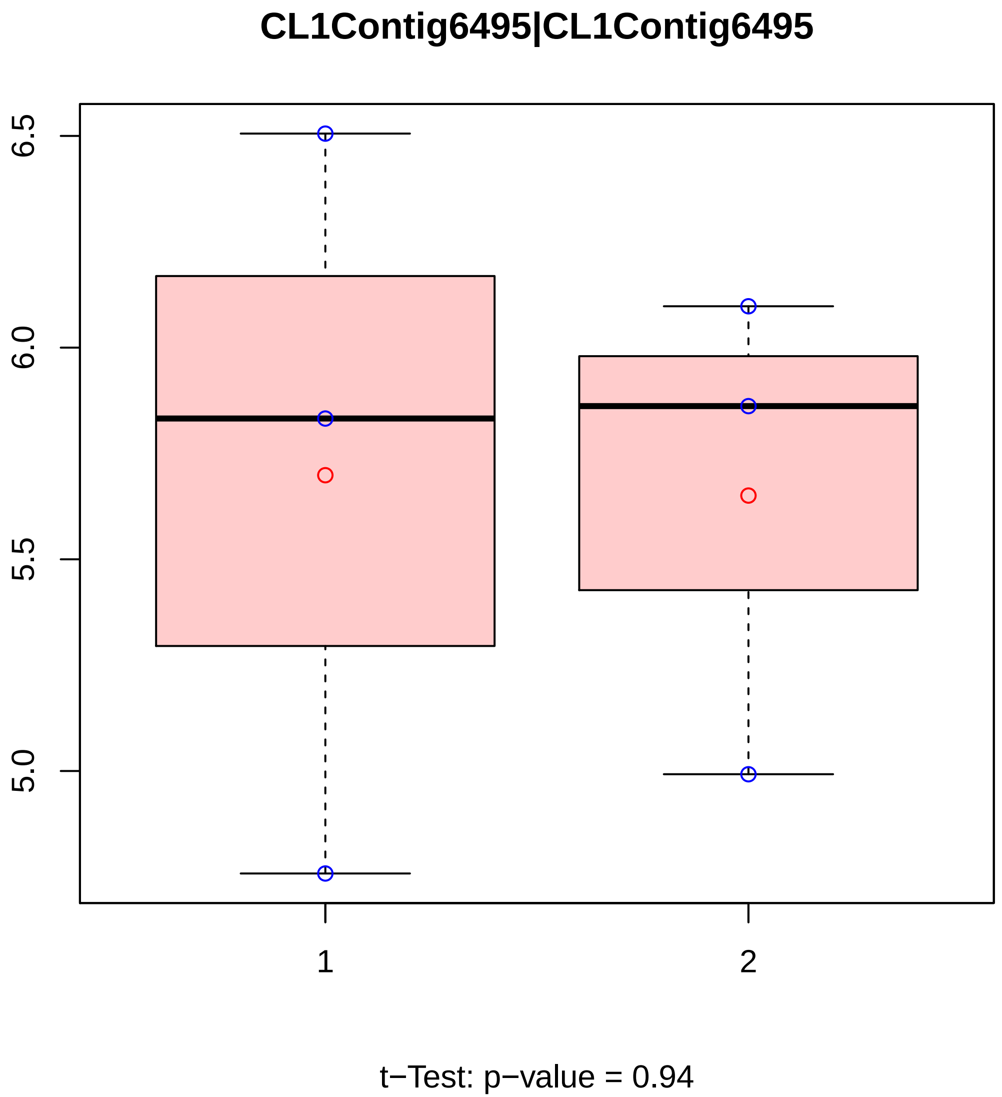
CL1Contig6495|CL1Contig6495
6.5
6.0
5.5
5.0
1
2
t−Test: p−value = 0.94

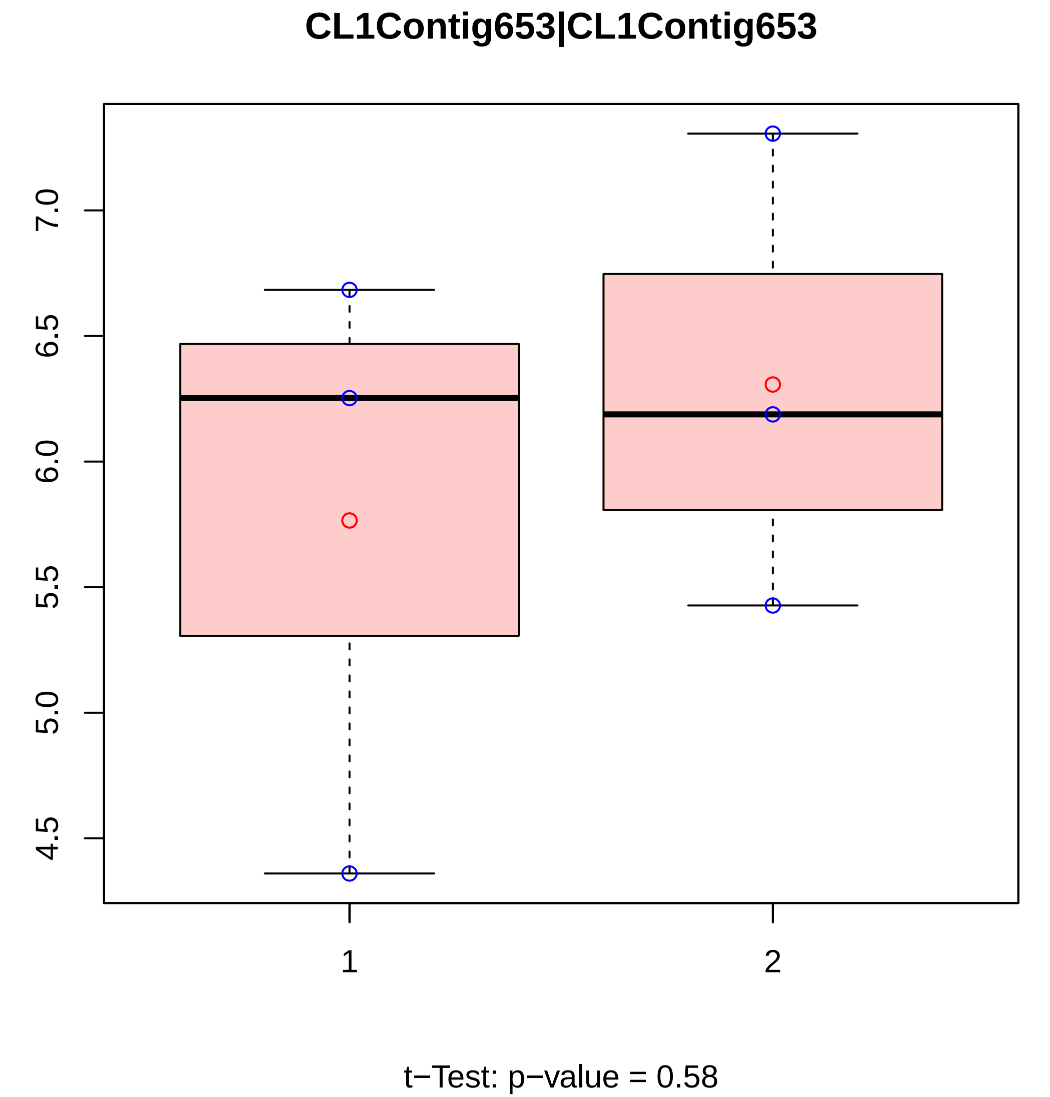
CL1Contig653|CL1Contig653
7.0
6.5
6.0
5.5
5.0
4.5
1
2
t−Test: p−value = 0.58

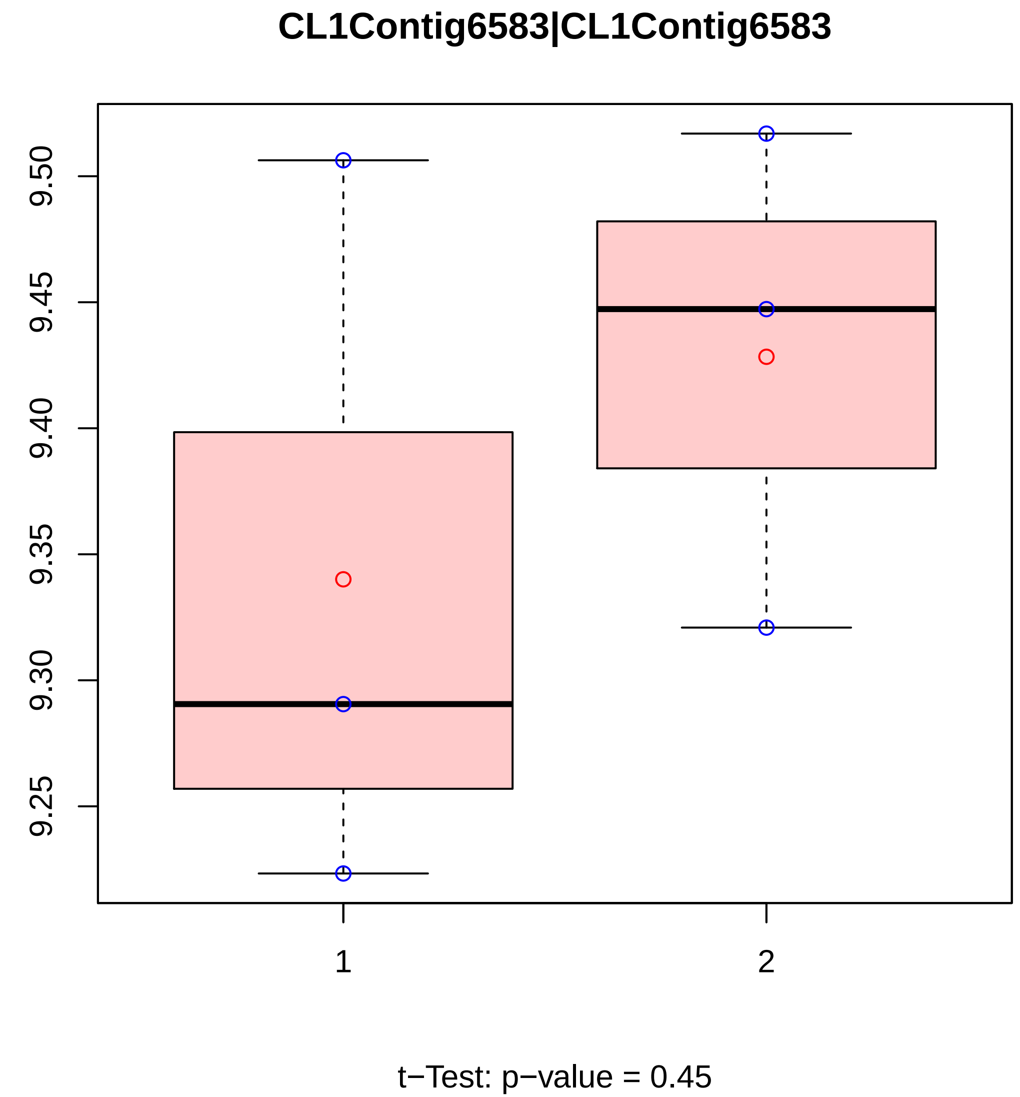
CL1Contig6583|CL1Contig6583
9.25
9.30
9.35
9.40
9.45
9.50
1
2
t−Test: p−value = 0.45

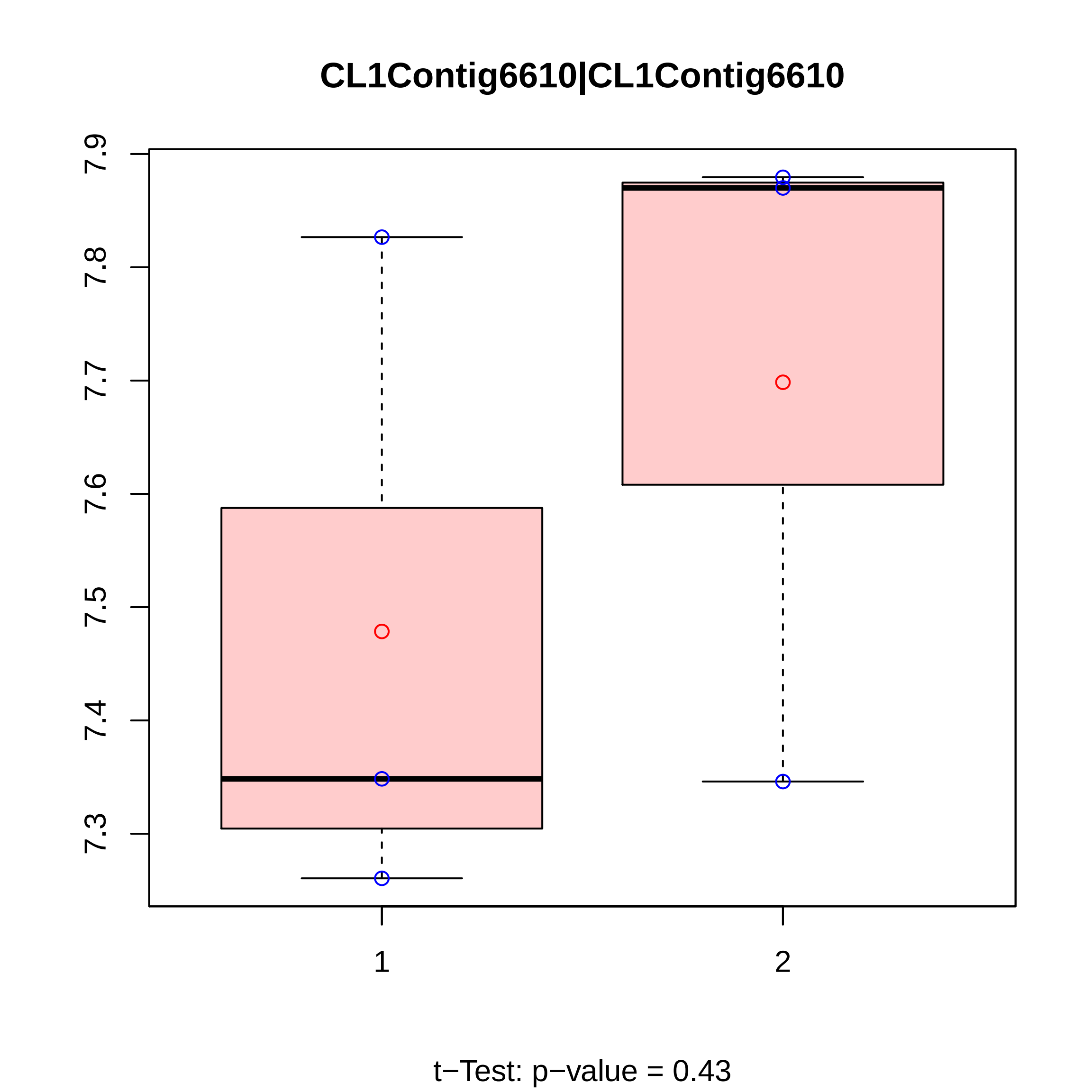

CL1Contig6610|CL1Contig6610
7.3
7.4
7.5
7.6
7.7
7.8
7.9
1
2
t−Test: p−value = 0.43

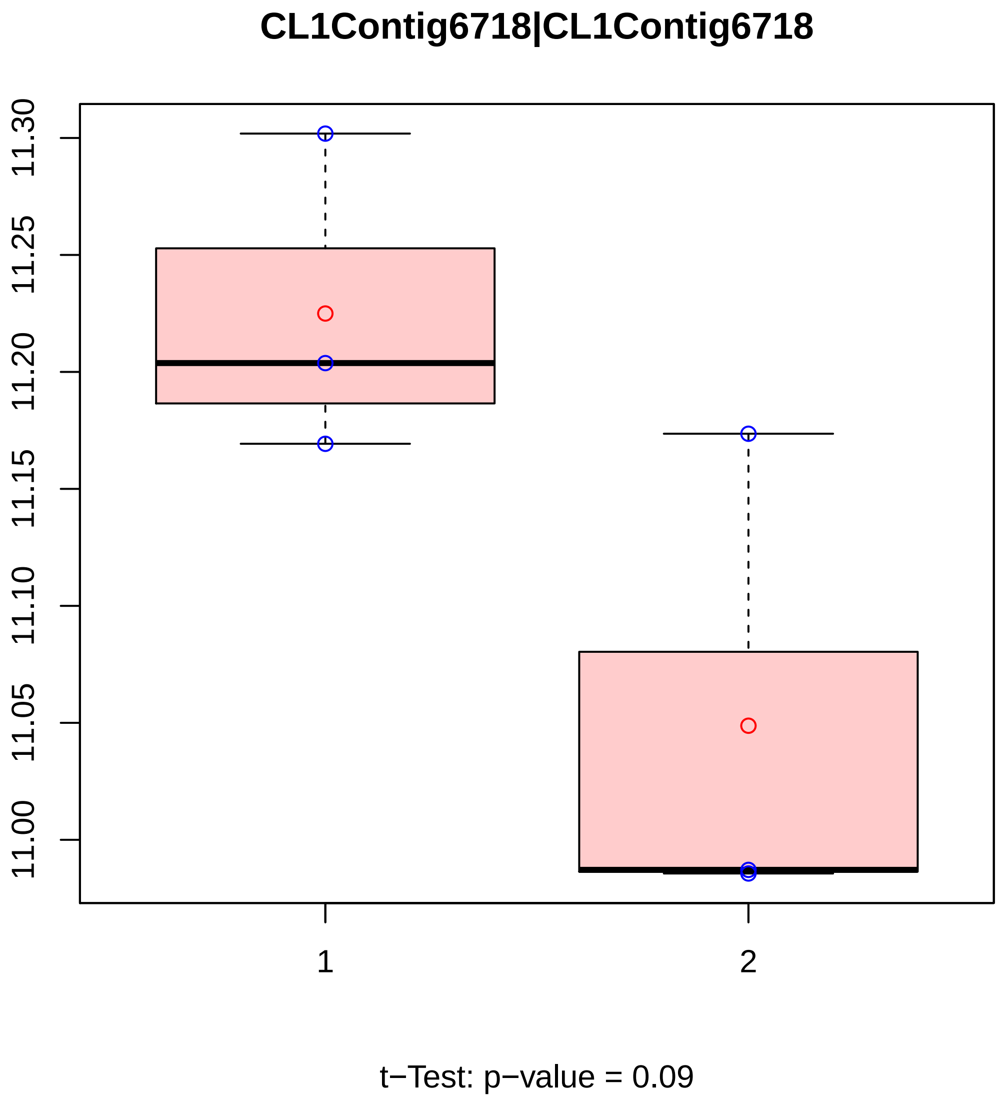

CL1Contig6718|CL1Contig6718
11.00
11.05
11.10
11.15
11.20
11.25
11.30
1
2
t−Test: p−value = 0.09

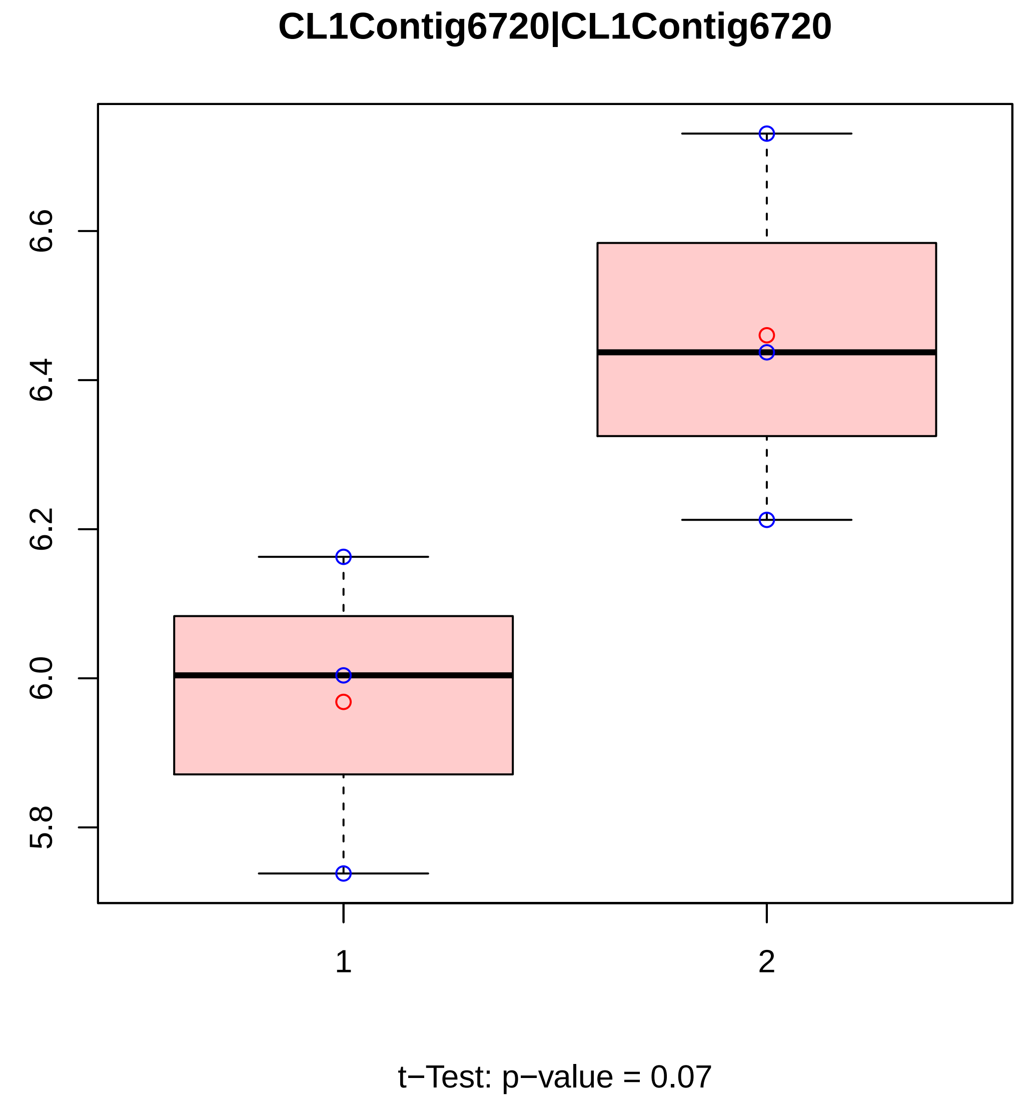
CL1Contig6720|CL1Contig6720
6.6
6.4
6.2
6.0
5.8
1
2
t−Test: p−value = 0.07

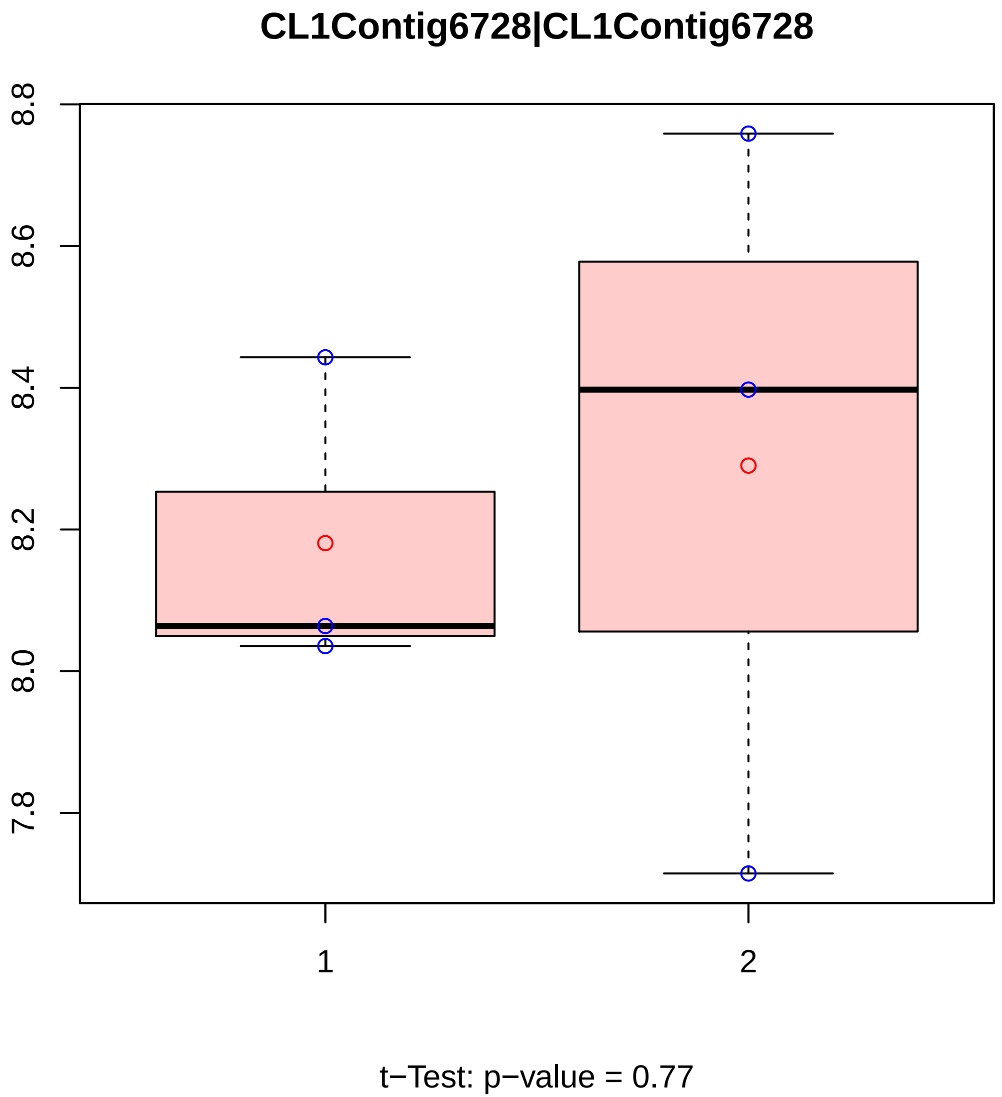
CL1Contig6728|CL1Contig6728
8.8
8.6
8.4
8.2
8.0
7.8
1
2
t−Test: p−value = 0.77

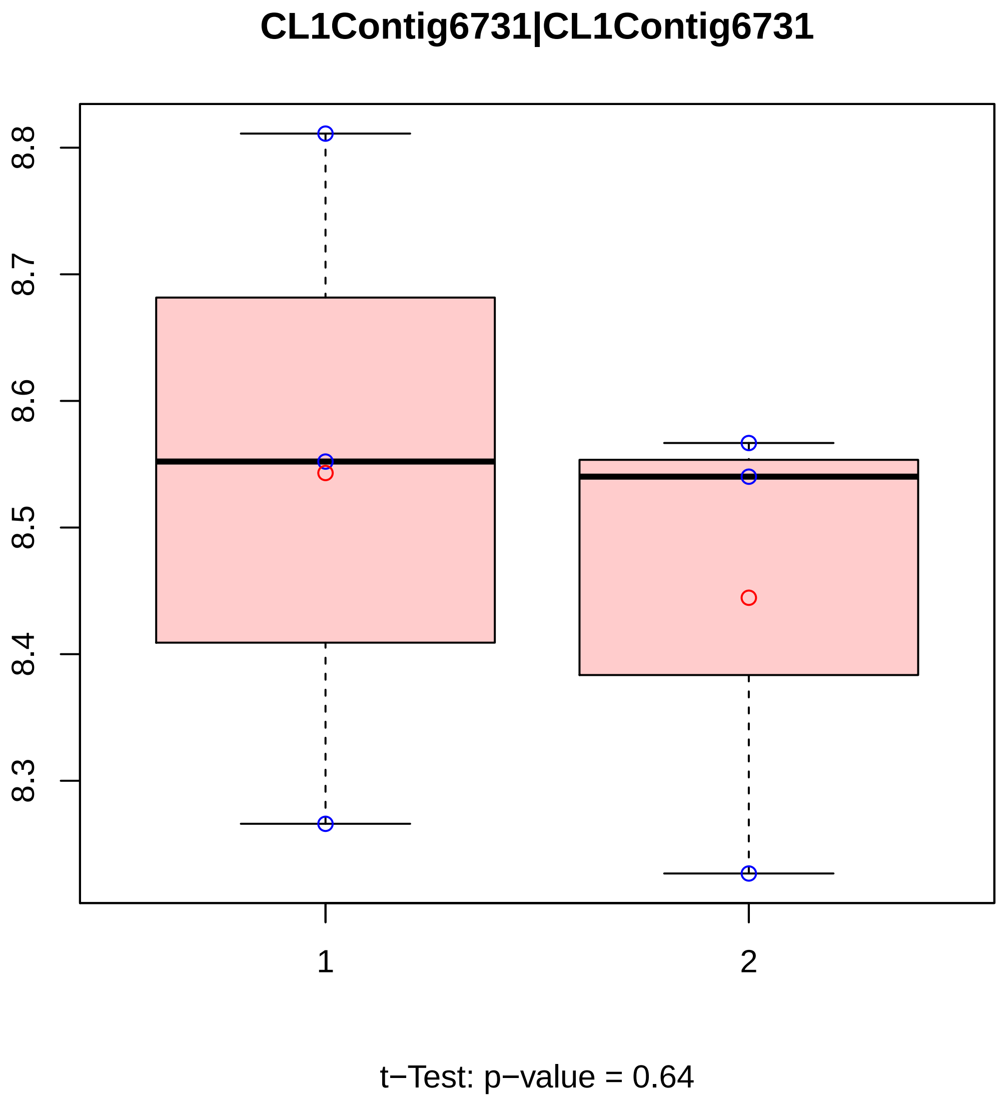
CL1Contig6731|CL1Contig6731
8.8
8.7
8.6
8.5
8.4
8.3
1
2
t−Test: p−value = 0.64

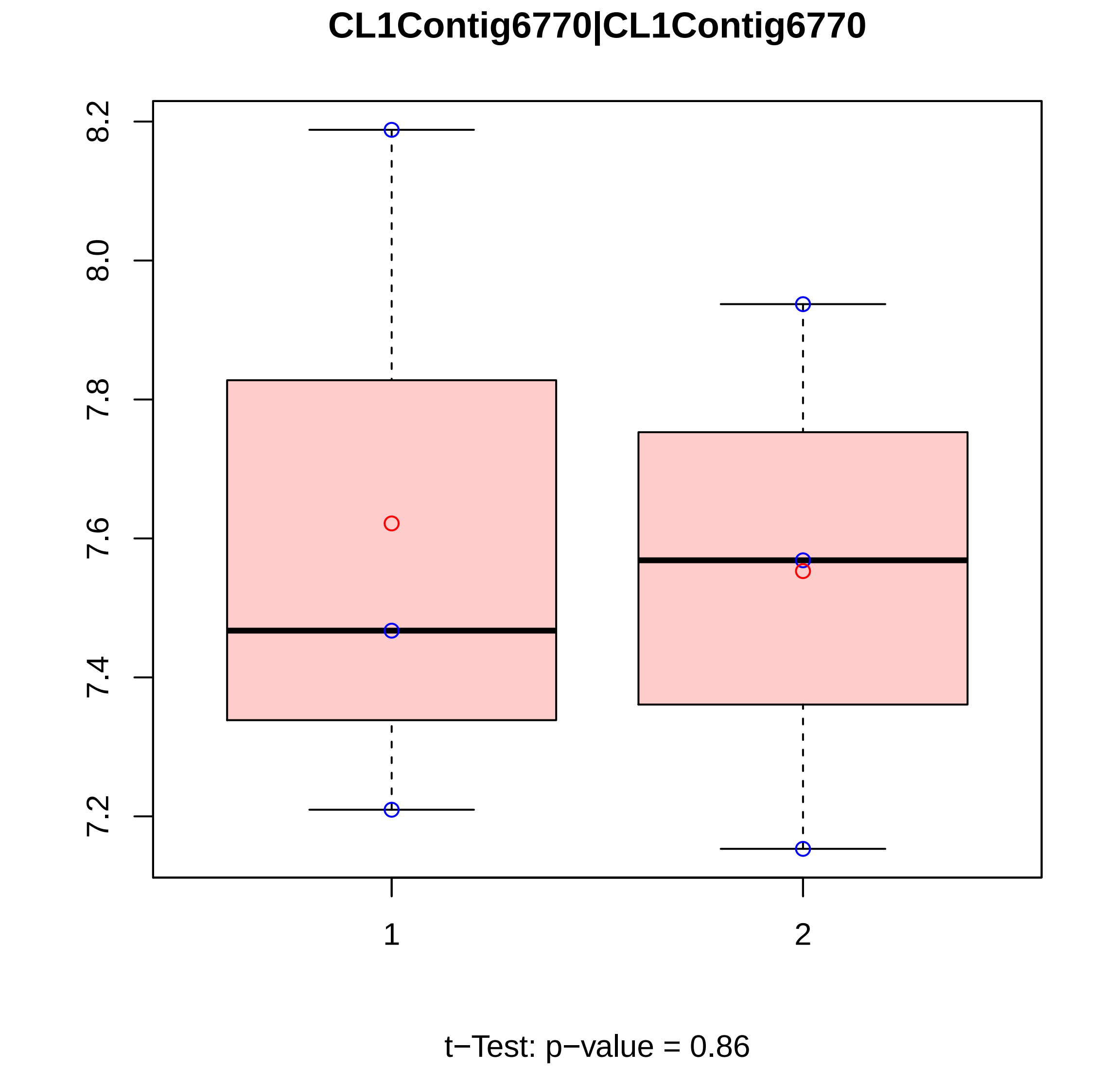
CL1Contig6770|CL1Contig6770
8.2
8.0
7.8
7.6
7.4
7.2
1
2
t−Test: p−value = 0.86

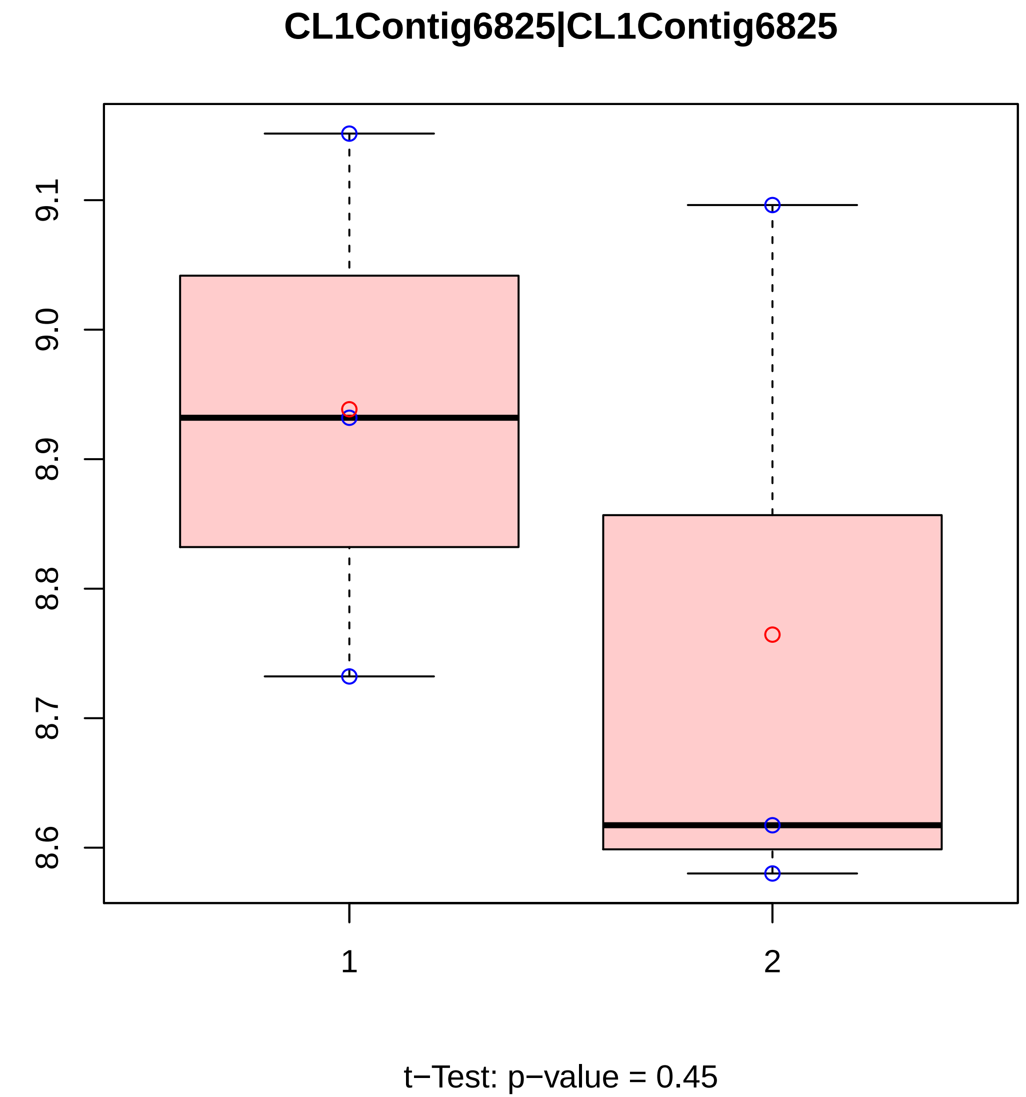

CL1Contig6825|CL1Contig6825
9.1
9.0
8.9
8.8
8.7
8.6
1
2
t−Test: p−value = 0.45

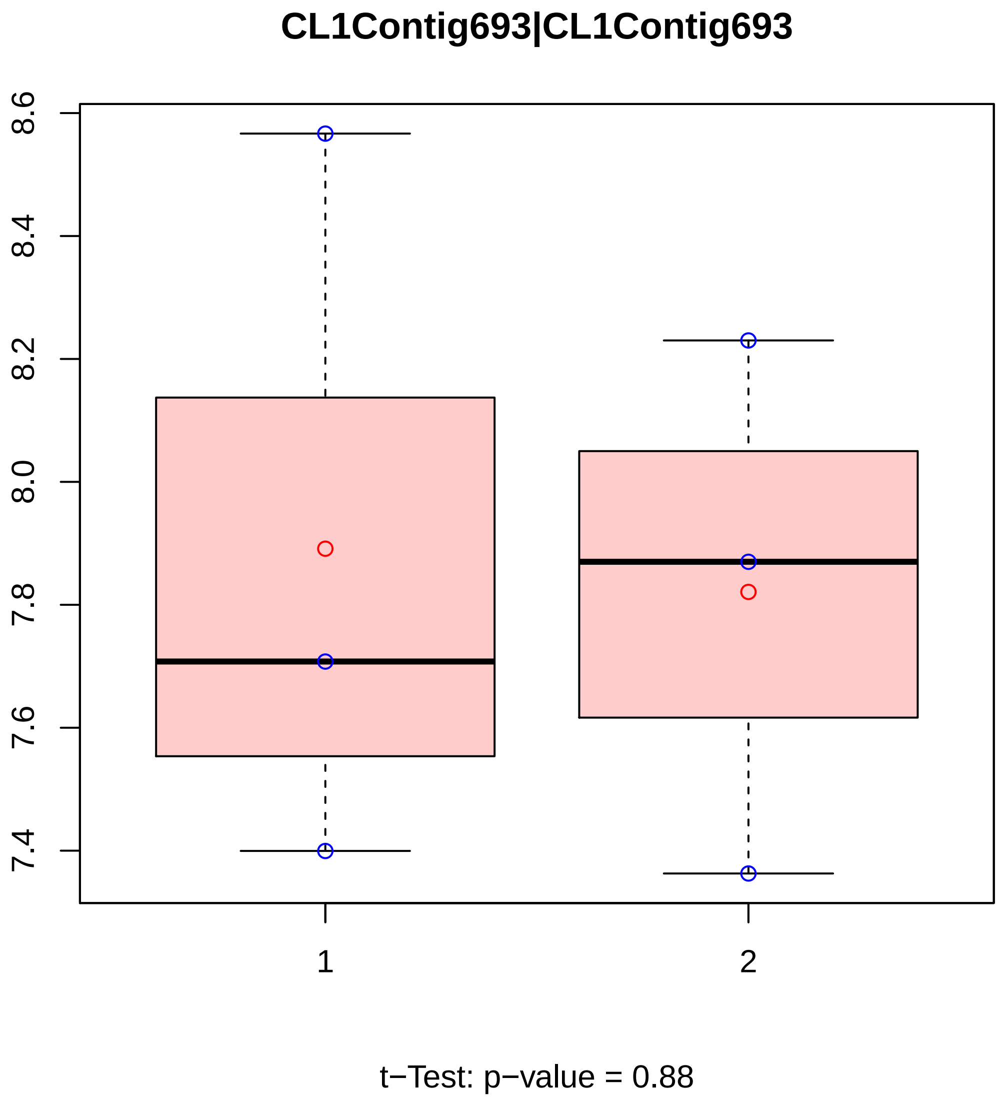
CL1Contig693|CL1Contig693
8.6
8.4
8.2
8.0
7.8
7.6
7.4
1
2
t−Test: p−value = 0.88

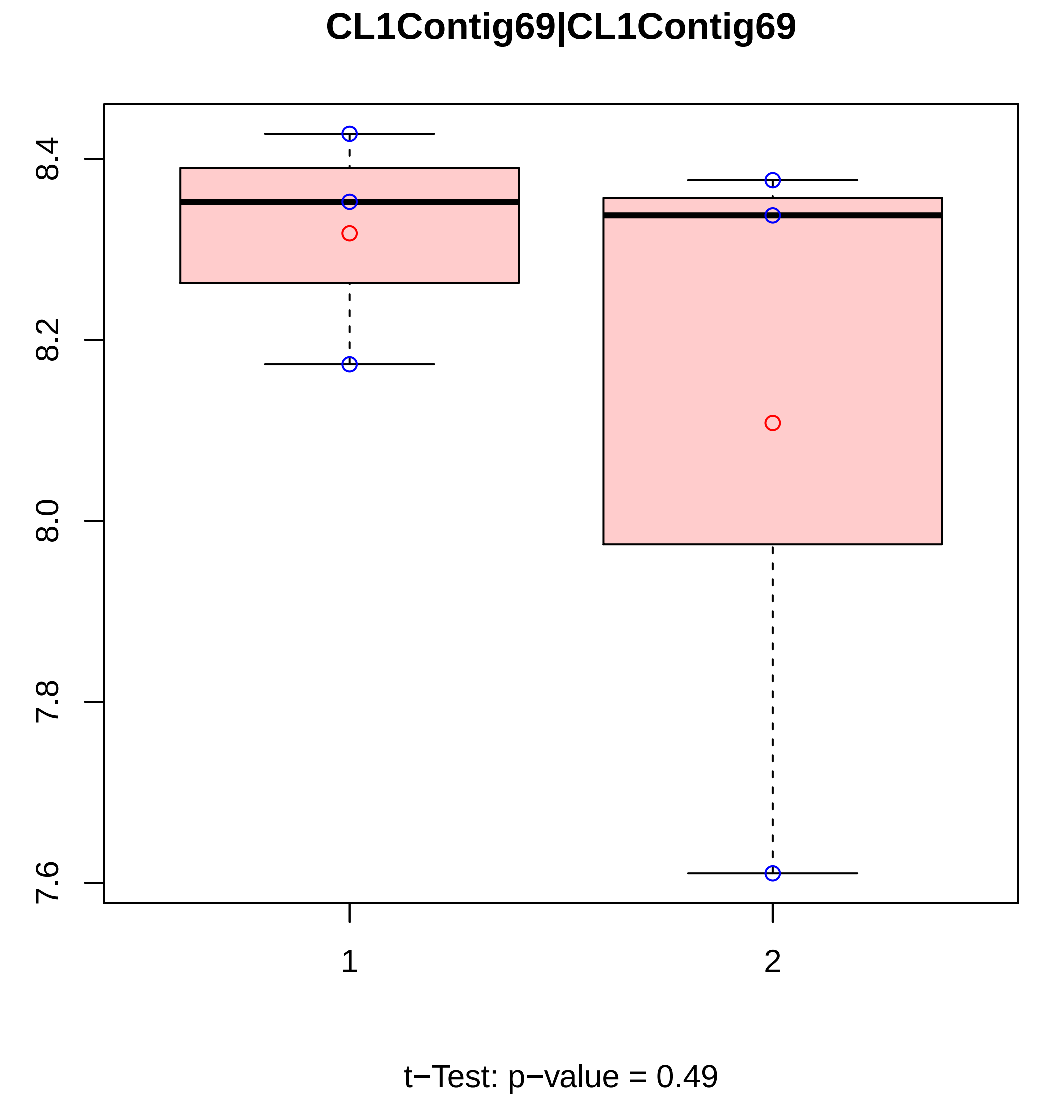

CL1Contig69|CL1Contig69
8.4
8.2
8.0
7.8
7.6
1
2
t−Test: p−value = 0.49

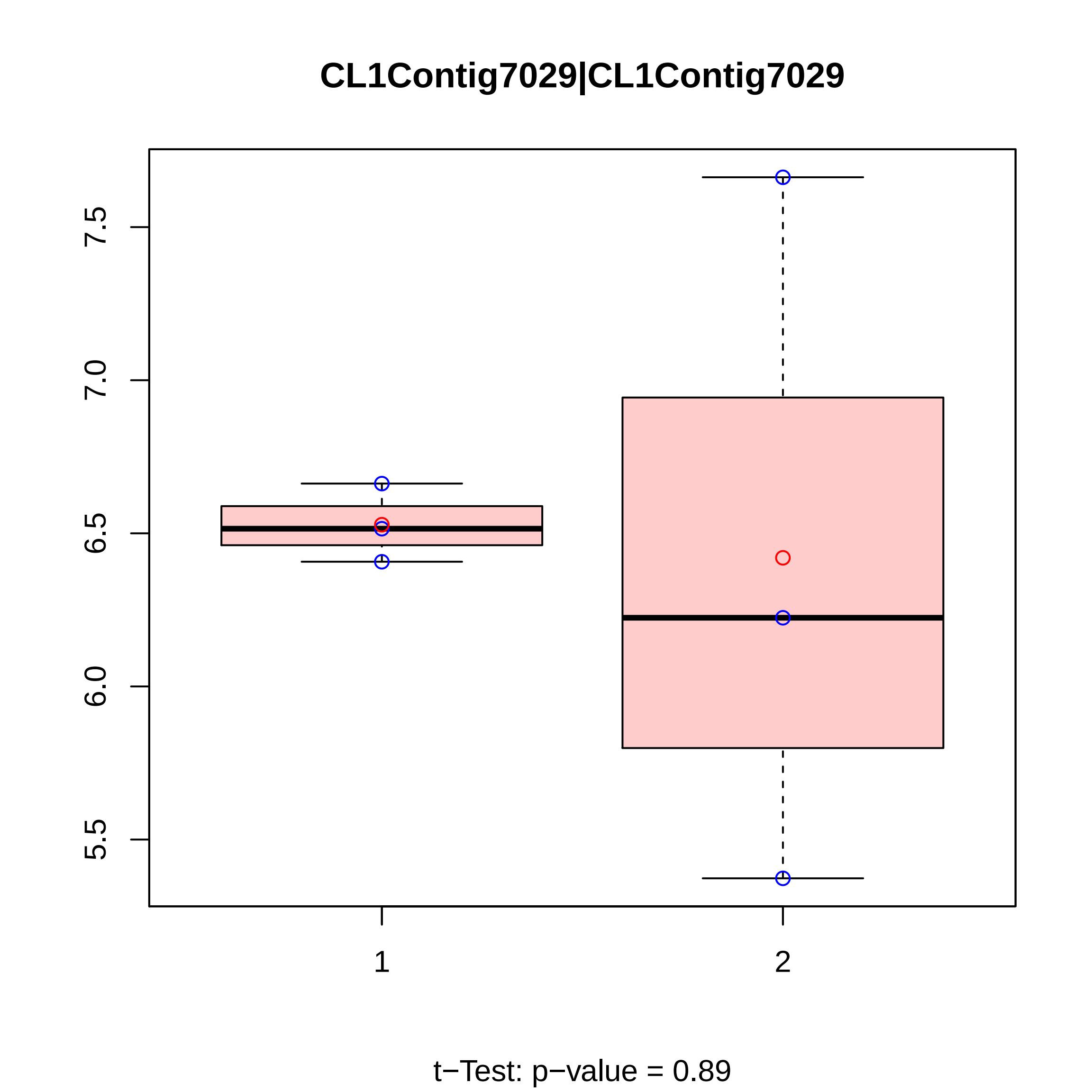
CL1Contig7029|CL1Contig7029
7.5
7.0
6.5
6.0
5.5
1
2
t−Test: p−value = 0.89

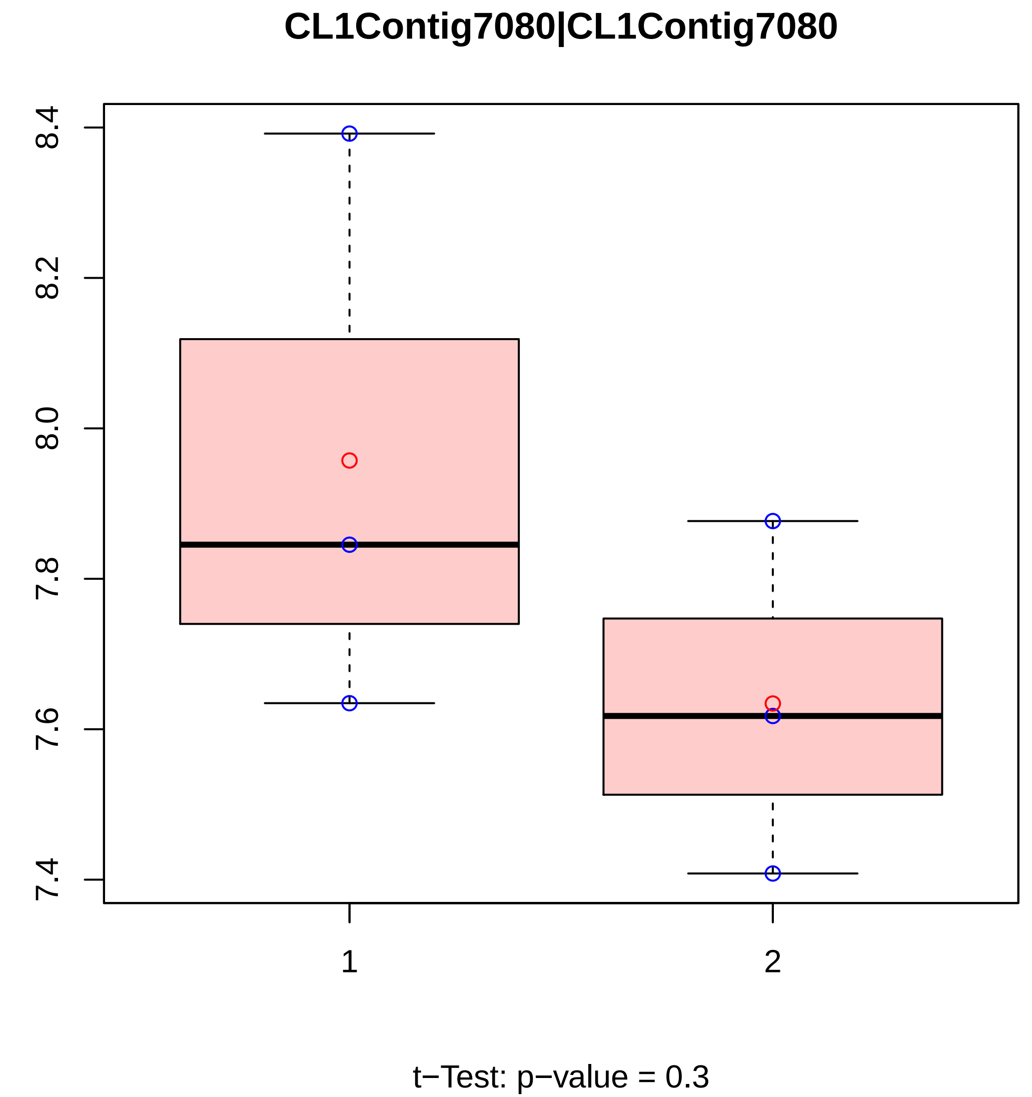
CL1Contig7080|CL1Contig7080
8.4
8.2
8.0
7.8
7.6
7.4
1
2
t−Test: p−value = 0.3

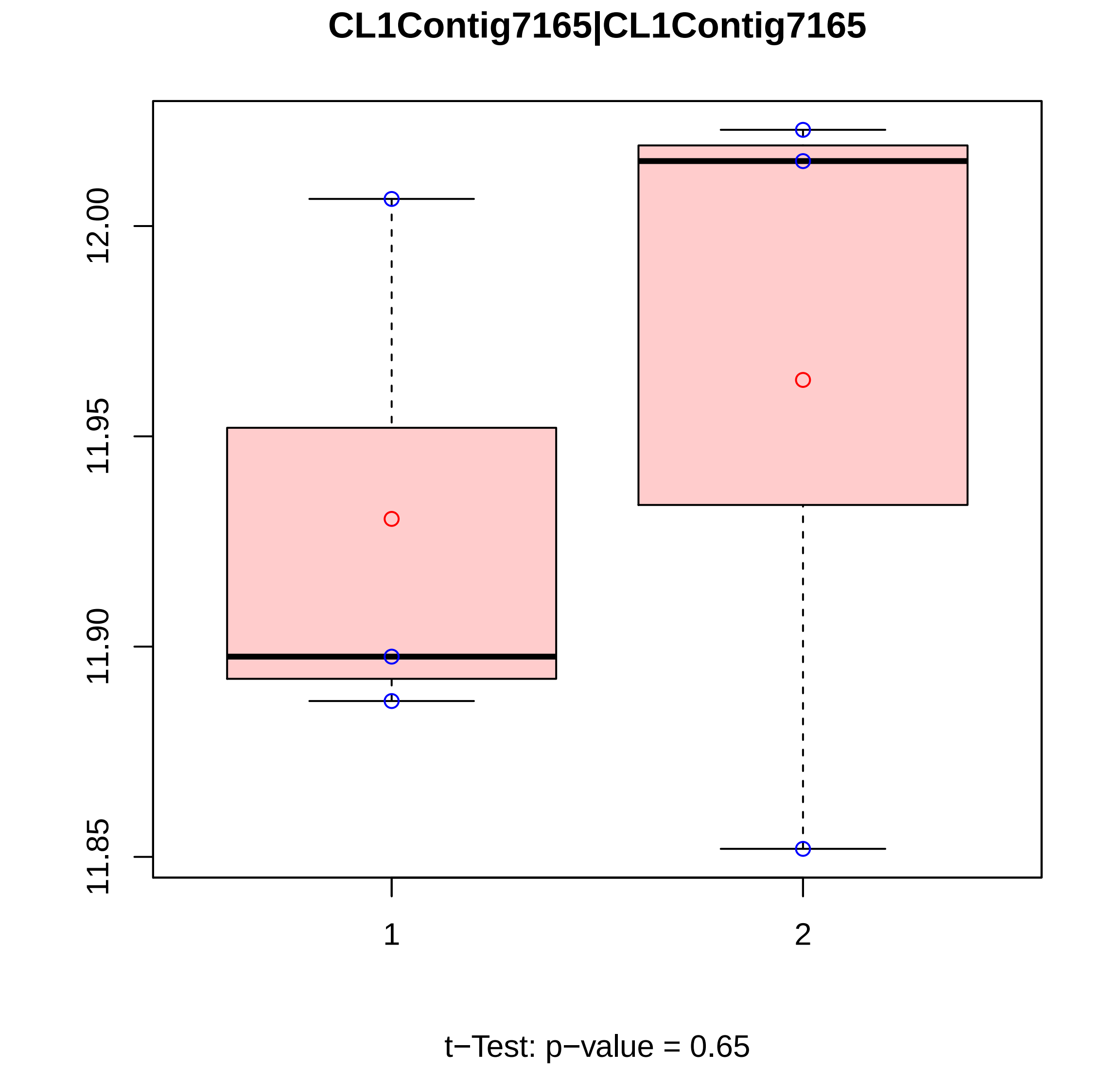
CL1Contig7165|CL1Contig7165
12.00
11.95
11.90
11.85
1
2
t−Test: p−value = 0.65

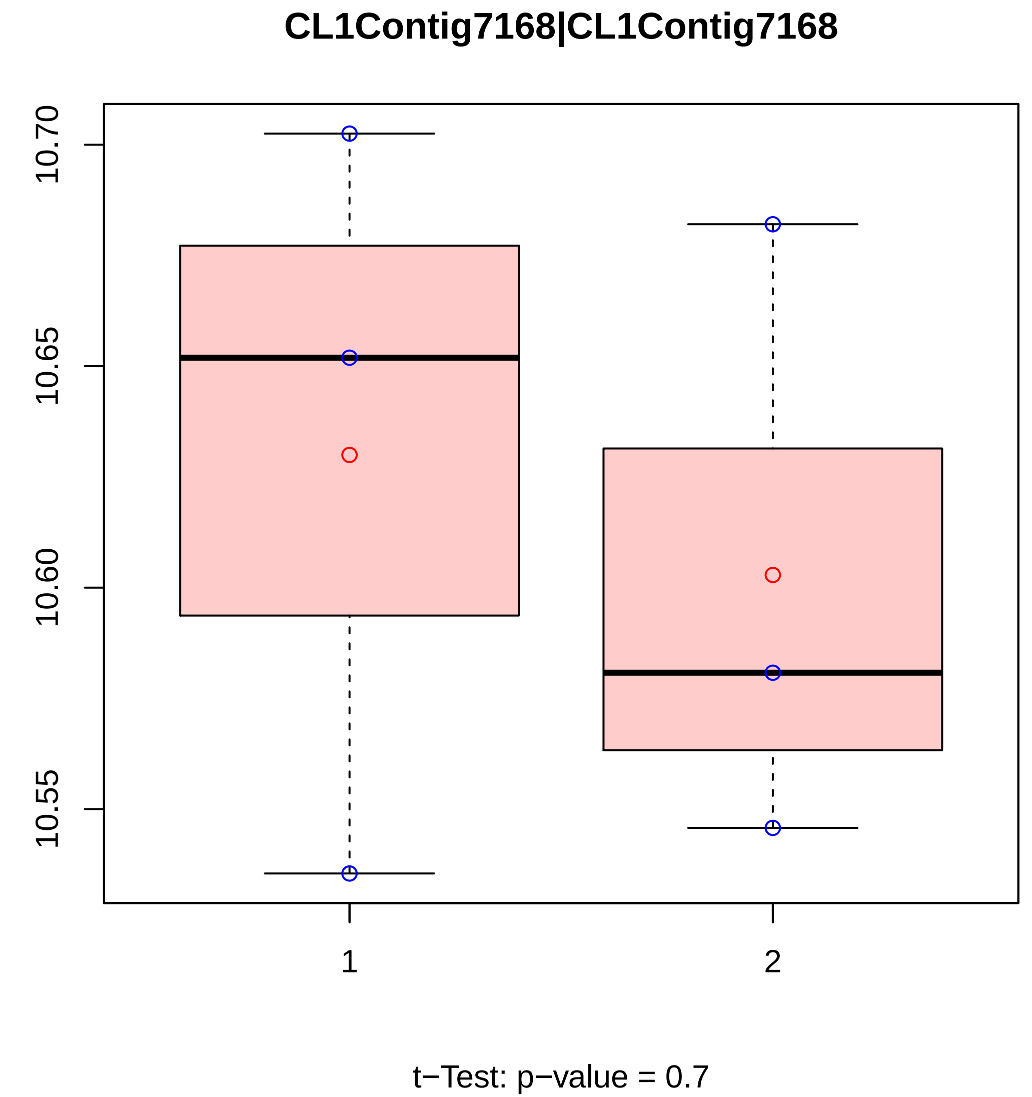
CL1Contig7168|CL1Contig7168
10.70
10.65
10.60
10.55
1
2
t−Test: p−value = 0.7

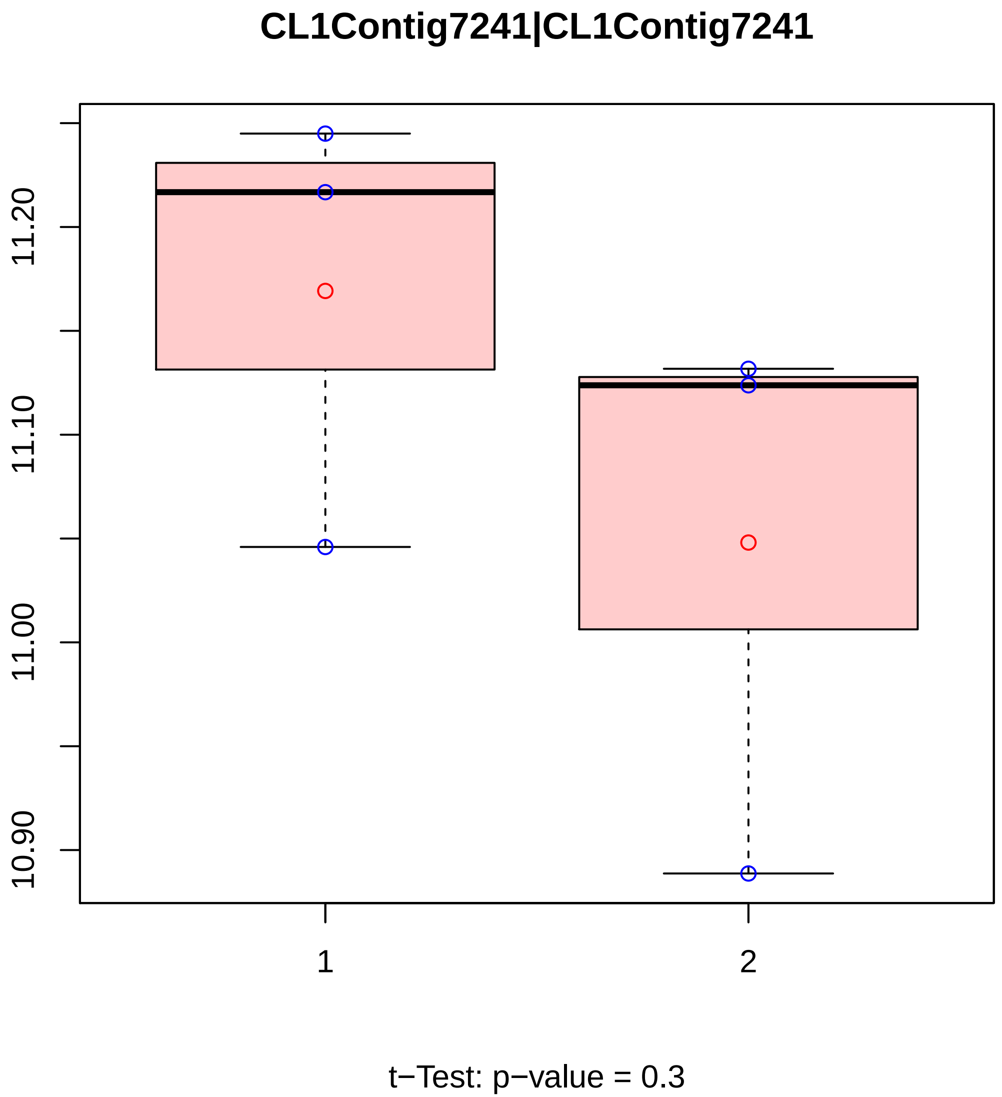
CL1Contig7241|CL1Contig7241
11.20
11.10
11.00
10.90
1
2
t−Test: p−value = 0.3

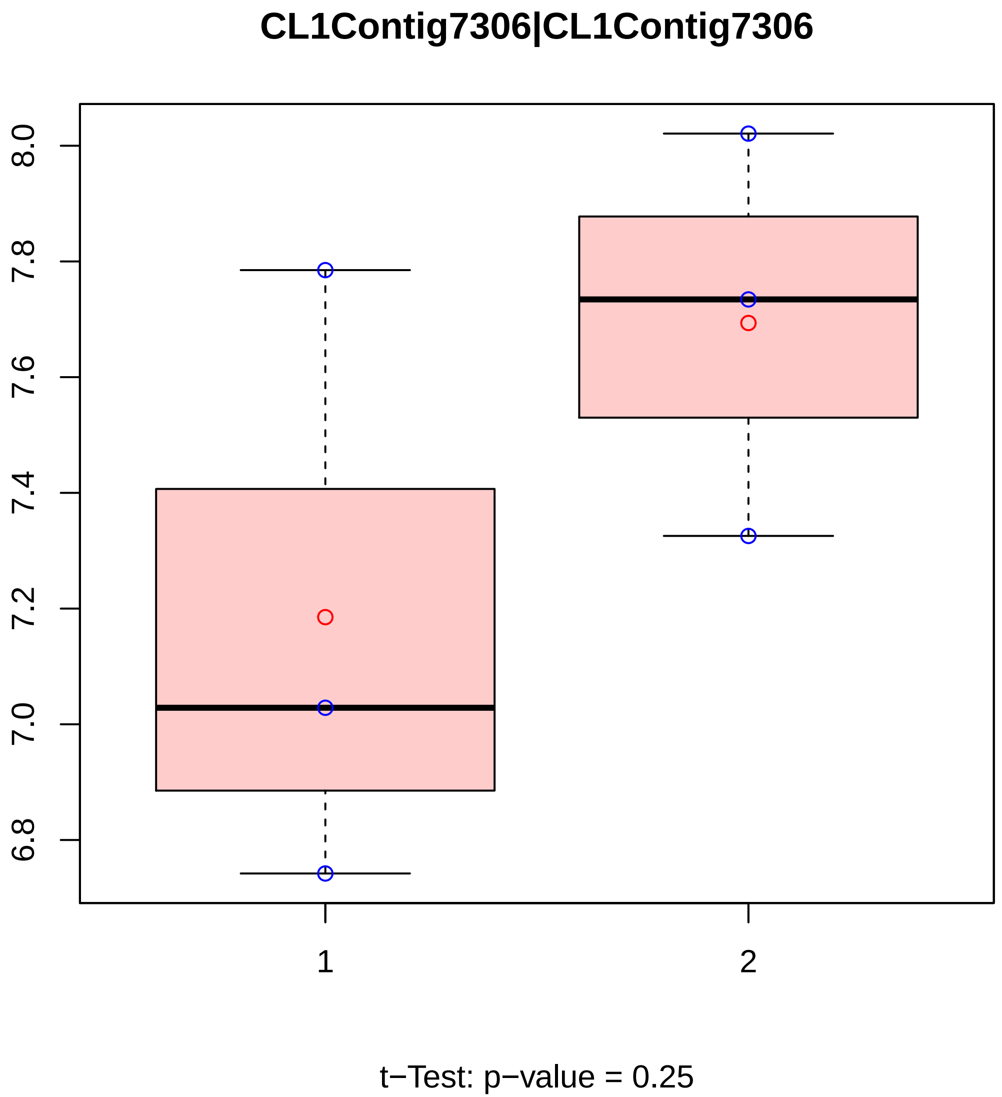
CL1Contig7306|CL1Contig7306
8.0
7.8
7.6
7.4
7.2
7.0
6.8
1
2
t−Test: p−value = 0.25

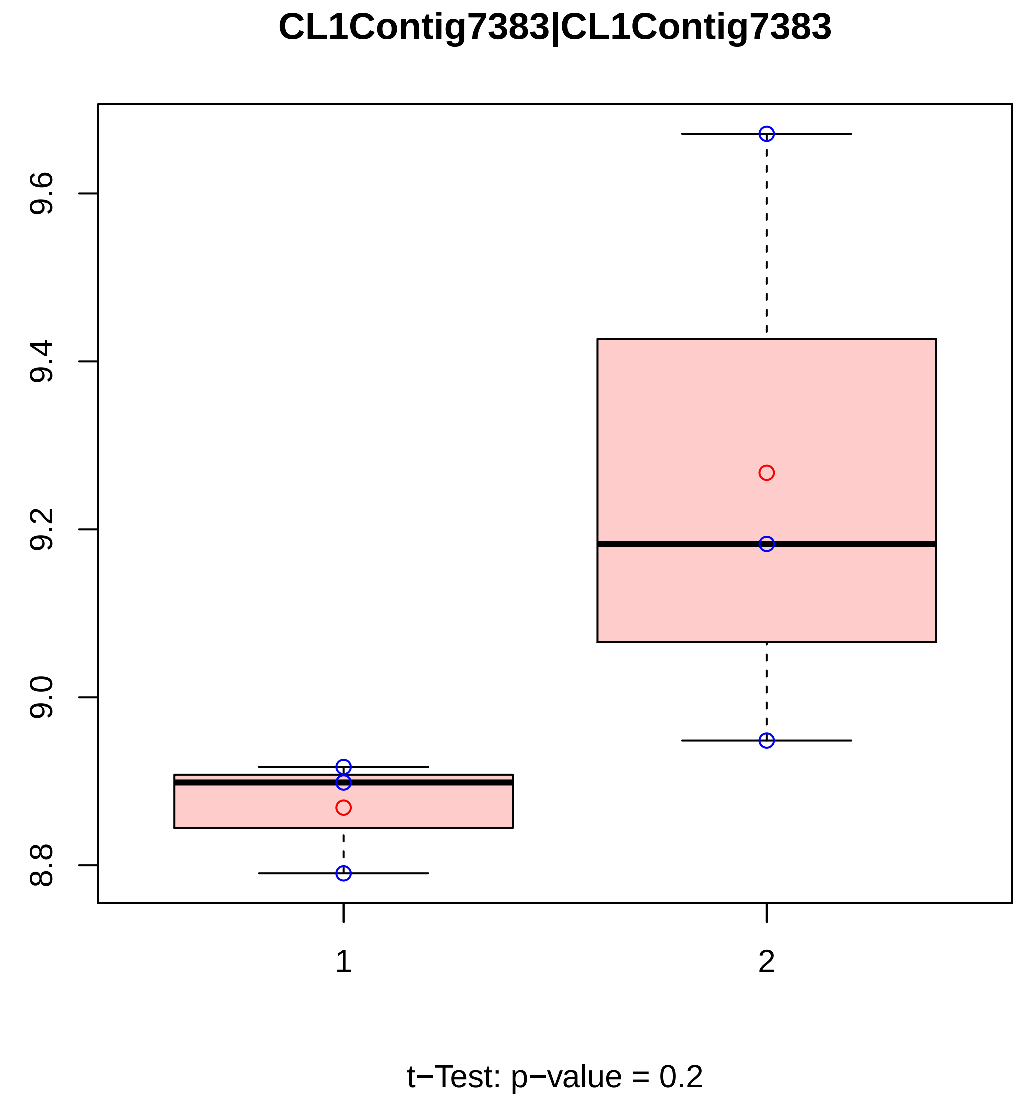
CL1Contig7383|CL1Contig7383
9.6
9.4
9.2
9.0
8.8
1
2
t−Test: p−value = 0.2

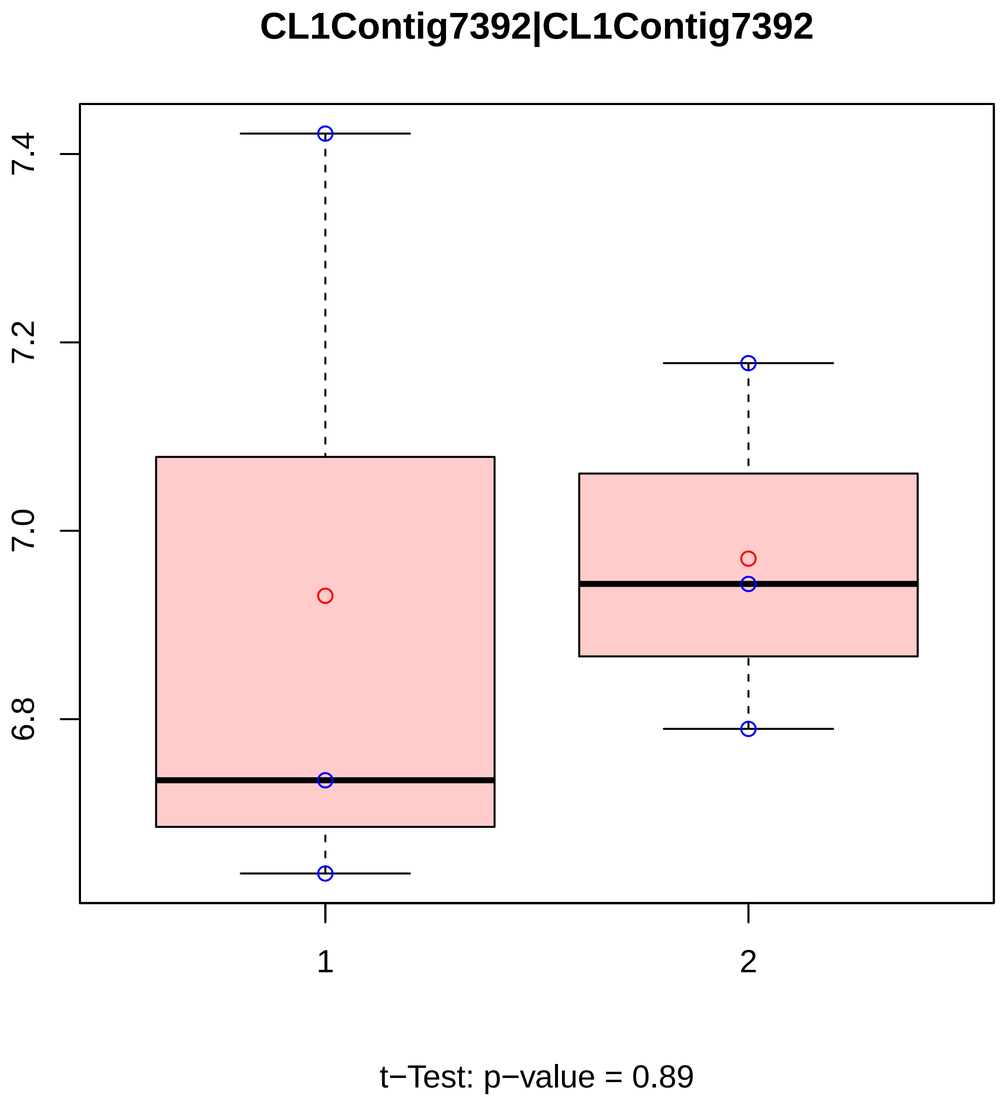
CL1Contig7392|CL1Contig7392
7.4
7.2
7.0
6.8
1
2
t−Test: p−value = 0.89

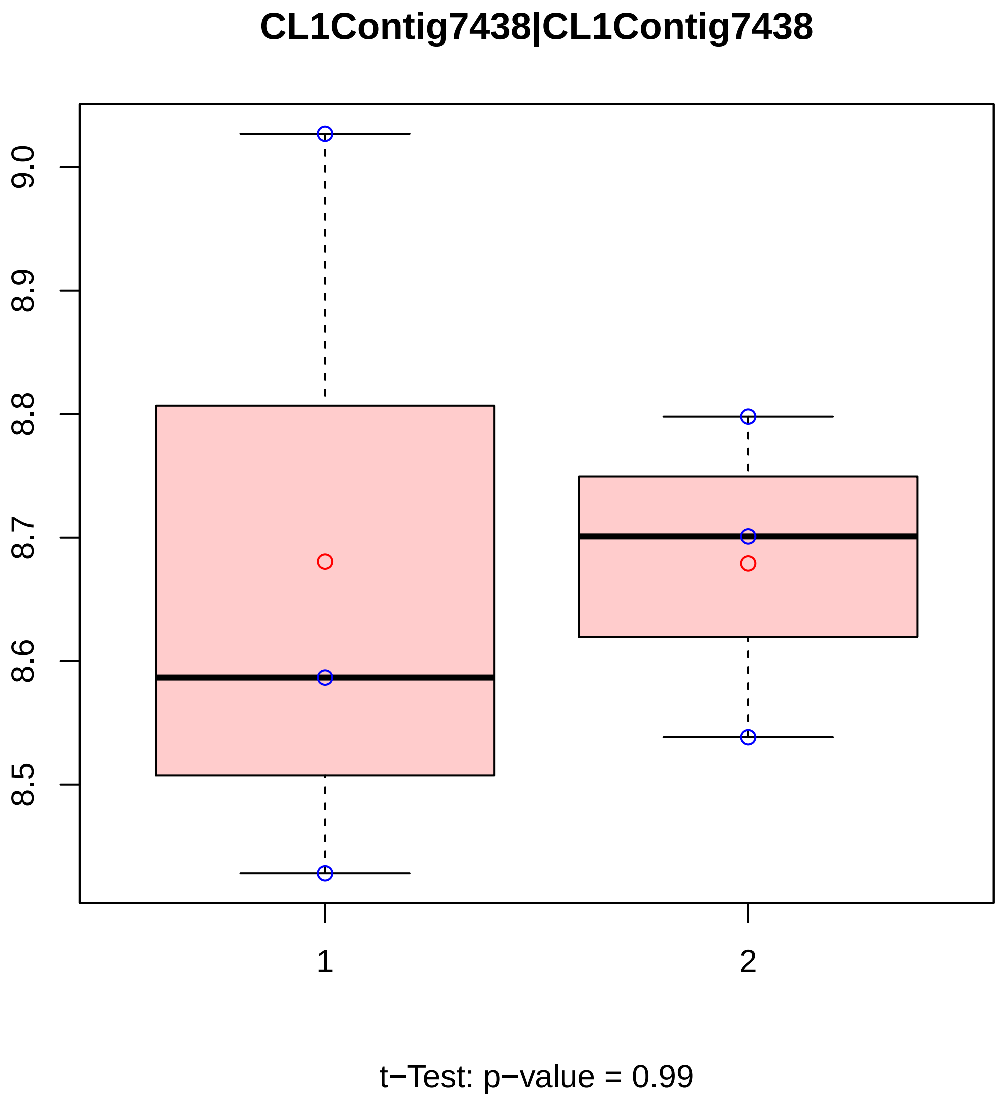

CL1Contig7438|CL1Contig7438
9.0
8.9
8.8
8.7
8.6
8.5
1
2
t−Test: p−value = 0.99

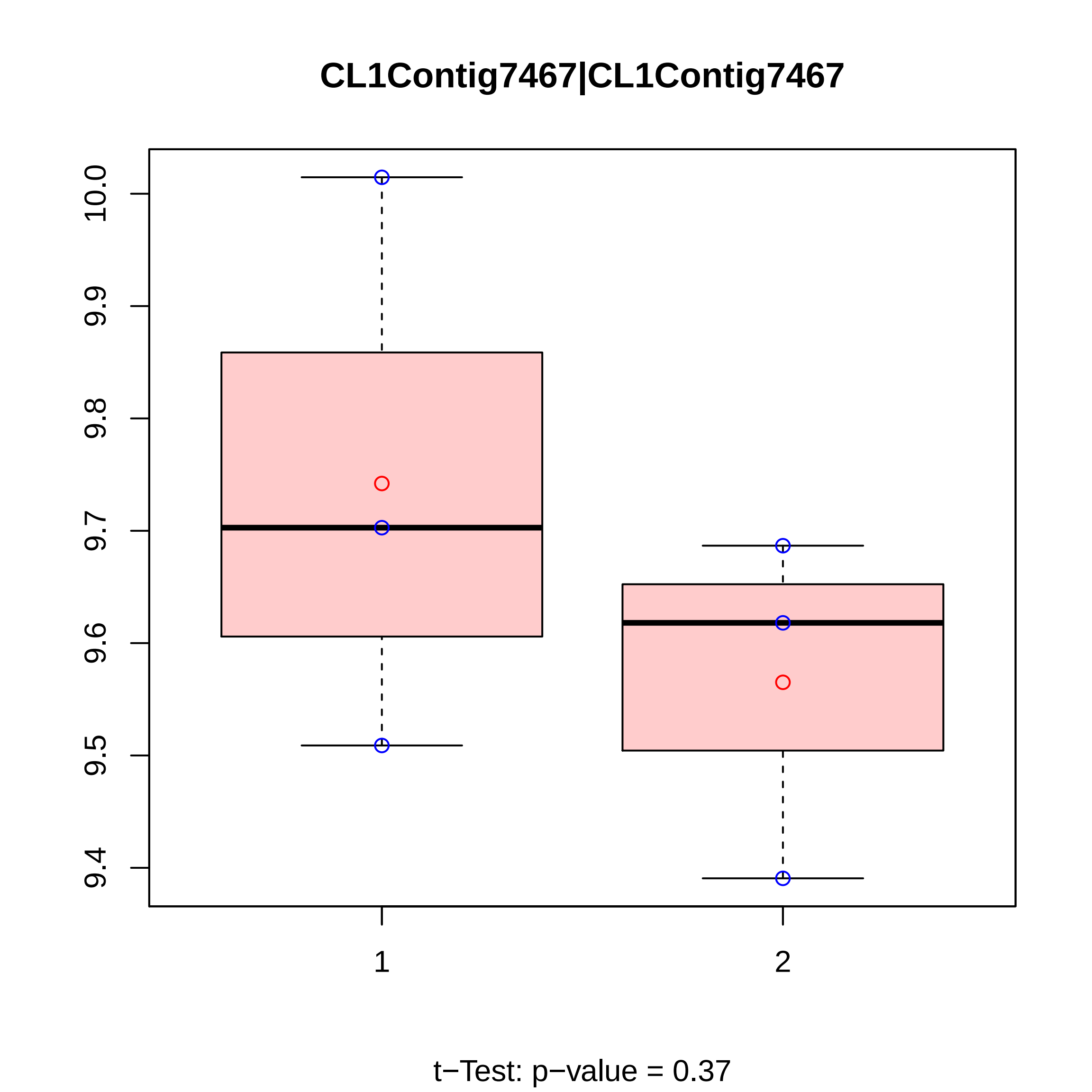
CL1Contig7467|CL1Contig7467
10.0
9.9
9.8
9.7
9.6
9.5
9.4
1
2
t−Test: p−value = 0.37

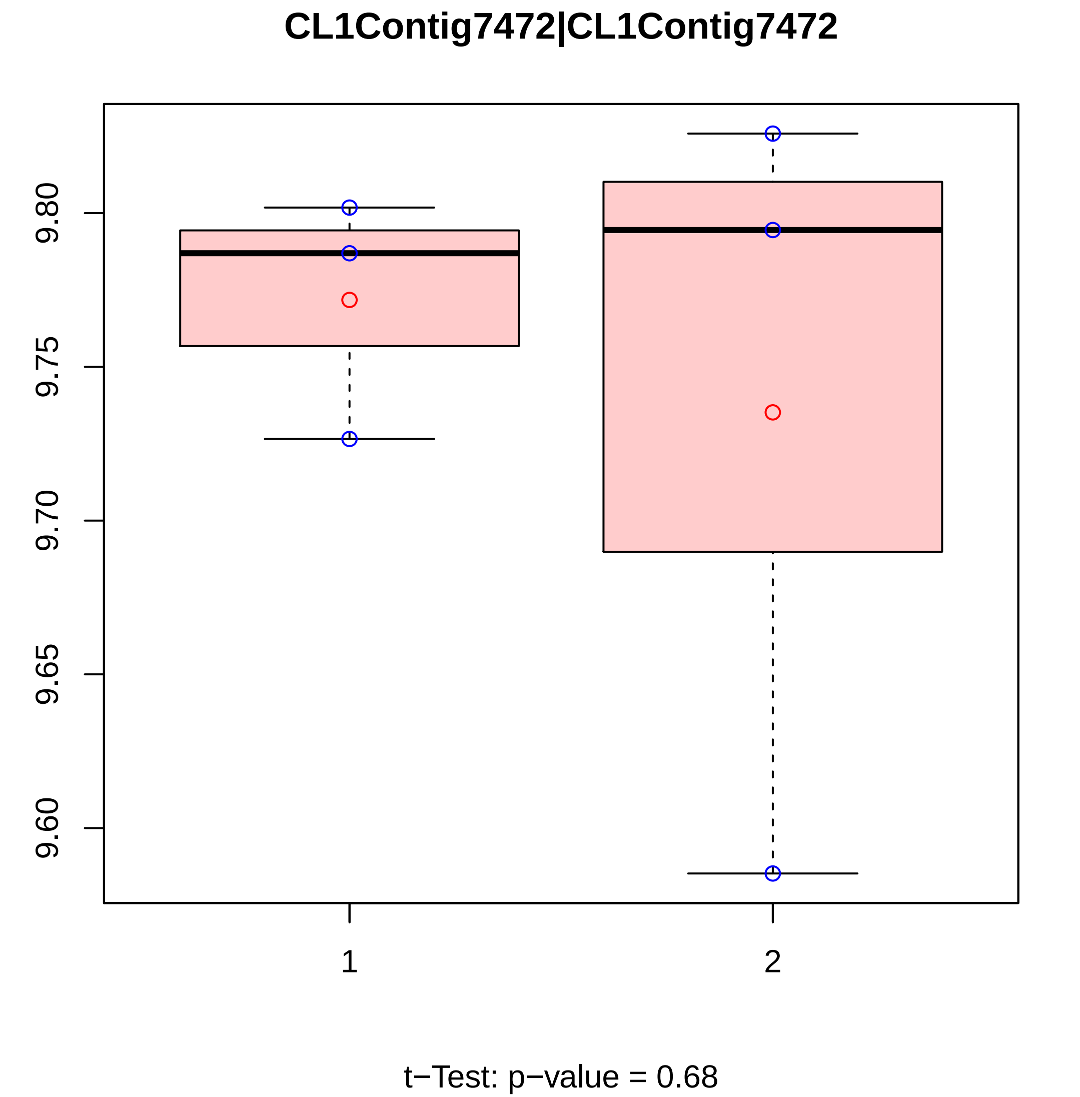
CL1Contig7472|CL1Contig7472
9.60
9.65
9.70
9.75
9.80
1
2
t−Test: p−value = 0.68

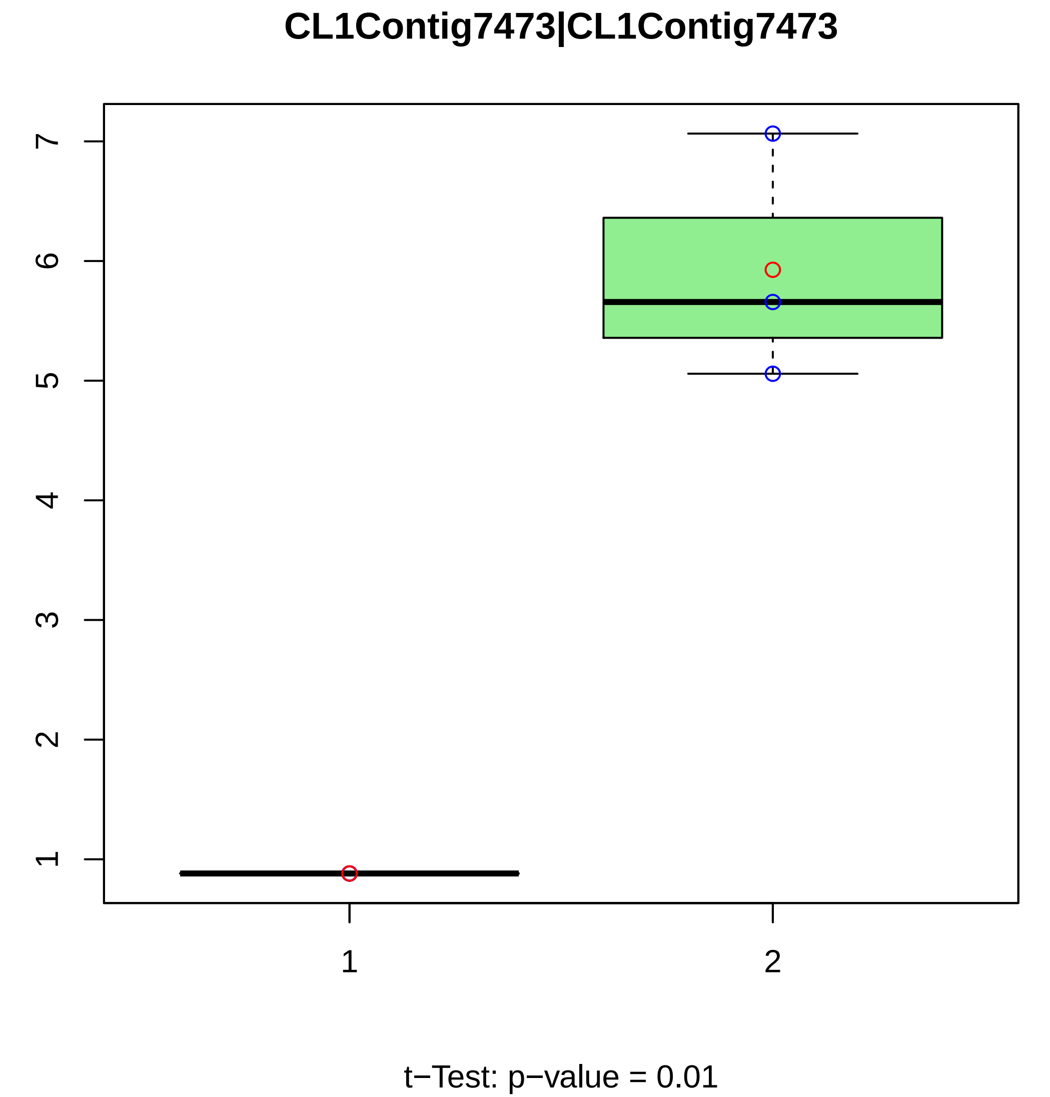
CL1Contig7473|CL1Contig7473
1
2
3
4
5
6
7
1
2
t−Test: p−value = 0.01

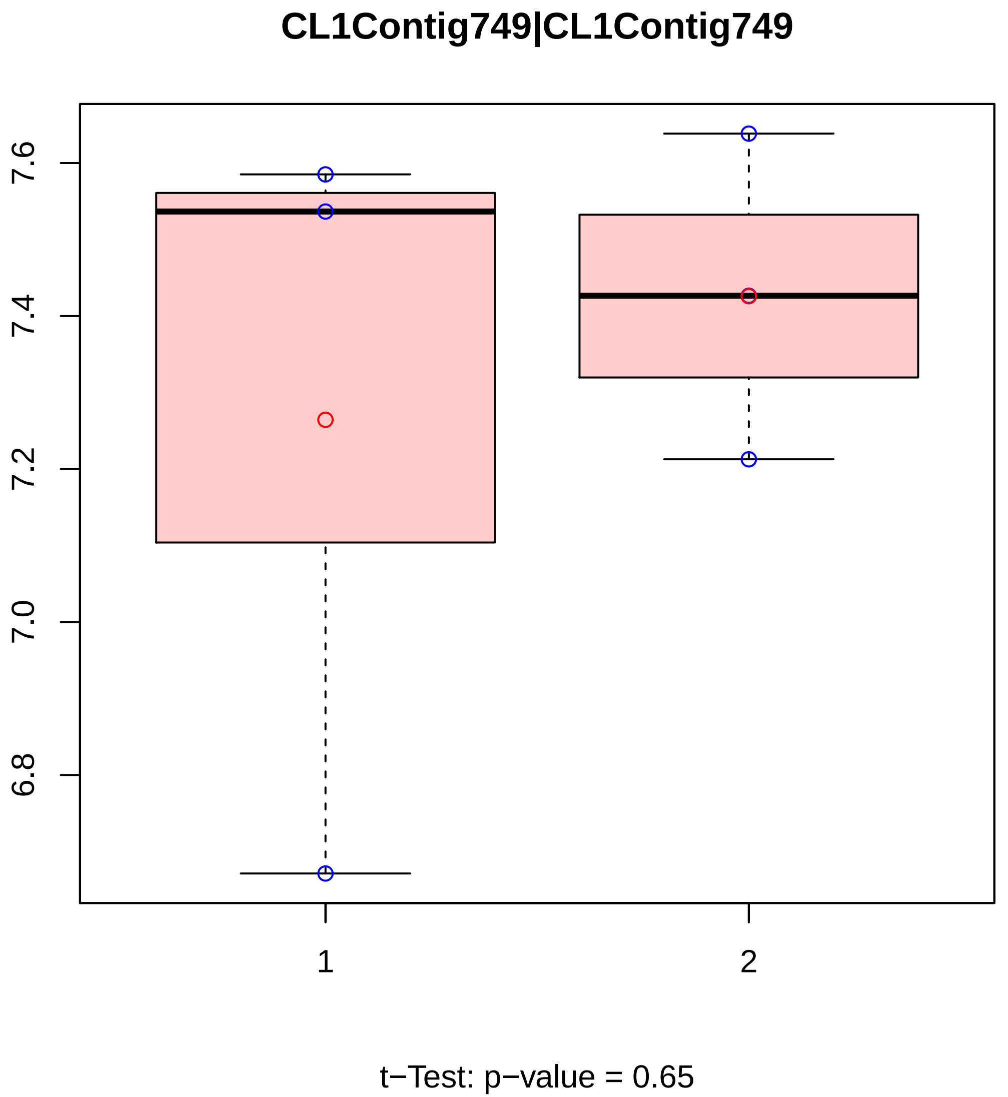
CL1Contig749|CL1Contig749
7.6
7.4
7.2
7.0
6.8
1
2
t−Test: p−value = 0.65

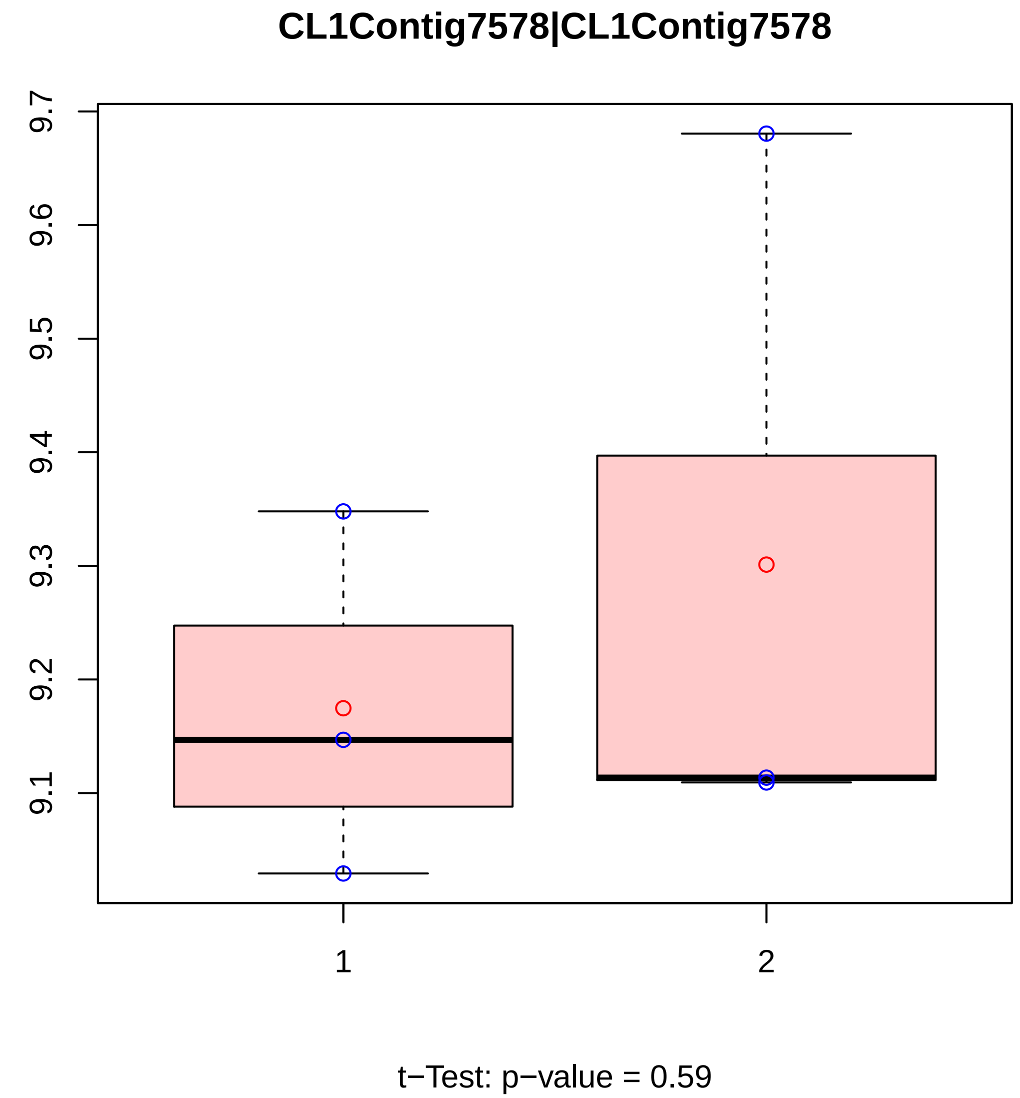

CL1Contig7578|CL1Contig7578
9.1
9.2
9.3
9.4
9.5
9.6
9.7
1
2
t−Test: p−value = 0.59

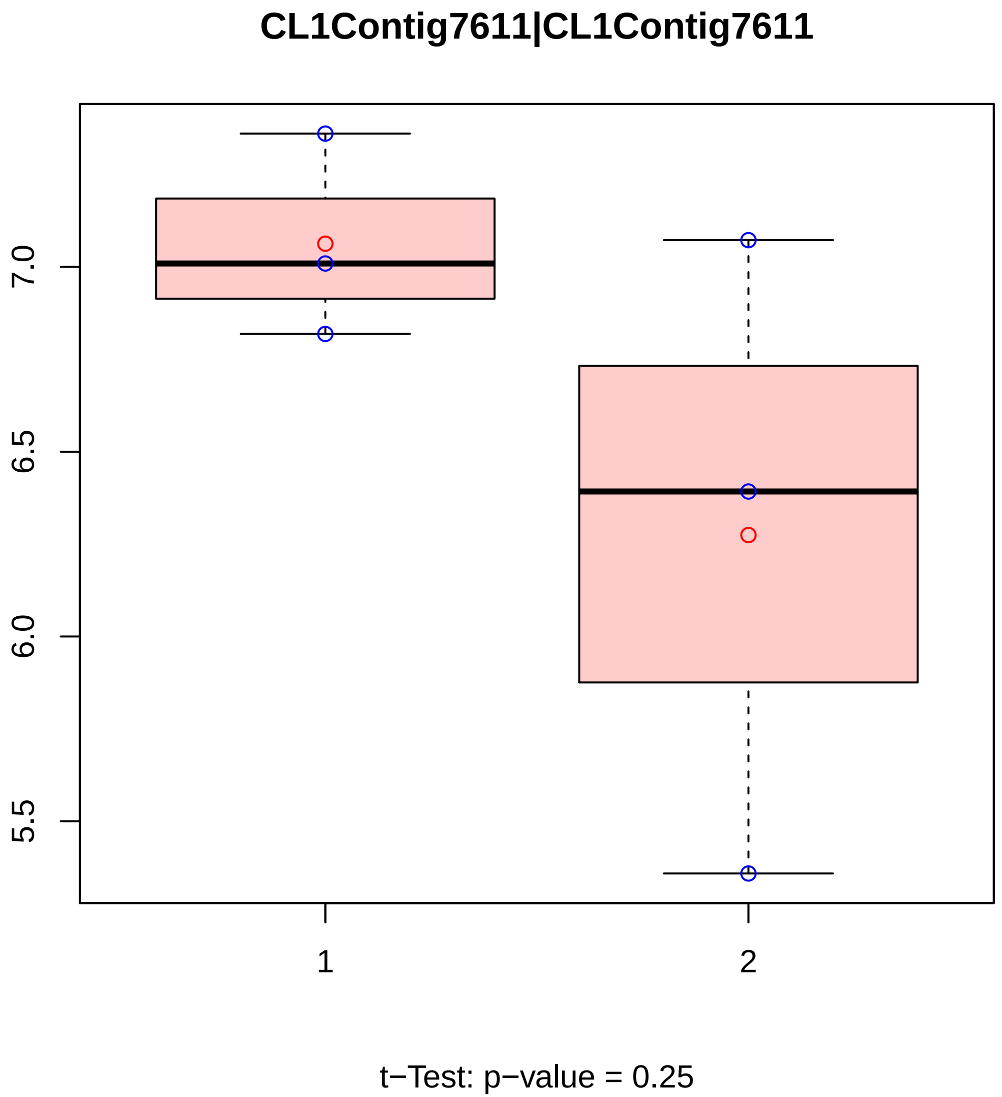
CL1Contig7611|CL1Contig7611
7.0
6.5
6.0
5.5
1
2
t−Test: p−value = 0.25

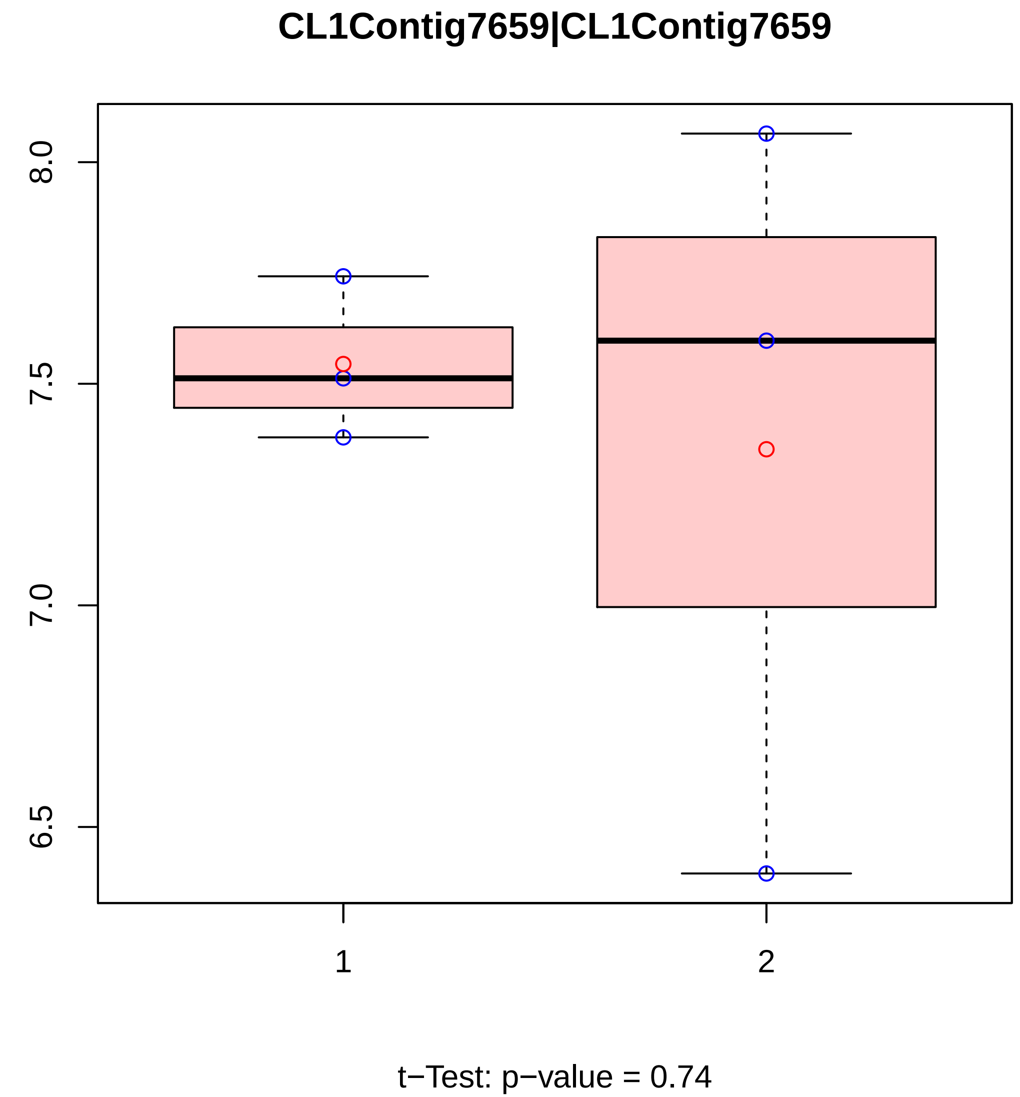
CL1Contig7659|CL1Contig7659
8.0
7.5
7.0
6.5
1
2
t−Test: p−value = 0.74

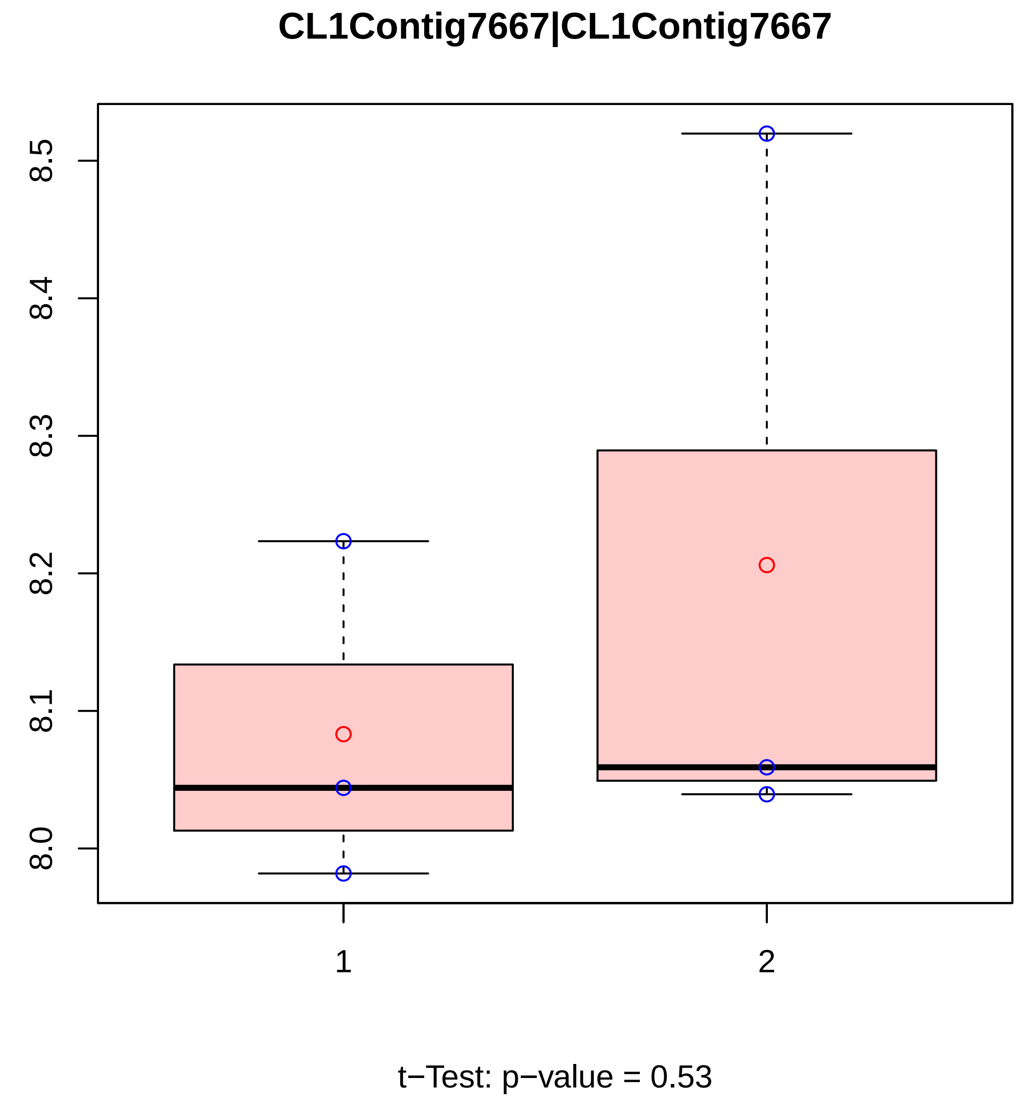
CL1Contig7667|CL1Contig7667
8.0
8.1
8.2
8.3
8.4
8.5
1
2
t−Test: p−value = 0.53

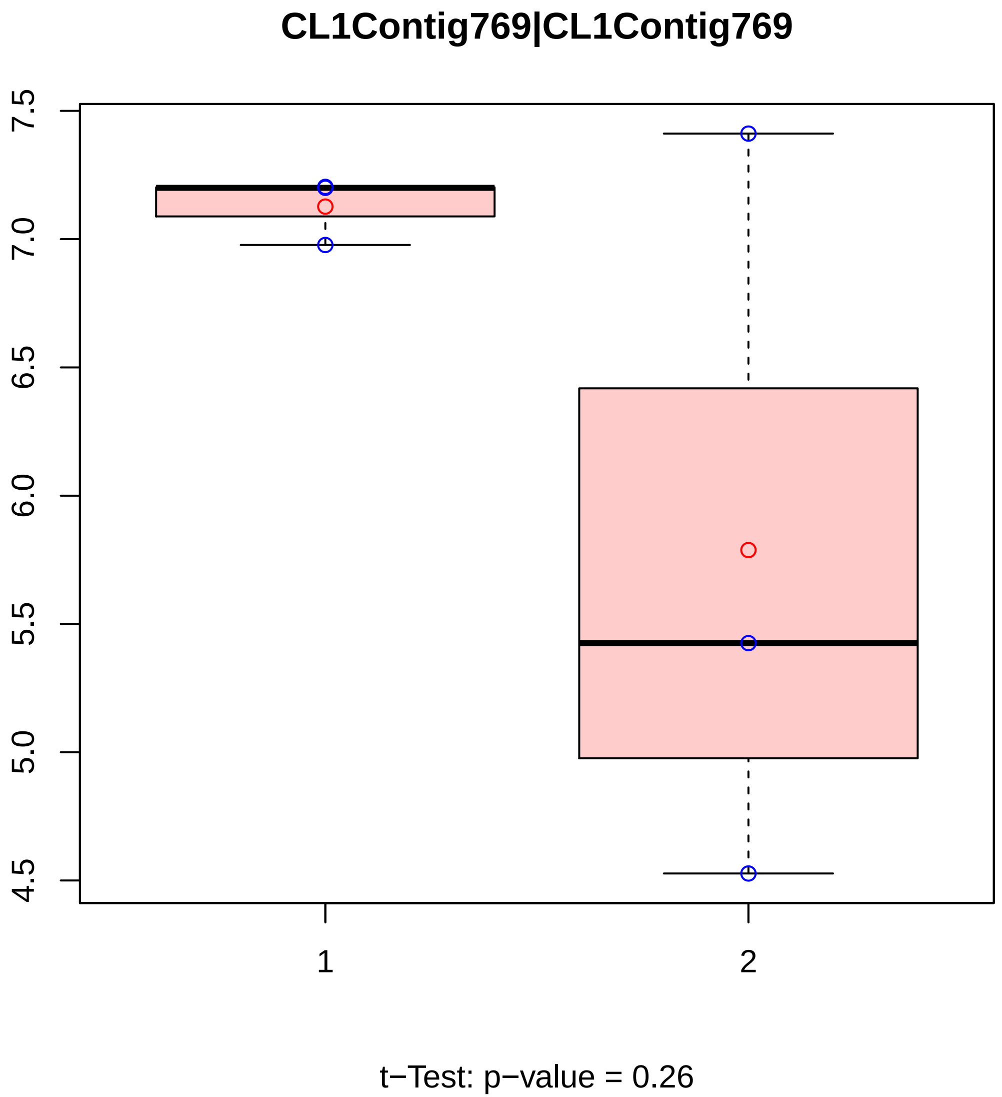
CL1Contig769|CL1Contig769
7.5
7.0
6.5
6.0
5.5
5.0
4.5
1
2
t−Test: p−value = 0.26

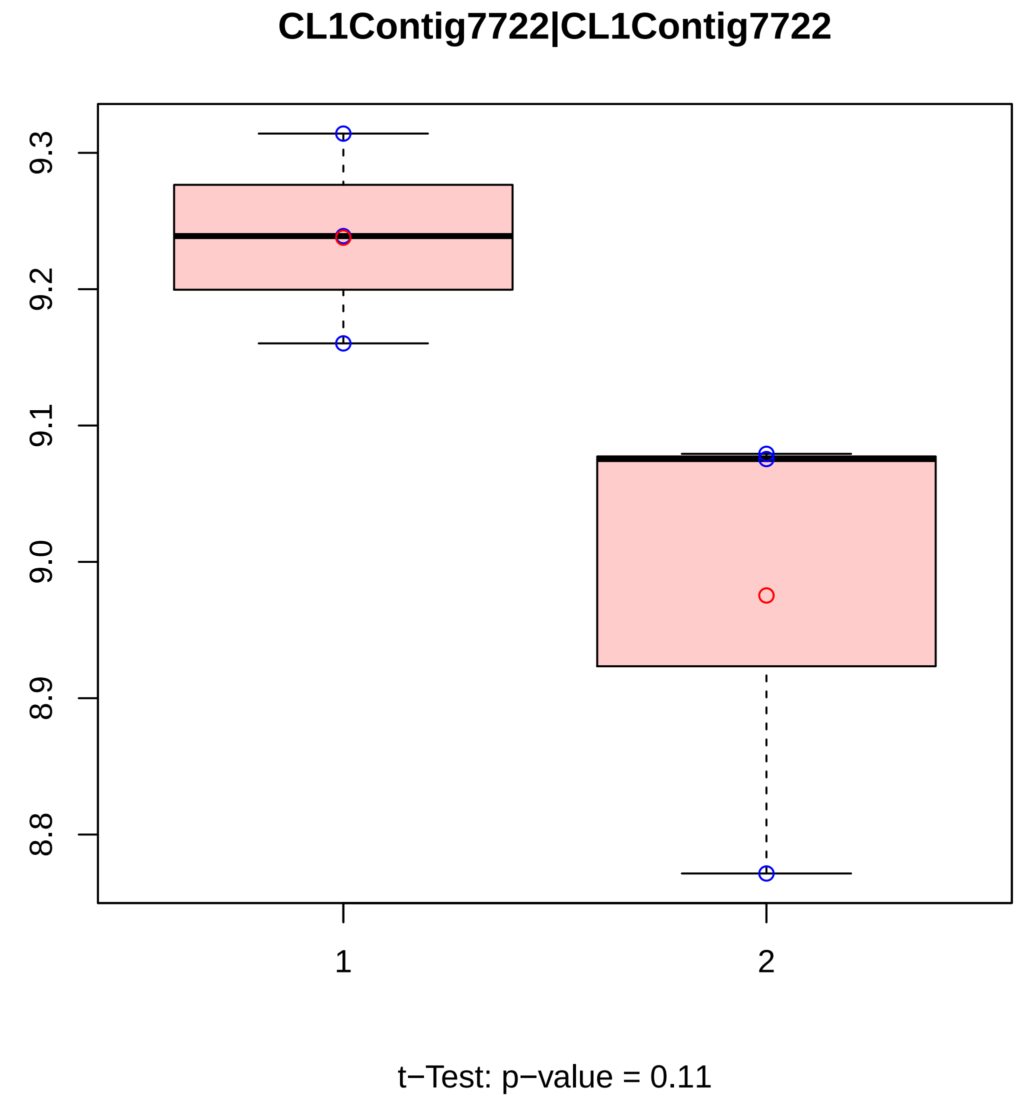
CL1Contig7722|CL1Contig7722
9.3
9.2
9.1
9.0
8.9
8.8
1
2
t−Test: p−value = 0.11

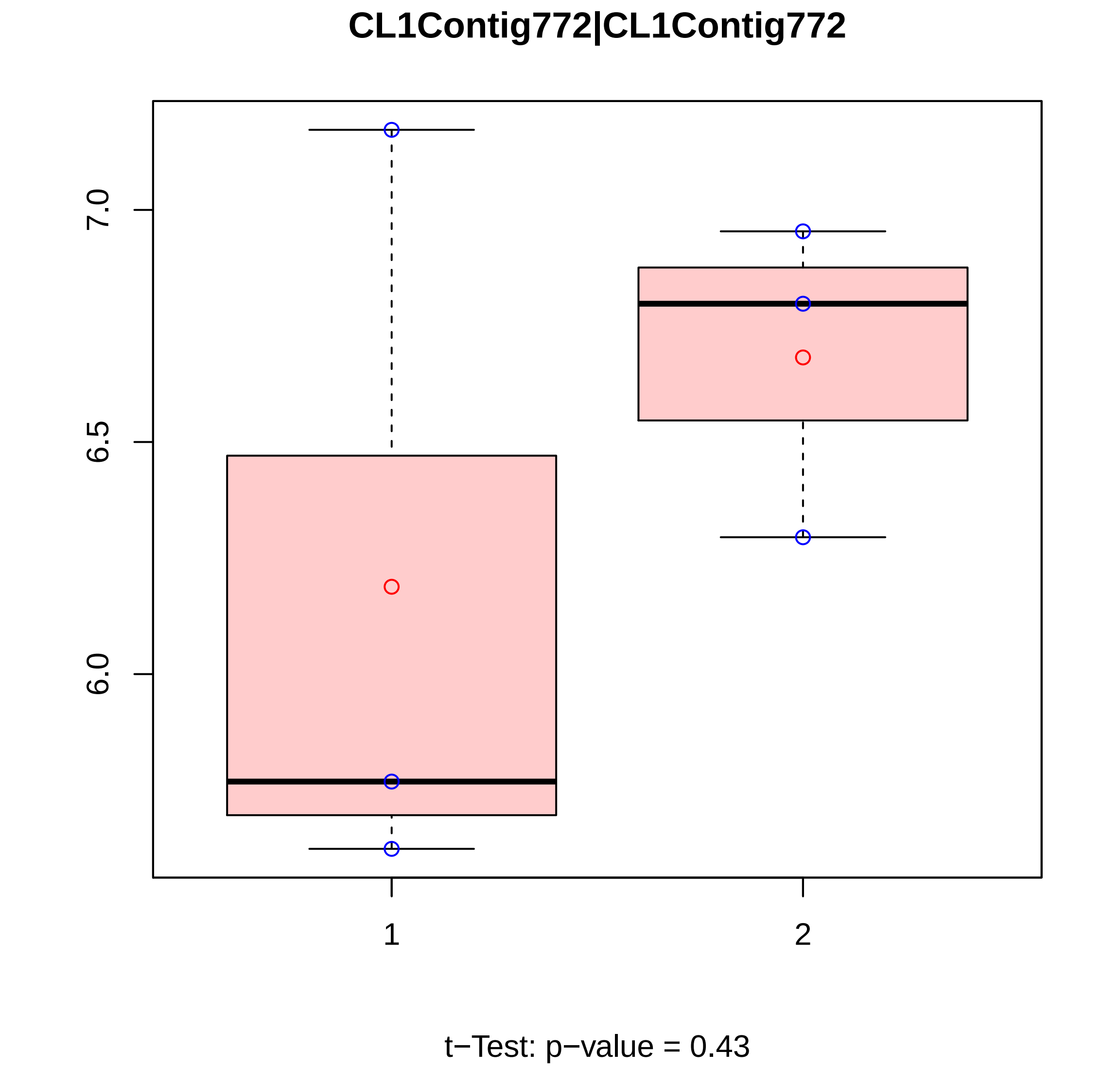
CL1Contig772|CL1Contig772
7.0
6.5
6.0
1
2
t−Test: p−value = 0.43

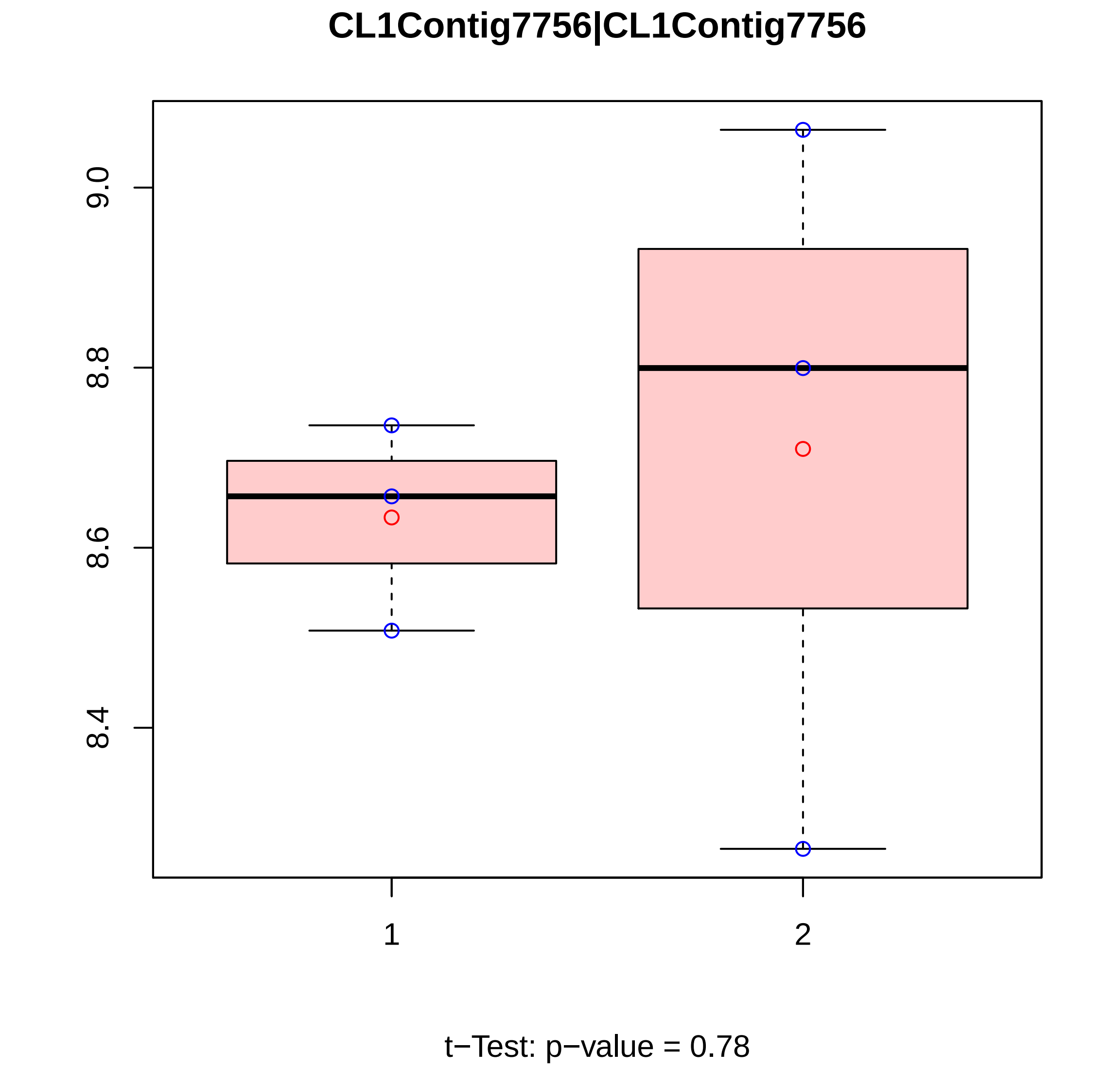
CL1Contig7756|CL1Contig7756
9.0
8.8
8.6
8.4
1
2
t−Test: p−value = 0.78

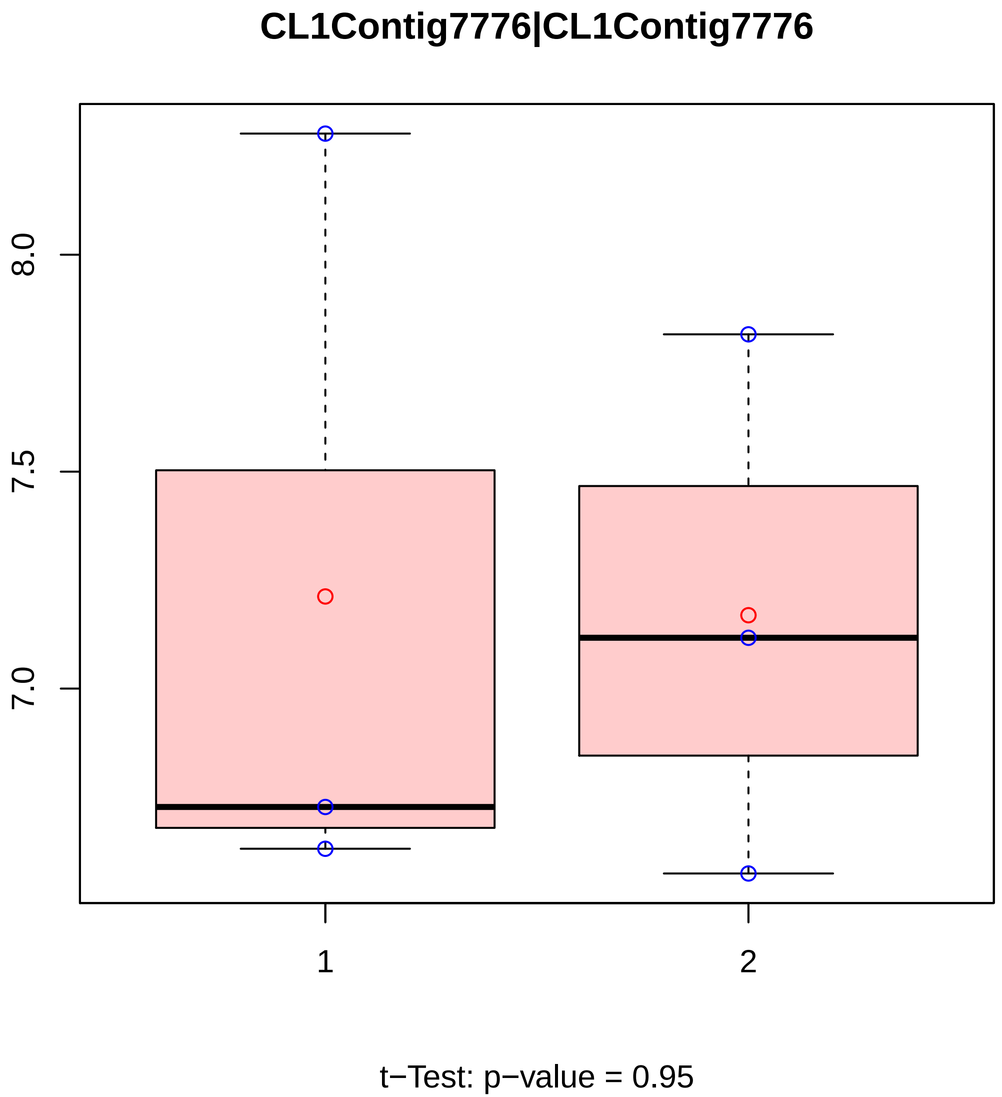
CL1Contig7776|CL1Contig7776
8.0
7.5
7.0
1
2
t−Test: p−value = 0.95

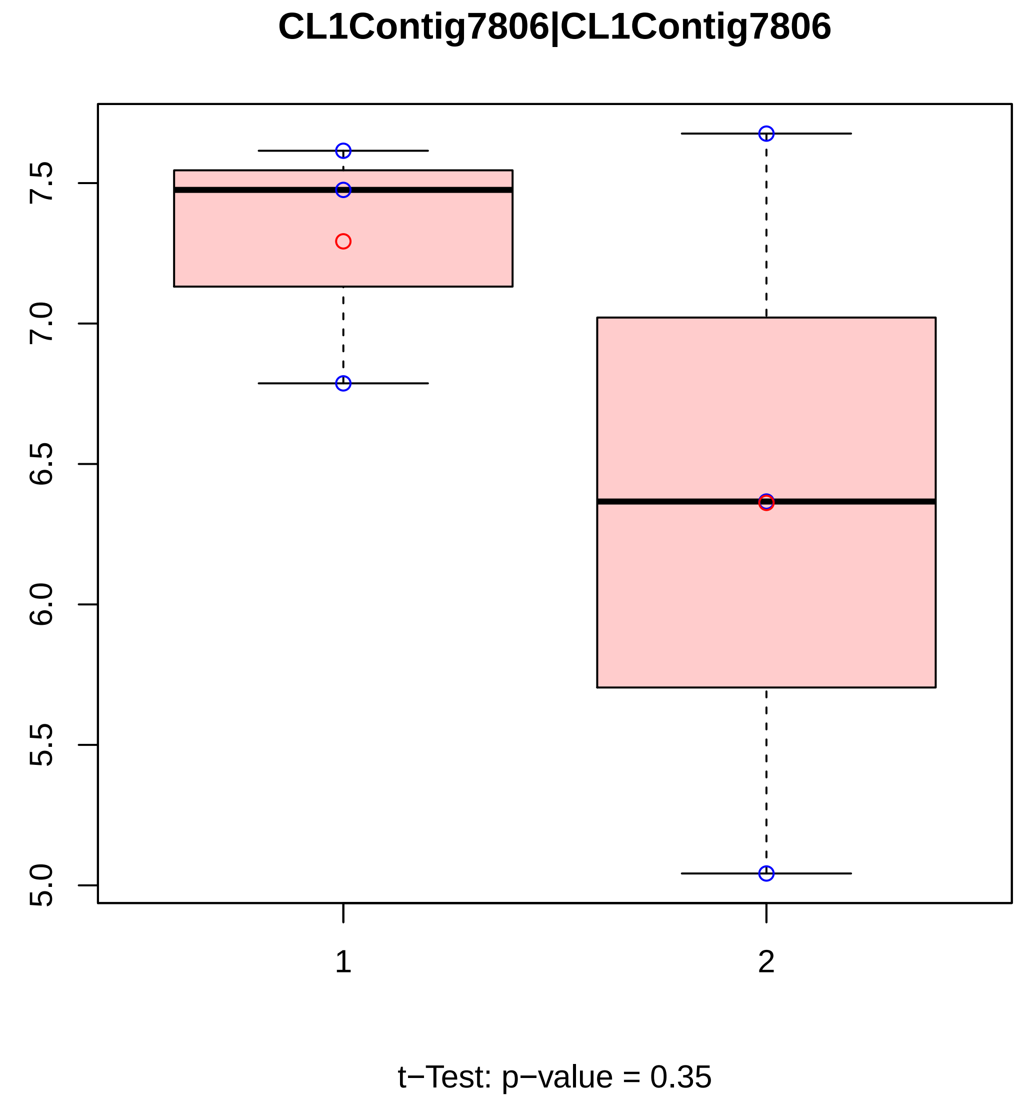
CL1Contig7806|CL1Contig7806
7.5
7.0
6.5
6.0
5.5
5.0
1
2
t−Test: p−value = 0.35

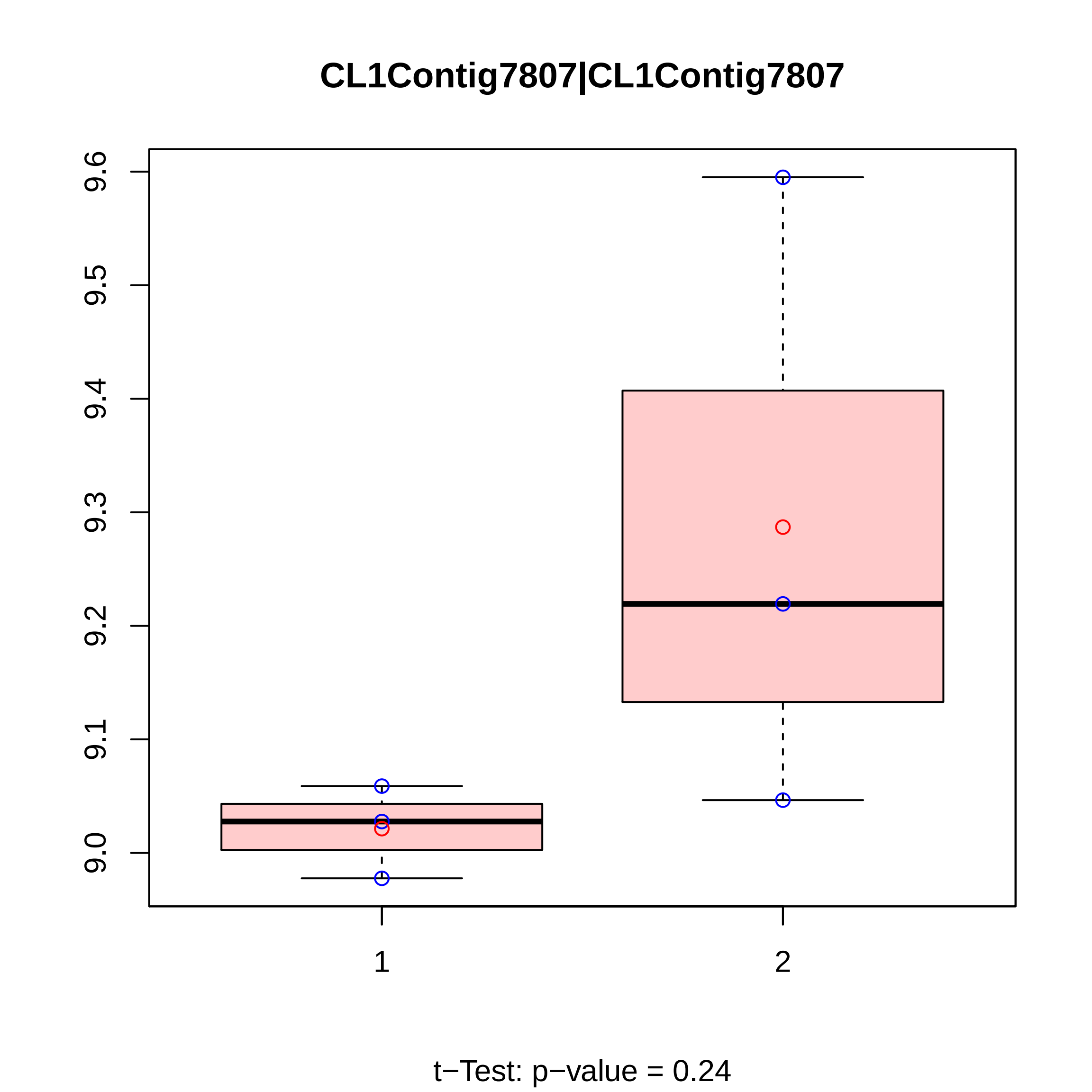
CL1Contig7807|CL1Contig7807
9.0
9.1
9.2
9.3
9.4
9.5
9.6
1
2
t−Test: p−value = 0.24

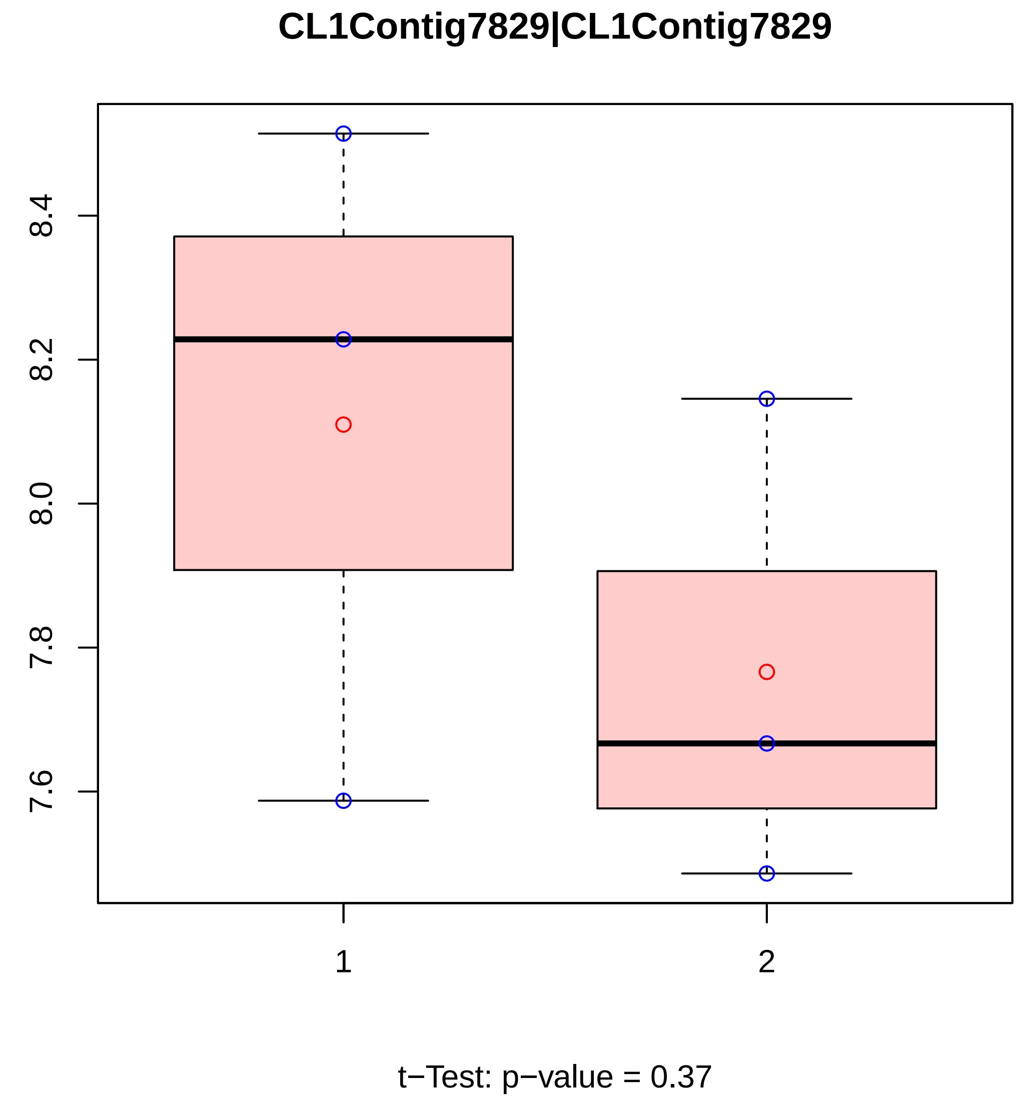
CL1Contig7829|CL1Contig7829
8.4
8.2
8.0
7.8
7.6
1
2
t−Test: p−value = 0.37

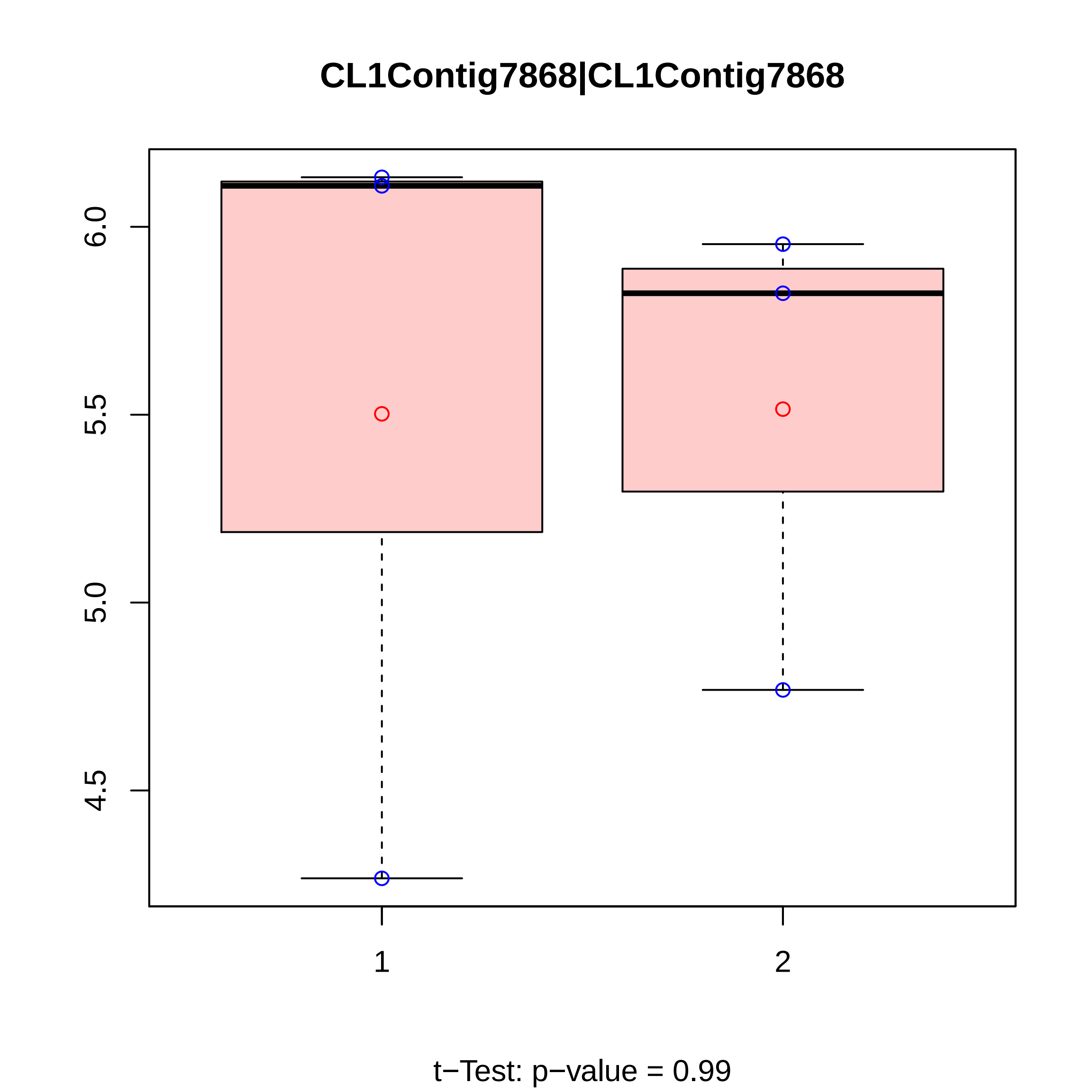

CL1Contig7868|CL1Contig7868
6.0
5.5
5.0
4.5
1
2
t−Test: p−value = 0.99

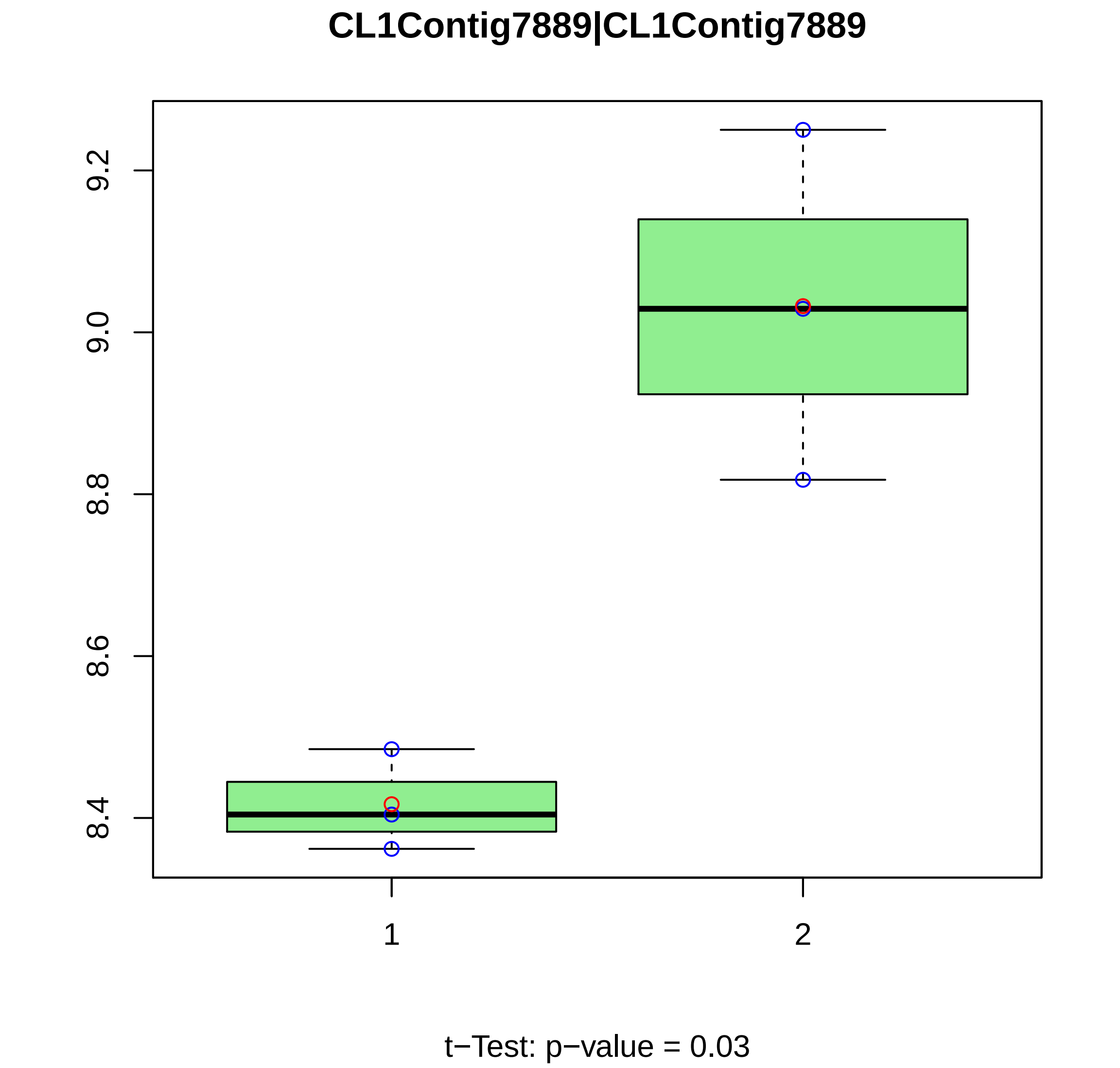

CL1Contig7889|CL1Contig7889
9.2
9.0
8.8
8.6
8.4
1
2
t−Test: p−value = 0.03

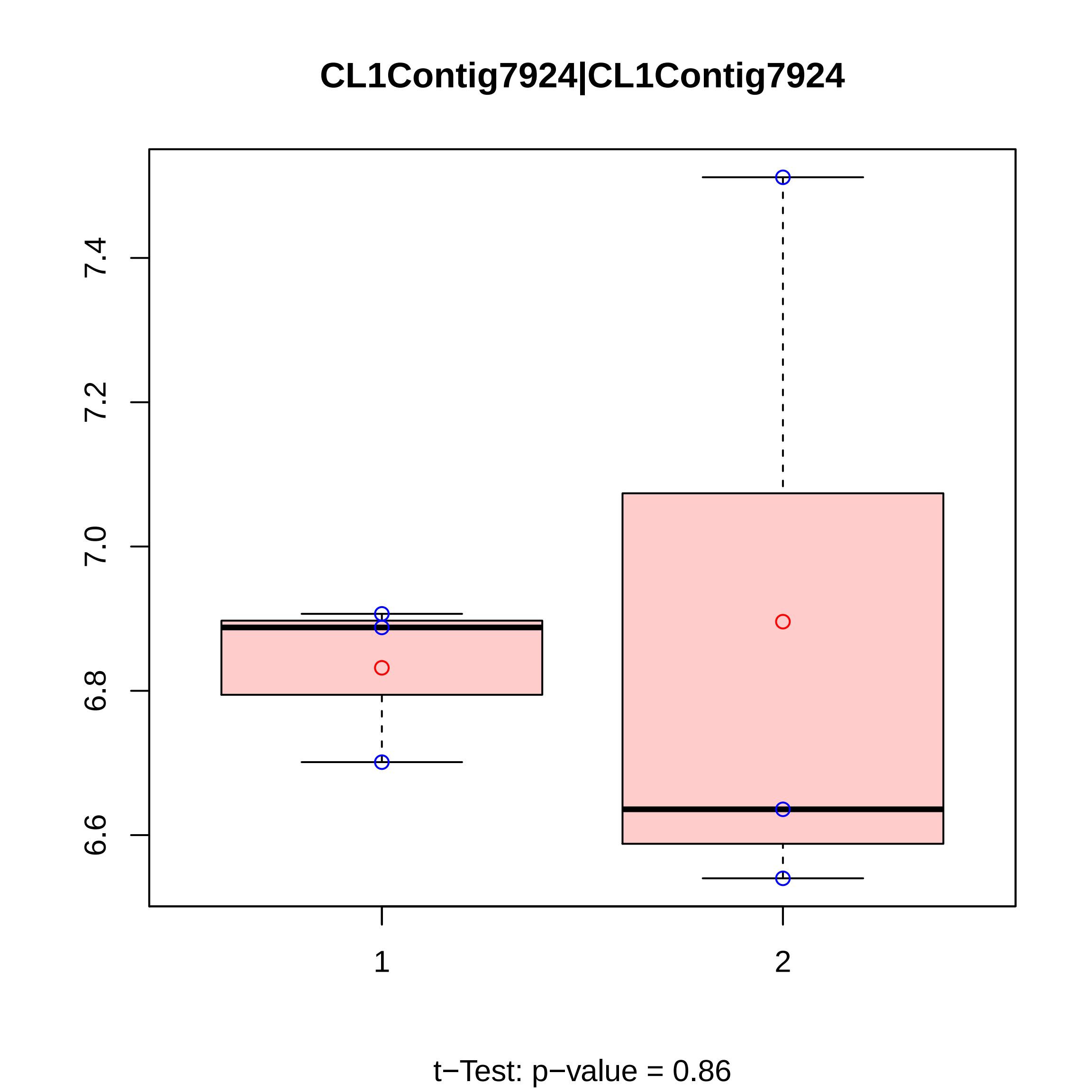

CL1Contig7924|CL1Contig7924
7.4
7.2
7.0
6.8
6.6
1
2
t−Test: p−value = 0.86

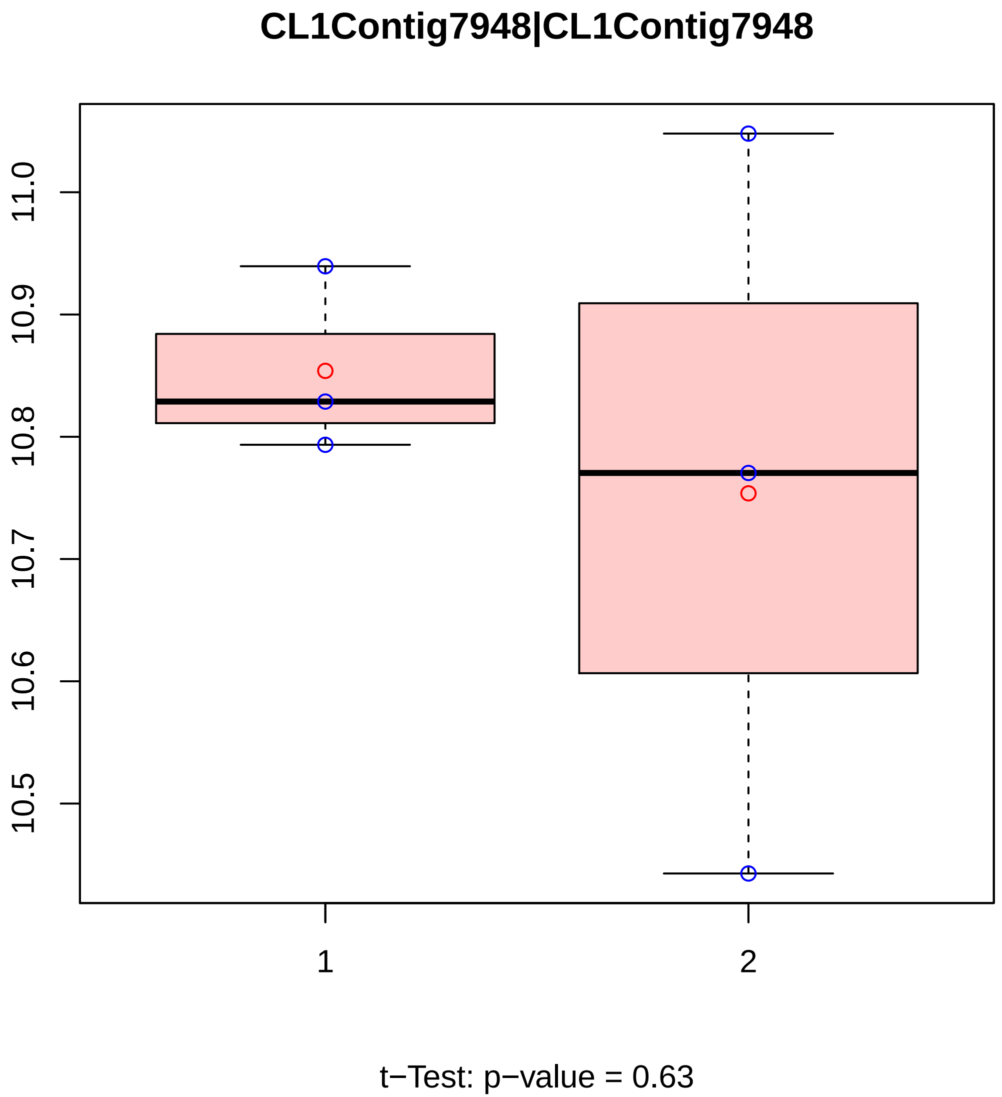

CL1Contig7948|CL1Contig7948
11.0
10.9
10.8
10.7
10.6
10.5
1
2
t−Test: p−value = 0.63

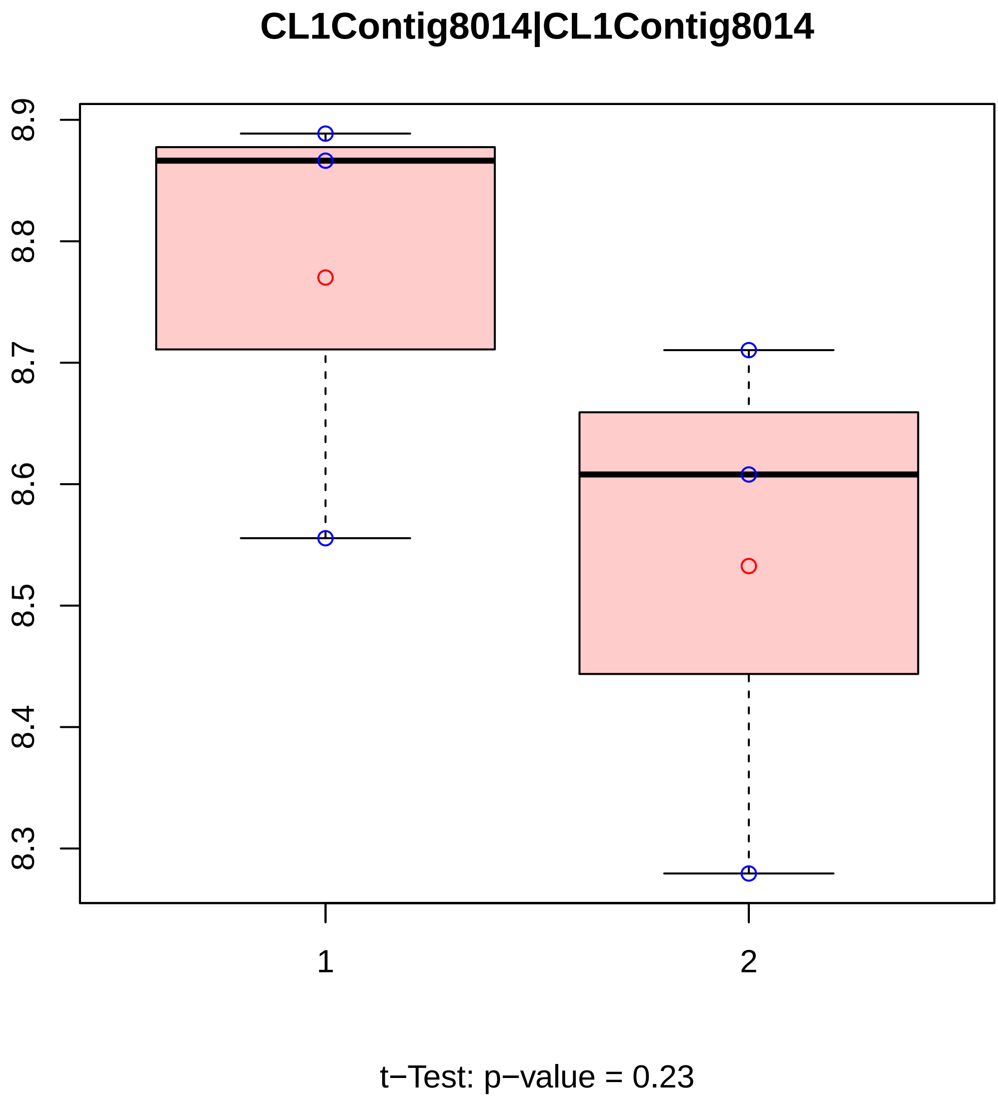

CL1Contig8014|CL1Contig8014
8.9
8.8
8.7
8.6
8.5
8.4
8.3
1
2
t−Test: p−value = 0.23

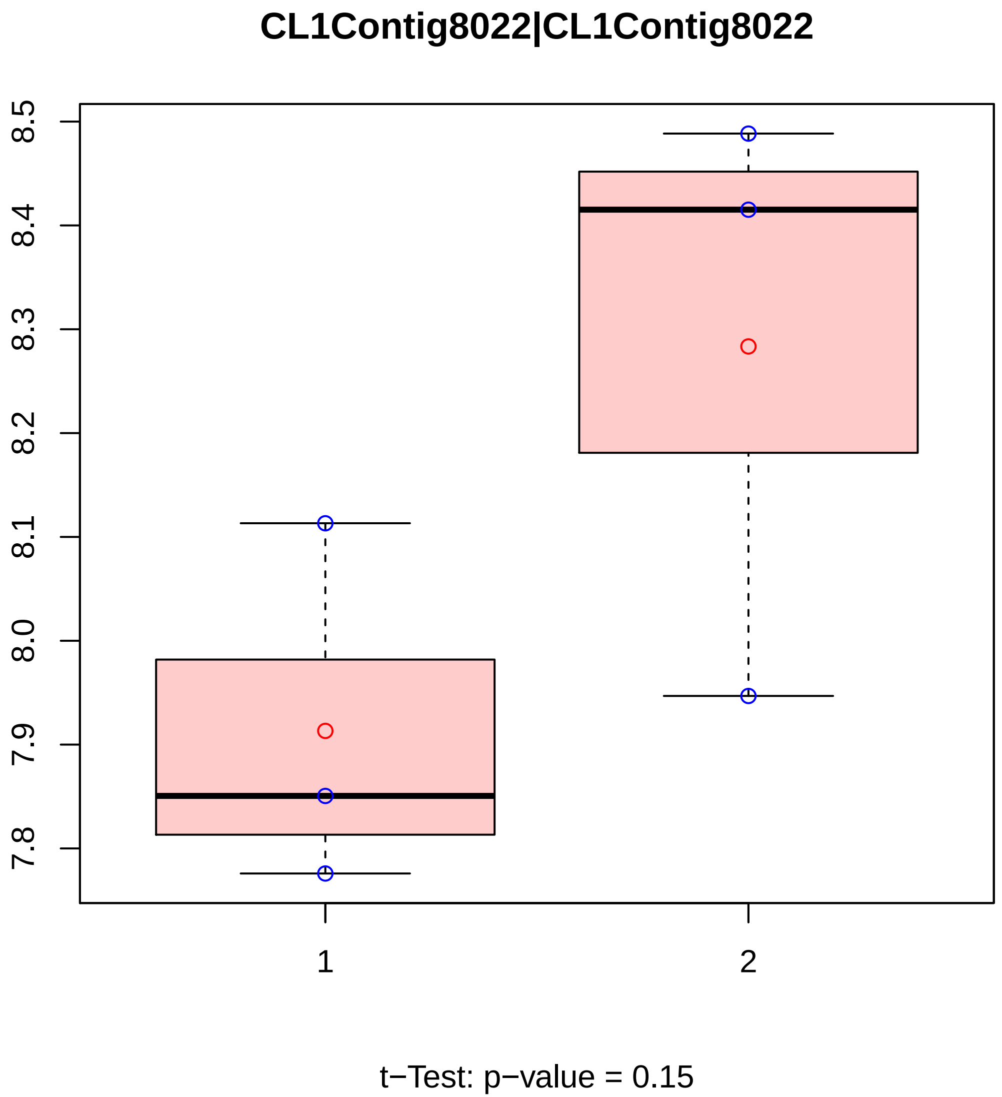

CL1Contig8022|CL1Contig8022
8.5
8.4
8.3
8.2
8.1
8.0
7.9
7.8
1
2
t−Test: p−value = 0.15

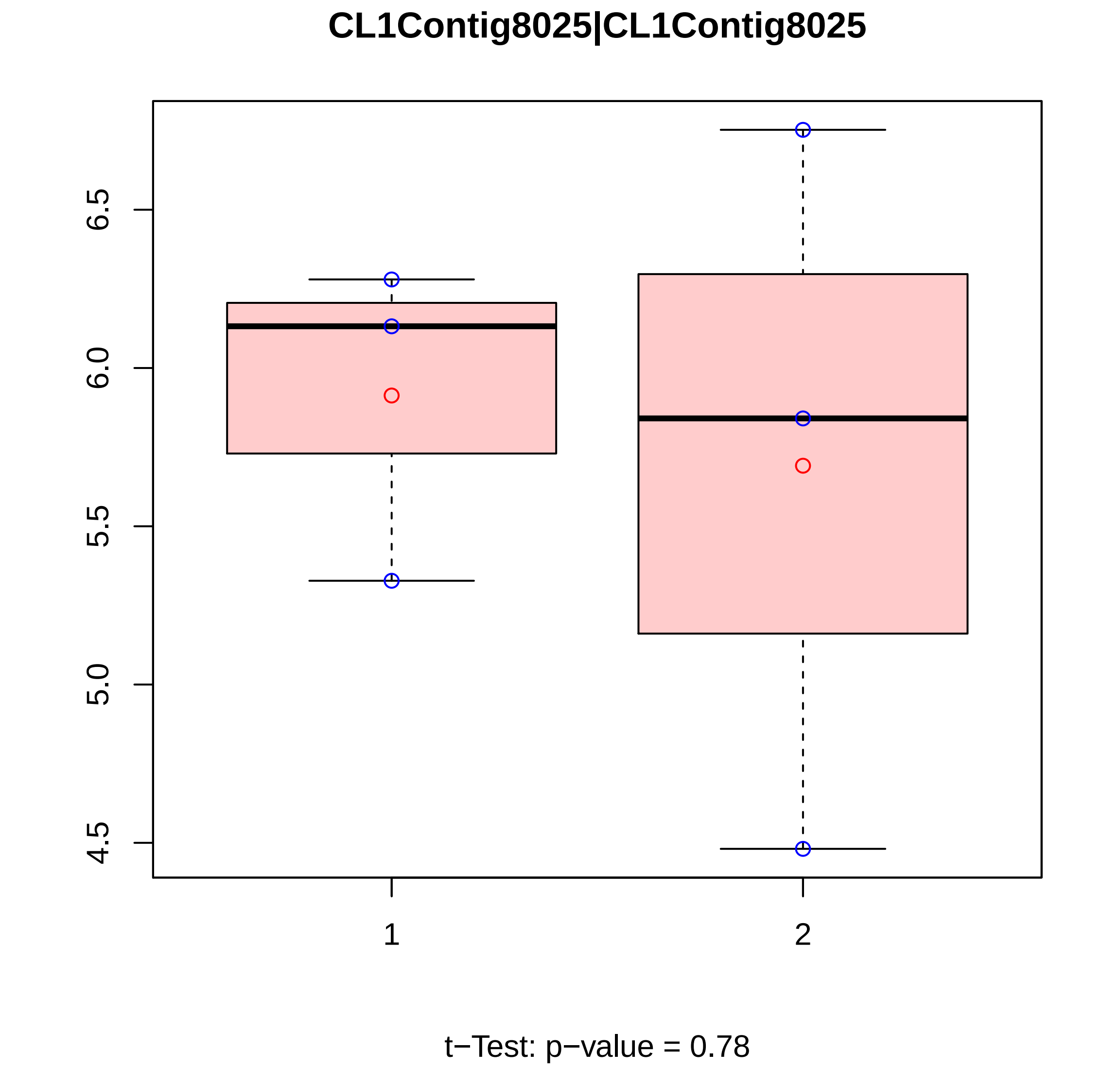
CL1Contig8025|CL1Contig8025
6.5
6.0
5.5
5.0
4.5
1
2
t−Test: p−value = 0.78

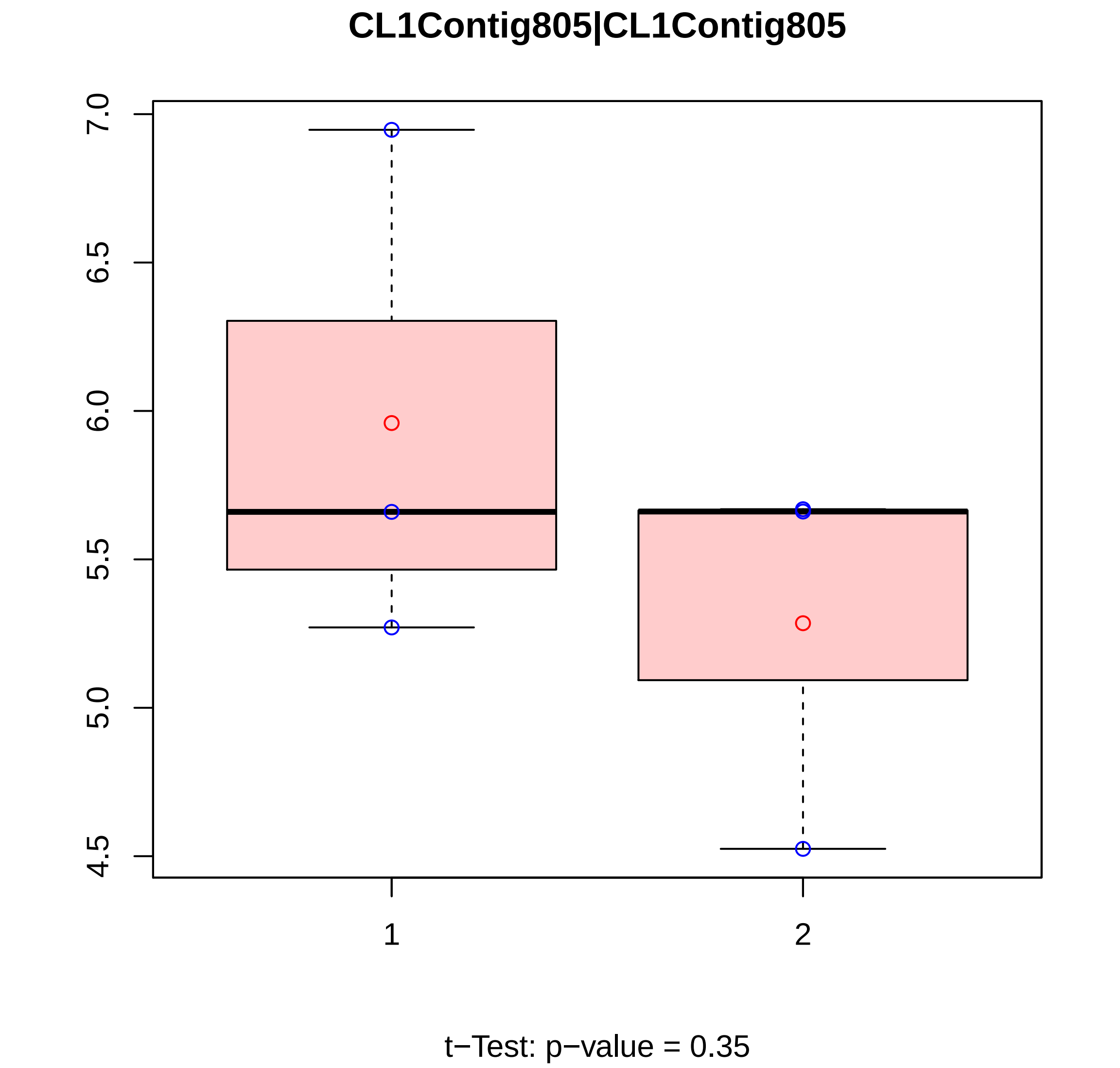
CL1Contig805|CL1Contig805
7.0
6.5
6.0
5.5
5.0
4.5
1
2
t−Test: p−value = 0.35

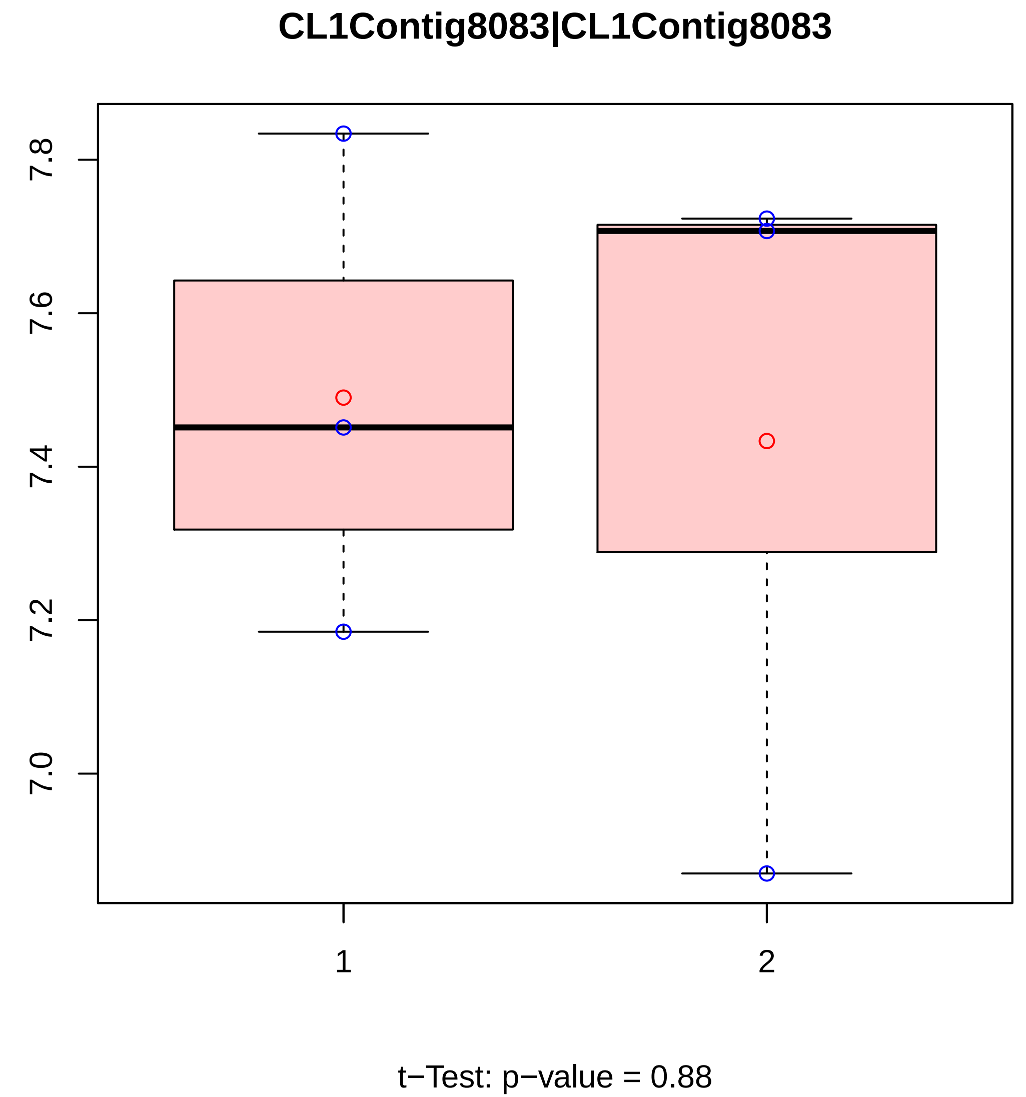
CL1Contig8083|CL1Contig8083
7.0
7.2
7.4
7.6
7.8
1
2
t−Test: p−value = 0.88

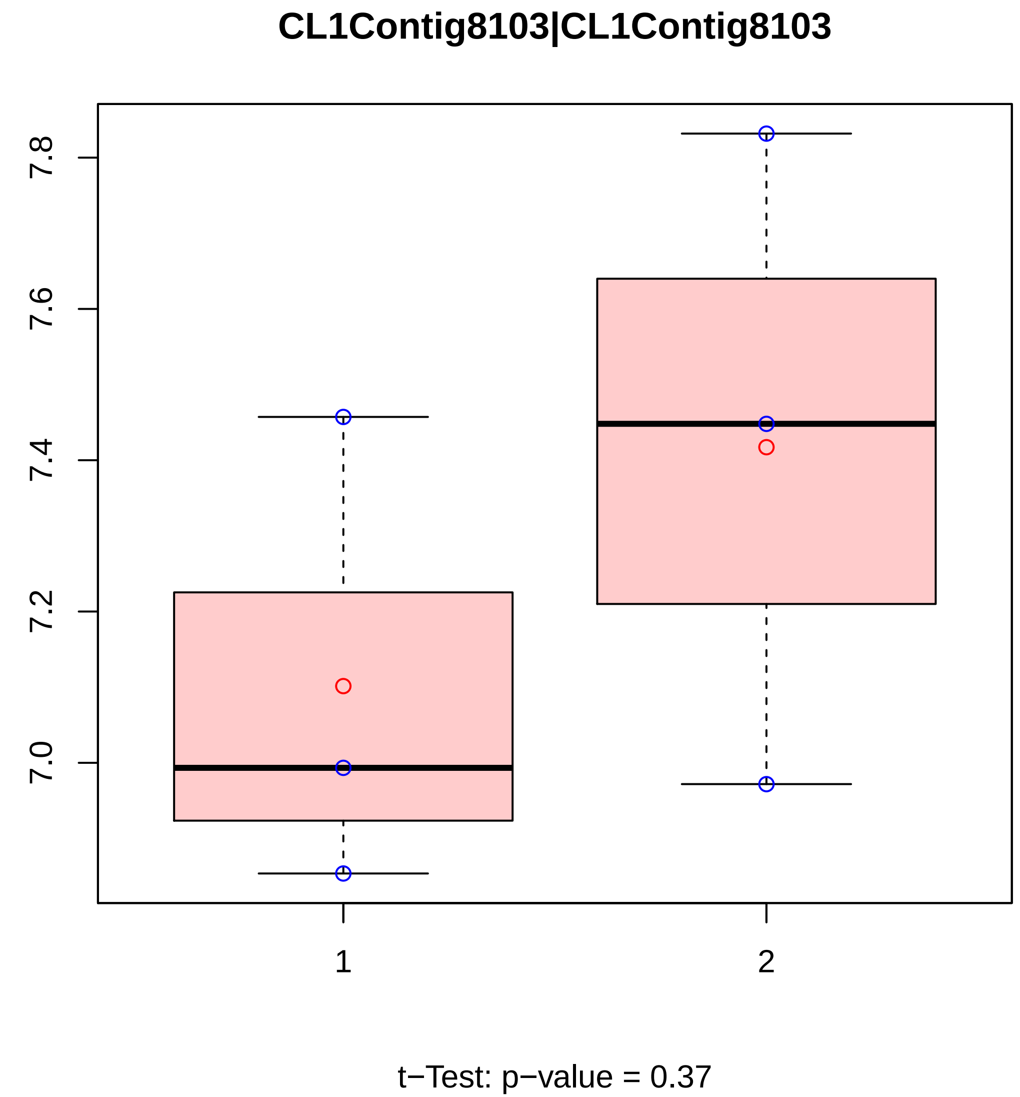

CL1Contig8103|CL1Contig8103
7.8
7.6
7.4
7.2
7.0
1
2
t−Test: p−value = 0.37

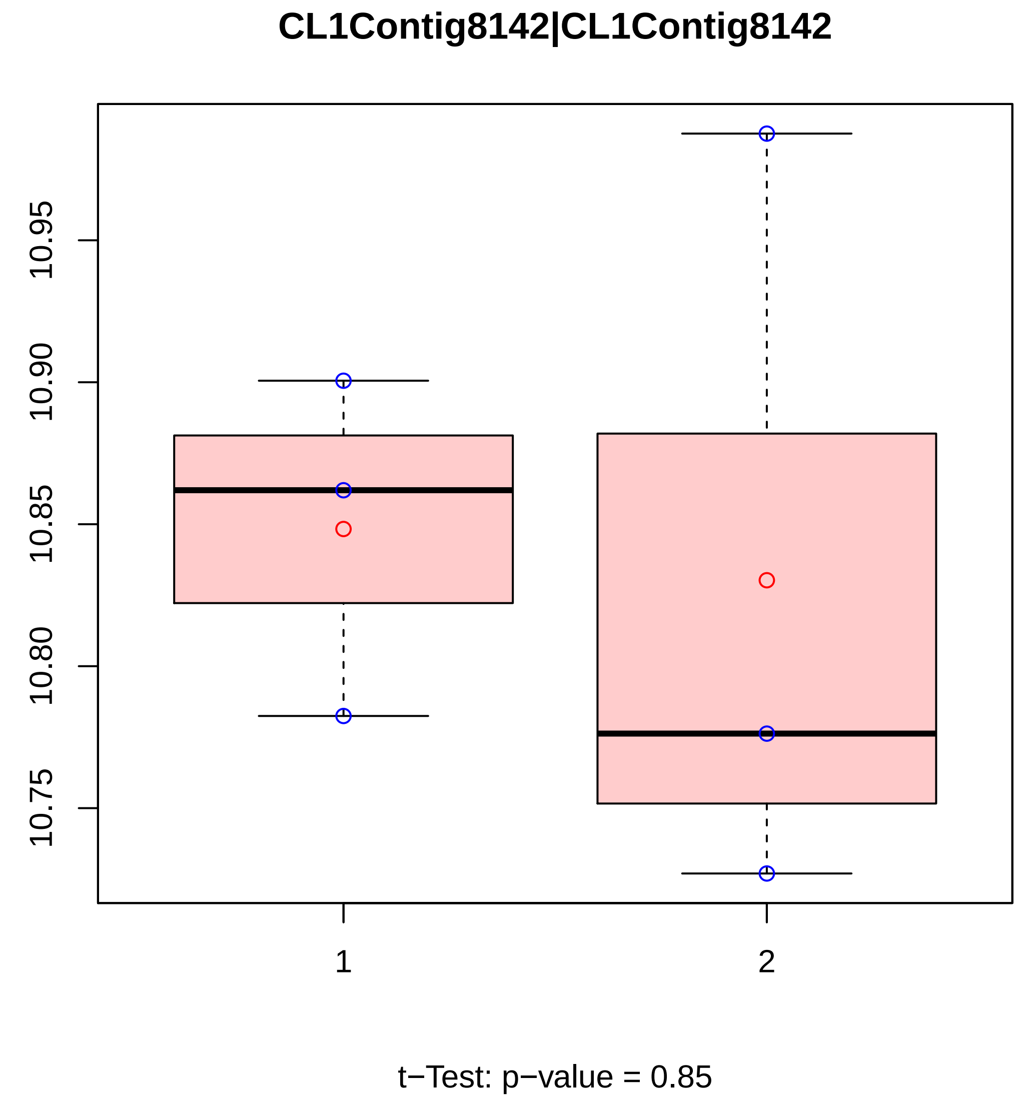
CL1Contig8142|CL1Contig8142
10.75
10.80
10.85
10.90
10.95
1
2
t−Test: p−value = 0.85

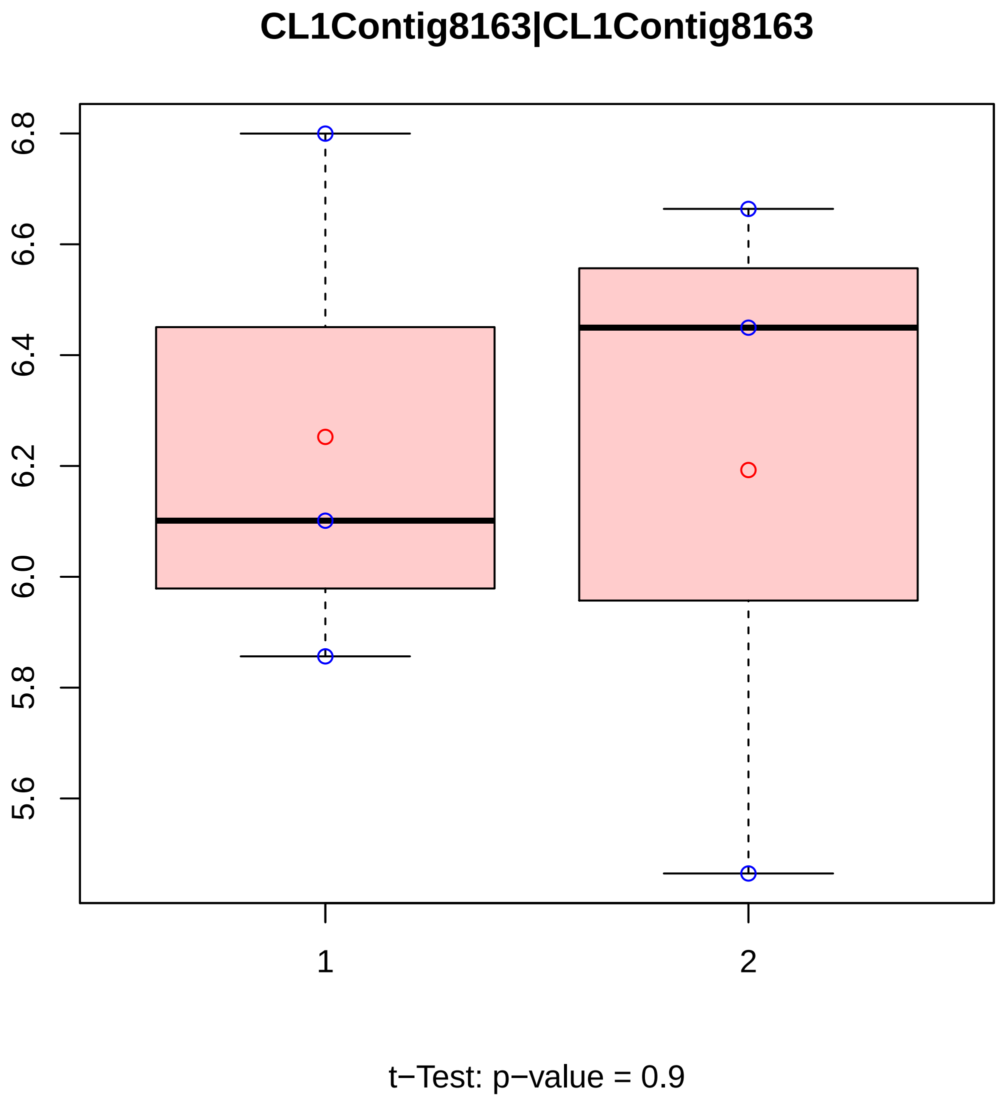
CL1Contig8163|CL1Contig8163
6.8
6.6
6.4
6.2
6.0
5.8
5.6
1
2
t−Test: p−value = 0.9

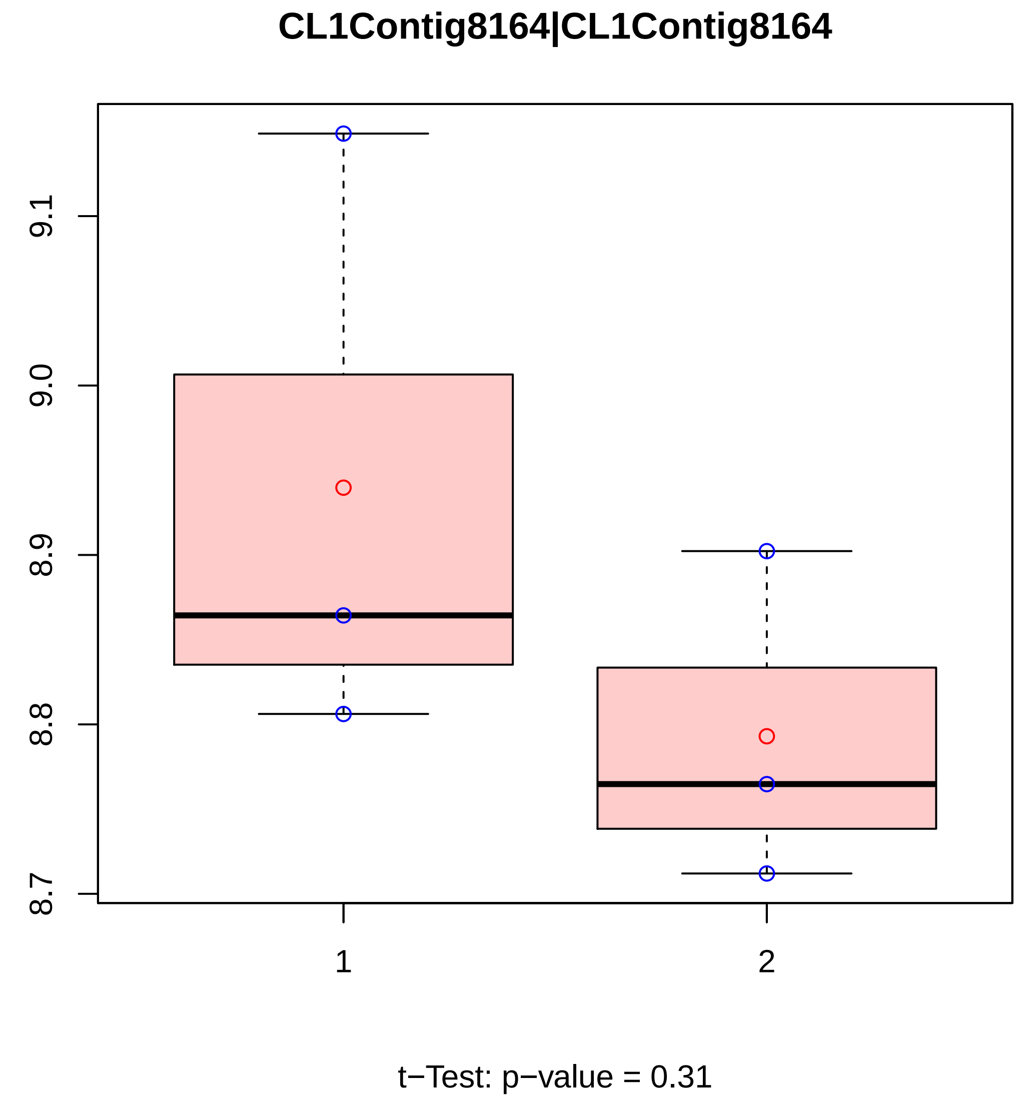
CL1Contig8164|CL1Contig8164
9.1
9.0
8.9
8.8
8.7
1
2
t−Test: p−value = 0.31

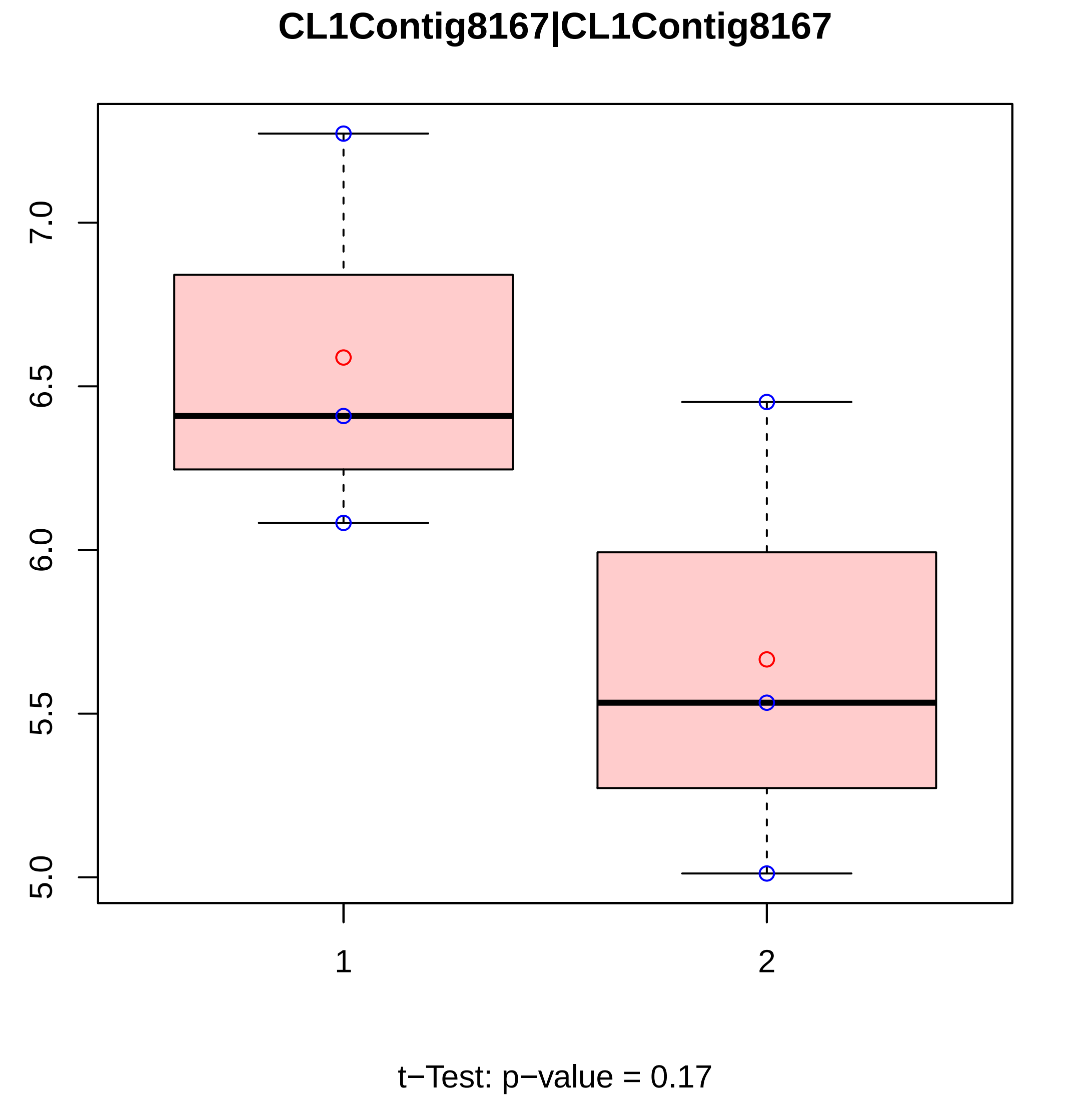
CL1Contig8167|CL1Contig8167
7.0
6.5
6.0
5.5
5.0
1
2
t−Test: p−value = 0.17

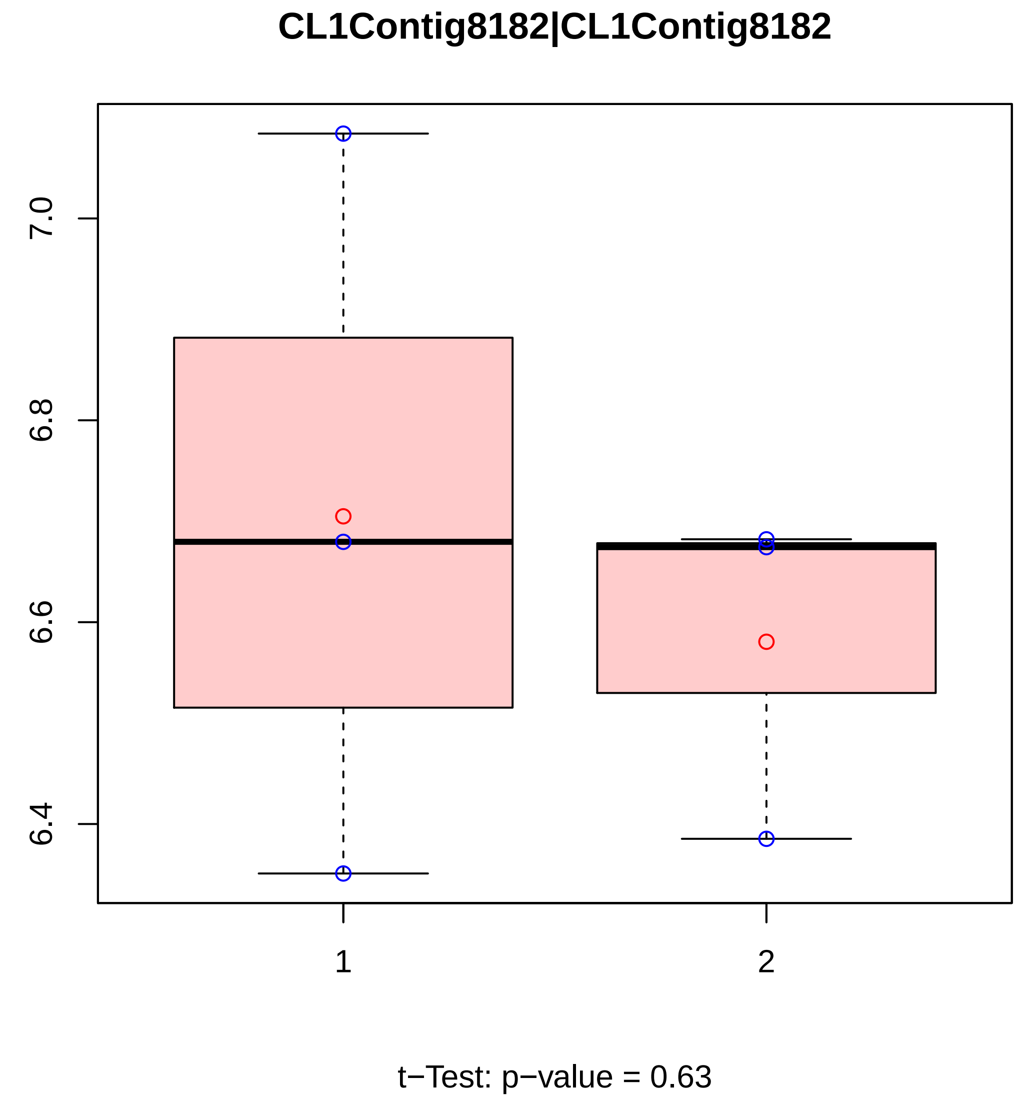

CL1Contig8182|CL1Contig8182
7.0
6.8
6.6
6.4
1
2
t−Test: p−value = 0.63

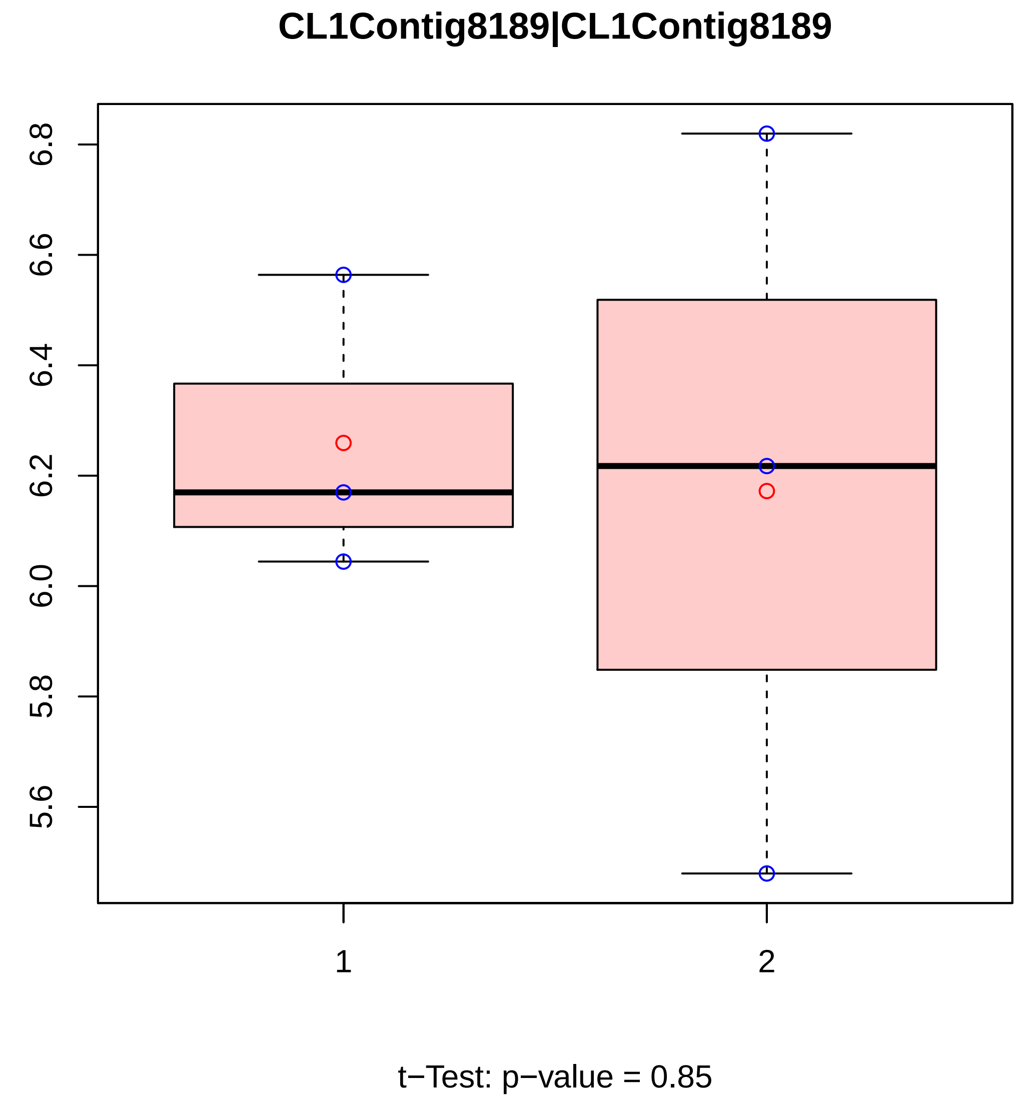
CL1Contig8189|CL1Contig8189
6.8
6.6
6.4
6.2
6.0
5.8
5.6
1
2
t−Test: p−value = 0.85

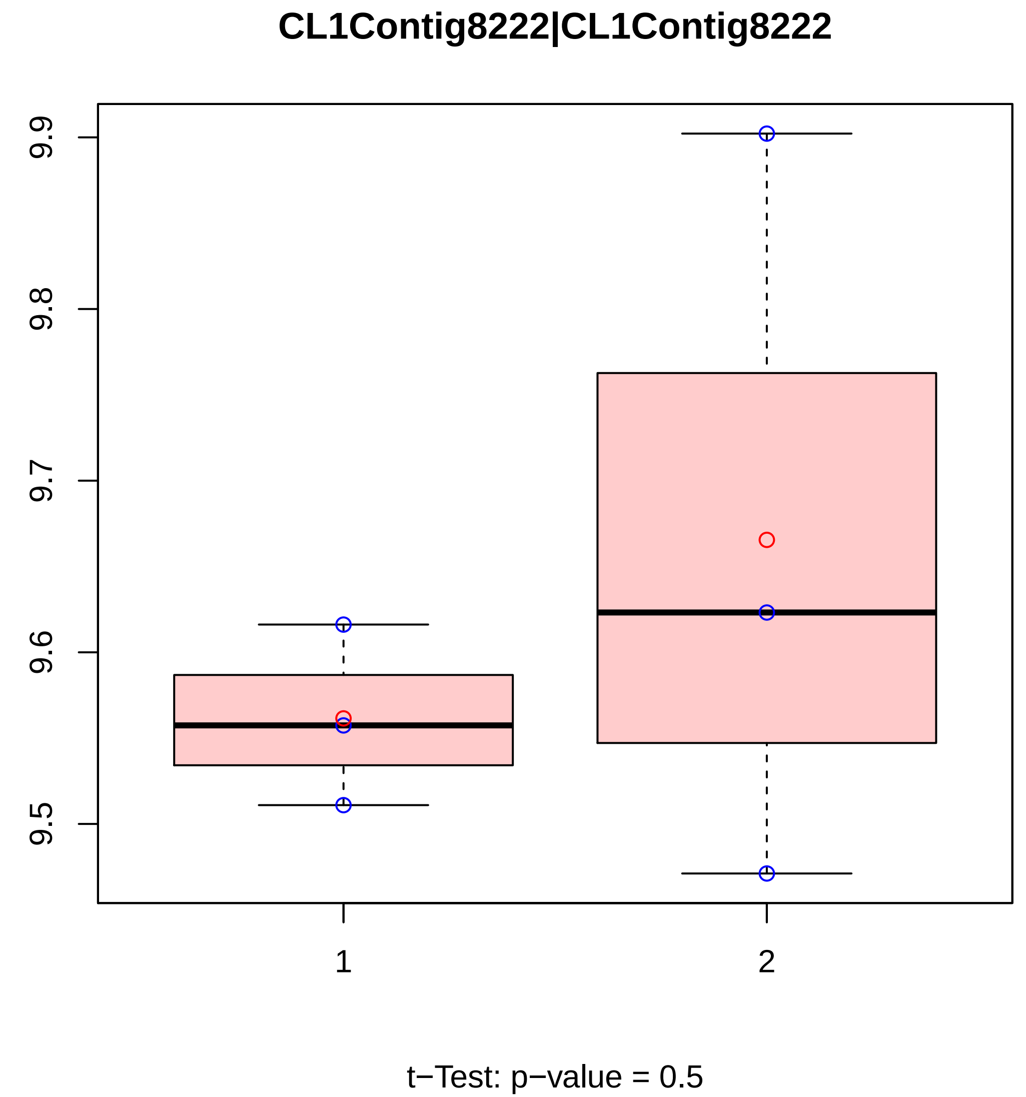
CL1Contig8222|CL1Contig8222
9.5
9.6
9.7
9.8
9.9
1
2
t−Test: p−value = 0.5

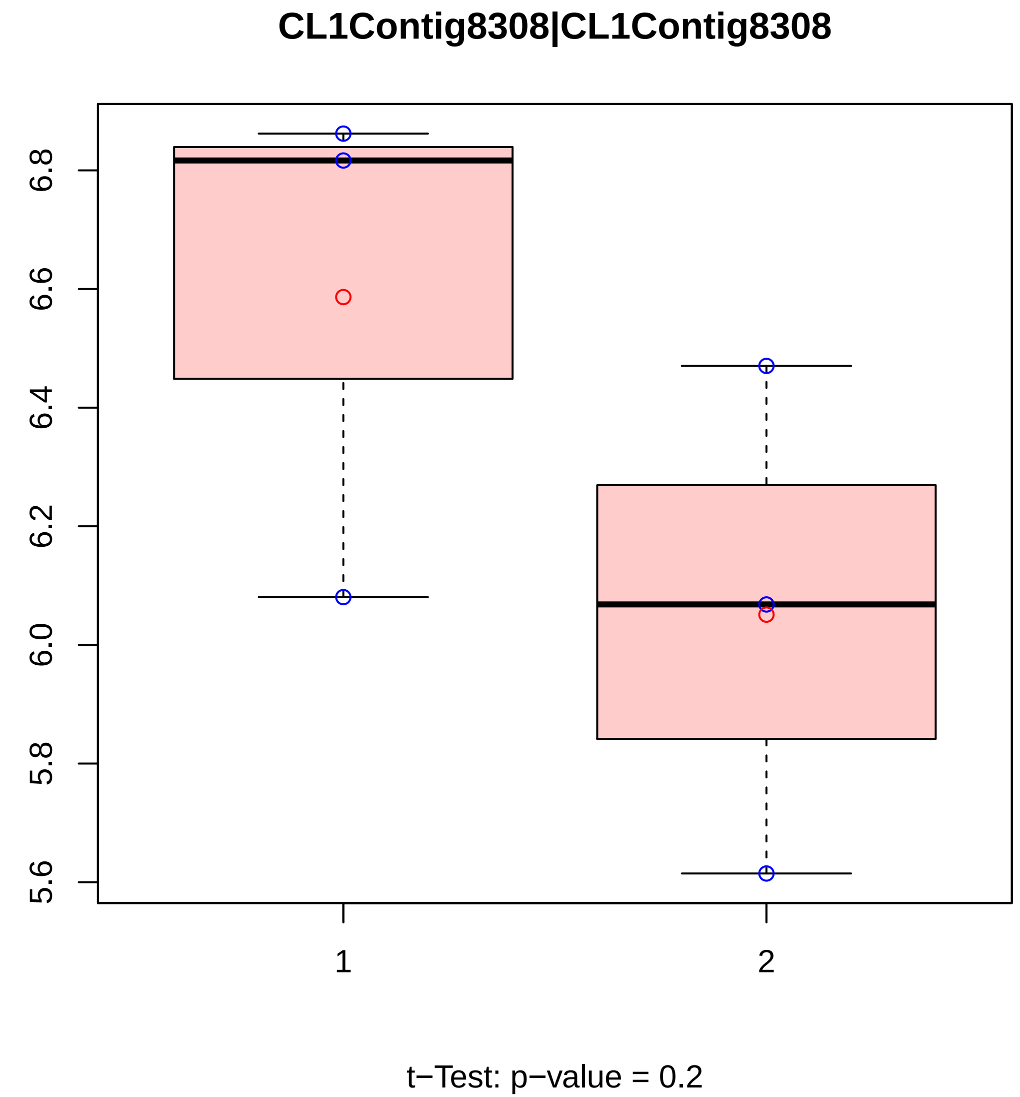

CL1Contig8308|CL1Contig8308
6.8
6.6
6.4
6.2
6.0
5.8
5.6
1
2
t−Test: p−value = 0.2

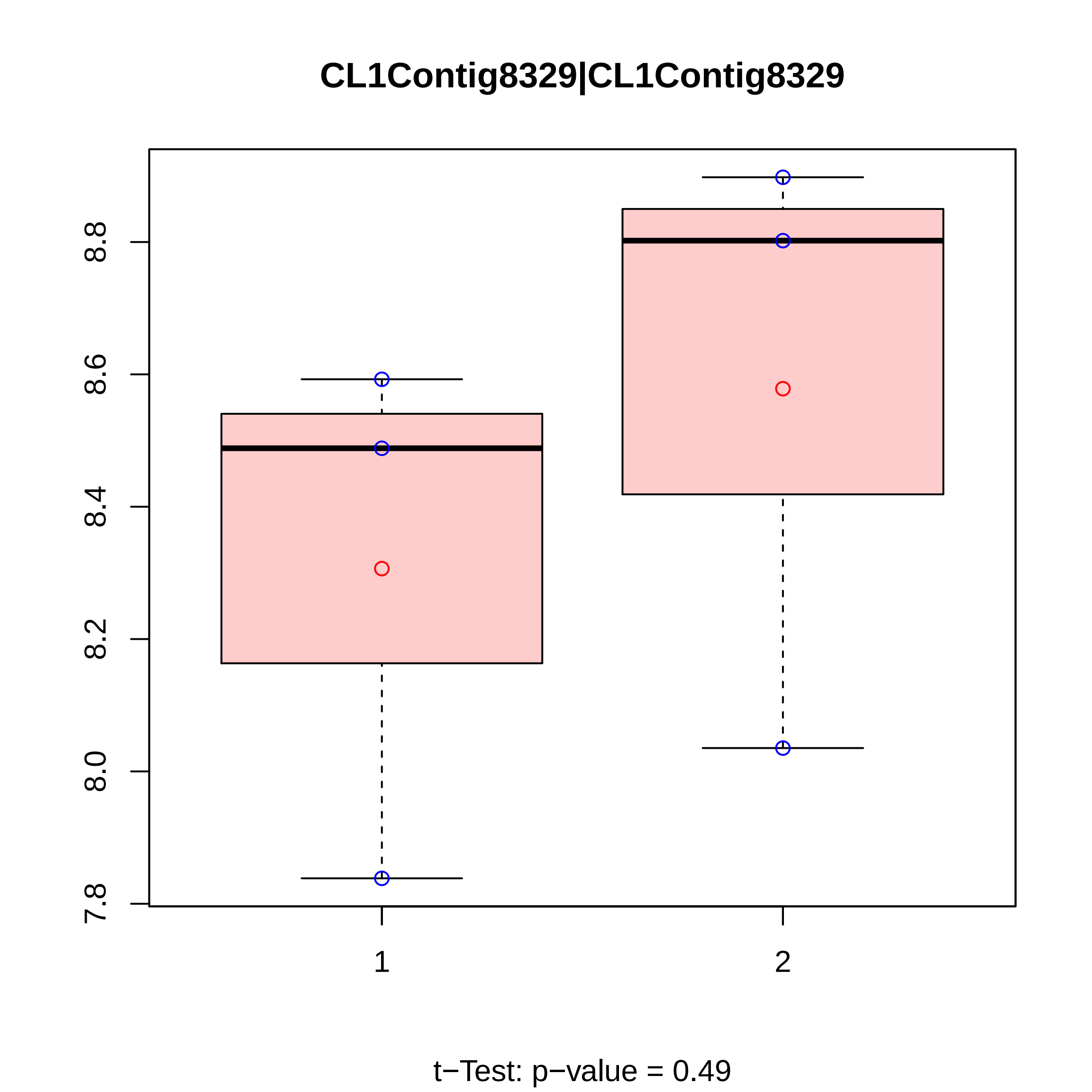

CL1Contig8329|CL1Contig8329
7.8
8.0
8.2
8.4
8.6
8.8
1
2
t−Test: p−value = 0.49

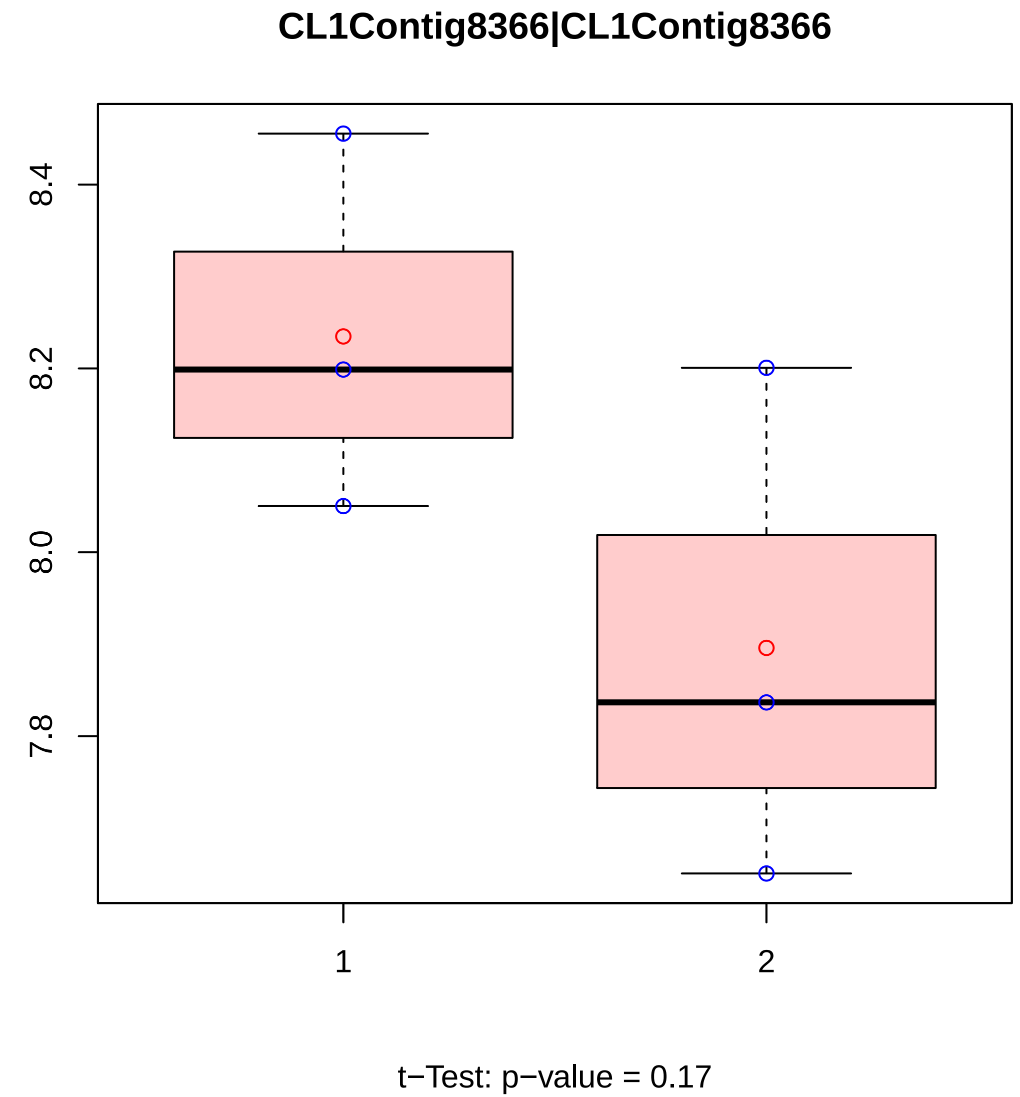
CL1Contig8366|CL1Contig8366
8.4
8.2
8.0
7.8
1
2
t−Test: p−value = 0.17

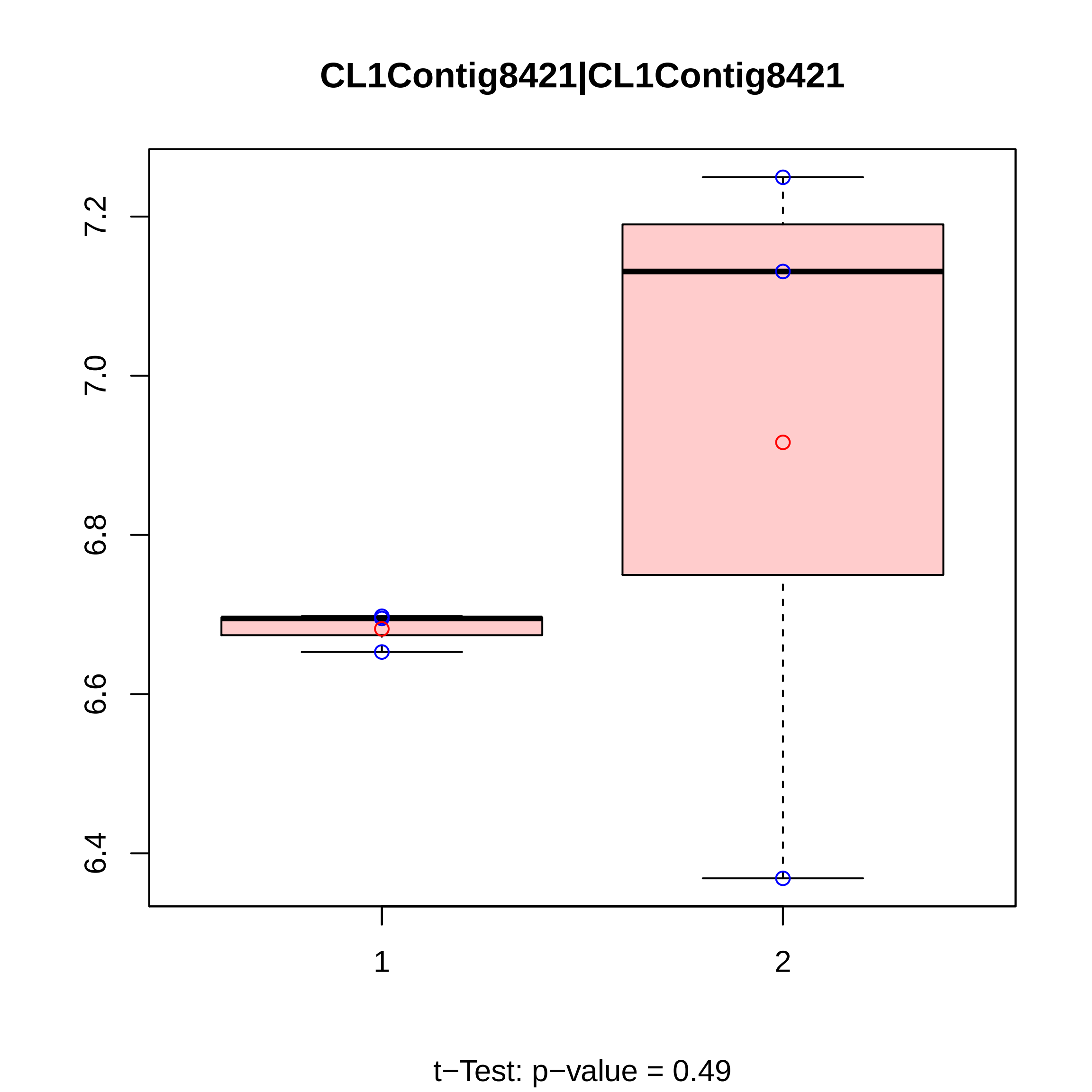
CL1Contig8421|CL1Contig8421
7.2
7.0
6.8
6.6
6.4
1
2
t−Test: p−value = 0.49

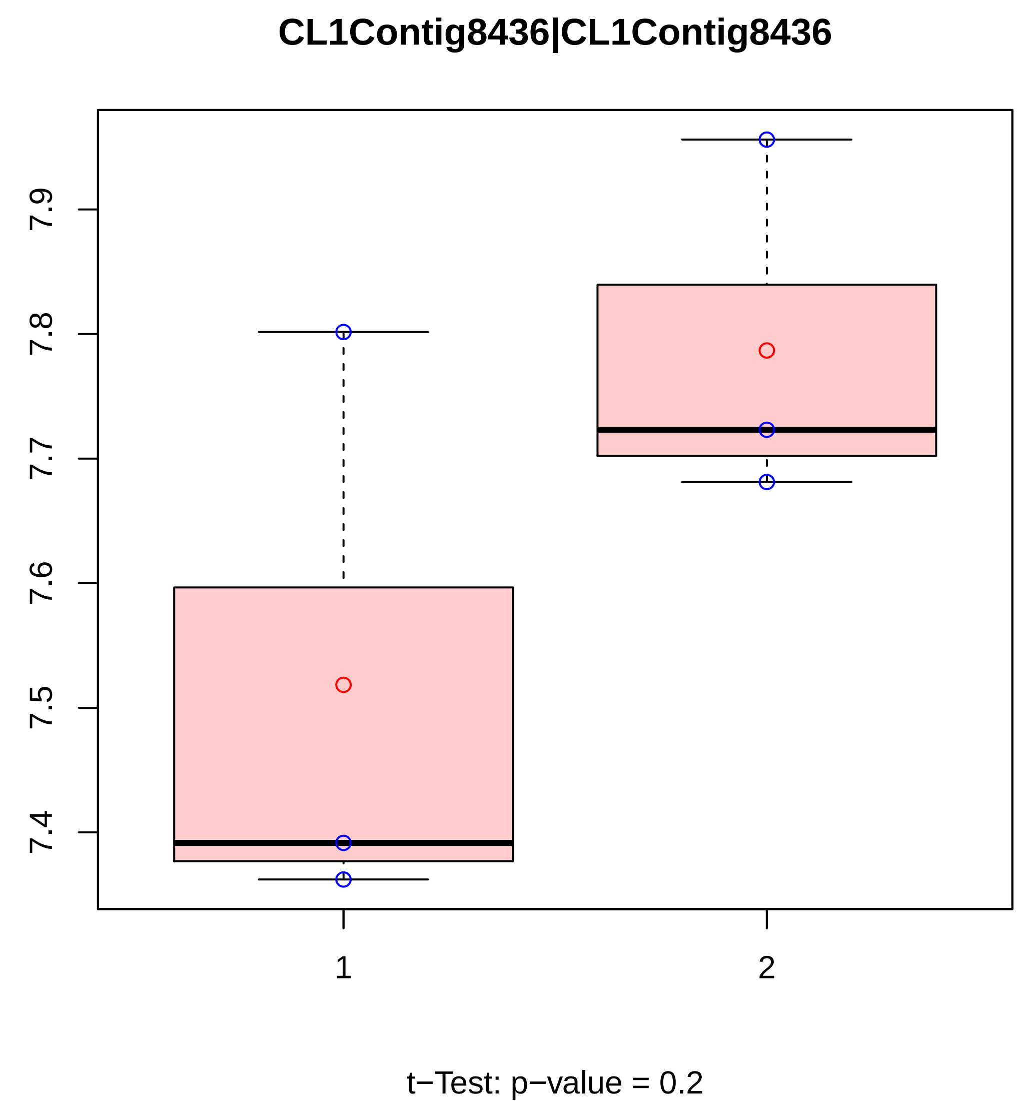
CL1Contig8436|CL1Contig8436
7.4
7.5
7.6
7.7
7.8
7.9
1
2
t−Test: p−value = 0.2

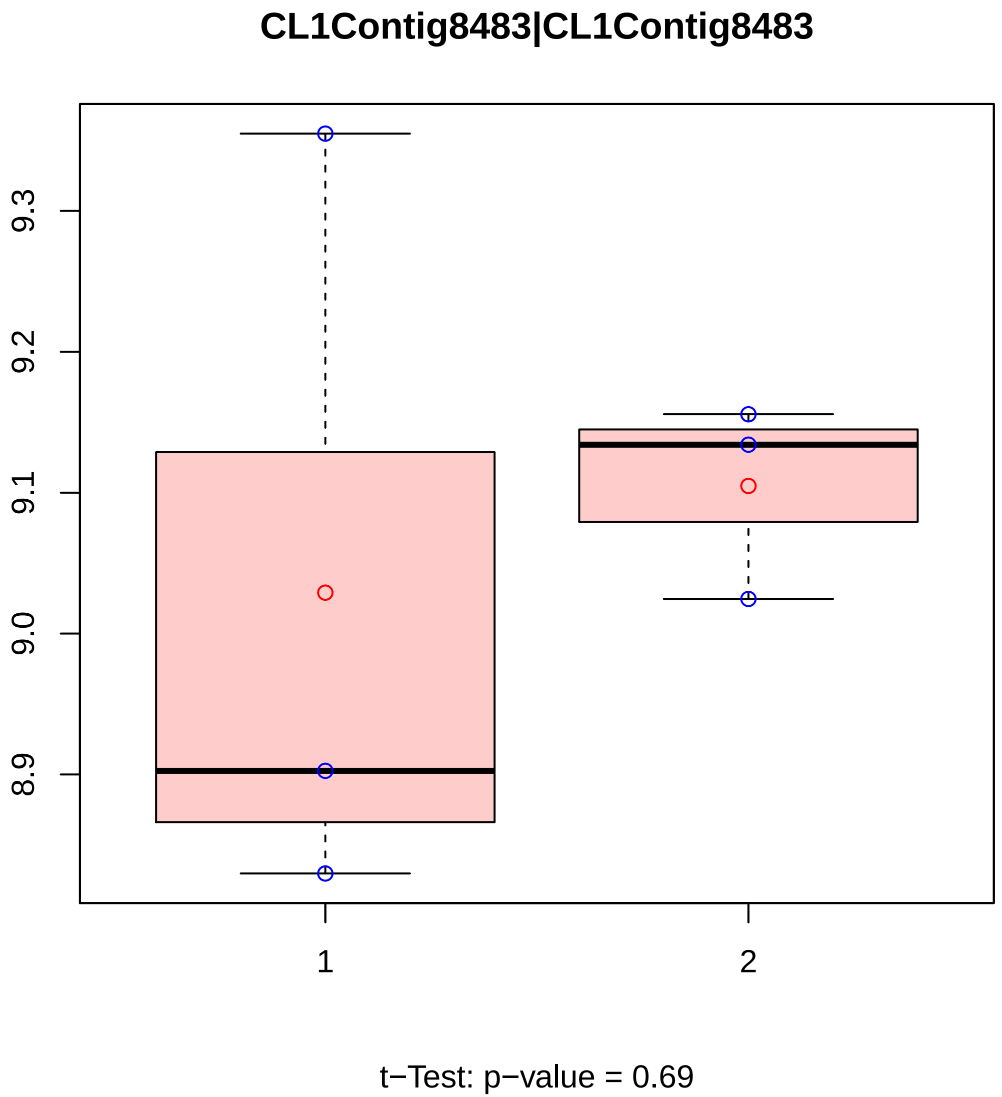

CL1Contig8483|CL1Contig8483
9.3
9.2
9.1
9.0
8.9
1
2
t−Test: p−value = 0.69

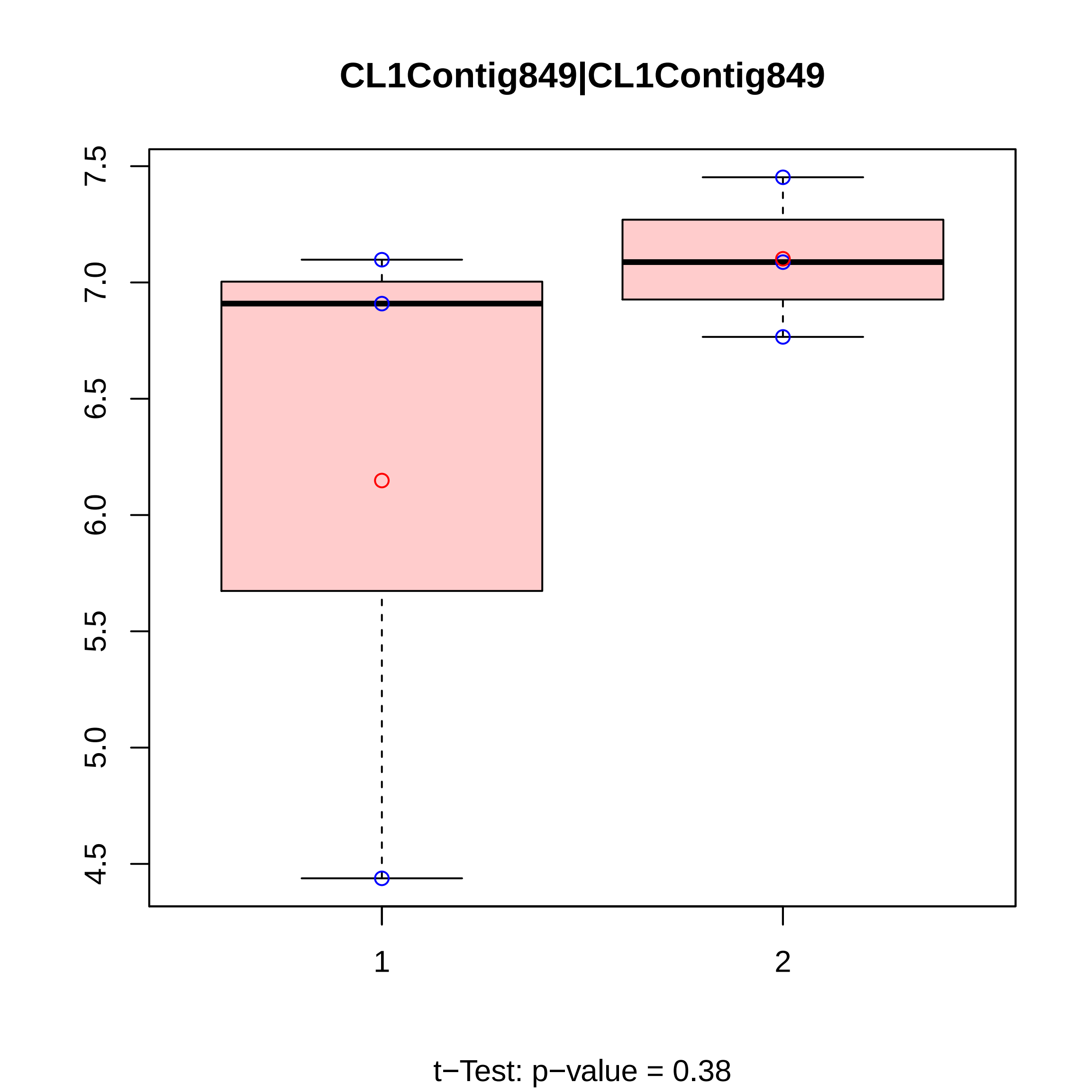
CL1Contig849|CL1Contig849
7.5
7.0
6.5
6.0
5.5
5.0
4.5
1
2
t−Test: p−value = 0.38

## CL1Contig8536|CL1Contig8536

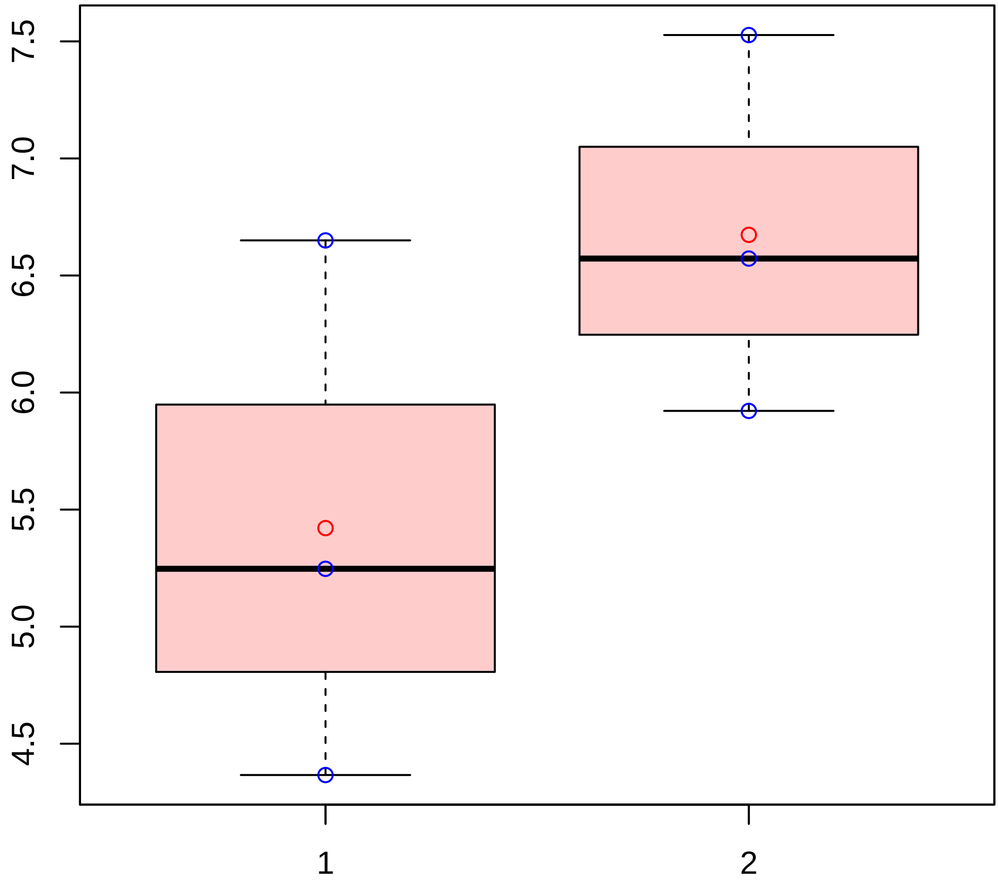


t−Test: p−value = 0.21

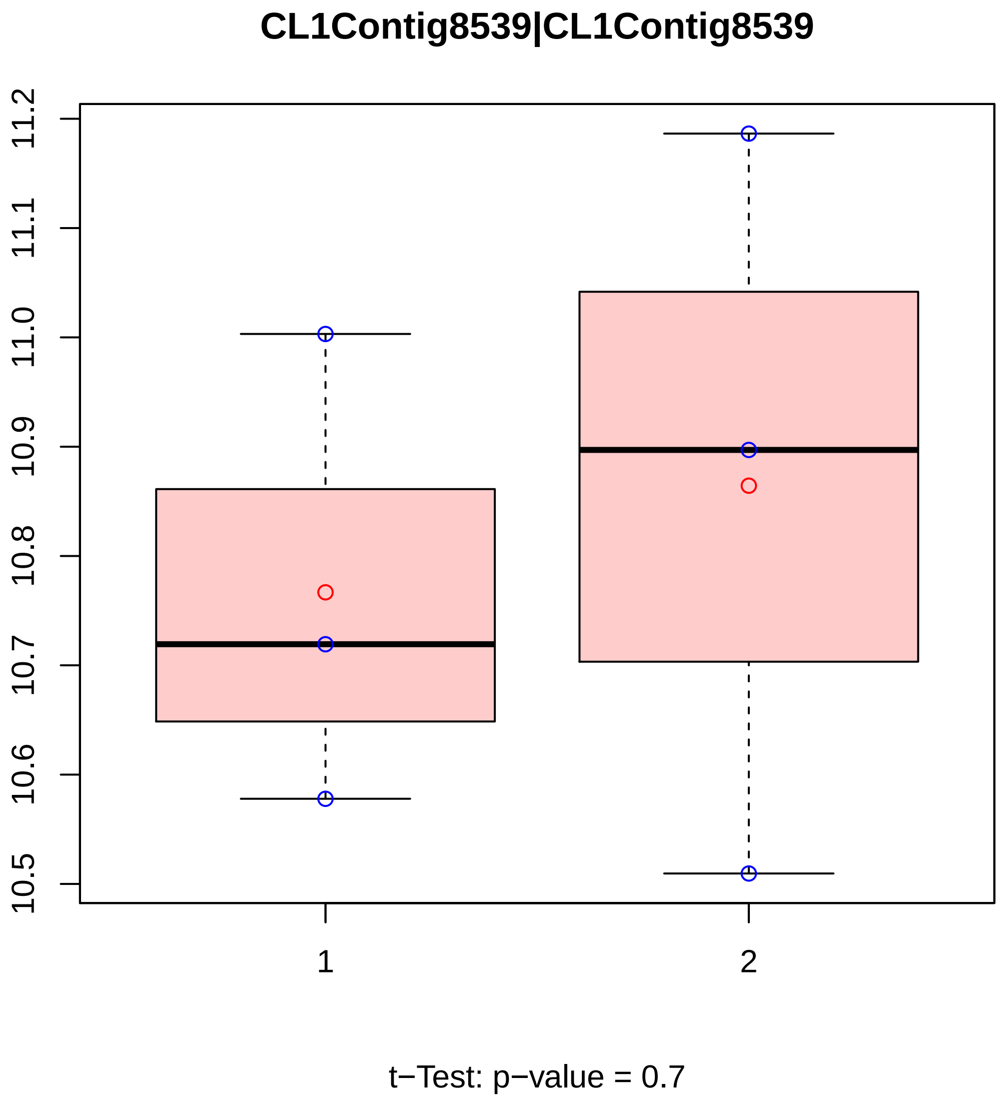
CL1Contig8539|CL1Contig8539
11.2
11.1
11.0
10.9
10.8
10.7
10.6
10.5
1
2
t−Test: p−value = 0.7

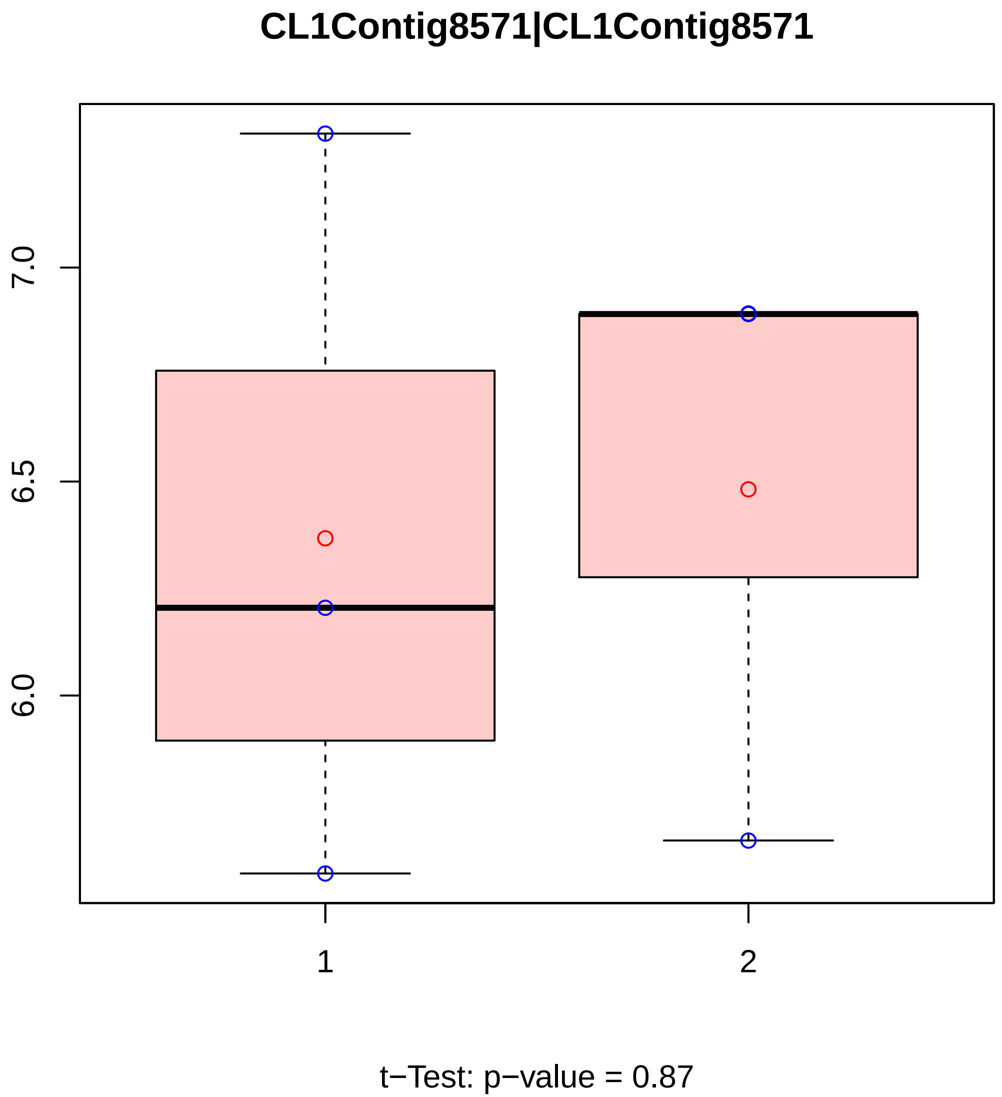
CL1Contig8571|CL1Contig8571
7.0
6.5
6.0
1
2
t−Test: p−value = 0.87

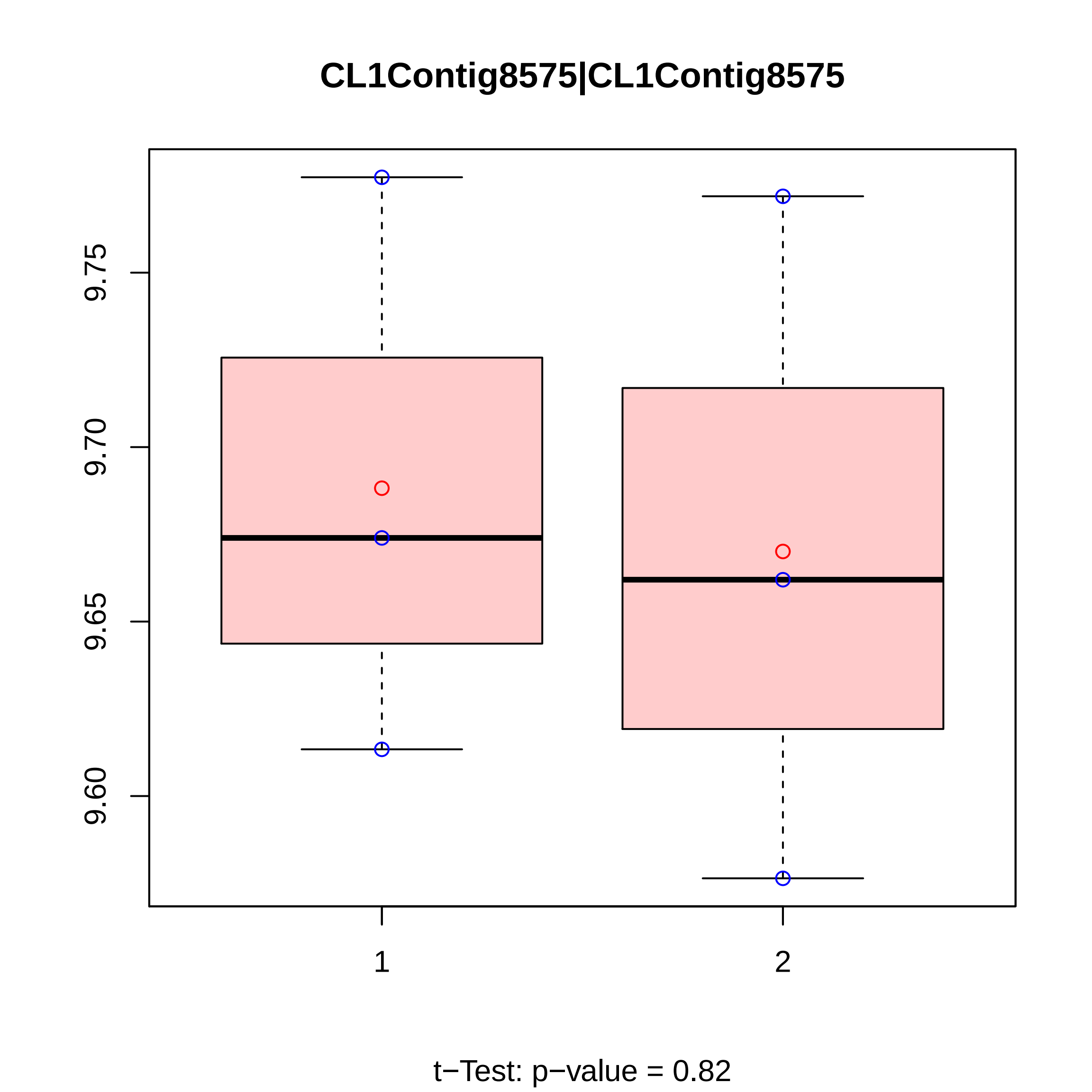
CL1Contig8575|CL1Contig8575
9.60
9.65
9.70
9.75
1
2
t−Test: p−value = 0.82

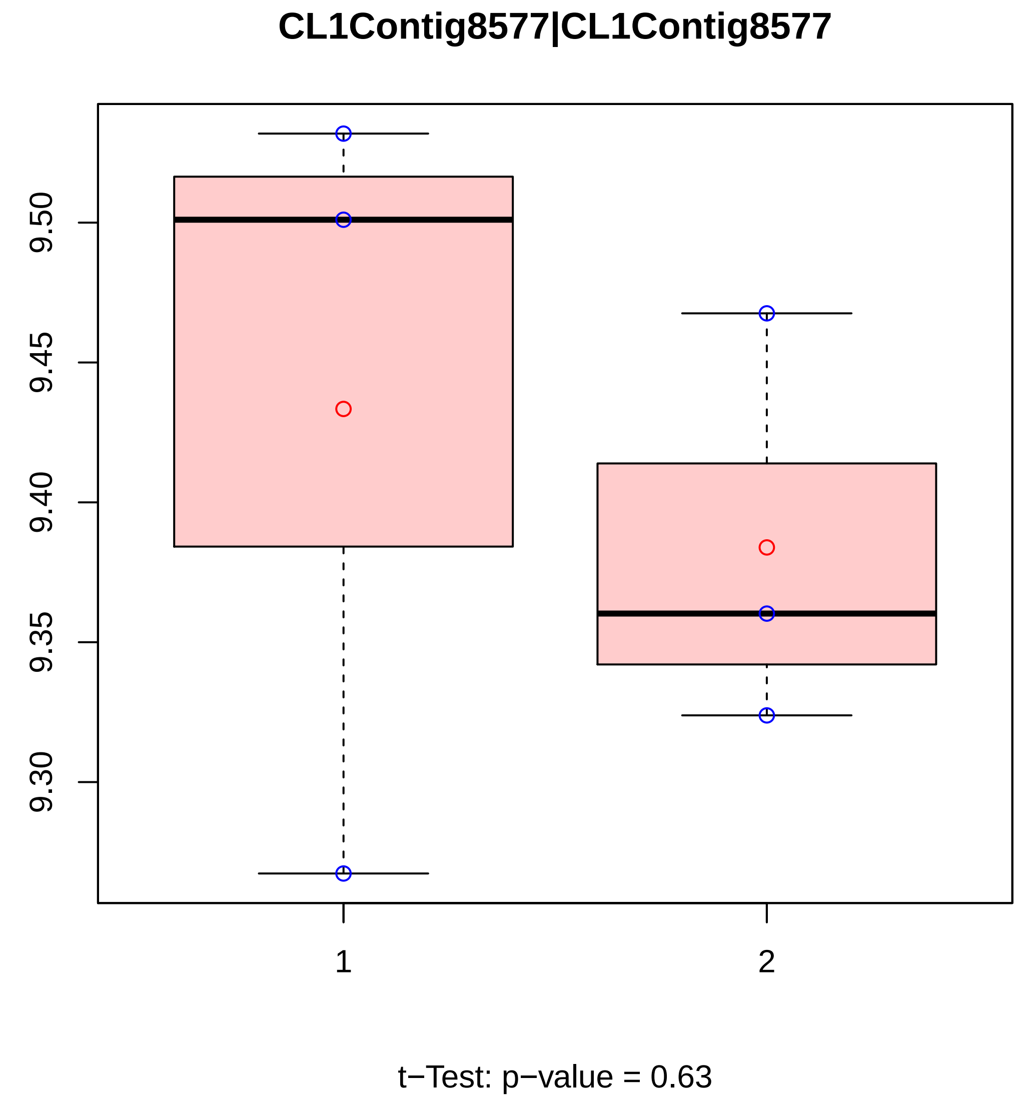
CL1Contig8577|CL1Contig8577
9.30
9.35
9.40
9.45
9.50
1
2
t−Test: p−value = 0.63

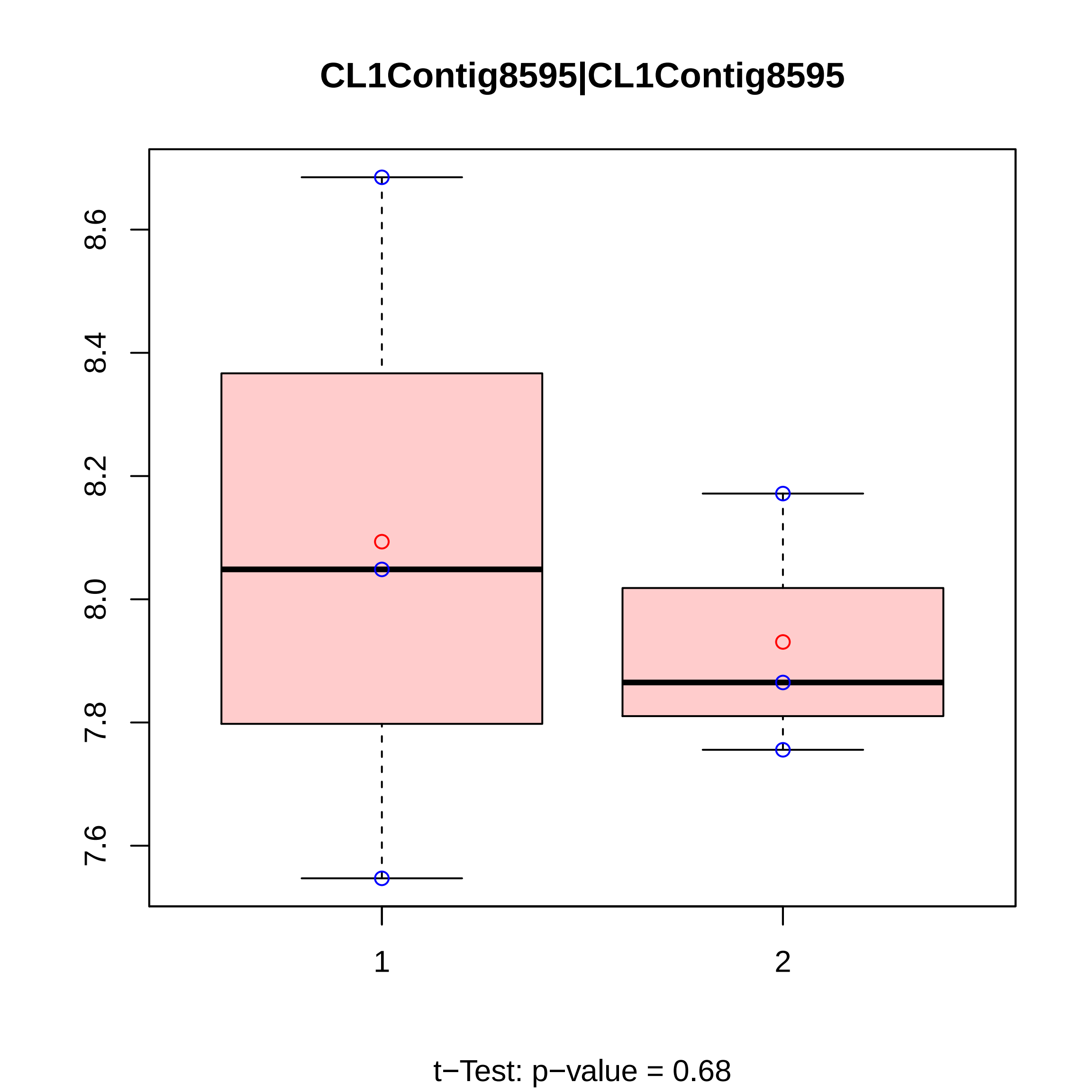
CL1Contig8595|CL1Contig8595
8.6
8.4
8.2
8.0
7.8
7.6
1
2
t−Test: p−value = 0.68

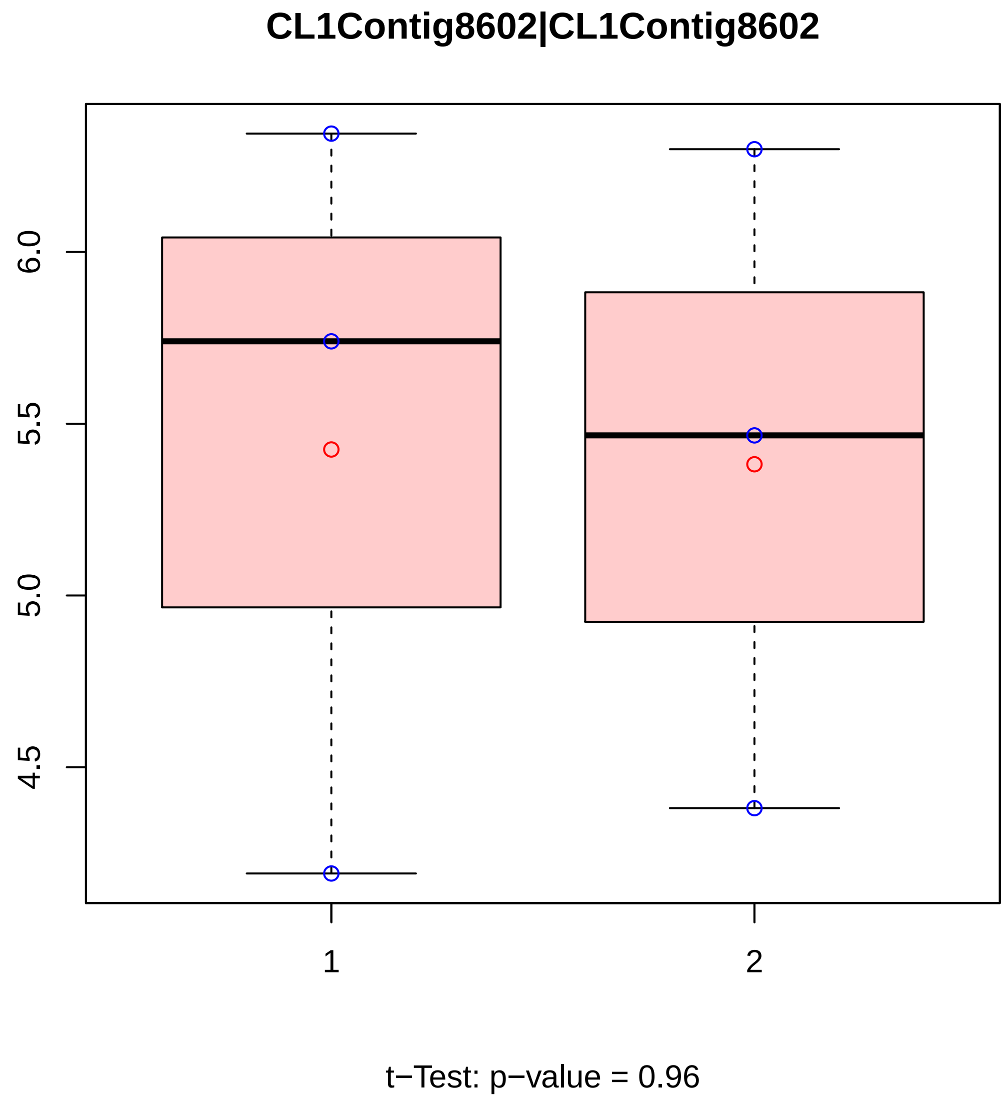
CL1Contig8602|CL1Contig8602
6.0
5.5
5.0
4.5
1
2
t−Test: p−value = 0.96

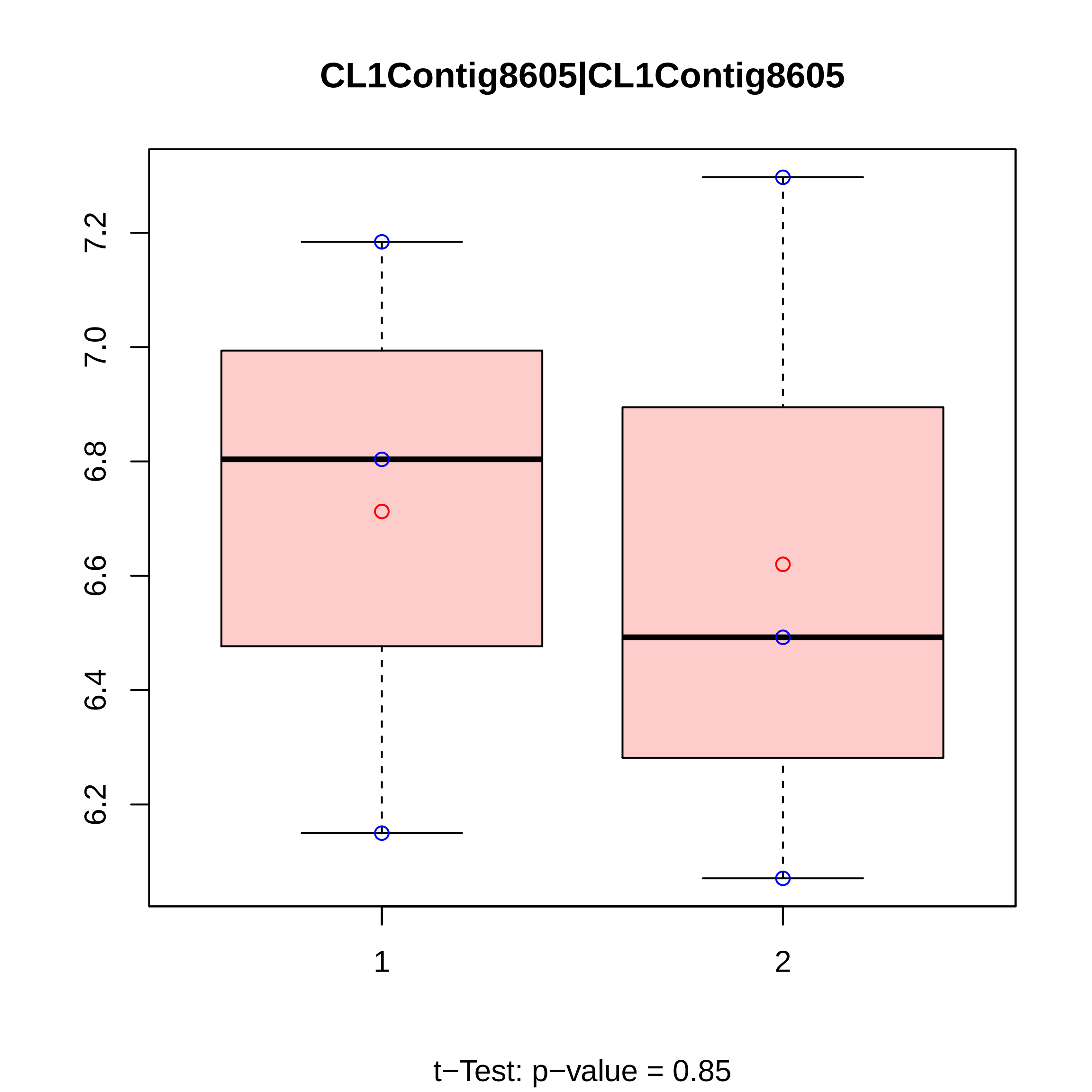
CL1Contig8605|CL1Contig8605
7.2
7.0
6.8
6.6
6.4
6.2
1
2
t−Test: p−value = 0.85

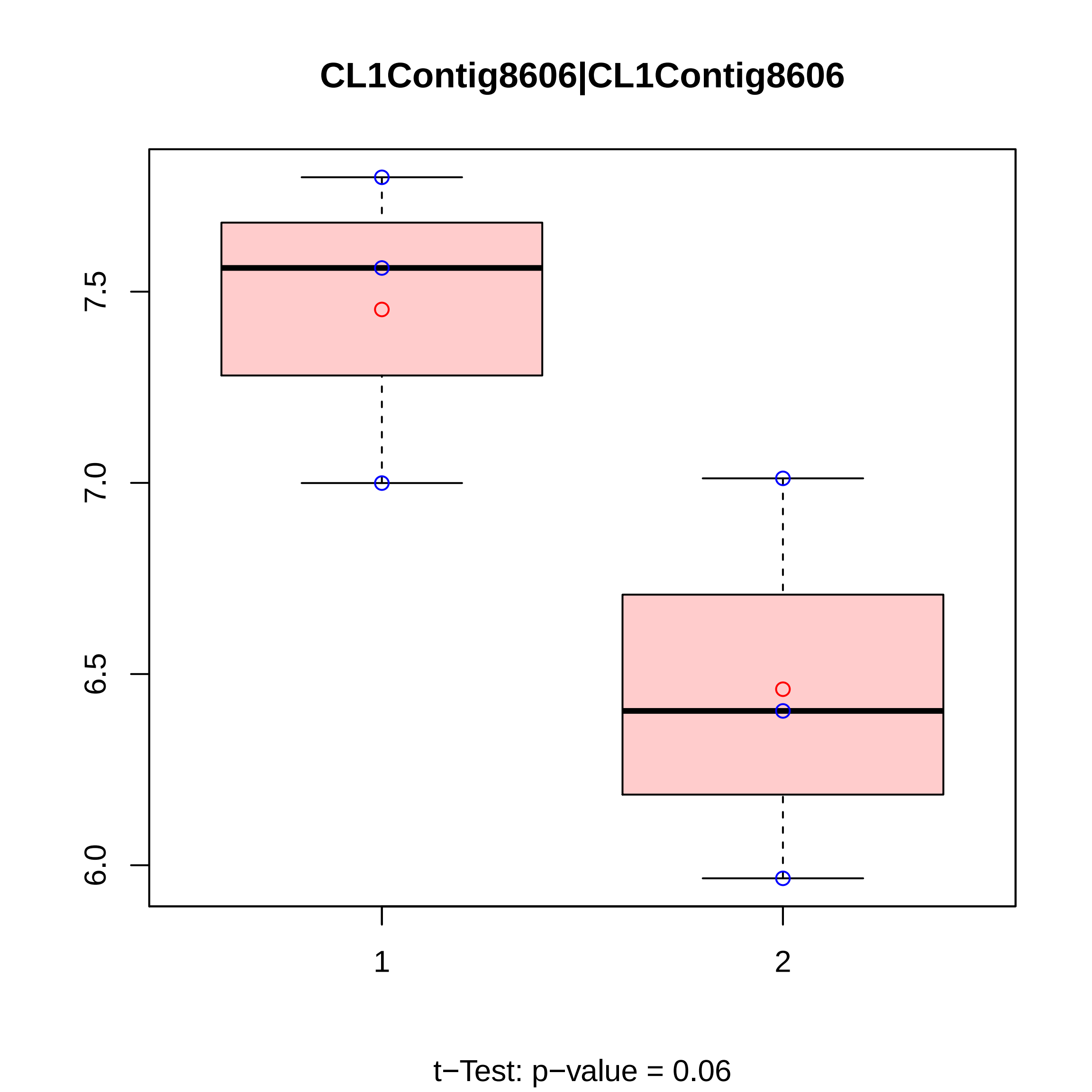
CL1Contig8606|CL1Contig8606
7.5
7.0
6.5
6.0
1
2
t−Test: p−value = 0.06

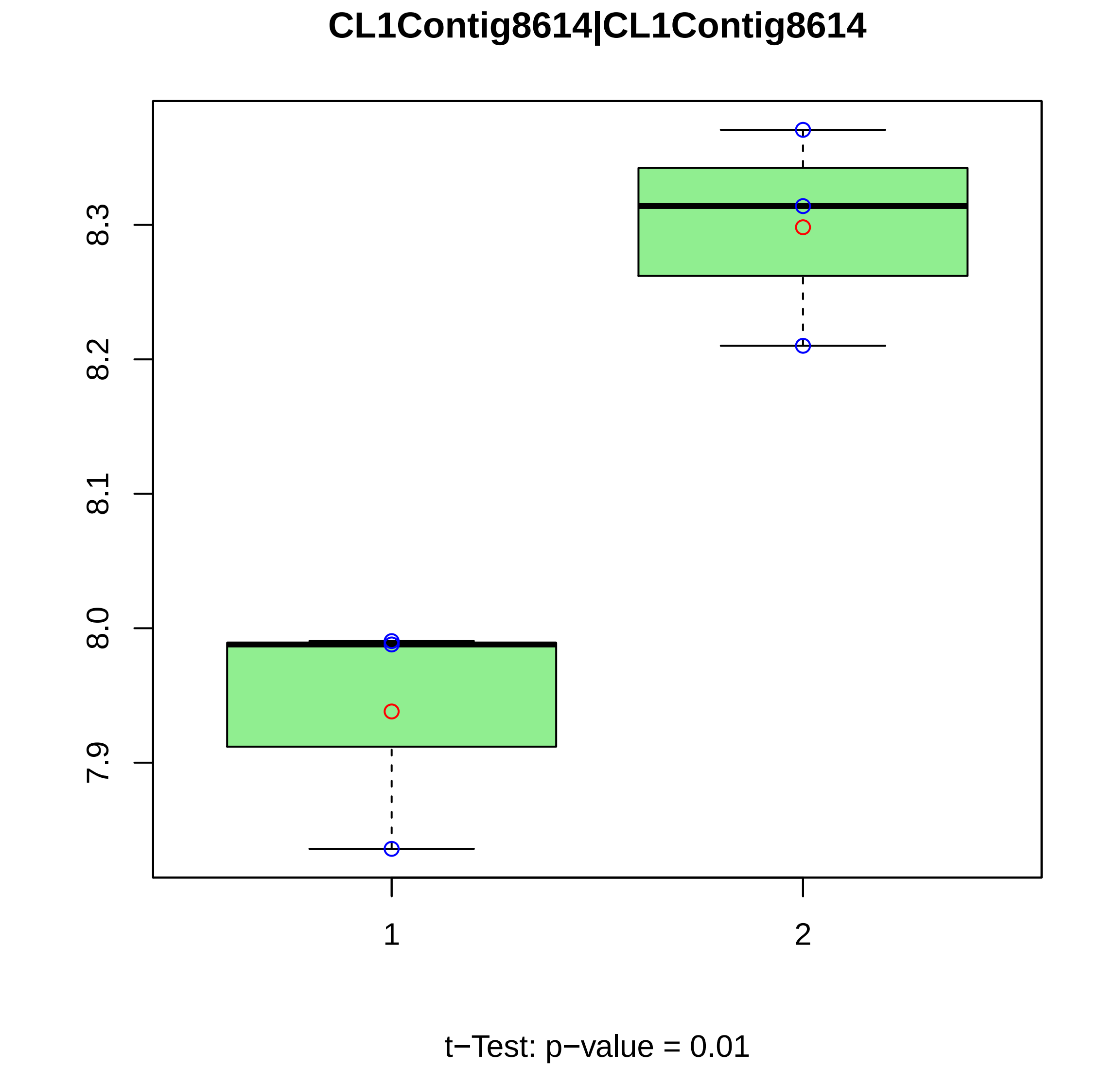
CL1Contig8614|CL1Contig8614
8.3
8.2
8.1
8.0
7.9
1
2
t−Test: p−value = 0.01

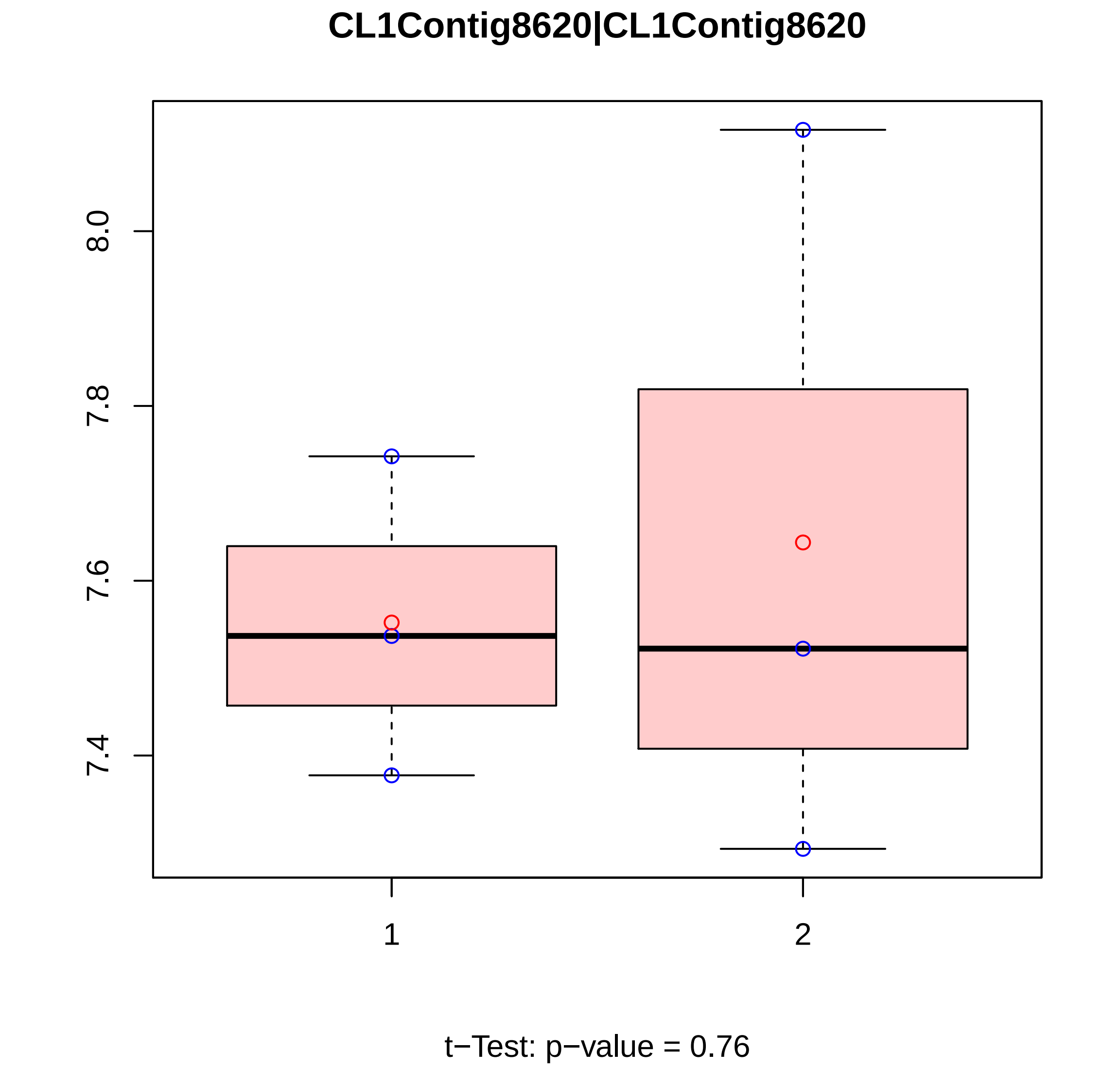
CL1Contig8620|CL1Contig8620
8.0
7.8
7.6
7.4
1
2
t−Test: p−value = 0.76

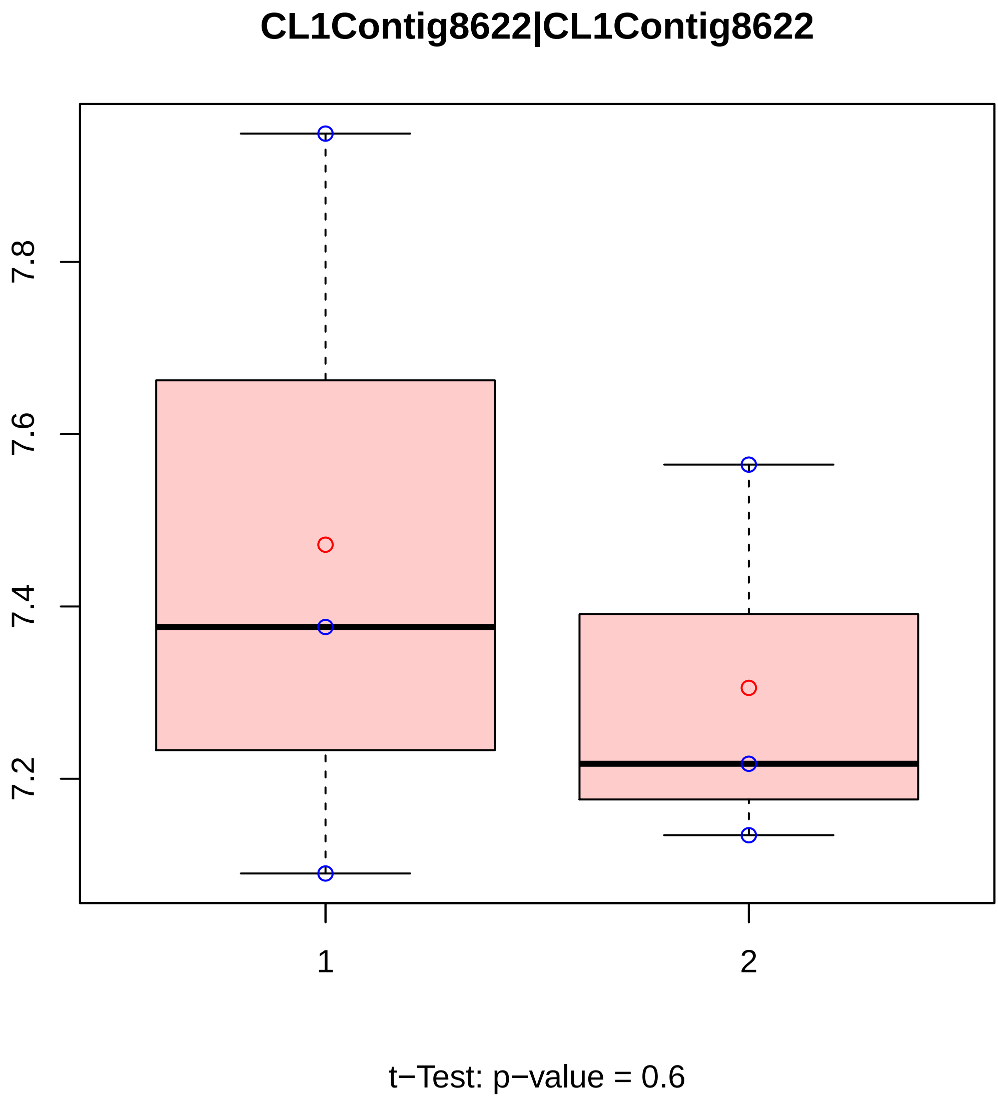

CL1Contig8622|CL1Contig8622
7.8
7.6
7.4
7.2
1
2
t−Test: p−value = 0.6

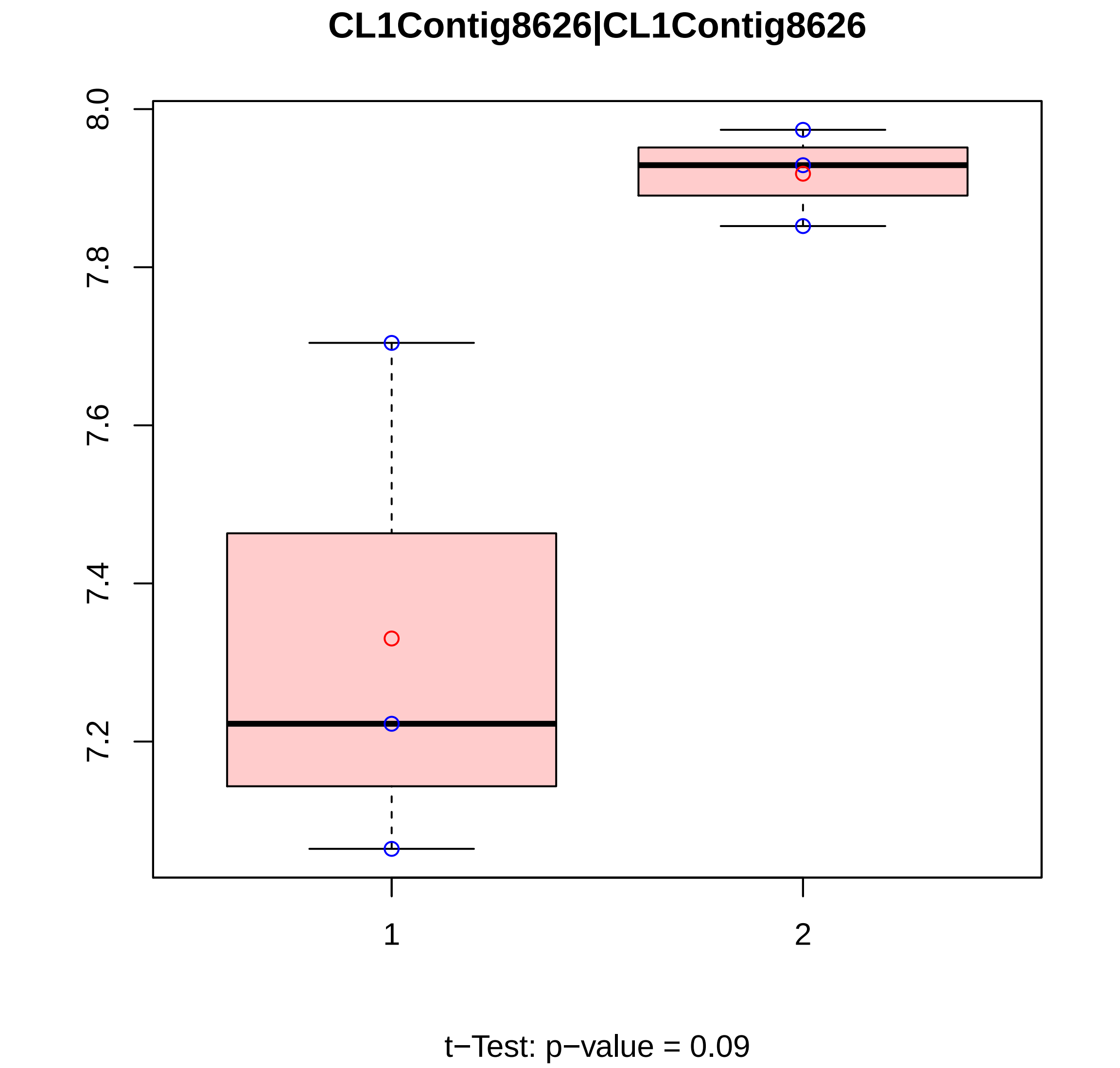
CL1Contig8626|CL1Contig8626
8.0
7.8
7.6
7.4
7.2
1
2
t−Test: p−value = 0.09

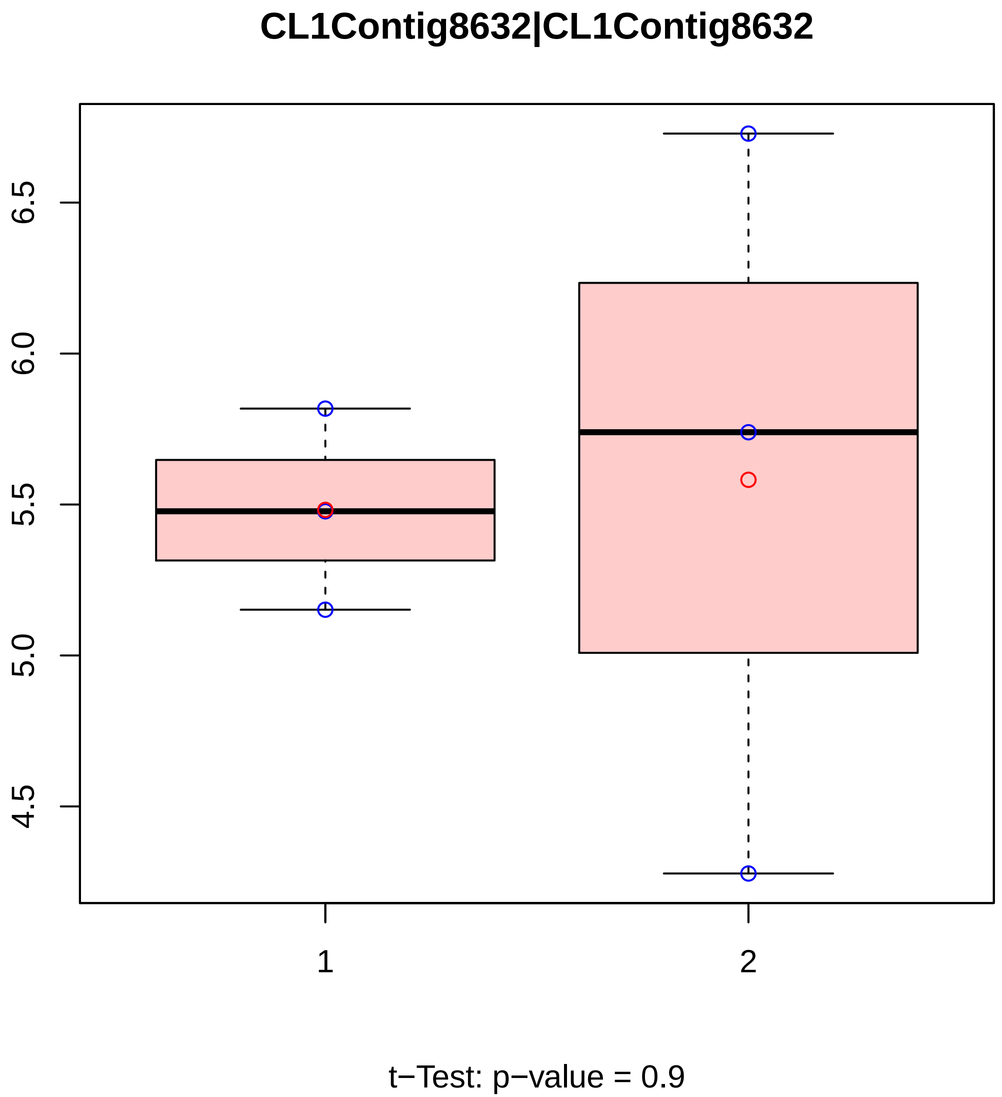
CL1Contig8632|CL1Contig8632
6.5
6.0
5.5
5.0
4.5
1
2
t−Test: p−value = 0.9

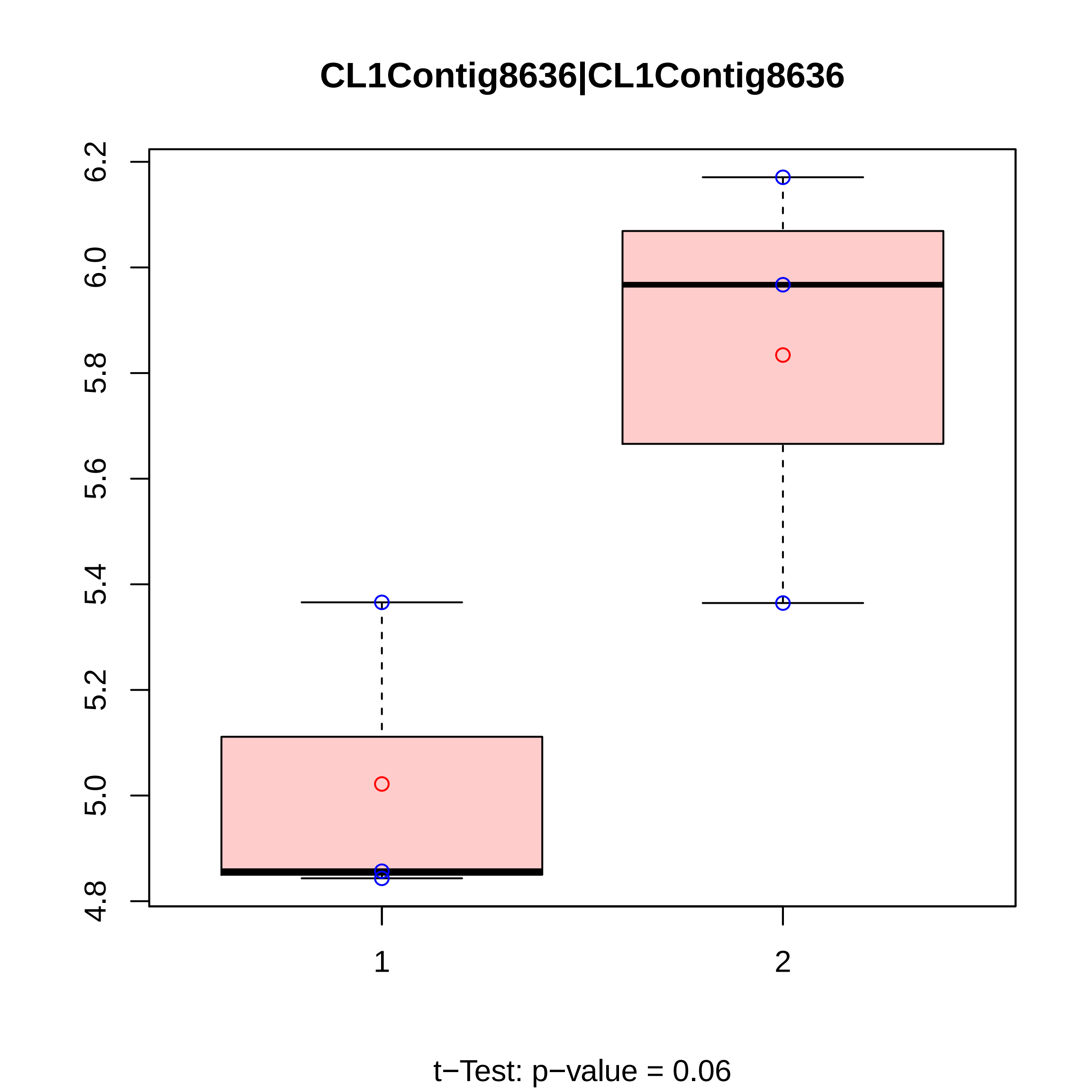

CL1Contig8636|CL1Contig8636
6.2
6.0
5.8
5.6
5.4
5.2
5.0
4.8
1
2
t−Test: p−value = 0.06

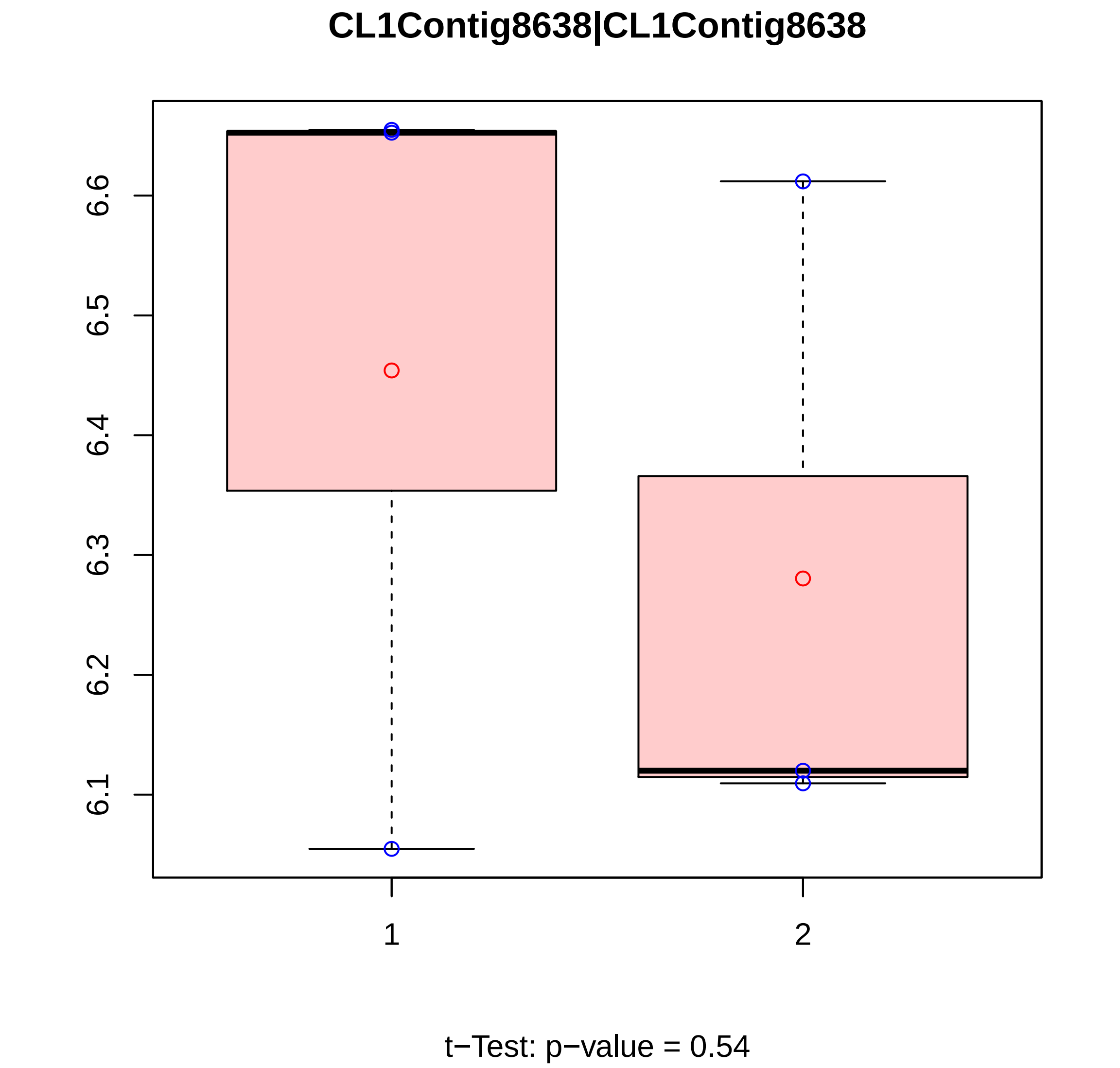
CL1Contig8638|CL1Contig8638
6.1
6.2
6.3
6.4
6.5
6.6
1
2
t−Test: p−value = 0.54

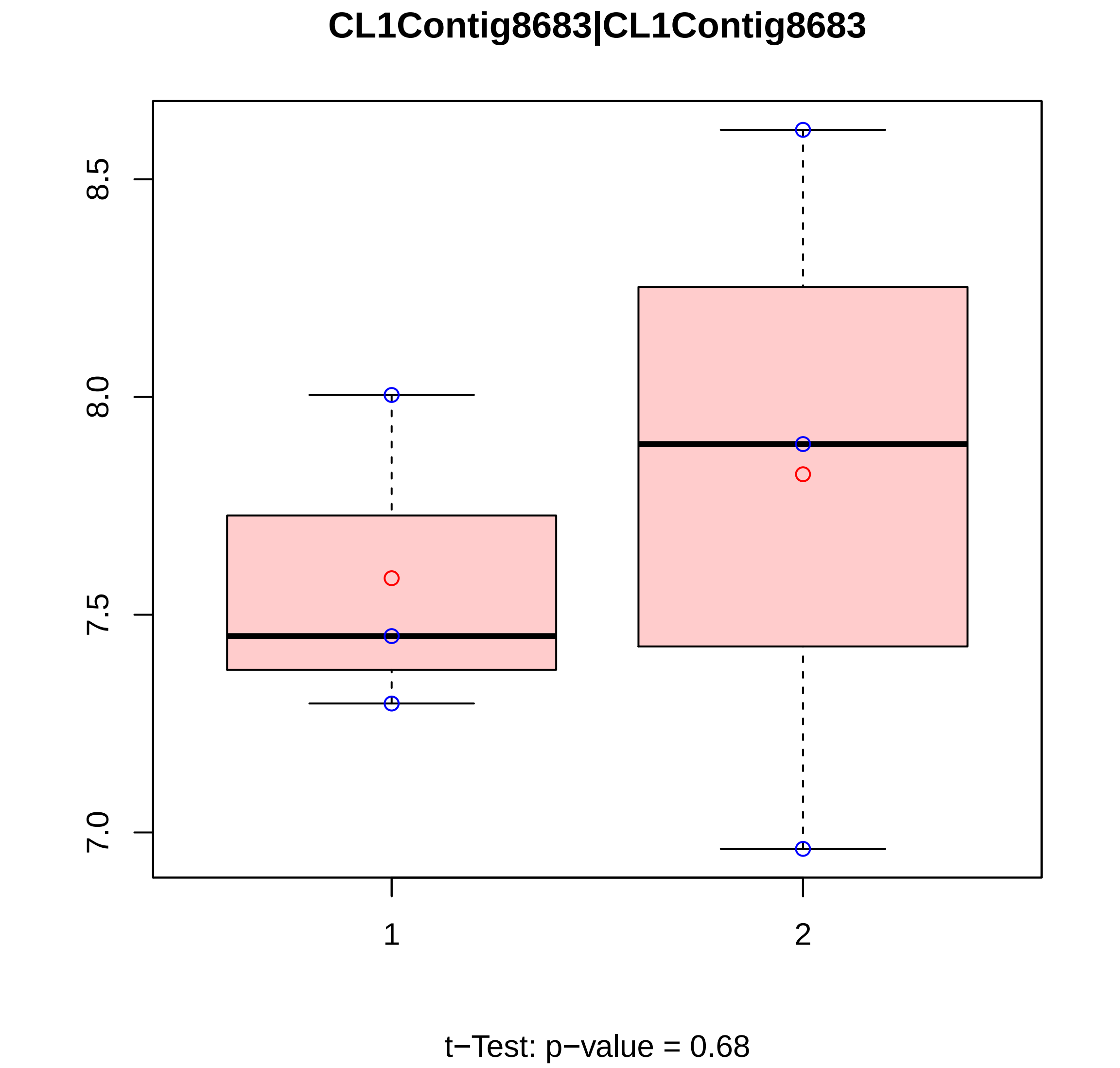
CL1Contig8683|CL1Contig8683
8.5
8.0
7.5
7.0
1
2
t−Test: p−value = 0.68

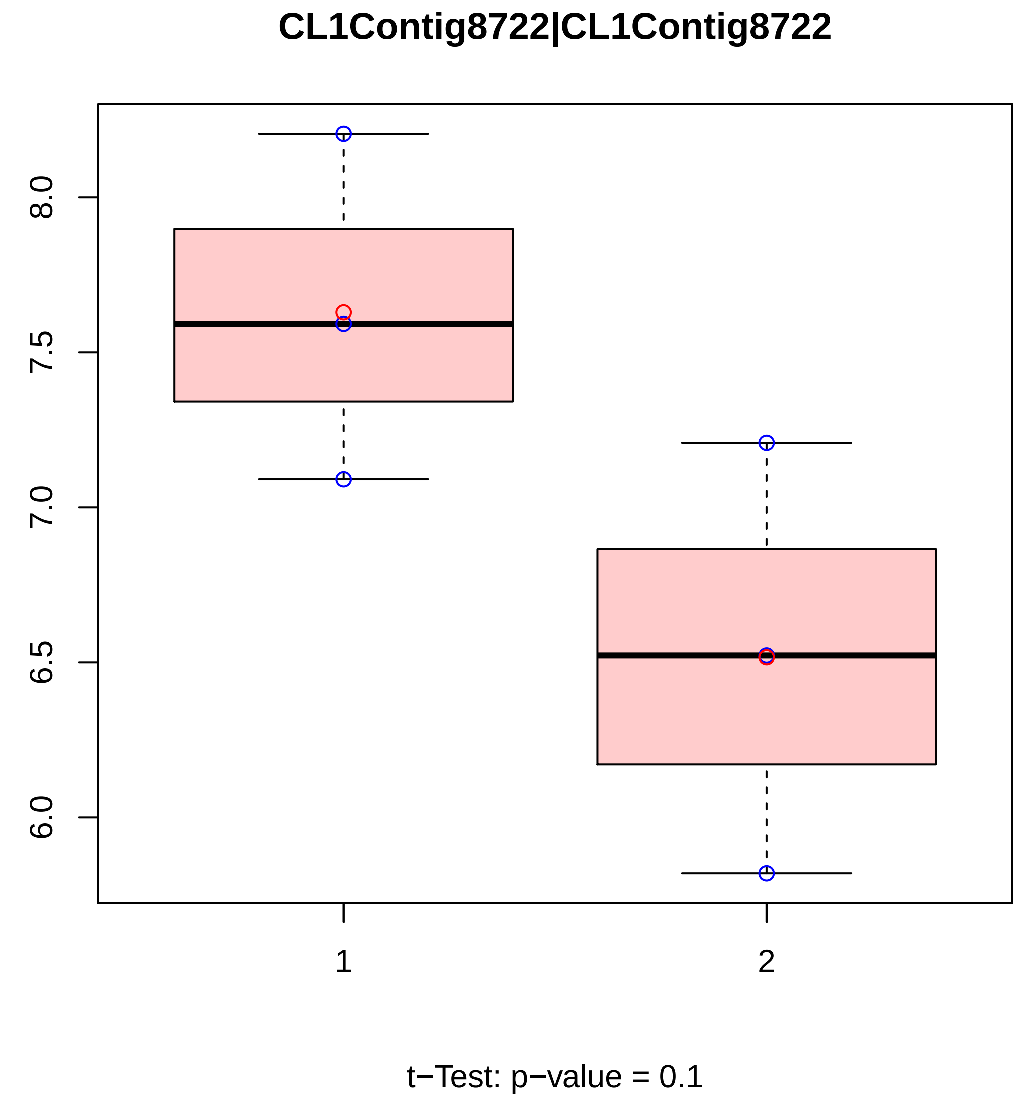
CL1Contig8722|CL1Contig8722
8.0
7.5
7.0
6.5
6.0
1
2
t−Test: p−value = 0.1

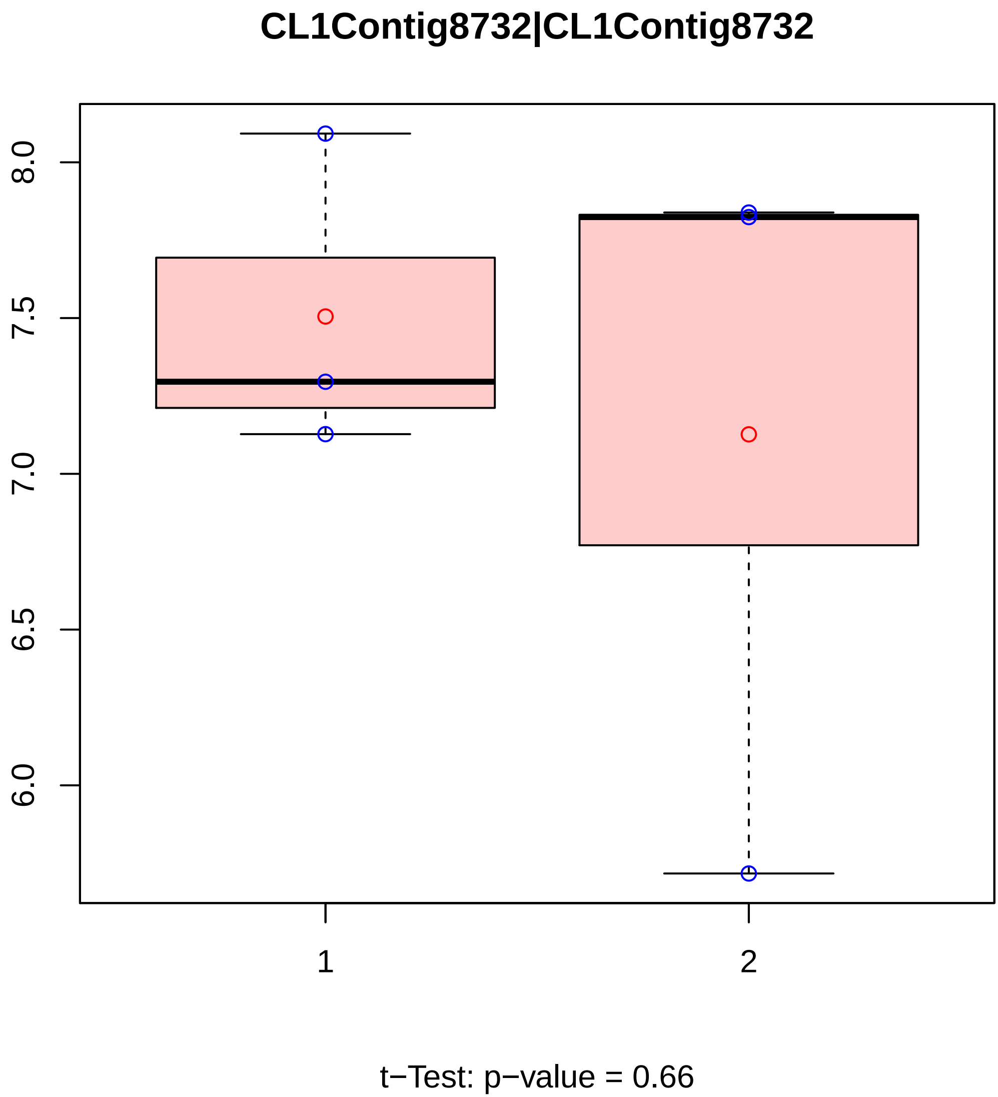

CL1Contig8732|CL1Contig8732
8.0
7.5
7.0
6.5
6.0
1
2
t−Test: p−value = 0.66

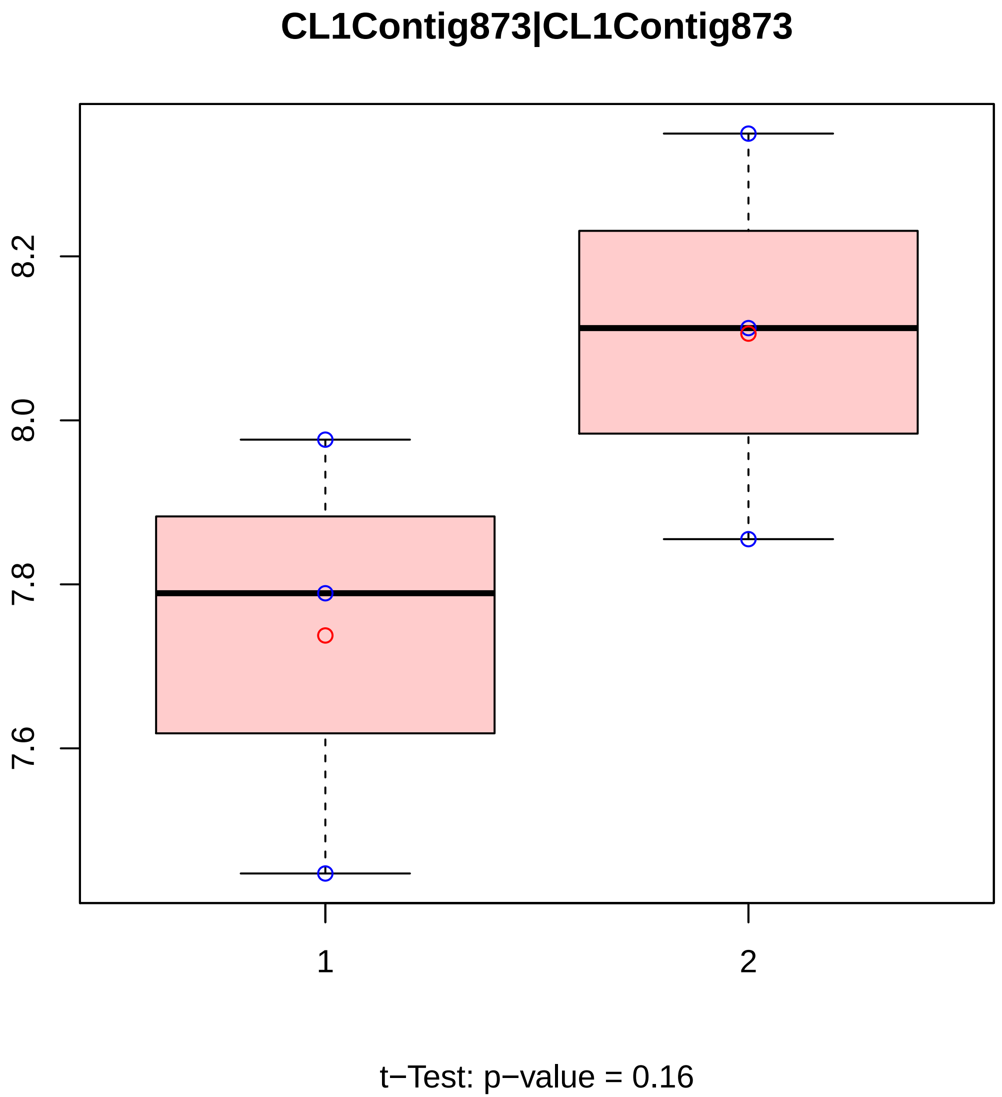
CL1Contig873|CL1Contig873
8.2
8.0
7.8
7.6
1
2
t−Test: p−value = 0.16

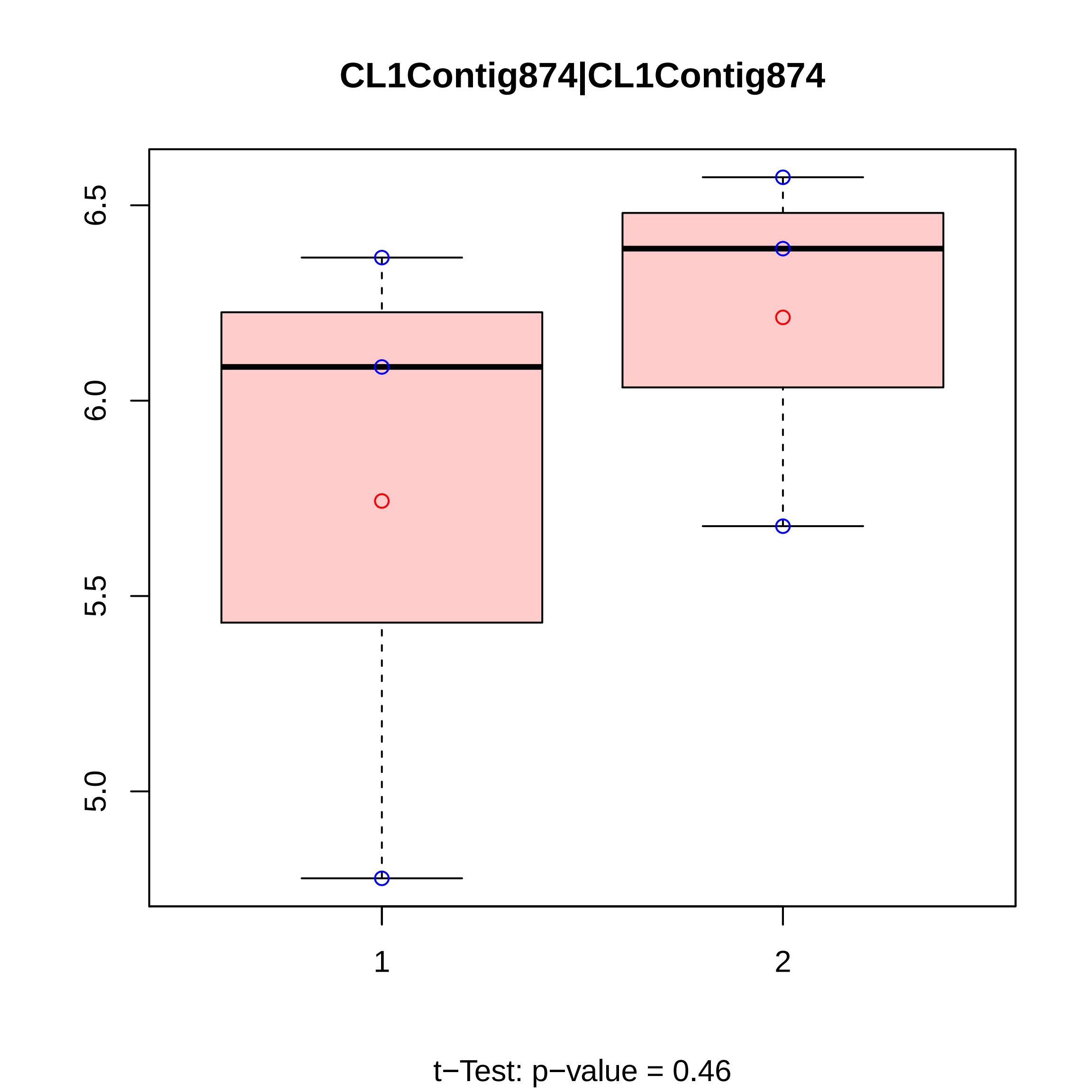
CL1Contig874|CL1Contig874
6.5
6.0
5.5
5.0
1
2
t−Test: p−value = 0.46

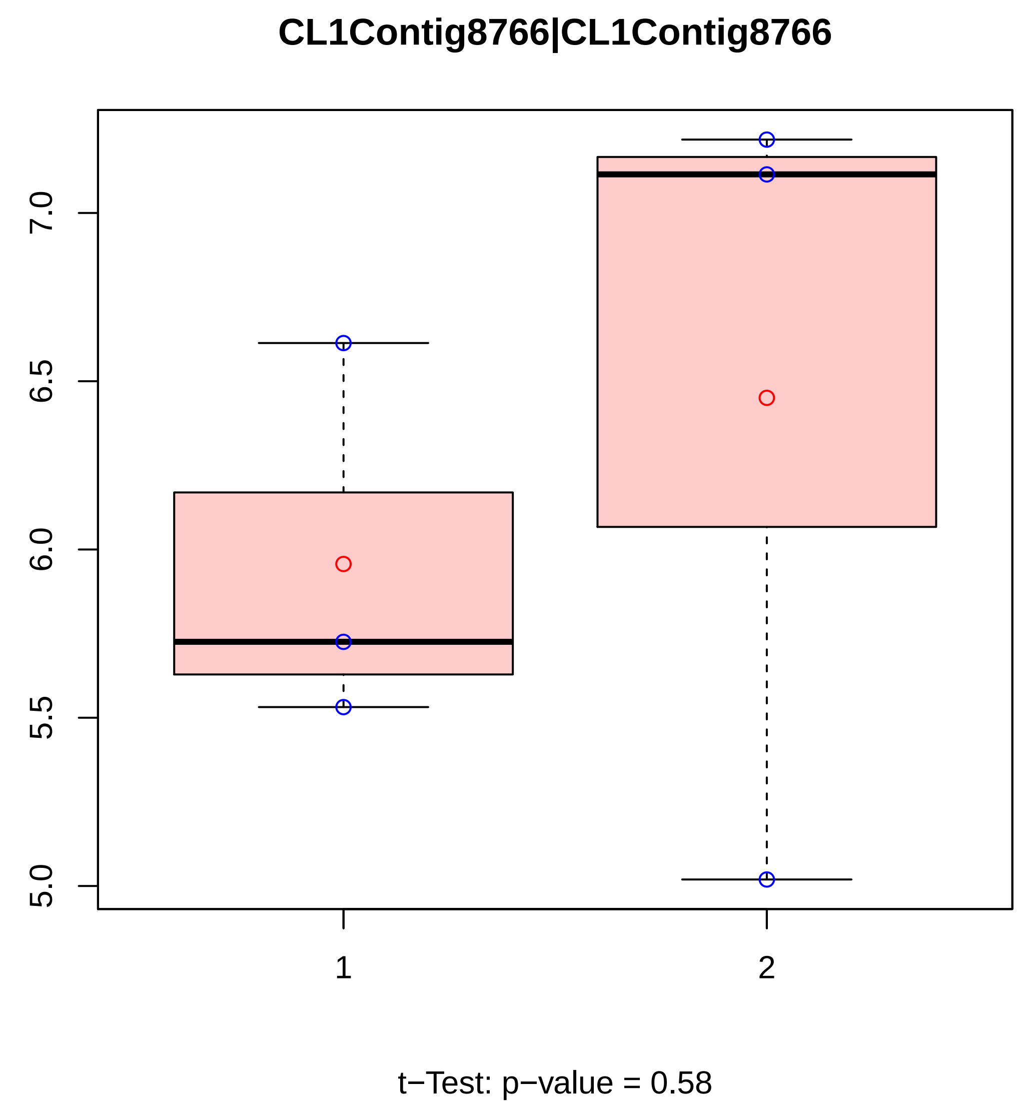
CL1Contig8766|CL1Contig8766
7.0
6.5
6.0
5.5
5.0
1
2
t−Test: p−value = 0.58

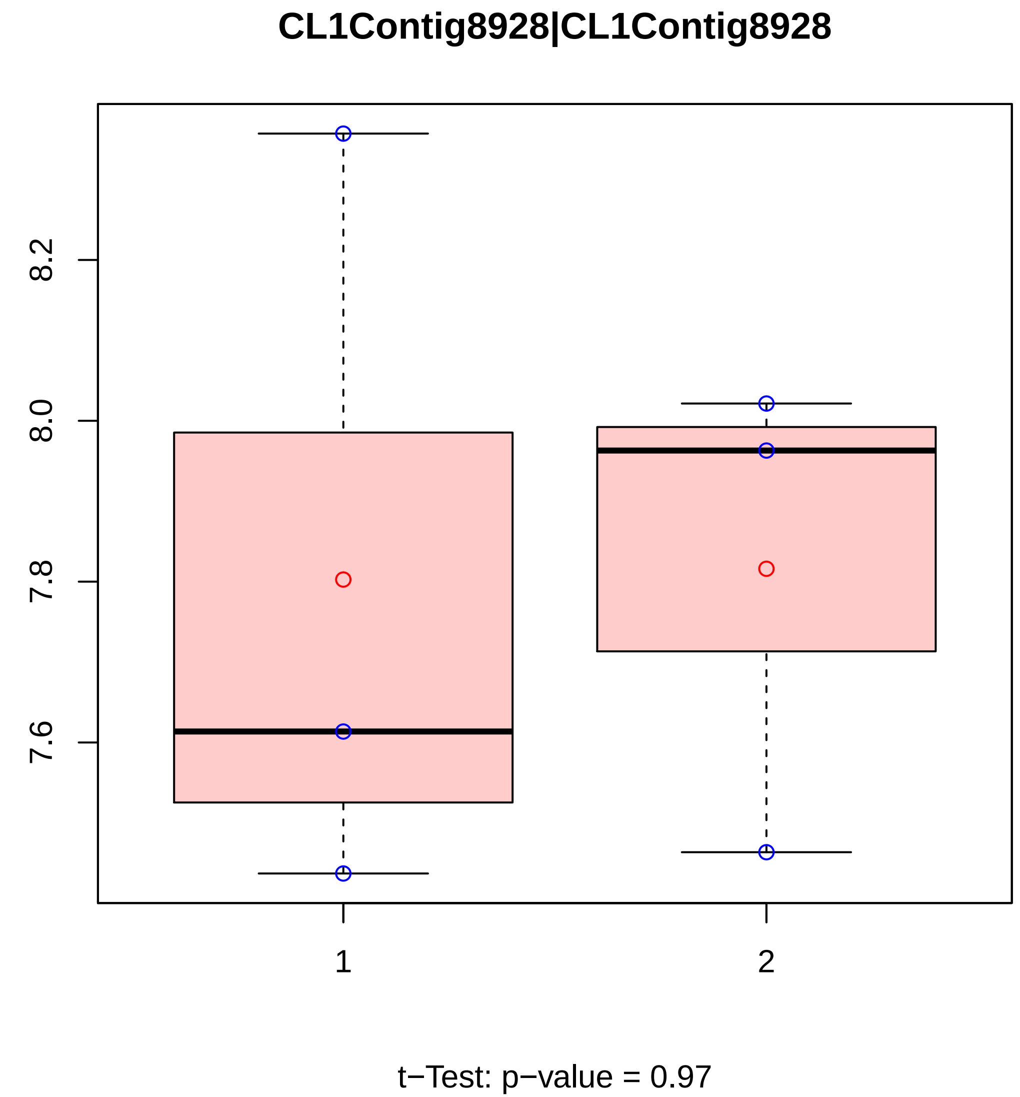
CL1Contig8928|CL1Contig8928
8.2
8.0
7.8
7.6
1
2
t−Test: p−value = 0.97

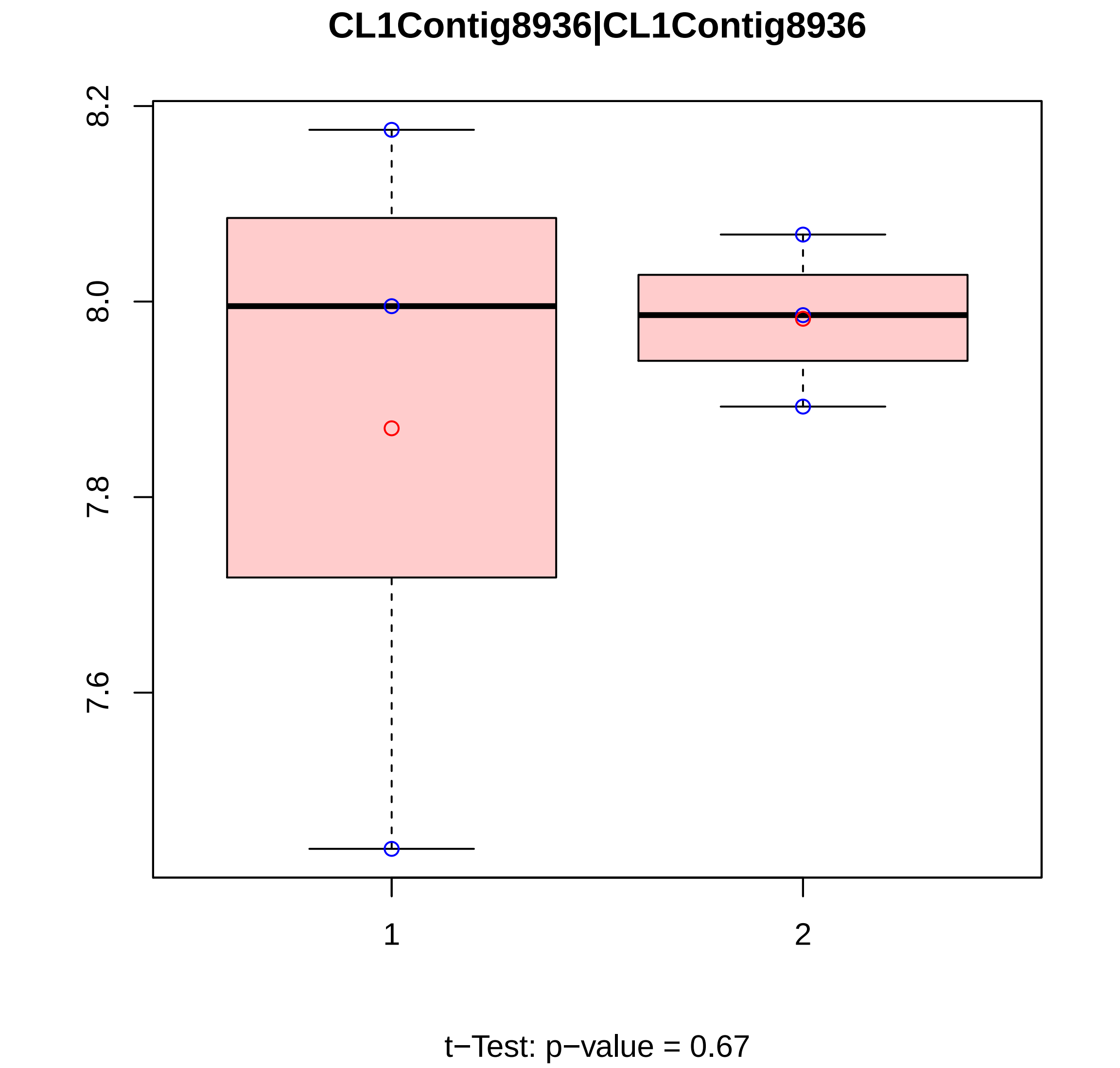
CL1Contig8936|CL1Contig8936
8.2
8.0
7.8
7.6
1
2
t−Test: p−value = 0.67

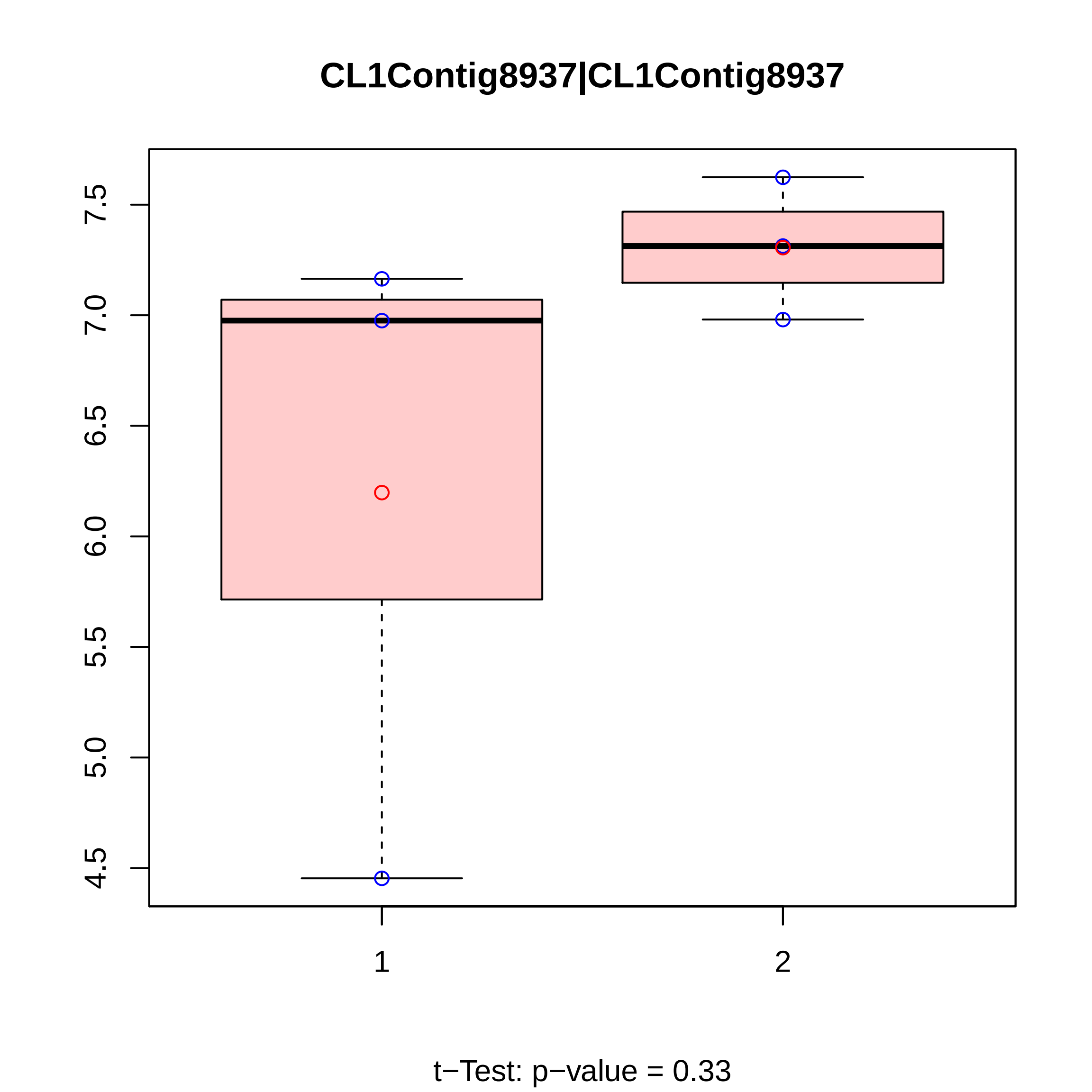

CL1Contig8937|CL1Contig8937
7.5
7.0
6.5
6.0
5.5
5.0
4.5
1
2
t−Test: p−value = 0.33

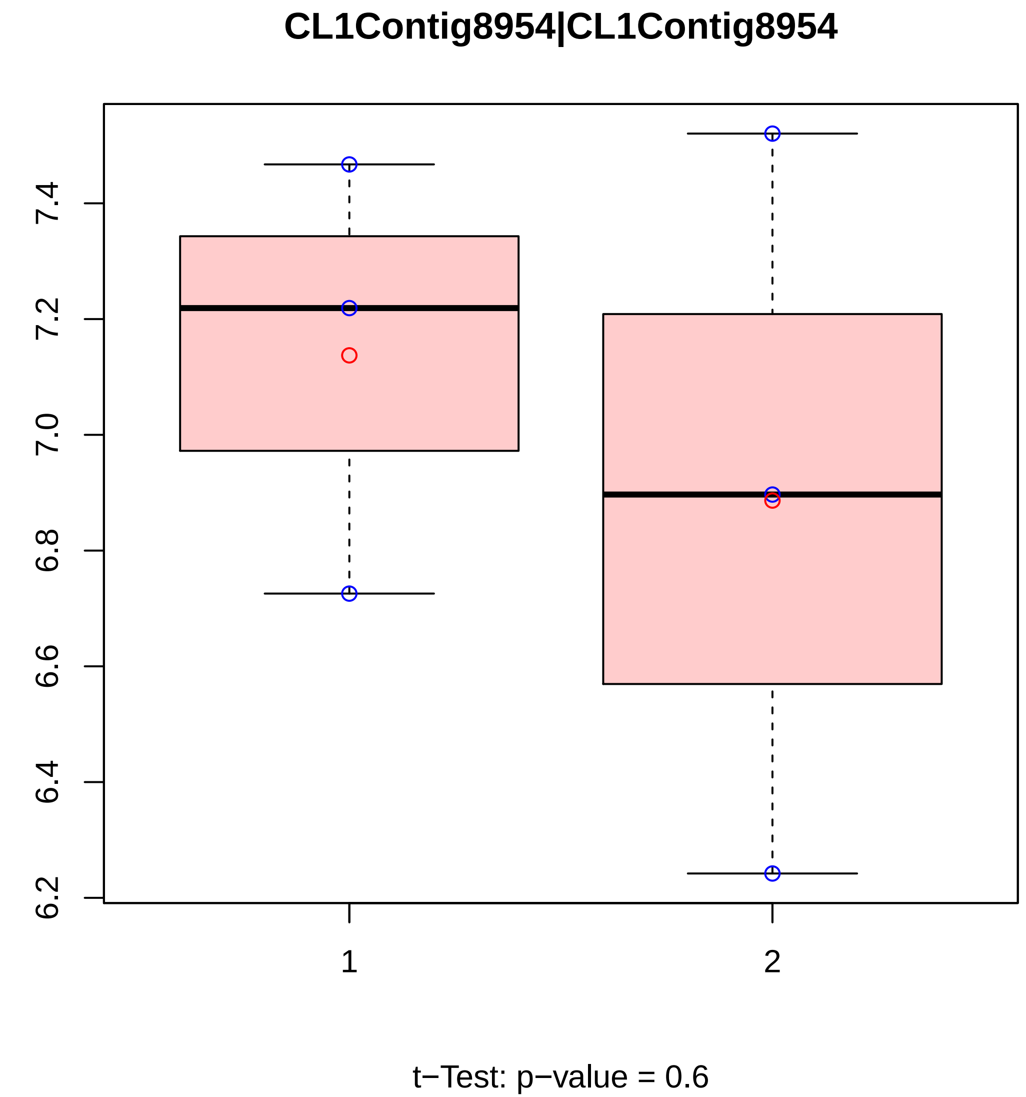
CL1Contig8954|CL1Contig8954
7.4
7.2
7.0
6.8
6.6
6.4
6.2
1
2
t−Test: p−value = 0.6

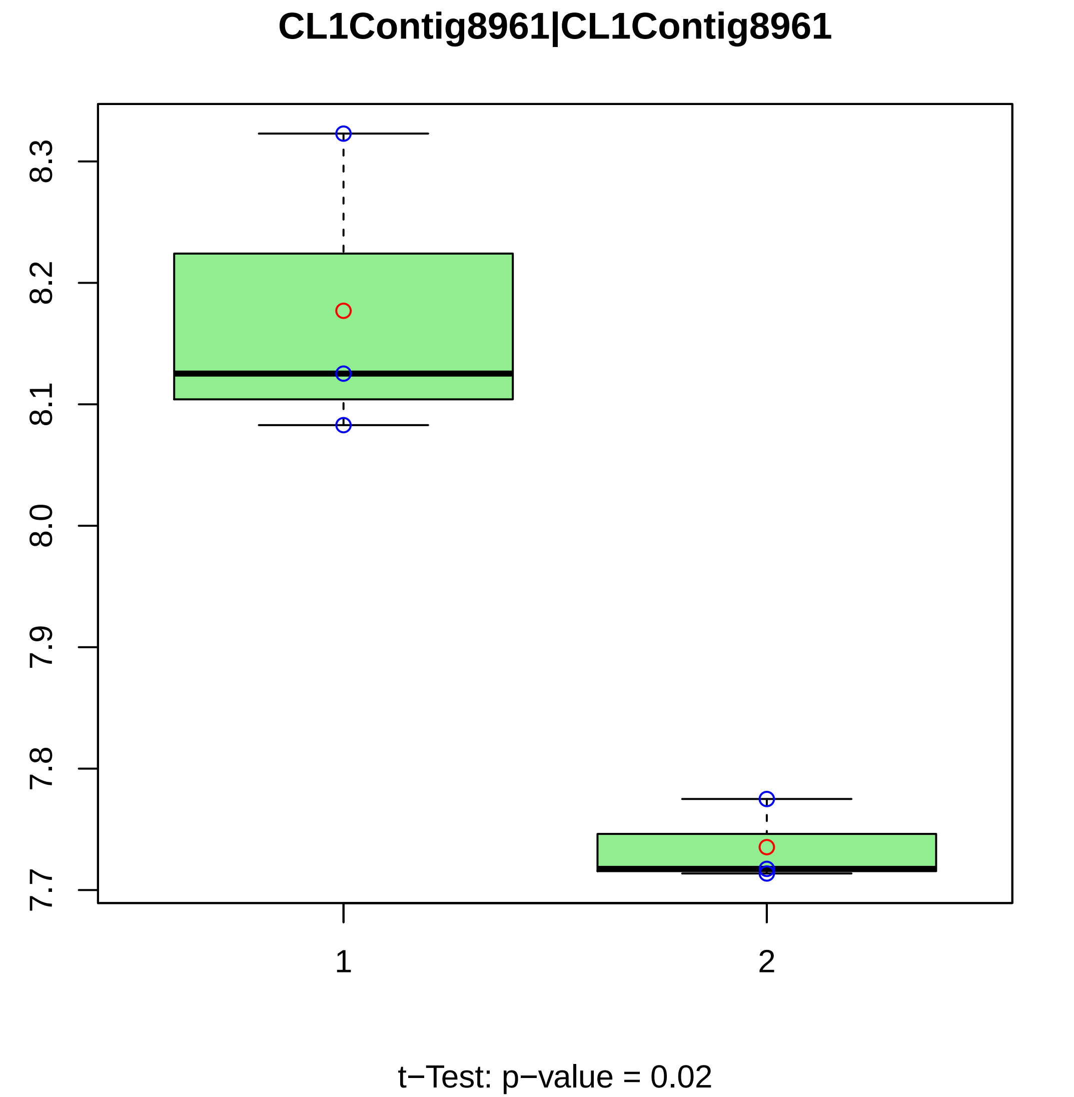
CL1Contig8961|CL1Contig8961
8.3
8.2
8.1
8.0
7.9
7.8
7.7
1
2
t−Test: p−value = 0.02

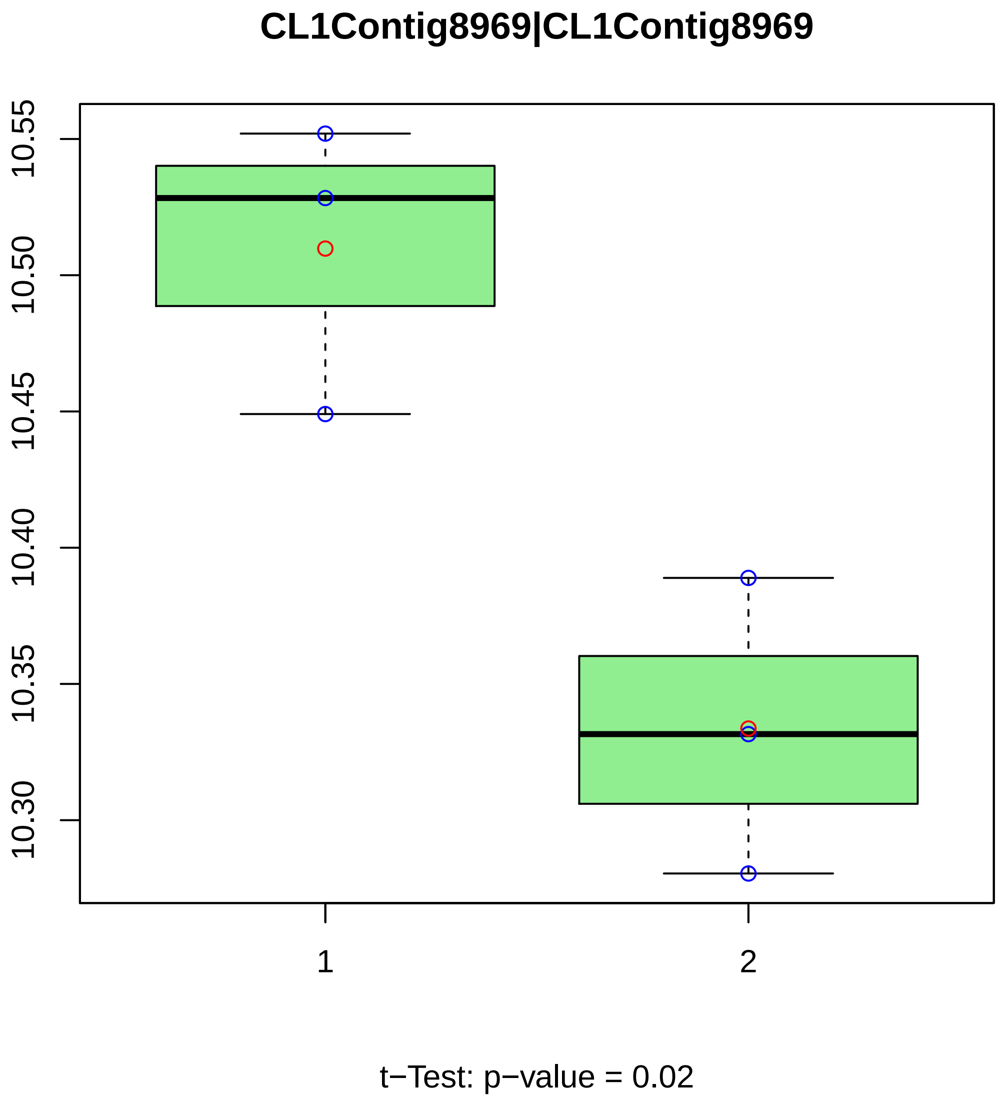

CL1Contig8969|CL1Contig8969
10.30
10.35
10.40
10.45
10.50
10.55
1
2
t−Test: p−value = 0.02

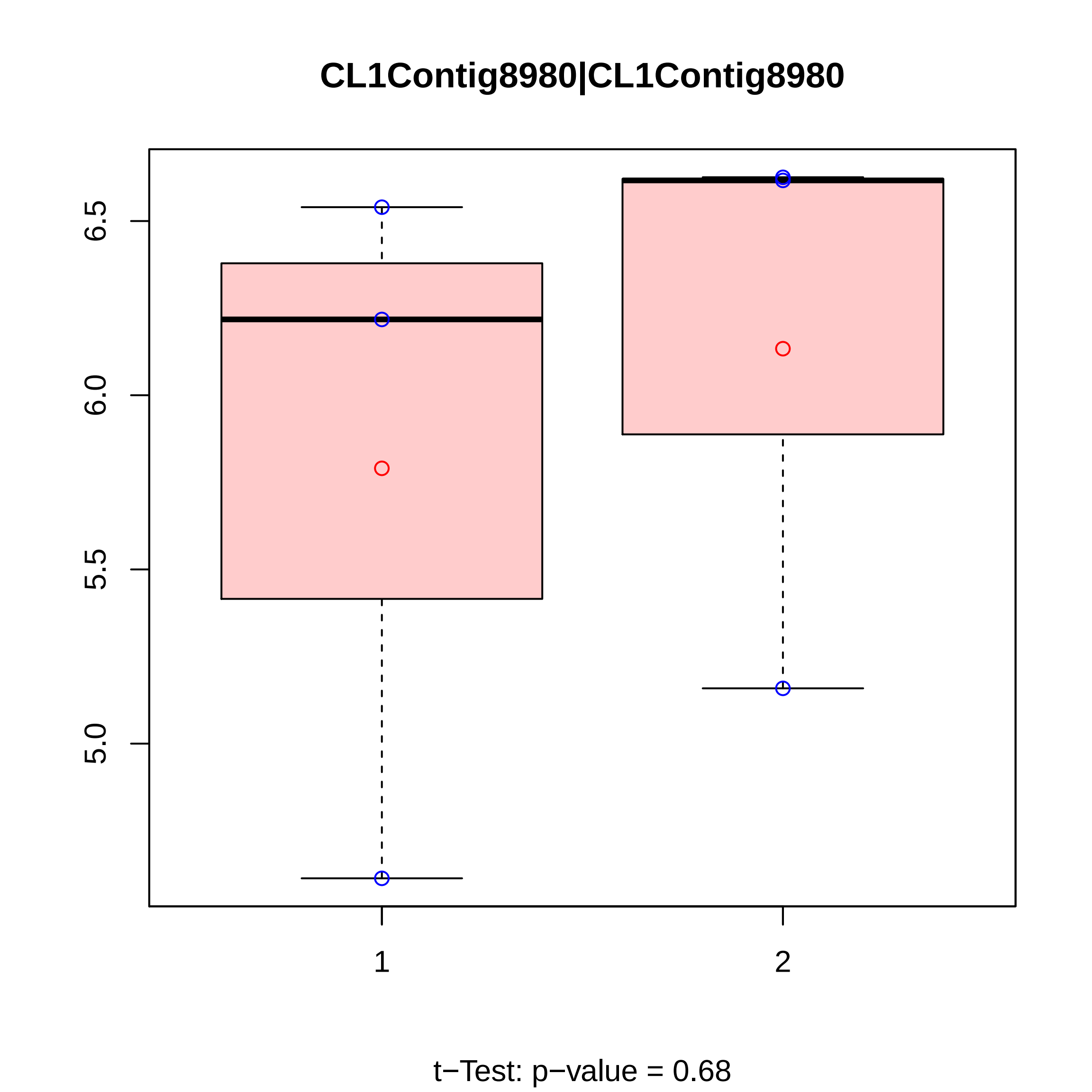

CL1Contig8980|CL1Contig8980
6.5
6.0
5.5
5.0
1
2
t−Test: p−value = 0.68

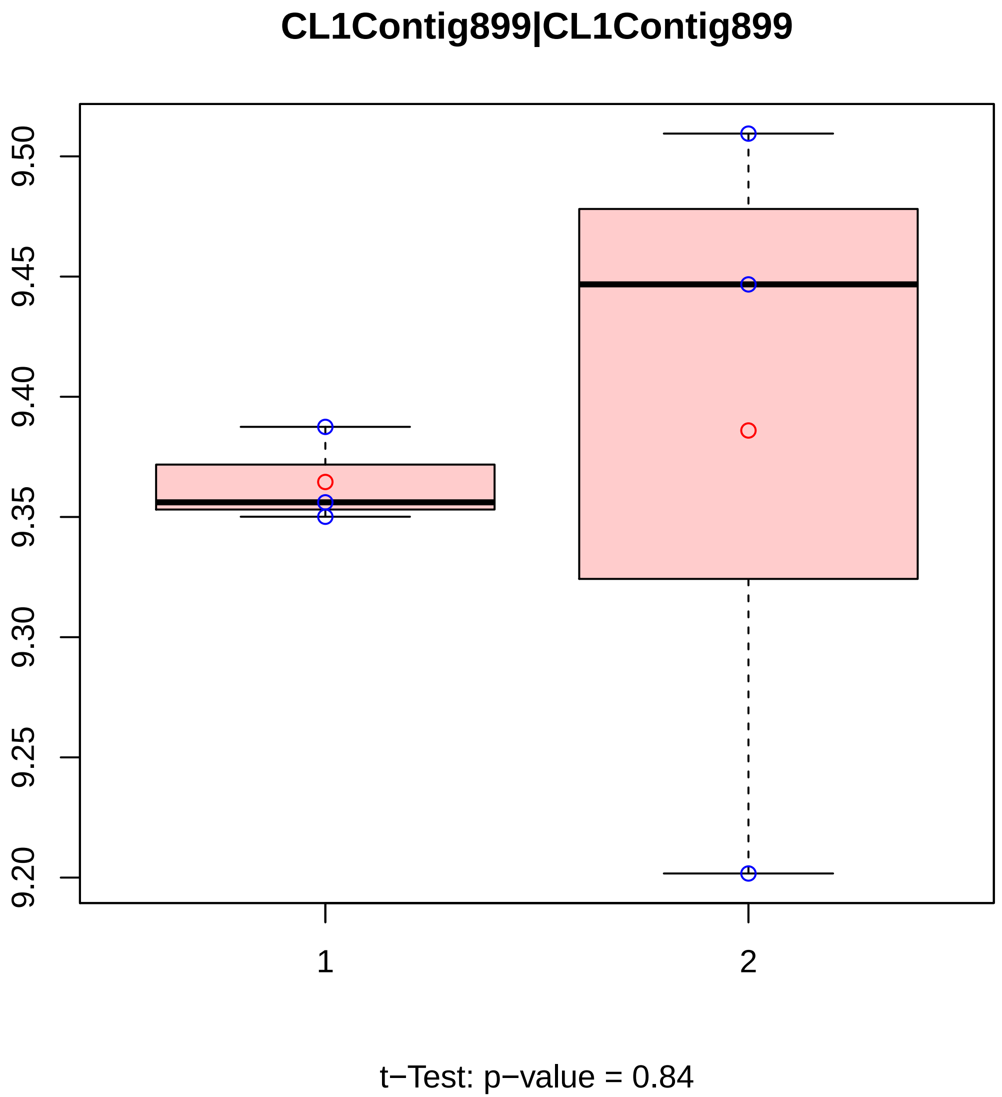

CL1Contig899|CL1Contig899
9.20
9.25
9.30
9.35
9.40
9.45
9.50
1
2
t−Test: p−value = 0.84

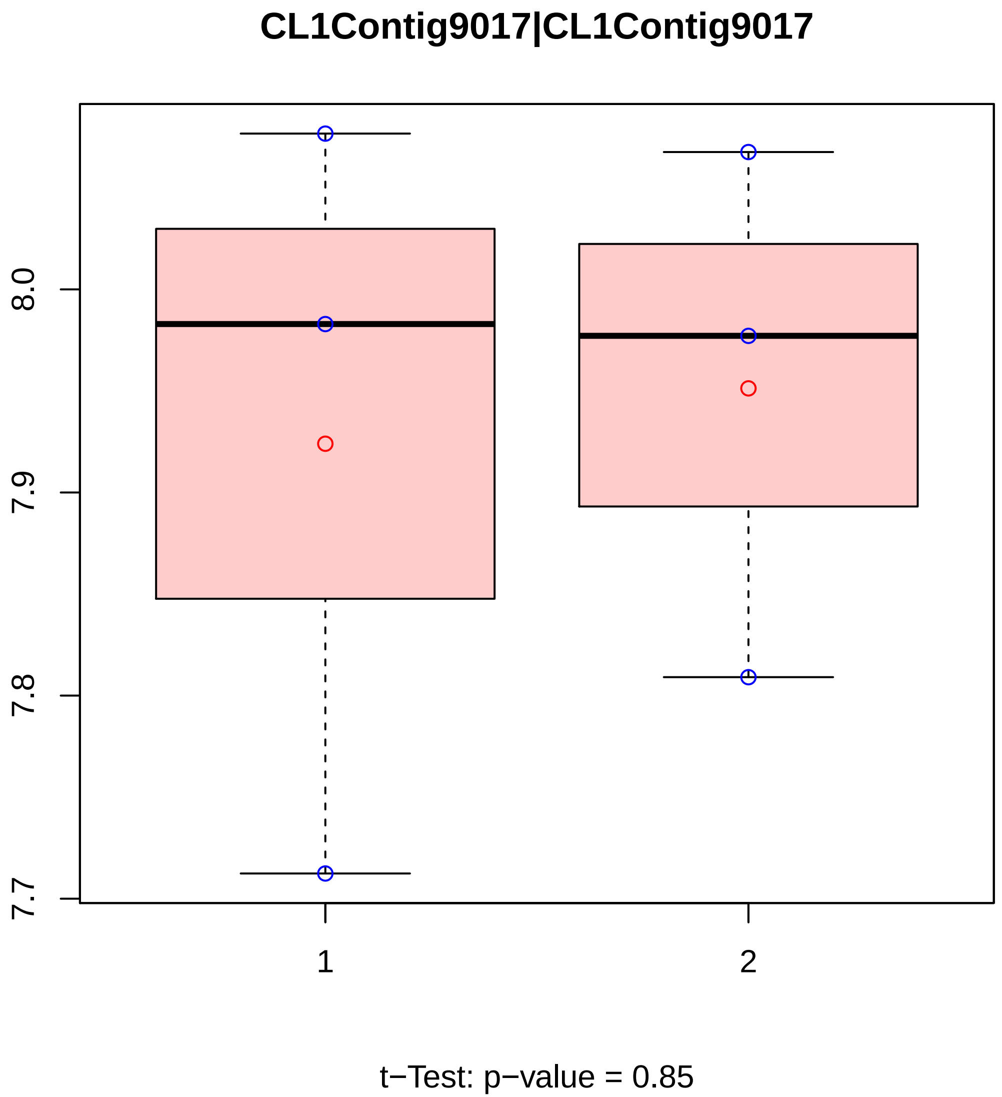
CL1Contig9017|CL1Contig9017
7.7
7.8
7.9
8.0
1
2
t−Test: p−value = 0.85

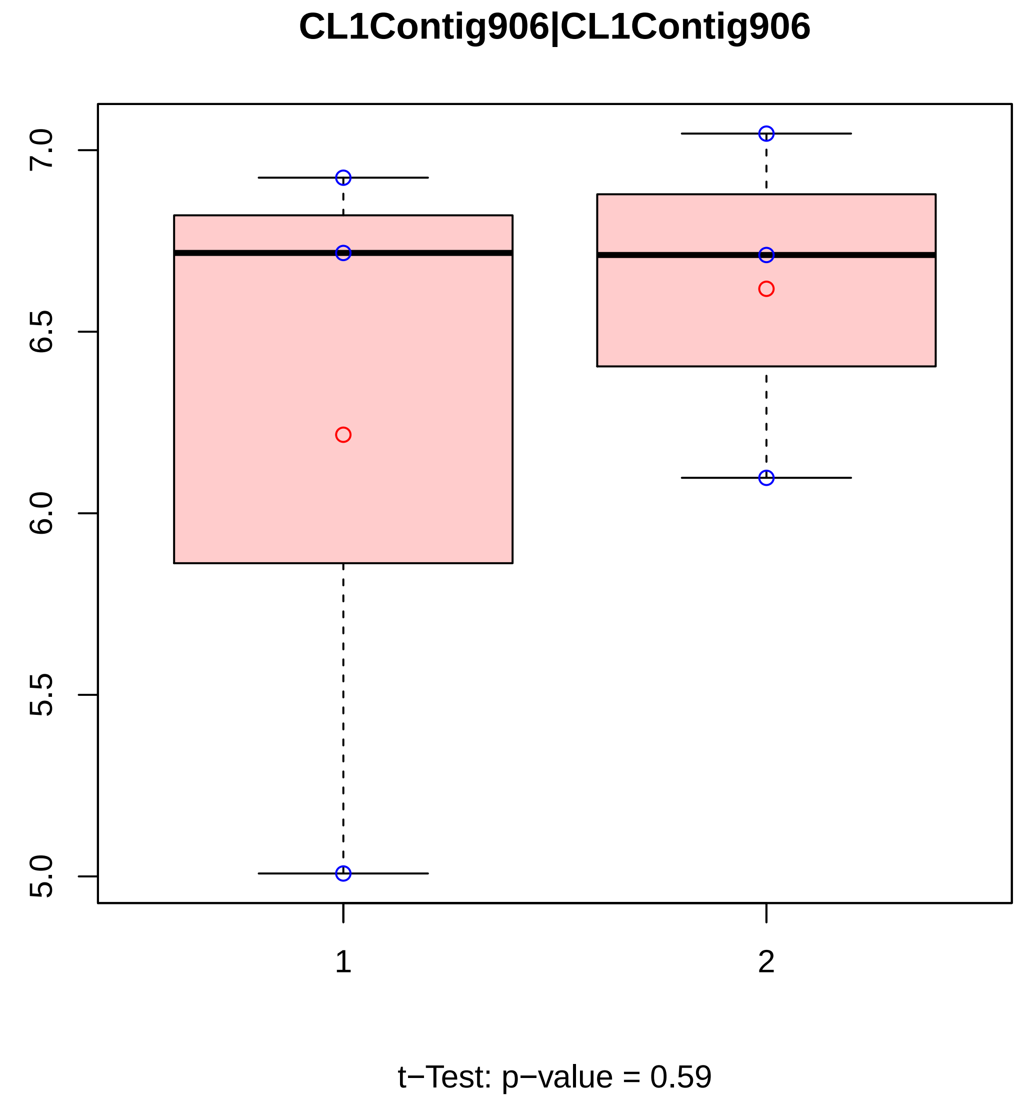
CL1Contig906|CL1Contig906
7.0
6.5
6.0
5.5
5.0
1
2
t−Test: p−value = 0.59

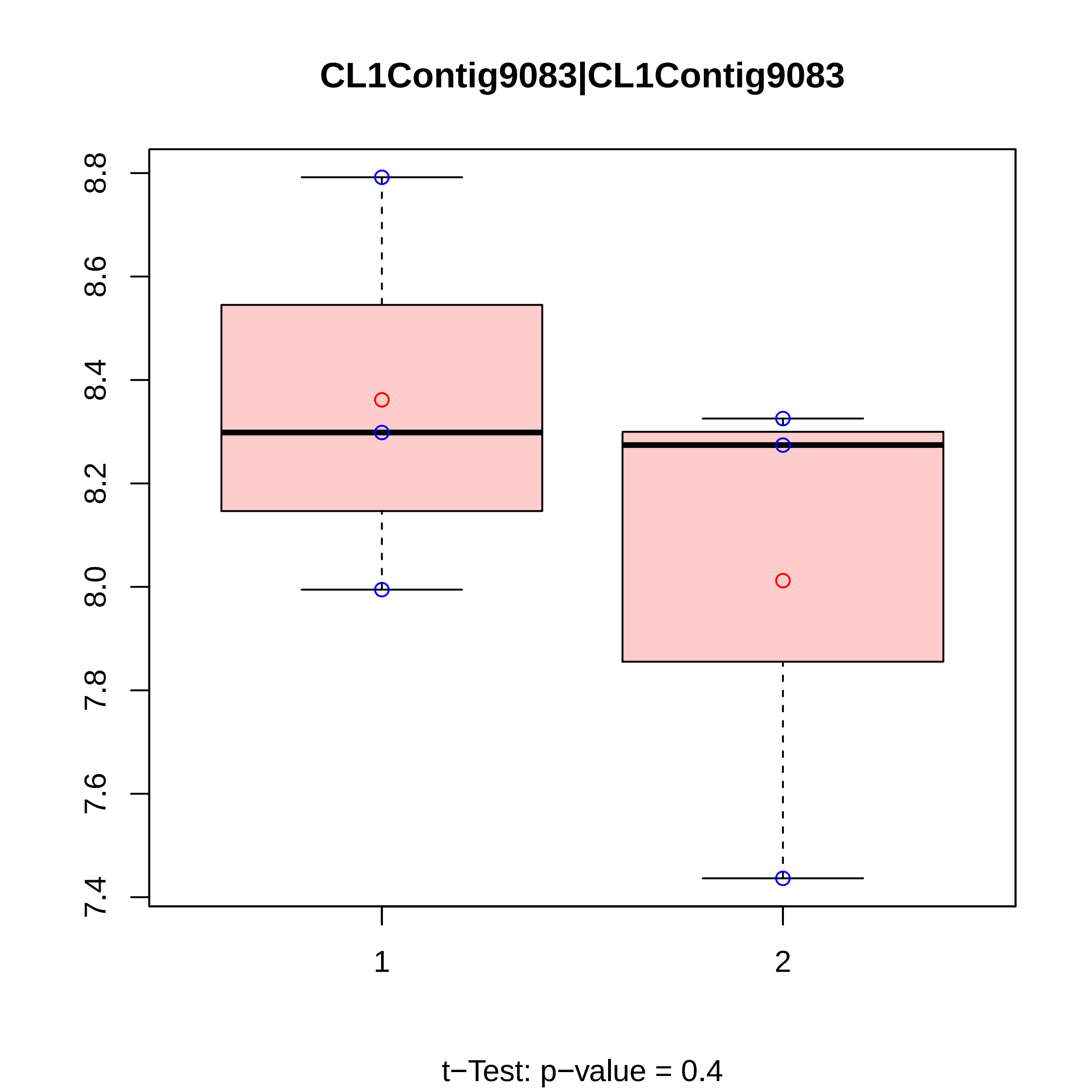

CL1Contig9083|CL1Contig9083
8.8
8.6
8.4
8.2
8.0
7.8
7.6
7.4
1
2
t−Test: p−value = 0.4

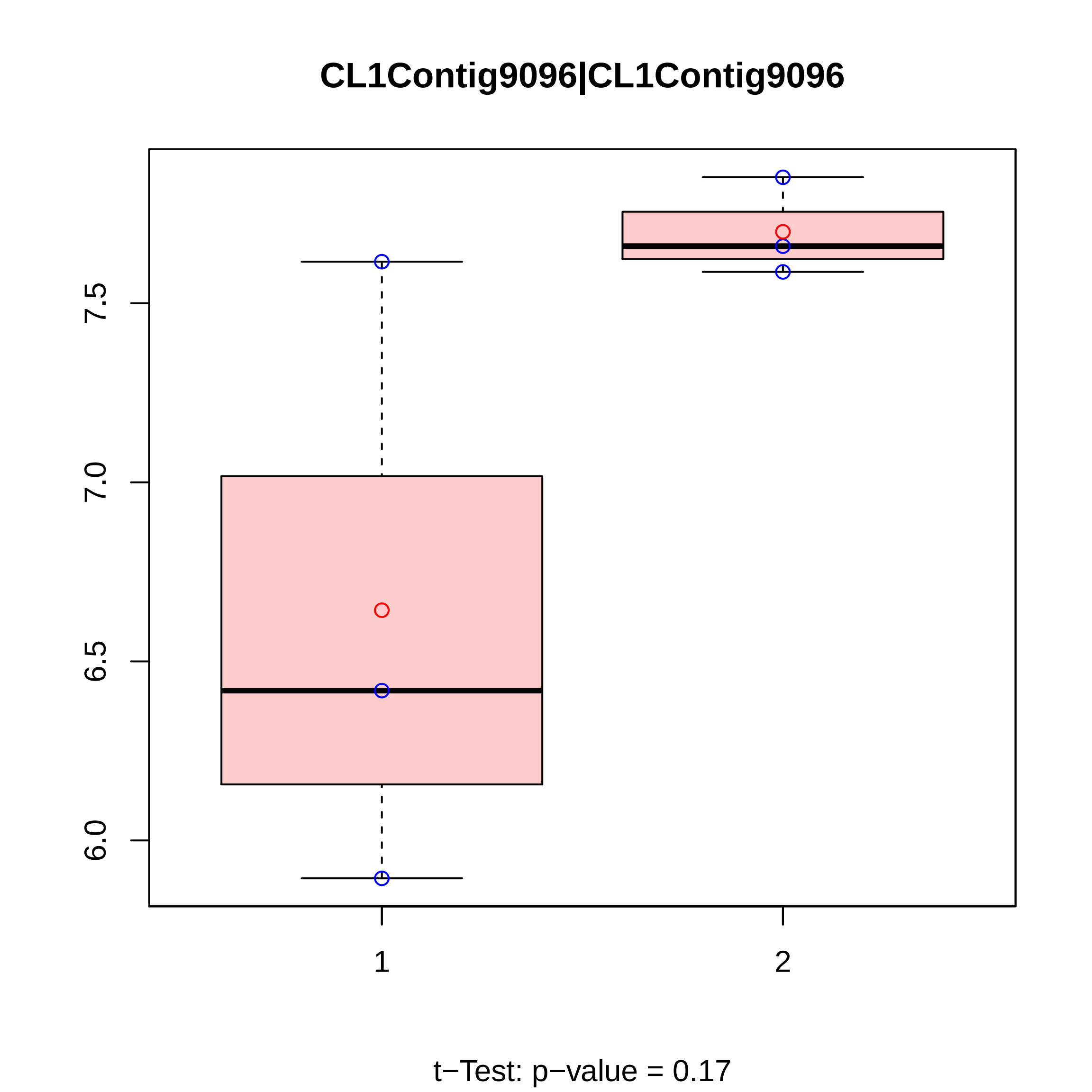
CL1Contig9096|CL1Contig9096
7.5
7.0
6.5
6.0
1
2
t−Test: p−value = 0.17

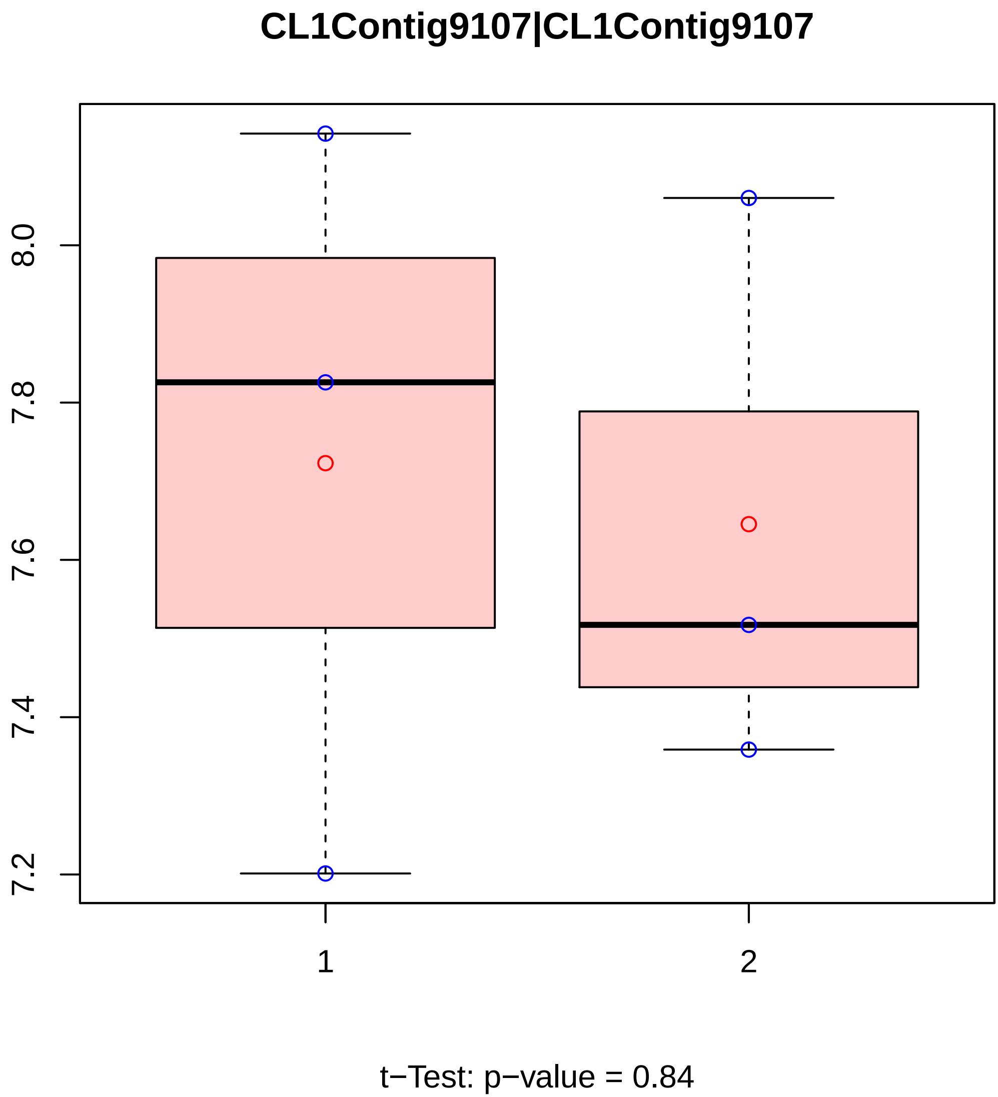

CL1Contig9107|CL1Contig9107
8.0
7.8
7.6
7.4
7.2
1
2
t−Test: p−value = 0.84

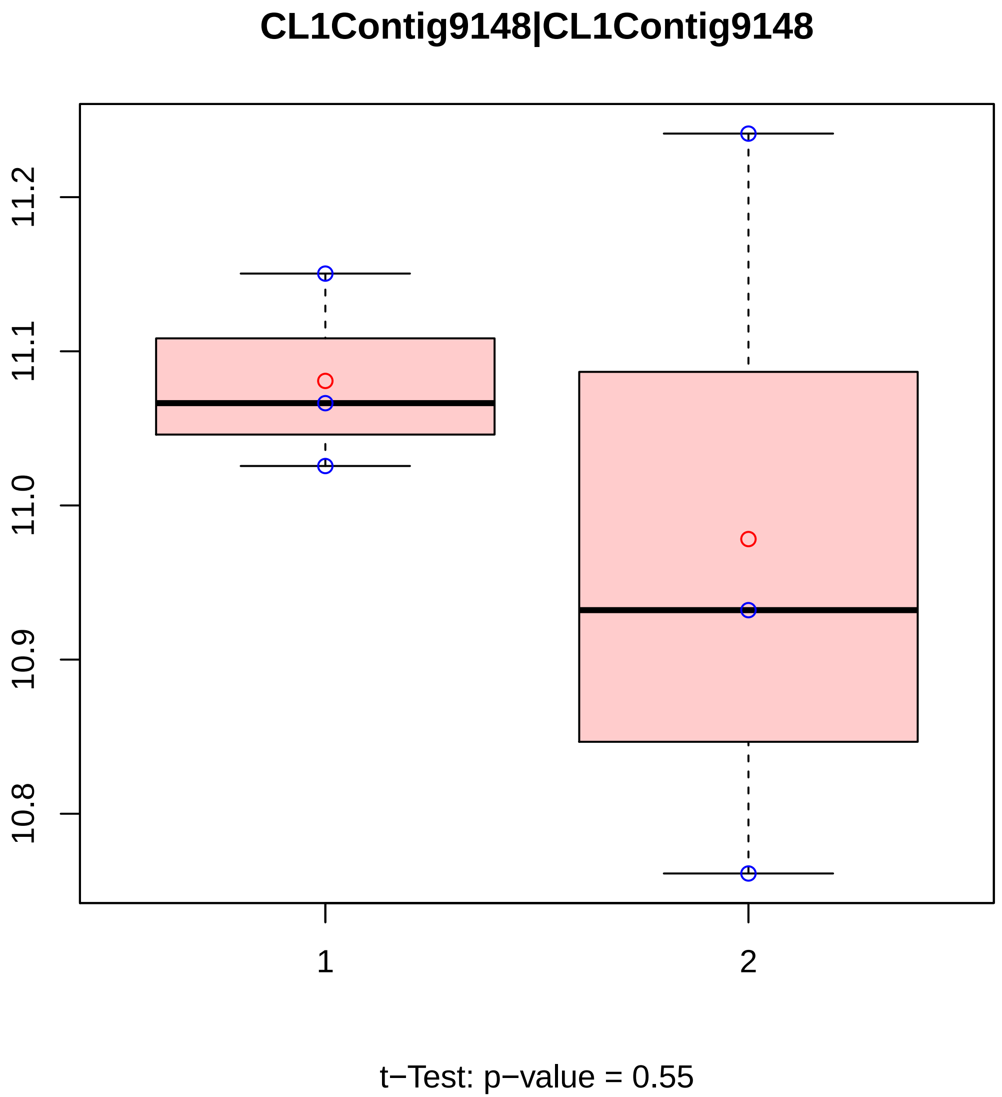
CL1Contig9148|CL1Contig9148
11.2
11.1
11.0
10.9
10.8
1
2
t−Test: p−value = 0.55

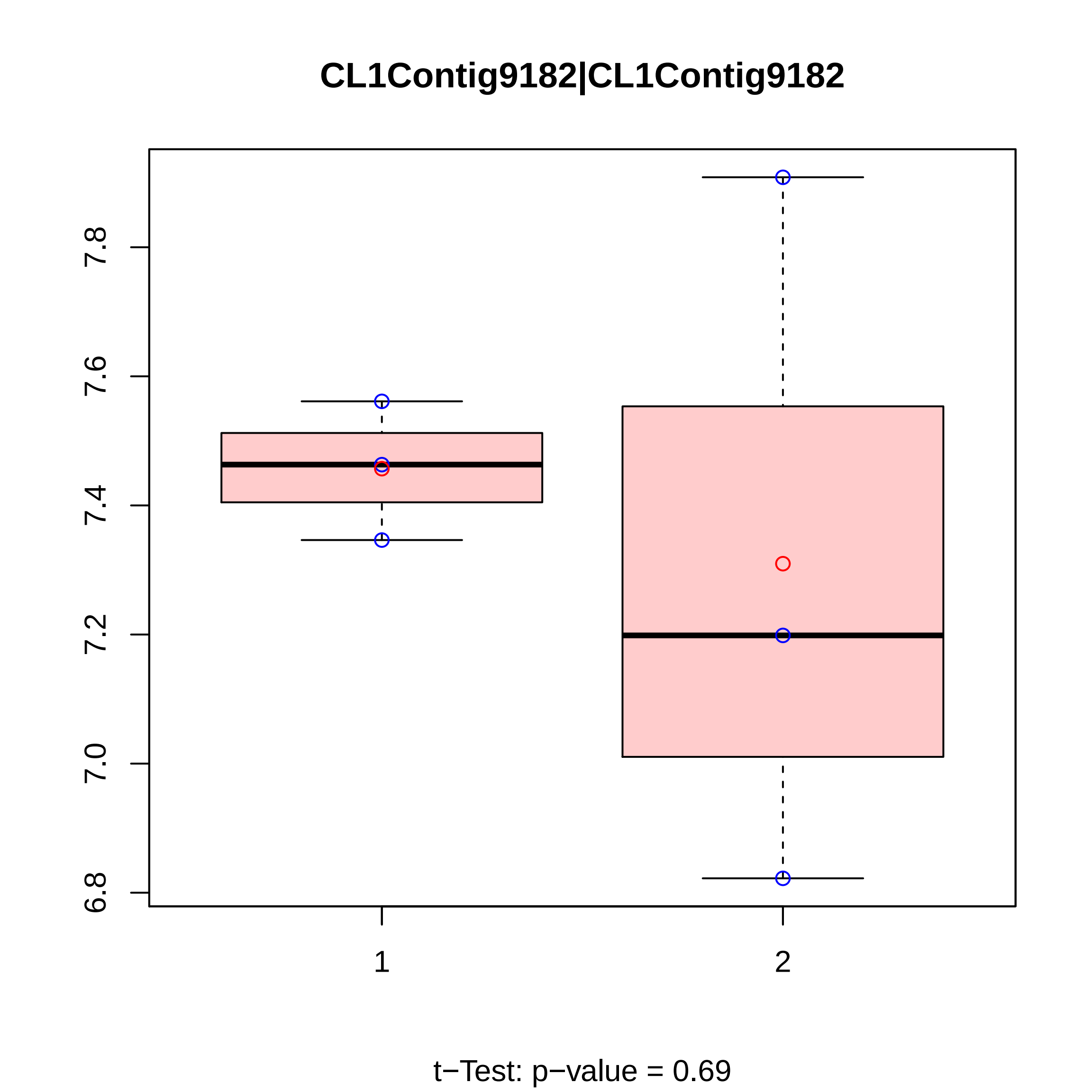

CL1Contig9182|CL1Contig9182
7.8
7.6
7.4
7.2
7.0
6.8
1
2
t−Test: p−value = 0.69

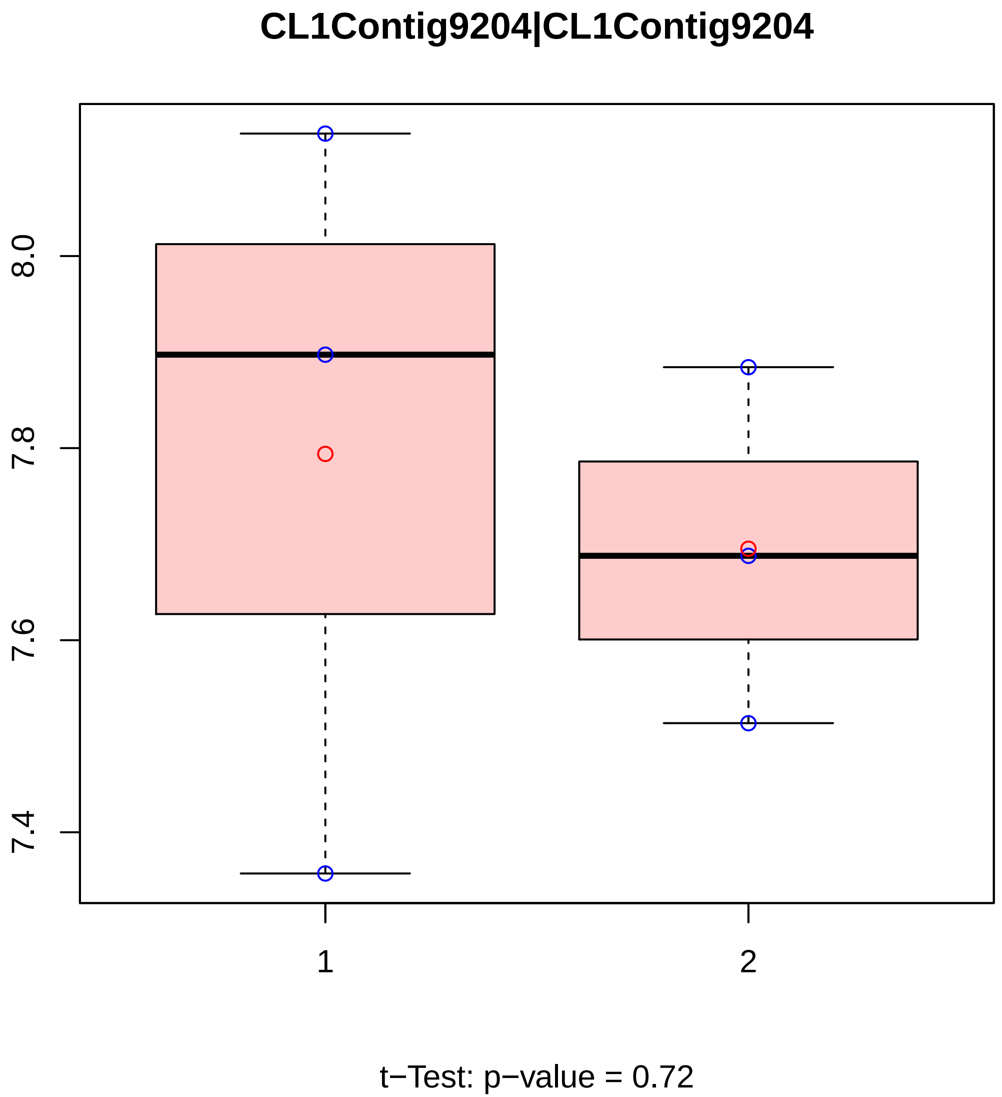
CL1Contig9204|CL1Contig9204
8.0
7.8
7.6
7.4
1
2
t−Test: p−value = 0.72

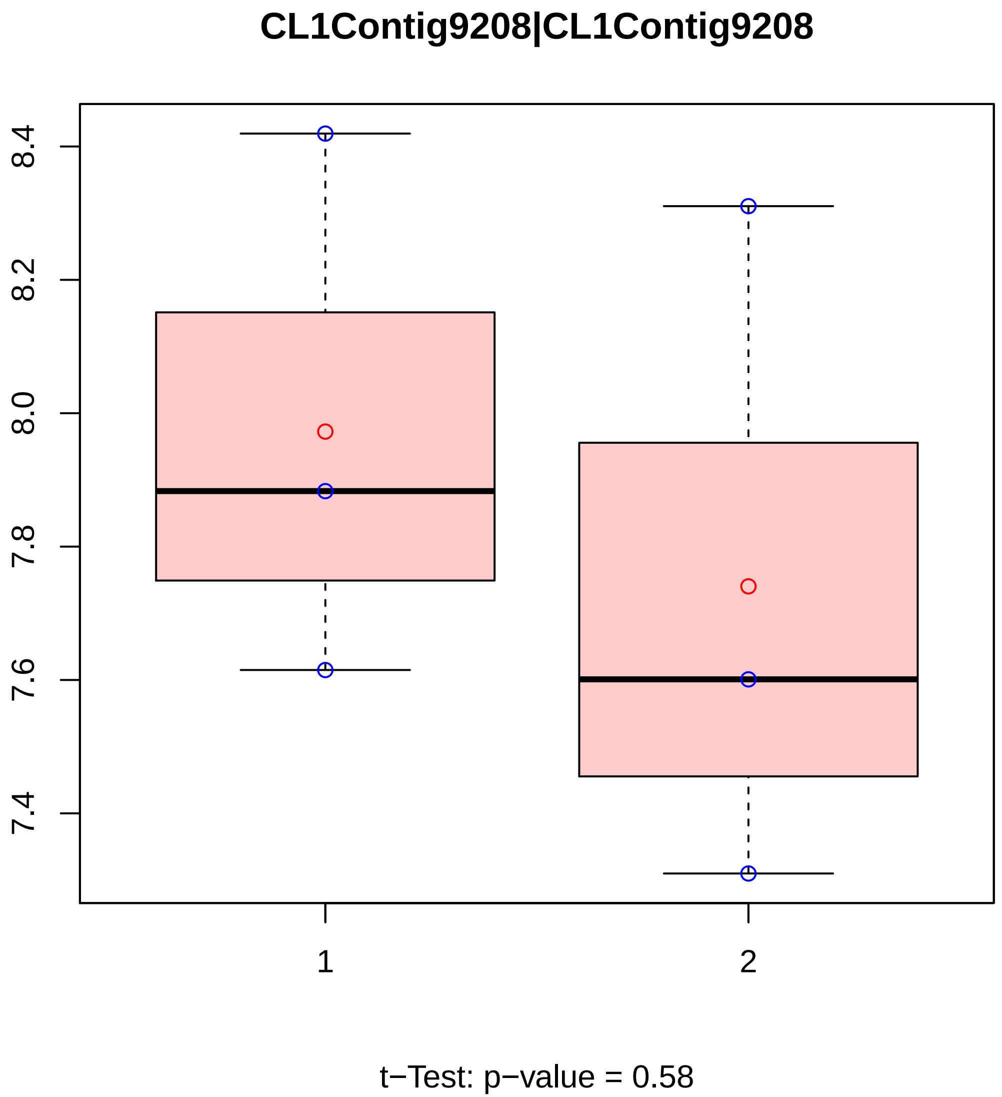
CL1Contig9208|CL1Contig9208
8.4
8.2
8.0
7.8
7.6
7.4
1
2
t−Test: p−value = 0.58

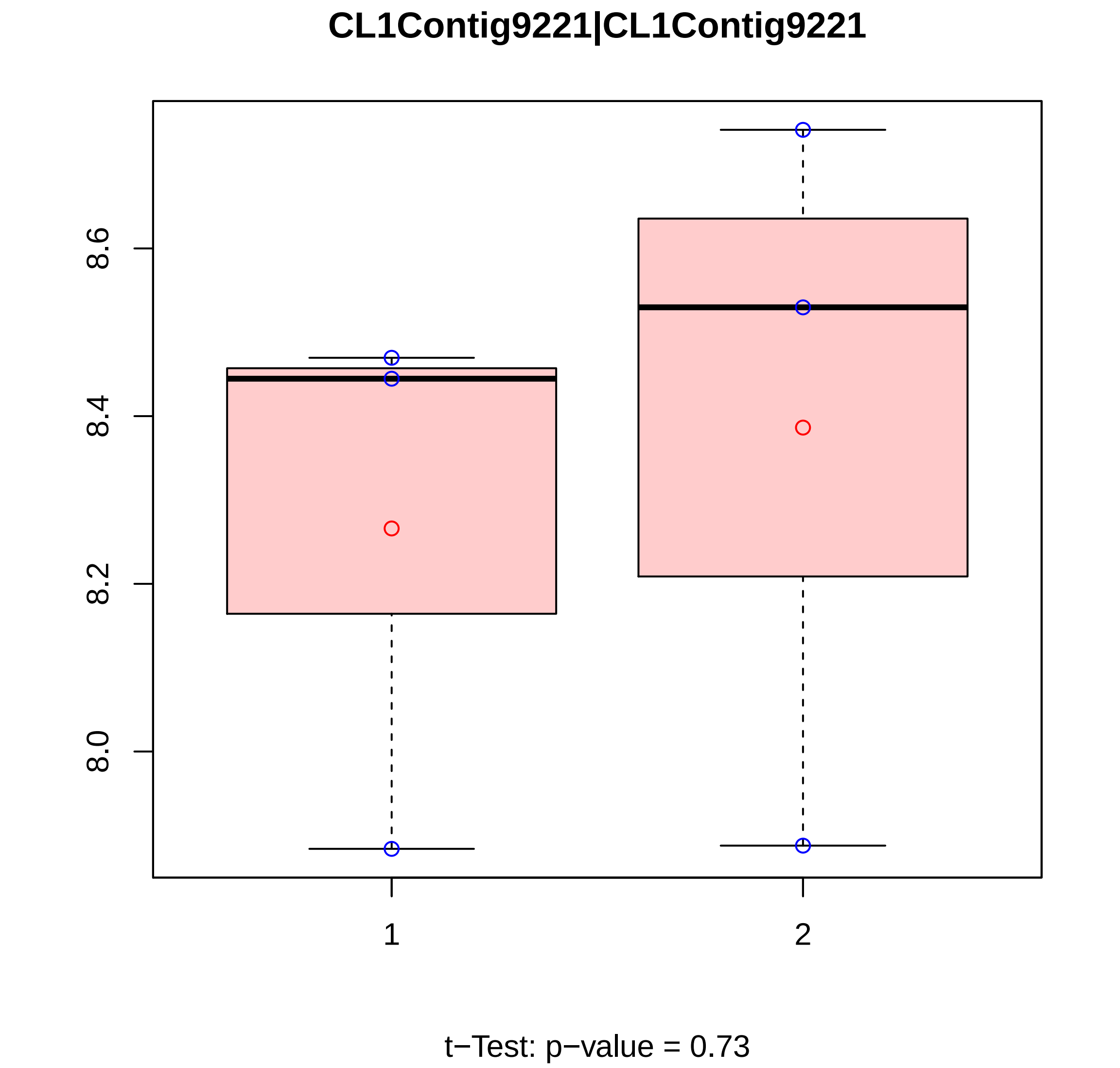
CL1Contig9221|CL1Contig9221
8.0
8.2
8.4
8.6
1
2
t−Test: p−value = 0.73

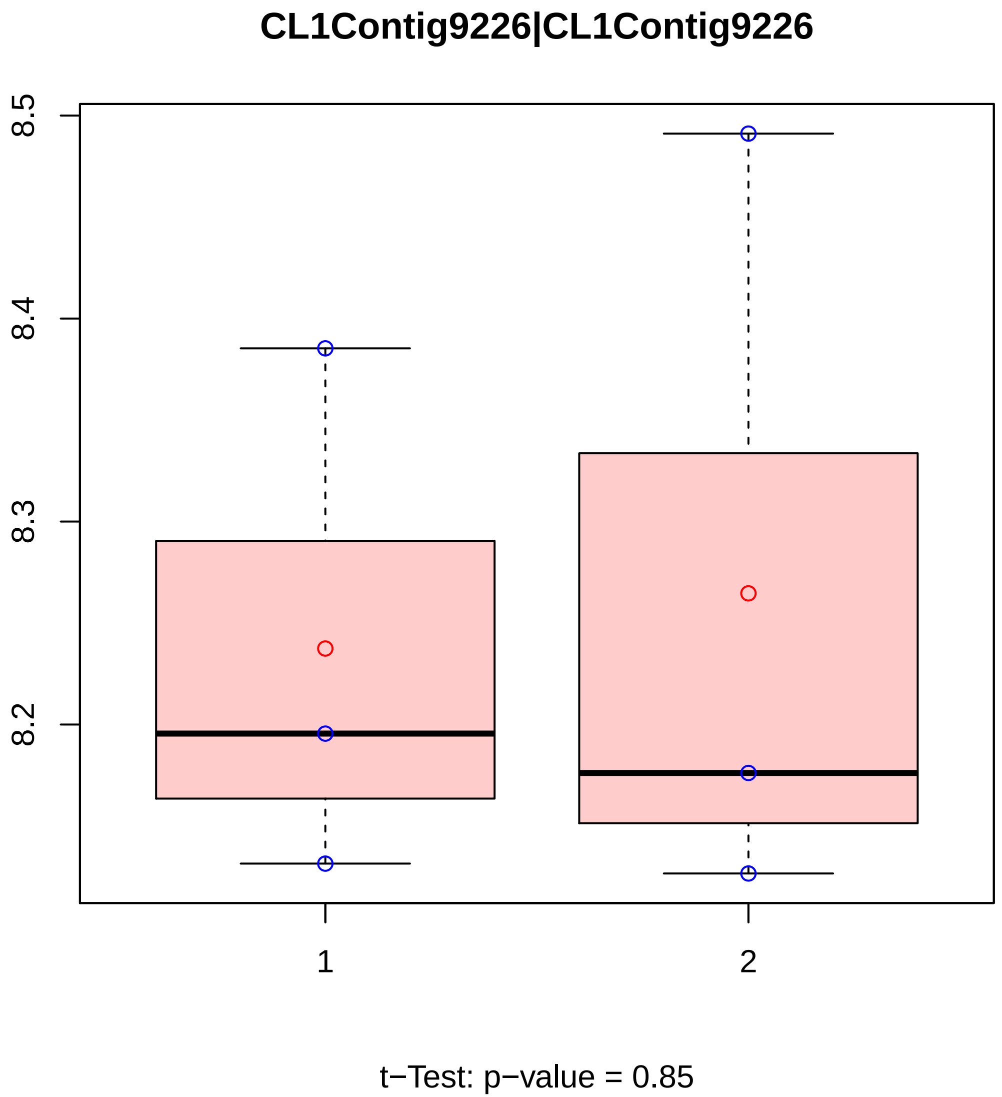
CL1Contig9226|CL1Contig9226
8.5
8.4
8.3
8.2
1
2
t−Test: p−value = 0.85

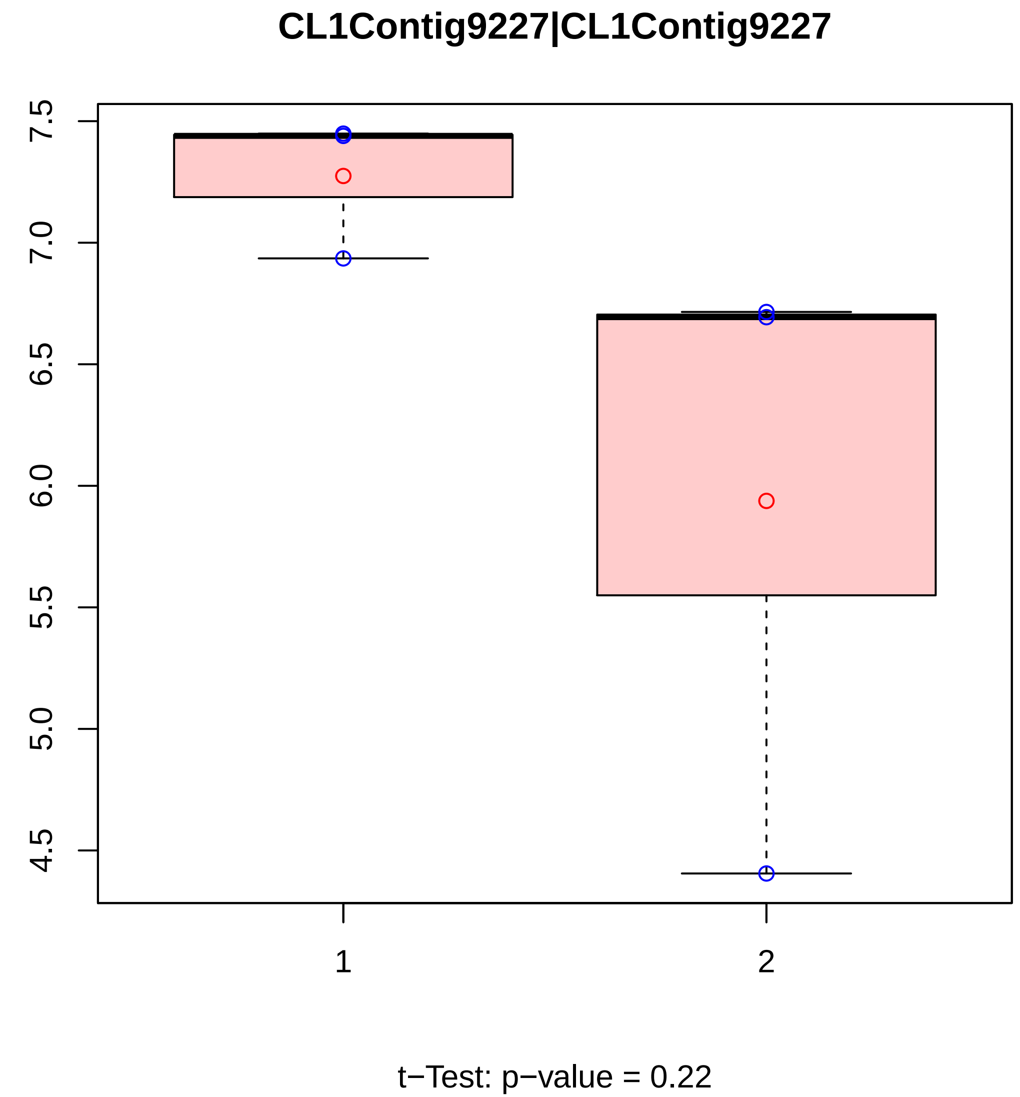

CL1Contig9227|CL1Contig9227
7.5
7.0
6.5
6.0
5.5
5.0
4.5
1
2
t−Test: p−value = 0.22

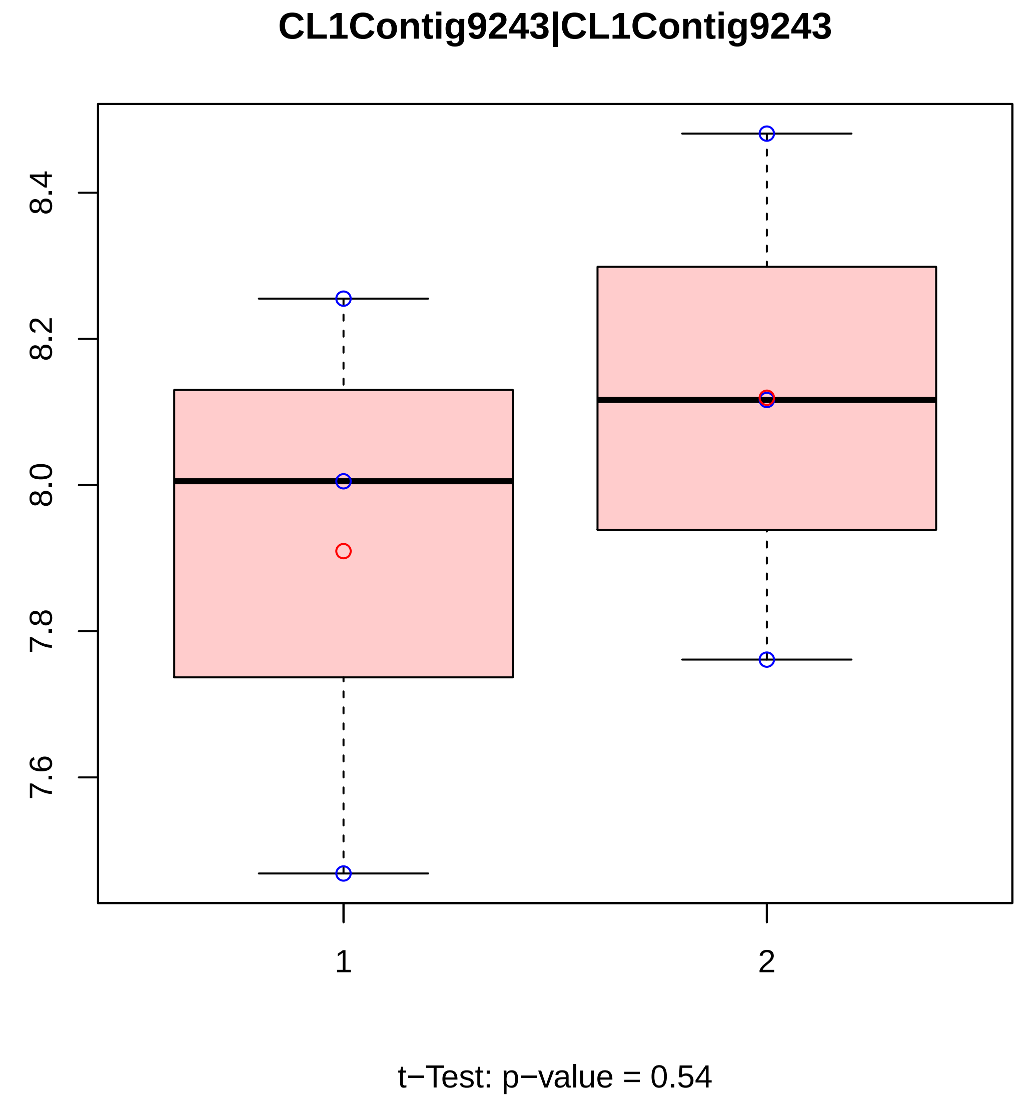

CL1Contig9243|CL1Contig9243
8.4
8.2
8.0
7.8
7.6
1
2
t−Test: p−value = 0.54

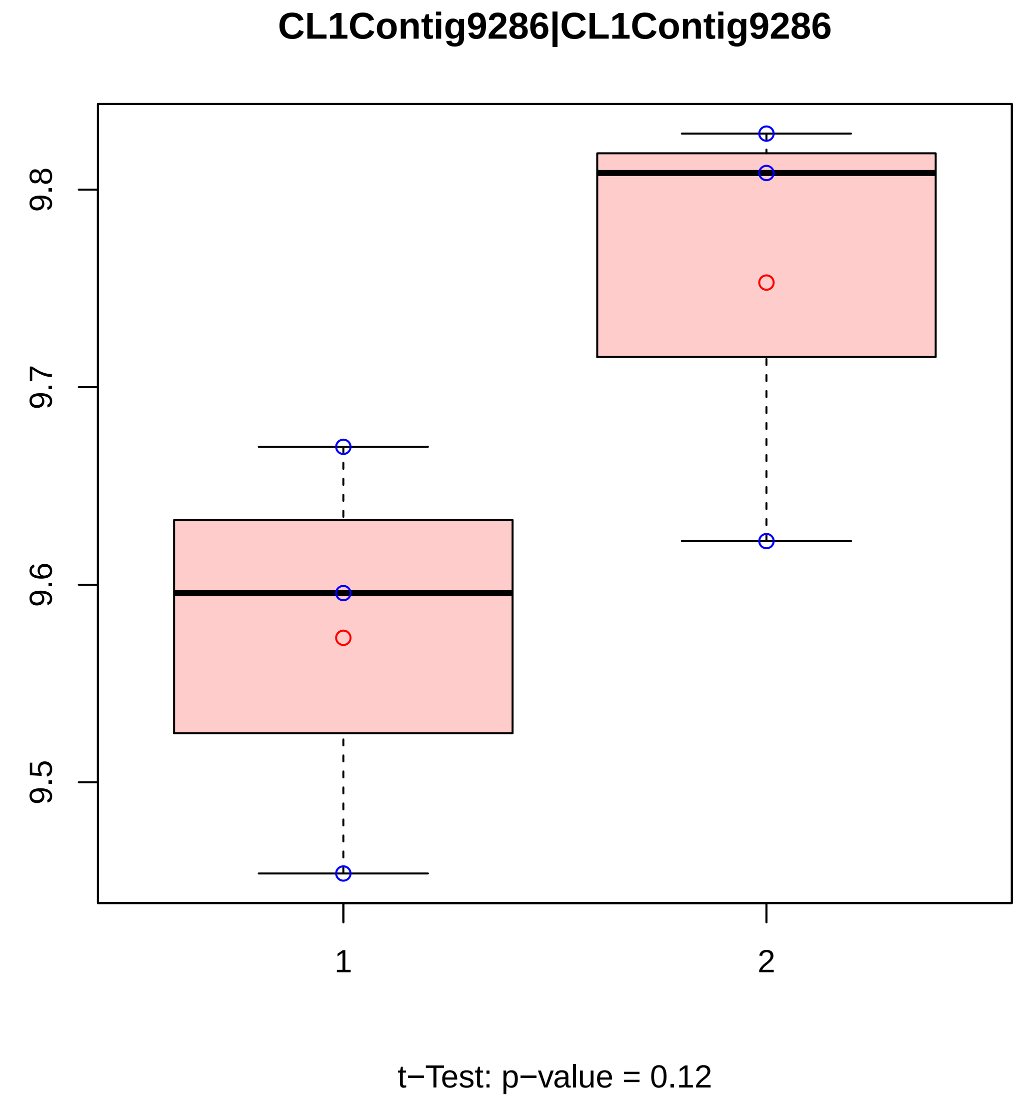
CL1Contig9286|CL1Contig9286
9.5
9.6
9.7
9.8
1
2
t−Test: p−value = 0.12

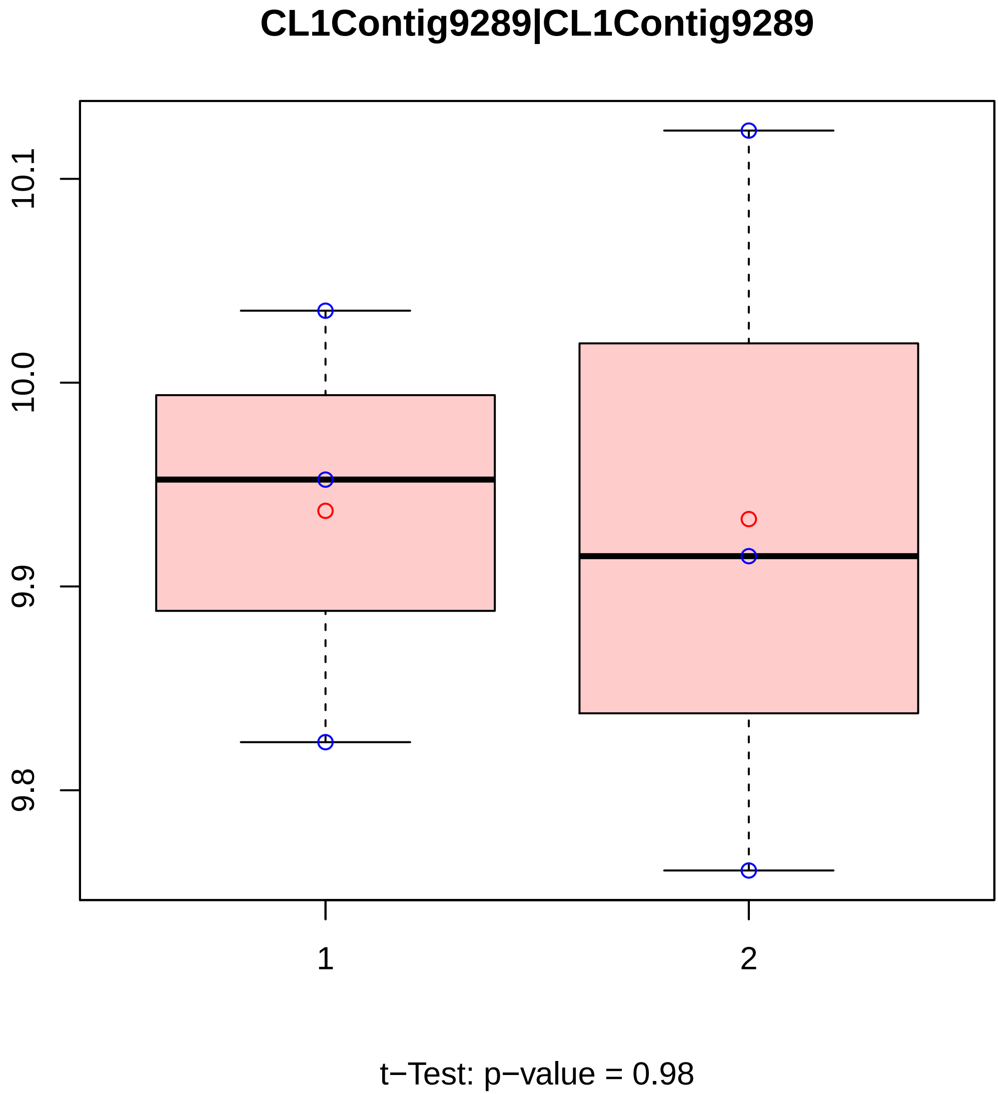
CL1Contig9289|CL1Contig9289
10.1
10.0
9.9
9.8
1
2
t−Test: p−value = 0.98

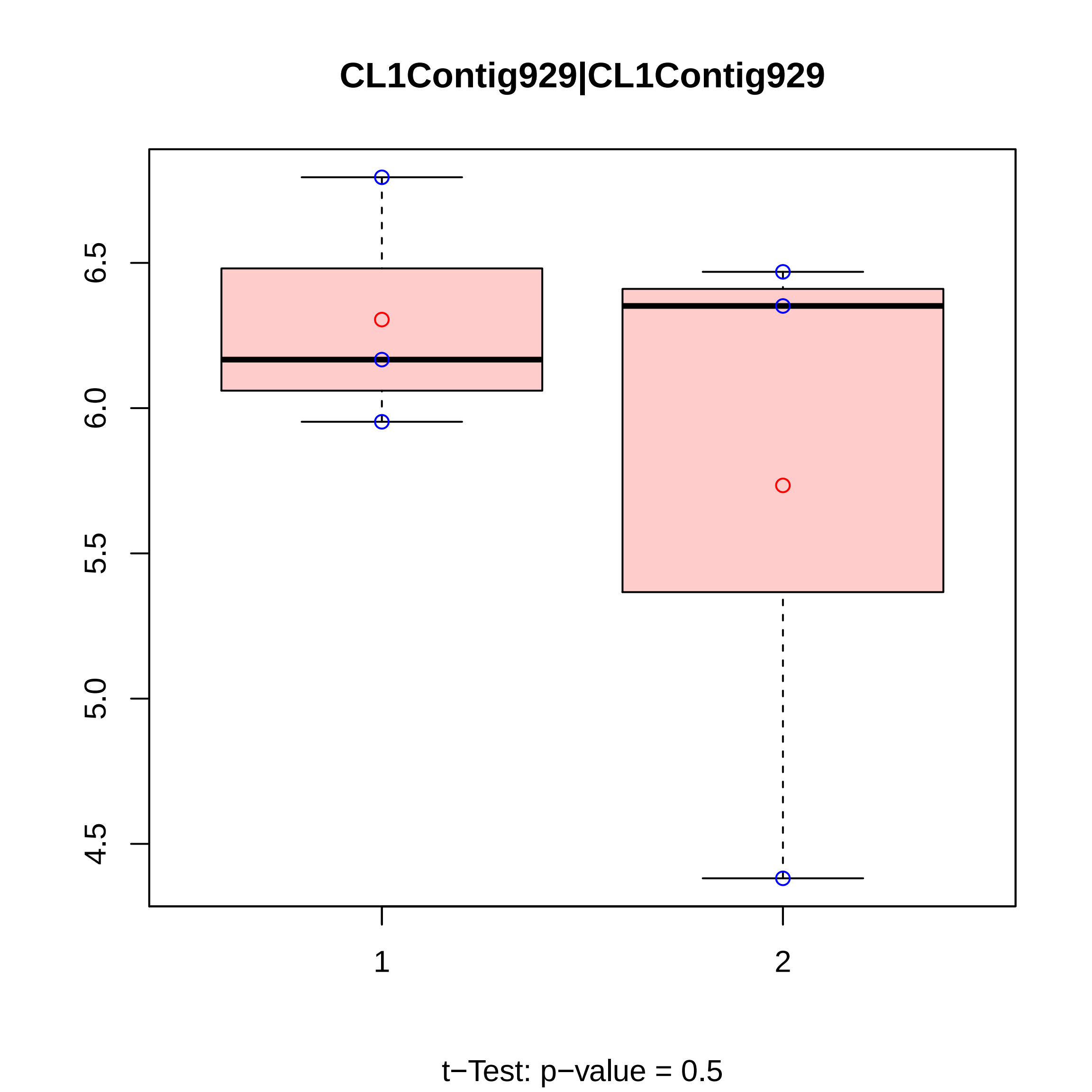
CL1Contig929|CL1Contig929
6.5
6.0
5.5
5.0
4.5
1
2
t−Test: p−value = 0.5

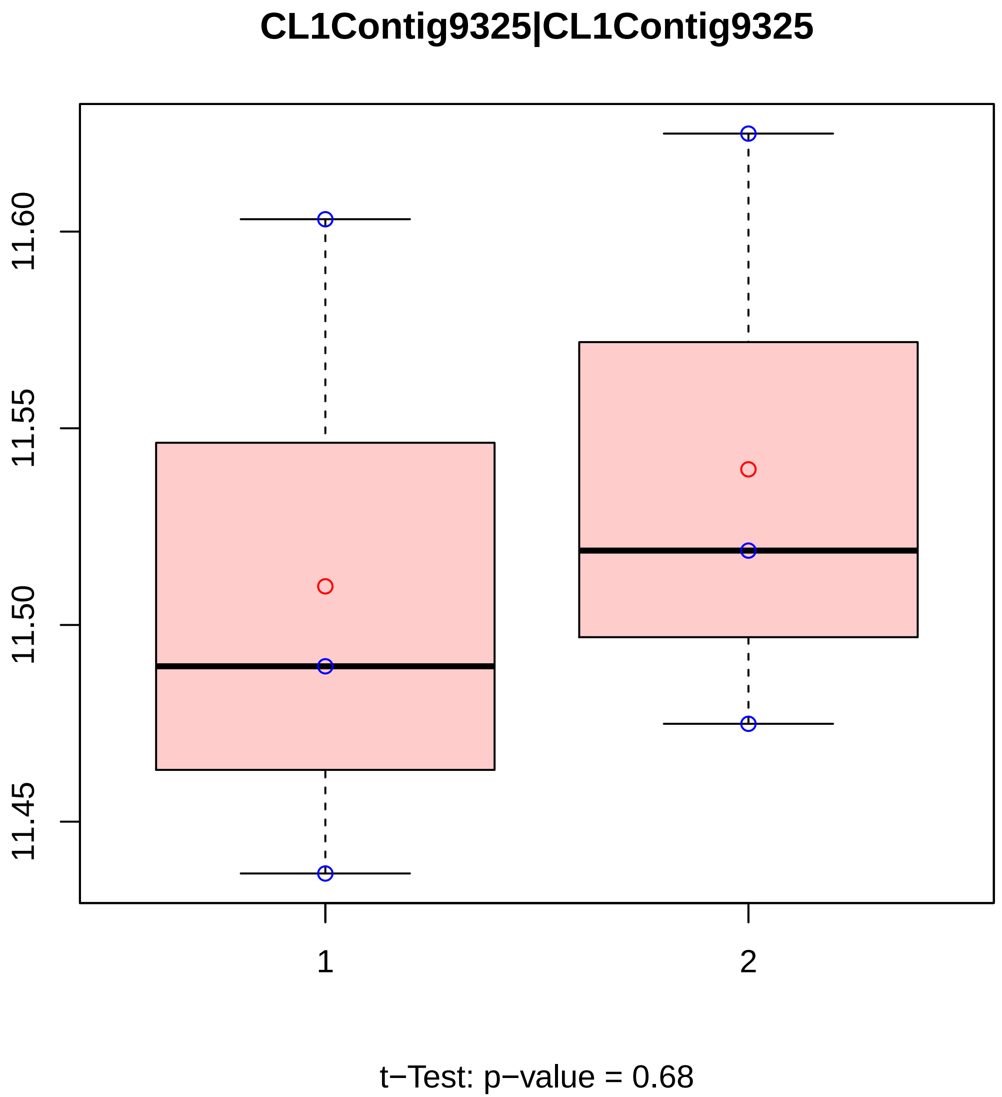
CL1Contig9325|CL1Contig9325
11.45
11.50
11.55
11.60
1
2
t−Test: p−value = 0.68

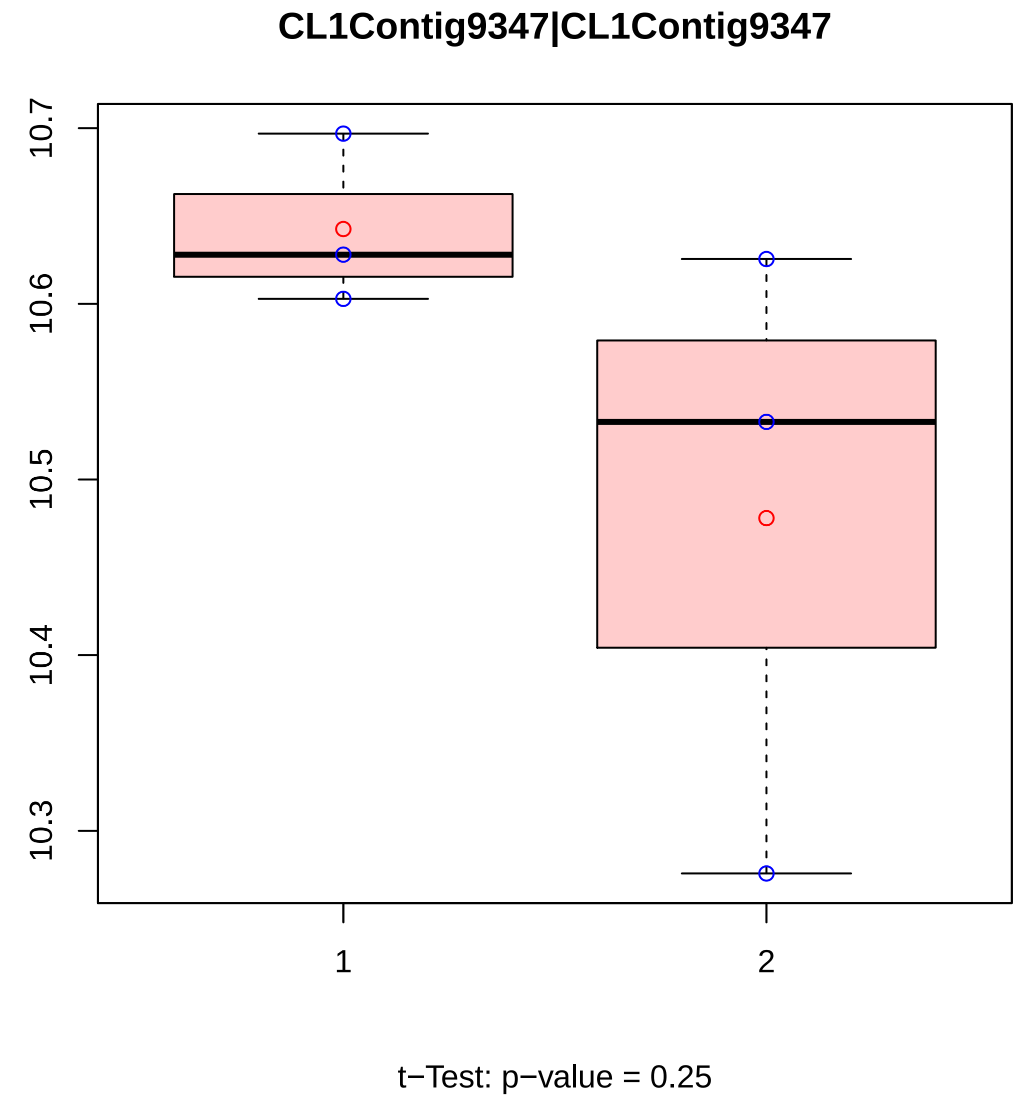

CL1Contig9347|CL1Contig9347
10.7
10.6
10.5
10.4
10.3
1
2
t−Test: p−value = 0.25

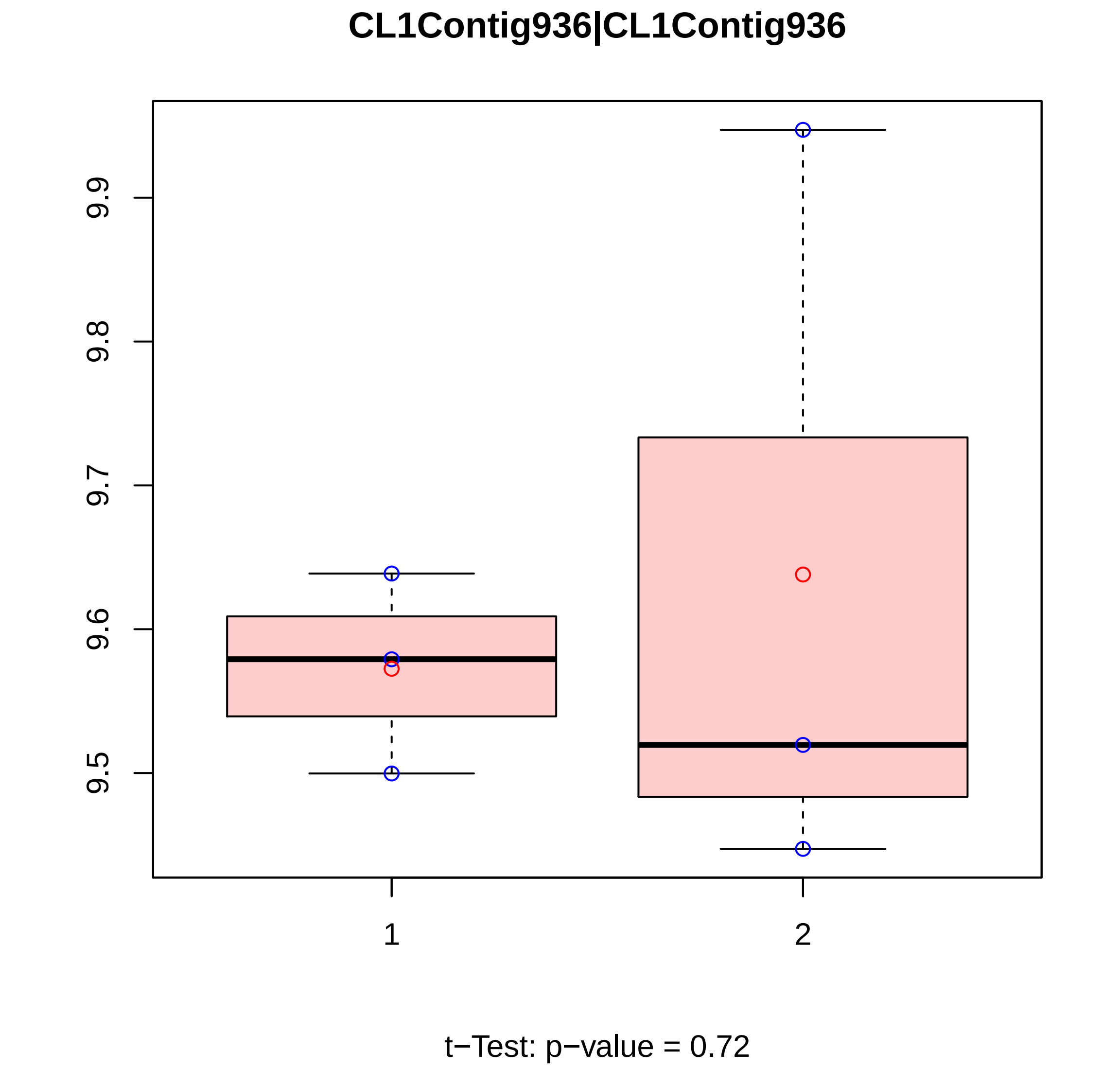
CL1Contig936|CL1Contig936
9.5
9.6
9.7
9.8
9.9
1
2
t−Test: p−value = 0.72

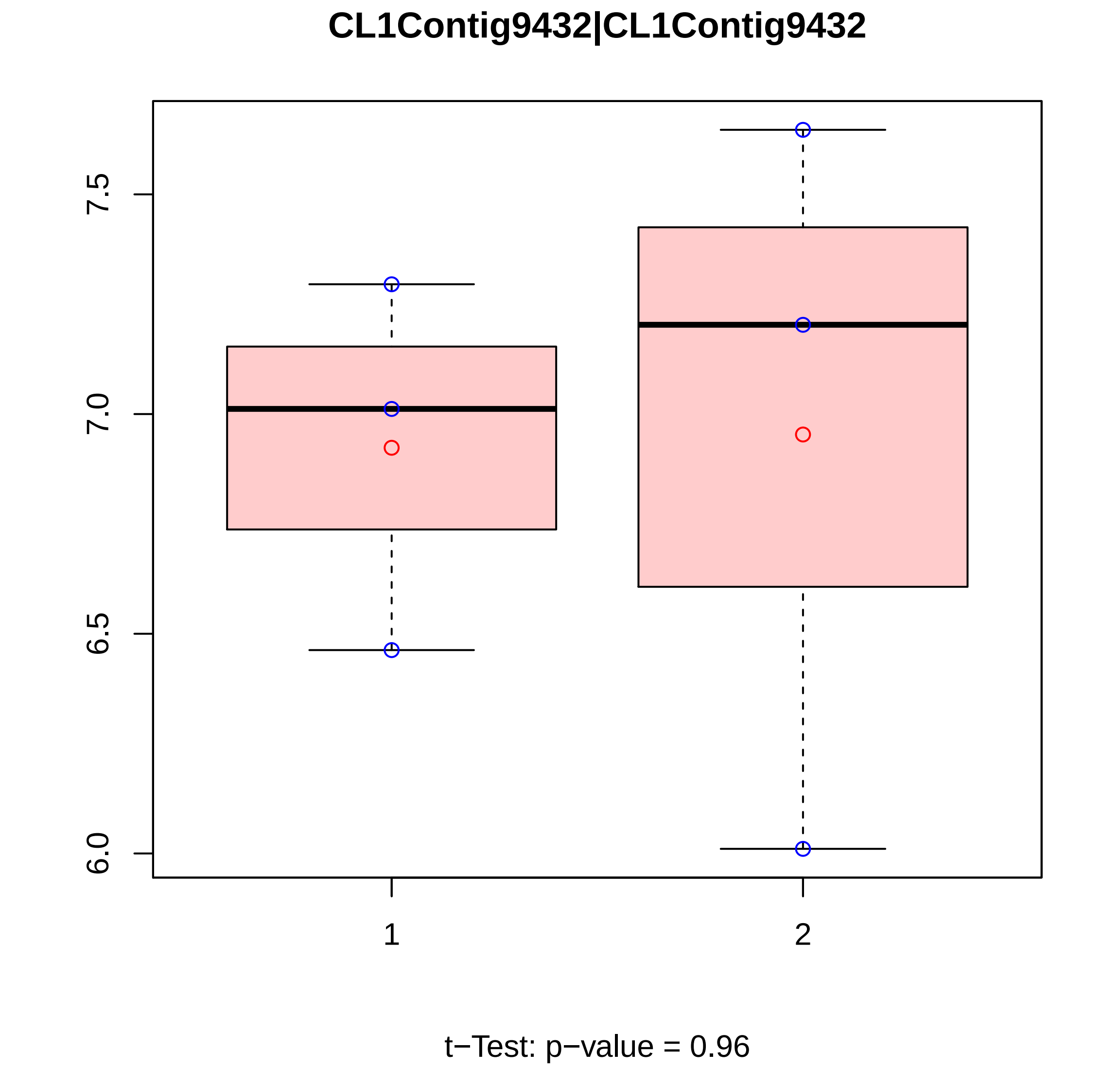
CL1Contig9432|CL1Contig9432
7.5
7.0
6.5
6.0
1
2
t−Test: p−value = 0.96

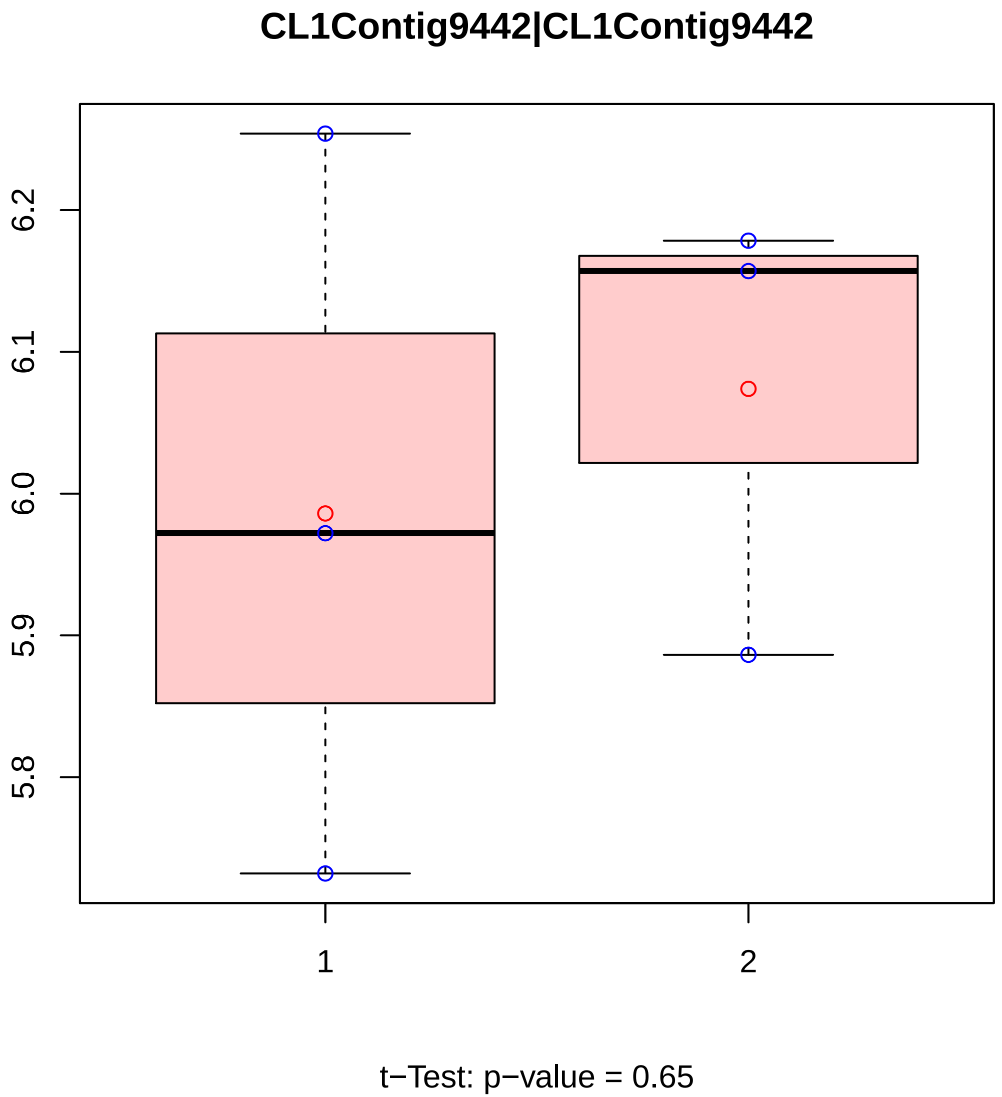
CL1Contig9442|CL1Contig9442
6.2
6.1
6.0
5.9
5.8
1
2
t−Test: p−value = 0.65

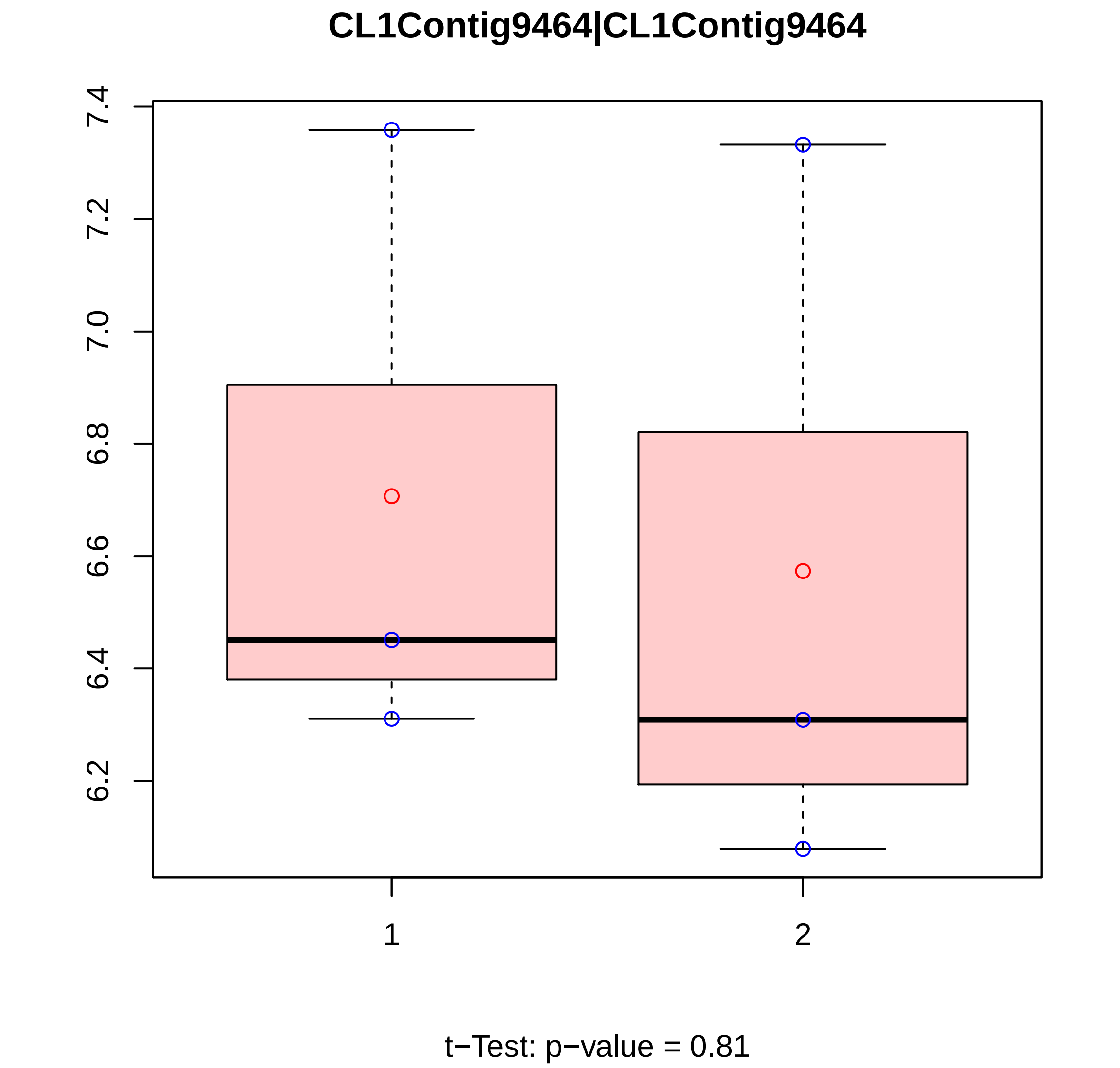

CL1Contig9464|CL1Contig9464
7.4
7.2
7.0
6.8
6.6
6.4
6.2
1
2
t−Test: p−value = 0.81

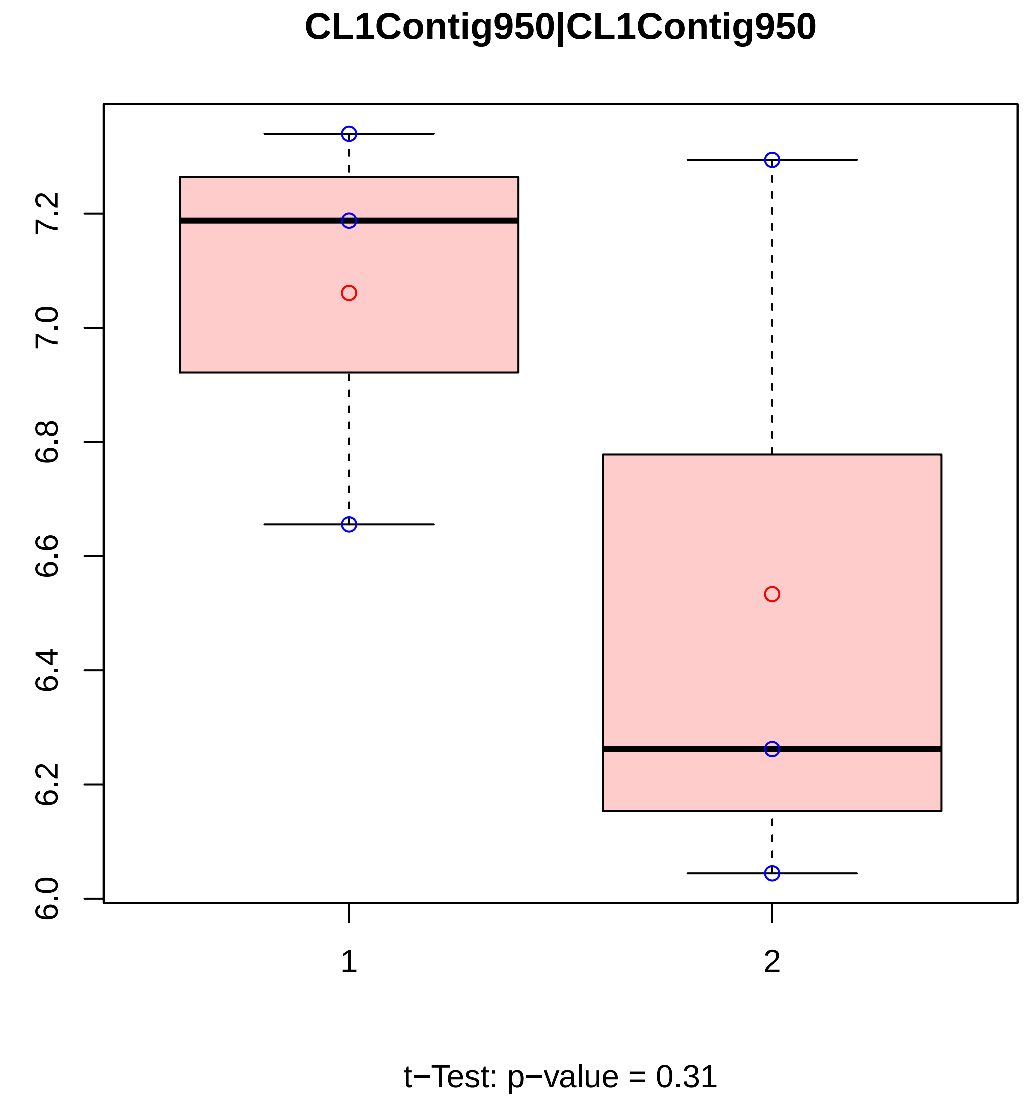
CL1Contig950|CL1Contig950
6.0
6.2
6.4
6.6
6.8
7.0
7.2
1
2
t−Test: p−value = 0.31

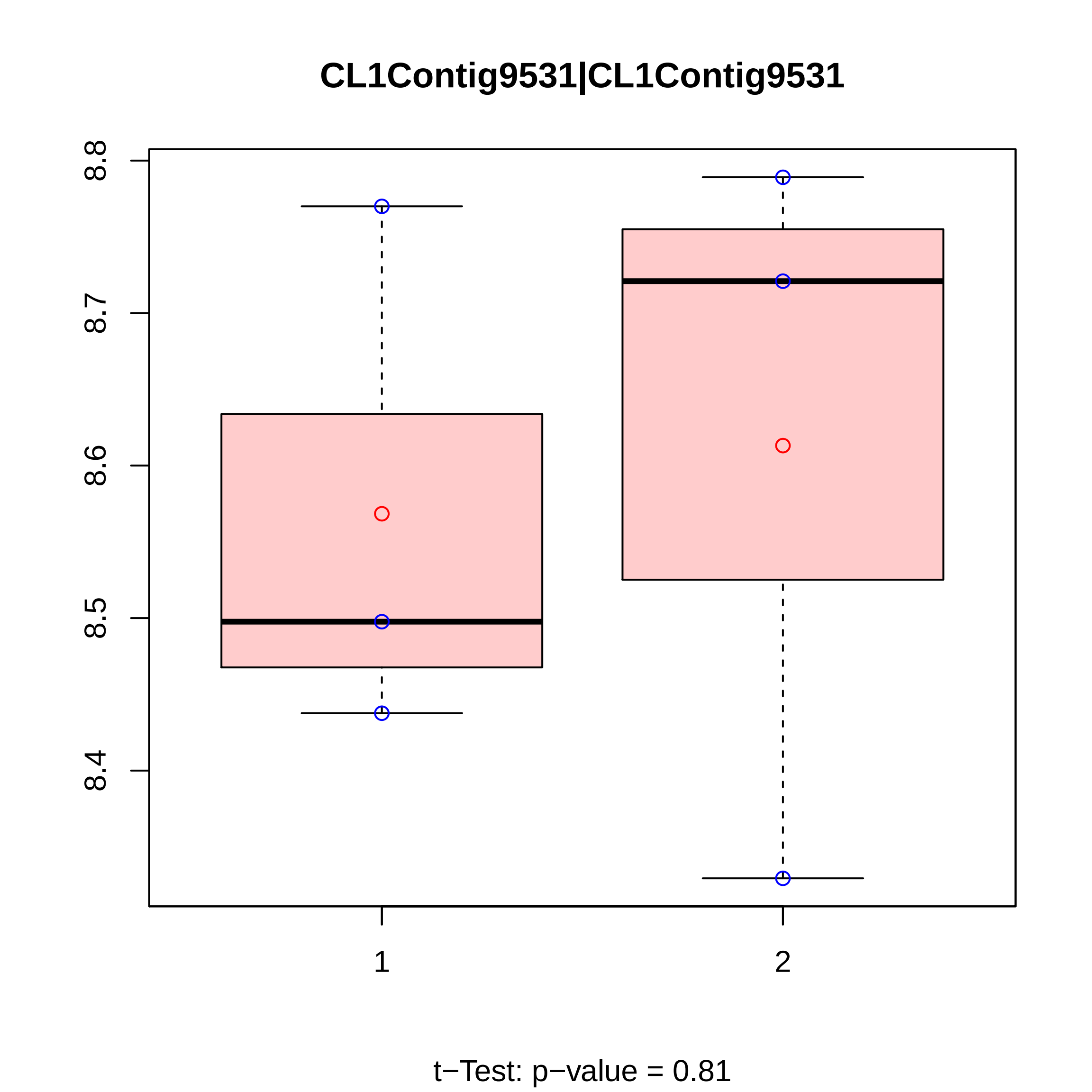
CL1Contig9531|CL1Contig9531
8.4
8.5
8.6
8.7
8.8
1
2
t−Test: p−value = 0.81

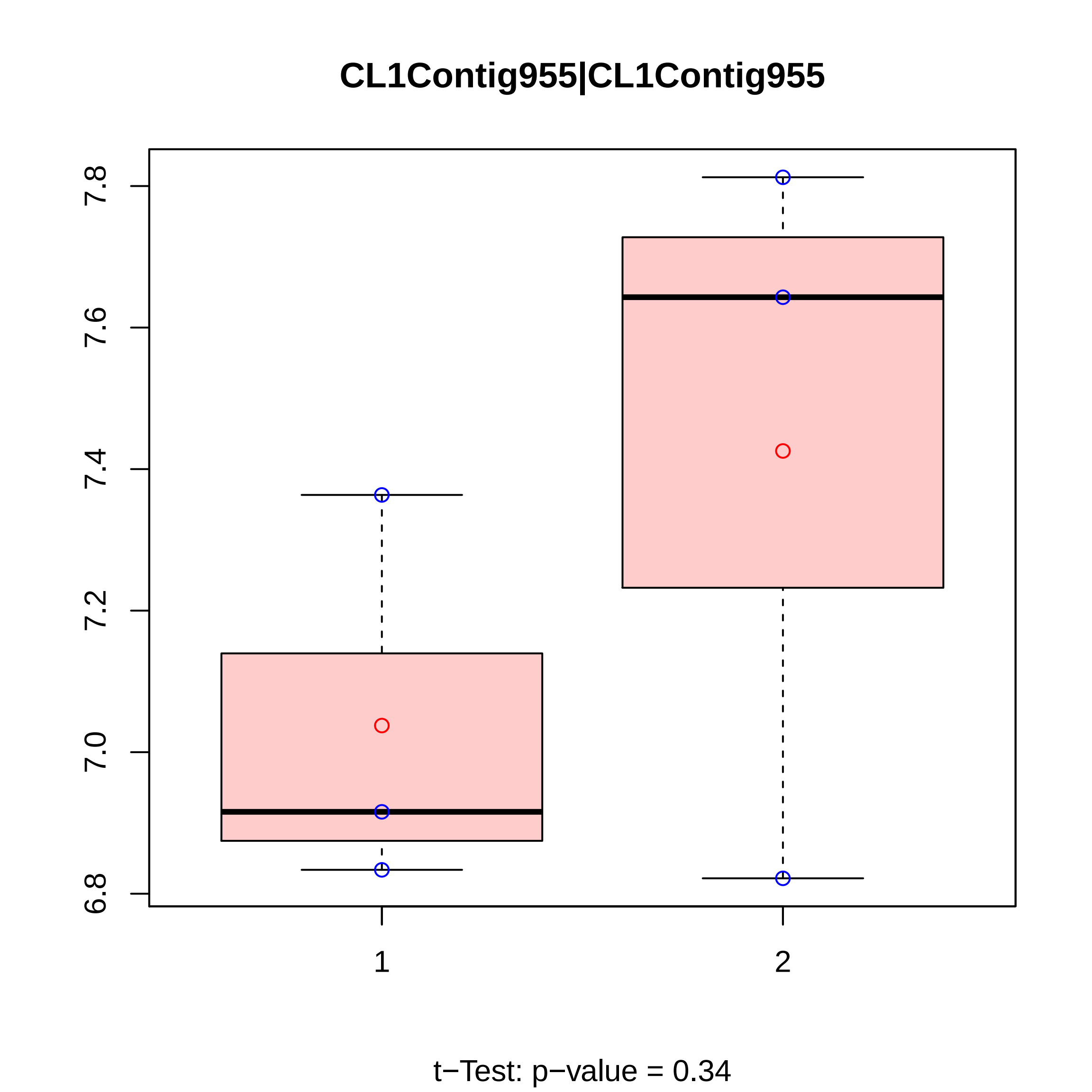
CL1Contig955|CL1Contig955
7.8
7.6
7.4
7.2
7.0
6.8
1
2
t−Test: p−value = 0.34

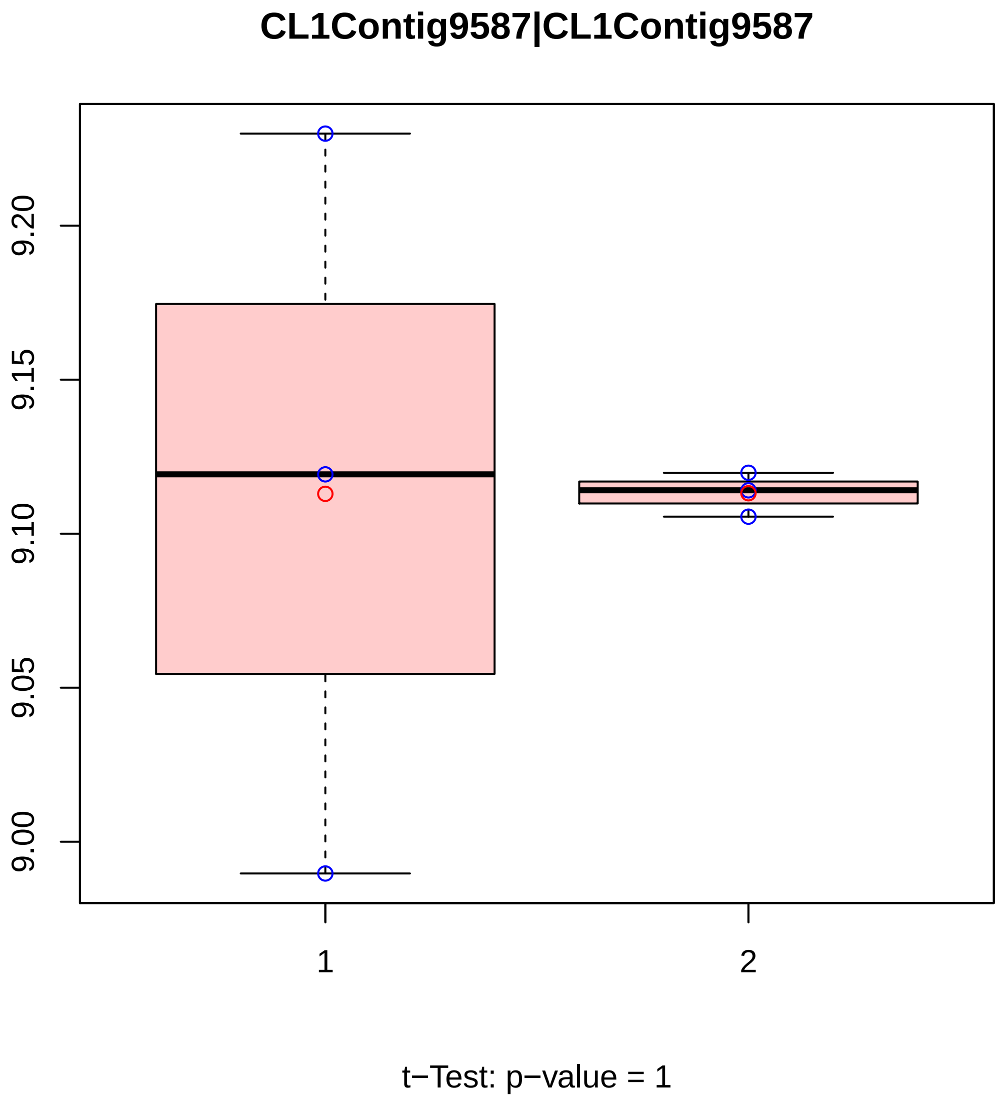

CL1Contig9587|CL1Contig9587
9.00
9.05
9.10
9.15
9.20
1
2
t−Test: p−value = 1

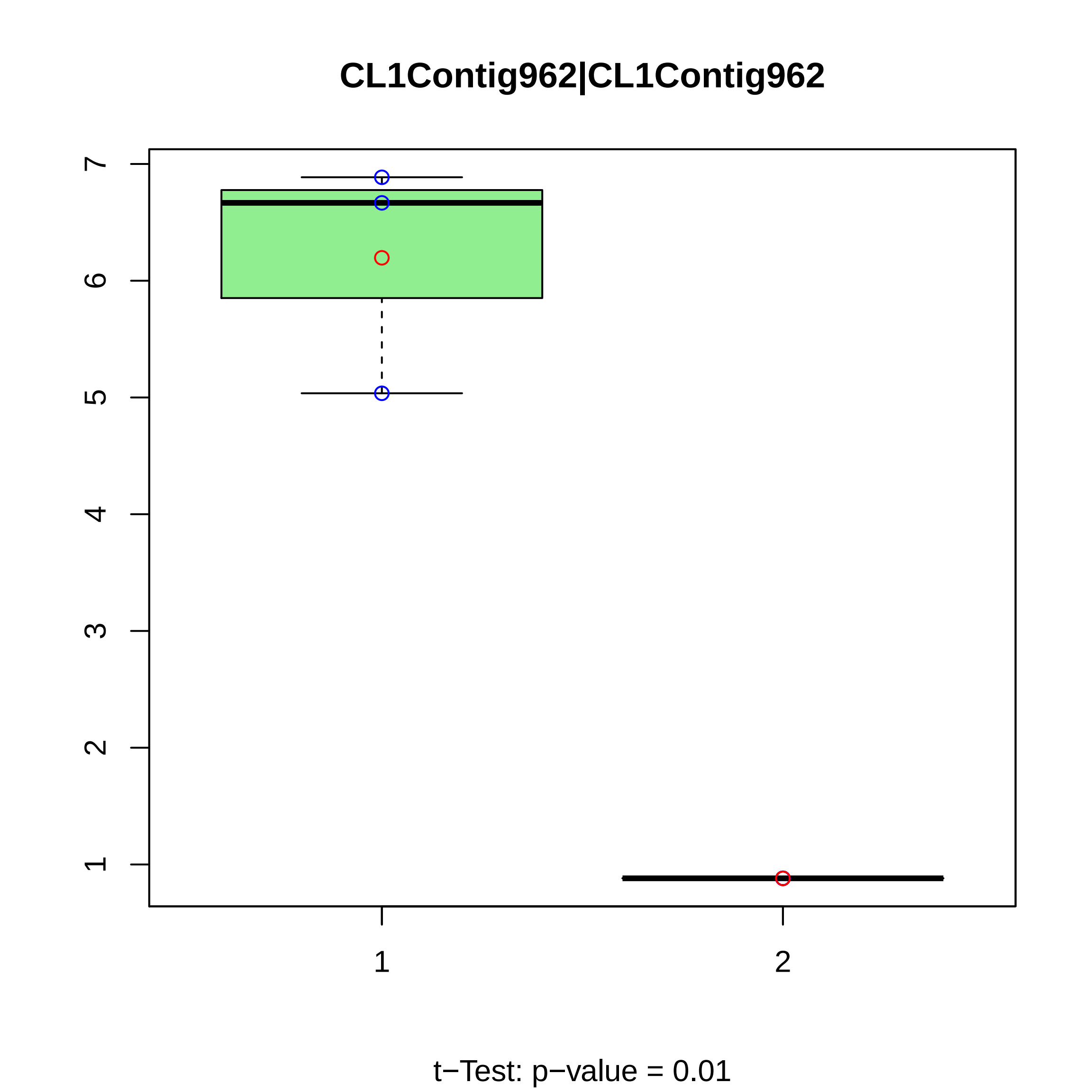
CL1Contig962|CL1Contig962
1
2
3
4
5
6
7
1
2
t−Test: p−value = 0.01

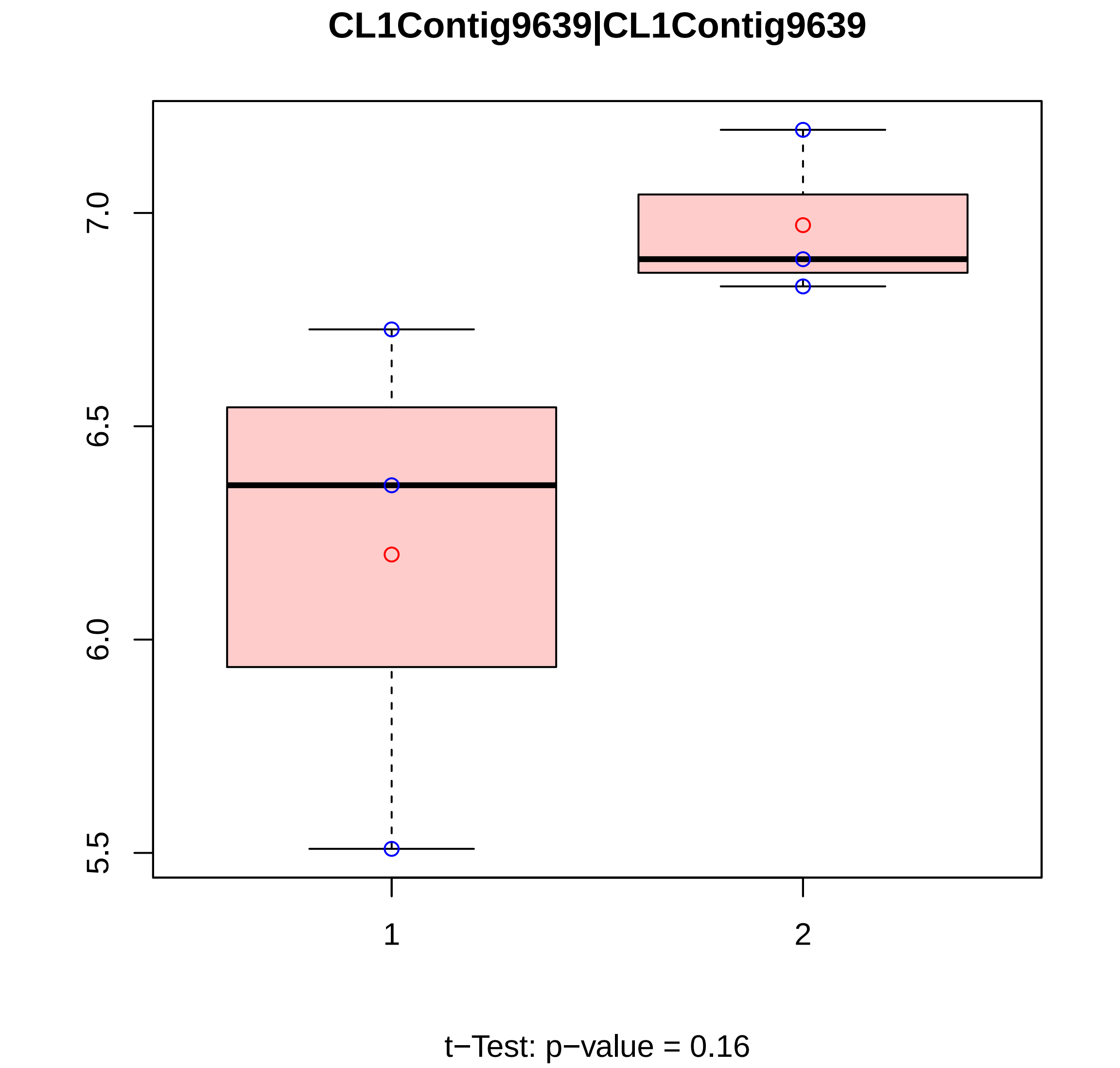
CL1Contig9639|CL1Contig9639
7.0
6.5
6.0
5.5
1
2
t−Test: p−value = 0.16

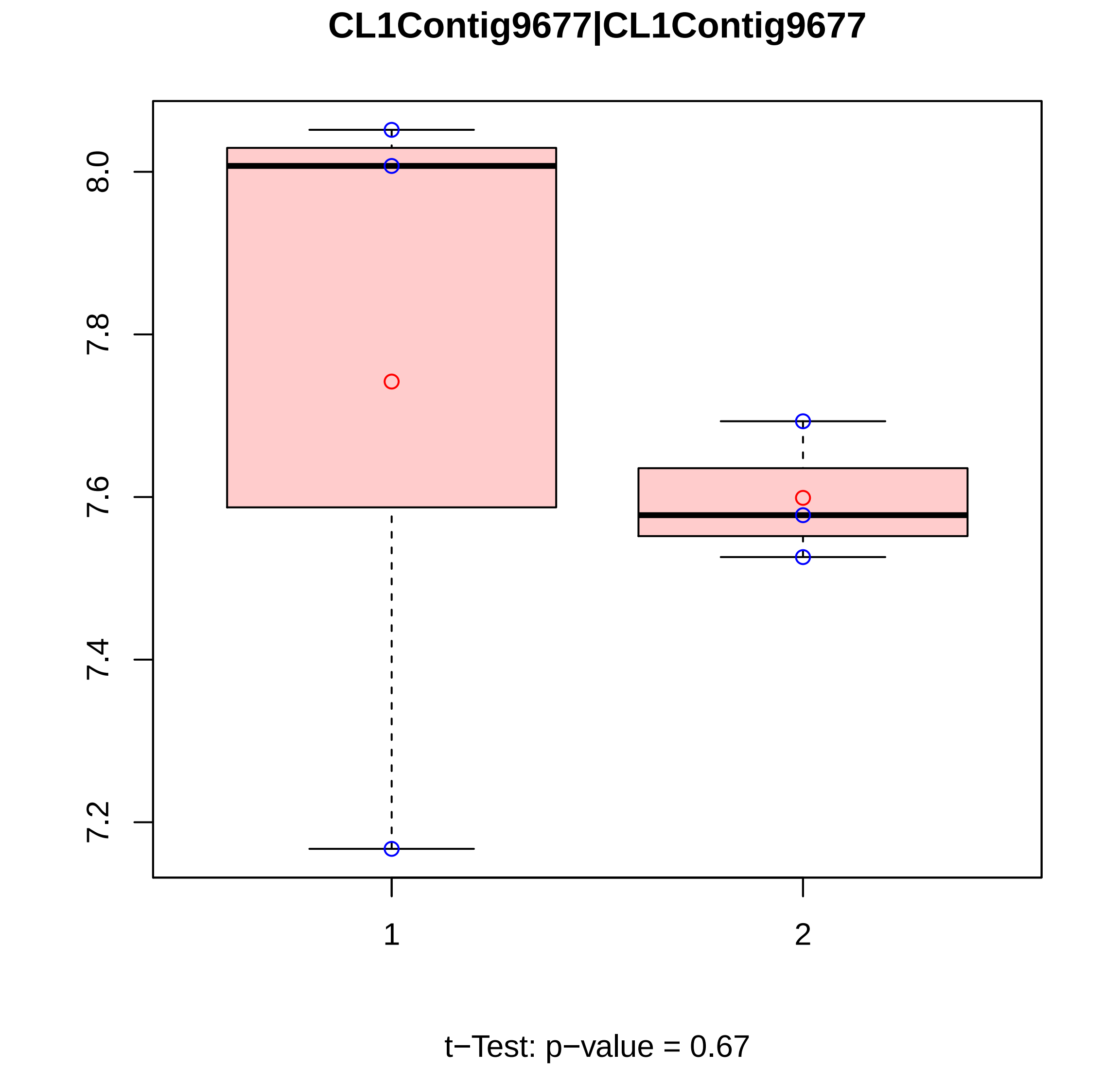
CL1Contig9677|CL1Contig9677
8.0
7.8
7.6
7.4
7.2
1
2
t−Test: p−value = 0.67

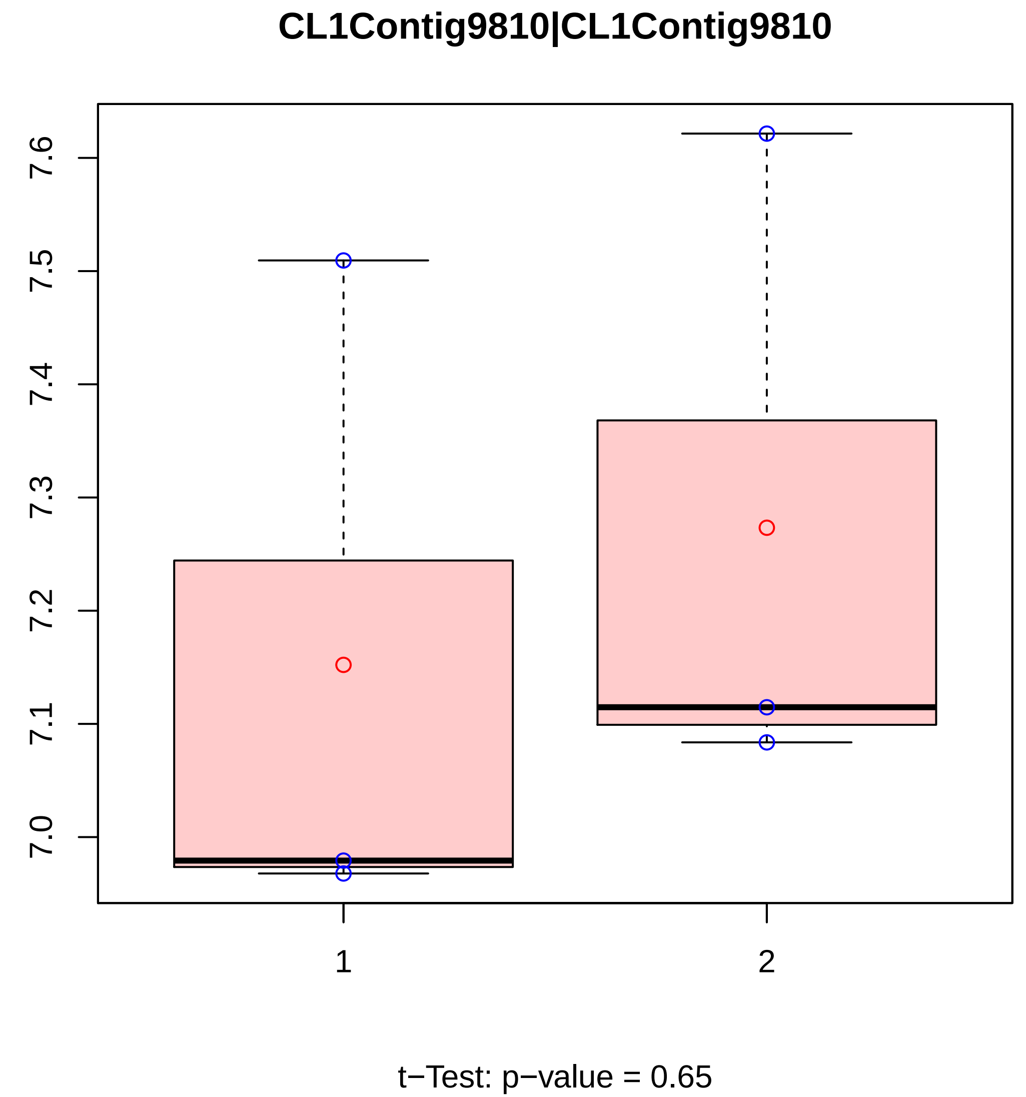
CL1Contig9810|CL1Contig9810
7.0
7.1
7.2
7.3
7.4
7.5
7.6
1
2
t−Test: p−value = 0.65

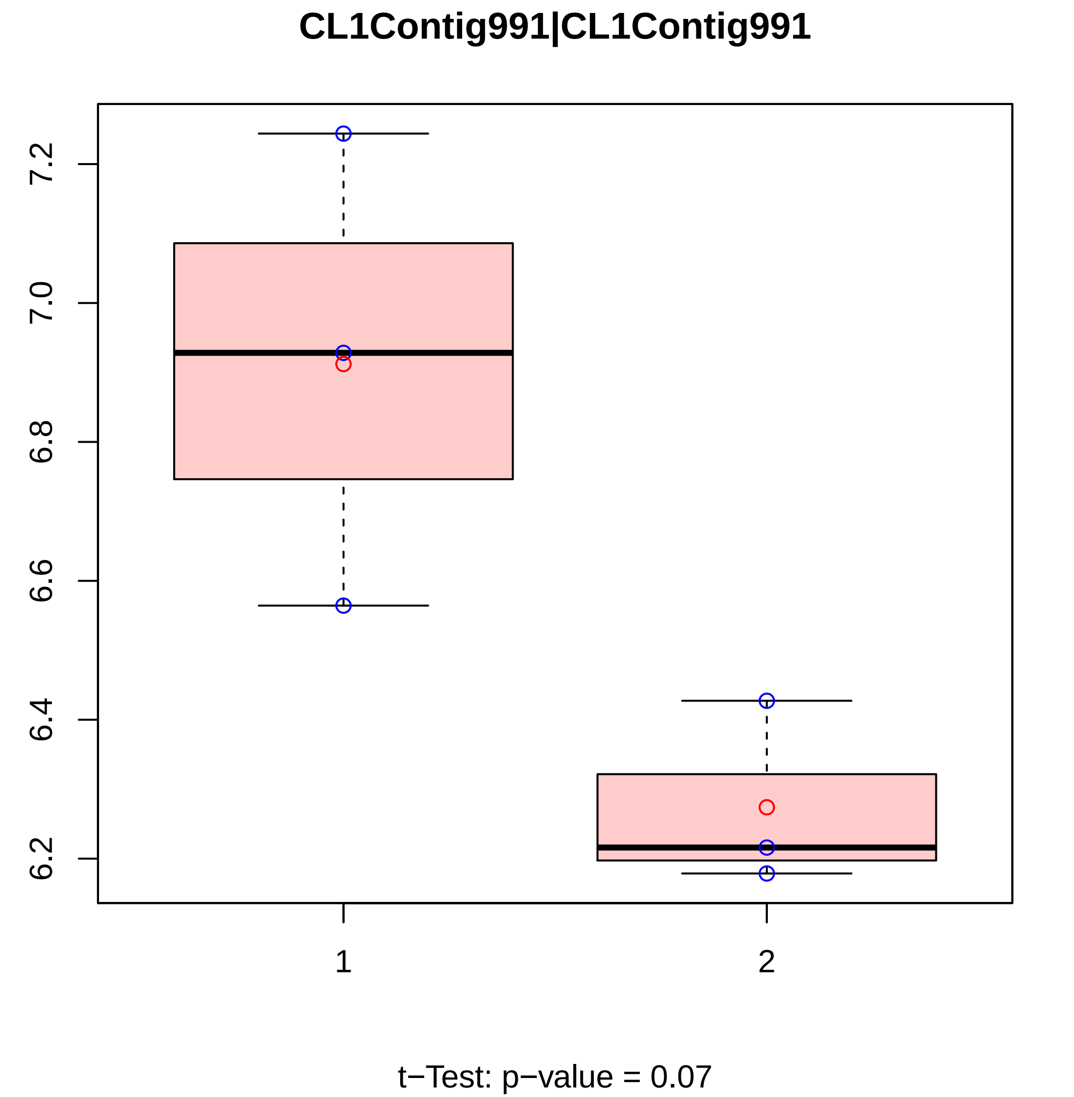

CL1Contig991|CL1Contig991
7.2
7.0
6.8
6.6
6.4
6.2
1
2
t−Test: p−value = 0.07

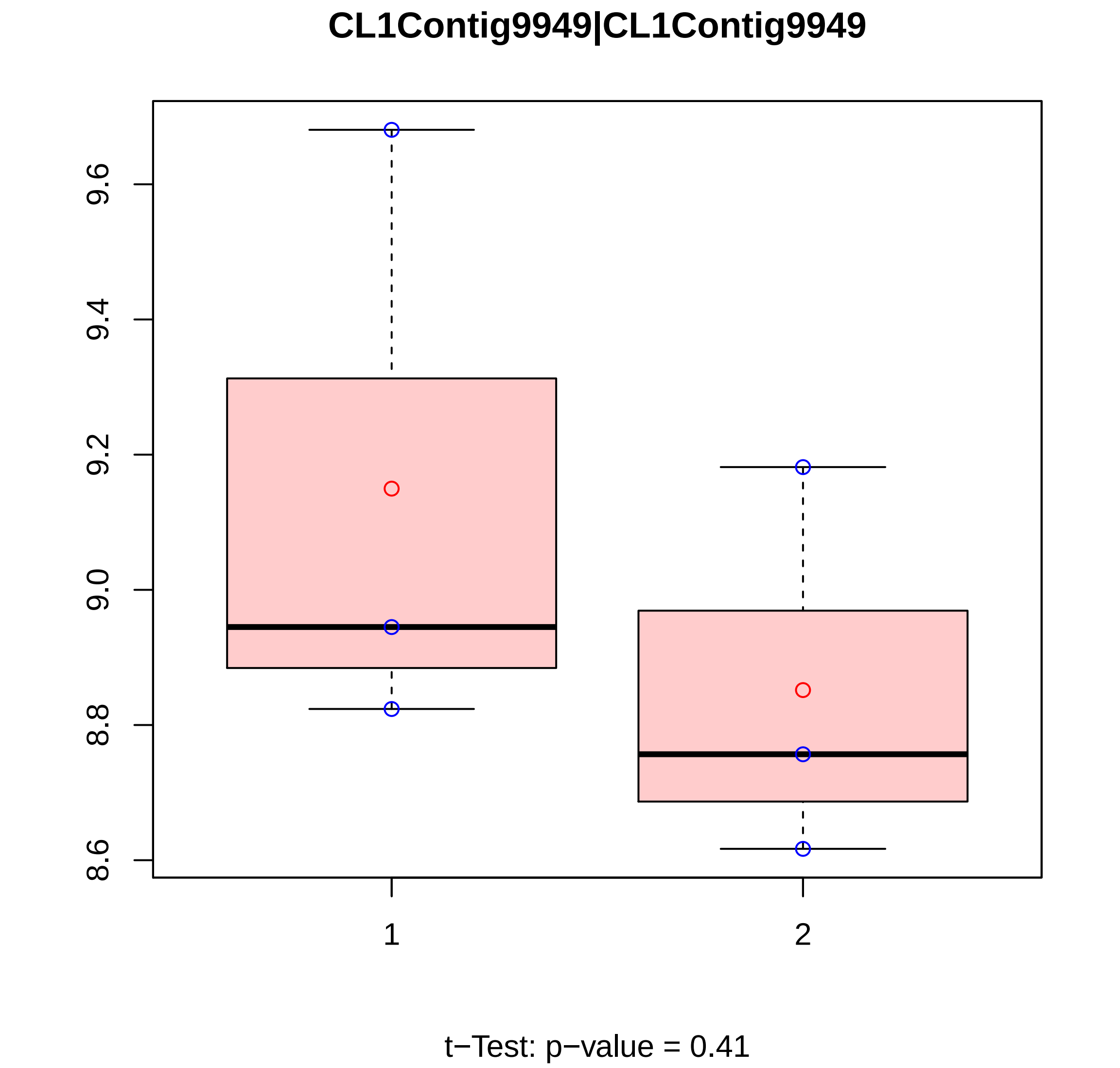
CL1Contig9949|CL1Contig9949
9.6
9.4
9.2
9.0
8.8
8.6
1
2
t−Test: p−value = 0.41

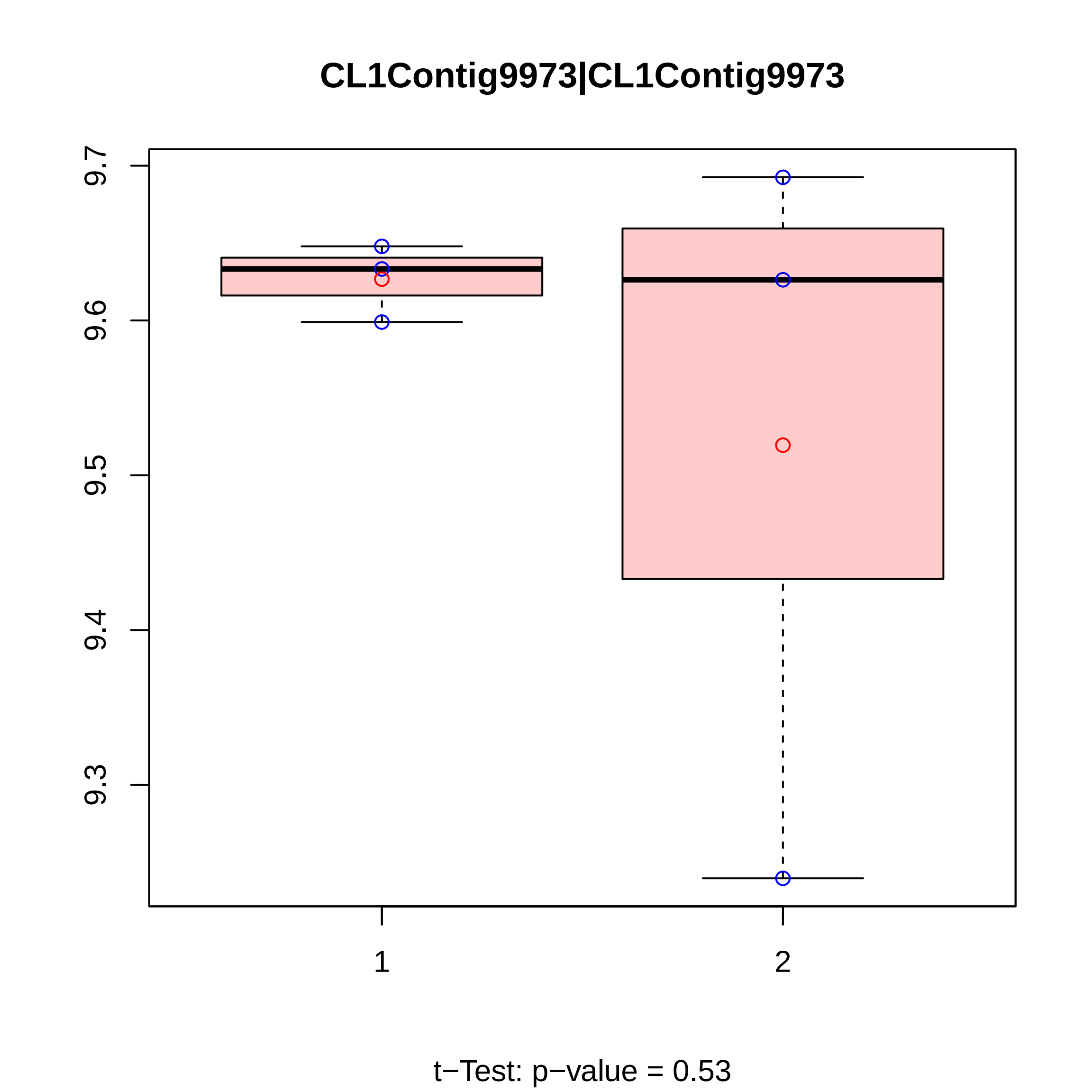

CL1Contig9973|CL1Contig9973
9.7
9.6
9.5
9.4
9.3
1
2
t−Test: p−value = 0.53

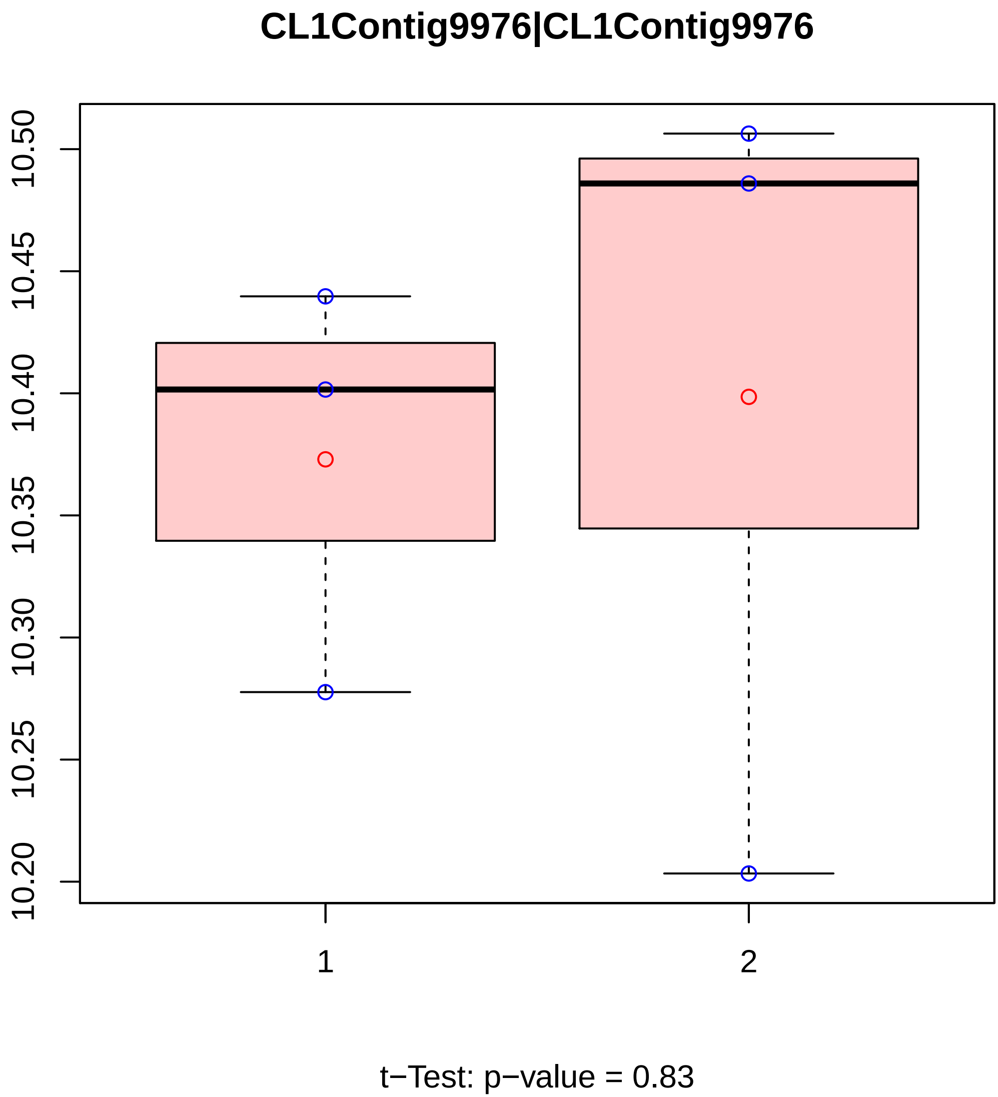
CL1Contig9976|CL1Contig9976
10.50
10.45
10.40
10.35
10.30
10.25
10.20
1
2
t−Test: p−value = 0.83

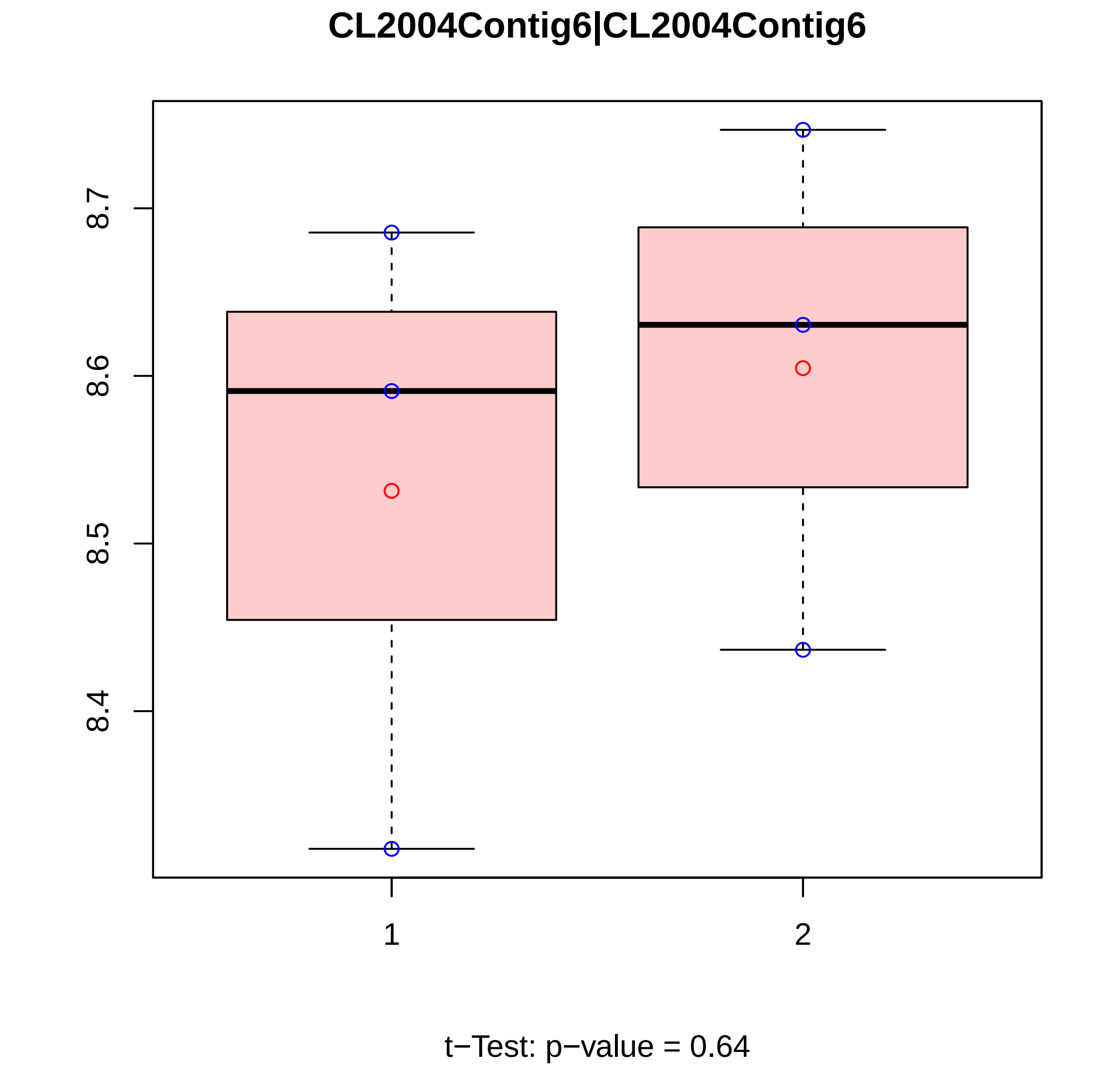
CL2004Contig6|CL2004Contig6
8.4
8.5
8.6
8.7
1
2
t−Test: p−value = 0.64

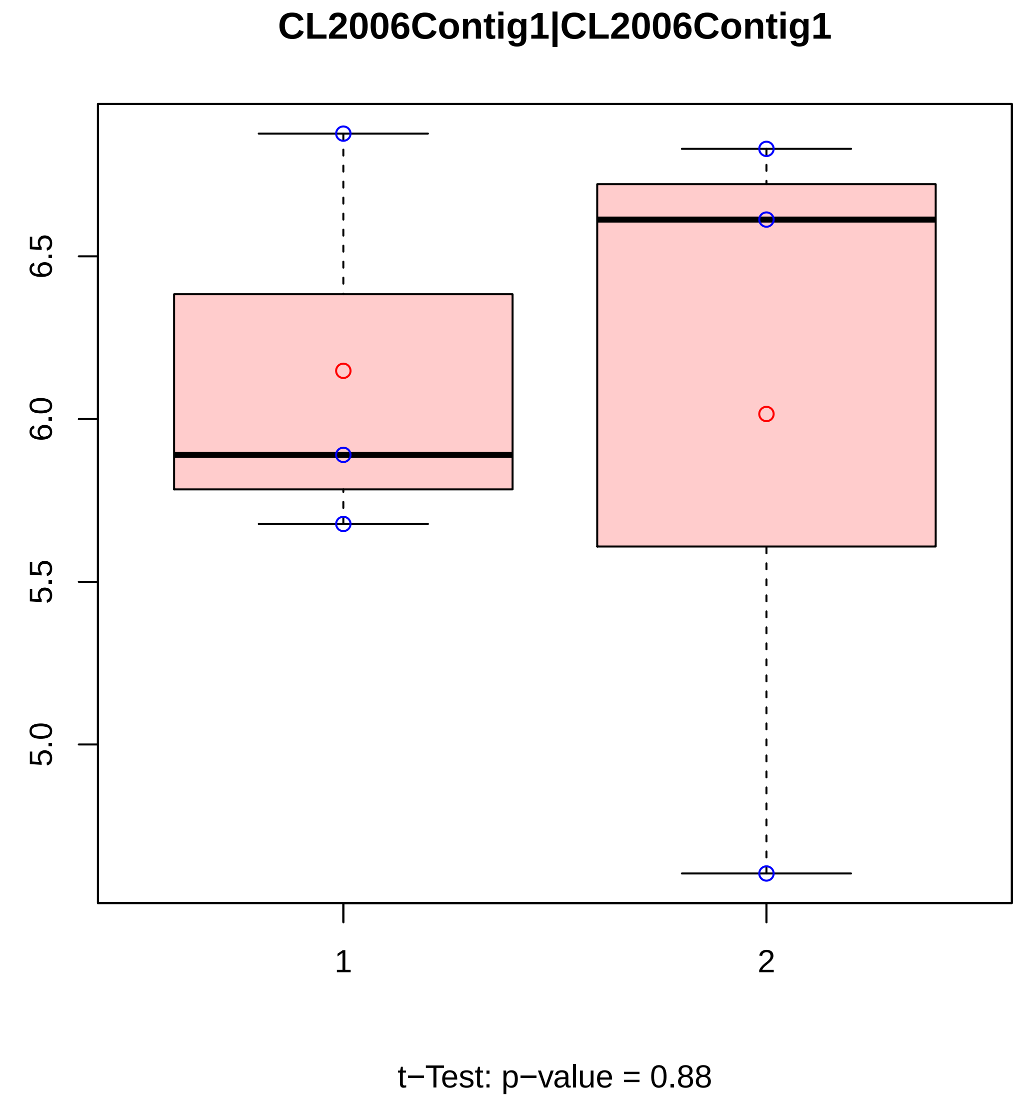
CL2006Contig1|CL2006Contig1
6.5
6.0
5.5
5.0
1
2
t−Test: p−value = 0.88

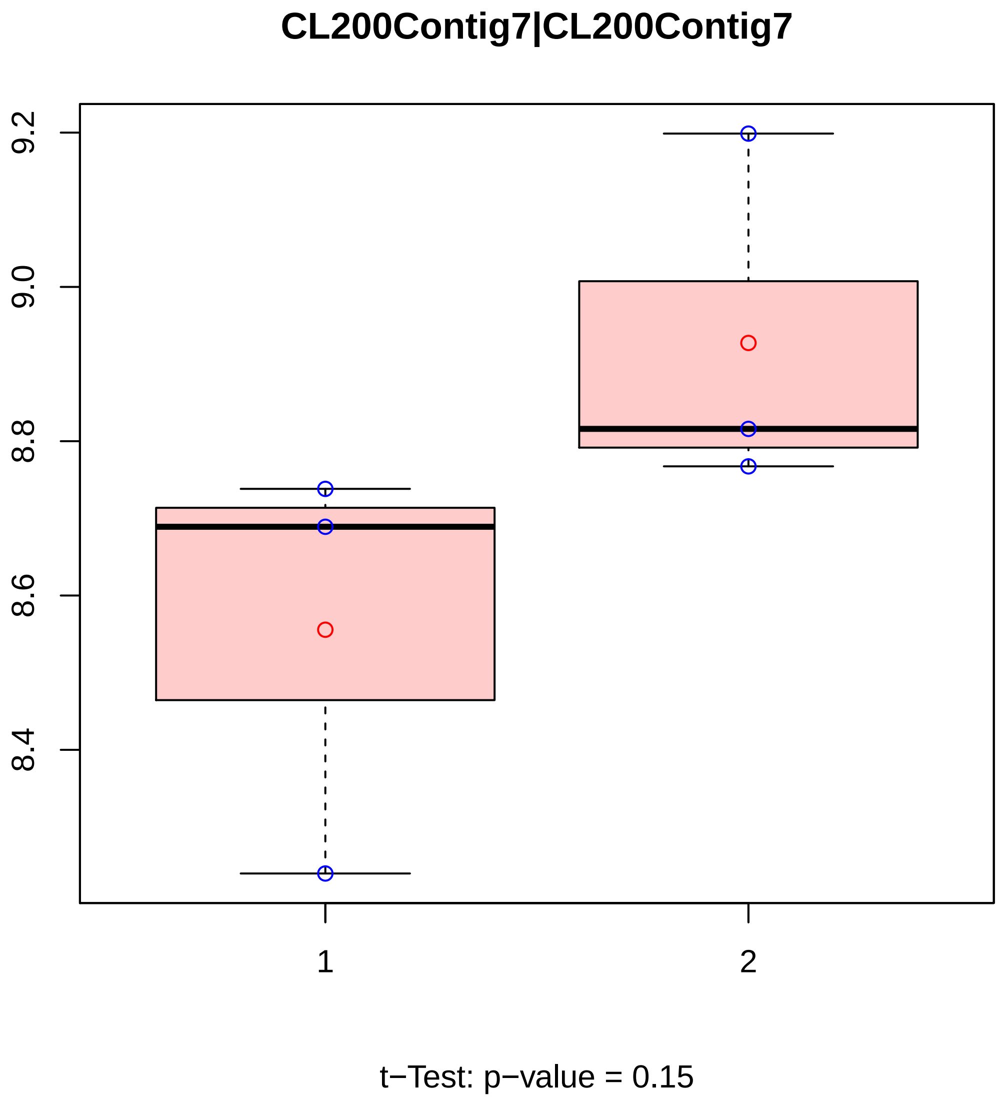
CL200Contig7|CL200Contig7
9.2
9.0
8.8
8.6
8.4
1
2
t−Test: p−value = 0.15

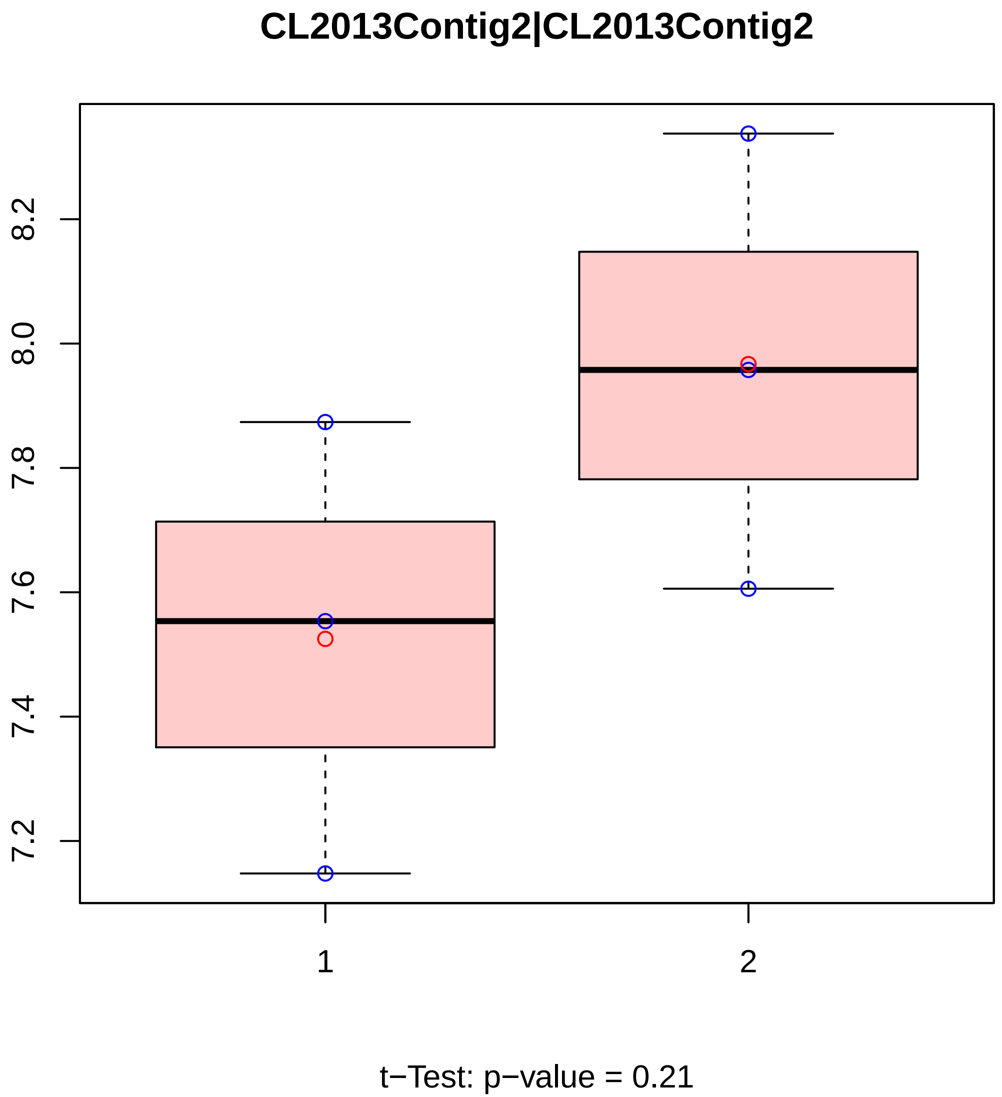
CL2013Contig2|CL2013Contig2
8.2
8.0
7.8
7.6
7.4
7.2
1
2
t−Test: p−value = 0.21

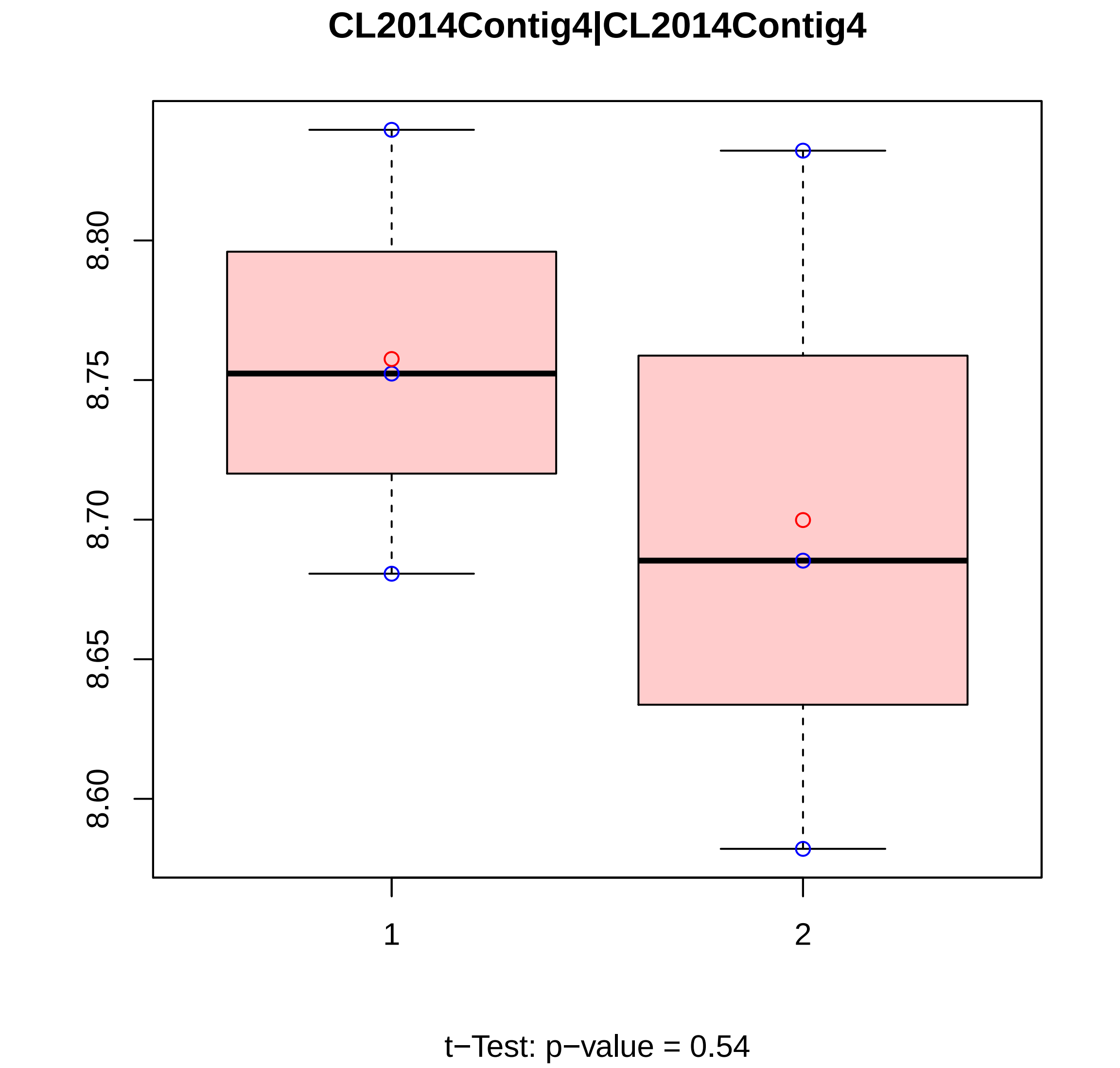
CL2014Contig4|CL2014Contig4
8.60
8.65
8.70
8.75
8.80
1
2
t−Test: p−value = 0.54

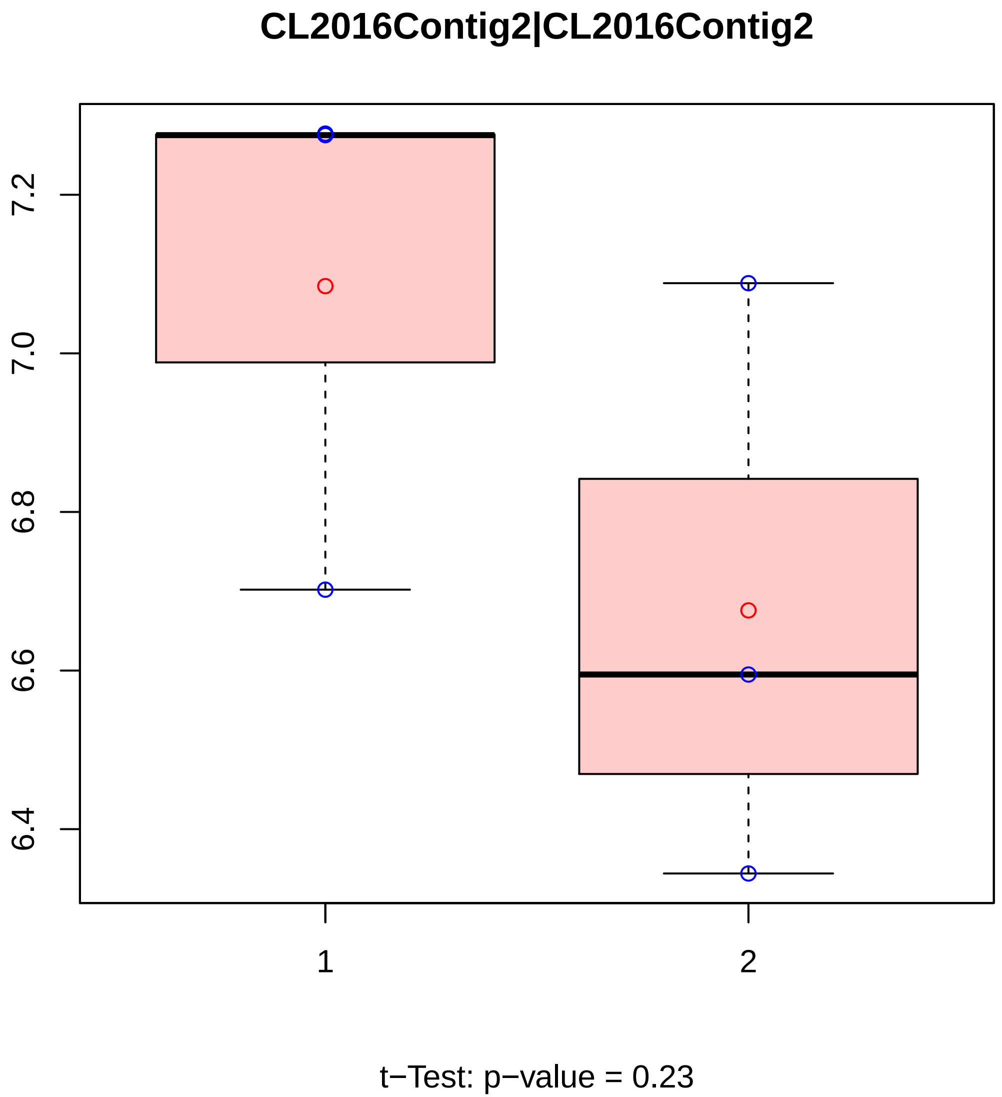

CL2016Contig2|CL2016Contig2
7.2
7.0
6.8
6.6
6.4
1
2
t−Test: p−value = 0.23

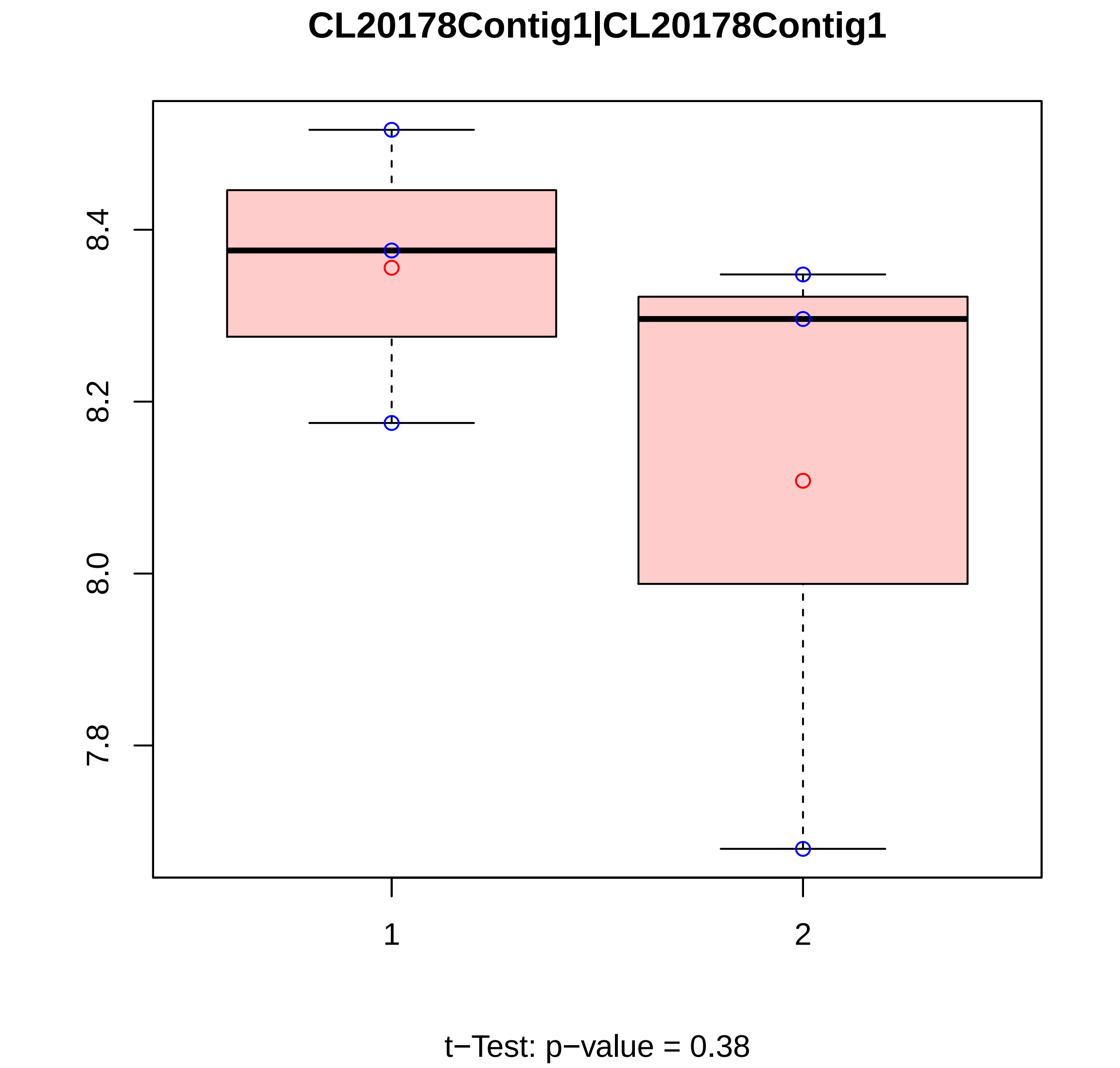
CL20178Contig1|CL20178Contig1
8.4
8.2
8.0
7.8
1
2
t−Test: p−value = 0.38

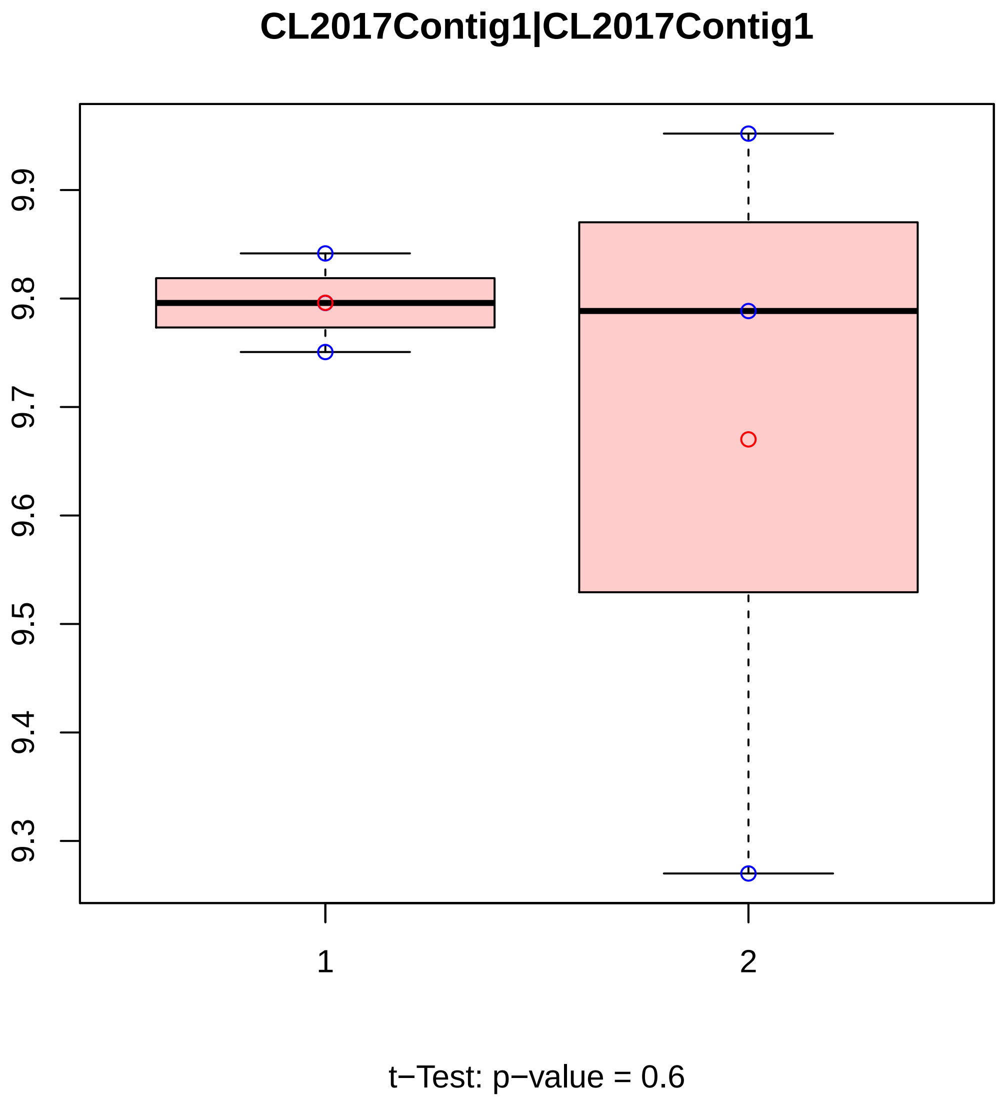
CL2017Contig1|CL2017Contig1
9.3
9.4
9.5
9.6
9.7
9.8
9.9
1
2
t−Test: p−value = 0.6

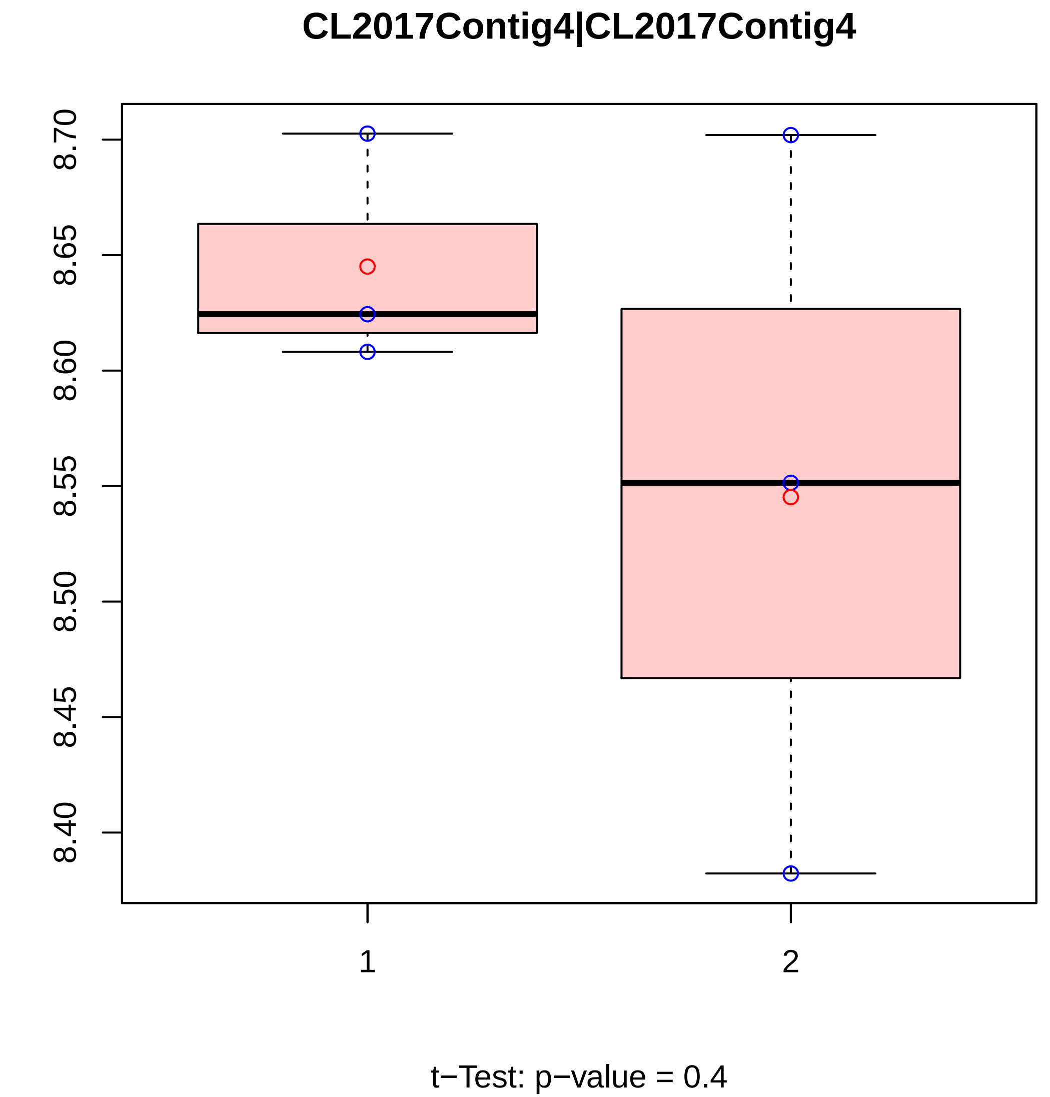
CL2017Contig4|CL2017Contig4
8.40
8.45
8.50
8.55
8.60
8.65
8.70
1
2
t−Test: p−value = 0.4

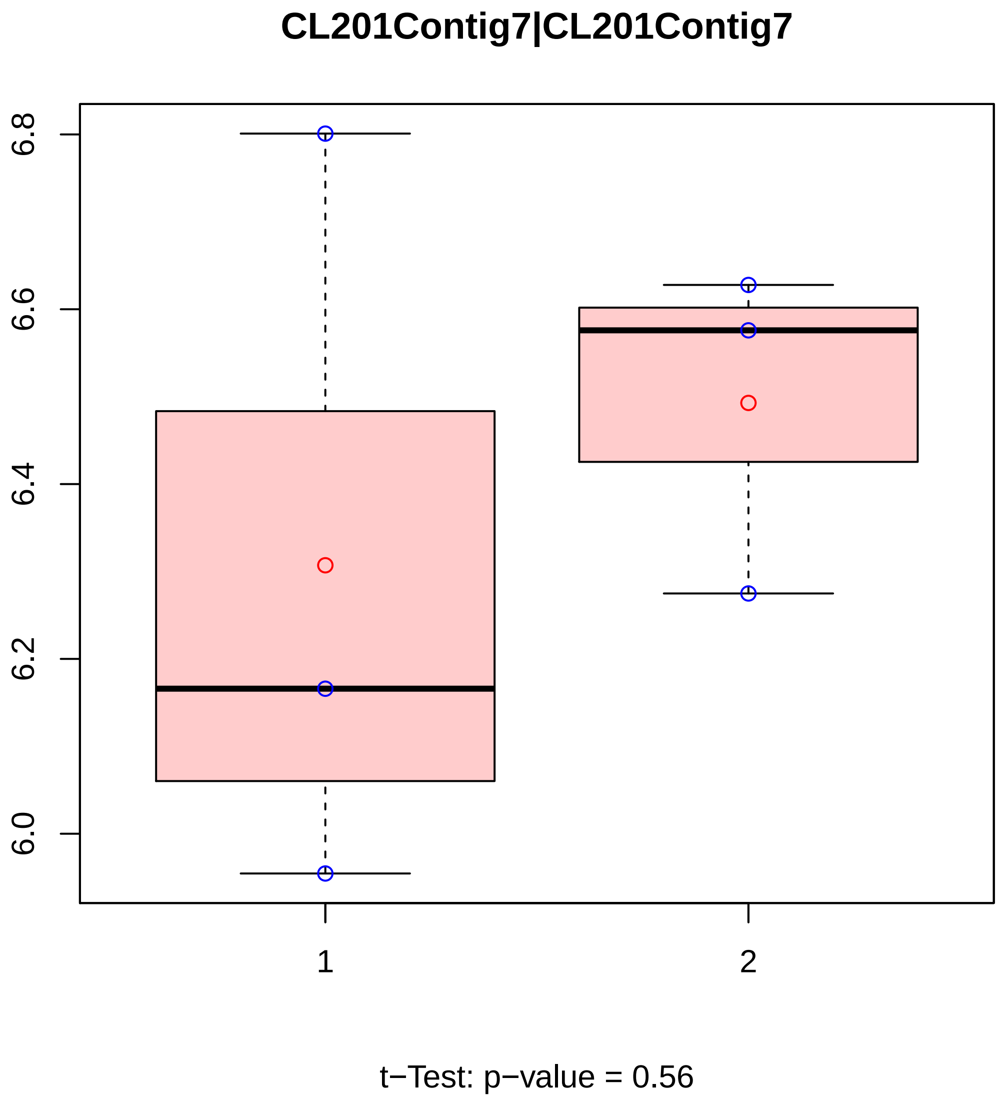
CL201Contig7|CL201Contig7
6.0
6.2
6.4
6.6
6.8
1
2
t−Test: p−value = 0.56

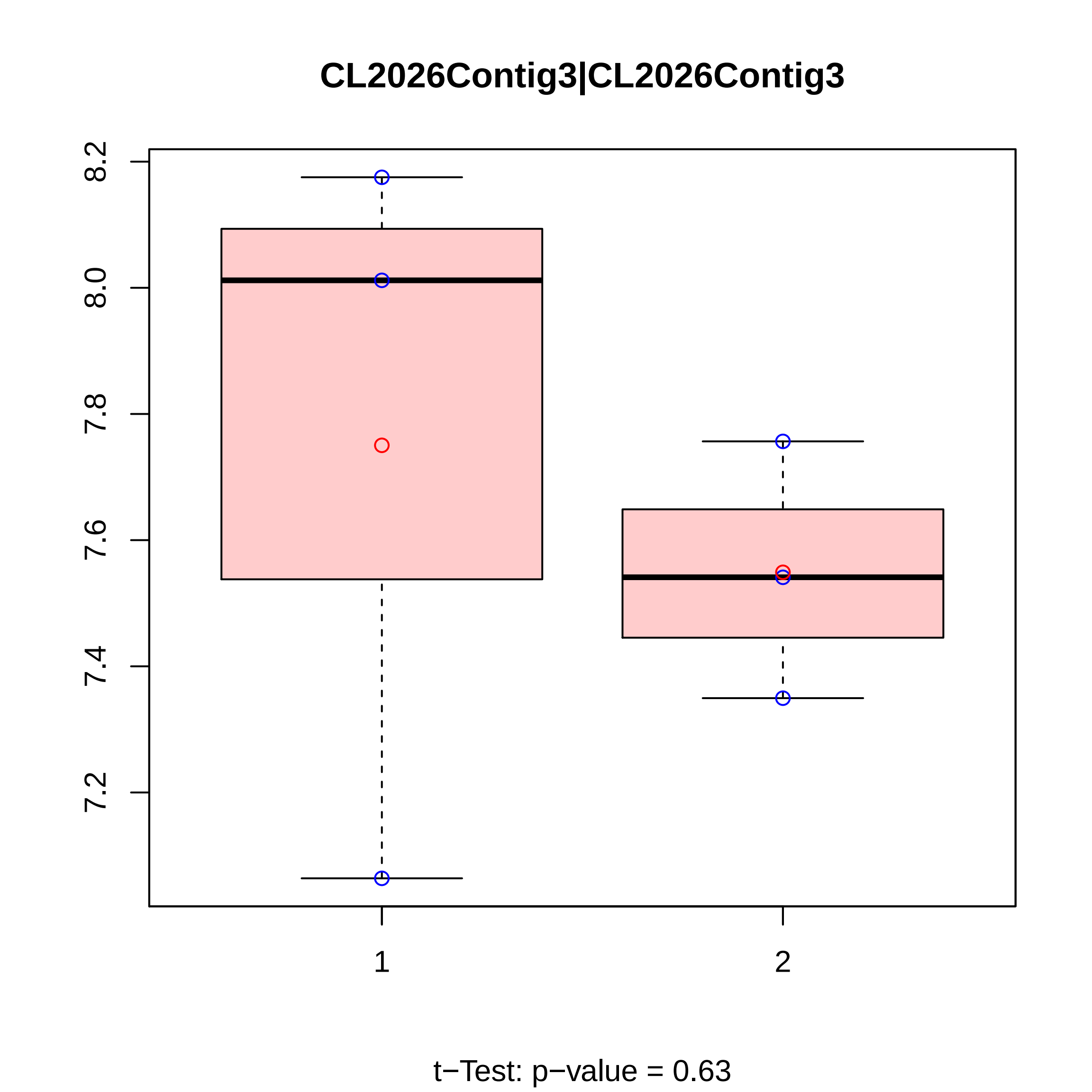
CL2026Contig3|CL2026Contig3
8.2
8.0
7.8
7.6
7.4
7.2
1
2
t−Test: p−value = 0.63

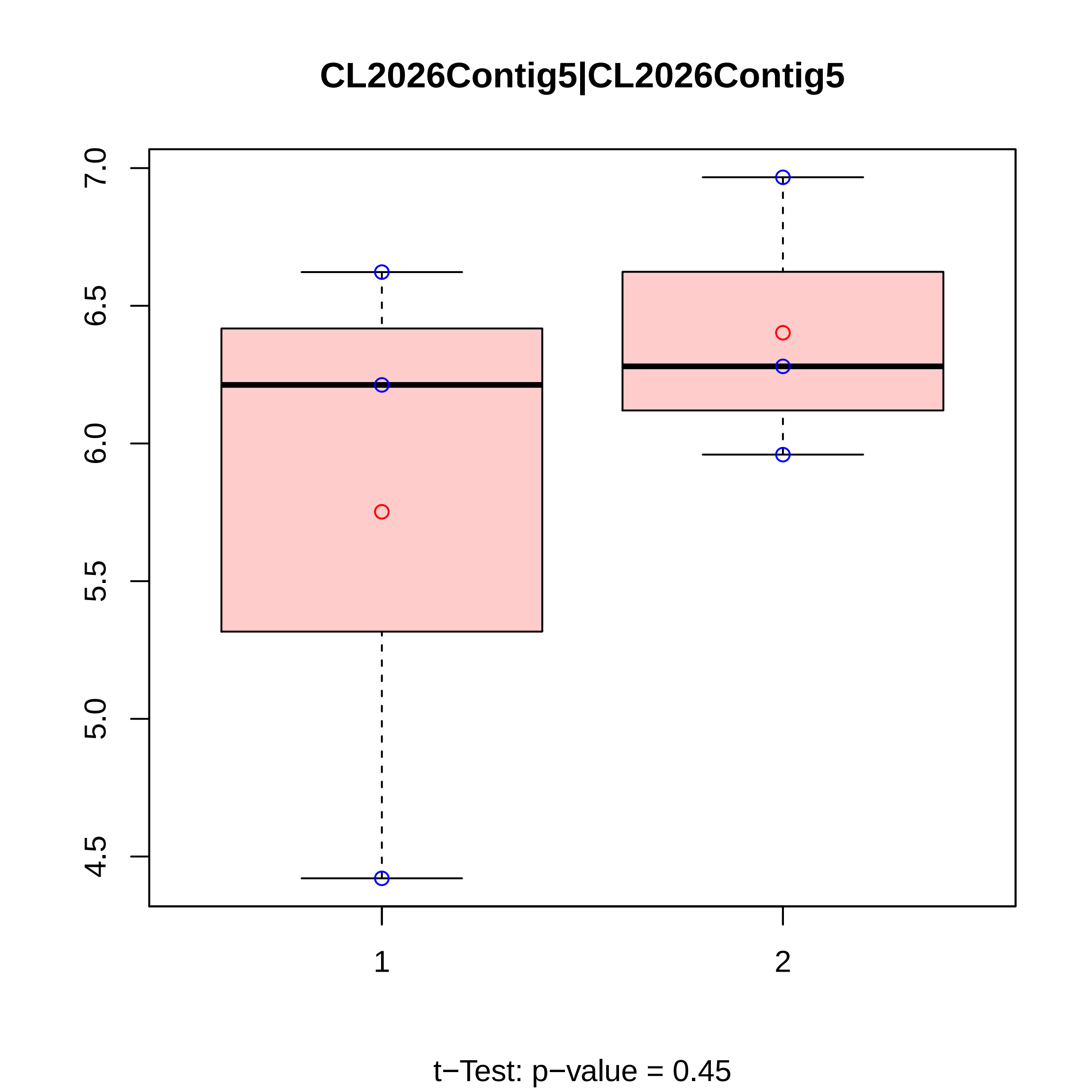
CL2026Contig5|CL2026Contig5
7.0
6.5
6.0
5.5
5.0
4.5
1
2
t−Test: p−value = 0.45

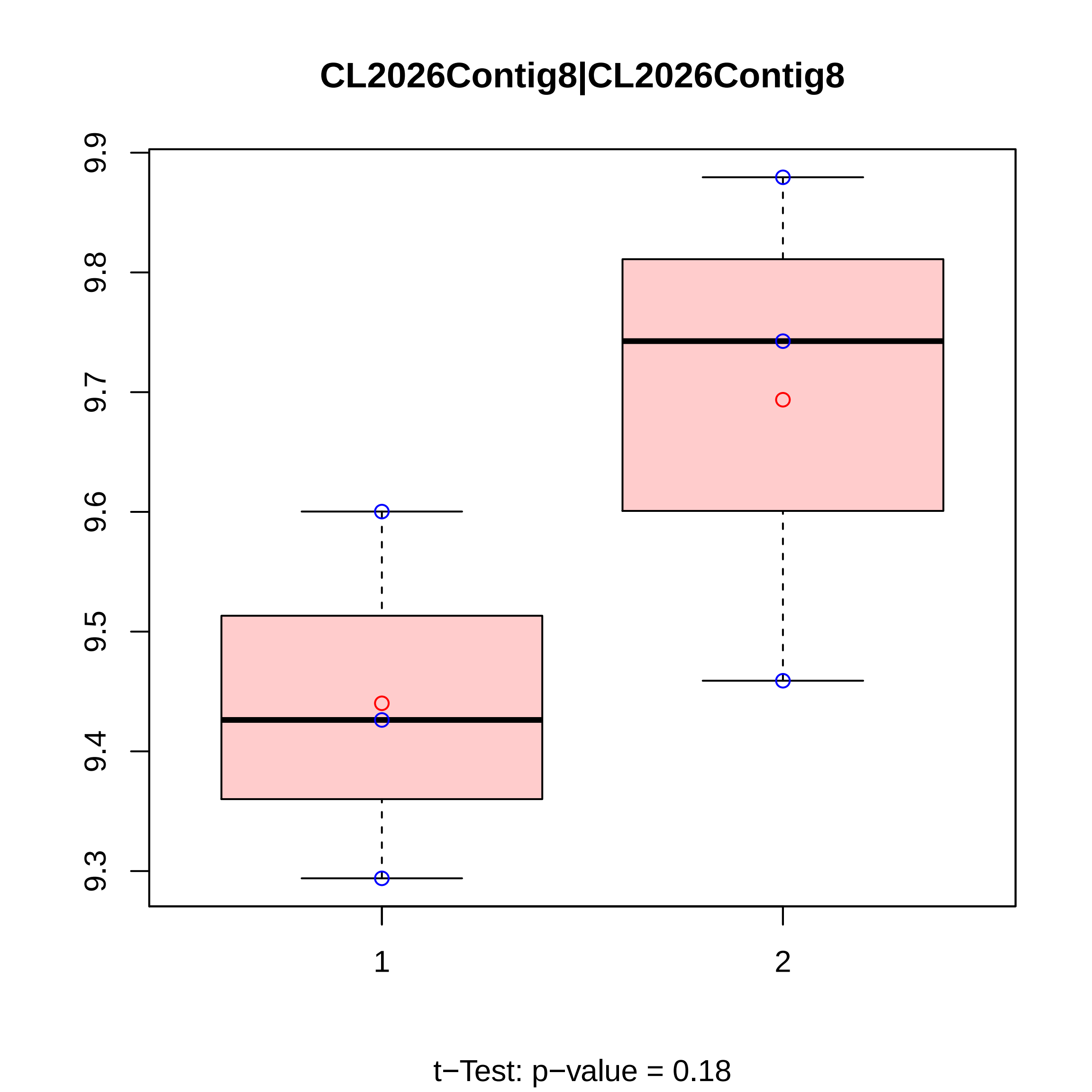

CL2026Contig8|CL2026Contig8
9.3
9.4
9.5
9.6
9.7
9.8
9.9
1
2
t−Test: p−value = 0.18

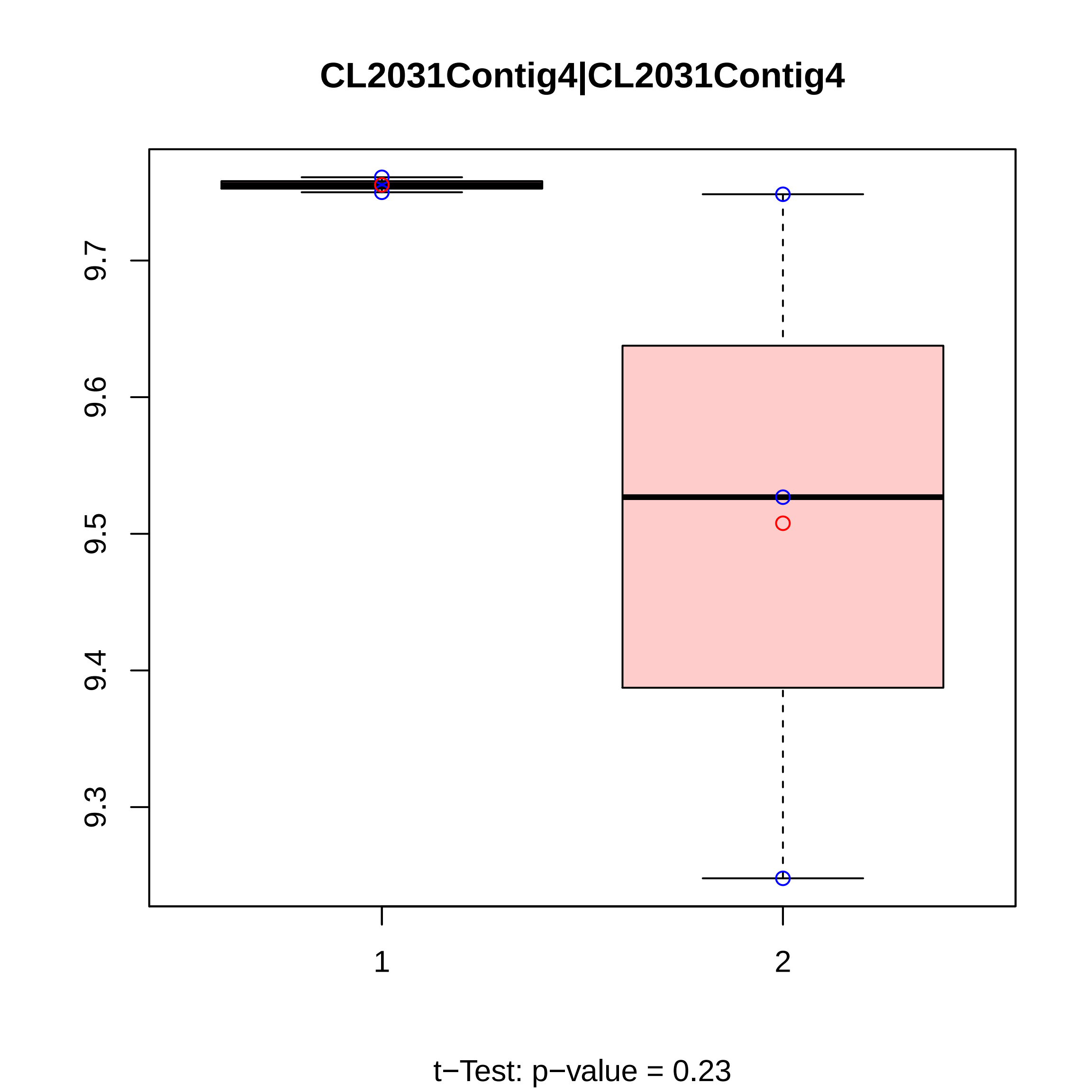

CL2031Contig4|CL2031Contig4
9.3
9.4
9.5
9.6
9.7
1
2
t−Test: p−value = 0.23

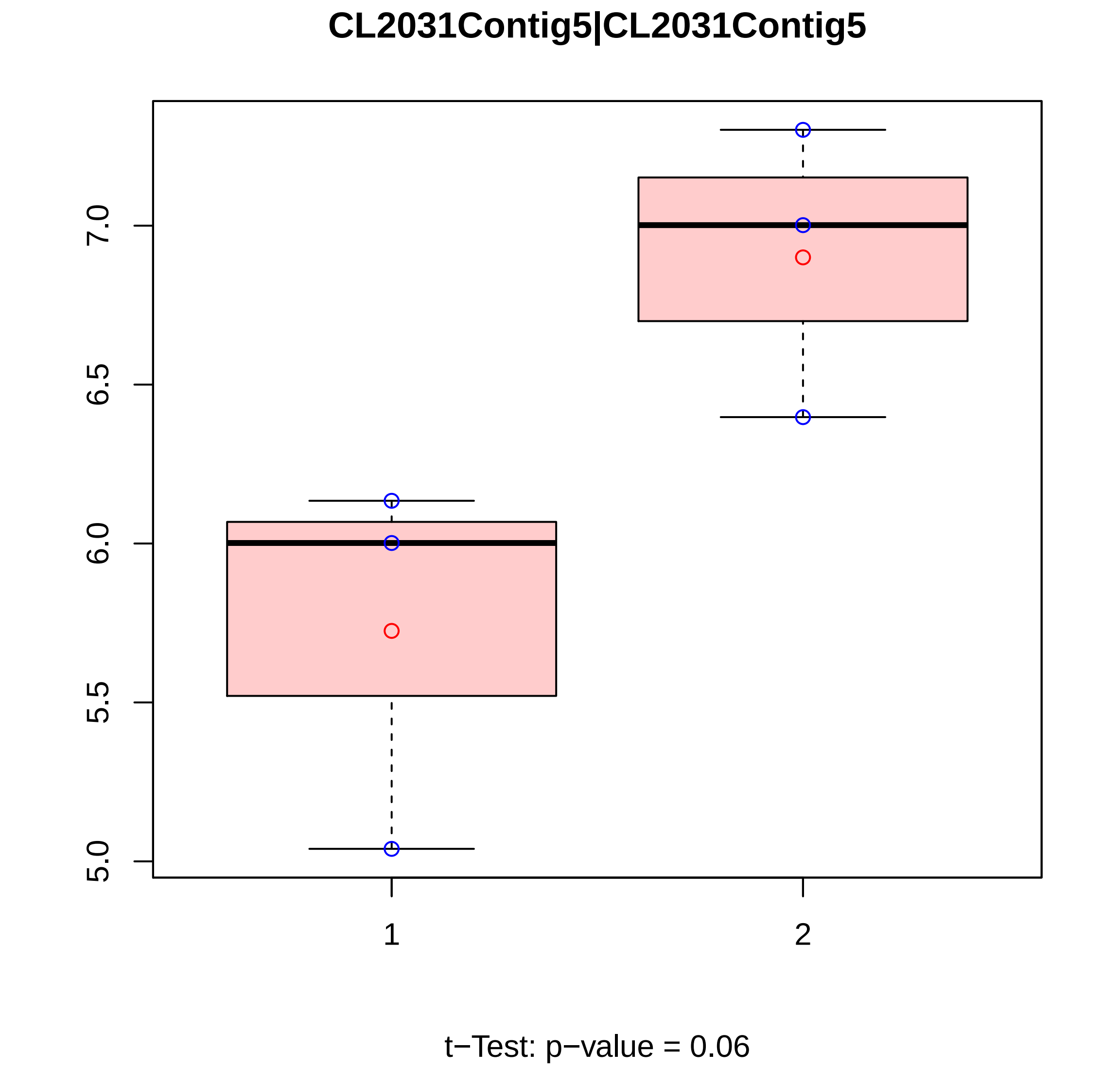
CL2031Contig5|CL2031Contig5
7.0
6.5
6.0
5.5
5.0
1
2
t−Test: p−value = 0.06

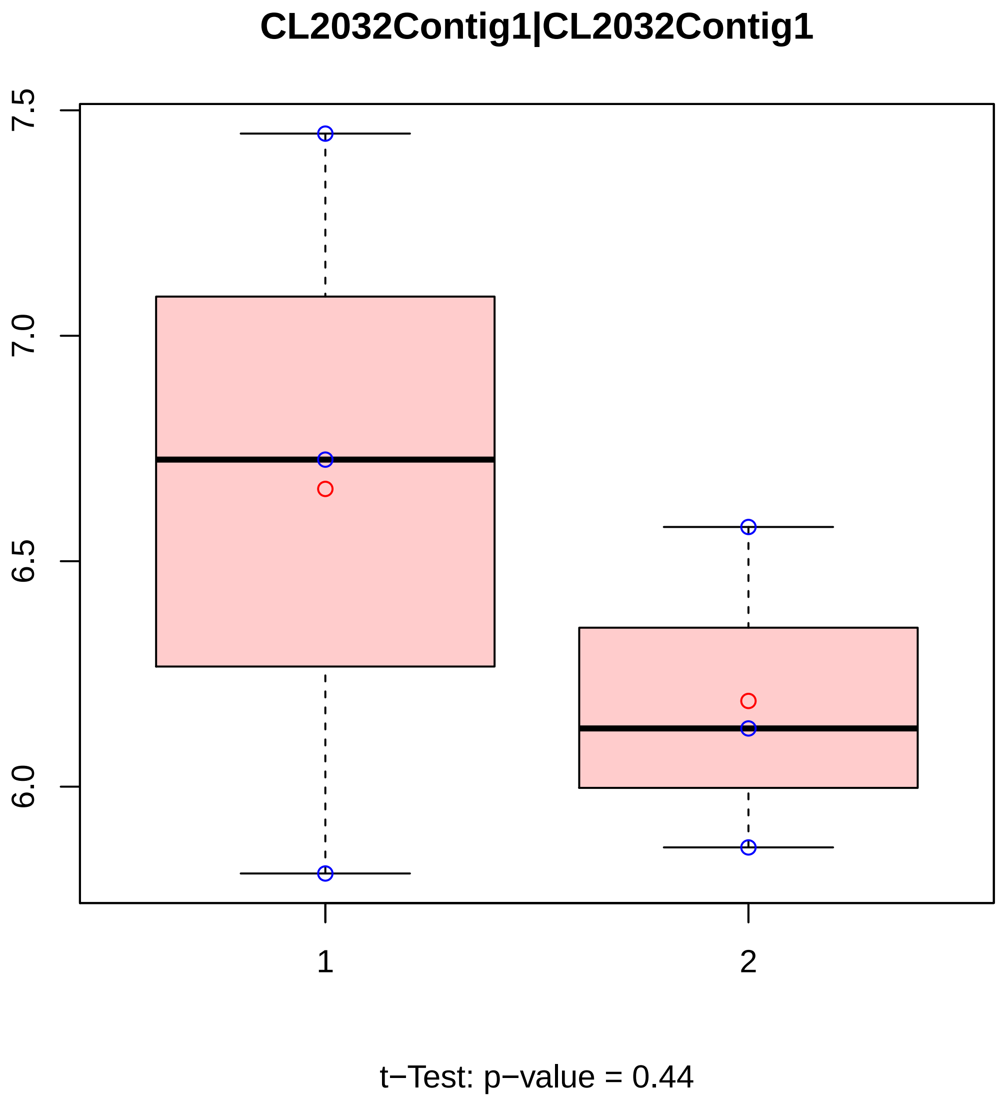
CL2032Contig1|CL2032Contig1
7.5
7.0
6.5
6.0
1
2
t−Test: p−value = 0.44

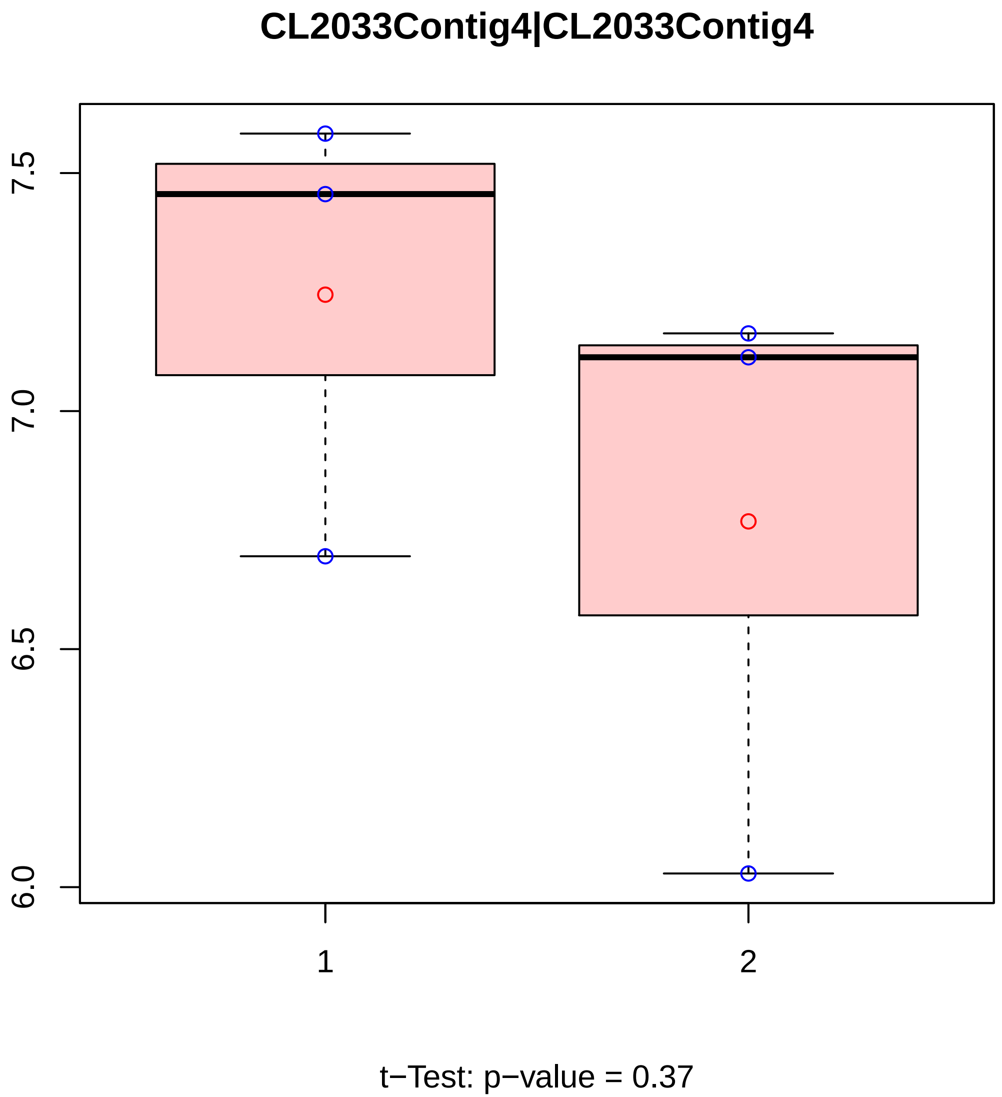
CL2033Contig4|CL2033Contig4
7.5
7.0
6.5
6.0
1
2
t−Test: p−value = 0.37

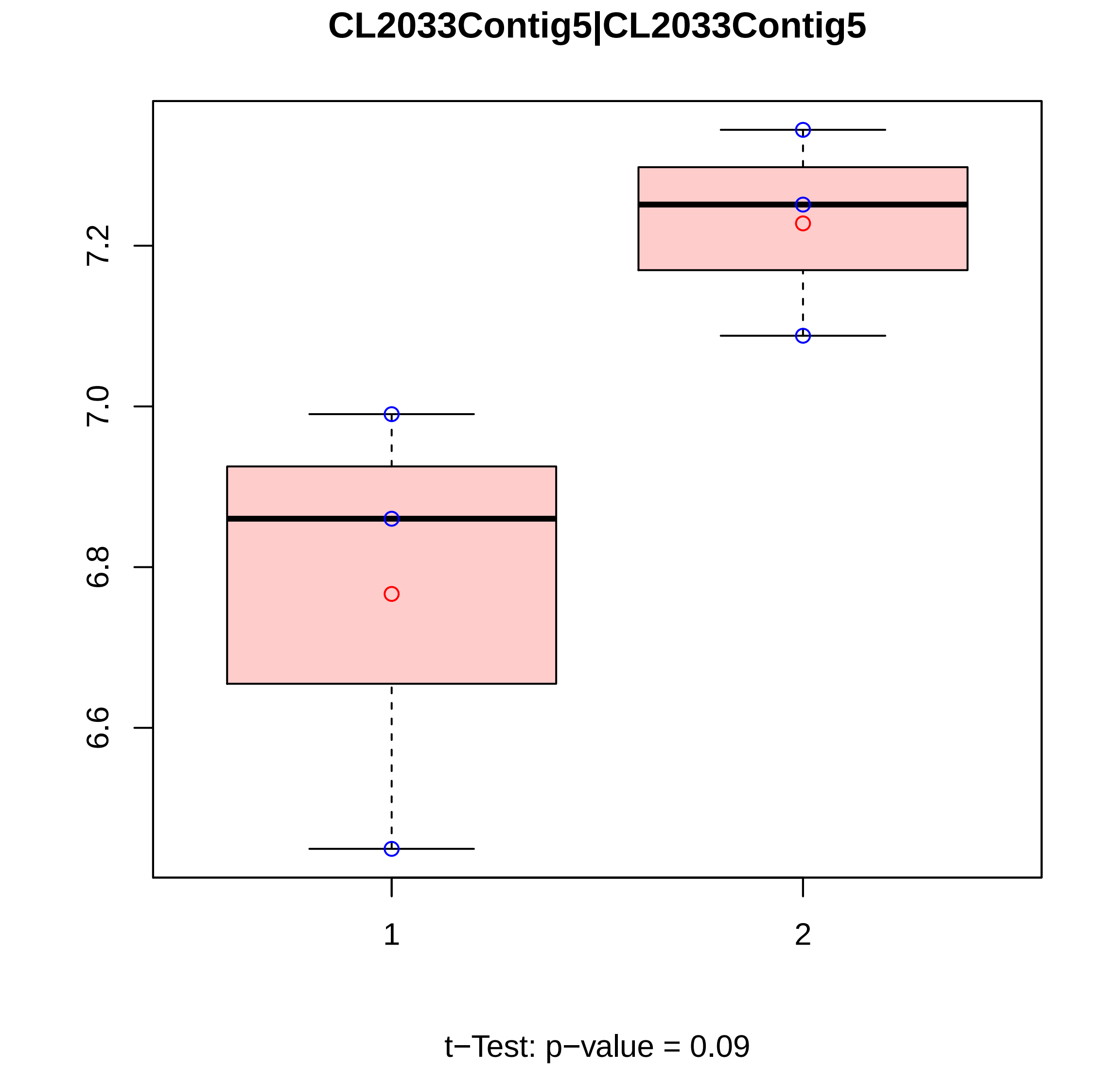
CL2033Contig5|CL2033Contig5
6.6
6.8
7.0
7.2
1
2
t−Test: p−value = 0.09

## CL2034Contig1|CL2034Contig1

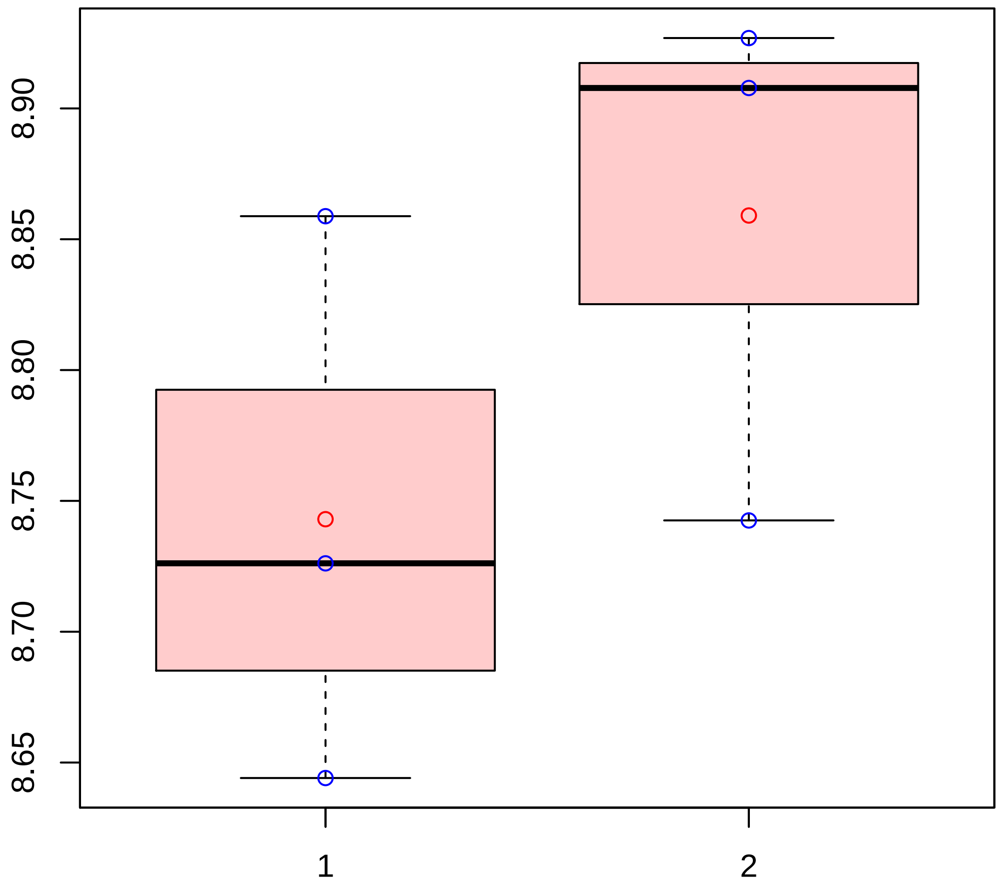


t−Test: p−value = 0.25

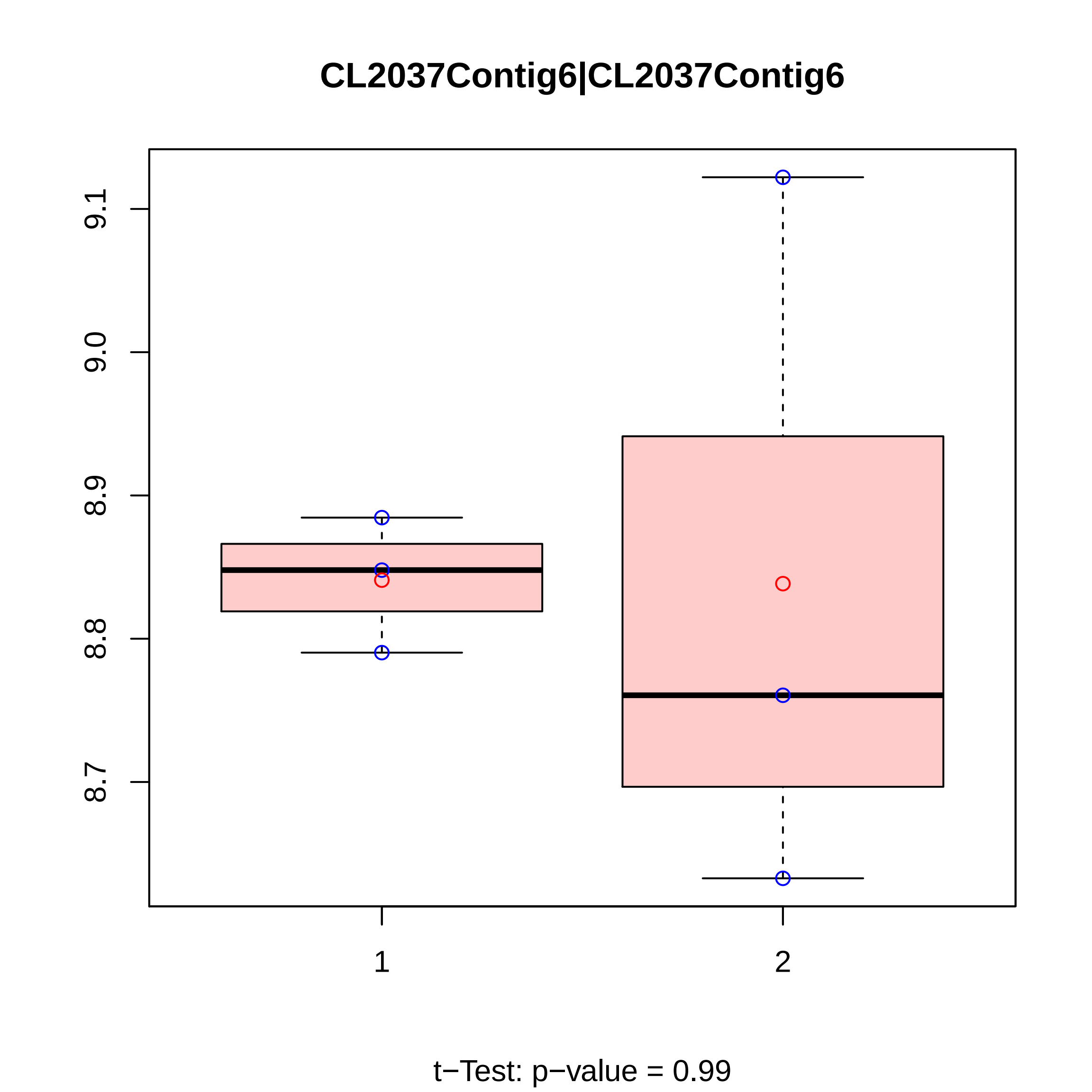
CL2037Contig6|CL2037Contig6
9.1
9.0
8.9
8.8
8.7
1
2
t−Test: p−value = 0.99

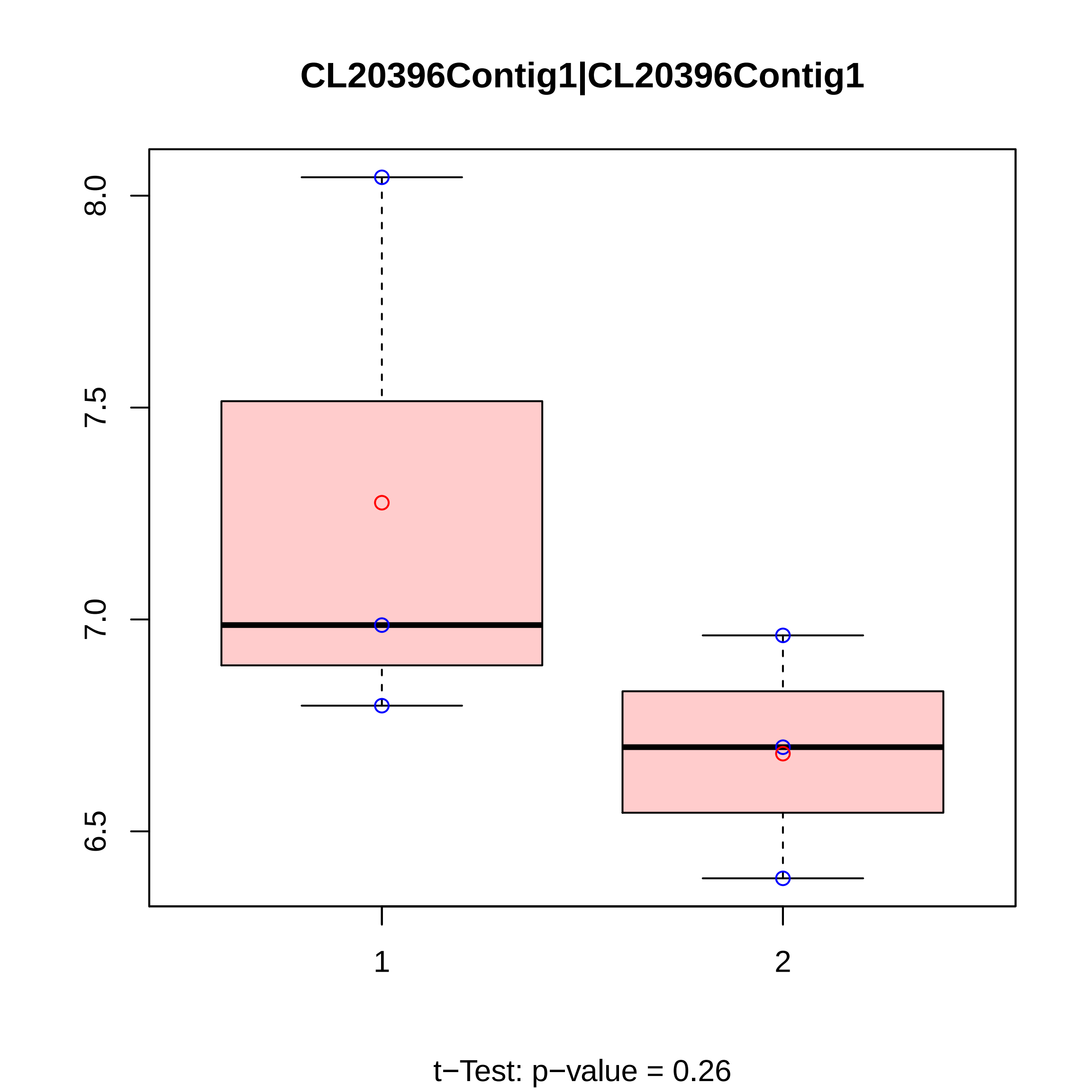

CL20396Contig1|CL20396Contig1
8.0
7.5
7.0
6.5
1
2
t−Test: p−value = 0.26

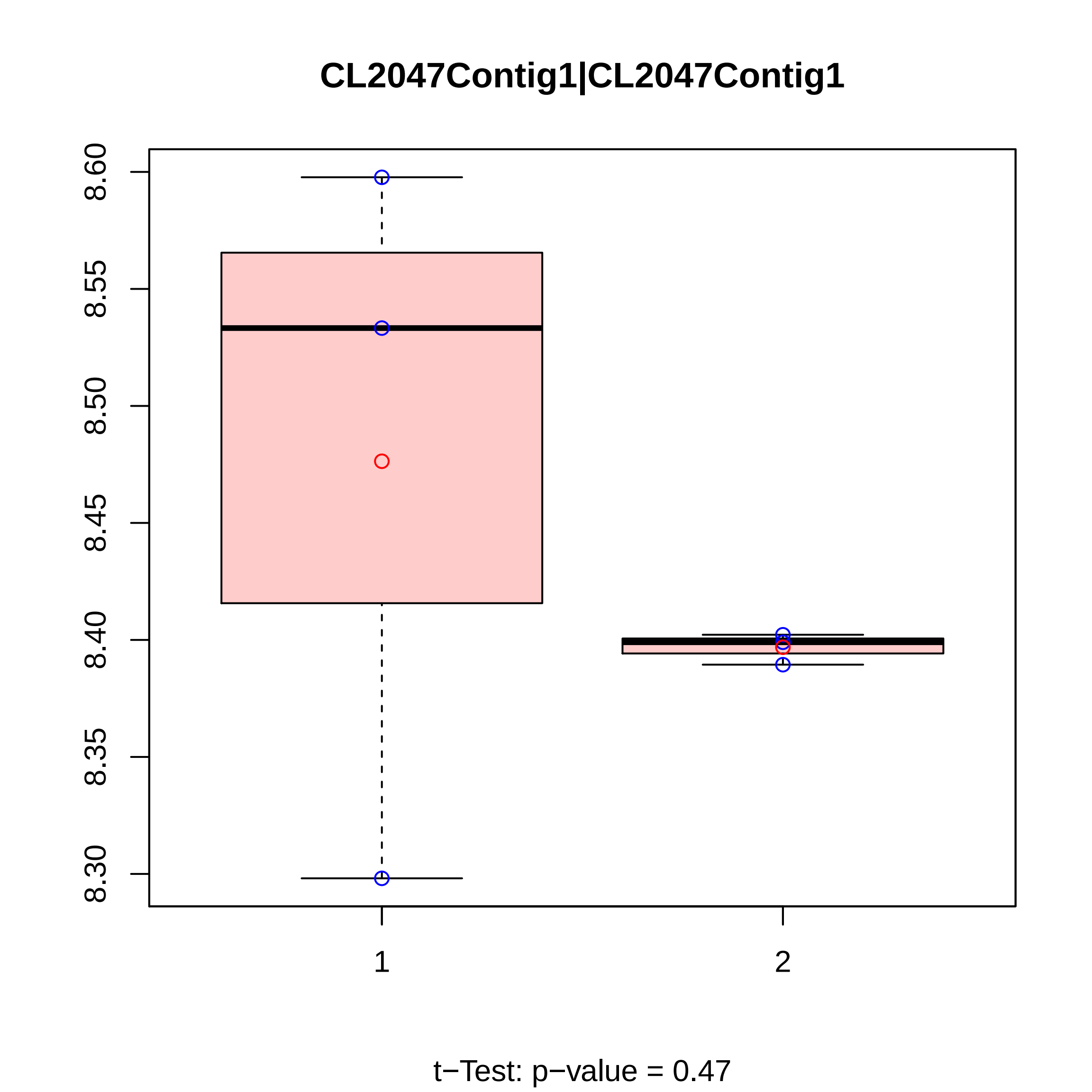
CL2047Contig1|CL2047Contig1
8.60
8.55
8.50
8.45
8.40
8.35
8.30
1
2
t−Test: p−value = 0.47

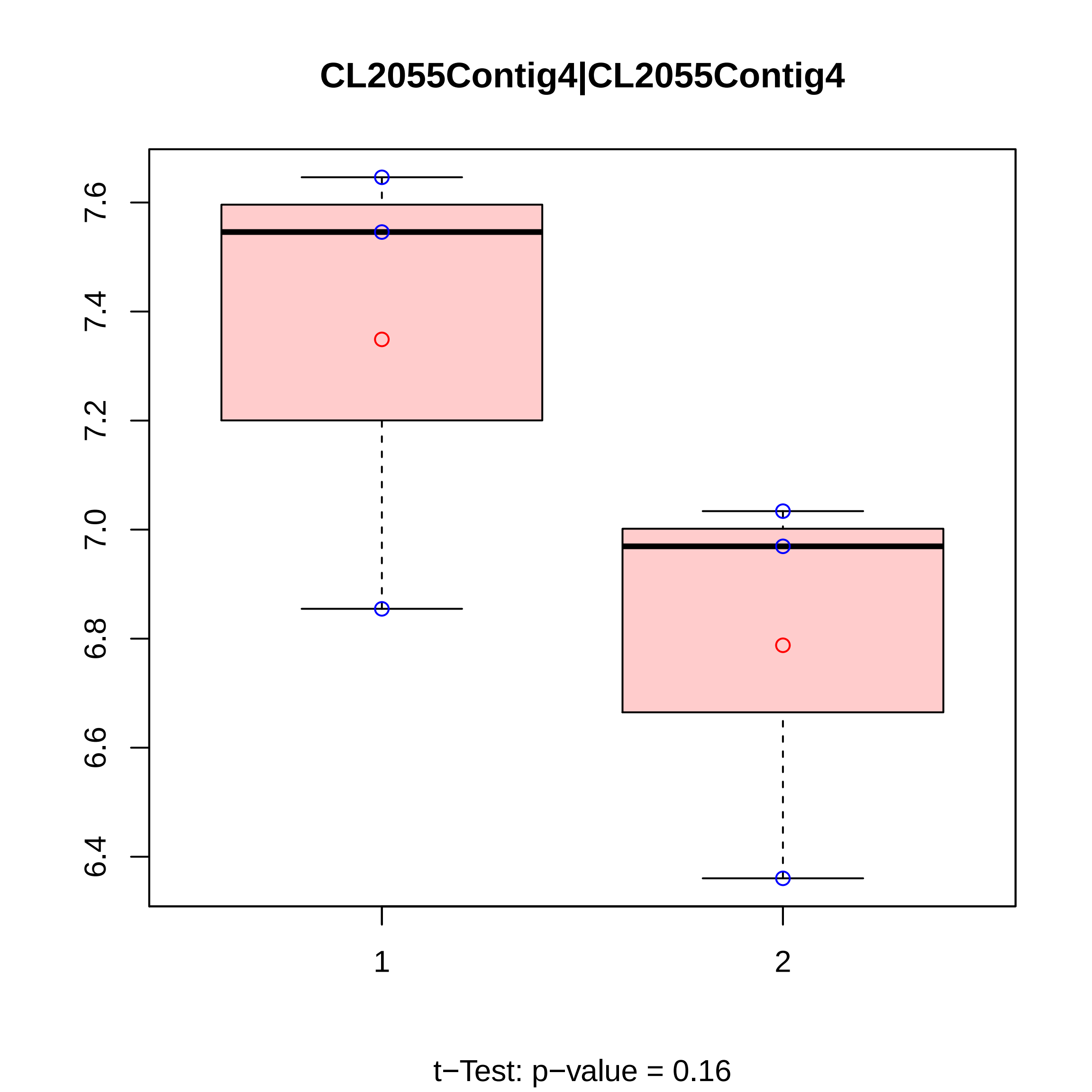

CL2055Contig4|CL2055Contig4
7.6
7.4
7.2
7.0
6.8
6.6
6.4
1
2
t−Test: p−value = 0.16

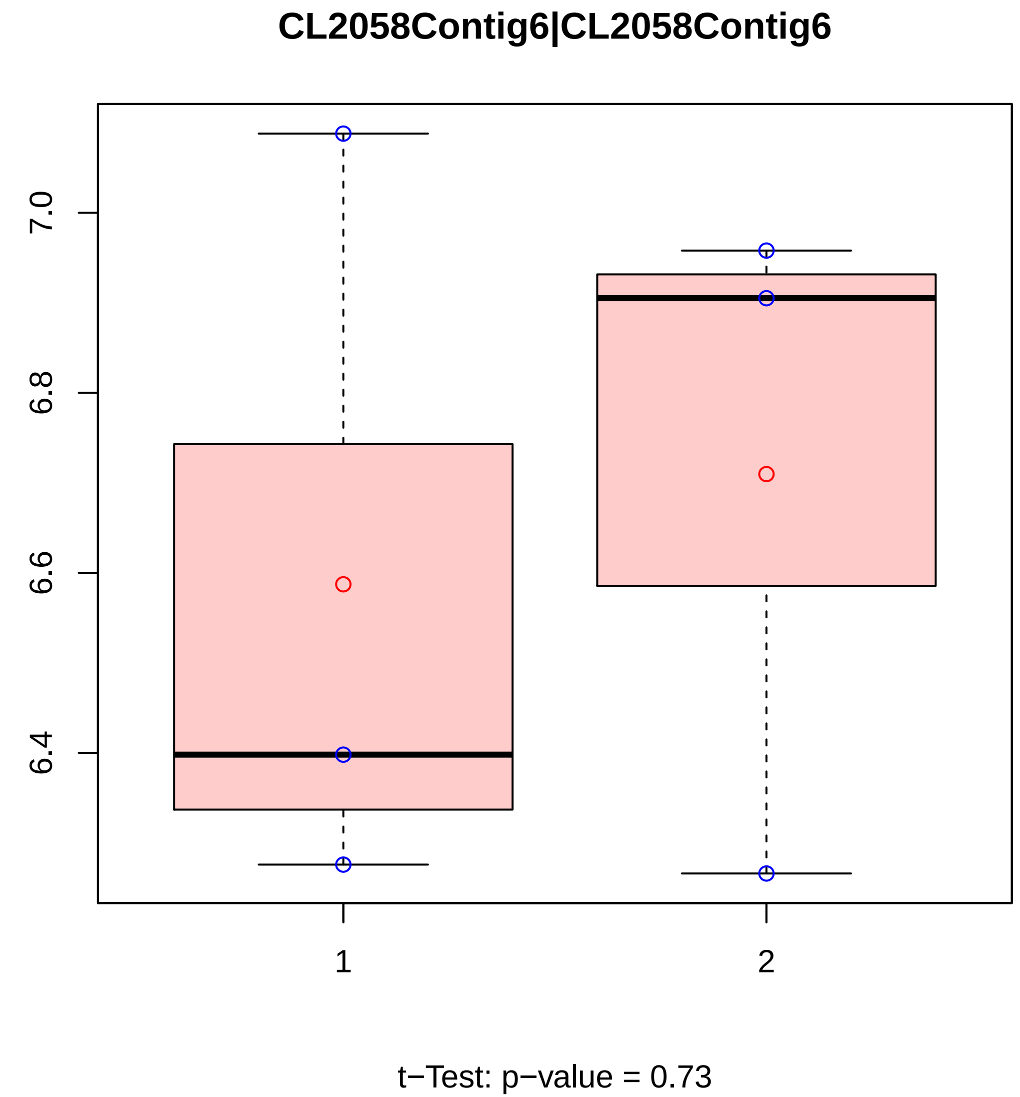
CL2058Contig6|CL2058Contig6
7.0
6.8
6.6
6.4
1
2
t−Test: p−value = 0.73

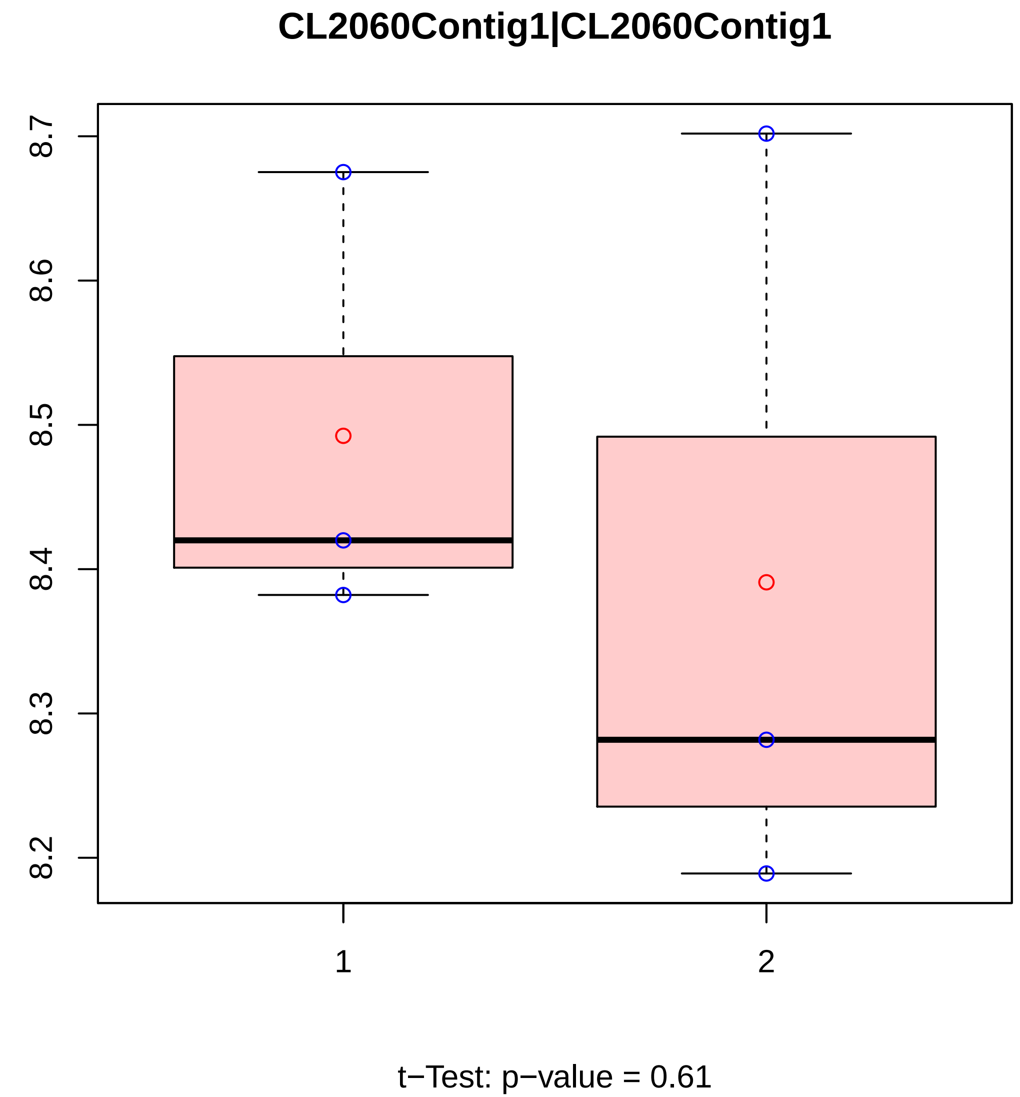
CL2060Contig1|CL2060Contig1
8.2
8.3
8.4
8.5
8.6
8.7
1
2
t−Test: p−value = 0.61

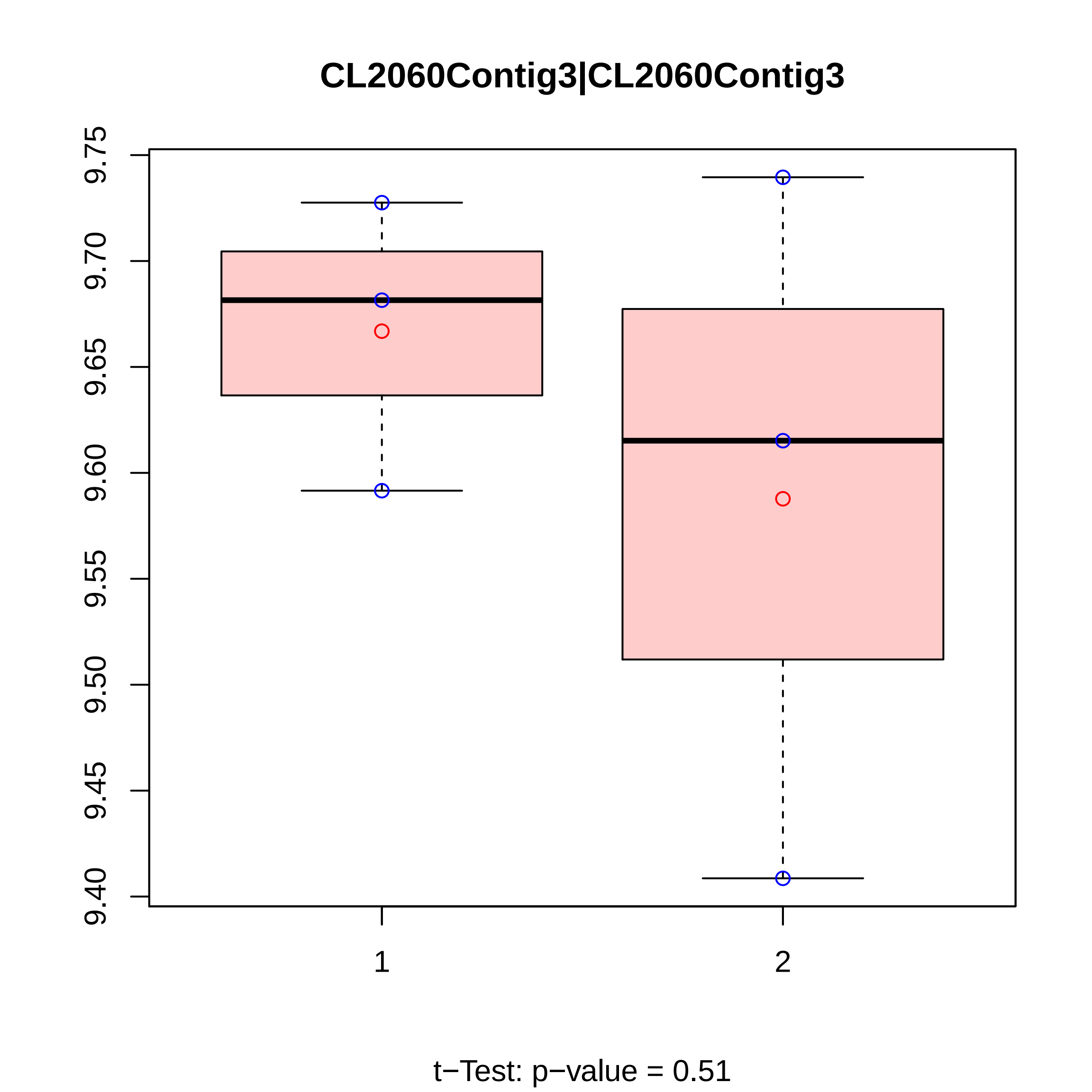
CL2060Contig3|CL2060Contig3
9.40
9.45
9.50
9.55
9.60
9.65
9.70
9.75
1
2
t−Test: p−value = 0.51

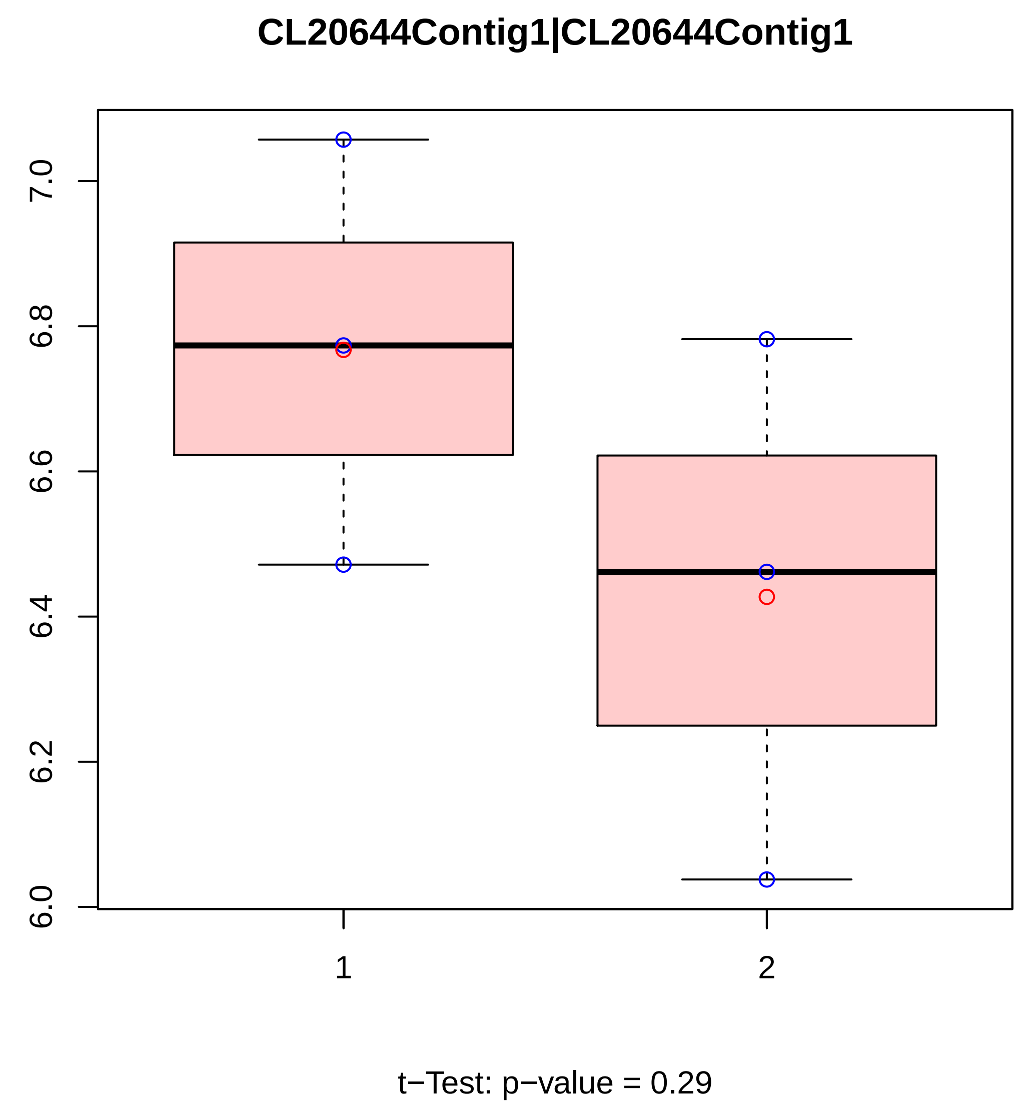
CL20644Contig1|CL20644Contig1
7.0
6.8
6.6
6.4
6.2
6.0
1
2
t−Test: p−value = 0.29

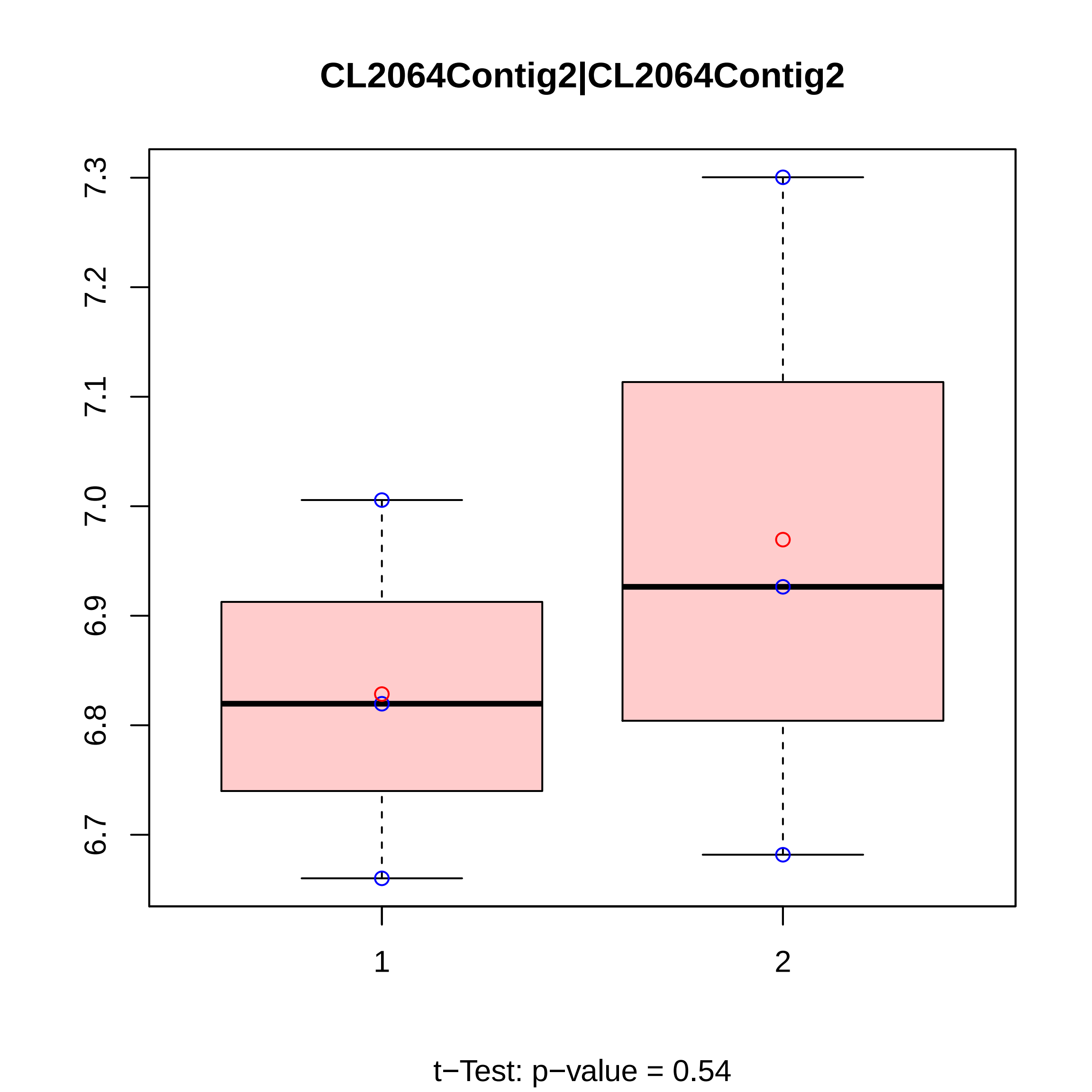

CL2064Contig2|CL2064Contig2
7.3
7.2
7.1
7.0
6.9
6.8
6.7
1
2
t−Test: p−value = 0.54

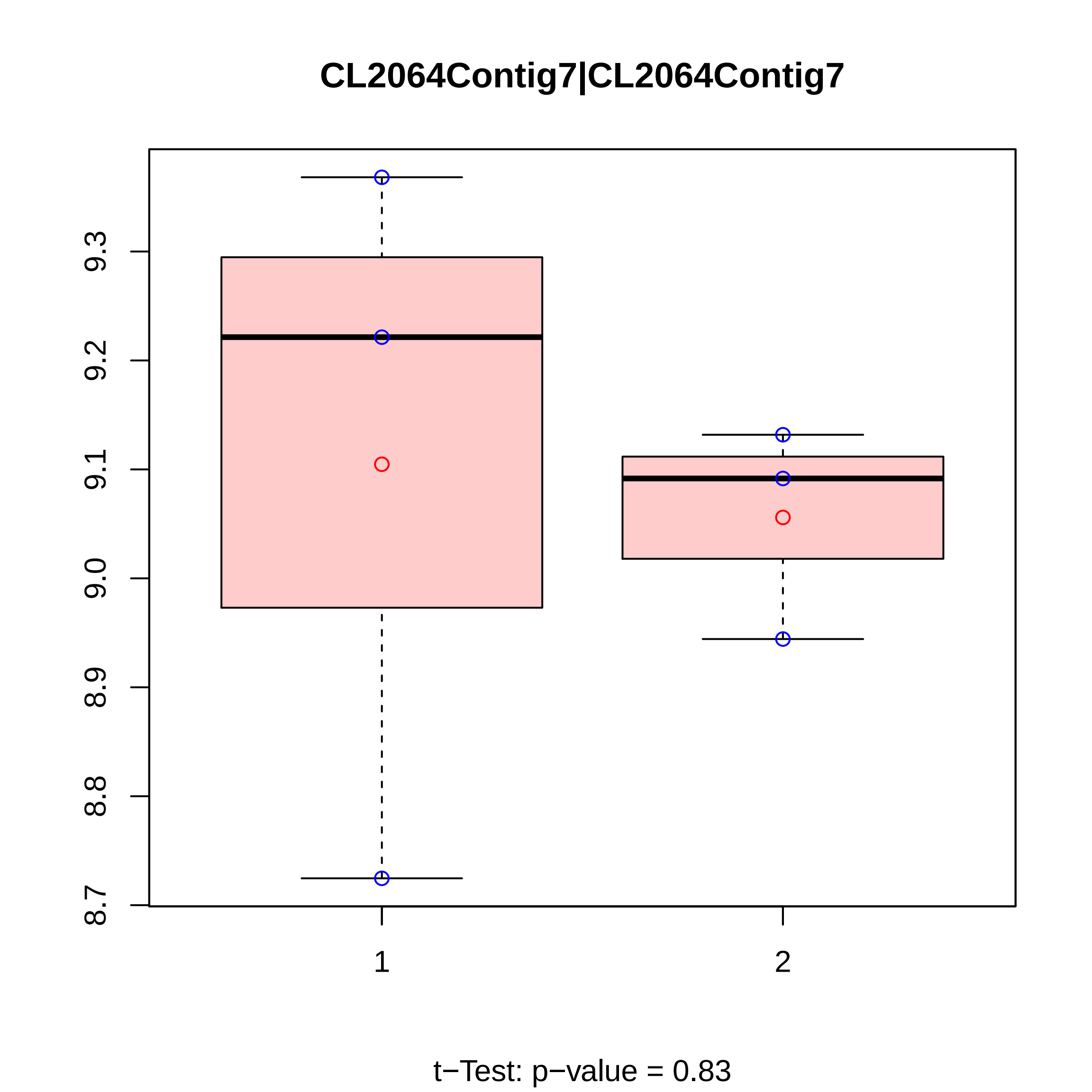
CL2064Contig7|CL2064Contig7
9.3
9.2
9.1
9.0
8.9
8.8
8.7
1
2
t−Test: p−value = 0.83

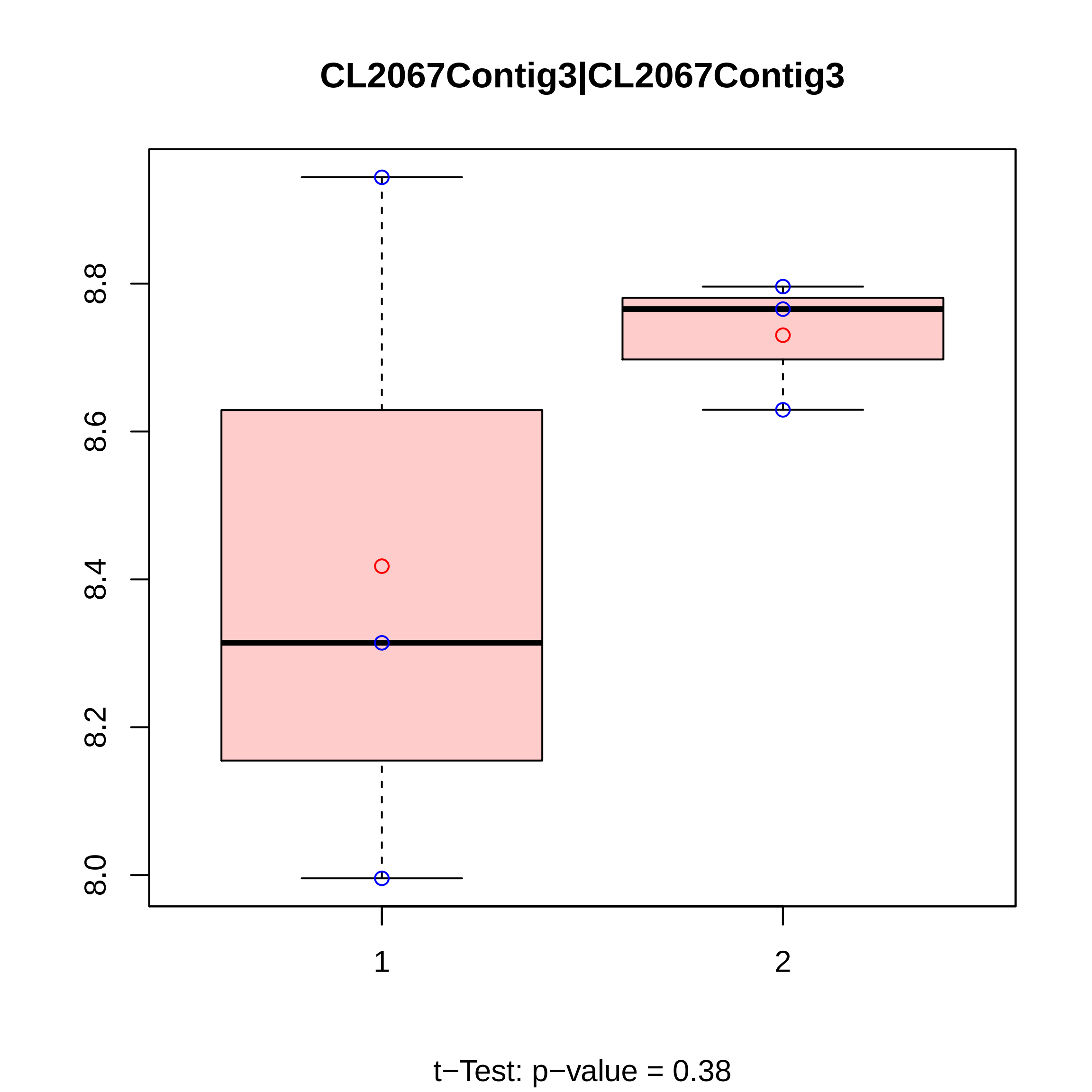
CL2067Contig3|CL2067Contig3
8.0
8.2
8.4
8.6
8.8
1
2
t−Test: p−value = 0.38

# CL206Contig8|CL206Contig8

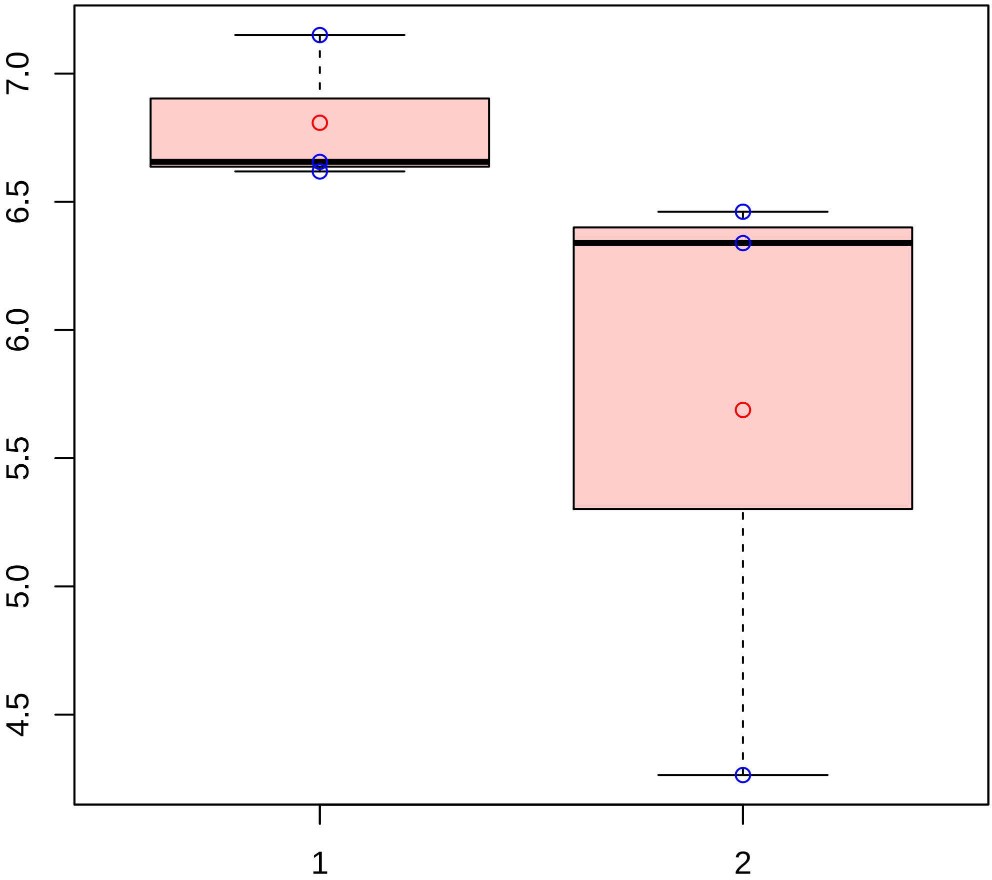


t−Test: p−value = 0.25

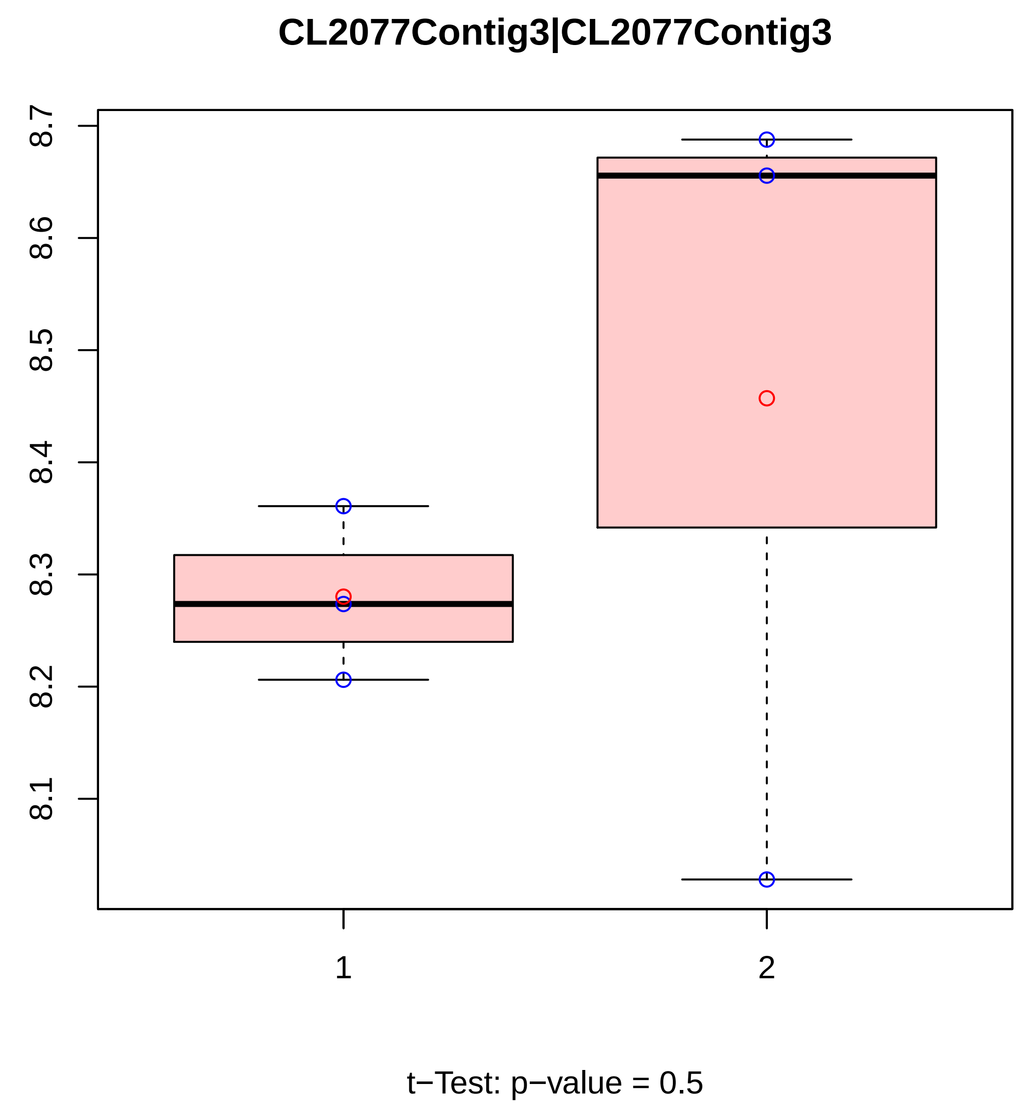
CL2077Contig3|CL2077Contig3
8.1
8.2
8.3
8.4
8.5
8.6
8.7
1
2
t−Test: p−value = 0.5

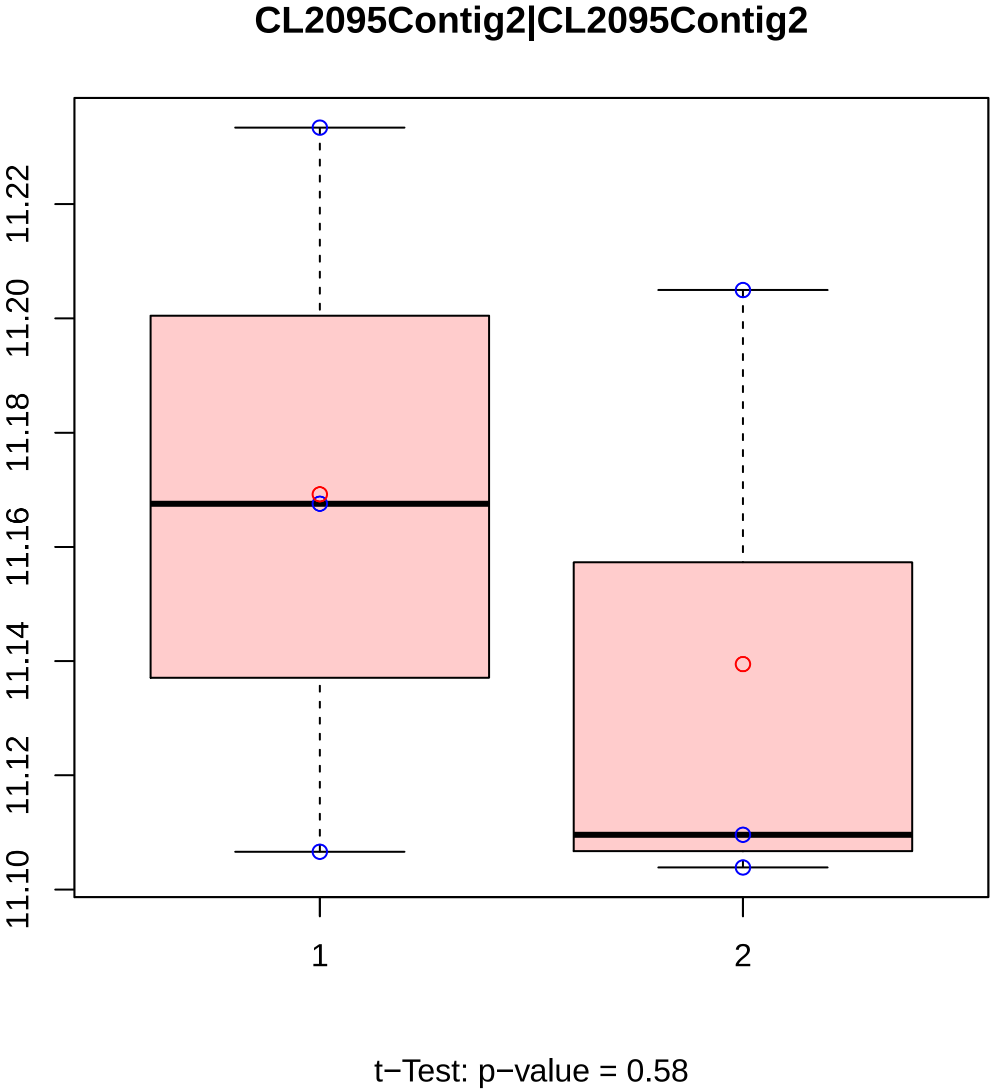

CL2095Contig2|CL2095Contig2
11.10
11.12
11.14
11.16
11.18
11.20
11.22
1
2
t−Test: p−value = 0.58

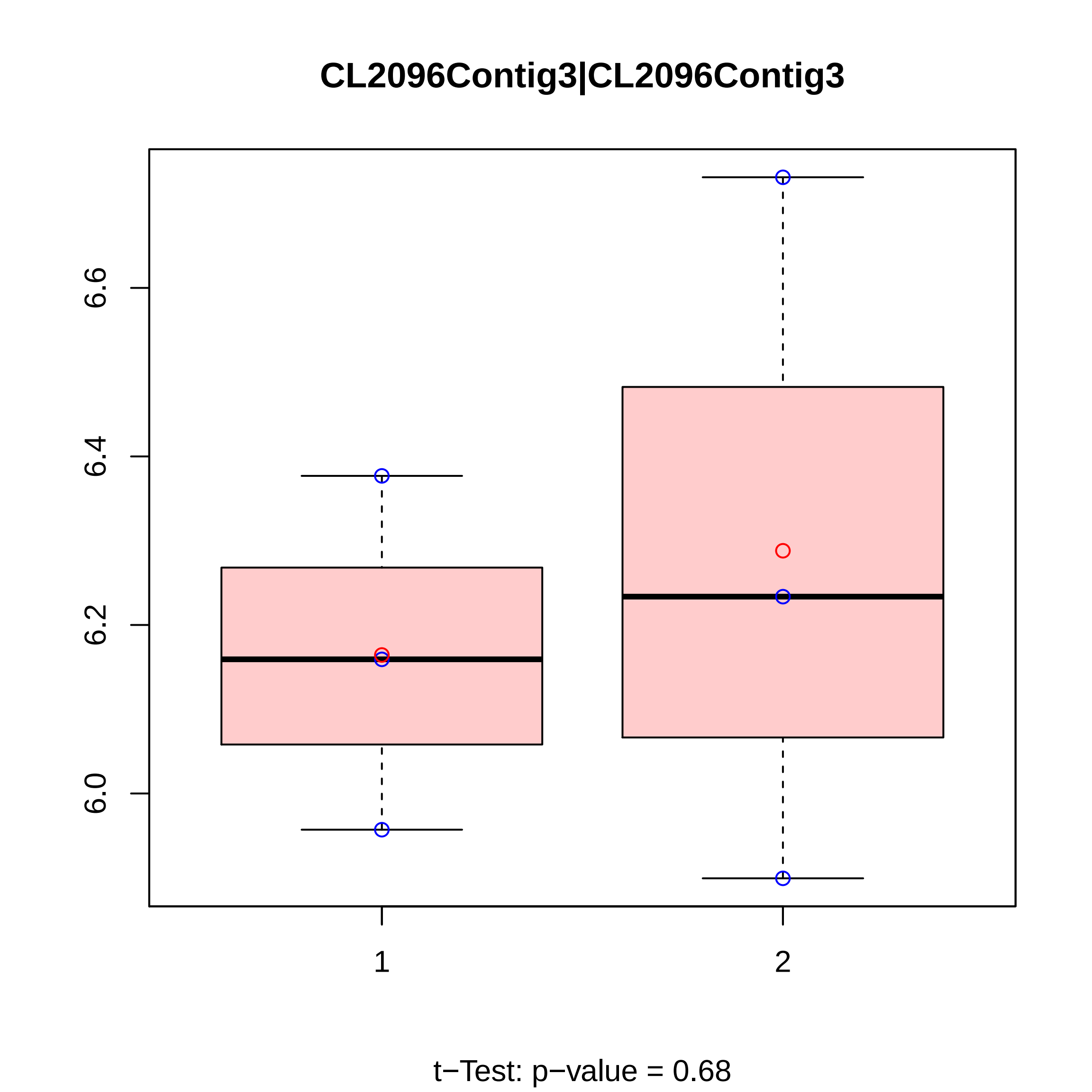

CL2096Contig3|CL2096Contig3
6.0
6.2
6.4
6.6
1
2
t−Test: p−value = 0.68

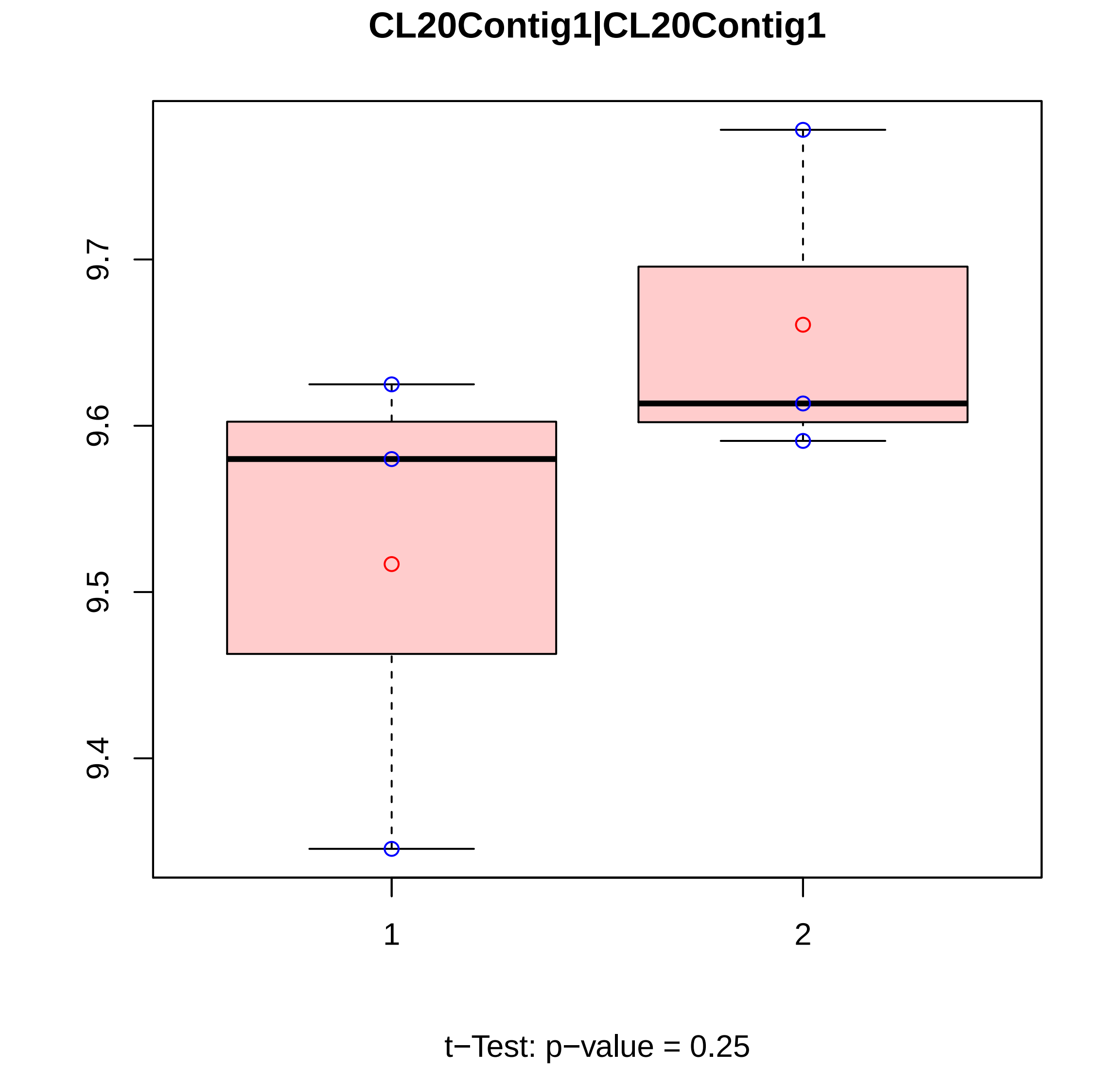

CL20Contig1|CL20Contig1
9.4
9.5
9.6
9.7
1
2
t−Test: p−value = 0.25

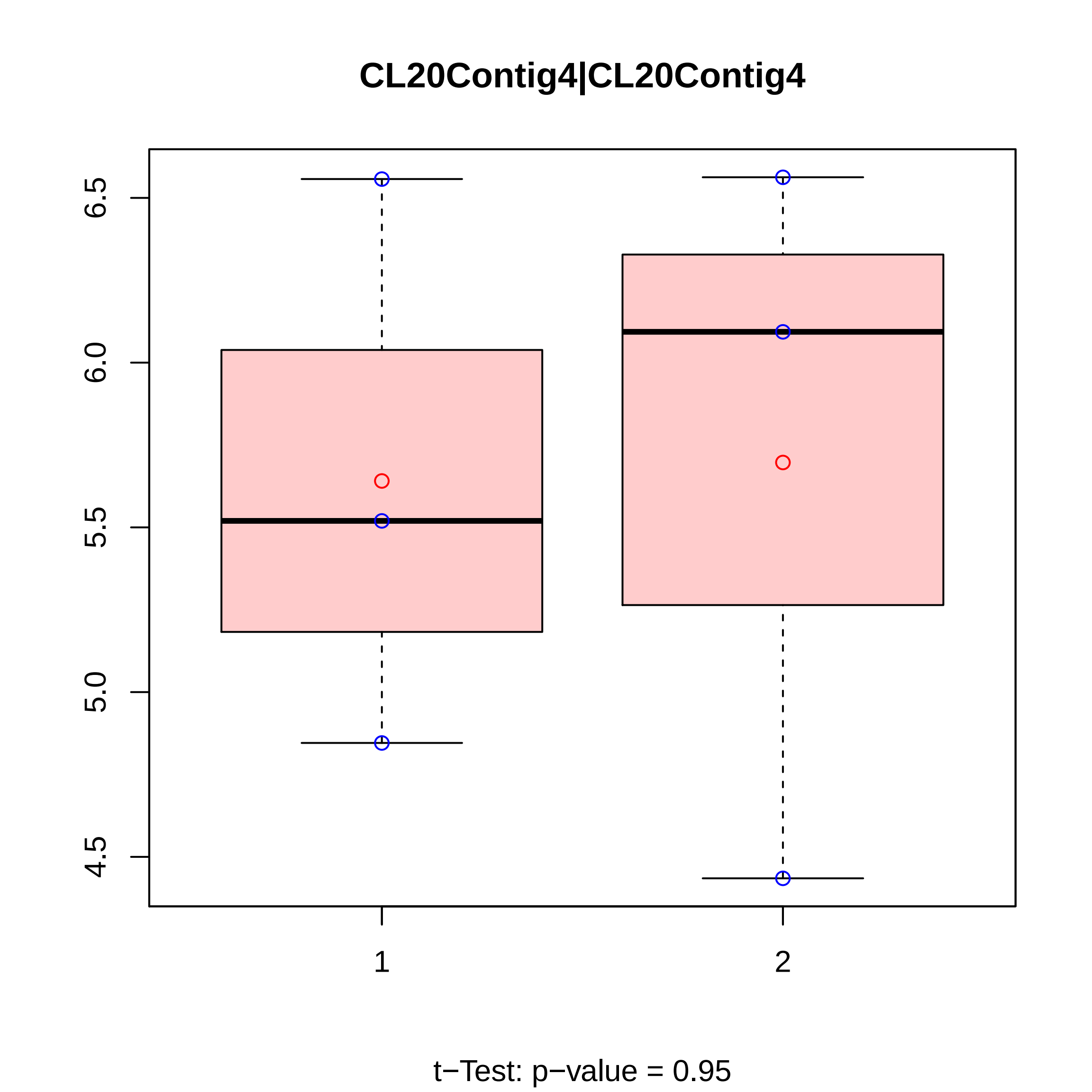

CL20Contig4|CL20Contig4
6.5
6.0
5.5
5.0
4.5
1
2
t−Test: p−value = 0.95

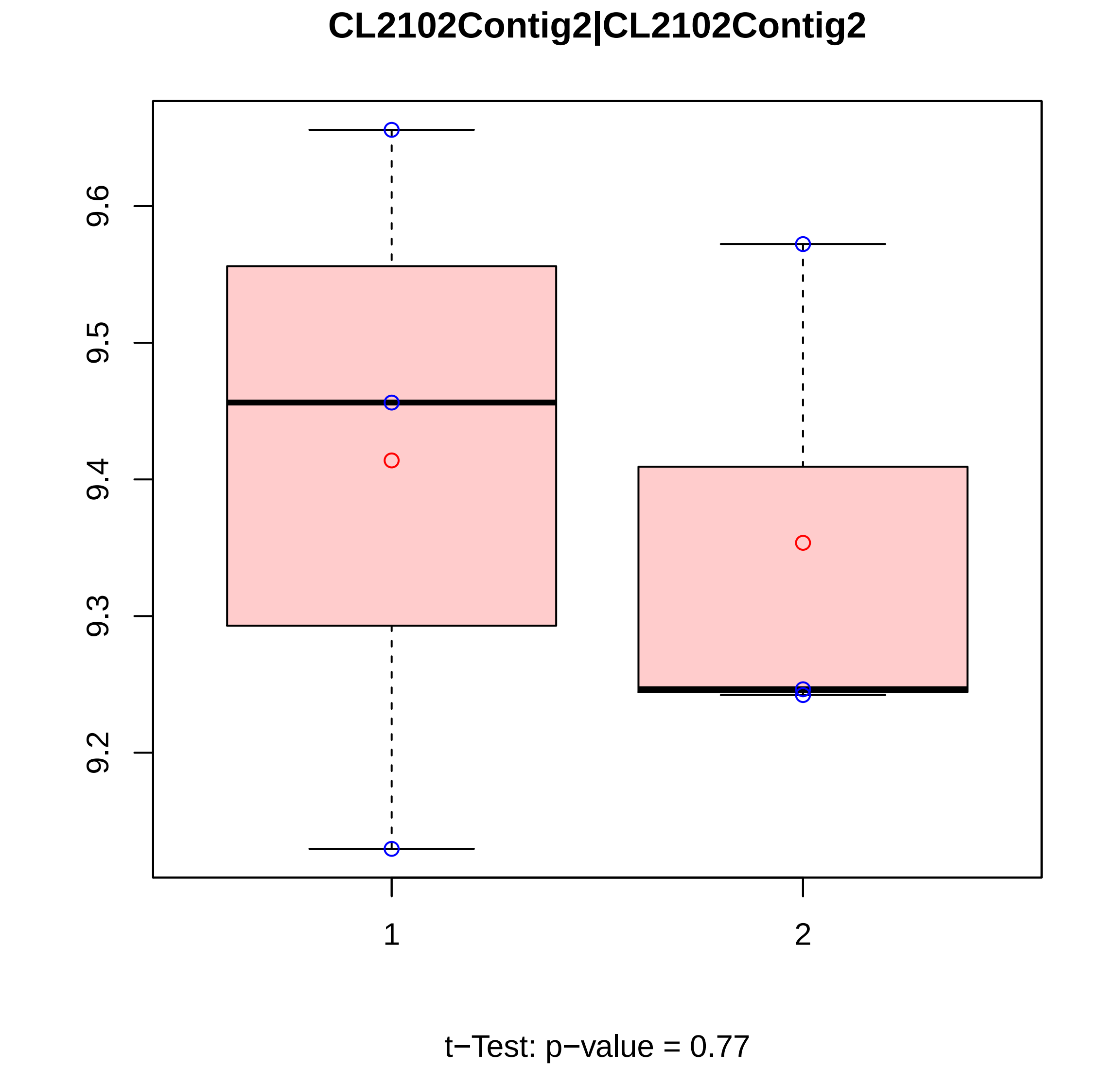
CL2102Contig2|CL2102Contig2
9.2
9.3
9.4
9.5
9.6
1
2
t−Test: p−value = 0.77

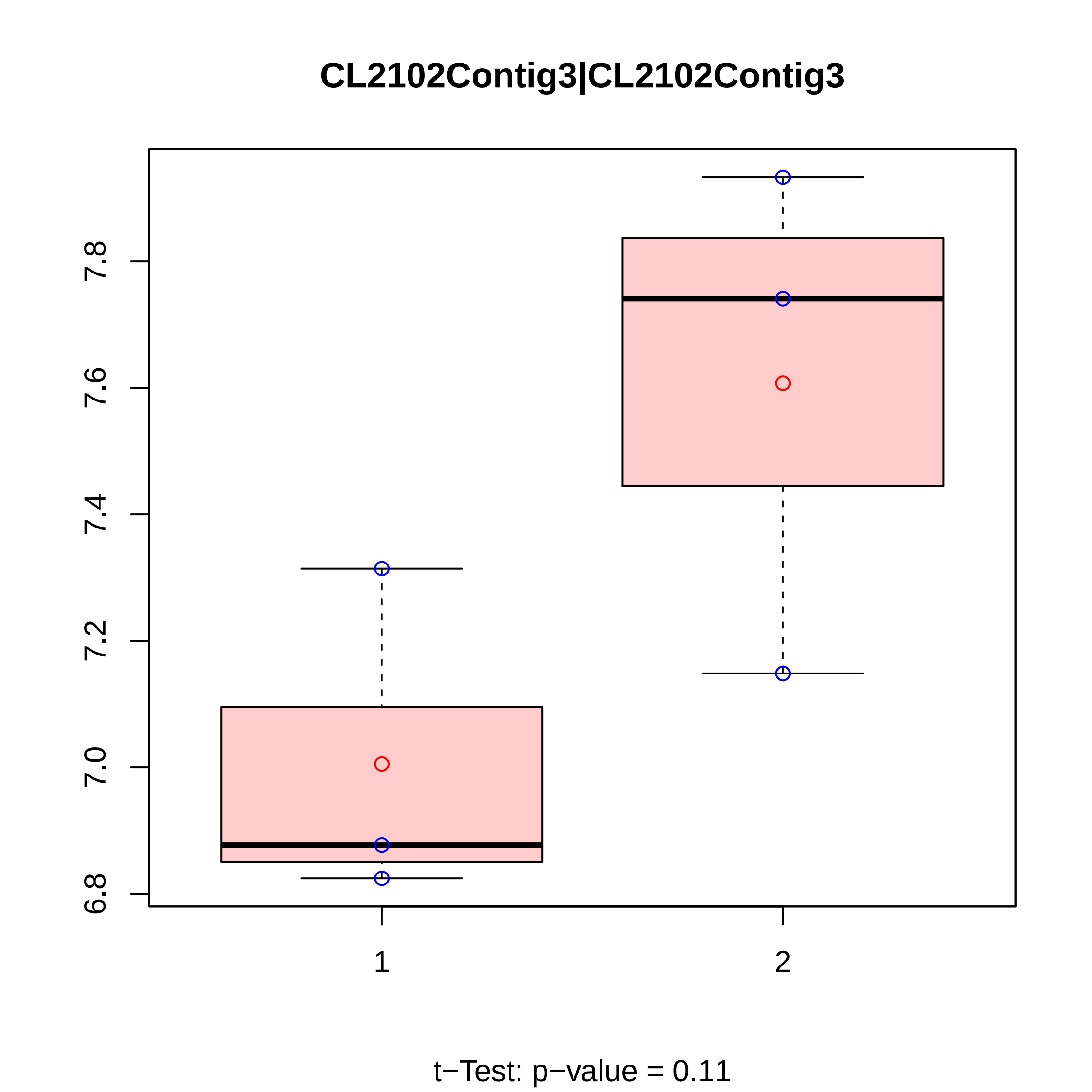

CL2102Contig3|CL2102Contig3
7.8
7.6
7.4
7.2
7.0
6.8
1
2
t−Test: p−value = 0.11

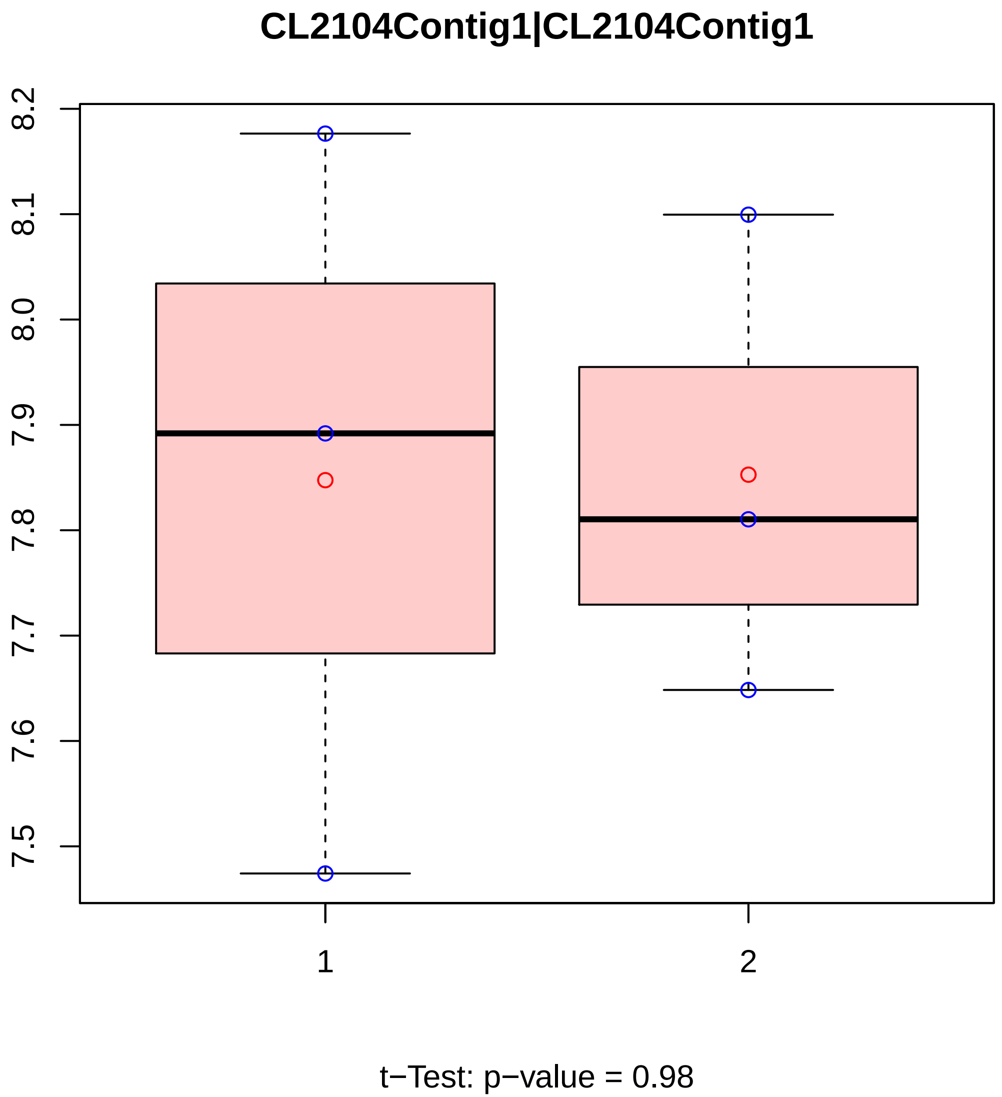
CL2104Contig1|CL2104Contig1
8.2
8.1
8.0
7.9
7.8
7.7
7.6
7.5
1
2
t−Test: p−value = 0.98

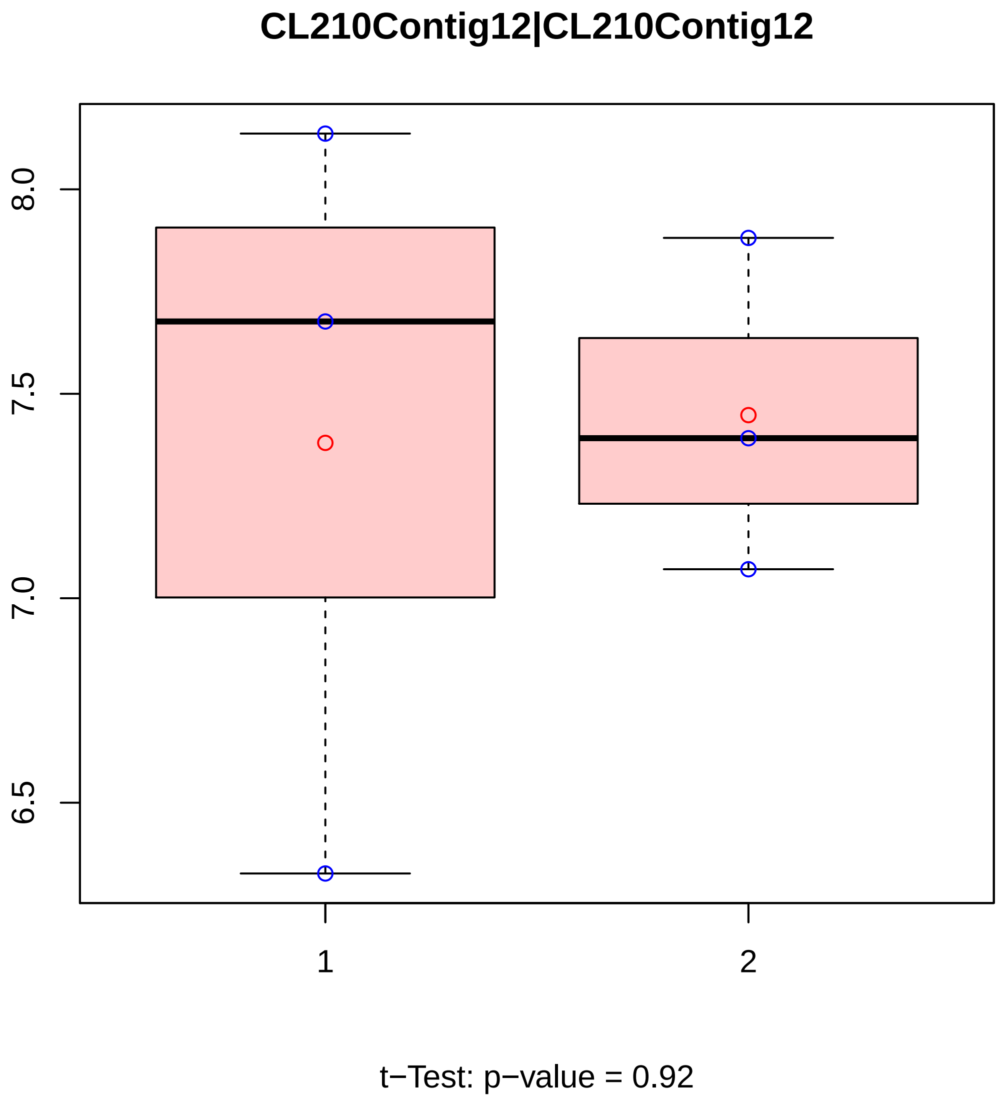
CL210Contig12|CL210Contig12
8.0
7.5
7.0
6.5
1
2
t−Test: p−value = 0.92

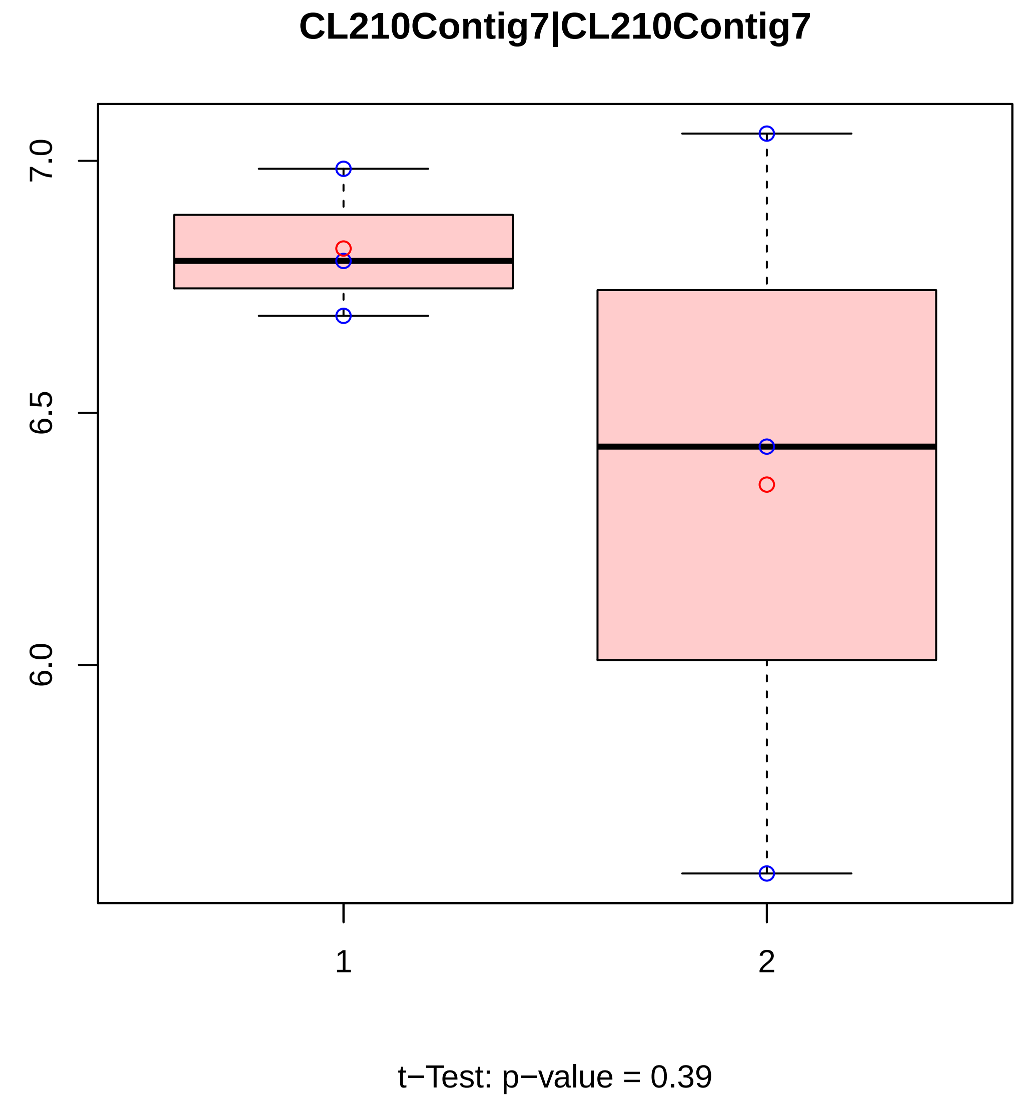
CL210Contig7|CL210Contig7
7.0
6.5
6.0
1
2
t−Test: p−value = 0.39

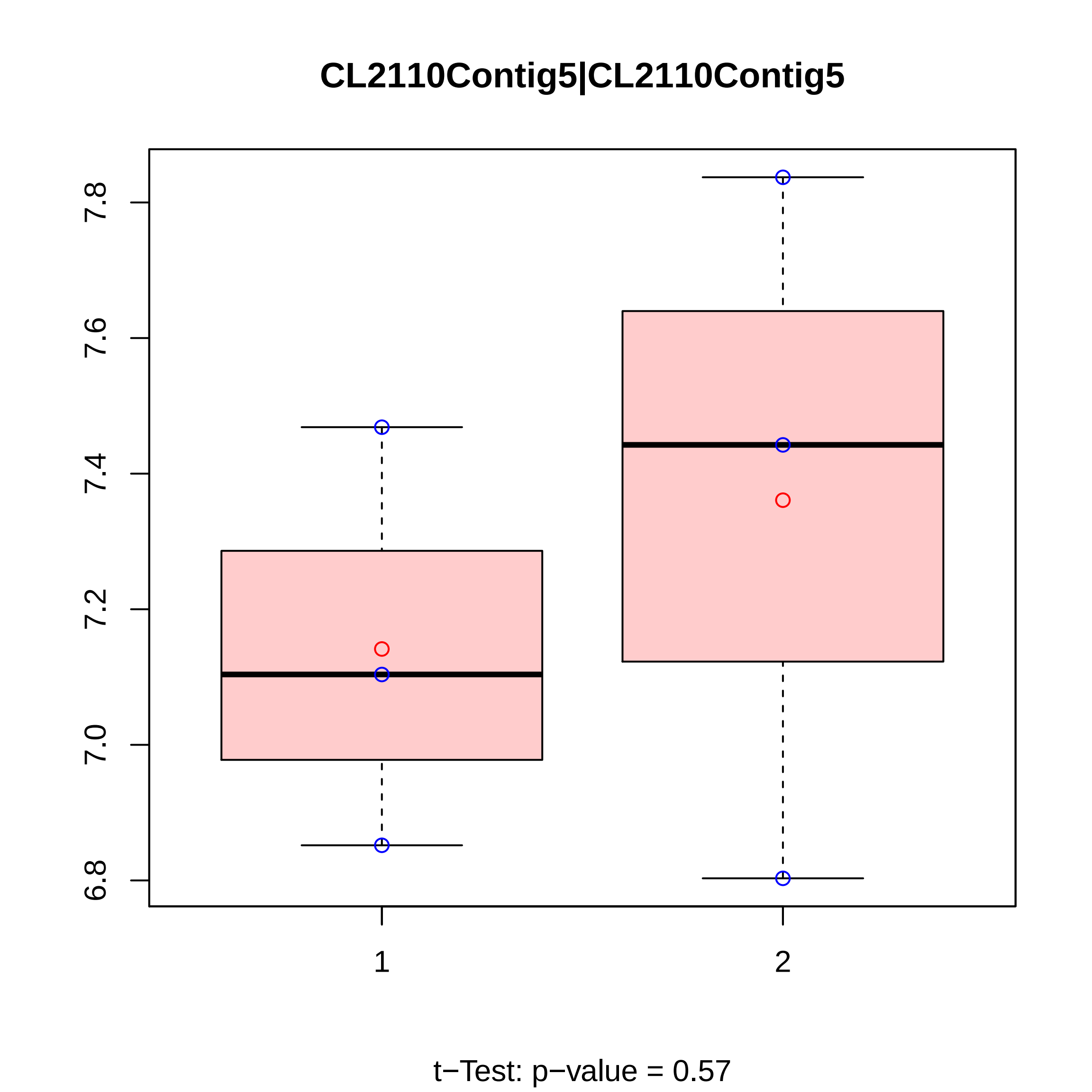
CL2110Contig5|CL2110Contig5
7.8
7.6
7.4
7.2
7.0
6.8
1
2
t−Test: p−value = 0.57

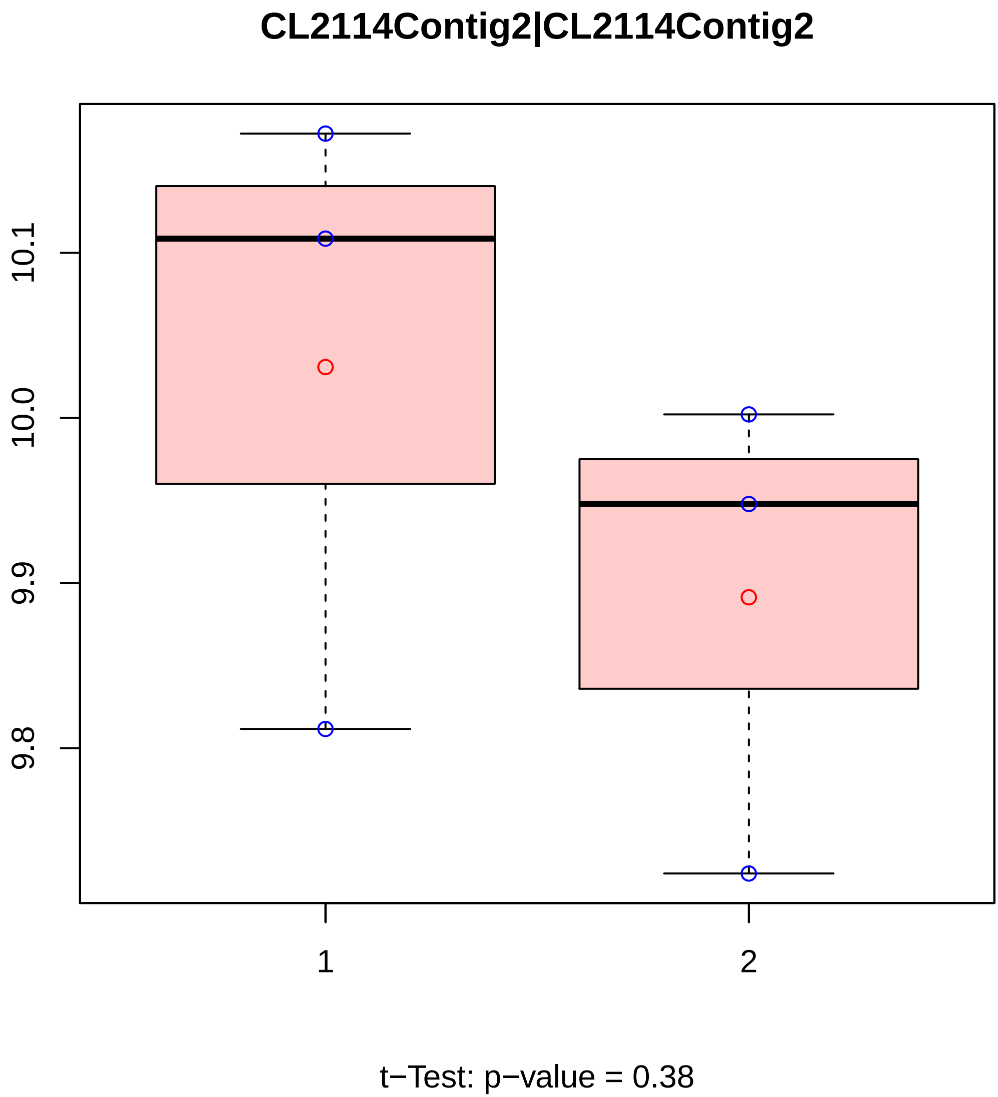
CL2114Contig2|CL2114Contig2
10.1
10.0
9.9
9.8
1
2
t−Test: p−value = 0.38

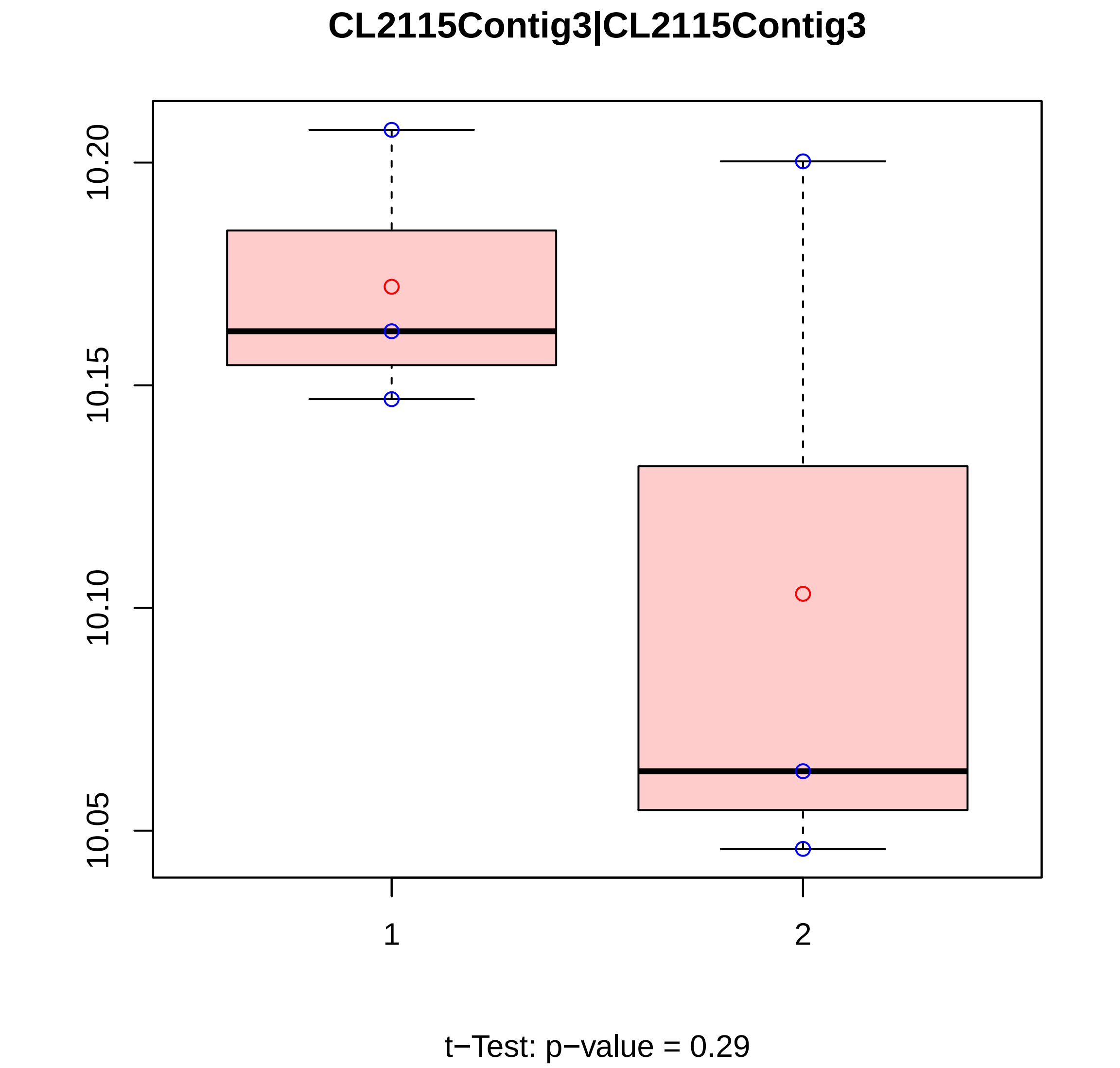
CL2115Contig3|CL2115Contig3
10.20
10.15
10.10
10.05
1
2
t−Test: p−value = 0.29

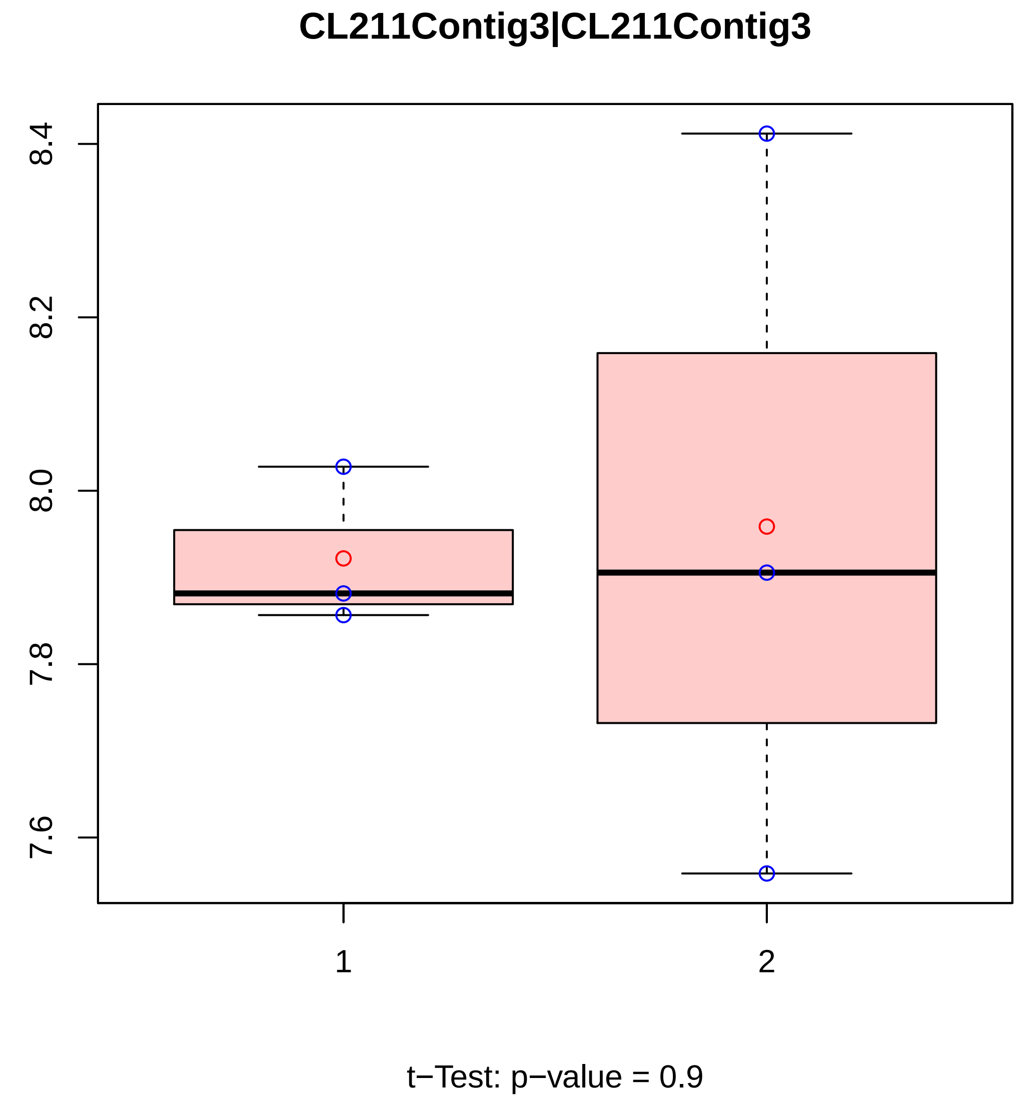
CL211Contig3|CL211Contig3
8.4
8.2
8.0
7.8
7.6
1
2
t−Test: p−value = 0.9

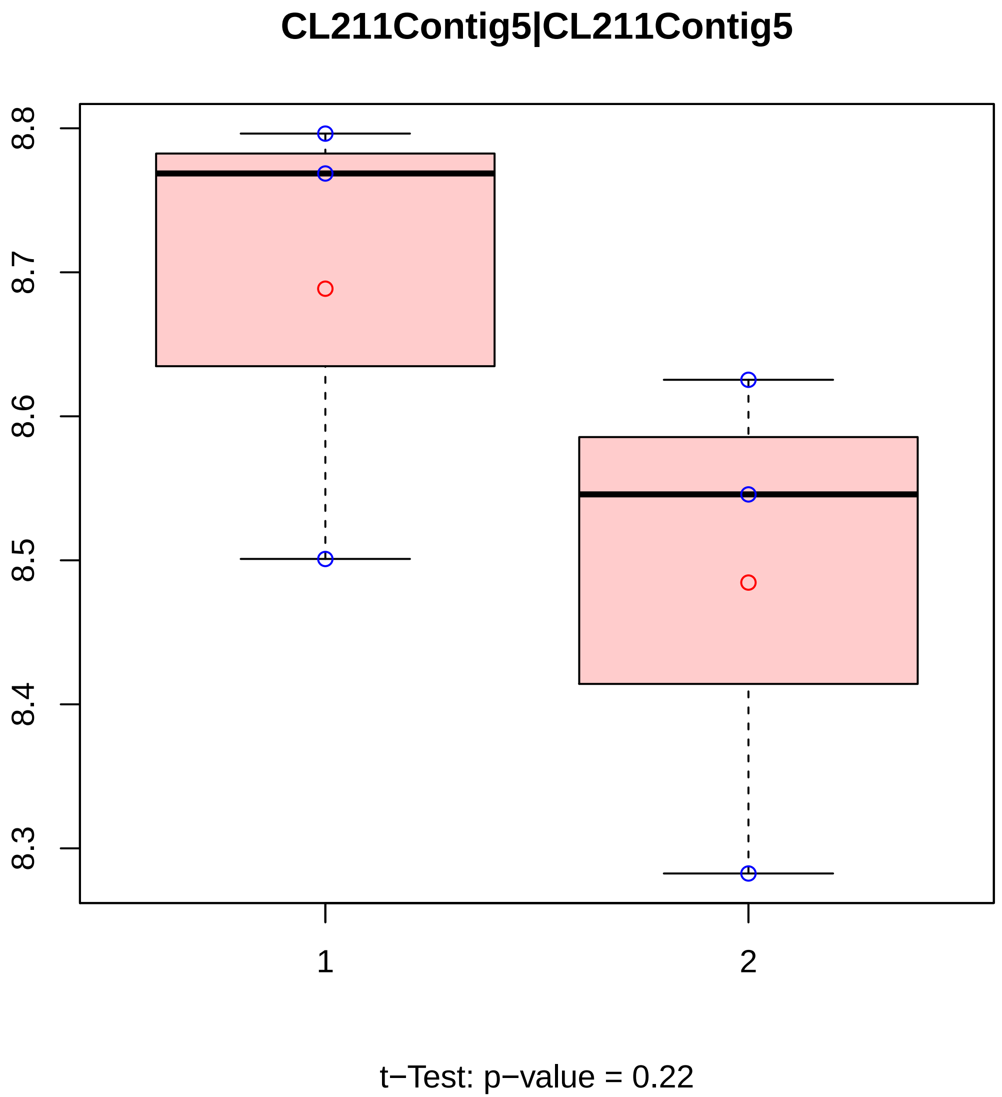
CL211Contig5|CL211Contig5
8.8
8.7
8.6
8.5
8.4
8.3
1
2
t−Test: p−value = 0.22

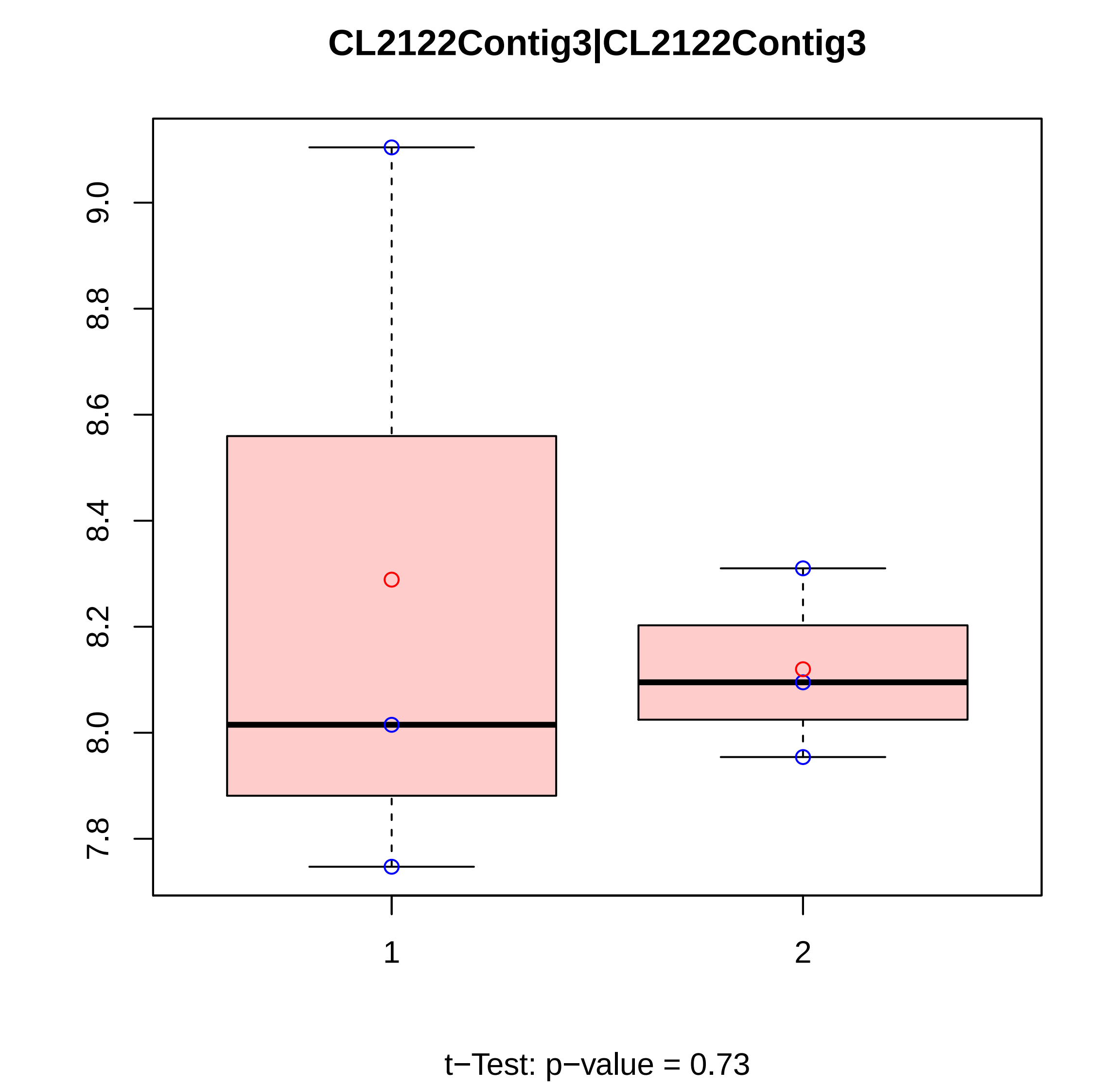
CL2122Contig3|CL2122Contig3
9.0
8.8
8.6
8.4
8.2
8.0
7.8
1
2
t−Test: p−value = 0.73

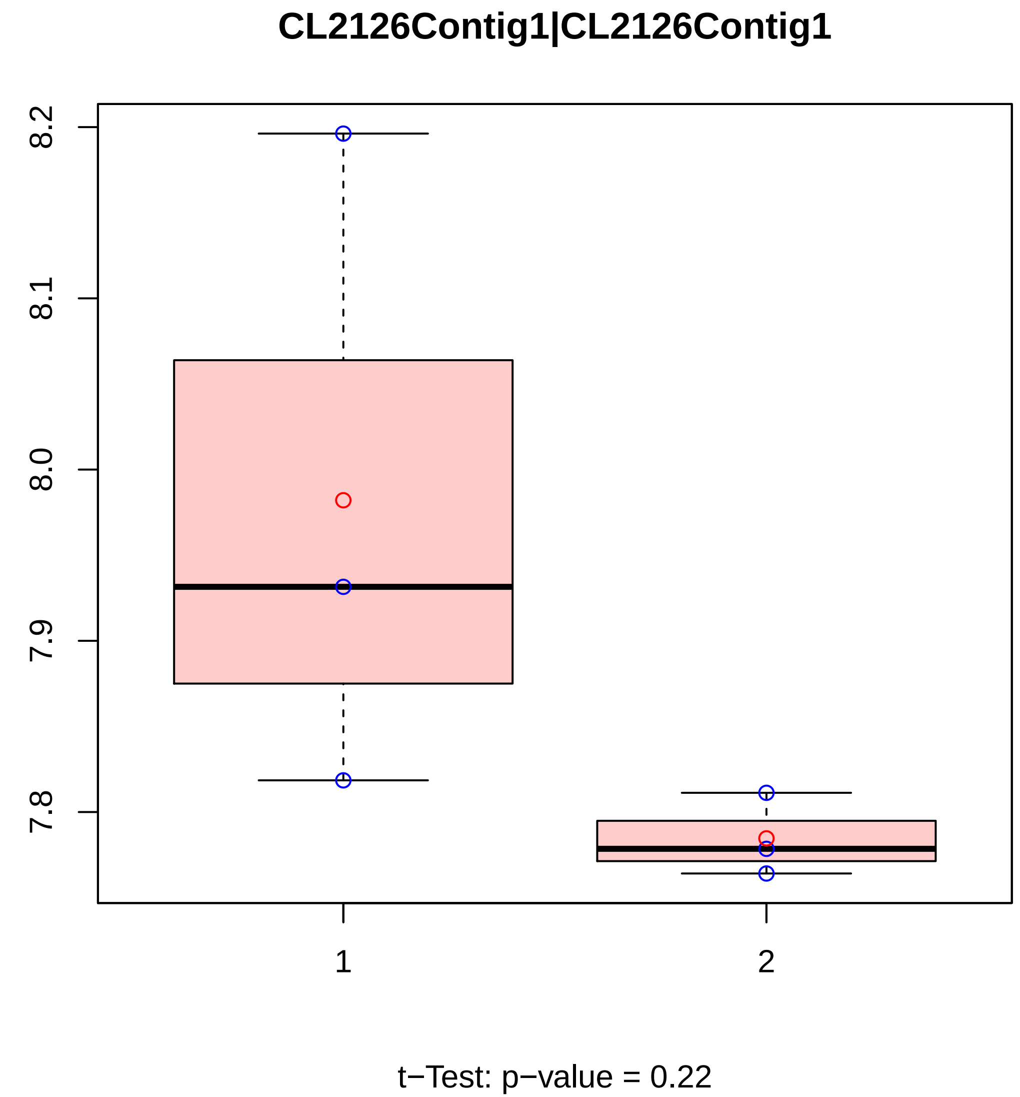
CL2126Contig1|CL2126Contig1
8.2
8.1
8.0
7.9
7.8
1
2
t−Test: p−value = 0.22

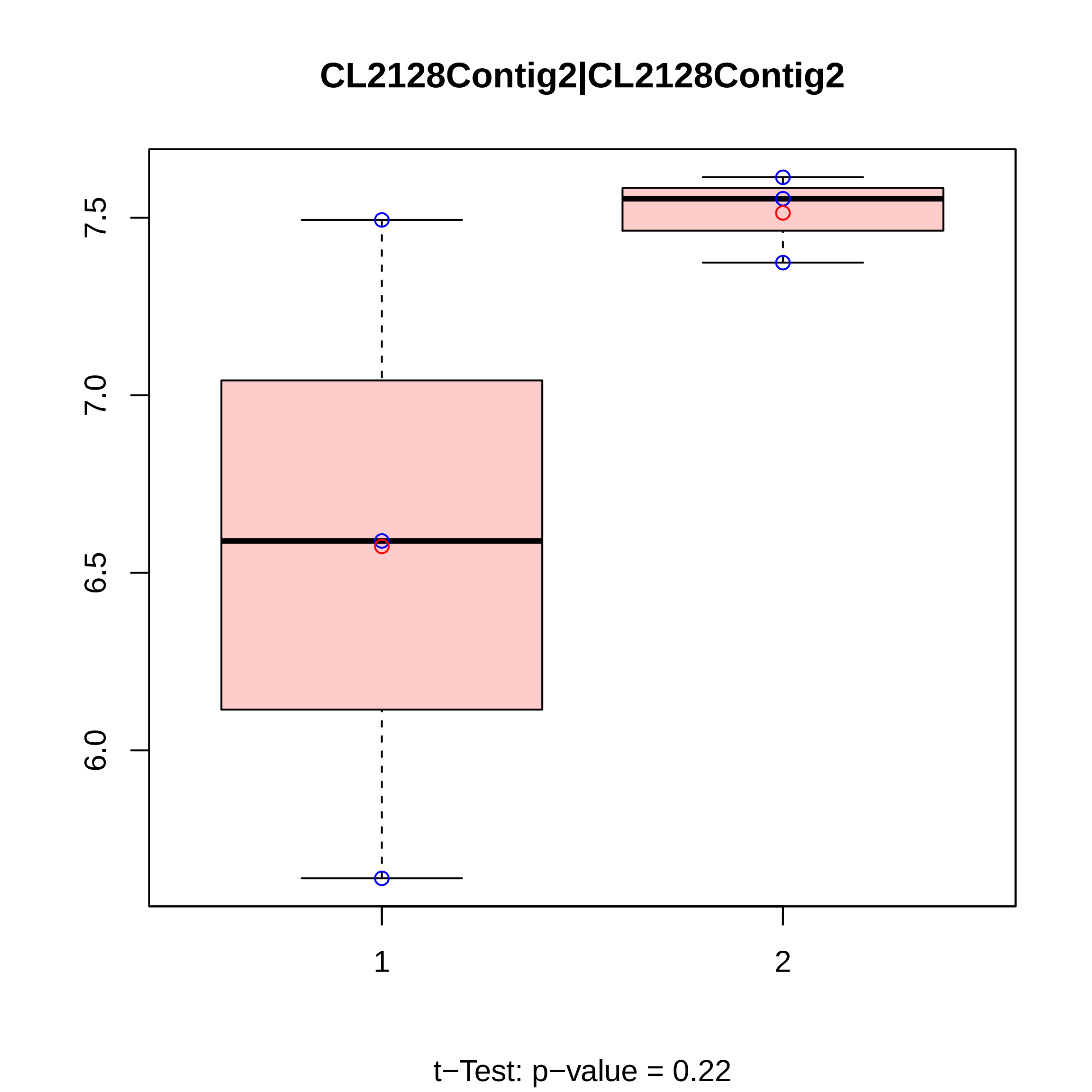
CL2128Contig2|CL2128Contig2
7.5
7.0
6.5
6.0
1
2
t−Test: p−value = 0.22

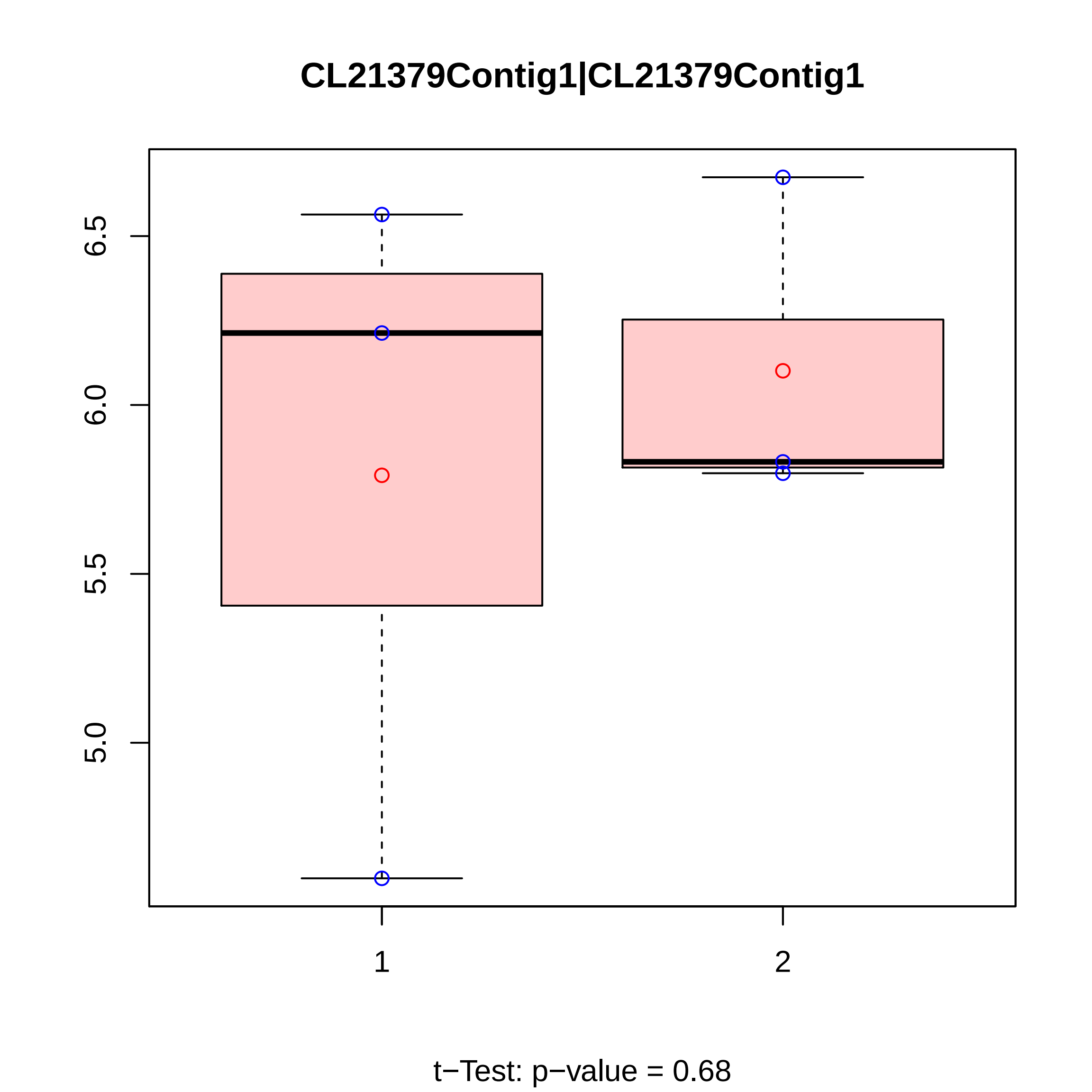
CL21379Contig1|CL21379Contig1
6.5
6.0
5.5
5.0
1
2
t−Test: p−value = 0.68

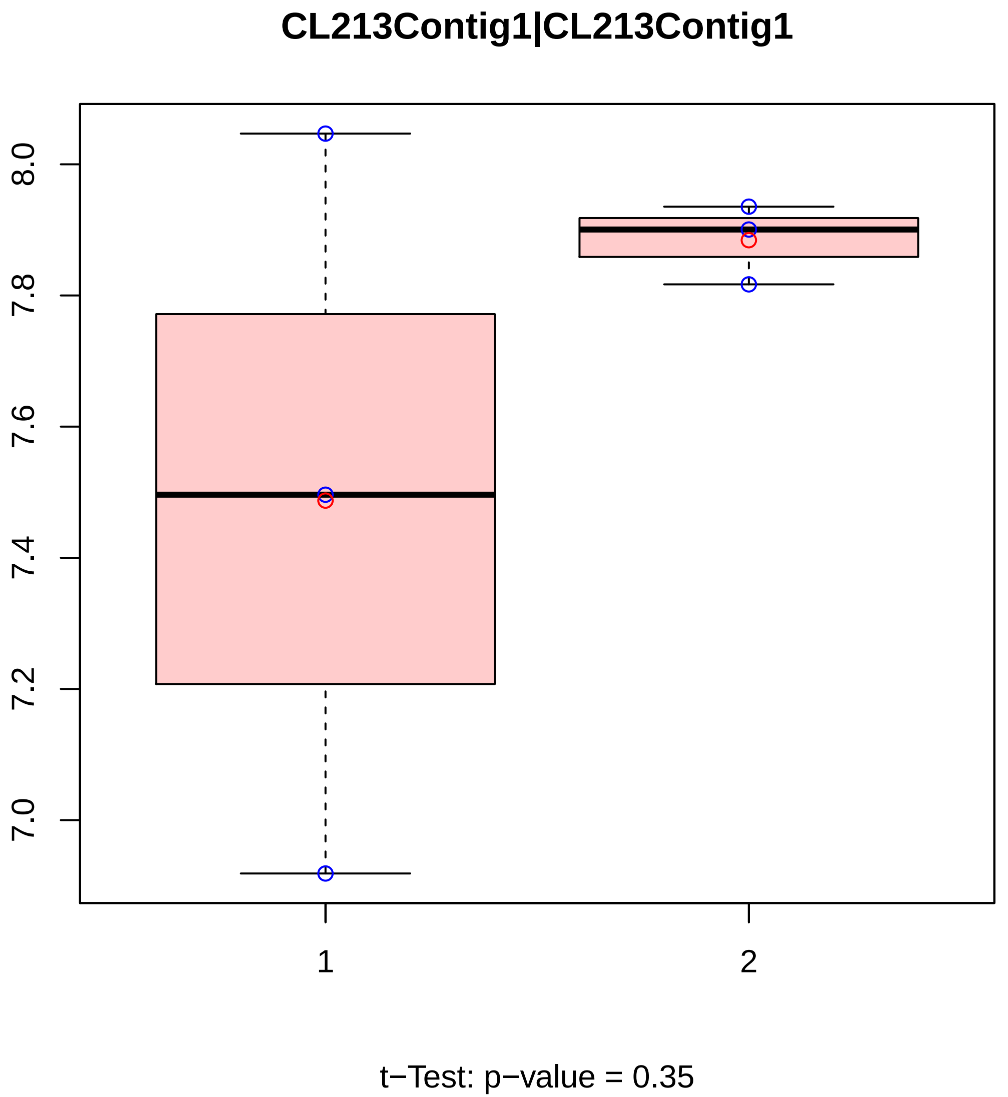
CL213Contig1|CL213Contig1
8.0
7.8
7.6
7.4
7.2
7.0
1
2
t−Test: p−value = 0.35

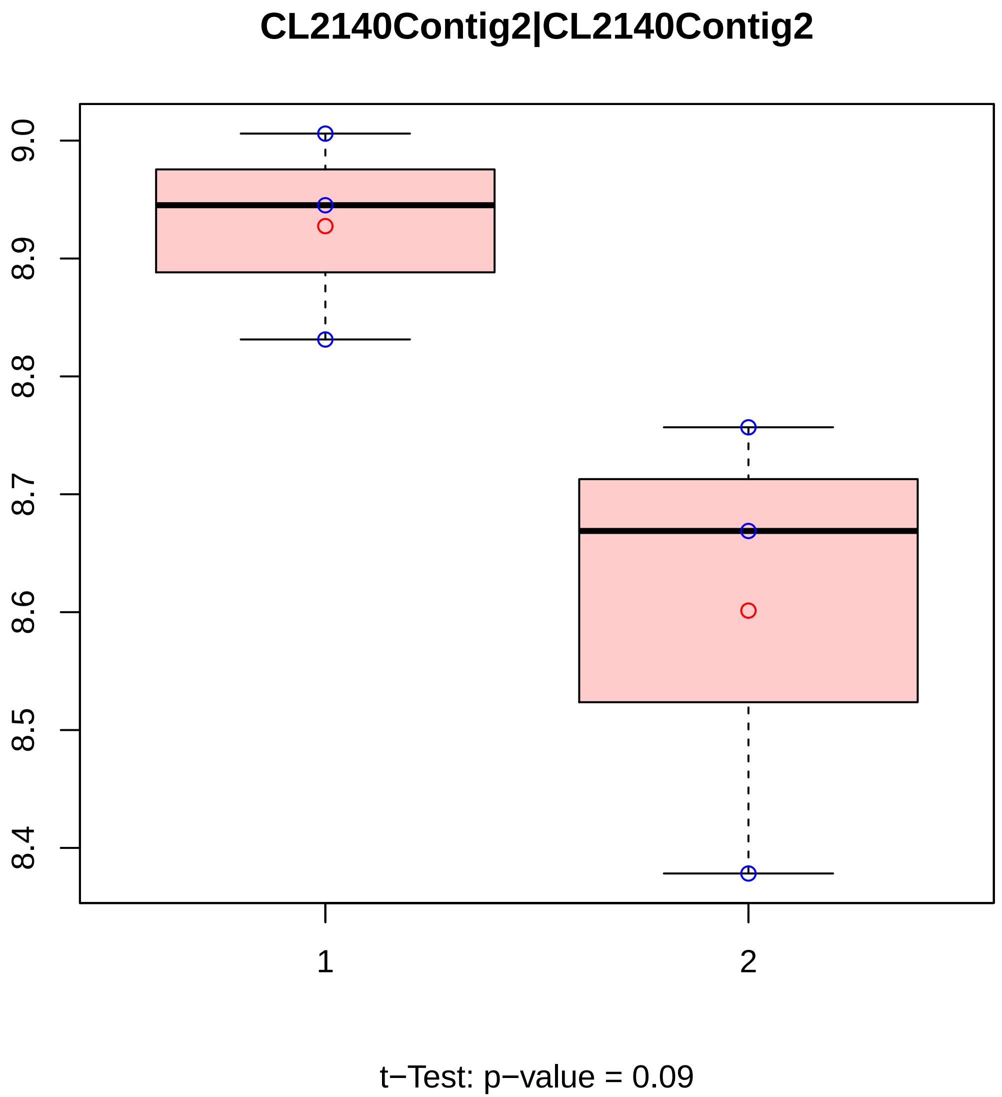

CL2140Contig2|CL2140Contig2
9.0
8.9
8.8
8.7
8.6
8.5
8.4
1
2
t−Test: p−value = 0.09

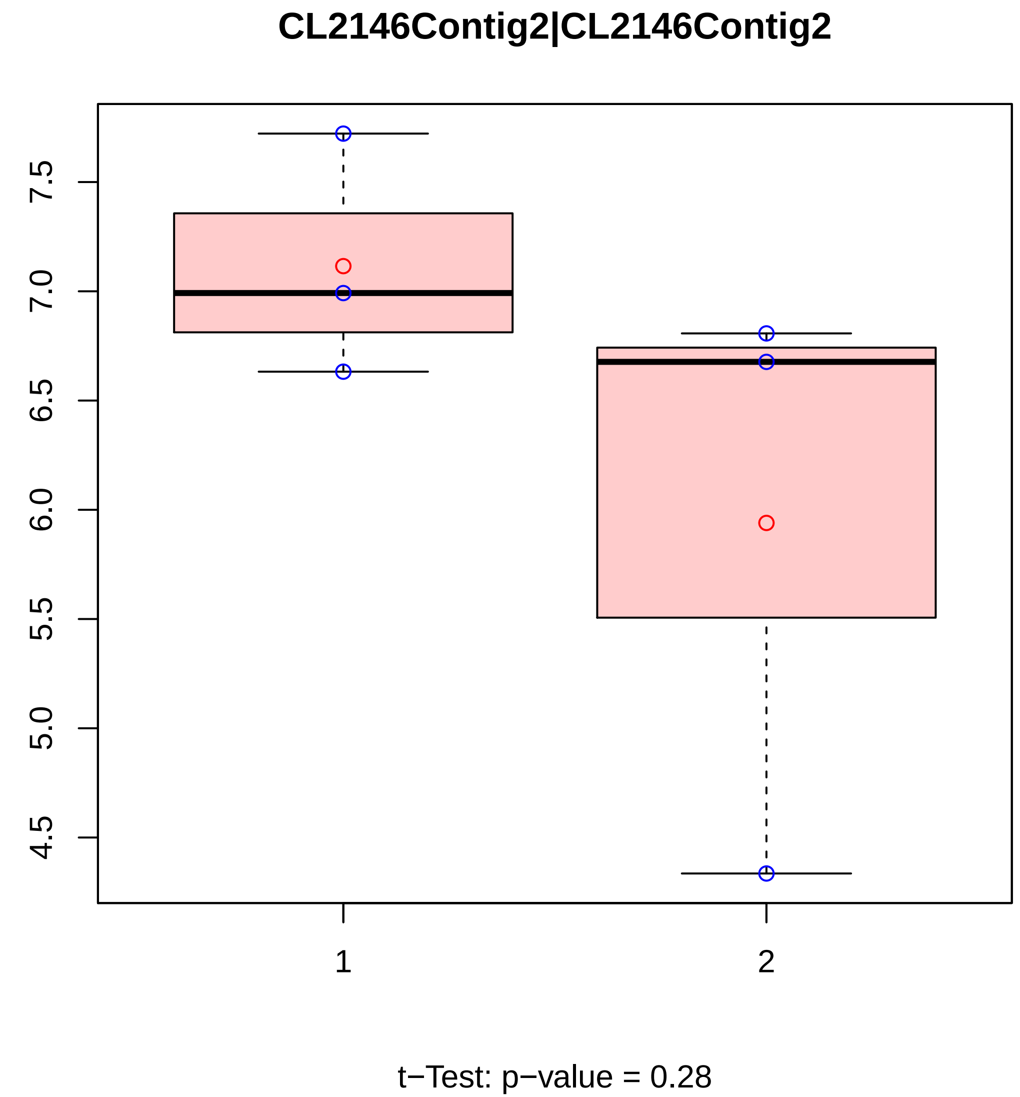
CL2146Contig2|CL2146Contig2
7.5
7.0
6.5
6.0
5.5
5.0
4.5
1
2
t−Test: p−value = 0.28

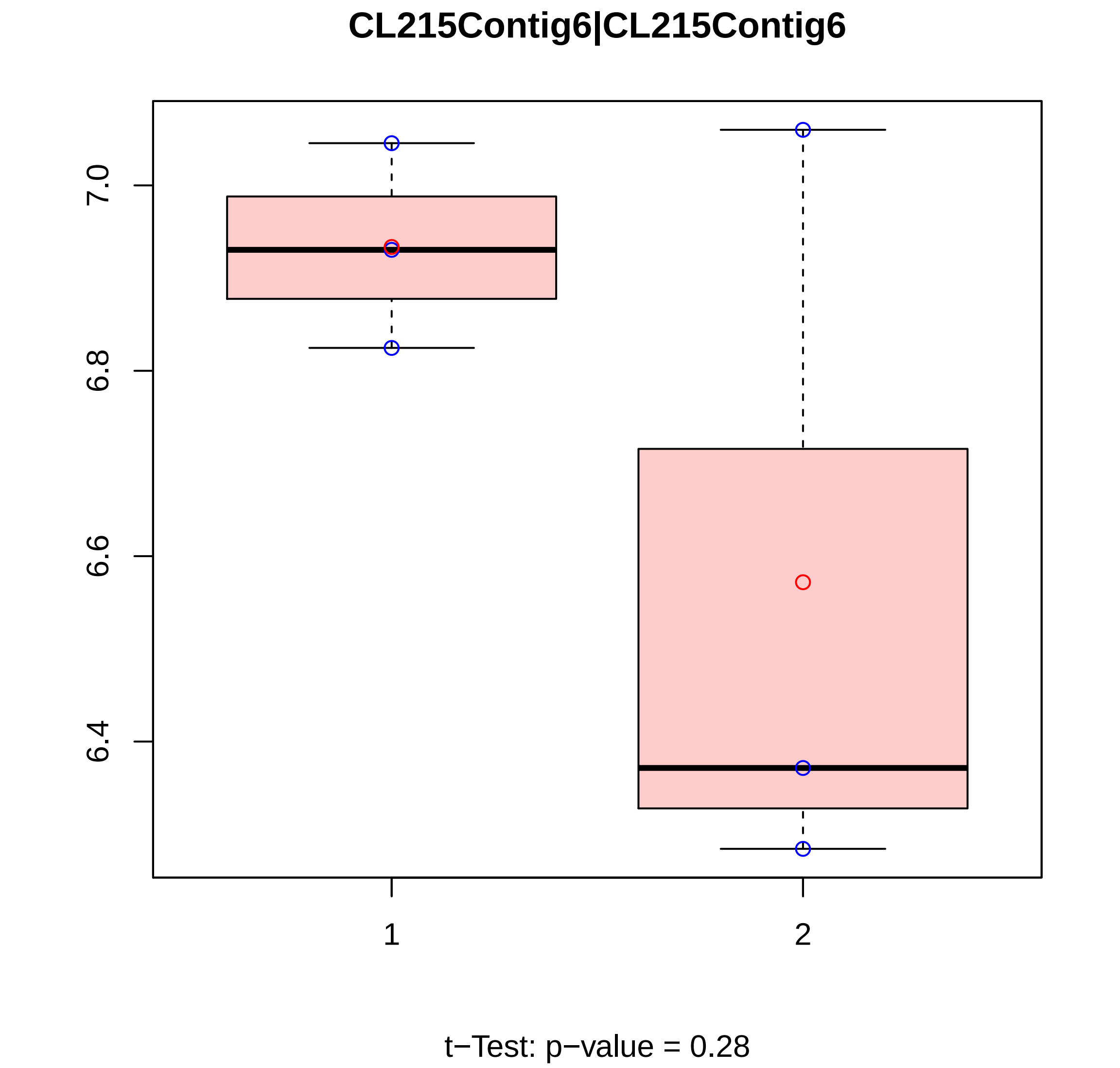
CL215Contig6|CL215Contig6
7.0
6.8
6.6
6.4
1
2
t−Test: p−value = 0.28

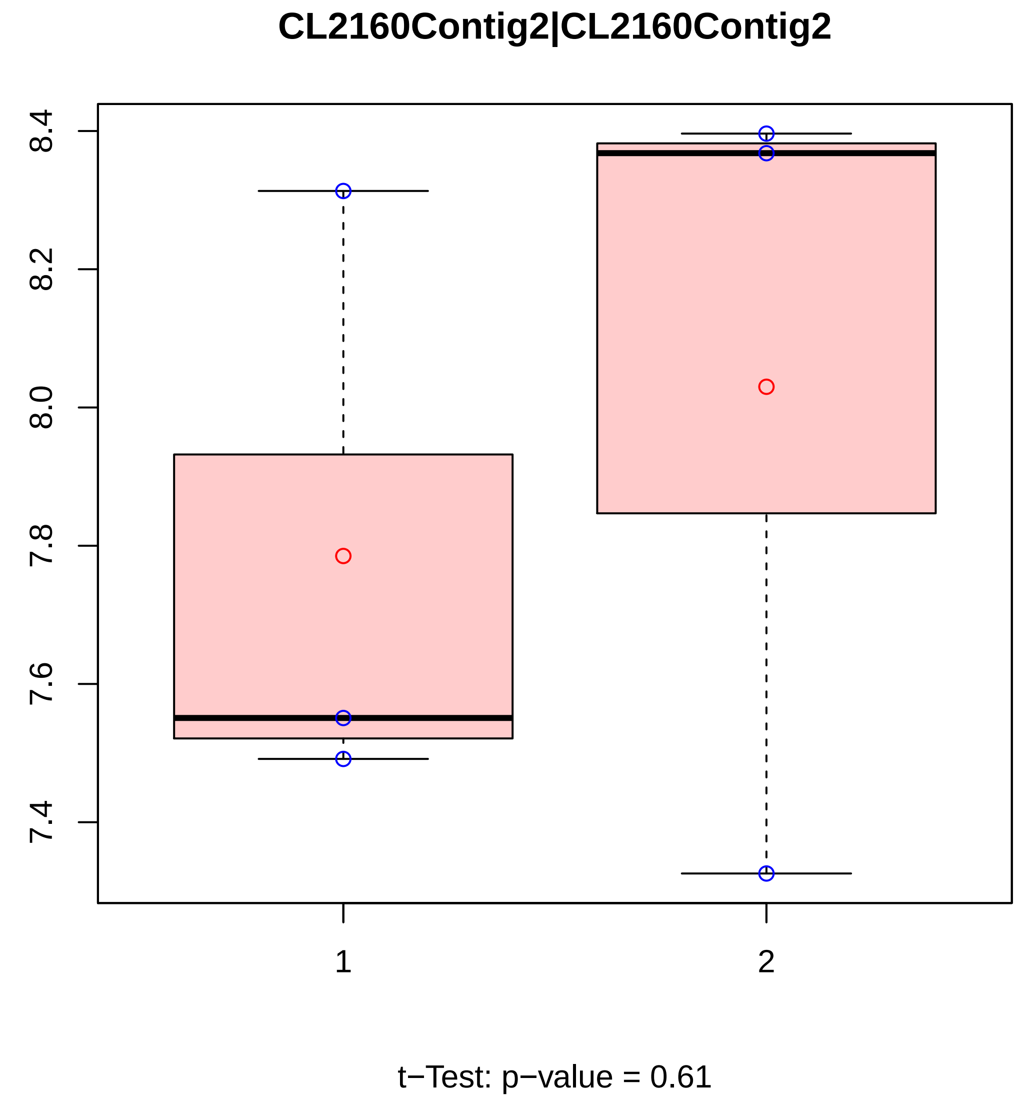
CL2160Contig2|CL2160Contig2
8.4
8.2
8.0
7.8
7.6
7.4
1
2
t−Test: p−value = 0.61

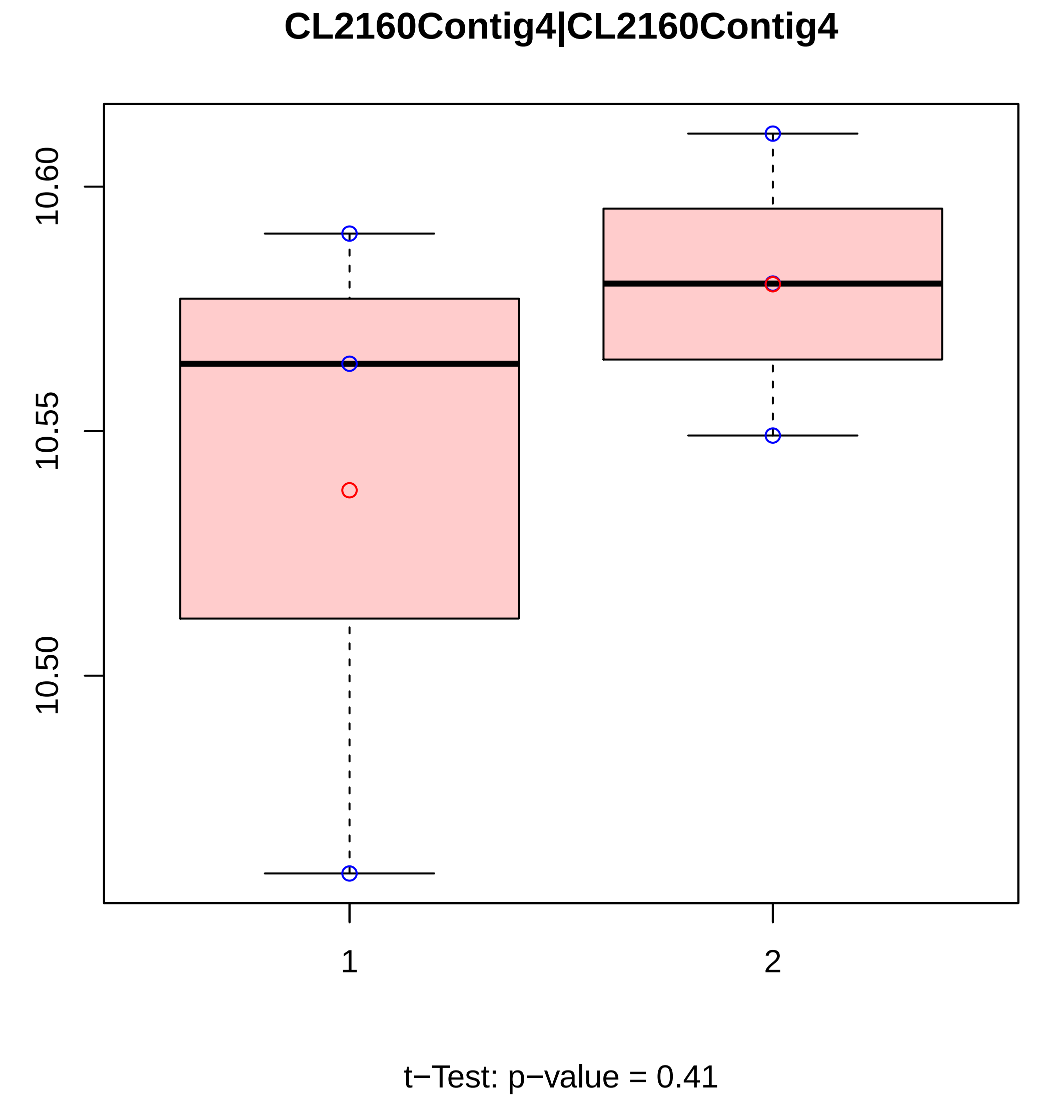
CL2160Contig4|CL2160Contig4
10.60
10.55
10.50
1
2
t−Test: p−value = 0.41

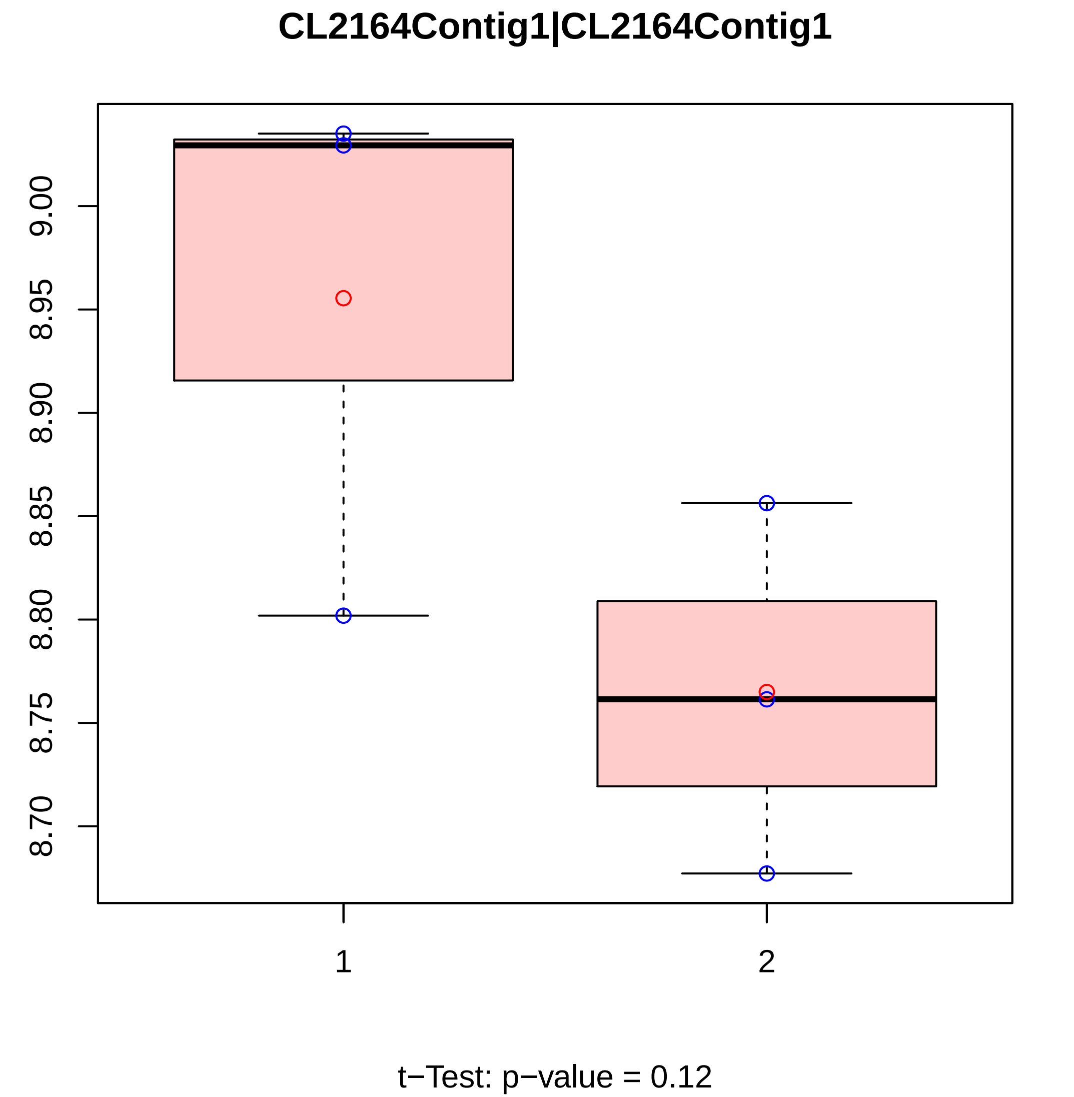
CL2164Contig1|CL2164Contig1
9.00
8.95
8.90
8.85
8.80
8.75
8.70
1
2
t−Test: p−value = 0.12

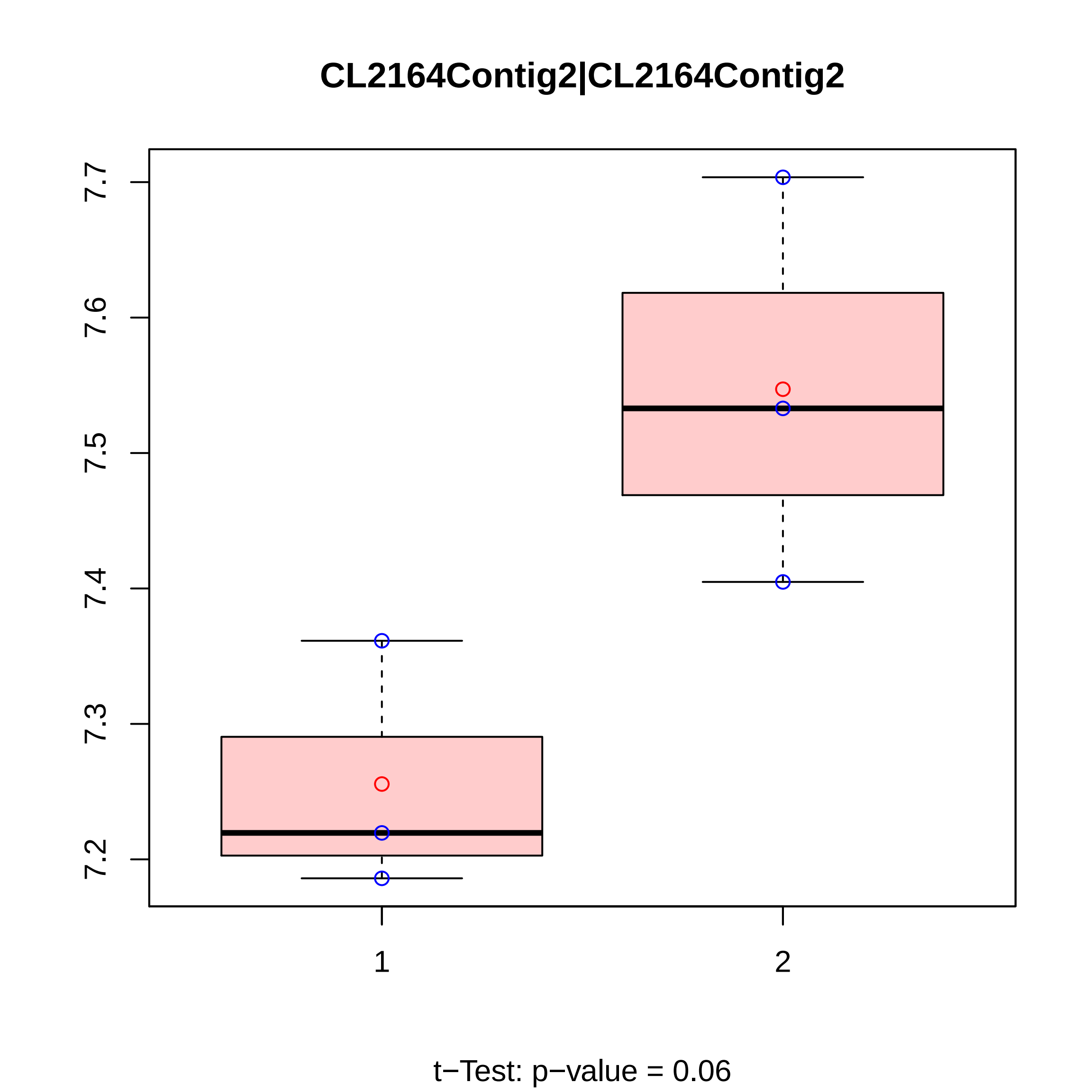

CL2164Contig2|CL2164Contig2
7.2
7.3
7.4
7.5
7.6
7.7
1
2
t−Test: p−value = 0.06

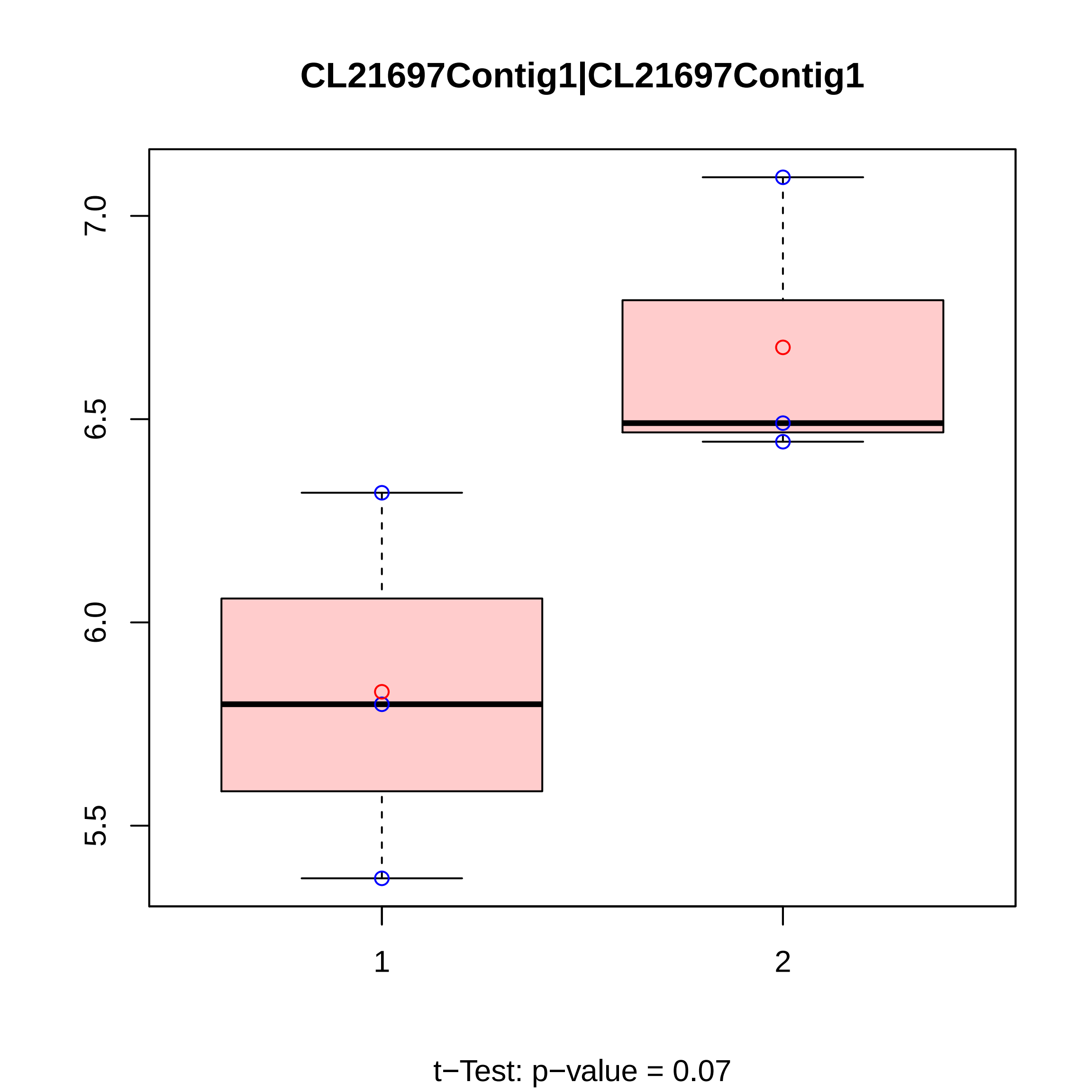
CL21697Contig1|CL21697Contig1
7.0
6.5
6.0
5.5
1
2
t−Test: p−value = 0.07

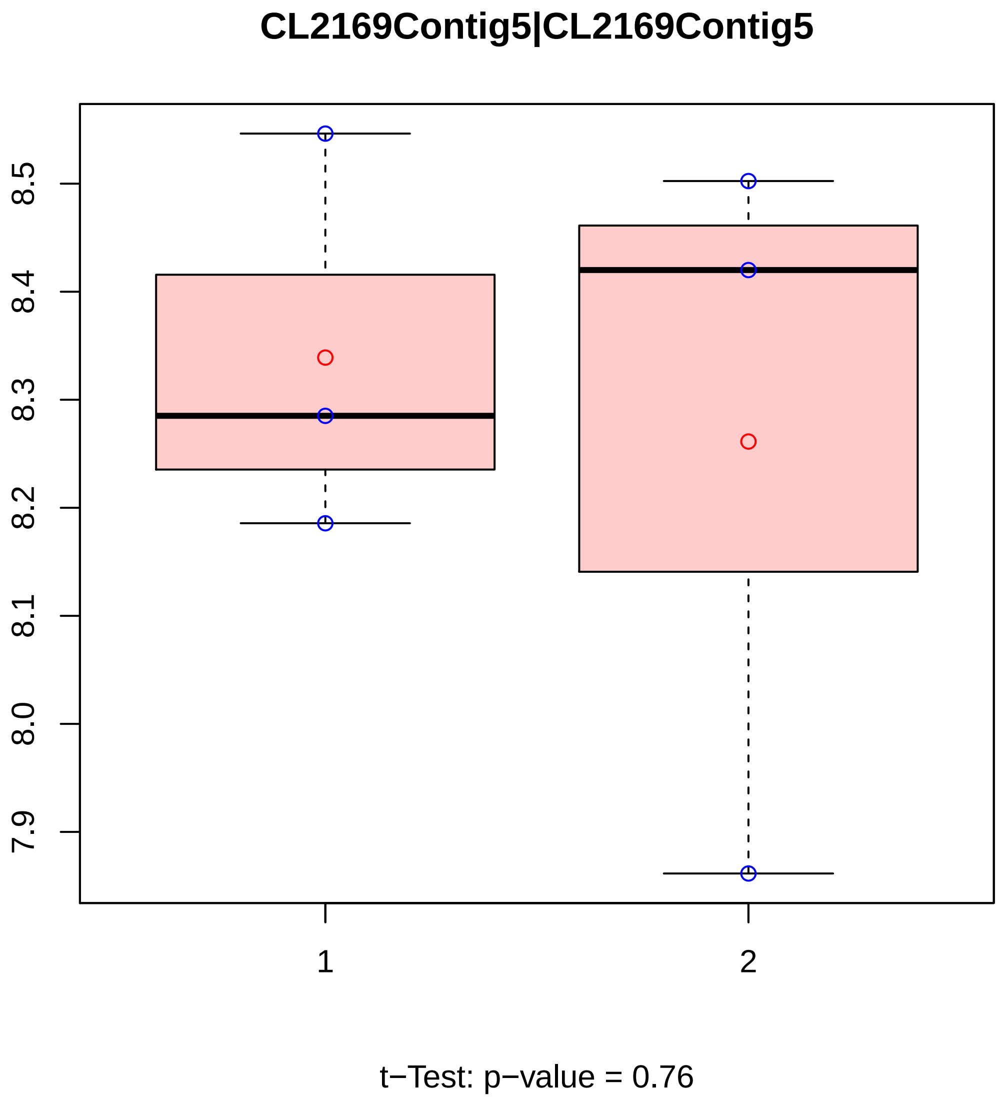

CL2169Contig5|CL2169Contig5
8.5
8.4
8.3
8.2
8.1
8.0
7.9
1
2
t−Test: p−value = 0.76

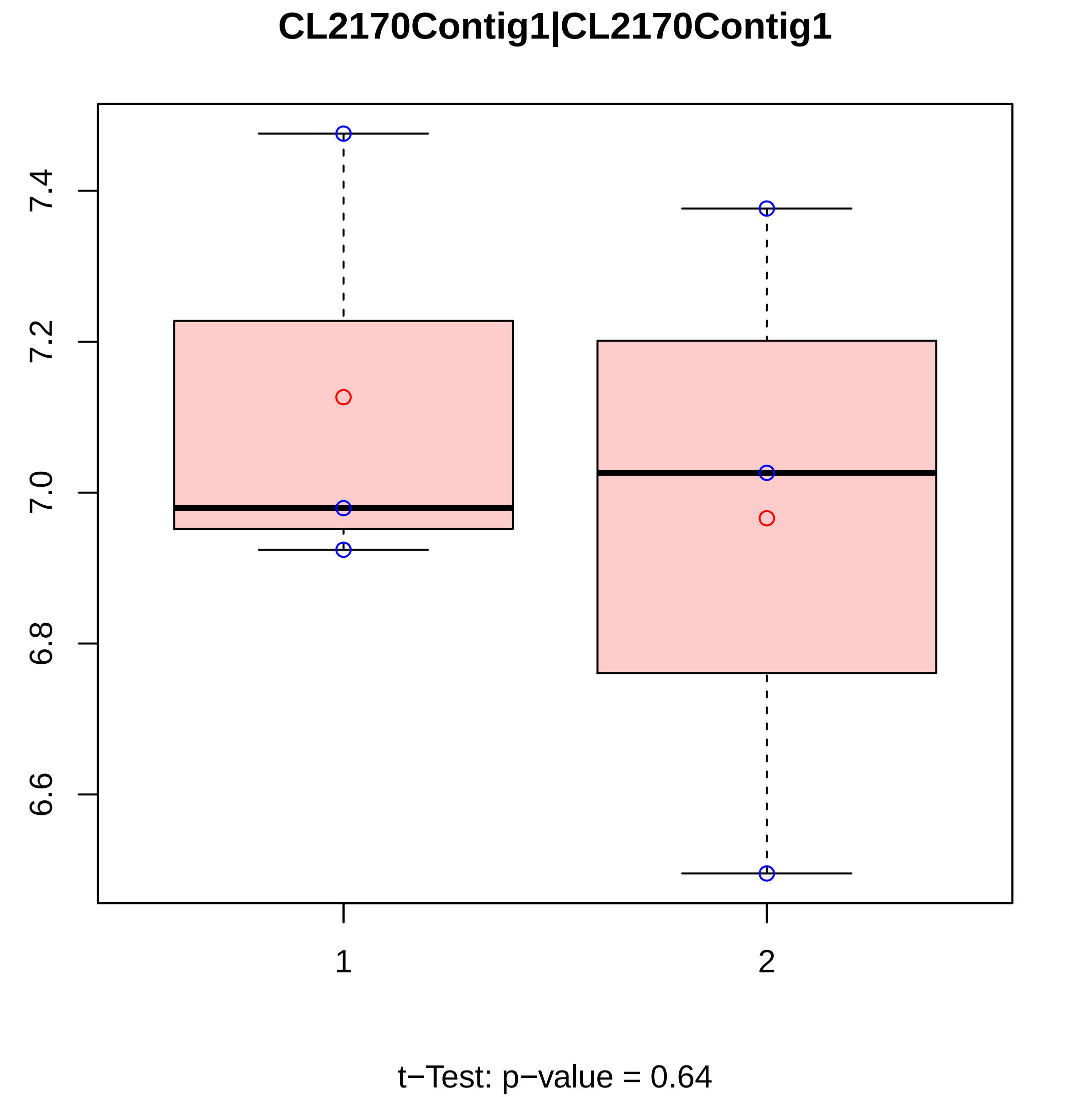
CL2170Contig1|CL2170Contig1
7.4
7.2
7.0
6.8
6.6
1
2
t−Test: p−value = 0.64

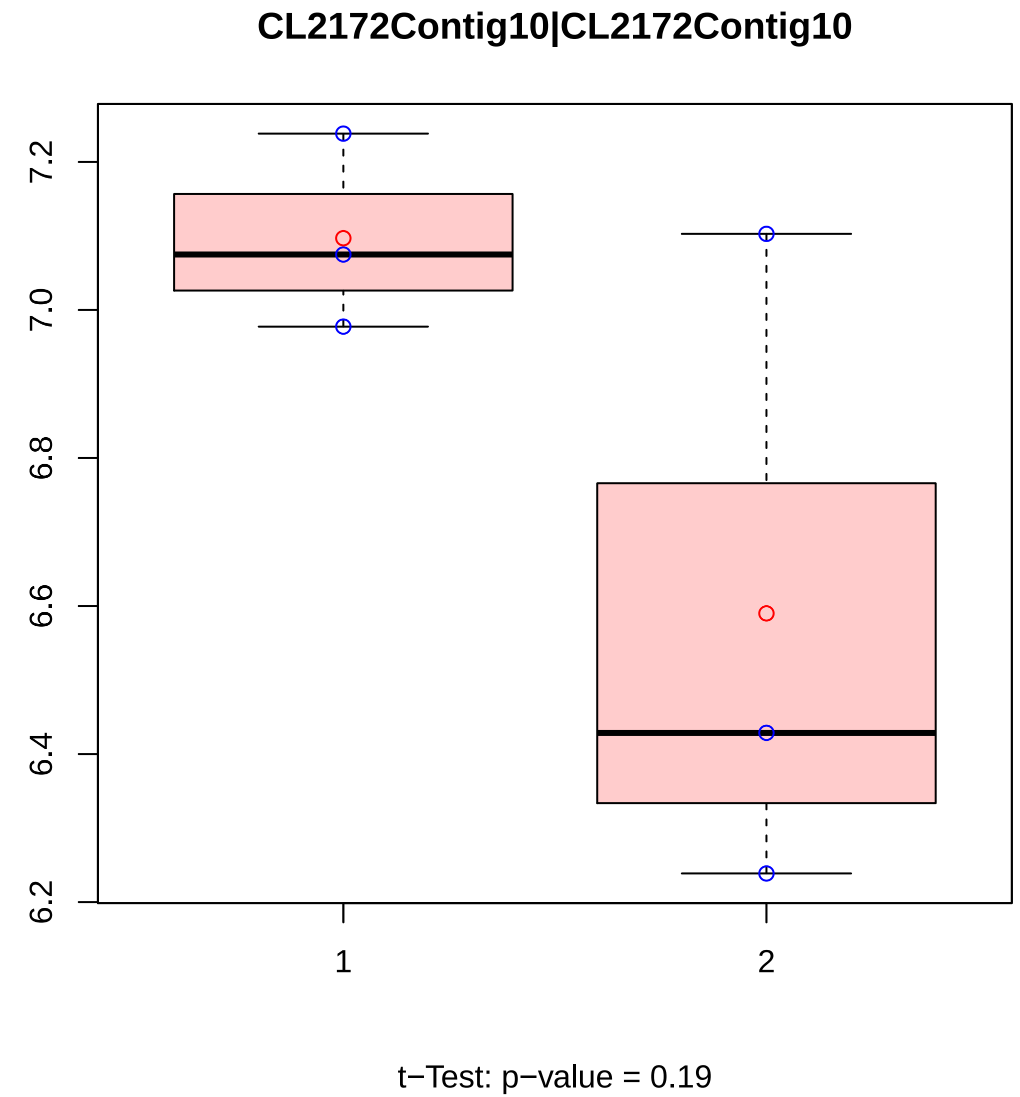
CL2172Contig10|CL2172Contig10
7.2
7.0
6.8
6.6
6.4
6.2
1
2
t−Test: p−value = 0.19

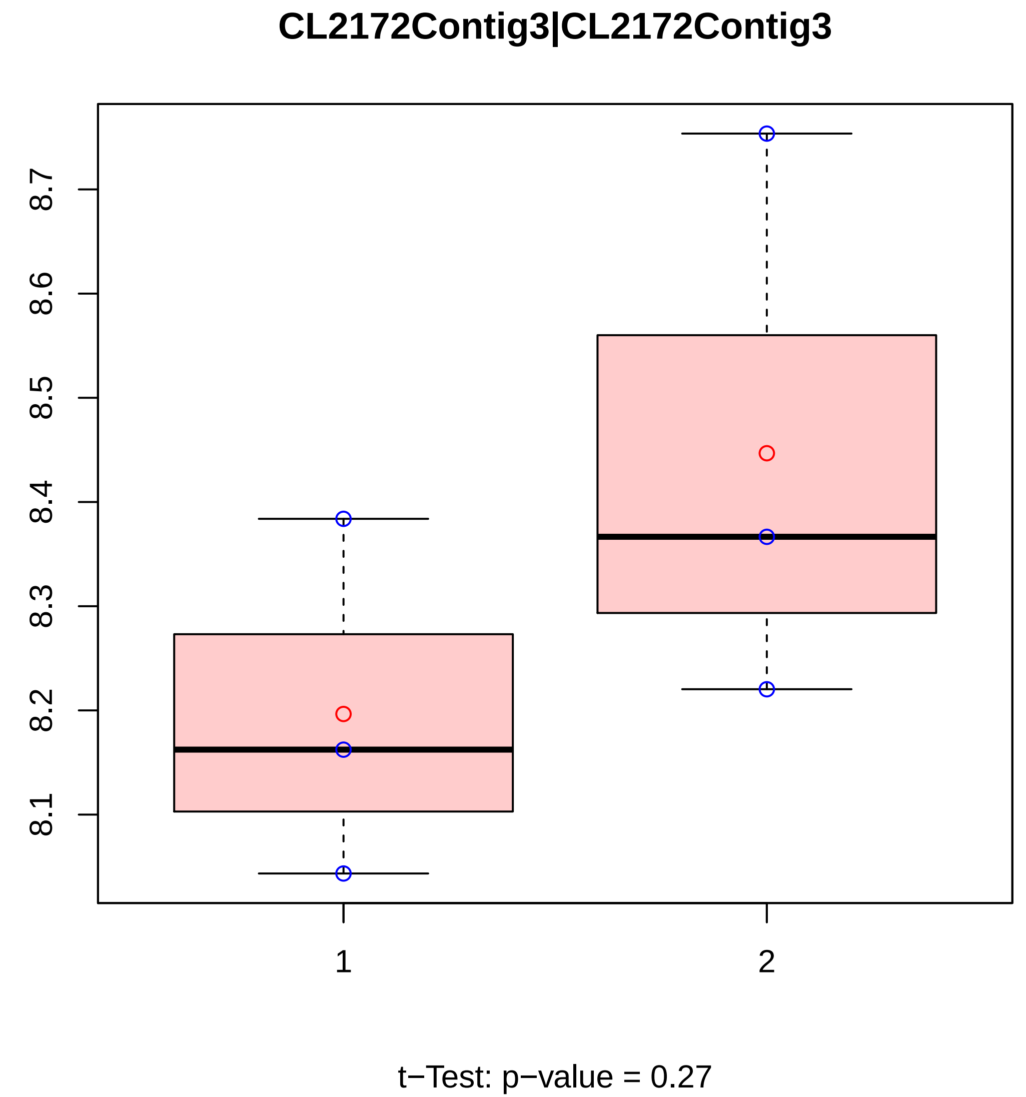

CL2172Contig3|CL2172Contig3
8.1
8.2
8.3
8.4
8.5
8.6
8.7
1
2
t−Test: p−value = 0.27

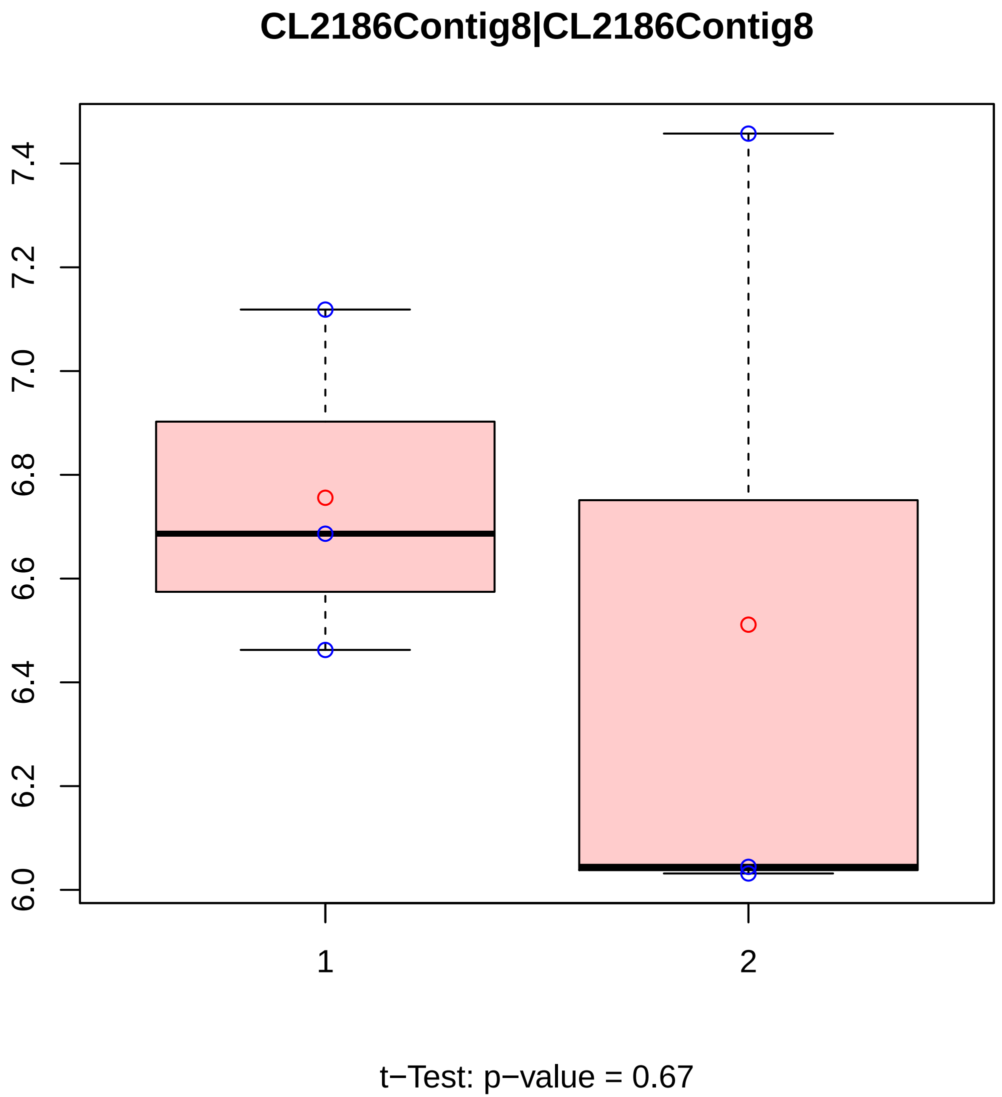

CL2186Contig8|CL2186Contig8
7.4
7.2
7.0
6.8
6.6
6.4
6.2
6.0
1
2
t−Test: p−value = 0.67

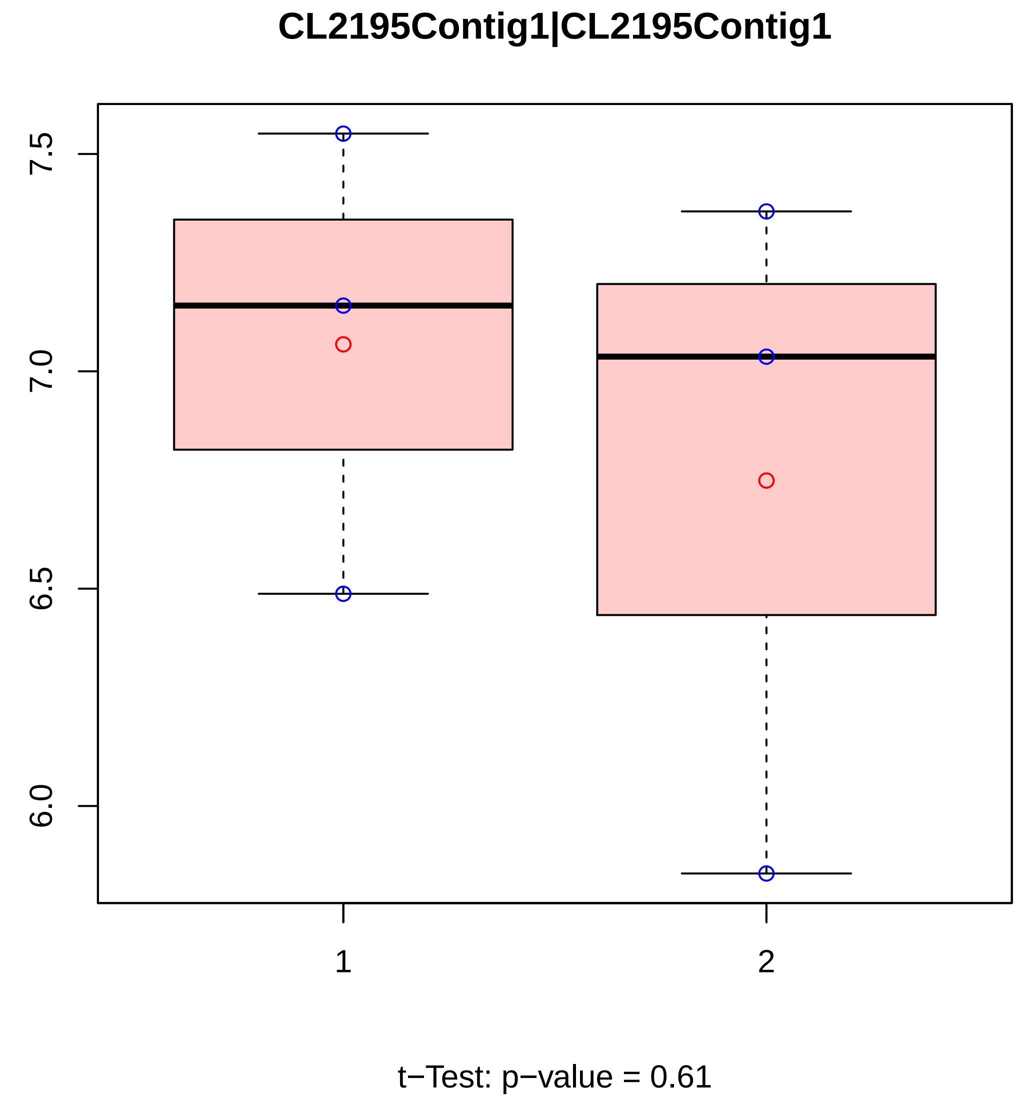

CL2195Contig1|CL2195Contig1
7.5
7.0
6.5
6.0
1
2
t−Test: p−value = 0.61

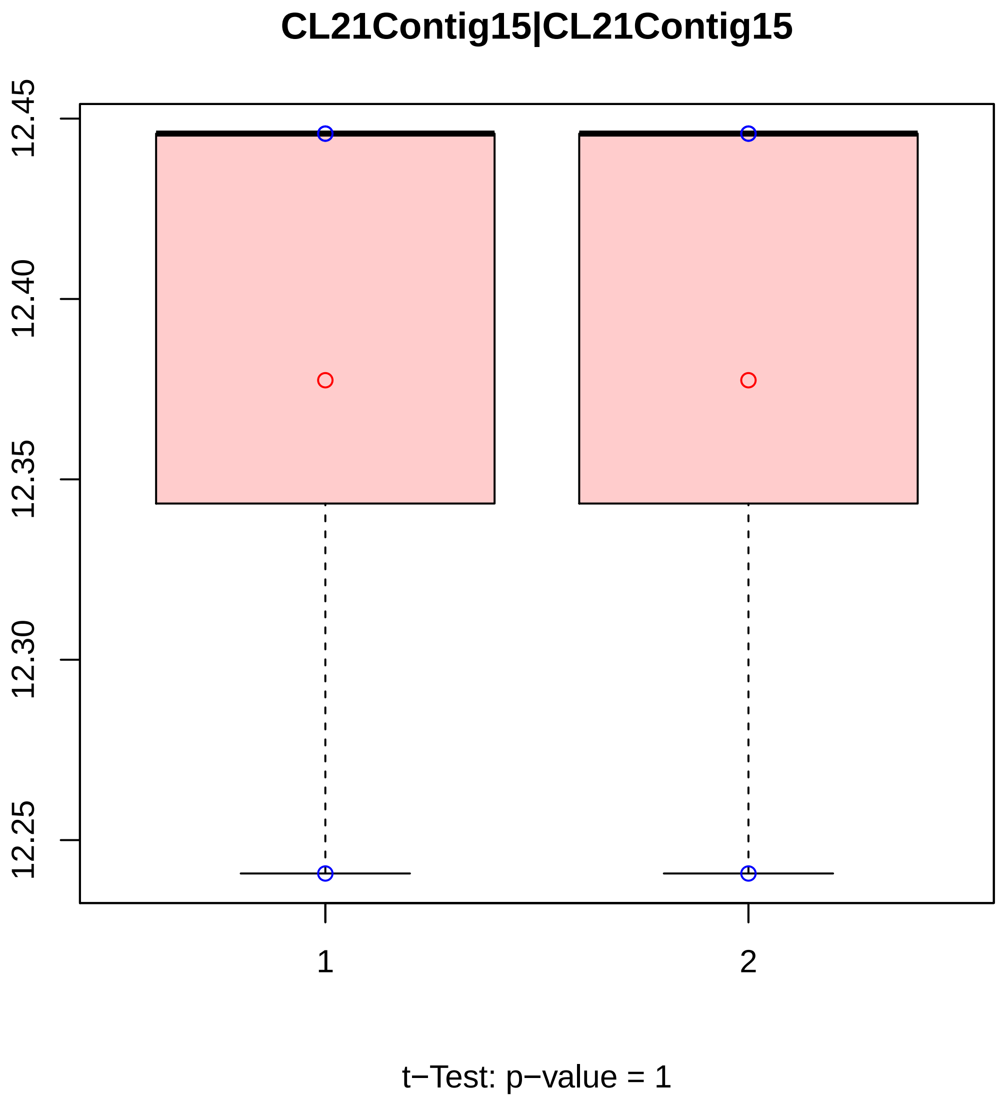

CL21Contig15|CL21Contig15
12.45
12.40
12.35
12.30
12.25
1
2
t−Test: p−value = 1

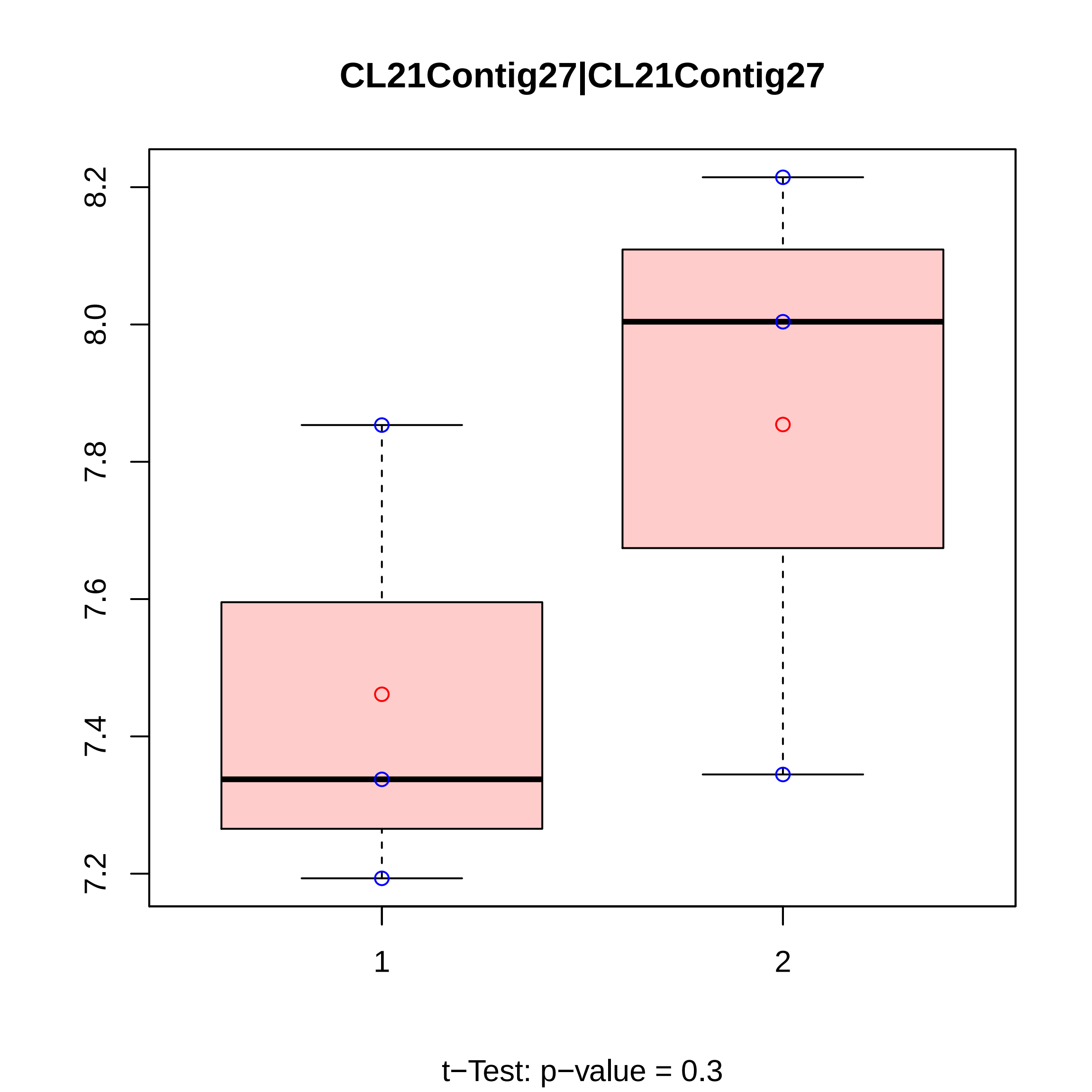

CL21Contig27|CL21Contig27
8.2
8.0
7.8
7.6
7.4
7.2
1
2
t−Test: p−value = 0.3

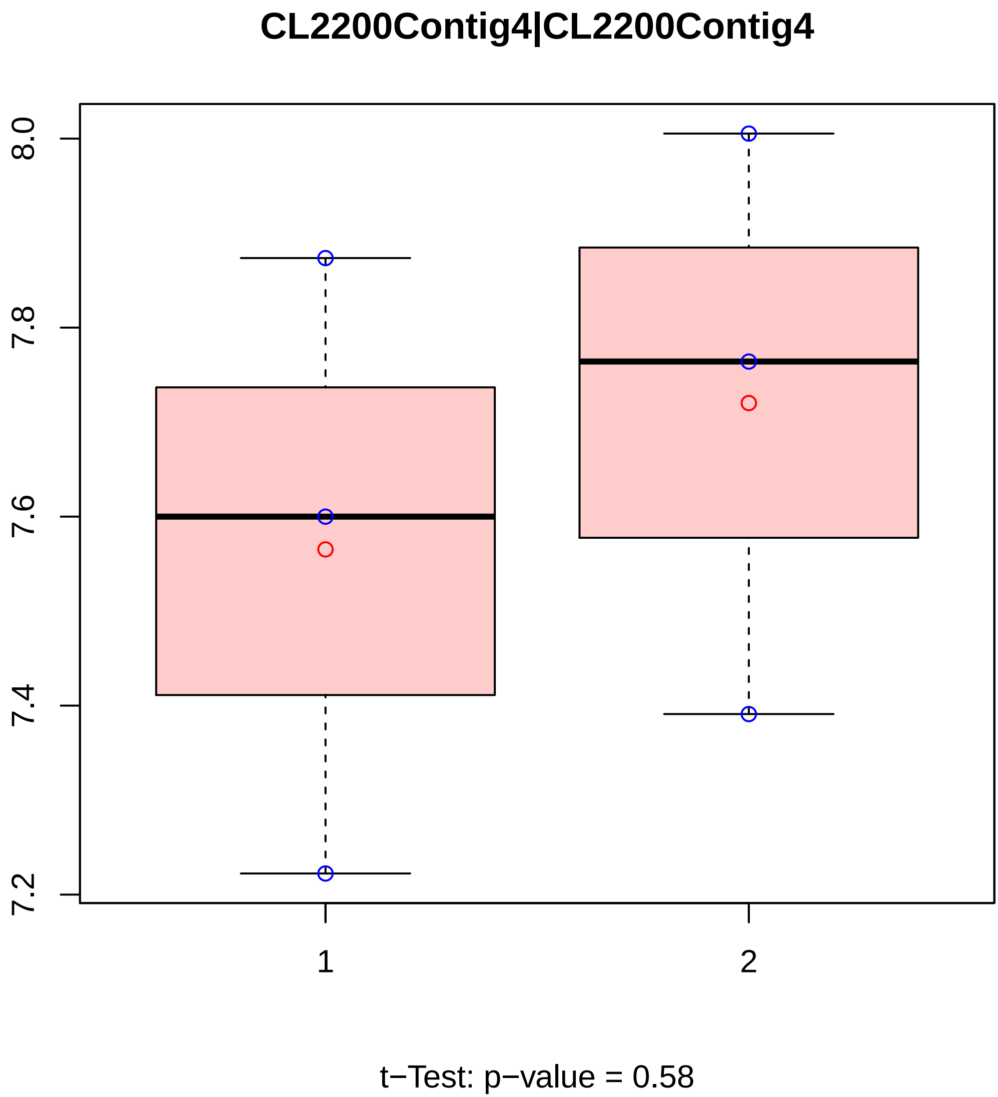

CL2200Contig4|CL2200Contig4
8.0
7.8
7.6
7.4
7.2
1
2
t−Test: p−value = 0.58

## CL2206Contig4|CL2206Contig4

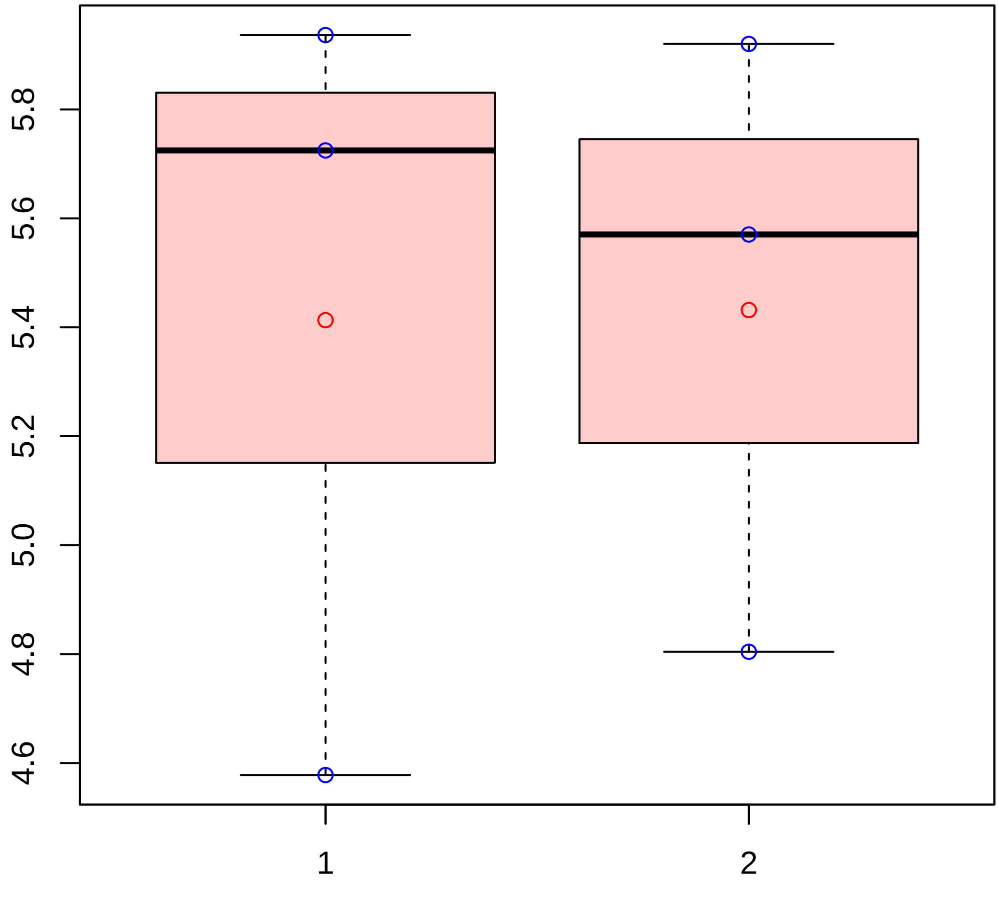


t−Test: p−value = 0.97

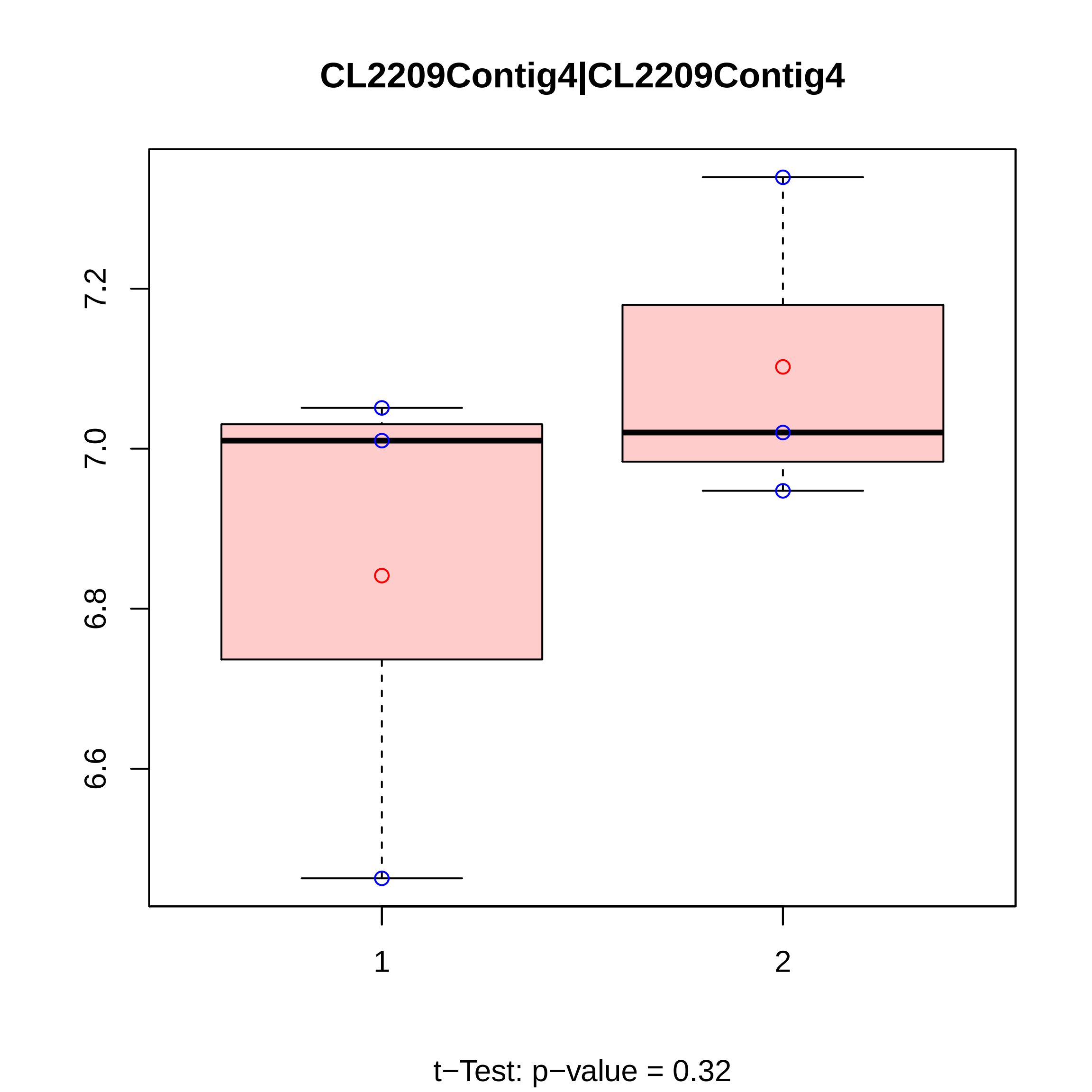
CL2209Contig4|CL2209Contig4
7.2
7.0
6.8
6.6
1
2
t−Test: p−value = 0.32

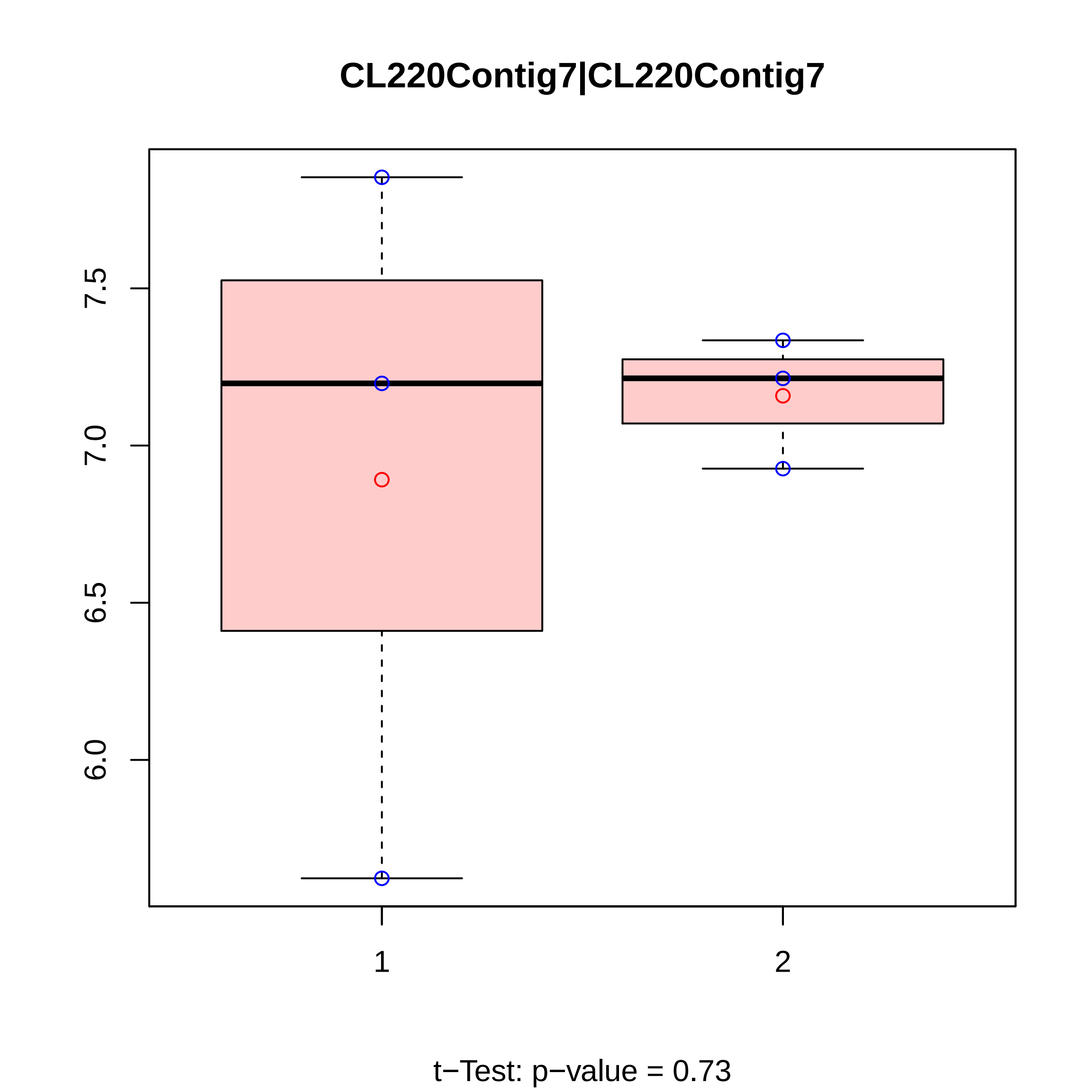

CL220Contig7|CL220Contig7
7.5
7.0
6.5
6.0
1
2
t−Test: p−value = 0.73

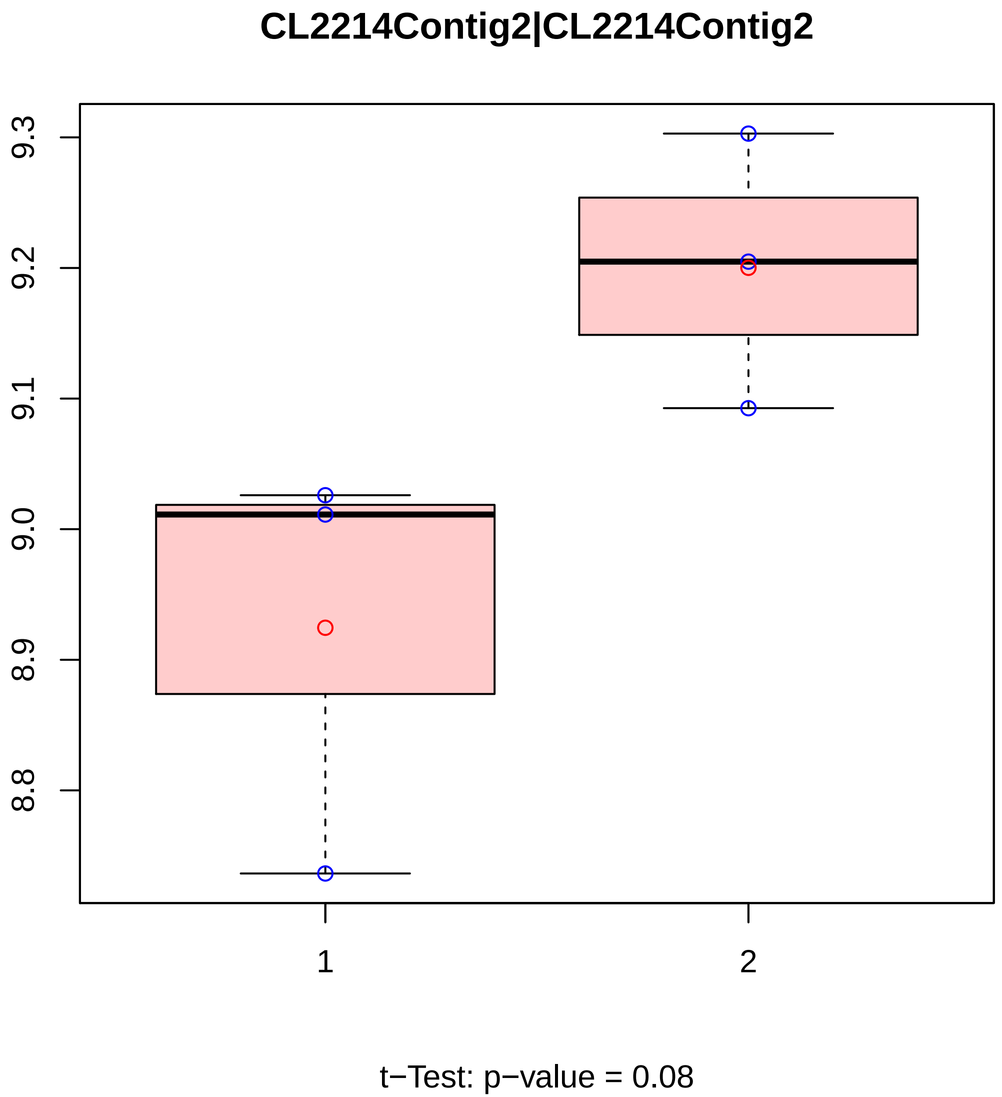
CL2214Contig2|CL2214Contig2
9.3
9.2
9.1
9.0
8.9
8.8
1
2
t−Test: p−value = 0.08

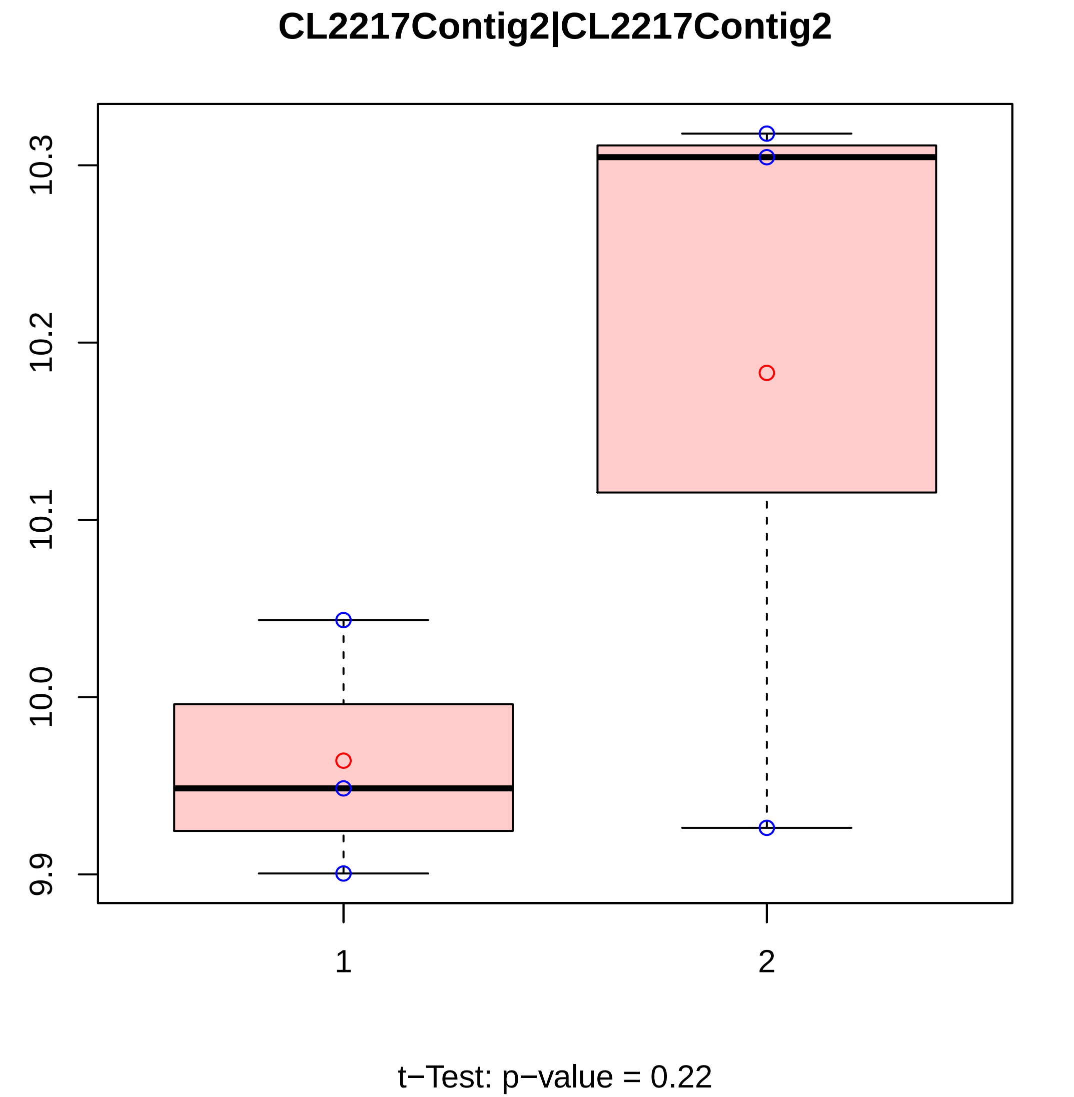
CL2217Contig2|CL2217Contig2
10.3
10.2
10.1
10.0
9.9
1
2
t−Test: p−value = 0.22

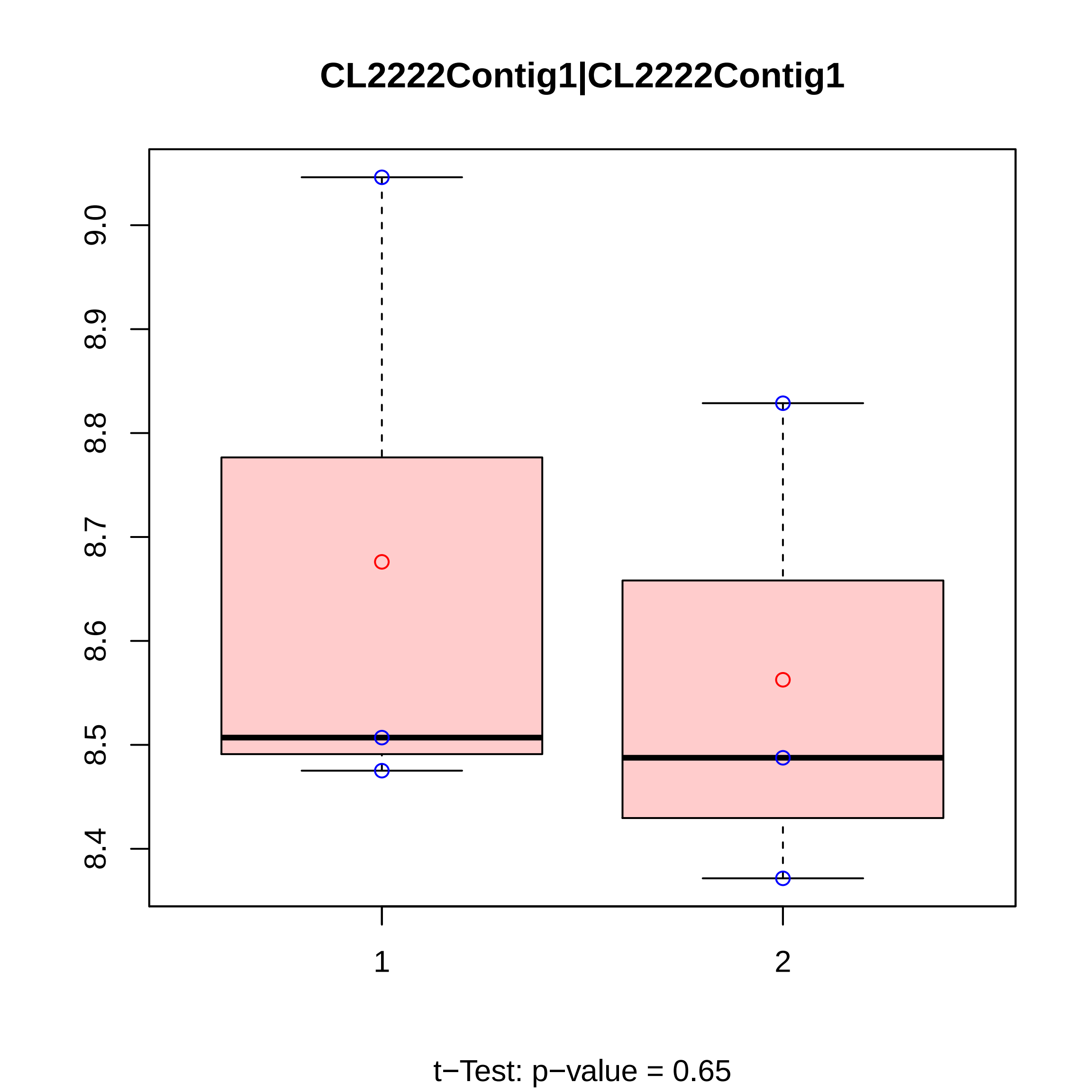
CL2222Contig1|CL2222Contig1
9.0
8.9
8.8
8.7
8.6
8.5
8.4
1
2
t−Test: p−value = 0.65

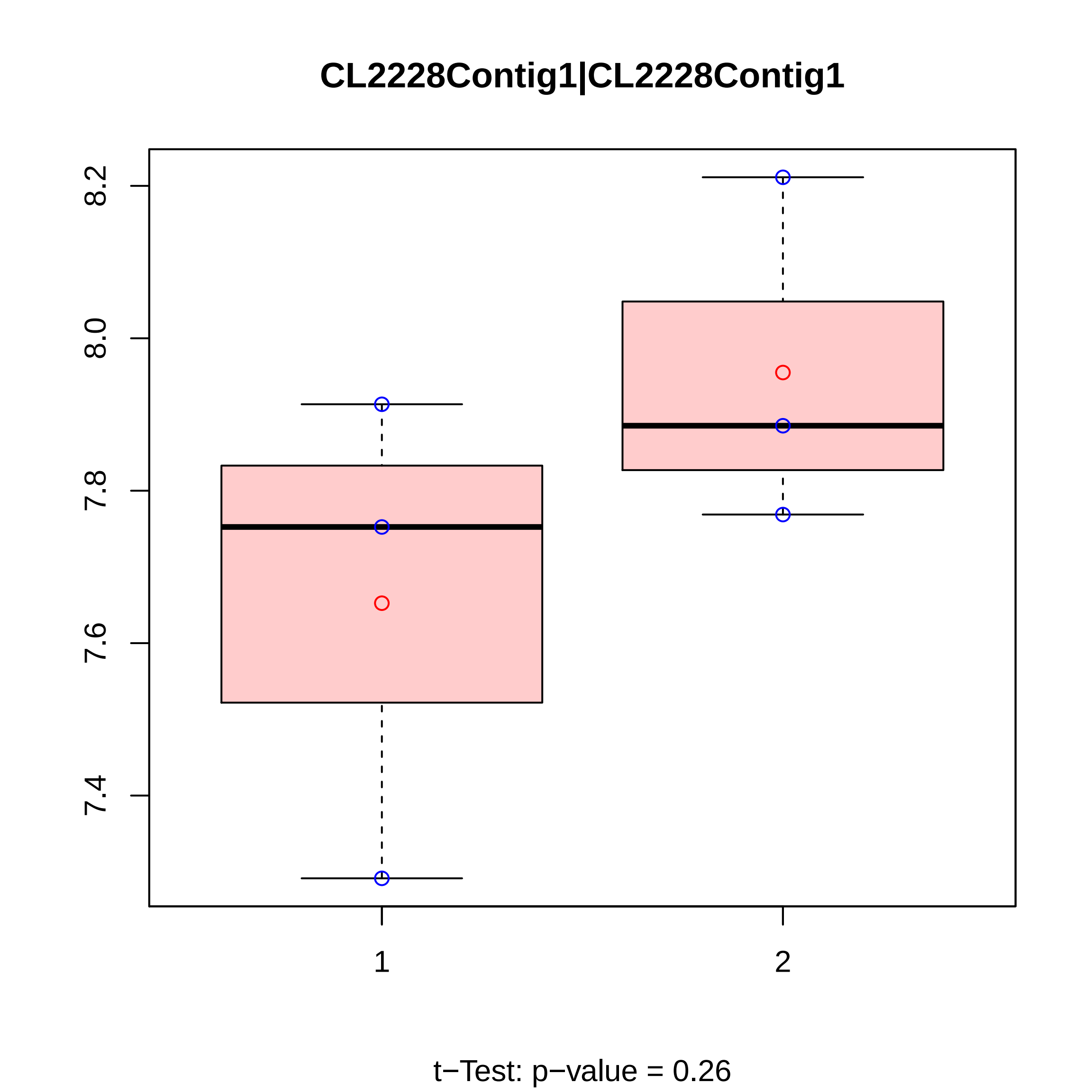

CL2228Contig1|CL2228Contig1
8.2
8.0
7.8
7.6
7.4
1
2
t−Test: p−value = 0.26

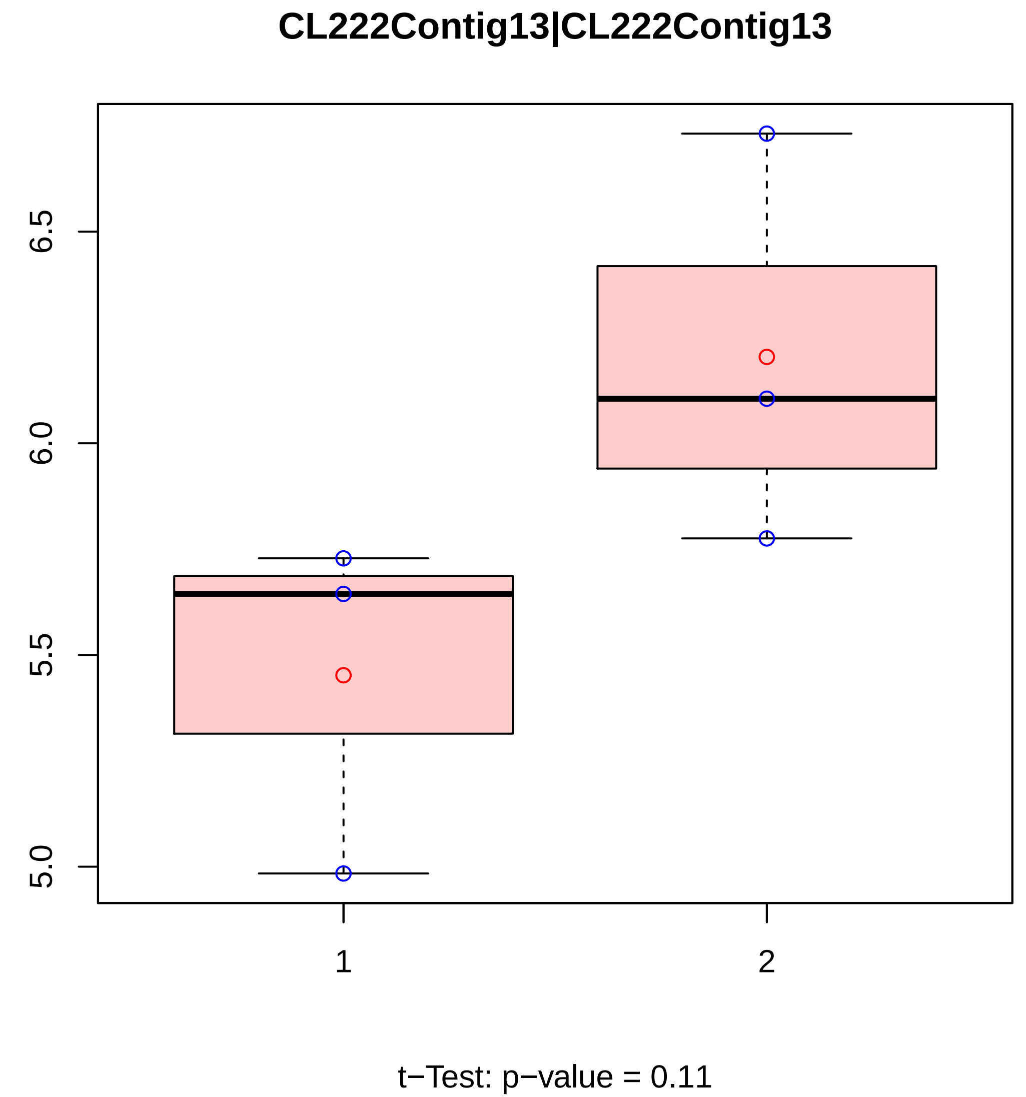
CL222Contig13|CL222Contig13
6.5
6.0
5.5
5.0
1
2
t−Test: p−value = 0.11

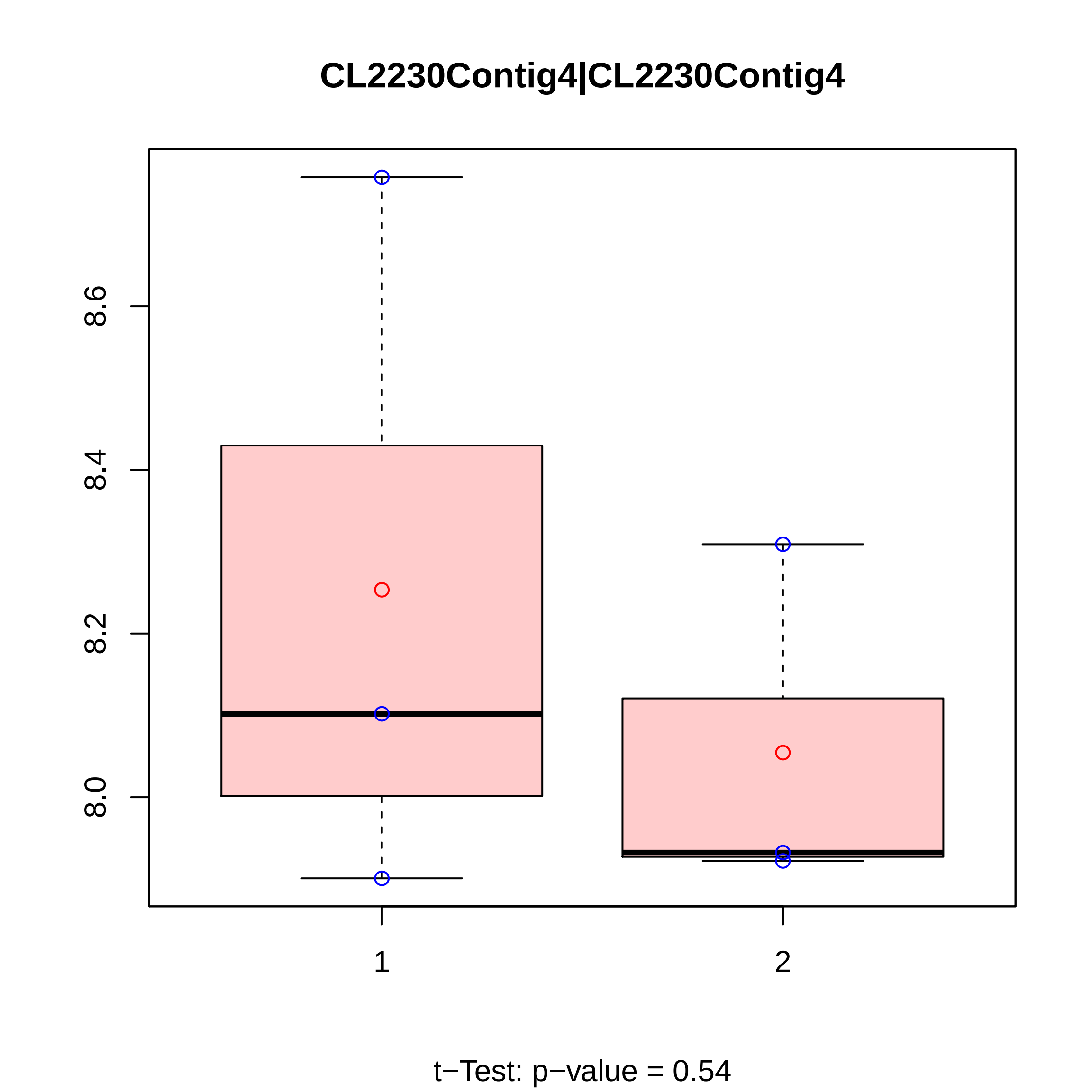
CL2230Contig4|CL2230Contig4
8.0
8.2
8.4
8.6
1
2
t−Test: p−value = 0.54

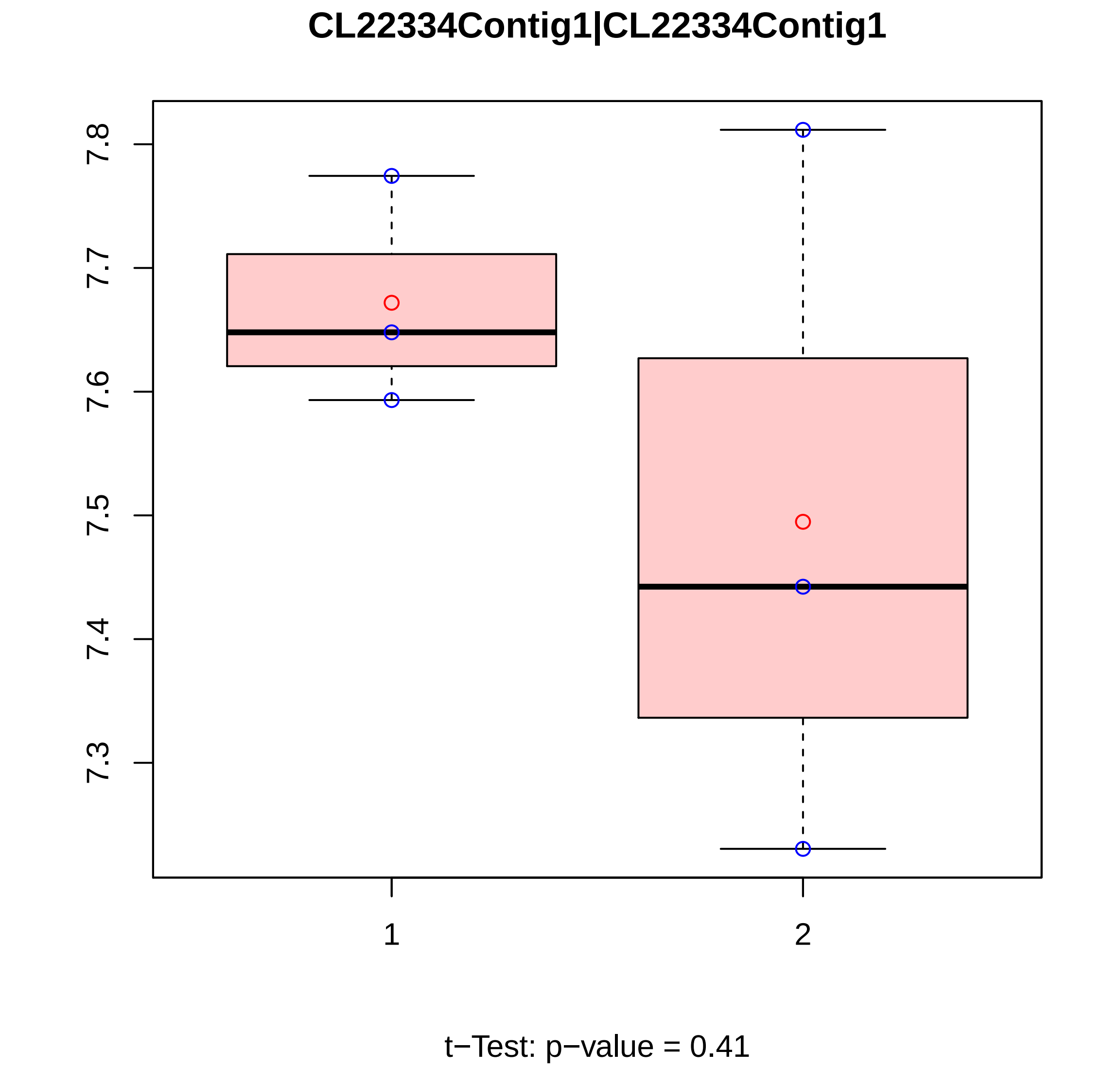
CL22334Contig1|CL22334Contig1
7.3
7.4
7.5
7.6
7.7
7.8
1
2
t−Test: p−value = 0.41

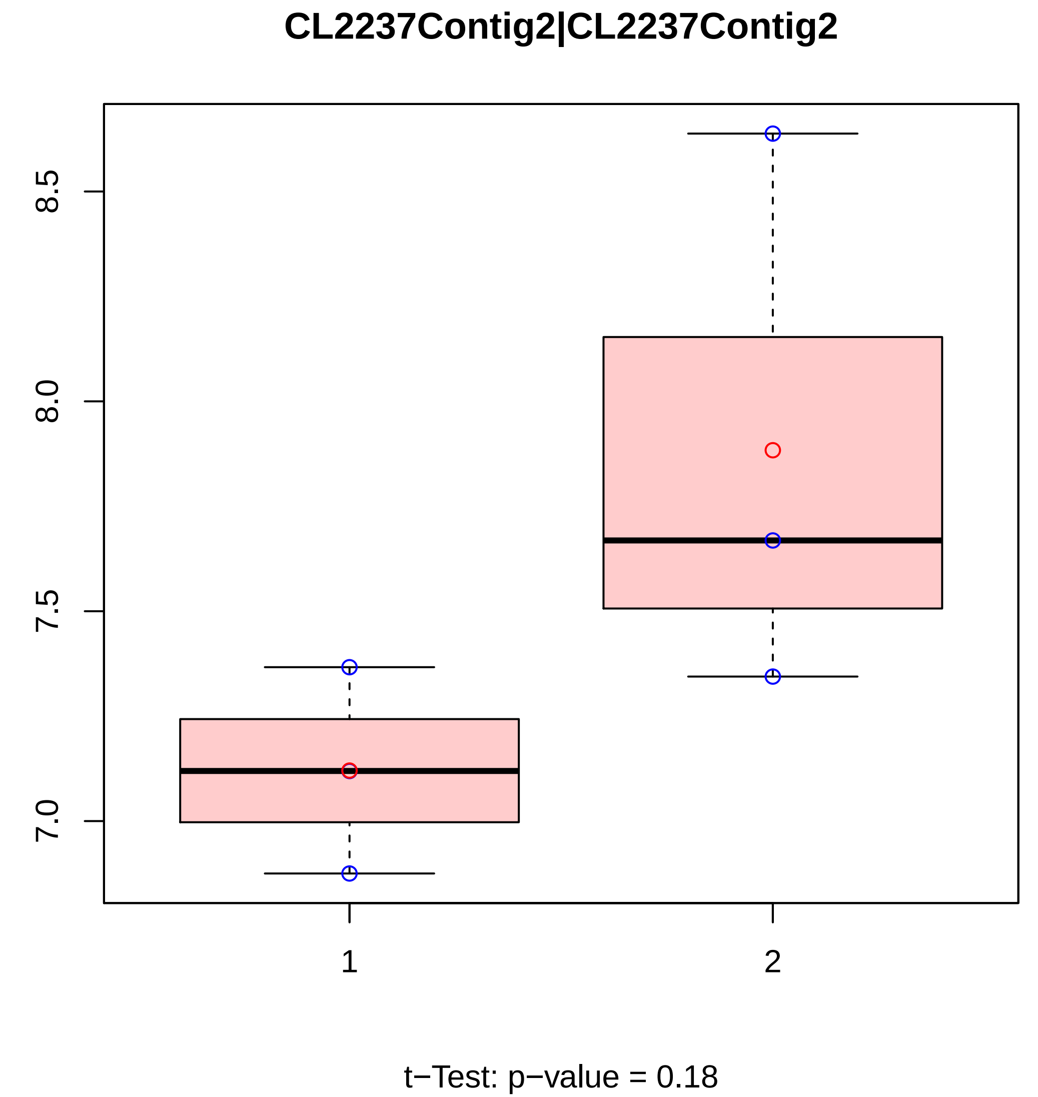

CL2237Contig2|CL2237Contig2
8.5
8.0
7.5
7.0
1
2
t−Test: p−value = 0.18

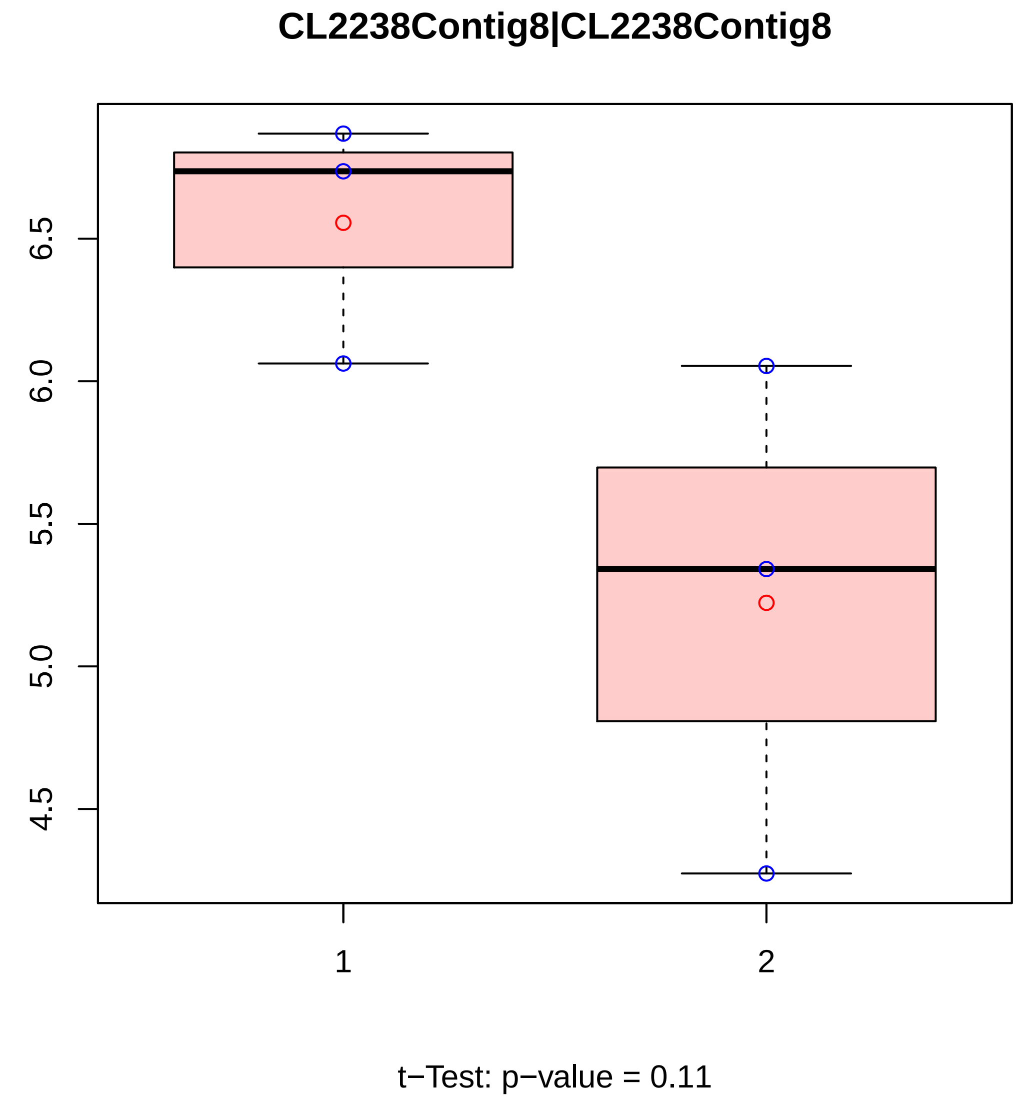
CL2238Contig8|CL2238Contig8
6.5
6.0
5.5
5.0
4.5
1
2
t−Test: p−value = 0.11

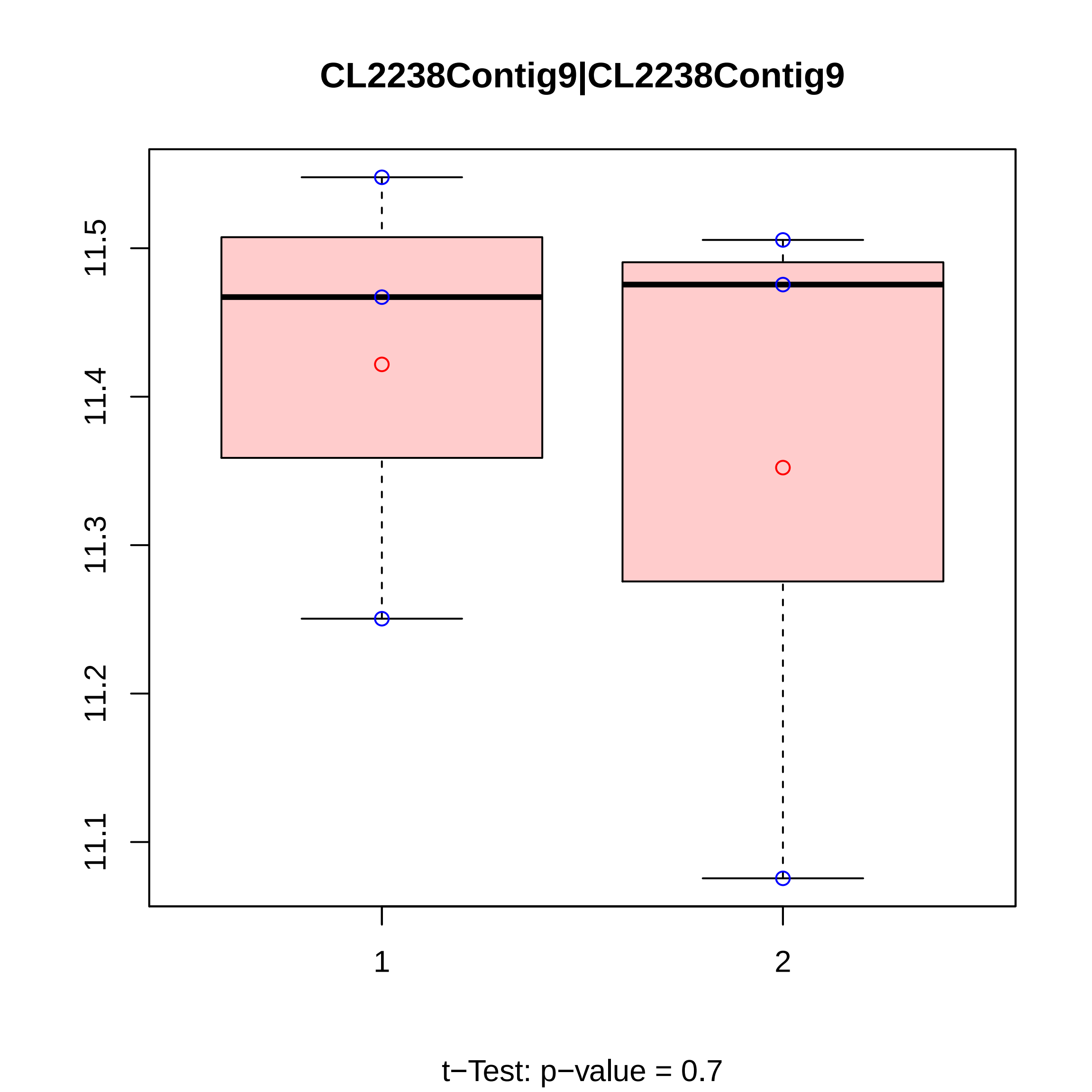

CL2238Contig9|CL2238Contig9
11.5
11.4
11.3
11.2
11.1
1
2
t−Test: p−value = 0.7

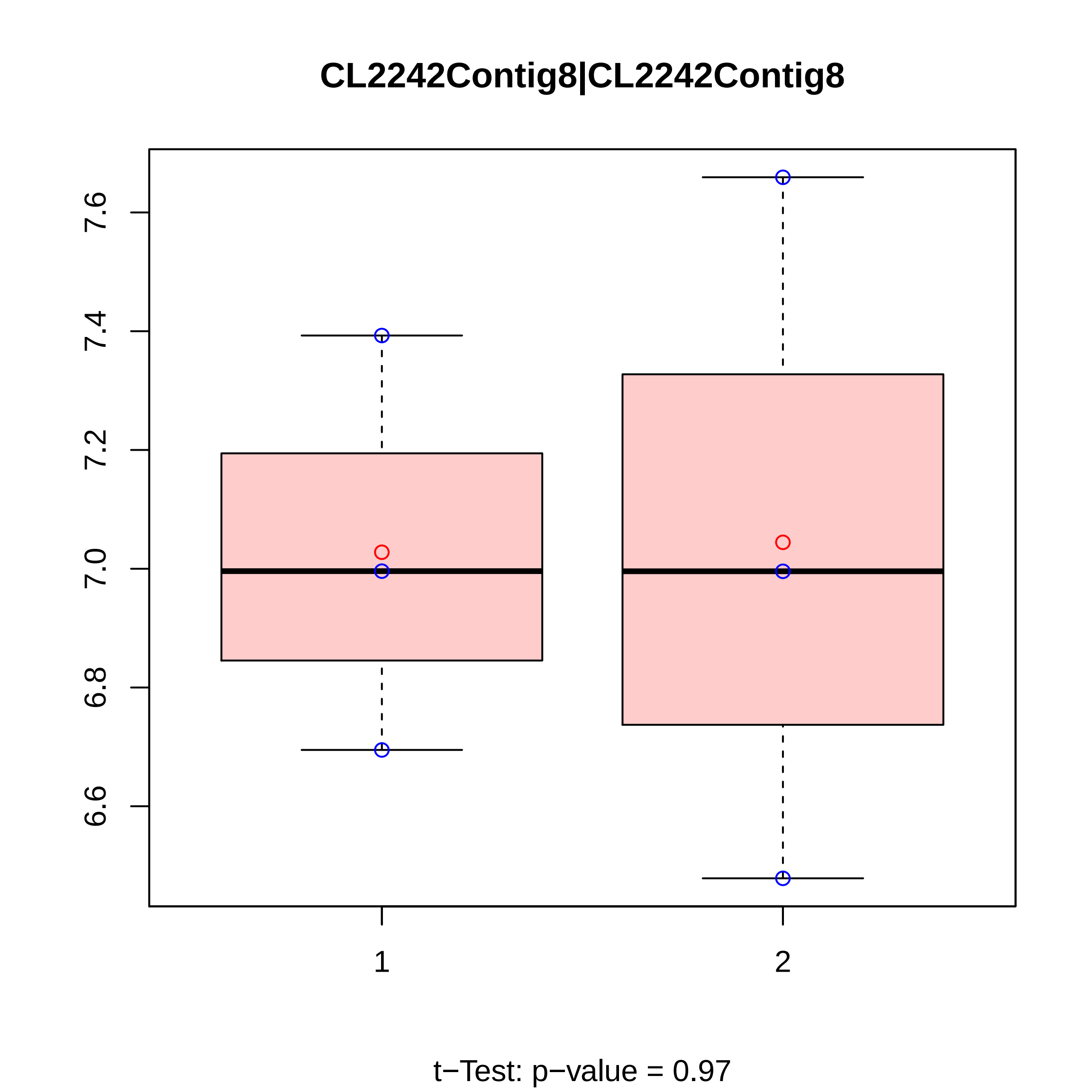
CL2242Contig8|CL2242Contig8
7.6
7.4
7.2
7.0
6.8
6.6
1
2
t−Test: p−value = 0.97

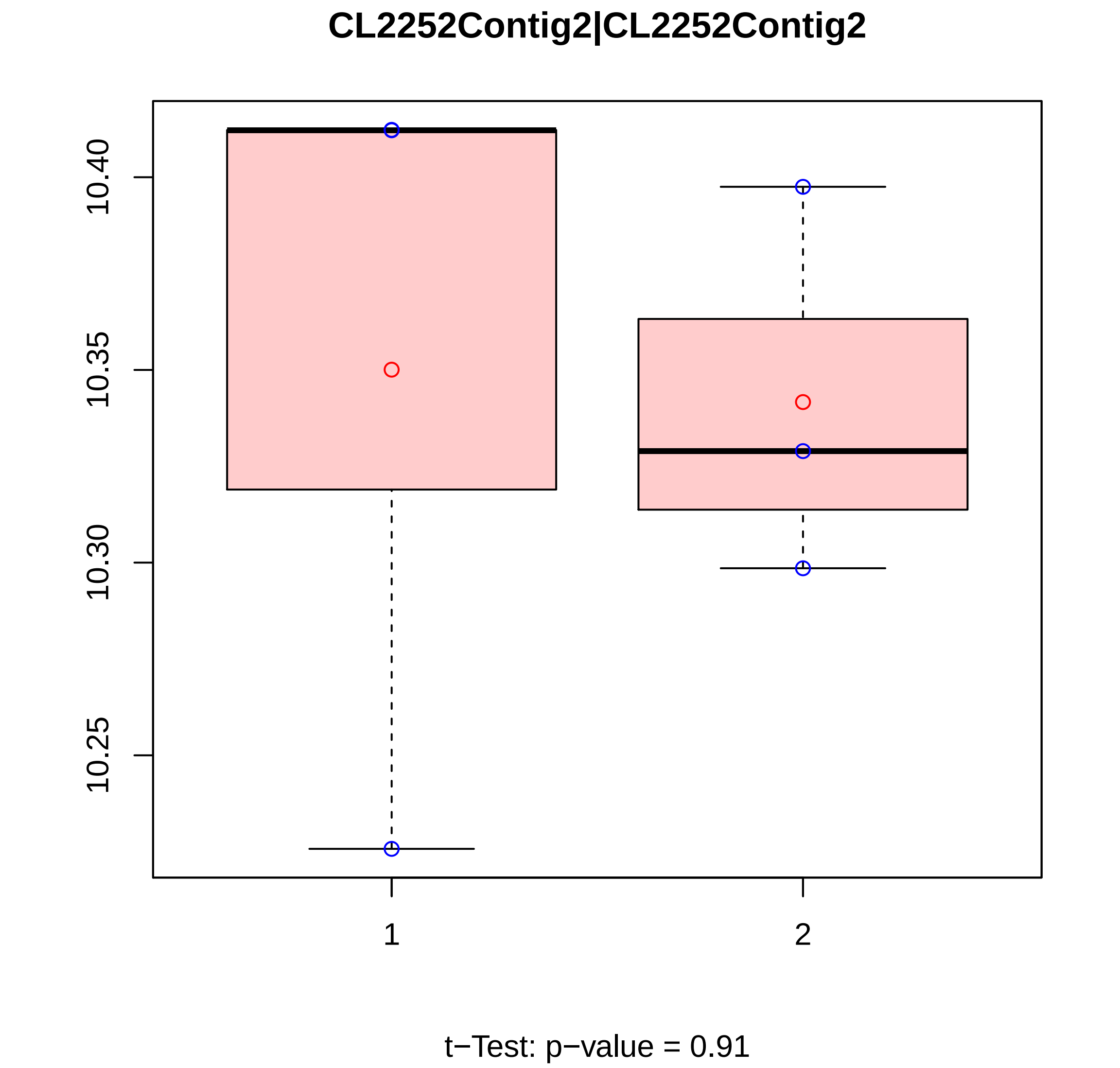
CL2252Contig2|CL2252Contig2
10.25
10.30
10.35
10.40
1
2
t−Test: p−value = 0.91

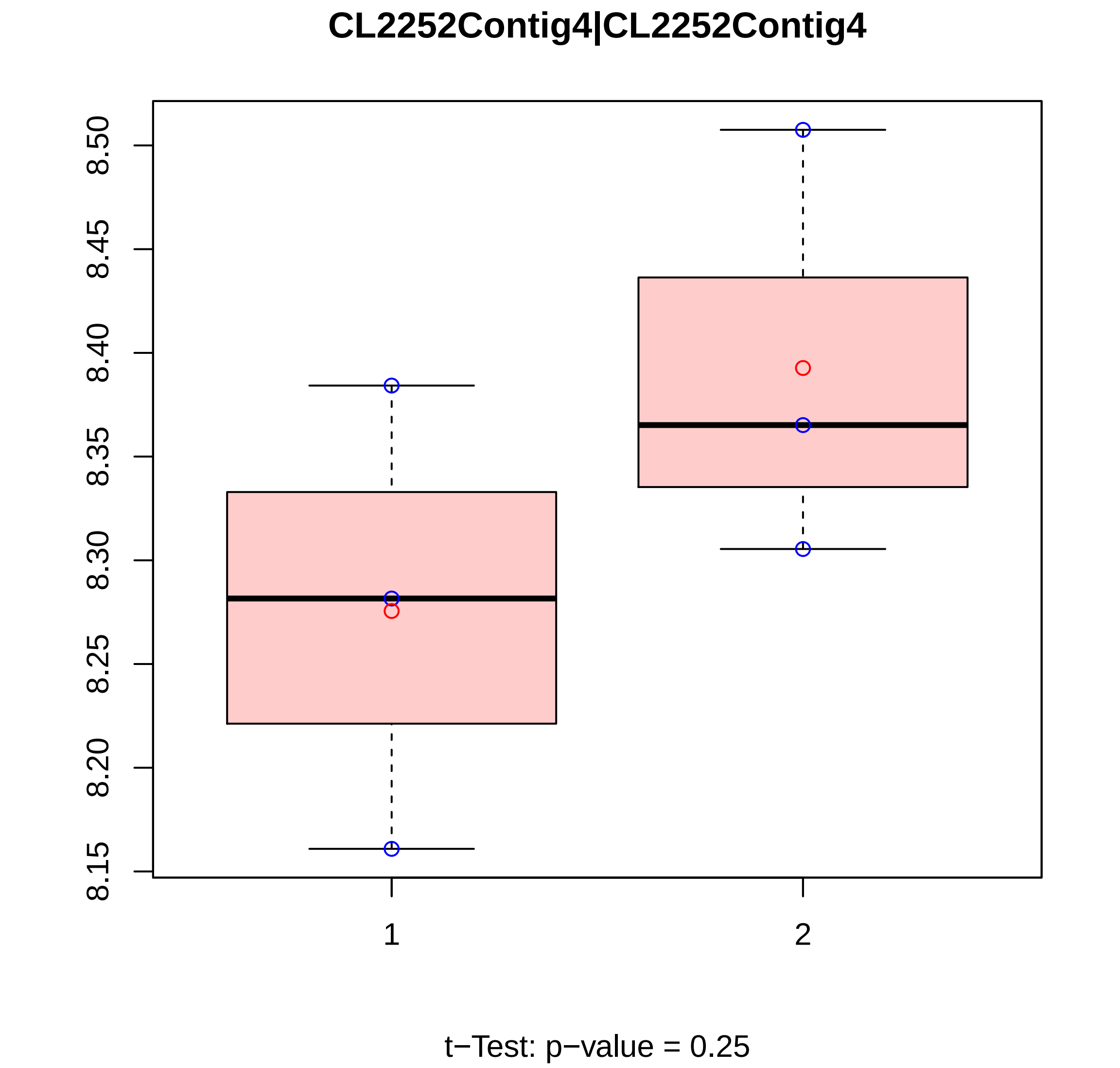
CL2252Contig4|CL2252Contig4
8.15
8.20
8.25
8.30
8.35
8.40
8.45
8.50
1
2
t−Test: p−value = 0.25

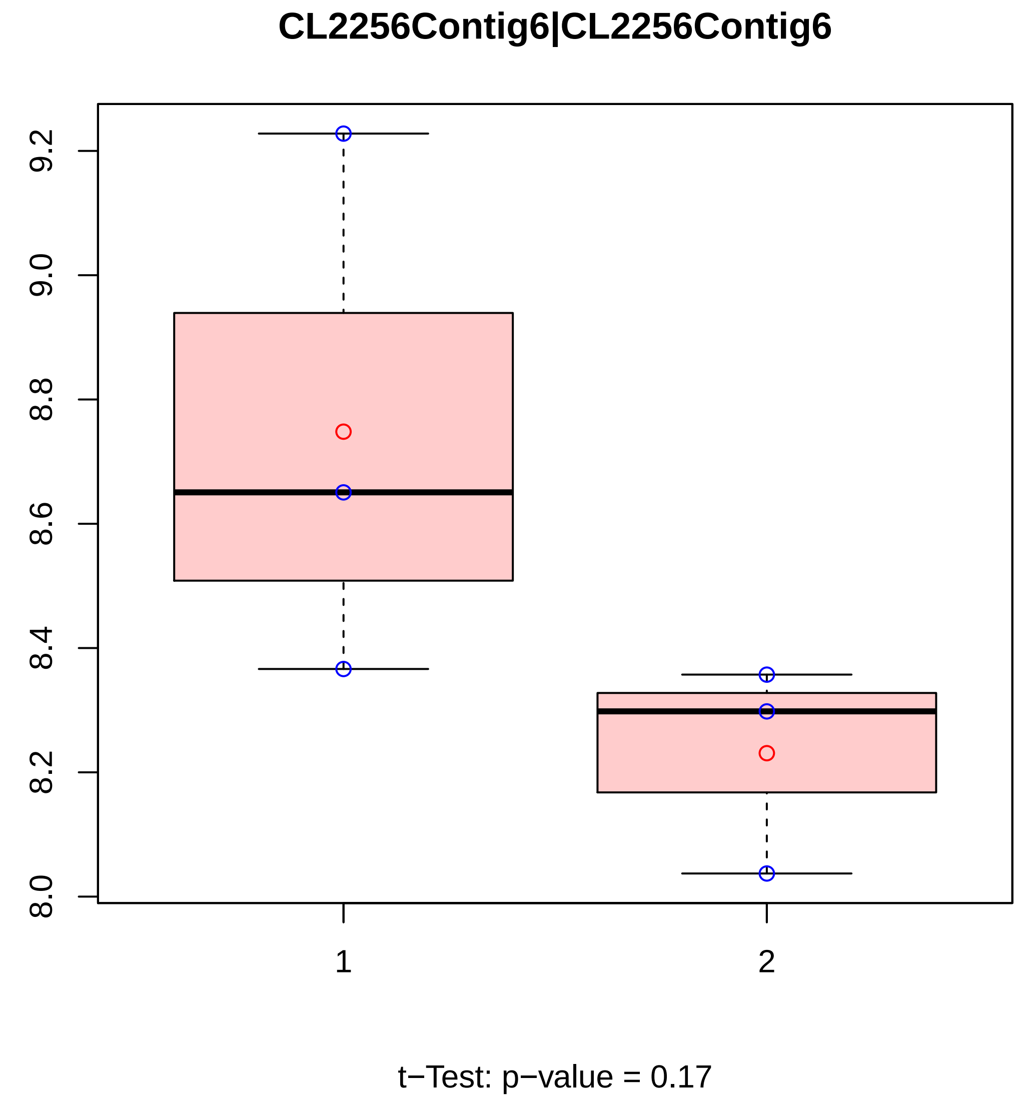
CL2256Contig6|CL2256Contig6
9.2
9.0
8.8
8.6
8.4
8.2
8.0
1
2
t−Test: p−value = 0.17

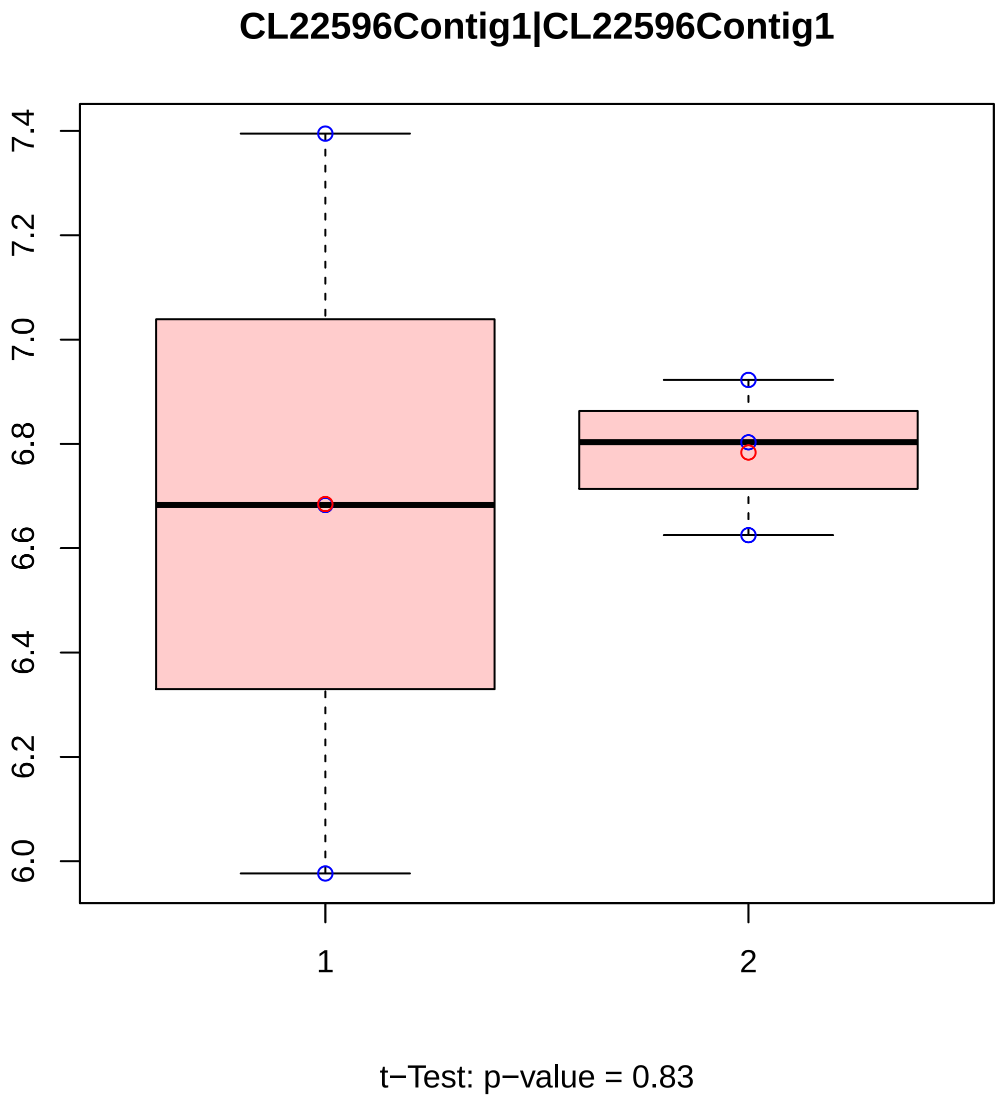

CL22596Contig1|CL22596Contig1
7.4
7.2
7.0
6.8
6.6
6.4
6.2
6.0
1
2
t−Test: p−value = 0.83

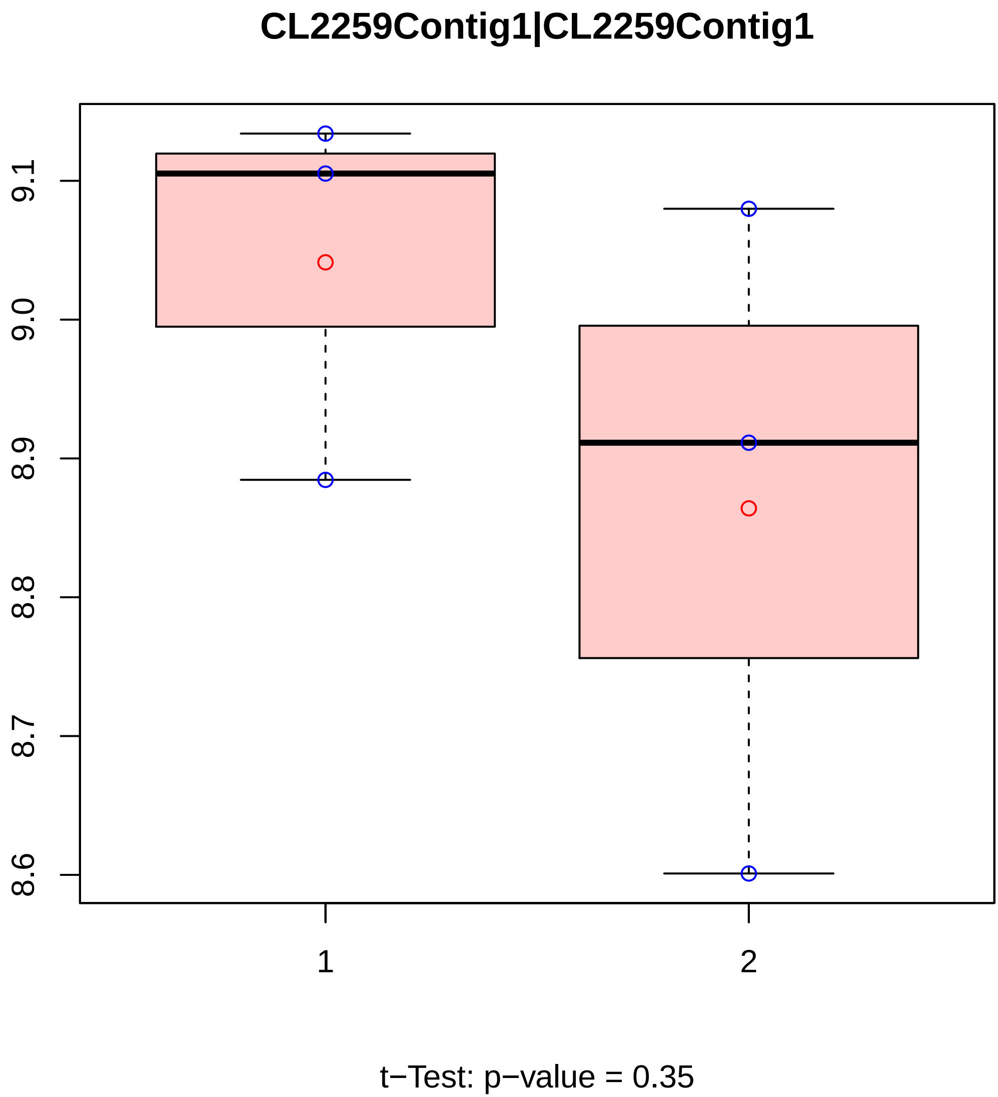
CL2259Contig1|CL2259Contig1
9.1
9.0
8.9
8.8
8.7
8.6
1
2
t−Test: p−value = 0.35

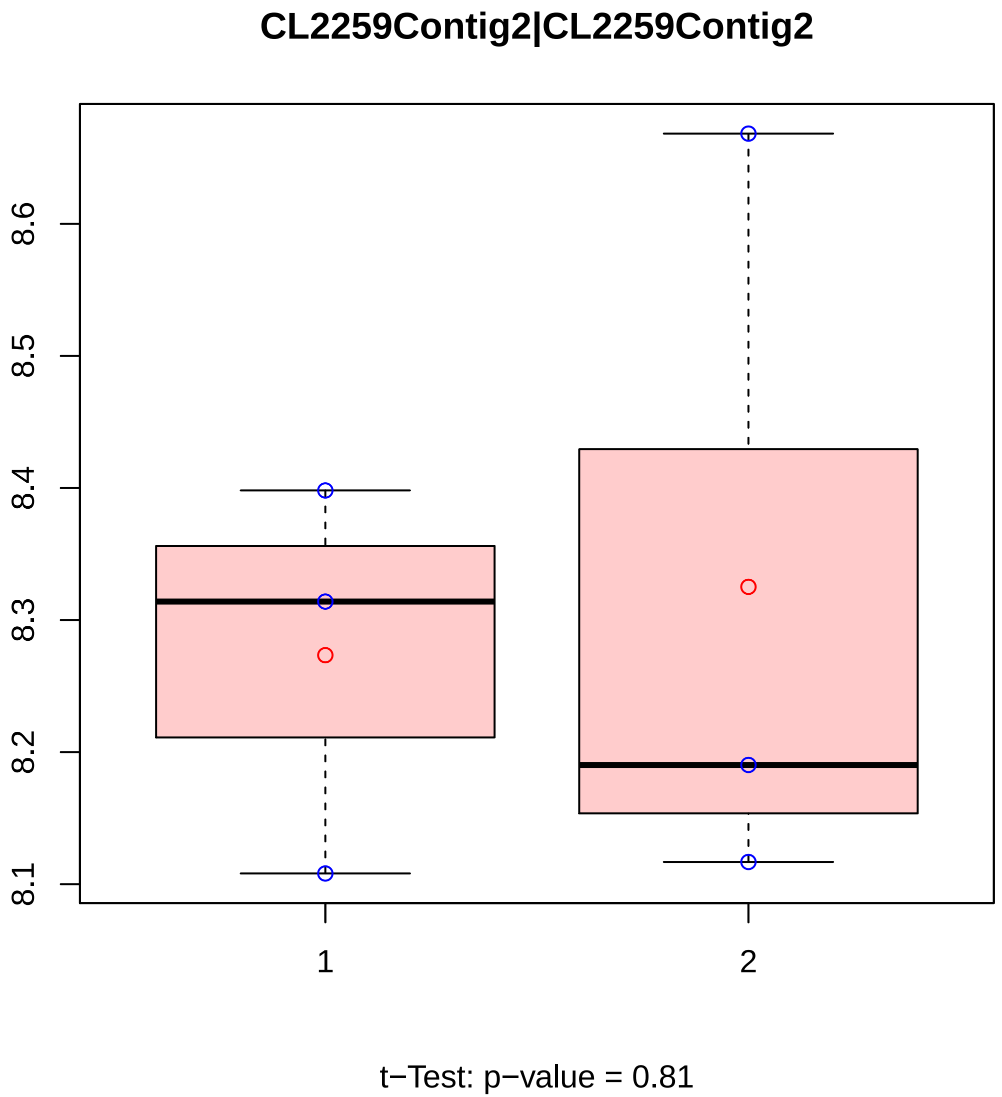
CL2259Contig2|CL2259Contig2
8.1
8.2
8.3
8.4
8.5
8.6
1
2
t−Test: p−value = 0.81

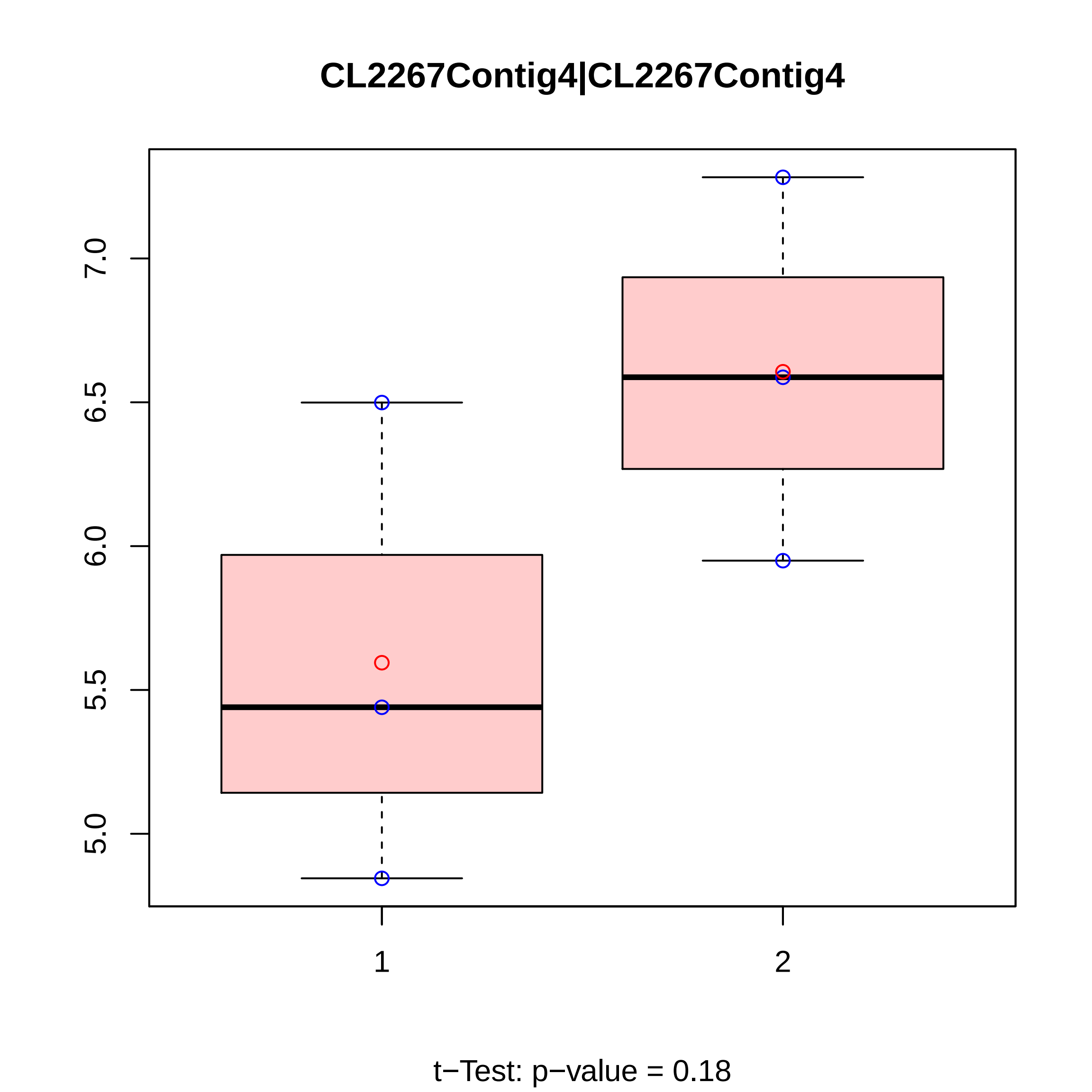

CL2267Contig4|CL2267Contig4
7.0
6.5
6.0
5.5
5.0
1
2
t−Test: p−value = 0.18

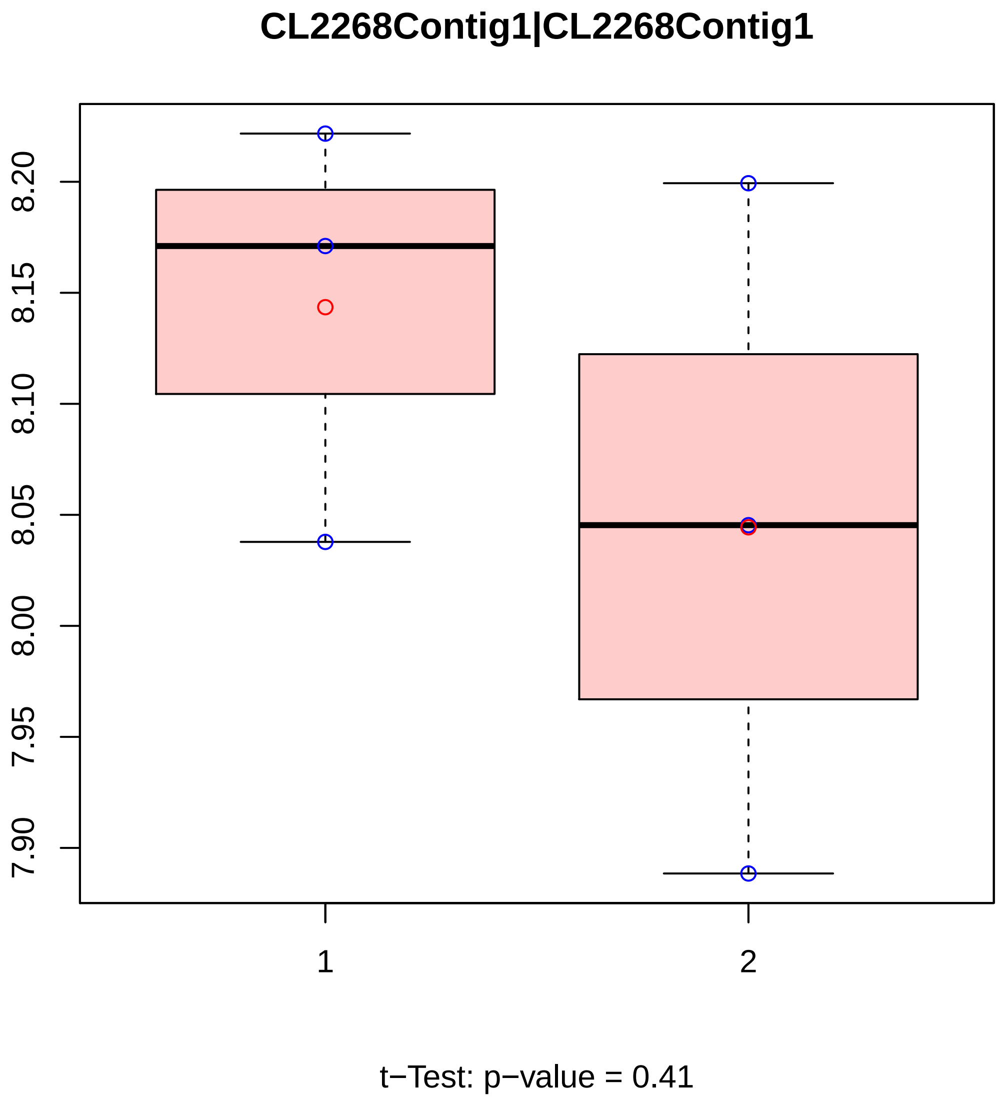

CL2268Contig1|CL2268Contig1
8.20
8.15
8.10
8.05
8.00
7.95
7.90
1
2
t−Test: p−value = 0.41

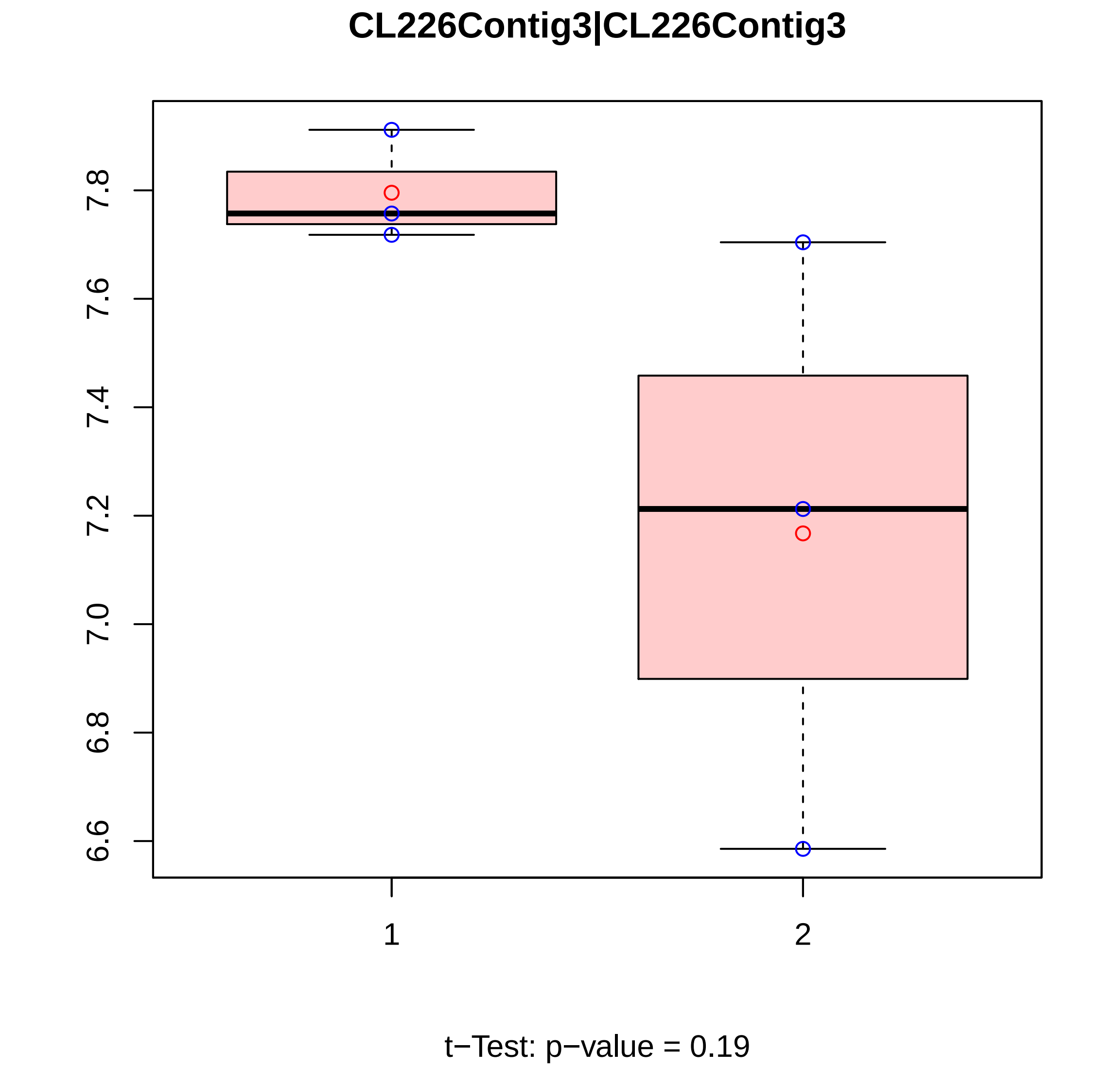

CL226Contig3|CL226Contig3
7.8
7.6
7.4
7.2
7.0
6.8
6.6
1
2
t−Test: p−value = 0.19

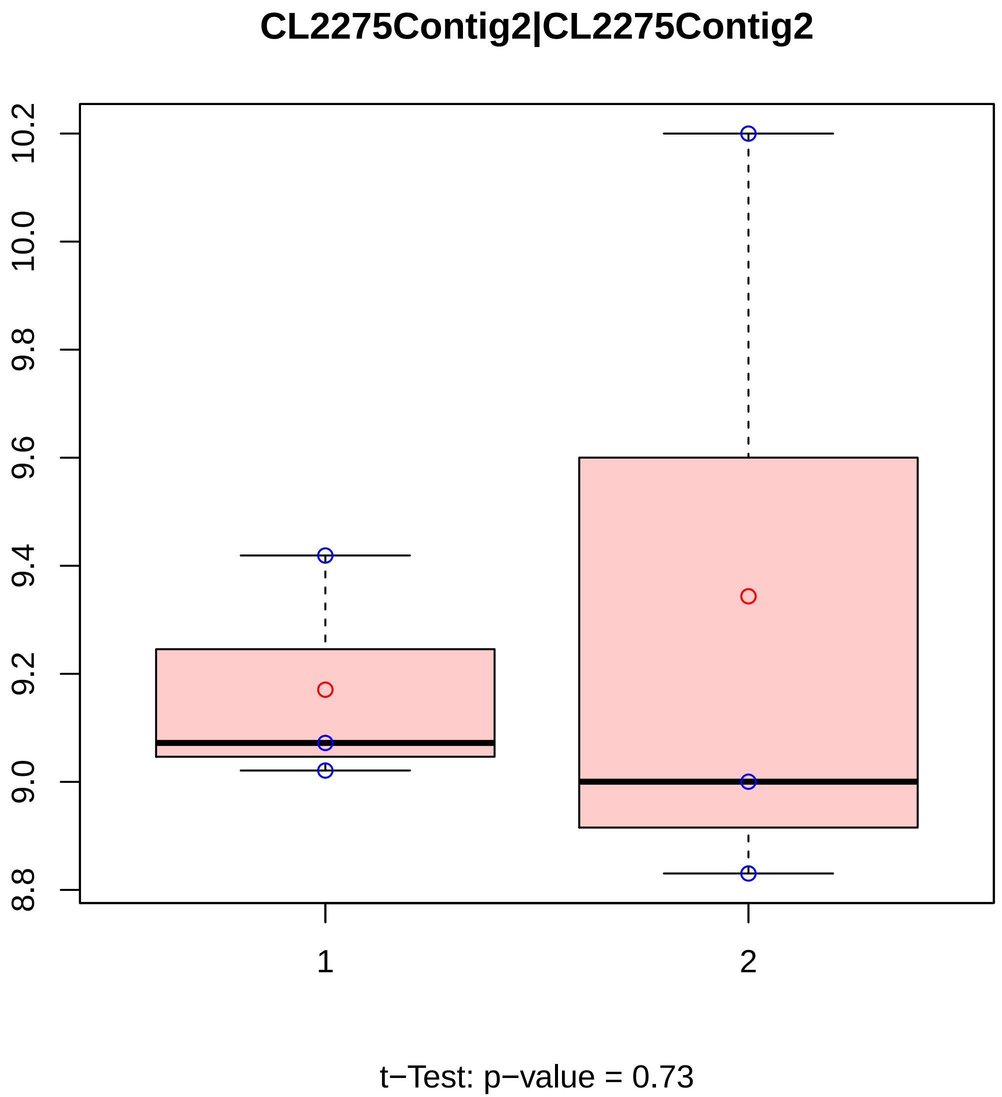
CL2275Contig2|CL2275Contig2
10.2
10.0
9.8
9.6
9.4
9.2
9.0
8.8
1
2
t−Test: p−value = 0.73

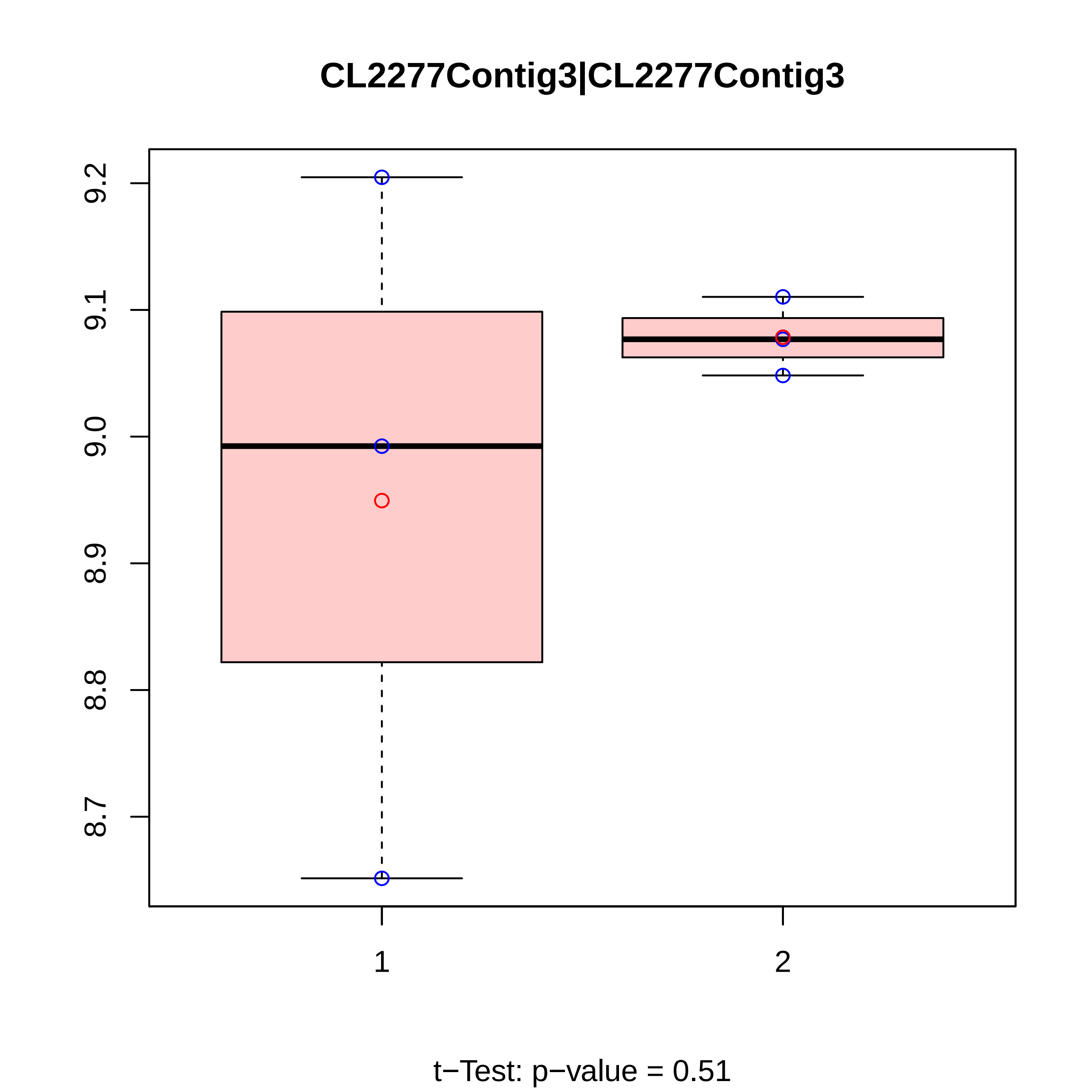
CL2277Contig3|CL2277Contig3
9.2
9.1
9.0
8.9
8.8
8.7
1
2
t−Test: p−value = 0.51

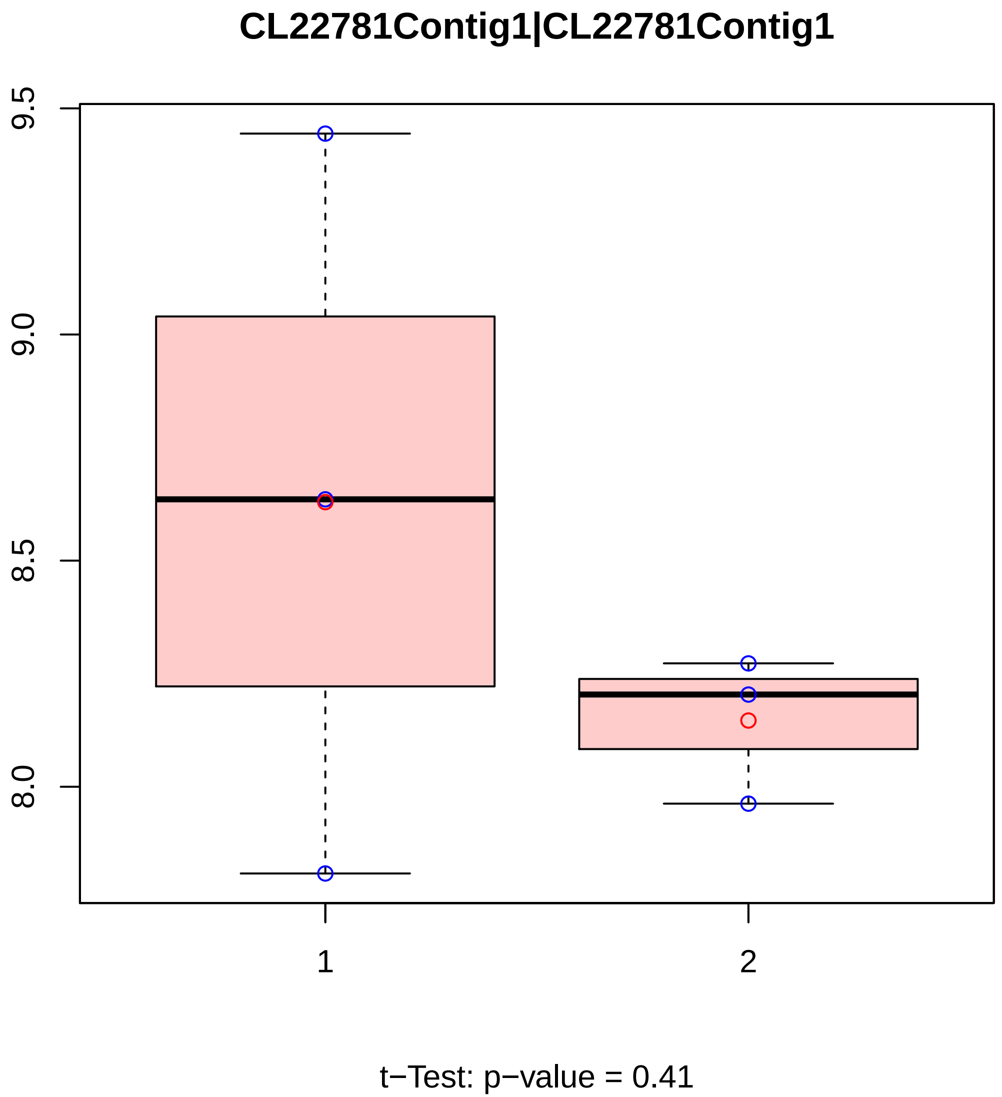

CL22781Contig1|CL22781Contig1
9.5
9.0
8.5
8.0
1
2
t−Test: p−value = 0.41

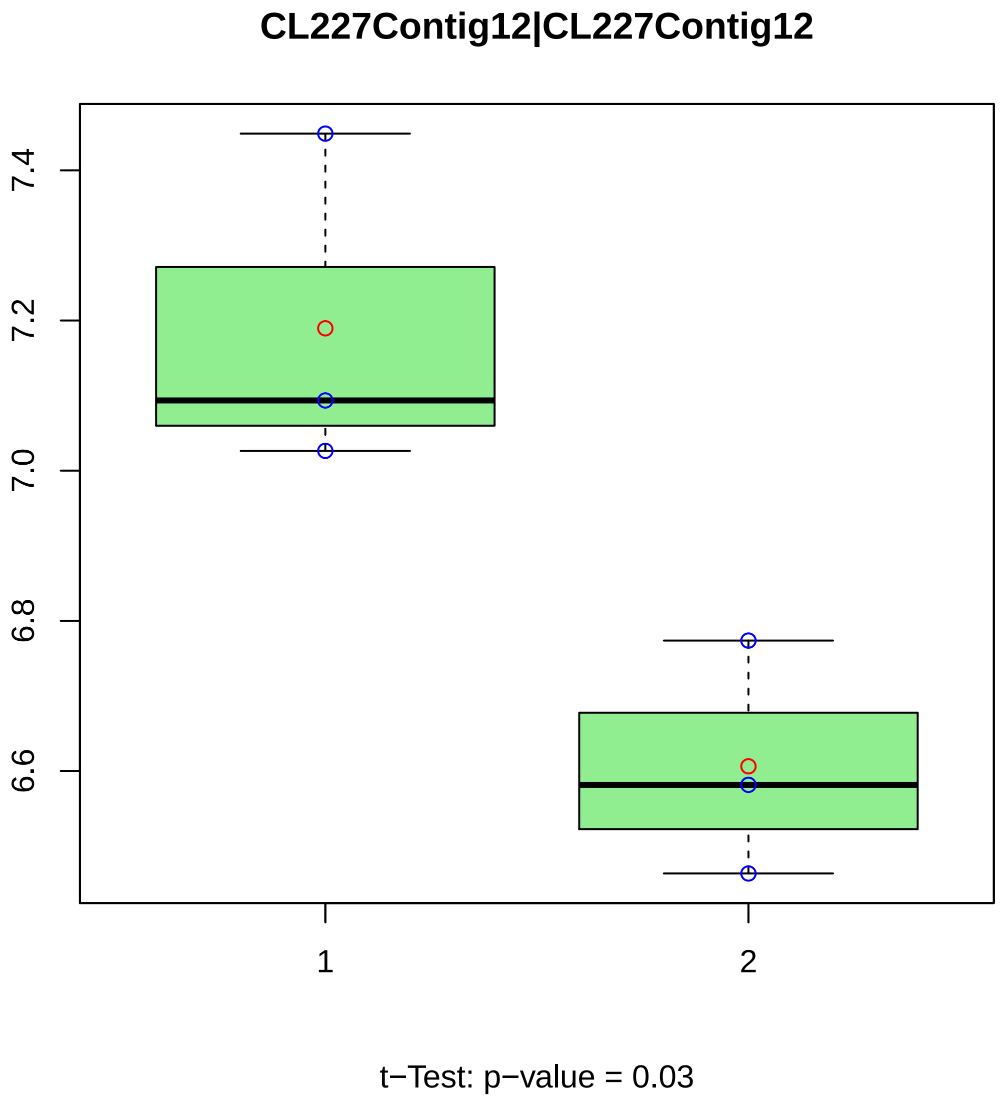
CL227Contig12|CL227Contig12
7.4
7.2
7.0
6.8
6.6
1
2
t−Test: p−value = 0.03

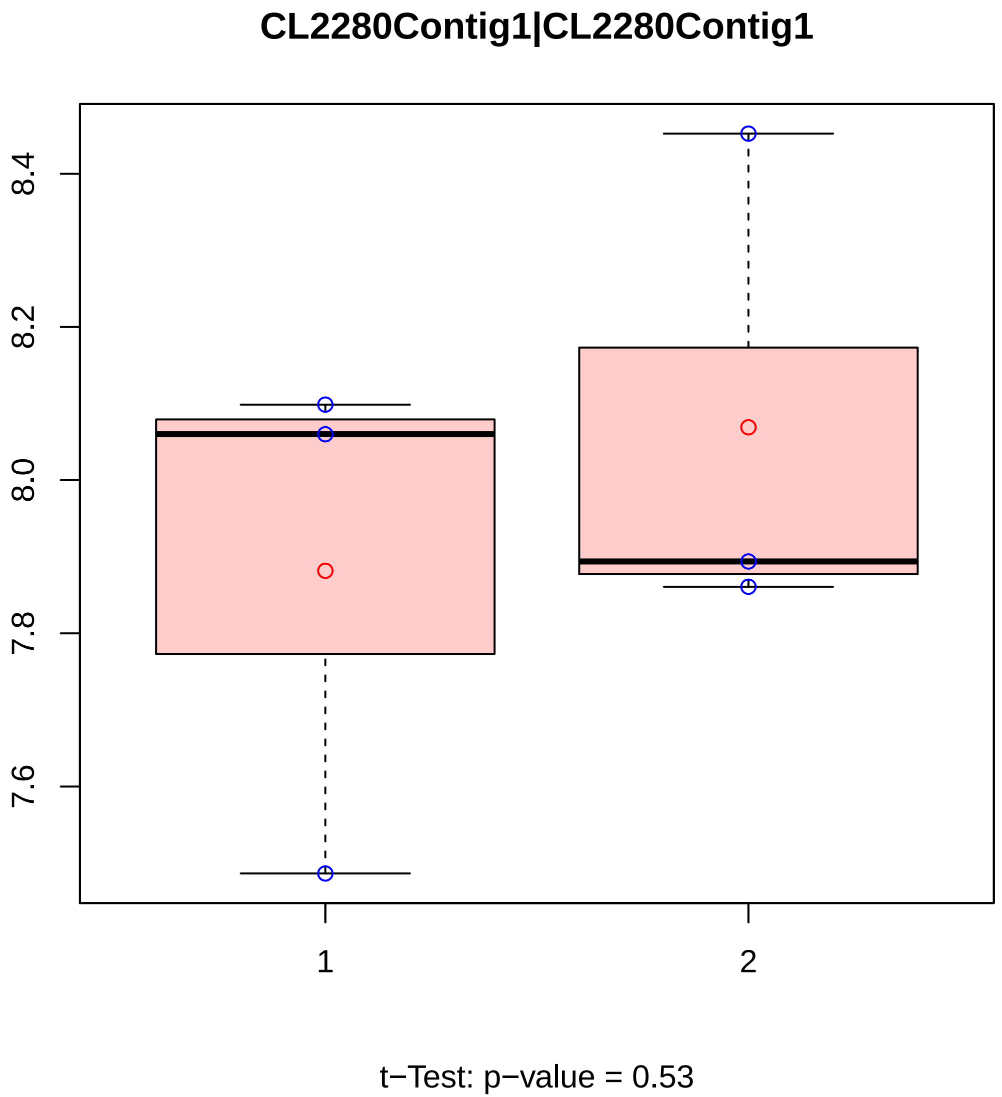

CL2280Contig1|CL2280Contig1
8.4
8.2
8.0
7.8
7.6
1
2
t−Test: p−value = 0.53

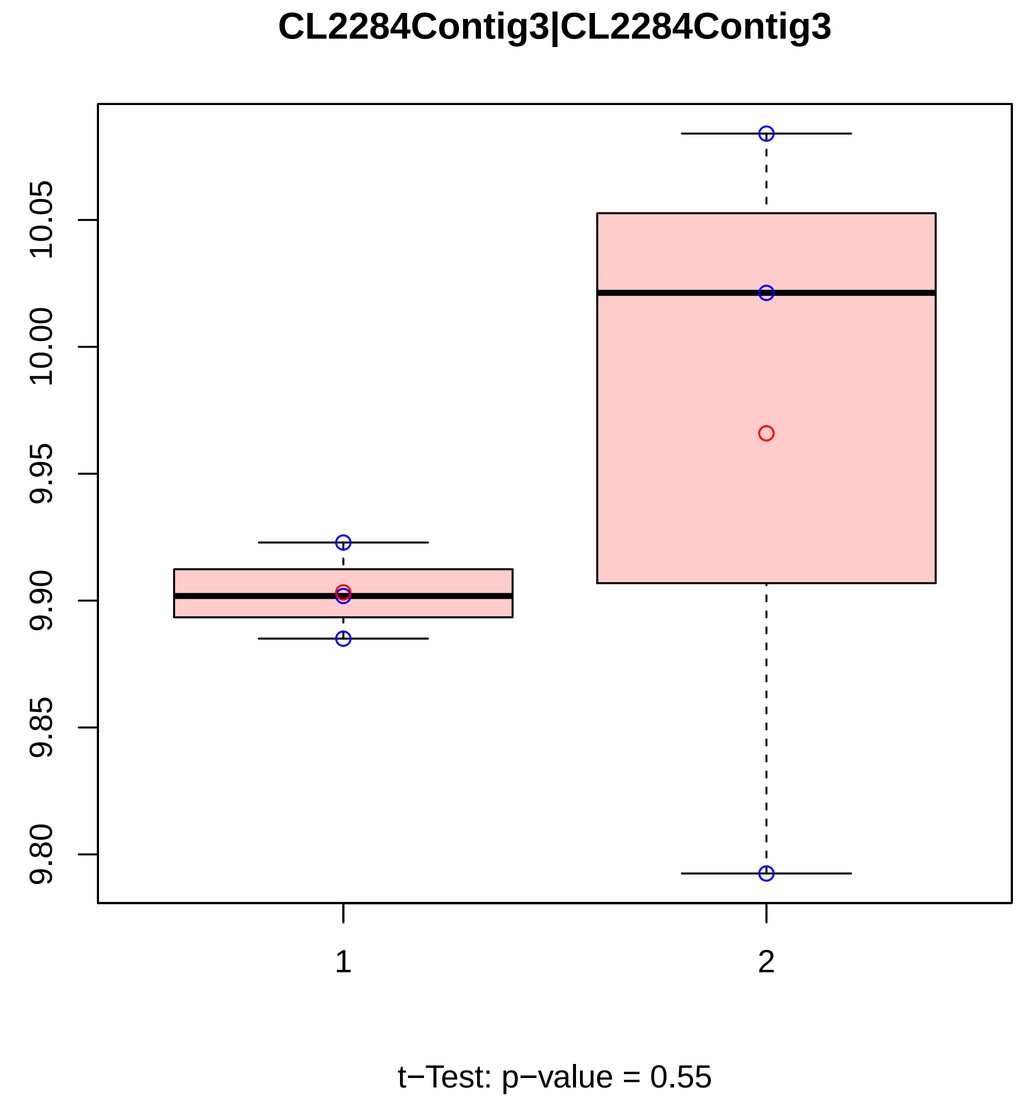
CL2284Contig3|CL2284Contig3
10.05
10.00
9.95
9.90
9.85
9.80
1
2
t−Test: p−value = 0.55

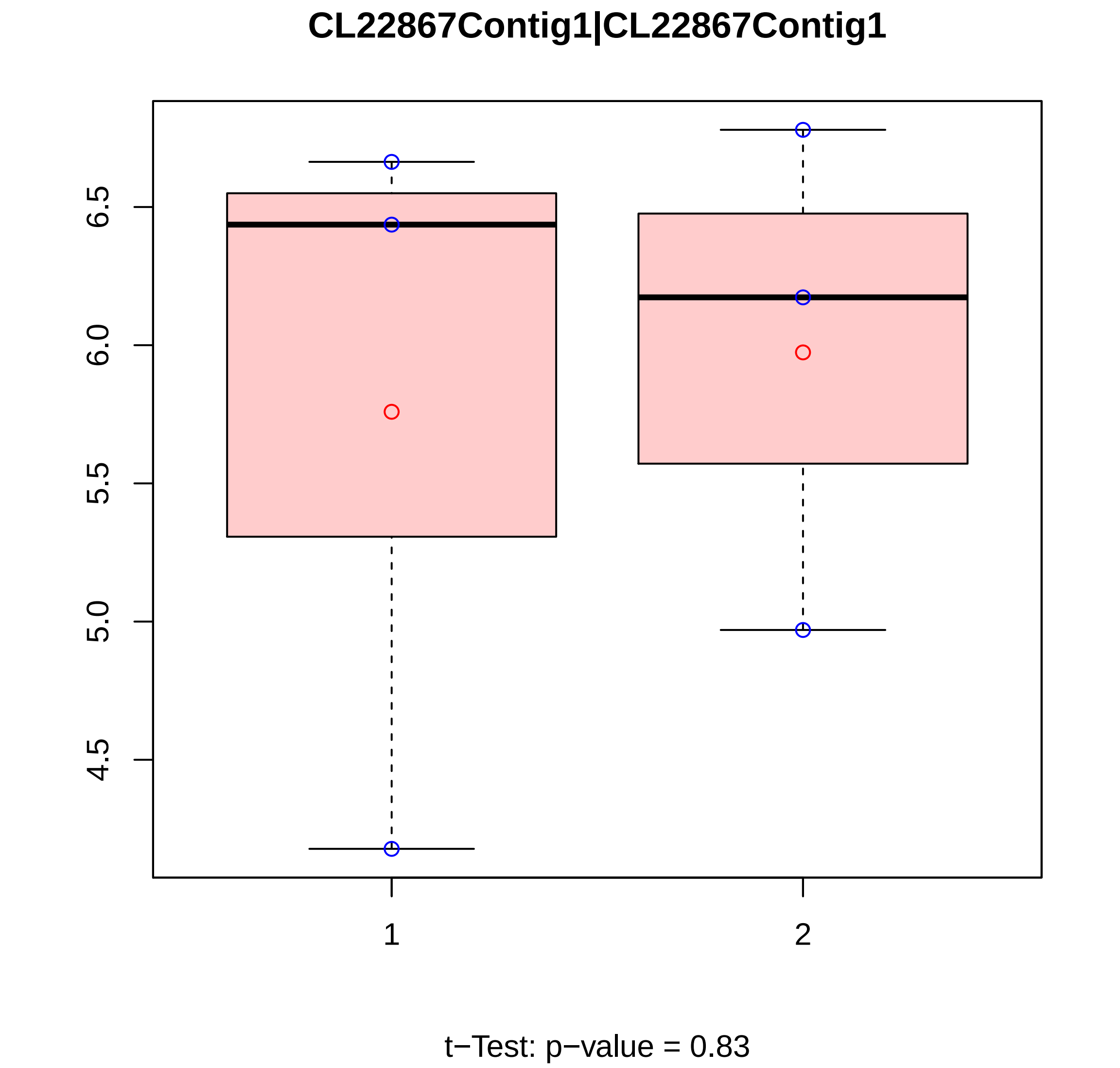
CL22867Contig1|CL22867Contig1
6.5
6.0
5.5
5.0
4.5
1
2
t−Test: p−value = 0.83

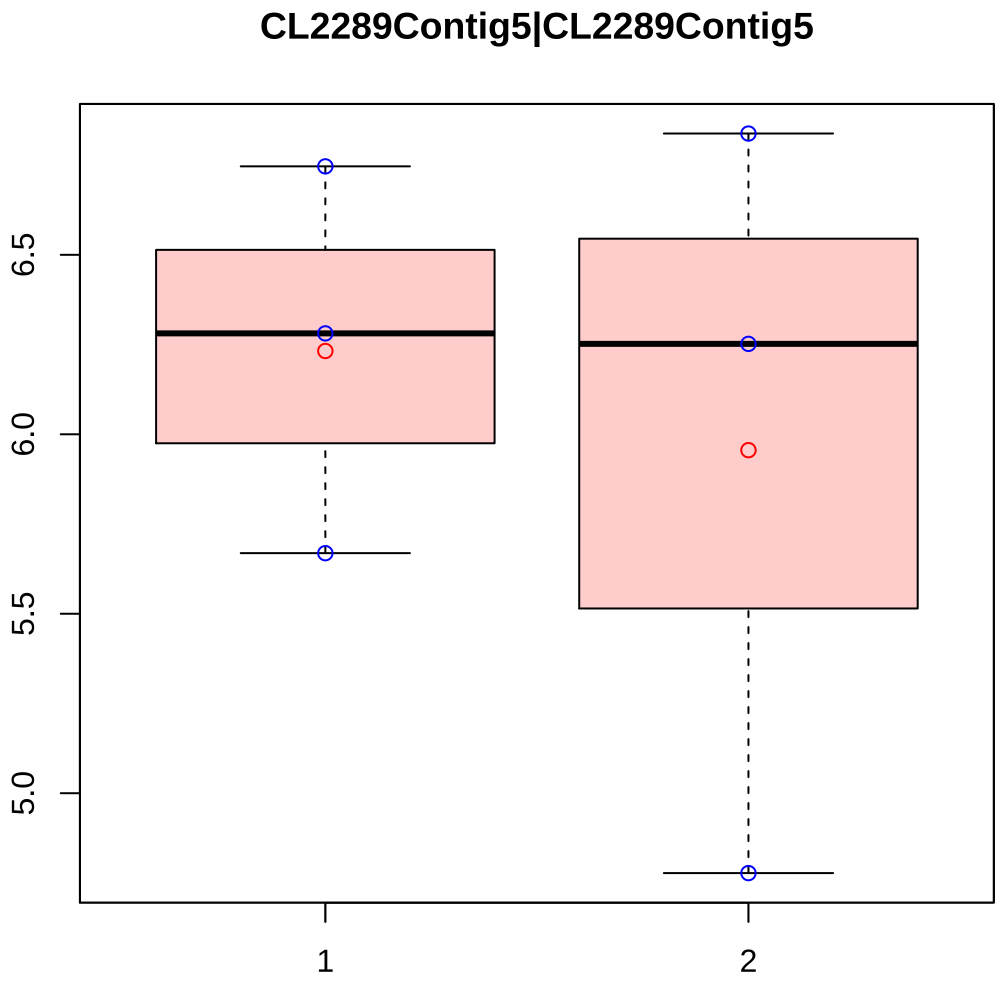


t−Test: p−value = 0.71

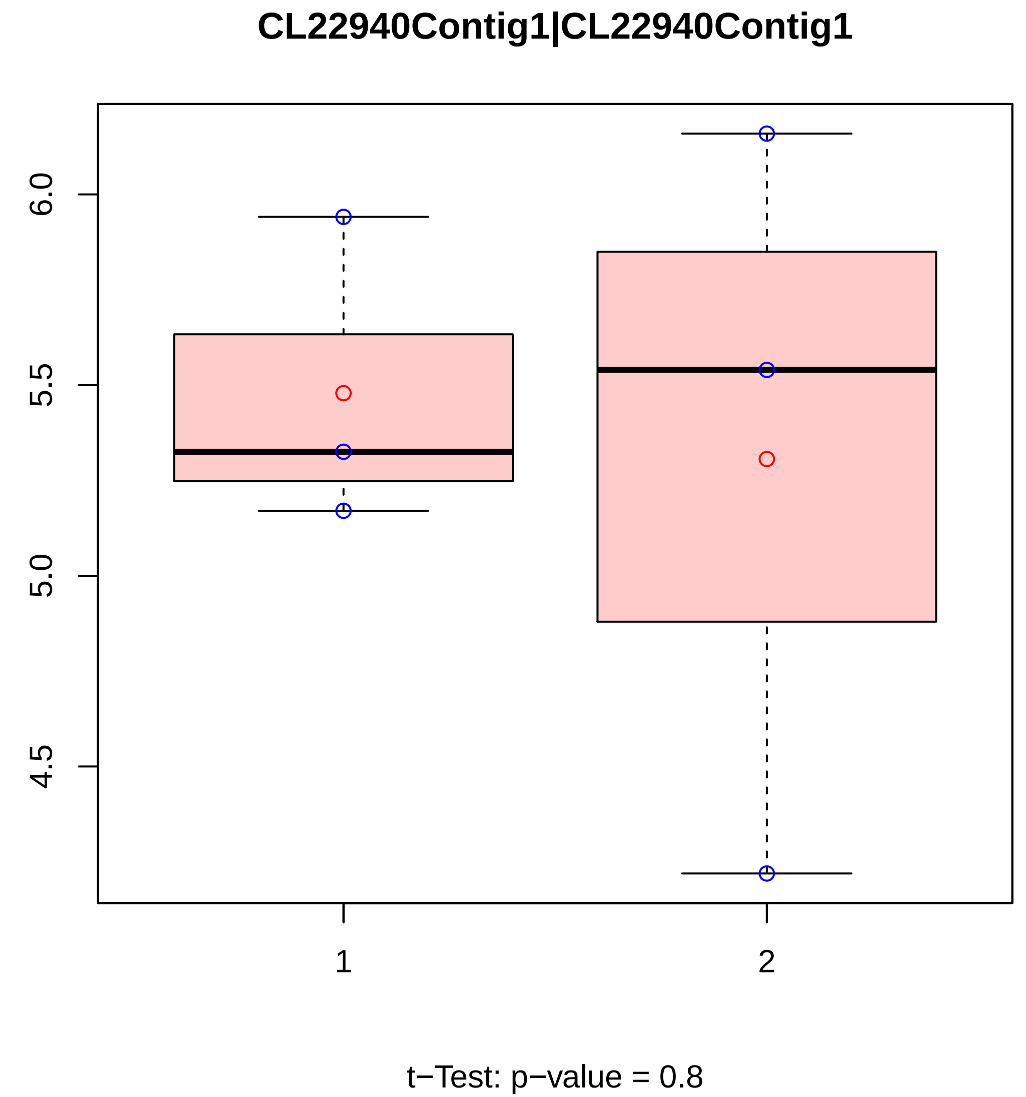
CL22940Contig1|CL22940Contig1
6.0
5.5
5.0
4.5
1
2
t−Test: p−value = 0.8

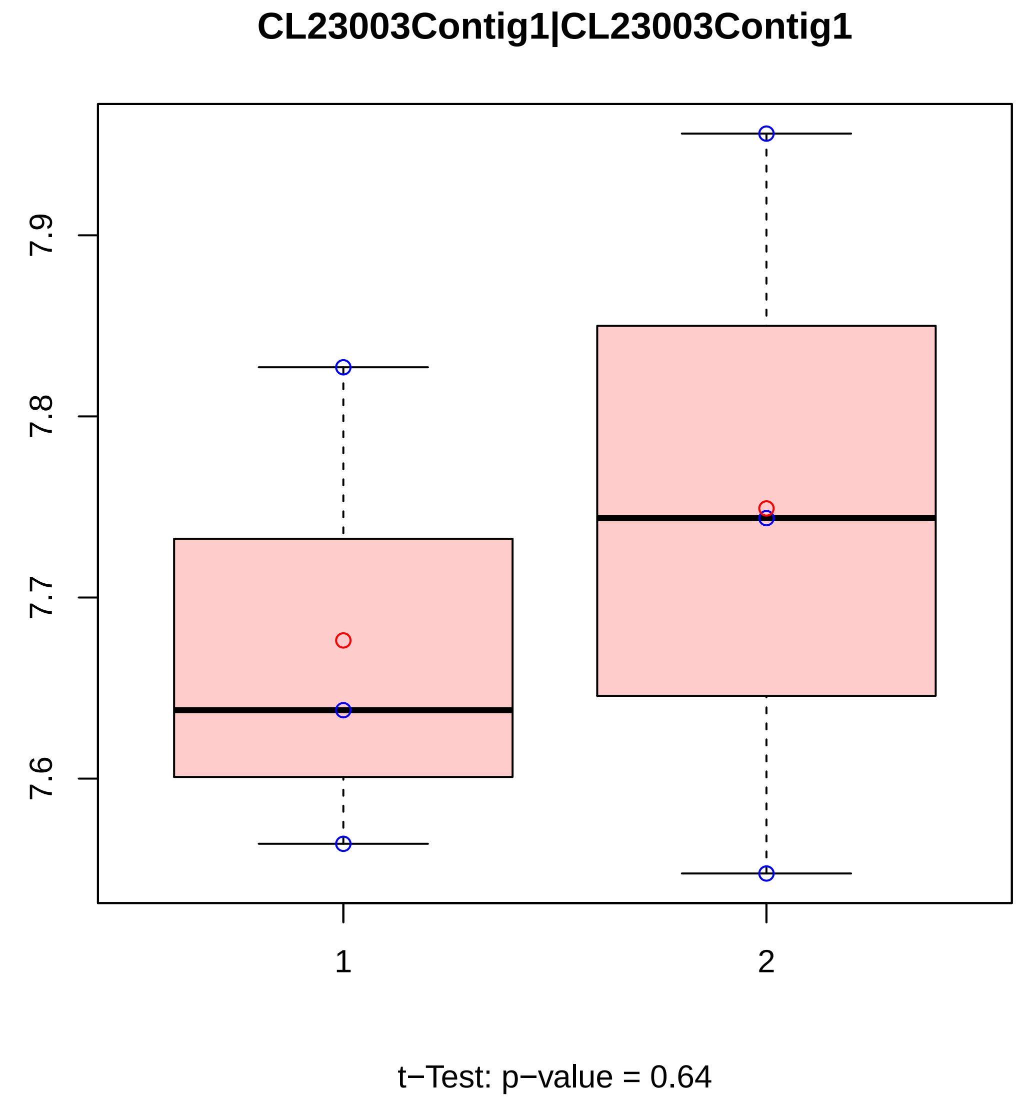
CL23003Contig1|CL23003Contig1
7.6
7.7
7.8
7.9
1
2
t−Test: p−value = 0.64

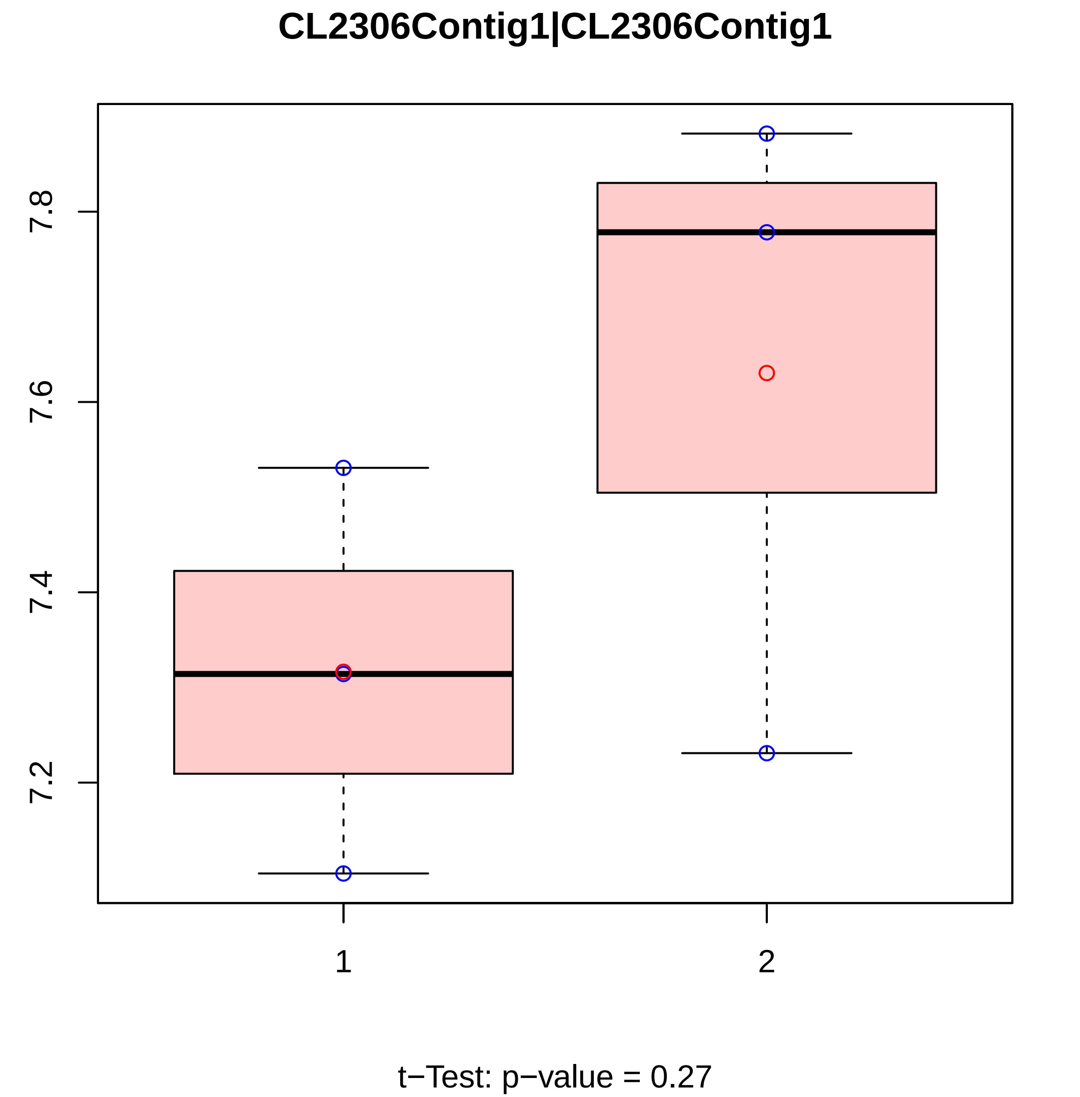

CL2306Contig1|CL2306Contig1
7.2
7.4
7.6
7.8
1
2
t−Test: p−value = 0.27

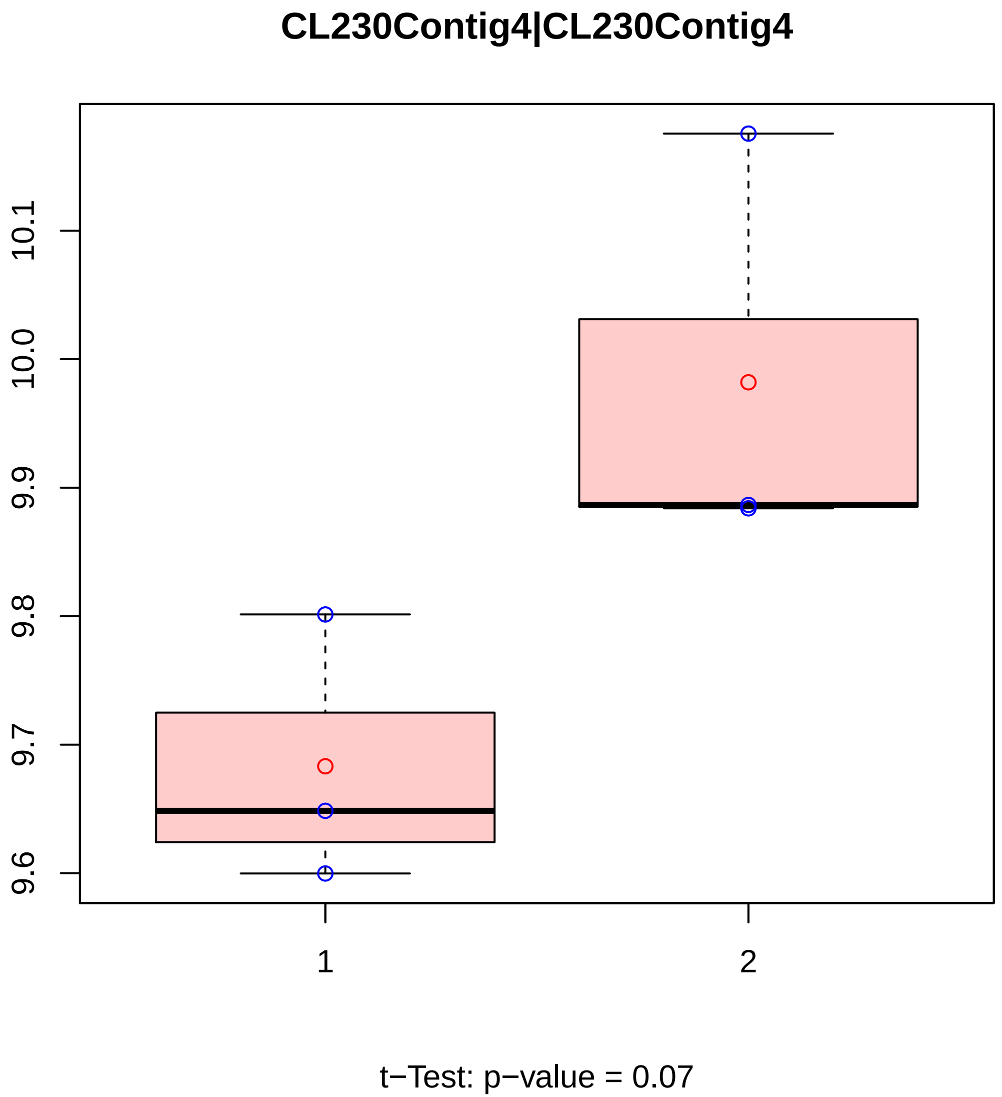

CL230Contig4|CL230Contig4
9.6
9.7
9.8
9.9
10.0
10.1
1
2
t−Test: p−value = 0.07

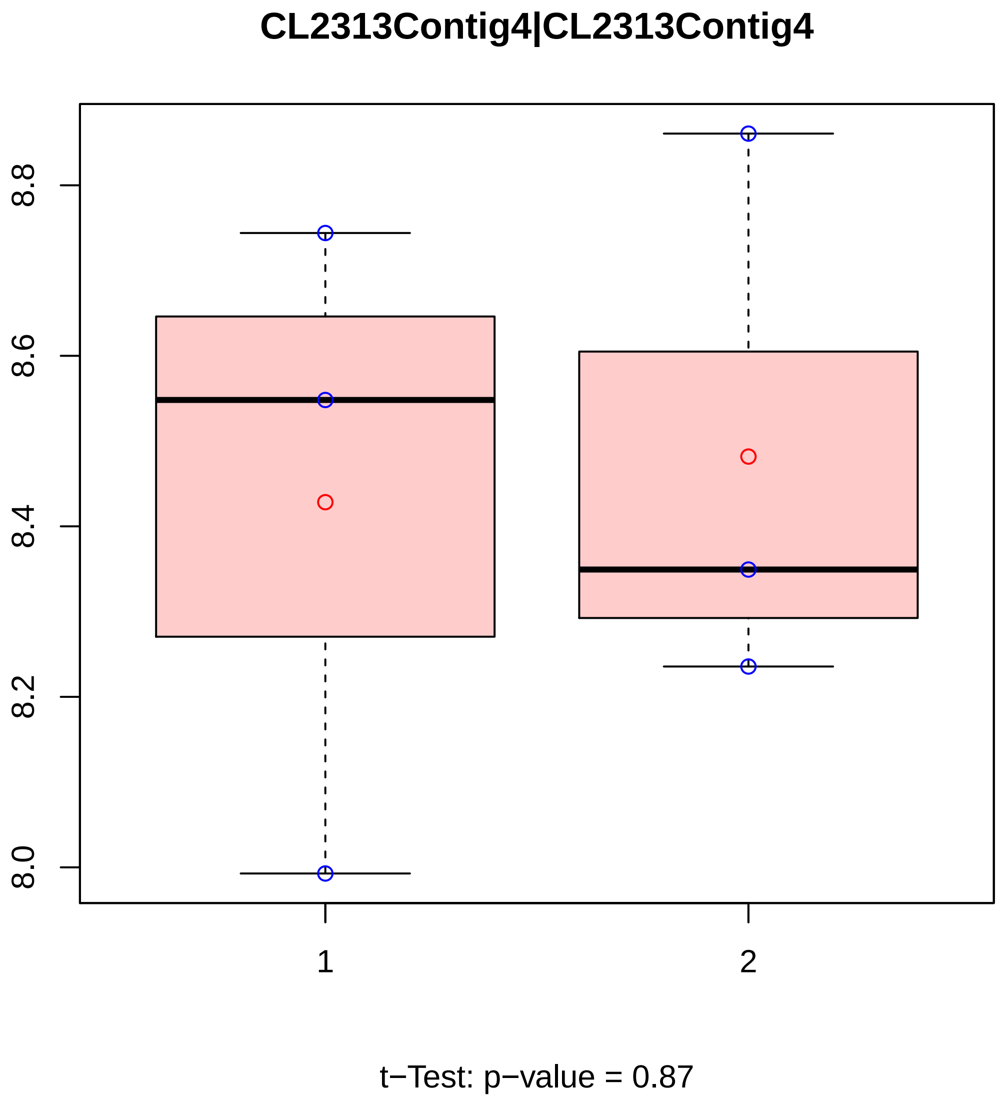
CL2313Contig4|CL2313Contig4
8.0
8.2
8.4
8.6
8.8
1
2
t−Test: p−value = 0.87

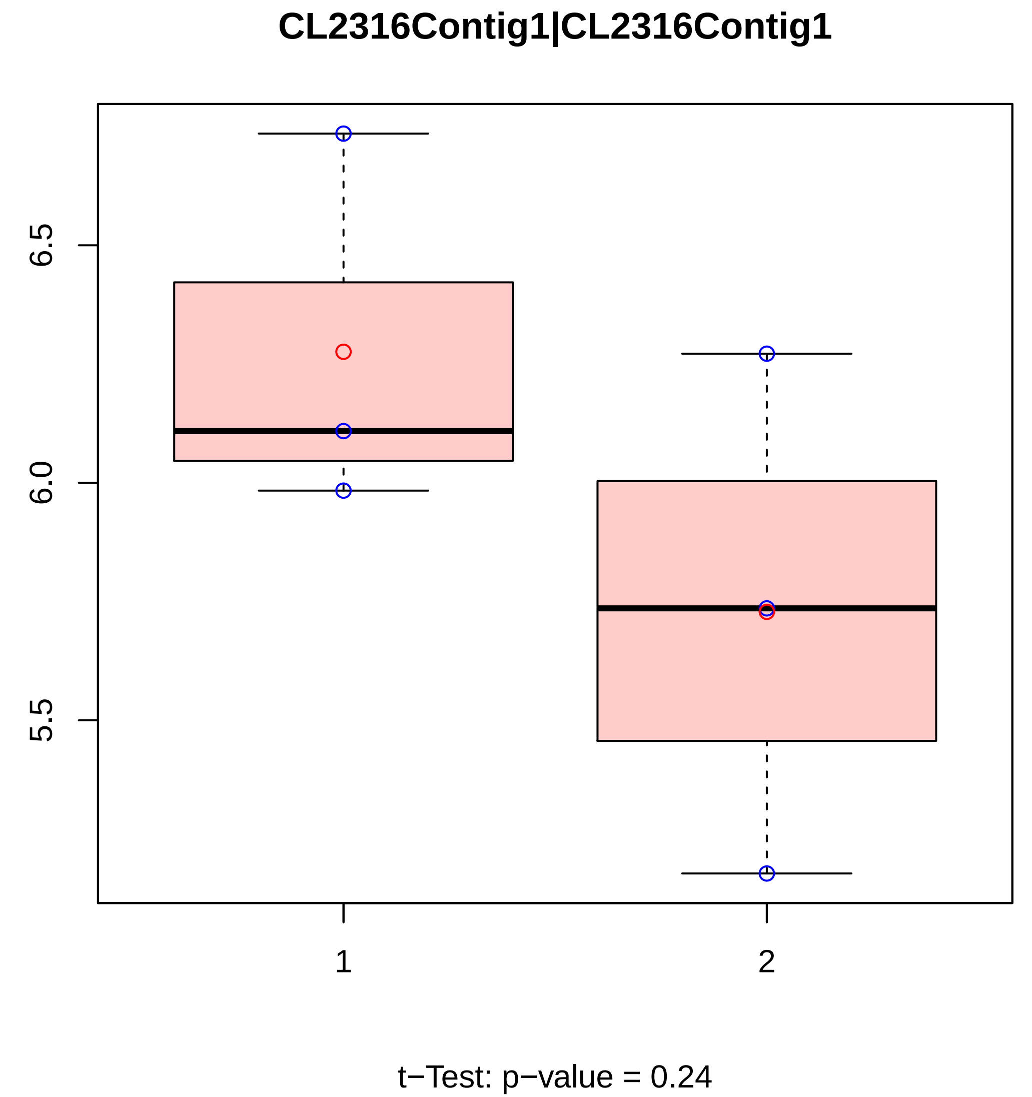
CL2316Contig1|CL2316Contig1
6.5
6.0
5.5
1
2
t−Test: p−value = 0.24

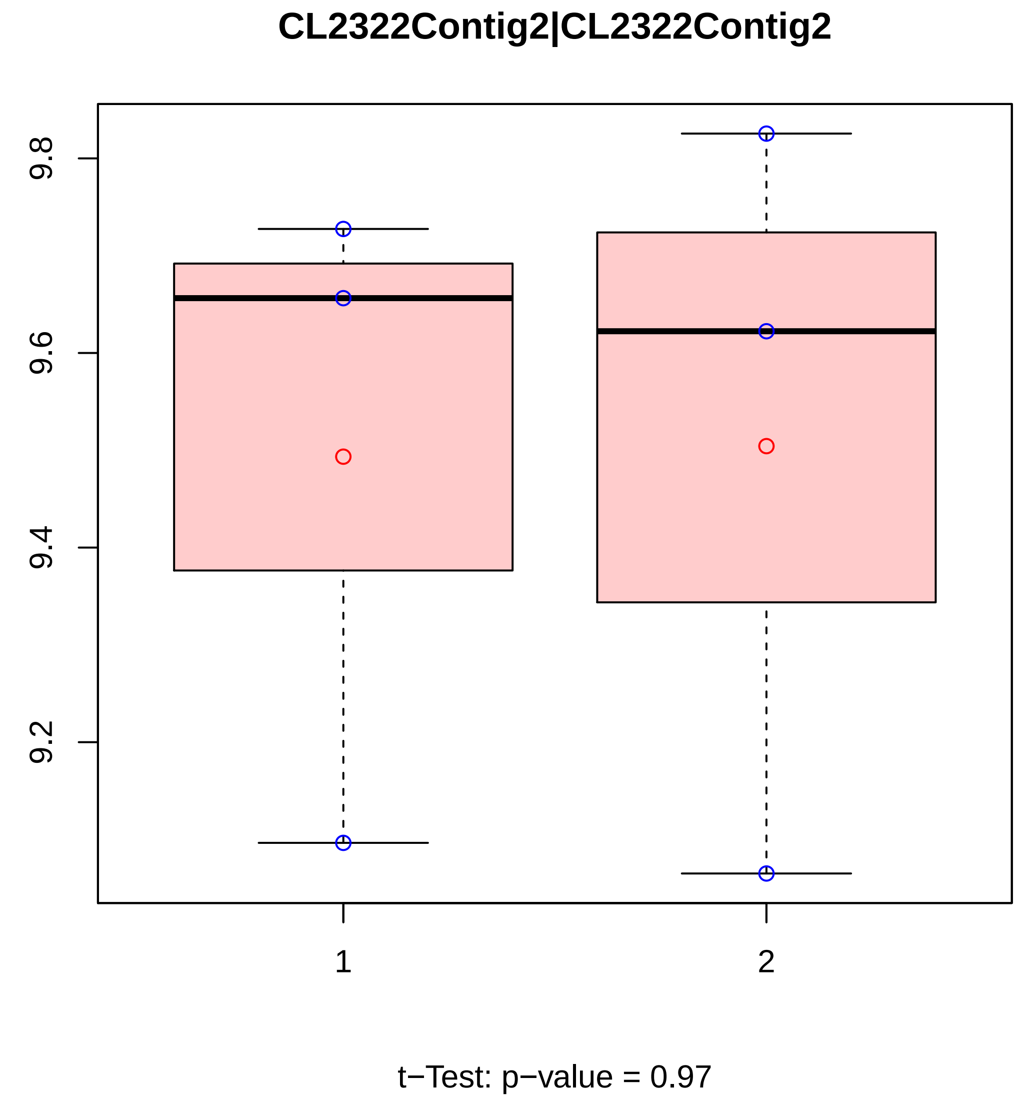
CL2322Contig2|CL2322Contig2
9.2
9.4
9.6
9.8
1
2
t−Test: p−value = 0.97

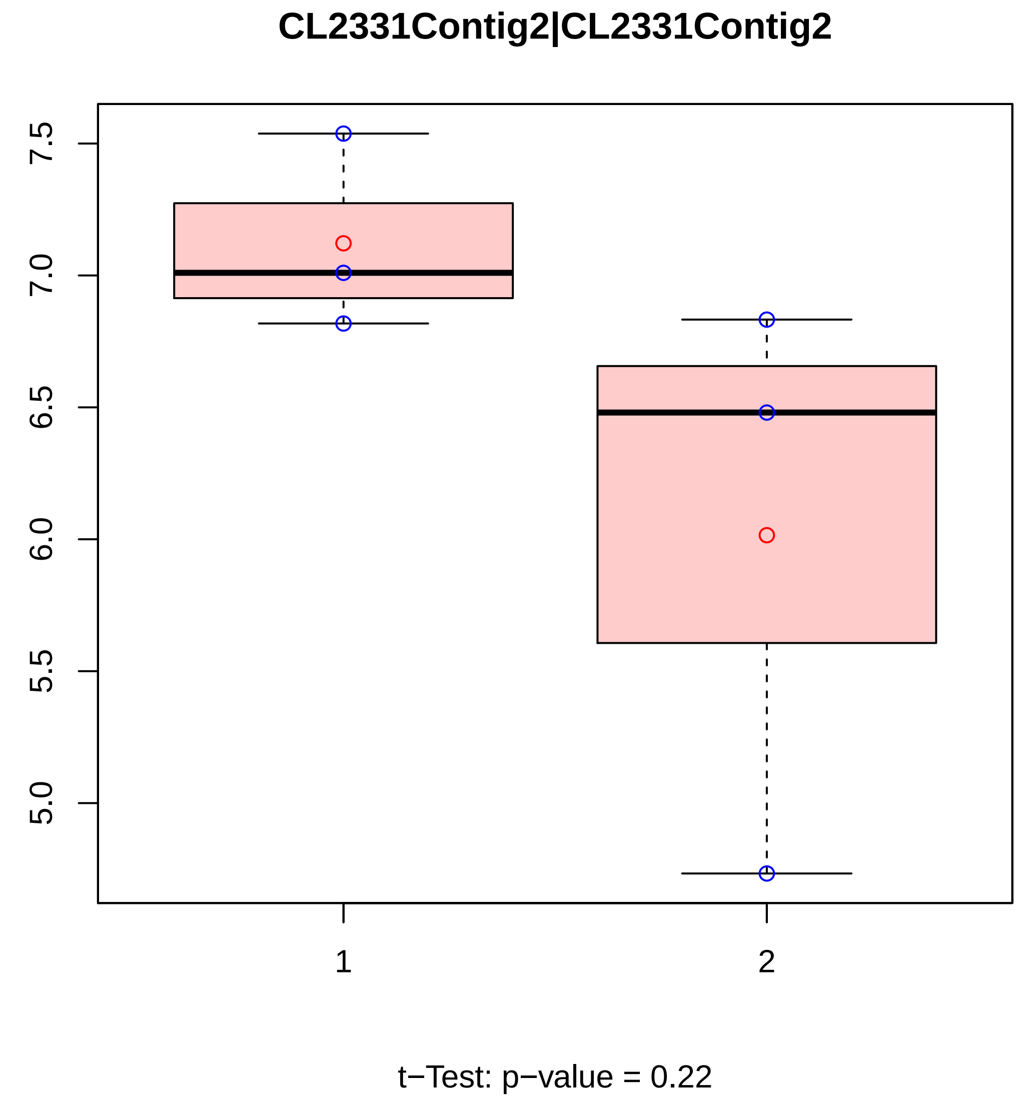
CL2331Contig2|CL2331Contig2
7.5
7.0
6.5
6.0
5.5
5.0
1
2
t−Test: p−value = 0.22

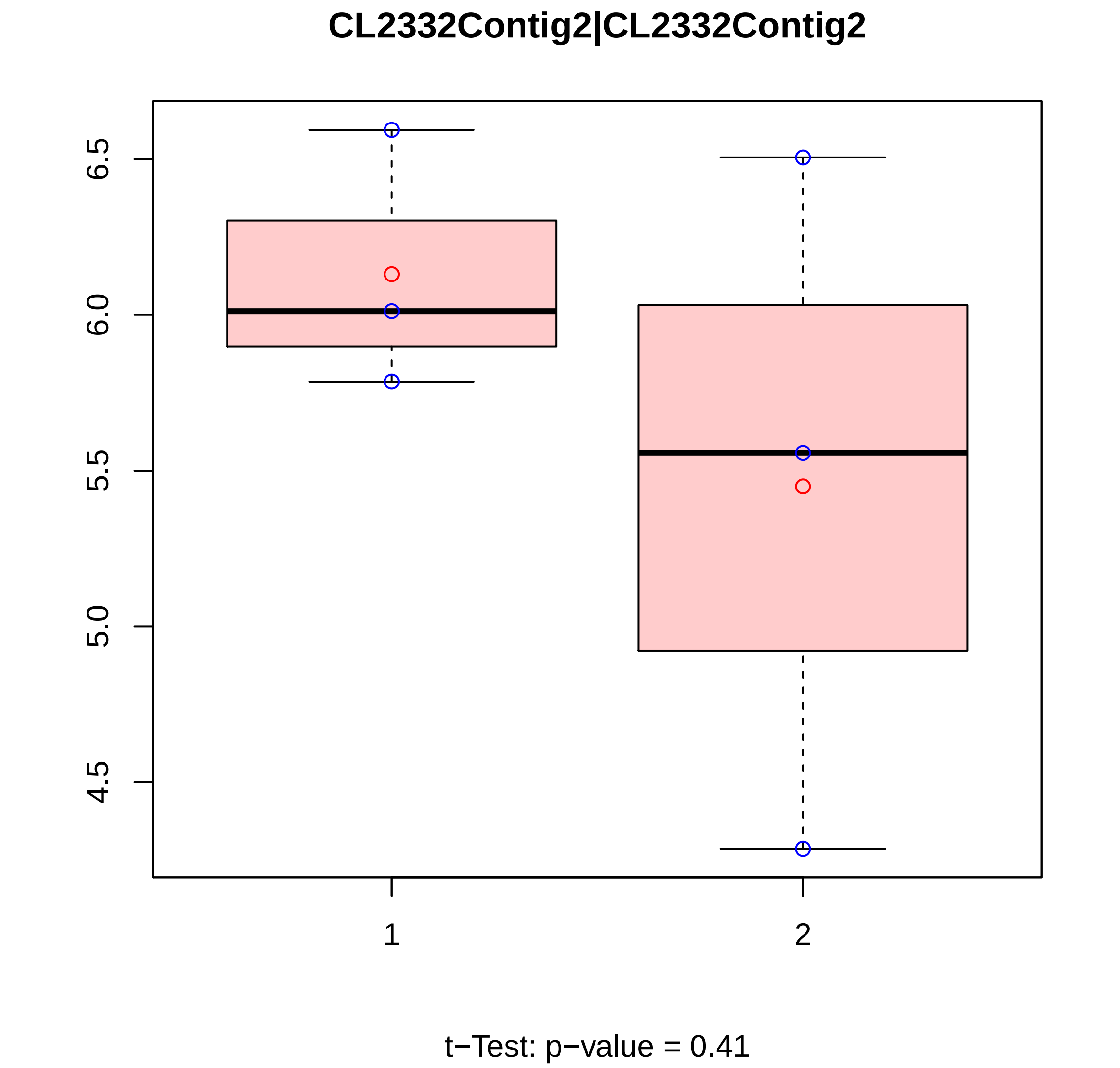
CL2332Contig2|CL2332Contig2
6.5
6.0
5.5
5.0
4.5
1
2
t−Test: p−value = 0.41

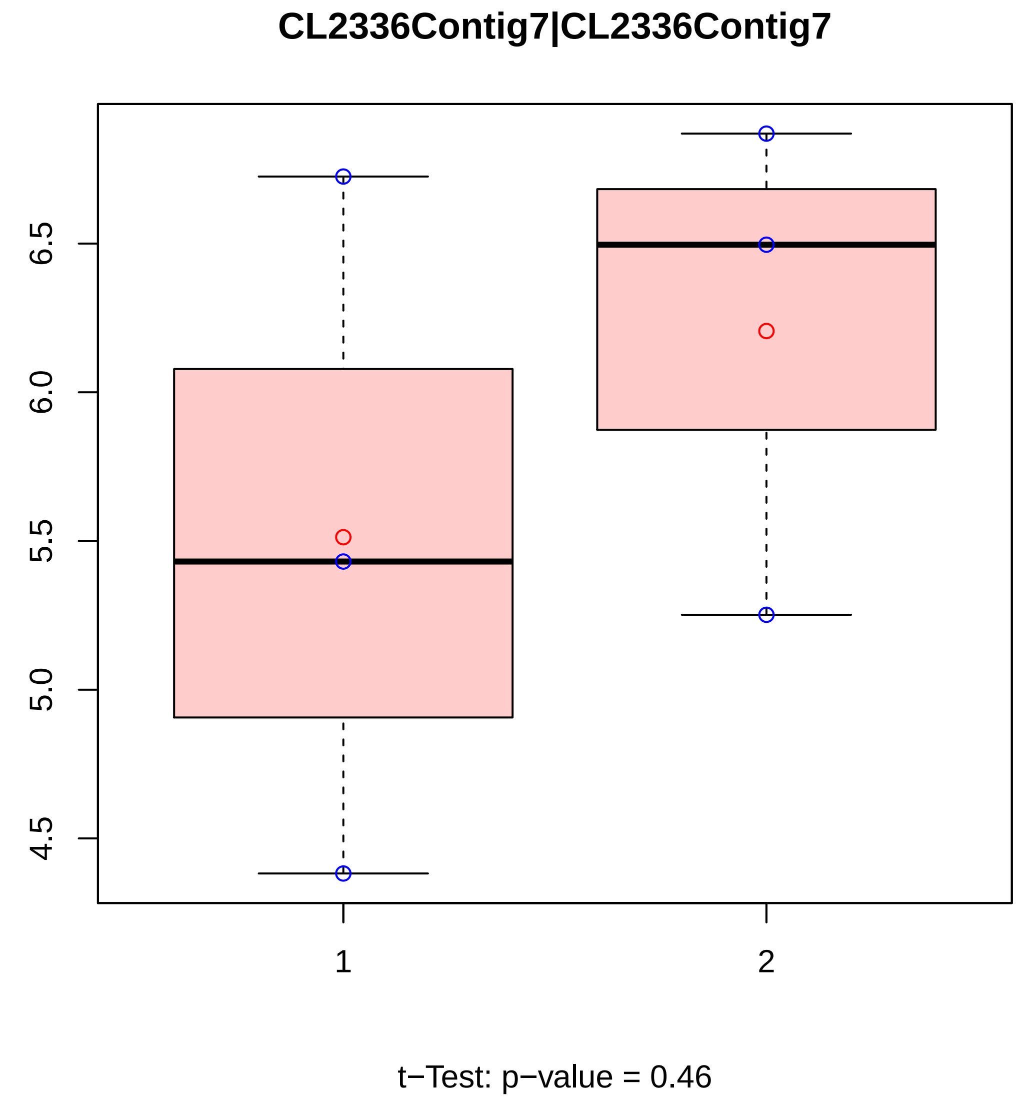

CL2336Contig7|CL2336Contig7
4.5
5.0
5.5
6.0
6.5
1
2
t−Test: p−value = 0.46

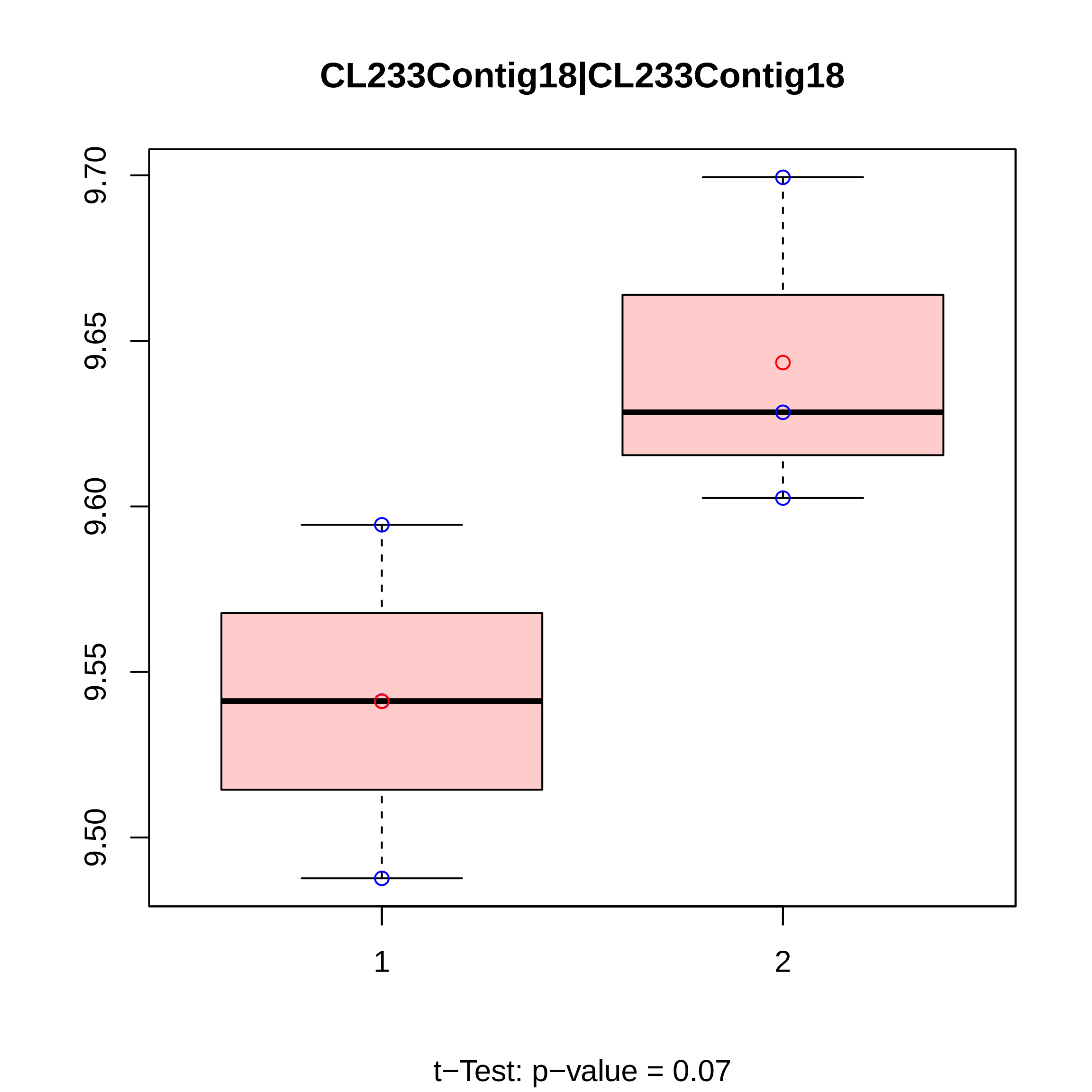
CL233Contig18|CL233Contig18
9.70
9.65
9.60
9.55
9.50
1
2
t−Test: p−value = 0.07

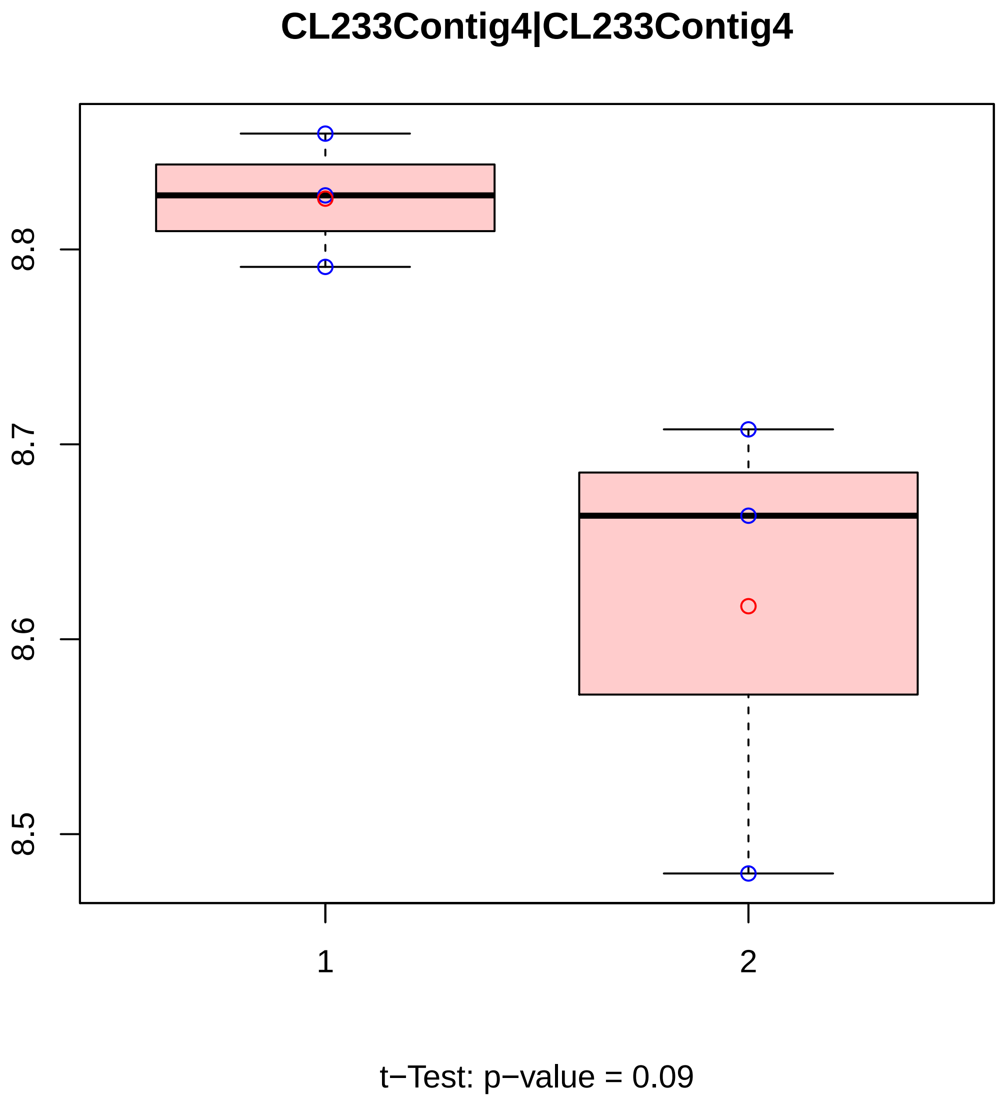

CL233Contig4|CL233Contig4
8.5
8.6
8.7
8.8
1
2
t−Test: p−value = 0.09

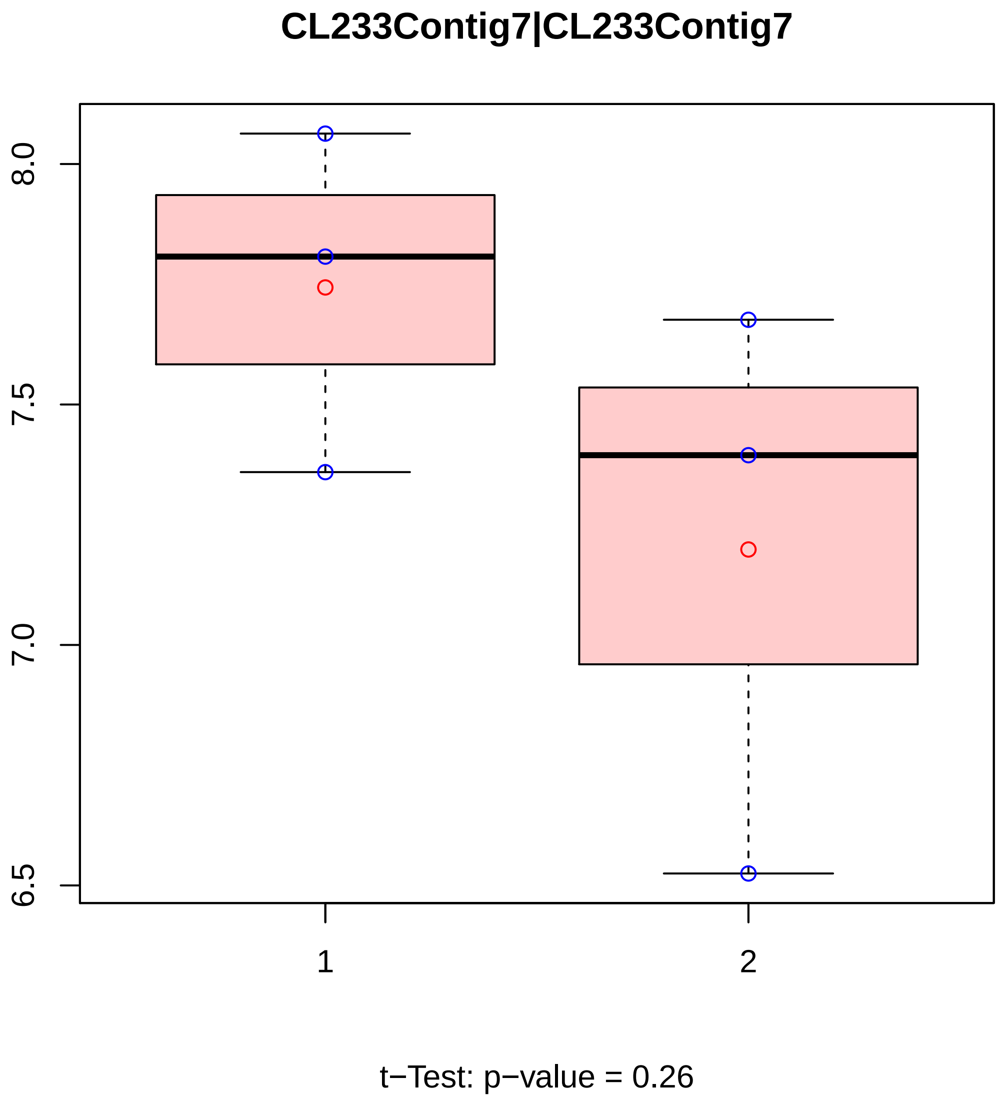

CL233Contig7|CL233Contig7
8.0
7.5
7.0
6.5
1
2
t−Test: p−value = 0.26

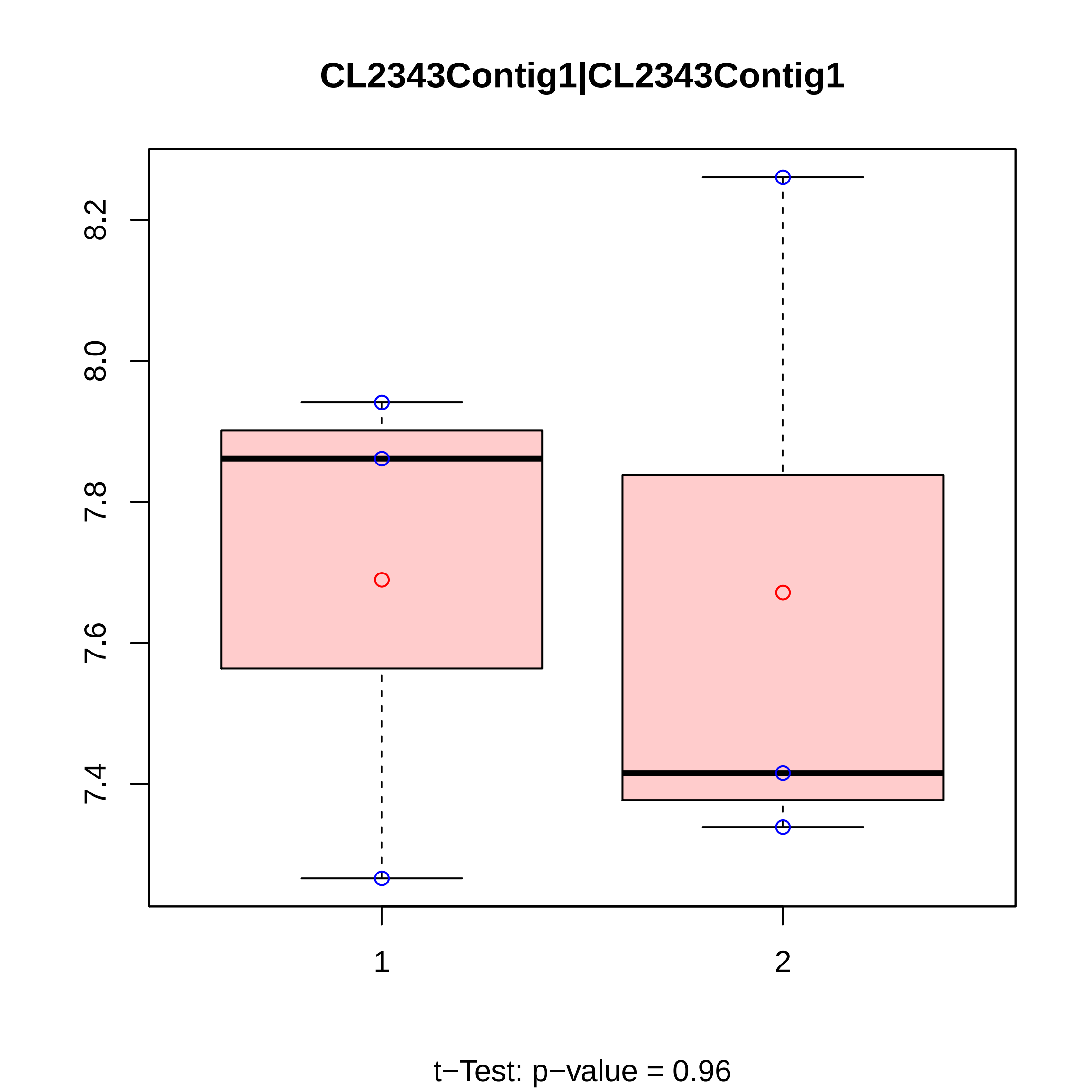
CL2343Contig1|CL2343Contig1
8.2
8.0
7.8
7.6
7.4
1
2
t−Test: p−value = 0.96

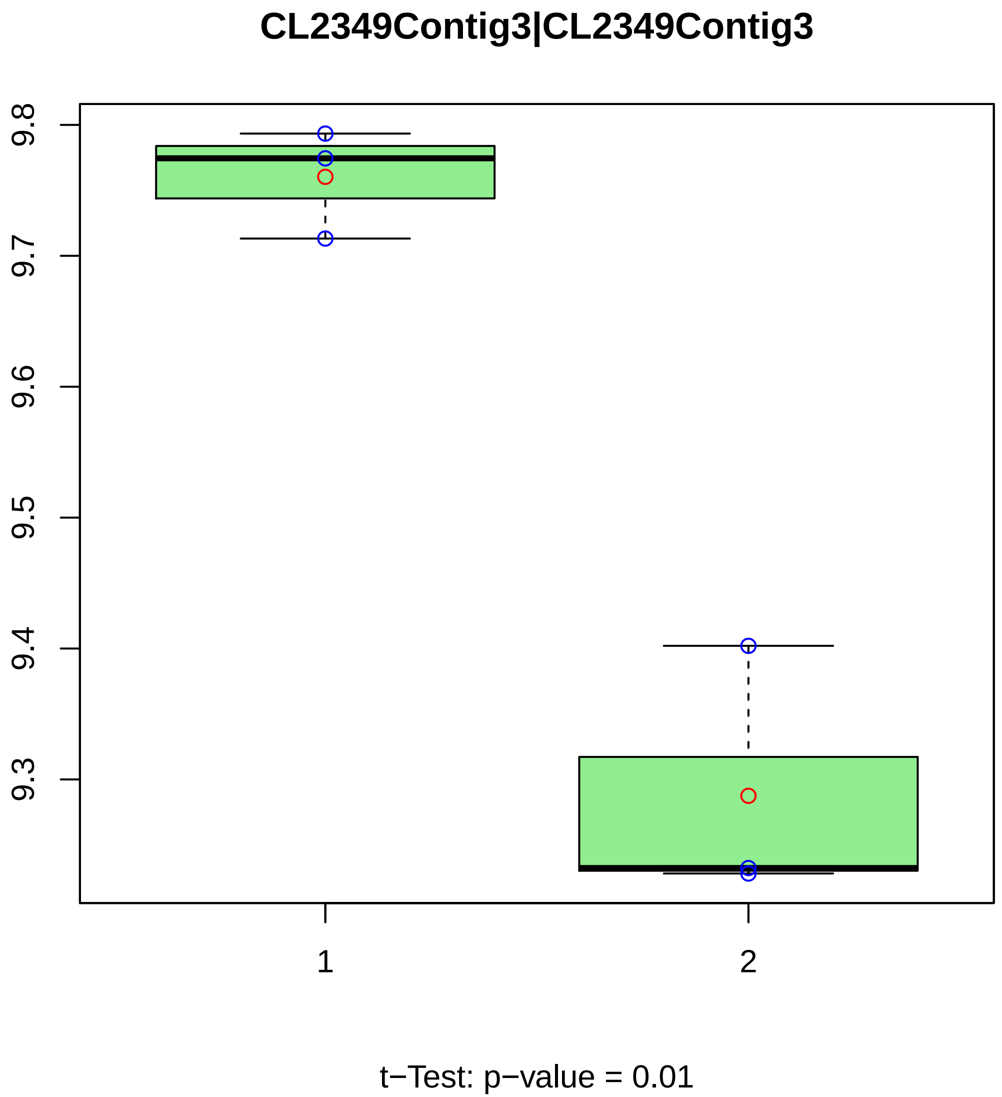
CL2349Contig3|CL2349Contig3
9.8
9.7
9.6
9.5
9.4
9.3
1
2
t−Test: p−value = 0.01

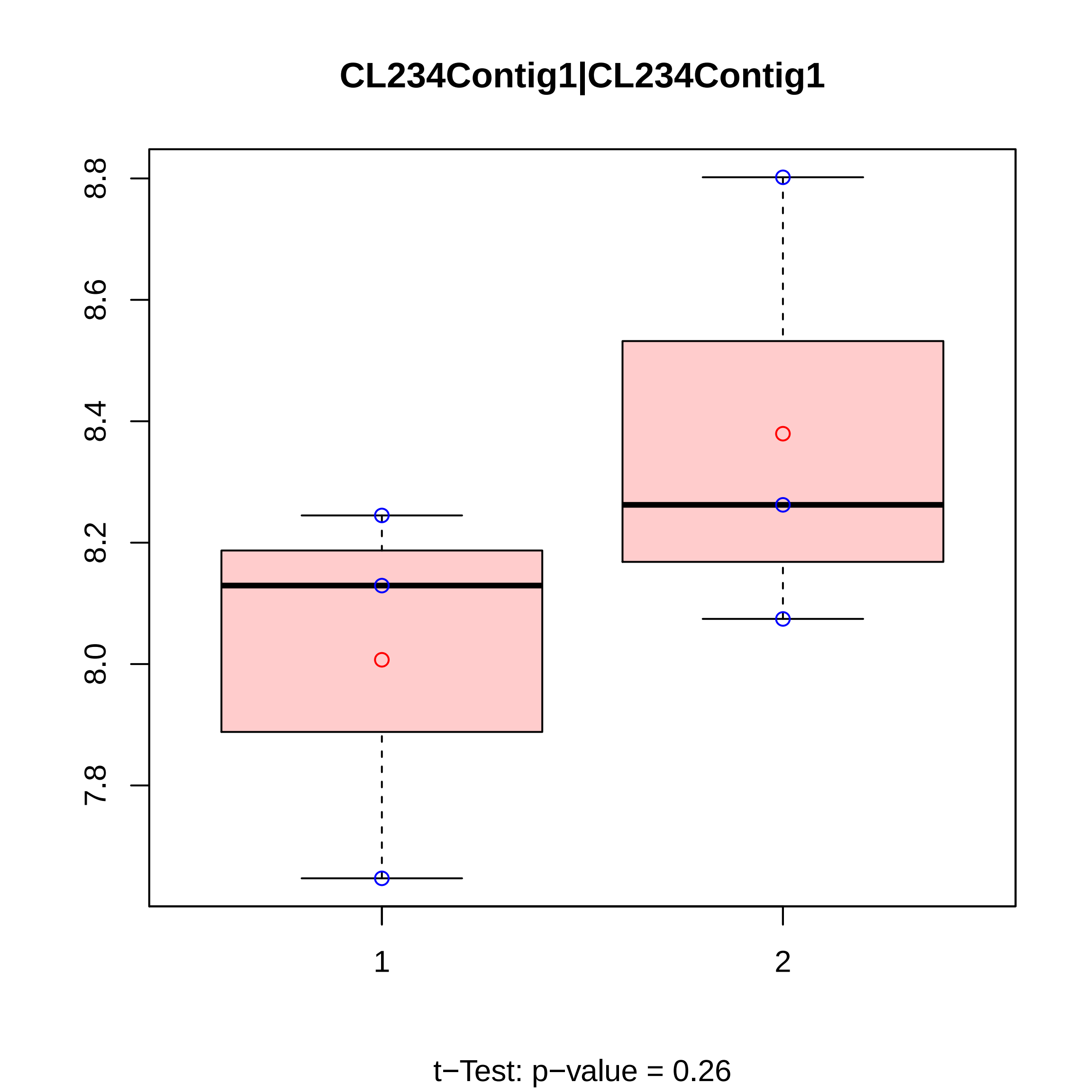

CL234Contig1|CL234Contig1
8.8
8.6
8.4
8.2
8.0
7.8
1
2
t−Test: p−value = 0.26

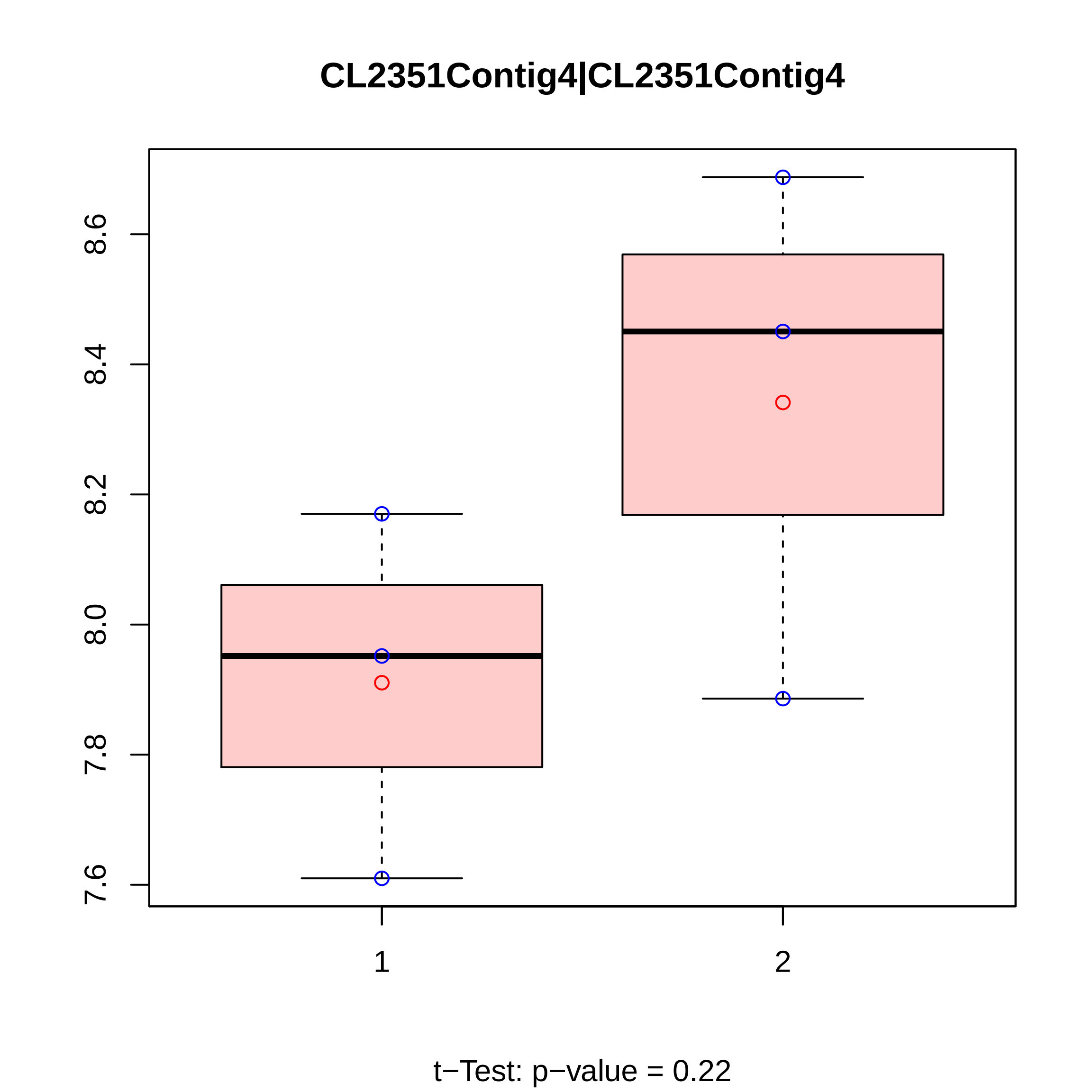
CL2351Contig4|CL2351Contig4
8.6
8.4
8.2
8.0
7.8
7.6
1
2
t−Test: p−value = 0.22

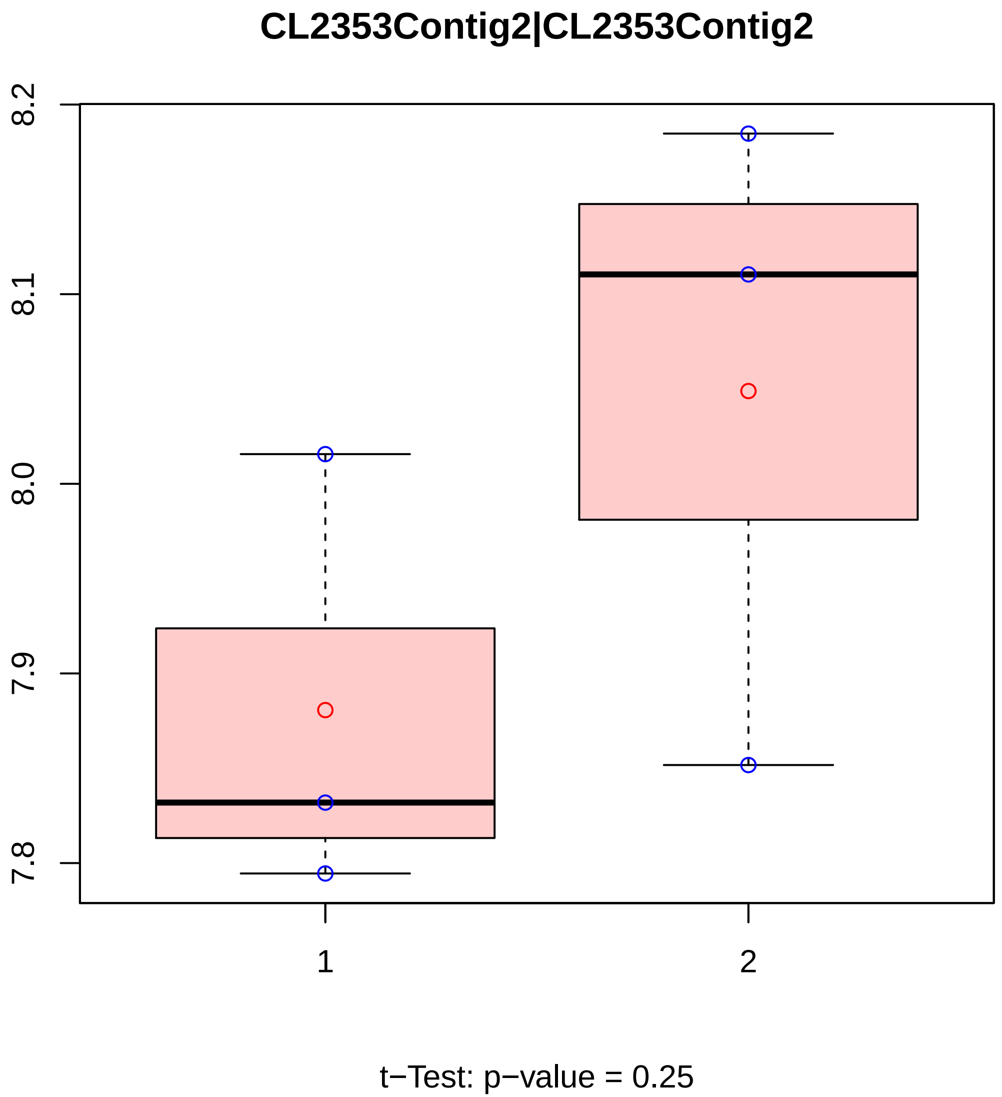

CL2353Contig2|CL2353Contig2
8.2
8.1
8.0
7.9
7.8
1
2
t−Test: p−value = 0.25

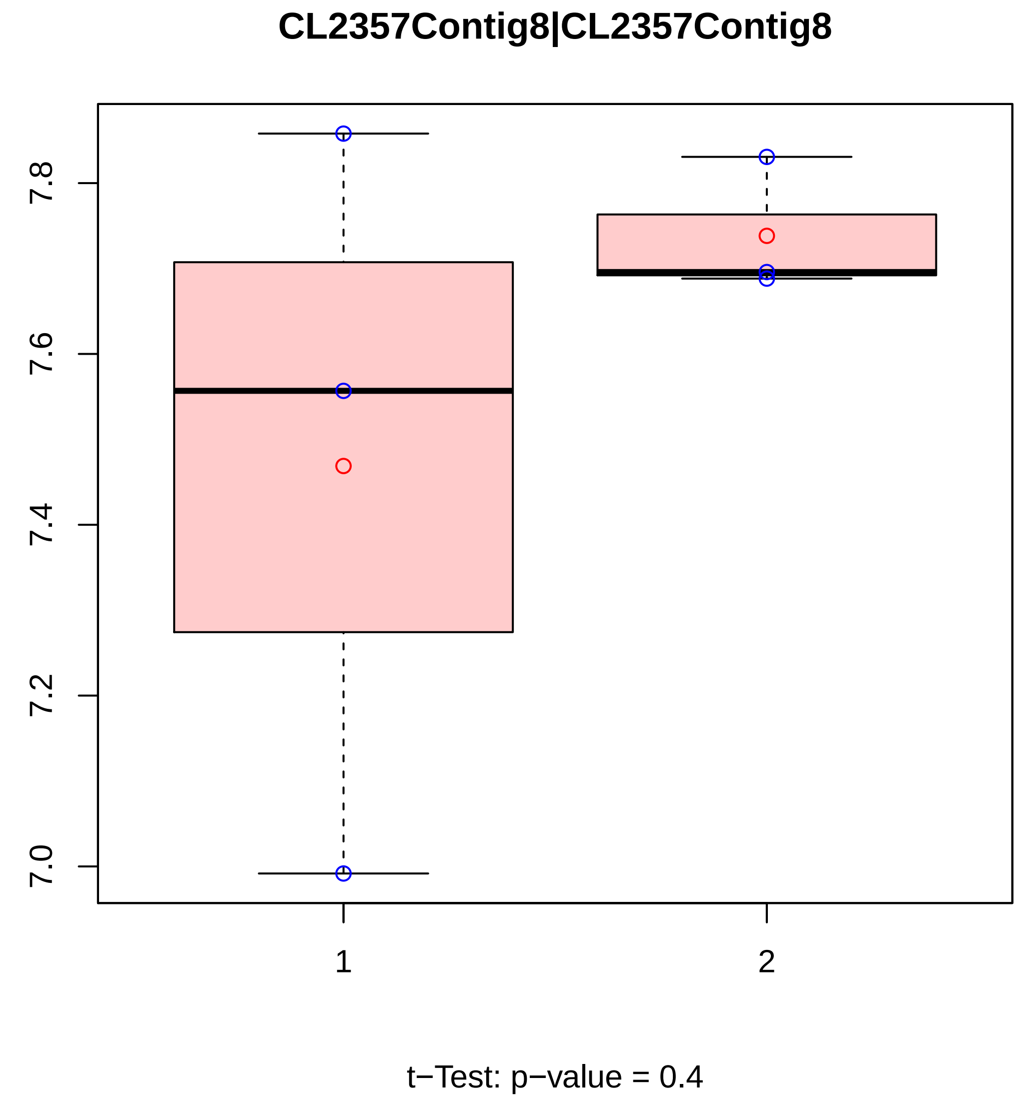
CL2357Contig8|CL2357Contig8
7.0
7.2
7.4
7.6
7.8
1
2
t−Test: p−value = 0.4

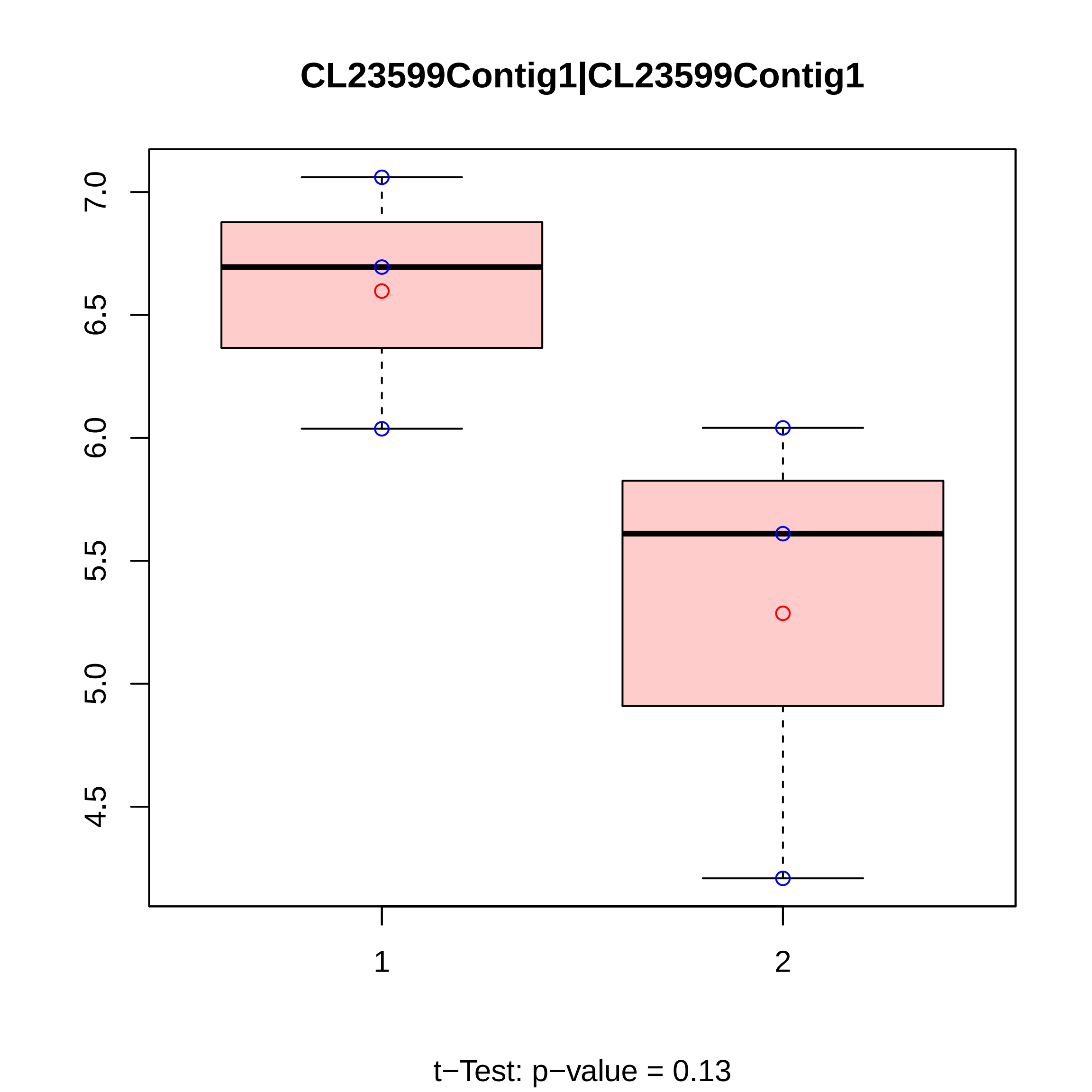

CL23599Contig1|CL23599Contig1
7.0
6.5
6.0
5.5
5.0
4.5
1
2
t−Test: p−value = 0.13

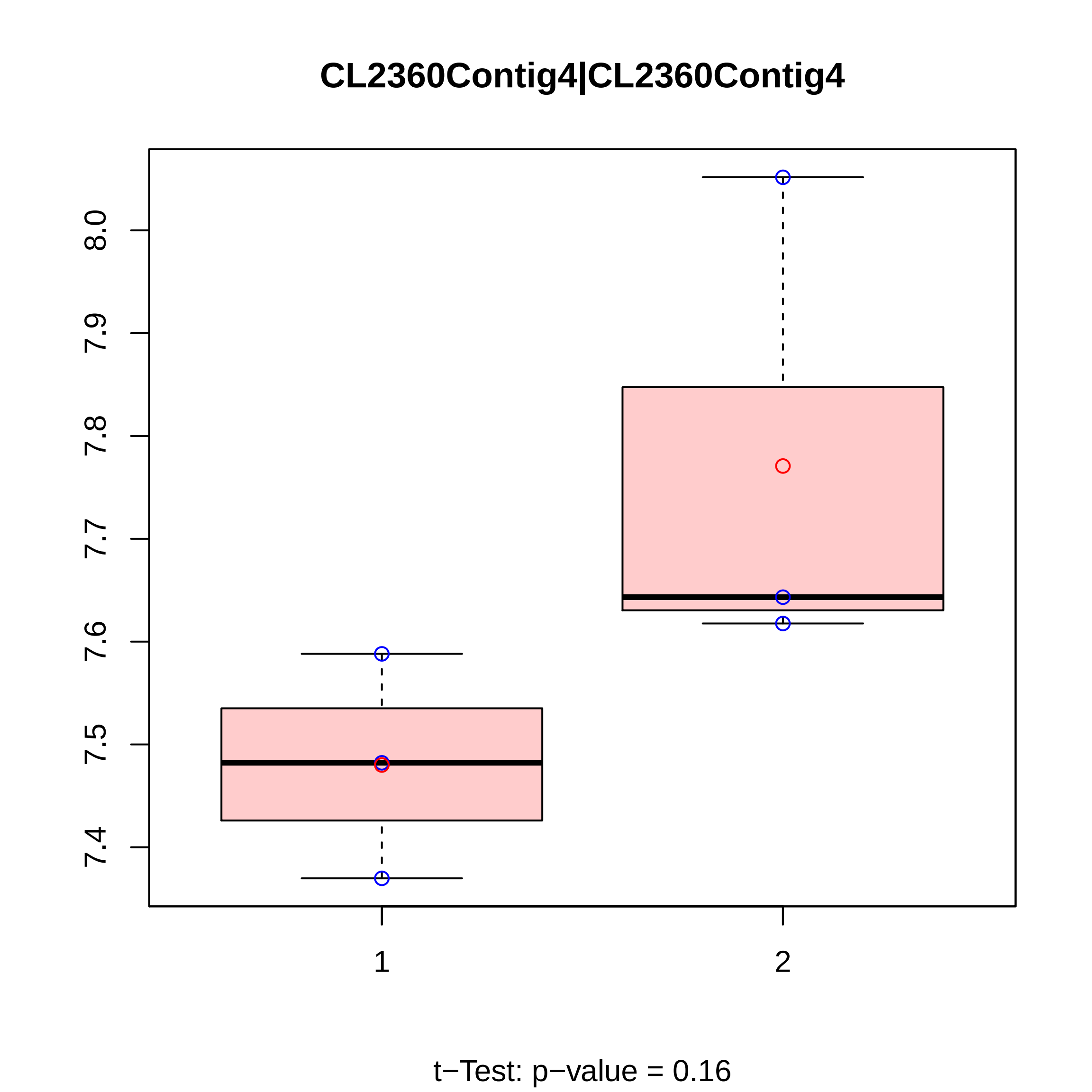

CL2360Contig4|CL2360Contig4
8.0
7.9
7.8
7.7
7.6
7.5
7.4
1
2
t−Test: p−value = 0.16

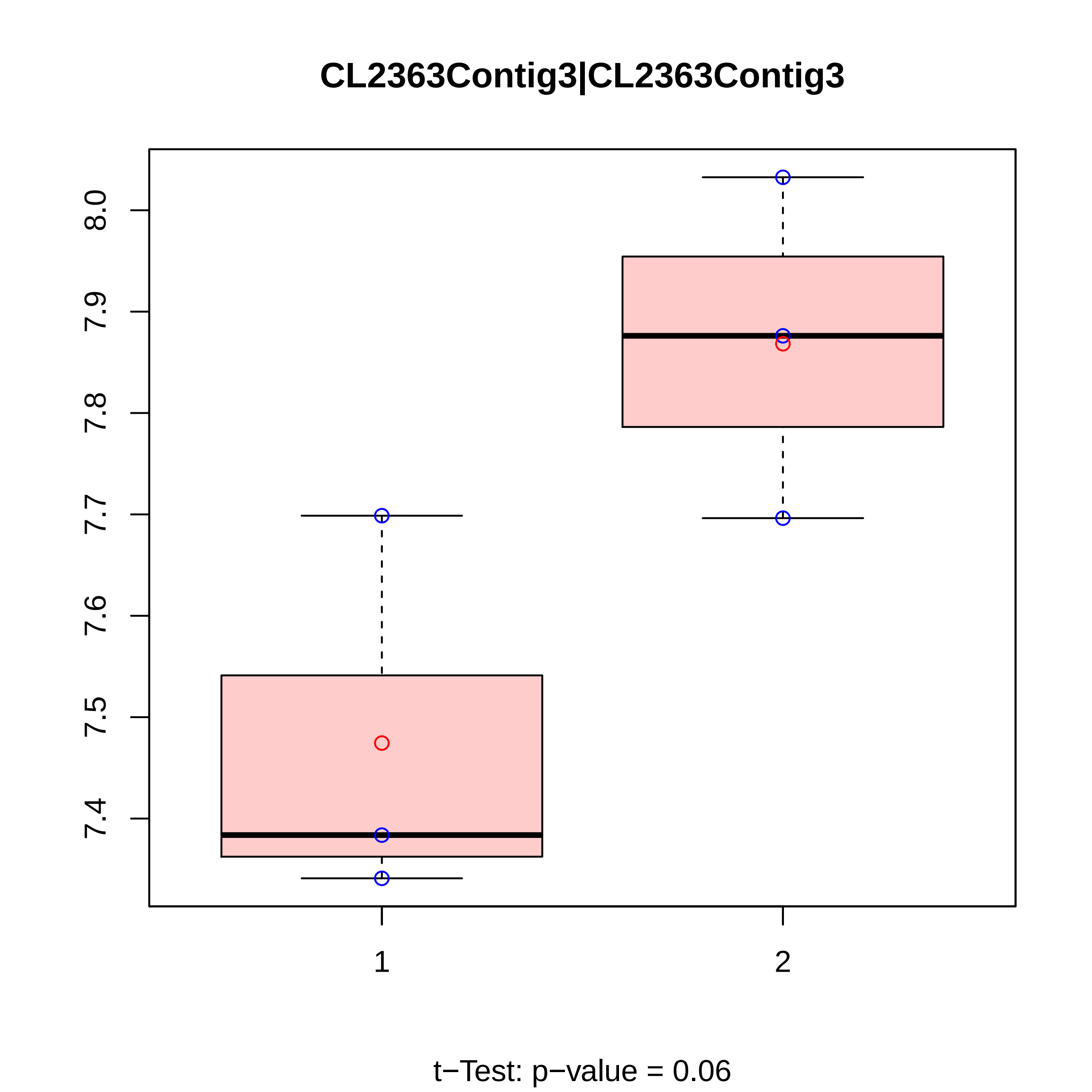

CL2363Contig3|CL2363Contig3
8.0
7.9
7.8
7.7
7.6
7.5
7.4
1
2
t−Test: p−value = 0.06

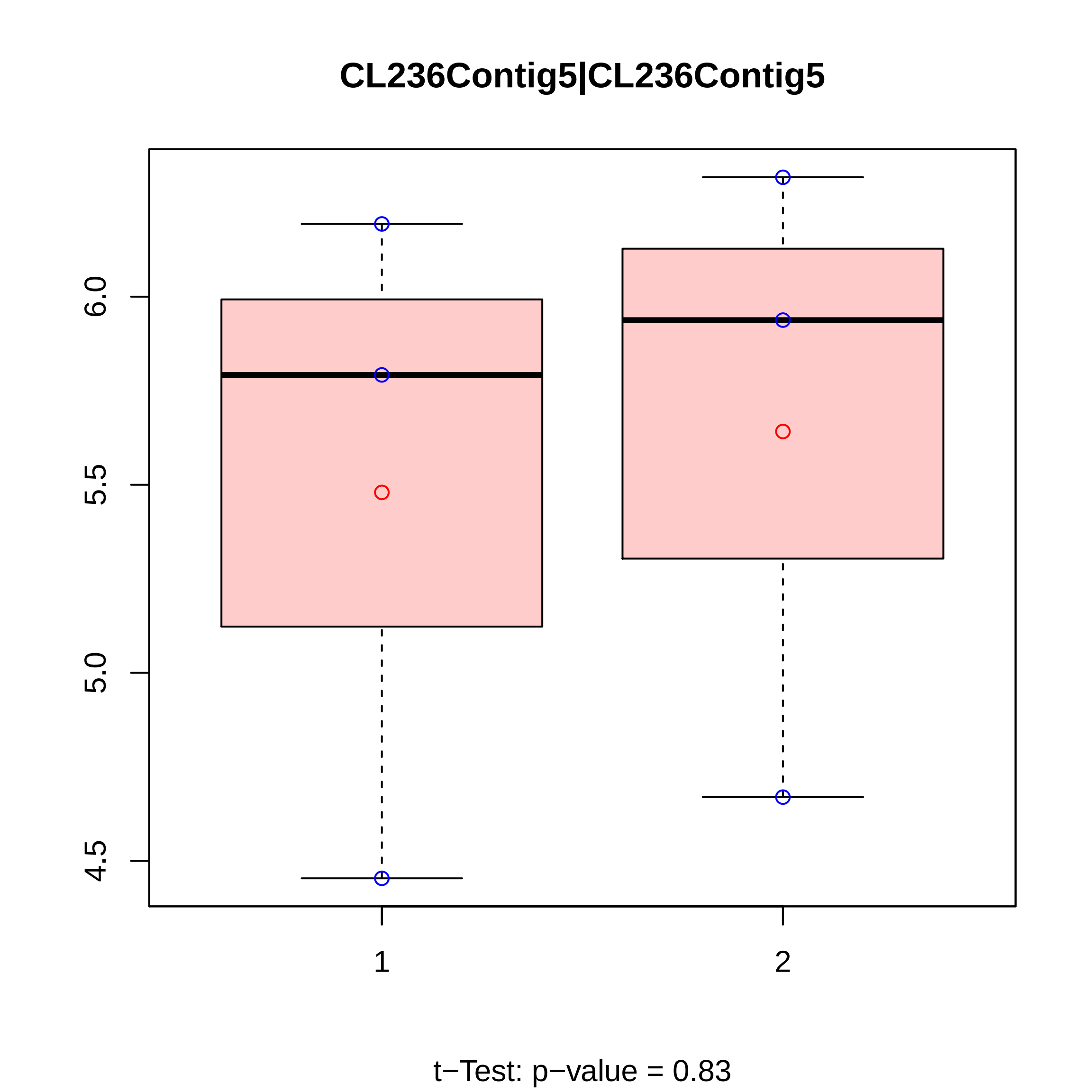
CL236Contig5|CL236Contig5
6.0
5.5
5.0
4.5
1
2
t−Test: p−value = 0.83

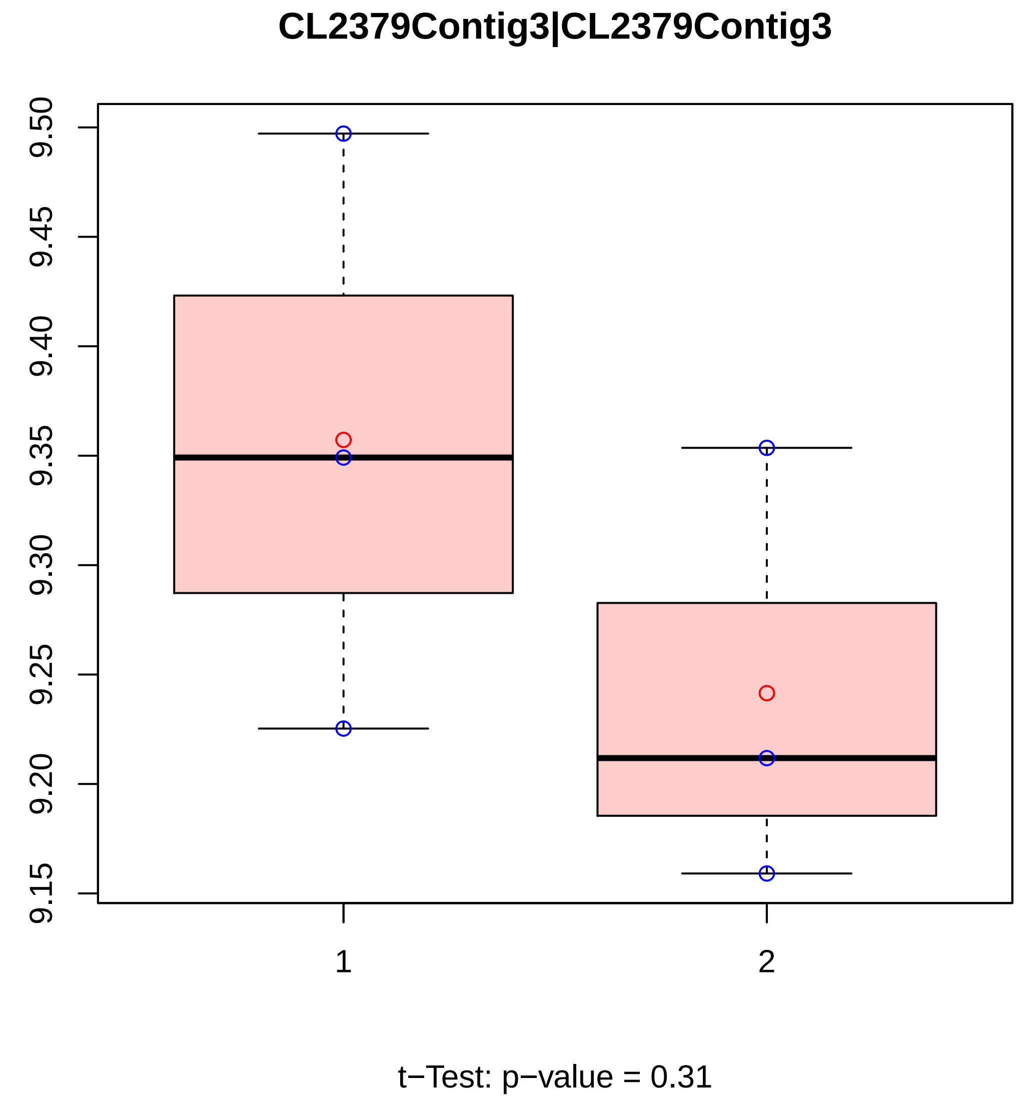
CL2379Contig3|CL2379Contig3
9.50
9.45
9.40
9.35
9.30
9.25
9.20
9.15
1
2
t−Test: p−value = 0.31

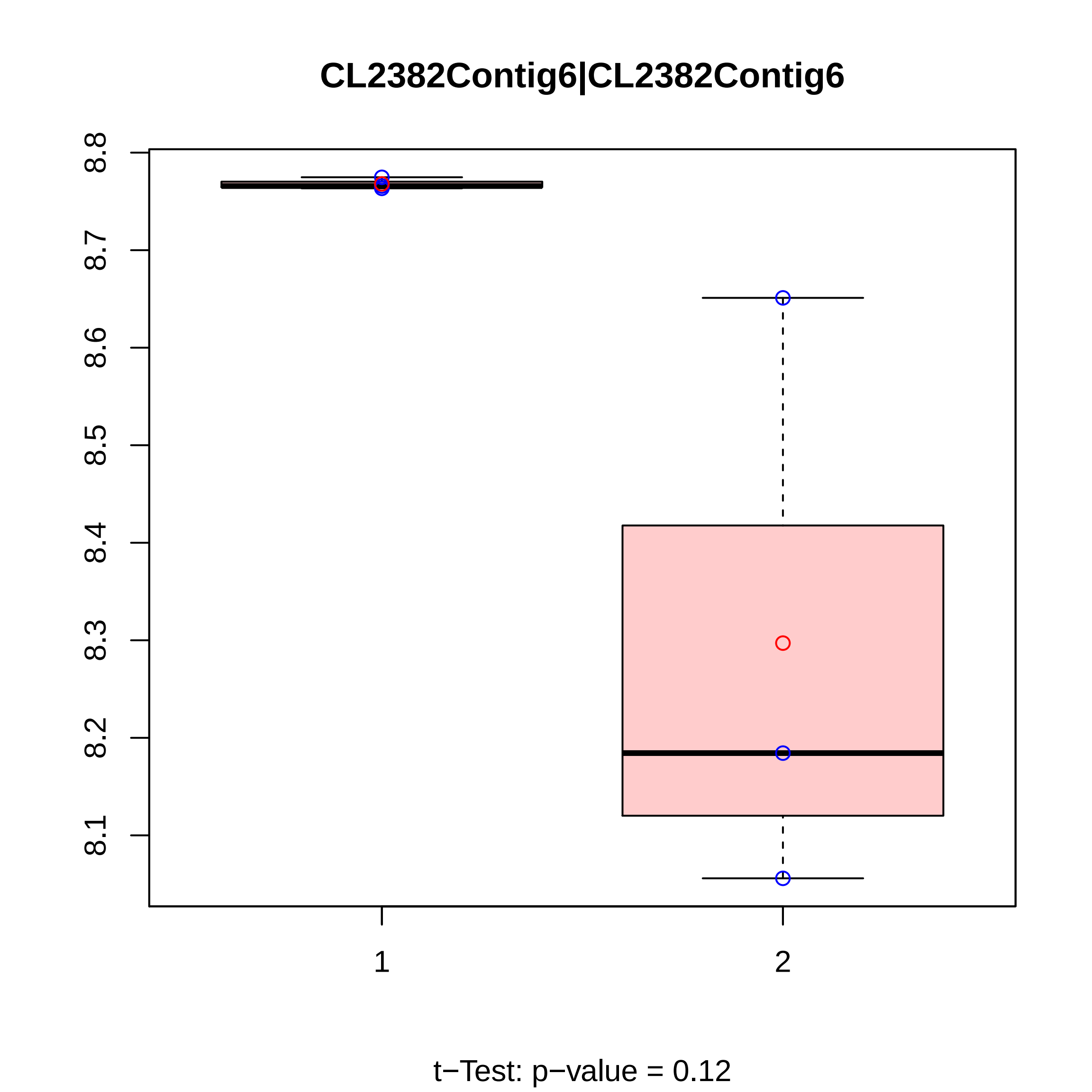
CL2382Contig6|CL2382Contig6
8.8
8.7
8.6
8.5
8.4
8.3
8.2
8.1
1
2
t−Test: p−value = 0.12

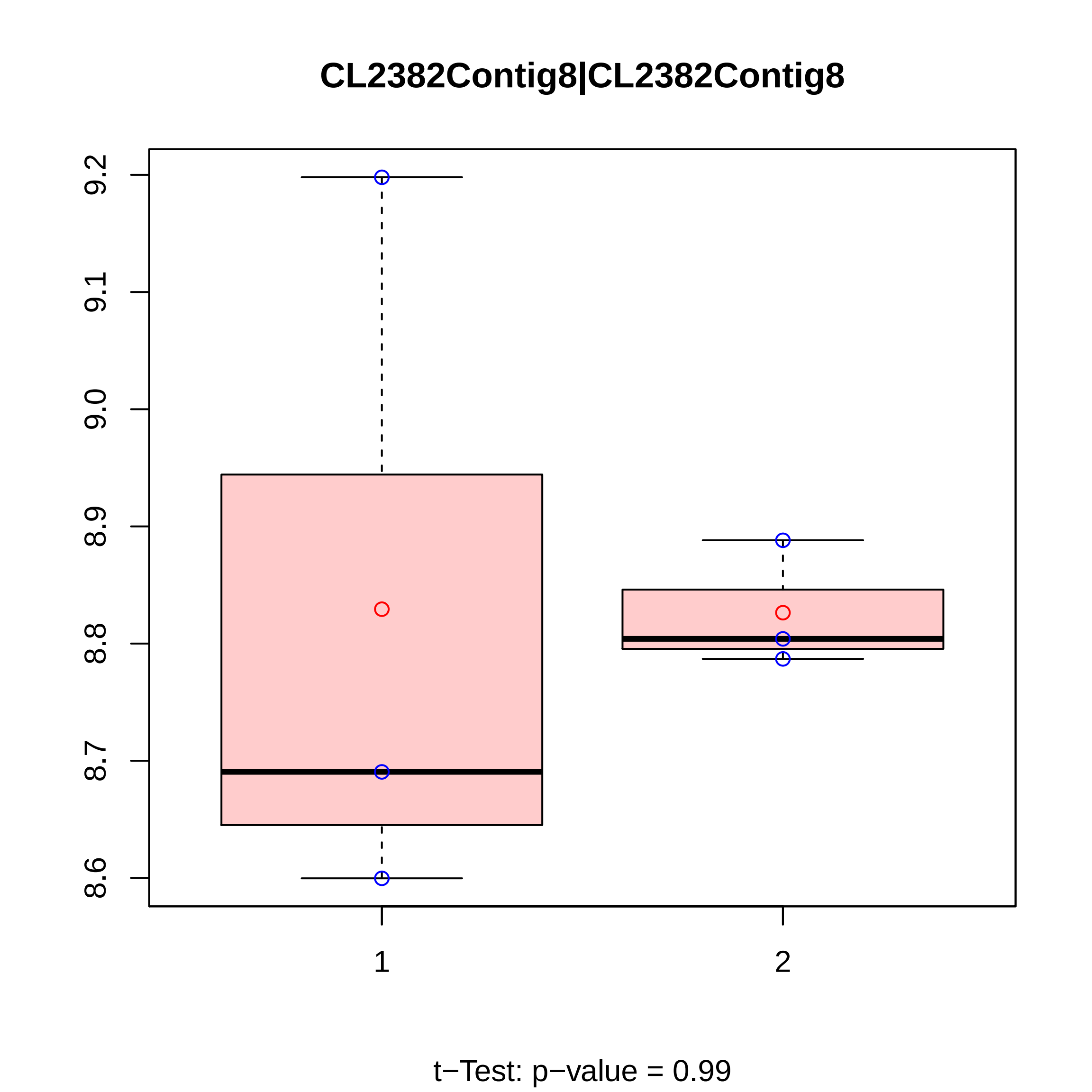
CL2382Contig8|CL2382Contig8
9.2
9.1
9.0
8.9
8.8
8.7
8.6
1
2
t−Test: p−value = 0.99

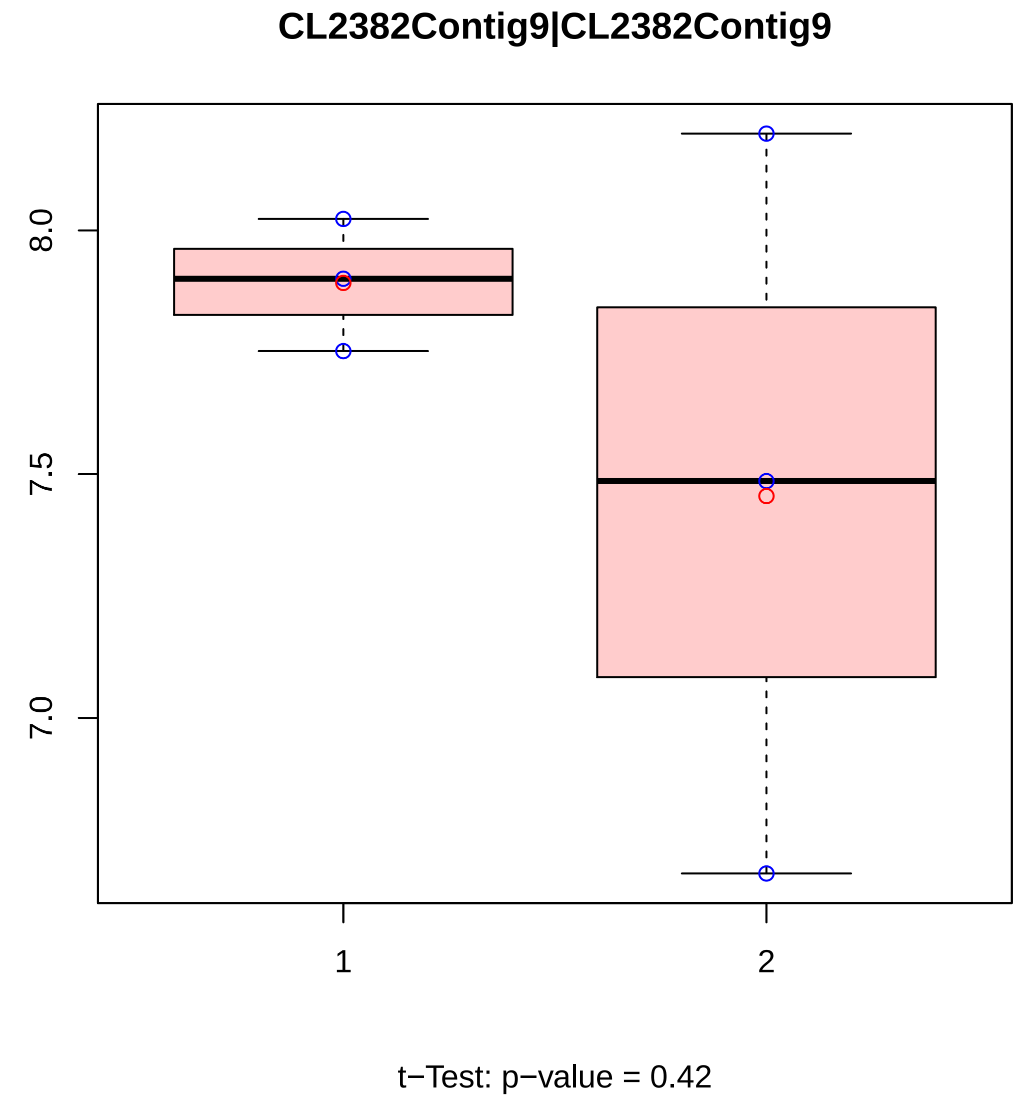
CL2382Contig9|CL2382Contig9
8.0
7.5
7.0
1
2
t−Test: p−value = 0.42

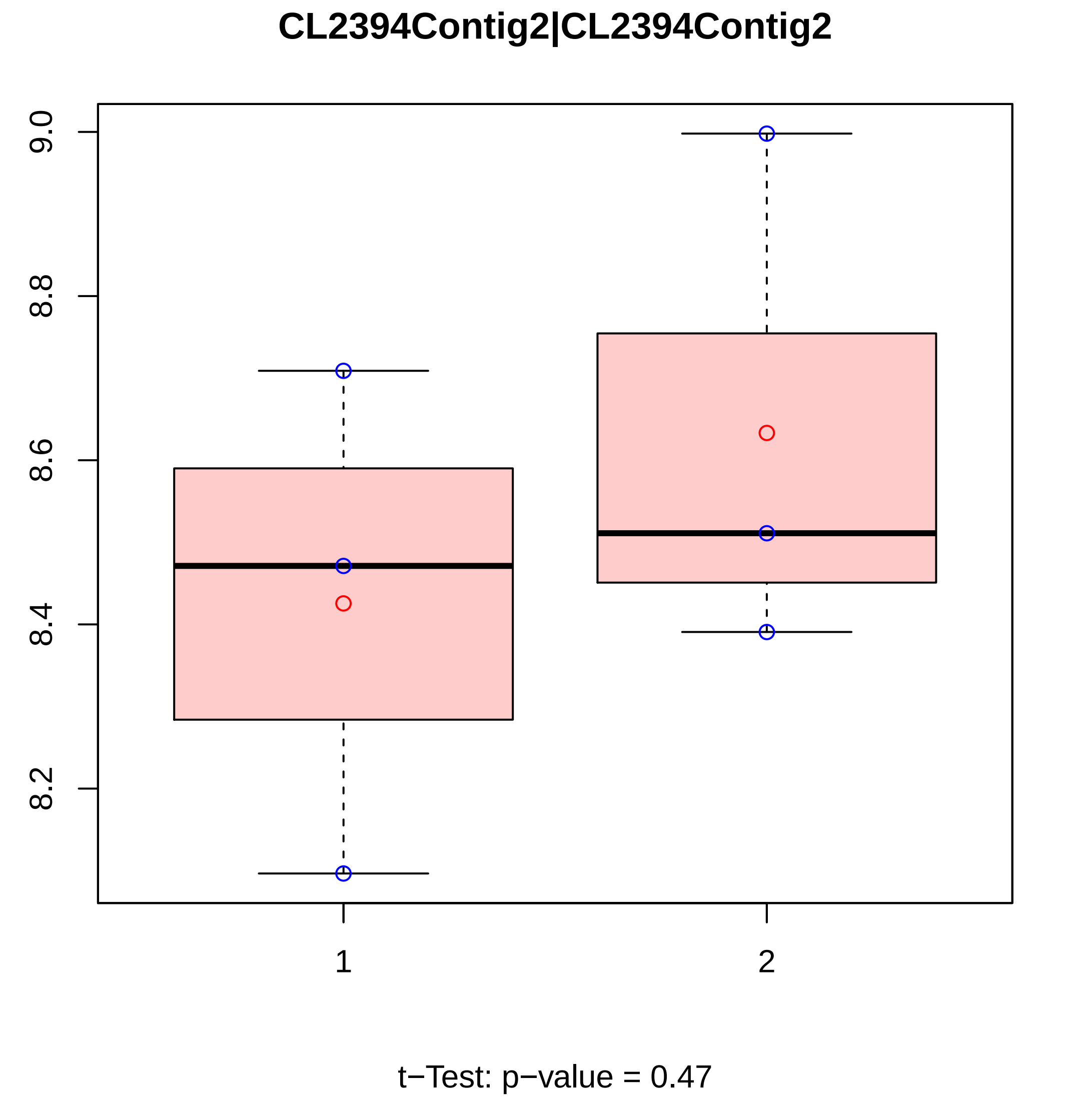
CL2394Contig2|CL2394Contig2
9.0
8.8
8.6
8.4
8.2
1
2
t−Test: p−value = 0.47

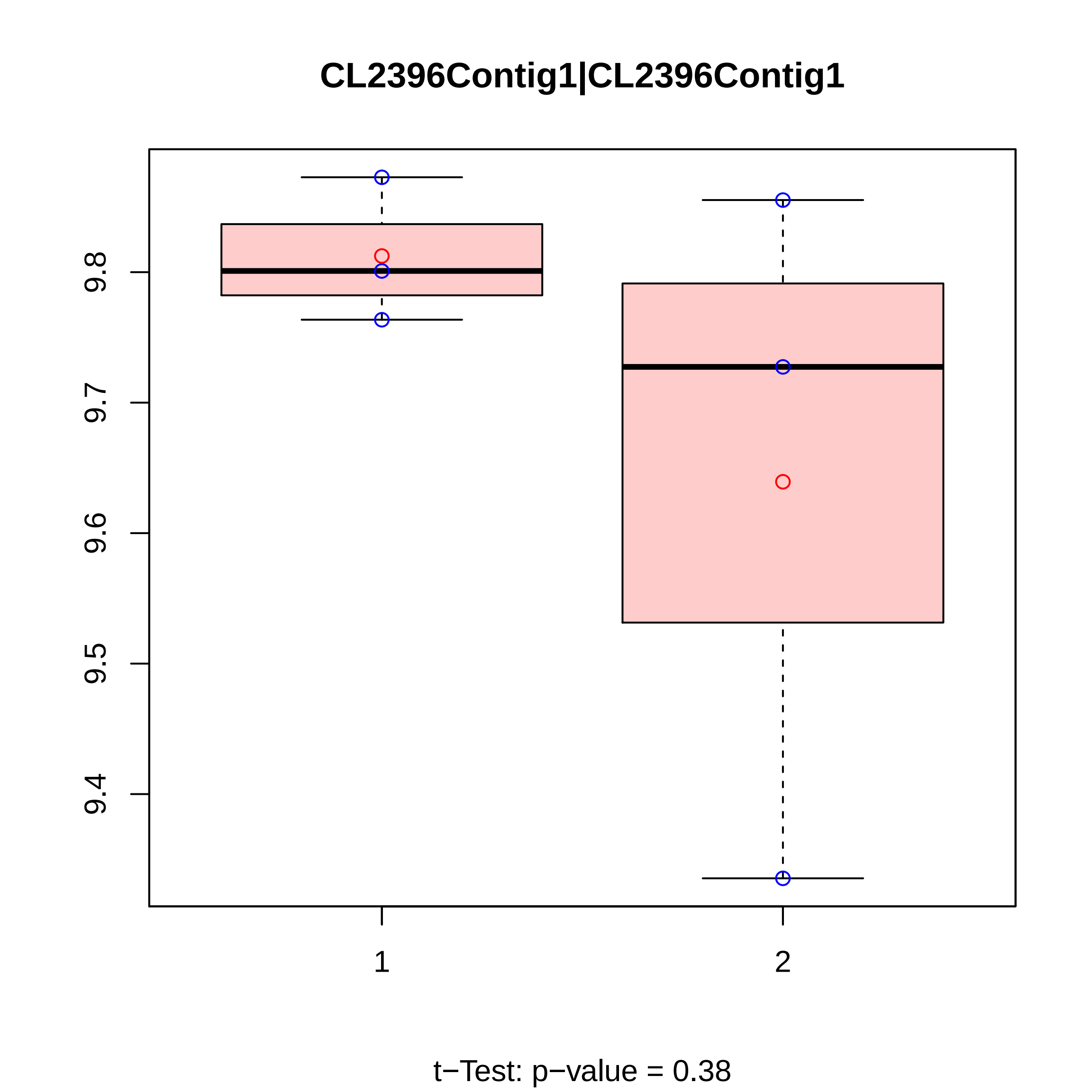
CL2396Contig1|CL2396Contig1
9.4
9.5
9.6
9.7
9.8
1
2
t−Test: p−value = 0.38

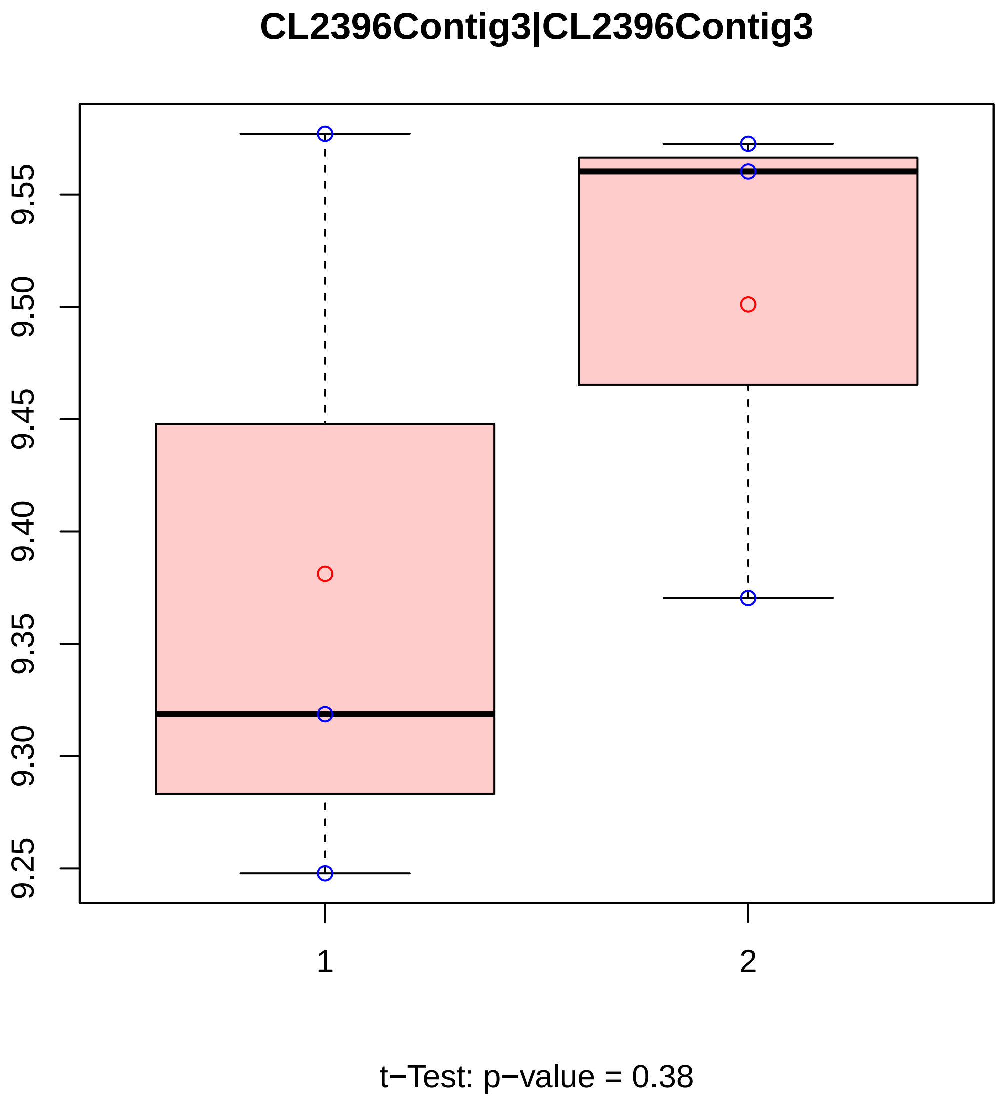

CL2396Contig3|CL2396Contig3
9.25
9.30
9.35
9.40
9.45
9.50
9.55
1
2
t−Test: p−value = 0.38

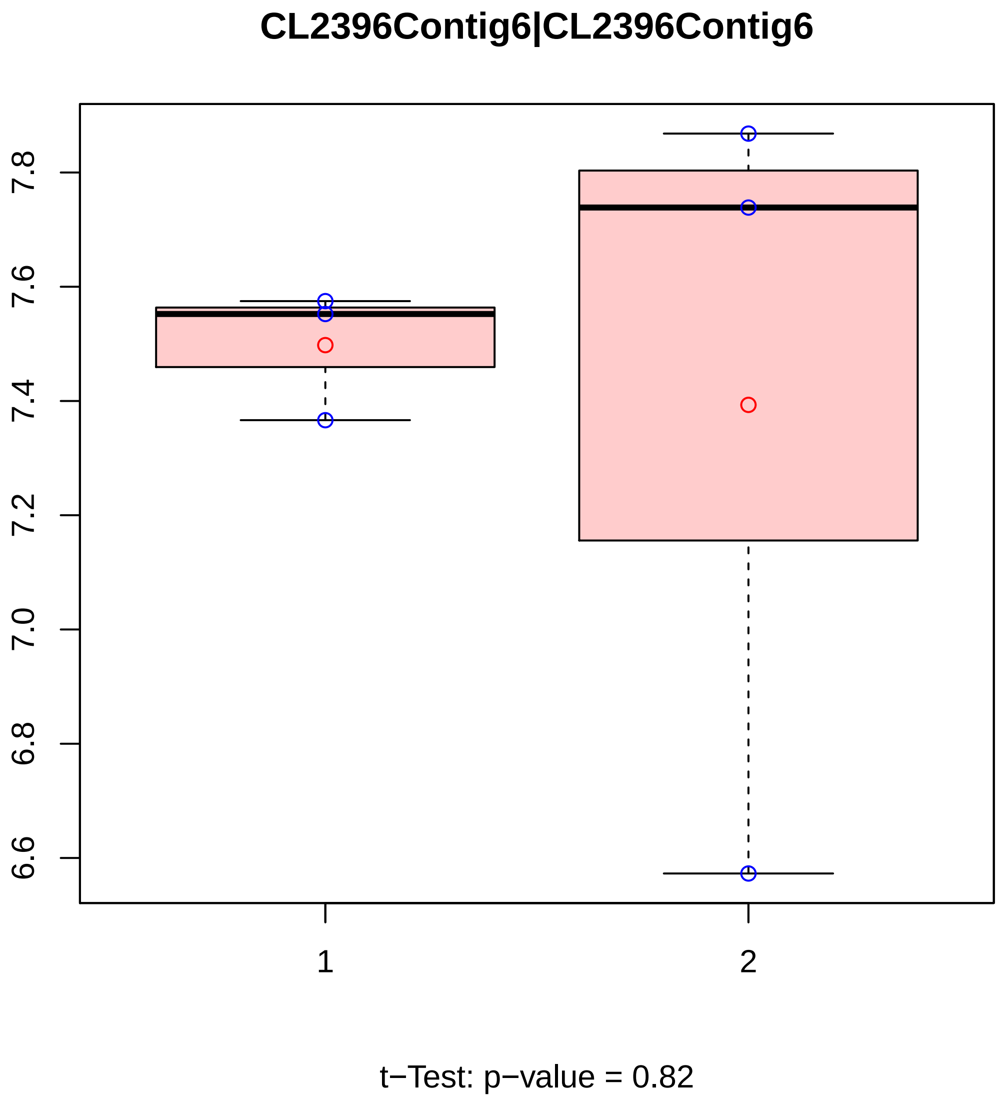
CL2396Contig6|CL2396Contig6
7.8
7.6
7.4
7.2
7.0
6.8
6.6
1
2
t−Test: p−value = 0.82

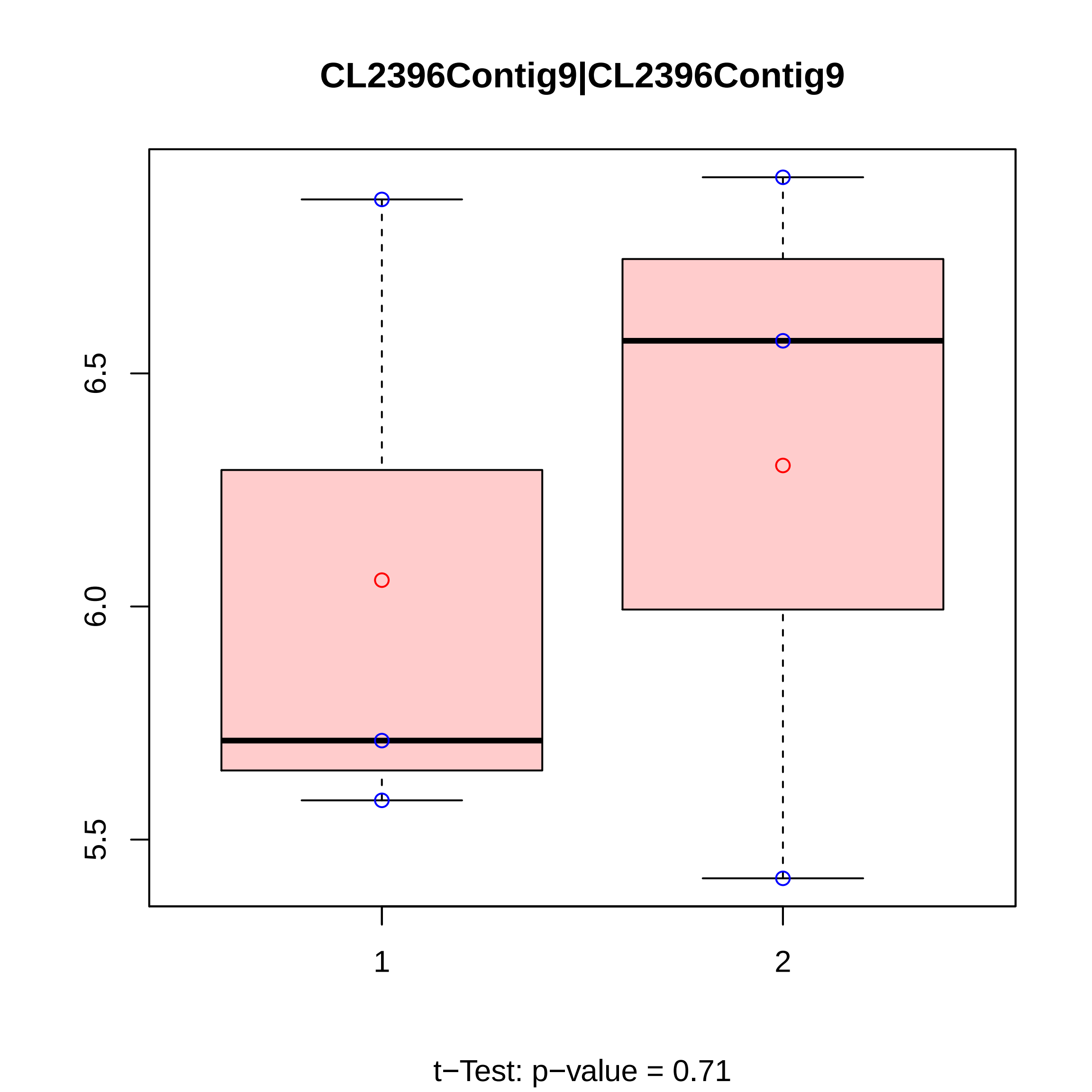

CL2396Contig9|CL2396Contig9
6.5
6.0
5.5
1
2
t−Test: p−value = 0.71

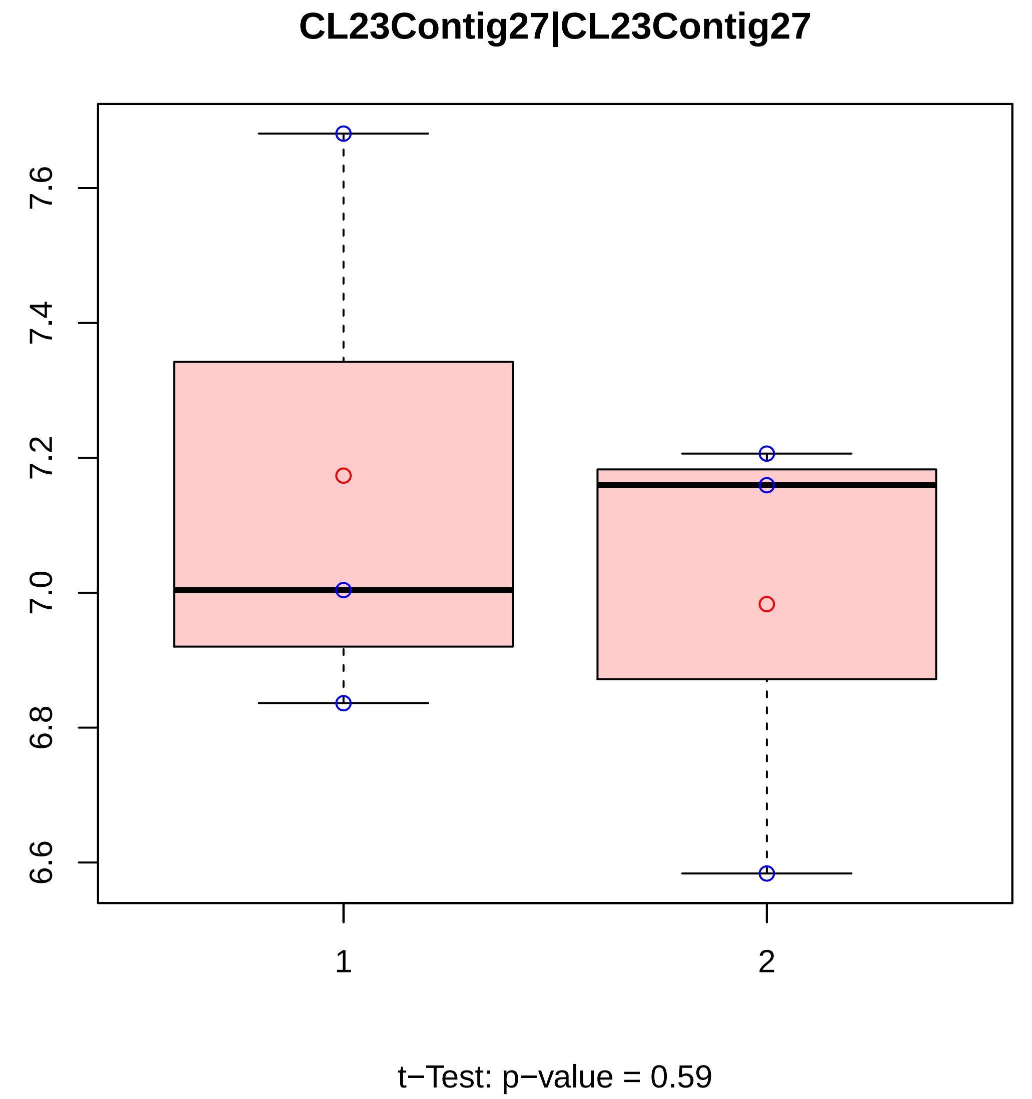

CL23Contig27|CL23Contig27
7.6
7.4
7.2
7.0
6.8
6.6
1
2
t−Test: p−value = 0.59

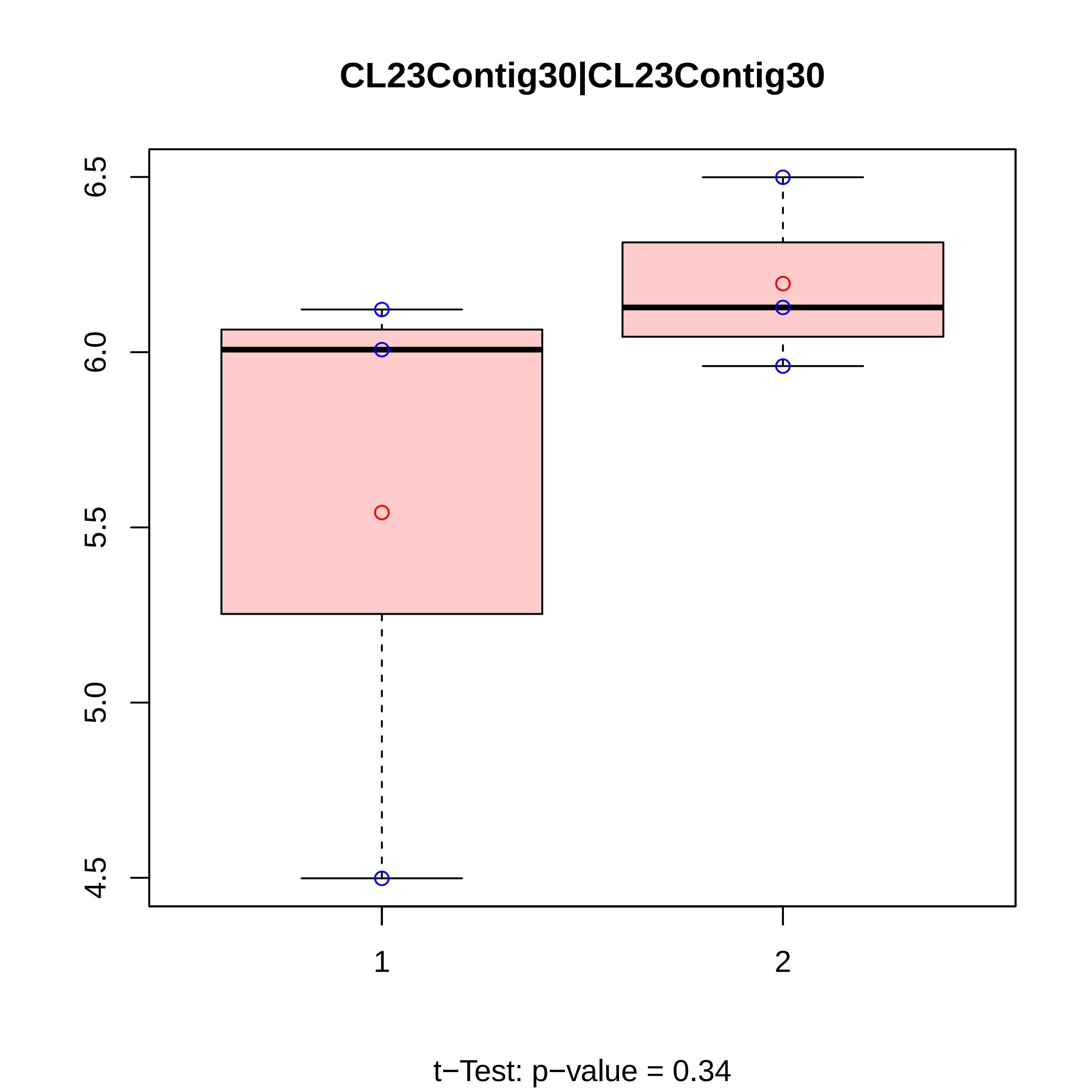
CL23Contig30|CL23Contig30
6.5
6.0
5.5
5.0
4.5
1
2
t−Test: p−value = 0.34

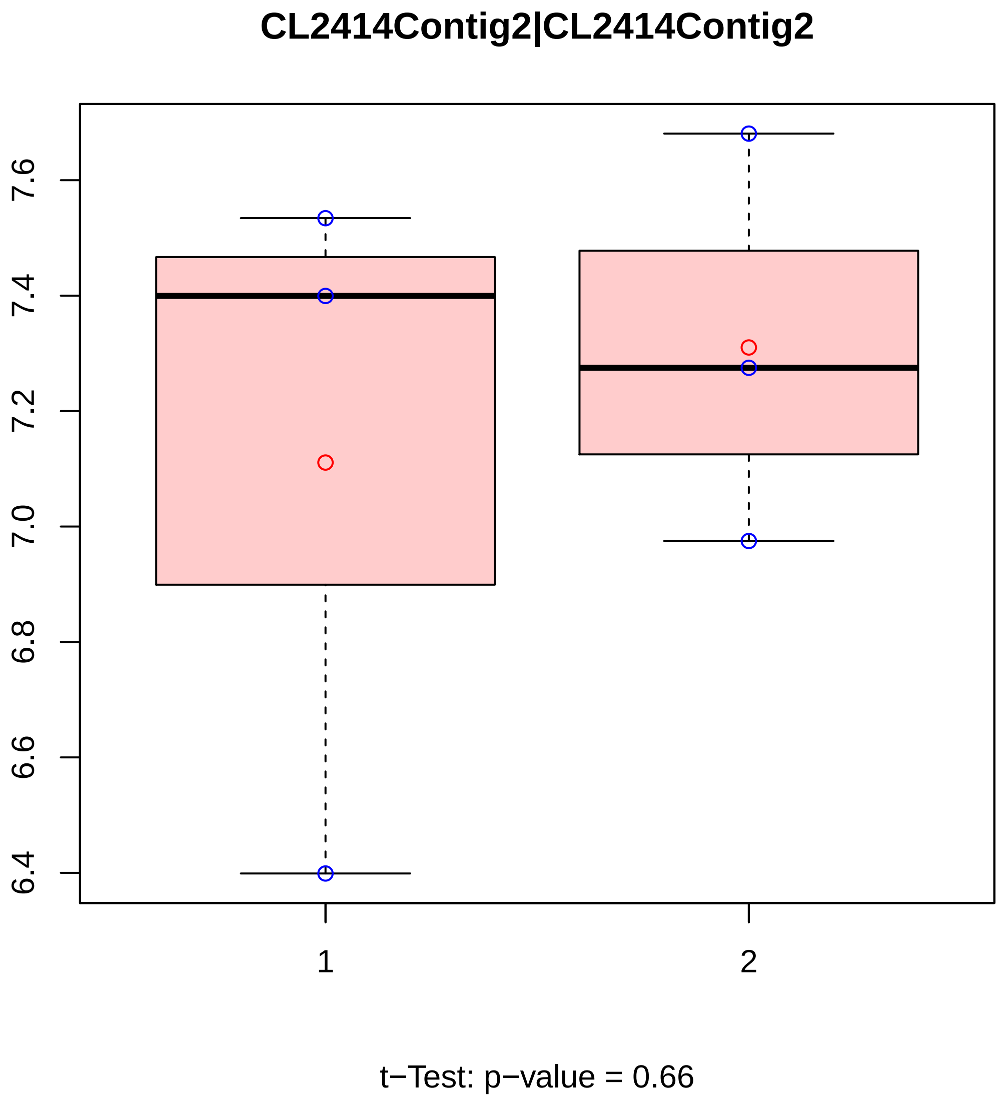
CL2414Contig2|CL2414Contig2
7.6
7.4
7.2
7.0
6.8
6.6
6.4
1
2
t−Test: p−value = 0.66

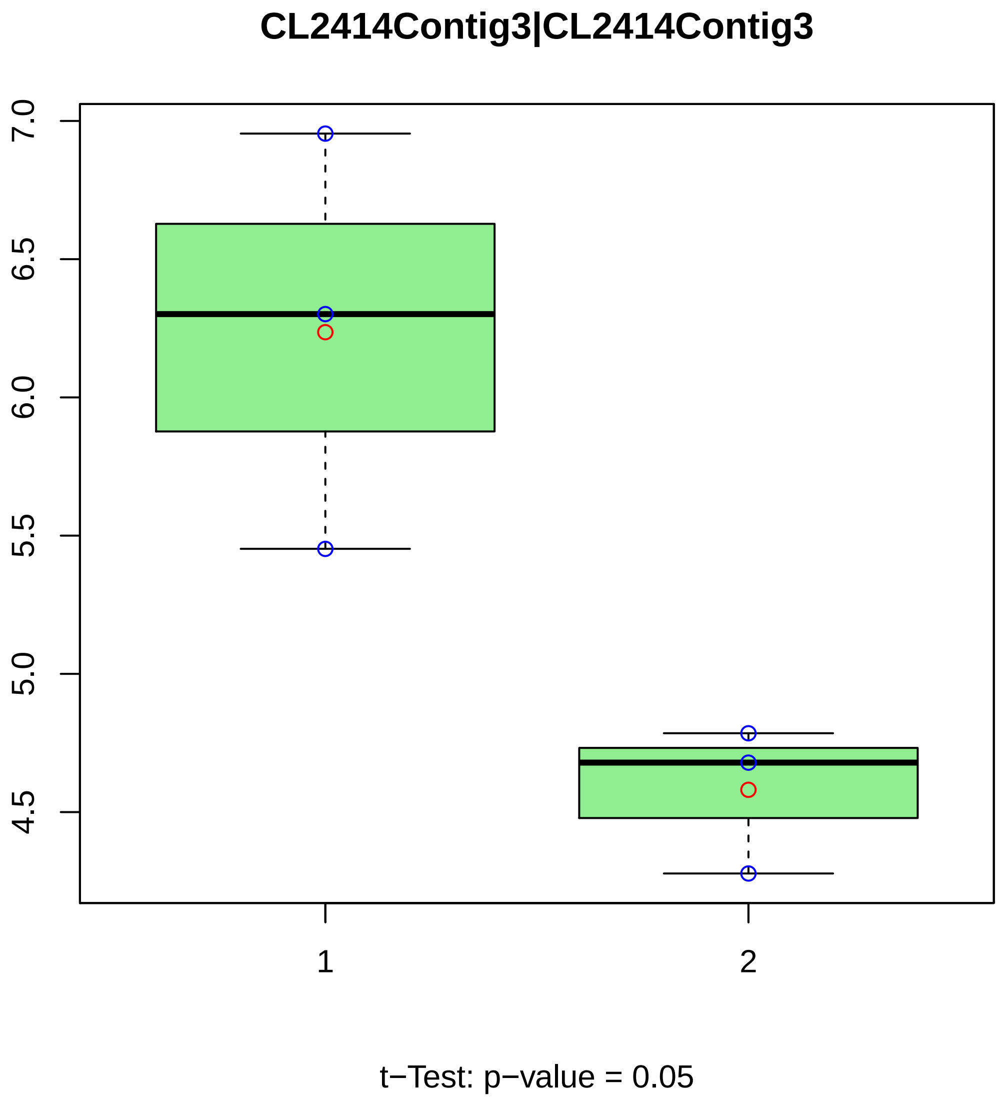
CL2414Contig3|CL2414Contig3
7.0
6.5
6.0
5.5
5.0
4.5
1
2
t−Test: p−value = 0.05

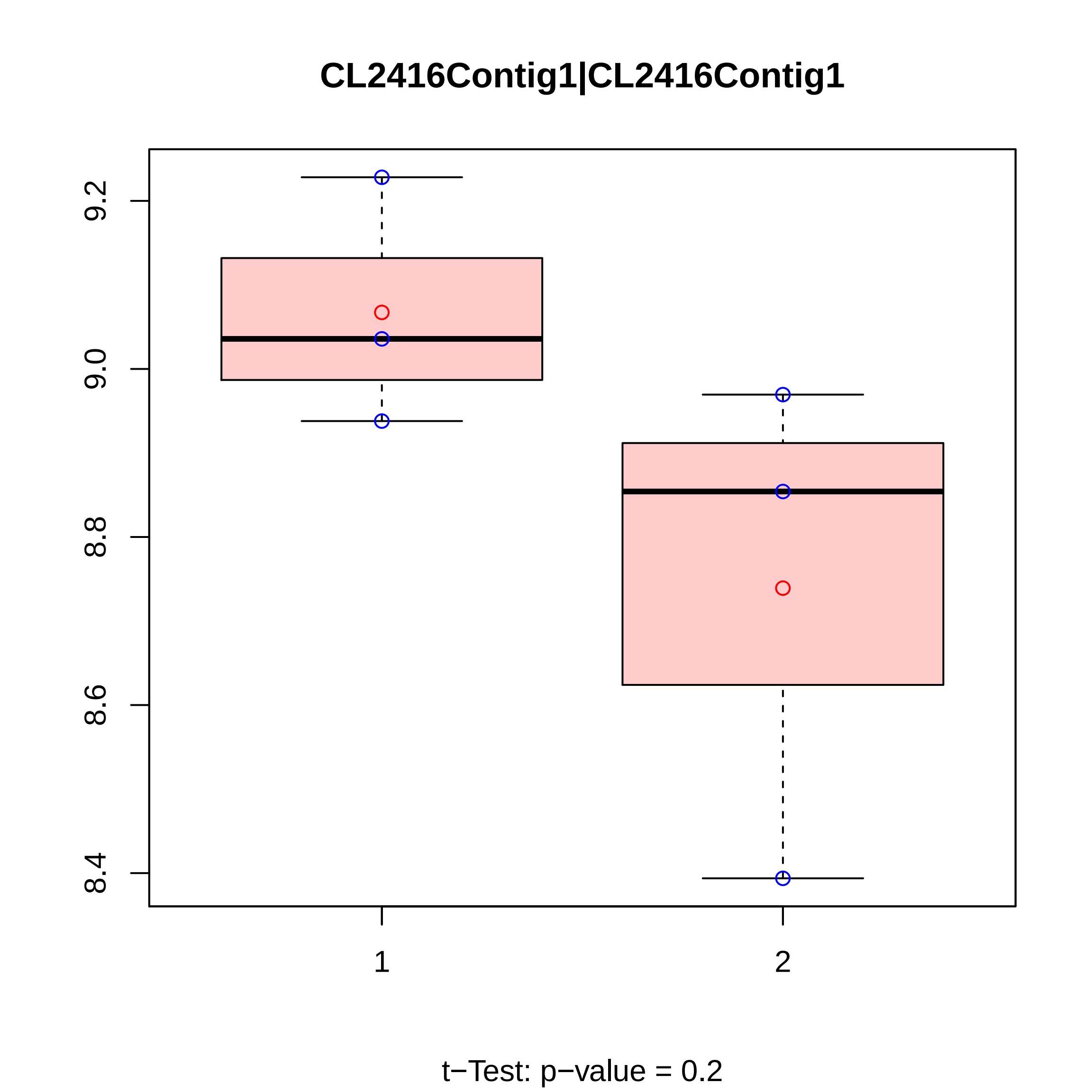

CL2416Contig1|CL2416Contig1
9.2
9.0
8.8
8.6
8.4
1
2
t−Test: p−value = 0.2

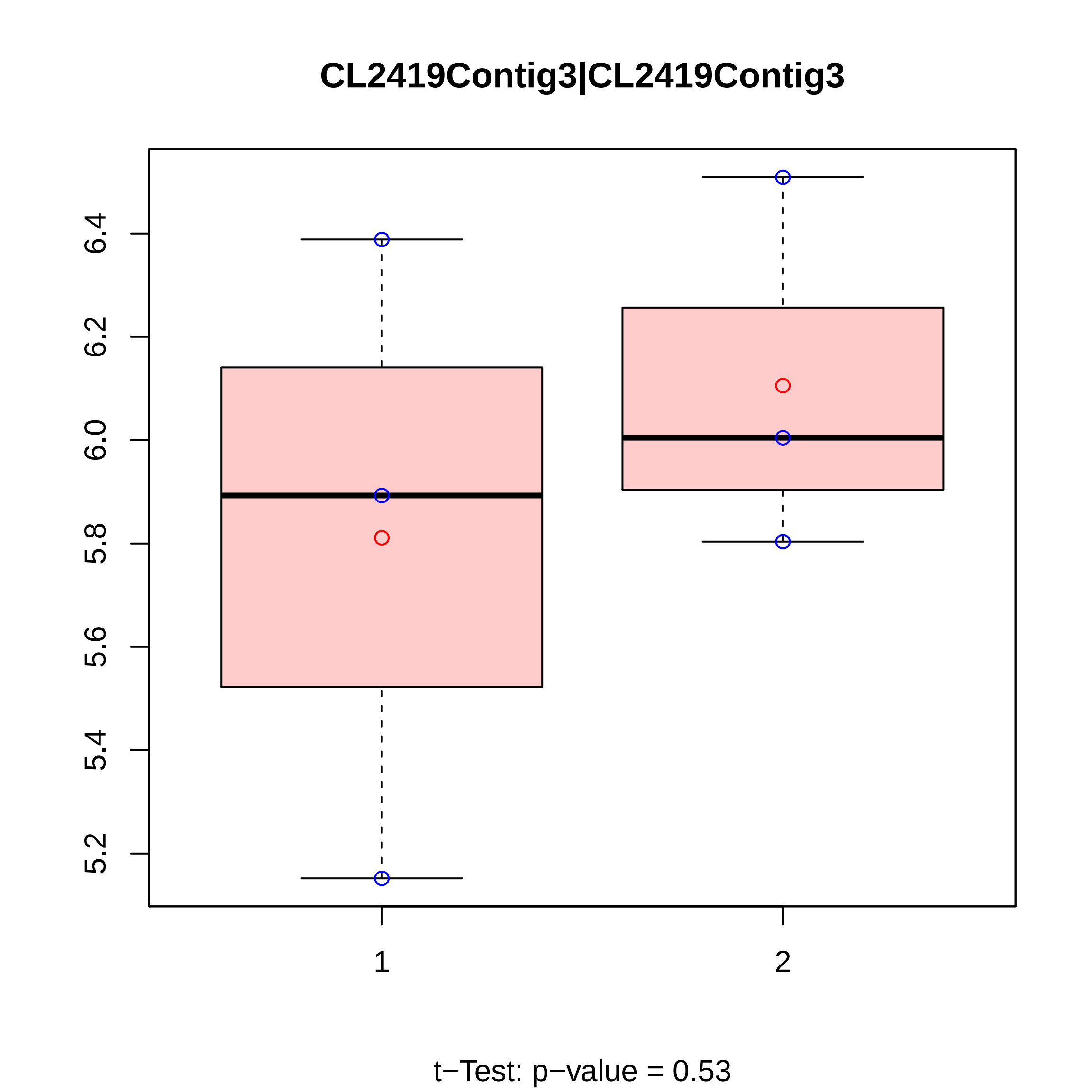

CL2419Contig3|CL2419Contig3
6.4
6.2
6.0
5.8
5.6
5.4
5.2
1
2
t−Test: p−value = 0.53

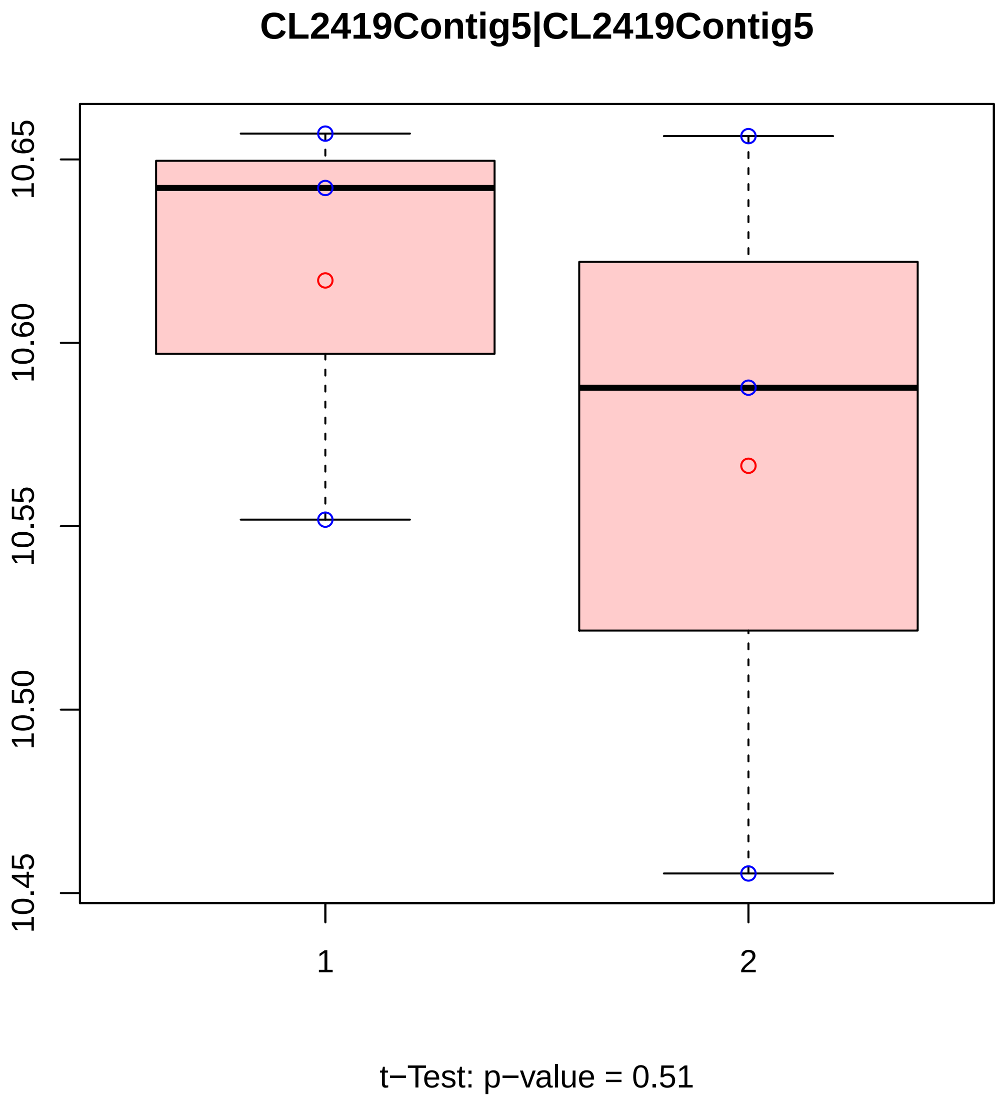
CL2419Contig5|CL2419Contig5
10.65
10.60
10.55
10.50
10.45
1
2
t−Test: p−value = 0.51

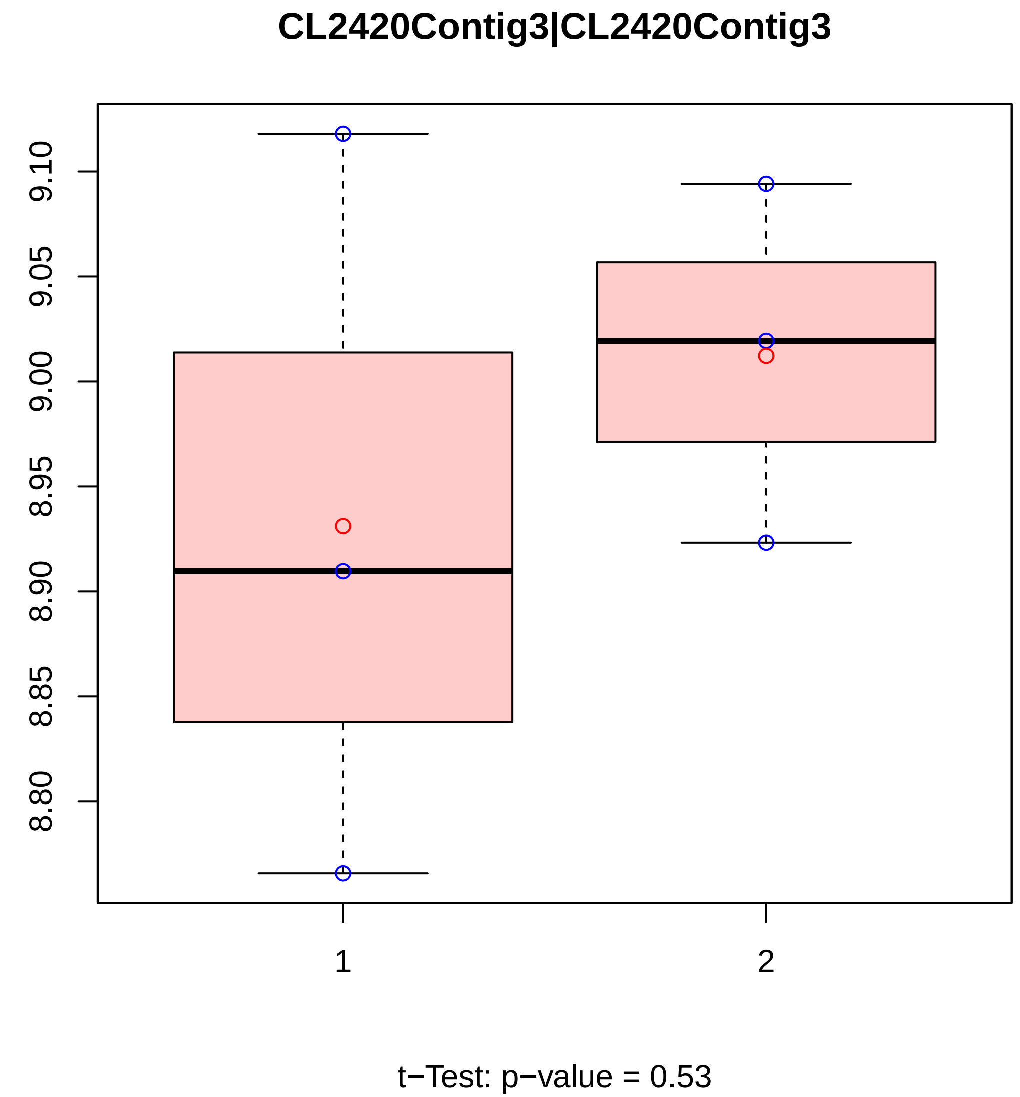
CL2420Contig3|CL2420Contig3
9.10
9.05
9.00
8.95
8.90
8.85
8.80
1
2
t−Test: p−value = 0.53

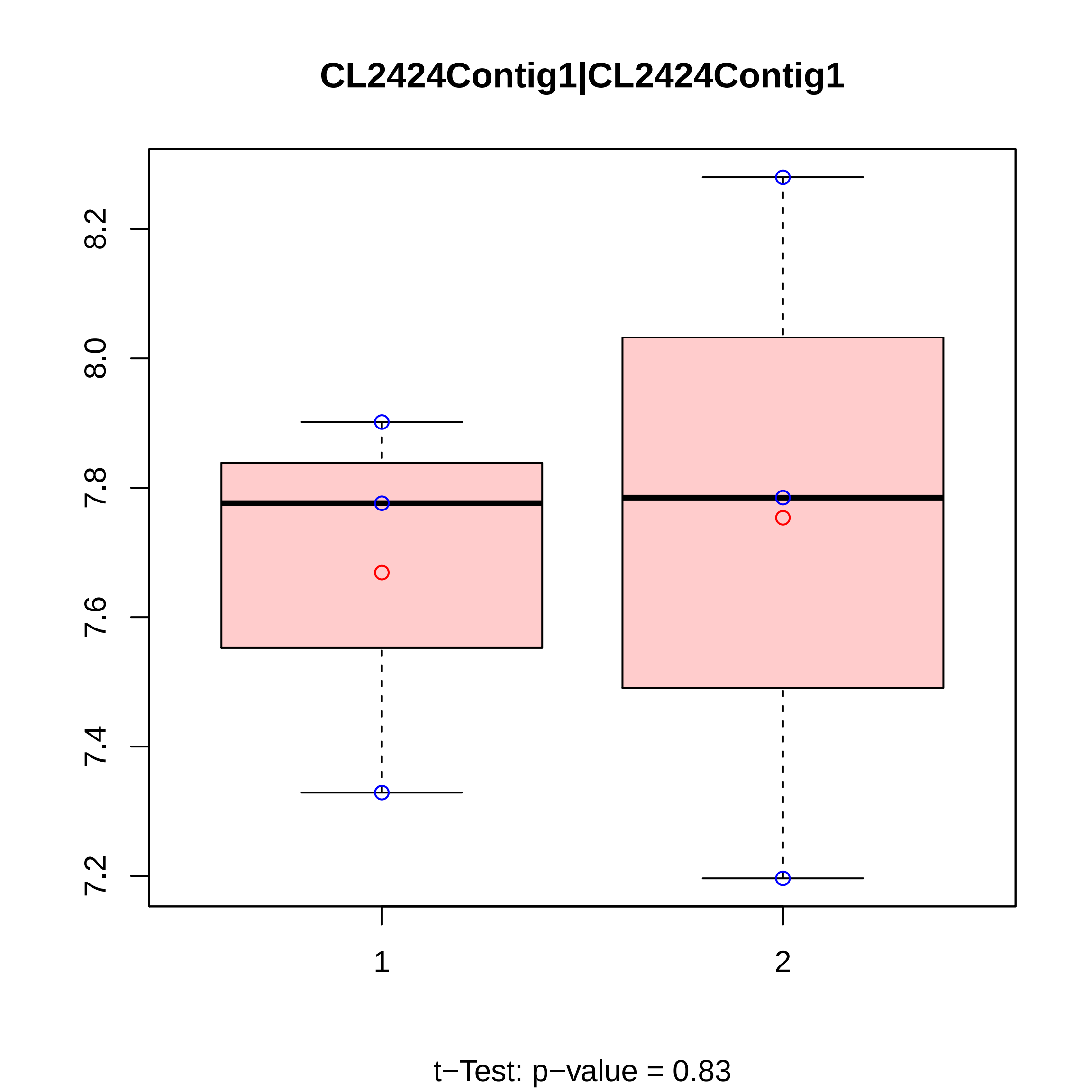
CL2424Contig1|CL2424Contig1
8.2
8.0
7.8
7.6
7.4
7.2
1
2
t−Test: p−value = 0.83

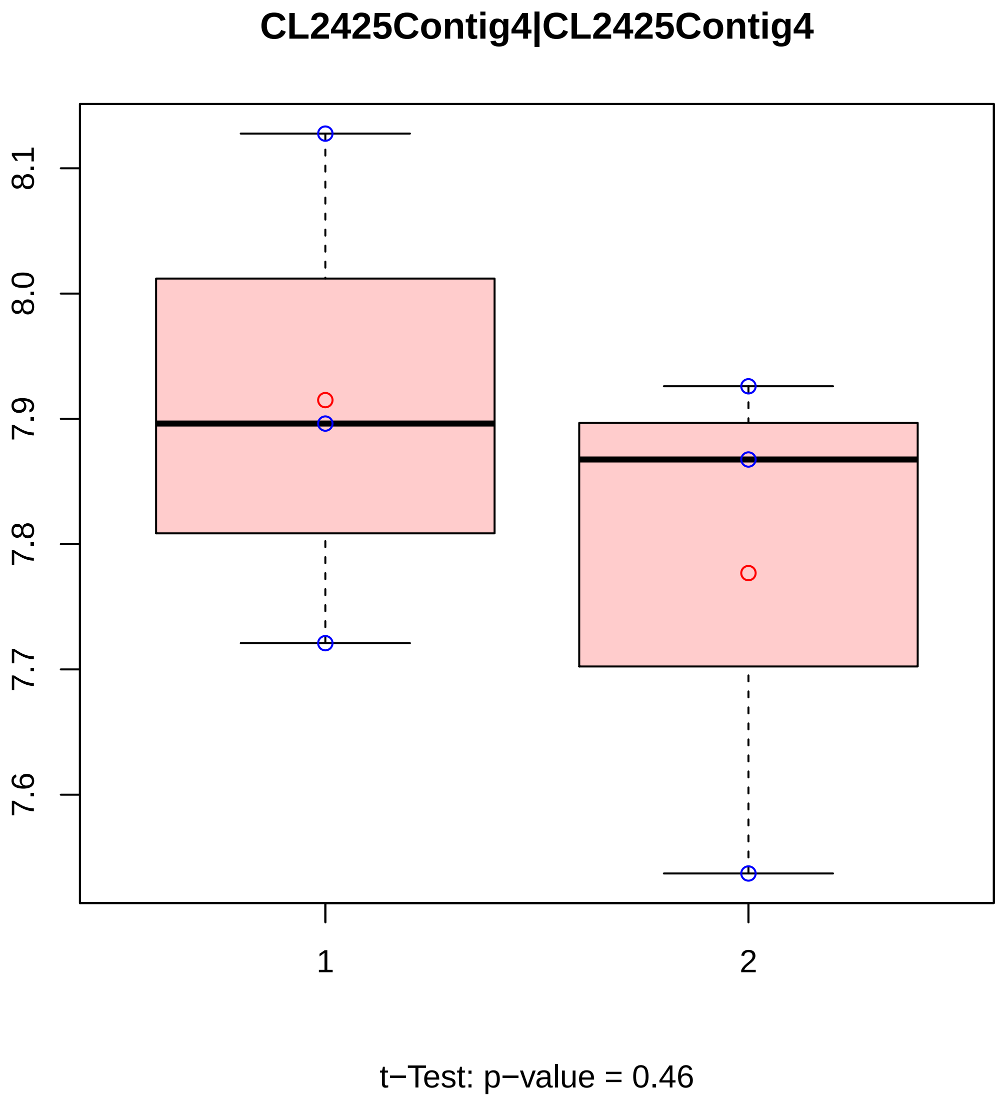

CL2425Contig4|CL2425Contig4
8.1
8.0
7.9
7.8
7.7
7.6
1
2
t−Test: p−value = 0.46

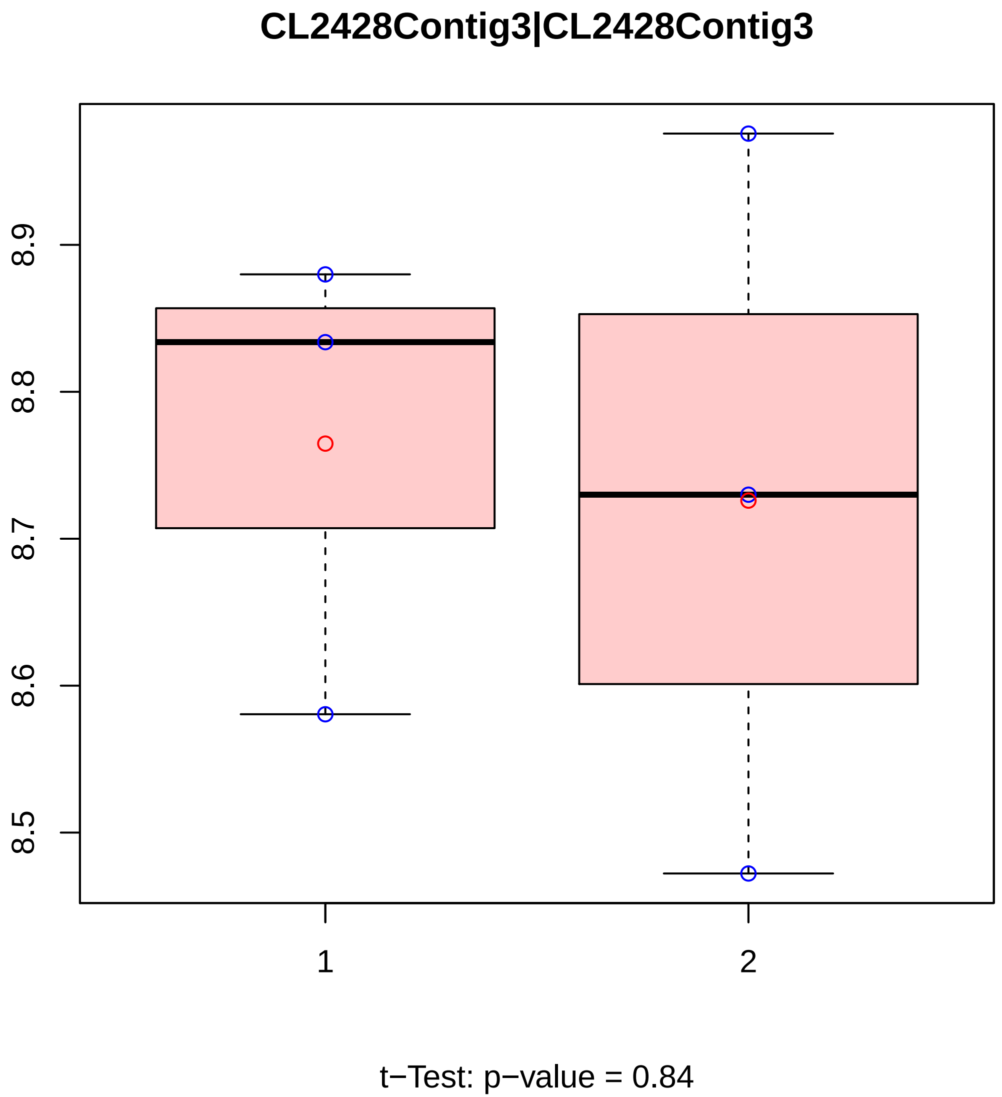
CL2428Contig3|CL2428Contig3
8.5
8.6
8.7
8.8
8.9
1
2
t−Test: p−value = 0.84

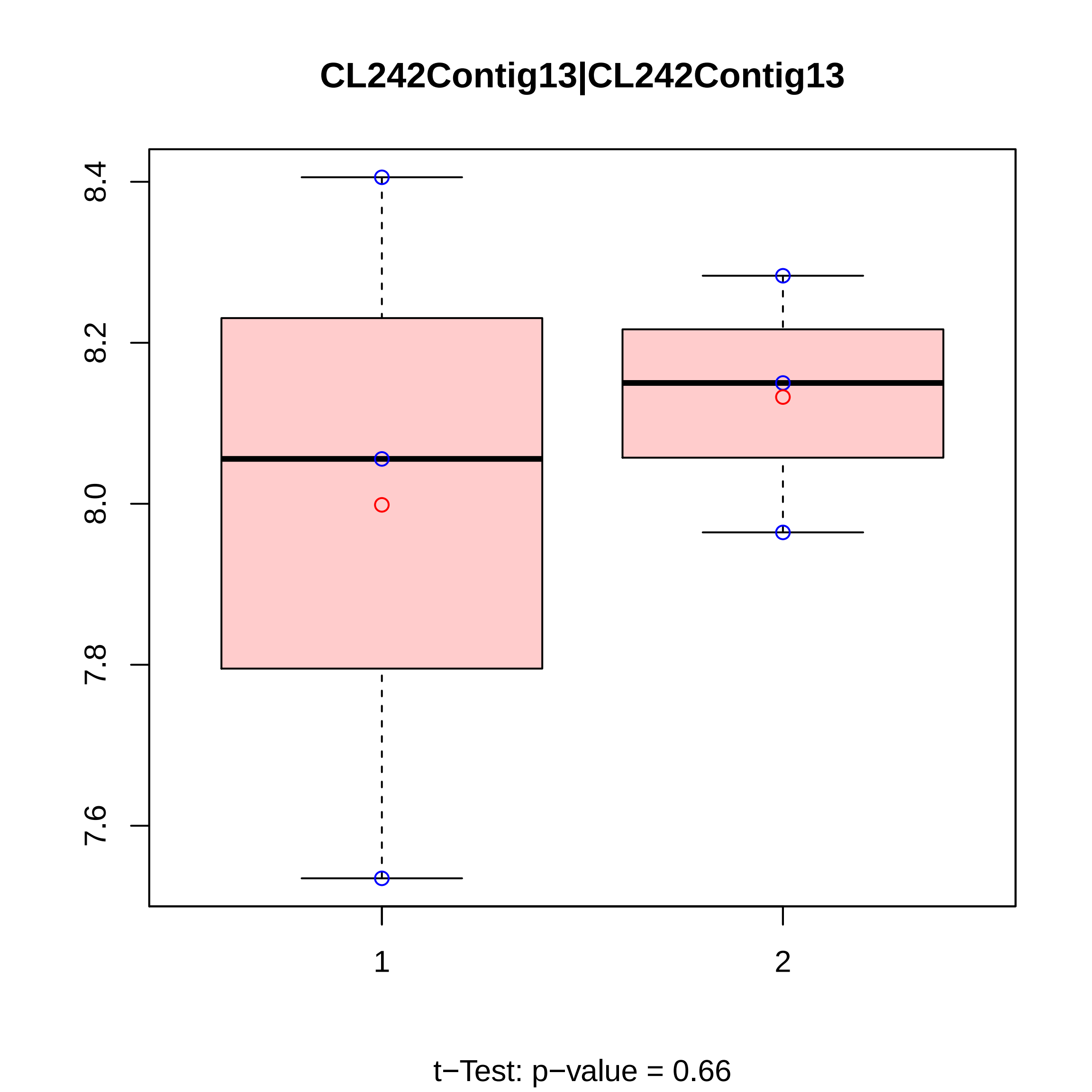

CL242Contig13|CL242Contig13
8.4
8.2
8.0
7.8
7.6
1
2
t−Test: p−value = 0.66

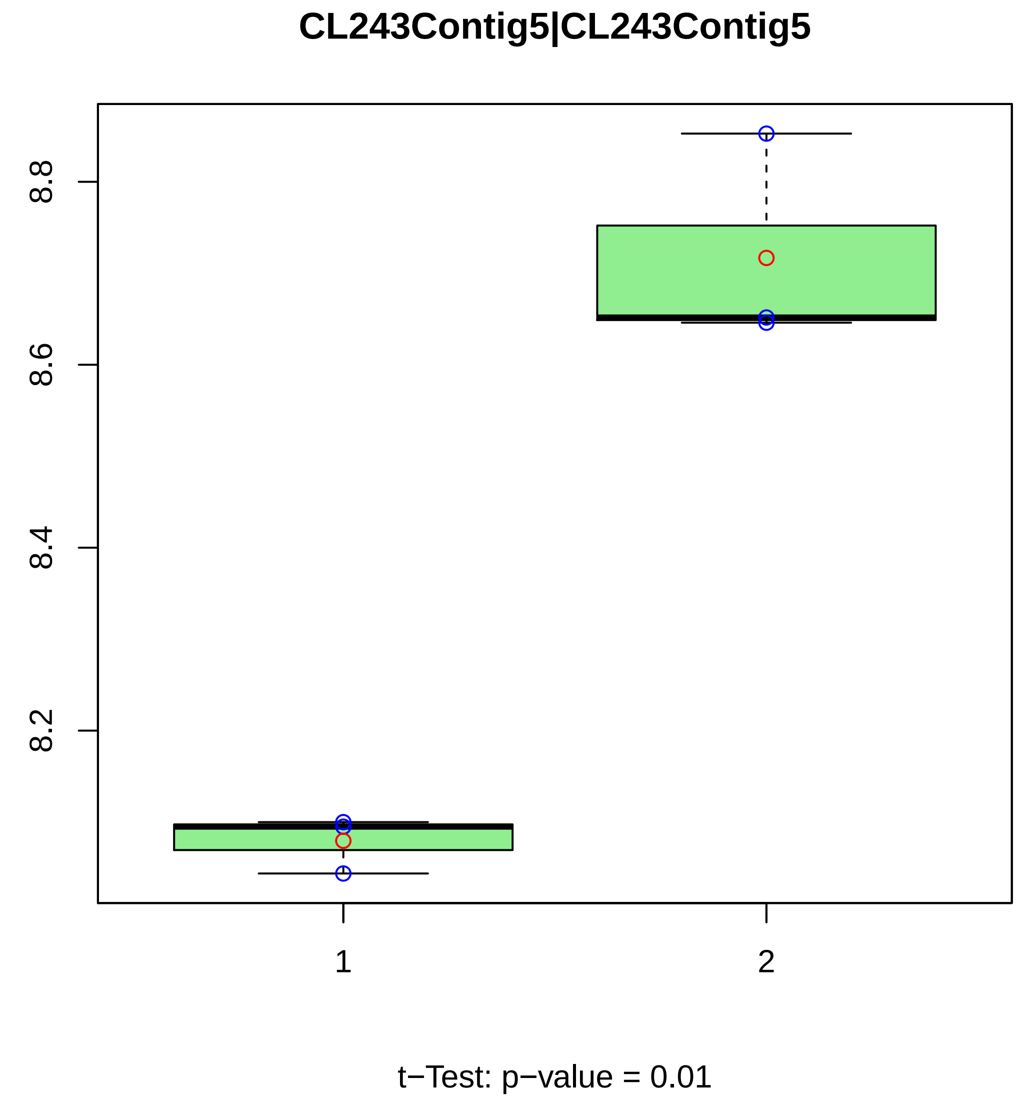
CL243Contig5|CL243Contig5
8.2
8.4
8.6
8.8
1
2
t−Test: p−value = 0.01

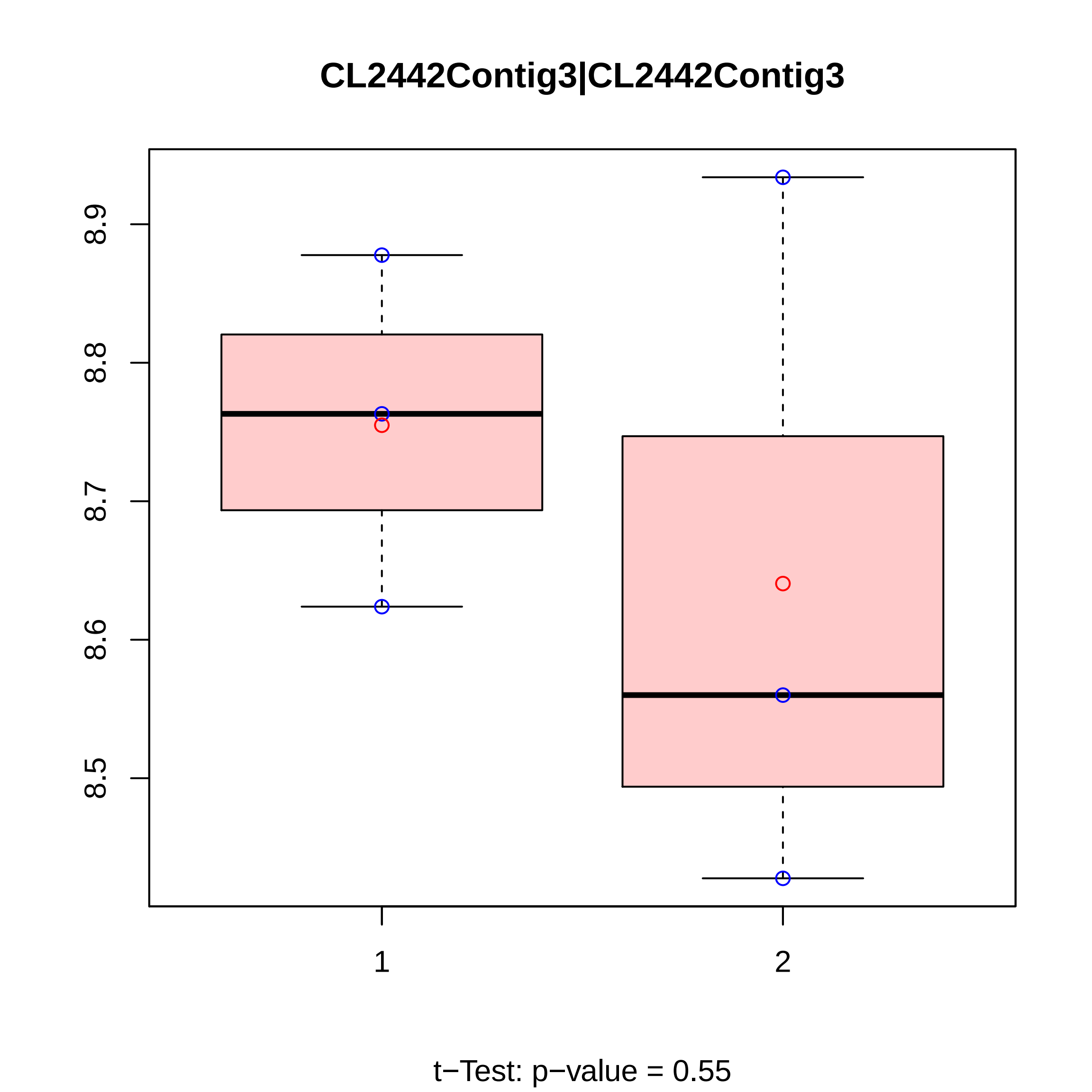

CL2442Contig3|CL2442Contig3
8.5
8.6
8.7
8.8
8.9
1
2
t−Test: p−value = 0.55

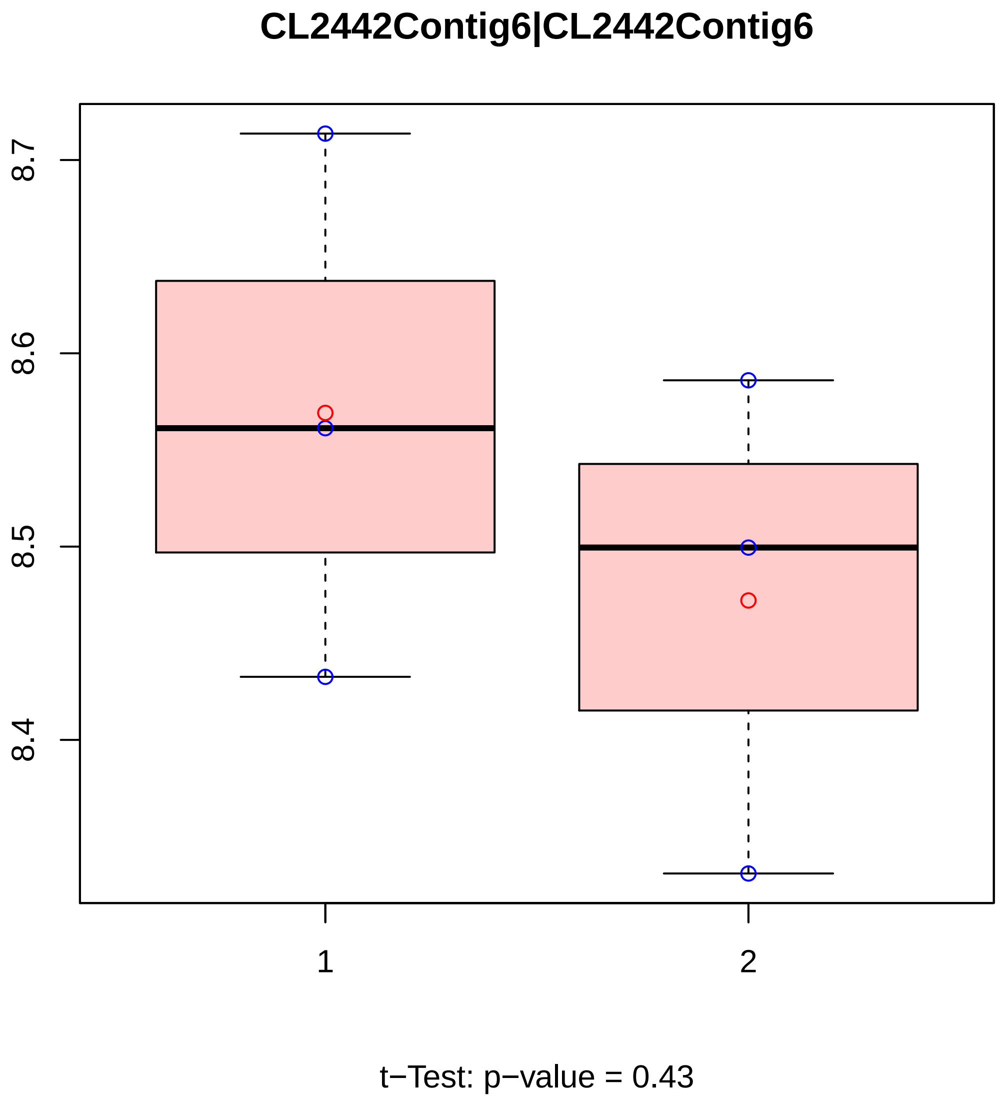
CL2442Contig6|CL2442Contig6
8.7
8.6
8.5
8.4
1
2
t−Test: p−value = 0.43

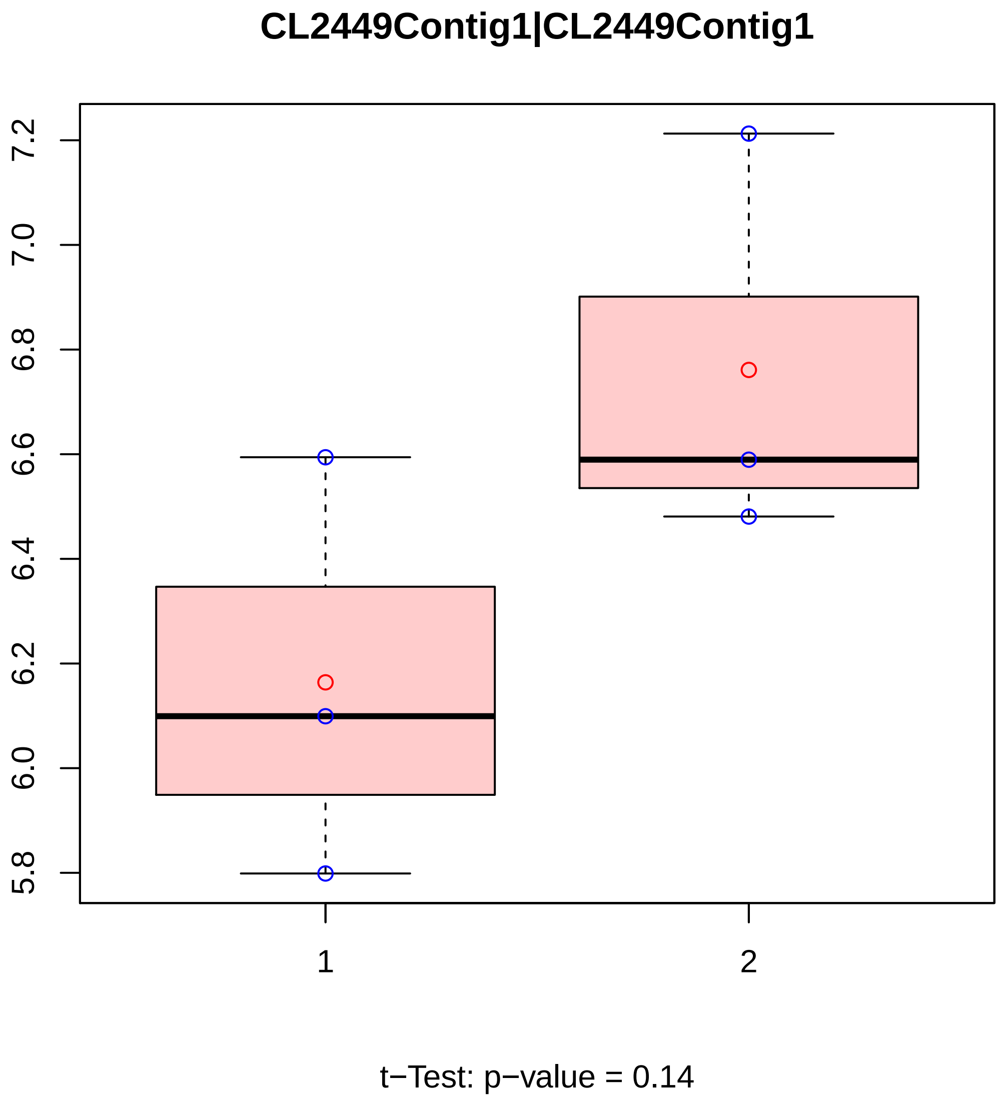
CL2449Contig1|CL2449Contig1
7.2
7.0
6.8
6.6
6.4
6.2
6.0
5.8
1
2
t−Test: p−value = 0.14

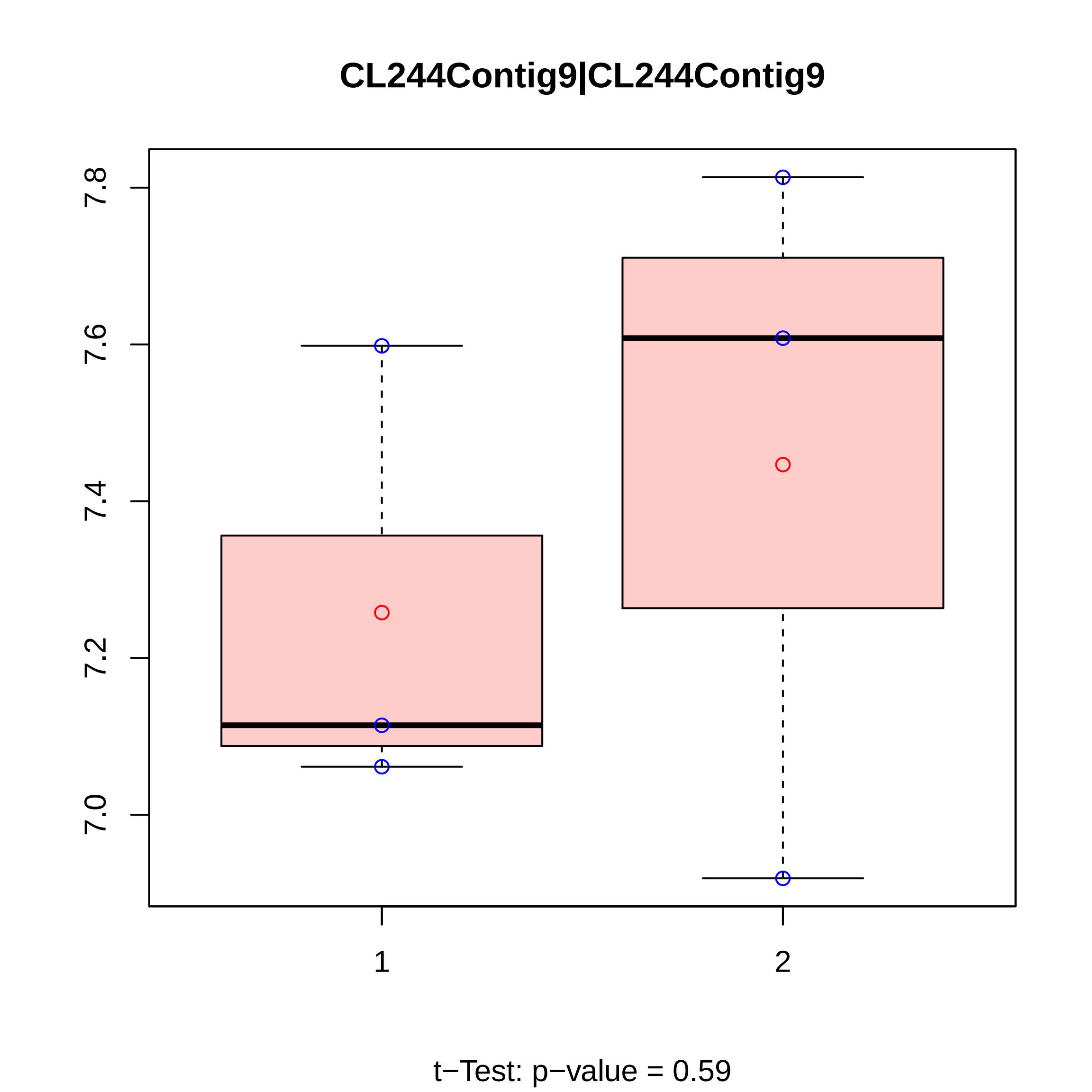

CL244Contig9|CL244Contig9
7.0
7.2
7.4
7.6
7.8
1
2
t−Test: p−value = 0.59

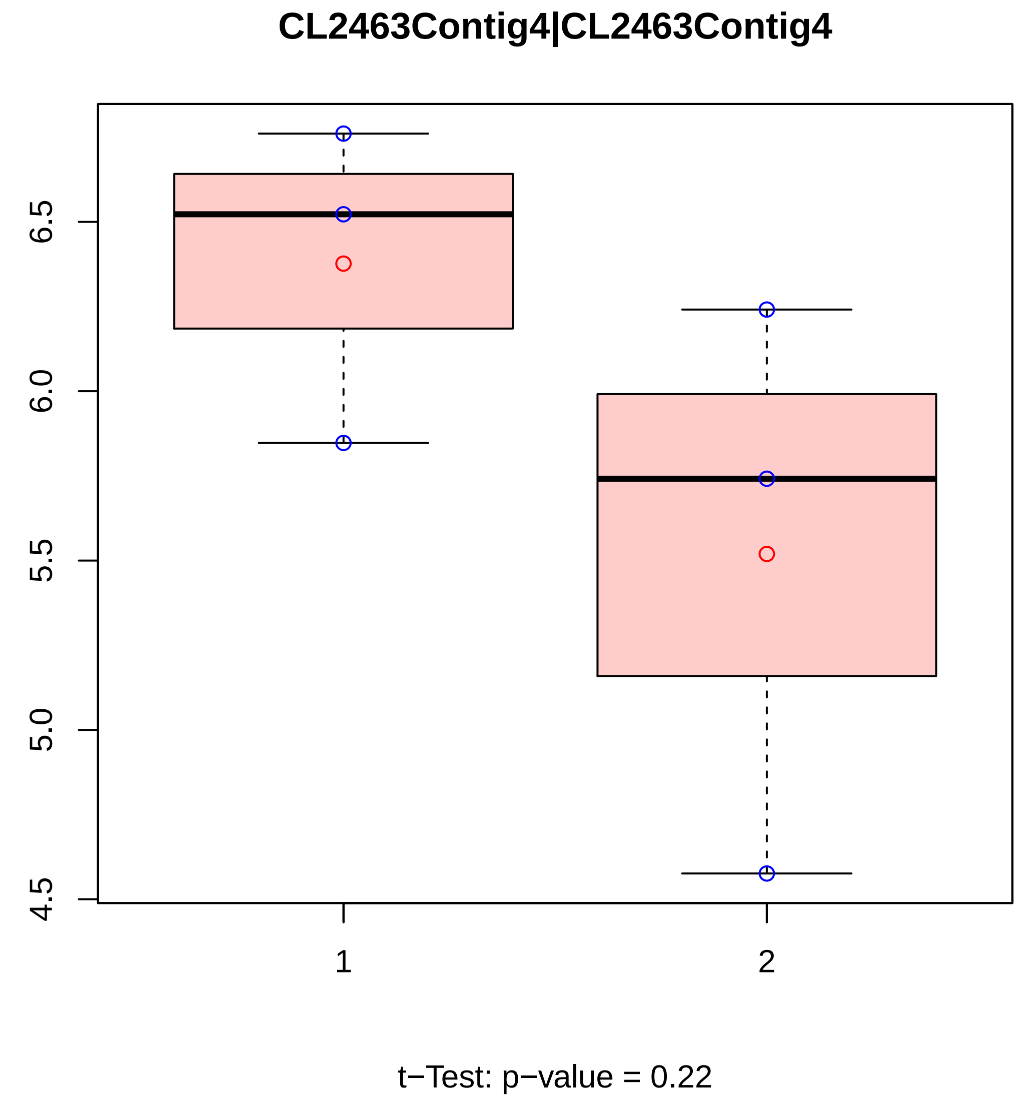
CL2463Contig4|CL2463Contig4
6.5
6.0
5.5
5.0
4.5
1
2
t−Test: p−value = 0.22

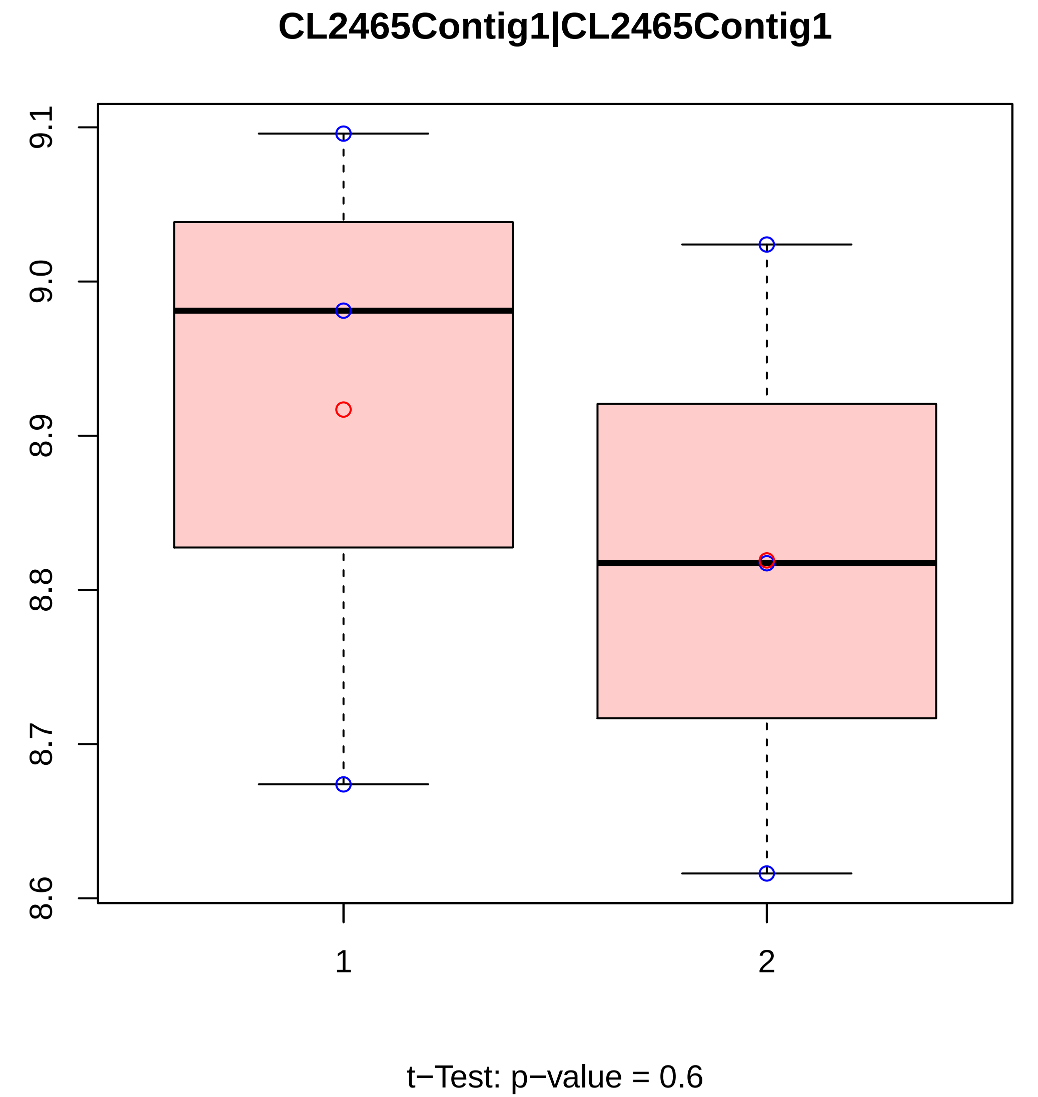
CL2465Contig1|CL2465Contig1
9.1
9.0
8.9
8.8
8.7
8.6
1
2
t−Test: p−value = 0.6

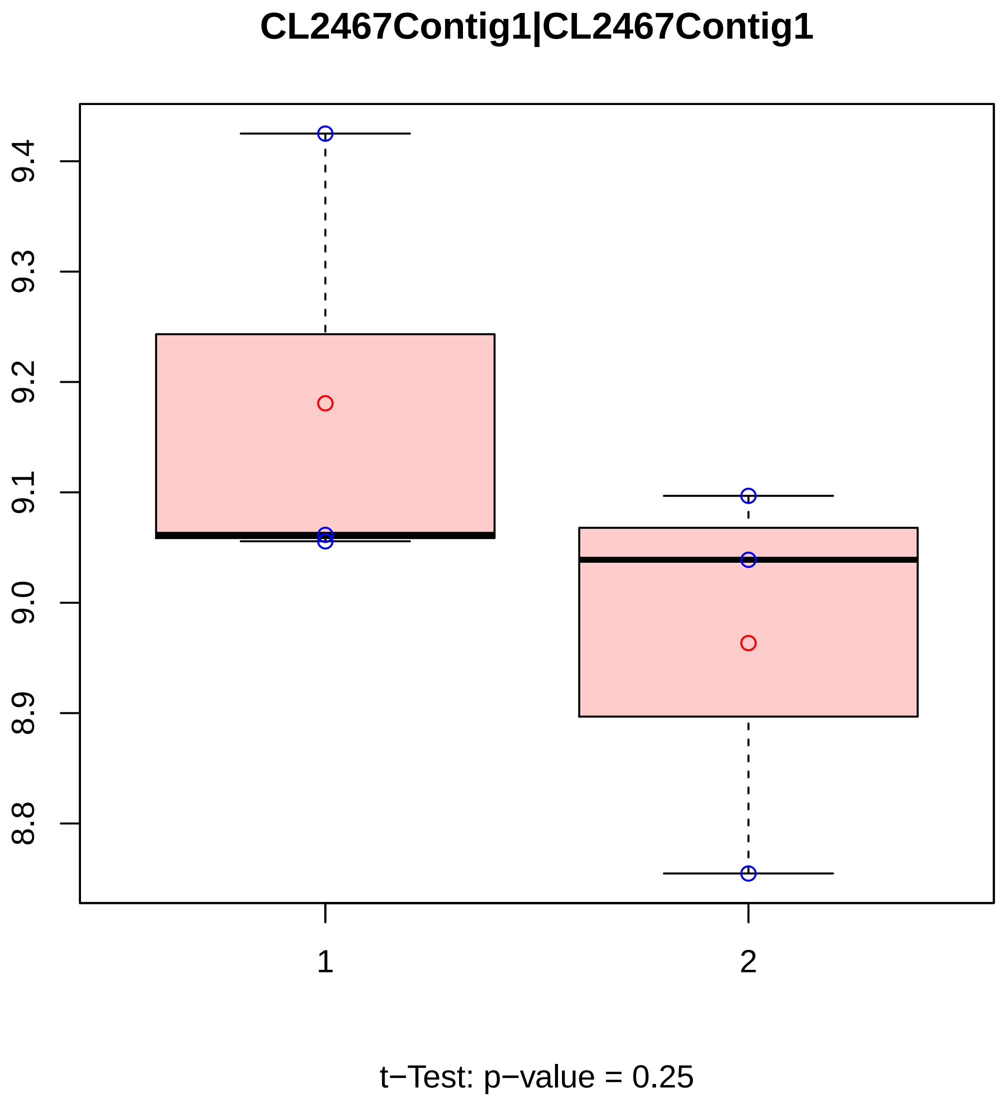
CL2467Contig1|CL2467Contig1
9.4
9.3
9.2
9.1
9.0
8.9
8.8
1
2
t−Test: p−value = 0.25

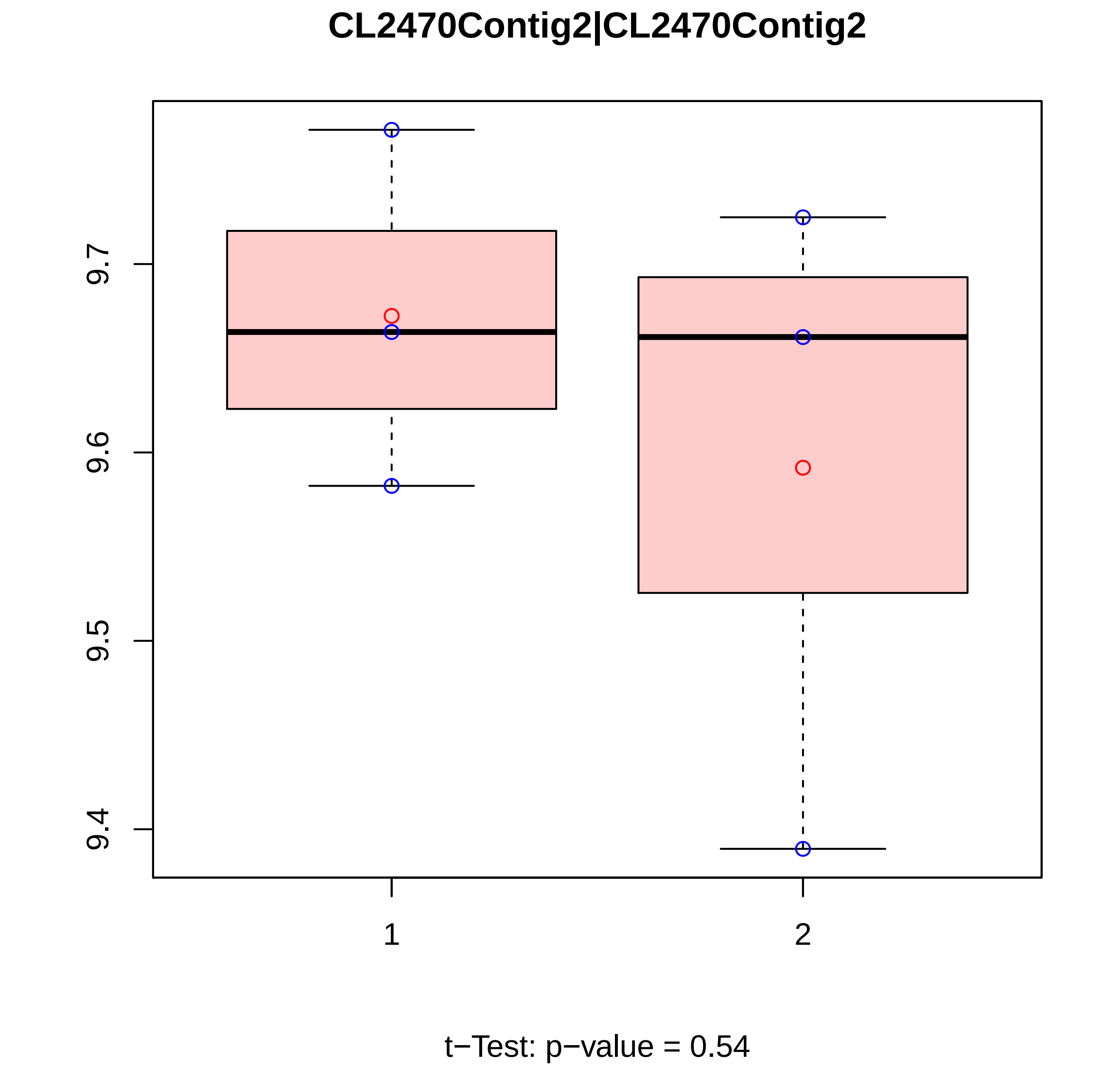
CL2470Contig2|CL2470Contig2
9.4
9.5
9.6
9.7
1
2
t−Test: p−value = 0.54

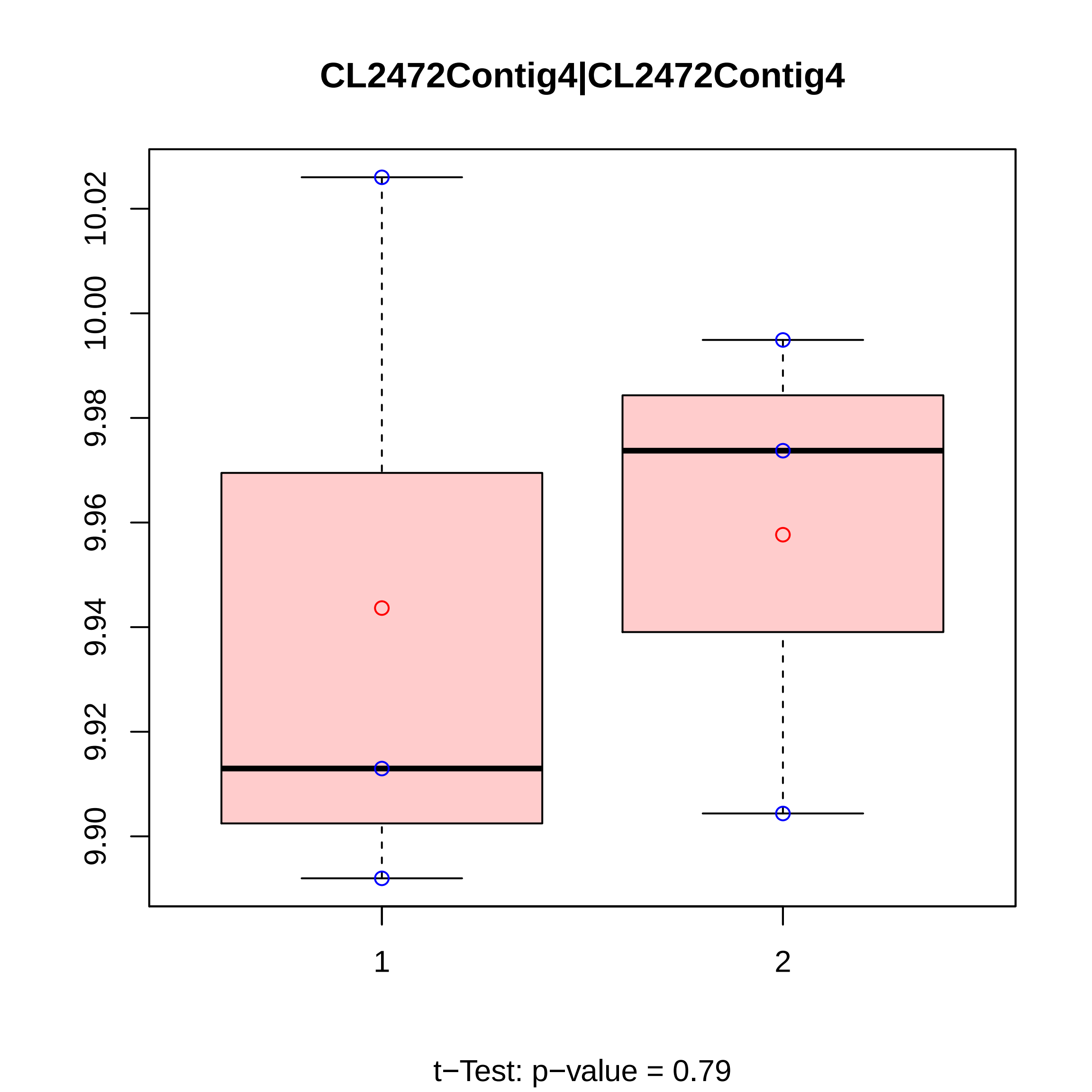
CL2472Contig4|CL2472Contig4
10.02
10.00
9.98
9.96
9.94
9.92
9.90
1
2
t−Test: p−value = 0.79

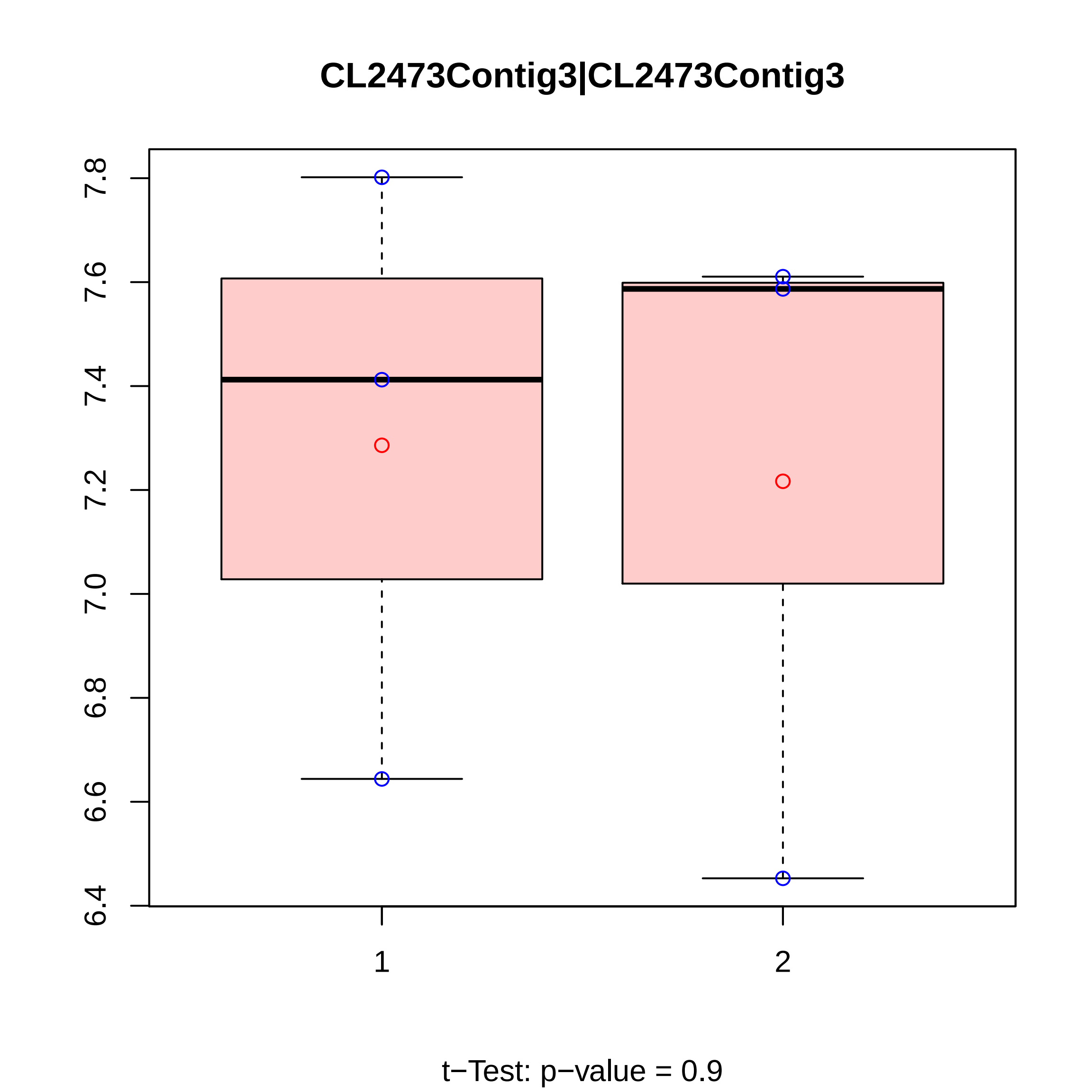
CL2473Contig3|CL2473Contig3
7.8
7.6
7.4
7.2
7.0
6.8
6.6
6.4
1
2
t−Test: p−value = 0.9

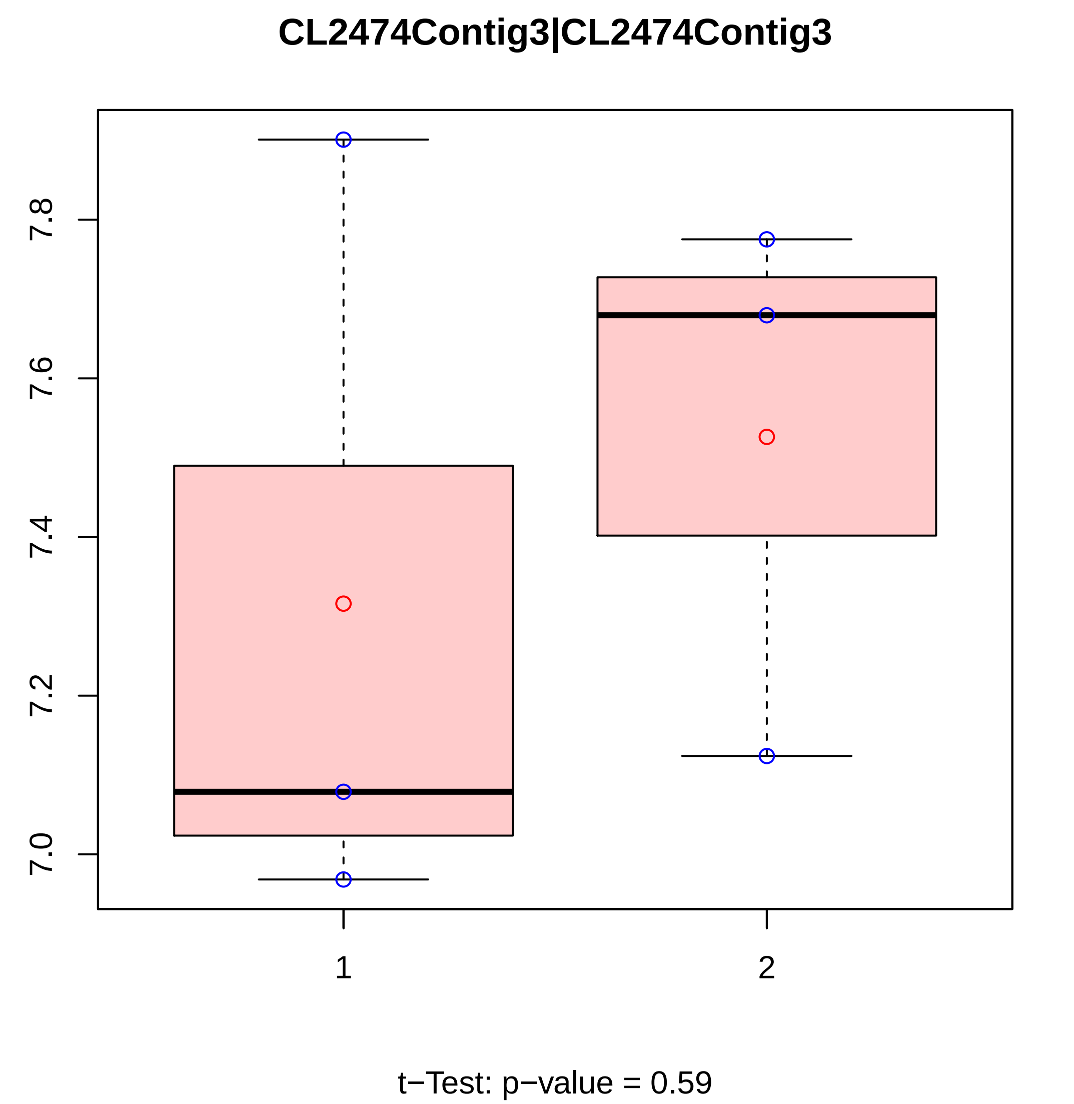
CL2474Contig3|CL2474Contig3
7.0
7.2
7.4
7.6
7.8
1
2
t−Test: p−value = 0.59

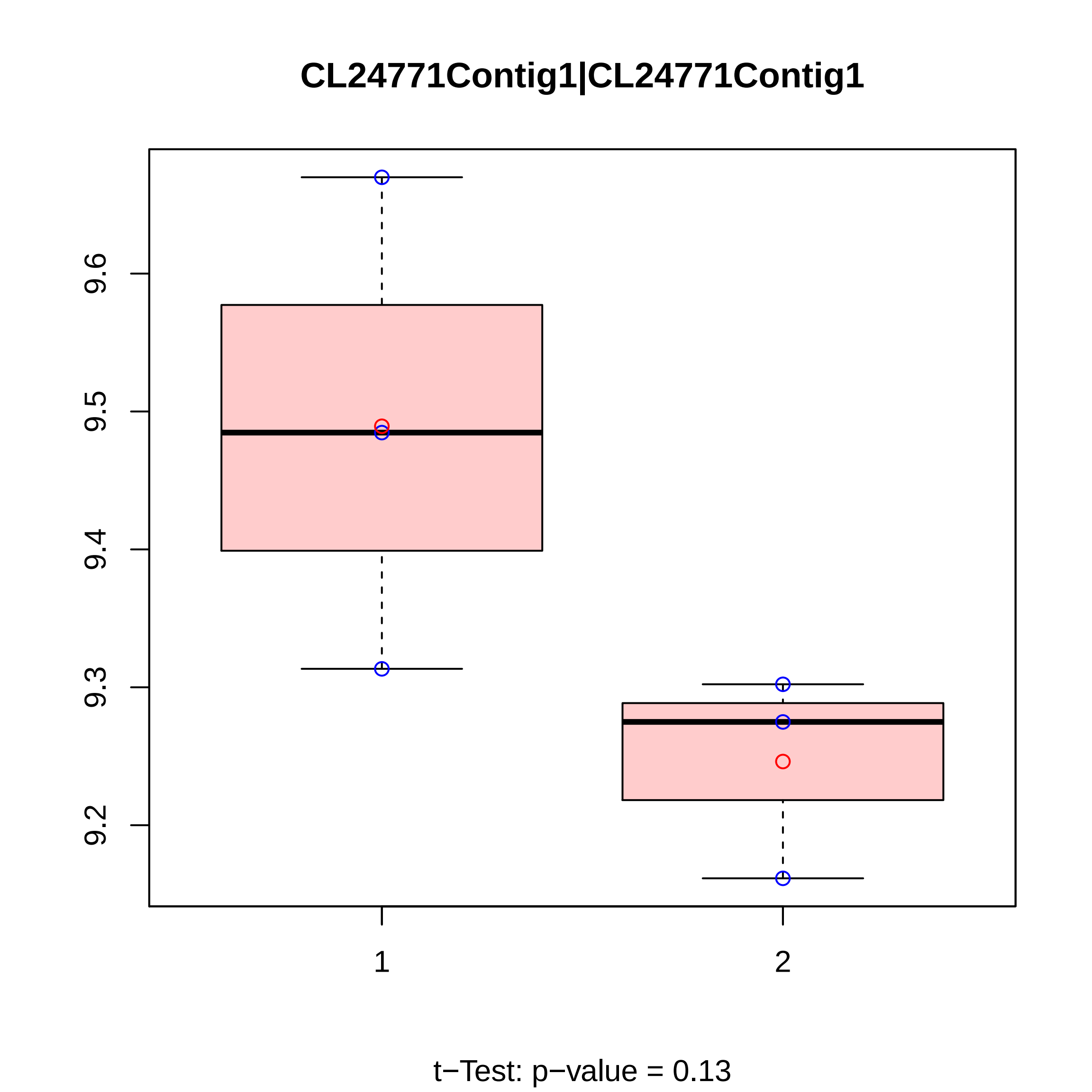
CL24771Contig1|CL24771Contig1
9.2
9.3
9.4
9.5
9.6
1
2
t−Test: p−value = 0.13

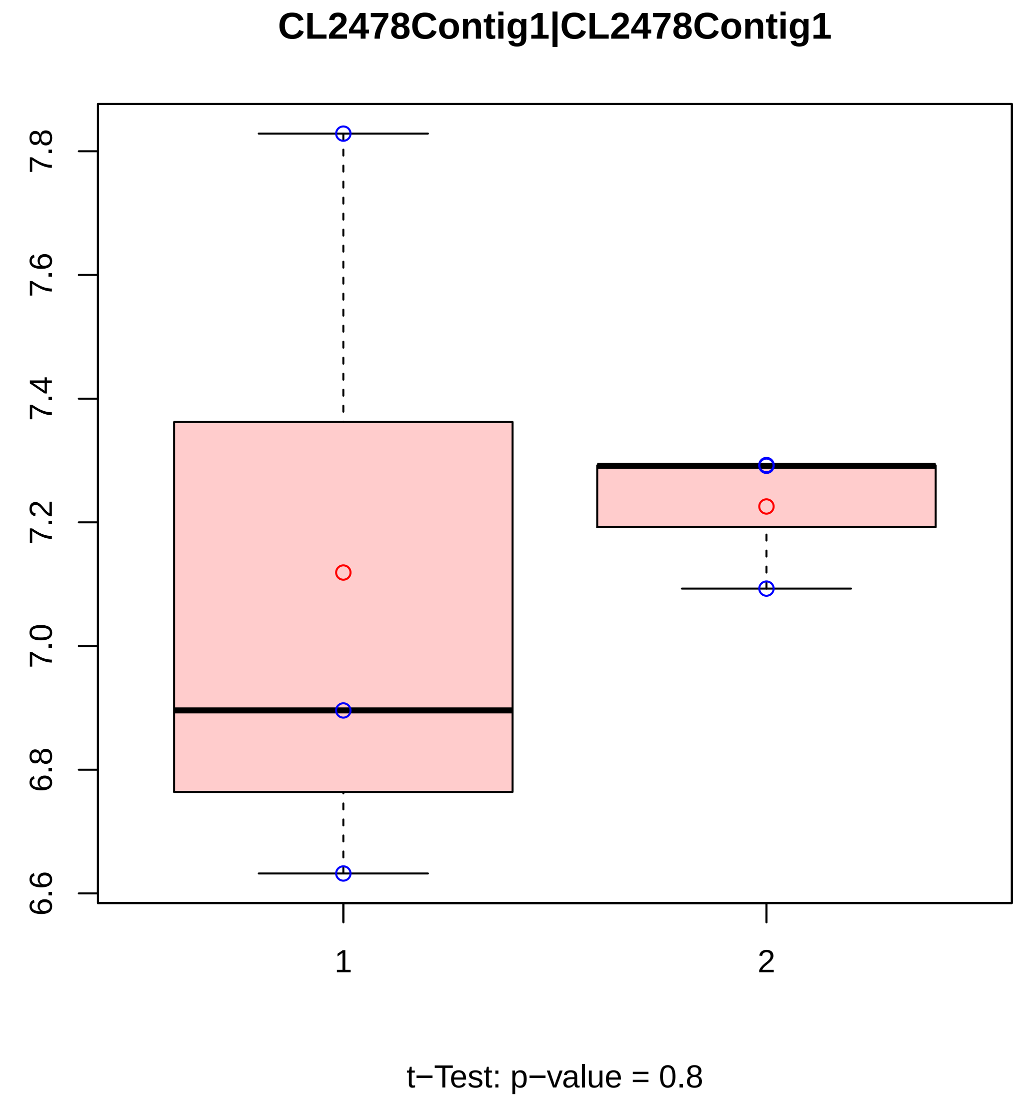
CL2478Contig1|CL2478Contig1
7.8
7.6
7.4
7.2
7.0
6.8
6.6
1
2
t−Test: p−value = 0.8

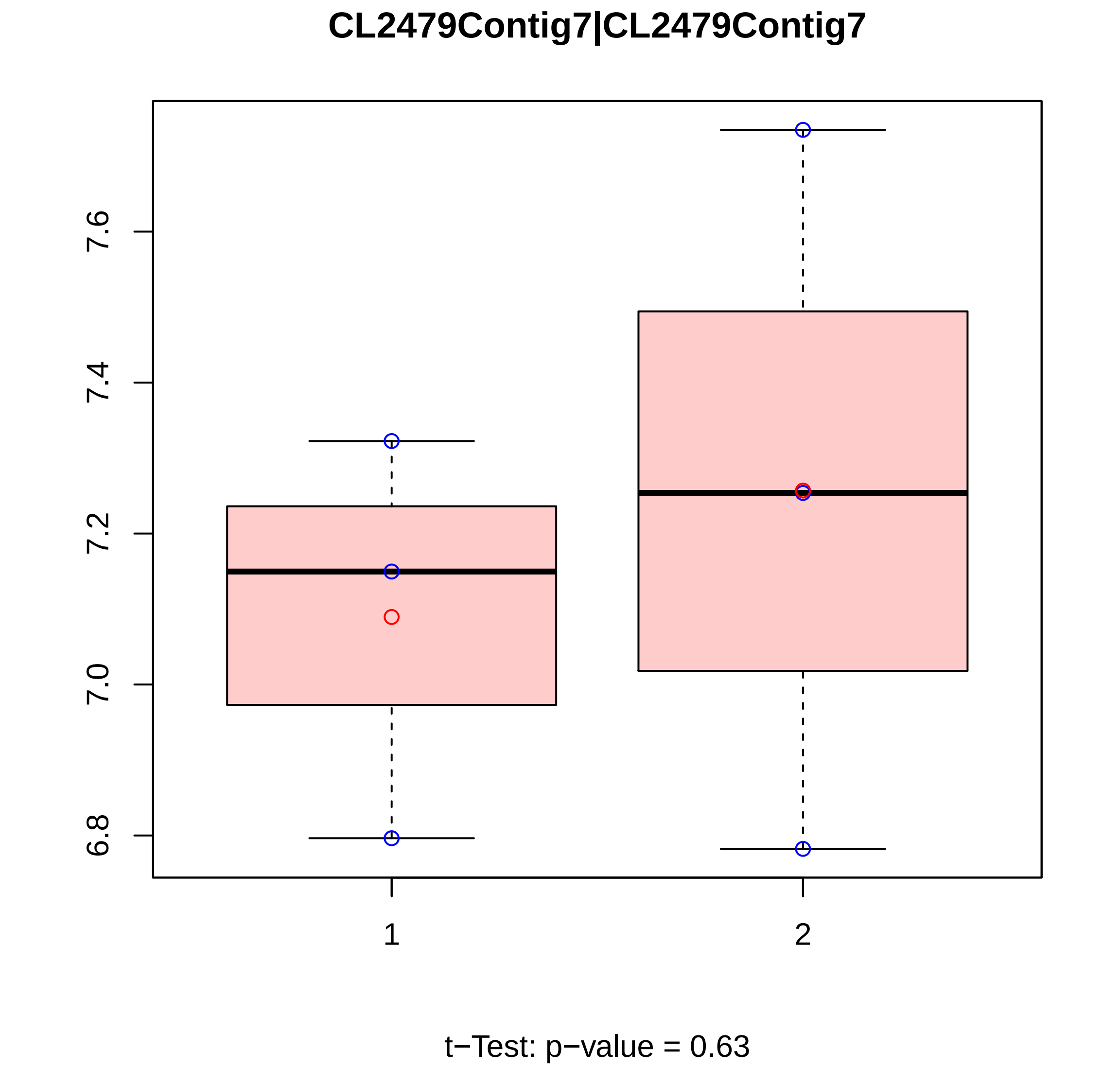

CL2479Contig7|CL2479Contig7
7.6
7.4
7.2
7.0
6.8
1
2
t−Test: p−value = 0.63

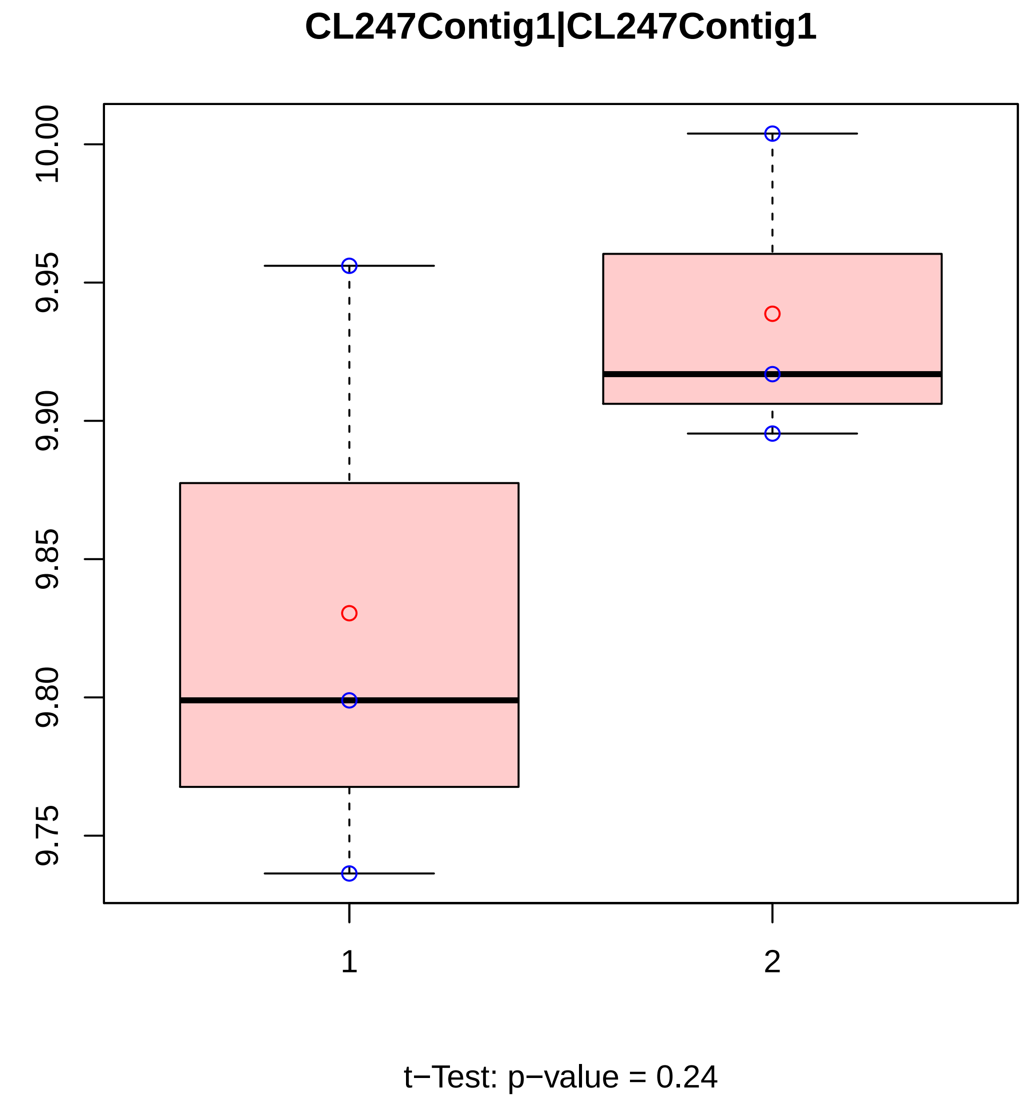
CL247Contig1|CL247Contig1
10.00
9.95
9.90
9.85
9.80
9.75
1
2
t−Test: p−value = 0.24

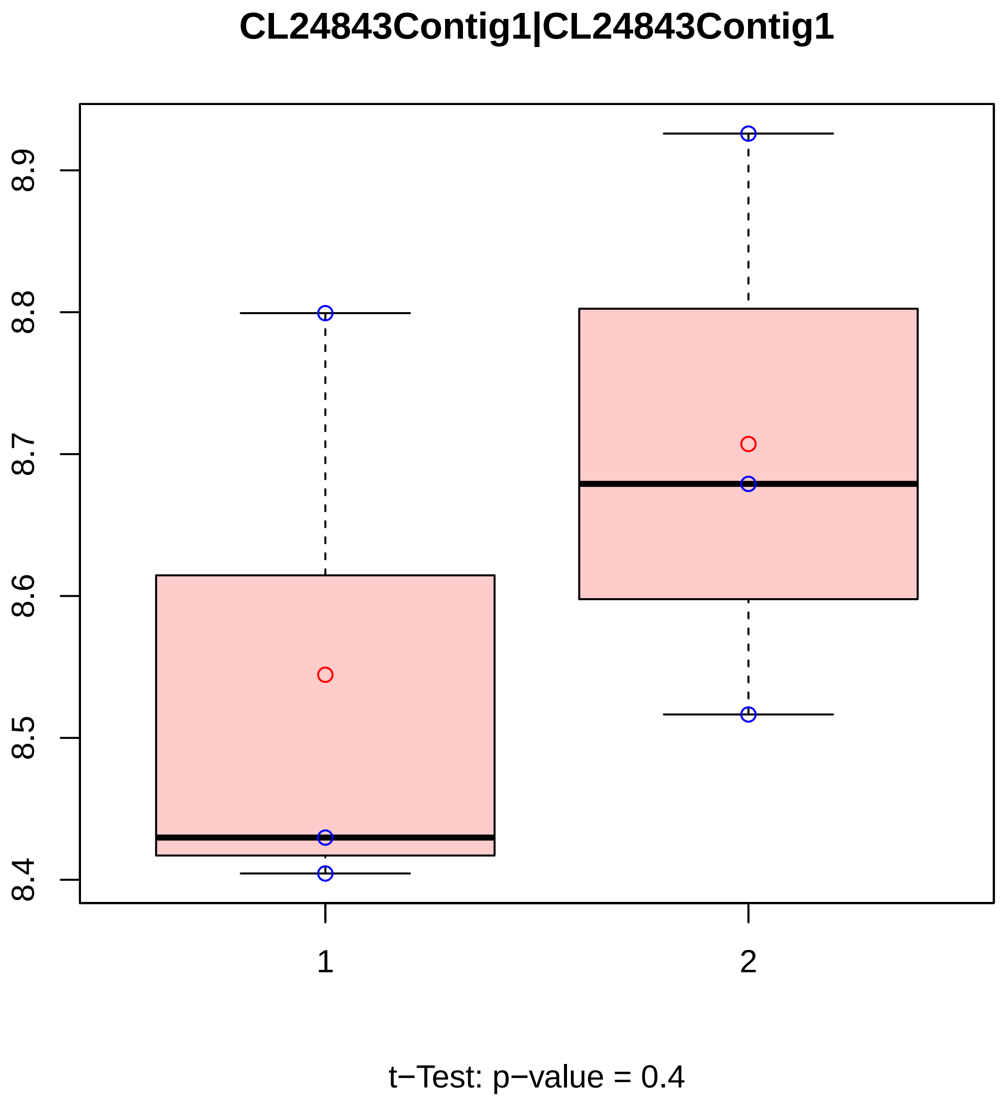
CL24843Contig1|CL24843Contig1
8.4
8.5
8.6
8.7
8.8
8.9
1
2
t−Test: p−value = 0.4

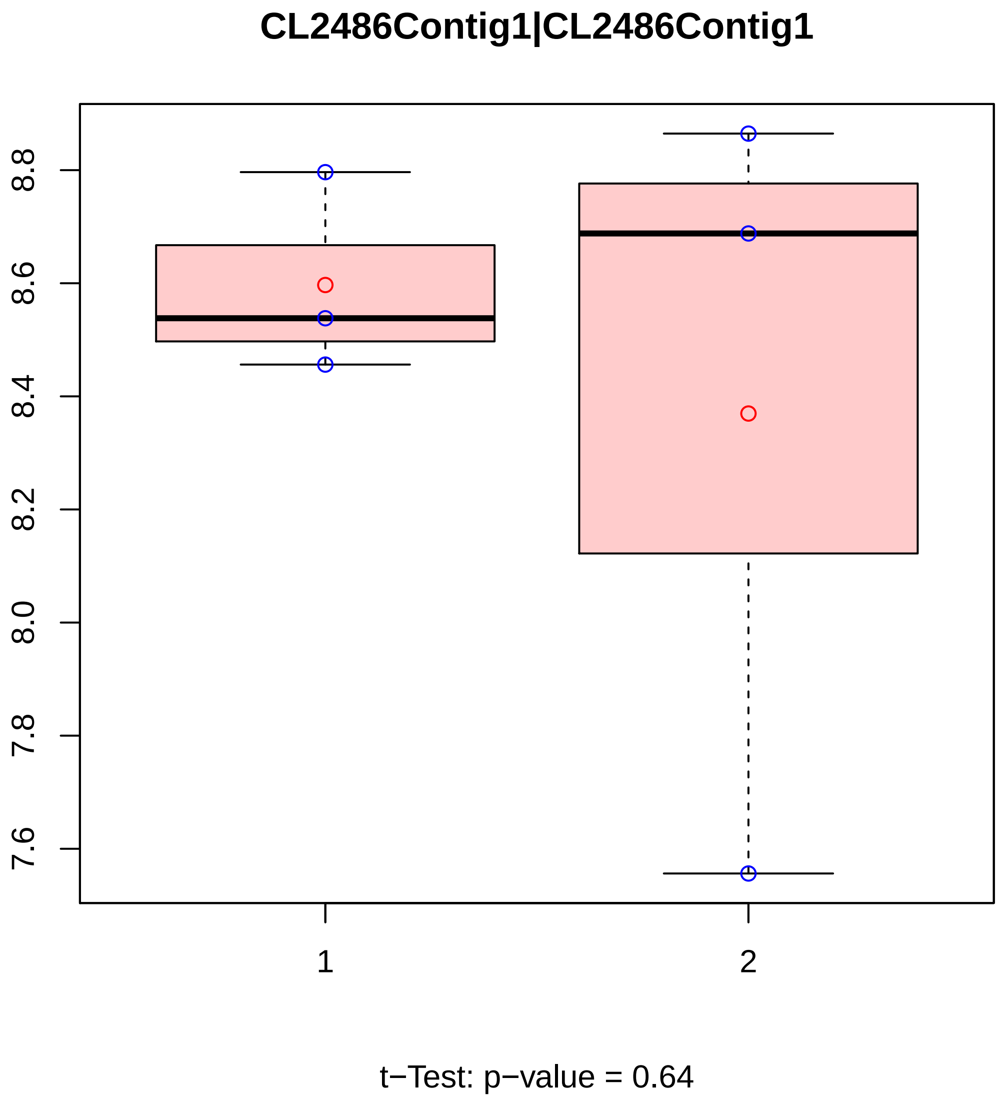

CL2486Contig1|CL2486Contig1
8.8
8.6
8.4
8.2
8.0
7.8
7.6
1
2
t−Test: p−value = 0.64

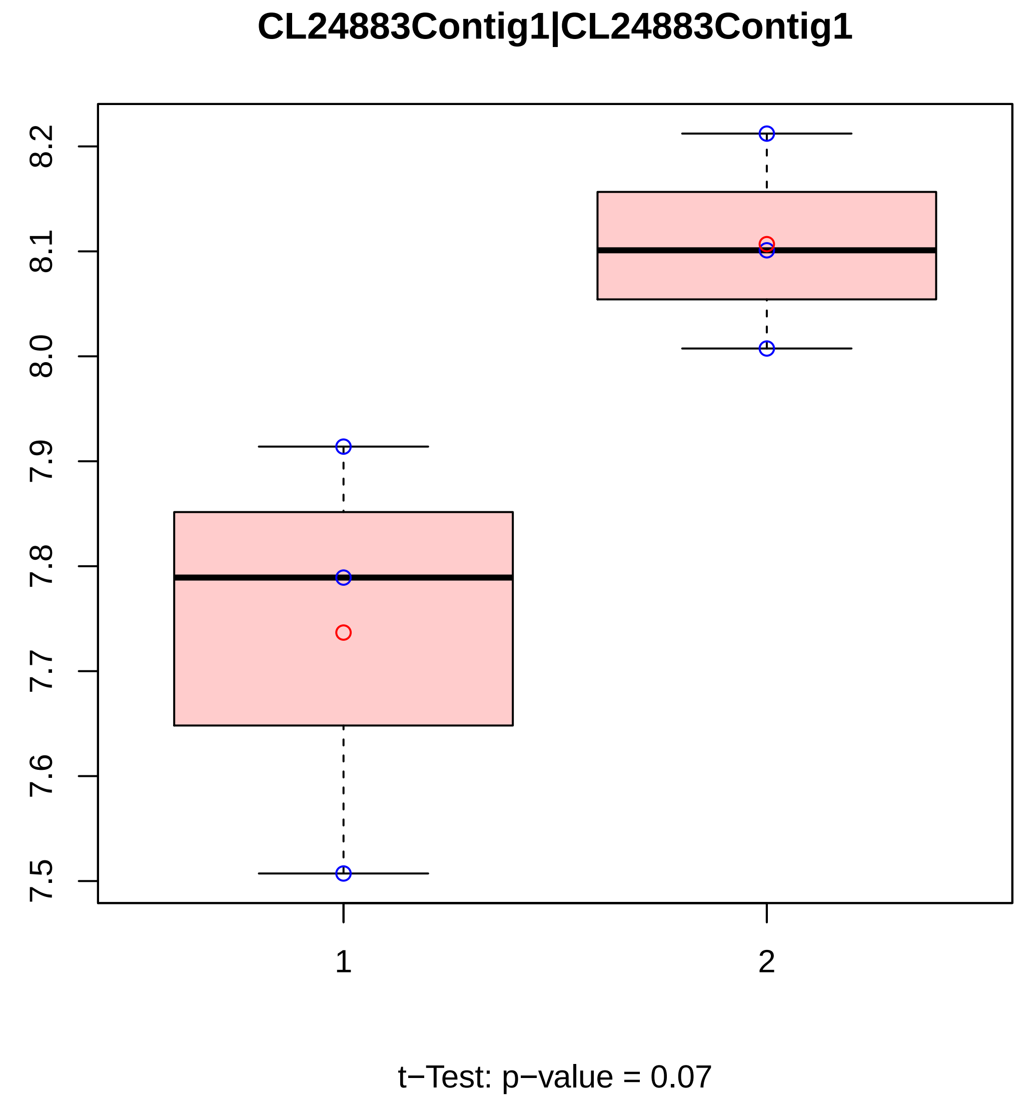
CL24883Contig1|CL24883Contig1
8.2
8.1
8.0
7.9
7.8
7.7
7.6
7.5
1
2
t−Test: p−value = 0.07

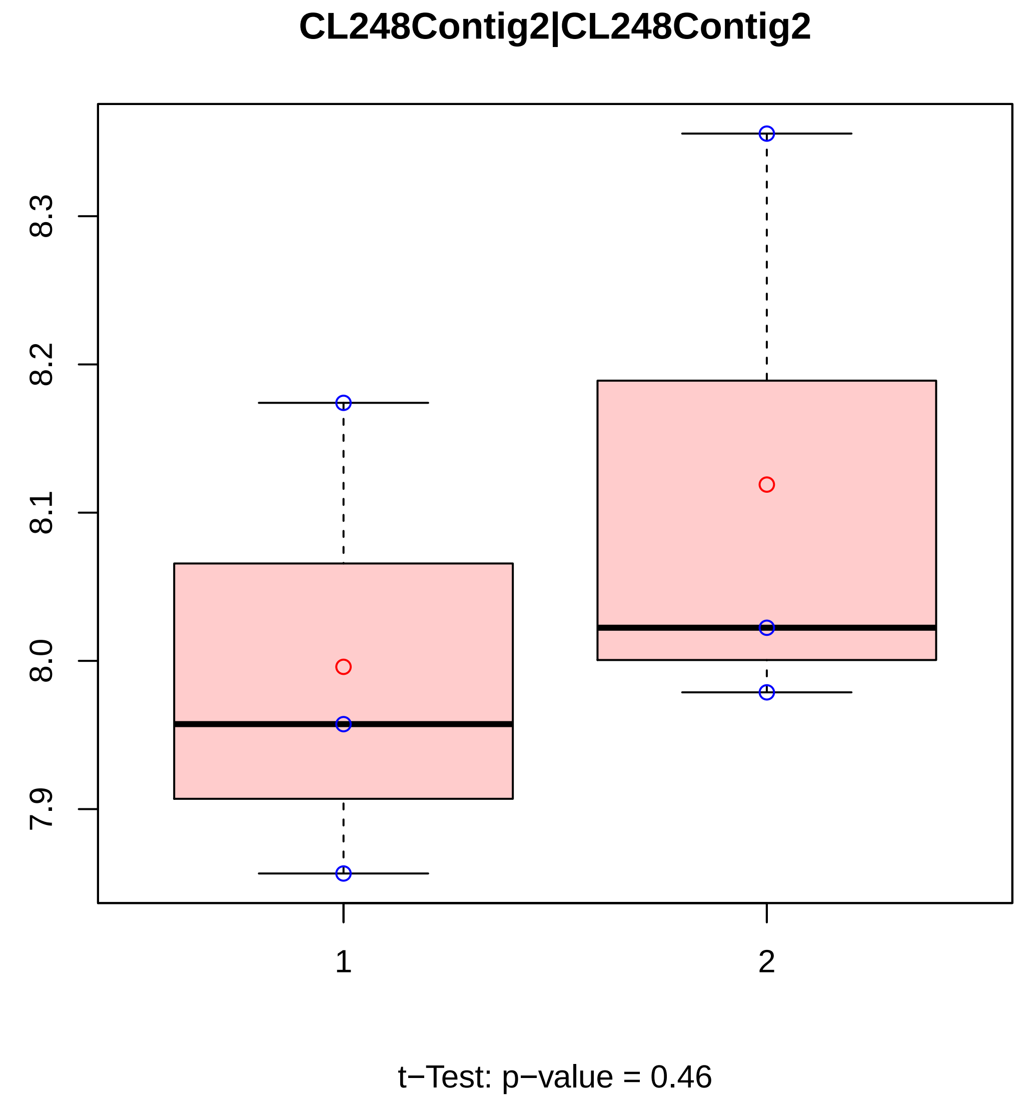
CL248Contig2|CL248Contig2
8.3
8.2
8.1
8.0
7.9
1
2
t−Test: p−value = 0.46

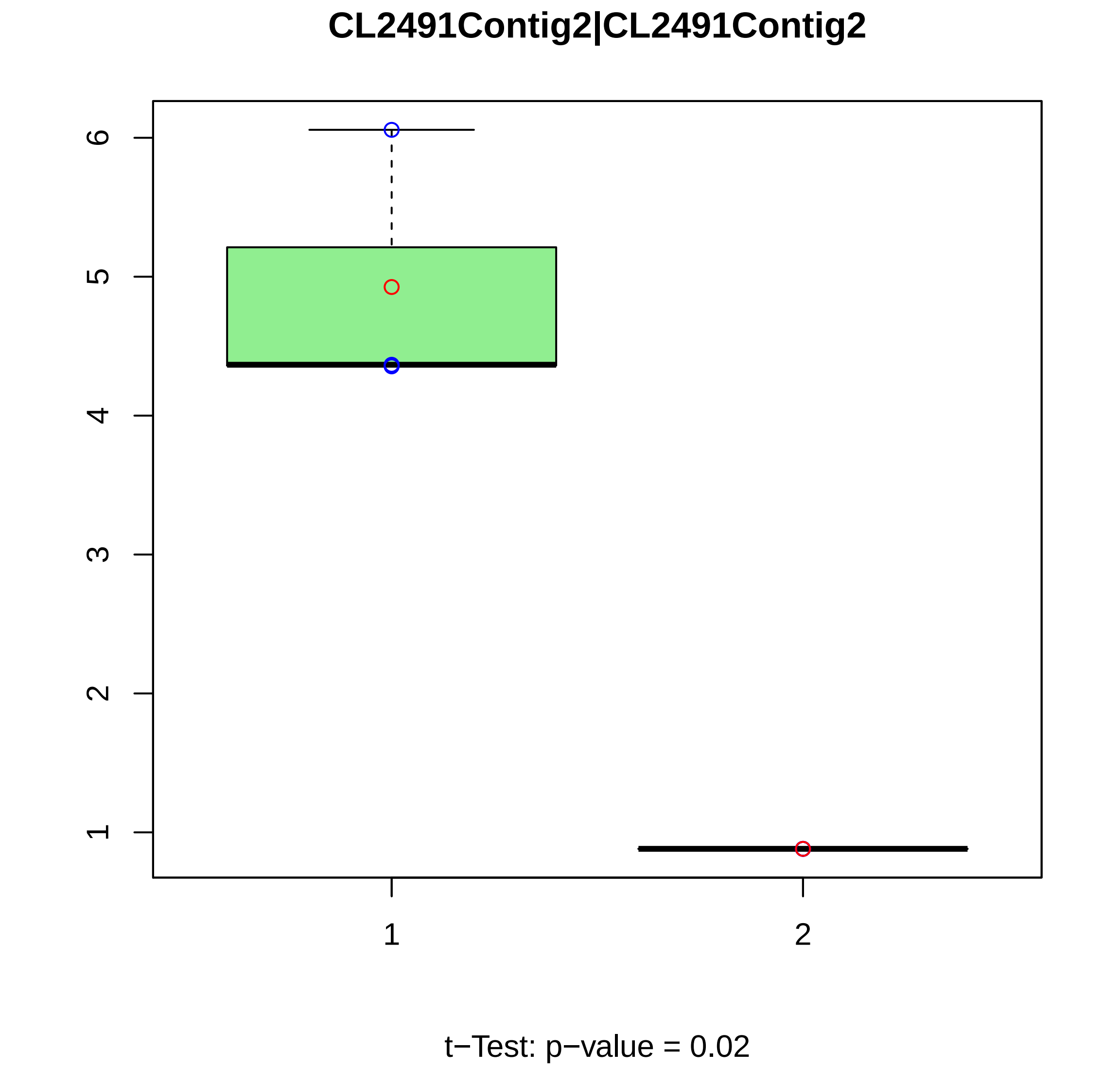
CL2491Contig2|CL2491Contig2
1
2
3
4
5
6
1
2
t−Test: p−value = 0.02

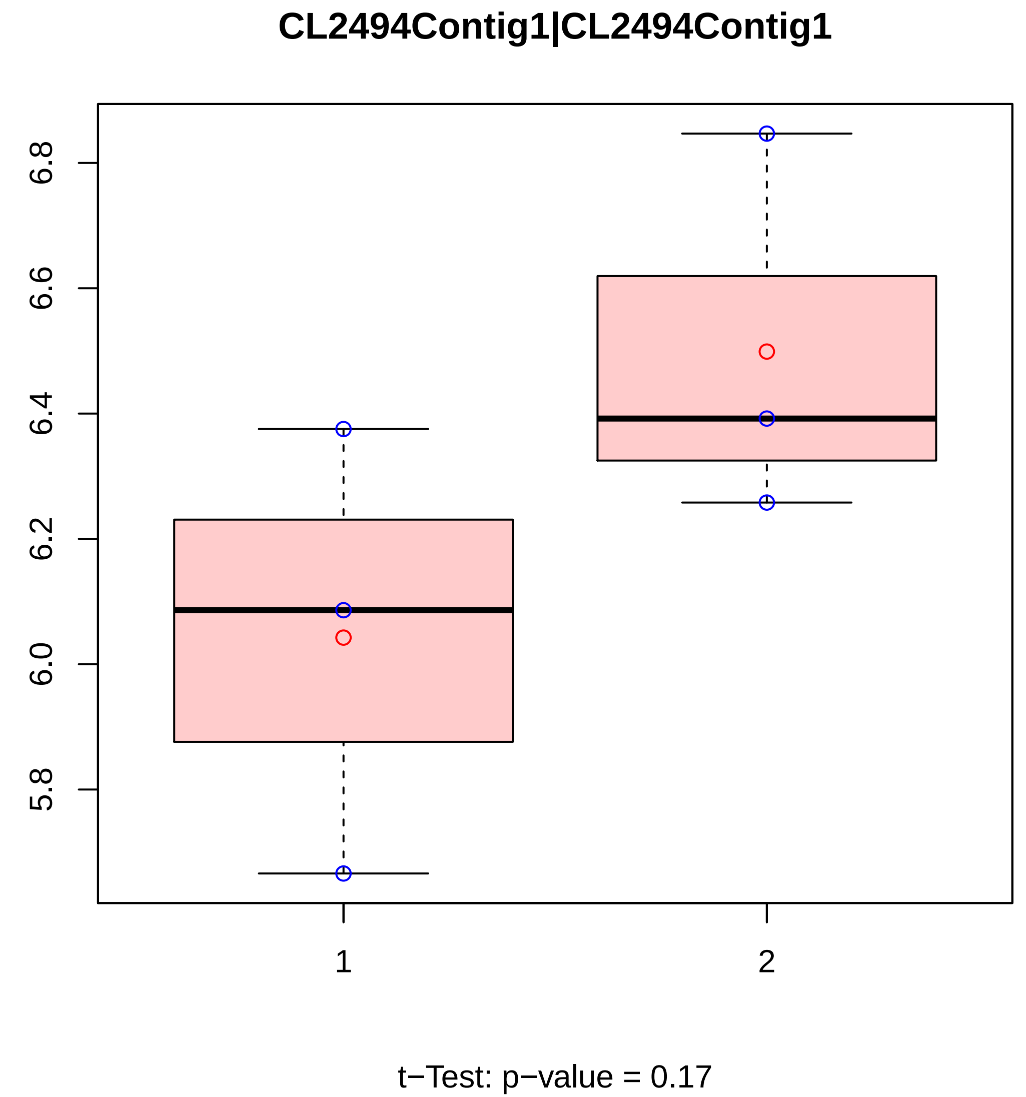

CL2494Contig1|CL2494Contig1
6.8
6.6
6.4
6.2
6.0
5.8
1
2
t−Test: p−value = 0.17

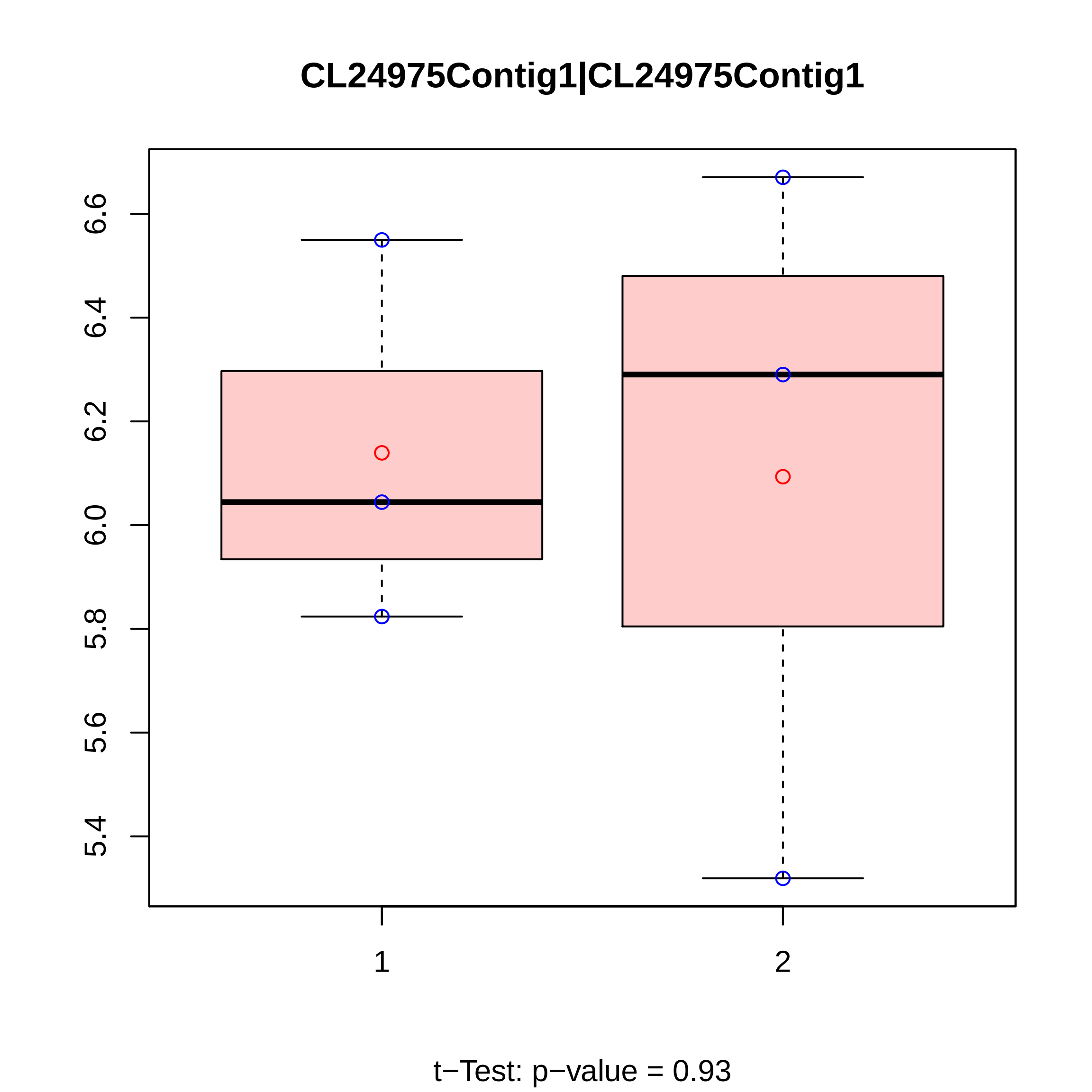
CL24975Contig1|CL24975Contig1
6.6
6.4
6.2
6.0
5.8
5.6
5.4
1
2
t−Test: p−value = 0.93

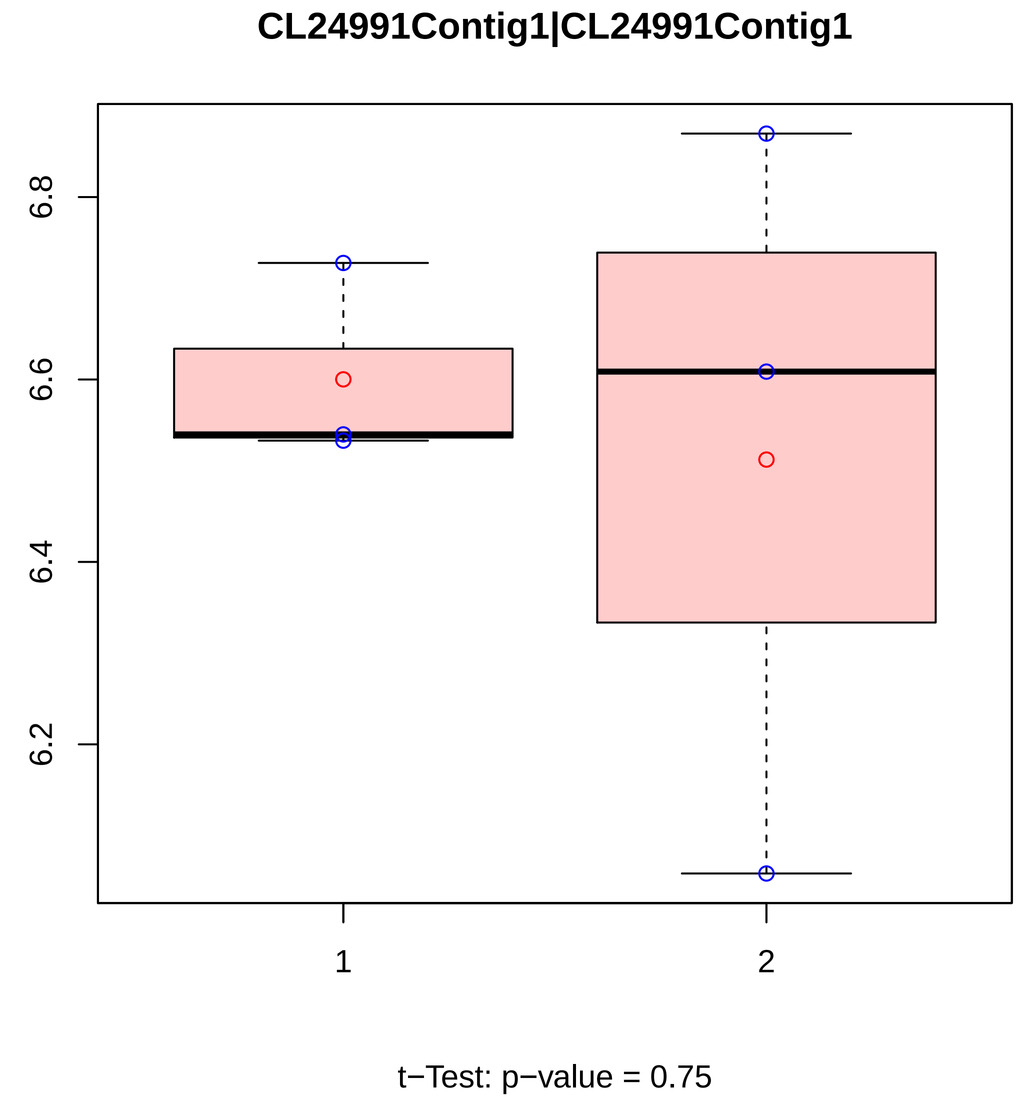
CL24991Contig1|CL24991Contig1
6.2
6.4
6.6
6.8
1
2
t−Test: p−value = 0.75

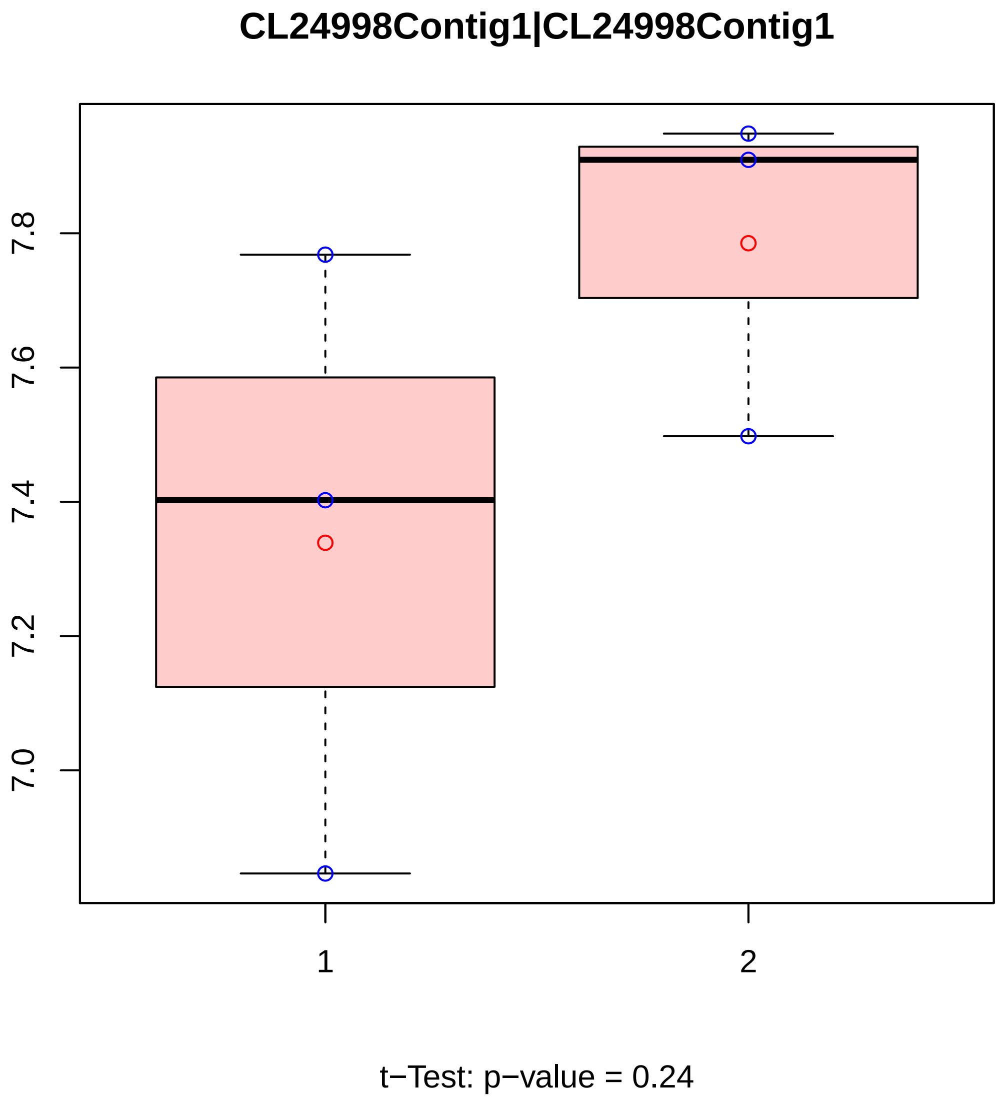
CL24998Contig1|CL24998Contig1
7.0
7.2
7.4
7.6
7.8
1
2
t−Test: p−value = 0.24

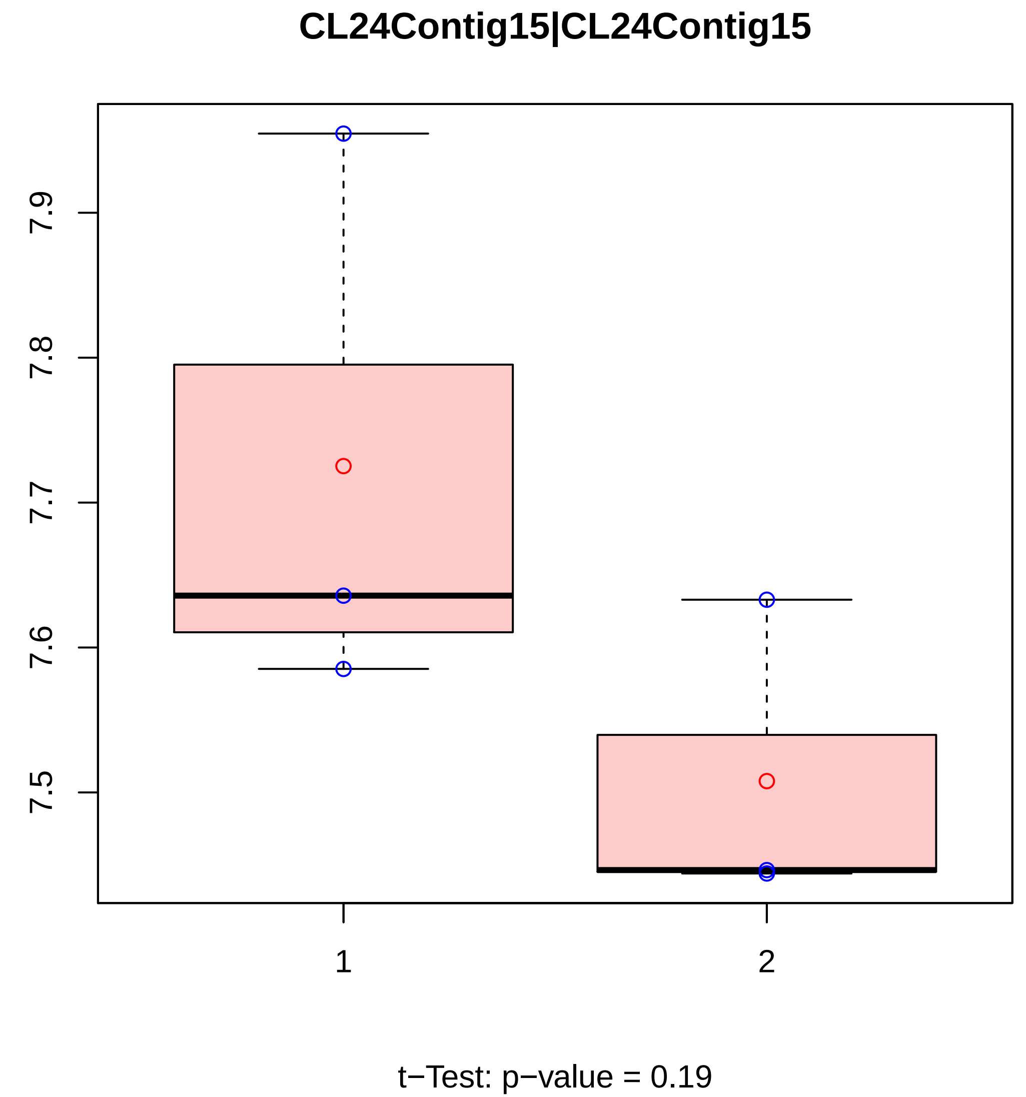

CL24Contig15|CL24Contig15
7.5
7.6
7.7
7.8
7.9
1
2
t−Test: p−value = 0.19

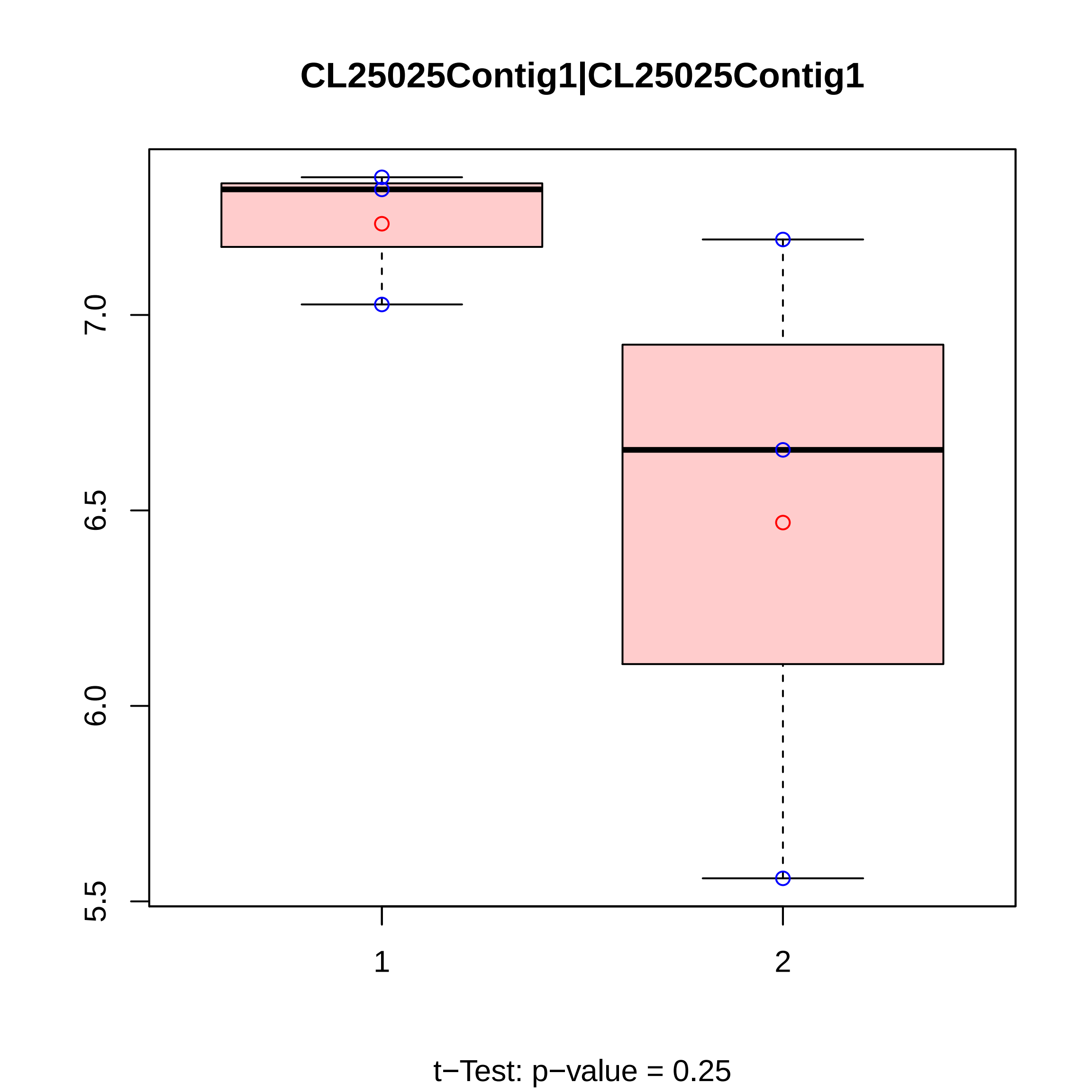

CL25025Contig1|CL25025Contig1
7.0
6.5
6.0
5.5
1
2
t−Test: p−value = 0.25

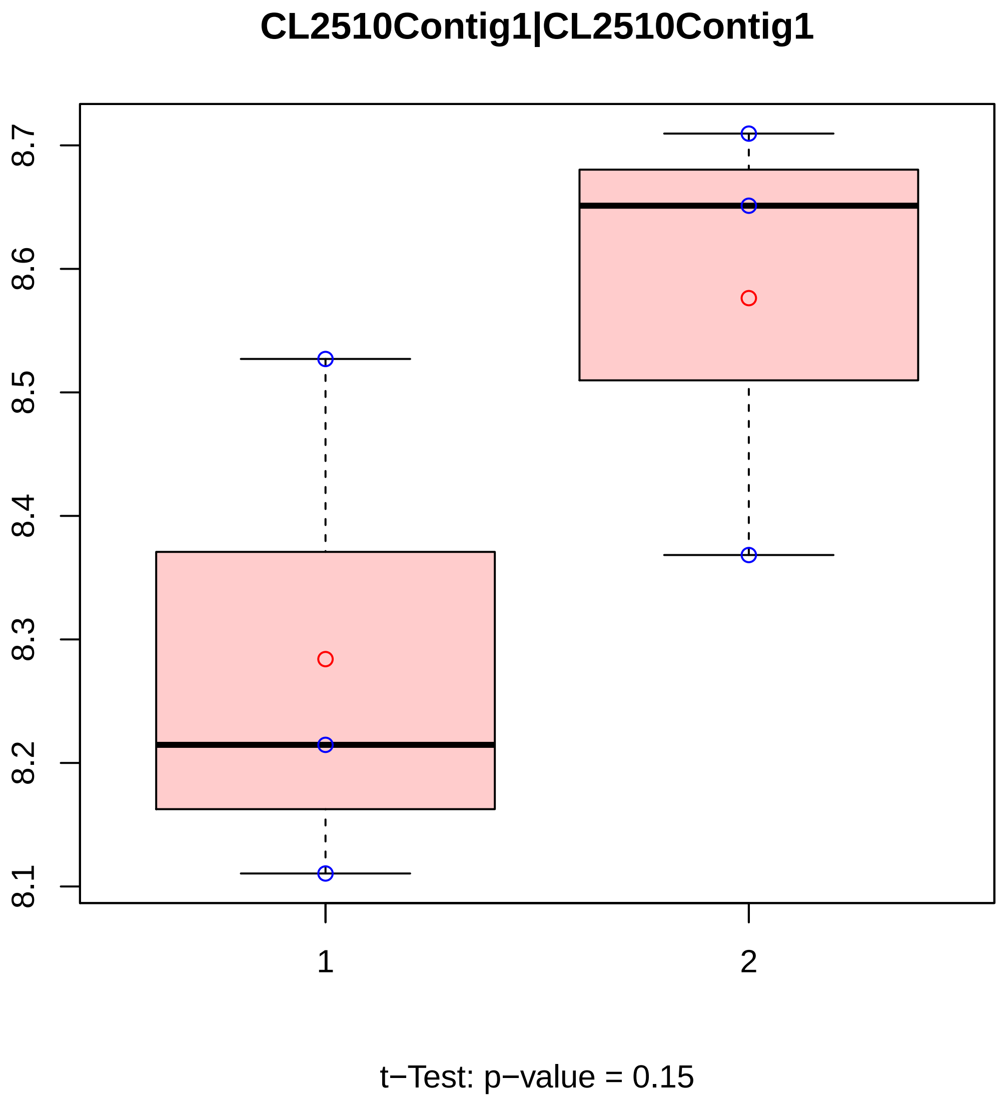

CL2510Contig1|CL2510Contig1
8.1
8.2
8.3
8.4
8.5
8.6
8.7
1
2
t−Test: p−value = 0.15

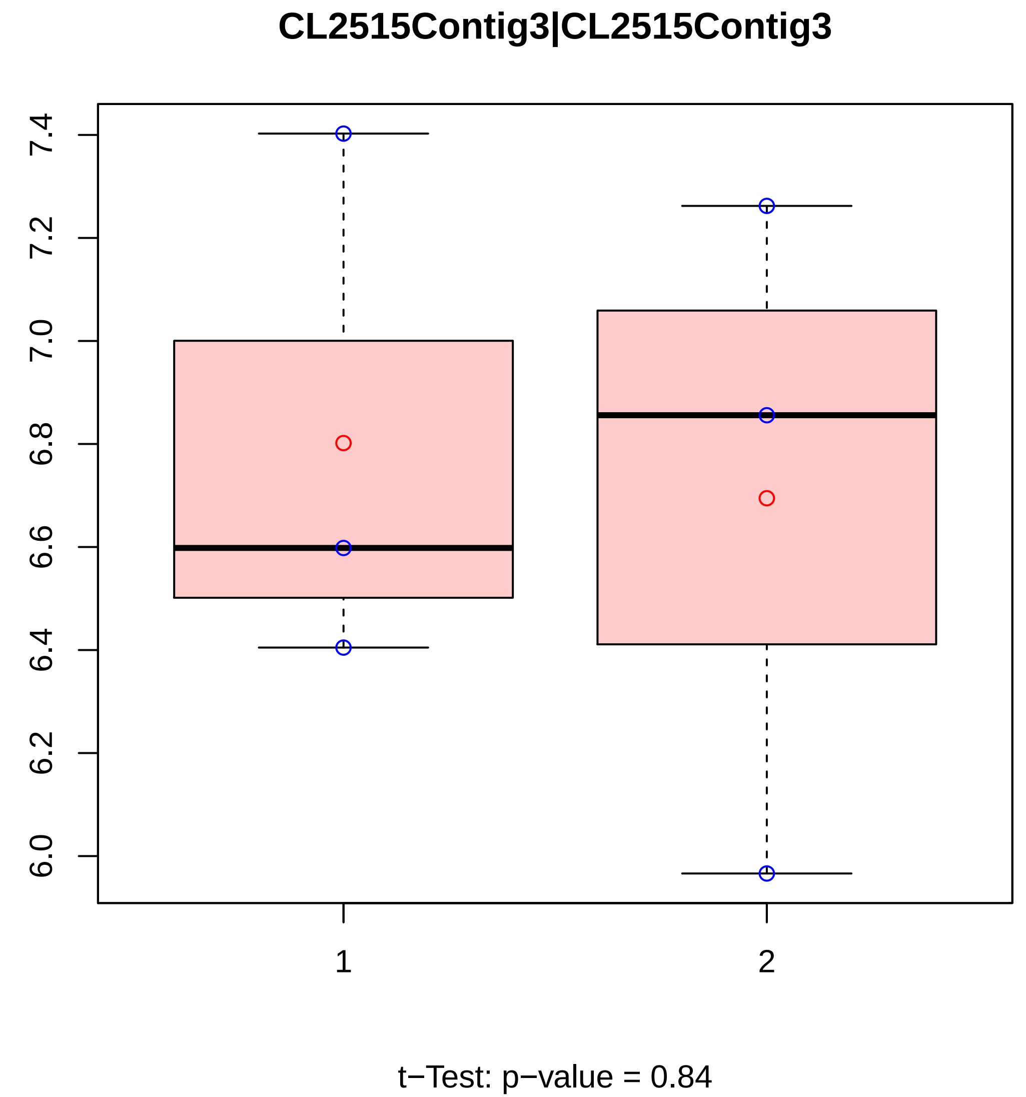
CL2515Contig3|CL2515Contig3
7.4
7.2
7.0
6.8
6.6
6.4
6.2
6.0
1
2
t−Test: p−value = 0.84

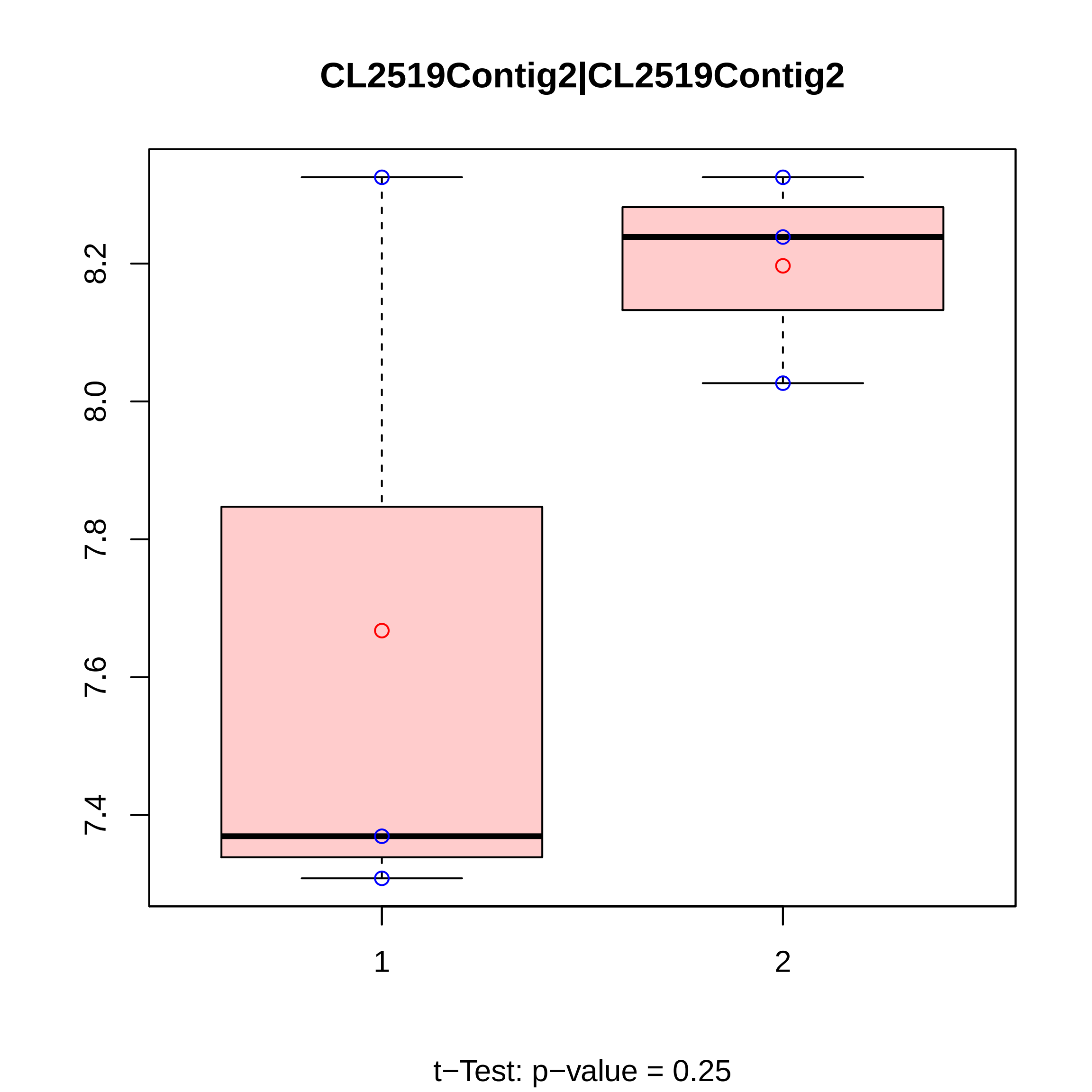
CL2519Contig2|CL2519Contig2
8.2
8.0
7.8
7.6
7.4
1
2
t−Test: p−value = 0.25

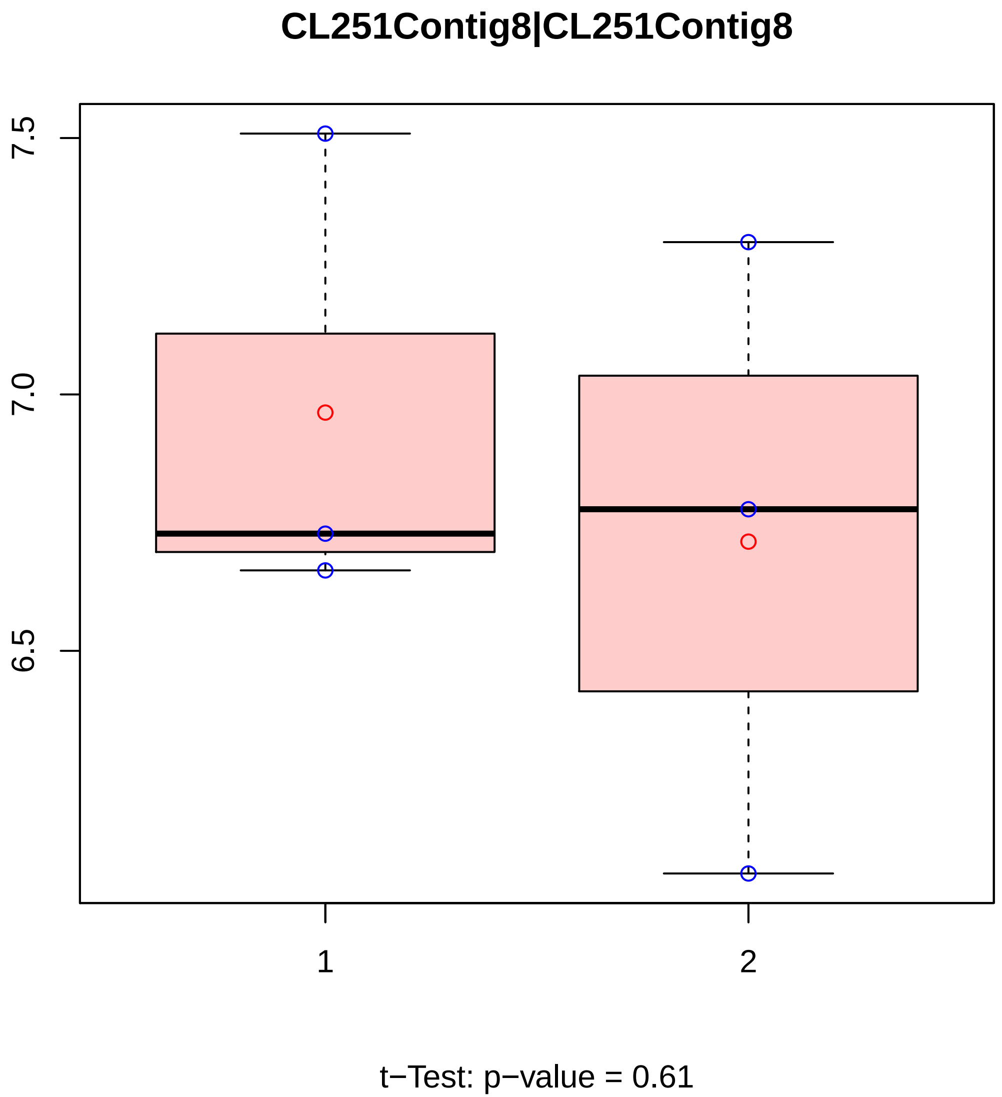

CL251Contig8|CL251Contig8
7.5
7.0
6.5
1
2
t−Test: p−value = 0.61

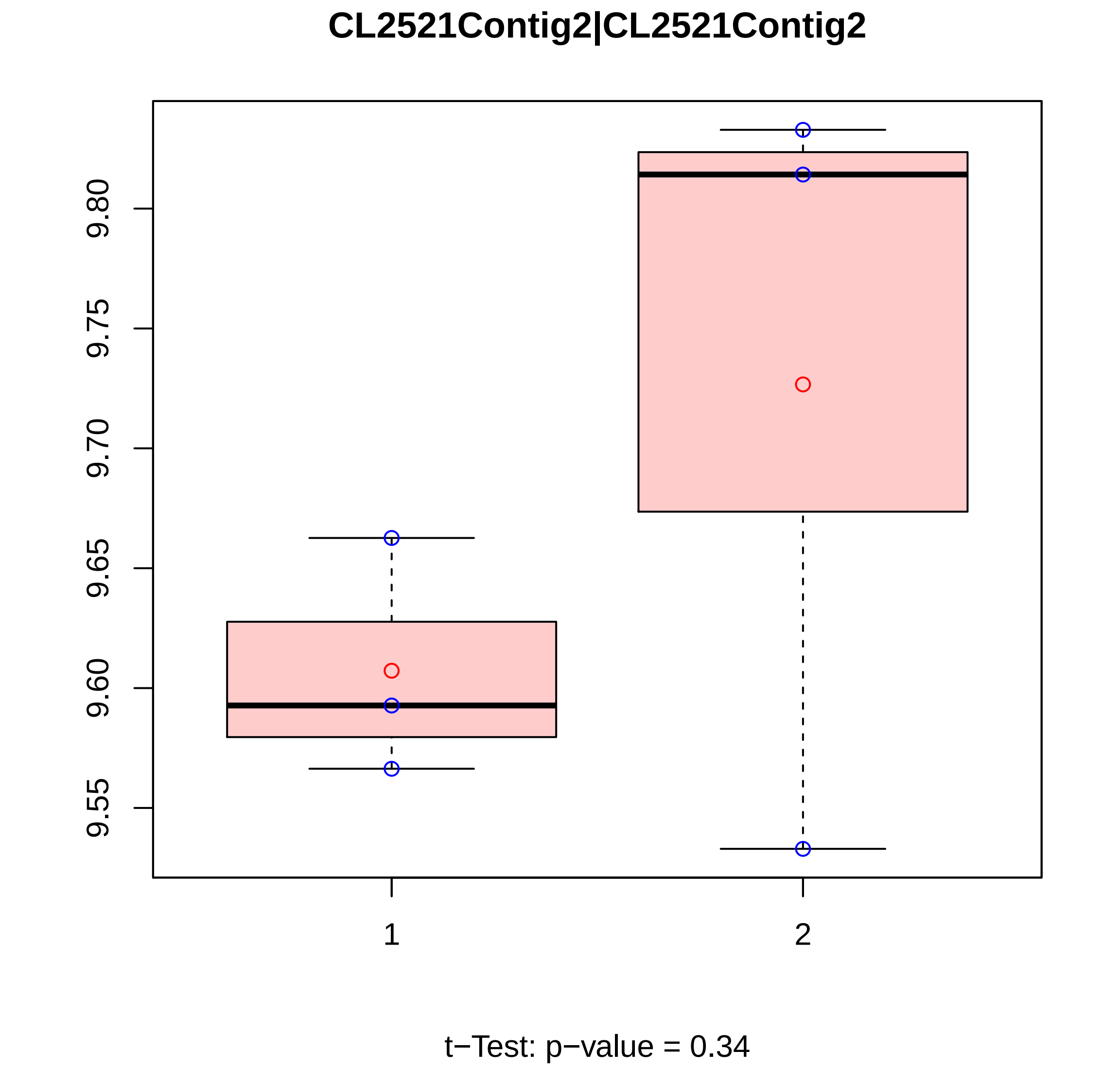

CL2521Contig2|CL2521Contig2
9.55
9.60
9.65
9.70
9.75
9.80
1
2
t−Test: p−value = 0.34

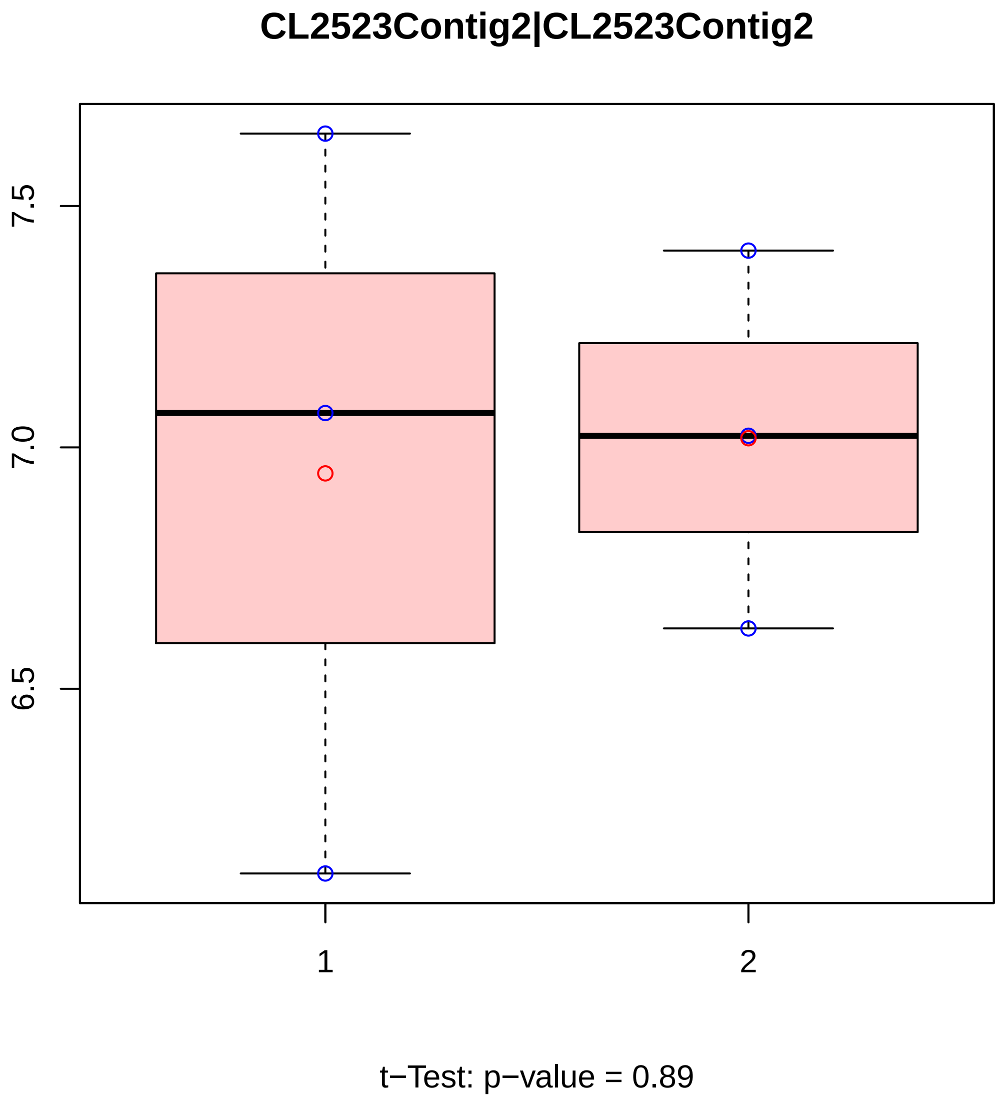
CL2523Contig2|CL2523Contig2
7.5
7.0
6.5
1
2
t−Test: p−value = 0.89

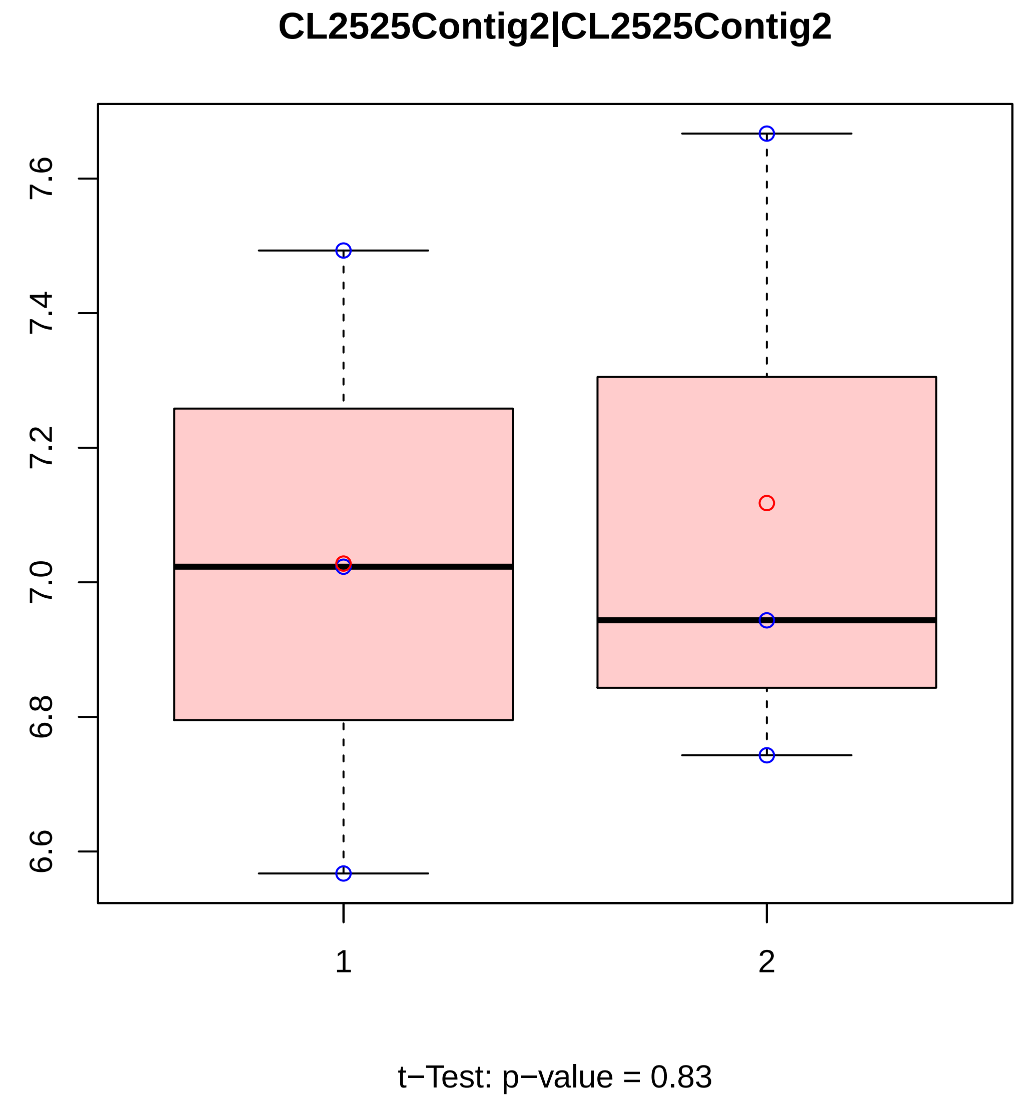
CL2525Contig2|CL2525Contig2
7.6
7.4
7.2
7.0
6.8
6.6
1
2
t−Test: p−value = 0.83

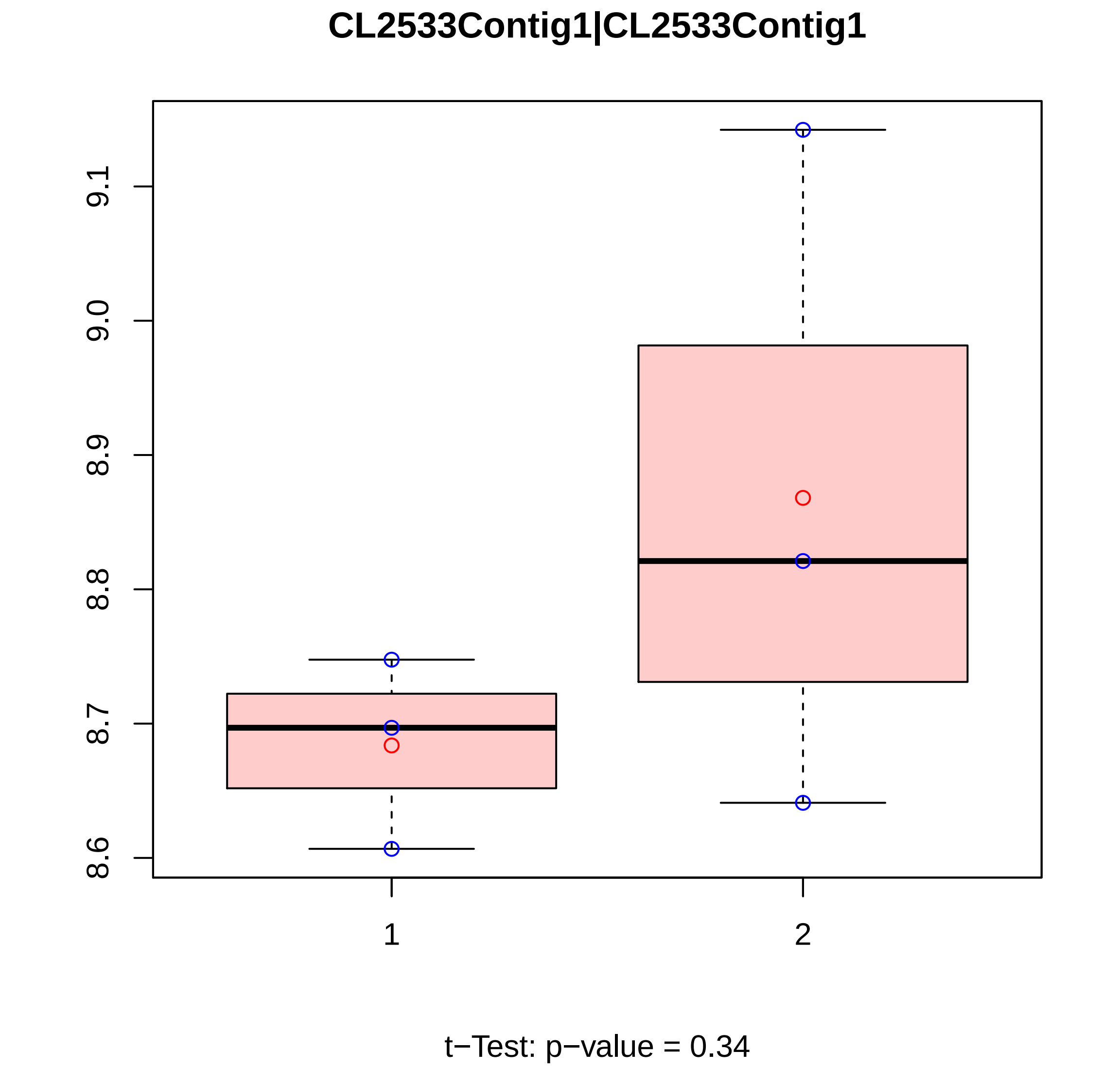

CL2533Contig1|CL2533Contig1
9.1
9.0
8.9
8.8
8.7
8.6
1
2
t−Test: p−value = 0.34

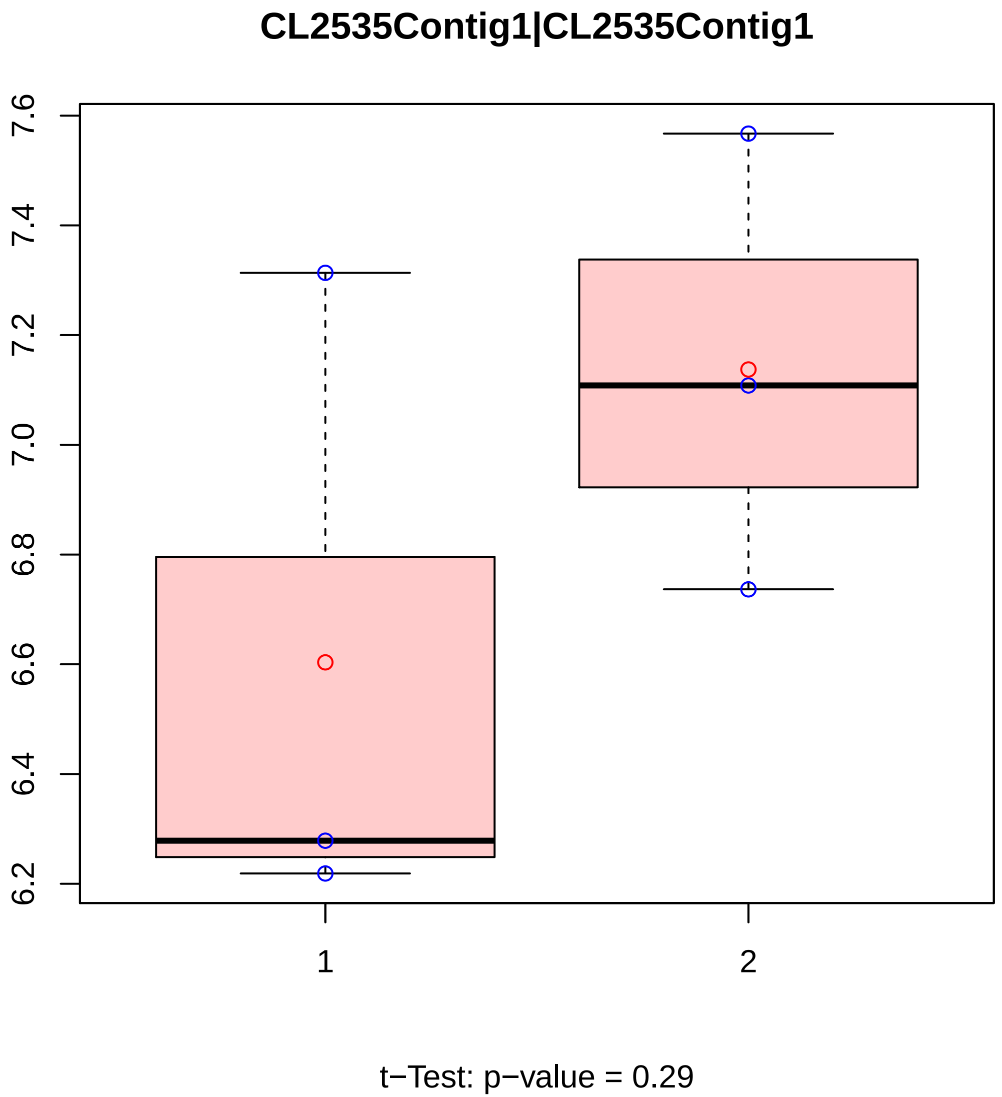
CL2535Contig1|CL2535Contig1
6.2
6.4
6.6
6.8
7.0
7.2
7.4
7.6
1
2
t−Test: p−value = 0.29

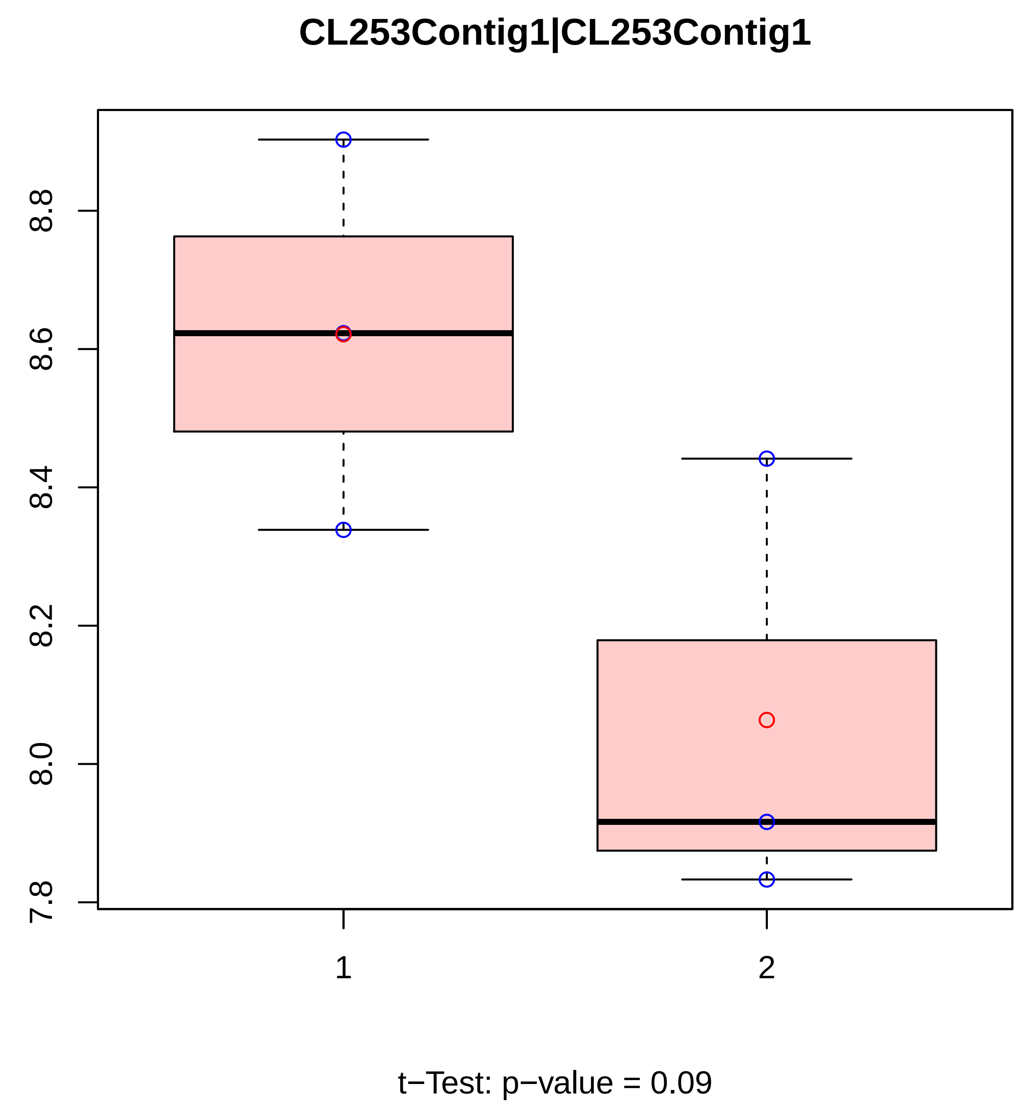
CL253Contig1|CL253Contig1
8.8
8.6
8.4
8.2
8.0
7.8
1
2
t−Test: p−value = 0.09

## CL253Contig5|CL253Contig5

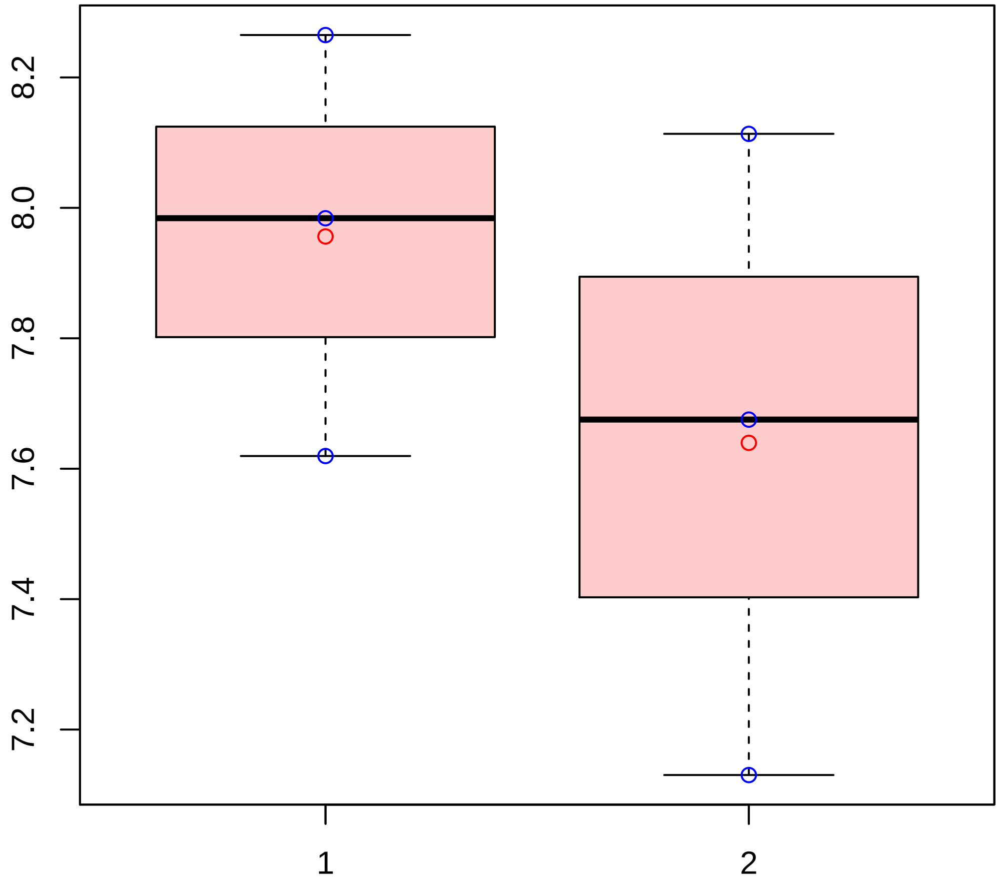


t−Test: p−value = 0.41

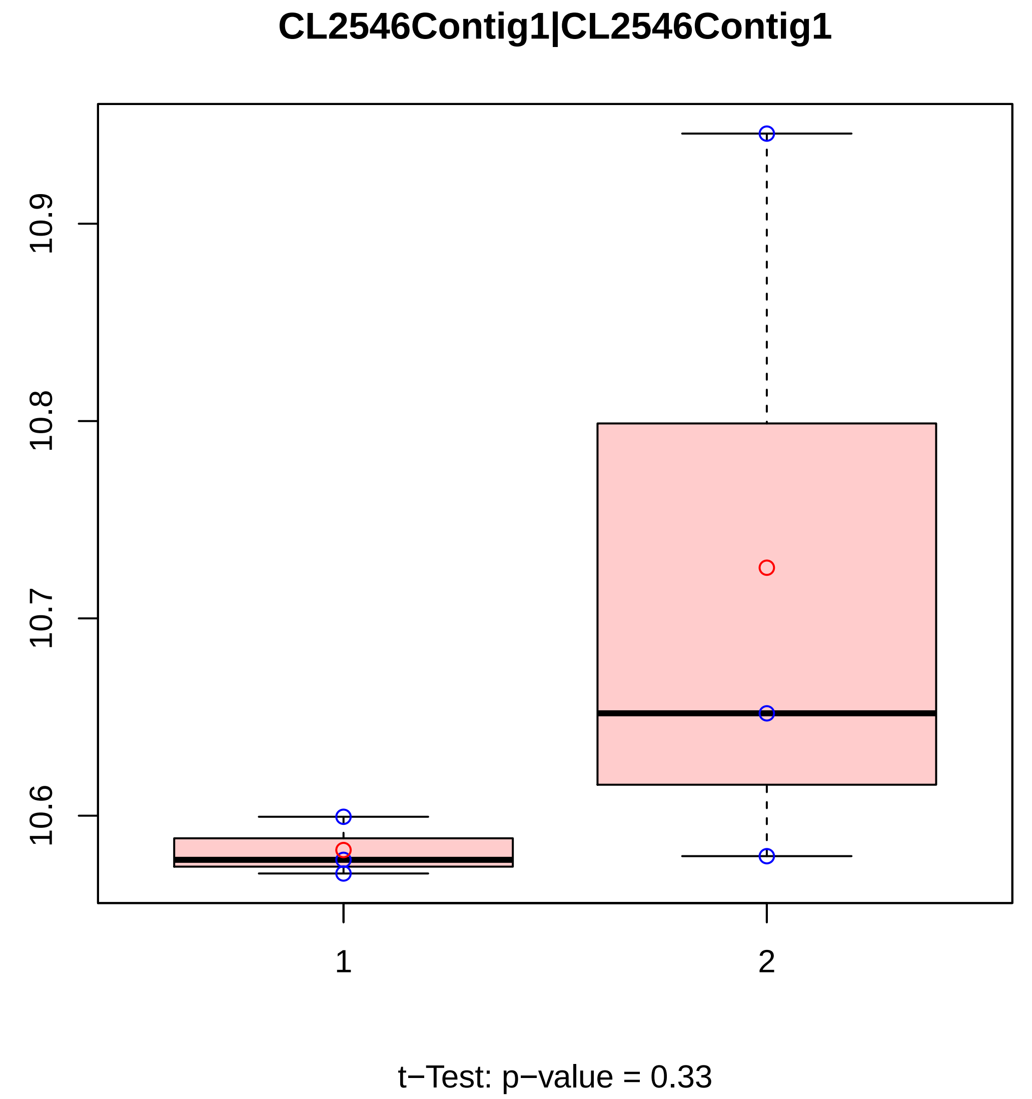
CL2546Contig1|CL2546Contig1
10.9
10.8
10.7
10.6
1
2
t−Test: p−value = 0.33

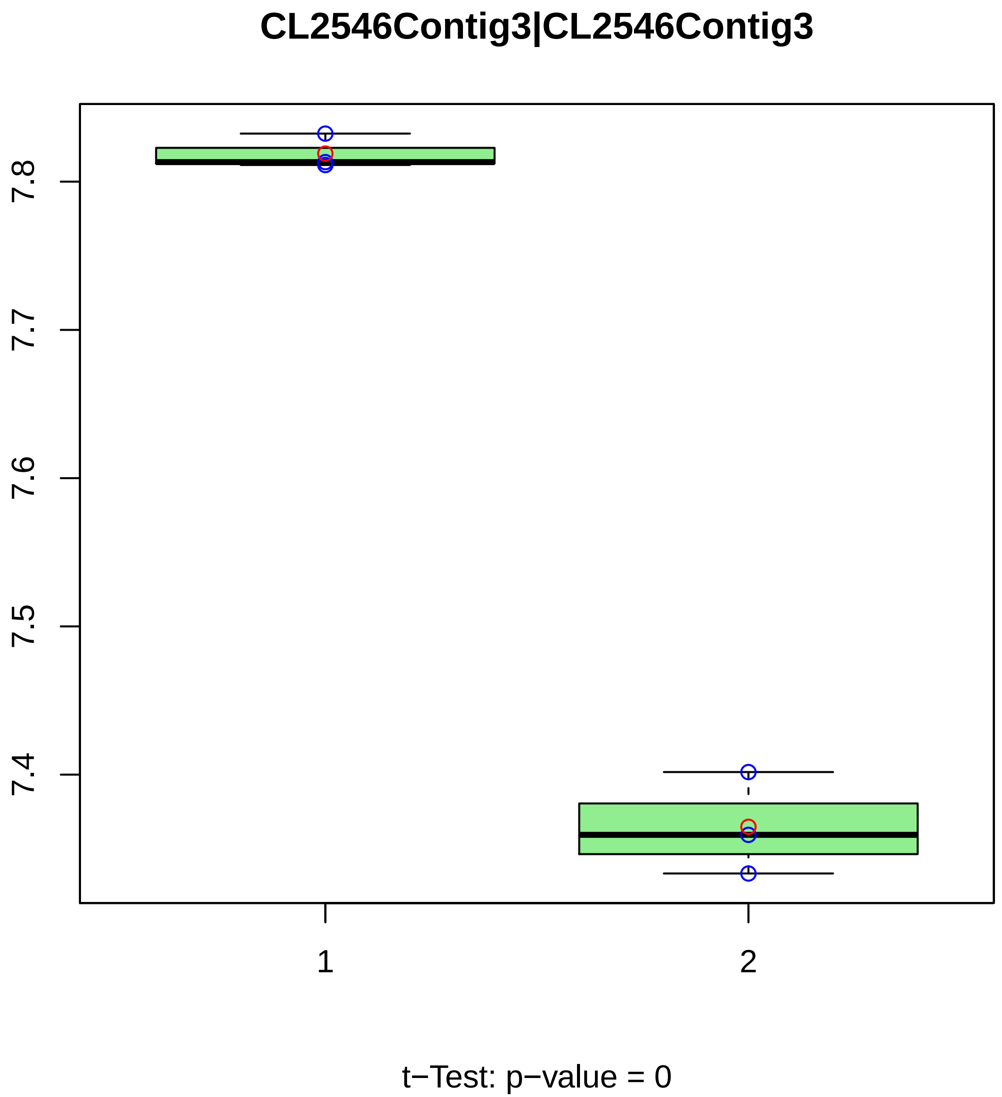
CL2546Contig3|CL2546Contig3
7.8
7.7
7.6
7.5
7.4
1
2
t−Test: p−value = 0

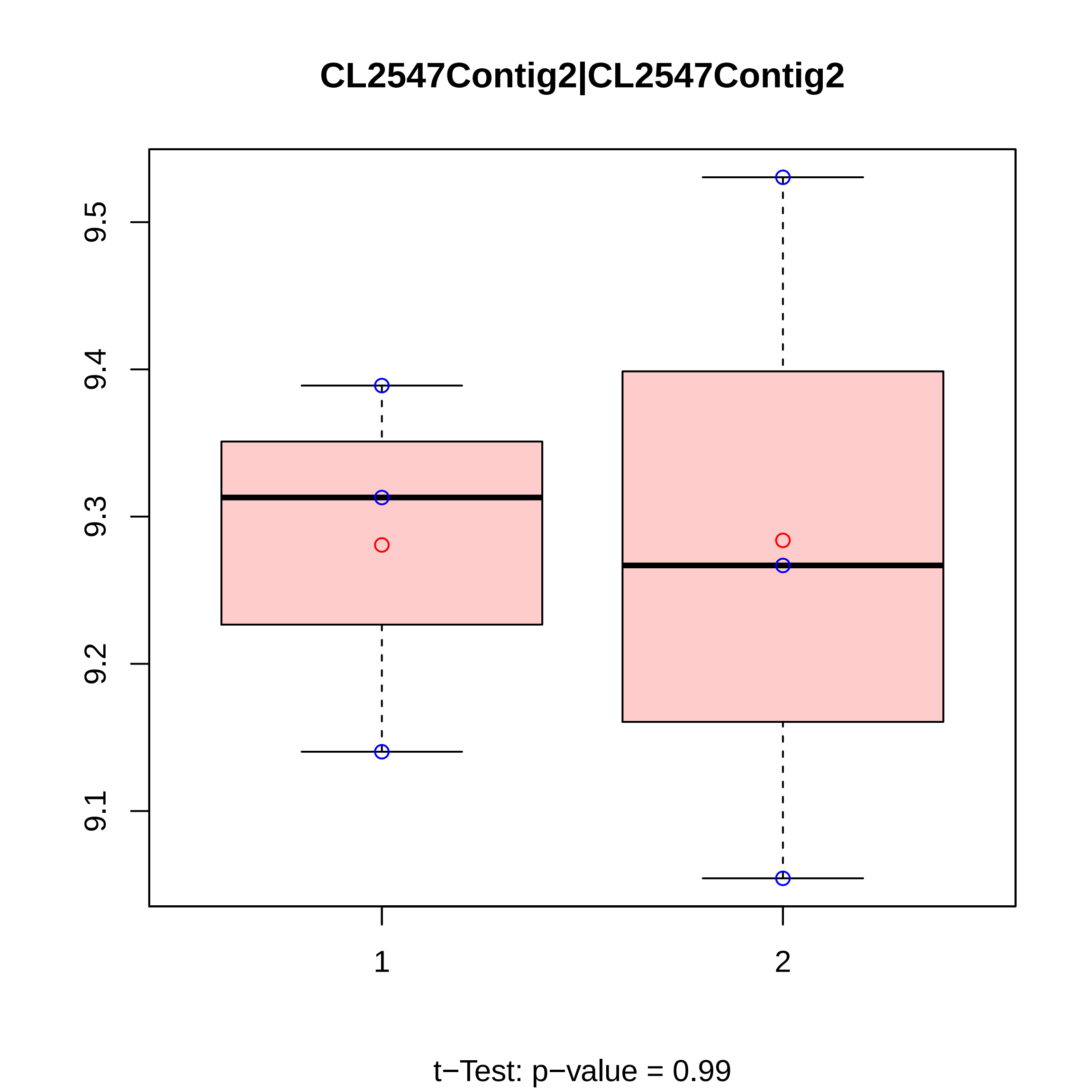

CL2547Contig2|CL2547Contig2
9.1
9.2
9.3
9.4
9.5
1
2
t−Test: p−value = 0.99

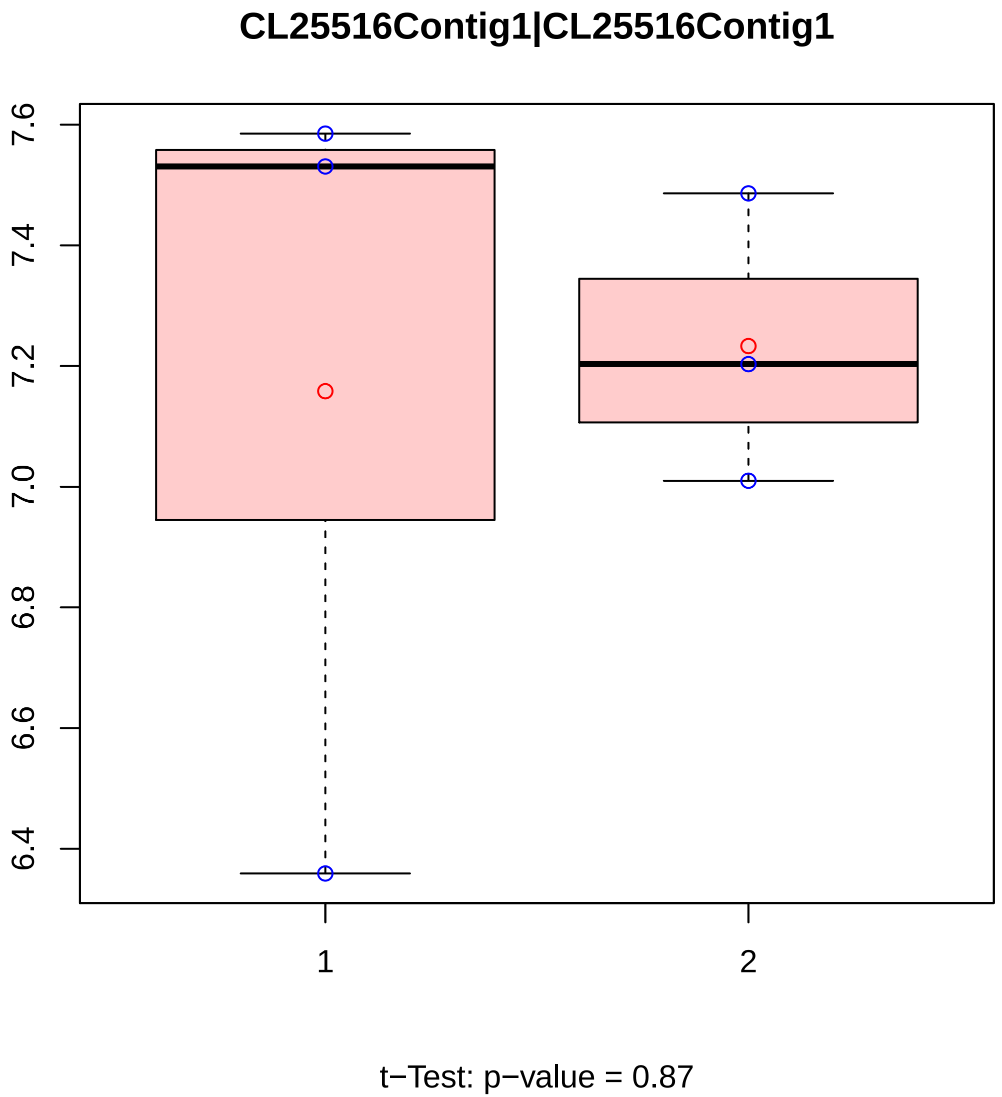
CL25516Contig1|CL25516Contig1
7.6
7.4
7.2
7.0
6.8
6.6
6.4
1
2
t−Test: p−value = 0.87

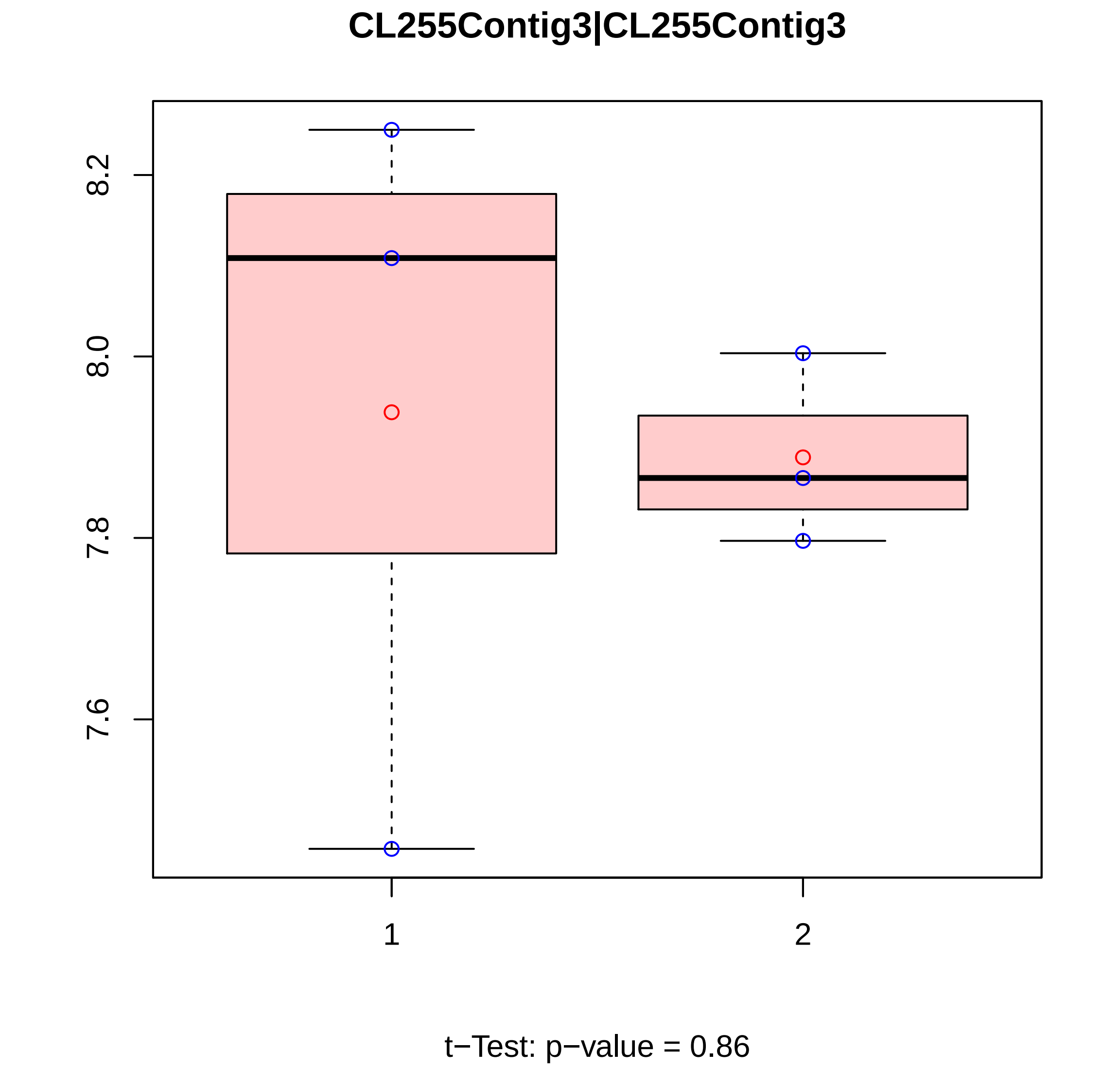
CL255Contig3|CL255Contig3
8.2
8.0
7.8
7.6
1
2
t−Test: p−value = 0.86

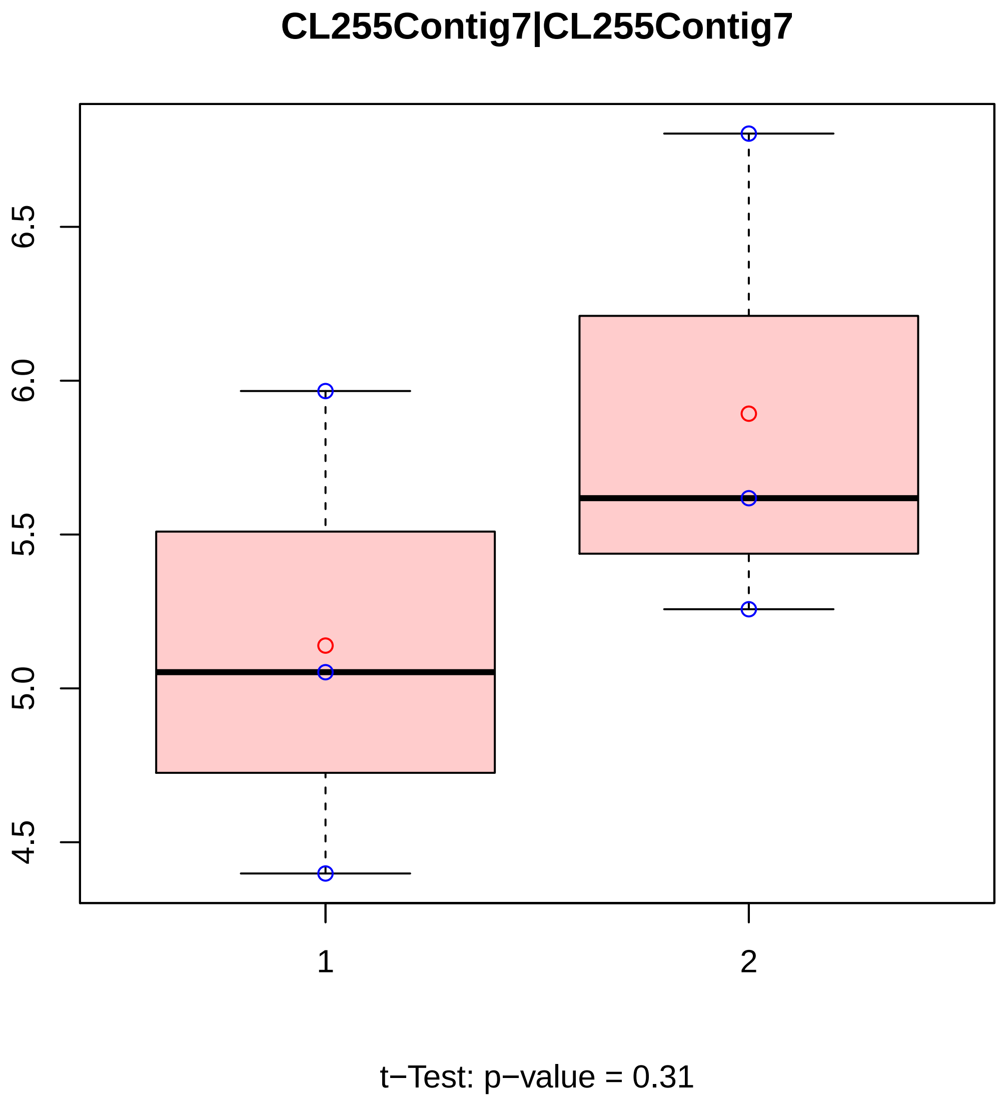
CL255Contig7|CL255Contig7
6.5
6.0
5.5
5.0
4.5
1
2
t−Test: p−value = 0.31

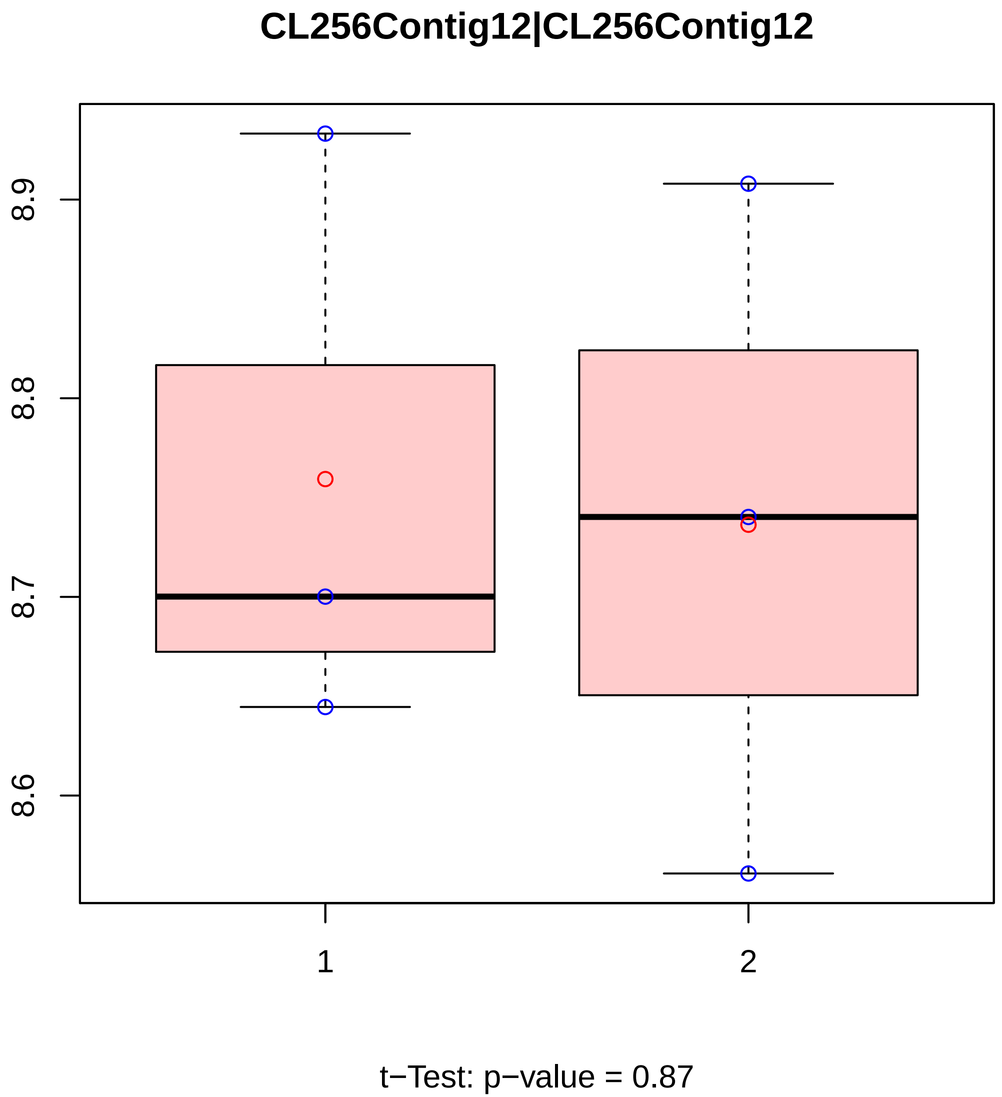

CL256Contig12|CL256Contig12
8.6
8.7
8.8
8.9
1
2
t−Test: p−value = 0.87

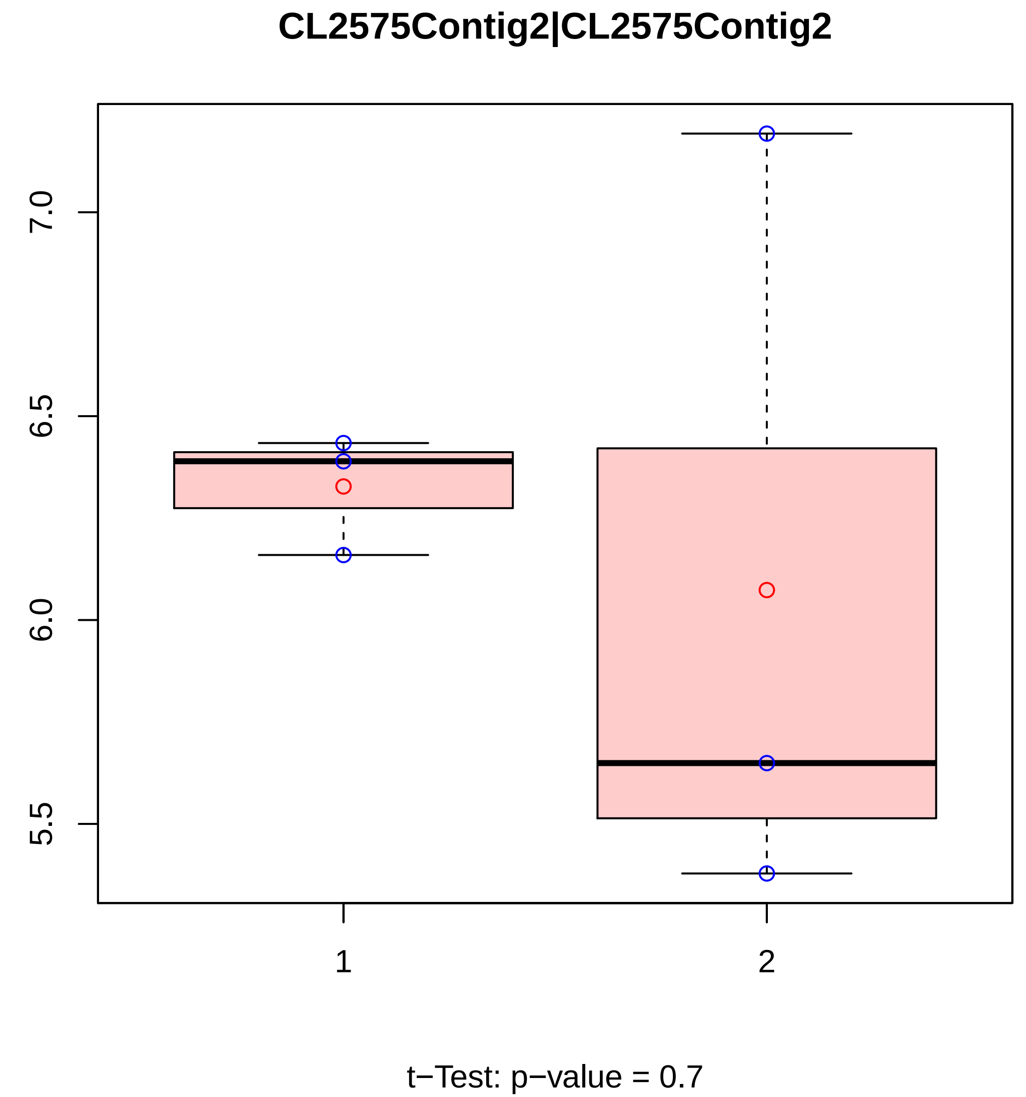
CL2575Contig2|CL2575Contig2
7.0
6.5
6.0
5.5
1
2
t−Test: p−value = 0.7

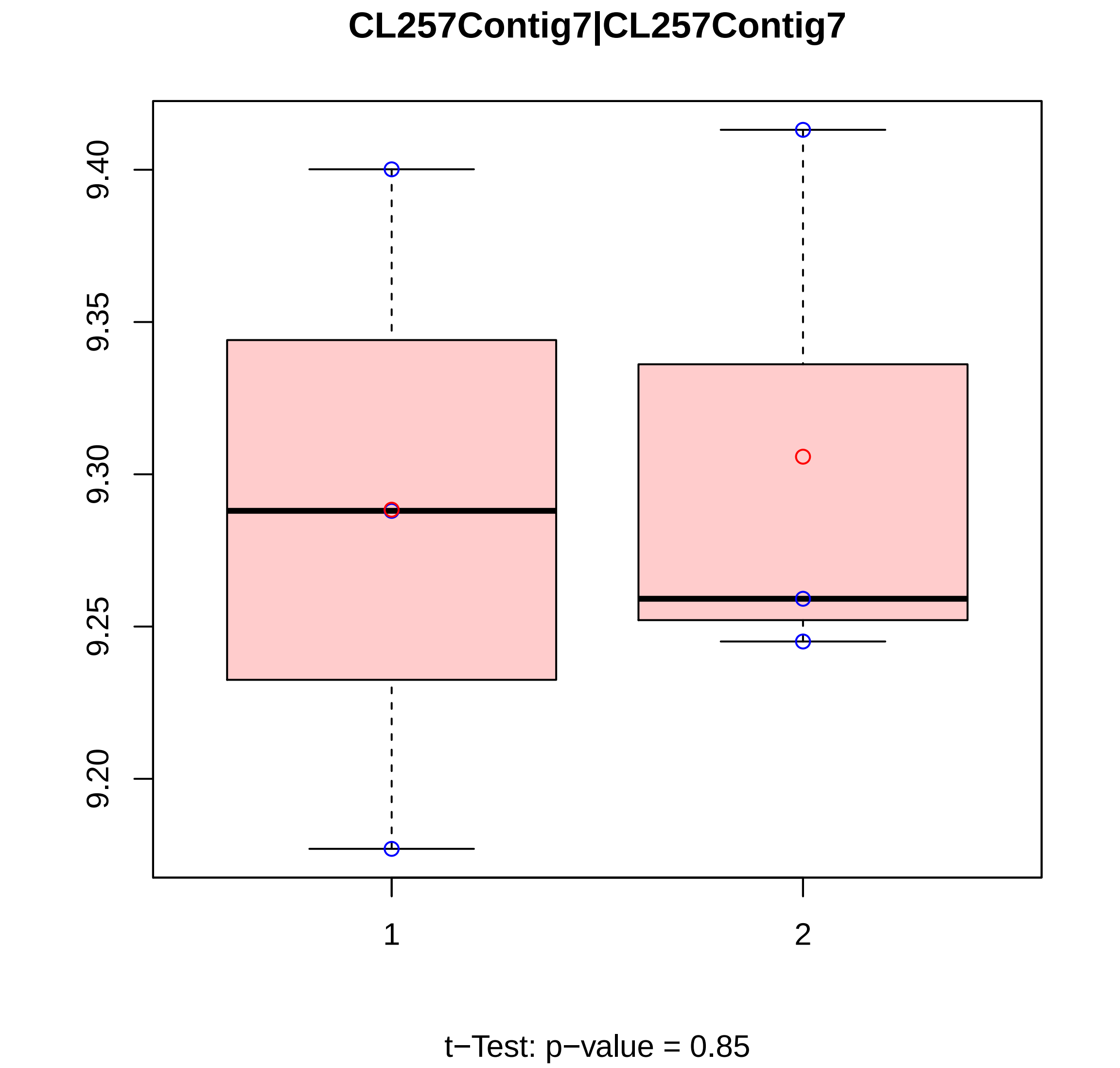
CL257Contig7|CL257Contig7
9.20
9.25
9.30
9.35
9.40
1
2
t−Test: p−value = 0.85

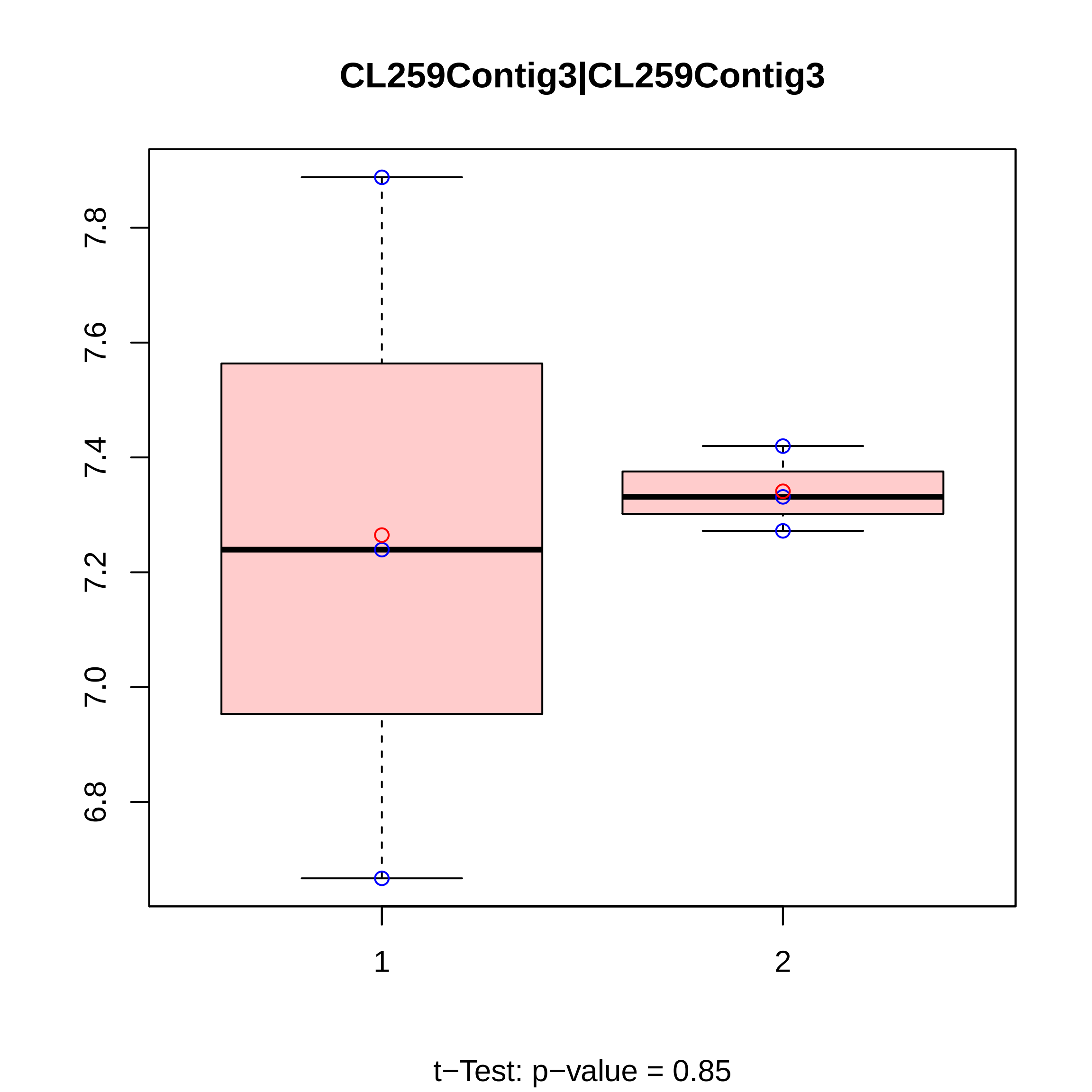
CL259Contig3|CL259Contig3
7.8
7.6
7.4
7.2
7.0
6.8
1
2
t−Test: p−value = 0.85

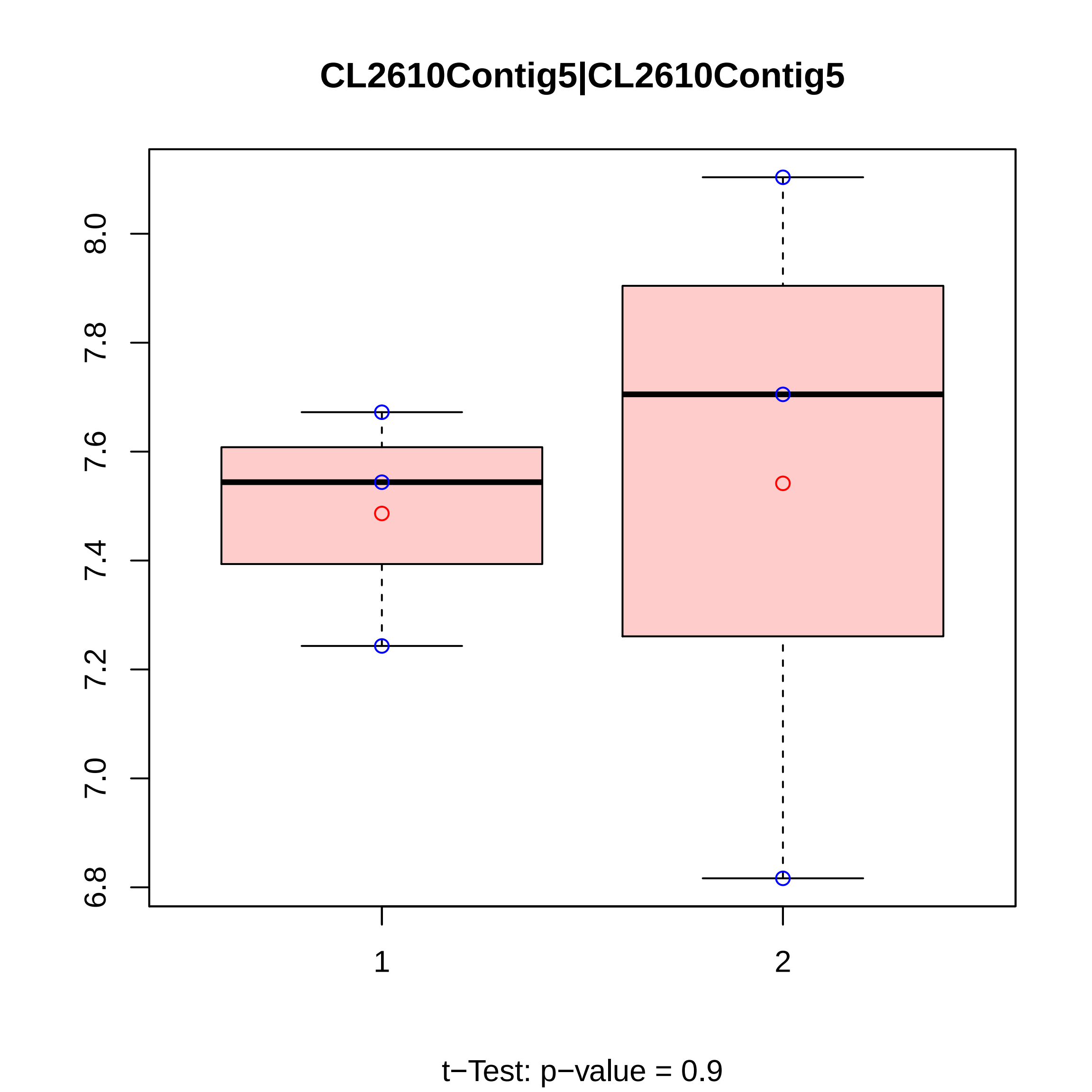
CL2610Contig5|CL2610Contig5
8.0
7.8
7.6
7.4
7.2
7.0
6.8
1
2
t−Test: p−value = 0.9

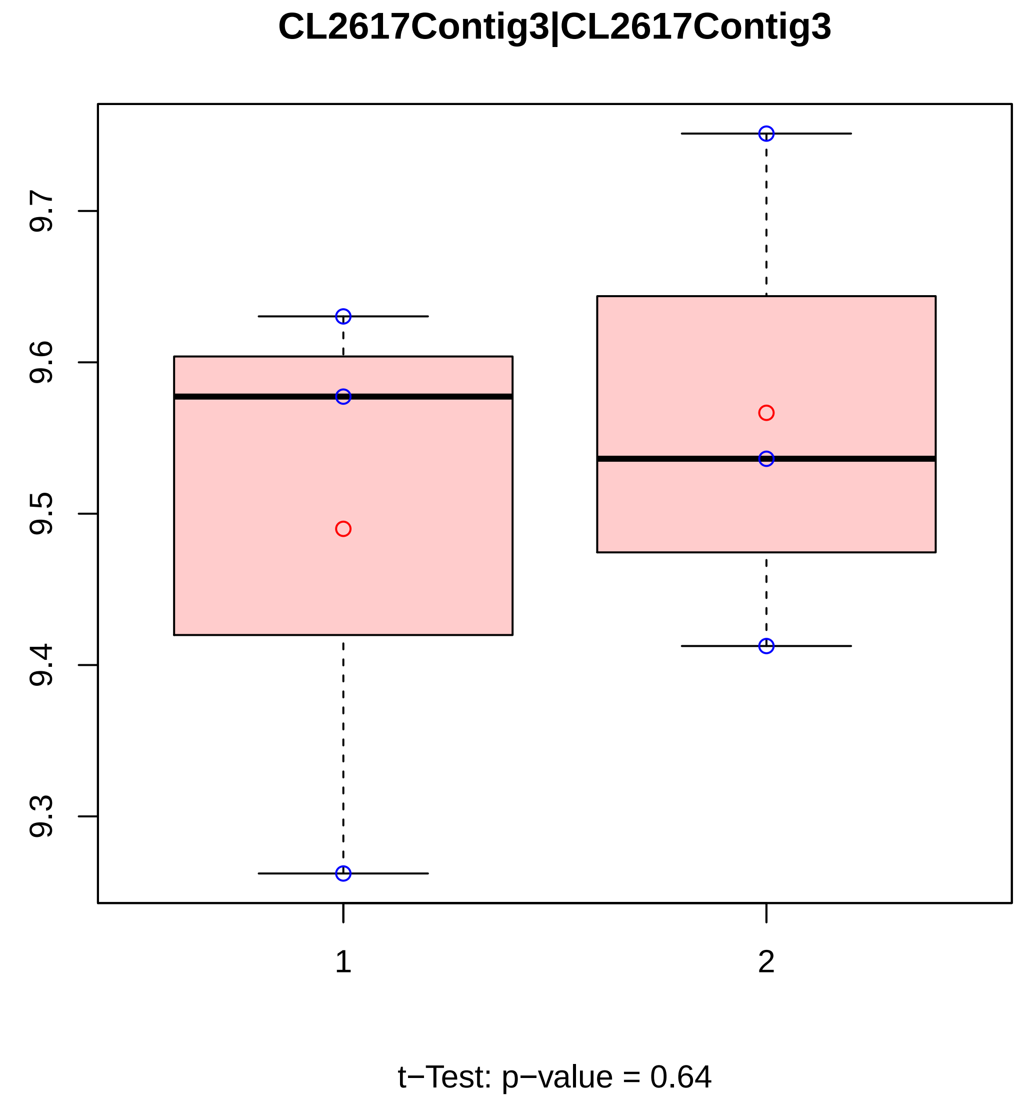
CL2617Contig3|CL2617Contig3
9.3
9.4
9.5
9.6
9.7
1
2
t−Test: p−value = 0.64

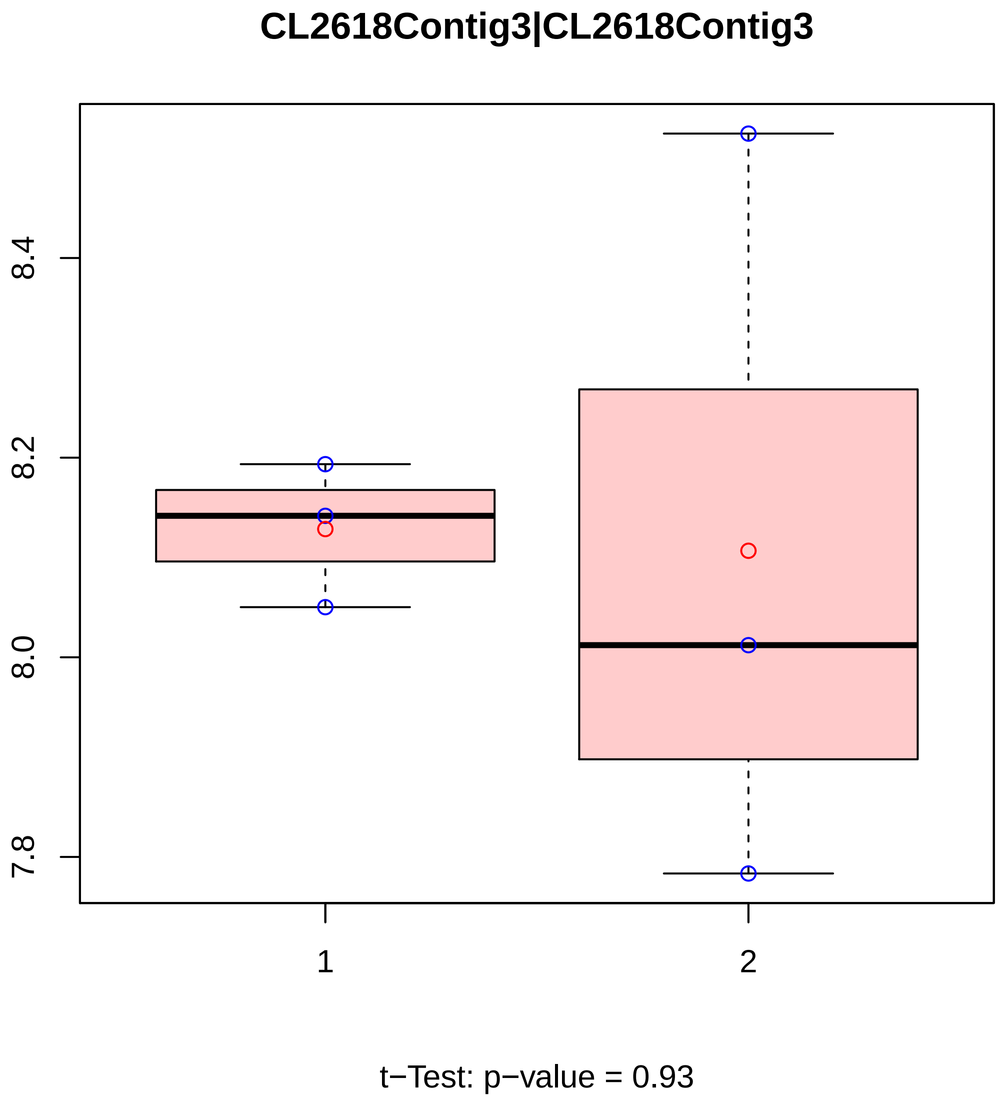
CL2618Contig3|CL2618Contig3
8.4
8.2
8.0
7.8
1
2
t−Test: p−value = 0.93

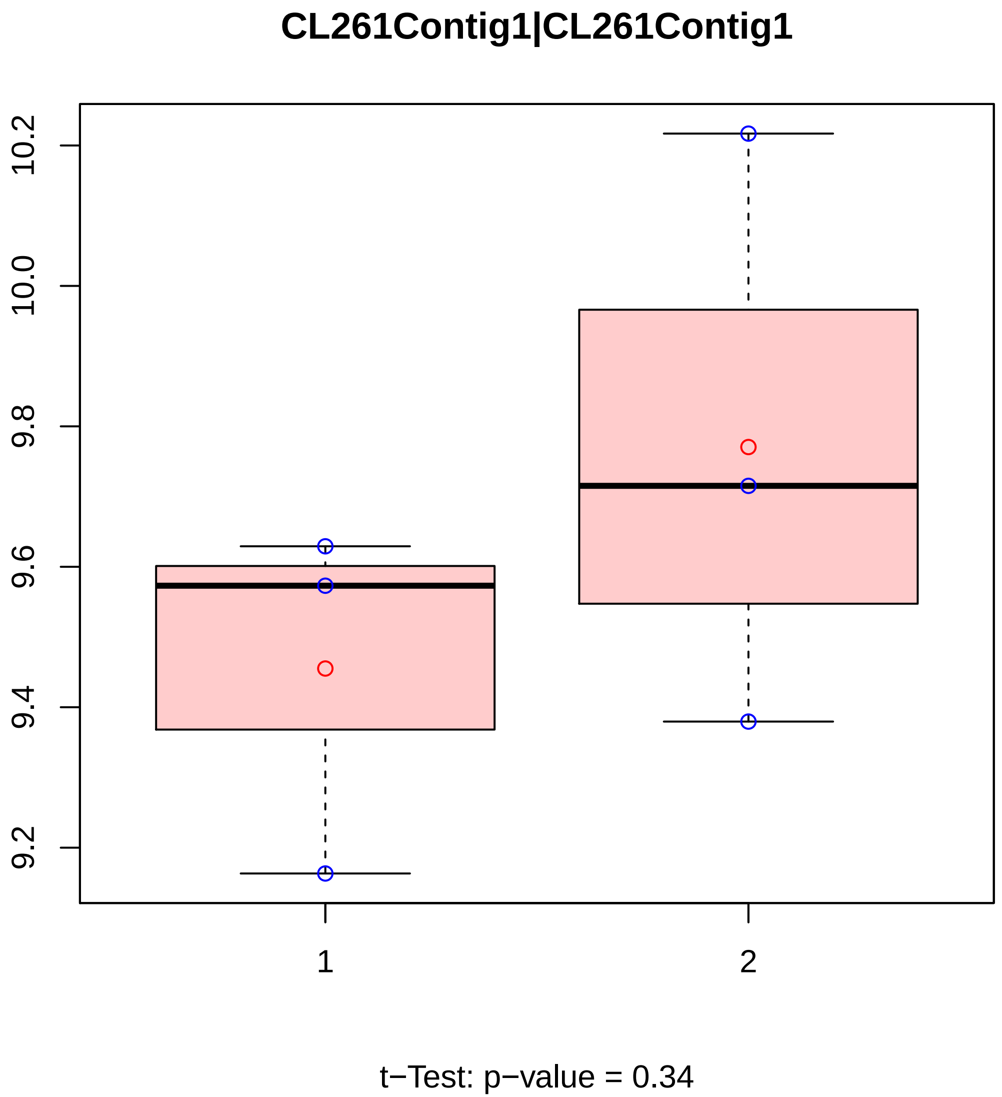

CL261Contig1|CL261Contig1
10.2
10.0
9.8
9.6
9.4
9.2
1
2
t−Test: p−value = 0.34

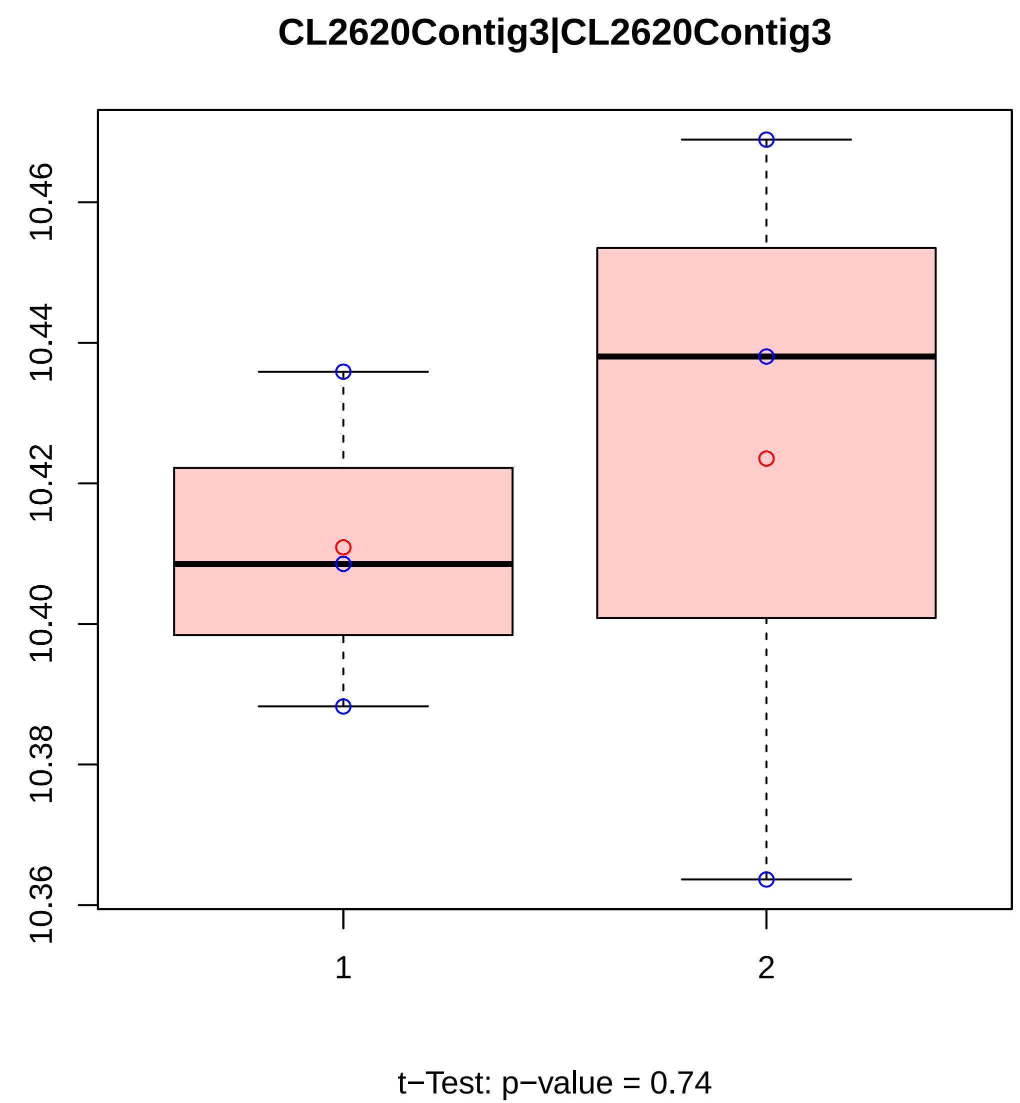

CL2620Contig3|CL2620Contig3
10.36
10.38
10.40
10.42
10.44
10.46
1
2
t−Test: p−value = 0.74

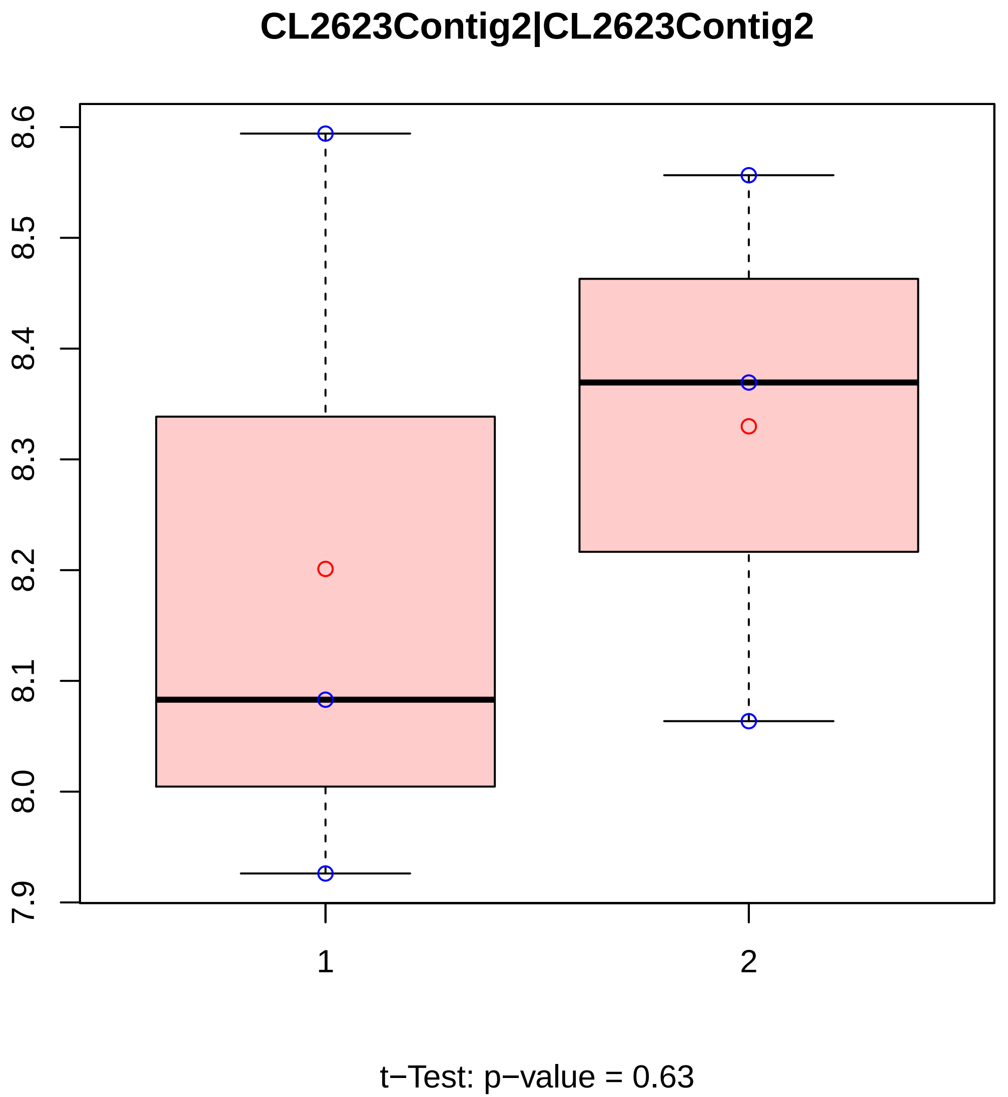
CL2623Contig2|CL2623Contig2
8.6
8.5
8.4
8.3
8.2
8.1
8.0
7.9
1
2
t−Test: p−value = 0.63

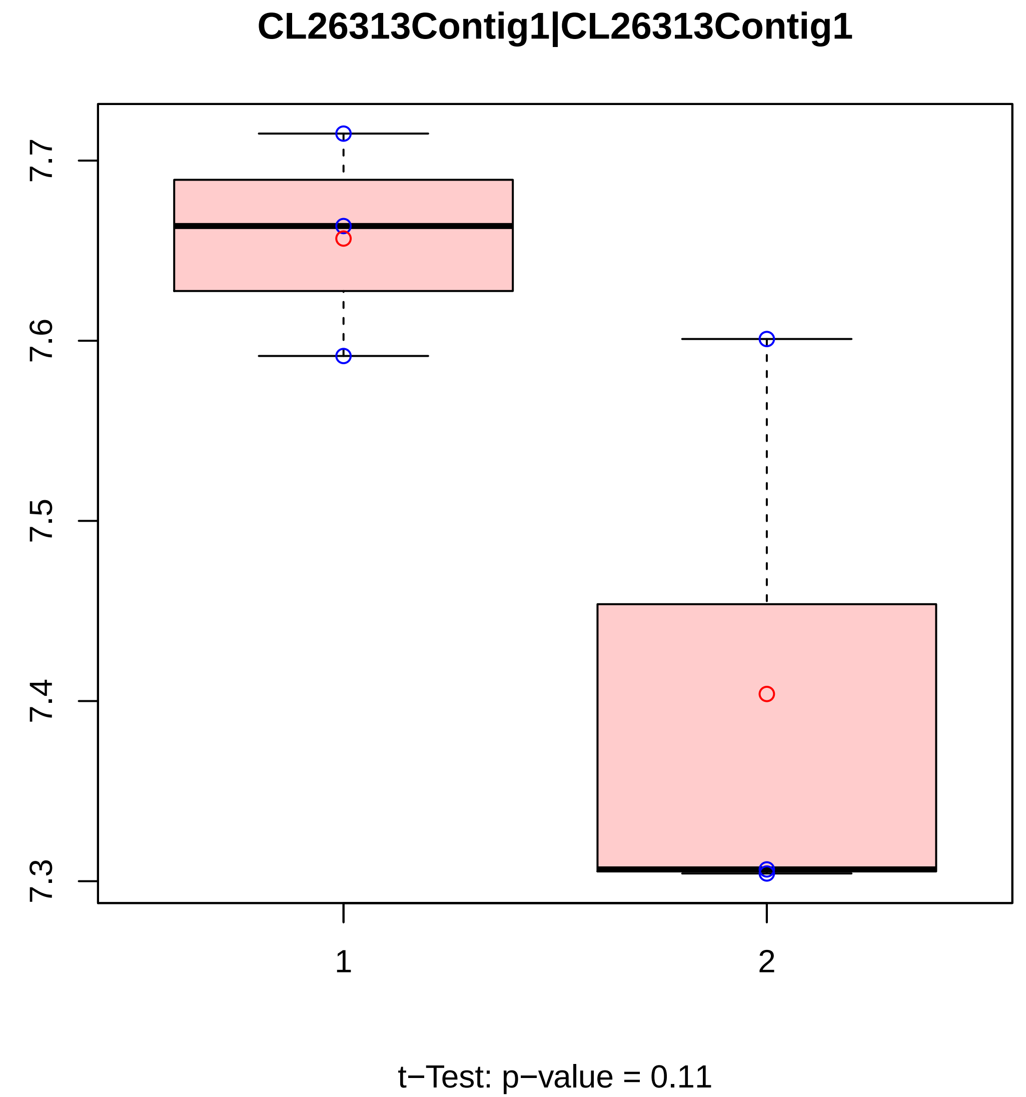

CL26313Contig1|CL26313Contig1
7.3
7.4
7.5
7.6
7.7
1
2
t−Test: p−value = 0.11

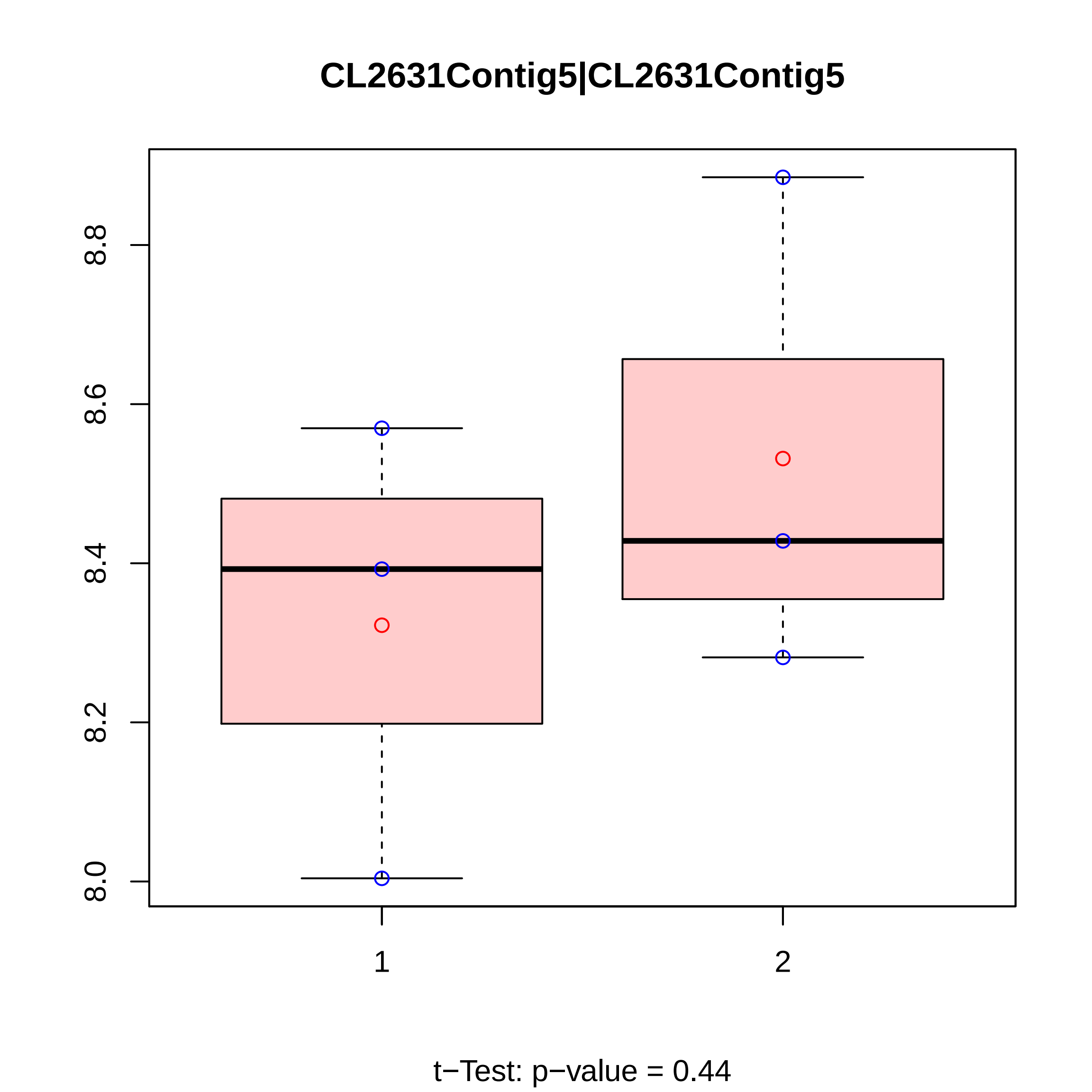
CL2631Contig5|CL2631Contig5
8.0
8.2
8.4
8.6
8.8
1
2
t−Test: p−value = 0.44

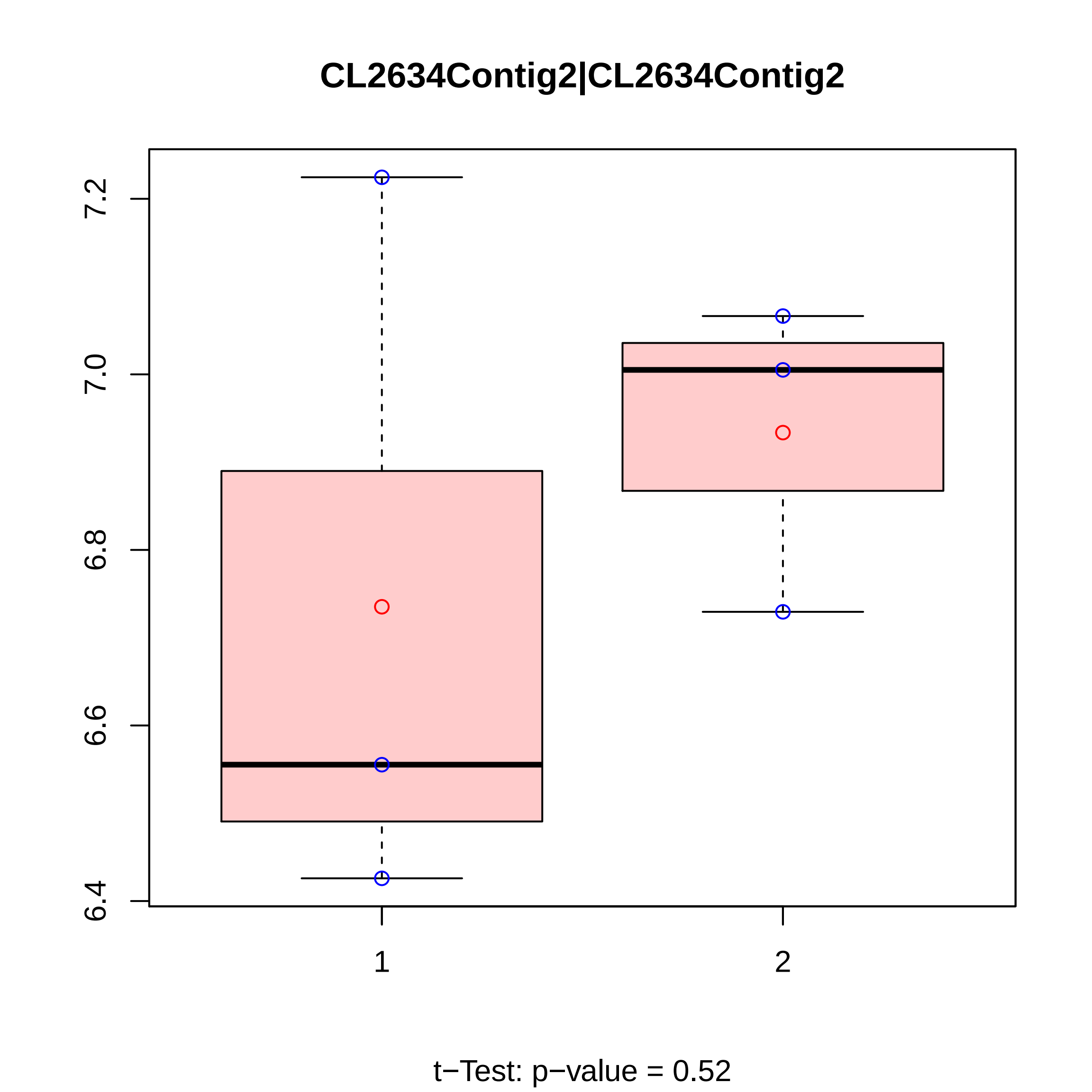
CL2634Contig2|CL2634Contig2
7.2
7.0
6.8
6.6
6.4
1
2
t−Test: p−value = 0.52

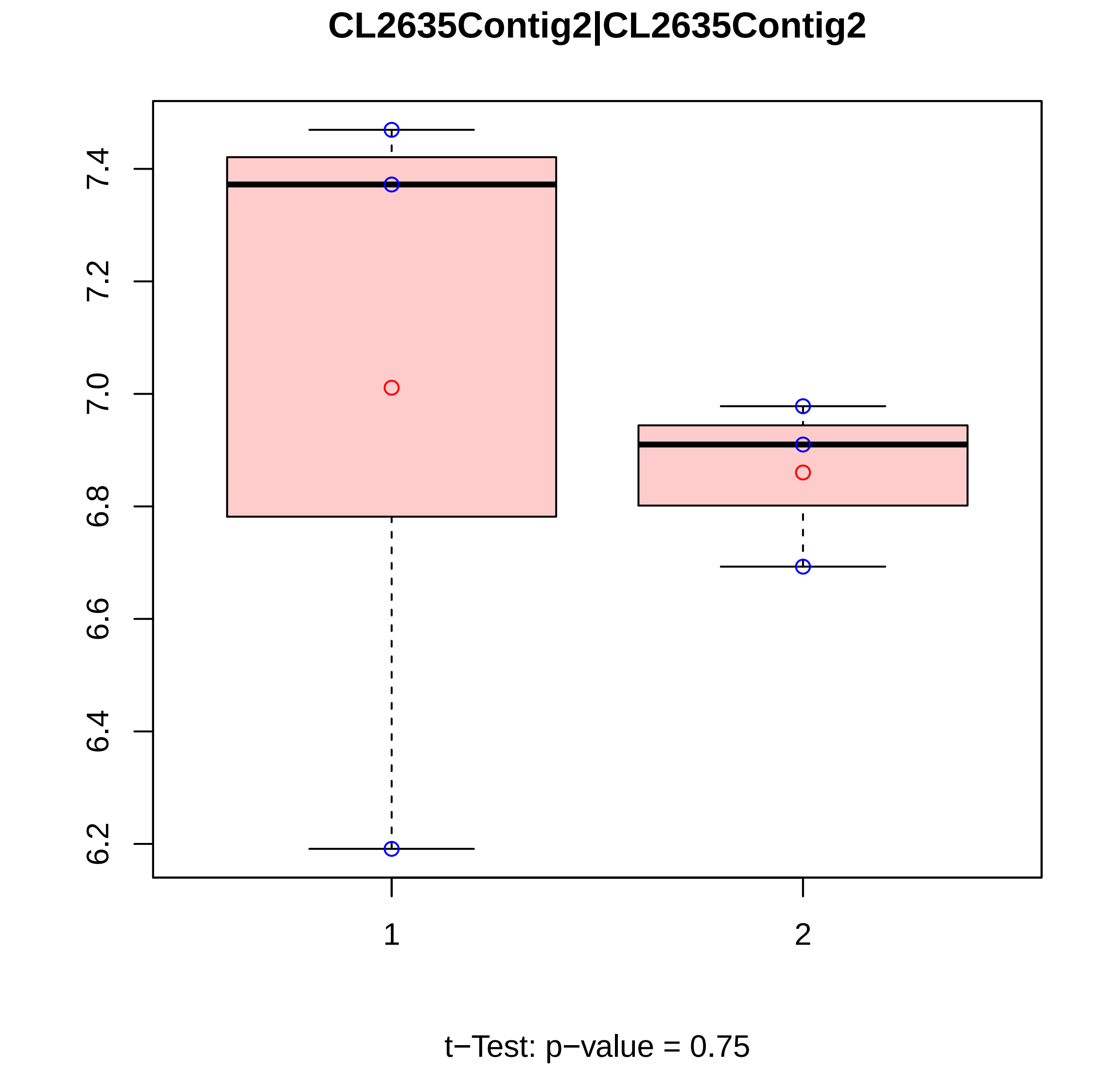

CL2635Contig2|CL2635Contig2
7.4
7.2
7.0
6.8
6.6
6.4
6.2
1
2
t−Test: p−value = 0.75

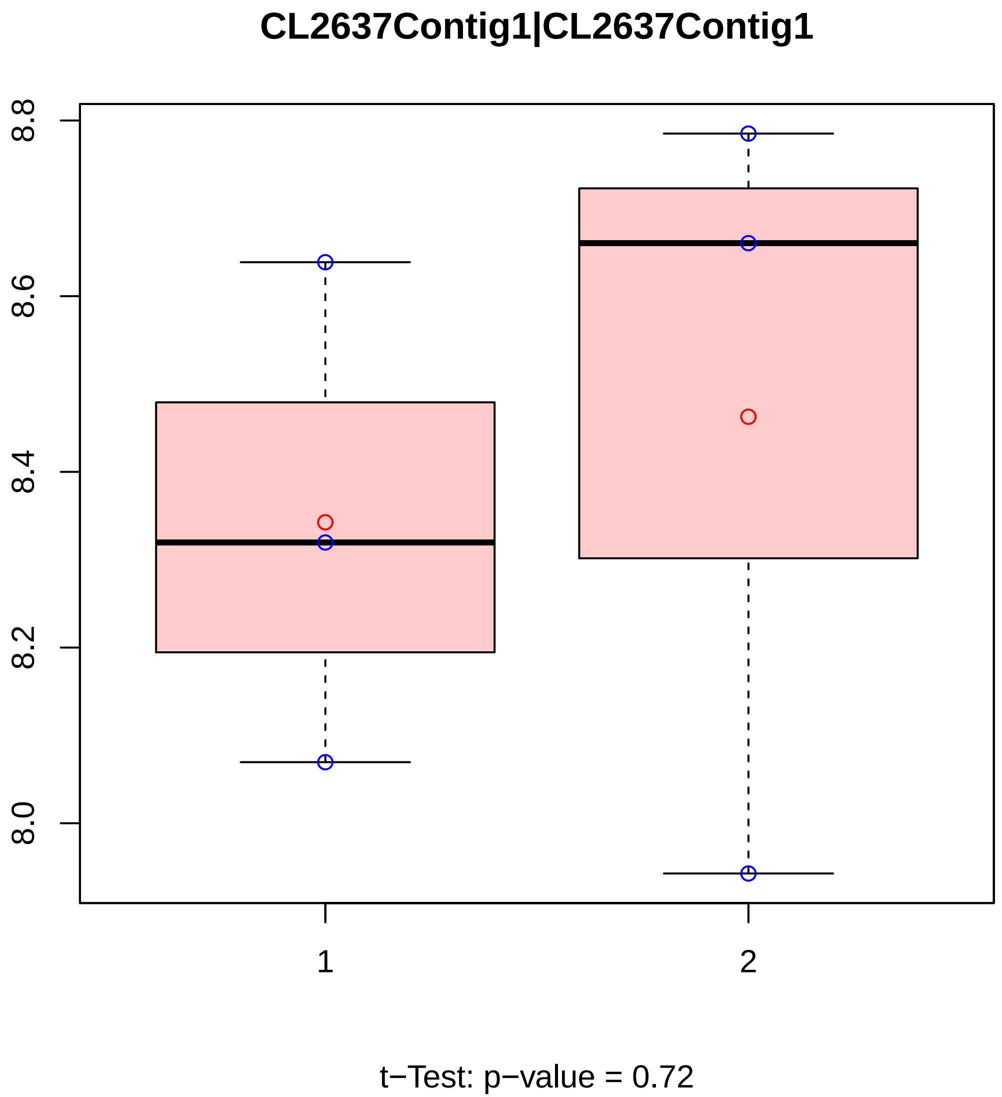
CL2637Contig1|CL2637Contig1
8.0
8.2
8.4
8.6
8.8
1
2
t−Test: p−value = 0.72

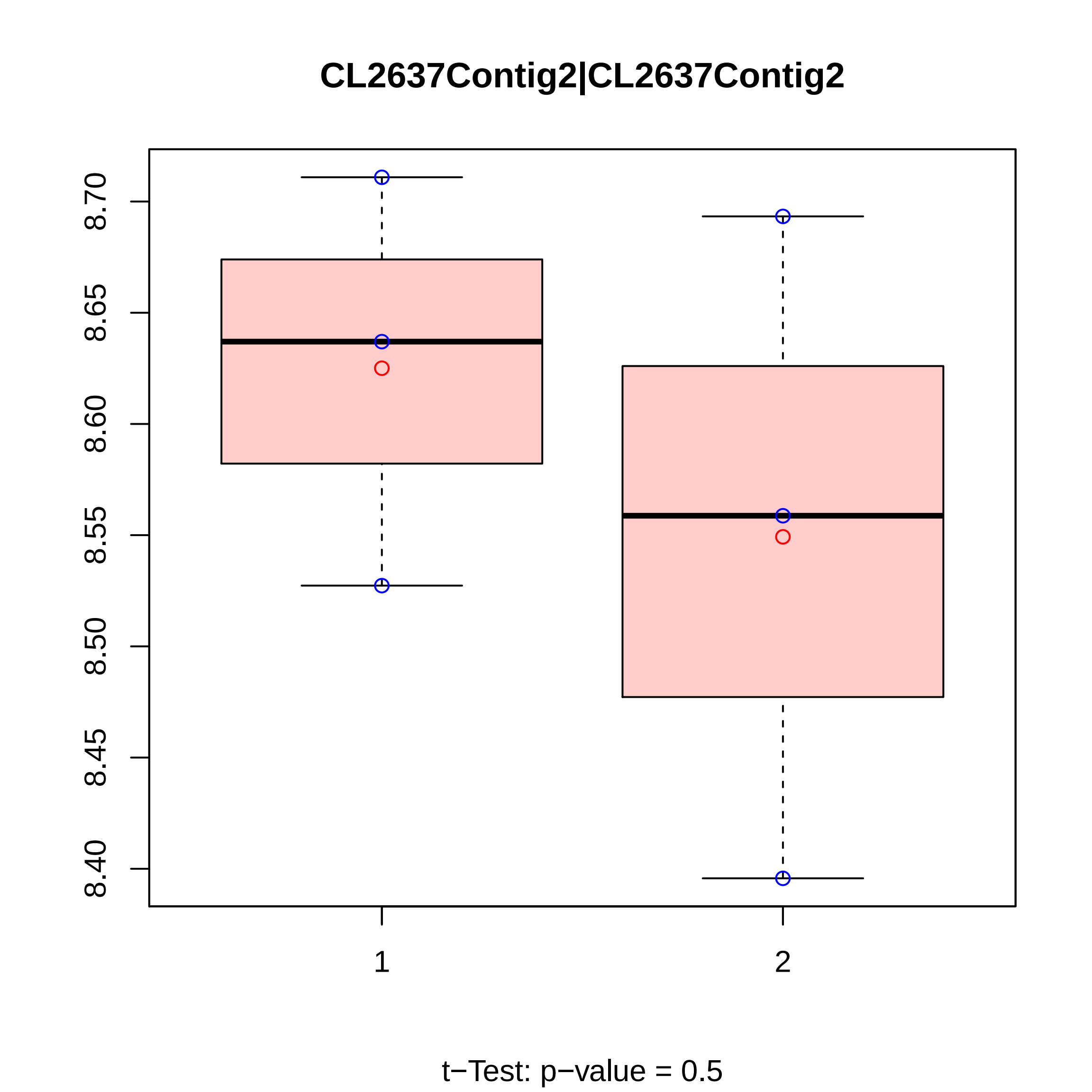
CL2637Contig2|CL2637Contig2
8.70
8.65
8.60
8.55
8.50
8.45
8.40
1
2
t−Test: p−value = 0.5

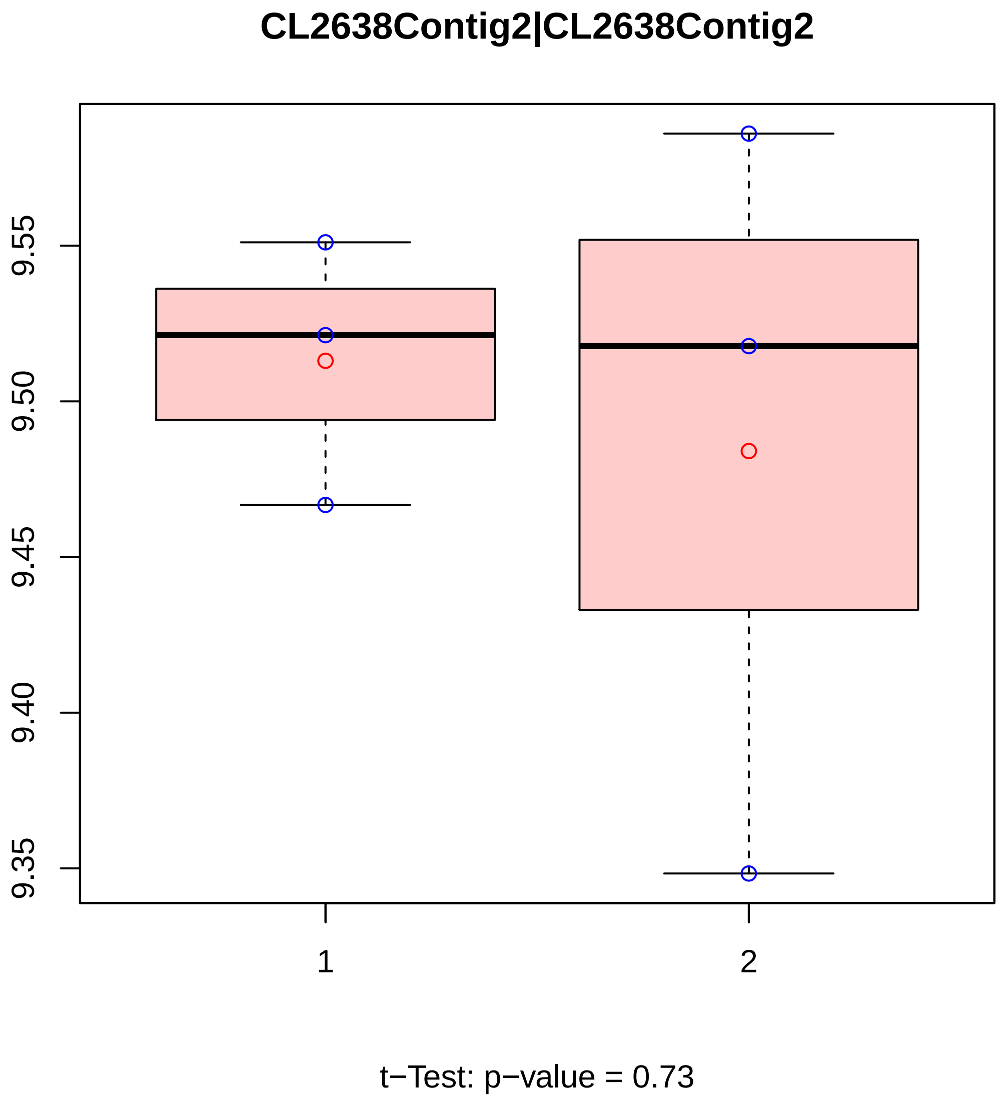

CL2638Contig2|CL2638Contig2
9.35
9.40
9.45
9.50
9.55
1
2
t−Test: p−value = 0.73

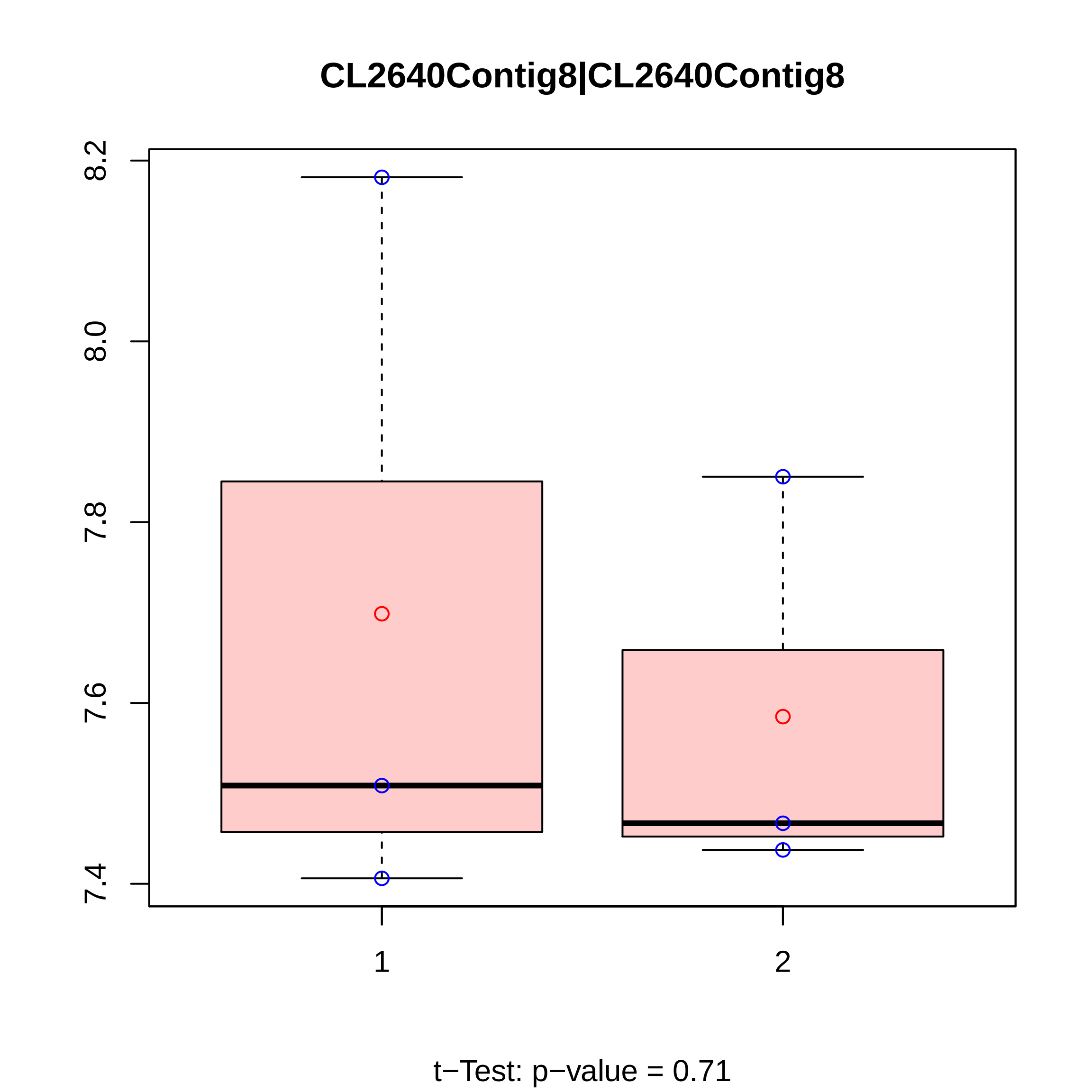

CL2640Contig8|CL2640Contig8
8.2
8.0
7.8
7.6
7.4
1
2
t−Test: p−value = 0.71

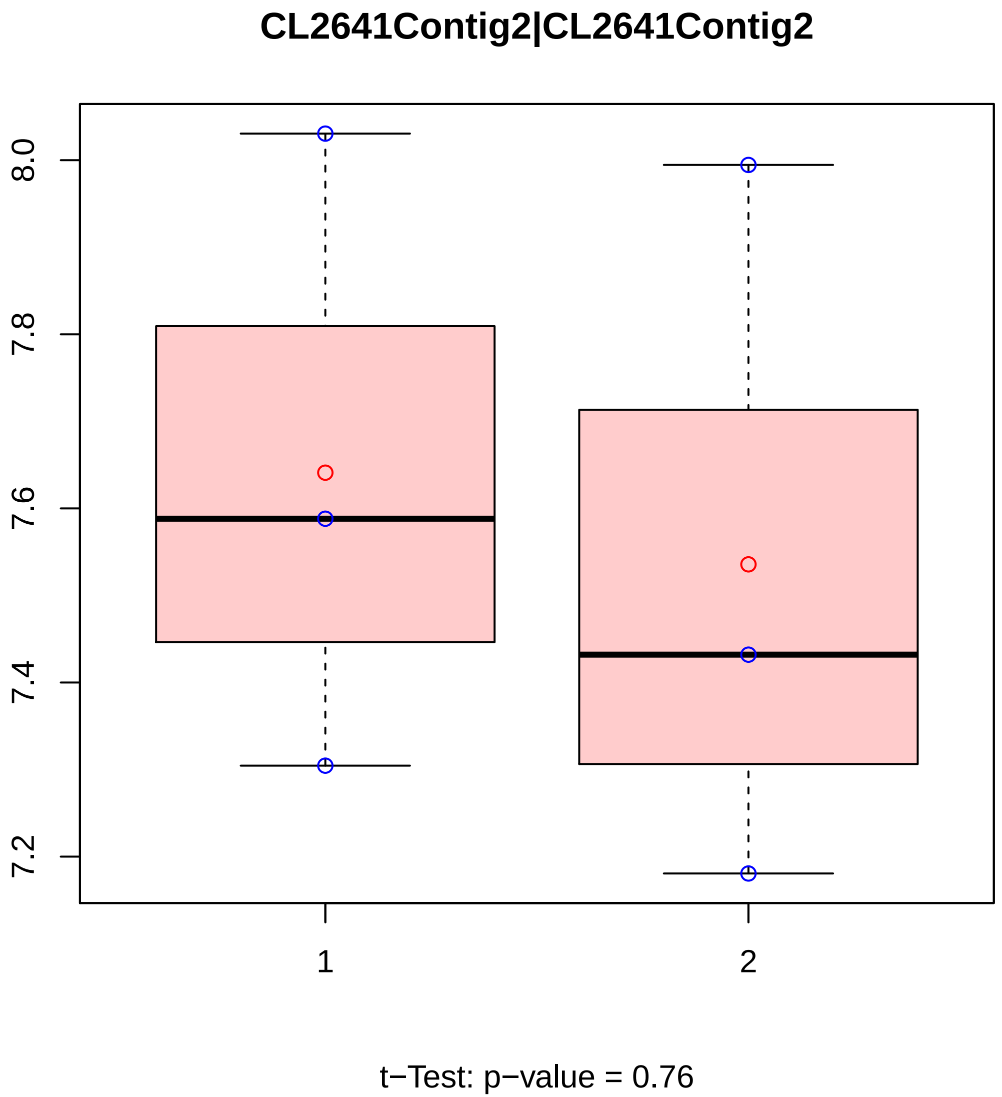

CL2641Contig2|CL2641Contig2
8.0
7.8
7.6
7.4
7.2
1
2
t−Test: p−value = 0.76

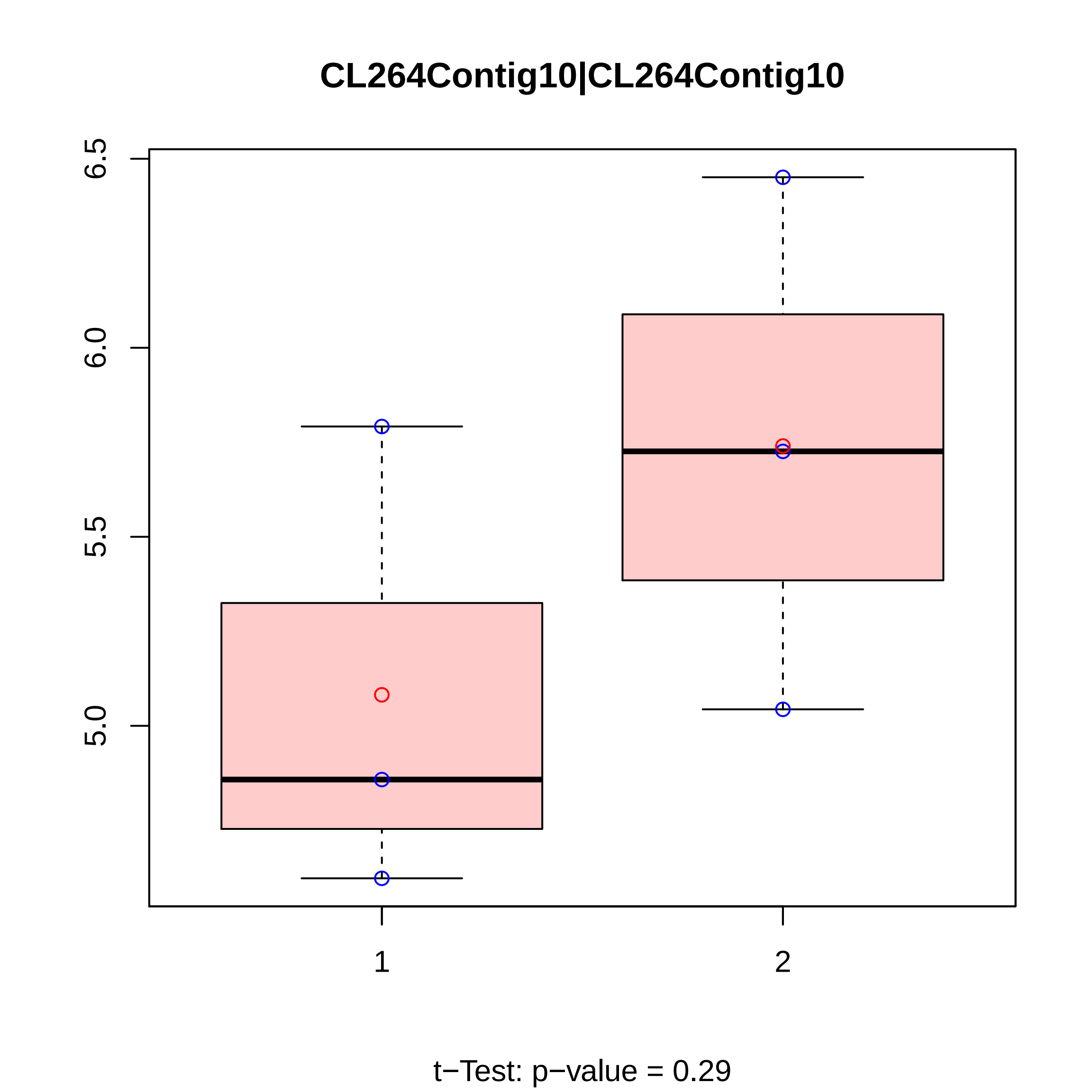

CL264Contig10|CL264Contig10
6.5
6.0
5.5
5.0
1
2
t−Test: p−value = 0.29

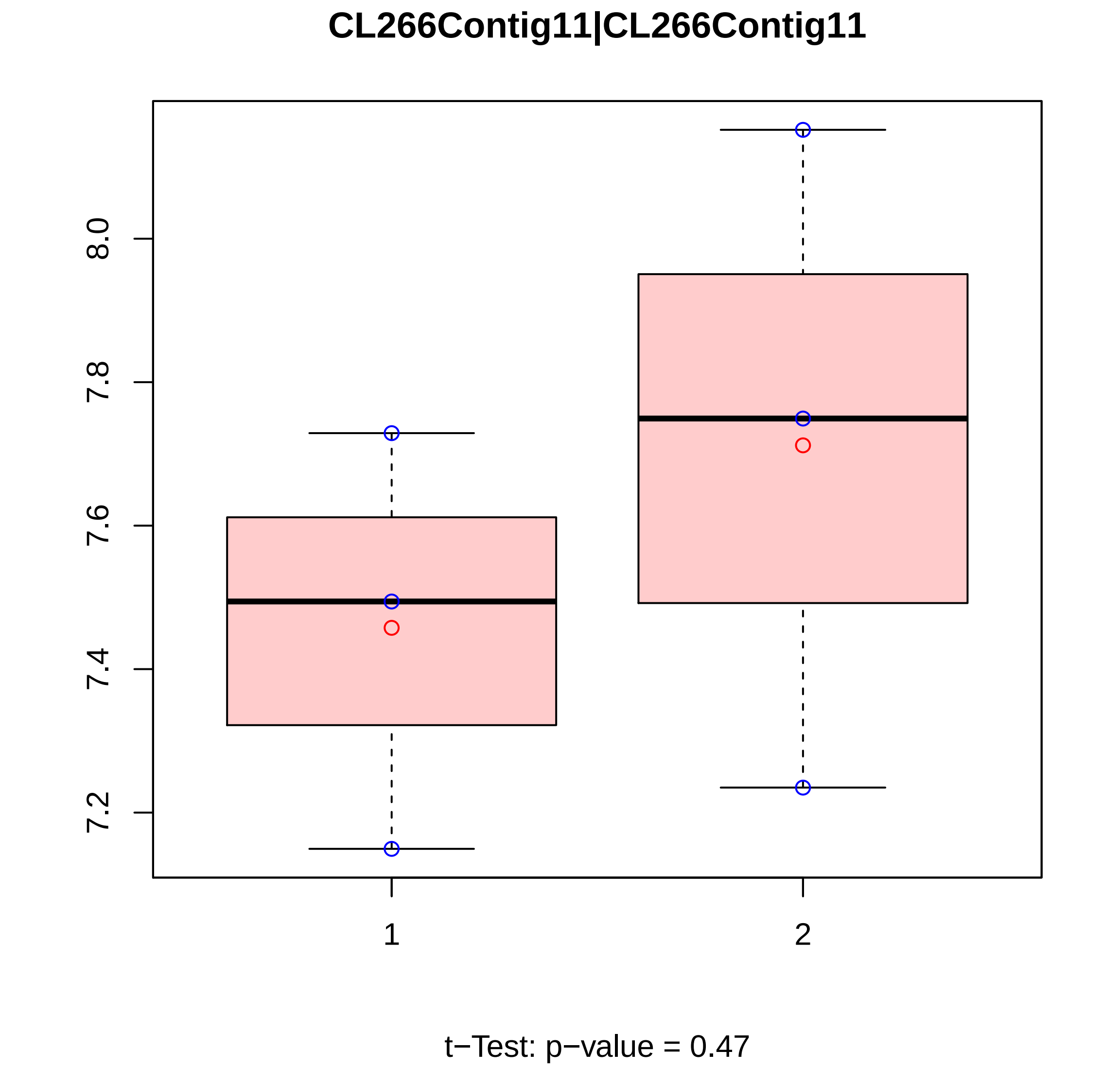
CL266Contig11|CL266Contig11
7.2
7.4
7.6
7.8
8.0
1
2
t−Test: p−value = 0.47

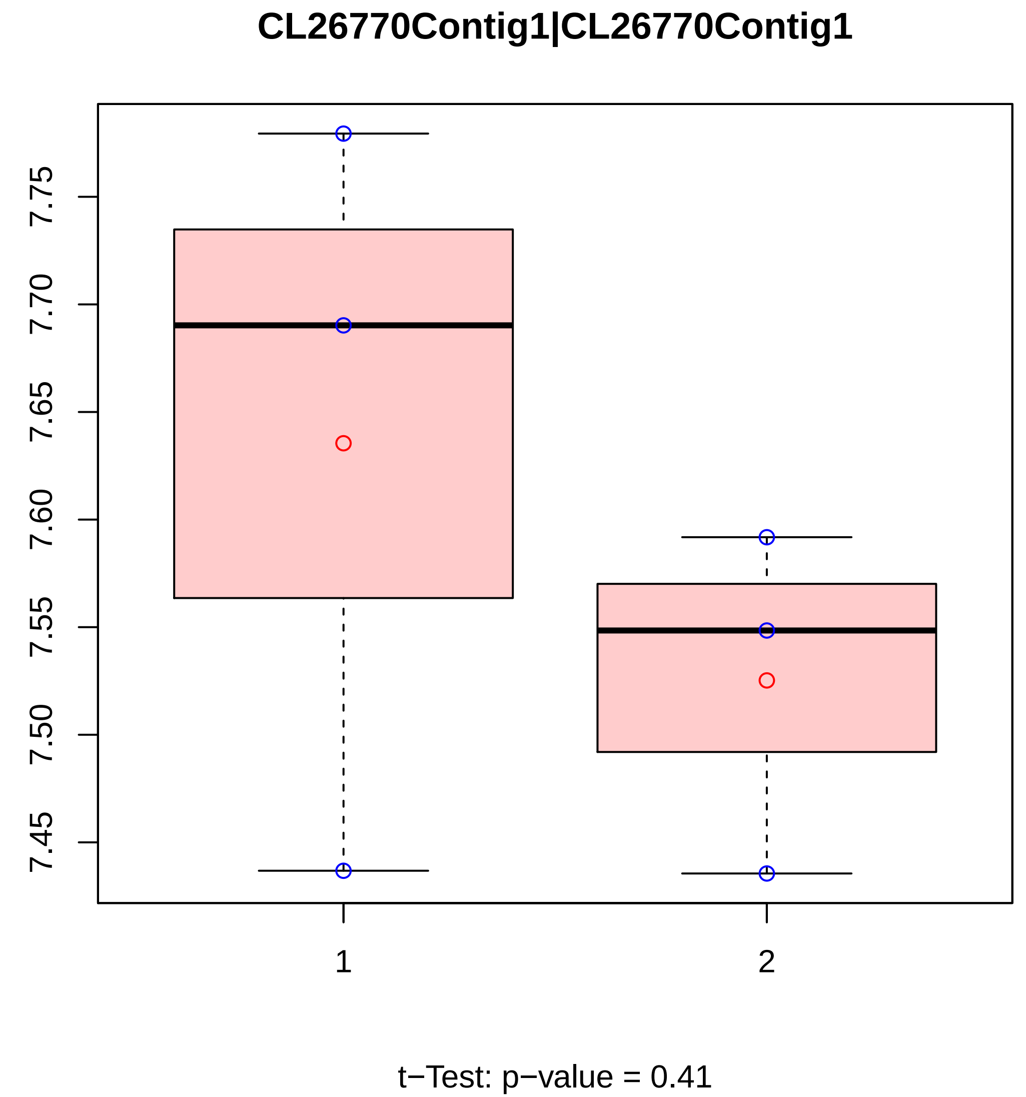

CL26770Contig1|CL26770Contig1
7.45
7.50
7.55
7.60
7.65
7.70
7.75
1
2
t−Test: p−value = 0.41

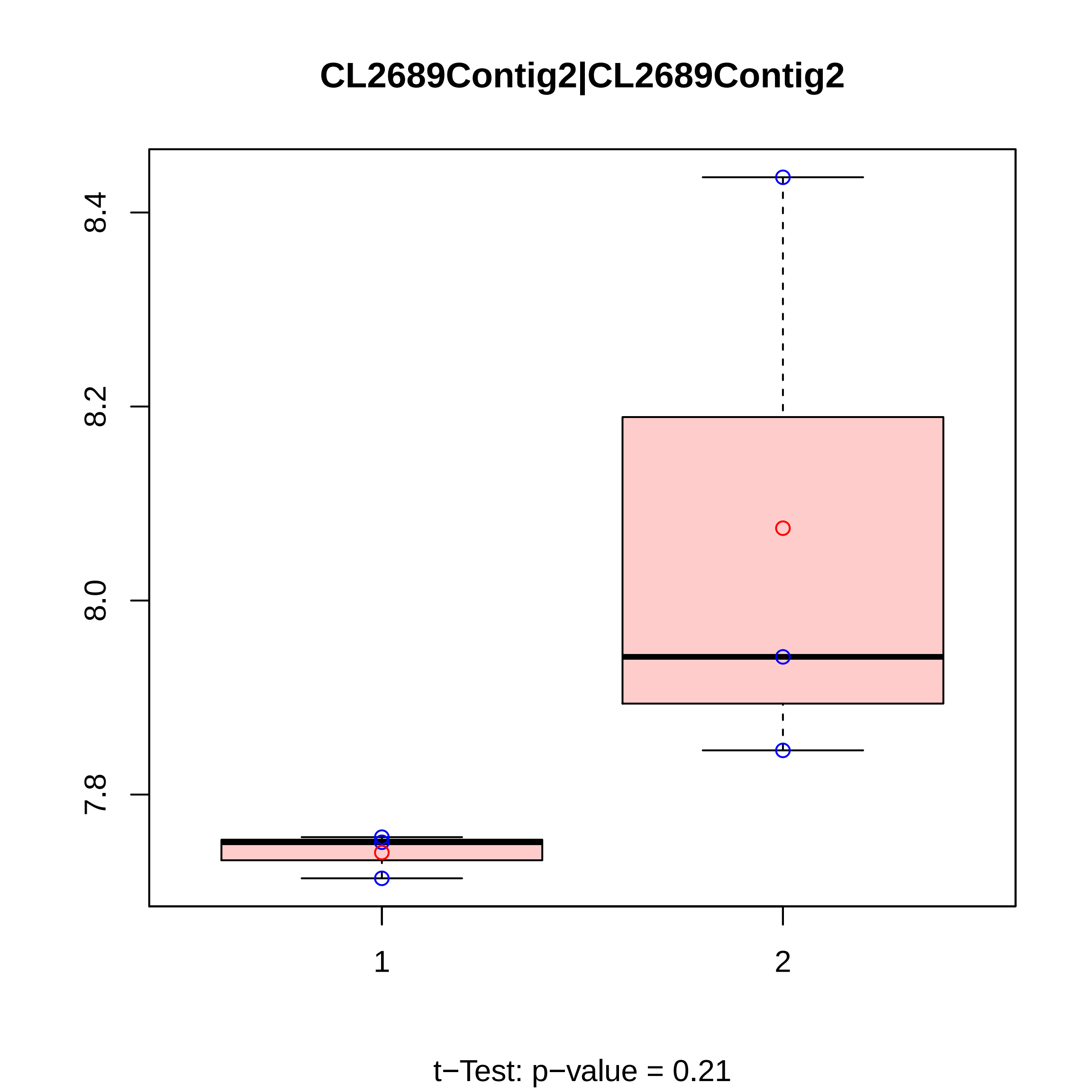
CL2689Contig2|CL2689Contig2
8.4
8.2
8.0
7.8
1
2
t−Test: p−value = 0.21

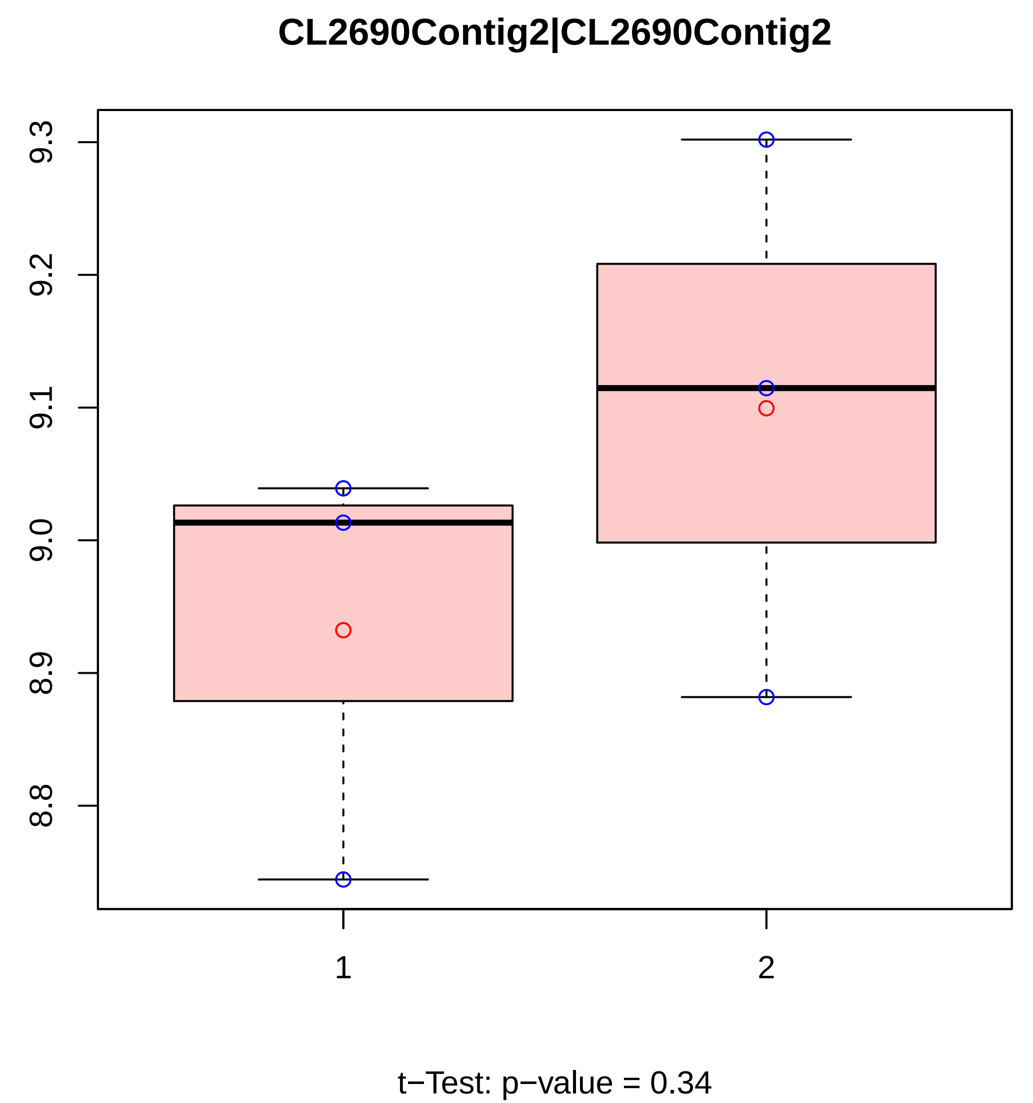
CL2690Contig2|CL2690Contig2
9.3
9.2
9.1
9.0
8.9
8.8
1
2
t−Test: p−value = 0.34

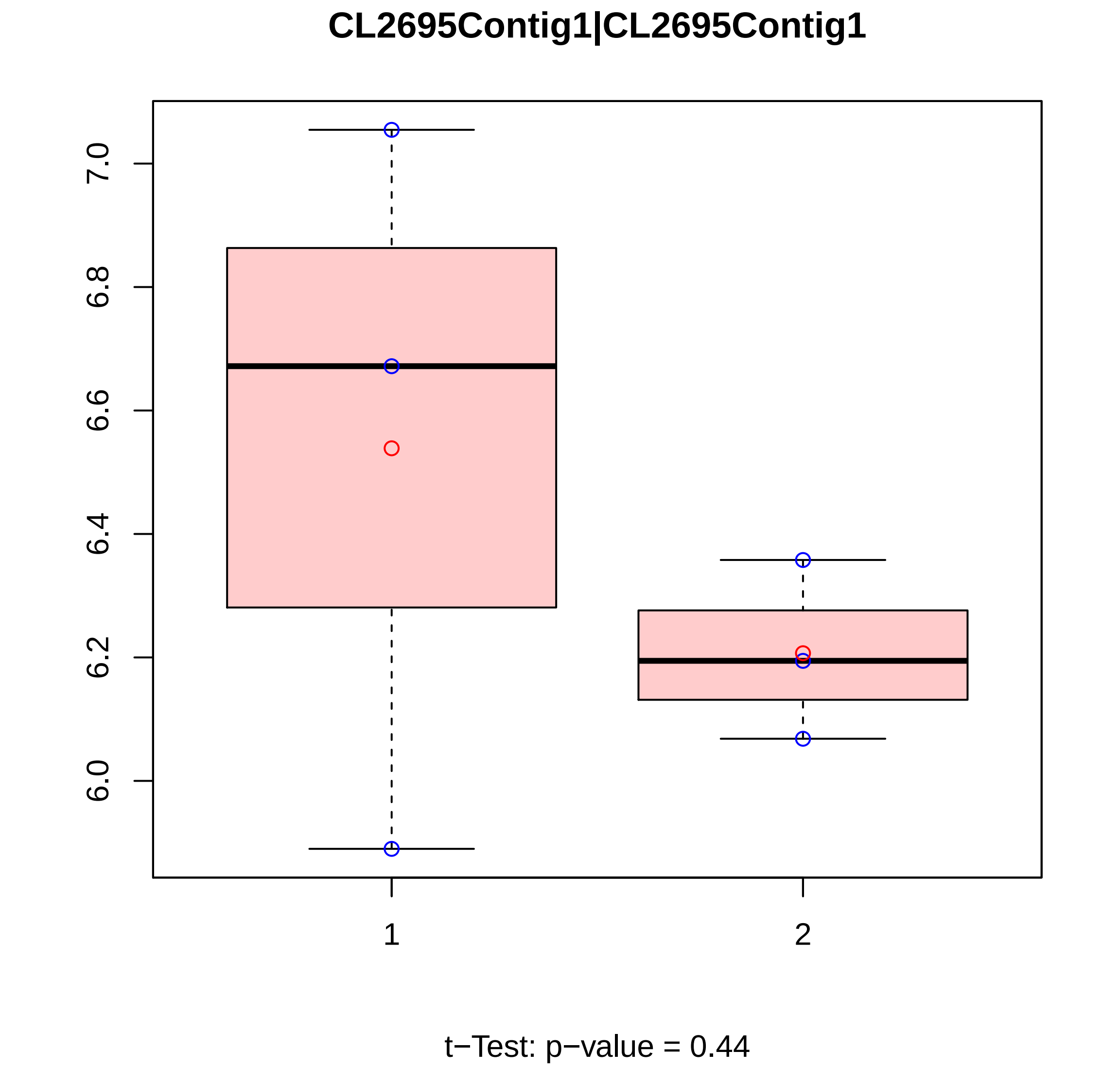
CL2695Contig1|CL2695Contig1
7.0
6.8
6.6
6.4
6.2
6.0
1
2
t−Test: p−value = 0.44

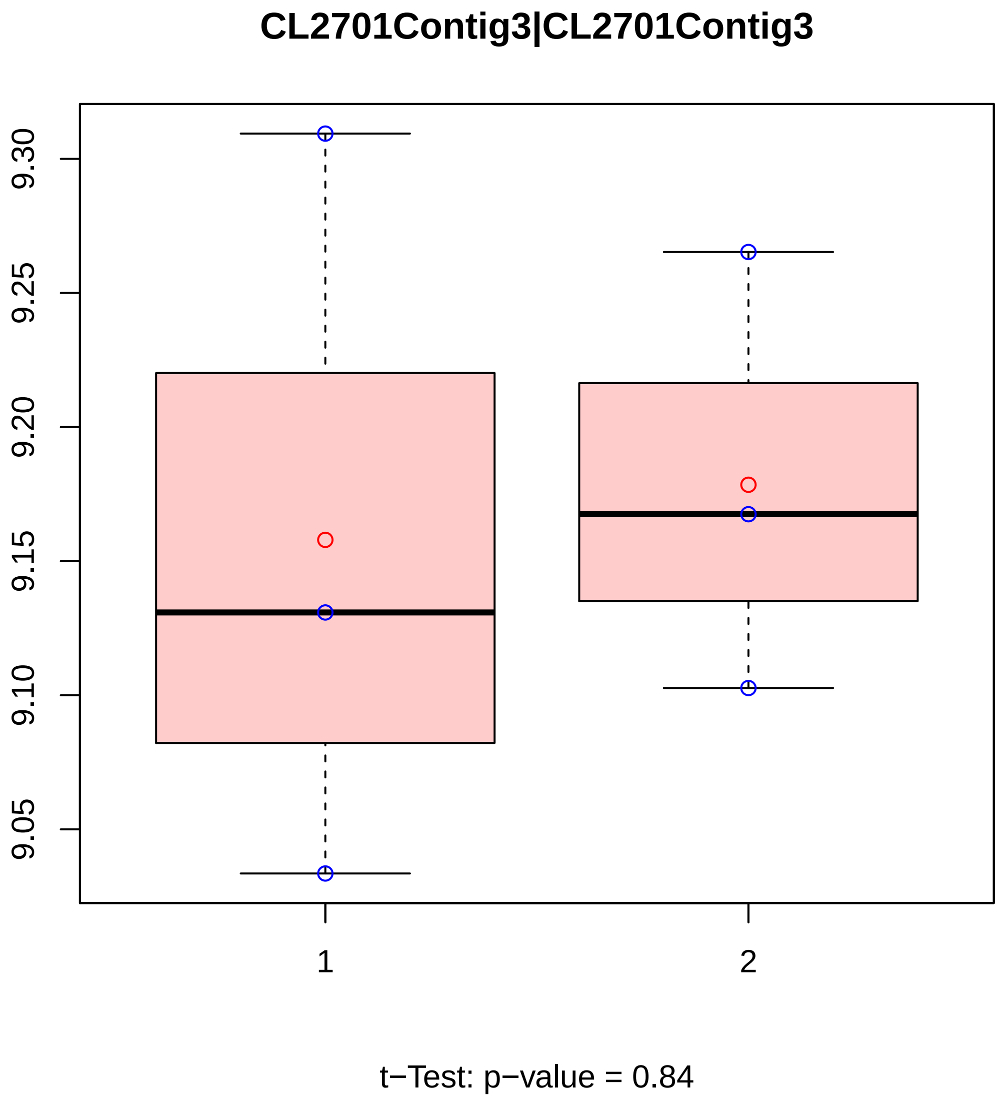
CL2701Contig3|CL2701Contig3
9.30
9.25
9.20
9.15
9.10
9.05
1
2
t−Test: p−value = 0.84

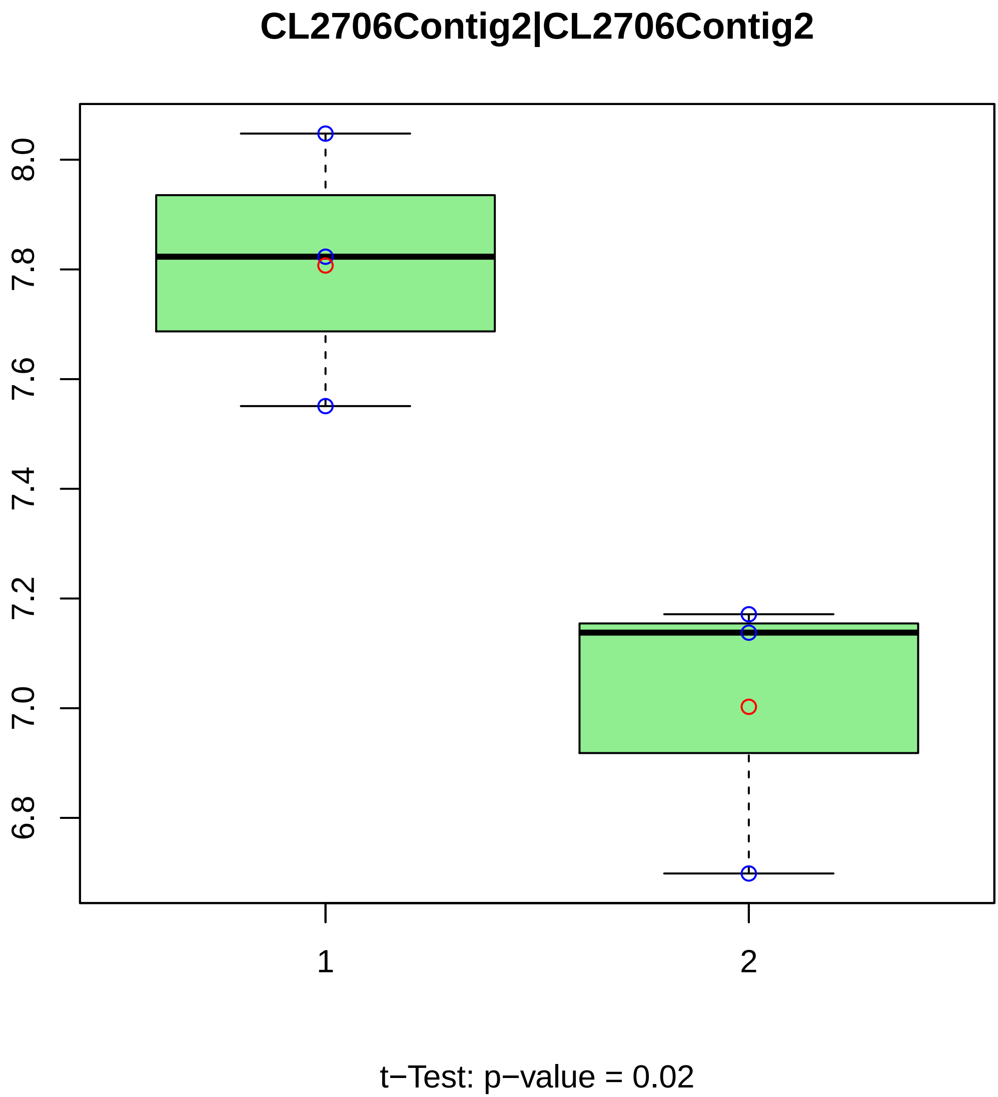

CL2706Contig2|CL2706Contig2
8.0
7.8
7.6
7.4
7.2
7.0
6.8
1
2
t−Test: p−value = 0.02

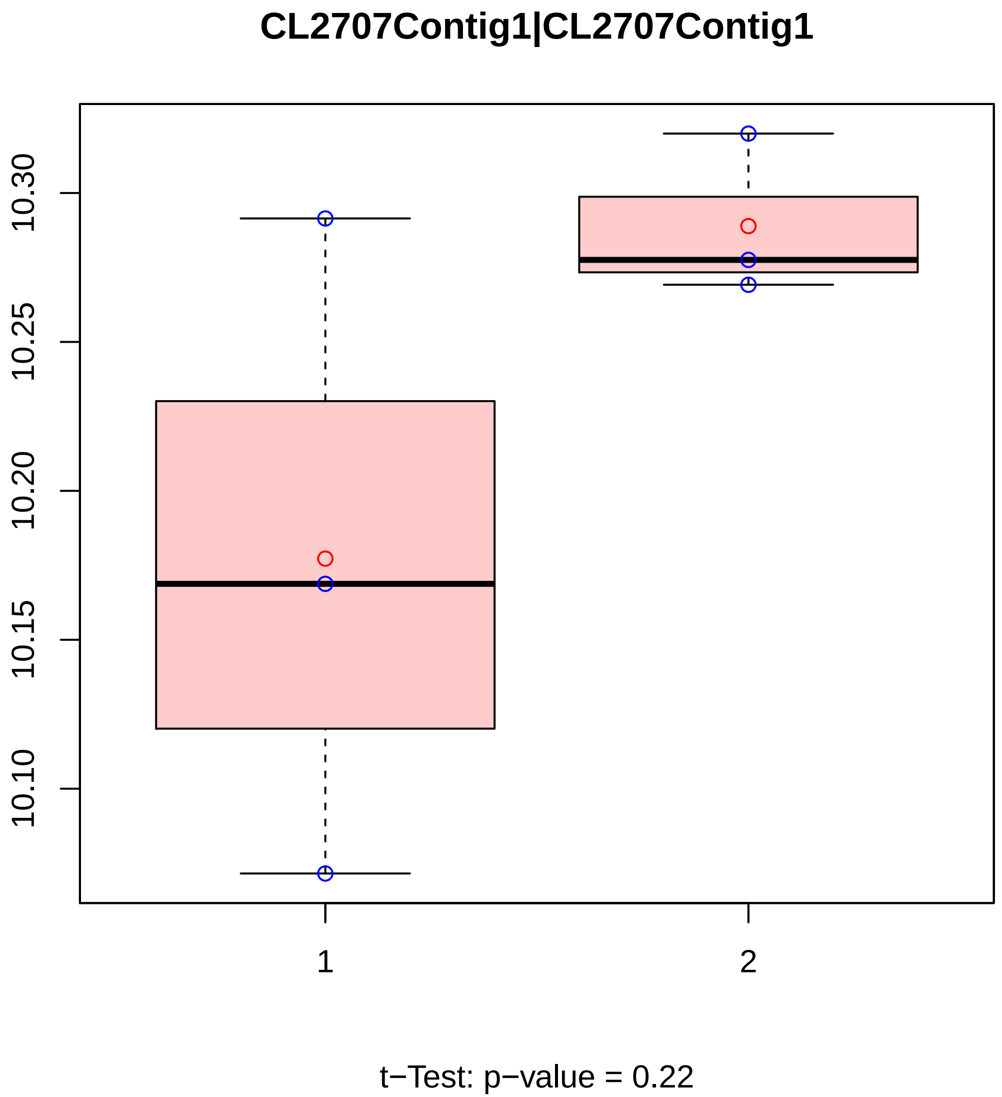

CL2707Contig1|CL2707Contig1
10.10
10.15
10.20
10.25
10.30
1
2
t−Test: p−value = 0.22

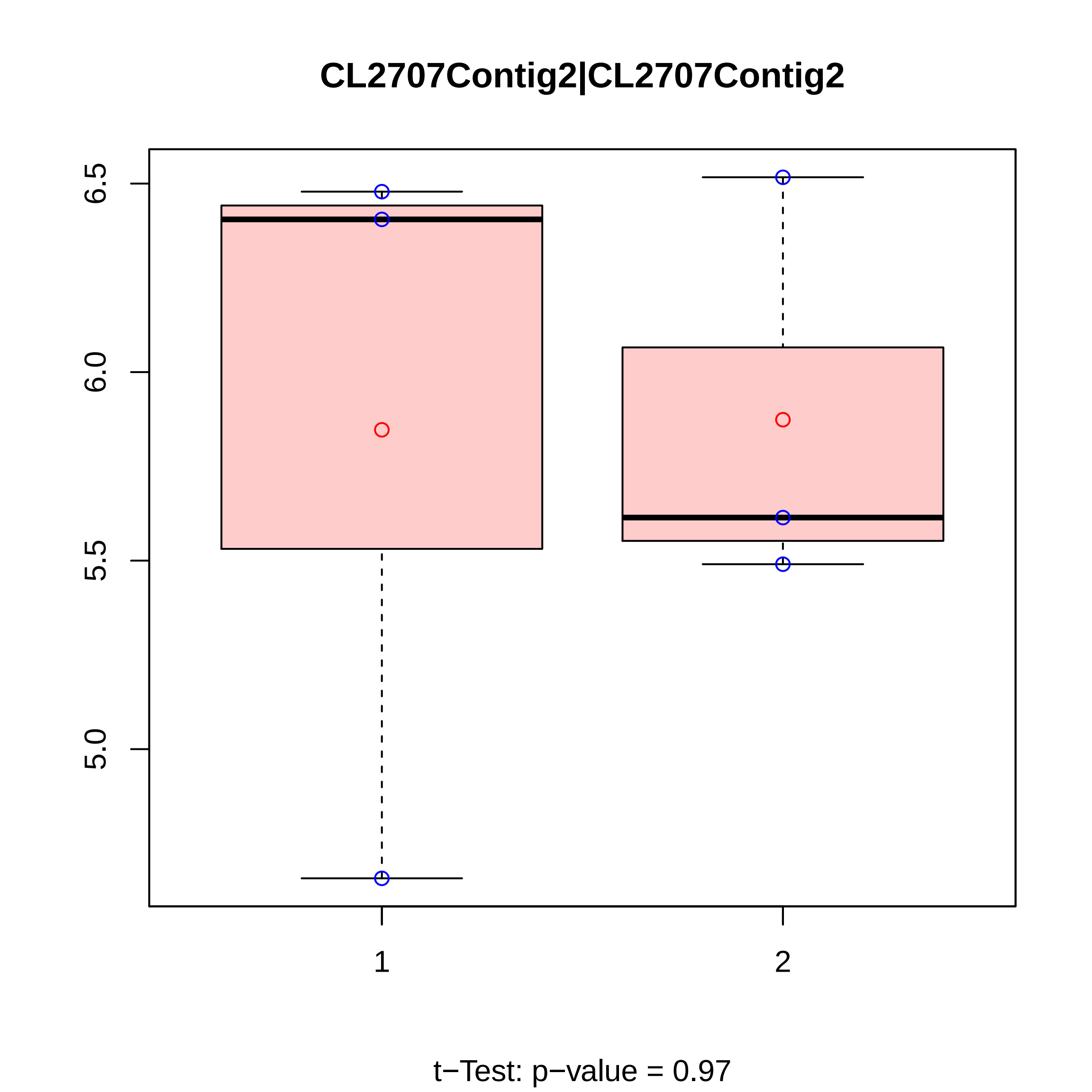

CL2707Contig2|CL2707Contig2
6.5
6.0
5.5
5.0
1
2
t−Test: p−value = 0.97

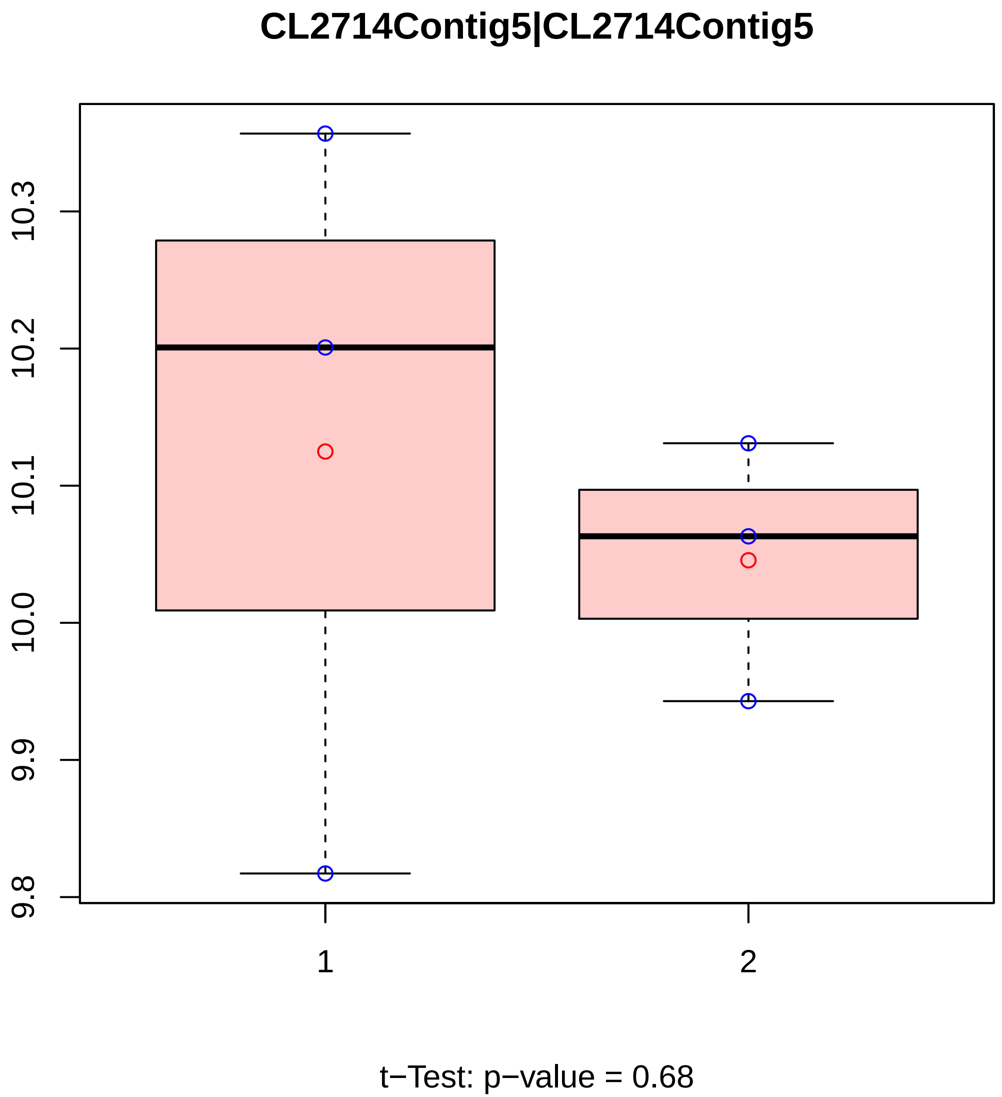
CL2714Contig5|CL2714Contig5
9.8
9.9
10.0
10.1
10.2
10.3
1
2
t−Test: p−value = 0.68

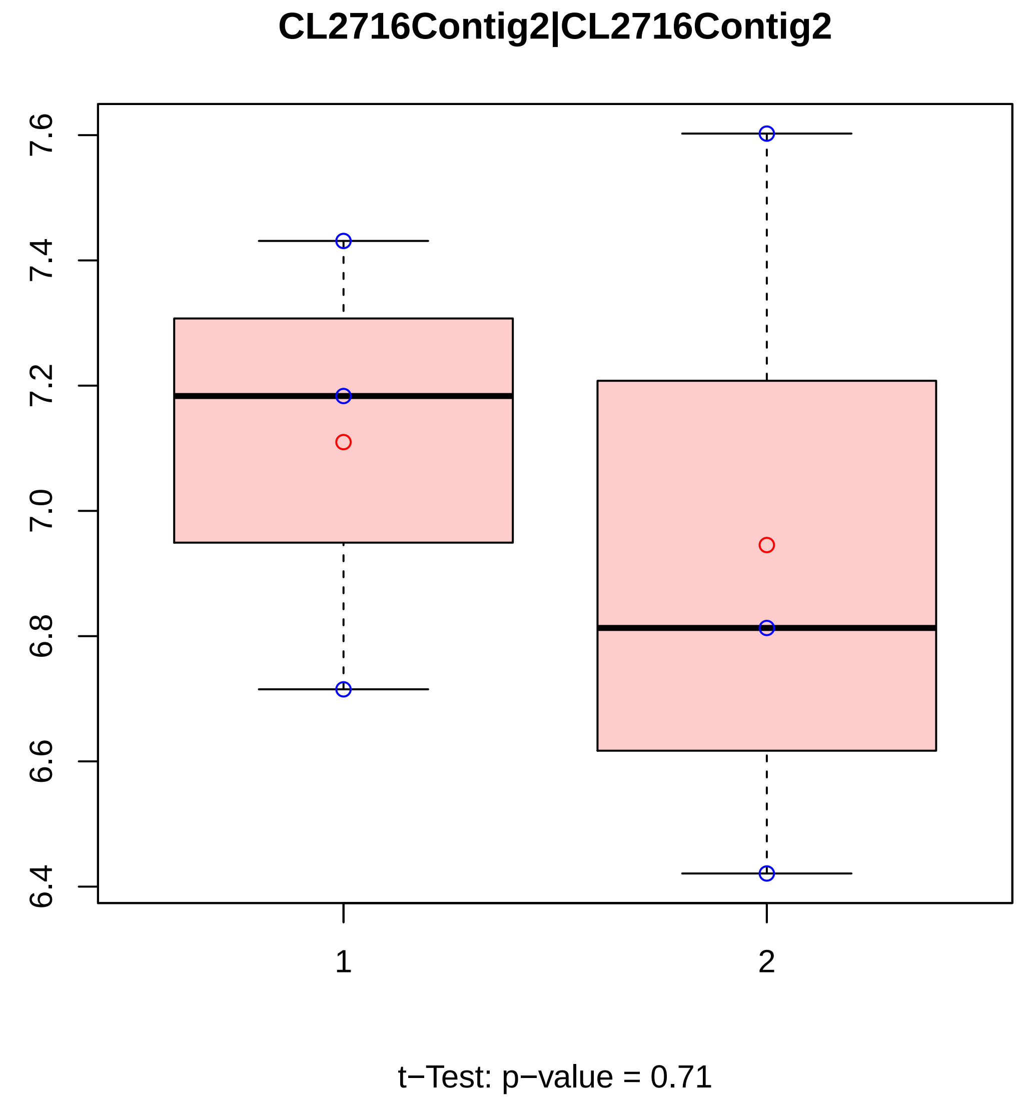
CL2716Contig2|CL2716Contig2
7.6
7.4
7.2
7.0
6.8
6.6
6.4
1
2
t−Test: p−value = 0.71

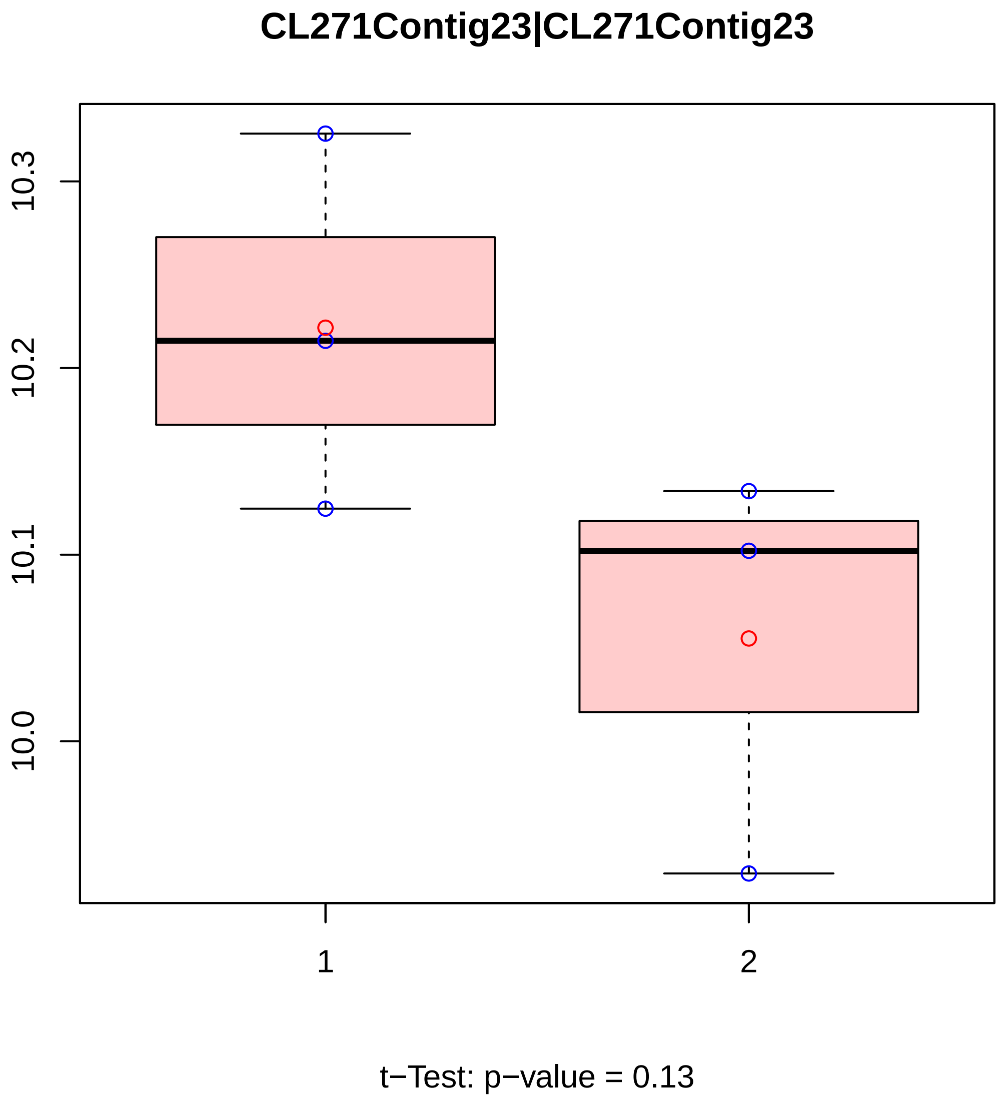
CL271Contig23|CL271Contig23
10.0
10.1
10.2
10.3
1
2
t−Test: p−value = 0.13

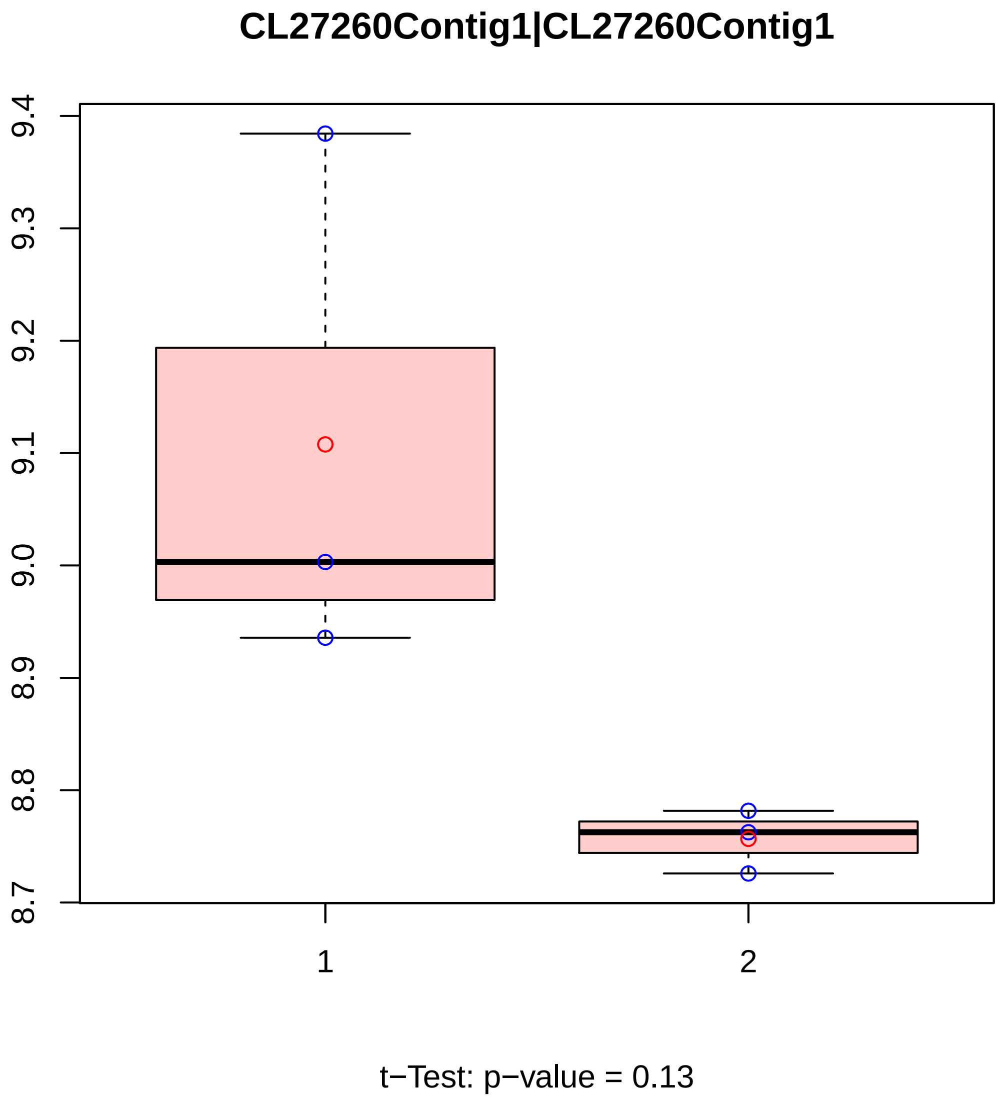
CL27260Contig1|CL27260Contig1
9.4
9.3
9.2
9.1
9.0
8.9
8.8
8.7
1
2
t−Test: p−value = 0.13

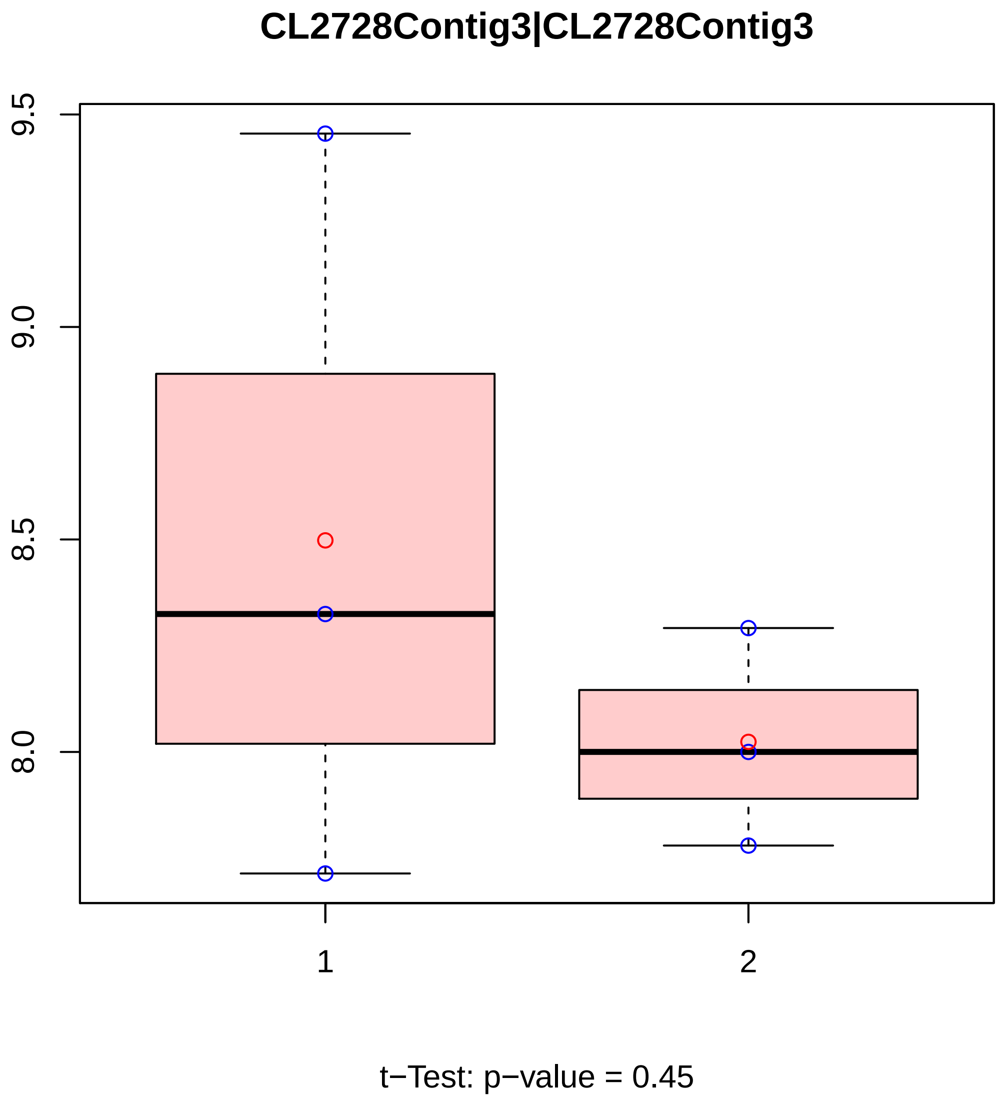

CL2728Contig3|CL2728Contig3
9.5
9.0
8.5
8.0
1
2
t−Test: p−value = 0.45

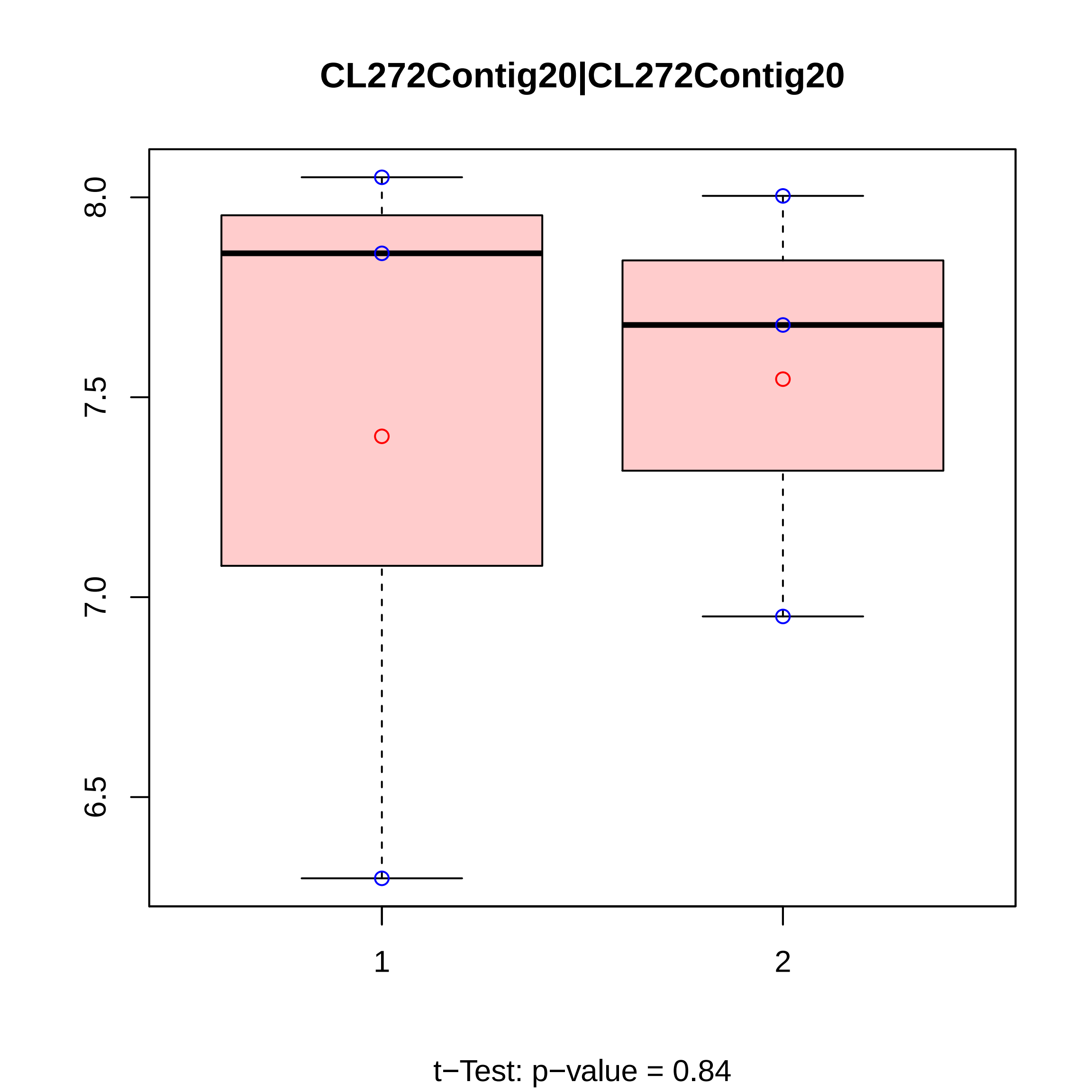
CL272Contig20|CL272Contig20
8.0
7.5
7.0
6.5
1
2
t−Test: p−value = 0.84

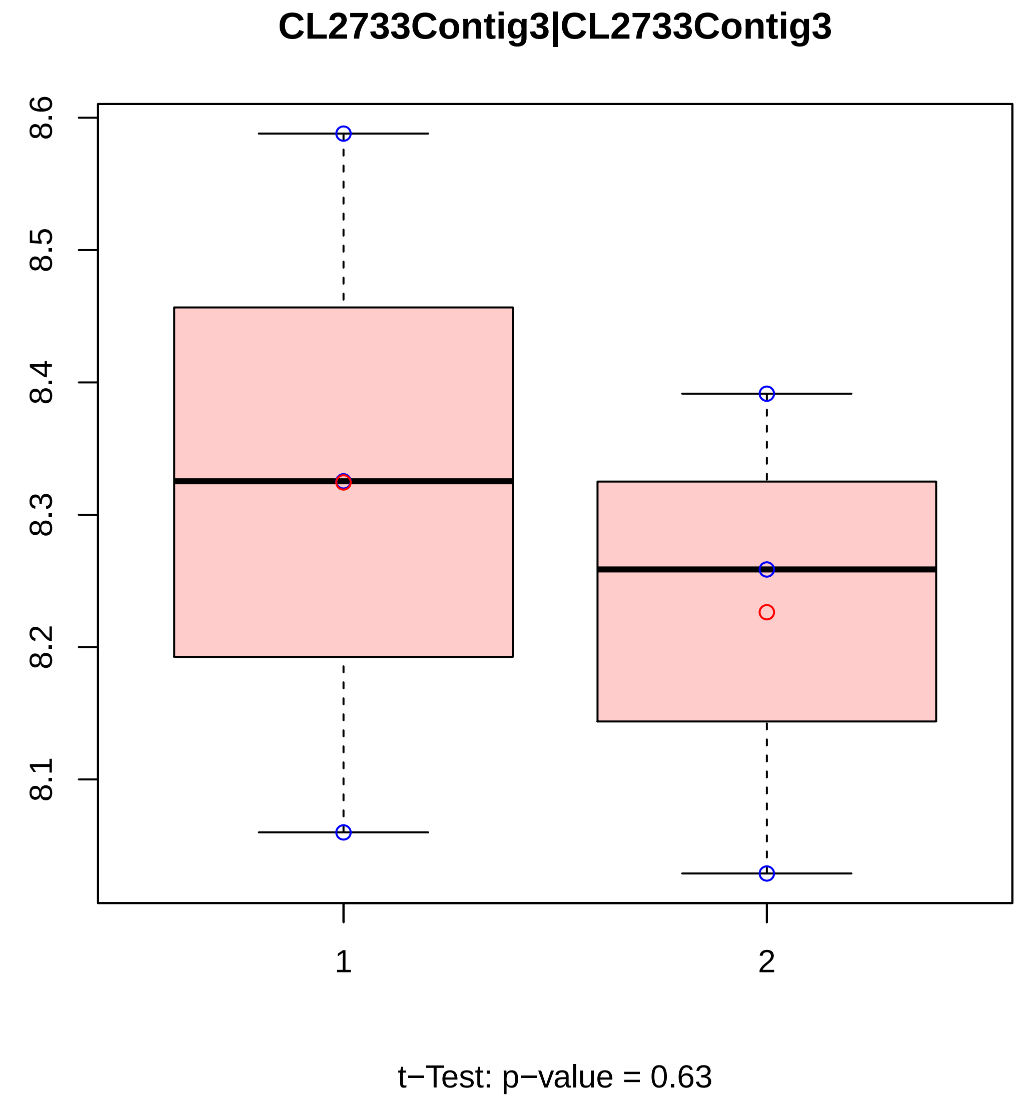
CL2733Contig3|CL2733Contig3
8.6
8.5
8.4
8.3
8.2
8.1
1
2
t−Test: p−value = 0.63

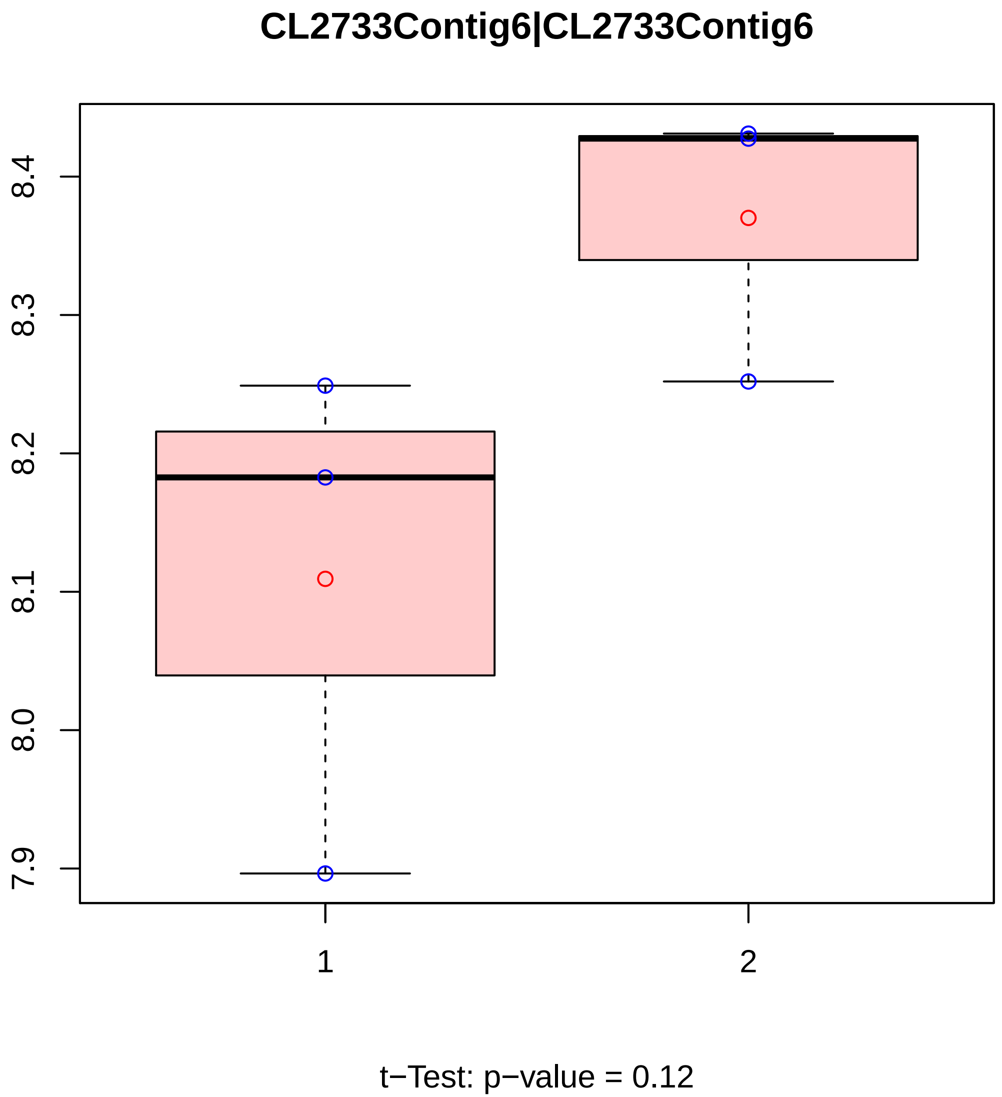

CL2733Contig6|CL2733Contig6
8.4
8.3
8.2
8.1
8.0
7.9
1
2
t−Test: p−value = 0.12

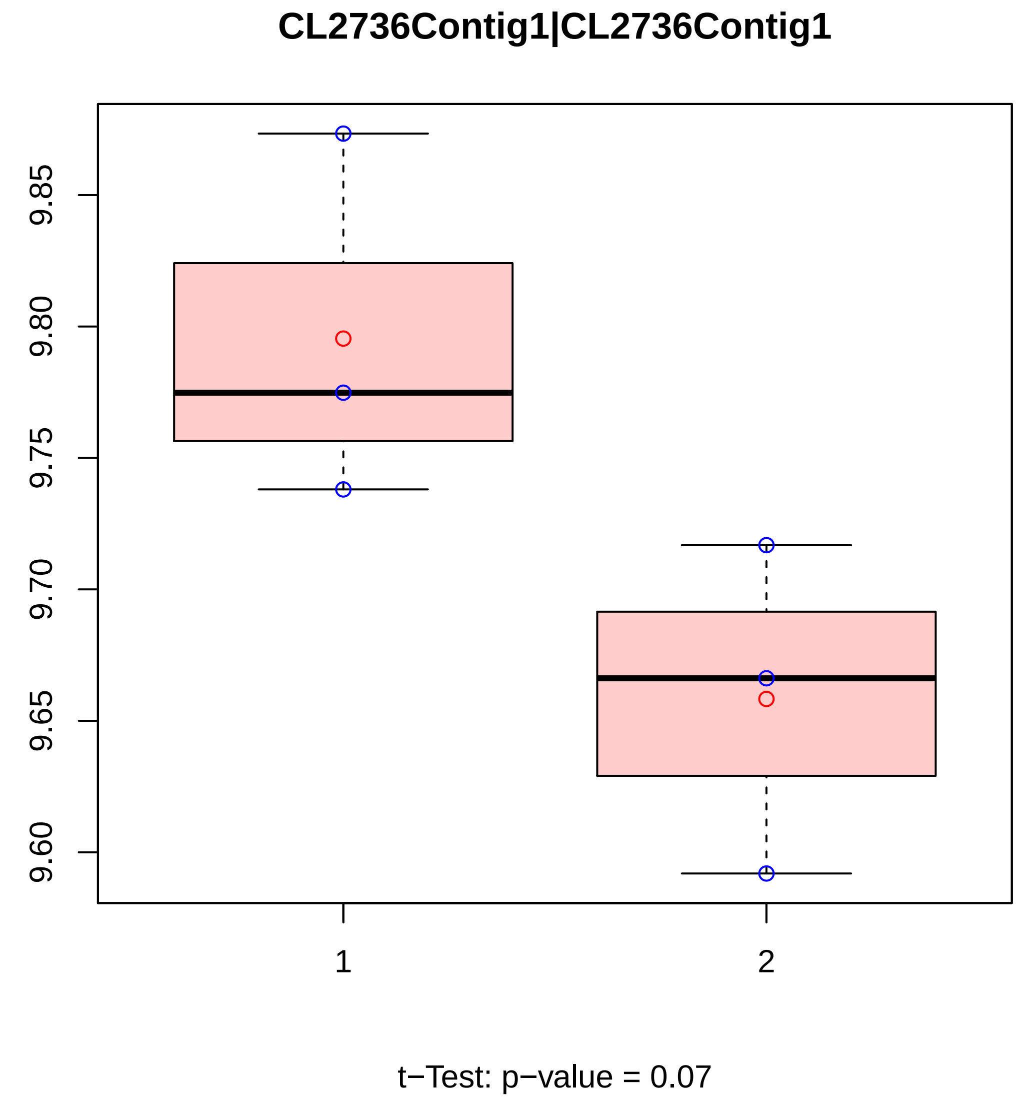

CL2736Contig1|CL2736Contig1
9.60
9.65
9.70
9.75
9.80
9.85
1
2
t−Test: p−value = 0.07

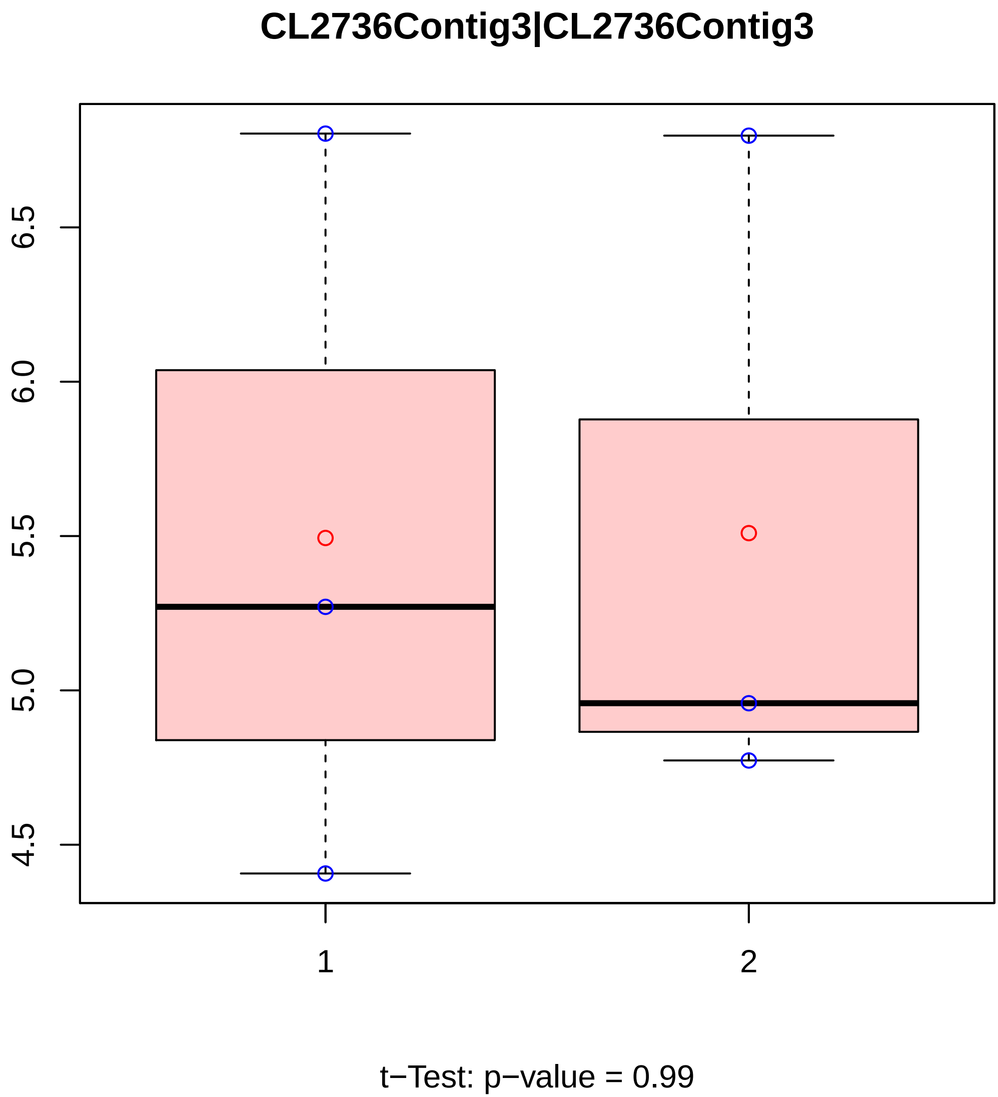

CL2736Contig3|CL2736Contig3
6.5
6.0
5.5
5.0
4.5
1
2
t−Test: p−value = 0.99

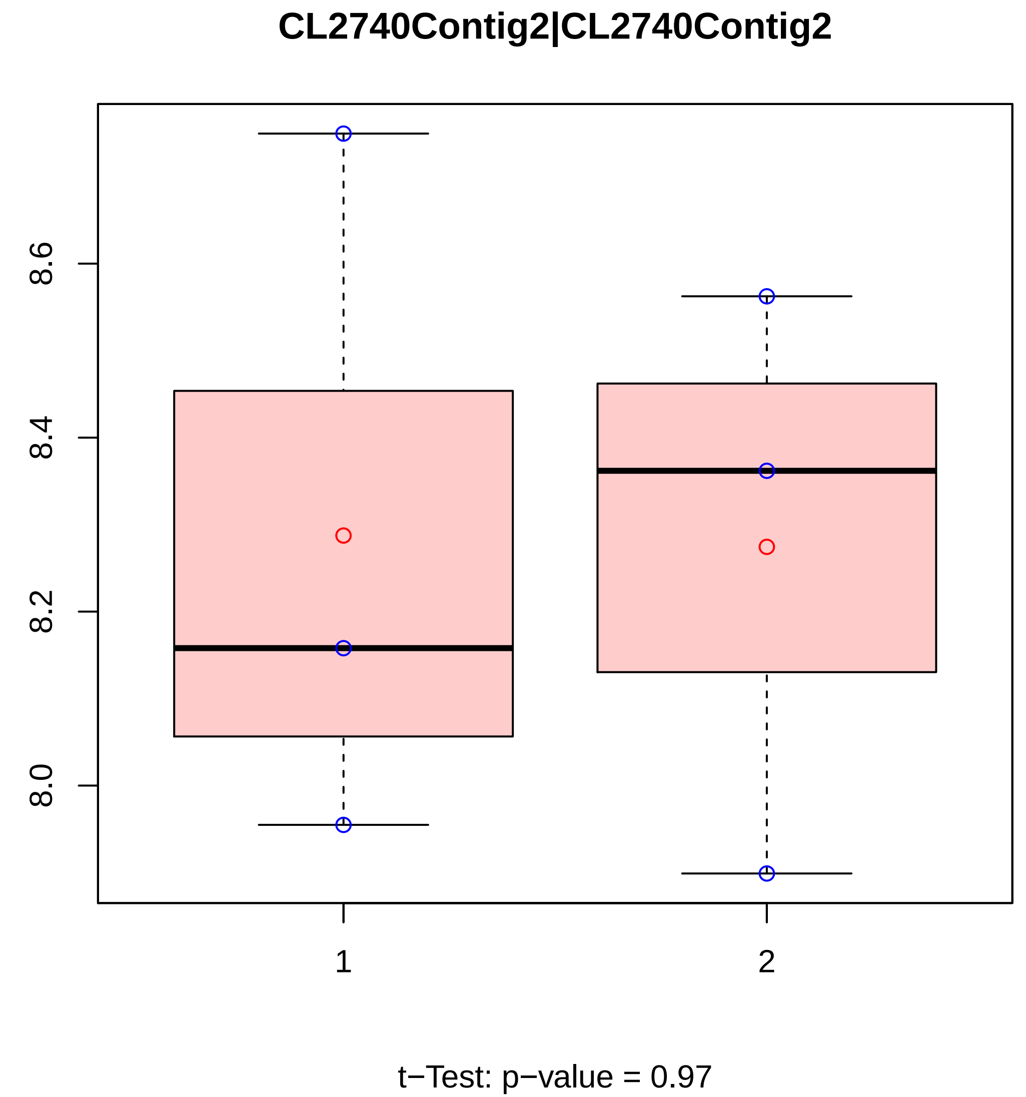
CL2740Contig2|CL2740Contig2
8.0
8.2
8.4
8.6
1
2
t−Test: p−value = 0.97

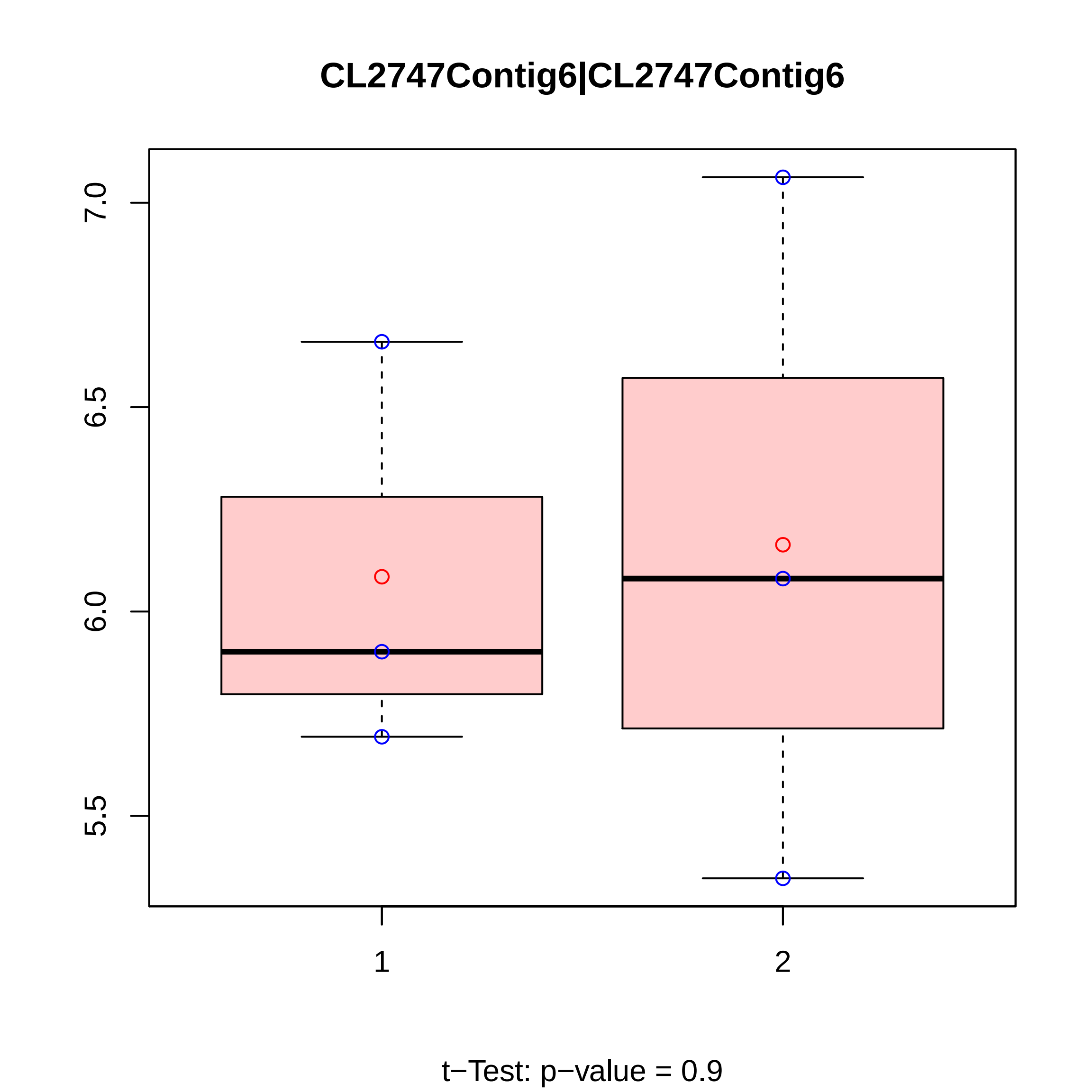
CL2747Contig6|CL2747Contig6
7.0
6.5
6.0
5.5
1
2
t−Test: p−value = 0.9

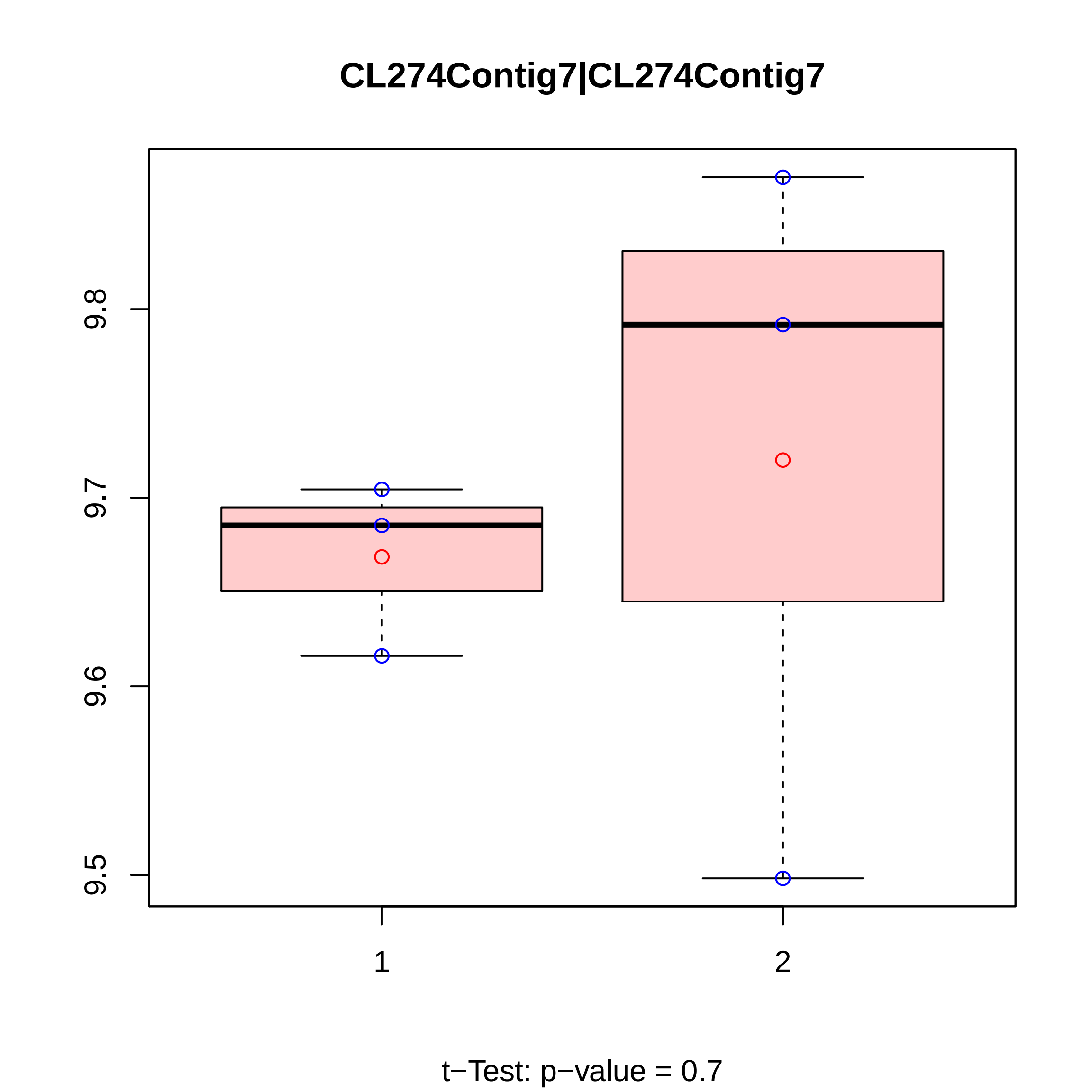

CL274Contig7|CL274Contig7
9.5
9.6
9.7
9.8
1
2
t−Test: p−value = 0.7

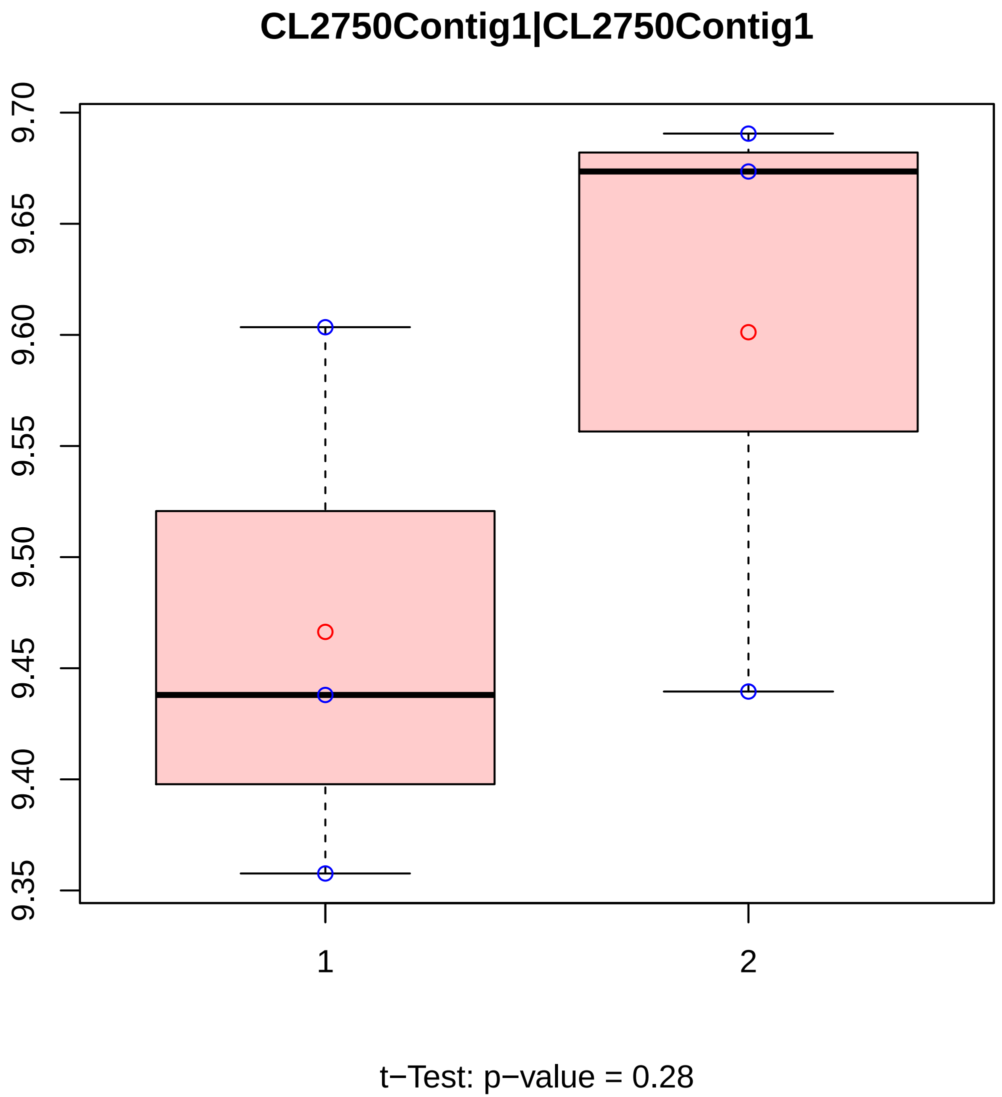
CL2750Contig1|CL2750Contig1
9.70
9.65
9.60
9.55
9.50
9.45
9.40
9.35
1
2
t−Test: p−value = 0.28

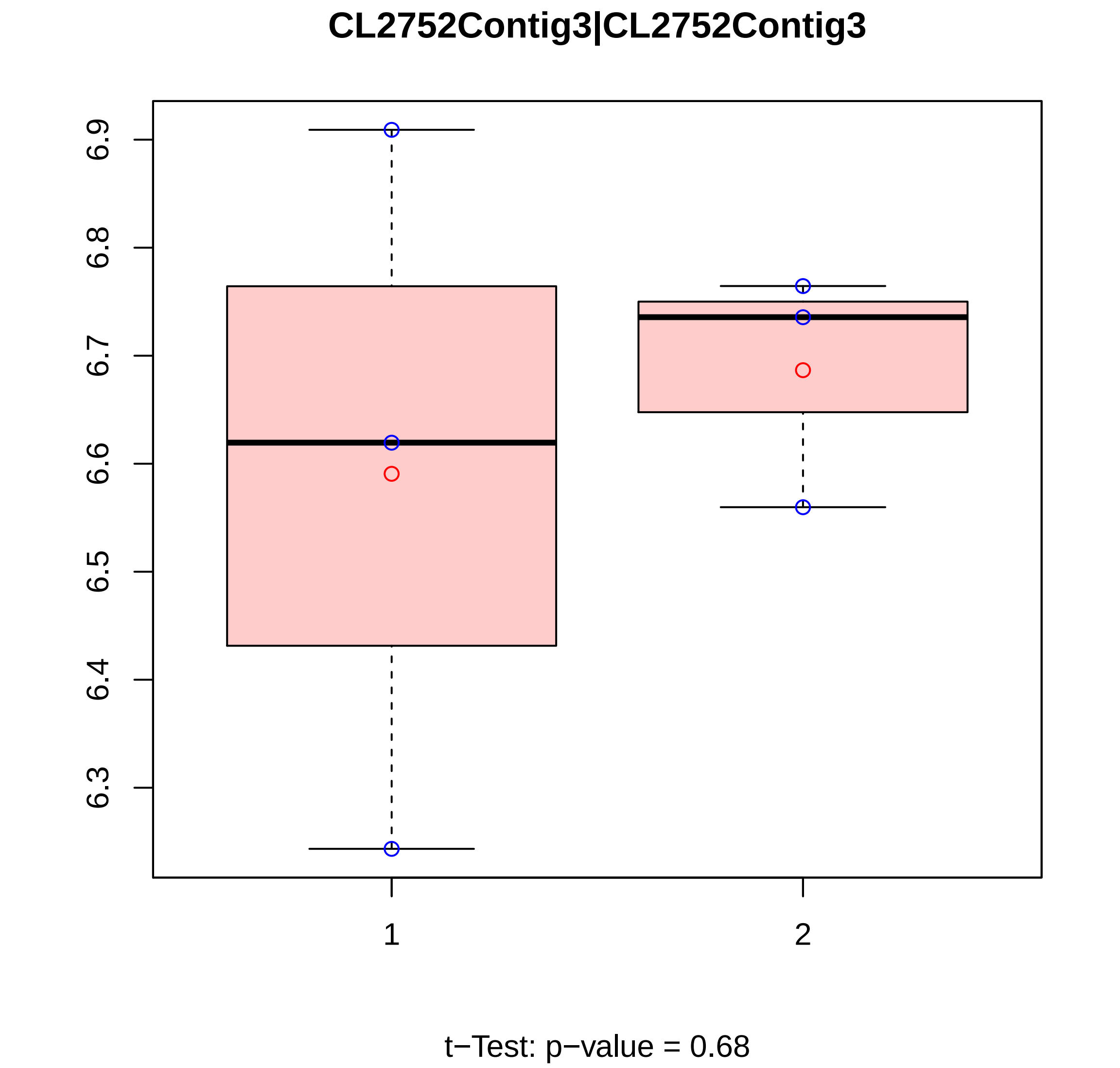
CL2752Contig3|CL2752Contig3
6.9
6.8
6.7
6.6
6.5
6.4
6.3
1
2
t−Test: p−value = 0.68

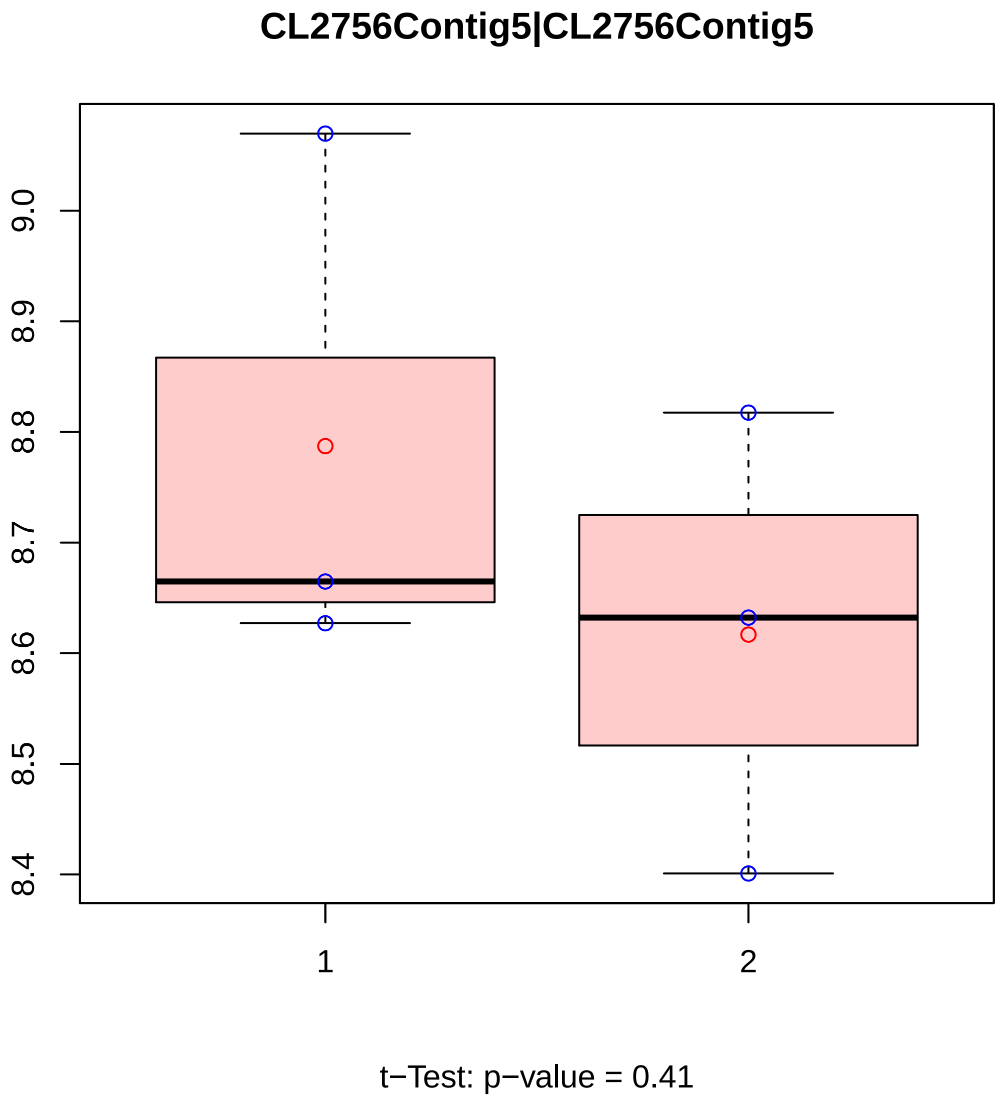

CL2756Contig5|CL2756Contig5
9.0
8.9
8.8
8.7
8.6
8.5
8.4
1
2
t−Test: p−value = 0.41

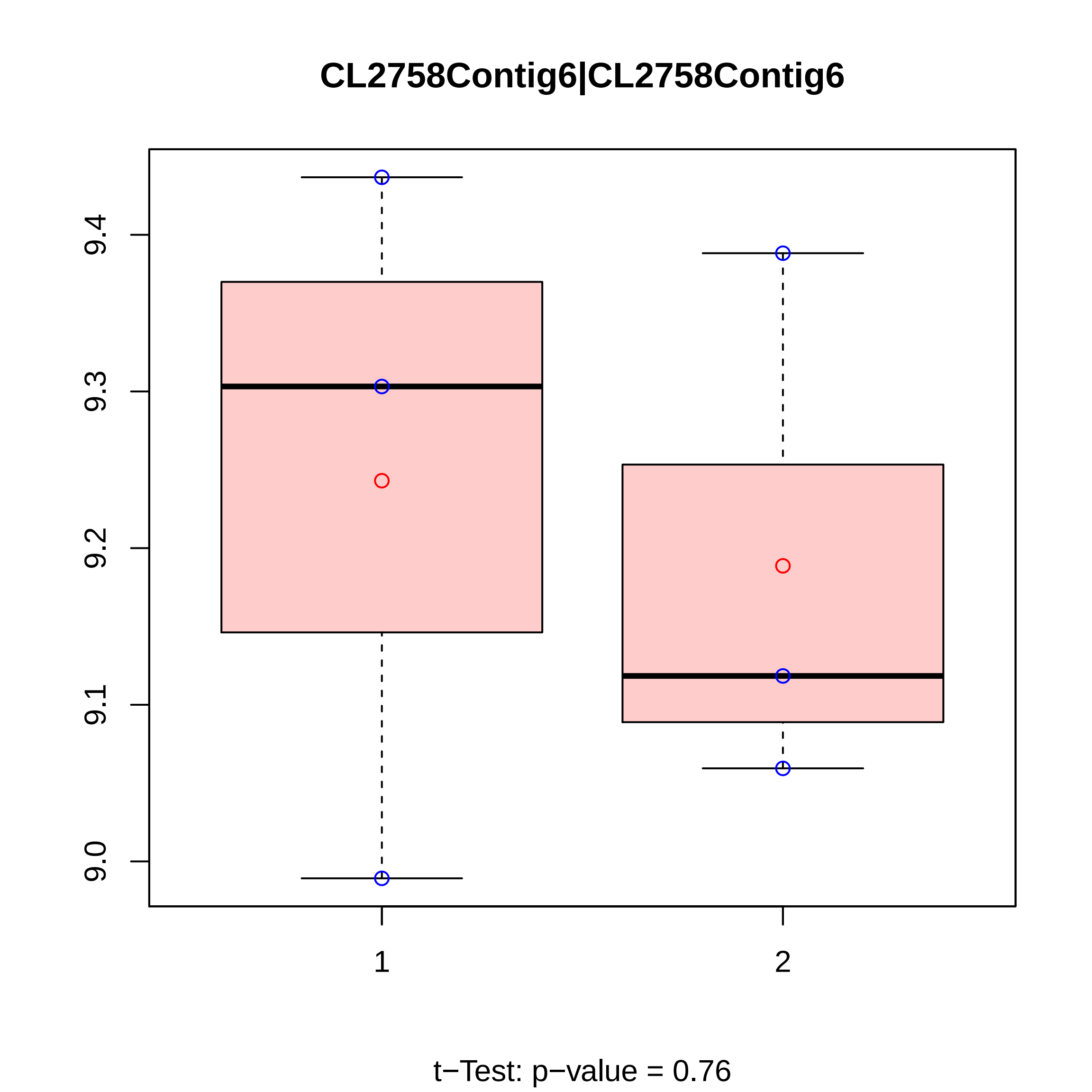

CL2758Contig6|CL2758Contig6
9.0
9.1
9.2
9.3
9.4
1
2
t−Test: p−value = 0.76

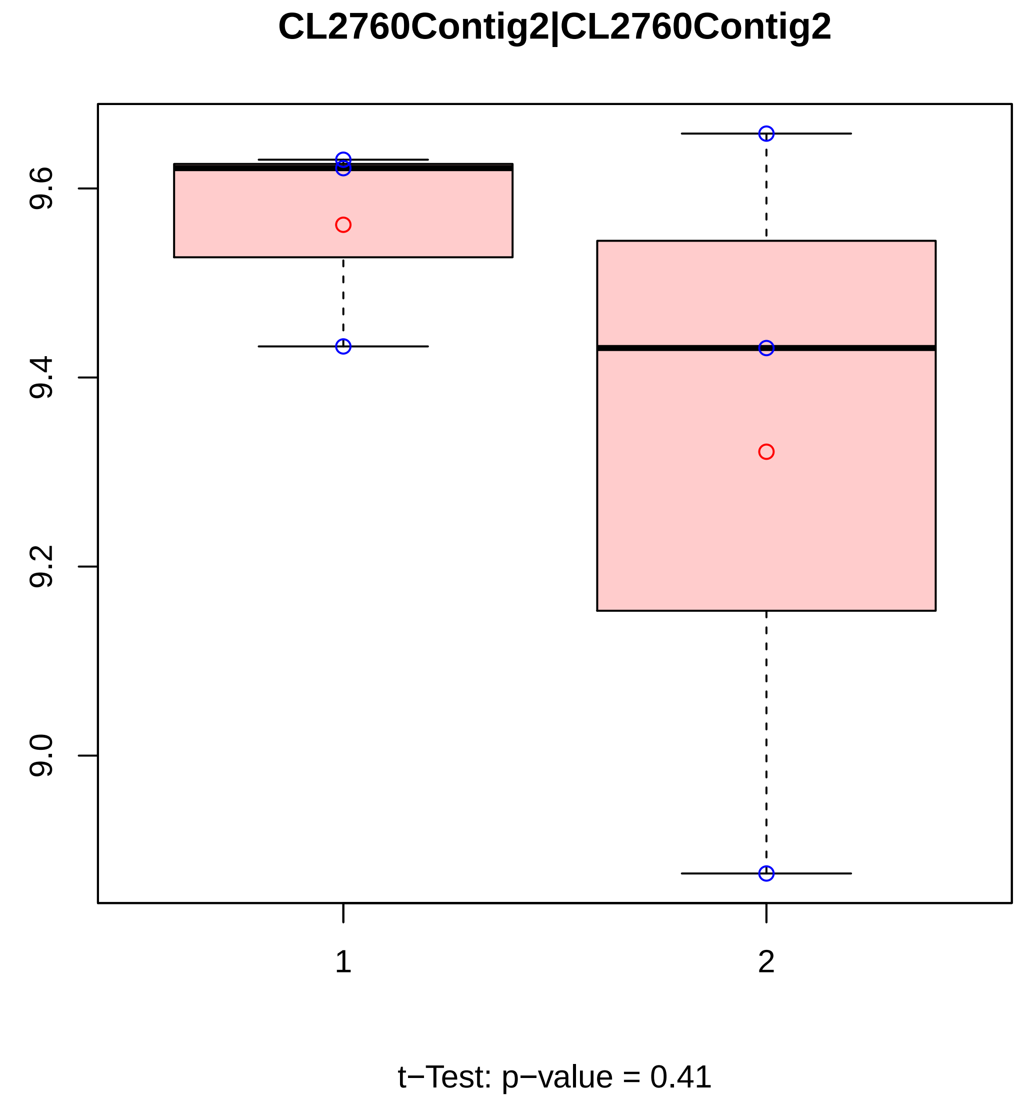
CL2760Contig2|CL2760Contig2
9.0
9.2
9.4
9.6
1
2
t−Test: p−value = 0.41

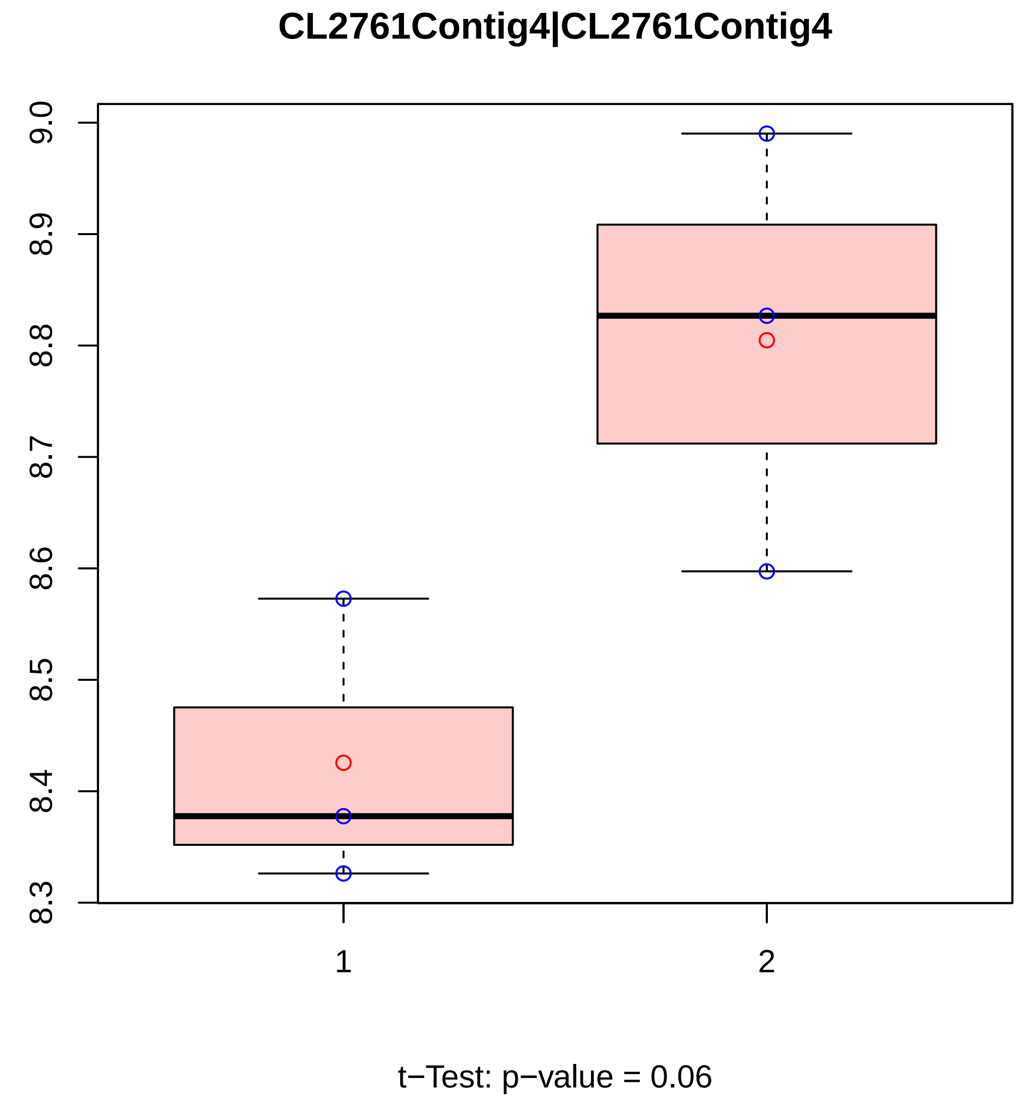
CL2761Contig4|CL2761Contig4
9.0
8.9
8.8
8.7
8.6
8.5
8.4
8.3
1
2
t−Test: p−value = 0.06

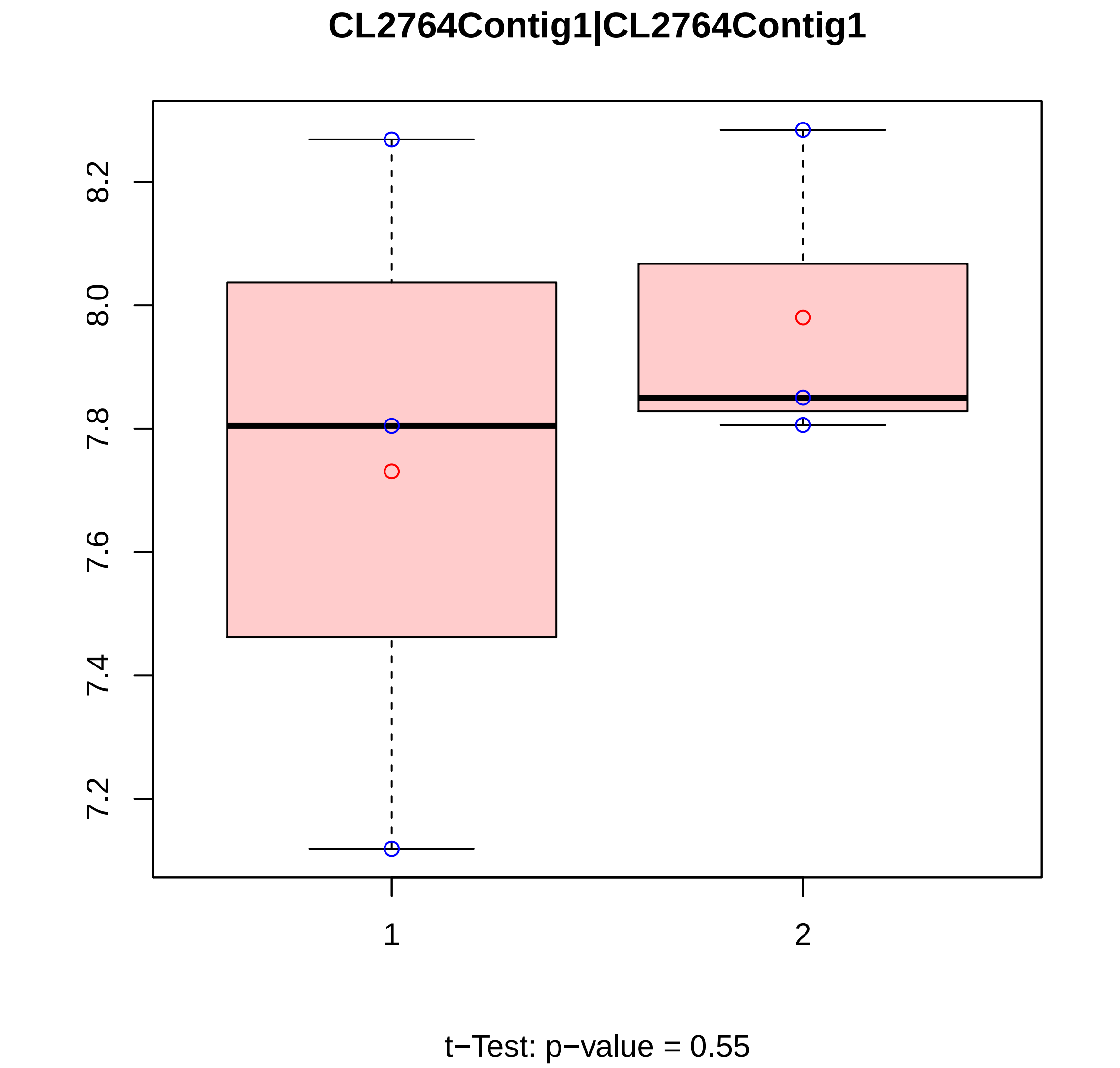
CL2764Contig1|CL2764Contig1
8.2
8.0
7.8
7.6
7.4
7.2
1
2
t−Test: p−value = 0.55

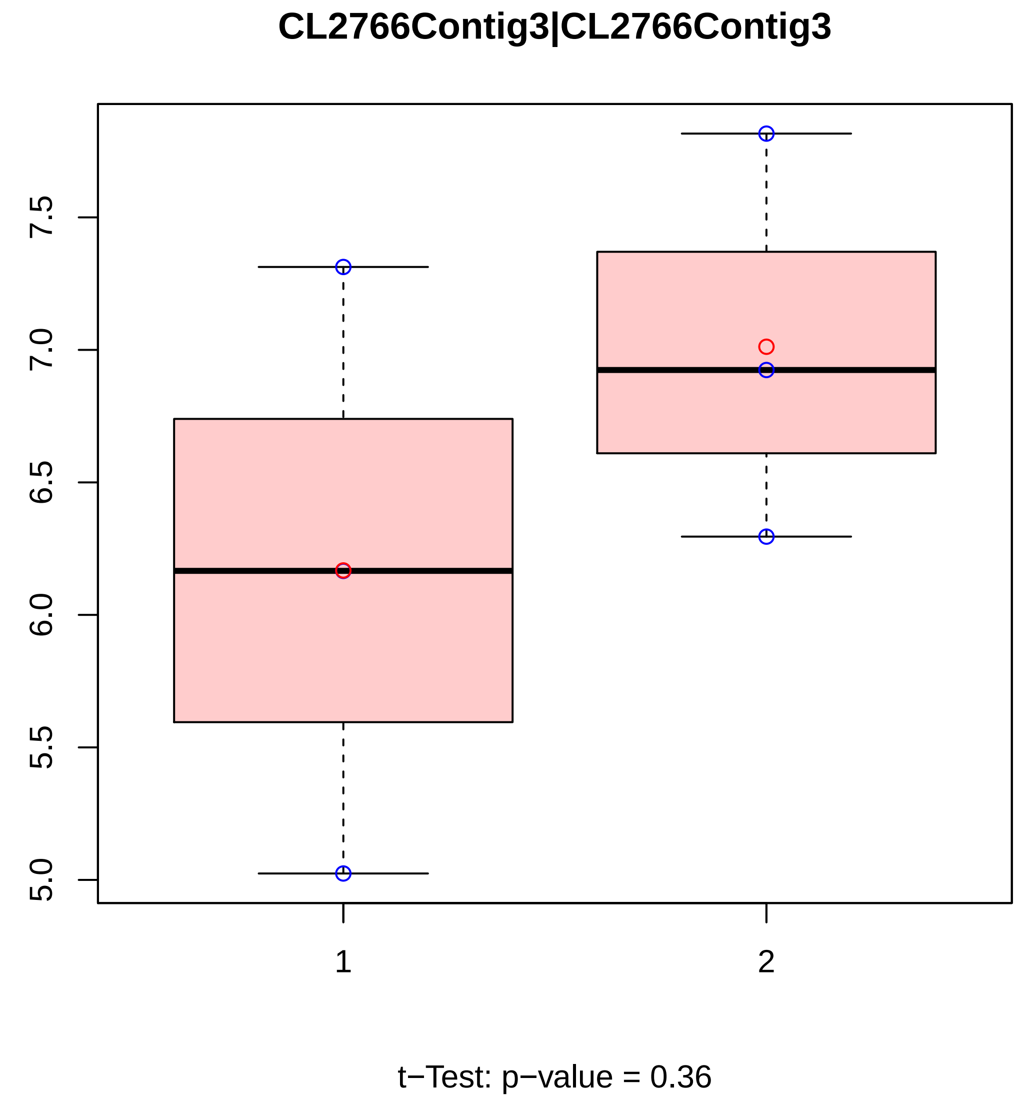
CL2766Contig3|CL2766Contig3
7.5
7.0
6.5
6.0
5.5
5.0
1
2
t−Test: p−value = 0.36

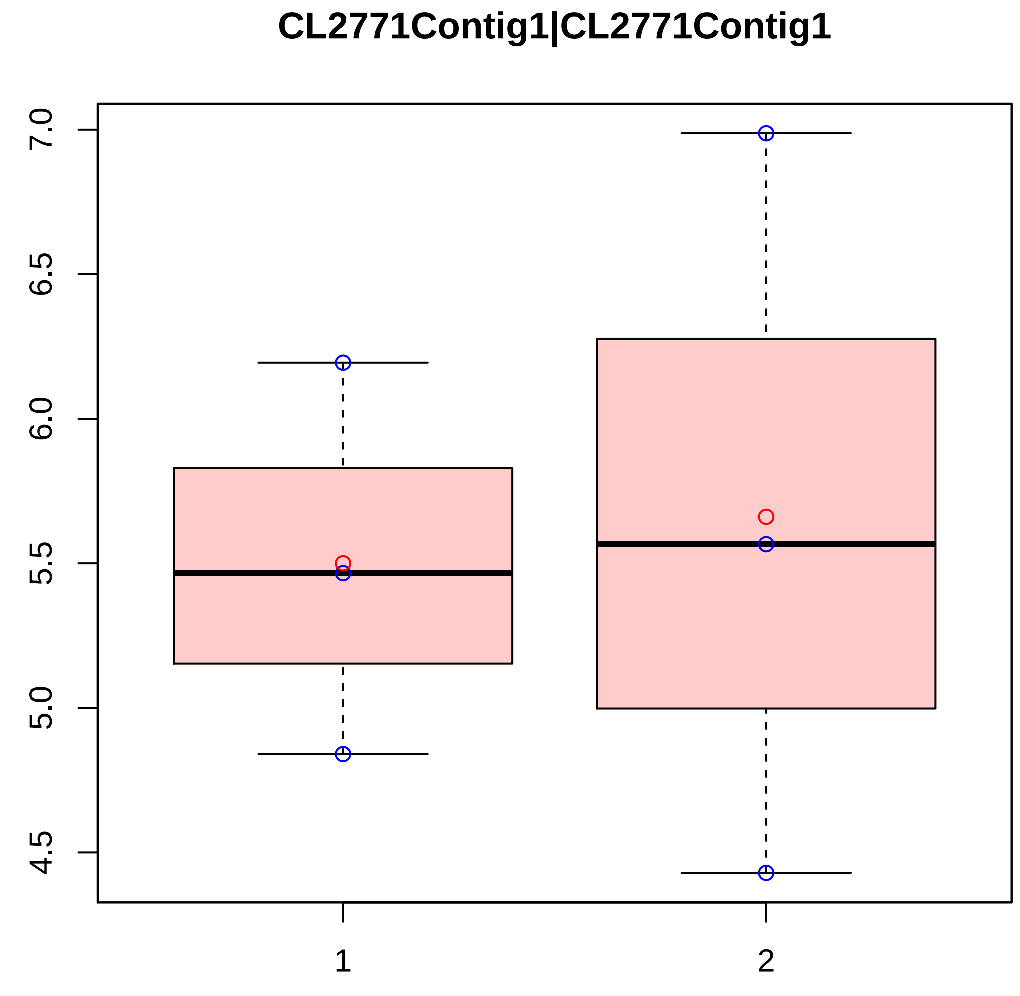


t−Test: p−value = 0.86

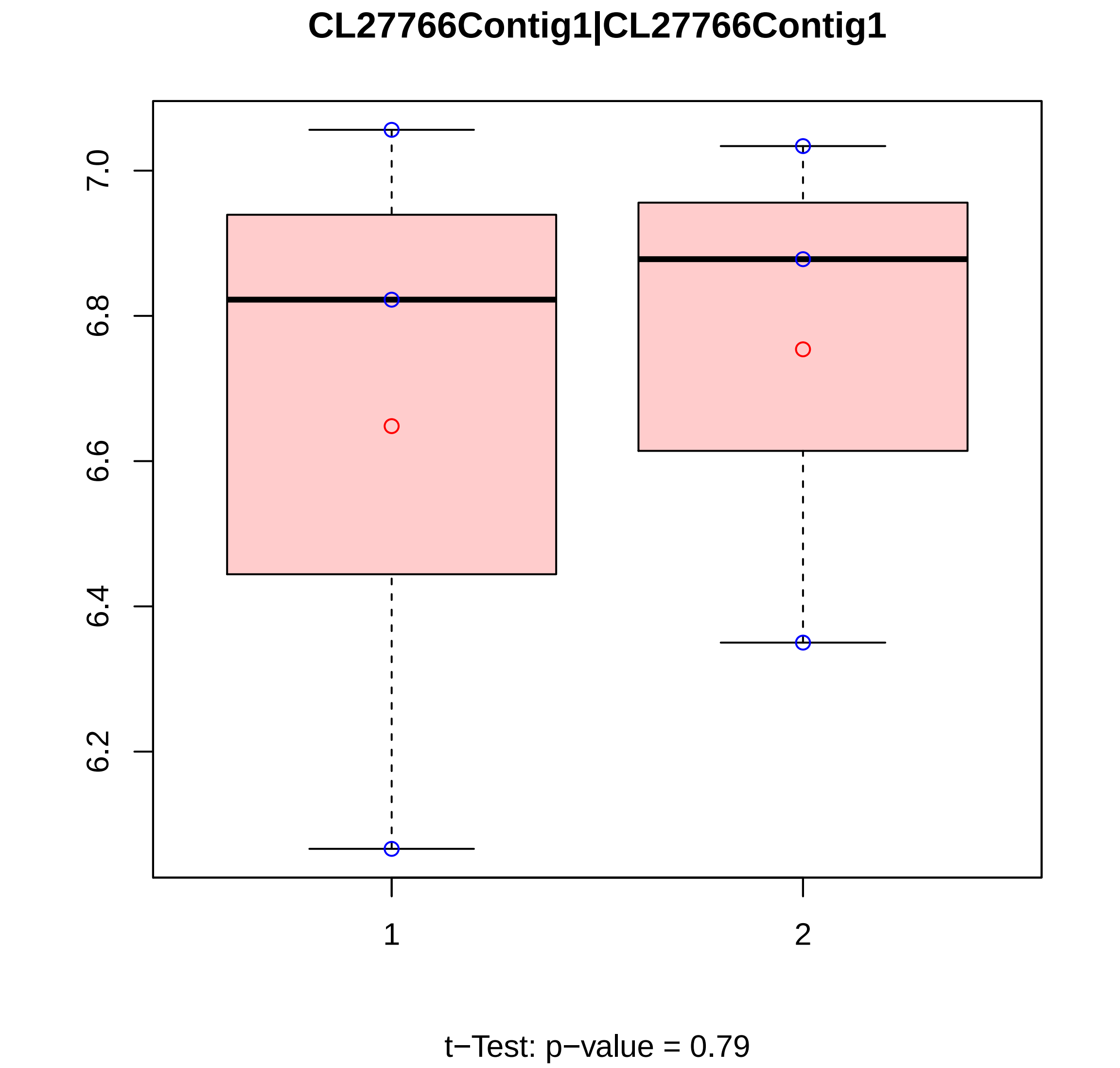

CL27766Contig1|CL27766Contig1
7.0
6.8
6.6
6.4
6.2
1
2
t−Test: p−value = 0.79

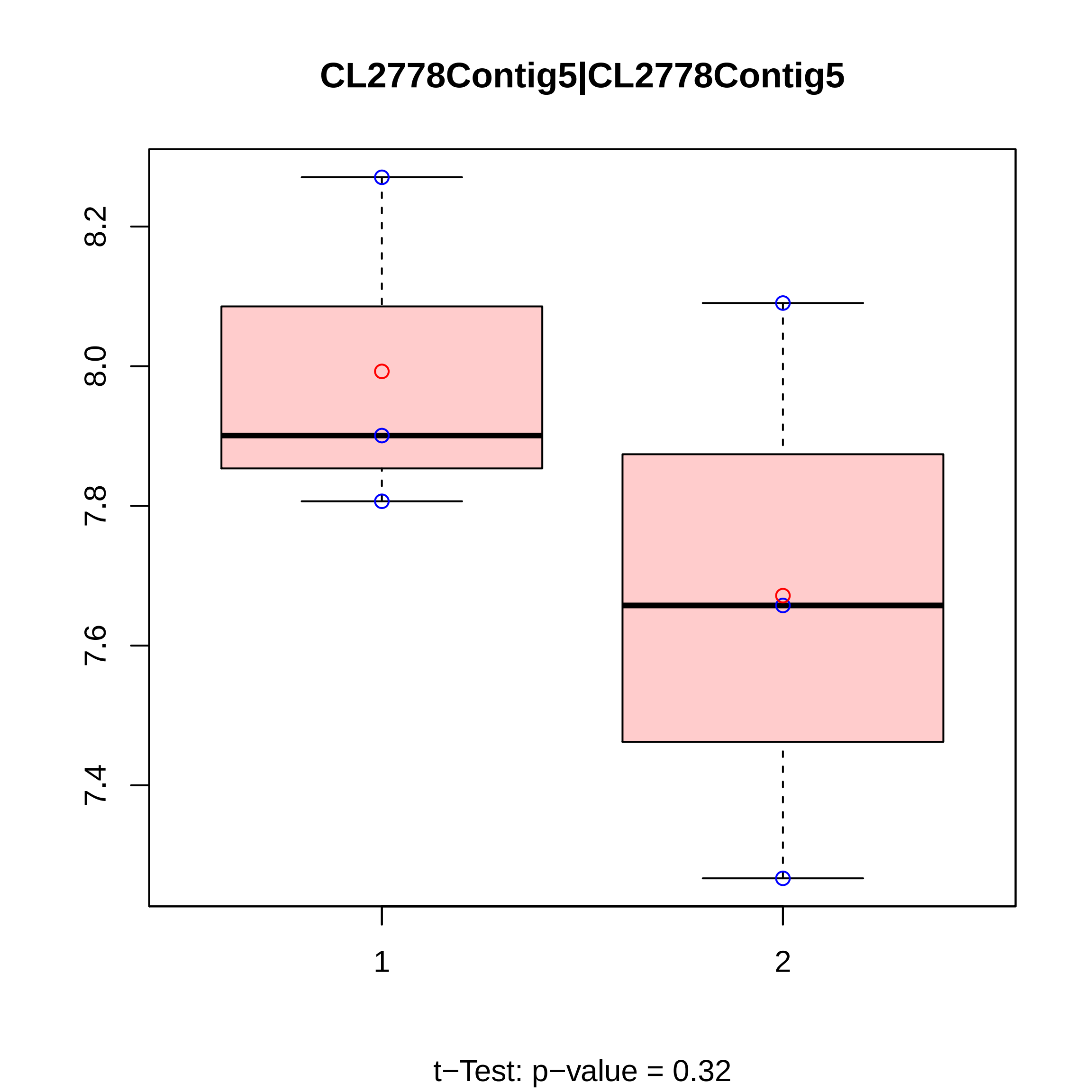
CL2778Contig5|CL2778Contig5
8.2
8.0
7.8
7.6
7.4
1
2
t−Test: p−value = 0.32

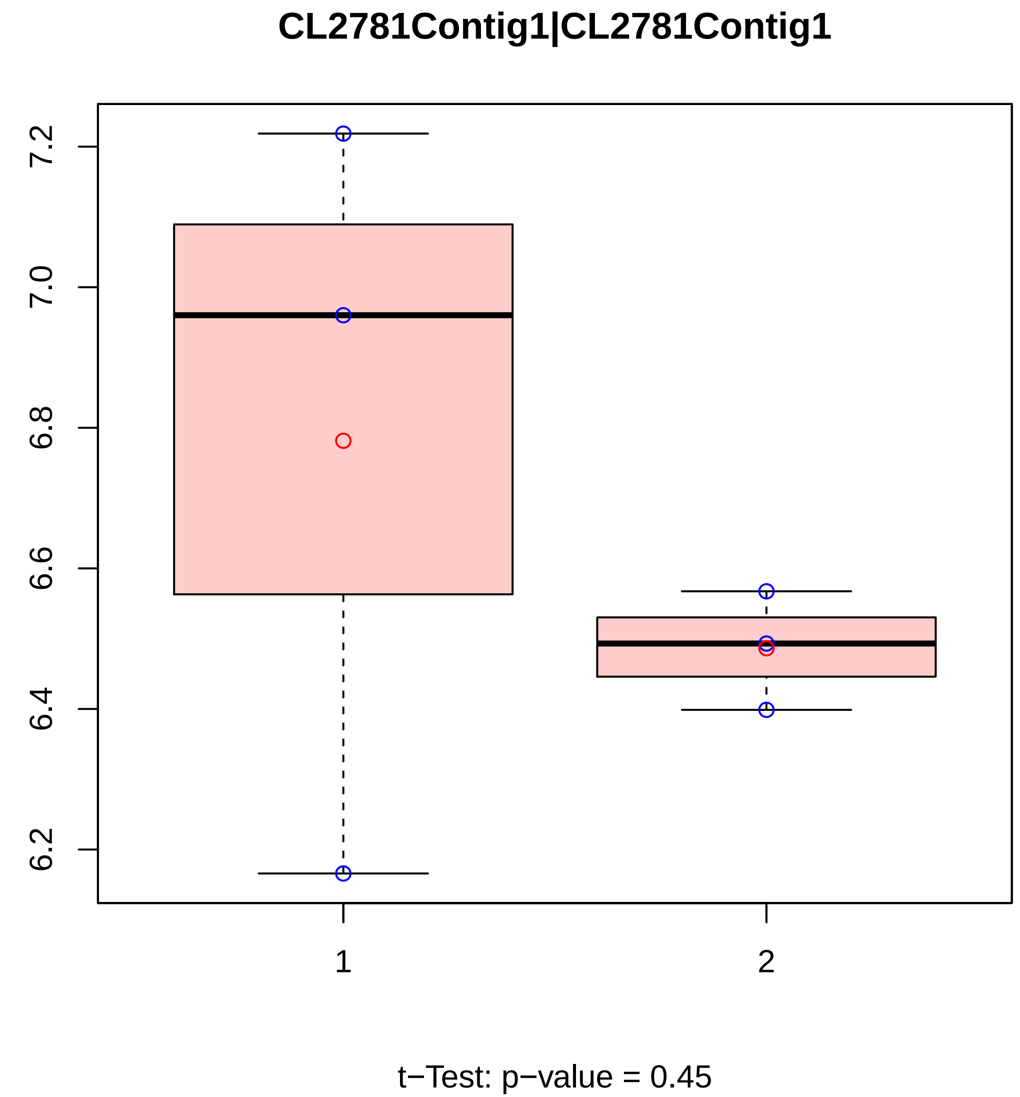
CL2781Contig1|CL2781Contig1
7.2
7.0
6.8
6.6
6.4
6.2
1
2
t−Test: p−value = 0.45

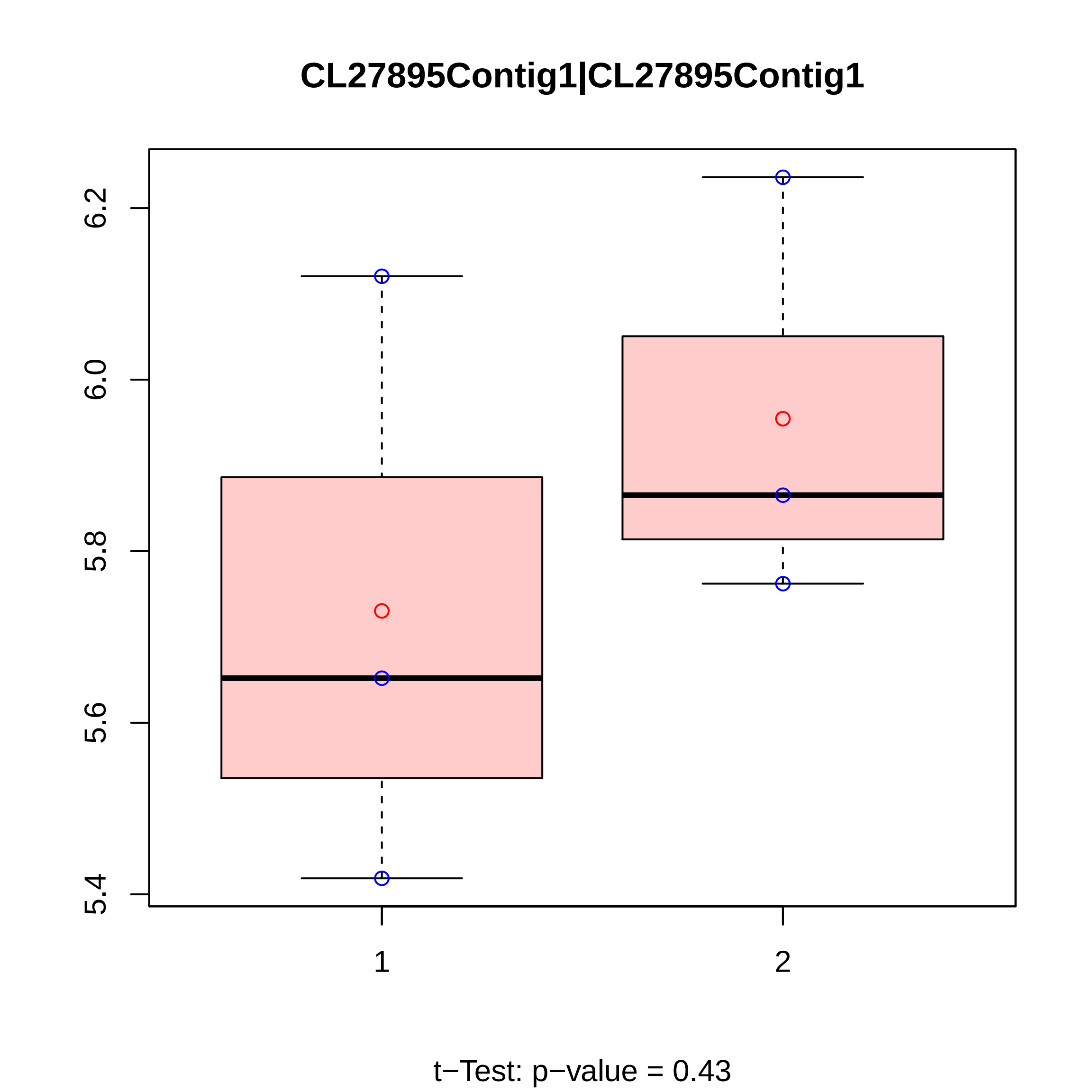

CL27895Contig1|CL27895Contig1
6.2
6.0
5.8
5.6
5.4
1
2
t−Test: p−value = 0.43

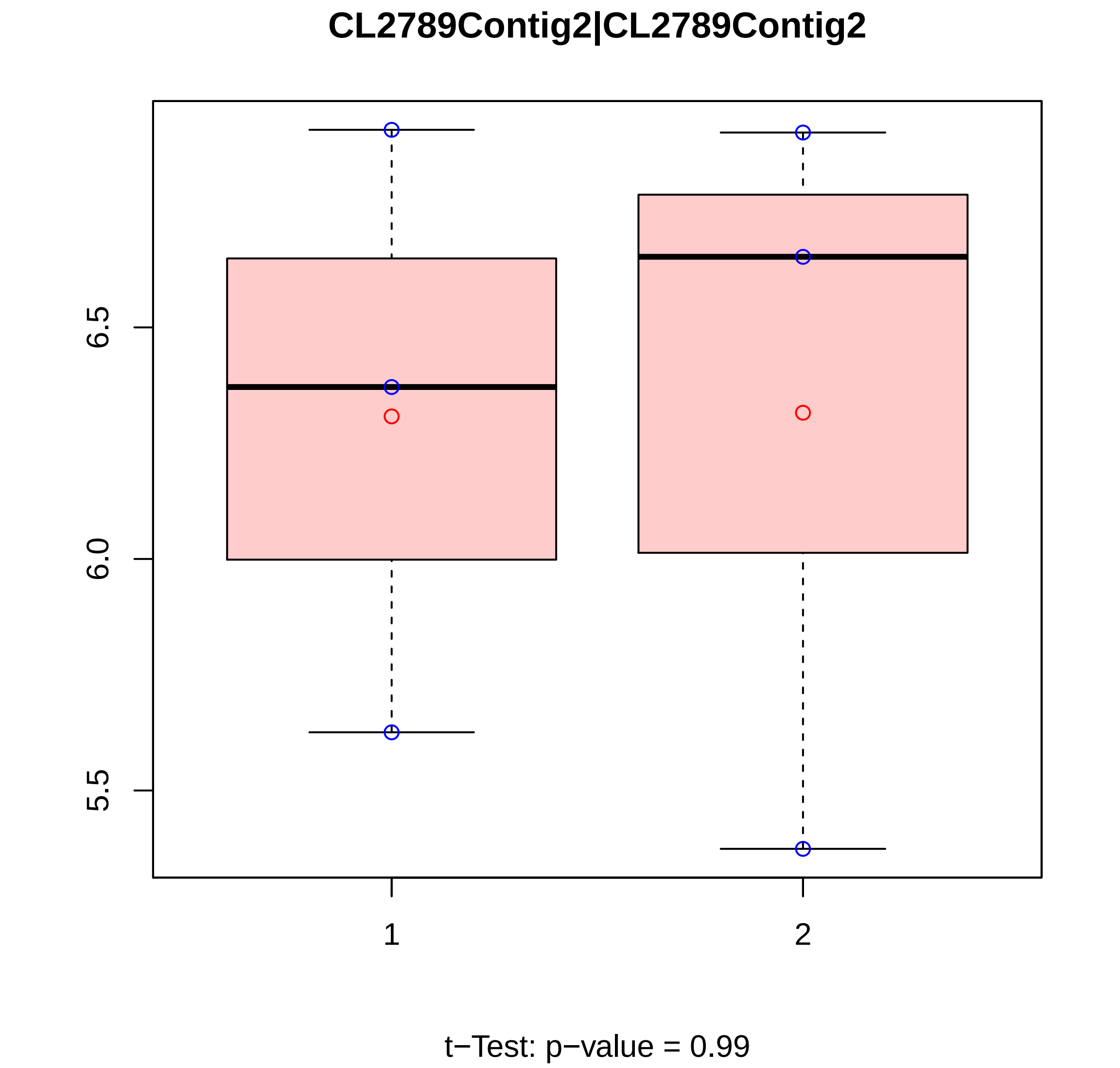
CL2789Contig2|CL2789Contig2
6.5
6.0
5.5
1
2
t−Test: p−value = 0.99

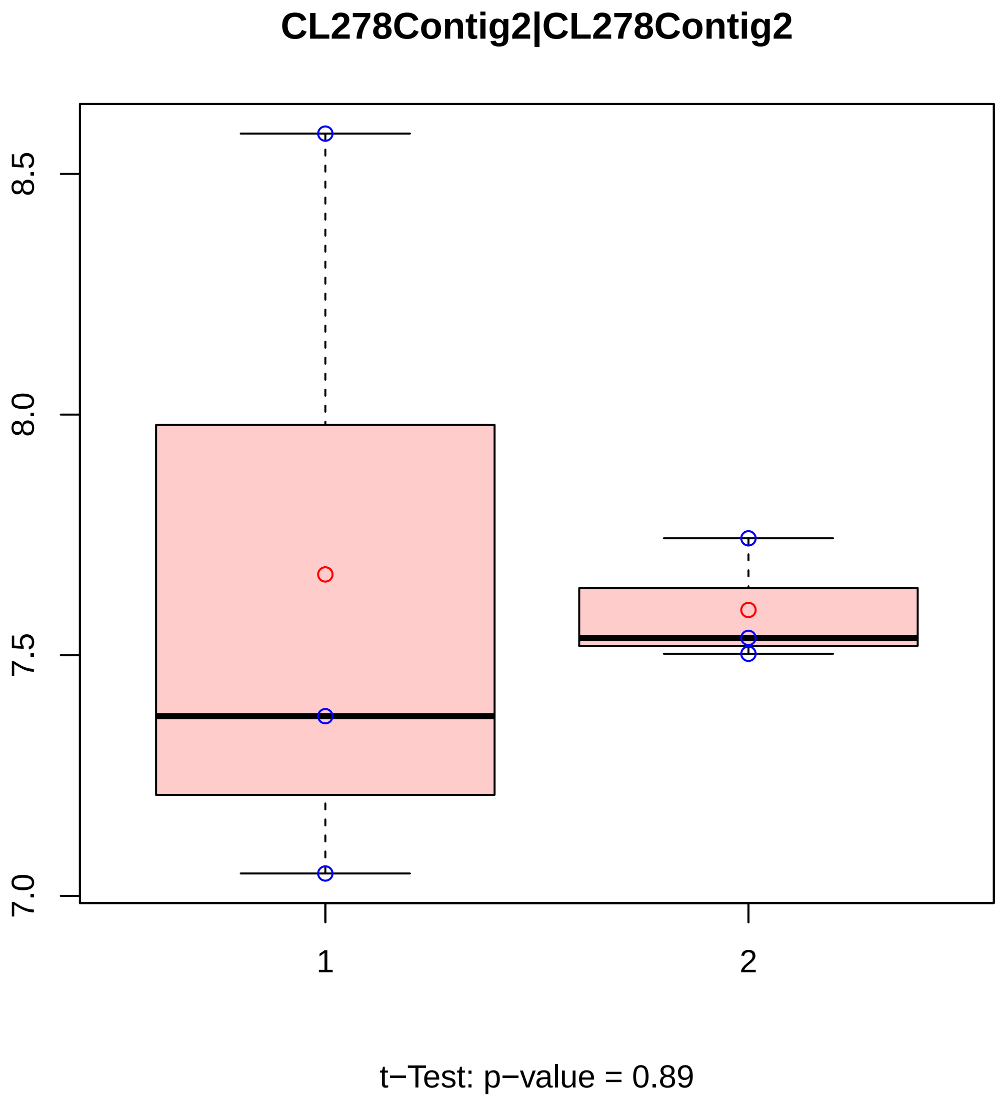
CL278Contig2|CL278Contig2
8.5
8.0
7.5
7.0
1
2
t−Test: p−value = 0.89

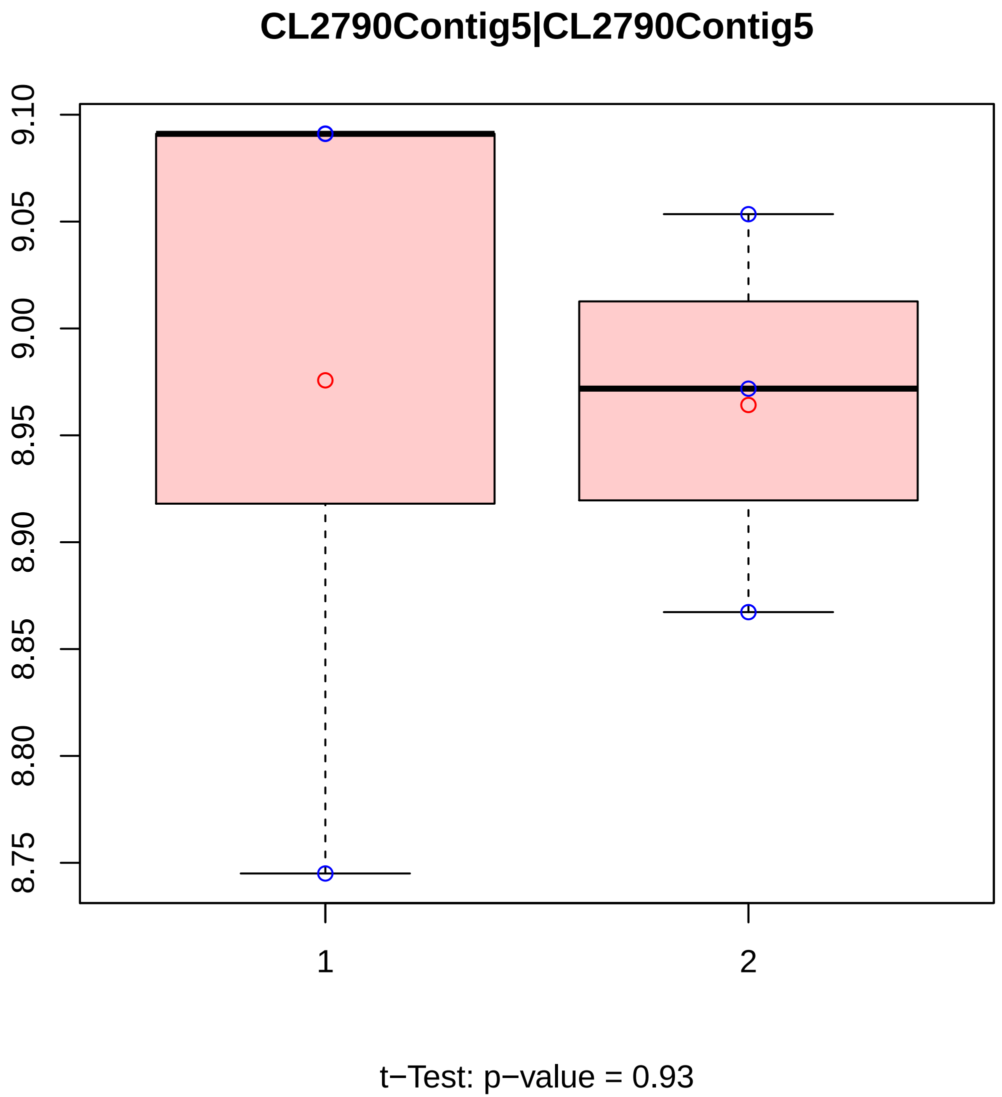

CL2790Contig5|CL2790Contig5
9.10
9.05
9.00
8.95
8.90
8.85
8.80
8.75
1
2
t−Test: p−value = 0.93

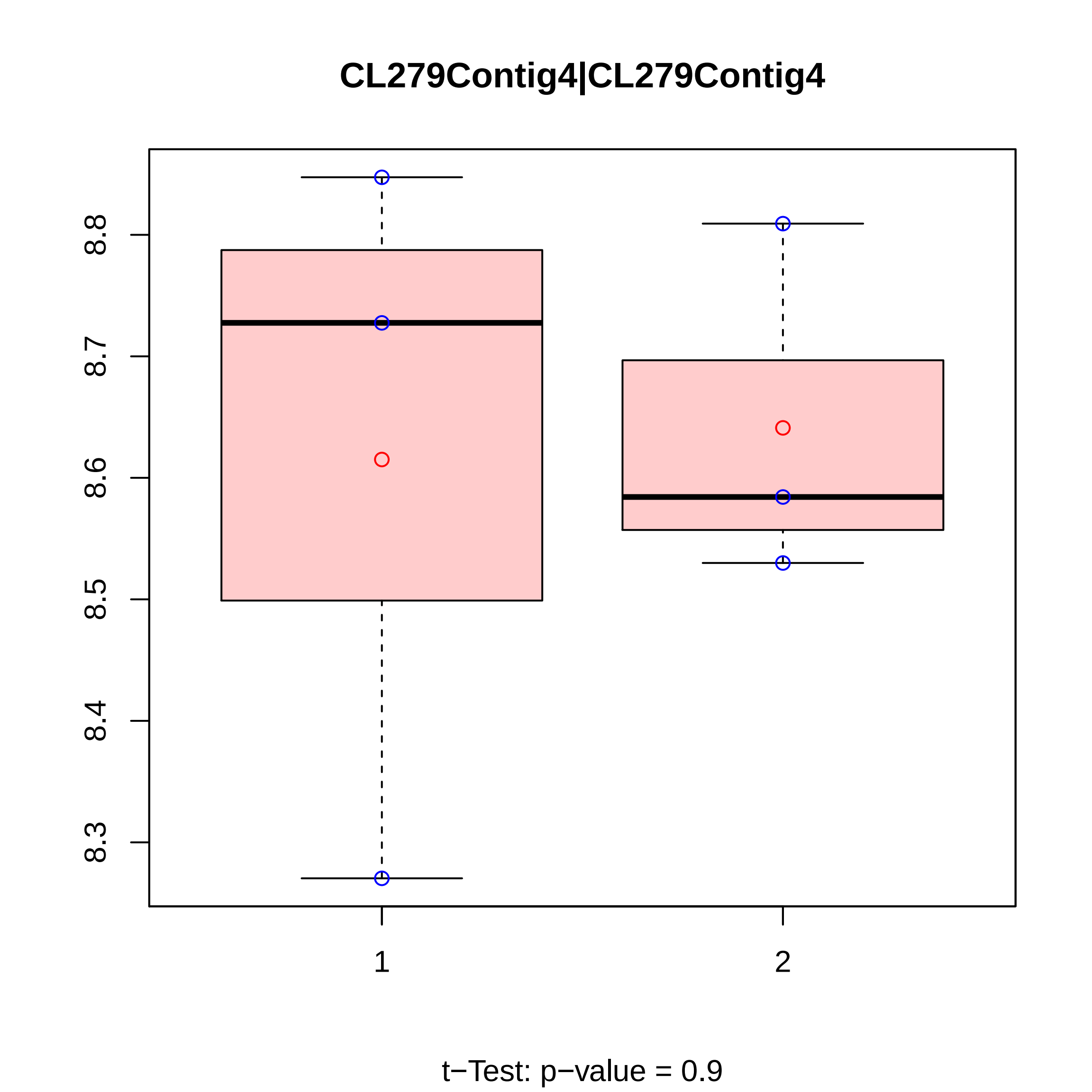

CL279Contig4|CL279Contig4
8.3
8.4
8.5
8.6
8.7
8.8
1
2
t−Test: p−value = 0.9

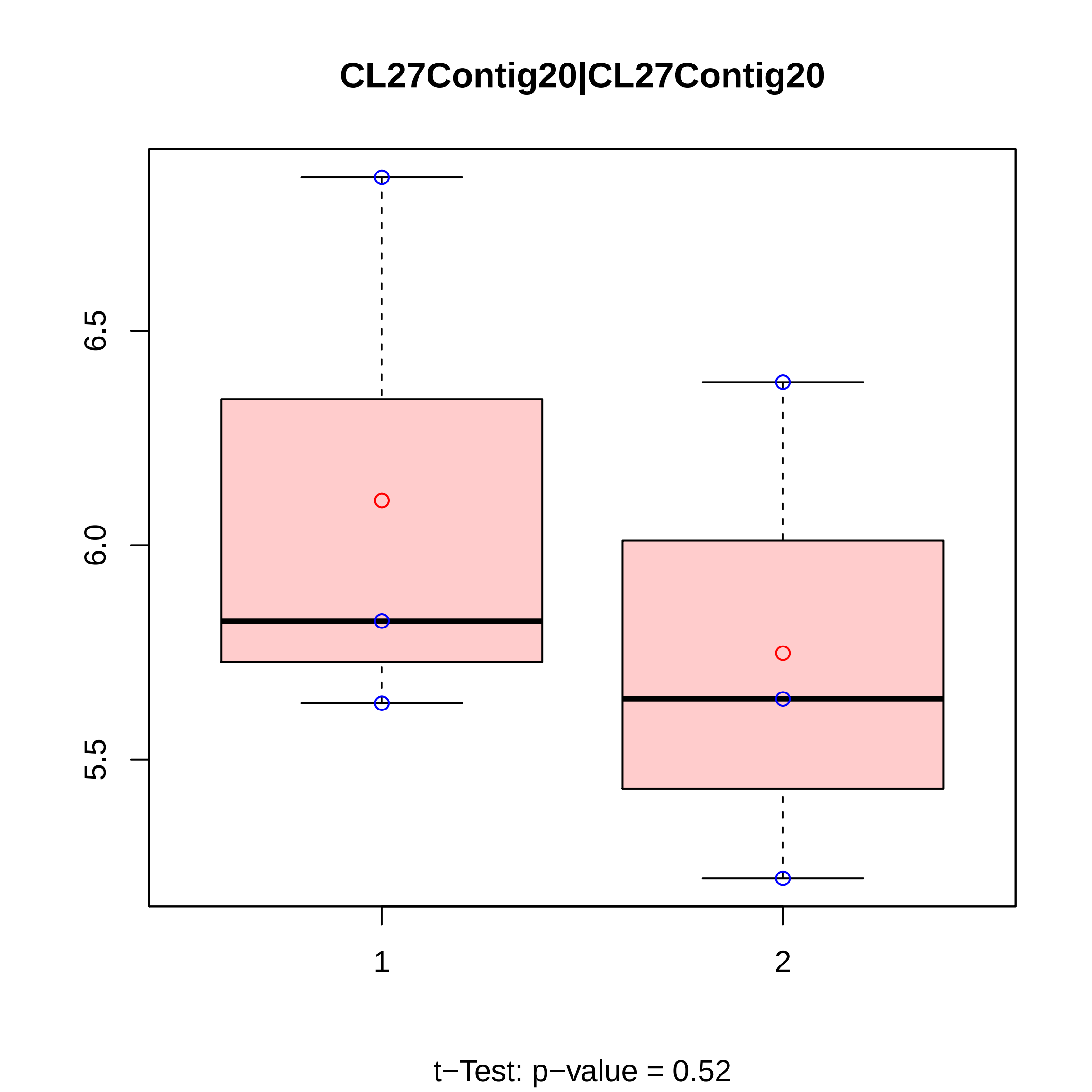

CL27Contig20|CL27Contig20
6.5
6.0
5.5
1
2
t−Test: p−value = 0.52

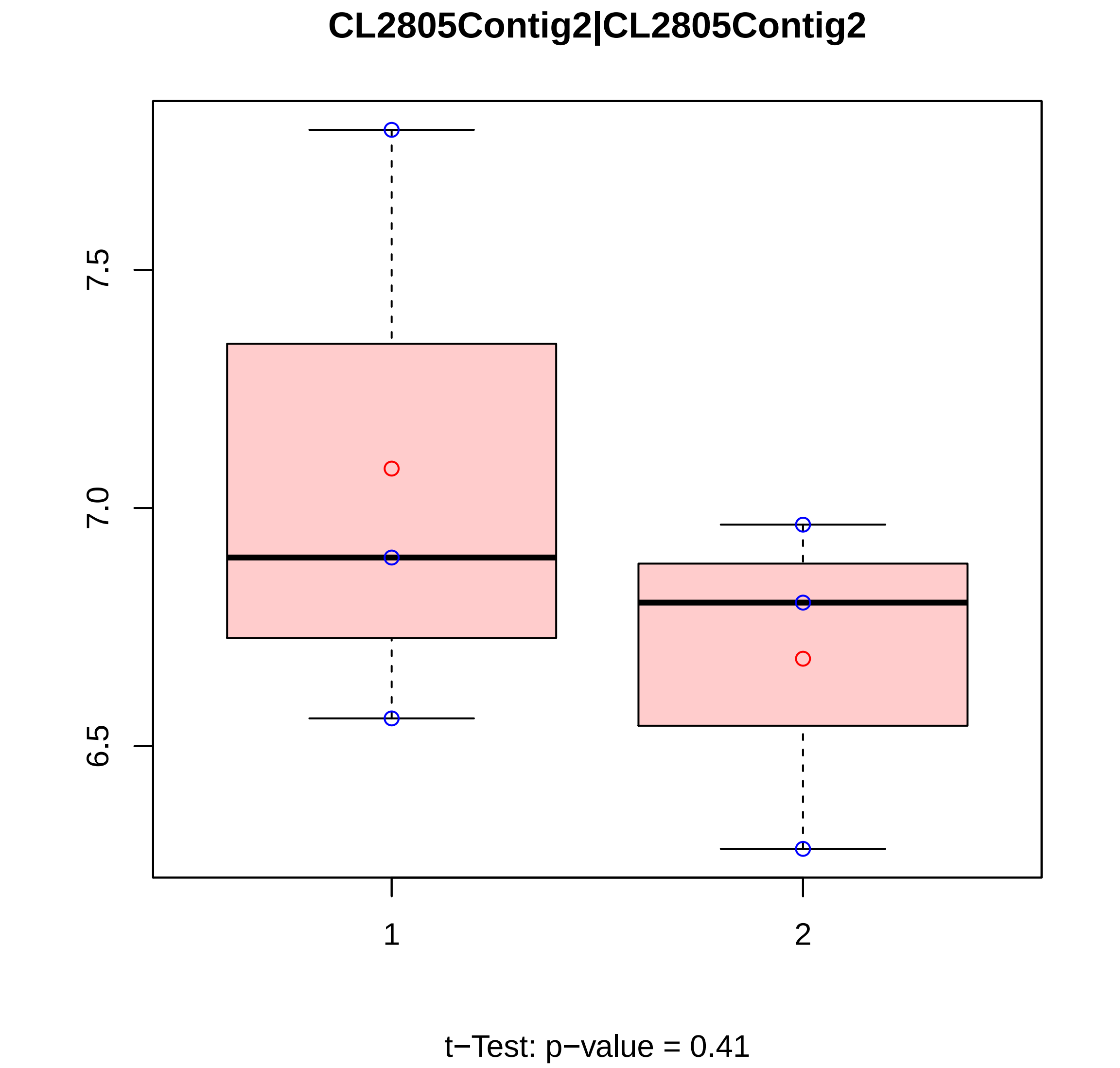
CL2805Contig2|CL2805Contig2
7.5
7.0
6.5
1
2
t−Test: p−value = 0.41

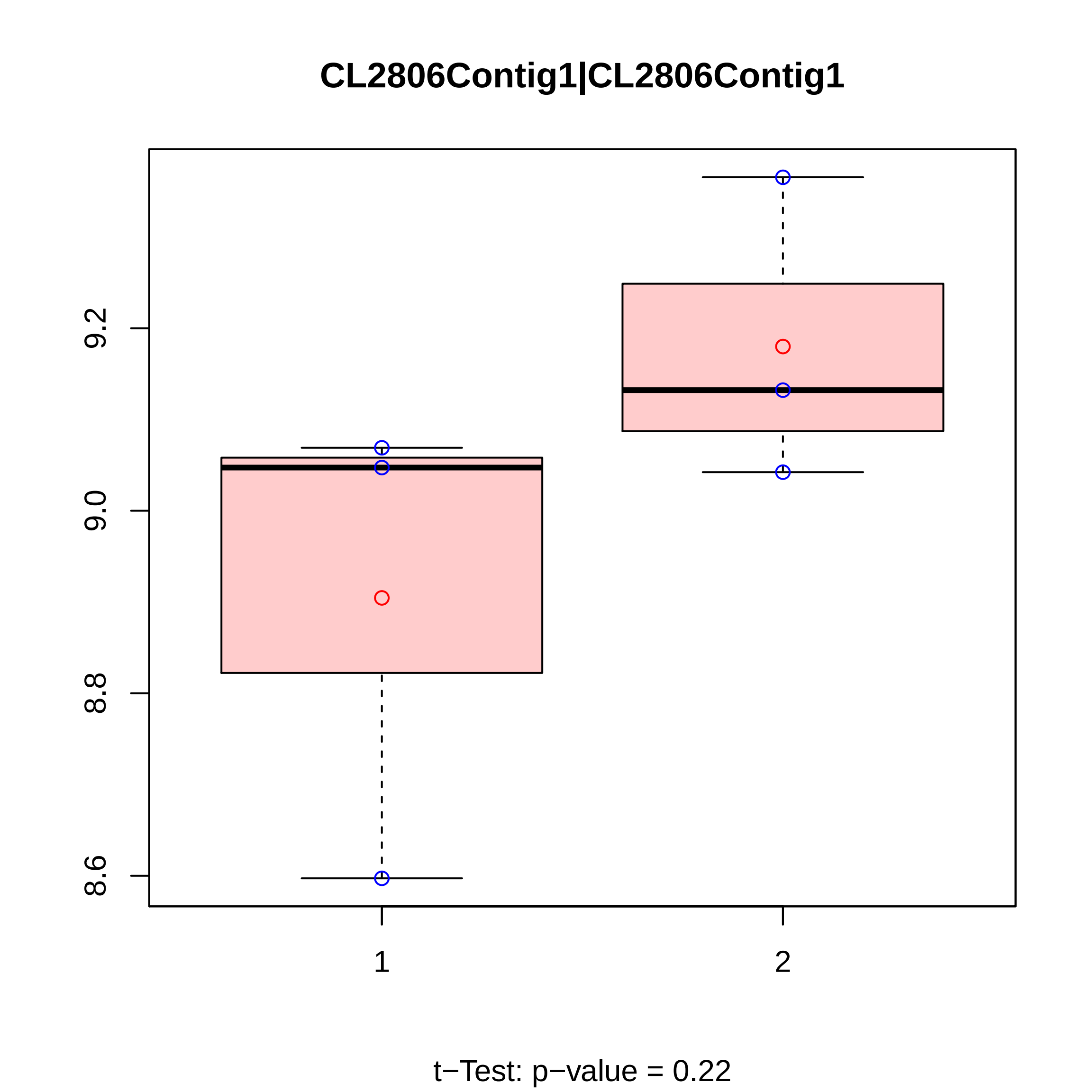
CL2806Contig1|CL2806Contig1
9.2
9.0
8.8
8.6
1
2
t−Test: p−value = 0.22

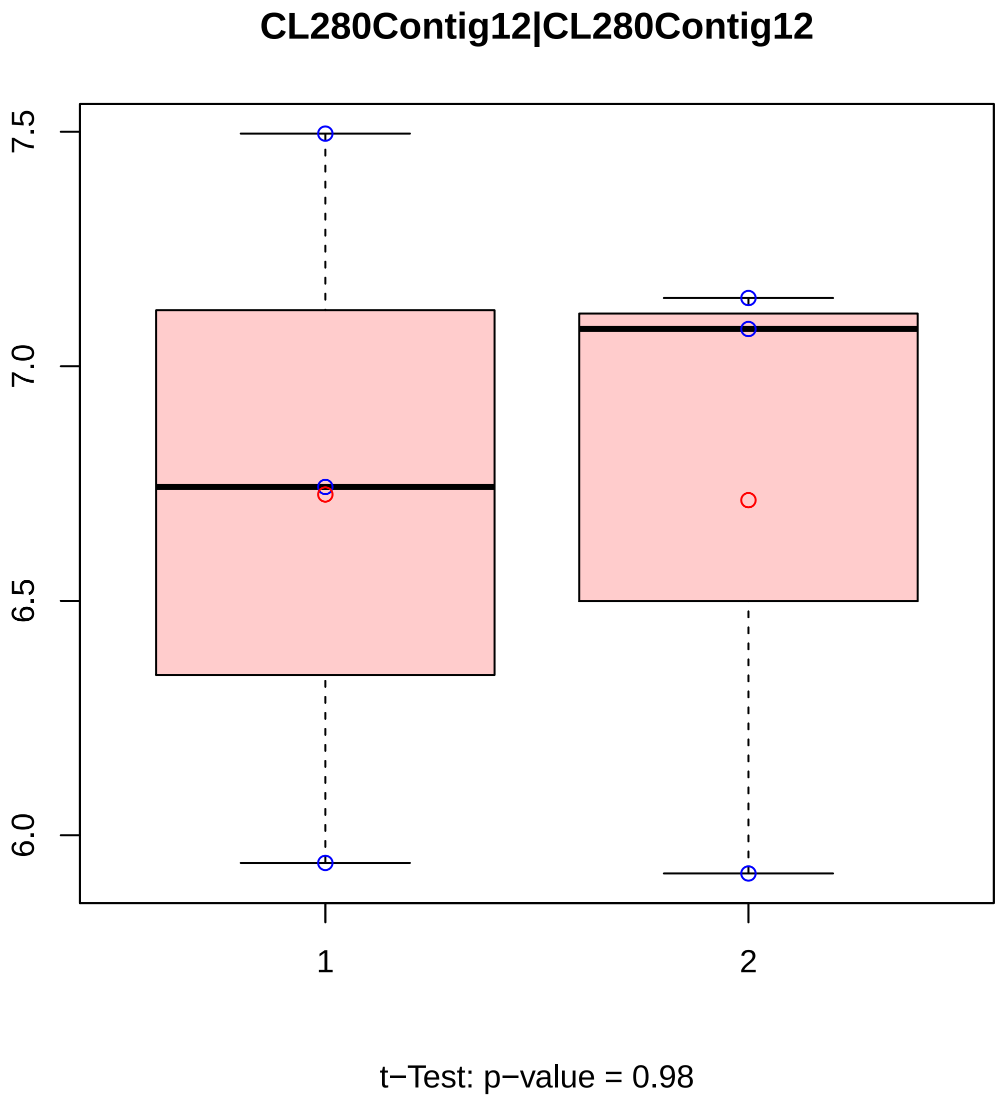
CL280Contig12|CL280Contig12
7.5
7.0
6.5
6.0
1
2
t−Test: p−value = 0.98

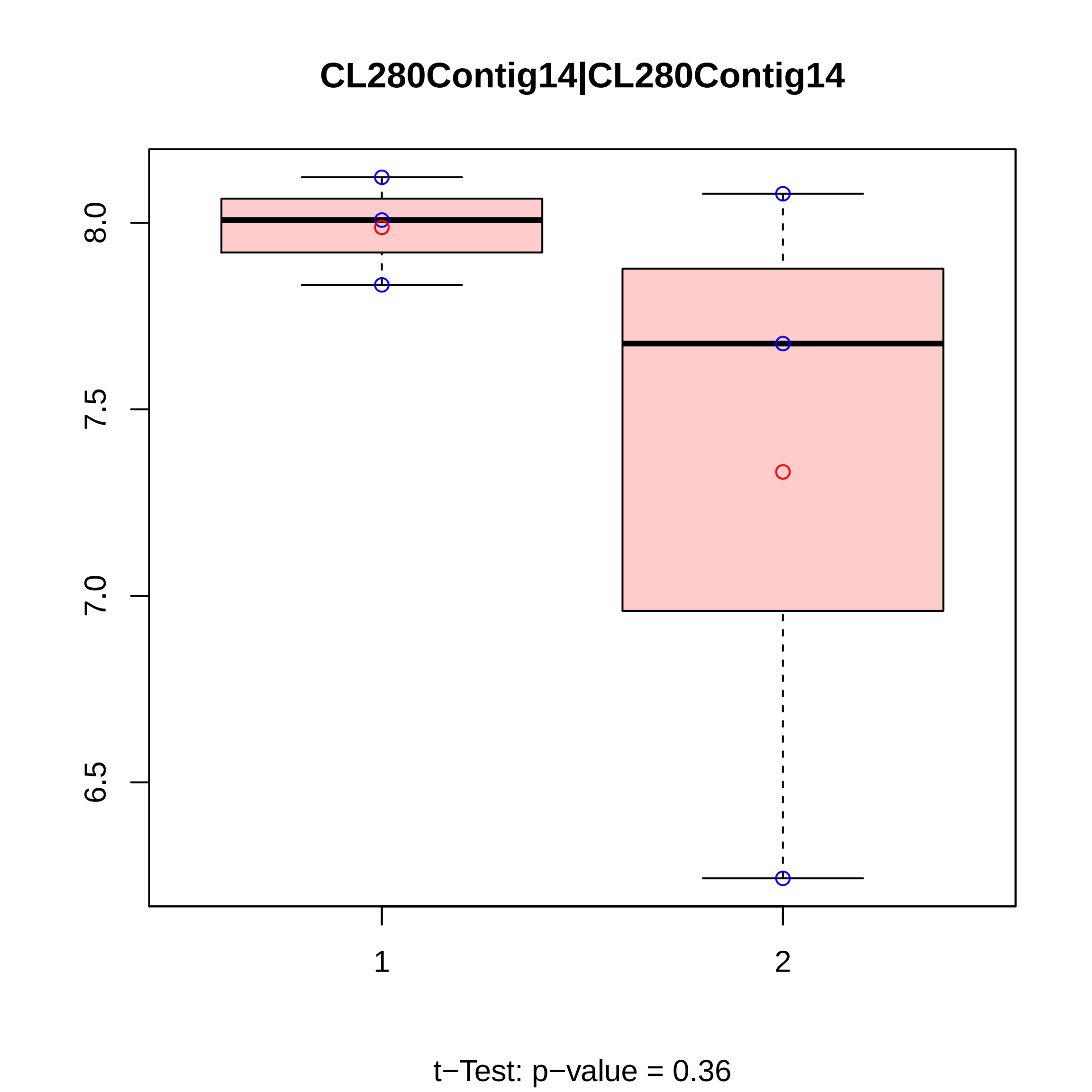
CL280Contig14|CL280Contig14
8.0
7.5
7.0
6.5
1
2
t−Test: p−value = 0.36

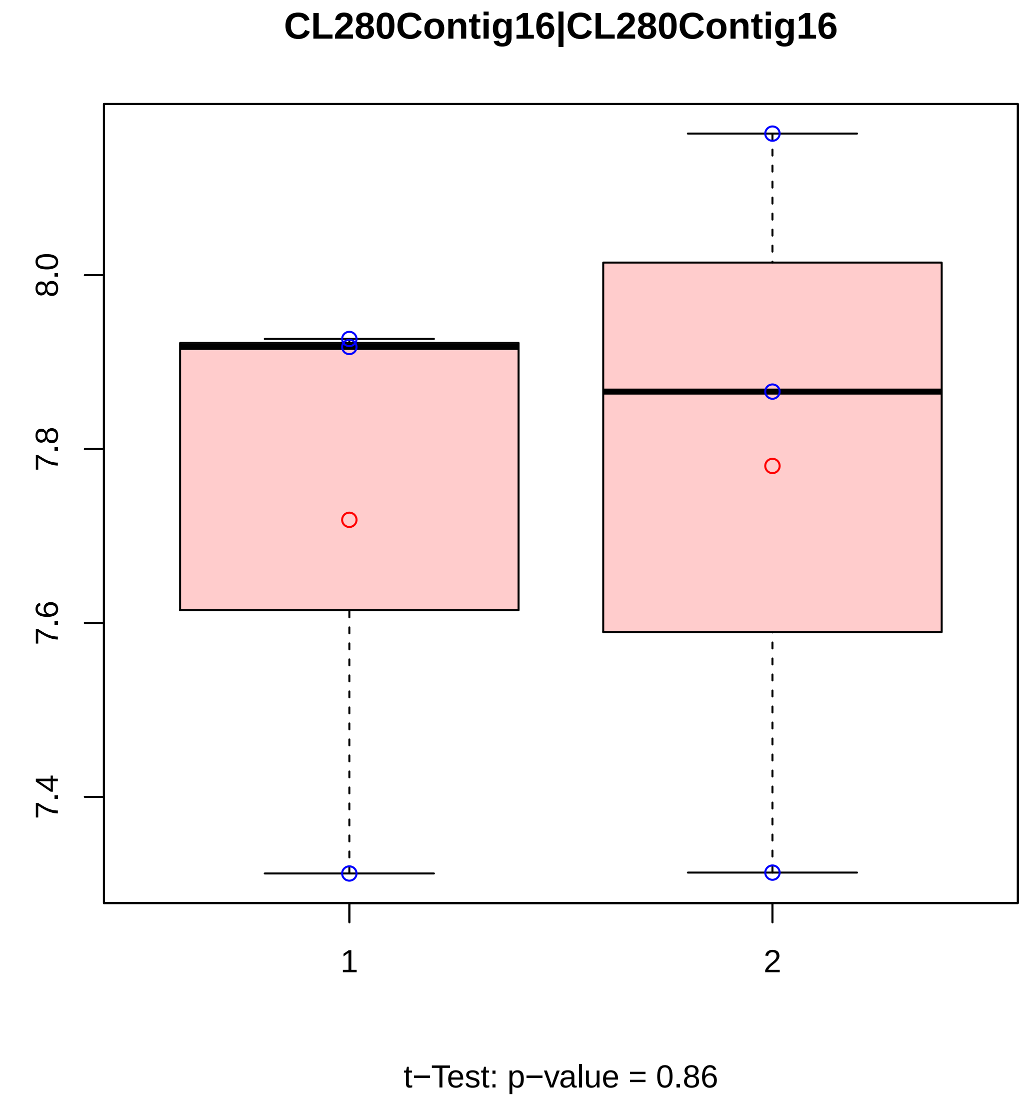

CL280Contig16|CL280Contig16
8.0
7.8
7.6
7.4
1
2
t−Test: p−value = 0.86

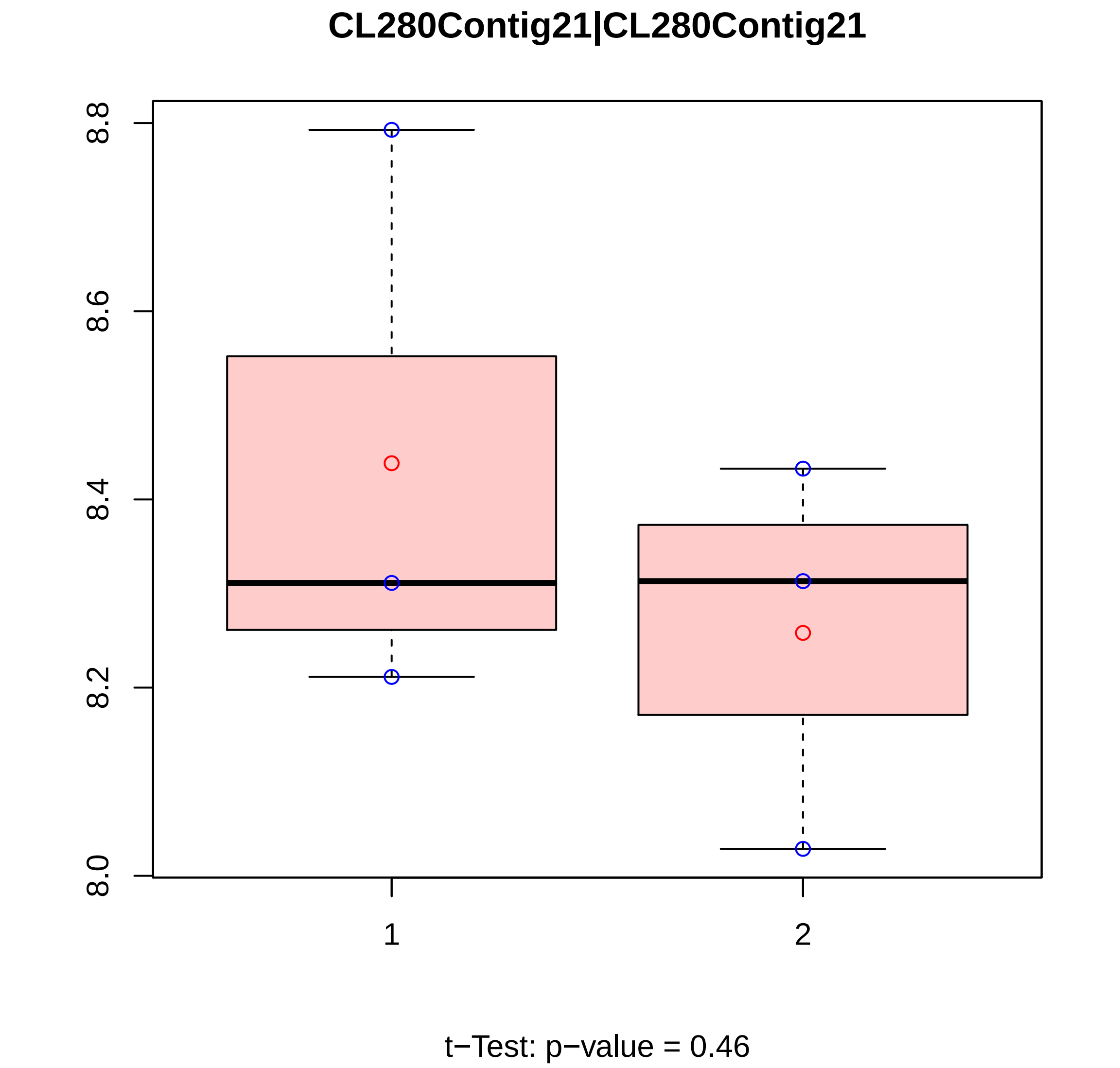
CL280Contig21|CL280Contig21
8.0
8.2
8.4
8.6
8.8
1
2
t−Test: p−value = 0.46

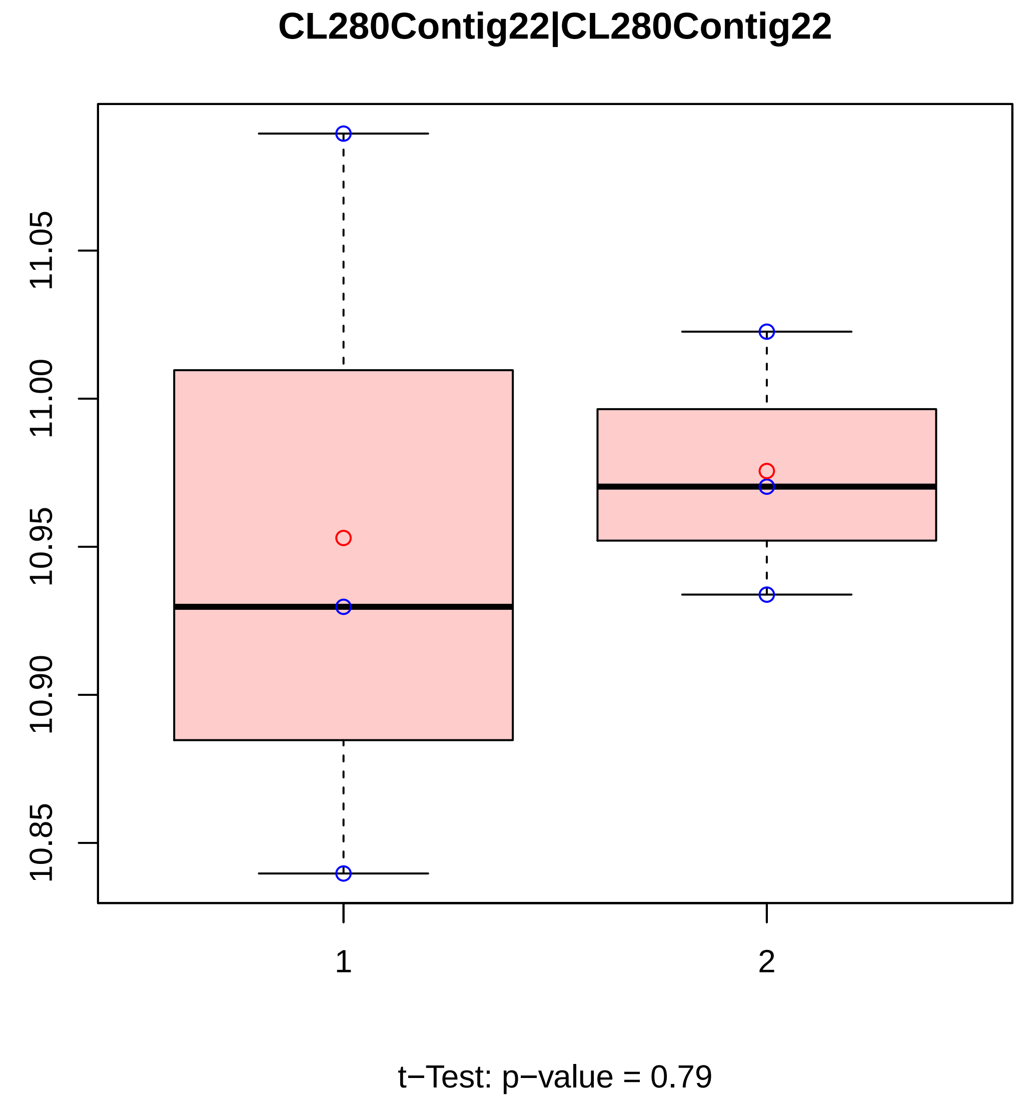
CL280Contig22|CL280Contig22
11.05
11.00
10.95
10.90
10.85
1
2
t−Test: p−value = 0.79

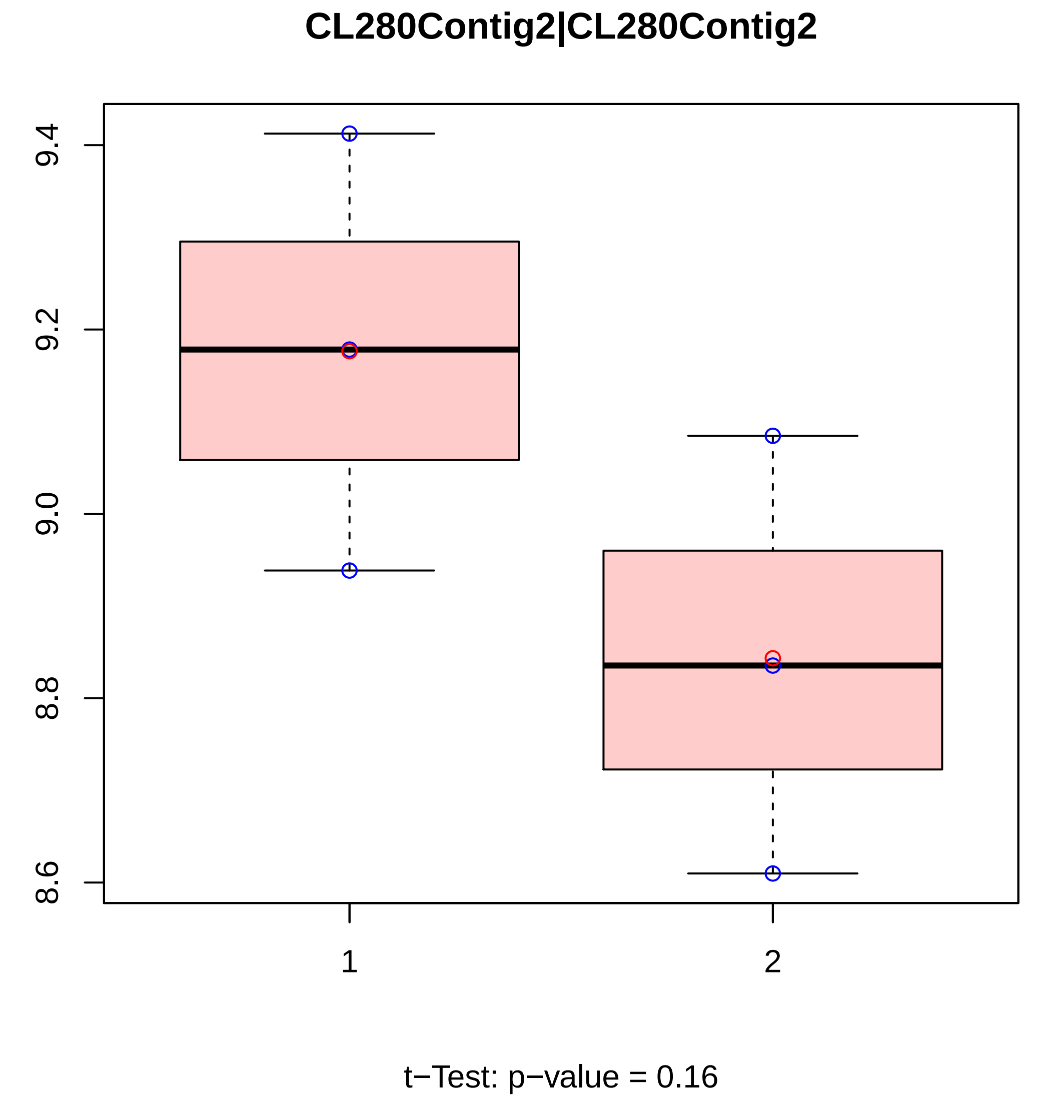
CL280Contig2|CL280Contig2
9.4
9.2
9.0
8.8
8.6
1
2
t−Test: p−value = 0.16

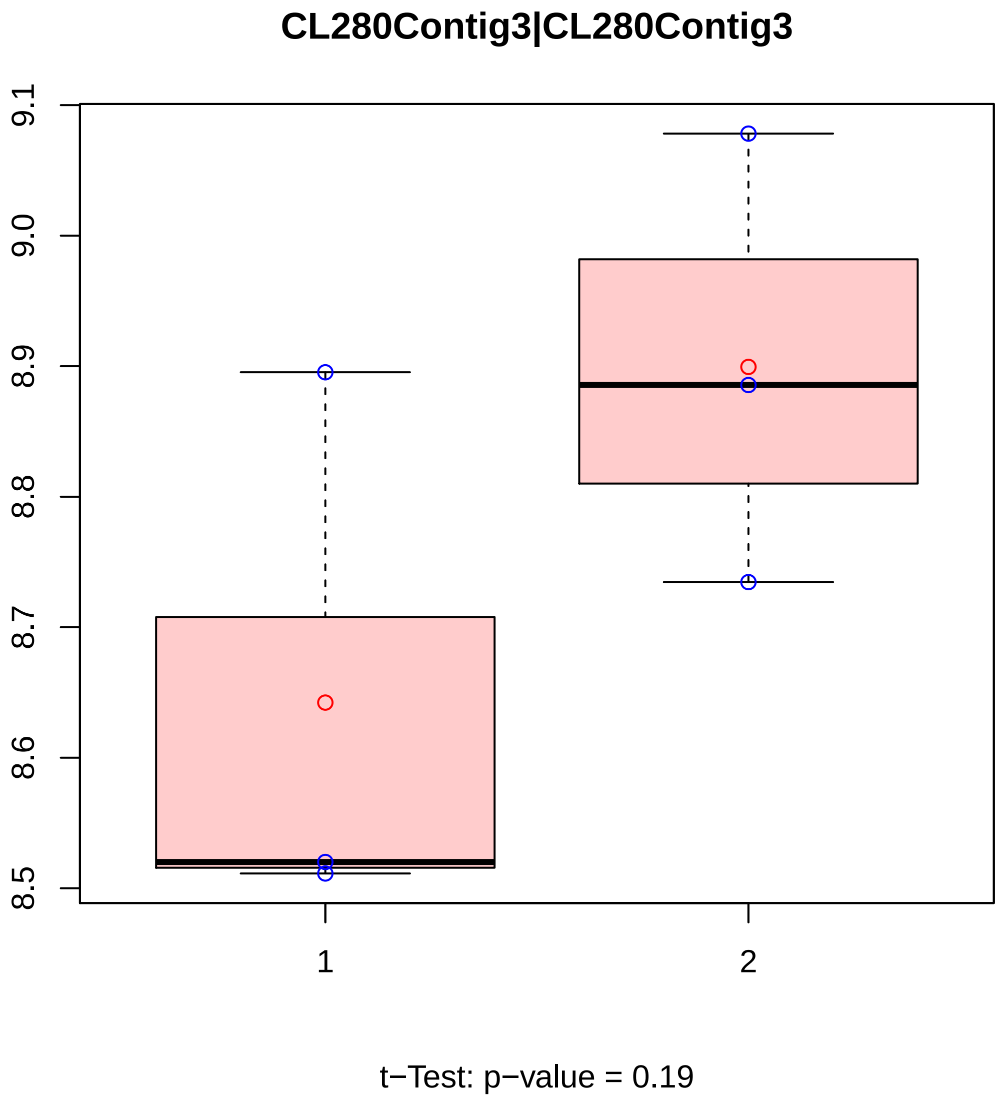
CL280Contig3|CL280Contig3
9.1
9.0
8.9
8.8
8.7
8.6
8.5
1
2
t−Test: p−value = 0.19

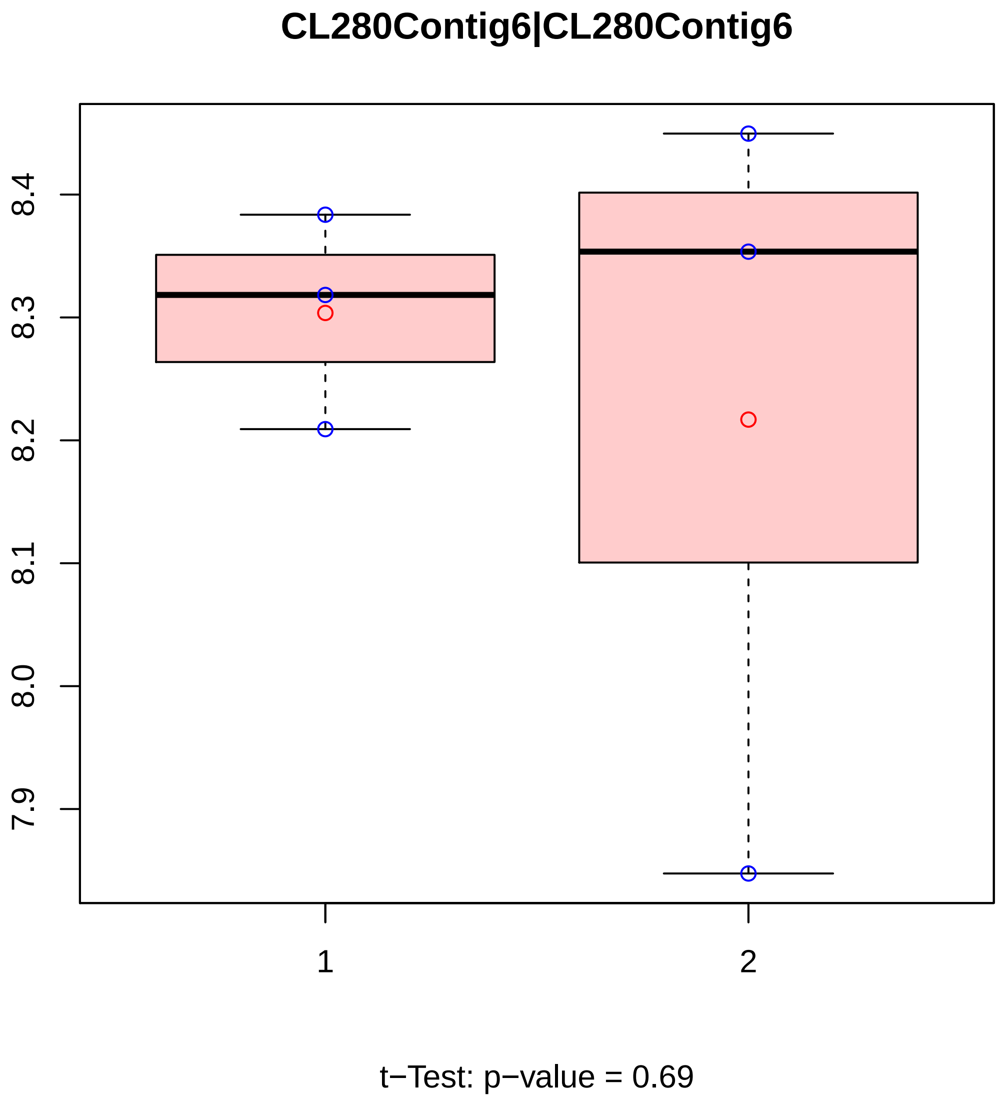

CL280Contig6|CL280Contig6
8.4
8.3
8.2
8.1
8.0
7.9
1
2
t−Test: p−value = 0.69

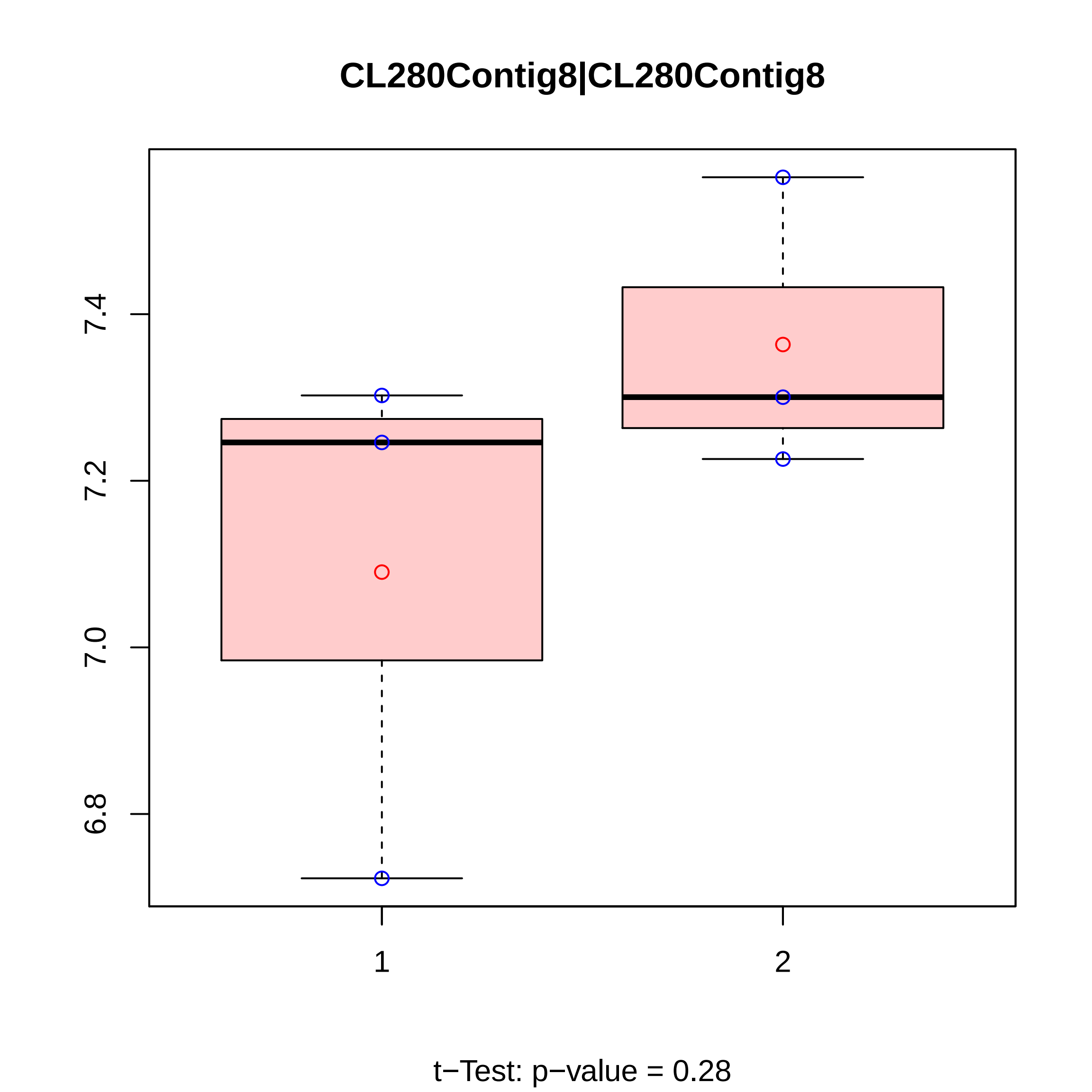
CL280Contig8|CL280Contig8
7.4
7.2
7.0
6.8
1
2
t−Test: p−value = 0.28

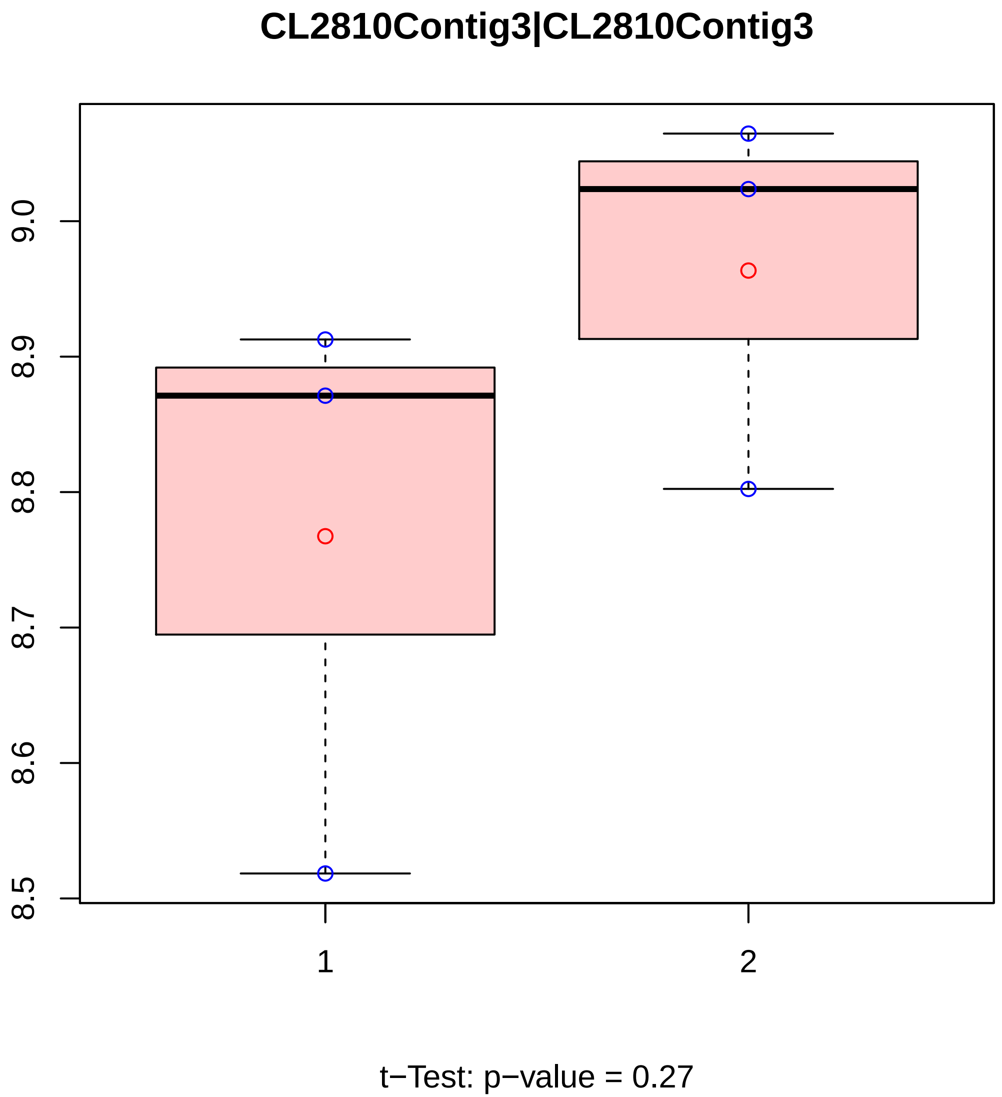
CL2810Contig3|CL2810Contig3
9.0
8.9
8.8
8.7
8.6
8.5
1
2
t−Test: p−value = 0.27

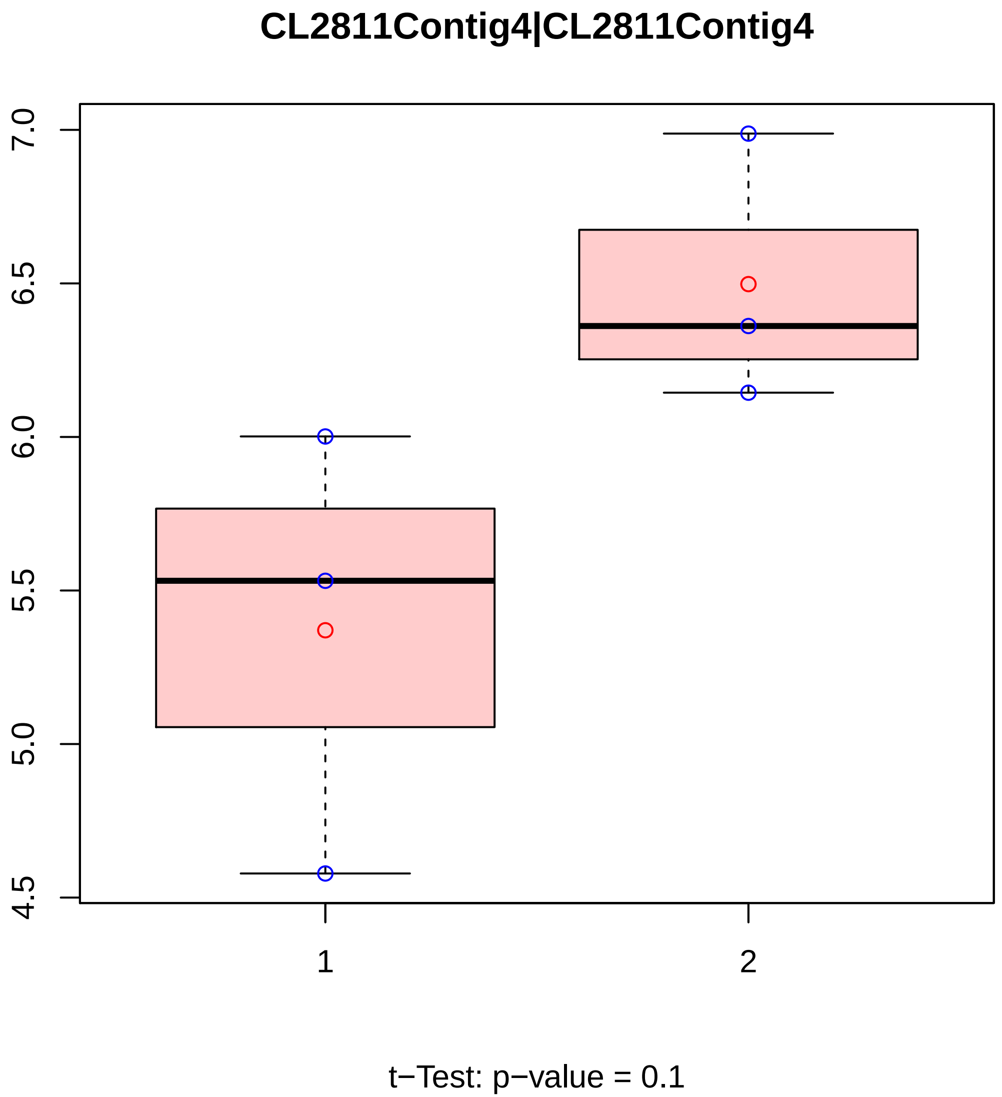
CL2811Contig4|CL2811Contig4
7.0
6.5
6.0
5.5
5.0
4.5
1
2
t−Test: p−value = 0.1

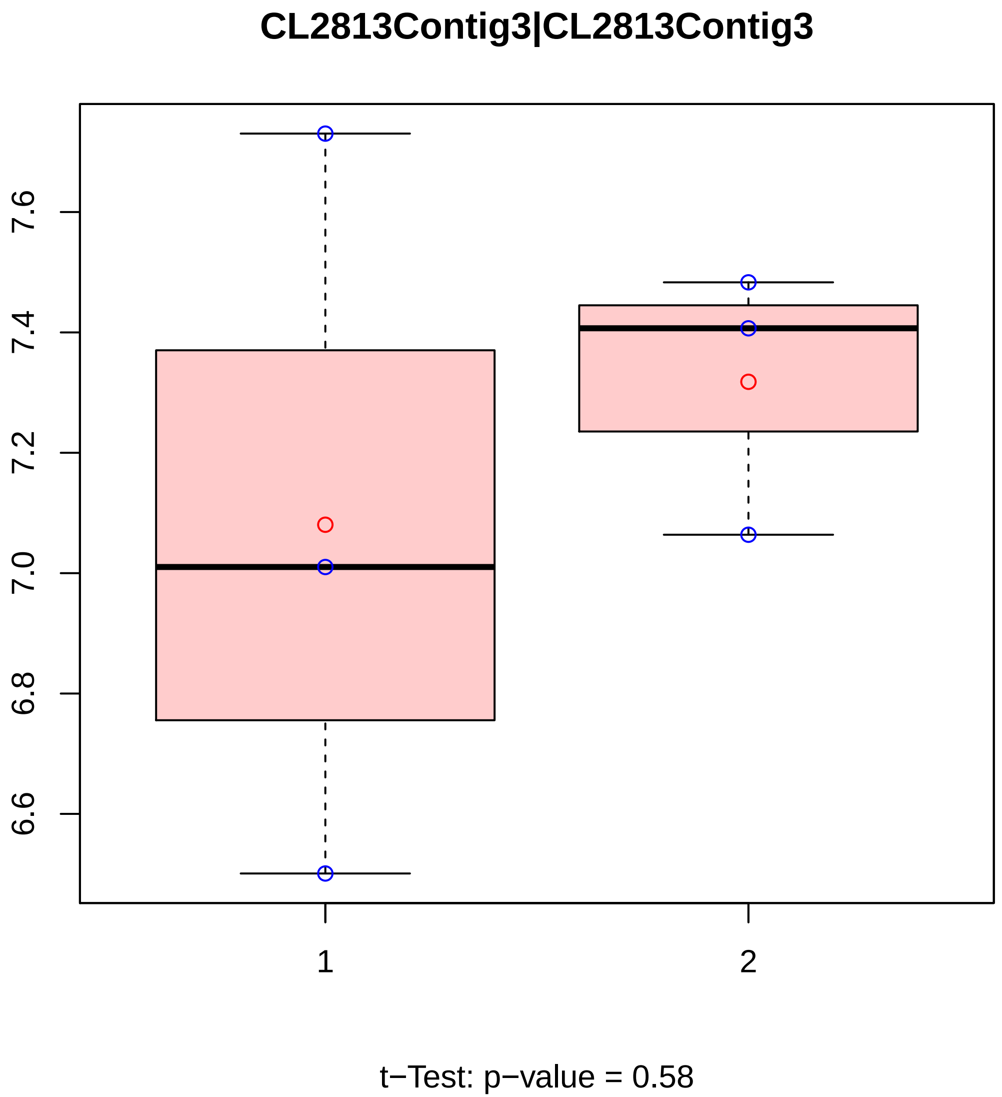

CL2813Contig3|CL2813Contig3
7.6
7.4
7.2
7.0
6.8
6.6
1
2
t−Test: p−value = 0.58

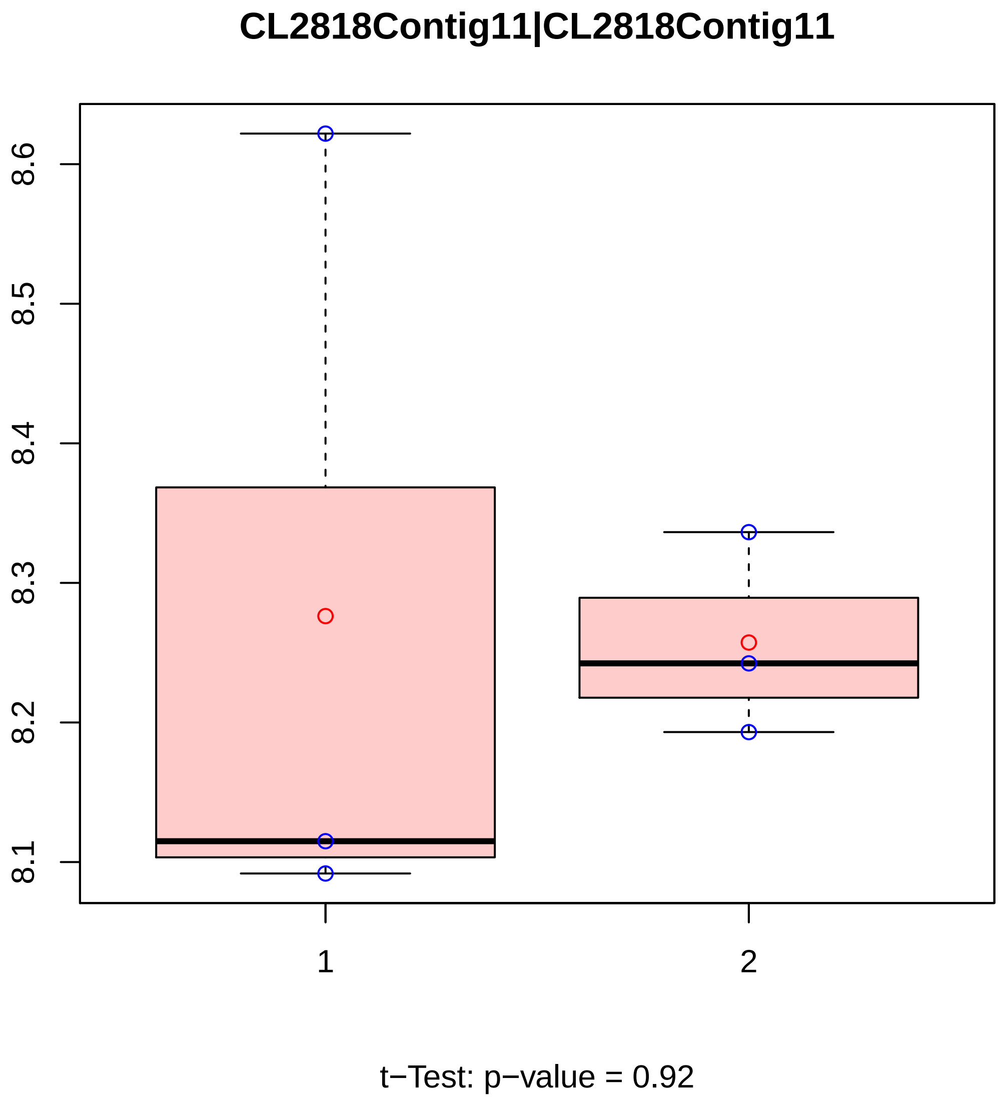

CL2818Contig11|CL2818Contig11
8.1
8.2
8.3
8.4
8.5
8.6
1
2
t−Test: p−value = 0.92

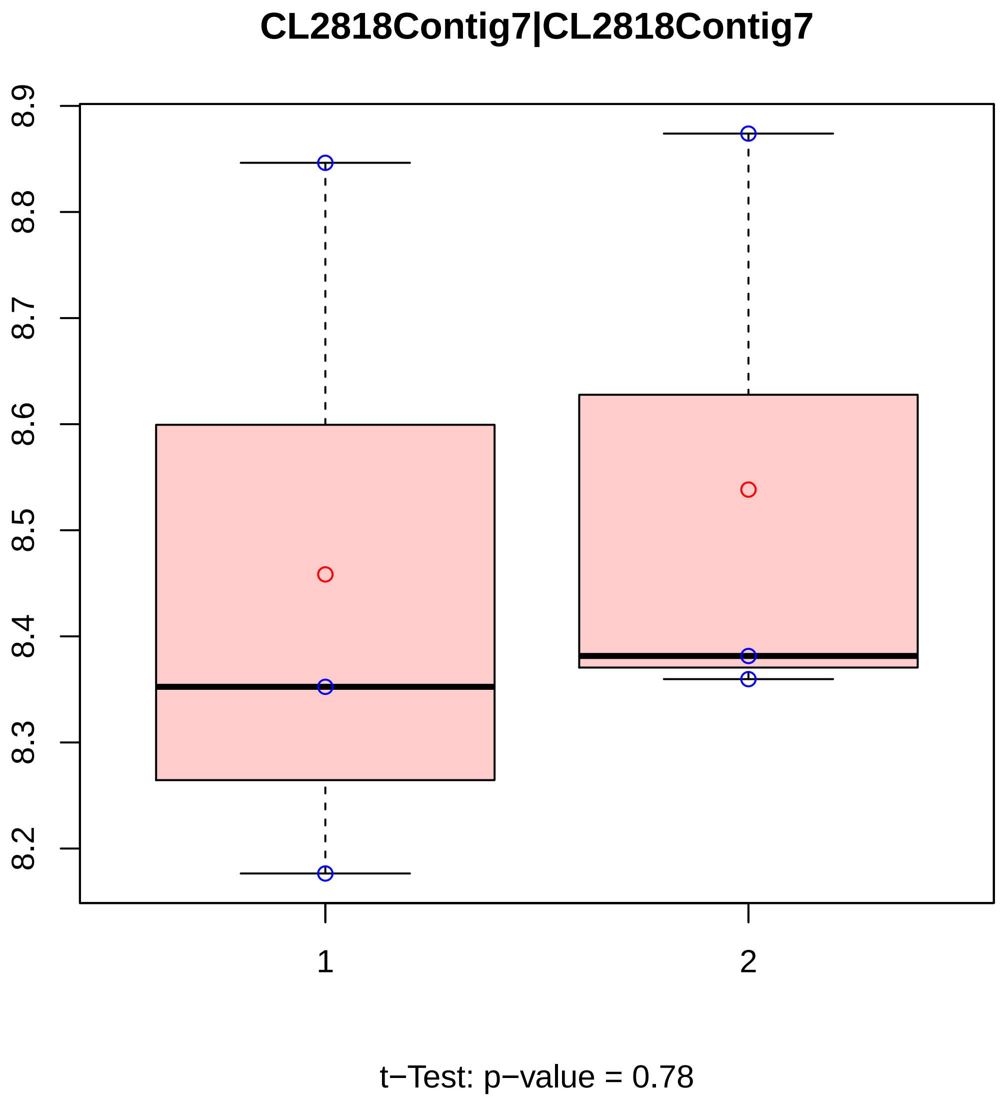
CL2818Contig7|CL2818Contig7
8.2
8.3
8.4
8.5
8.6
8.7
8.8
8.9
1
2
t−Test: p−value = 0.78

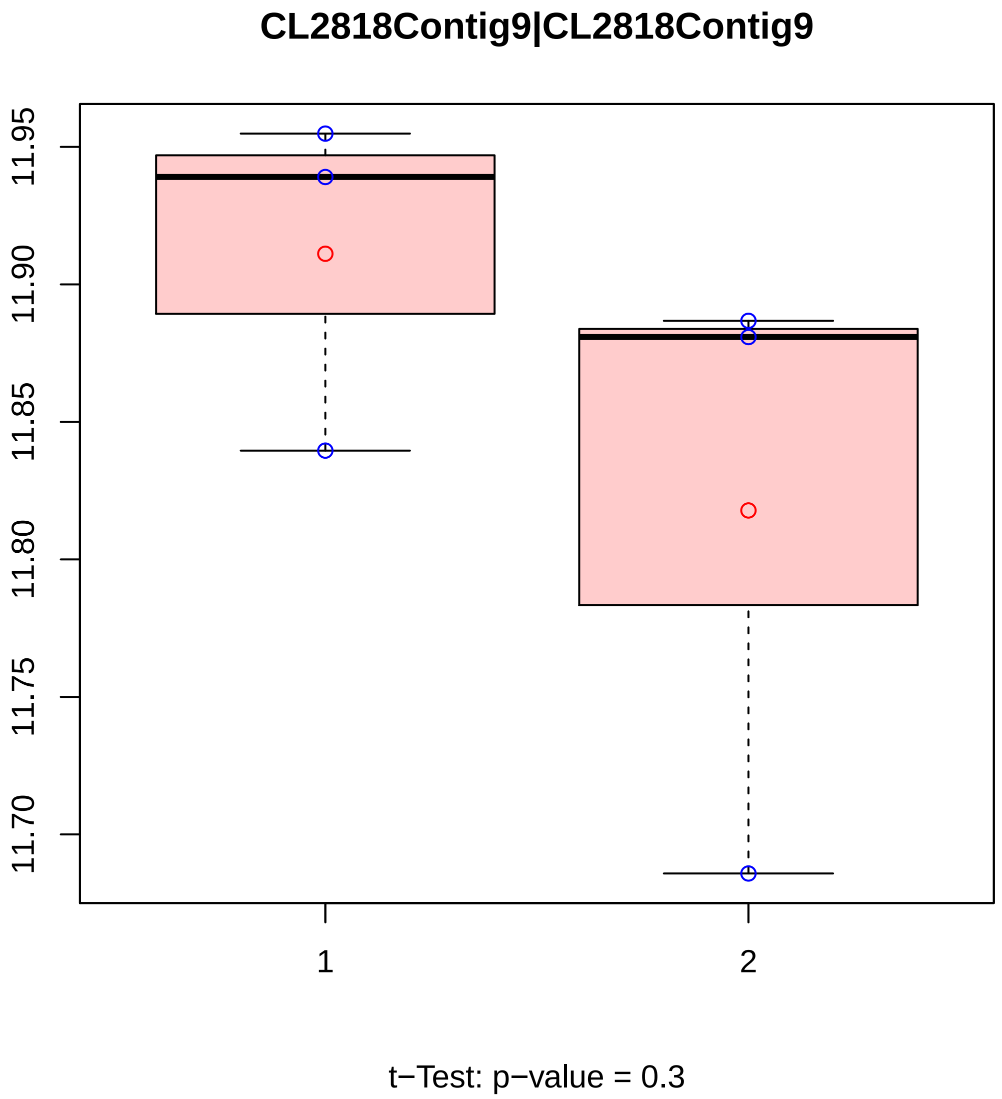
CL2818Contig9|CL2818Contig9
11.95
11.90
11.85
11.80
11.75
11.70
1
2
t−Test: p−value = 0.3

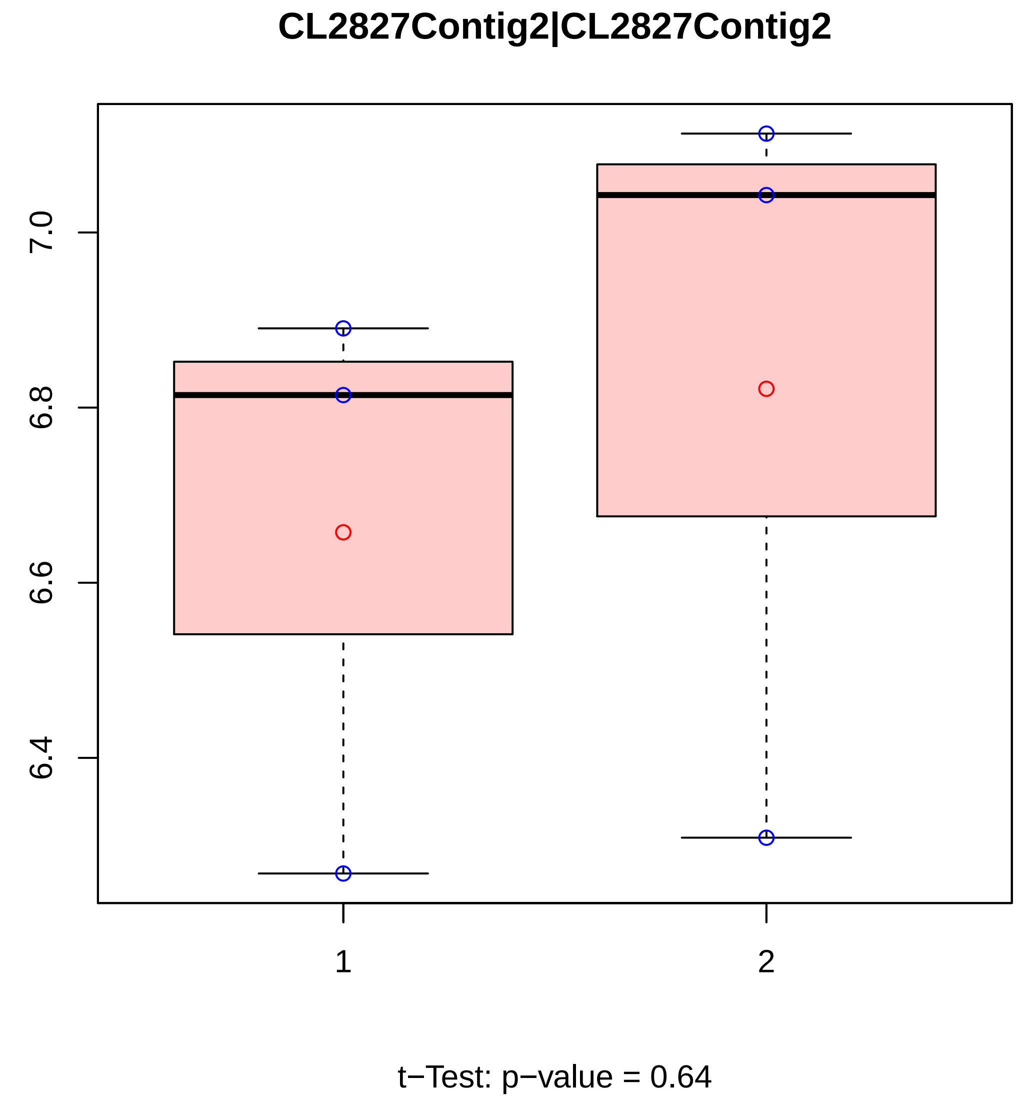

CL2827Contig2|CL2827Contig2
7.0
6.8
6.6
6.4
1
2
t−Test: p−value = 0.64

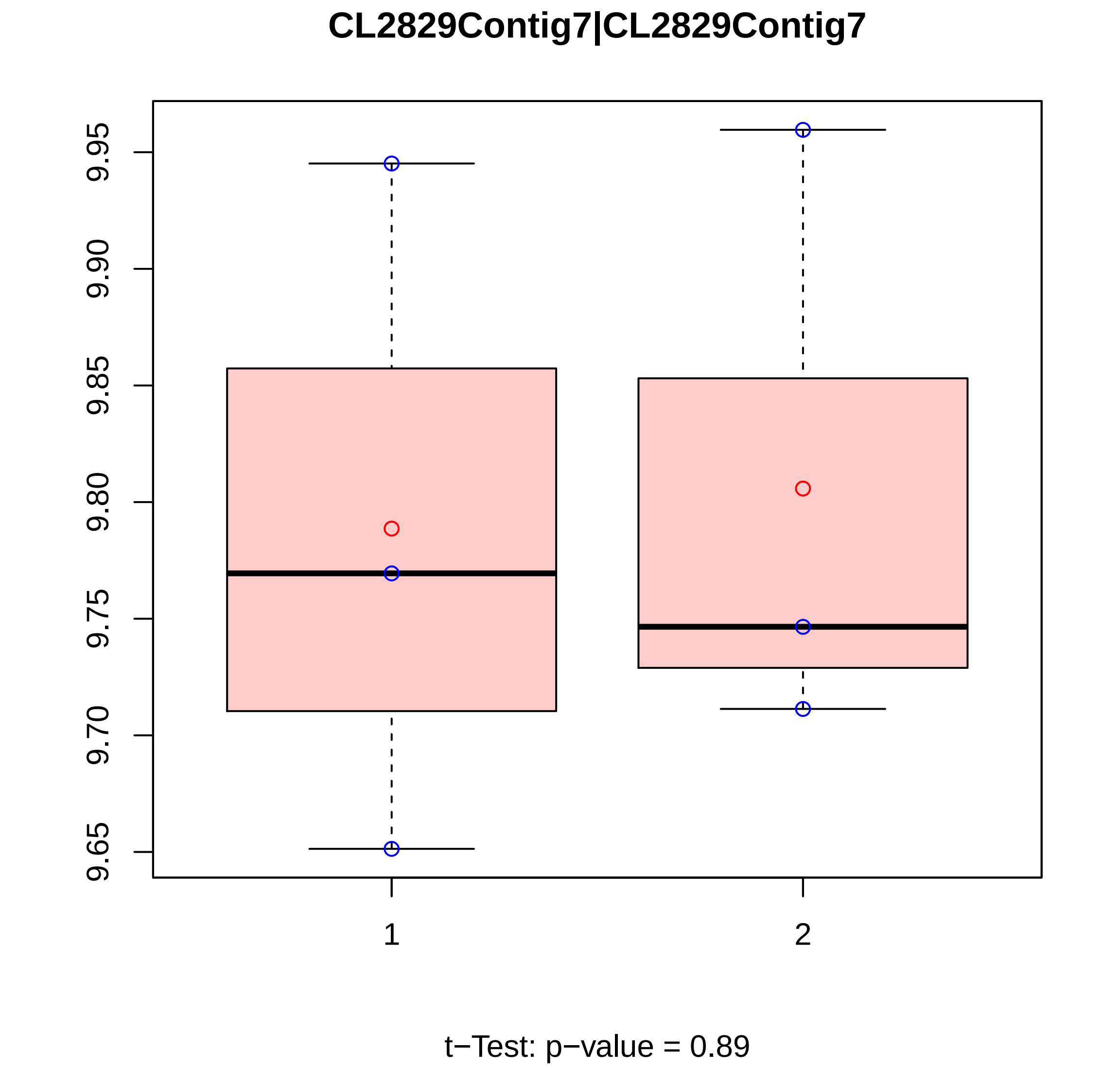
CL2829Contig7|CL2829Contig7
9.65
9.70
9.75
9.80
9.85
9.90
9.95
1
2
t−Test: p−value = 0.89

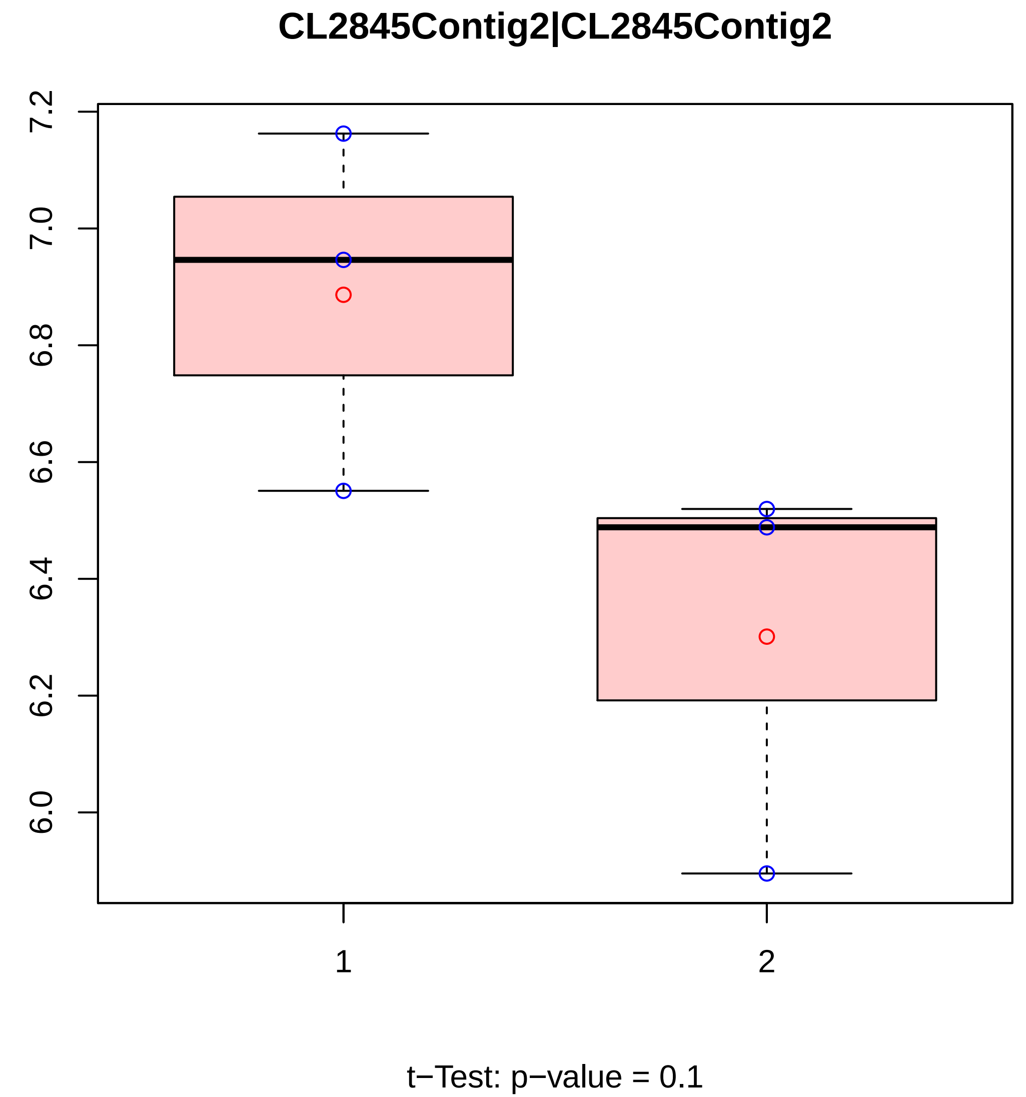
CL2845Contig2|CL2845Contig2
7.2
7.0
6.8
6.6
6.4
6.2
6.0
1
2
t−Test: p−value = 0.1

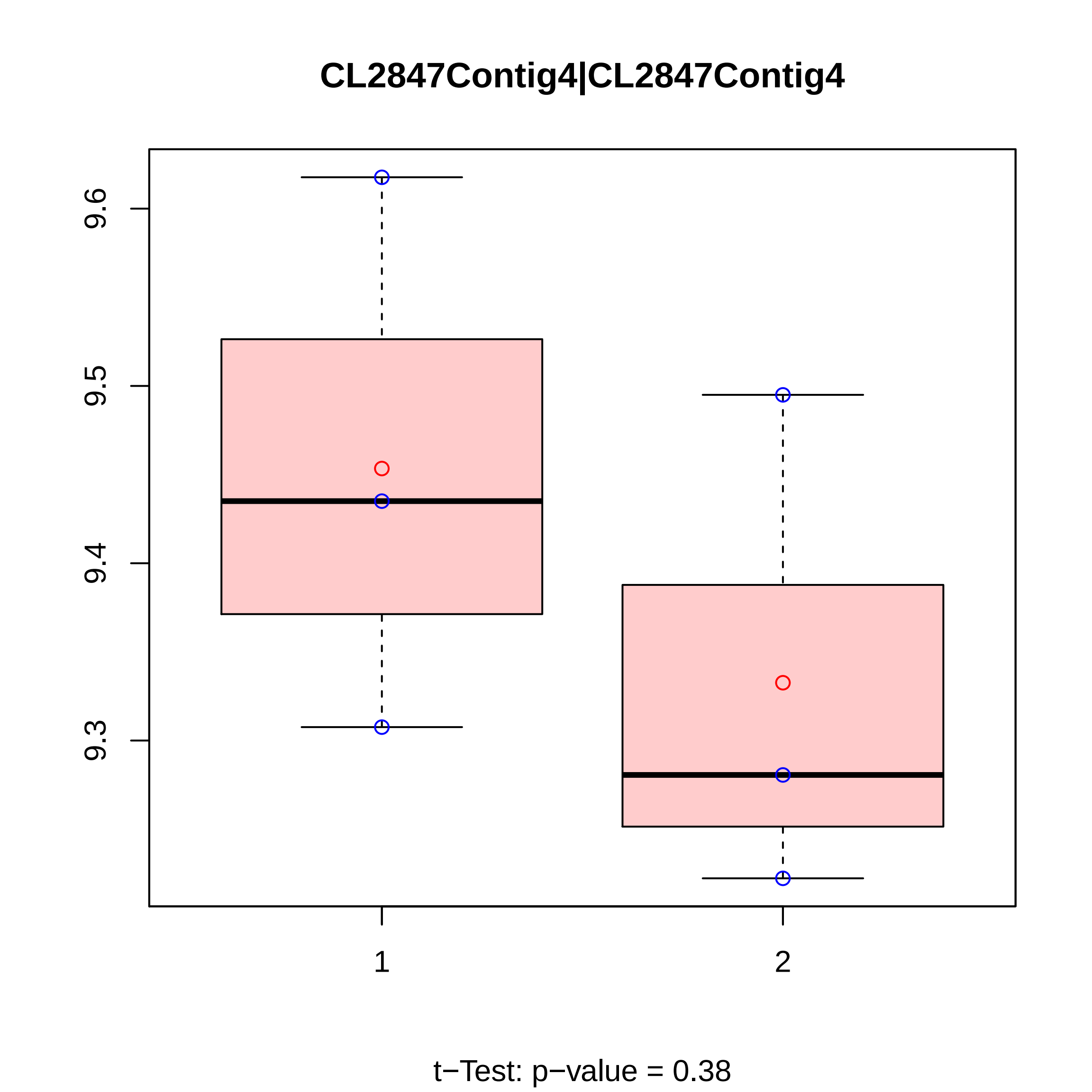

CL2847Contig4|CL2847Contig4
9.6
9.5
9.4
9.3
1
2
t−Test: p−value = 0.38

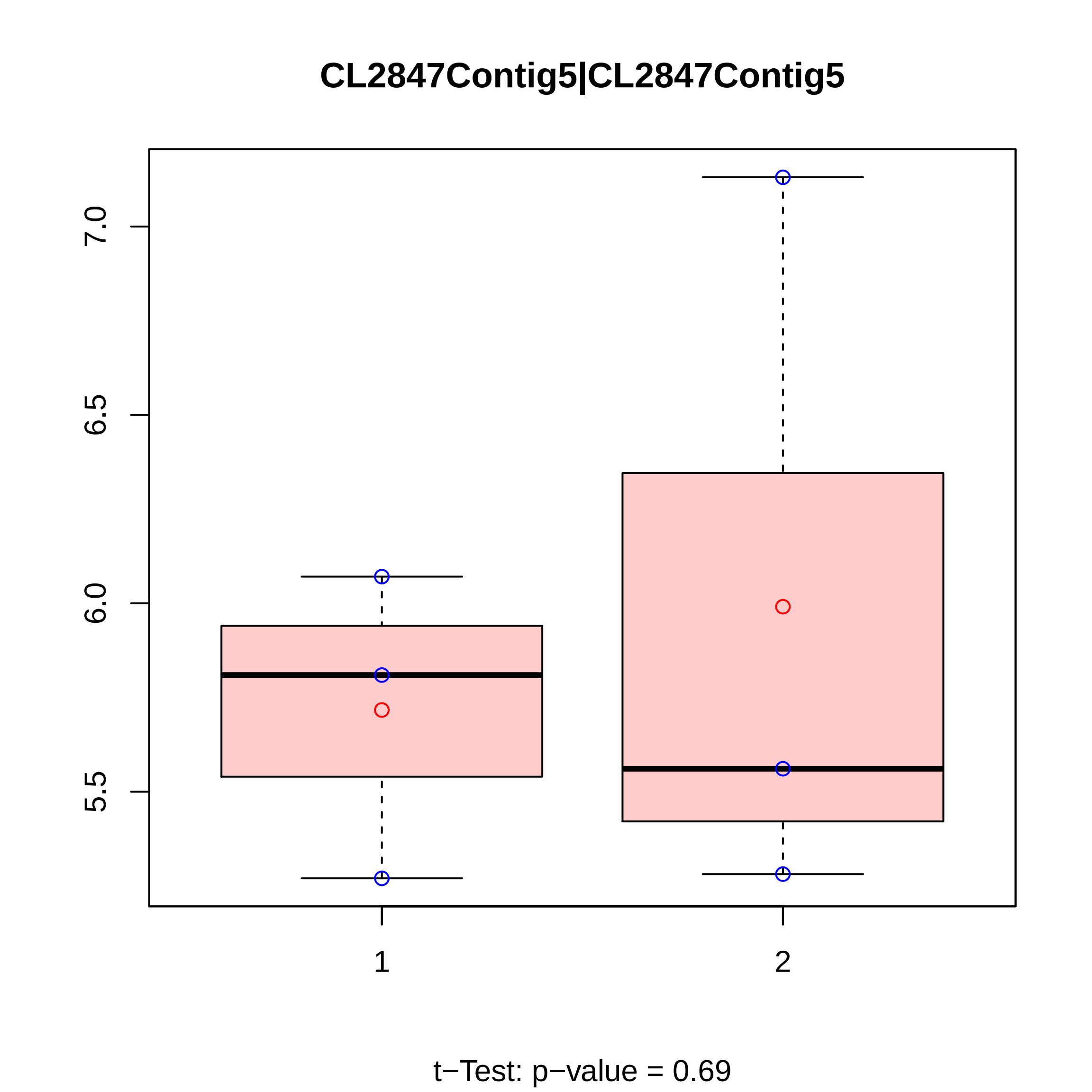
CL2847Contig5|CL2847Contig5
7.0
6.5
6.0
5.5
1
2
t−Test: p−value = 0.69

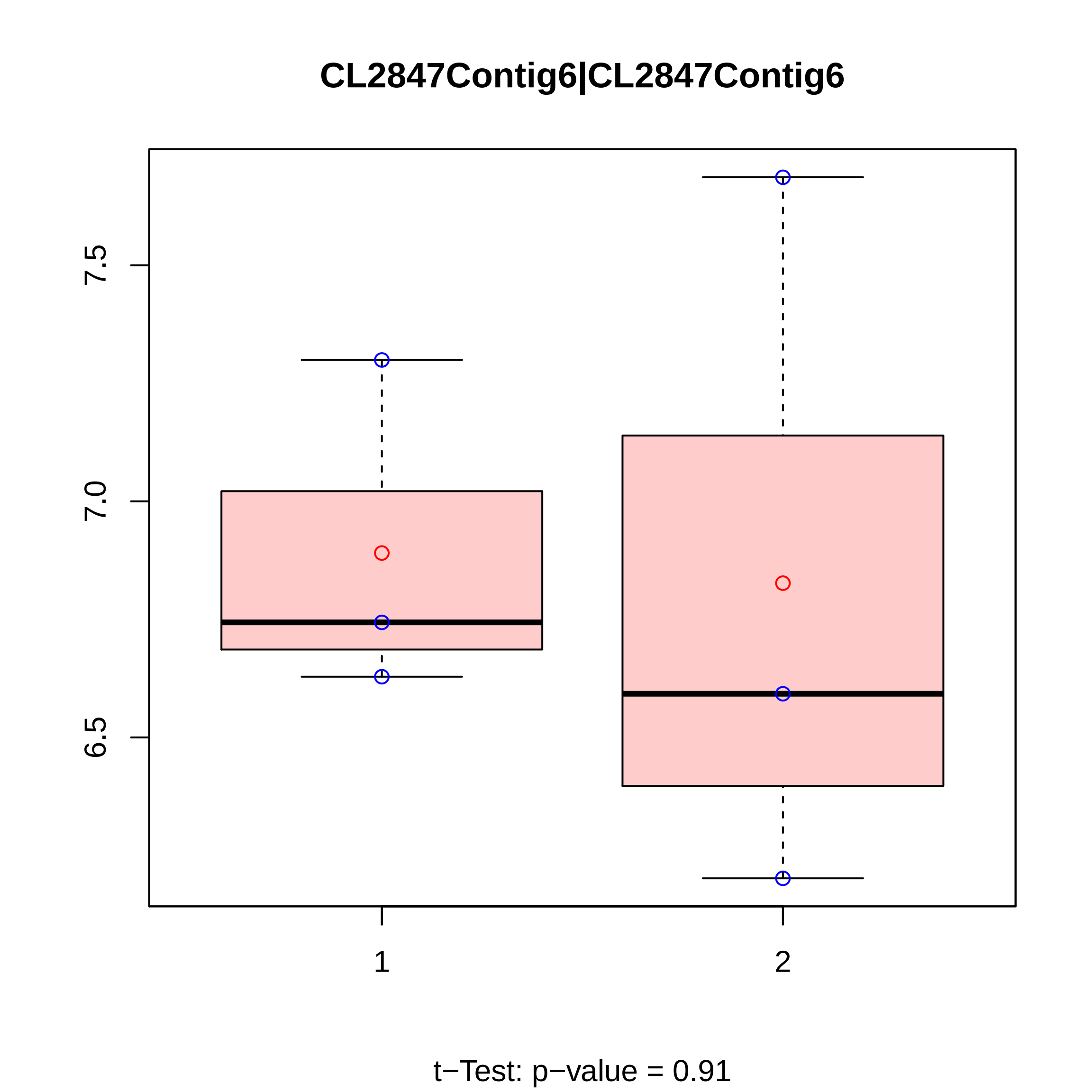

CL2847Contig6|CL2847Contig6
7.5
7.0
6.5
1
2
t−Test: p−value = 0.91

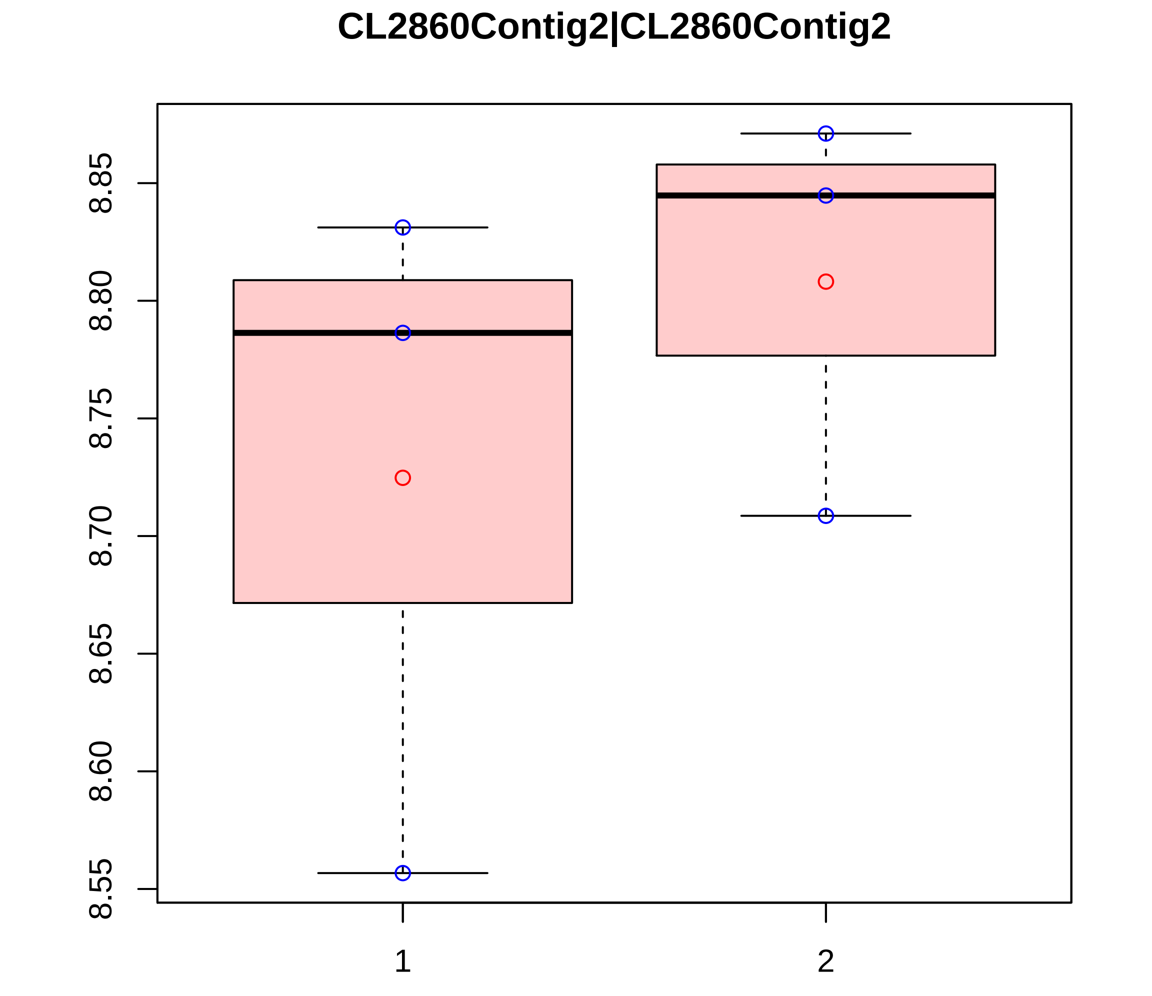


t−Test: p−value = 0.46

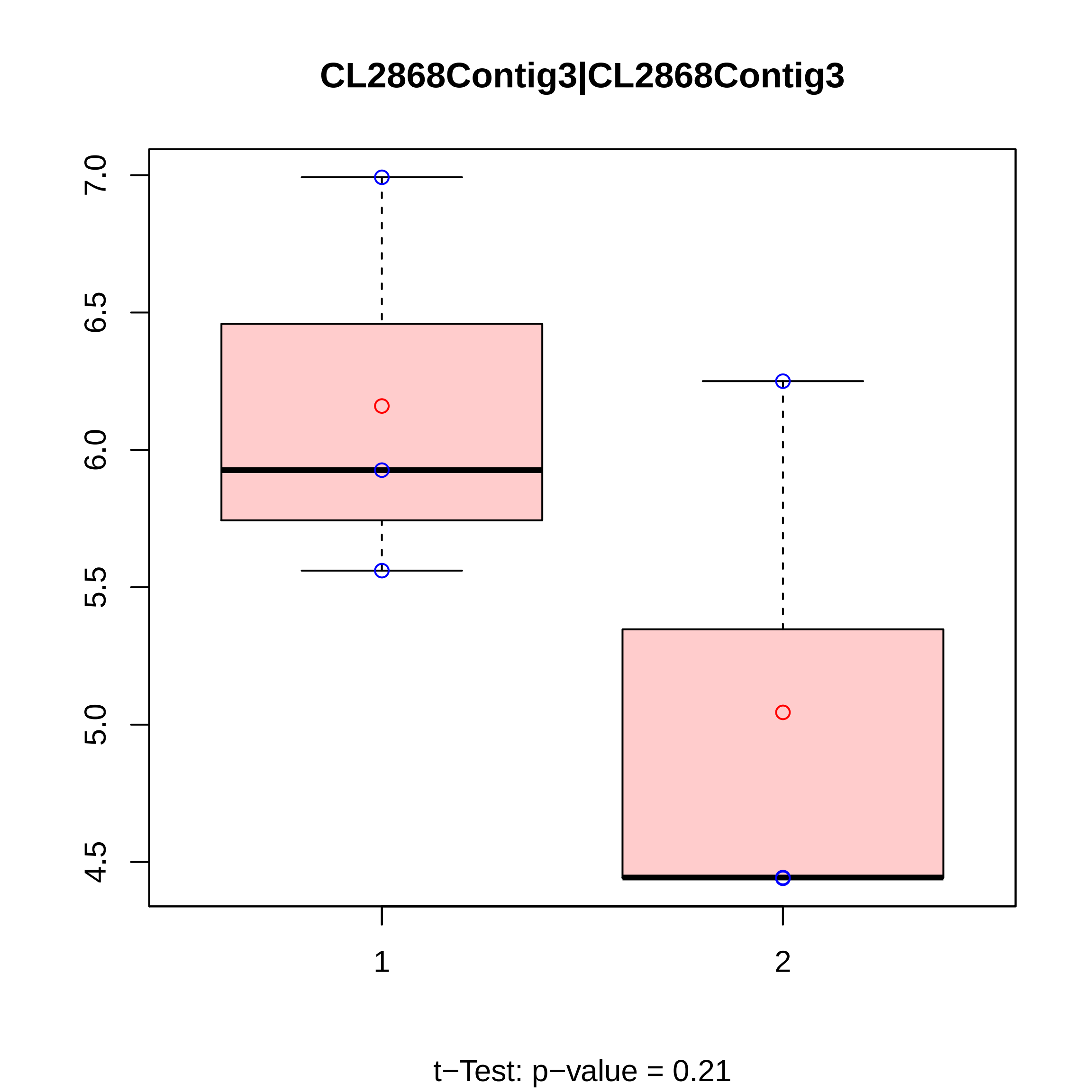
CL2868Contig3|CL2868Contig3
7.0
6.5
6.0
5.5
5.0
4.5
1
2
t−Test: p−value = 0.21

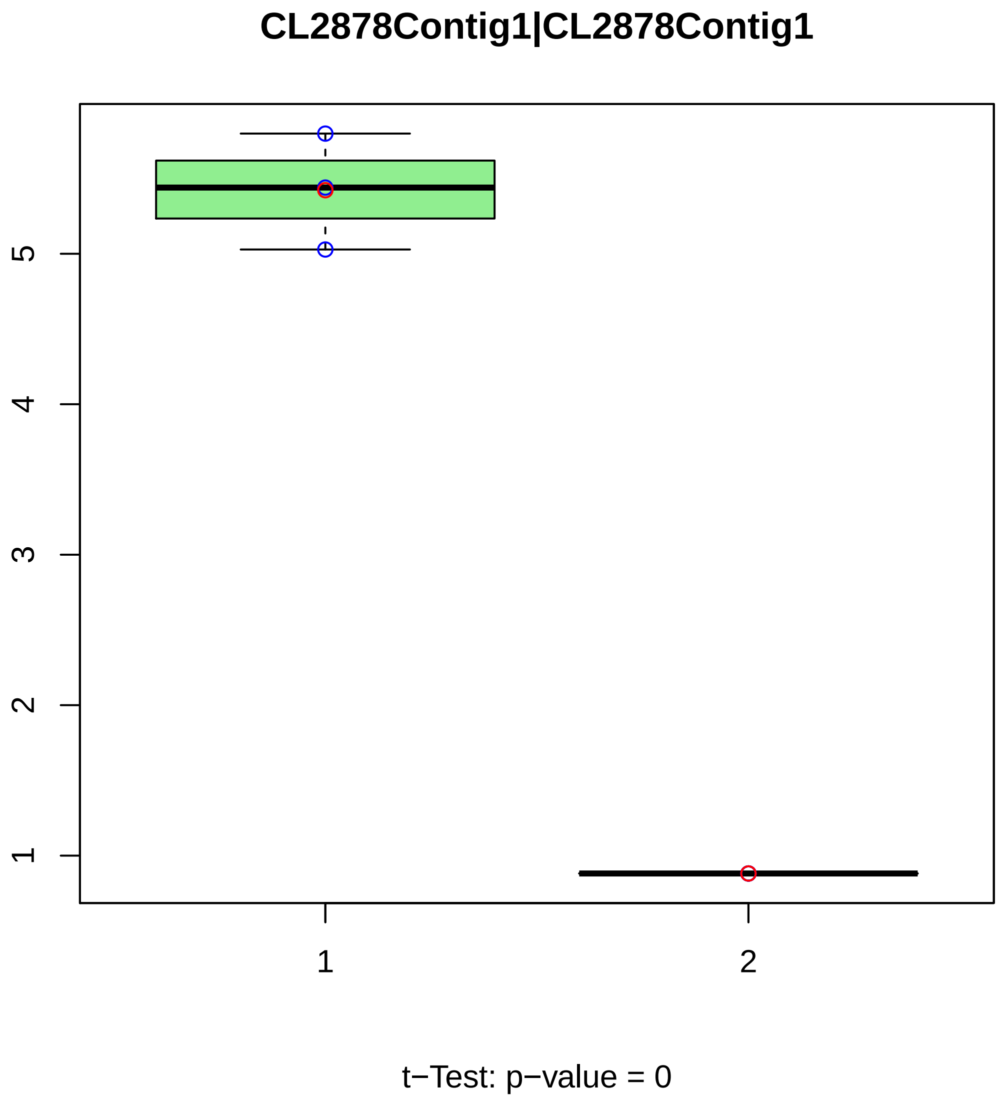

CL2878Contig1|CL2878Contig1
1
2
3
4
5
1
2
t−Test: p−value = 0

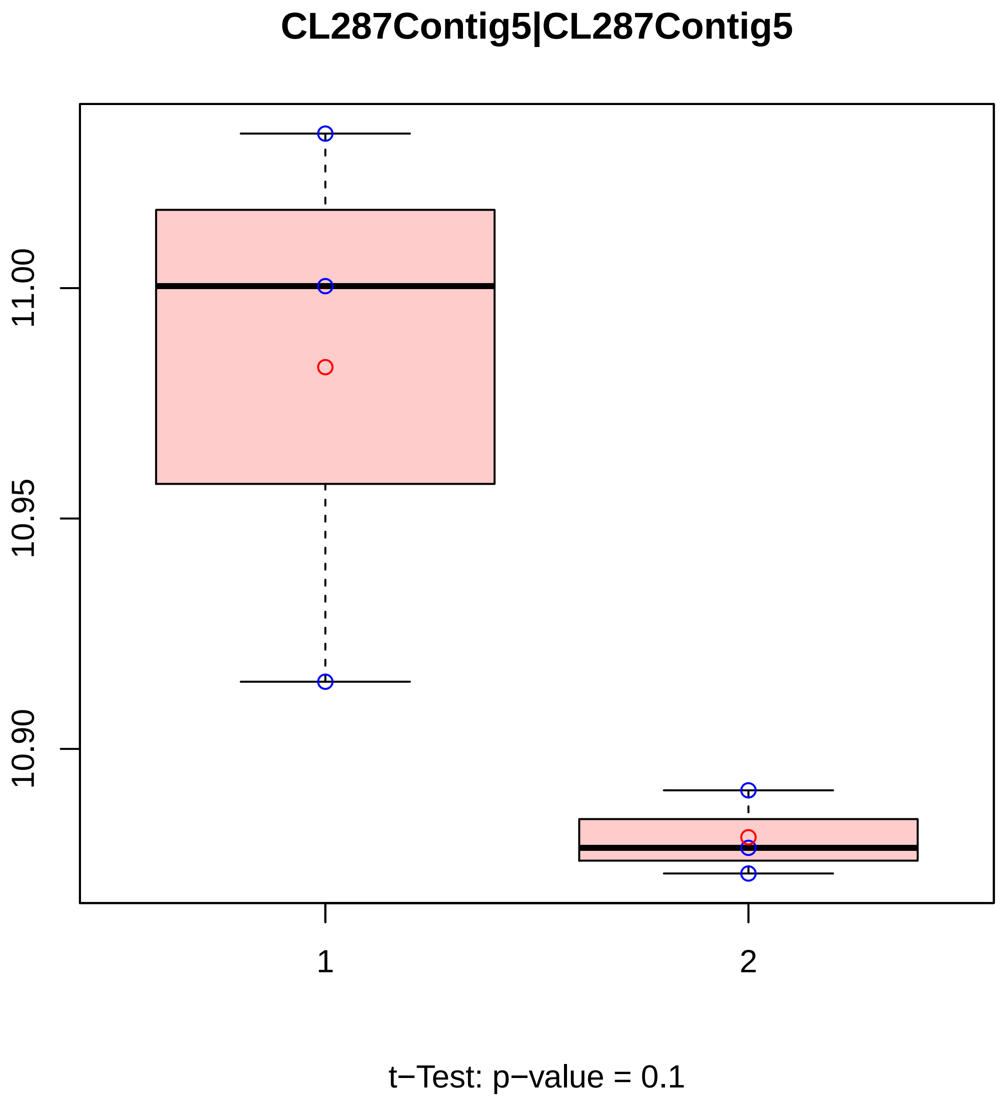
CL287Contig5|CL287Contig5
11.00
10.95
10.90
1
2
t−Test: p−value = 0.1

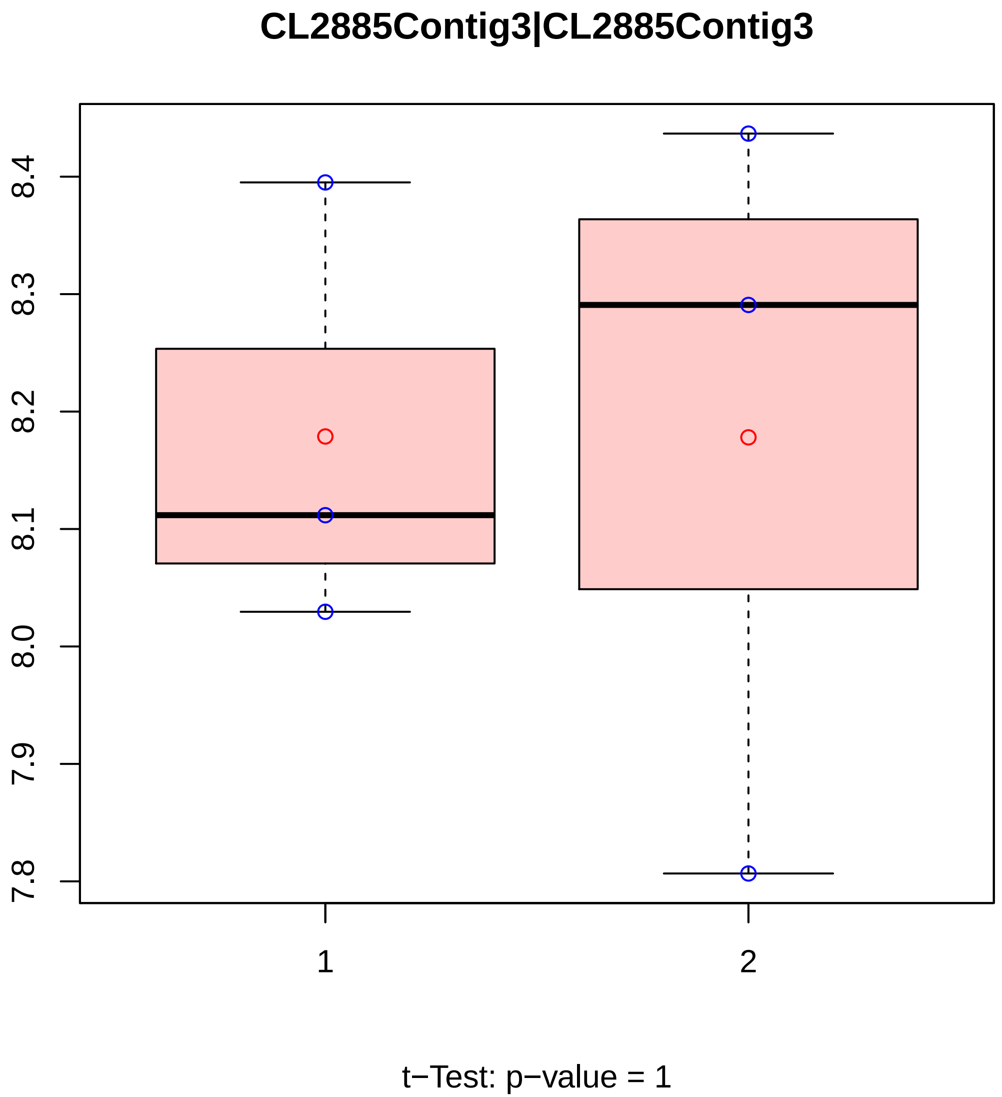

CL2885Contig3|CL2885Contig3
8.4
8.3
8.2
8.1
8.0
7.9
7.8
1
2
t−Test: p−value = 1

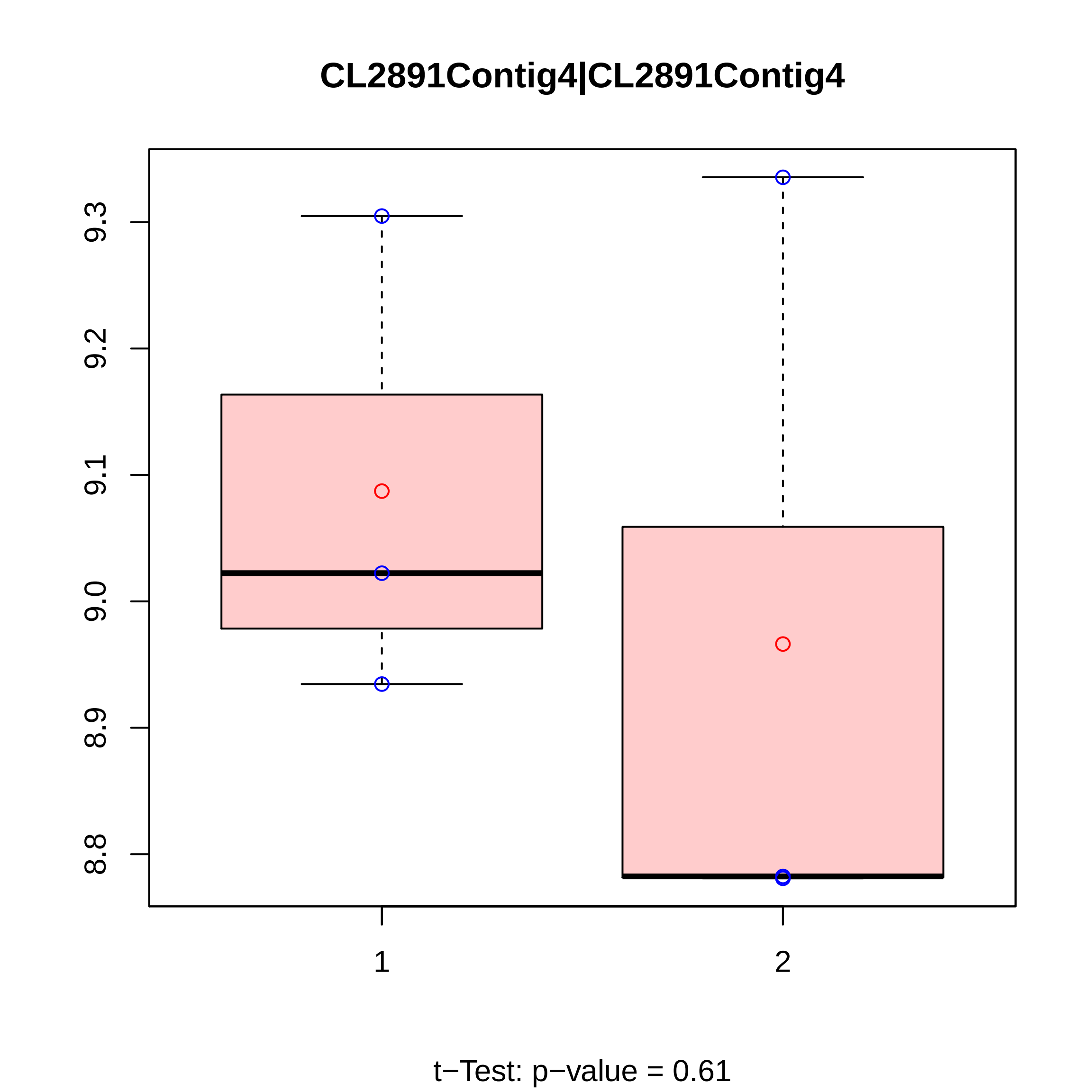

CL2891Contig4|CL2891Contig4
9.3
9.2
9.1
9.0
8.9
8.8
1
2
t−Test: p−value = 0.61

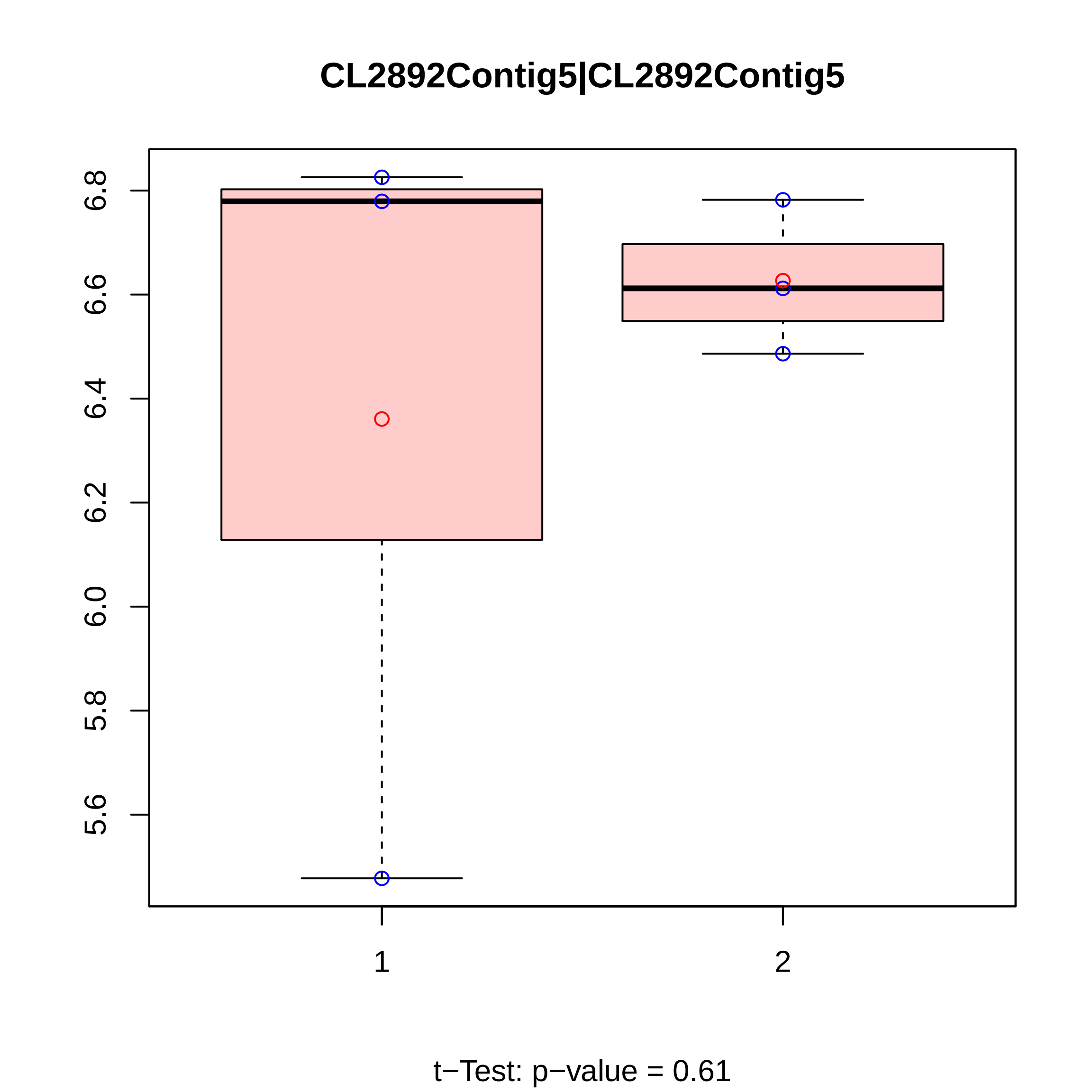
CL2892Contig5|CL2892Contig5
6.8
6.6
6.4
6.2
6.0
5.8
5.6
1
2
t−Test: p−value = 0.61

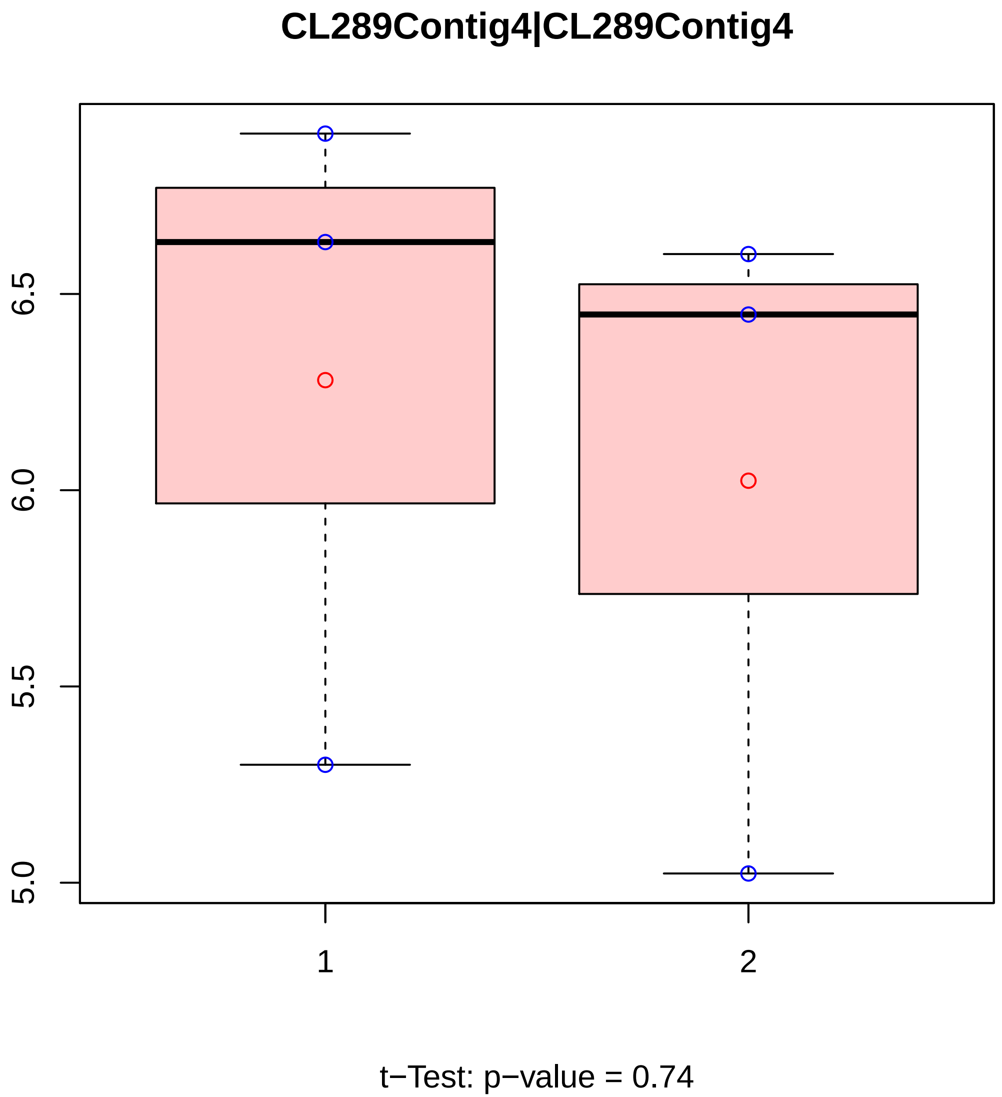
CL289Contig4|CL289Contig4
6.5
6.0
5.5
5.0
1
2
t−Test: p−value = 0.74

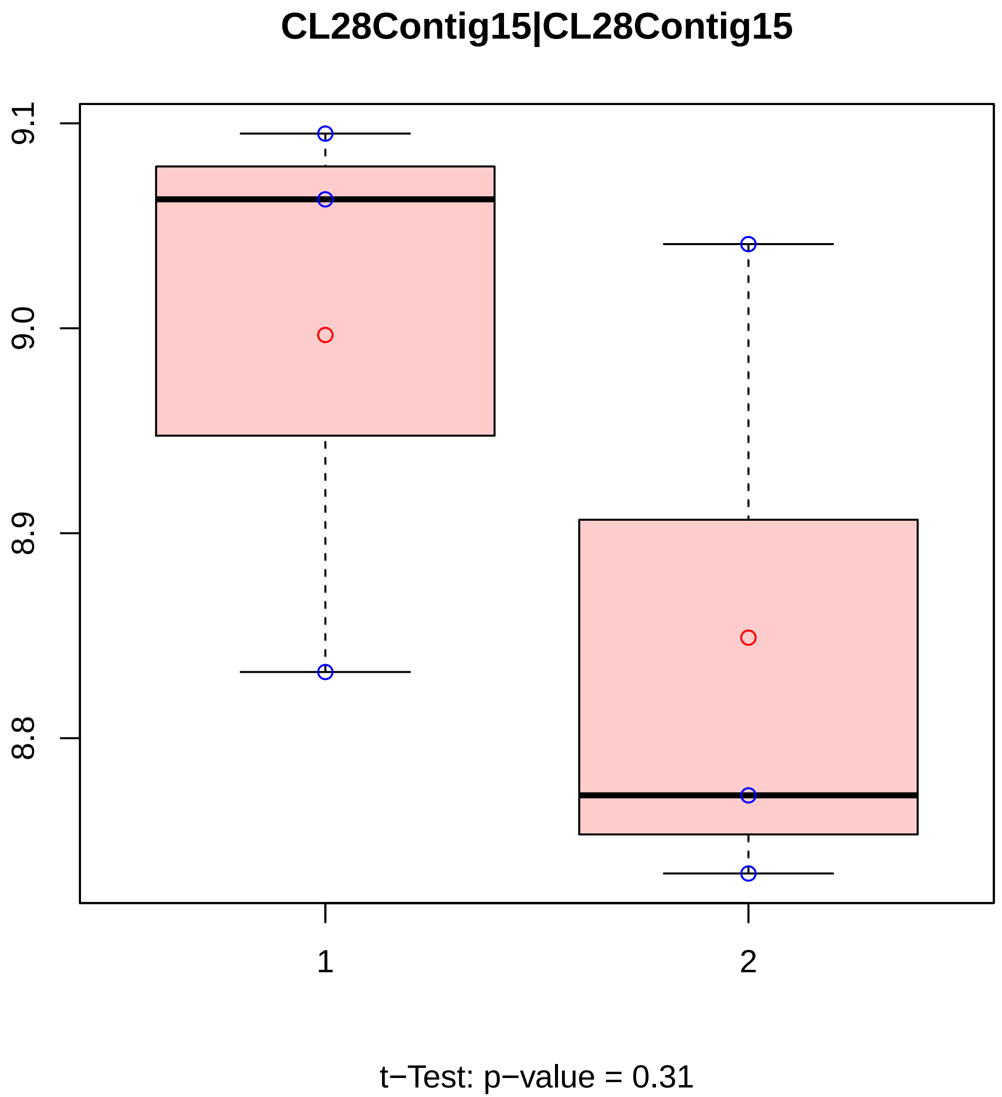

CL28Contig15|CL28Contig15
9.1
9.0
8.9
8.8
1
2
t−Test: p−value = 0.31

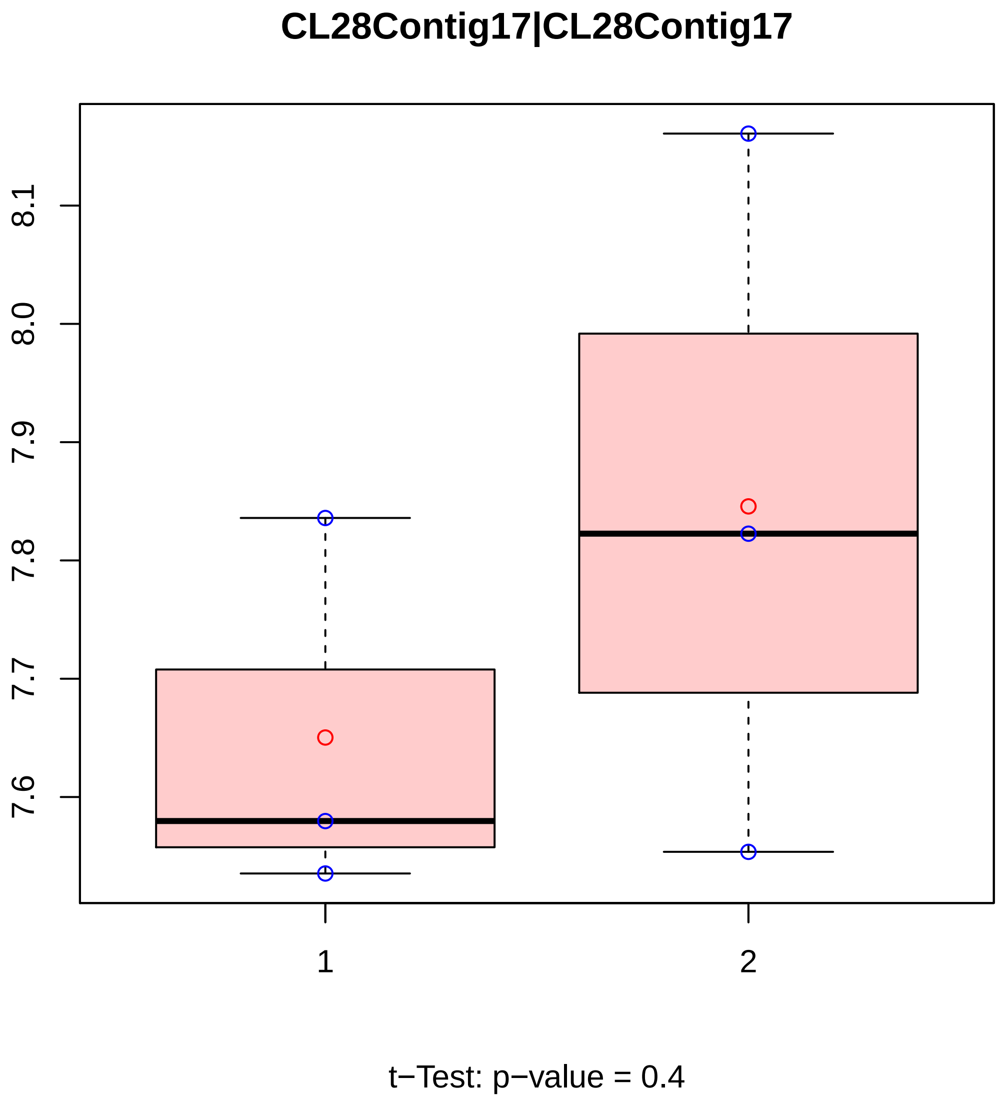
CL28Contig17|CL28Contig17
8.1
8.0
7.9
7.8
7.7
7.6
1
2
t−Test: p−value = 0.4

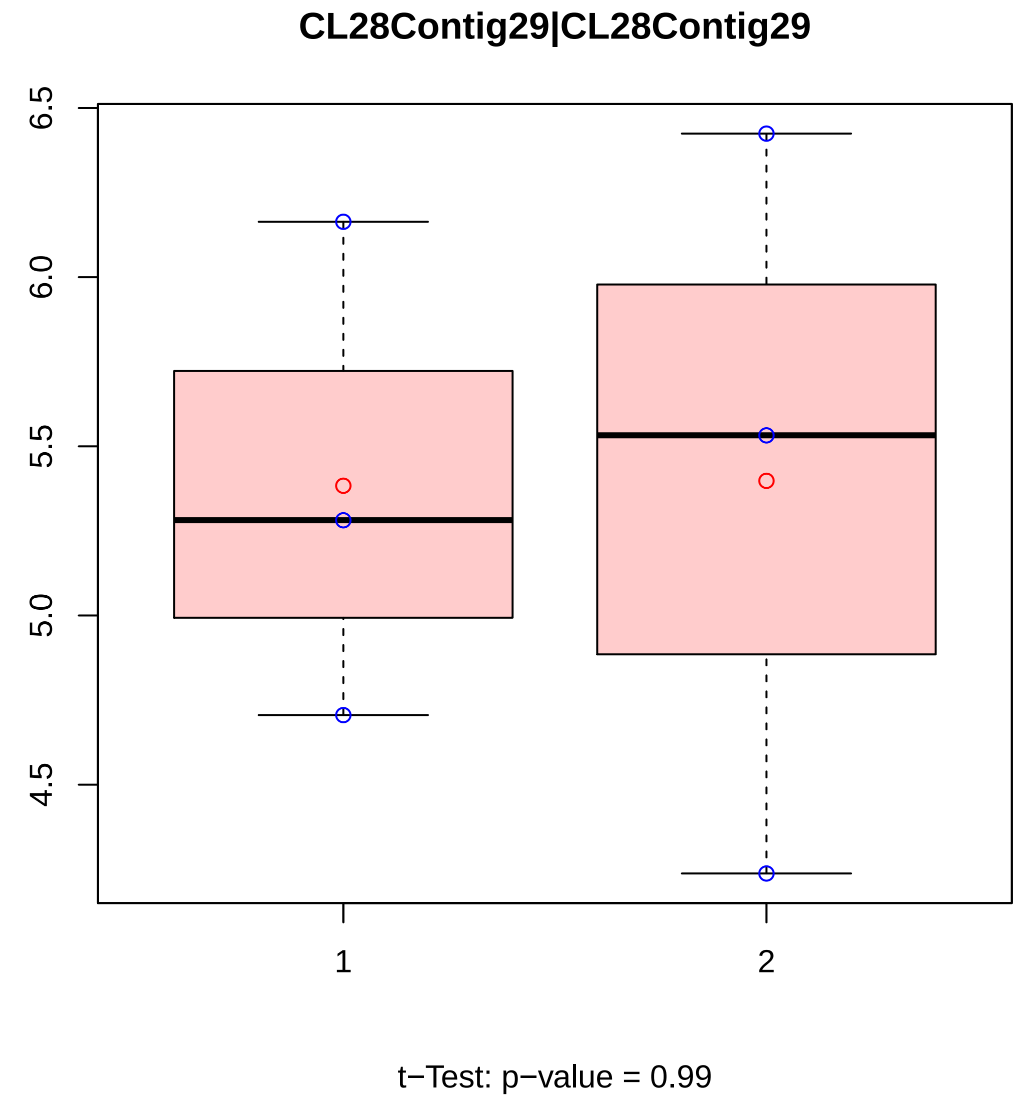

CL28Contig29|CL28Contig29
6.5
6.0
5.5
5.0
4.5
1
2
t−Test: p−value = 0.99

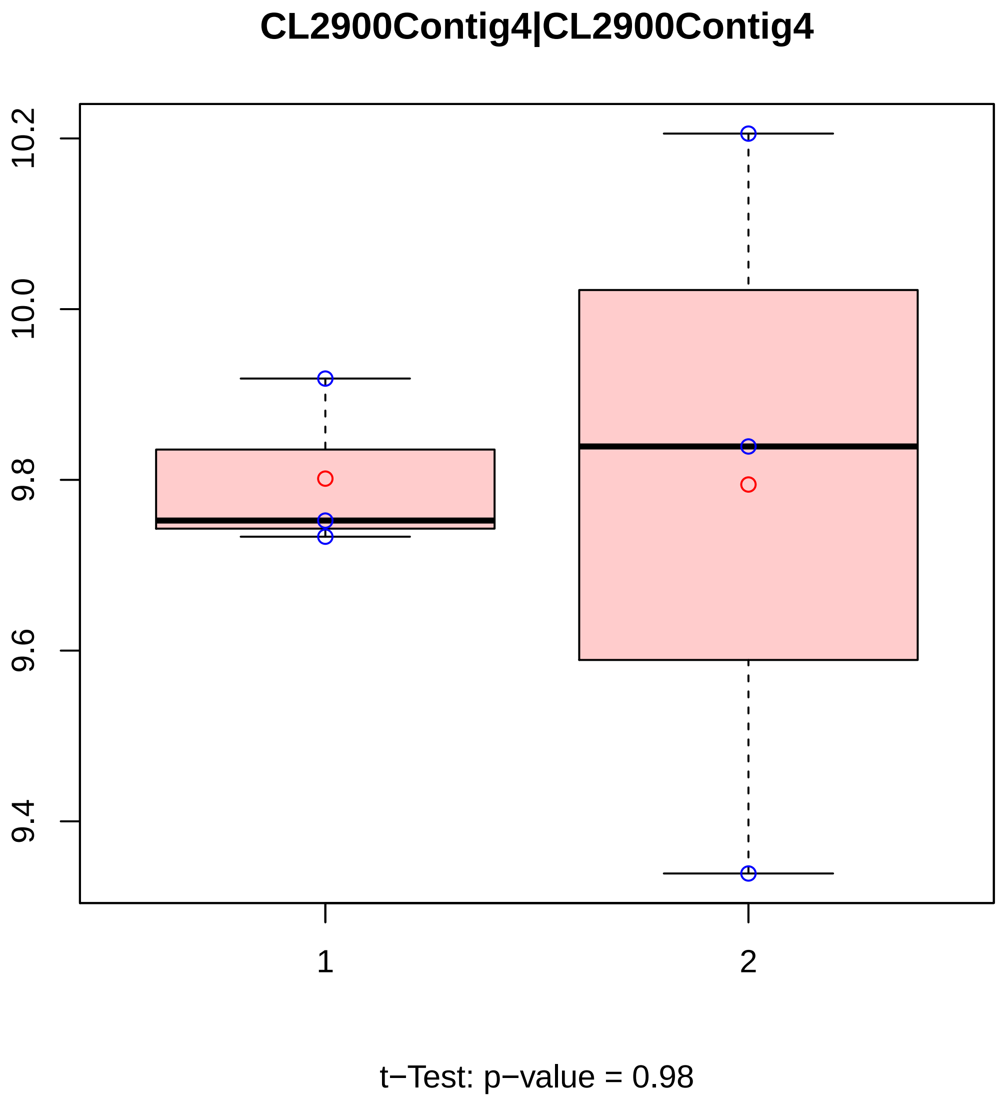
CL2900Contig4|CL2900Contig4
10.2
10.0
9.8
9.6
9.4
1
2
t−Test: p−value = 0.98

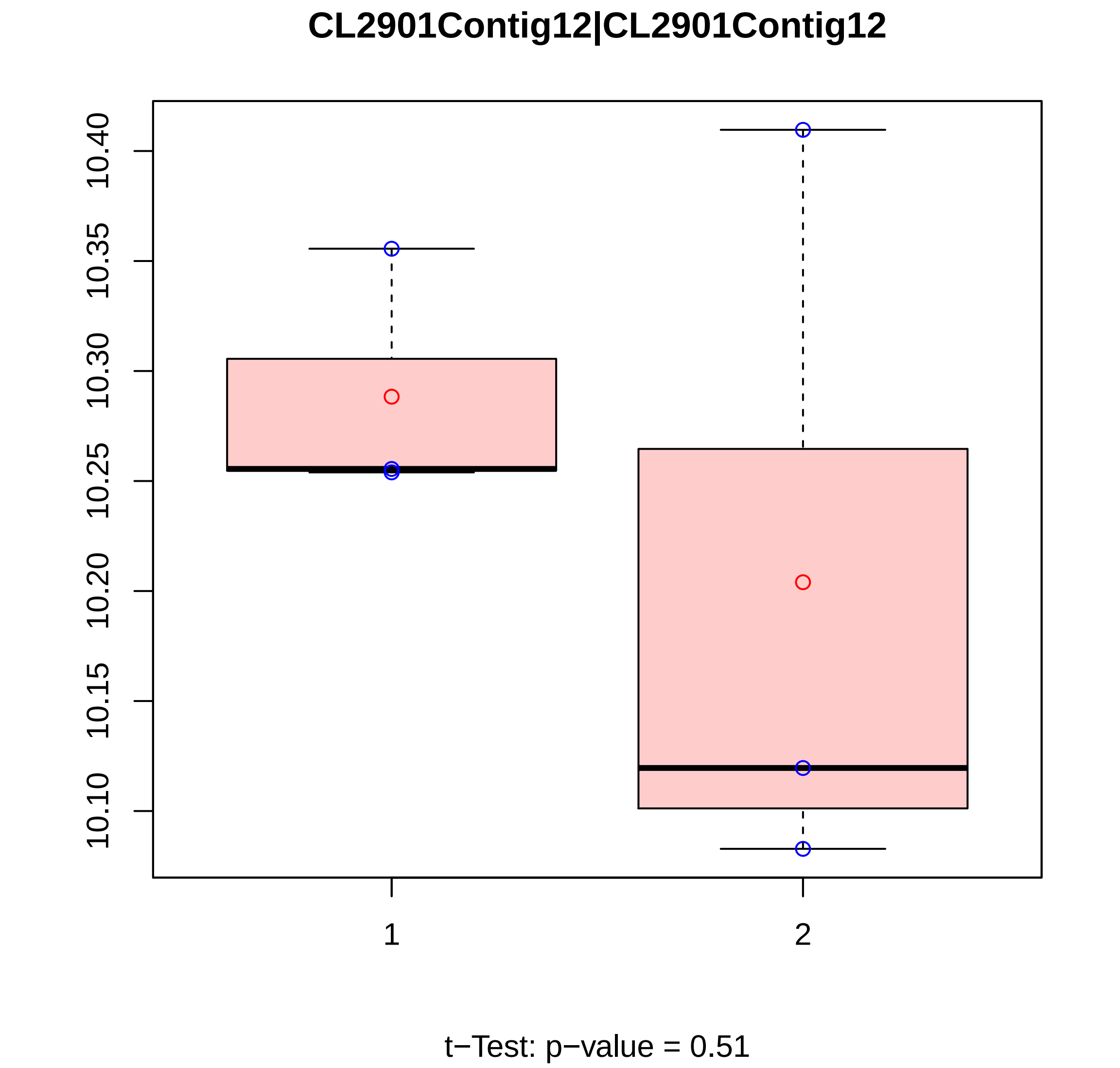
CL2901Contig12|CL2901Contig12
10.10
10.15
10.20
10.25
10.30
10.35
10.40
1
2
t−Test: p−value = 0.51

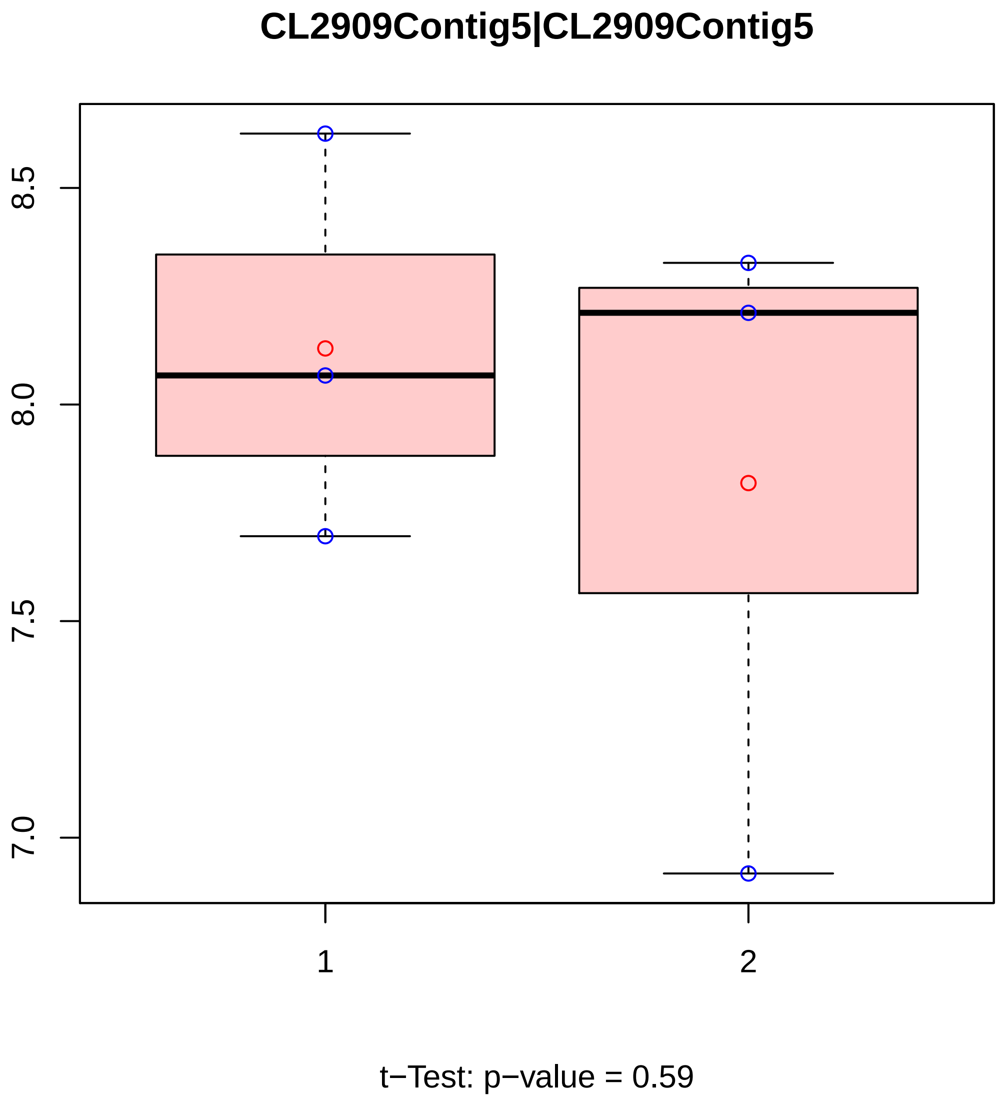
CL2909Contig5|CL2909Contig5
8.5
8.0
7.5
7.0
1
2
t−Test: p−value = 0.59

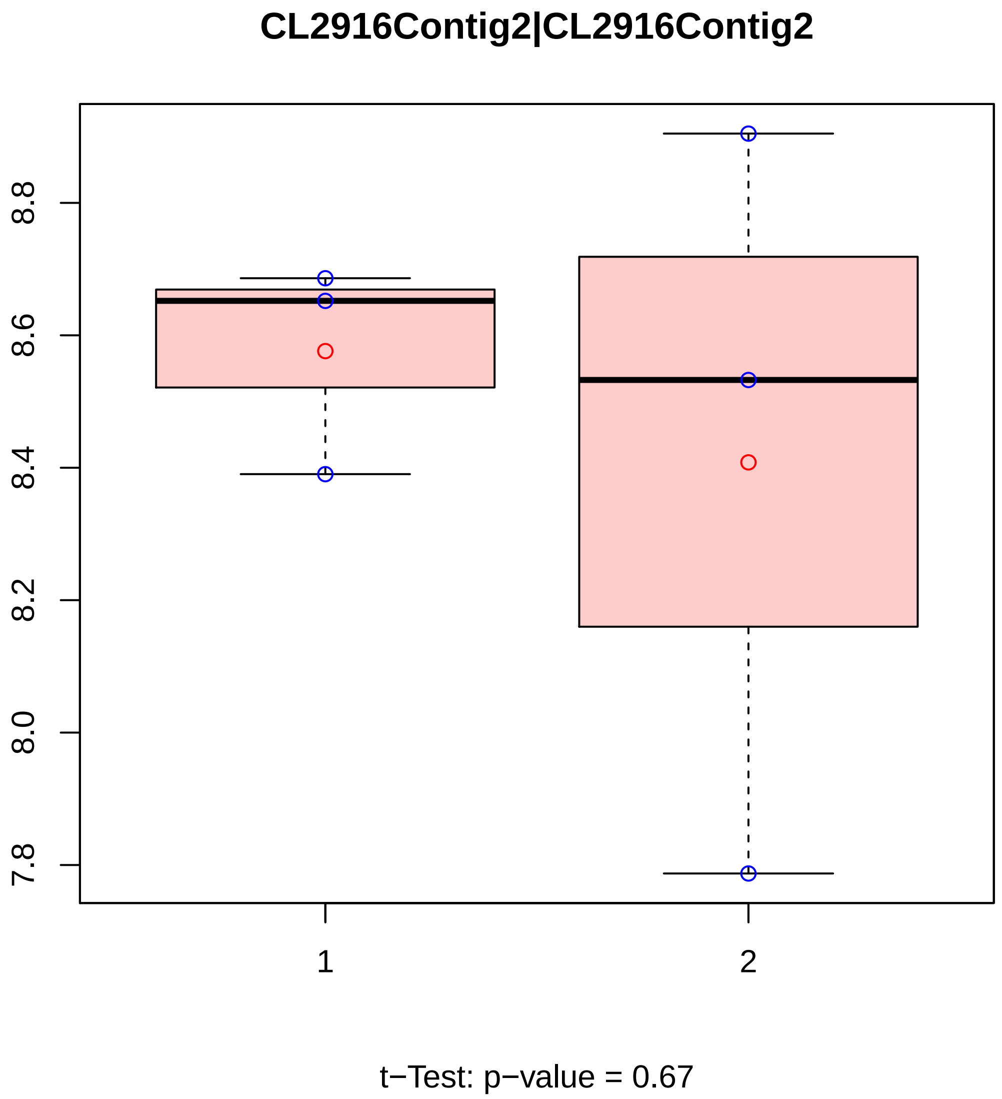
CL2916Contig2|CL2916Contig2
8.8
8.6
8.4
8.2
8.0
7.8
1
2
t−Test: p−value = 0.67

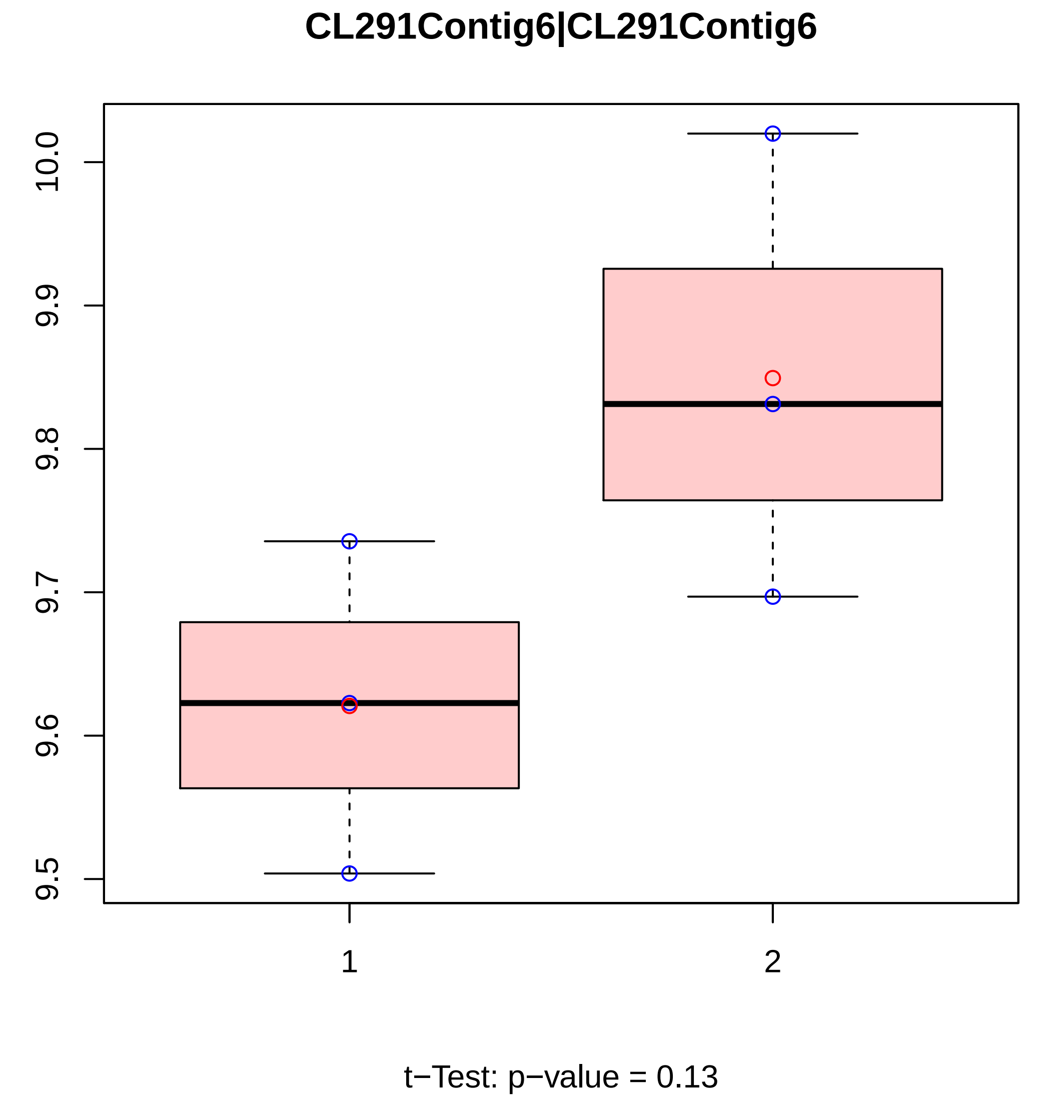
CL291Contig6|CL291Contig6
10.0
9.9
9.8
9.7
9.6
9.5
1
2
t−Test: p−value = 0.13

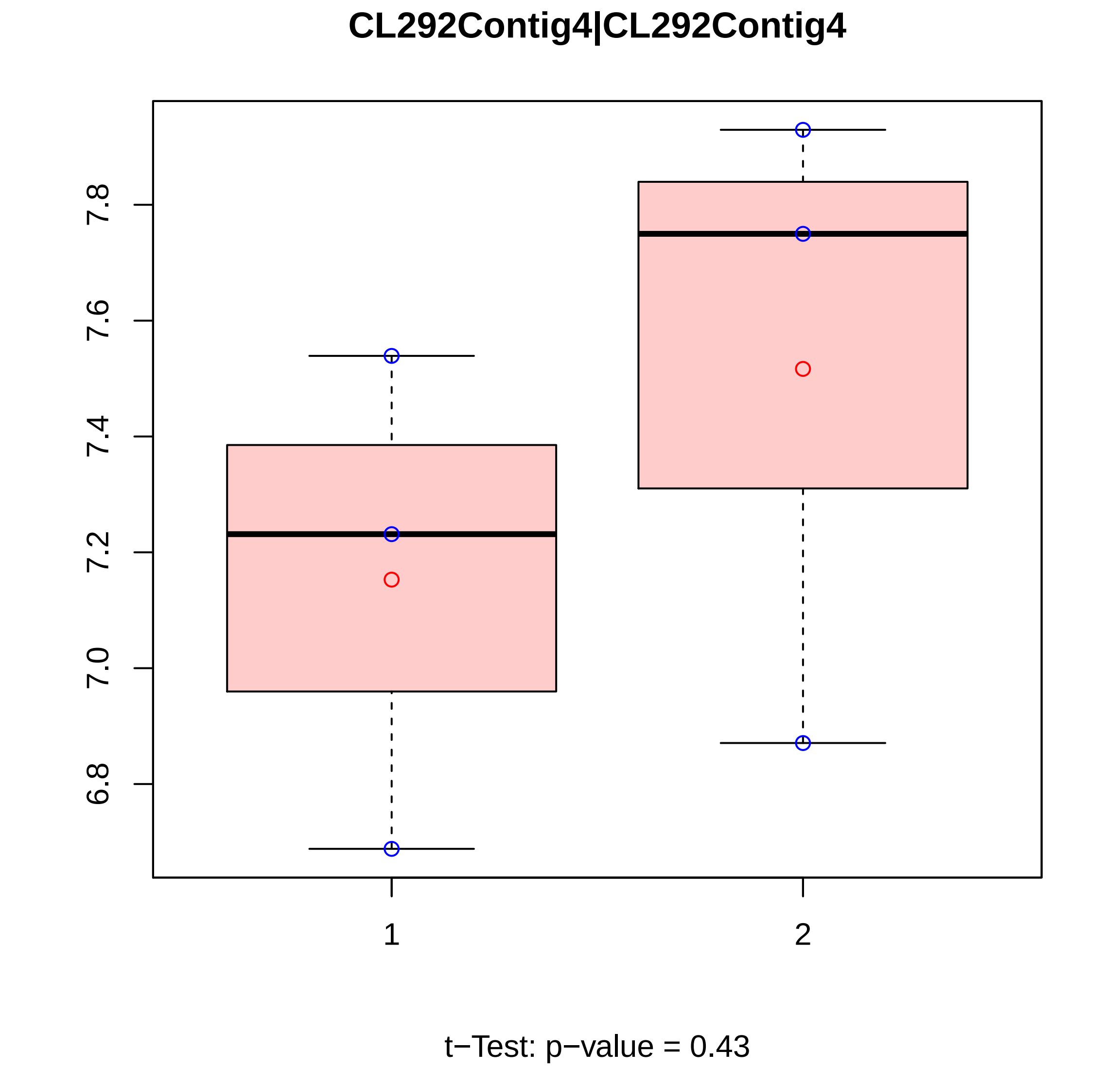

CL292Contig4|CL292Contig4
7.8
7.6
7.4
7.2
7.0
6.8
1
2
t−Test: p−value = 0.43

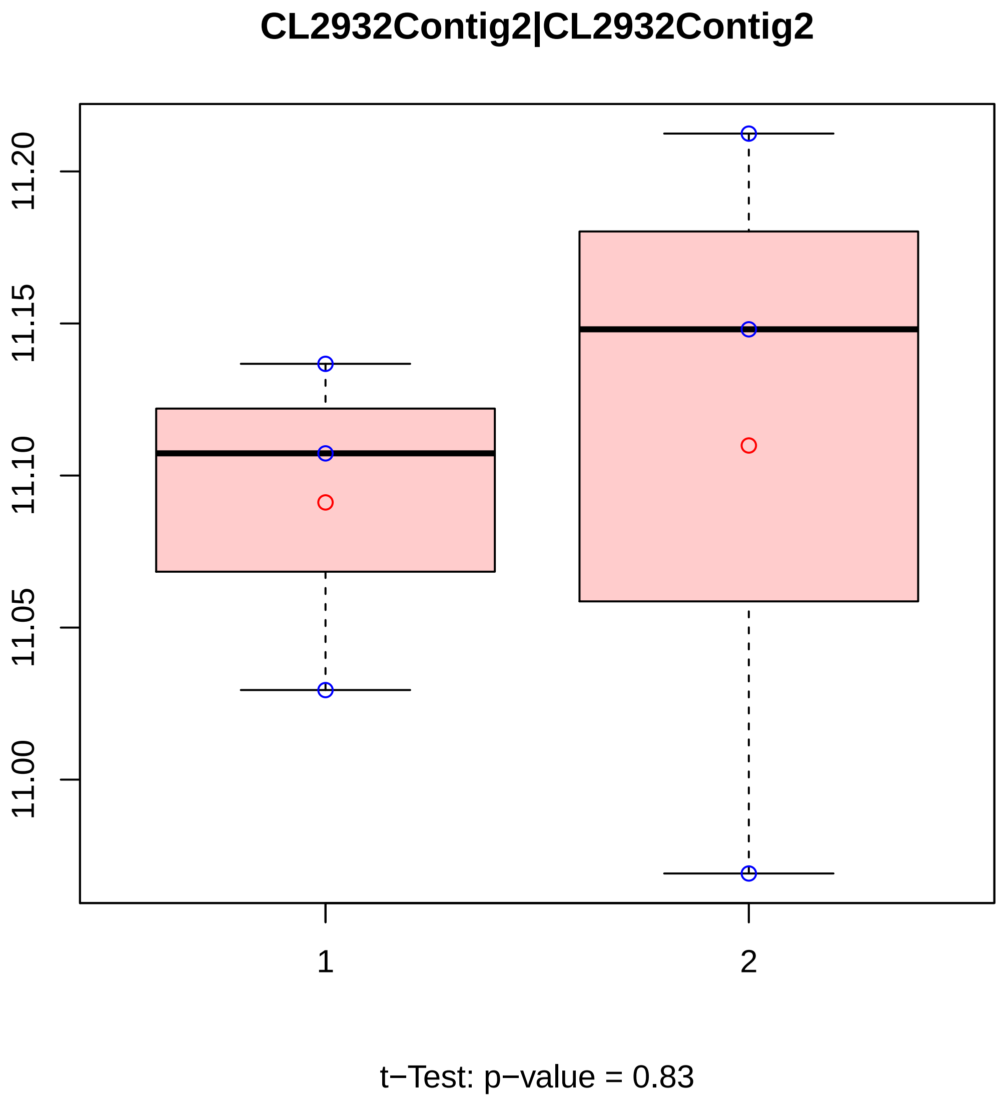
CL2932Contig2|CL2932Contig2
11.00
11.05
11.10
11.15
11.20
1
2
t−Test: p−value = 0.83

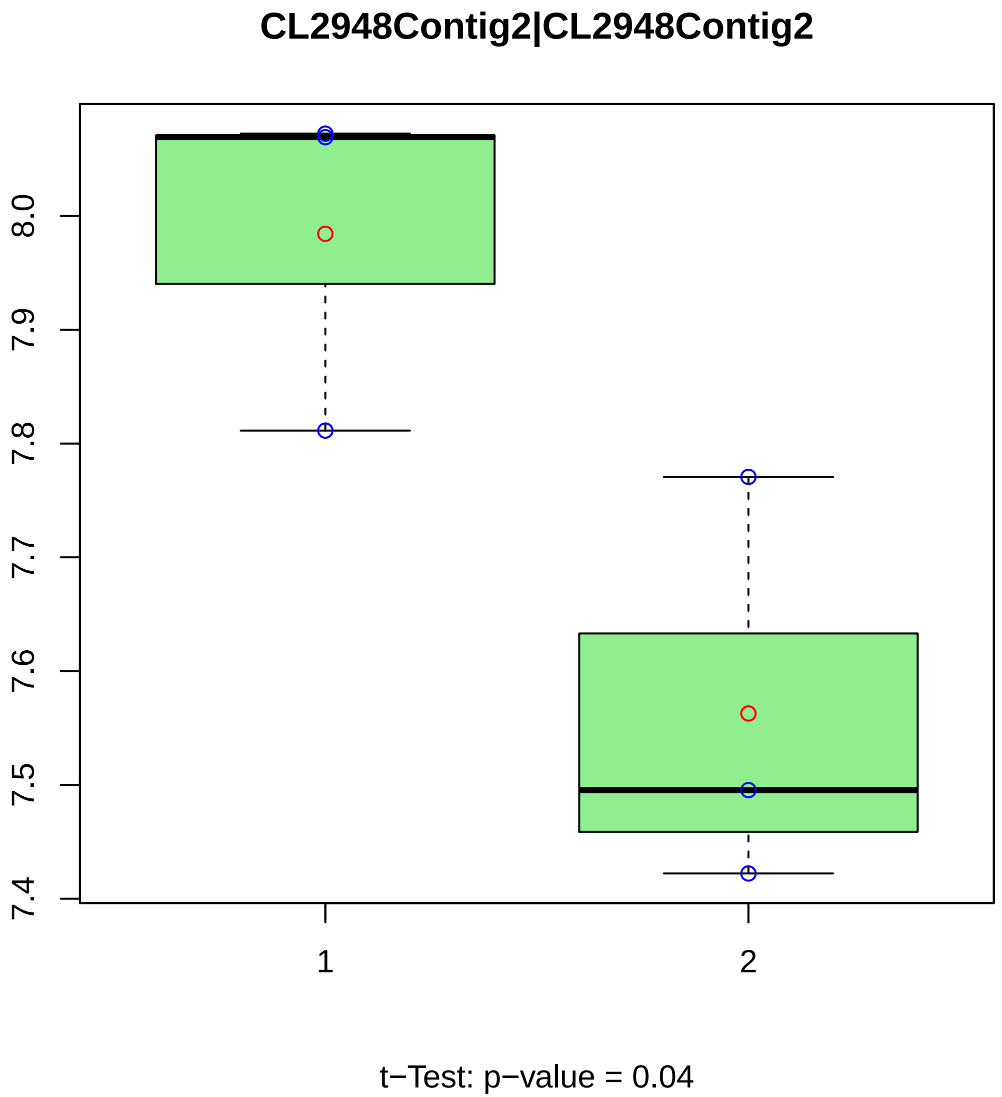

CL2948Contig2|CL2948Contig2
8.0
7.9
7.8
7.7
7.6
7.5
7.4
1
2
t−Test: p−value = 0.04

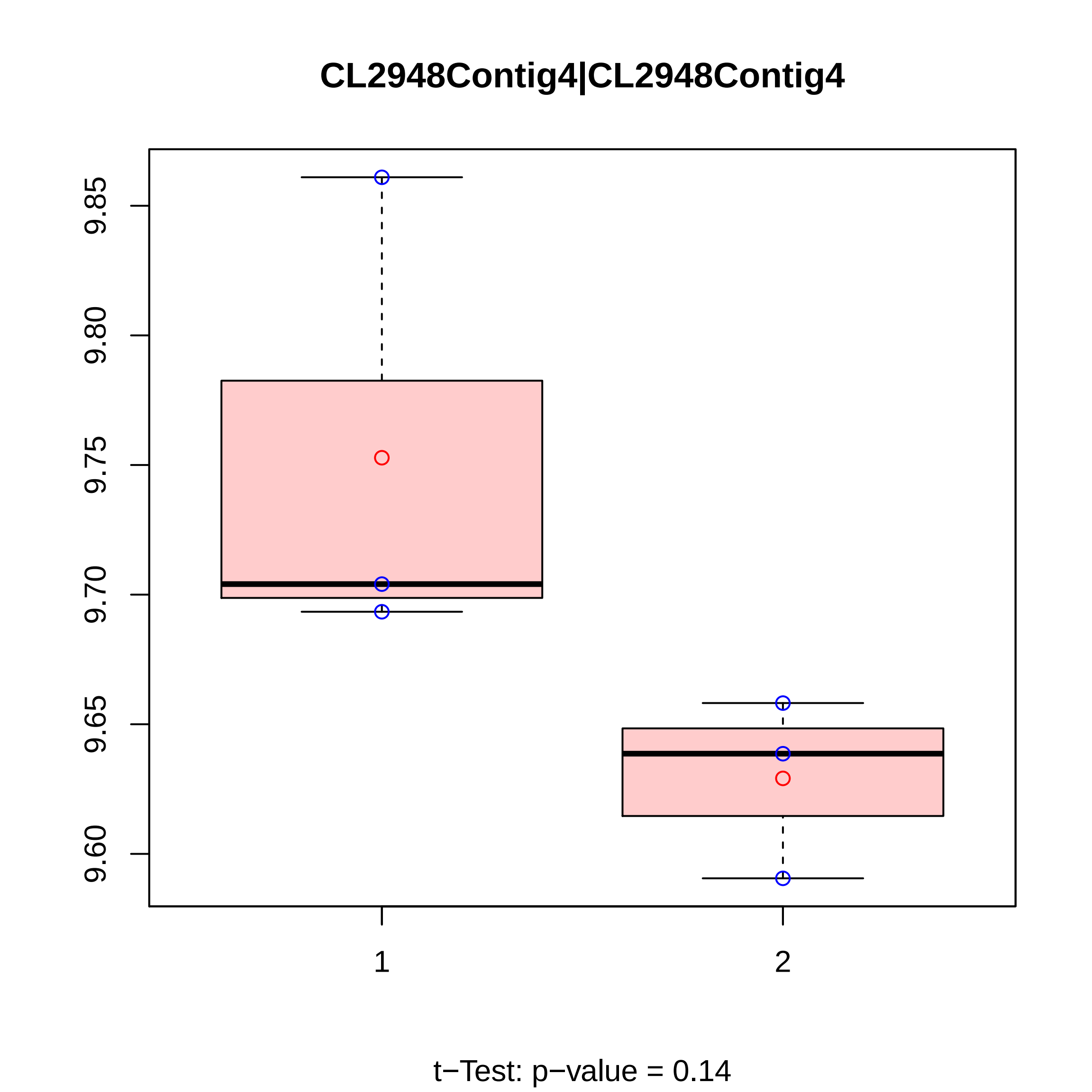

CL2948Contig4|CL2948Contig4
9.60
9.65
9.70
9.75
9.80
9.85
1
2
t−Test: p−value = 0.14

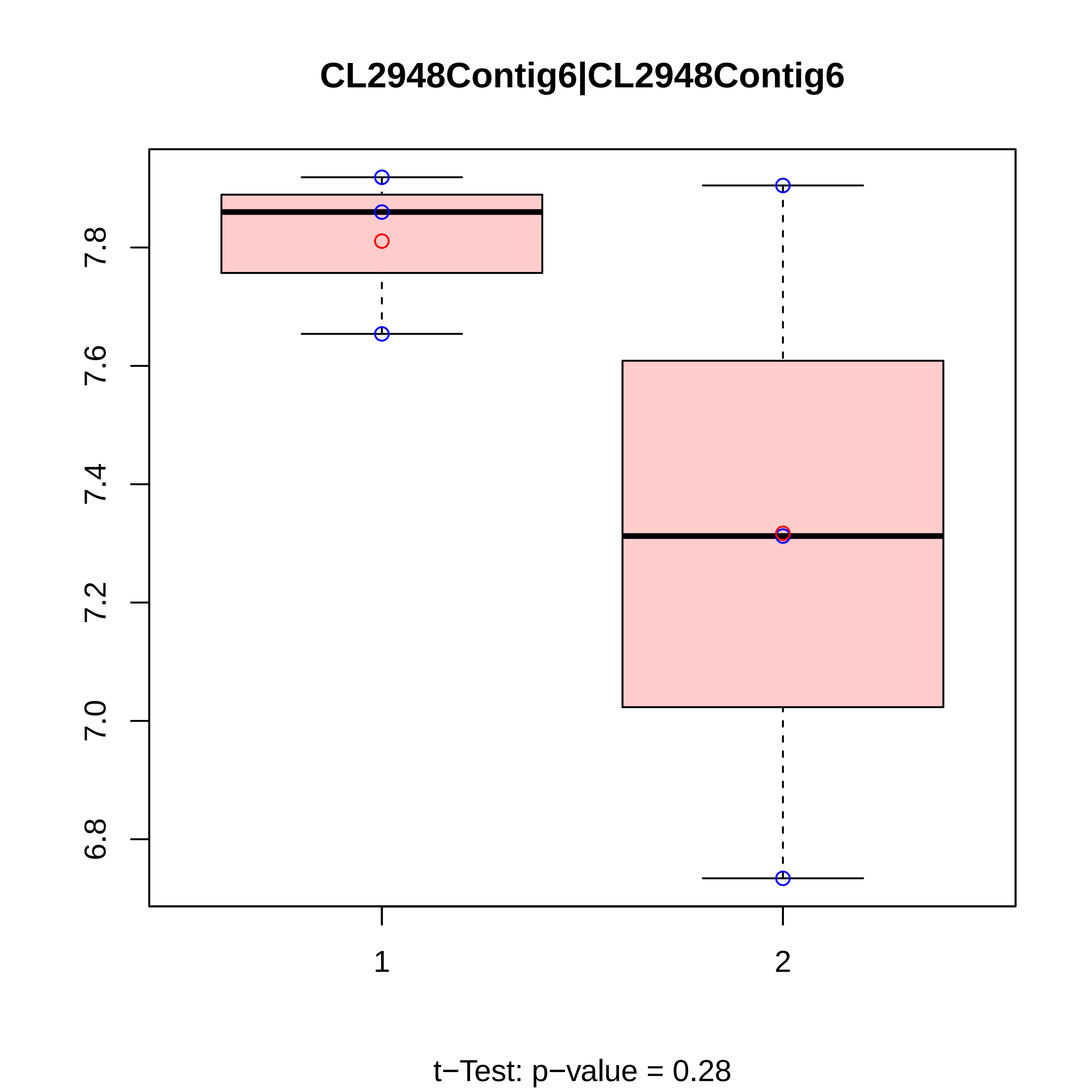
CL2948Contig6|CL2948Contig6
7.8
7.6
7.4
7.2
7.0
6.8
1
2
t−Test: p−value = 0.28

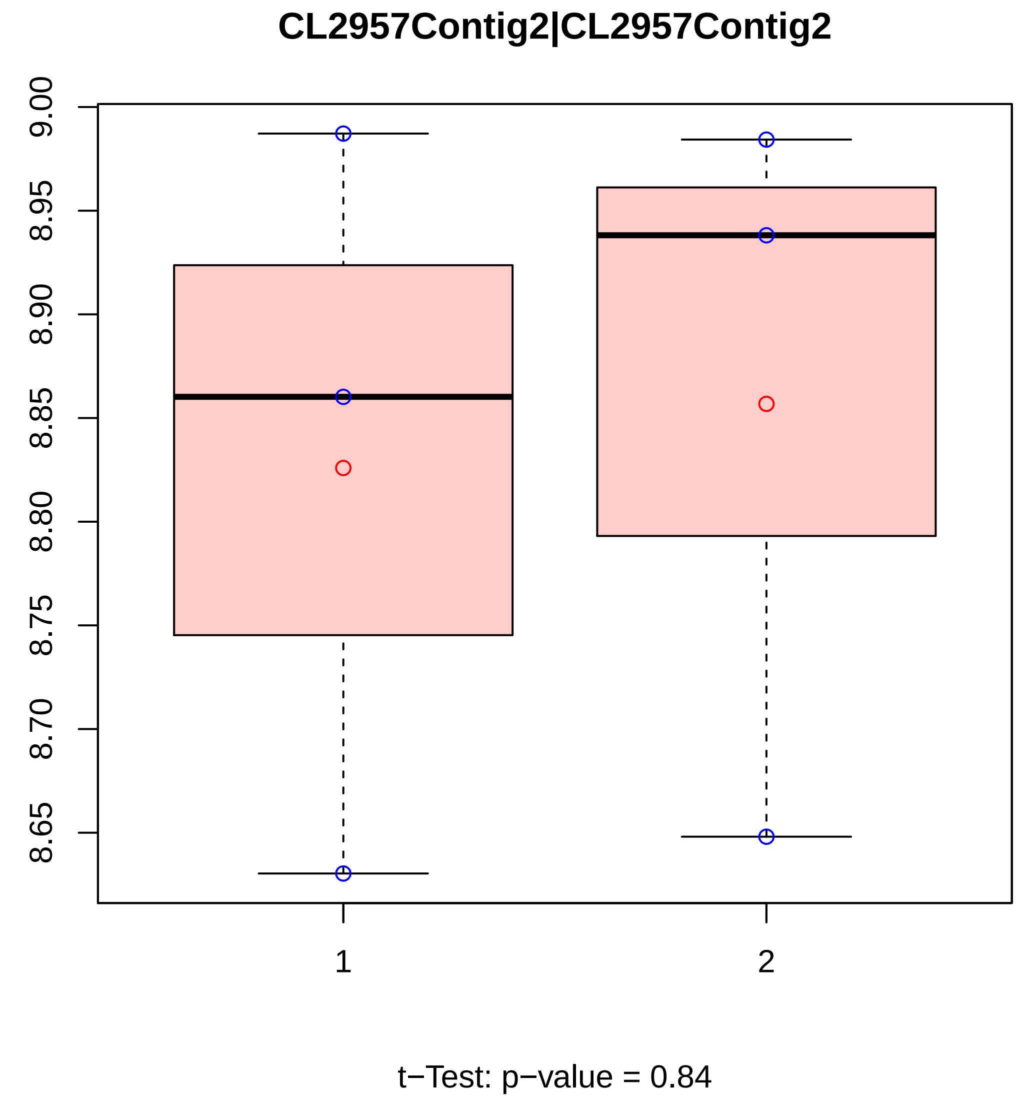
CL2957Contig2|CL2957Contig2
9.00
8.95
8.90
8.85
8.80
8.75
8.70
8.65
1
2
t−Test: p−value = 0.84

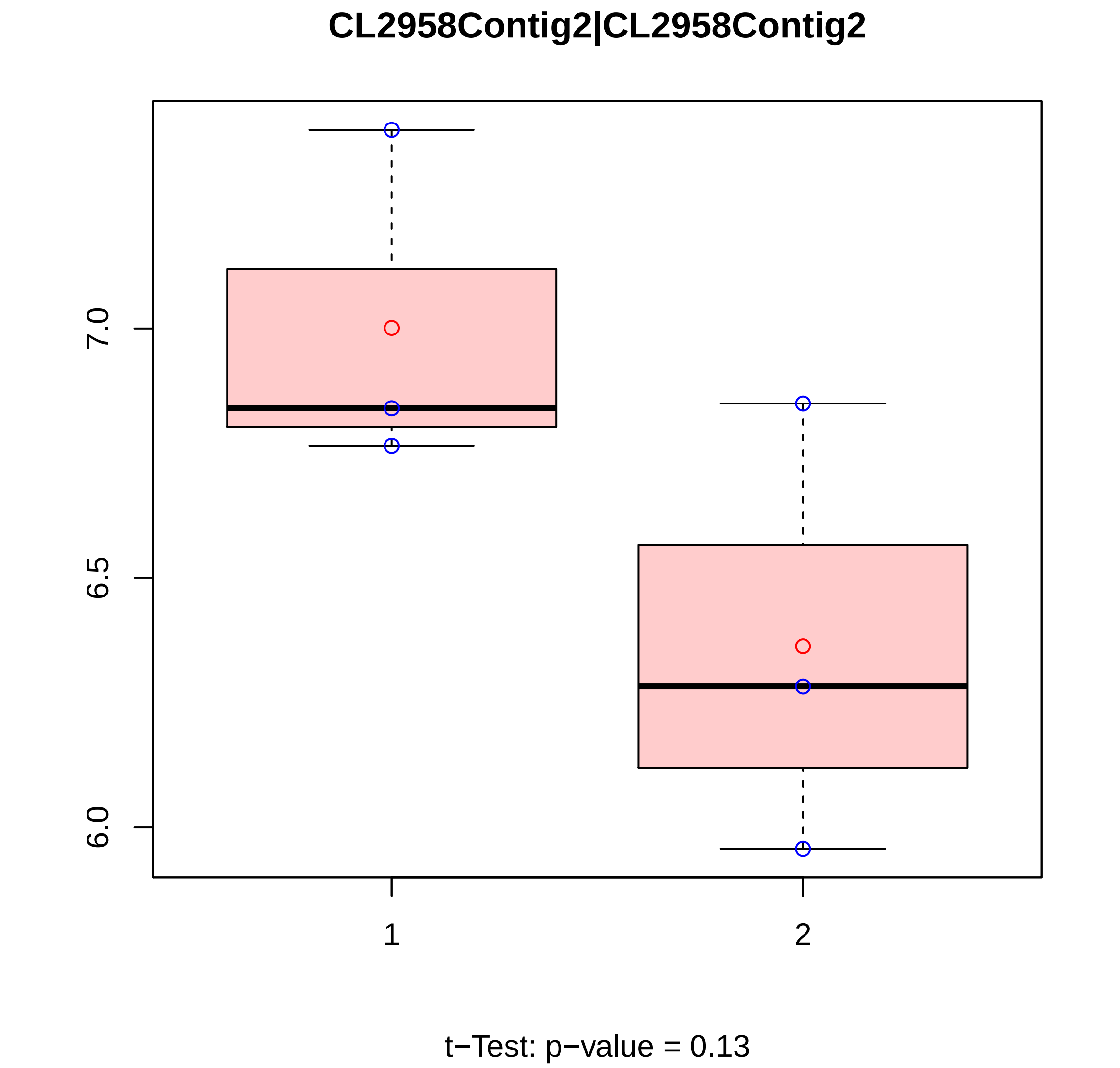

CL2958Contig2|CL2958Contig2
7.0
6.5
6.0
1
2
t−Test: p−value = 0.13

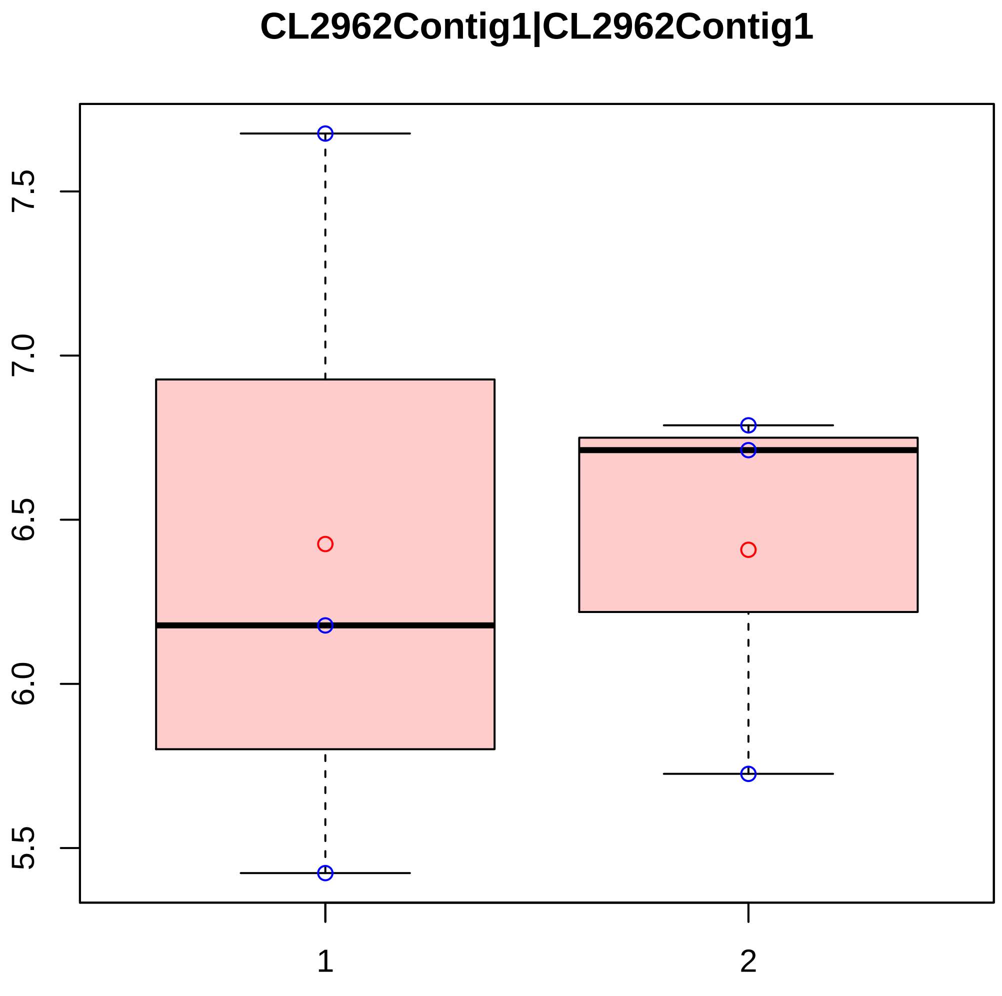


t−Test: p−value = 0.98

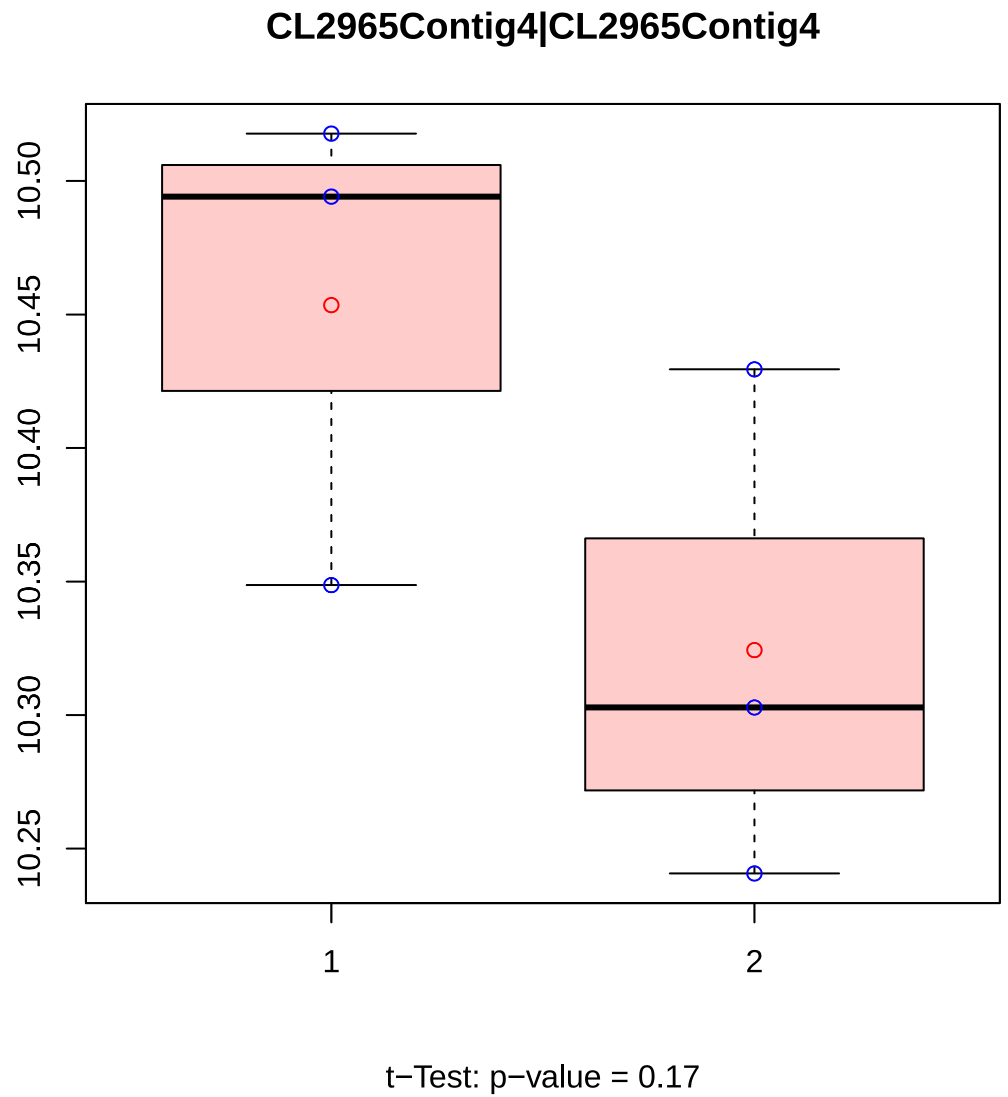

CL2965Contig4|CL2965Contig4
10.25
10.30
10.35
10.40
10.45
10.50
1
2
t−Test: p−value = 0.17

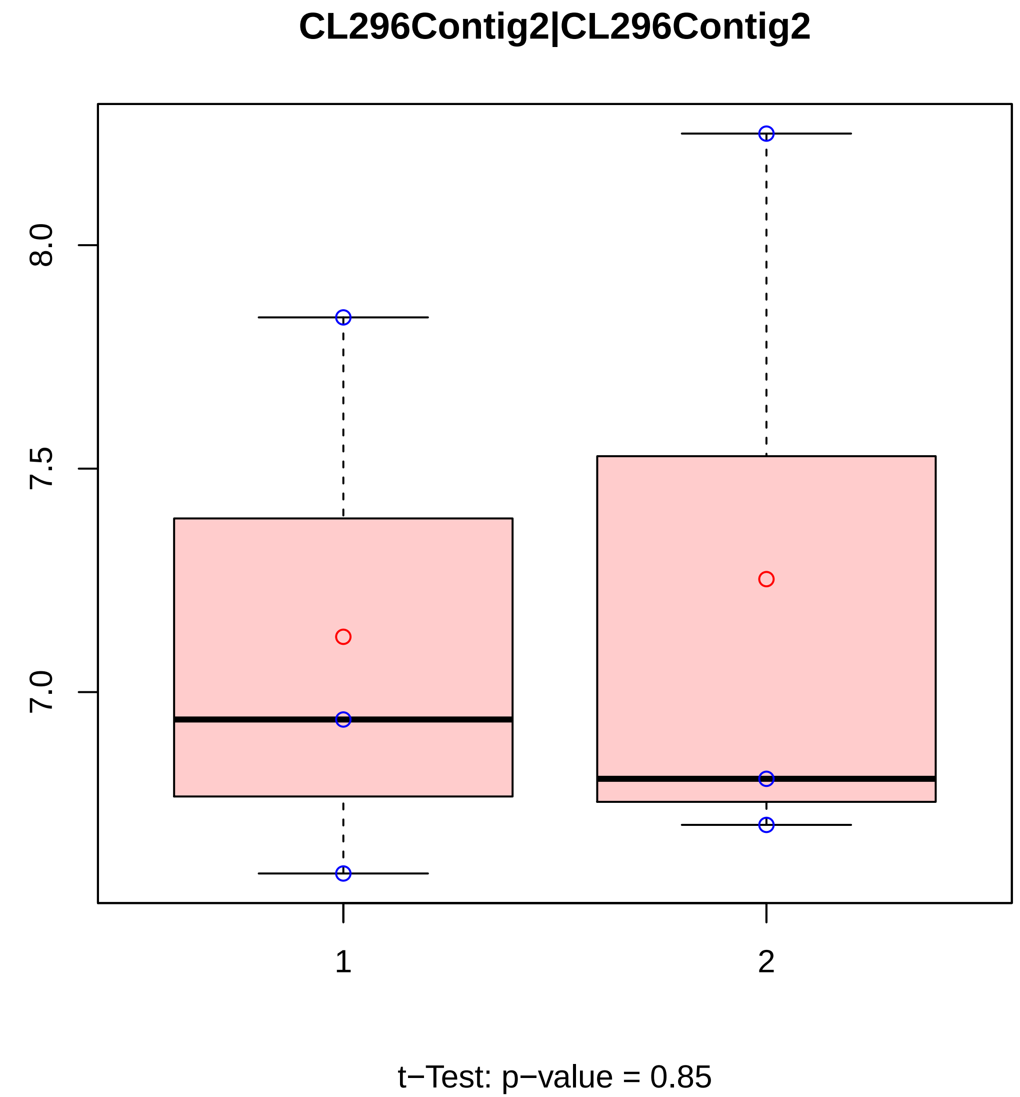
CL296Contig2|CL296Contig2
8.0
7.5
7.0
1
2
t−Test: p−value = 0.85

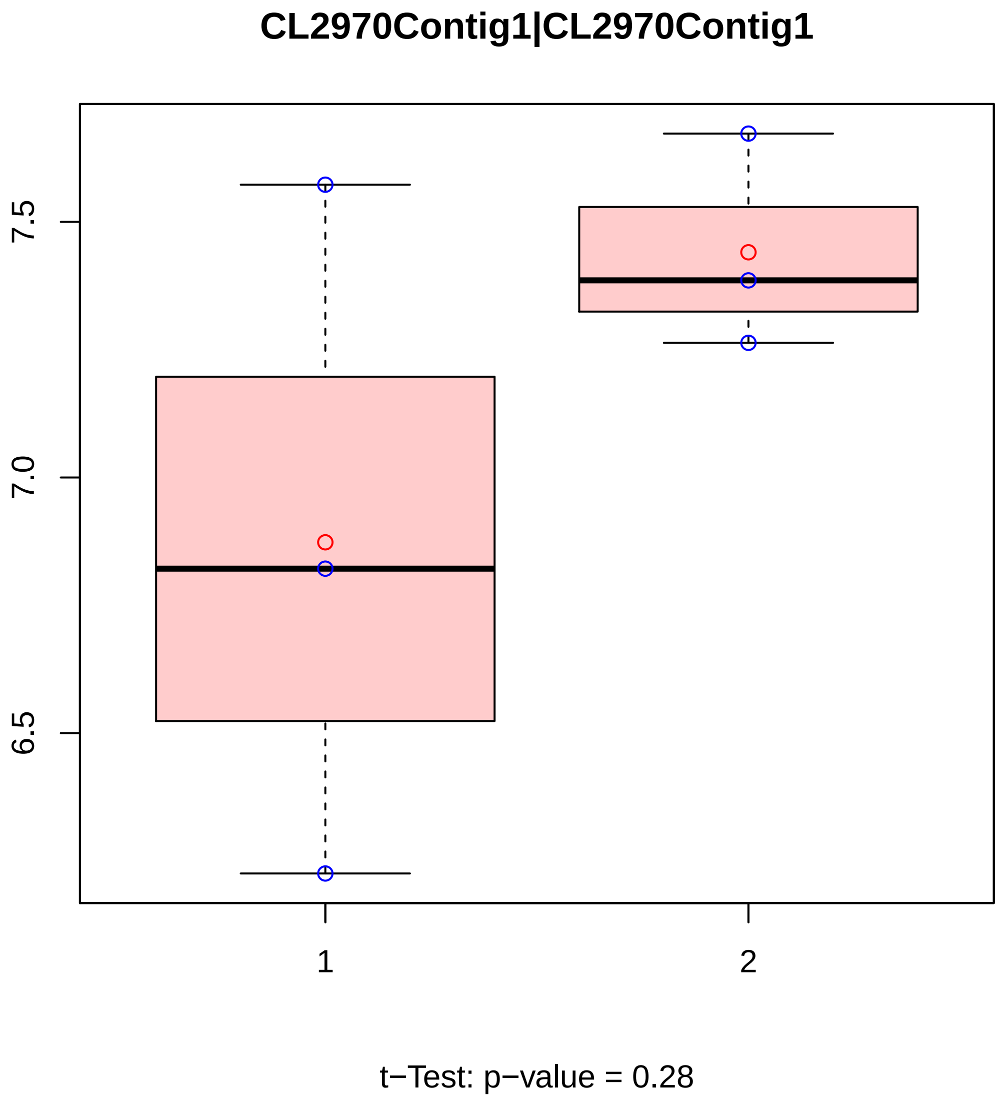

CL2970Contig1|CL2970Contig1
7.5
7.0
6.5
1
2
t−Test: p−value = 0.28

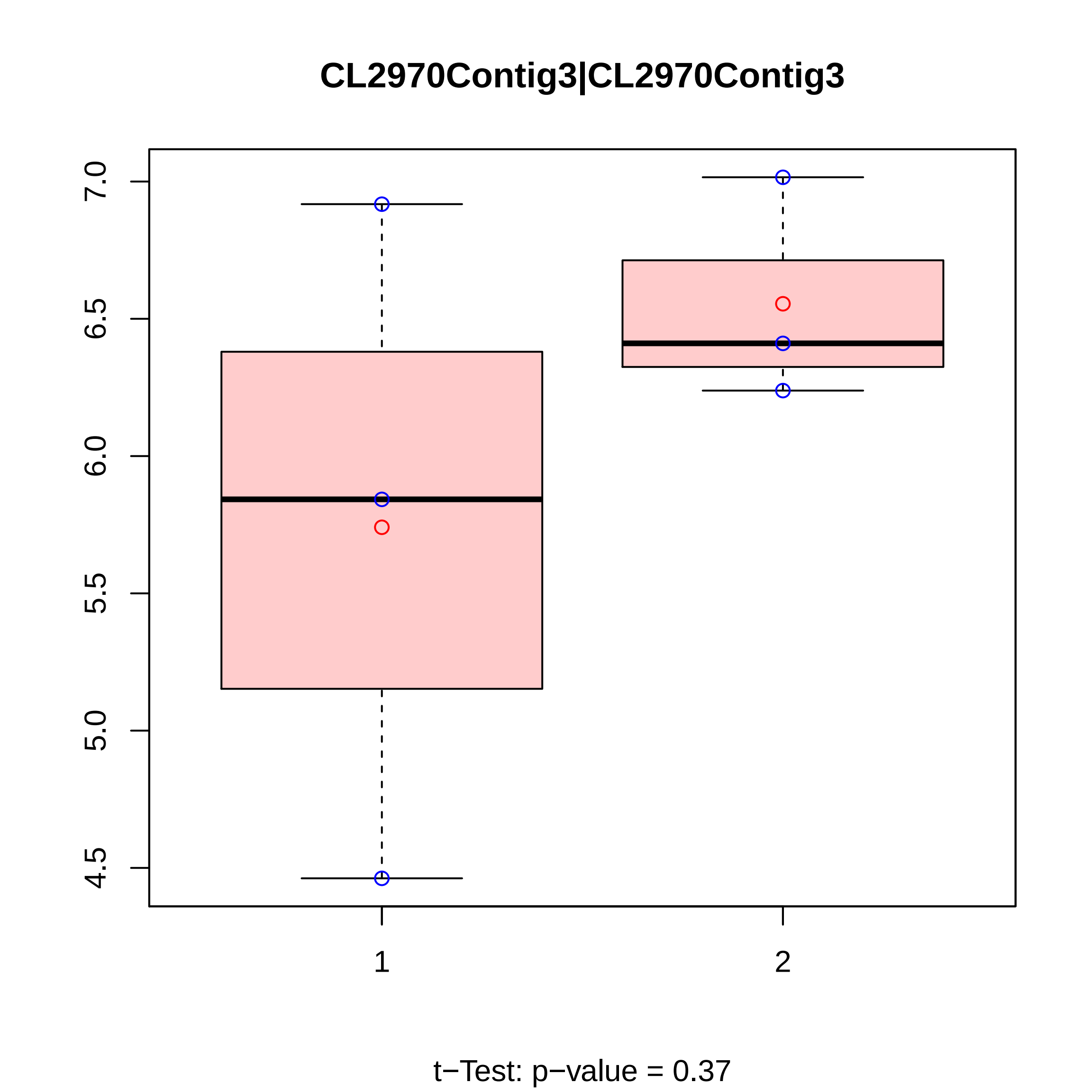
CL2970Contig3|CL2970Contig3
7.0
6.5
6.0
5.5
5.0
4.5
1
2
t−Test: p−value = 0.37

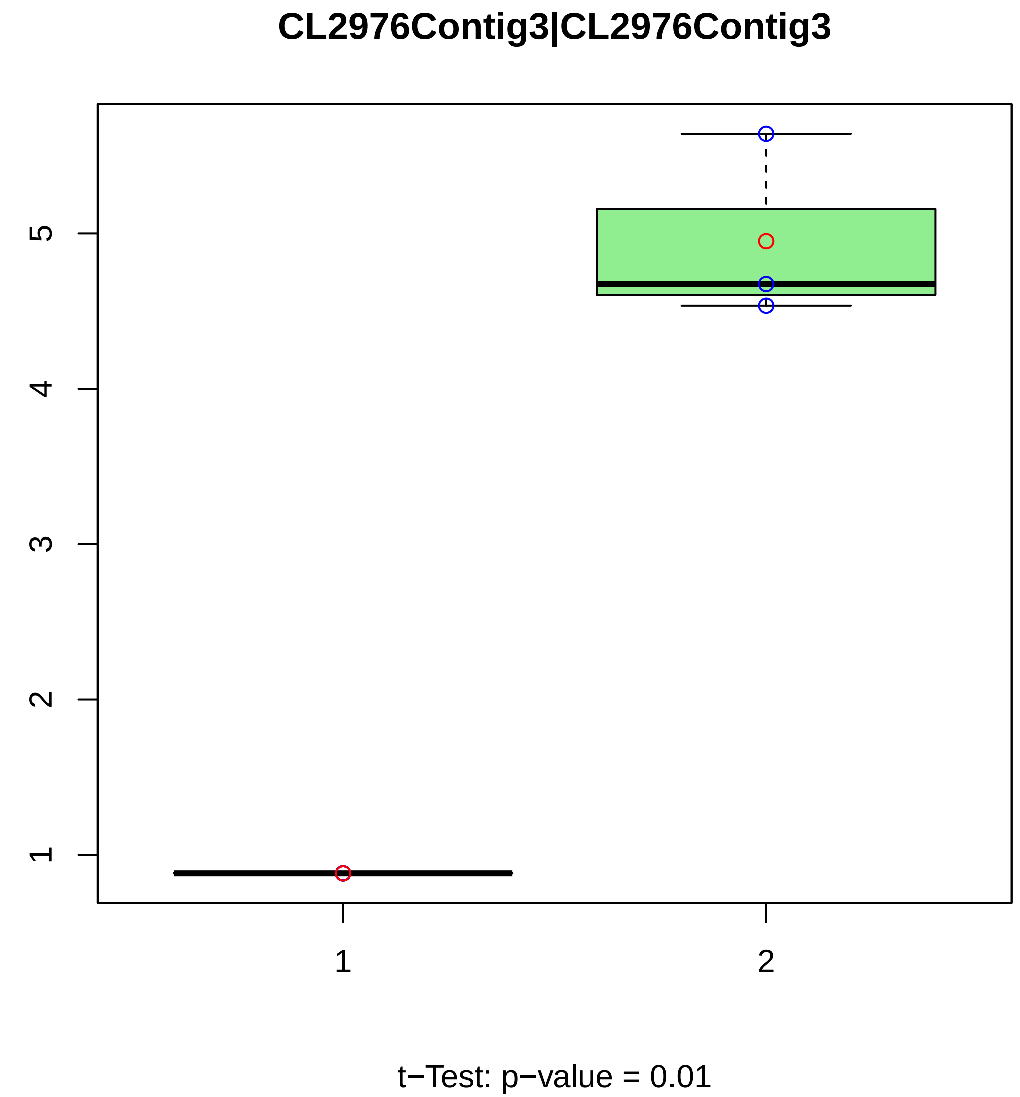

CL2976Contig3|CL2976Contig3
1
2
3
4
5
1
2
t−Test: p−value = 0.01

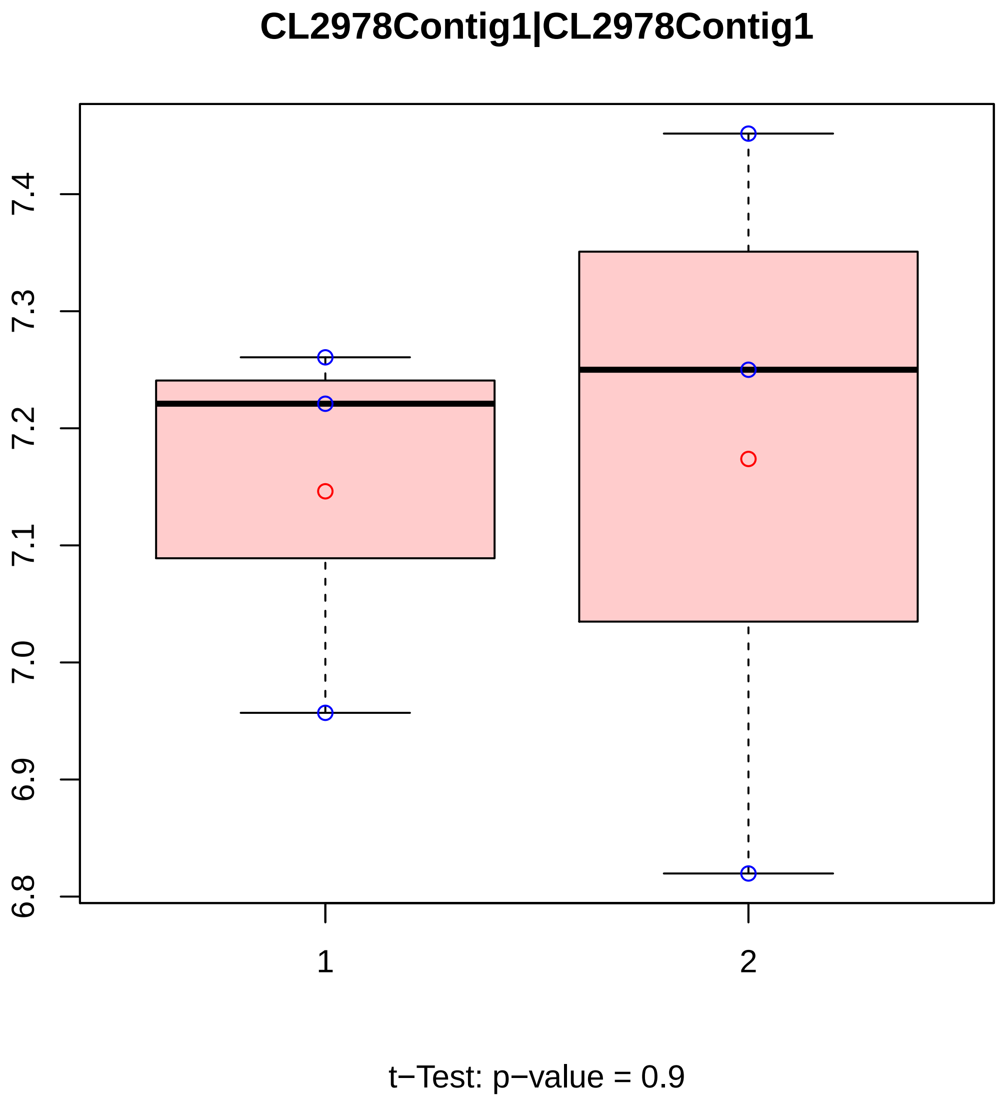
CL2978Contig1|CL2978Contig1
7.4
7.3
7.2
7.1
7.0
6.9
6.8
1
2
t−Test: p−value = 0.9

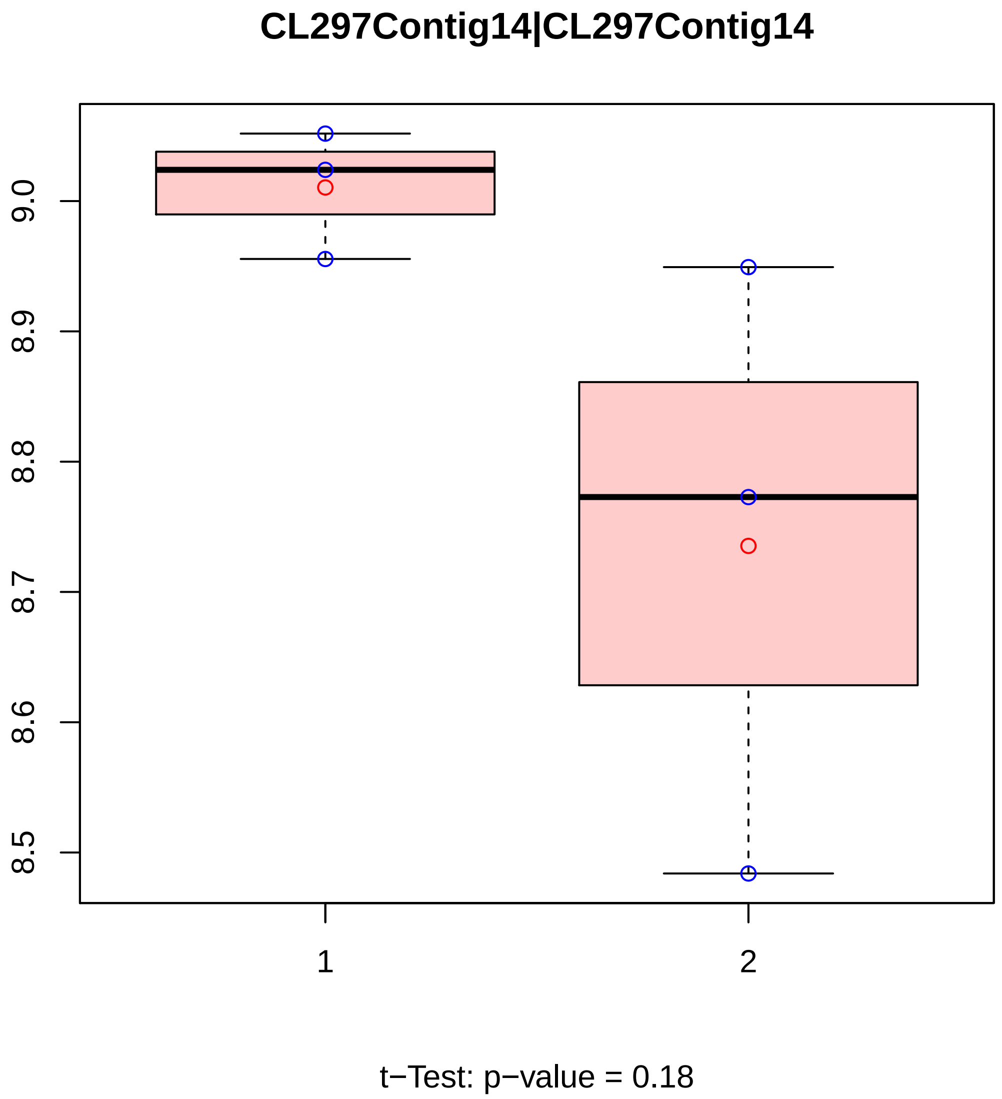
CL297Contig14|CL297Contig14
9.0
8.9
8.8
8.7
8.6
8.5
1
2
t−Test: p−value = 0.18

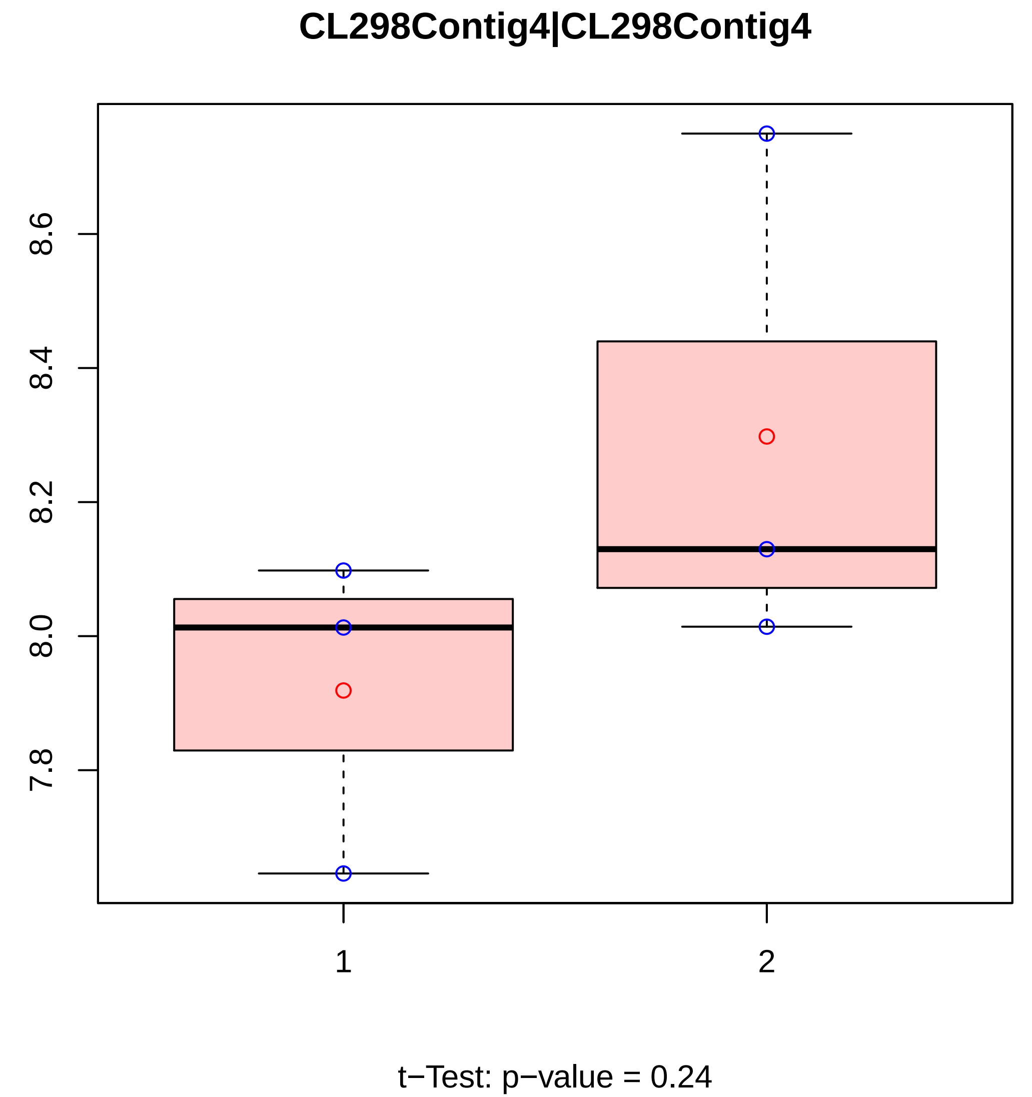
CL298Contig4|CL298Contig4
8.6
8.4
8.2
8.0
7.8
1
2
t−Test: p−value = 0.24

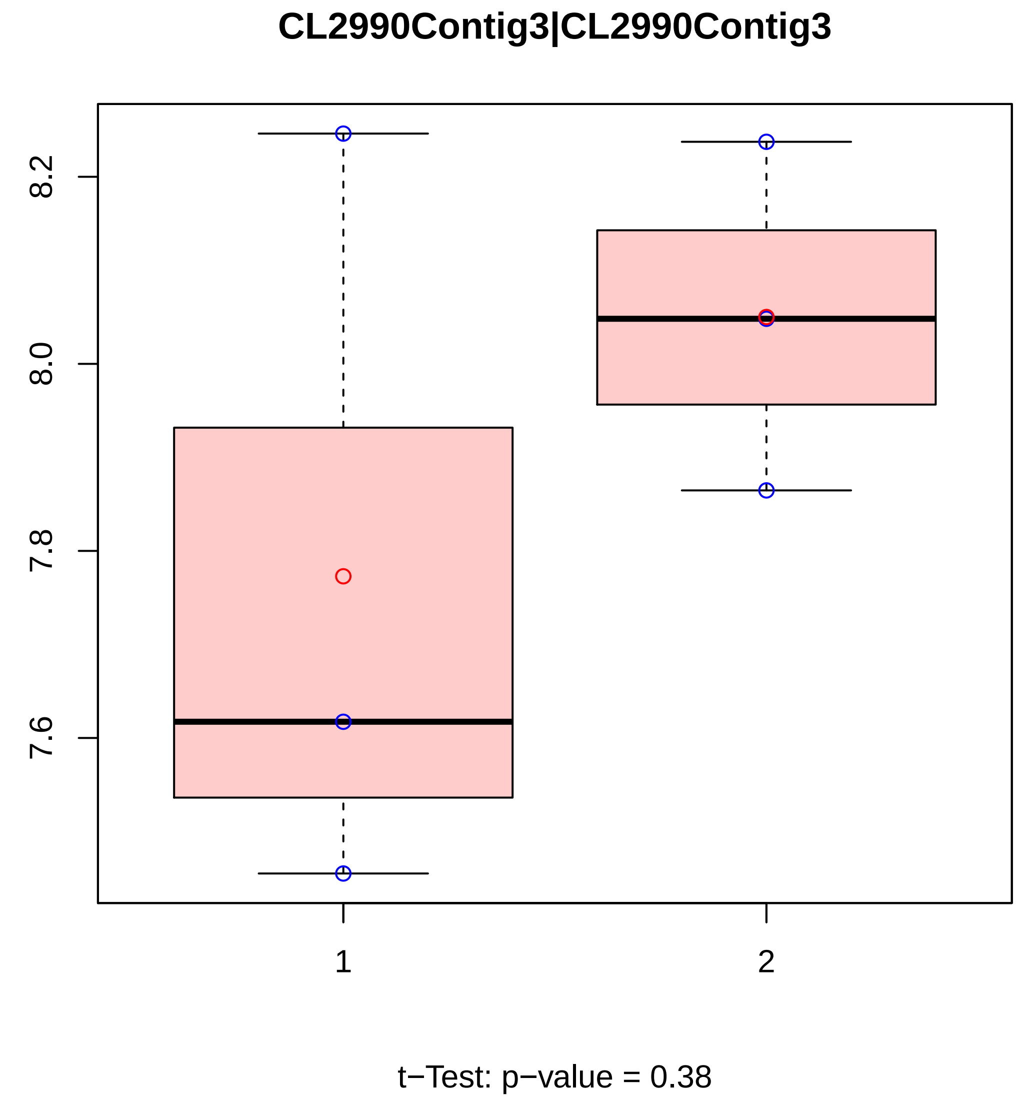
CL2990Contig3|CL2990Contig3
8.2
8.0
7.8
7.6
1
2
t−Test: p−value = 0.38

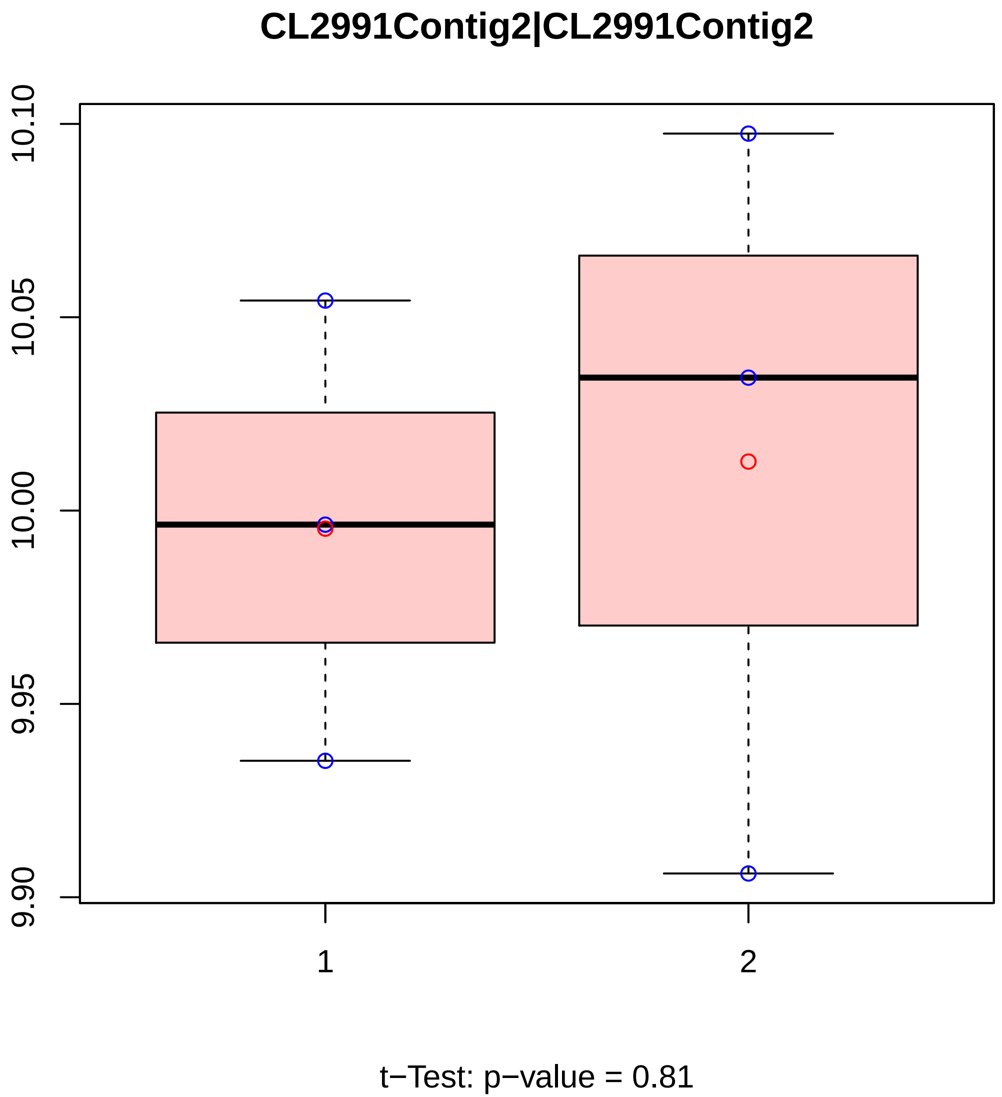

CL2991Contig2|CL2991Contig2
10.10
10.05
10.00
9.95
9.90
1
2
t−Test: p−value = 0.81

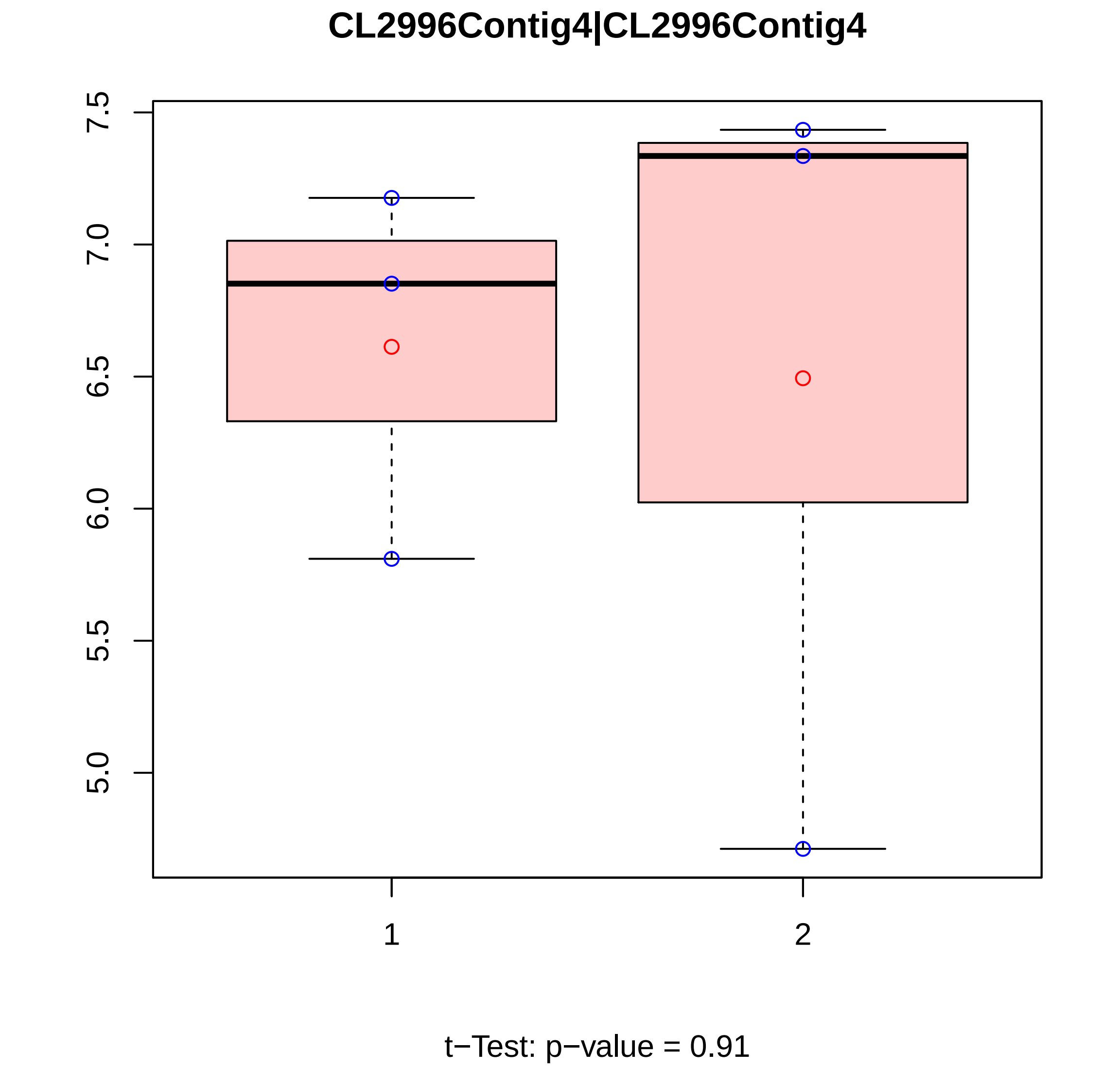

CL2996Contig4|CL2996Contig4
7.5
7.0
6.5
6.0
5.5
5.0
1
2
t−Test: p−value = 0.91

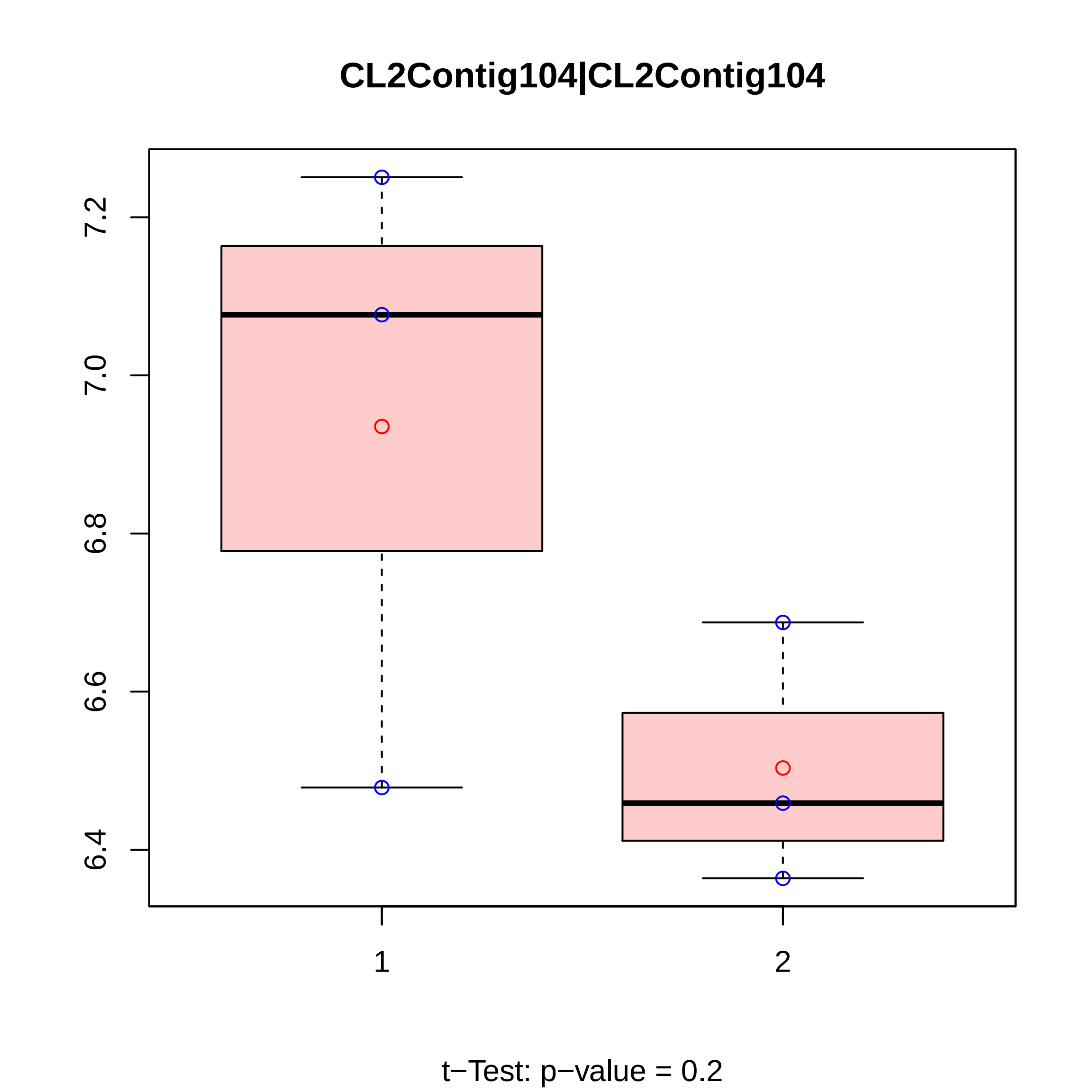
CL2Contig104|CL2Contig104
7.2
7.0
6.8
6.6
6.4
1
2
t−Test: p−value = 0.2

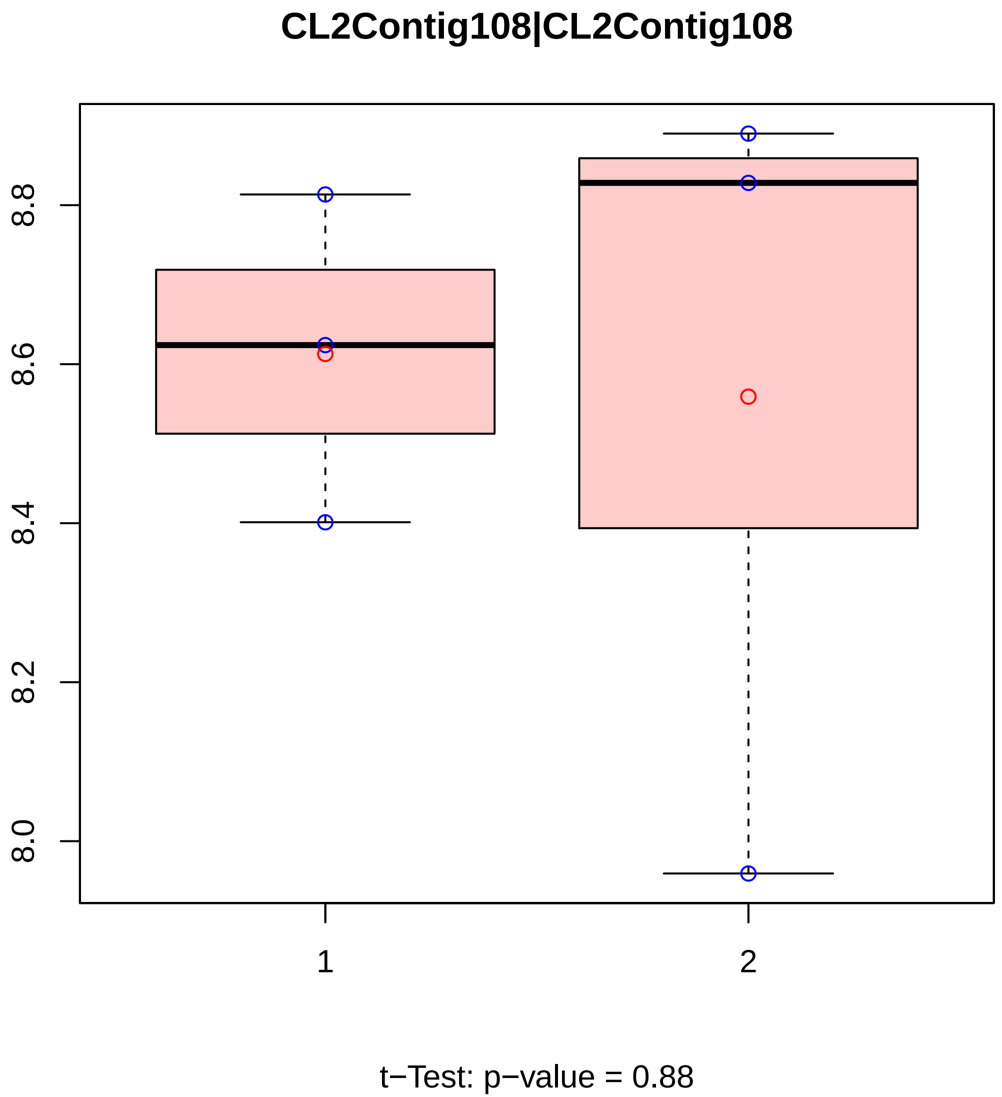
CL2Contig108|CL2Contig108
8.0
8.2
8.4
8.6
8.8
1
2
t−Test: p−value = 0.88

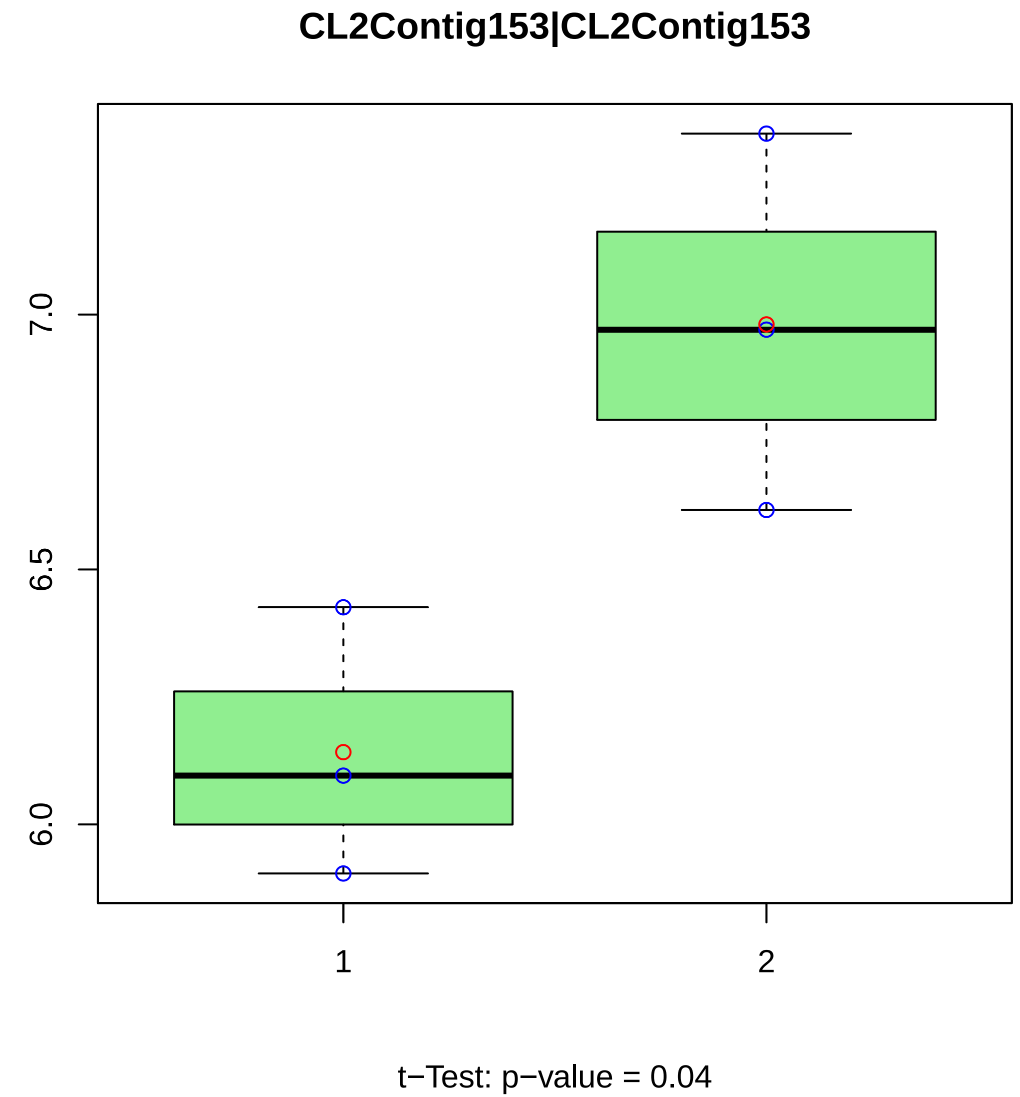
CL2Contig153|CL2Contig153
7.0
6.5
6.0
1
2
t−Test: p−value = 0.04

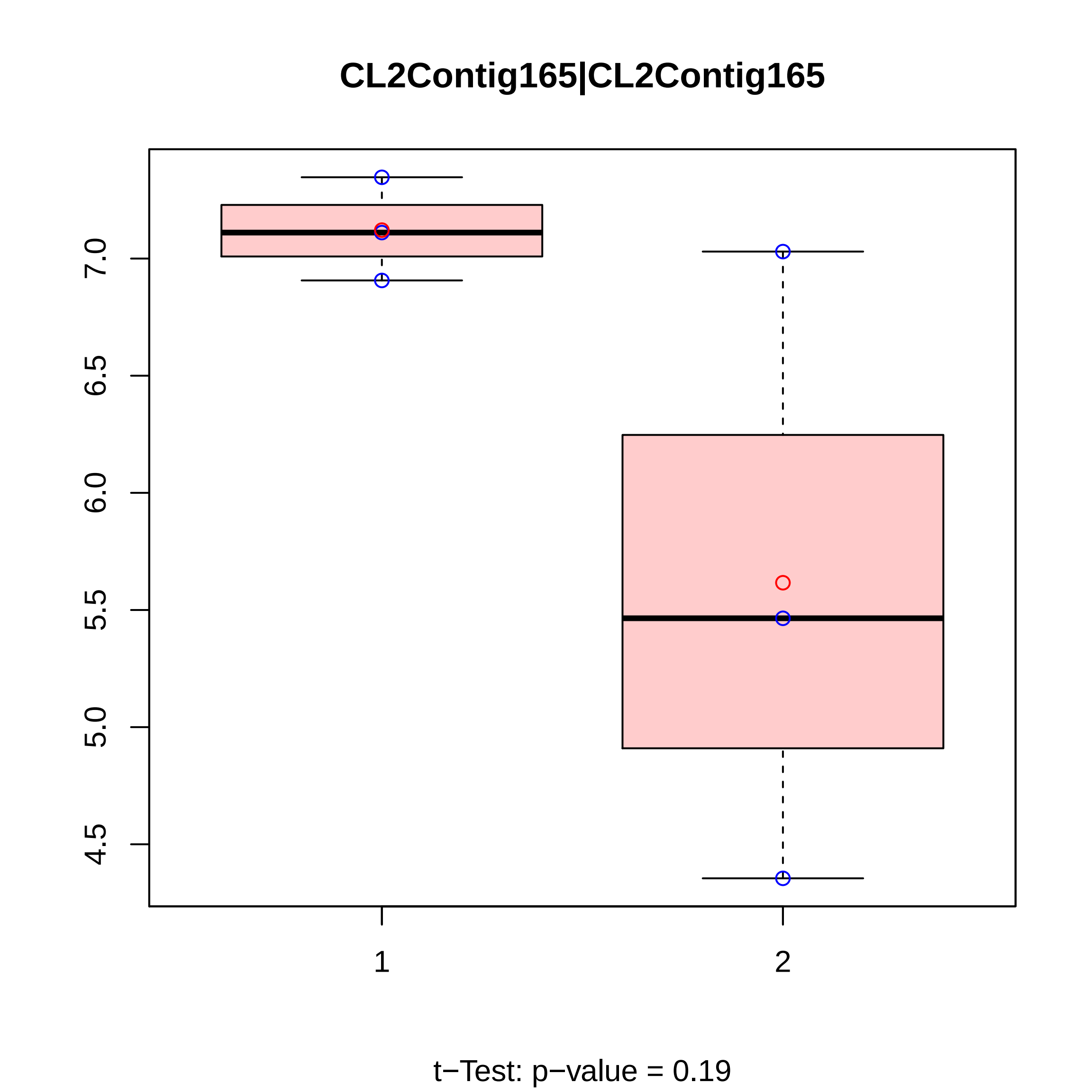

CL2Contig165|CL2Contig165
7.0
6.5
6.0
5.5
5.0
4.5
1
2
t−Test: p−value = 0.19

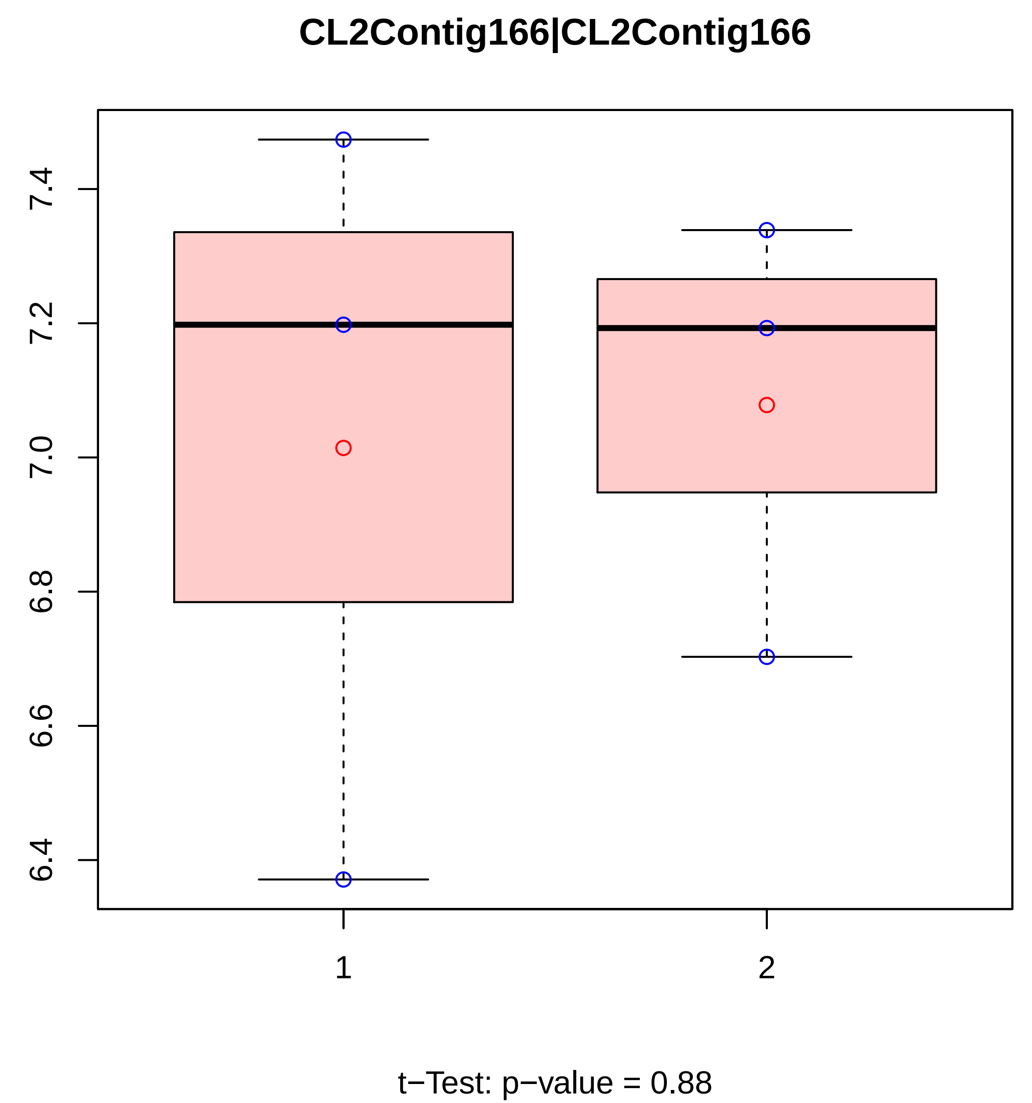
CL2Contig166|CL2Contig166
7.4
7.2
7.0
6.8
6.6
6.4
1
2
t−Test: p−value = 0.88

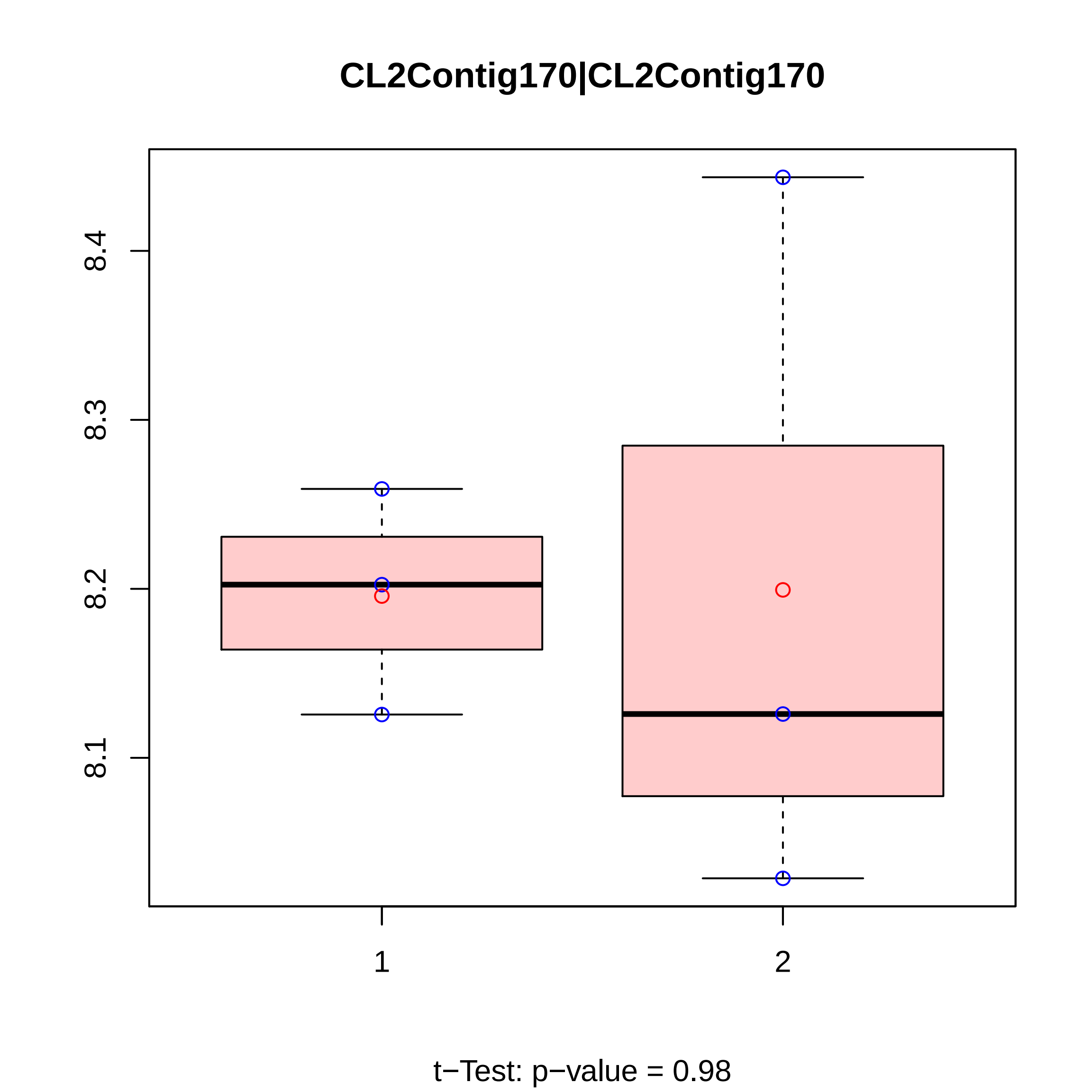

CL2Contig170|CL2Contig170
8.1
8.2
8.3
8.4
1
2
t−Test: p−value = 0.98

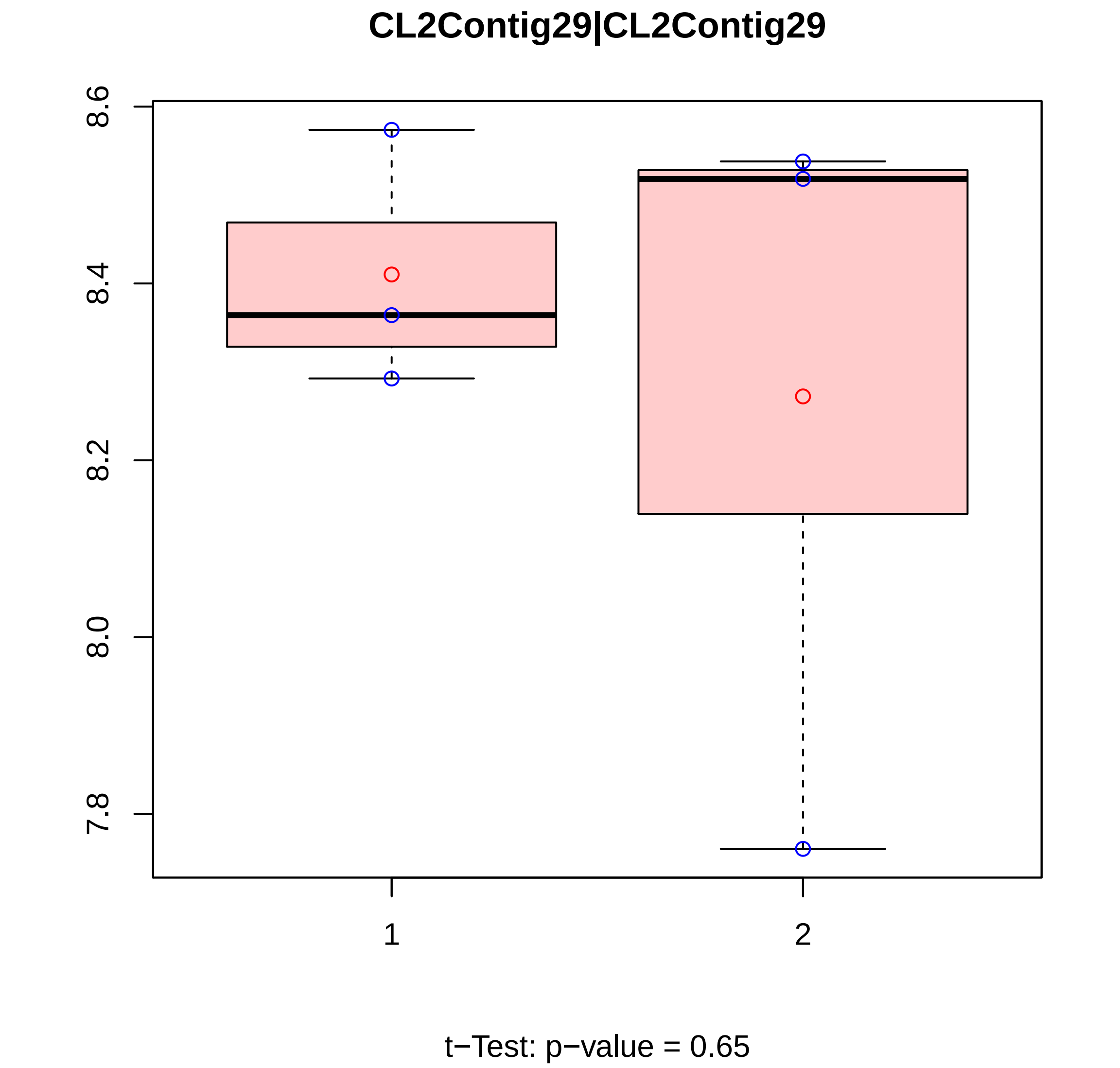
CL2Contig29|CL2Contig29
8.6
8.4
8.2
8.0
7.8
1
2
t−Test: p−value = 0.65

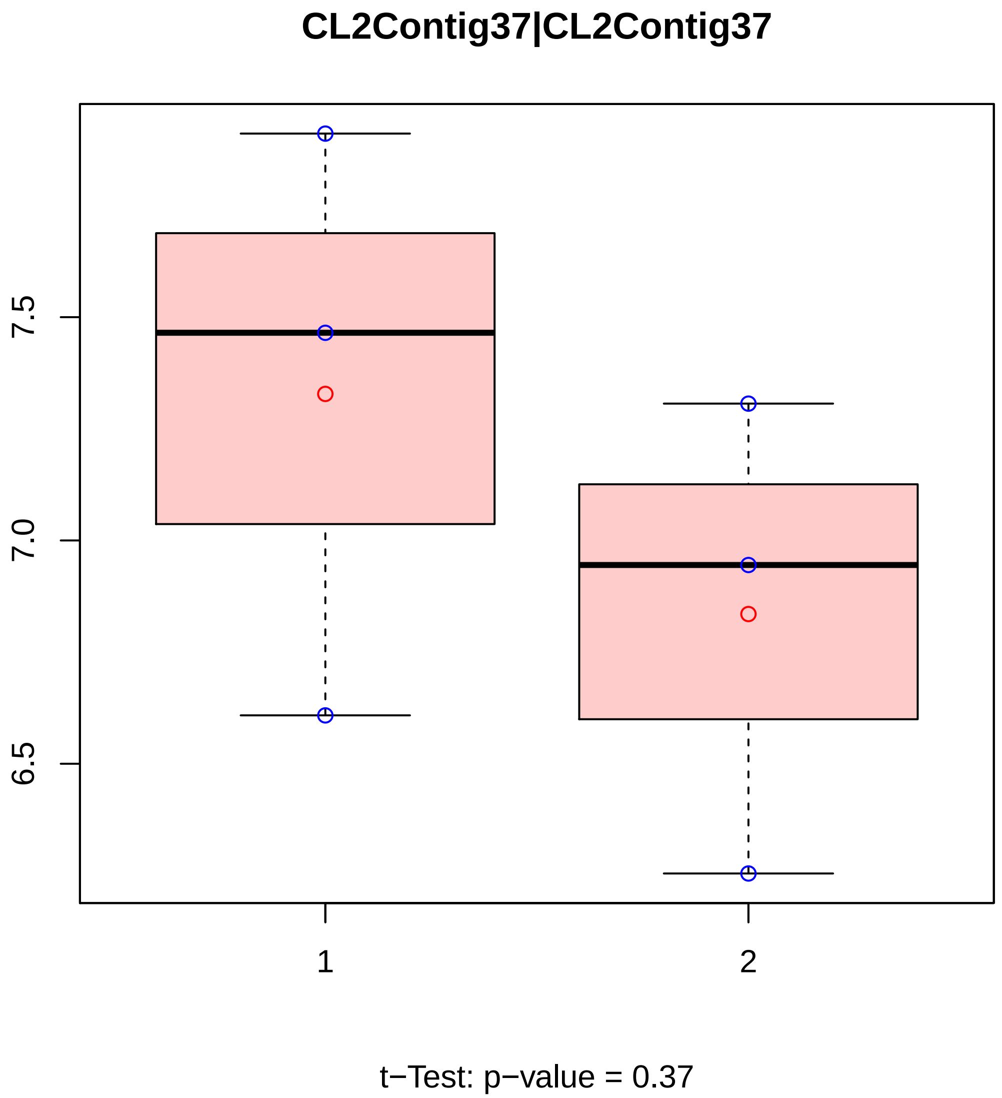
CL2Contig37|CL2Contig37
7.5
7.0
6.5
1
2
t−Test: p−value = 0.37

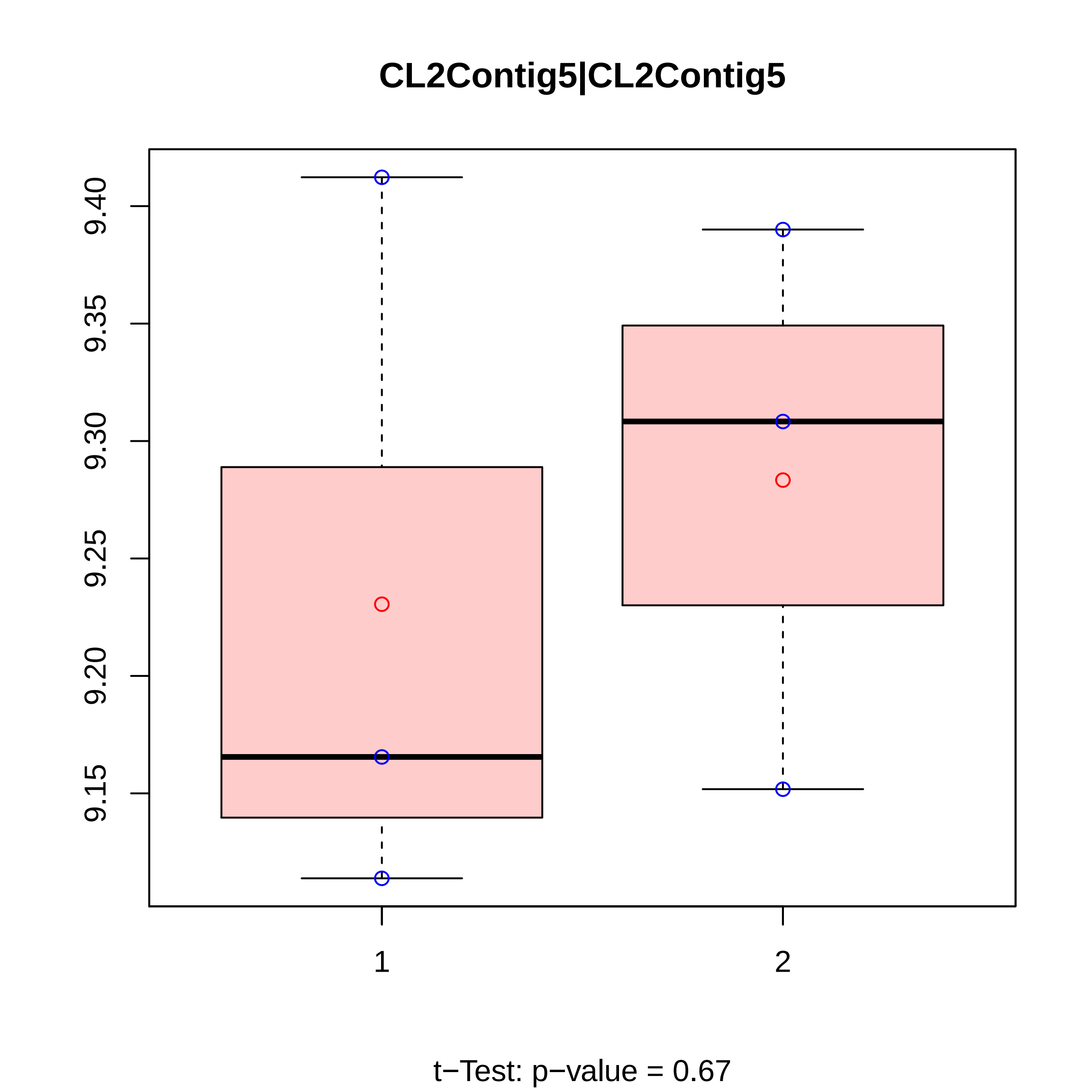
CL2Contig5|CL2Contig5
9.40
9.35
9.30
9.25
9.20
9.15
1
2
t−Test: p−value = 0.67

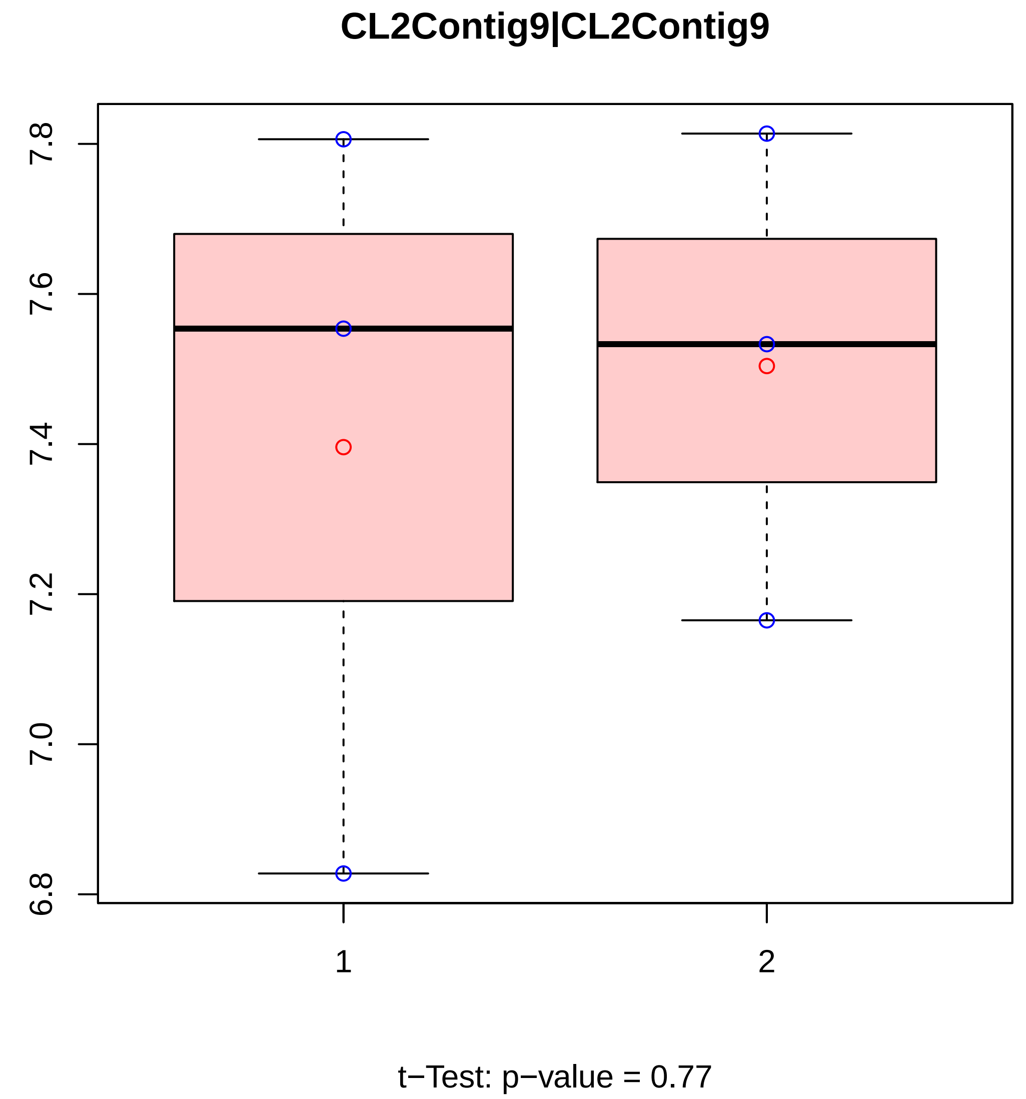
CL2Contig9|CL2Contig9
7.8
7.6
7.4
7.2
7.0
6.8
1
2
t−Test: p−value = 0.77

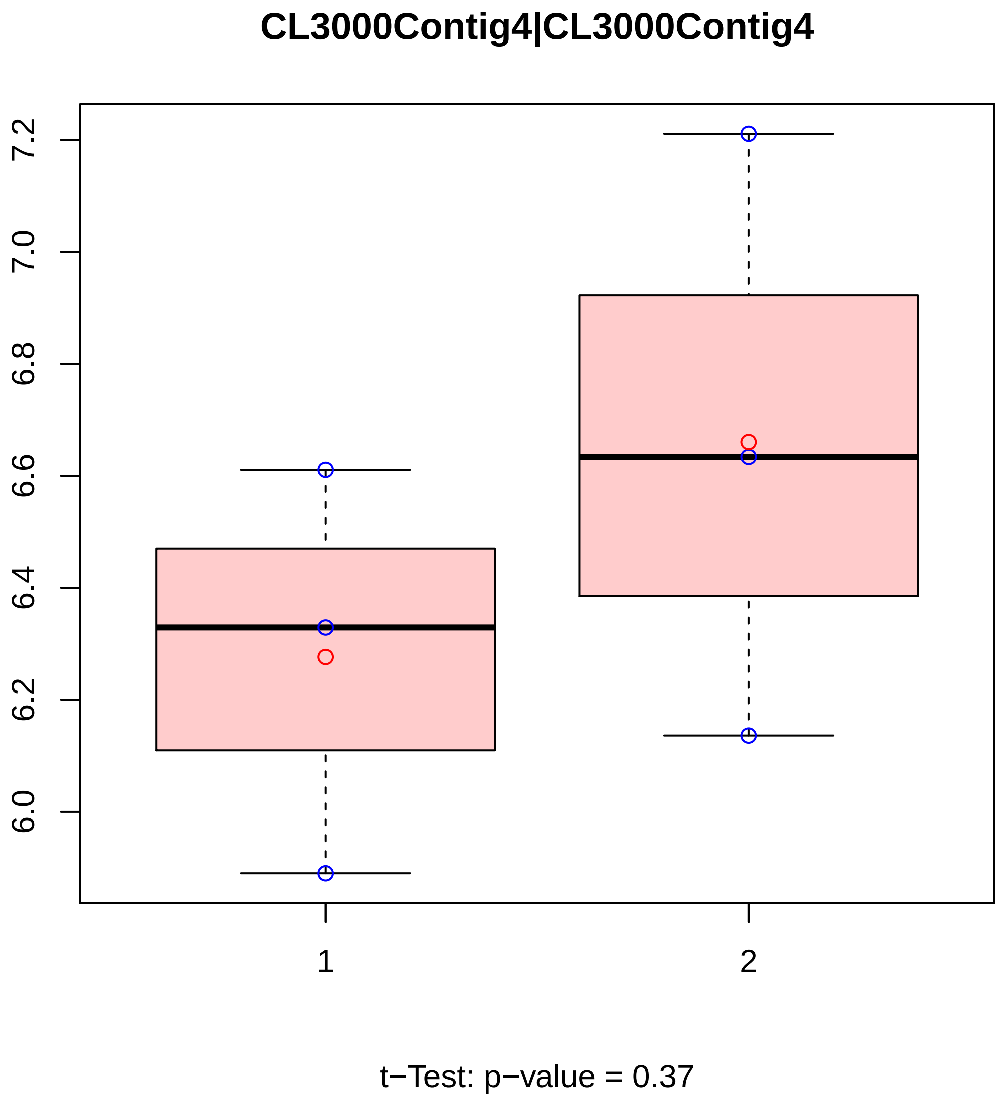
CL3000Contig4|CL3000Contig4
7.2
7.0
6.8
6.6
6.4
6.2
6.0
1
2
t−Test: p−value = 0.37

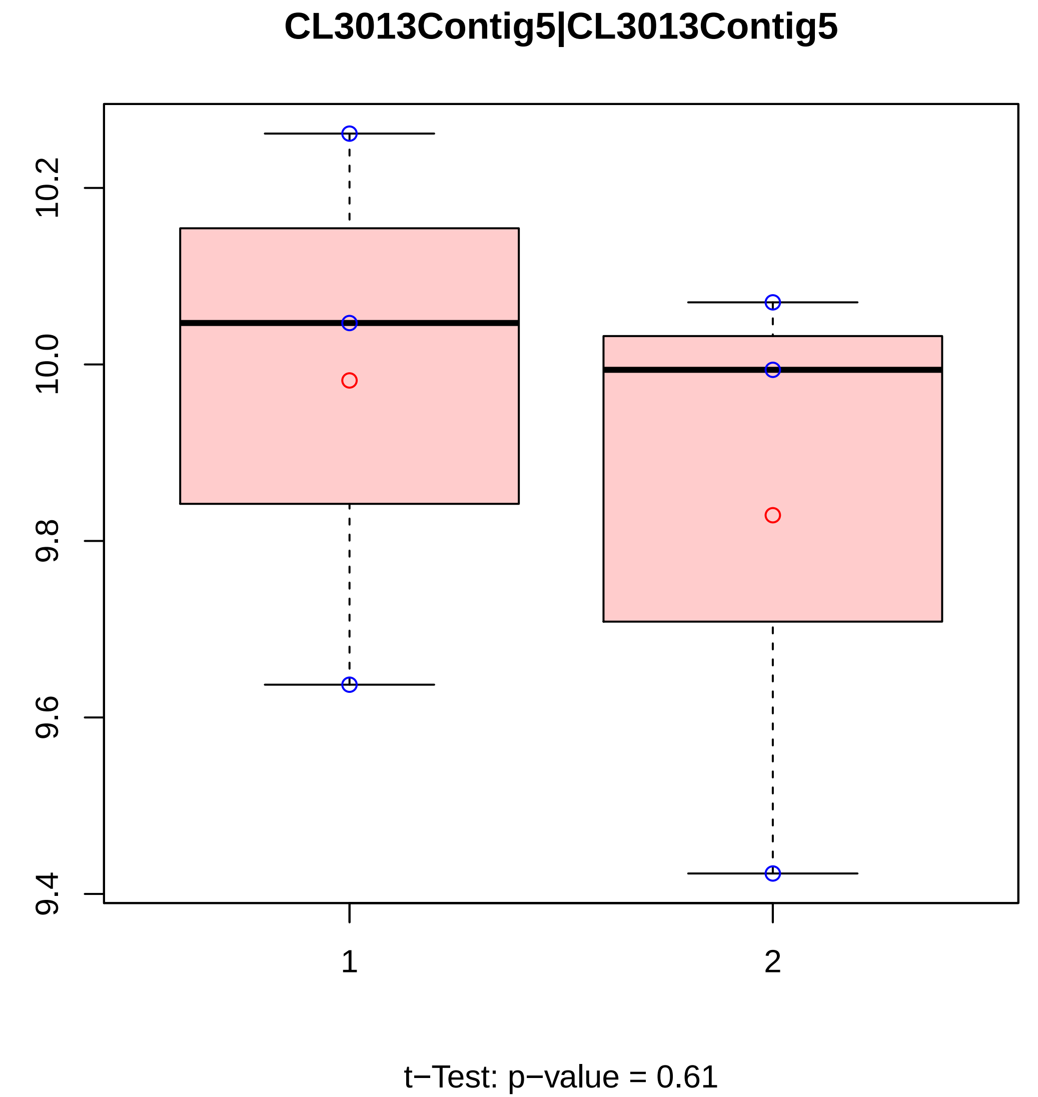
CL3013Contig5|CL3013Contig5
10.2
10.0
9.8
9.6
9.4
1
2
t−Test: p−value = 0.61

# CL3017Contig4|CL3017Contig4

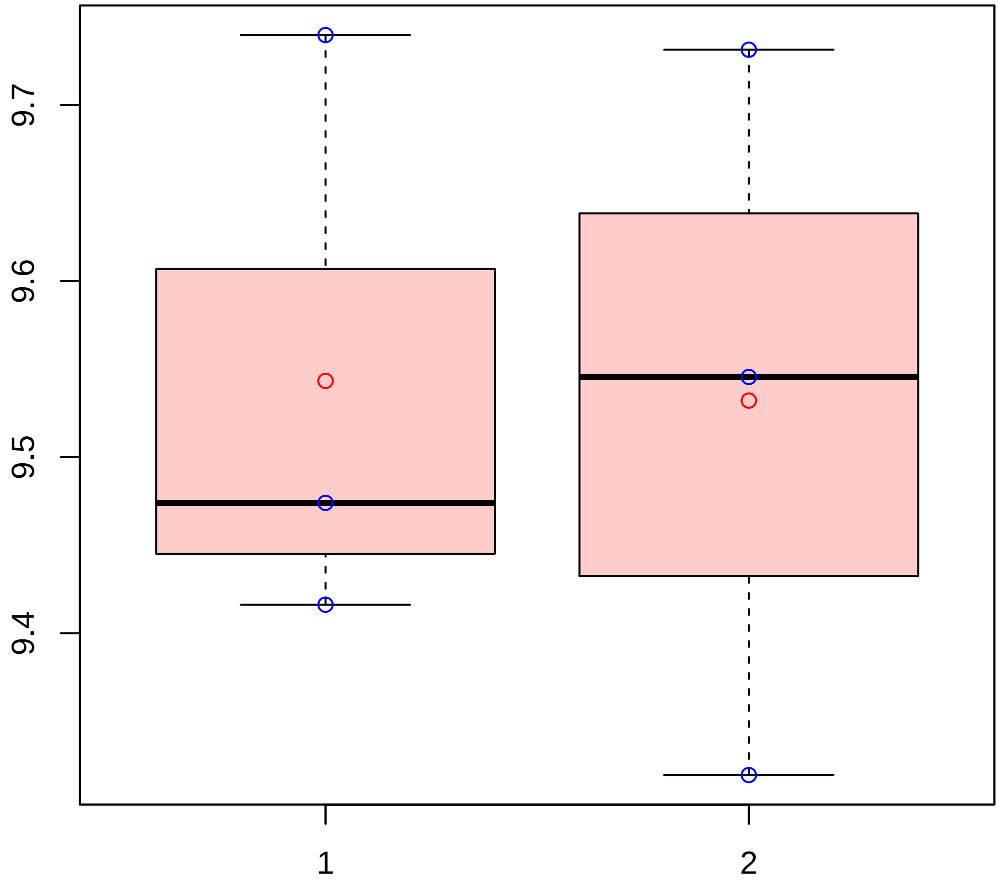


t−Test: p−value = 0.95

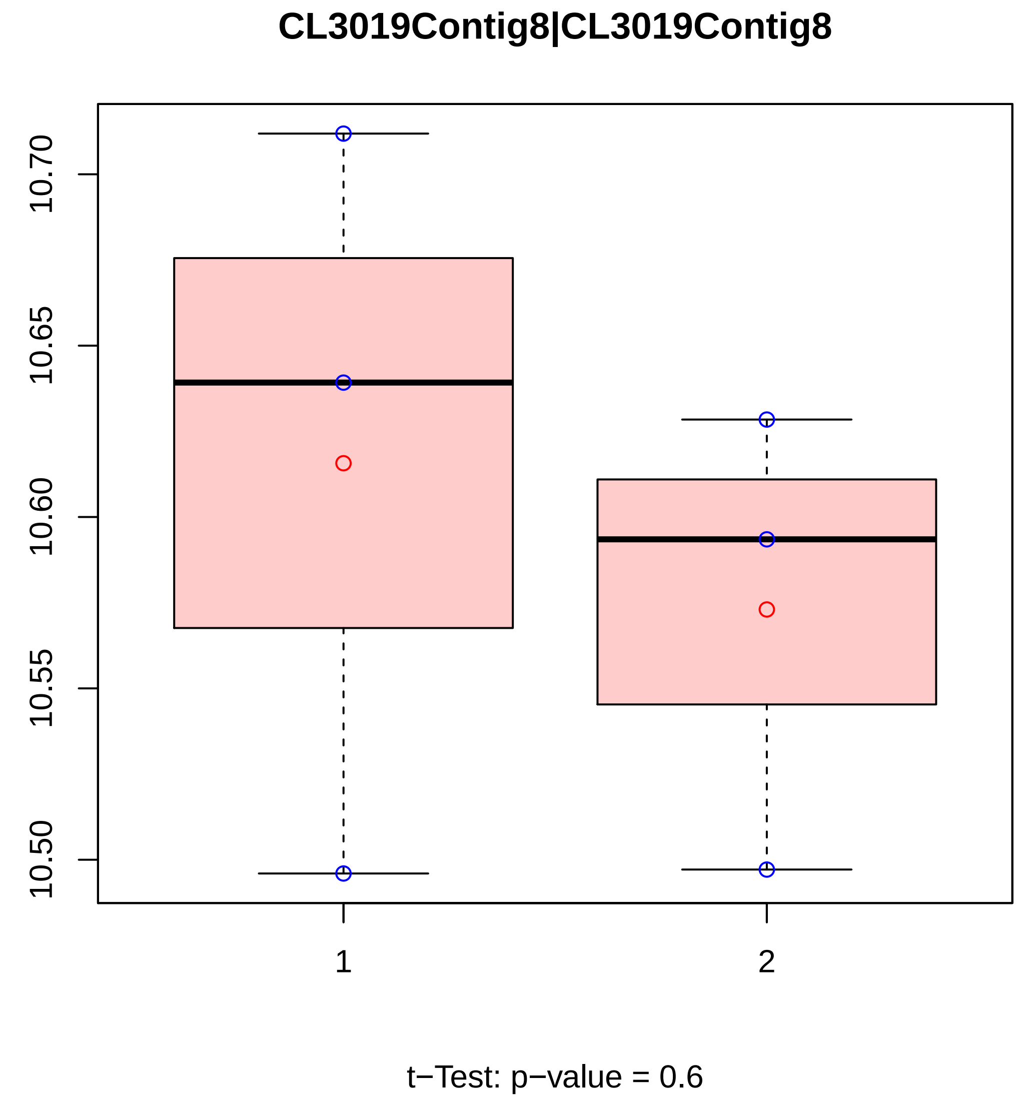

CL3019Contig8|CL3019Contig8
10.70
10.65
10.60
10.55
10.50
1
2
t−Test: p−value = 0.6

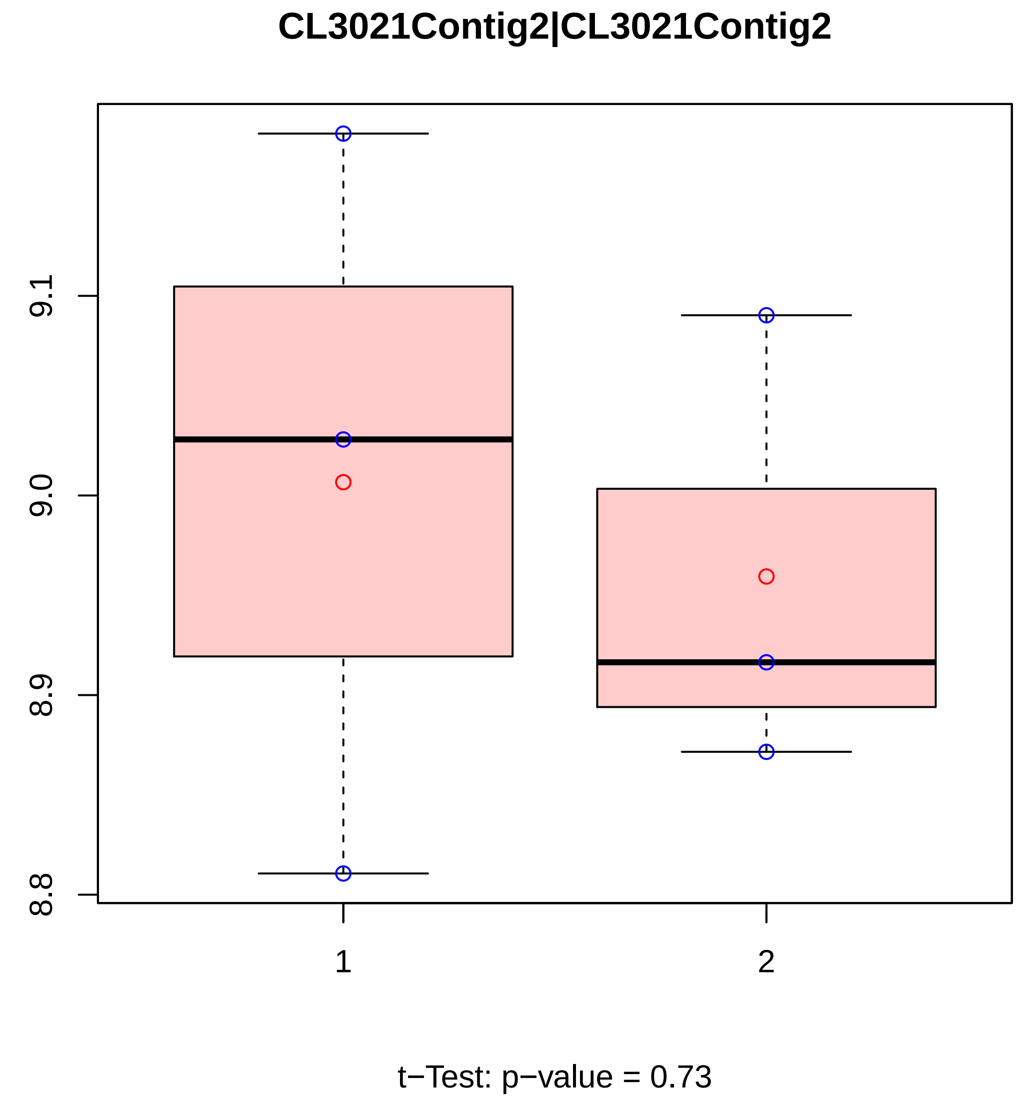

CL3021Contig2|CL3021Contig2
9.1
9.0
8.9
8.8
1
2
t−Test: p−value = 0.73

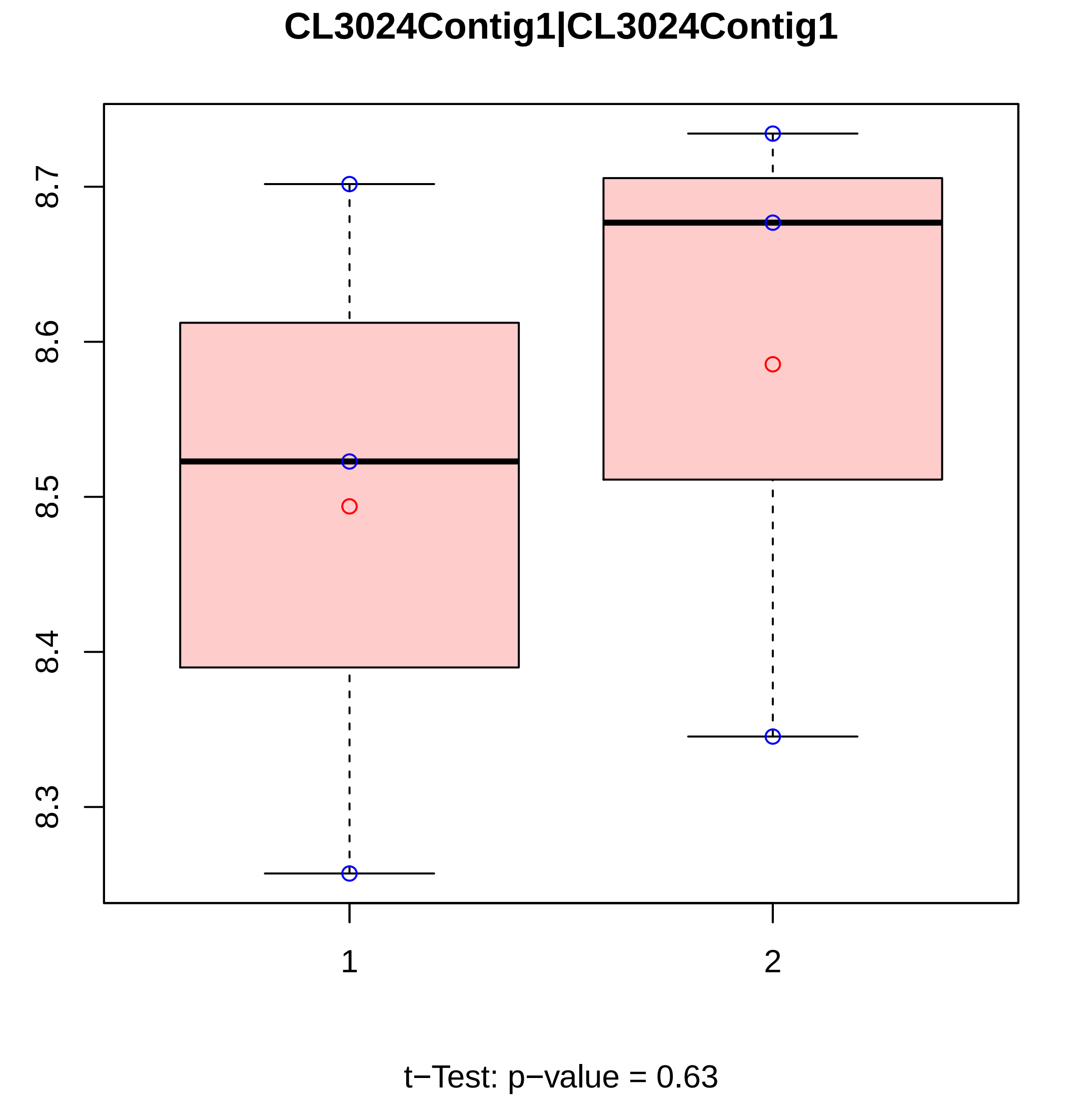
CL3024Contig1|CL3024Contig1
8.3
8.4
8.5
8.6
8.7
1
2
t−Test: p−value = 0.63

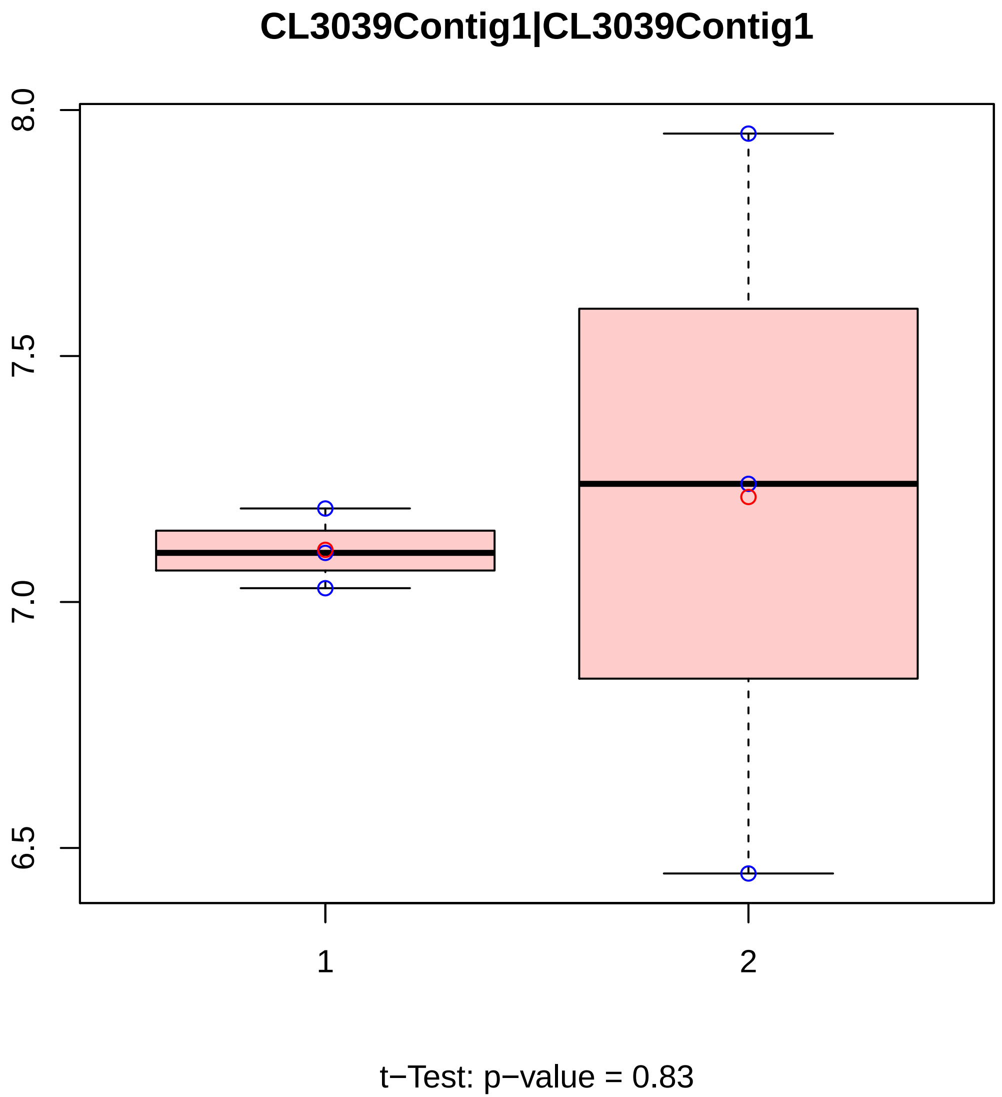
CL3039Contig1|CL3039Contig1
8.0
7.5
7.0
6.5
1
2
t−Test: p−value = 0.83

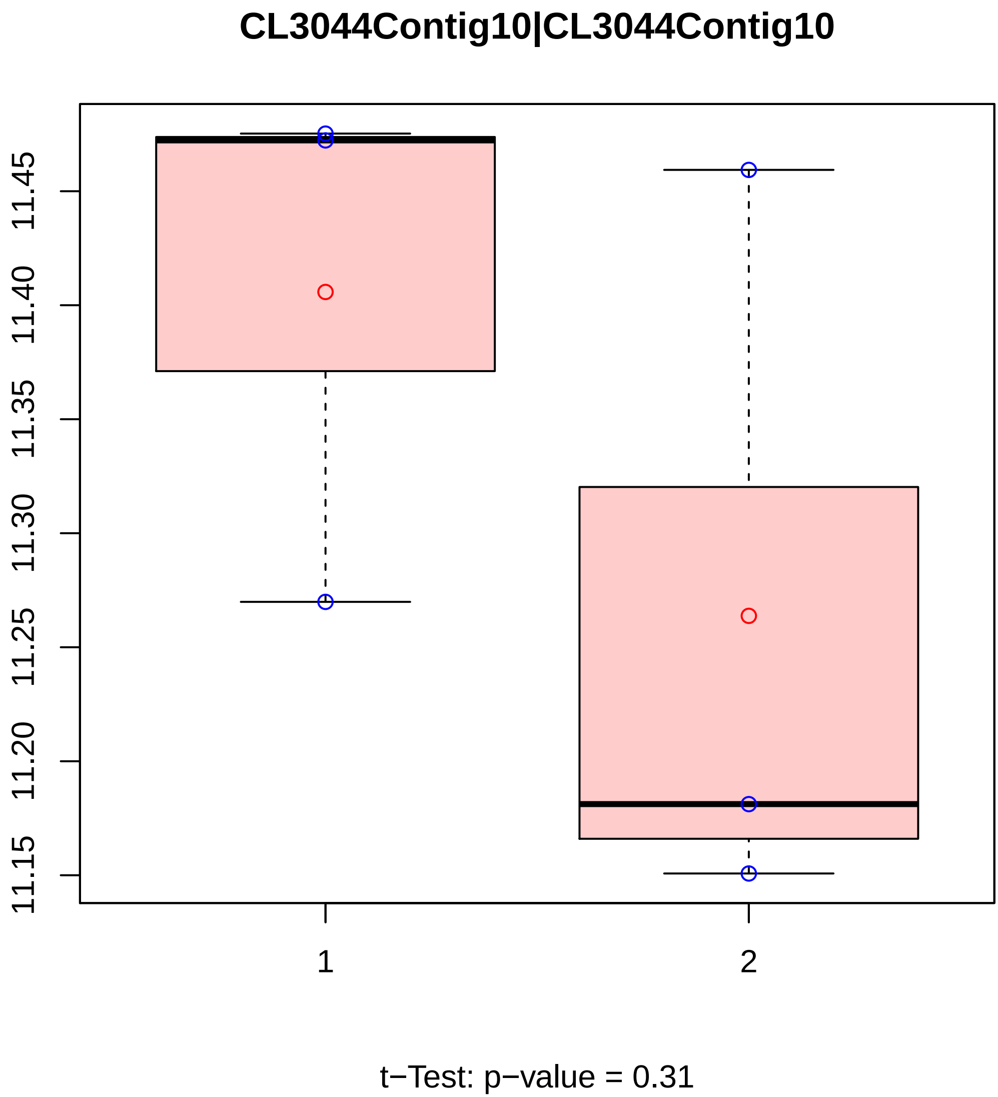

CL3044Contig10|CL3044Contig10
11.15
11.20
11.25
11.30
11.35
11.40
11.45
1
2
t−Test: p−value = 0.31

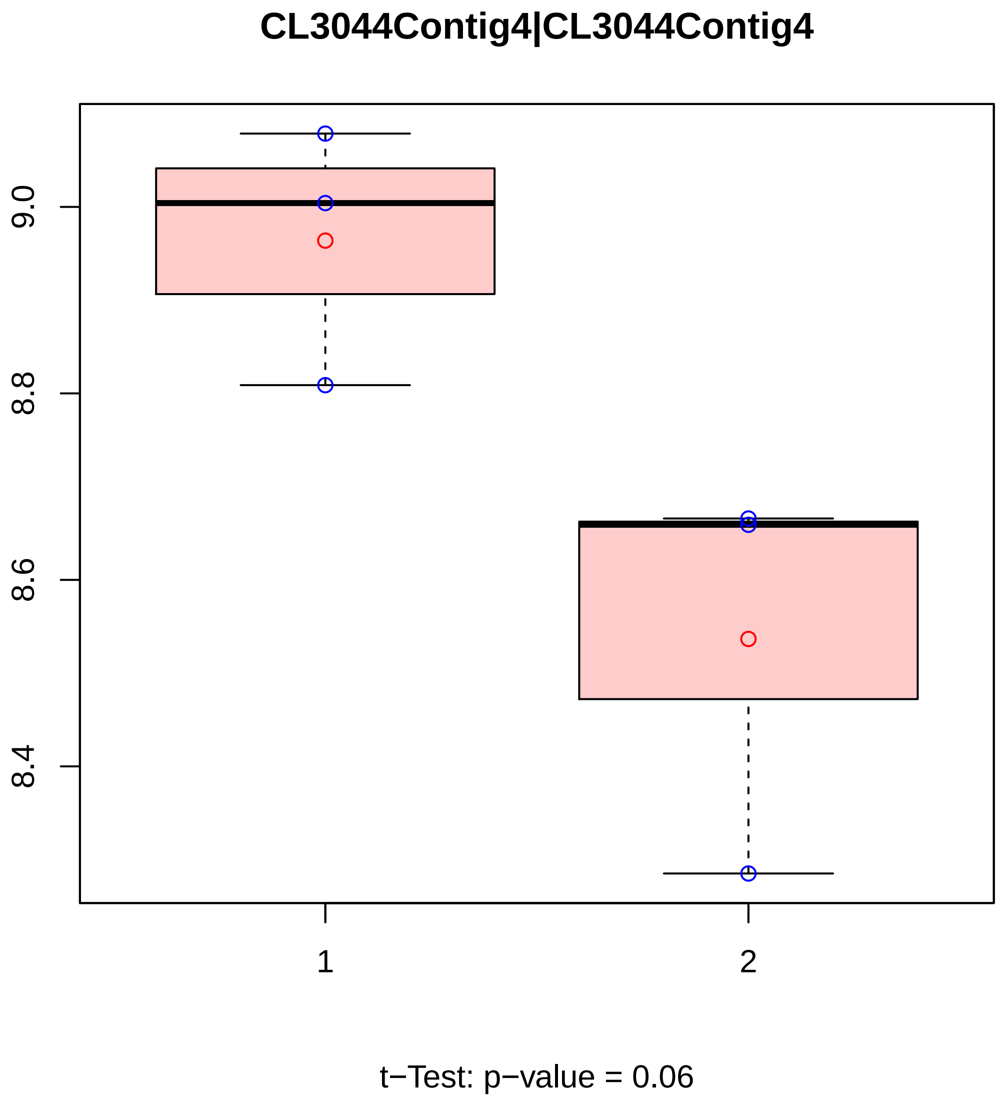

CL3044Contig4|CL3044Contig4
9.0
8.8
8.6
8.4
1
2
t−Test: p−value = 0.06

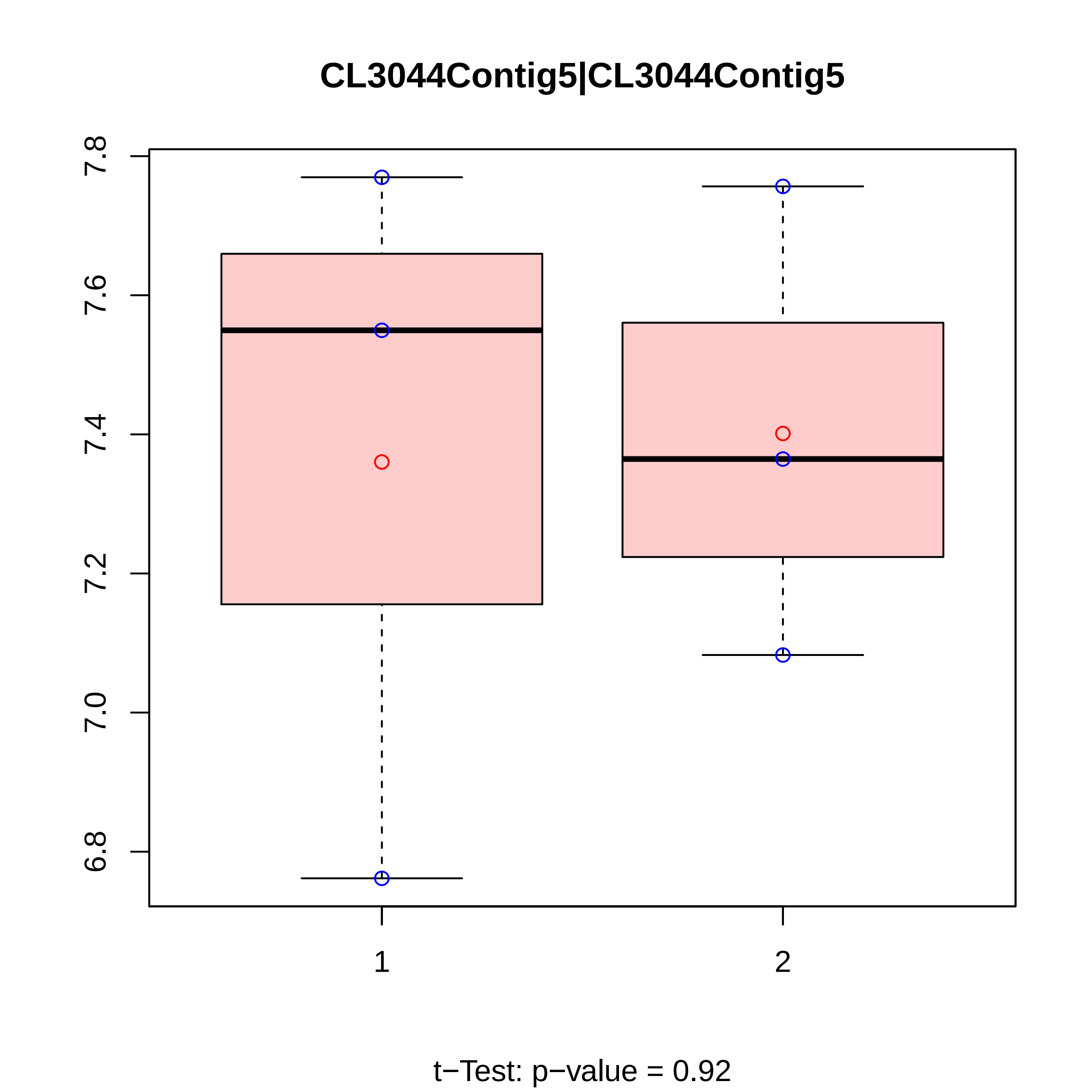
CL3044Contig5|CL3044Contig5
7.8
7.6
7.4
7.2
7.0
6.8
1
2
t−Test: p−value = 0.92

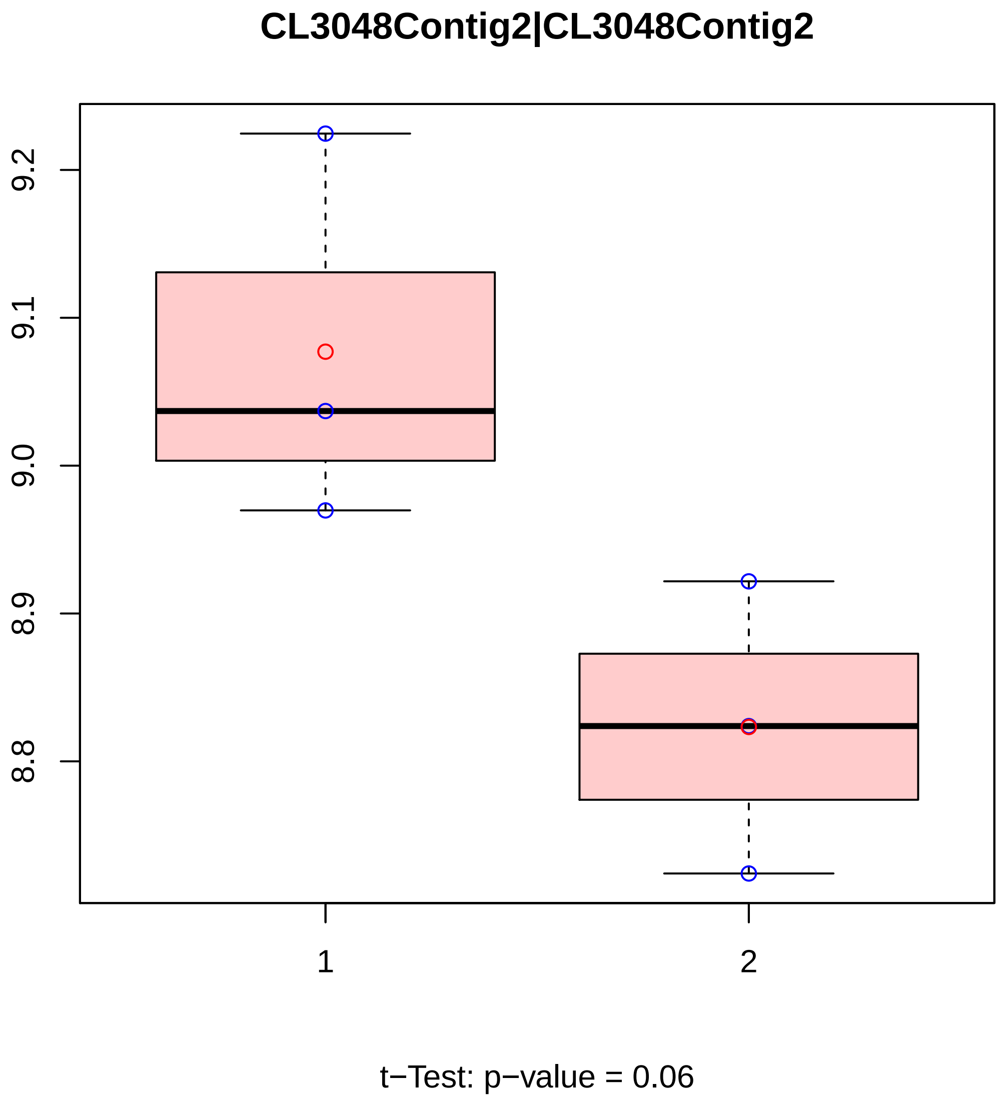
CL3048Contig2|CL3048Contig2
9.2
9.1
9.0
8.9
8.8
1
2
t−Test: p−value = 0.06

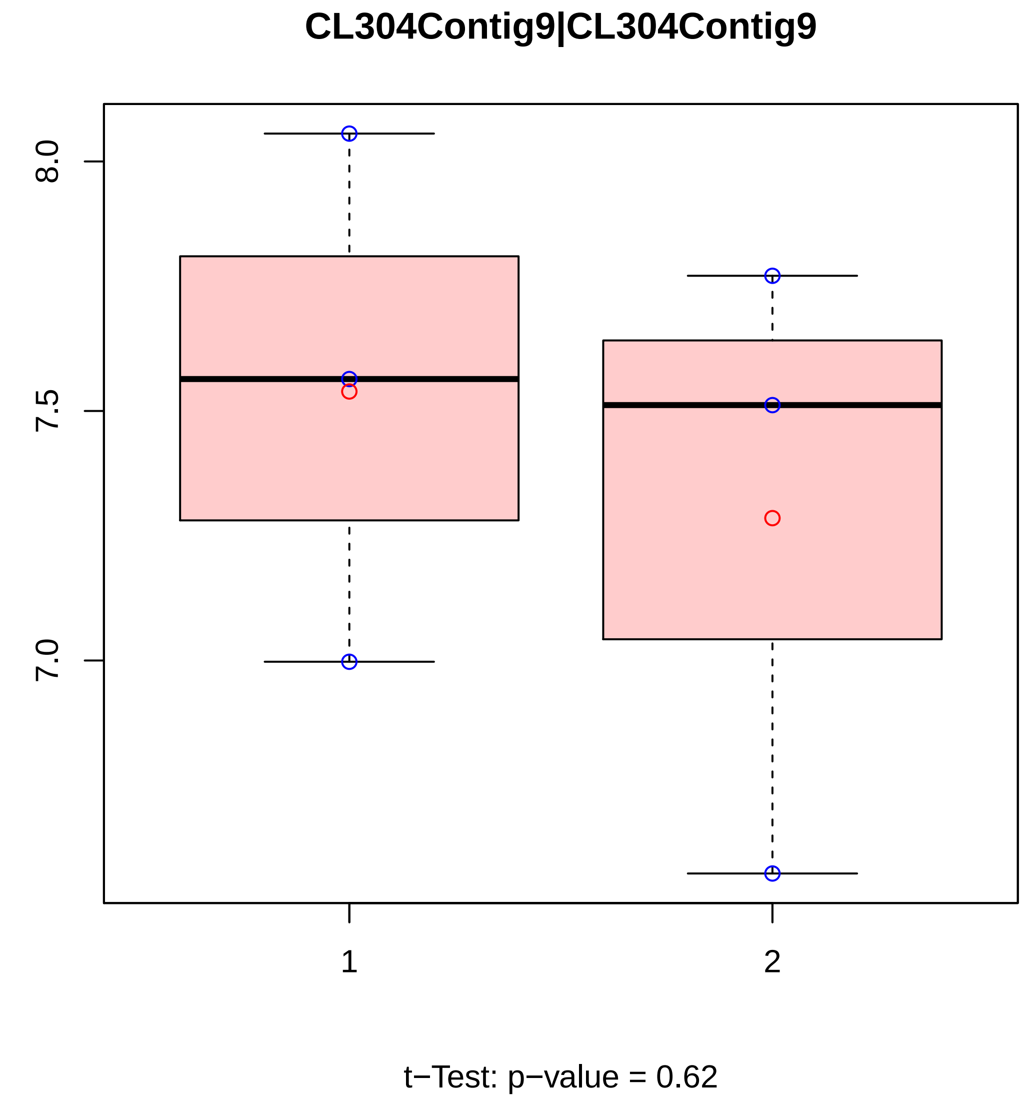
CL304Contig9|CL304Contig9
8.0
7.5
7.0
1
2
t−Test: p−value = 0.62

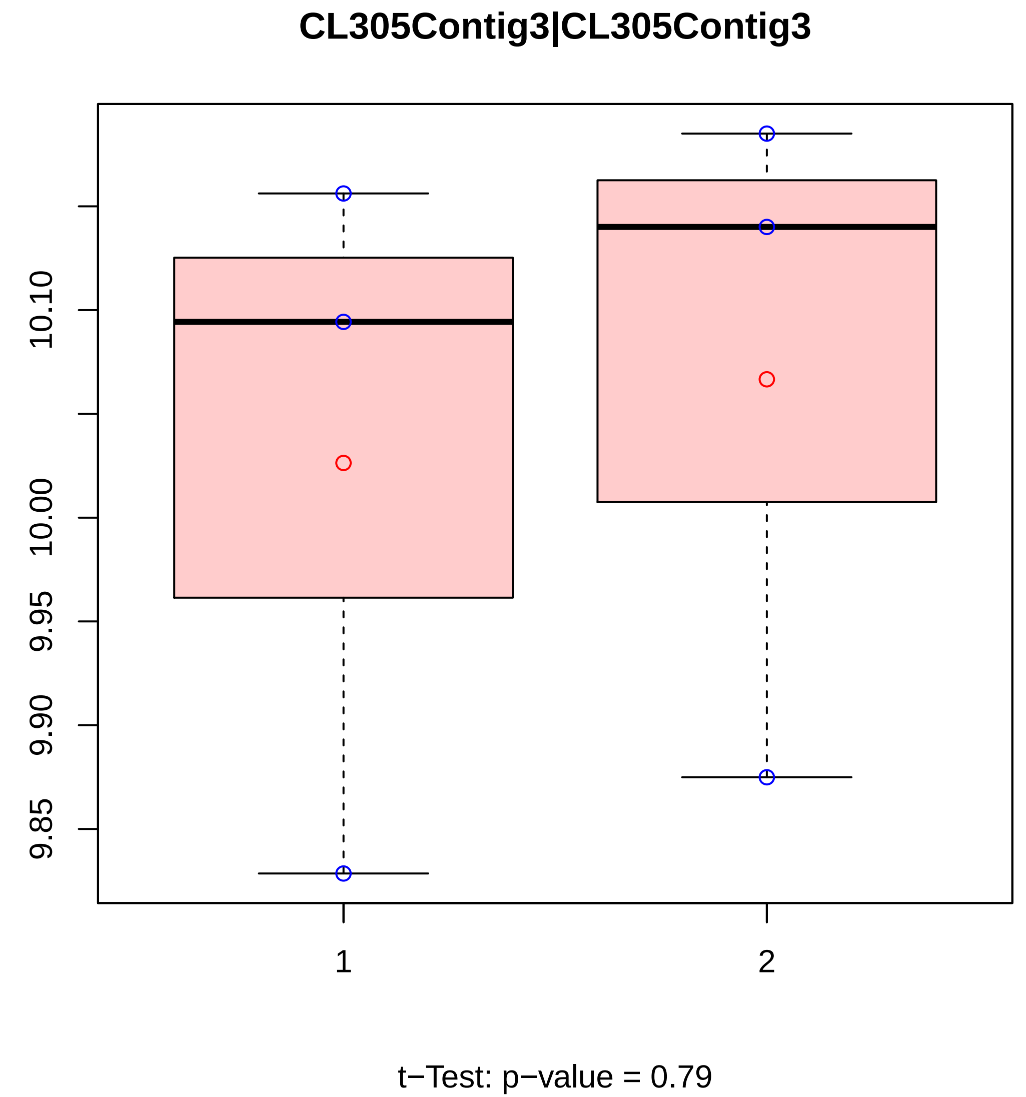

CL305Contig3|CL305Contig3
9.85
9.90
9.95
10.00
10.10
1
2
t−Test: p−value = 0.79

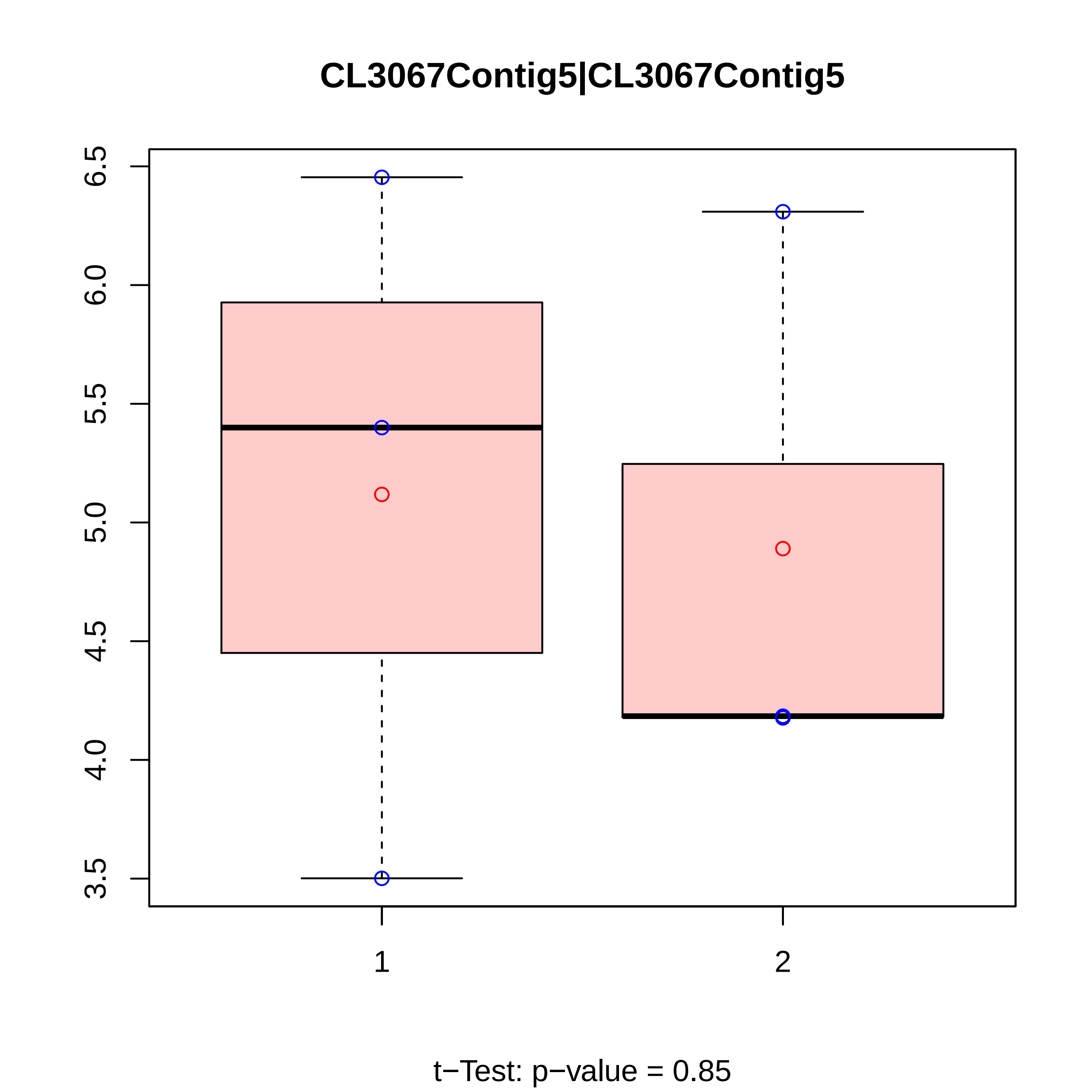

CL3067Contig5|CL3067Contig5
6.5
6.0
5.5
5.0
4.5
4.0
3.5
1
2
t−Test: p−value = 0.85

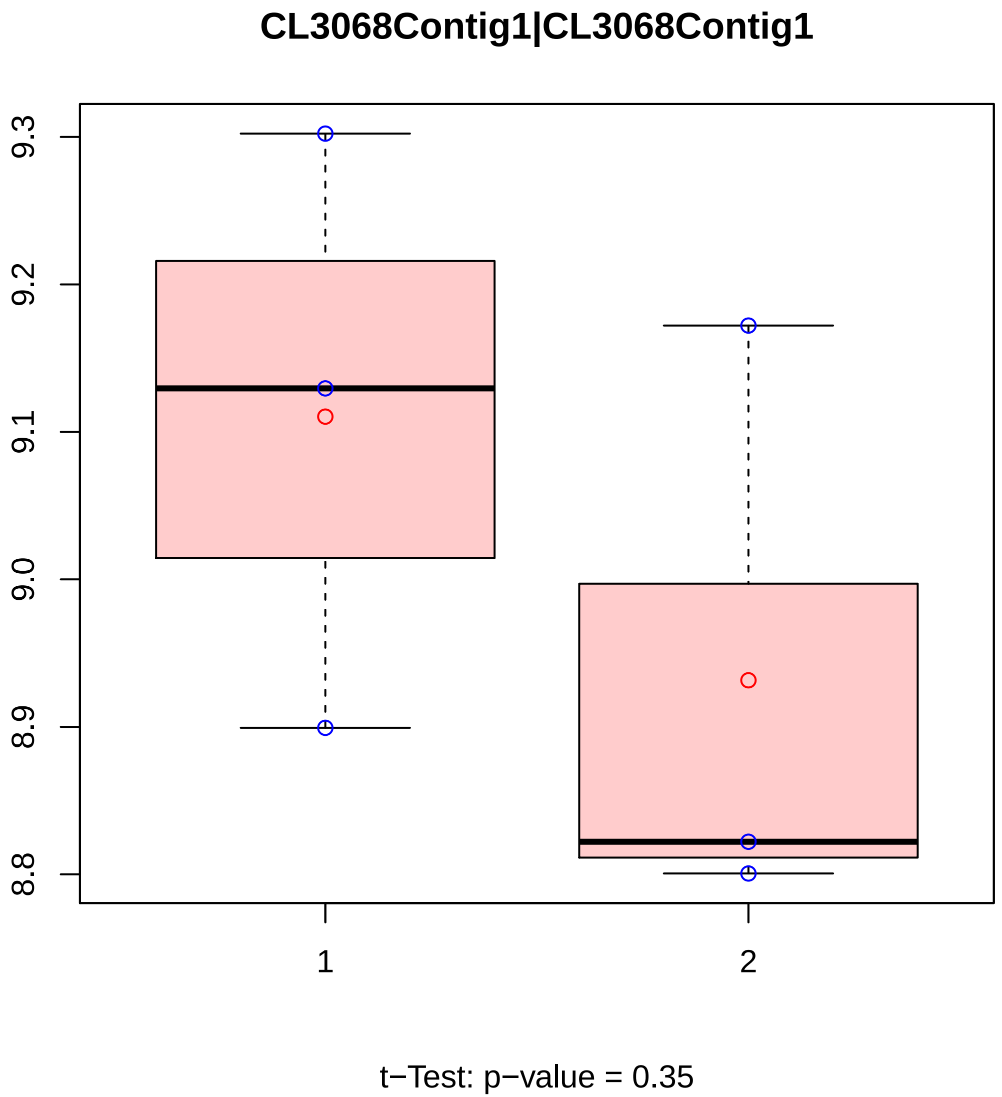
CL3068Contig1|CL3068Contig1
9.3
9.2
9.1
9.0
8.9
8.8
1
2
t−Test: p−value = 0.35

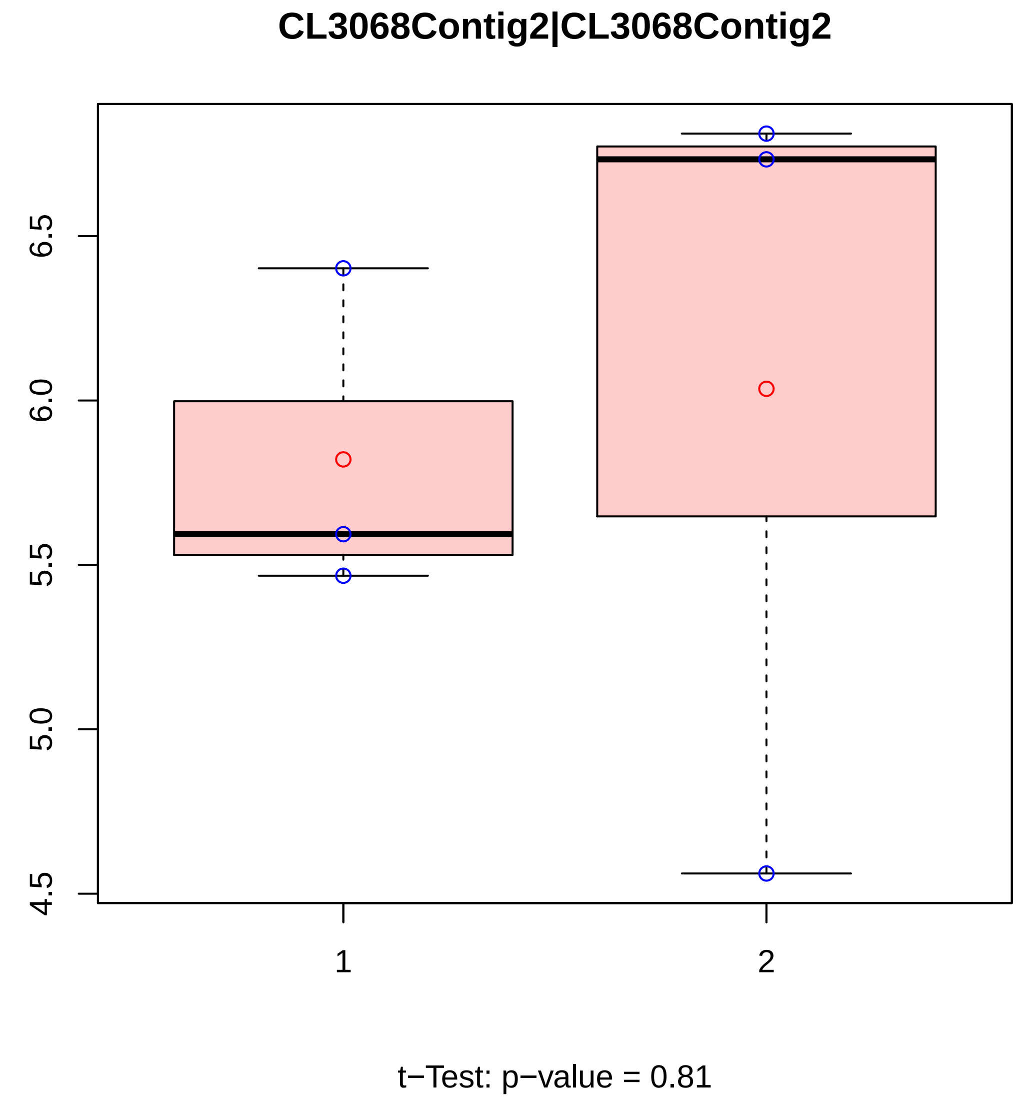

CL3068Contig2|CL3068Contig2
4.5
5.0
5.5
6.0
6.5
1
2
t−Test: p−value = 0.81

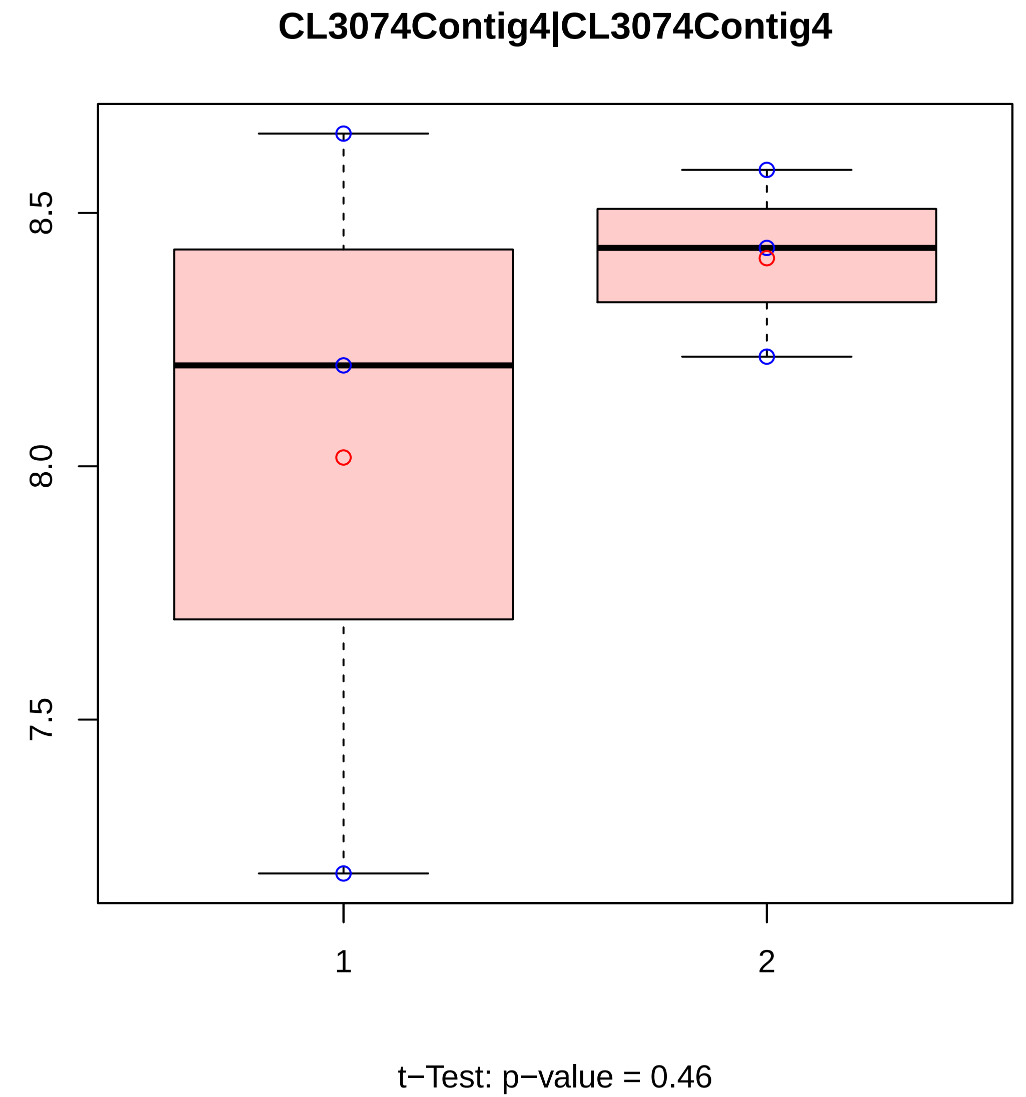

CL3074Contig4|CL3074Contig4
8.5
8.0
7.5
1
2
t−Test: p−value = 0.46

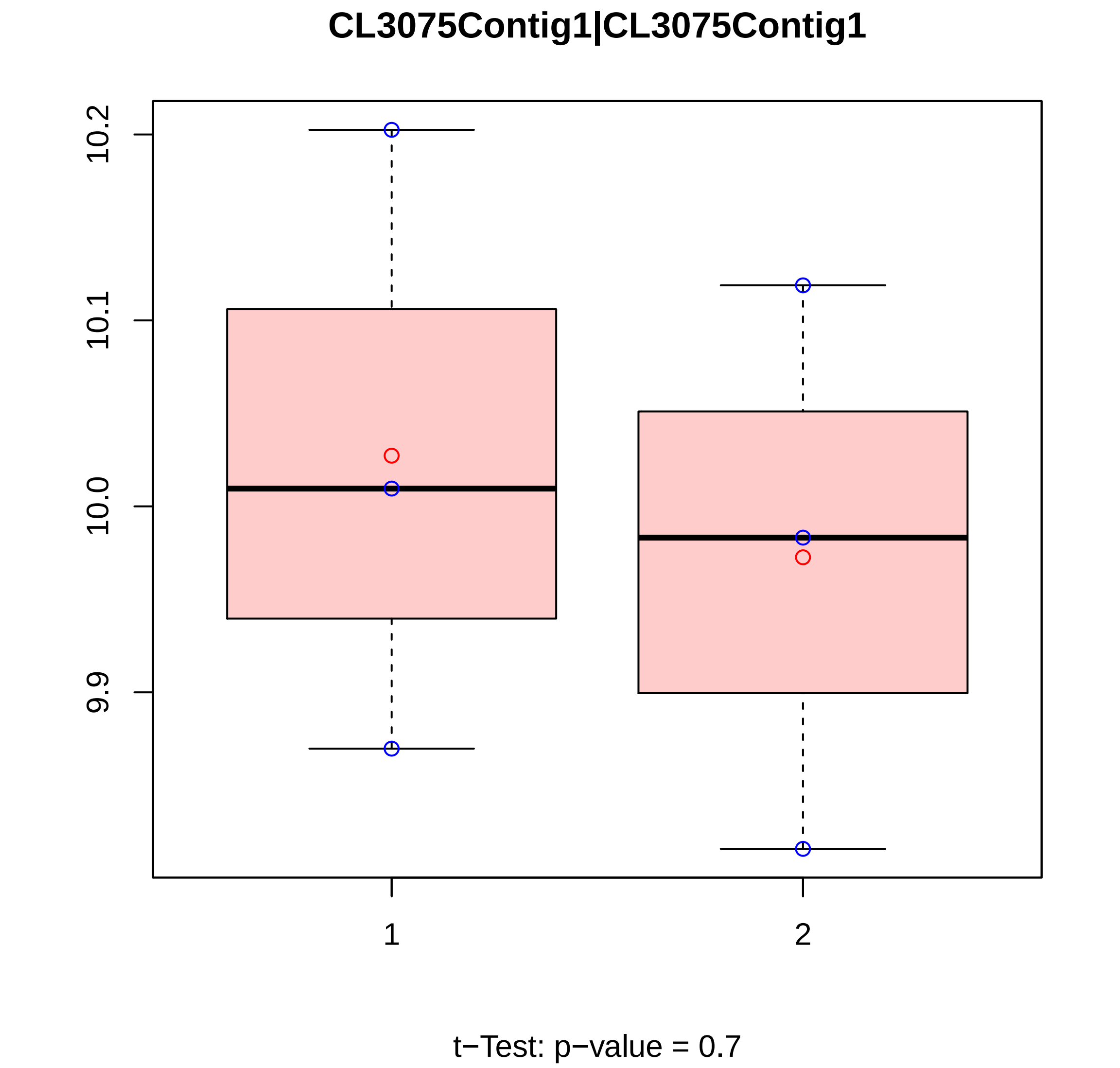
CL3075Contig1|CL3075Contig1
10.2
10.1
10.0
9.9
1
2
t−Test: p−value = 0.7

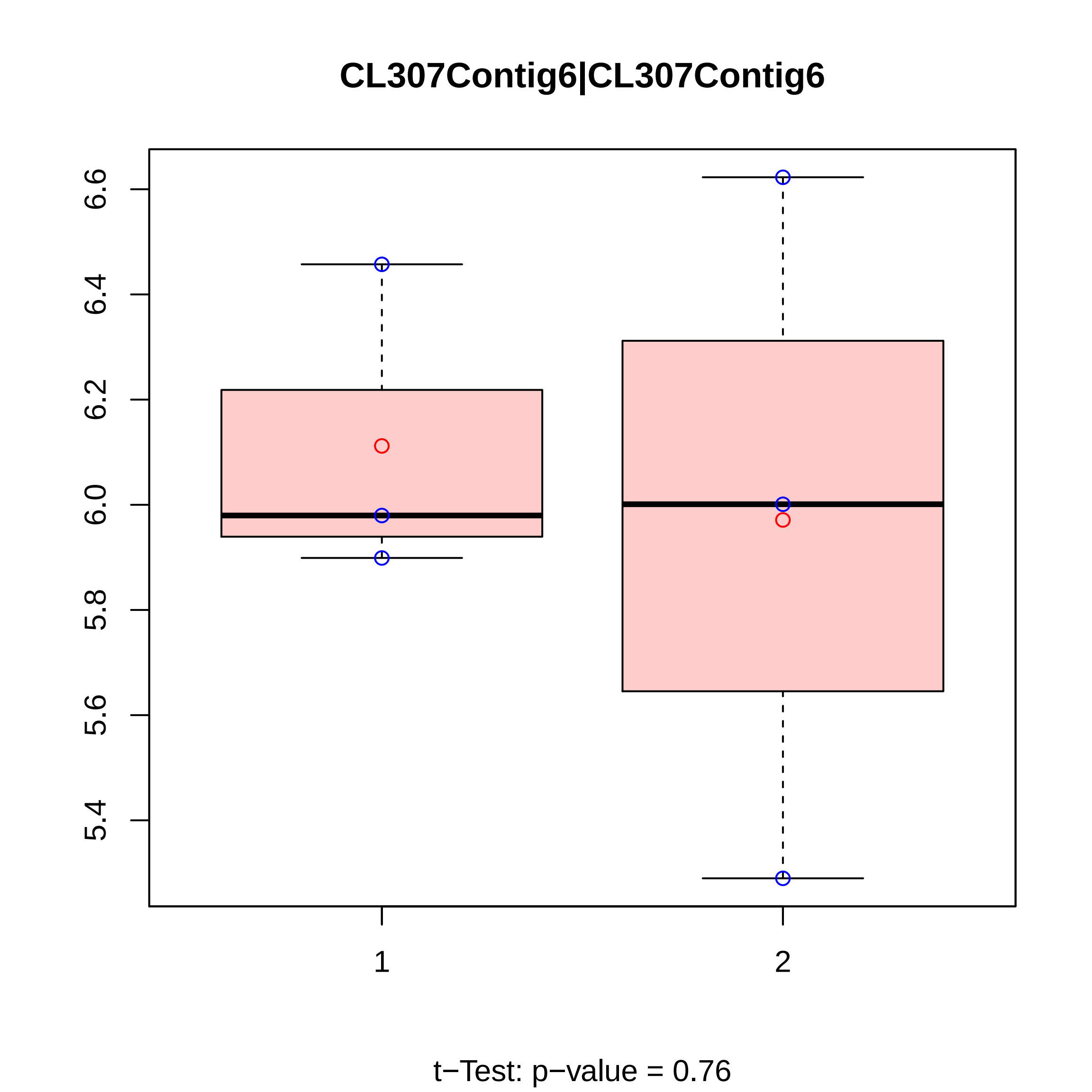

CL307Contig6|CL307Contig6
6.6
6.4
6.2
6.0
5.8
5.6
5.4
1
2
t−Test: p−value = 0.76

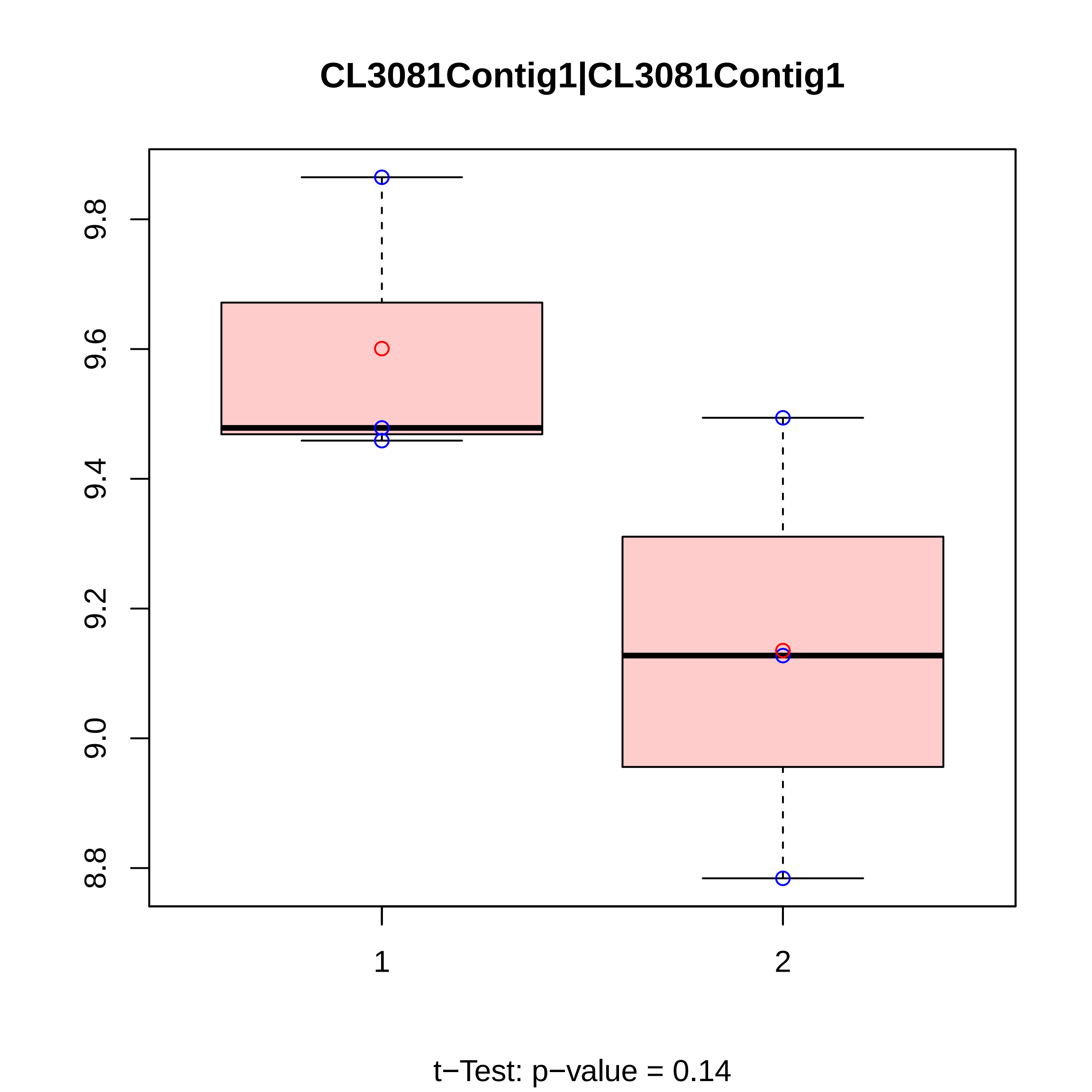

CL3081Contig1|CL3081Contig1
9.8
9.6
9.4
9.2
9.0
8.8
1
2
t−Test: p−value = 0.14

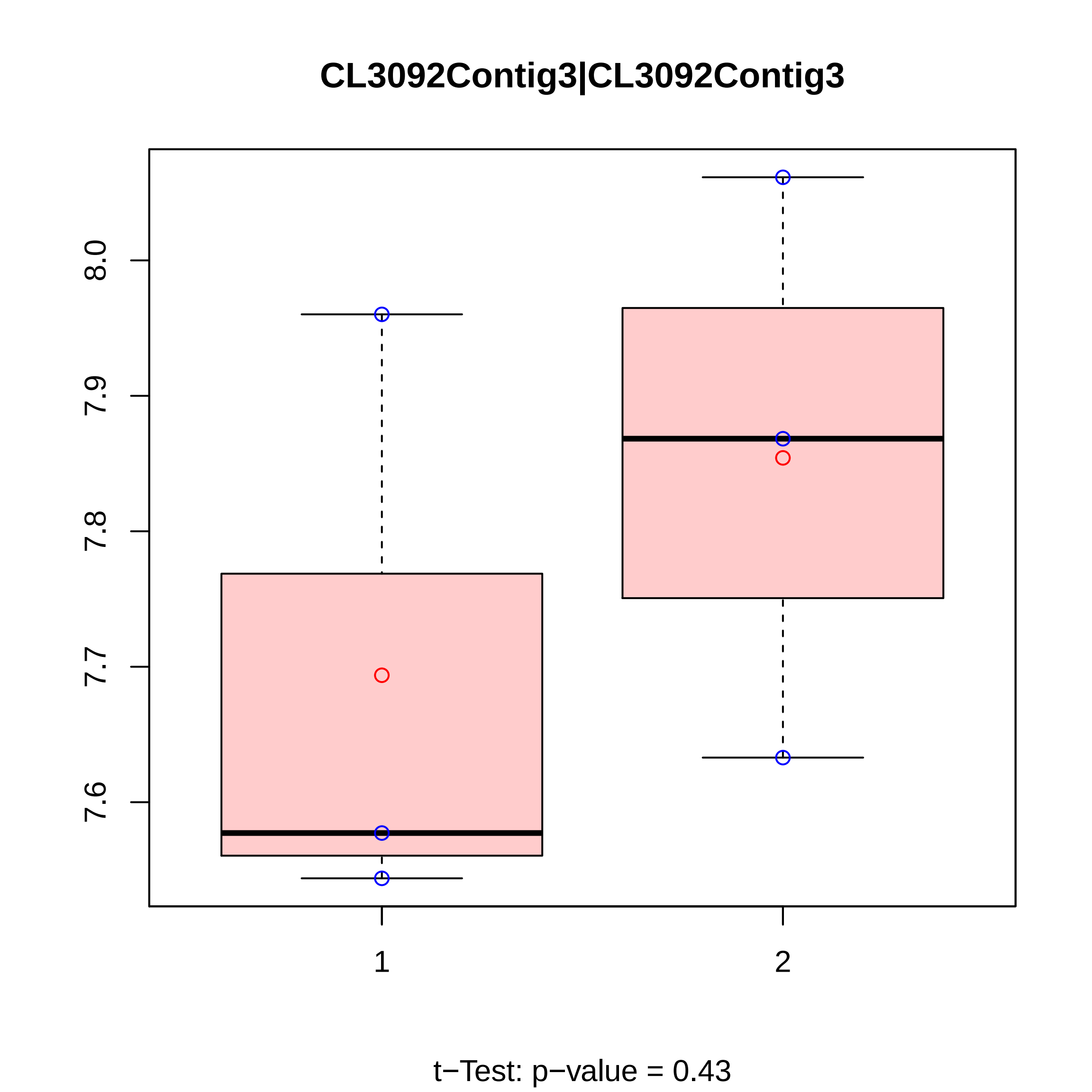
CL3092Contig3|CL3092Contig3
8.0
7.9
7.8
7.7
7.6
1
2
t−Test: p−value = 0.43

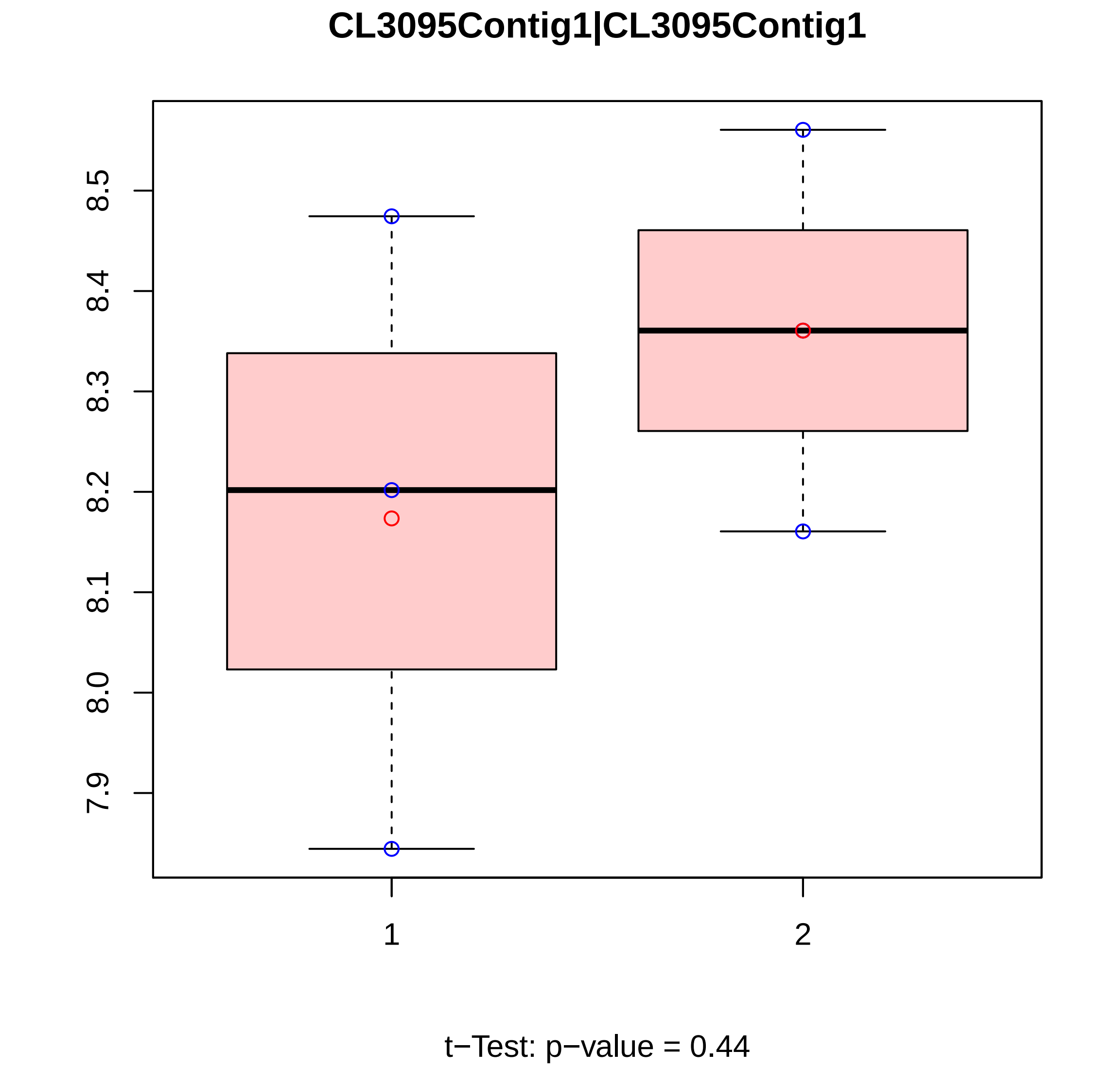

CL3095Contig1|CL3095Contig1
8.5
8.4
8.3
8.2
8.1
8.0
7.9
1
2
t−Test: p−value = 0.44

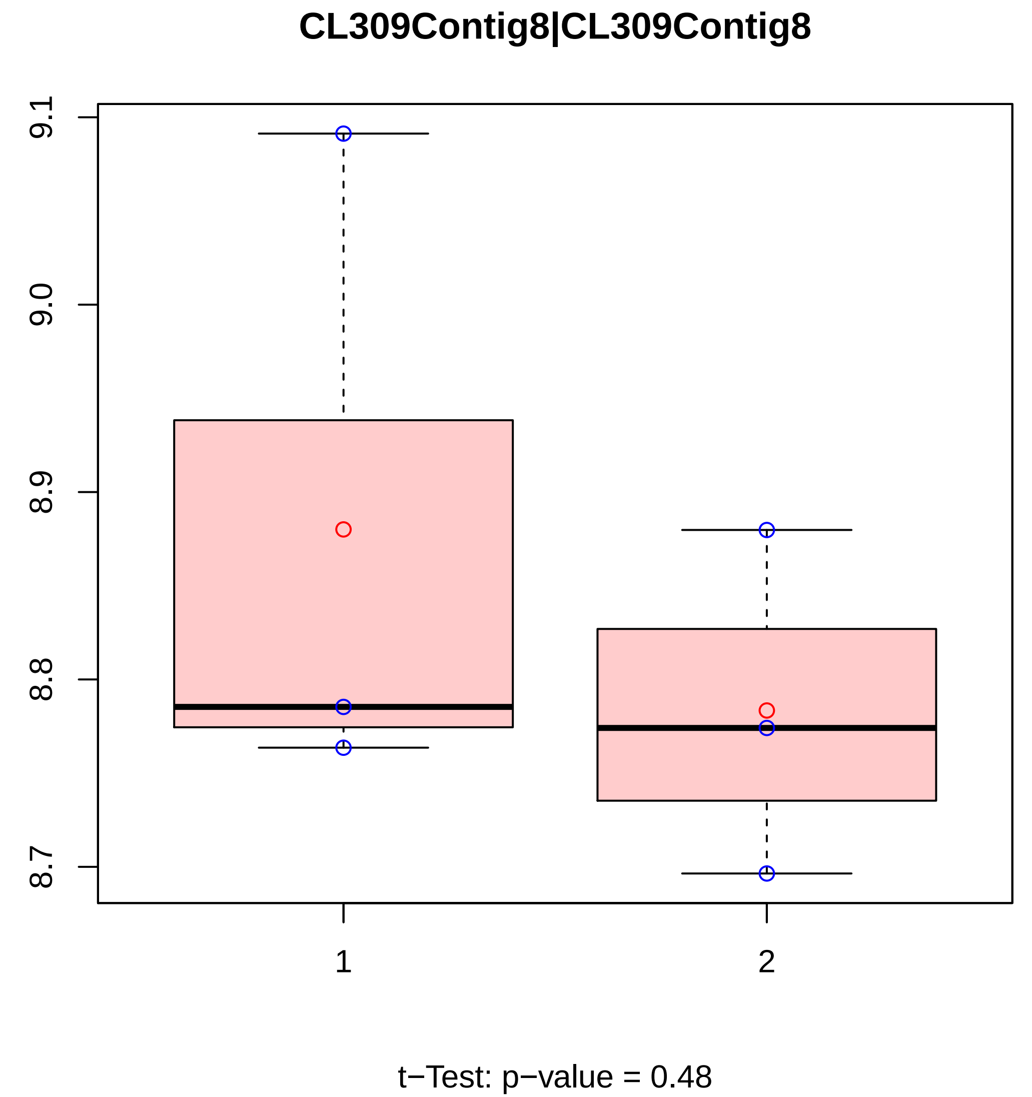
CL309Contig8|CL309Contig8
9.1
9.0
8.9
8.8
8.7
1
2
t−Test: p−value = 0.48

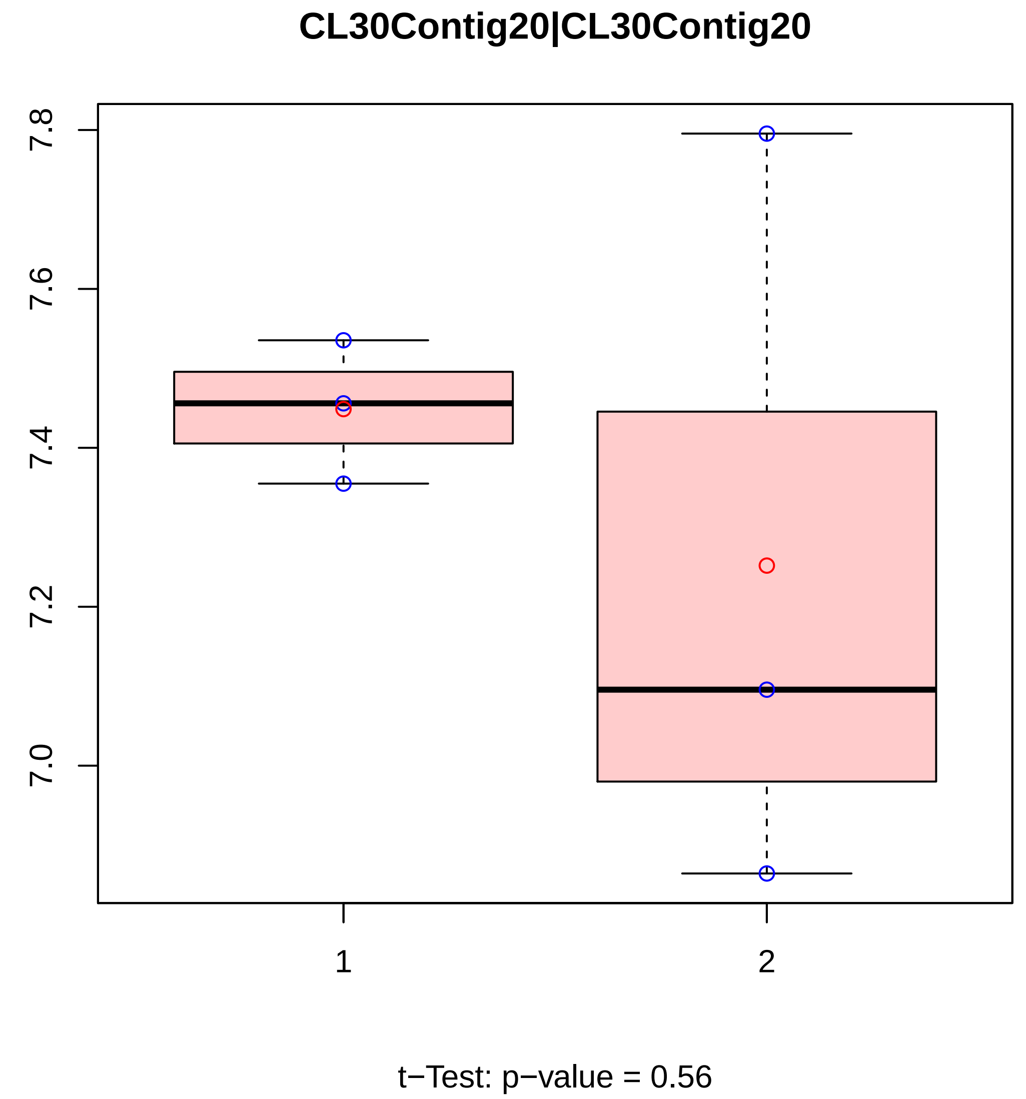
CL30Contig20|CL30Contig20
7.8
7.6
7.4
7.2
7.0
1
2
t−Test: p−value = 0.56

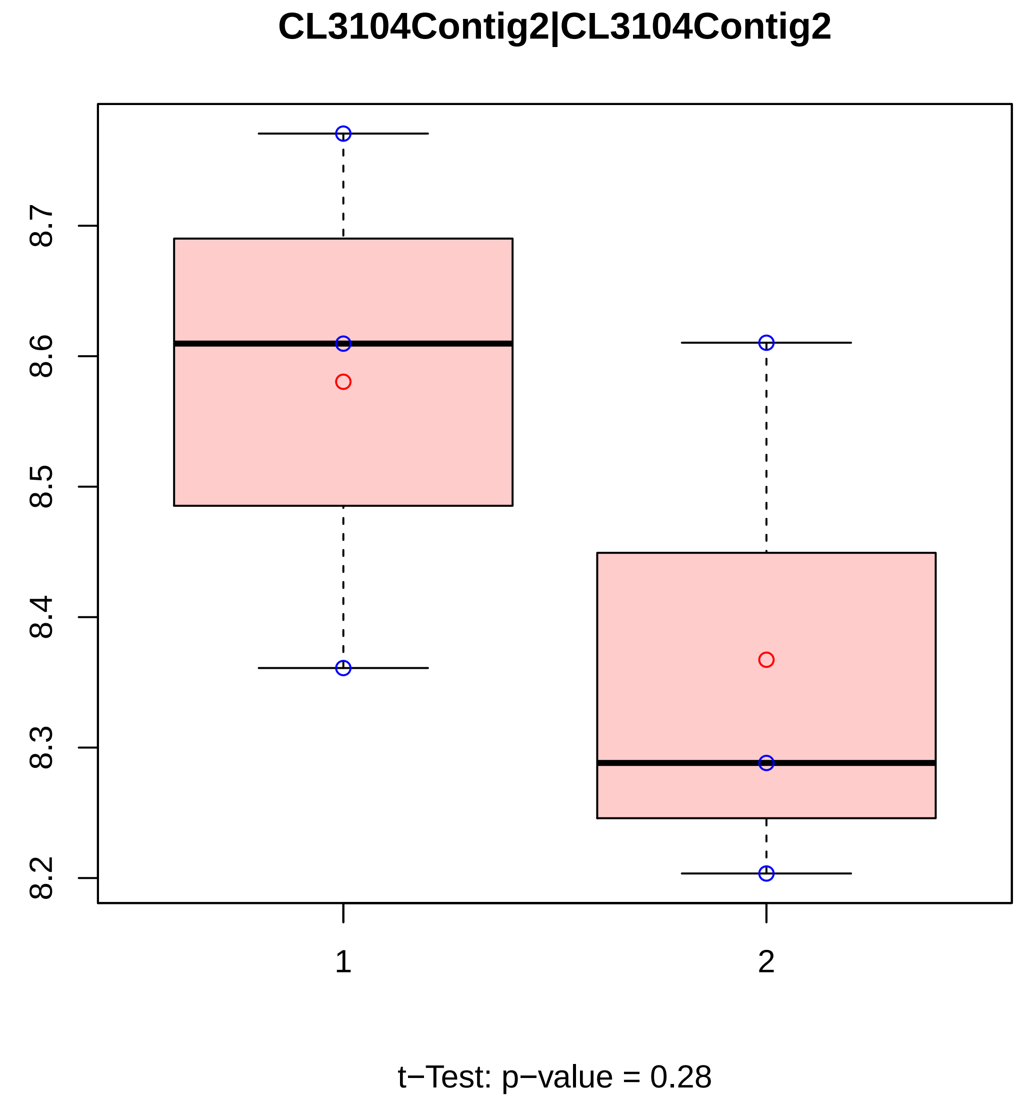
CL3104Contig2|CL3104Contig2
8.2
8.3
8.4
8.5
8.6
8.7
1
2
t−Test: p−value = 0.28

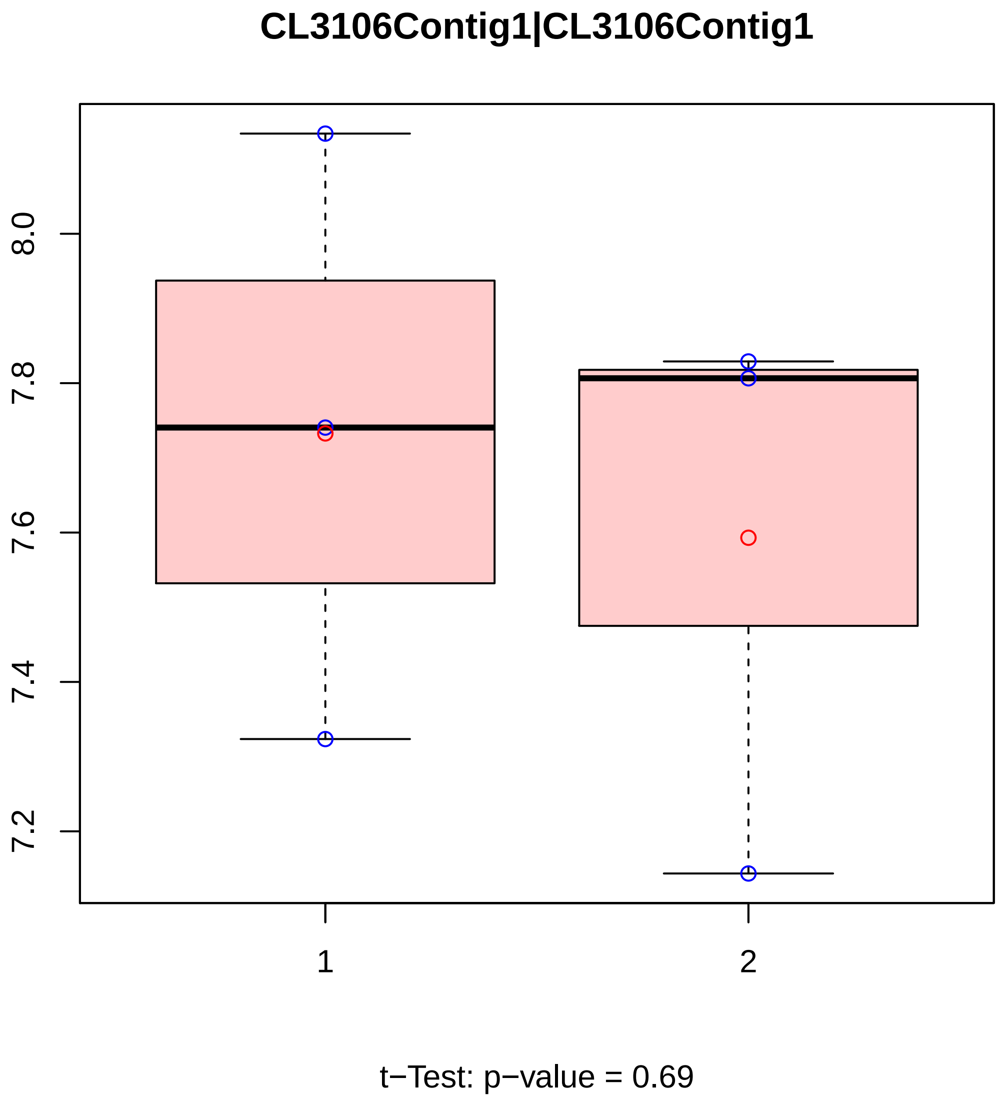

CL3106Contig1|CL3106Contig1
8.0
7.8
7.6
7.4
7.2
1
2
t−Test: p−value = 0.69

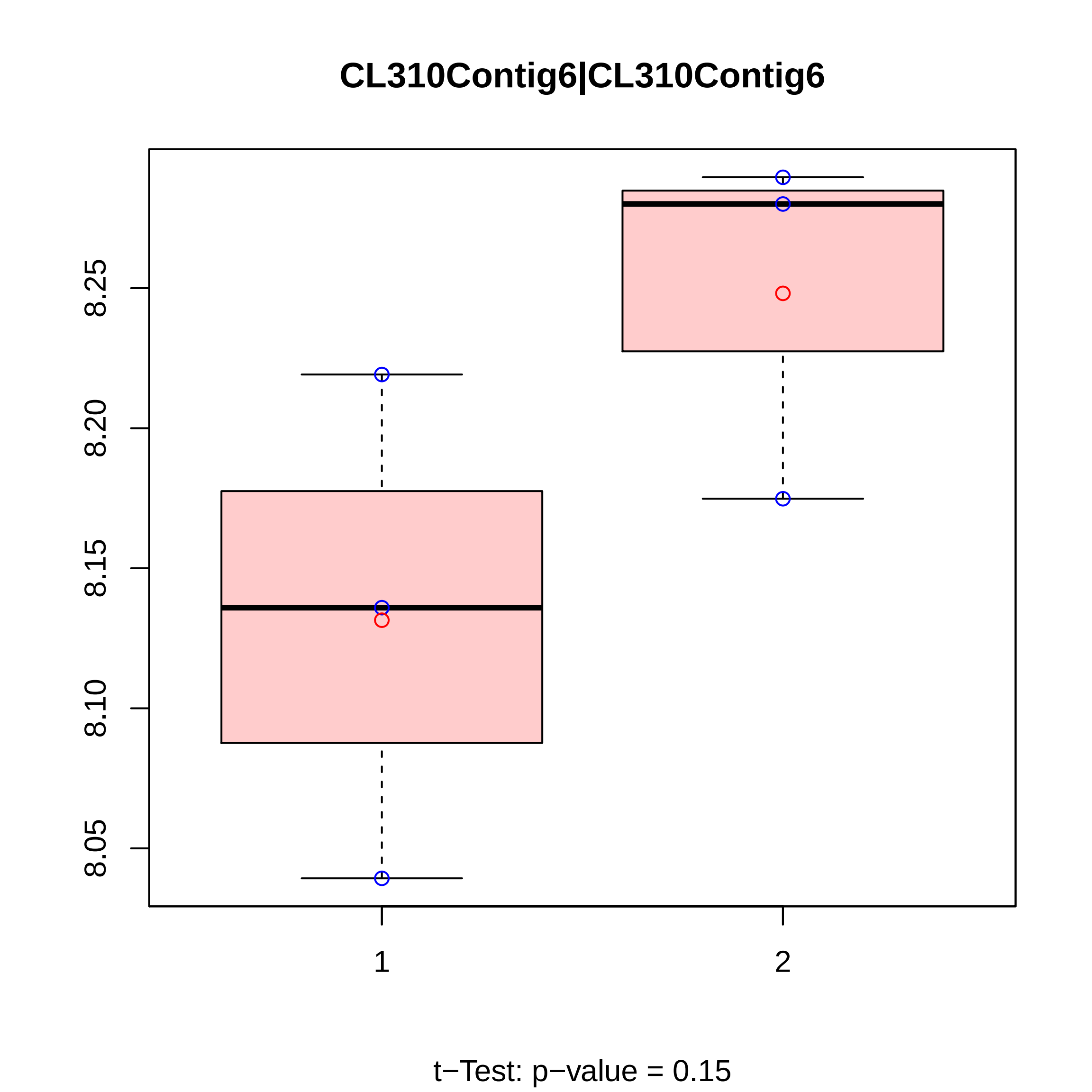

CL310Contig6|CL310Contig6
8.05
8.10
8.15
8.20
8.25
1
2
t−Test: p−value = 0.15

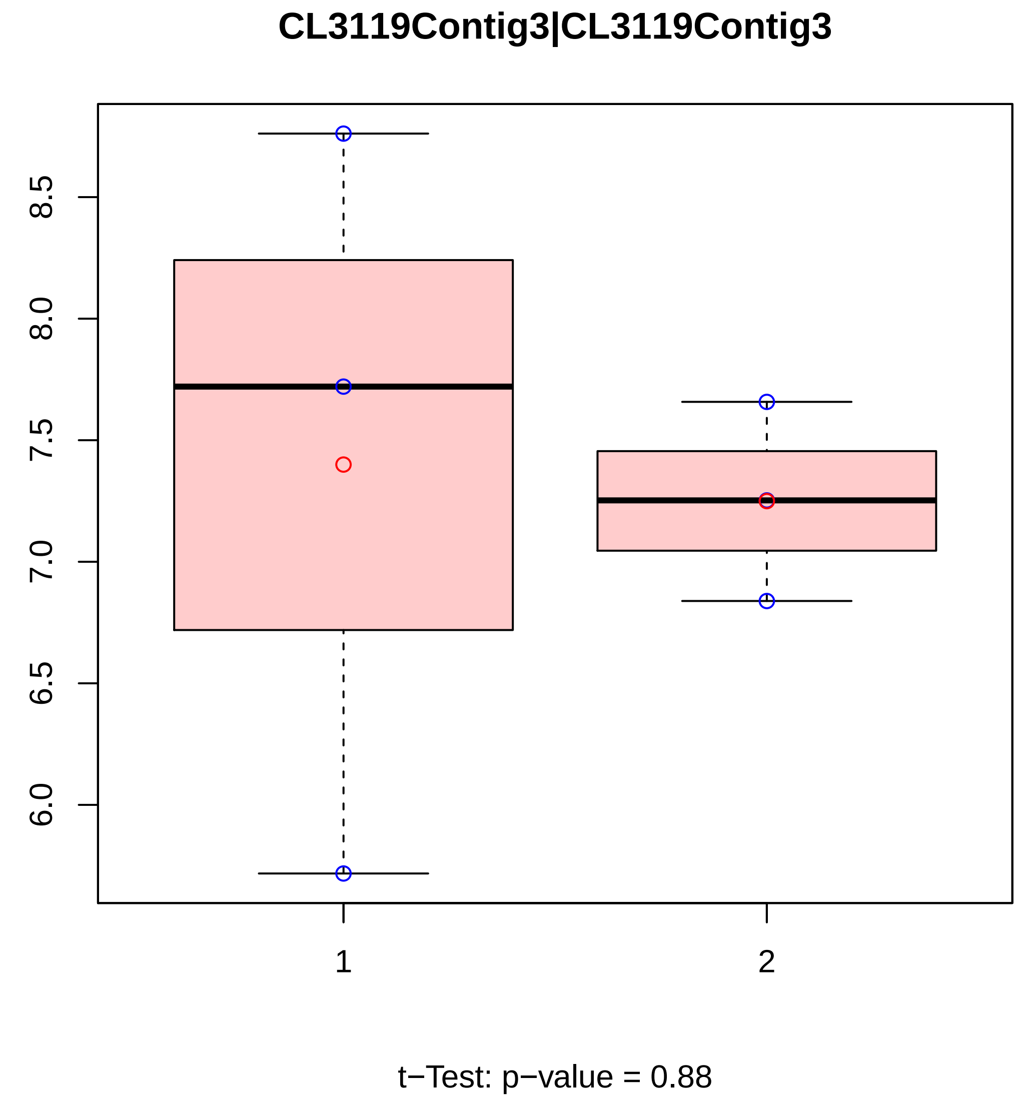
CL3119Contig3|CL3119Contig3
8.5
8.0
7.5
7.0
6.5
6.0
1
2
t−Test: p−value = 0.88

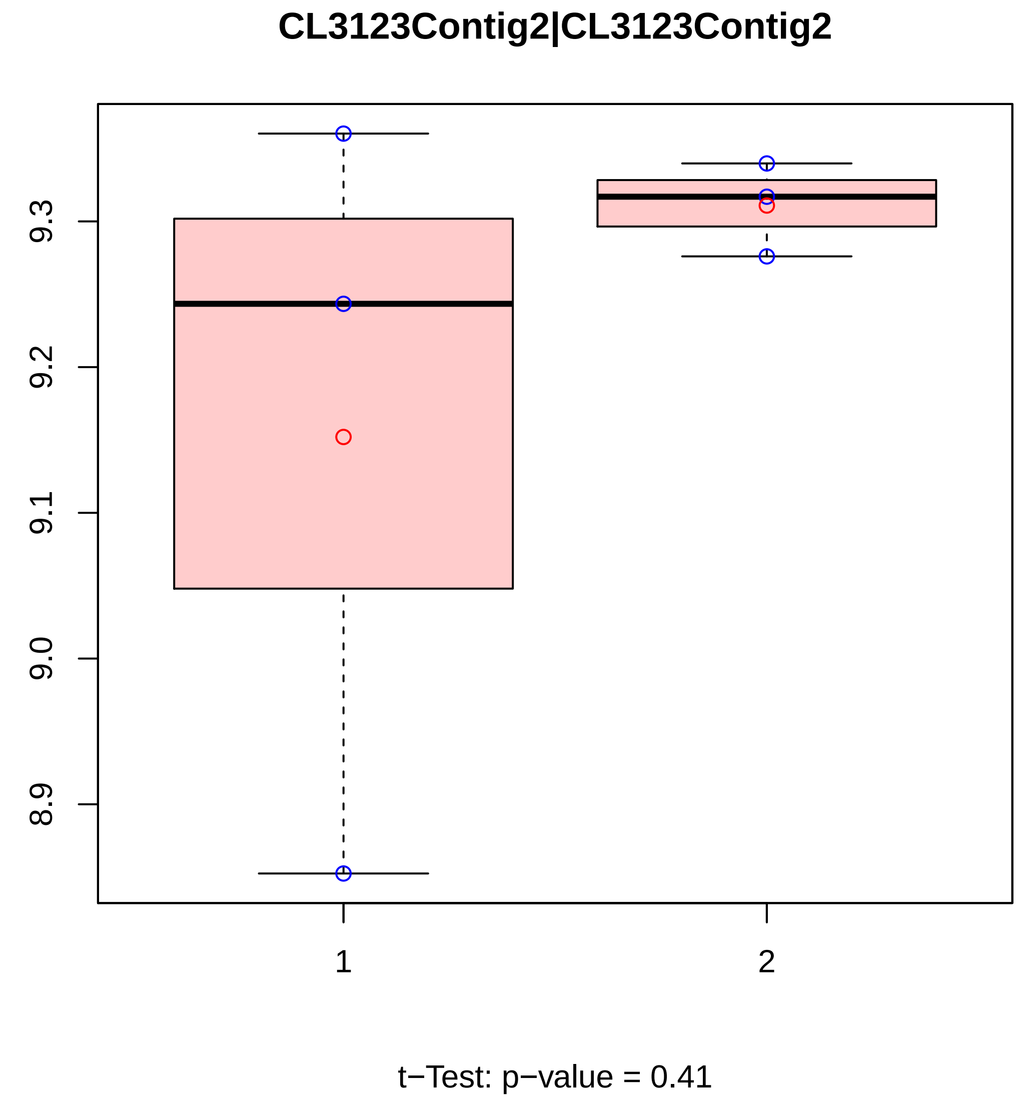

CL3123Contig2|CL3123Contig2
9.3
9.2
9.1
9.0
8.9
1
2
t−Test: p−value = 0.41

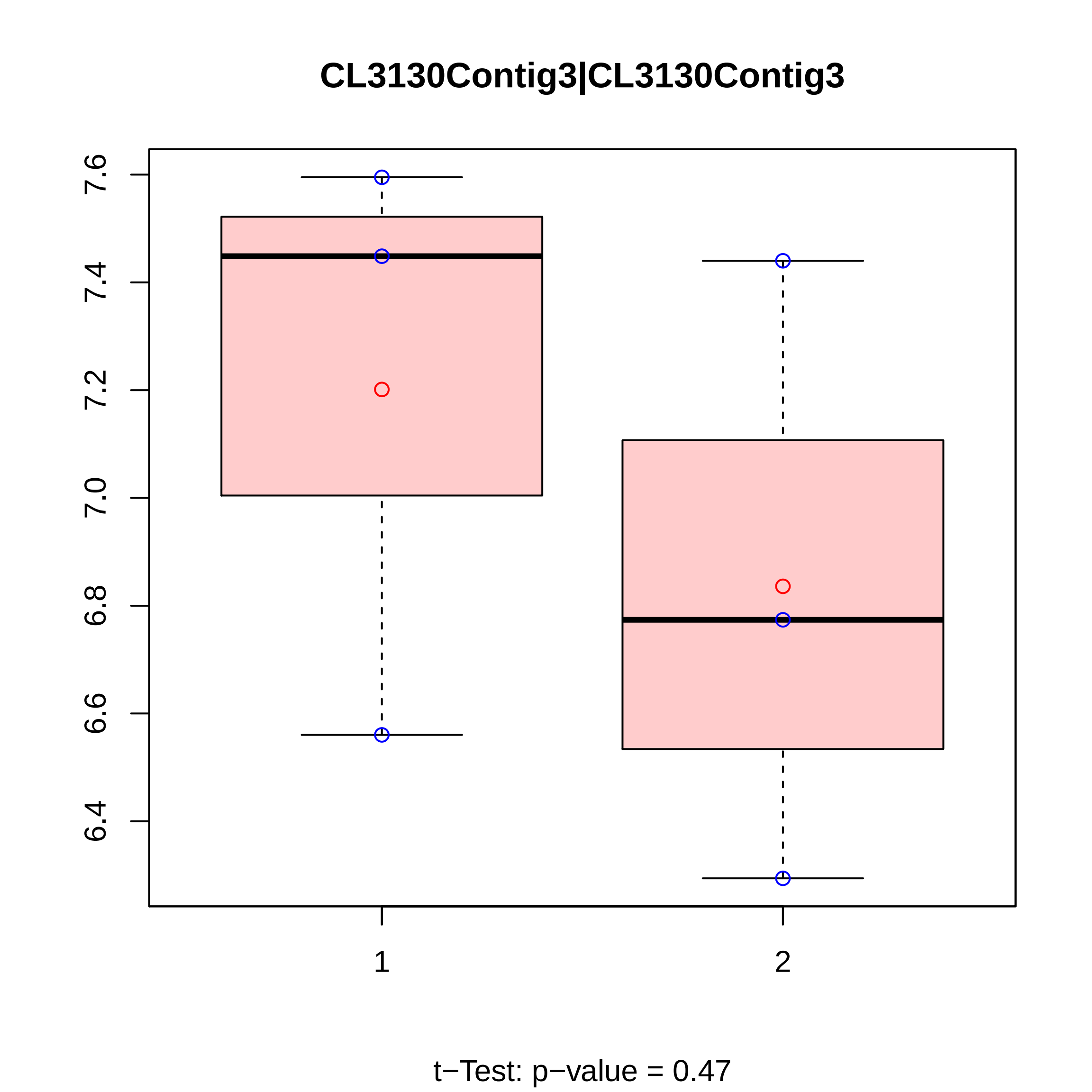
CL3130Contig3|CL3130Contig3
7.6
7.4
7.2
7.0
6.8
6.6
6.4
1
2
t−Test: p−value = 0.47

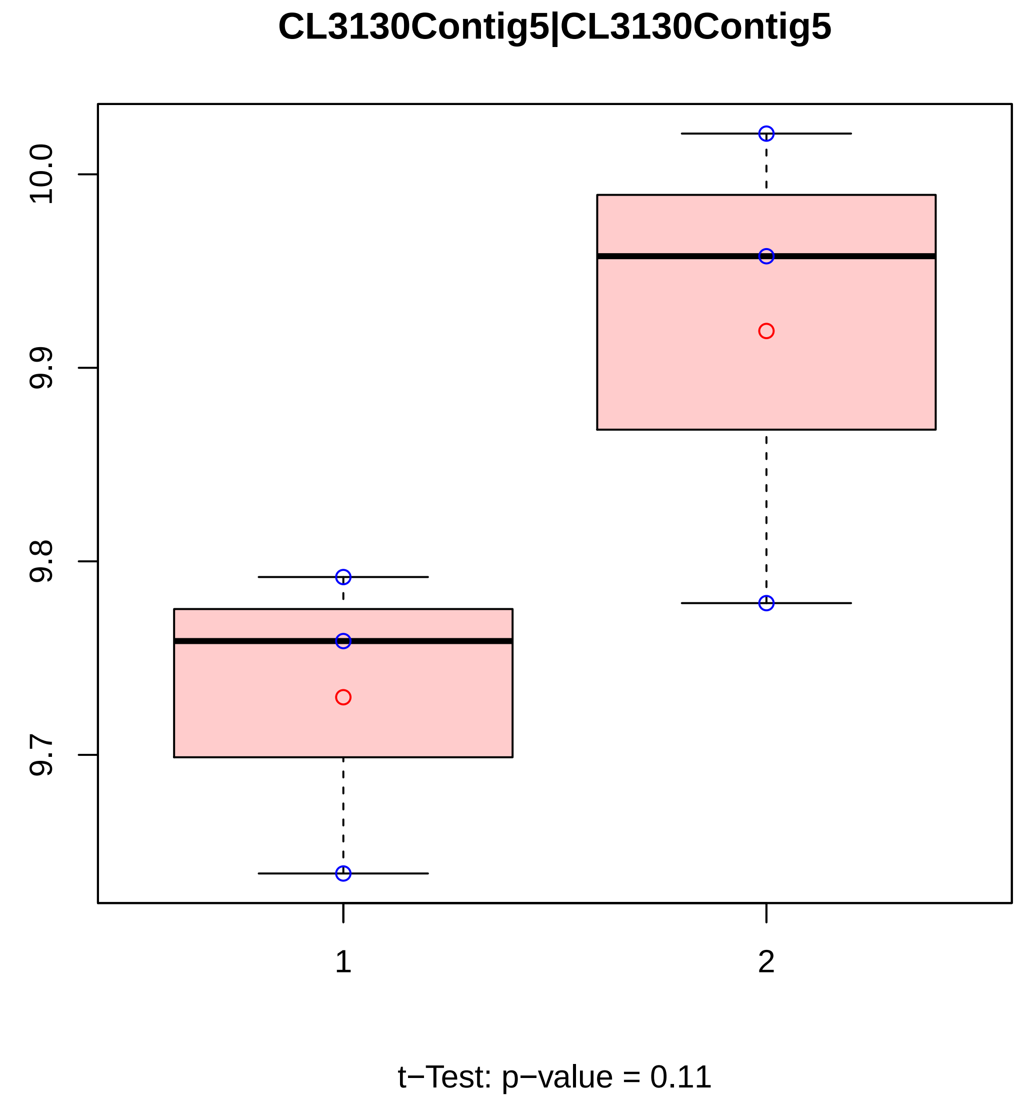
CL3130Contig5|CL3130Contig5
10.0
9.9
9.8
9.7
1
2
t−Test: p−value = 0.11

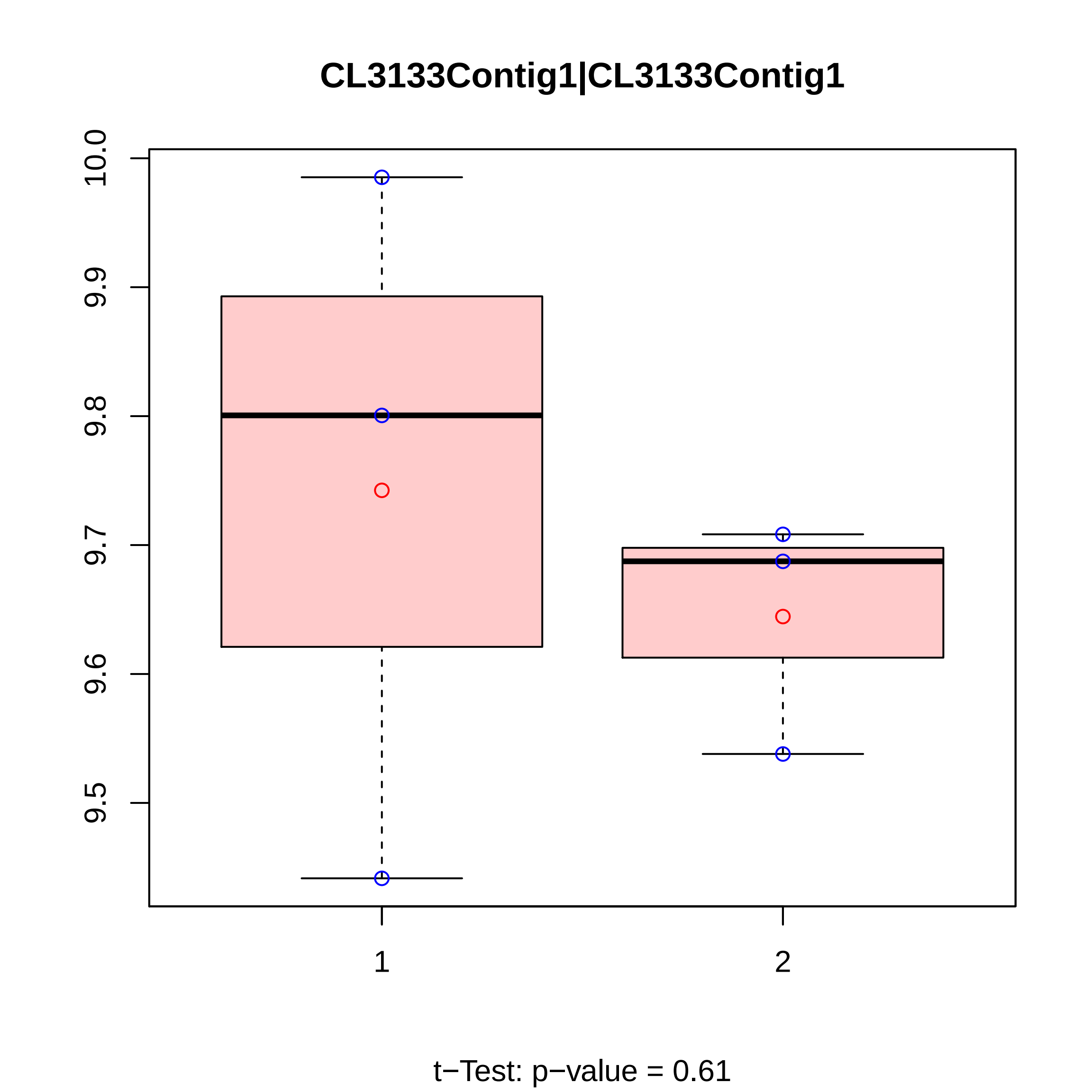
CL3133Contig1|CL3133Contig1
10.0
9.9
9.8
9.7
9.6
9.5
1
2
t−Test: p−value = 0.61

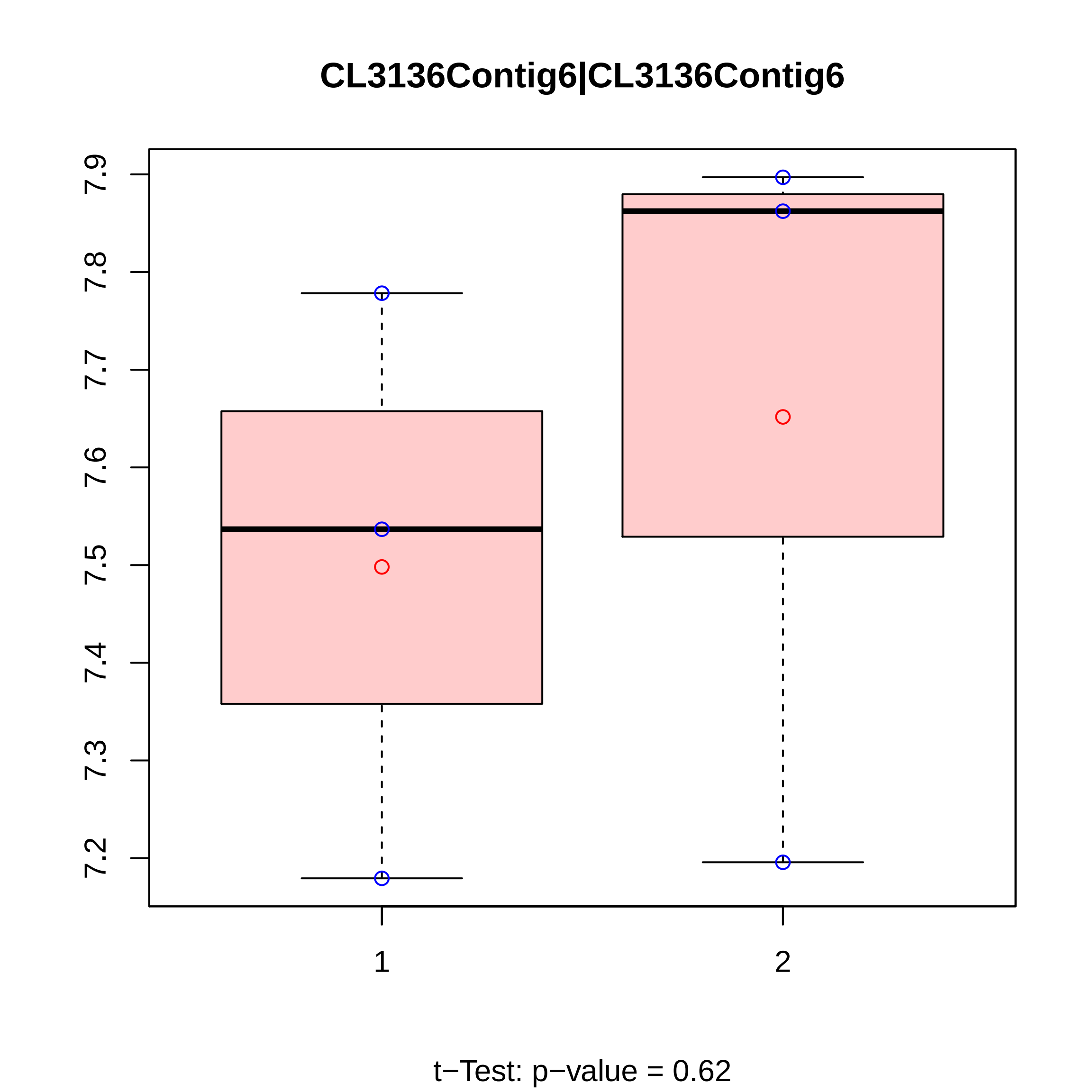
CL3136Contig6|CL3136Contig6
7.2
7.3
7.4
7.5
7.6
7.7
7.8
7.9
1
2
t−Test: p−value = 0.62

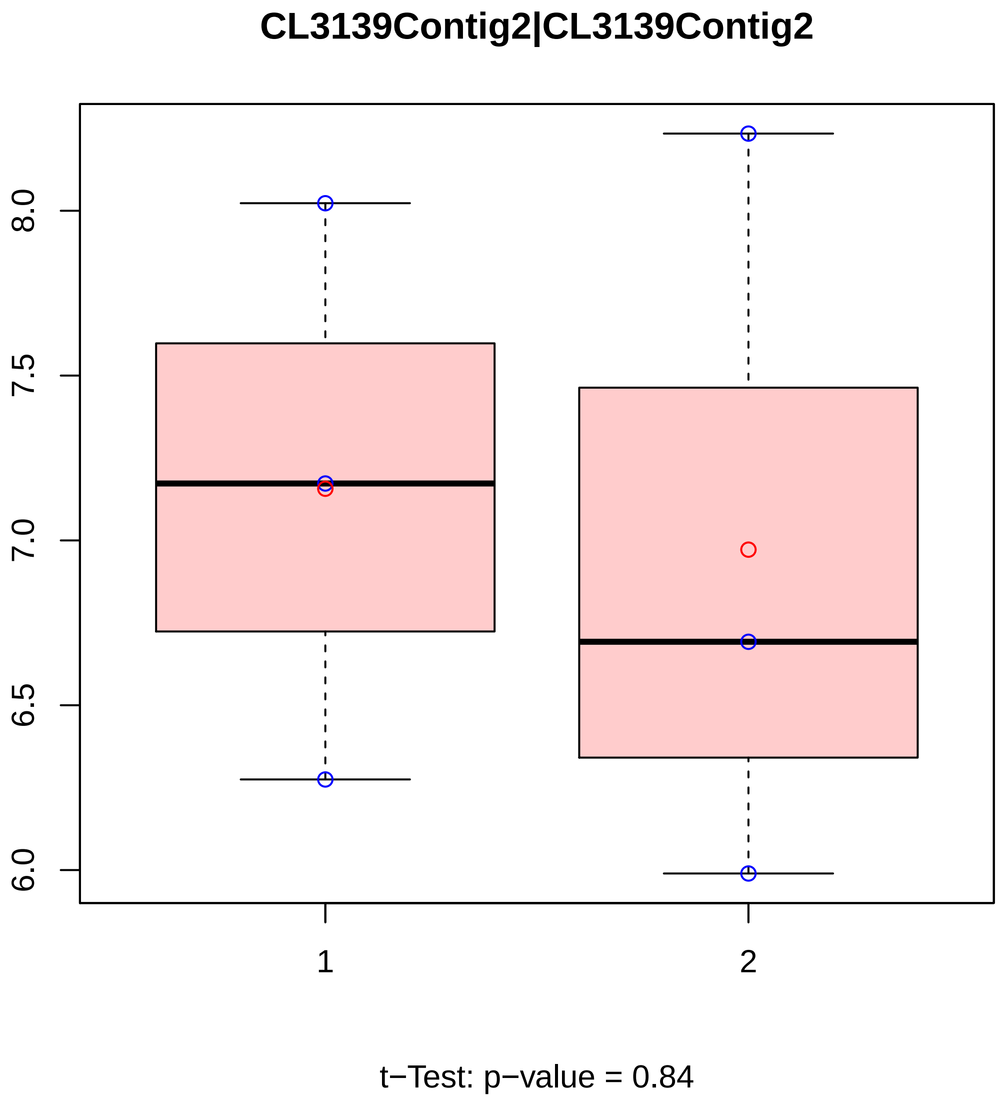

CL3139Contig2|CL3139Contig2
8.0
7.5
7.0
6.5
6.0
1
2
t−Test: p−value = 0.84

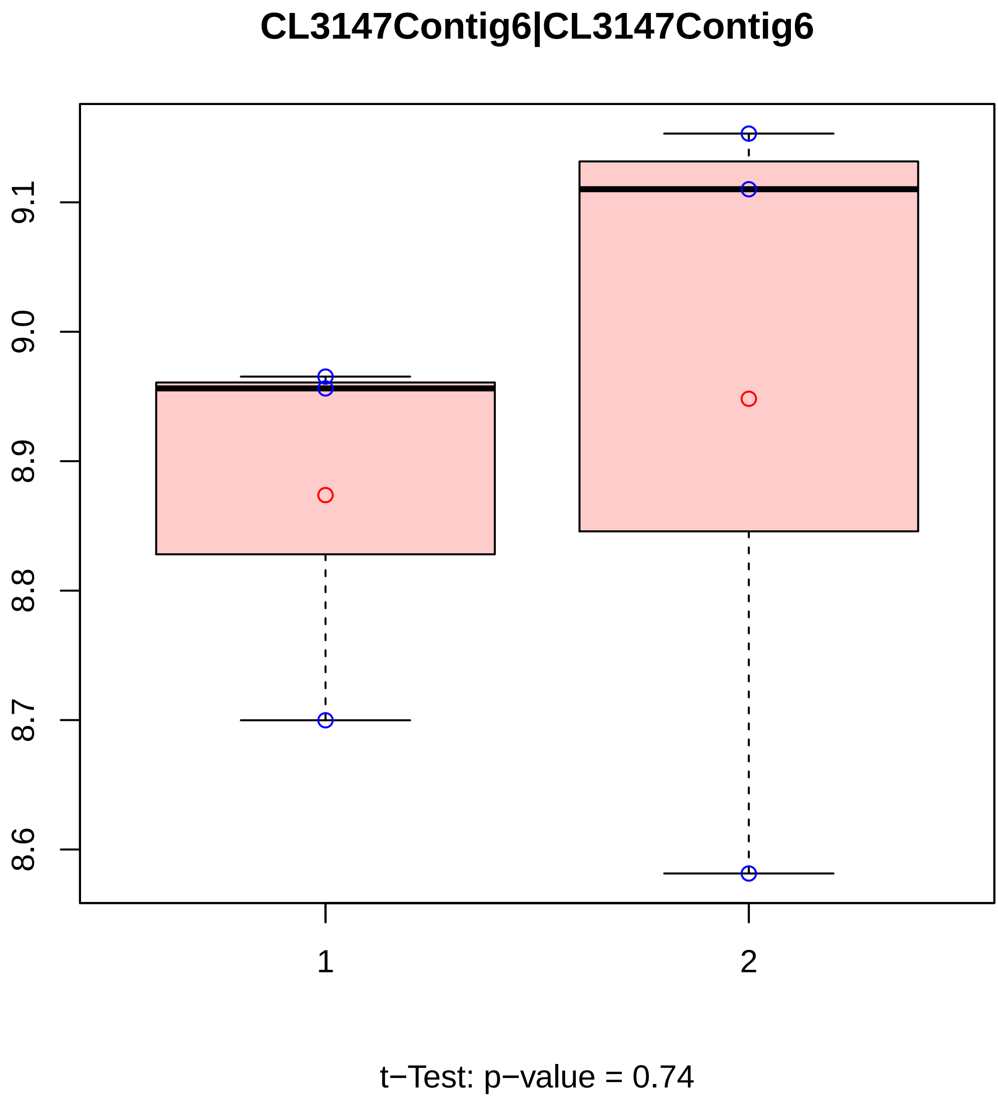
CL3147Contig6|CL3147Contig6
9.1
9.0
8.9
8.8
8.7
8.6
1
2
t−Test: p−value = 0.74

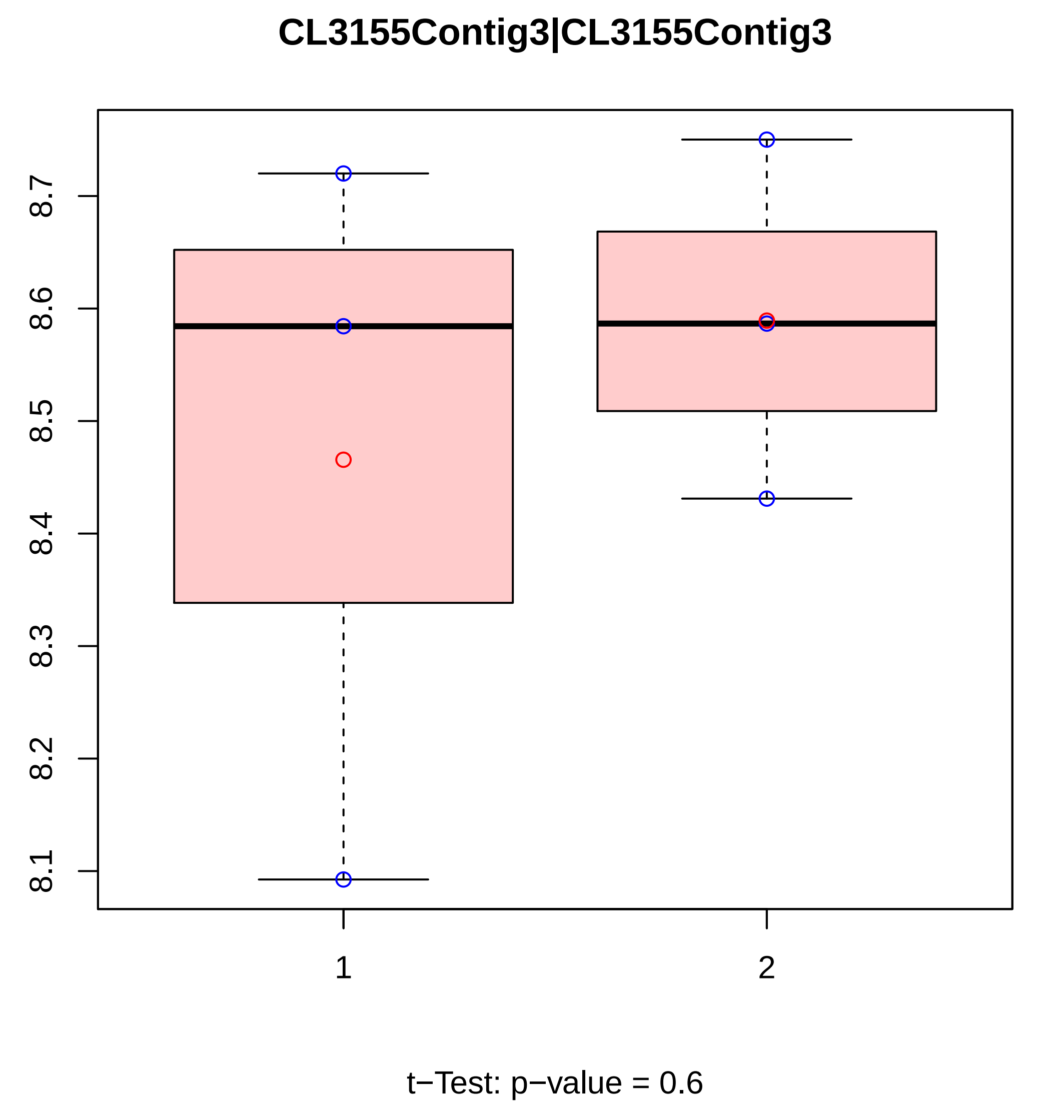
CL3155Contig3|CL3155Contig3
8.1
8.2
8.3
8.4
8.5
8.6
8.7
1
2
t−Test: p−value = 0.6

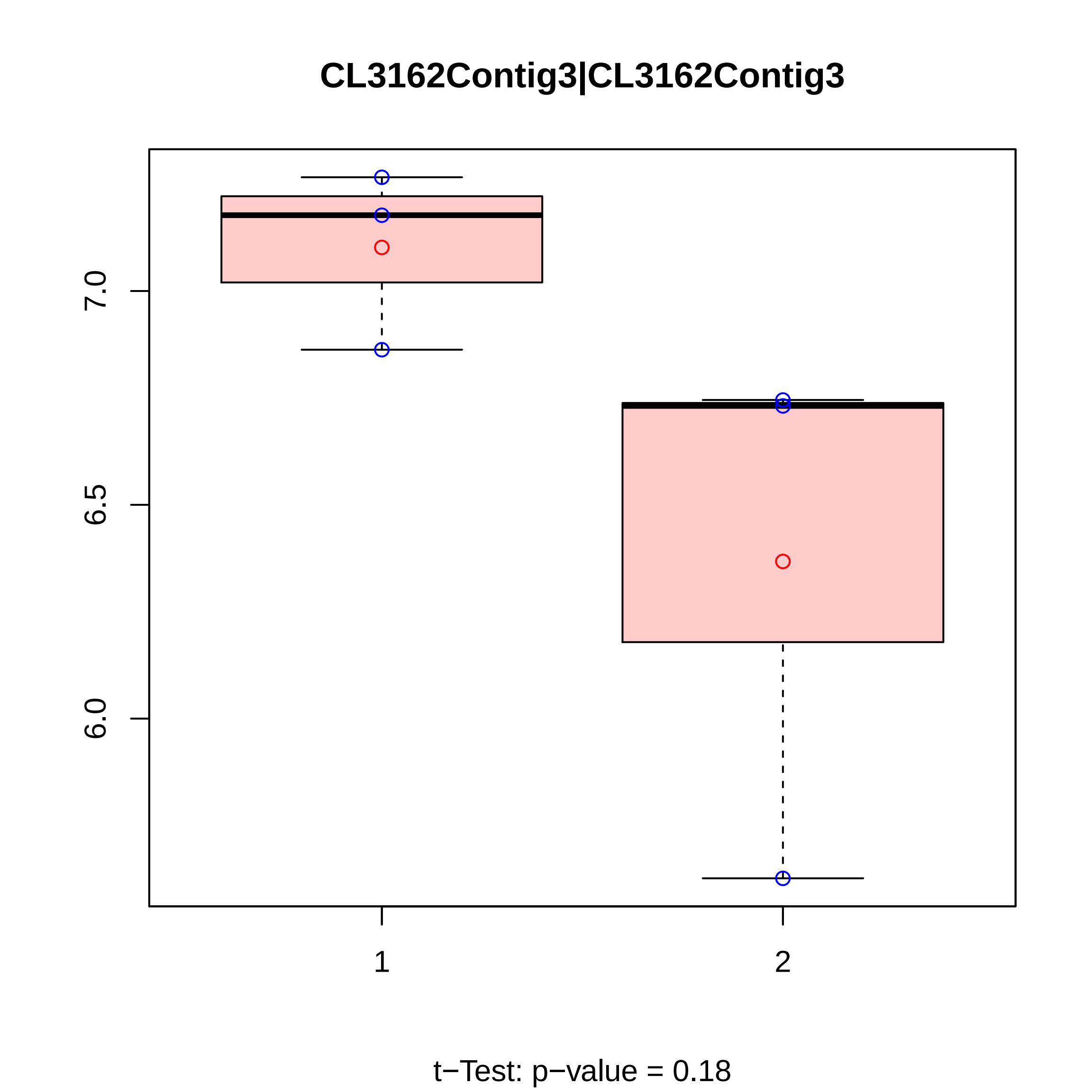

CL3162Contig3|CL3162Contig3
7.0
6.5
6.0
1
2
t−Test: p−value = 0.18

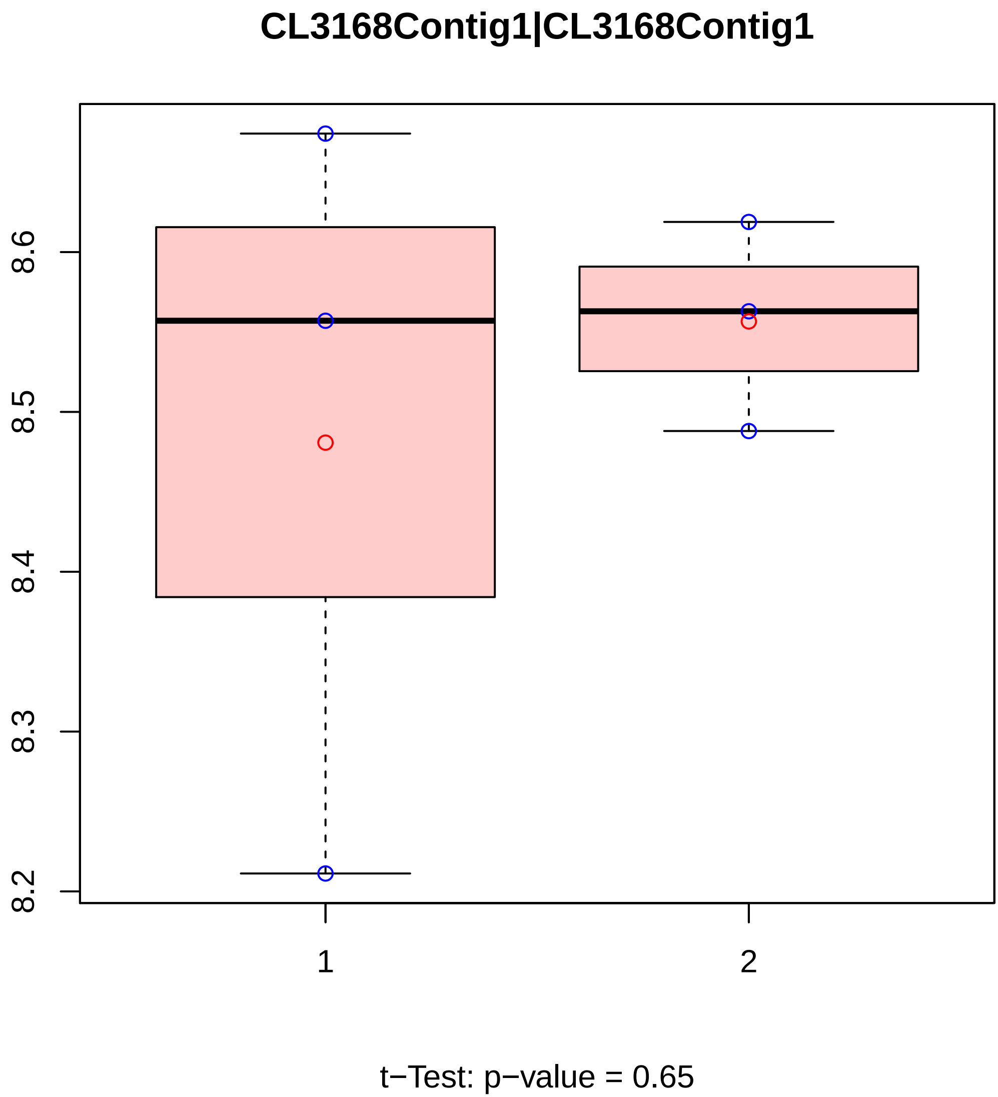
CL3168Contig1|CL3168Contig1
8.2
8.3
8.4
8.5
8.6
1
2
t−Test: p−value = 0.65

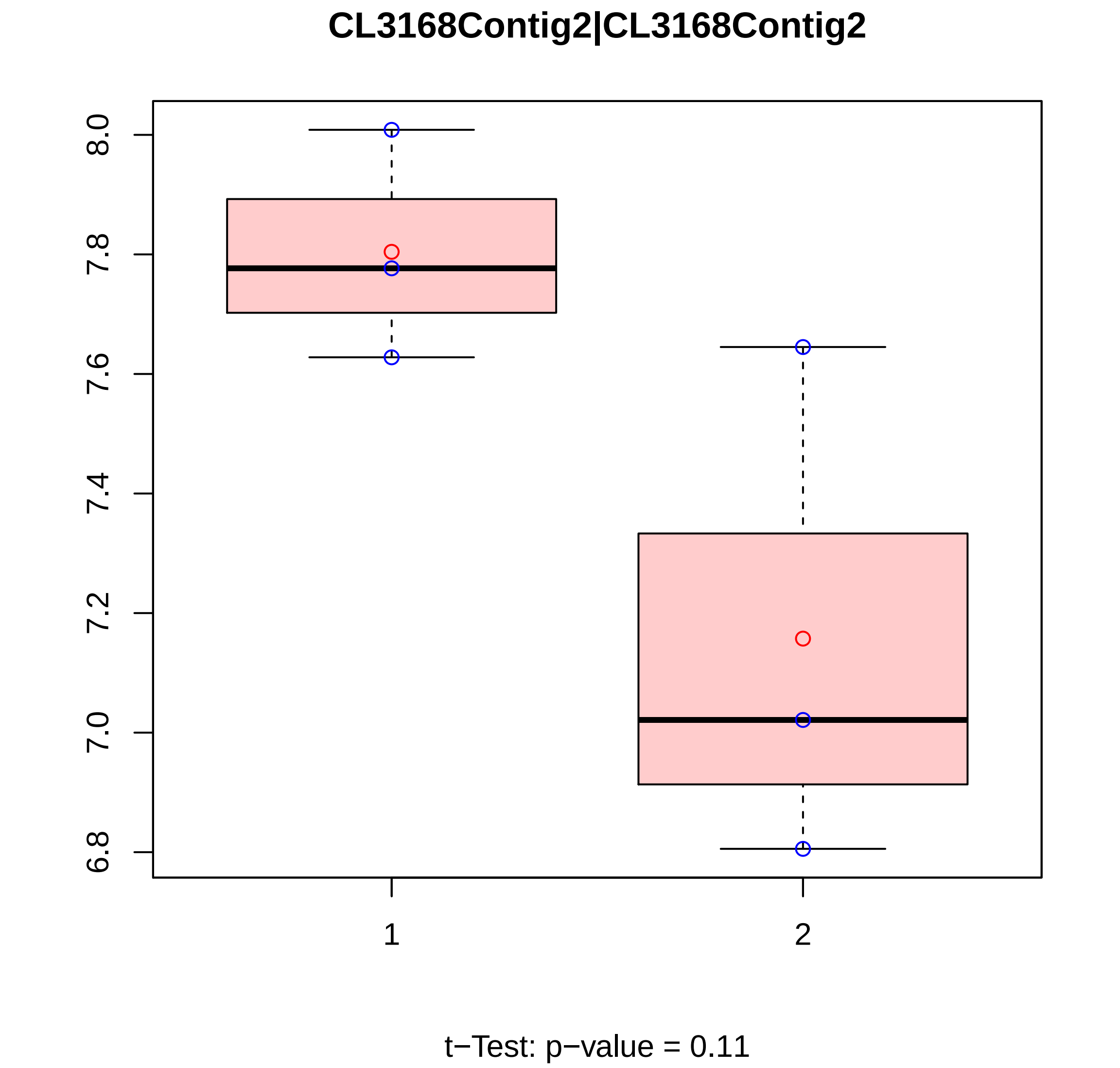
CL3168Contig2|CL3168Contig2
8.0
7.8
7.6
7.4
7.2
7.0
6.8
1
2
t−Test: p−value = 0.11

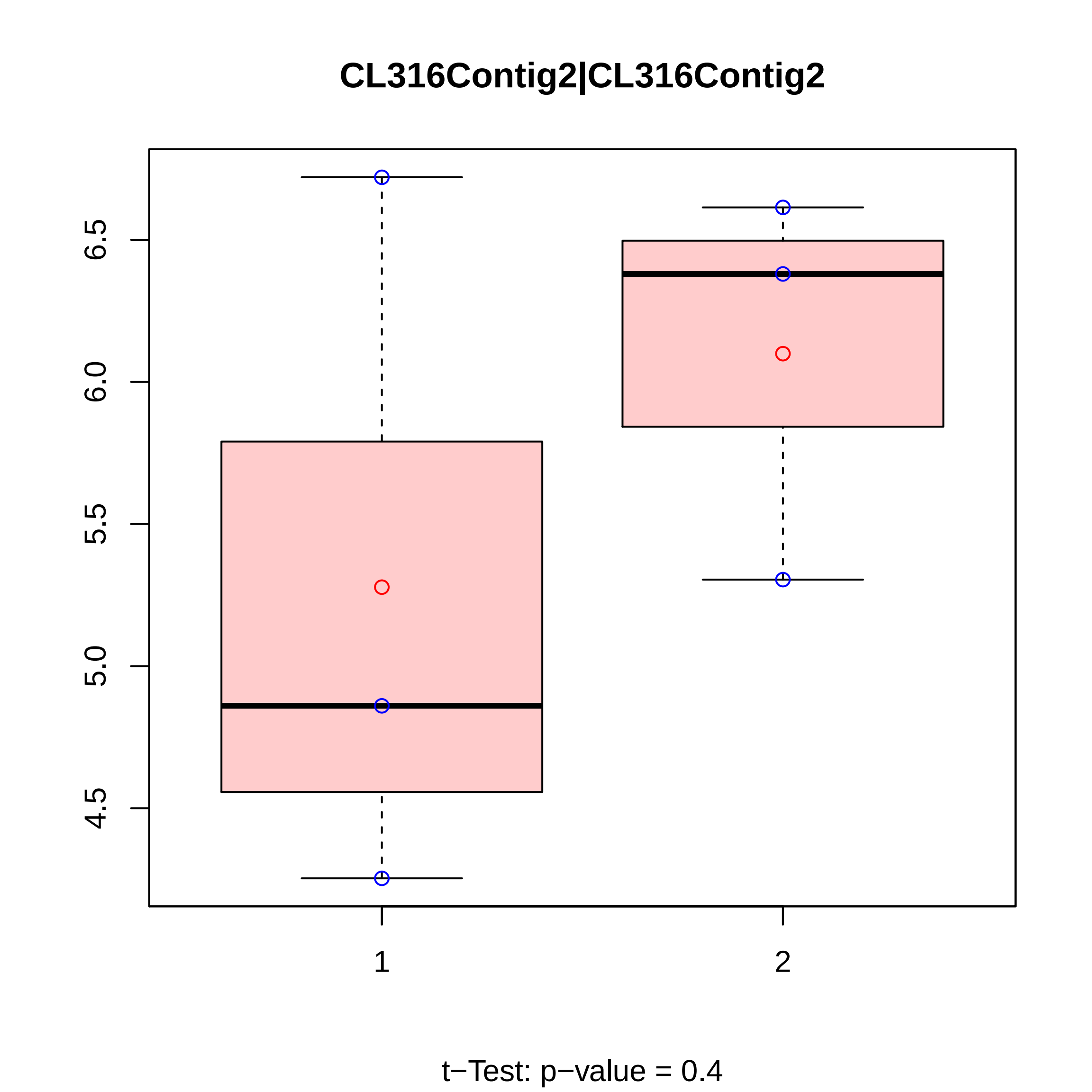

CL316Contig2|CL316Contig2
4.5
5.0
5.5
6.0
6.5
1
2
t−Test: p−value = 0.4

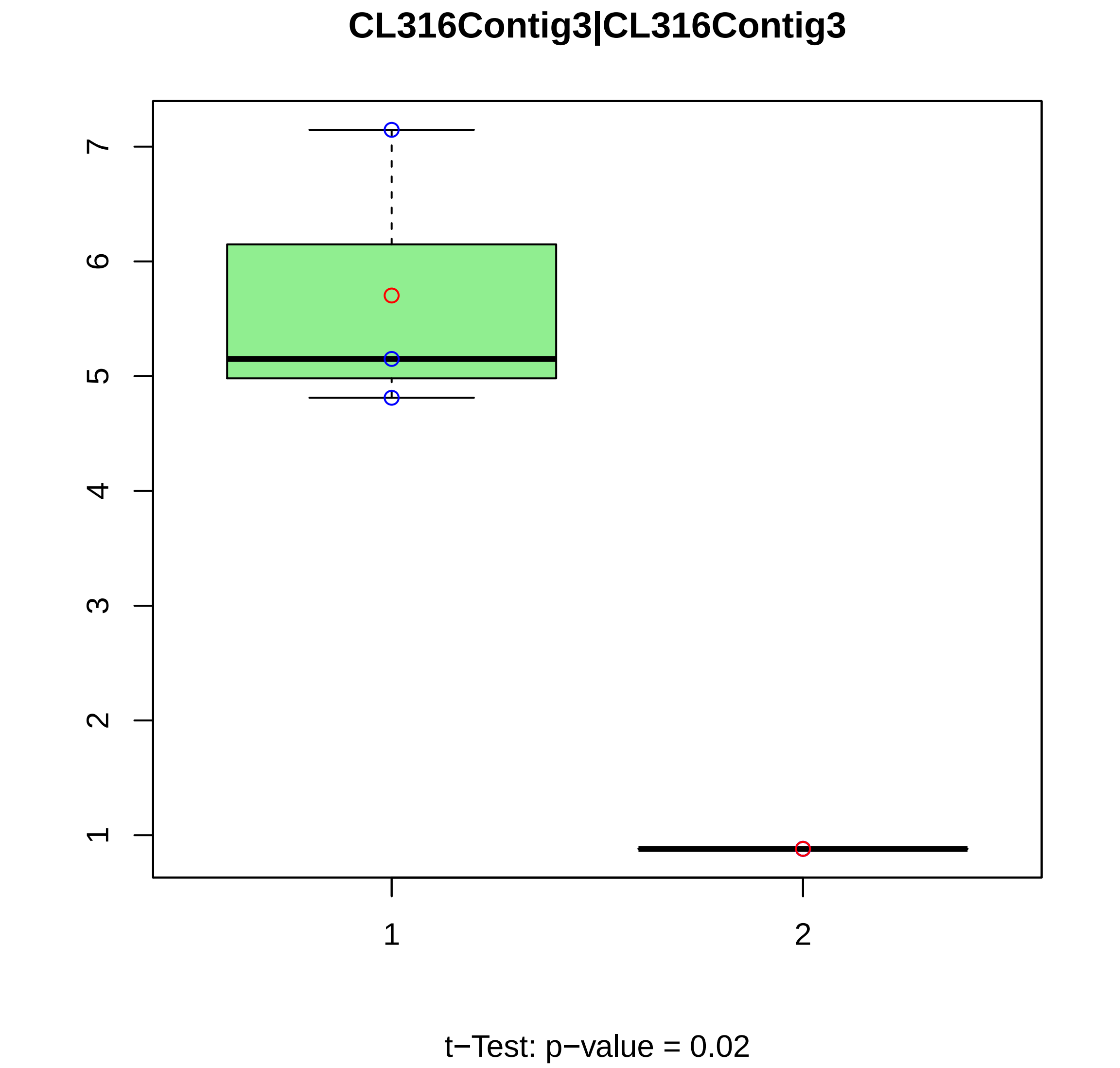
CL316Contig3|CL316Contig3
1
2
3
4
5
6
7
1
2
t−Test: p−value = 0.02

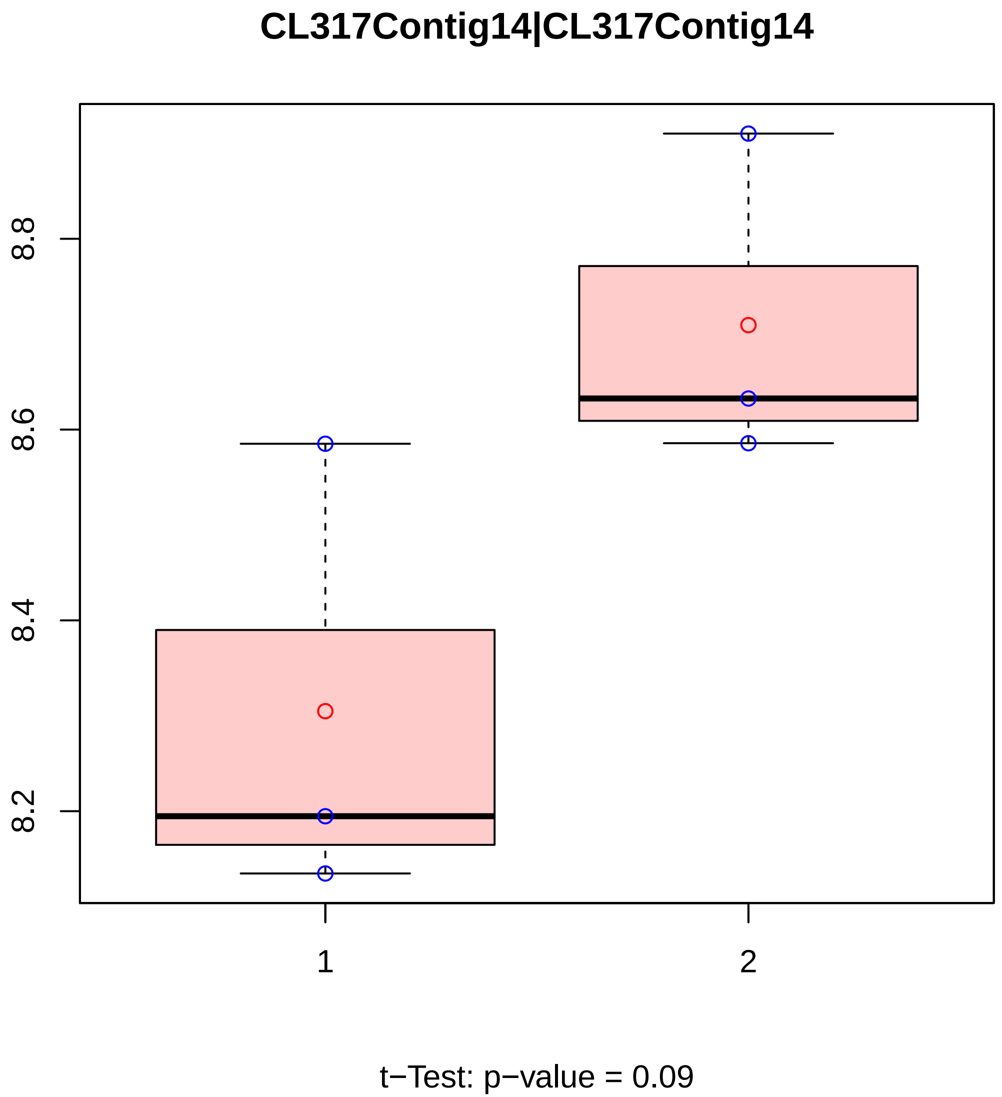
CL317Contig14|CL317Contig14
8.2
8.4
8.6
8.8
1
2
t−Test: p−value = 0.09

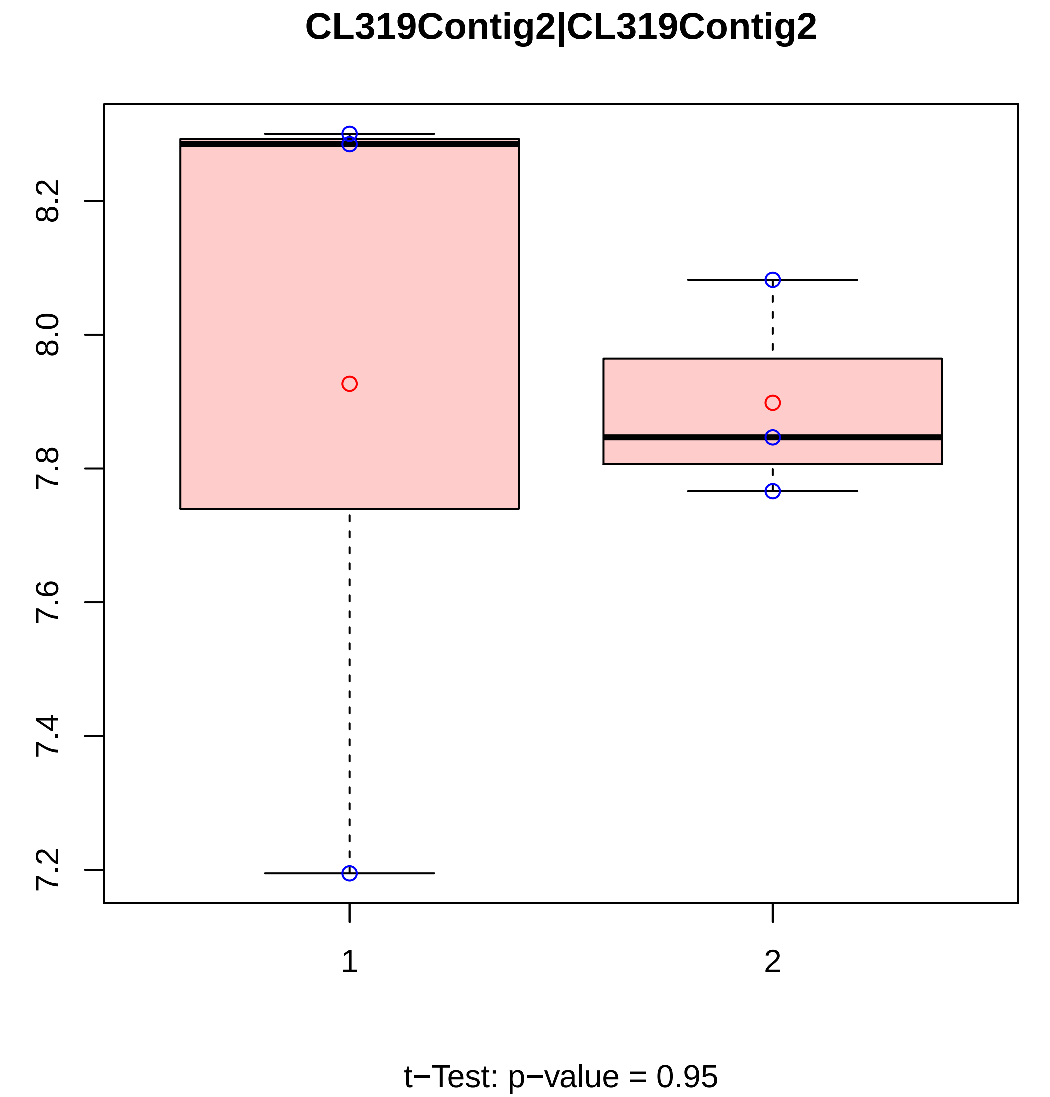
CL319Contig2|CL319Contig2
7.2
7.4
7.6
7.8
8.0
8.2
1
2
t−Test: p−value = 0.95

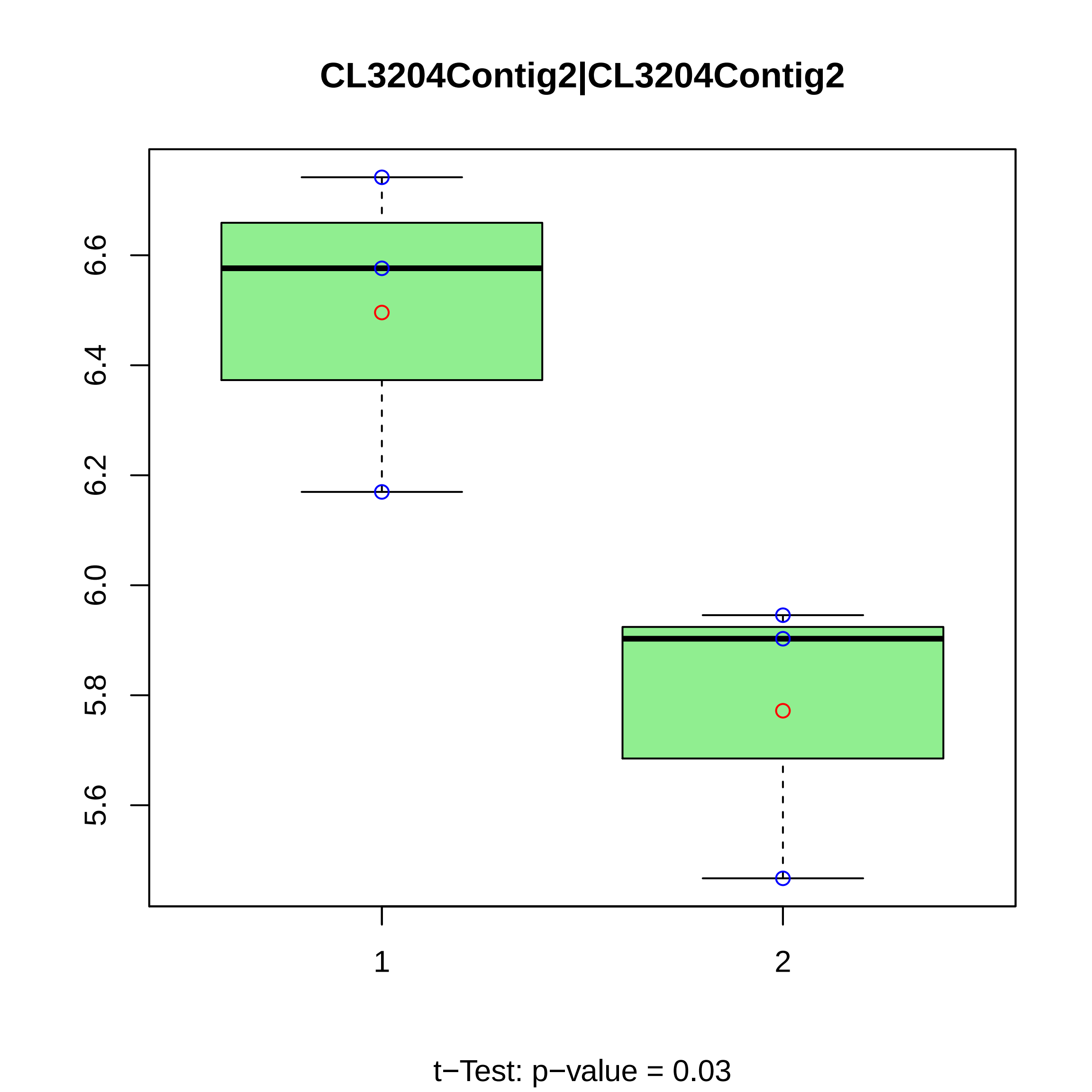
CL3204Contig2|CL3204Contig2
6.6
6.4
6.2
6.0
5.8
5.6
1
2
t−Test: p−value = 0.03

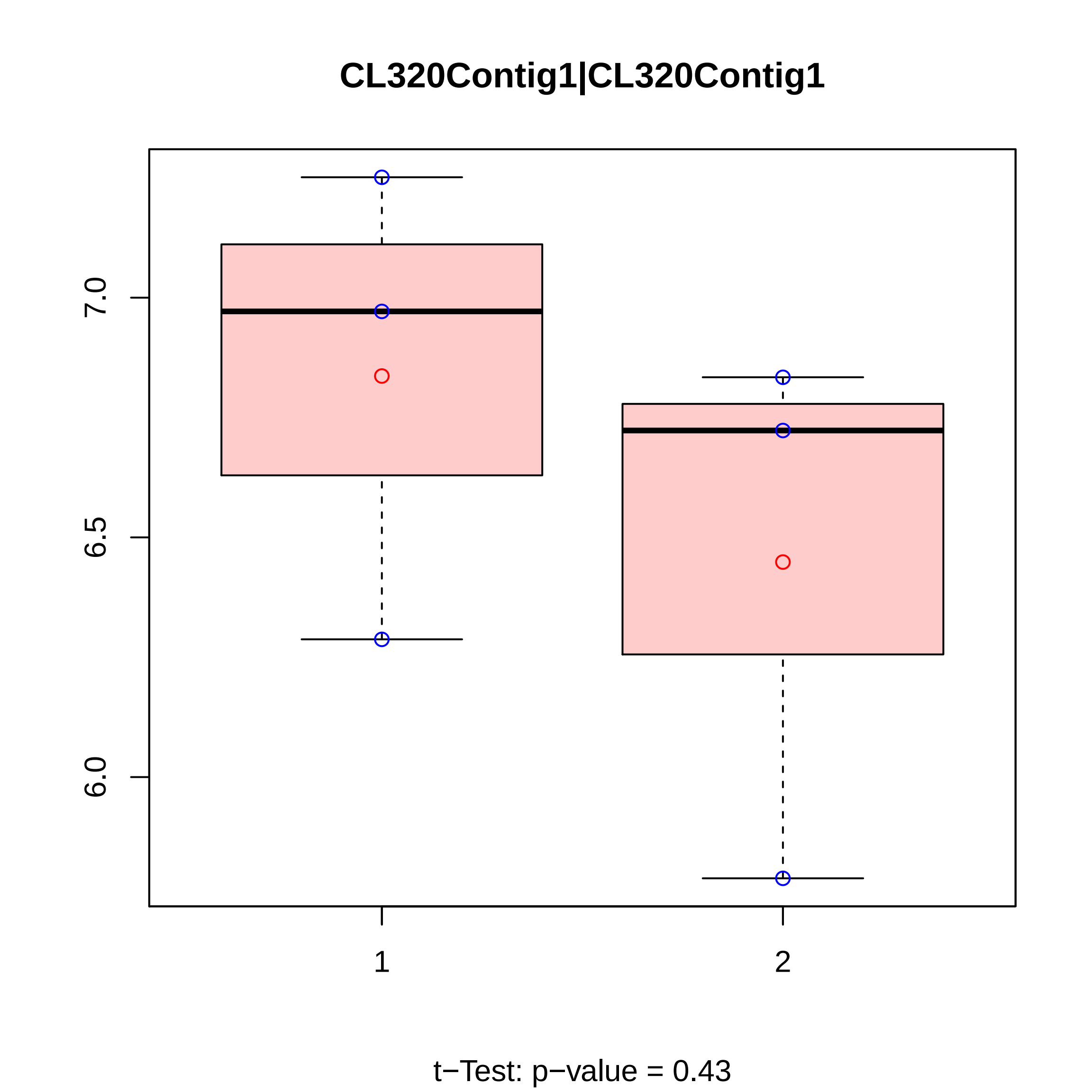
CL320Contig1|CL320Contig1
7.0
6.5
6.0
1
2
t−Test: p−value = 0.43

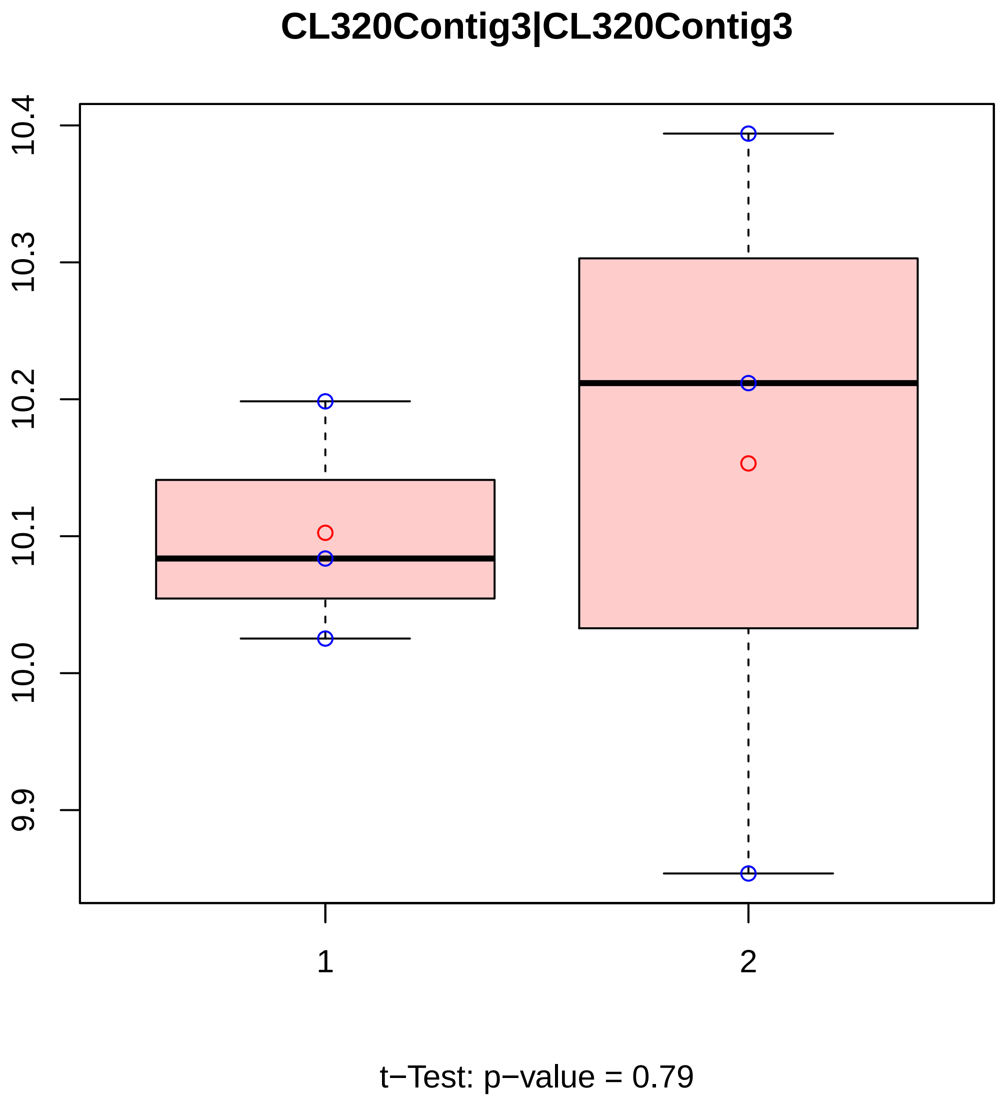
CL320Contig3|CL320Contig3
10.4
10.3
10.2
10.1
10.0
9.9
1
2
t−Test: p−value = 0.79

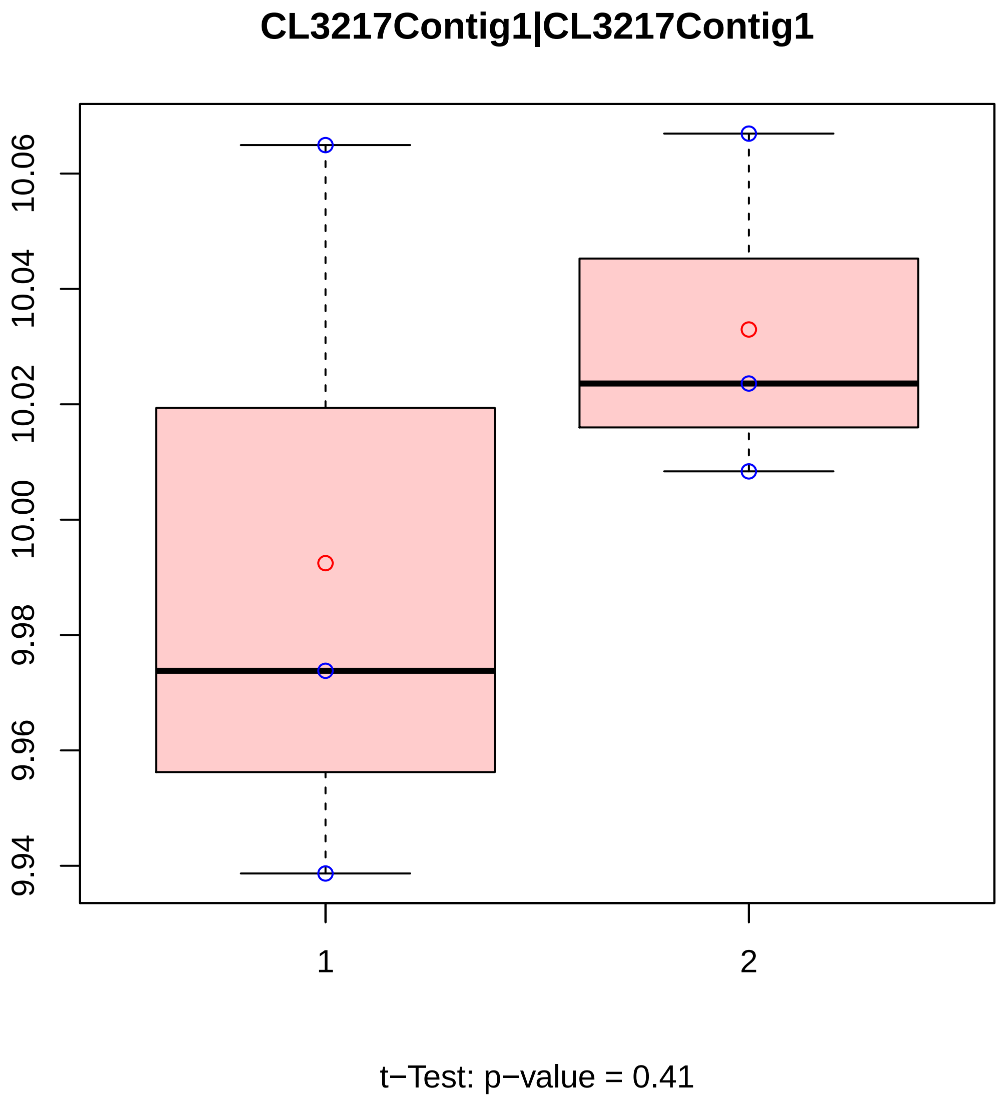

CL3217Contig1|CL3217Contig1
10.06
10.04
10.02
10.00
9.98
9.96
9.94
1
2
t−Test: p−value = 0.41

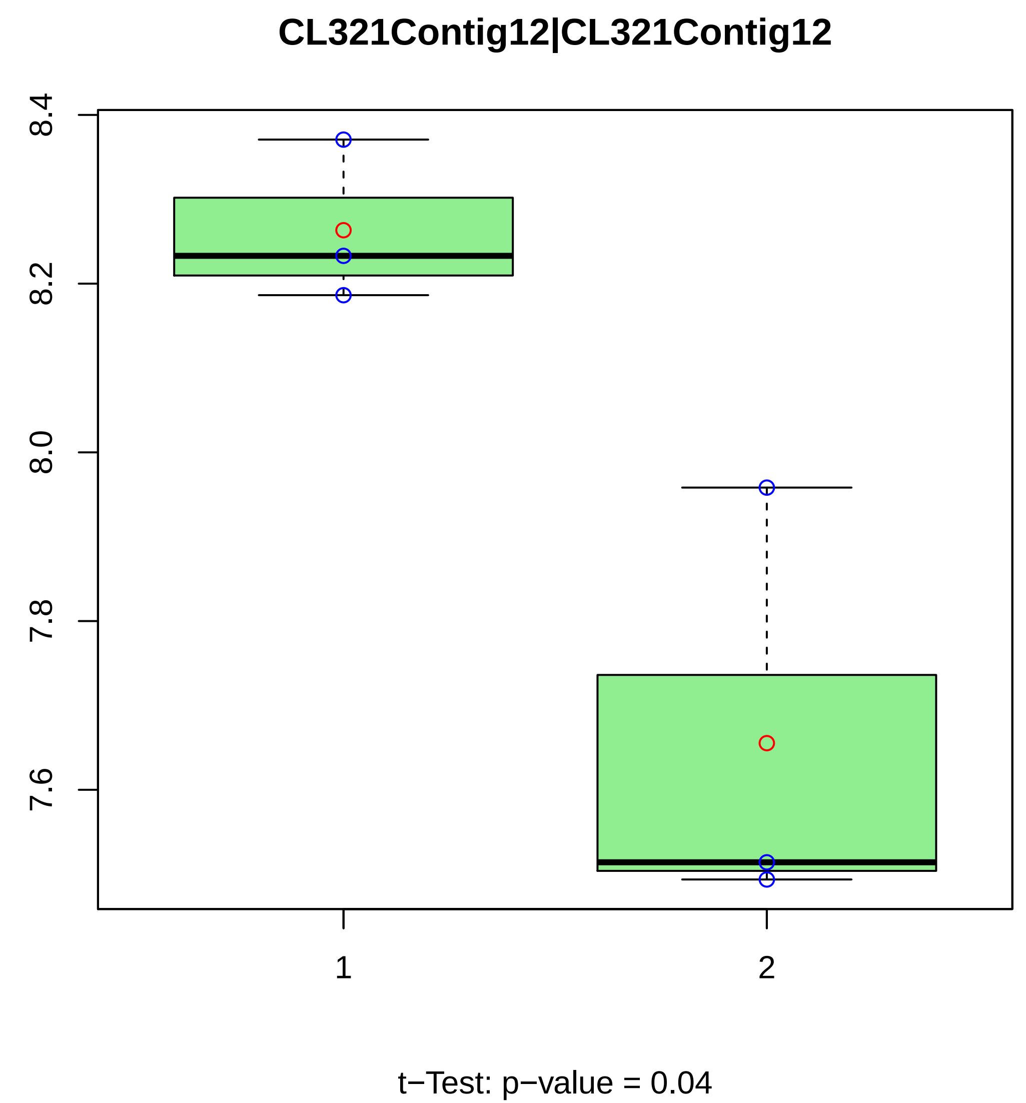
CL321Contig12|CL321Contig12
8.4
8.2
8.0
7.8
7.6
1
2
t−Test: p−value = 0.04

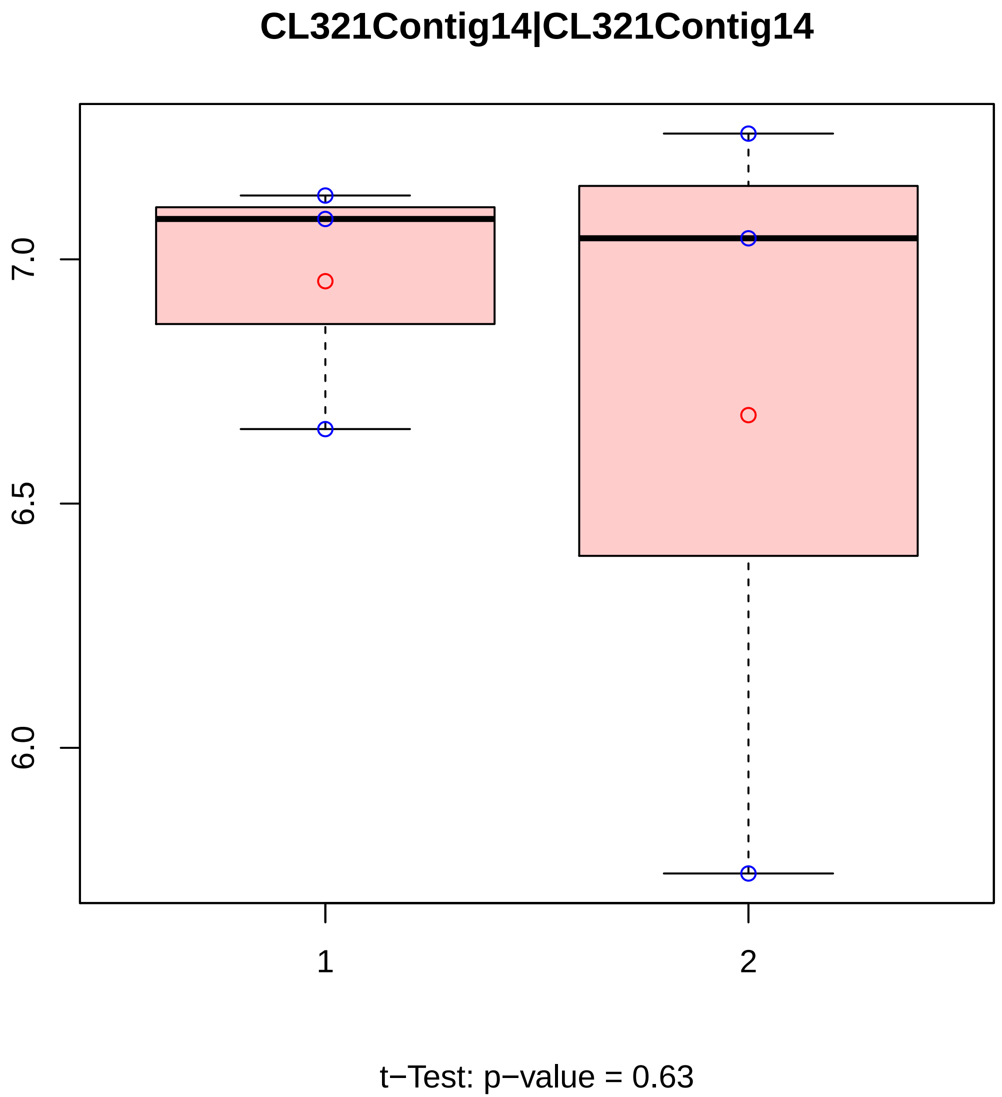

CL321Contig14|CL321Contig14
7.0
6.5
6.0
1
2
t−Test: p−value = 0.63

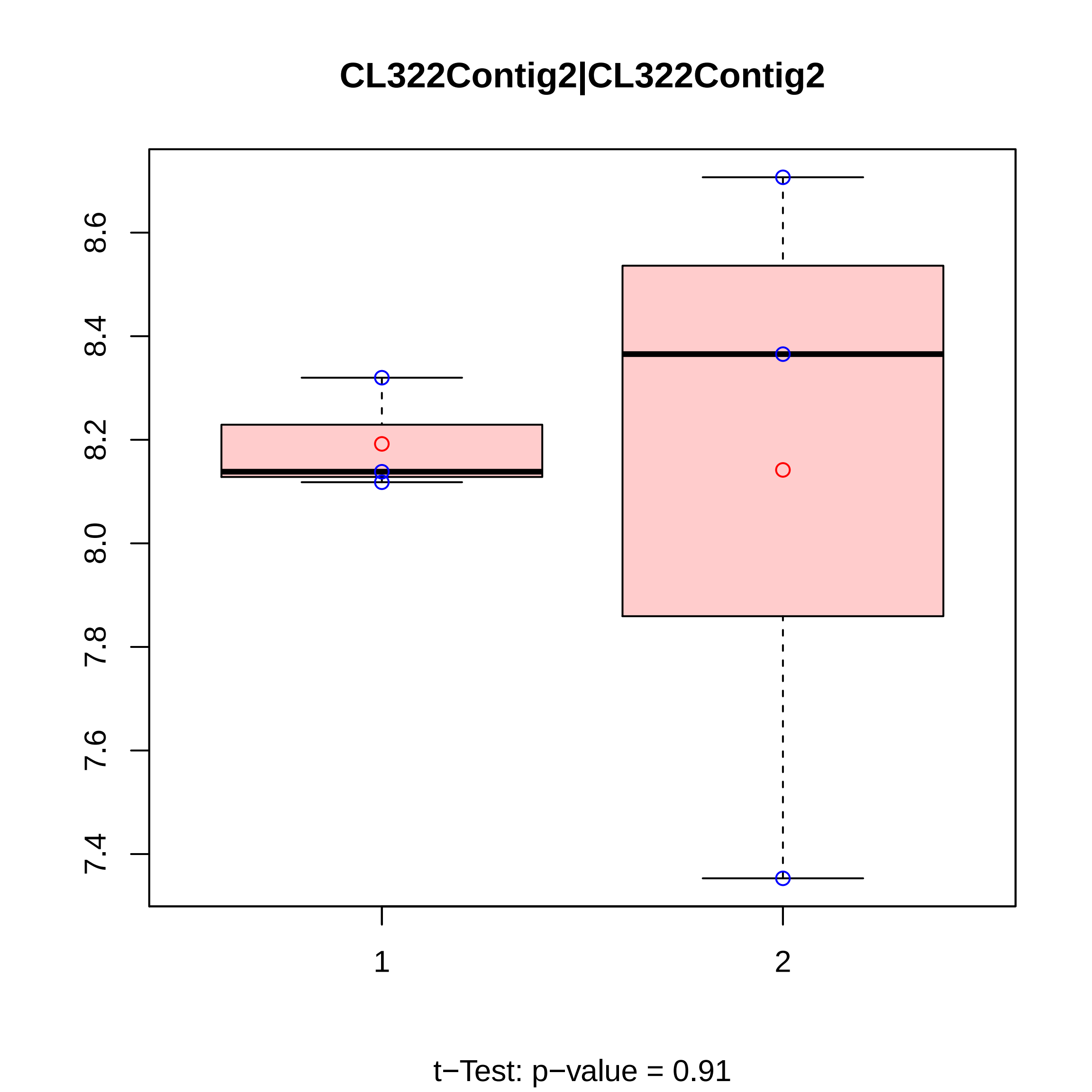

CL322Contig2|CL322Contig2
8.6
8.4
8.2
8.0
7.8
7.6
7.4
1
2
t−Test: p−value = 0.91

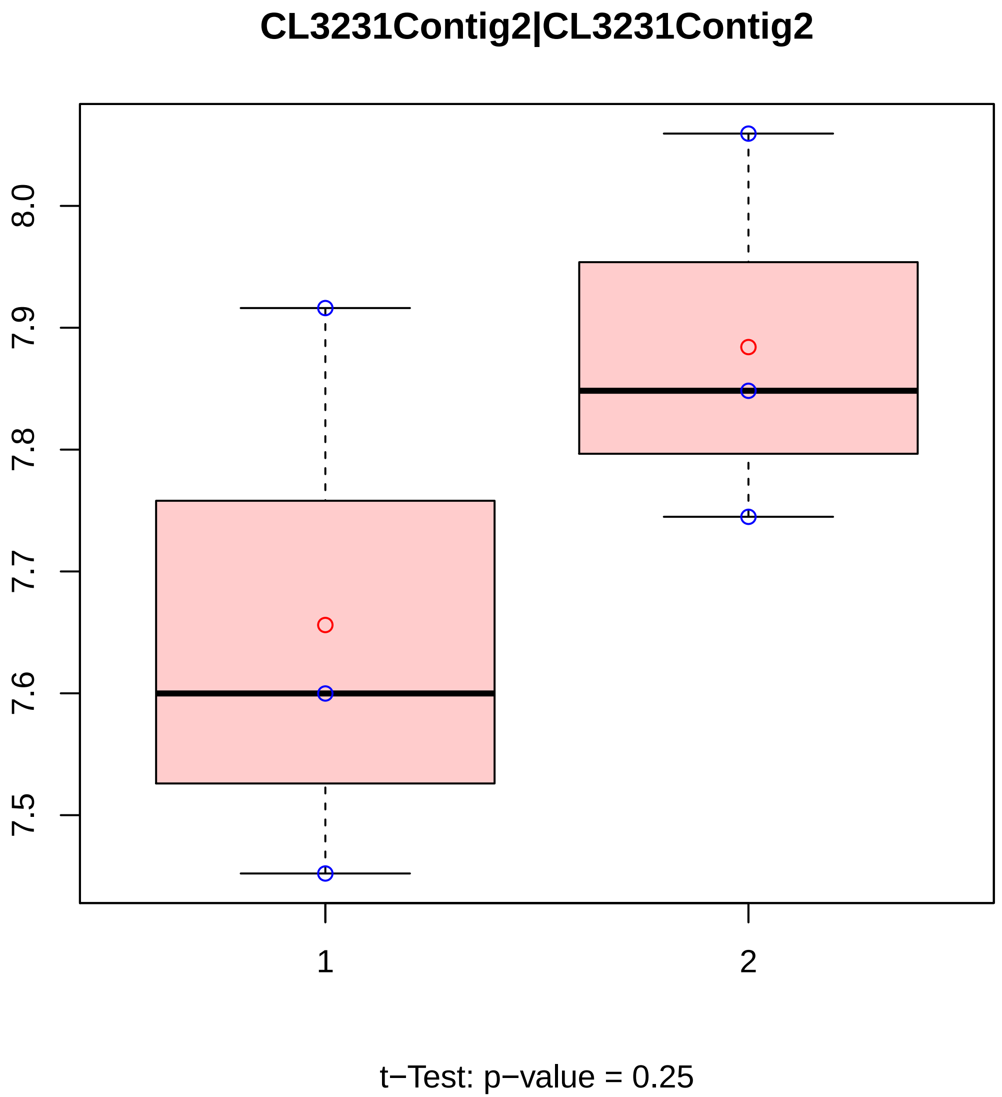

CL3231Contig2|CL3231Contig2
8.0
7.9
7.8
7.7
7.6
7.5
1
2
t−Test: p−value = 0.25

# CL3239Contig2|CL3239Contig2

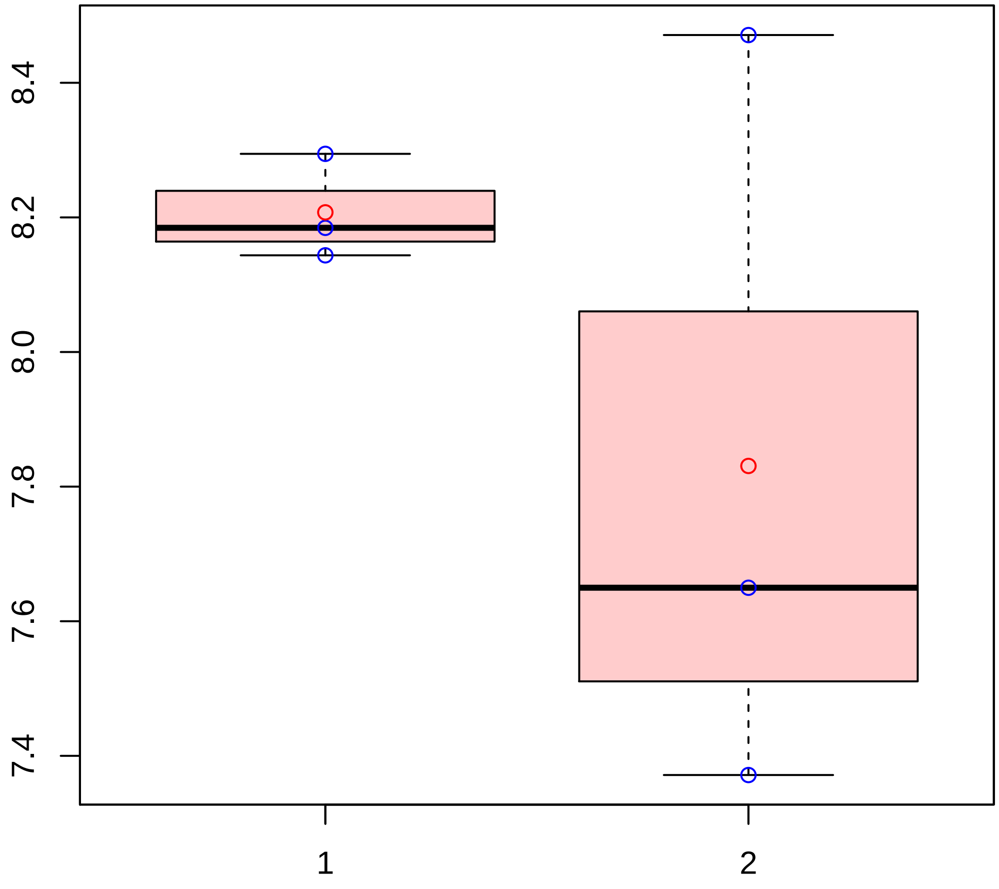


t−Test: p−value = 0.37

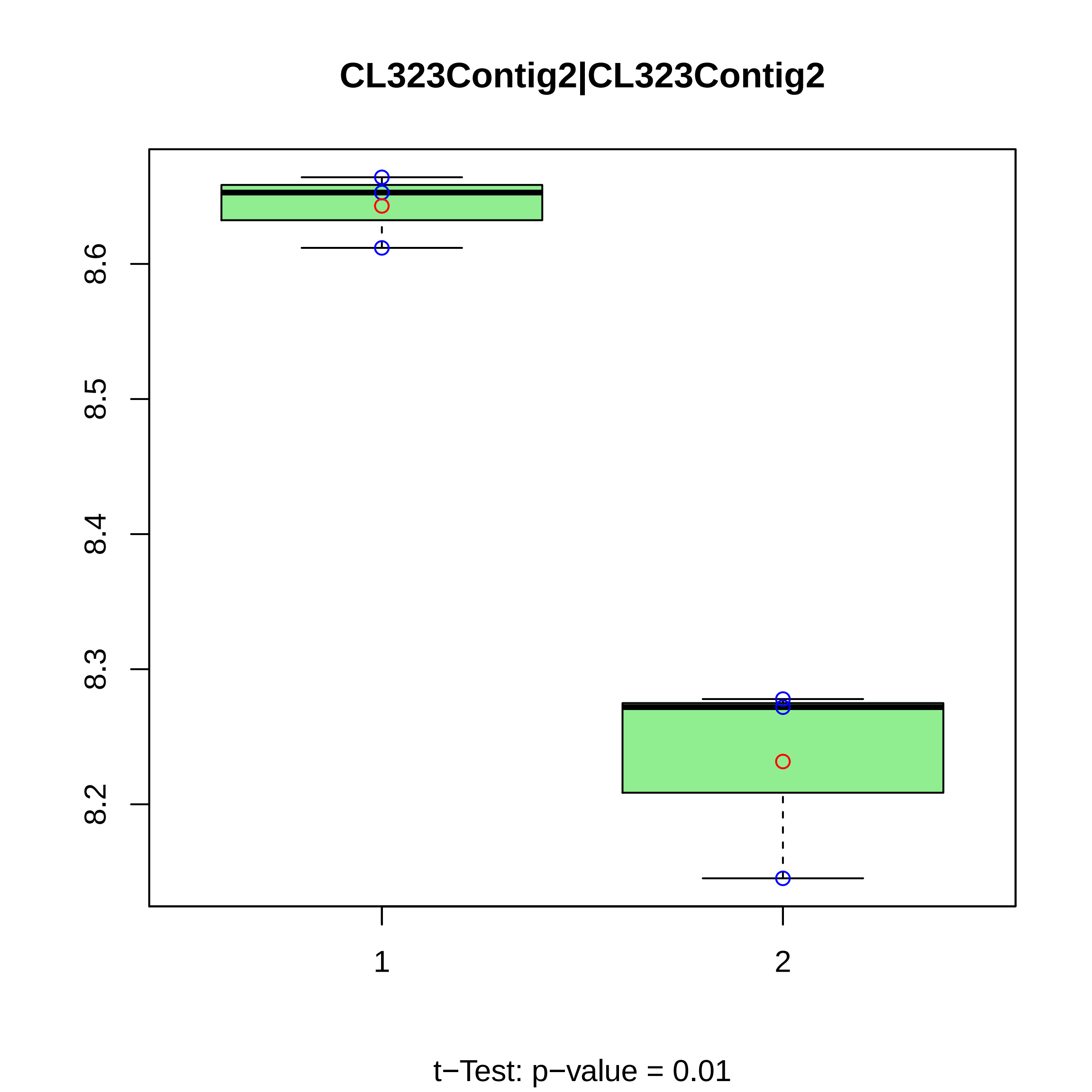
CL323Contig2|CL323Contig2
8.2
8.3
8.4
8.5
8.6
1
2
t−Test: p−value = 0.01

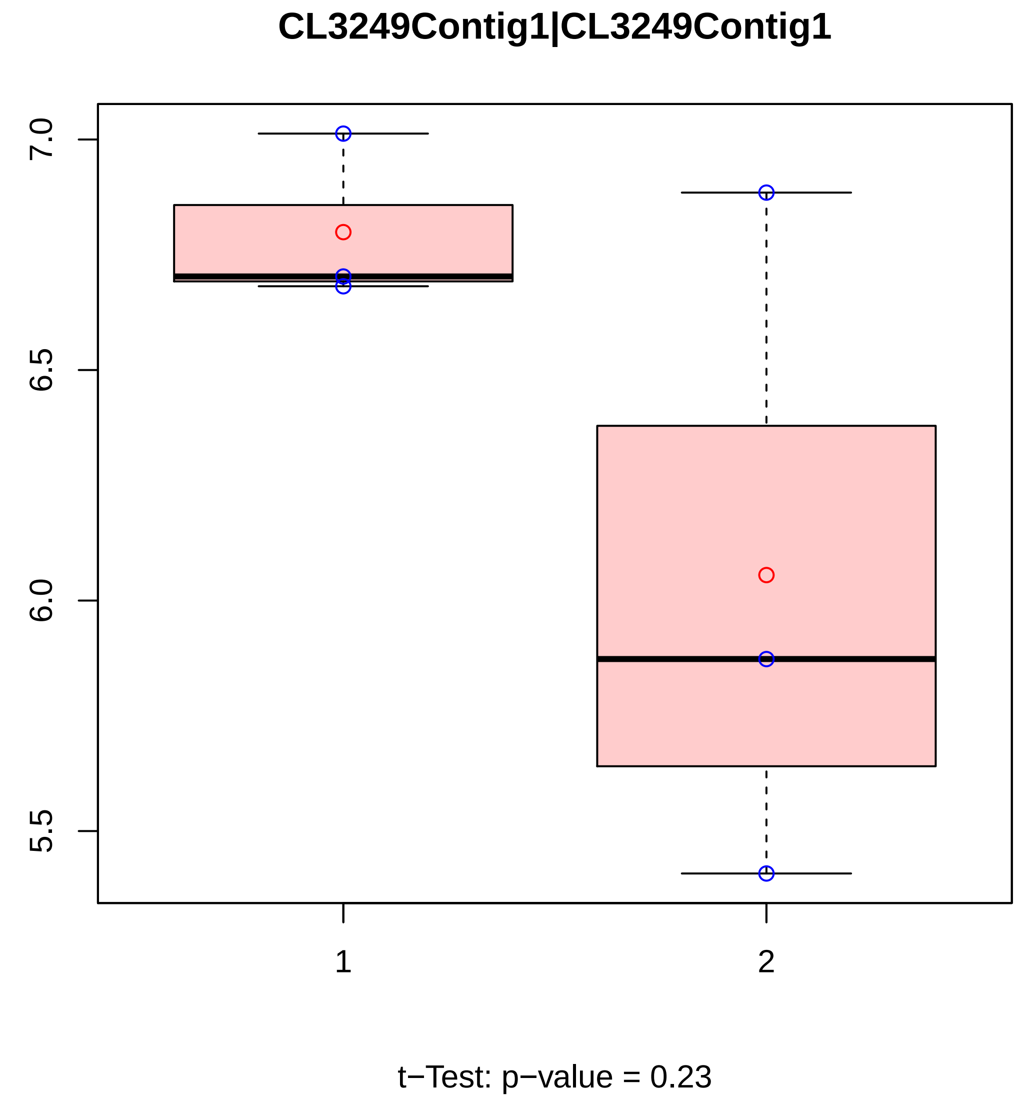

CL3249Contig1|CL3249Contig1
7.0
6.5
6.0
5.5
1
2
t−Test: p−value = 0.23

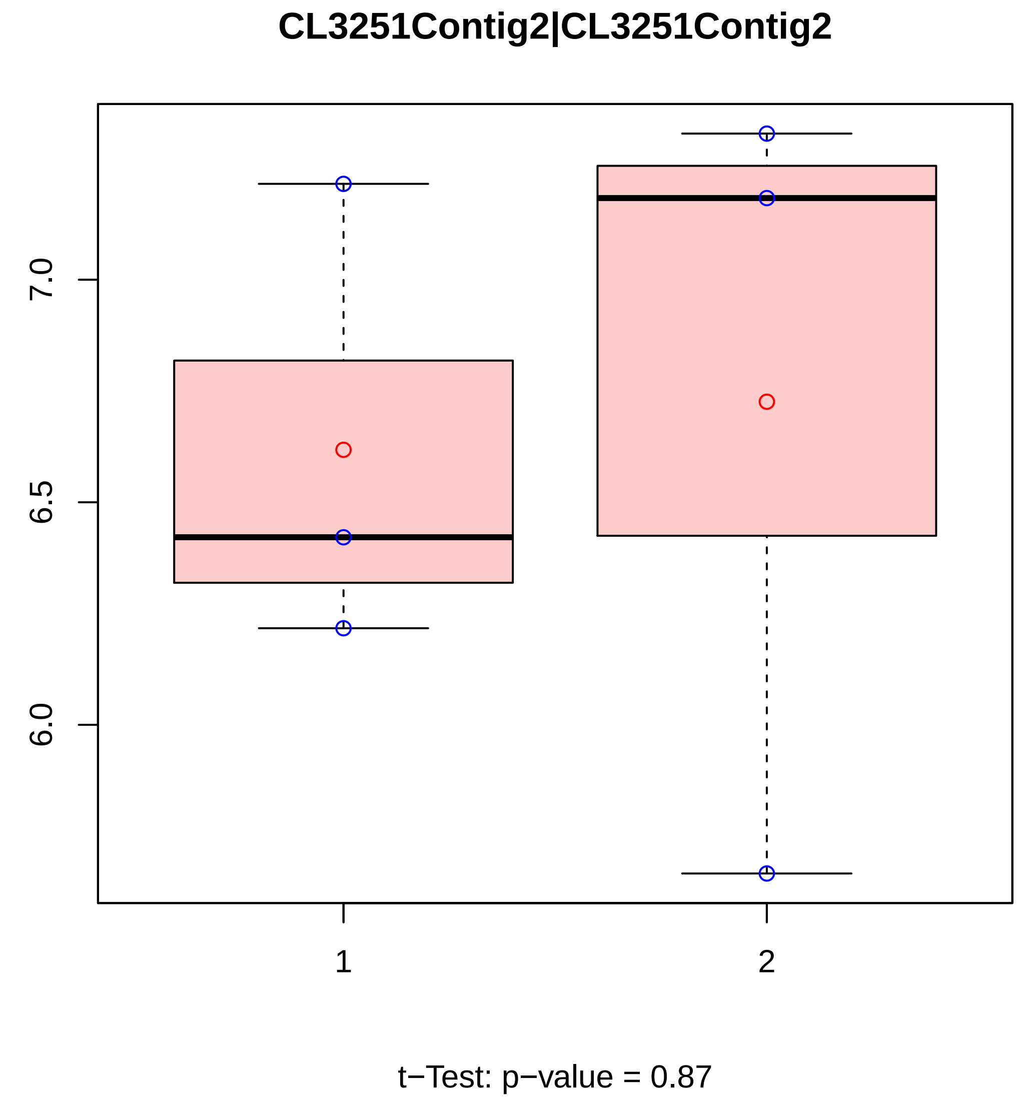

CL3251Contig2|CL3251Contig2
7.0
6.5
6.0
1
2
t−Test: p−value = 0.87

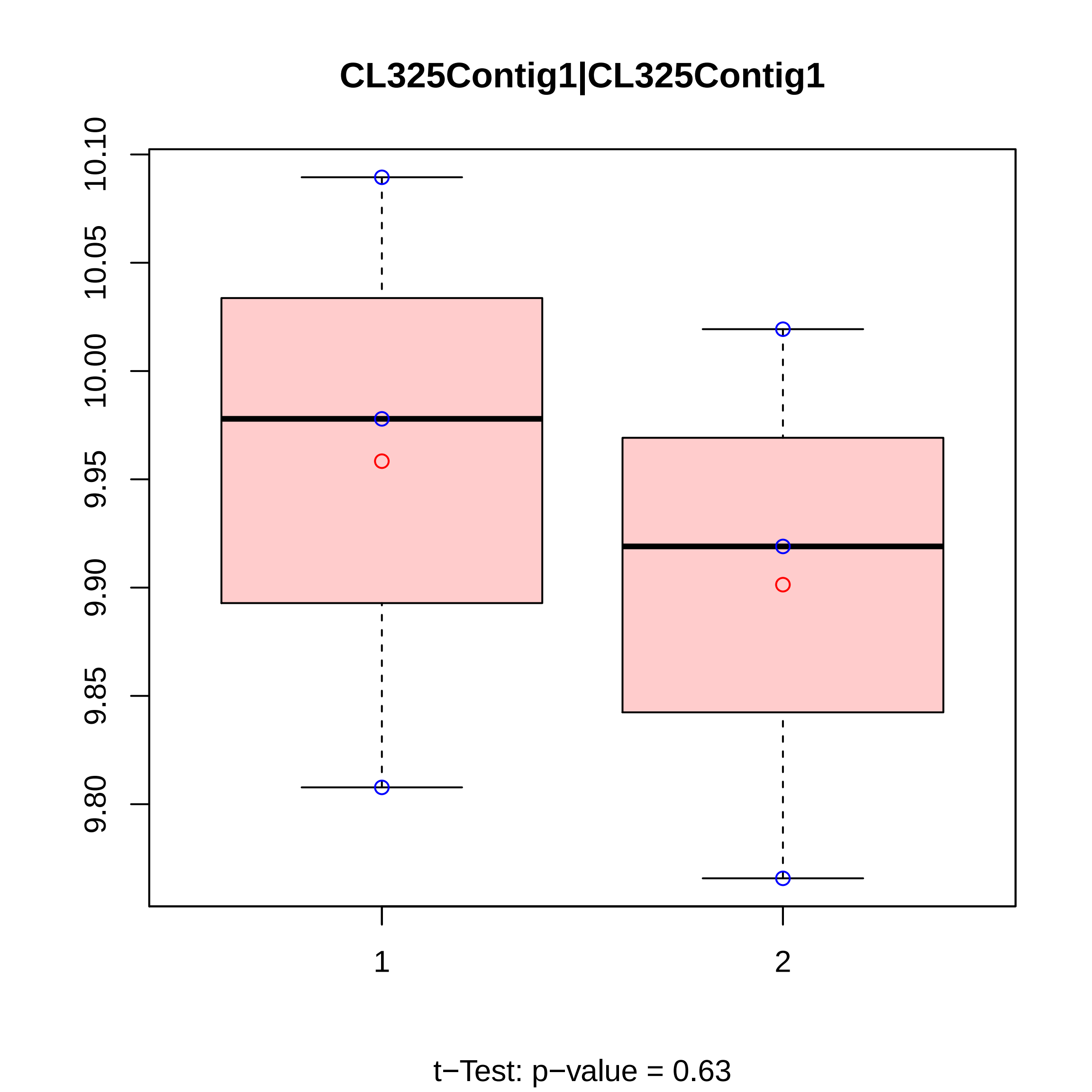
CL325Contig1|CL325Contig1
10.10
10.05
10.00
9.95
9.90
9.85
9.80
1
2
t−Test: p−value = 0.63

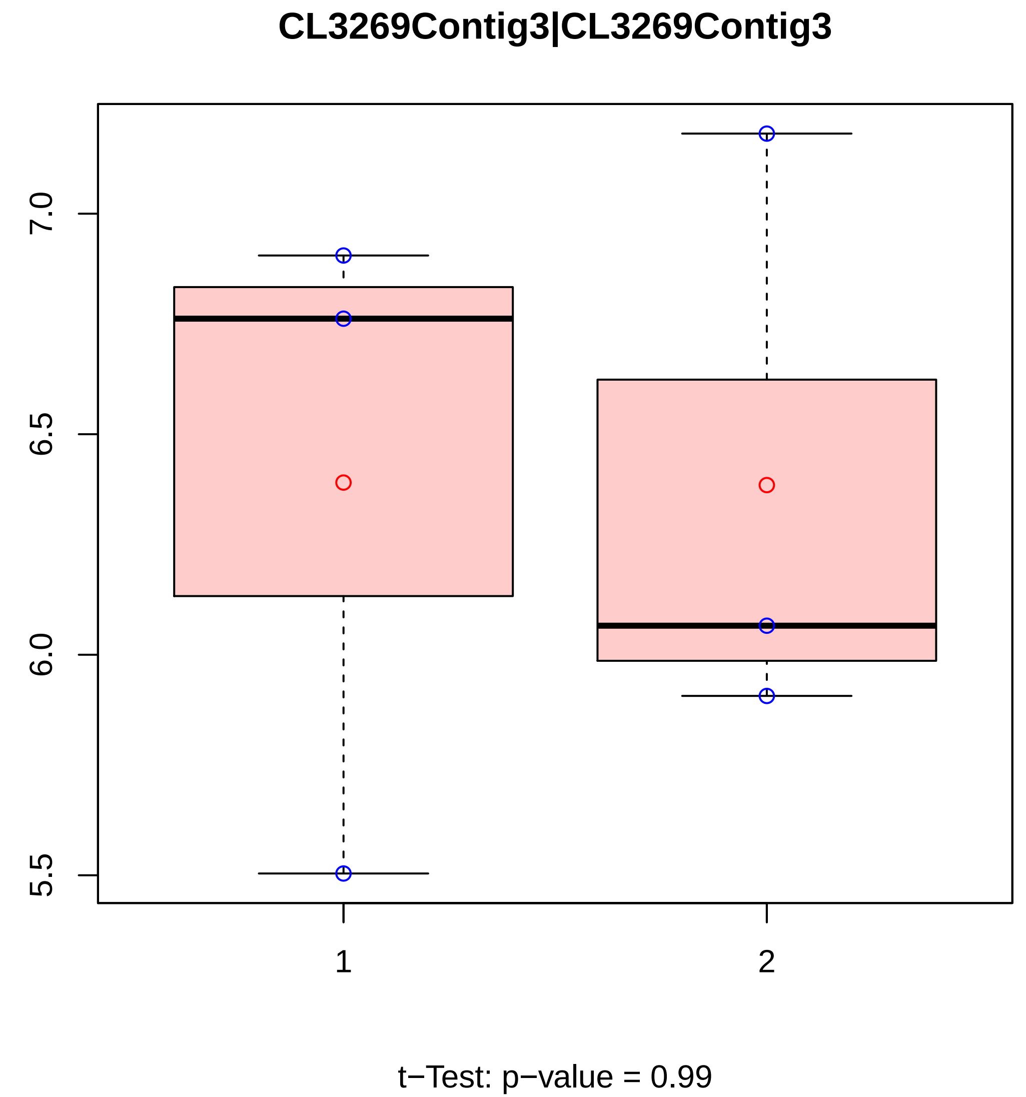

CL3269Contig3|CL3269Contig3
7.0
6.5
6.0
5.5
1
2
t−Test: p−value = 0.99

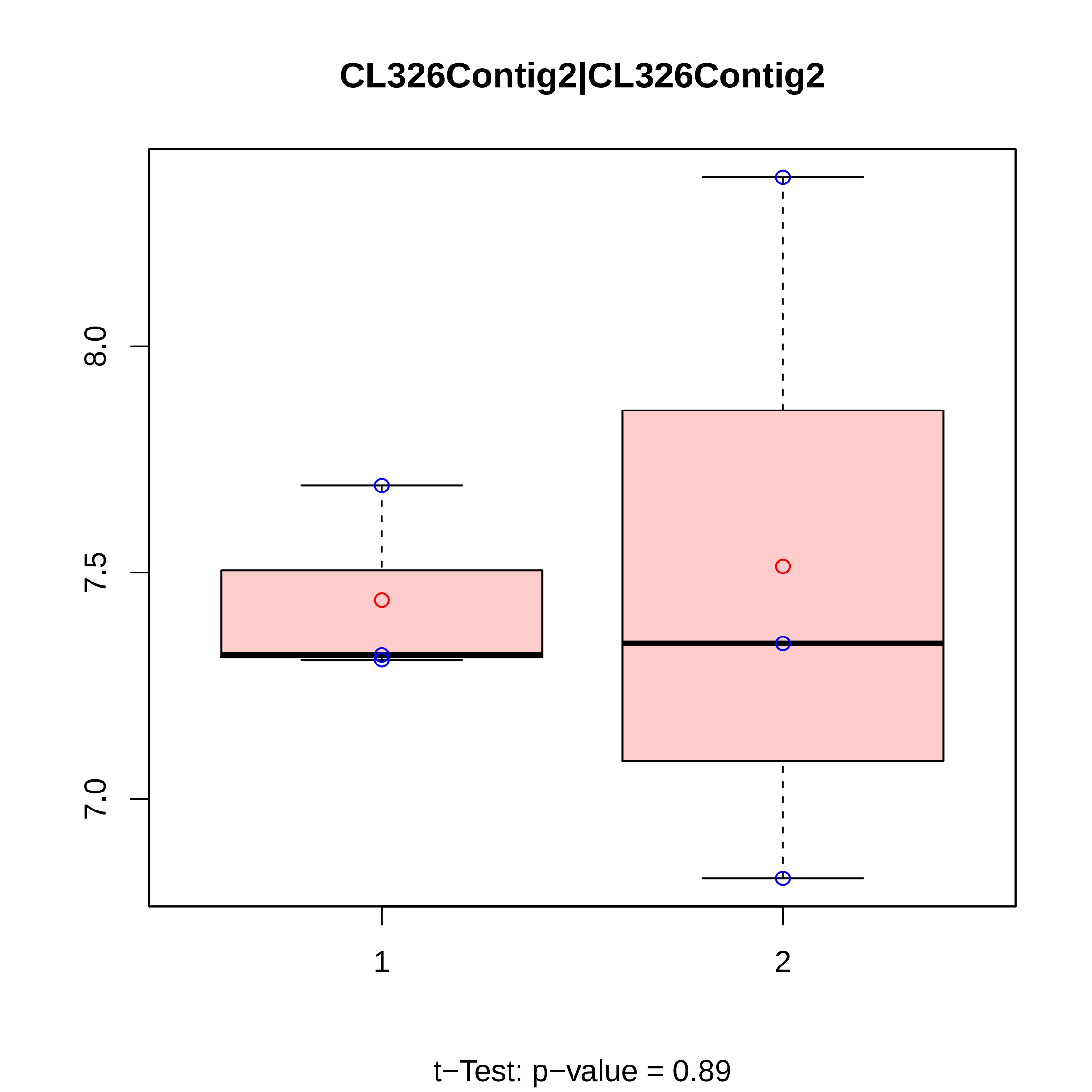
CL326Contig2|CL326Contig2
8.0
7.5
7.0
1
2
t−Test: p−value = 0.89

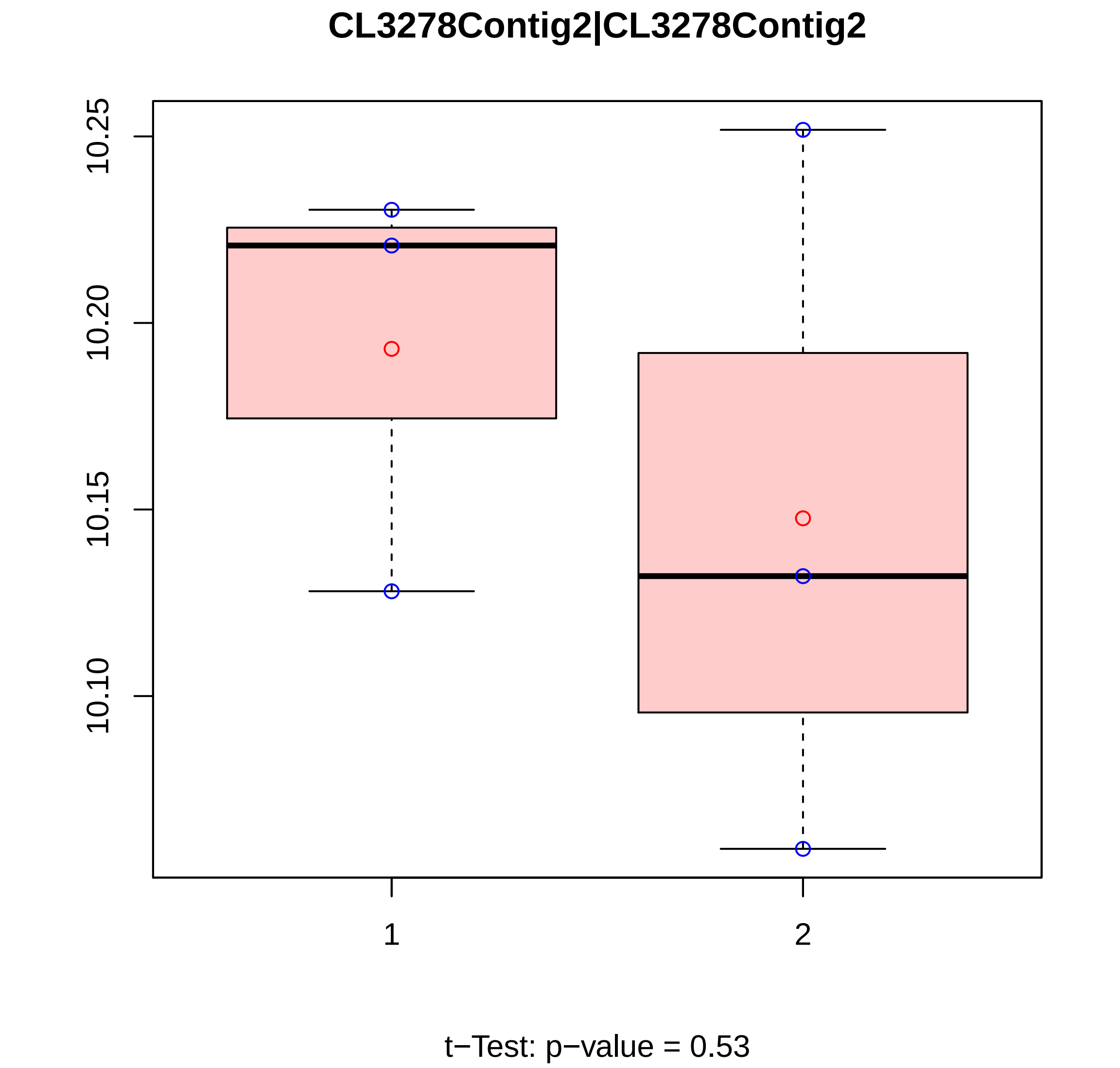

CL3278Contig2|CL3278Contig2
10.25
10.20
10.15
10.10
1
2
t−Test: p−value = 0.53

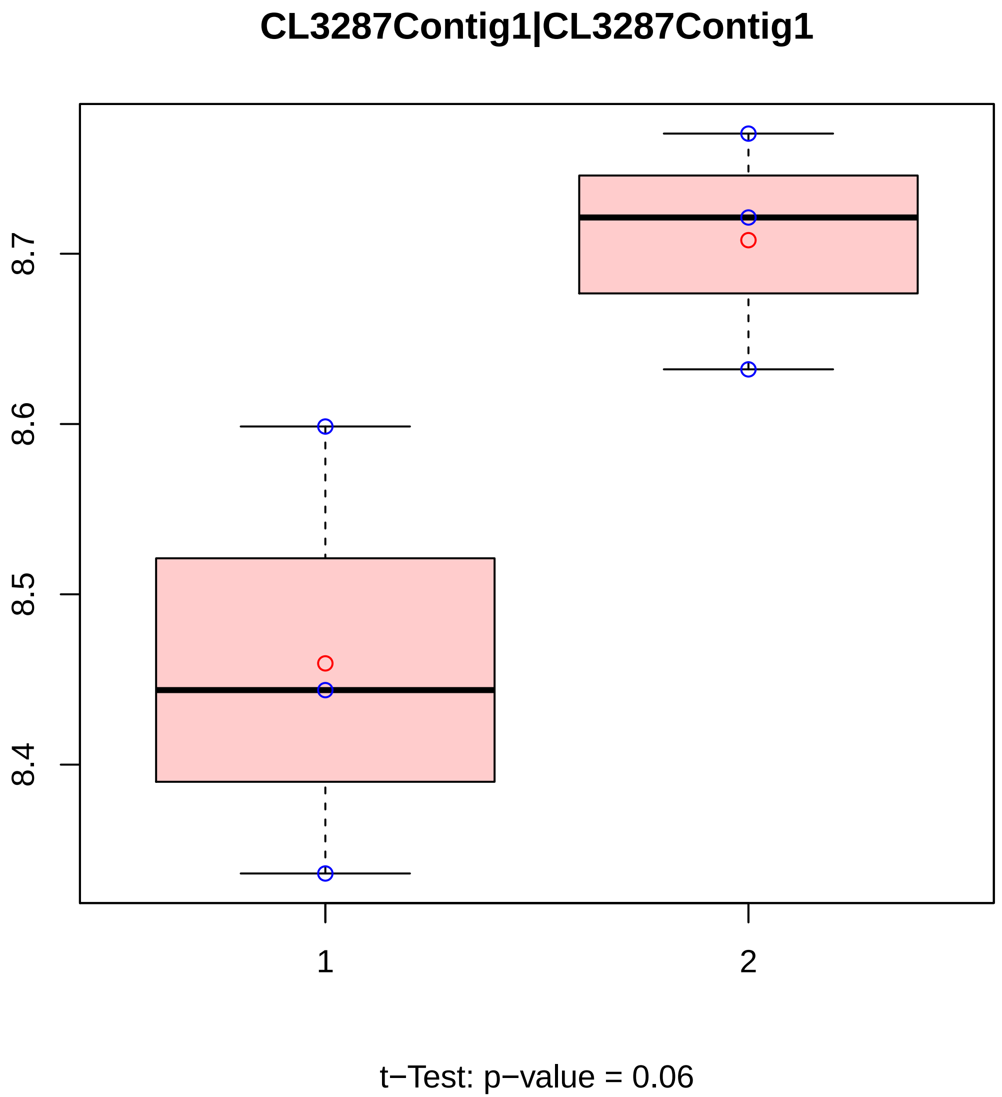

CL3287Contig1|CL3287Contig1
8.4
8.5
8.6
8.7
1
2
t−Test: p−value = 0.06

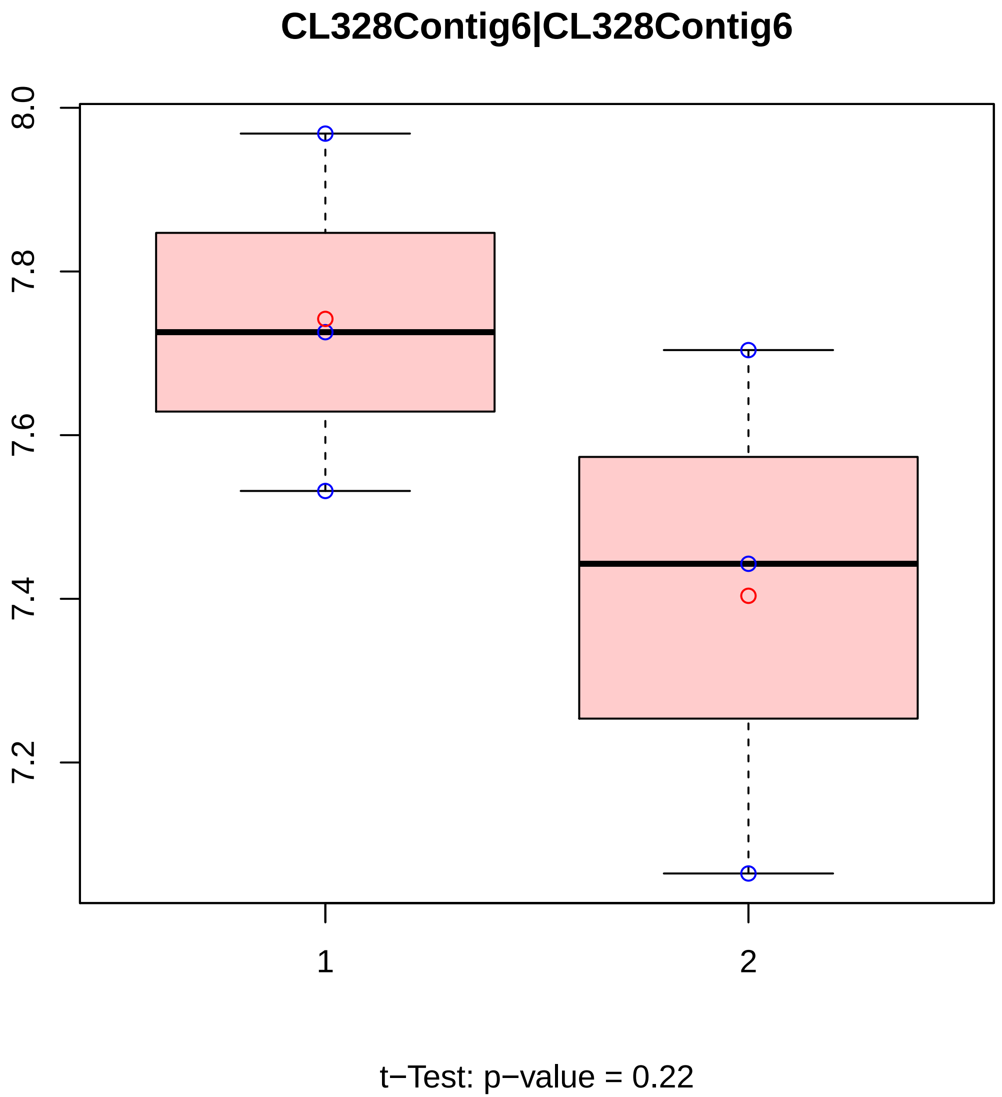
CL328Contig6|CL328Contig6
8.0
7.8
7.6
7.4
7.2
1
2
t−Test: p−value = 0.22

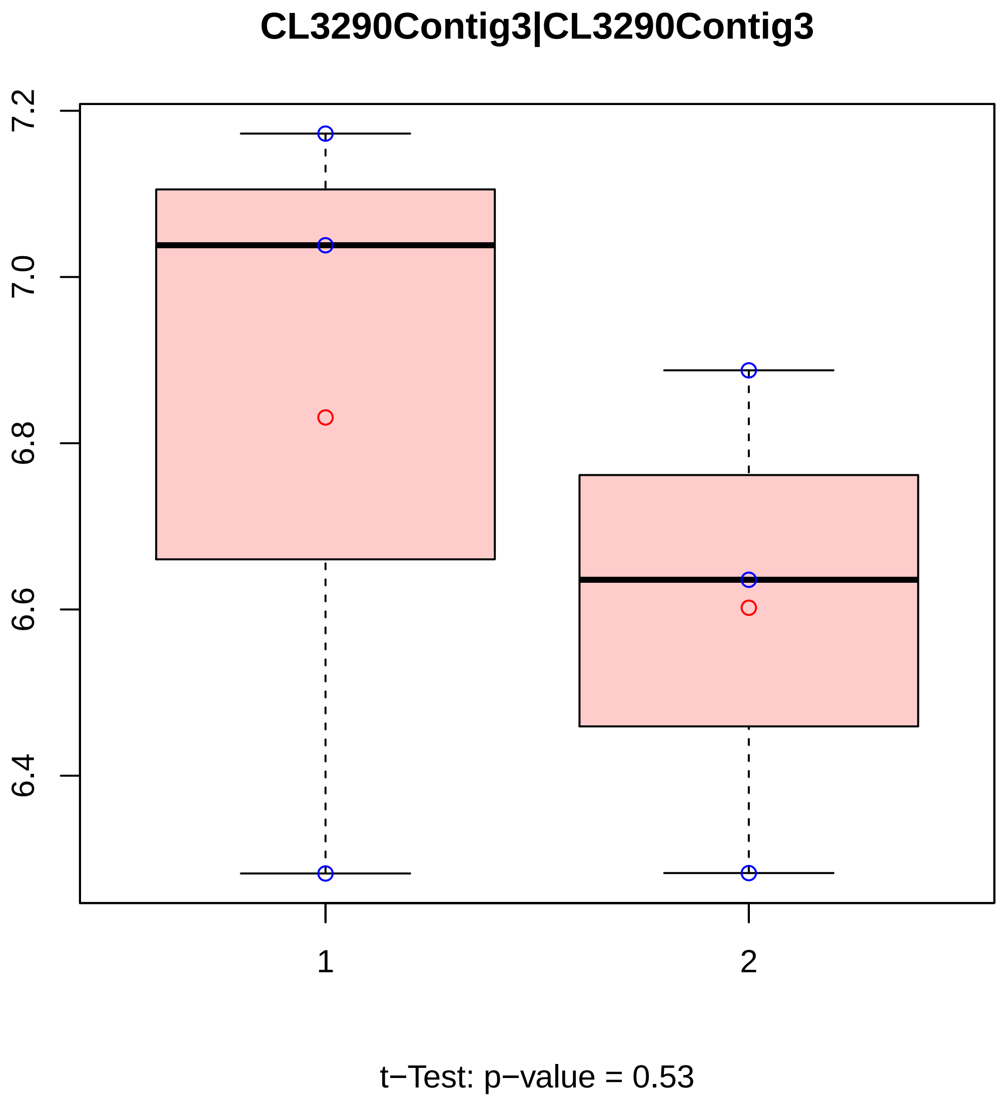
CL3290Contig3|CL3290Contig3
7.2
7.0
6.8
6.6
6.4
1
2
t−Test: p−value = 0.53

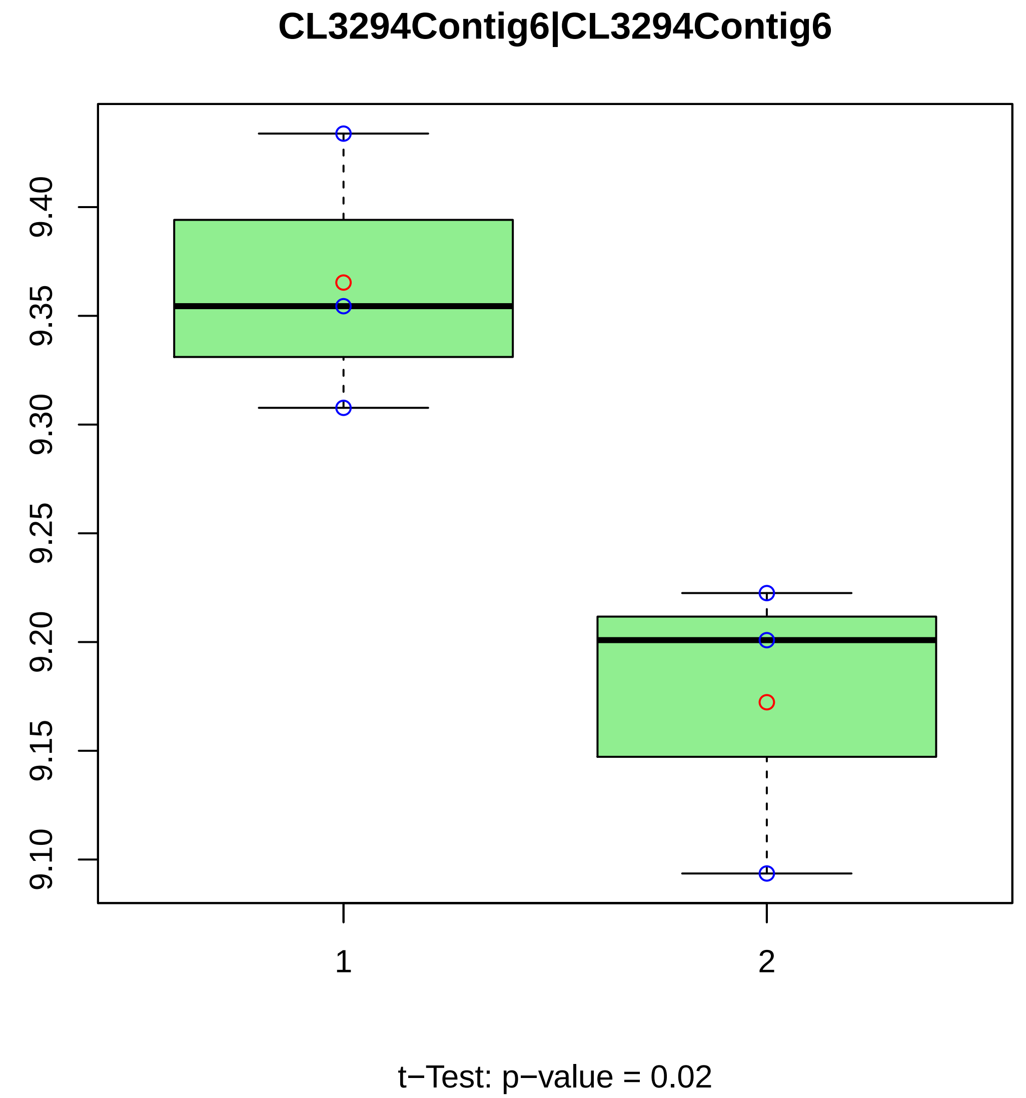
CL3294Contig6|CL3294Contig6
9.10
9.15
9.20
9.25
9.30
9.35
9.40
1
2
t−Test: p−value = 0.02

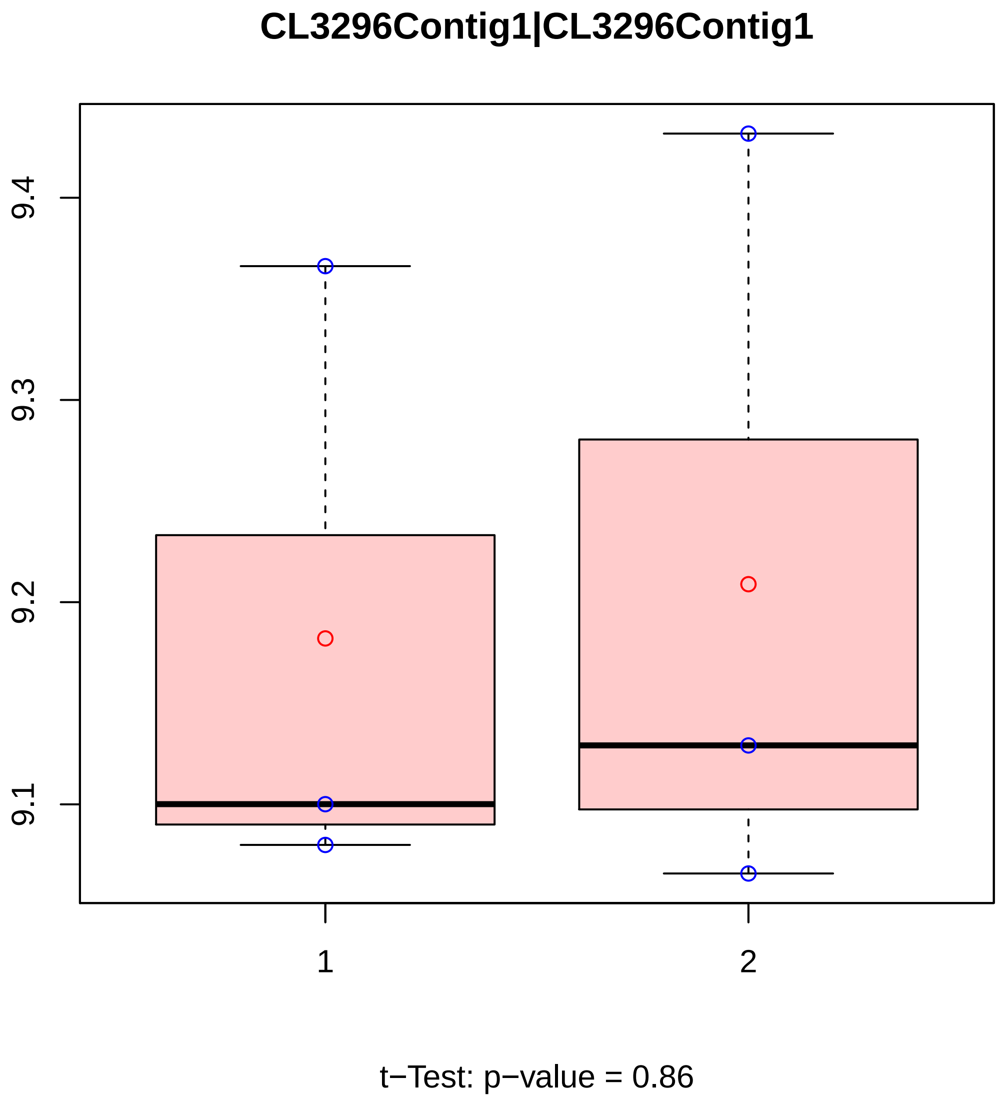
CL3296Contig1|CL3296Contig1
9.1
9.2
9.3
9.4
1
2
t−Test: p−value = 0.86

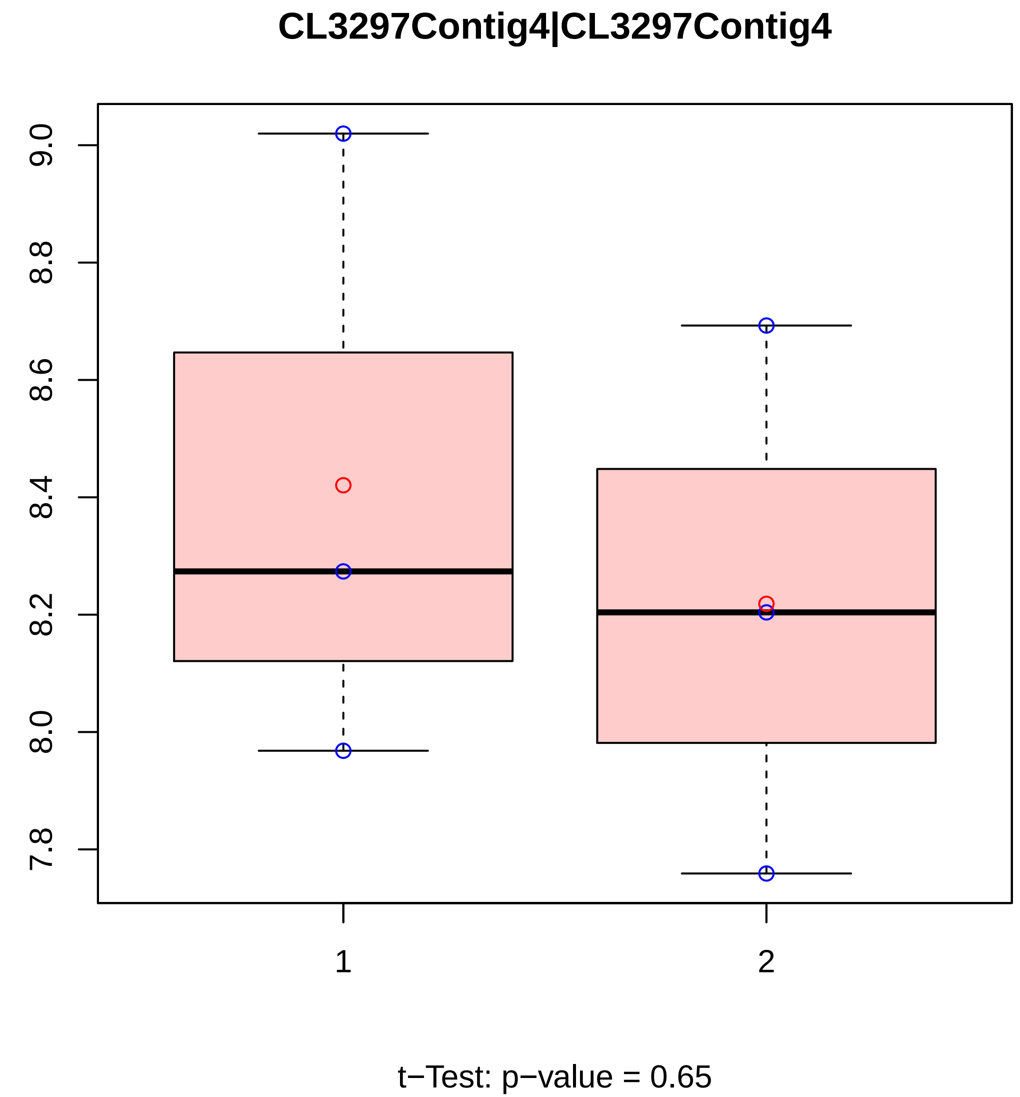
CL3297Contig4|CL3297Contig4
9.0
8.8
8.6
8.4
8.2
8.0
7.8
1
2
t−Test: p−value = 0.65

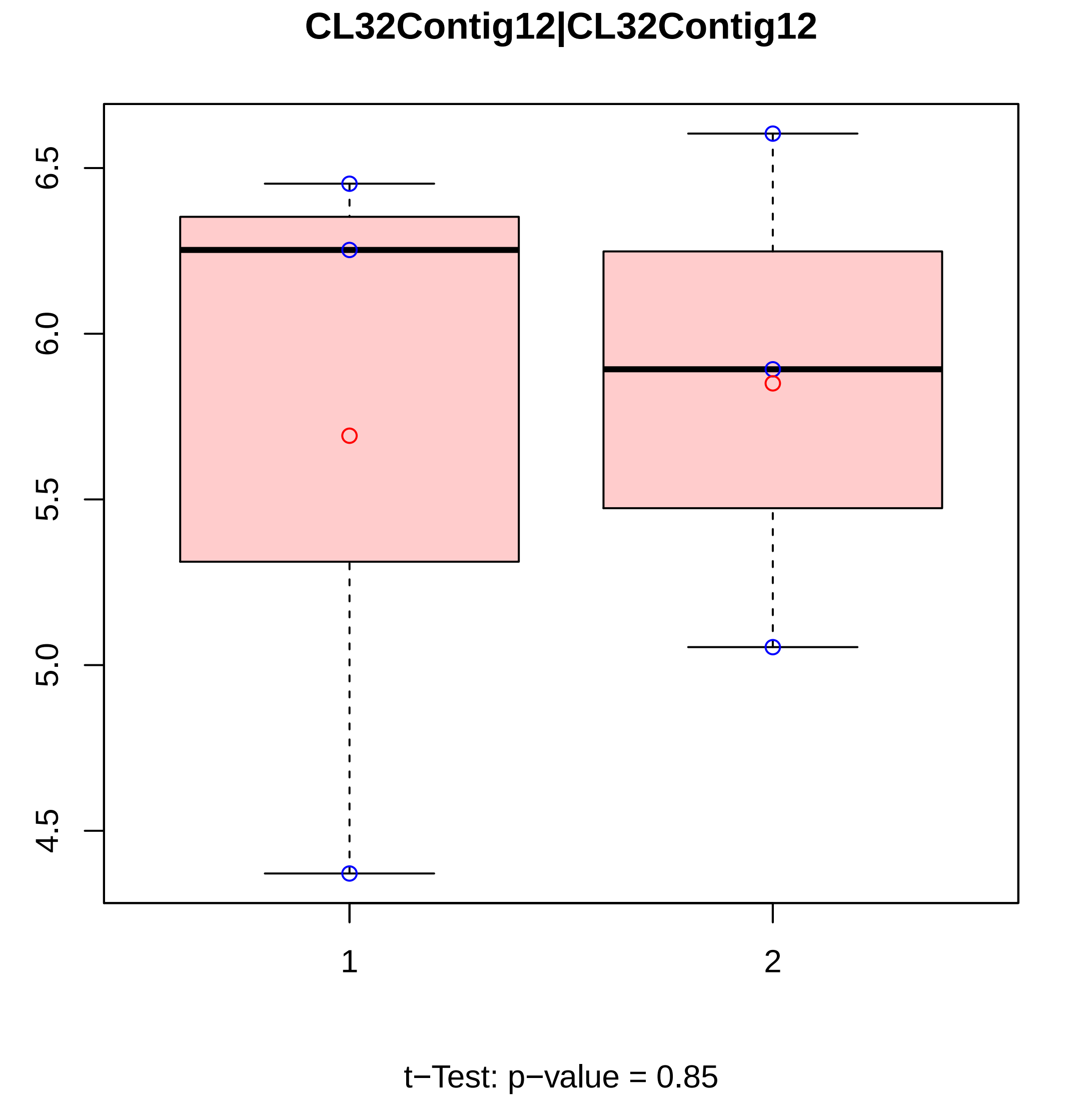

CL32Contig12|CL32Contig12
6.5
6.0
5.5
5.0
4.5
1
2
t−Test: p−value = 0.85

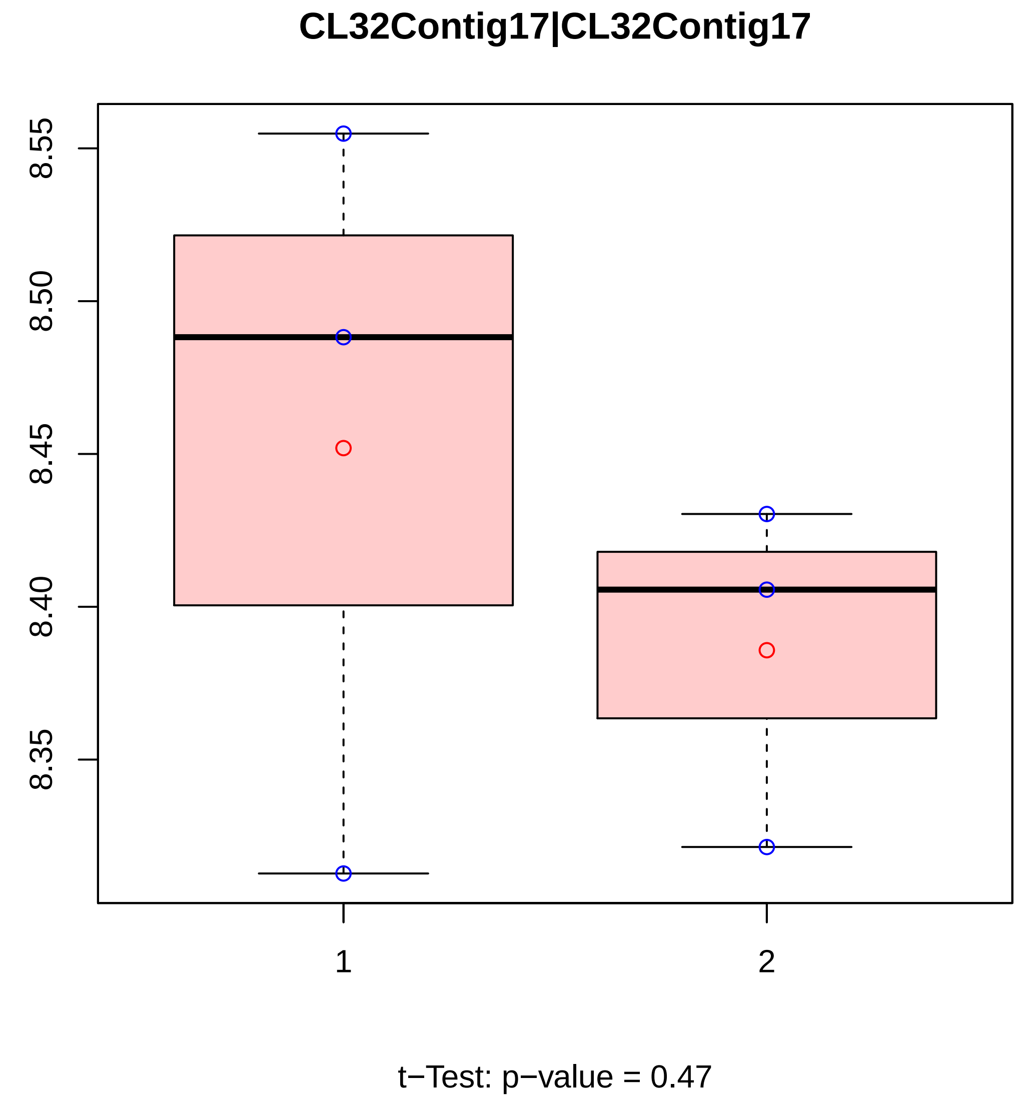
CL32Contig17|CL32Contig17
8.55
8.50
8.45
8.40
8.35
1
2
t−Test: p−value = 0.47

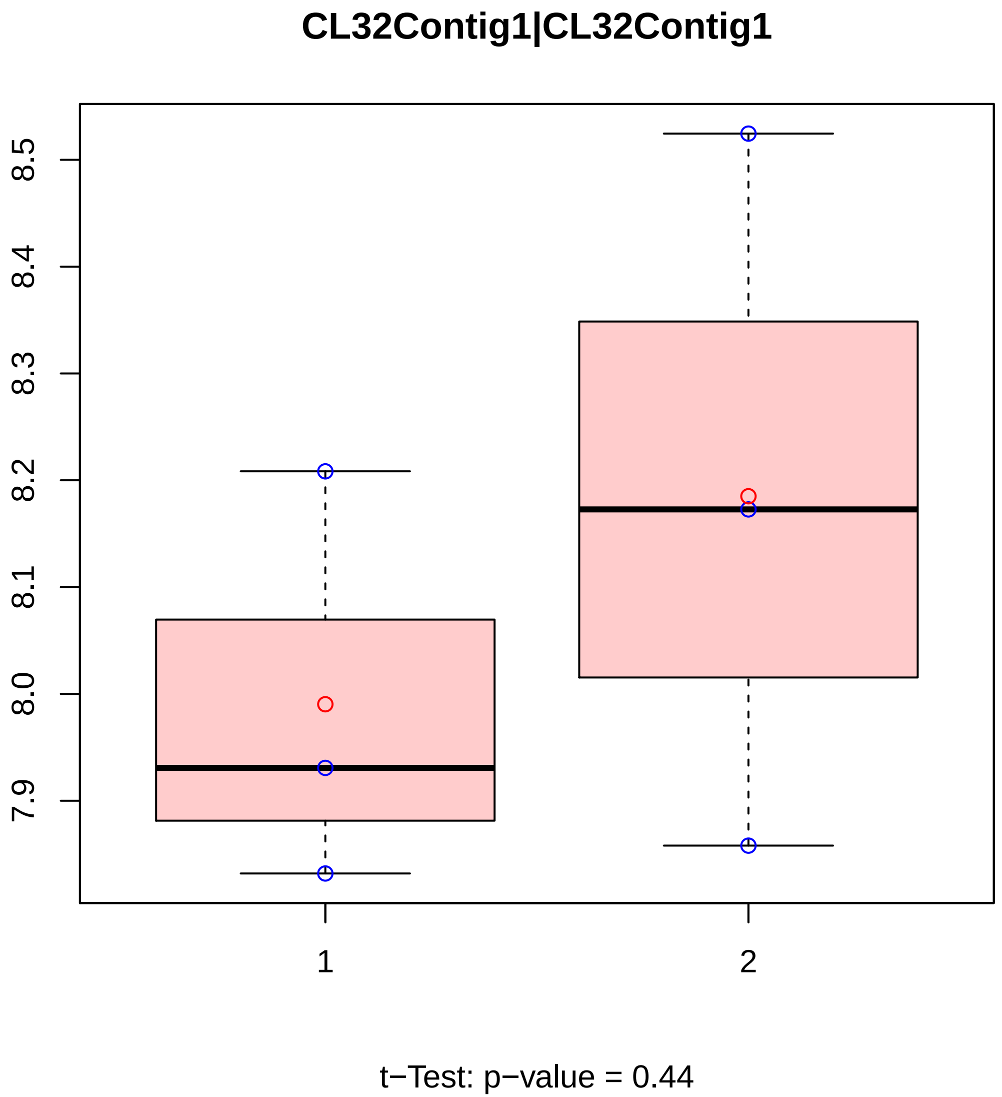
CL32Contig1|CL32Contig1
8.5
8.4
8.3
8.2
8.1
8.0
7.9
1
2
t−Test: p−value = 0.44

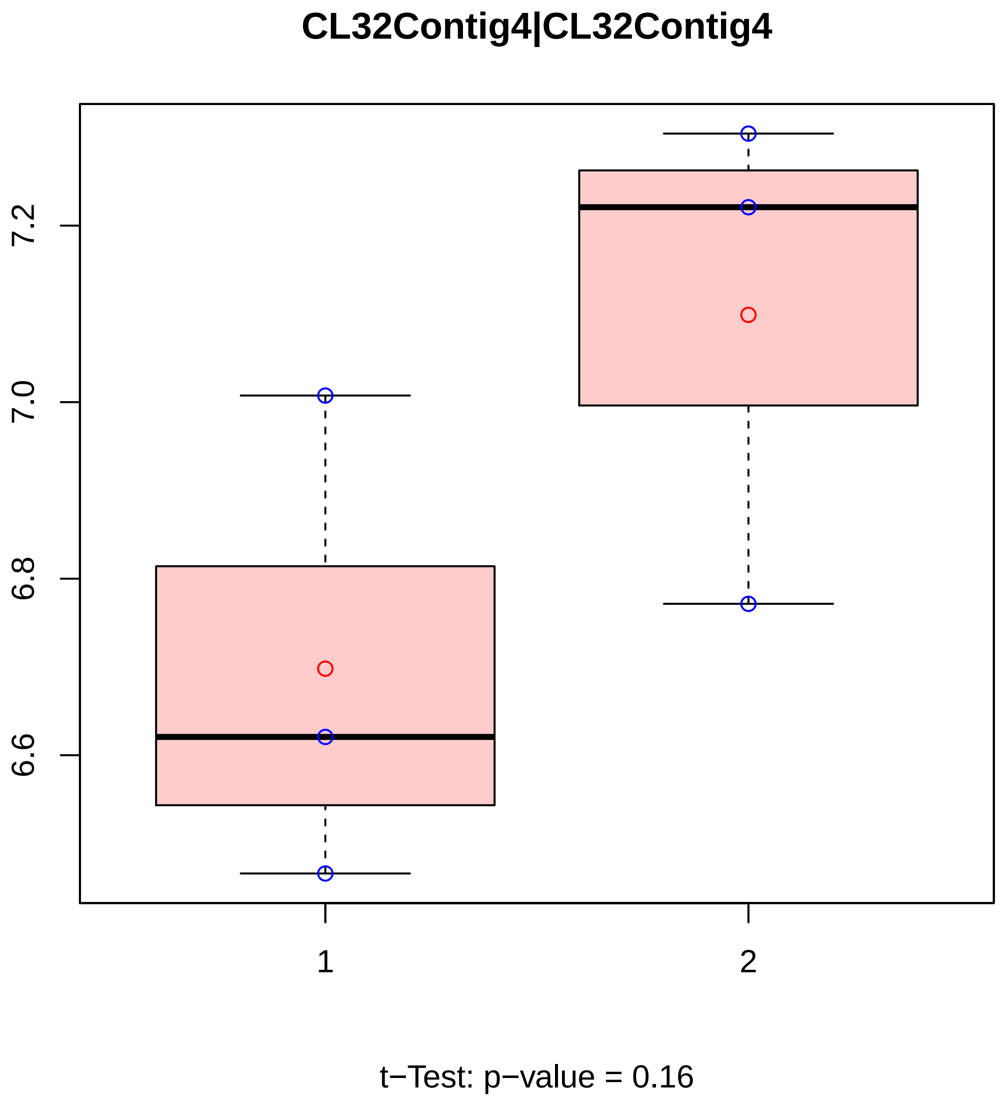

CL32Contig4|CL32Contig4
7.2
7.0
6.8
6.6
1
2
t−Test: p−value = 0.16

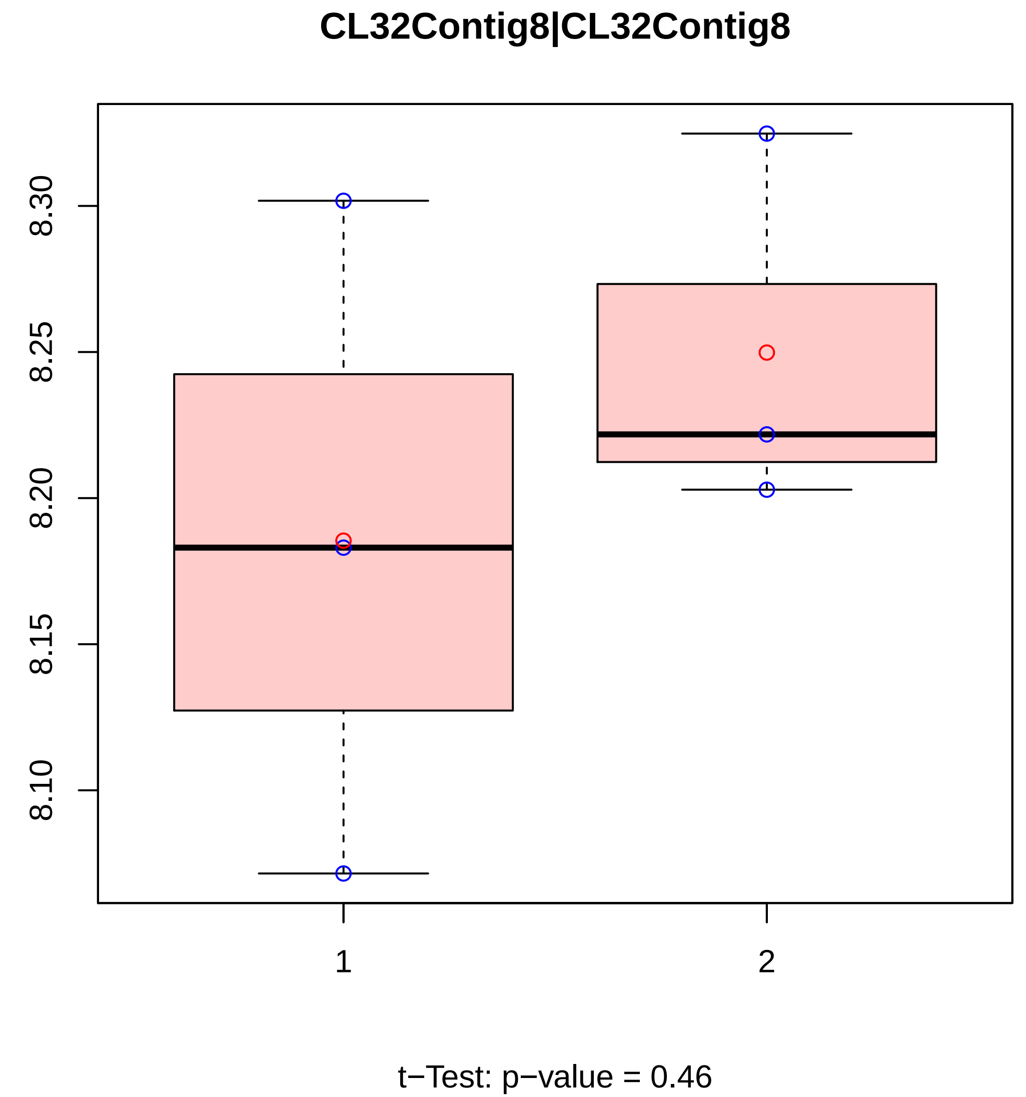
CL32Contig8|CL32Contig8
8.10
8.15
8.20
8.25
8.30
1
2
t−Test: p−value = 0.46

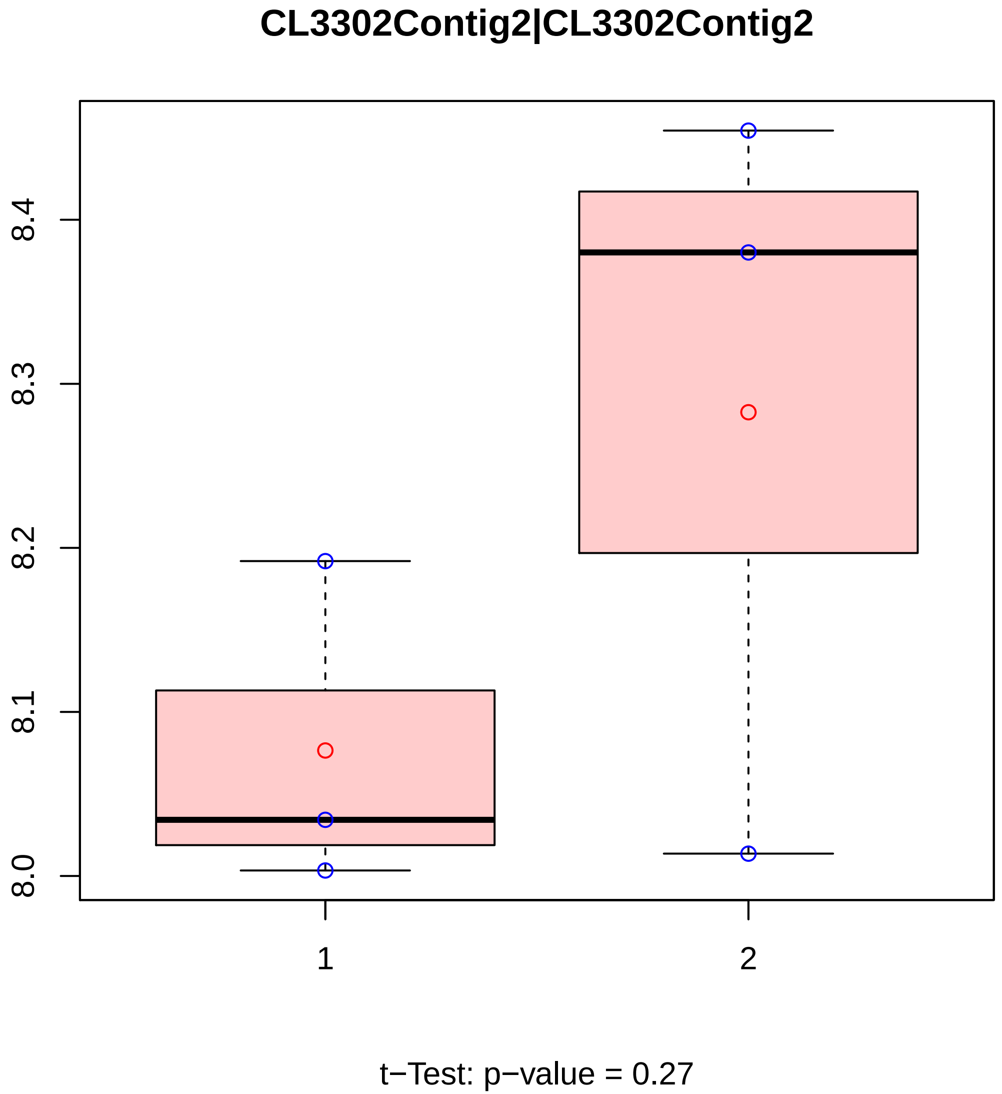

CL3302Contig2|CL3302Contig2
8.0
8.1
8.2
8.3
8.4
1
2
t−Test: p−value = 0.27

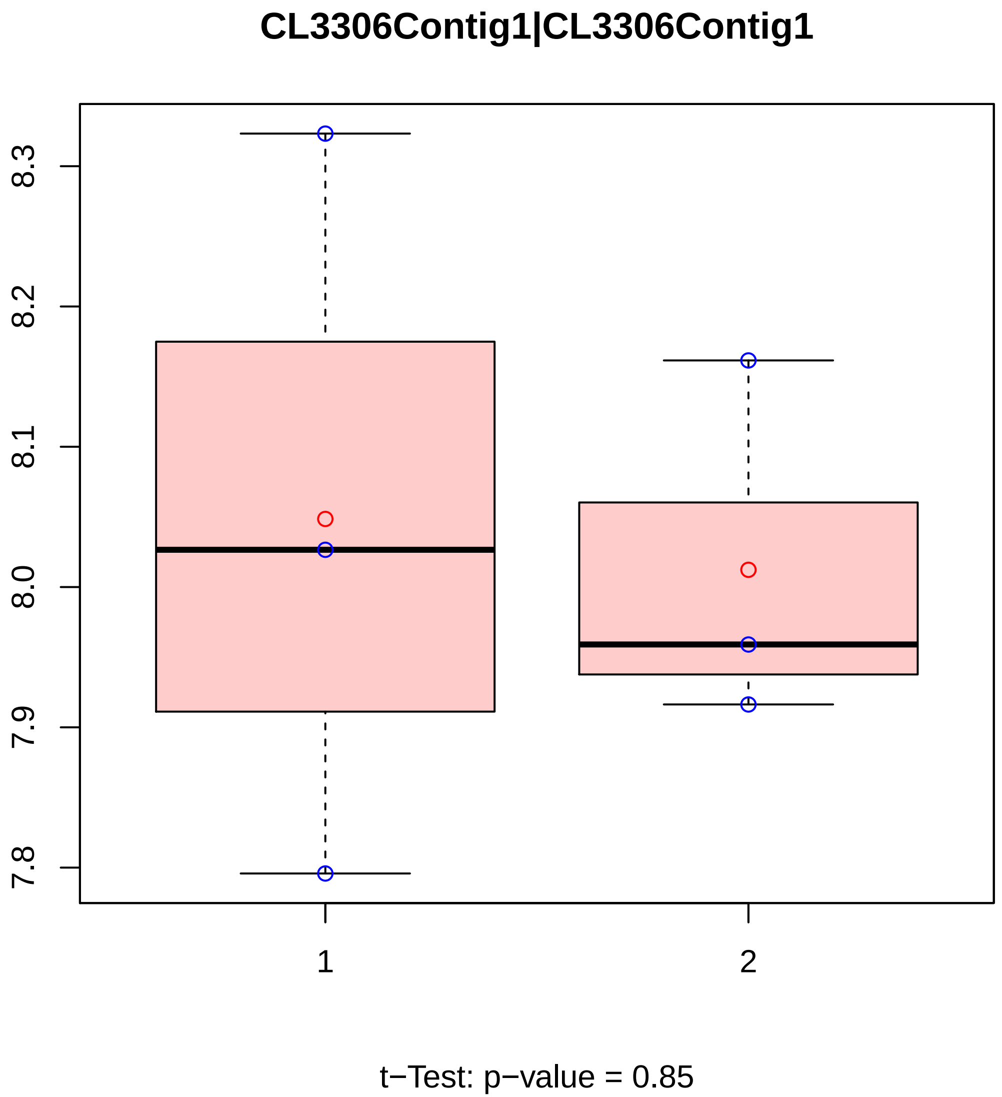

CL3306Contig1|CL3306Contig1
8.3
8.2
8.1
8.0
7.9
7.8
1
2
t−Test: p−value = 0.85

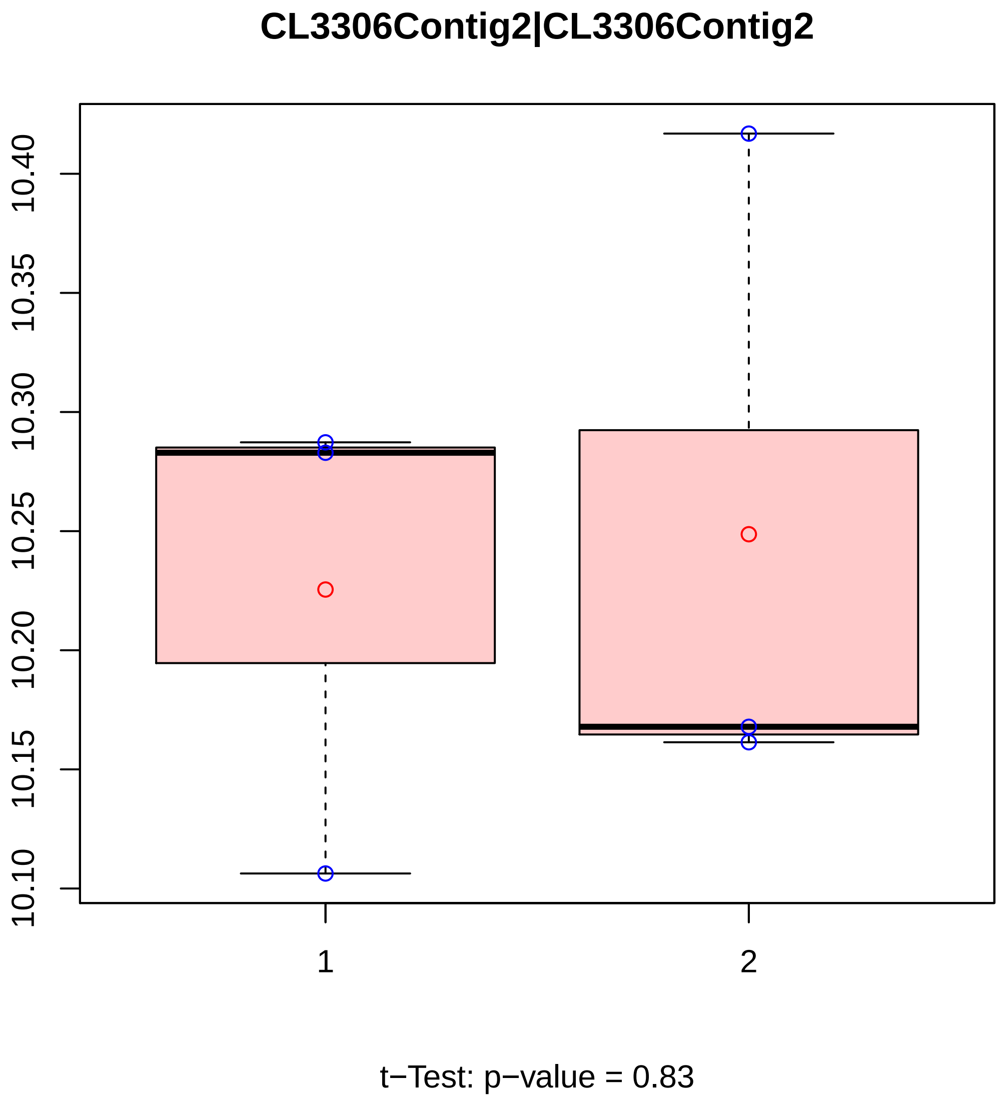

CL3306Contig2|CL3306Contig2
10.10
10.15
10.20
10.25
10.30
10.35
10.40
1
2
t−Test: p−value = 0.83

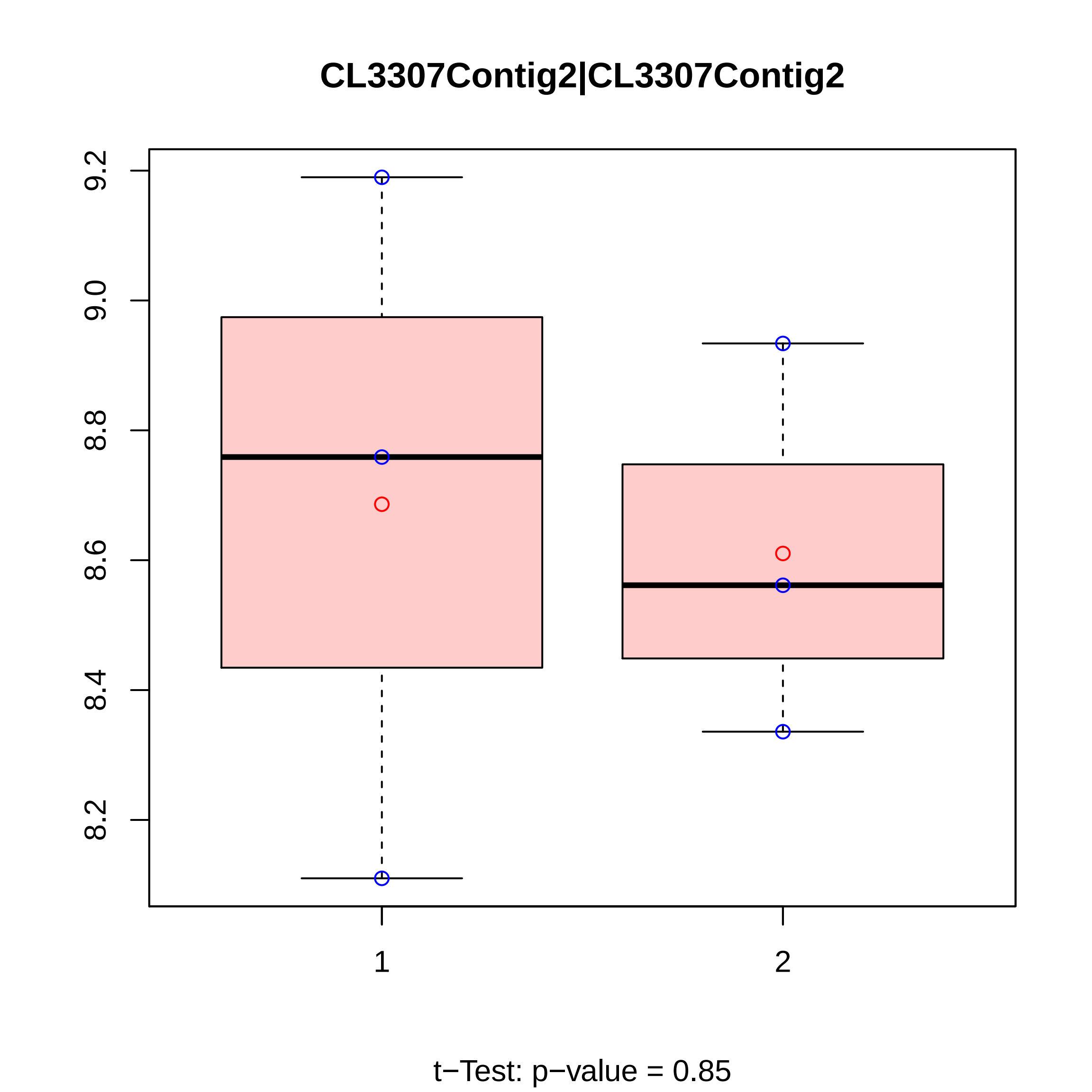
CL3307Contig2|CL3307Contig2
9.2
9.0
8.8
8.6
8.4
8.2
1
2
t−Test: p−value = 0.85

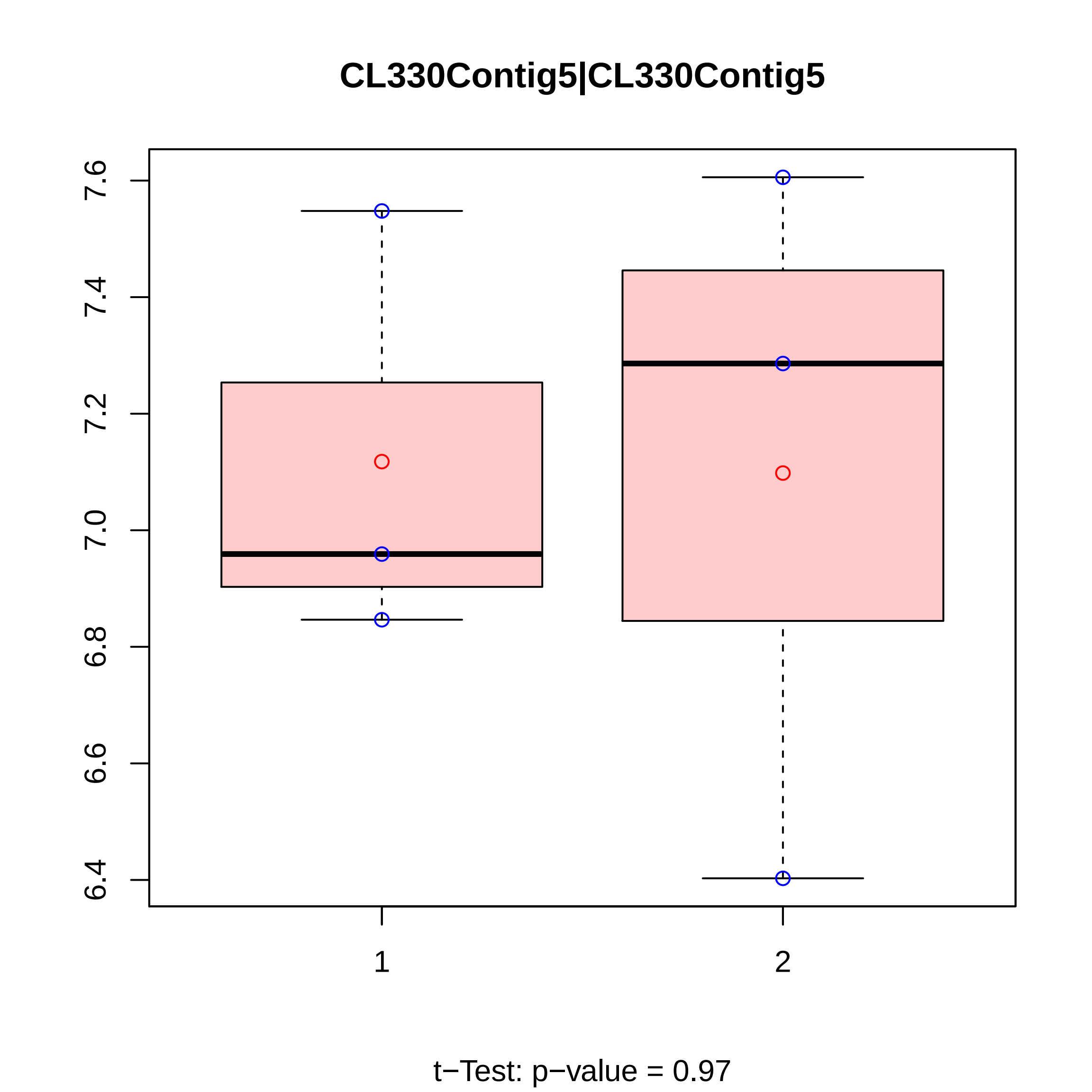
CL330Contig5|CL330Contig5
7.6
7.4
7.2
7.0
6.8
6.6
6.4
1
2
t−Test: p−value = 0.97

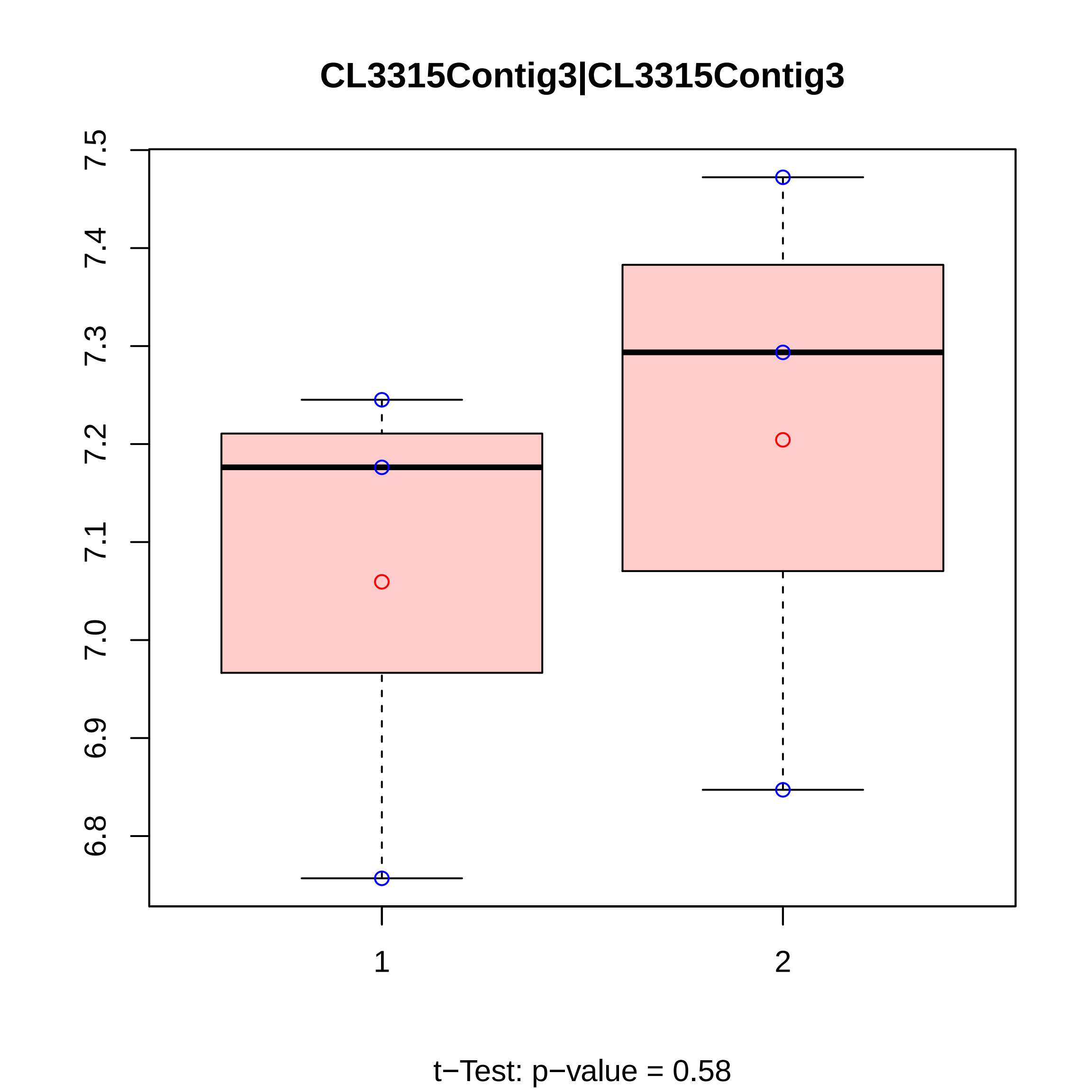

CL3315Contig3|CL3315Contig3
7.5
7.4
7.3
7.2
7.1
7.0
6.9
6.8
1
2
t−Test: p−value = 0.58

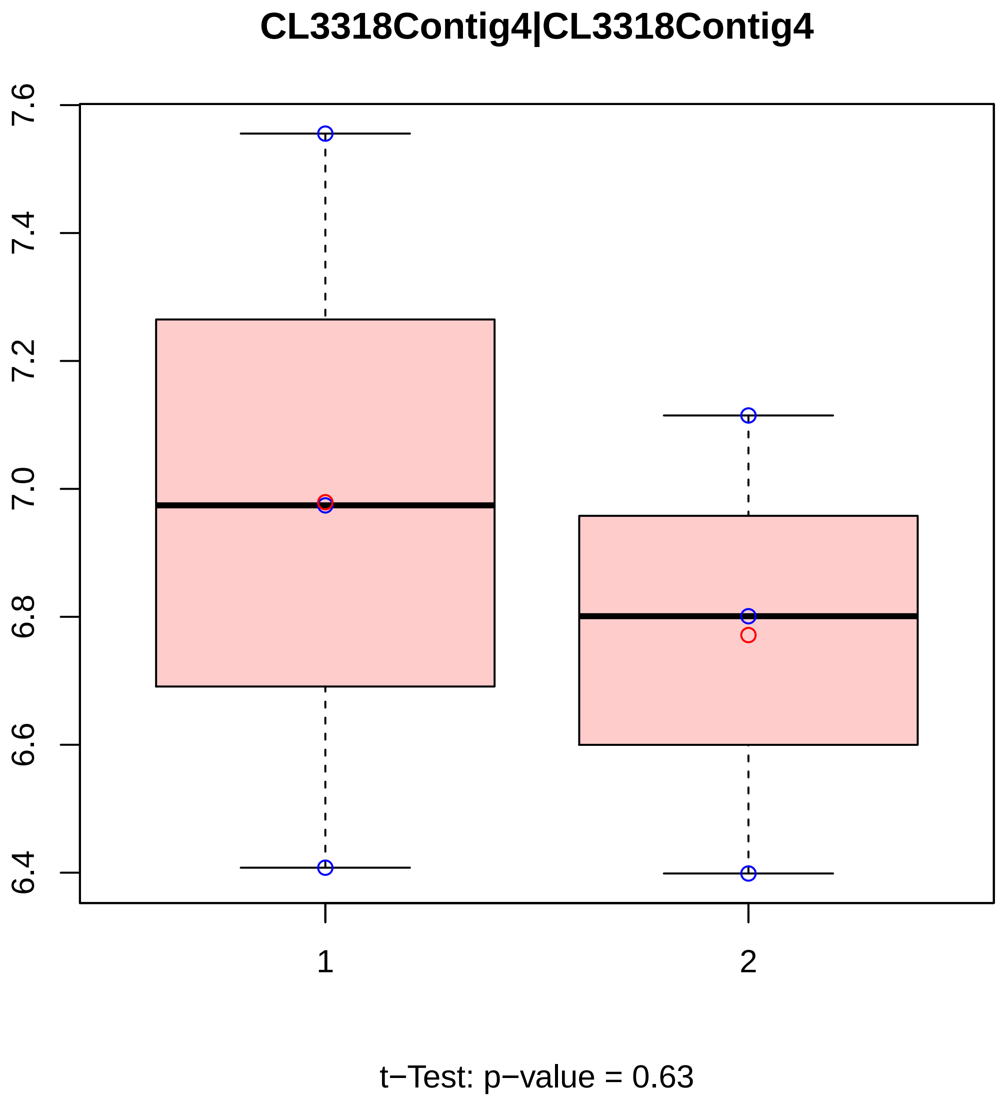
CL3318Contig4|CL3318Contig4
7.6
7.4
7.2
7.0
6.8
6.6
6.4
1
2
t−Test: p−value = 0.63

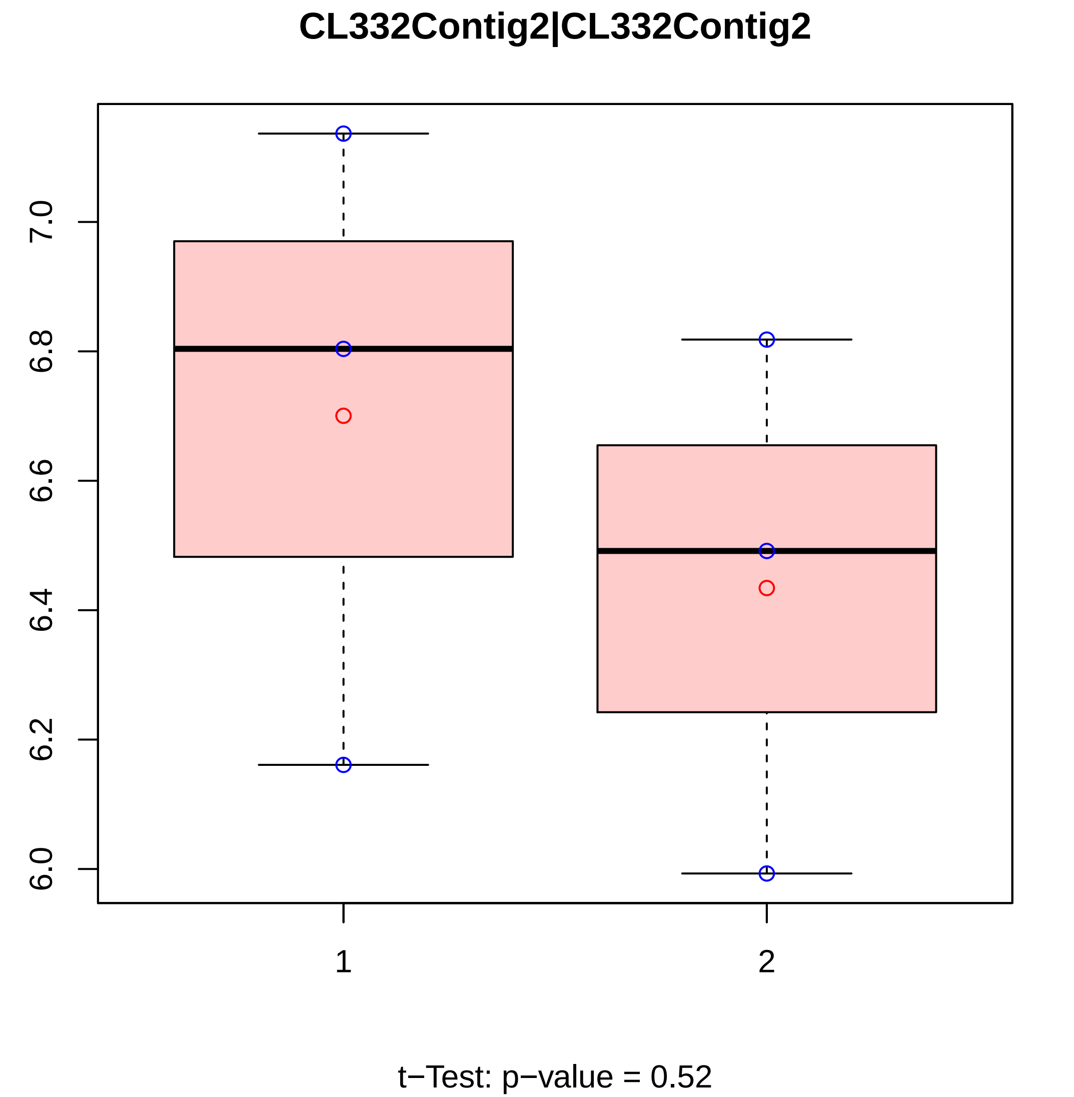

CL332Contig2|CL332Contig2
7.0
6.8
6.6
6.4
6.2
6.0
1
2
t−Test: p−value = 0.52

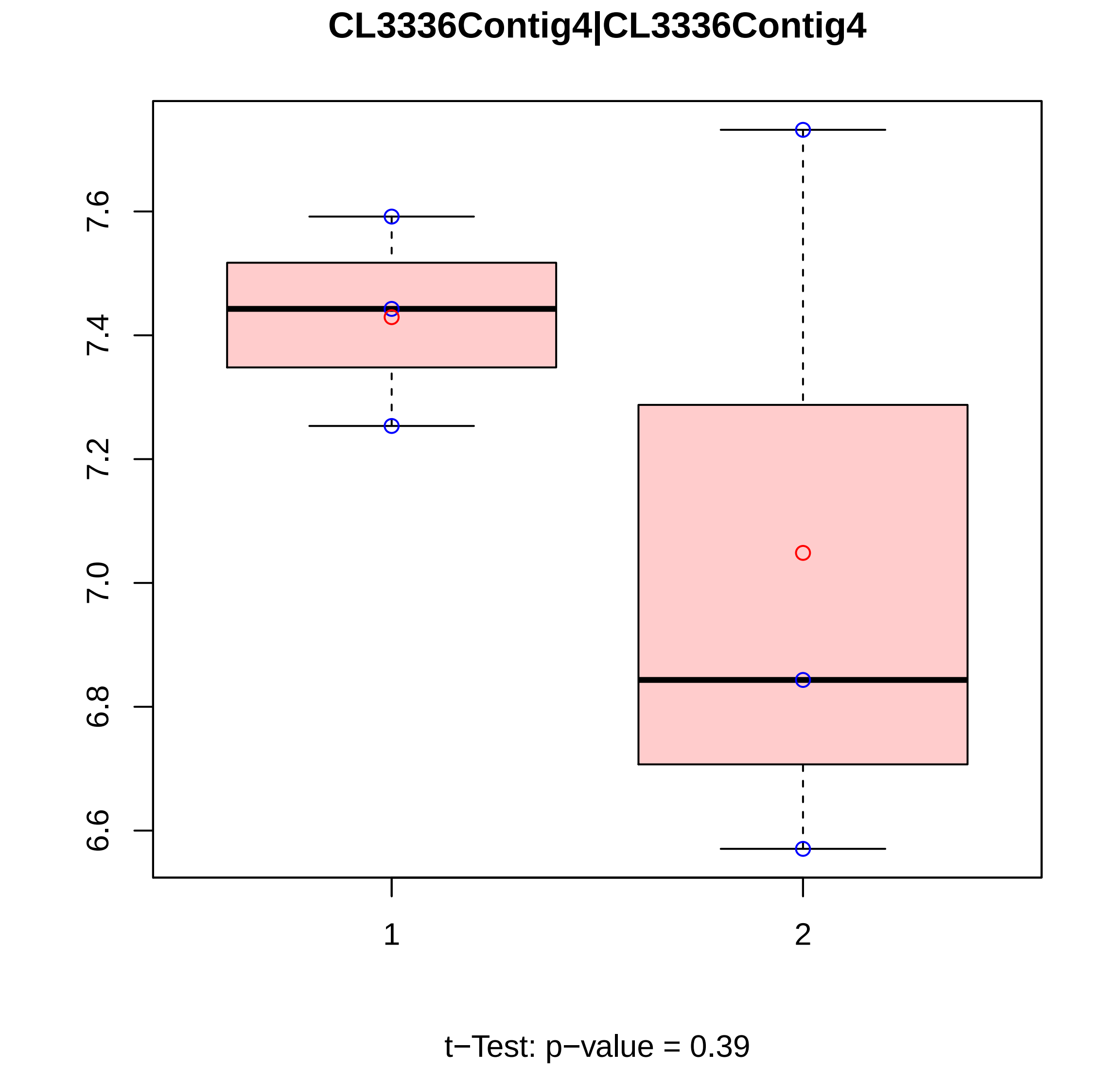
CL3336Contig4|CL3336Contig4
7.6
7.4
7.2
7.0
6.8
6.6
1
2
t−Test: p−value = 0.39

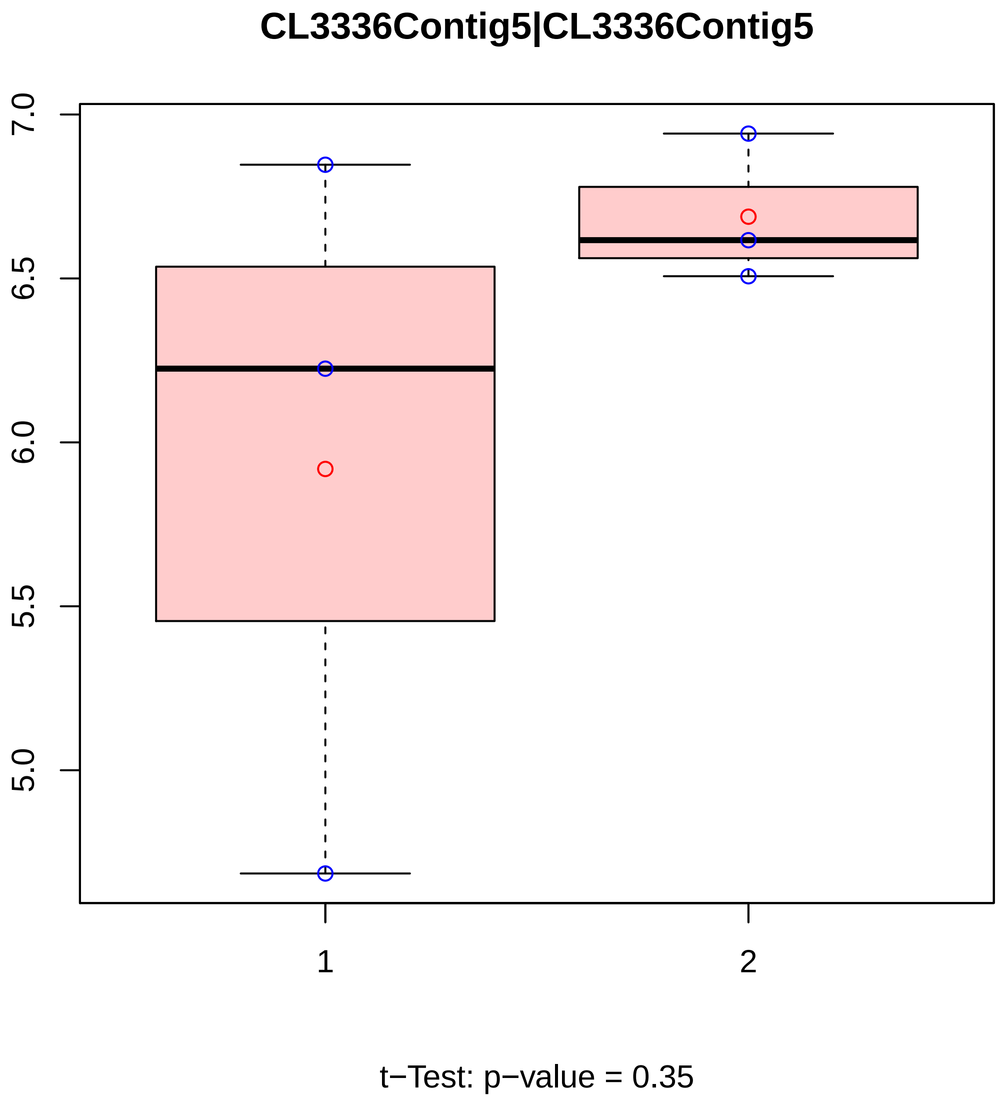

CL3336Contig5|CL3336Contig5
7.0
6.5
6.0
5.5
5.0
1
2
t−Test: p−value = 0.35

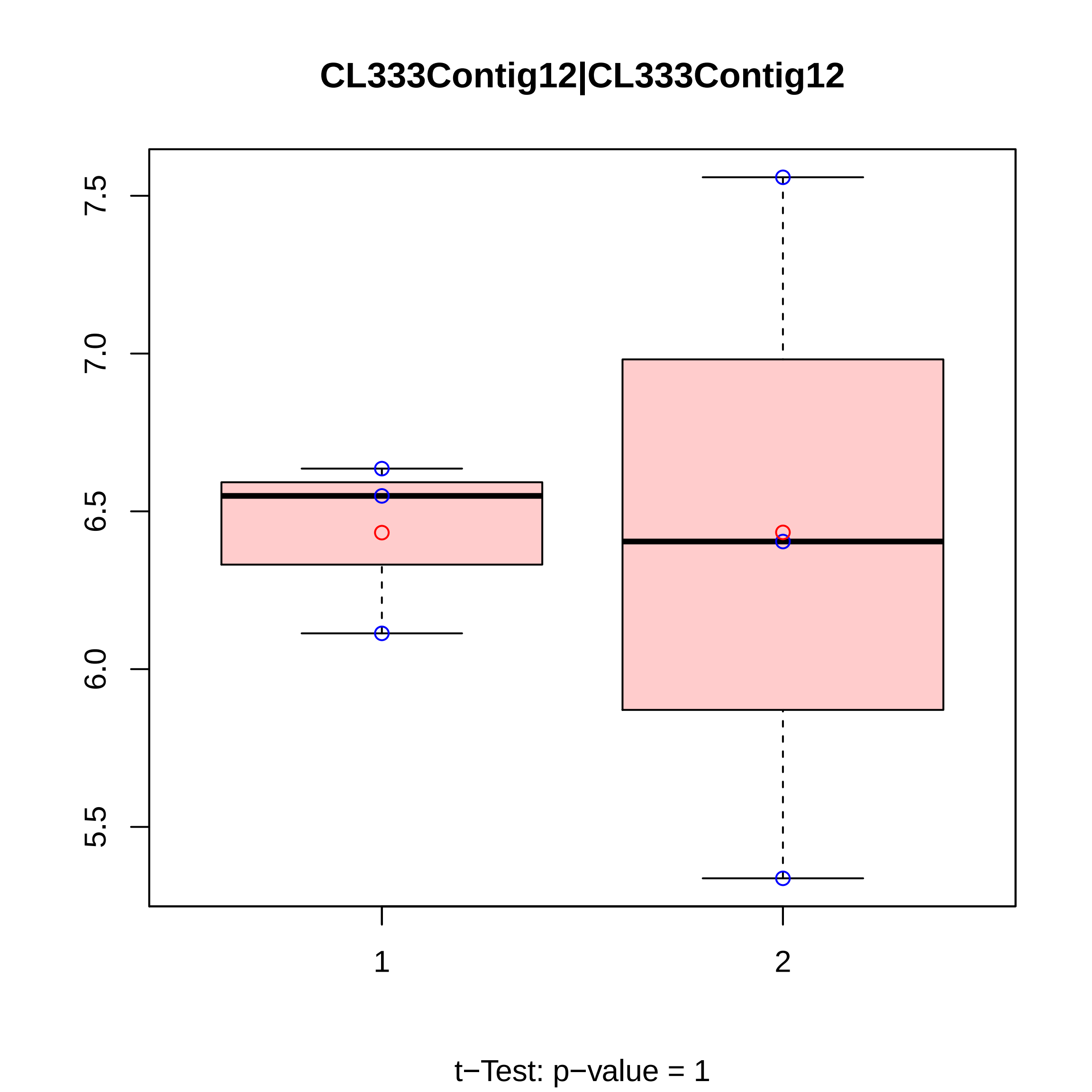
CL333Contig12|CL333Contig12
7.5
7.0
6.5
6.0
5.5
1
2
t−Test: p−value = 1

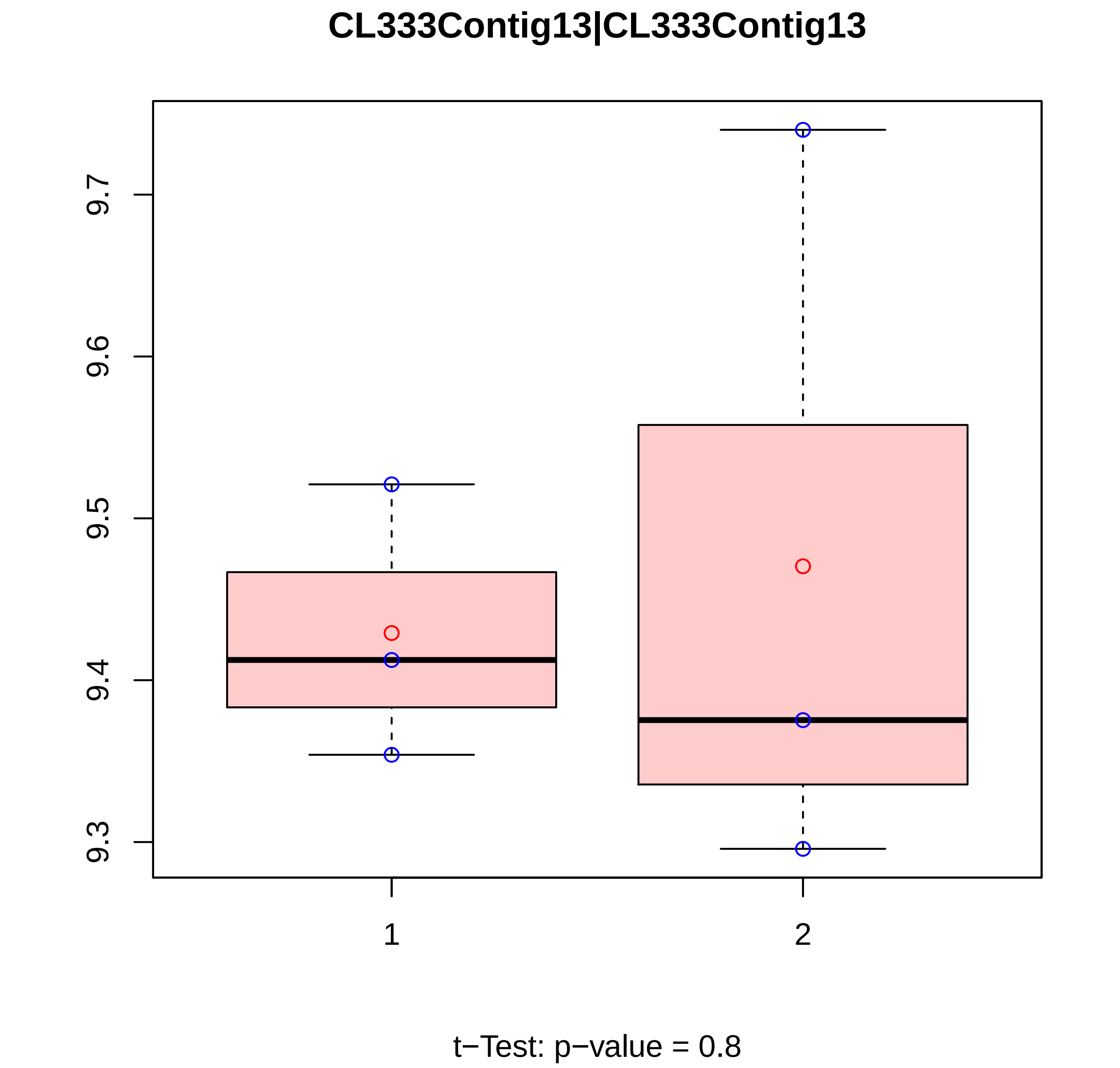
CL333Contig13|CL333Contig13
9.3
9.4
9.5
9.6
9.7
1
2
t−Test: p−value = 0.8

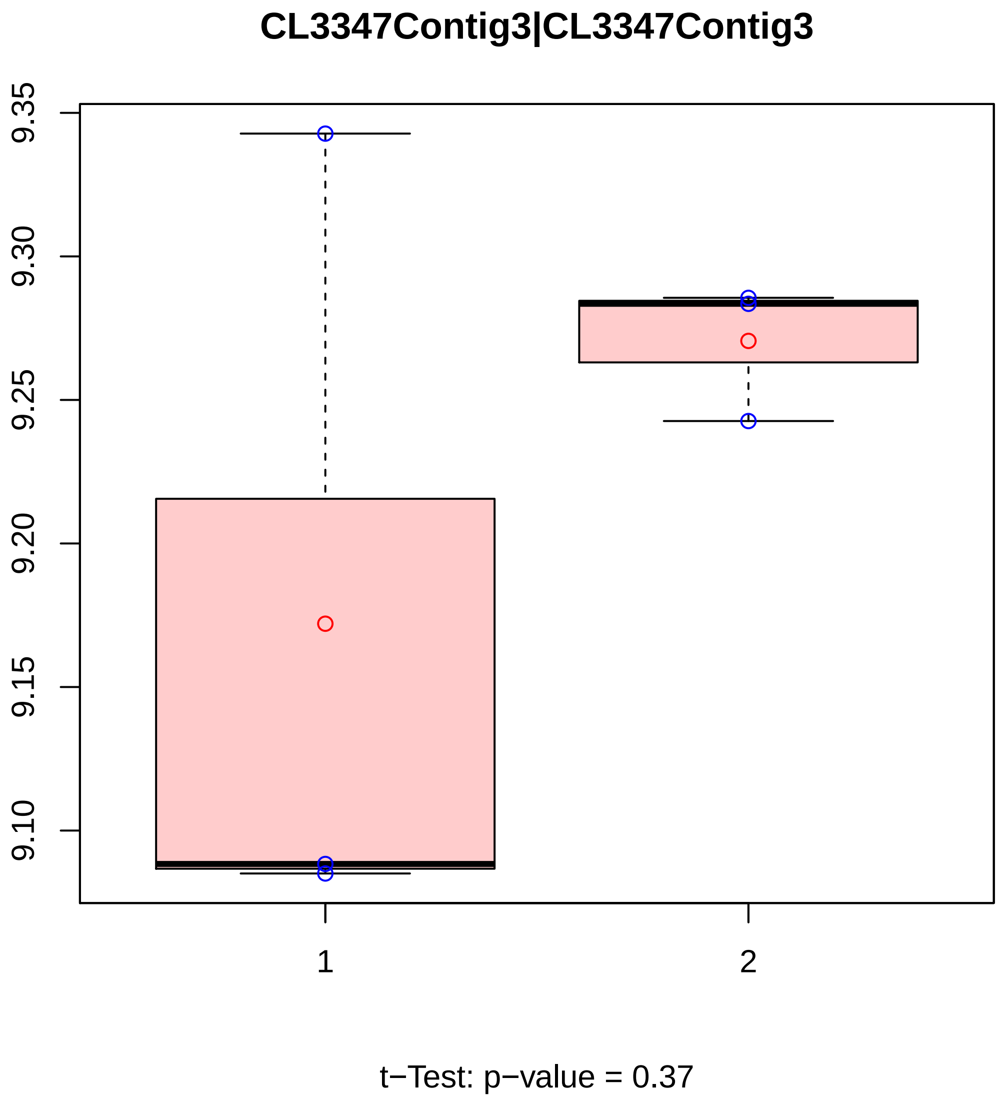
CL3347Contig3|CL3347Contig3
9.35
9.30
9.25
9.20
9.15
9.10
1
2
t−Test: p−value = 0.37

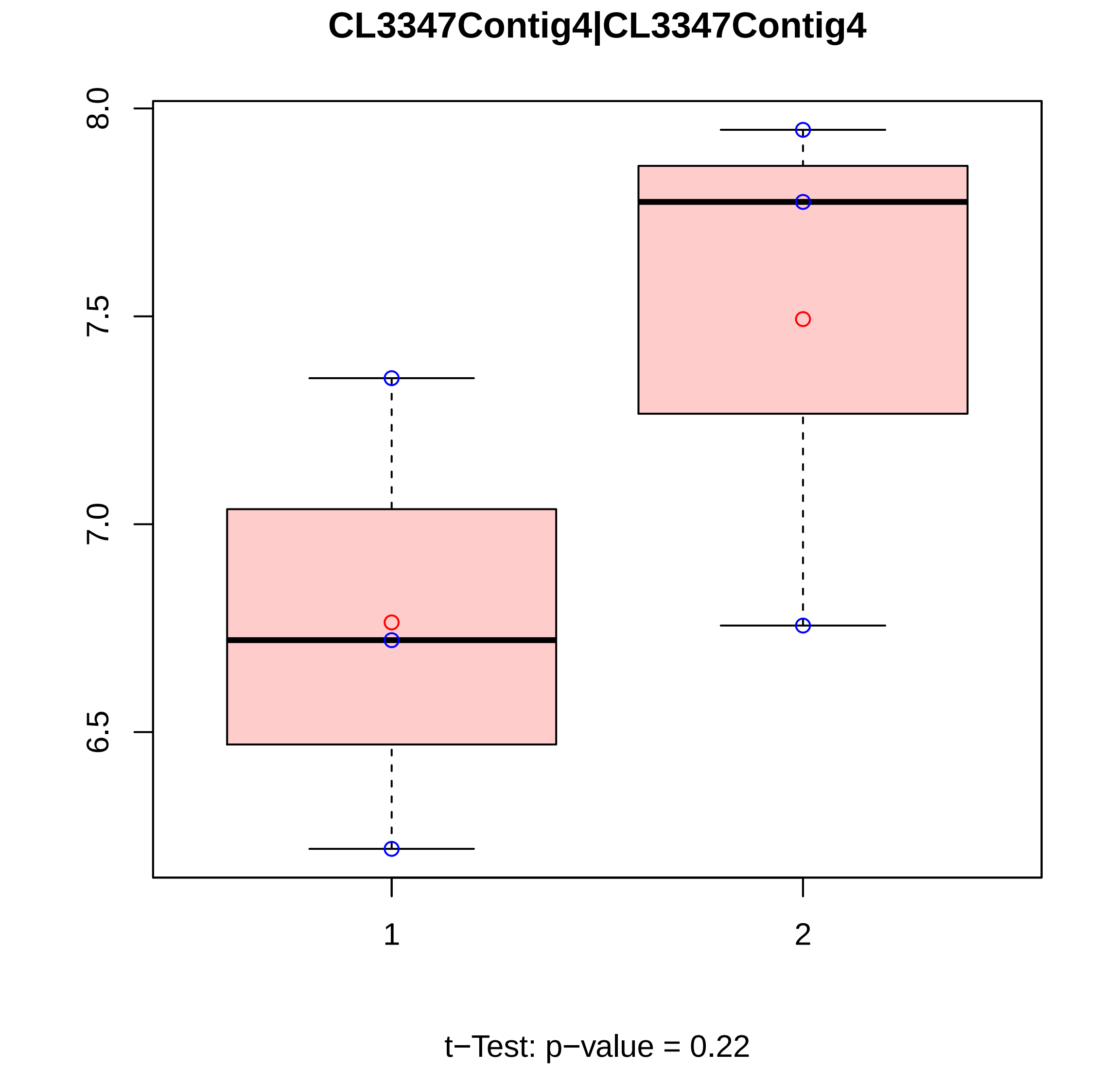
CL3347Contig4|CL3347Contig4
8.0
7.5
7.0
6.5
1
2
t−Test: p−value = 0.22

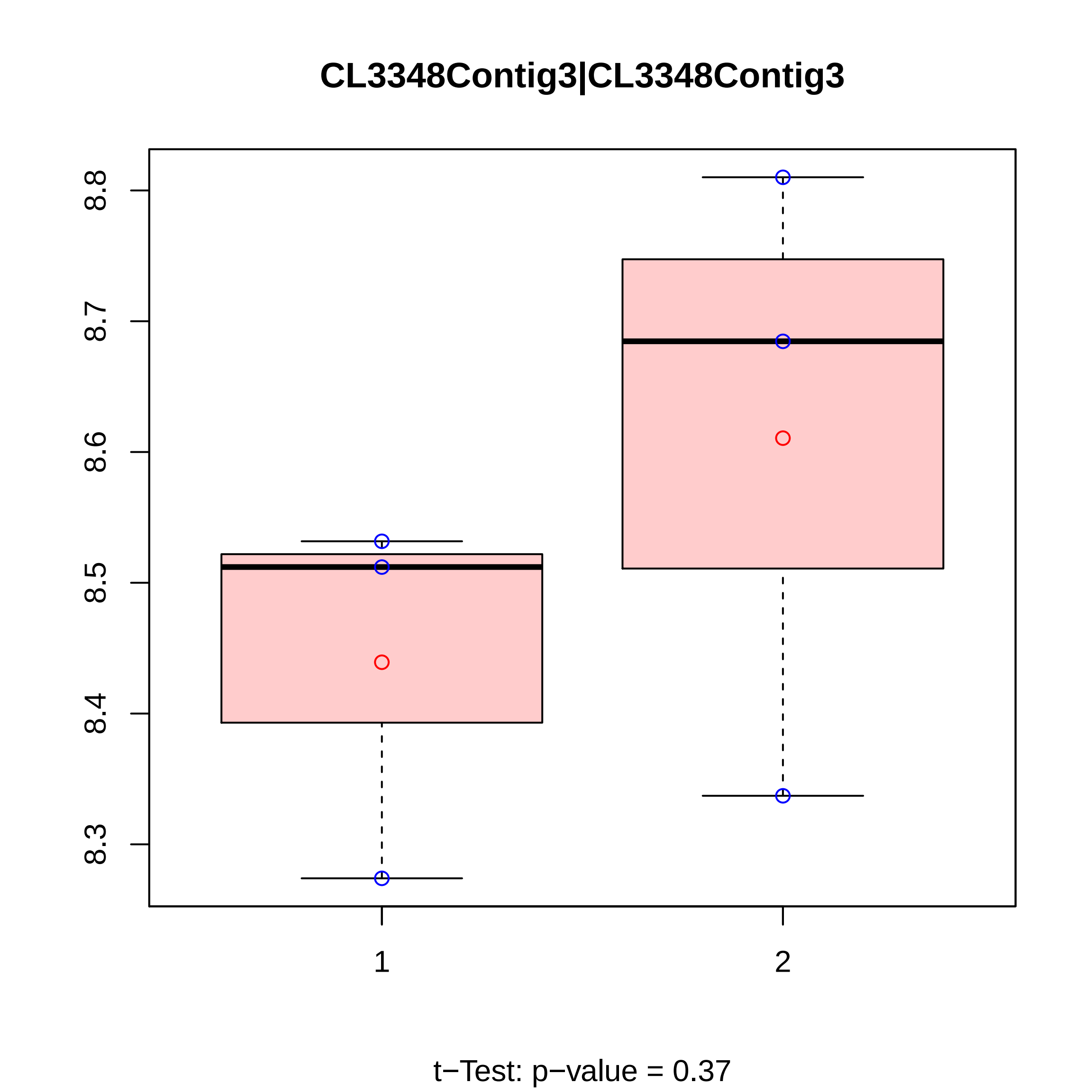
CL3348Contig3|CL3348Contig3
8.3
8.4
8.5
8.6
8.7
8.8
1
2
t−Test: p−value = 0.37

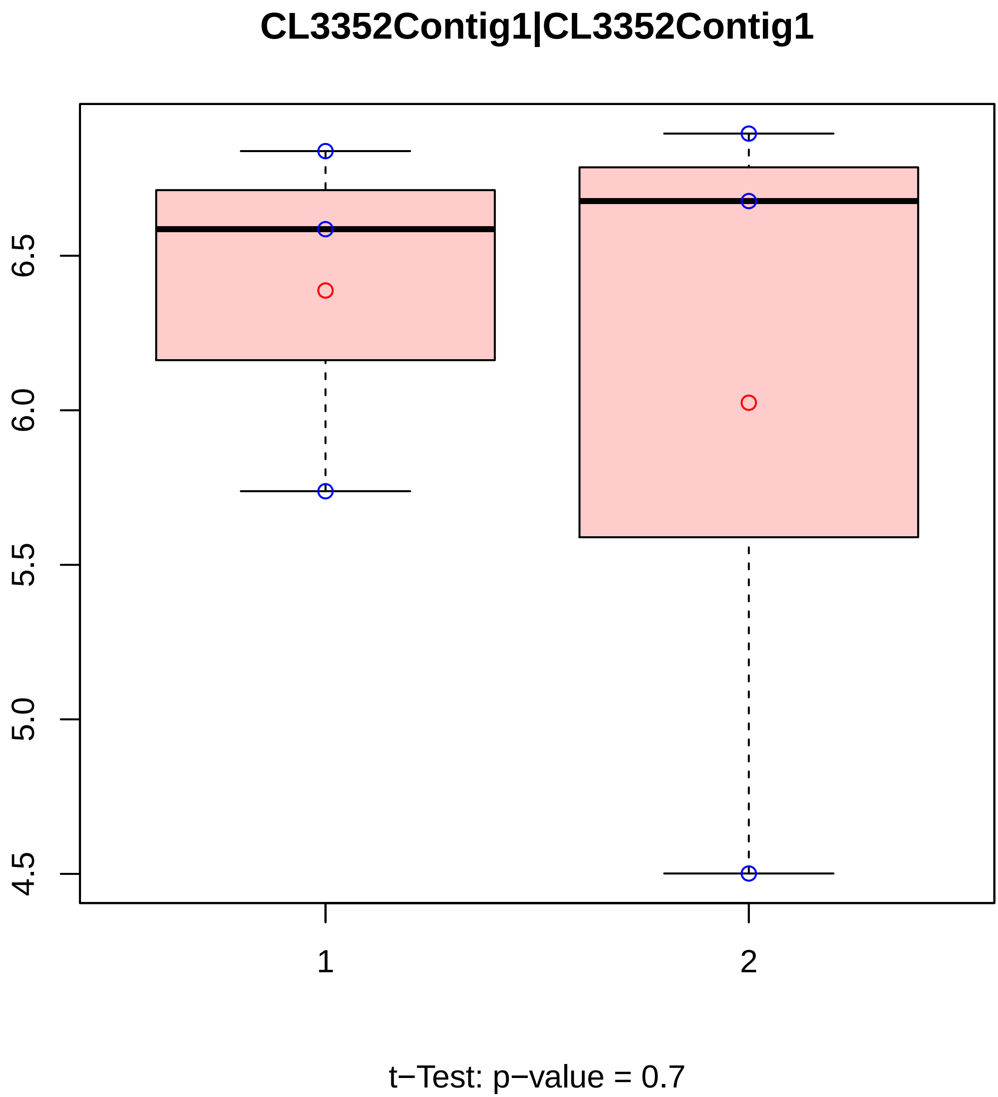

CL3352Contig1|CL3352Contig1
6.5
6.0
5.5
5.0
4.5
1
2
t−Test: p−value = 0.7

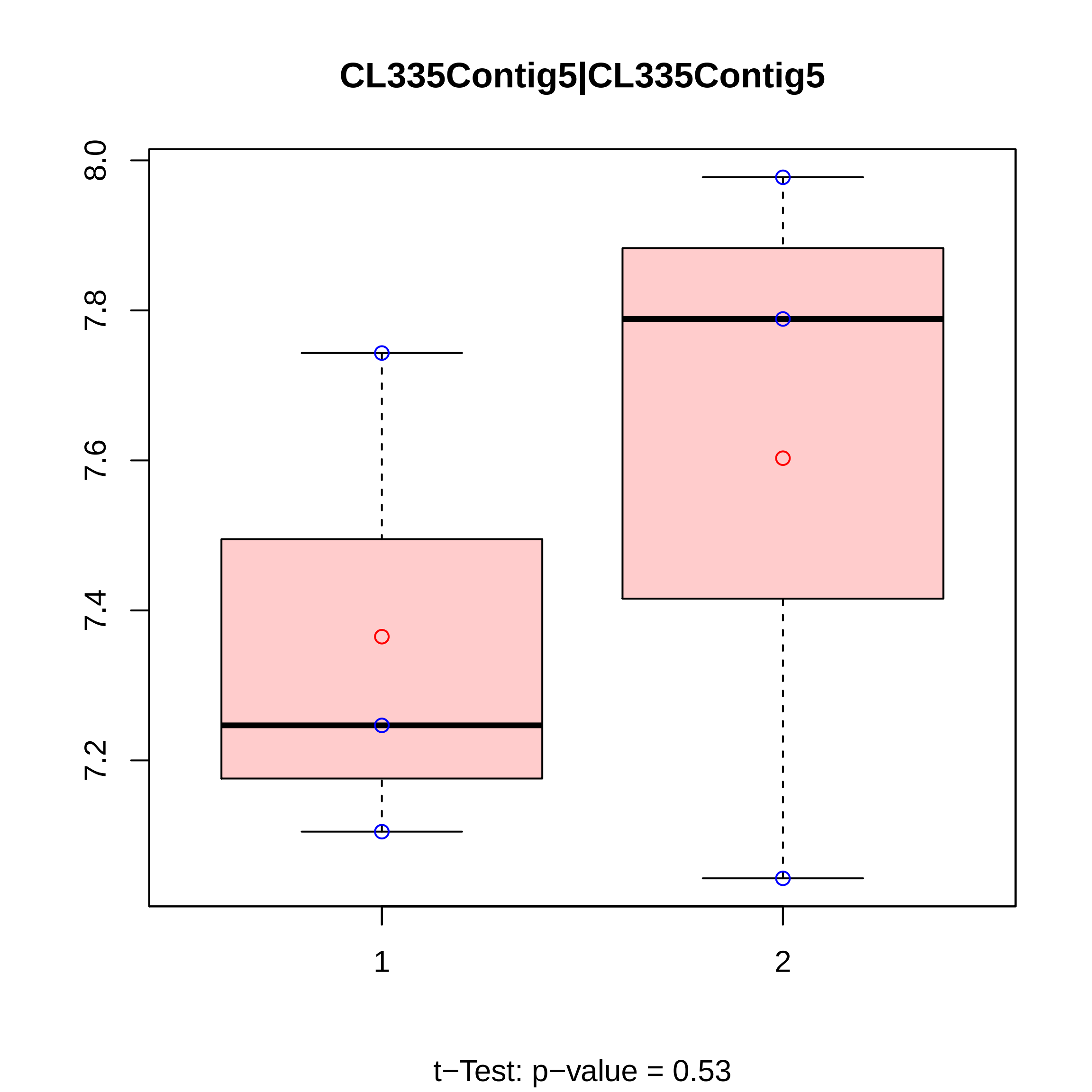
CL335Contig5|CL335Contig5
8.0
7.8
7.6
7.4
7.2
1
2
t−Test: p−value = 0.53

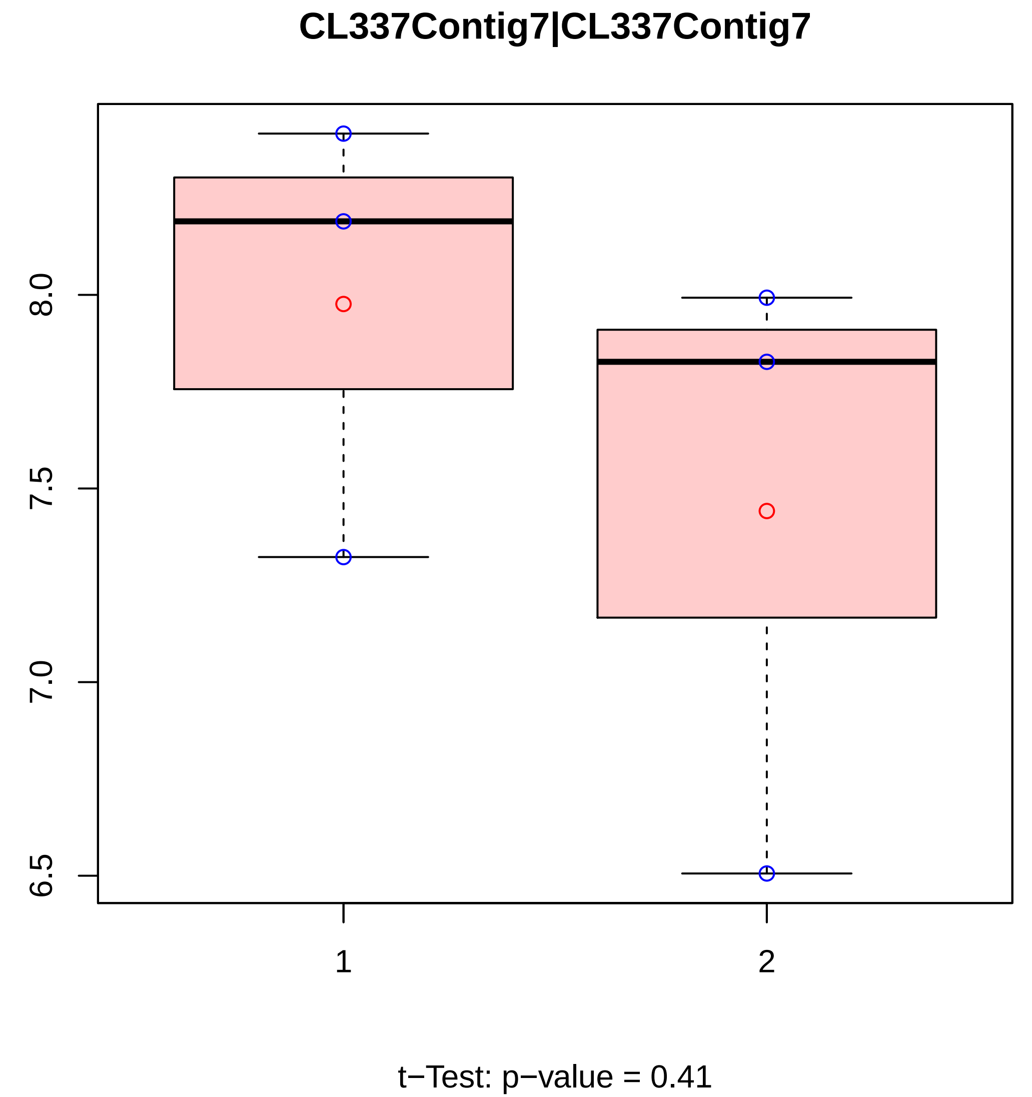

CL337Contig7|CL337Contig7
8.0
7.5
7.0
6.5
1
2
t−Test: p−value = 0.41

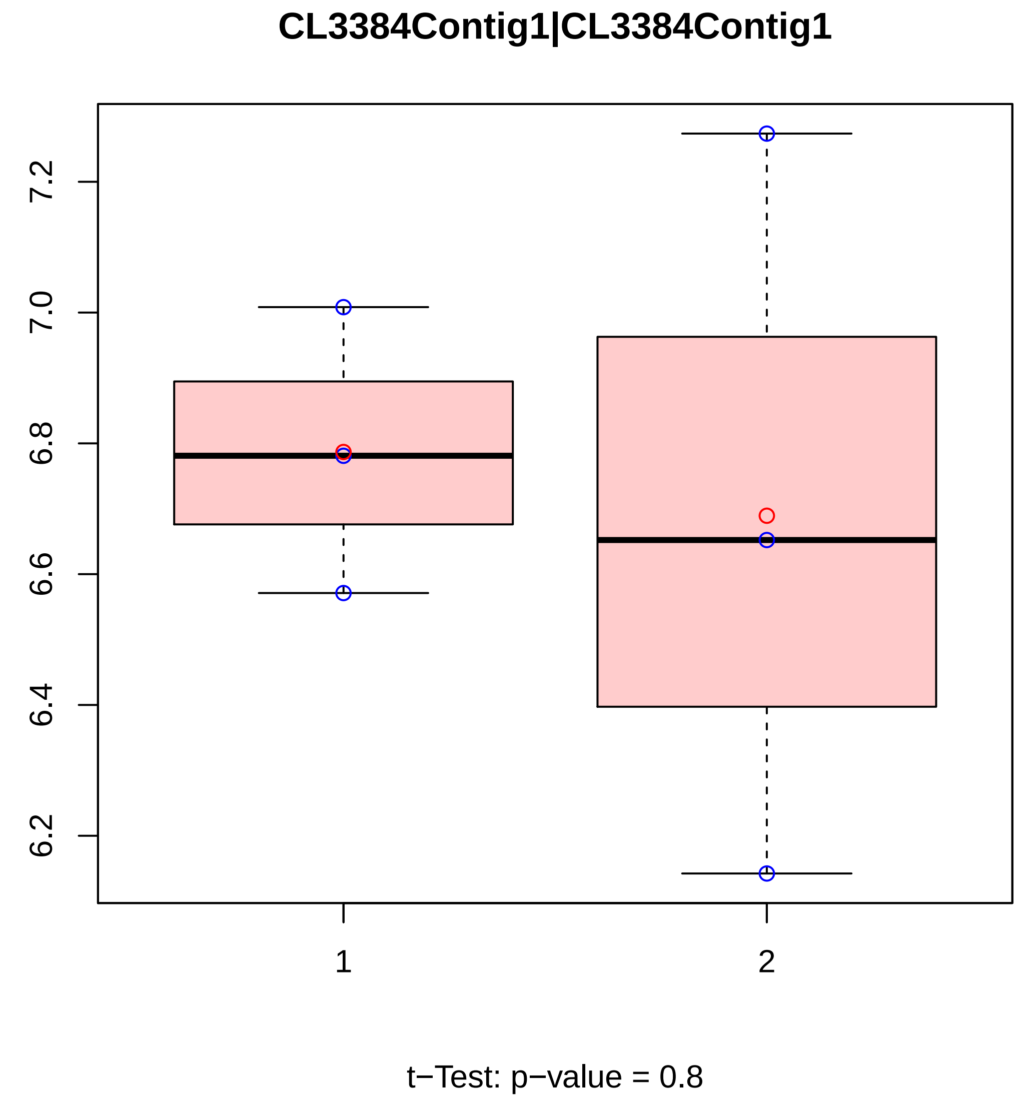
CL3384Contig1|CL3384Contig1
7.2
7.0
6.8
6.6
6.4
6.2
1
2
t−Test: p−value = 0.8

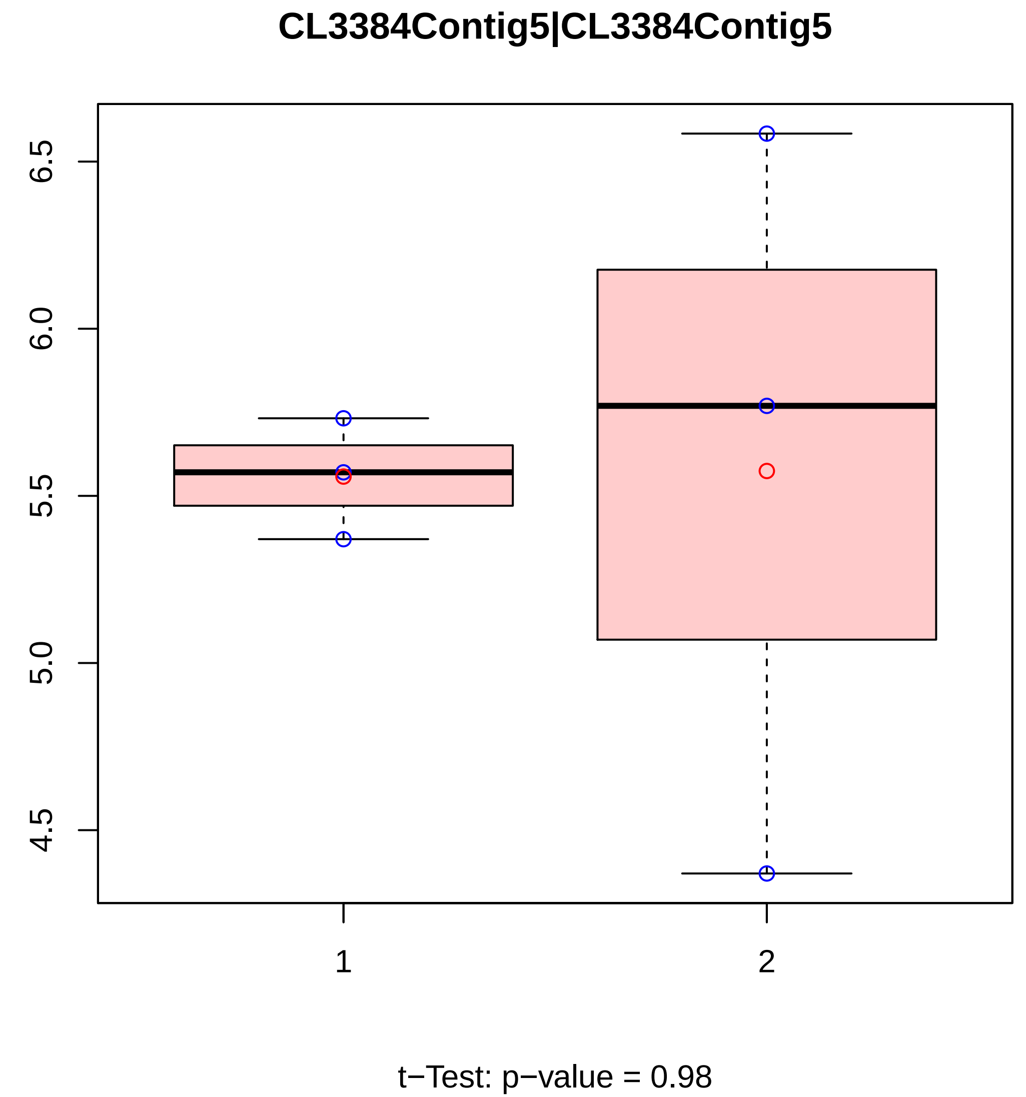
CL3384Contig5|CL3384Contig5
6.5
6.0
5.5
5.0
4.5
1
2
t−Test: p−value = 0.98

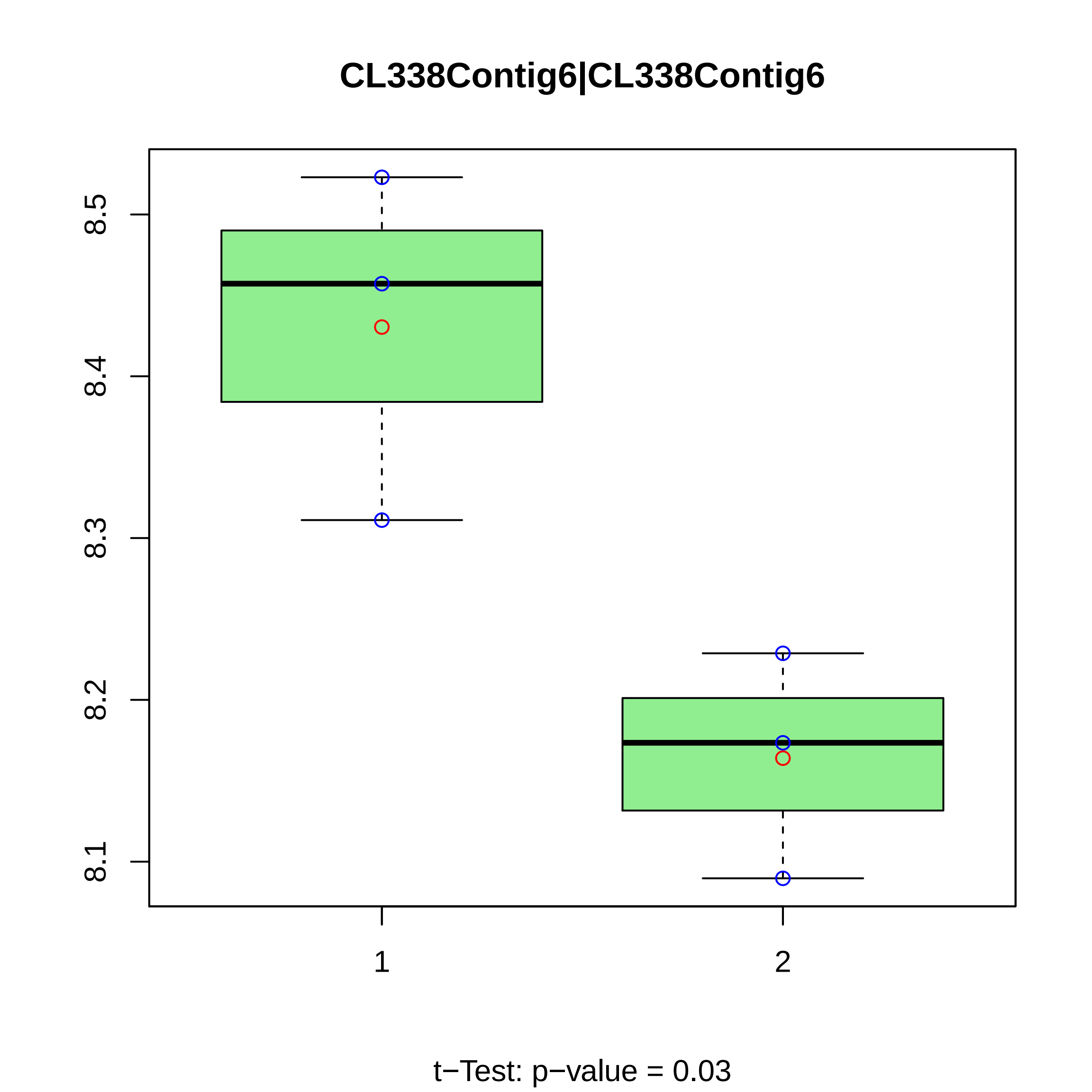
CL338Contig6|CL338Contig6
8.1
8.2
8.3
8.4
8.5
1
2
t−Test: p−value = 0.03

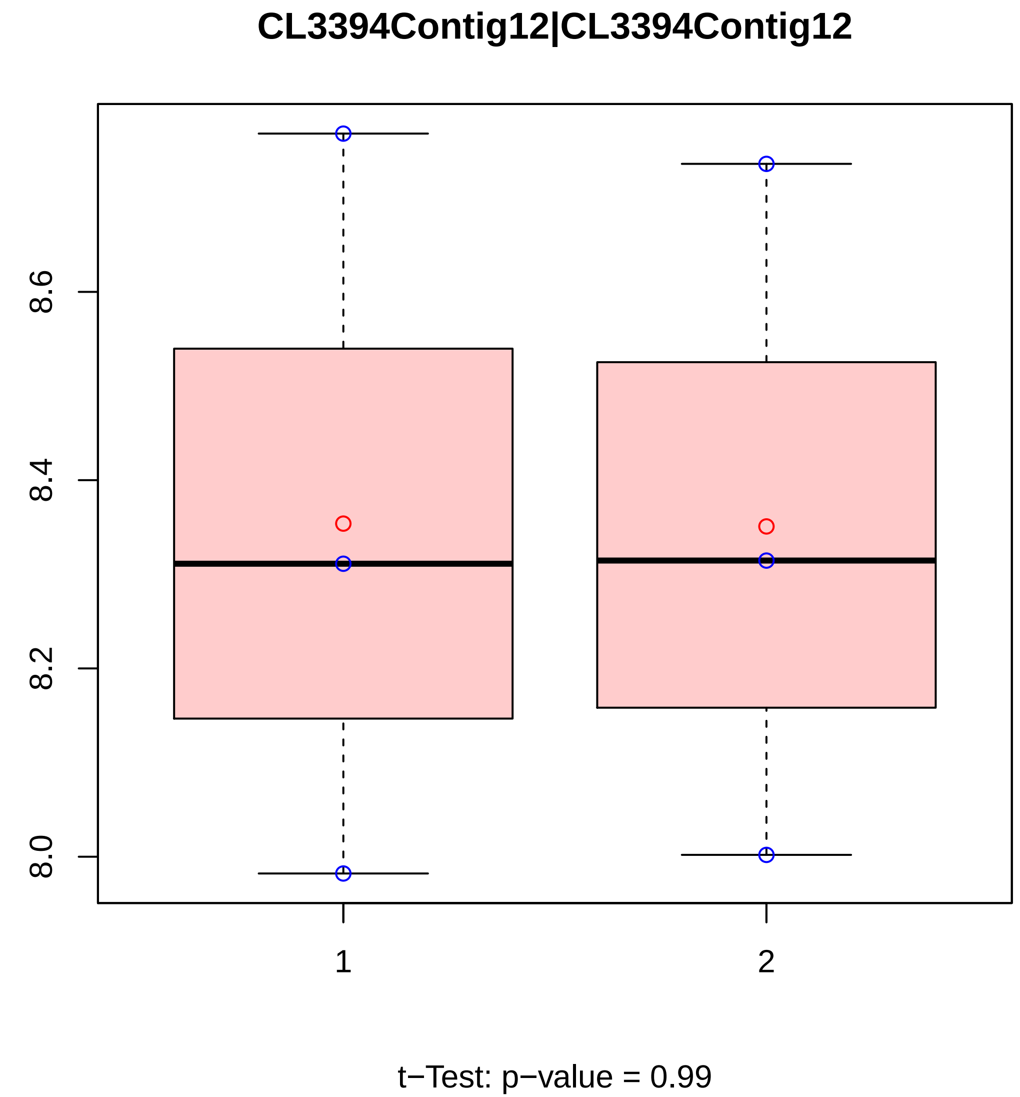
CL3394Contig12|CL3394Contig12
8.0
8.2
8.4
8.6
1
2
t−Test: p−value = 0.99

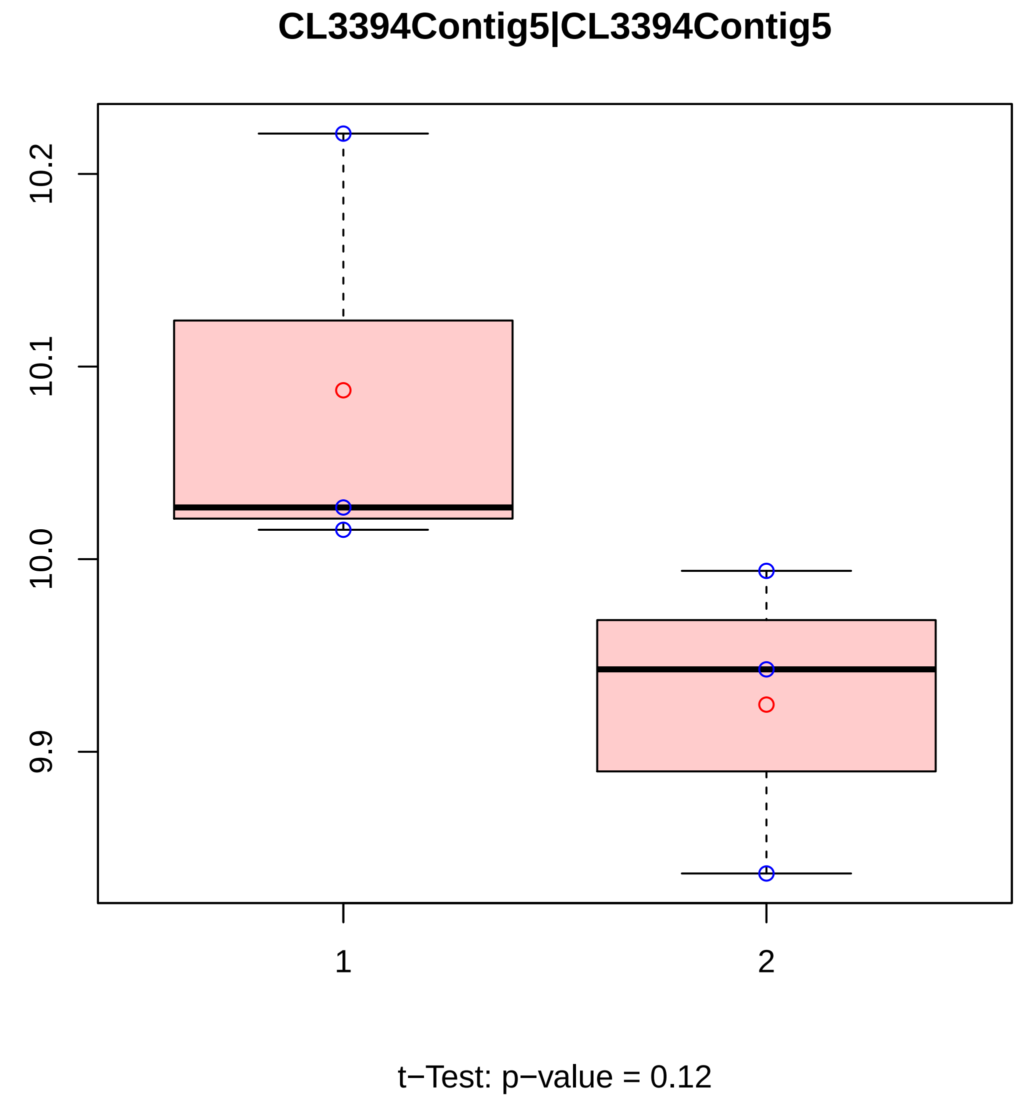
CL3394Contig5|CL3394Contig5
10.2
10.1
10.0
9.9
1
2
t−Test: p−value = 0.12

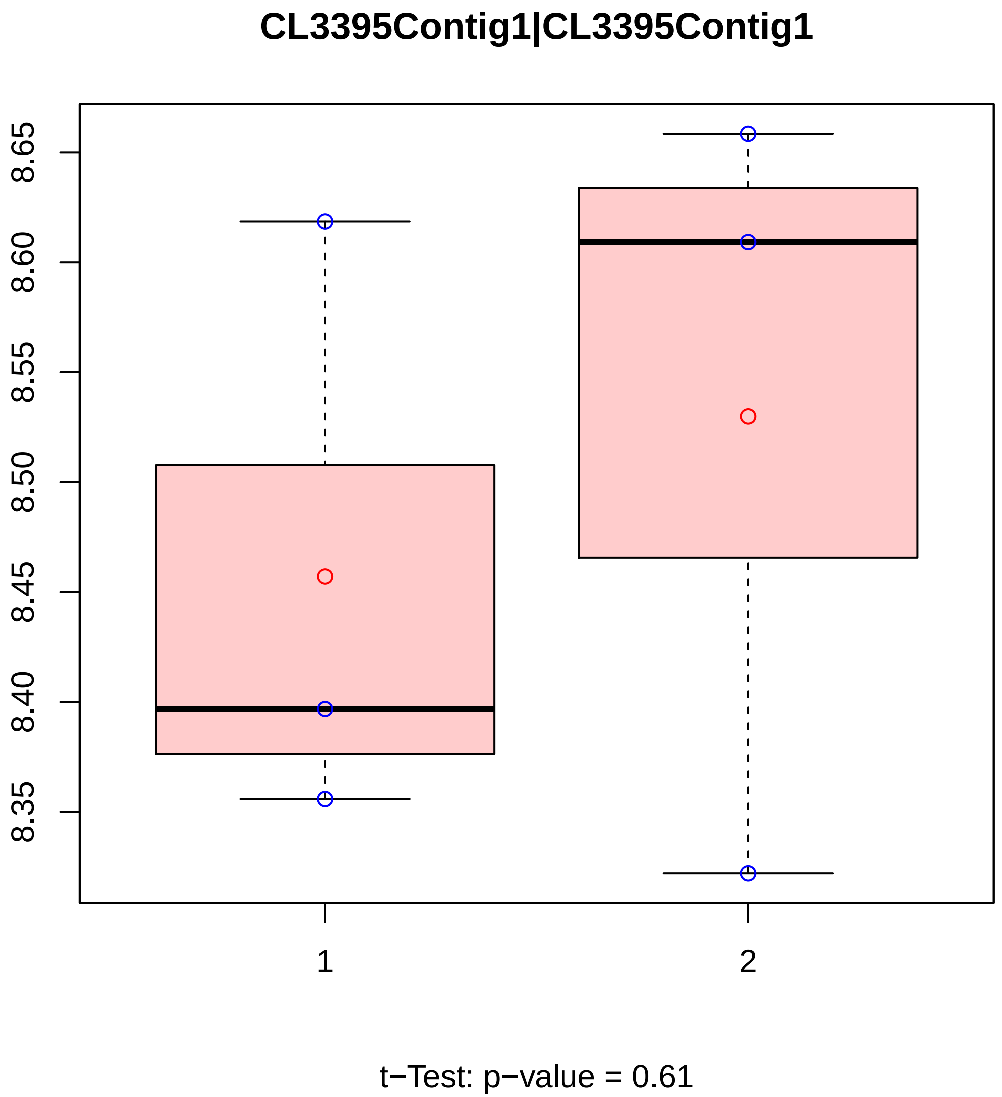
CL3395Contig1|CL3395Contig1
8.35
8.40
8.45
8.50
8.55
8.60
8.65
1
2
t−Test: p−value = 0.61

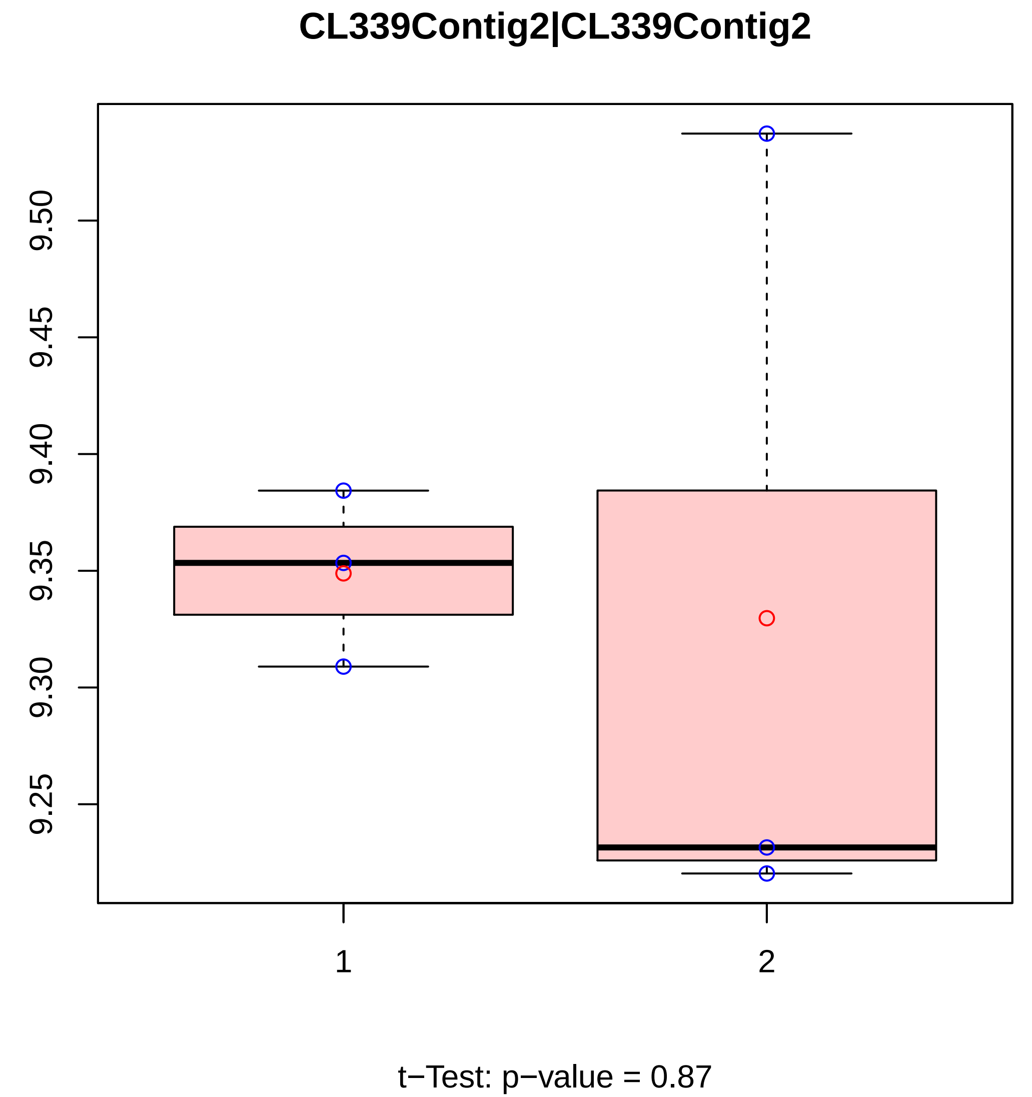

CL339Contig2|CL339Contig2
9.25
9.30
9.35
9.40
9.45
9.50
1
2
t−Test: p−value = 0.87

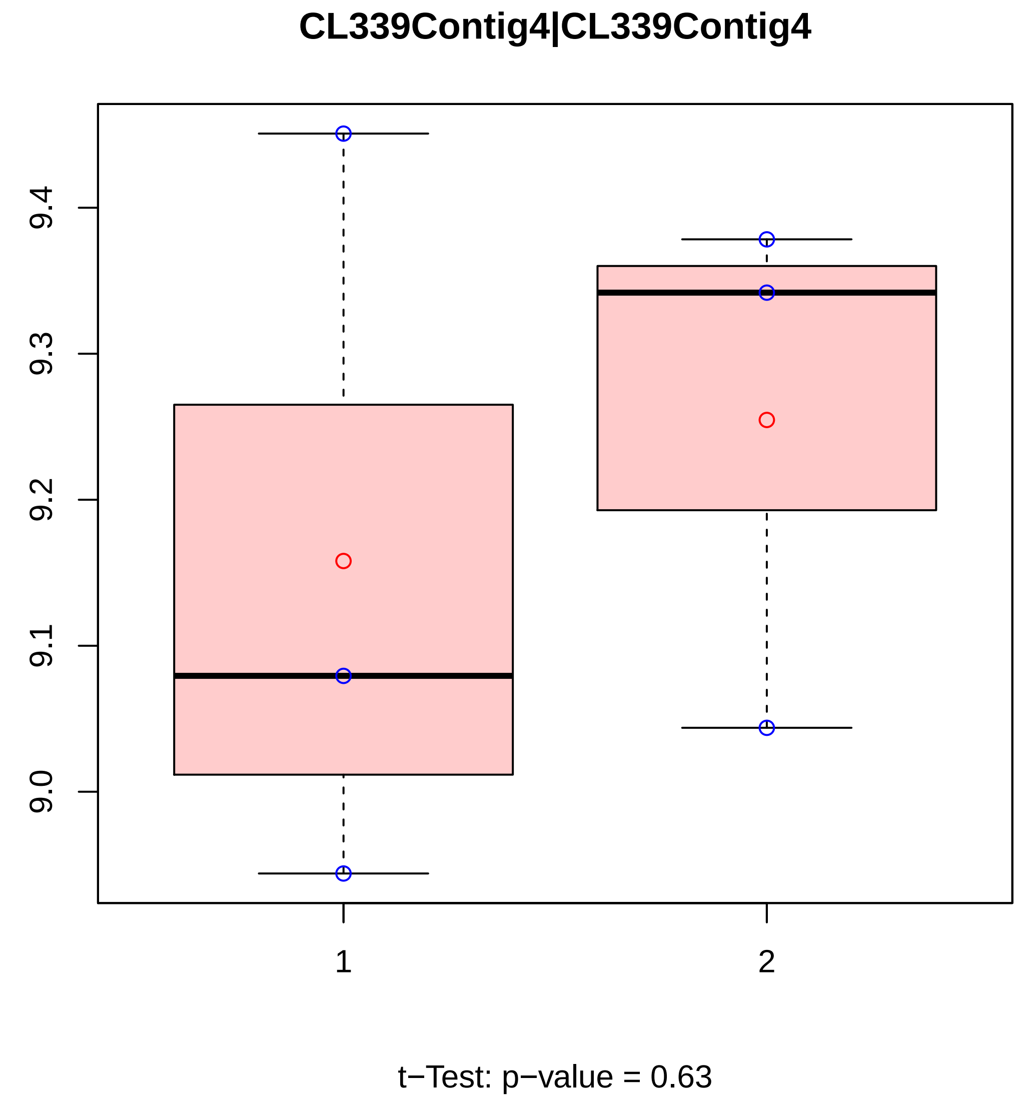
CL339Contig4|CL339Contig4
9.0
9.1
9.2
9.3
9.4
1
2
t−Test: p−value = 0.63

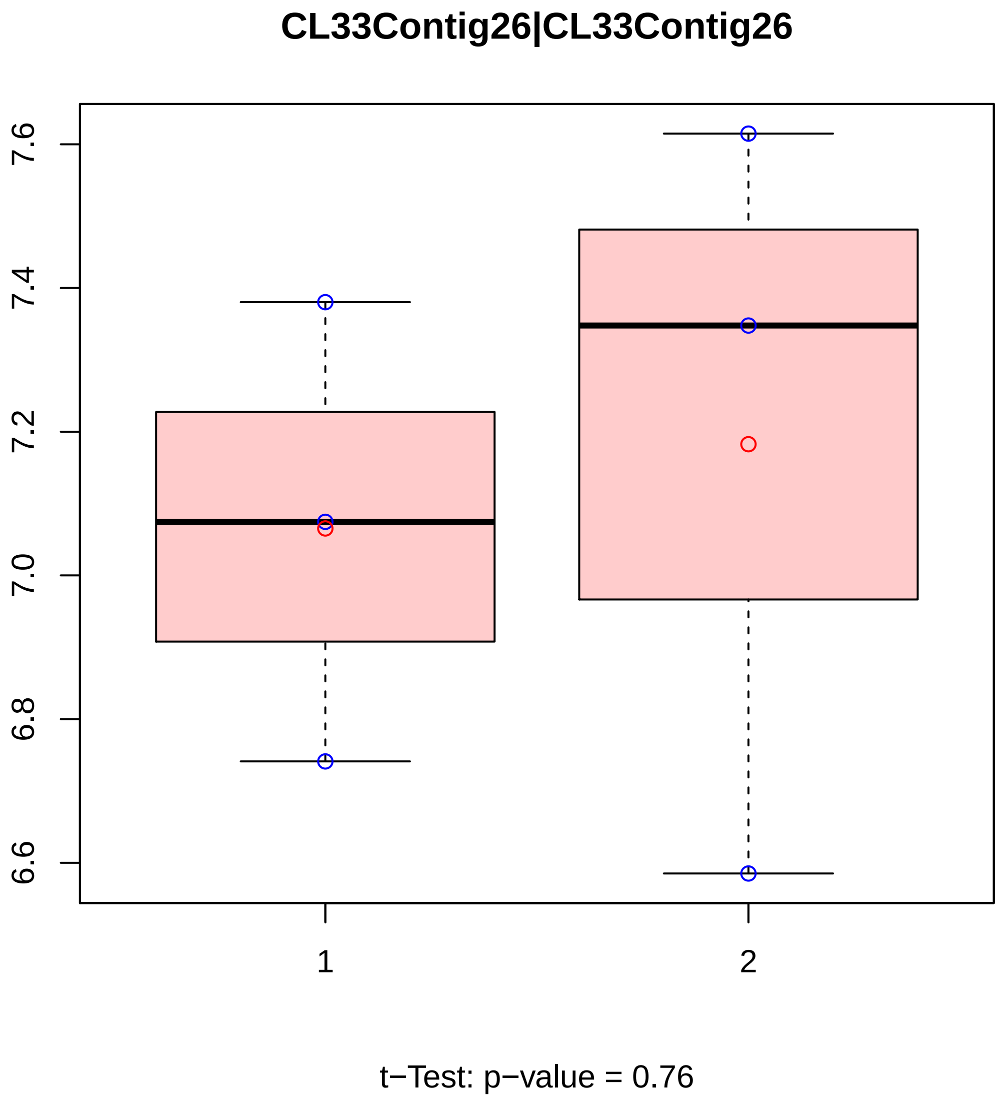
CL33Contig26|CL33Contig26
7.6
7.4
7.2
7.0
6.8
6.6
1
2
t−Test: p−value = 0.76

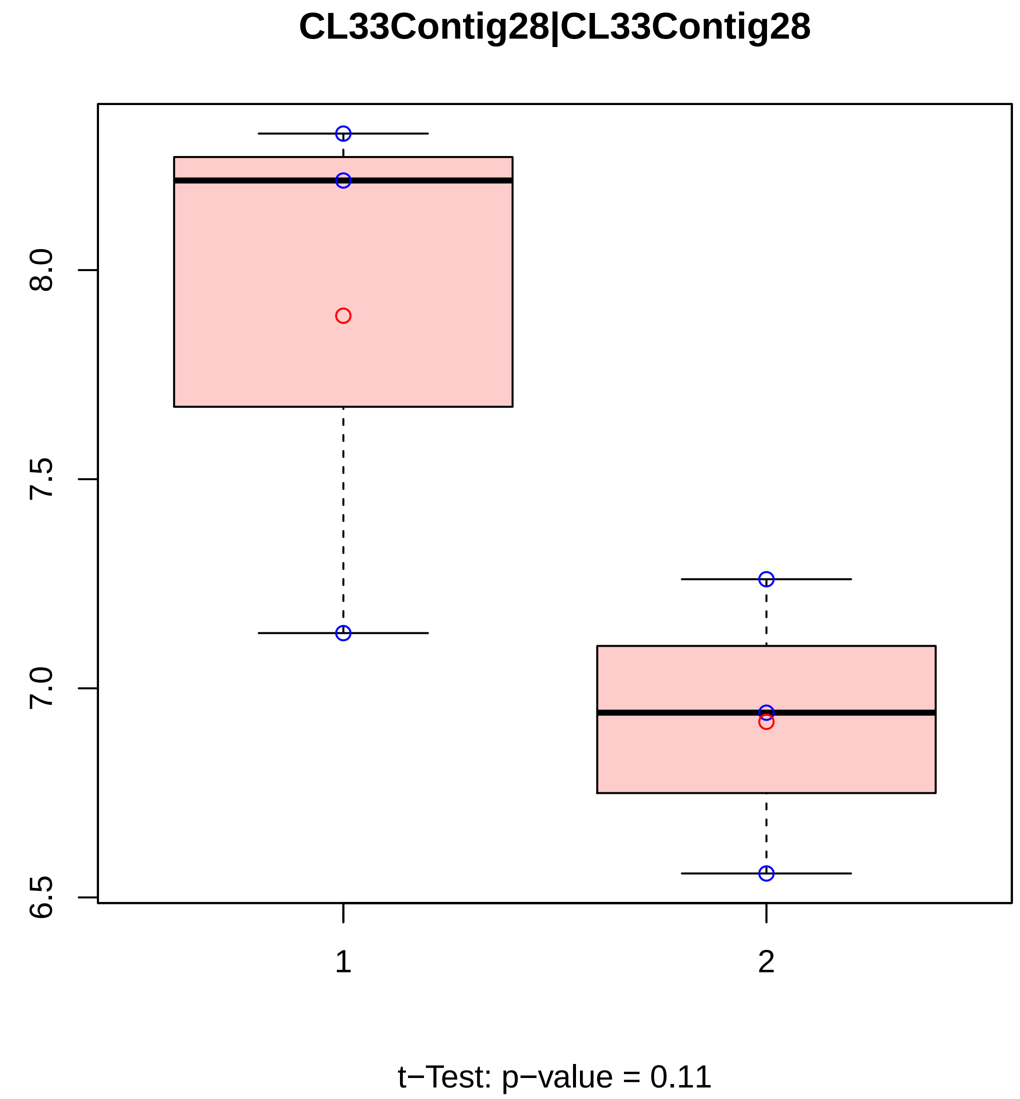
CL33Contig28|CL33Contig28
8.0
7.5
7.0
6.5
1
2
t−Test: p−value = 0.11

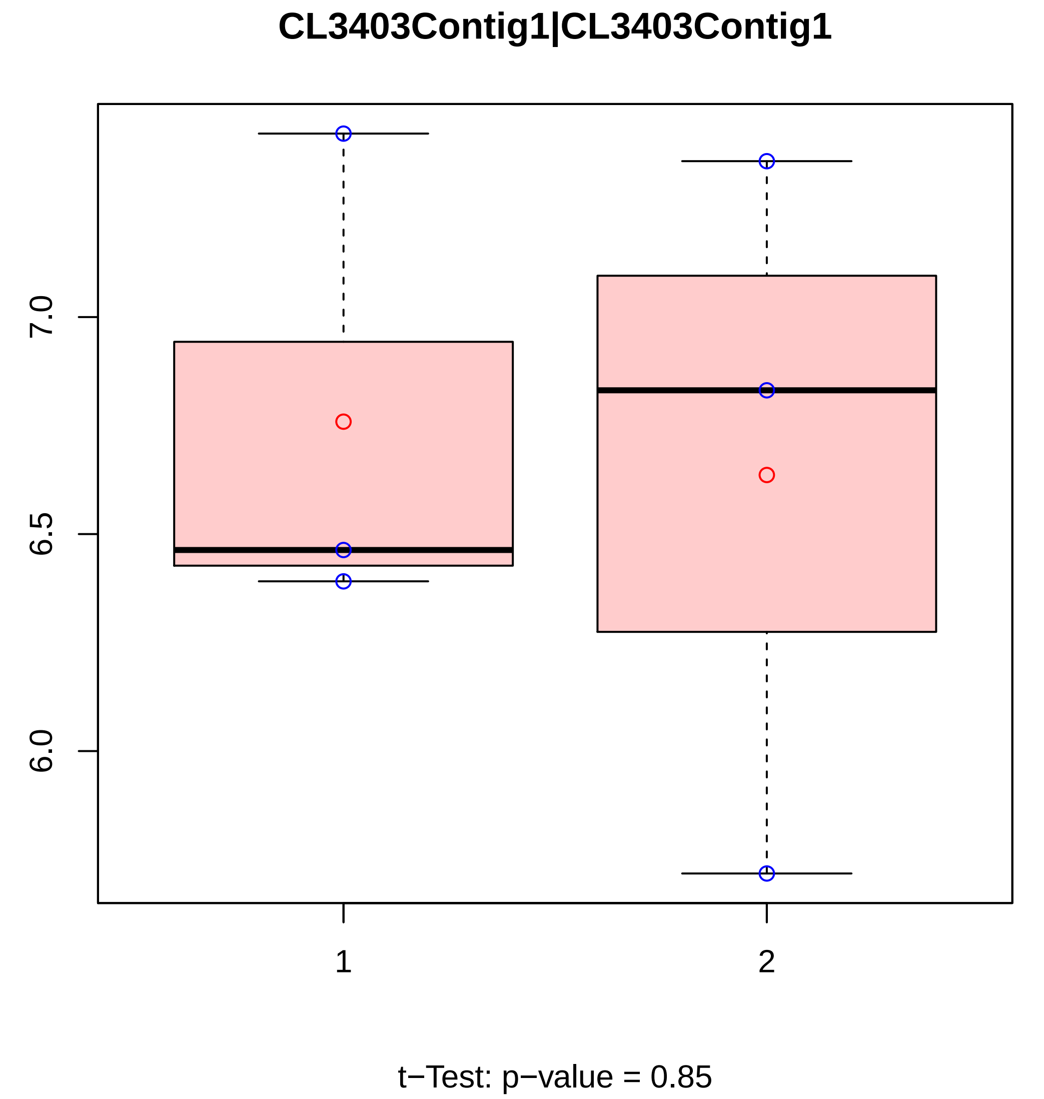
CL3403Contig1|CL3403Contig1
7.0
6.5
6.0
1
2
t−Test: p−value = 0.85

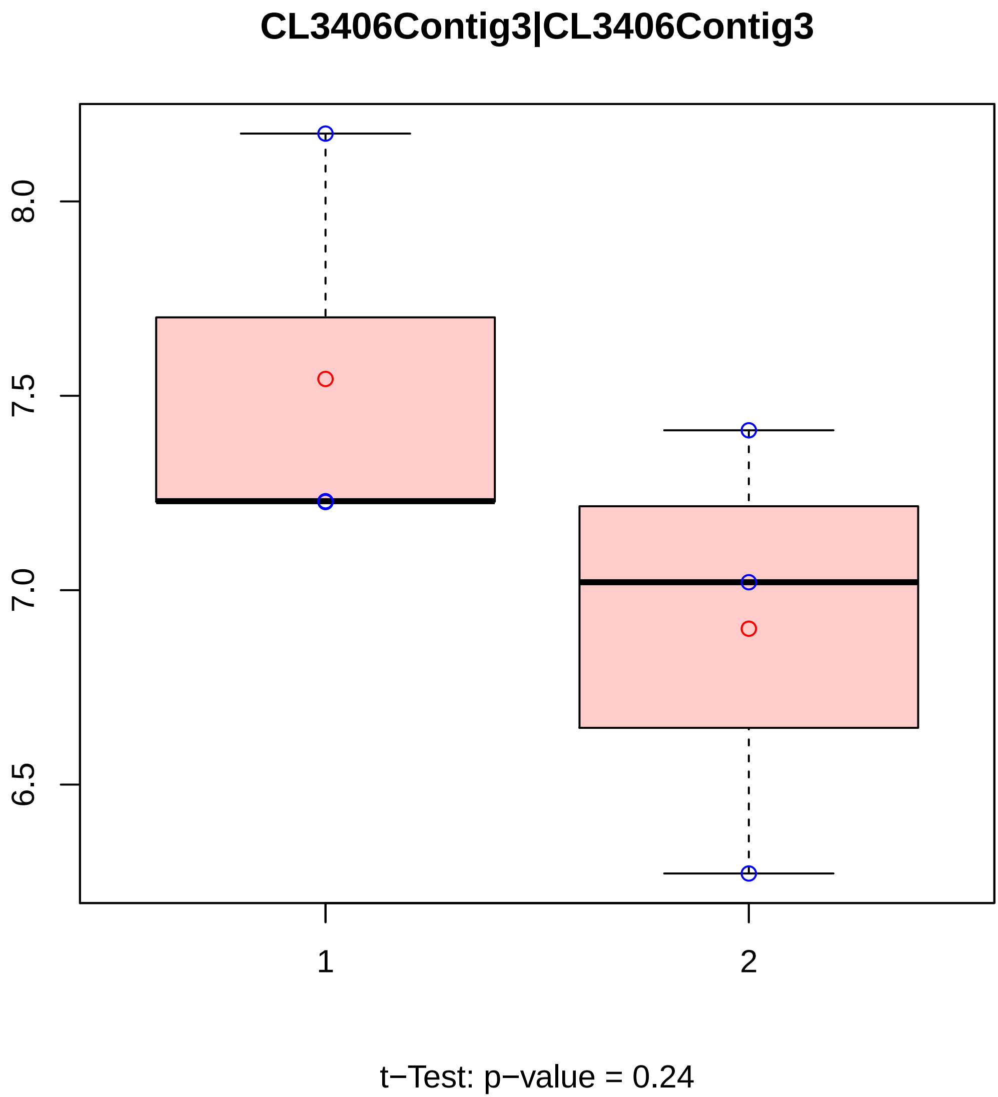
CL3406Contig3|CL3406Contig3
8.0
7.5
7.0
6.5
1
2
t−Test: p−value = 0.24

# CL3442Contig1|CL3442Contig1

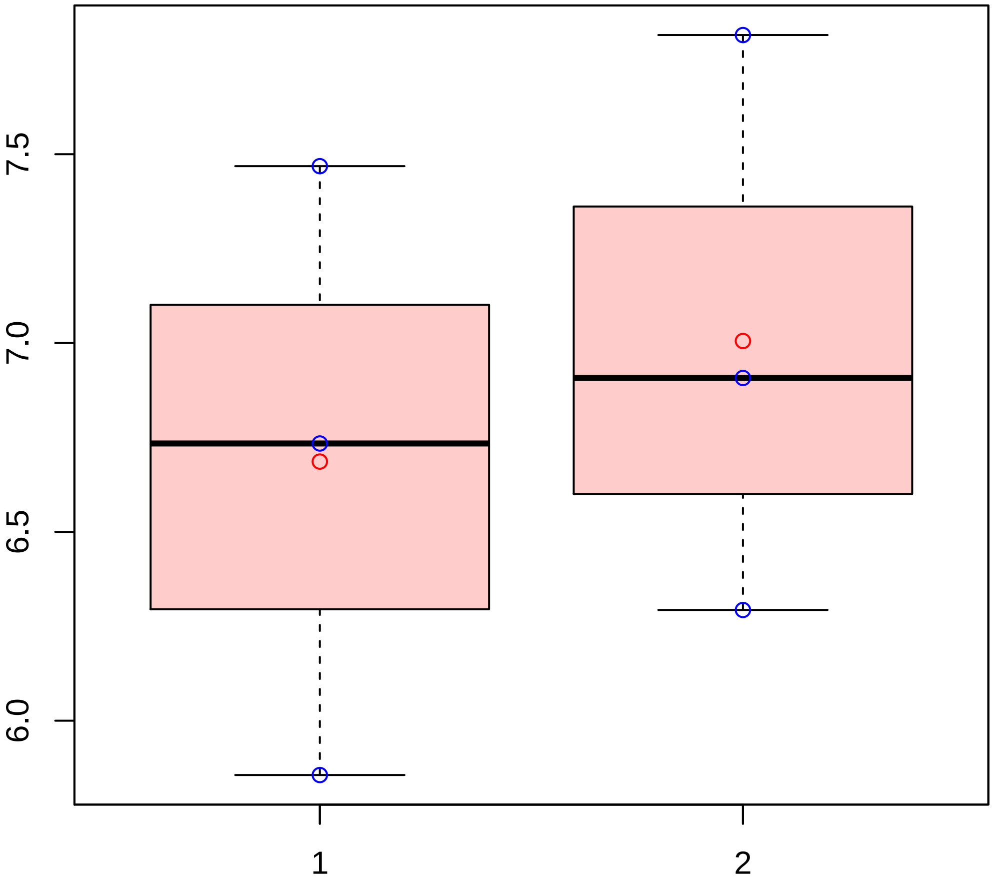


t−Test: p−value = 0.65

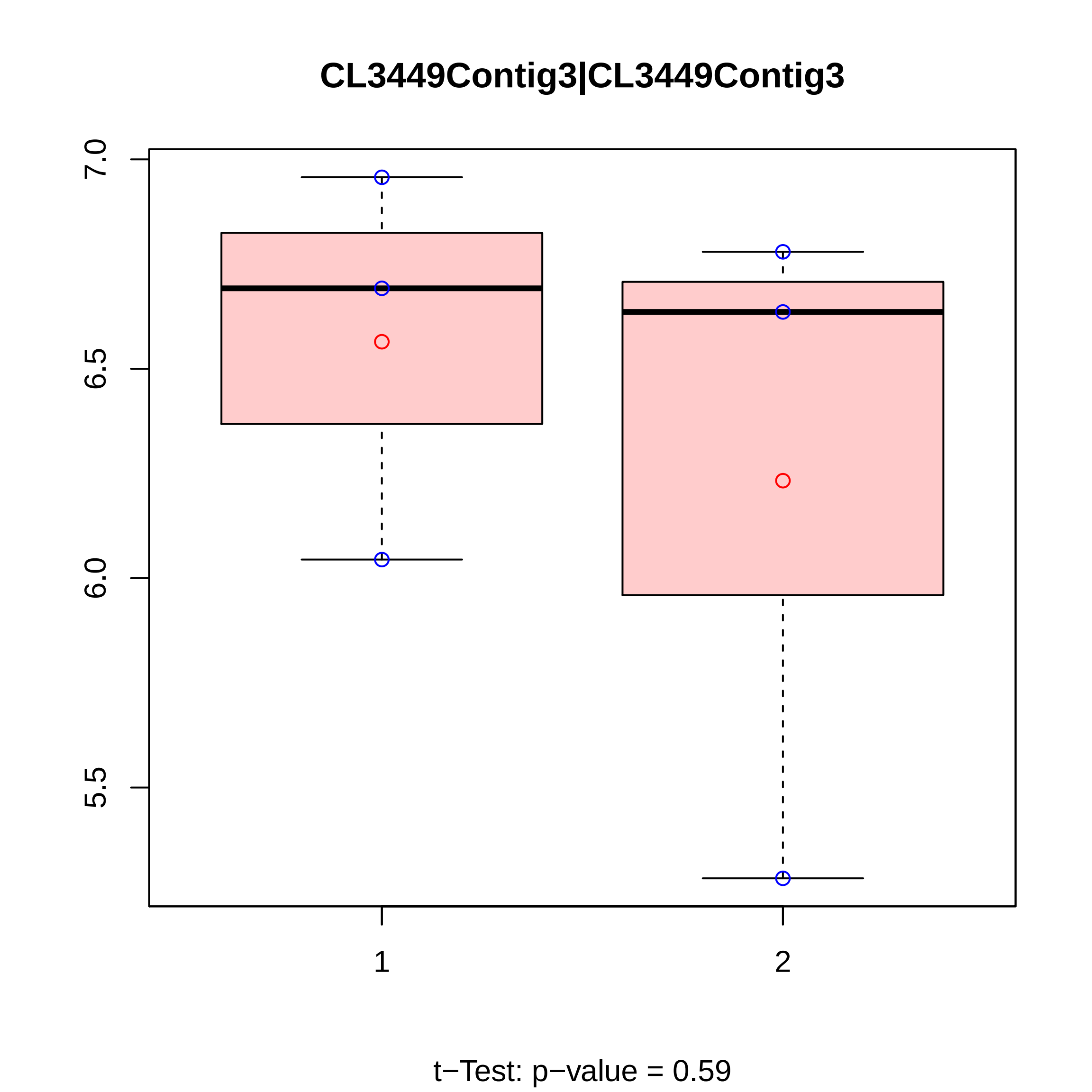

CL3449Contig3|CL3449Contig3
7.0
6.5
6.0
5.5
1
2
t−Test: p−value = 0.59

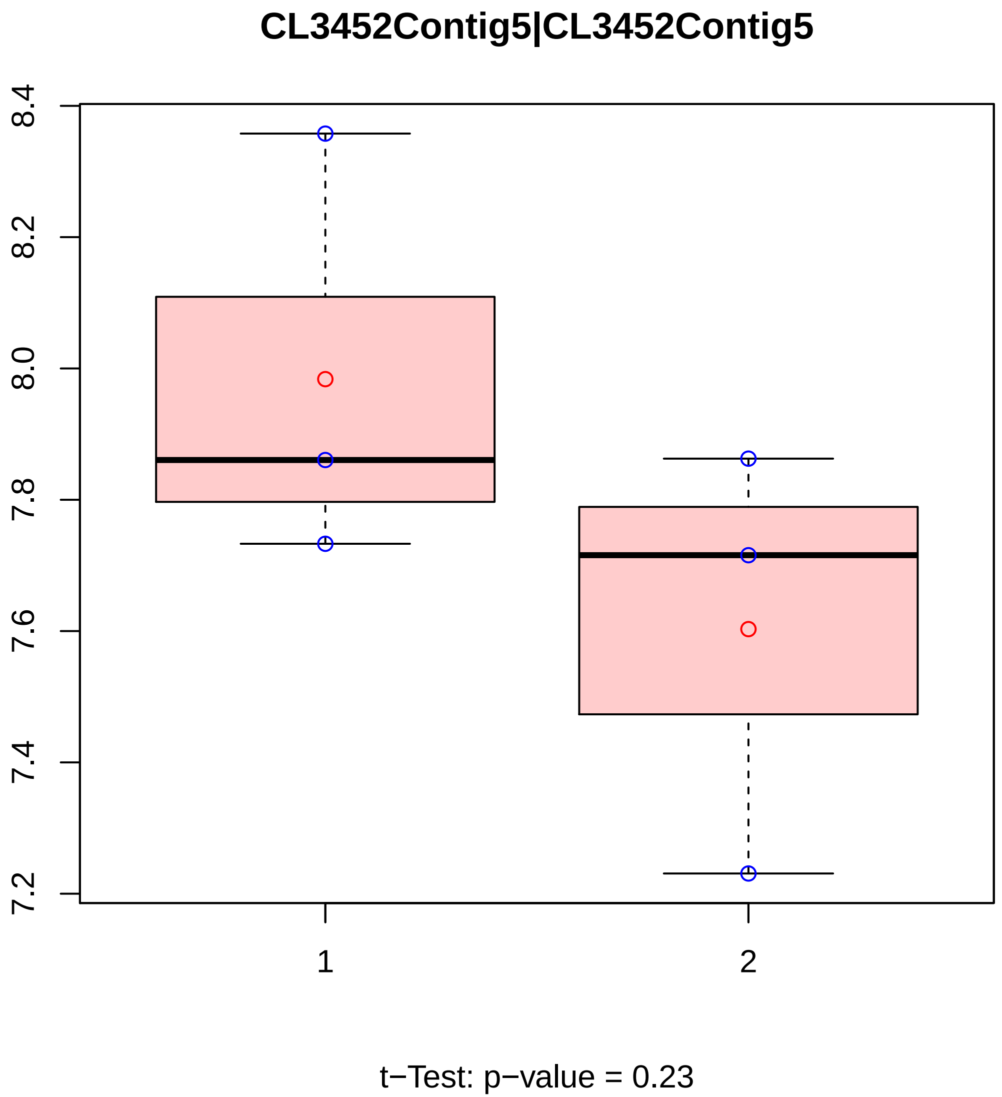
CL3452Contig5|CL3452Contig5
8.4
8.2
8.0
7.8
7.6
7.4
7.2
1
2
t−Test: p−value = 0.23

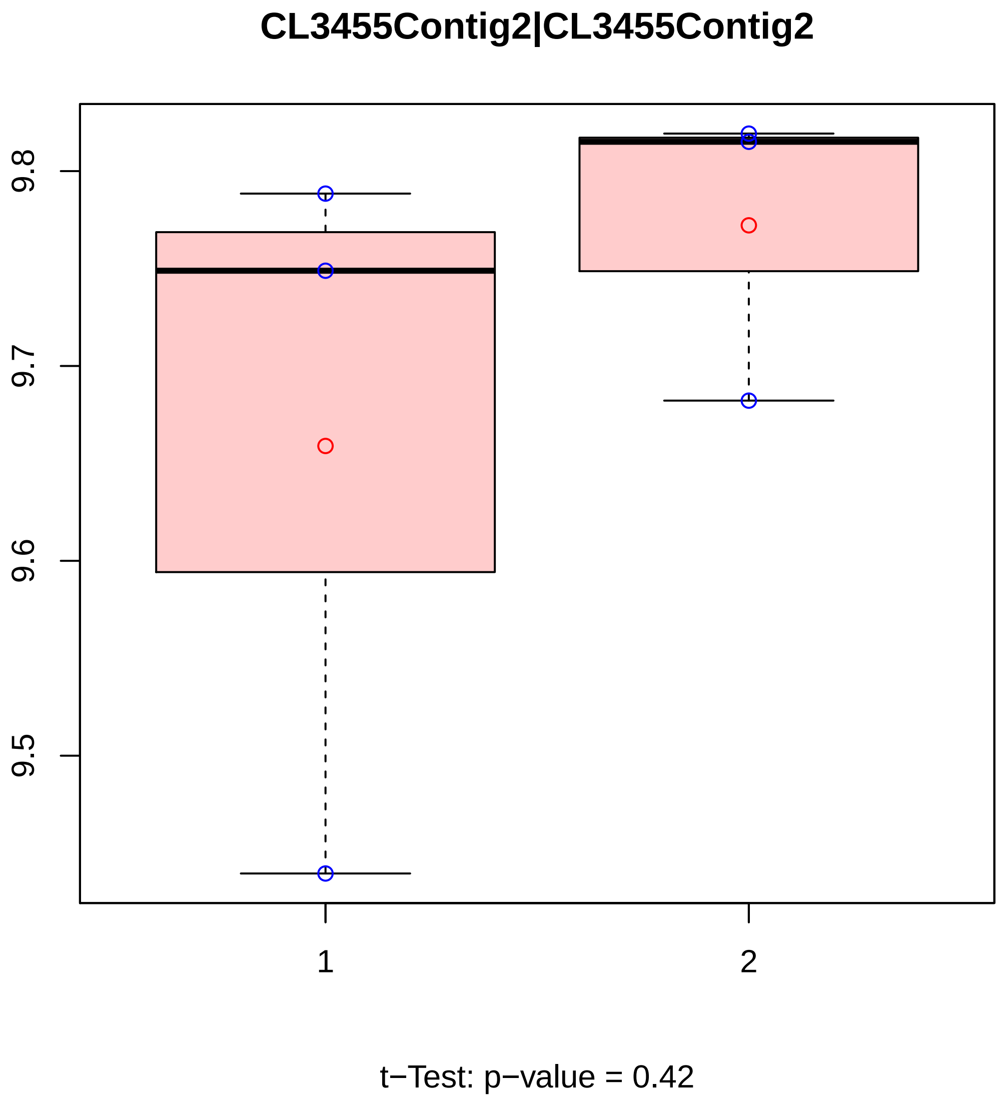
CL3455Contig2|CL3455Contig2
9.8
9.7
9.6
9.5
1
2
t−Test: p−value = 0.42

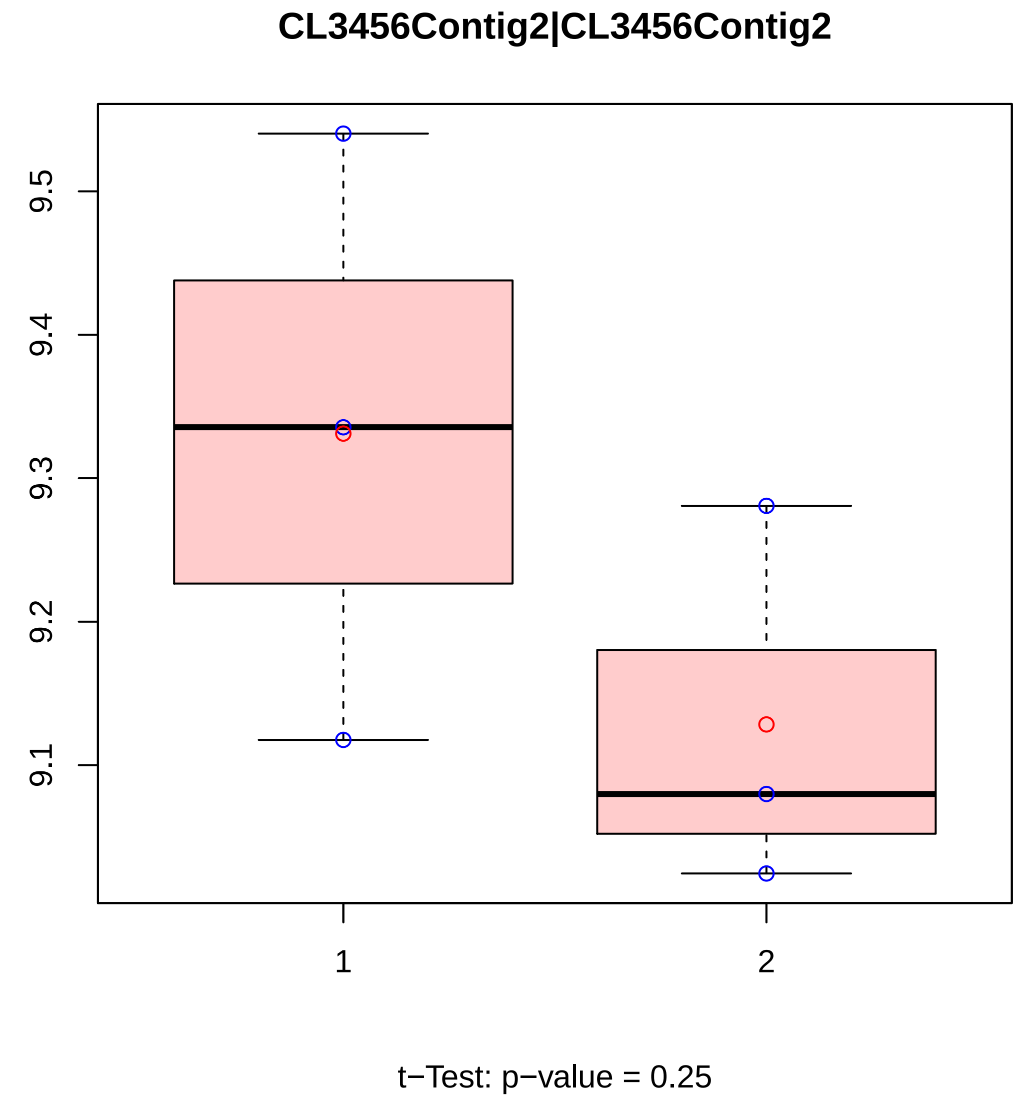

CL3456Contig2|CL3456Contig2
9.1
9.2
9.3
9.4
9.5
1
2
t−Test: p−value = 0.25

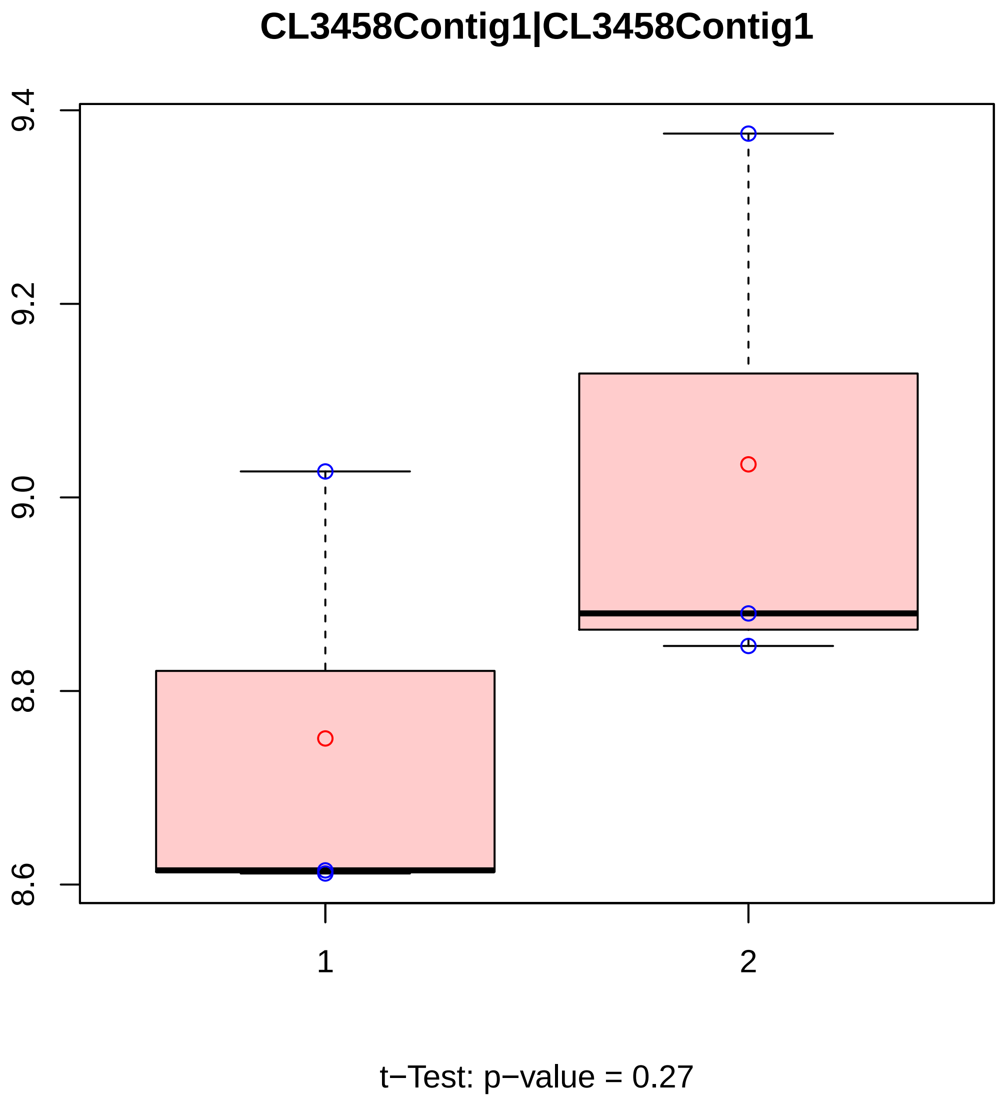
CL3458Contig1|CL3458Contig1
9.4
9.2
9.0
8.8
8.6
1
2
t−Test: p−value = 0.27

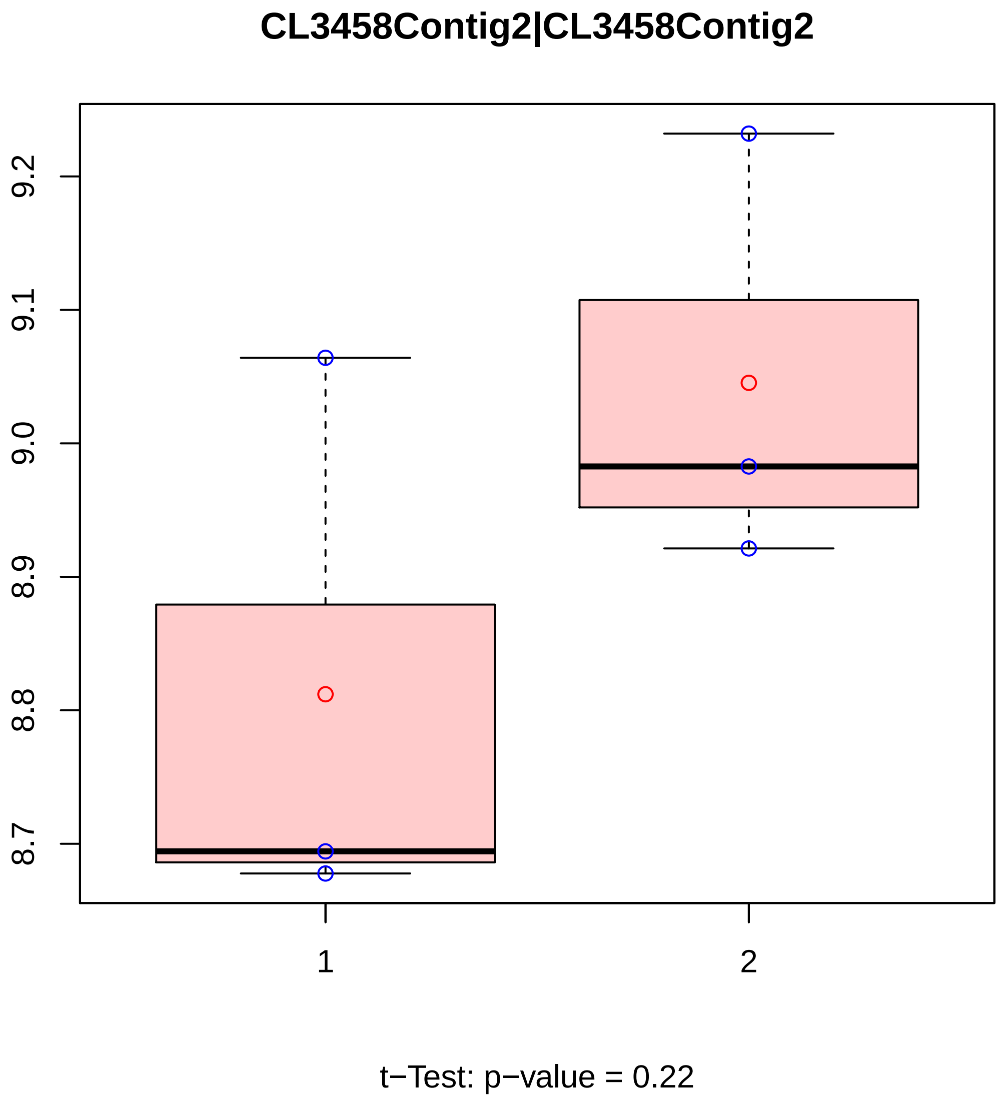
CL3458Contig2|CL3458Contig2
9.2
9.1
9.0
8.9
8.8
8.7
1
2
t−Test: p−value = 0.22

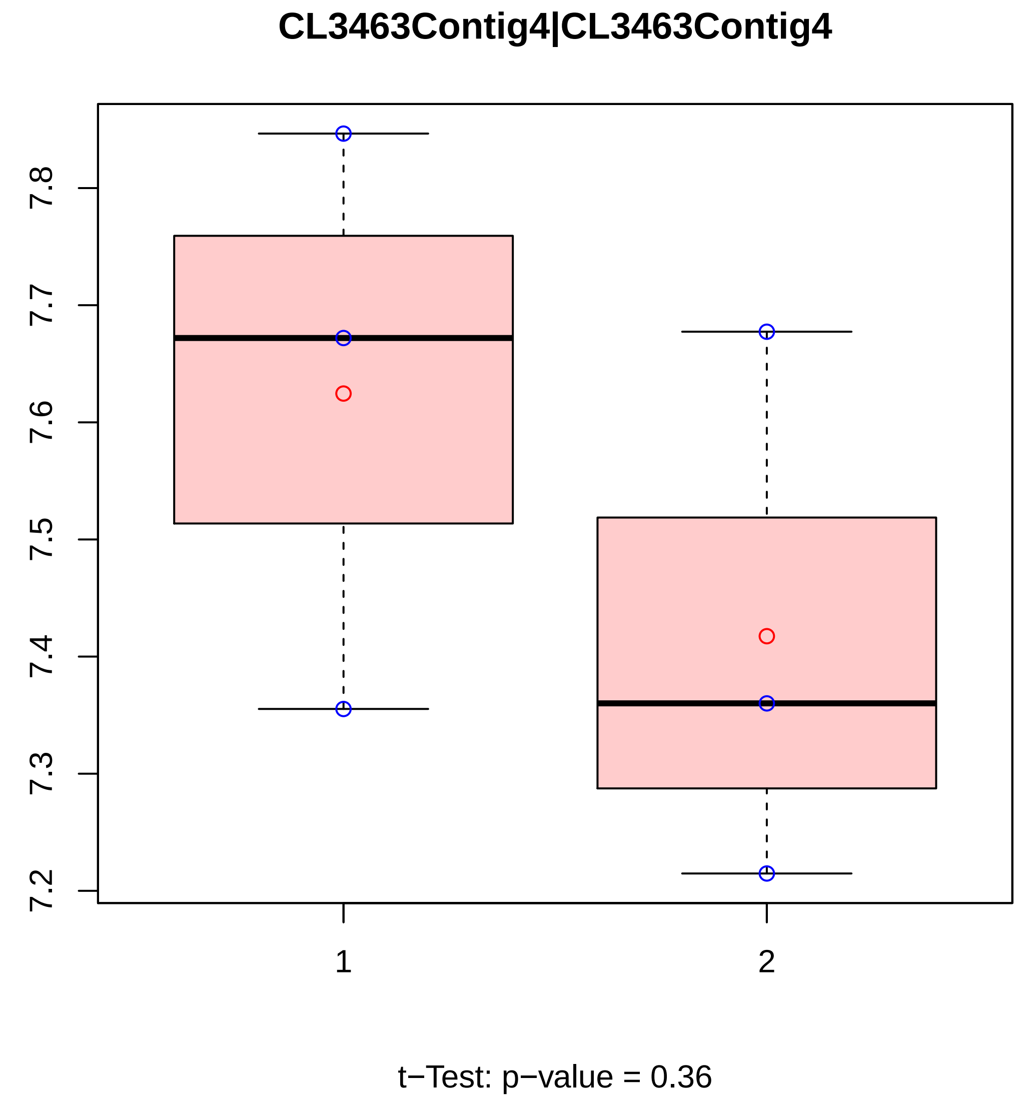

CL3463Contig4|CL3463Contig4
7.2
7.3
7.4
7.5
7.6
7.7
7.8
1
2
t−Test: p−value = 0.36

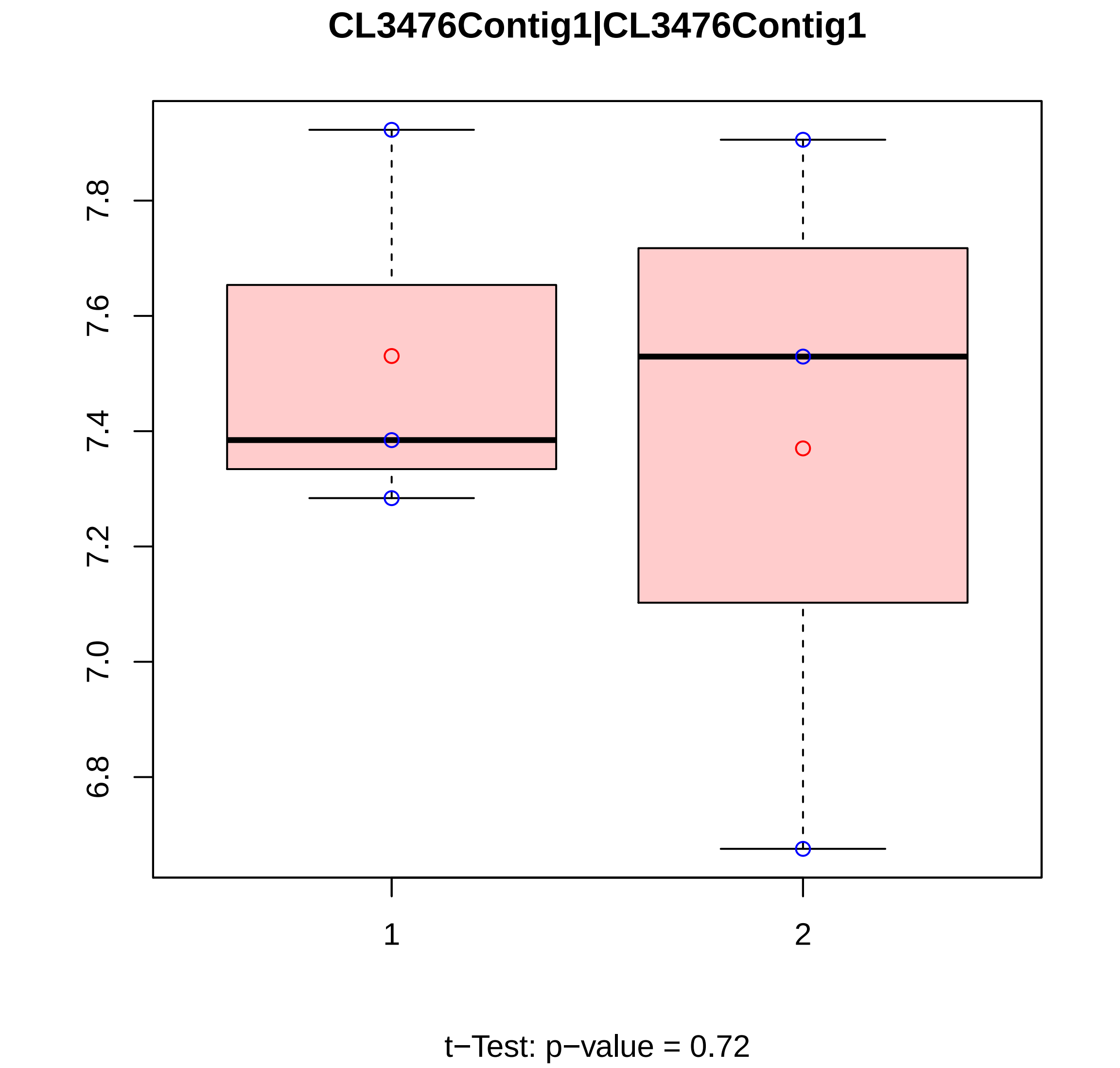

CL3476Contig1|CL3476Contig1
7.8
7.6
7.4
7.2
7.0
6.8
1
2
t−Test: p−value = 0.72

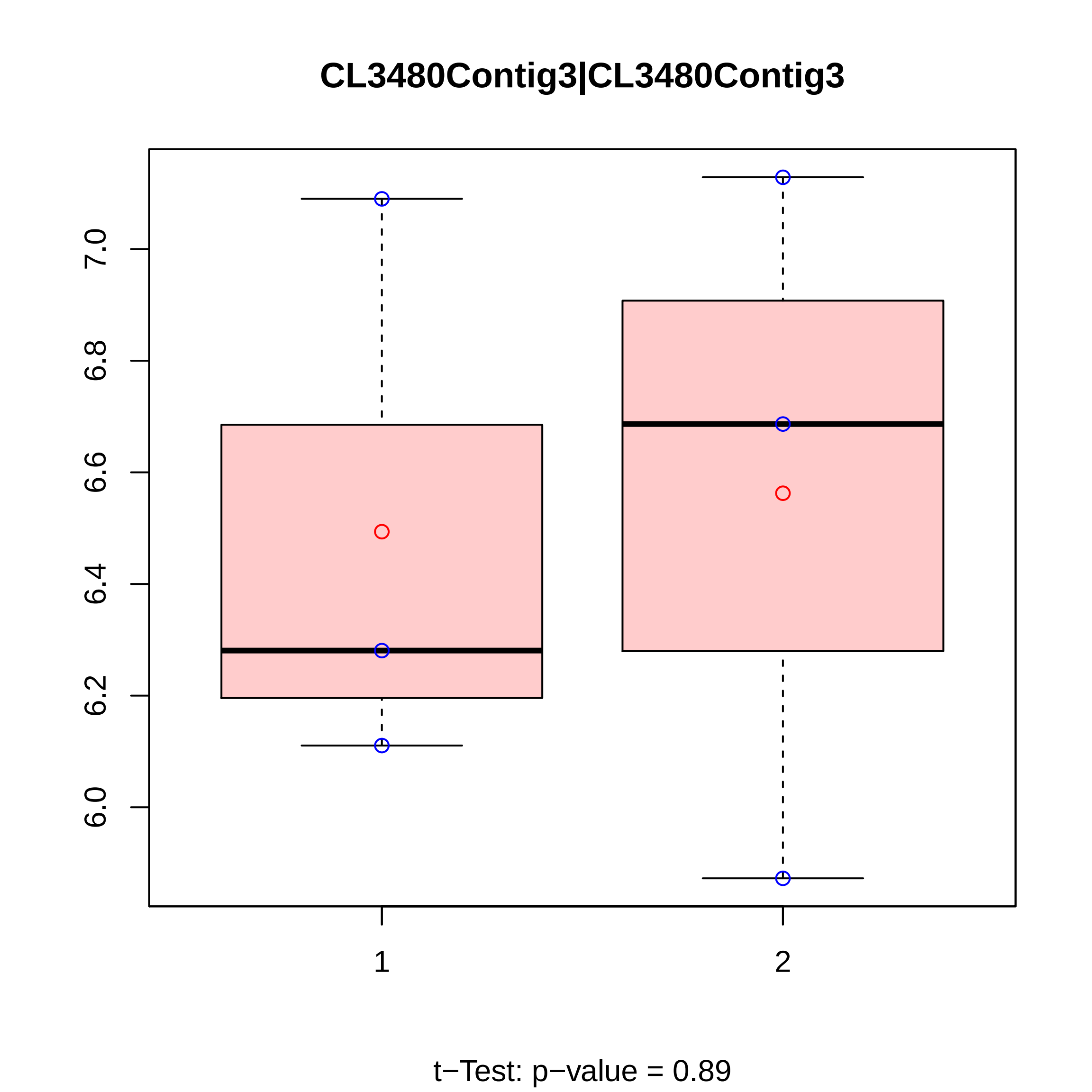

CL3480Contig3|CL3480Contig3
7.0
6.8
6.6
6.4
6.2
6.0
1
2
t−Test: p−value = 0.89

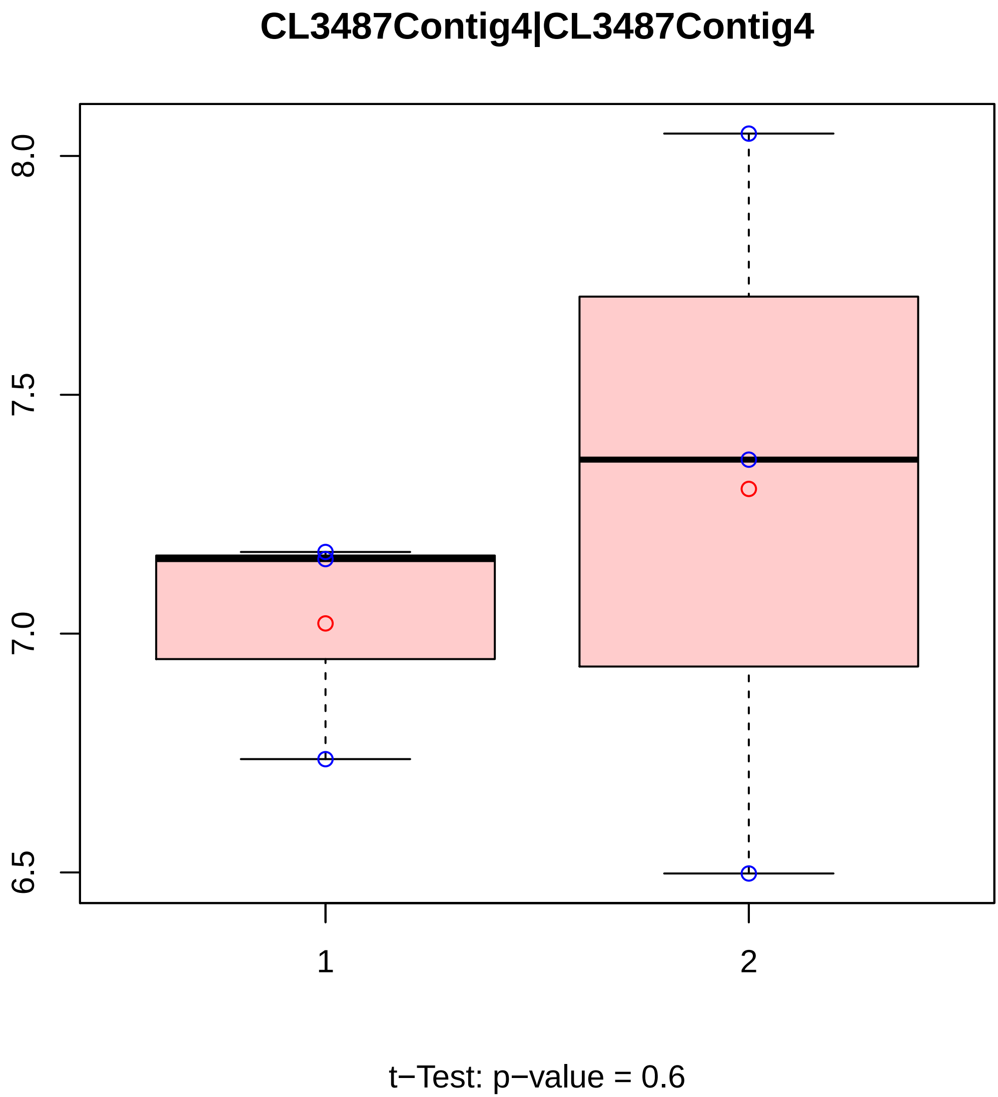
CL3487Contig4|CL3487Contig4
8.0
7.5
7.0
6.5
1
2
t−Test: p−value = 0.6

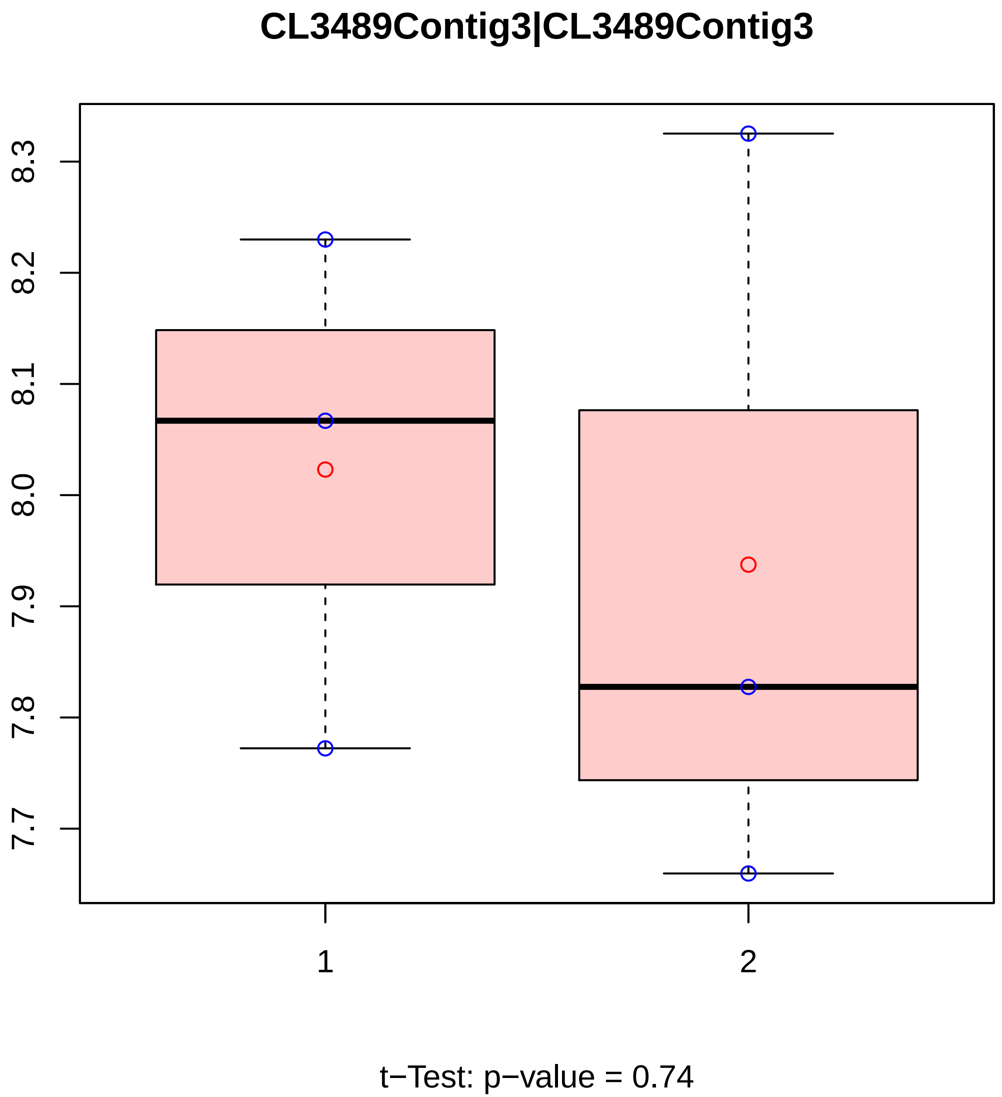
CL3489Contig3|CL3489Contig3
8.3
8.2
8.1
8.0
7.9
7.8
7.7
1
2
t−Test: p−value = 0.74

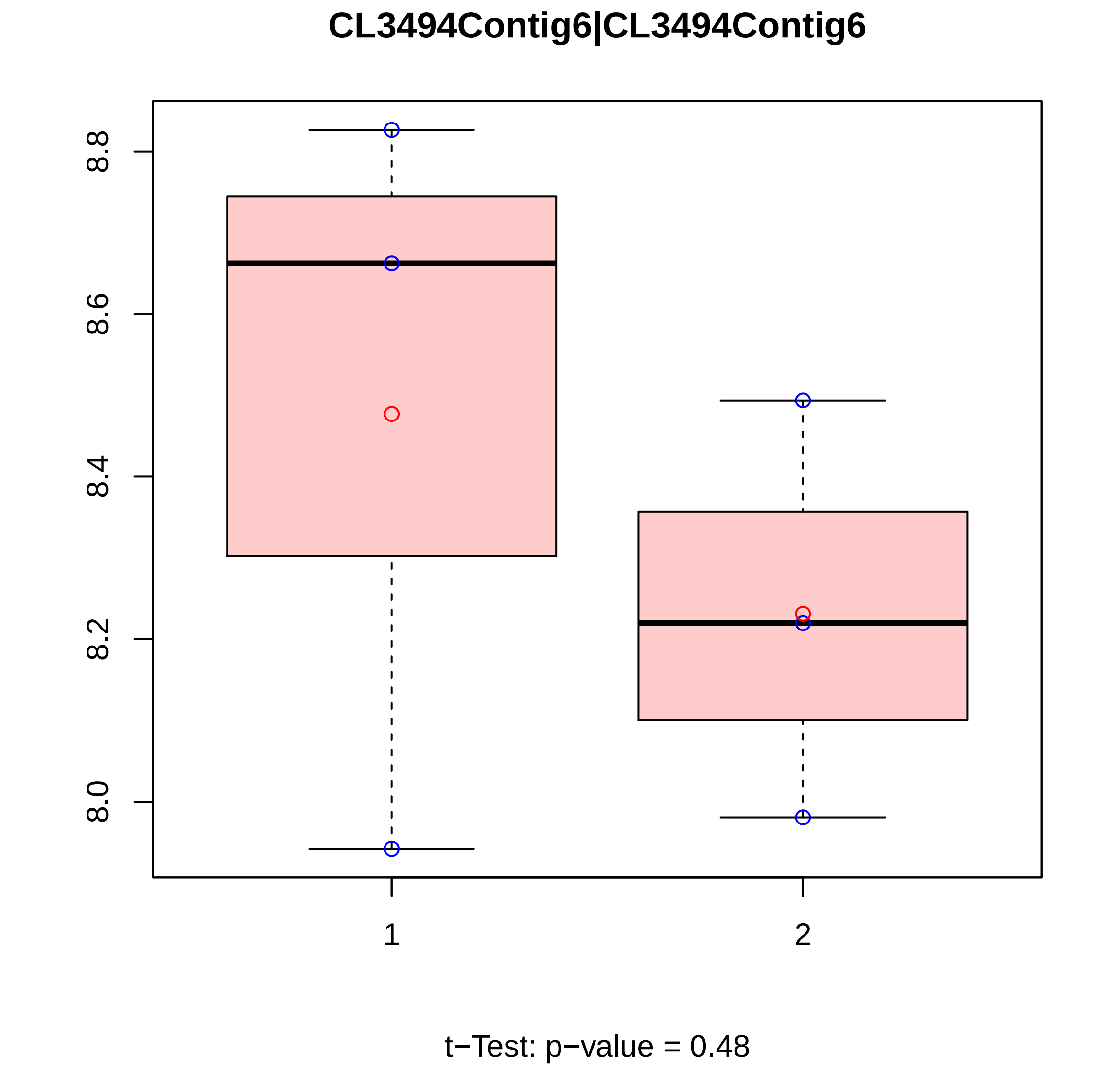

CL3494Contig6|CL3494Contig6
8.0
8.2
8.4
8.6
8.8
1
2
t−Test: p−value = 0.48

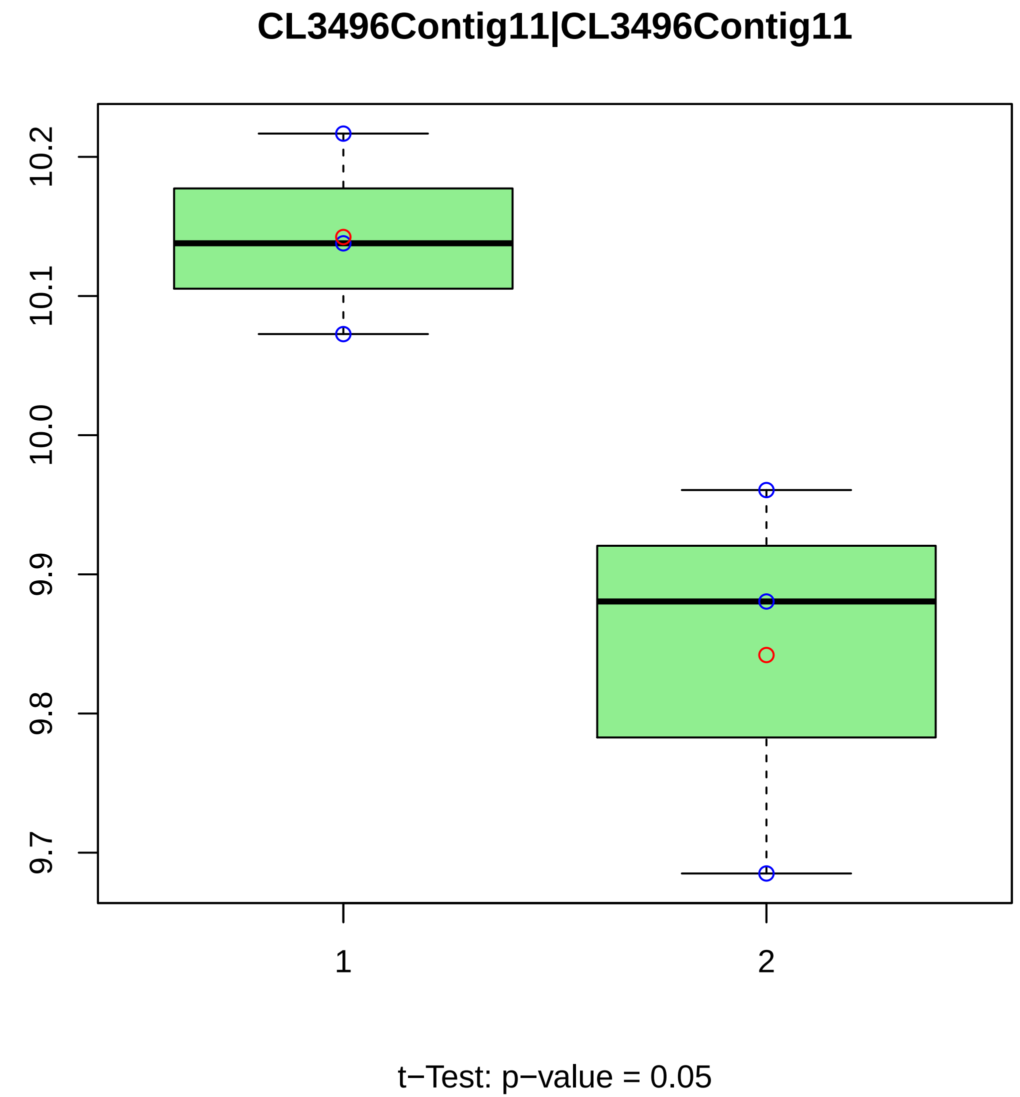
CL3496Contig11|CL3496Contig11
10.2
10.1
10.0
9.9
9.8
9.7
1
2
t−Test: p−value = 0.05

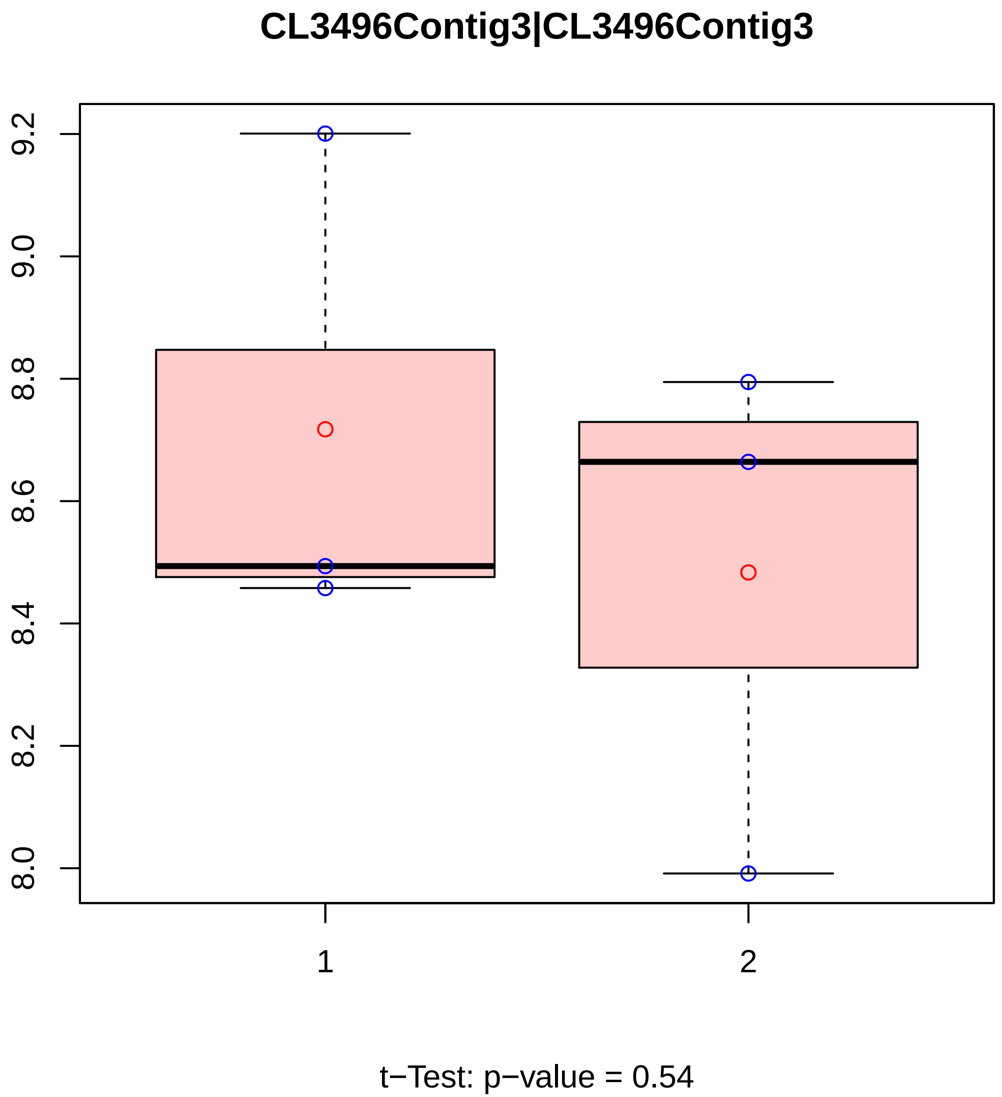
CL3496Contig3|CL3496Contig3
9.2
9.0
8.8
8.6
8.4
8.2
8.0
1
2
t−Test: p−value = 0.54

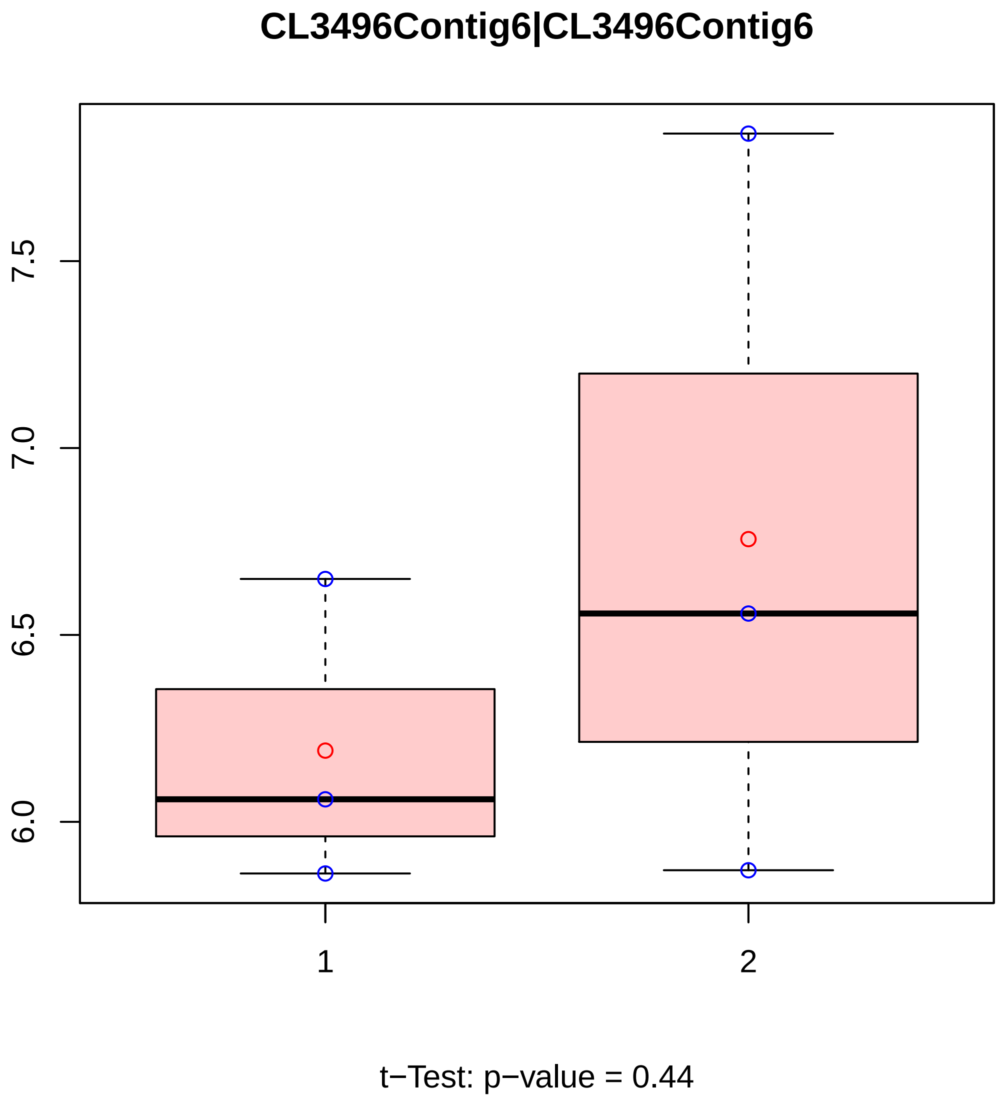
CL3496Contig6|CL3496Contig6
7.5
7.0
6.5
6.0
1
2
t−Test: p−value = 0.44

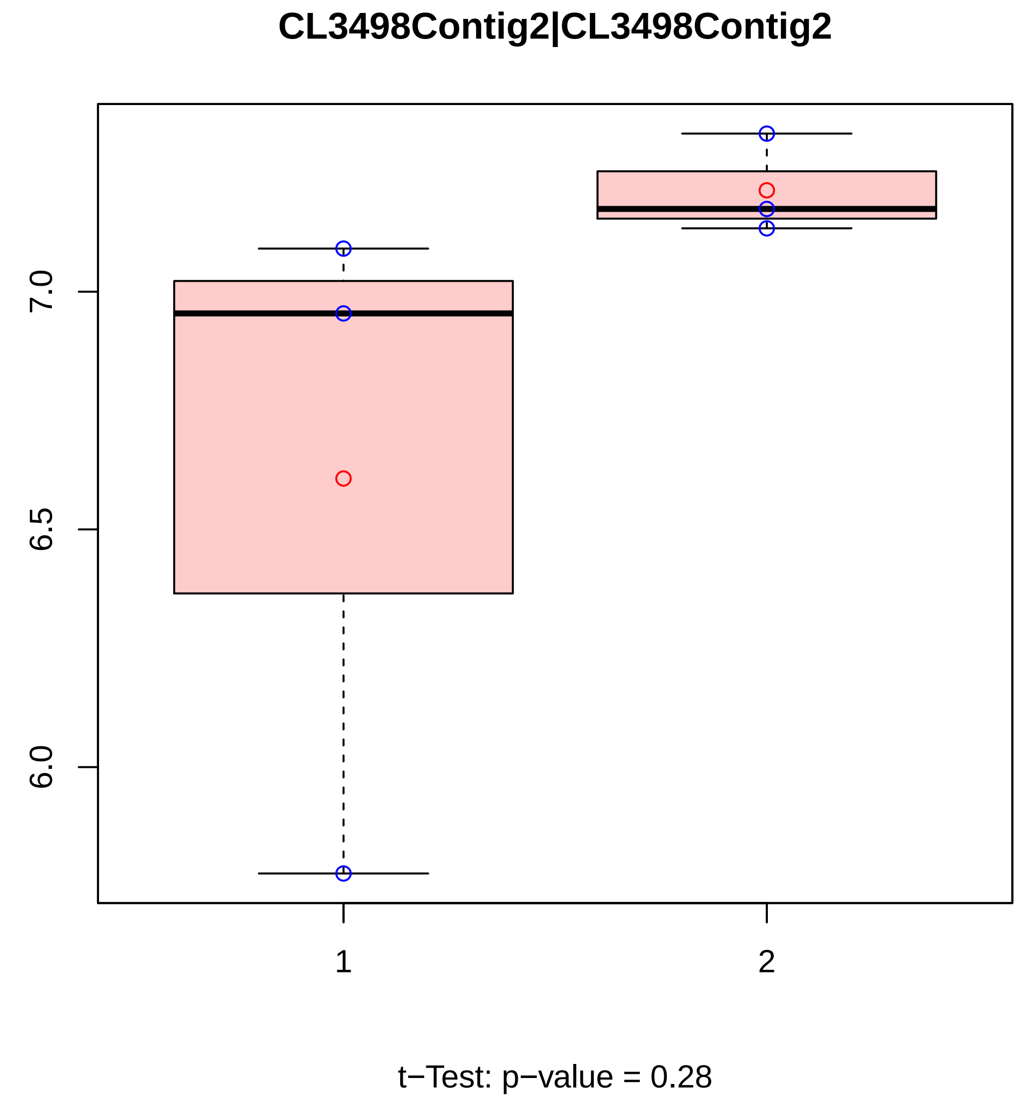
CL3498Contig2|CL3498Contig2
7.0
6.5
6.0
1
2
t−Test: p−value = 0.28

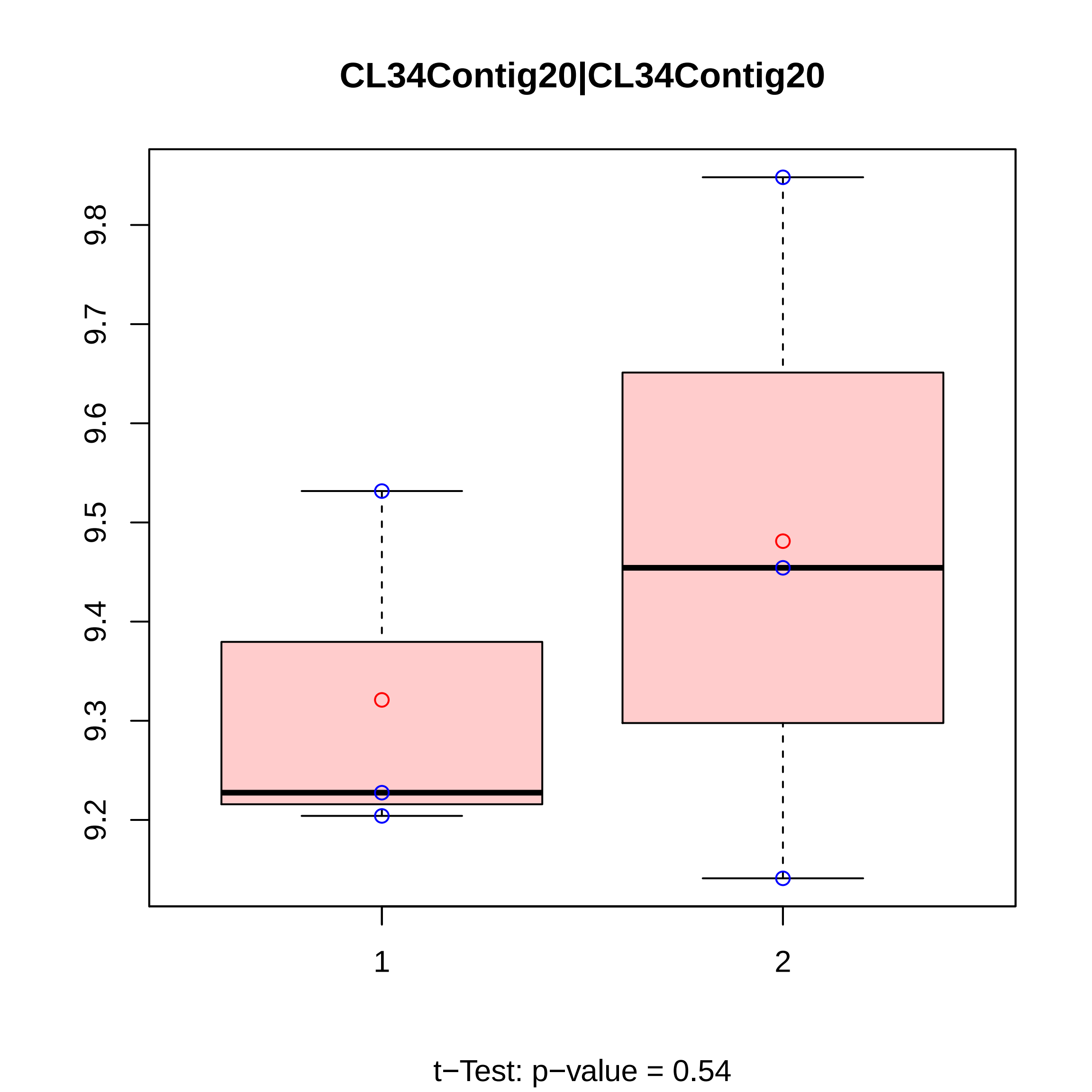
CL34Contig20|CL34Contig20
9.2
9.3
9.4
9.5
9.6
9.7
9.8
1
2
t−Test: p−value = 0.54

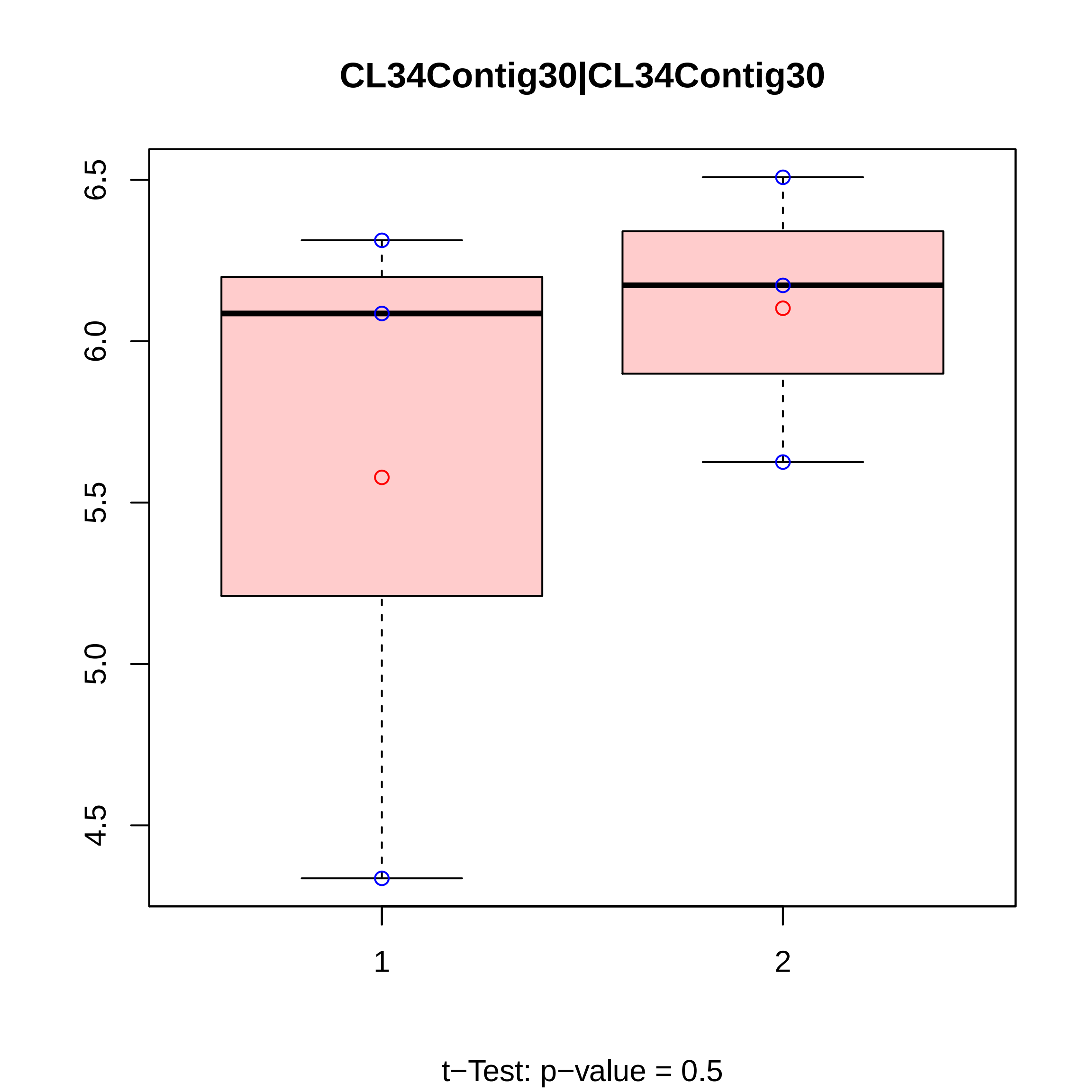

CL34Contig30|CL34Contig30
6.5
6.0
5.5
5.0
4.5
1
2
t−Test: p−value = 0.5

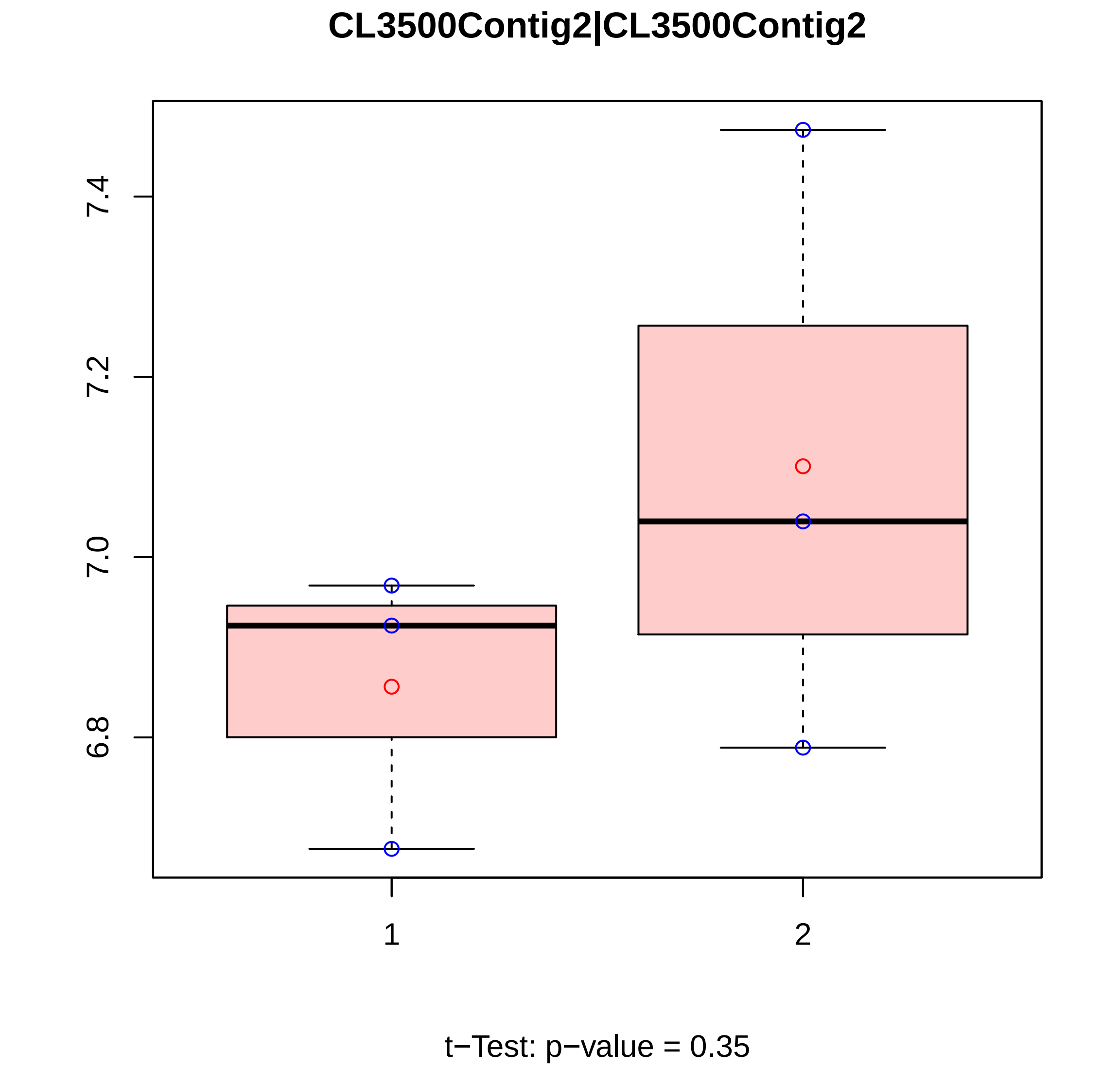
CL3500Contig2|CL3500Contig2
7.4
7.2
7.0
6.8
1
2
t−Test: p−value = 0.35

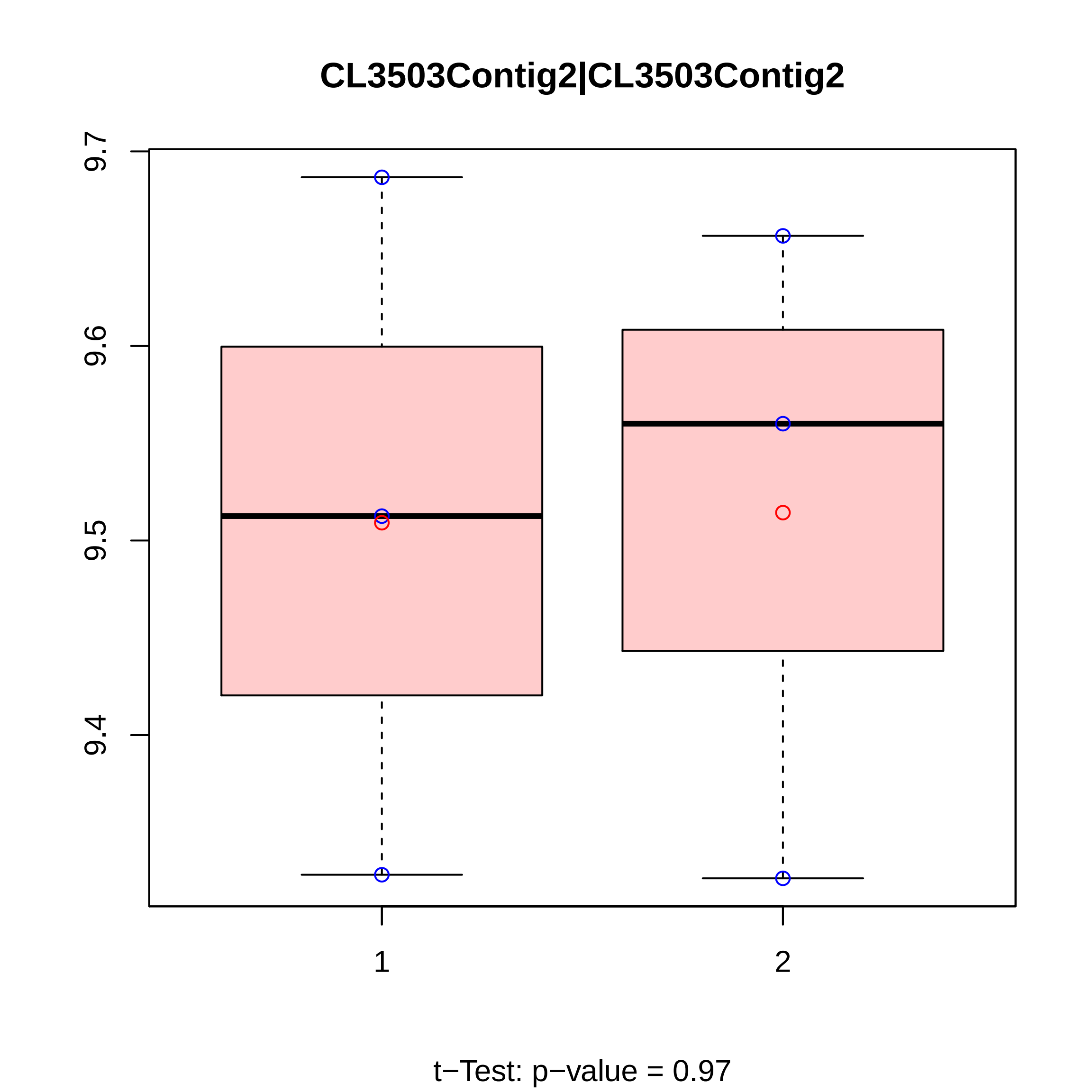
CL3503Contig2|CL3503Contig2
9.4
9.5
9.6
9.7
1
2
t−Test: p−value = 0.97

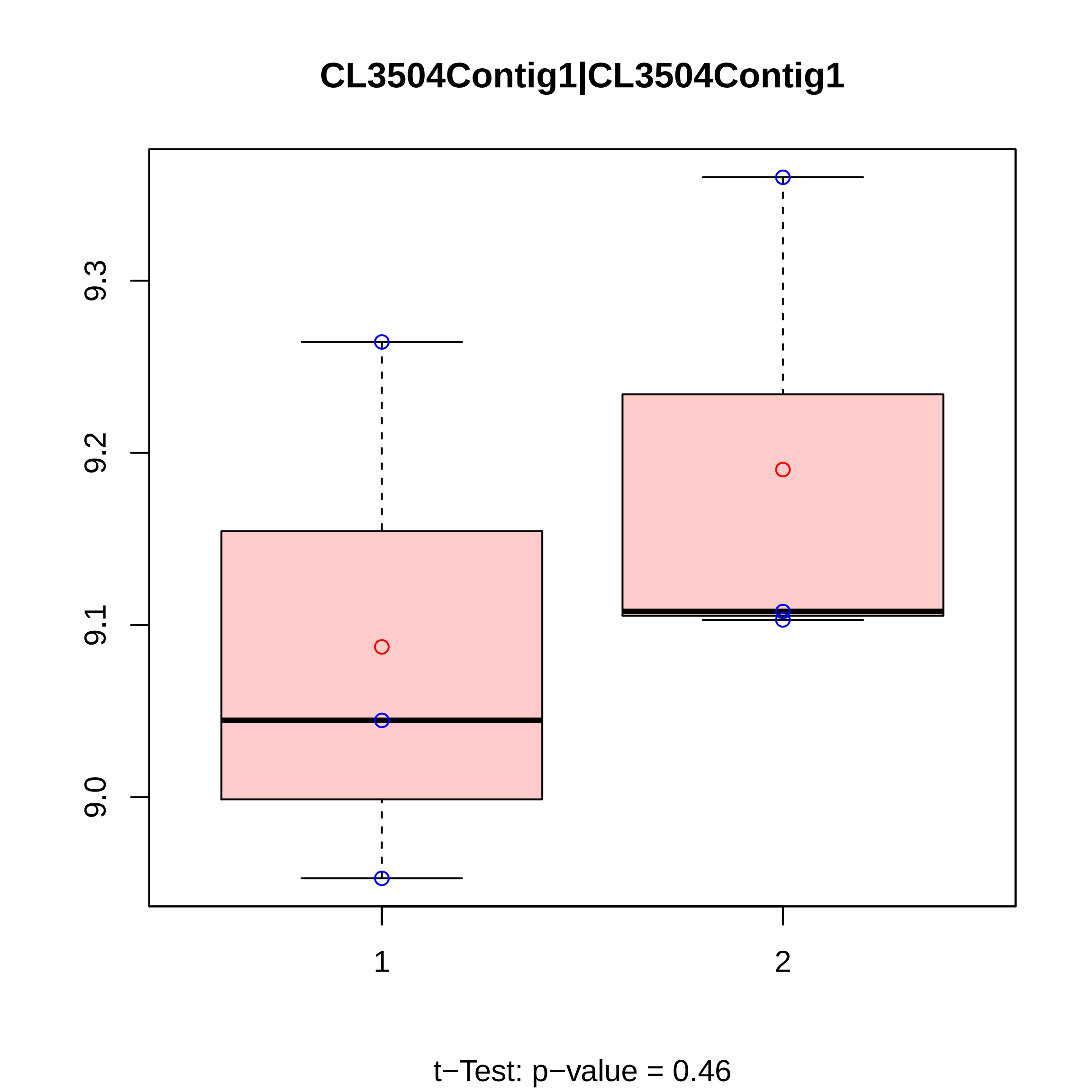
CL3504Contig1|CL3504Contig1
9.0
9.1
9.2
9.3
1
2
t−Test: p−value = 0.46

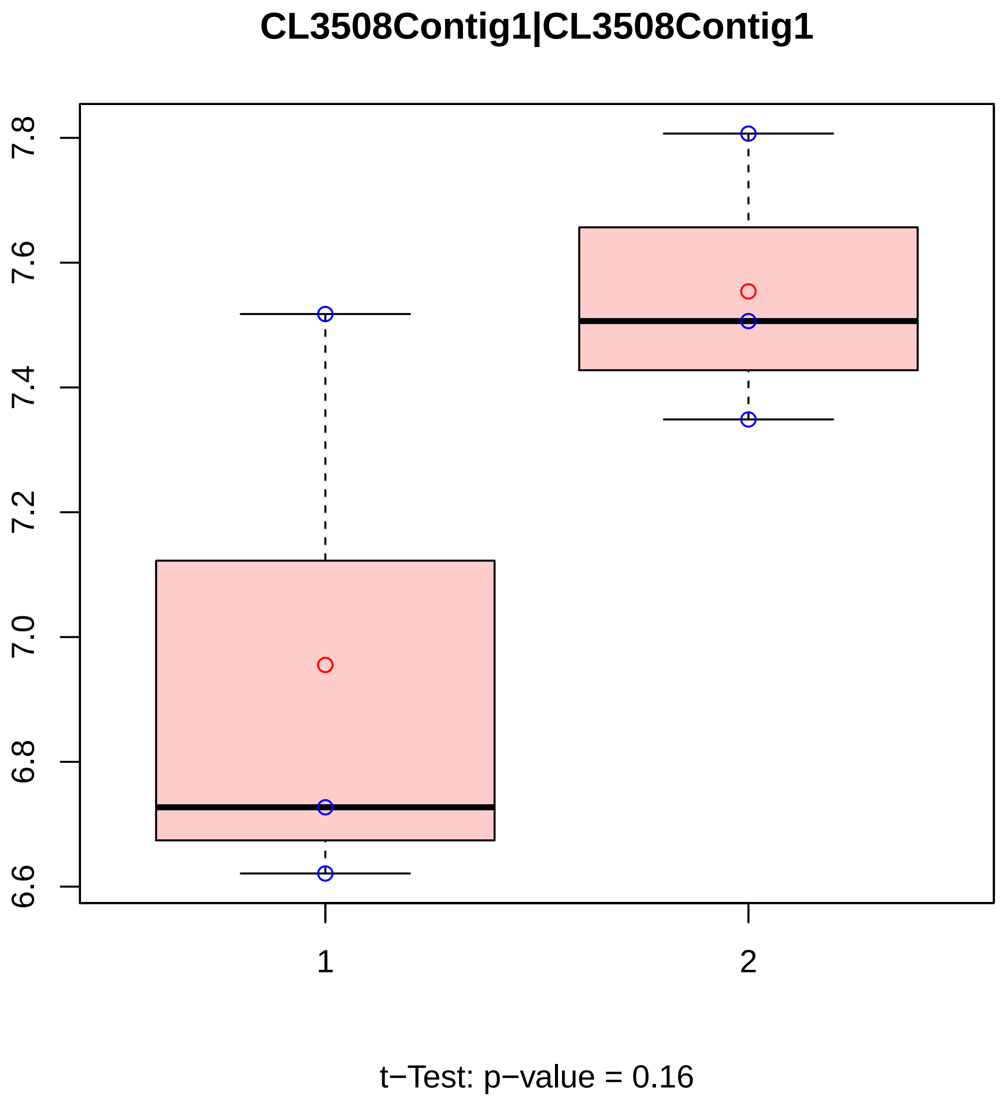
CL3508Contig1|CL3508Contig1
7.8
7.6
7.4
7.2
7.0
6.8
6.6
1
2
t−Test: p−value = 0.16

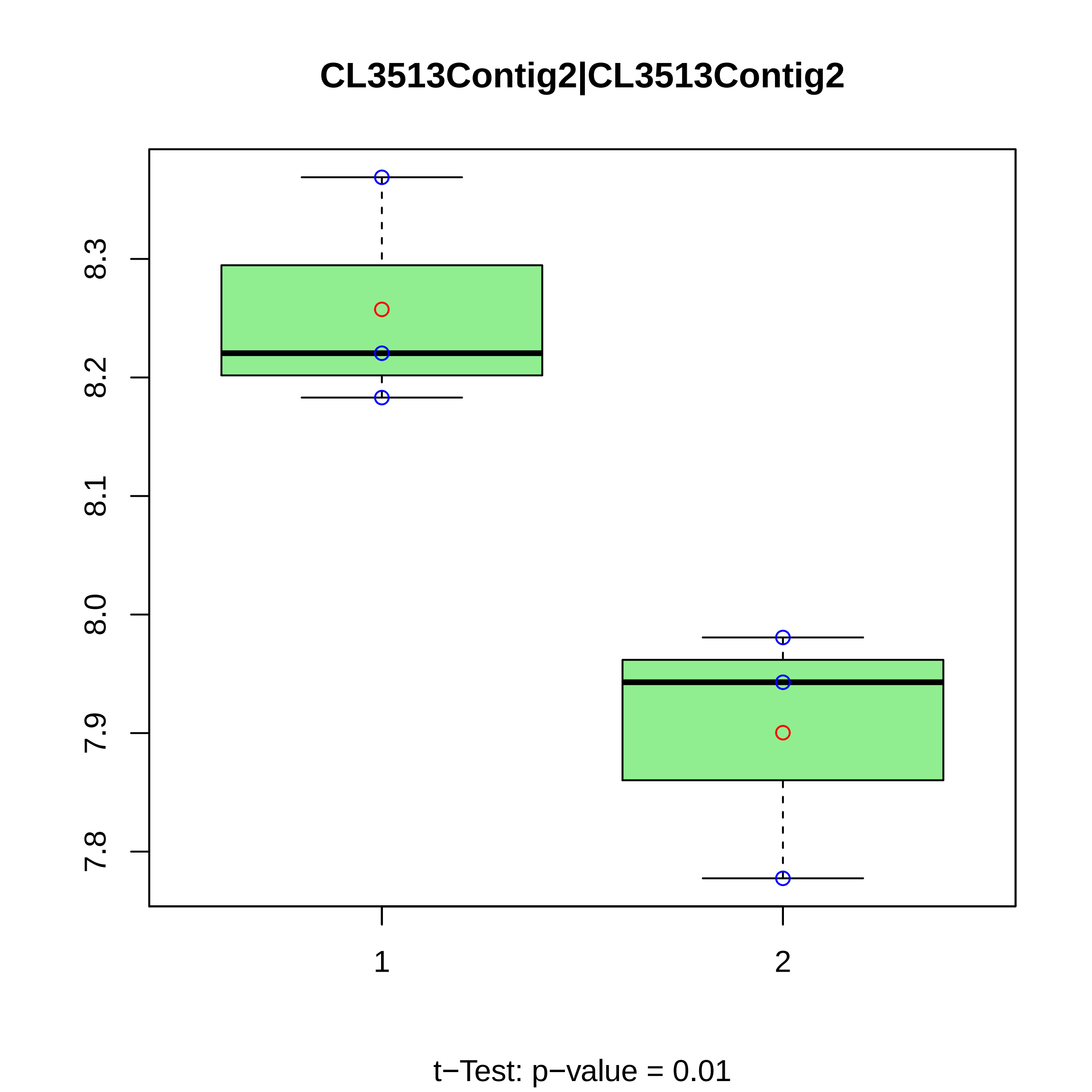

CL3513Contig2|CL3513Contig2
8.3
8.2
8.1
8.0
7.9
7.8
1
2
t−Test: p−value = 0.01

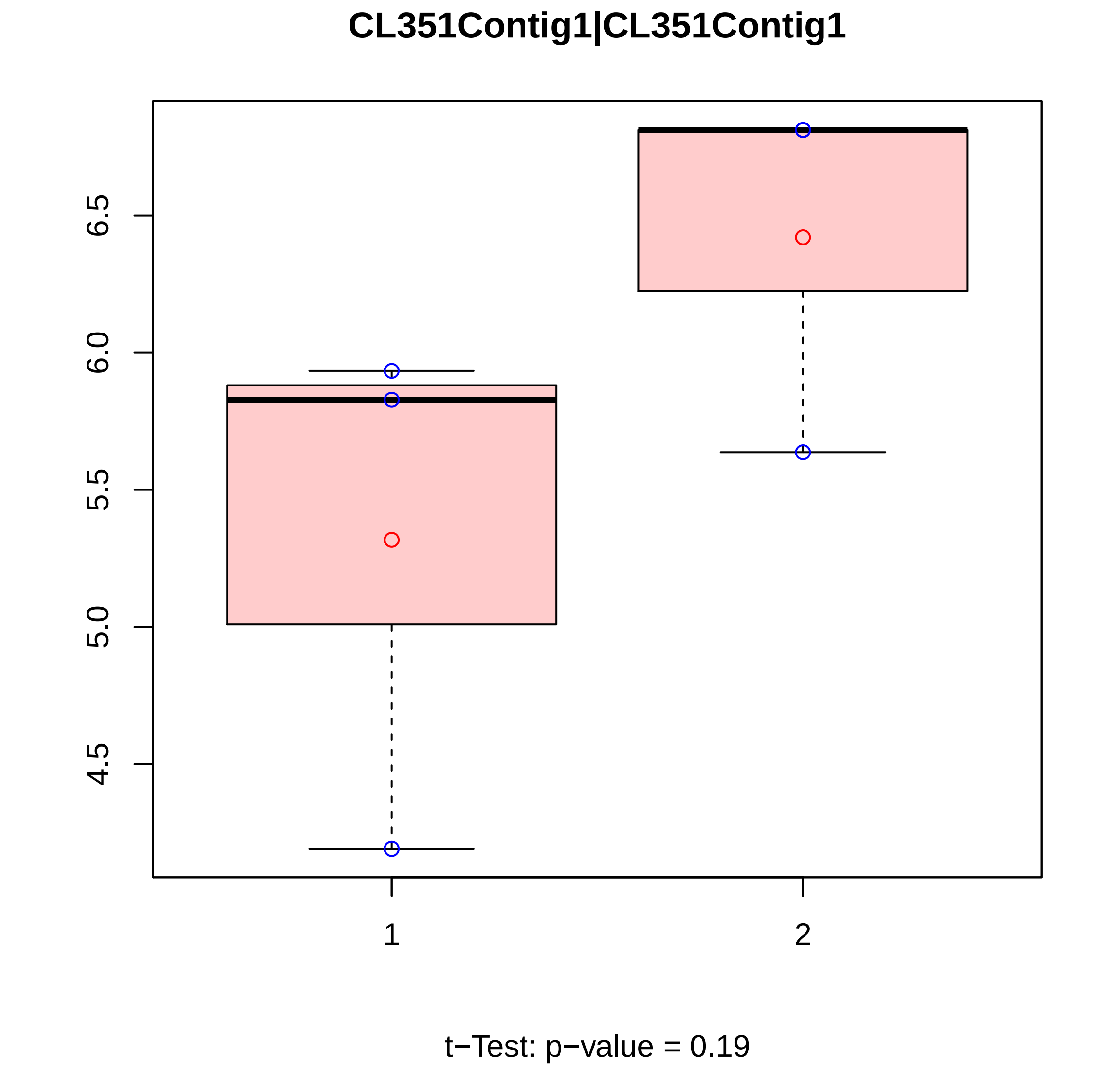

CL351Contig1|CL351Contig1
6.5
6.0
5.5
5.0
4.5
1
2
t−Test: p−value = 0.19

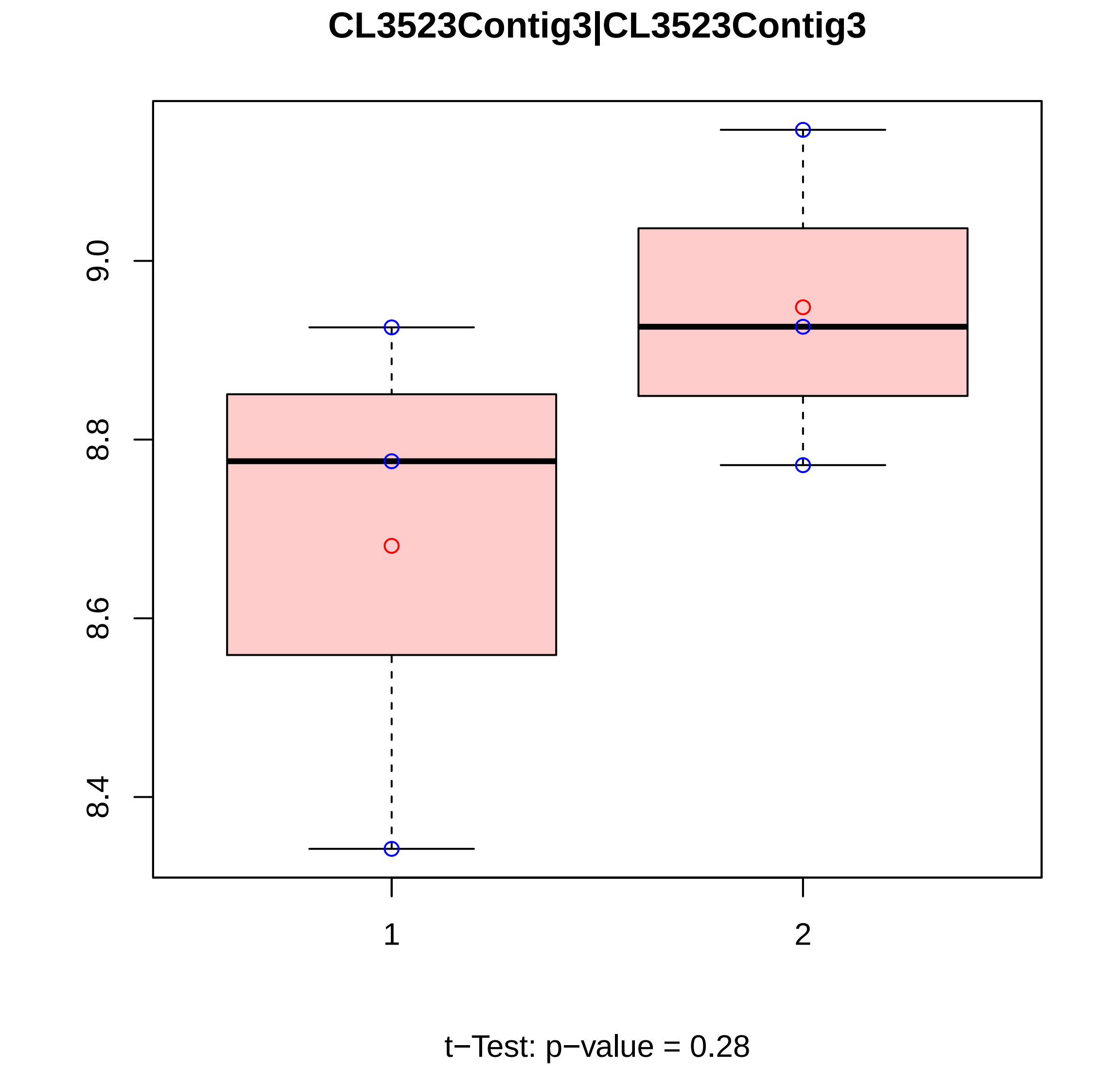
CL3523Contig3|CL3523Contig3
9.0
8.8
8.6
8.4
1
2
t−Test: p−value = 0.28

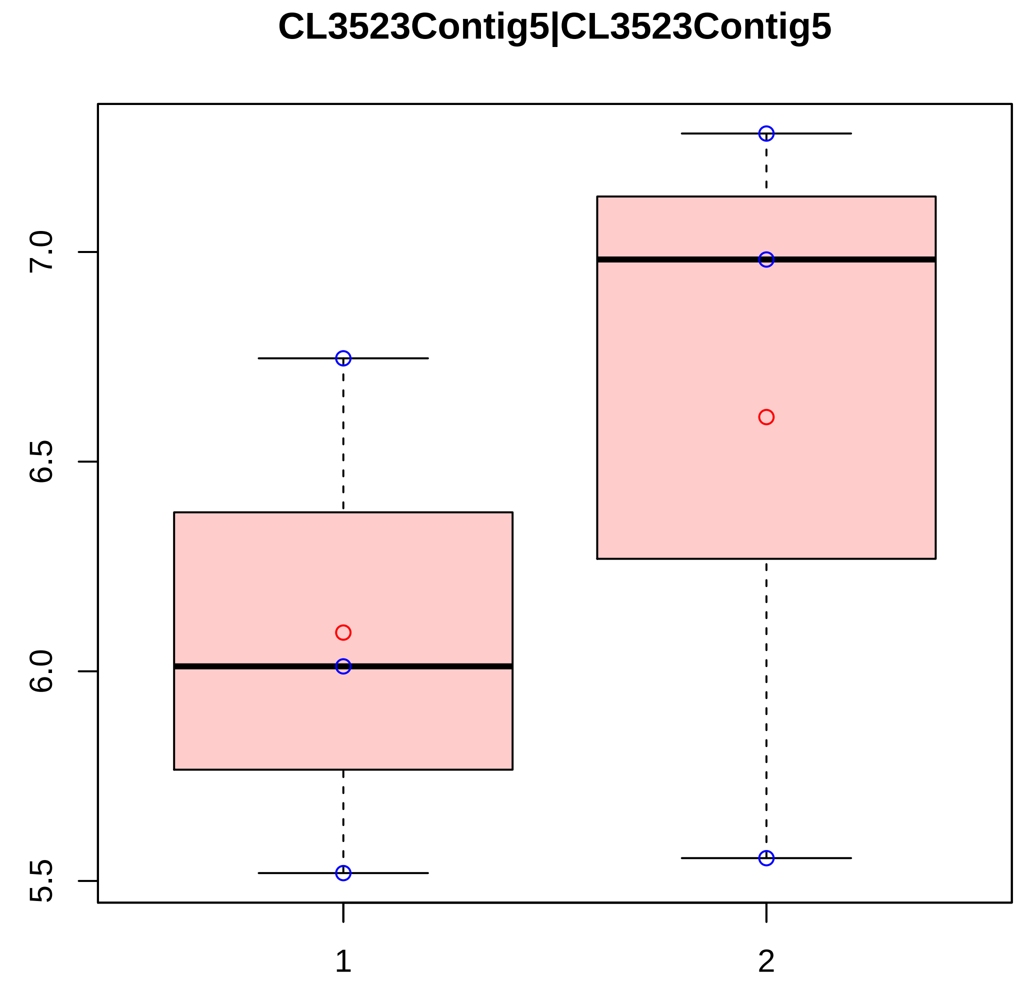


t−Test: p−value = 0.47

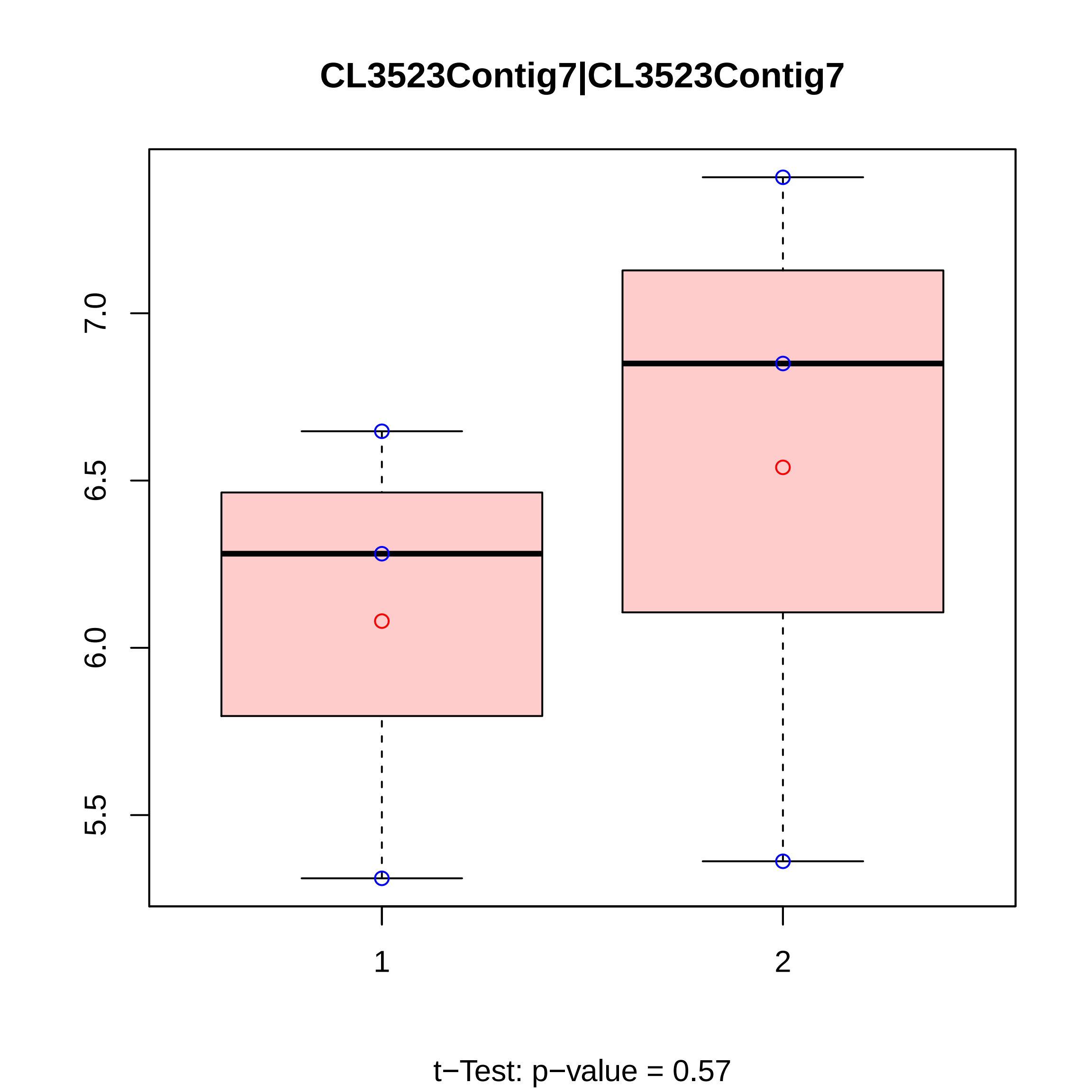
CL3523Contig7|CL3523Contig7
7.0
6.5
6.0
5.5
1
2
t−Test: p−value = 0.57

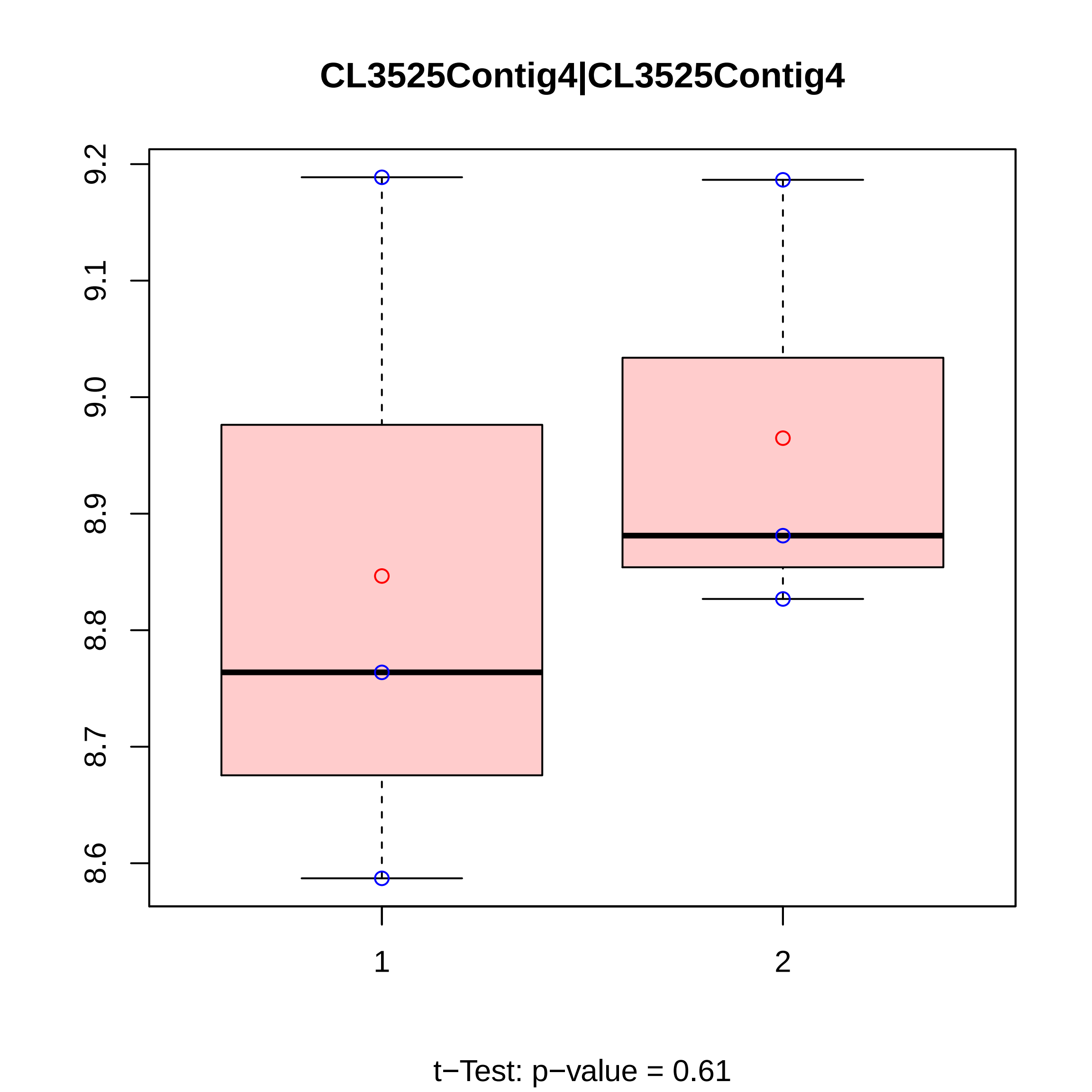

CL3525Contig4|CL3525Contig4
9.2
9.1
9.0
8.9
8.8
8.7
8.6
1
2
t−Test: p−value = 0.61

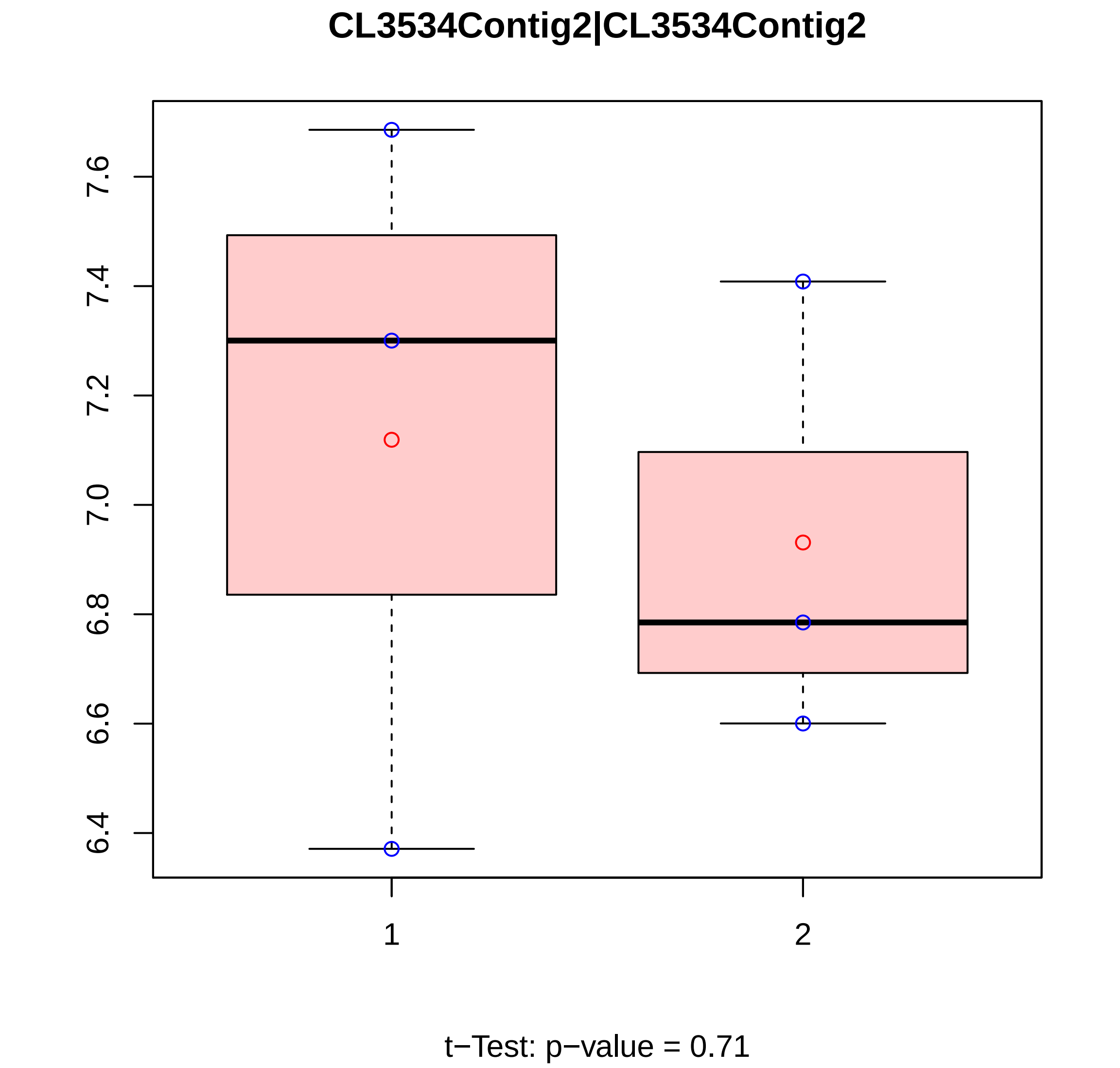
CL3534Contig2|CL3534Contig2
7.6
7.4
7.2
7.0
6.8
6.6
6.4
1
2
t−Test: p−value = 0.71

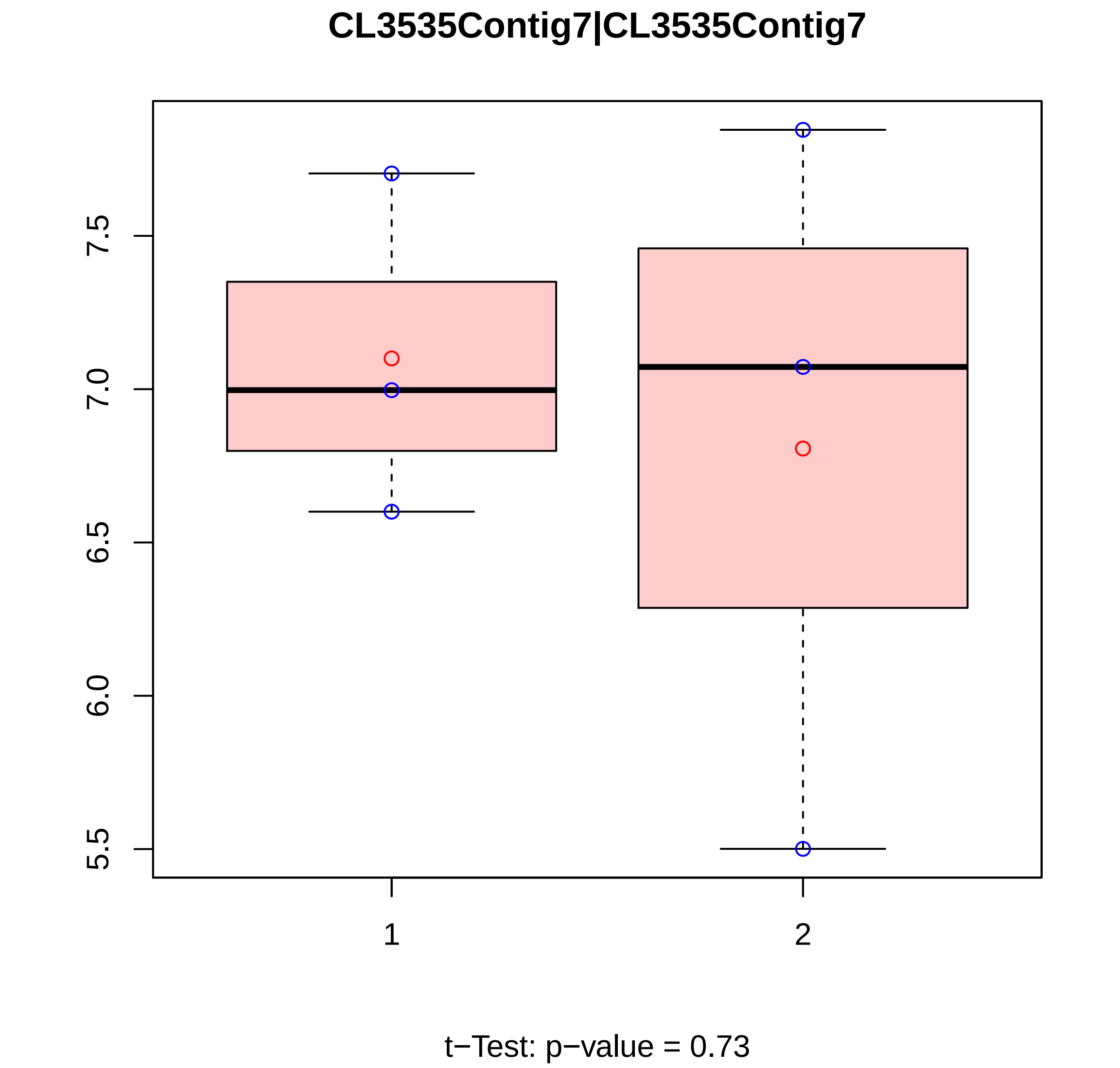
CL3535Contig7|CL3535Contig7
7.5
7.0
6.5
6.0
5.5
1
2
t−Test: p−value = 0.73

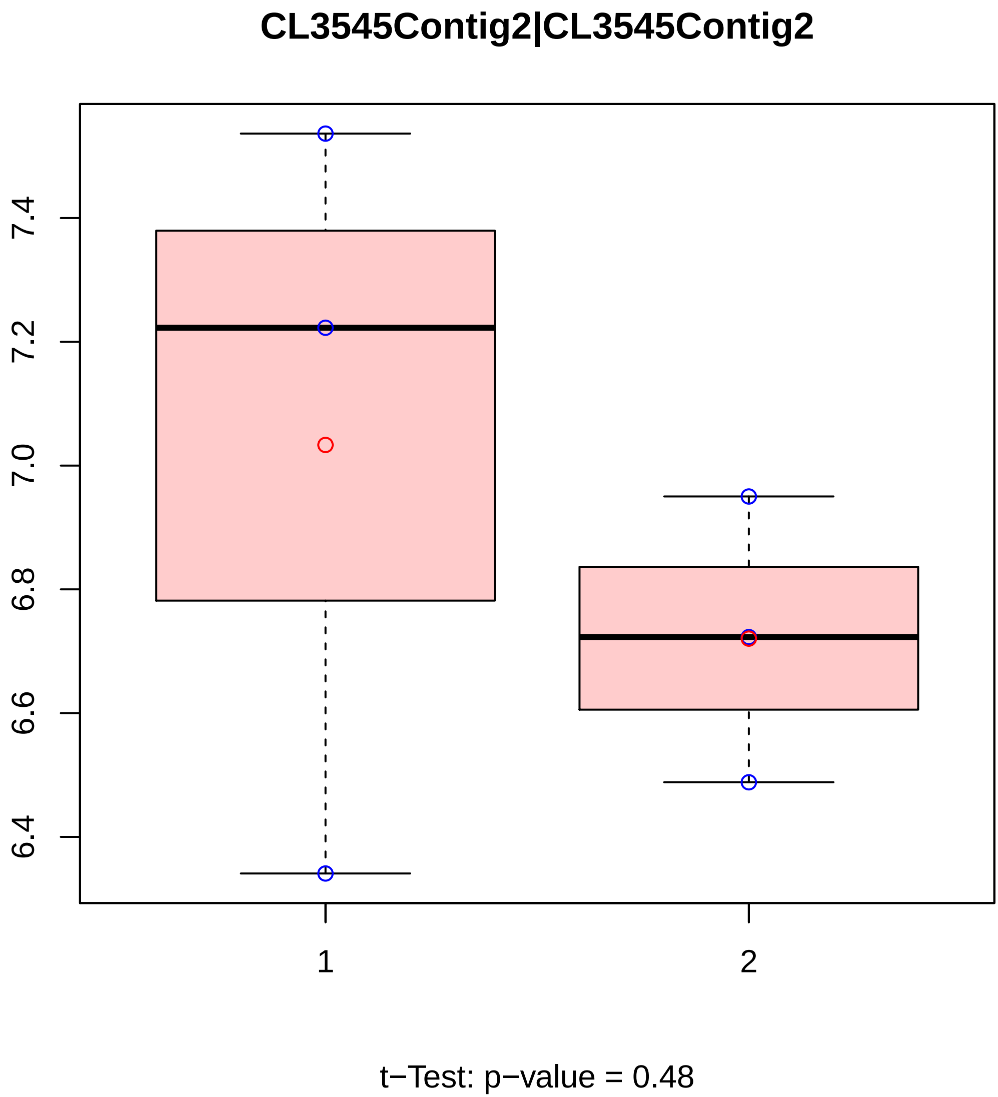
CL3545Contig2|CL3545Contig2
7.4
7.2
7.0
6.8
6.6
6.4
1
2
t−Test: p−value = 0.48

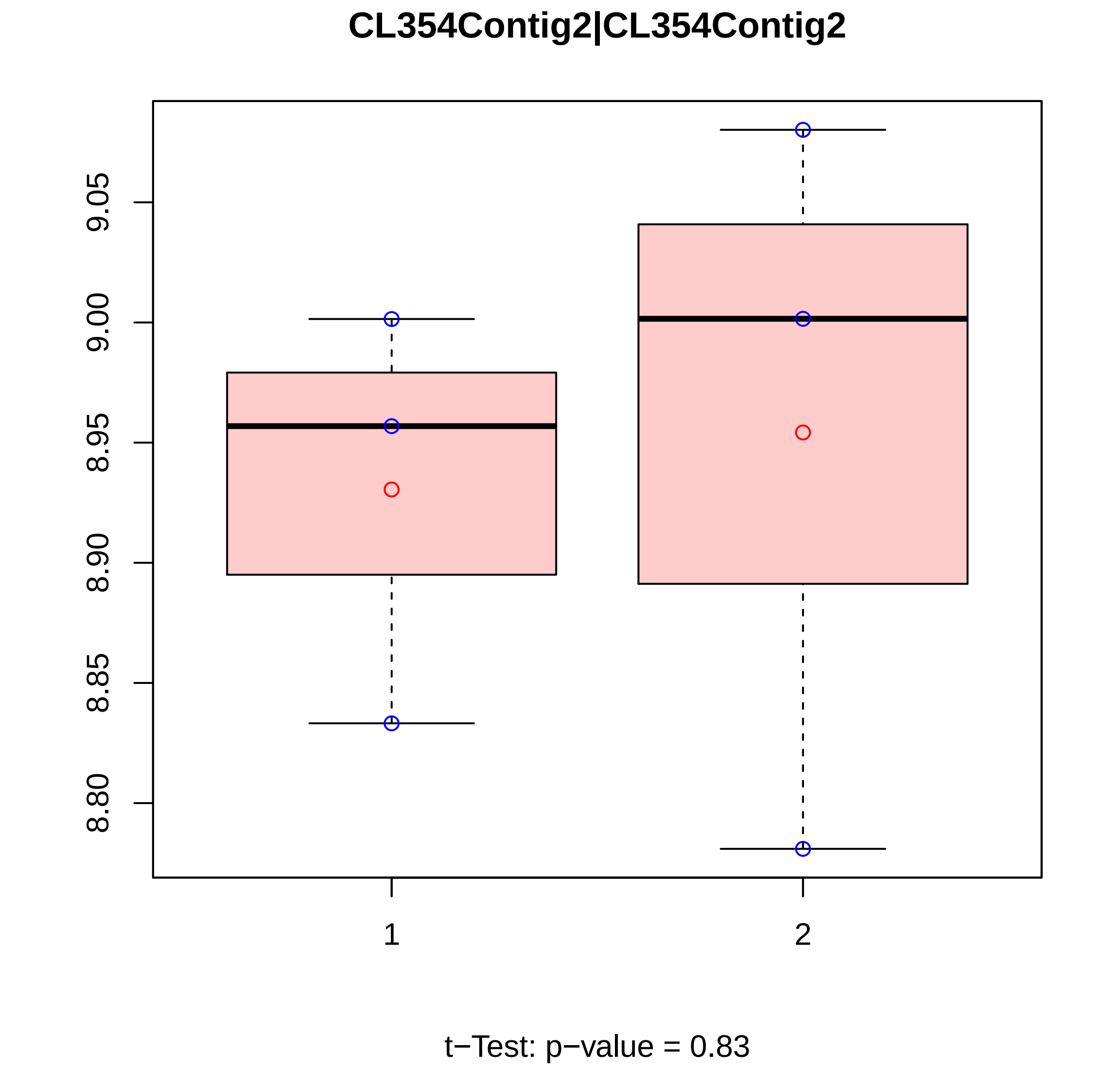
CL354Contig2|CL354Contig2
9.05
9.00
8.95
8.90
8.85
8.80
1
2
t−Test: p−value = 0.83

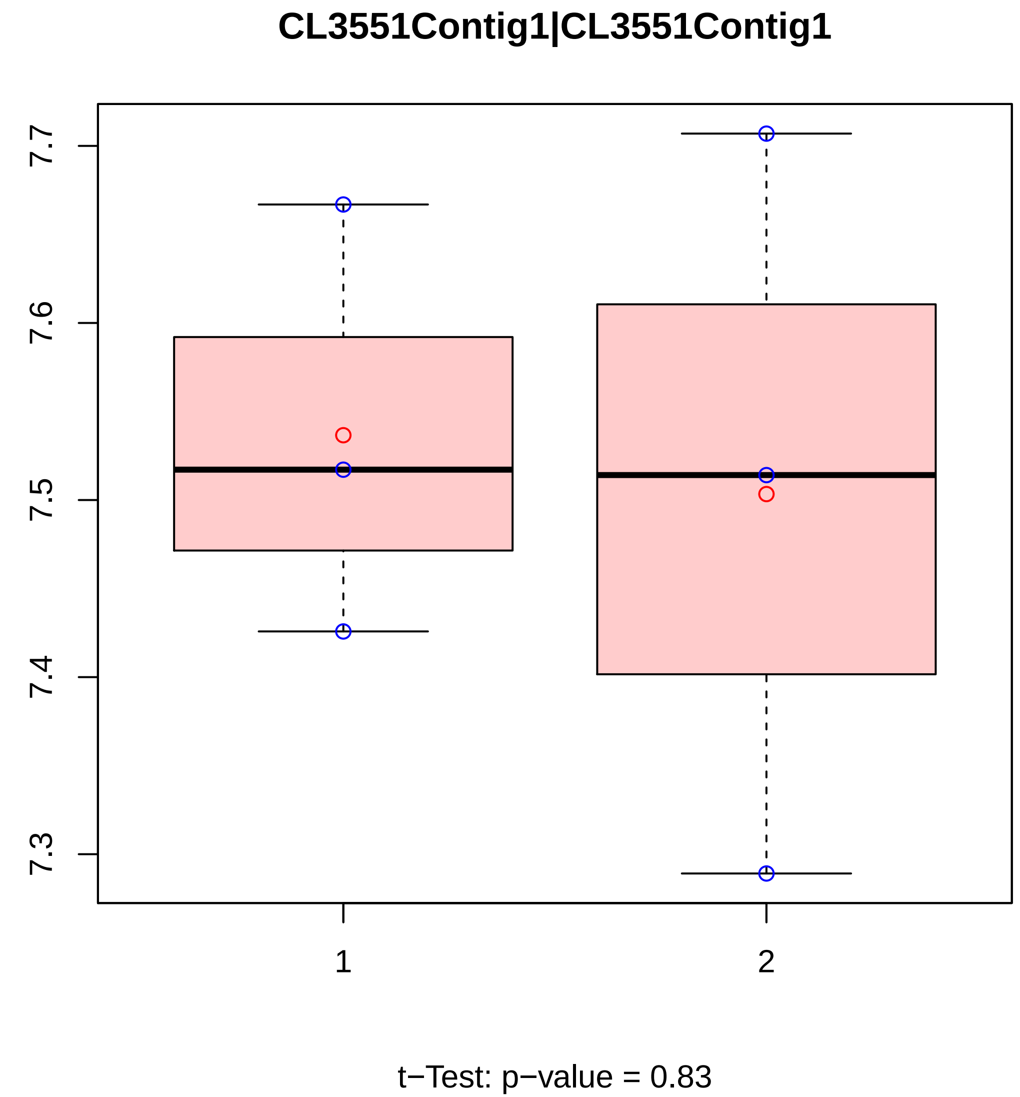
CL3551Contig1|CL3551Contig1
7.3
7.4
7.5
7.6
7.7
1
2
t−Test: p−value = 0.83

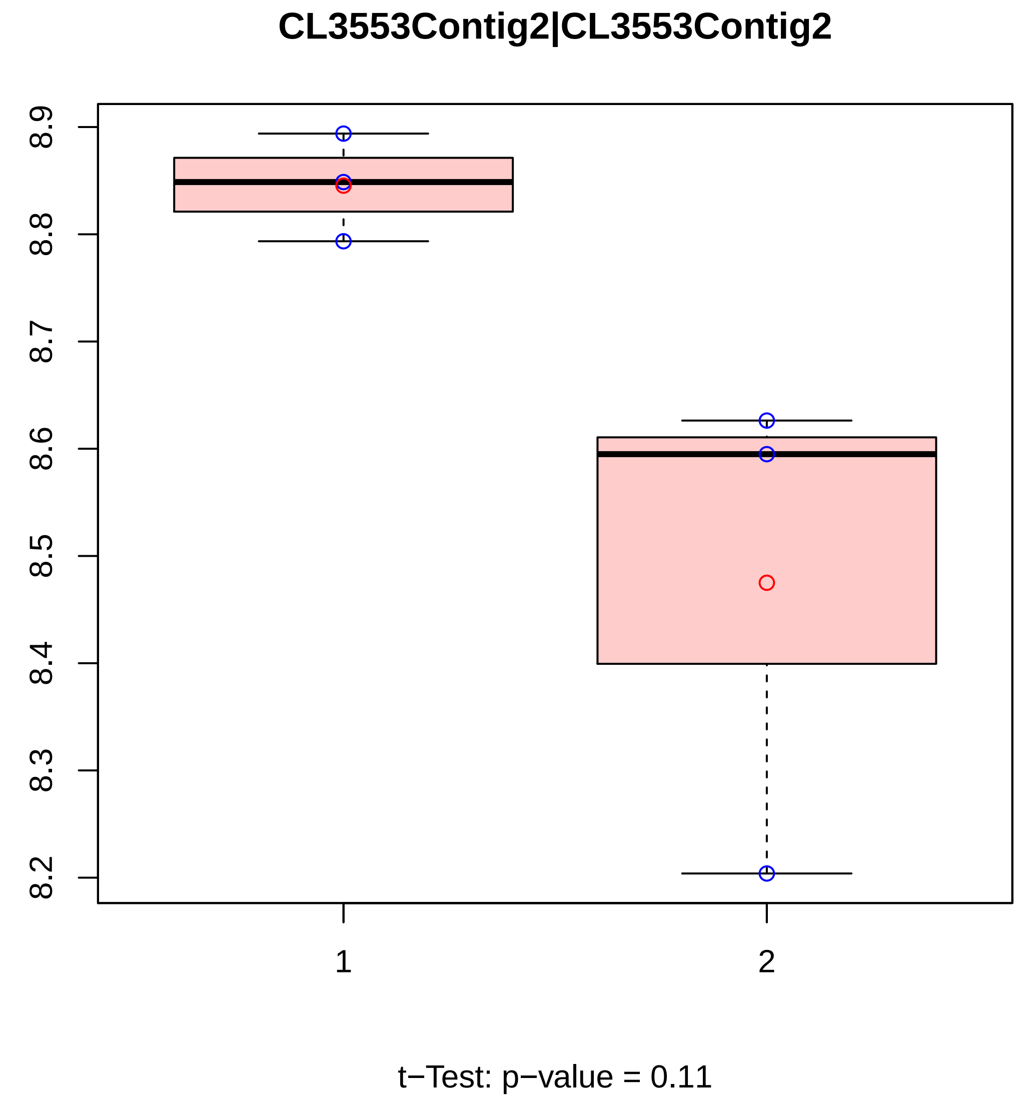

CL3553Contig2|CL3553Contig2
8.9
8.8
8.7
8.6
8.5
8.4
8.3
8.2
1
2
t−Test: p−value = 0.11

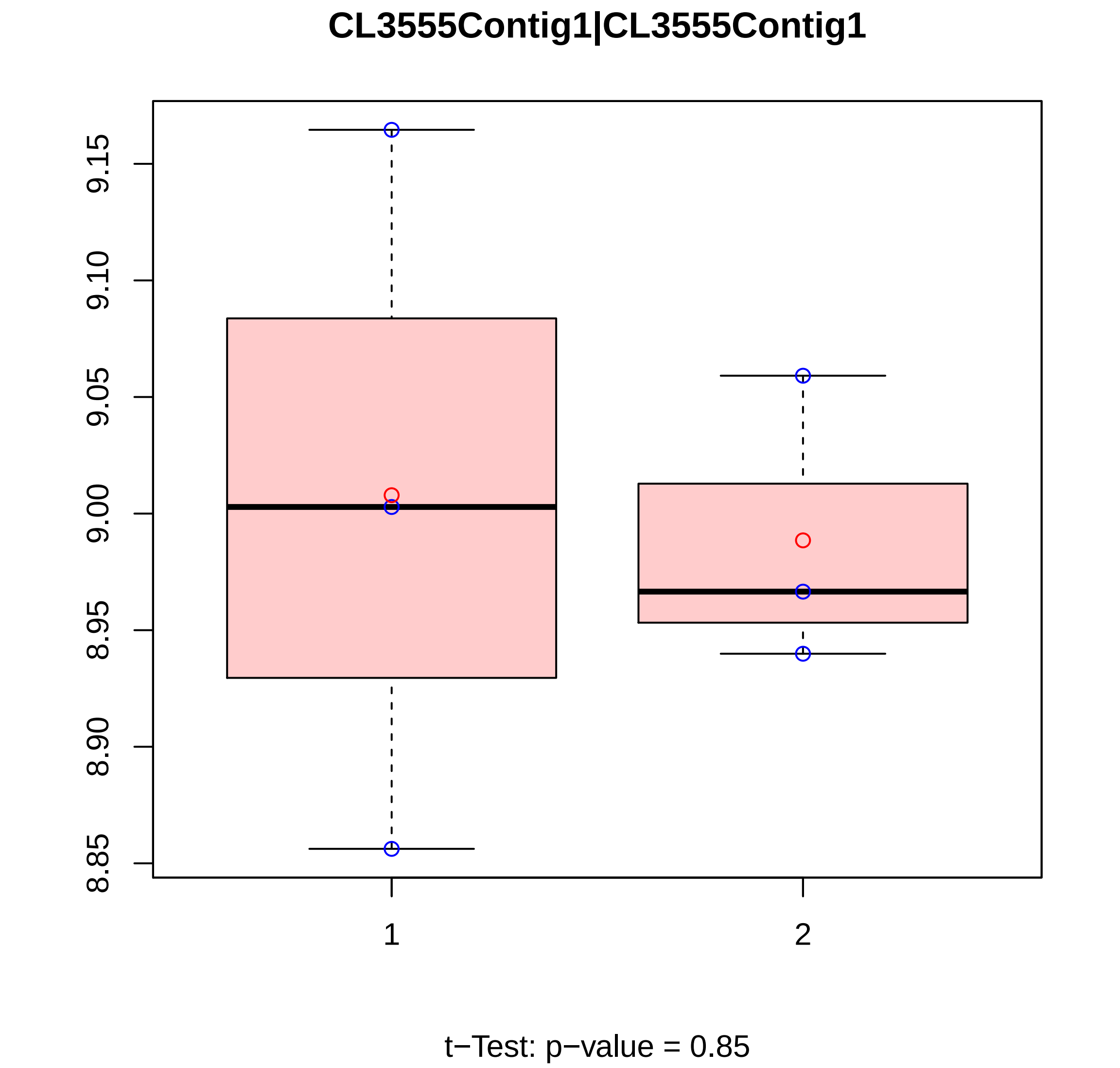
CL3555Contig1|CL3555Contig1
9.15
9.10
9.05
9.00
8.95
8.90
8.85
1
2
t−Test: p−value = 0.85

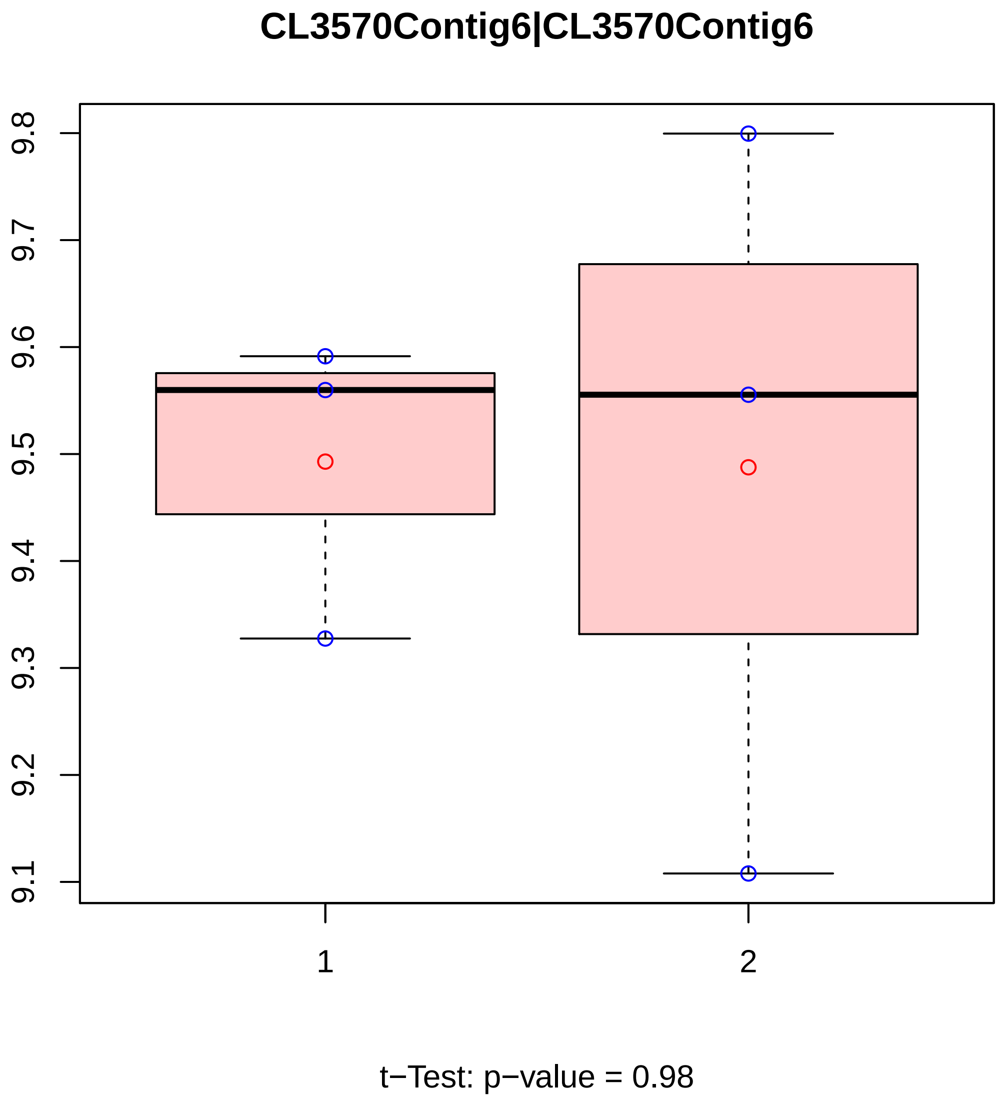
CL3570Contig6|CL3570Contig6
9.8
9.7
9.6
9.5
9.4
9.3
9.2
9.1
1
2
t−Test: p−value = 0.98

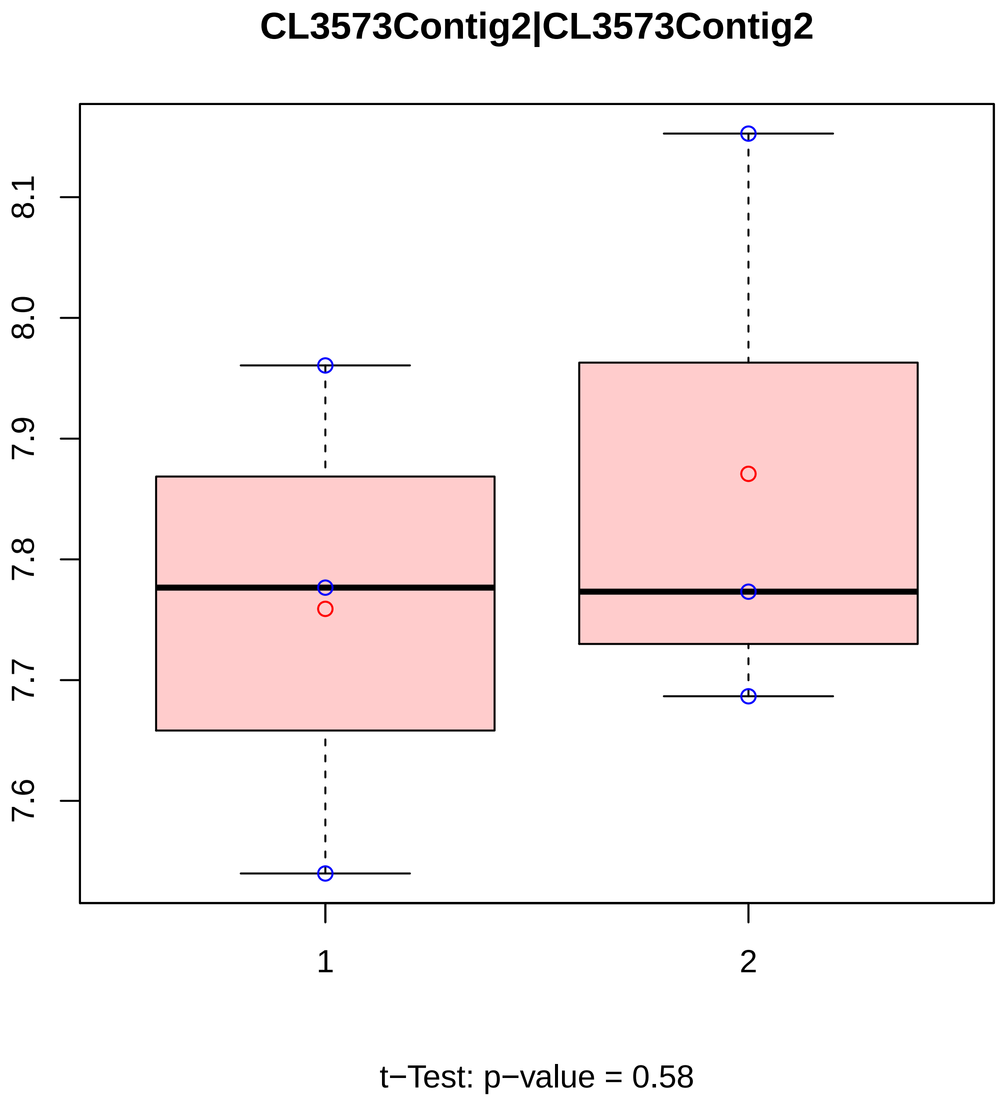

CL3573Contig2|CL3573Contig2
8.1
8.0
7.9
7.8
7.7
7.6
1
2
t−Test: p−value = 0.58

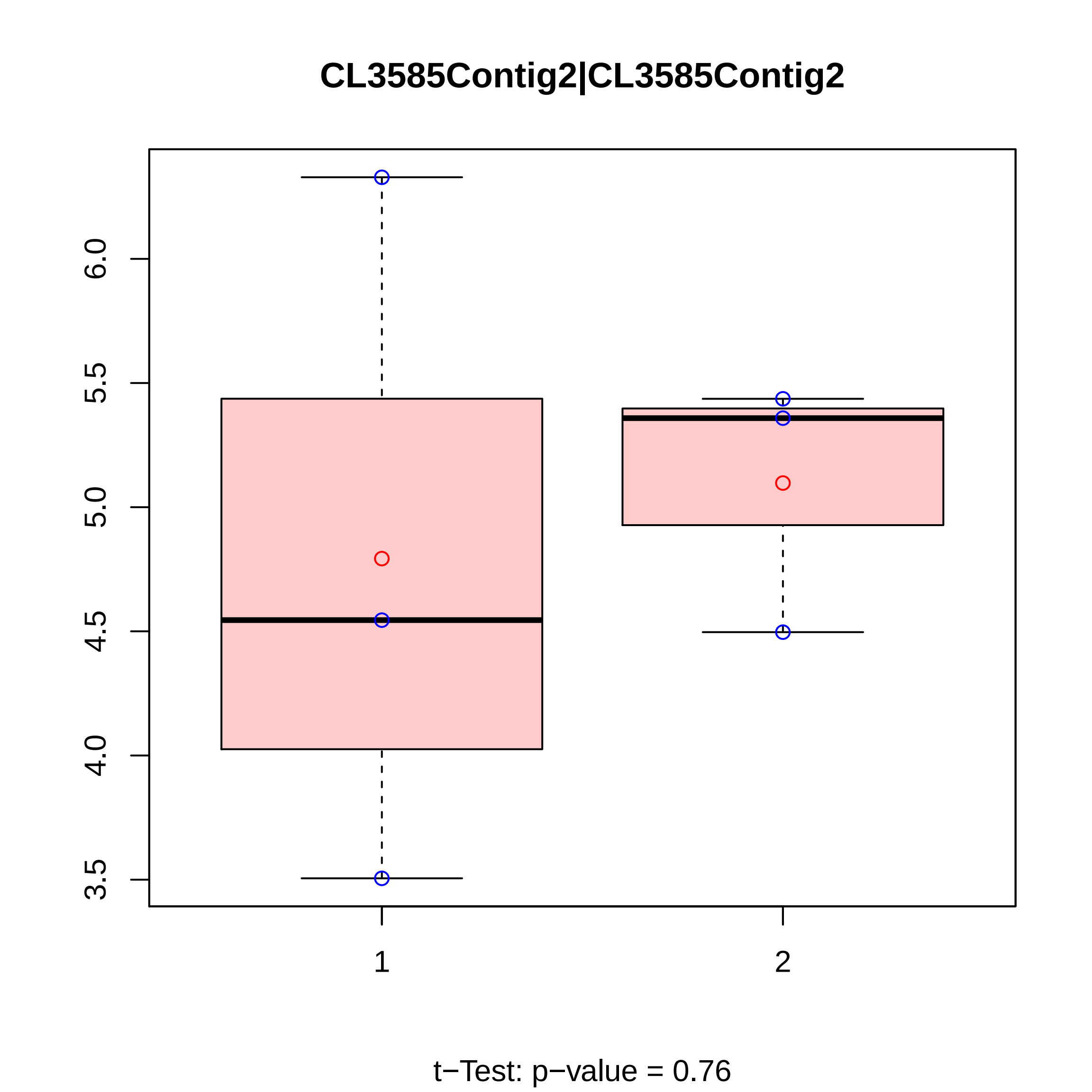
CL3585Contig2|CL3585Contig2
6.0
5.5
5.0
4.5
4.0
3.5
1
2
t−Test: p−value = 0.76

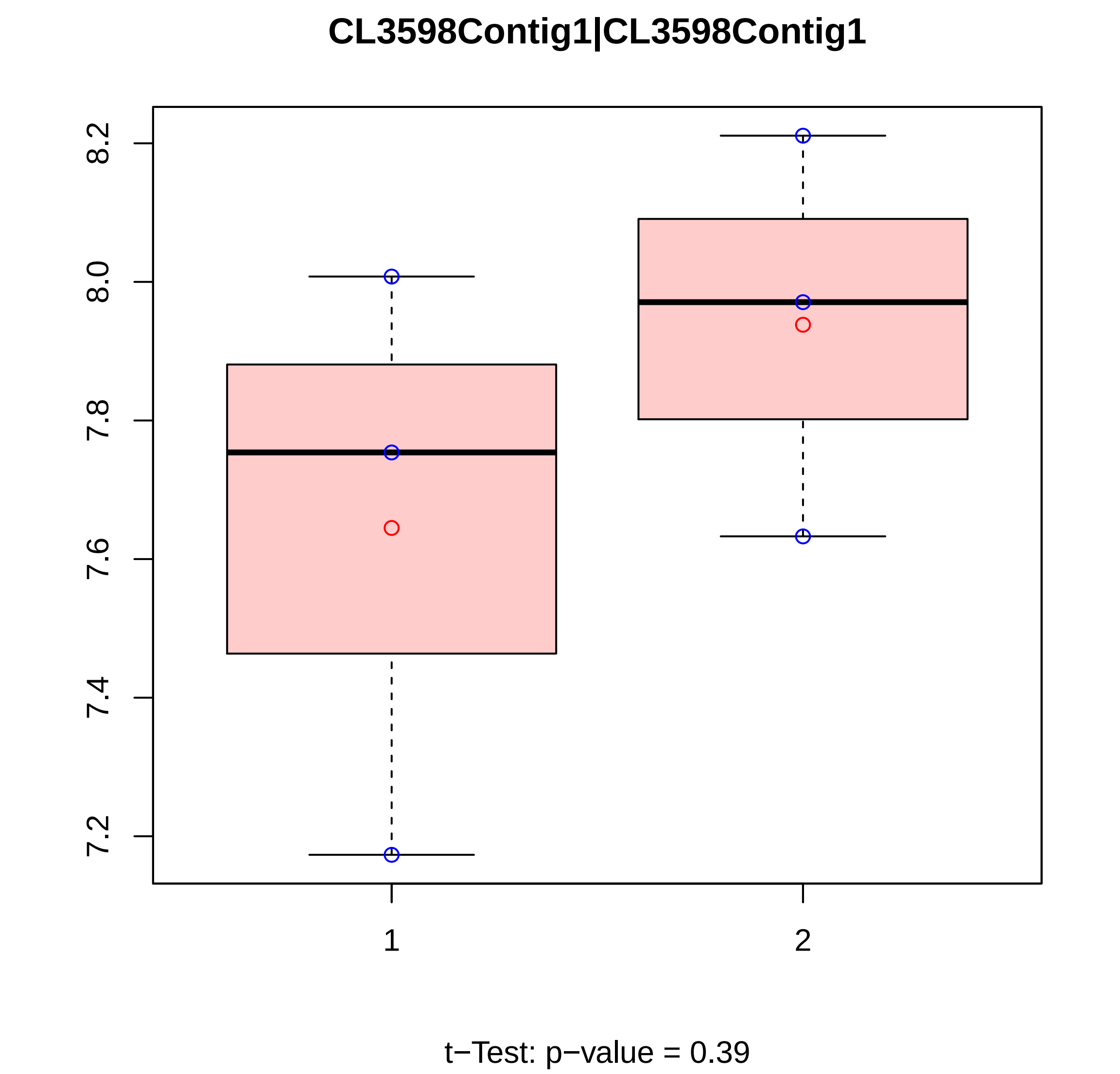
CL3598Contig1|CL3598Contig1
8.2
8.0
7.8
7.6
7.4
7.2
1
2
t−Test: p−value = 0.39

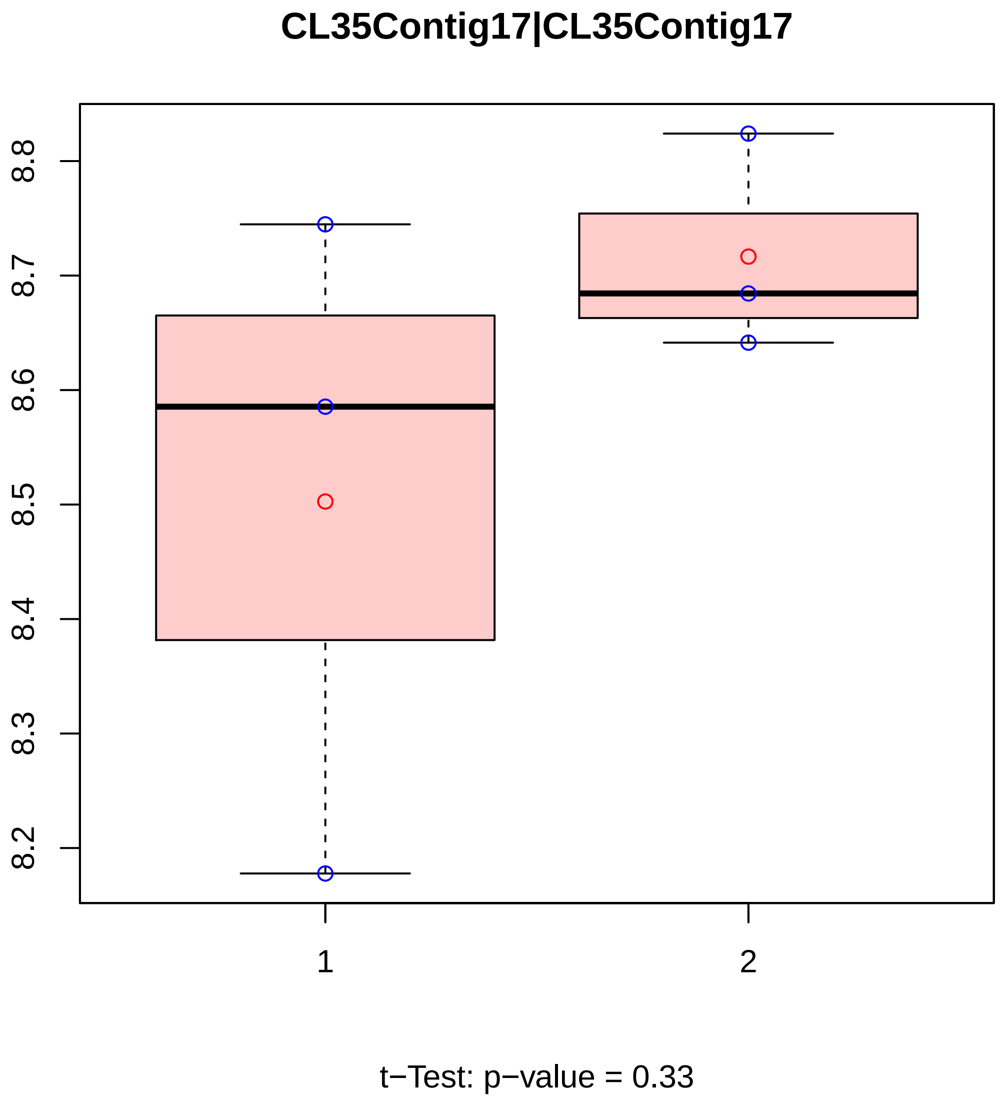
CL35Contig17|CL35Contig17
8.2
8.3
8.4
8.5
8.6
8.7
8.8
1
2
t−Test: p−value = 0.33

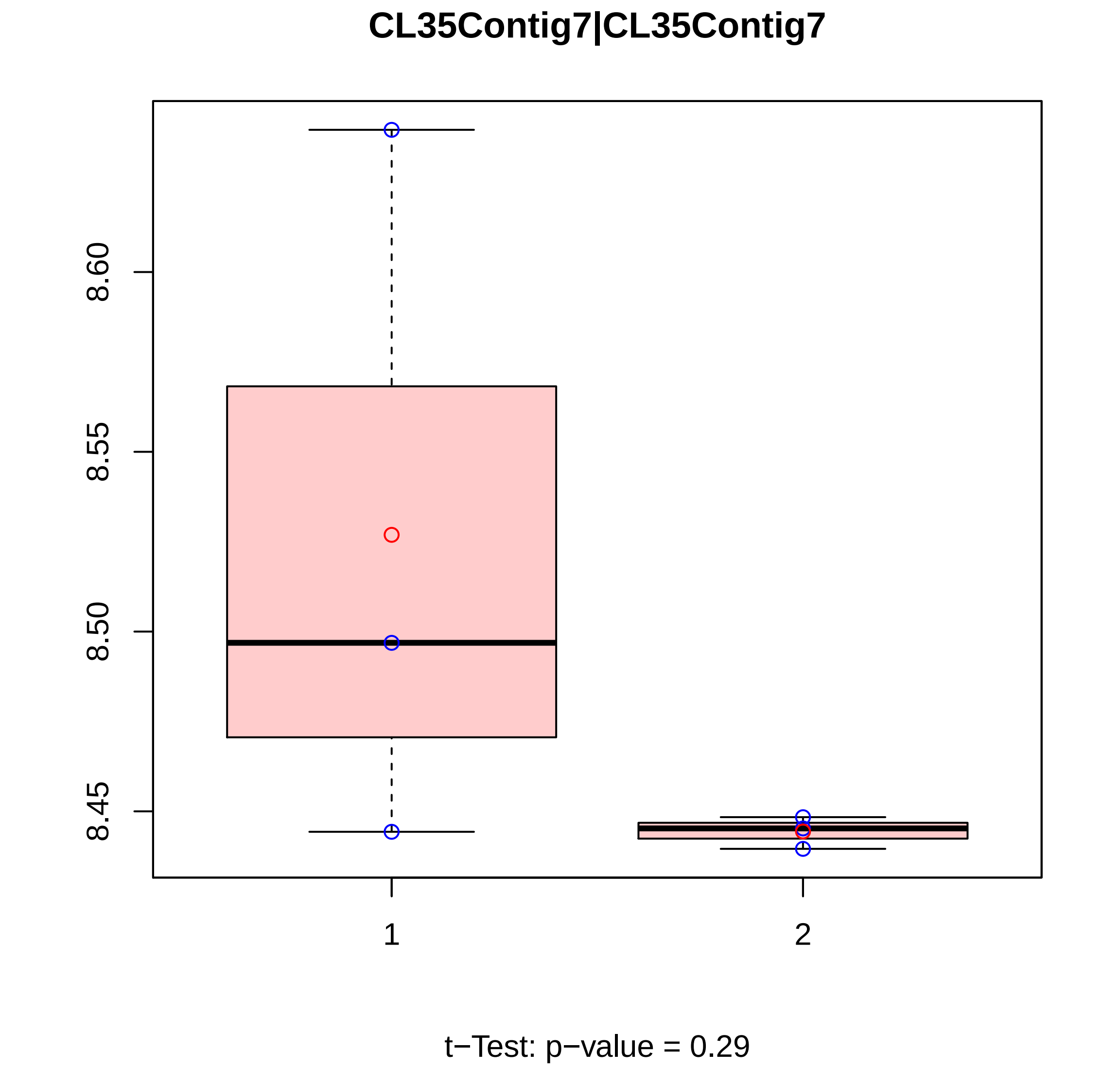

CL35Contig7|CL35Contig7
8.45
8.50
8.55
8.60
1
2
t−Test: p−value = 0.29

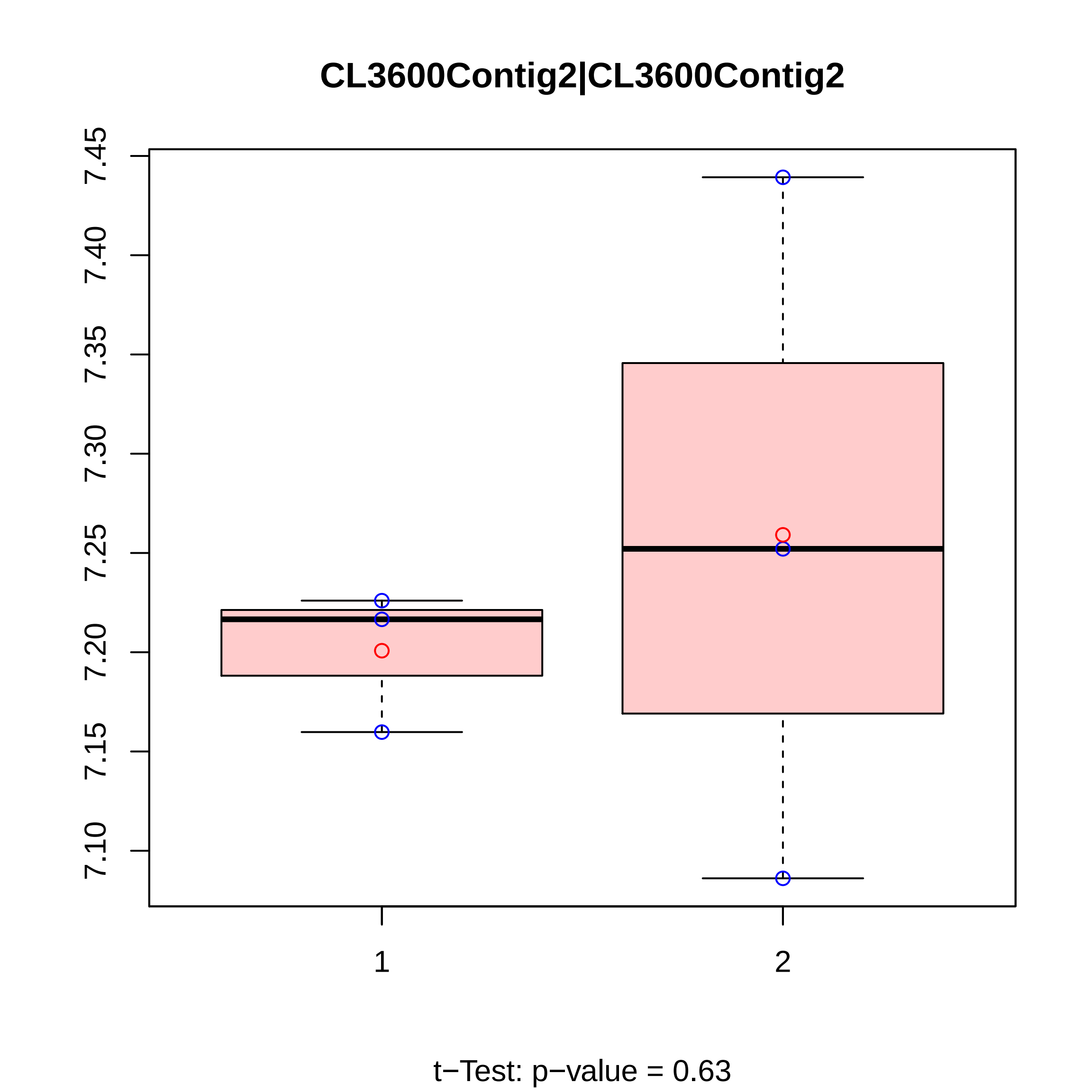
CL3600Contig2|CL3600Contig2
7.45
7.40
7.35
7.30
7.25
7.20
7.15
7.10
1
2
t−Test: p−value = 0.63

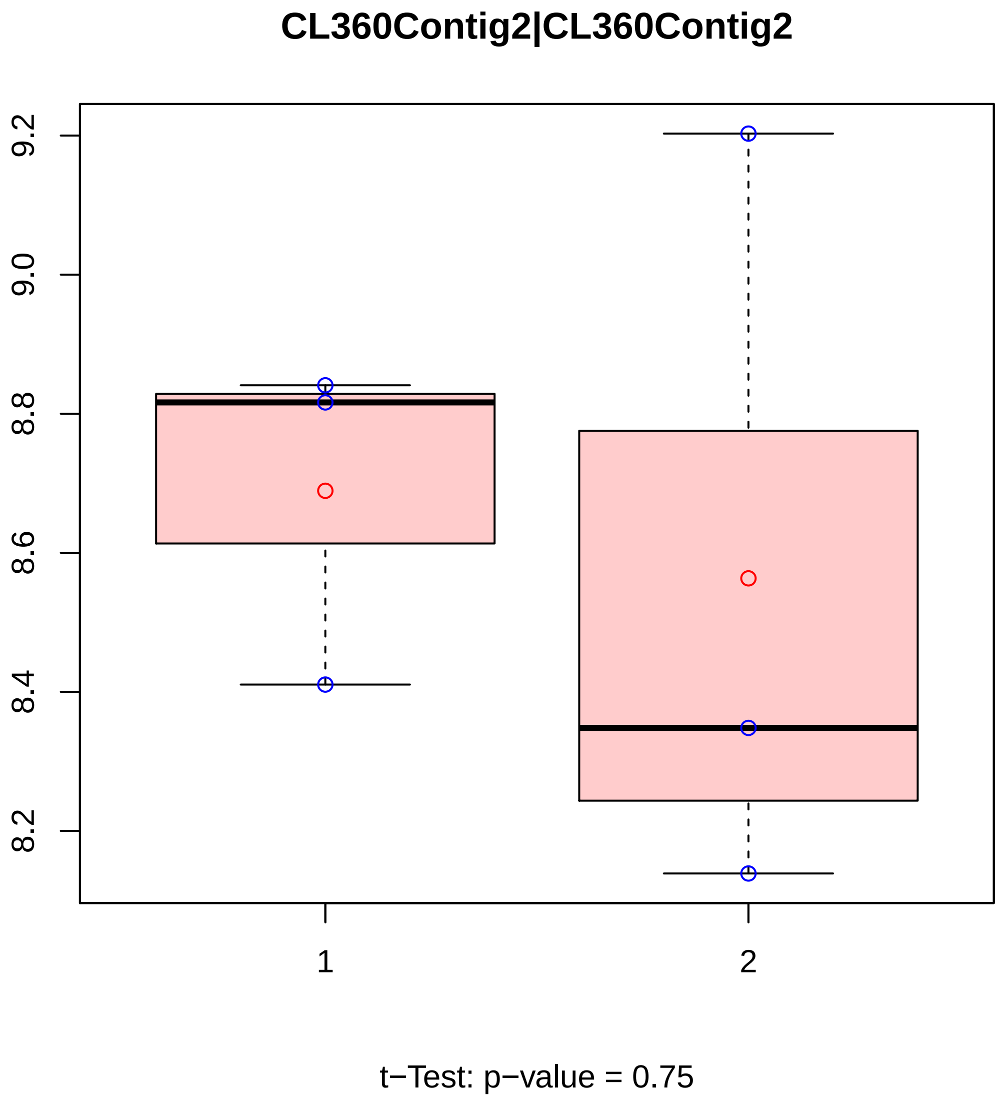

CL360Contig2|CL360Contig2
9.2
9.0
8.8
8.6
8.4
8.2
1
2
t−Test: p−value = 0.75

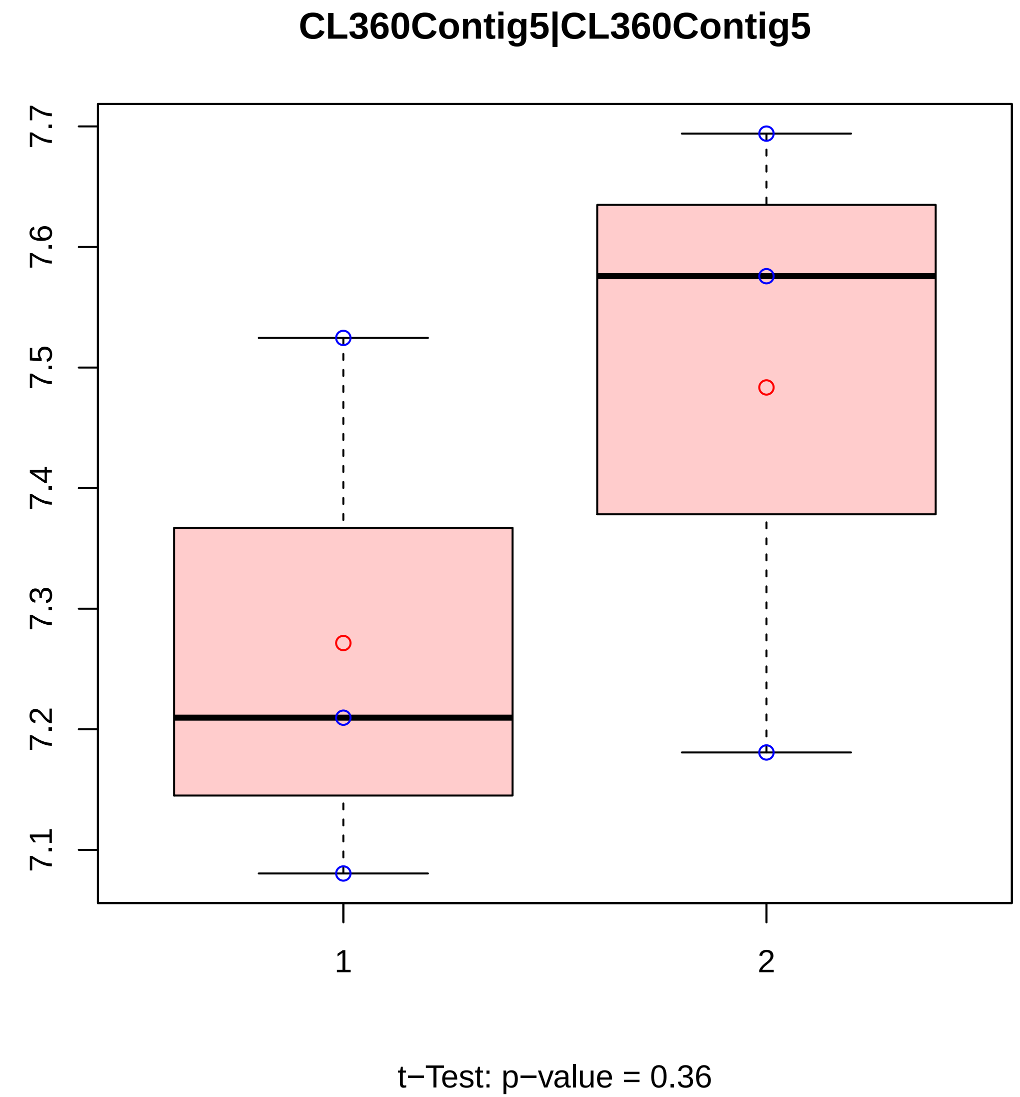
CL360Contig5|CL360Contig5
7.1
7.2
7.3
7.4
7.5
7.6
7.7
1
2
t−Test: p−value = 0.36

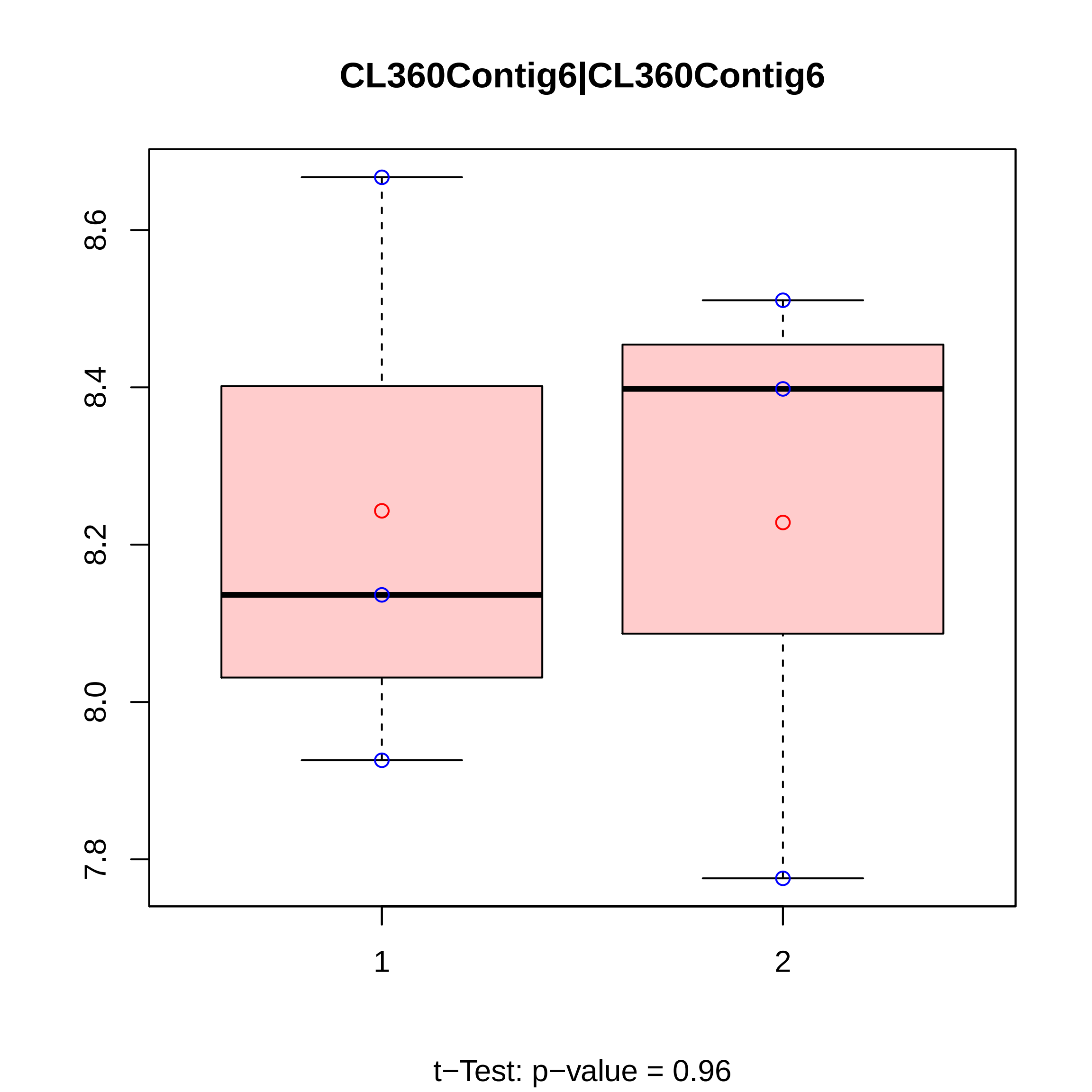

CL360Contig6|CL360Contig6
8.6
8.4
8.2
8.0
7.8
1
2
t−Test: p−value = 0.96

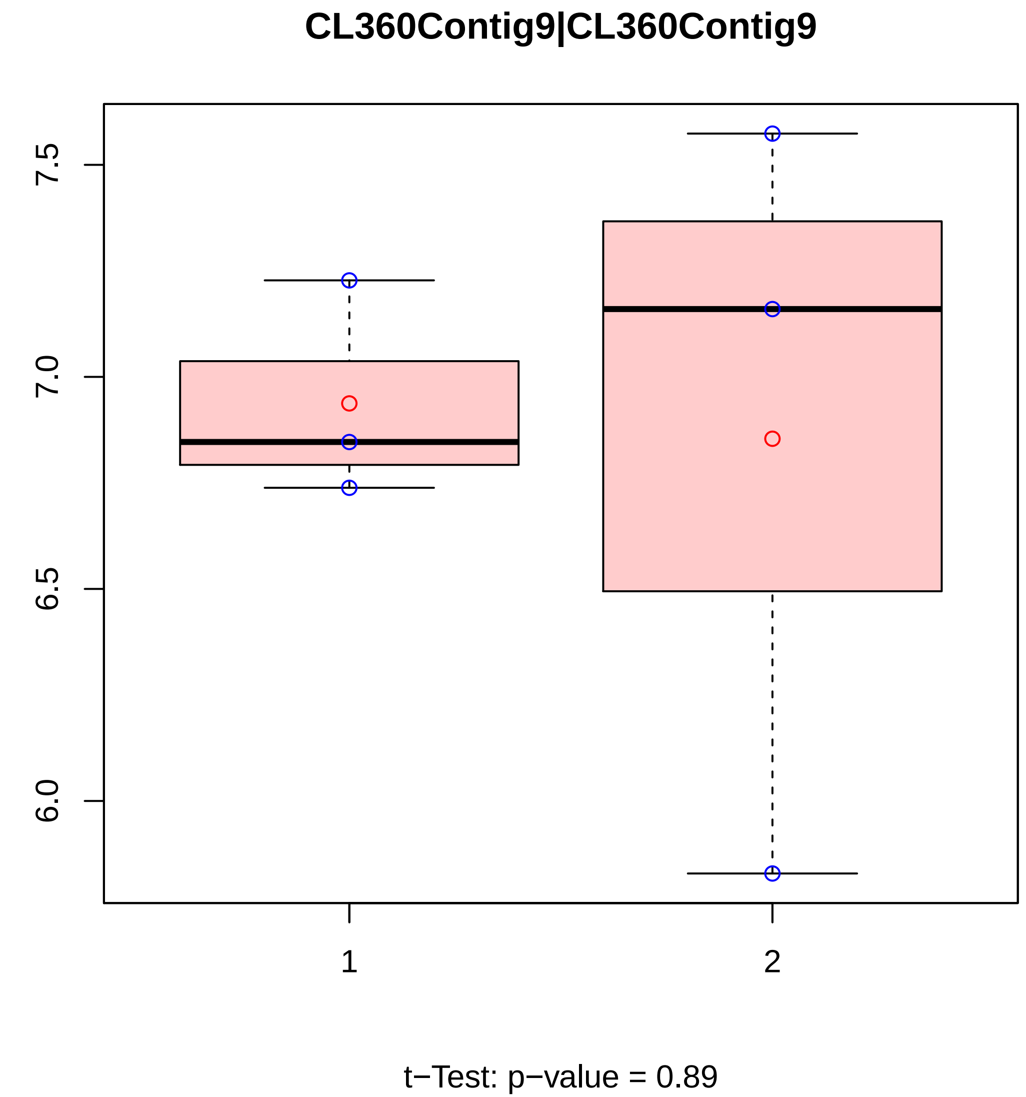

CL360Contig9|CL360Contig9
7.5
7.0
6.5
6.0
1
2
t−Test: p−value = 0.89

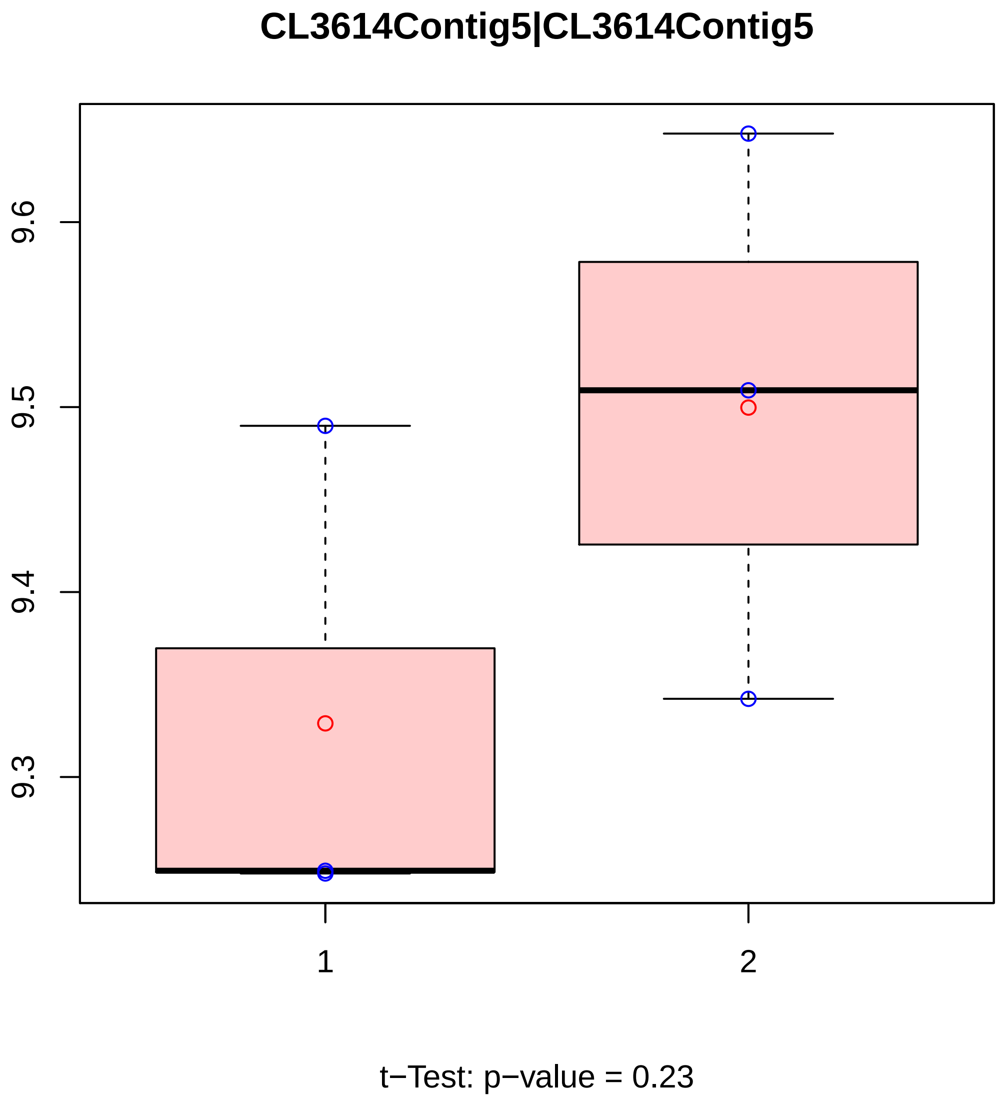

CL3614Contig5|CL3614Contig5
9.3
9.4
9.5
9.6
1
2
t−Test: p−value = 0.23

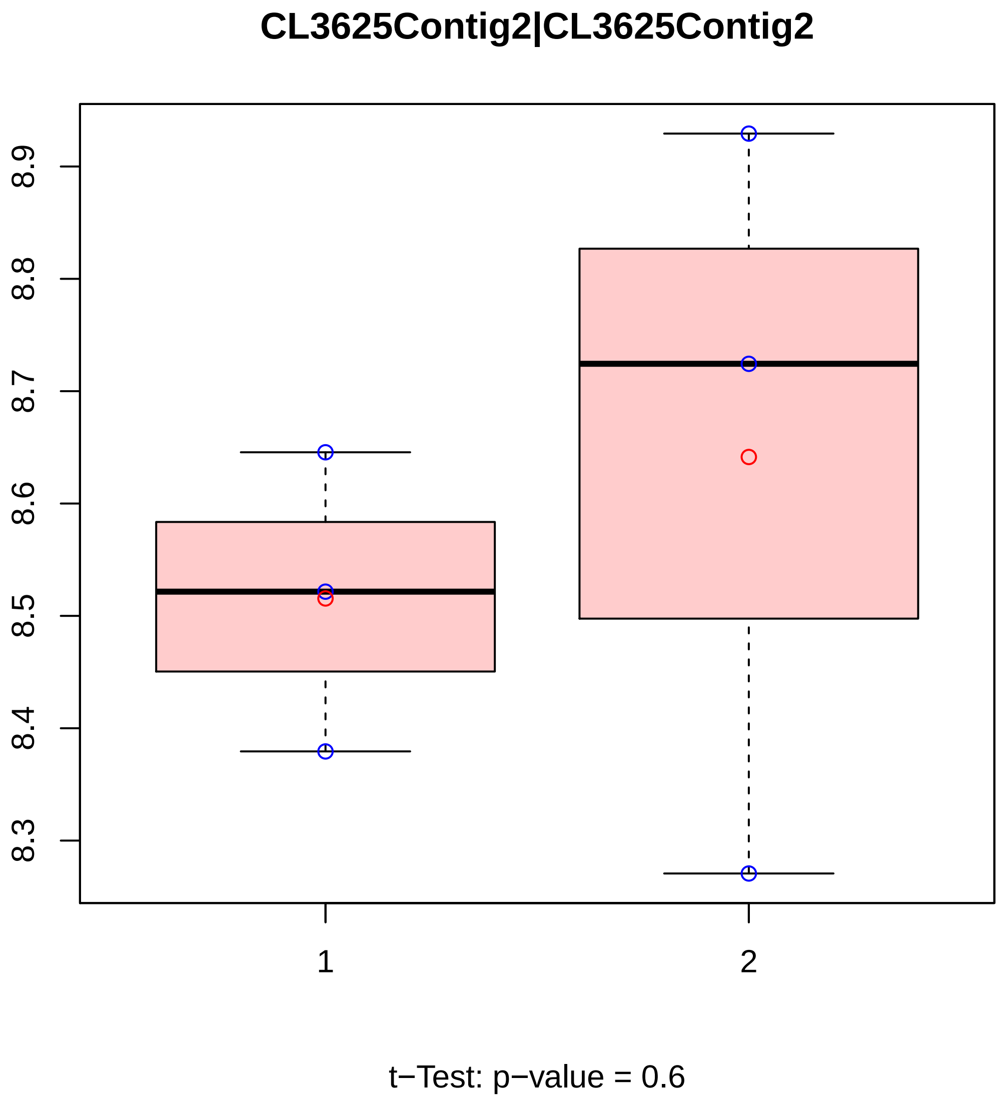
CL3625Contig2|CL3625Contig2
8.3
8.4
8.5
8.6
8.7
8.8
8.9
1
2
t−Test: p−value = 0.6

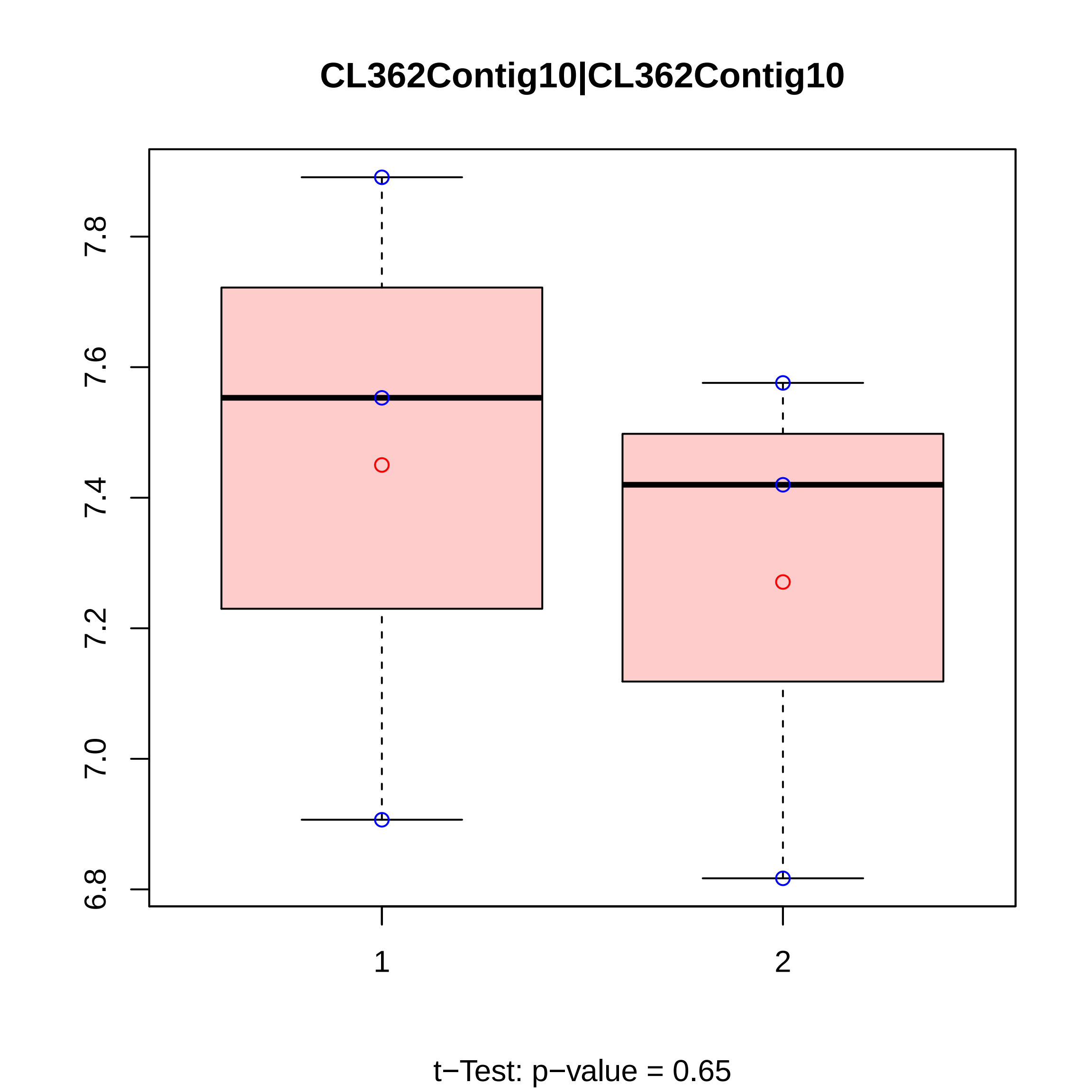
CL362Contig10|CL362Contig10
7.8
7.6
7.4
7.2
7.0
6.8
1
2
t−Test: p−value = 0.65

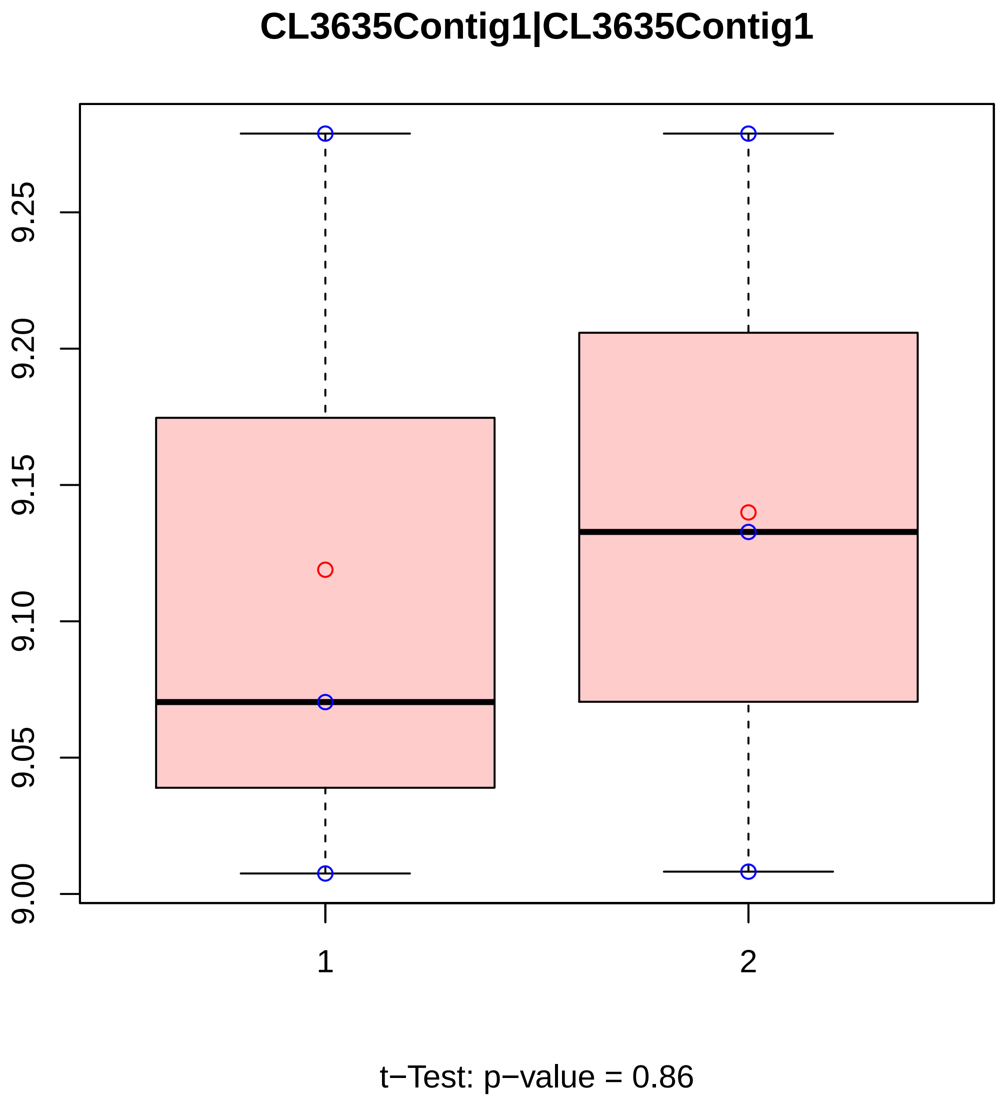
CL3635Contig1|CL3635Contig1
9.25
9.20
9.15
9.10
9.05
9.00
1
2
t−Test: p−value = 0.86

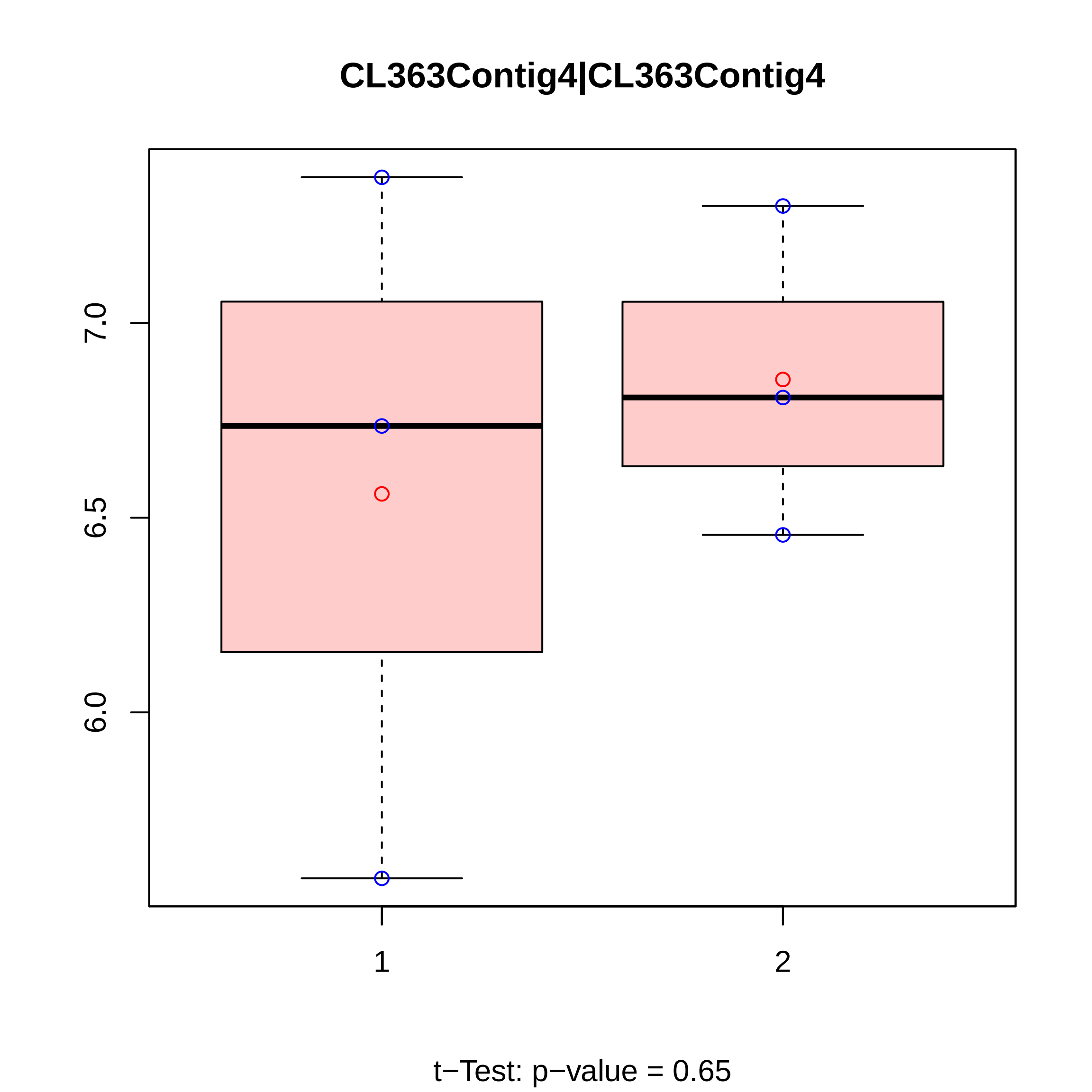

CL363Contig4|CL363Contig4
7.0
6.5
6.0
1
2
t−Test: p−value = 0.65

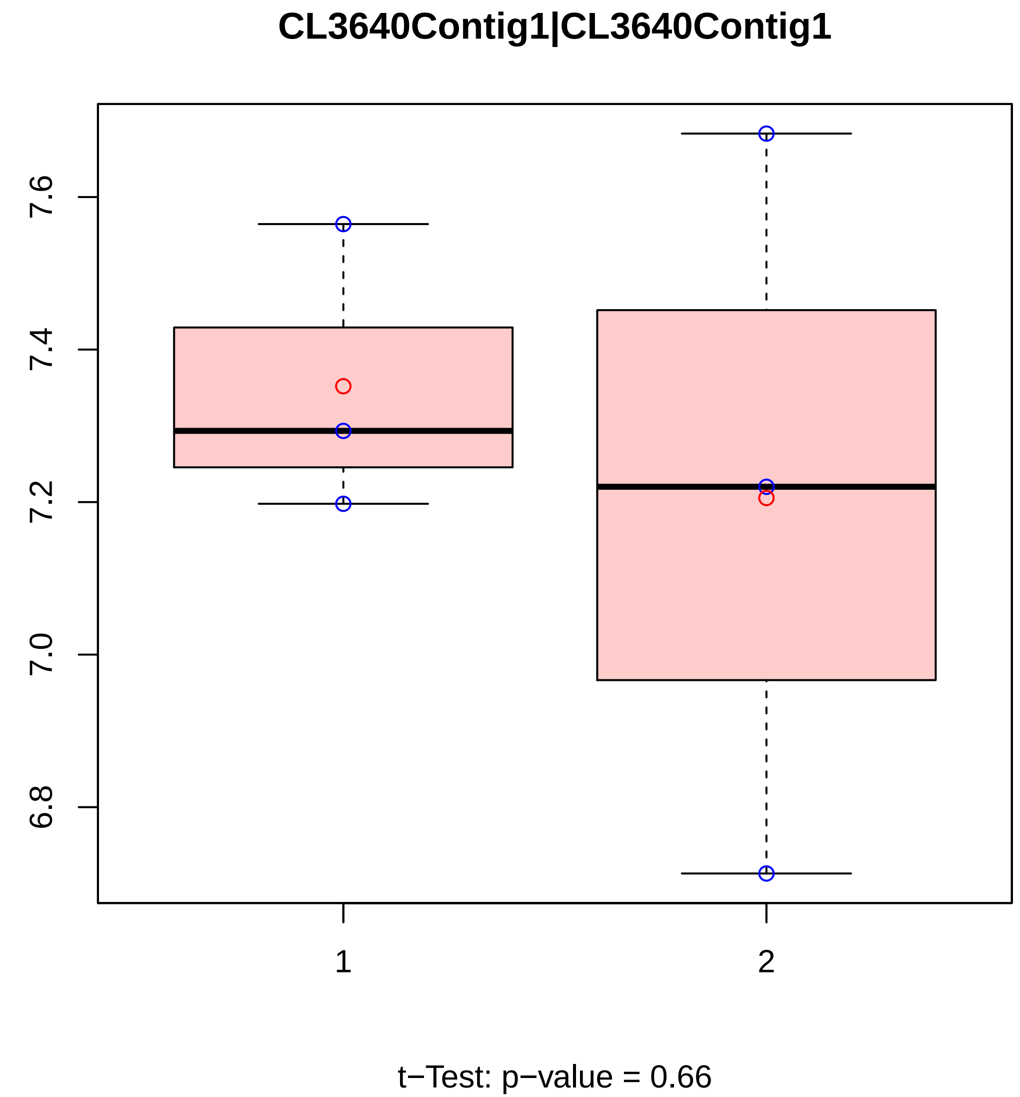
CL3640Contig1|CL3640Contig1
7.6
7.4
7.2
7.0
6.8
1
2
t−Test: p−value = 0.66

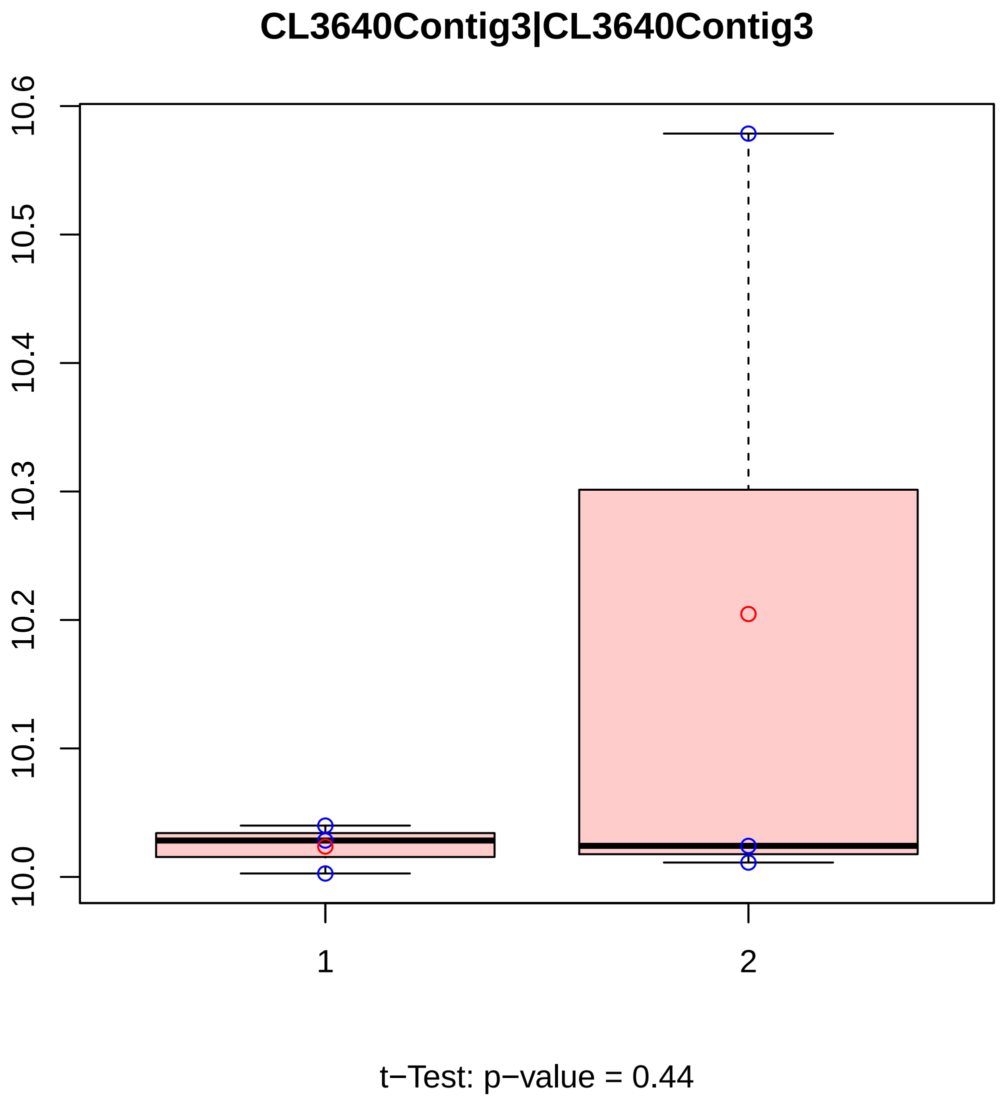

CL3640Contig3|CL3640Contig3
10.0
10.1
10.2
10.3
10.4
10.5
10.6
1
2
t−Test: p−value = 0.44

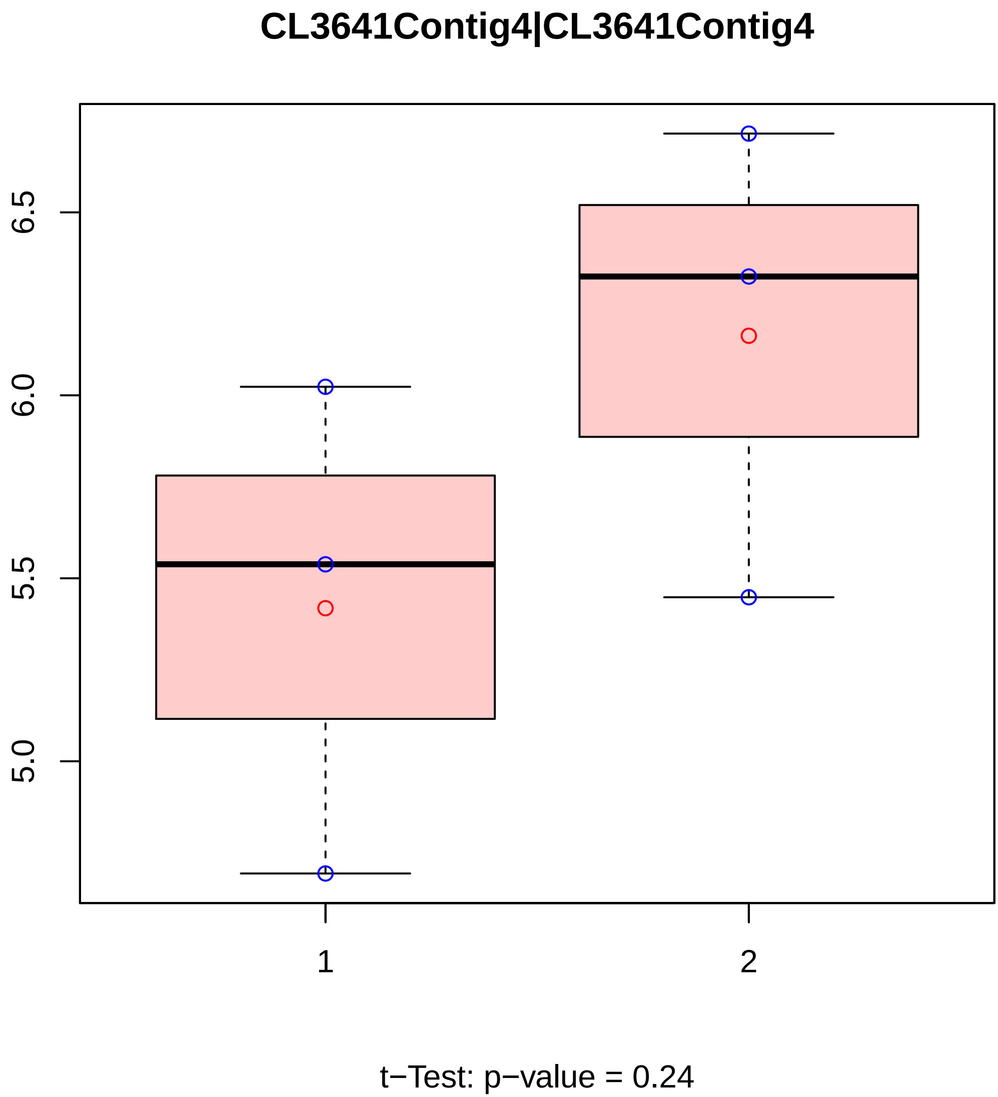

CL3641Contig4|CL3641Contig4
6.5
6.0
5.5
5.0
1
2
t−Test: p−value = 0.24

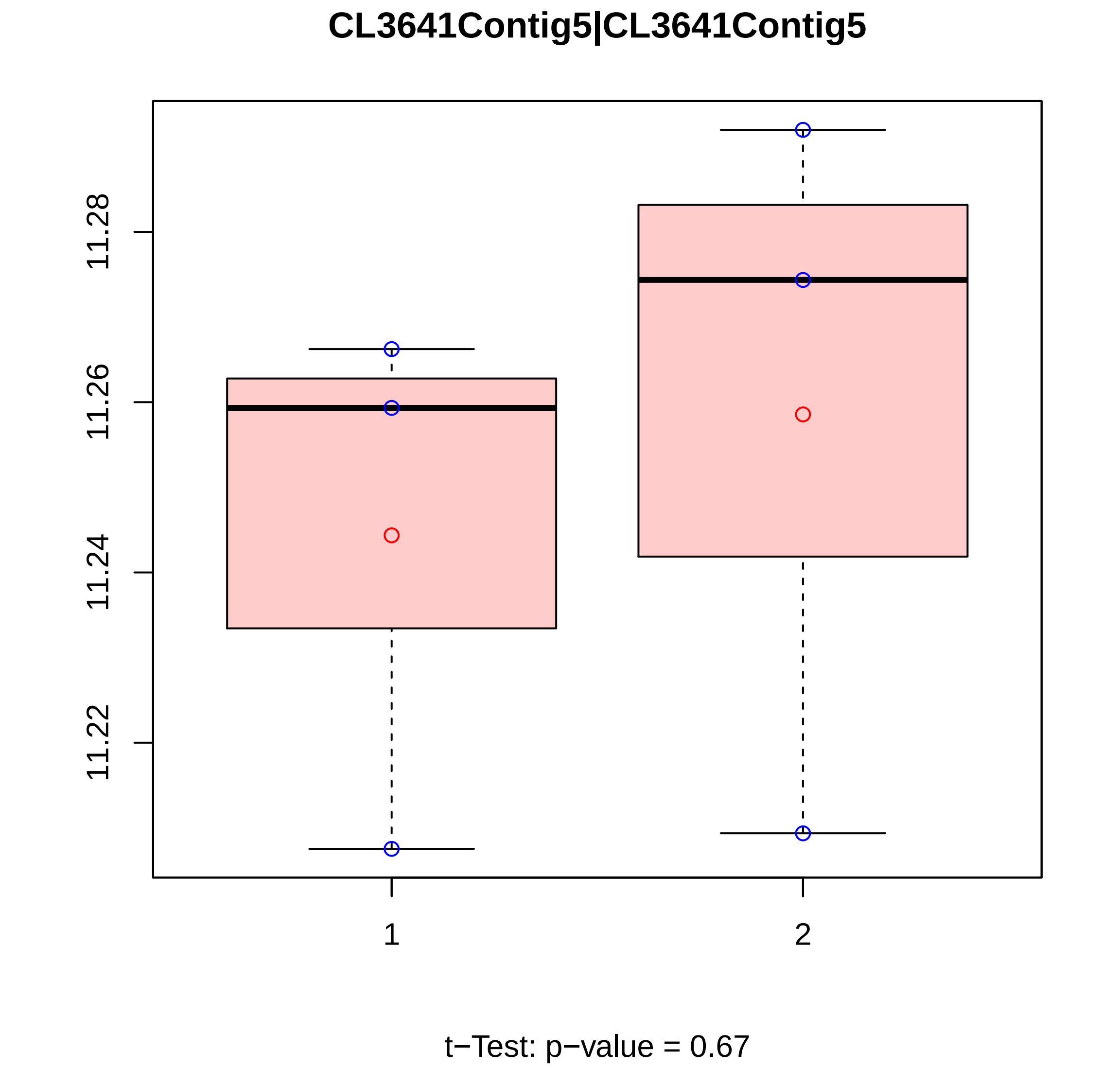

CL3641Contig5|CL3641Contig5
11.22
11.24
11.26
11.28
1
2
t−Test: p−value = 0.67

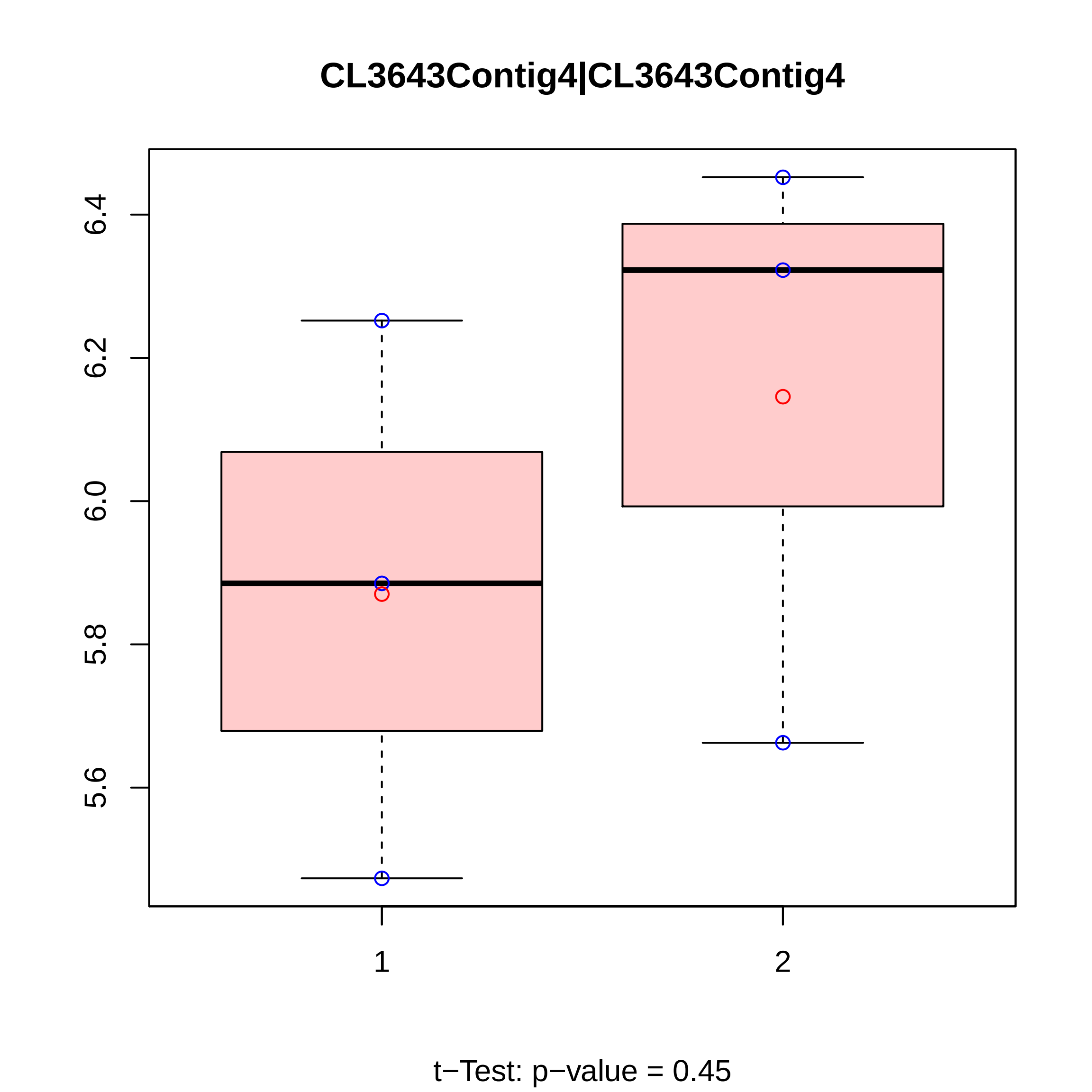

CL3643Contig4|CL3643Contig4
6.4
6.2
6.0
5.8
5.6
1
2
t−Test: p−value = 0.45

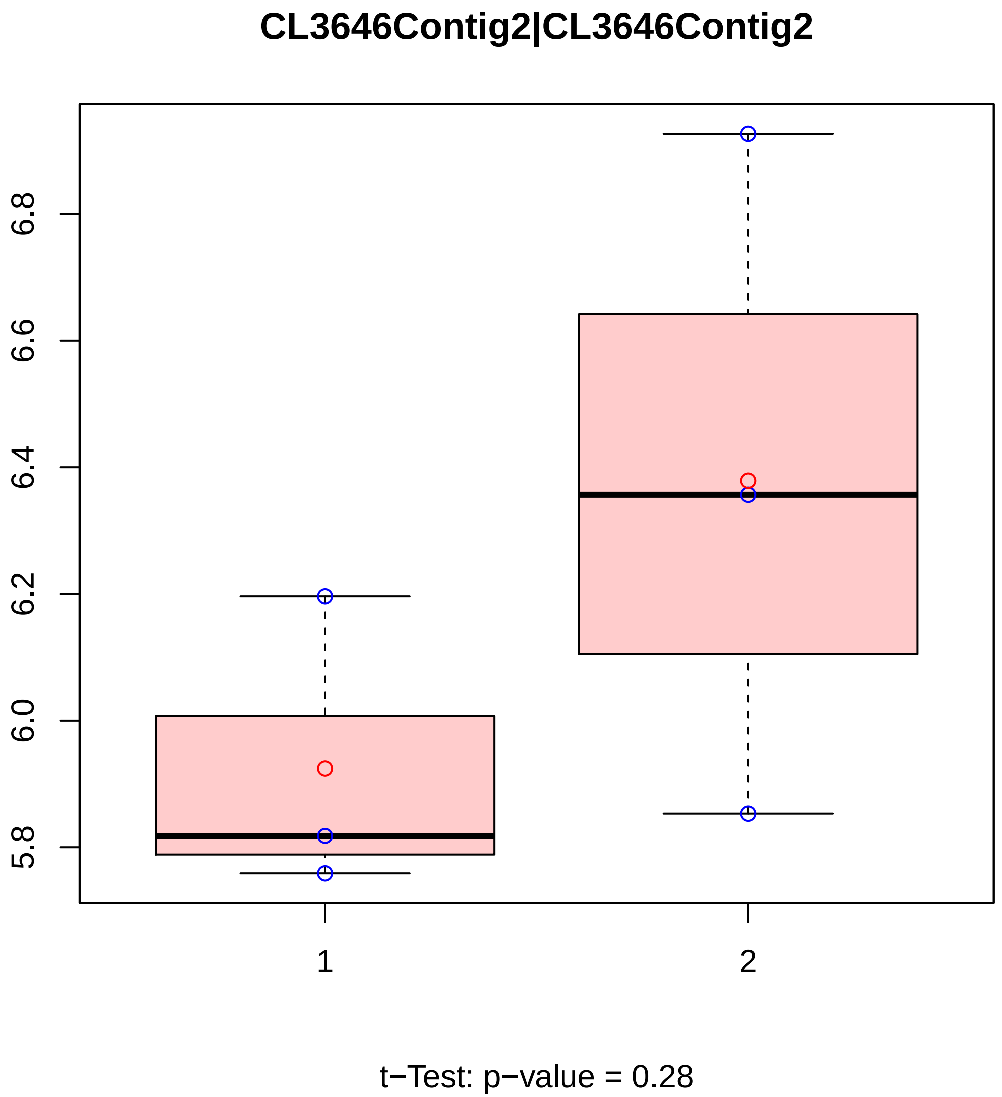
CL3646Contig2|CL3646Contig2
6.8
6.6
6.4
6.2
6.0
5.8
1
2
t−Test: p−value = 0.28

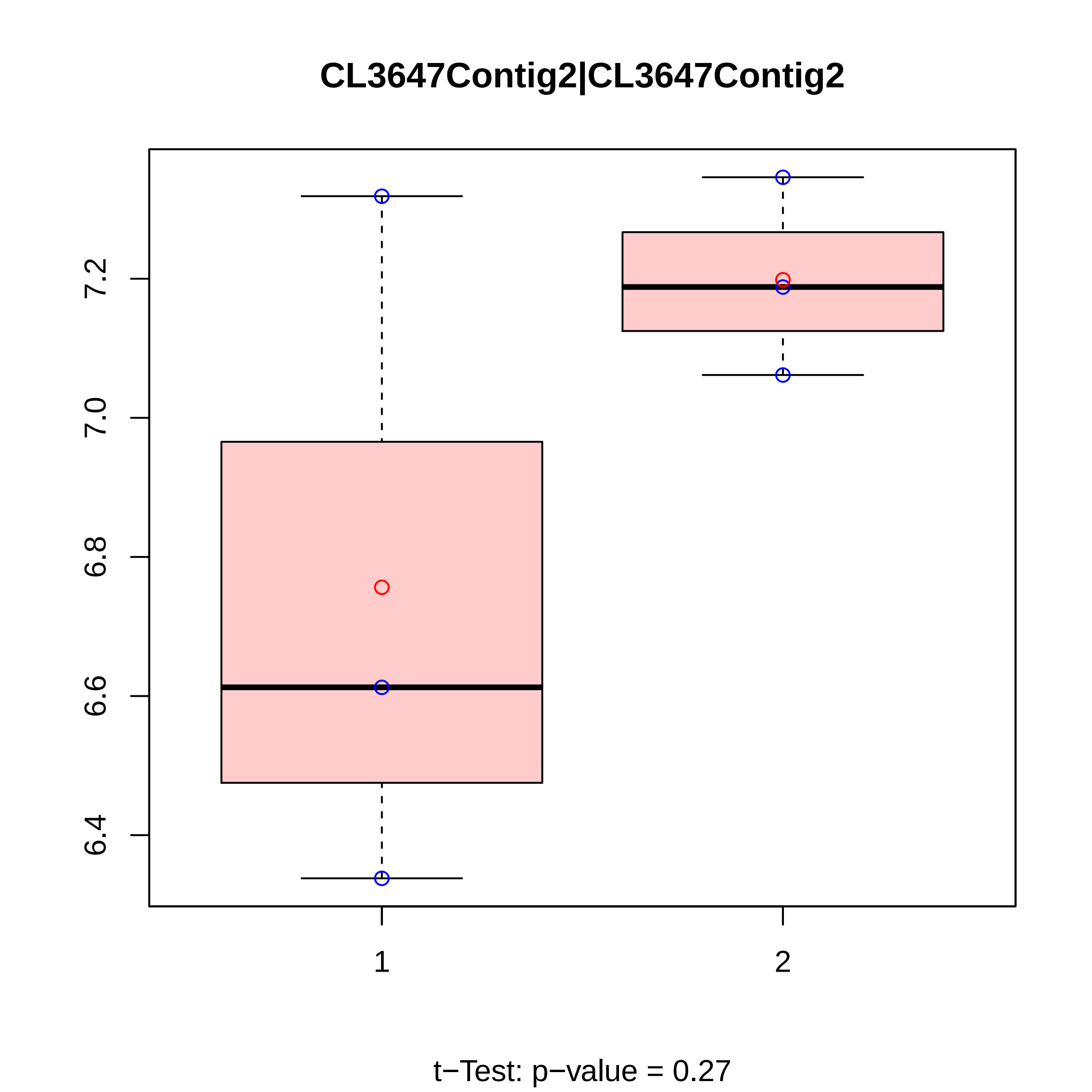

CL3647Contig2|CL3647Contig2
6.4
6.6
6.8
7.0
7.2
1
2
t−Test: p−value = 0.27

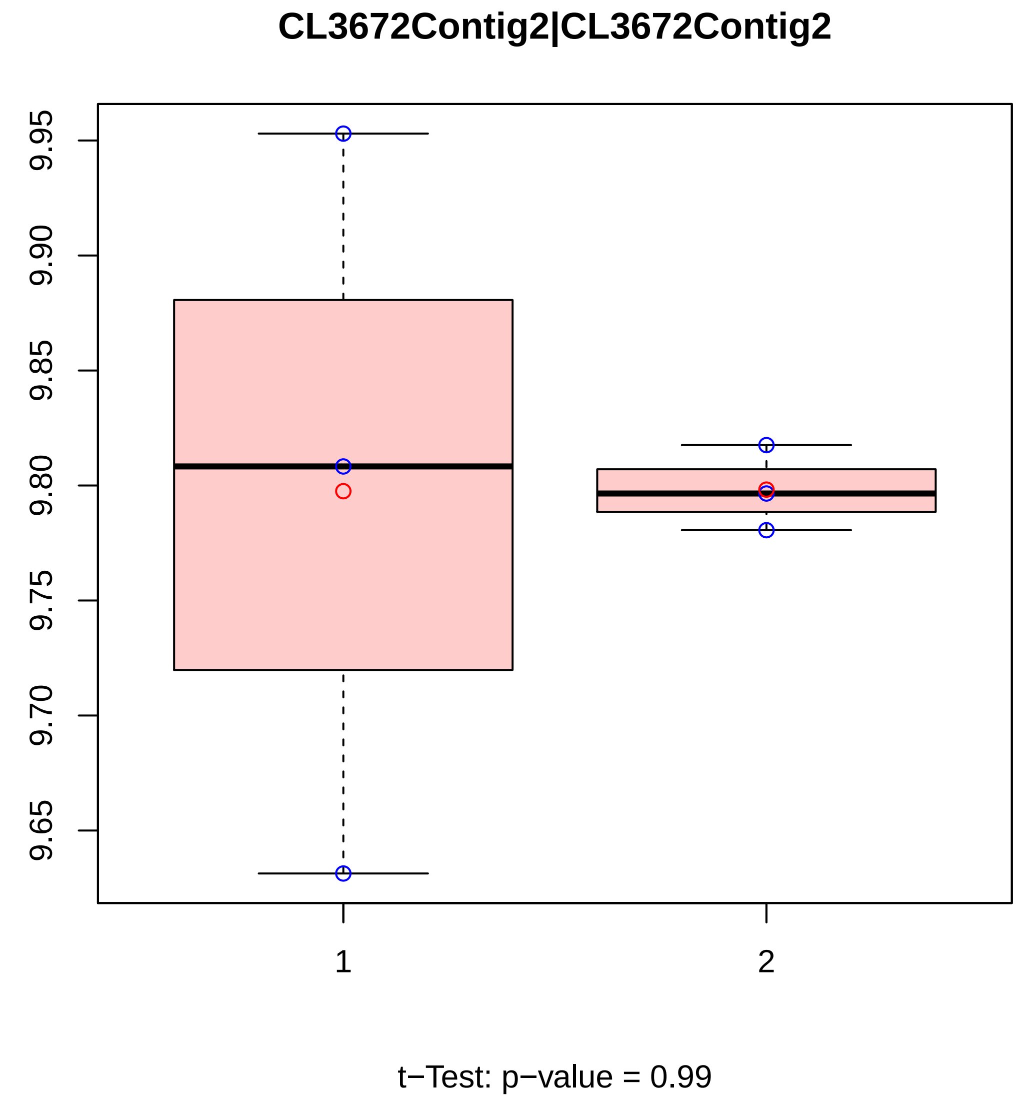

CL3672Contig2|CL3672Contig2
9.95
9.90
9.85
9.80
9.75
9.70
9.65
1
2
t−Test: p−value = 0.99

# CL367Contig10|CL367Contig10

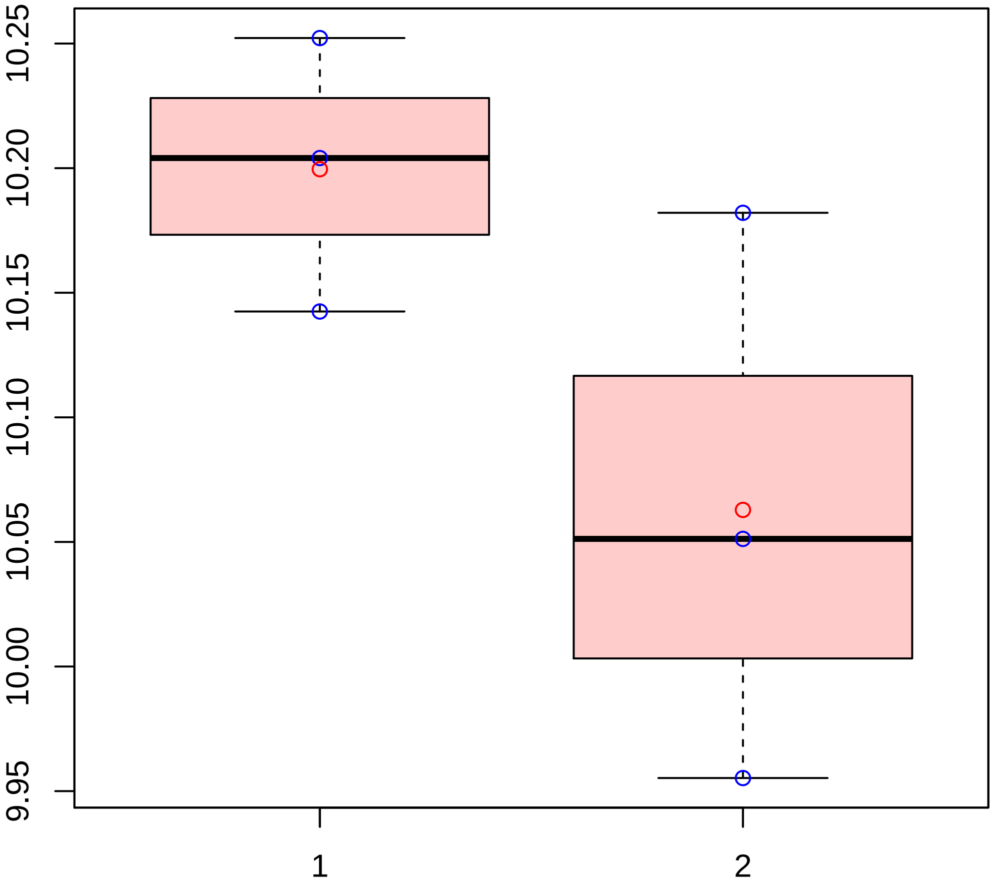


t−Test: p−value = 0.16

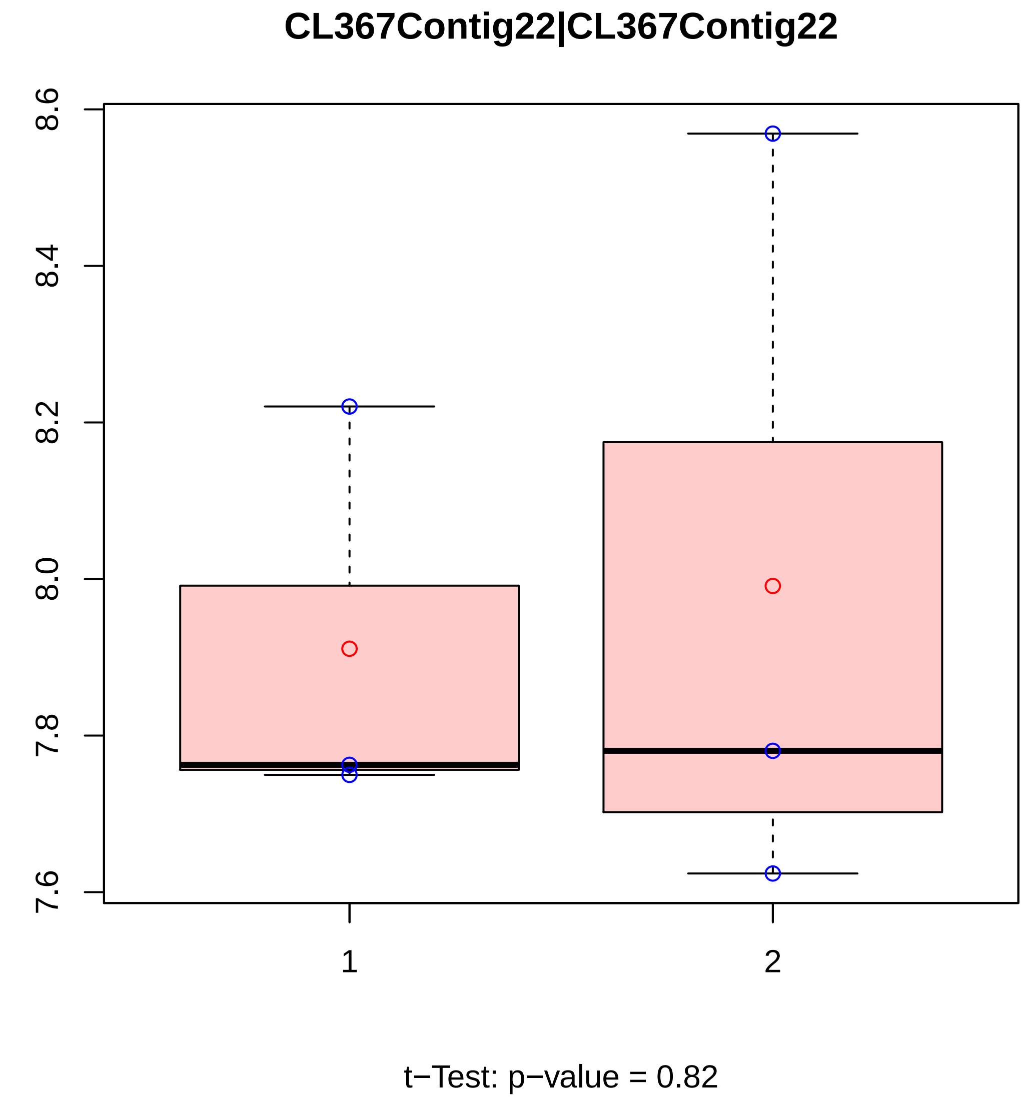

CL367Contig22|CL367Contig22
8.6
8.4
8.2
8.0
7.8
7.6
1
2
t−Test: p−value = 0.82

# CL367Contig23|CL367Contig23

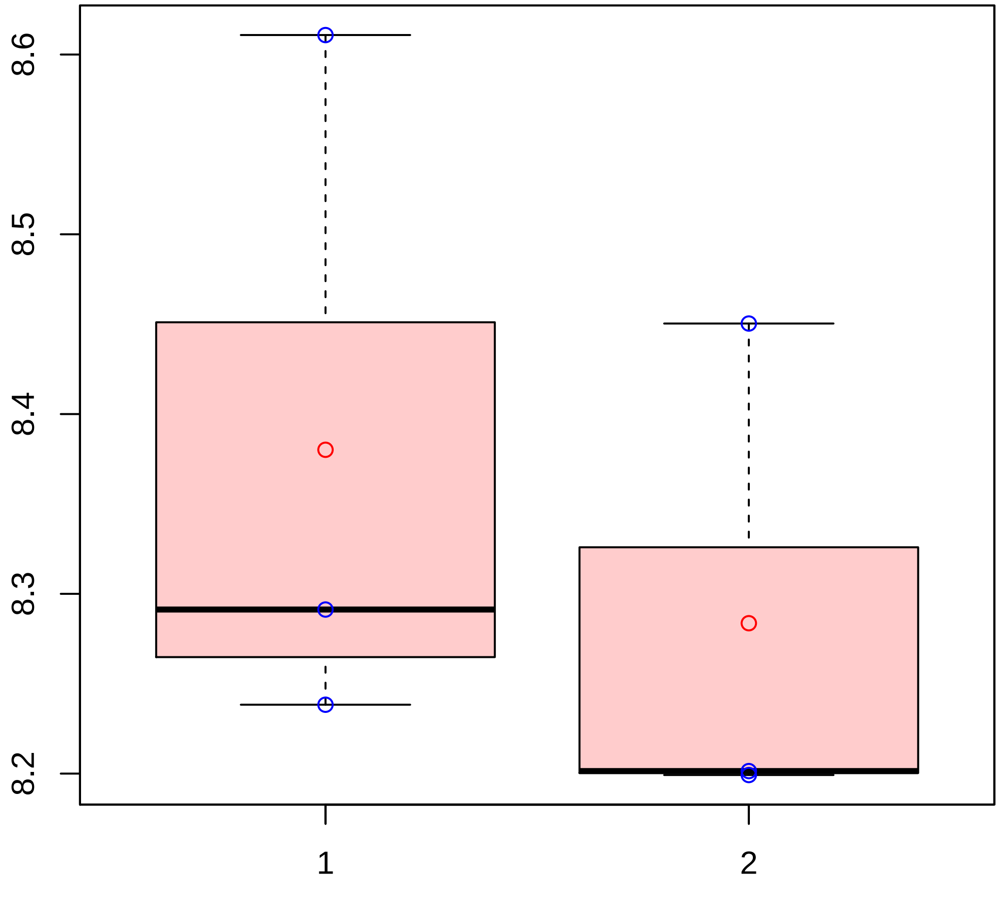


t−Test: p−value = 0.54

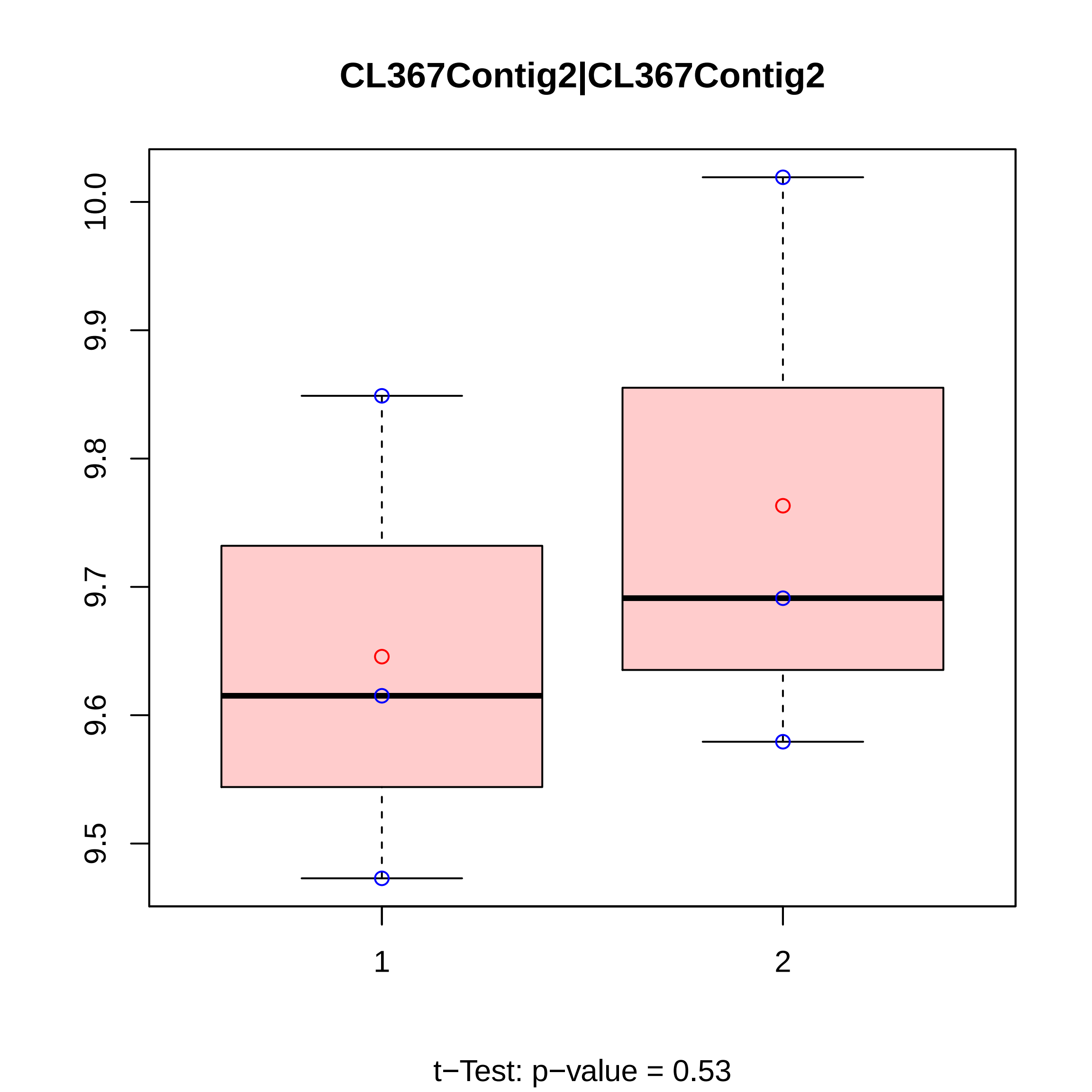
CL367Contig2|CL367Contig2
10.0
9.9
9.8
9.7
9.6
9.5
1
2
t−Test: p−value = 0.53

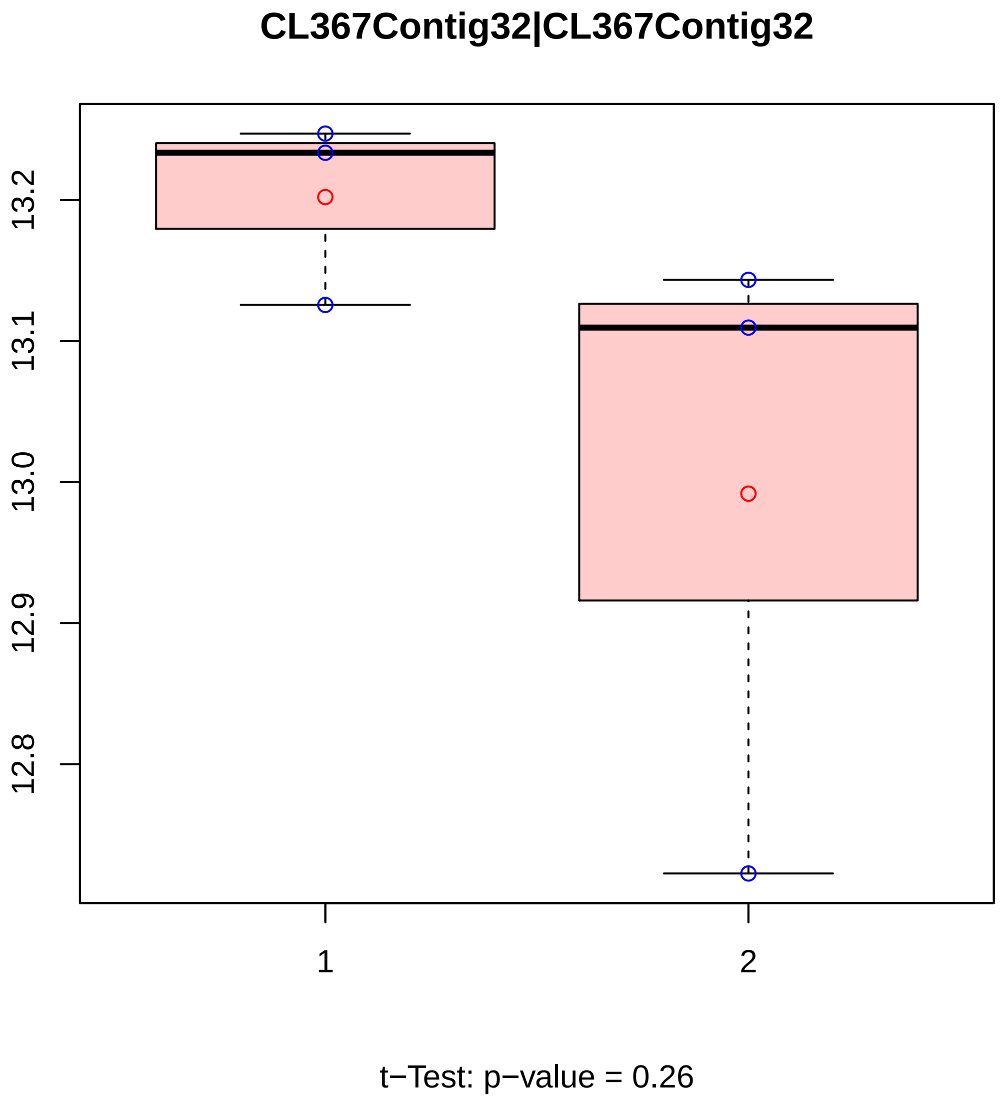
CL367Contig32|CL367Contig32
13.2
13.1
13.0
12.9
12.8
1
2
t−Test: p−value = 0.26

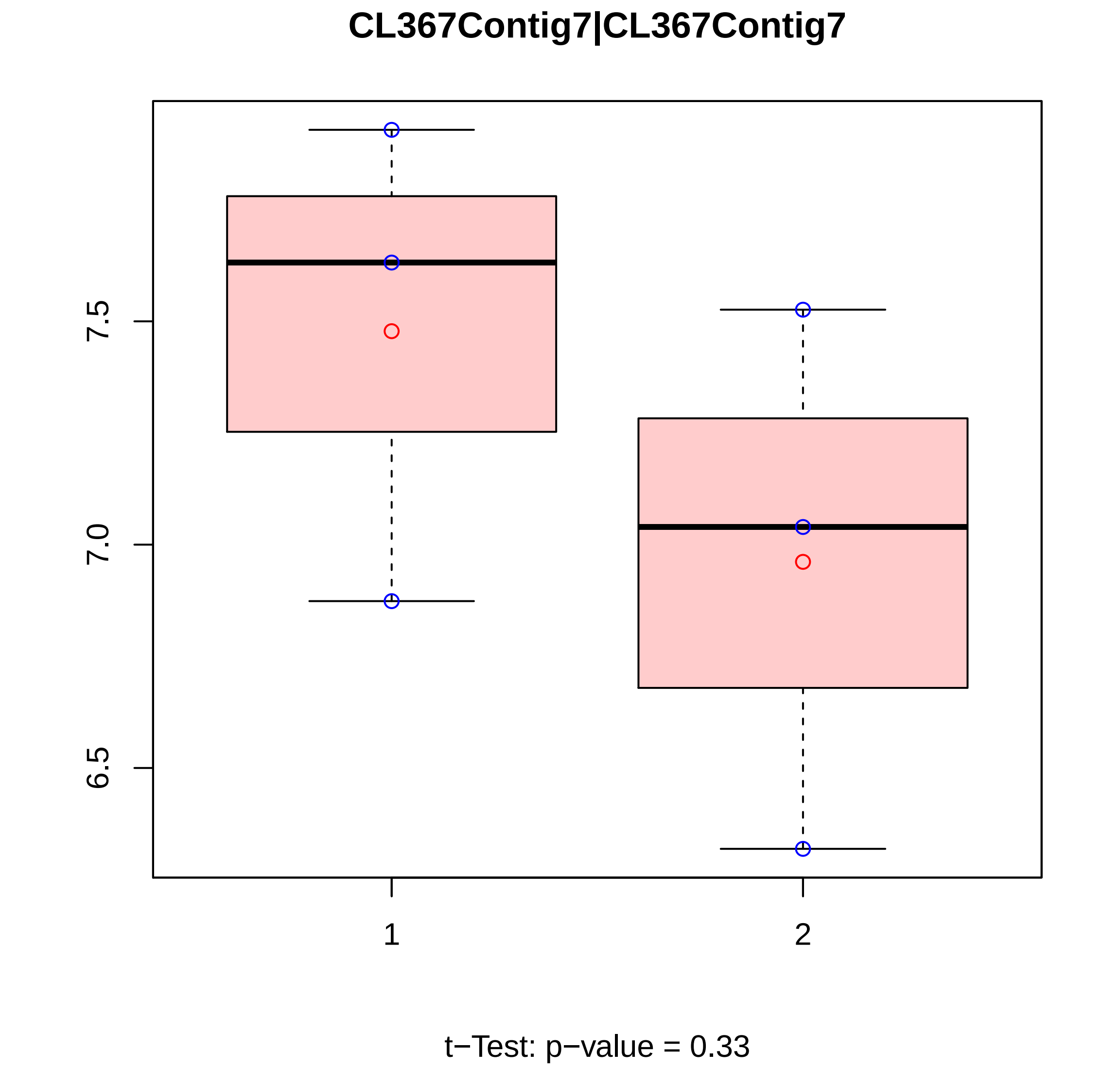
CL367Contig7|CL367Contig7
7.5
7.0
6.5
1
2
t−Test: p−value = 0.33

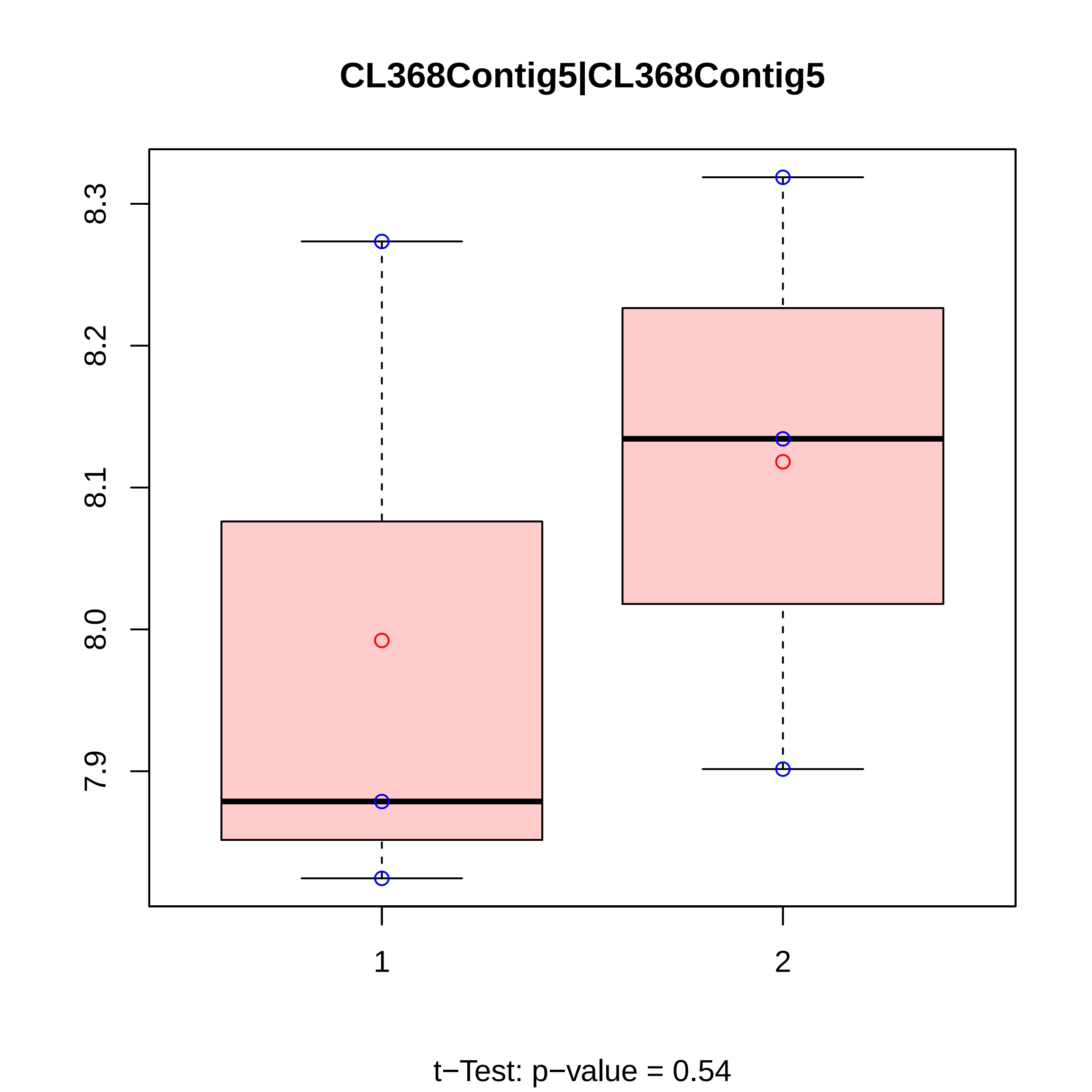
CL368Contig5|CL368Contig5
8.3
8.2
8.1
8.0
7.9
1
2
t−Test: p−value = 0.54

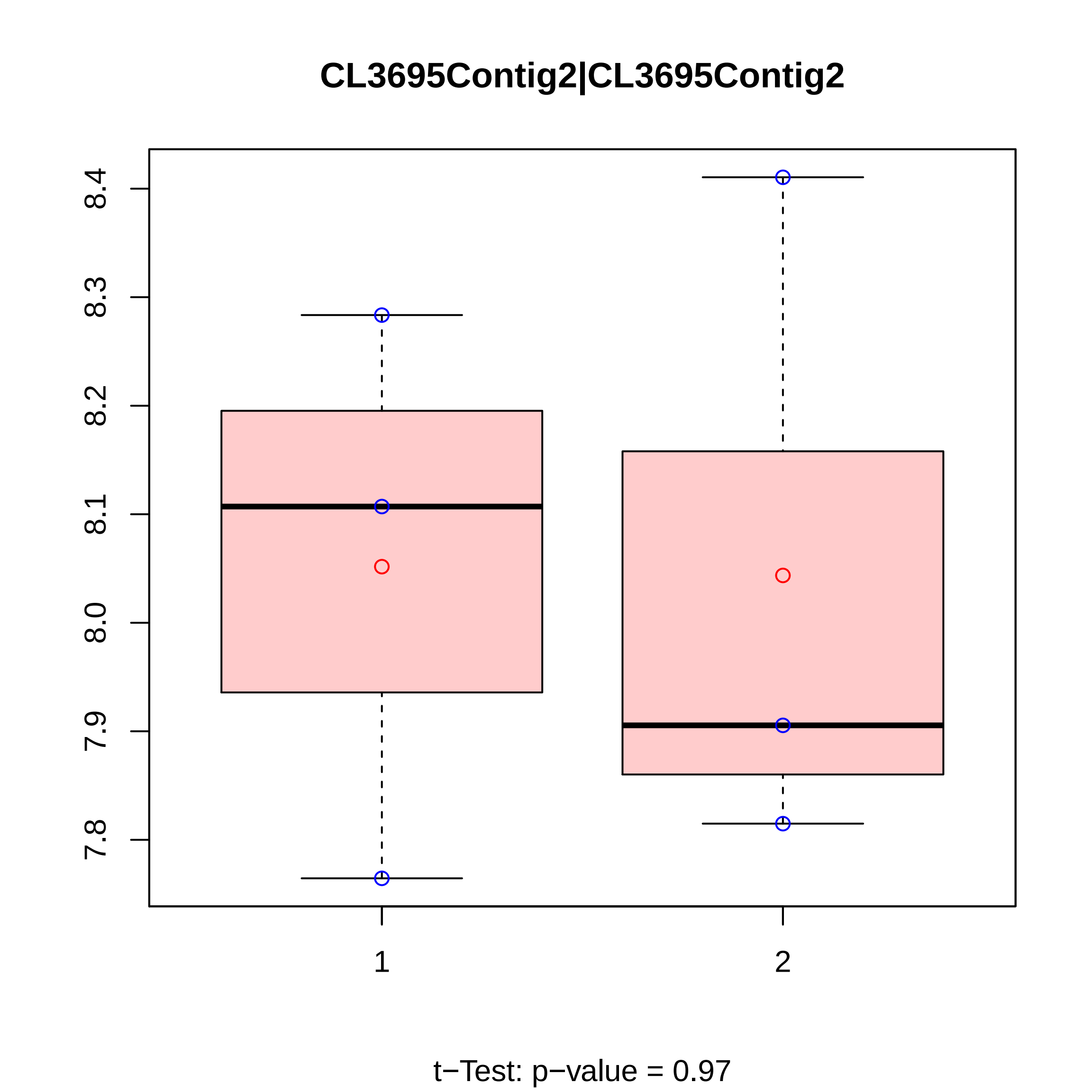

CL3695Contig2|CL3695Contig2
8.4
8.3
8.2
8.1
8.0
7.9
7.8
1
2
t−Test: p−value = 0.97

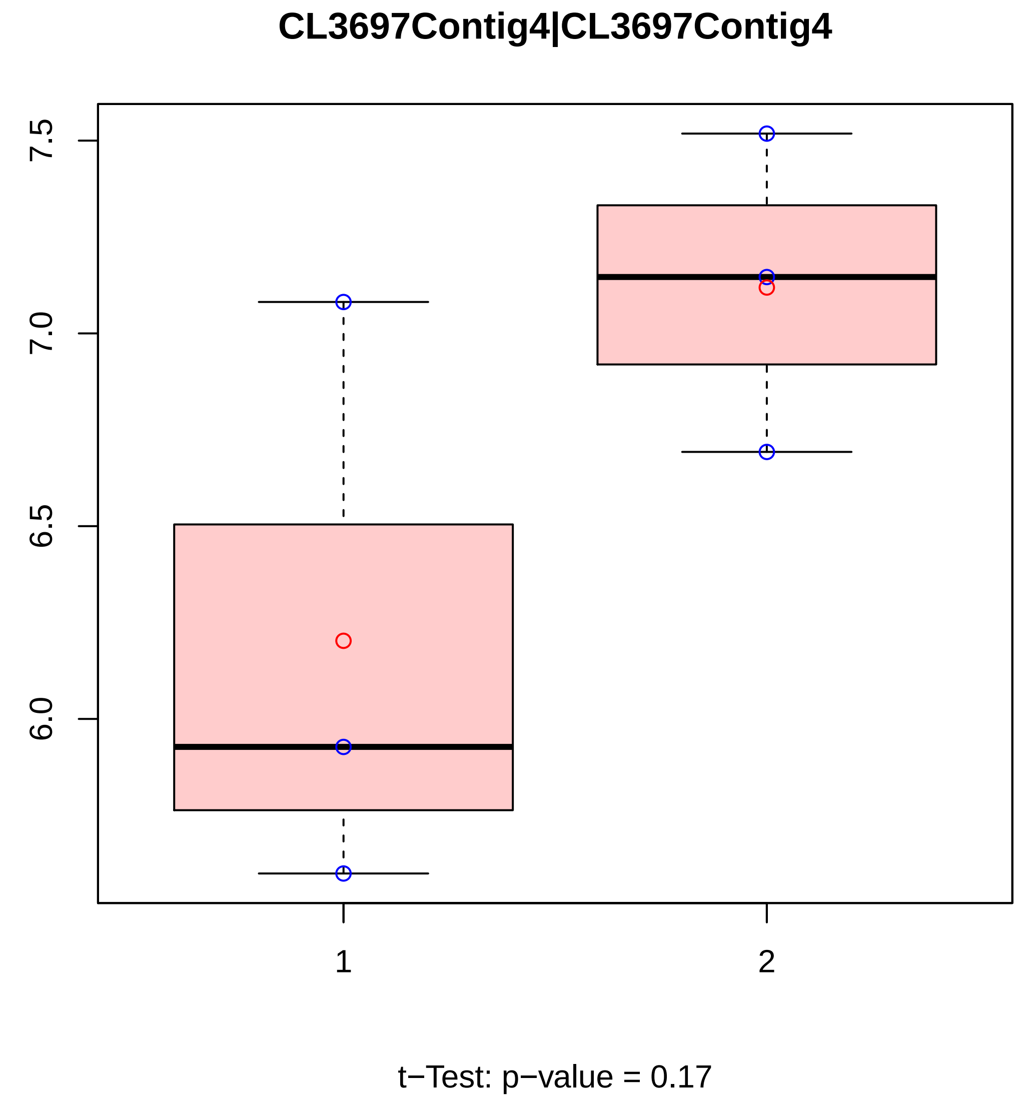

CL3697Contig4|CL3697Contig4
7.5
7.0
6.5
6.0
1
2
t−Test: p−value = 0.17

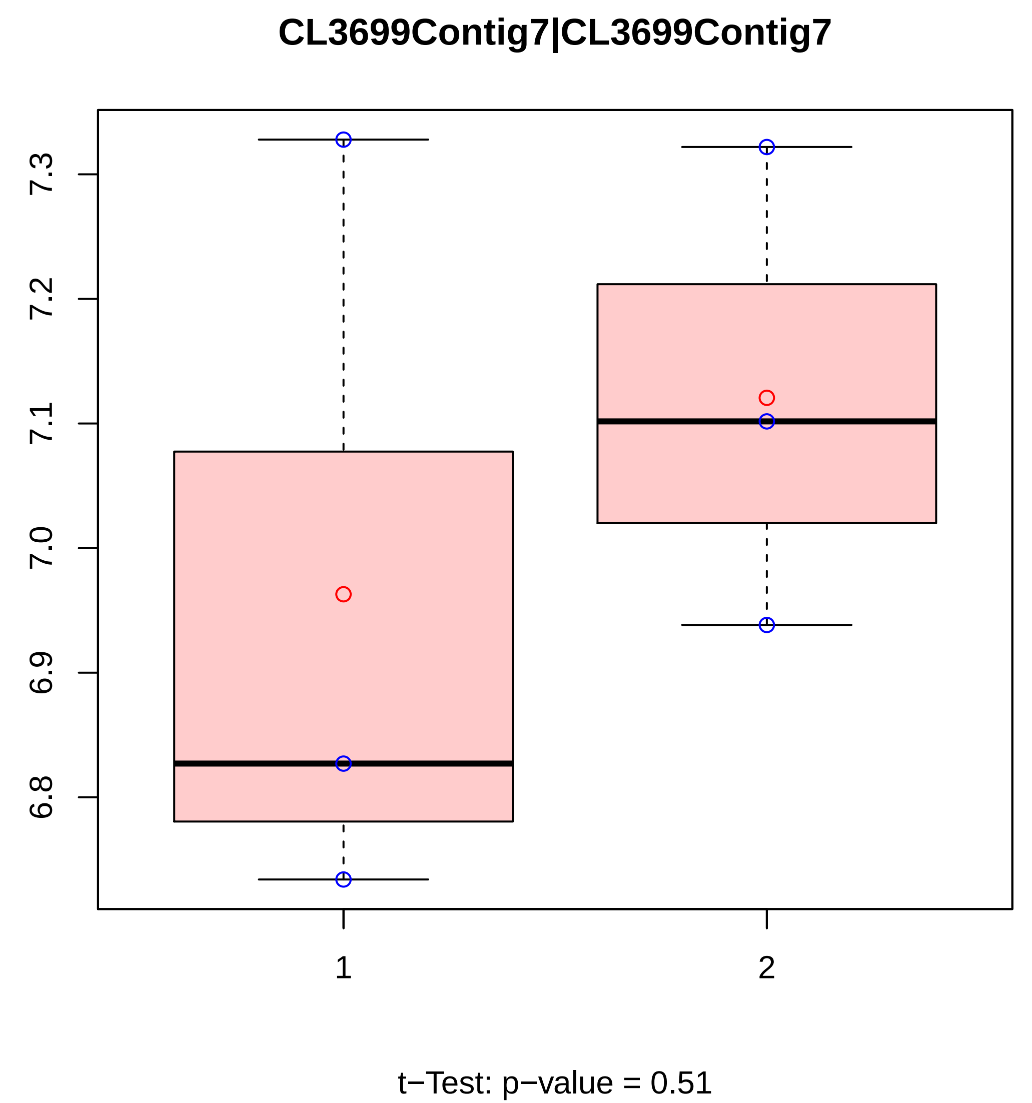

CL3699Contig7|CL3699Contig7
7.3
7.2
7.1
7.0
6.9
6.8
1
2
t−Test: p−value = 0.51

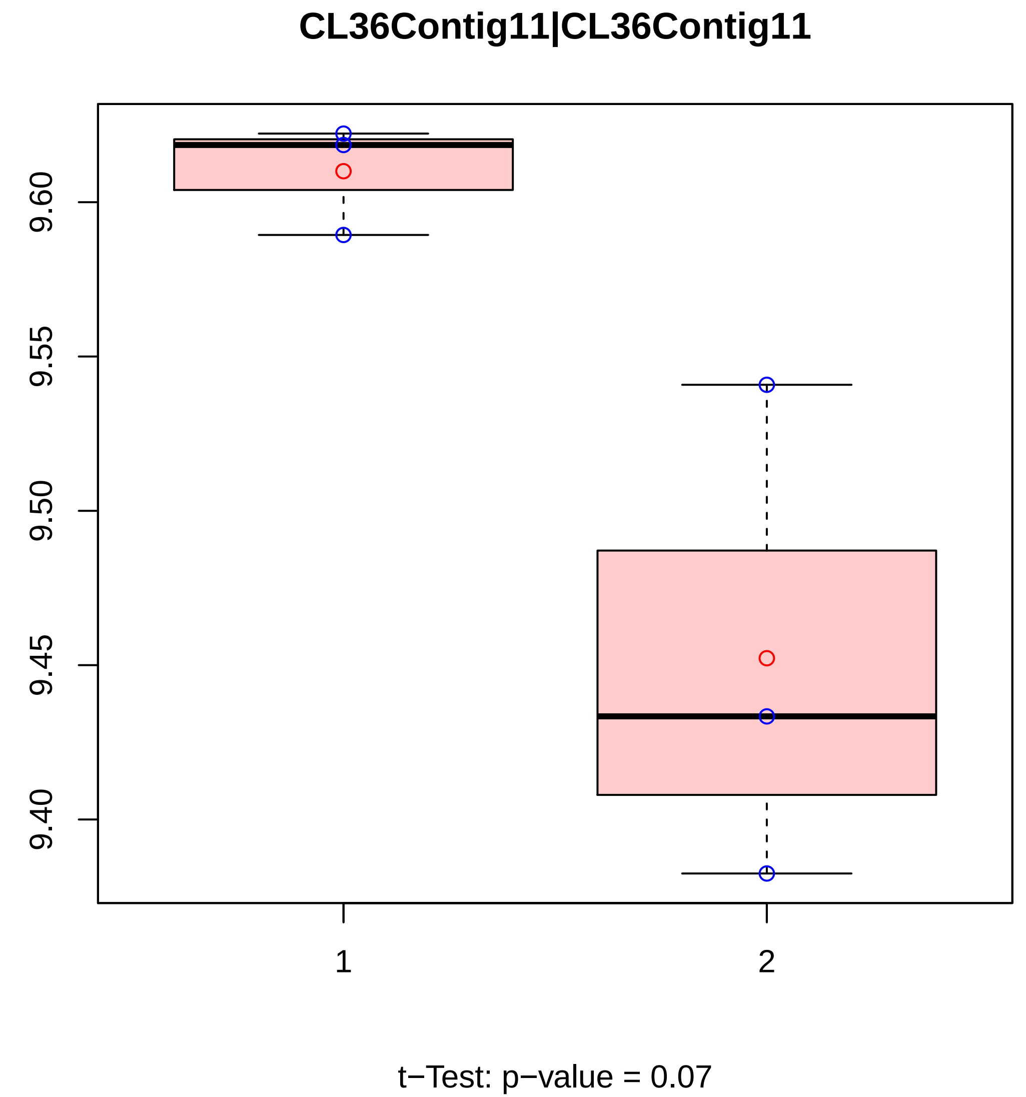

CL36Contig11|CL36Contig11
9.40
9.45
9.50
9.55
9.60
1
2
t−Test: p−value = 0.07

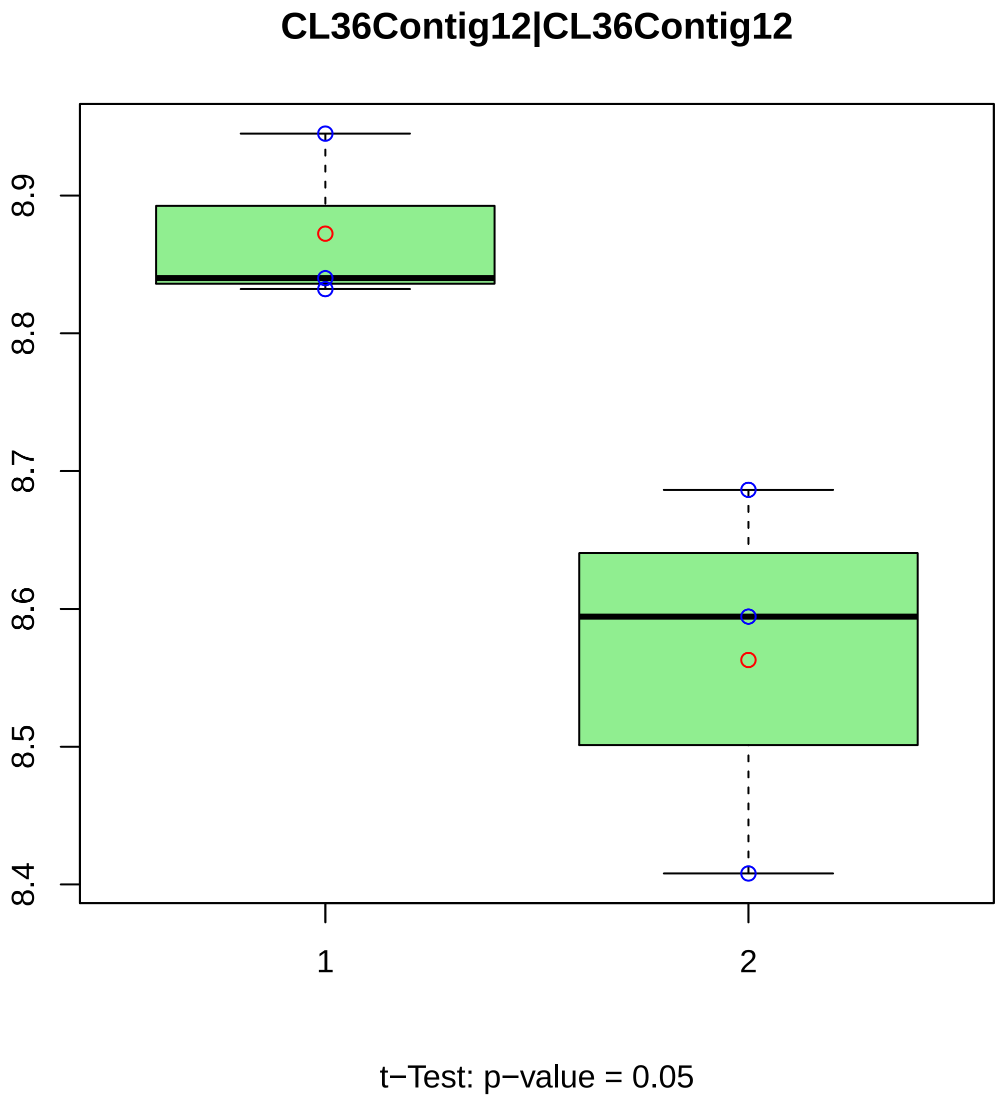
CL36Contig12|CL36Contig12
8.4
8.5
8.6
8.7
8.8
8.9
1
2
t−Test: p−value = 0.05

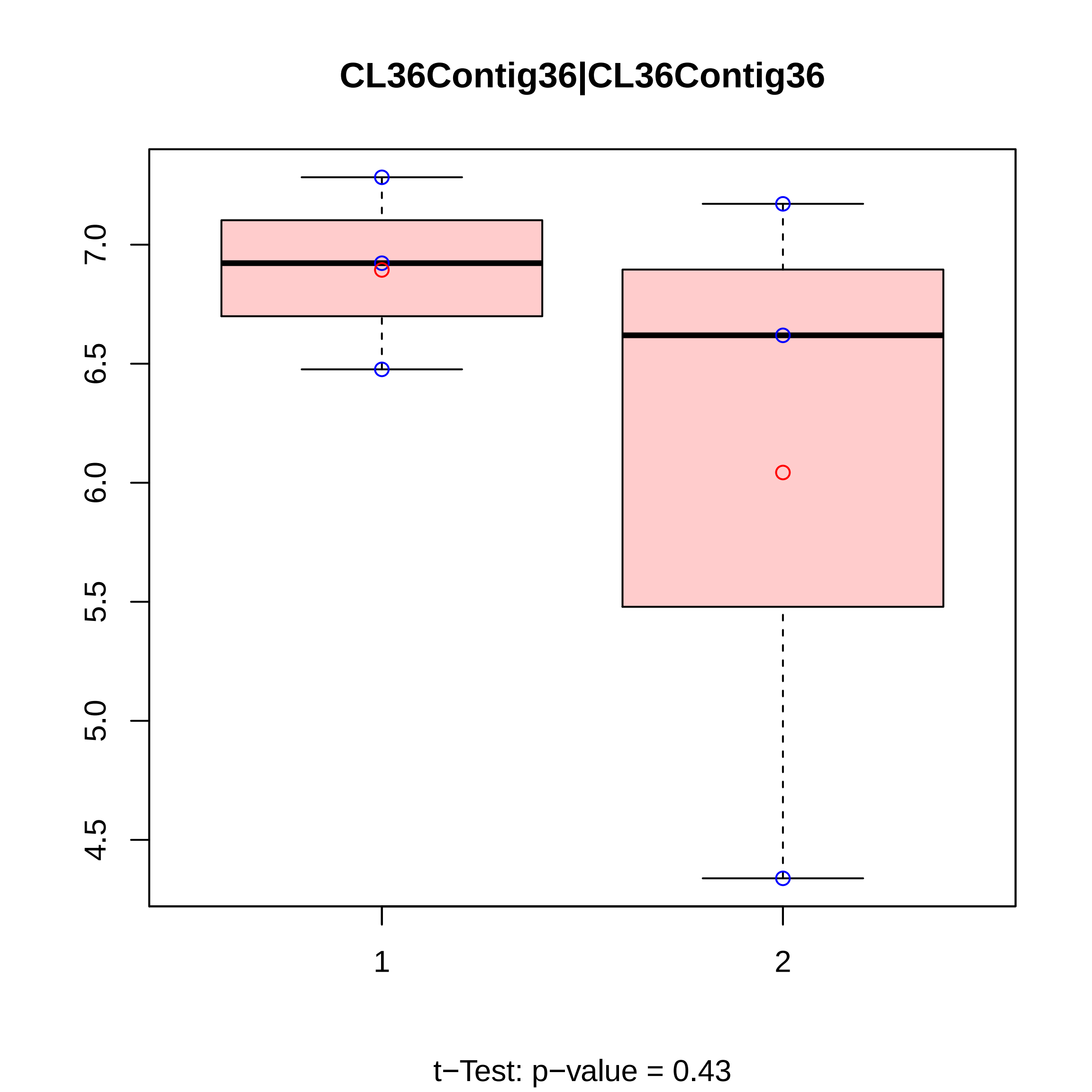

CL36Contig36|CL36Contig36
7.0
6.5
6.0
5.5
5.0
4.5
1
2
t−Test: p−value = 0.43

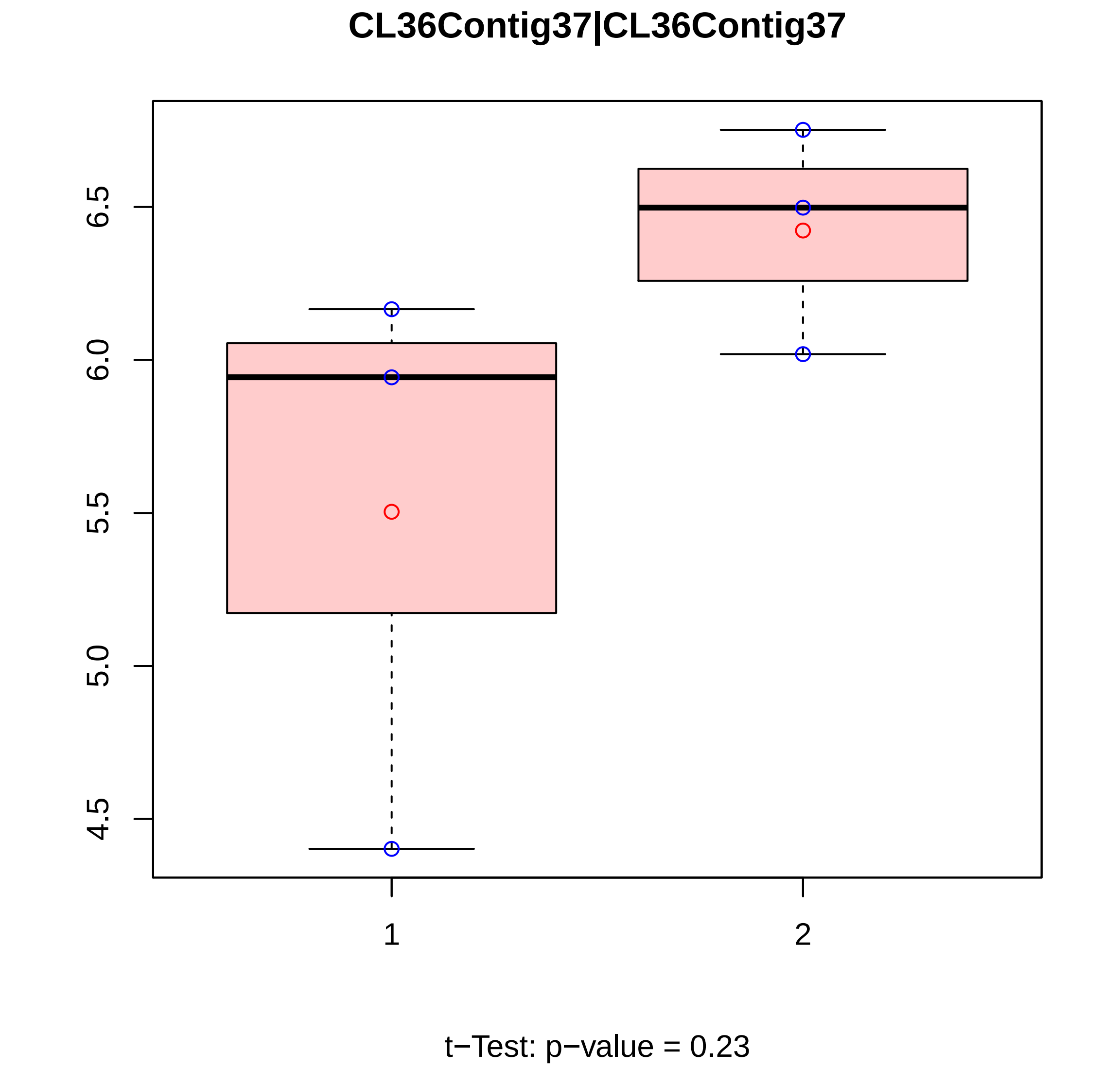

CL36Contig37|CL36Contig37
6.5
6.0
5.5
5.0
4.5
1
2
t−Test: p−value = 0.23

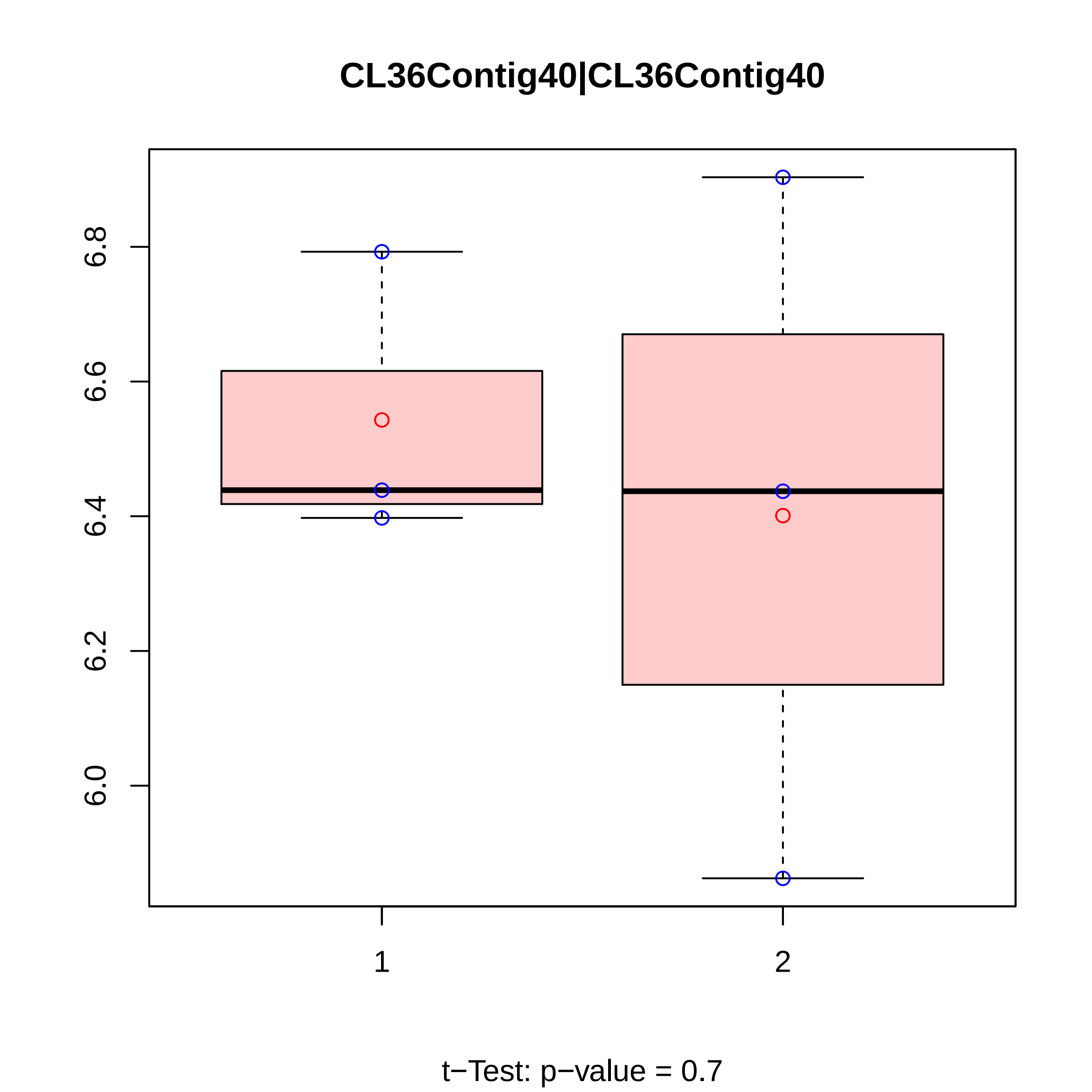
CL36Contig40|CL36Contig40
6.0
6.2
6.4
6.6
6.8
1
2
t−Test: p−value = 0.7

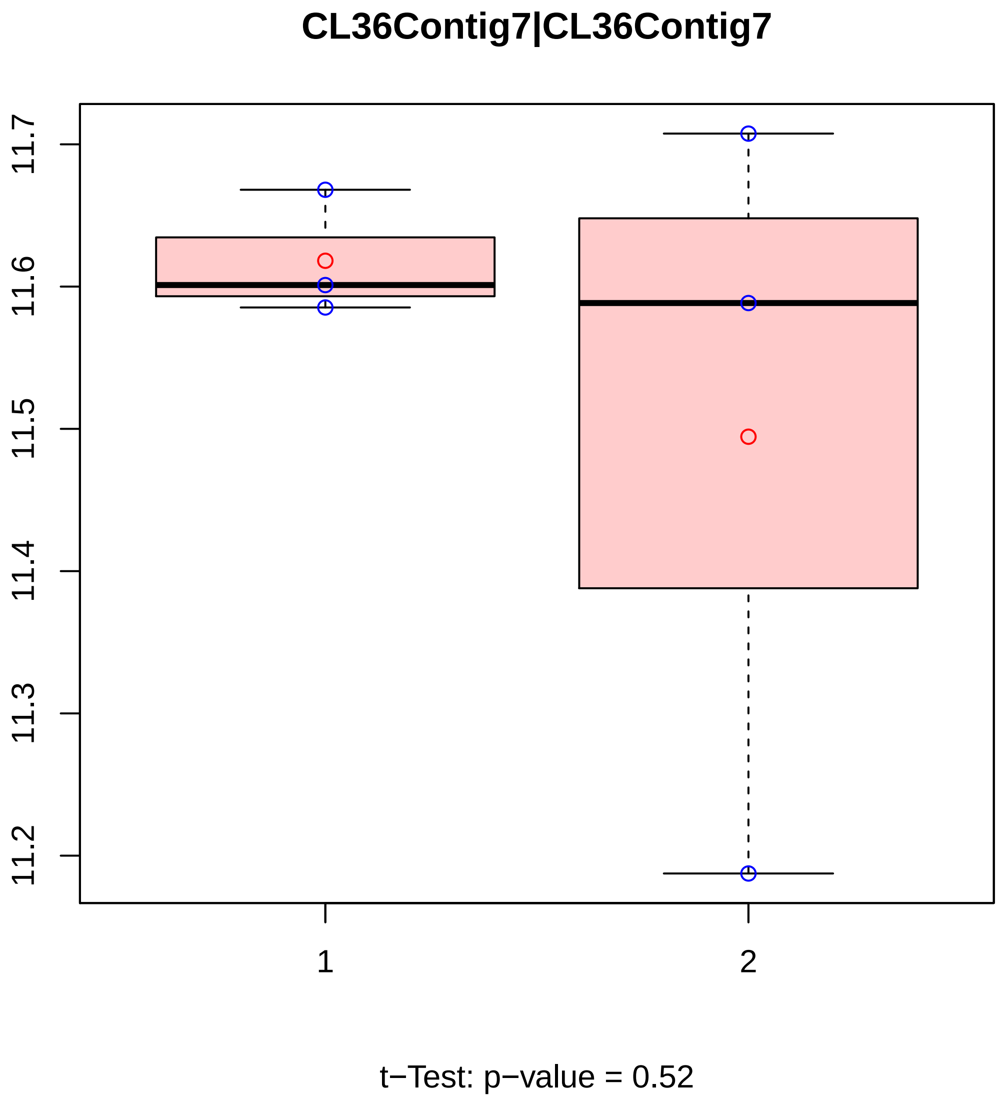
CL36Contig7|CL36Contig7
11.7
11.6
11.5
11.4
11.3
11.2
1
2
t−Test: p−value = 0.52

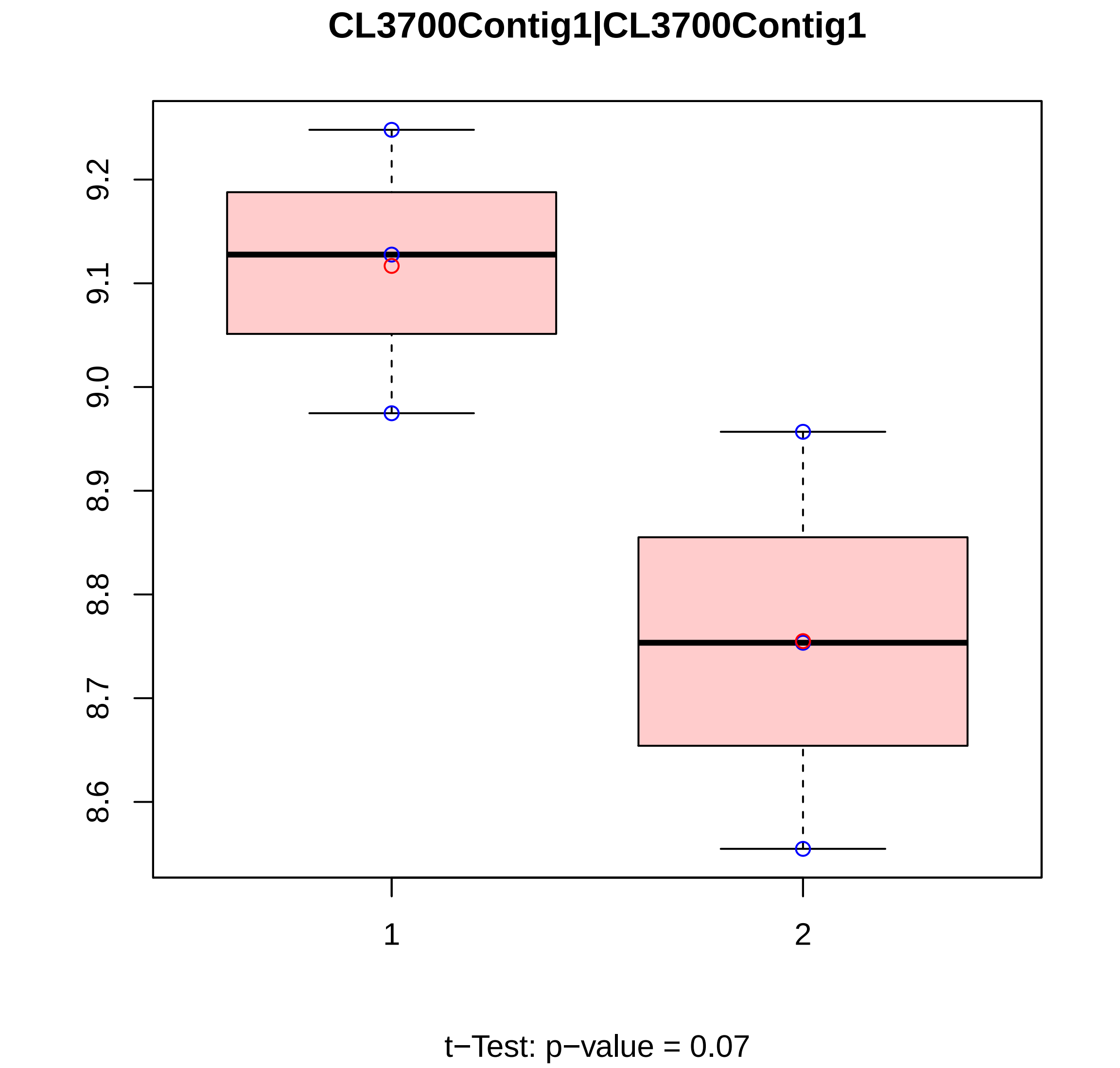
CL3700Contig1|CL3700Contig1
9.2
9.1
9.0
8.9
8.8
8.7
8.6
1
2
t−Test: p−value = 0.07

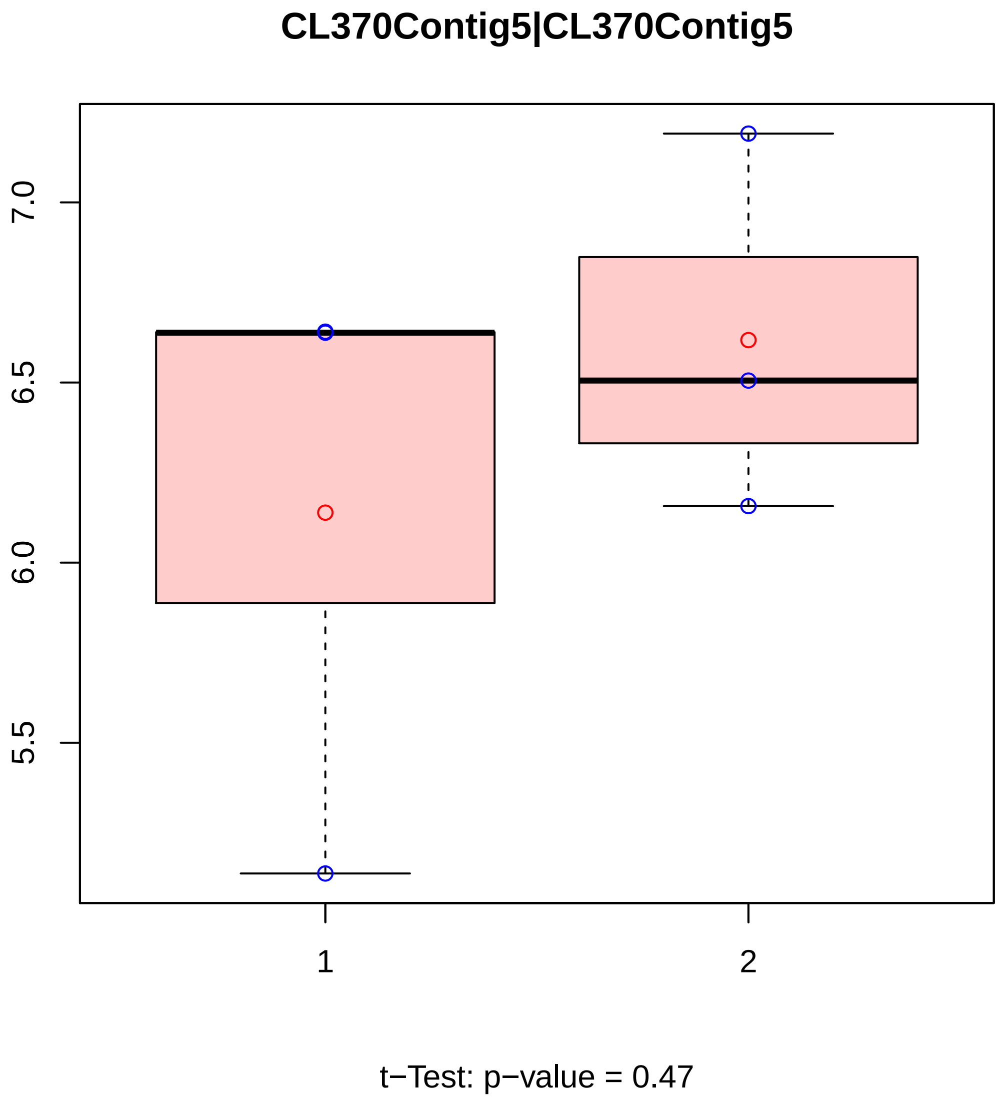

CL370Contig5|CL370Contig5
7.0
6.5
6.0
5.5
1
2
t−Test: p−value = 0.47

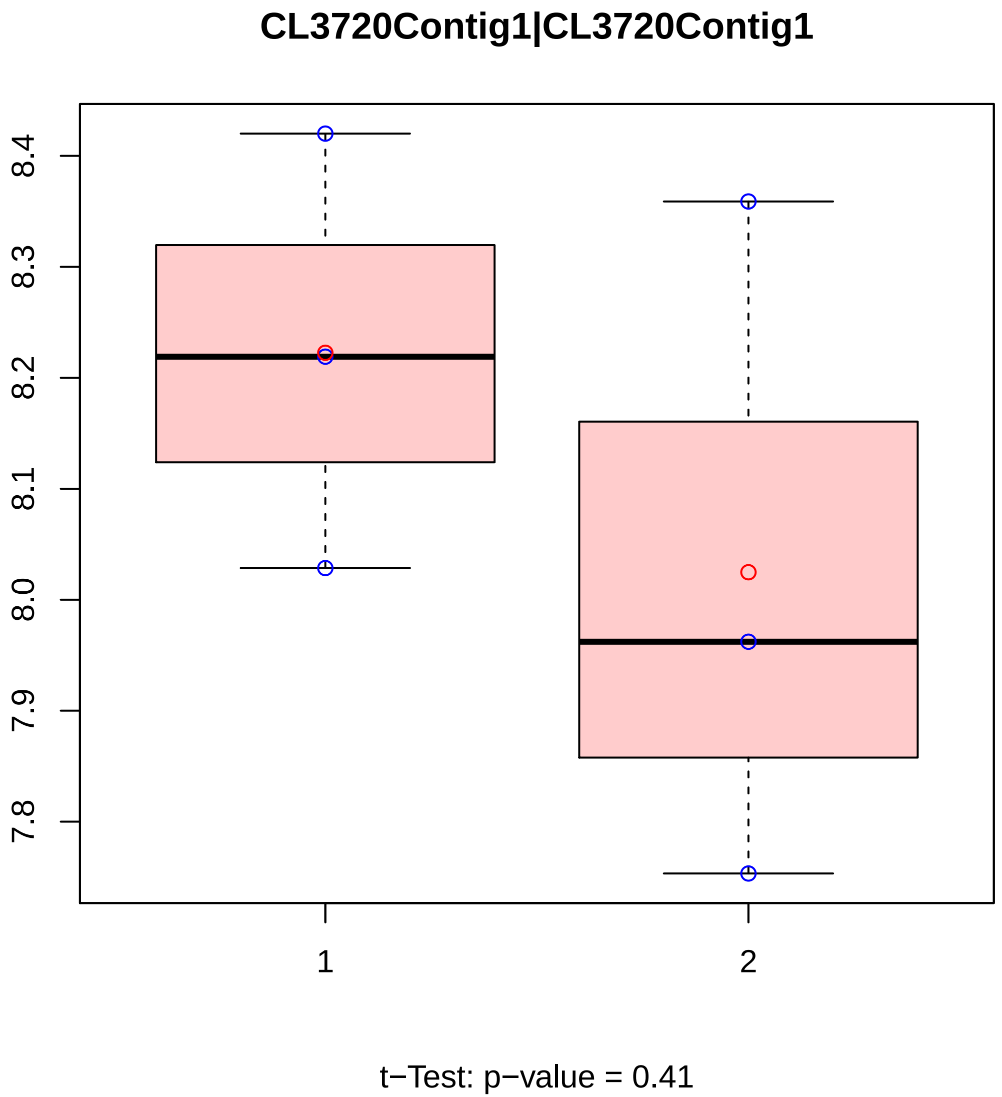
CL3720Contig1|CL3720Contig1
8.4
8.3
8.2
8.1
8.0
7.9
7.8
1
2
t−Test: p−value = 0.41

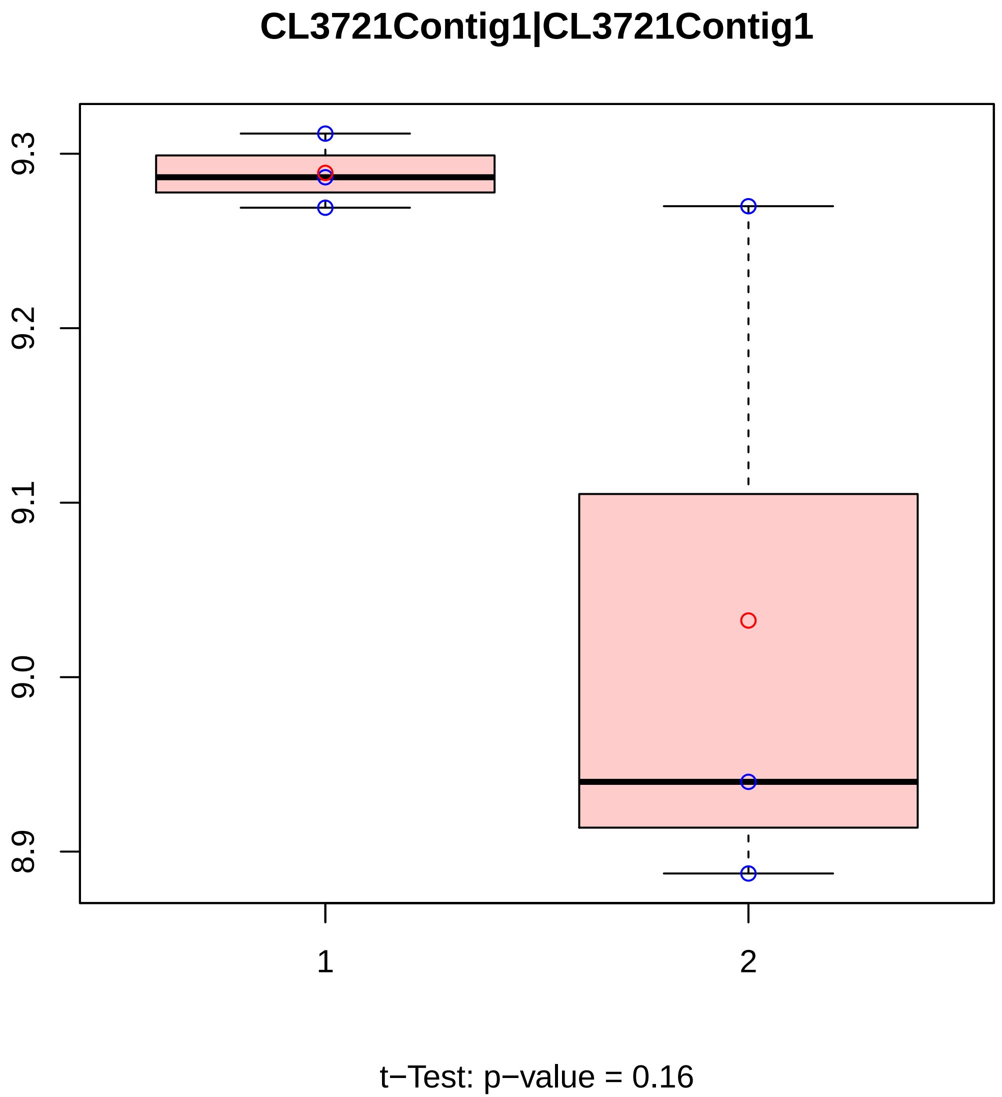

CL3721Contig1|CL3721Contig1
9.3
9.2
9.1
9.0
8.9
1
2
t−Test: p−value = 0.16

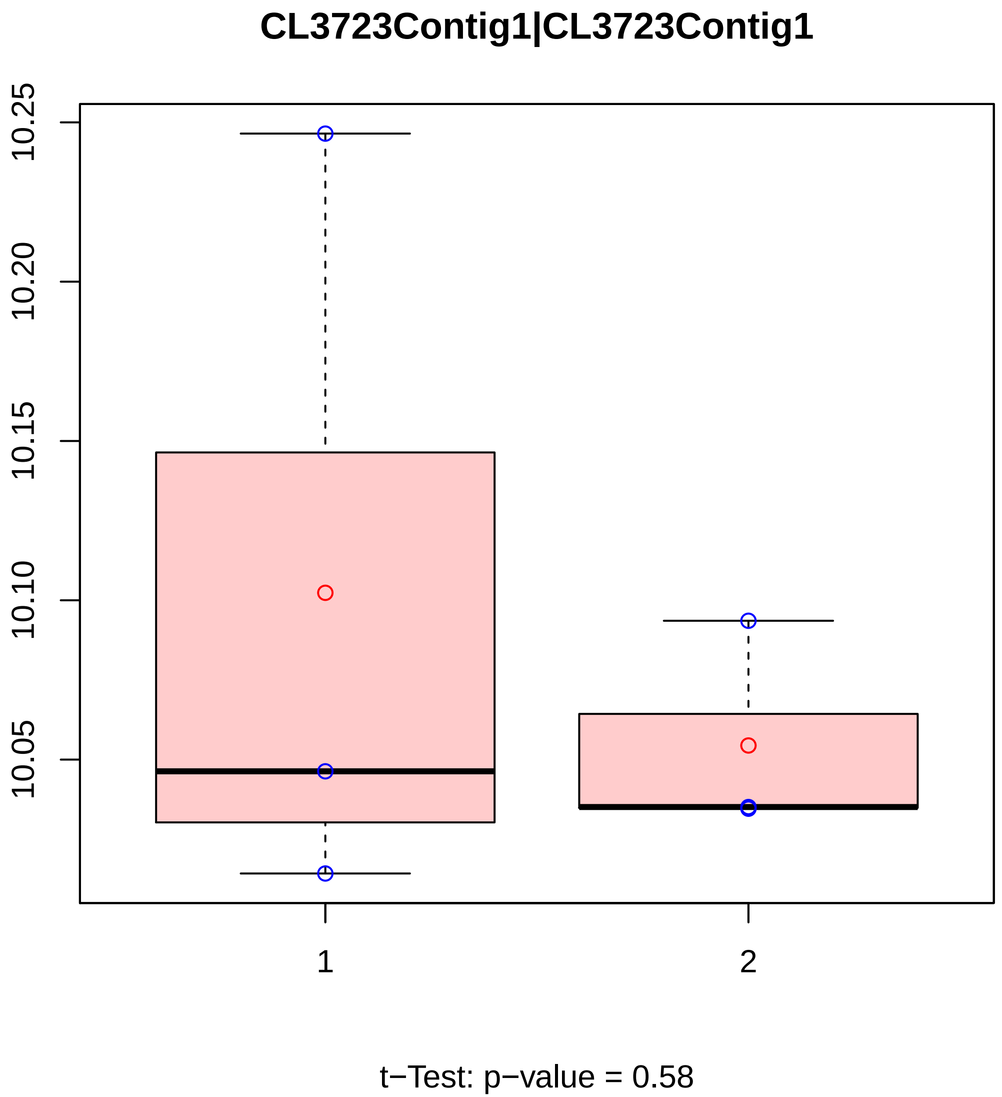
CL3723Contig1|CL3723Contig1
10.25
10.20
10.15
10.10
10.05
1
2
t−Test: p−value = 0.58

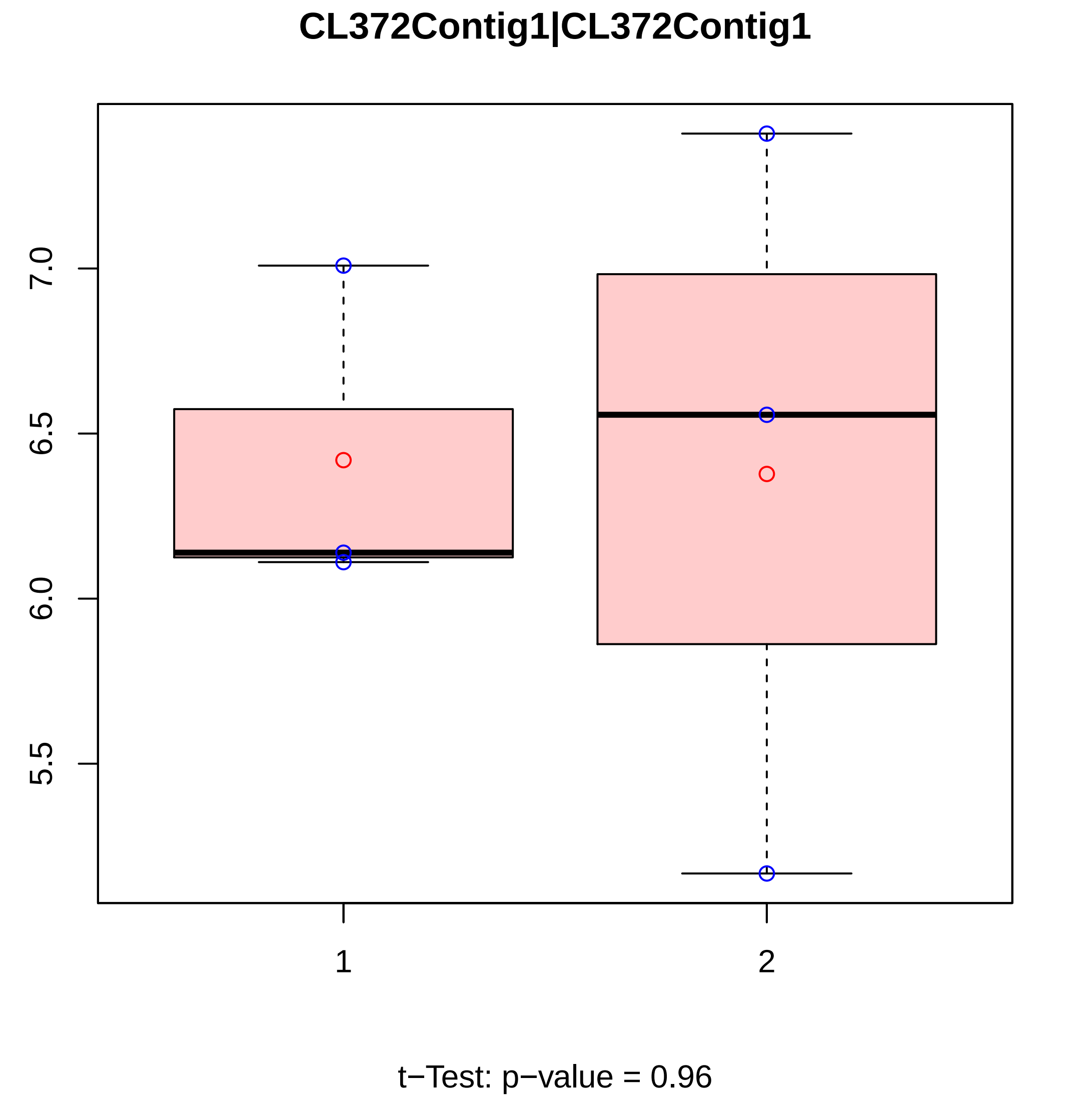
CL372Contig1|CL372Contig1
7.0
6.5
6.0
5.5
1
2
t−Test: p−value = 0.96

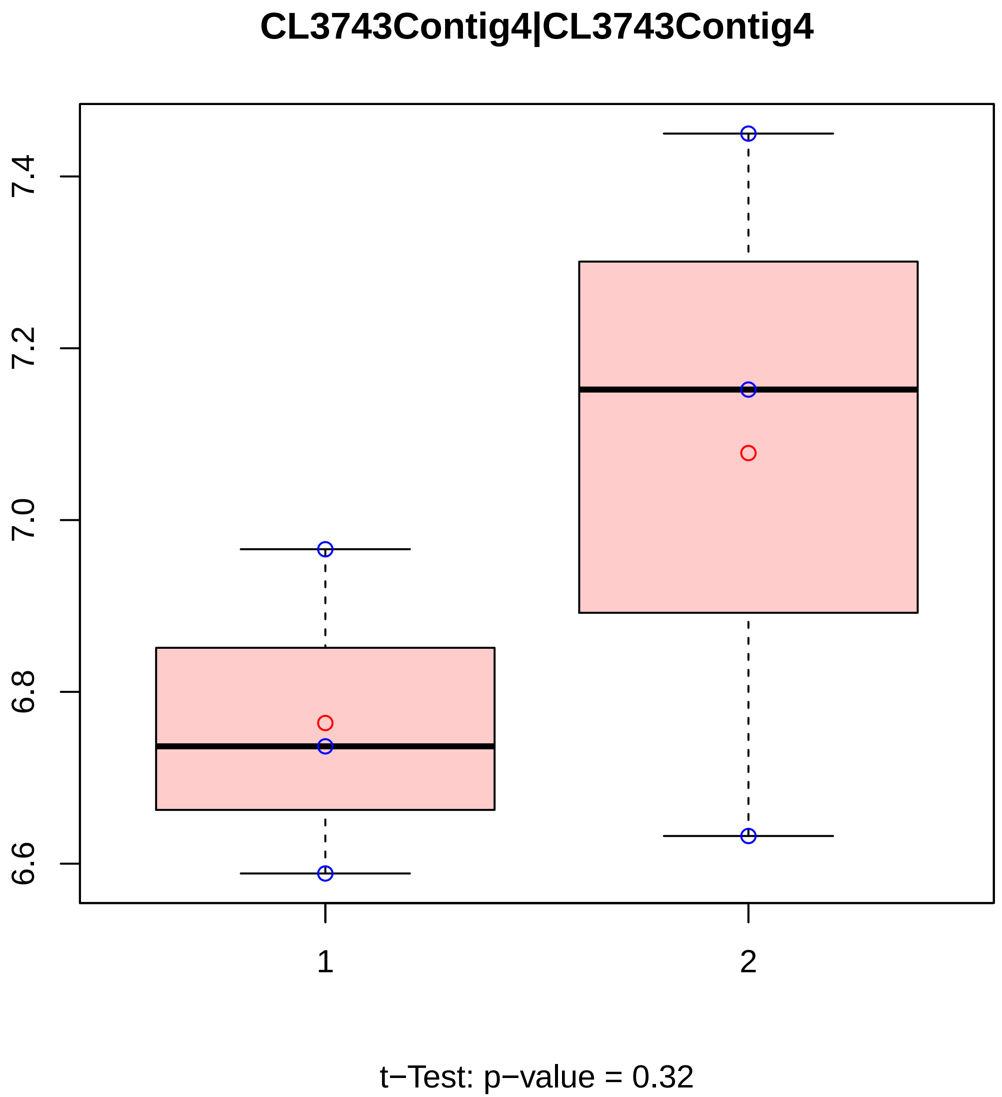
CL3743Contig4|CL3743Contig4
7.4
7.2
7.0
6.8
6.6
1
2
t−Test: p−value = 0.32

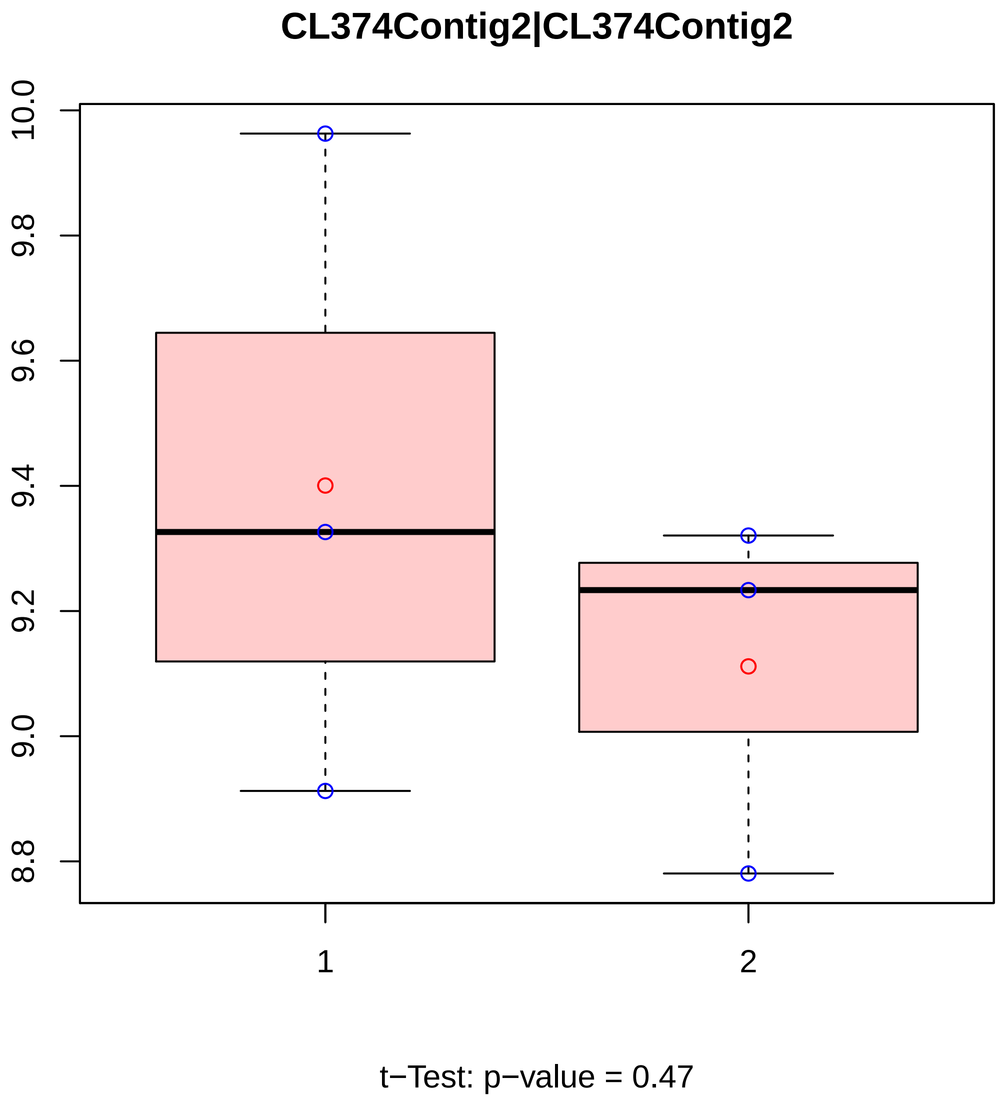
CL374Contig2|CL374Contig2
10.0
9.8
9.6
9.4
9.2
9.0
8.8
1
2
t−Test: p−value = 0.47

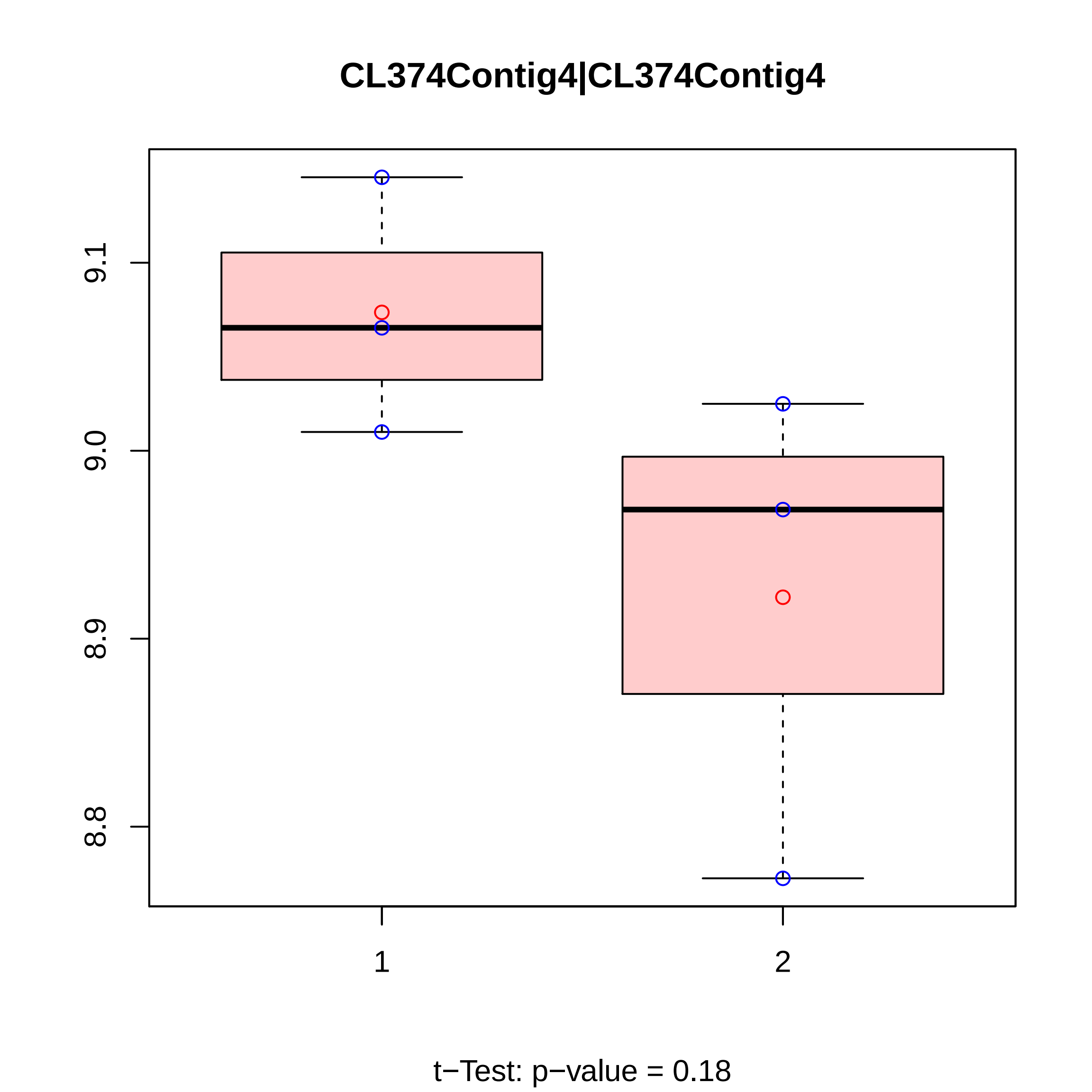
CL374Contig4|CL374Contig4
9.1
9.0
8.9
8.8
1
2
t−Test: p−value = 0.18

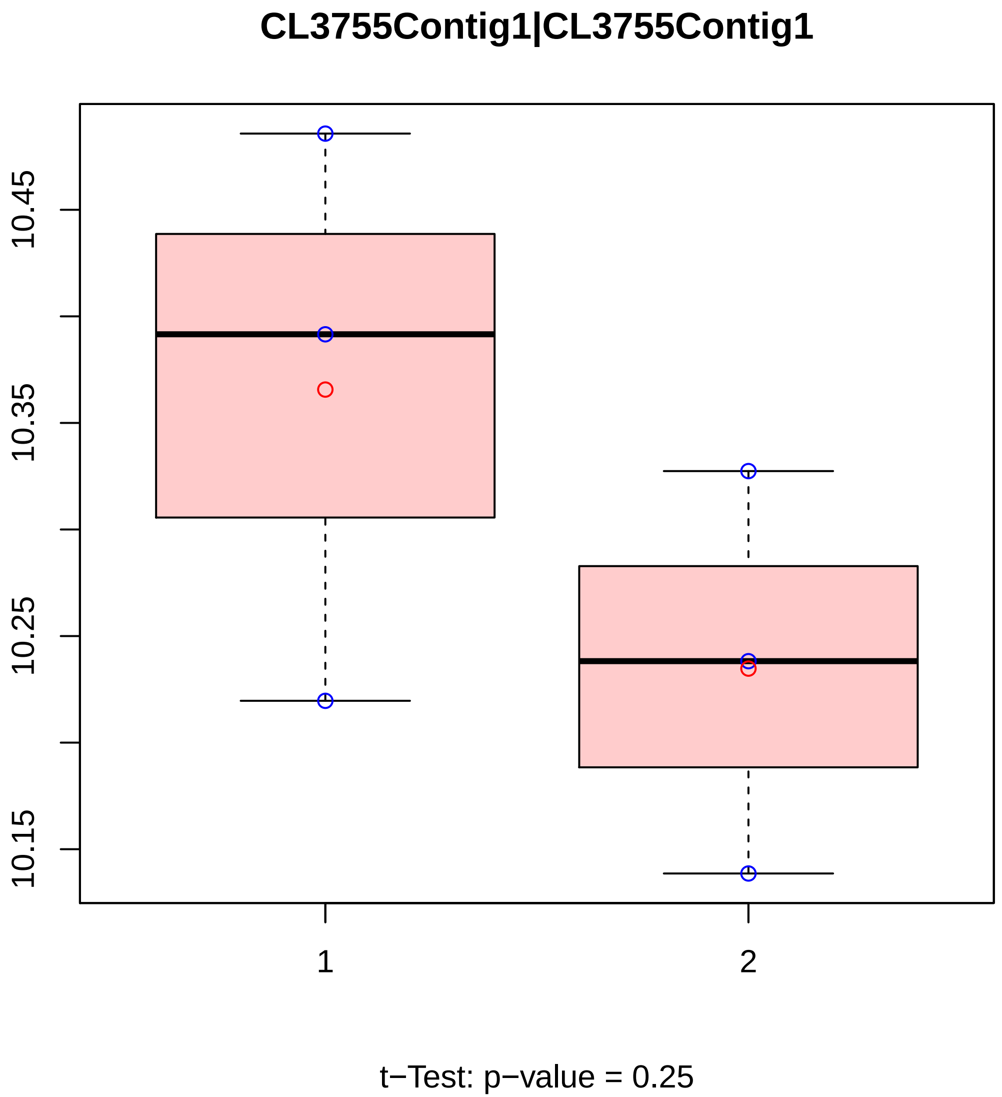

CL3755Contig1|CL3755Contig1
10.45
10.35
10.25
10.15
1
2
t−Test: p−value = 0.25

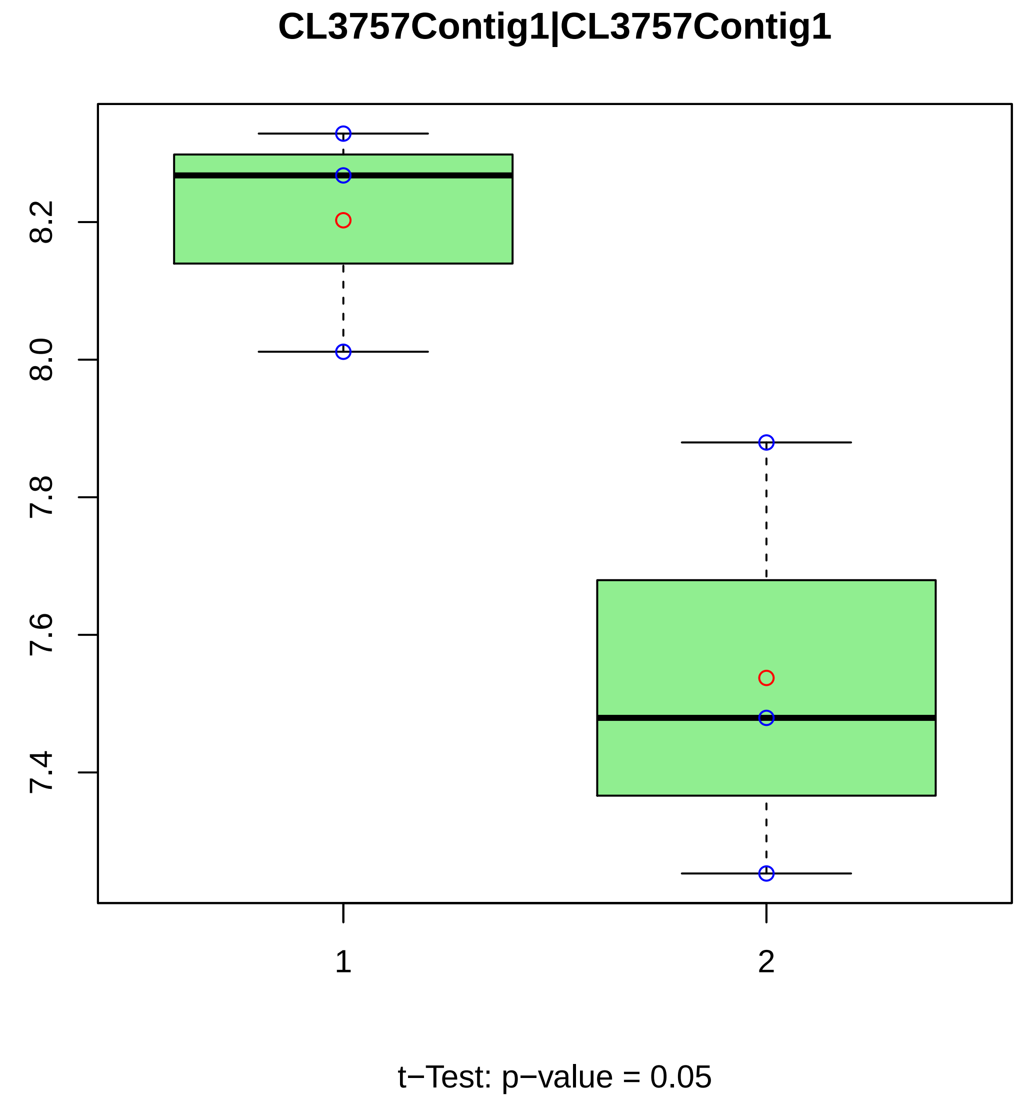
CL3757Contig1|CL3757Contig1
8.2
8.0
7.8
7.6
7.4
1
2
t−Test: p−value = 0.05

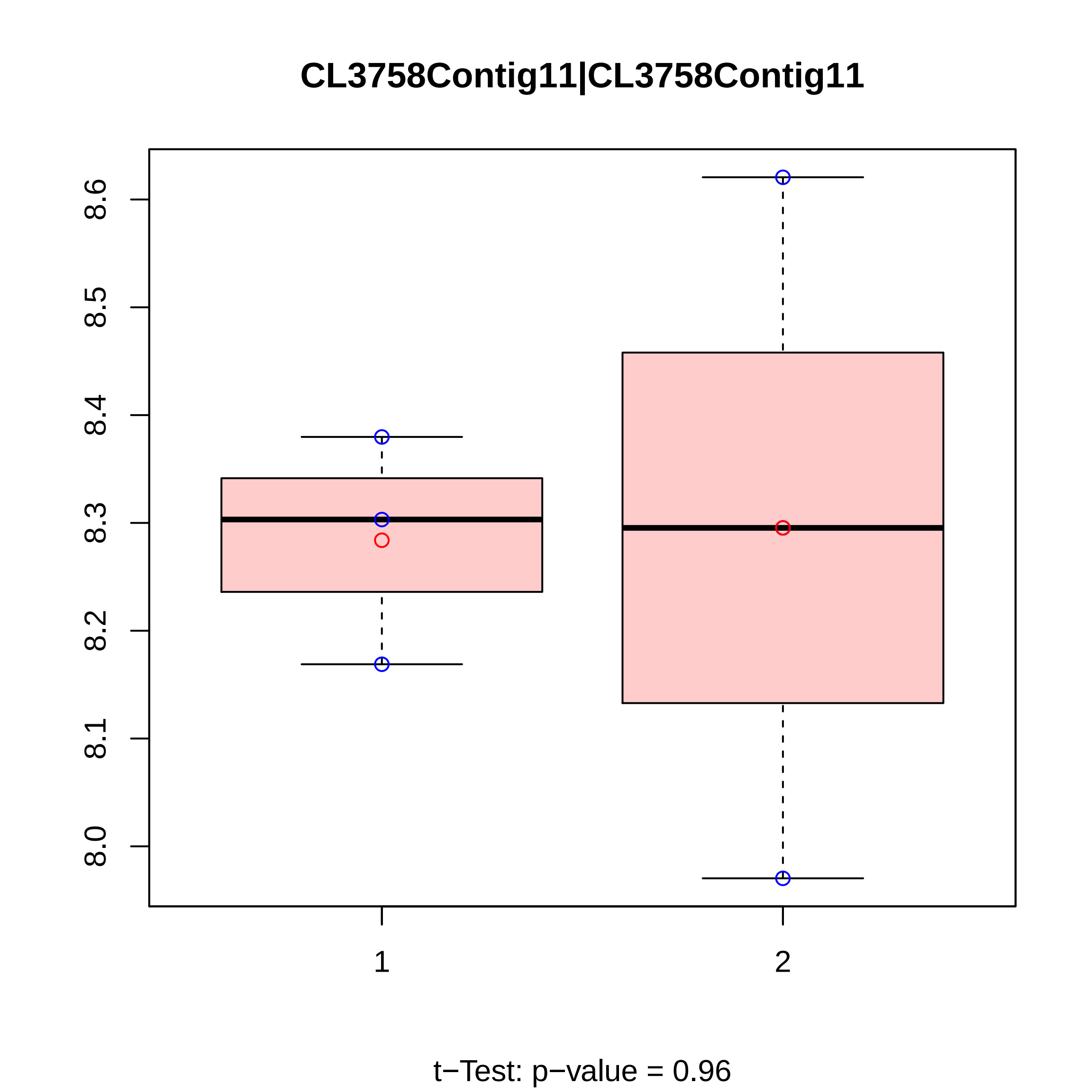
CL3758Contig11|CL3758Contig11
8.0
8.1
8.2
8.3
8.4
8.5
8.6
1
2
t−Test: p−value = 0.96

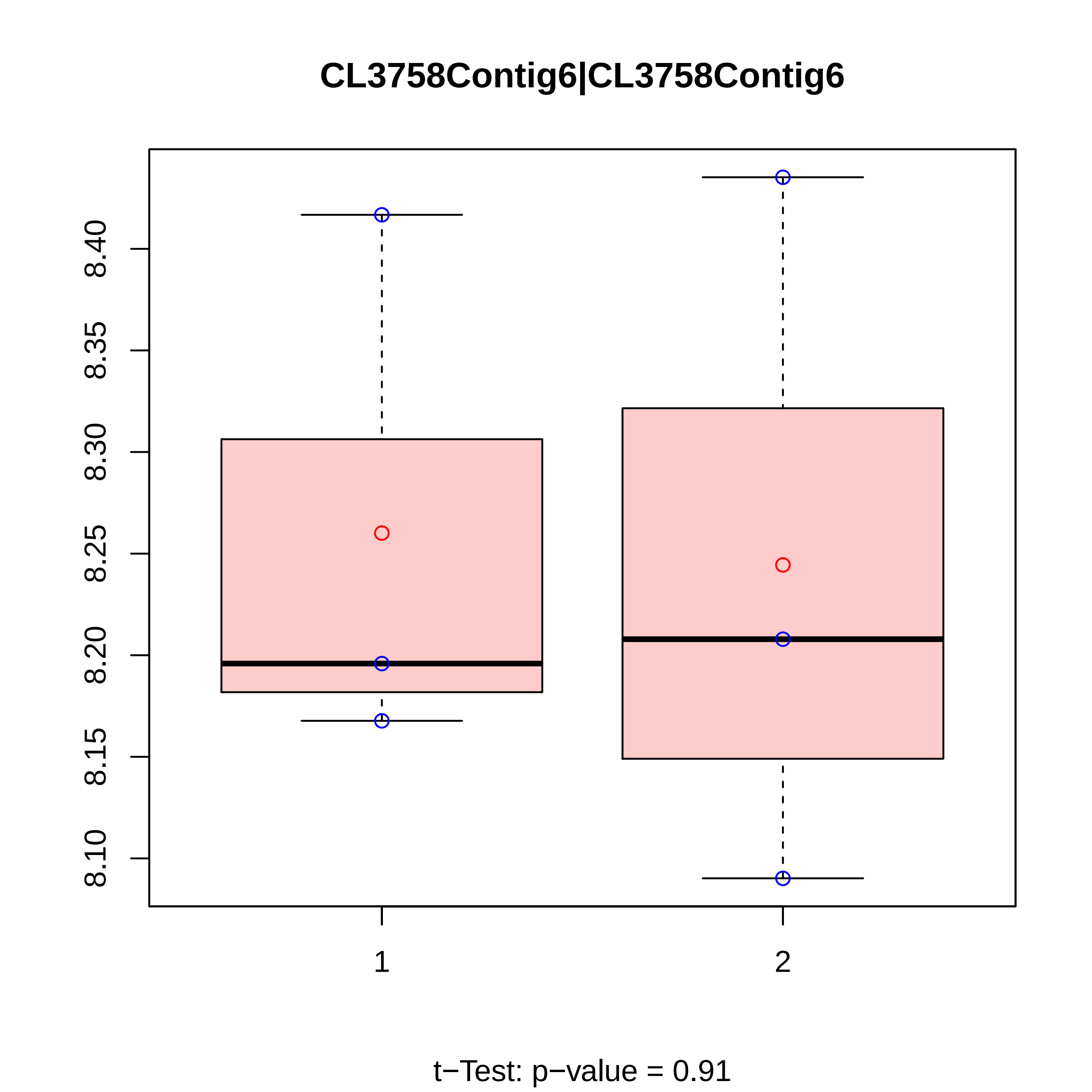
CL3758Contig6|CL3758Contig6
8.10
8.15
8.20
8.25
8.30
8.35
8.40
1
2
t−Test: p−value = 0.91

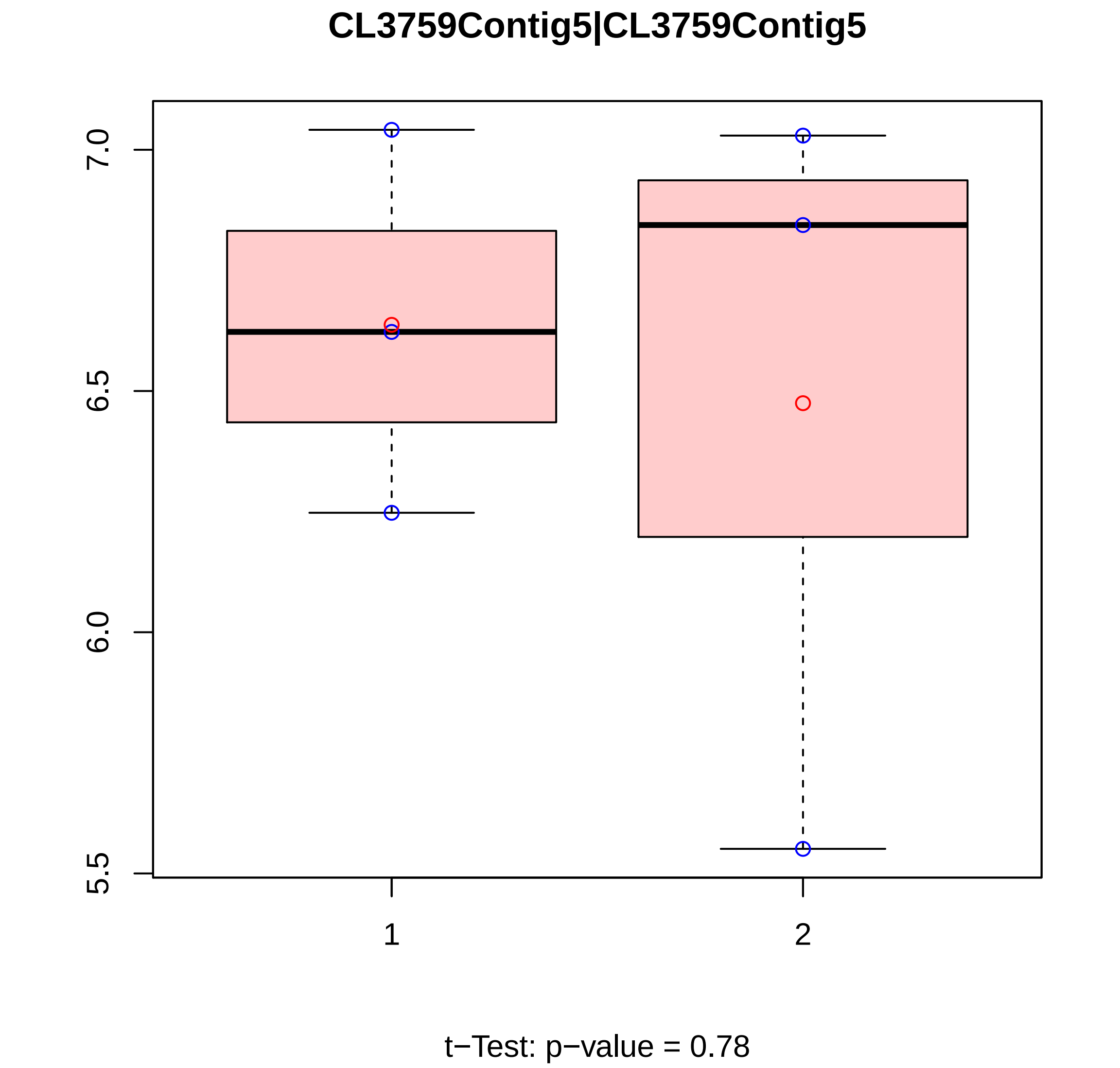
CL3759Contig5|CL3759Contig5
7.0
6.5
6.0
5.5
1
2
t−Test: p−value = 0.78

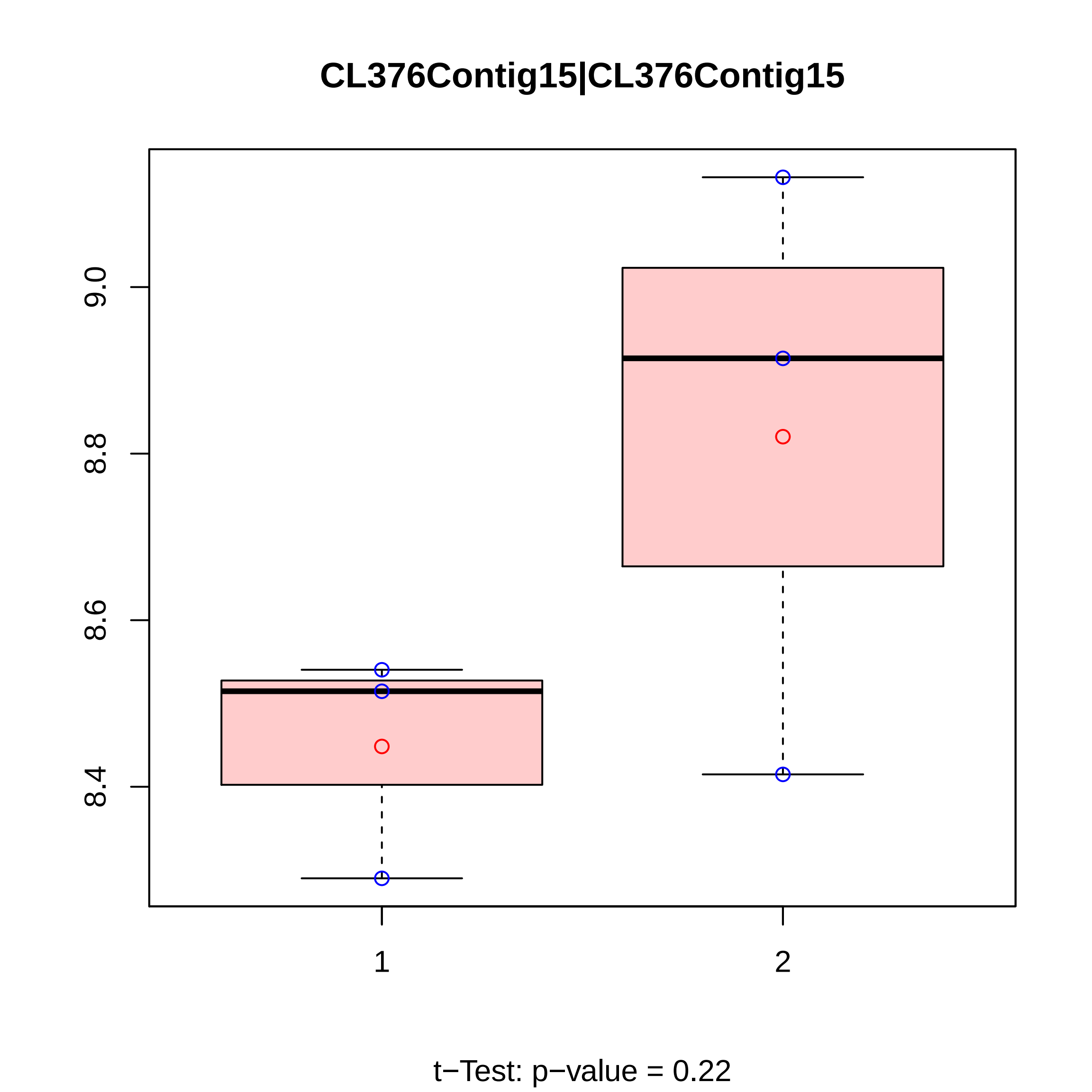

CL376Contig15|CL376Contig15
9.0
8.8
8.6
8.4
1
2
t−Test: p−value = 0.22

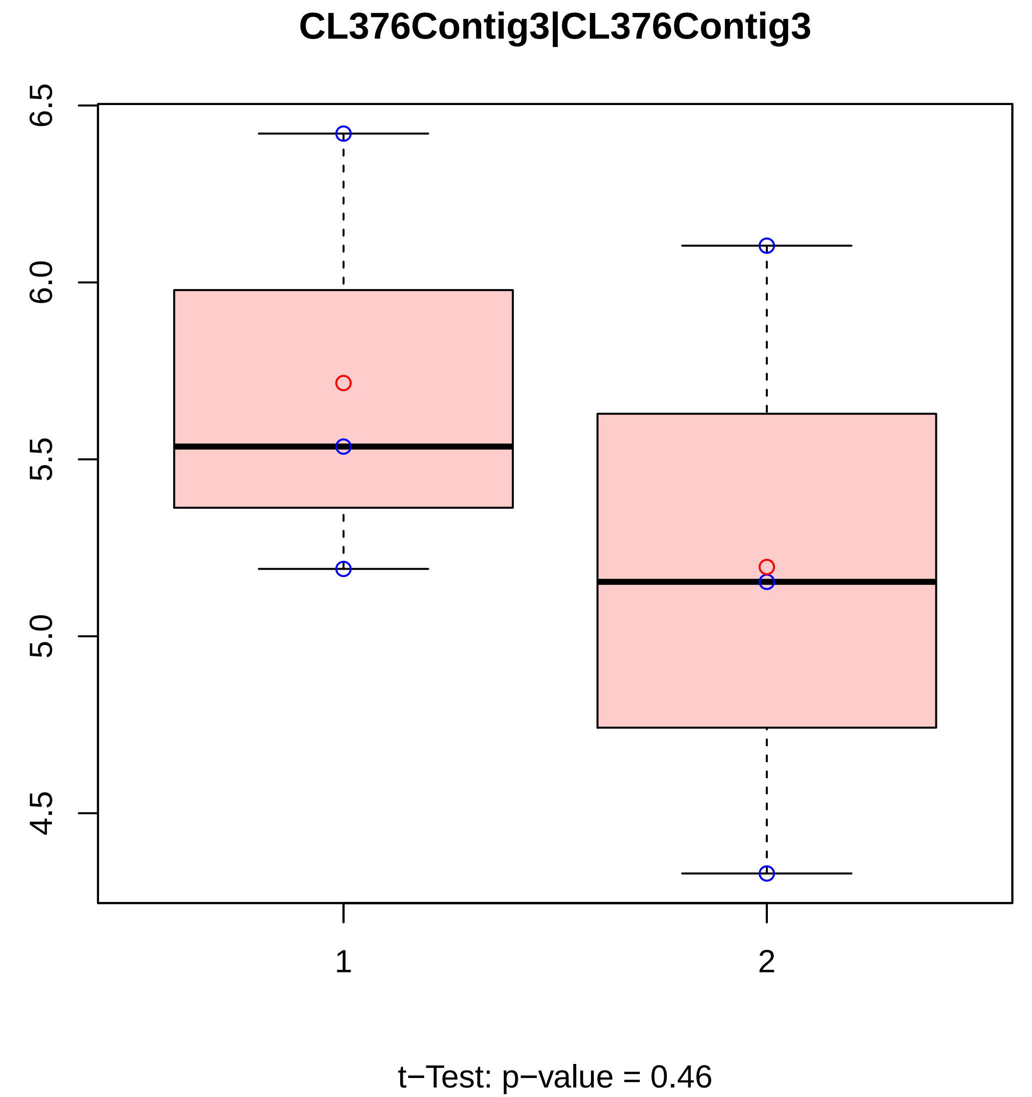
CL376Contig3|CL376Contig3
6.5
6.0
5.5
5.0
4.5
1
2
t−Test: p−value = 0.46

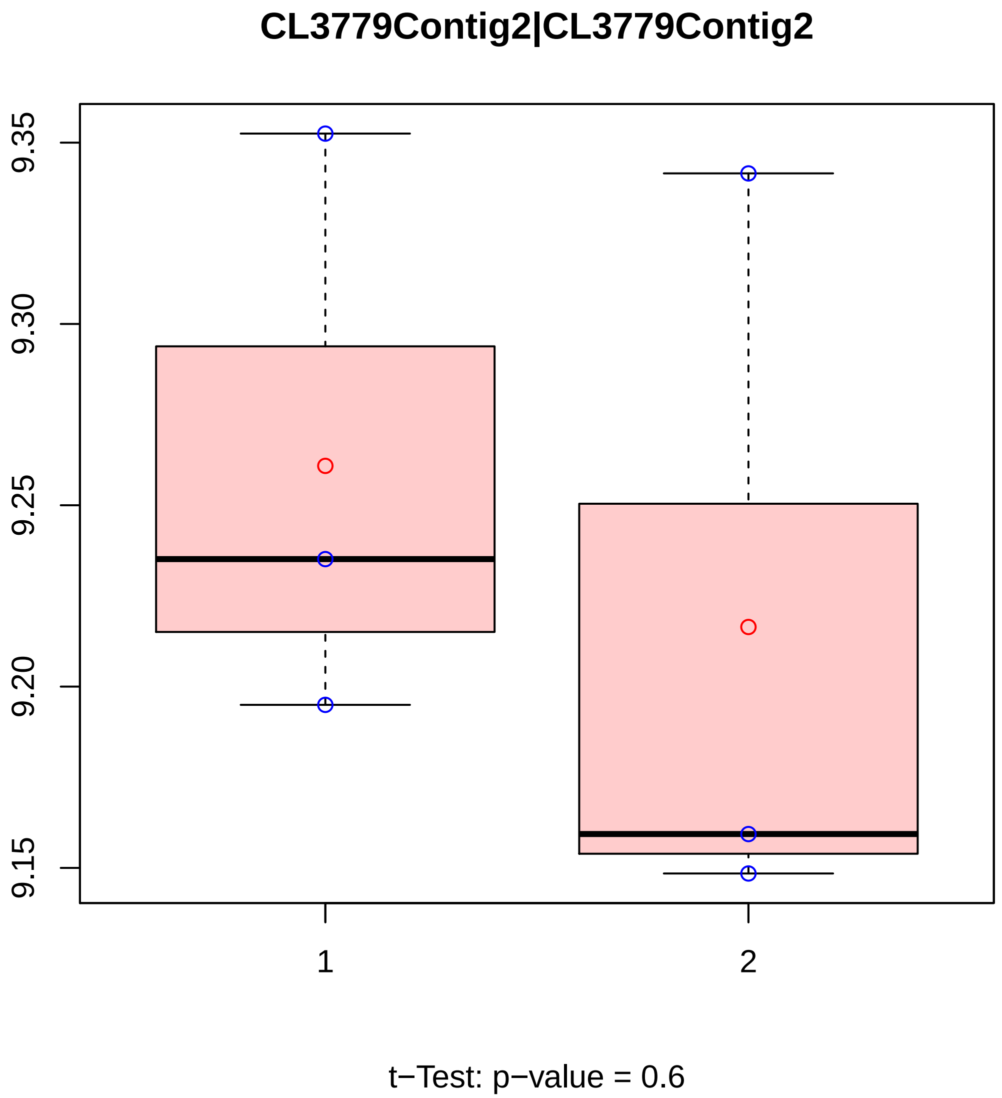

CL3779Contig2|CL3779Contig2
9.35
9.30
9.25
9.20
9.15
1
2
t−Test: p−value = 0.6

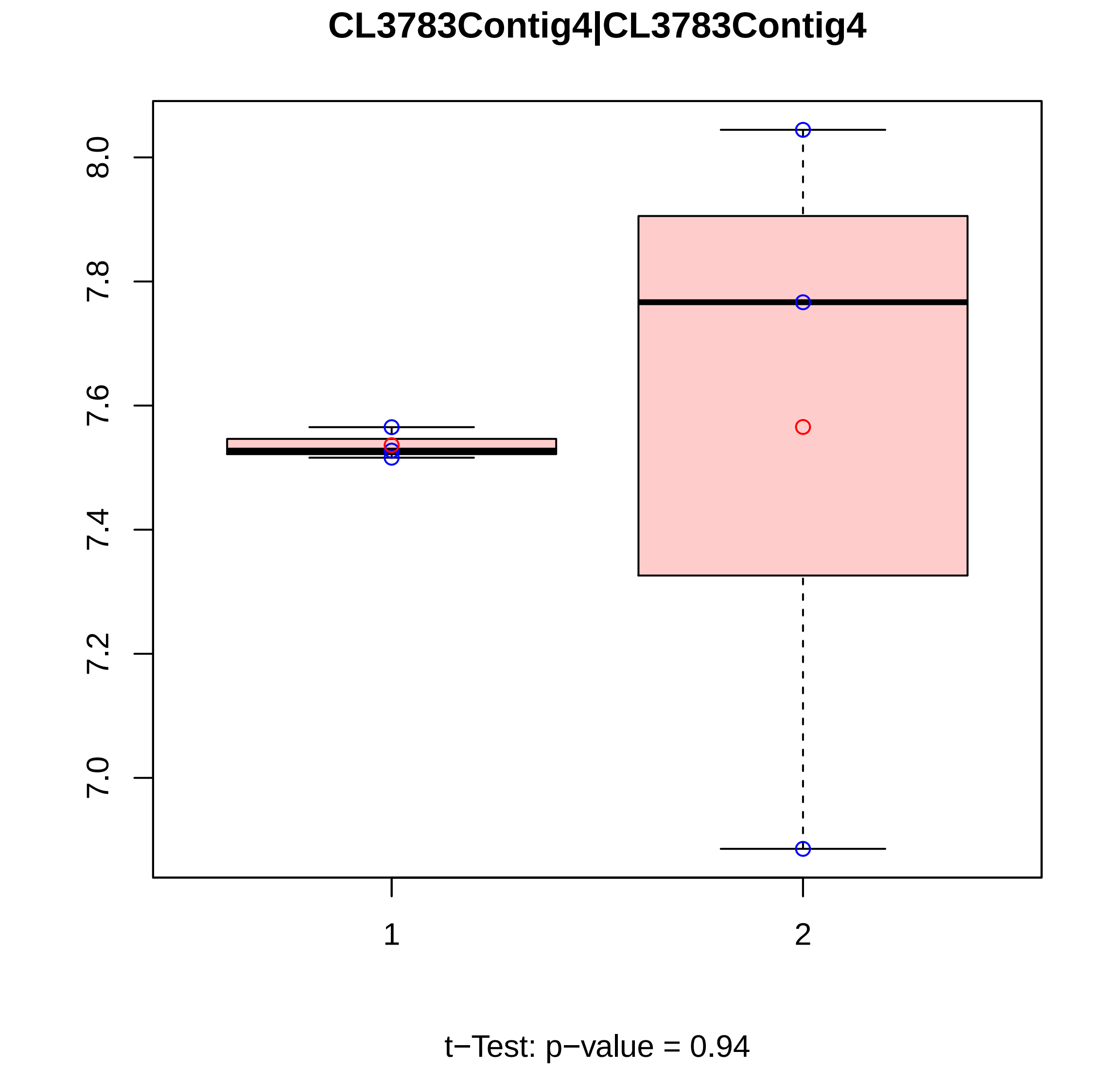
CL3783Contig4|CL3783Contig4
8.0
7.8
7.6
7.4
7.2
7.0
1
2
t−Test: p−value = 0.94

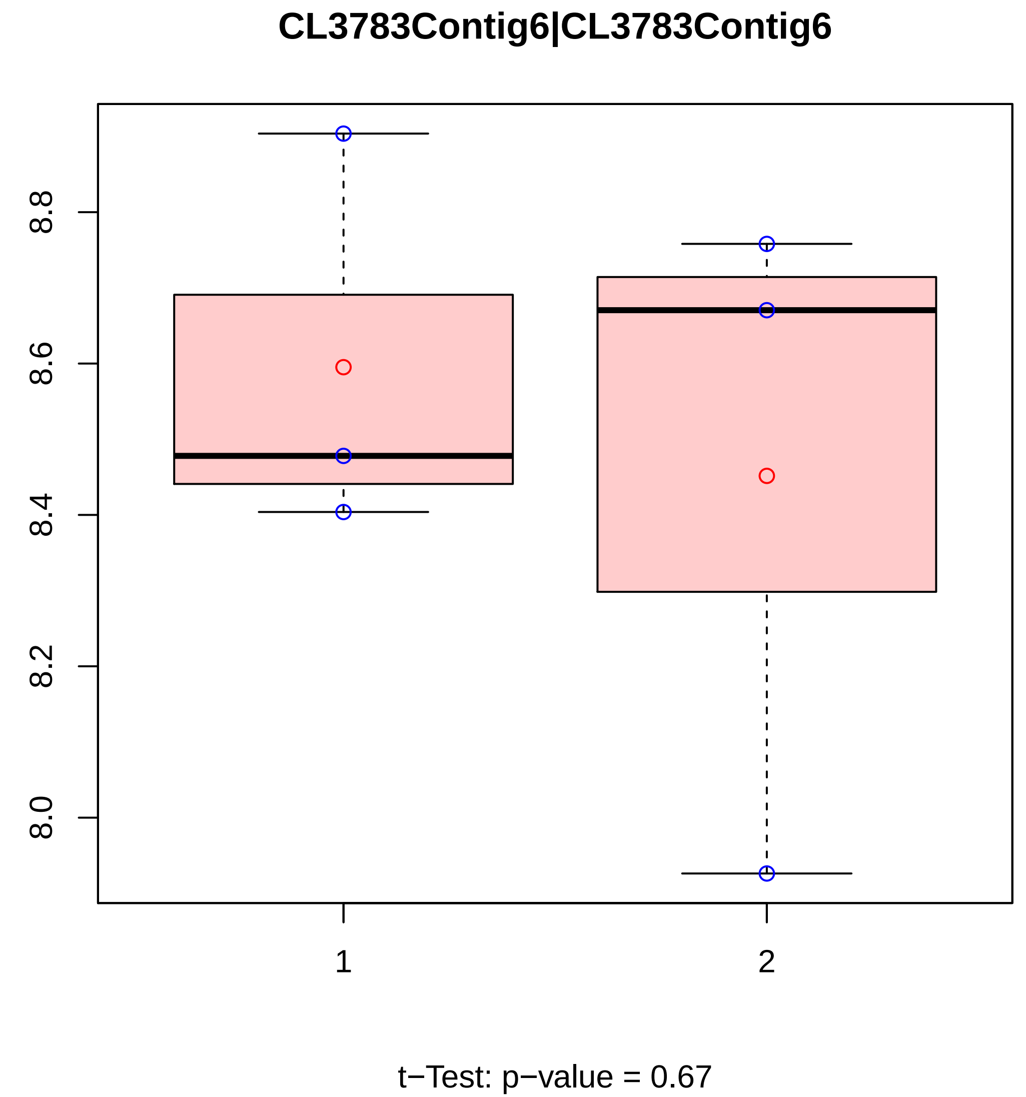
CL3783Contig6|CL3783Contig6
8.0
8.2
8.4
8.6
8.8
1
2
t−Test: p−value = 0.67

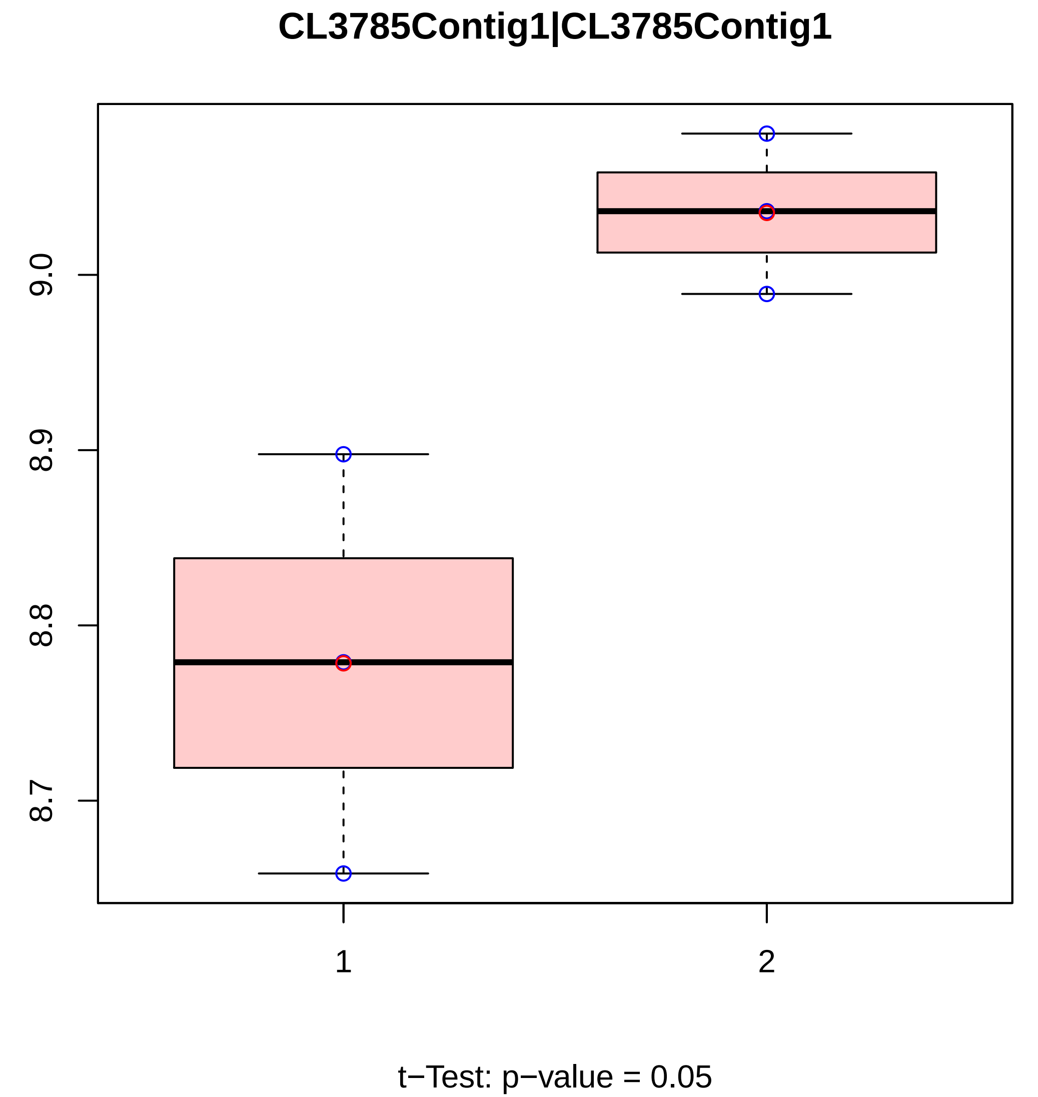
CL3785Contig1|CL3785Contig1
9.0
8.9
8.8
8.7
1
2
t−Test: p−value = 0.05

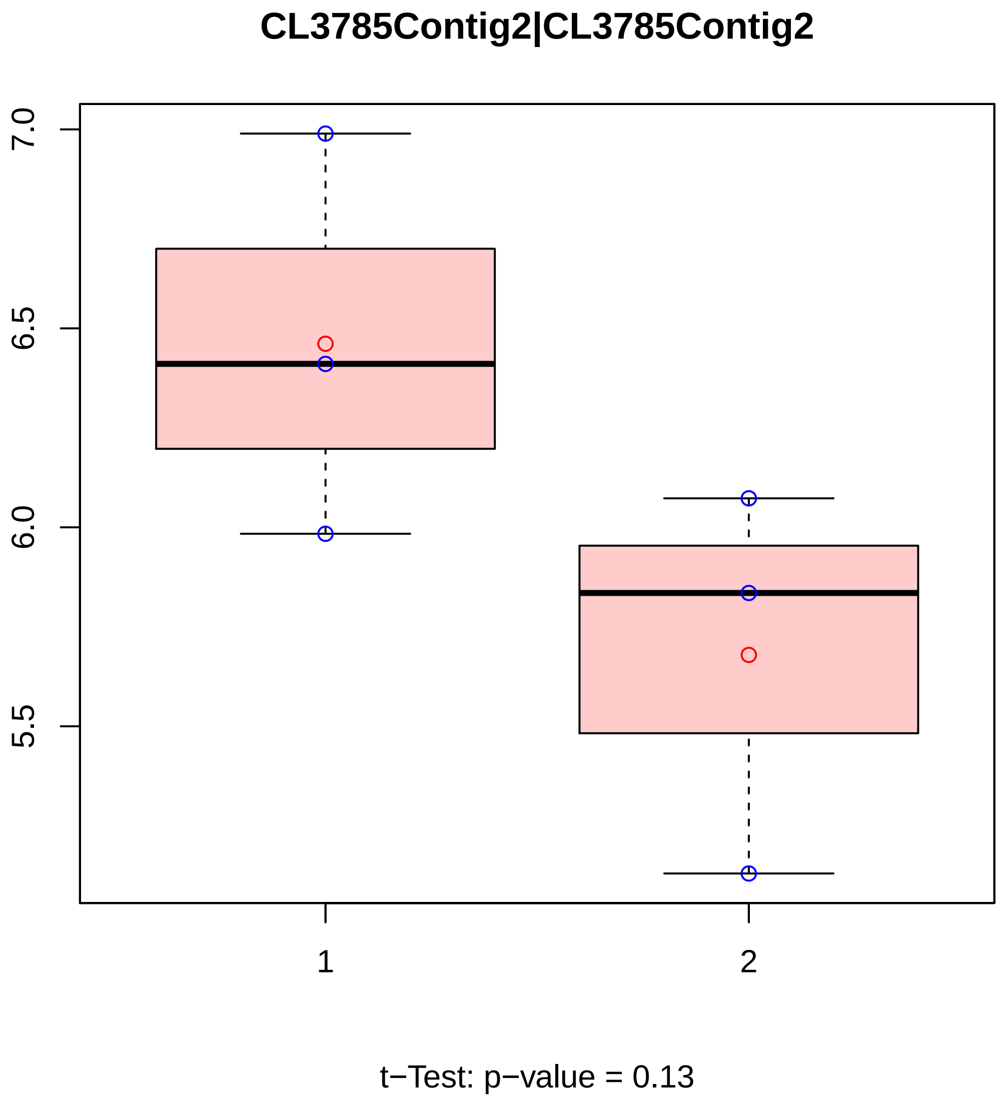
CL3785Contig2|CL3785Contig2
7.0
6.5
6.0
5.5
1
2
t−Test: p−value = 0.13

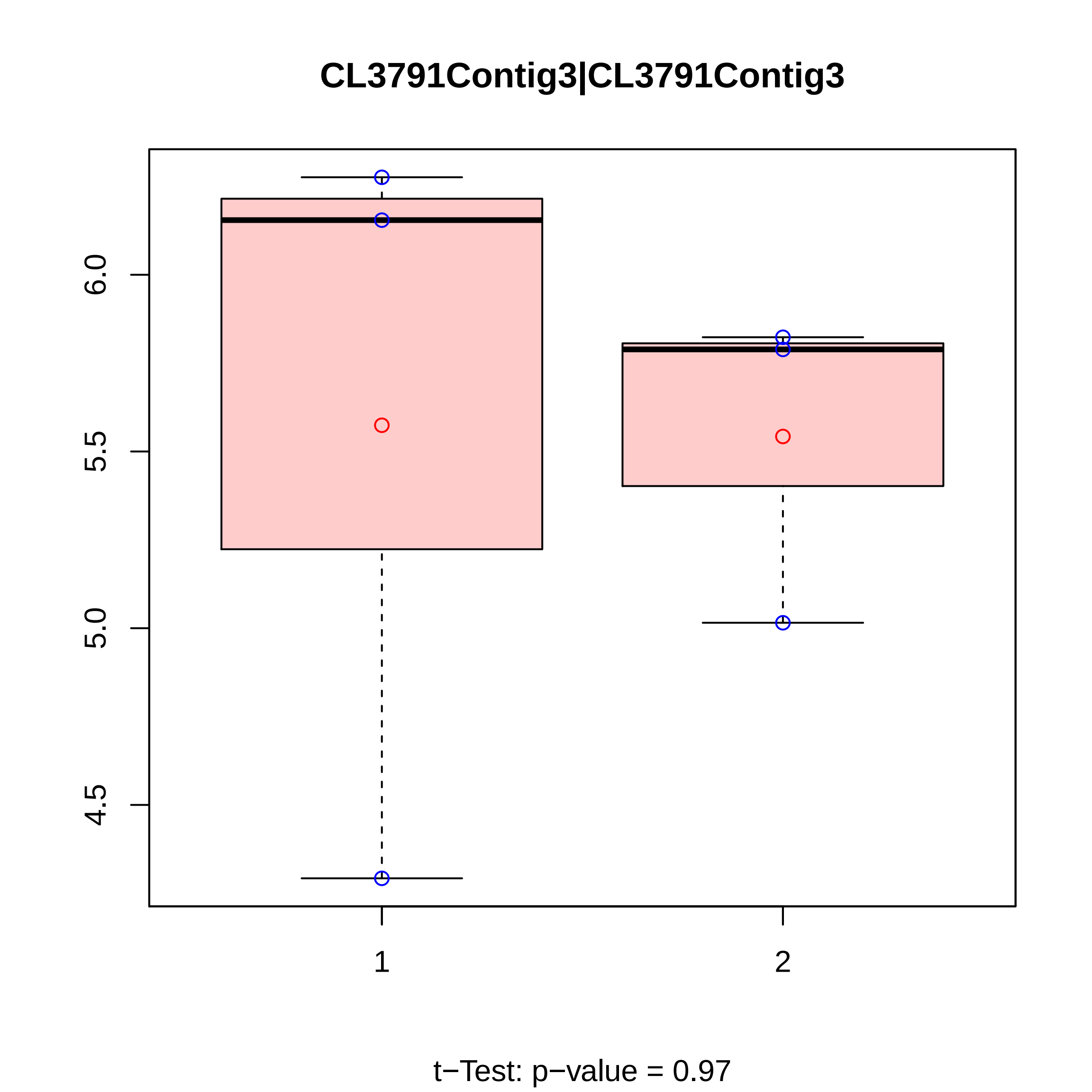
CL3791Contig3|CL3791Contig3
6.0
5.5
5.0
4.5
1
2
t−Test: p−value = 0.97

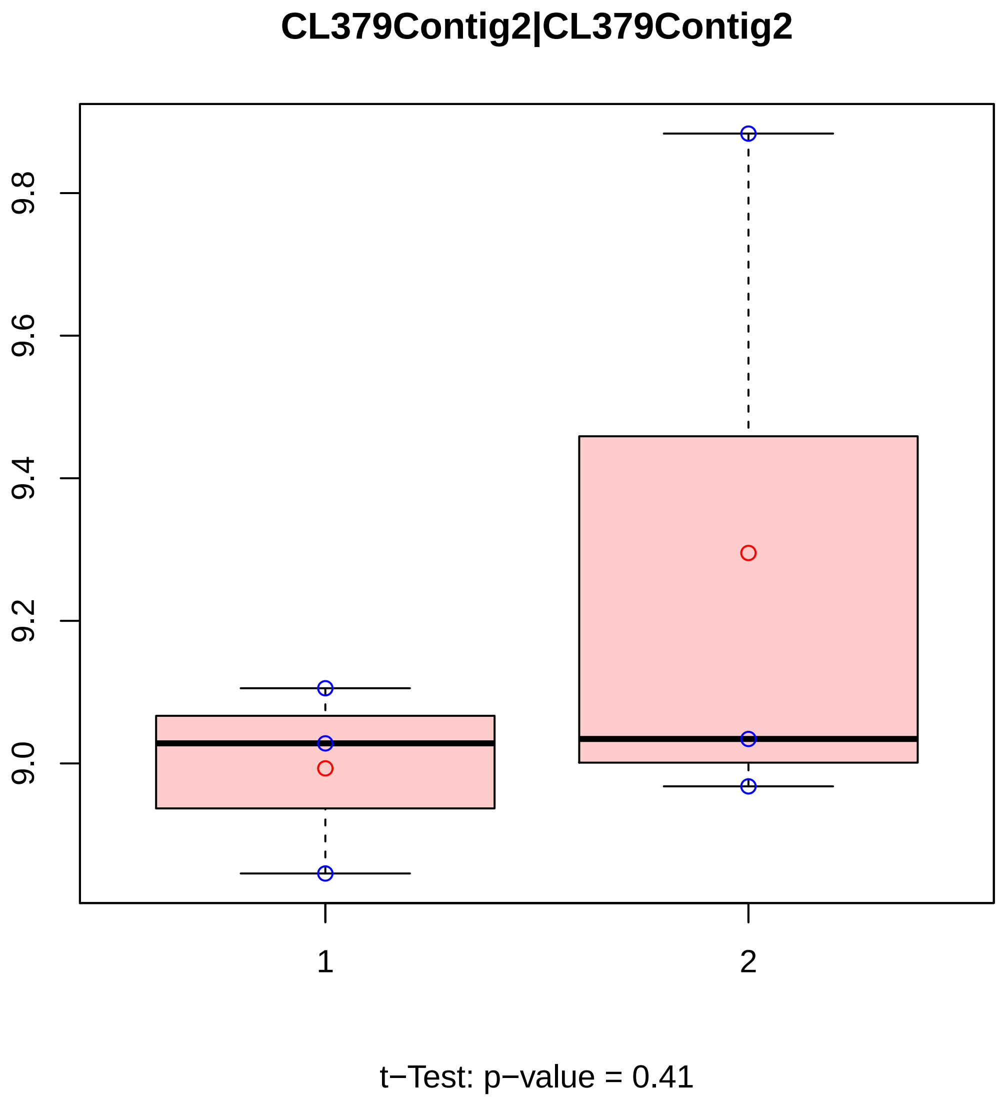

CL379Contig2|CL379Contig2
9.0
9.2
9.4
9.6
9.8
1
2
t−Test: p−value = 0.41

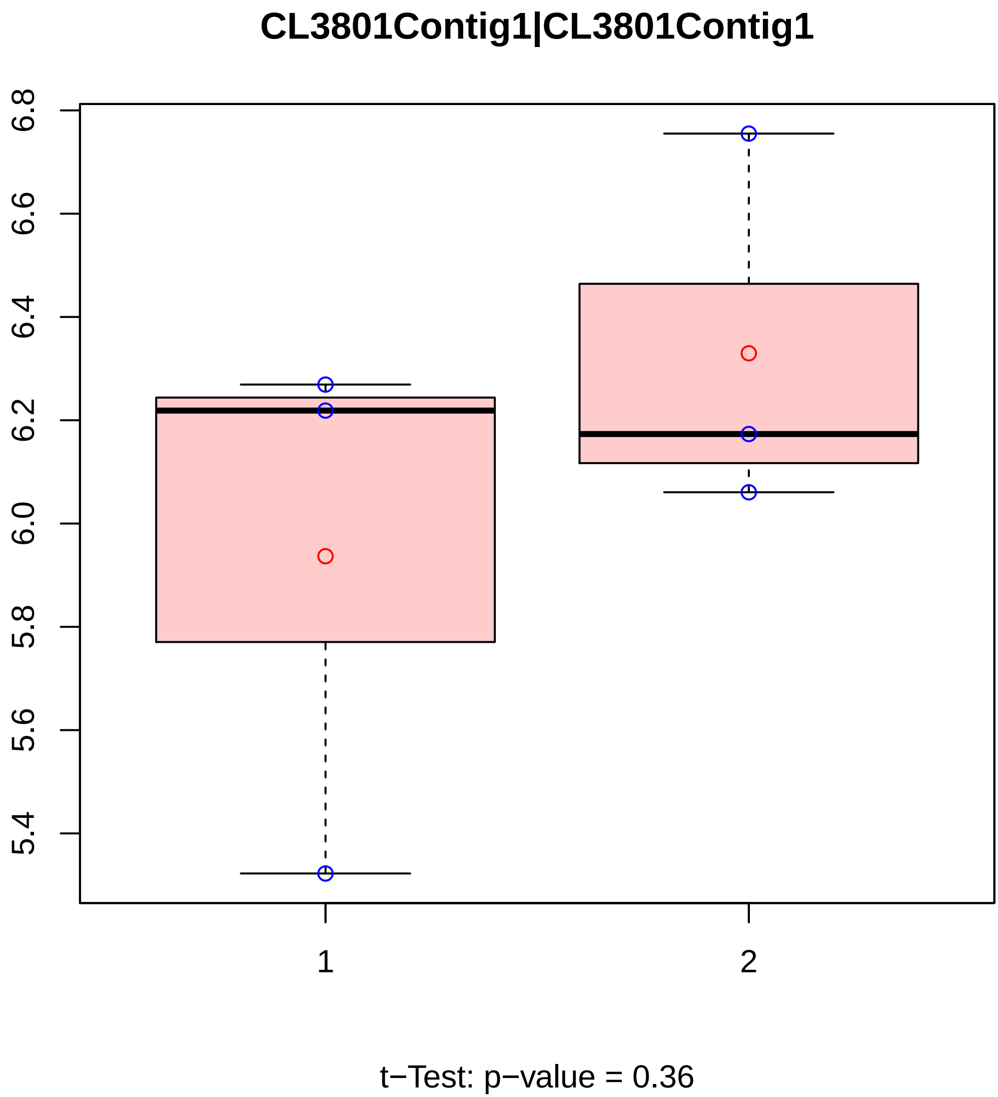
CL3801Contig1|CL3801Contig1
6.8
6.6
6.4
6.2
6.0
5.8
5.6
5.4
1
2
t−Test: p−value = 0.36

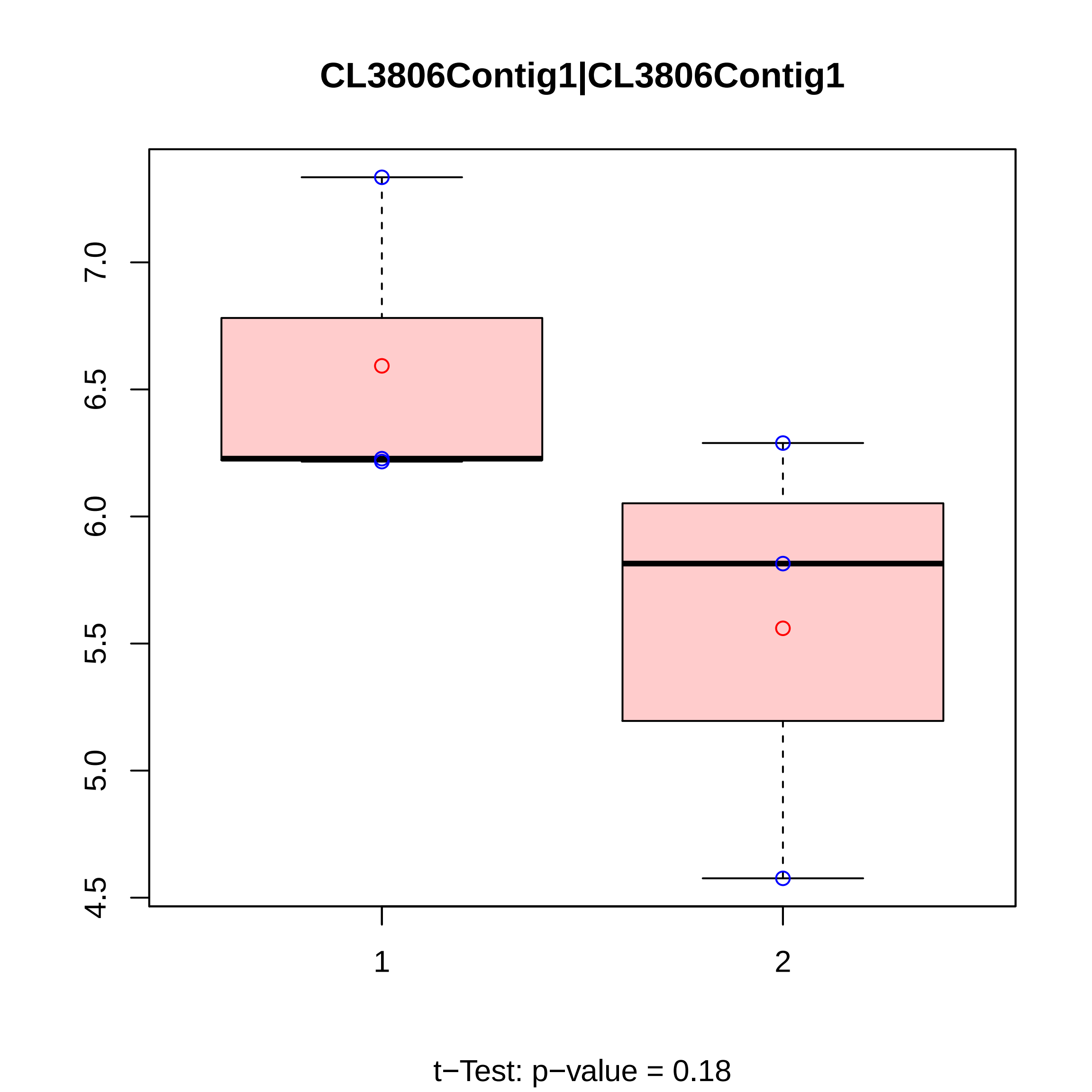

CL3806Contig1|CL3806Contig1
4.5
5.0
5.5
6.0
6.5
7.0
1
2
t−Test: p−value = 0.18

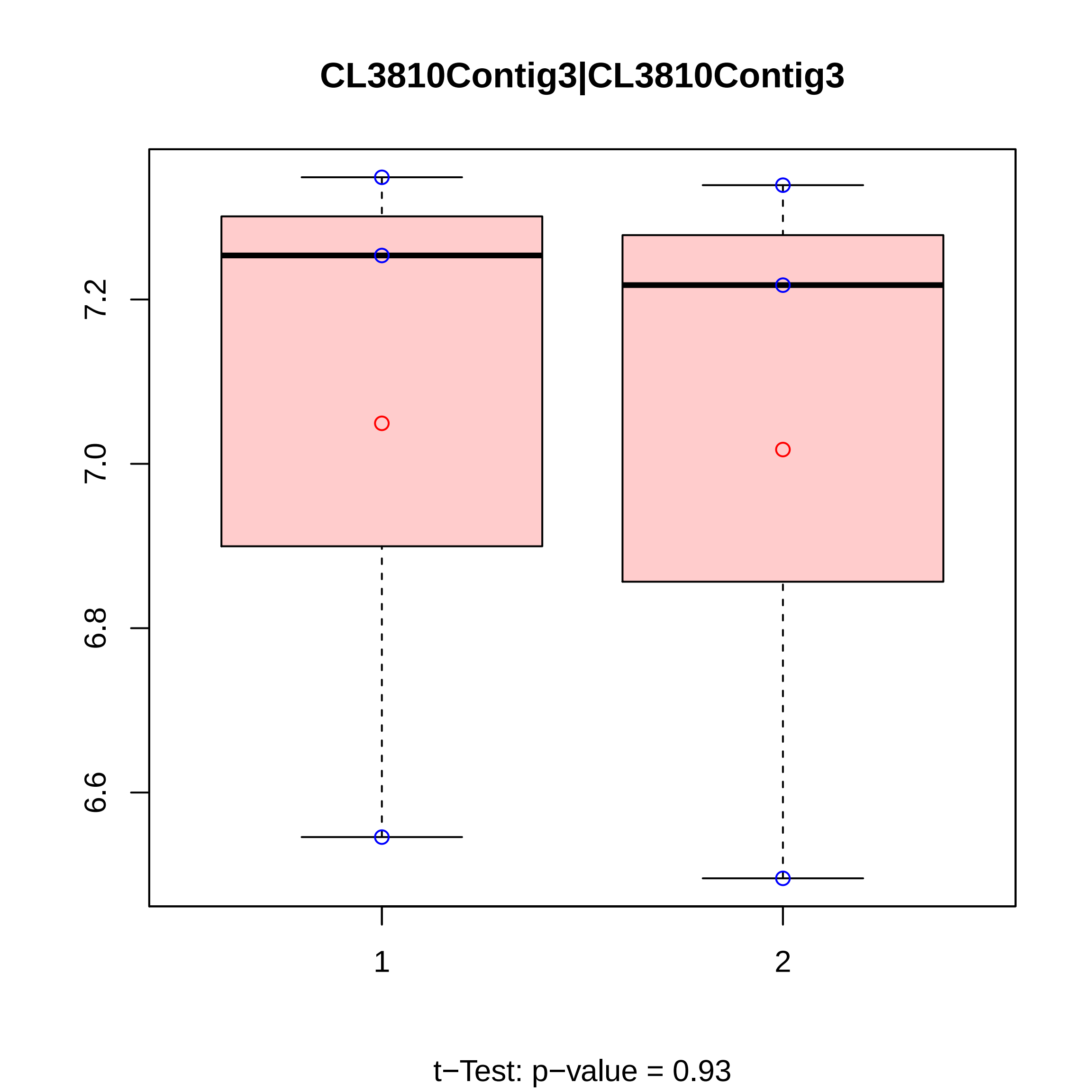

CL3810Contig3|CL3810Contig3
7.2
7.0
6.8
6.6
1
2
t−Test: p−value = 0.93

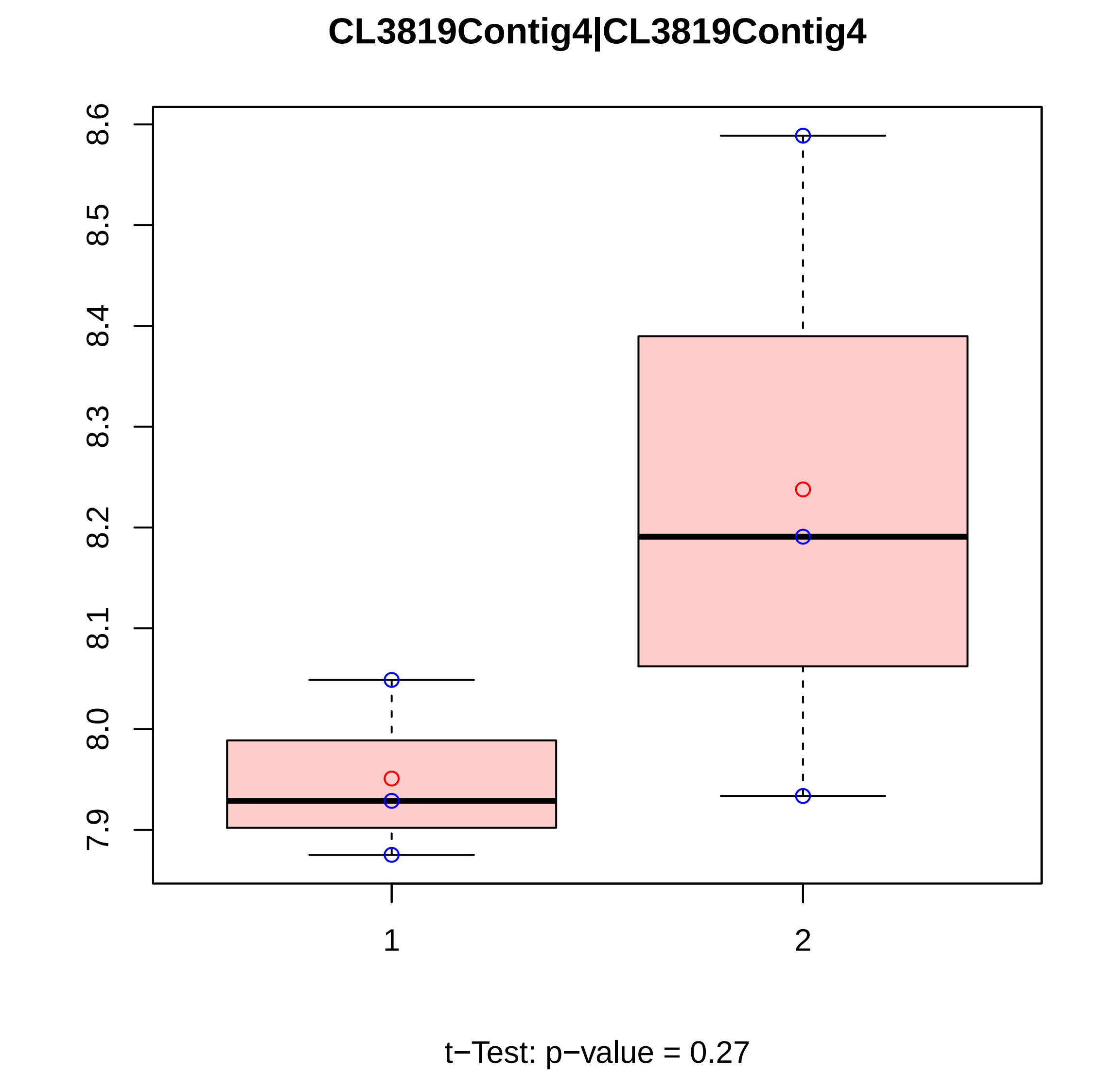

CL3819Contig4|CL3819Contig4
8.6
8.5
8.4
8.3
8.2
8.1
8.0
7.9
1
2
t−Test: p−value = 0.27

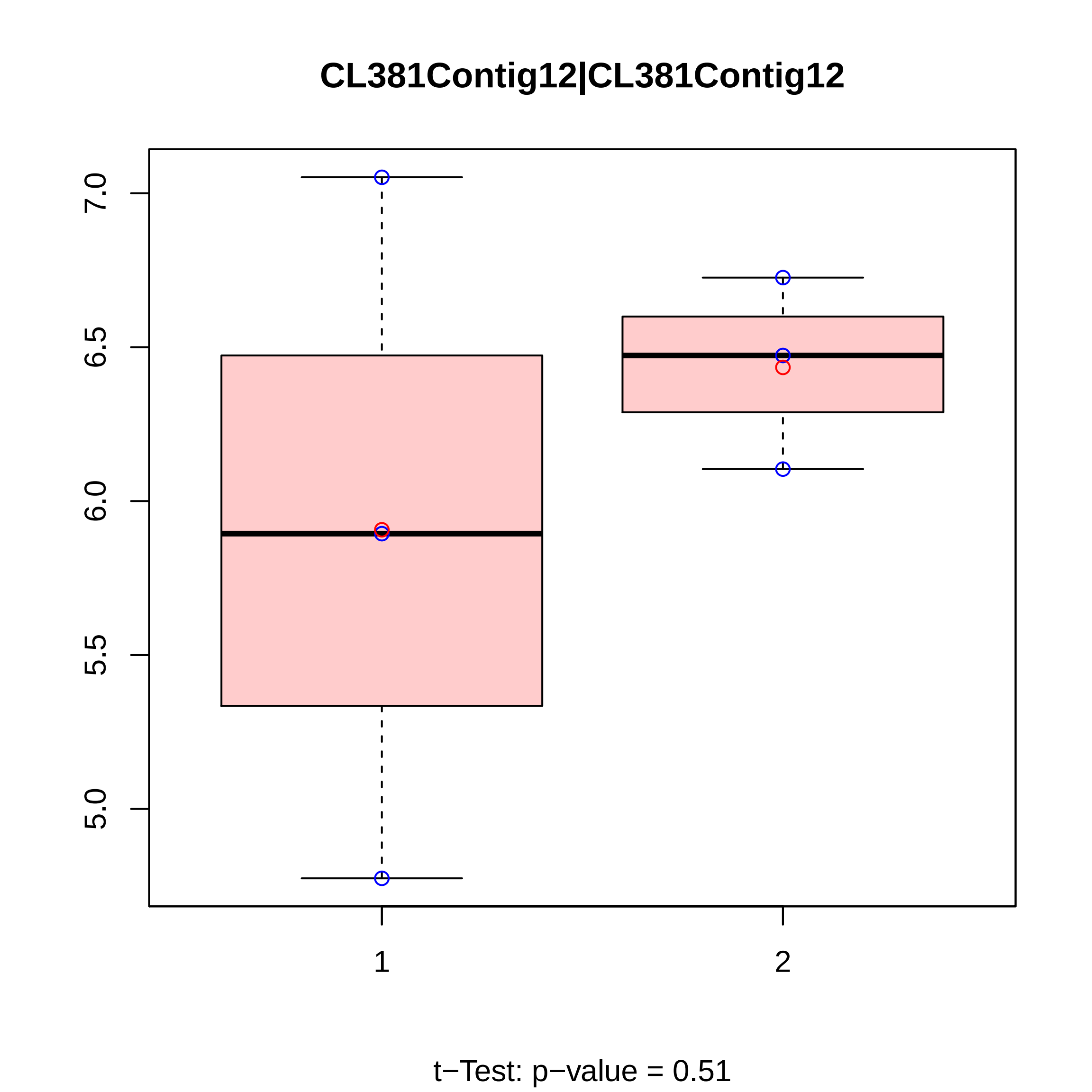

CL381Contig12|CL381Contig12
7.0
6.5
6.0
5.5
5.0
1
2
t−Test: p−value = 0.51

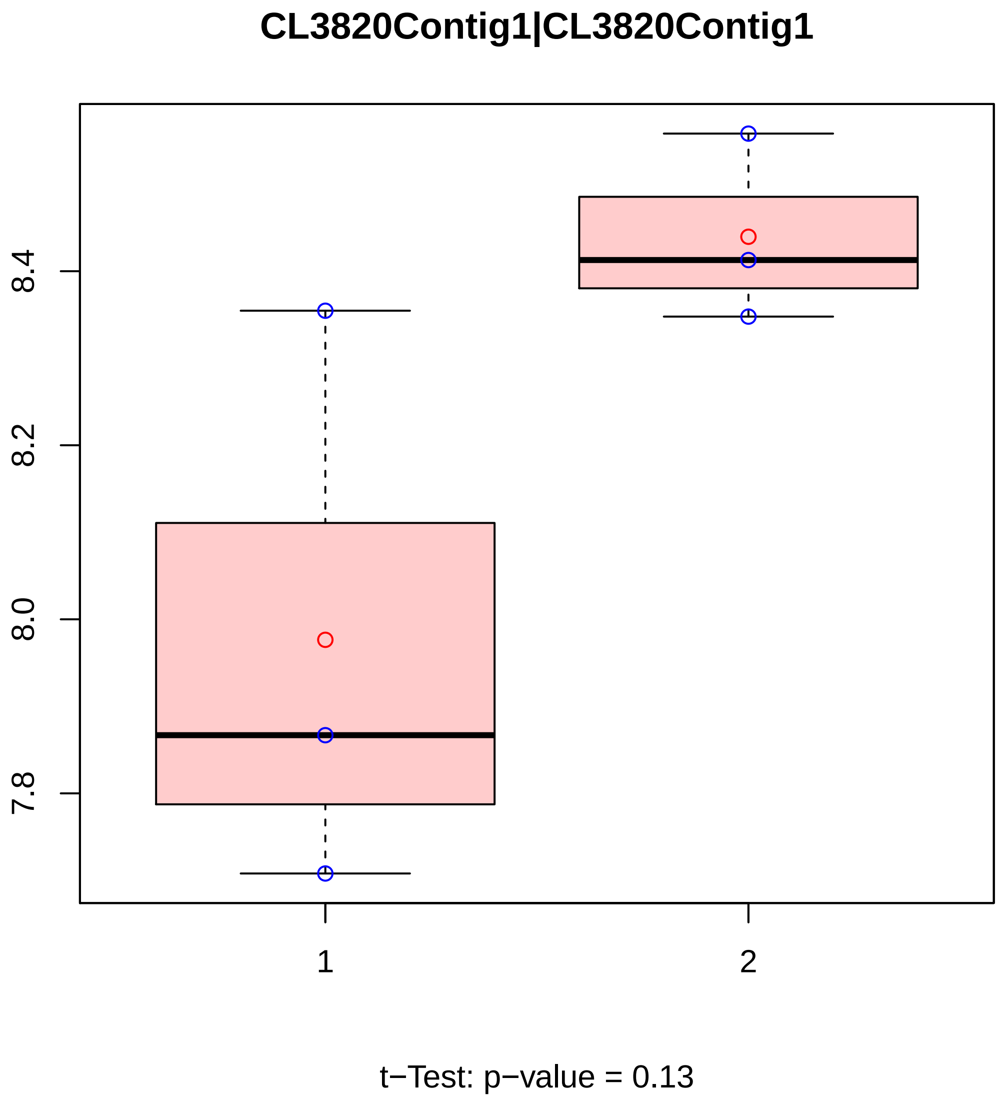
CL3820Contig1|CL3820Contig1
8.4
8.2
8.0
7.8
1
2
t−Test: p−value = 0.13

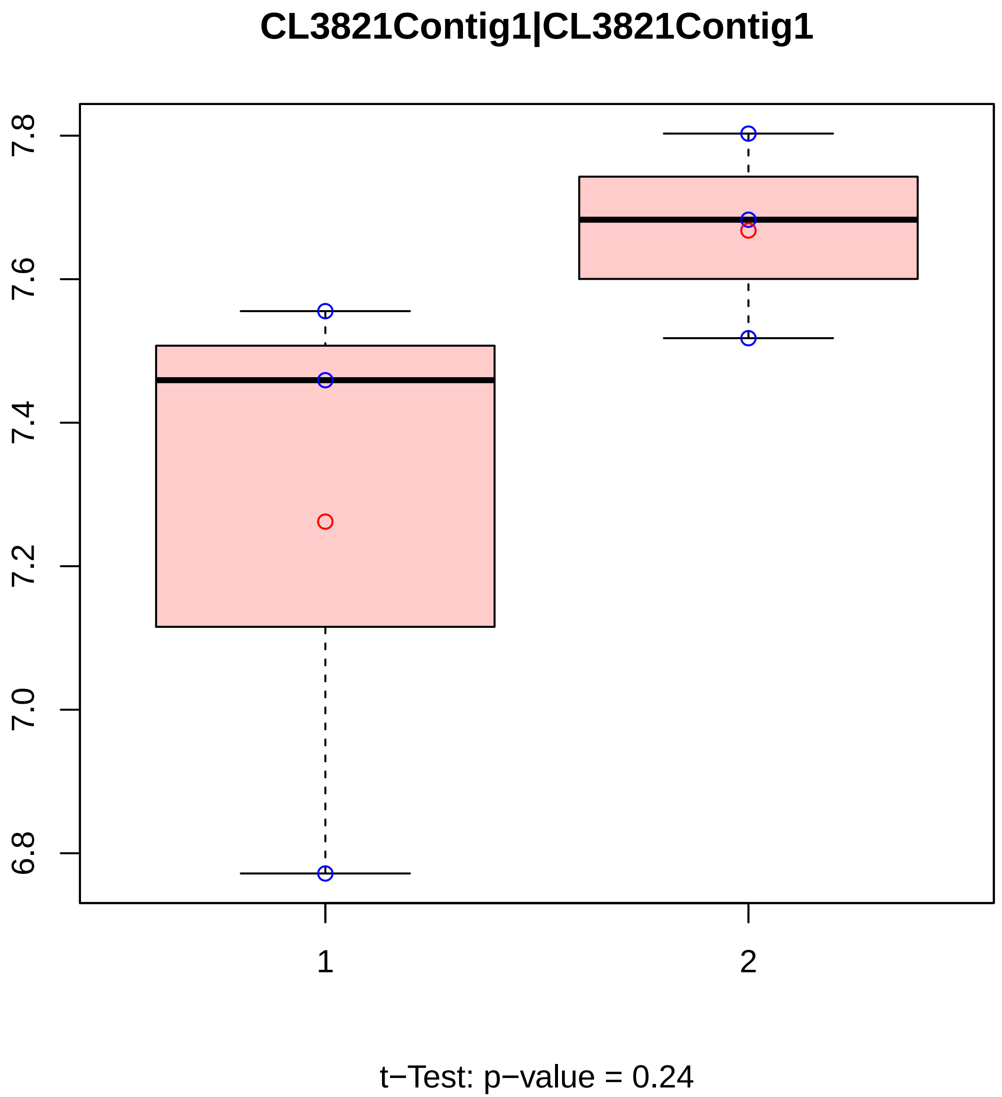

CL3821Contig1|CL3821Contig1
7.8
7.6
7.4
7.2
7.0
6.8
1
2
t−Test: p−value = 0.24

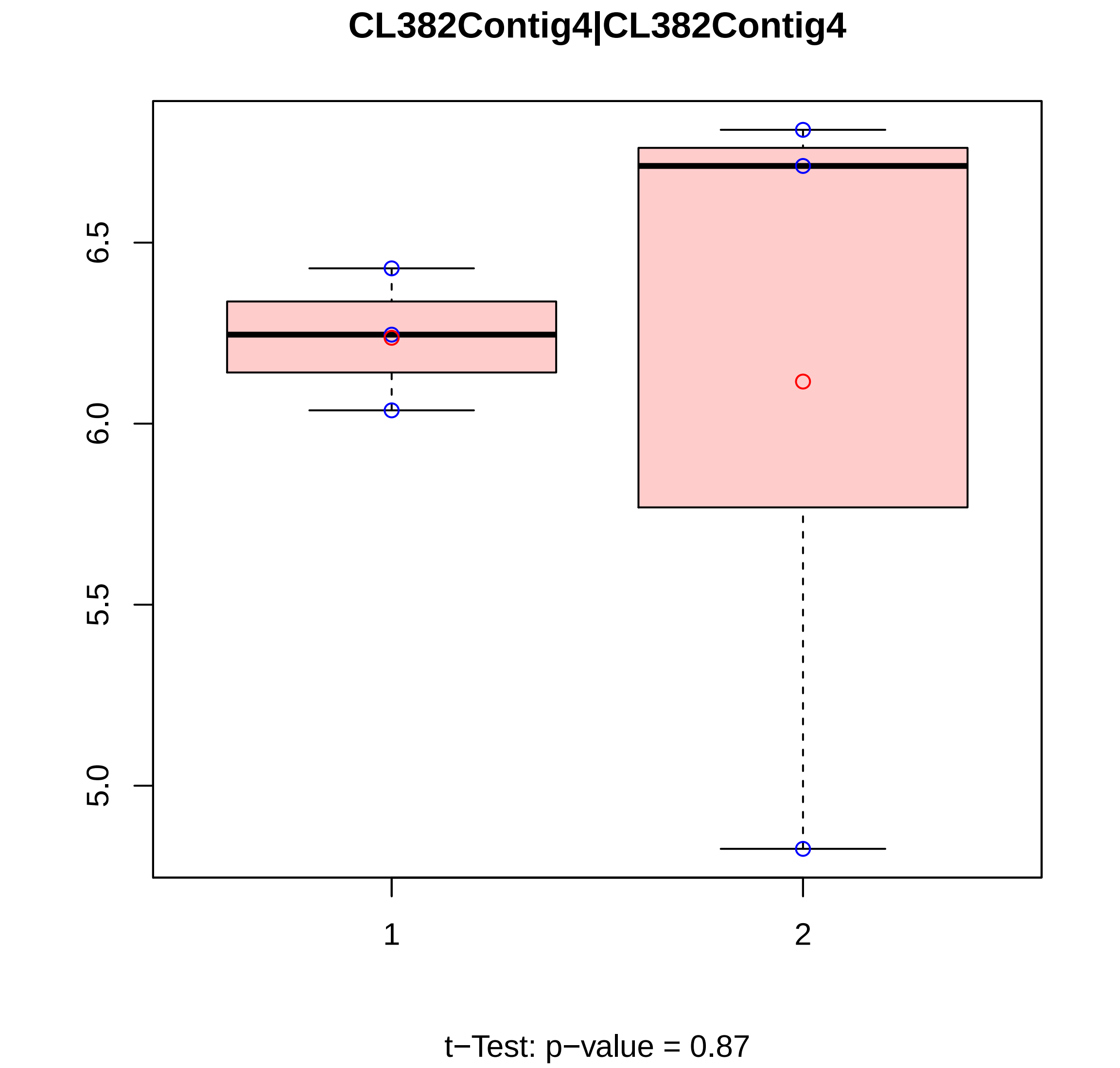
CL382Contig4|CL382Contig4
6.5
6.0
5.5
5.0
1
2
t−Test: p−value = 0.87

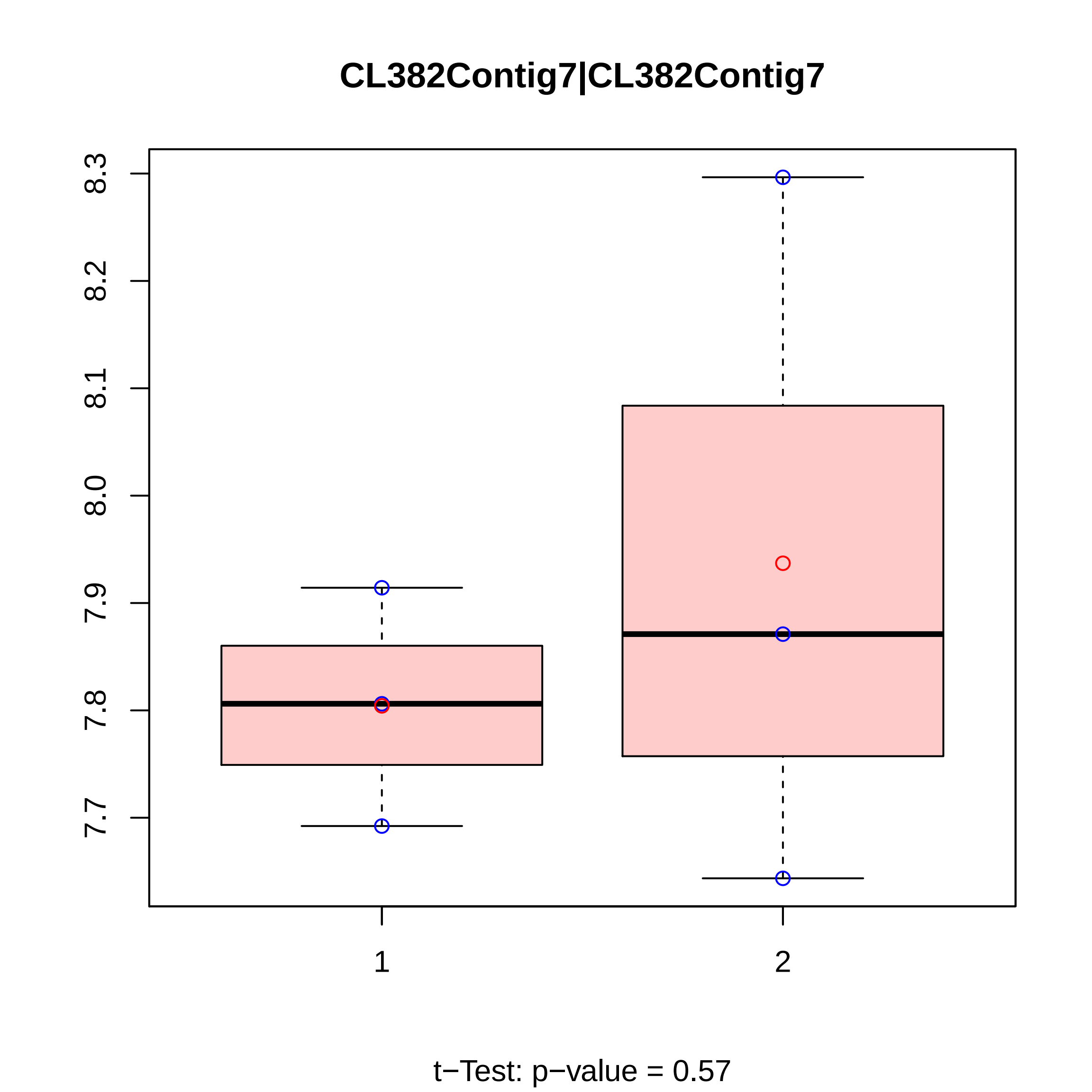

CL382Contig7|CL382Contig7
8.3
8.2
8.1
8.0
7.9
7.8
7.7
1
2
t−Test: p−value = 0.57

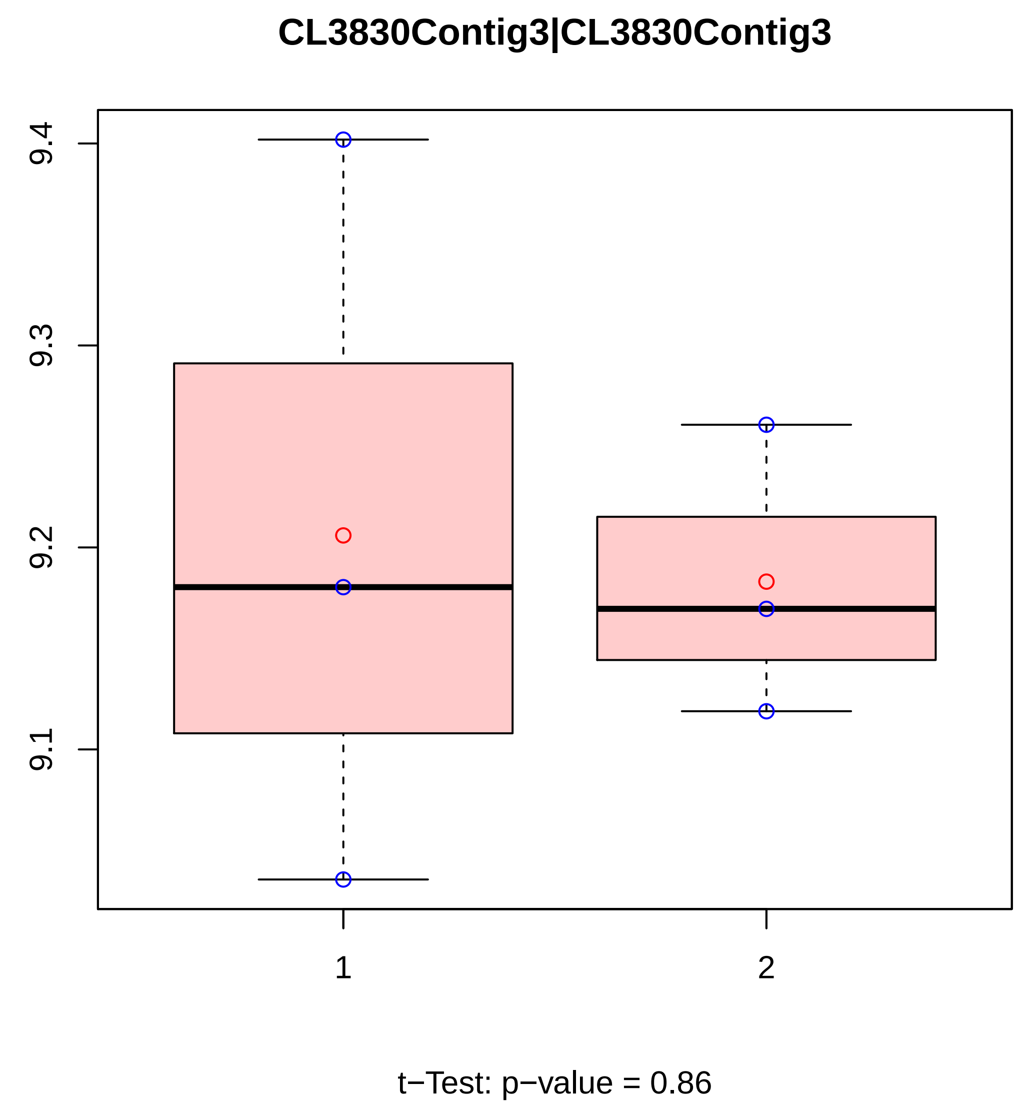
CL3830Contig3|CL3830Contig3
9.4
9.3
9.2
9.1
1
2
t−Test: p−value = 0.86

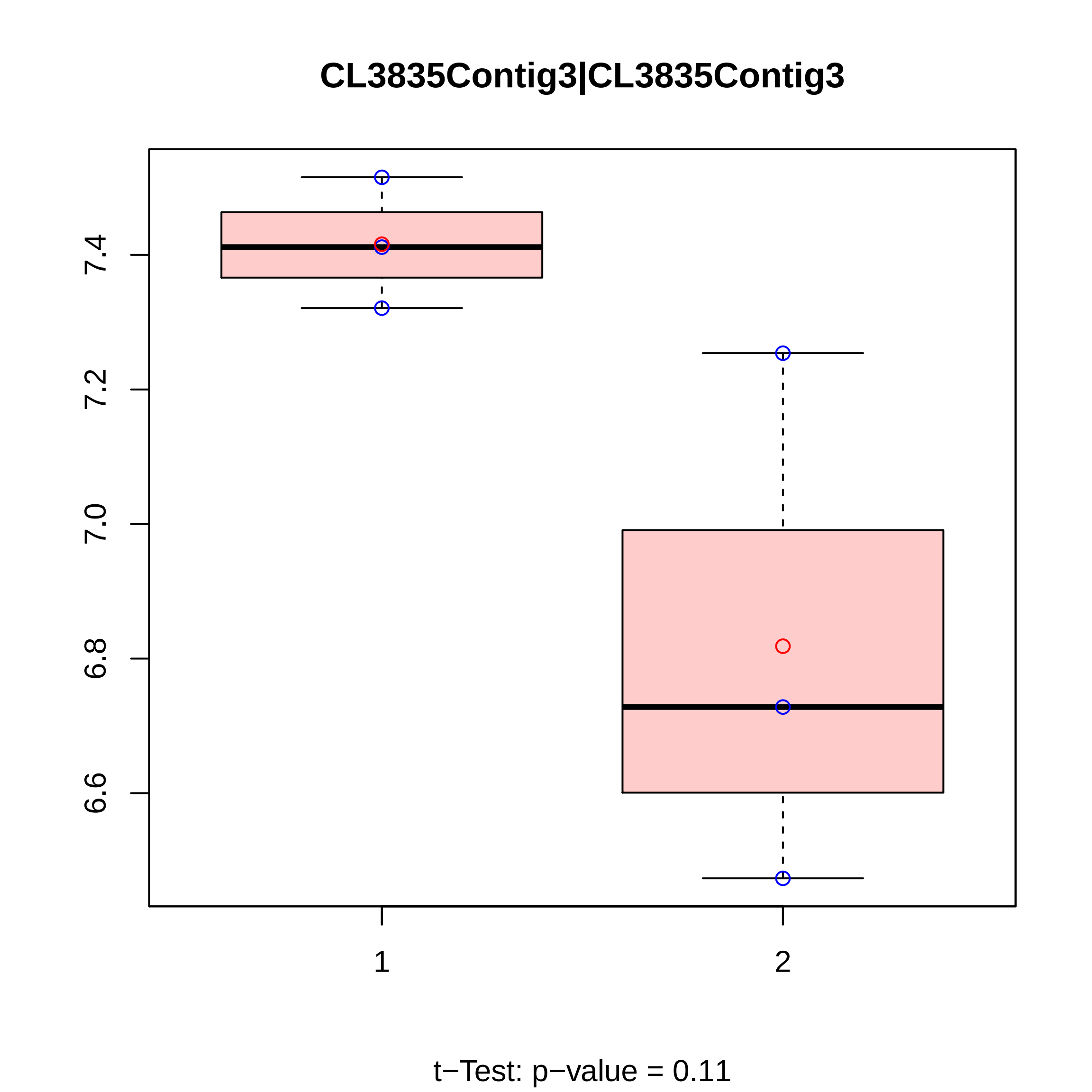
CL3835Contig3|CL3835Contig3
7.4
7.2
7.0
6.8
6.6
1
2
t−Test: p−value = 0.11

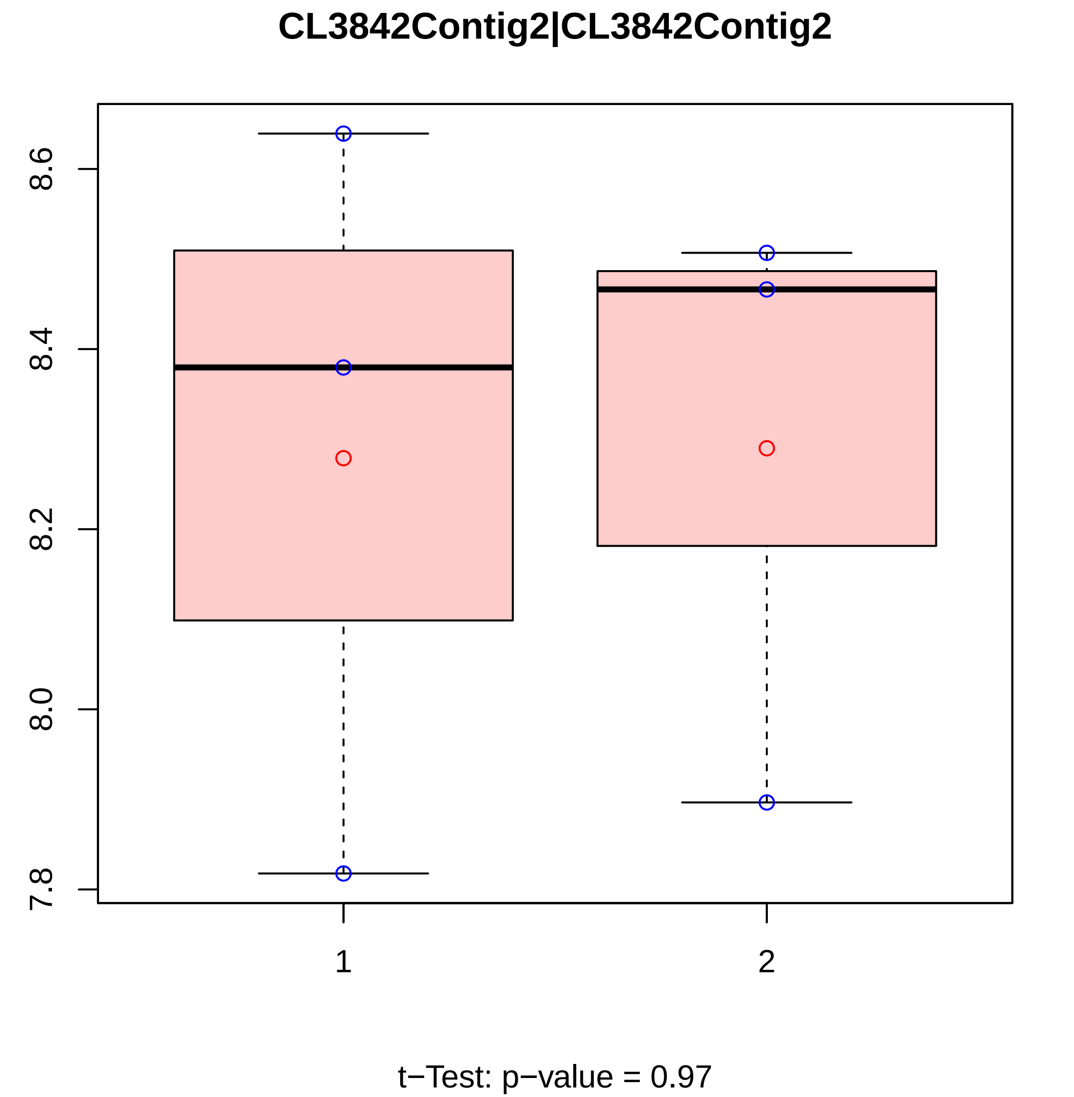

CL3842Contig2|CL3842Contig2
8.6
8.4
8.2
8.0
7.8
1
2
t−Test: p−value = 0.97

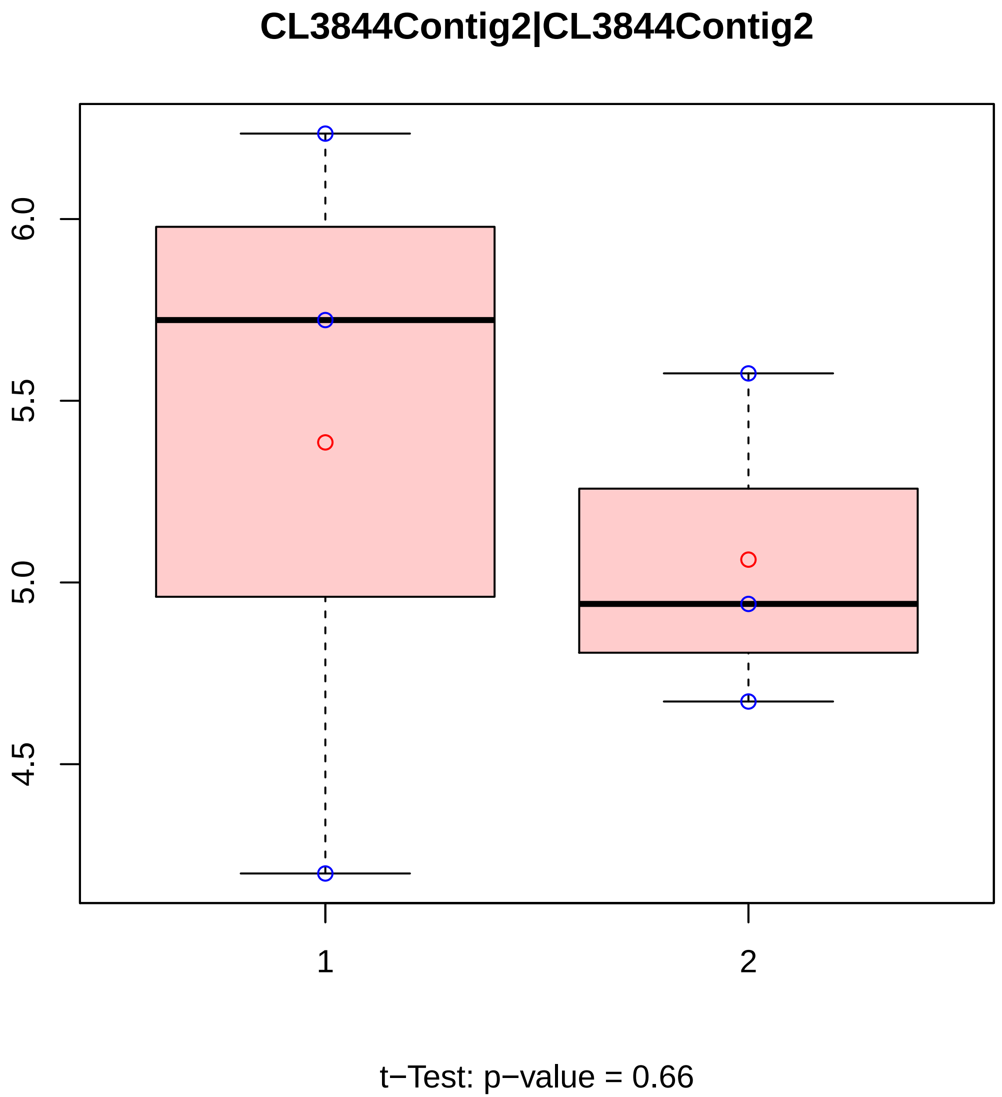
CL3844Contig2|CL3844Contig2
6.0
5.5
5.0
4.5
1
2
t−Test: p−value = 0.66

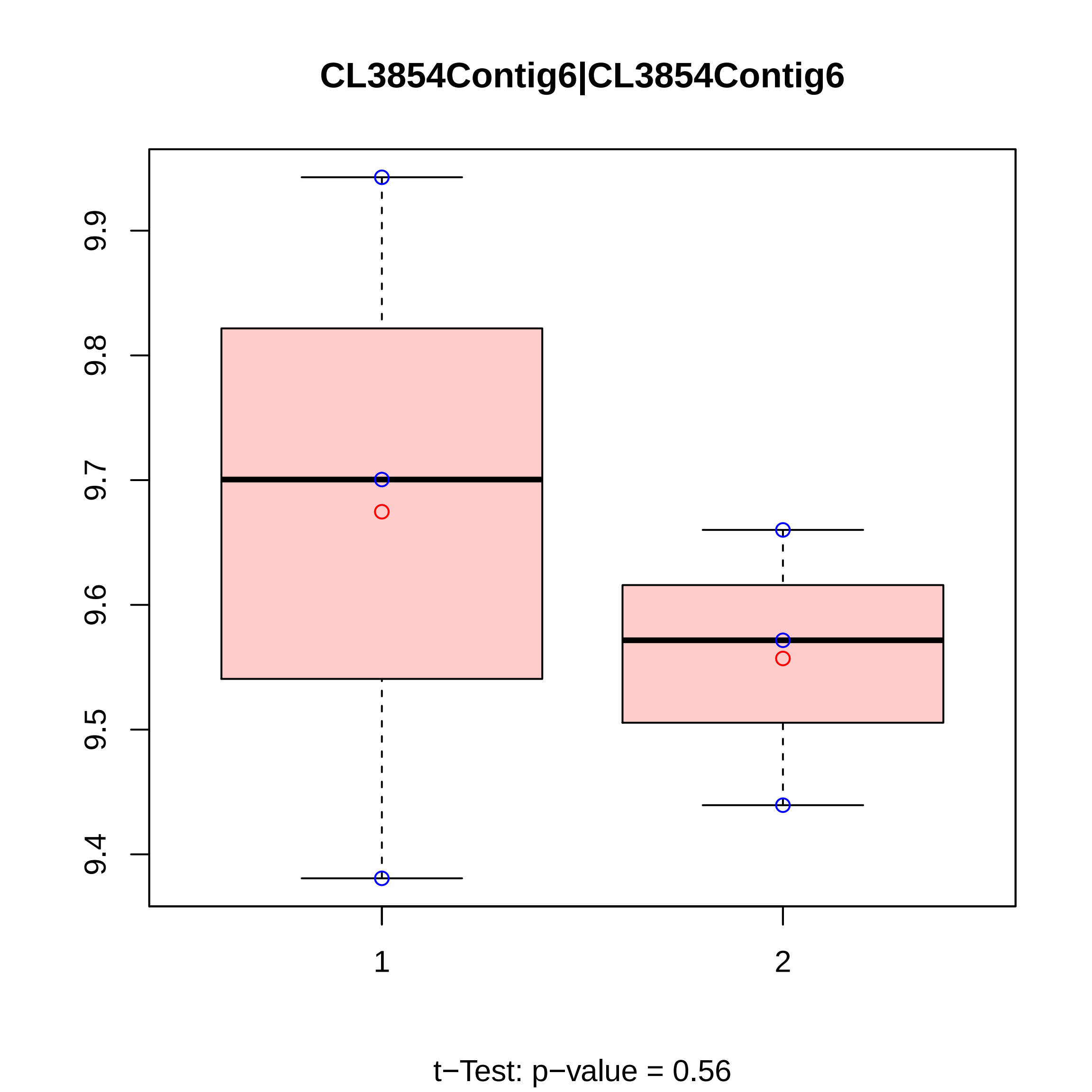

CL3854Contig6|CL3854Contig6
9.4
9.5
9.6
9.7
9.8
9.9
1
2
t−Test: p−value = 0.56

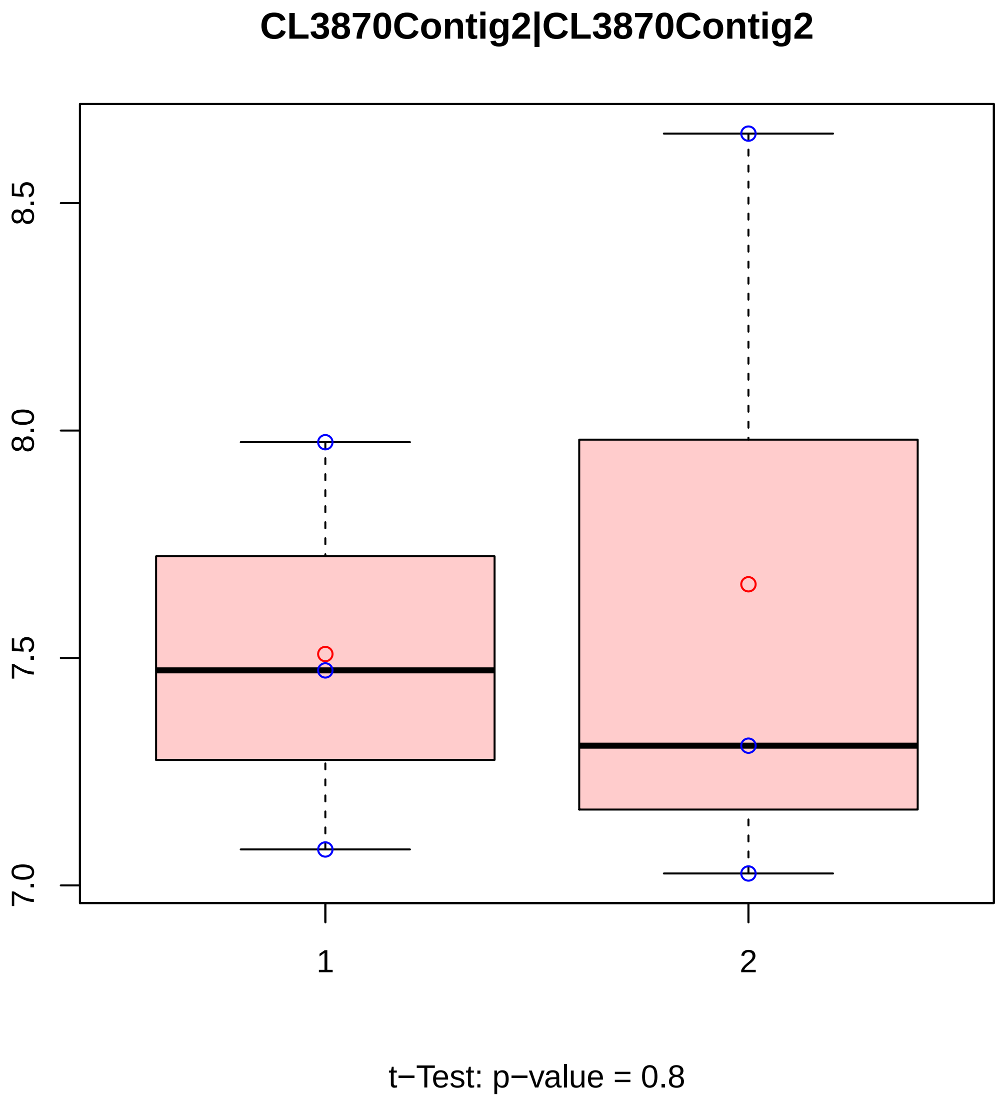

CL3870Contig2|CL3870Contig2
8.5
8.0
7.5
7.0
1
2
t−Test: p−value = 0.8

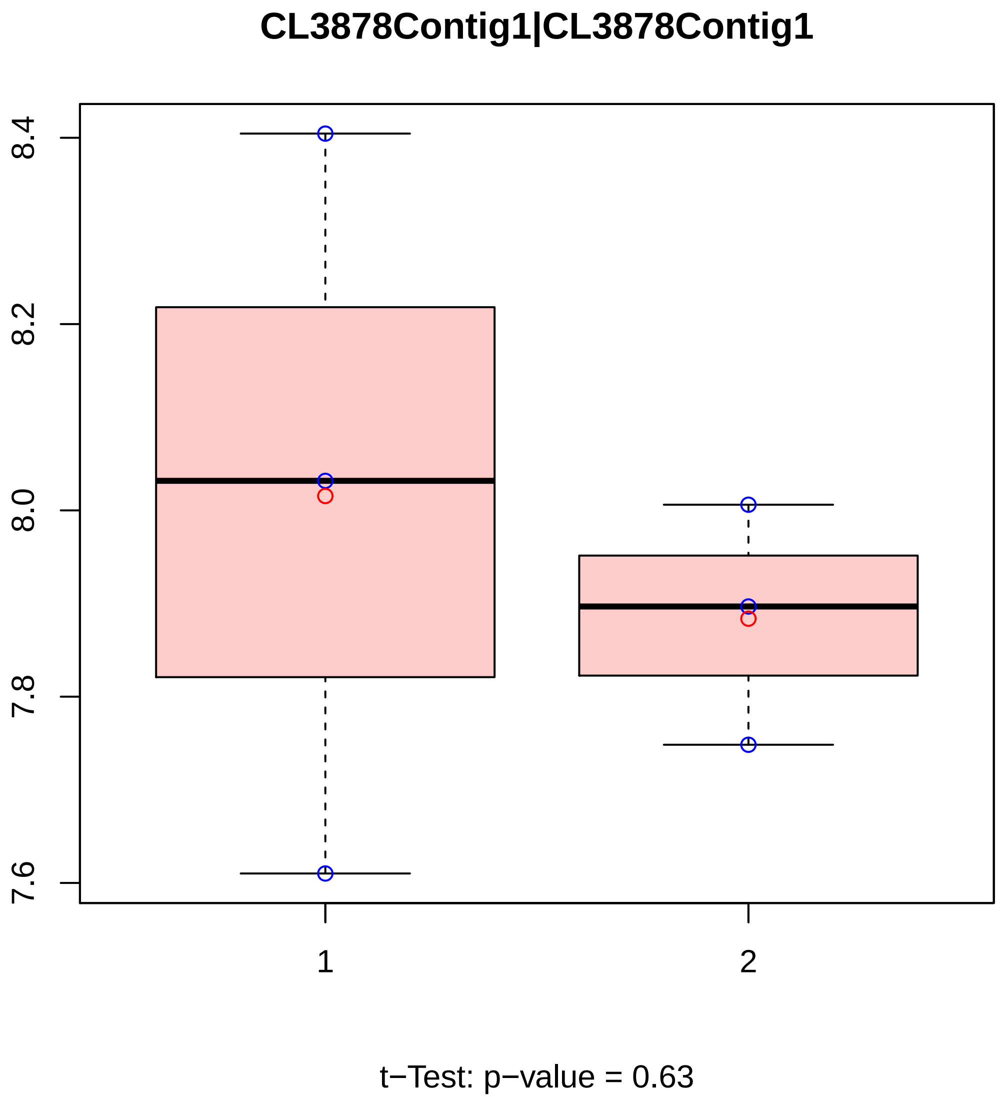
CL3878Contig1|CL3878Contig1
8.4
8.2
8.0
7.8
7.6
1
2
t−Test: p−value = 0.63

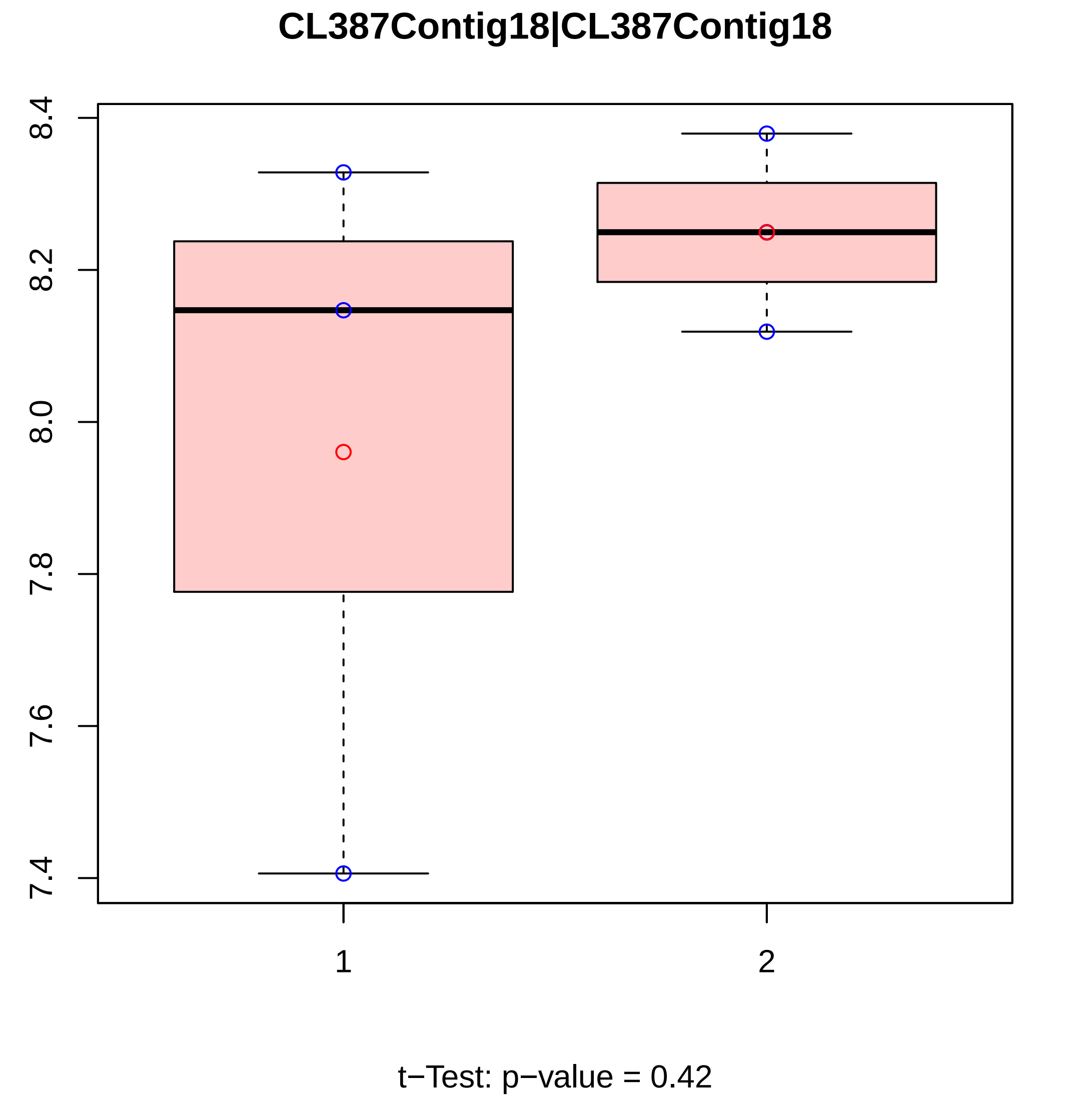

CL387Contig18|CL387Contig18
8.4
8.2
8.0
7.8
7.6
7.4
1
2
t−Test: p−value = 0.42

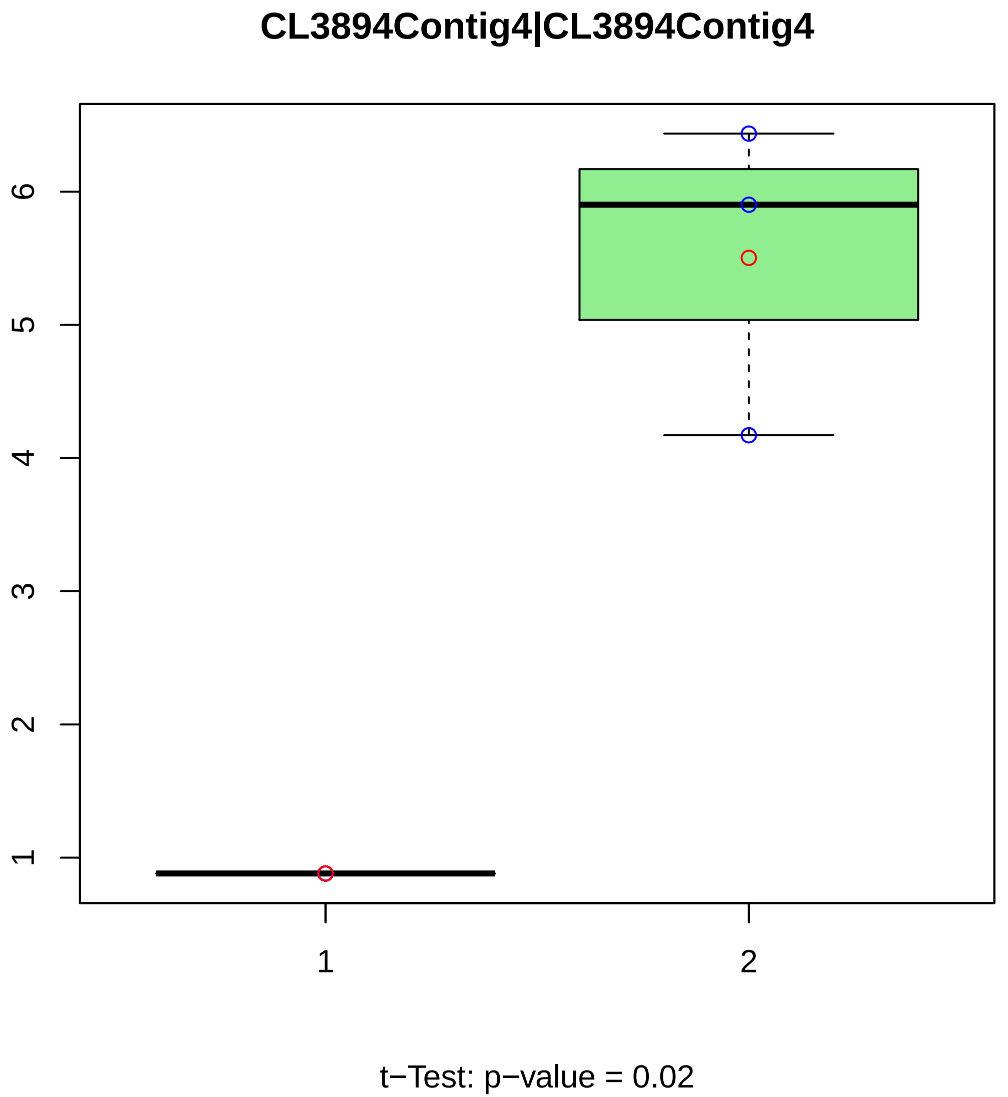

CL3894Contig4|CL3894Contig4
1
2
3
4
5
6
1
2
t−Test: p−value = 0.02

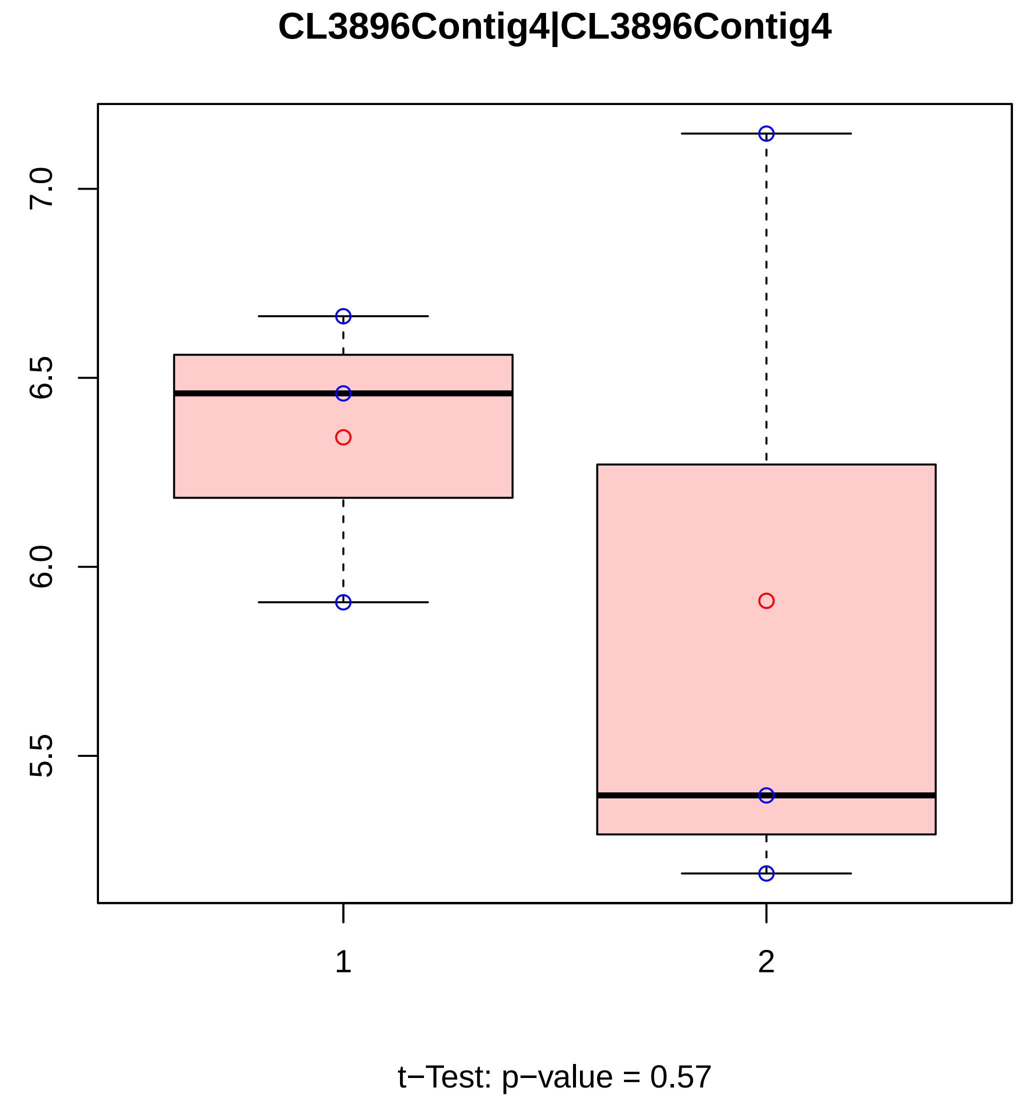
CL3896Contig4|CL3896Contig4
7.0
6.5
6.0
5.5
1
2
t−Test: p−value = 0.57

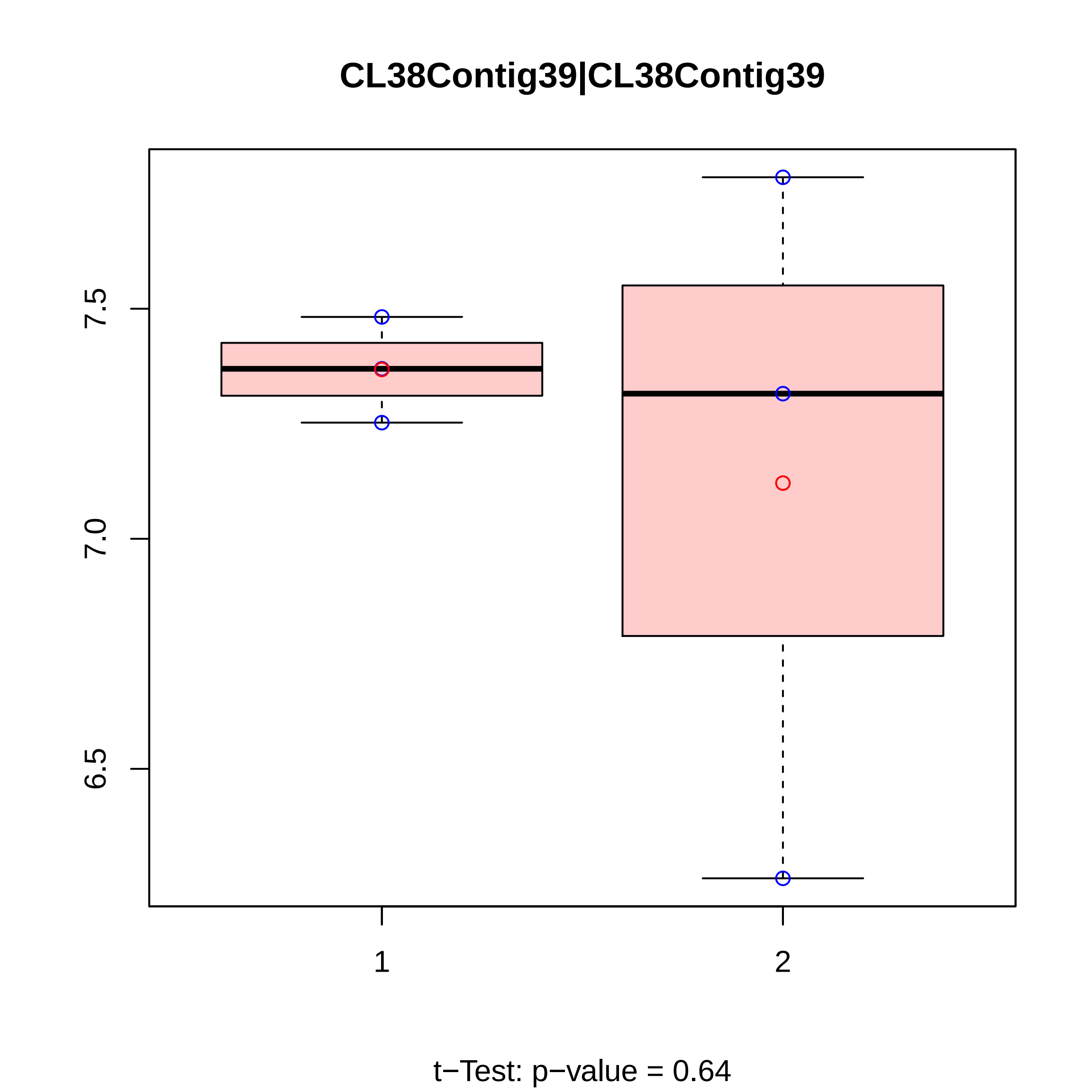

CL38Contig39|CL38Contig39
7.5
7.0
6.5
1
2
t−Test: p−value = 0.64

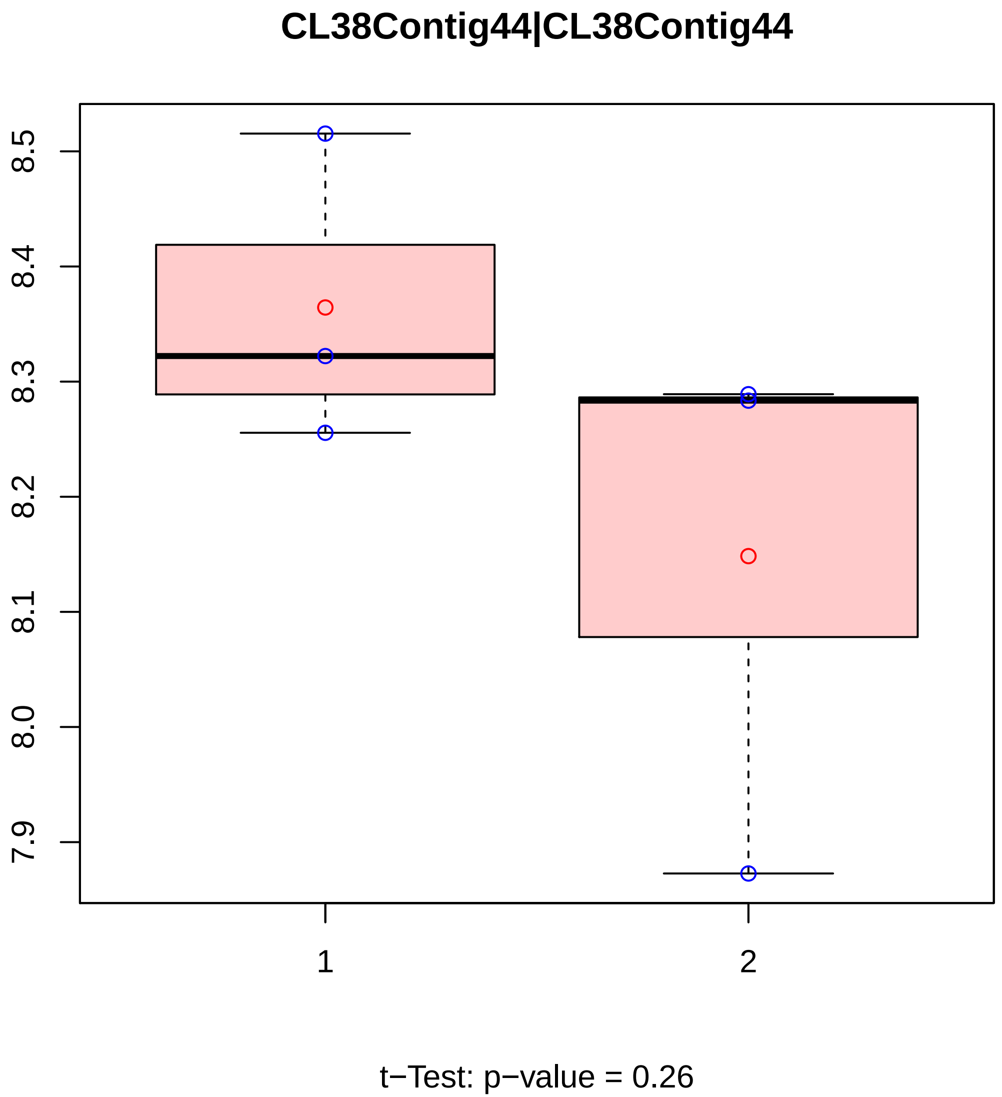
CL38Contig44|CL38Contig44
8.5
8.4
8.3
8.2
8.1
8.0
7.9
1
2
t−Test: p−value = 0.26

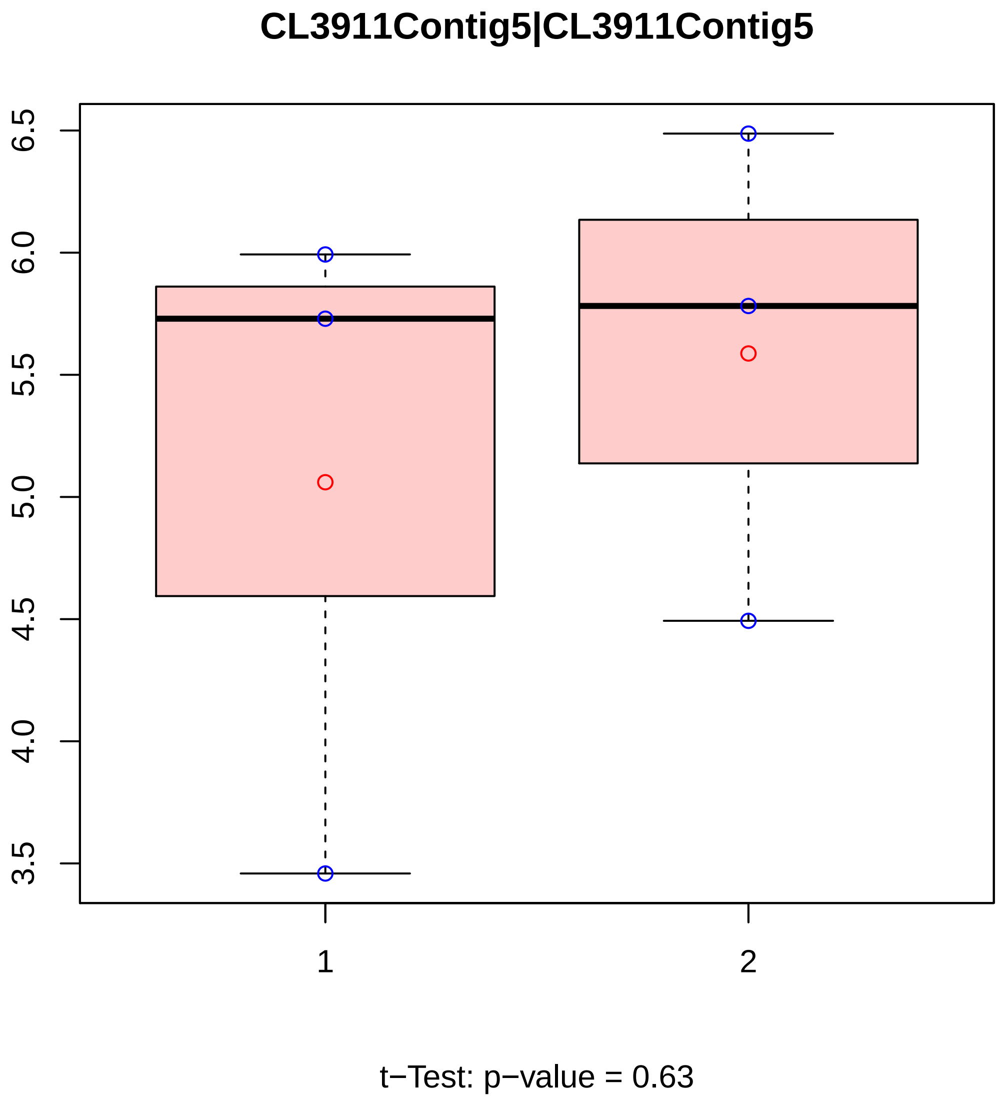
CL3911Contig5|CL3911Contig5
6.5
6.0
5.5
5.0
4.5
4.0
3.5
1
2
t−Test: p−value = 0.63

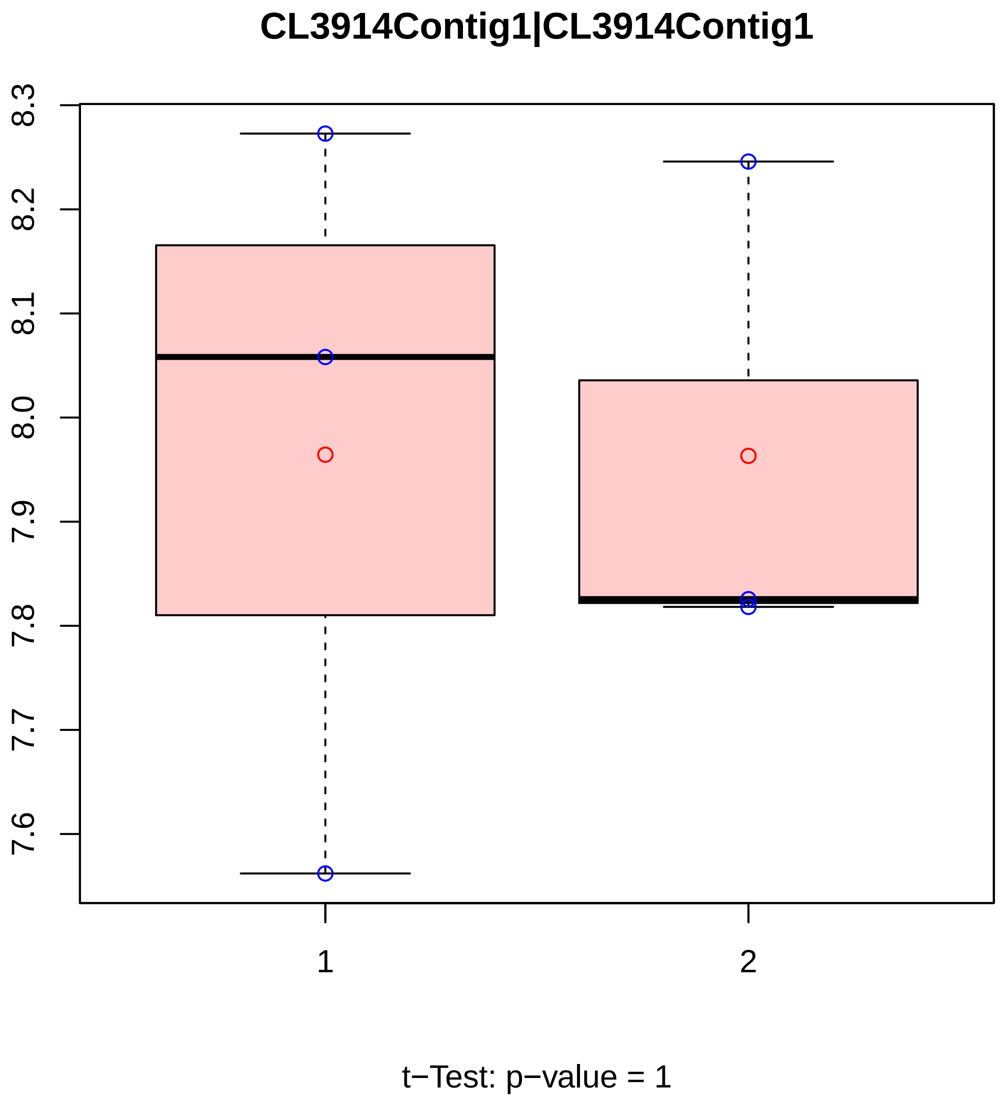
CL3914Contig1|CL3914Contig1
8.3
8.2
8.1
8.0
7.9
7.8
7.7
7.6
1
2
t−Test: p−value = 1

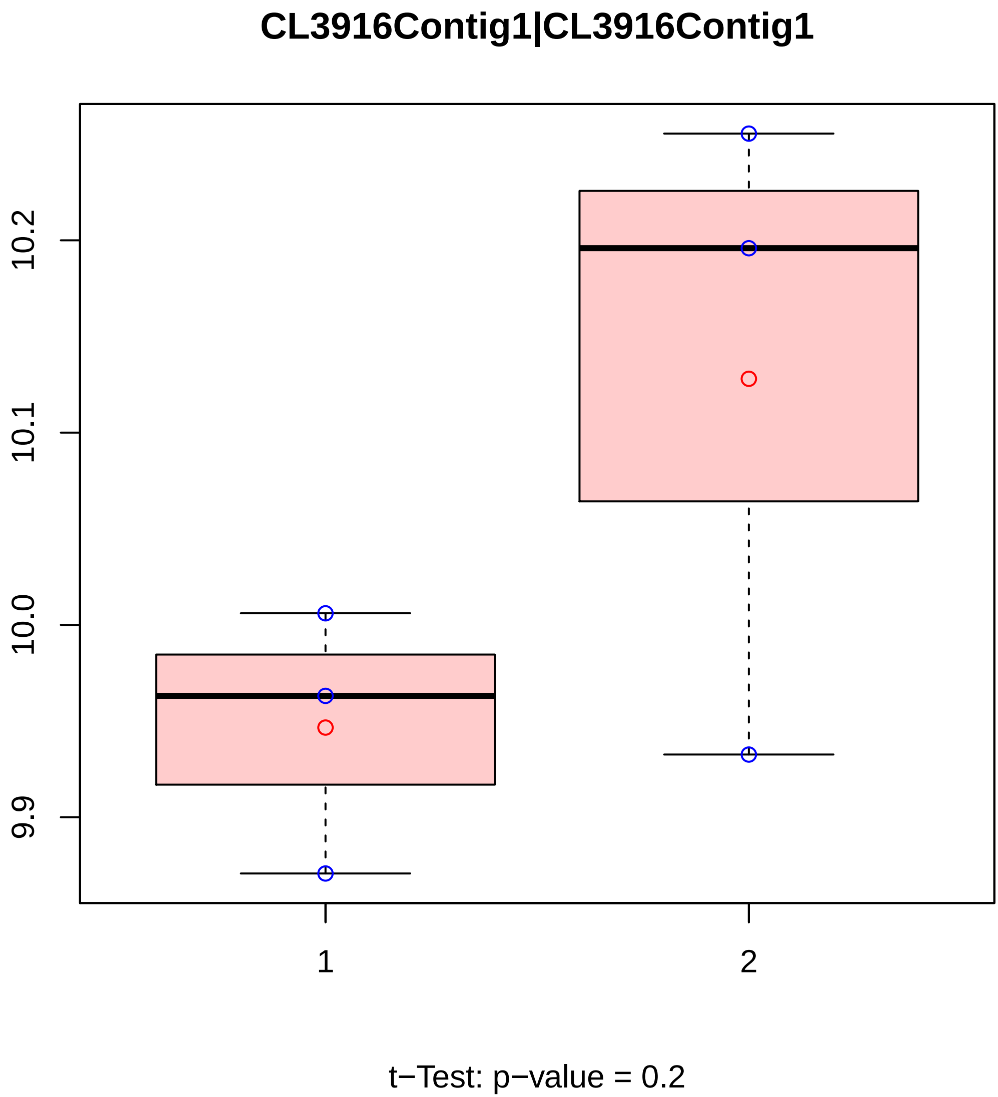

CL3916Contig1|CL3916Contig1
10.2
10.1
10.0
9.9
1
2
t−Test: p−value = 0.2

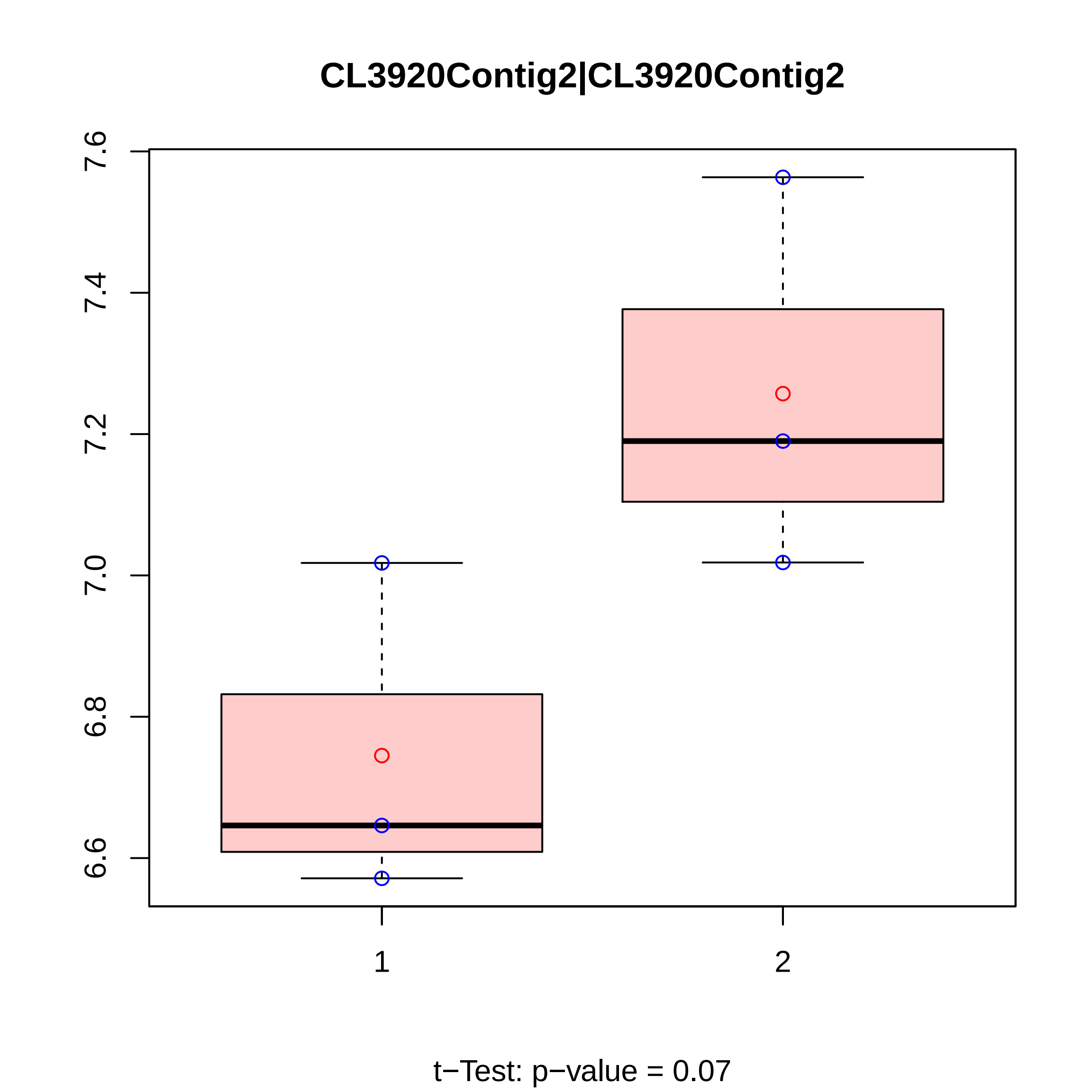
CL3920Contig2|CL3920Contig2
6.6
6.8
7.0
7.2
7.4
7.6
1
2
t−Test: p−value = 0.07

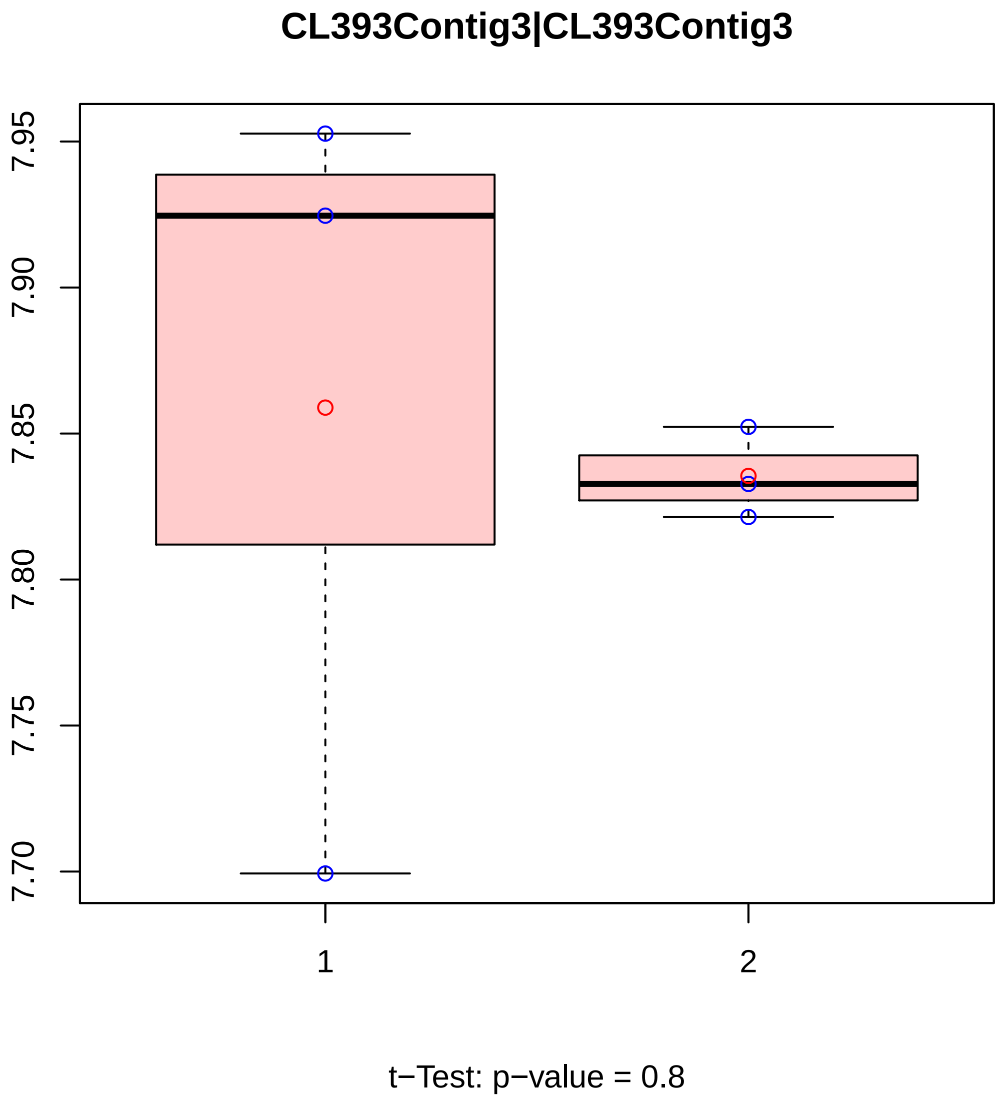

CL393Contig3|CL393Contig3
7.95
7.90
7.85
7.80
7.75
7.70
1
2
t−Test: p−value = 0.8

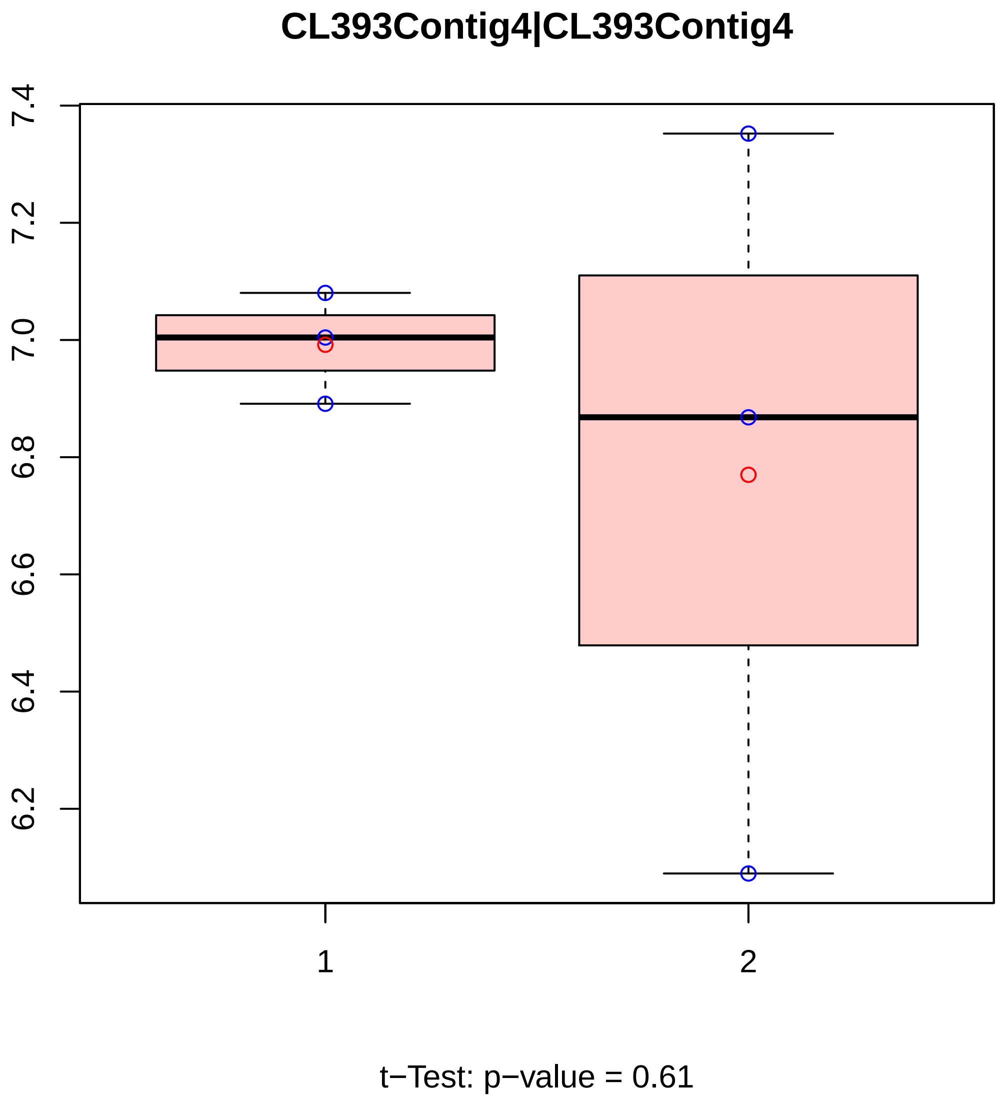
CL393Contig4|CL393Contig4
7.4
7.2
7.0
6.8
6.6
6.4
6.2
1
2
t−Test: p−value = 0.61

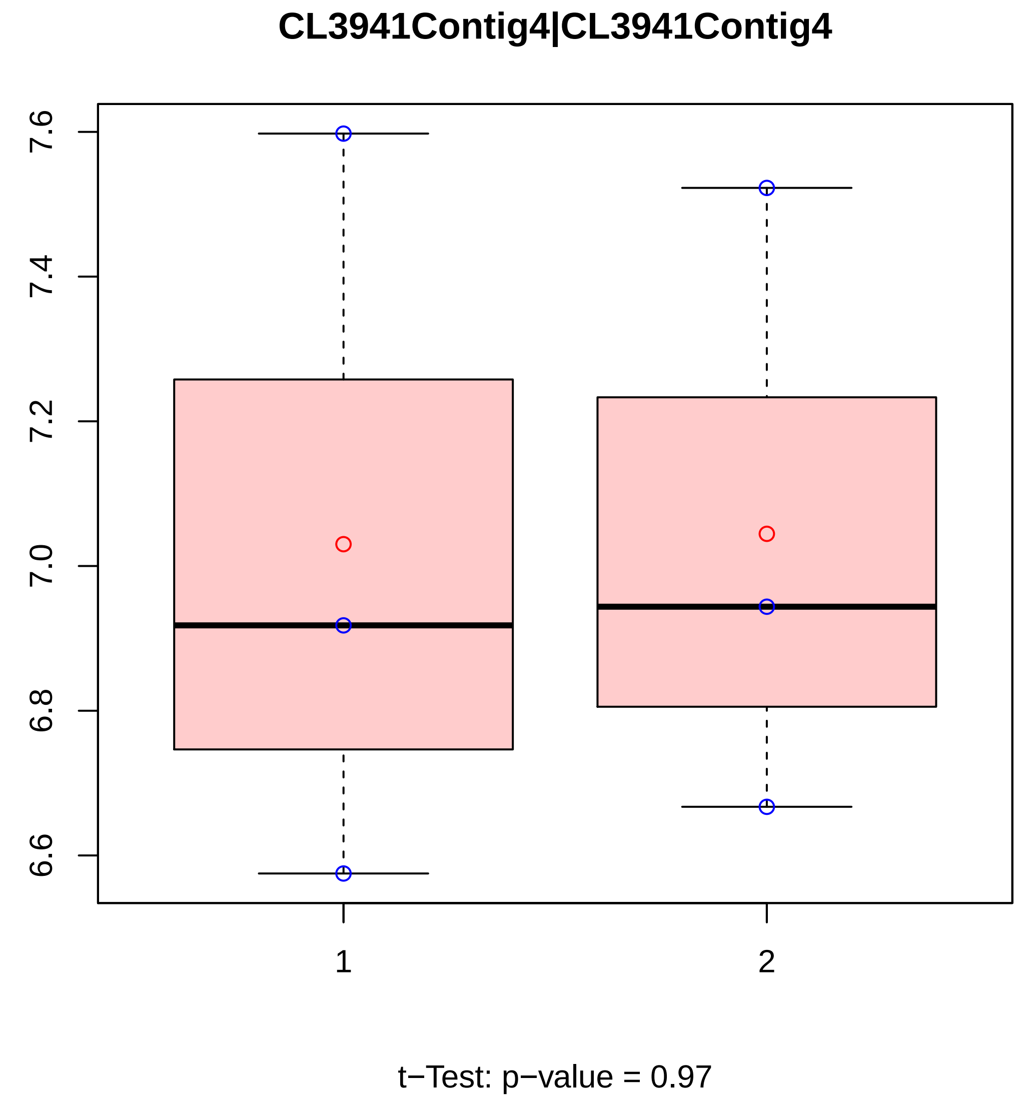
CL3941Contig4|CL3941Contig4
7.6
7.4
7.2
7.0
6.8
6.6
1
2
t−Test: p−value = 0.97

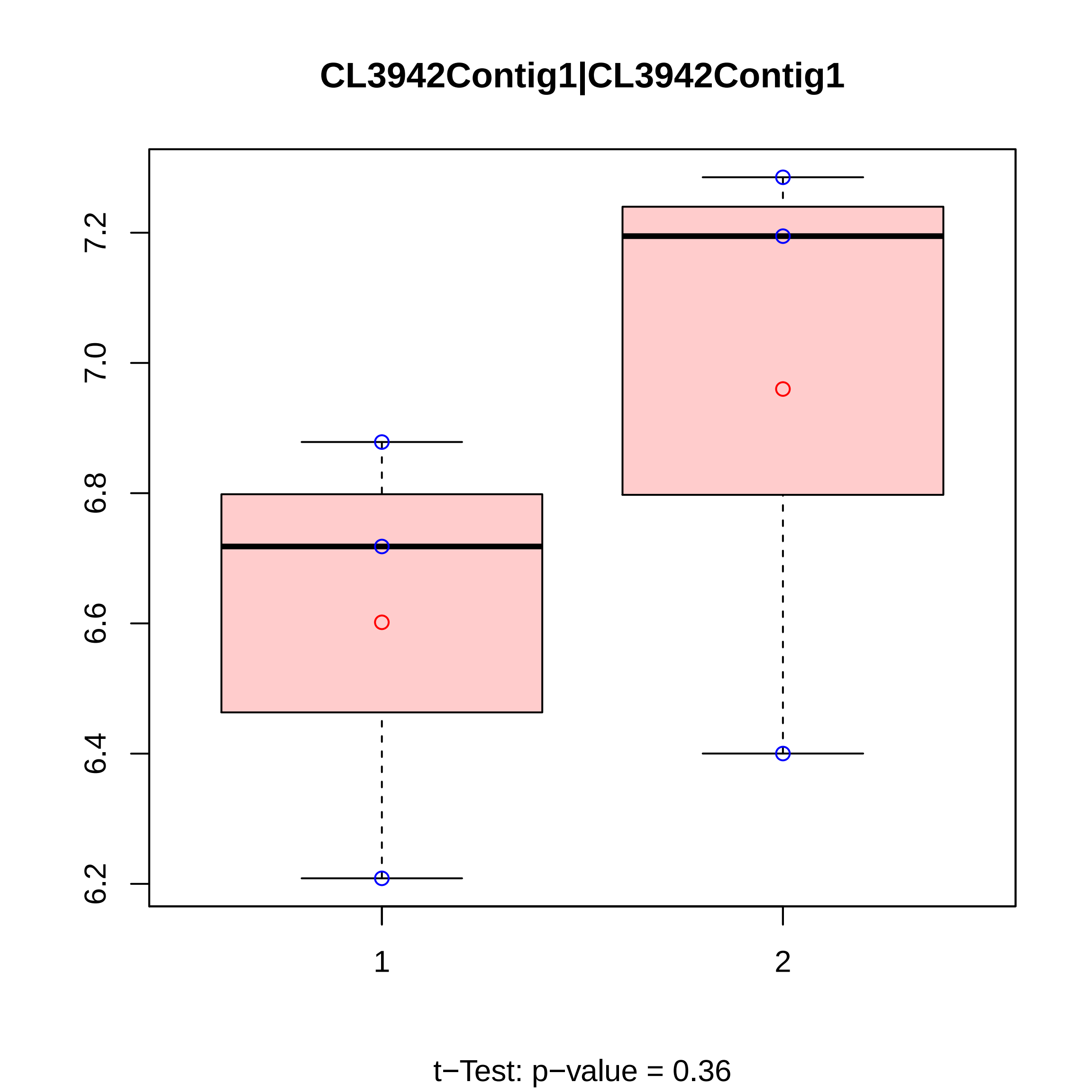
CL3942Contig1|CL3942Contig1
6.2
6.4
6.6
6.8
7.0
7.2
1
2
t−Test: p−value = 0.36

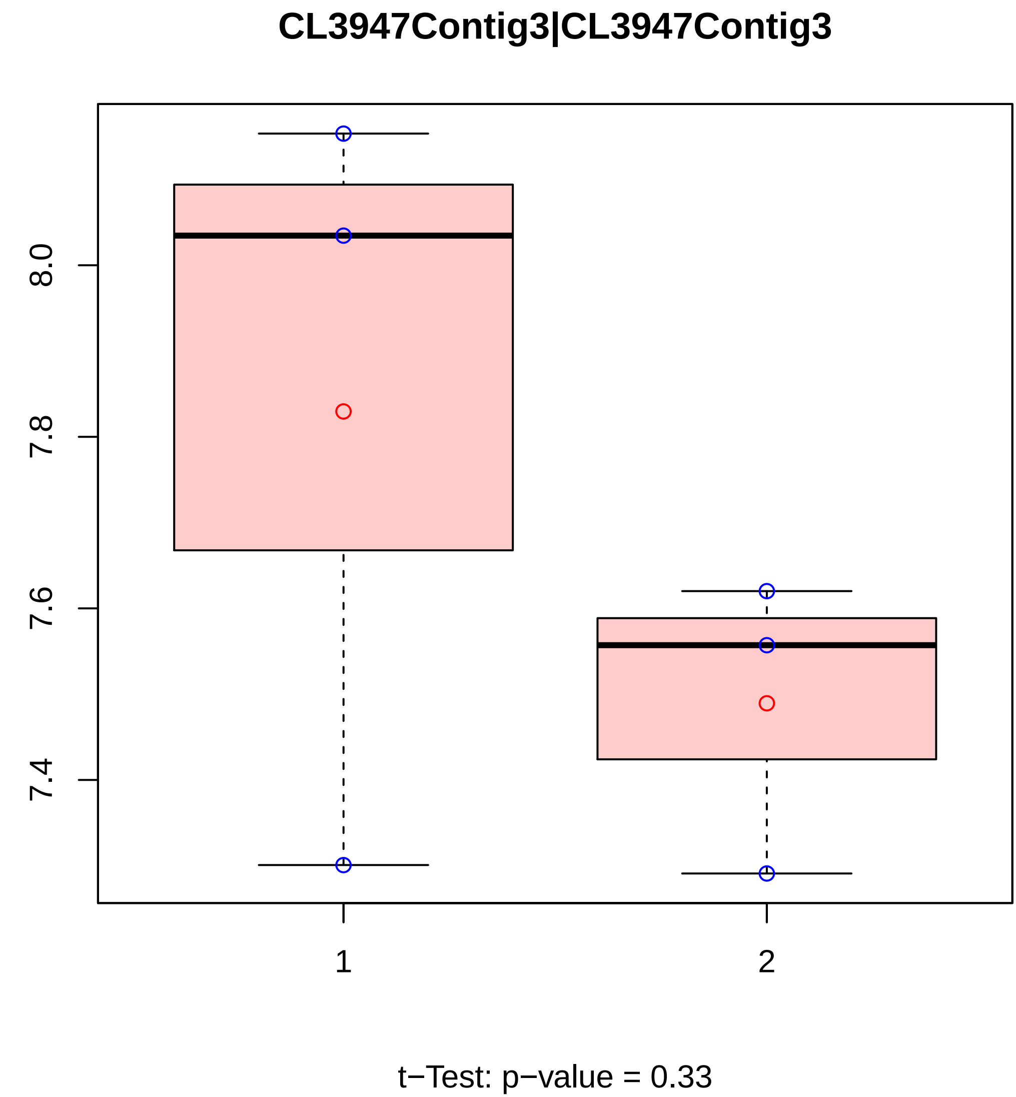
CL3947Contig3|CL3947Contig3
8.0
7.8
7.6
7.4
1
2
t−Test: p−value = 0.33

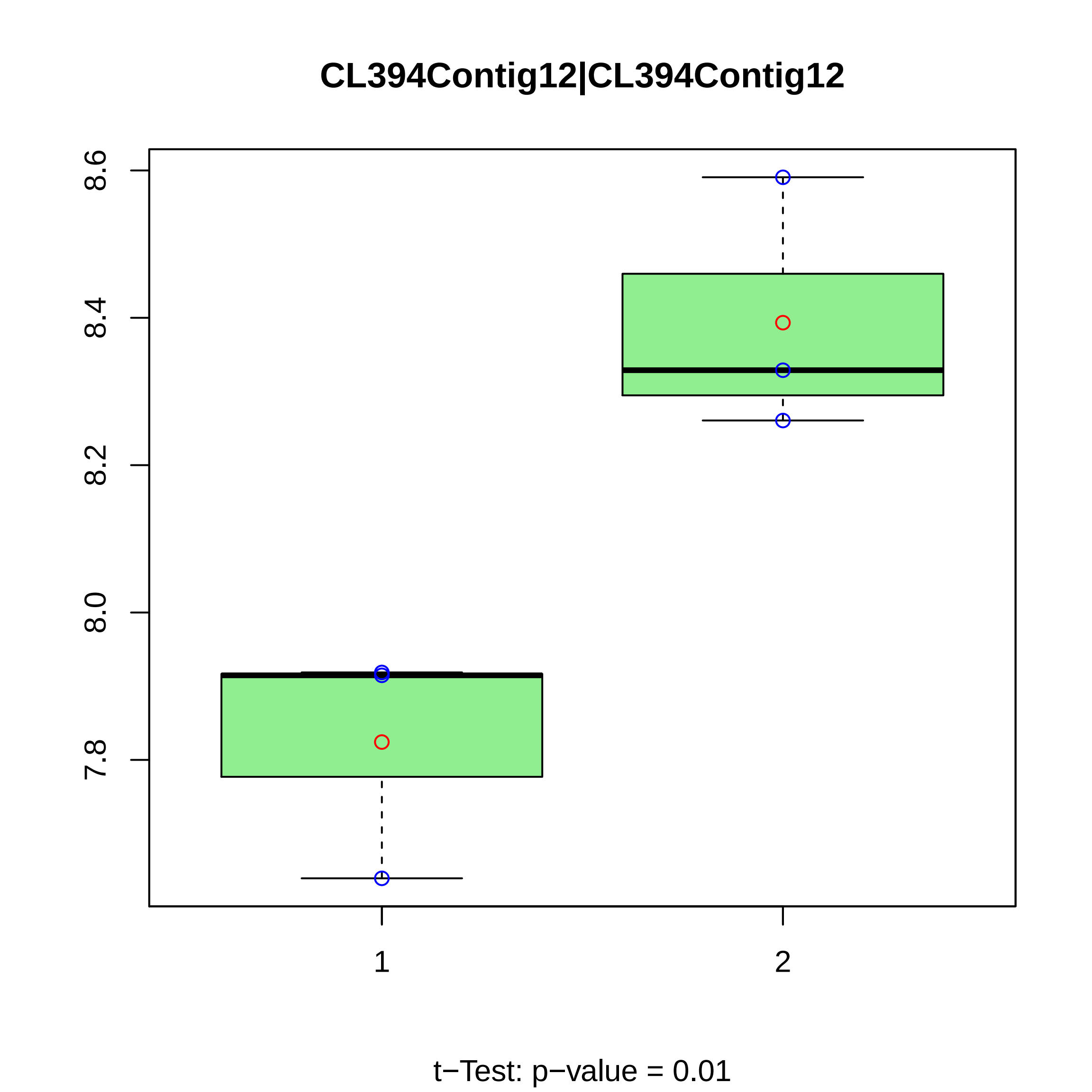
CL394Contig12|CL394Contig12
8.6
8.4
8.2
8.0
7.8
1
2
t−Test: p−value = 0.01

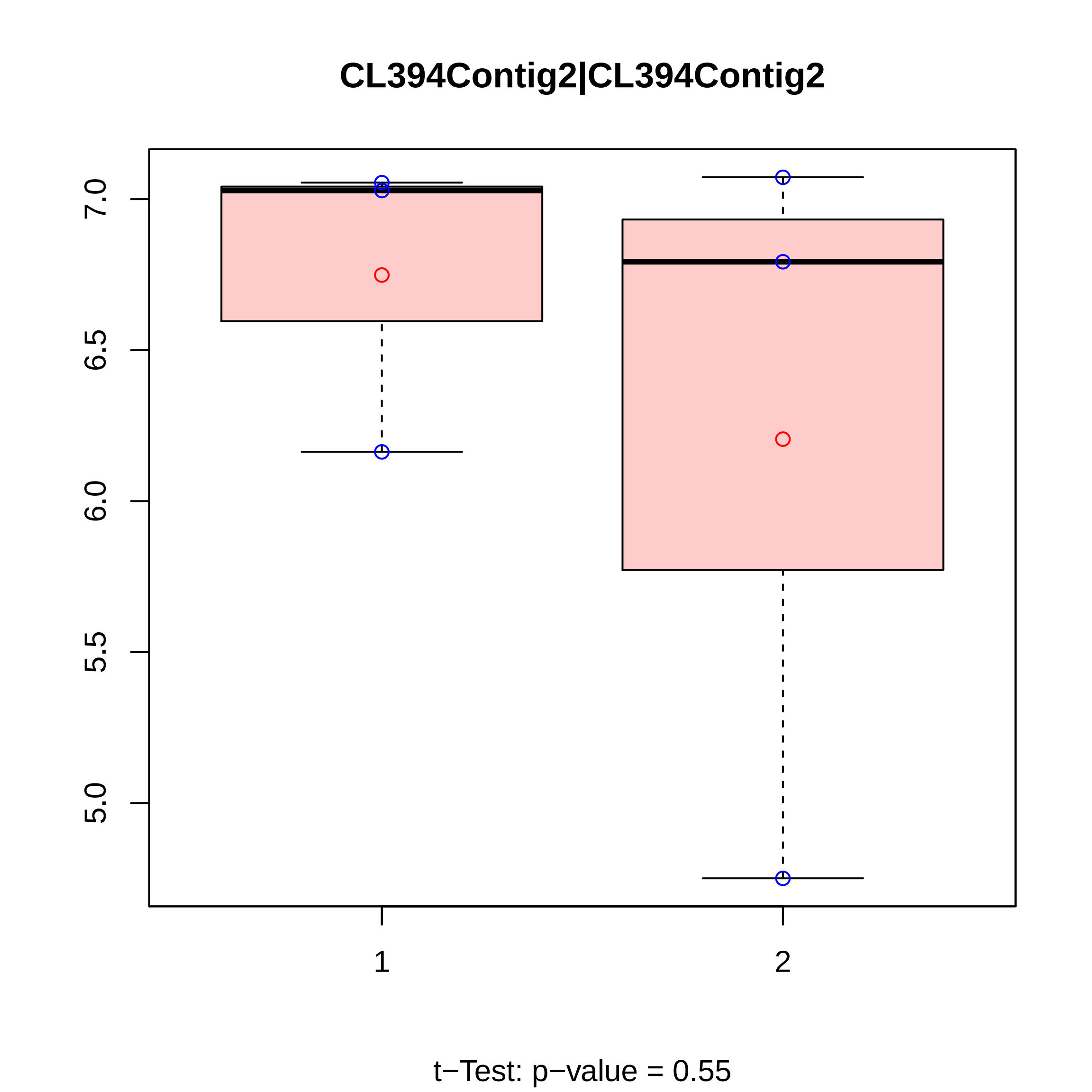
CL394Contig2|CL394Contig2
7.0
6.5
6.0
5.5
5.0
1
2
t−Test: p−value = 0.55

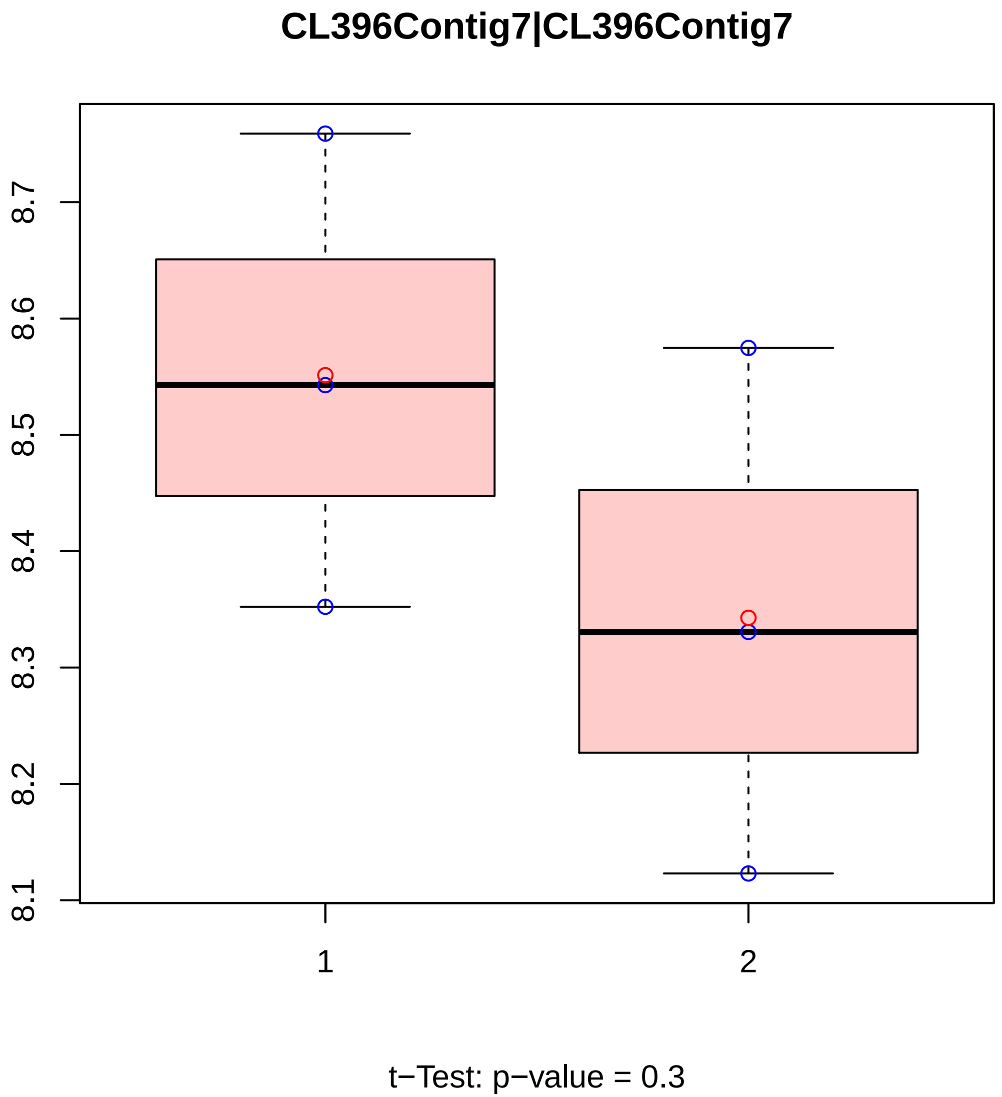

CL396Contig7|CL396Contig7
8.1
8.2
8.3
8.4
8.5
8.6
8.7
1
2
t−Test: p−value = 0.3

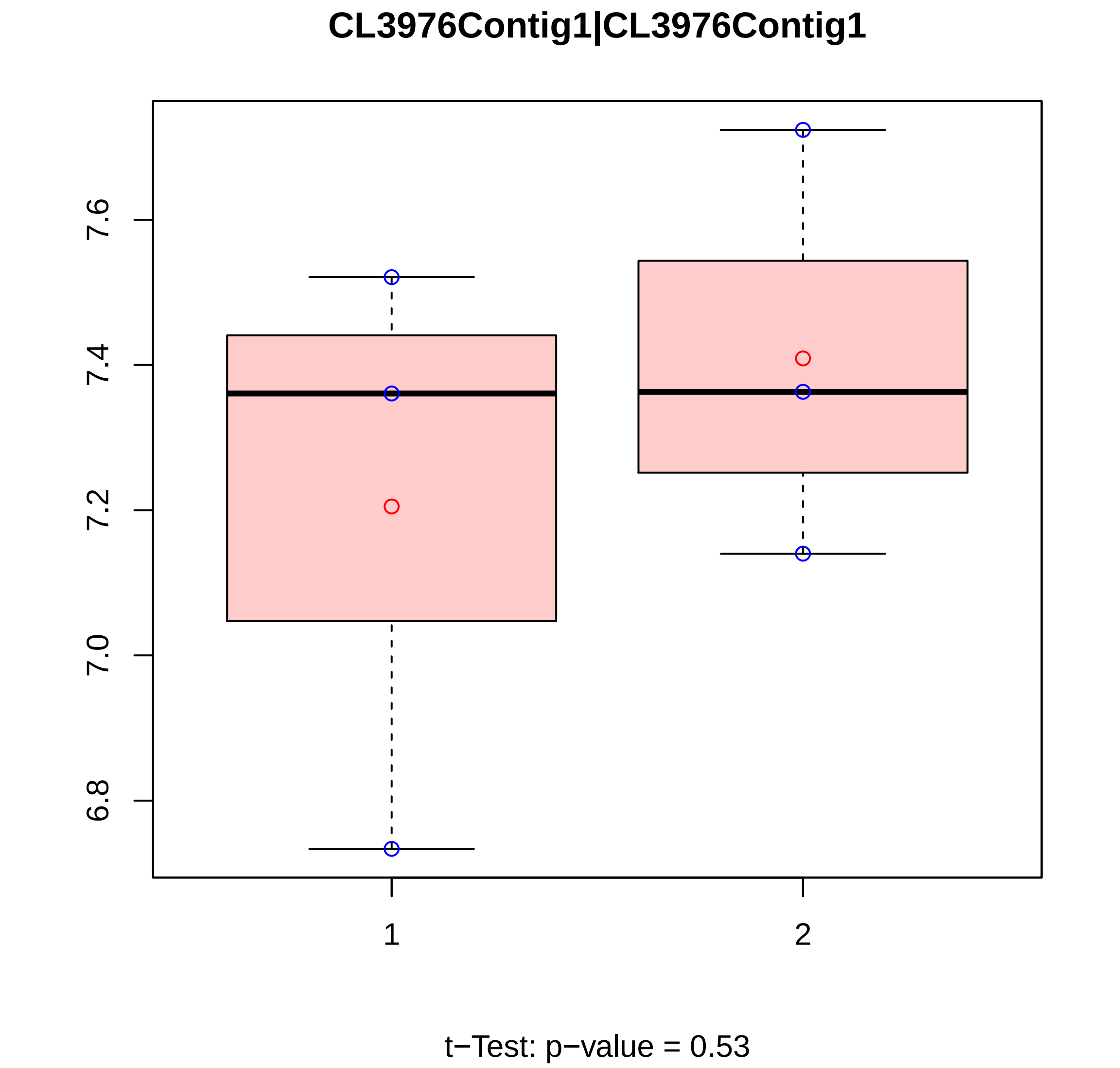
CL3976Contig1|CL3976Contig1
7.6
7.4
7.2
7.0
6.8
1
2
t−Test: p−value = 0.53

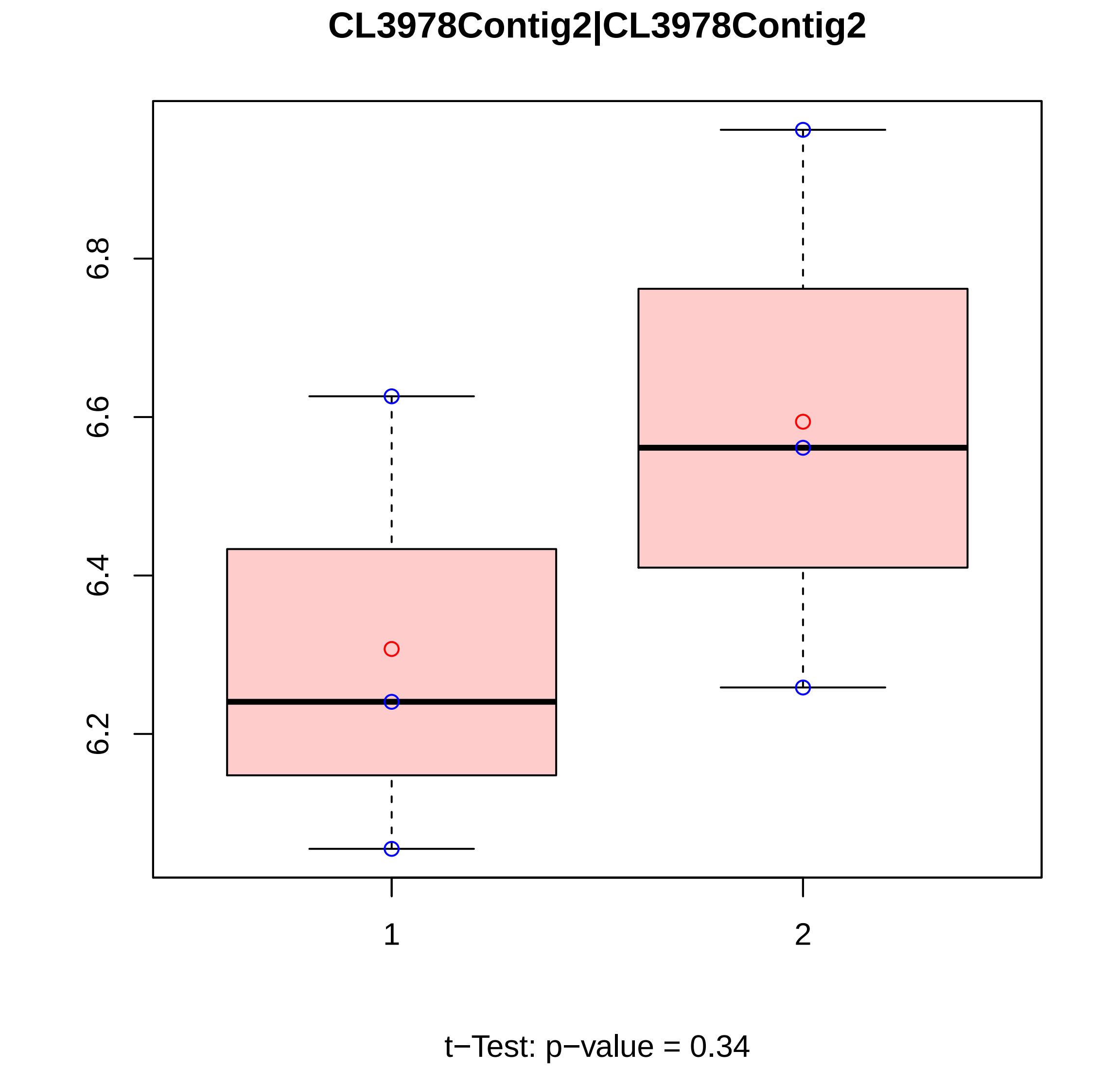
CL3978Contig2|CL3978Contig2
6.2
6.4
6.6
6.8
1
2
t−Test: p−value = 0.34

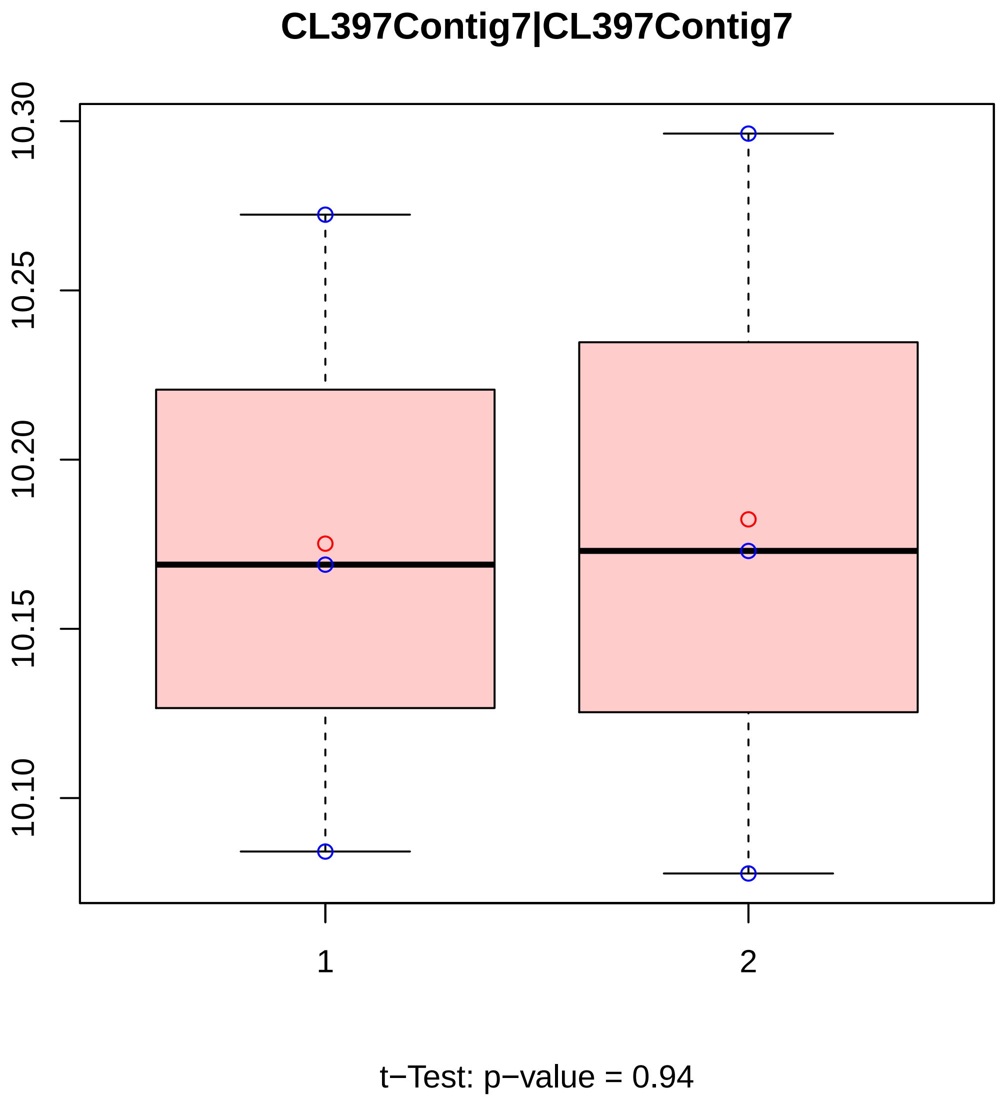
CL397Contig7|CL397Contig7
10.30
10.25
10.20
10.15
10.10
1
2
t−Test: p−value = 0.94

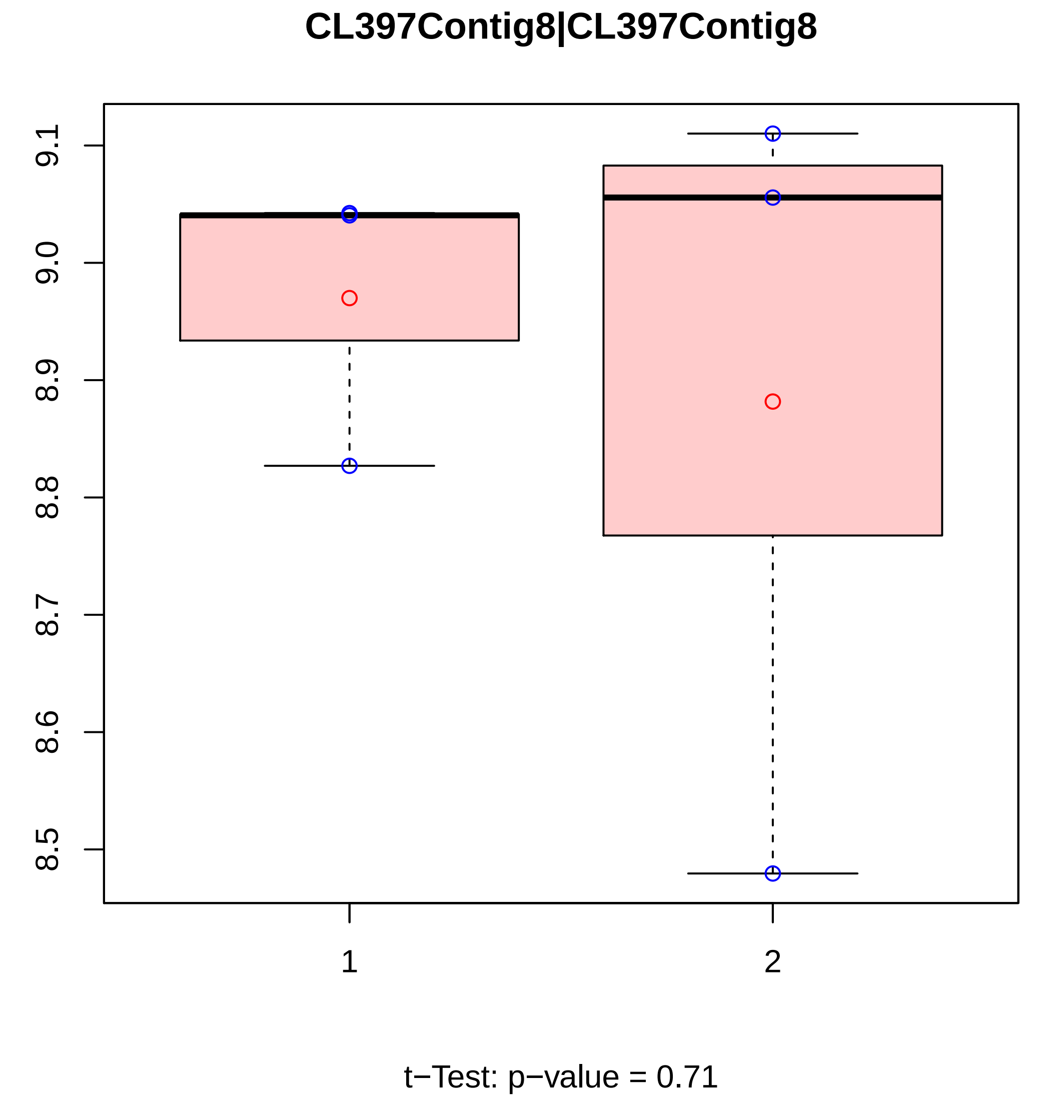

CL397Contig8|CL397Contig8
9.1
9.0
8.9
8.8
8.7
8.6
8.5
1
2
t−Test: p−value = 0.71

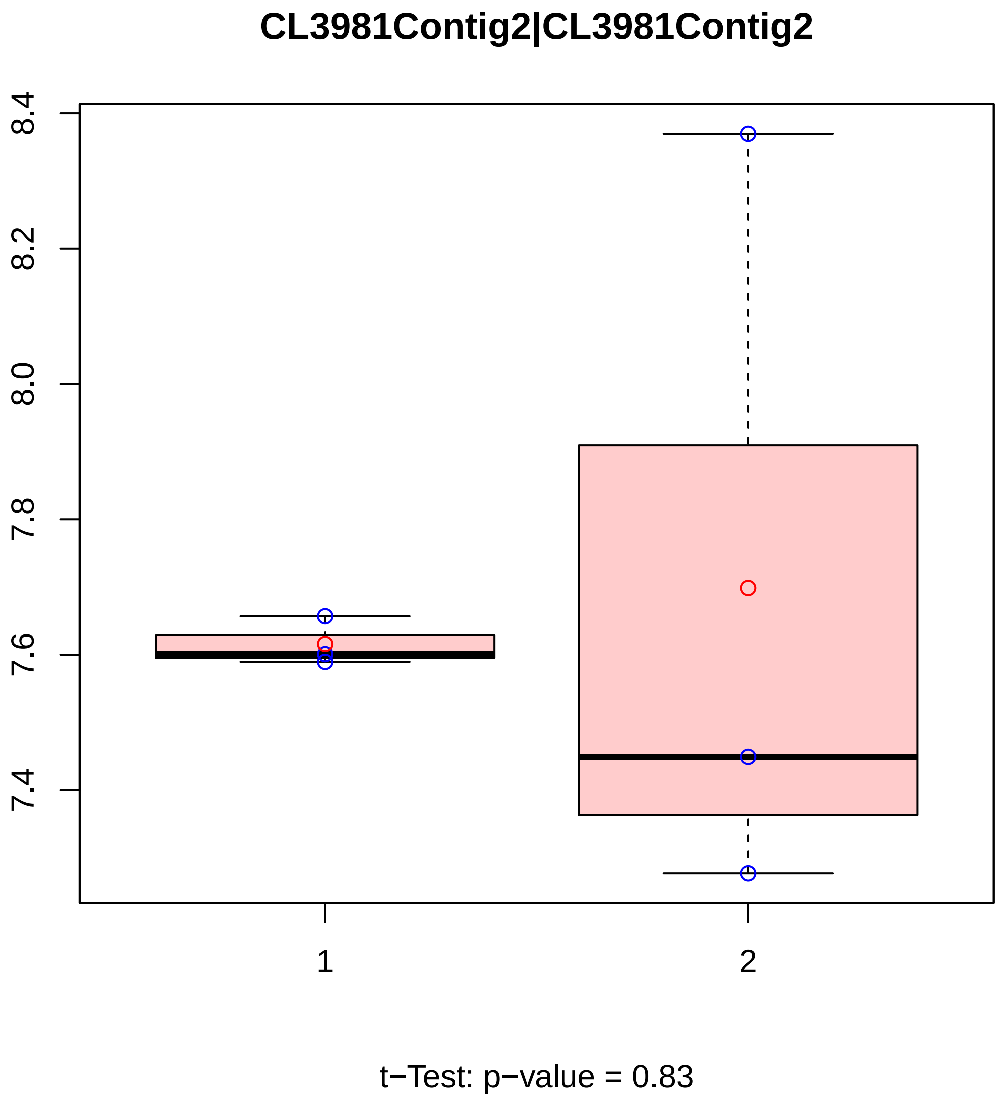
CL3981Contig2|CL3981Contig2
8.4
8.2
8.0
7.8
7.6
7.4
1
2
t−Test: p−value = 0.83

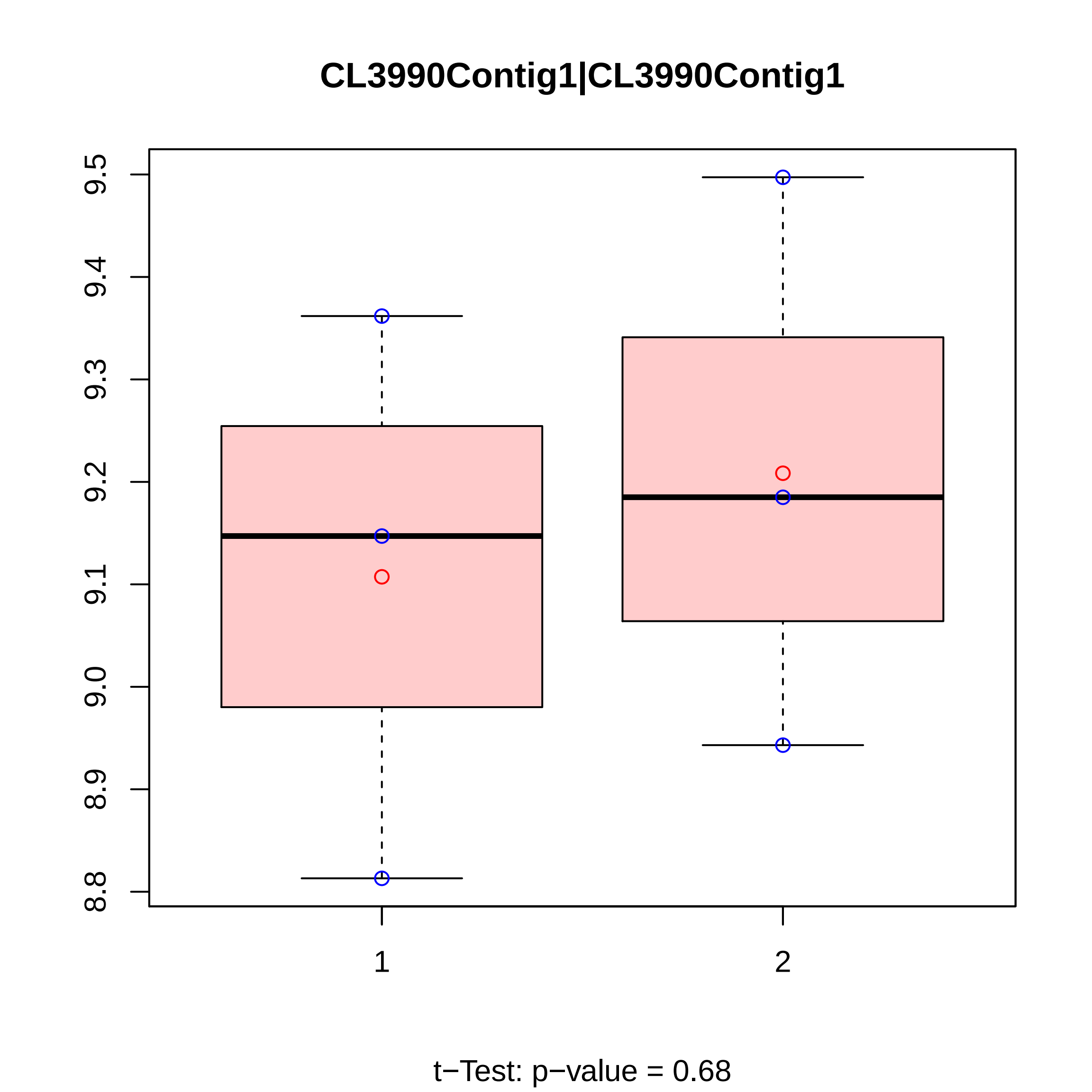
CL3990Contig1|CL3990Contig1
9.5
9.4
9.3
9.2
9.1
9.0
8.9
8.8
1
2
t−Test: p−value = 0.68

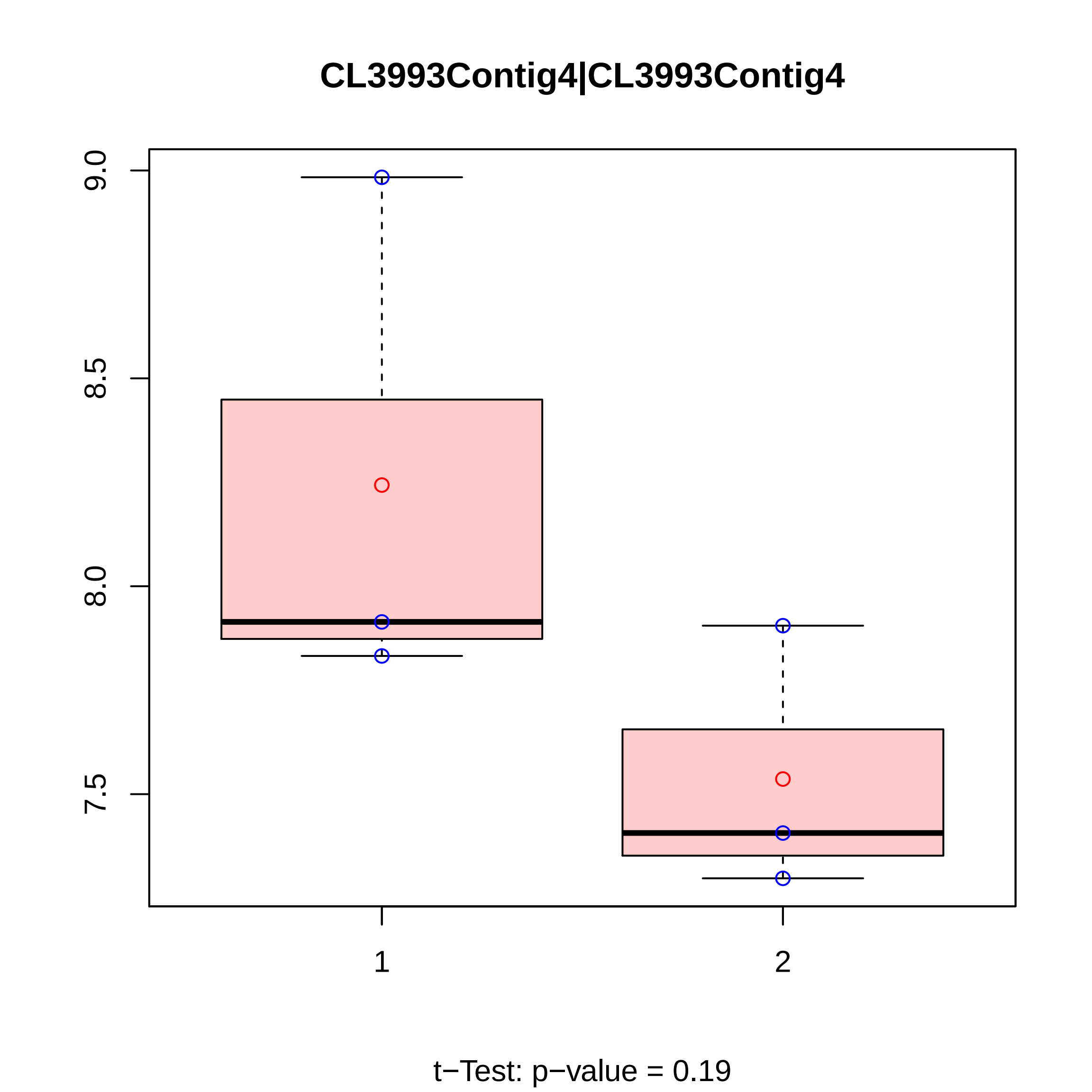

CL3993Contig4|CL3993Contig4
9.0
8.5
8.0
7.5
1
2
t−Test: p−value = 0.19

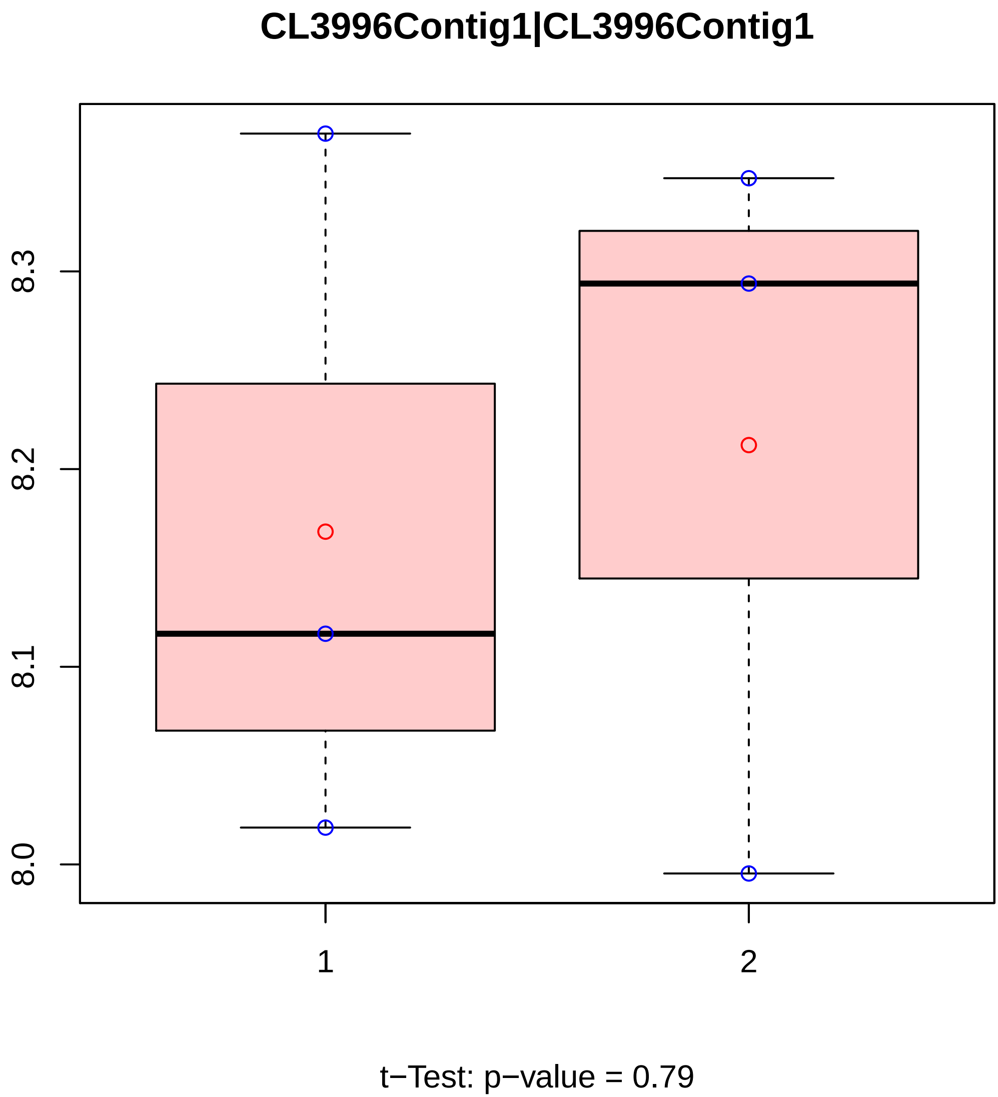

CL3996Contig1|CL3996Contig1
8.0
8.1
8.2
8.3
1
2
t−Test: p−value = 0.79

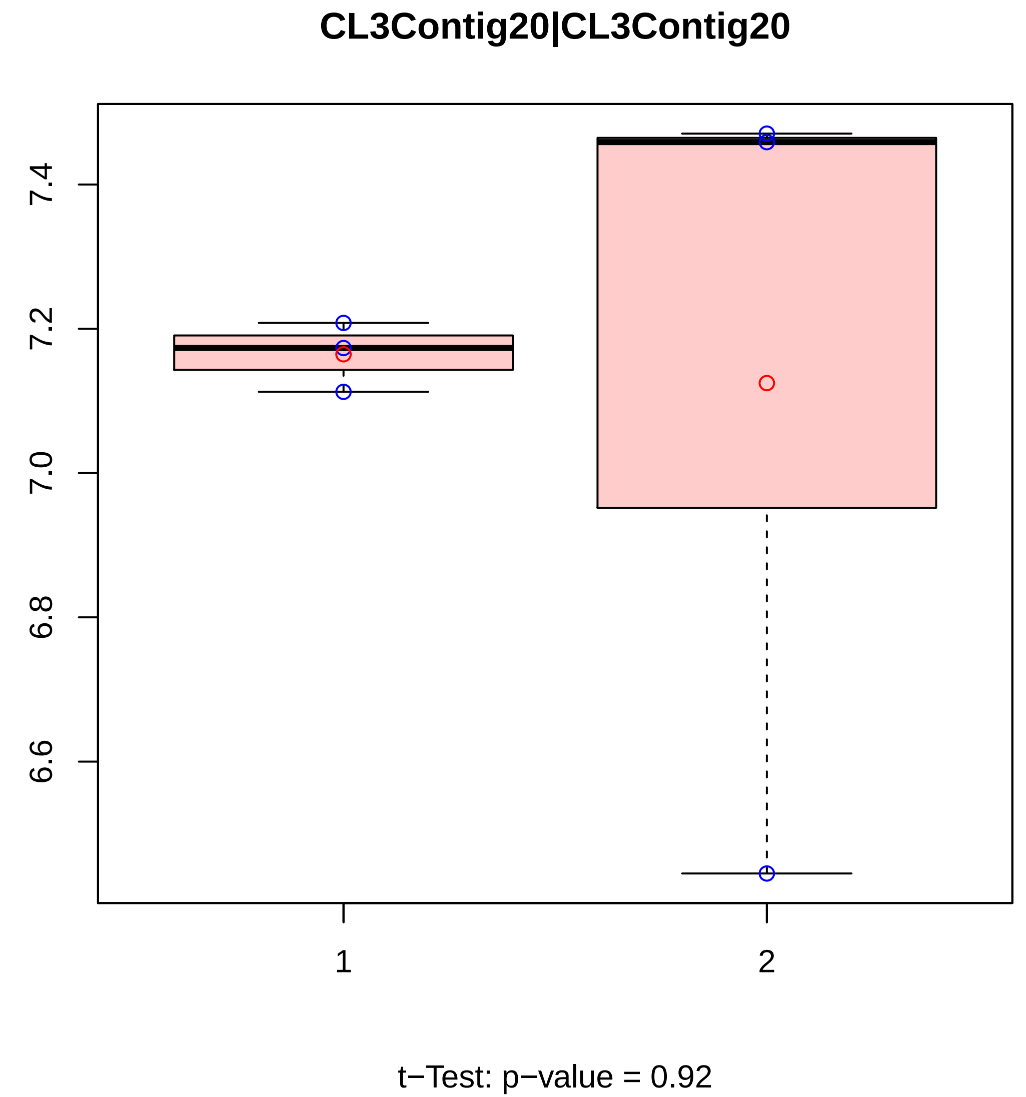

CL3Contig20|CL3Contig20
7.4
7.2
7.0
6.8
6.6
1
2
t−Test: p−value = 0.92

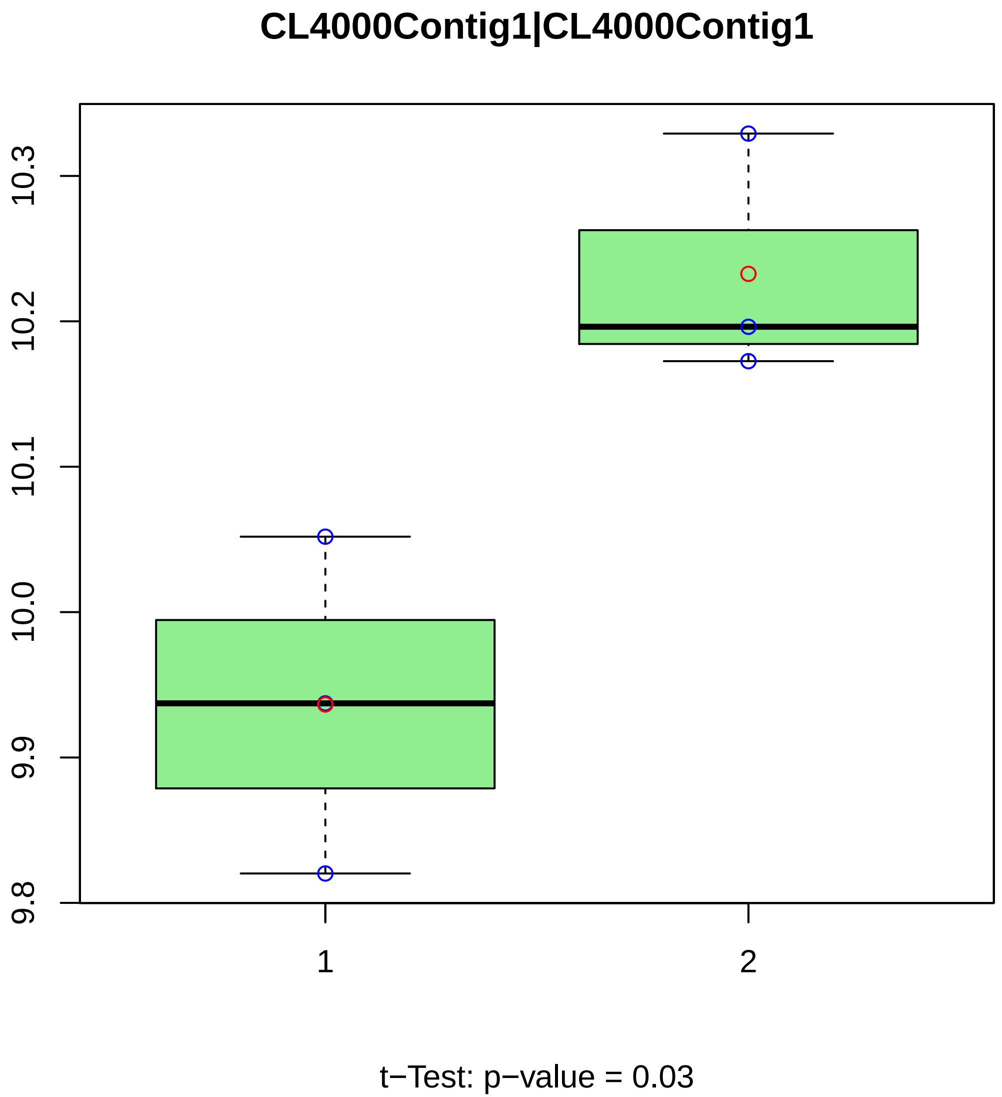

CL4000Contig1|CL4000Contig1
10.3
10.2
10.1
10.0
9.9
9.8
1
2
t−Test: p−value = 0.03

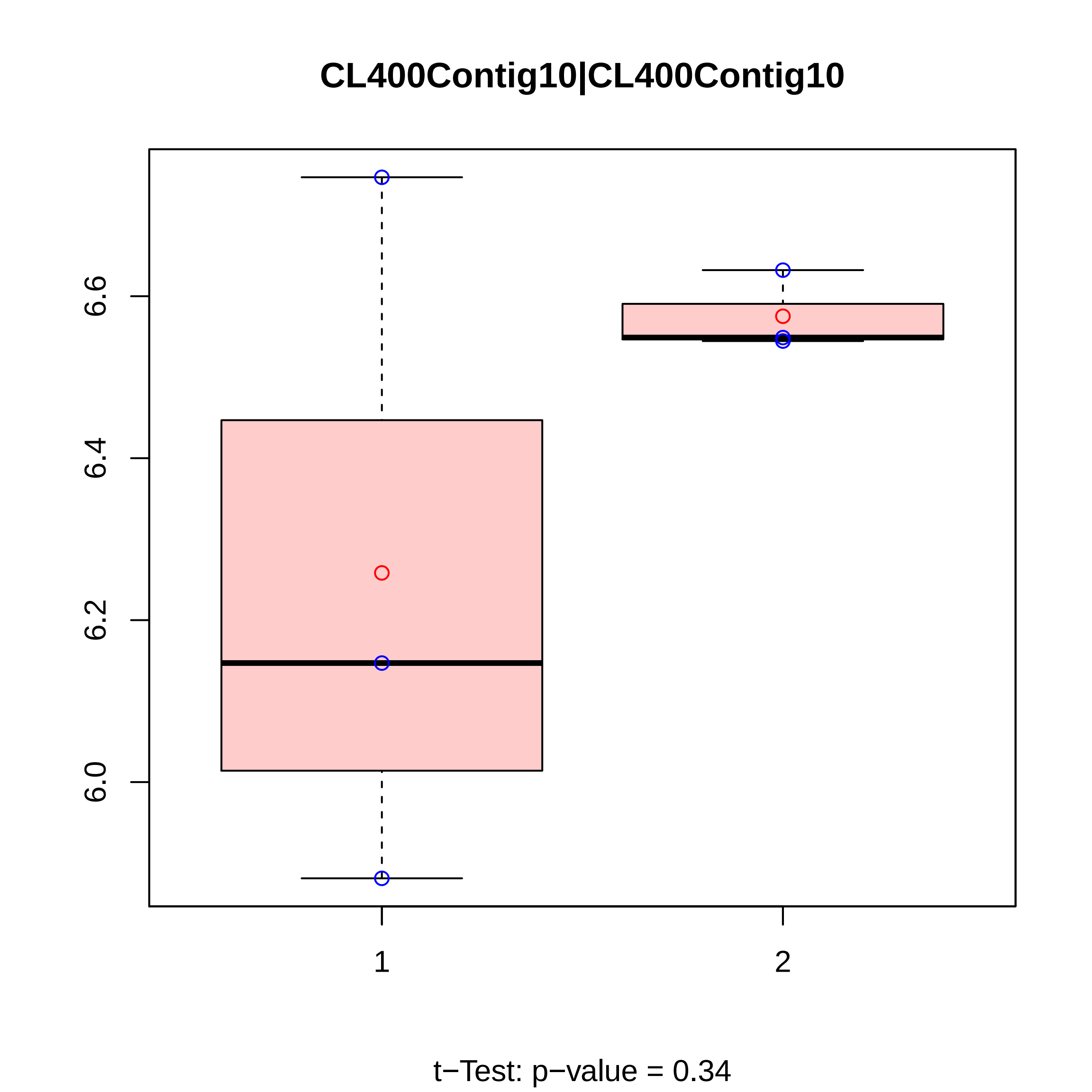

CL400Contig10|CL400Contig10
6.0
6.2
6.4
6.6
1
2
t−Test: p−value = 0.34

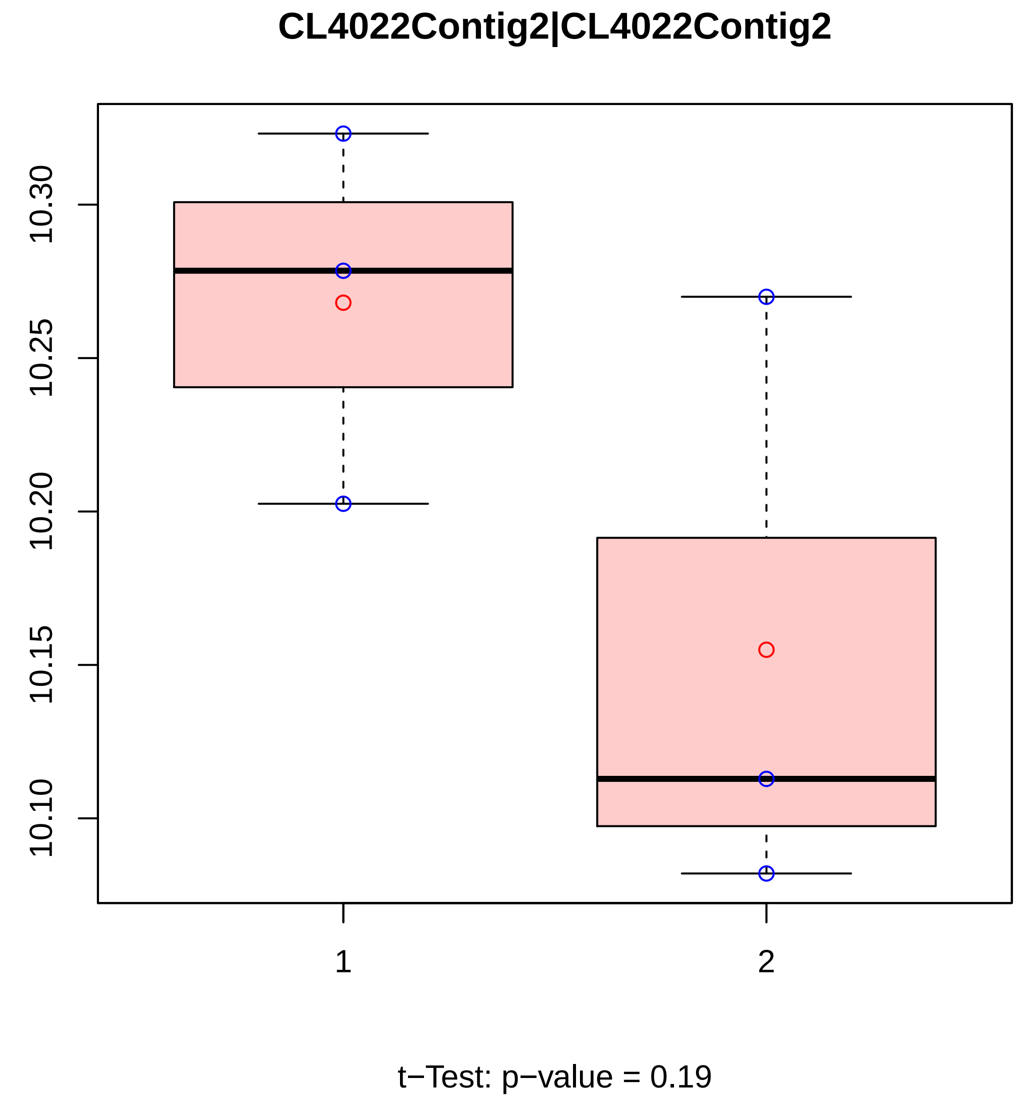
CL4022Contig2|CL4022Contig2
10.10
10.15
10.20
10.25
10.30
1
2
t−Test: p−value = 0.19

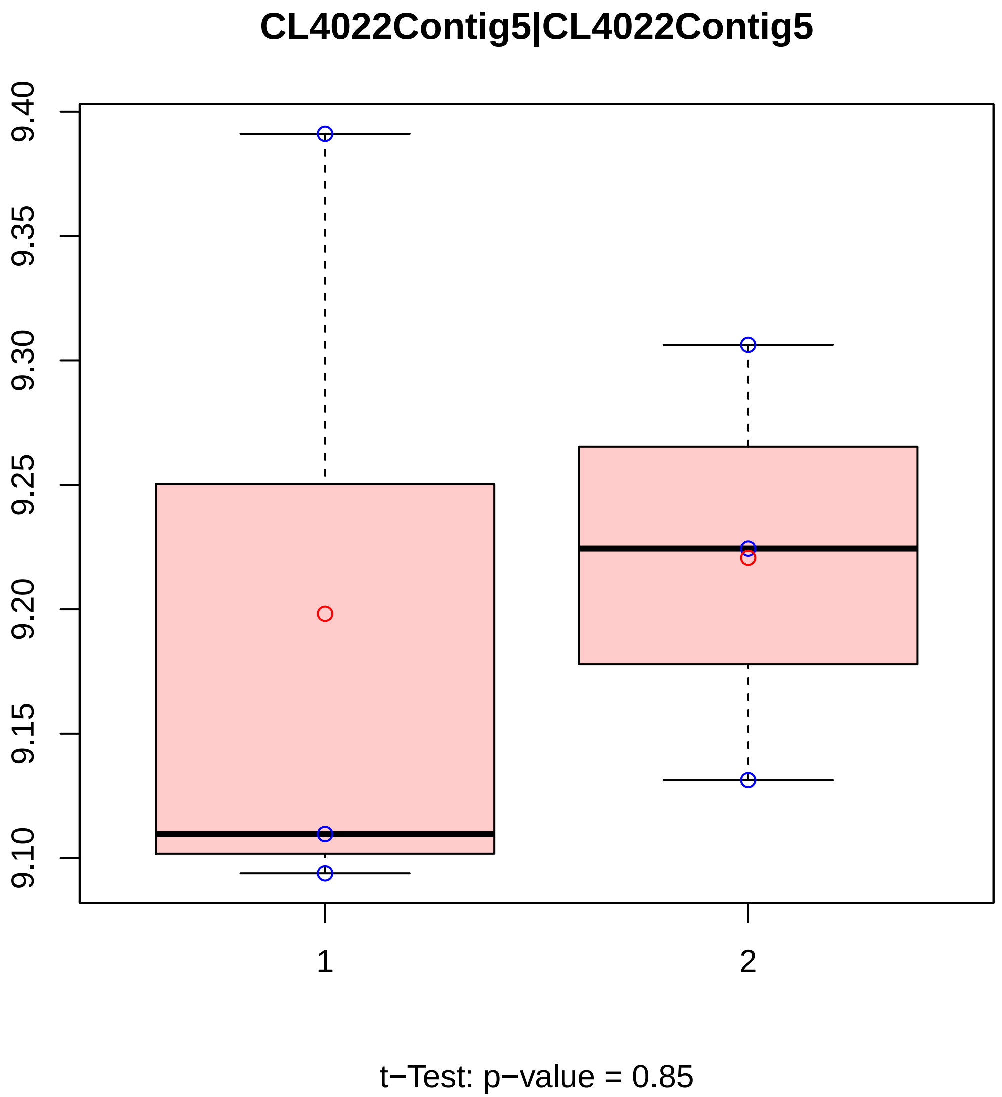

CL4022Contig5|CL4022Contig5
9.40
9.35
9.30
9.25
9.20
9.15
9.10
1
2
t−Test: p−value = 0.85

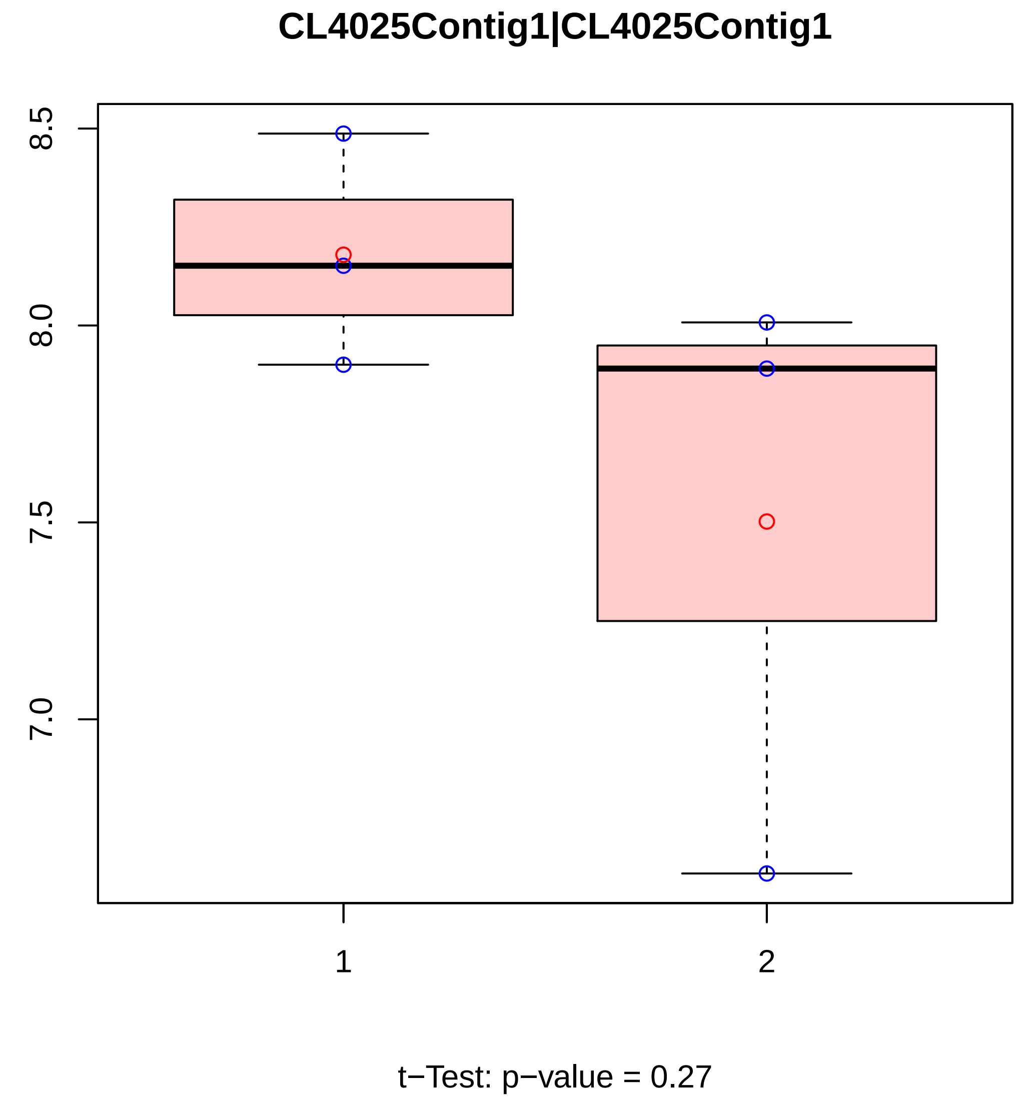
CL4025Contig1|CL4025Contig1
8.5
8.0
7.5
7.0
1
2
t−Test: p−value = 0.27

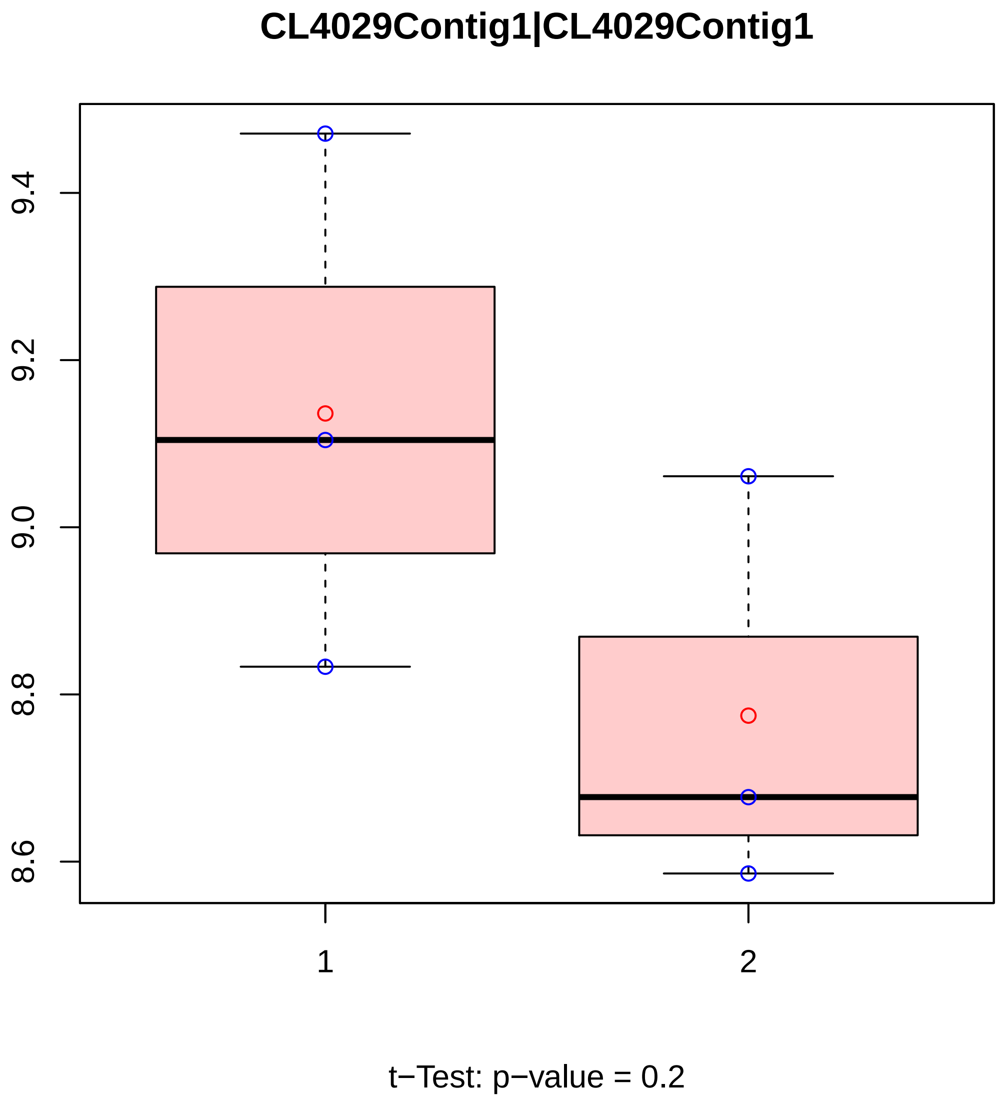
CL4029Contig1|CL4029Contig1
9.4
9.2
9.0
8.8
8.6
1
2
t−Test: p−value = 0.2

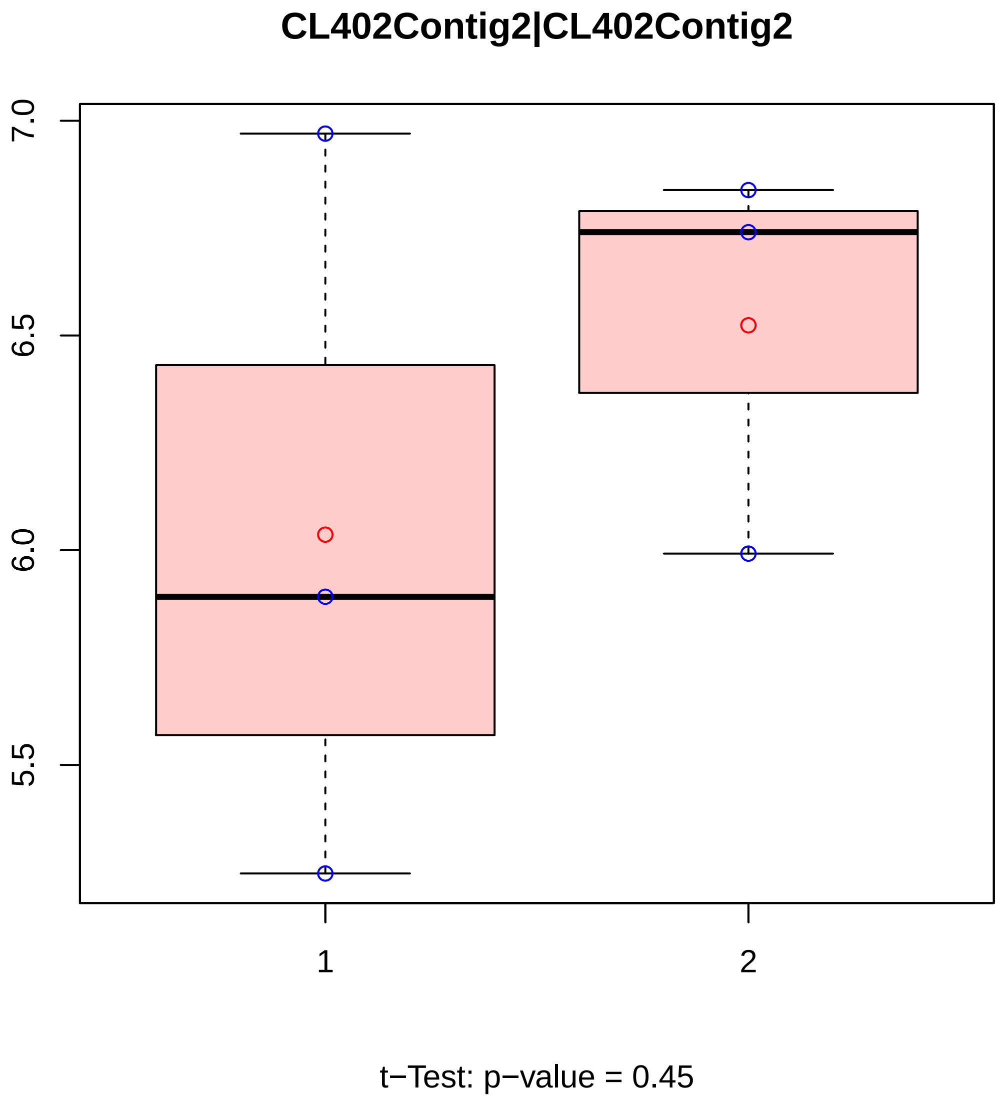
CL402Contig2|CL402Contig2
7.0
6.5
6.0
5.5
1
2
t−Test: p−value = 0.45

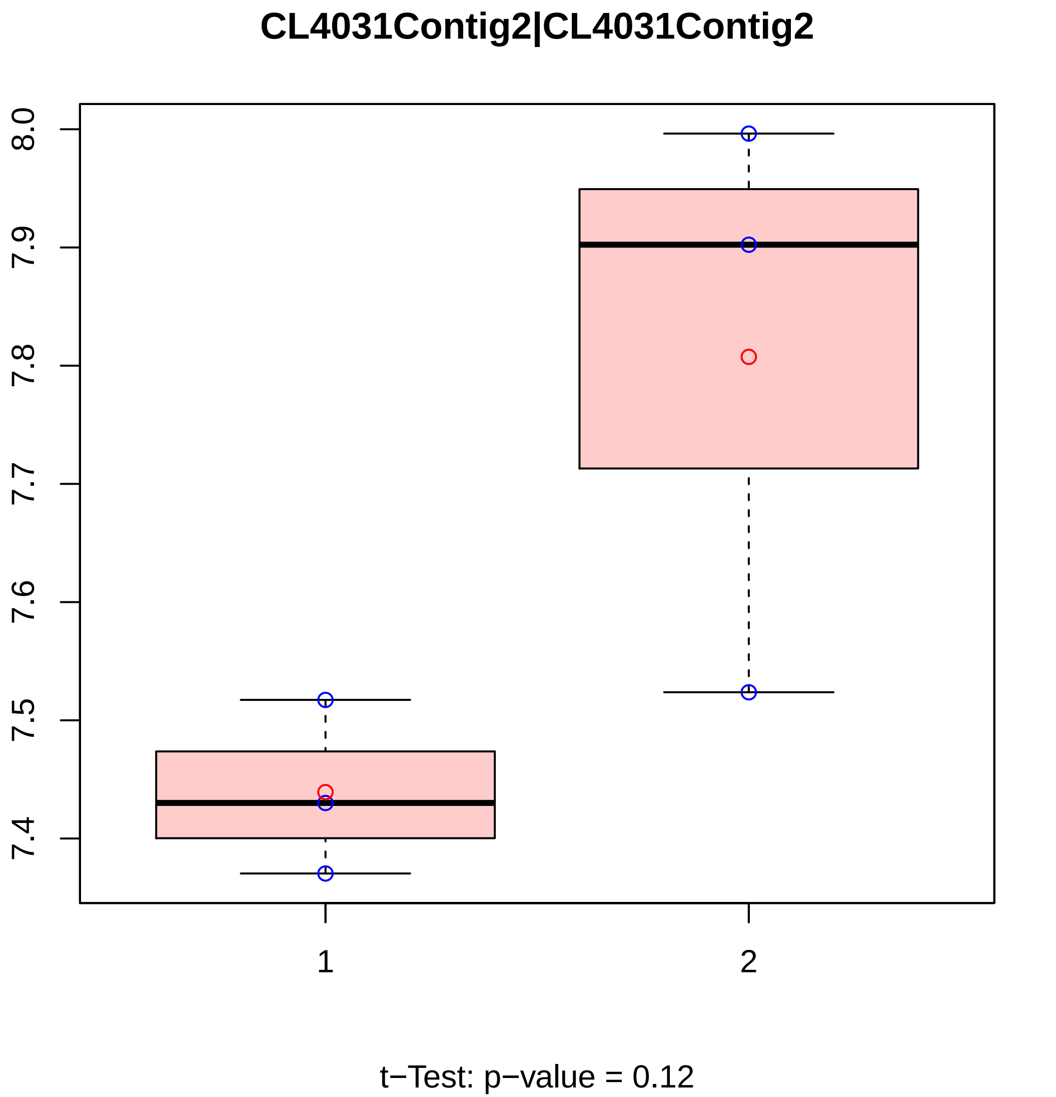

CL4031Contig2|CL4031Contig2
8.0
7.9
7.8
7.7
7.6
7.5
7.4
1
2
t−Test: p−value = 0.12

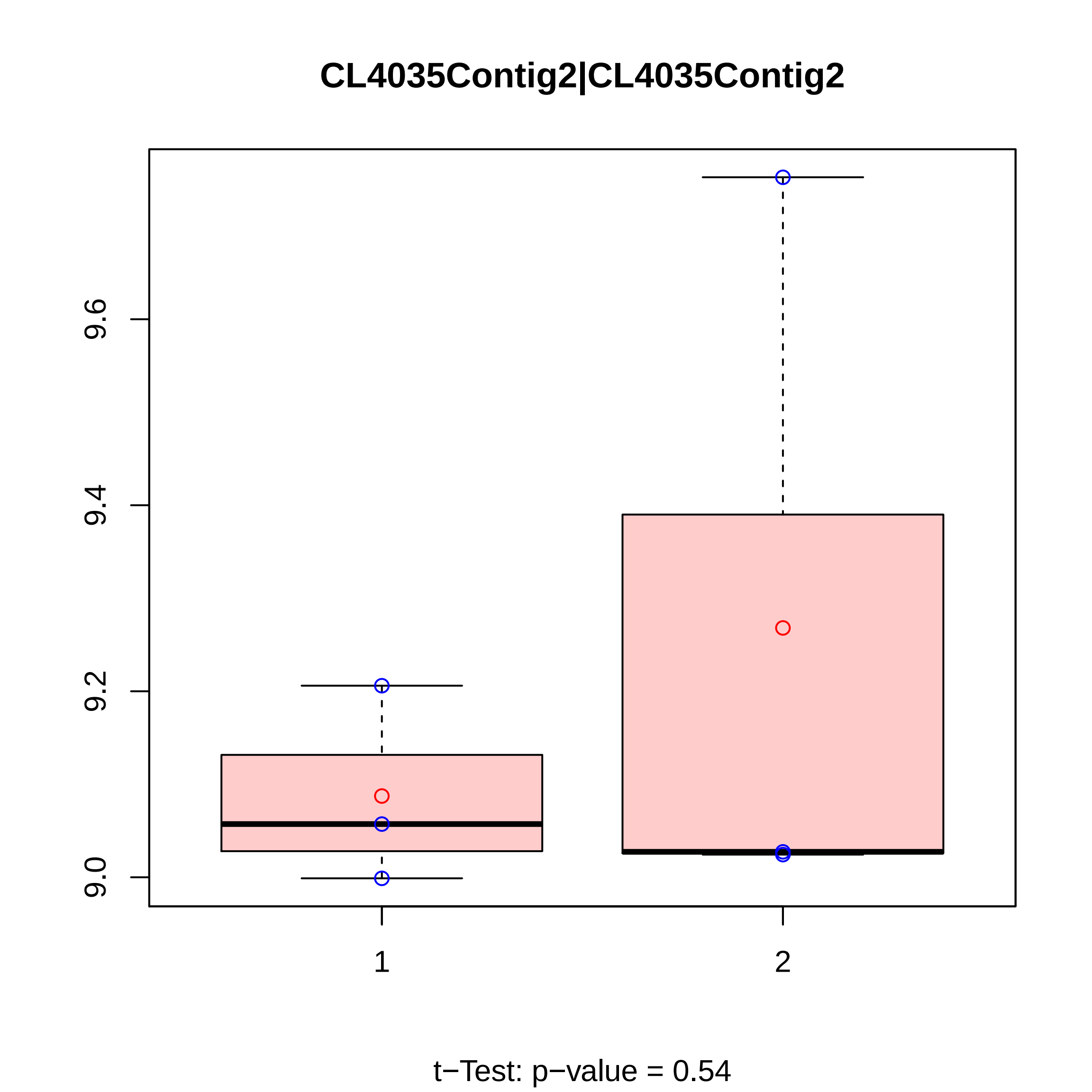

CL4035Contig2|CL4035Contig2
9.0
9.2
9.4
9.6
1
2
t−Test: p−value = 0.54

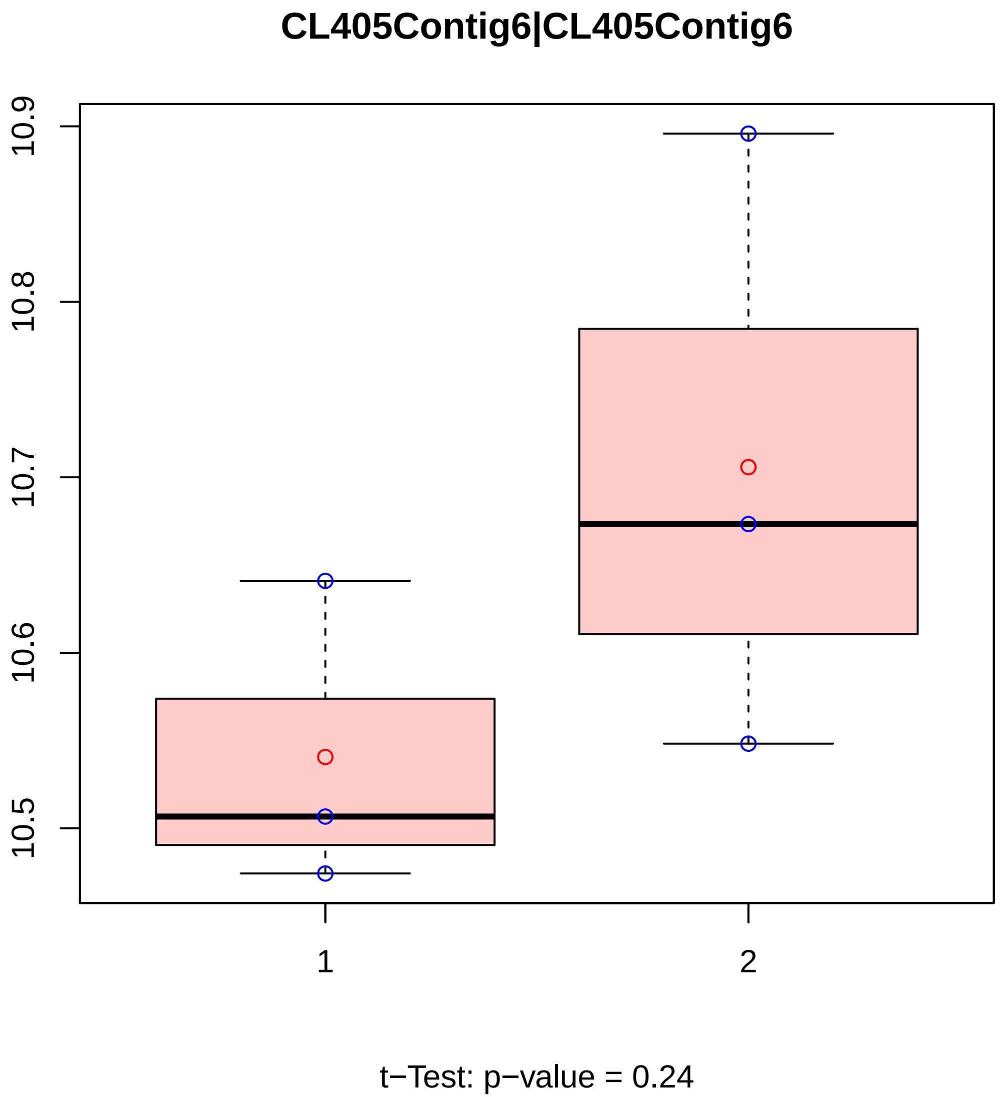
CL405Contig6|CL405Contig6
10.9
10.8
10.7
10.6
10.5
1
2
t−Test: p−value = 0.24

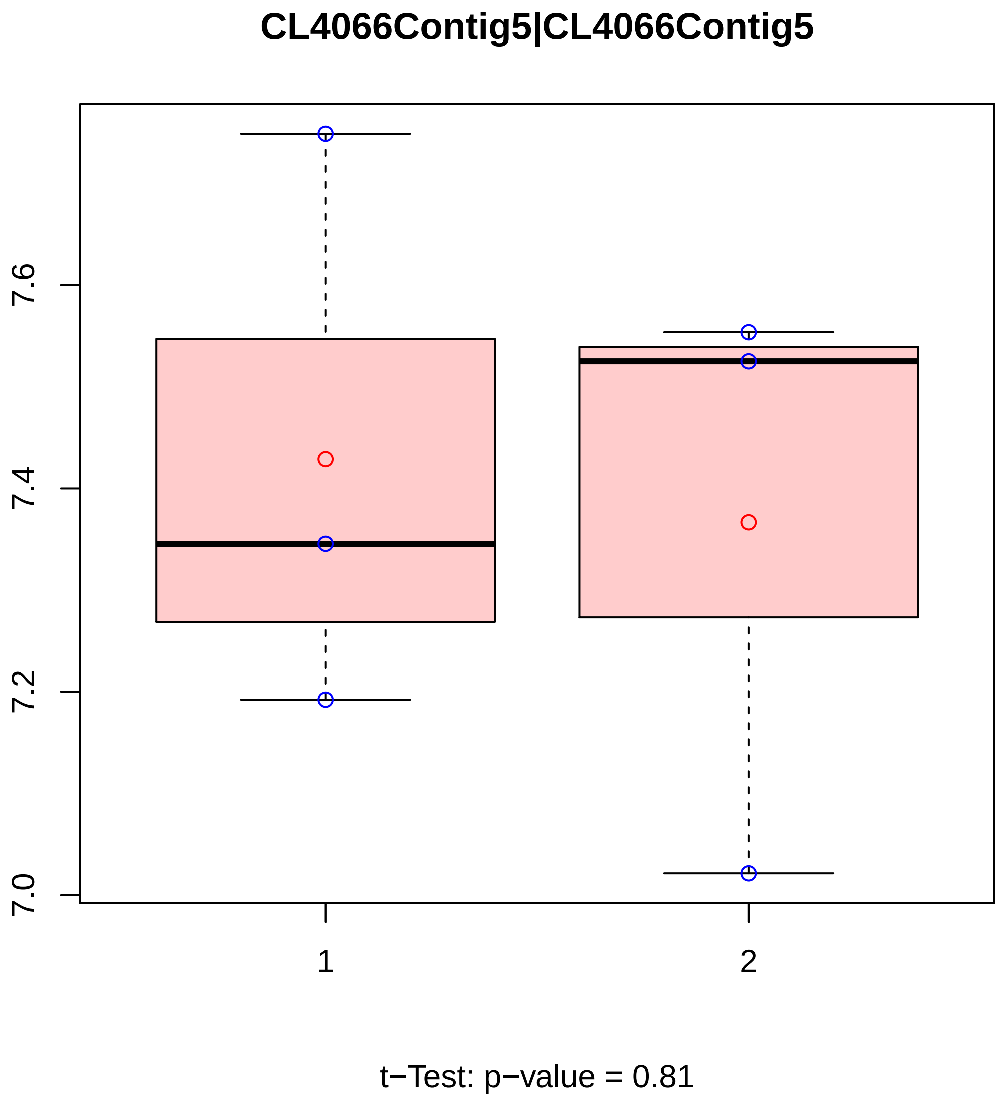
CL4066Contig5|CL4066Contig5
7.0
7.2
7.4
7.6
1
2
t−Test: p−value = 0.81

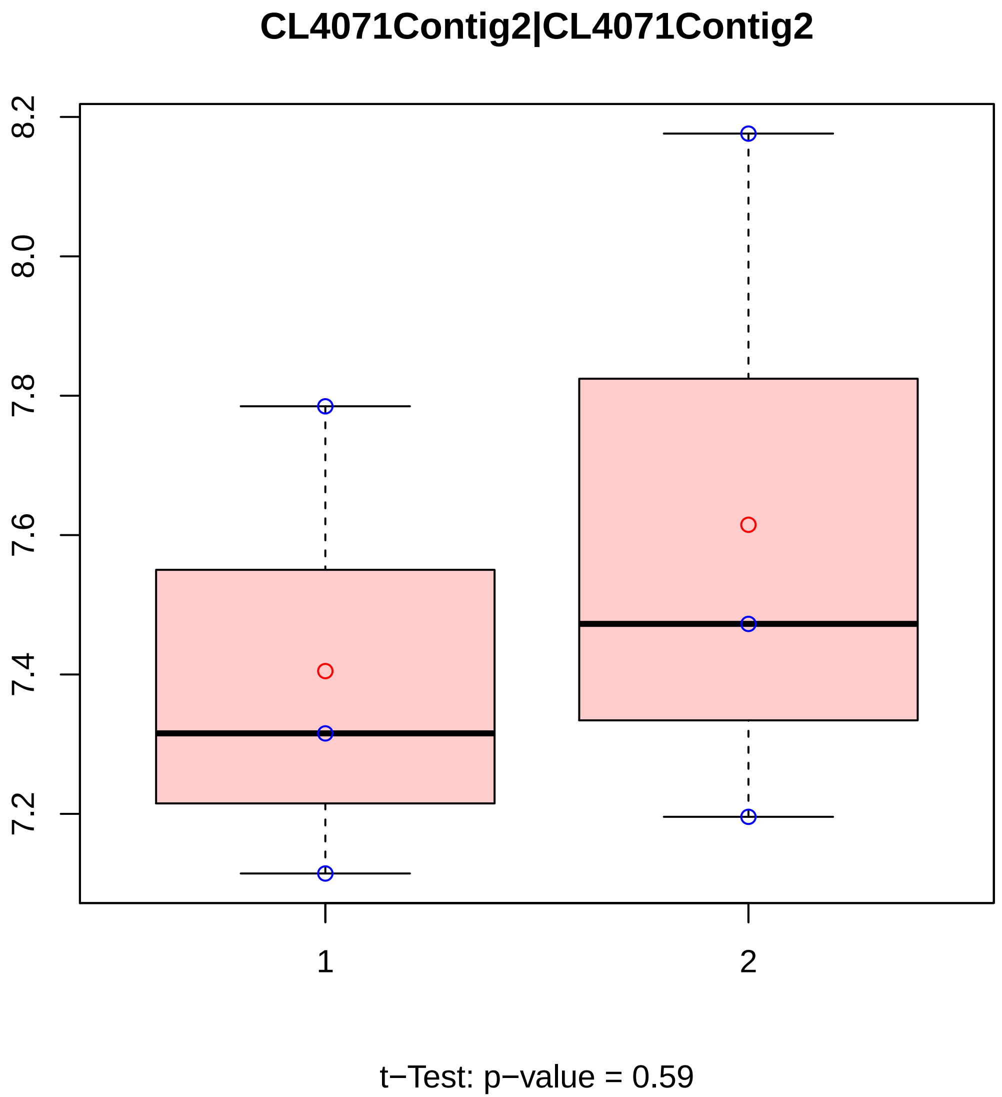

CL4071Contig2|CL4071Contig2
8.2
8.0
7.8
7.6
7.4
7.2
1
2
t−Test: p−value = 0.59

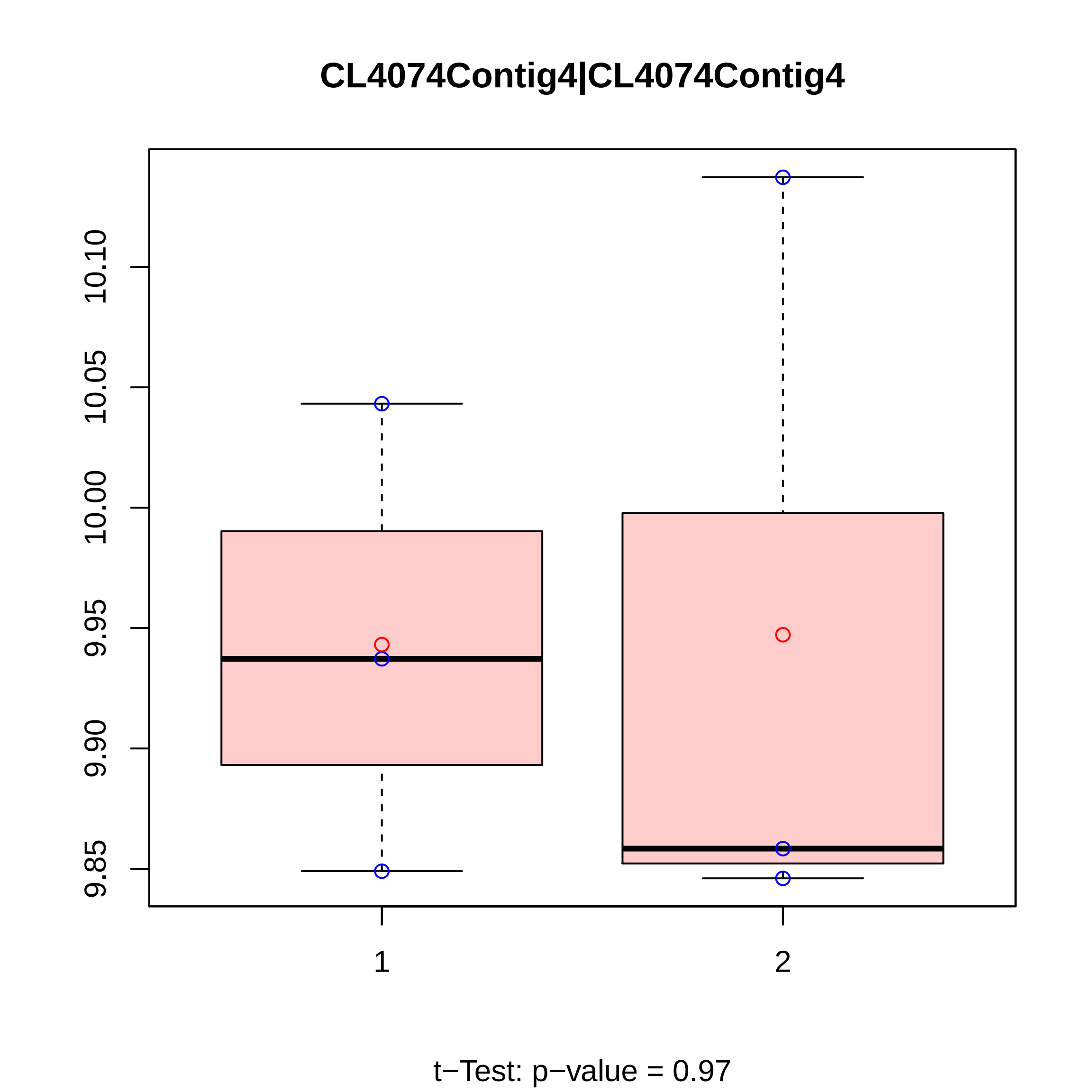
CL4074Contig4|CL4074Contig4
10.10
10.05
10.00
9.95
9.90
9.85
1
2
t−Test: p−value = 0.97

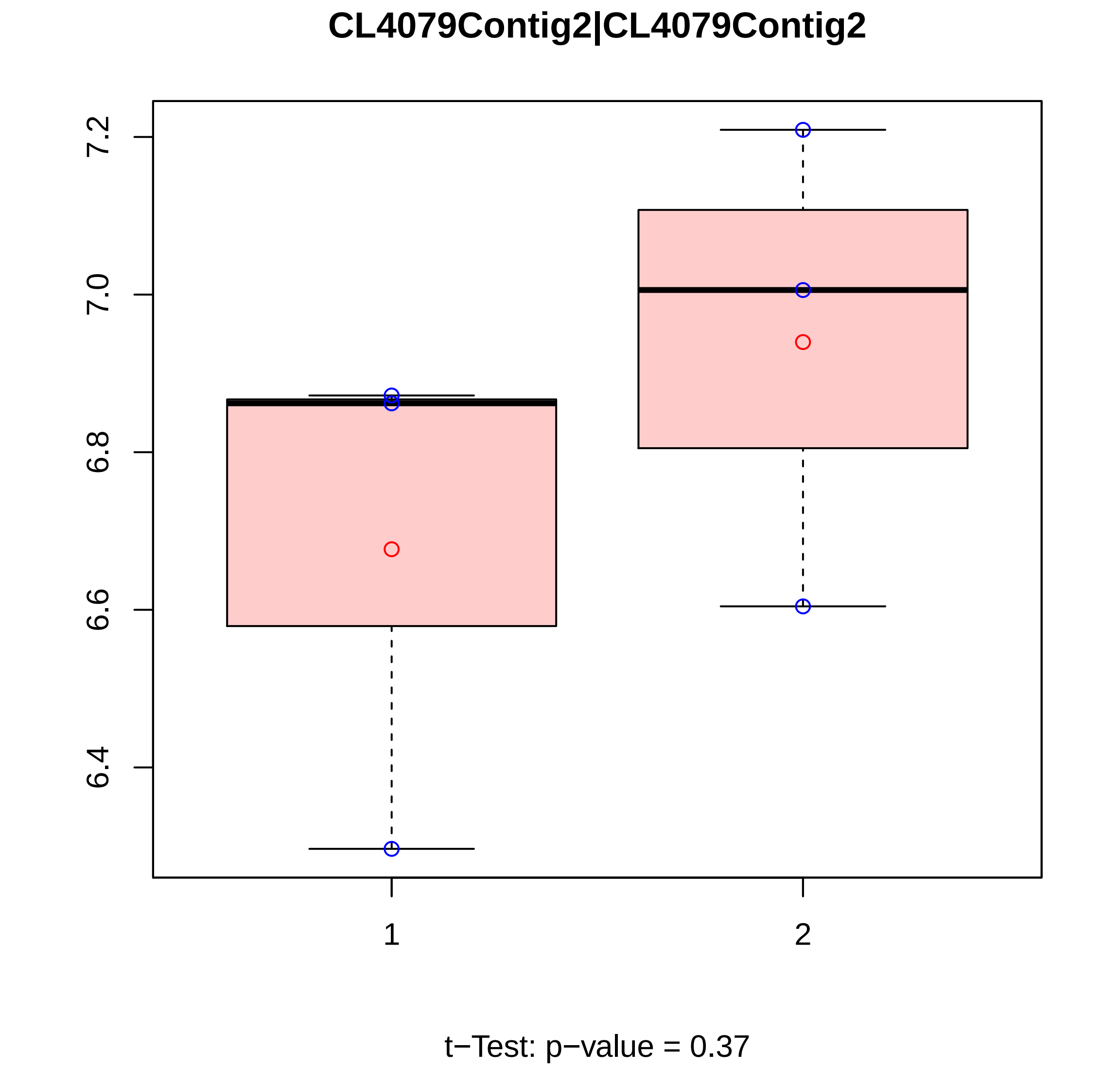
CL4079Contig2|CL4079Contig2
7.2
7.0
6.8
6.6
6.4
1
2
t−Test: p−value = 0.37

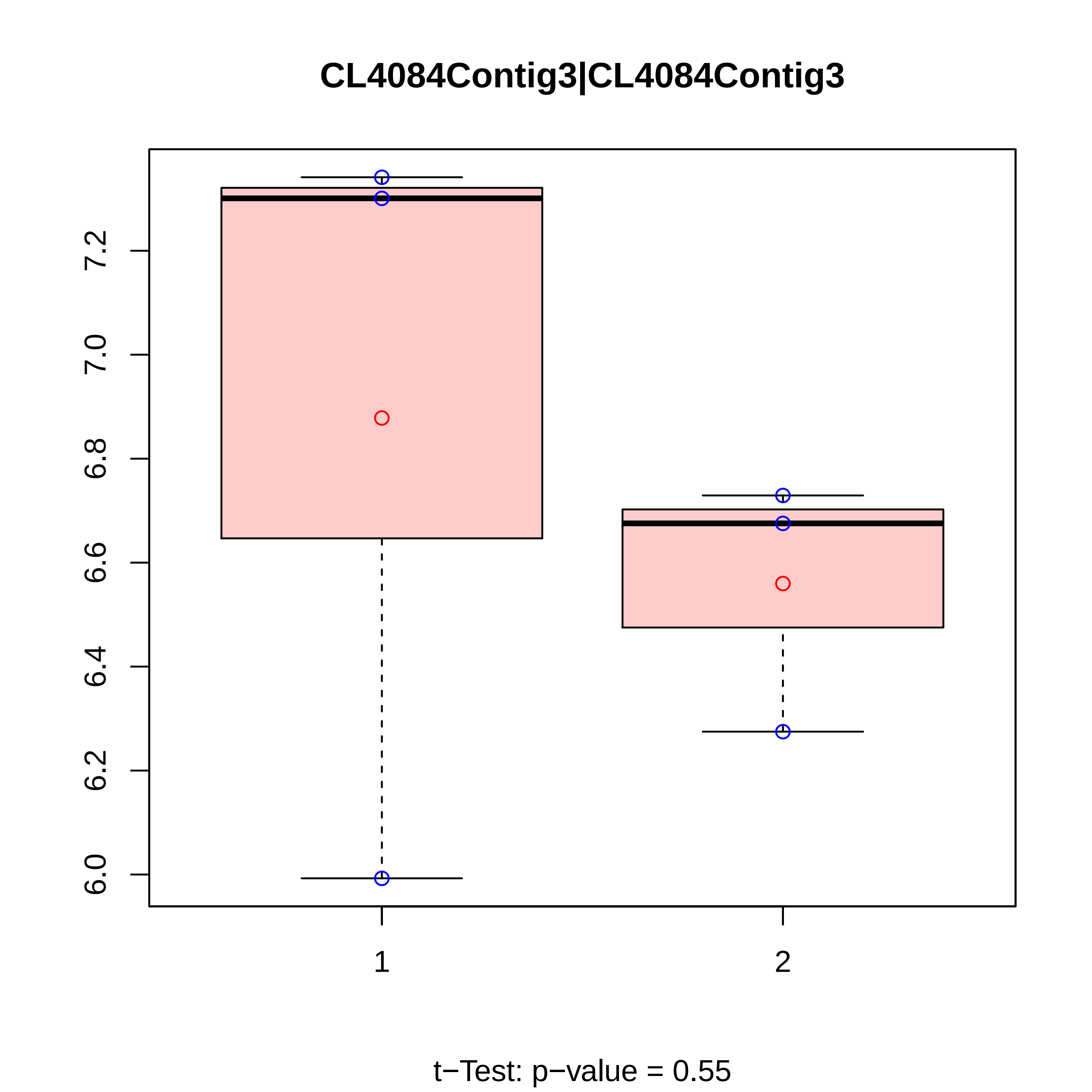
CL4084Contig3|CL4084Contig3
7.2
7.0
6.8
6.6
6.4
6.2
6.0
1
2
t−Test: p−value = 0.55

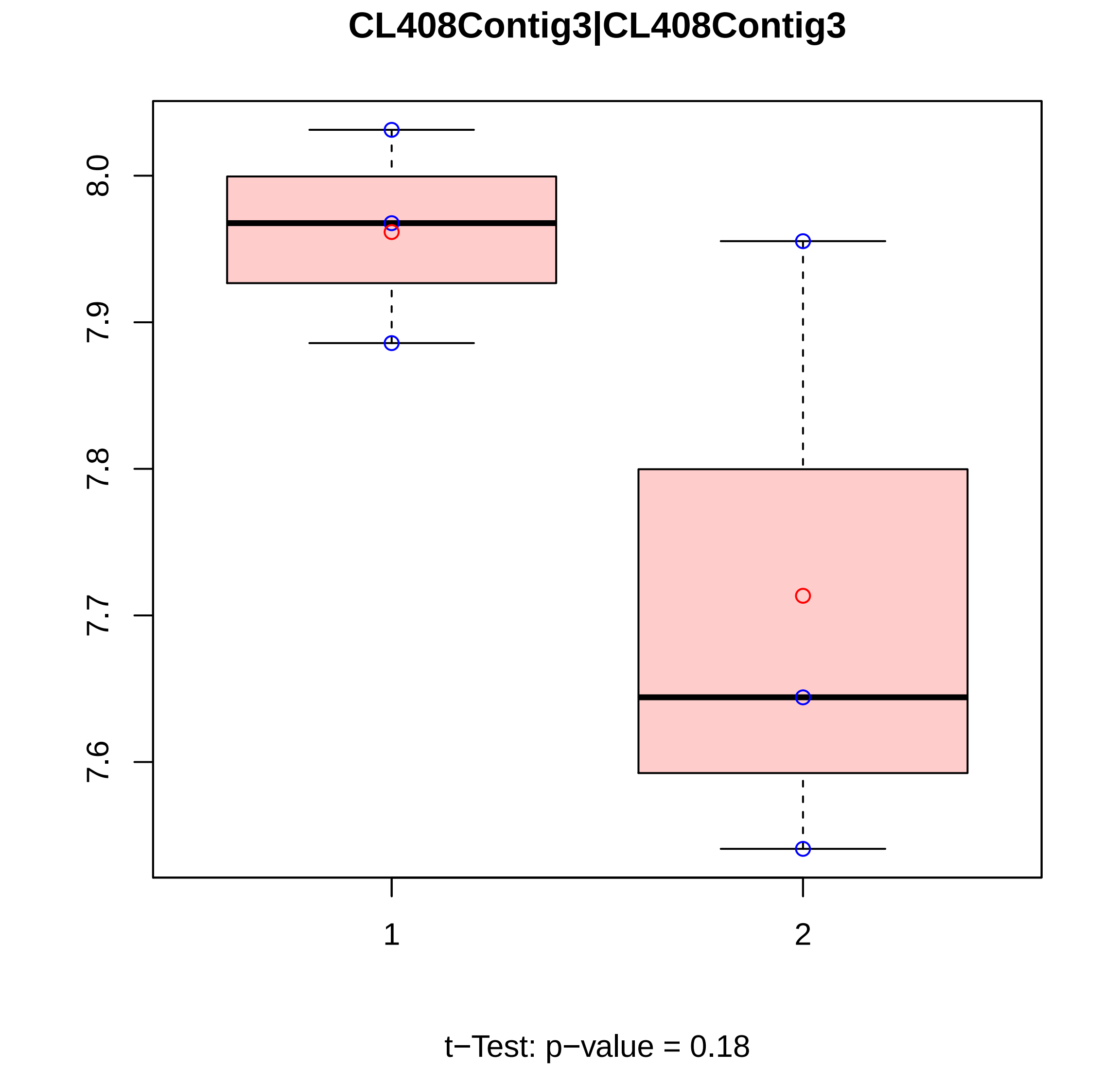
CL408Contig3|CL408Contig3
8.0
7.9
7.8
7.7
7.6
1
2
t−Test: p−value = 0.18

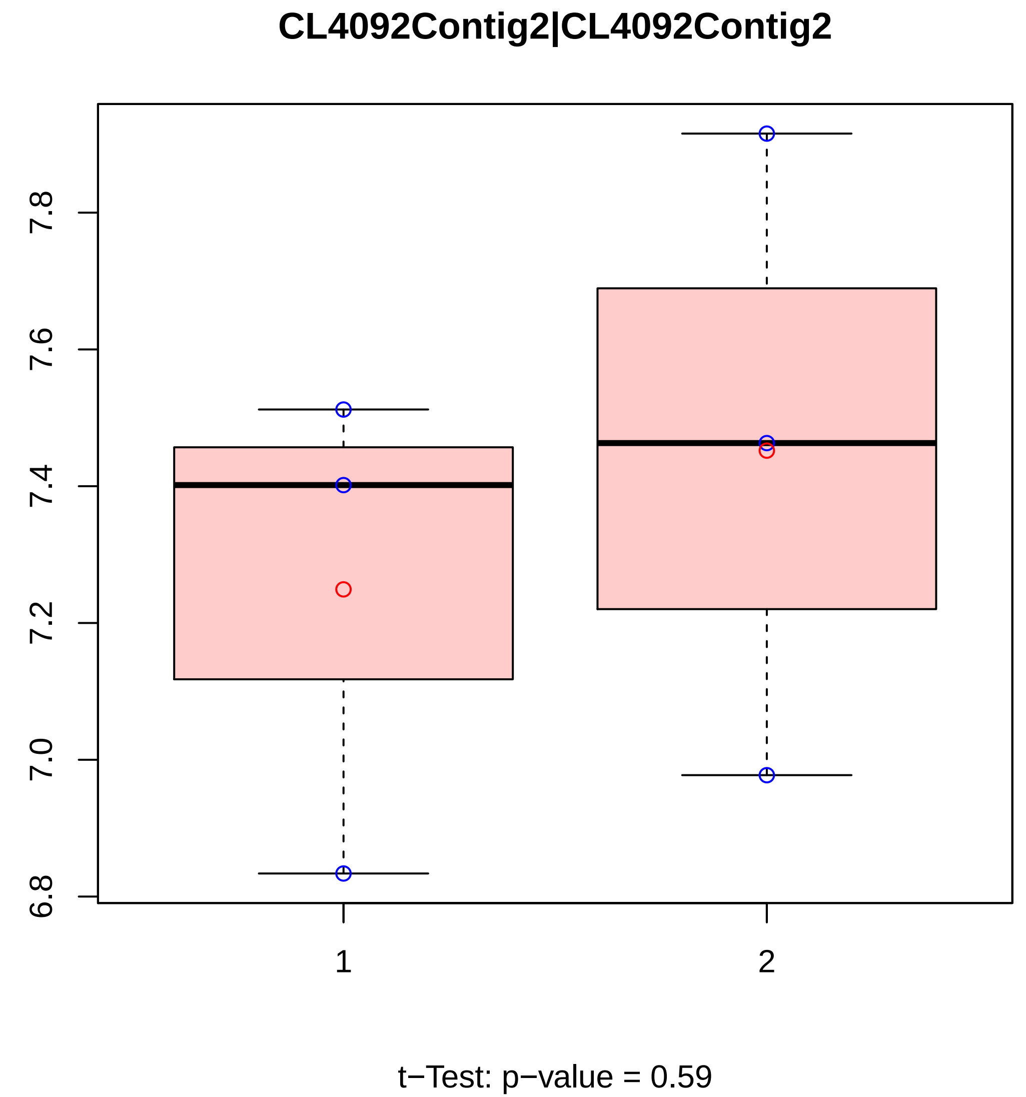
CL4092Contig2|CL4092Contig2
7.8
7.6
7.4
7.2
7.0
6.8
1
2
t−Test: p−value = 0.59

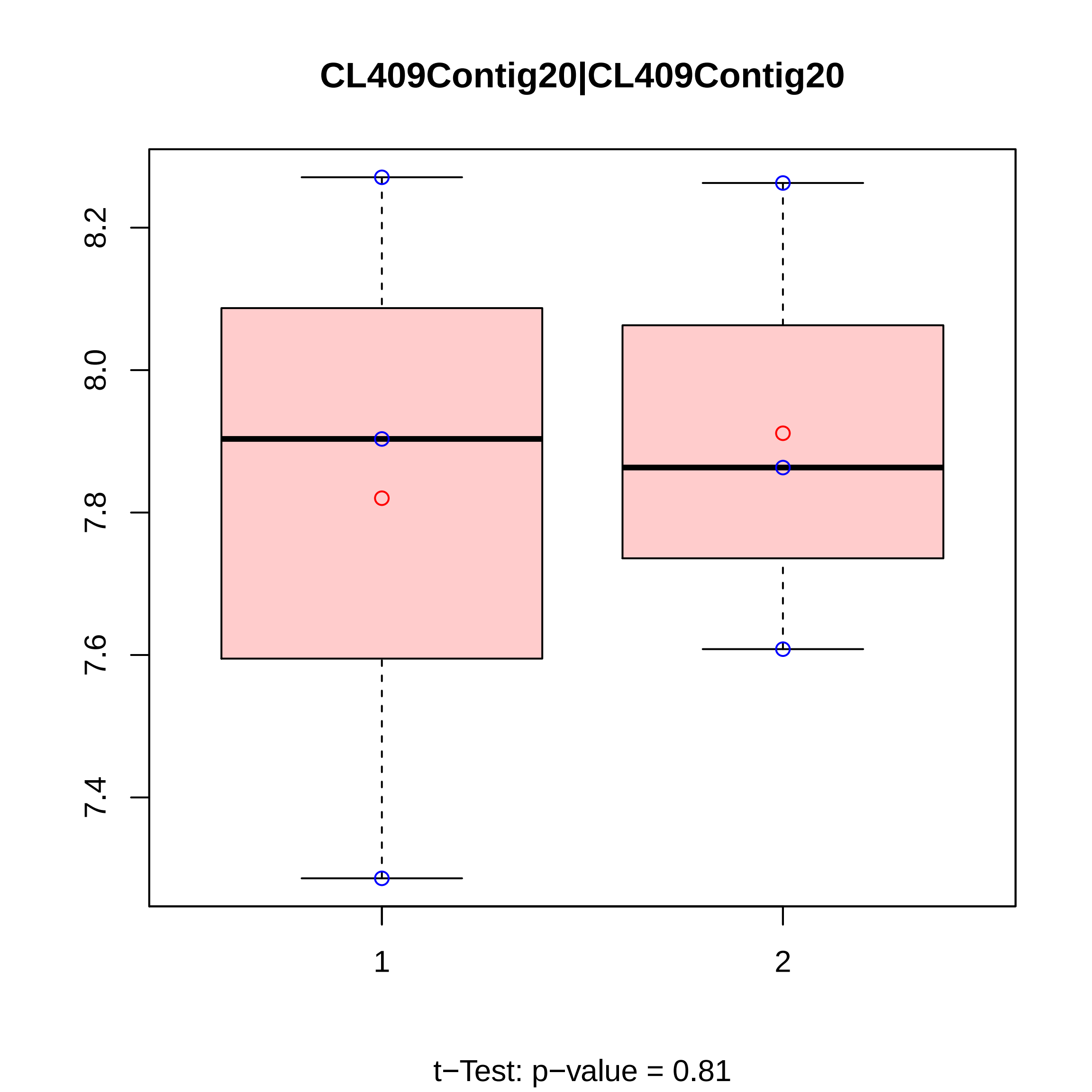
CL409Contig20|CL409Contig20
8.2
8.0
7.8
7.6
7.4
1
2
t−Test: p−value = 0.81

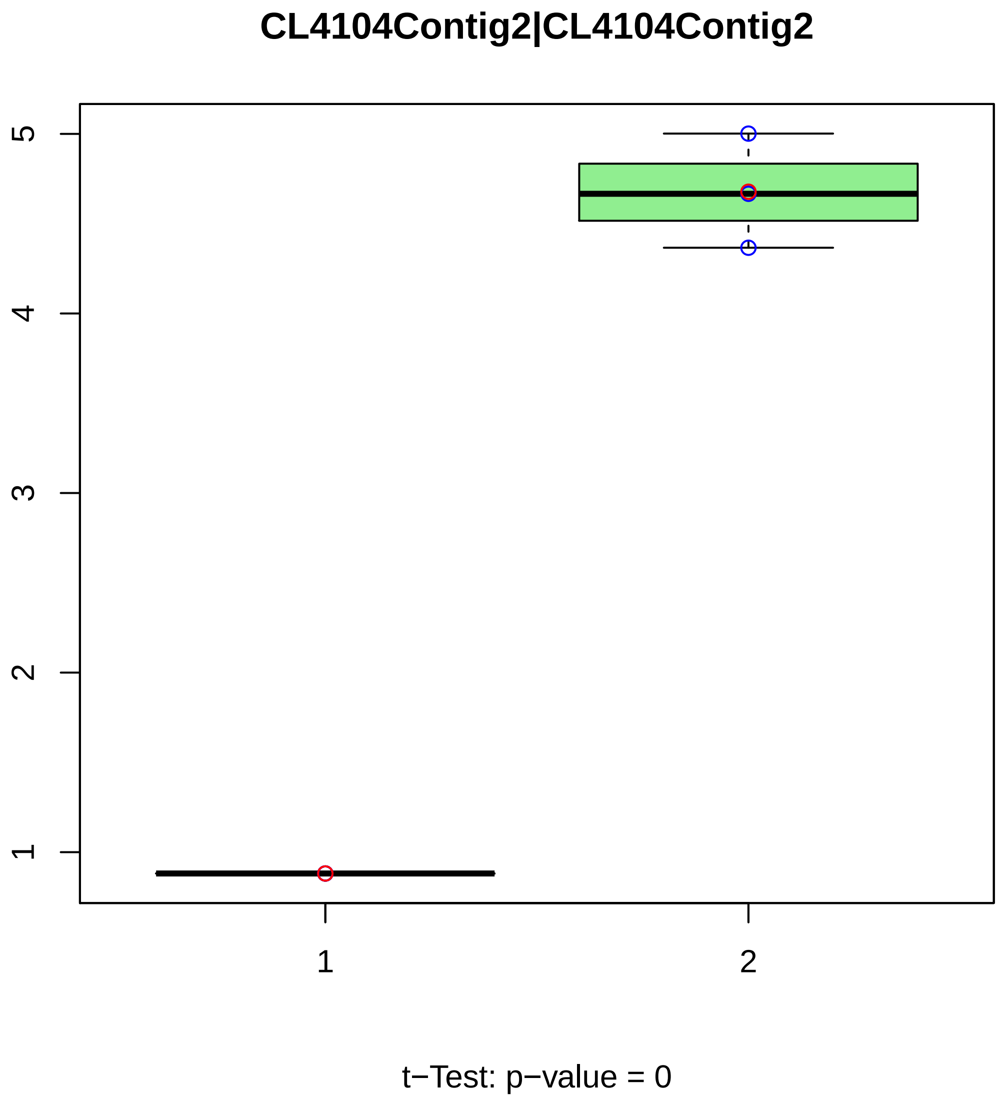
CL4104Contig2|CL4104Contig2
1
2
3
4
5
1
2
t−Test: p−value = 0

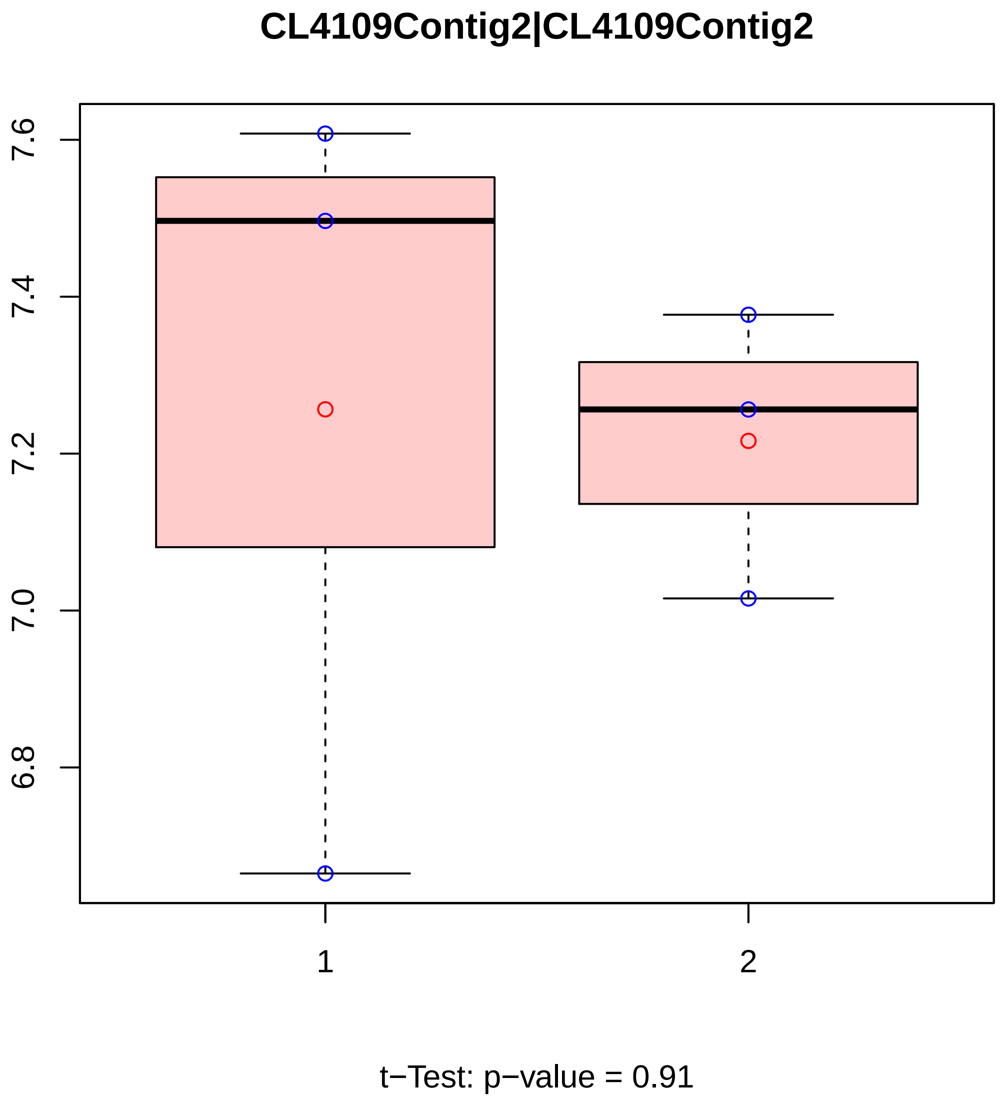
CL4109Contig2|CL4109Contig2
7.6
7.4
7.2
7.0
6.8
1
2
t−Test: p−value = 0.91

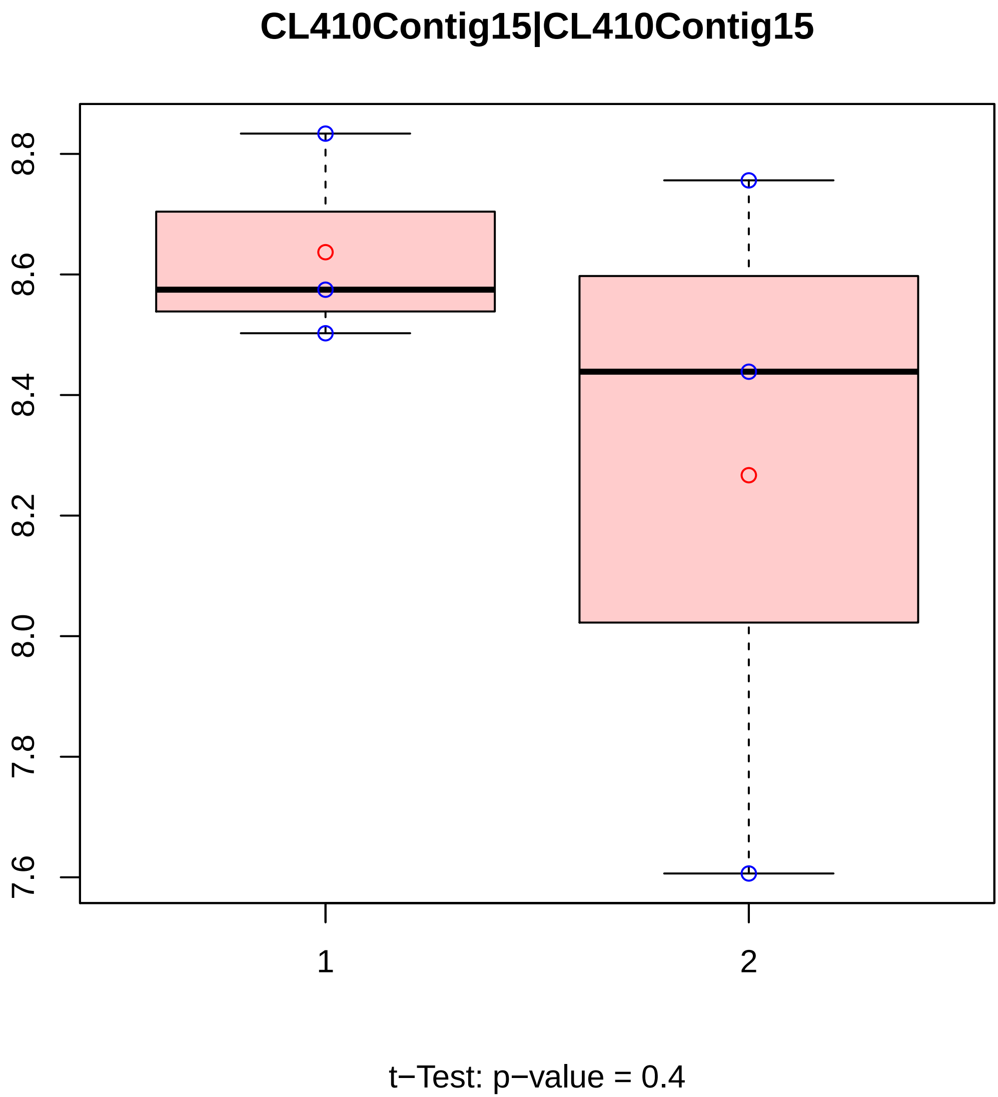

CL410Contig15|CL410Contig15
8.8
8.6
8.4
8.2
8.0
7.8
7.6
1
2
t−Test: p−value = 0.4

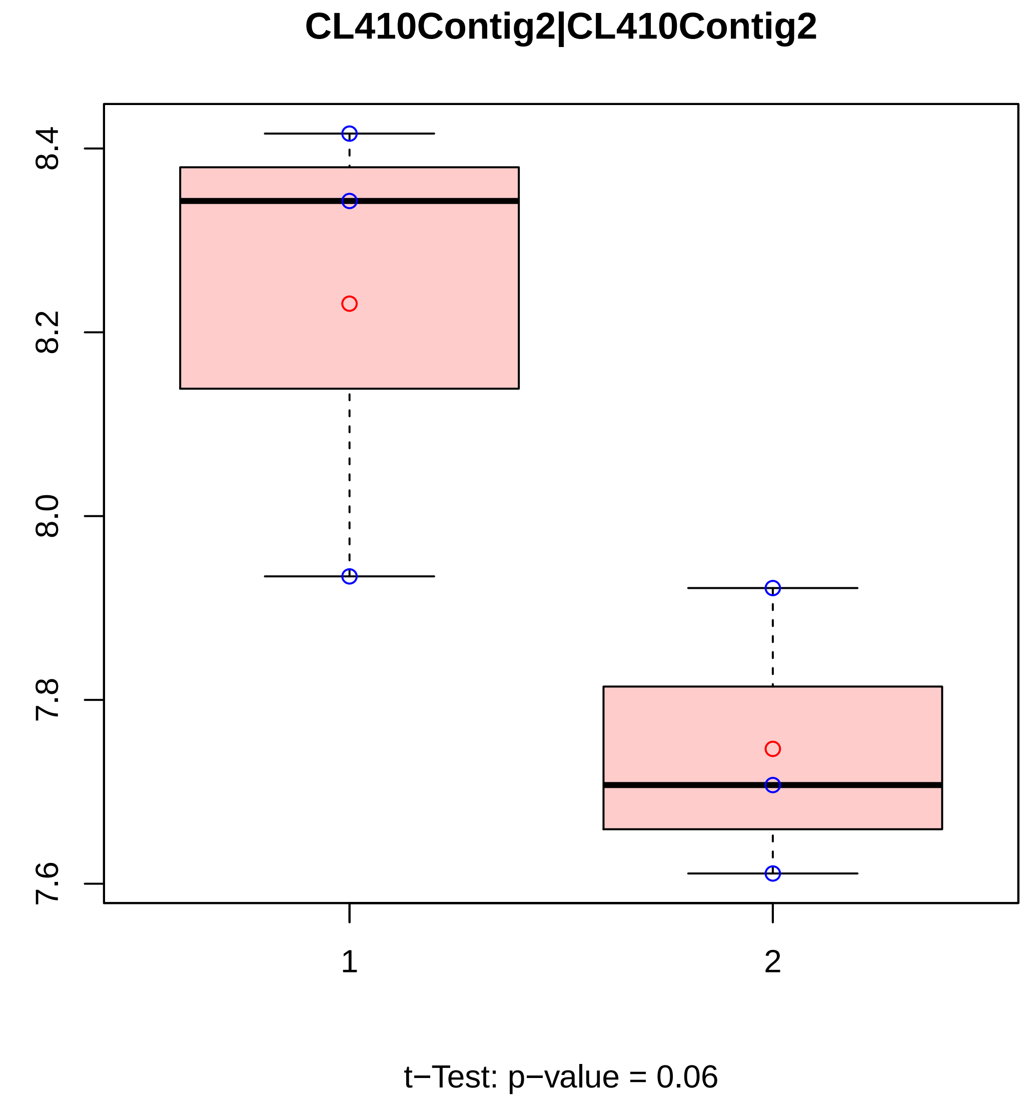
CL410Contig2|CL410Contig2
8.4
8.2
8.0
7.8
7.6
1
2
t−Test: p−value = 0.06

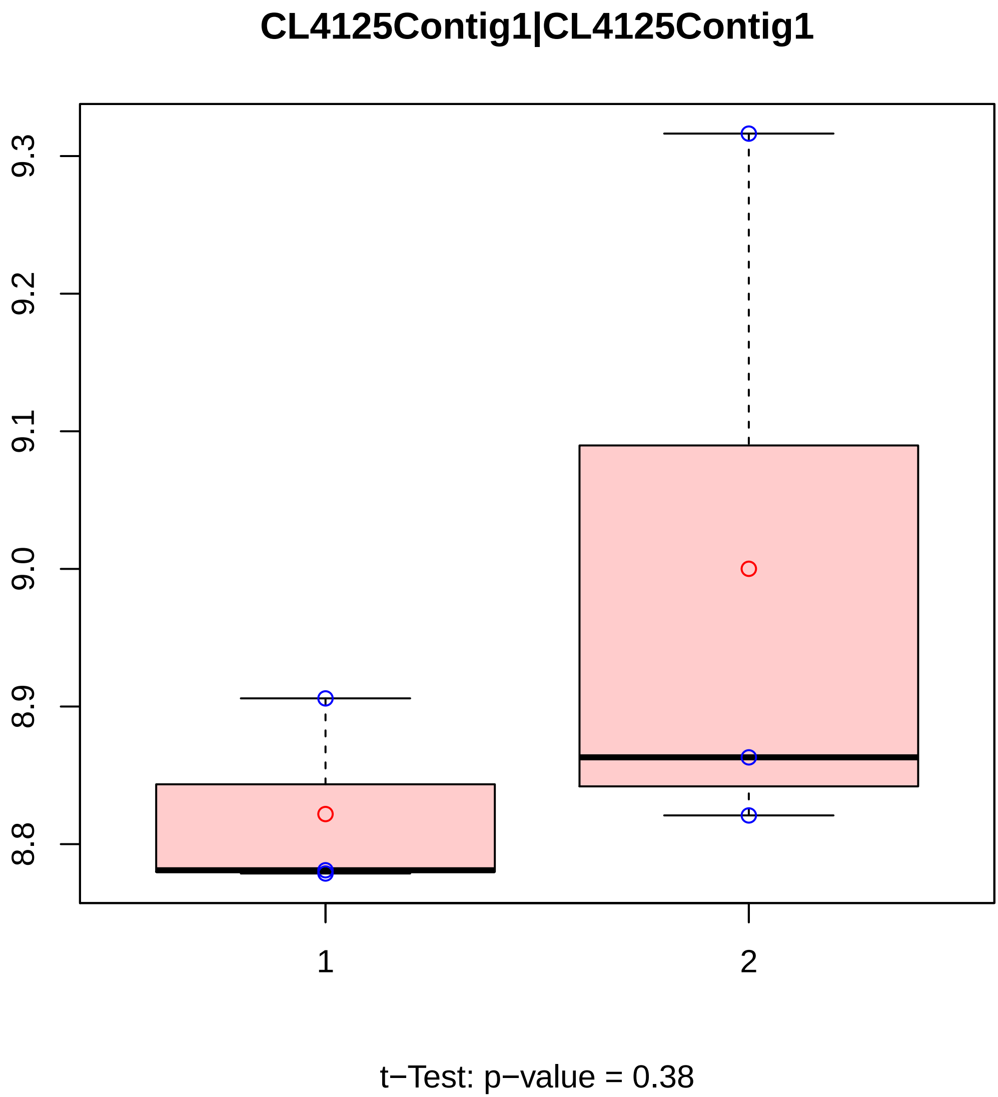
CL4125Contig1|CL4125Contig1
9.3
9.2
9.1
9.0
8.9
8.8
1
2
t−Test: p−value = 0.38

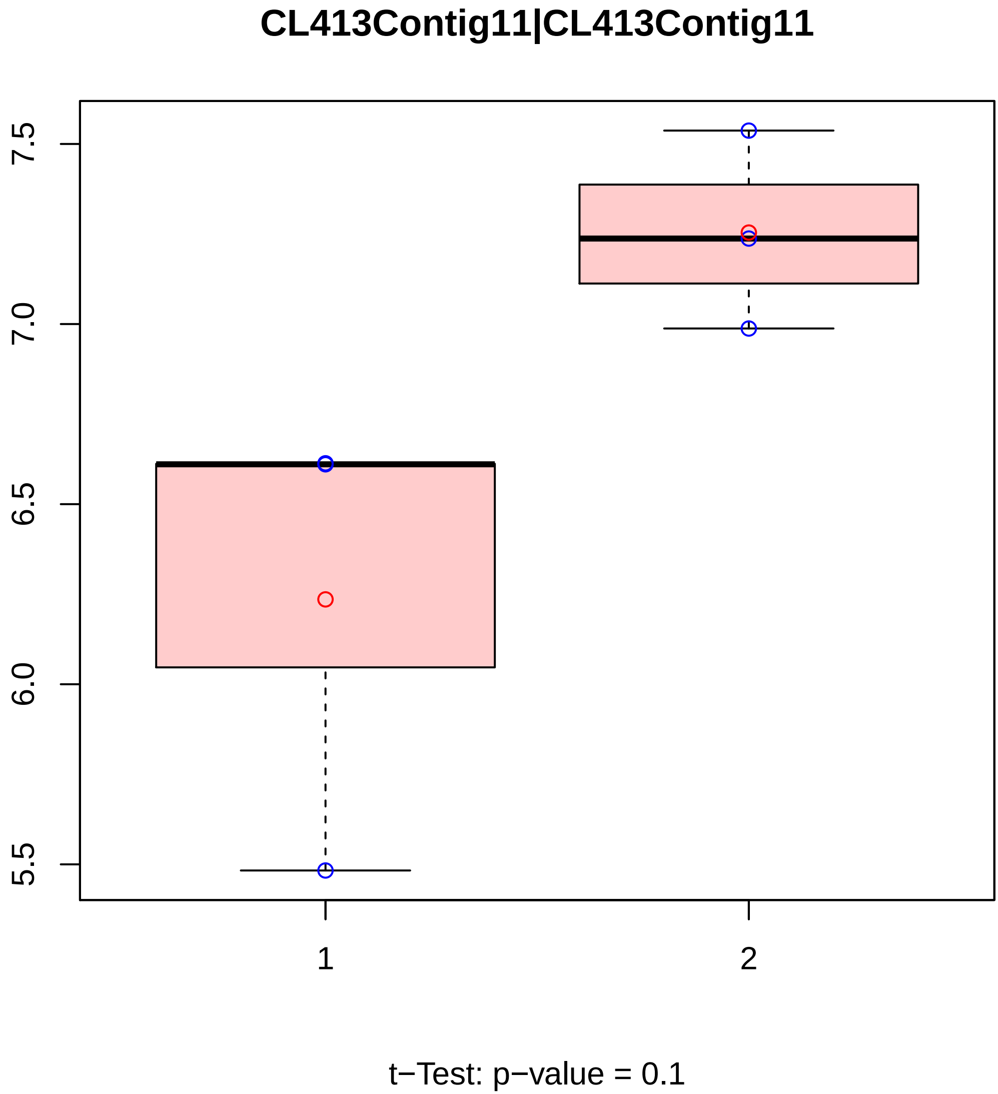
CL413Contig11|CL413Contig11
7.5
7.0
6.5
6.0
5.5
1
2
t−Test: p−value = 0.1

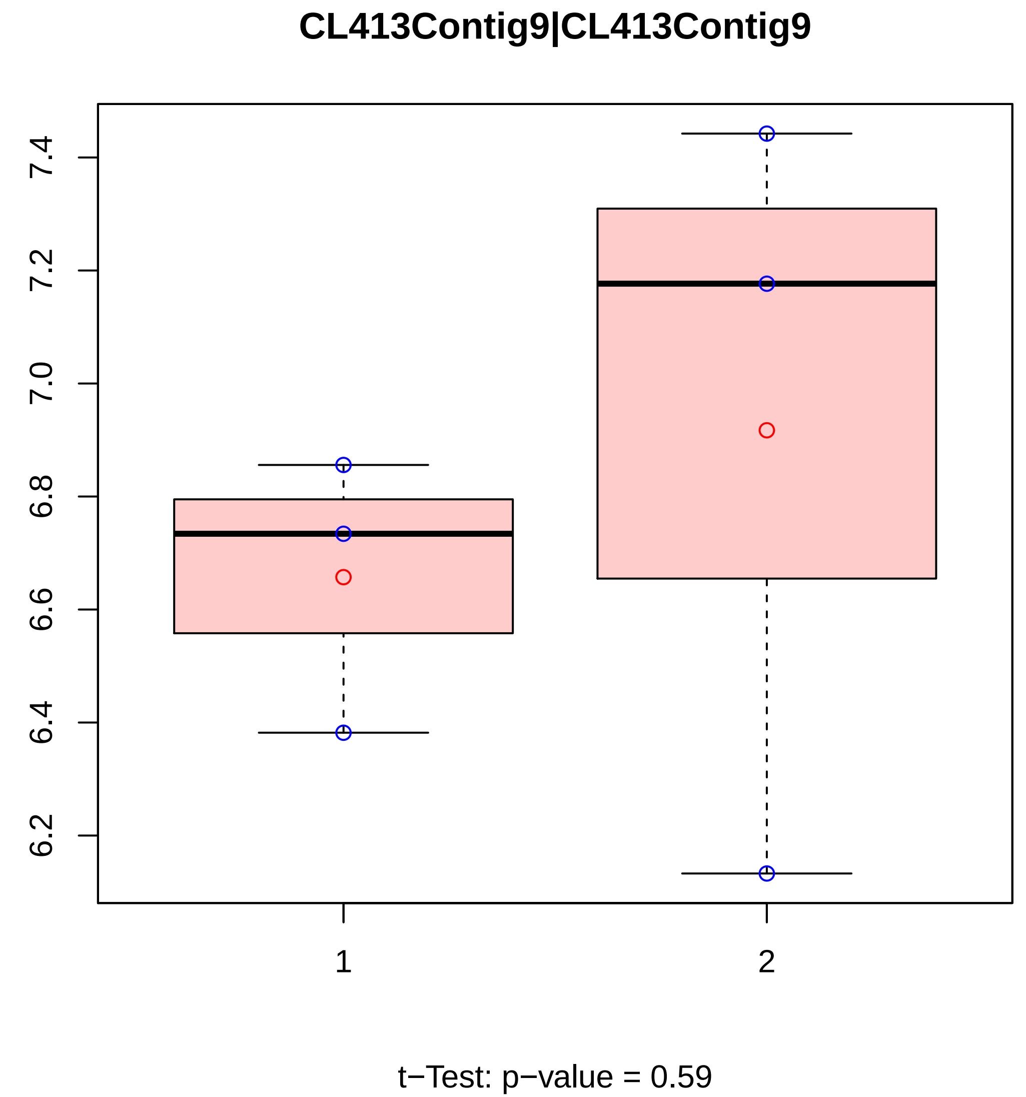
CL413Contig9|CL413Contig9
7.4
7.2
7.0
6.8
6.6
6.4
6.2
1
2
t−Test: p−value = 0.59

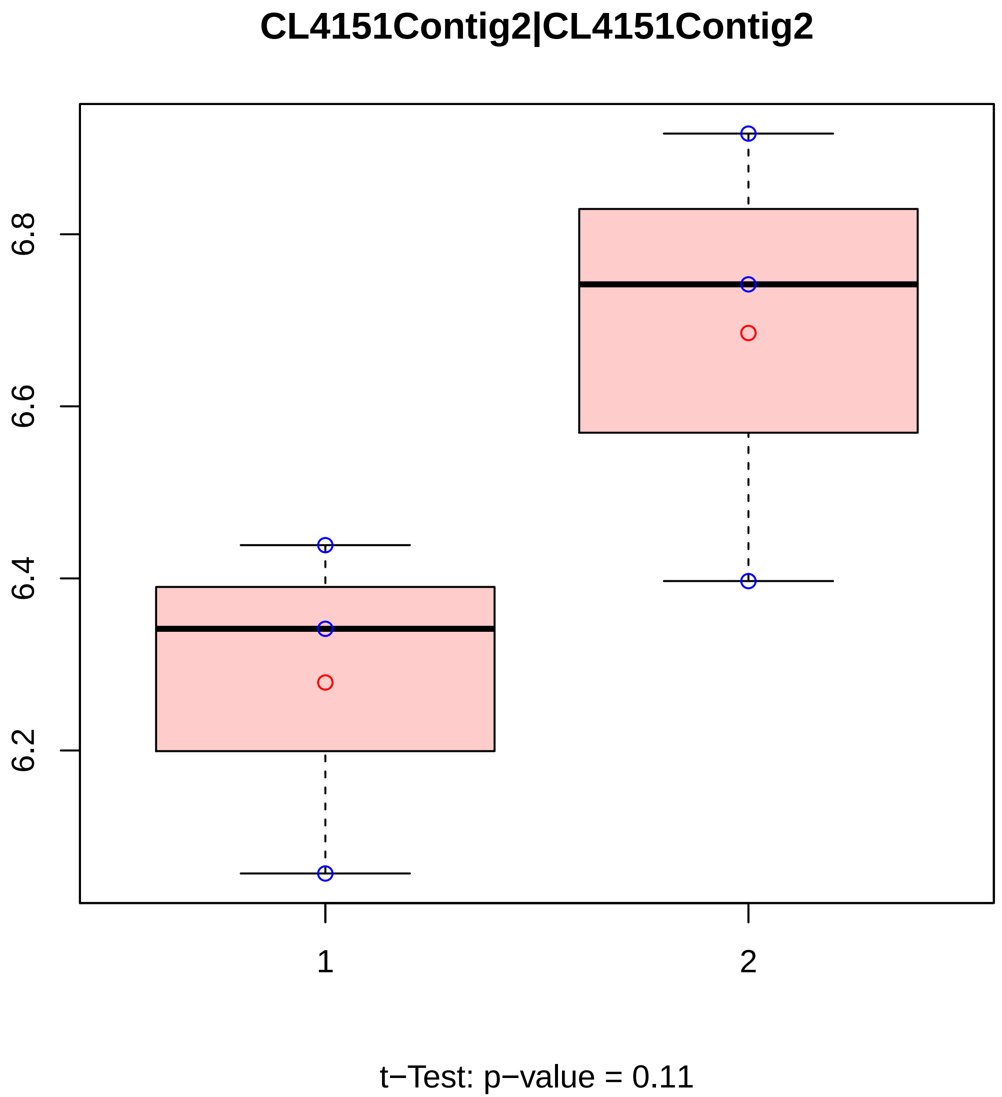
CL4151Contig2|CL4151Contig2
6.2
6.4
6.6
6.8
1
2
t−Test: p−value = 0.11

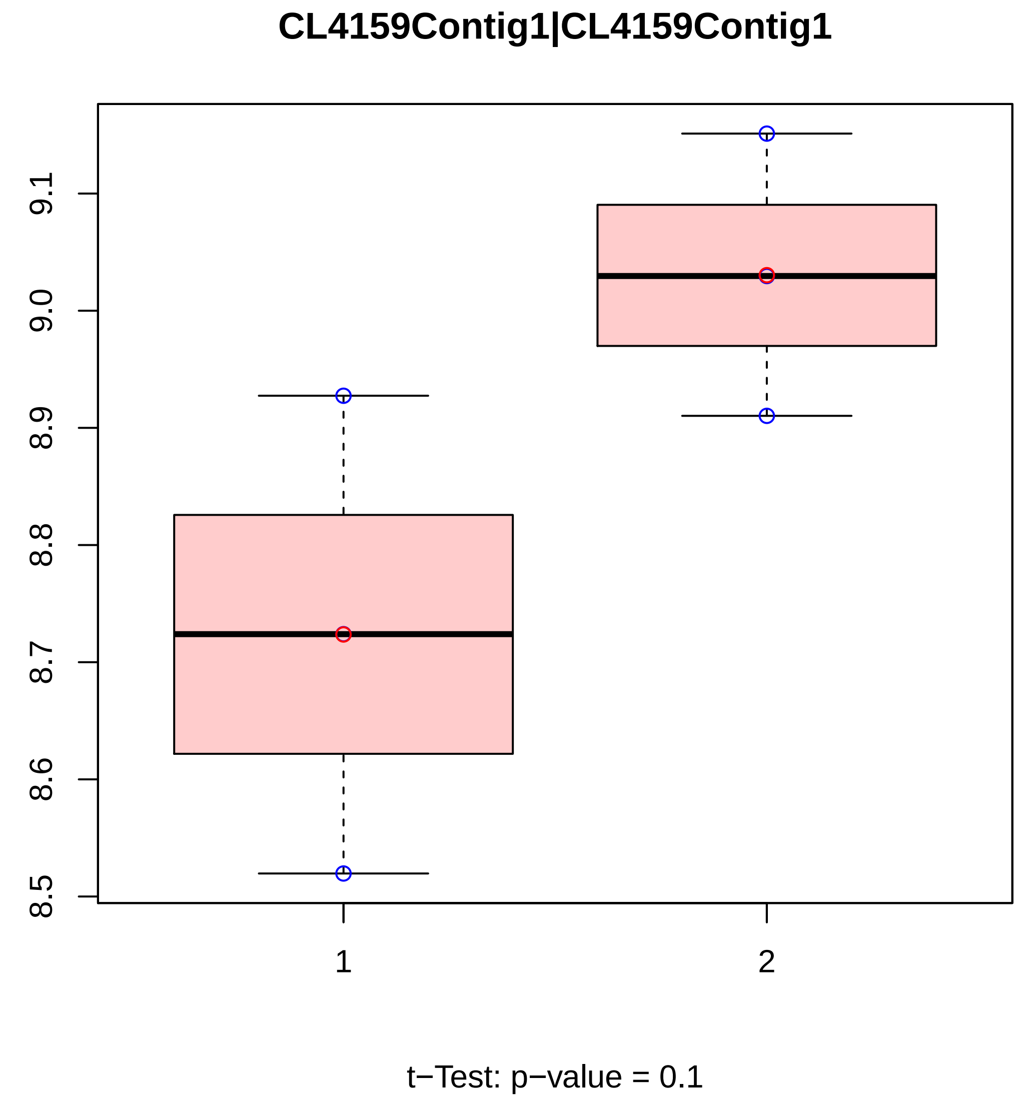

CL4159Contig1|CL4159Contig1
9.1
9.0
8.9
8.8
8.7
8.6
8.5
1
2
t−Test: p−value = 0.1

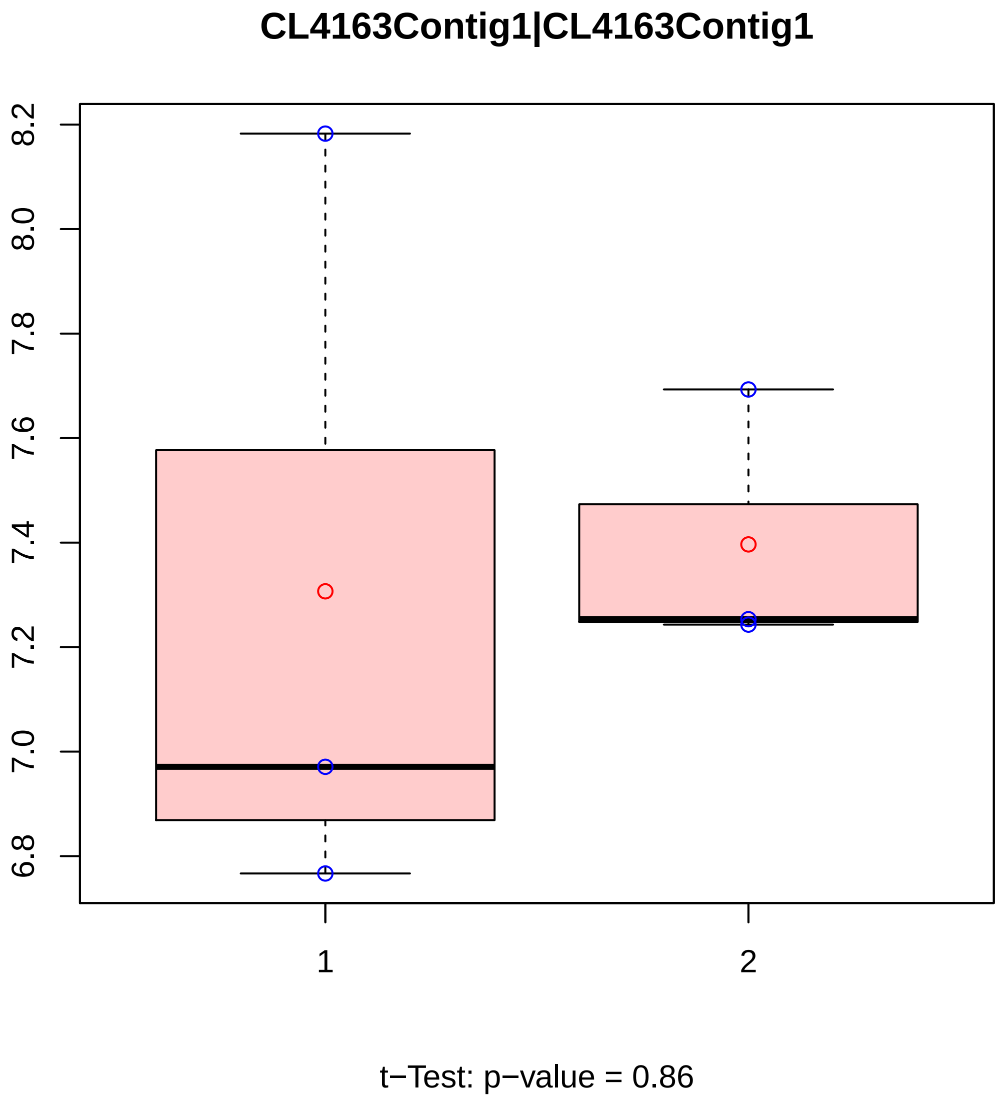
CL4163Contig1|CL4163Contig1
6.8
7.0
7.2
7.4
7.6
7.8
8.0
8.2
1
2
t−Test: p−value = 0.86

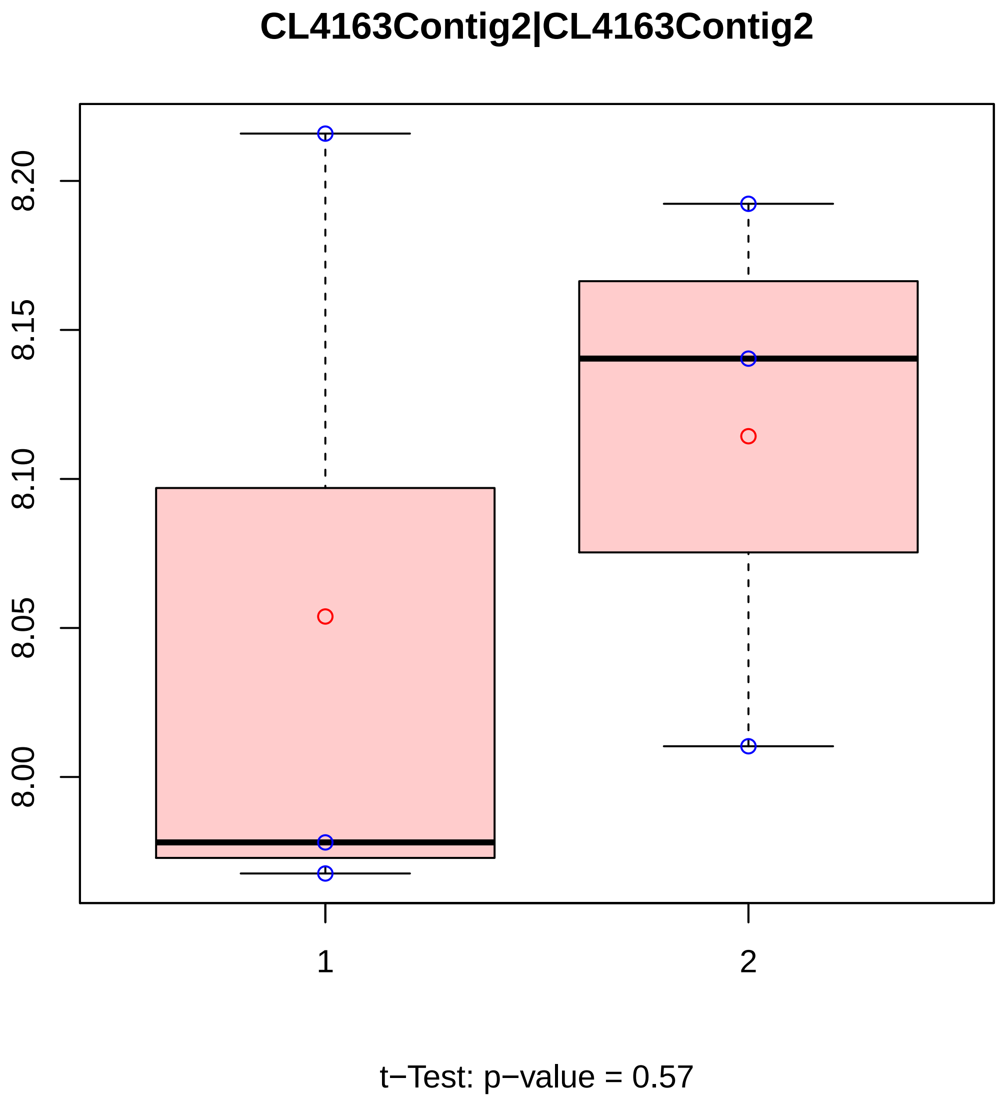
CL4163Contig2|CL4163Contig2
8.20
8.15
8.10
8.05
8.00
1
2
t−Test: p−value = 0.57

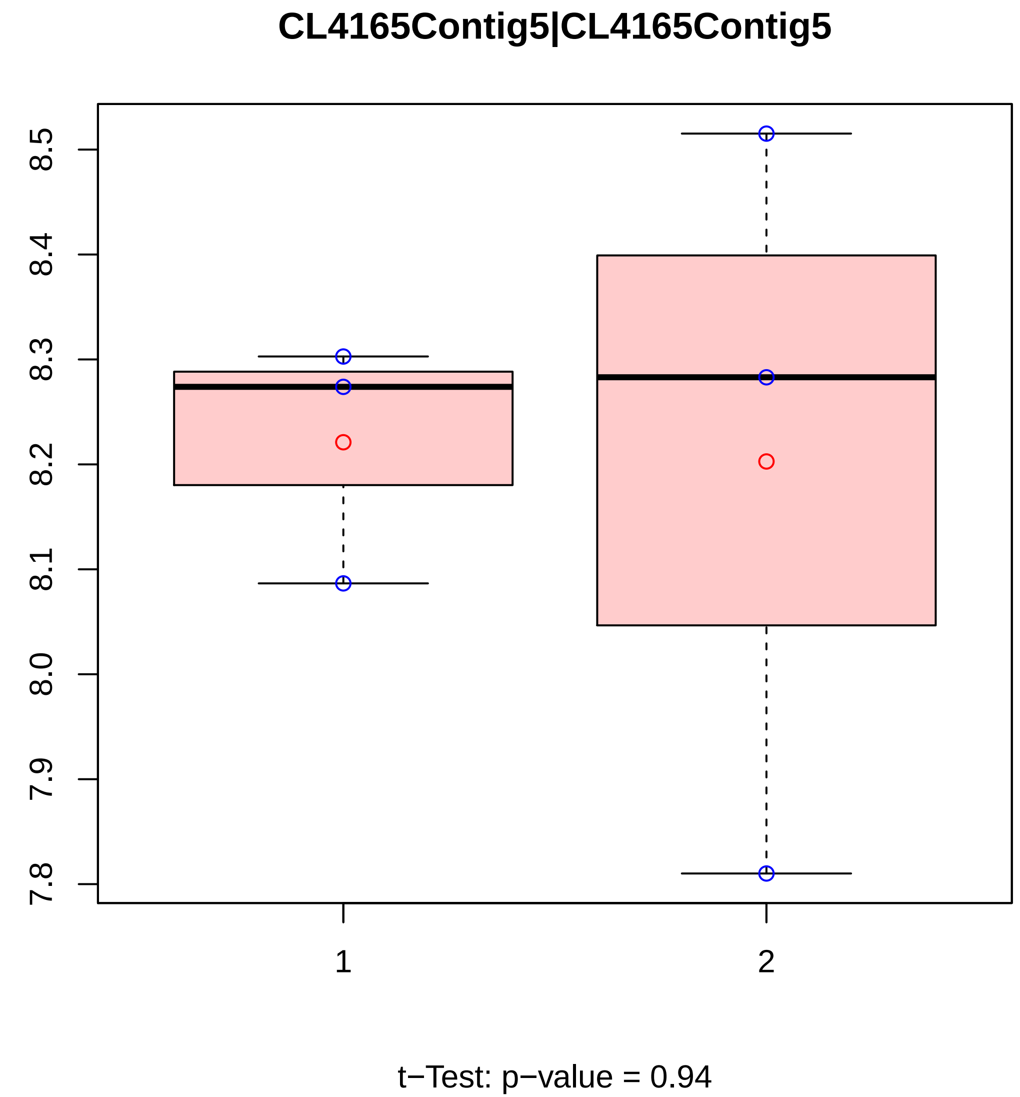
CL4165Contig5|CL4165Contig5
8.5
8.4
8.3
8.2
8.1
8.0
7.9
7.8
1
2
t−Test: p−value = 0.94

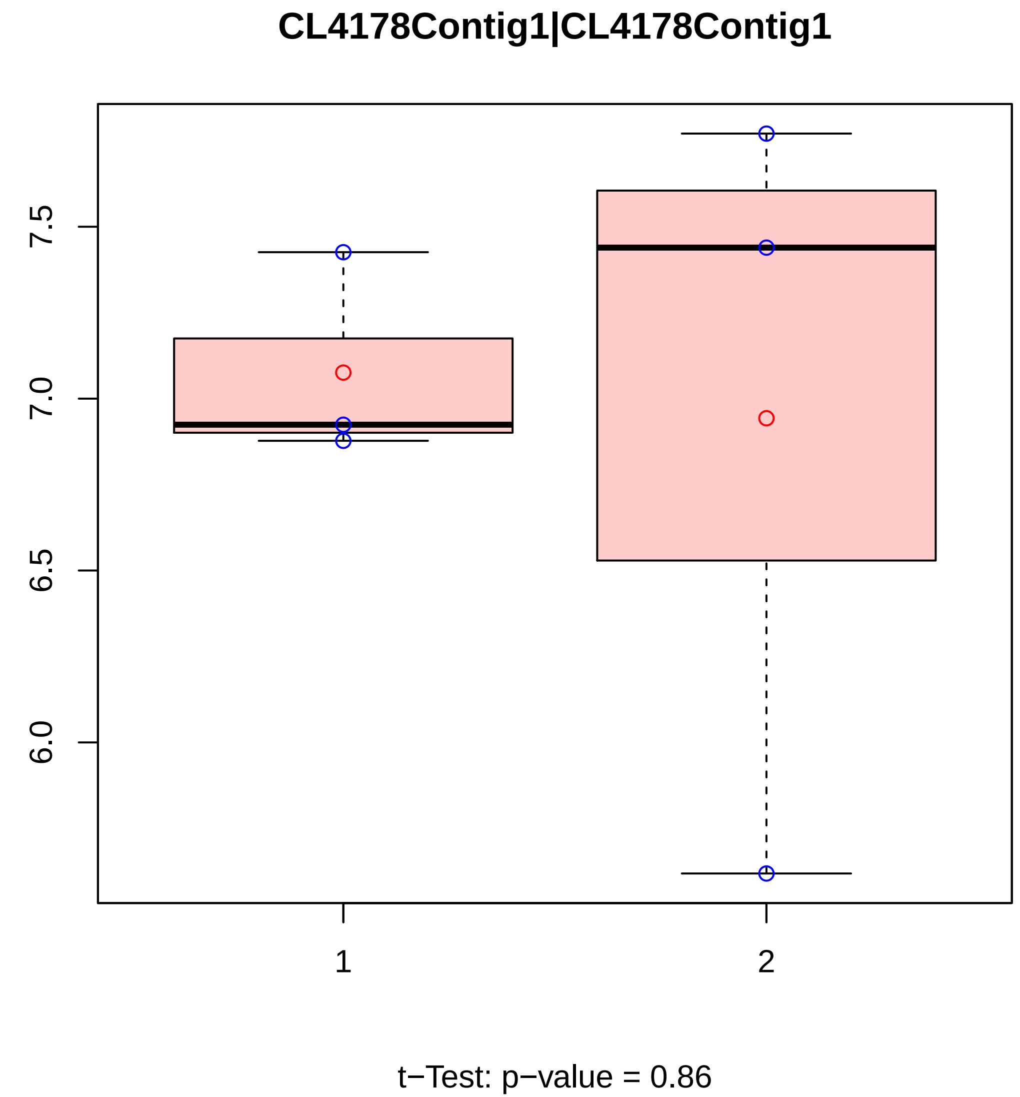
CL4178Contig1|CL4178Contig1
7.5
7.0
6.5
6.0
1
2
t−Test: p−value = 0.86

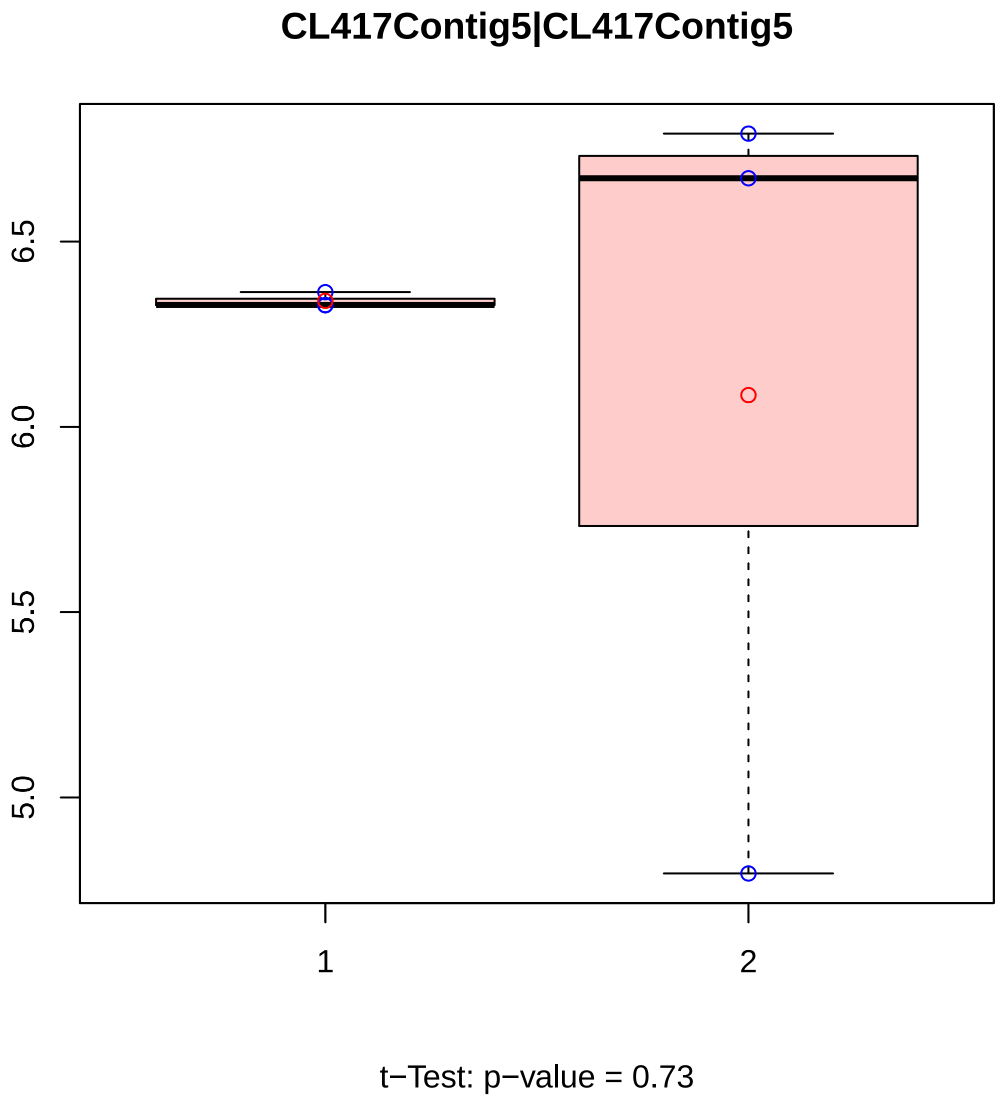

CL417Contig5|CL417Contig5
6.5
6.0
5.5
5.0
1
2
t−Test: p−value = 0.73

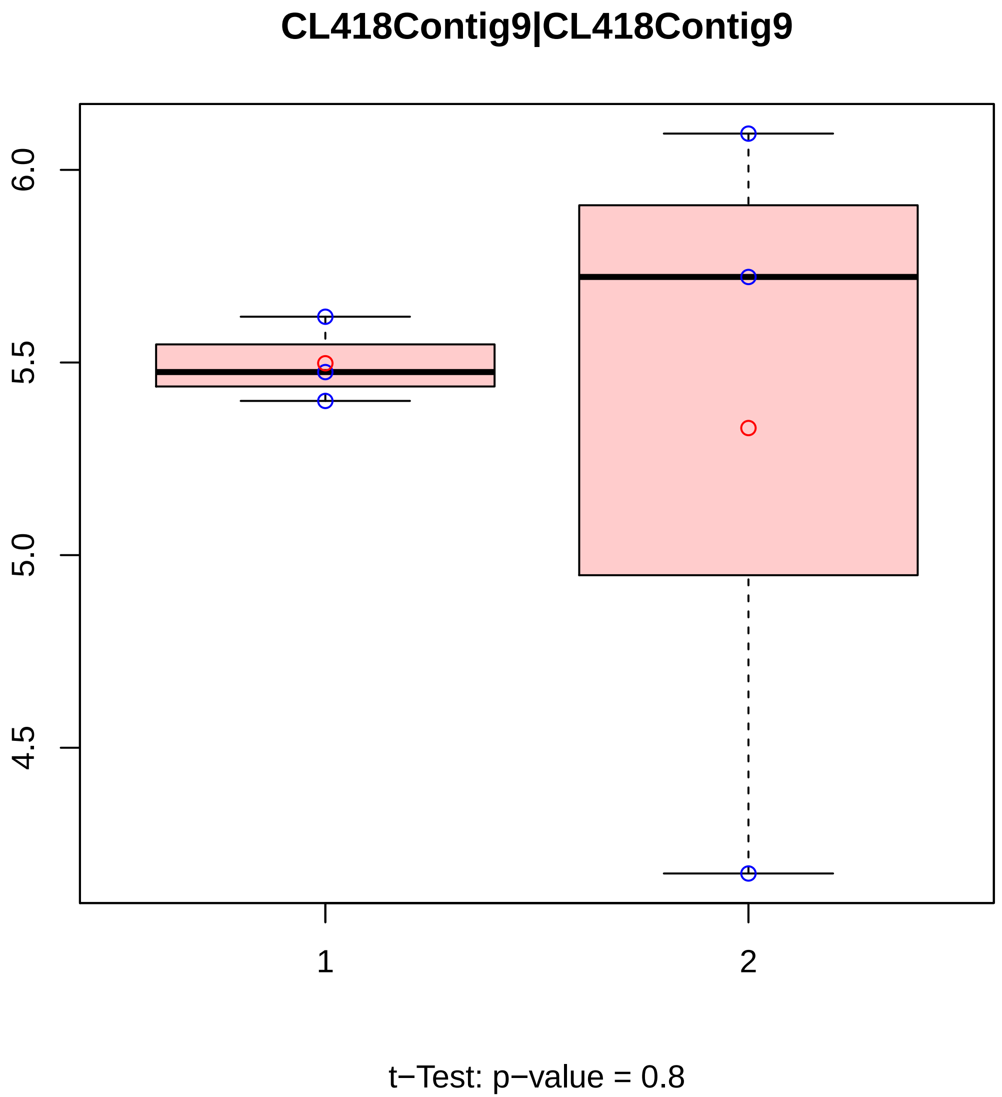
CL418Contig9|CL418Contig9
6.0
5.5
5.0
4.5
1
2
t−Test: p−value = 0.8

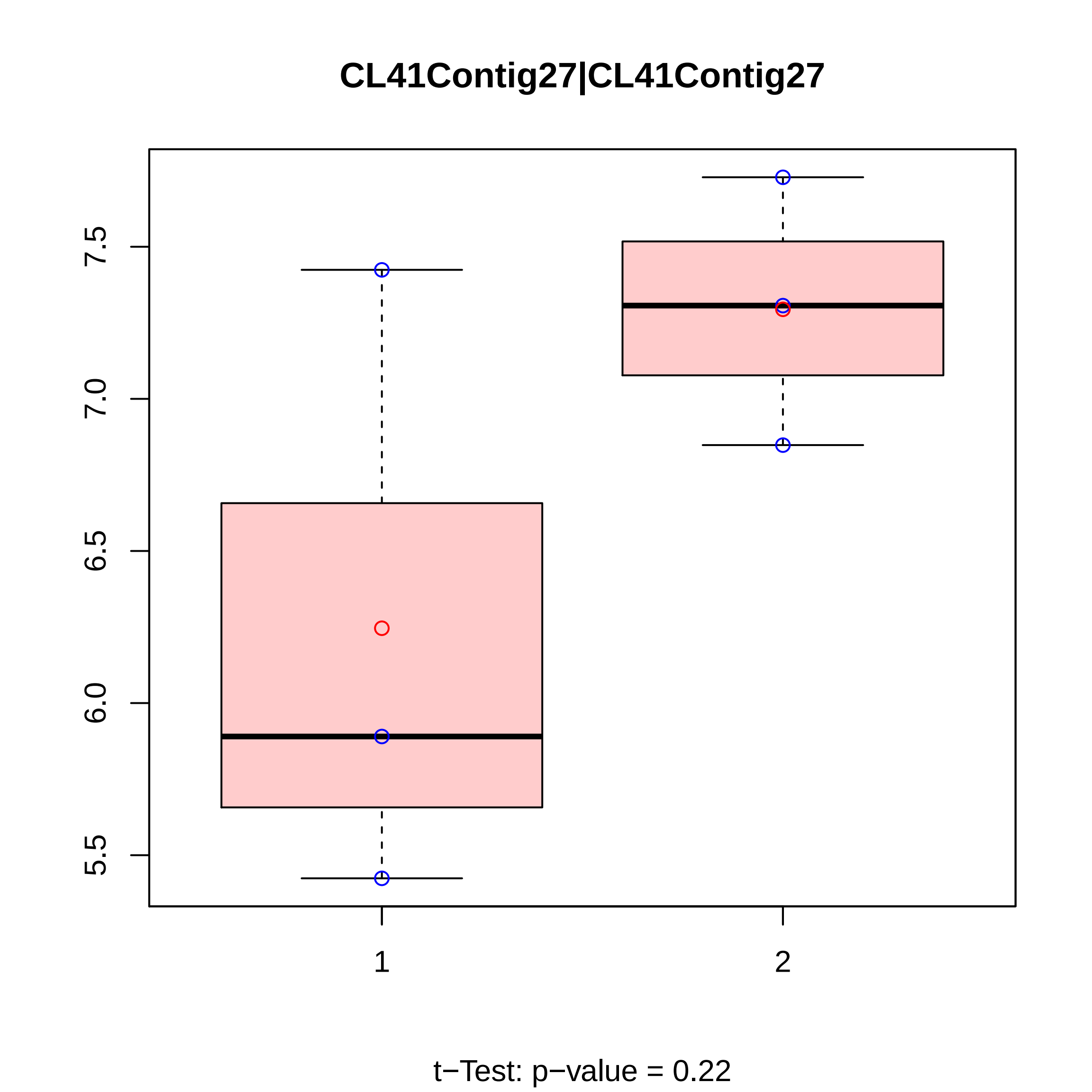
CL41Contig27|CL41Contig27
7.5
7.0
6.5
6.0
5.5
1
2
t−Test: p−value = 0.22

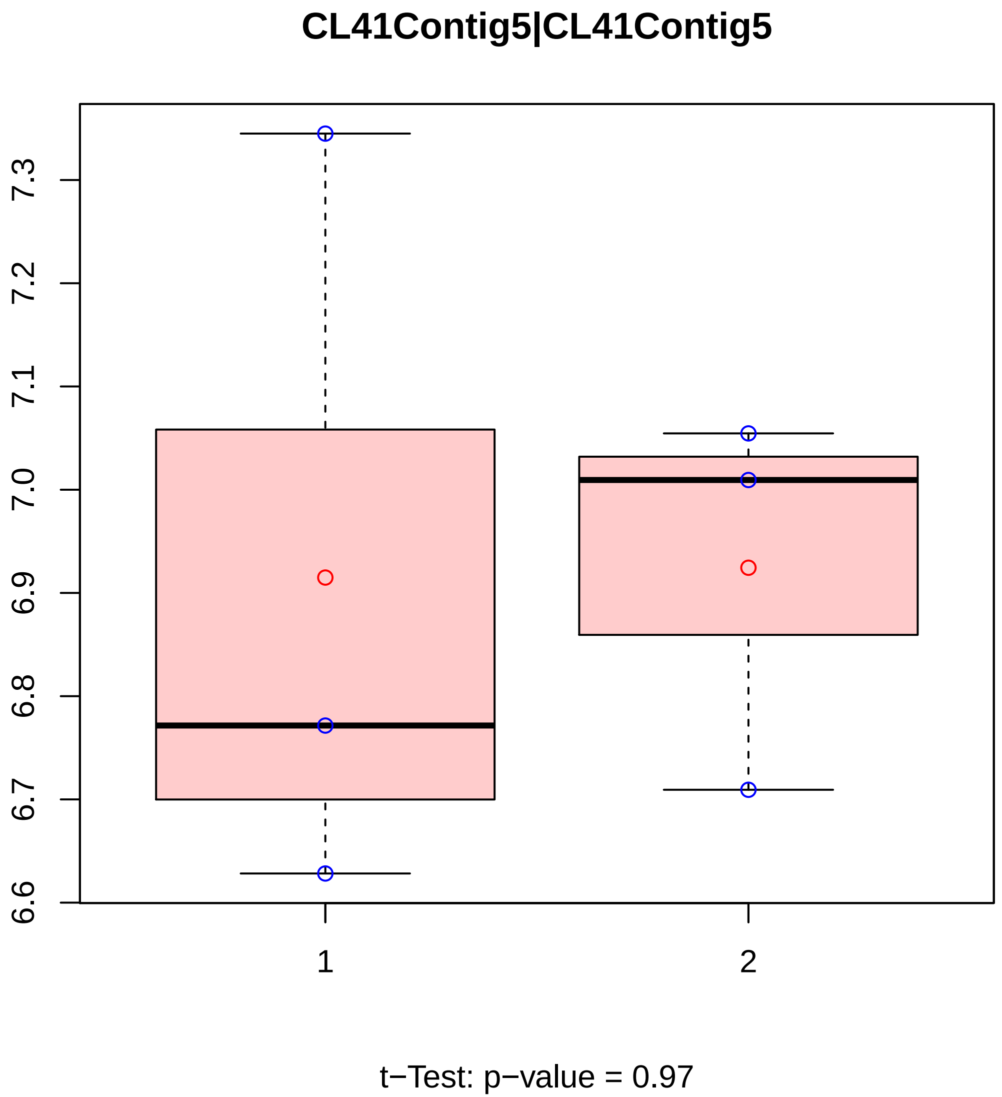
CL41Contig5|CL41Contig5
7.3
7.2
7.1
7.0
6.9
6.8
6.7
6.6
1
2
t−Test: p−value = 0.97

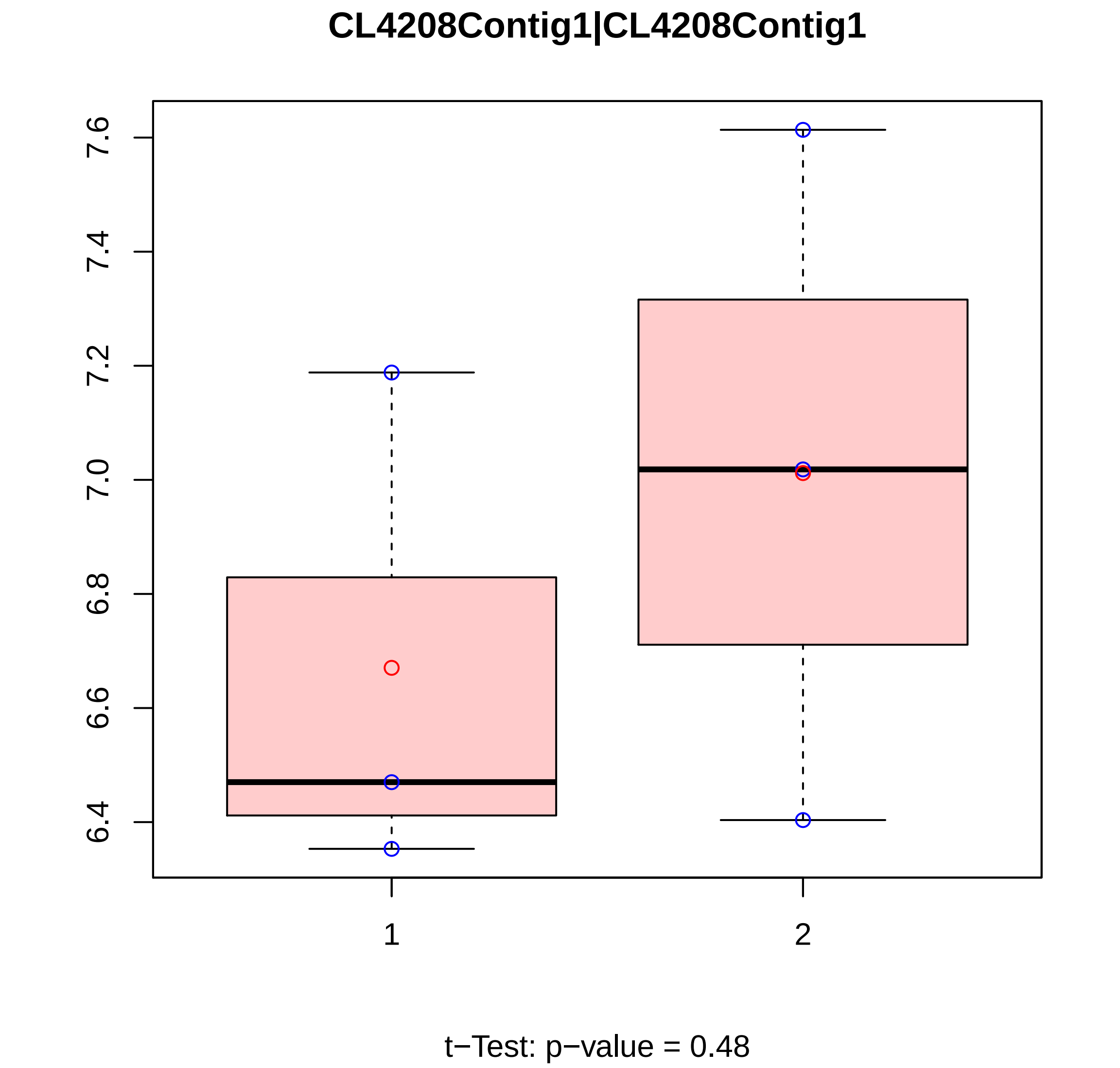
CL4208Contig1|CL4208Contig1
7.6
7.4
7.2
7.0
6.8
6.6
6.4
1
2
t−Test: p−value = 0.48

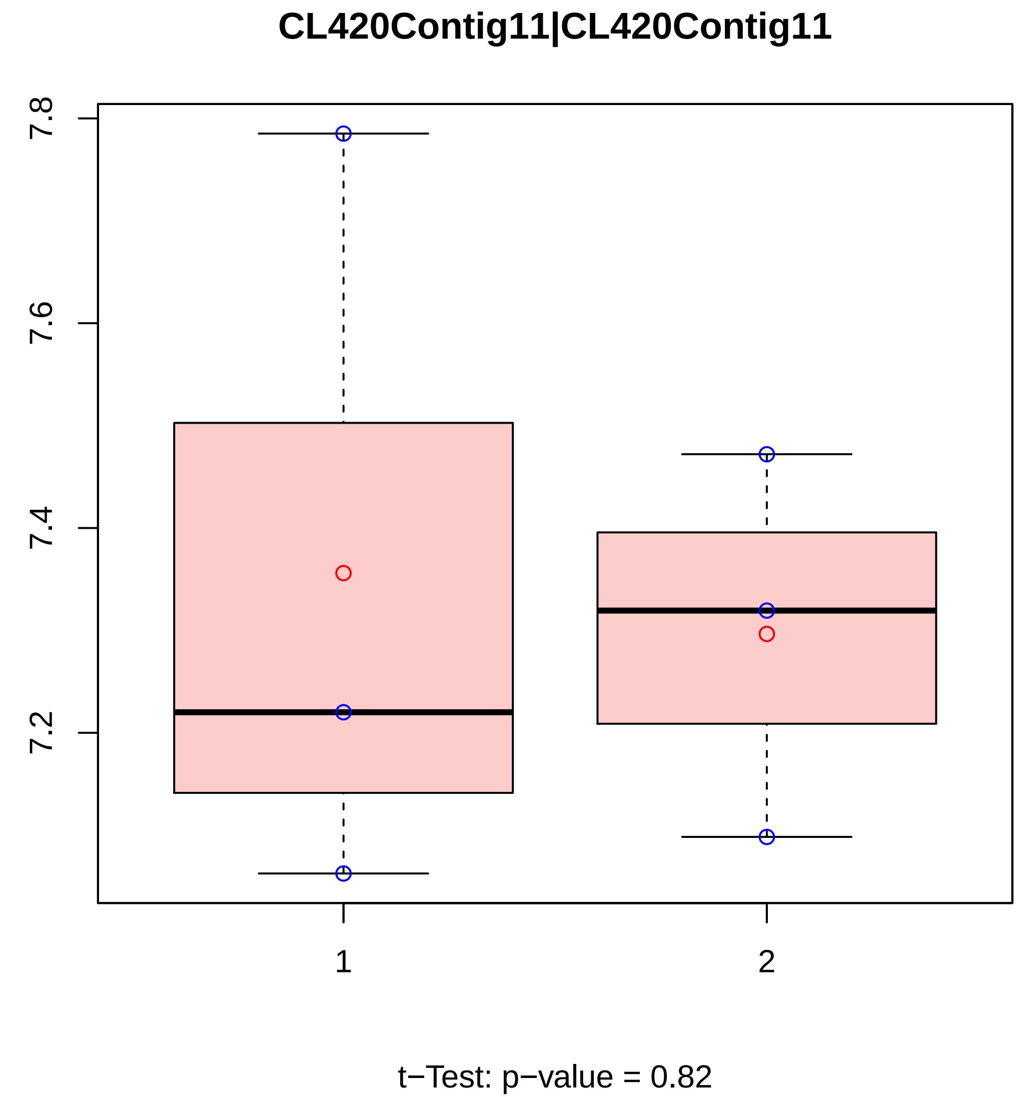
CL420Contig11|CL420Contig11
7.8
7.6
7.4
7.2
1
2
t−Test: p−value = 0.82

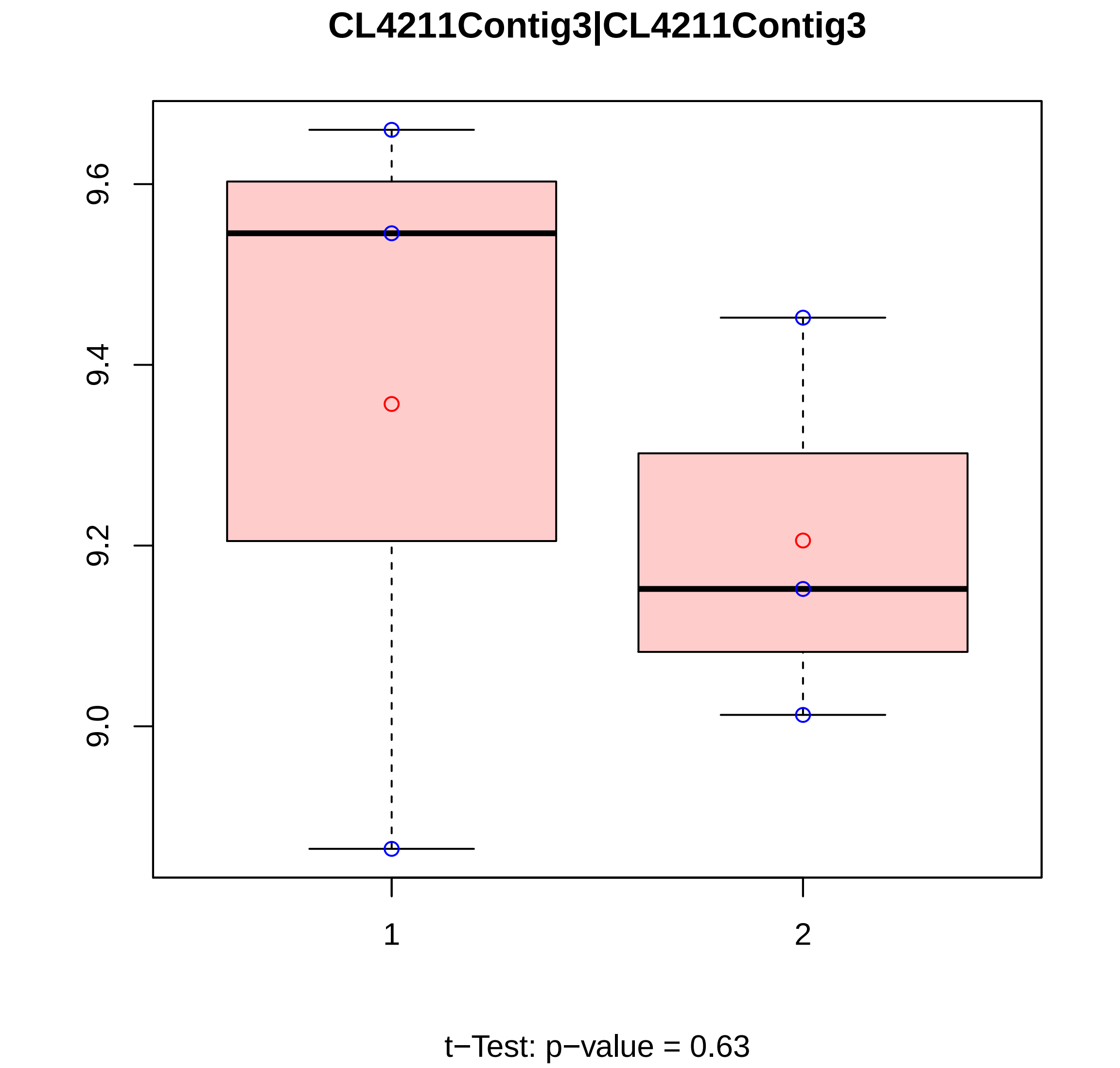
CL4211Contig3|CL4211Contig3
9.0
9.2
9.4
9.6
1
2
t−Test: p−value = 0.63

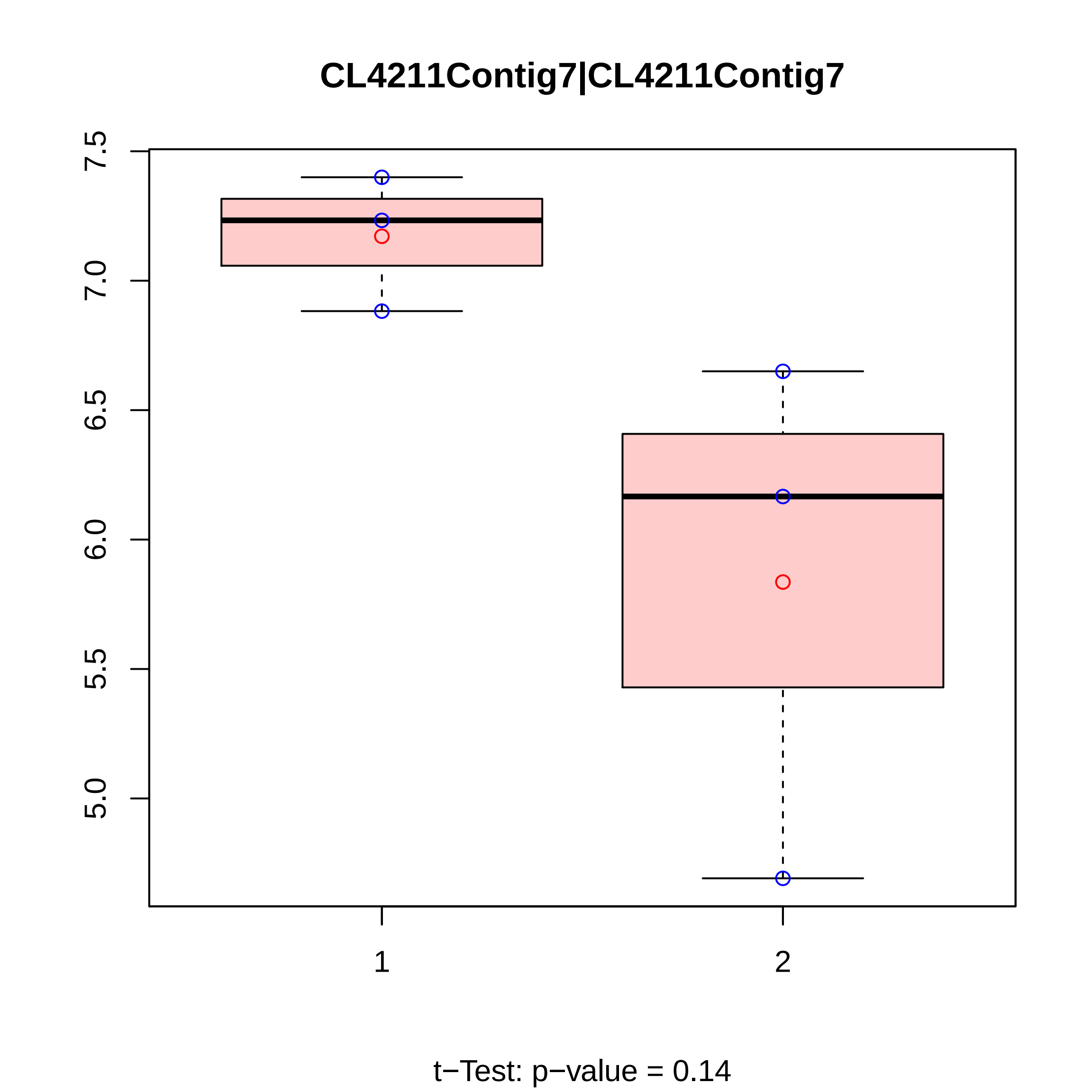
CL4211Contig7|CL4211Contig7
7.5
7.0
6.5
6.0
5.5
5.0
1
2
t−Test: p−value = 0.14

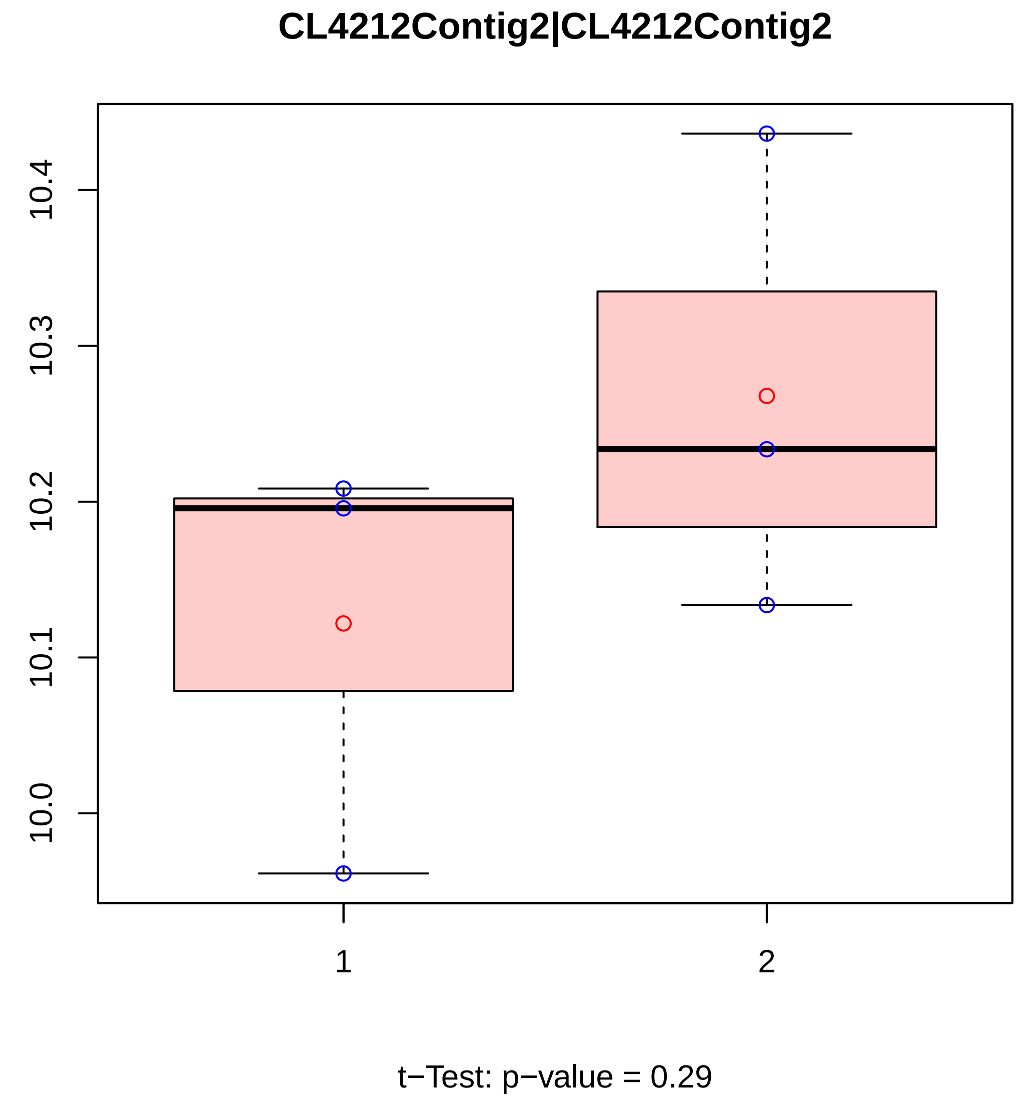
CL4212Contig2|CL4212Contig2
10.0
10.1
10.2
10.3
10.4
1
2
t−Test: p−value = 0.29

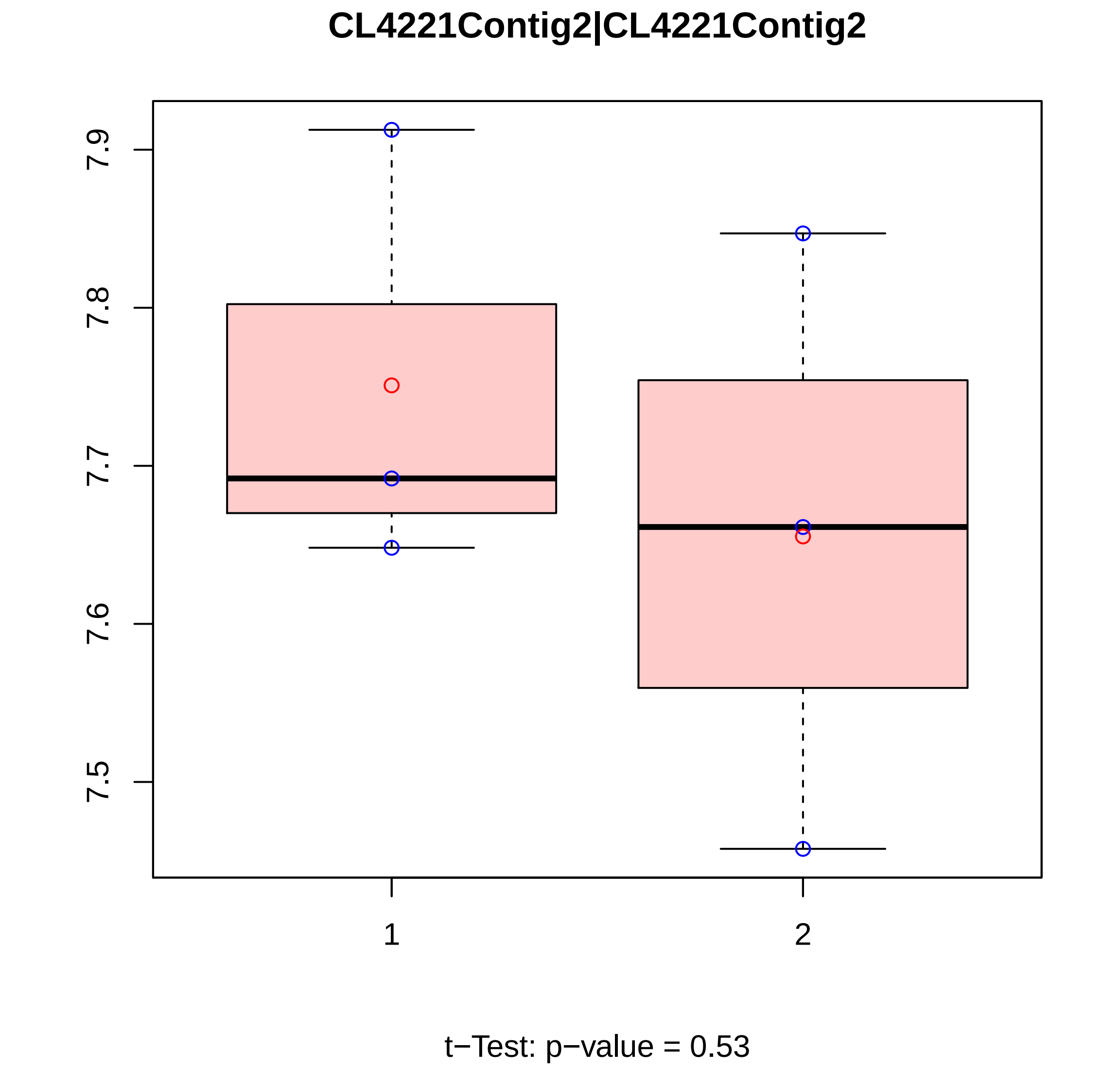

CL4221Contig2|CL4221Contig2
7.5
7.6
7.7
7.8
7.9
1
2
t−Test: p−value = 0.53

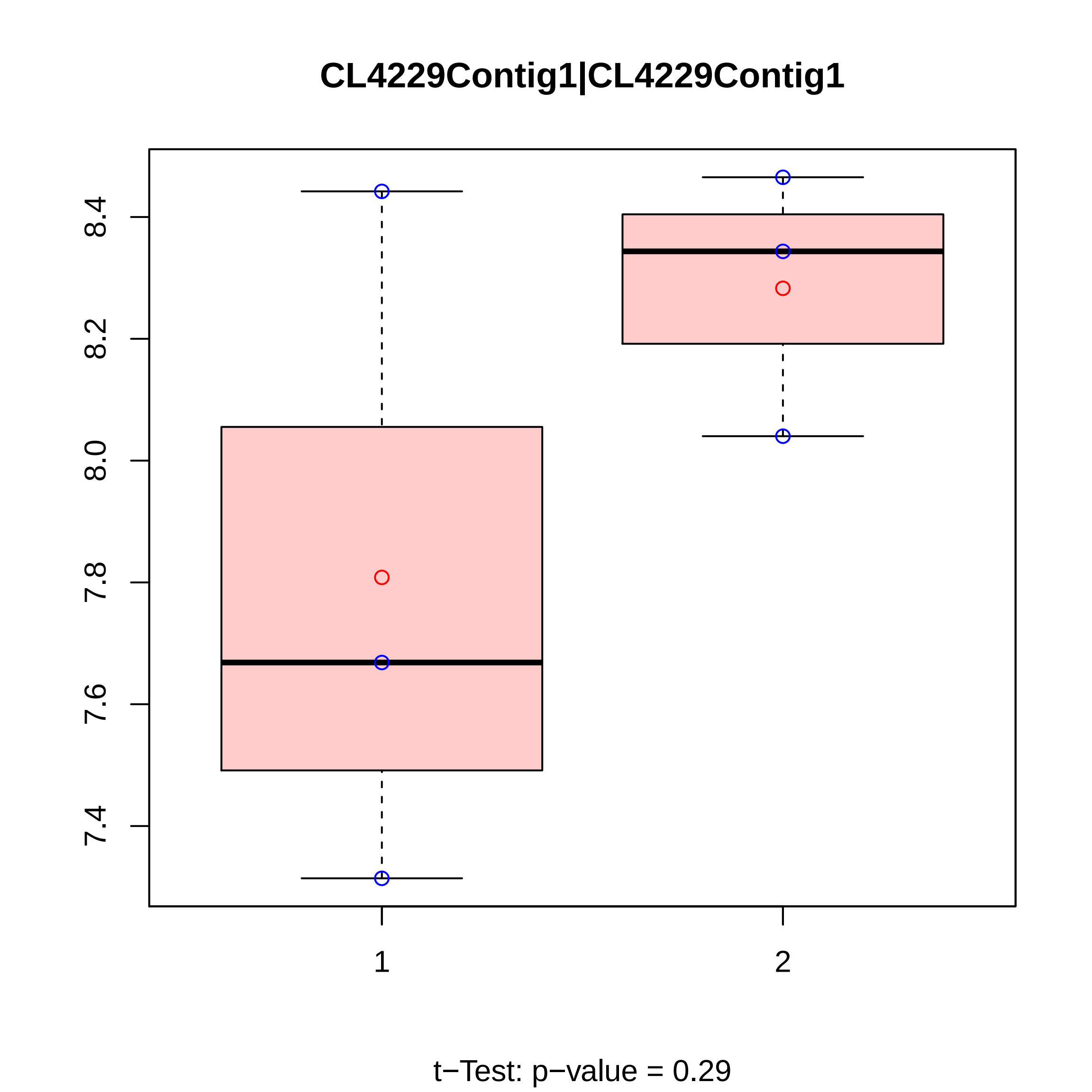

CL4229Contig1|CL4229Contig1
8.4
8.2
8.0
7.8
7.6
7.4
1
2
t−Test: p−value = 0.29

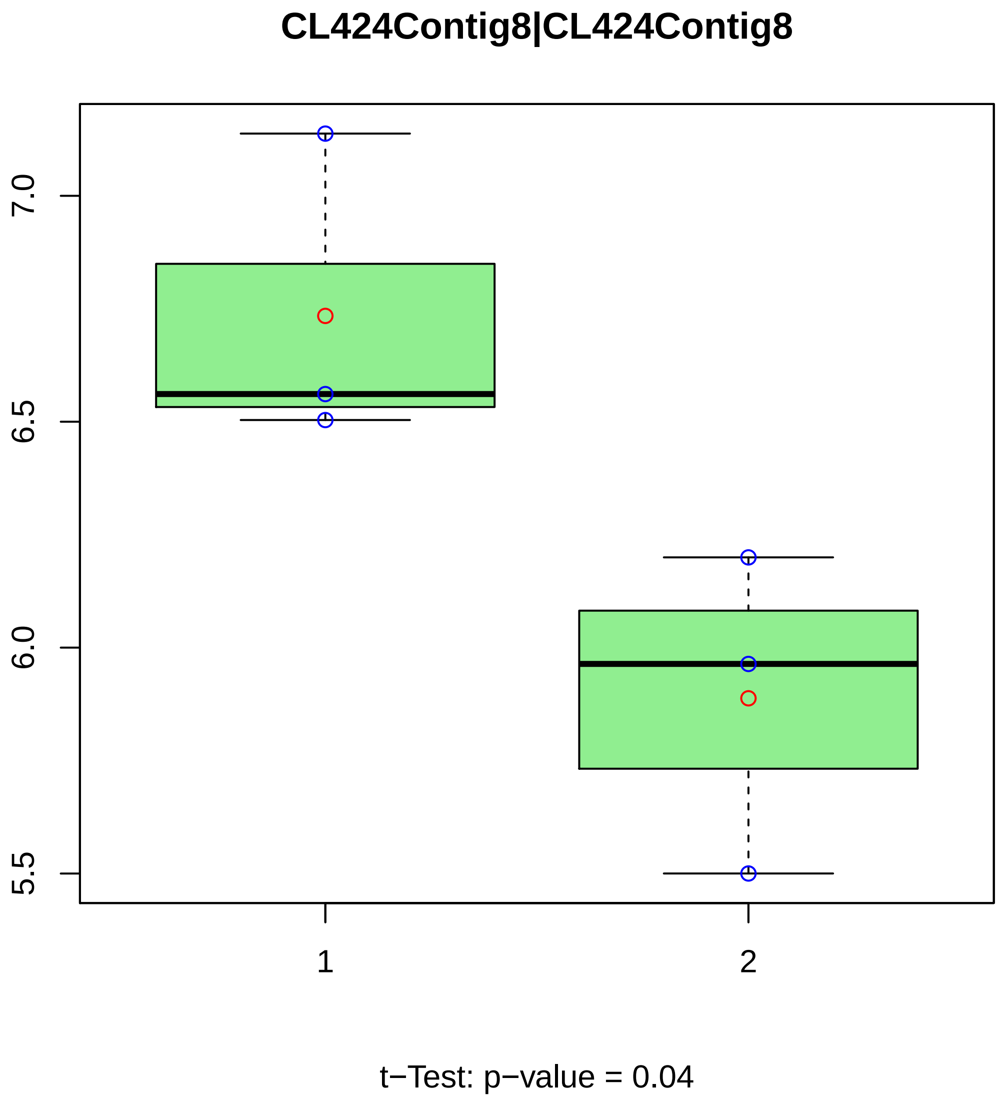

CL424Contig8|CL424Contig8
7.0
6.5
6.0
5.5
1
2
t−Test: p−value = 0.04

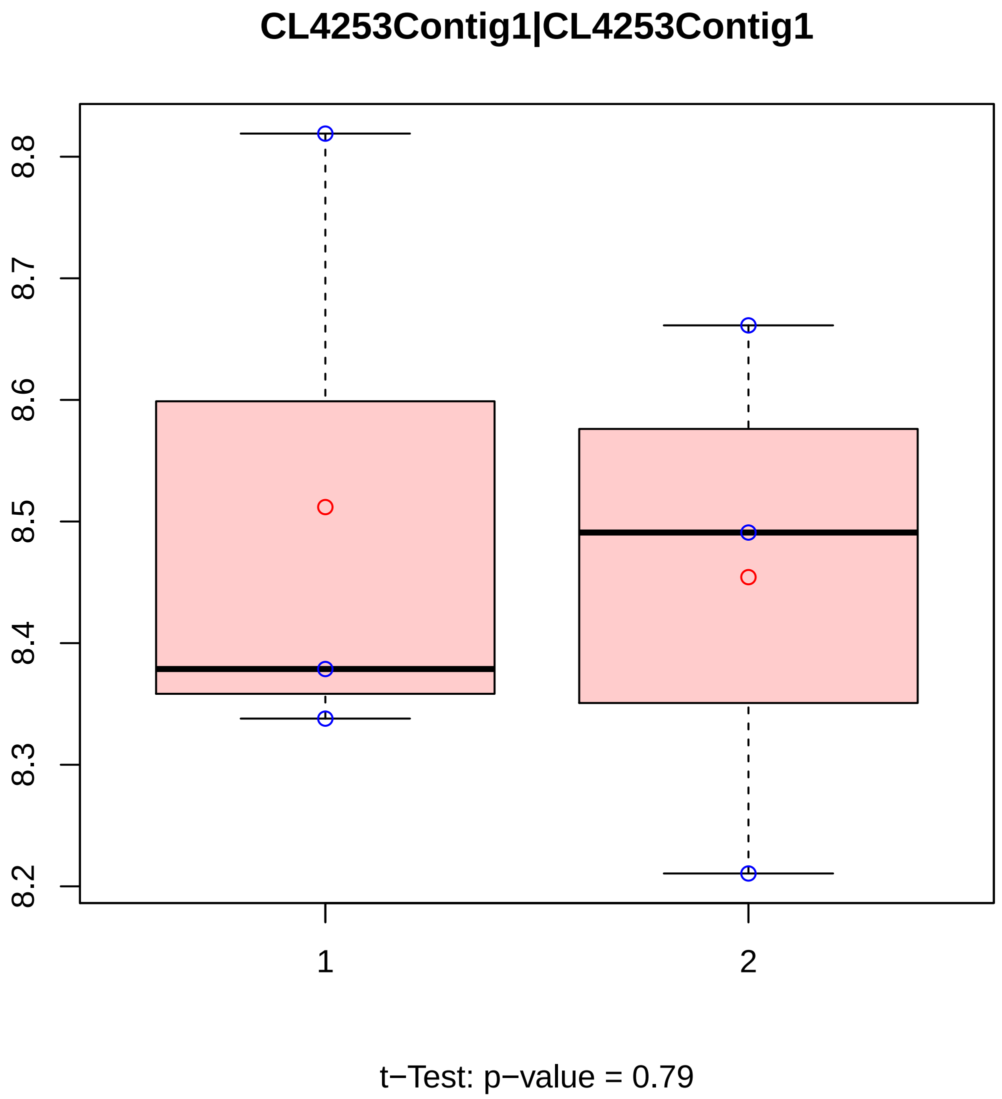
CL4253Contig1|CL4253Contig1
8.2
8.3
8.4
8.5
8.6
8.7
8.8
1
2
t−Test: p−value = 0.79

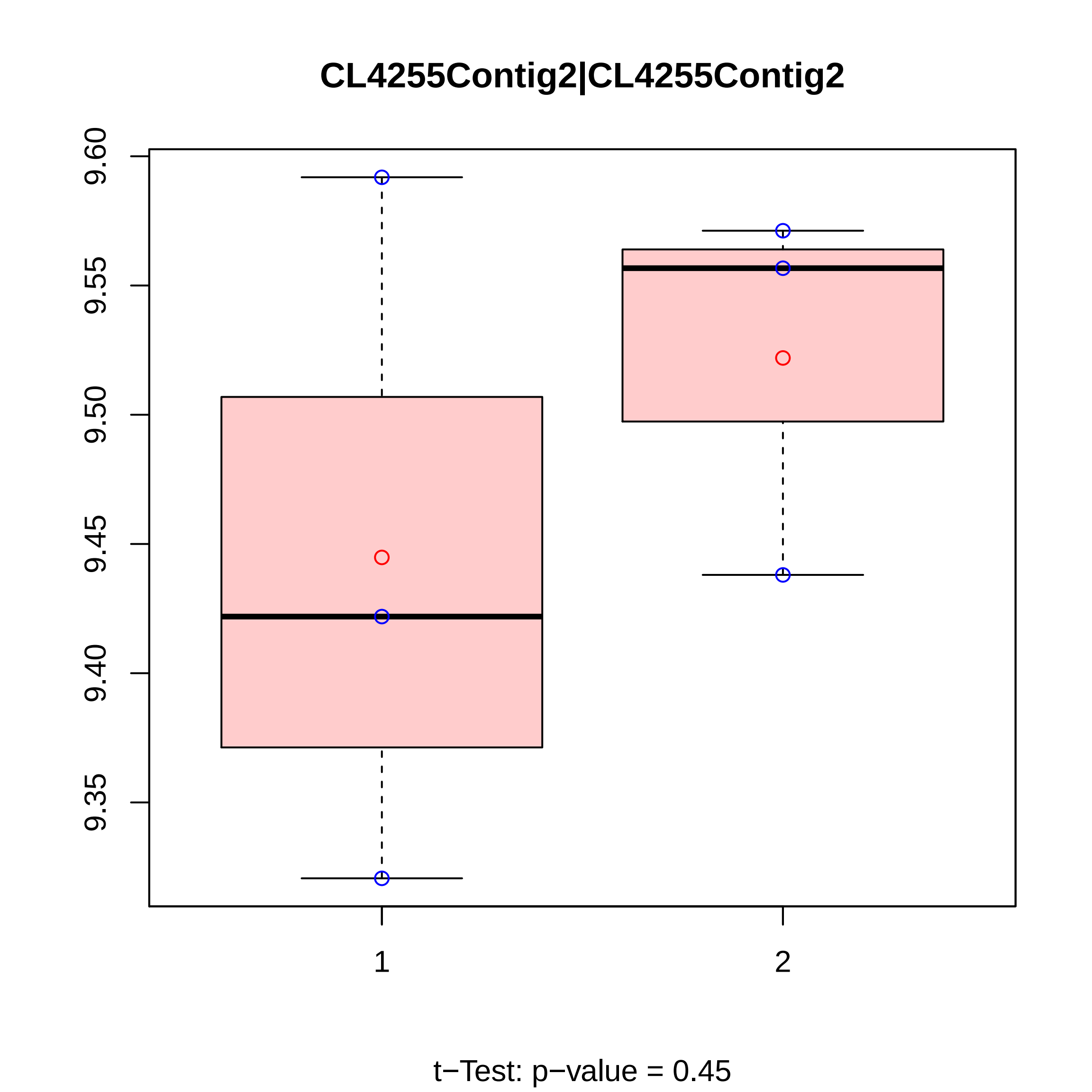
CL4255Contig2|CL4255Contig2
9.60
9.55
9.50
9.45
9.40
9.35
1
2
t−Test: p−value = 0.45

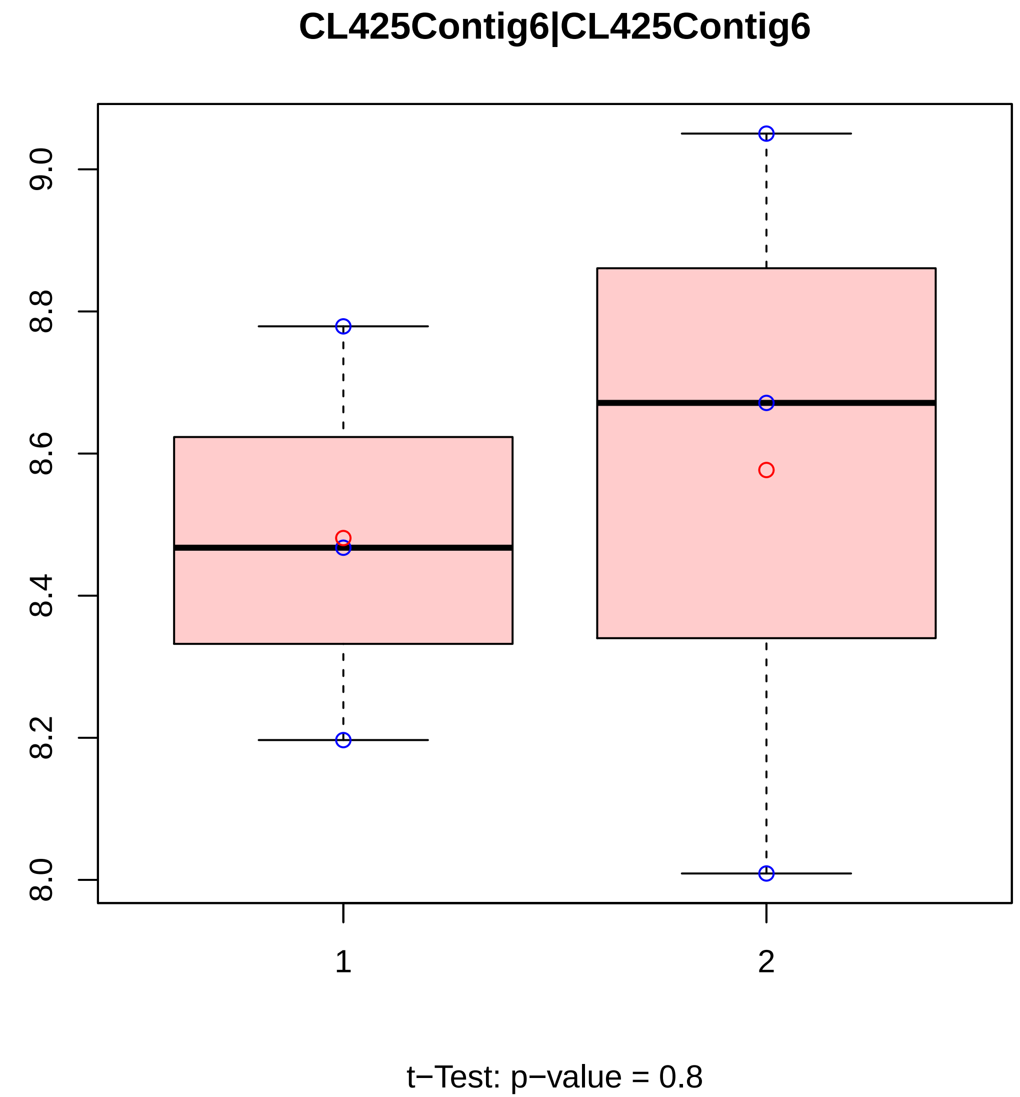
CL425Contig6|CL425Contig6
9.0
8.8
8.6
8.4
8.2
8.0
1
2
t−Test: p−value = 0.8

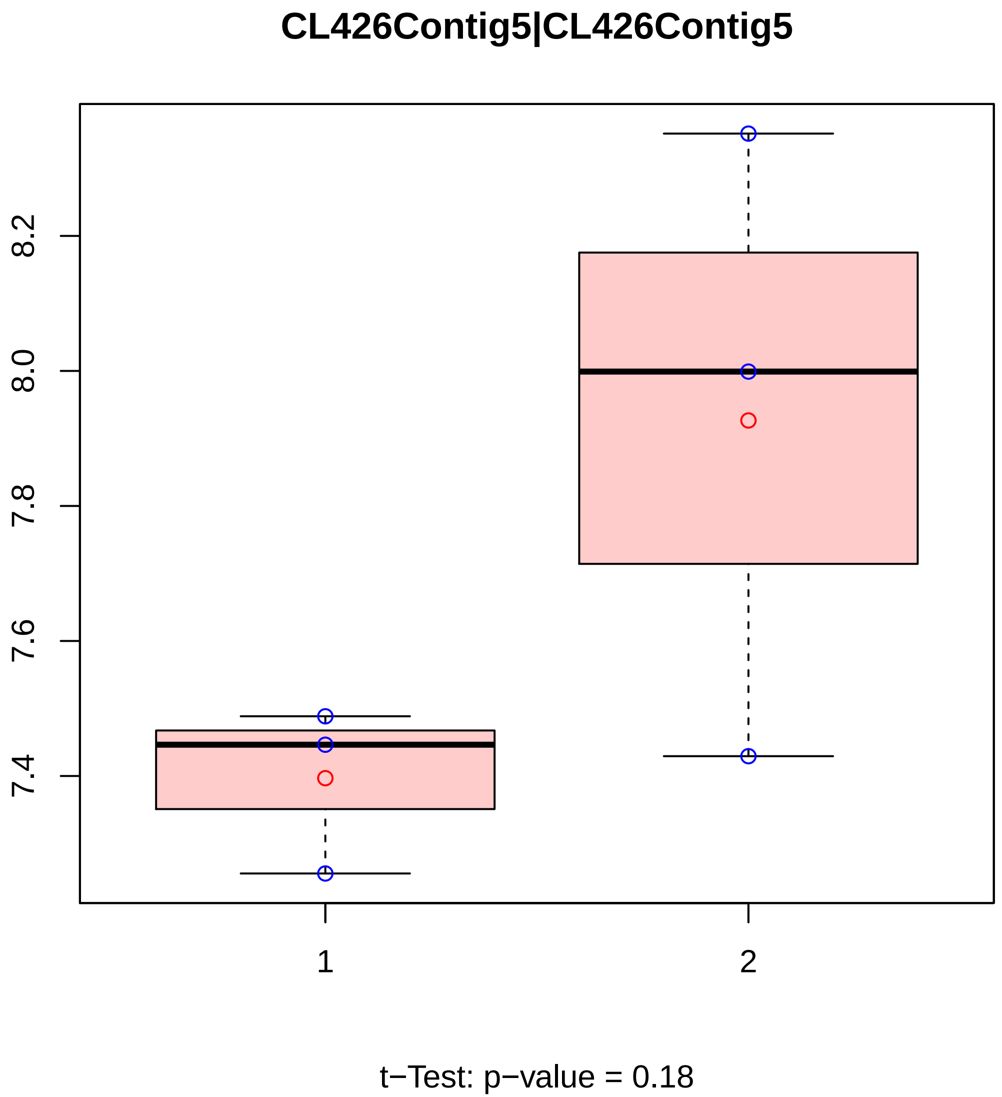
CL426Contig5|CL426Contig5
7.4
7.6
7.8
8.0
8.2
1
2
t−Test: p−value = 0.18

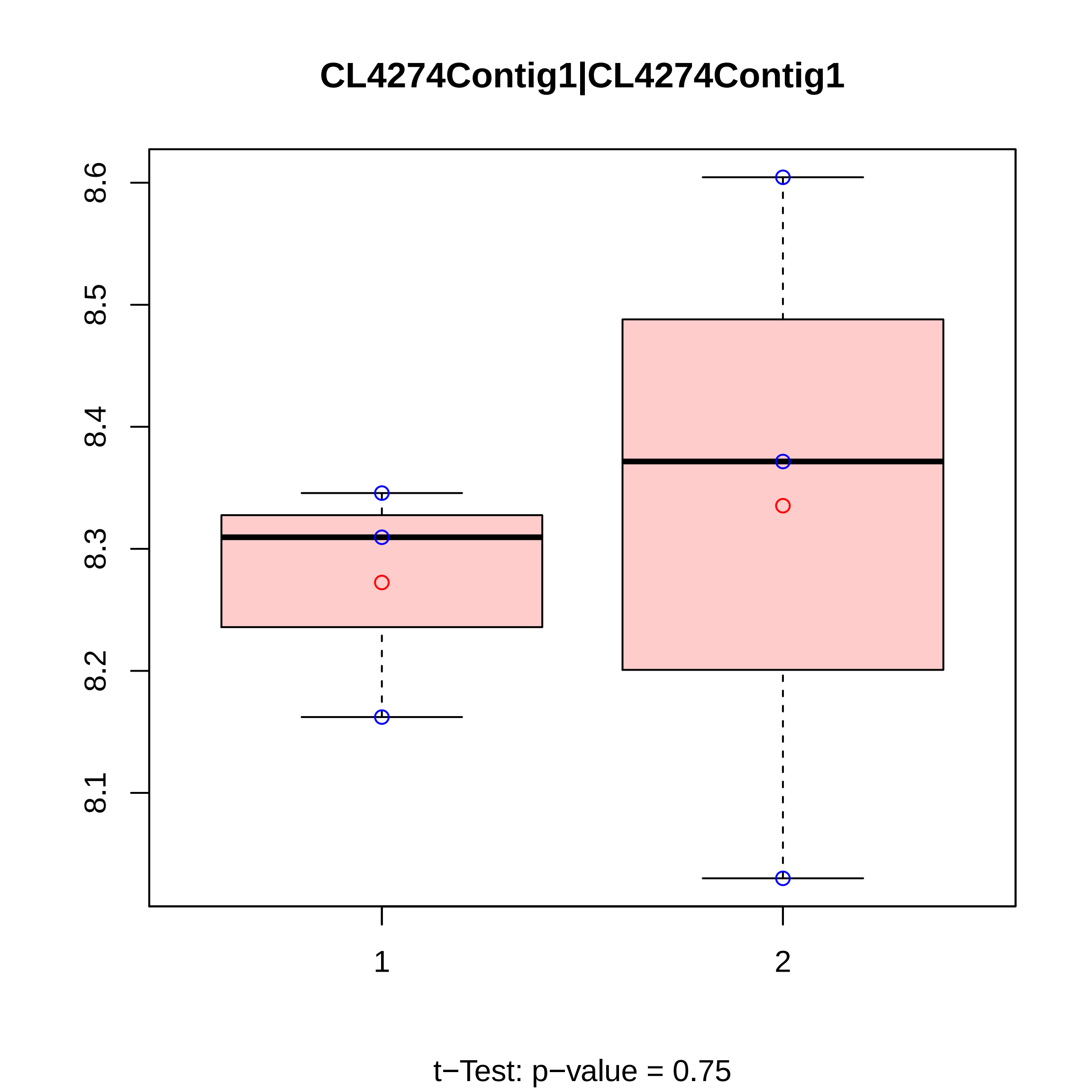
CL4274Contig1|CL4274Contig1
8.1
8.2
8.3
8.4
8.5
8.6
1
2
t−Test: p−value = 0.75

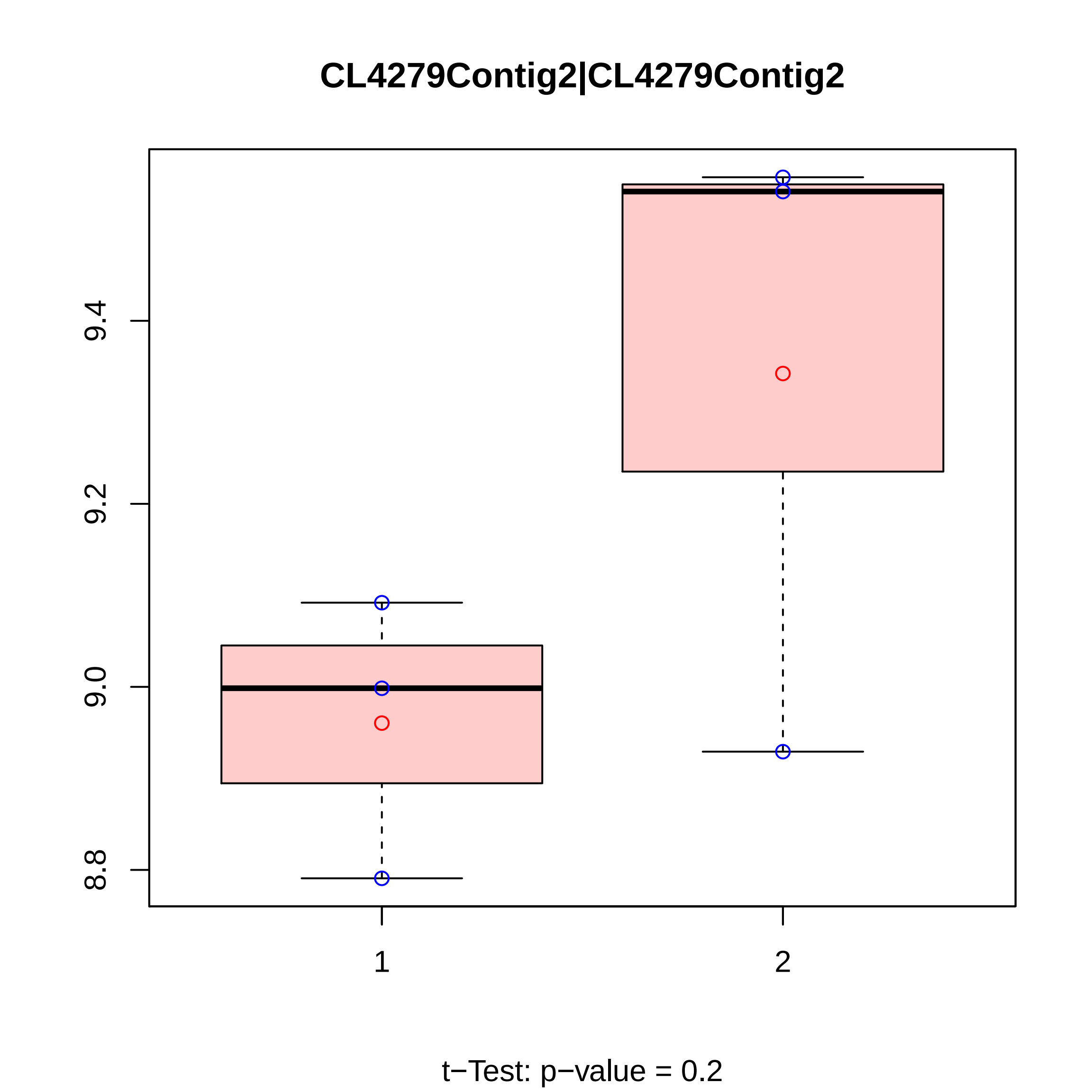
CL4279Contig2|CL4279Contig2
8.8
9.0
9.2
9.4
1
2
t−Test: p−value = 0.2

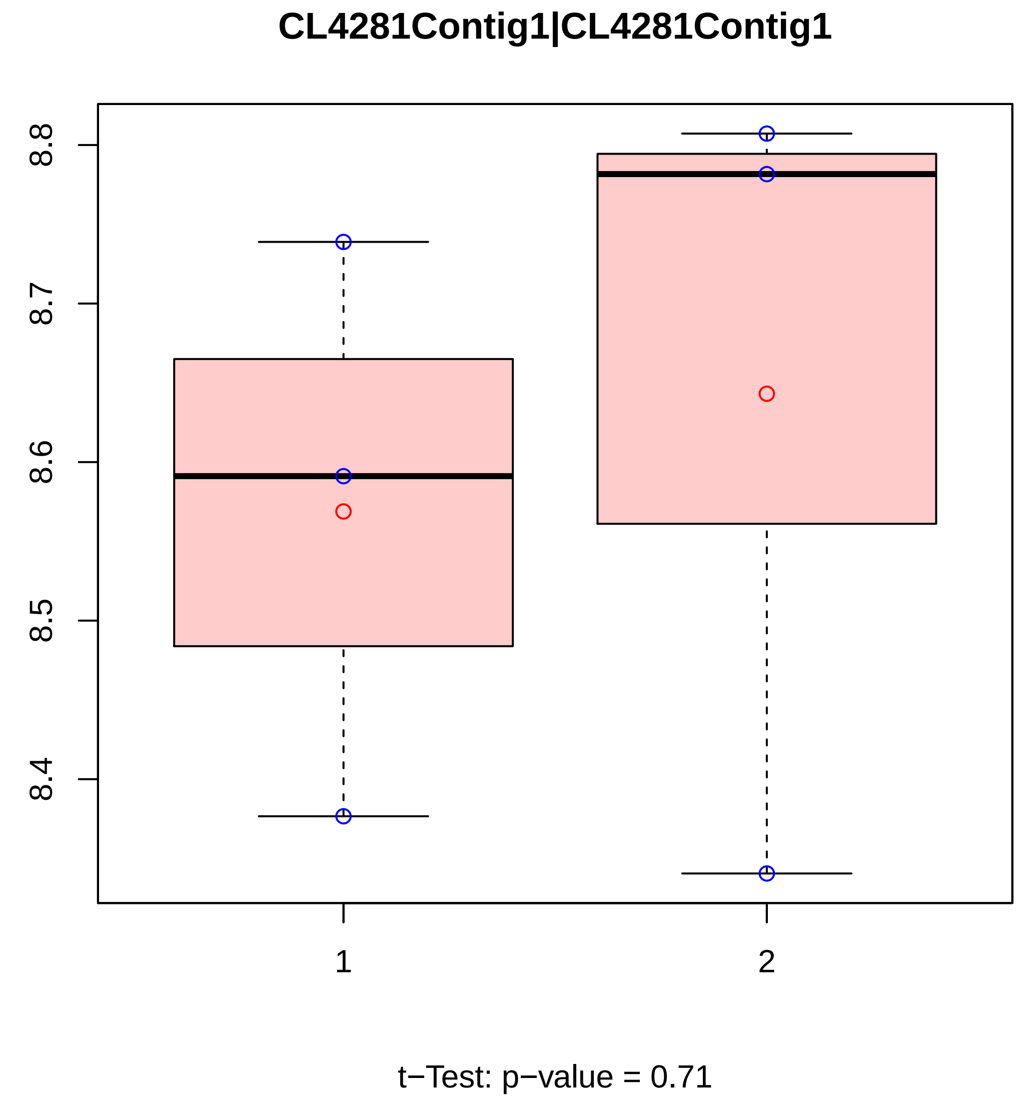

CL4281Contig1|CL4281Contig1
8.4
8.5
8.6
8.7
8.8
1
2
t−Test: p−value = 0.71

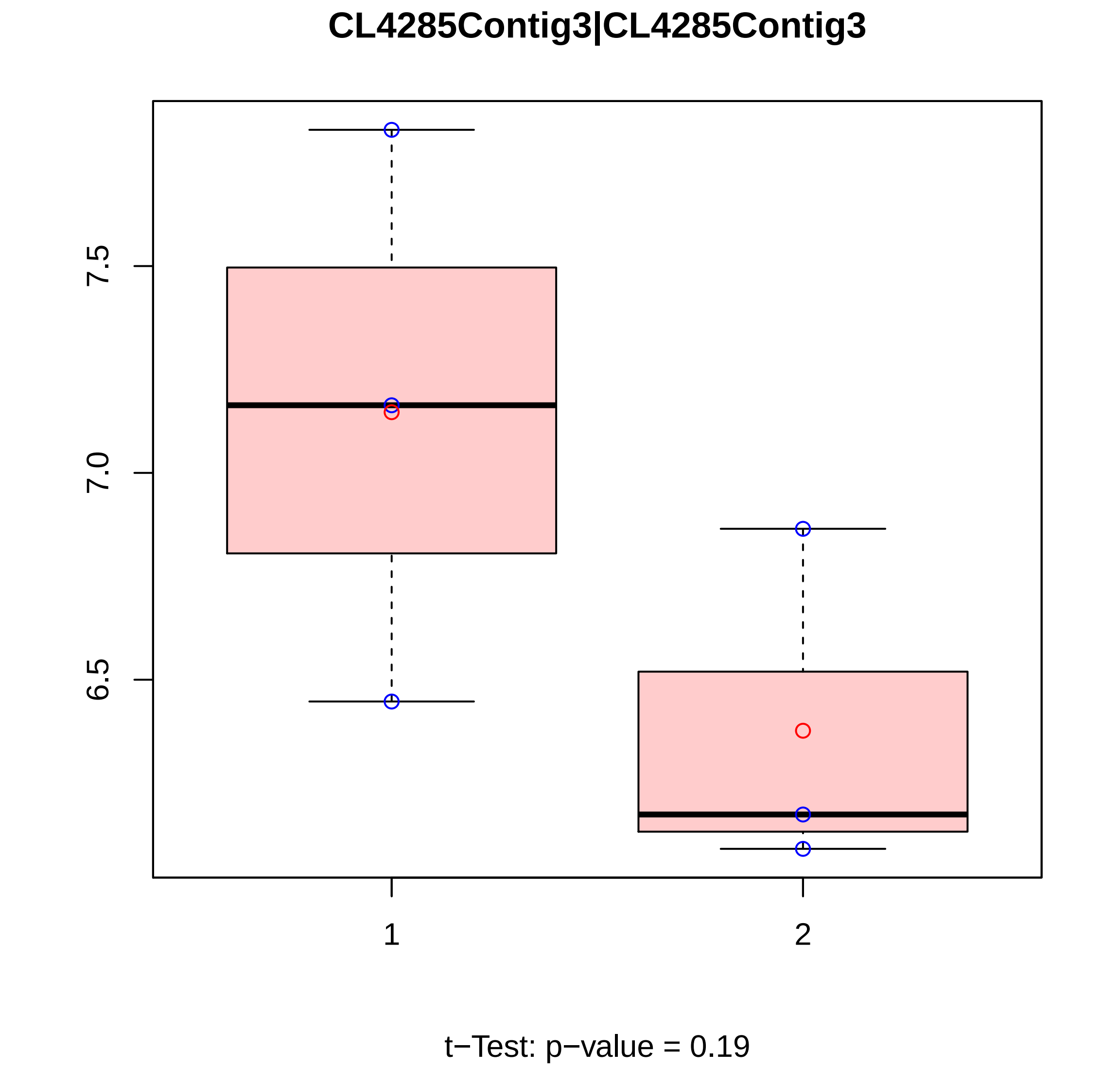

CL4285Contig3|CL4285Contig3
7.5
7.0
6.5
1
2
t−Test: p−value = 0.19

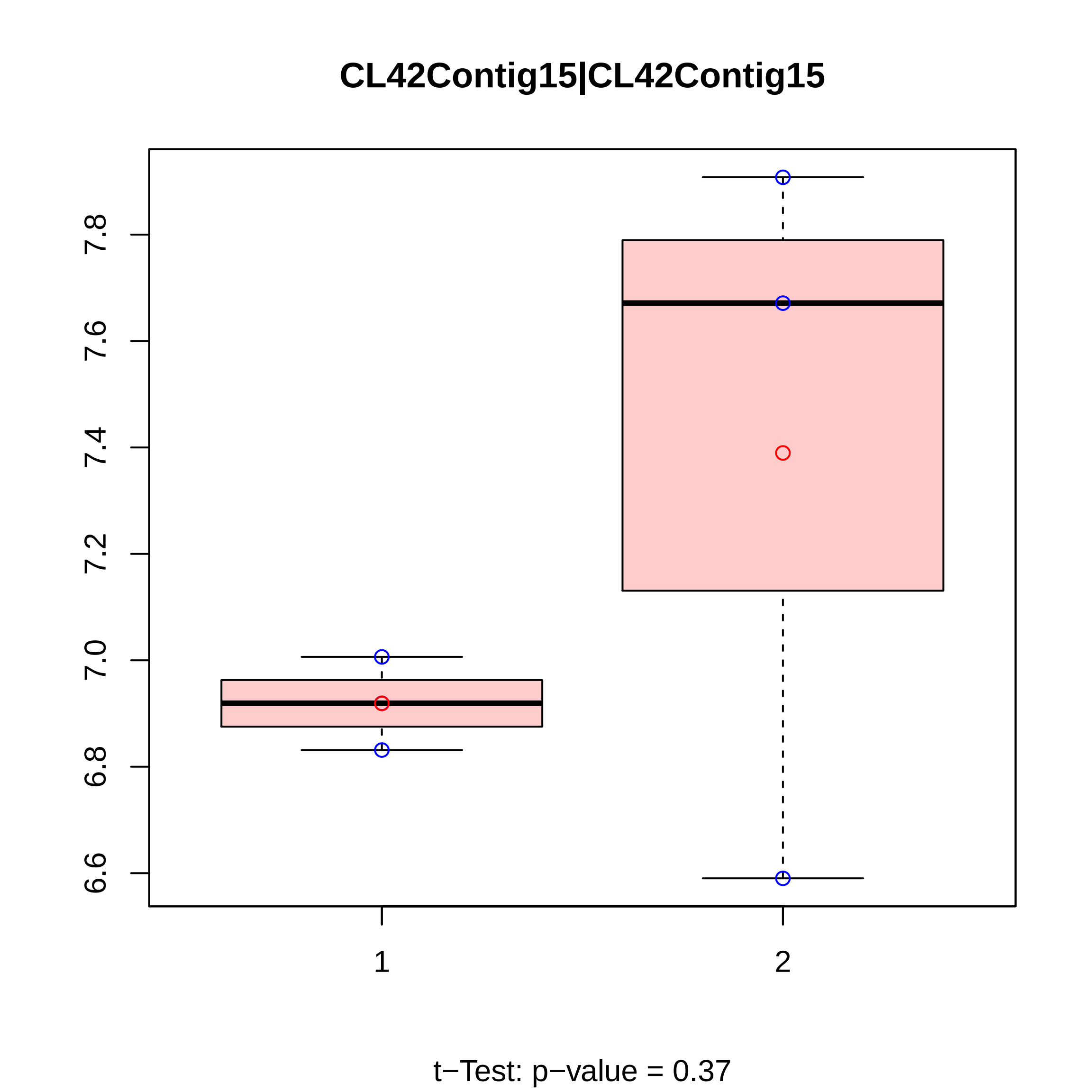

CL42Contig15|CL42Contig15
7.8
7.6
7.4
7.2
7.0
6.8
6.6
1
2
t−Test: p−value = 0.37

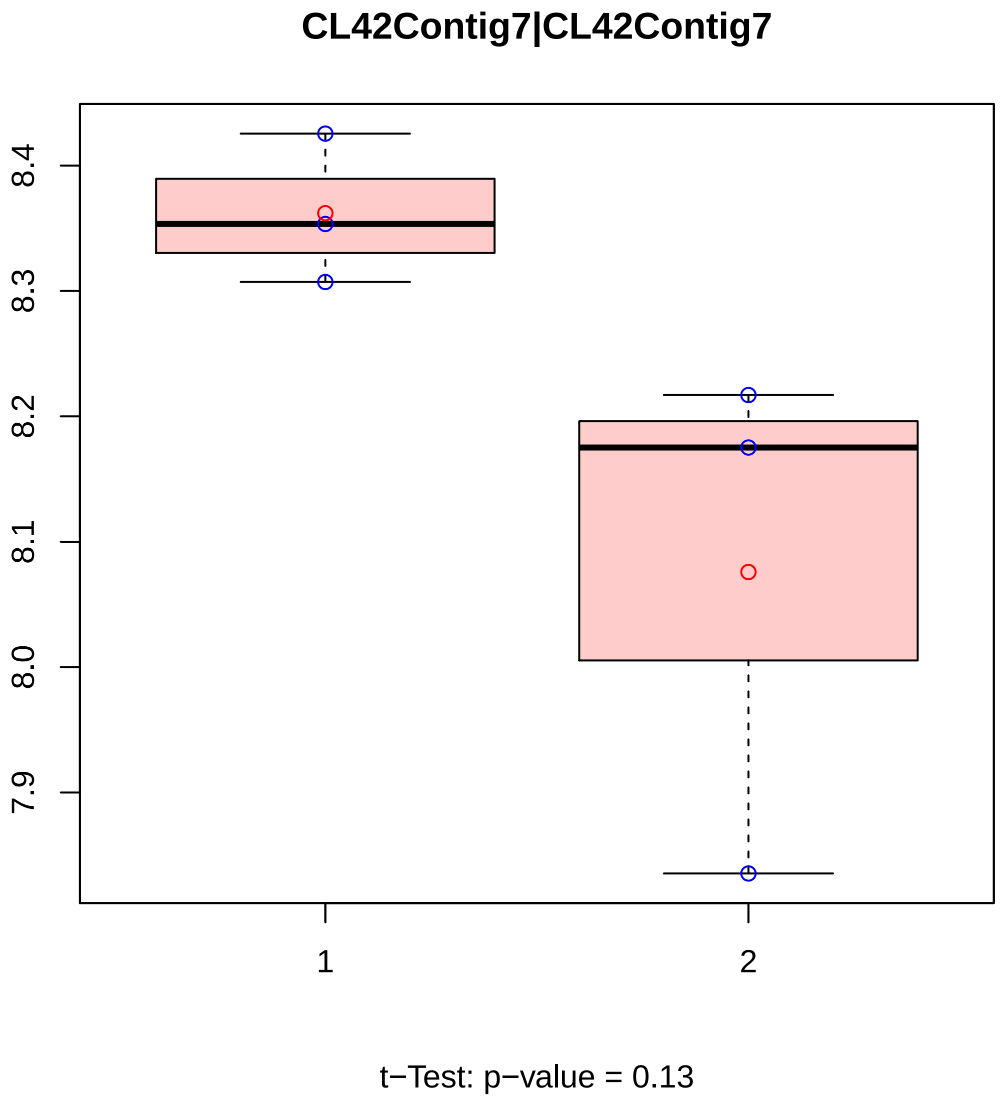

CL42Contig7|CL42Contig7
8.4
8.3
8.2
8.1
8.0
7.9
1
2
t−Test: p−value = 0.13

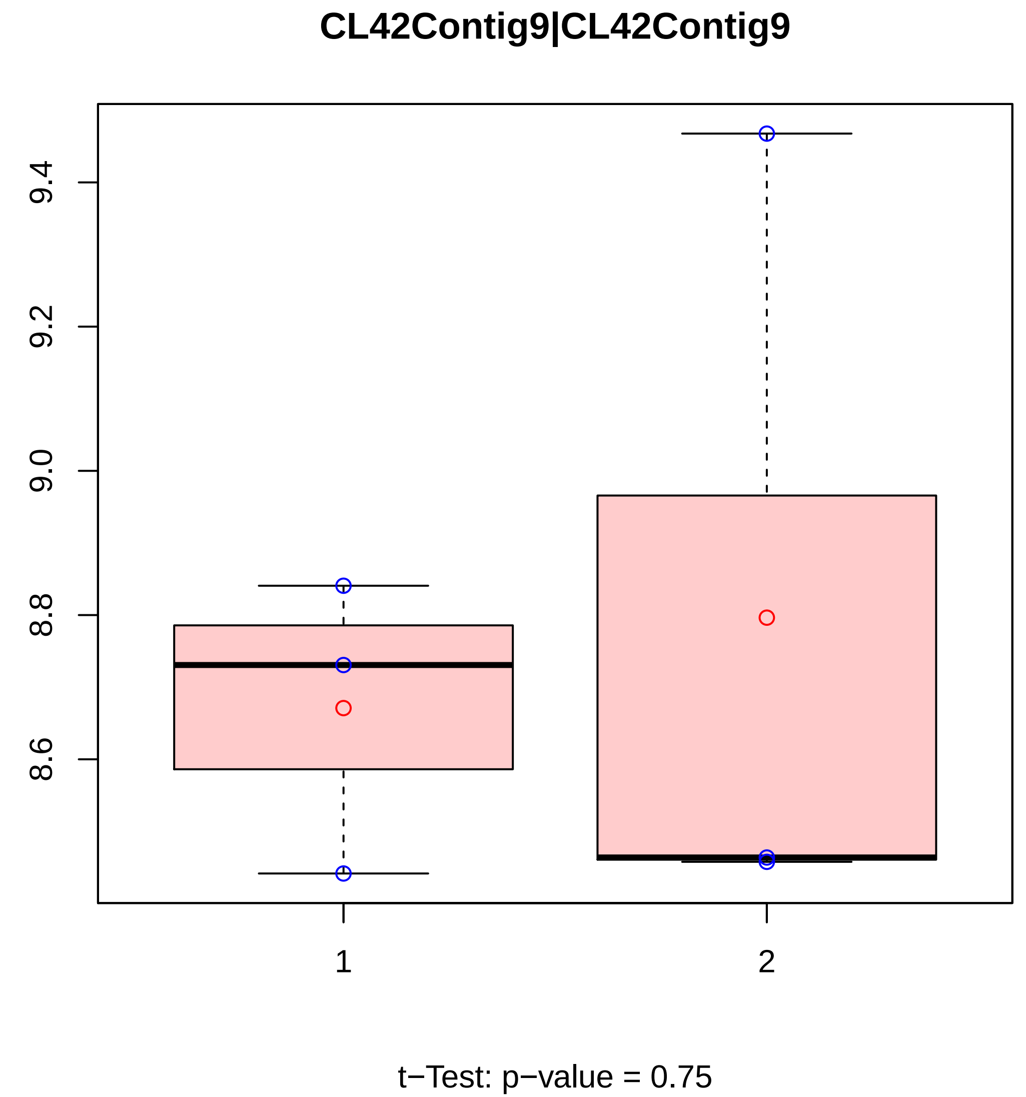

CL42Contig9|CL42Contig9
9.4
9.2
9.0
8.8
8.6
1
2
t−Test: p−value = 0.75

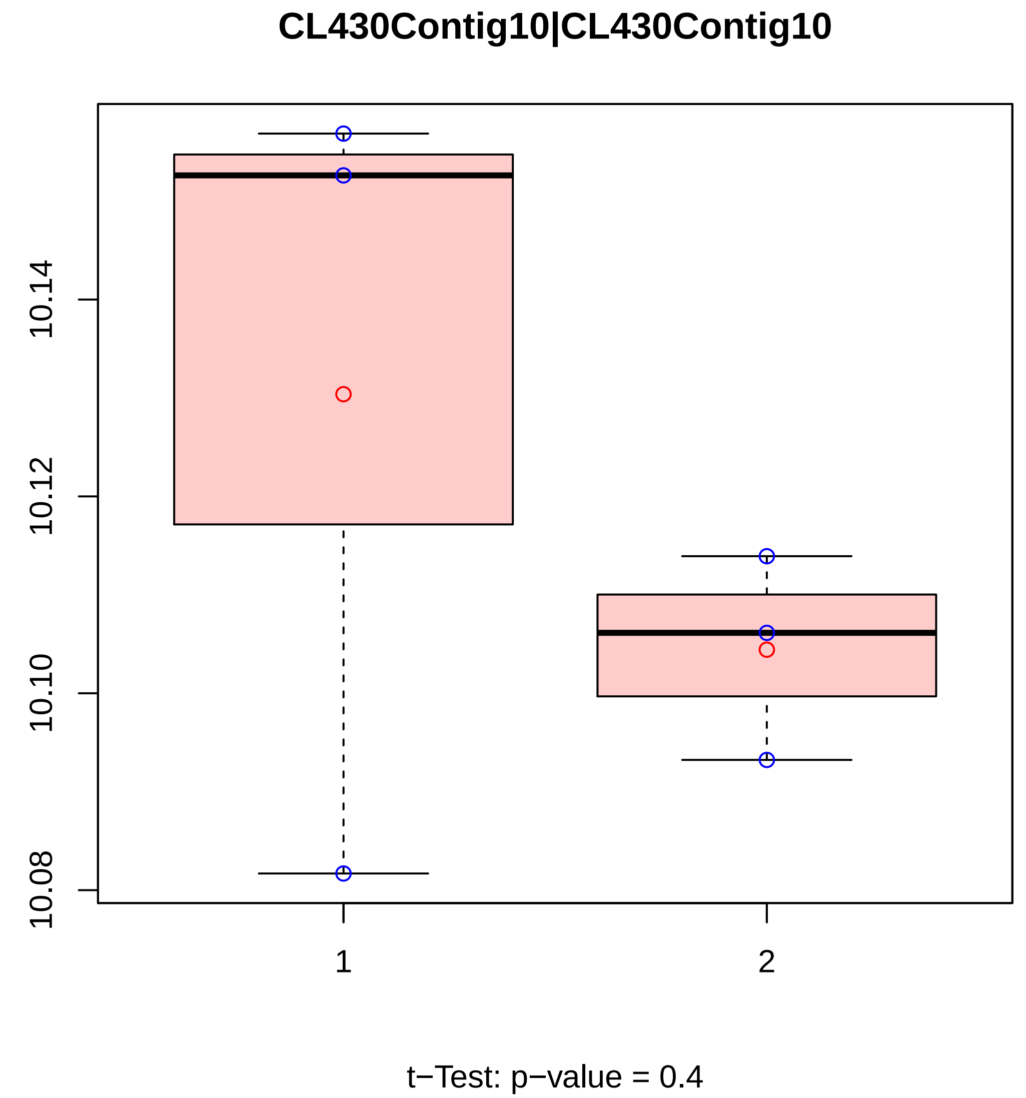

CL430Contig10|CL430Contig10
10.08
10.10
10.12
10.14
1
2
t−Test: p−value = 0.4

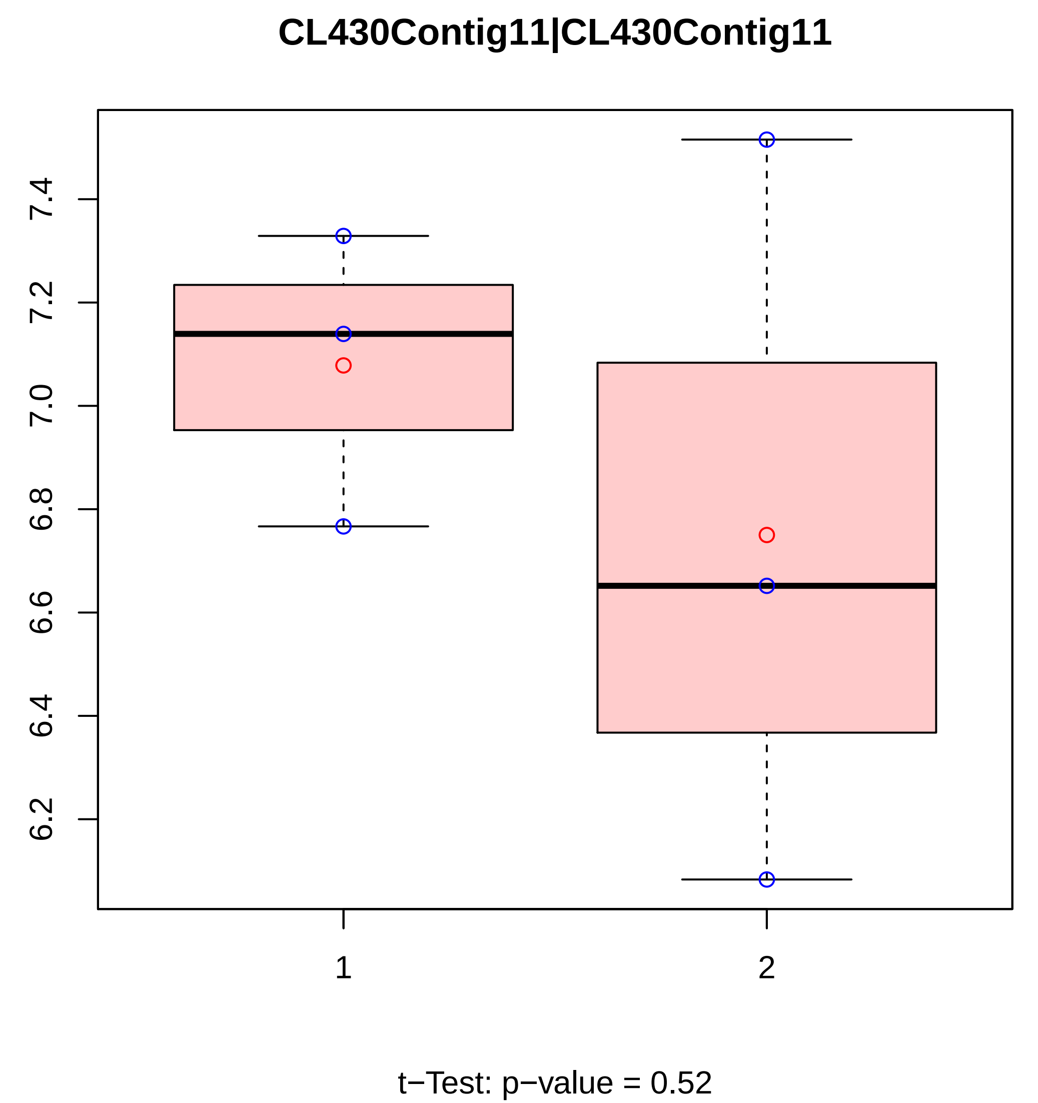
CL430Contig11|CL430Contig11
7.4
7.2
7.0
6.8
6.6
6.4
6.2
1
2
t−Test: p−value = 0.52

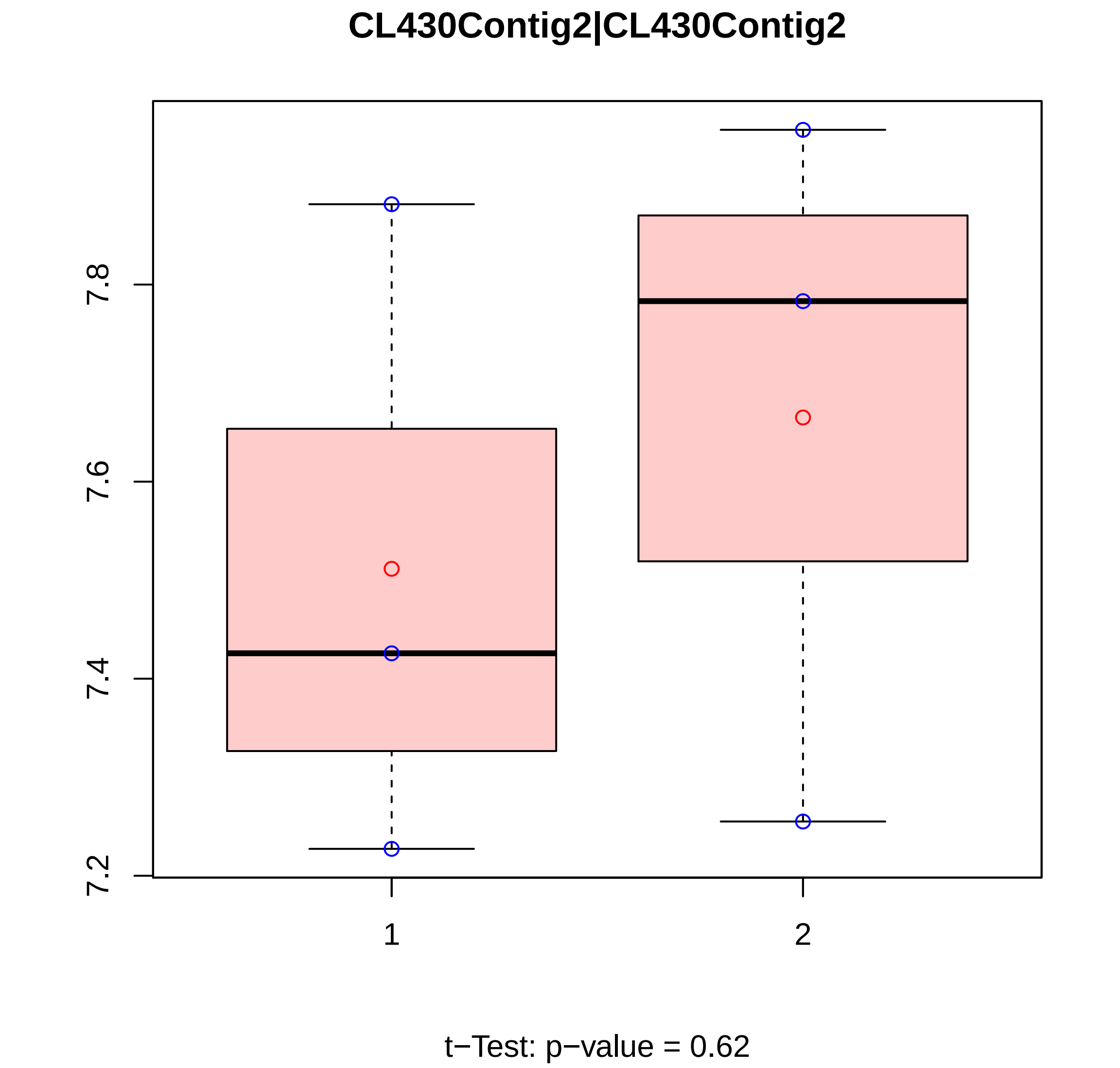

CL430Contig2|CL430Contig2
7.2
7.4
7.6
7.8
1
2
t−Test: p−value = 0.62

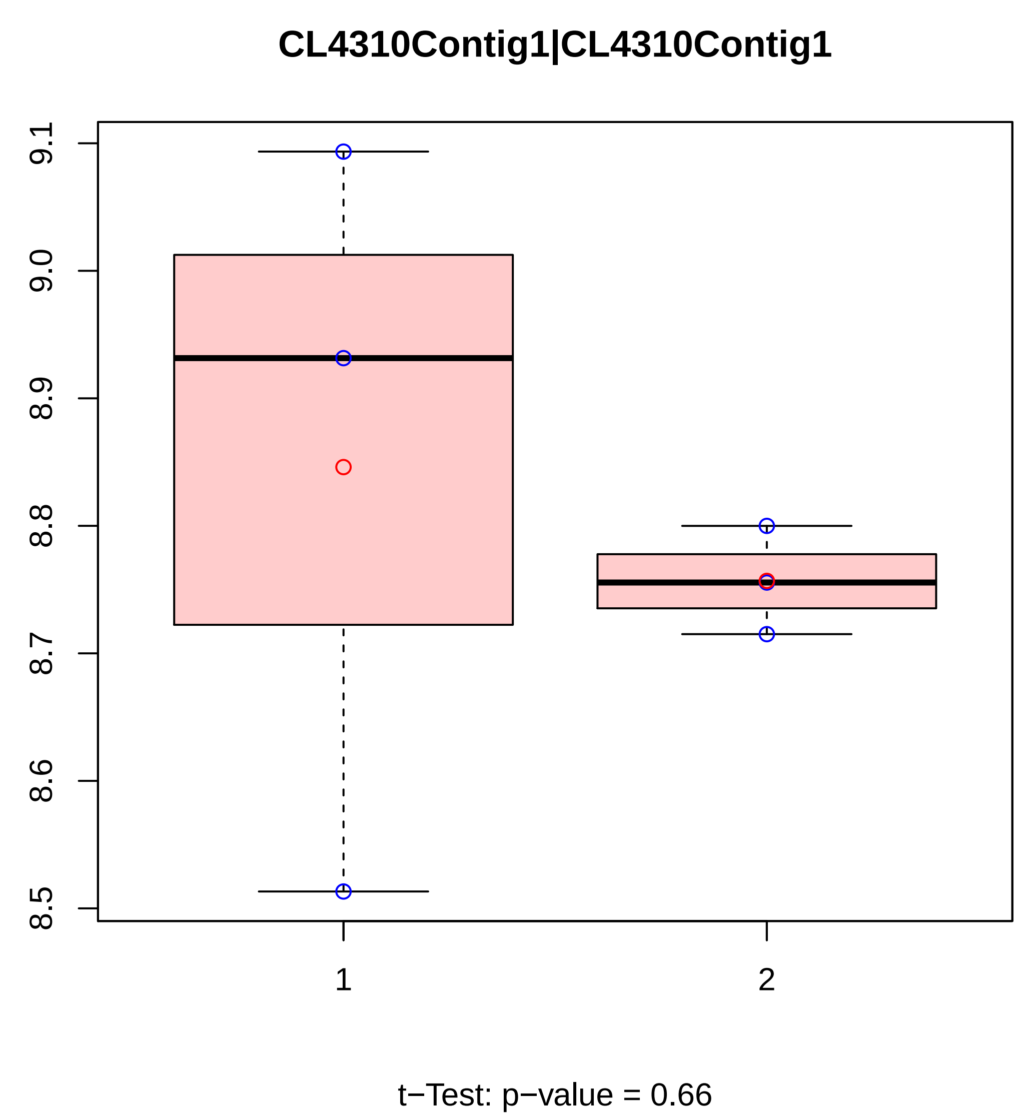

CL4310Contig1|CL4310Contig1
9.1
9.0
8.9
8.8
8.7
8.6
8.5
1
2
t−Test: p−value = 0.66

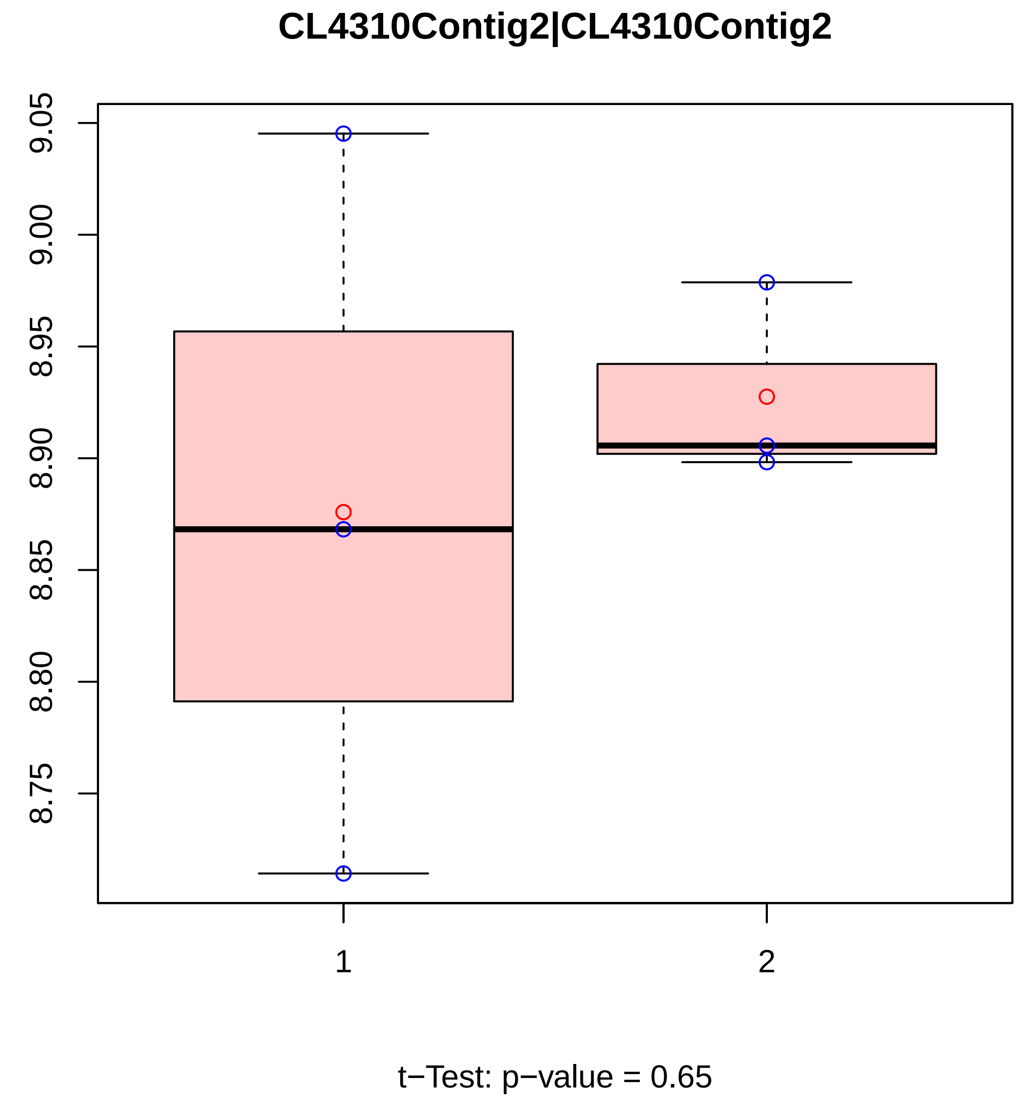
CL4310Contig2|CL4310Contig2
9.05
9.00
8.95
8.90
8.85
8.80
8.75
1
2
t−Test: p−value = 0.65

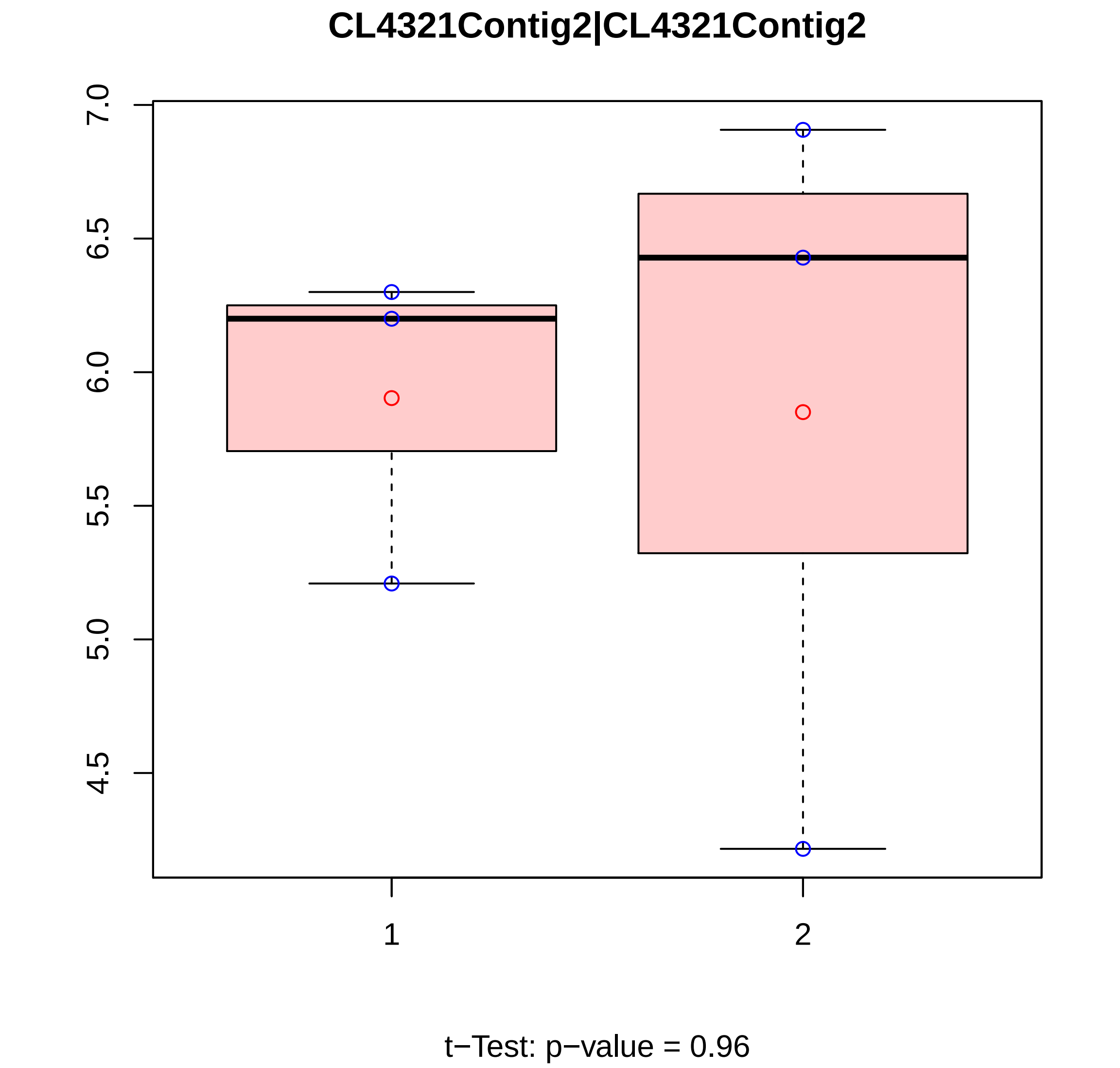
CL4321Contig2|CL4321Contig2
7.0
6.5
6.0
5.5
5.0
4.5
1
2
t−Test: p−value = 0.96

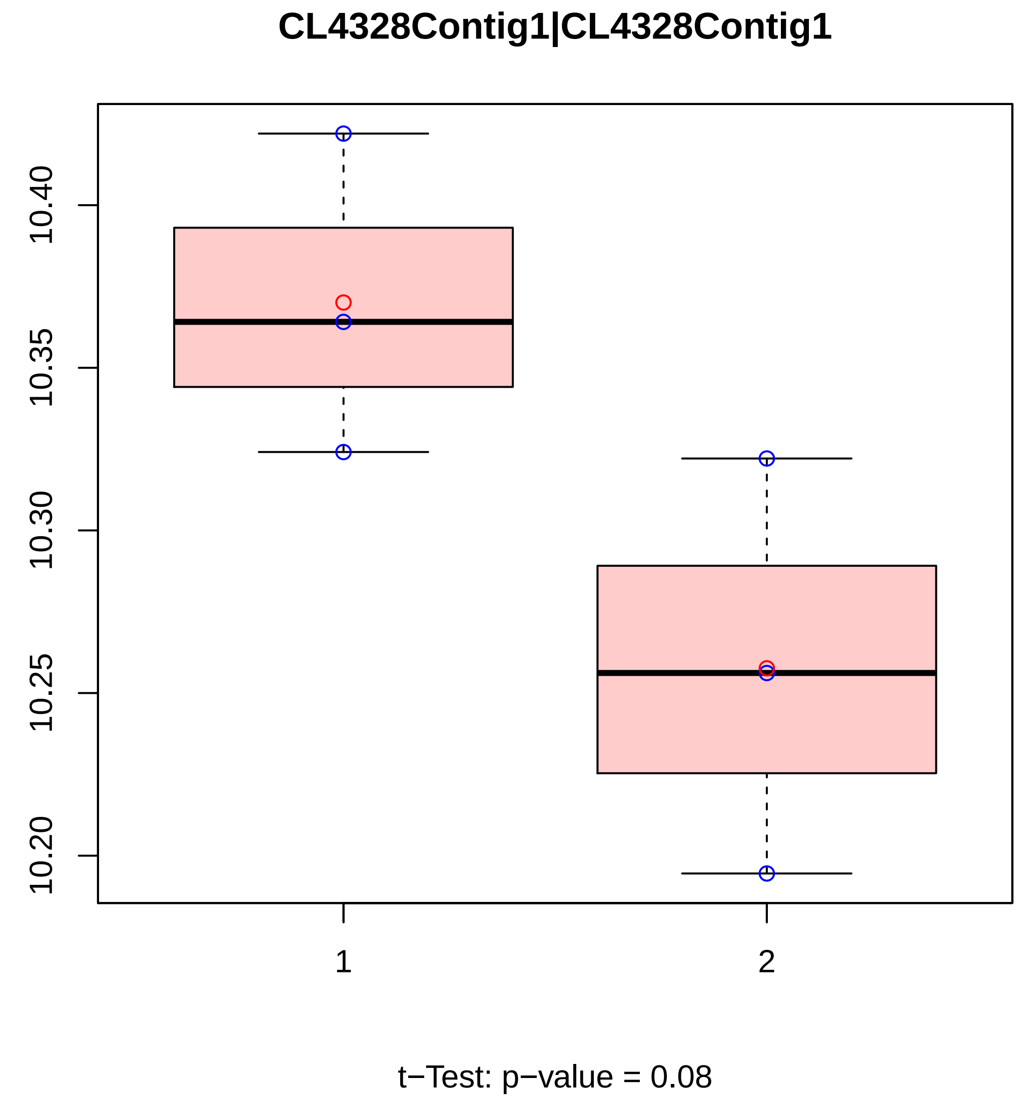
CL4328Contig1|CL4328Contig1
10.20
10.25
10.30
10.35
10.40
1
2
t−Test: p−value = 0.08

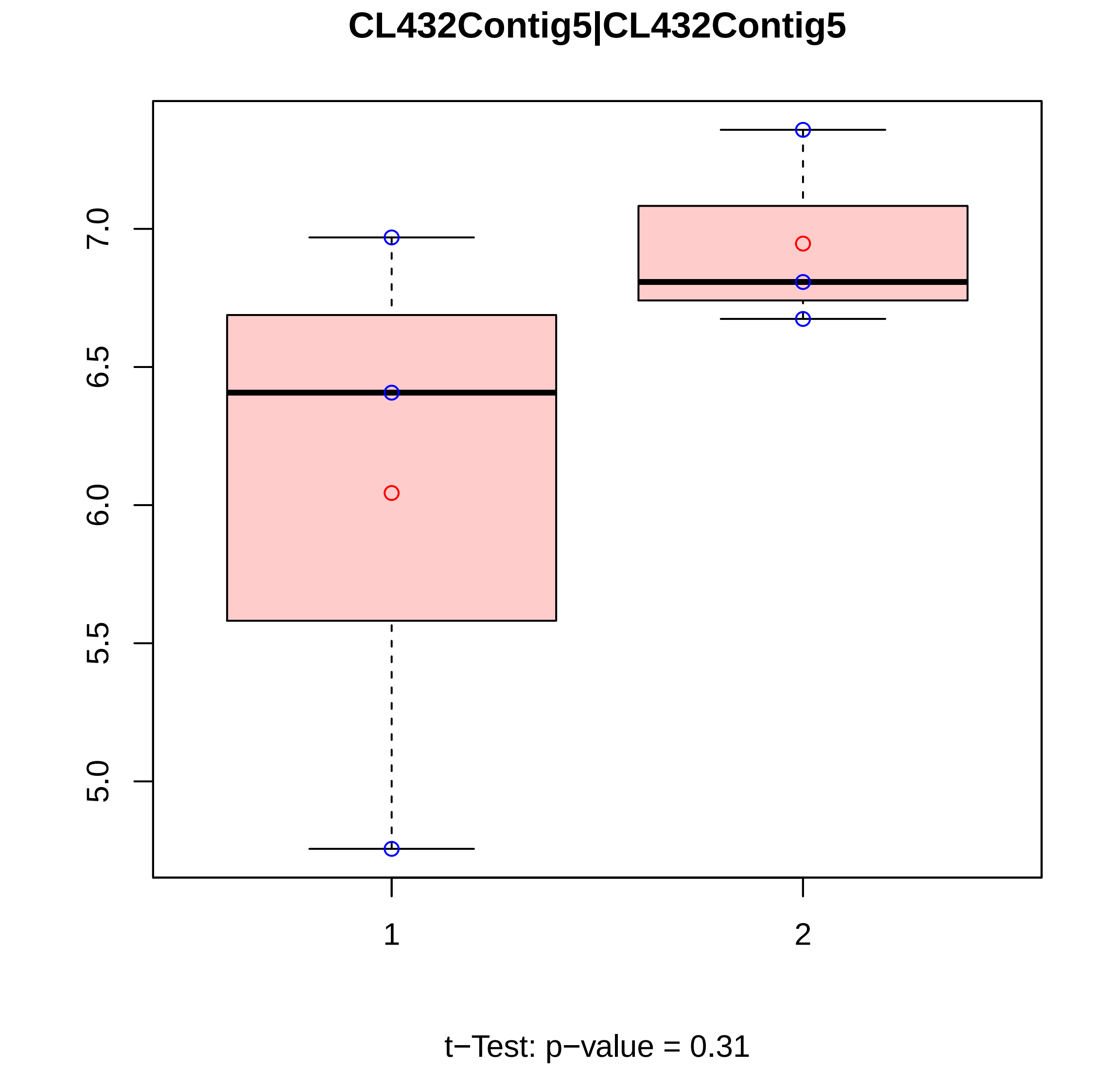
CL432Contig5|CL432Contig5
7.0
6.5
6.0
5.5
5.0
1
2
t−Test: p−value = 0.31

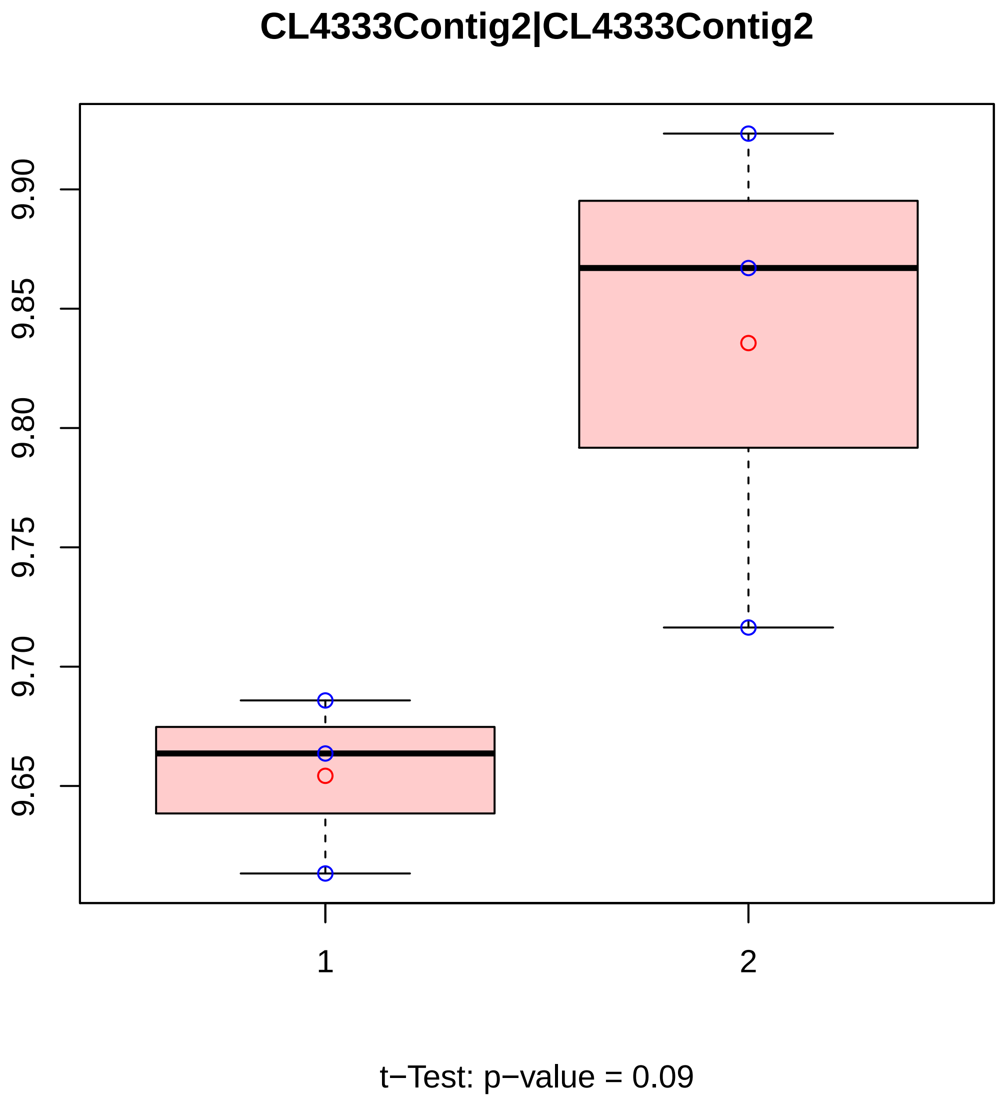

CL4333Contig2|CL4333Contig2
9.65
9.70
9.75
9.80
9.85
9.90
1
2
t−Test: p−value = 0.09

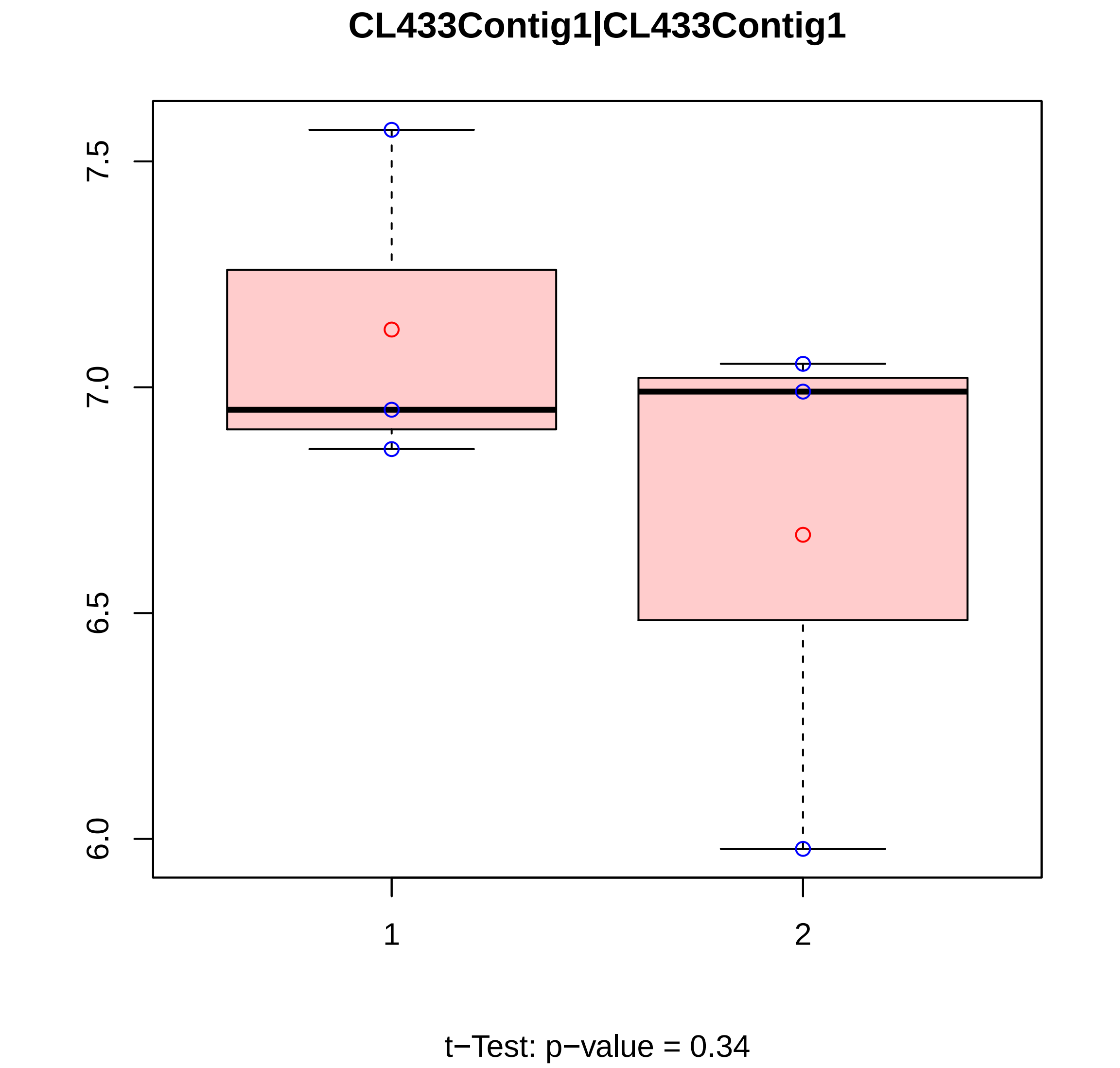
CL433Contig1|CL433Contig1
7.5
7.0
6.5
6.0
1
2
t−Test: p−value = 0.34

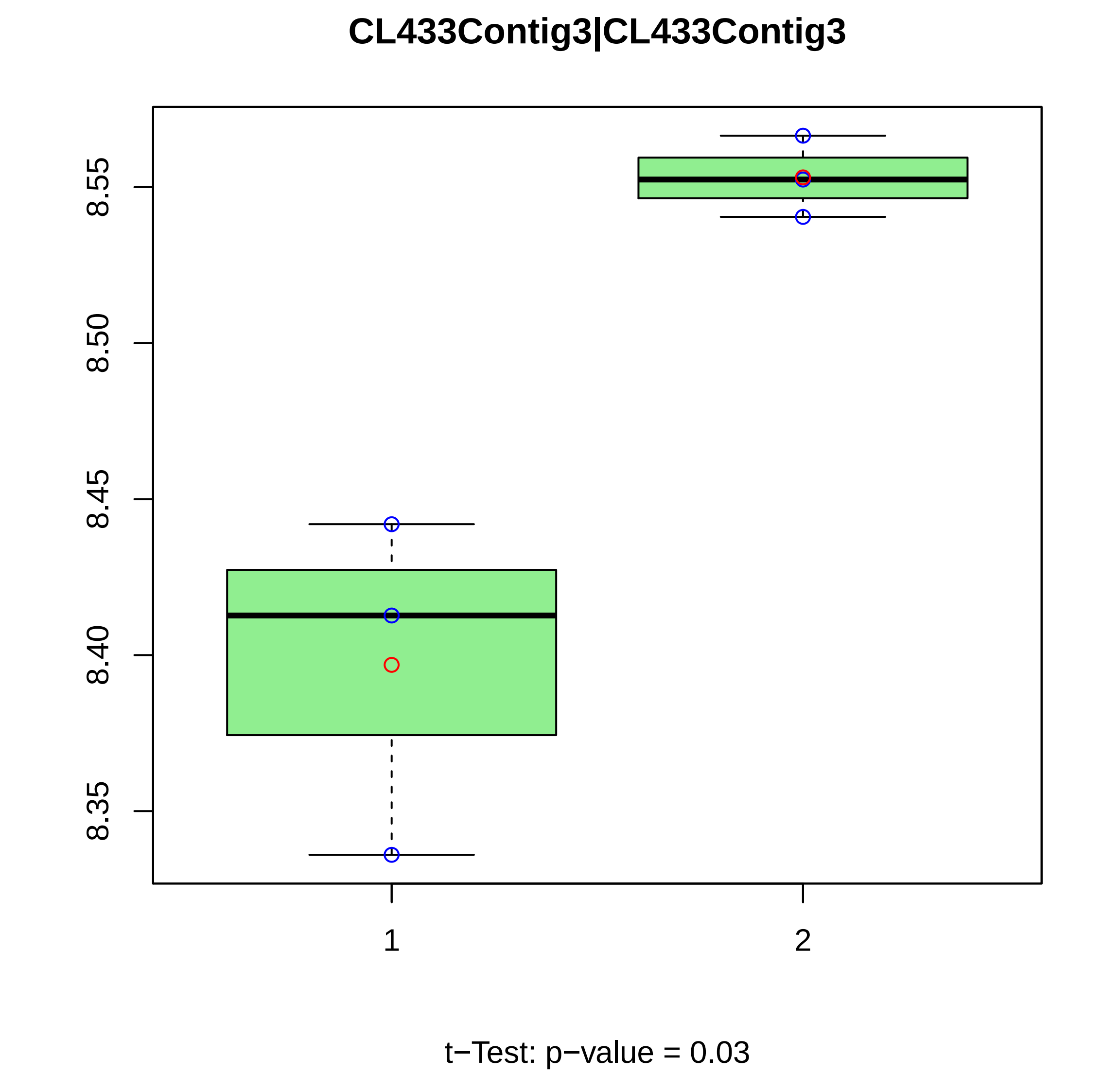
CL433Contig3|CL433Contig3
8.35
8.40
8.45
8.50
8.55
1
2
t−Test: p−value = 0.03

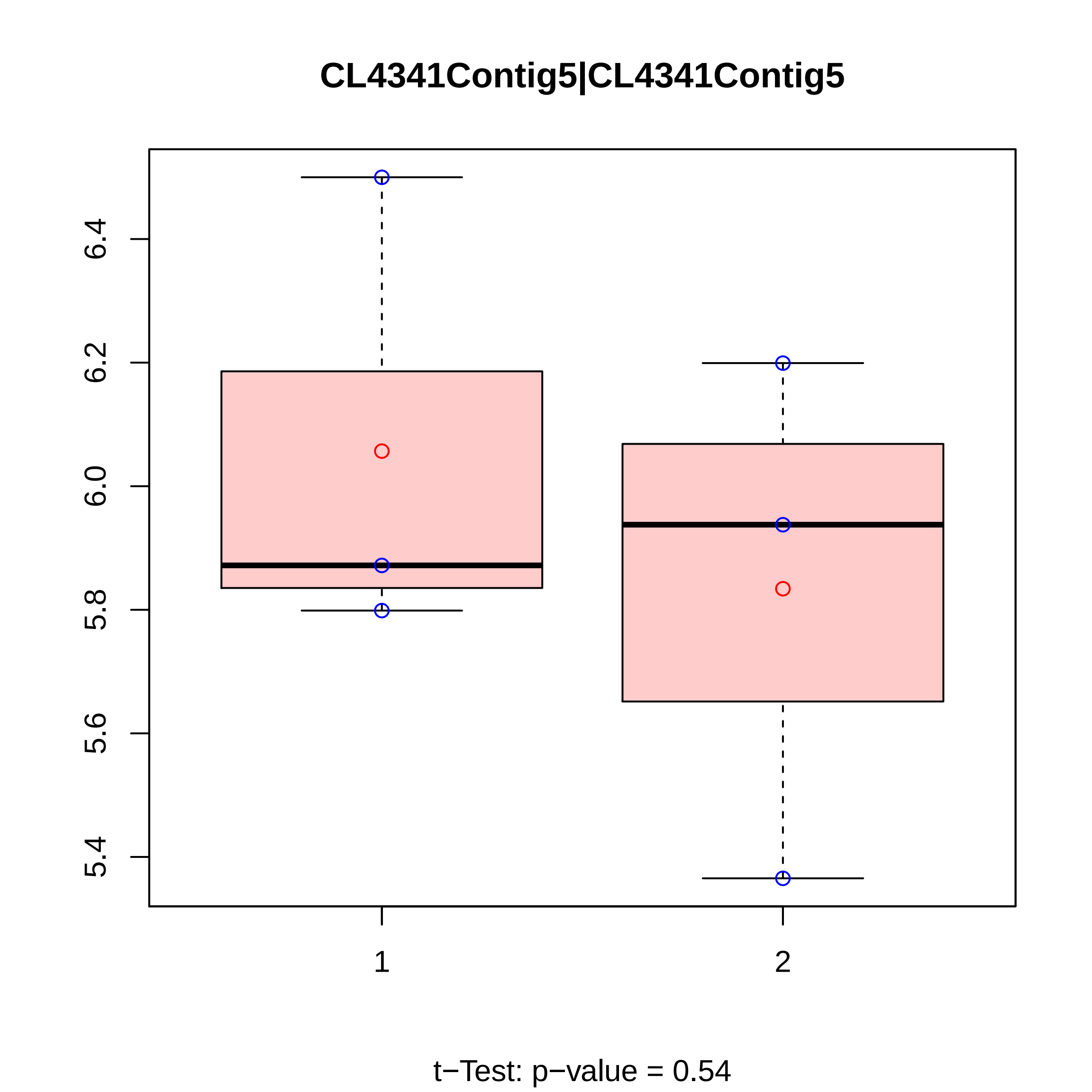

CL4341Contig5|CL4341Contig5
6.4
6.2
6.0
5.8
5.6
5.4
1
2
t−Test: p−value = 0.54

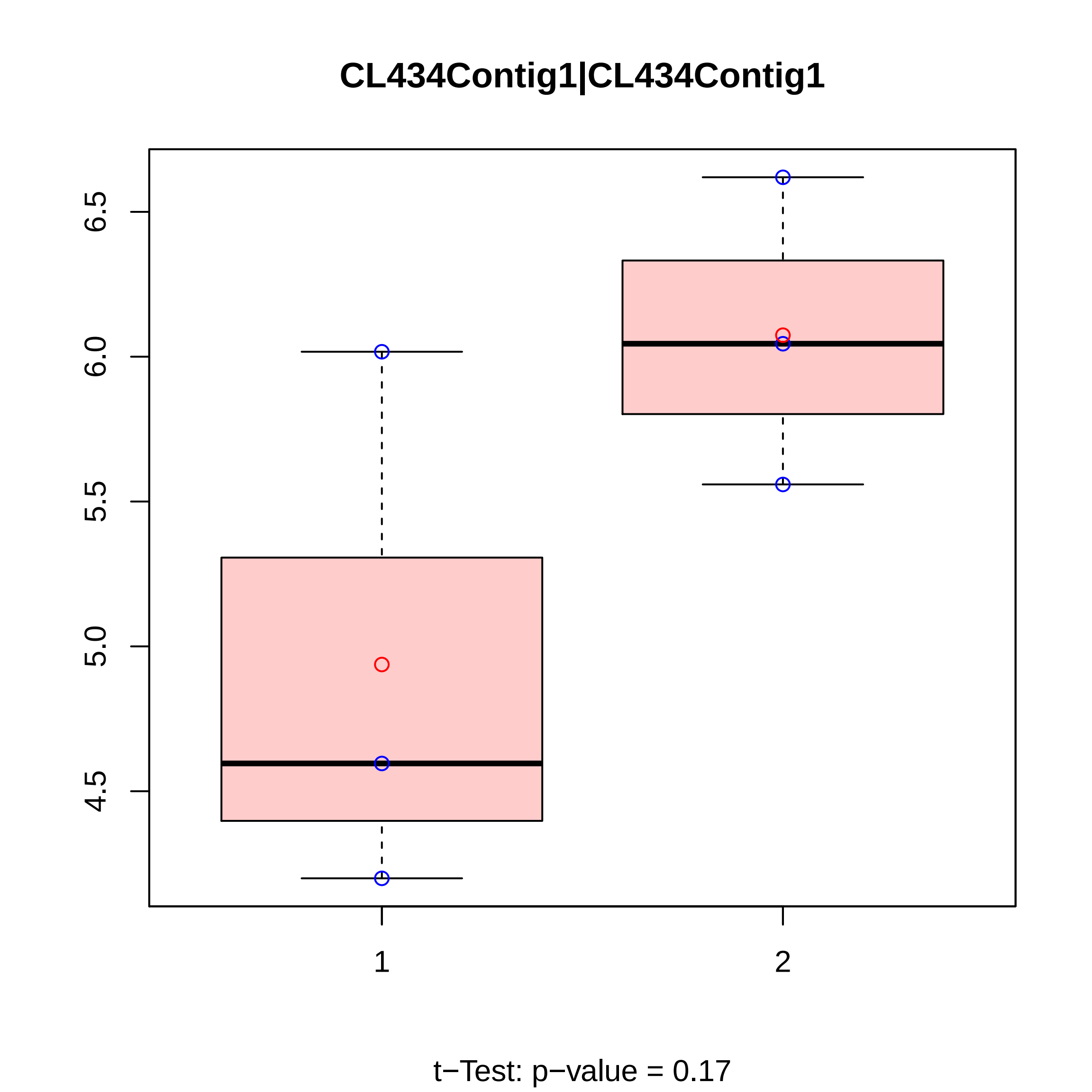

CL434Contig1|CL434Contig1
6.5
6.0
5.5
5.0
4.5
1
2
t−Test: p−value = 0.17

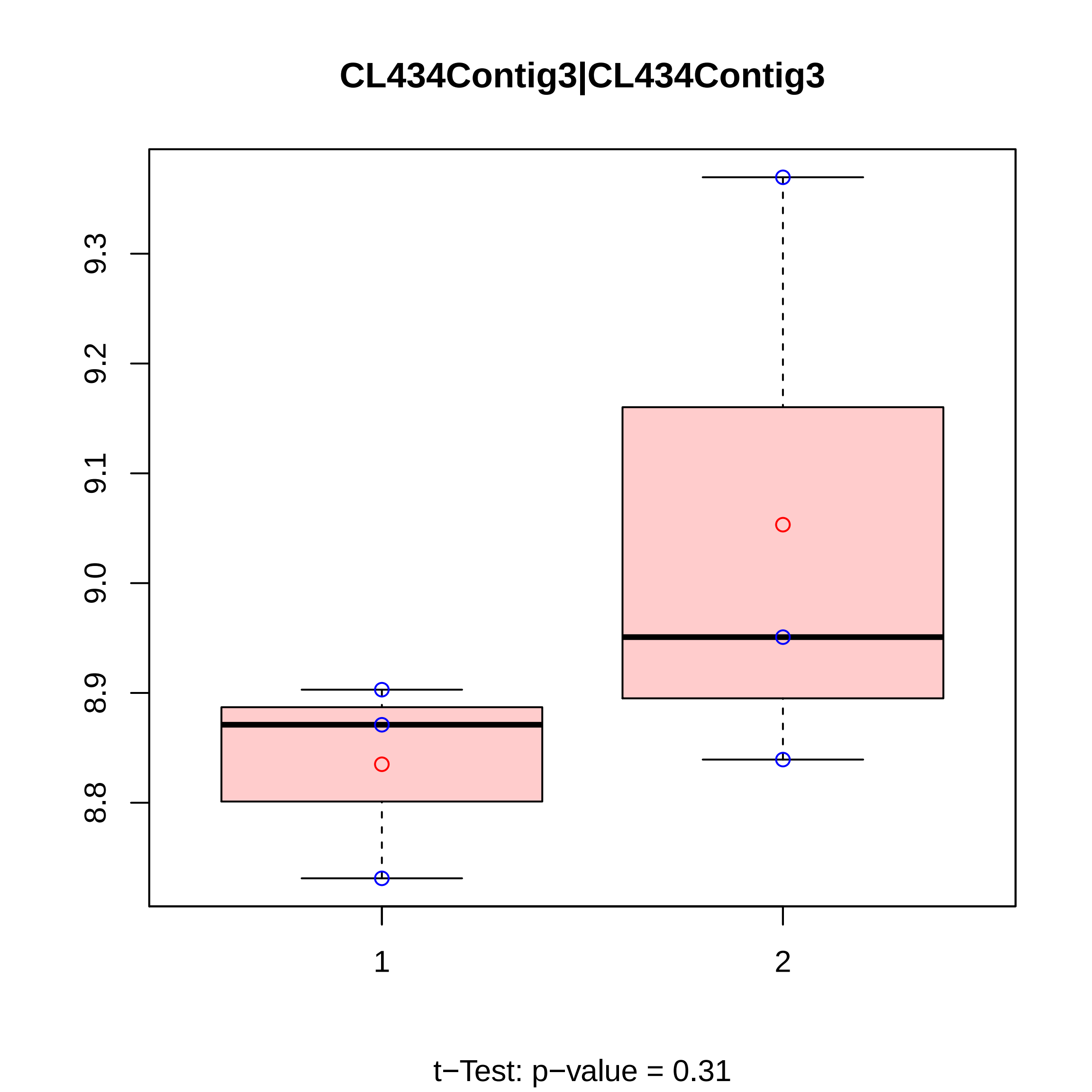

CL434Contig3|CL434Contig3
9.3
9.2
9.1
9.0
8.9
8.8
1
2
t−Test: p−value = 0.31

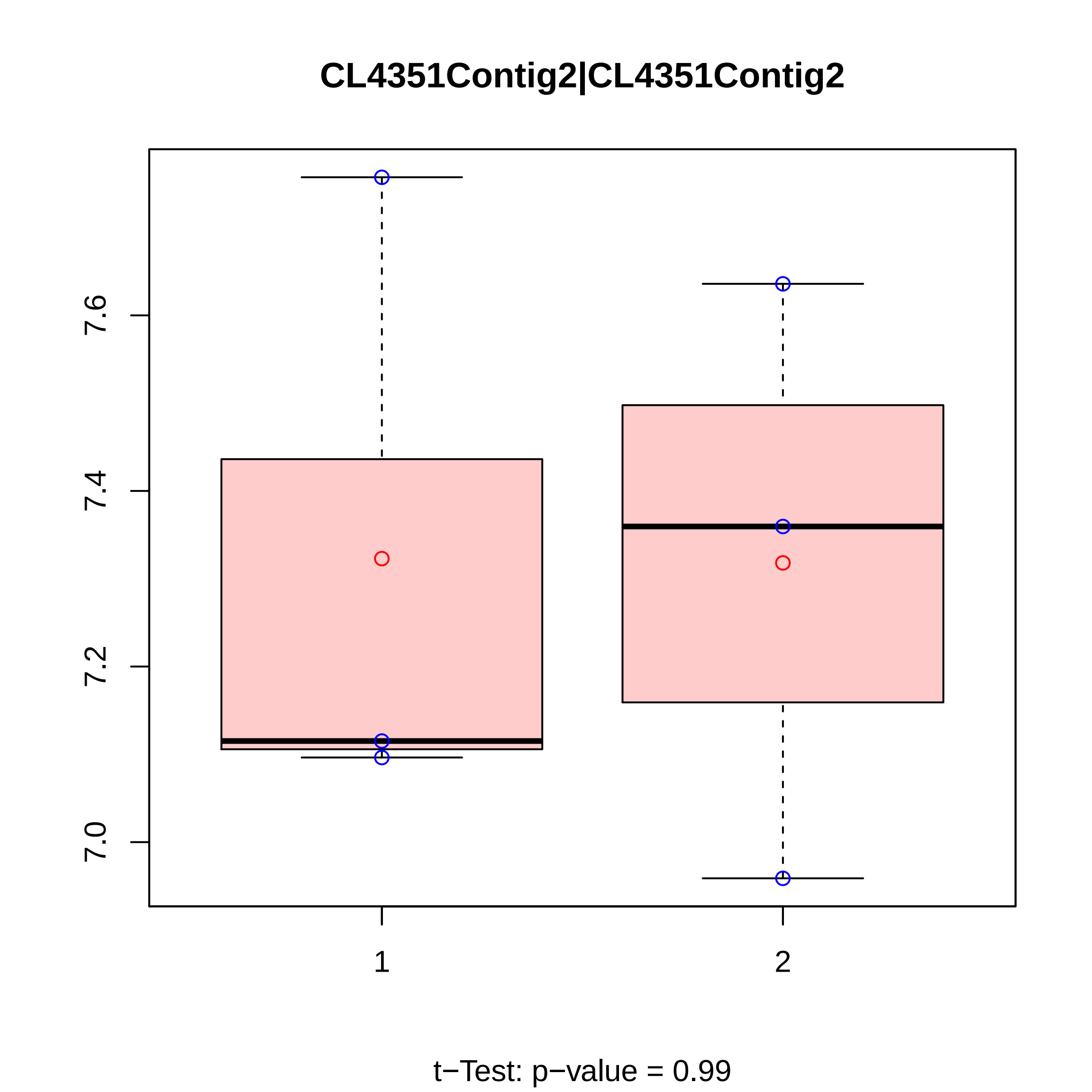
CL4351Contig2|CL4351Contig2
7.0
7.2
7.4
7.6
1
2
t−Test: p−value = 0.99

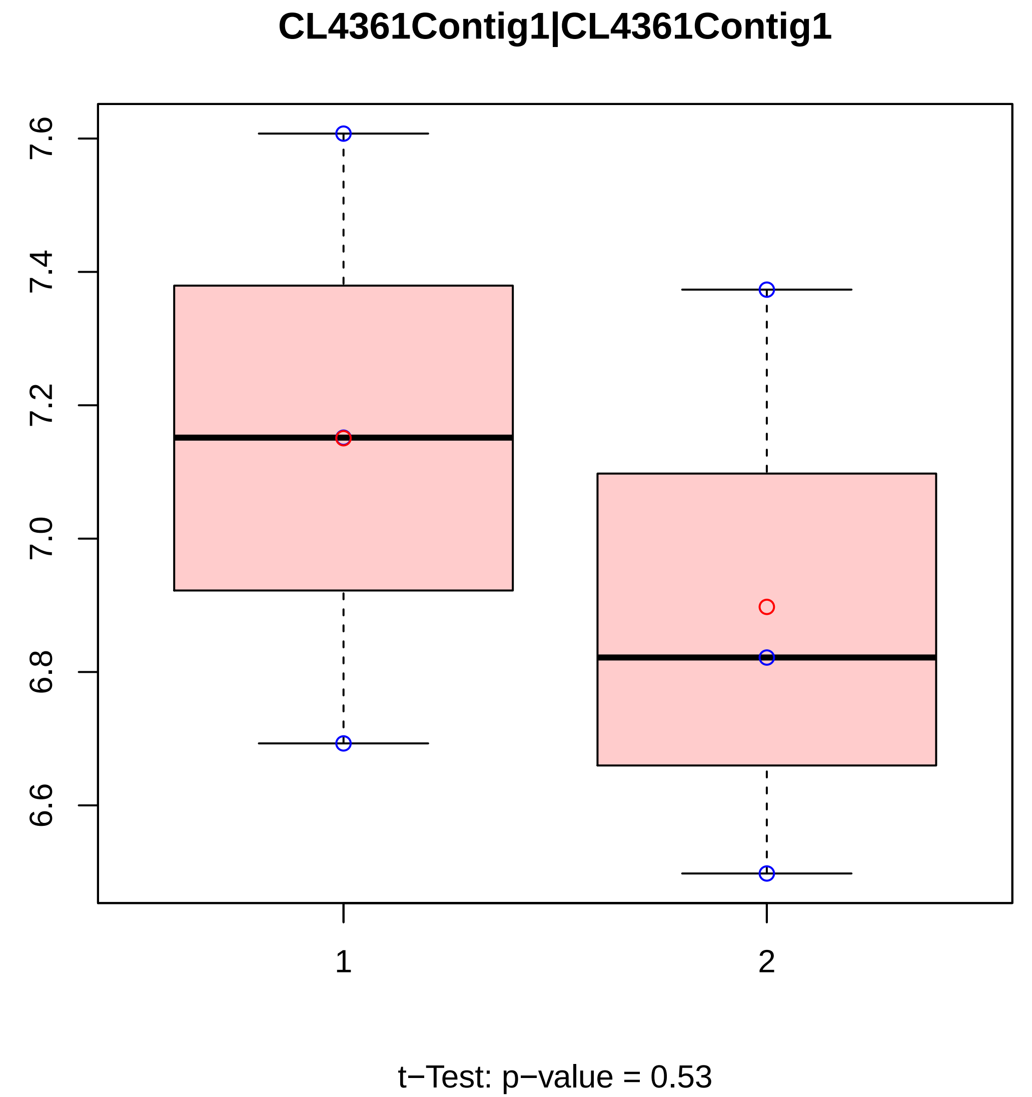

CL4361Contig1|CL4361Contig1
7.6
7.4
7.2
7.0
6.8
6.6
1
2
t−Test: p−value = 0.53

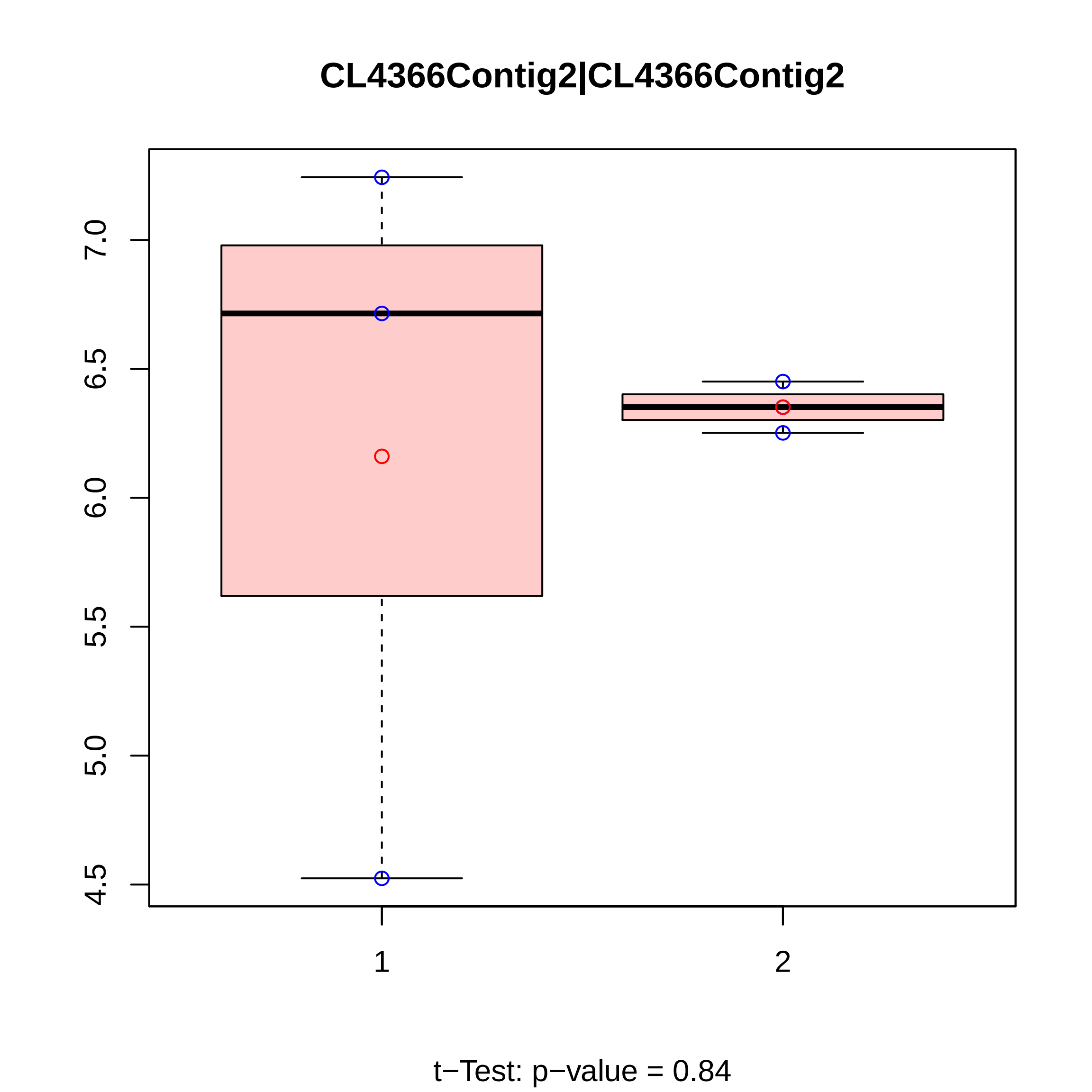
CL4366Contig2|CL4366Contig2
7.0
6.5
6.0
5.5
5.0
4.5
1
2
t−Test: p−value = 0.84

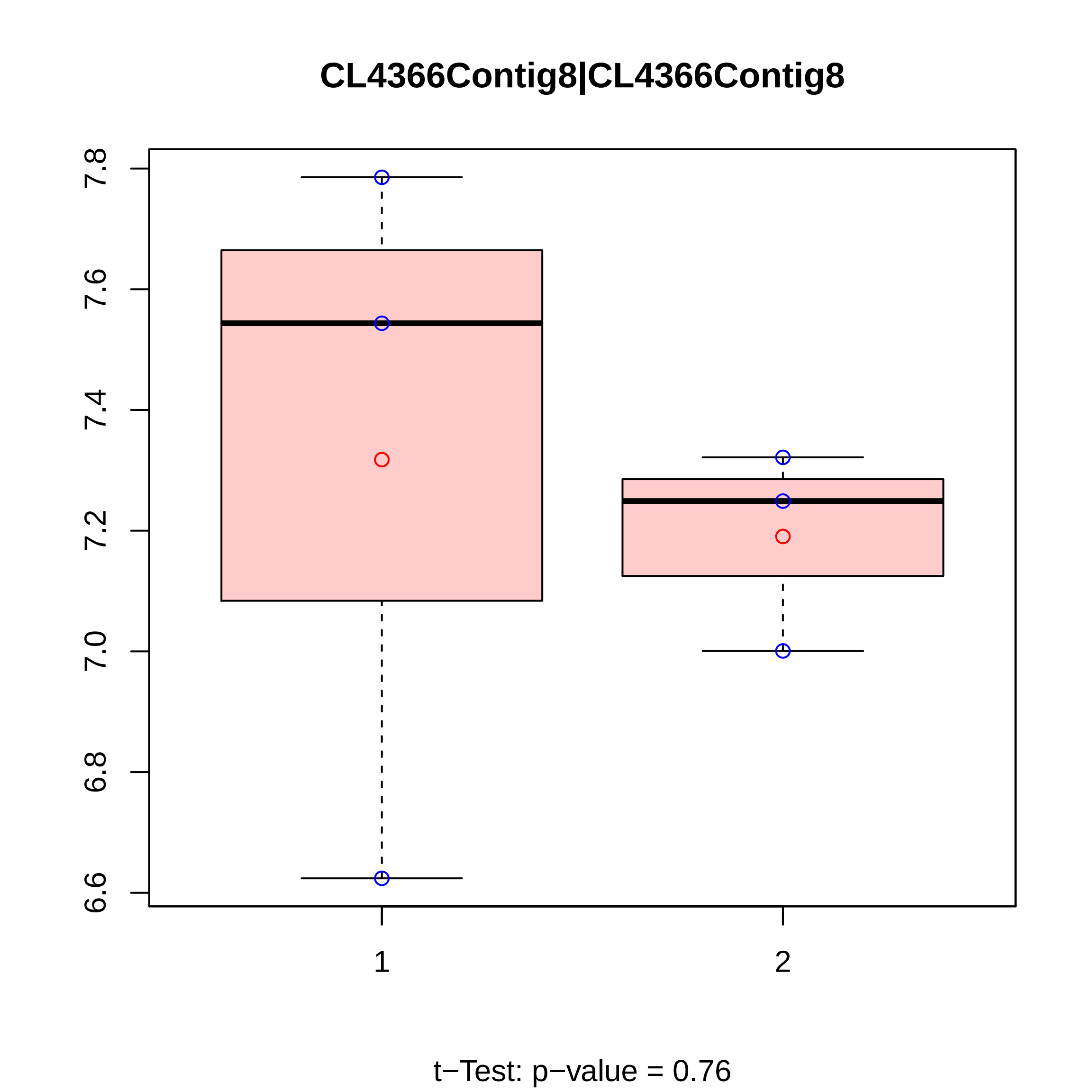

CL4366Contig8|CL4366Contig8
7.8
7.6
7.4
7.2
7.0
6.8
6.6
1
2
t−Test: p−value = 0.76

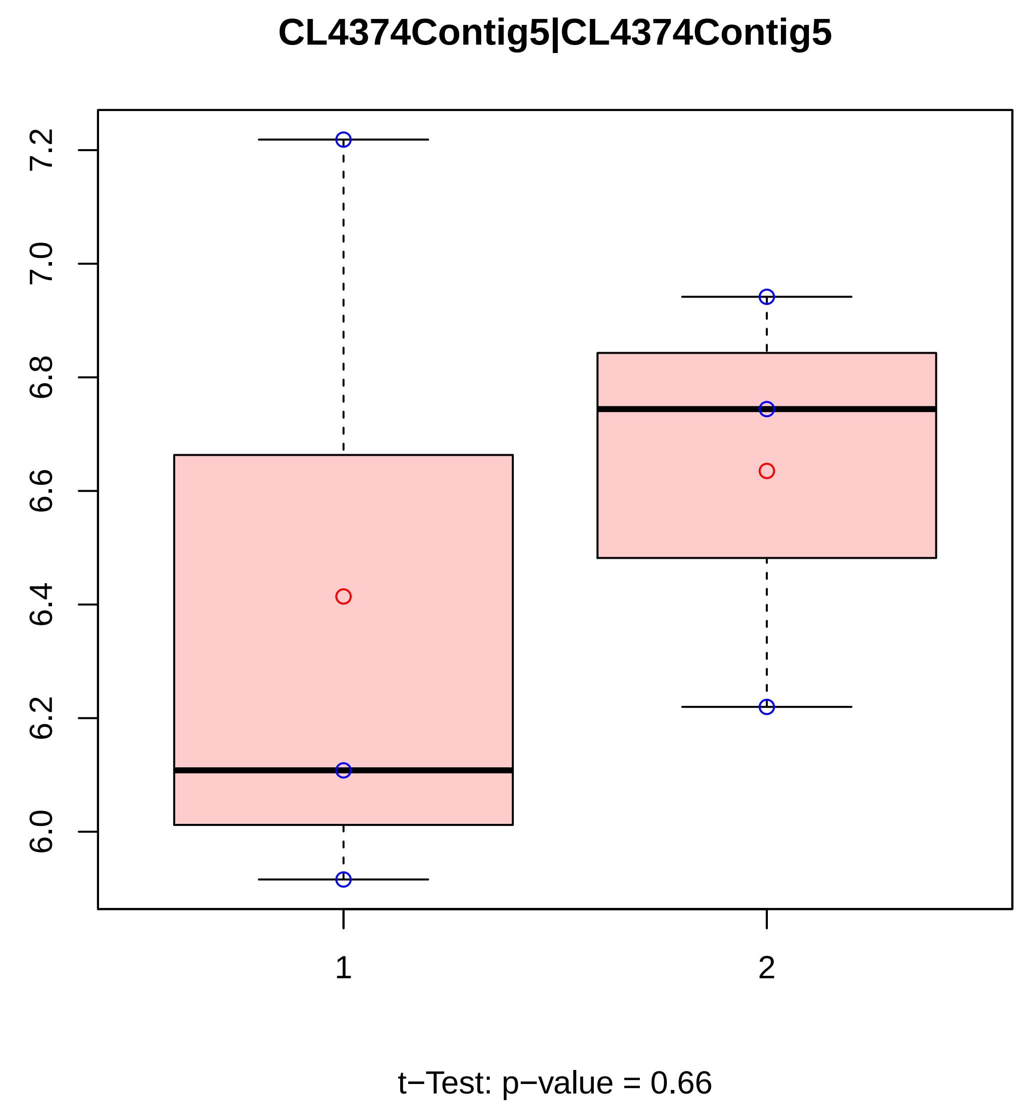

CL4374Contig5|CL4374Contig5
7.2
7.0
6.8
6.6
6.4
6.2
6.0
1
2
t−Test: p−value = 0.66

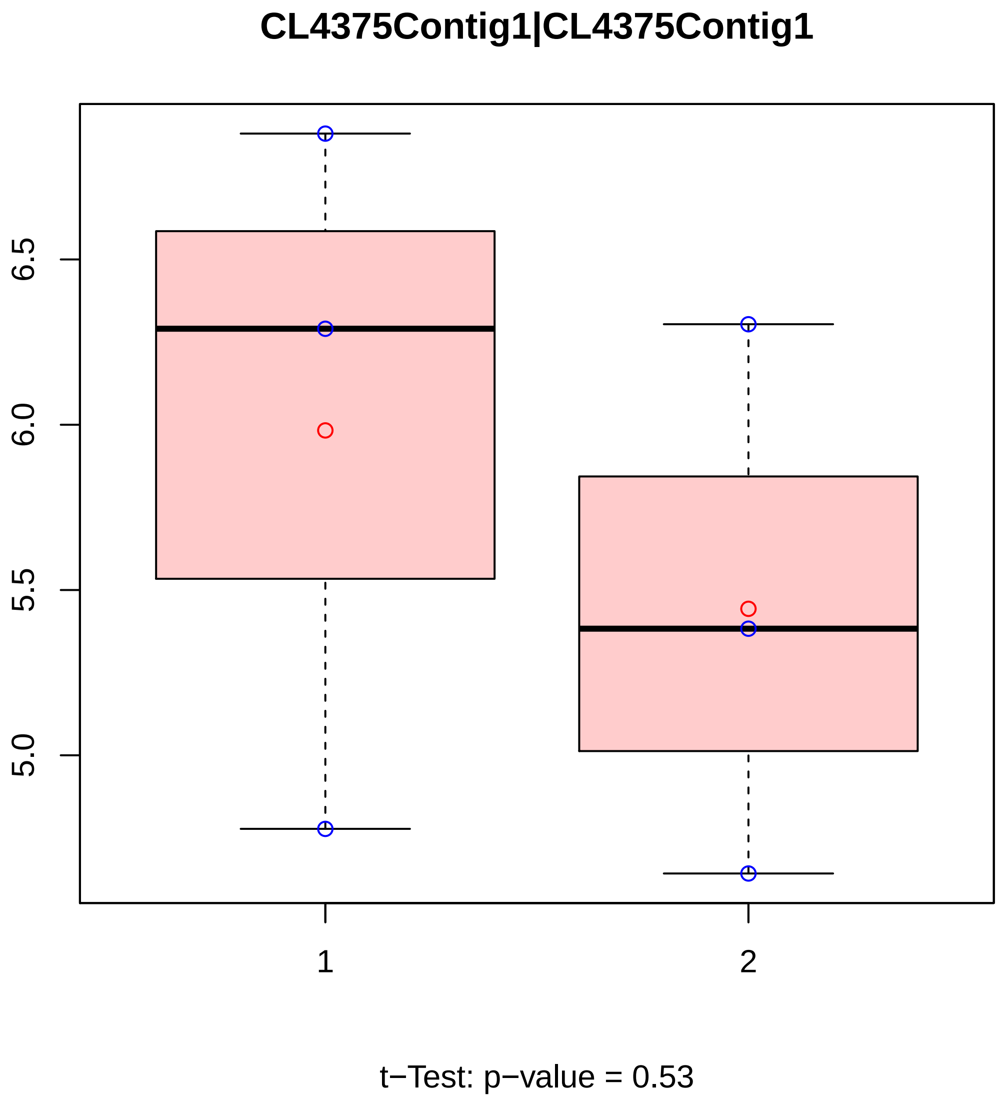

CL4375Contig1|CL4375Contig1
6.5
6.0
5.5
5.0
1
2
t−Test: p−value = 0.53

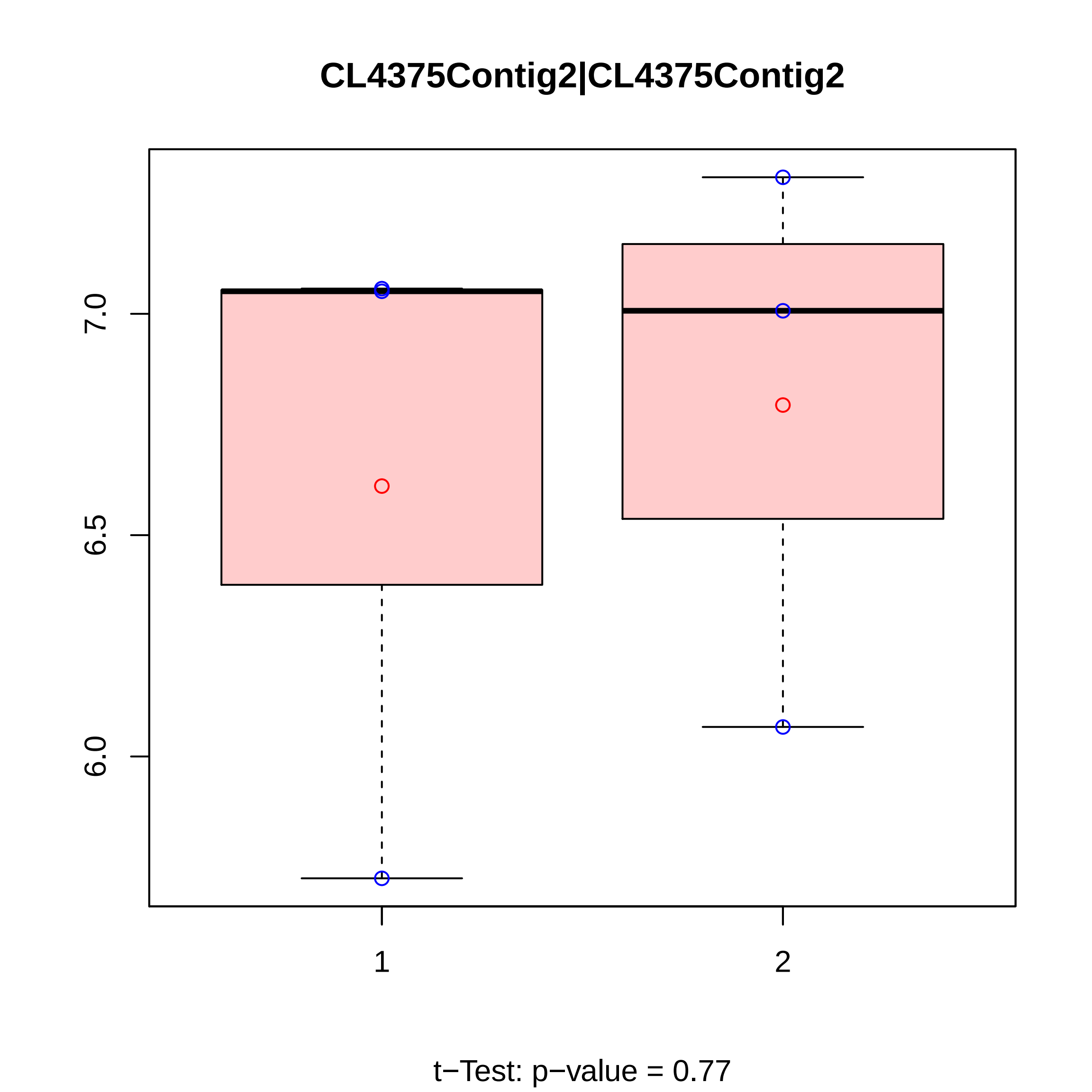

CL4375Contig2|CL4375Contig2
7.0
6.5
6.0
1
2
t−Test: p−value = 0.77

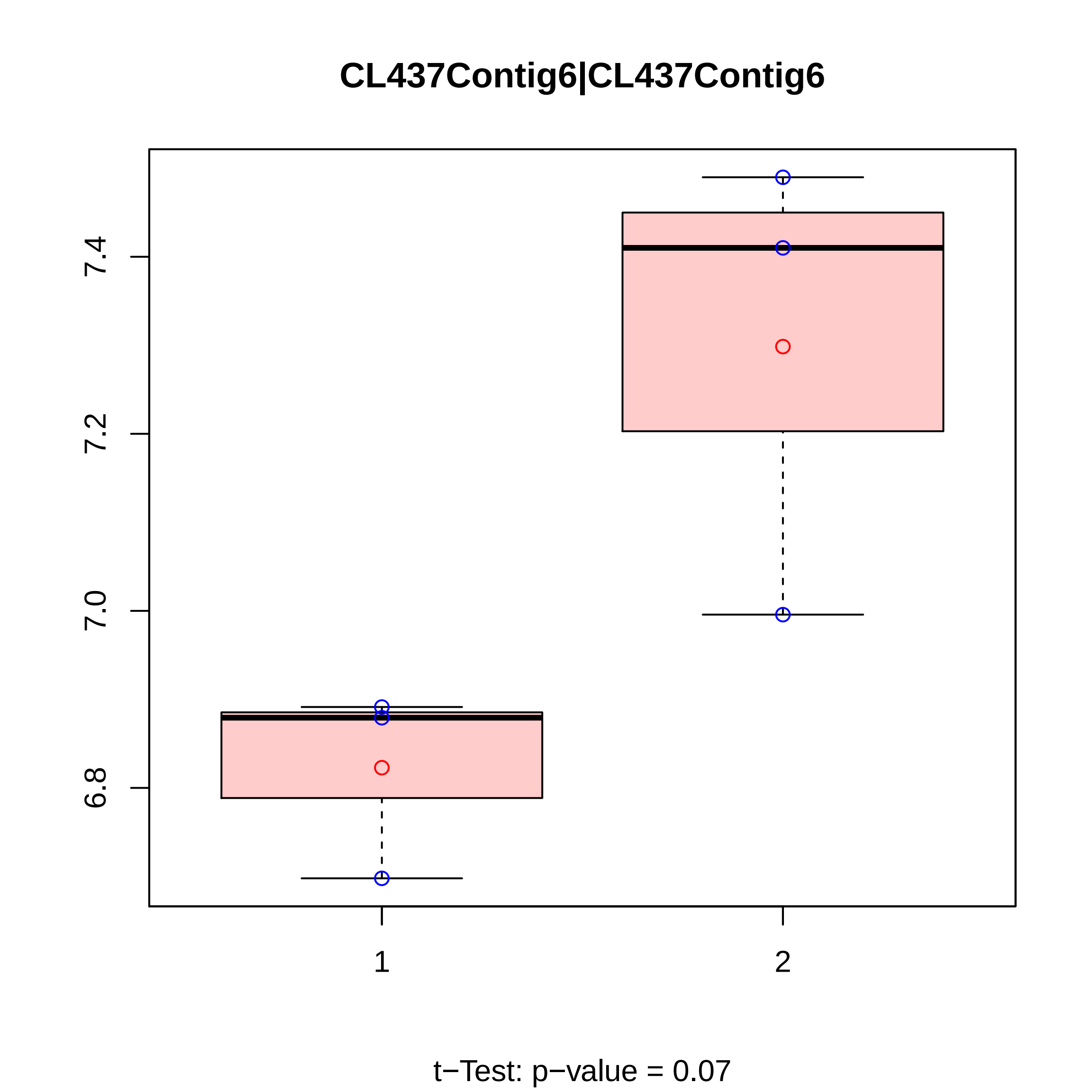
CL437Contig6|CL437Contig6
7.4
7.2
7.0
6.8
1
2
t−Test: p−value = 0.07

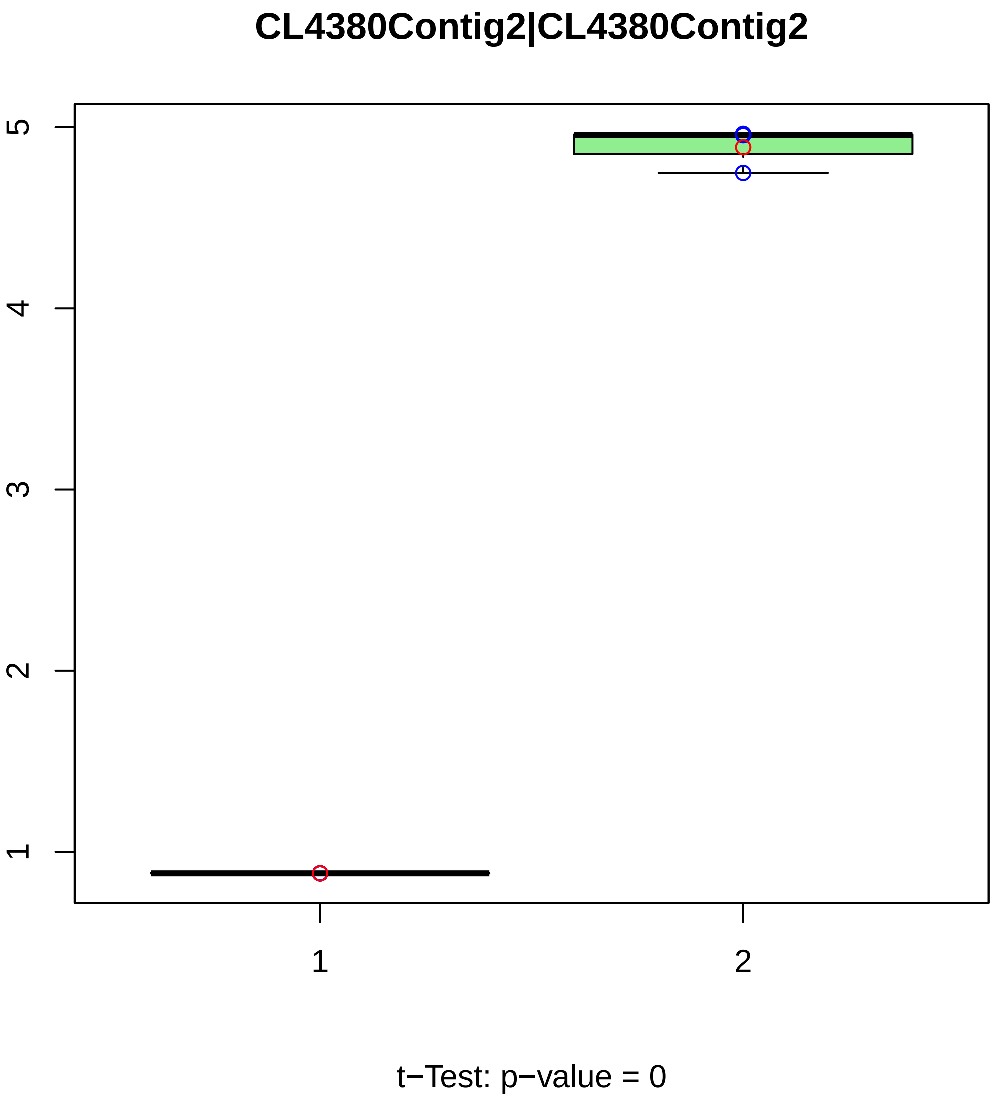
CL4380Contig2|CL4380Contig2
5
4
3
2
1
1
2
t−Test: p−value = 0

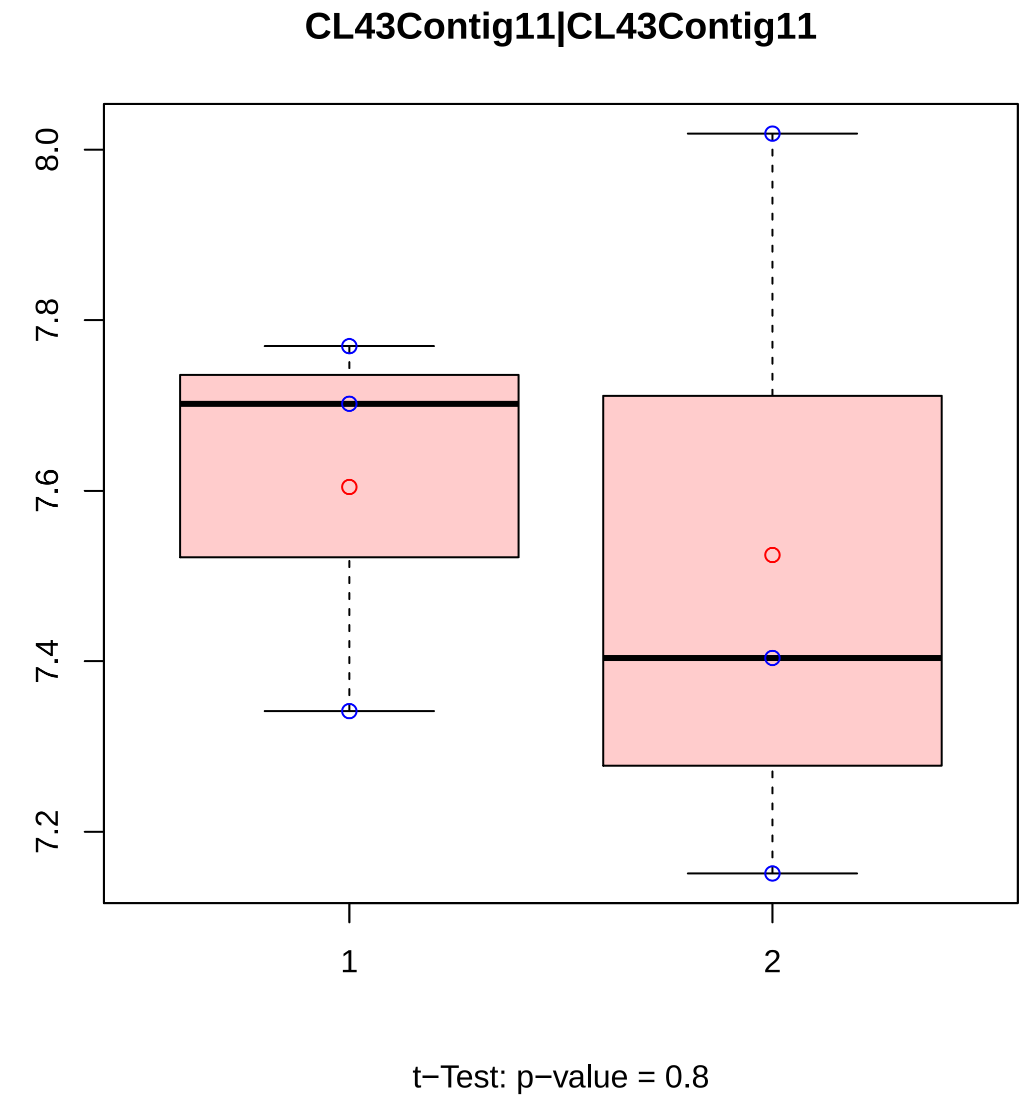

CL43Contig11|CL43Contig11
8.0
7.8
7.6
7.4
7.2
1
2
t−Test: p−value = 0.8

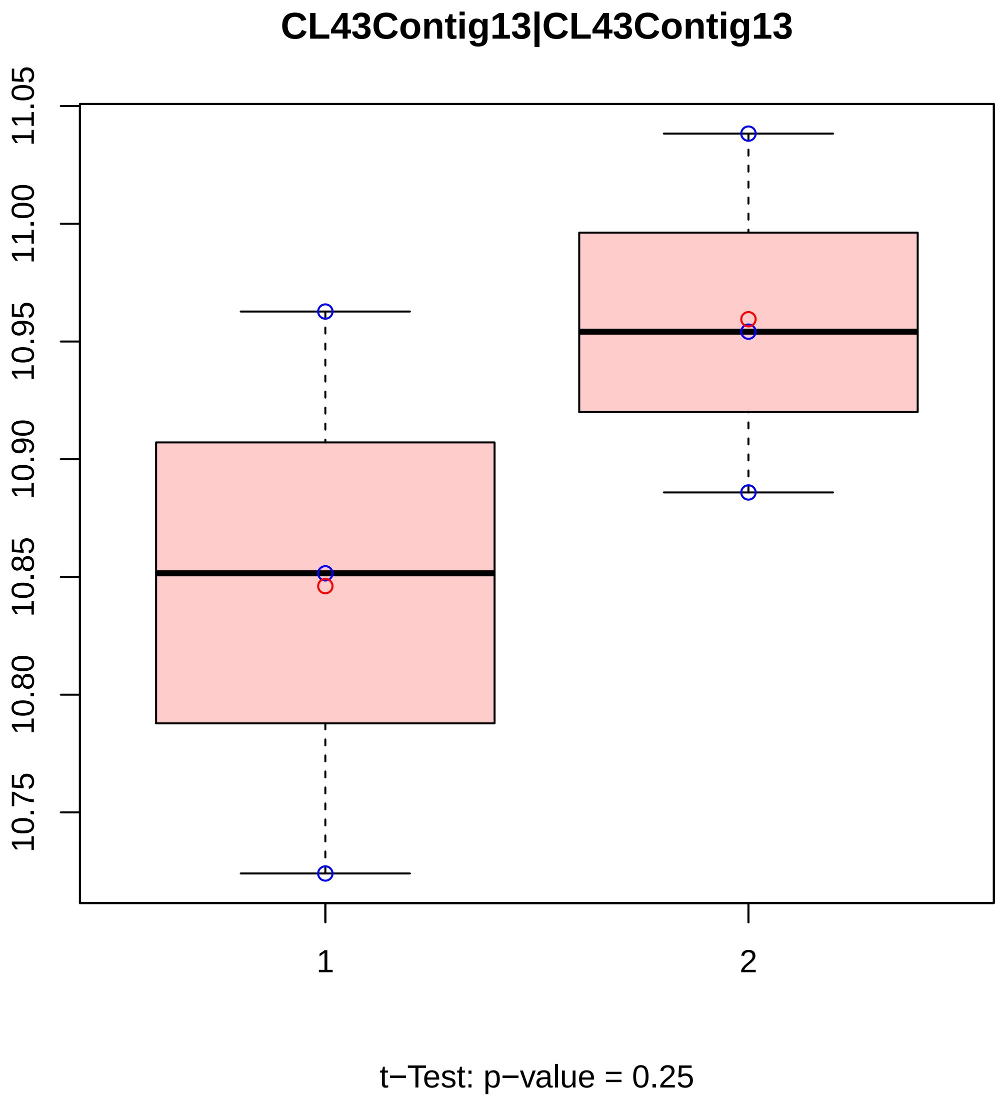
CL43Contig13|CL43Contig13
11.05
11.00
10.95
10.90
10.85
10.80
10.75
1
2
t−Test: p−value = 0.25

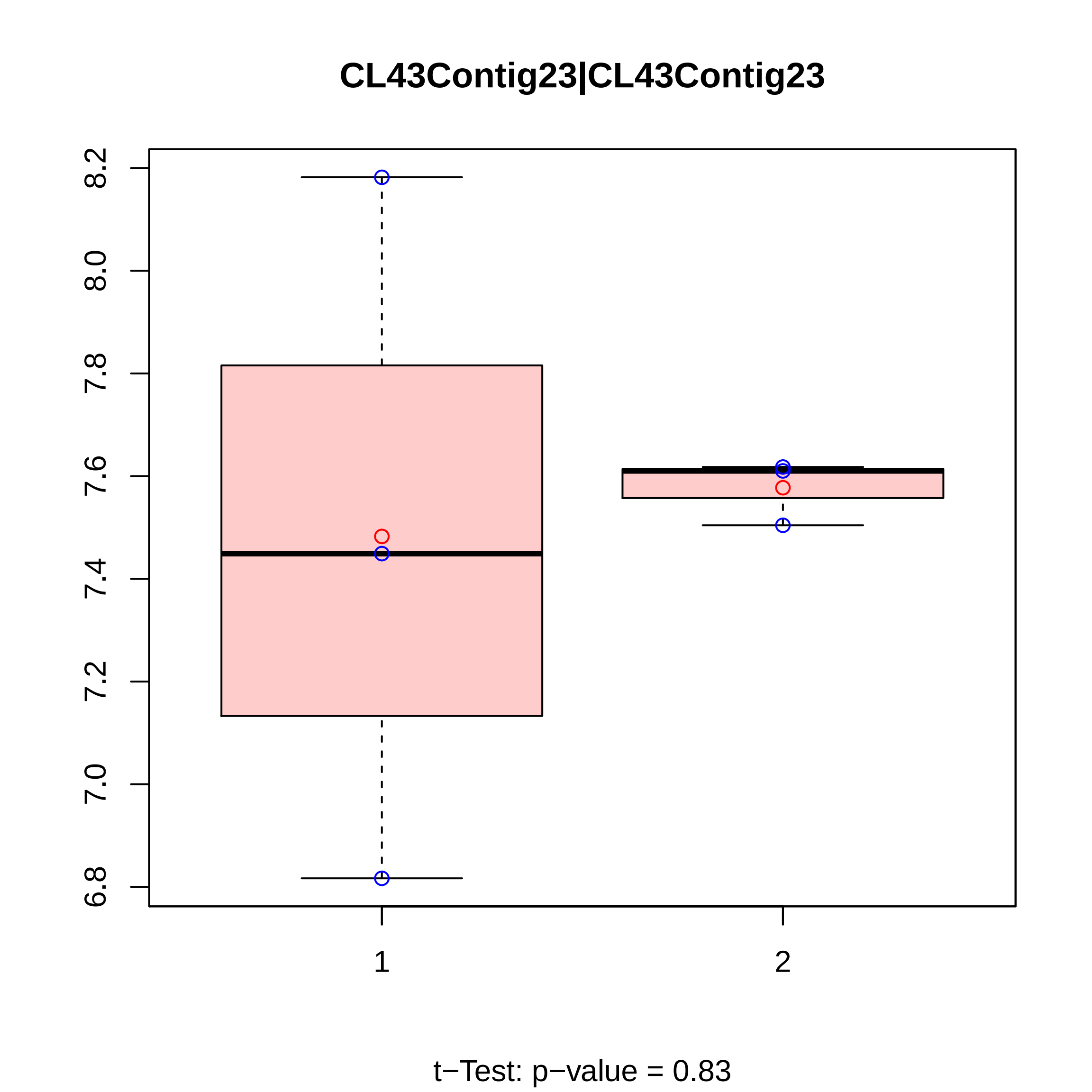

CL43Contig23|CL43Contig23
8.2
8.0
7.8
7.6
7.4
7.2
7.0
6.8
1
2
t−Test: p−value = 0.83

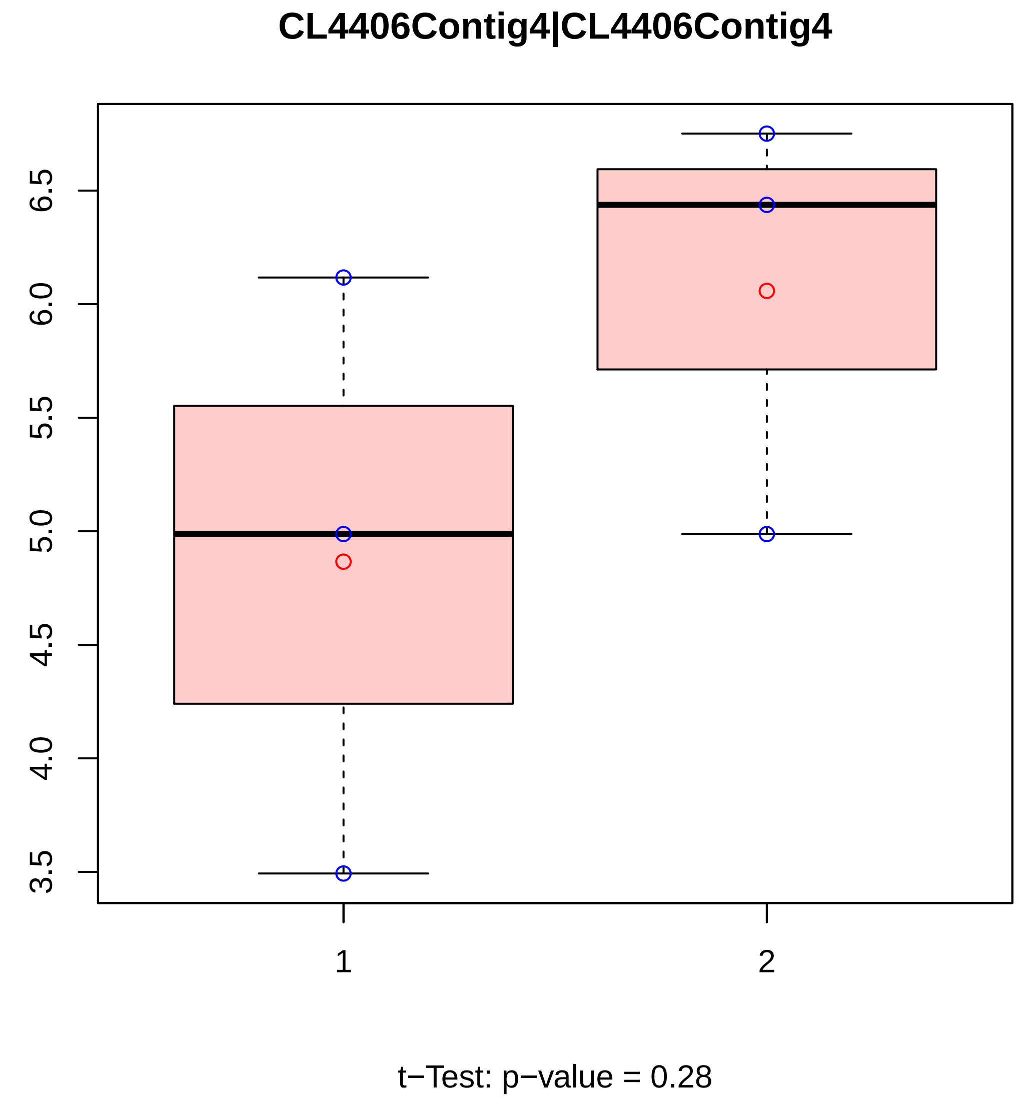
CL4406Contig4|CL4406Contig4
6.5
6.0
5.5
5.0
4.5
4.0
3.5
1
2
t−Test: p−value = 0.28

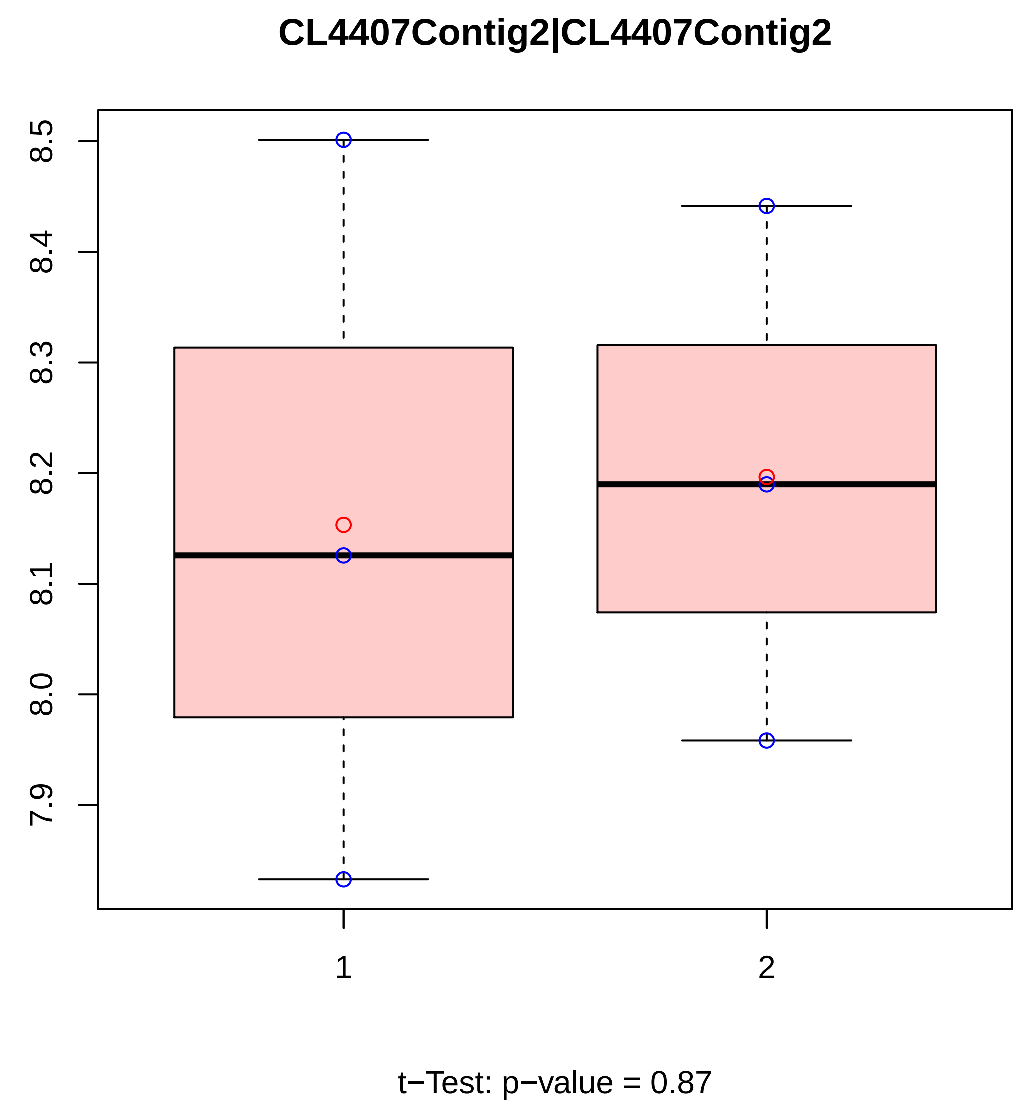

CL4407Contig2|CL4407Contig2
8.5
8.4
8.3
8.2
8.1
8.0
7.9
1
2
t−Test: p−value = 0.87

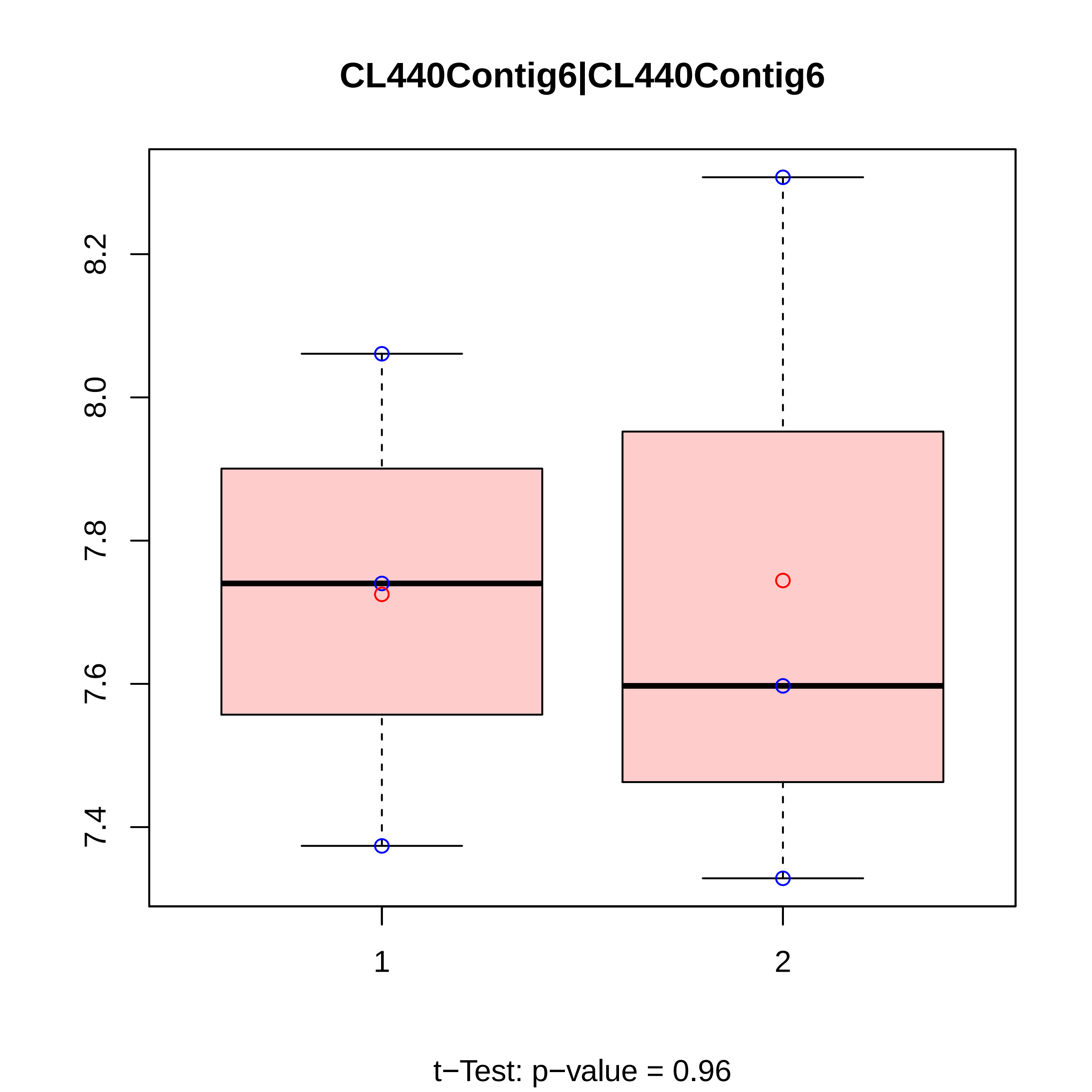

CL440Contig6|CL440Contig6
8.2
8.0
7.8
7.6
7.4
1
2
t−Test: p−value = 0.96

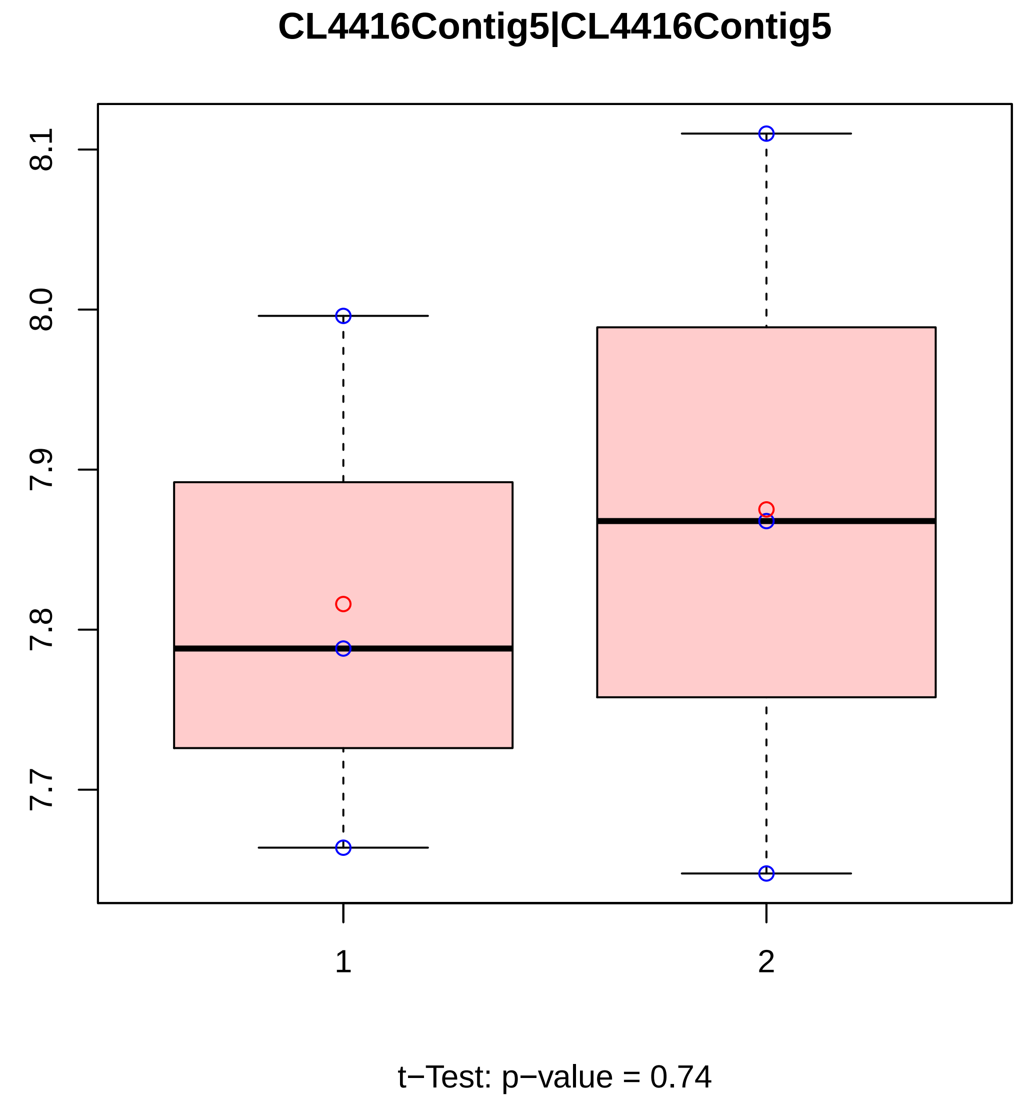
CL4416Contig5|CL4416Contig5
8.1
8.0
7.9
7.8
7.7
1
2
t−Test: p−value = 0.74

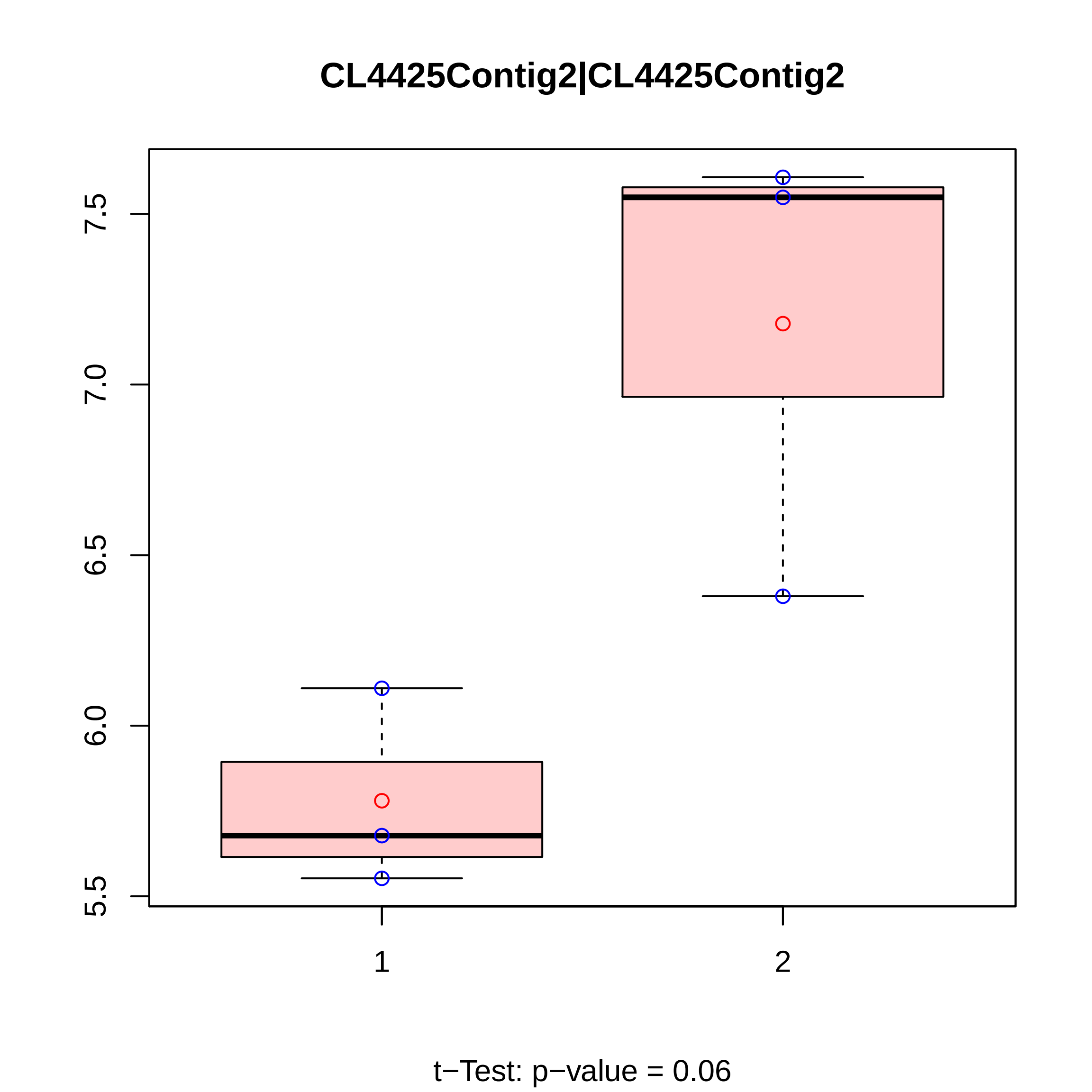

CL4425Contig2|CL4425Contig2
7.5
7.0
6.5
6.0
5.5
1
2
t−Test: p−value = 0.06

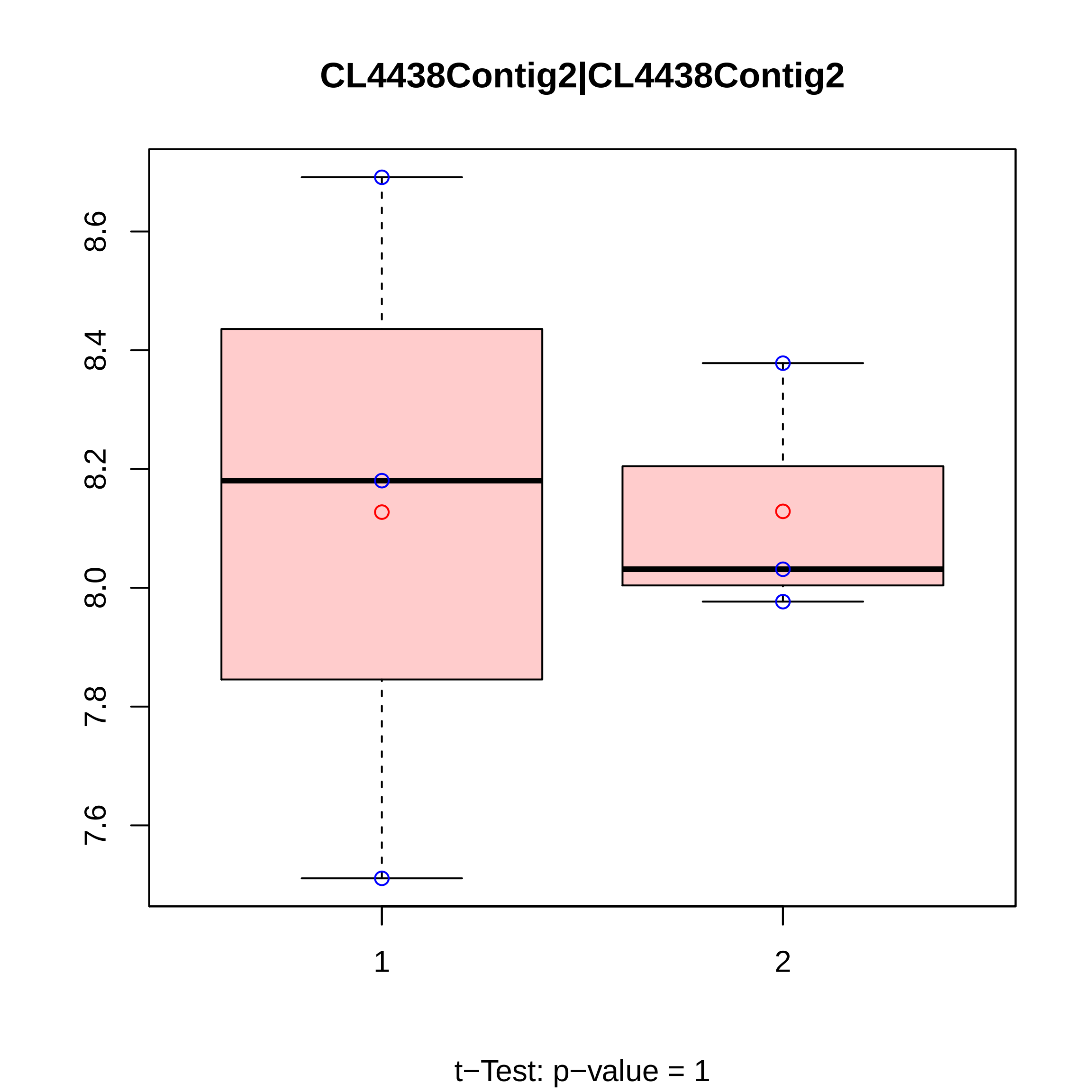
CL4438Contig2|CL4438Contig2
8.6
8.4
8.2
8.0
7.8
7.6
1
2
t−Test: p−value = 1

# CL443Contig10|CL443Contig10

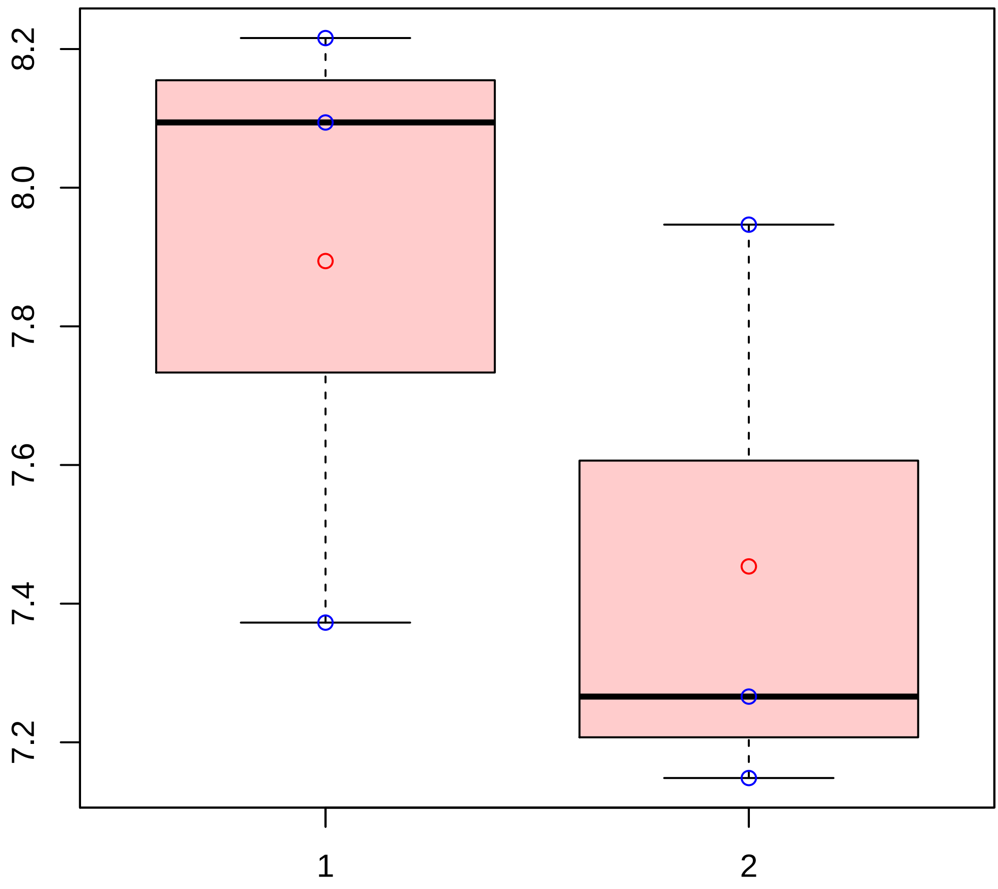


t−Test: p−value = 0.29

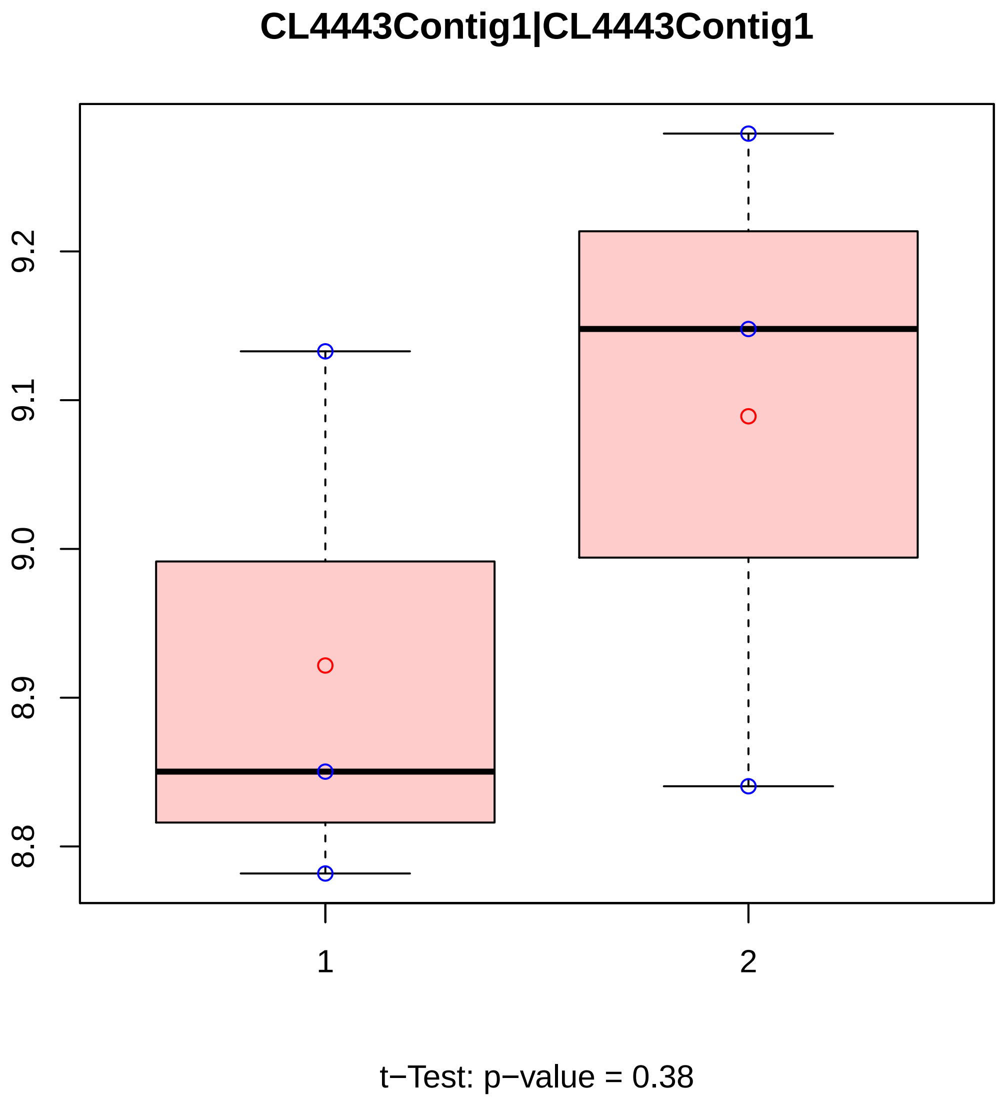
CL4443Contig1|CL4443Contig1
9.2
9.1
9.0
8.9
8.8
1
2
t−Test: p−value = 0.38

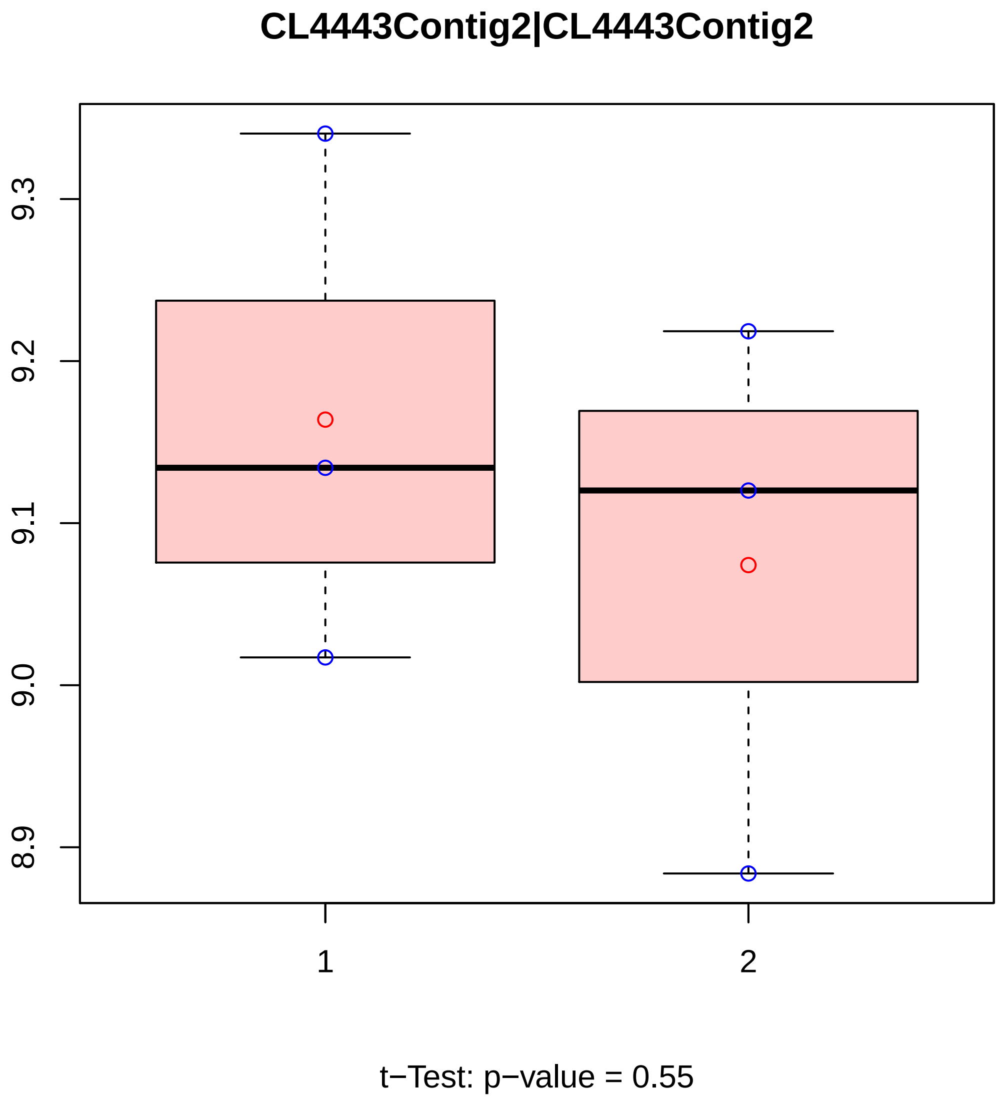
CL4443Contig2|CL4443Contig2
9.3
9.2
9.1
9.0
8.9
1
2
t−Test: p−value = 0.55

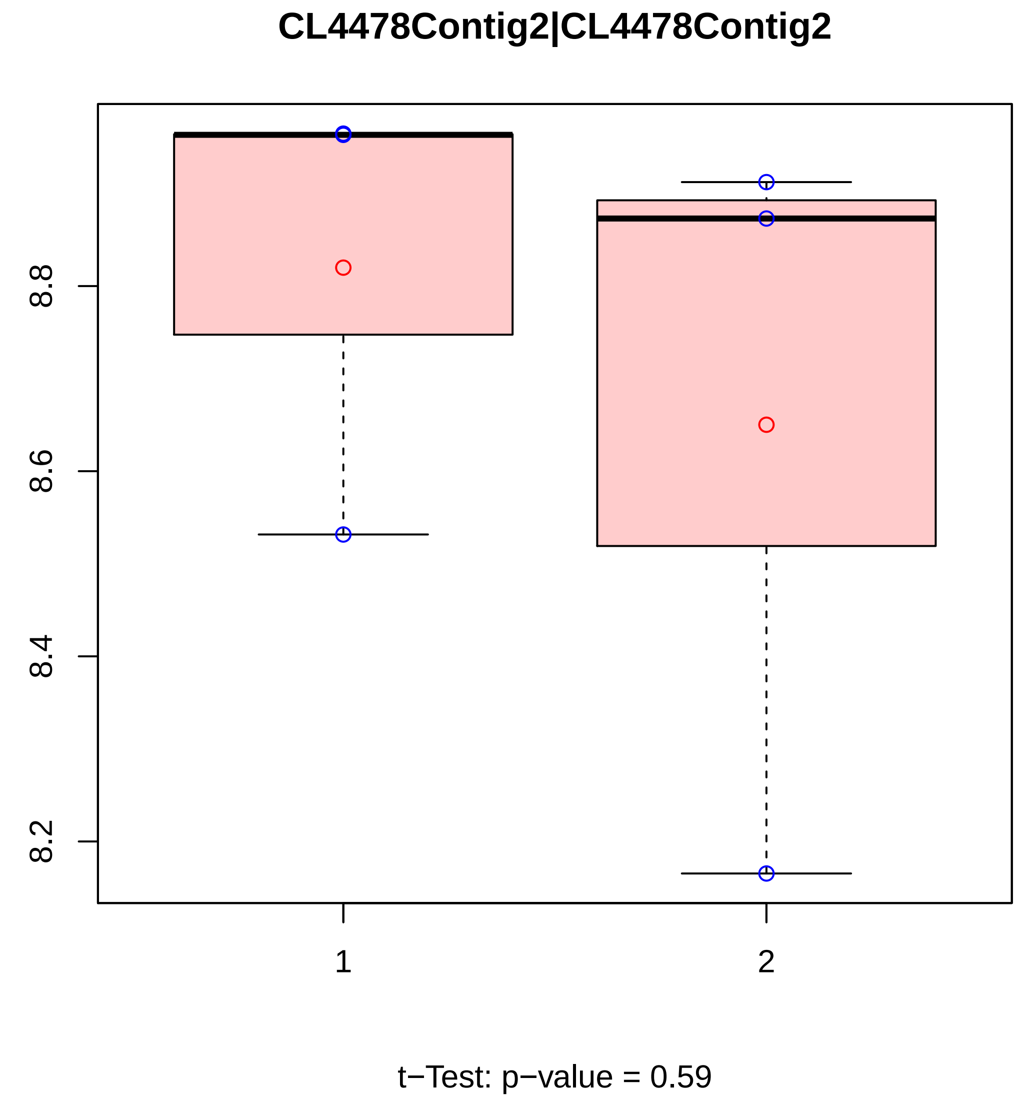
CL4478Contig2|CL4478Contig2
8.2
8.4
8.6
8.8
1
2
t−Test: p−value = 0.59

## CL448Contig7|CL448Contig7

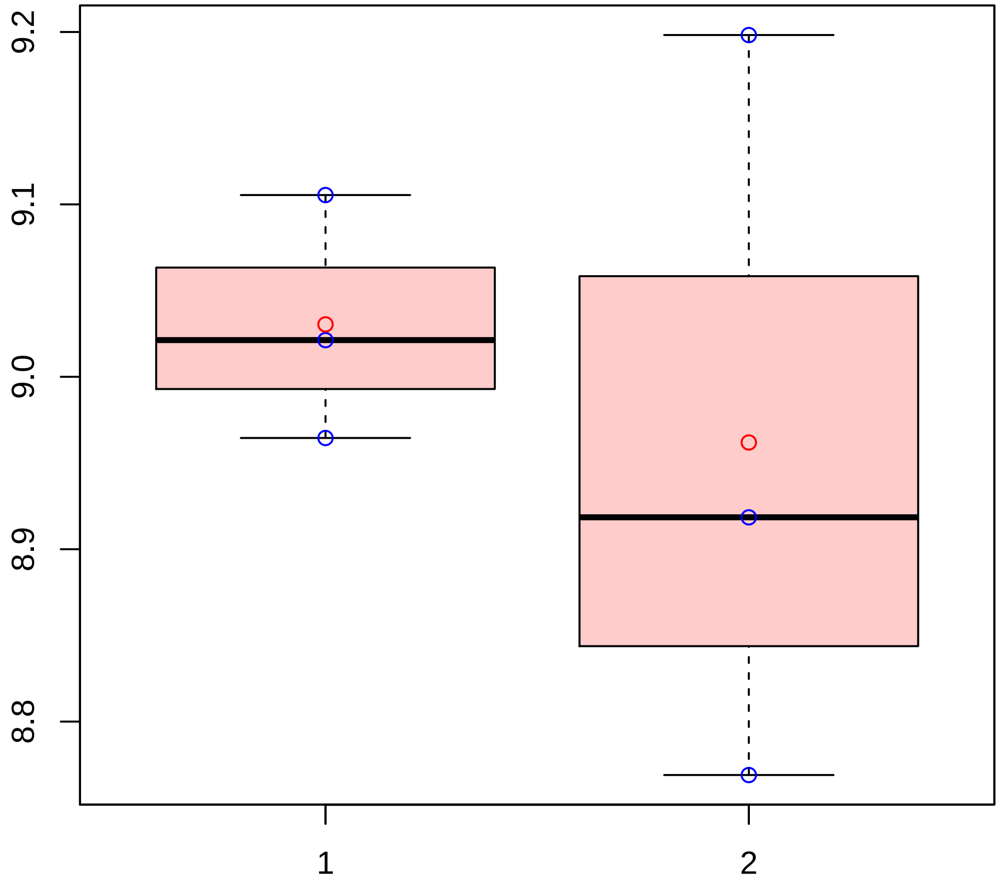


t−Test: p−value = 0.65

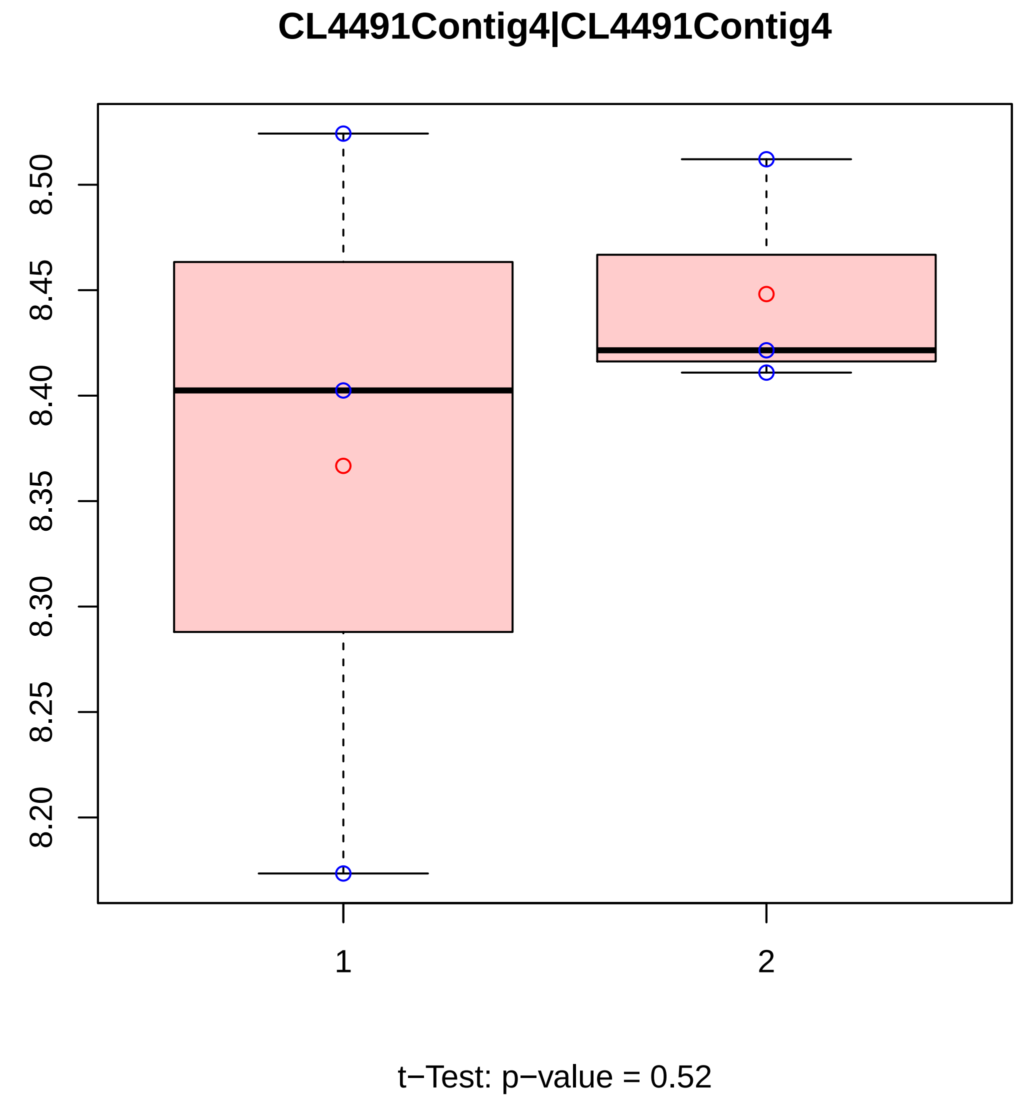

CL4491Contig4|CL4491Contig4
8.20
8.25
8.30
8.35
8.40
8.45
8.50
1
2
t−Test: p−value = 0.52

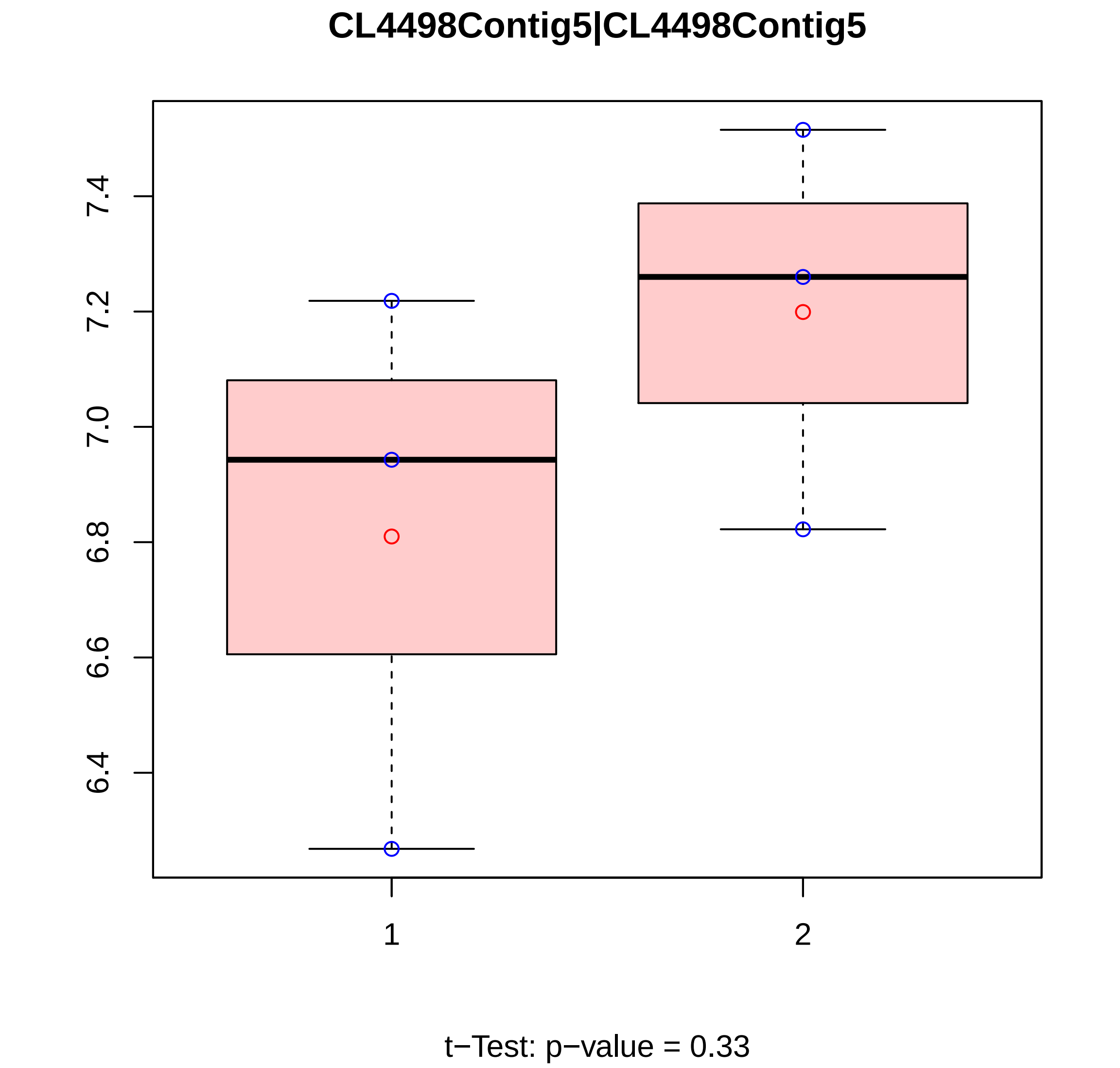

CL4498Contig5|CL4498Contig5
7.4
7.2
7.0
6.8
6.6
6.4
1
2
t−Test: p−value = 0.33

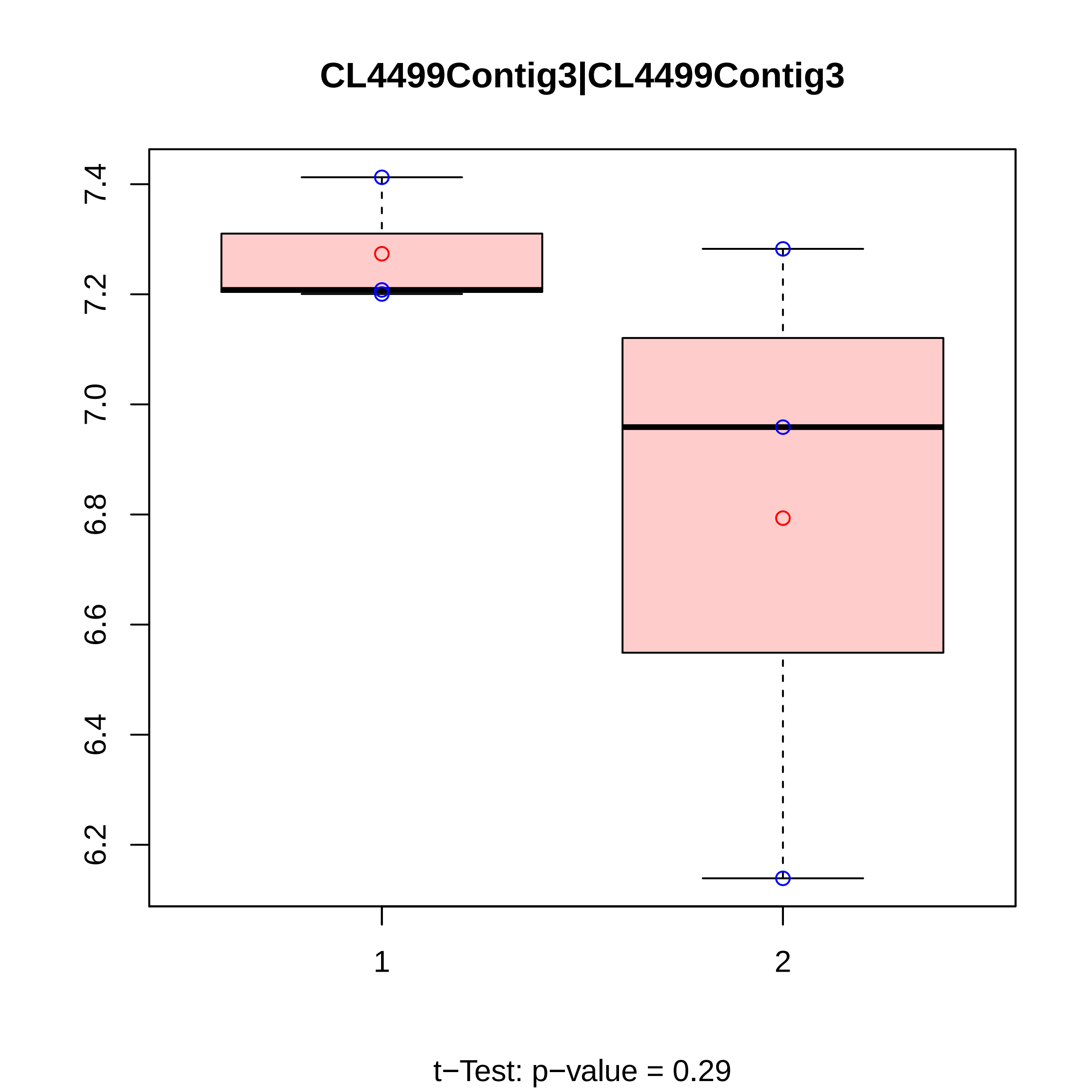

CL4499Contig3|CL4499Contig3
7.4
7.2
7.0
6.8
6.6
6.4
6.2
1
2
t−Test: p−value = 0.29

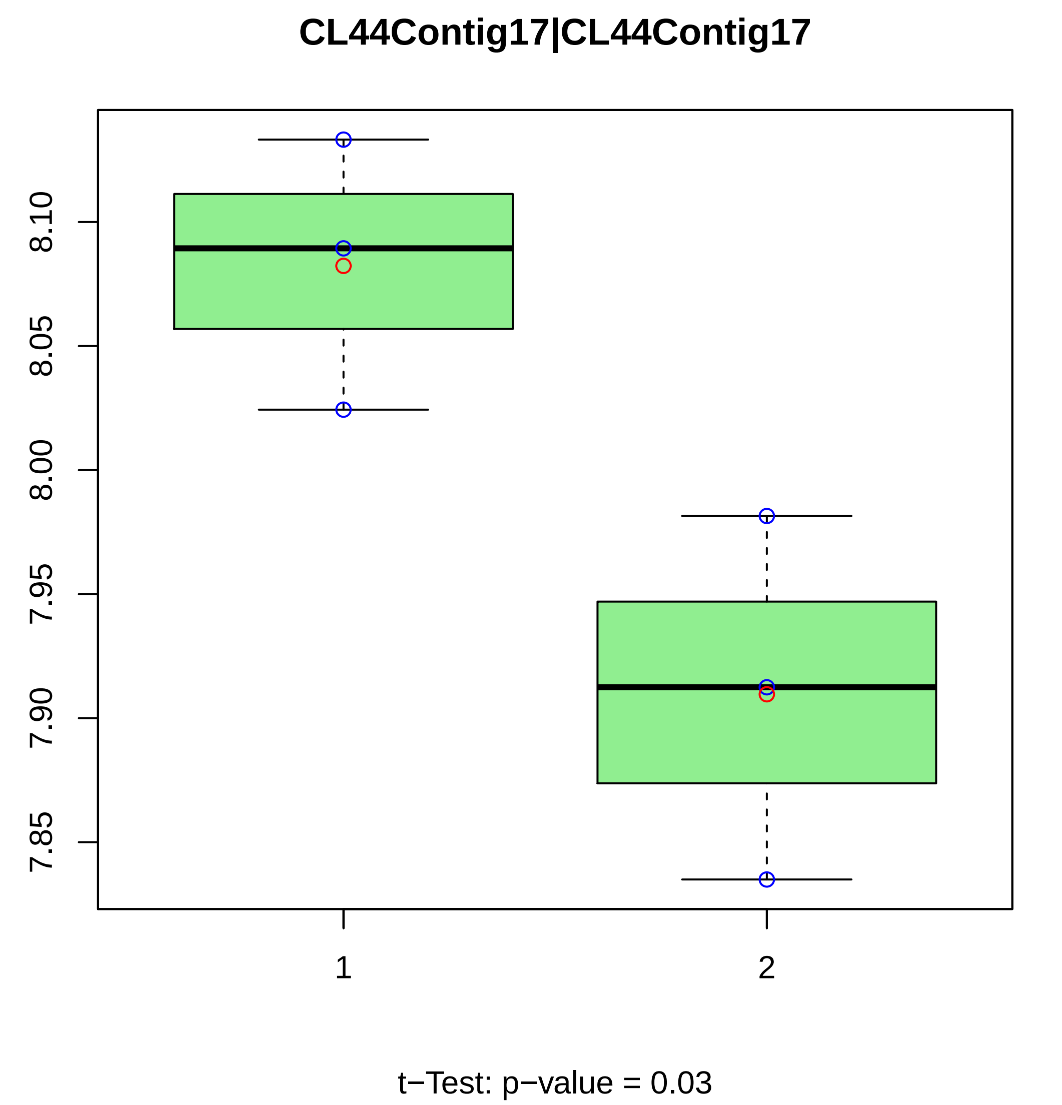
CL44Contig17|CL44Contig17
8.10
8.05
8.00
7.95
7.90
7.85
1
2
t−Test: p−value = 0.03

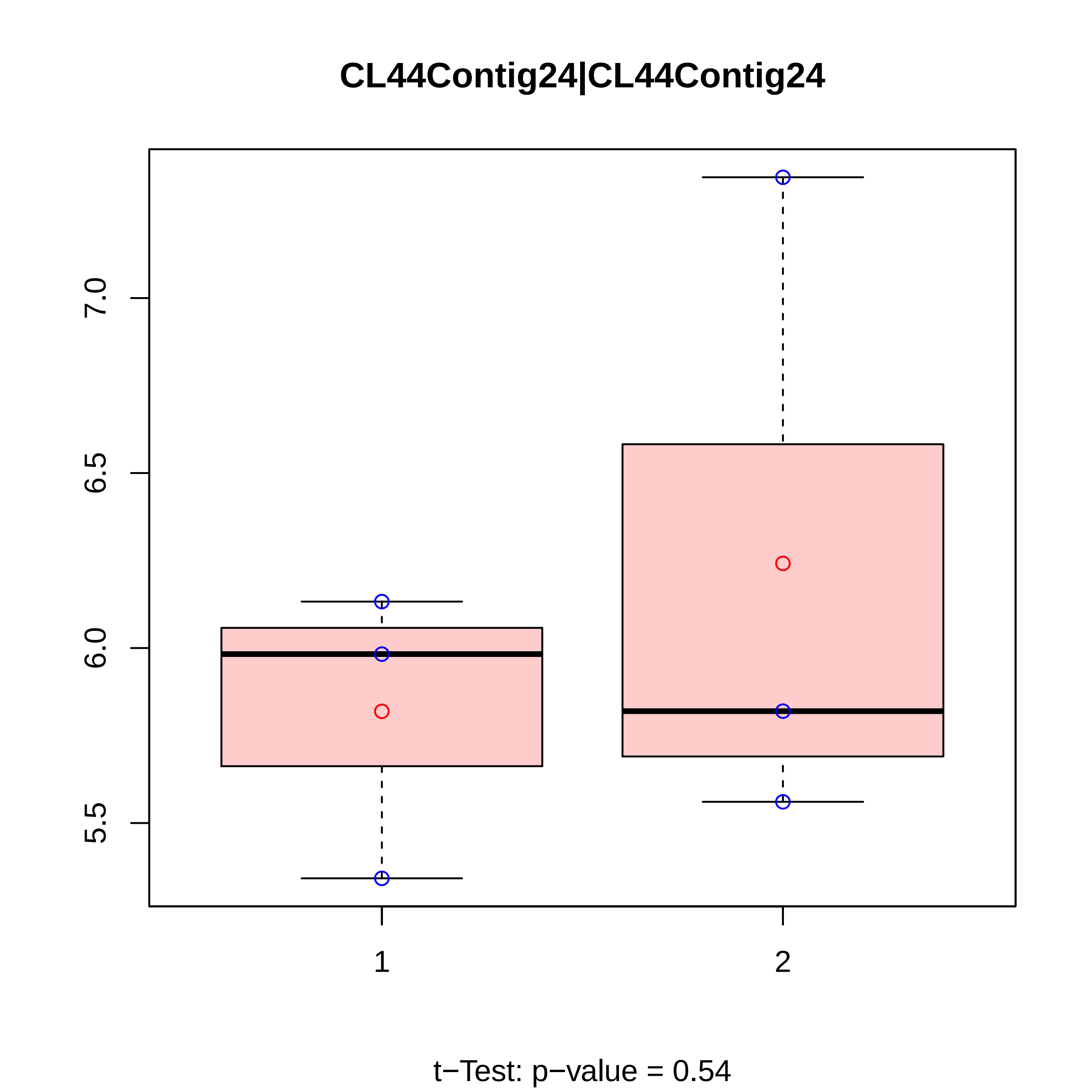
CL44Contig24|CL44Contig24
7.0
6.5
6.0
5.5
1
2
t−Test: p−value = 0.54

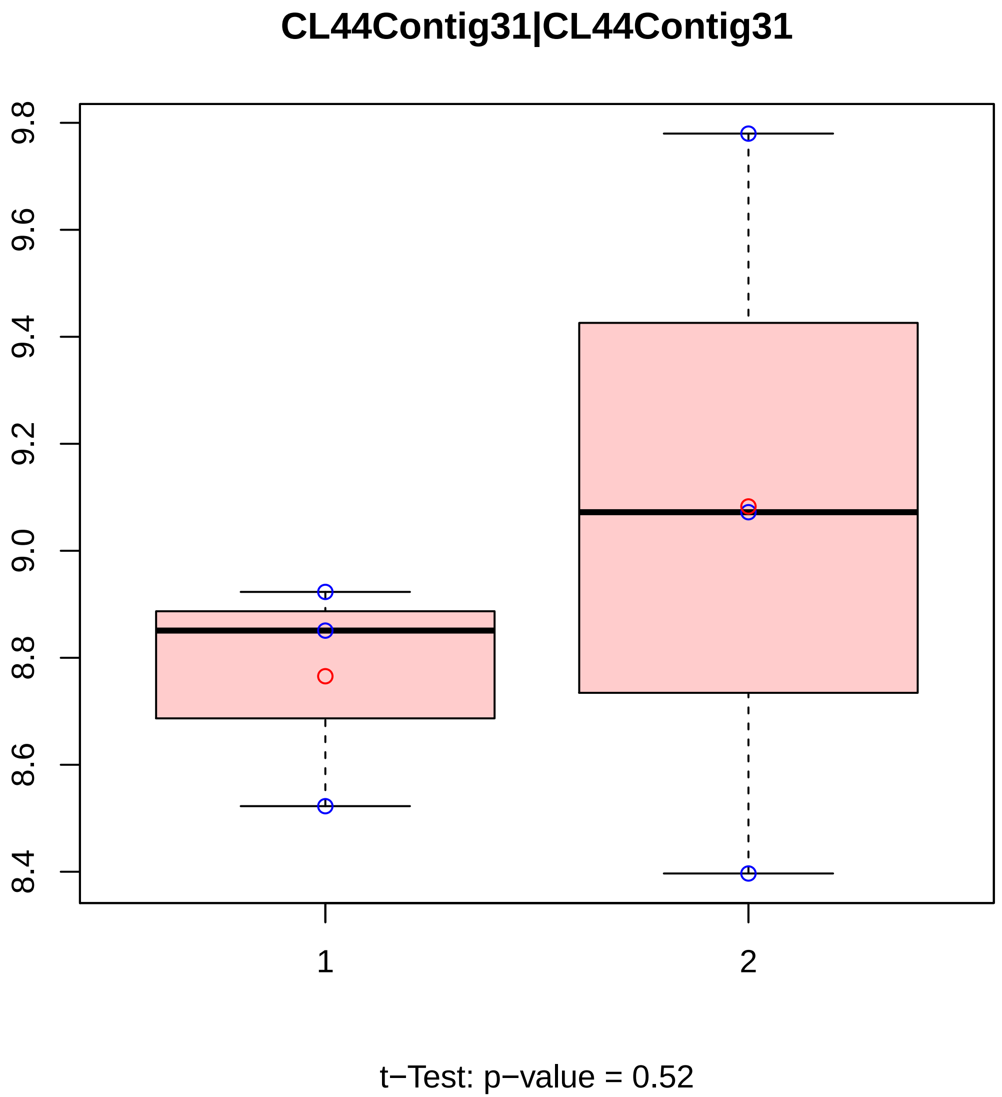
CL44Contig31|CL44Contig31
9.8
9.6
9.4
9.2
9.0
8.8
8.6
8.4
1
2
t−Test: p−value = 0.52

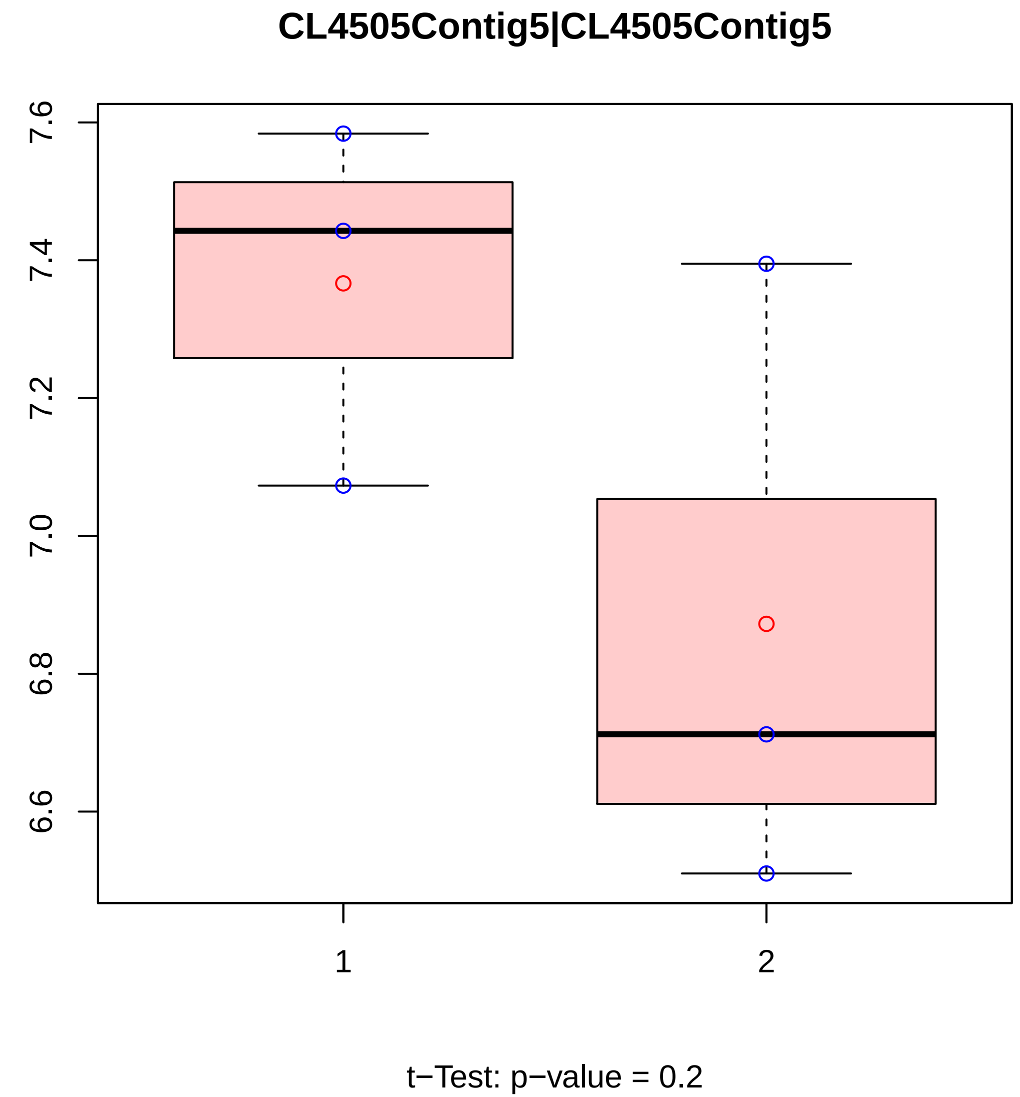
CL4505Contig5|CL4505Contig5
7.6
7.4
7.2
7.0
6.8
6.6
1
2
t−Test: p−value = 0.2

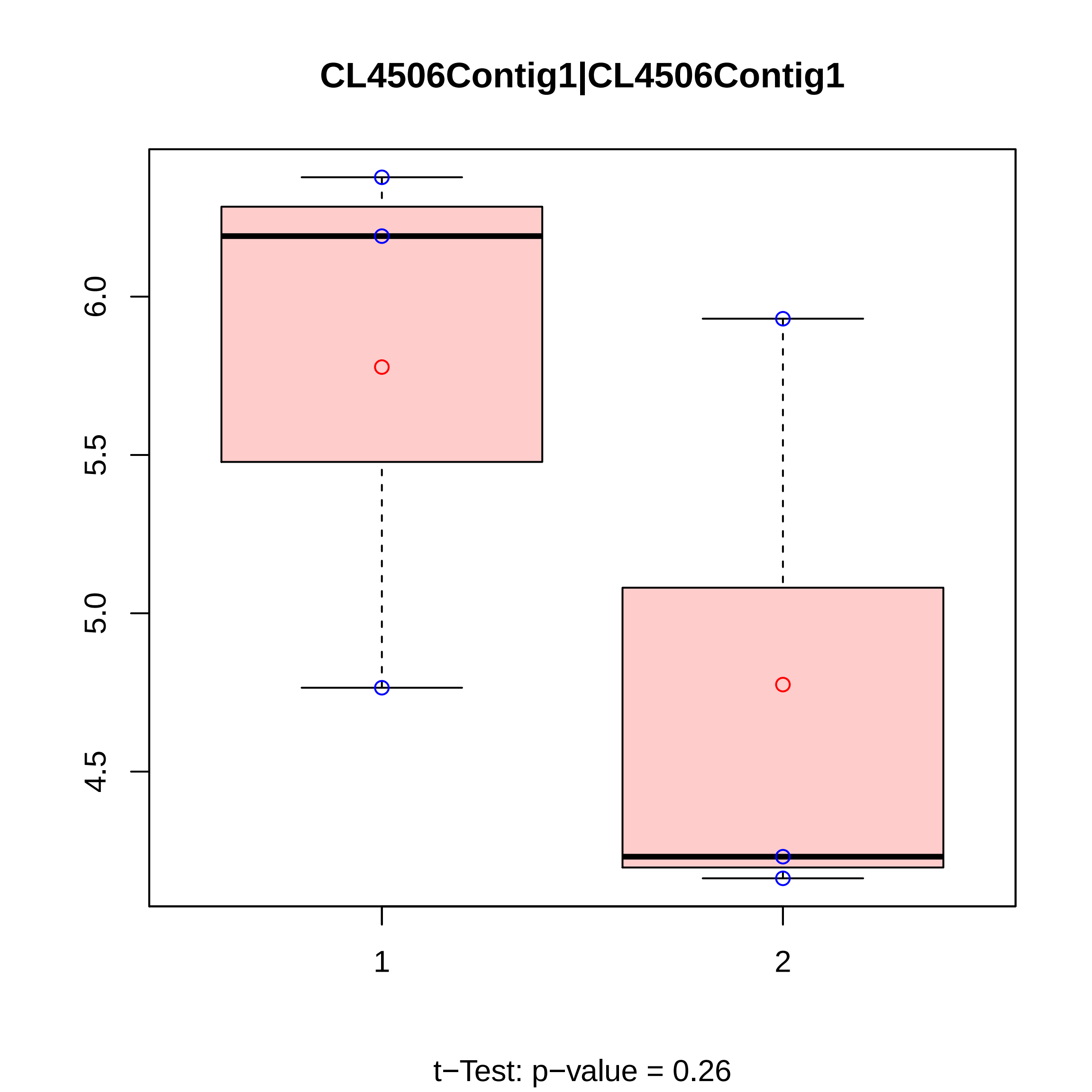

CL4506Contig1|CL4506Contig1
6.0
5.5
5.0
4.5
1
2
t−Test: p−value = 0.26

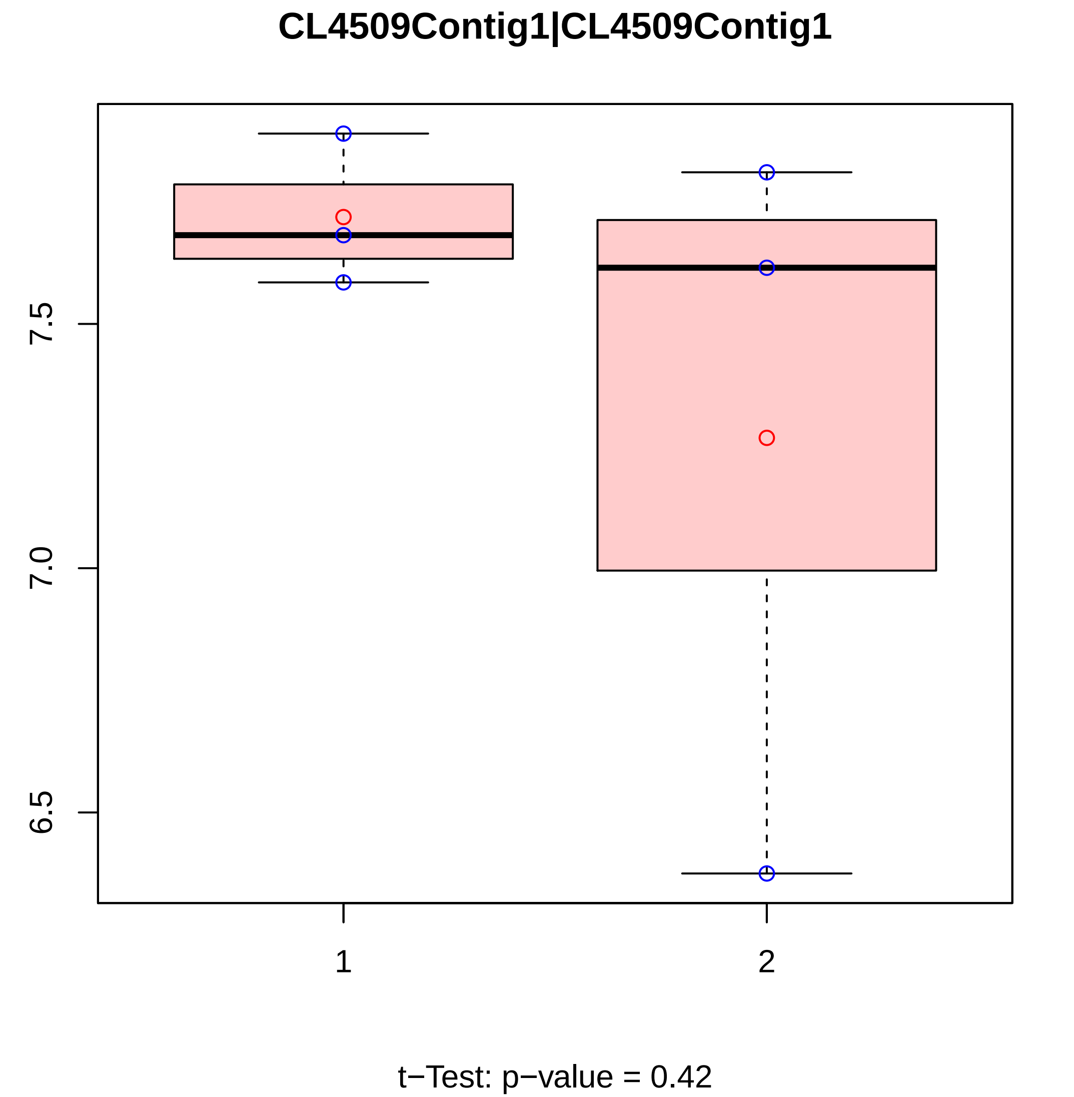
CL4509Contig1|CL4509Contig1
7.5
7.0
6.5
1
2
t−Test: p−value = 0.42

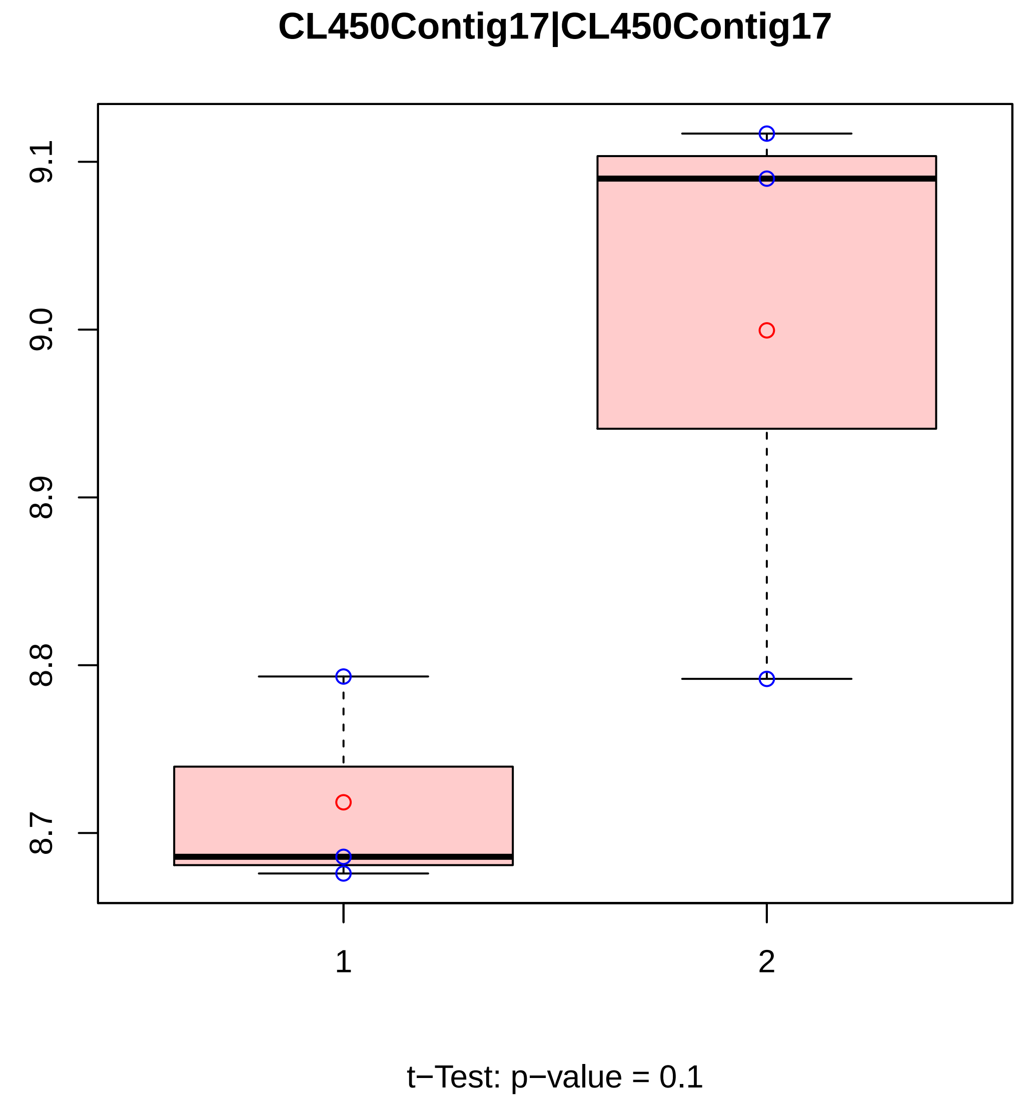
CL450Contig17|CL450Contig17
9.1
9.0
8.9
8.8
8.7
1
2
t−Test: p−value = 0.1

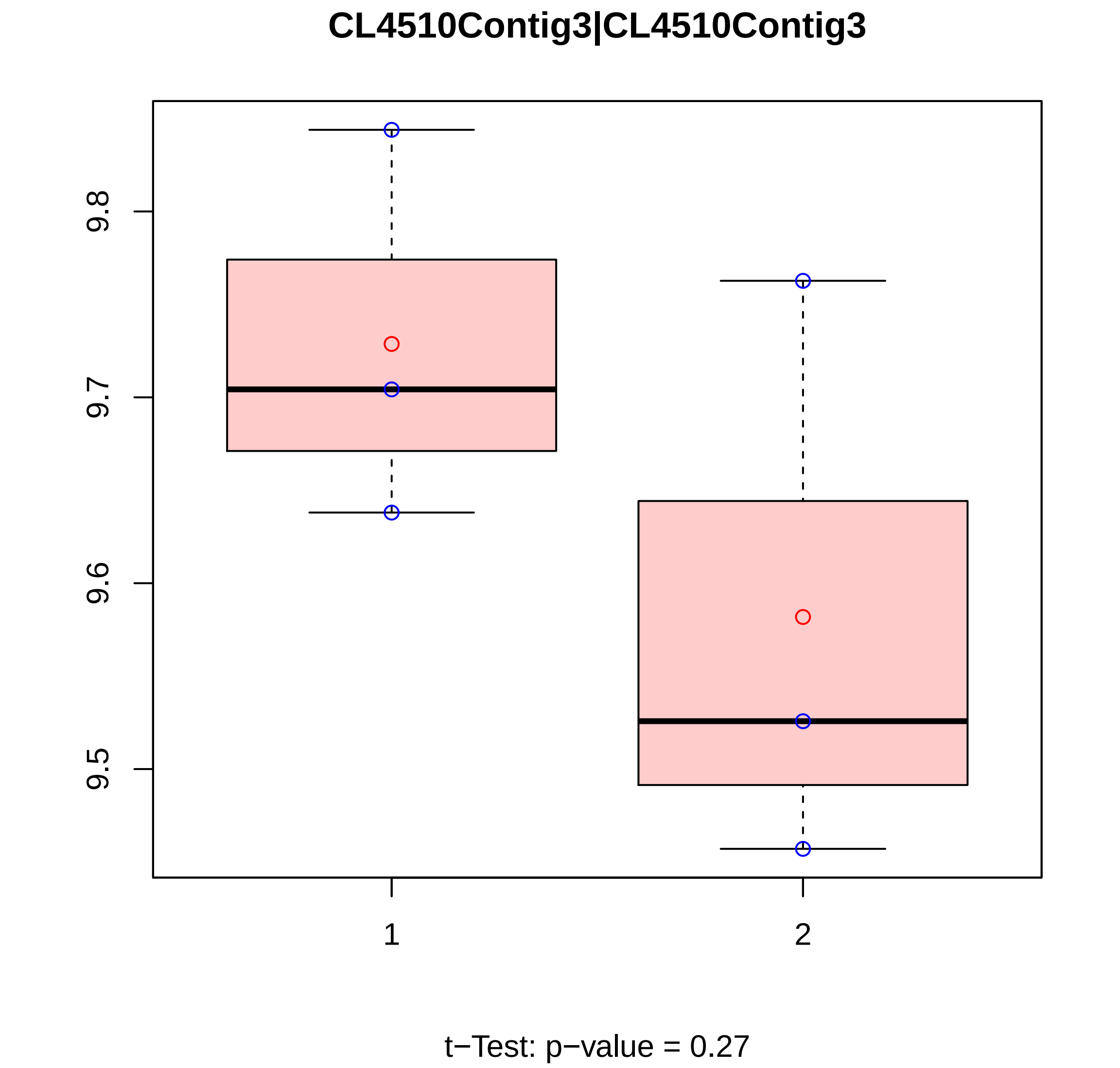

CL4510Contig3|CL4510Contig3
9.8
9.7
9.6
9.5
1
2
t−Test: p−value = 0.27

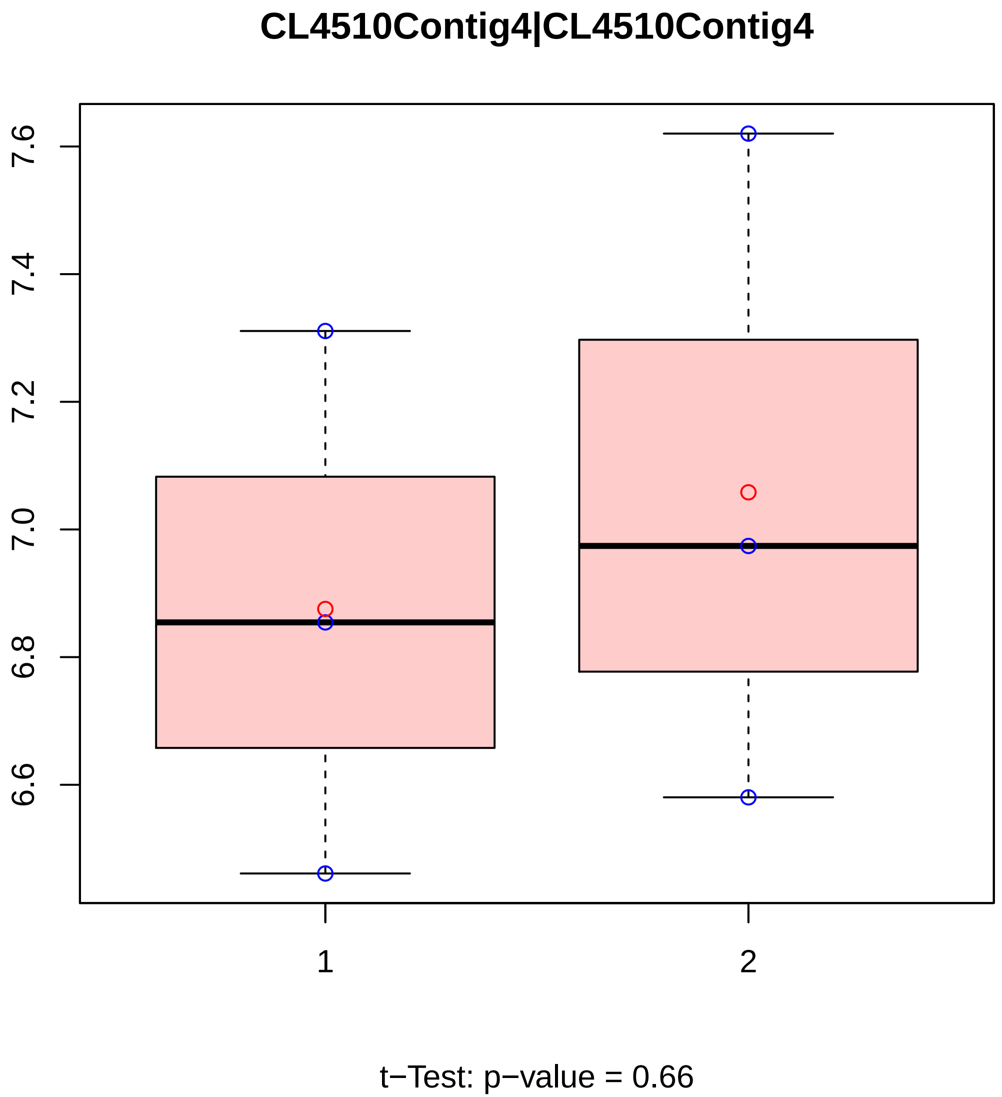
CL4510Contig4|CL4510Contig4
7.6
7.4
7.2
7.0
6.8
6.6
1
2
t−Test: p−value = 0.66

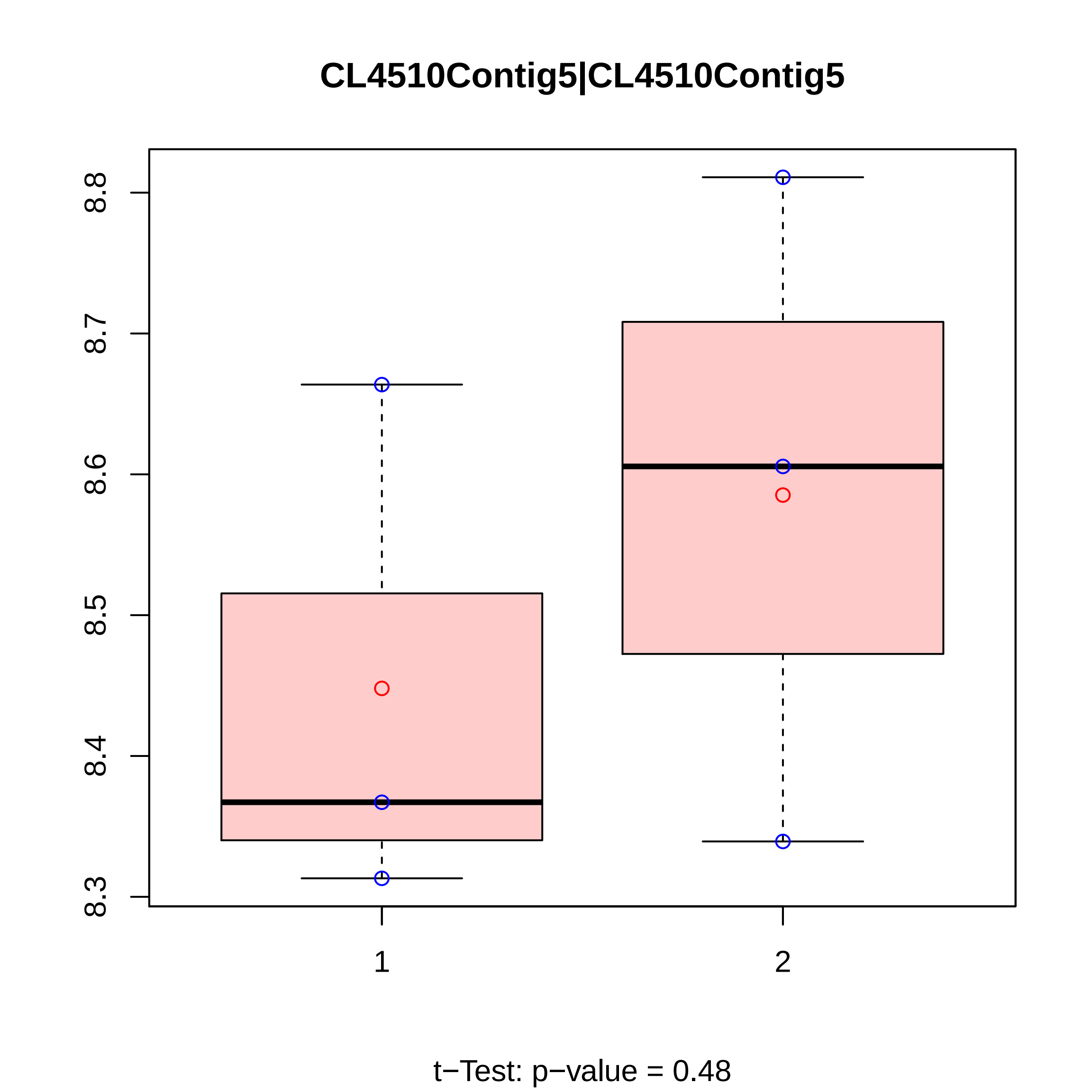

CL4510Contig5|CL4510Contig5
8.3
8.4
8.5
8.6
8.7
8.8
1
2
t−Test: p−value = 0.48

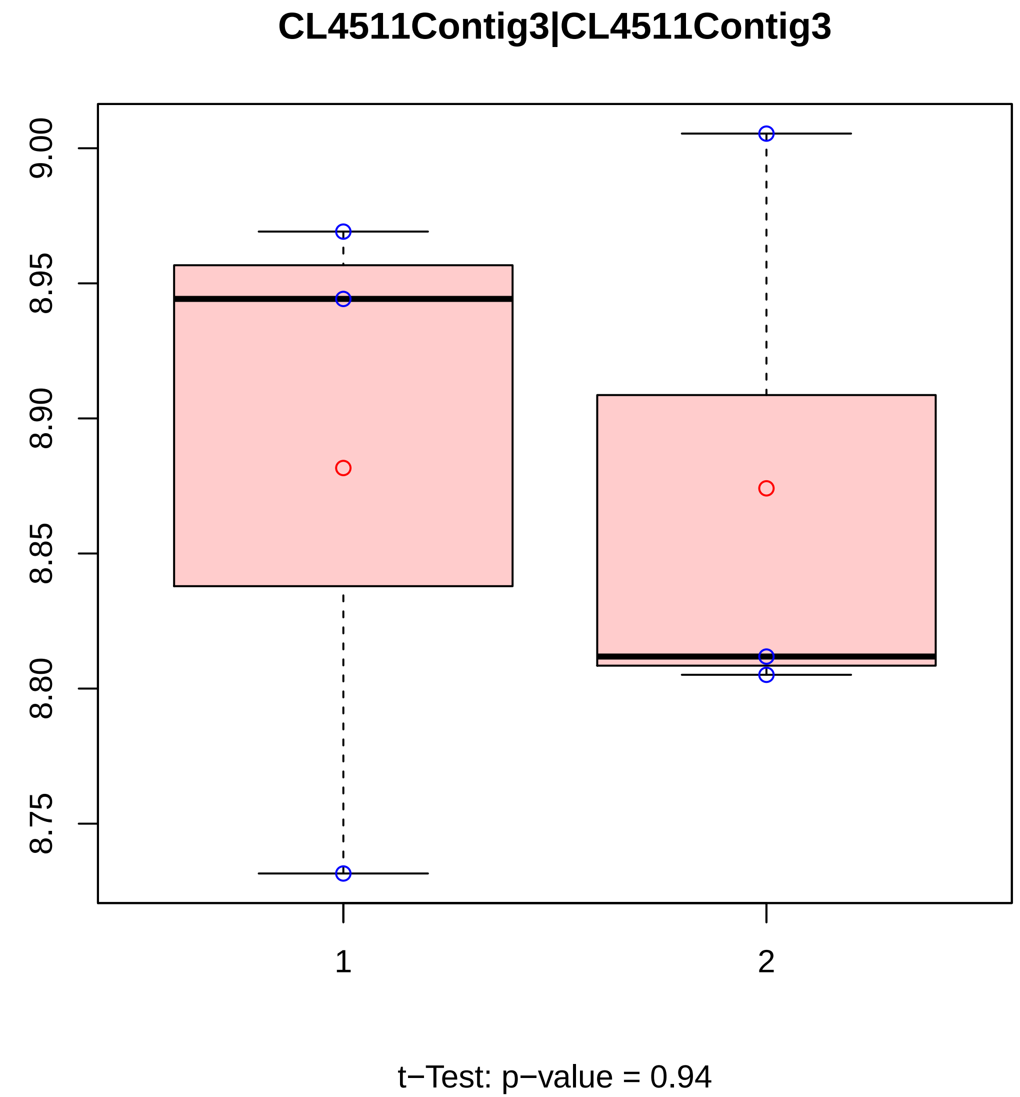
CL4511Contig3|CL4511Contig3
9.00
8.95
8.90
8.85
8.80
8.75
1
2
t−Test: p−value = 0.94

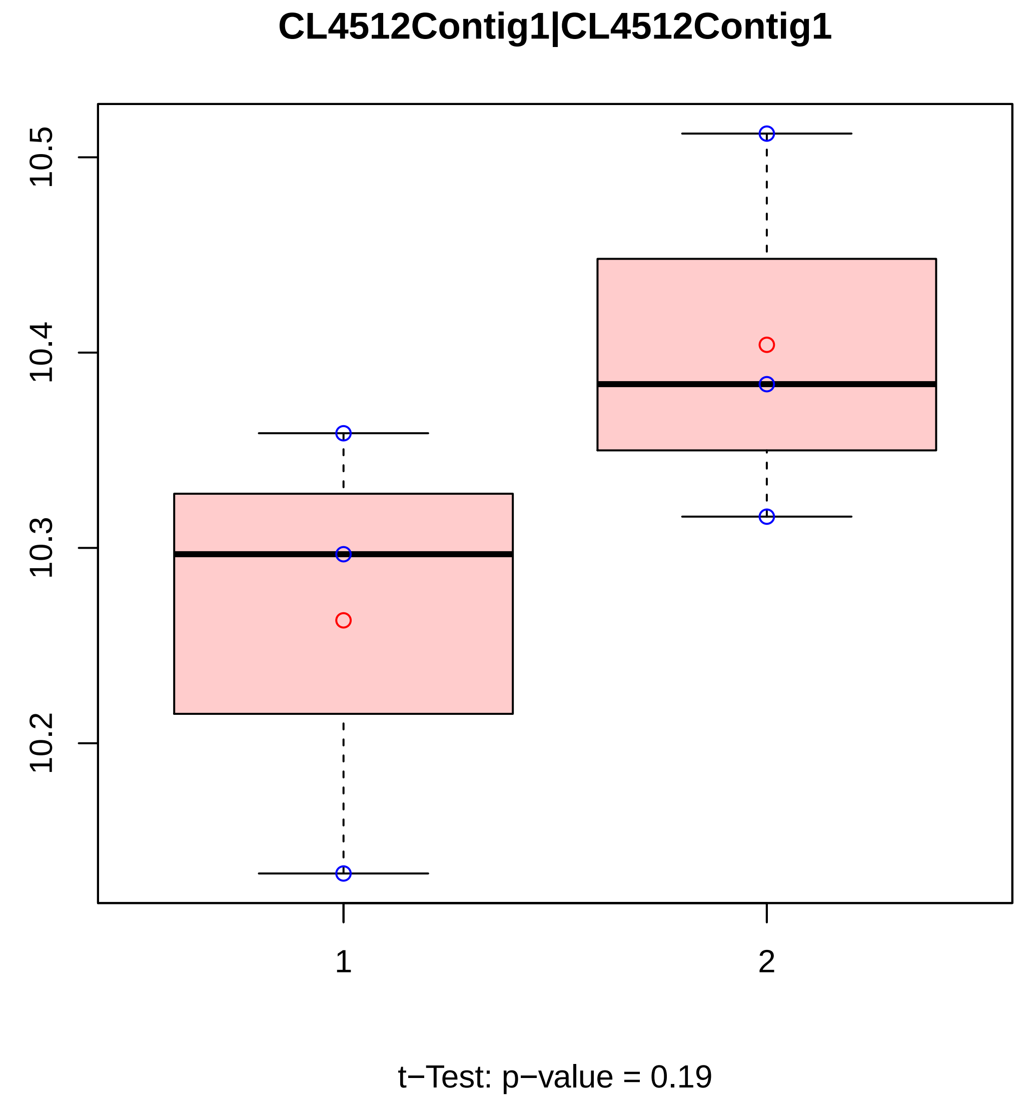
CL4512Contig1|CL4512Contig1
10.5
10.4
10.3
10.2
1
2
t−Test: p−value = 0.19

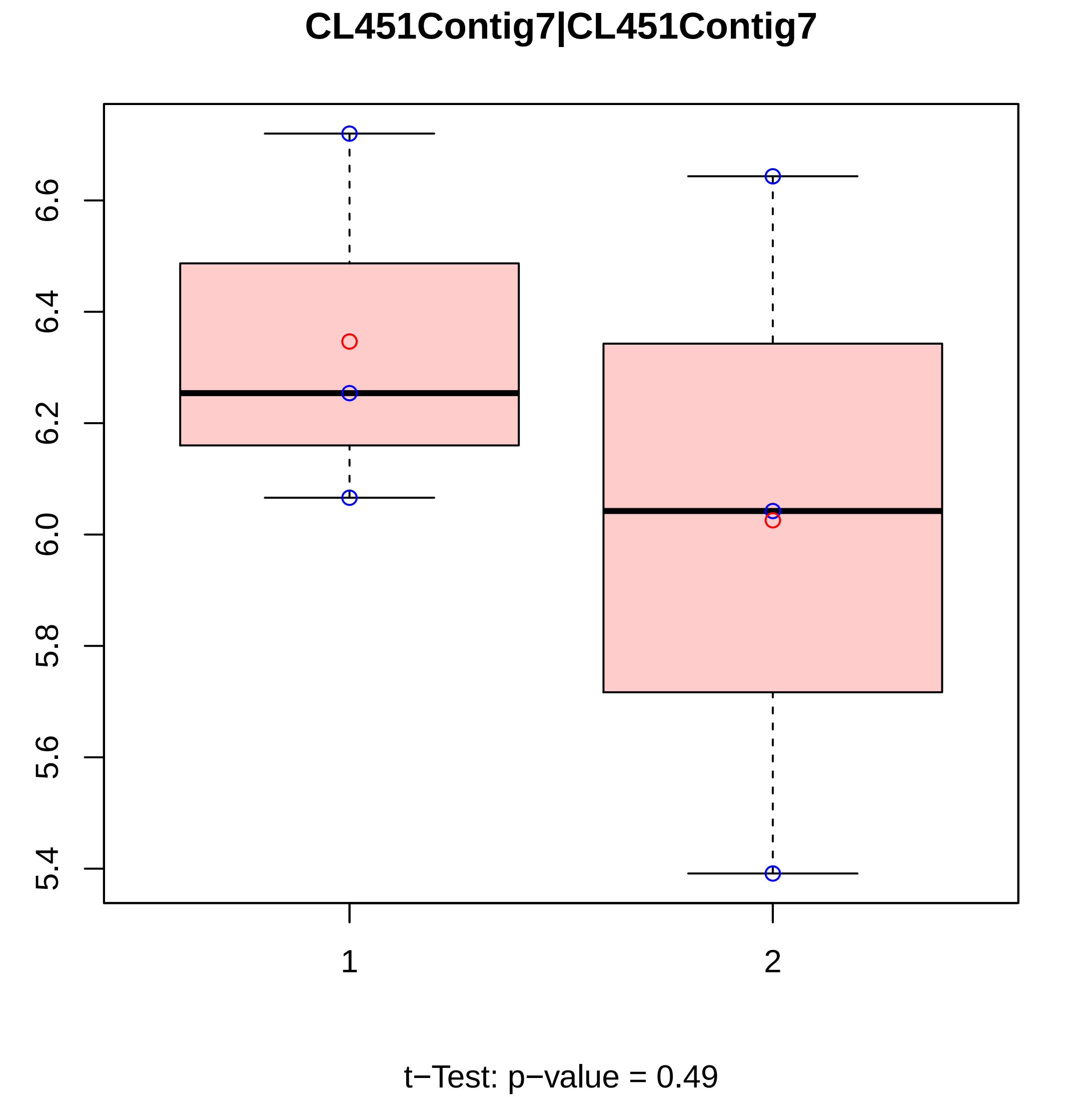

CL451Contig7|CL451Contig7
6.6
6.4
6.2
6.0
5.8
5.6
5.4
1
2
t−Test: p−value = 0.49

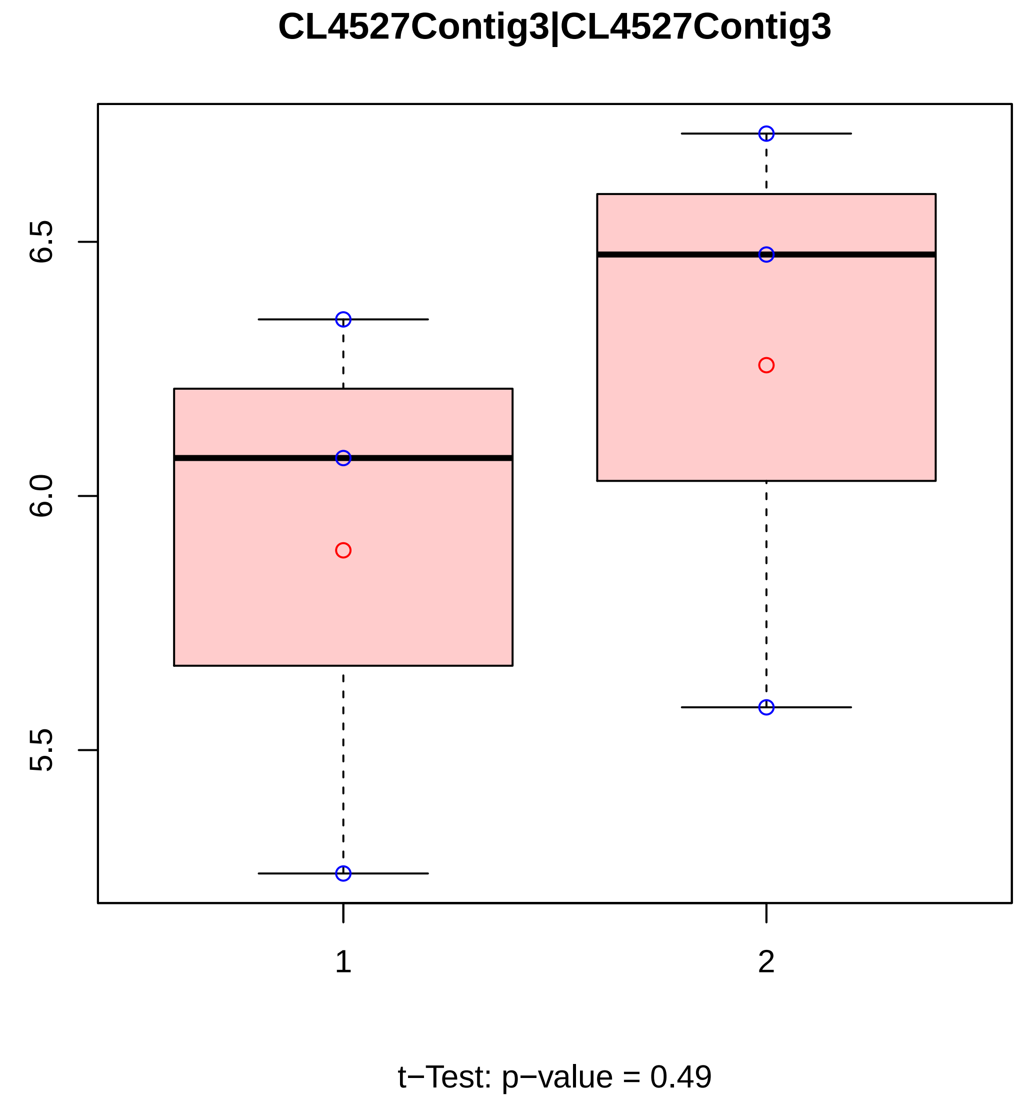
CL4527Contig3|CL4527Contig3
6.5
6.0
5.5
1
2
t−Test: p−value = 0.49

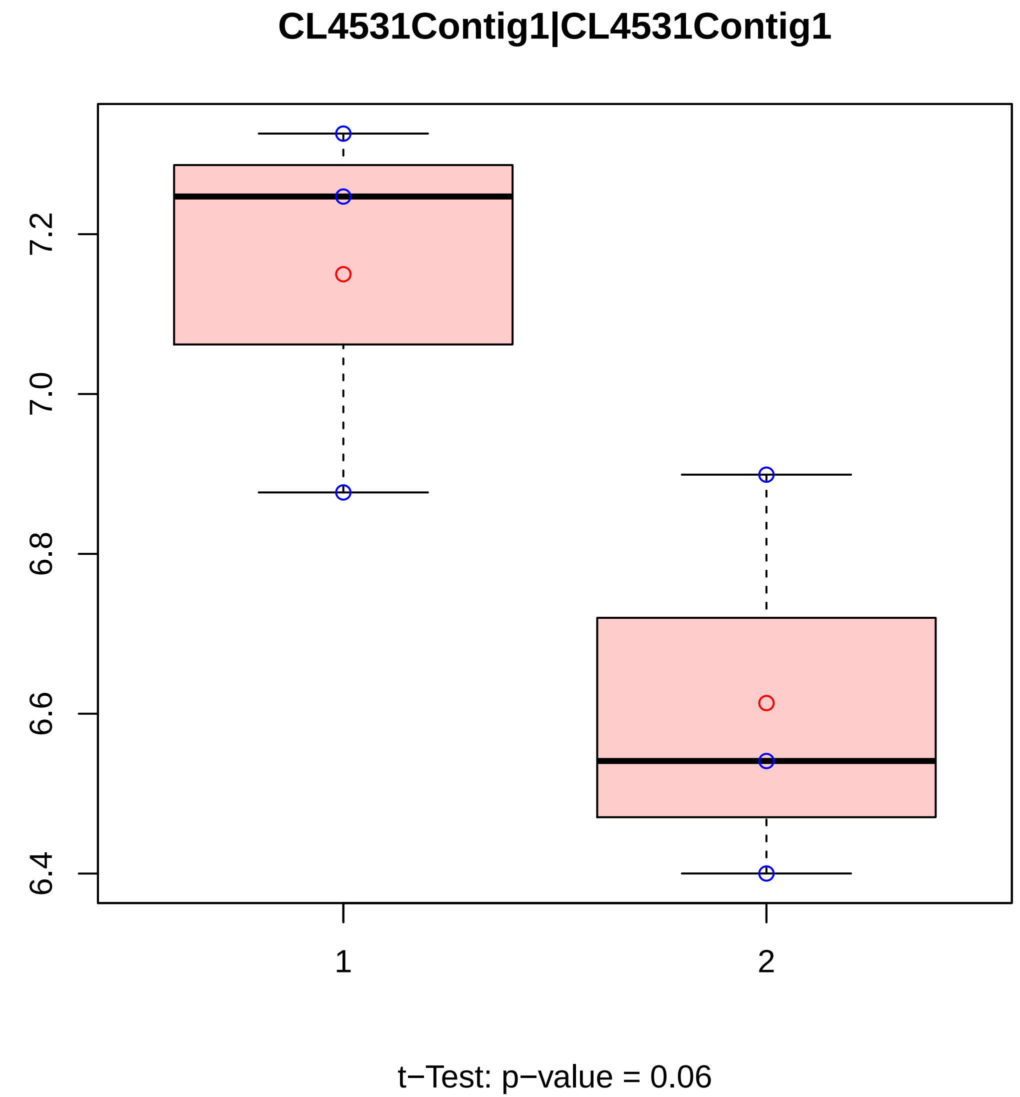

CL4531Contig1|CL4531Contig1
7.2
7.0
6.8
6.6
6.4
1
2
t−Test: p−value = 0.06

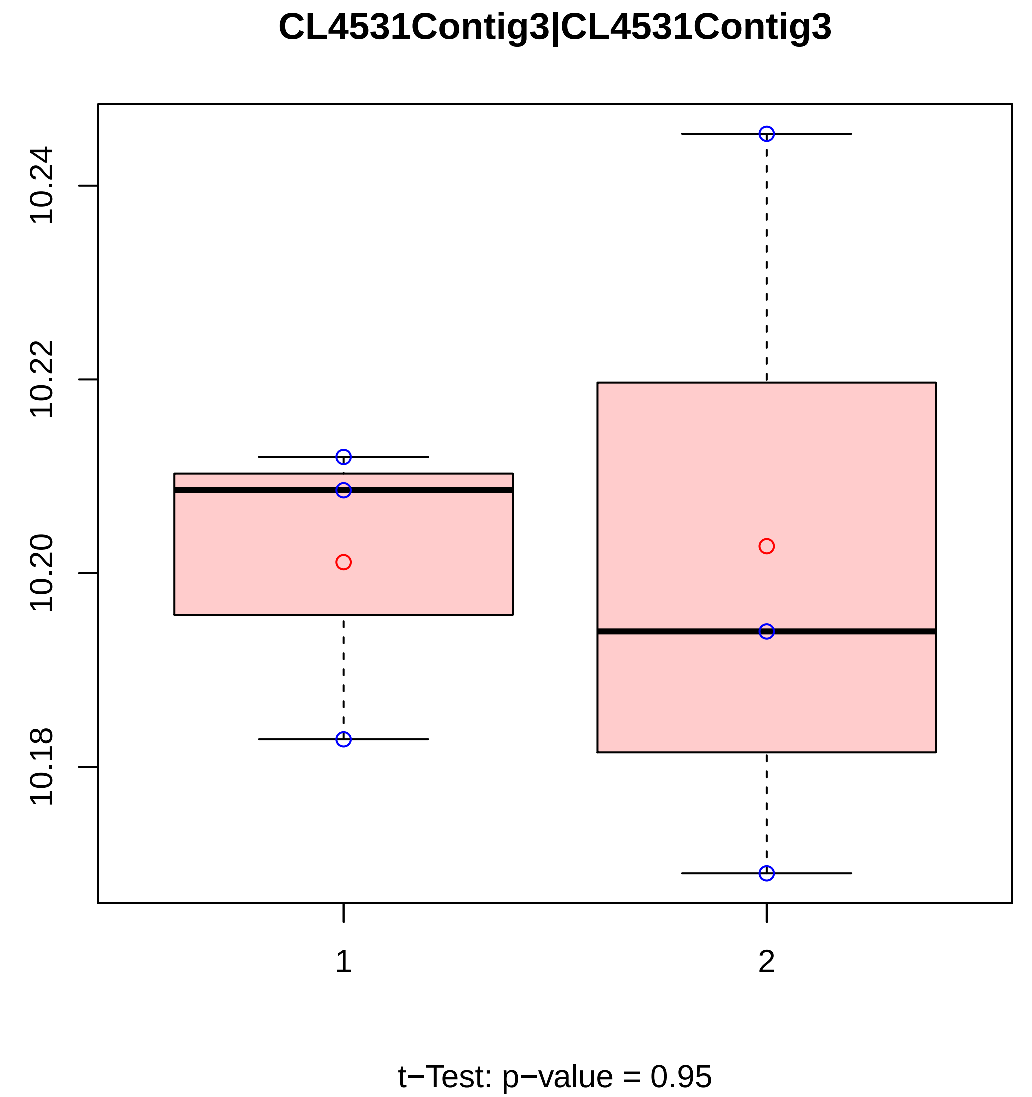
CL4531Contig3|CL4531Contig3
10.18
10.20
10.22
10.24
1
2
t−Test: p−value = 0.95

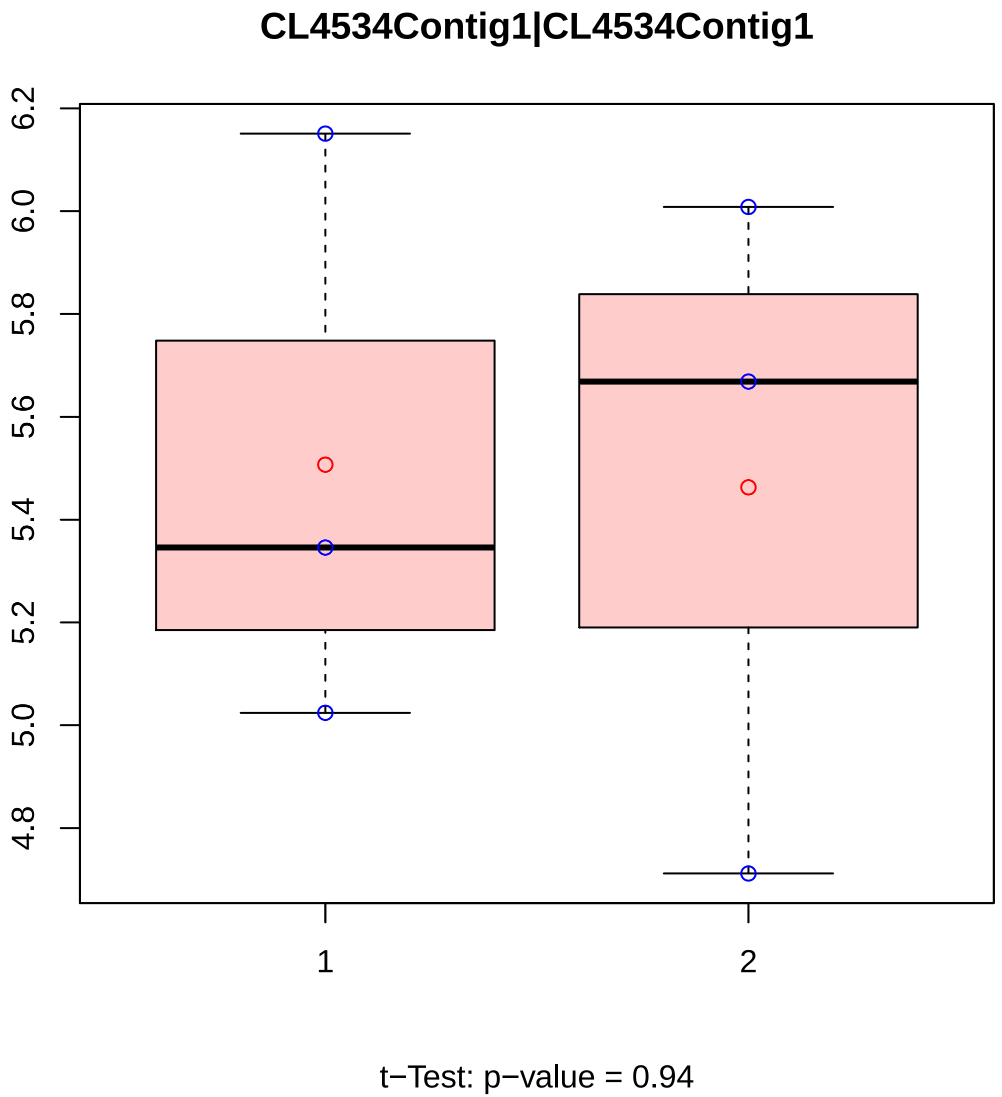
CL4534Contig1|CL4534Contig1
6.2
6.0
5.8
5.6
5.4
5.2
5.0
4.8
1
2
t−Test: p−value = 0.94

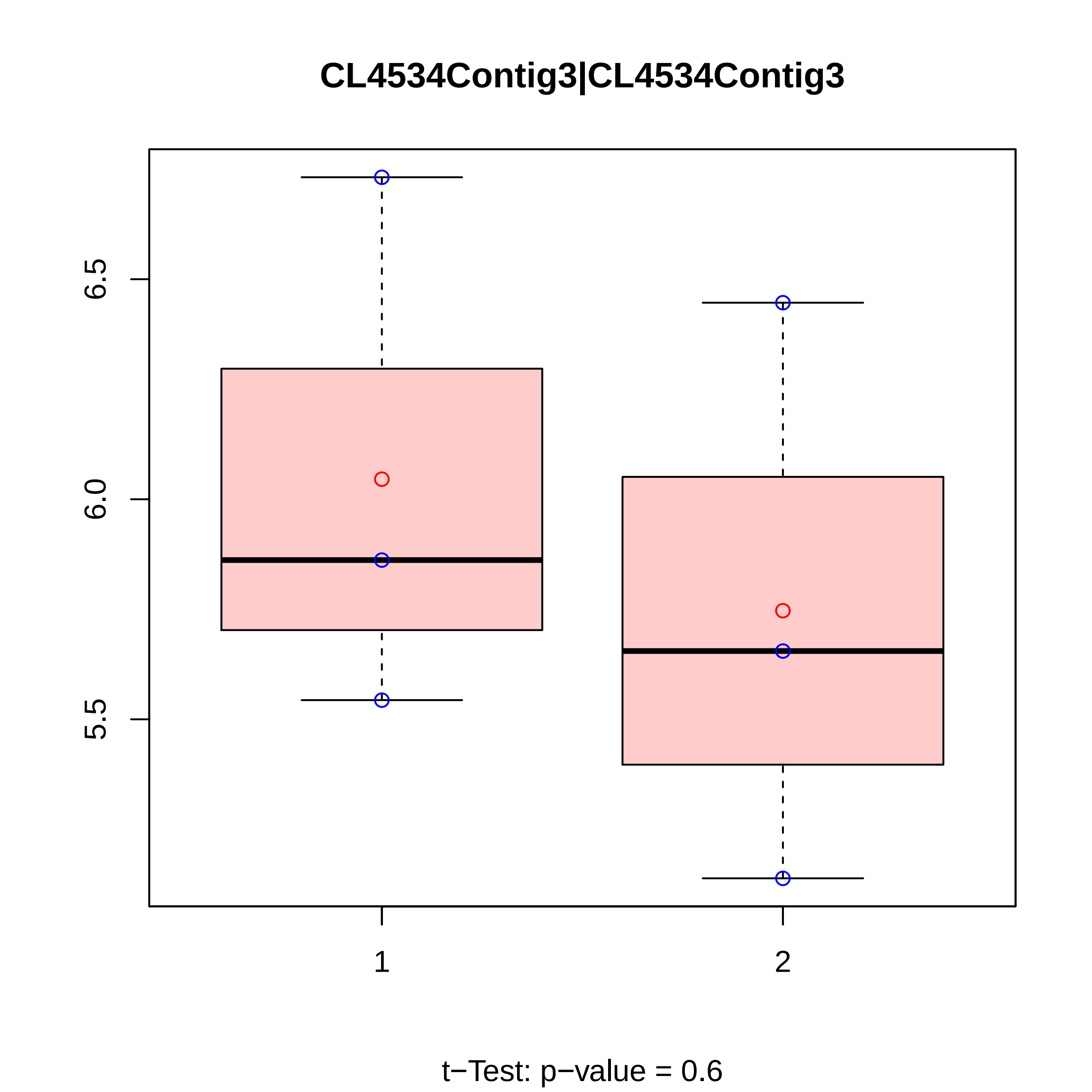

CL4534Contig3|CL4534Contig3
6.5
6.0
5.5
1
2
t−Test: p−value = 0.6

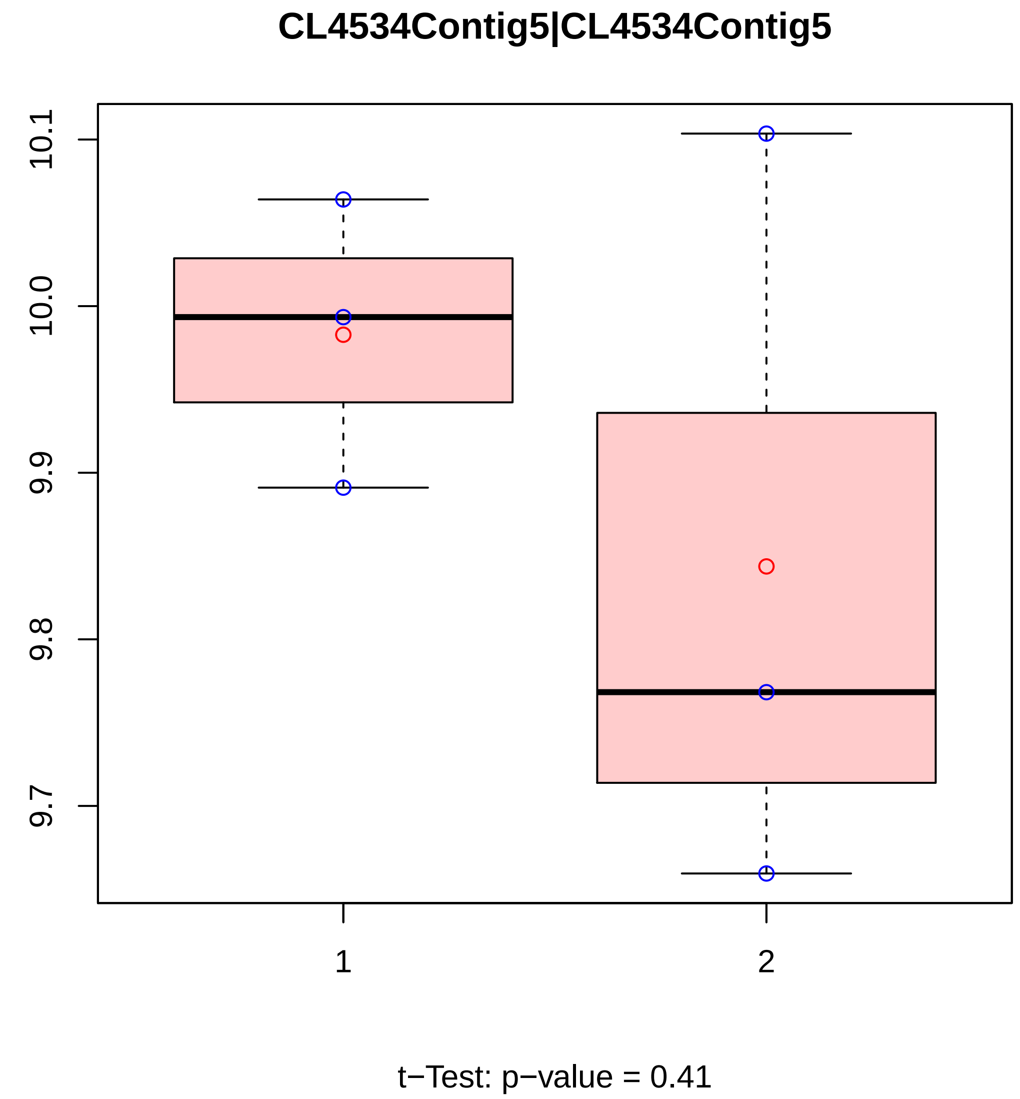

CL4534Contig5|CL4534Contig5
10.1
10.0
9.9
9.8
9.7
1
2
t−Test: p−value = 0.41

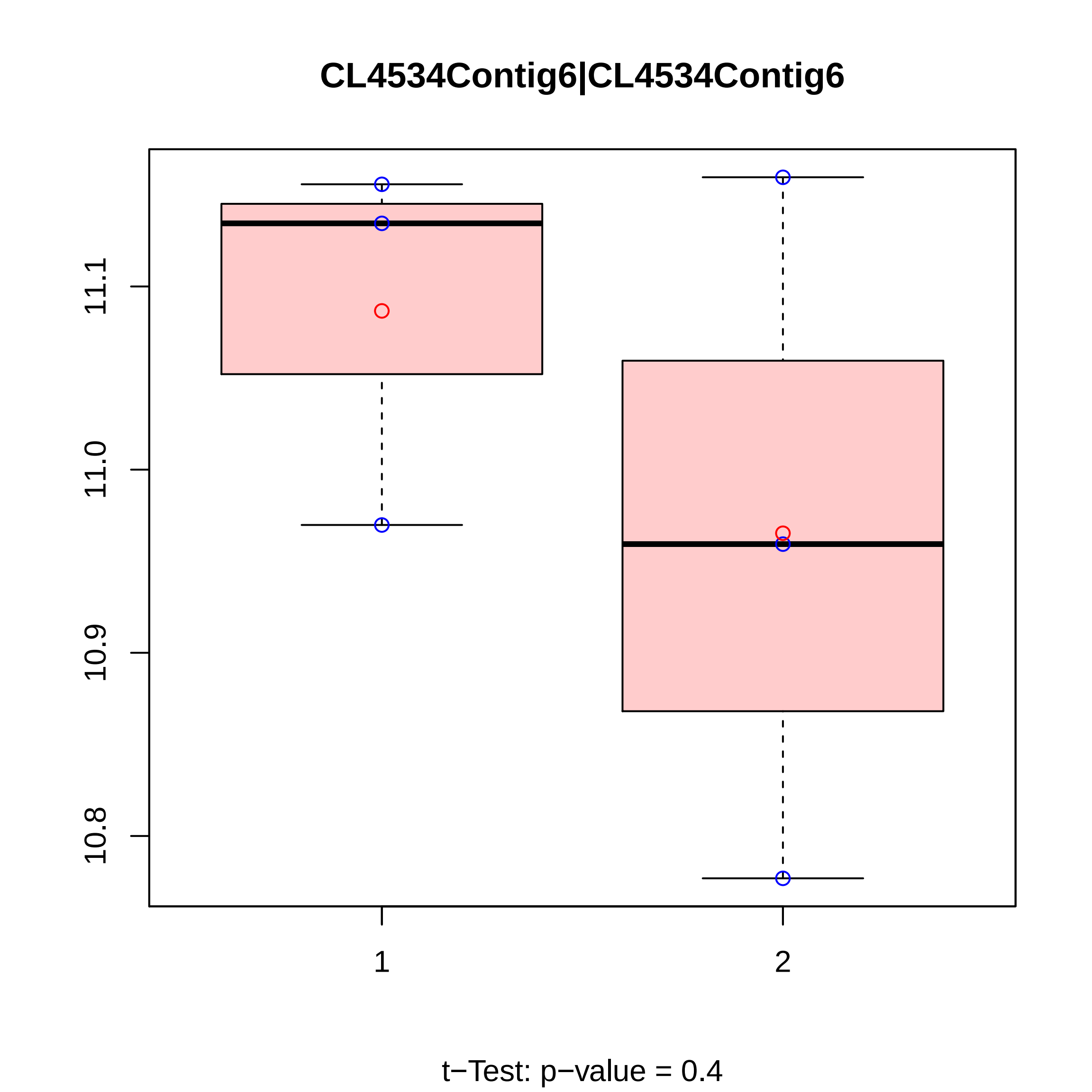

CL4534Contig6|CL4534Contig6
11.1
11.0
10.9
10.8
1
2
t−Test: p−value = 0.4

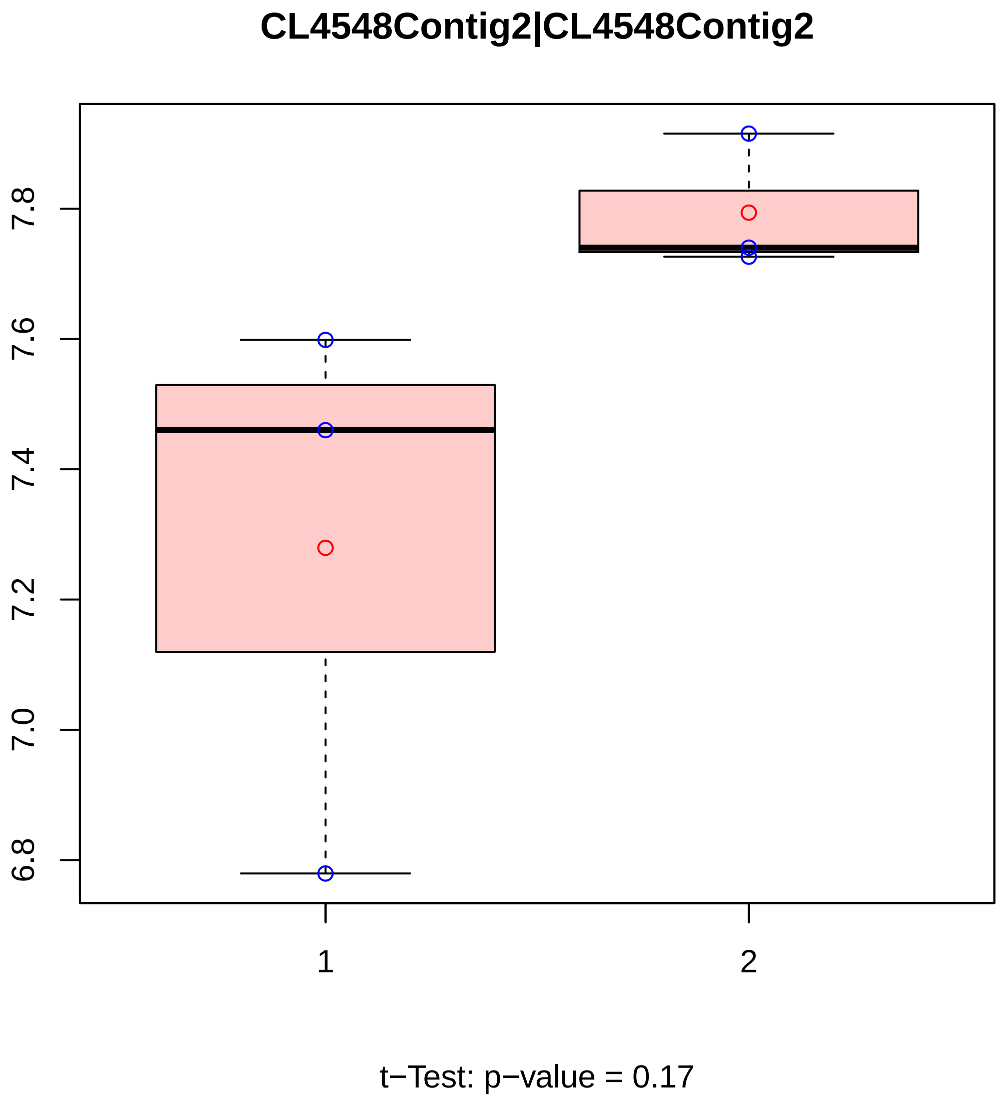
CL4548Contig2|CL4548Contig2
7.8
7.6
7.4
7.2
7.0
6.8
1
2
t−Test: p−value = 0.17

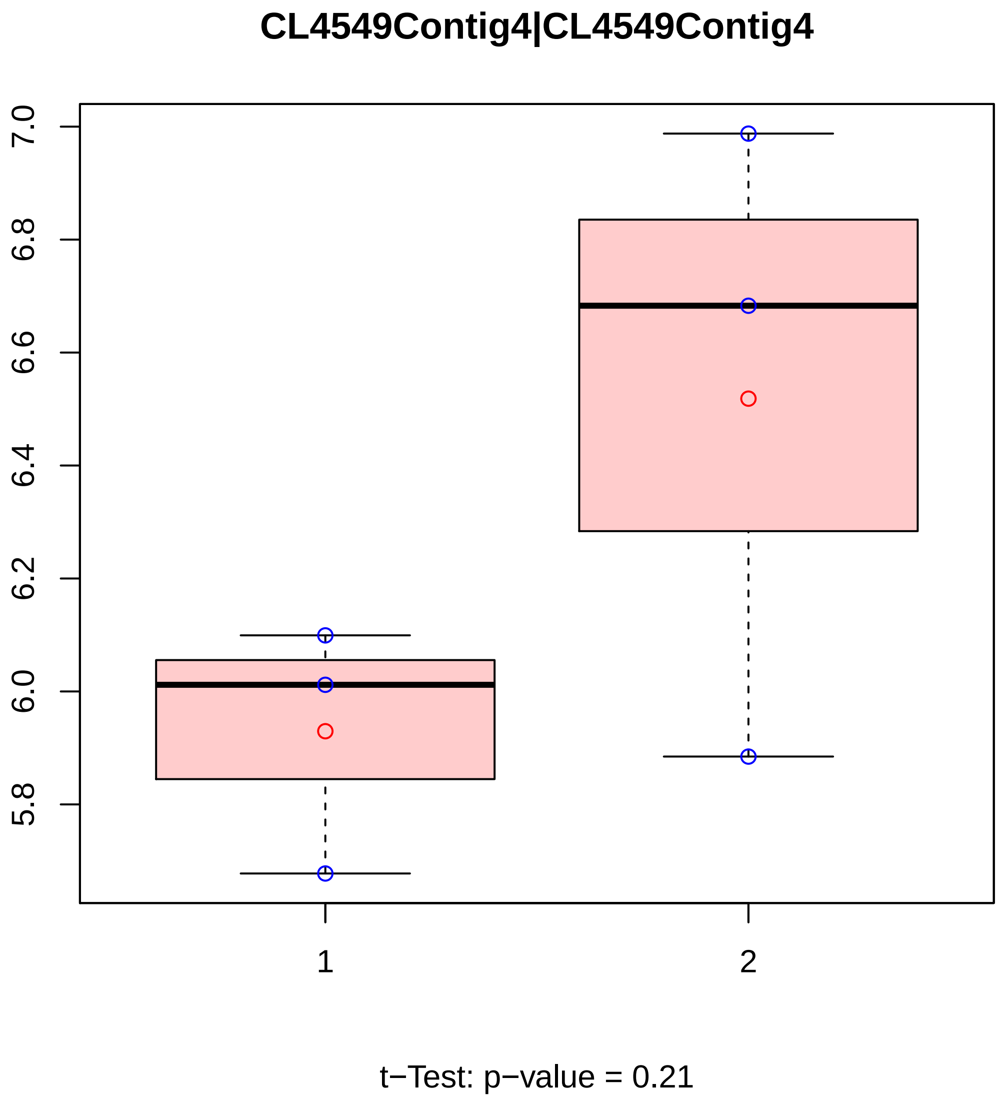
CL4549Contig4|CL4549Contig4
7.0
6.8
6.6
6.4
6.2
6.0
5.8
1
2
t−Test: p−value = 0.21

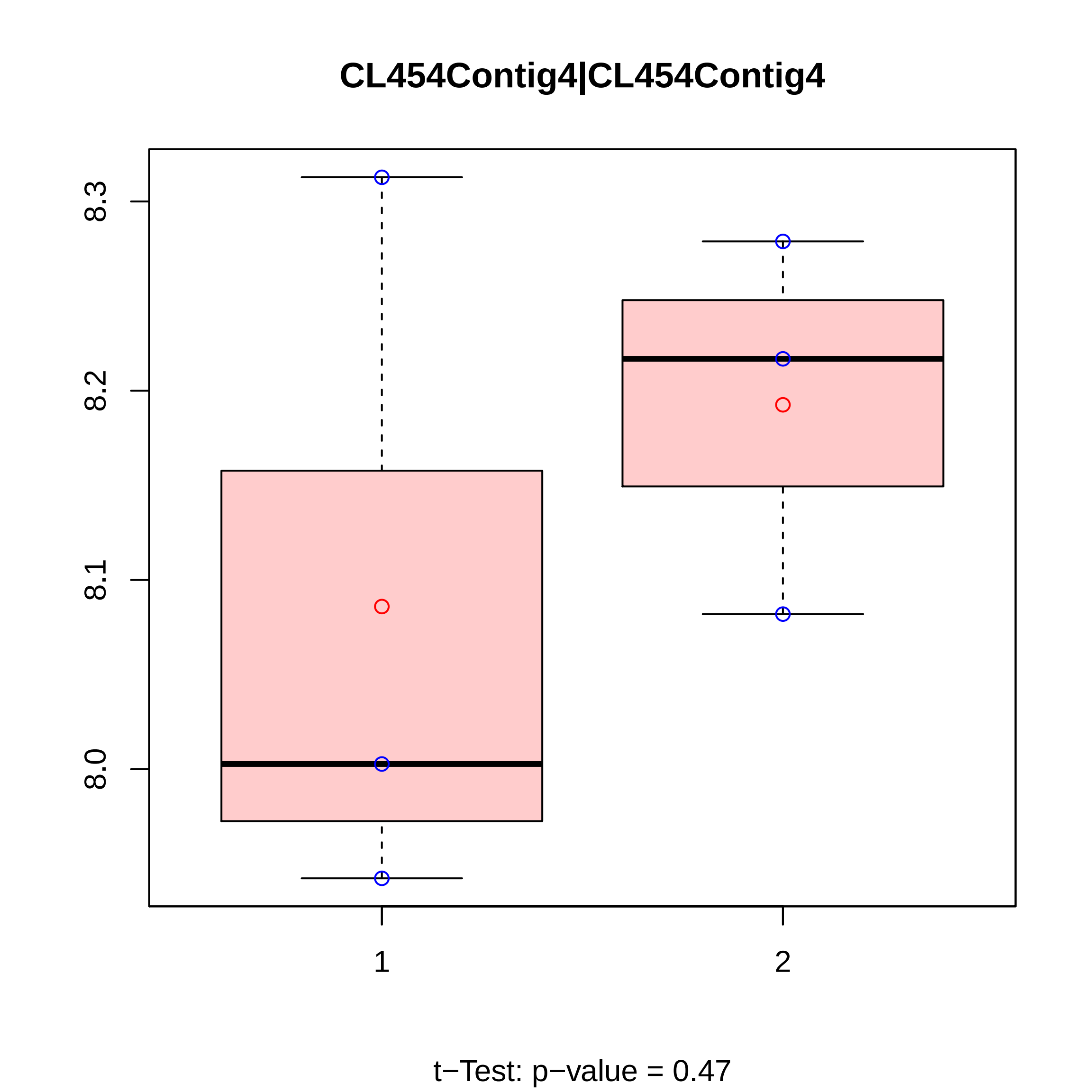
CL454Contig4|CL454Contig4
8.0
8.1
8.2
8.3
1
2
t−Test: p−value = 0.47

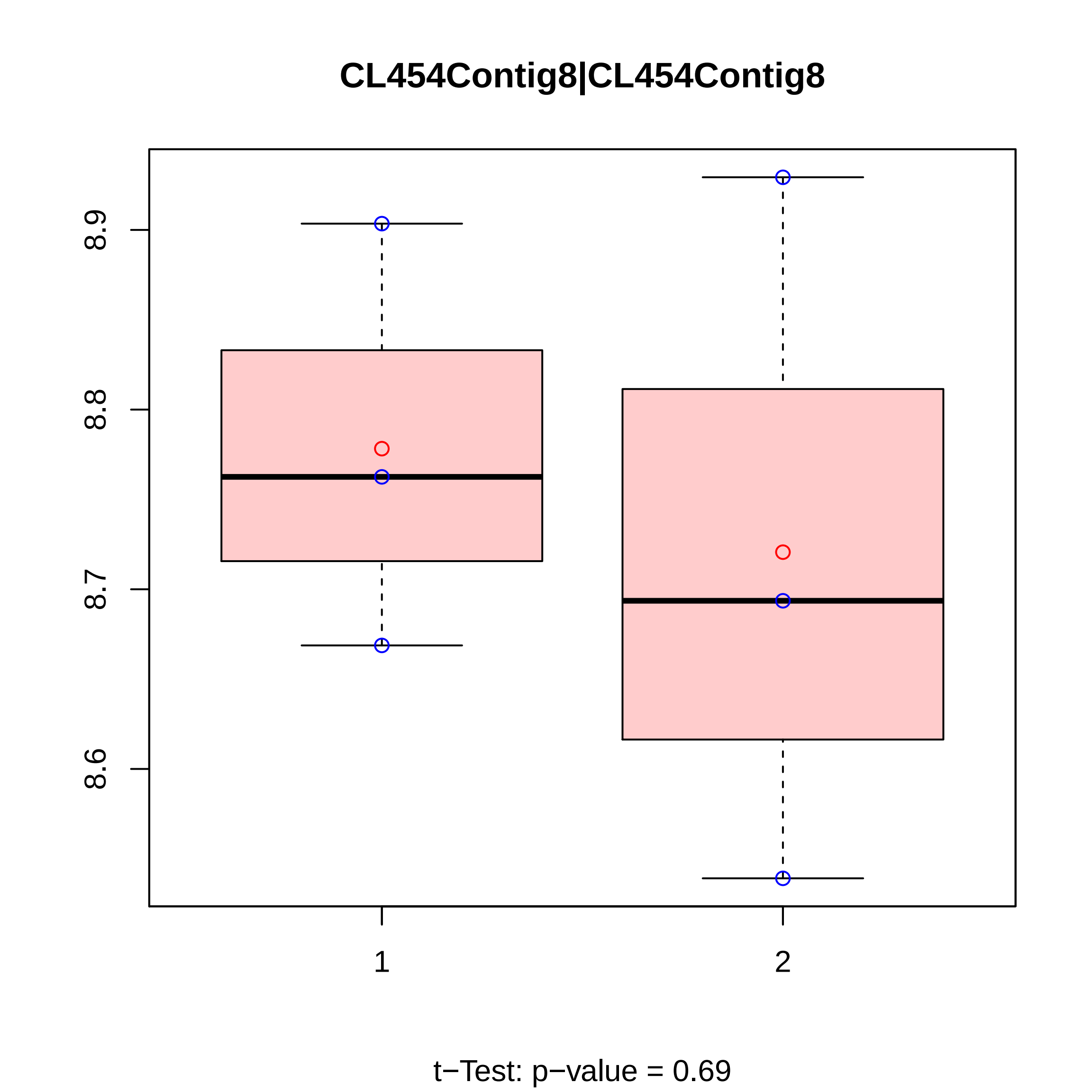
CL454Contig8|CL454Contig8
8.6
8.7
8.8
8.9
1
2
t−Test: p−value = 0.69

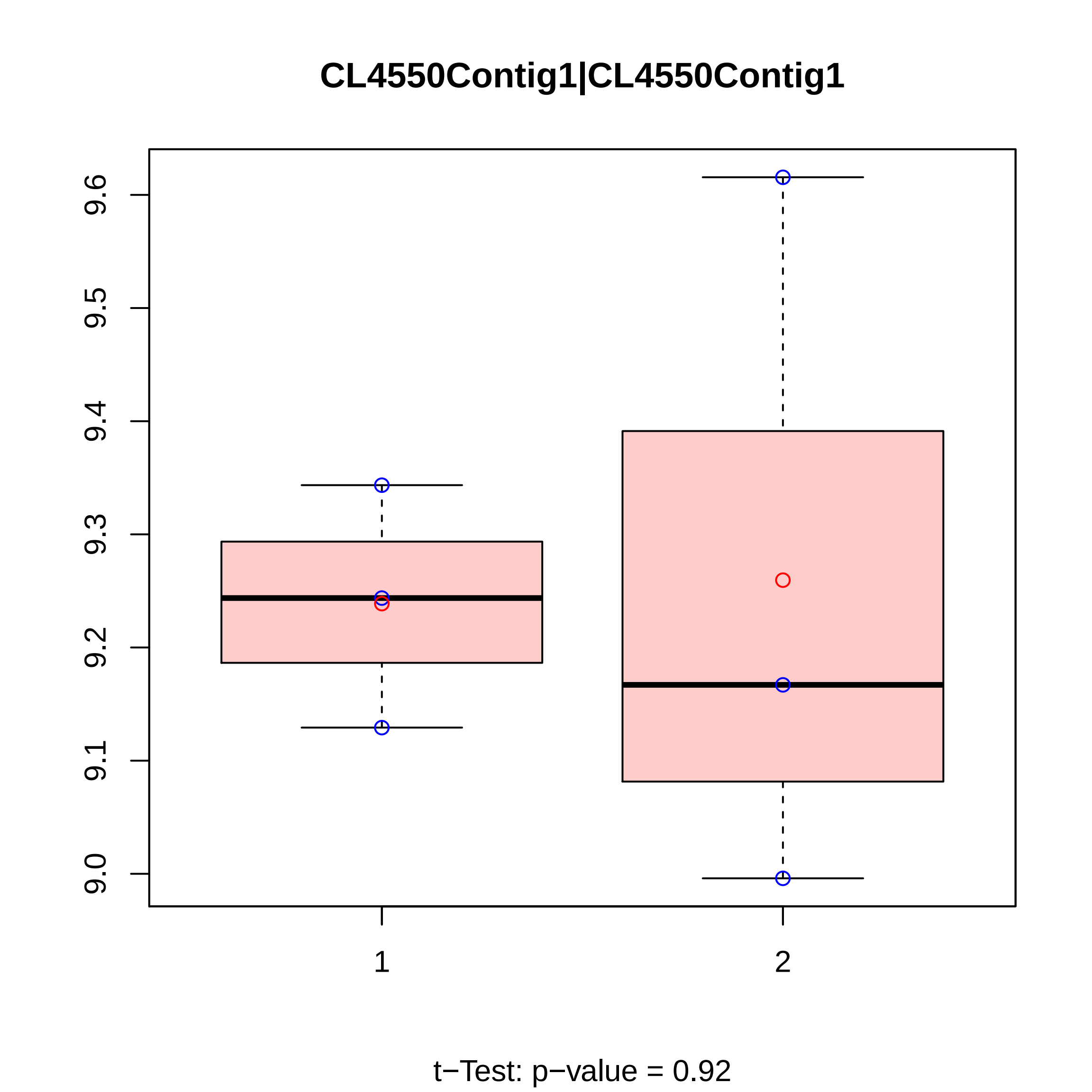
CL4550Contig1|CL4550Contig1
9.0
9.1
9.2
9.3
9.4
9.5
9.6
1
2
t−Test: p−value = 0.92

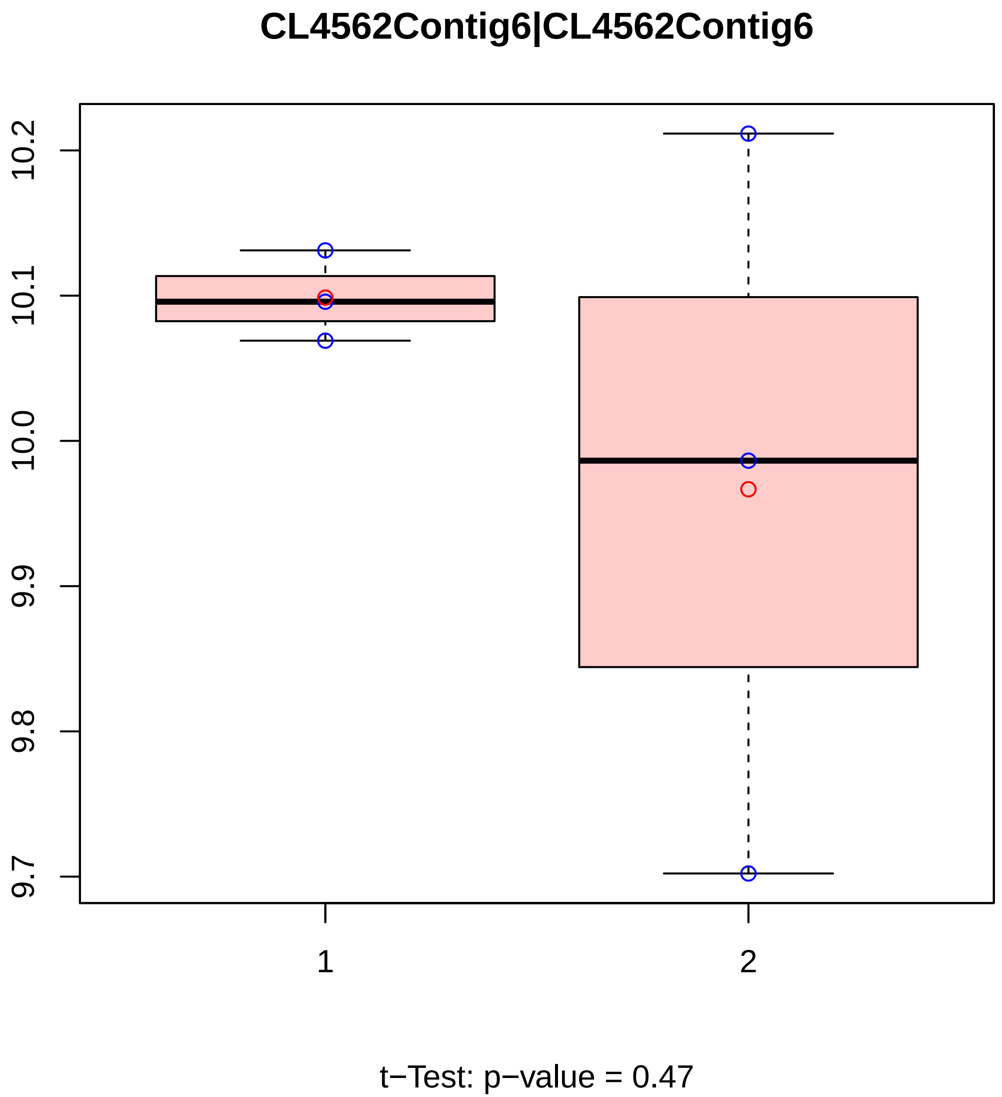

CL4562Contig6|CL4562Contig6
10.2
10.1
10.0
9.9
9.8
9.7
1
2
t−Test: p−value = 0.47

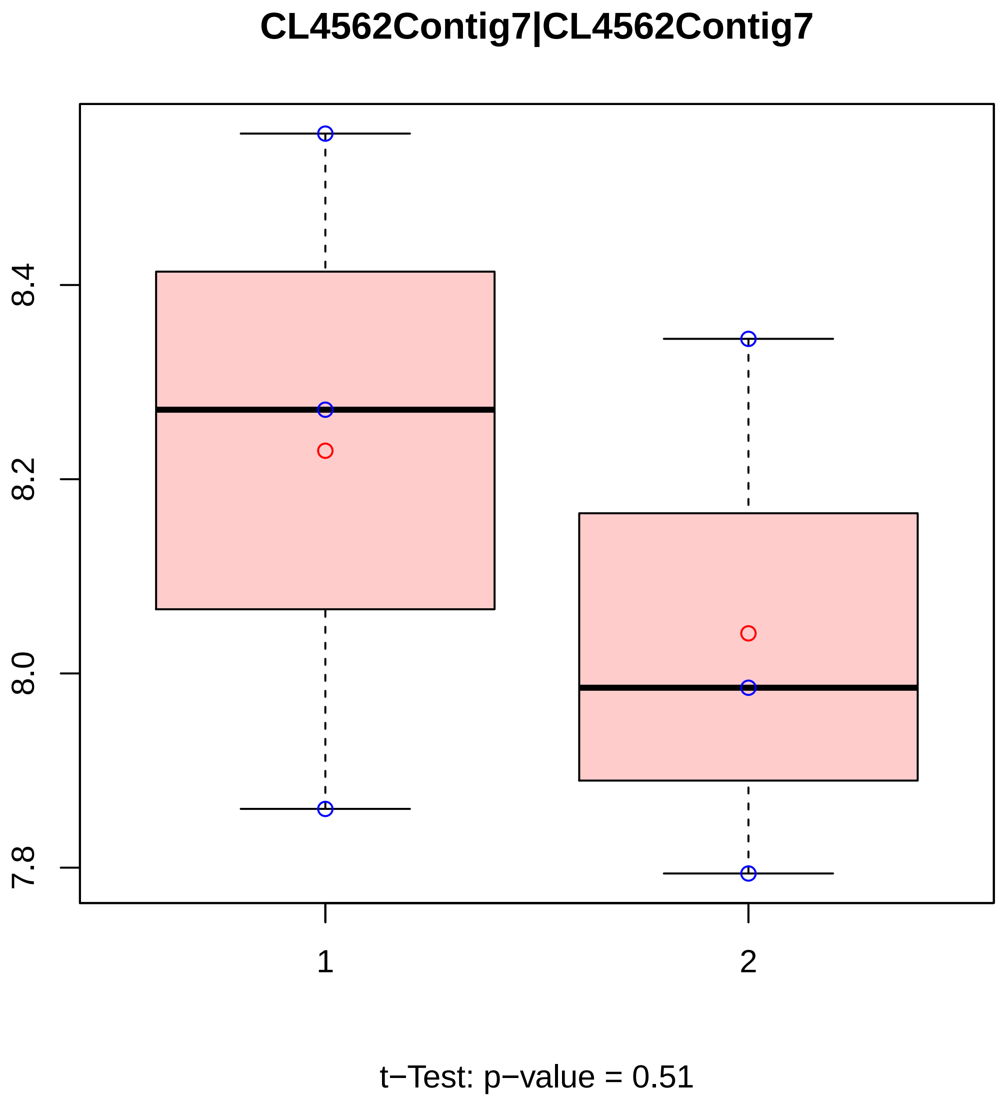

CL4562Contig7|CL4562Contig7
8.4
8.2
8.0
7.8
1
2
t−Test: p−value = 0.51

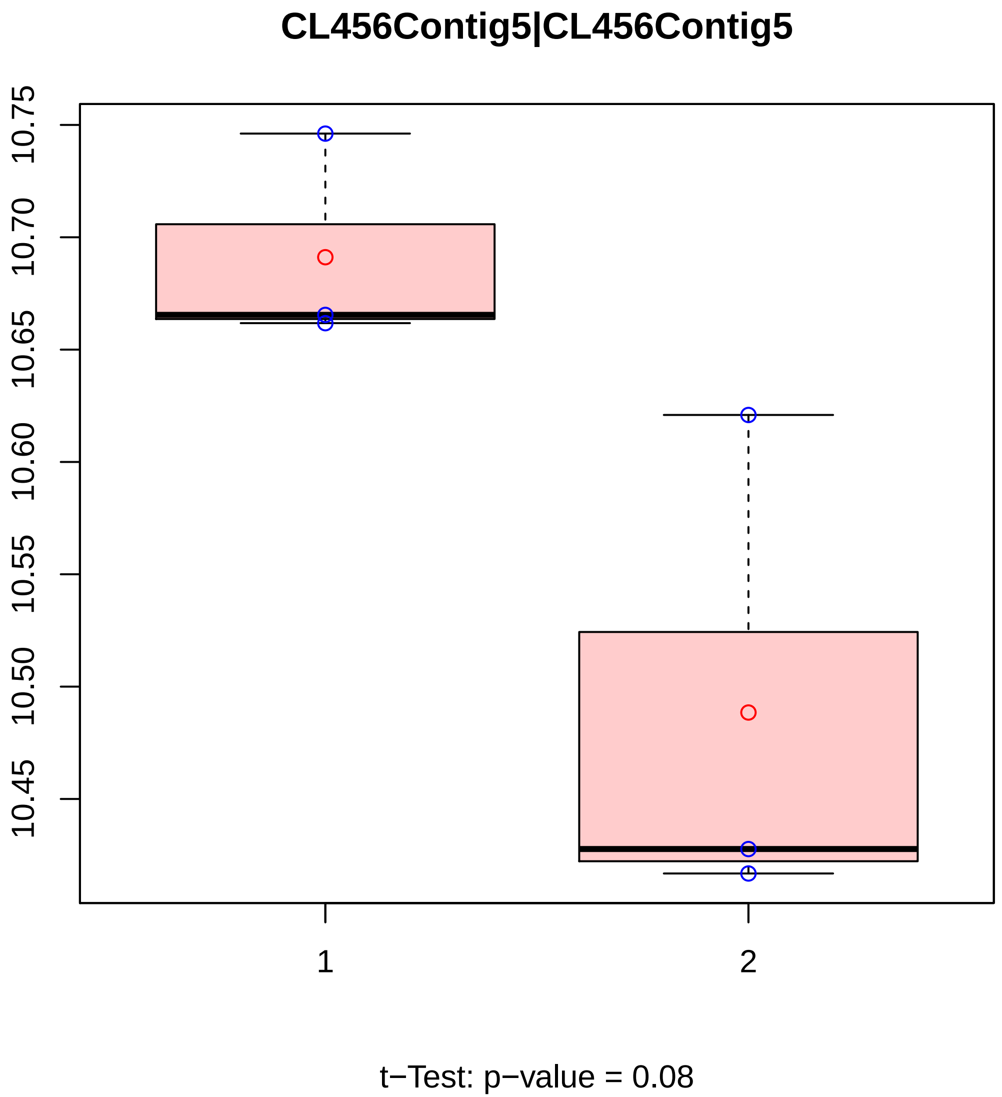
CL456Contig5|CL456Contig5
10.75
10.70
10.65
10.60
10.55
10.50
10.45
1
2
t−Test: p−value = 0.08

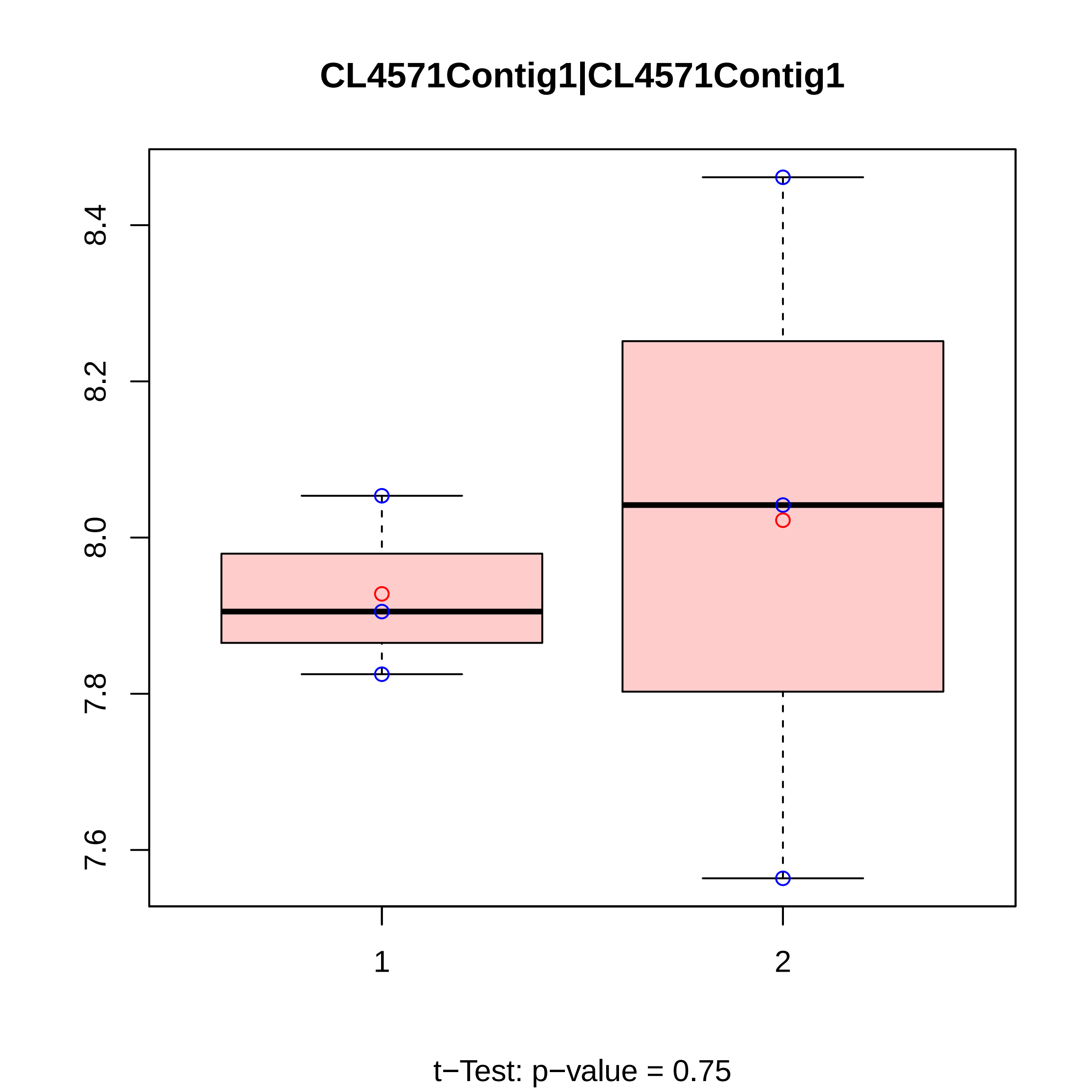

CL4571Contig1|CL4571Contig1
8.4
8.2
8.0
7.8
7.6
1
2
t−Test: p−value = 0.75

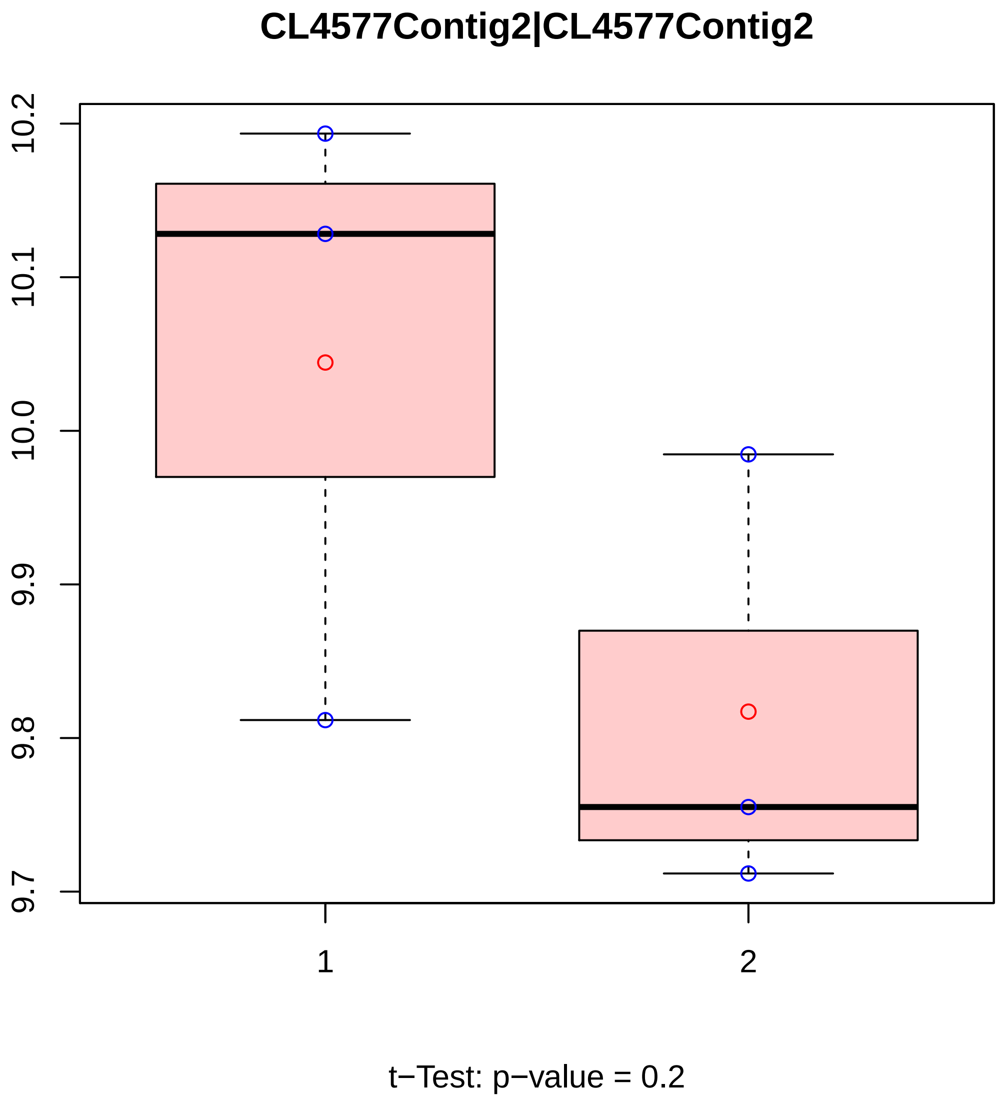
CL4577Contig2|CL4577Contig2
10.2
10.1
10.0
9.9
9.8
9.7
1
2
t−Test: p−value = 0.2

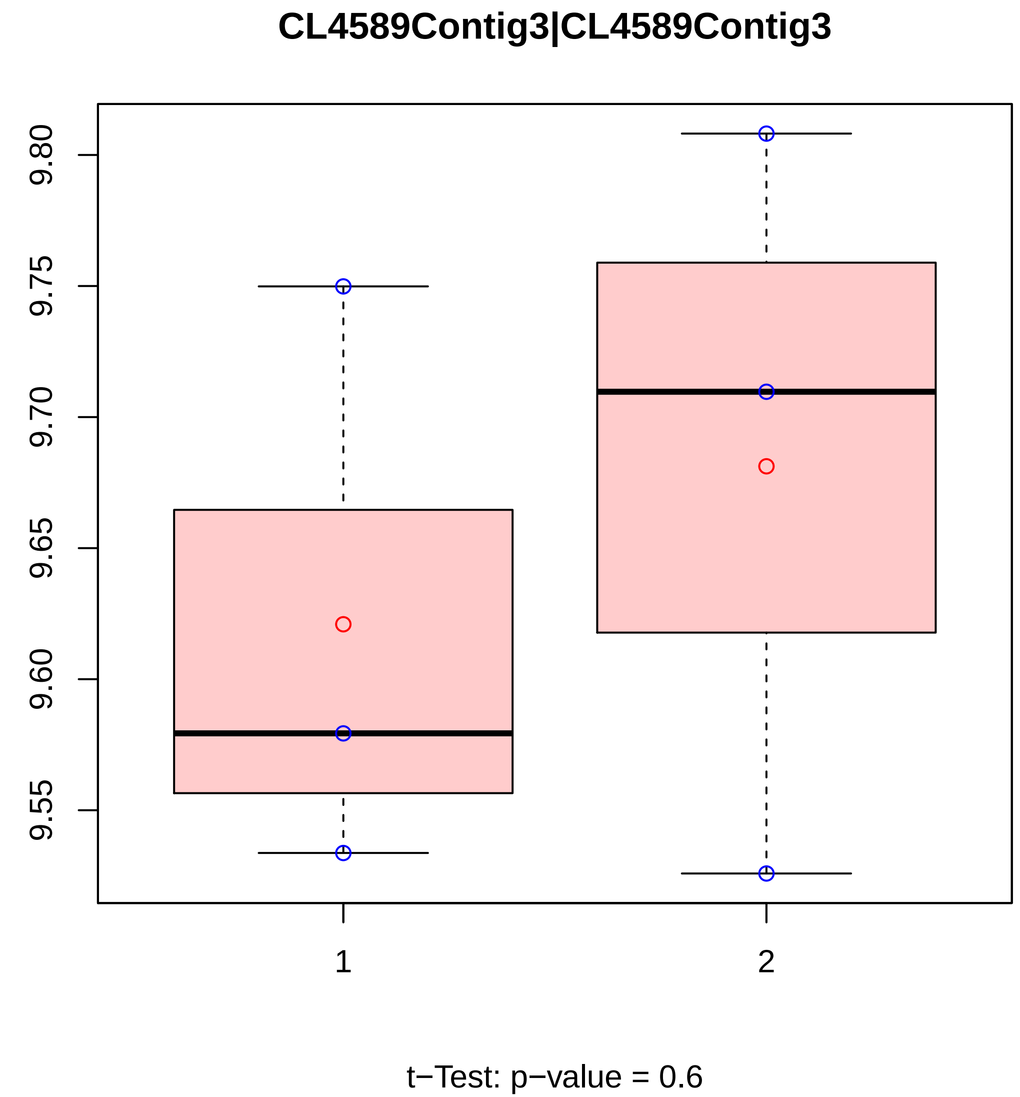

CL4589Contig3|CL4589Contig3
9.80
9.75
9.70
9.65
9.60
9.55
1
2
t−Test: p−value = 0.6

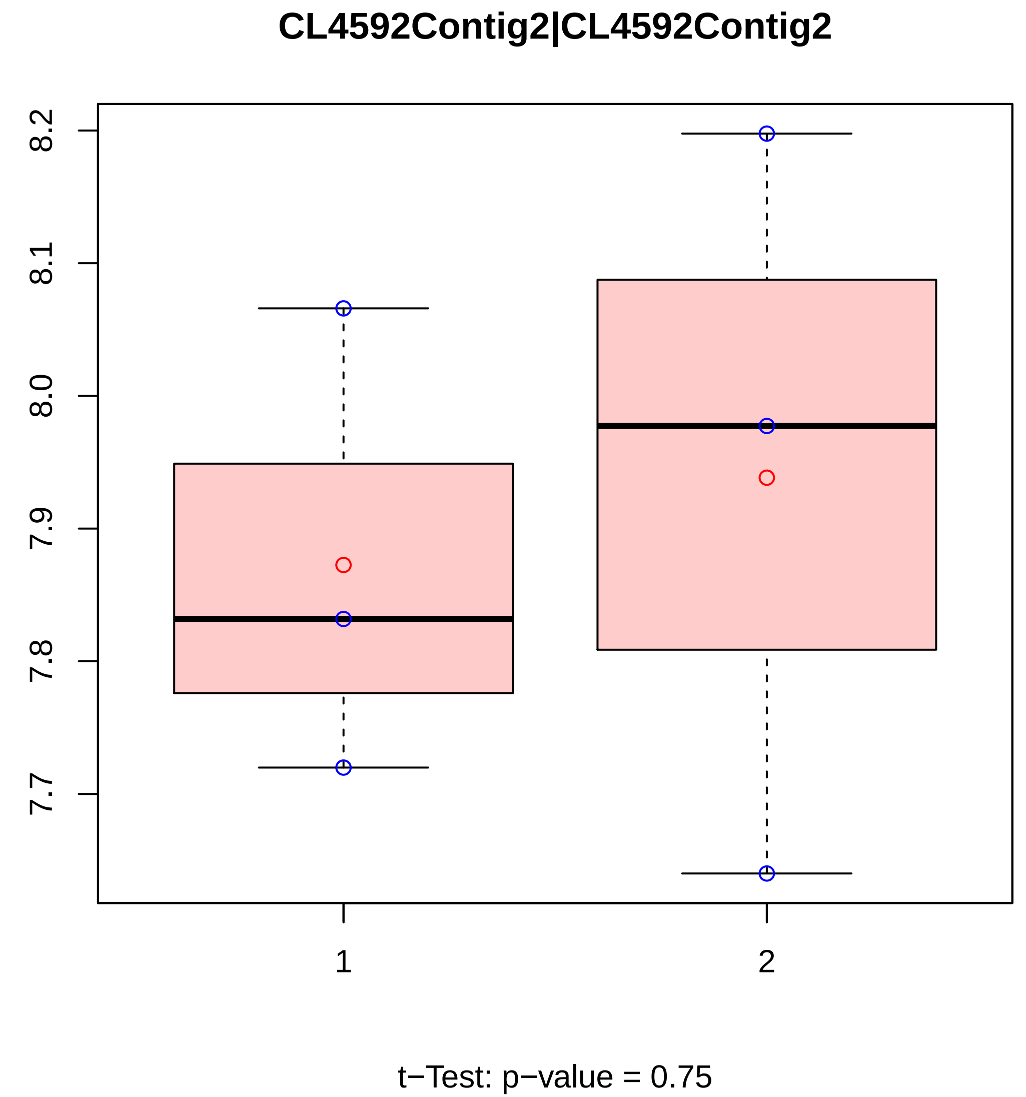
CL4592Contig2|CL4592Contig2
8.2
8.1
8.0
7.9
7.8
7.7
1
2
t−Test: p−value = 0.75

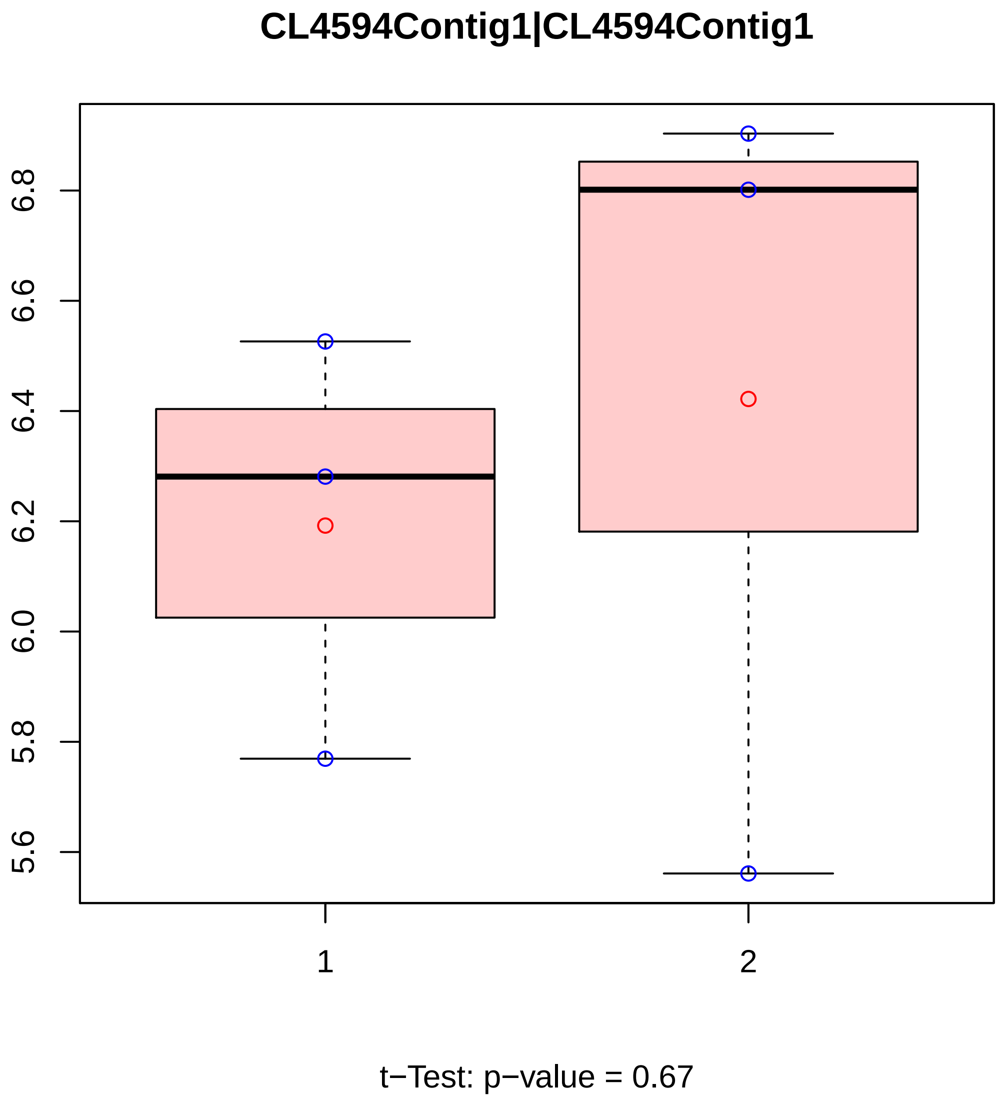
CL4594Contig1|CL4594Contig1
6.8
6.6
6.4
6.2
6.0
5.8
5.6
1
2
t−Test: p−value = 0.67

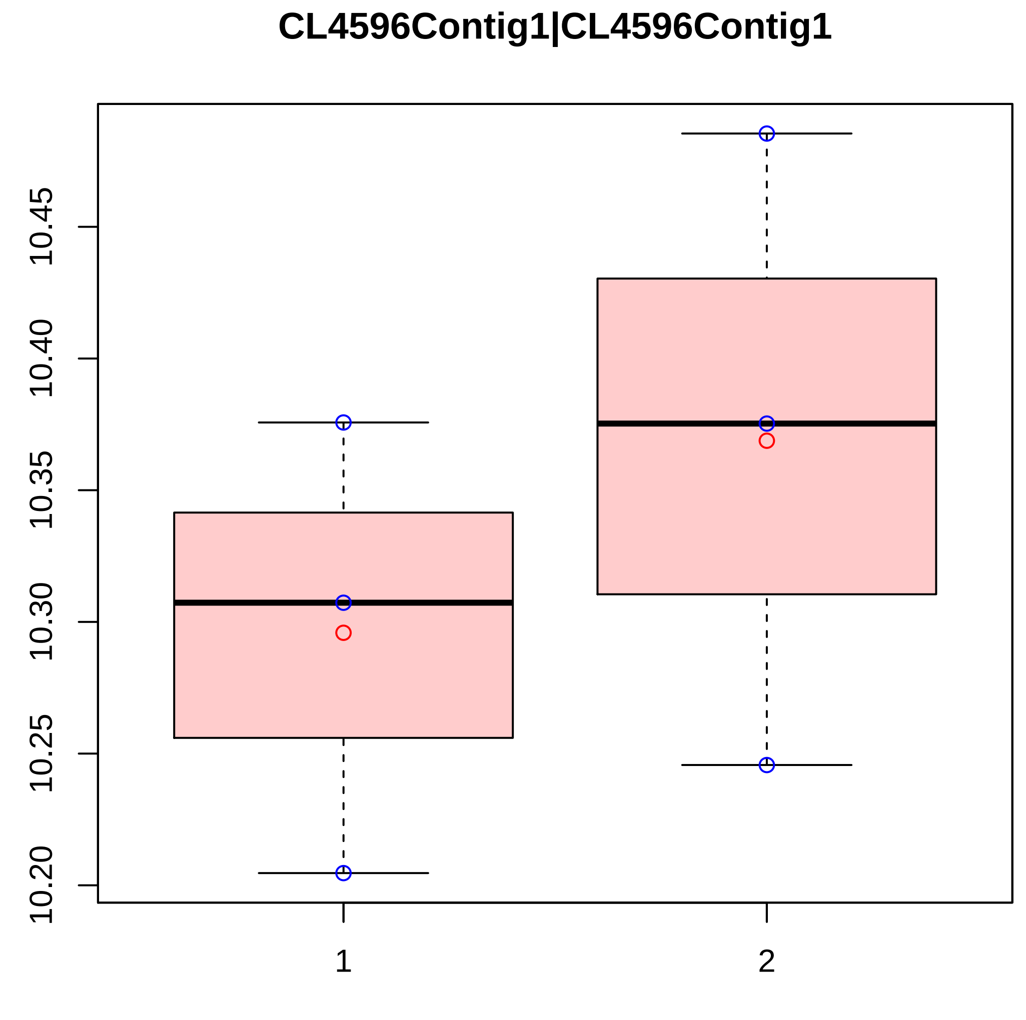


t−Test: p−value = 0.45

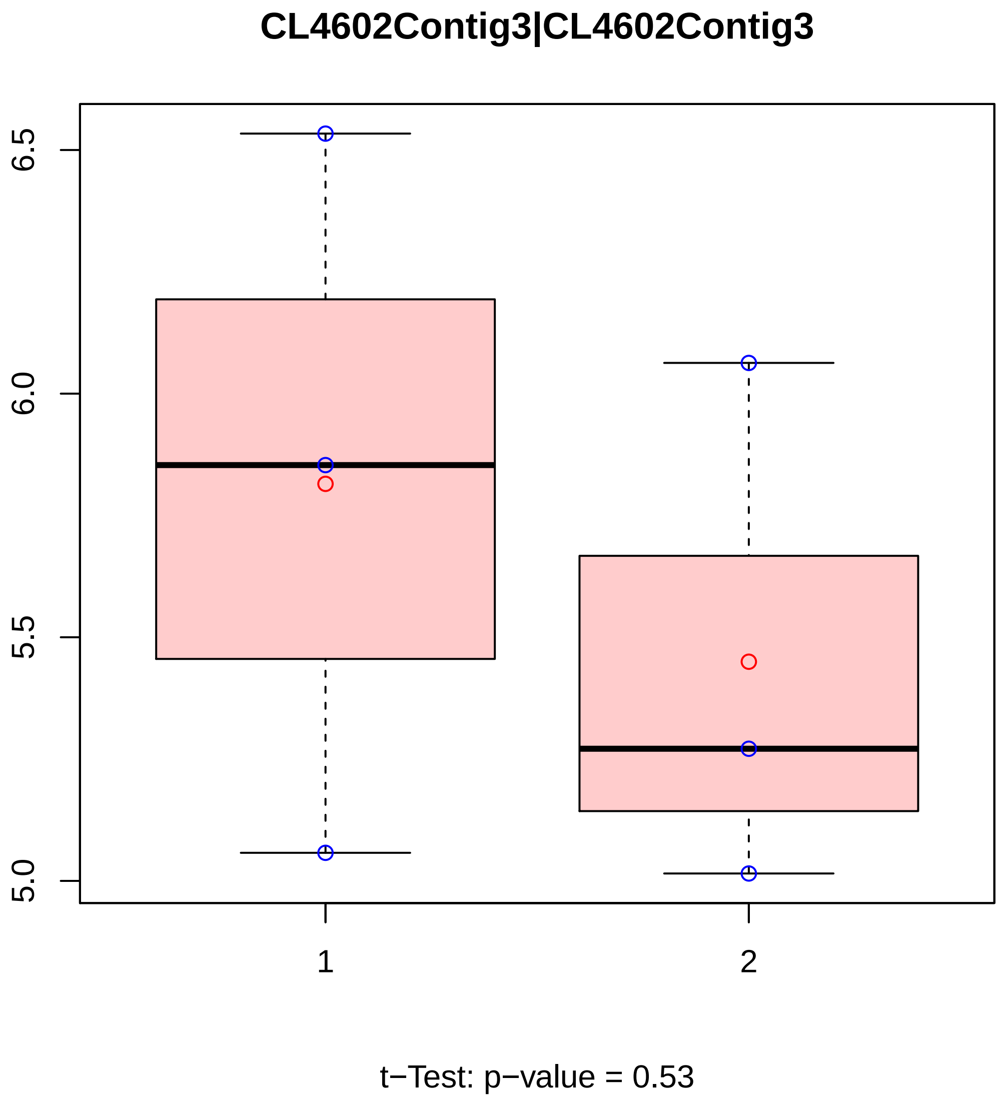
CL4602Contig3|CL4602Contig3
6.5
6.0
5.5
5.0
1
2
t−Test: p−value = 0.53

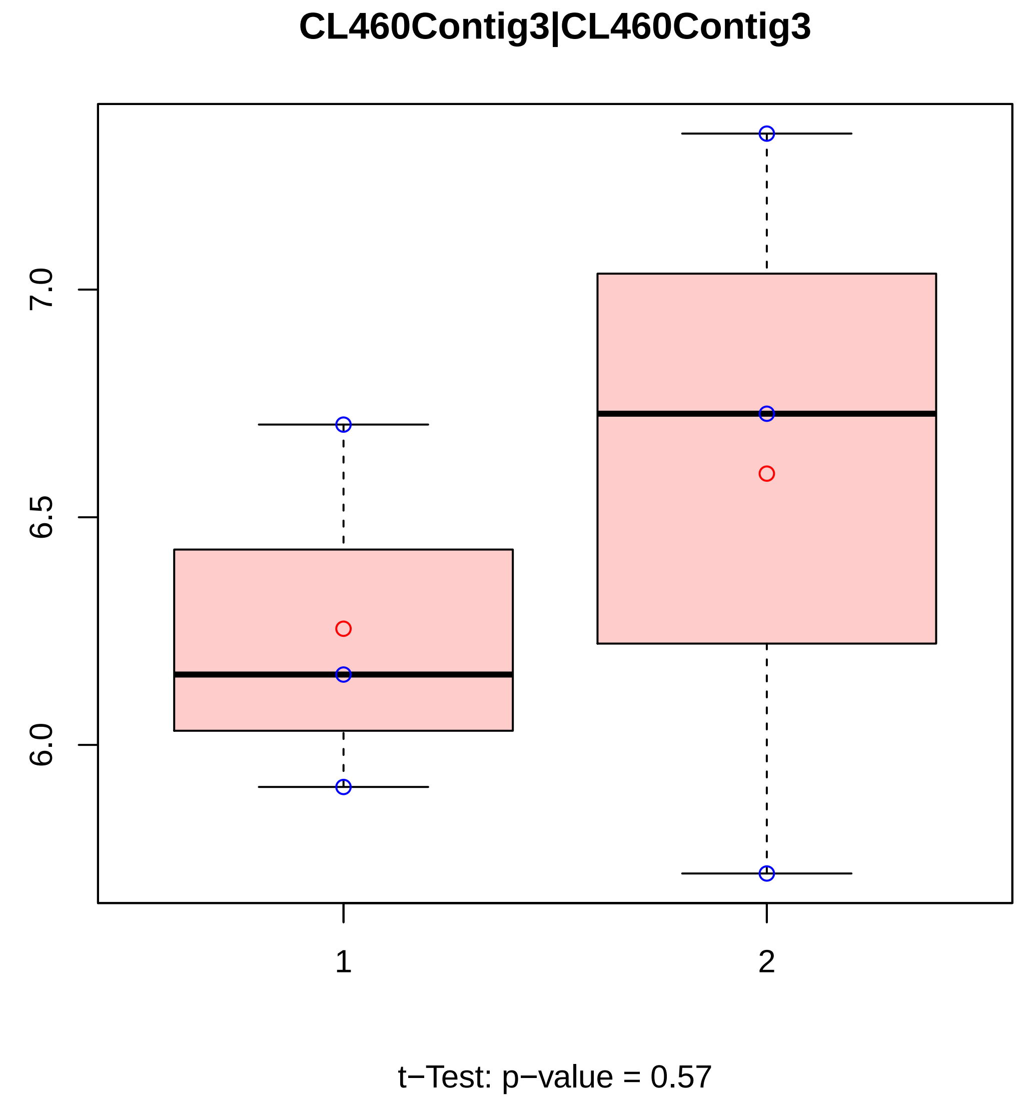
CL460Contig3|CL460Contig3
7.0
6.5
6.0
1
2
t−Test: p−value = 0.57

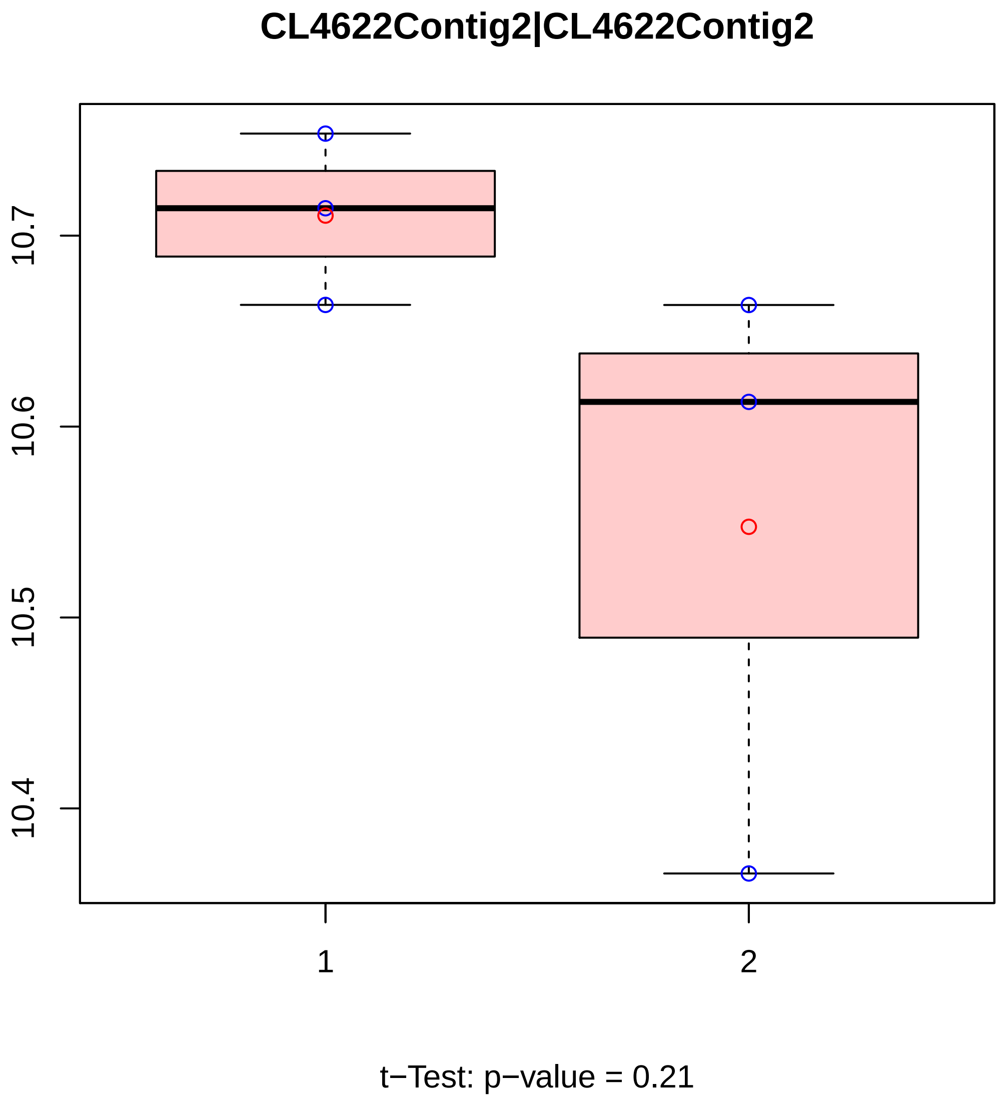
CL4622Contig2|CL4622Contig2
10.4
10.5
10.6
10.7
1
2
t−Test: p−value = 0.21

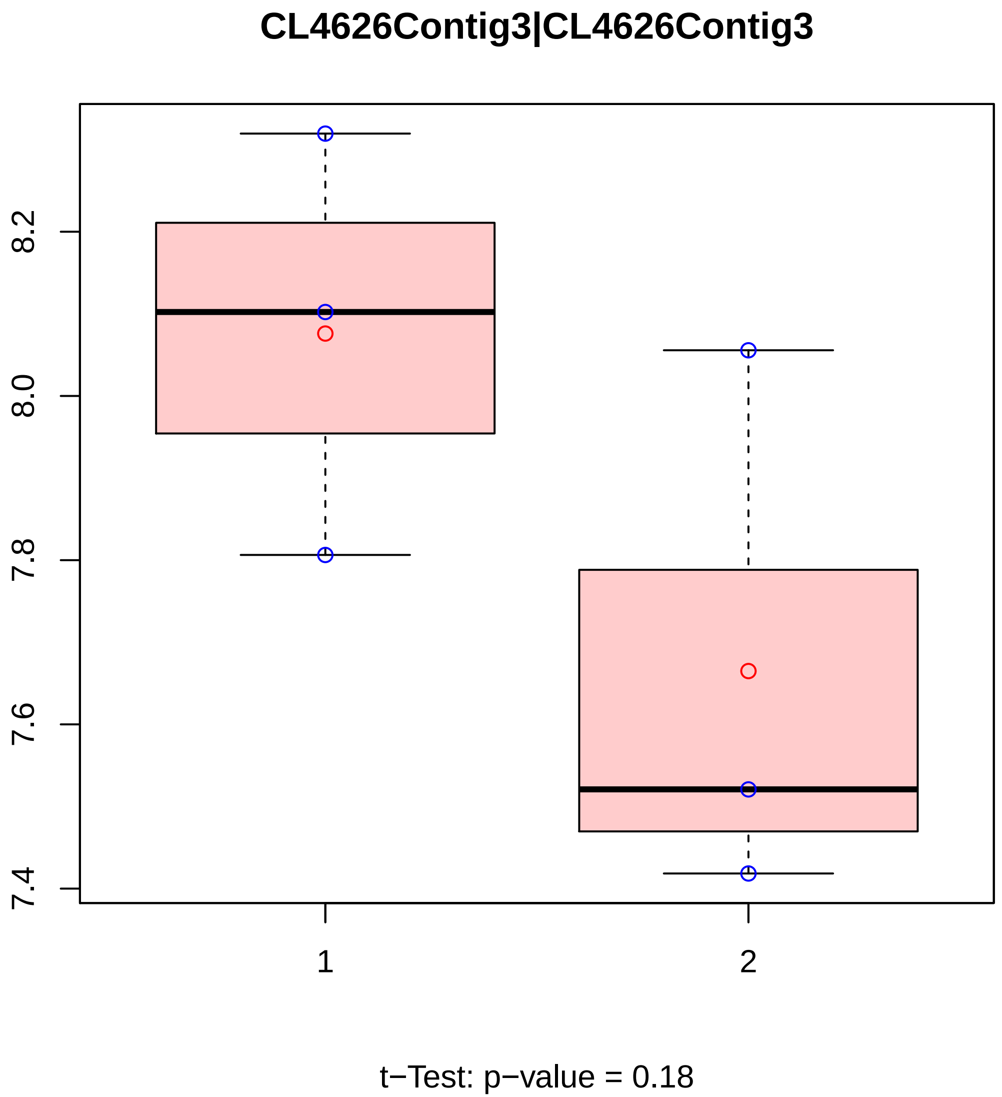
CL4626Contig3|CL4626Contig3
8.2
8.0
7.8
7.6
7.4
1
2
t−Test: p−value = 0.18

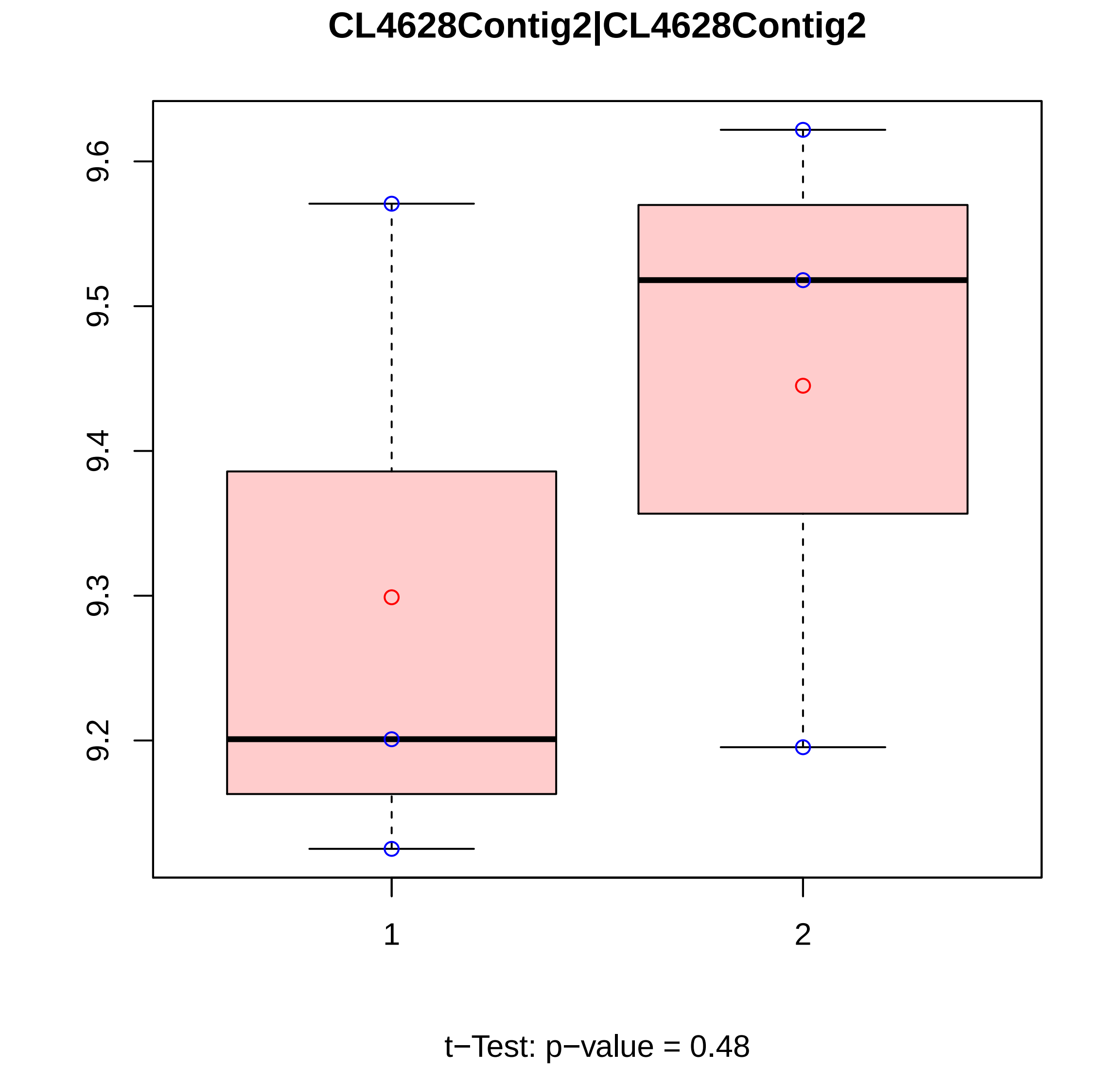
CL4628Contig2|CL4628Contig2
9.2
9.3
9.4
9.5
9.6
1
2
t−Test: p−value = 0.48

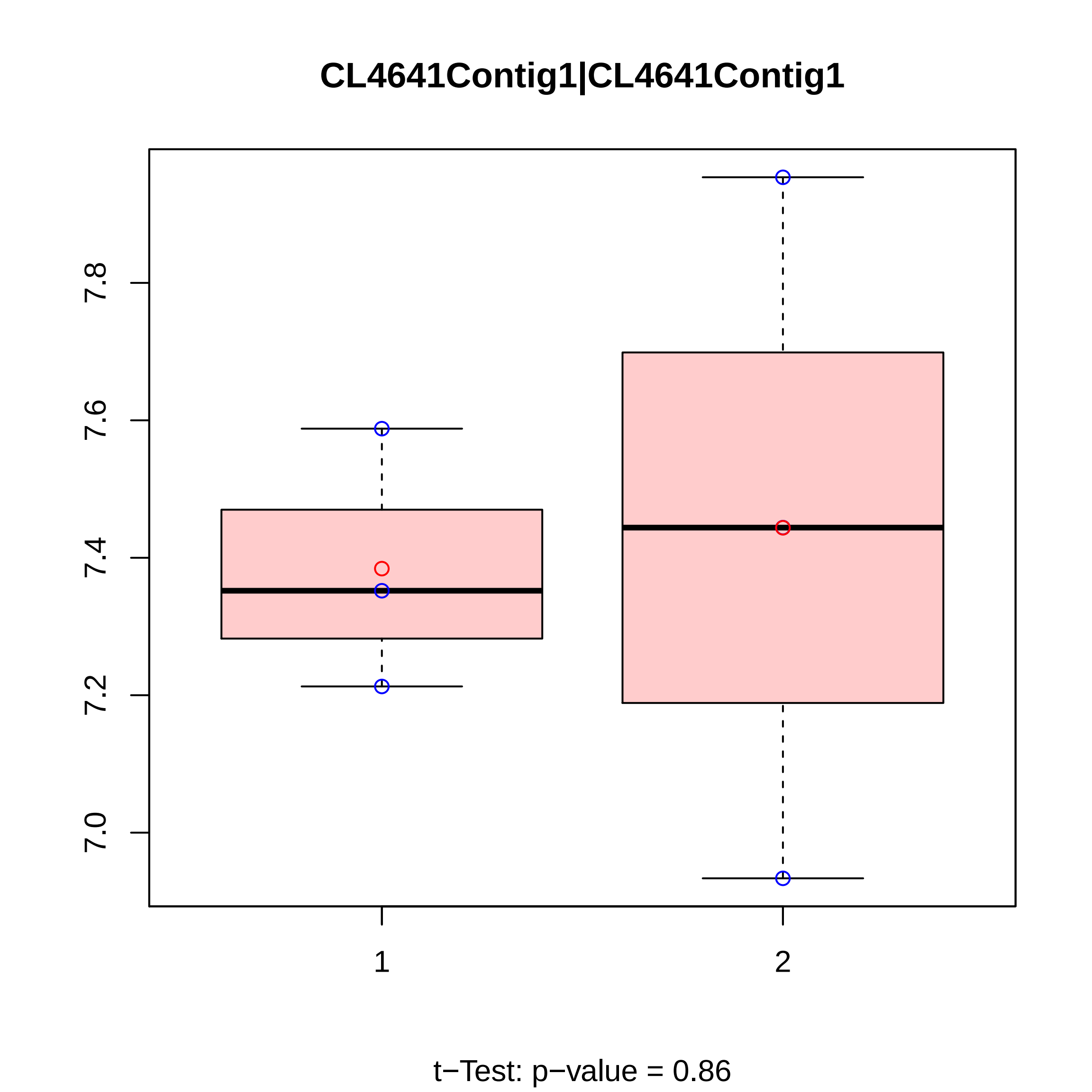
CL4641Contig1|CL4641Contig1
7.0
7.2
7.4
7.6
7.8
1
2
t−Test: p−value = 0.86

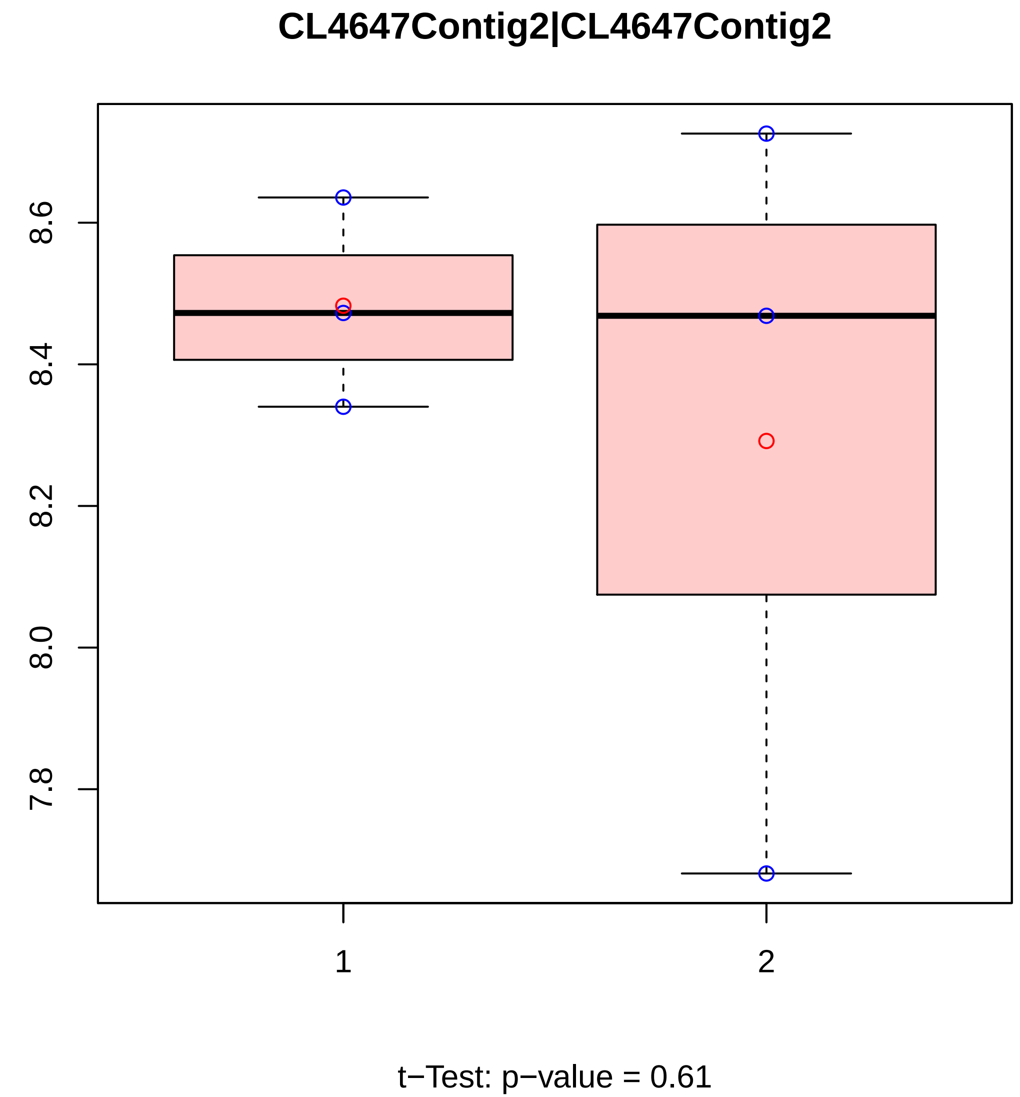

CL4647Contig2|CL4647Contig2
8.6
8.4
8.2
8.0
7.8
1
2
t−Test: p−value = 0.61

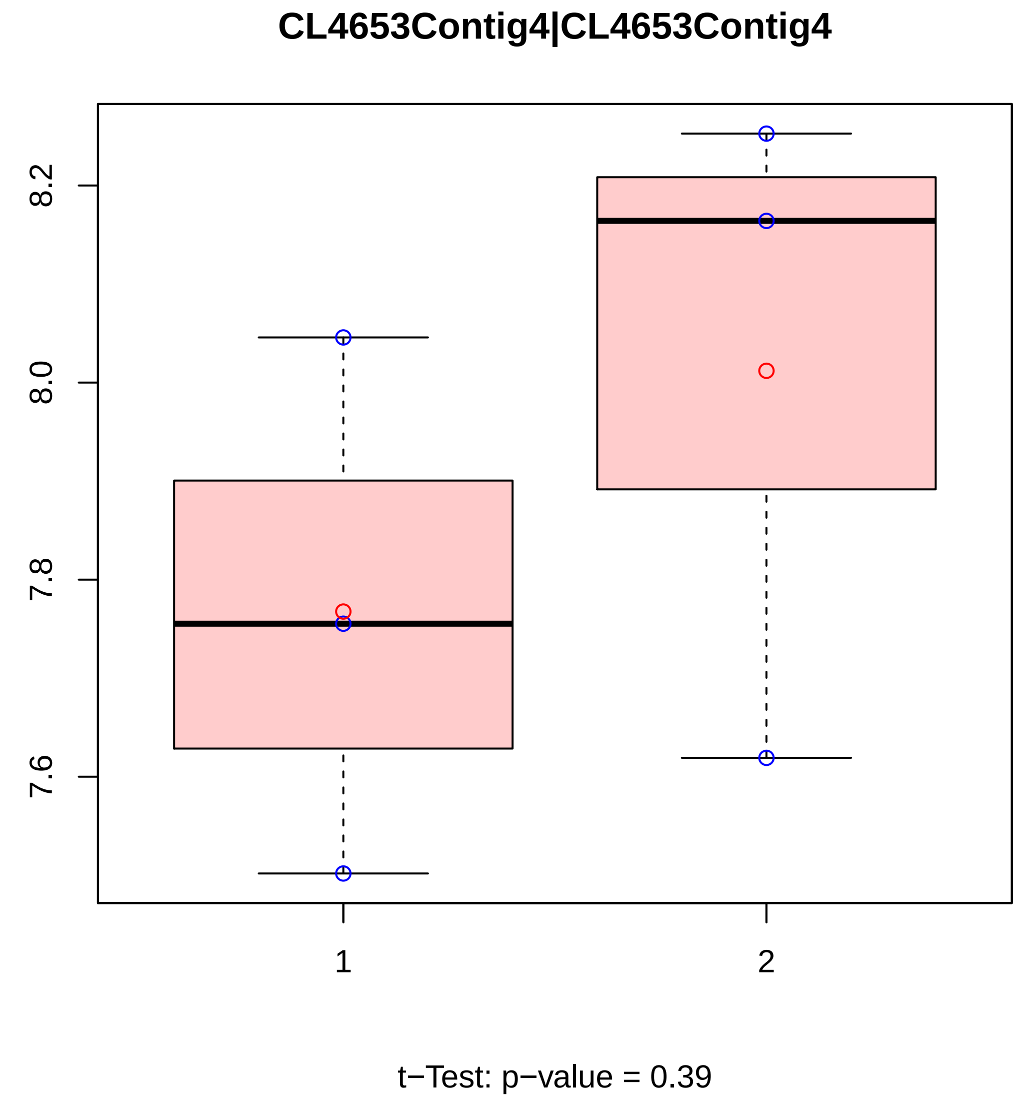
CL4653Contig4|CL4653Contig4
8.2
8.0
7.8
7.6
1
2
t−Test: p−value = 0.39

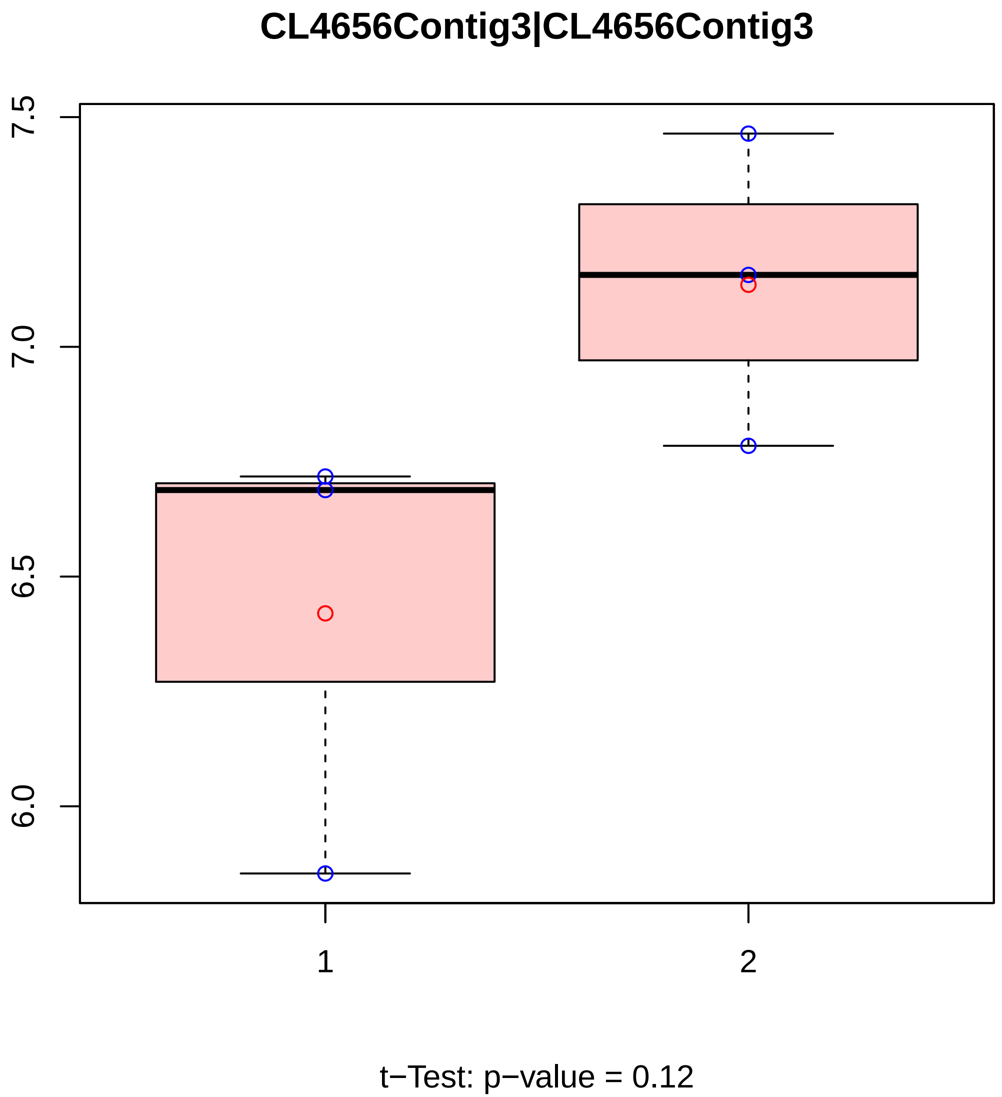
CL4656Contig3|CL4656Contig3
7.5
7.0
6.5
6.0
1
2
t−Test: p−value = 0.12

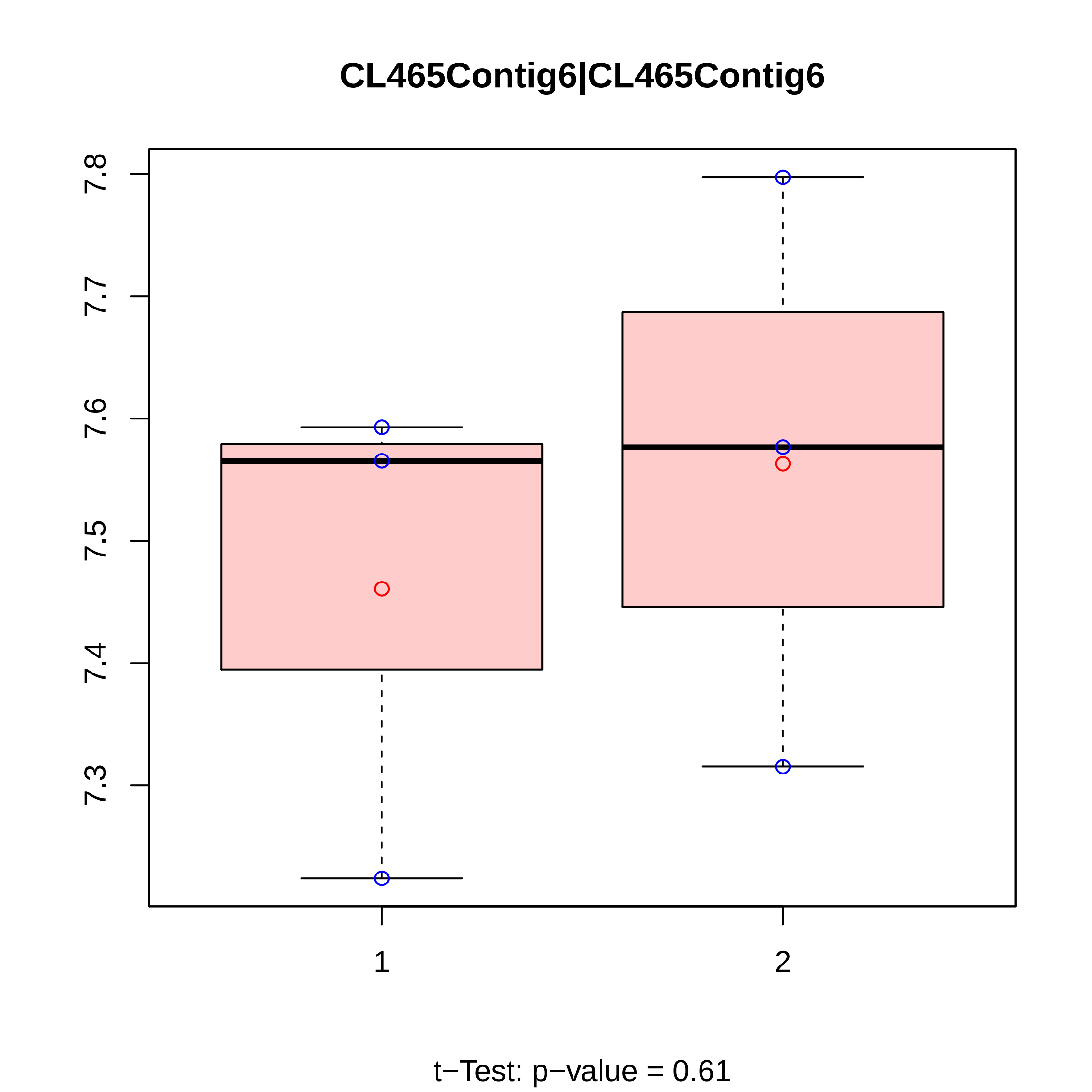

CL465Contig6|CL465Contig6
7.3
7.4
7.5
7.6
7.7
7.8
1
2
t−Test: p−value = 0.61

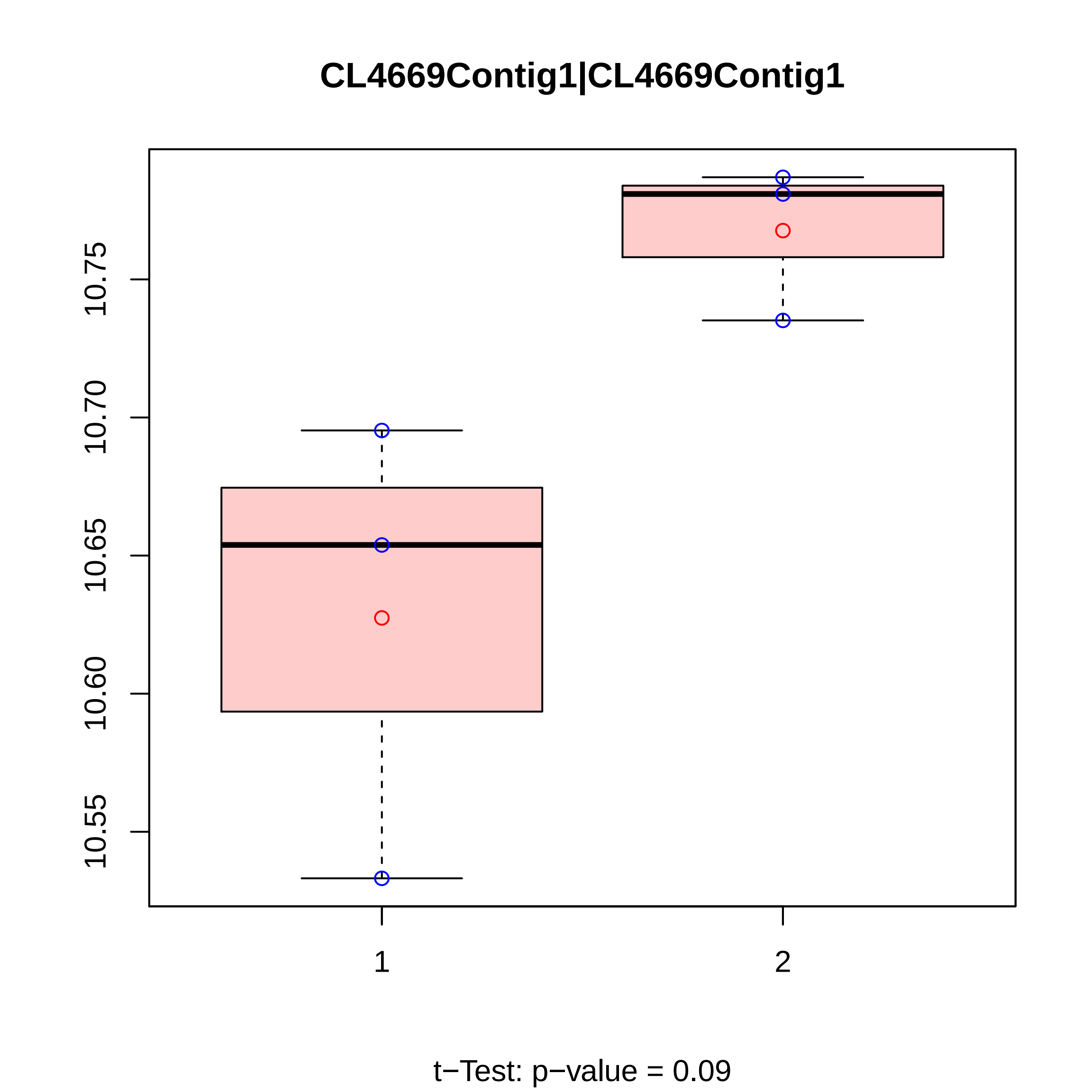
CL4669Contig1|CL4669Contig1
10.55
10.60
10.65
10.70
10.75
1
2
t−Test: p−value = 0.09

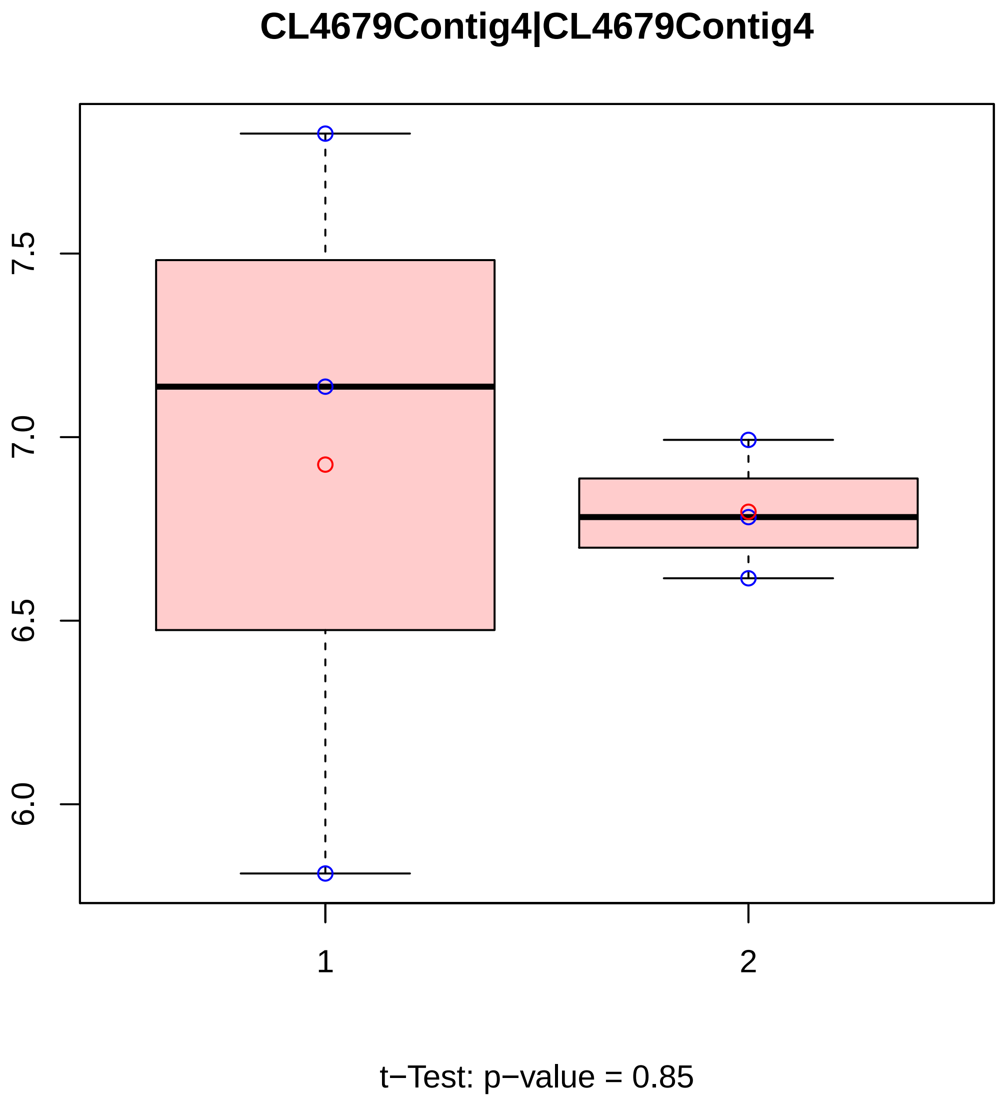
CL4679Contig4|CL4679Contig4
7.5
7.0
6.5
6.0
1
2
t−Test: p−value = 0.85

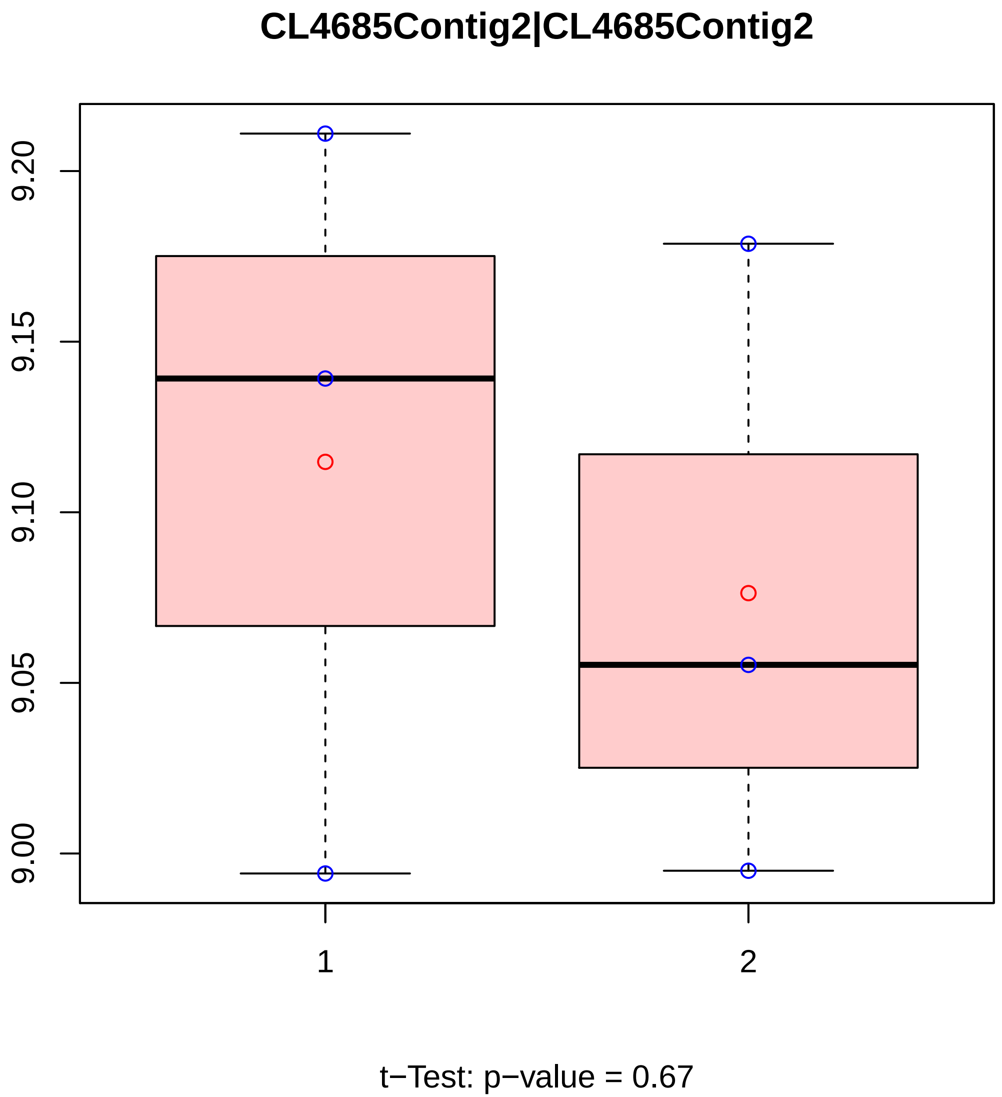

CL4685Contig2|CL4685Contig2
9.00
9.05
9.10
9.15
9.20
1
2
t−Test: p−value = 0.67

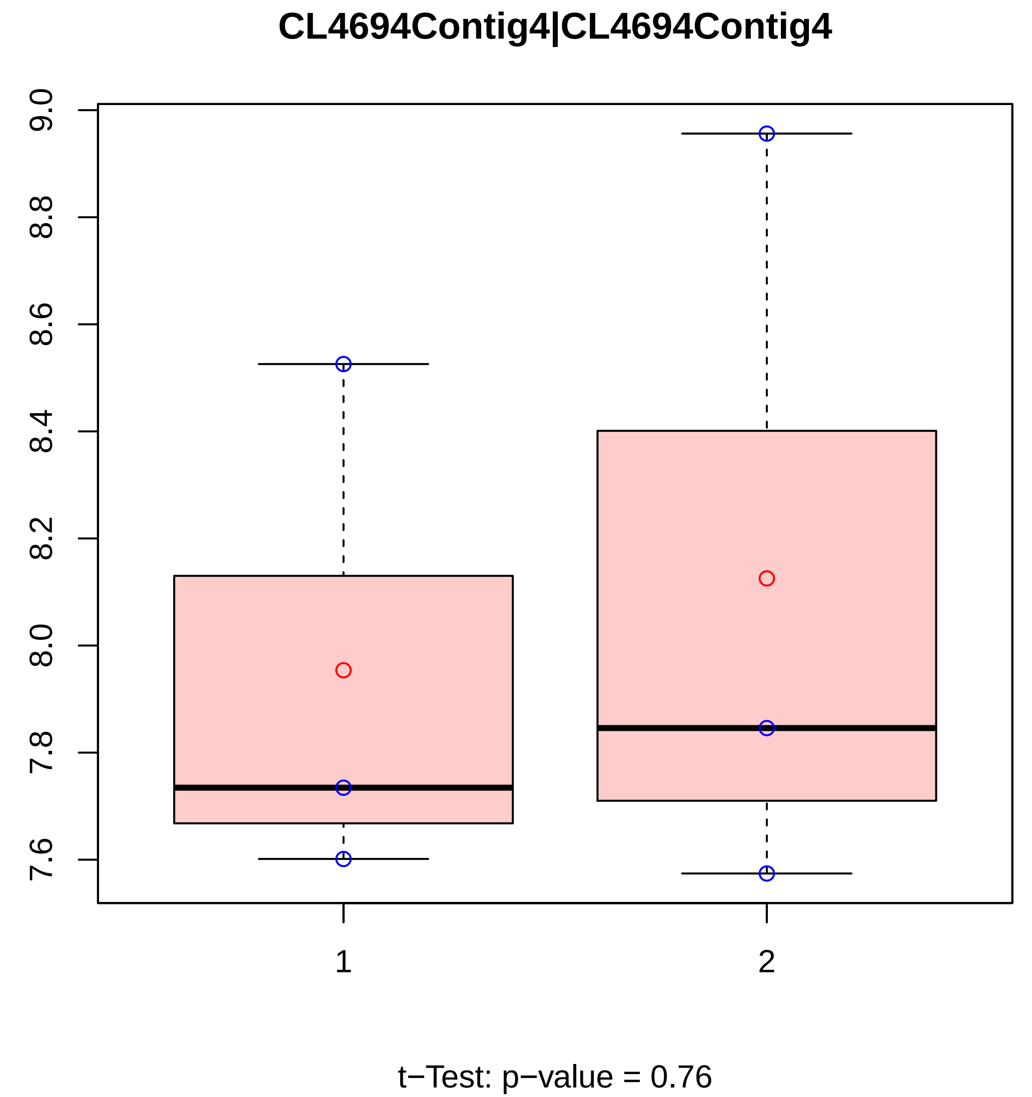

CL4694Contig4|CL4694Contig4
9.0
8.8
8.6
8.4
8.2
8.0
7.8
7.6
1
2
t−Test: p−value = 0.76

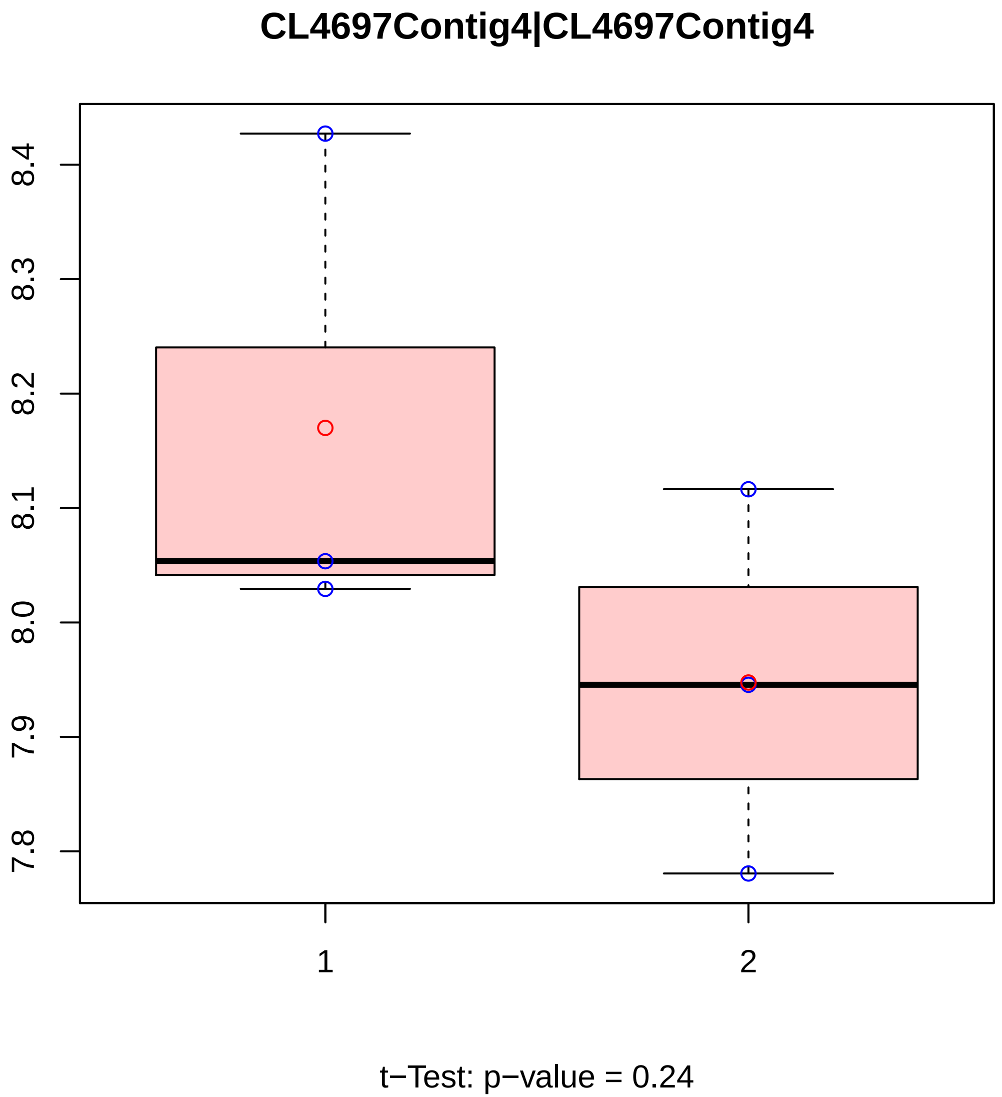
CL4697Contig4|CL4697Contig4
8.4
8.3
8.2
8.1
8.0
7.9
7.8
1
2
t−Test: p−value = 0.24

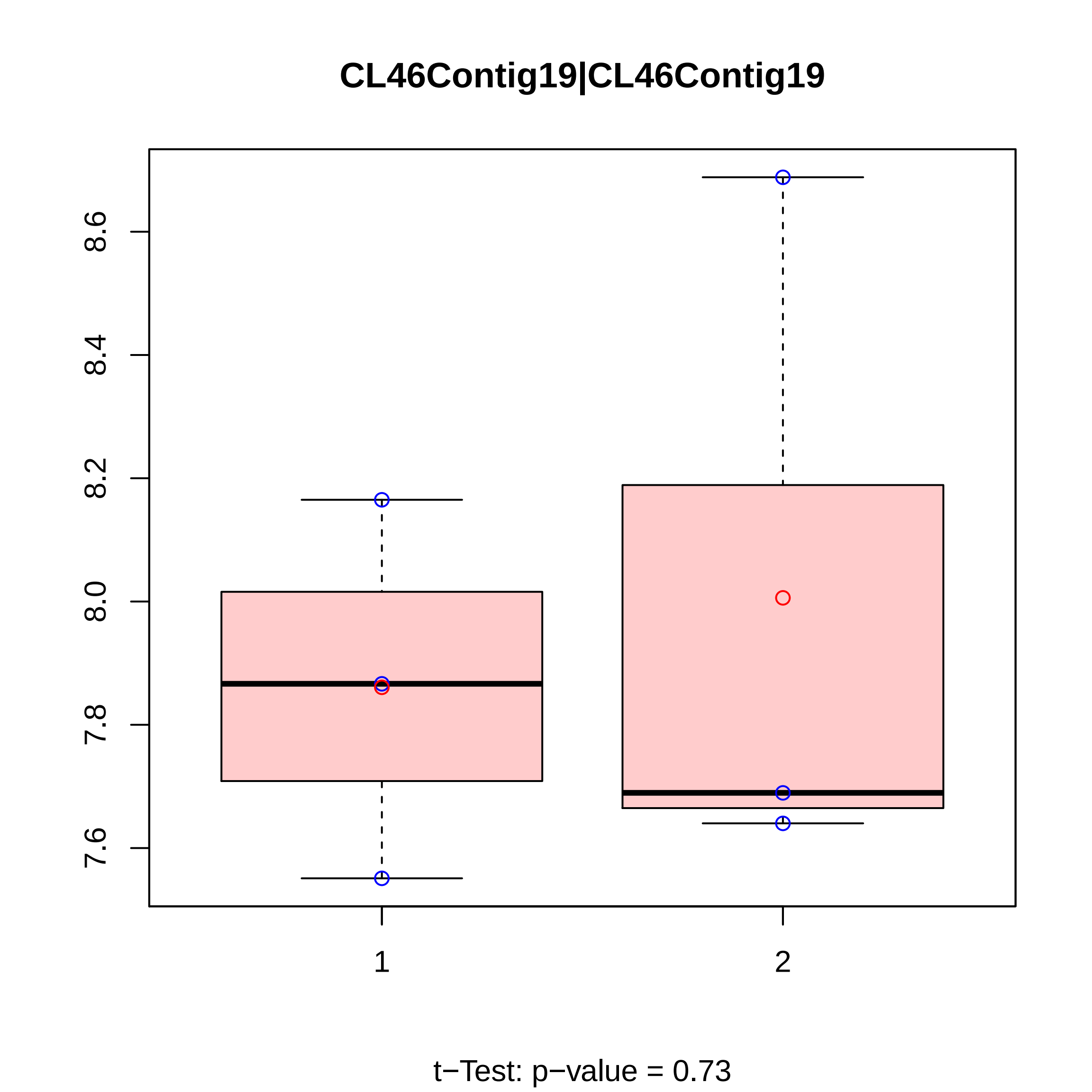

CL46Contig19|CL46Contig19
8.6
8.4
8.2
8.0
7.8
7.6
1
2
t−Test: p−value = 0.73

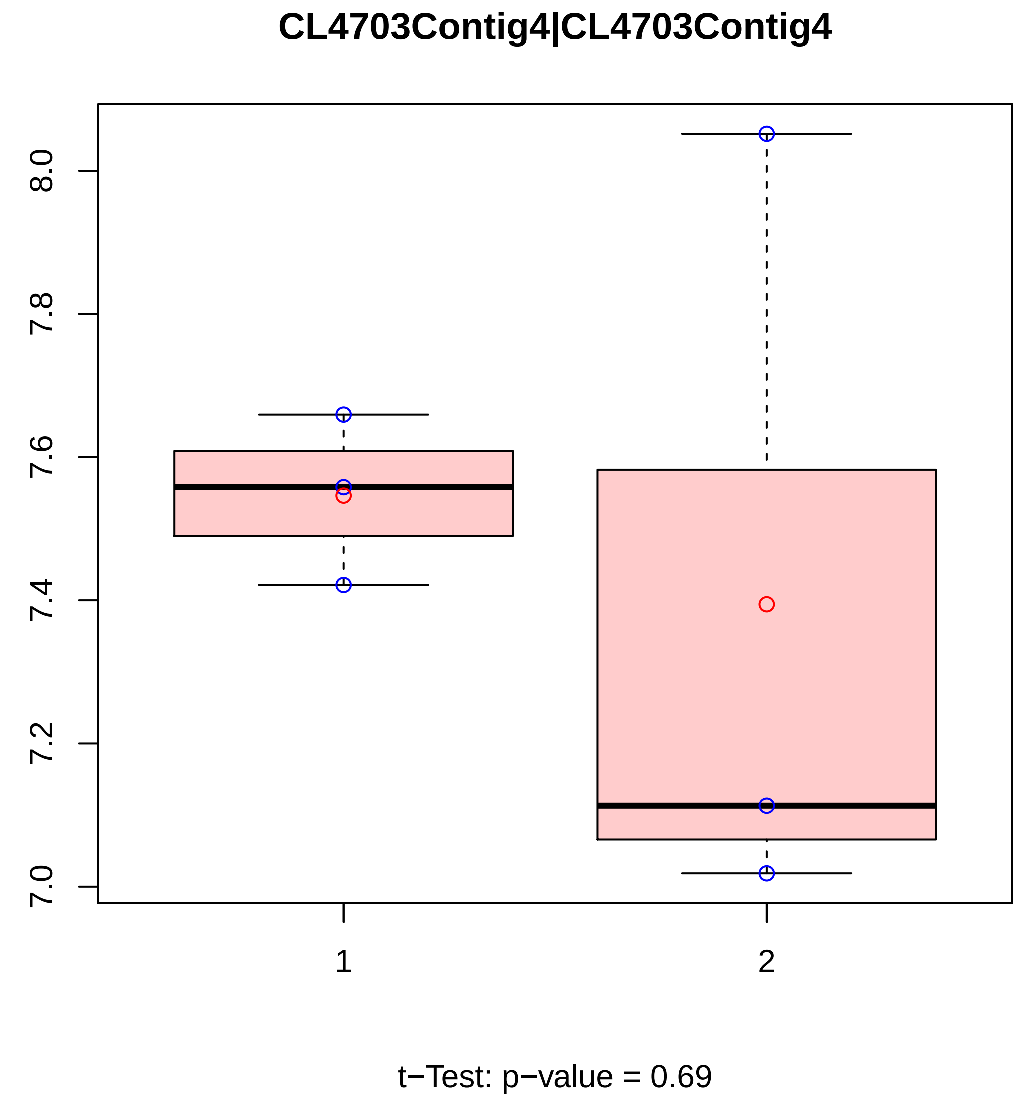
CL4703Contig4|CL4703Contig4
8.0
7.8
7.6
7.4
7.2
7.0
1
2
t−Test: p−value = 0.69

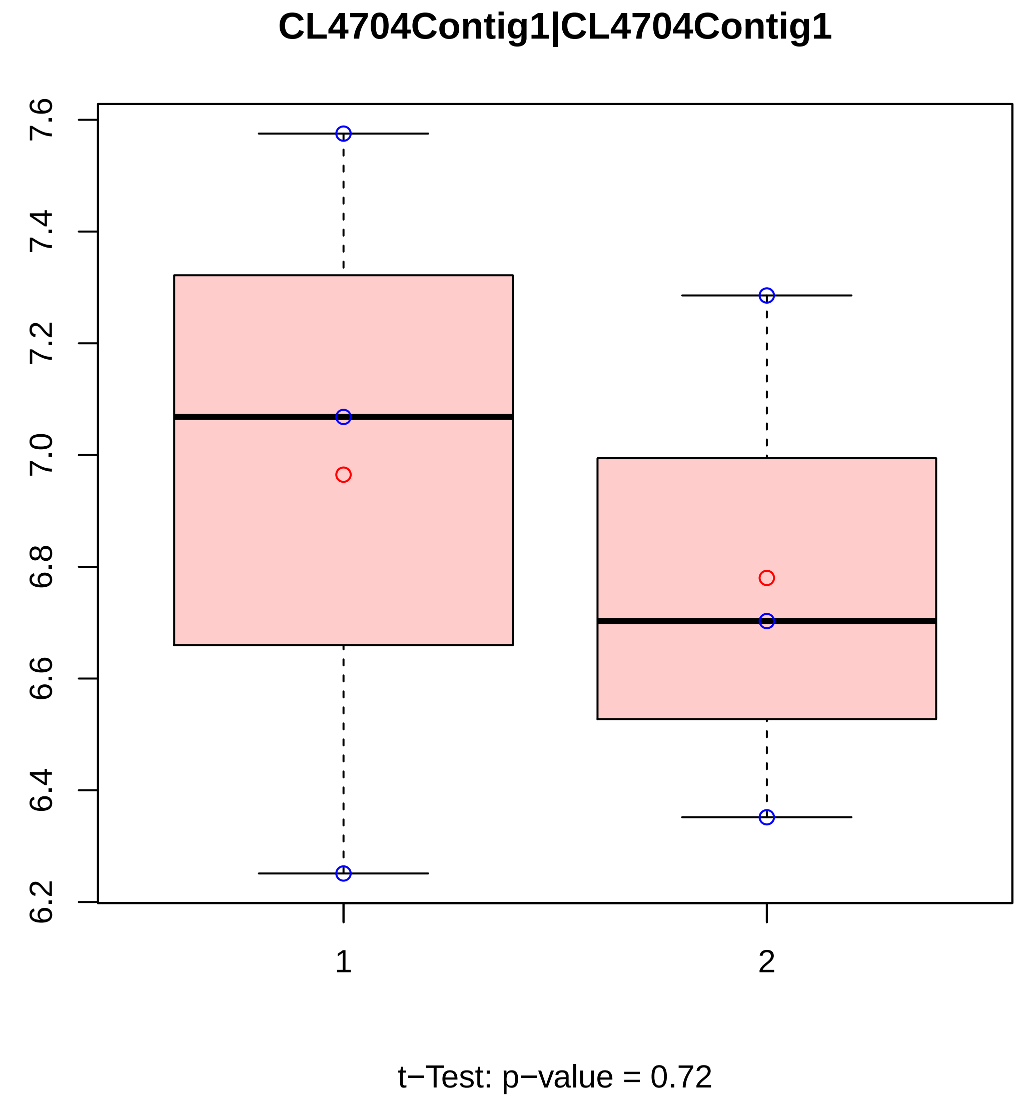

CL4704Contig1|CL4704Contig1
7.6
7.4
7.2
7.0
6.8
6.6
6.4
6.2
1
2
t−Test: p−value = 0.72

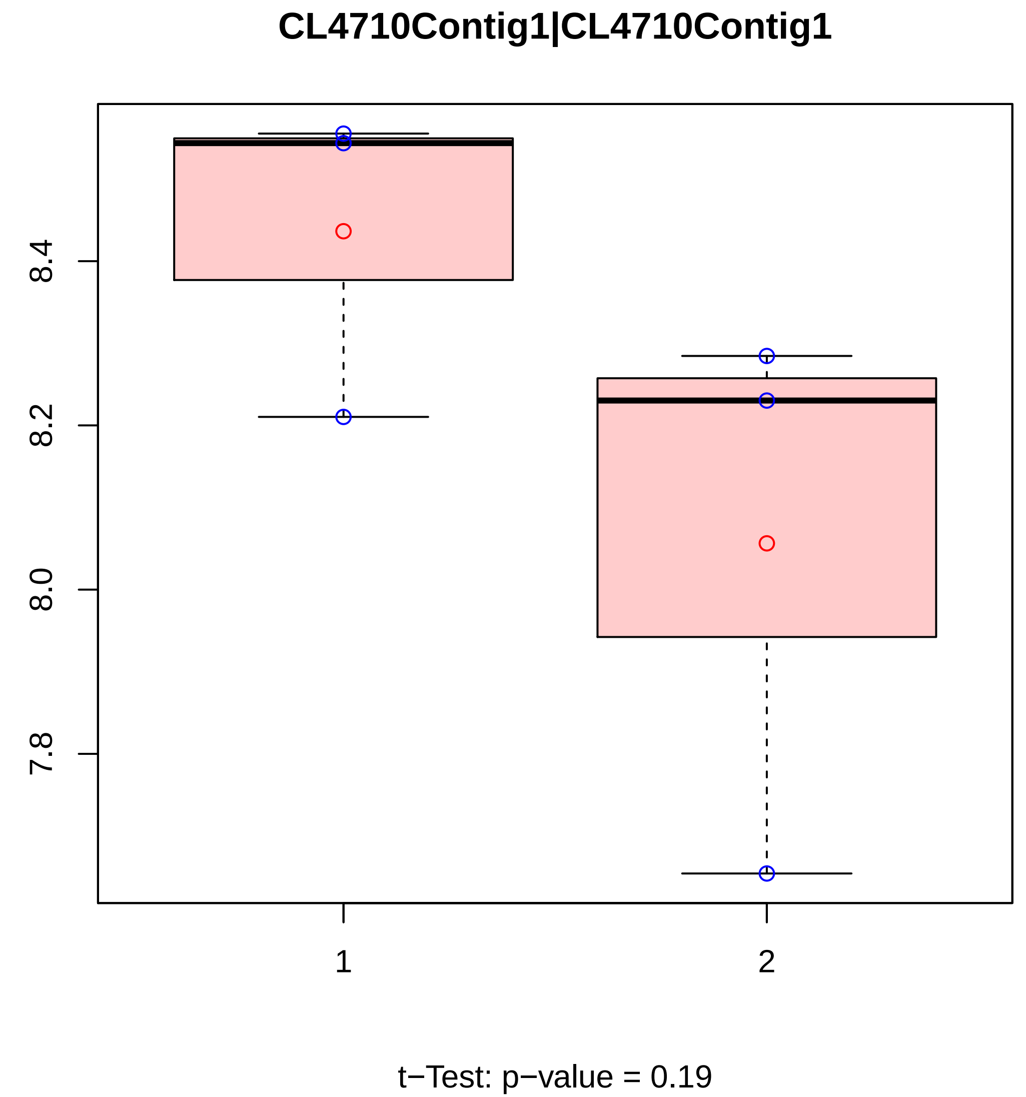
CL4710Contig1|CL4710Contig1
8.4
8.2
8.0
7.8
1
2
t−Test: p−value = 0.19

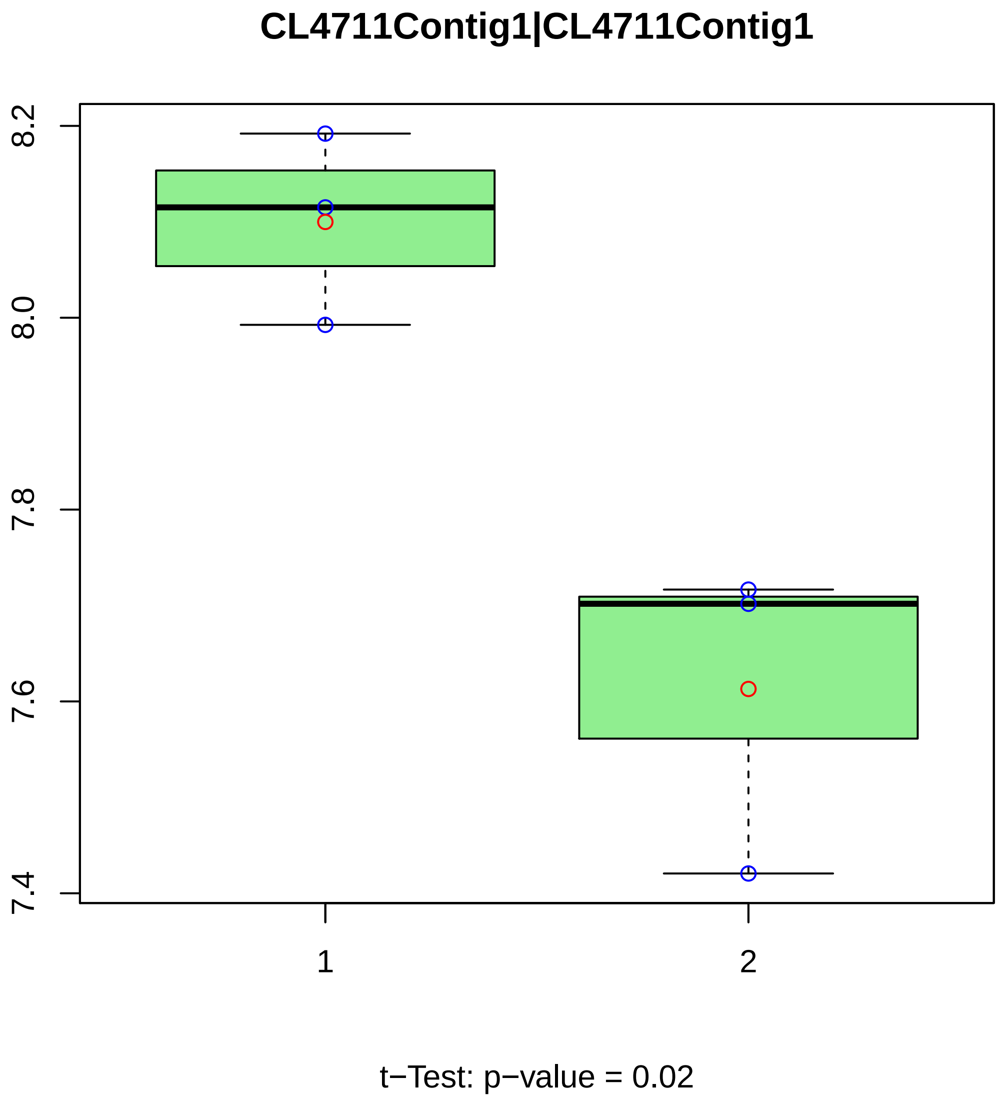
CL4711Contig1|CL4711Contig1
8.2
8.0
7.8
7.6
7.4
1
2
t−Test: p−value = 0.02

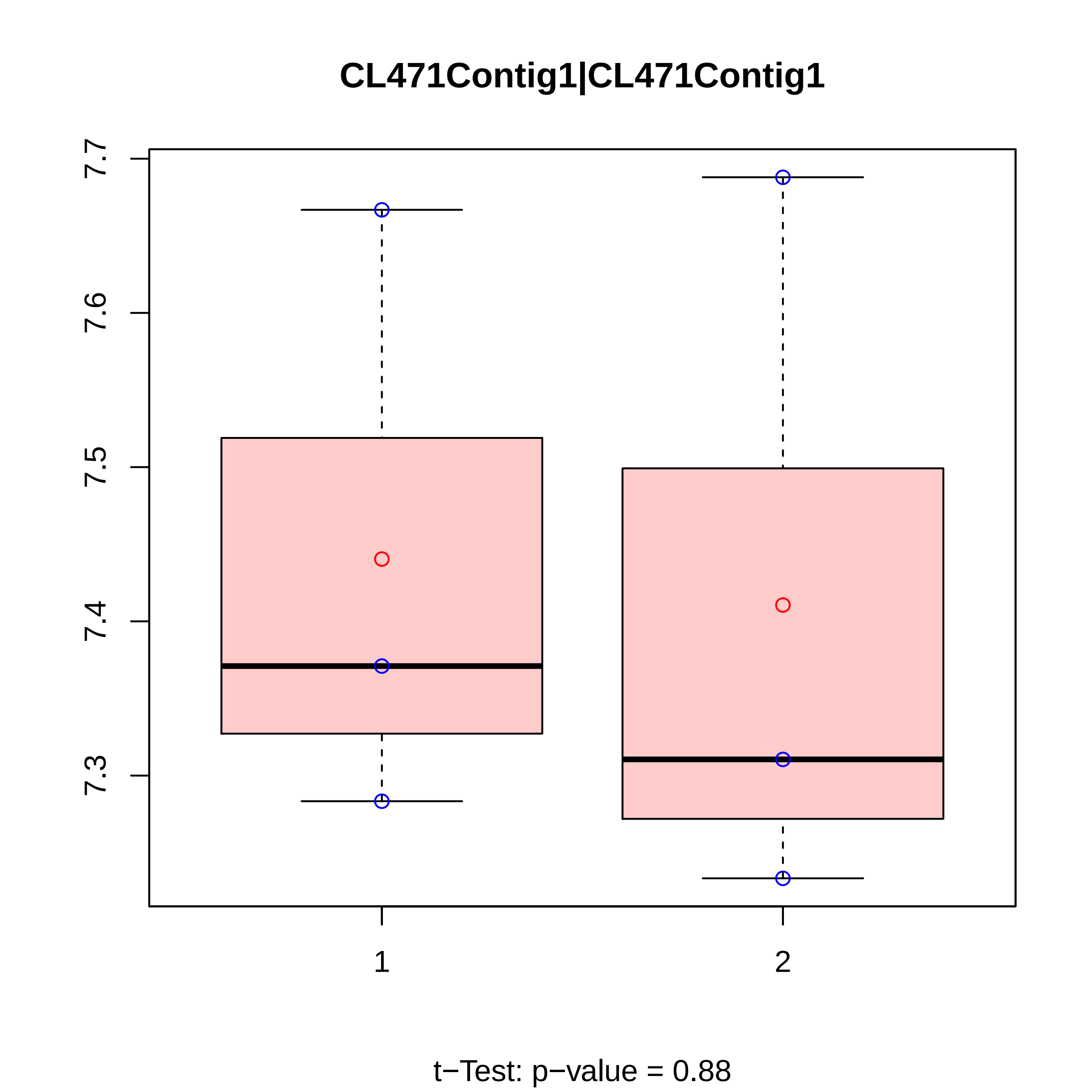
CL471Contig1|CL471Contig1
7.3
7.4
7.5
7.6
7.7
1
2
t−Test: p−value = 0.88

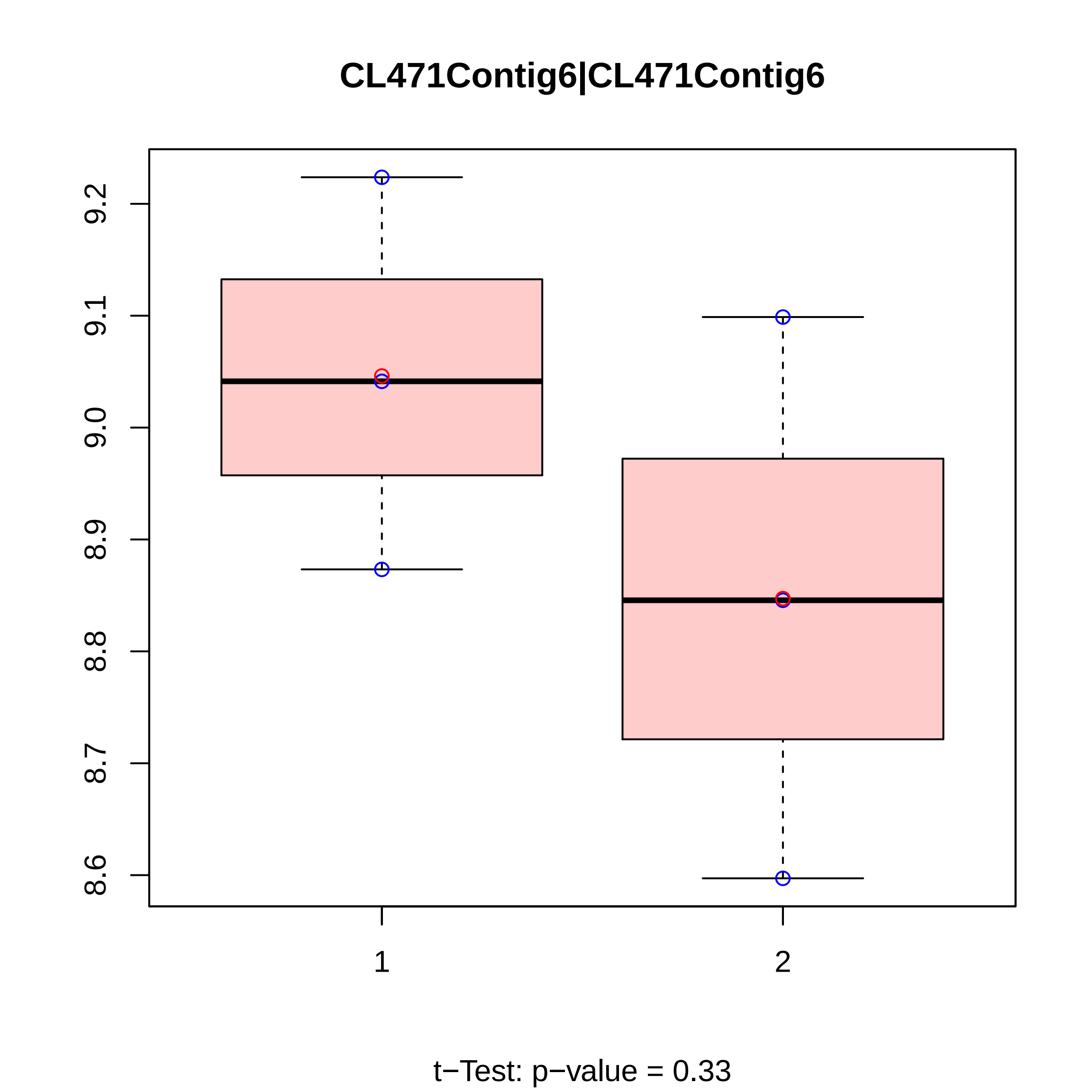
CL471Contig6|CL471Contig6
9.2
9.1
9.0
8.9
8.8
8.7
8.6
1
2
t−Test: p−value = 0.33

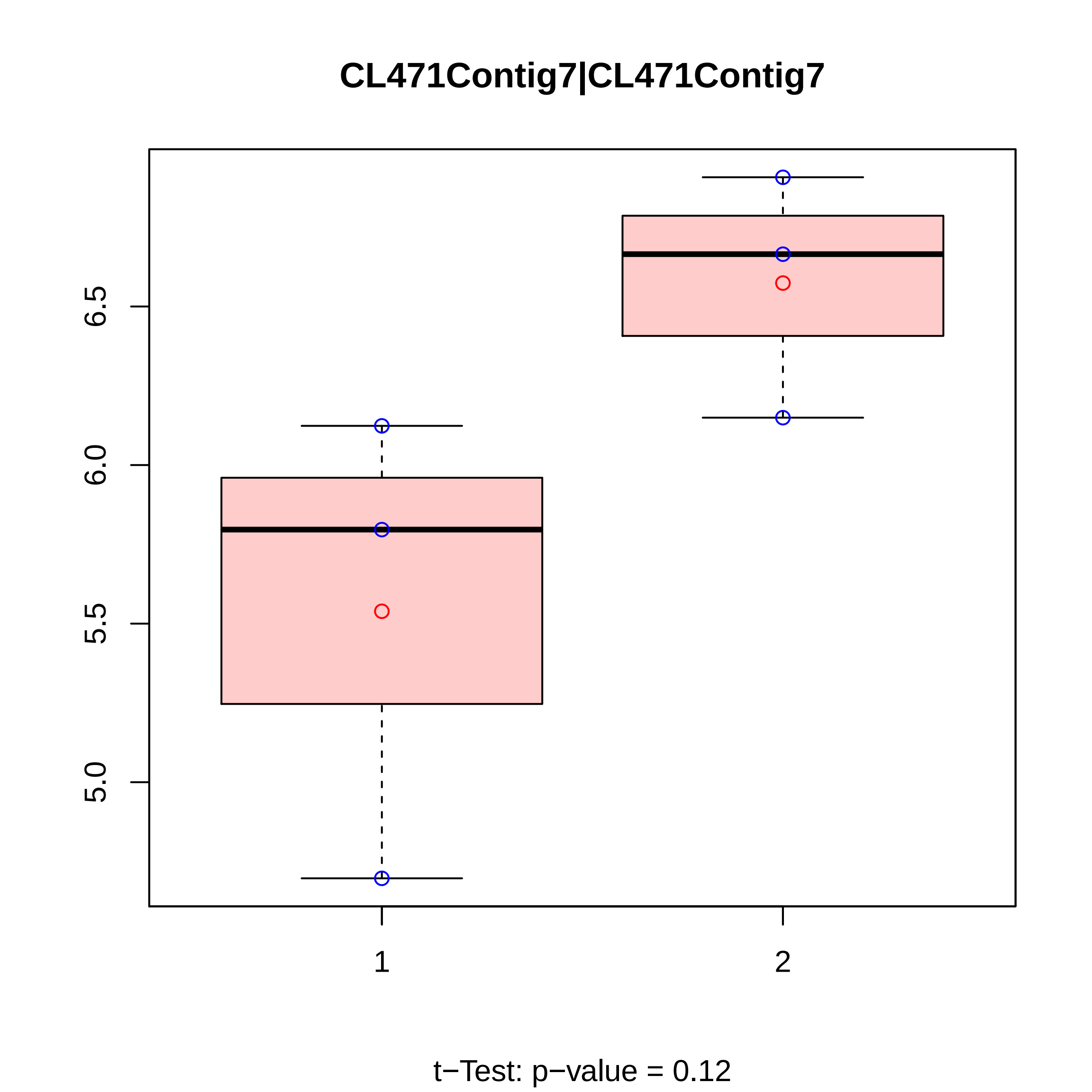
CL471Contig7|CL471Contig7
6.5
6.0
5.5
5.0
1
2
t−Test: p−value = 0.12

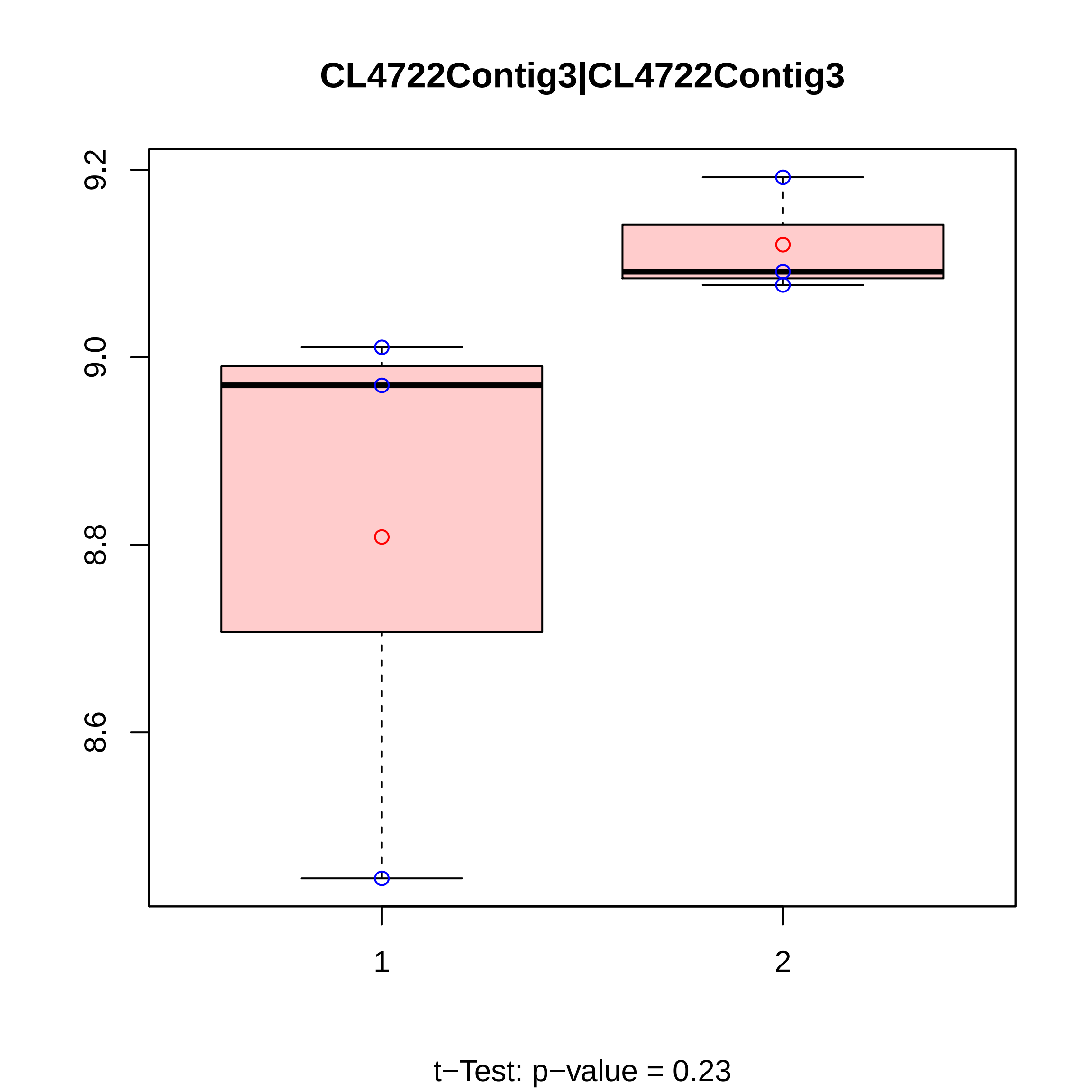

CL4722Contig3|CL4722Contig3
9.2
9.0
8.8
8.6
1
2
t−Test: p−value = 0.23

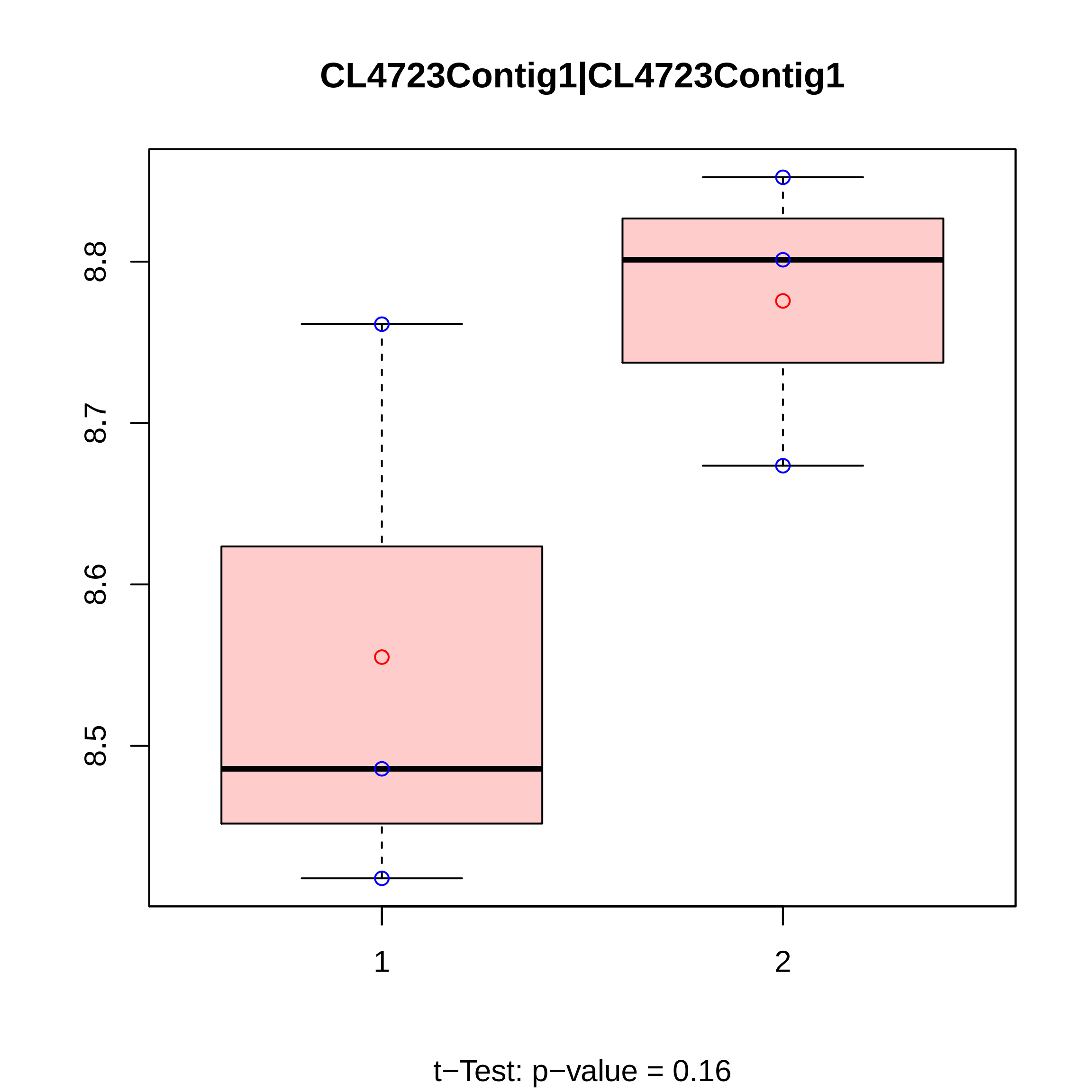
CL4723Contig1|CL4723Contig1
8.5
8.6
8.7
8.8
1
2
t−Test: p−value = 0.16

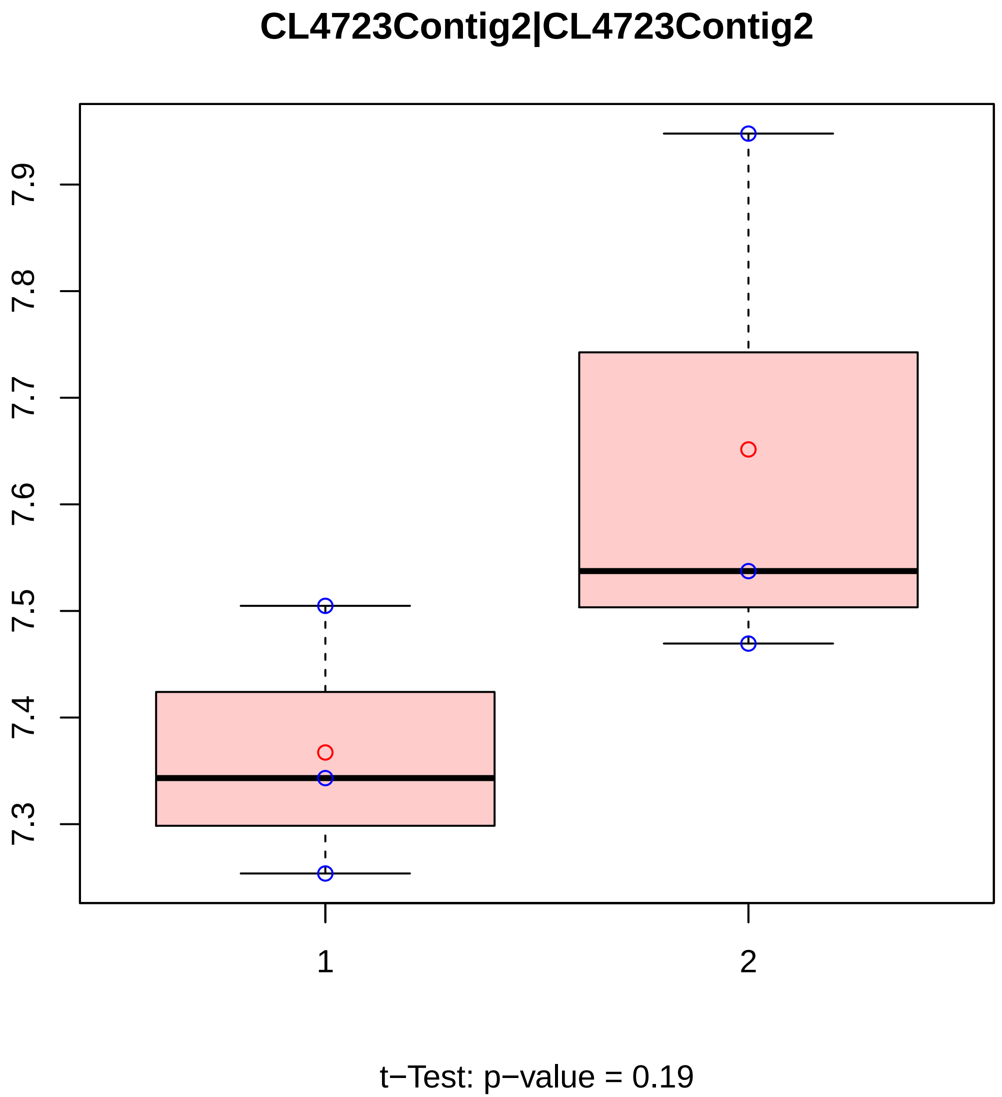

CL4723Contig2|CL4723Contig2
7.3
7.4
7.5
7.6
7.7
7.8
7.9
1
2
t−Test: p−value = 0.19

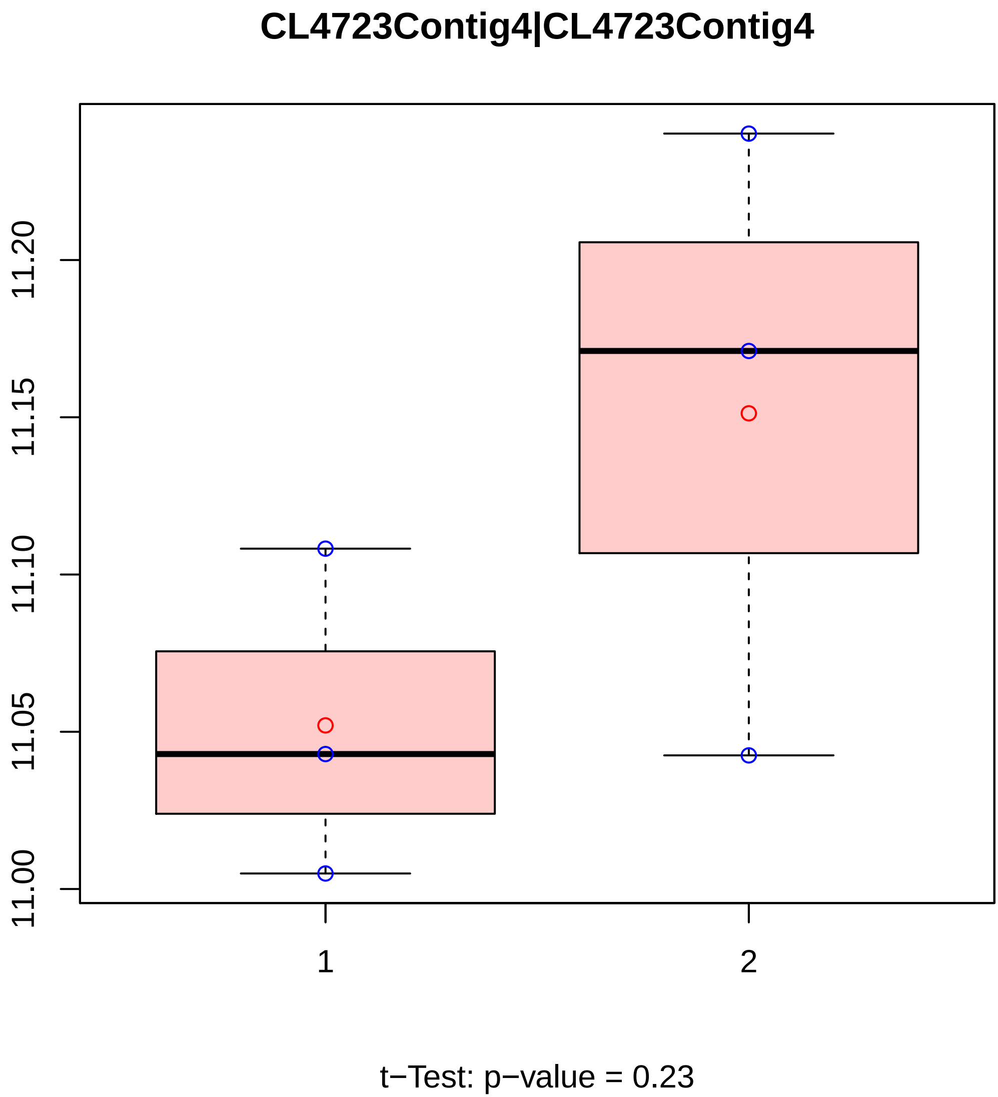

CL4723Contig4|CL4723Contig4
11.00
11.05
11.10
11.15
11.20
1
2
t−Test: p−value = 0.23

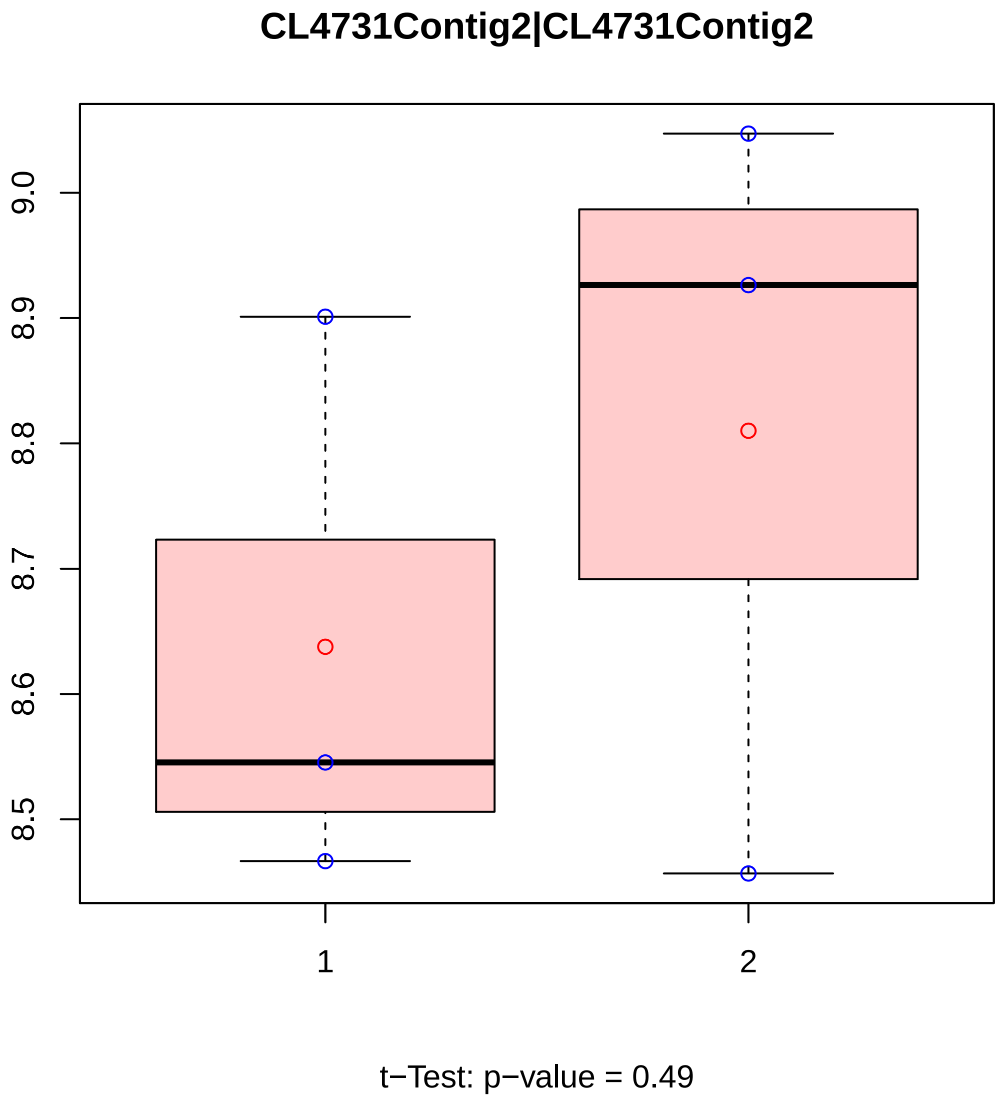
CL4731Contig2|CL4731Contig2
9.0
8.9
8.8
8.7
8.6
8.5
1
2
t−Test: p−value = 0.49

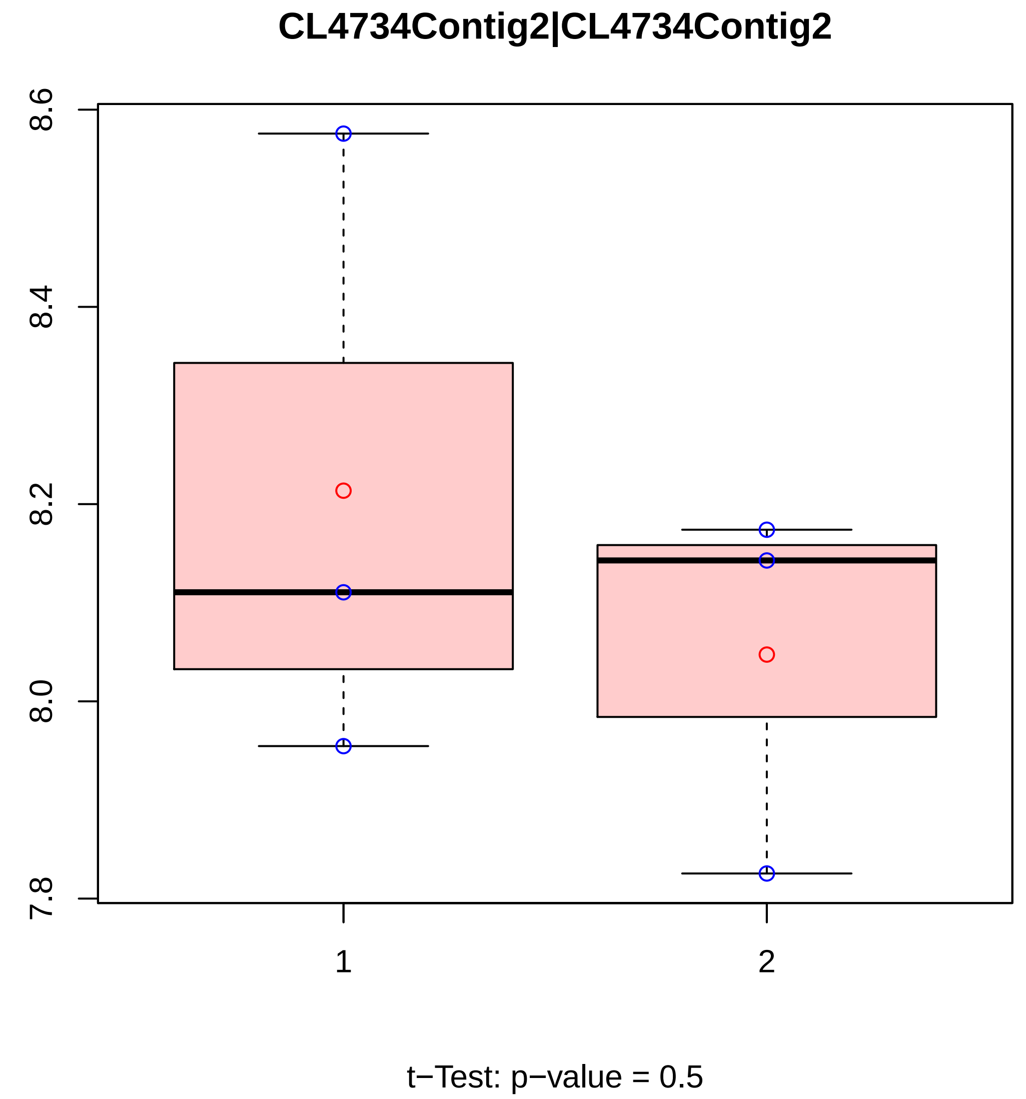

CL4734Contig2|CL4734Contig2
8.6
8.4
8.2
8.0
7.8
1
2
t−Test: p−value = 0.5

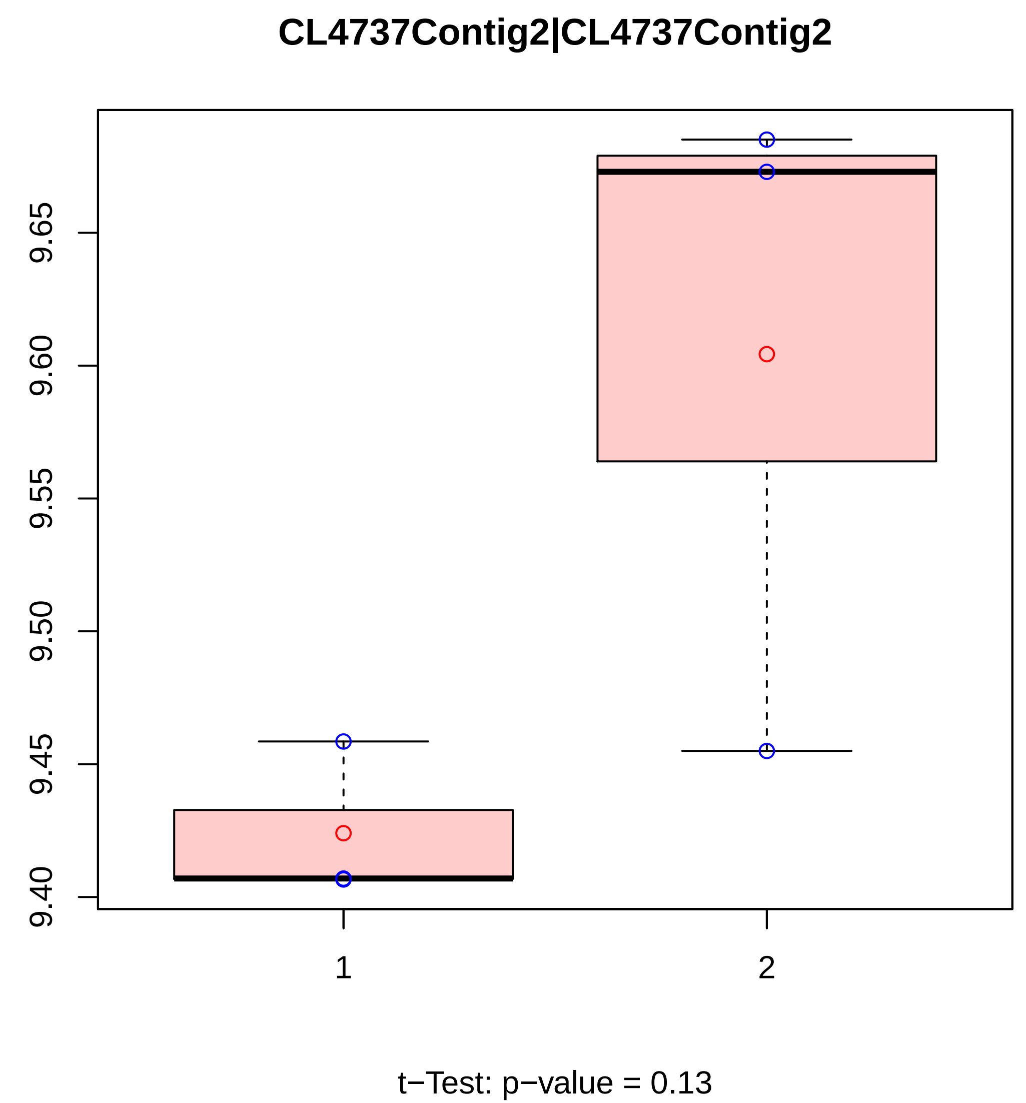
CL4737Contig2|CL4737Contig2
9.40
9.45
9.50
9.55
9.60
9.65
1
2
t−Test: p−value = 0.13

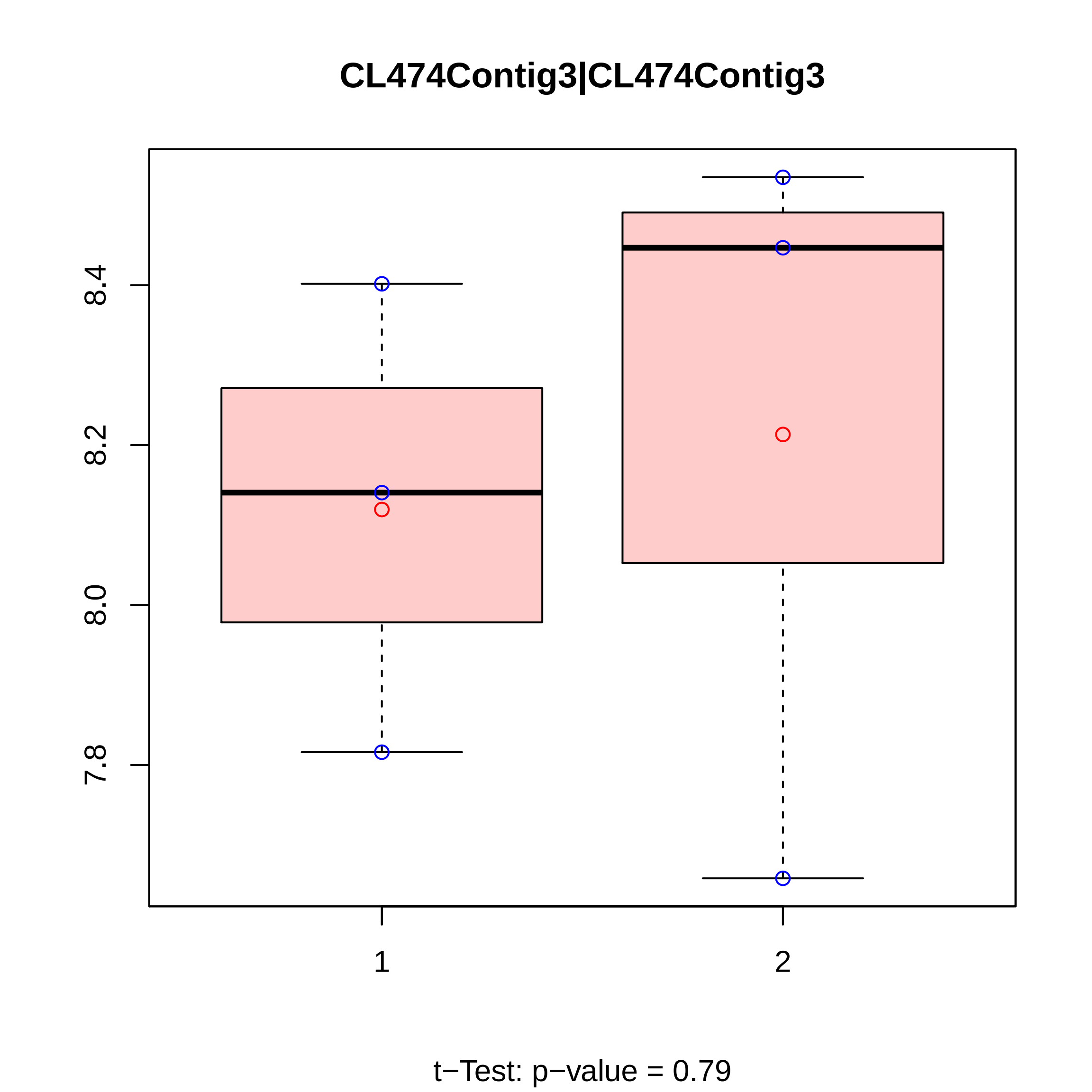

CL474Contig3|CL474Contig3
8.4
8.2
8.0
7.8
1
2
t−Test: p−value = 0.79

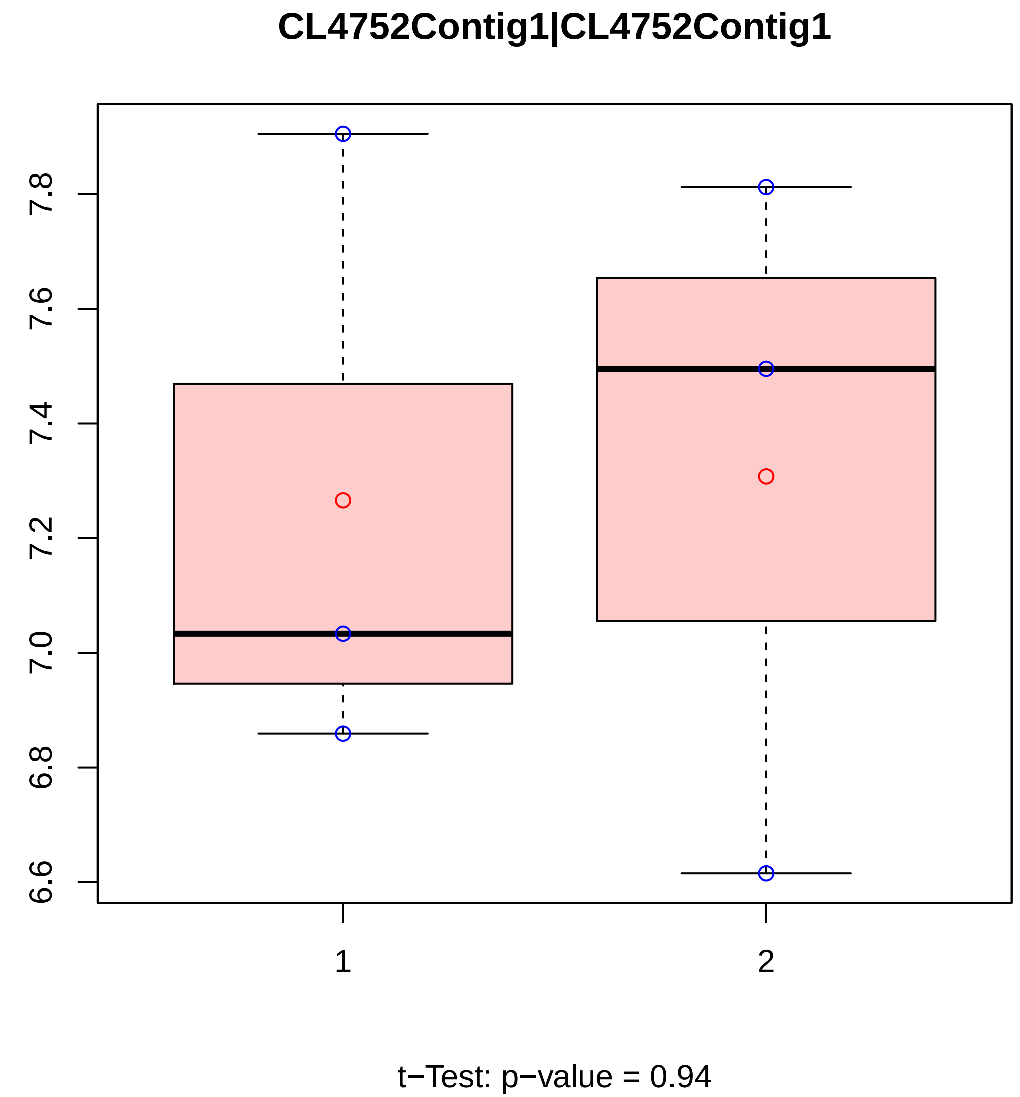
CL4752Contig1|CL4752Contig1
7.8
7.6
7.4
7.2
7.0
6.8
6.6
1
2
t−Test: p−value = 0.94

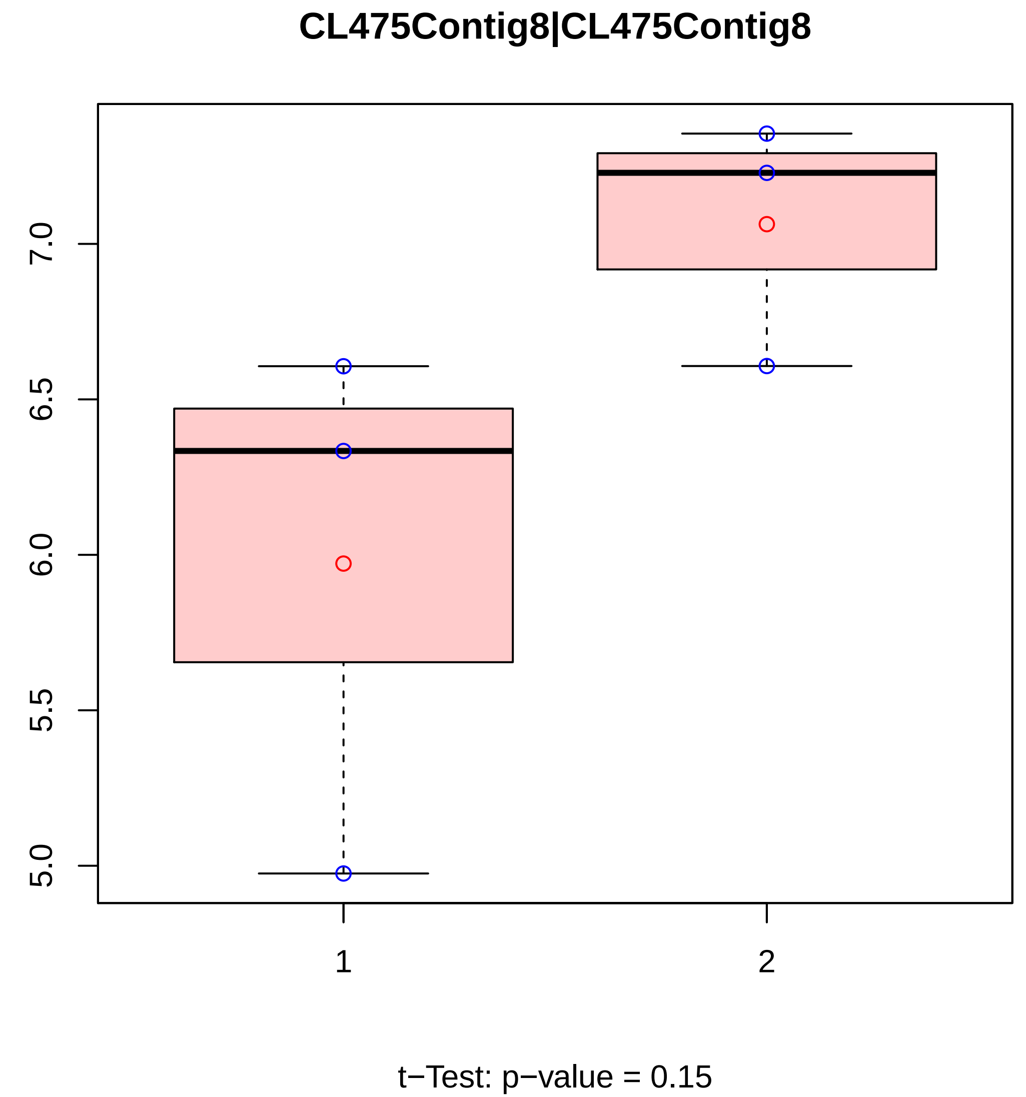

CL475Contig8|CL475Contig8
7.0
6.5
6.0
5.5
5.0
1
2
t−Test: p−value = 0.15

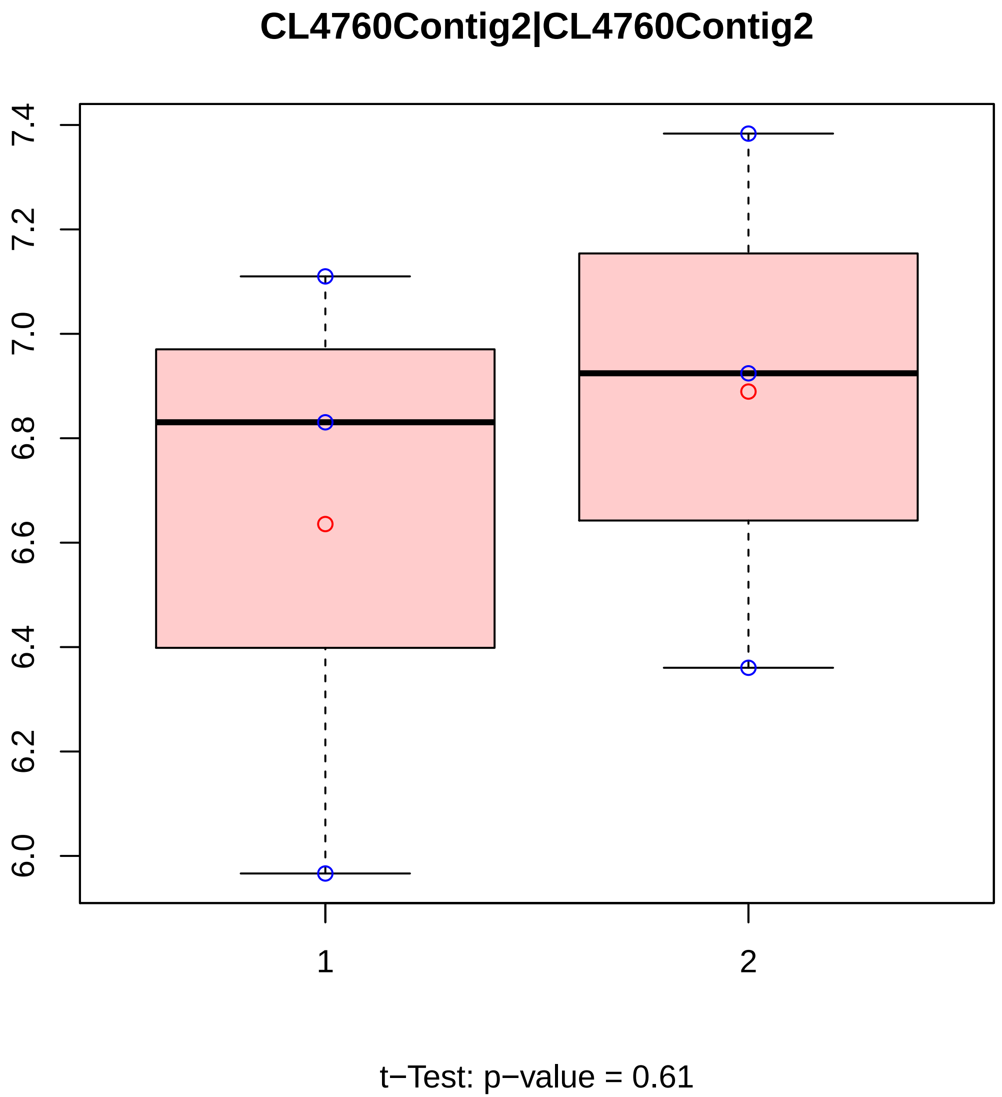

CL4760Contig2|CL4760Contig2
7.4
7.2
7.0
6.8
6.6
6.4
6.2
6.0
1
2
t−Test: p−value = 0.61

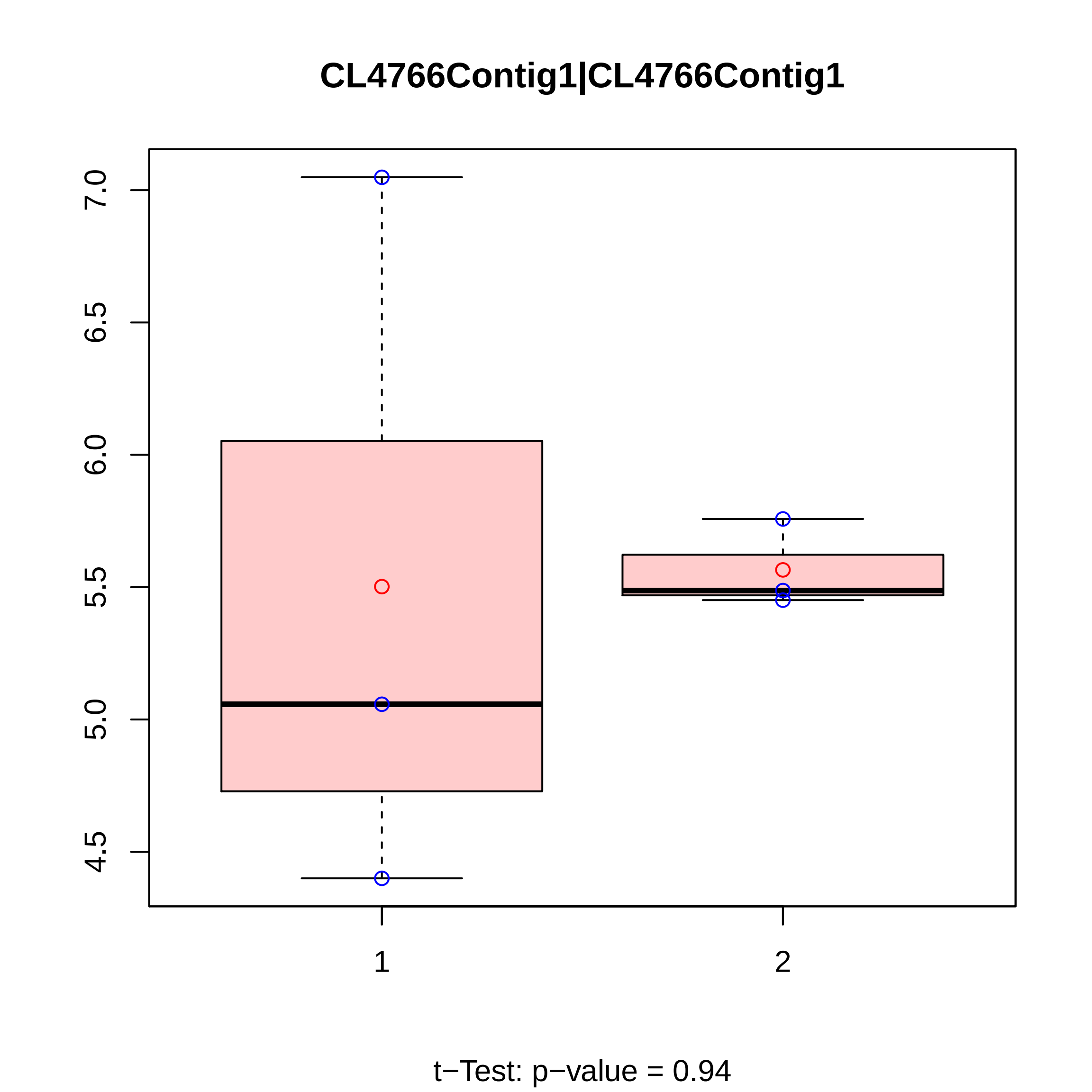

CL4766Contig1|CL4766Contig1
7.0
6.5
6.0
5.5
5.0
4.5
1
2
t−Test: p−value = 0.94

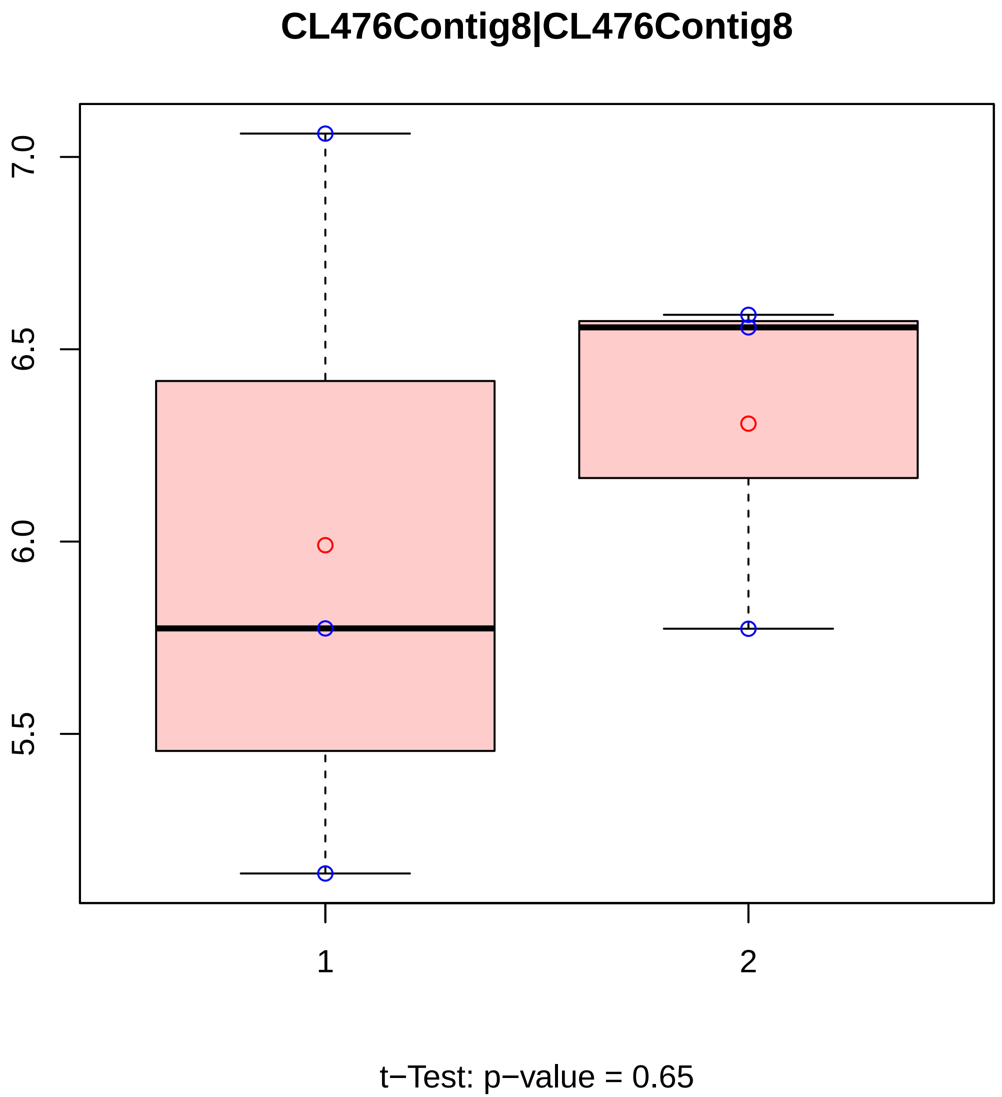
CL476Contig8|CL476Contig8
7.0
6.5
6.0
5.5
1
2
t−Test: p−value = 0.65

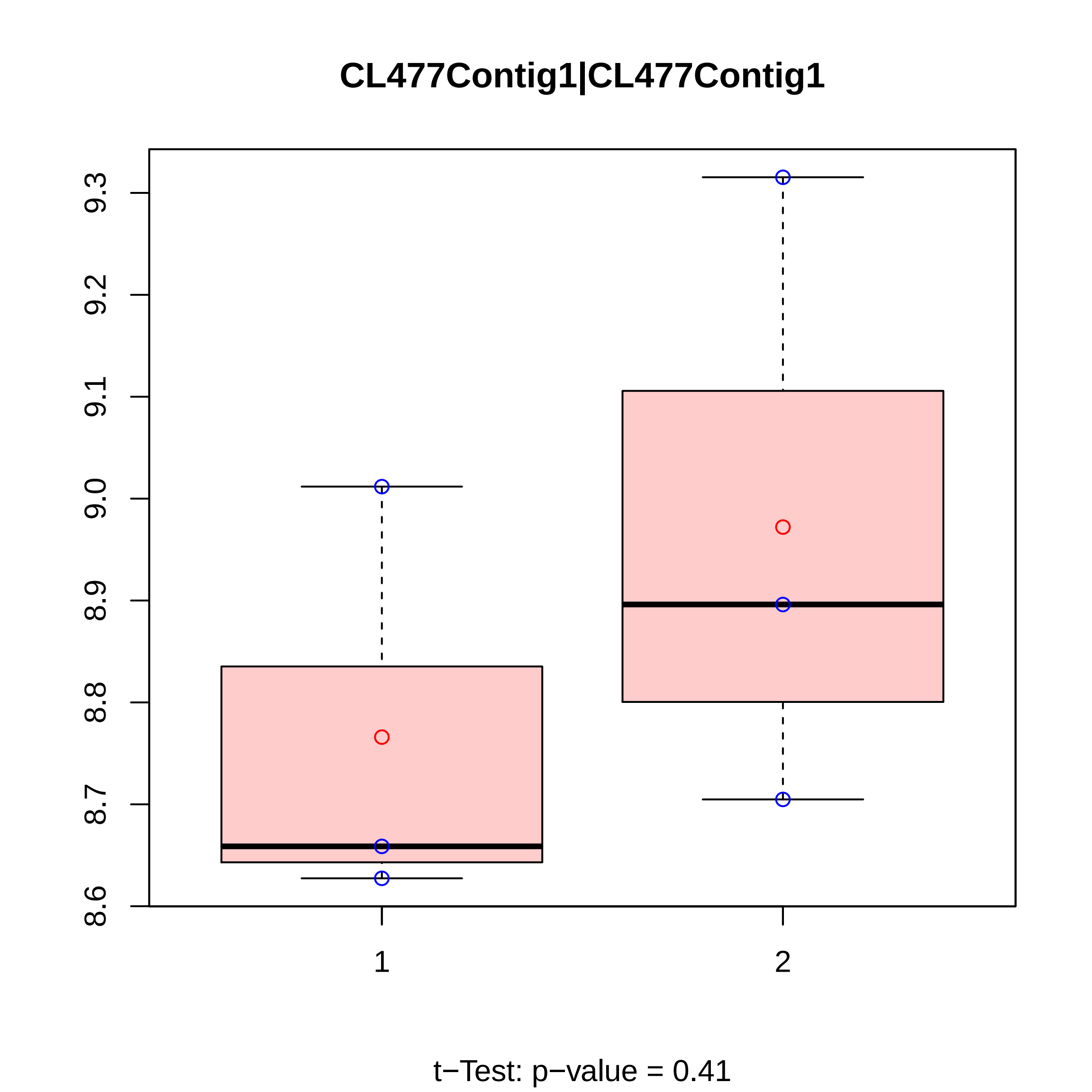

CL477Contig1|CL477Contig1
9.3
9.2
9.1
9.0
8.9
8.8
8.7
8.6
1
2
t−Test: p−value = 0.41

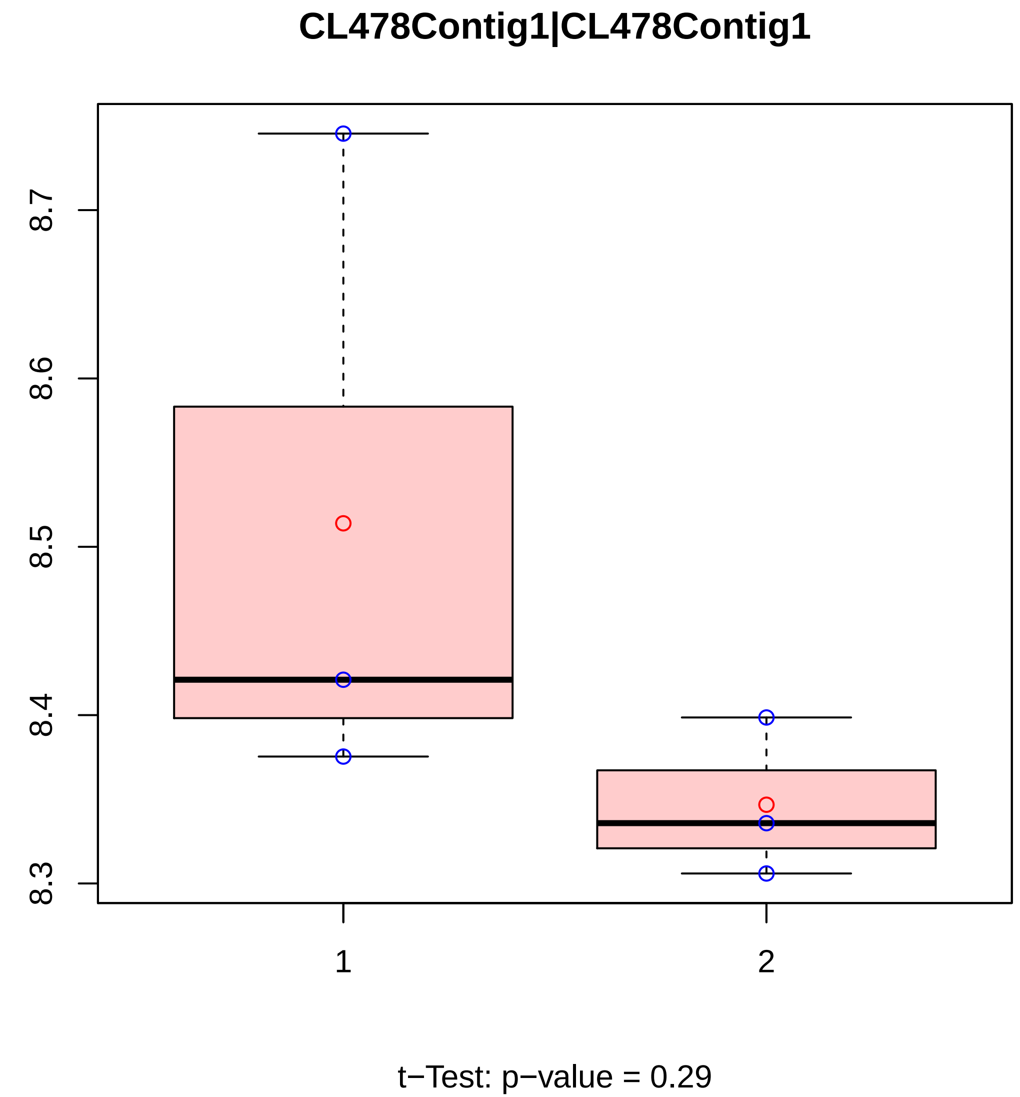
CL478Contig1|CL478Contig1
8.3
8.4
8.5
8.6
8.7
1
2
t−Test: p−value = 0.29

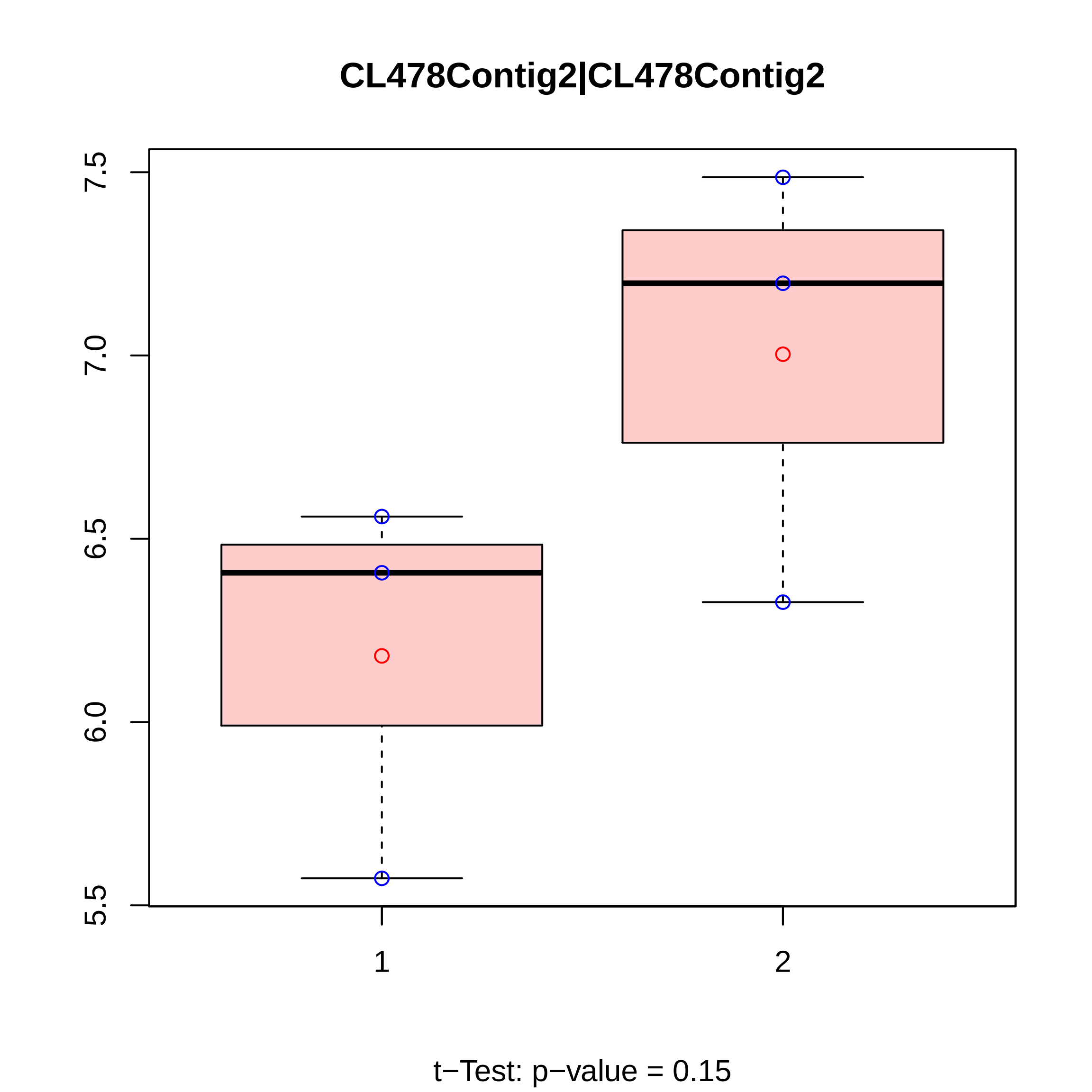

CL478Contig2|CL478Contig2
7.5
7.0
6.5
6.0
5.5
1
2
t−Test: p−value = 0.15

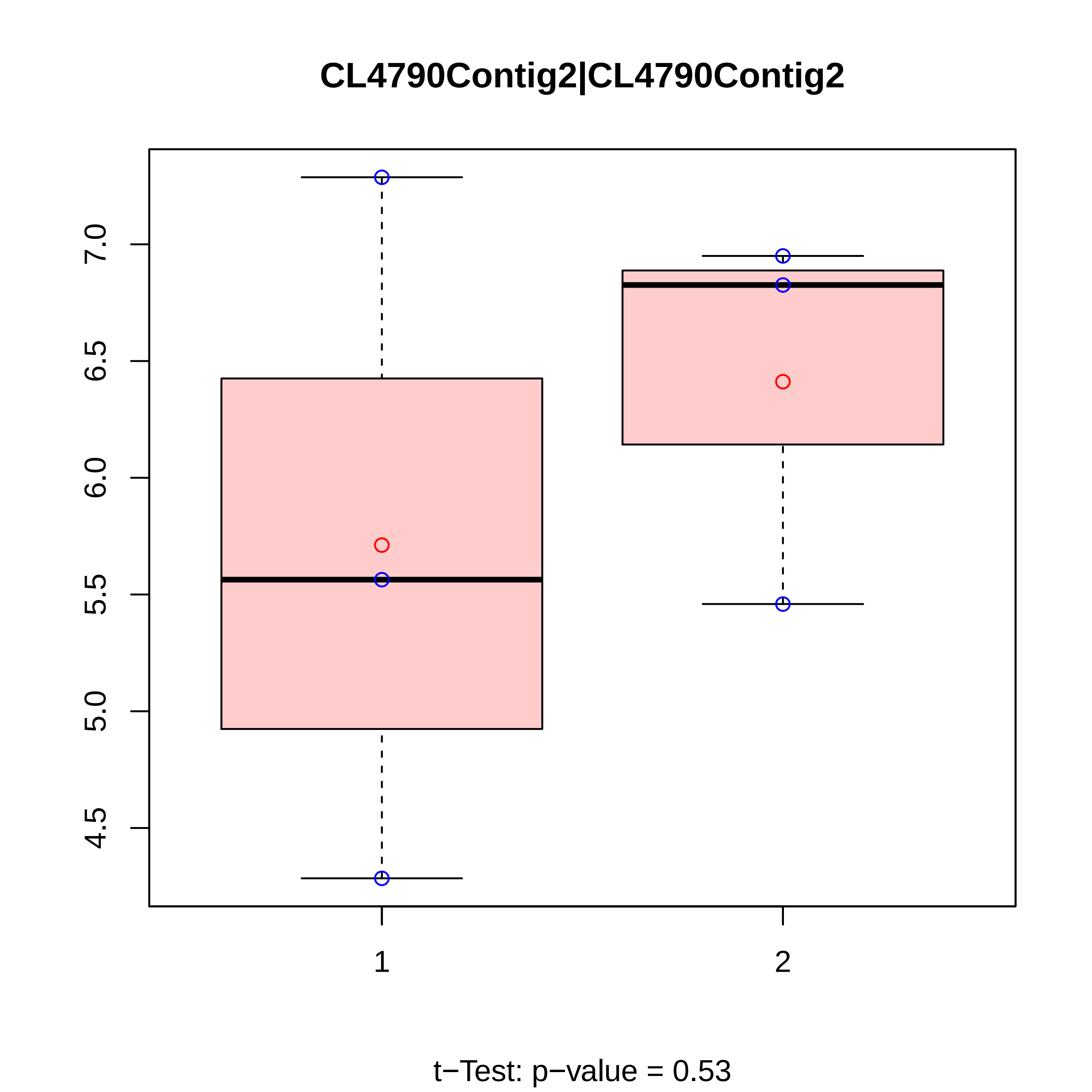

CL4790Contig2|CL4790Contig2
7.0
6.5
6.0
5.5
5.0
4.5
1
2
t−Test: p−value = 0.53

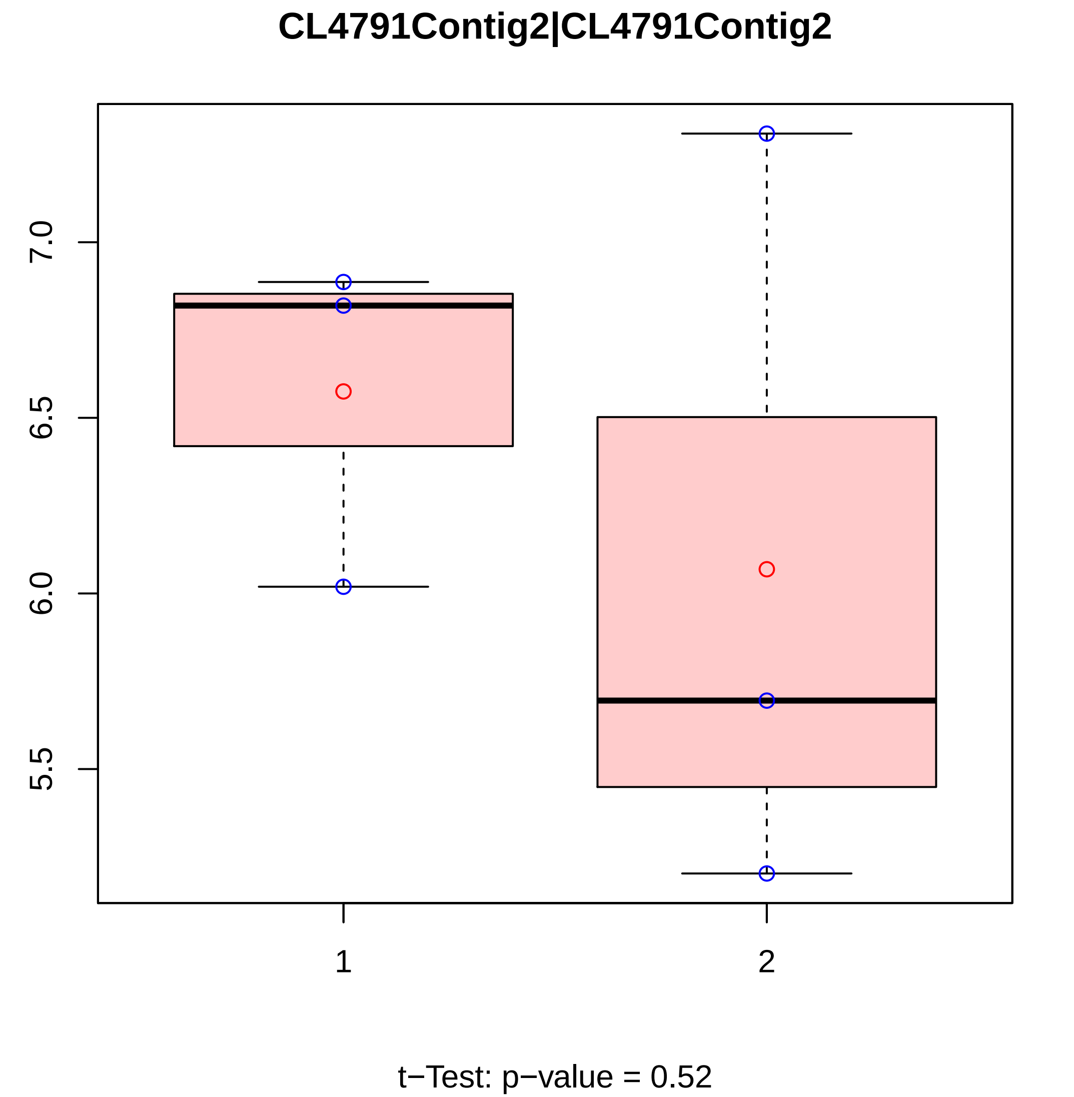
CL4791Contig2|CL4791Contig2
7.0
6.5
6.0
5.5
1
2
t−Test: p−value = 0.52

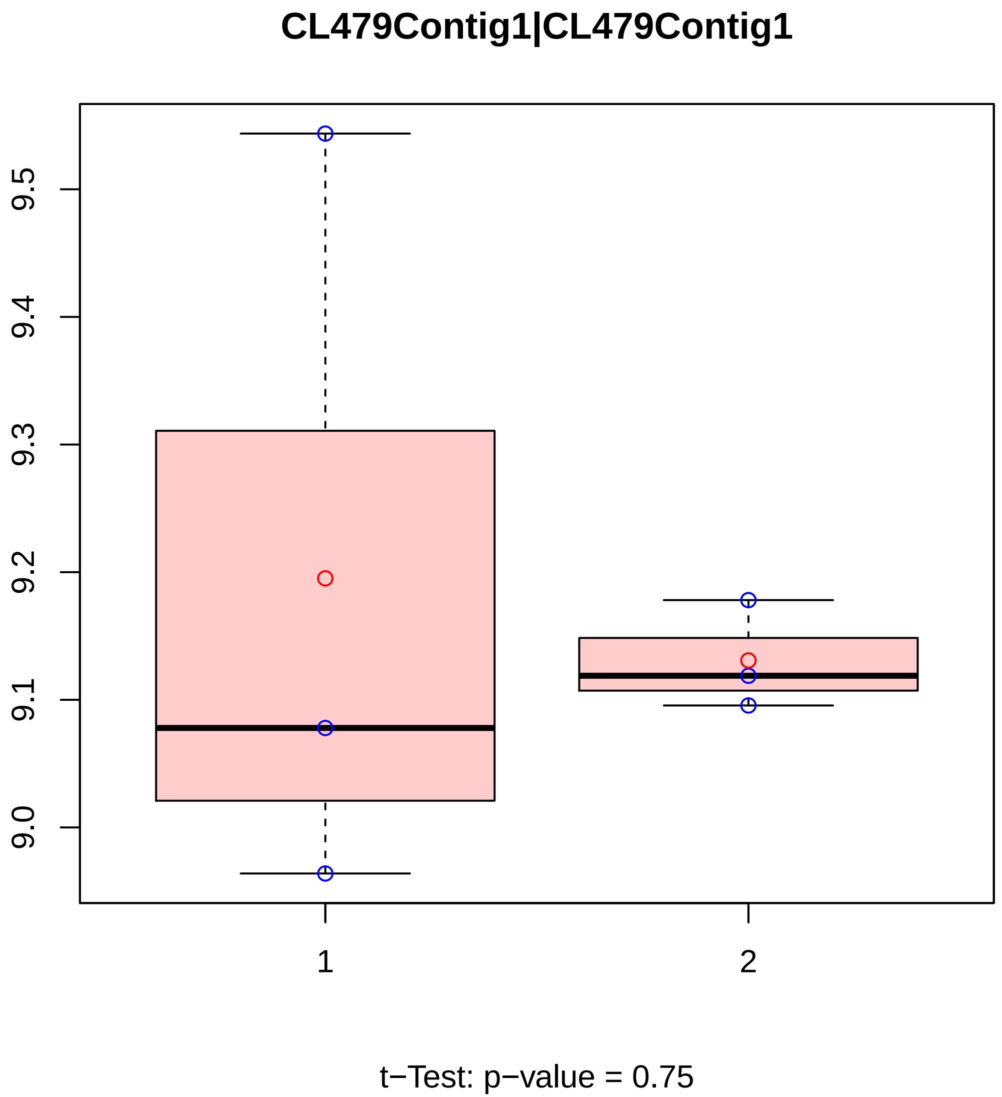
CL479Contig1|CL479Contig1
9.0
9.1
9.2
9.3
9.4
9.5
1
2
t−Test: p−value = 0.75

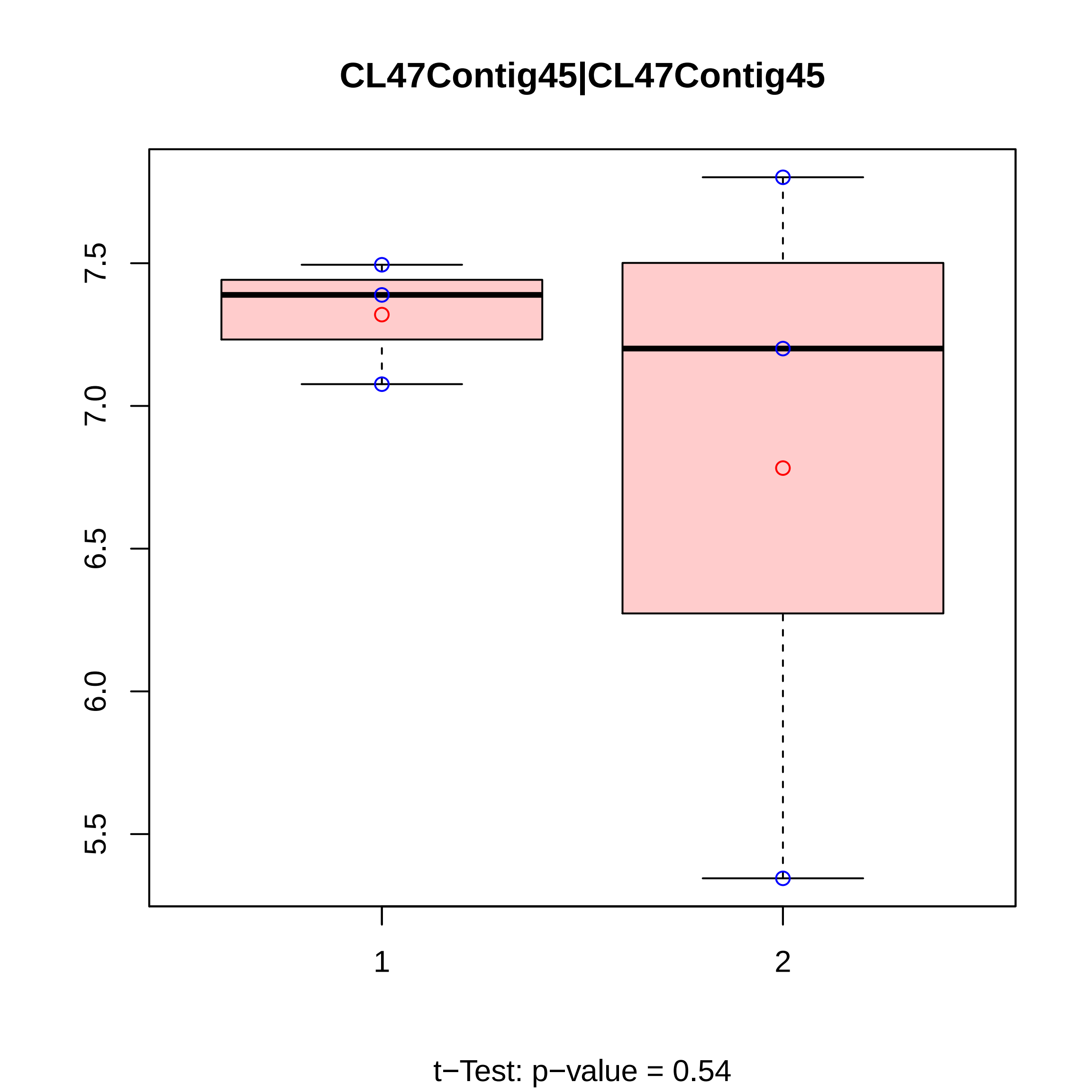
CL47Contig45|CL47Contig45
7.5
7.0
6.5
6.0
5.5
1
2
t−Test: p−value = 0.54

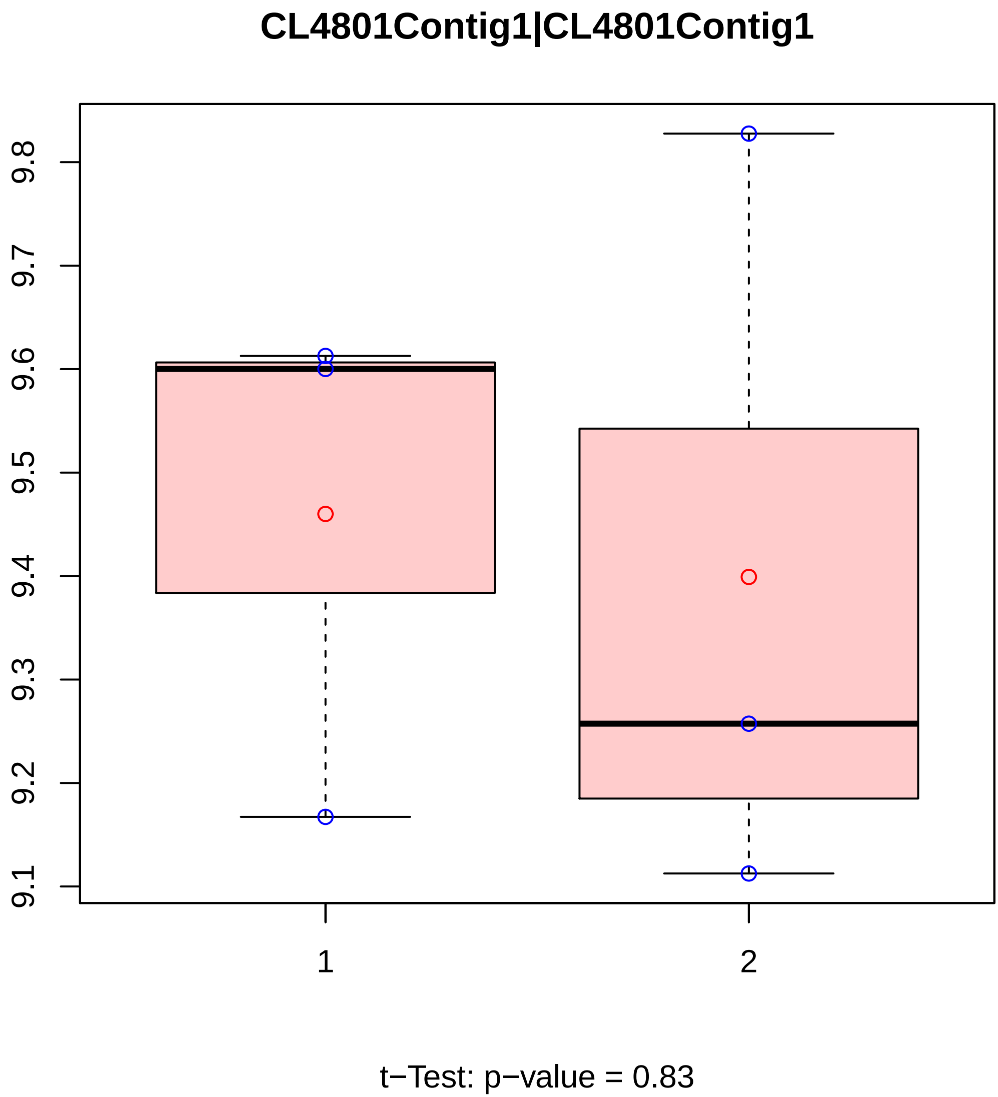
CL4801Contig1|CL4801Contig1
9.1
9.2
9.3
9.4
9.5
9.6
9.7
9.8
1
2
t−Test: p−value = 0.83

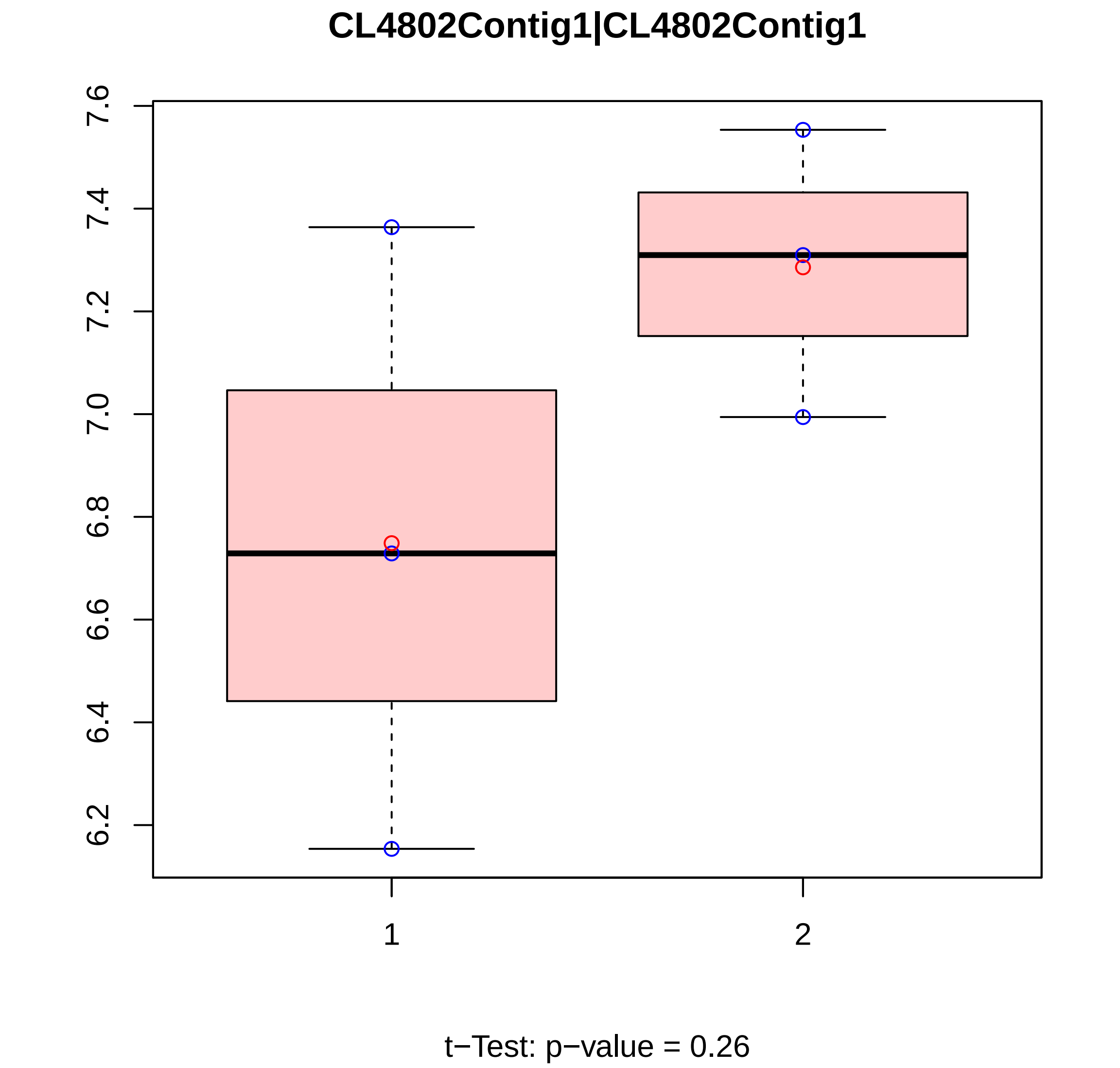
CL4802Contig1|CL4802Contig1
7.6
7.4
7.2
7.0
6.8
6.6
6.4
6.2
1
2
t−Test: p−value = 0.26

# CL482Contig7|CL482Contig7

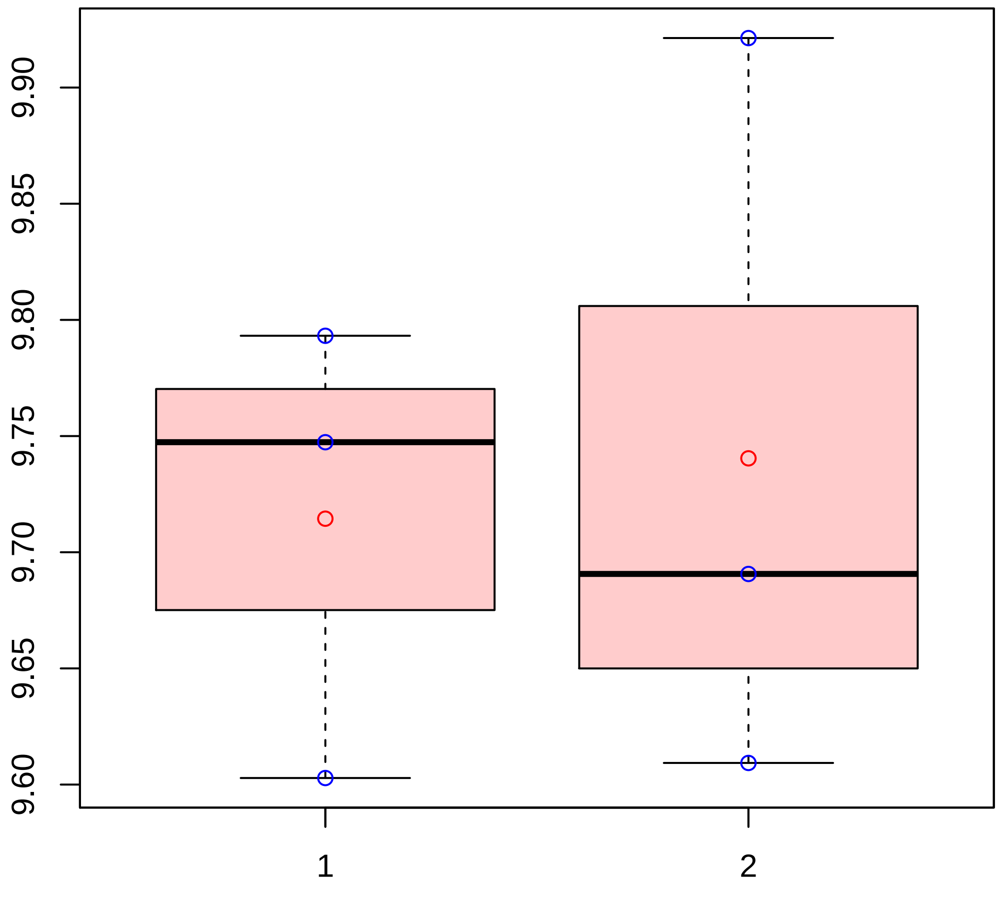


t−Test: p−value = 0.83

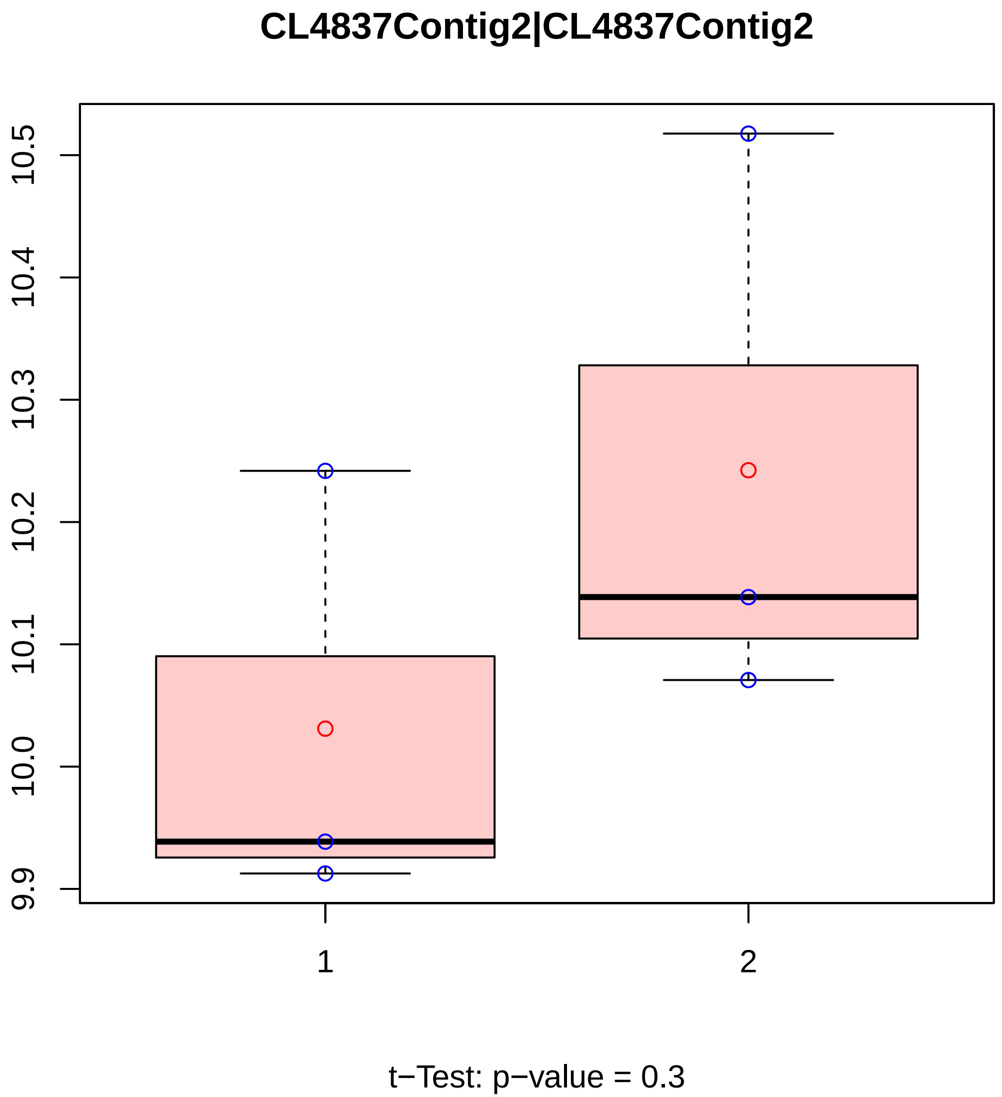
CL4837Contig2|CL4837Contig2
10.5
10.4
10.3
10.2
10.1
10.0
9.9
1
2
t−Test: p−value = 0.3

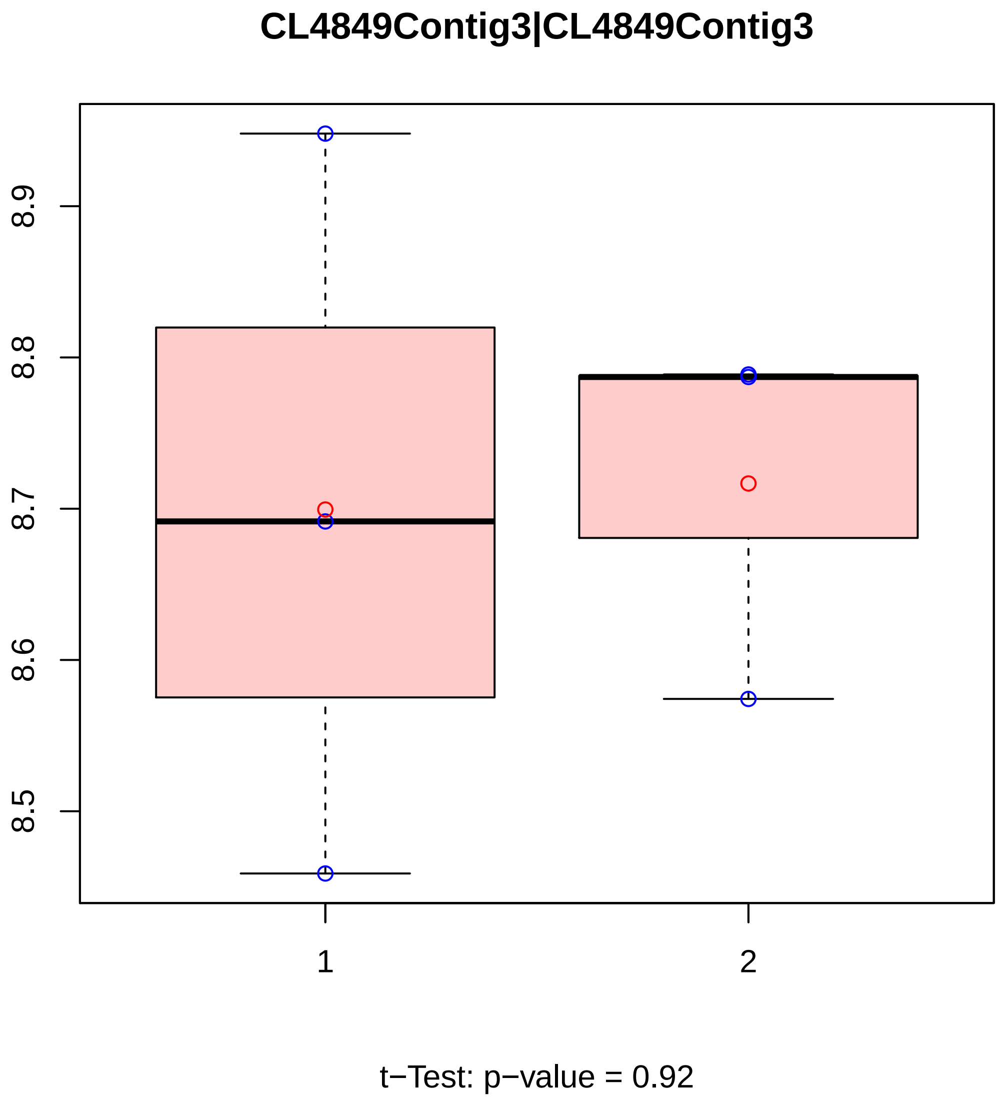

CL4849Contig3|CL4849Contig3
8.5
8.6
8.7
8.8
8.9
1
2
t−Test: p−value = 0.92

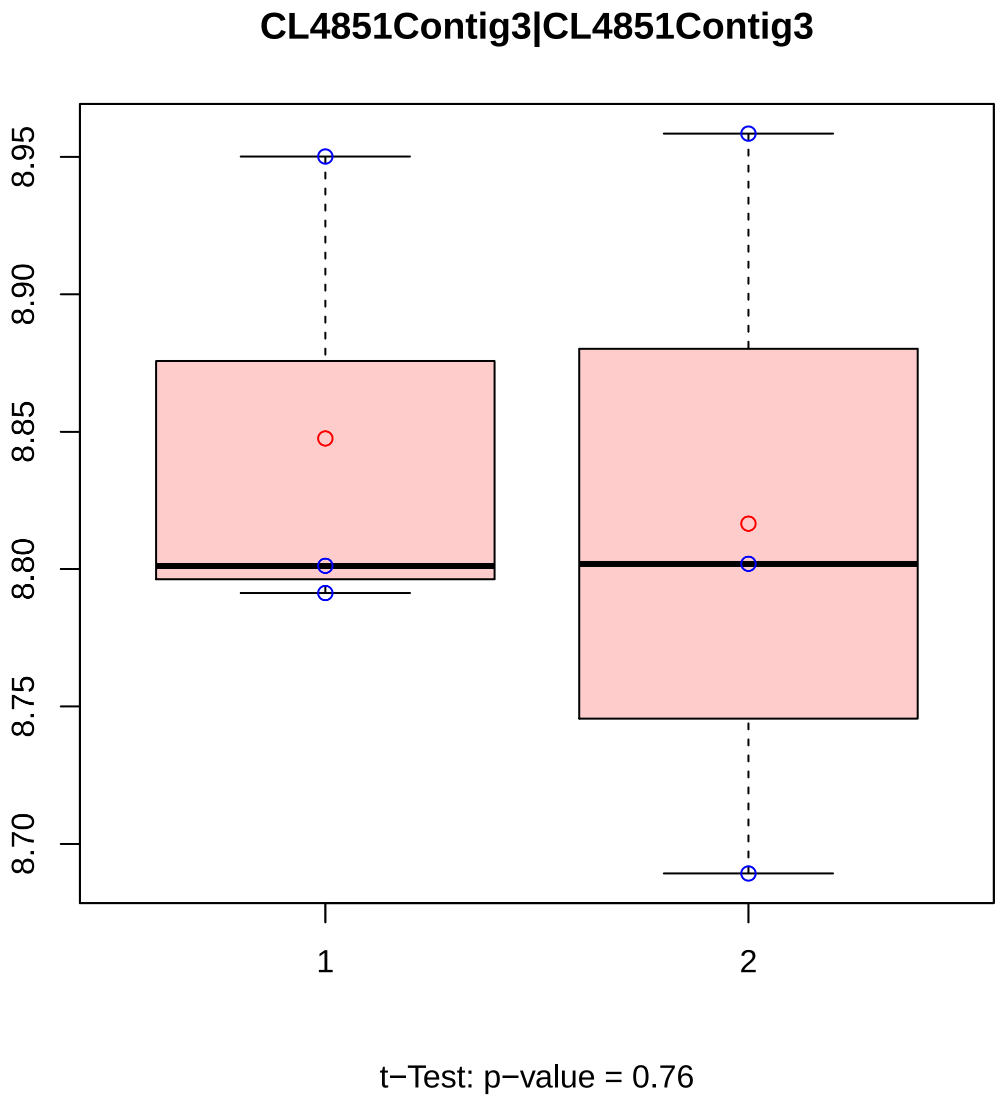
CL4851Contig3|CL4851Contig3
8.95
8.90
8.85
8.80
8.75
8.70
1
2
t−Test: p−value = 0.76

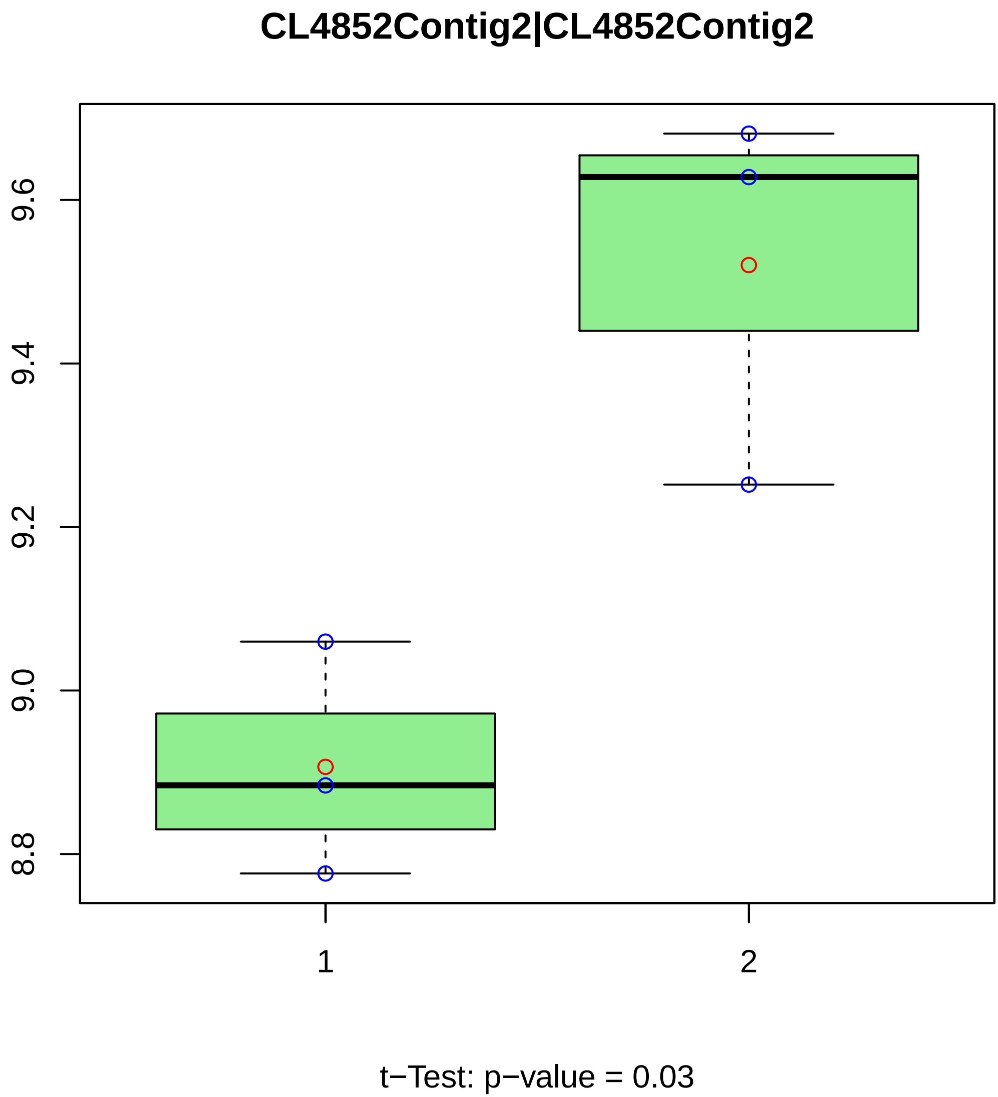

CL4852Contig2|CL4852Contig2
9.6
9.4
9.2
9.0
8.8
1
2
t−Test: p−value = 0.03

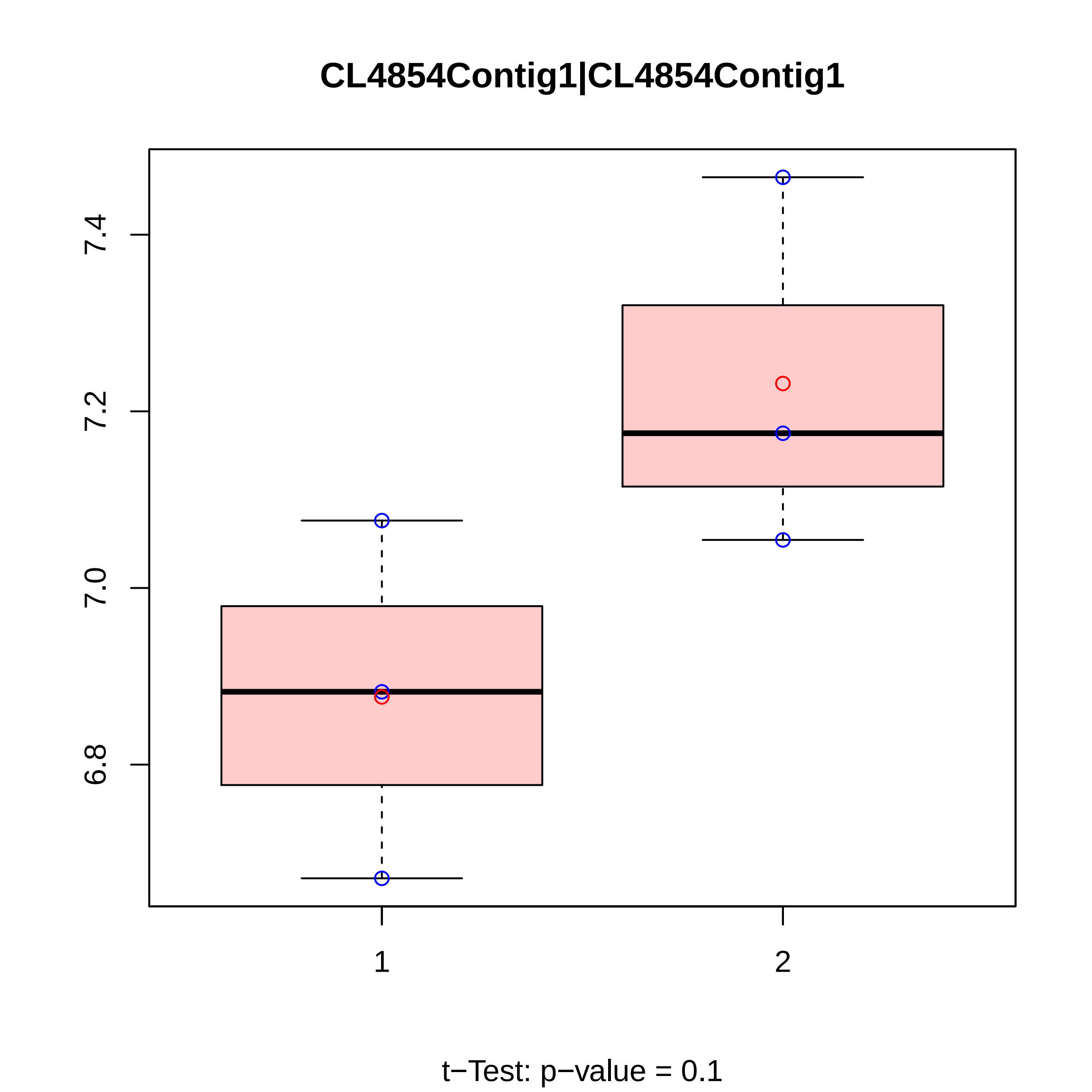

CL4854Contig1|CL4854Contig1
7.4
7.2
7.0
6.8
1
2
t−Test: p−value = 0.1

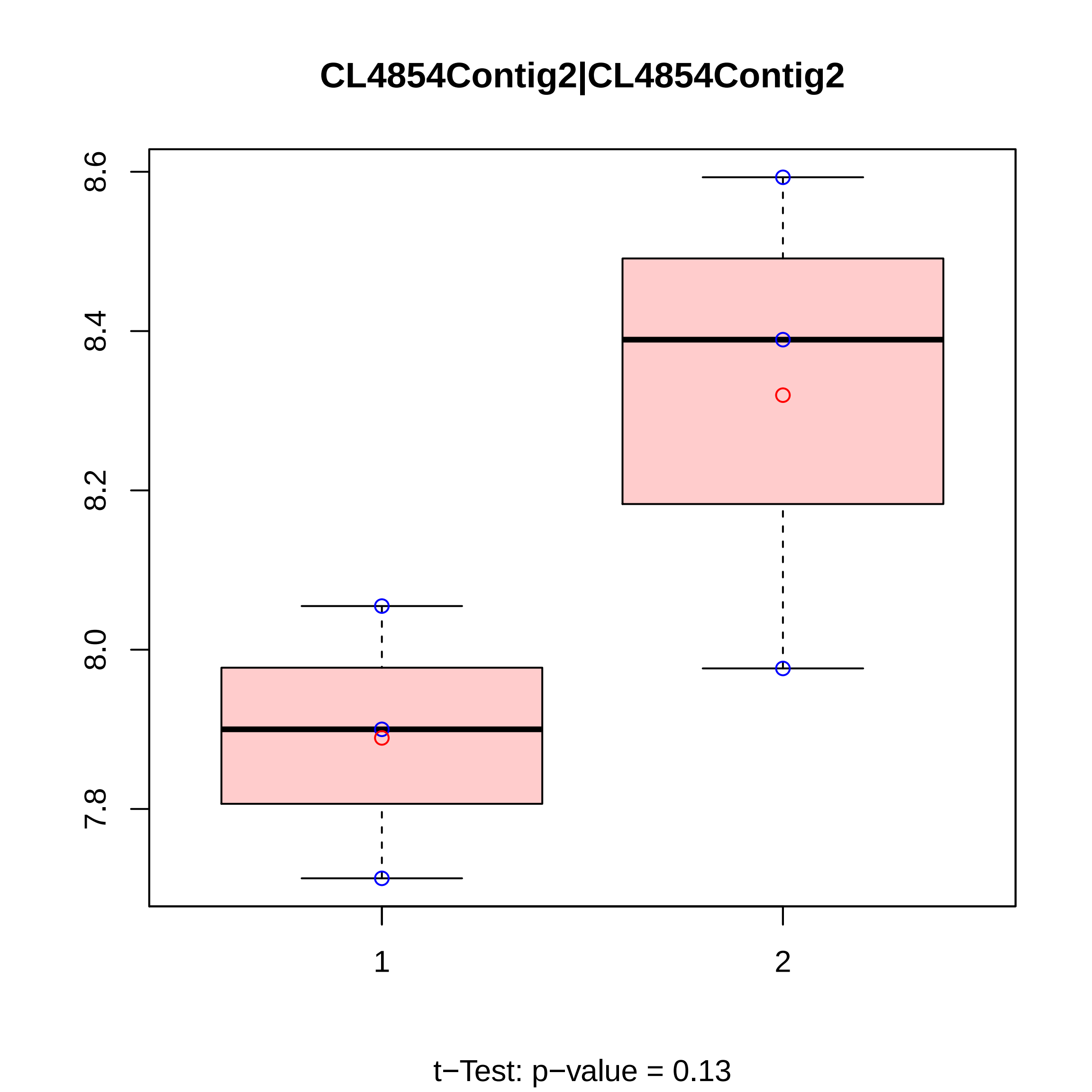
CL4854Contig2|CL4854Contig2
8.6
8.4
8.2
8.0
7.8
1
2
t−Test: p−value = 0.13

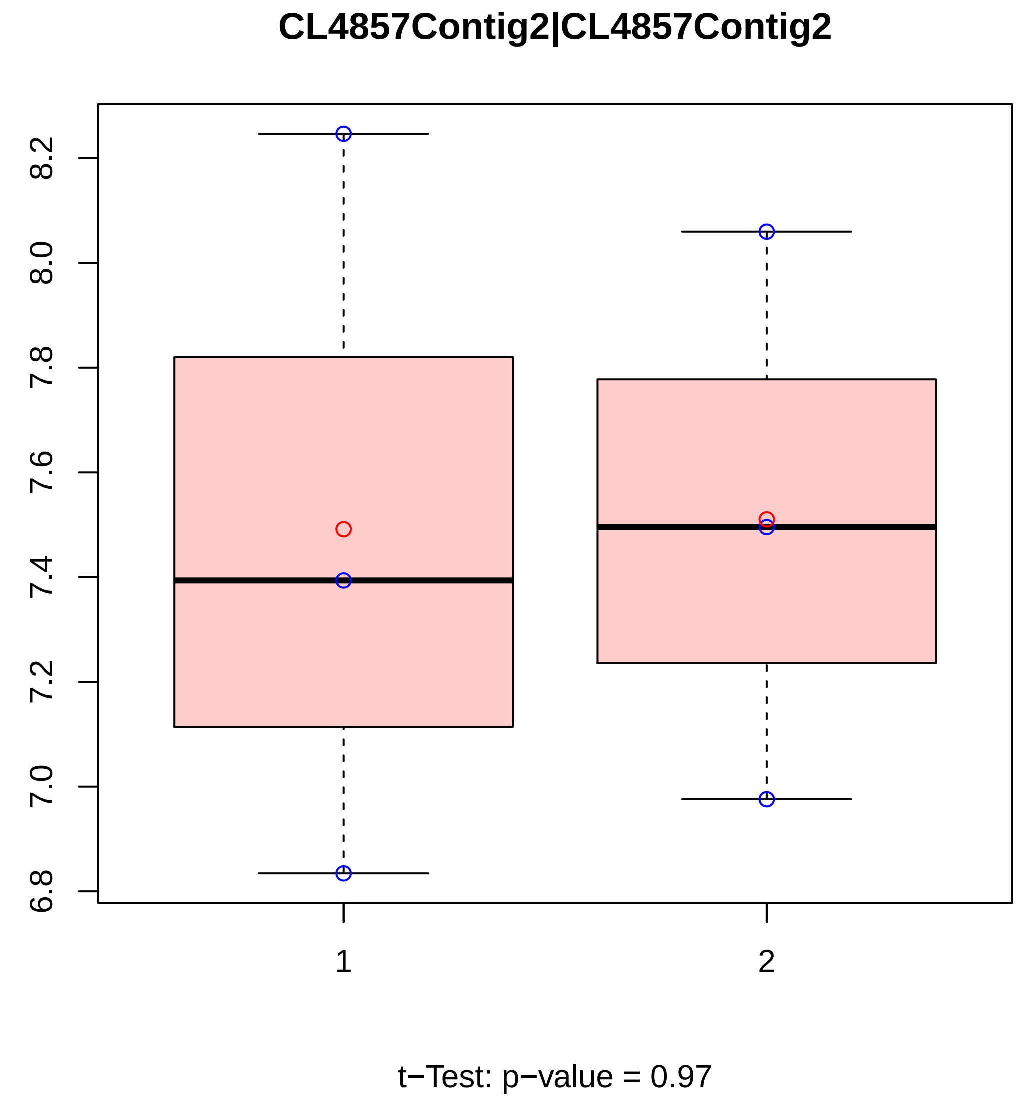
CL4857Contig2|CL4857Contig2
8.2
8.0
7.8
7.6
7.4
7.2
7.0
6.8
1
2
t−Test: p−value = 0.97

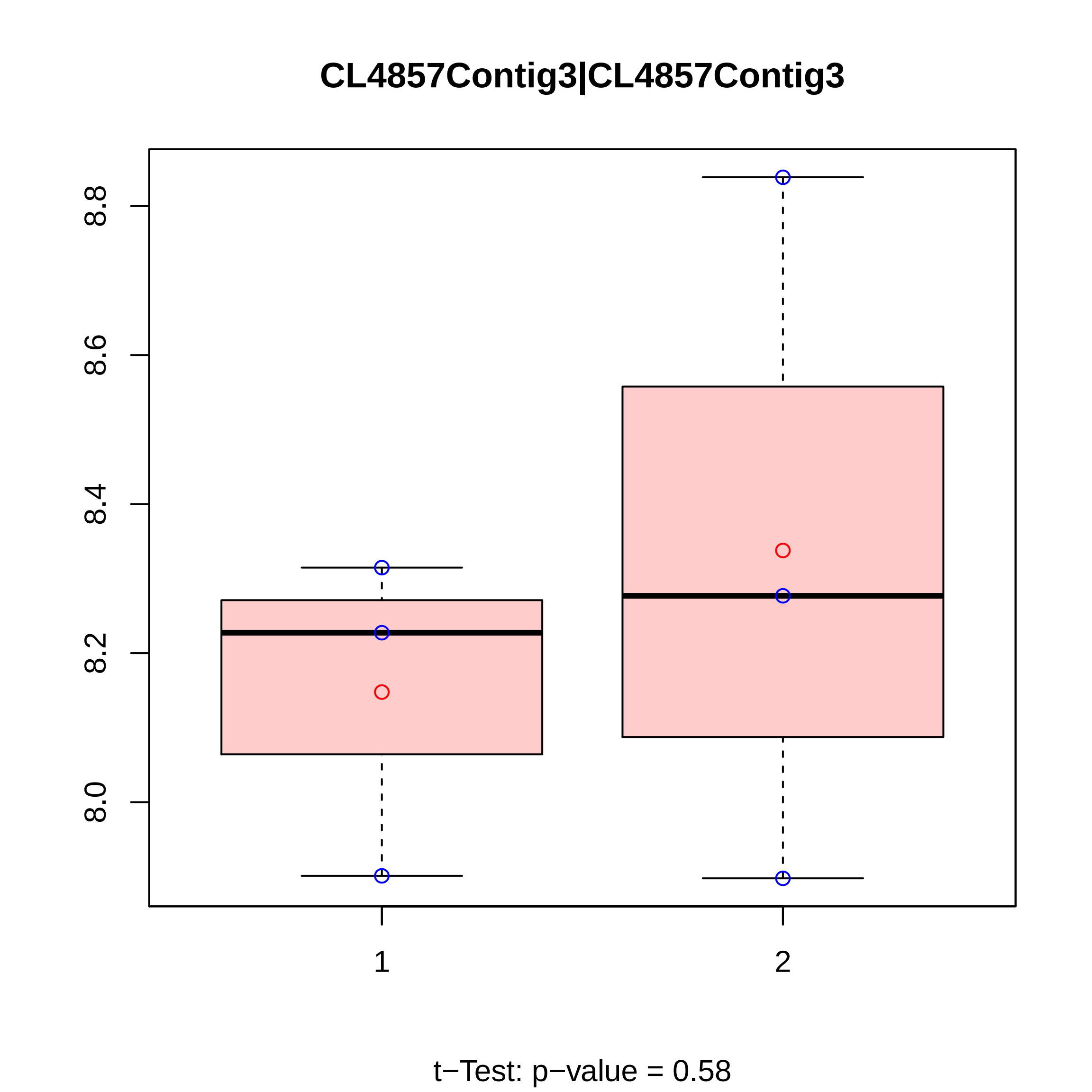
CL4857Contig3|CL4857Contig3
8.0
8.2
8.4
8.6
8.8
1
2
t−Test: p−value = 0.58

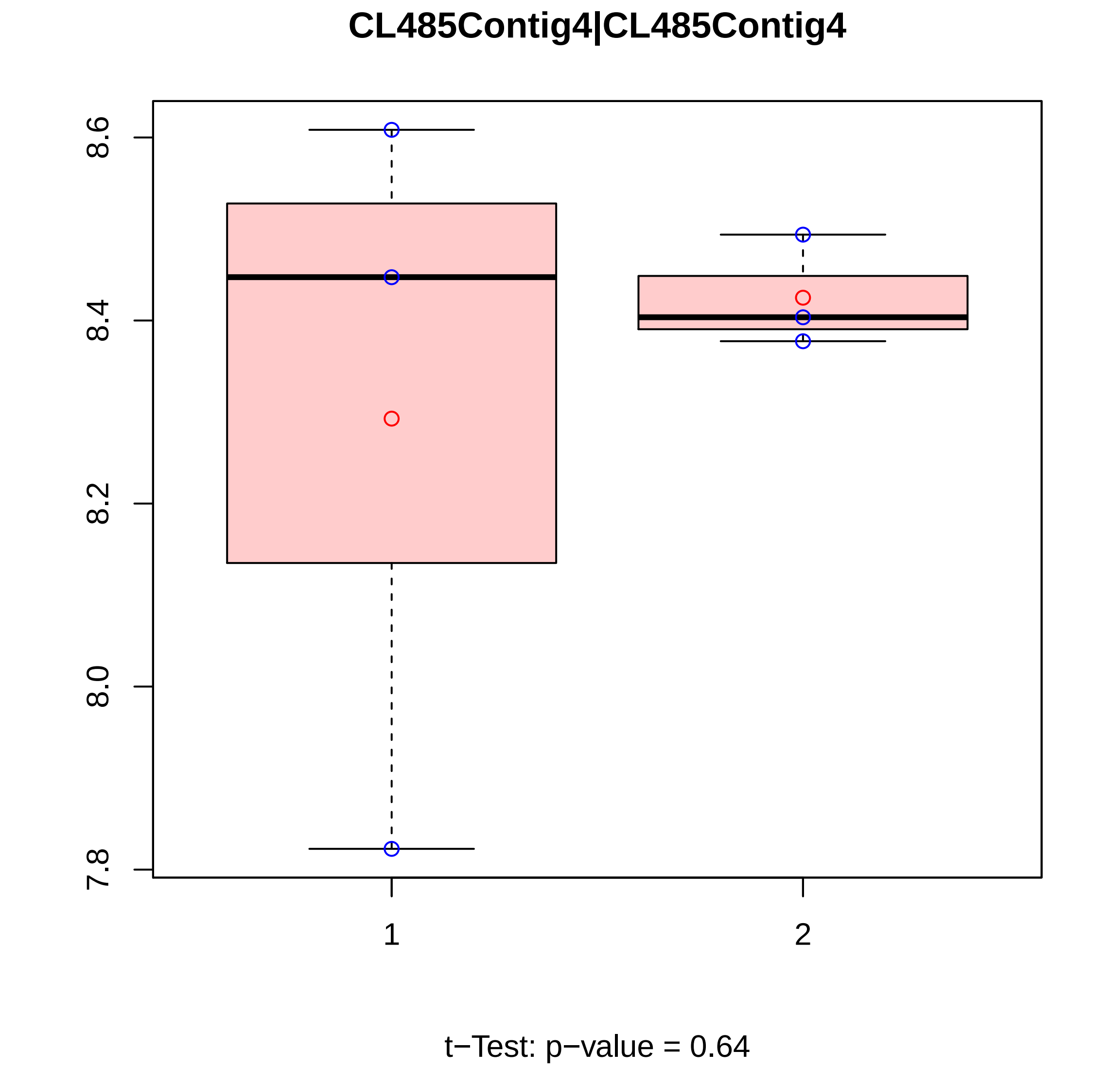
CL485Contig4|CL485Contig4
8.6
8.4
8.2
8.0
7.8
1
2
t−Test: p−value = 0.64

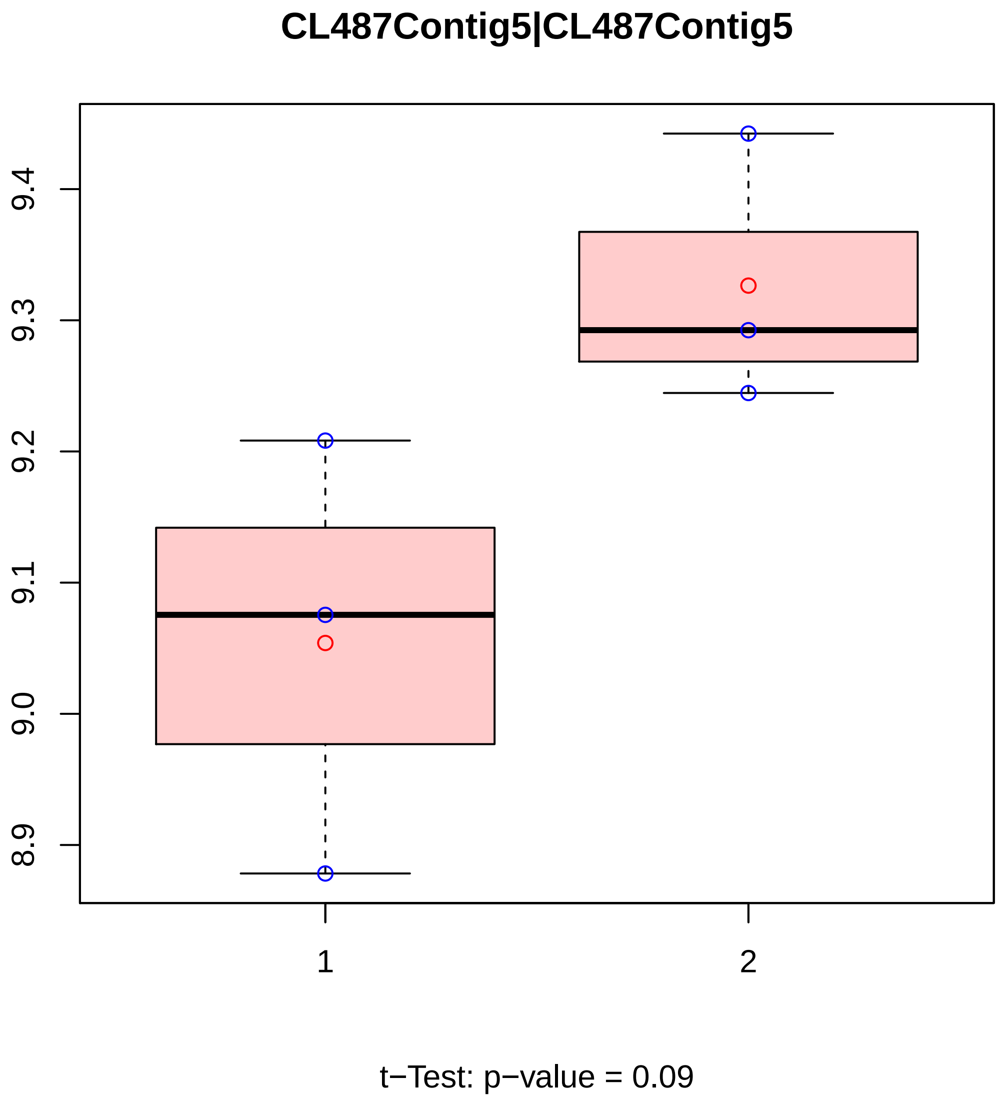

CL487Contig5|CL487Contig5
9.4
9.3
9.2
9.1
9.0
8.9
1
2
t−Test: p−value = 0.09

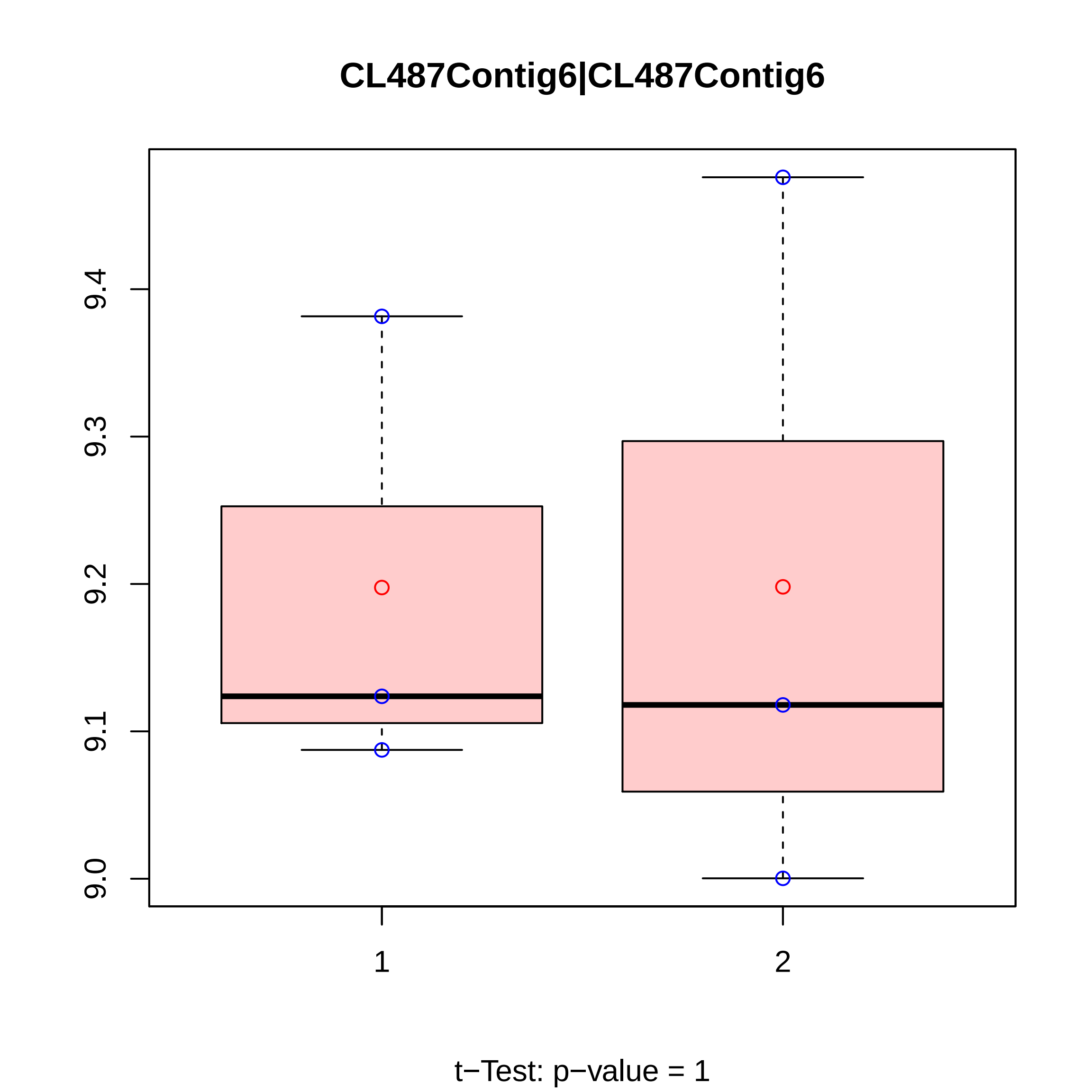
CL487Contig6|CL487Contig6
9.0
9.1
9.2
9.3
9.4
1
2
t−Test: p−value = 1

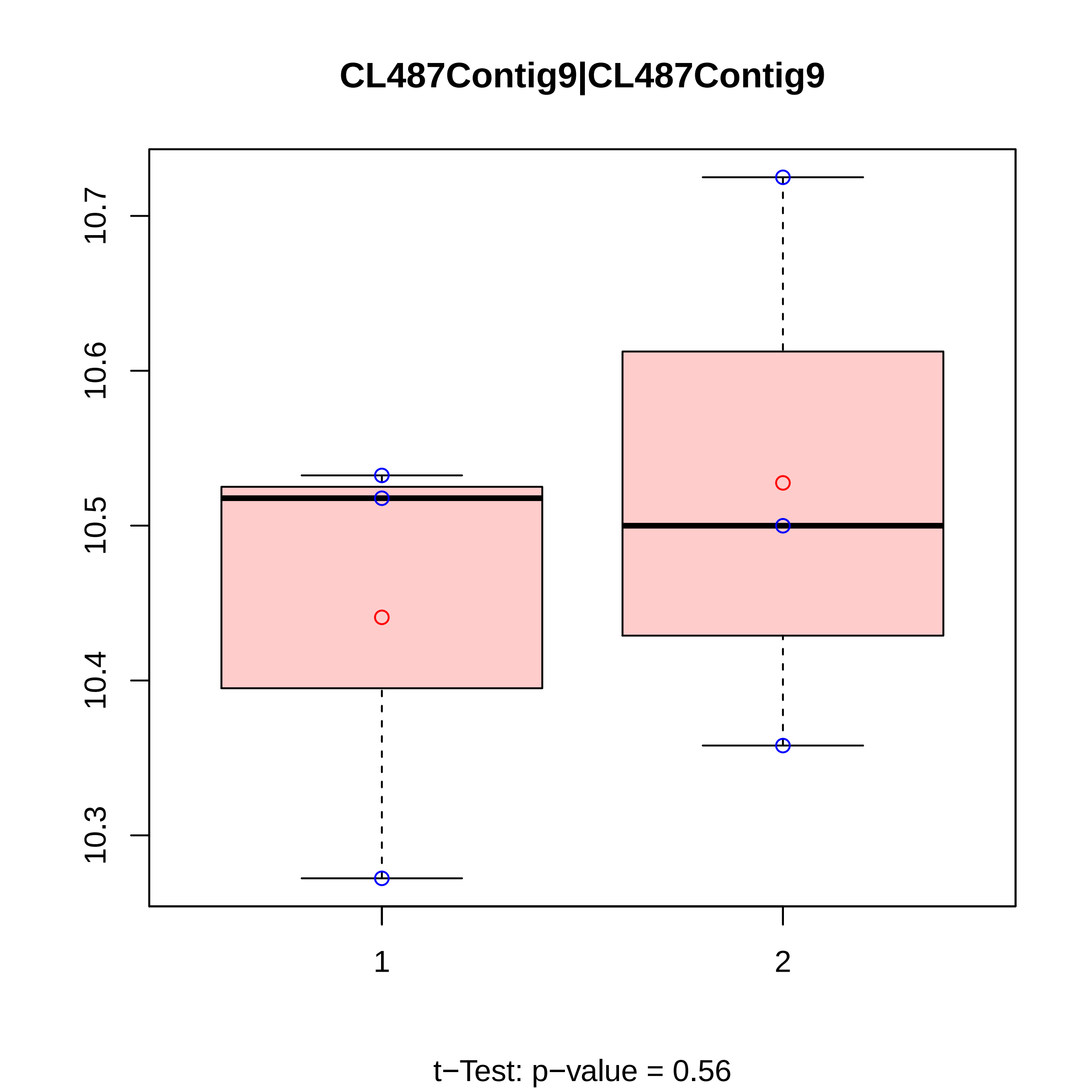

CL487Contig9|CL487Contig9
10.3
10.4
10.5
10.6
10.7
1
2
t−Test: p−value = 0.56

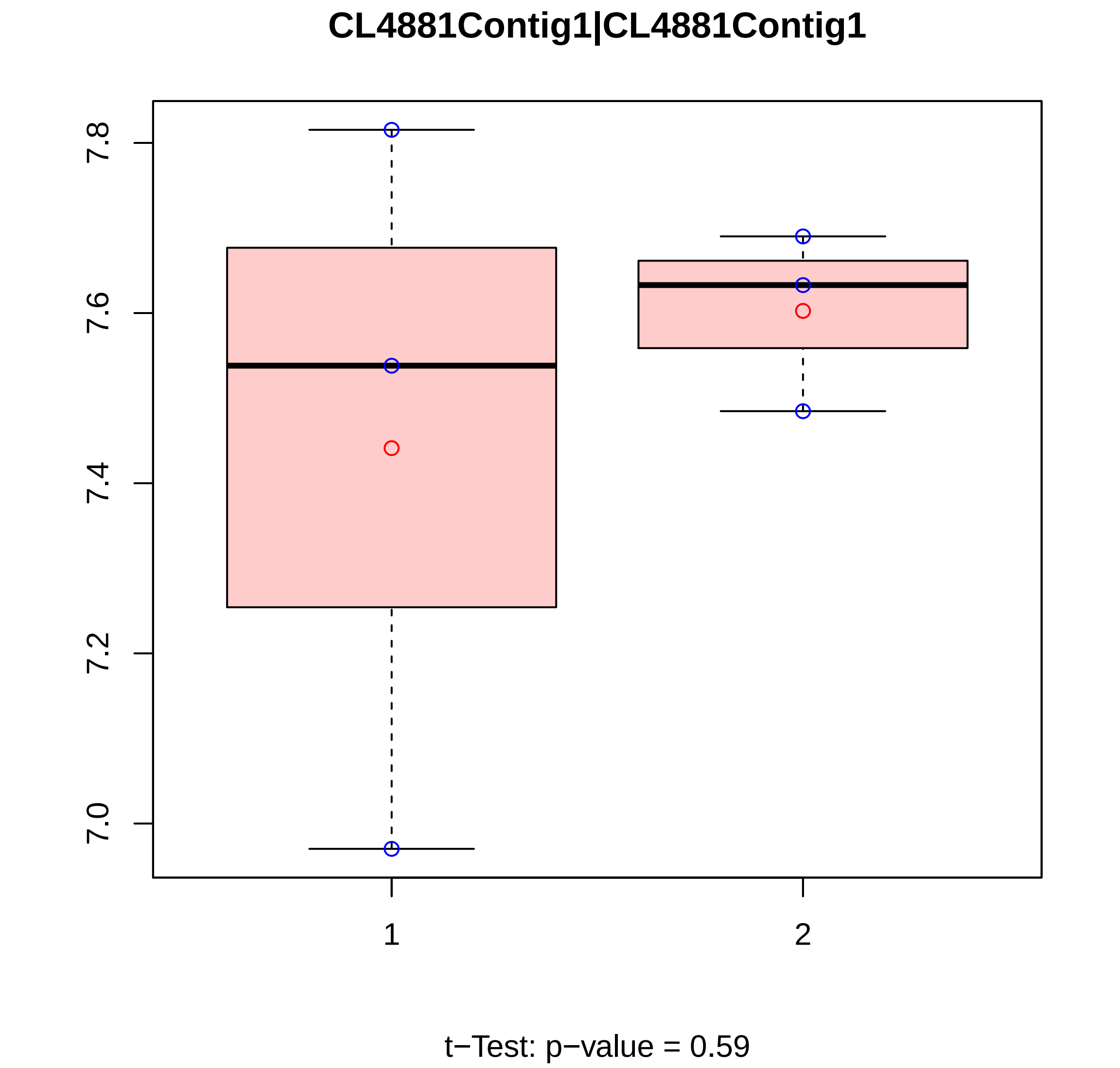
CL4881Contig1|CL4881Contig1
7.0
7.2
7.4
7.6
7.8
1
2
t−Test: p−value = 0.59

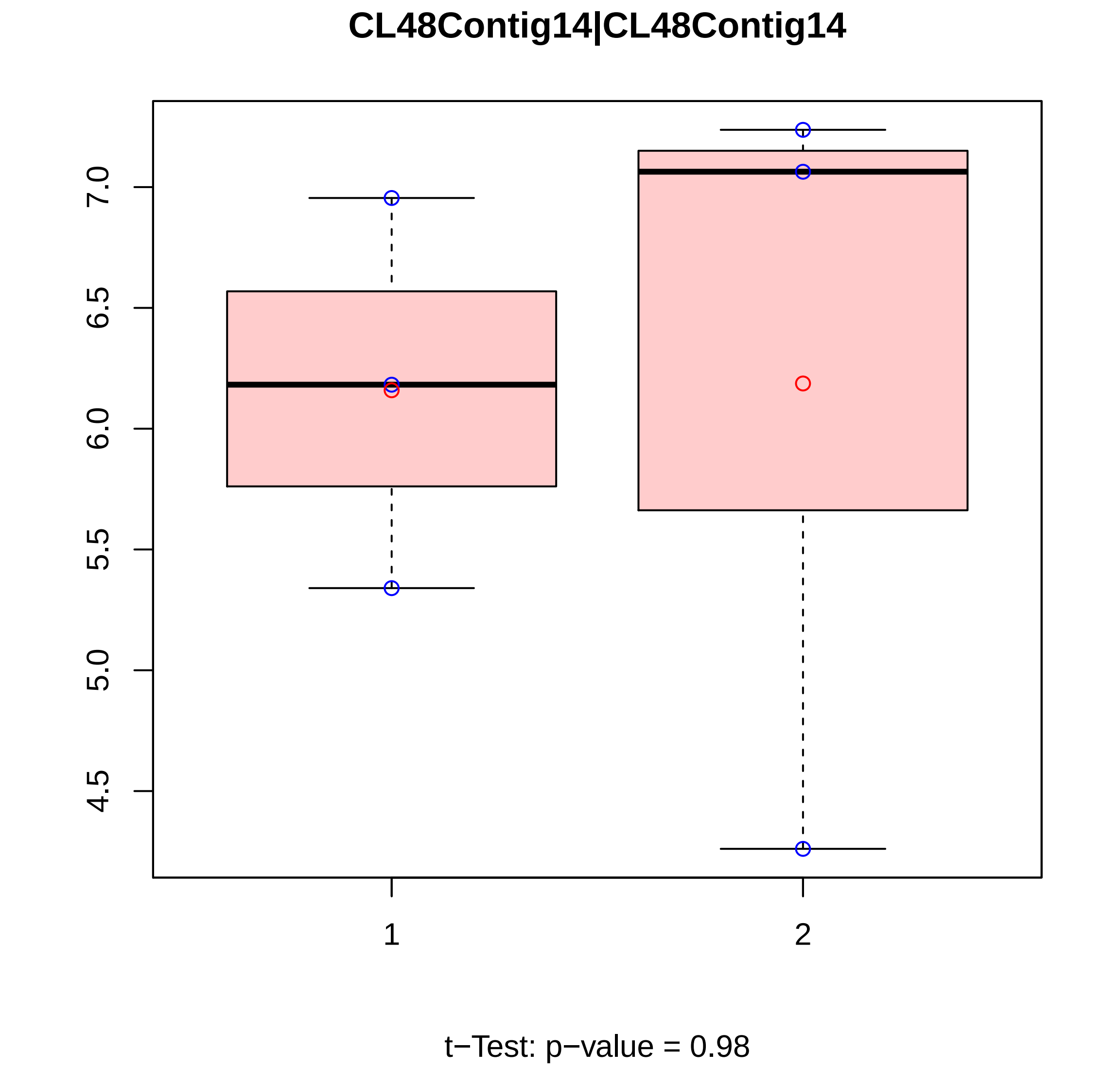
CL48Contig14|CL48Contig14
7.0
6.5
6.0
5.5
5.0
4.5
1
2
t−Test: p−value = 0.98

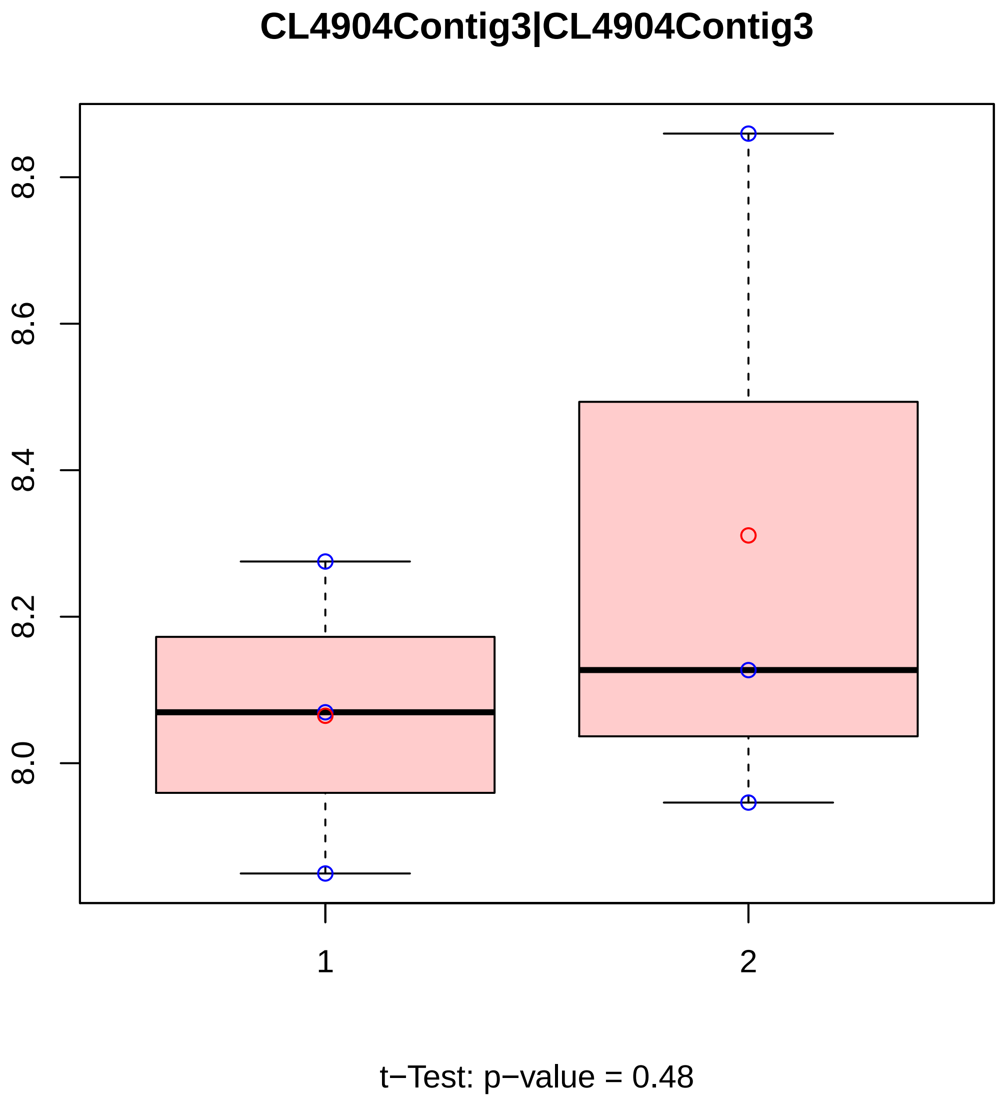
CL4904Contig3|CL4904Contig3
8.0
8.2
8.4
8.6
8.8
1
2
t−Test: p−value = 0.48

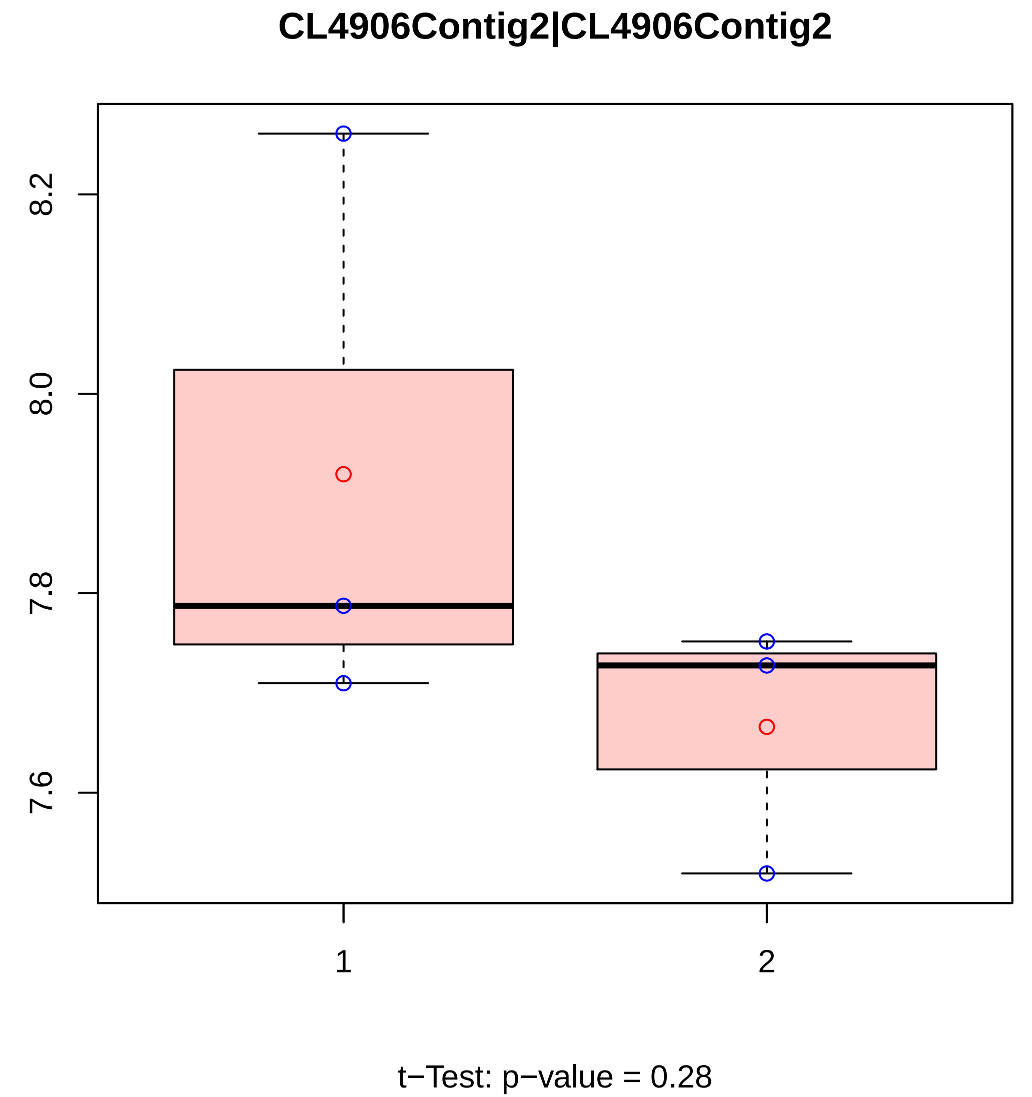
CL4906Contig2|CL4906Contig2
8.2
8.0
7.8
7.6
1
2
t−Test: p−value = 0.28

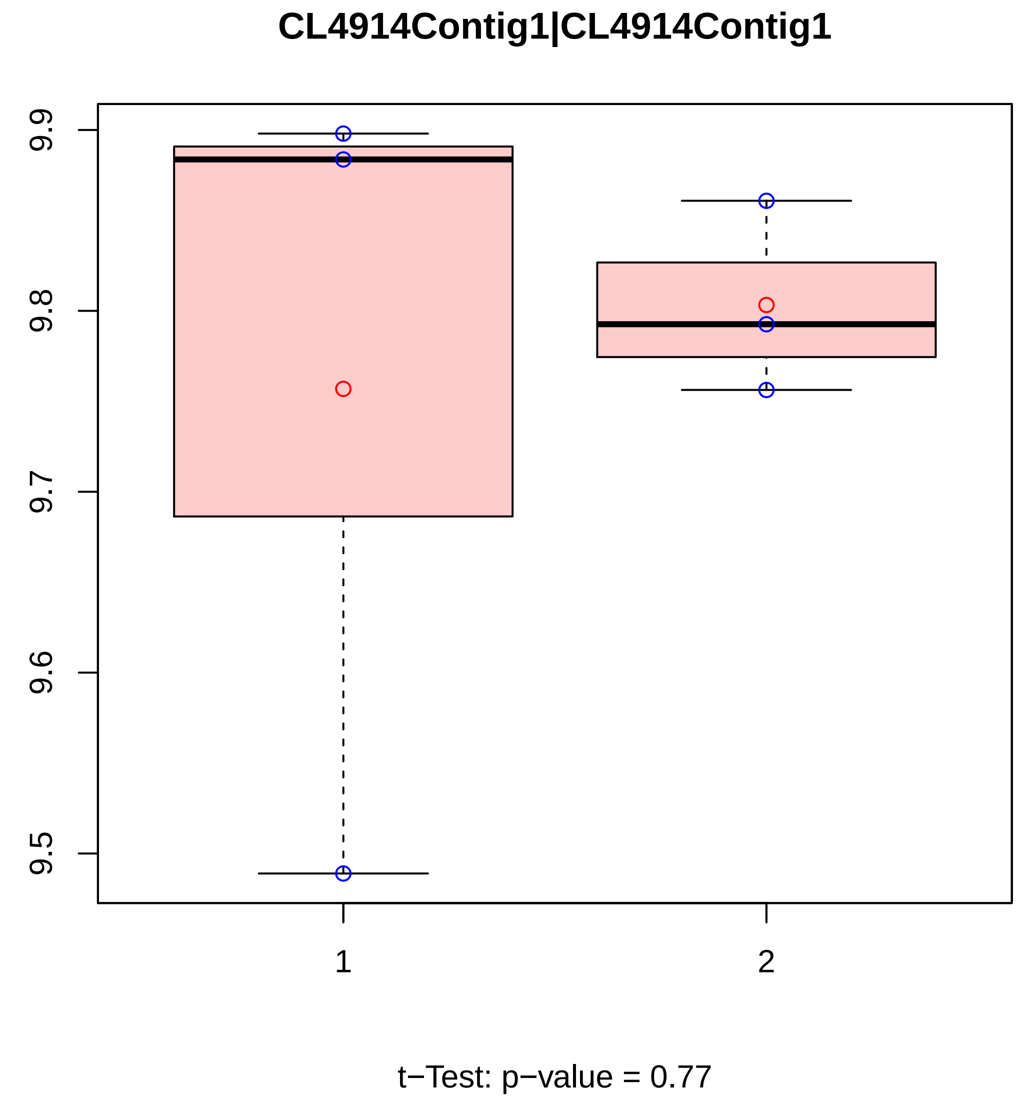

CL4914Contig1|CL4914Contig1
9.5
9.6
9.7
9.8
9.9
1
2
t−Test: p−value = 0.77

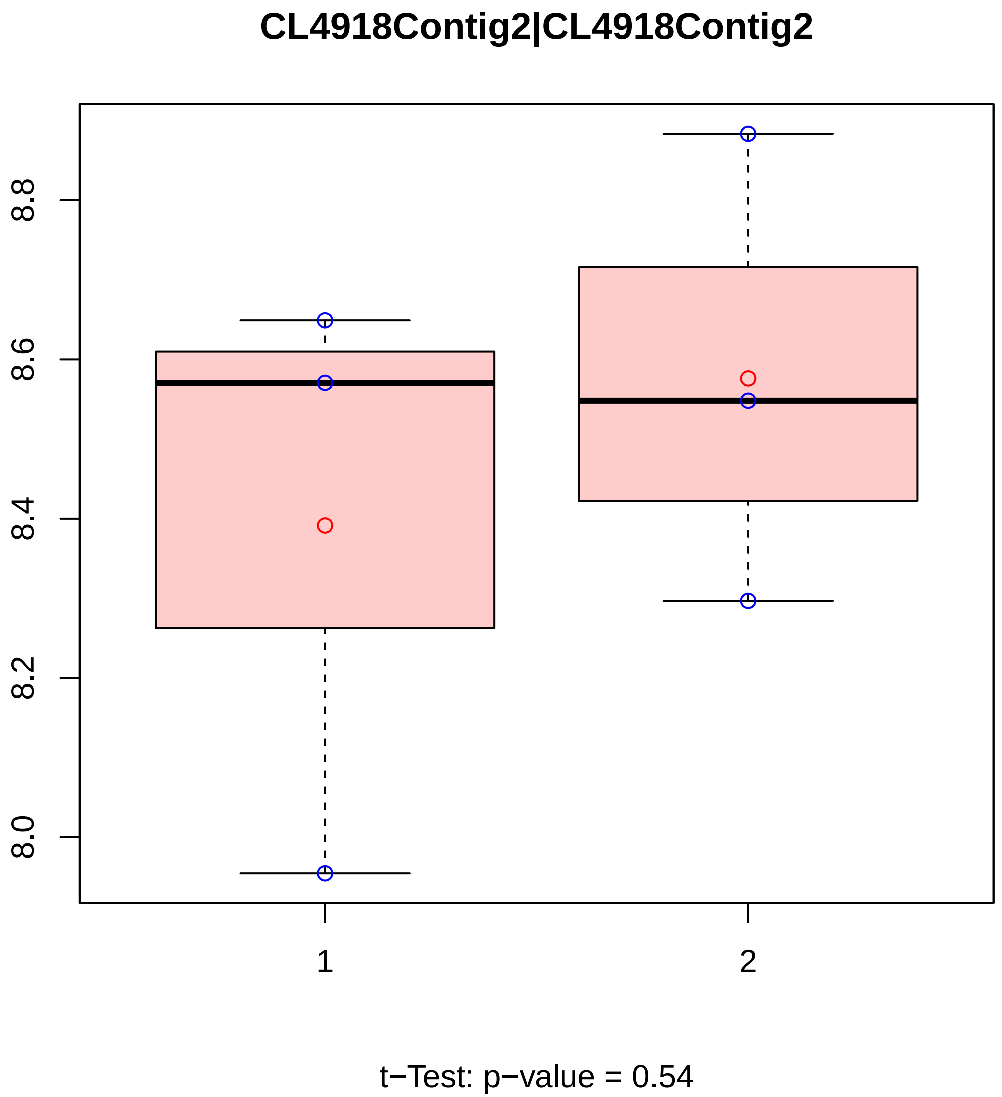

CL4918Contig2|CL4918Contig2
8.0
8.2
8.4
8.6
8.8
1
2
t−Test: p−value = 0.54

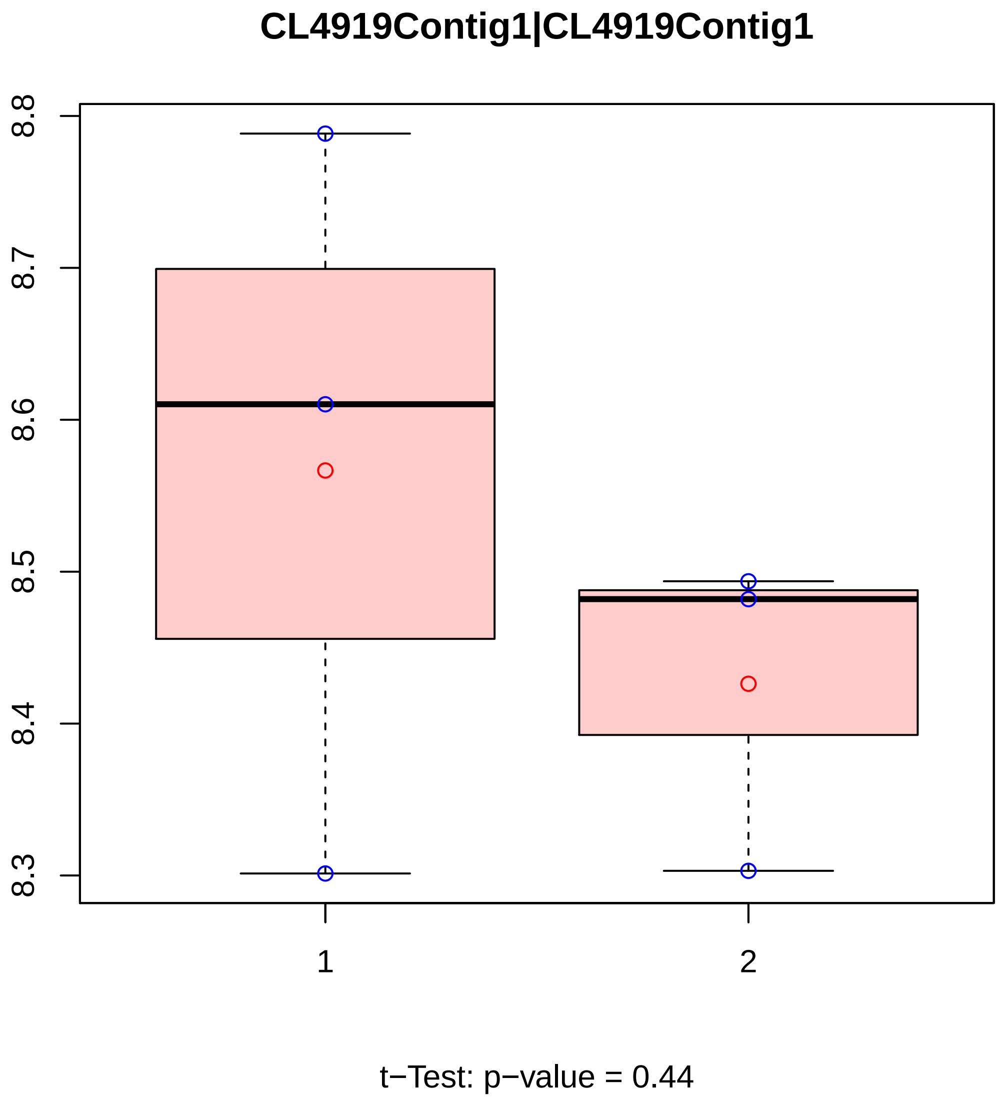
CL4919Contig1|CL4919Contig1
8.3
8.4
8.5
8.6
8.7
8.8
1
2
t−Test: p−value = 0.44

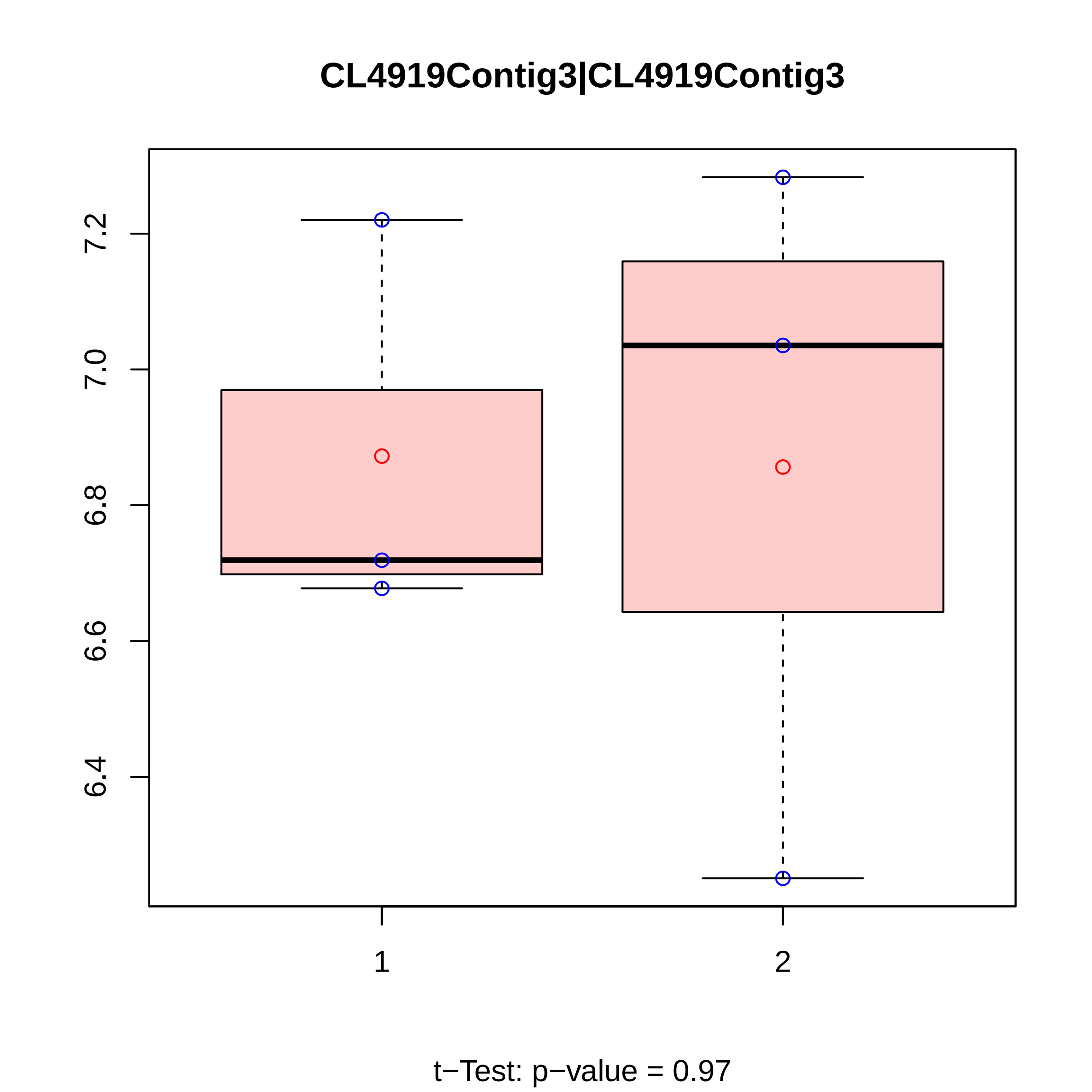
CL4919Contig3|CL4919Contig3
7.2
7.0
6.8
6.6
6.4
1
2
t−Test: p−value = 0.97

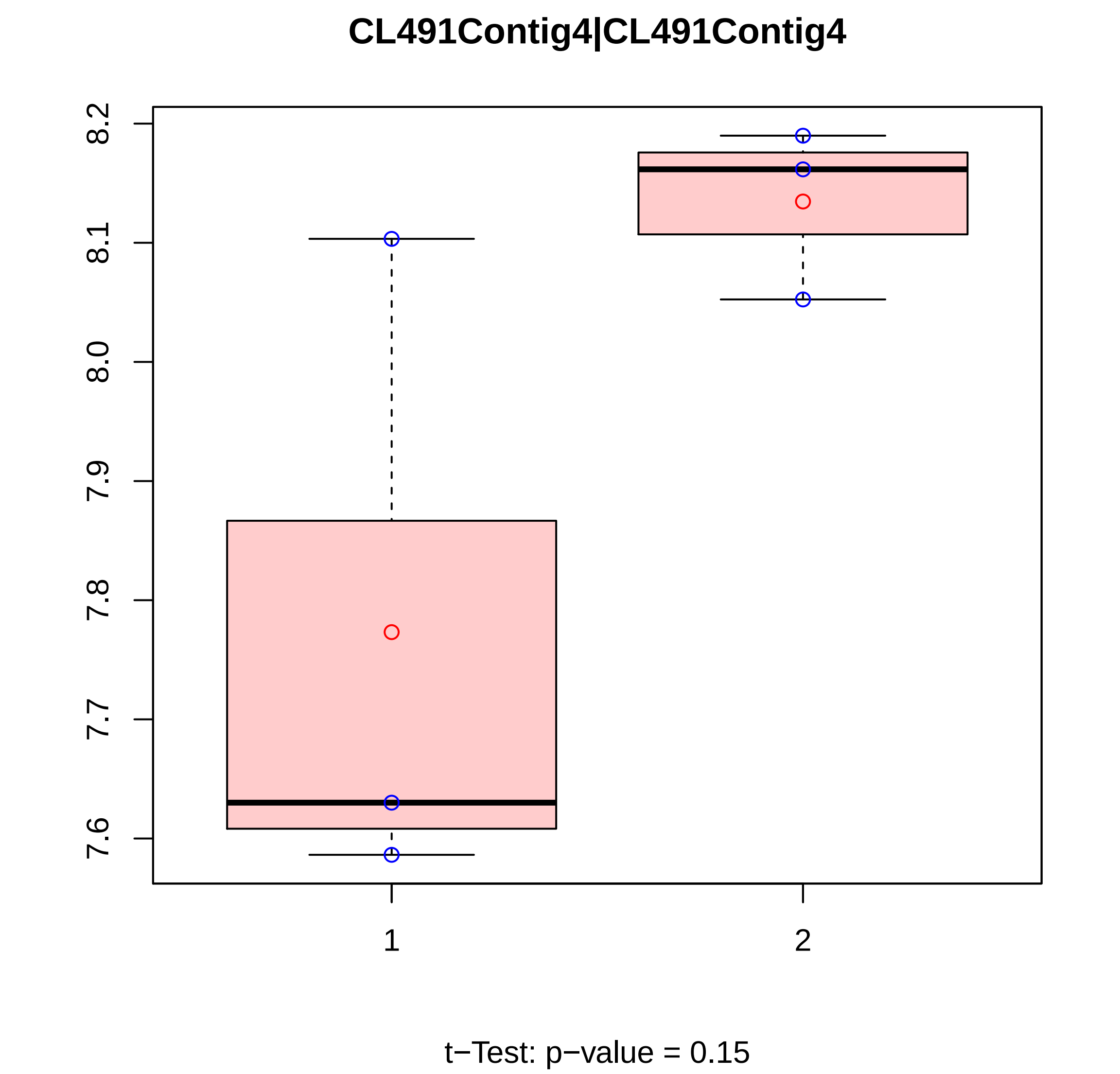
CL491Contig4|CL491Contig4
8.2
8.1
8.0
7.9
7.8
7.7
7.6
1
2
t−Test: p−value = 0.15

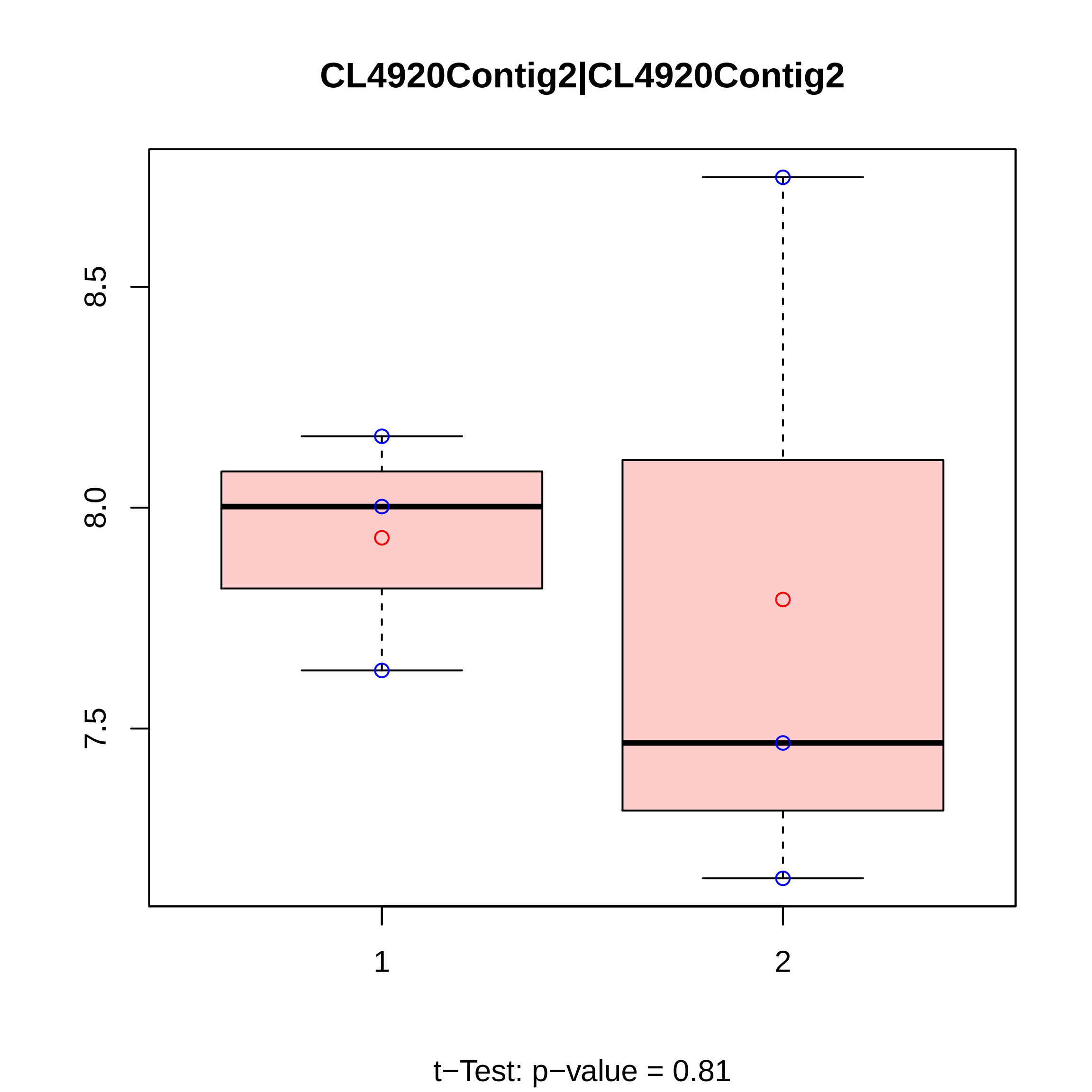
CL4920Contig2|CL4920Contig2
8.5
8.0
7.5
1
2
t−Test: p−value = 0.81

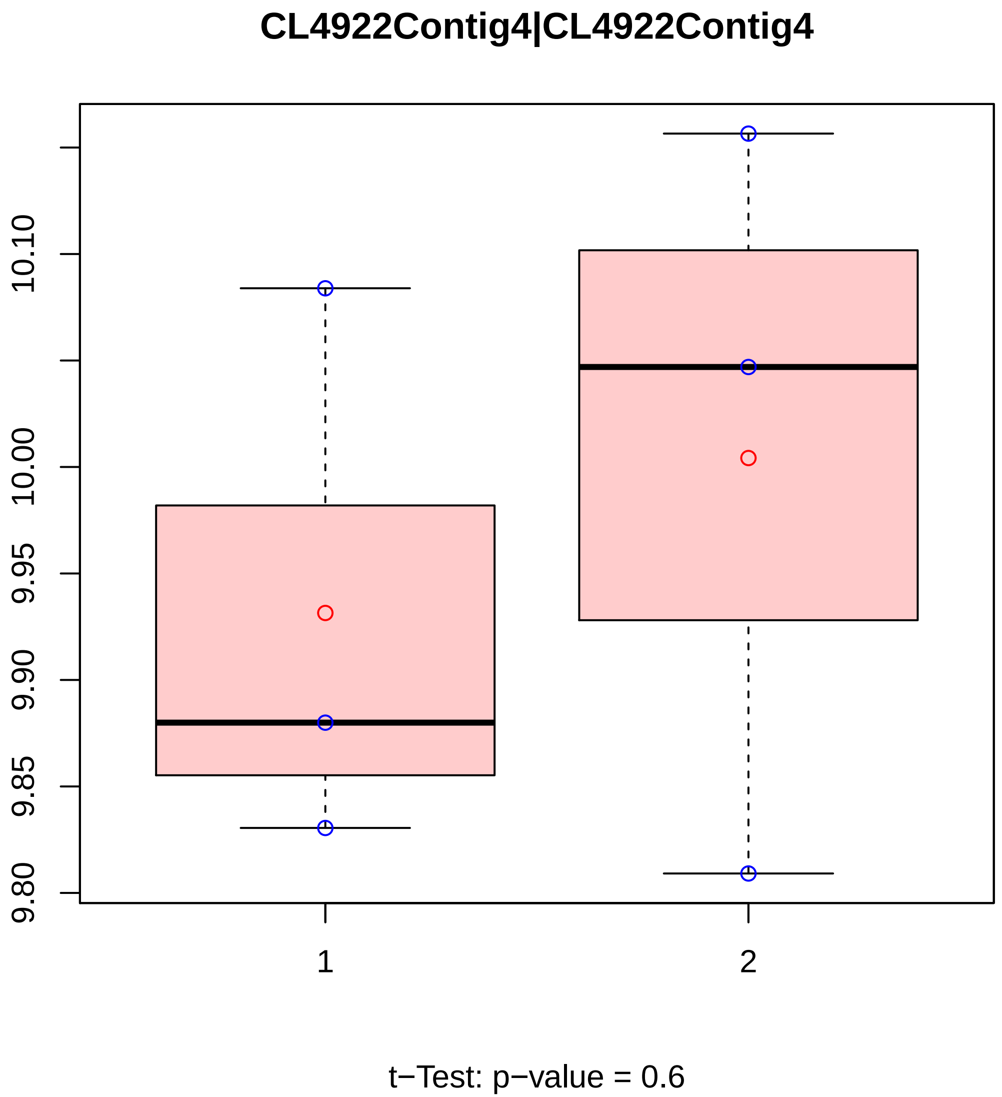

CL4922Contig4|CL4922Contig4
10.10
10.00
9.95
9.90
9.85
9.80
1
2
t−Test: p−value = 0.6

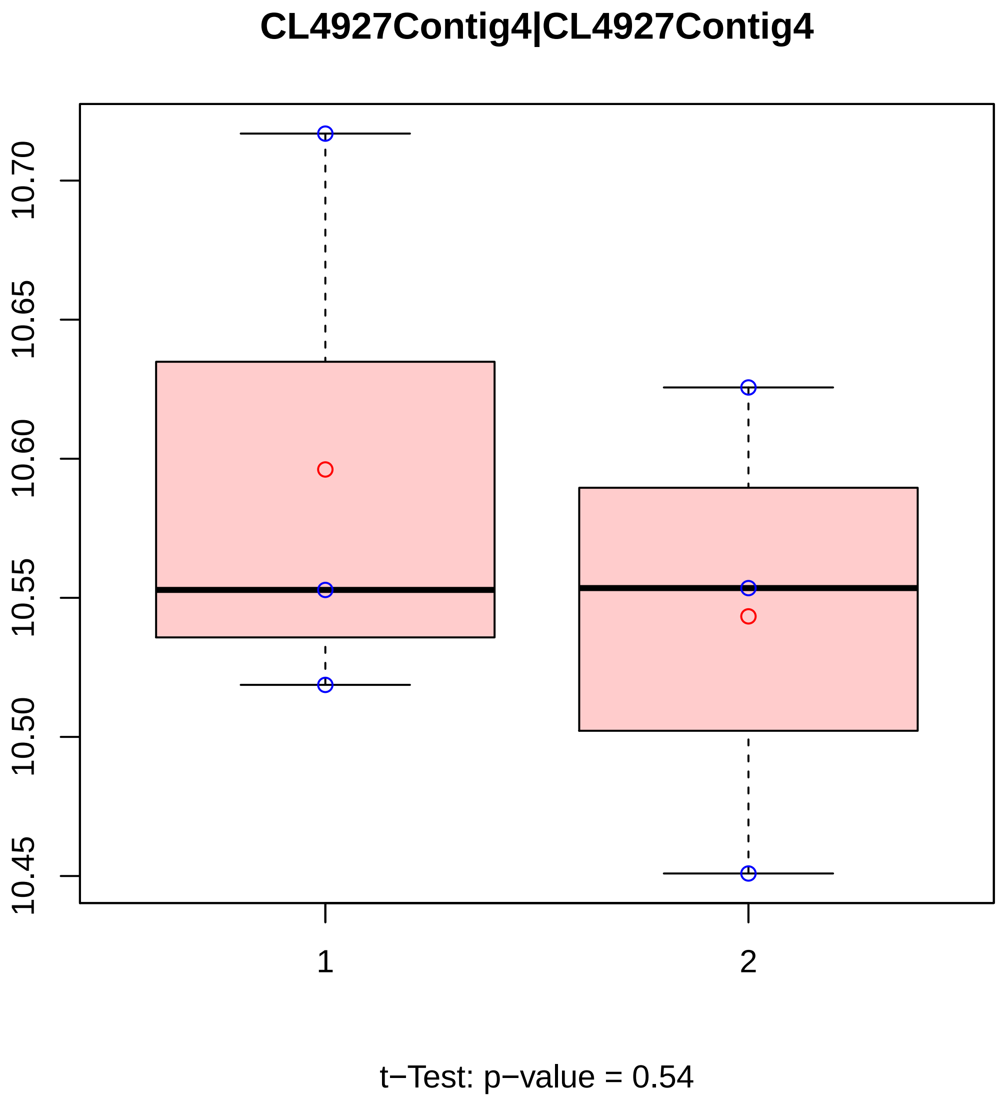

CL4927Contig4|CL4927Contig4
10.70
10.65
10.60
10.55
10.50
10.45
1
2
t−Test: p−value = 0.54

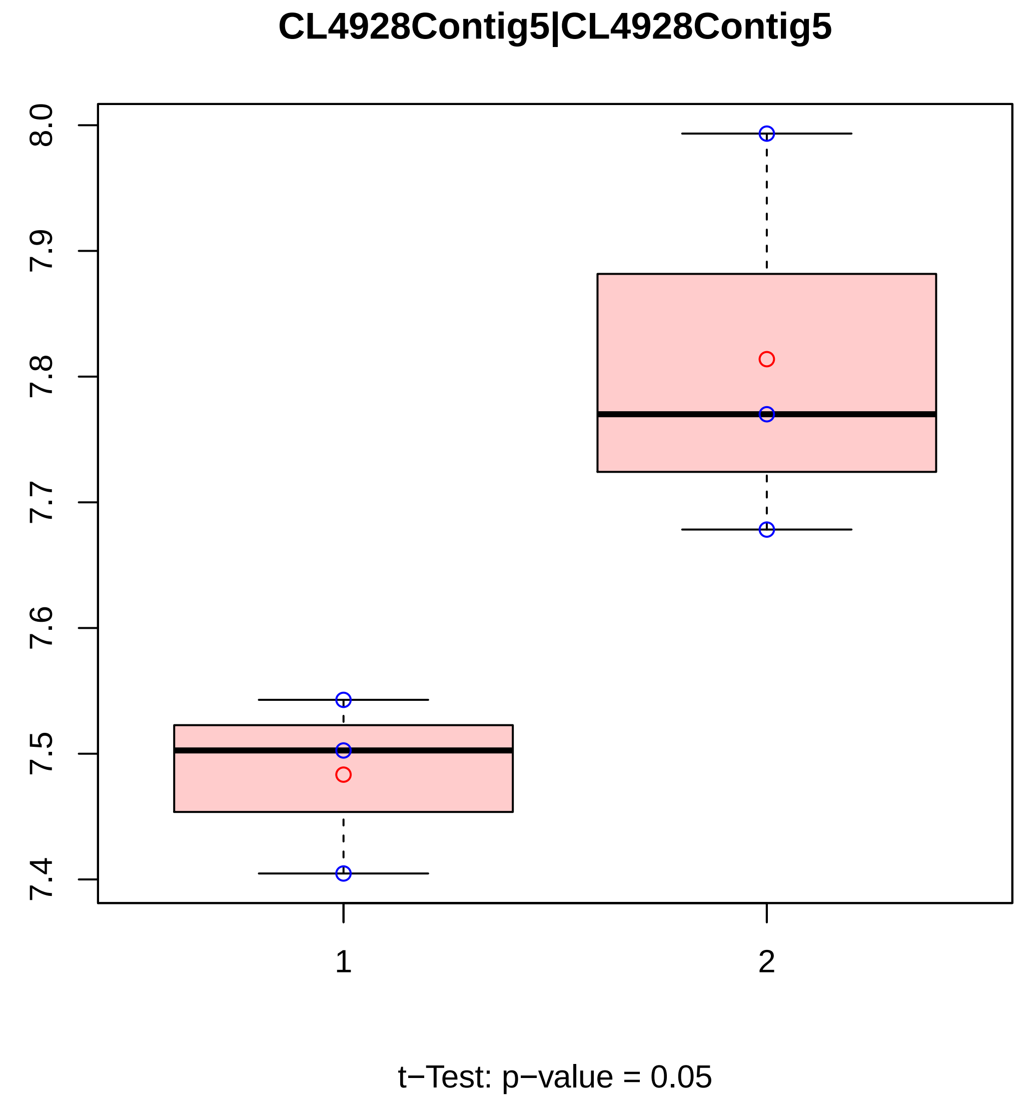
CL4928Contig5|CL4928Contig5
8.0
7.9
7.8
7.7
7.6
7.5
7.4
1
2
t−Test: p−value = 0.05

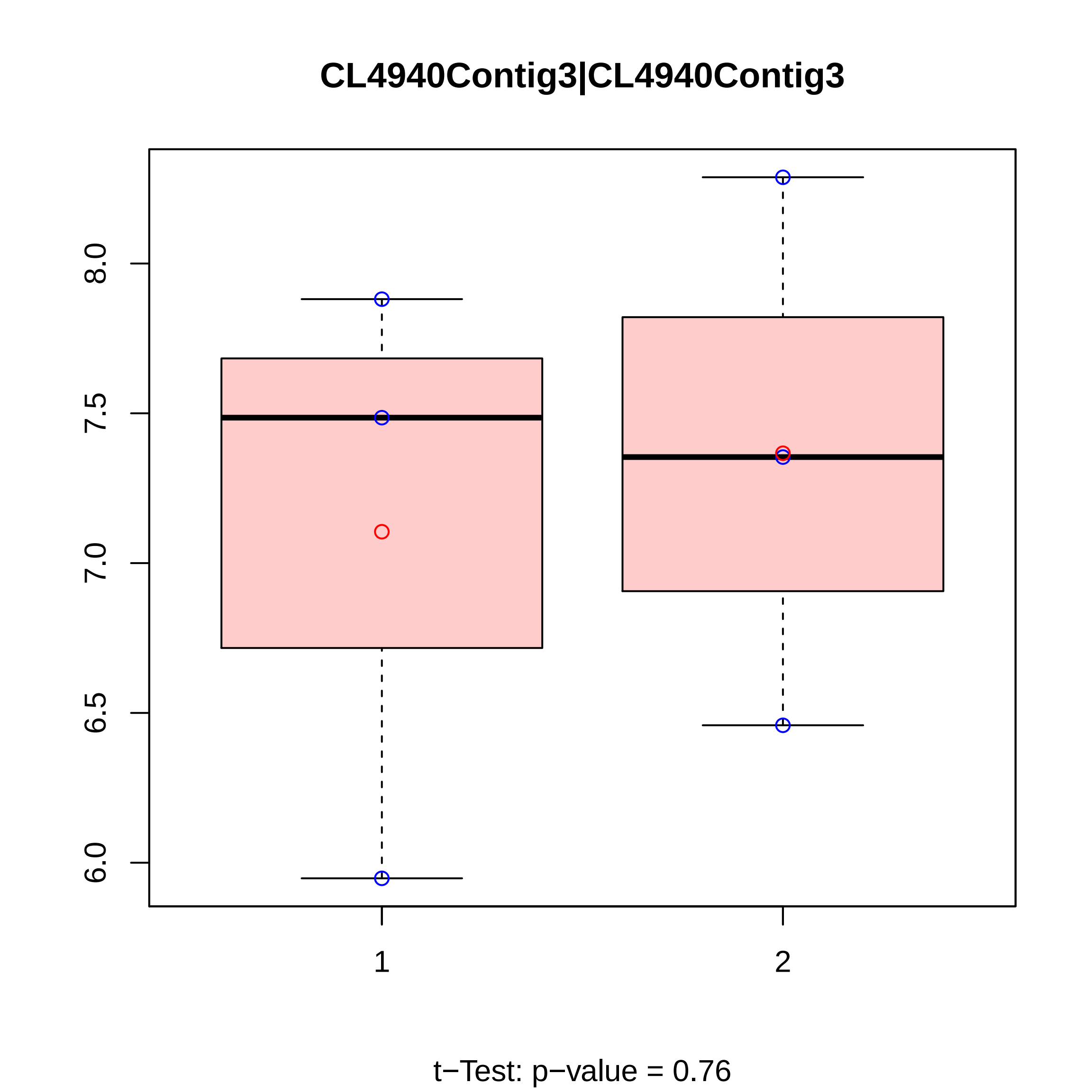
CL4940Contig3|CL4940Contig3
8.0
7.5
7.0
6.5
6.0
1
2
t−Test: p−value = 0.76

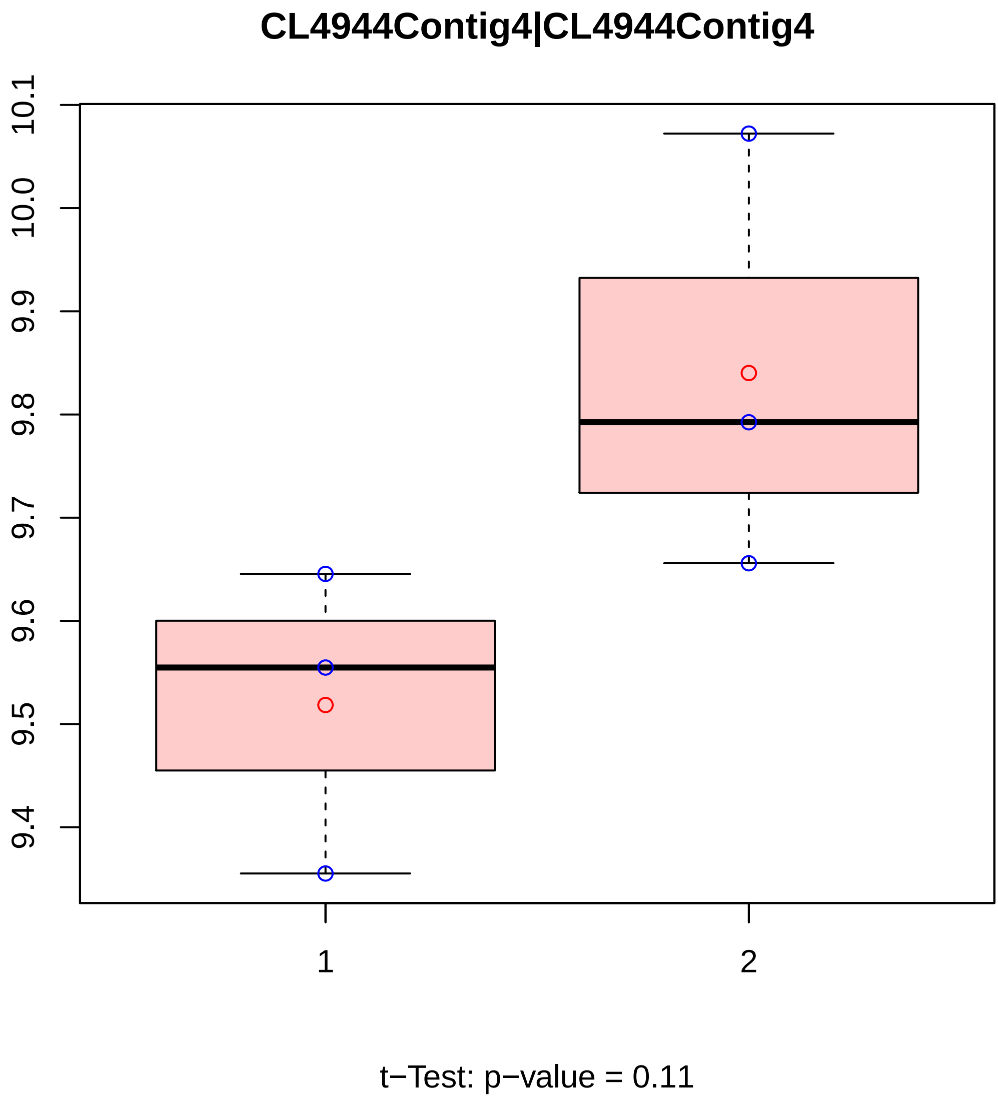
CL4944Contig4|CL4944Contig4
10.1
10.0
9.9
9.8
9.7
9.6
9.5
9.4
1
2
t−Test: p−value = 0.11

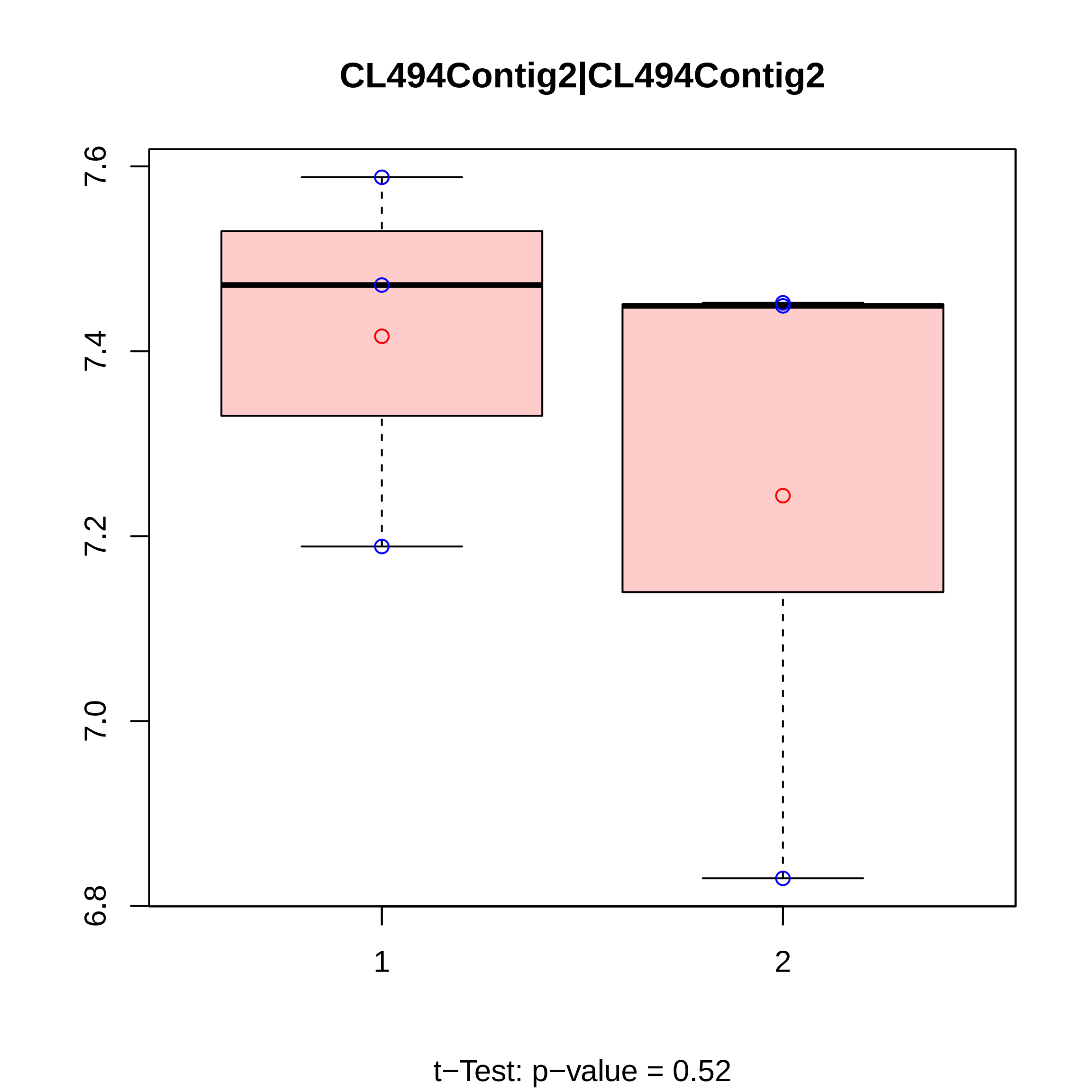
CL494Contig2|CL494Contig2
7.6
7.4
7.2
7.0
6.8
1
2
t−Test: p−value = 0.52

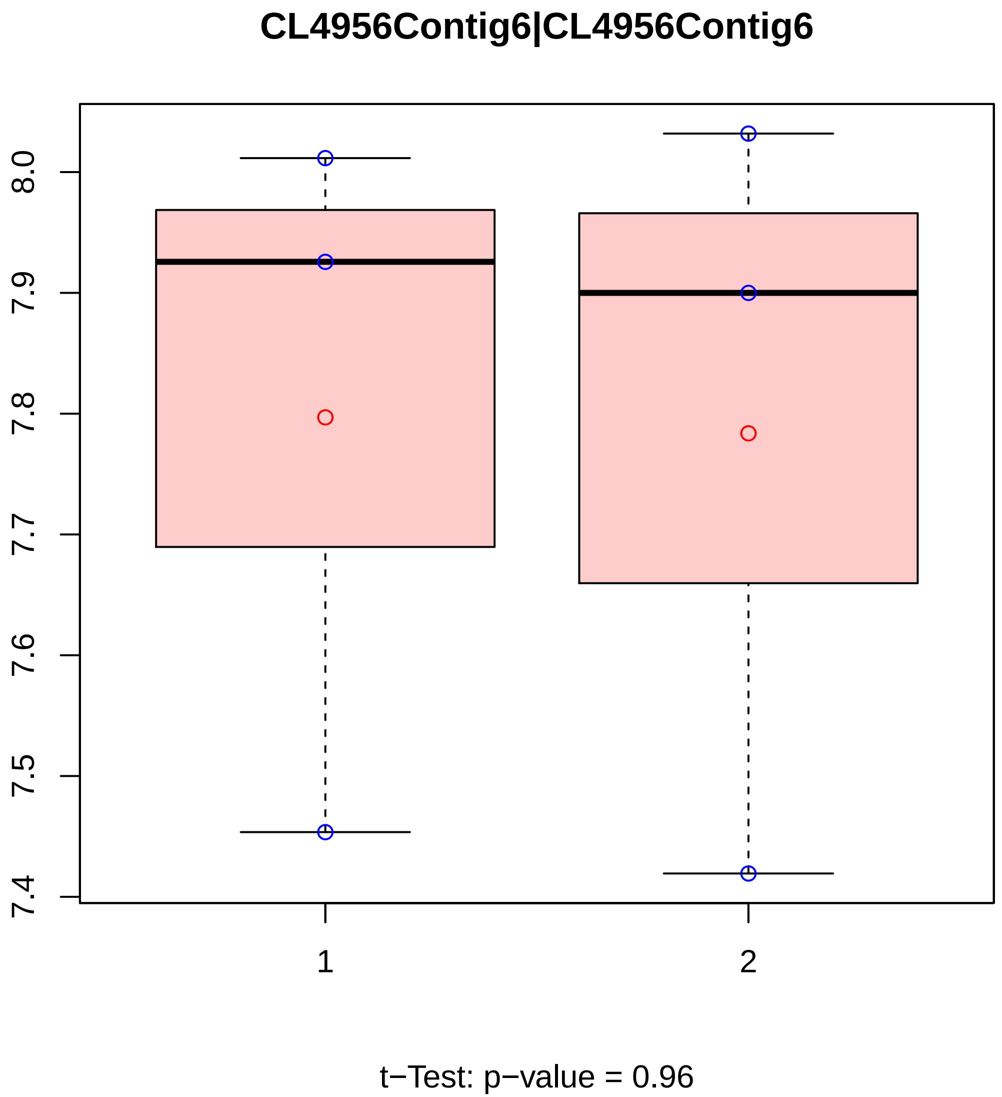
CL4956Contig6|CL4956Contig6
8.0
7.9
7.8
7.7
7.6
7.5
7.4
1
2
t−Test: p−value = 0.96

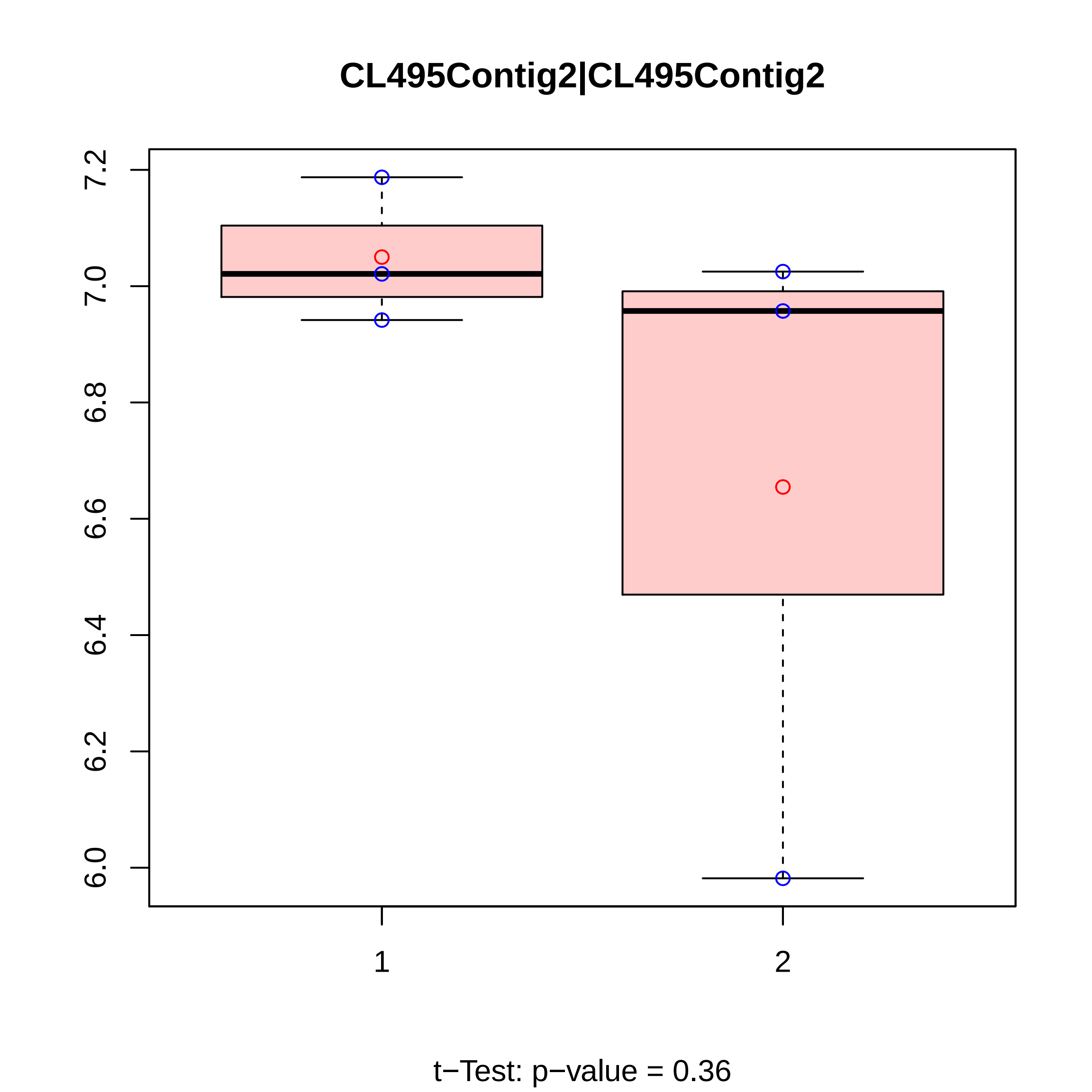
CL495Contig2|CL495Contig2
7.2
7.0
6.8
6.6
6.4
6.2
6.0
1
2
t−Test: p−value = 0.36

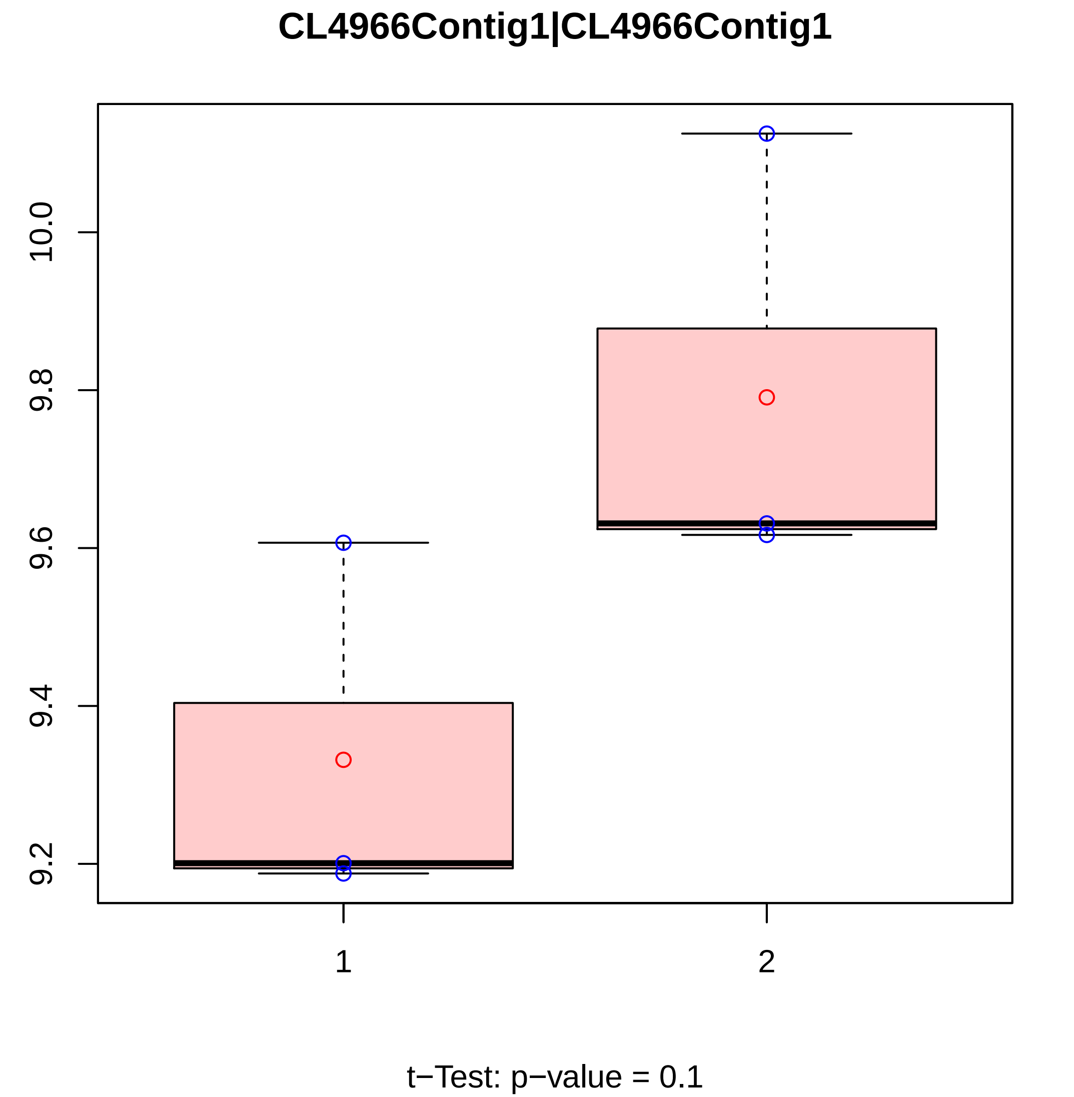

CL4966Contig1|CL4966Contig1
10.0
9.8
9.6
9.4
9.2
1
2
t−Test: p−value = 0.1

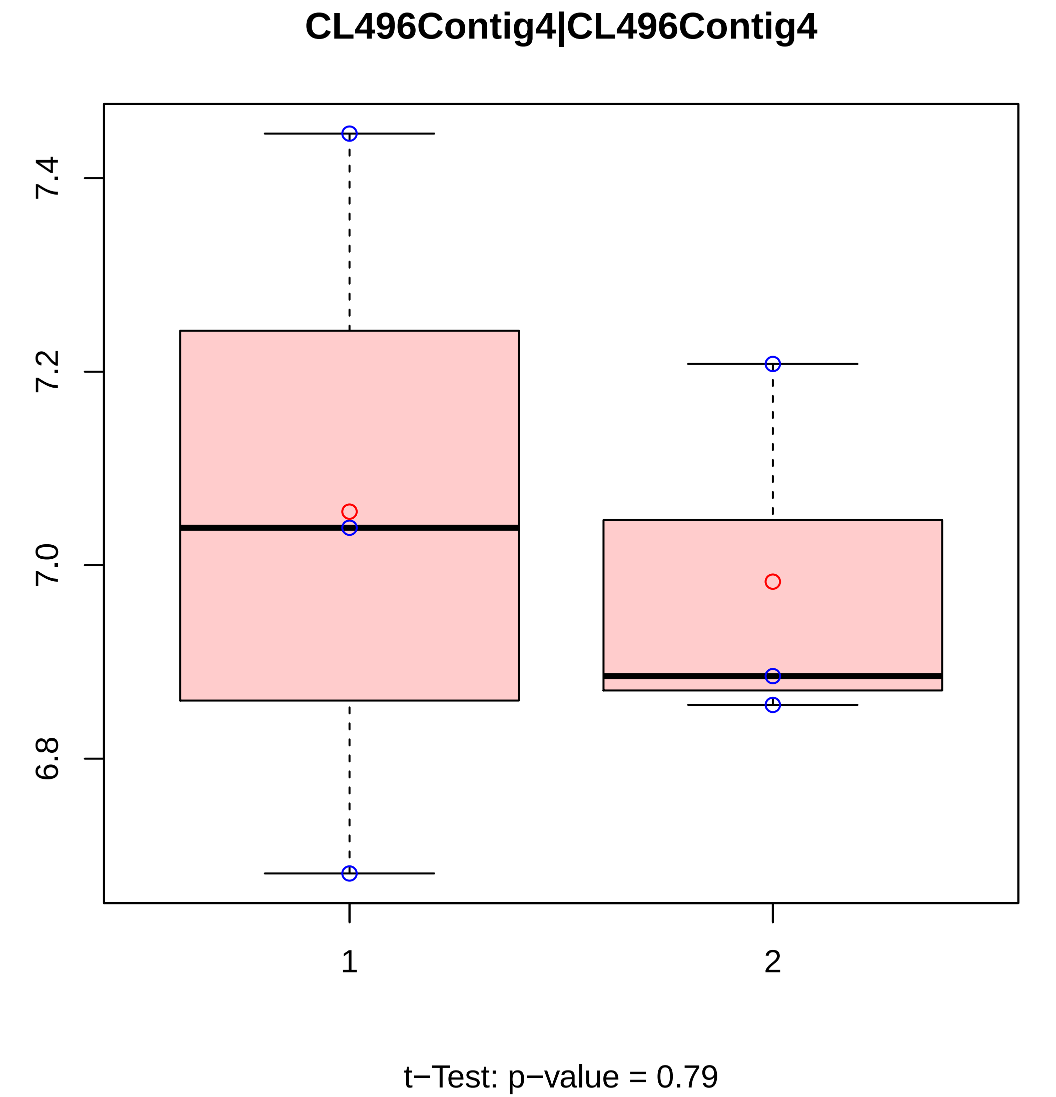
CL496Contig4|CL496Contig4
7.4
7.2
7.0
6.8
1
2
t−Test: p−value = 0.79

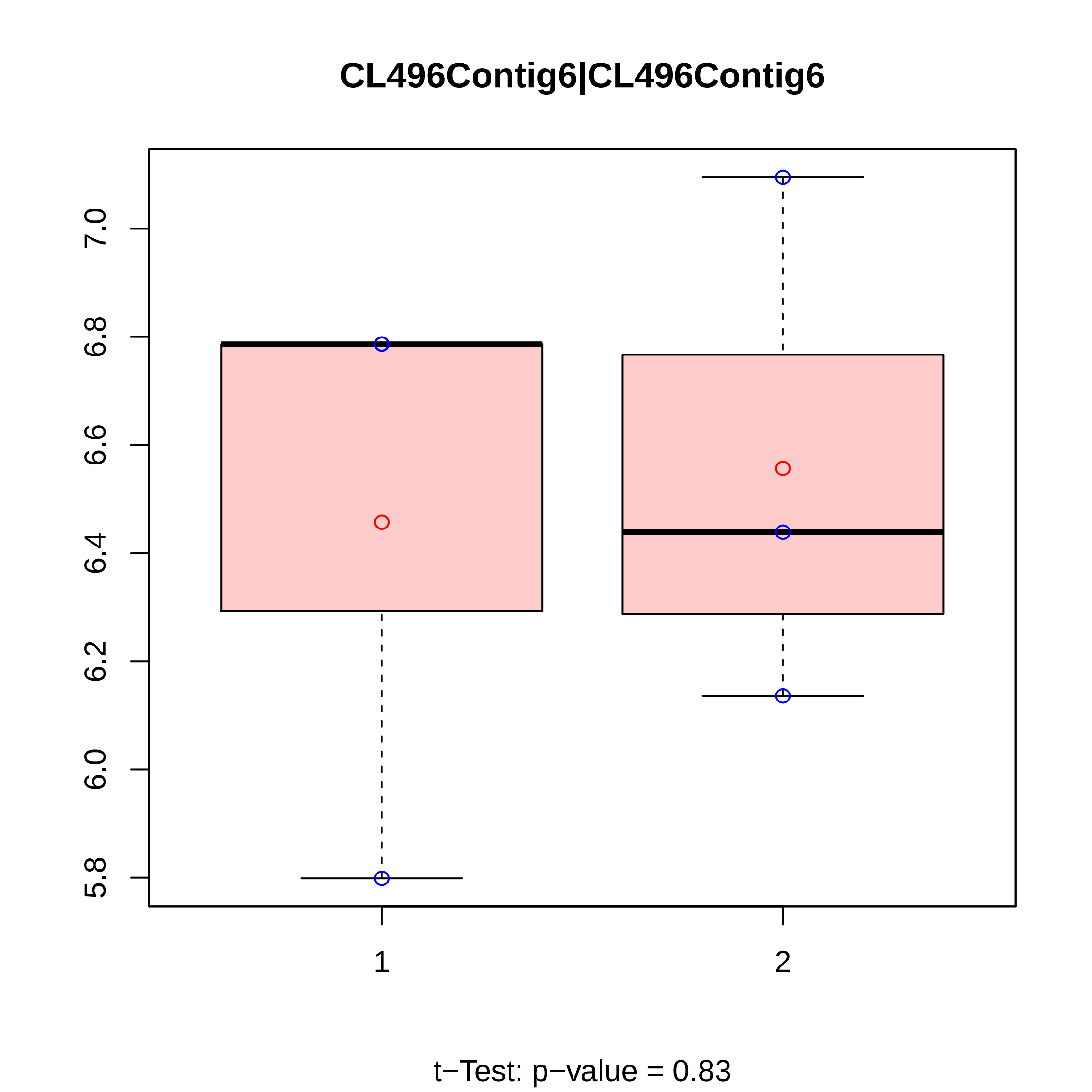
CL496Contig6|CL496Contig6
7.0
6.8
6.6
6.4
6.2
6.0
5.8
1
2
t−Test: p−value = 0.83

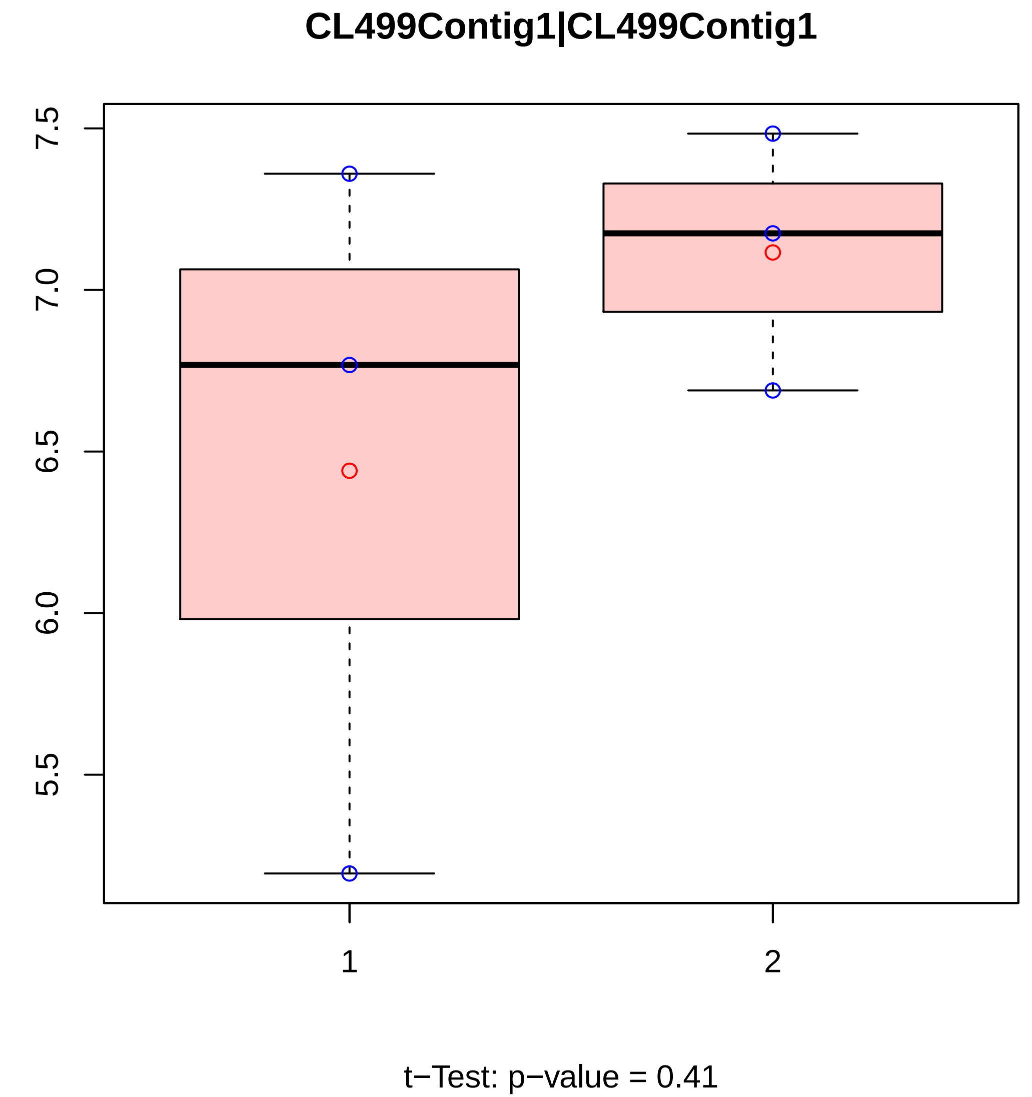
CL499Contig1|CL499Contig1
7.5
7.0
6.5
6.0
5.5
1
2
t−Test: p−value = 0.41

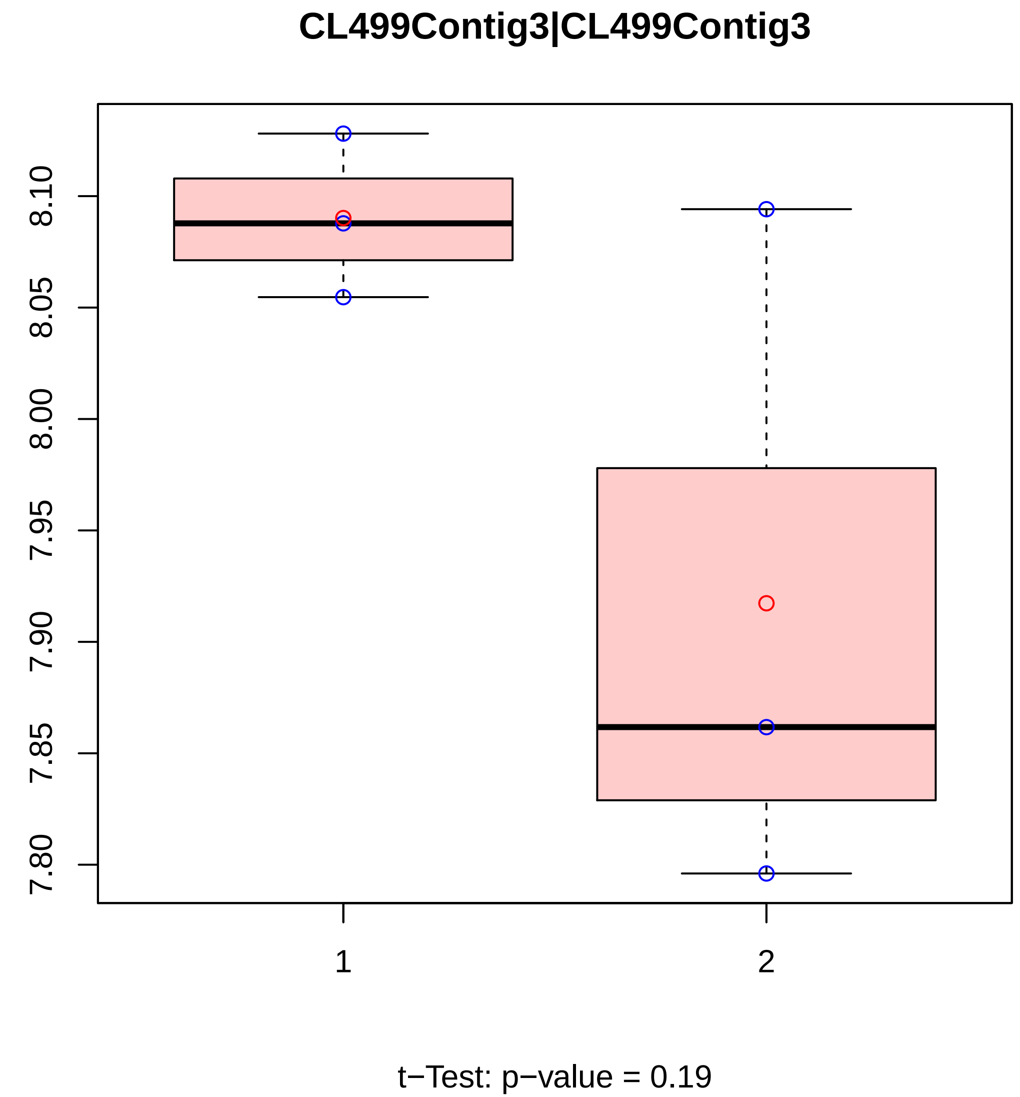
CL499Contig3|CL499Contig3
8.10
8.05
8.00
7.95
7.90
7.85
7.80
1
2
t−Test: p−value = 0.19

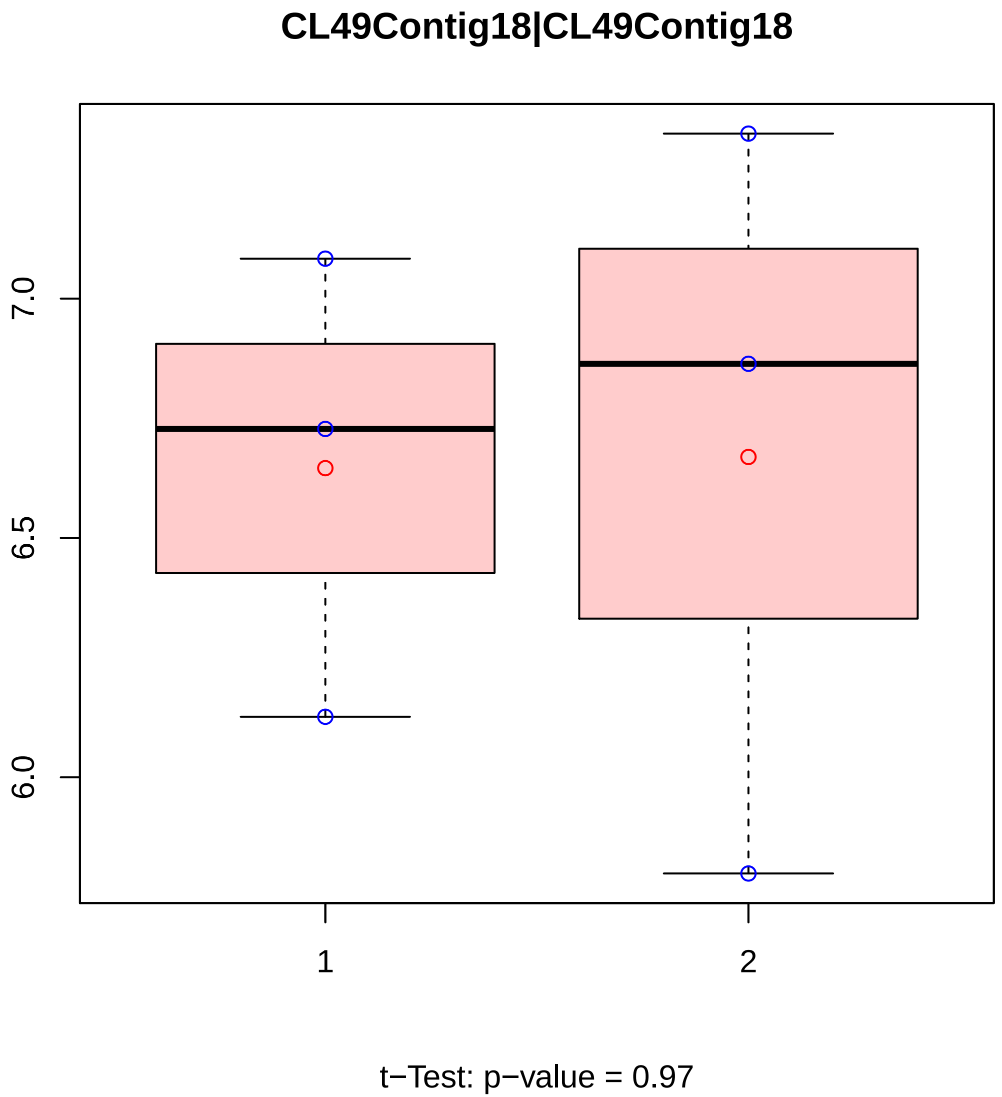
CL49Contig18|CL49Contig18
7.0
6.5
6.0
1
2
t−Test: p−value = 0.97

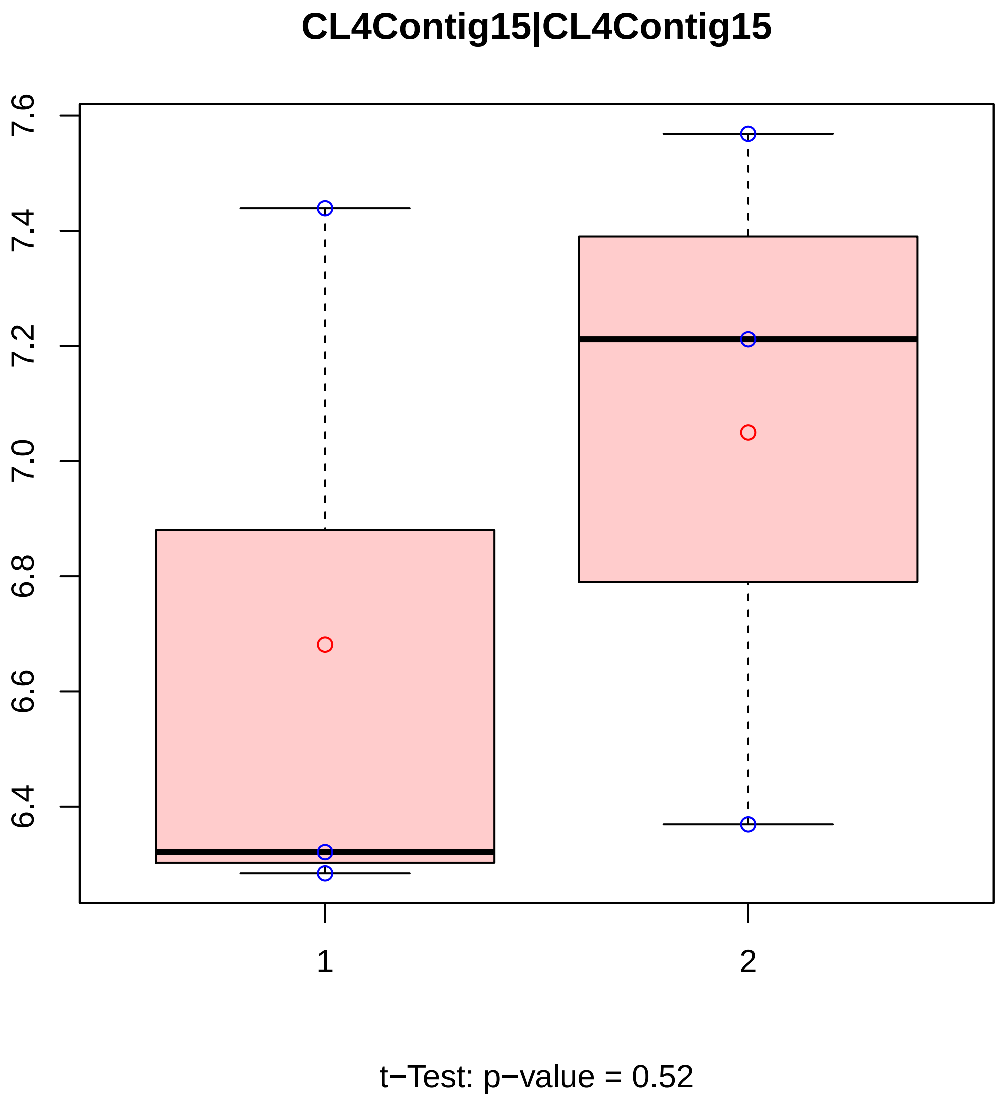
CL4Contig15|CL4Contig15
7.6
7.4
7.2
7.0
6.8
6.6
6.4
1
2
t−Test: p−value = 0.52

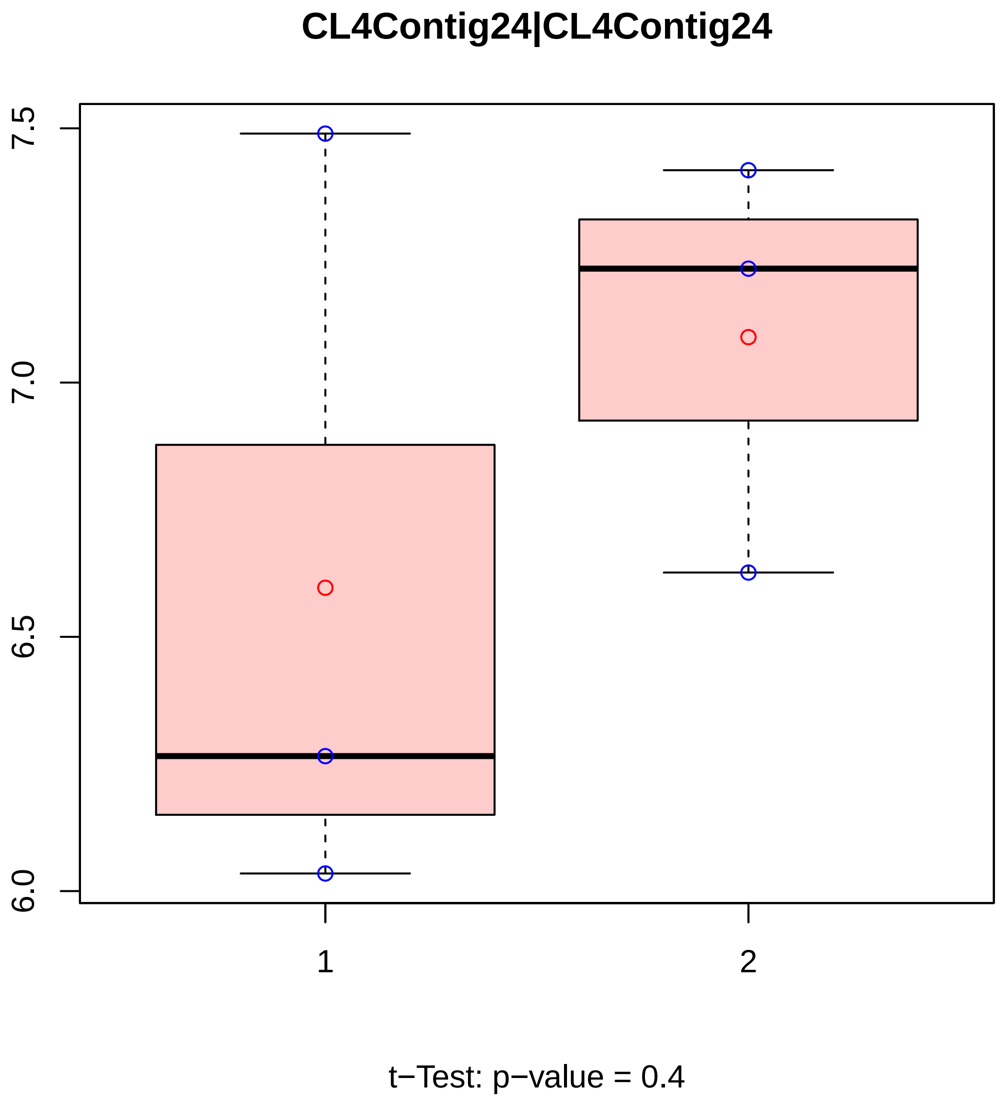

CL4Contig24|CL4Contig24
7.5
7.0
6.5
6.0
1
2
t−Test: p−value = 0.4

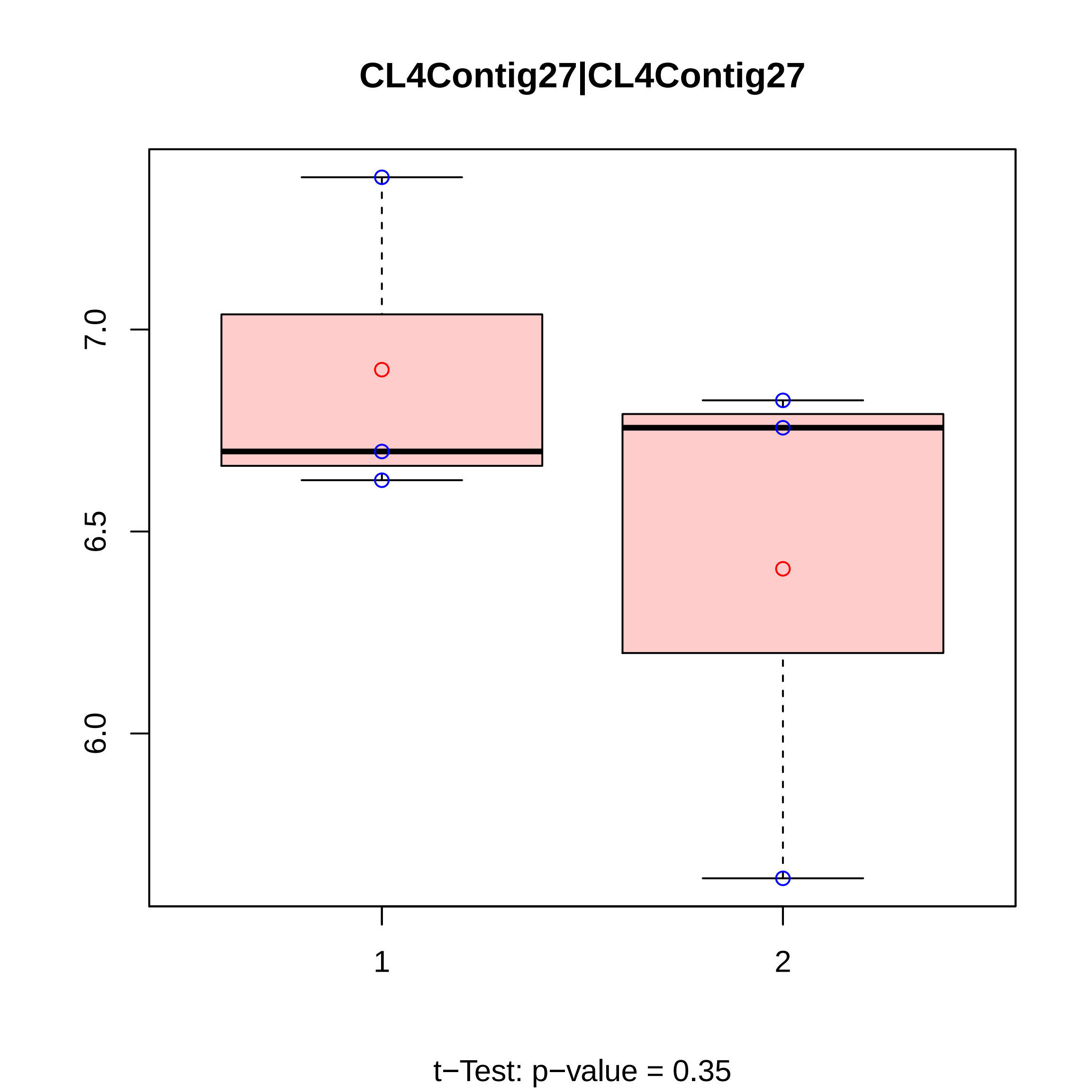

CL4Contig27|CL4Contig27
7.0
6.5
6.0
1
2
t−Test: p−value = 0.35

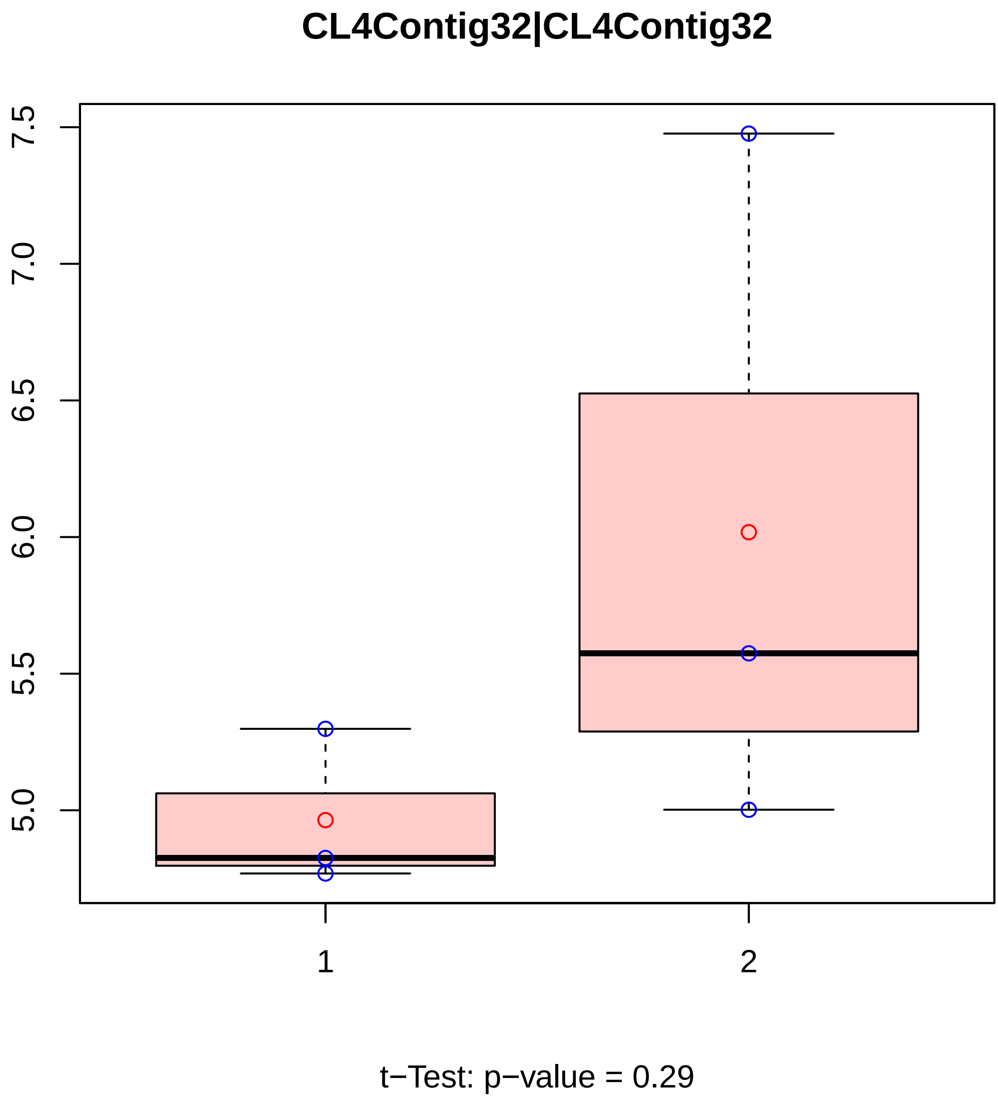
CL4Contig32|CL4Contig32
7.5
7.0
6.5
6.0
5.5
5.0
1
2
t−Test: p−value = 0.29

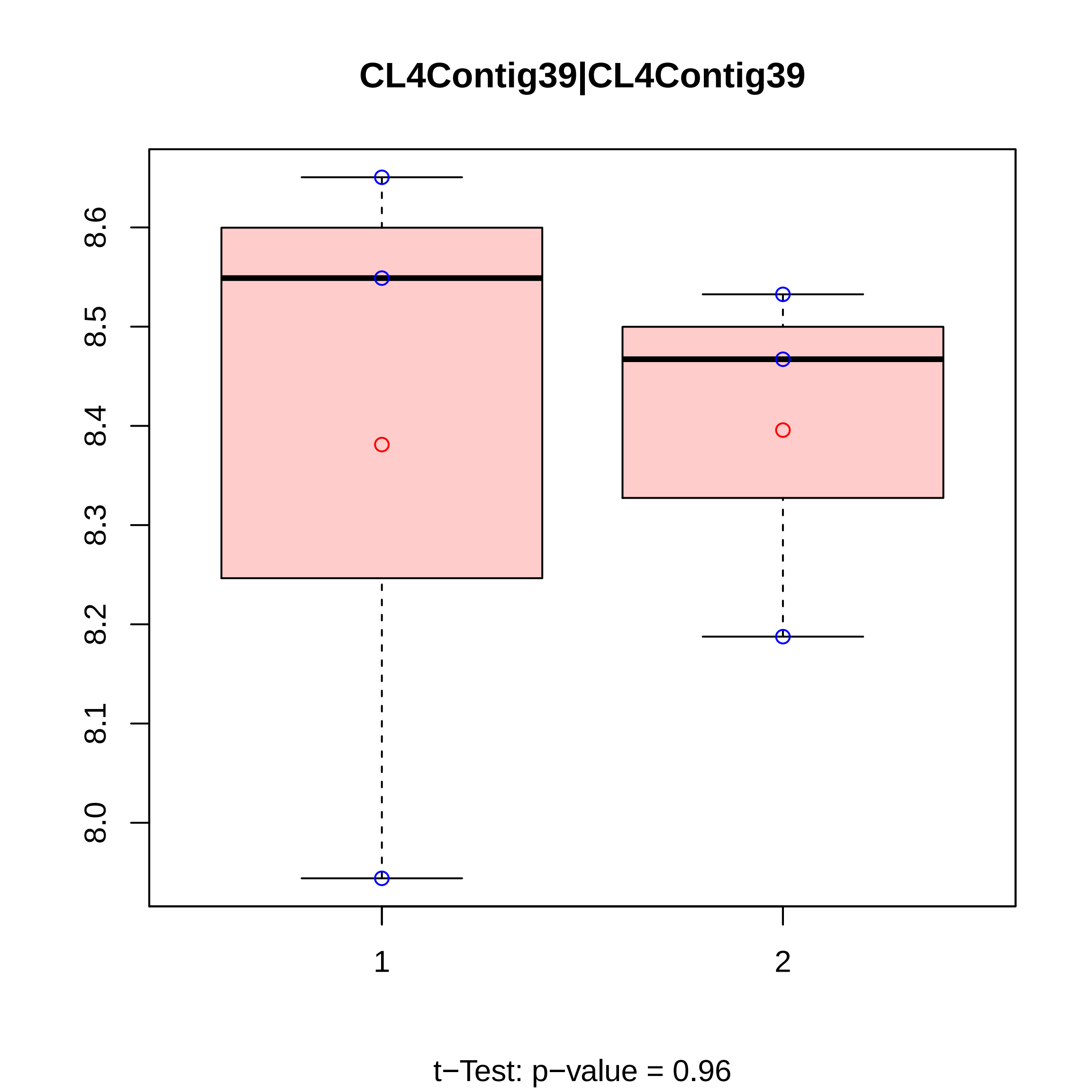

CL4Contig39|CL4Contig39
8.0
8.1
8.2
8.3
8.4
8.5
8.6
1
2
t−Test: p−value = 0.96

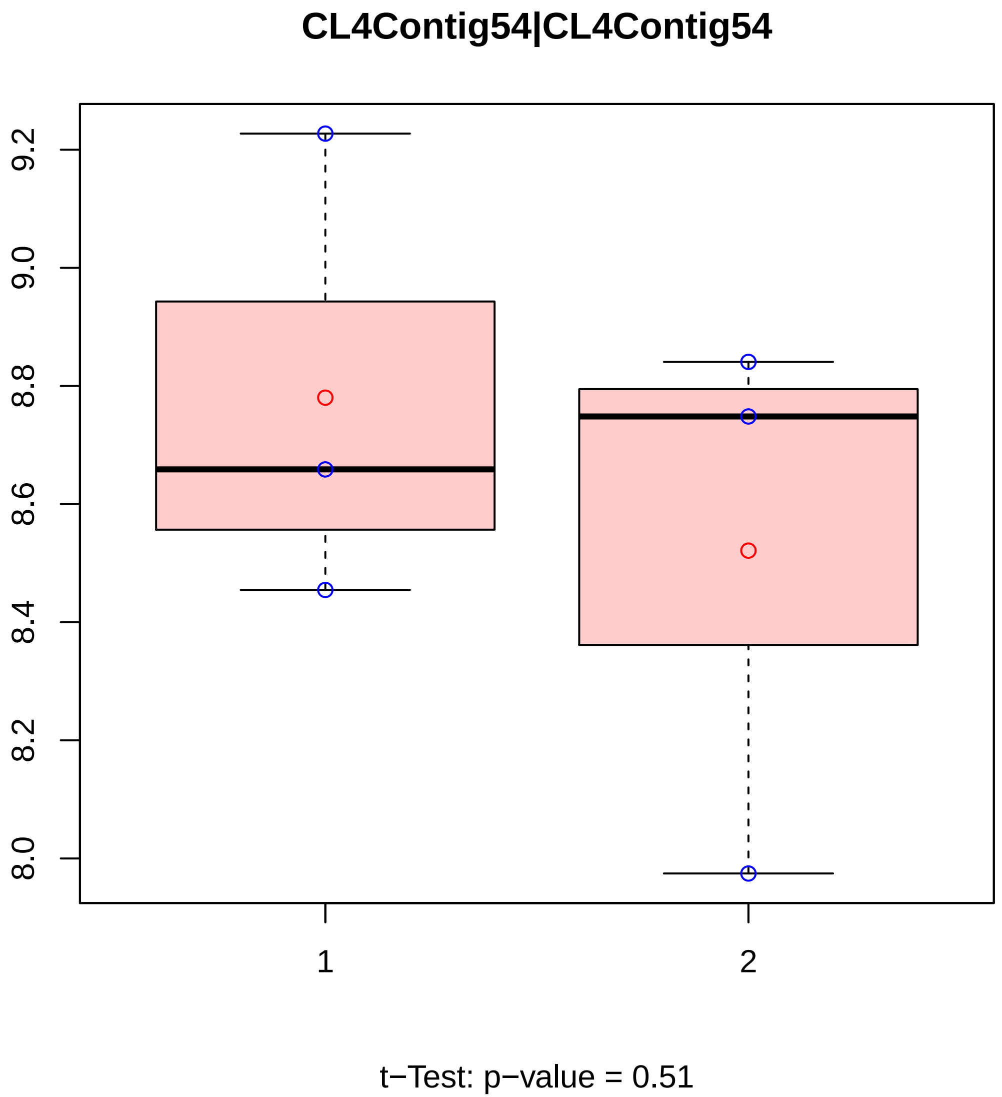
CL4Contig54|CL4Contig54
9.2
9.0
8.8
8.6
8.4
8.2
8.0
1
2
t−Test: p−value = 0.51

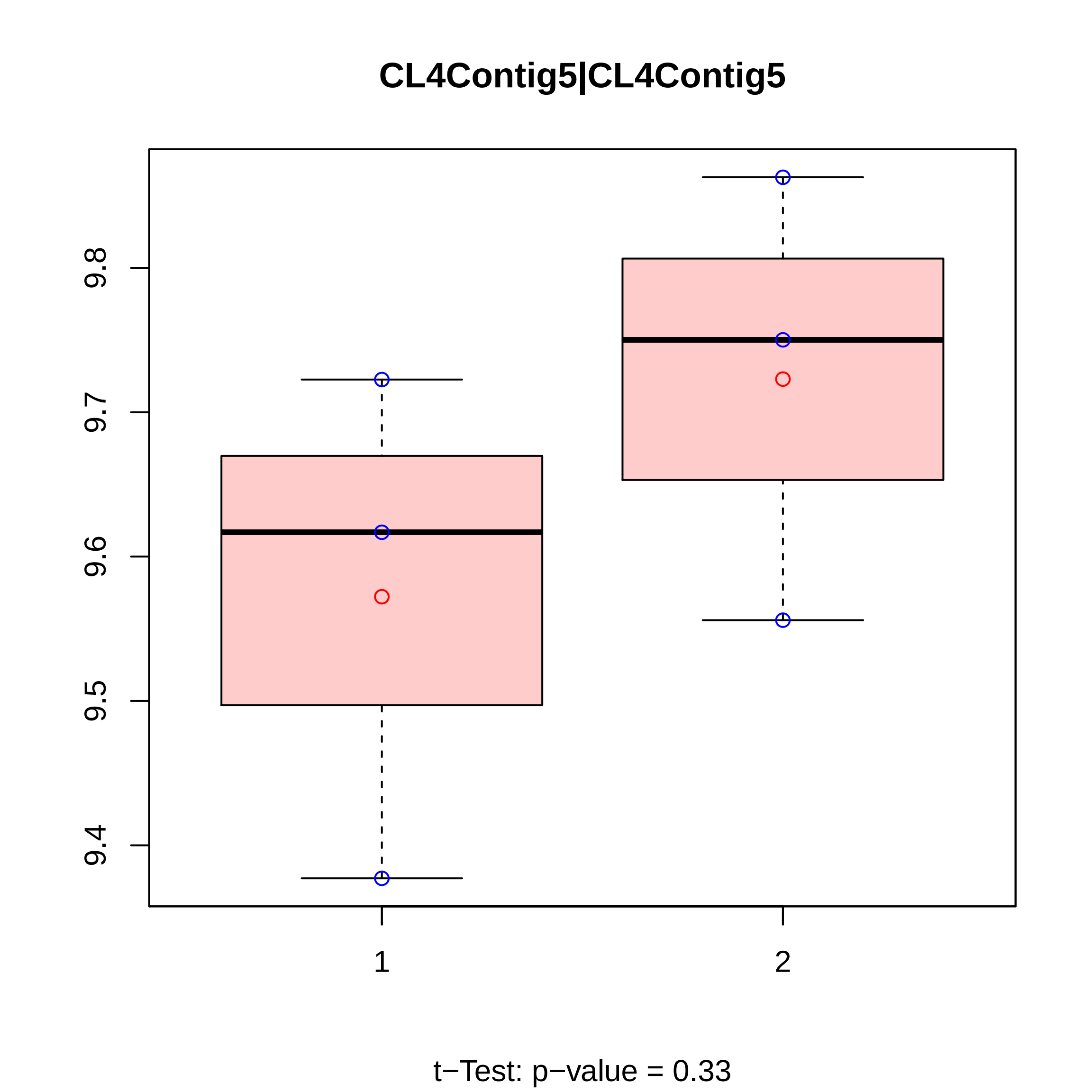

CL4Contig5|CL4Contig5
9.4
9.5
9.6
9.7
9.8
1
2
t−Test: p−value = 0.33

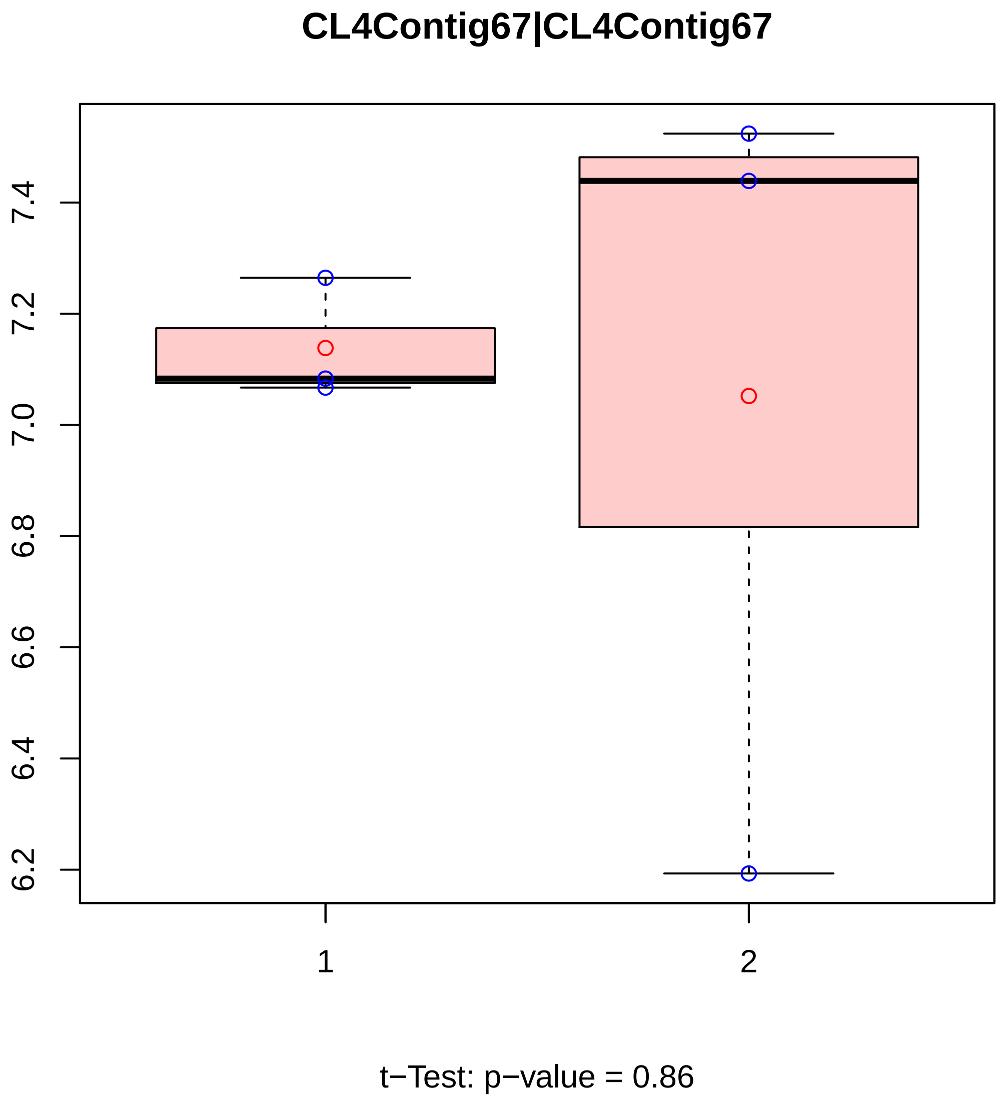
CL4Contig67|CL4Contig67
7.4
7.2
7.0
6.8
6.6
6.4
6.2
1
2
t−Test: p−value = 0.86

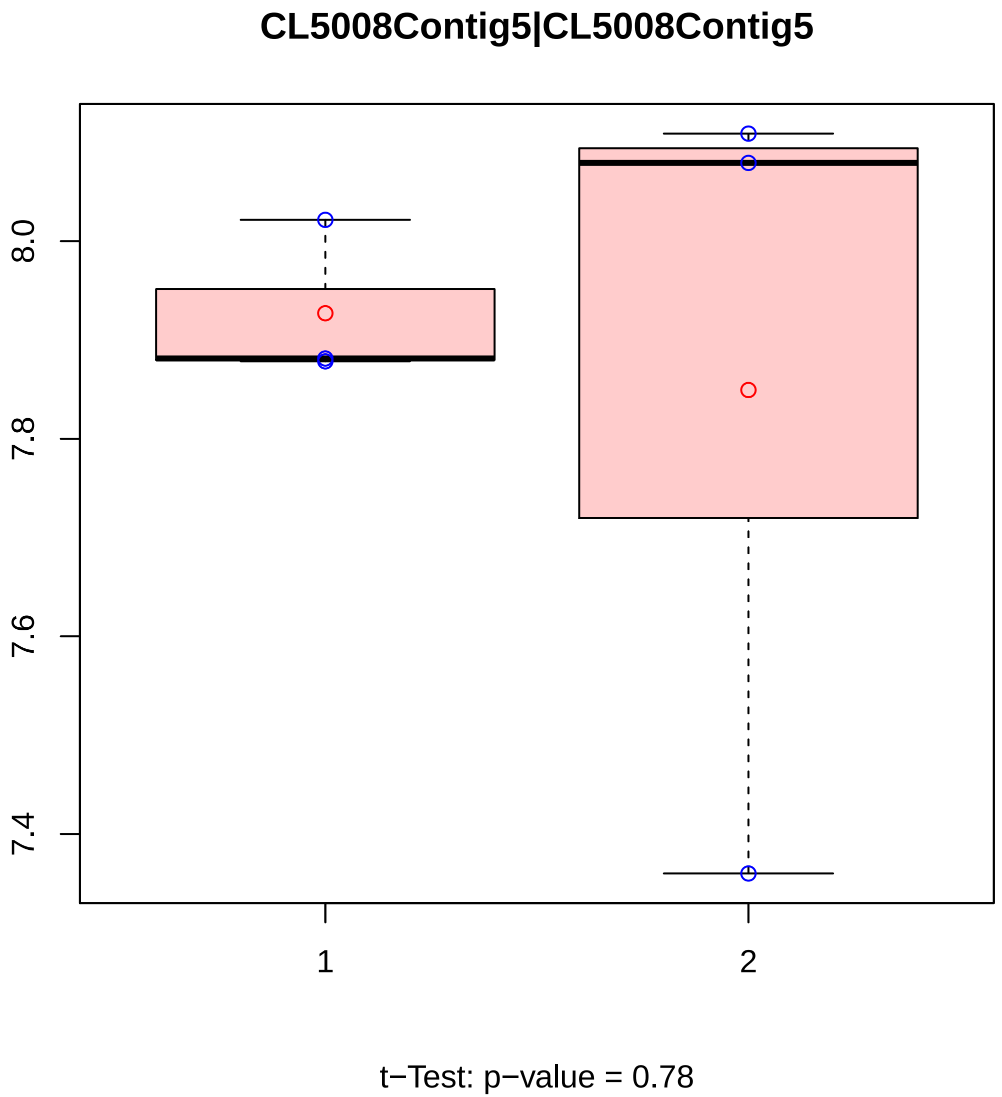

CL5008Contig5|CL5008Contig5
8.0
7.8
7.6
7.4
1
2
t−Test: p−value = 0.78

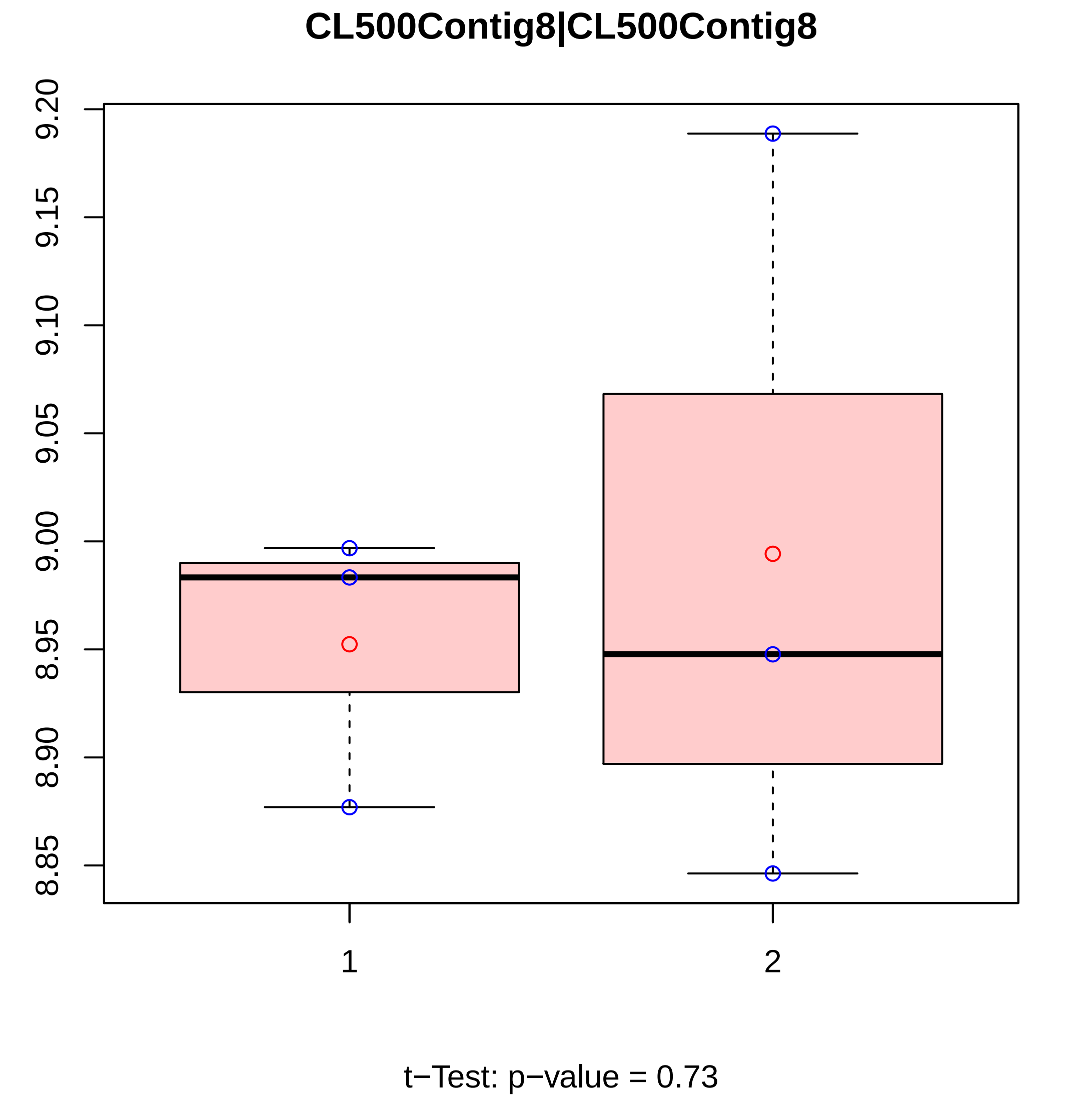
CL500Contig8|CL500Contig8
9.20
9.15
9.10
9.05
9.00
8.95
8.90
8.85
1
2
t−Test: p−value = 0.73

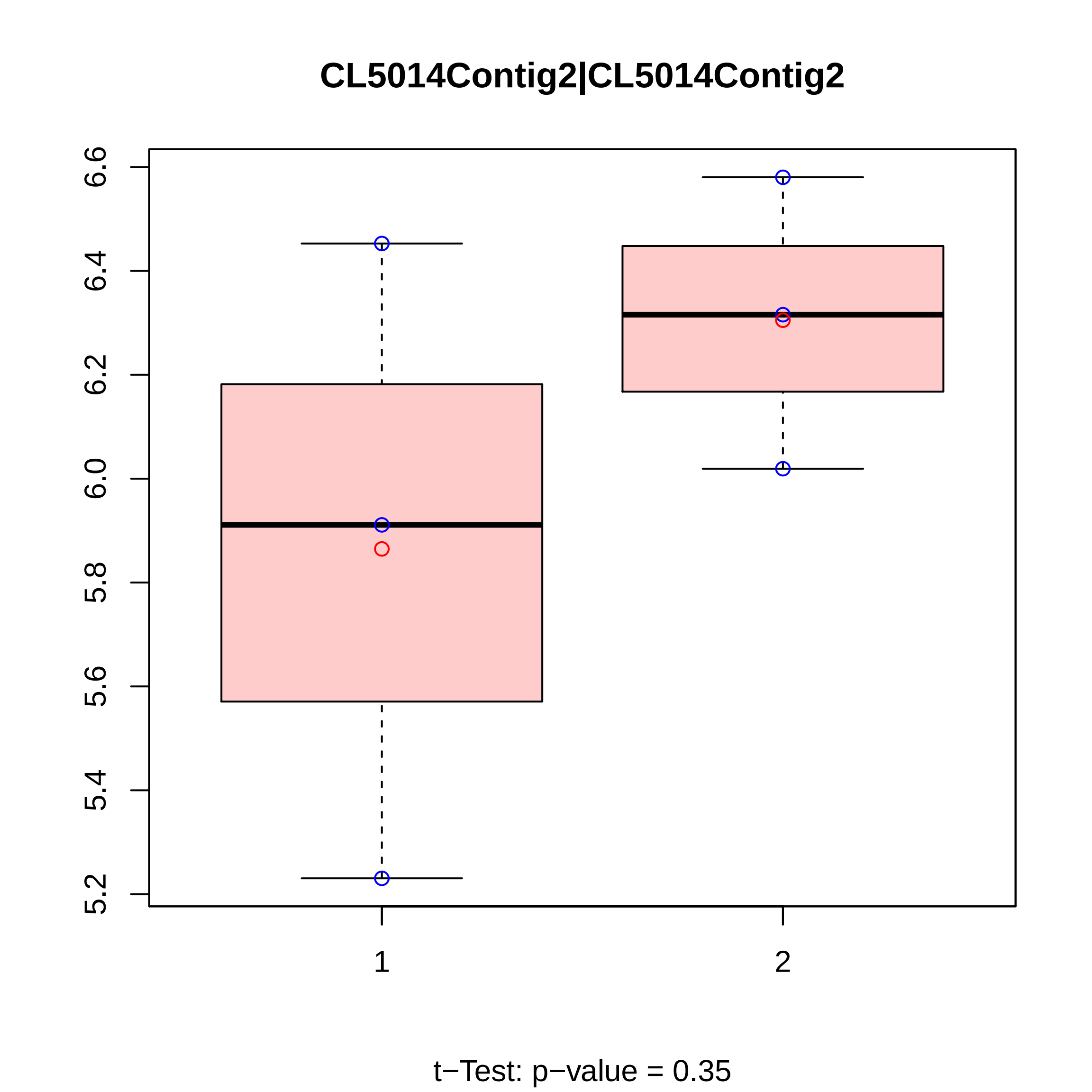
CL5014Contig2|CL5014Contig2
6.6
6.4
6.2
6.0
5.8
5.6
5.4
5.2
1
2
t−Test: p−value = 0.35

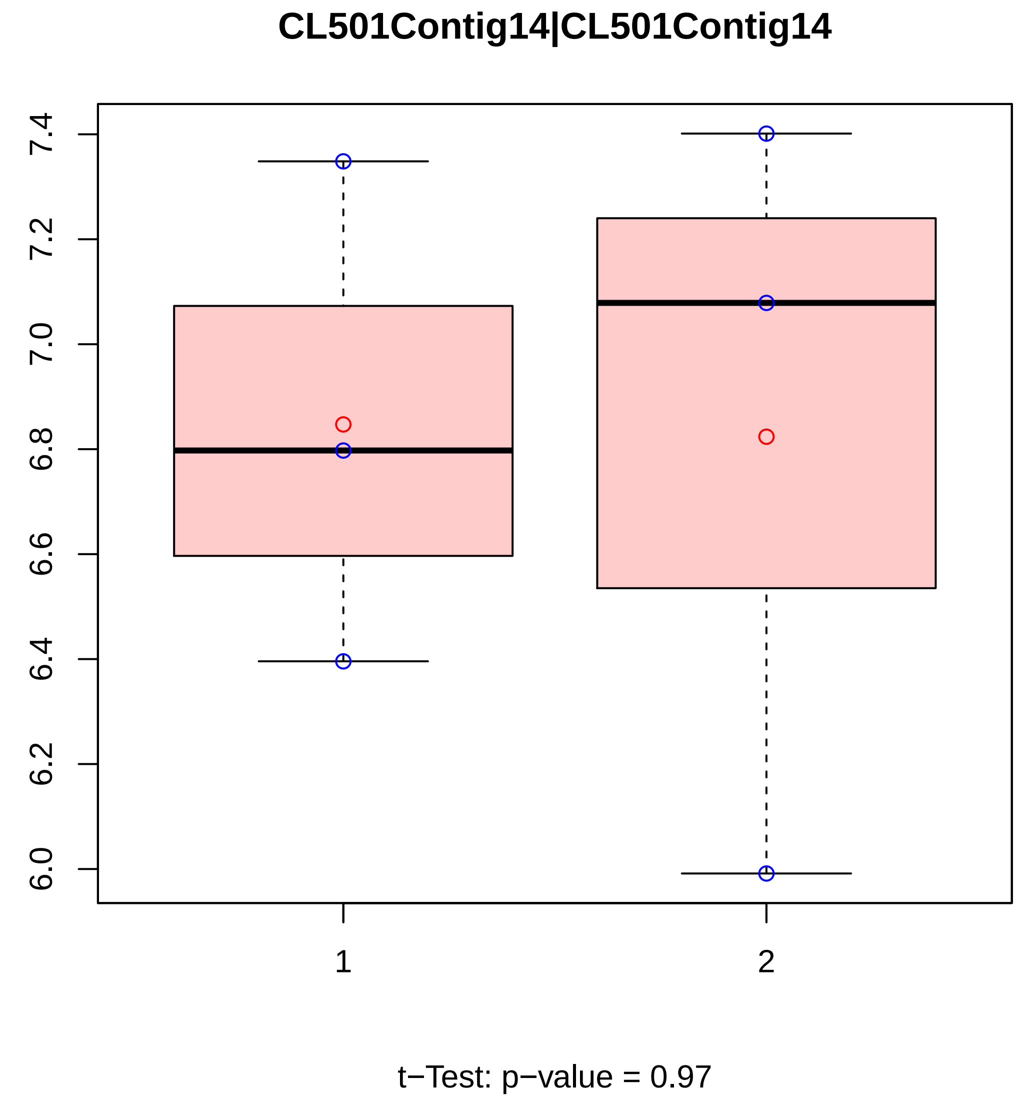
CL501Contig14|CL501Contig14
7.4
7.2
7.0
6.8
6.6
6.4
6.2
6.0
1
2
t−Test: p−value = 0.97

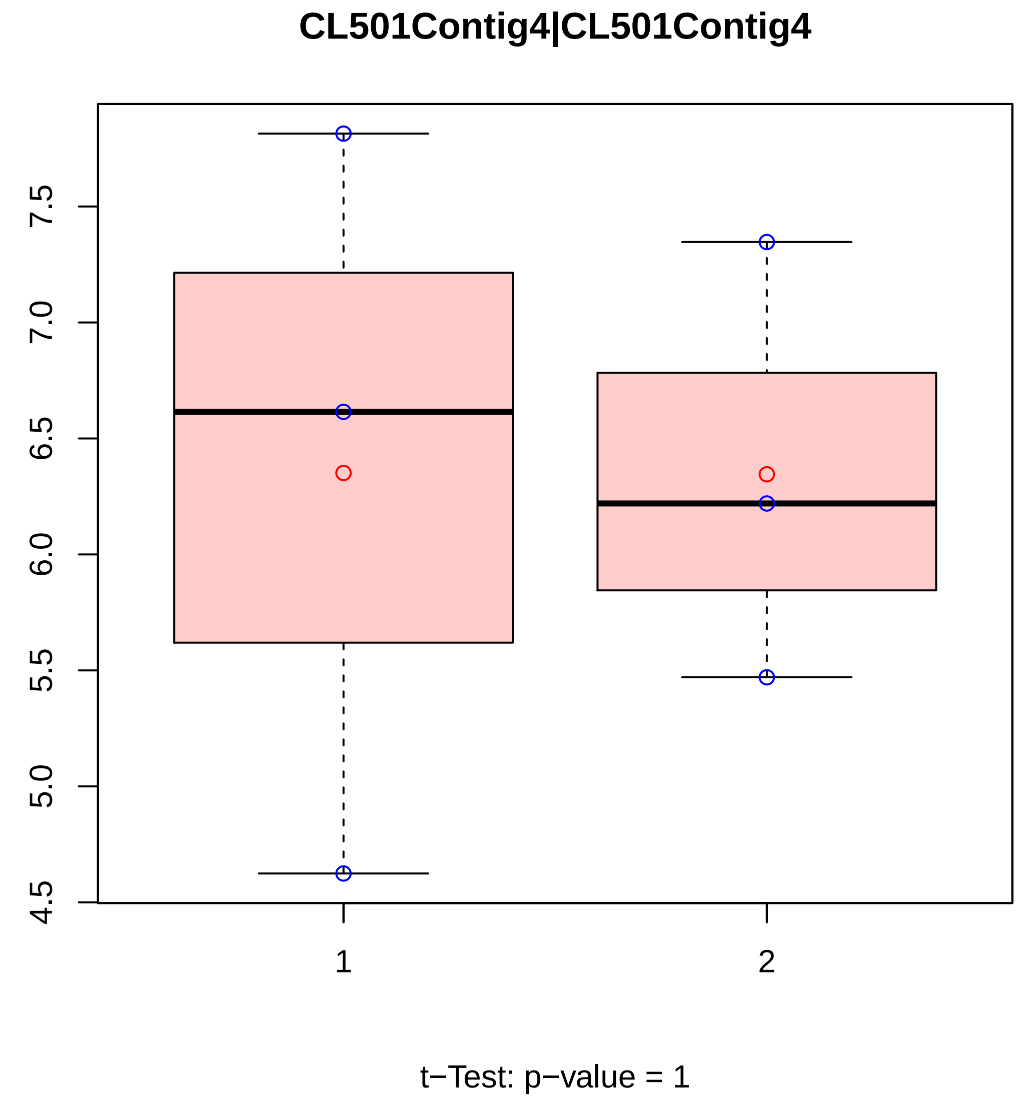
CL501Contig4|CL501Contig4
4.5
5.0
5.5
6.0
6.5
7.0
7.5
1
2
t−Test: p−value = 1

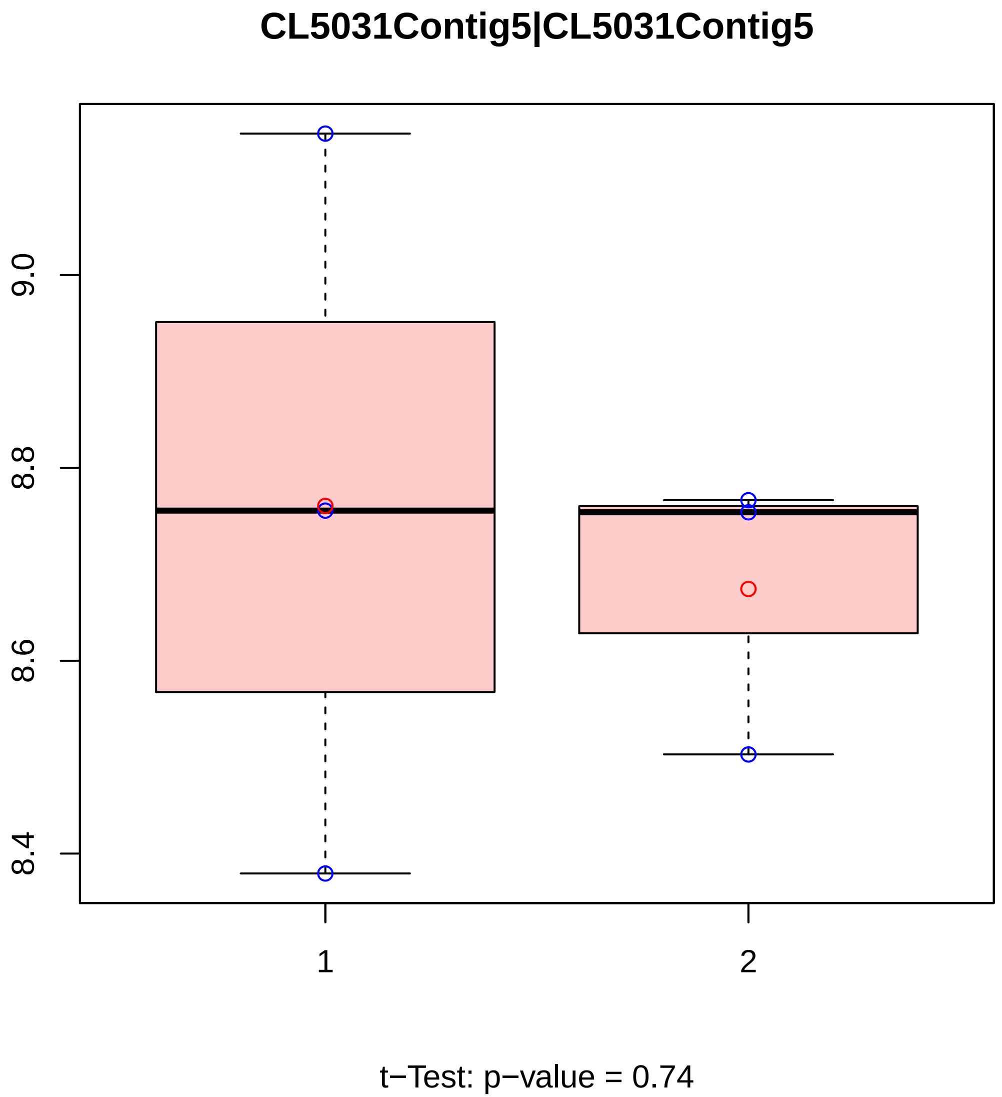

CL5031Contig5|CL5031Contig5
9.0
8.8
8.6
8.4
1
2
t−Test: p−value = 0.74

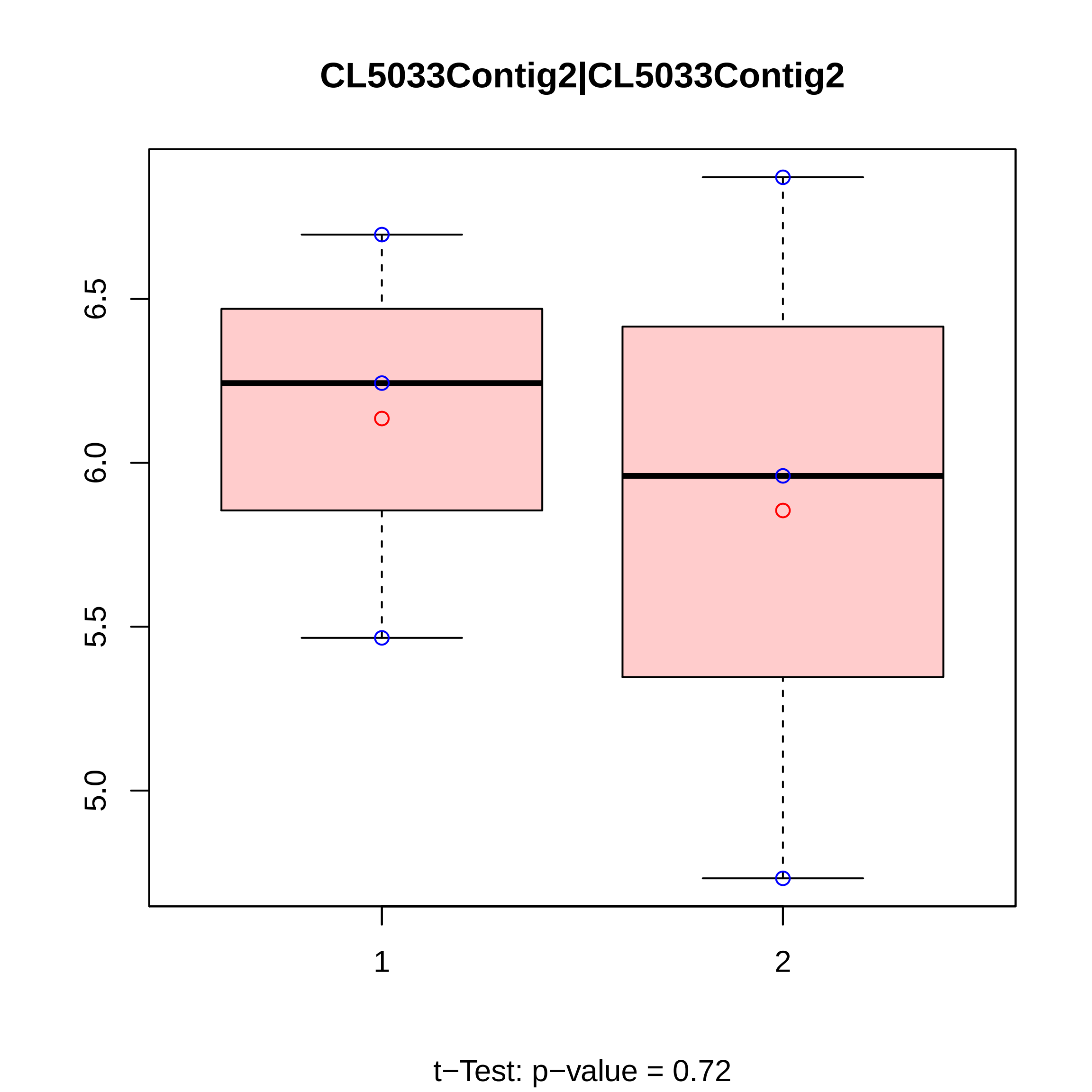

CL5033Contig2|CL5033Contig2
6.5
6.0
5.5
5.0
1
2
t−Test: p−value = 0.72

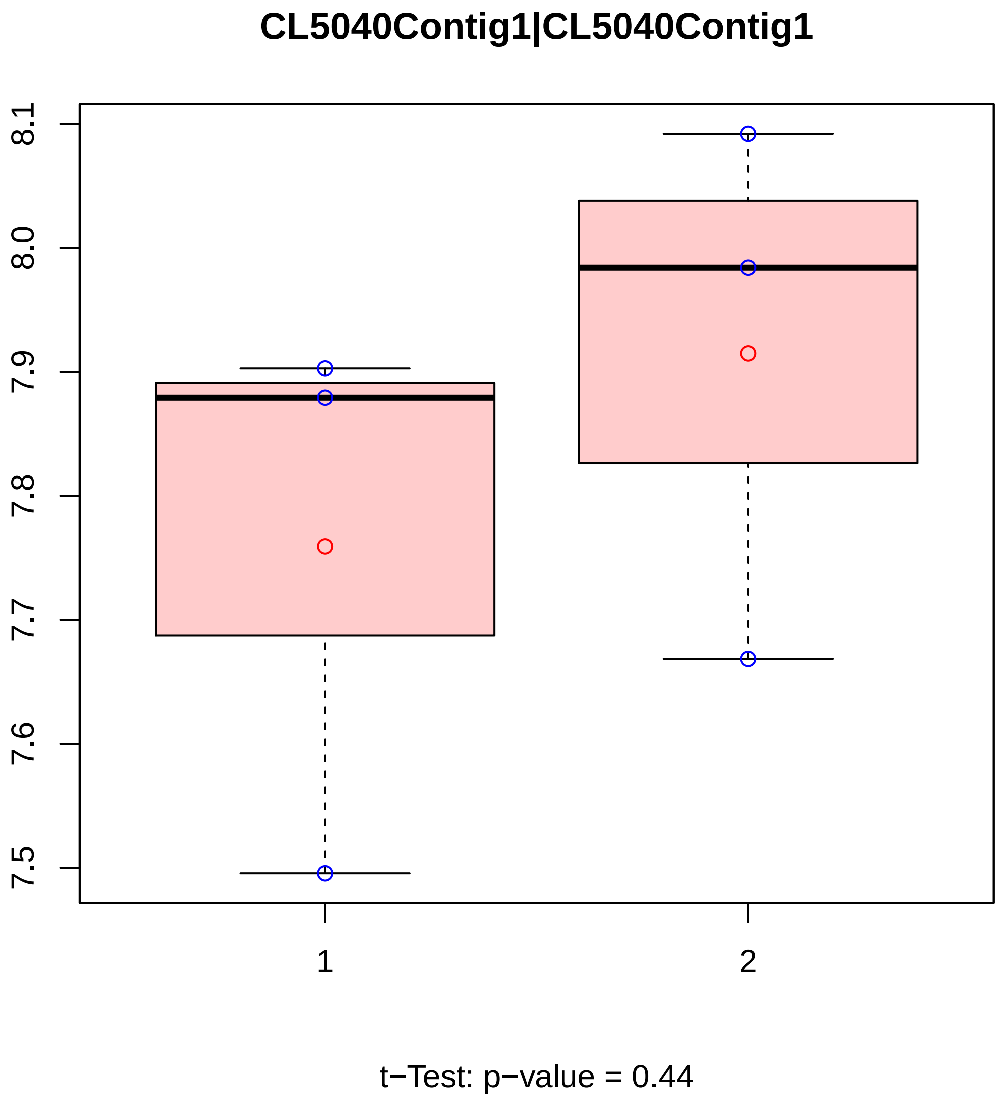

CL5040Contig1|CL5040Contig1
8.1
8.0
7.9
7.8
7.7
7.6
7.5
1
2
t−Test: p−value = 0.44

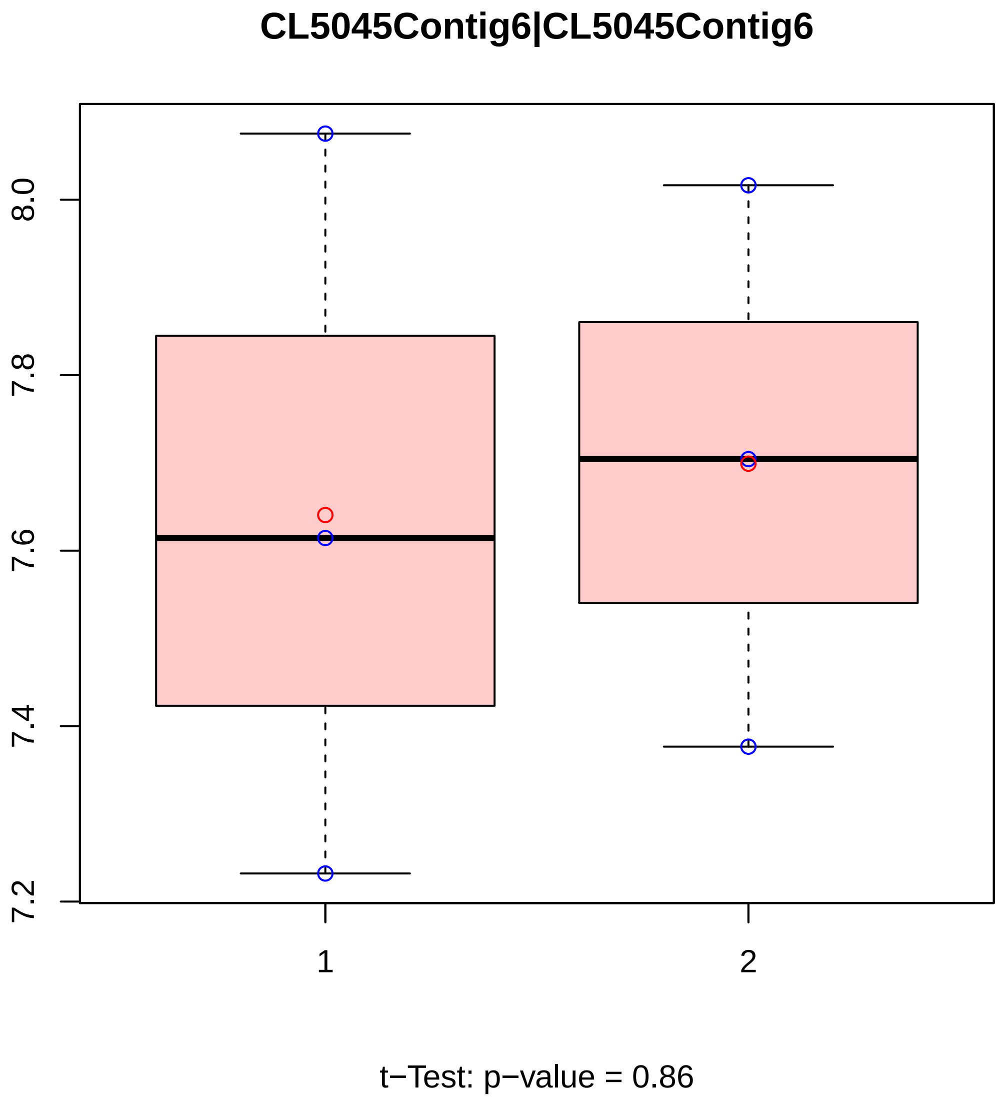

CL5045Contig6|CL5045Contig6
7.2
7.4
7.6
7.8
8.0
1
2
t−Test: p−value = 0.86

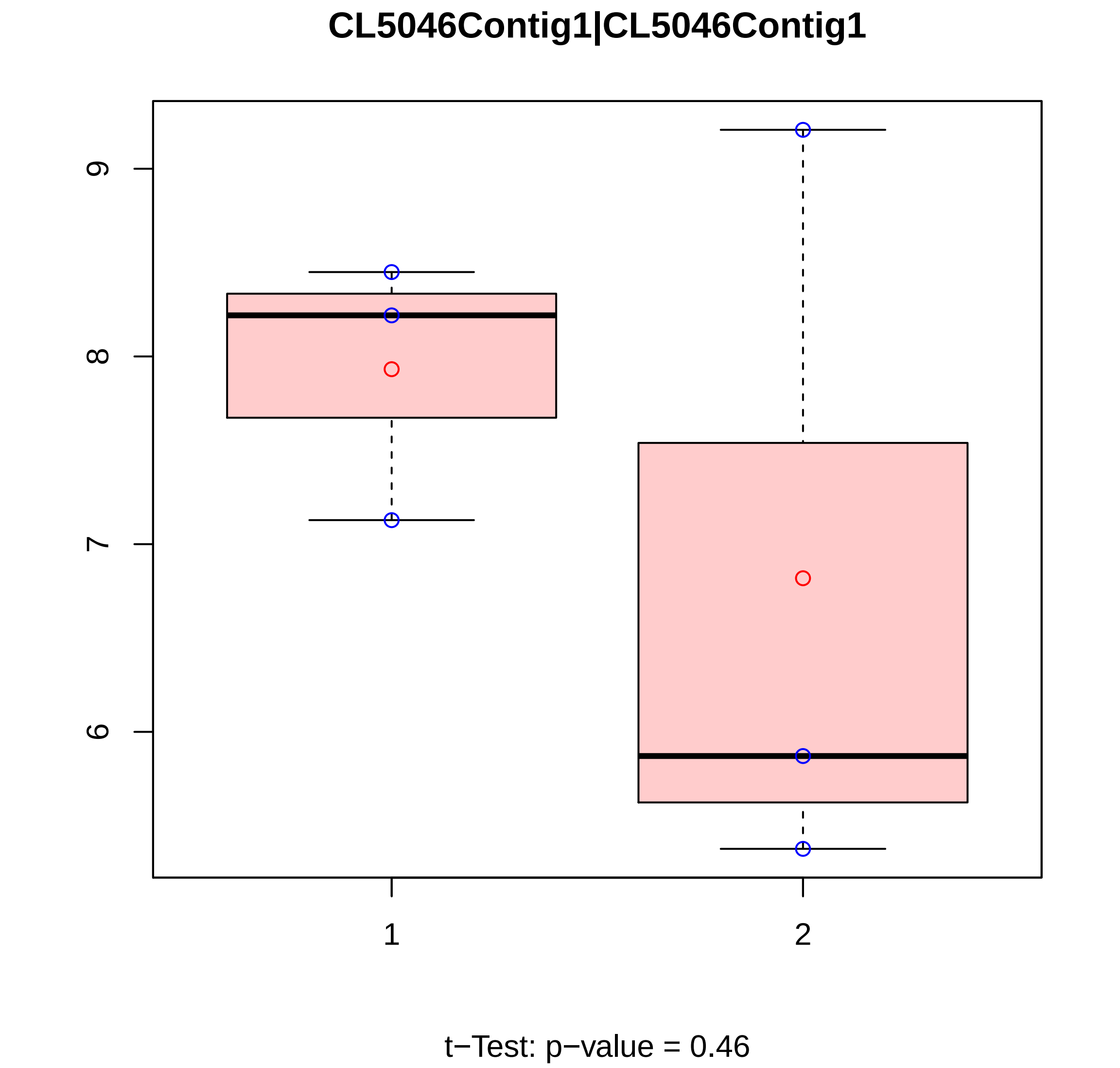

CL5046Contig1|CL5046Contig1
9
8
7
6
1
2
t−Test: p−value = 0.46

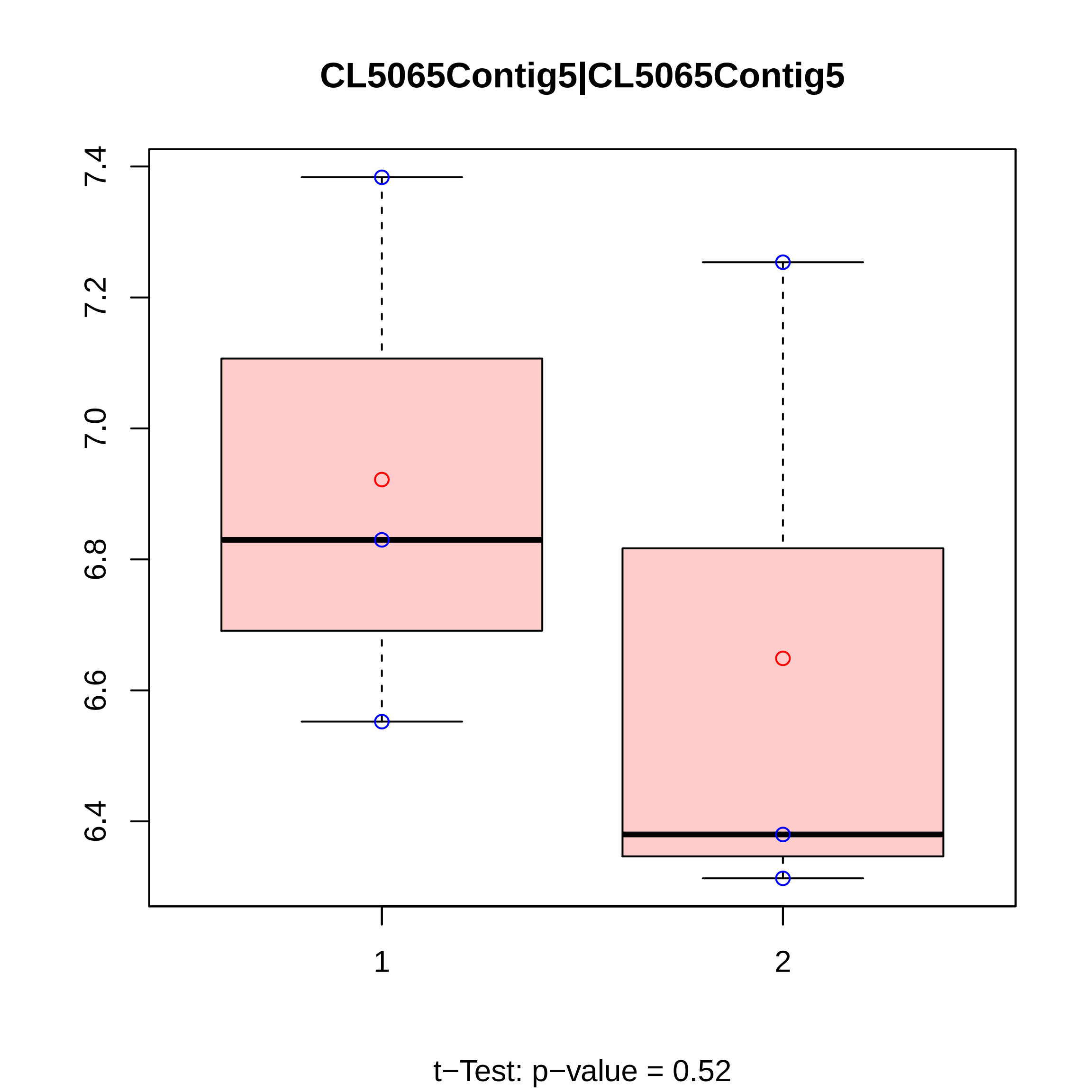
CL5065Contig5|CL5065Contig5
7.4
7.2
7.0
6.8
6.6
6.4
1
2
t−Test: p−value = 0.52

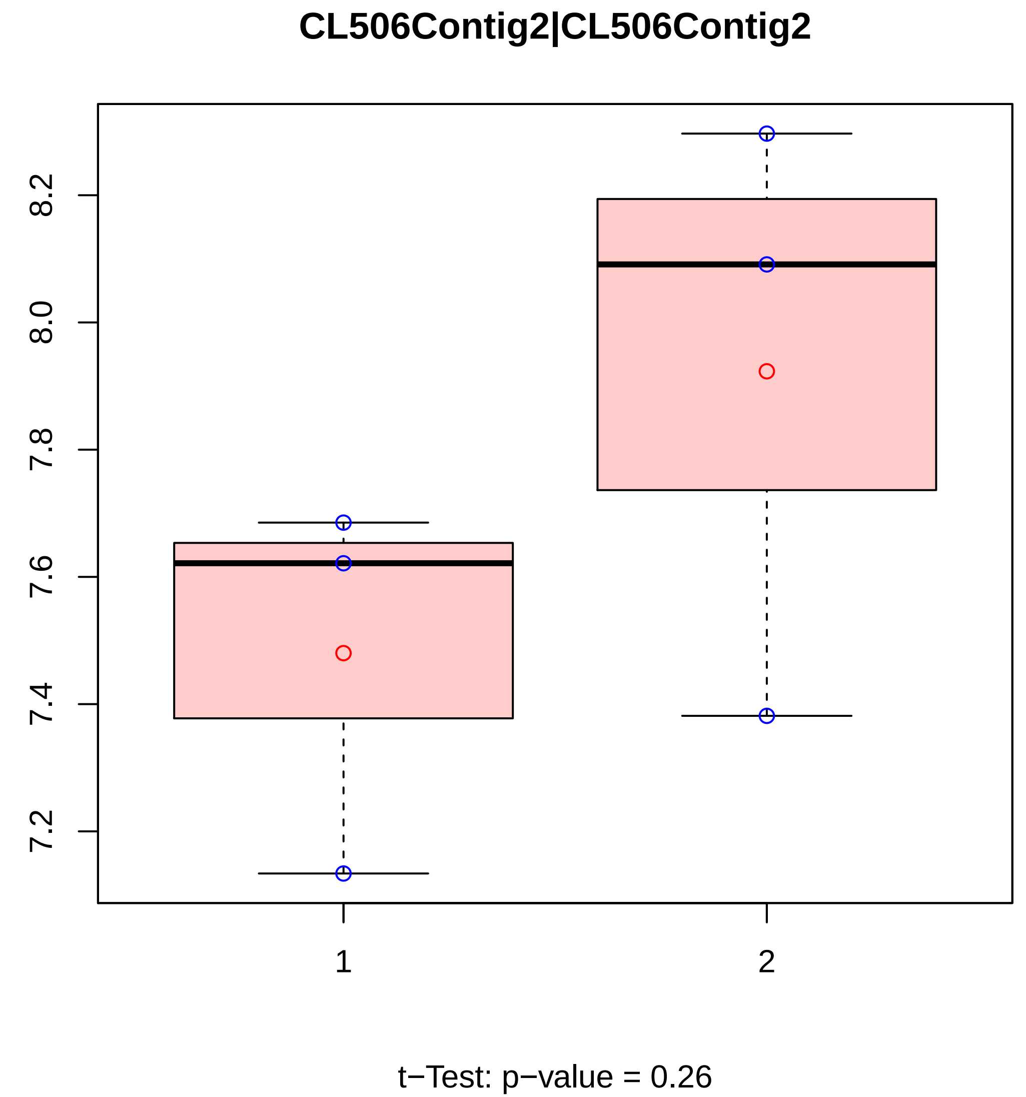
CL506Contig2|CL506Contig2
8.2
8.0
7.8
7.6
7.4
7.2
1
2
t−Test: p−value = 0.26

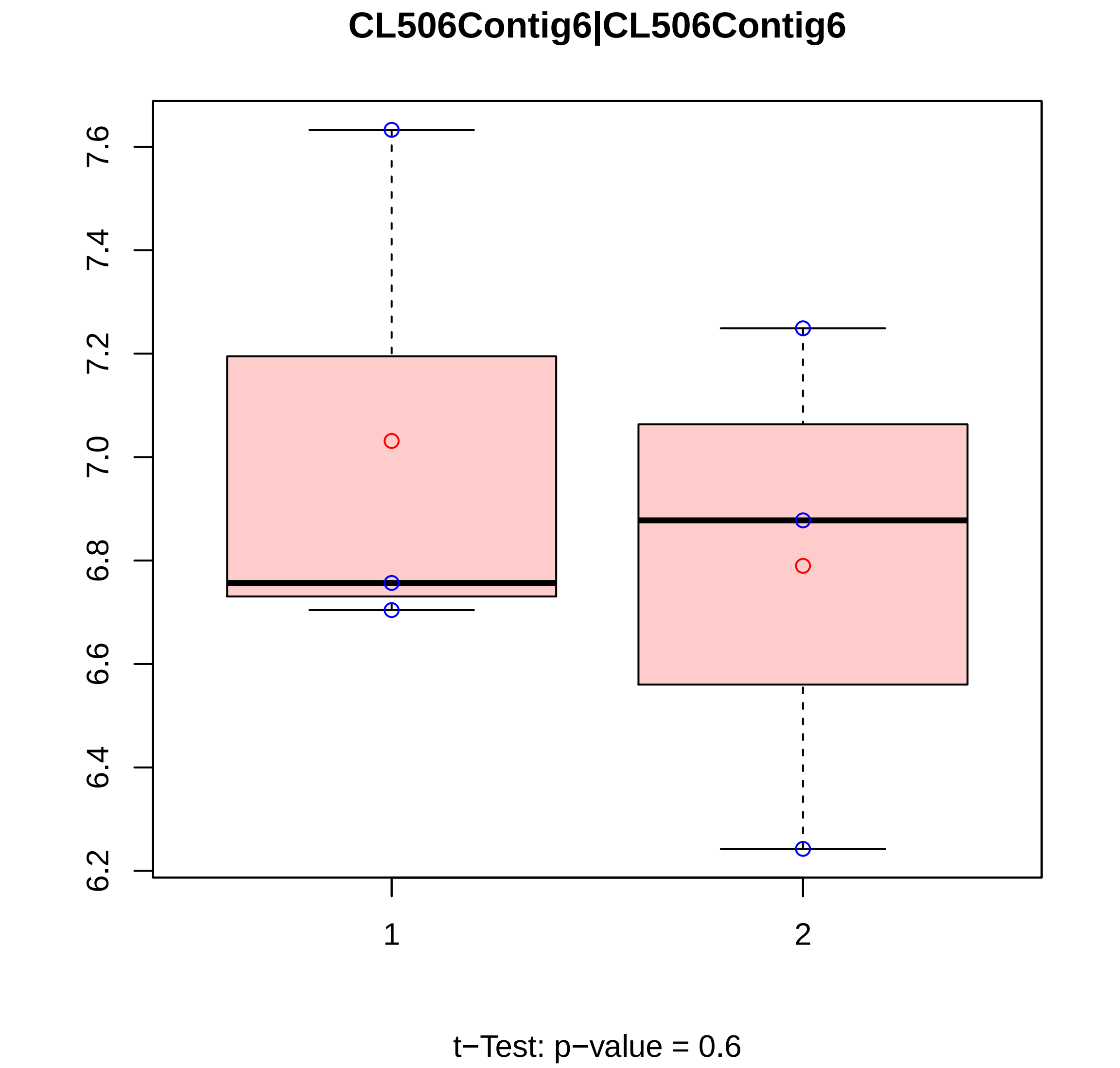
CL506Contig6|CL506Contig6
7.6
7.4
7.2
7.0
6.8
6.6
6.4
6.2
1
2
t−Test: p−value = 0.6

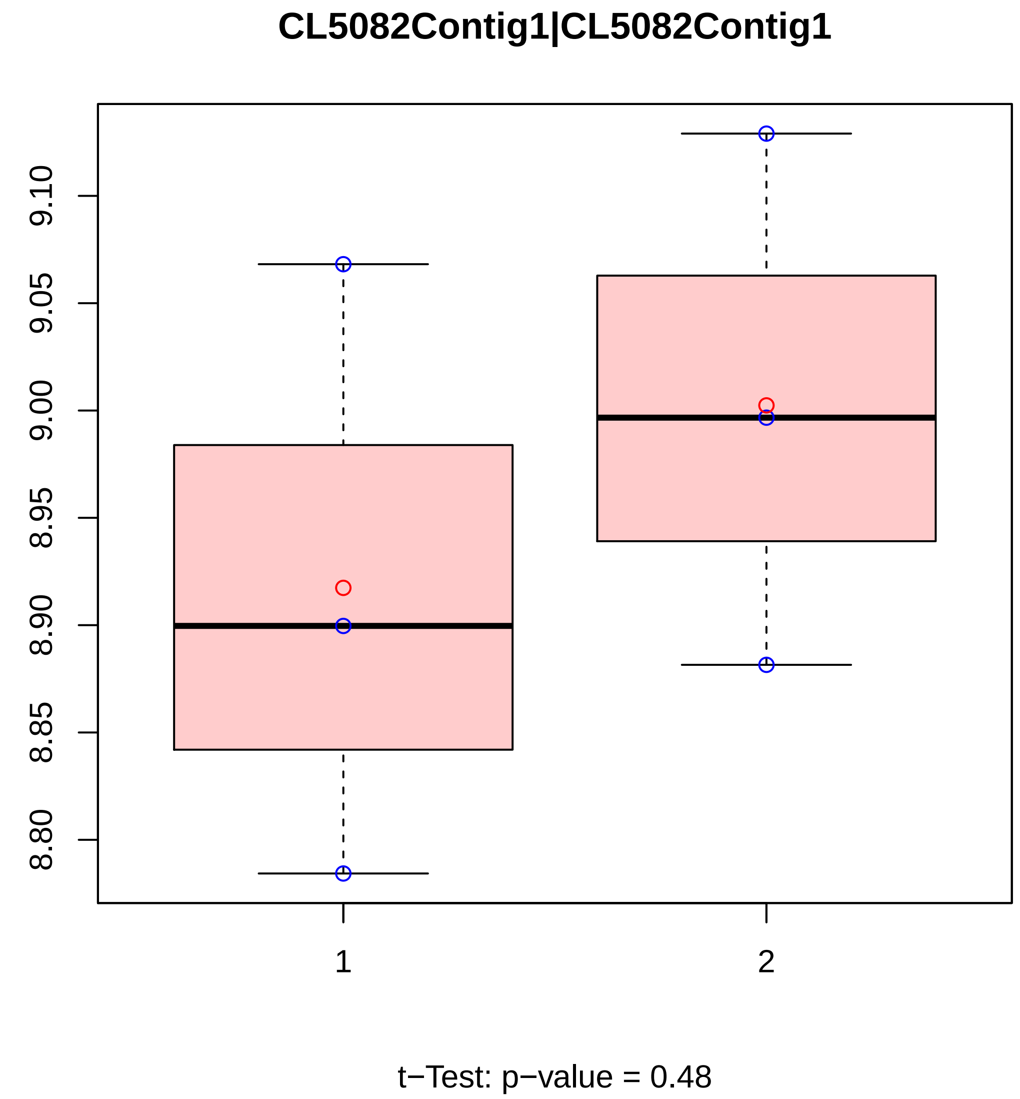
CL5082Contig1|CL5082Contig1
9.10
9.05
9.00
8.95
8.90
8.85
8.80
1
2
t−Test: p−value = 0.48

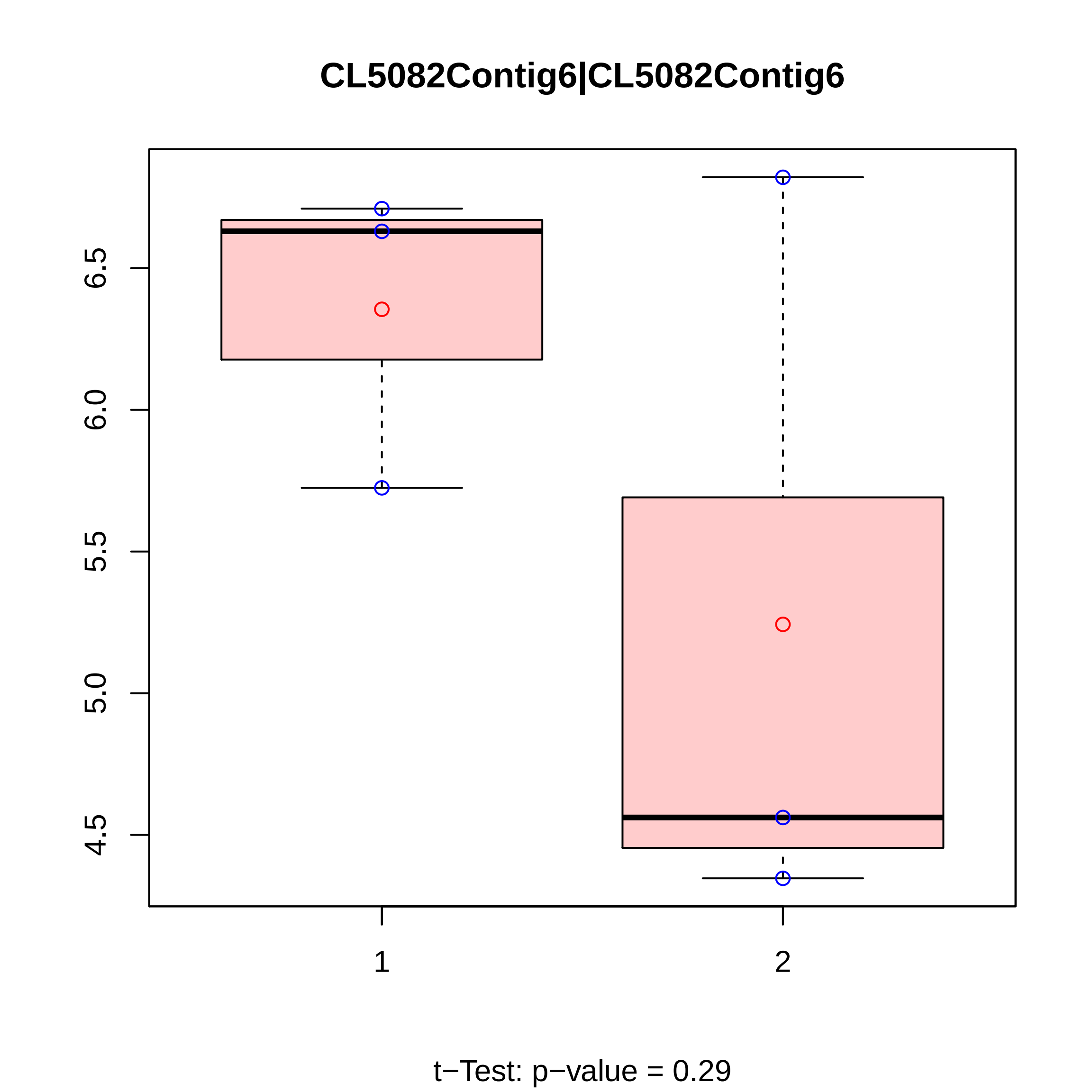
CL5082Contig6|CL5082Contig6
6.5
6.0
5.5
5.0
4.5
1
2
t−Test: p−value = 0.29

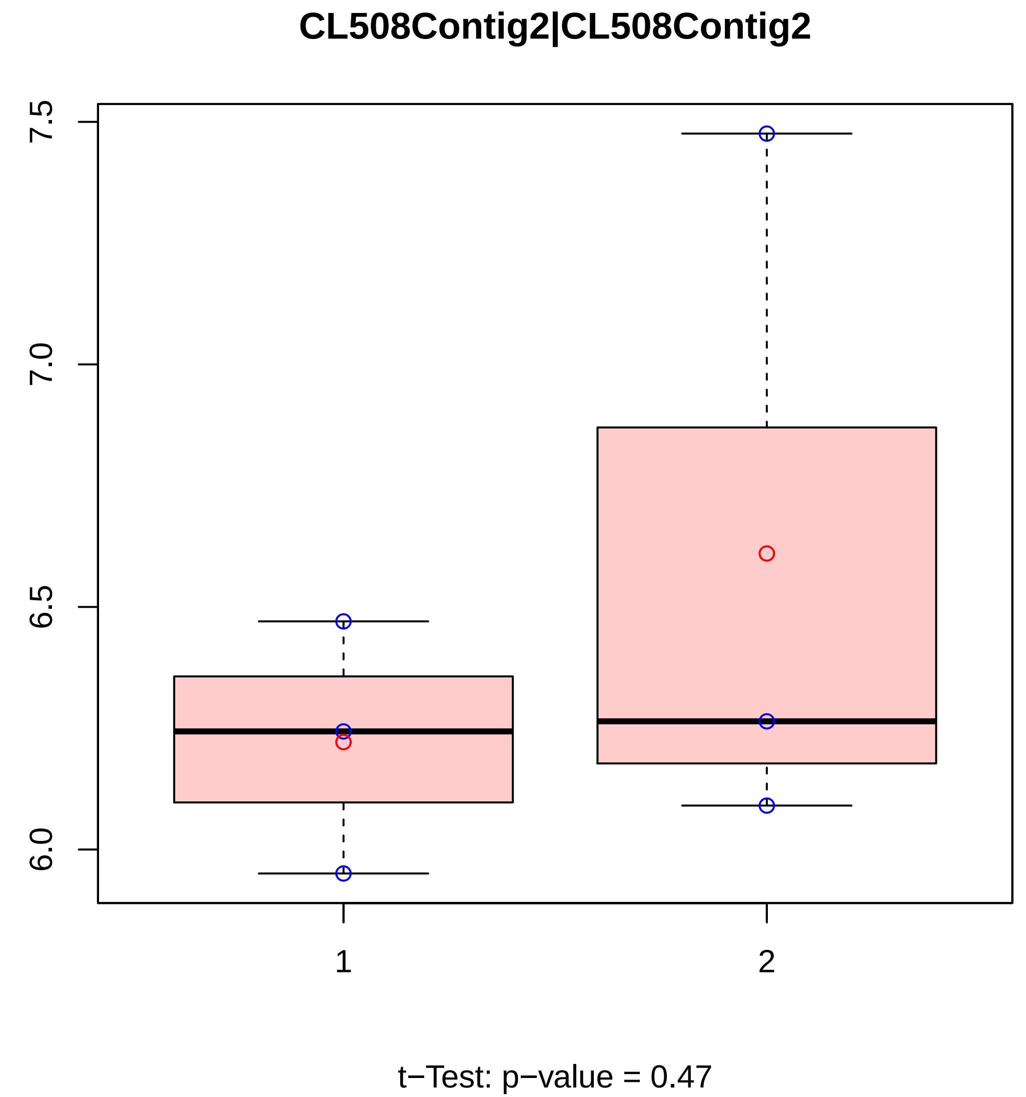

CL508Contig2|CL508Contig2
7.5
7.0
6.5
6.0
1
2
t−Test: p−value = 0.47

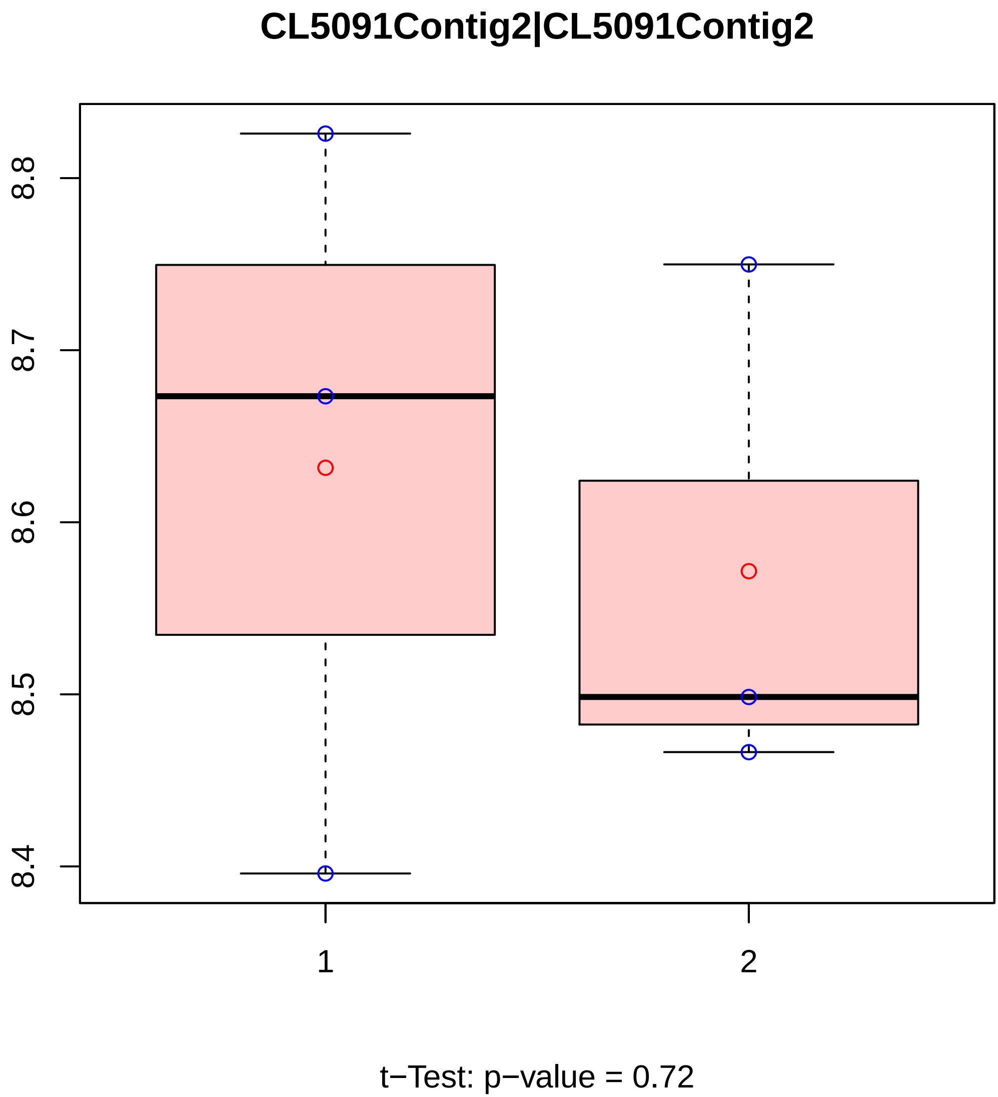

CL5091Contig2|CL5091Contig2
8.4
8.5
8.6
8.7
8.8
1
2
t−Test: p−value = 0.72

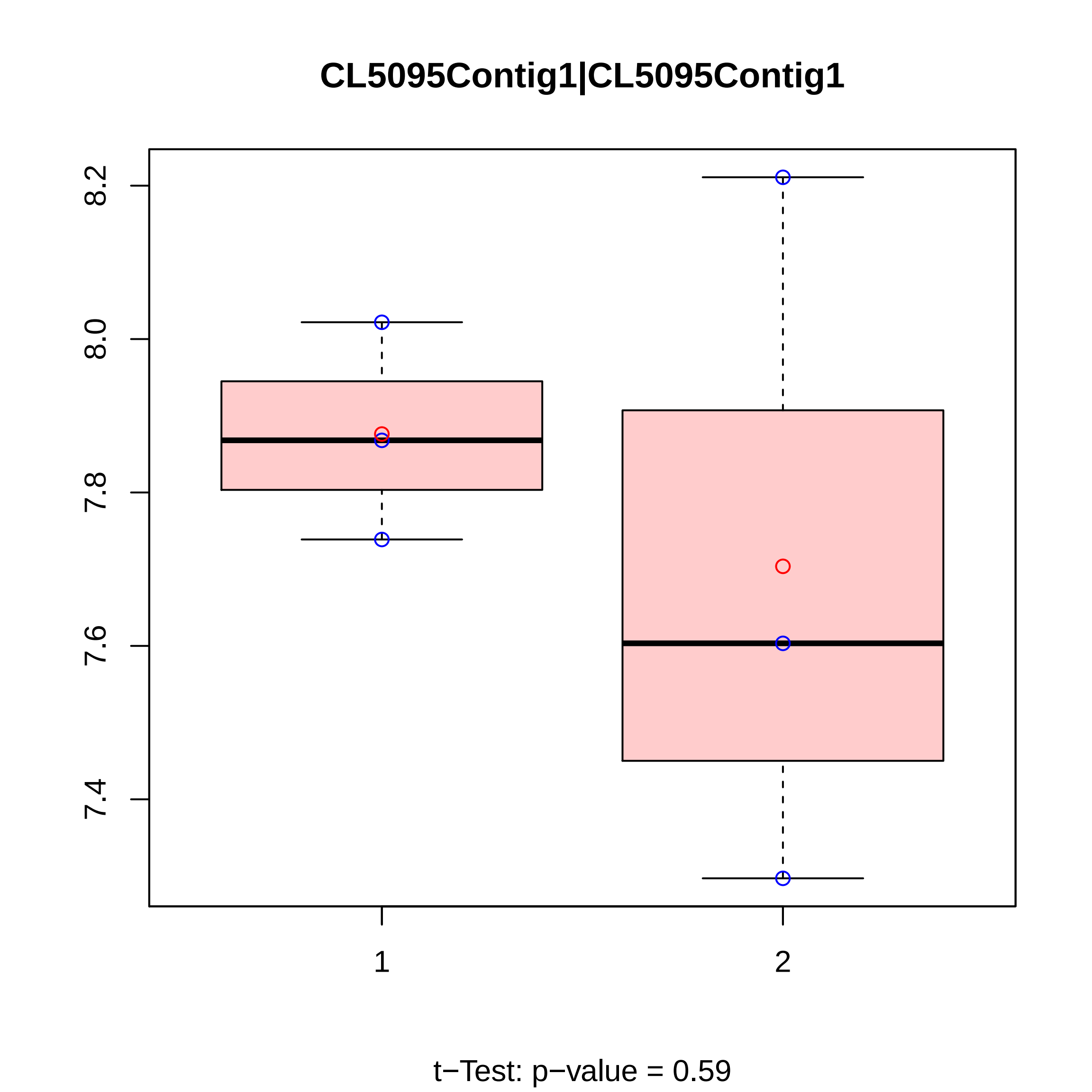
CL5095Contig1|CL5095Contig1
8.2
8.0
7.8
7.6
7.4
1
2
t−Test: p−value = 0.59

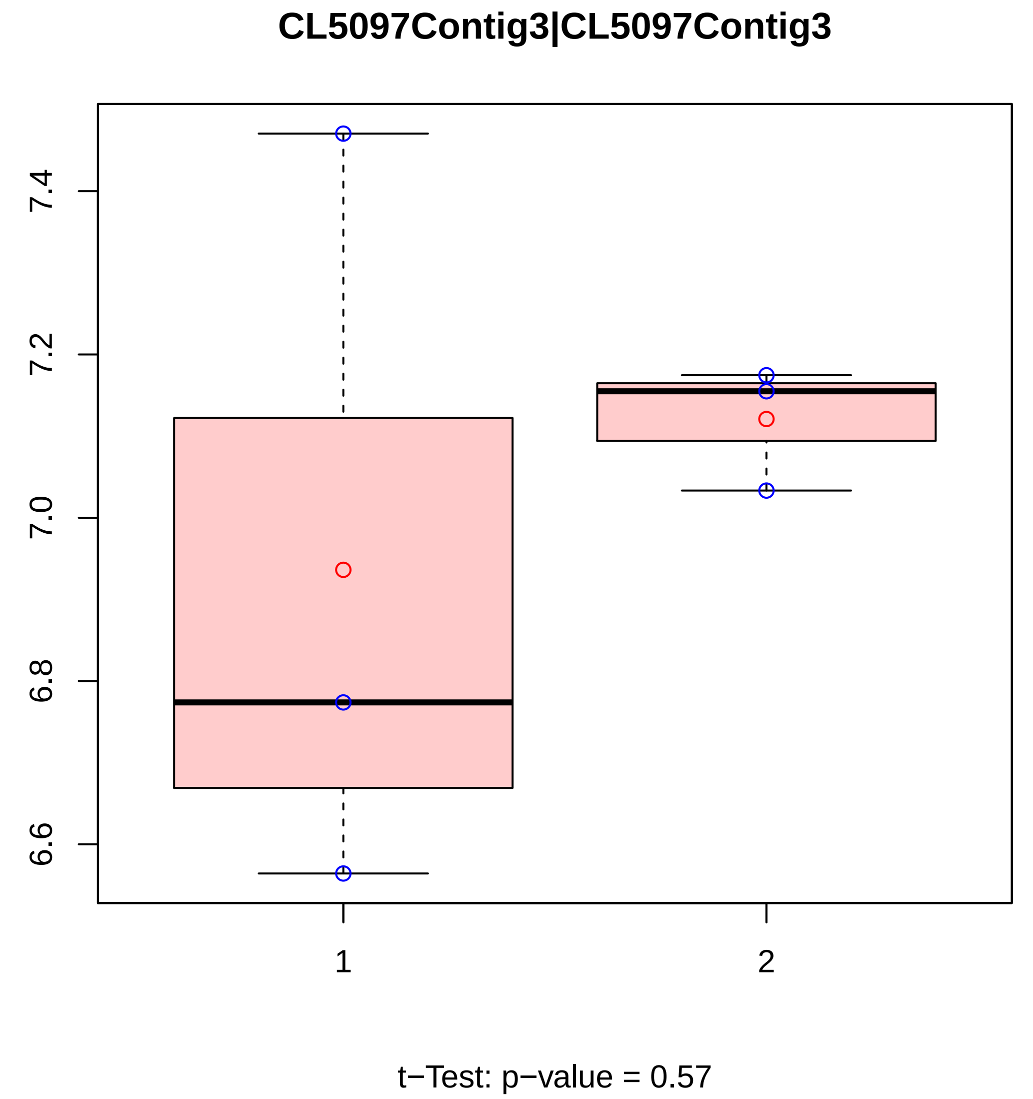
CL5097Contig3|CL5097Contig3
7.4
7.2
7.0
6.8
6.6
1
2
t−Test: p−value = 0.57

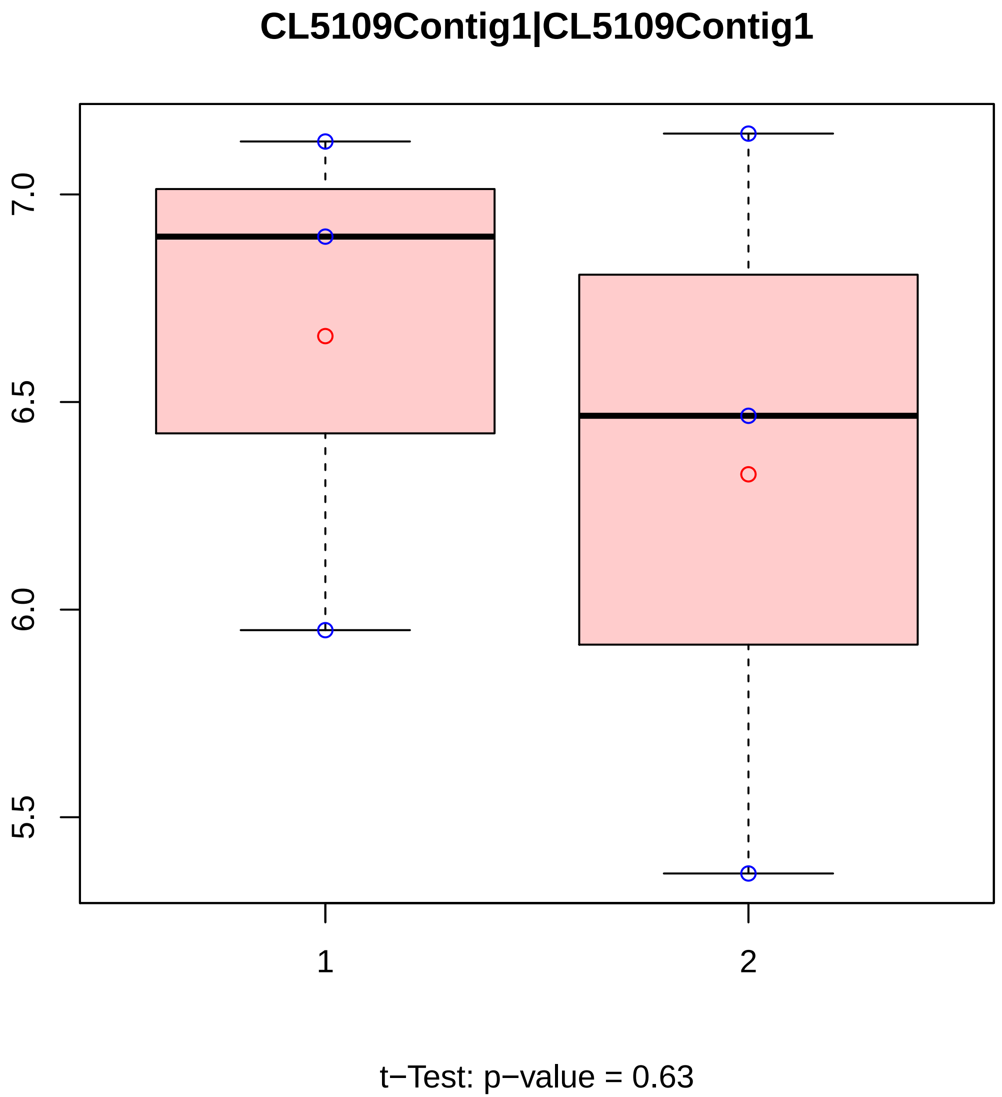
CL5109Contig1|CL5109Contig1
7.0
6.5
6.0
5.5
1
2
t−Test: p−value = 0.63

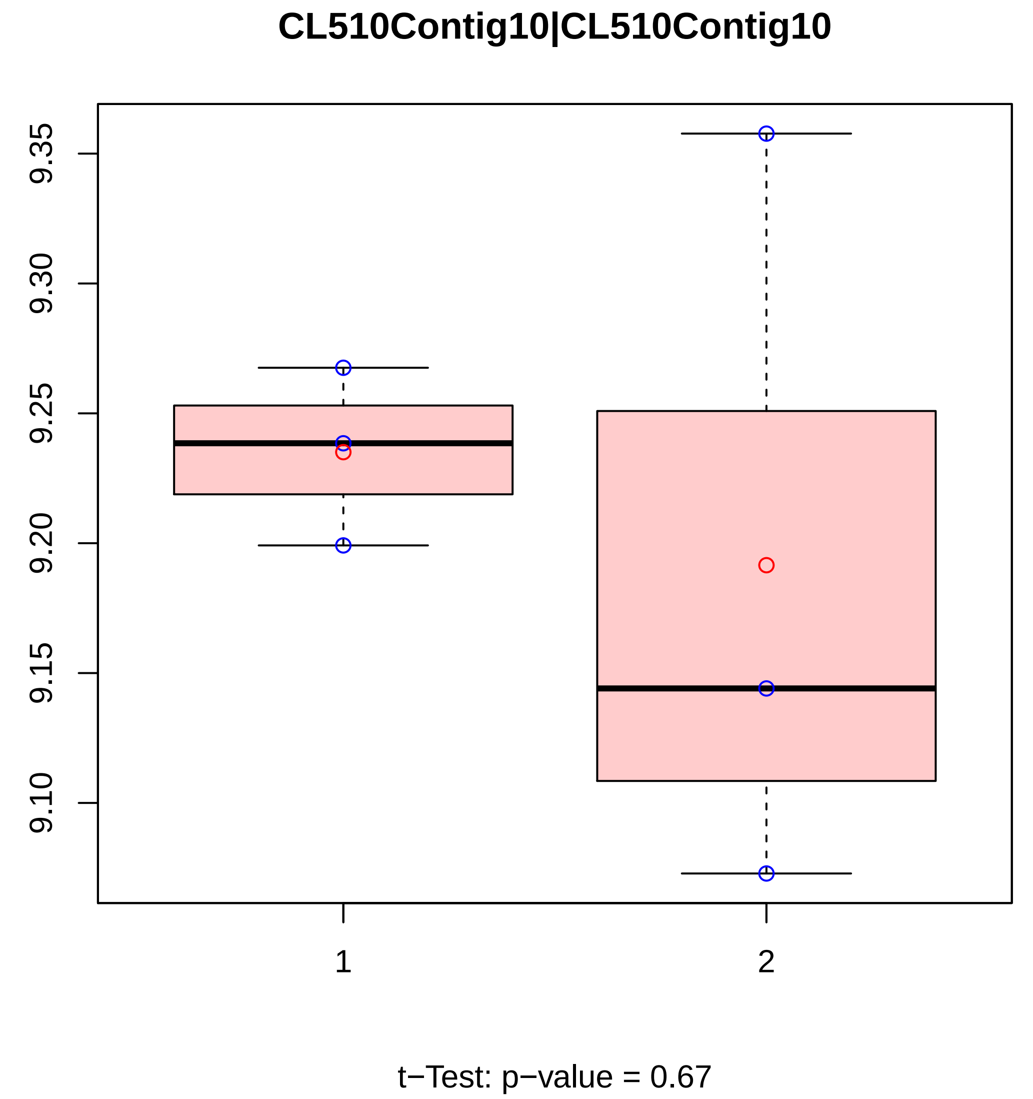
CL510Contig10|CL510Contig10
9.10
9.15
9.20
9.25
9.30
9.35
1
2
t−Test: p−value = 0.67

**CL5128Contig1|CL5128Contig1**

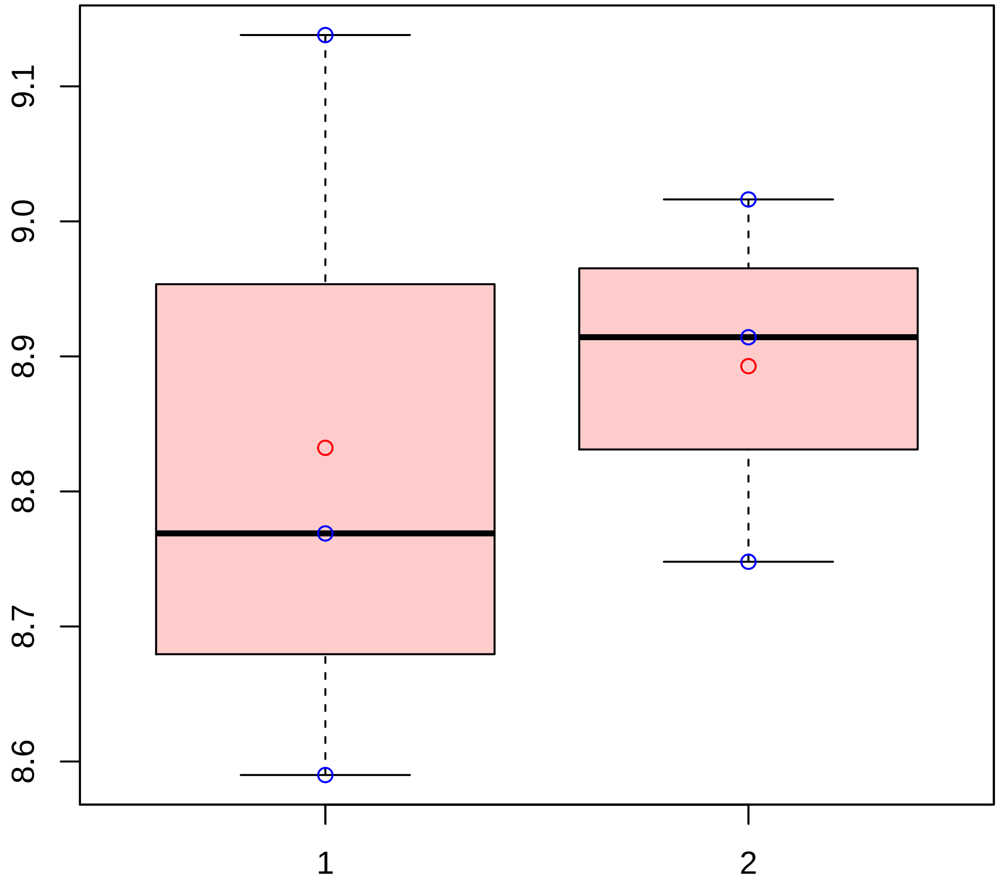


t−Test: p−value = 0.76

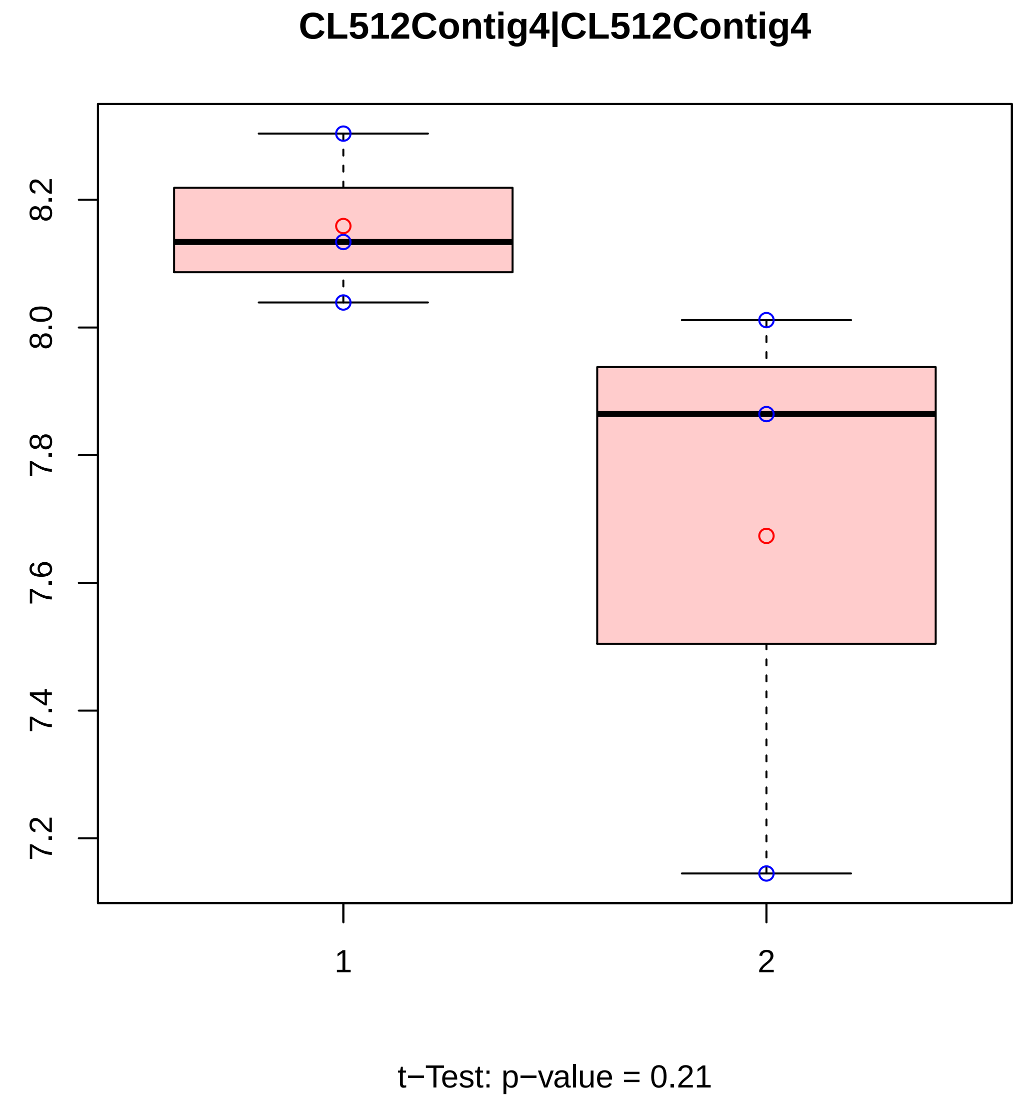

CL512Contig4|CL512Contig4
8.2
8.0
7.8
7.6
7.4
7.2
1
2
t−Test: p−value = 0.21

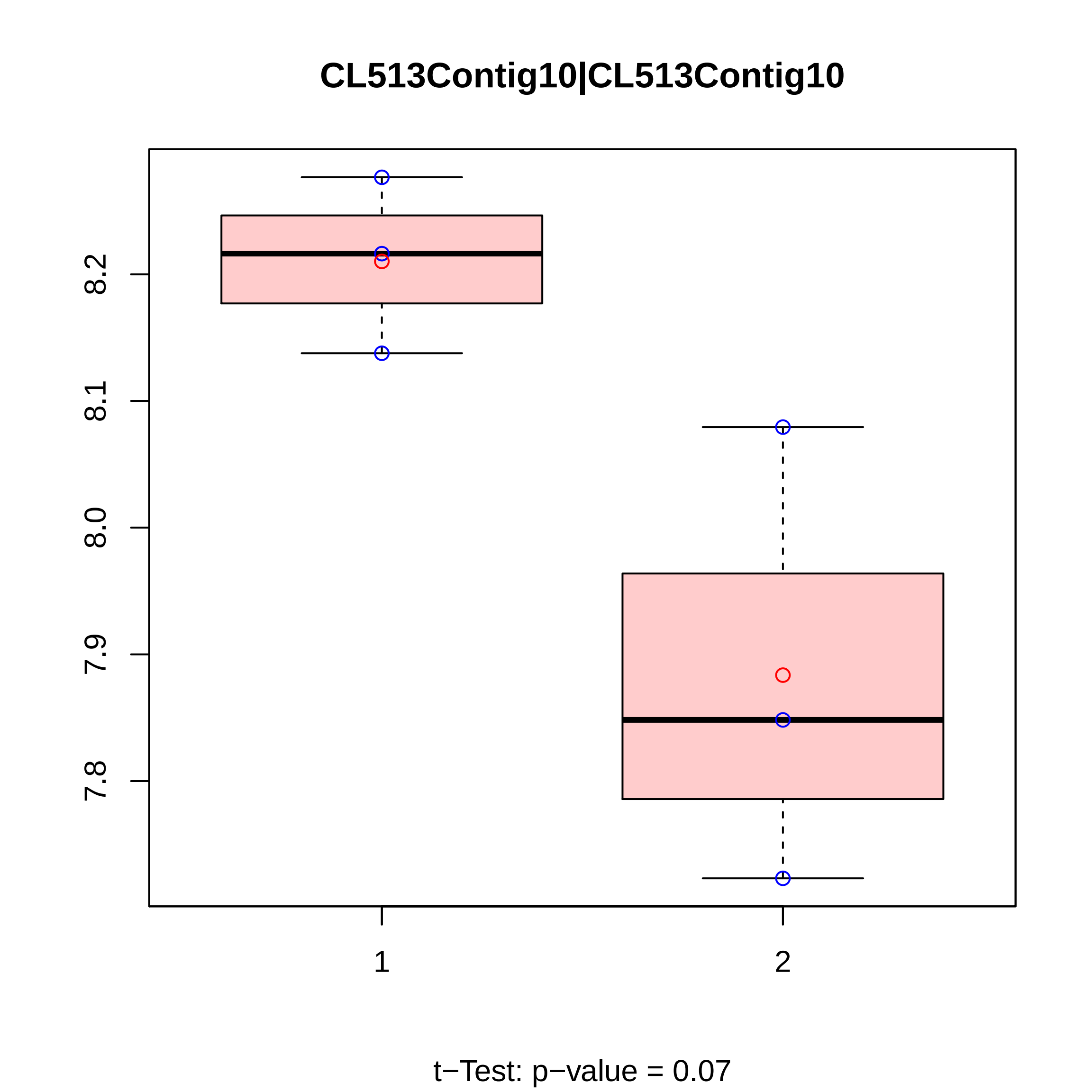

CL513Contig10|CL513Contig10
8.2
8.1
8.0
7.9
7.8
1
2
t−Test: p−value = 0.07

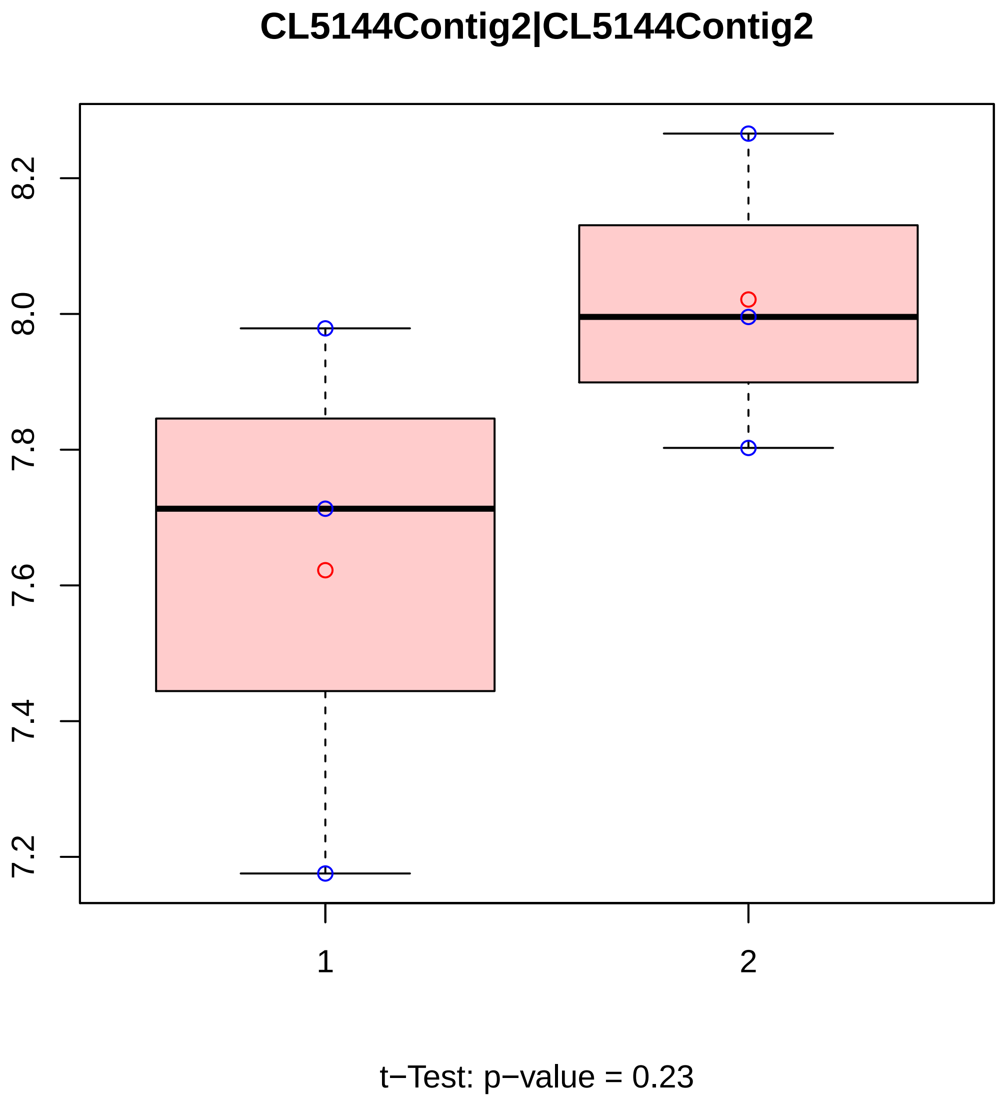

CL5144Contig2|CL5144Contig2
8.2
8.0
7.8
7.6
7.4
7.2
1
2
t−Test: p−value = 0.23

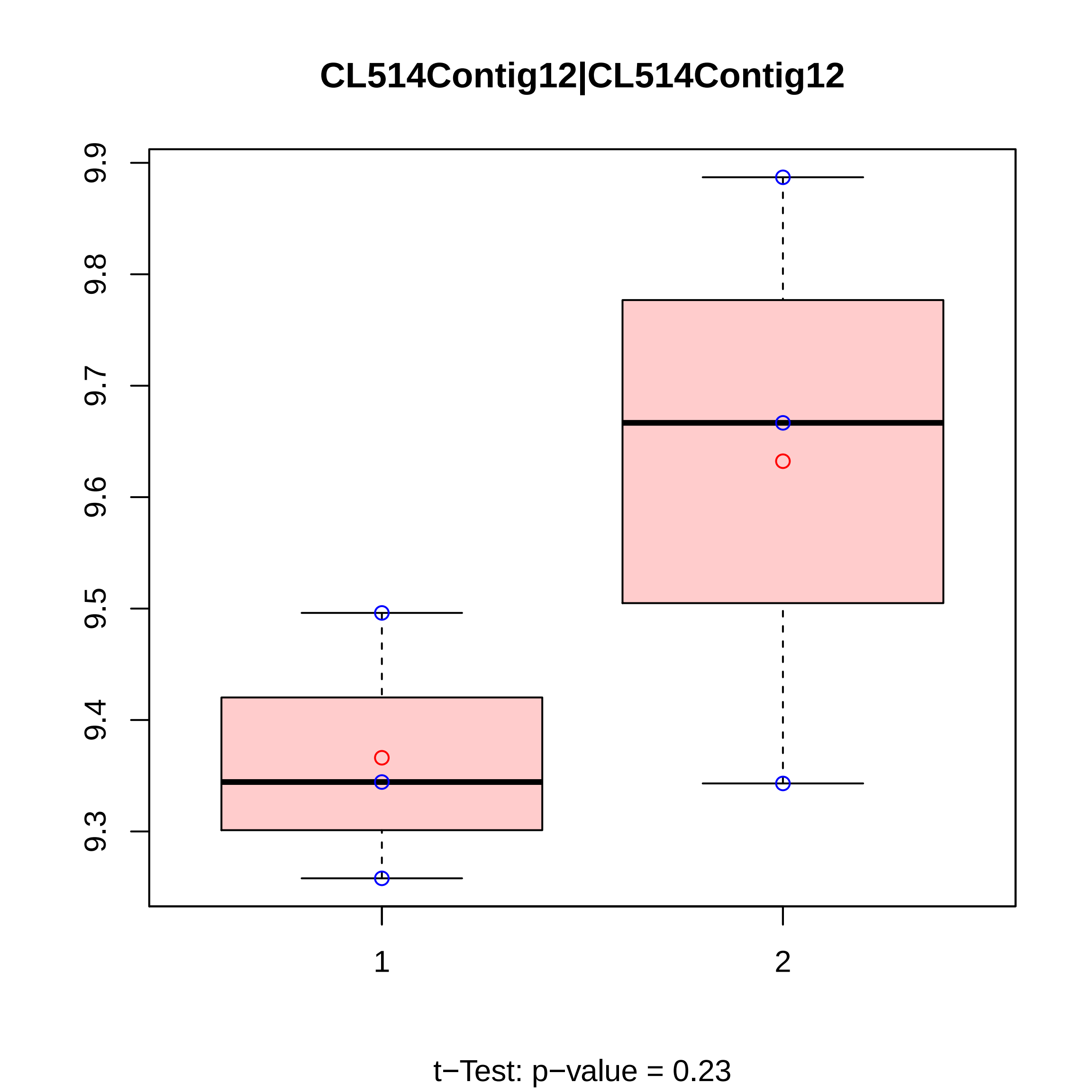
CL514Contig12|CL514Contig12
9.3
9.4
9.5
9.6
9.7
9.8
9.9
1
2
t−Test: p−value = 0.23

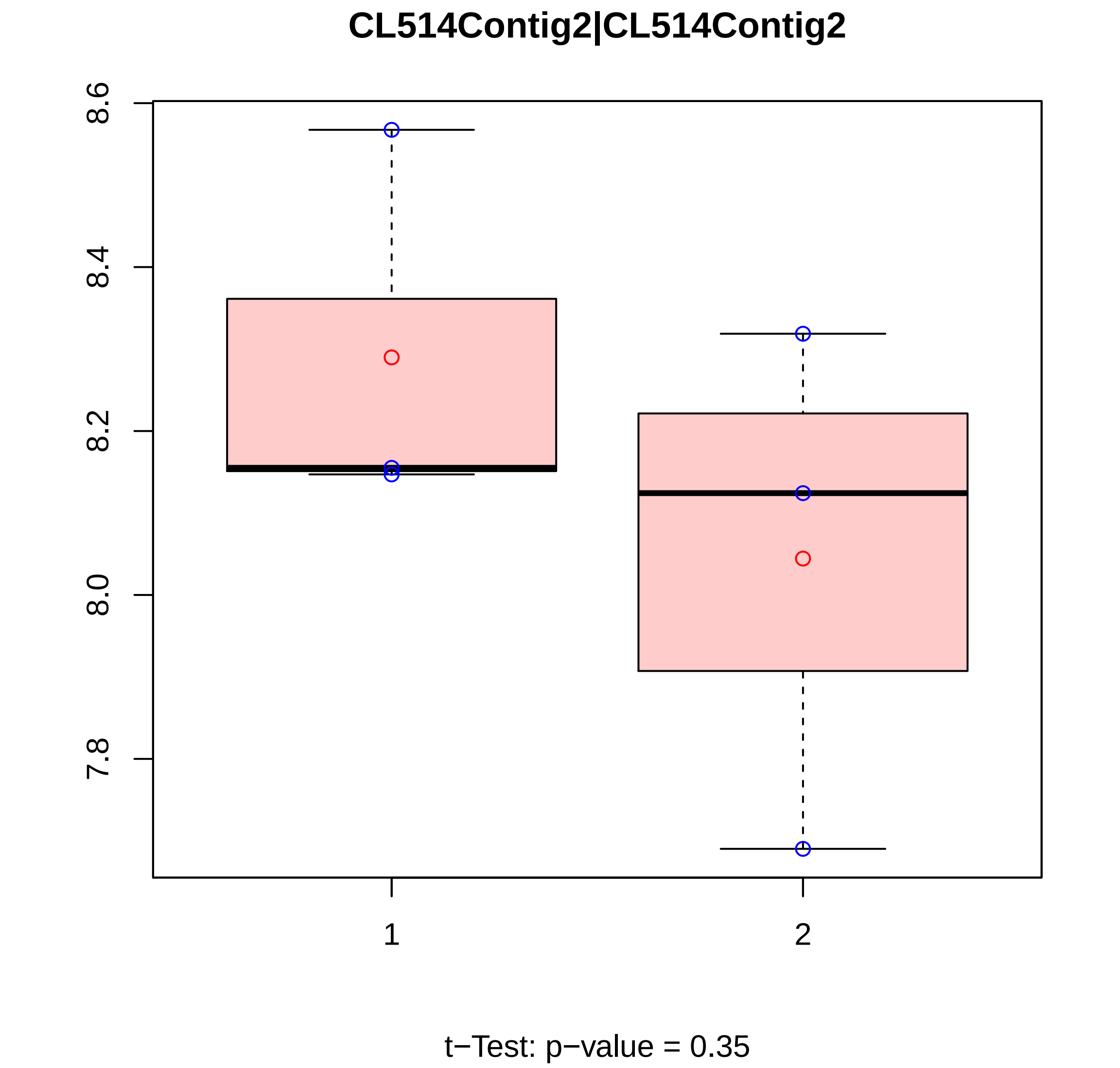
CL514Contig2|CL514Contig2
8.6
8.4
8.2
8.0
7.8
1
2
t−Test: p−value = 0.35

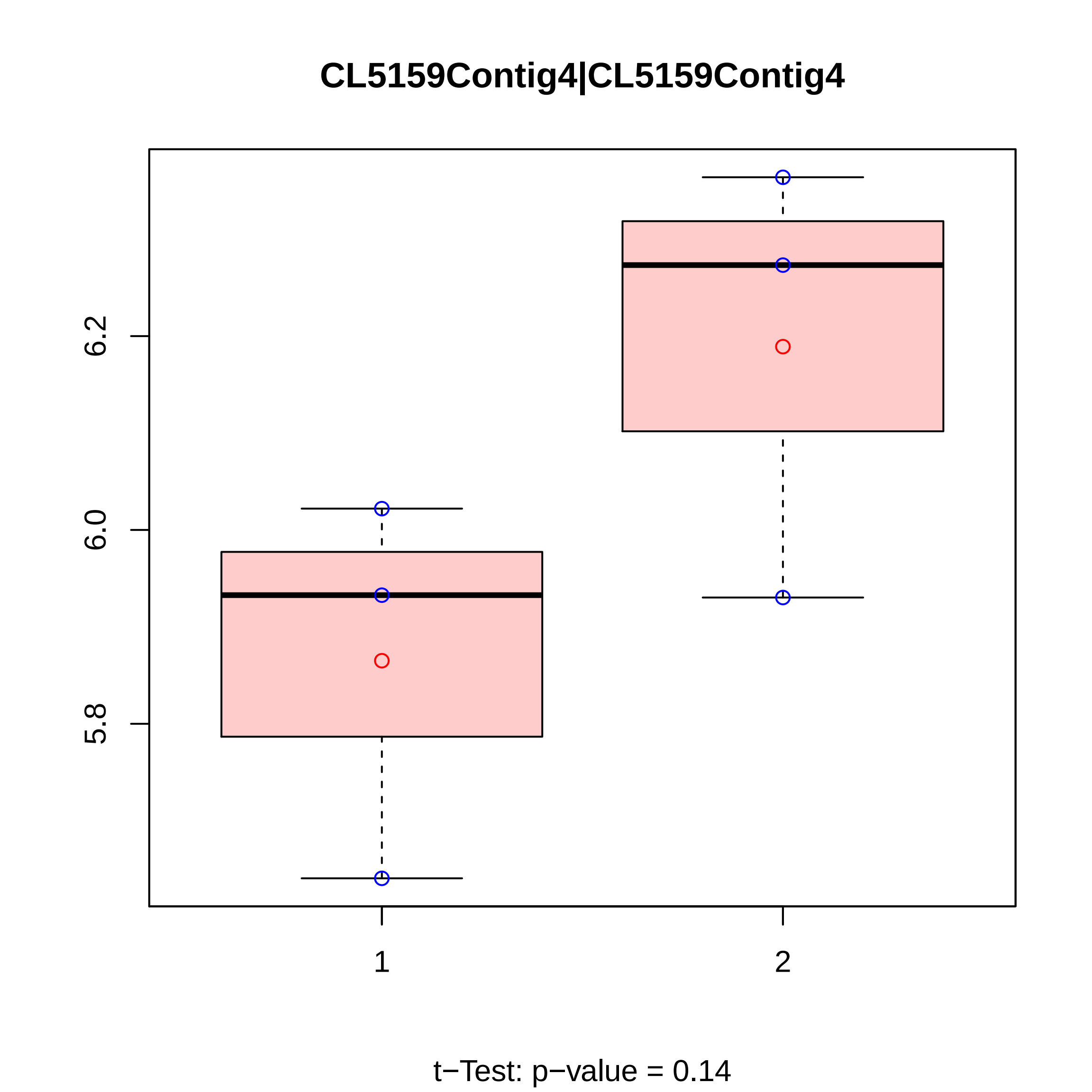
CL5159Contig4|CL5159Contig4
6.2
6.0
5.8
1
2
t−Test: p−value = 0.14

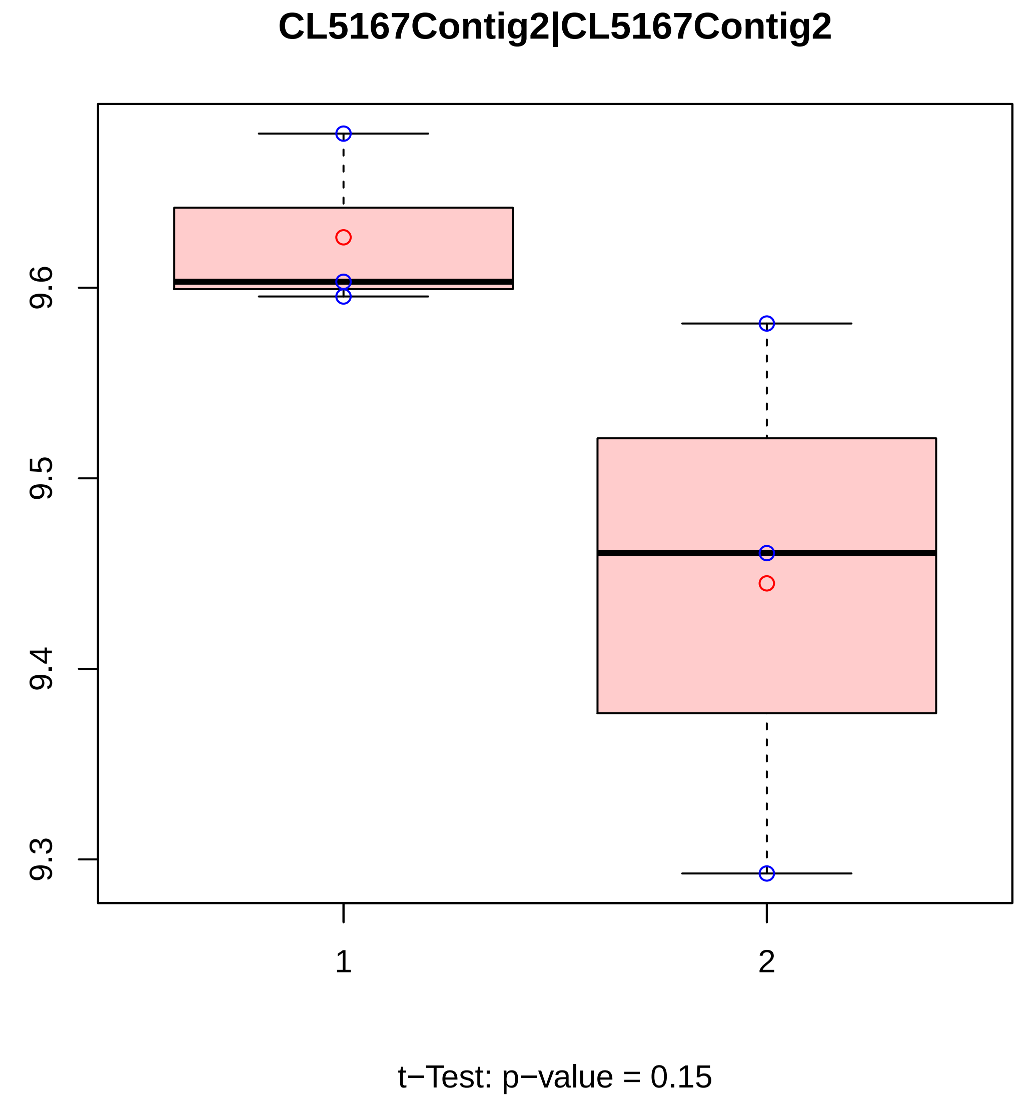
CL5167Contig2|CL5167Contig2
9.3
9.4
9.5
9.6
1
2
t−Test: p−value = 0.15

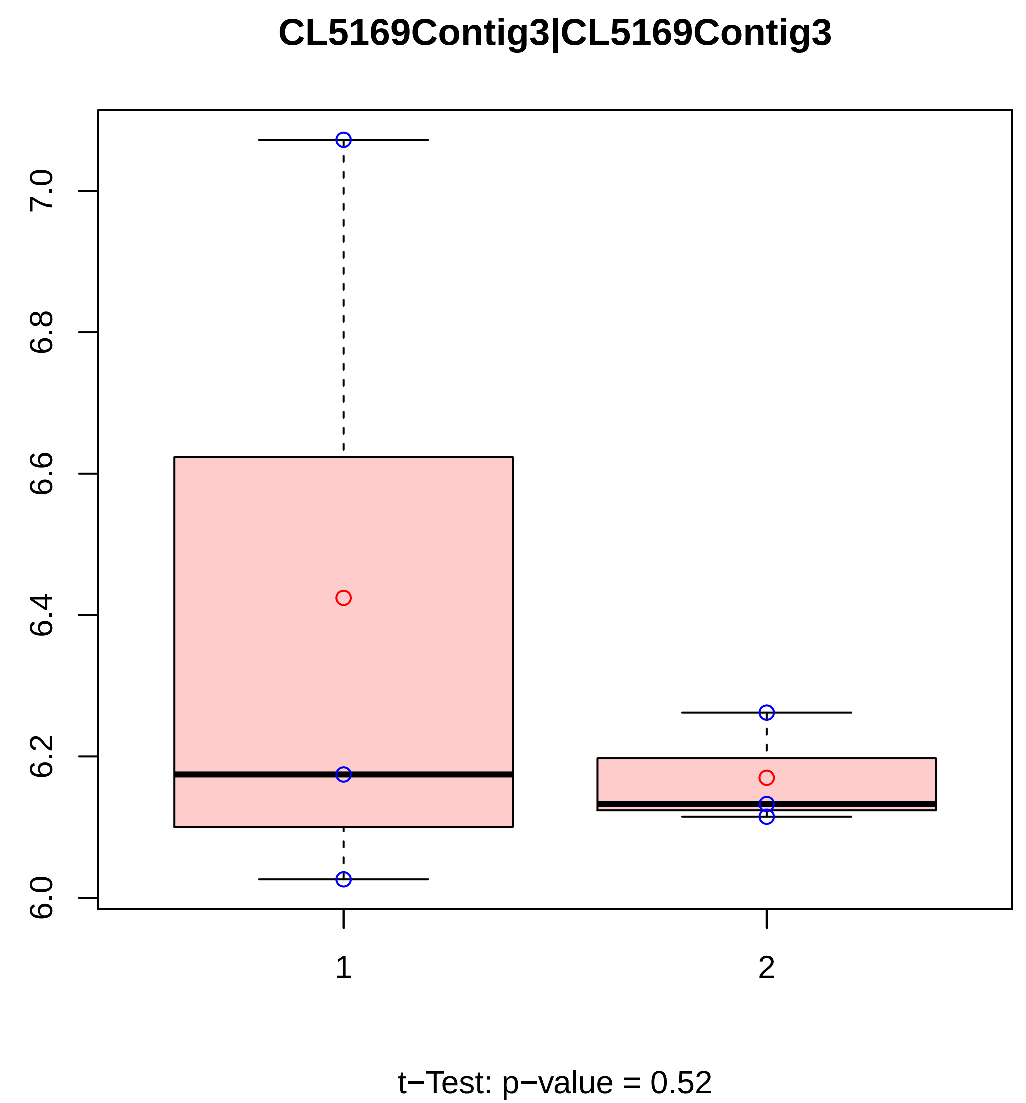
CL5169Contig3|CL5169Contig3
7.0
6.8
6.6
6.4
6.2
6.0
1
2
t−Test: p−value = 0.52

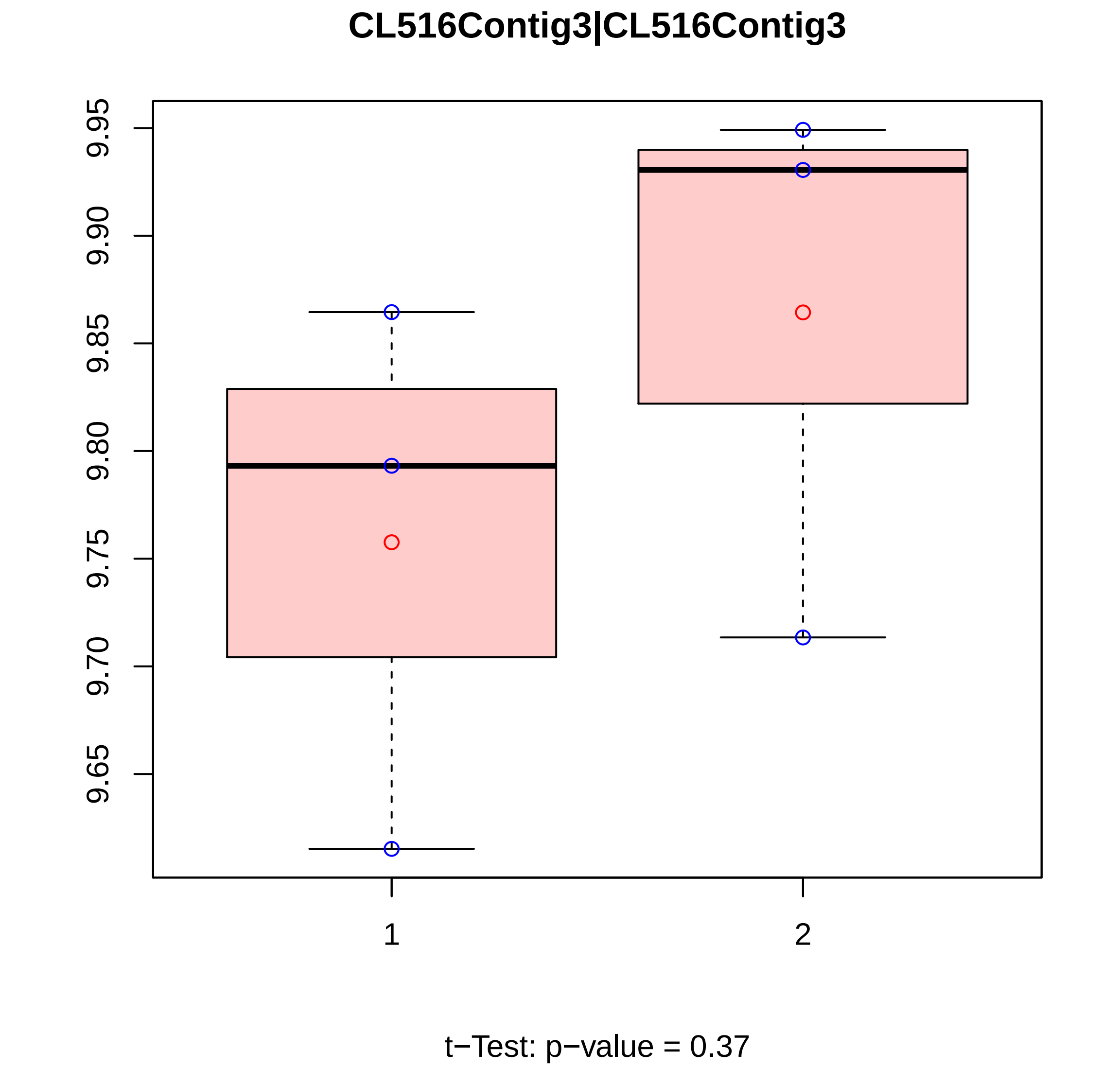
CL516Contig3|CL516Contig3
9.65
9.70
9.75
9.80
9.85
9.90
9.95
1
2
t−Test: p−value = 0.37

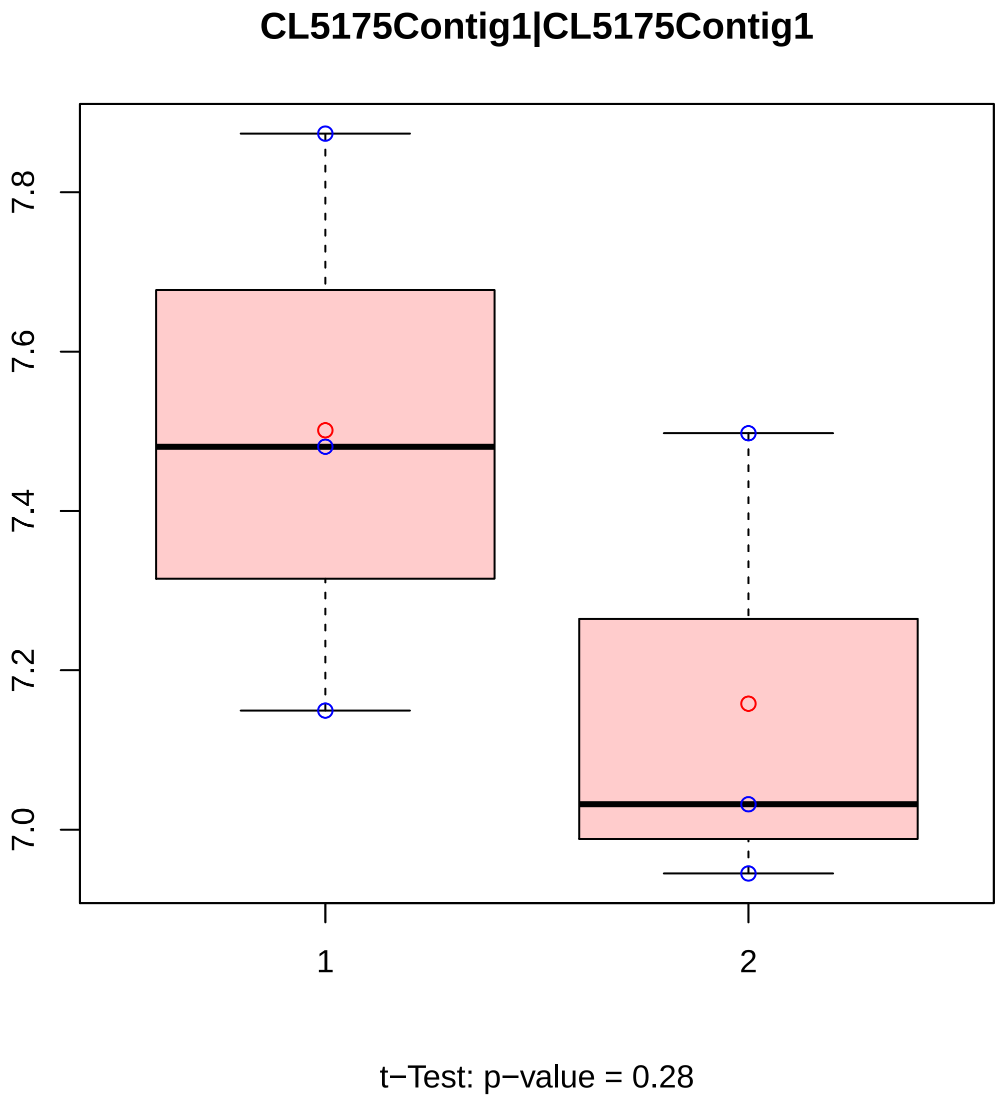
CL5175Contig1|CL5175Contig1
7.0
7.2
7.4
7.6
7.8
1
2
t−Test: p−value = 0.28

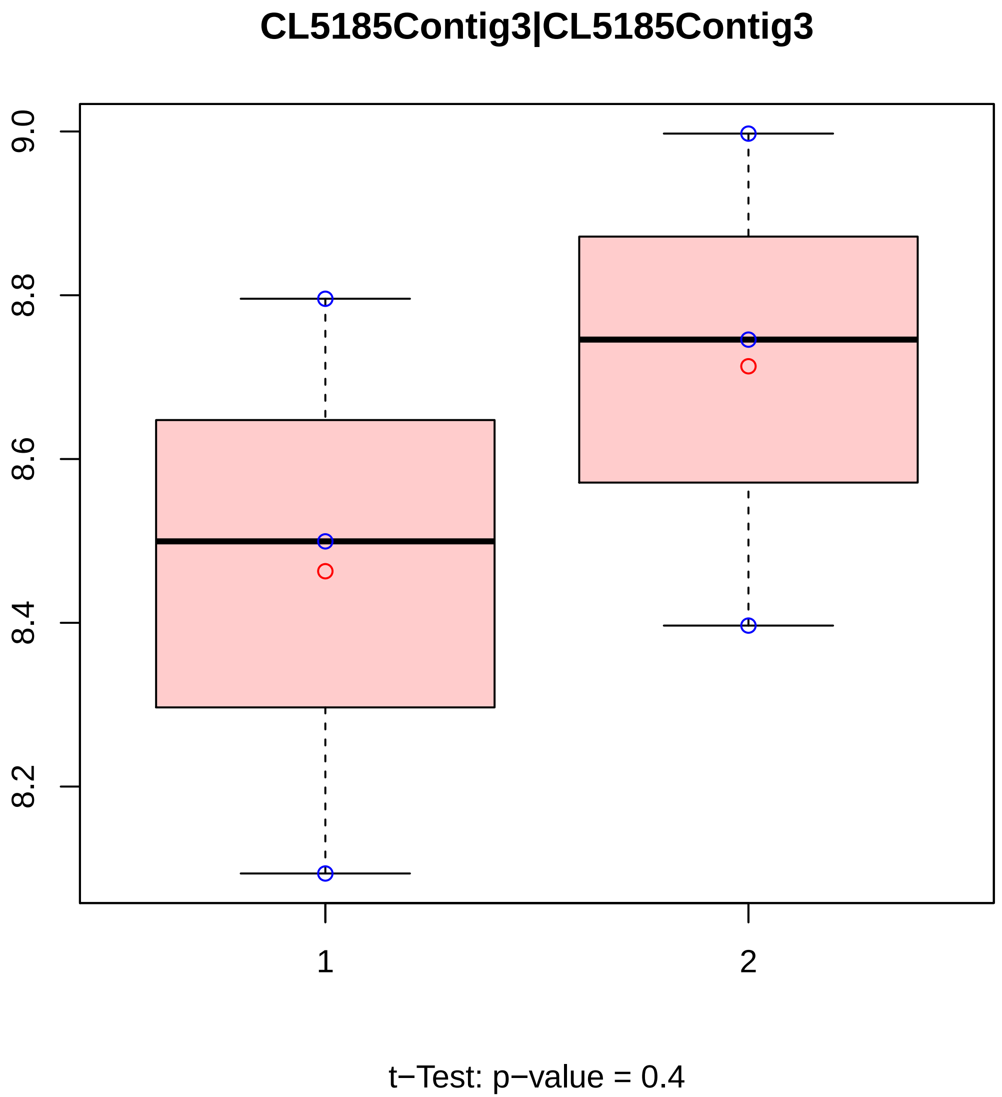
CL5185Contig3|CL5185Contig3
9.0
8.8
8.6
8.4
8.2
1
2
t−Test: p−value = 0.4

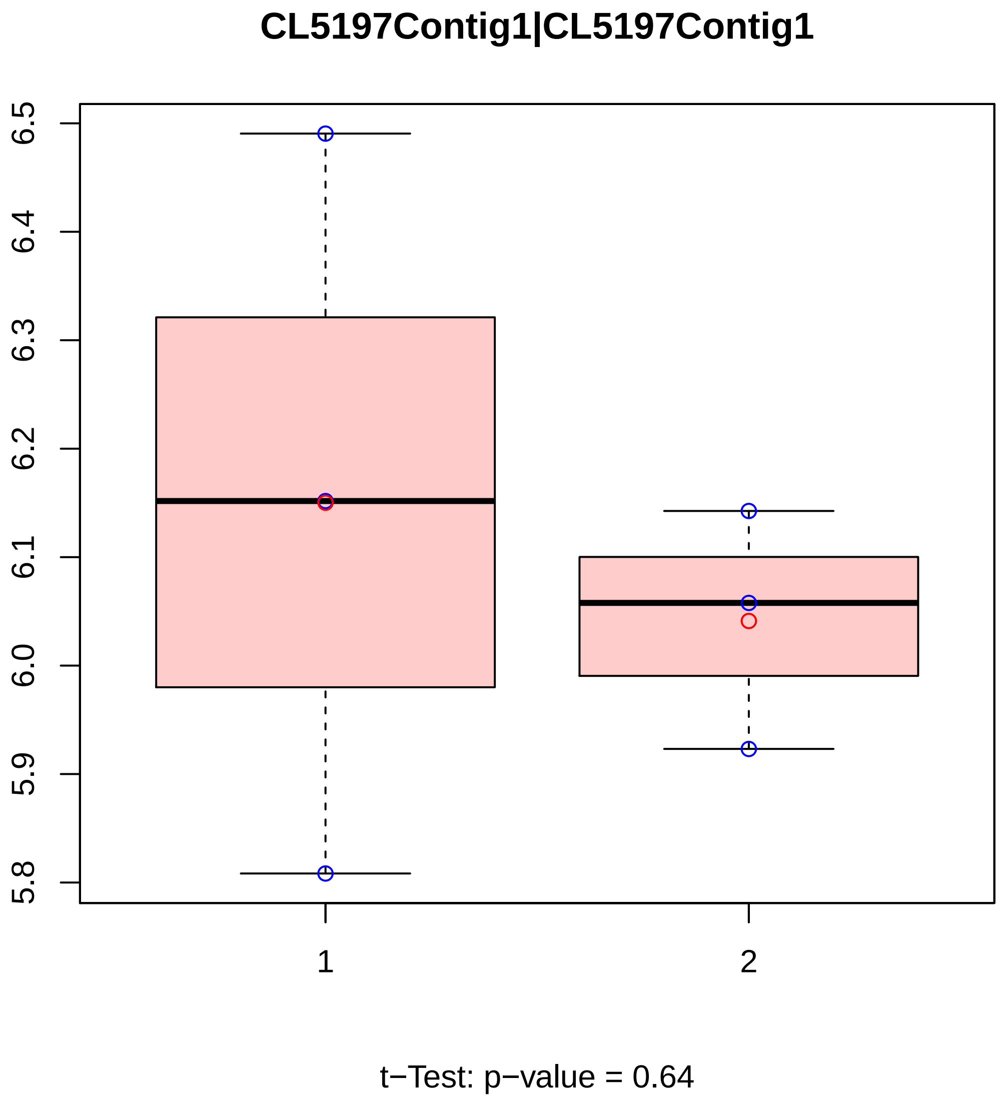
CL5197Contig1|CL5197Contig1
6.5
6.4
6.3
6.2
6.1
6.0
5.9
5.8
1
2
t−Test: p−value = 0.64

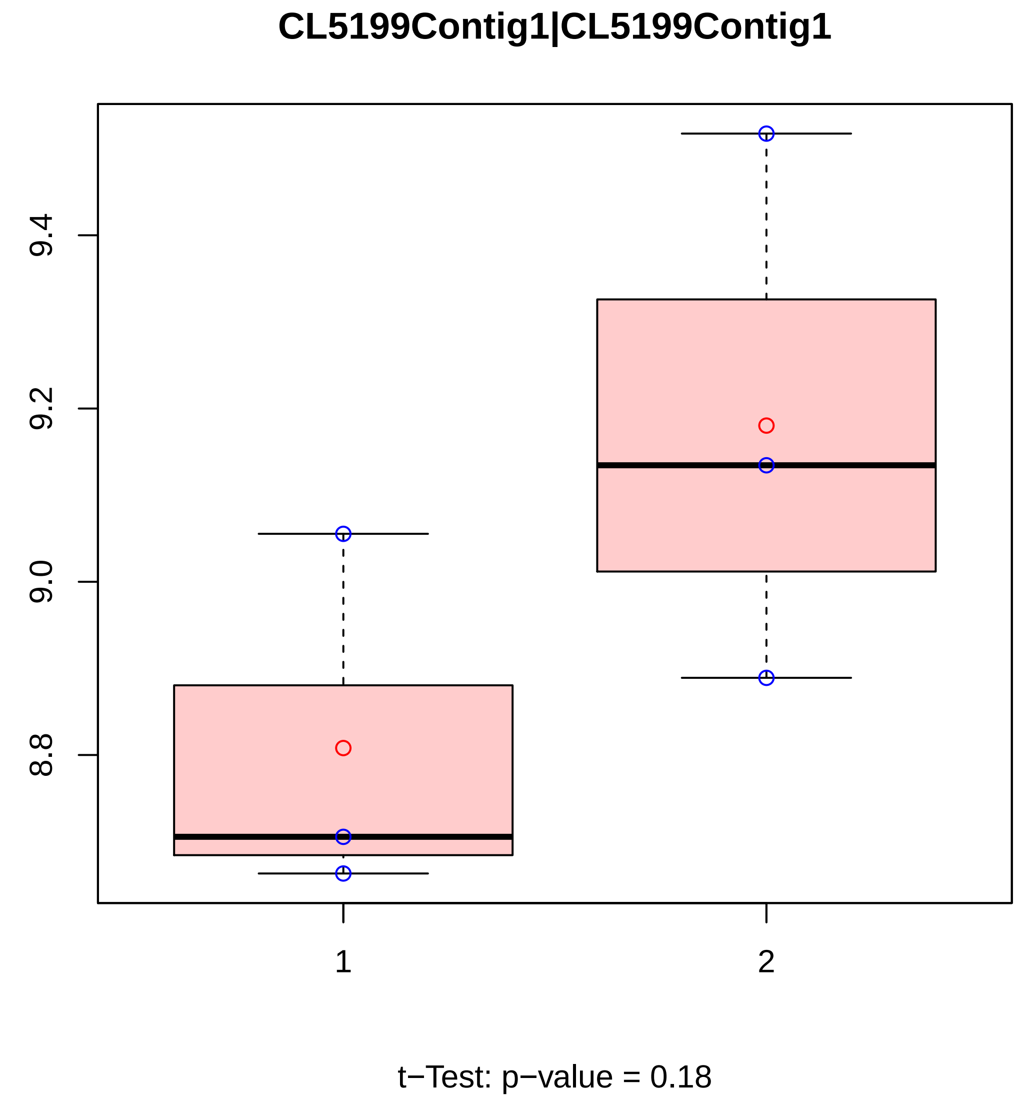

CL5199Contig1|CL5199Contig1
9.4
9.2
9.0
8.8
1
2
t−Test: p−value = 0.18

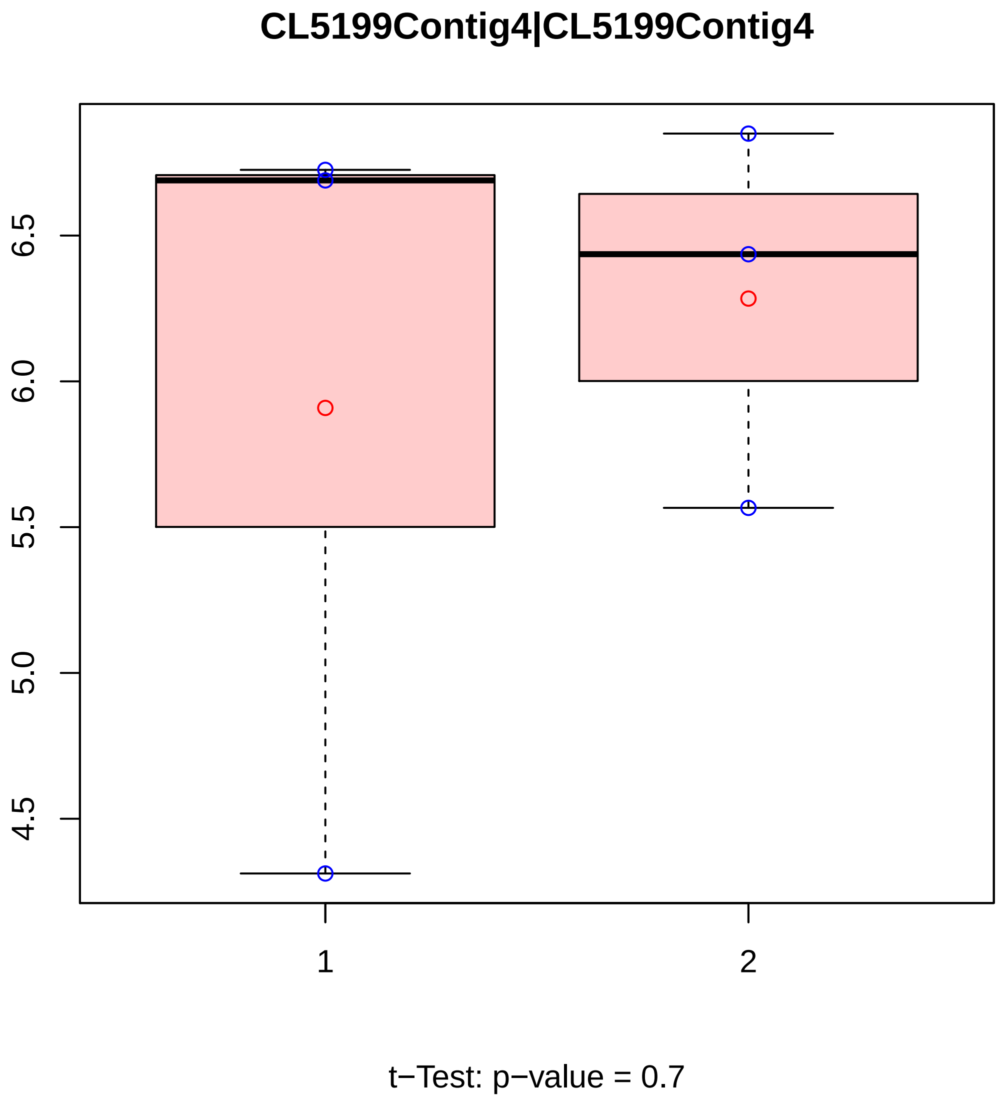
CL5199Contig4|CL5199Contig4
4.5
5.0
5.5
6.0
6.5
1
2
t−Test: p−value = 0.7

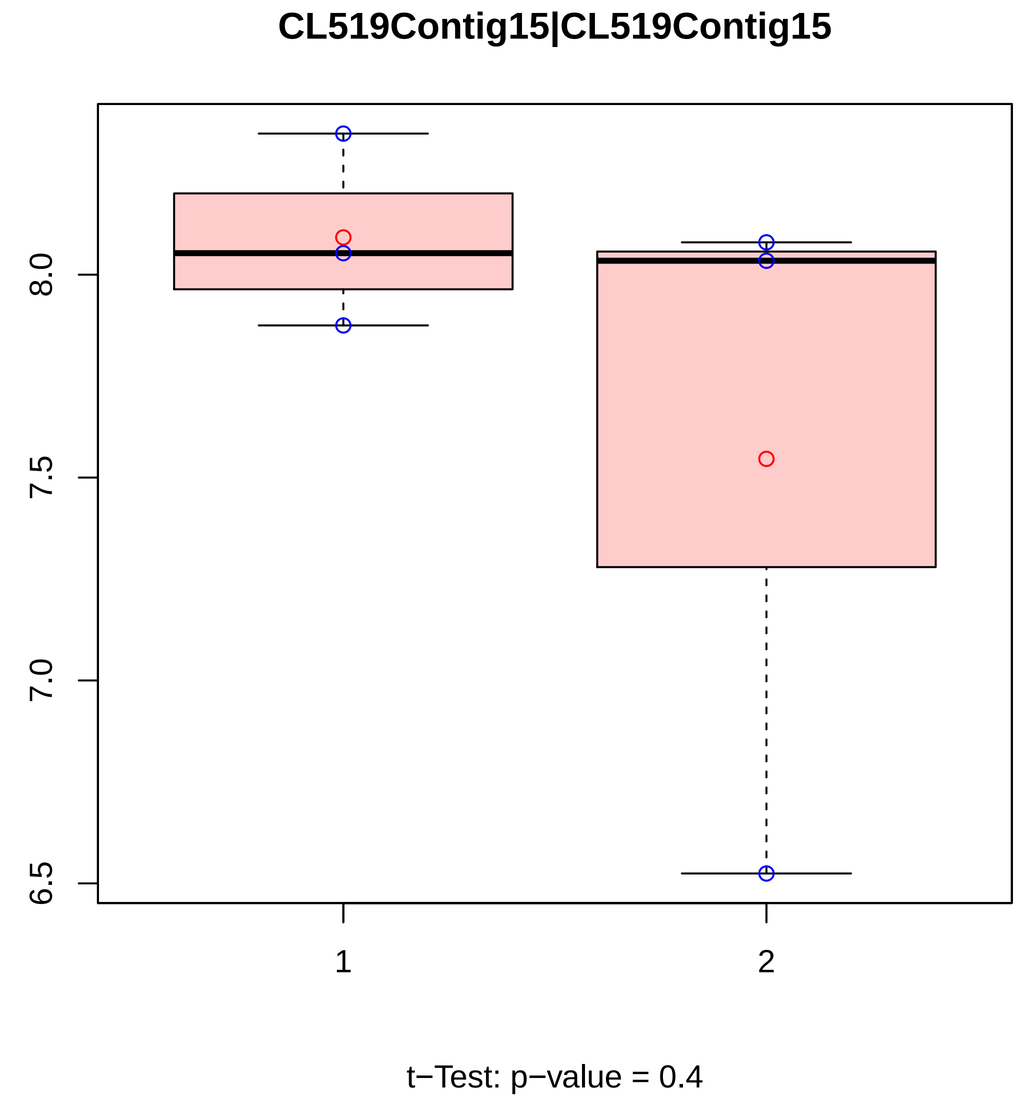
CL519Contig15|CL519Contig15
8.0
7.5
7.0
6.5
1
2
t−Test: p−value = 0.4

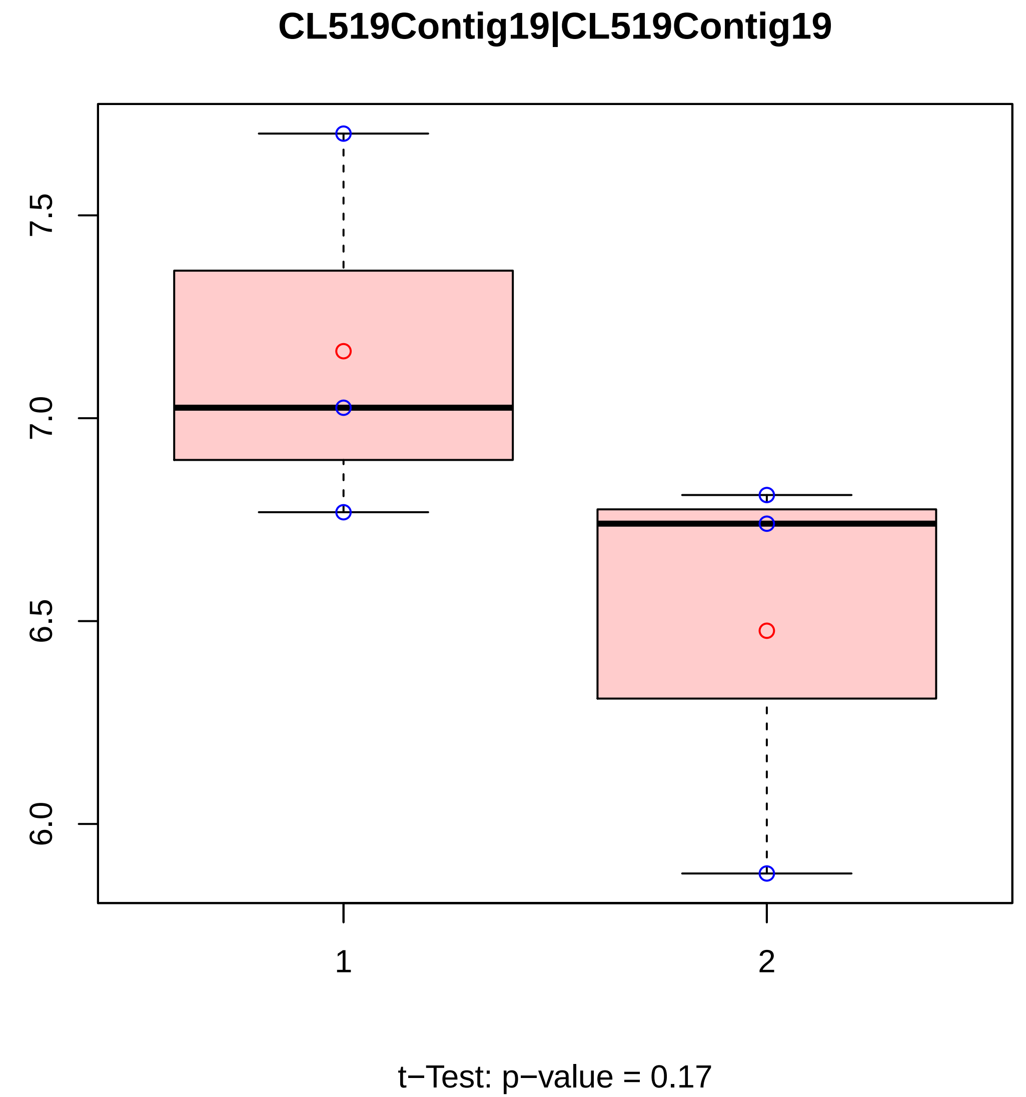

CL519Contig19|CL519Contig19
7.5
7.0
6.5
6.0
1
2
t−Test: p−value = 0.17

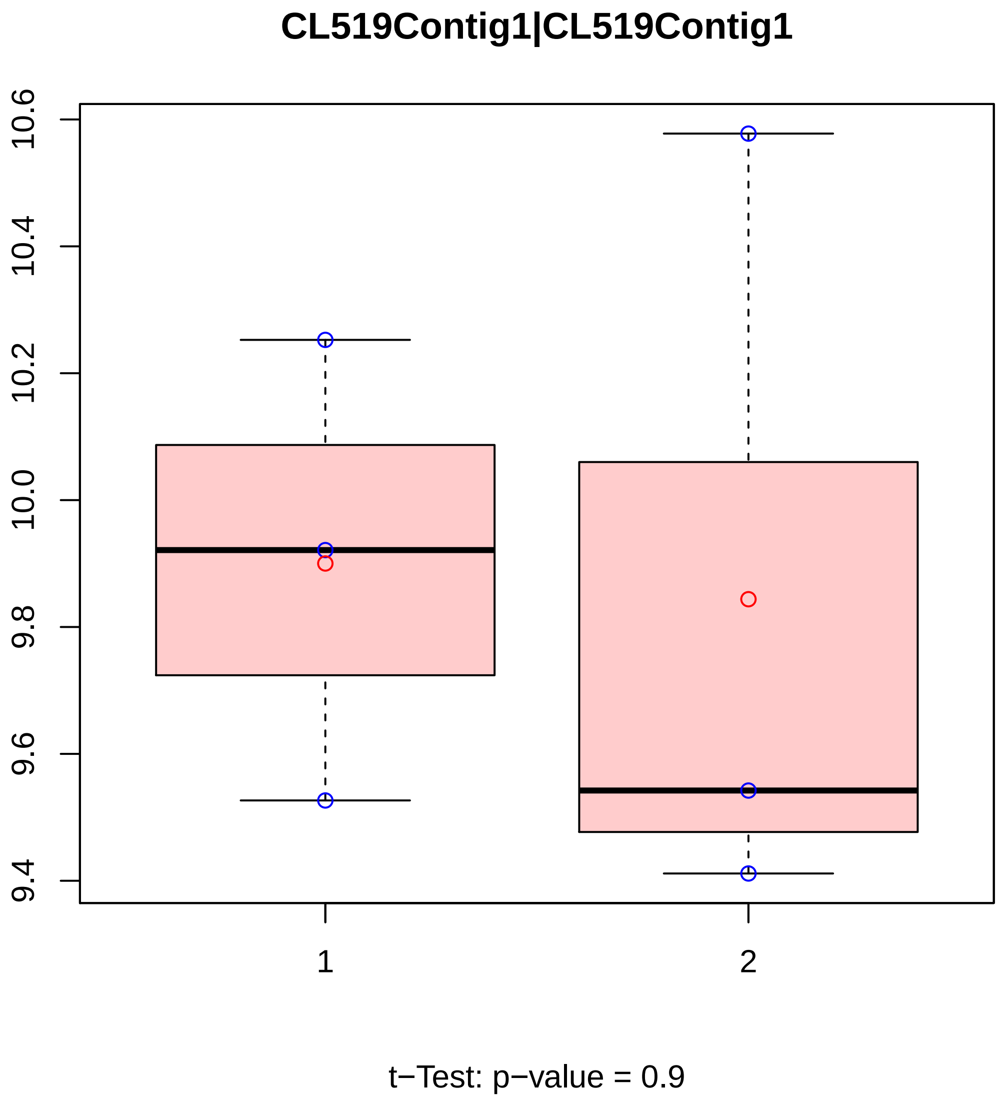

CL519Contig1|CL519Contig1
10.6
10.4
10.2
10.0
9.8
9.6
9.4
1
2
t−Test: p−value = 0.9

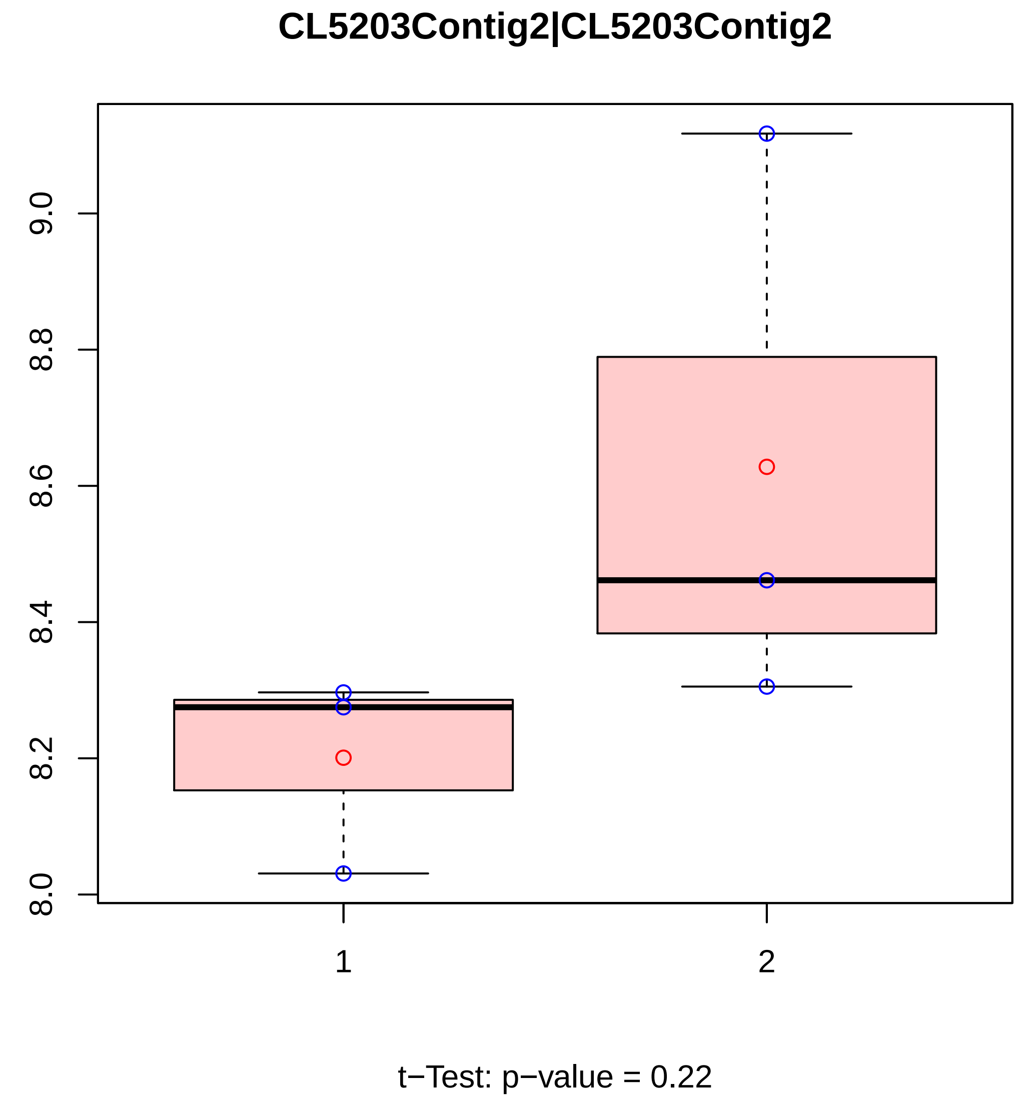
CL5203Contig2|CL5203Contig2
9.0
8.8
8.6
8.4
8.2
8.0
1
2
t−Test: p−value = 0.22

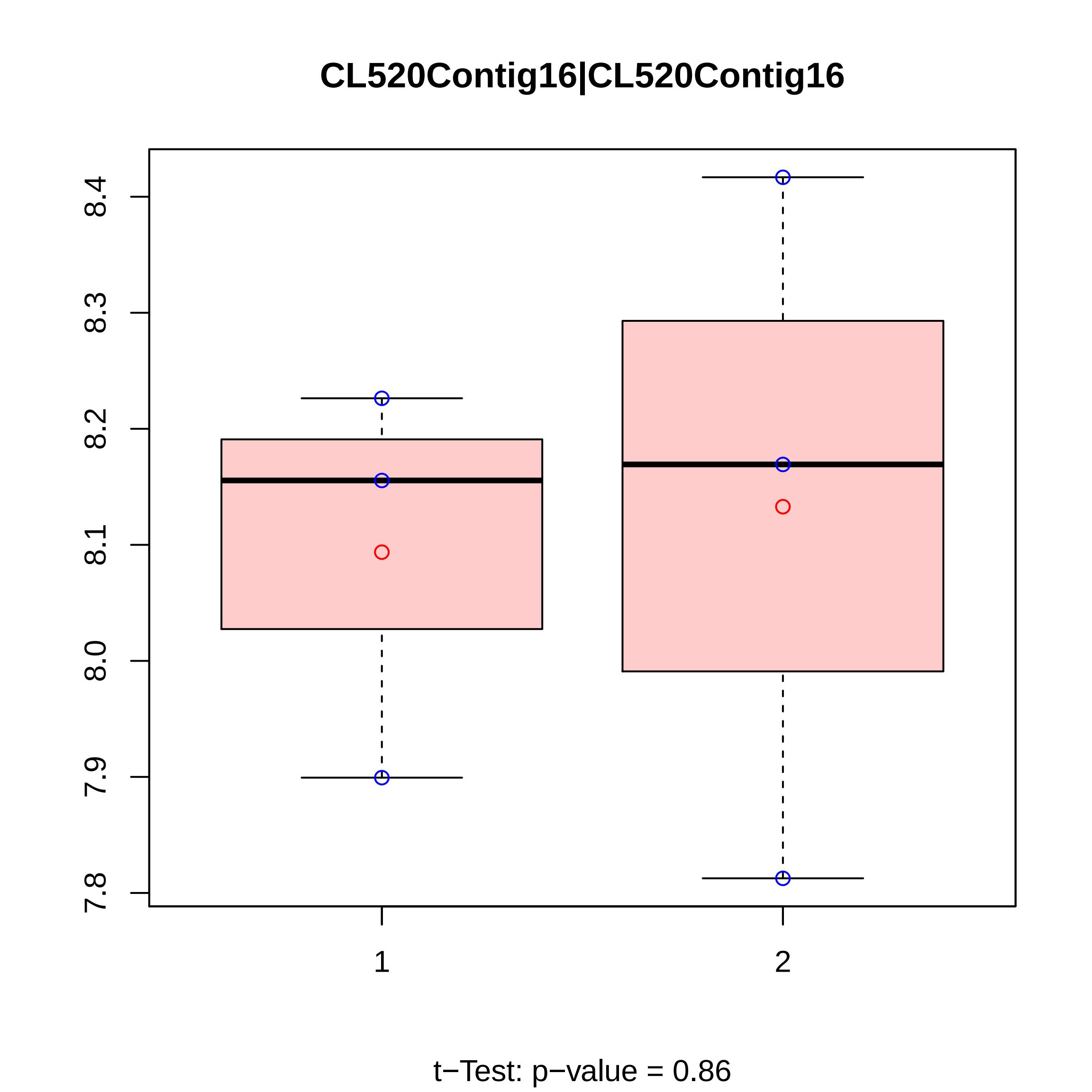

CL520Contig16|CL520Contig16
8.4
8.3
8.2
8.1
8.0
7.9
7.8
1
2
t−Test: p−value = 0.86

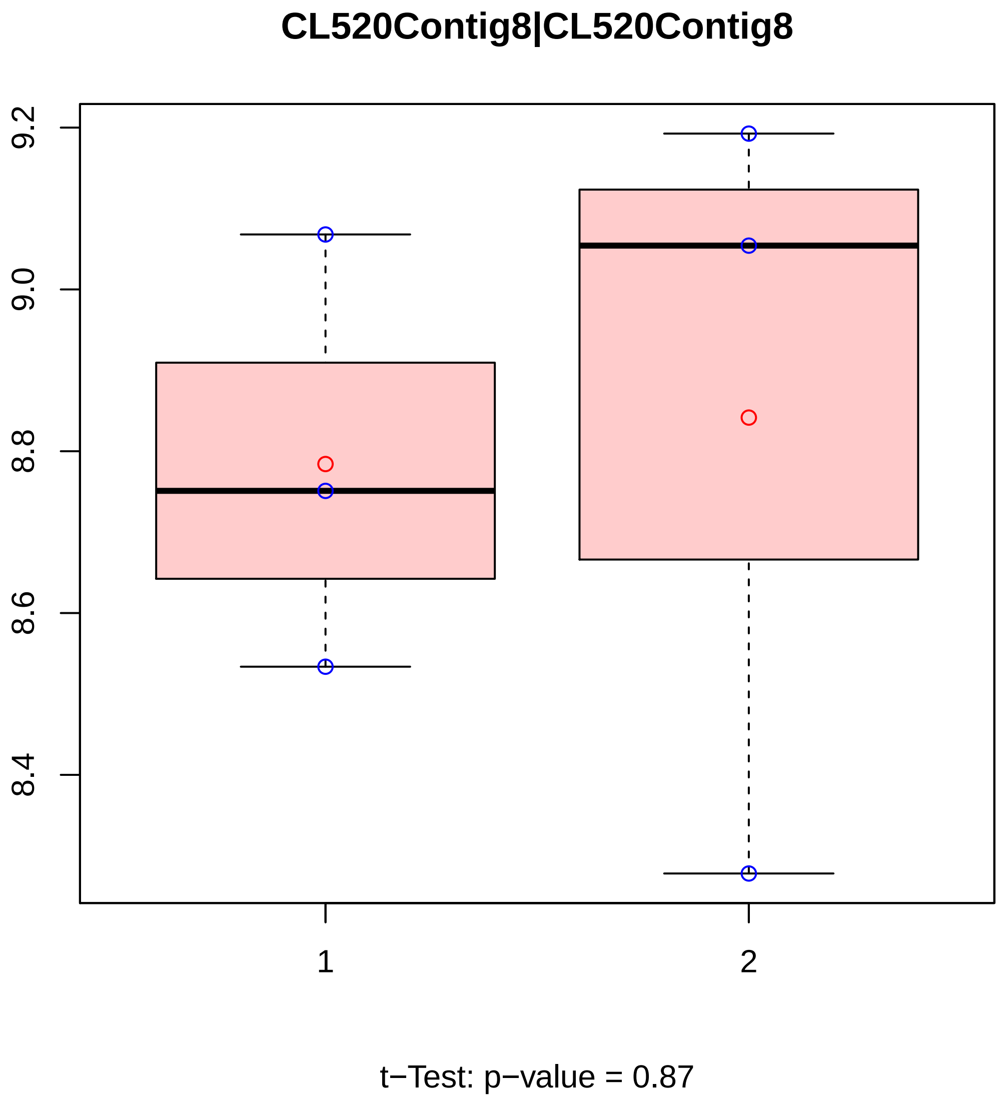
CL520Contig8|CL520Contig8
9.2
9.0
8.8
8.6
8.4
1
2
t−Test: p−value = 0.87

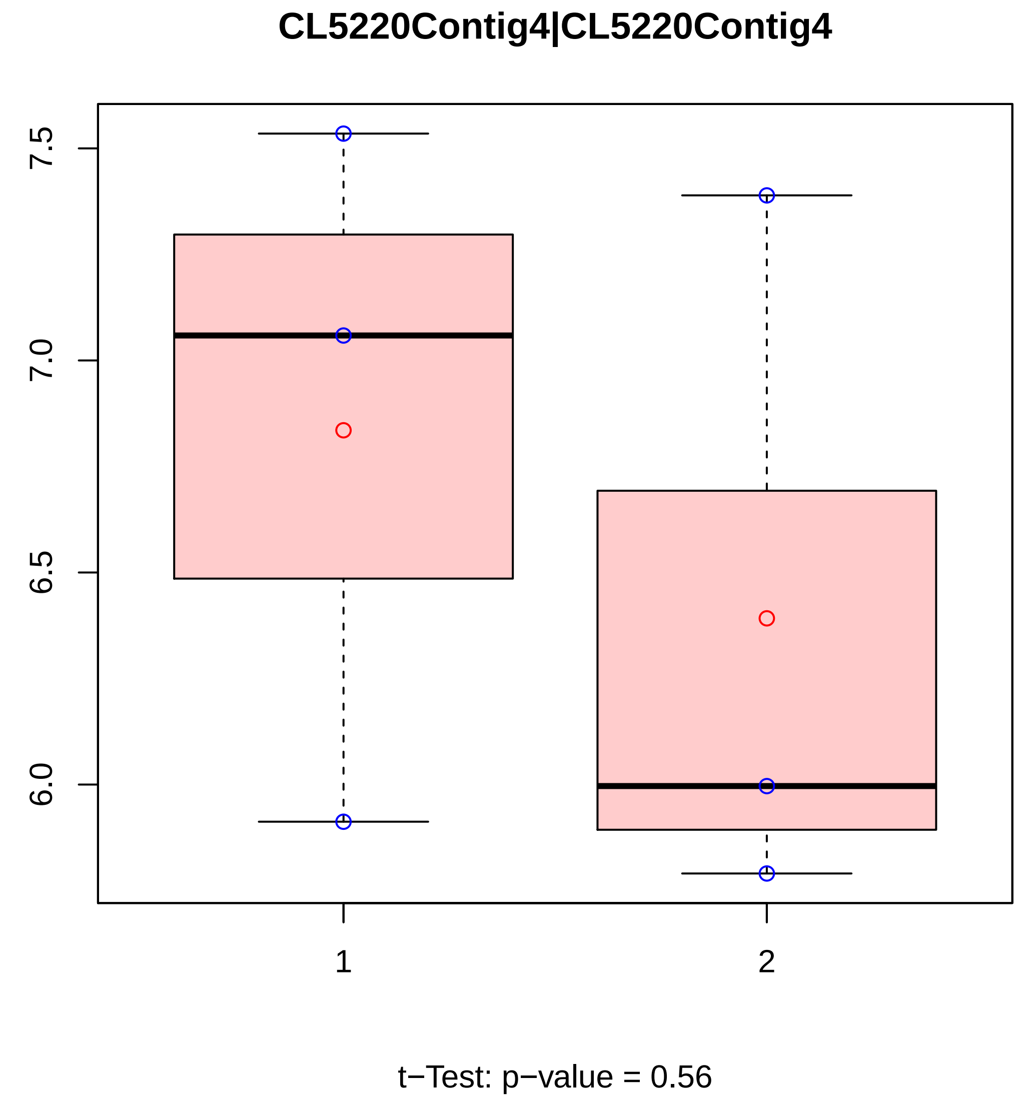
CL5220Contig4|CL5220Contig4
7.5
7.0
6.5
6.0
1
2
t−Test: p−value = 0.56

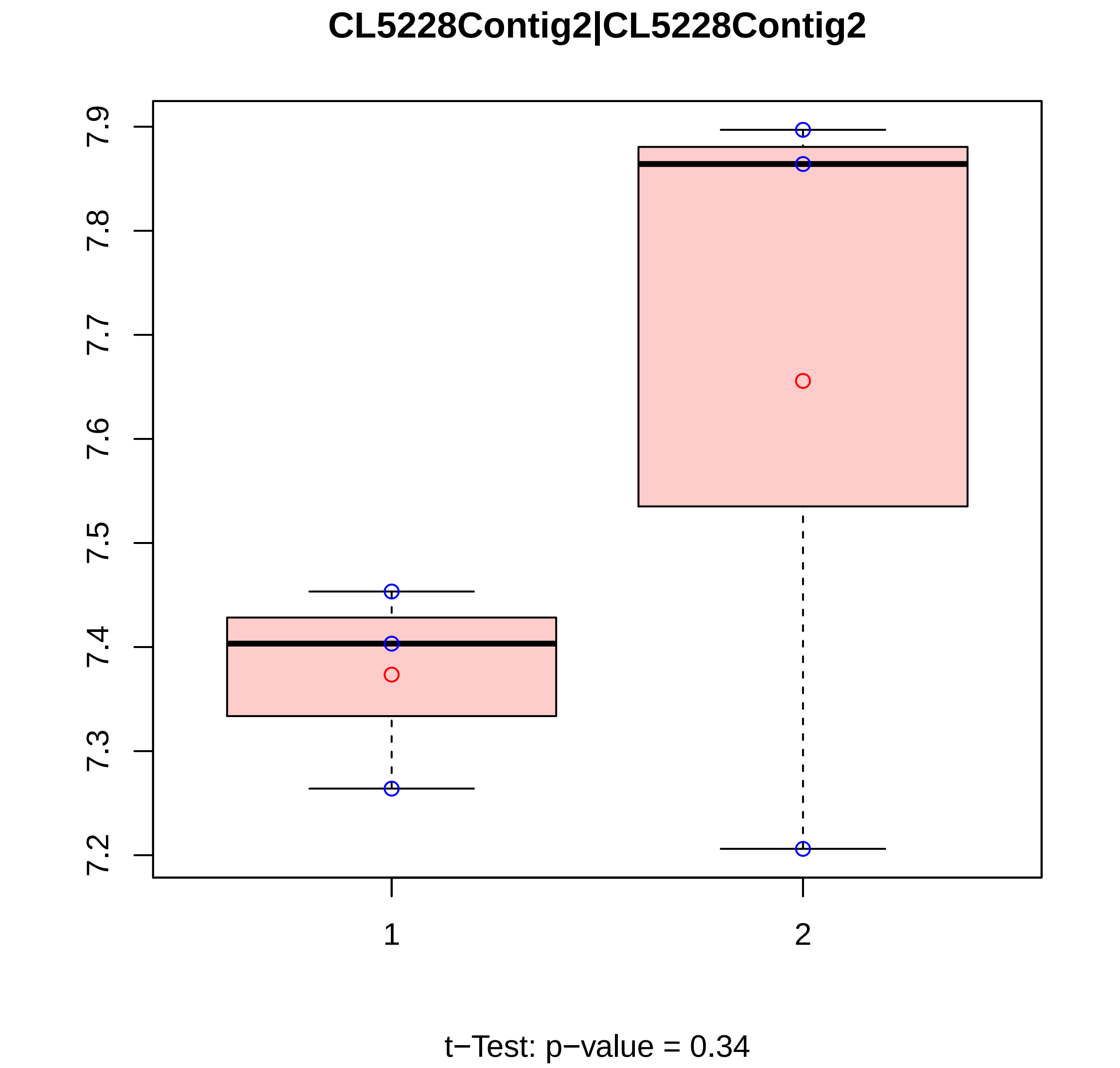
CL5228Contig2|CL5228Contig2
7.9
7.8
7.7
7.6
7.5
7.4
7.3
7.2
1
2
t−Test: p−value = 0.34

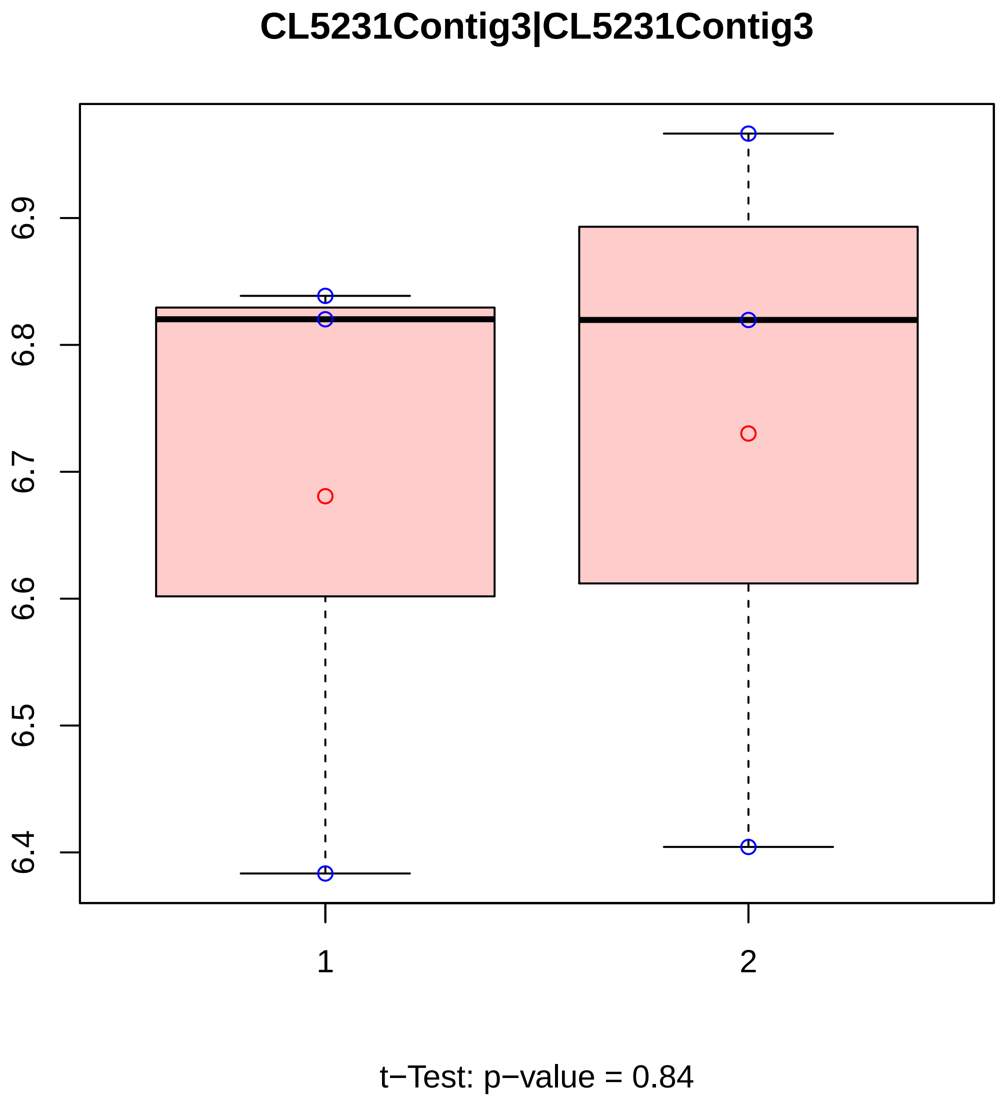
CL5231Contig3|CL5231Contig3
6.4
6.5
6.6
6.7
6.8
6.9
1
2
t−Test: p−value = 0.84

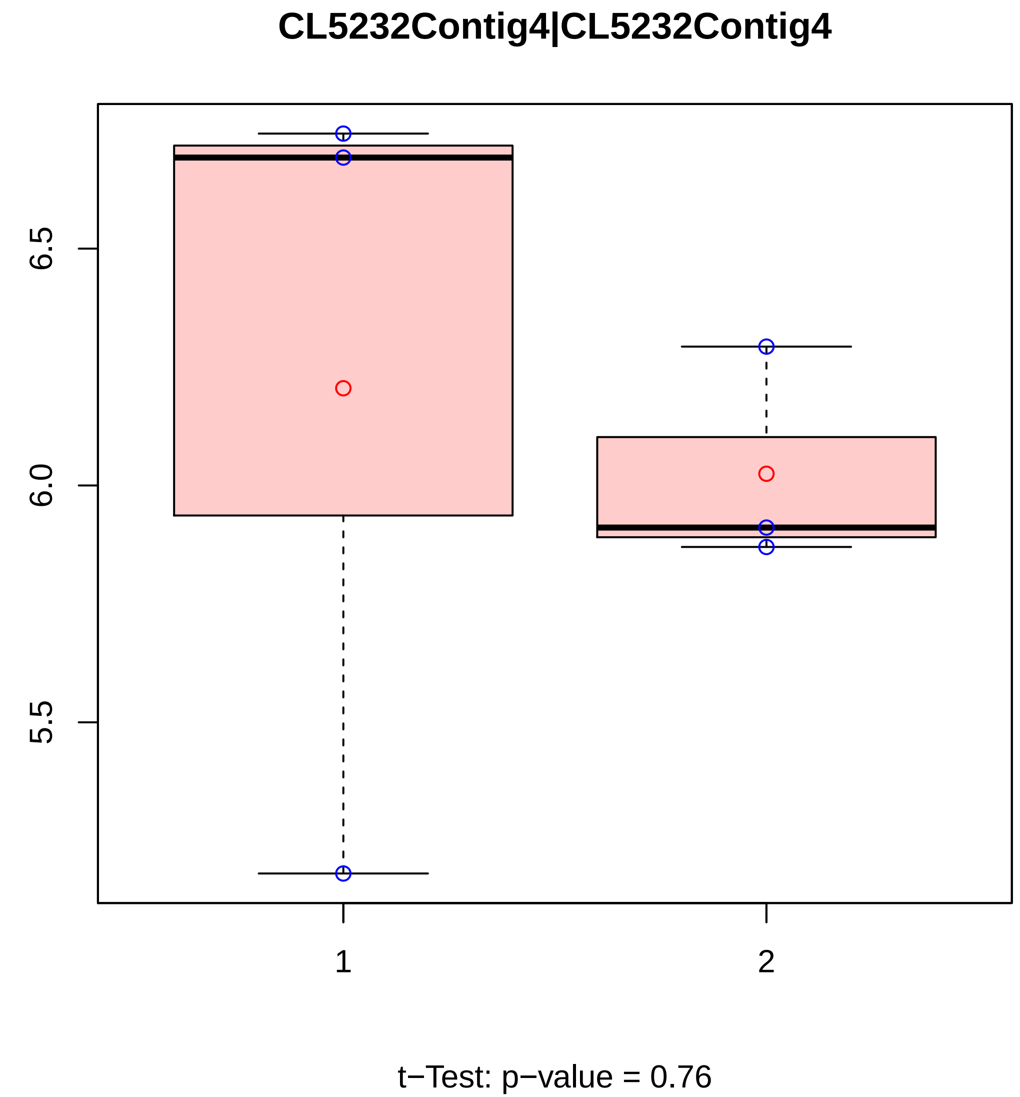
CL5232Contig4|CL5232Contig4
6.5
6.0
5.5
1
2
t−Test: p−value = 0.76

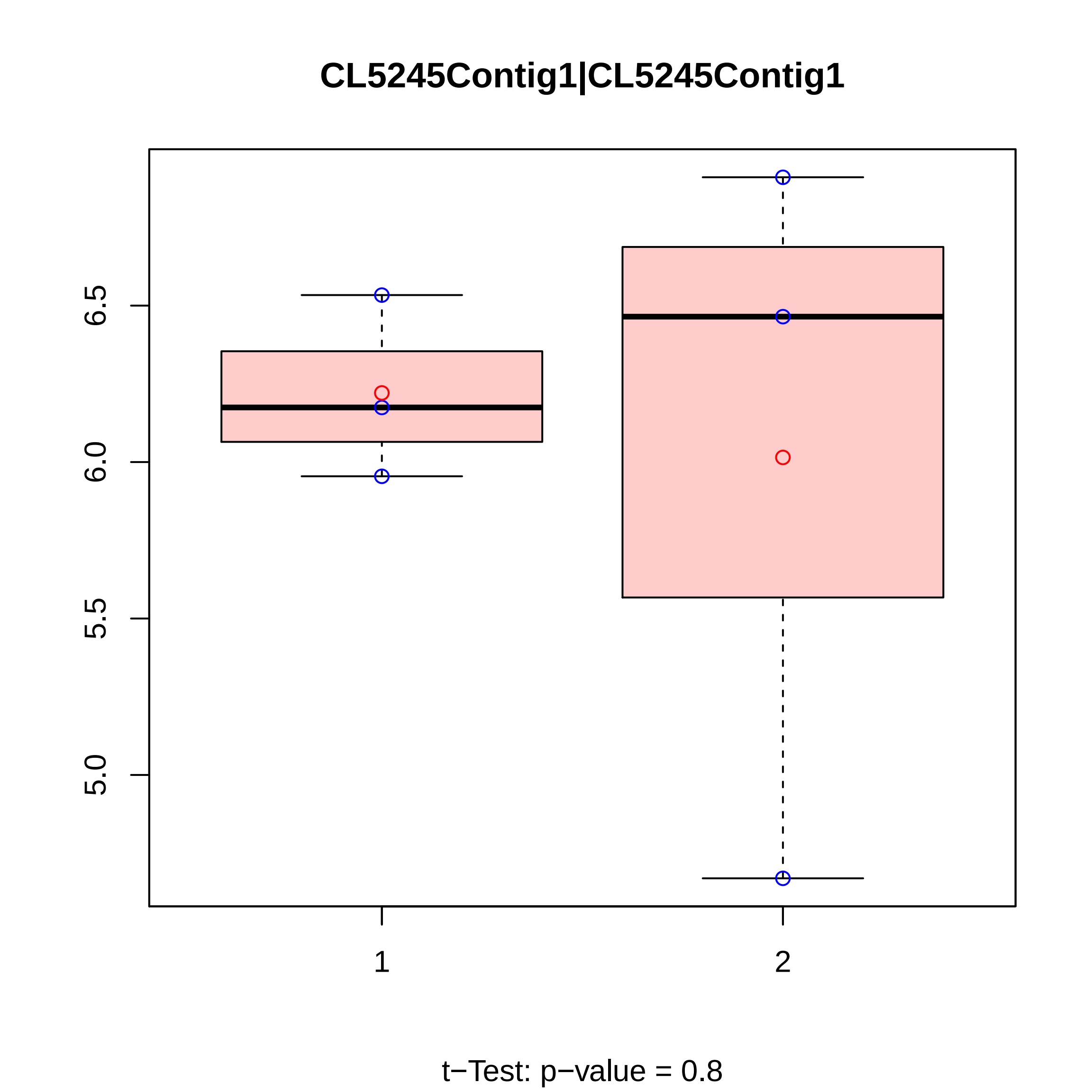

CL5245Contig1|CL5245Contig1
6.5
6.0
5.5
5.0
1
2
t−Test: p−value = 0.8

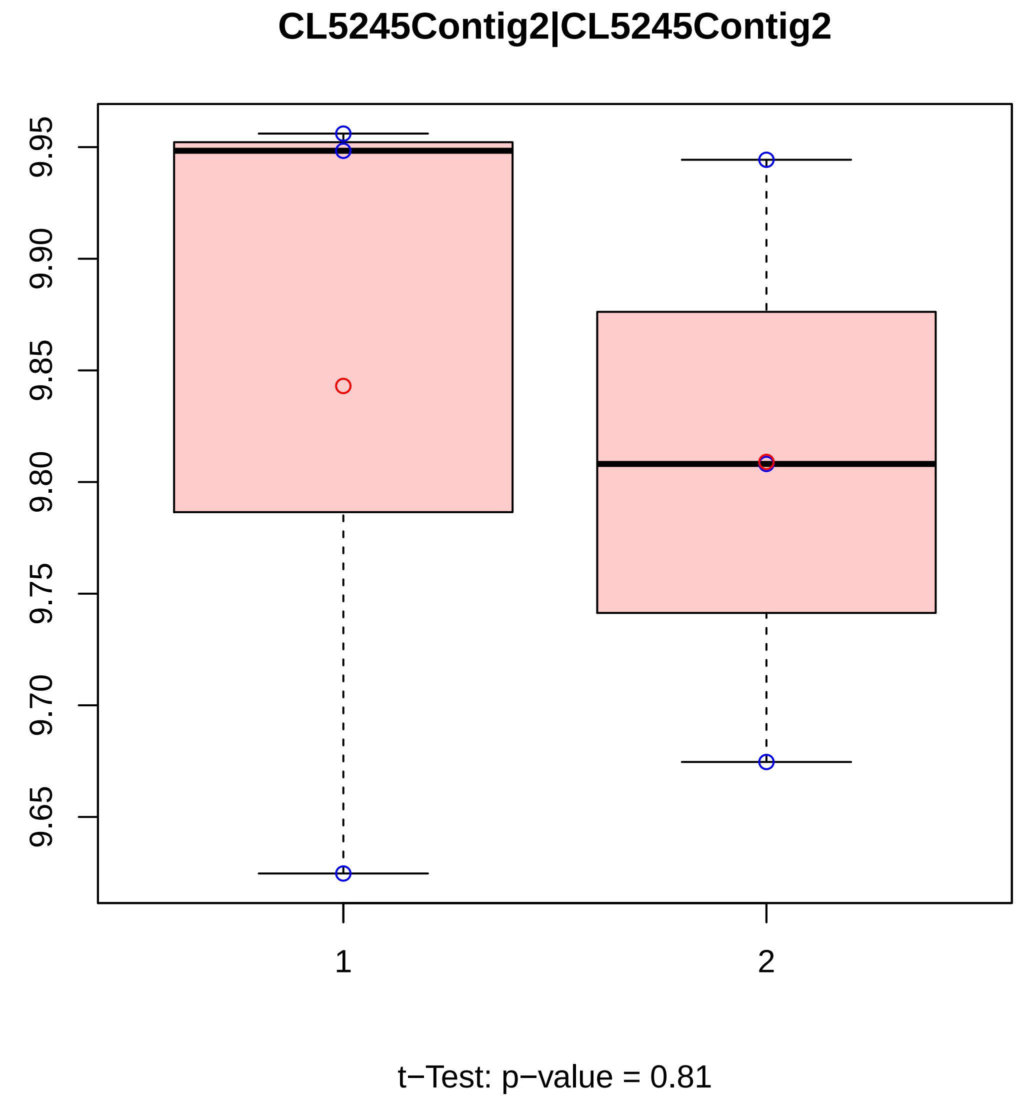
CL5245Contig2|CL5245Contig2
9.65
9.70
9.75
9.80
9.85
9.90
9.95
1
2
t−Test: p−value = 0.81

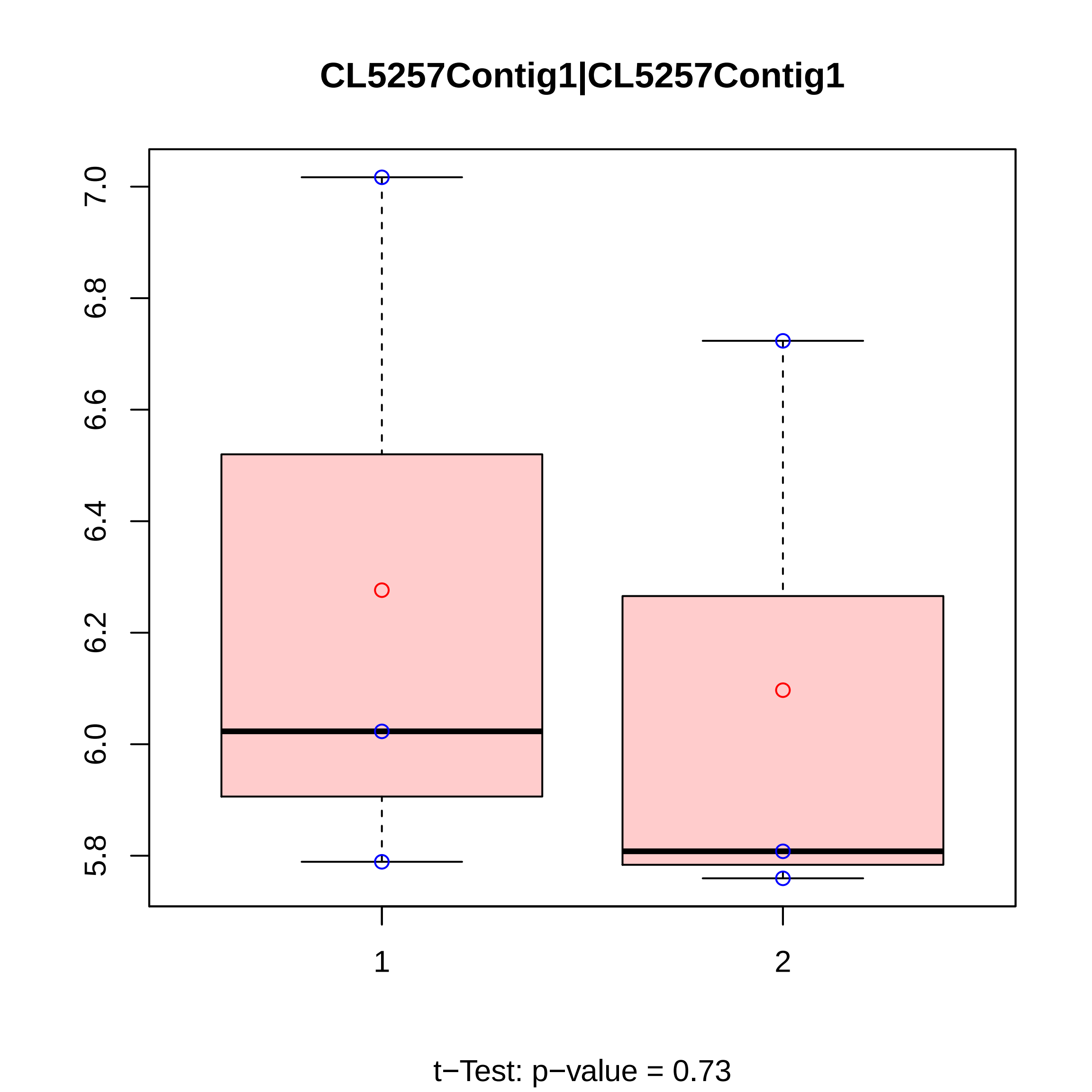

CL5257Contig1|CL5257Contig1
7.0
6.8
6.6
6.4
6.2
6.0
5.8
1
2
t−Test: p−value = 0.73

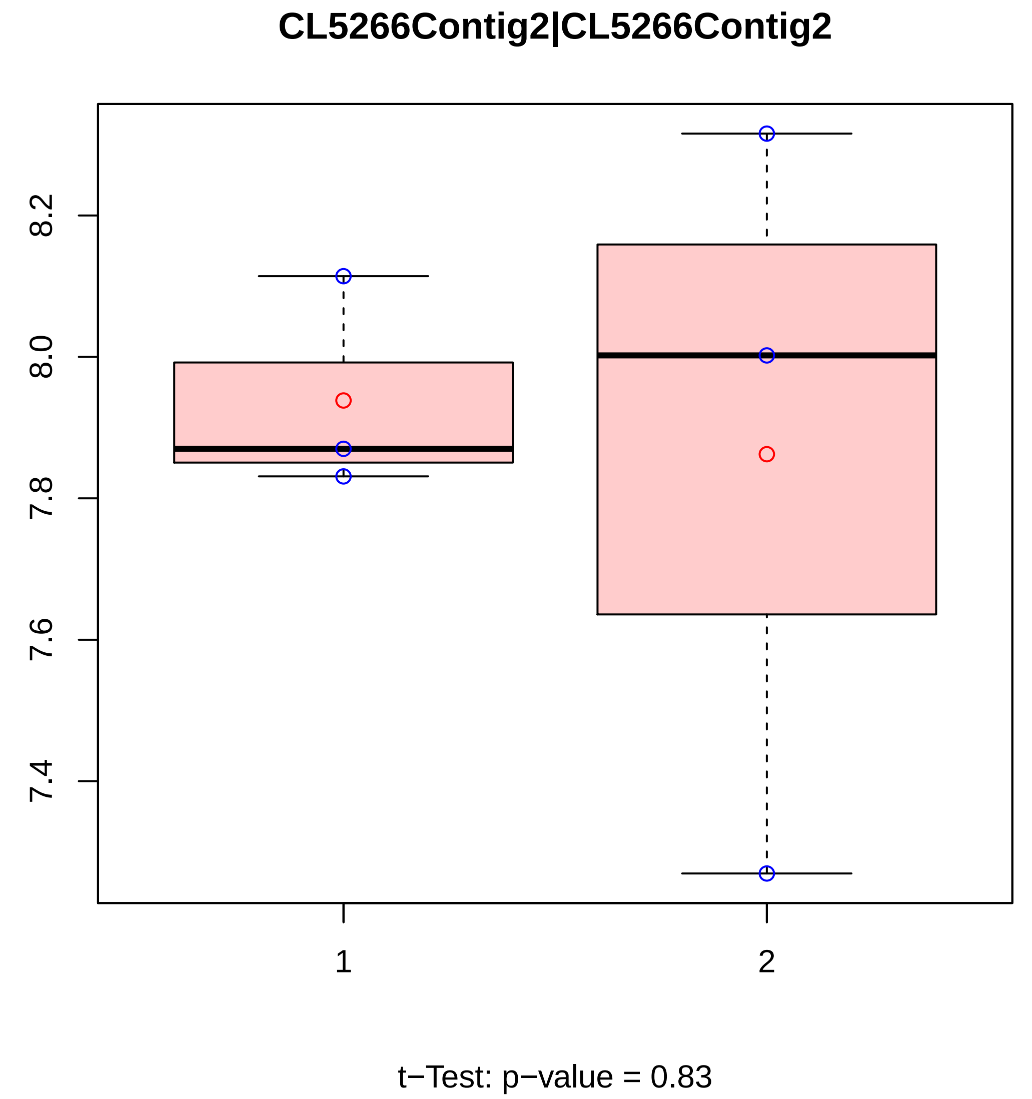
CL5266Contig2|CL5266Contig2
8.2
8.0
7.8
7.6
7.4
1
2
t−Test: p−value = 0.83

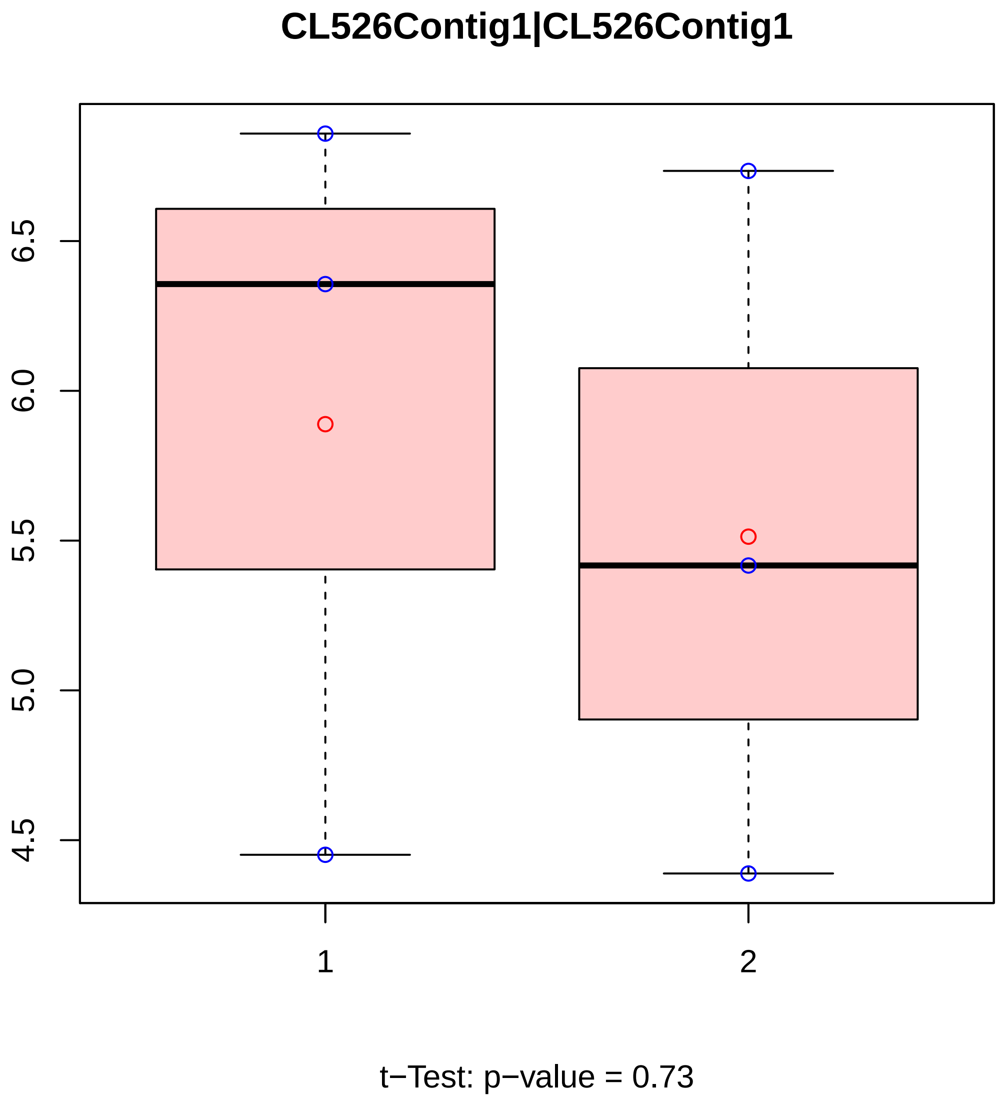
CL526Contig1|CL526Contig1
6.5
6.0
5.5
5.0
4.5
1
2
t−Test: p−value = 0.73

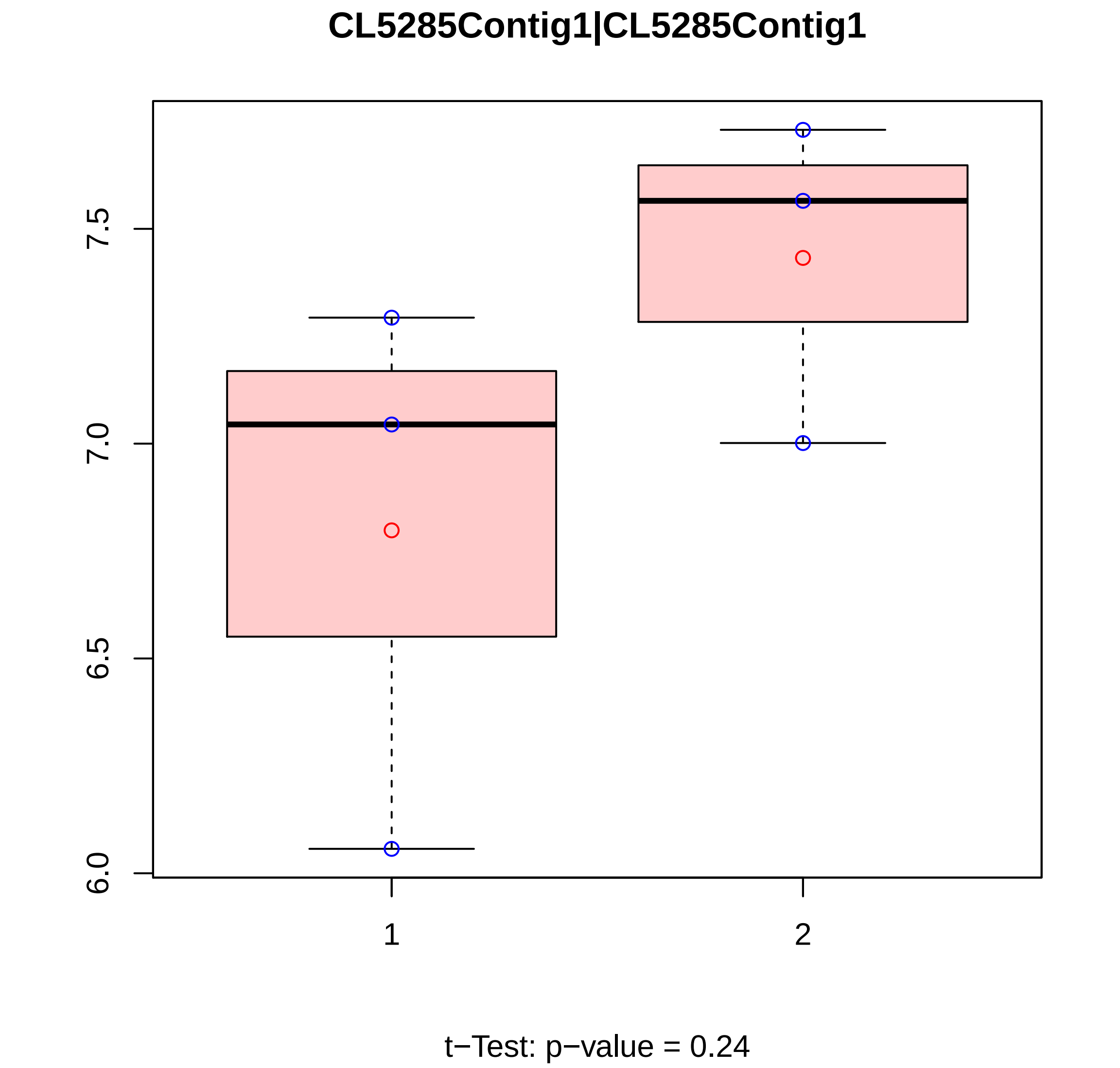

CL5285Contig1|CL5285Contig1
7.5
7.0
6.5
6.0
1
2
t−Test: p−value = 0.24

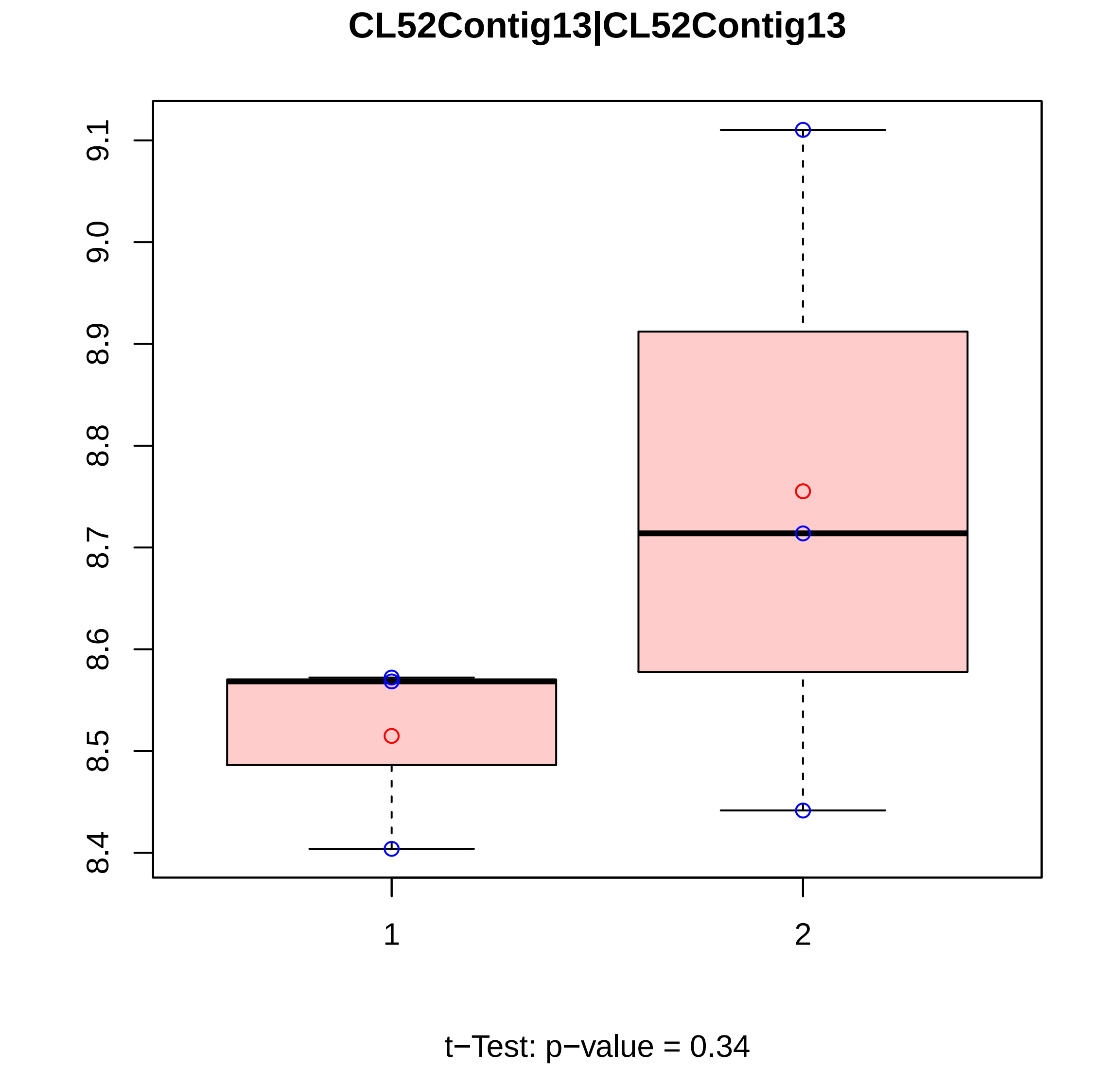

CL52Contig13|CL52Contig13
9.1
9.0
8.9
8.8
8.7
8.6
8.5
8.4
1
2
t−Test: p−value = 0.34

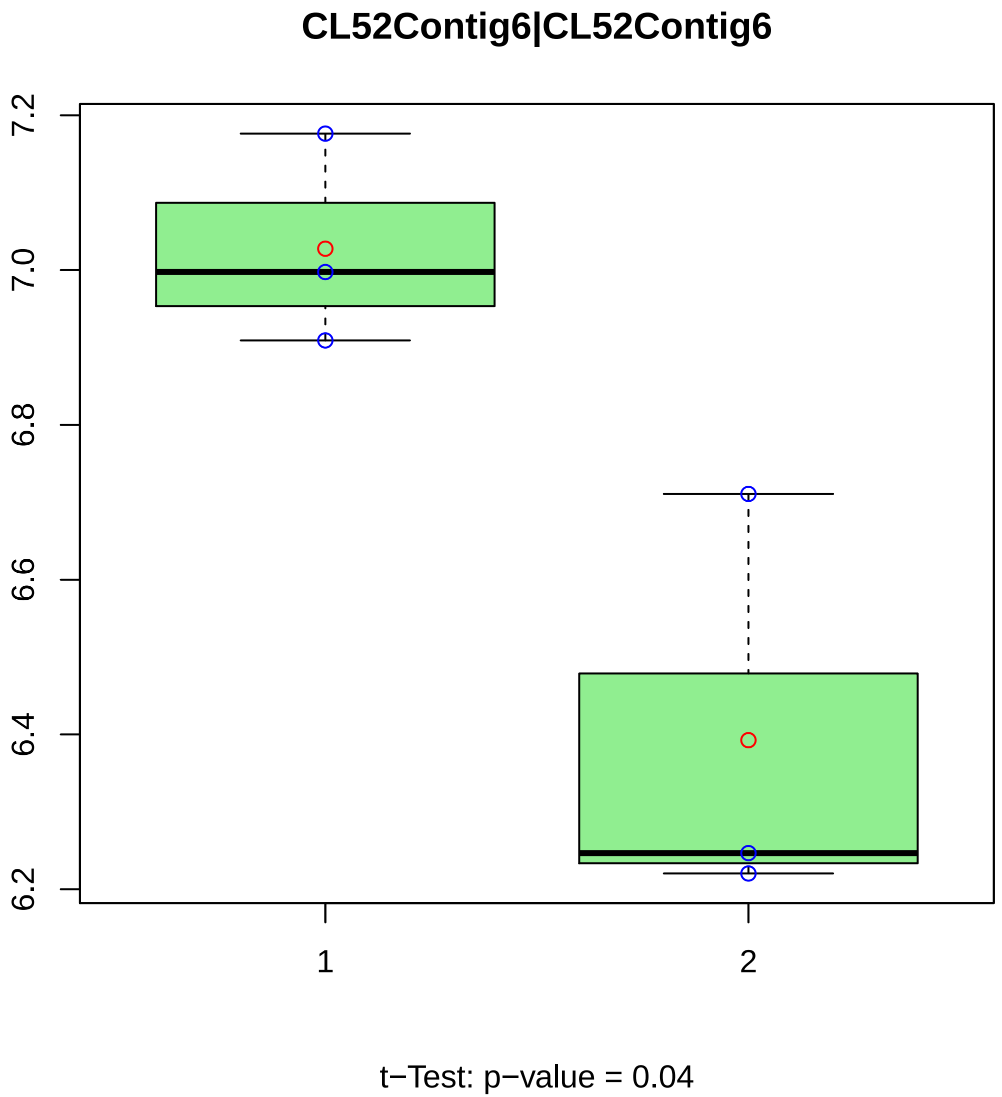
CL52Contig6|CL52Contig6
7.2
7.0
6.8
6.6
6.4
6.2
1
2
t−Test: p−value = 0.04

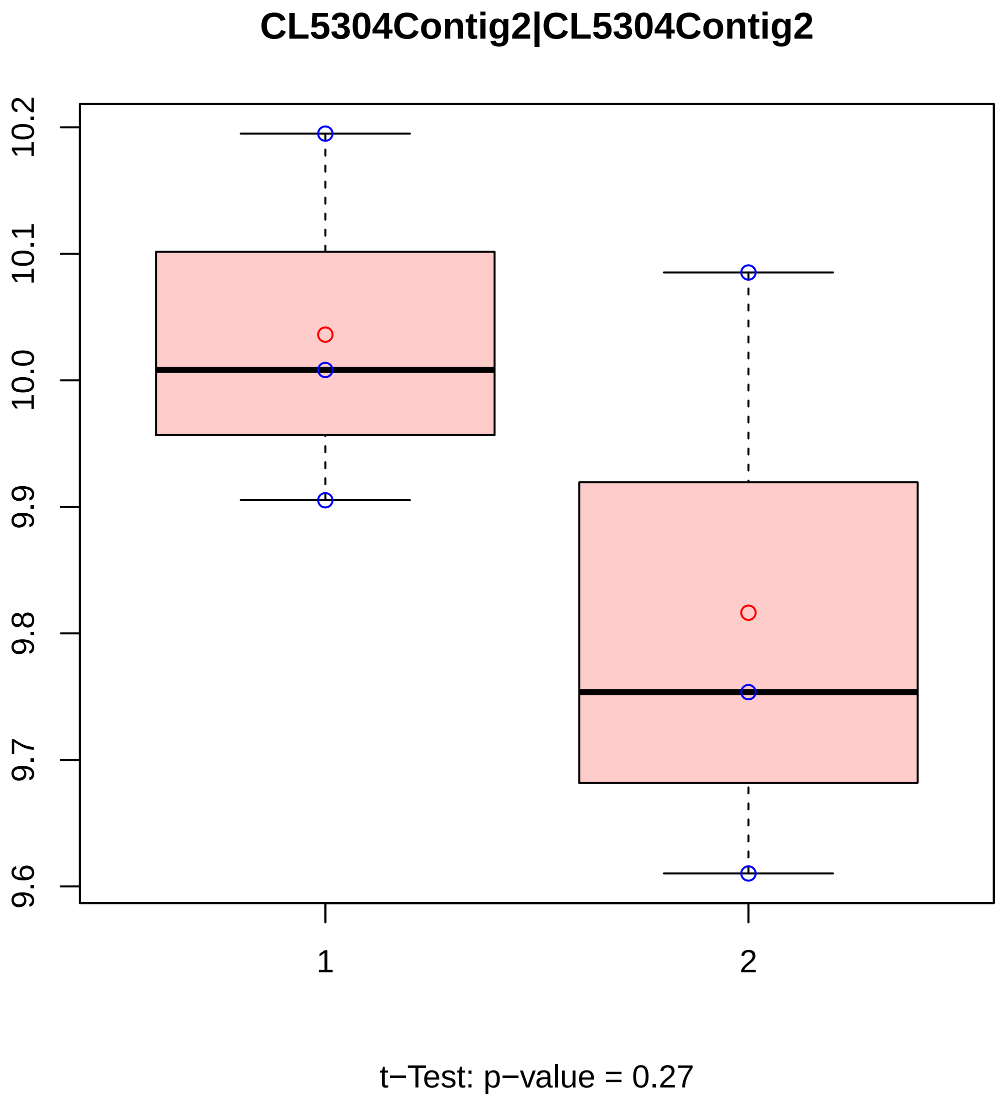
CL5304Contig2|CL5304Contig2
10.2
10.1
10.0
9.9
9.8
9.7
9.6
1
2
t−Test: p−value = 0.27

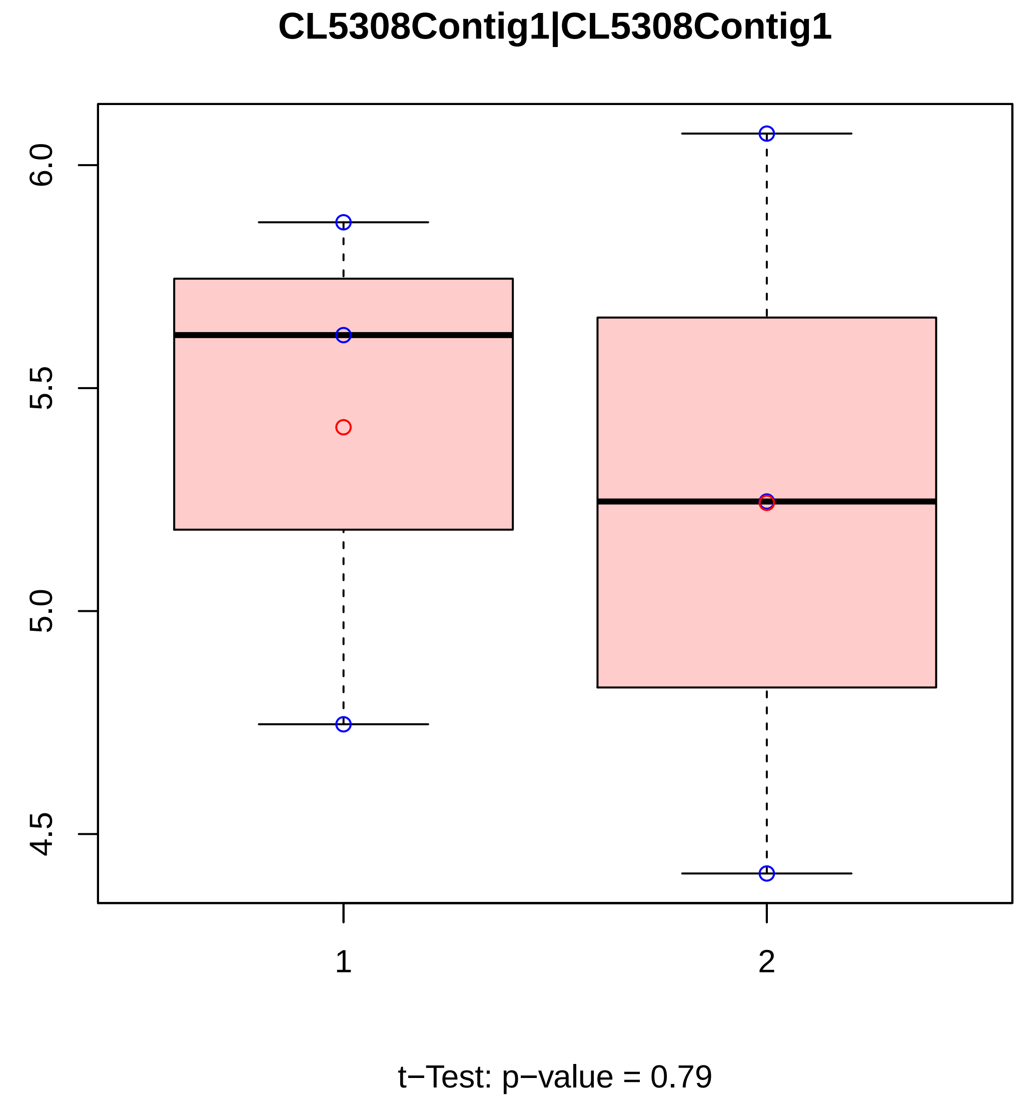
CL5308Contig1|CL5308Contig1
6.0
5.5
5.0
4.5
1
2
t−Test: p−value = 0.79

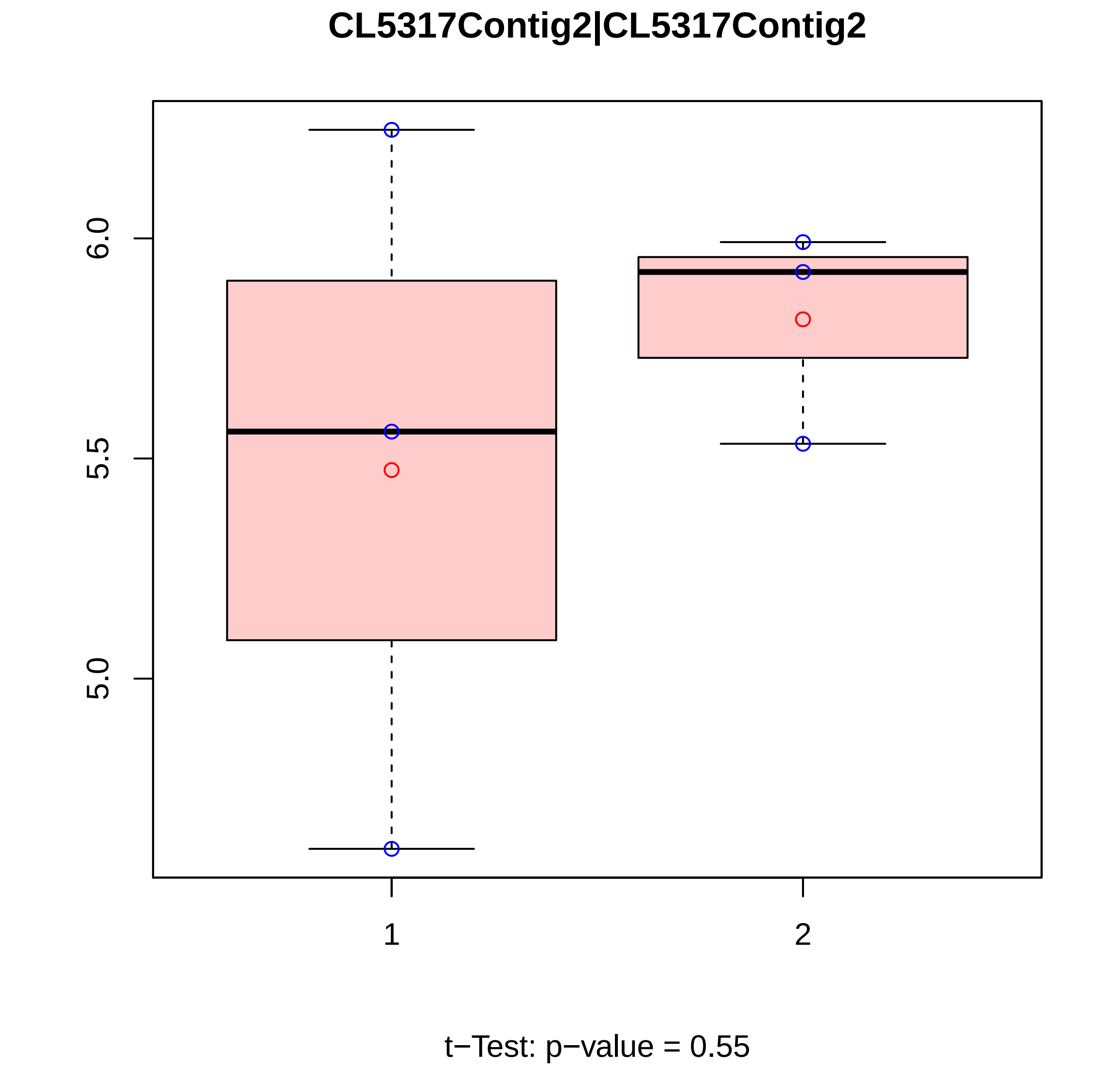
CL5317Contig2|CL5317Contig2
6.0
5.5
5.0
1
2
t−Test: p−value = 0.55

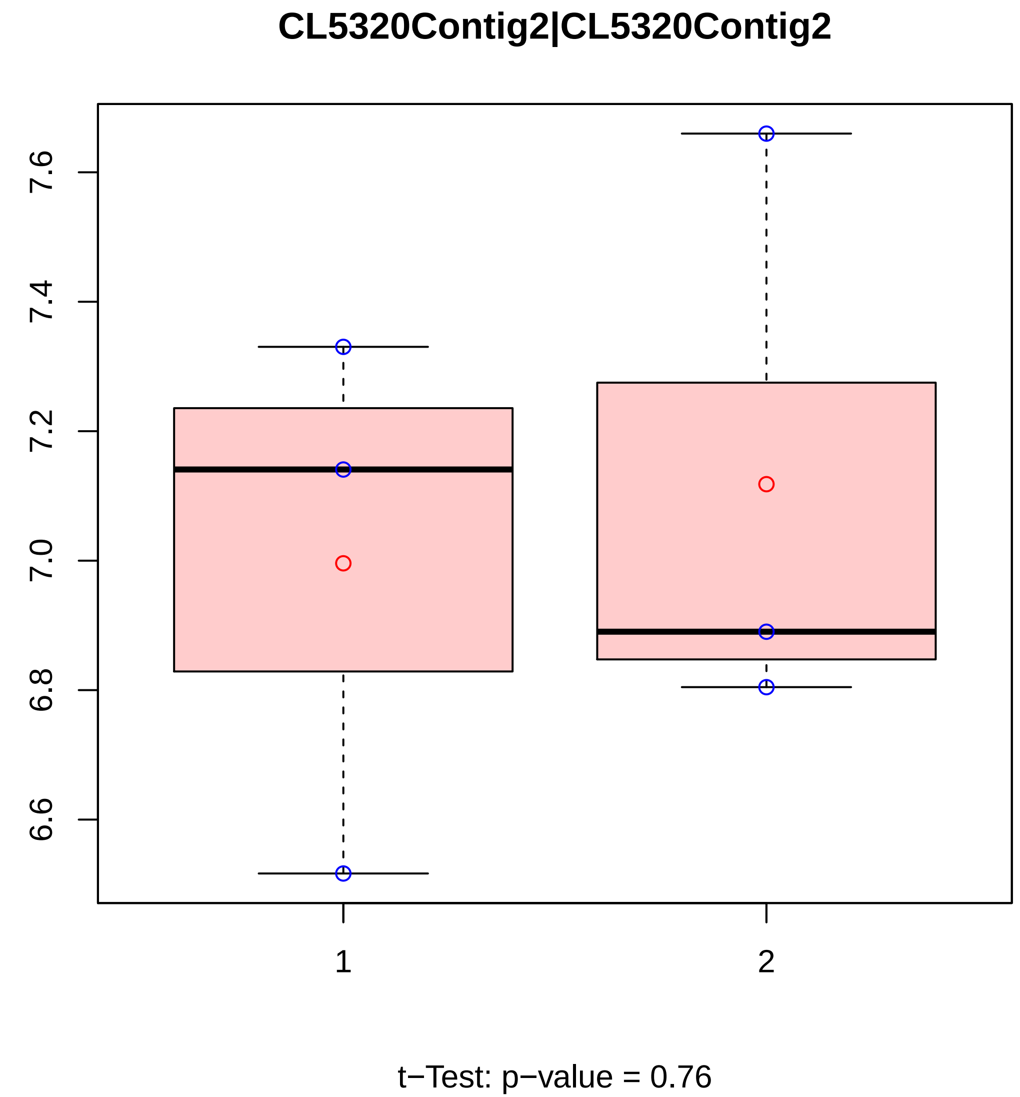
CL5320Contig2|CL5320Contig2
7.6
7.4
7.2
7.0
6.8
6.6
1
2
t−Test: p−value = 0.76

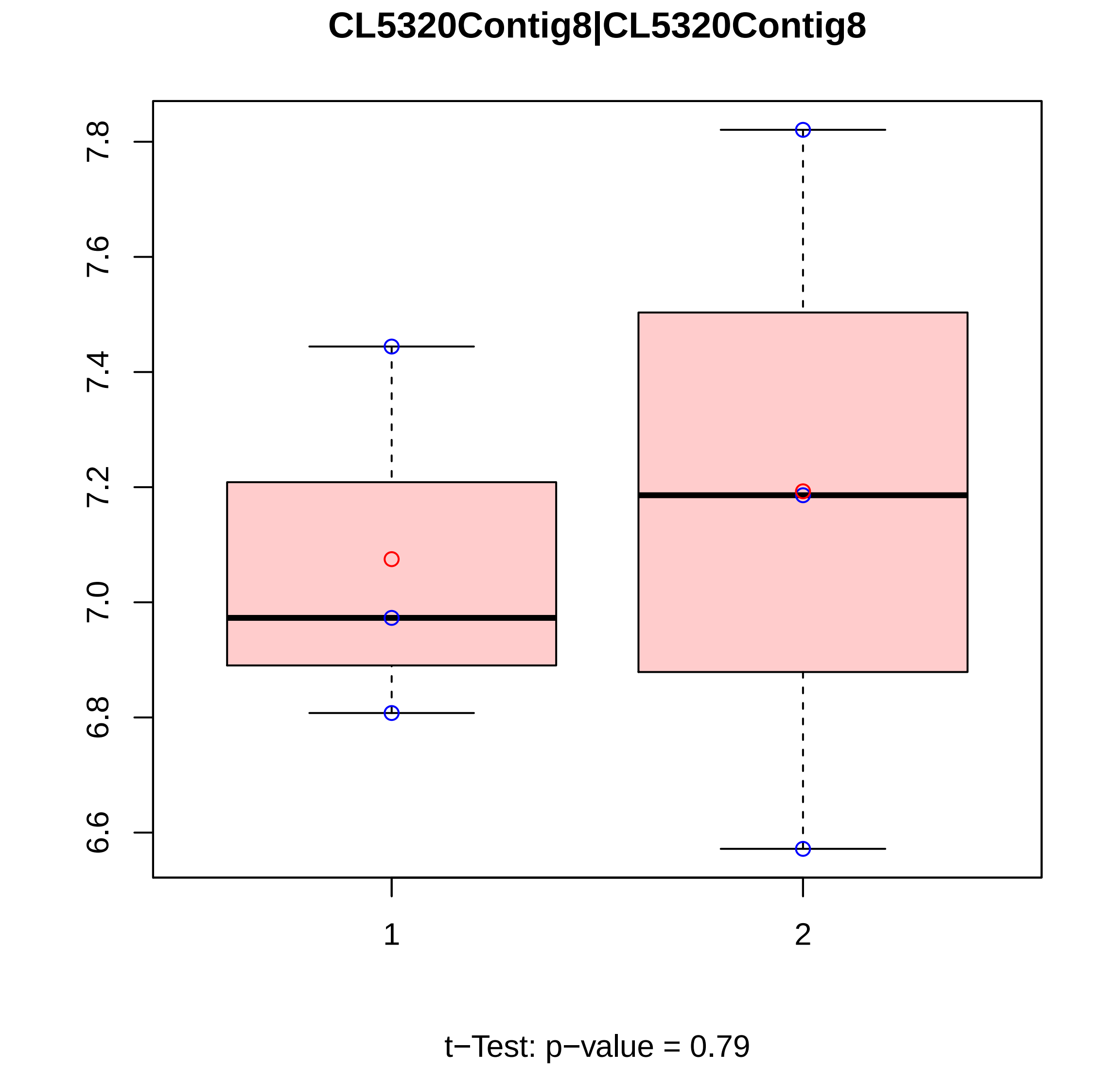
CL5320Contig8|CL5320Contig8
7.8
7.6
7.4
7.2
7.0
6.8
6.6
1
2
t−Test: p−value = 0.79

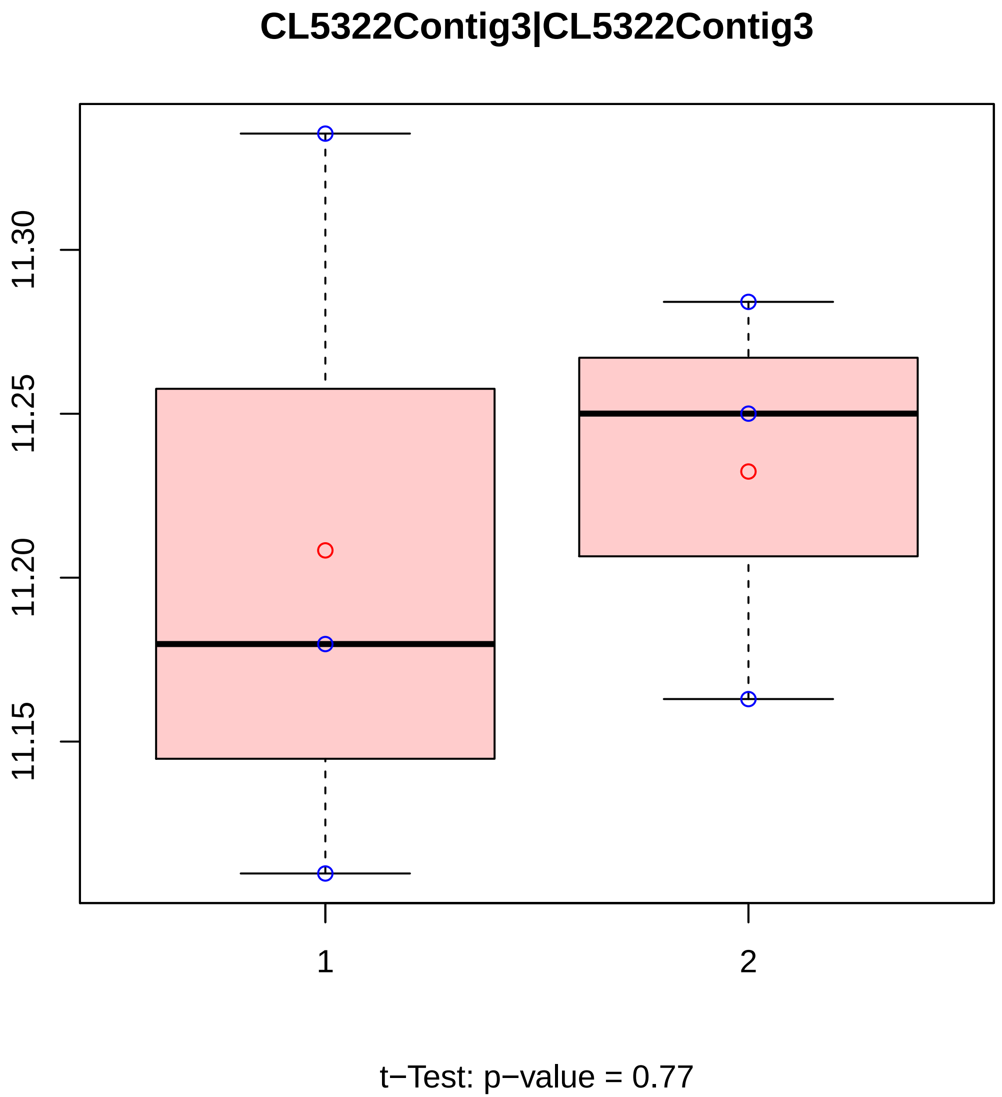
CL5322Contig3|CL5322Contig3
11.15
11.20
11.25
11.30
1
2
t−Test: p−value = 0.77

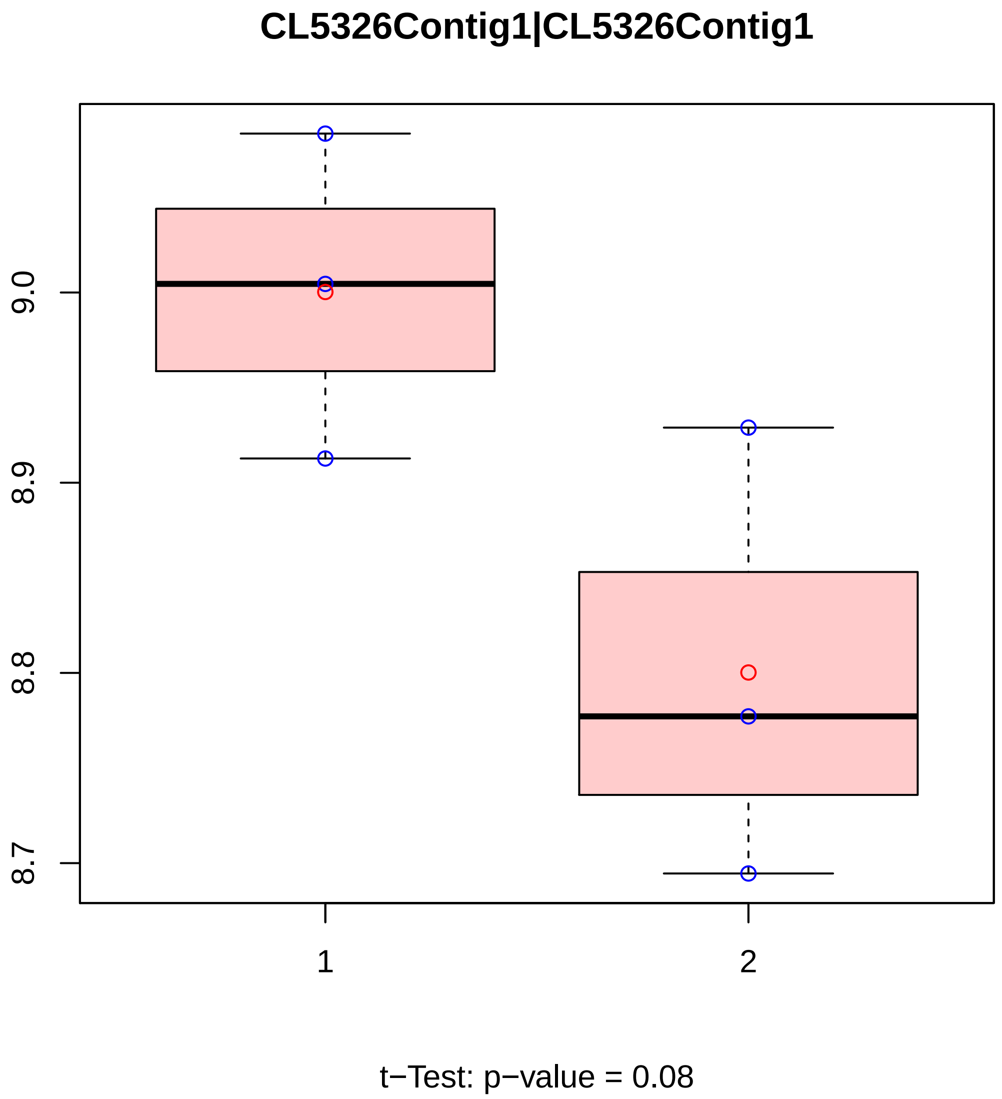

CL5326Contig1|CL5326Contig1
9.0
8.9
8.8
8.7
1
2
t−Test: p−value = 0.08

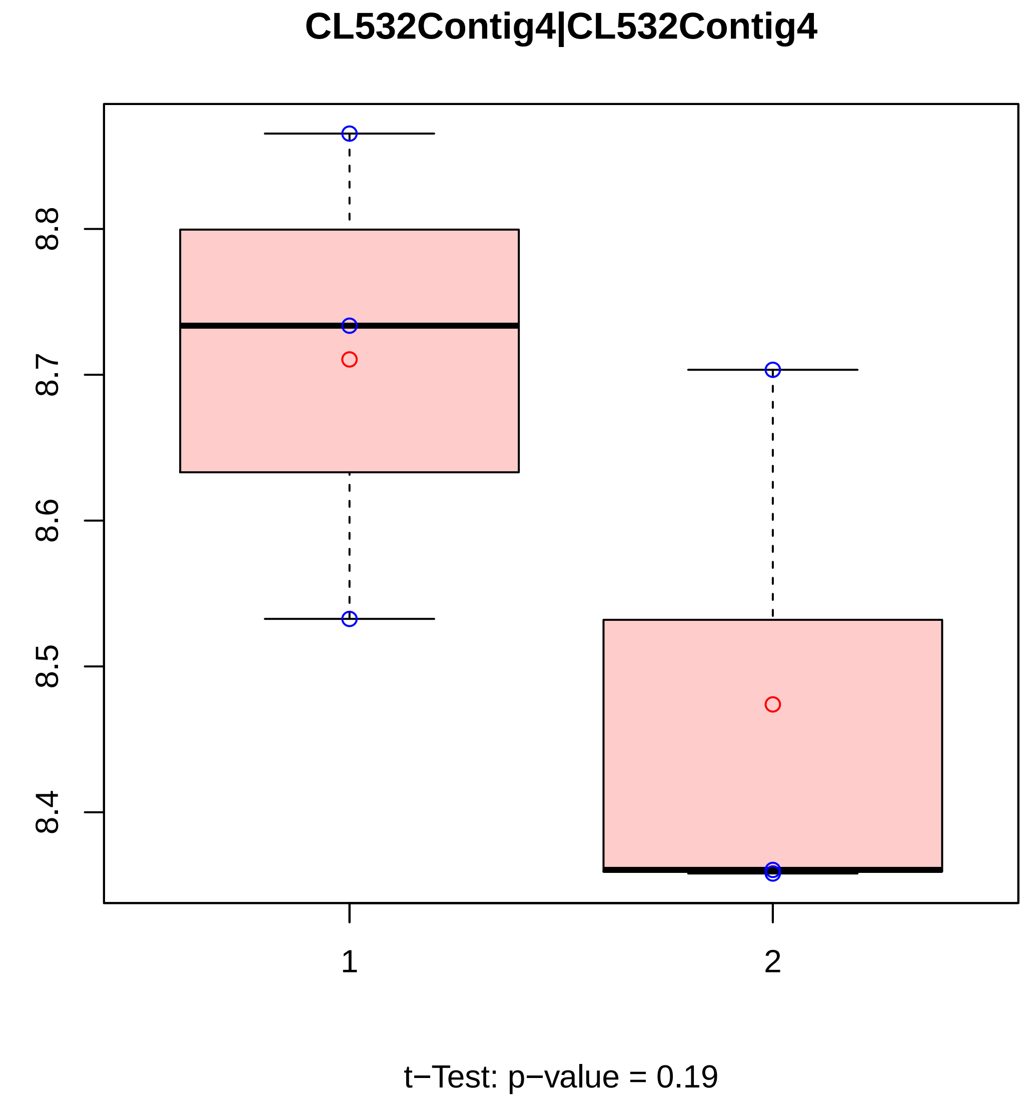

CL532Contig4|CL532Contig4
8.4
8.5
8.6
8.7
8.8
1
2
t−Test: p−value = 0.19

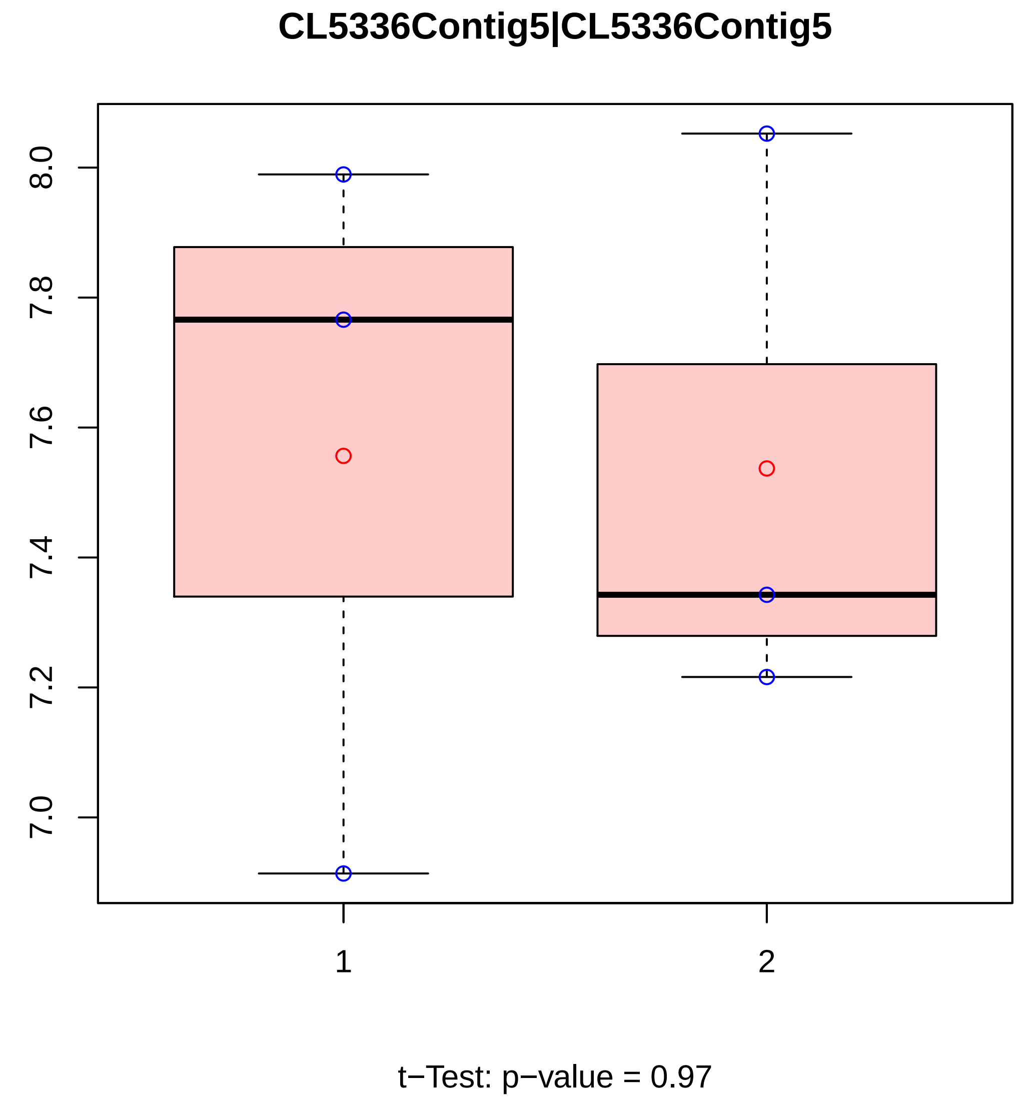
CL5336Contig5|CL5336Contig5
8.0
7.8
7.6
7.4
7.2
7.0
1
2
t−Test: p−value = 0.97

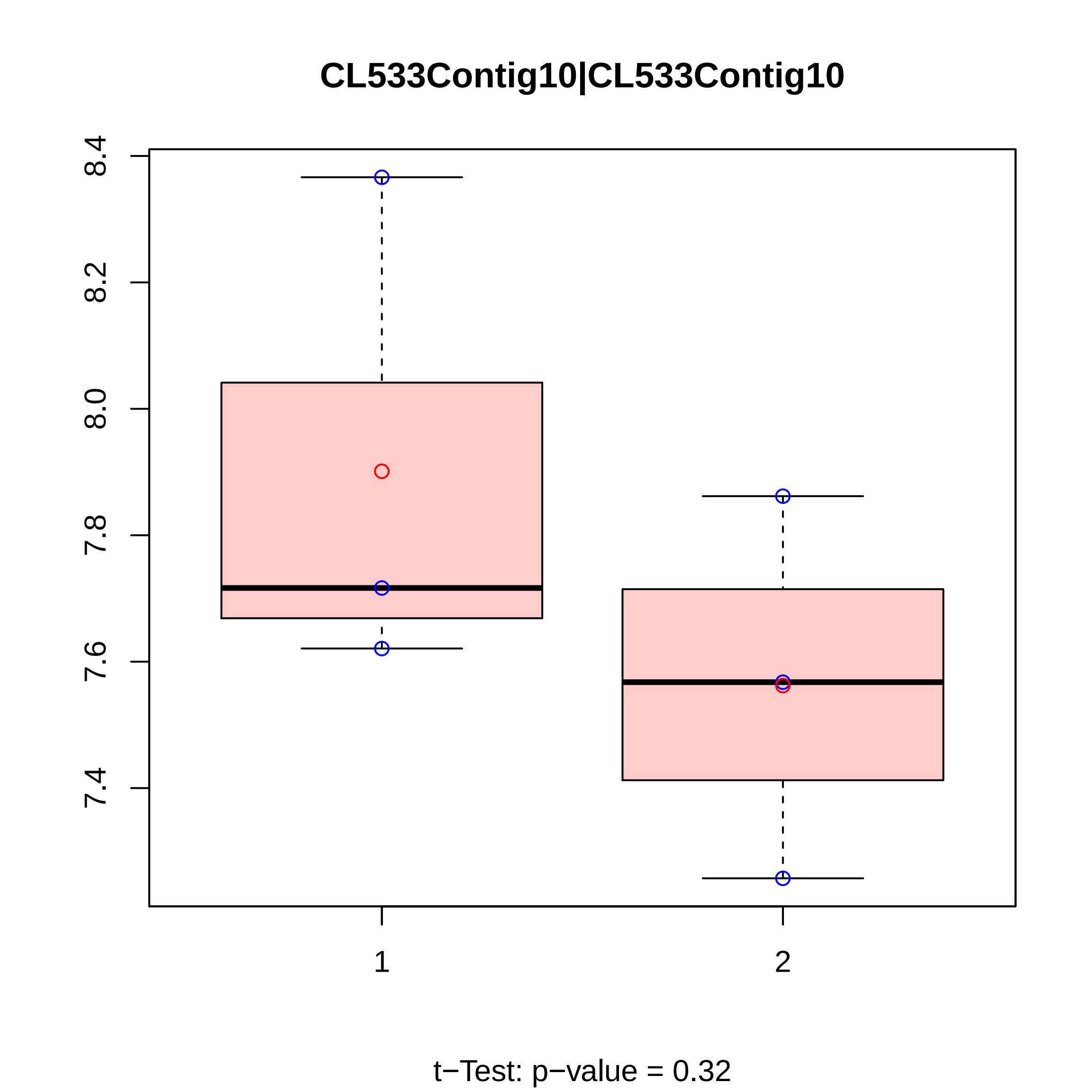
CL533Contig10|CL533Contig10
8.4
8.2
8.0
7.8
7.6
7.4
1
2
t−Test: p−value = 0.32

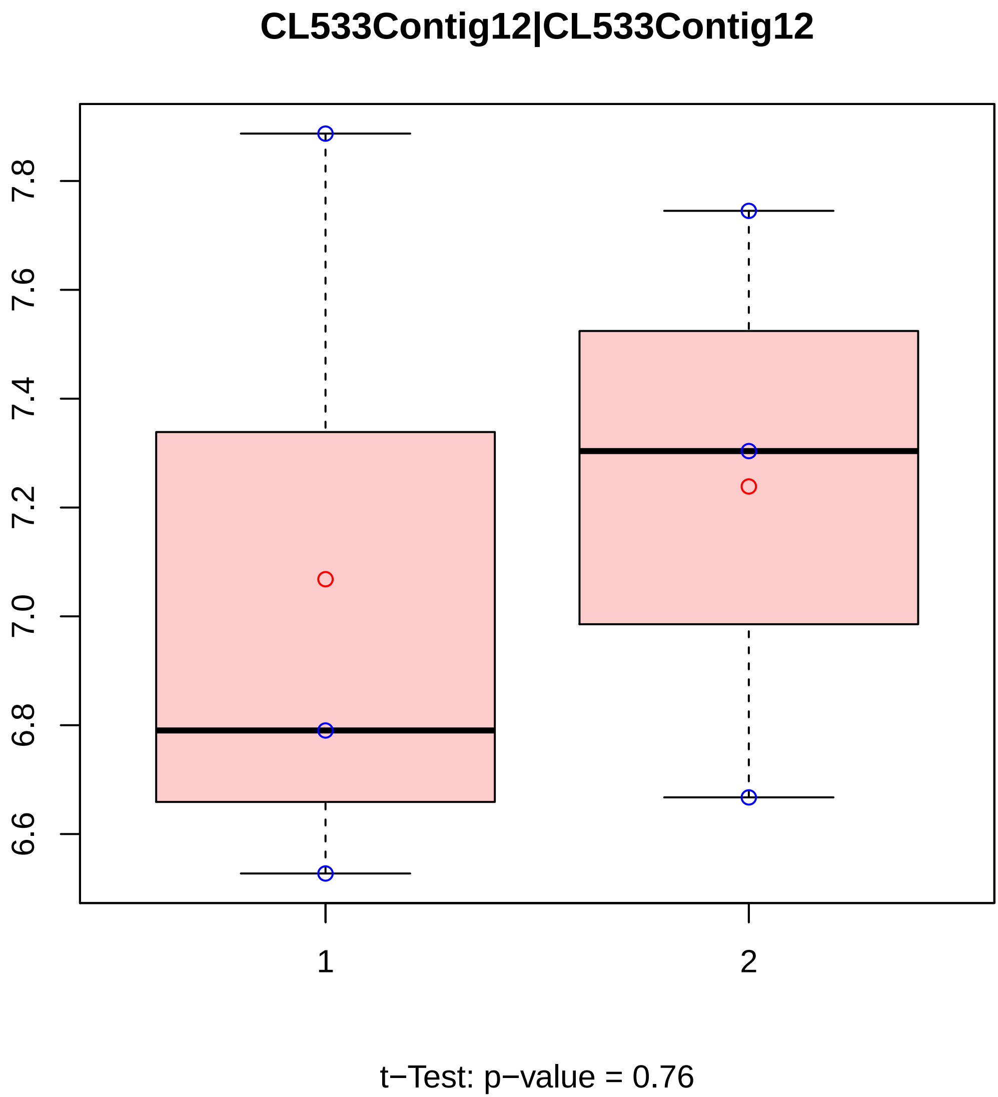

CL533Contig12|CL533Contig12
7.8
7.6
7.4
7.2
7.0
6.8
6.6
1
2
t−Test: p−value = 0.76

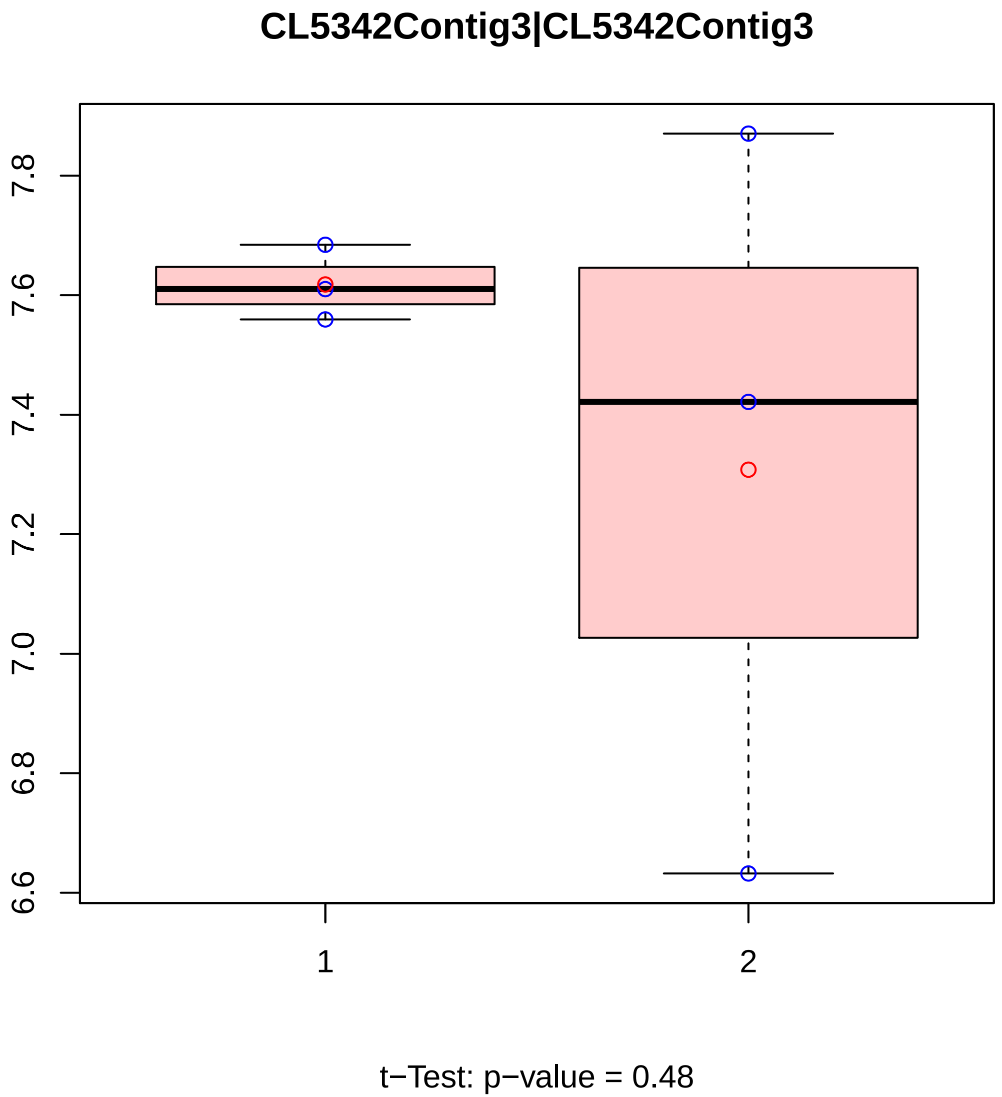

CL5342Contig3|CL5342Contig3
7.8
7.6
7.4
7.2
7.0
6.8
6.6
1
2
t−Test: p−value = 0.48

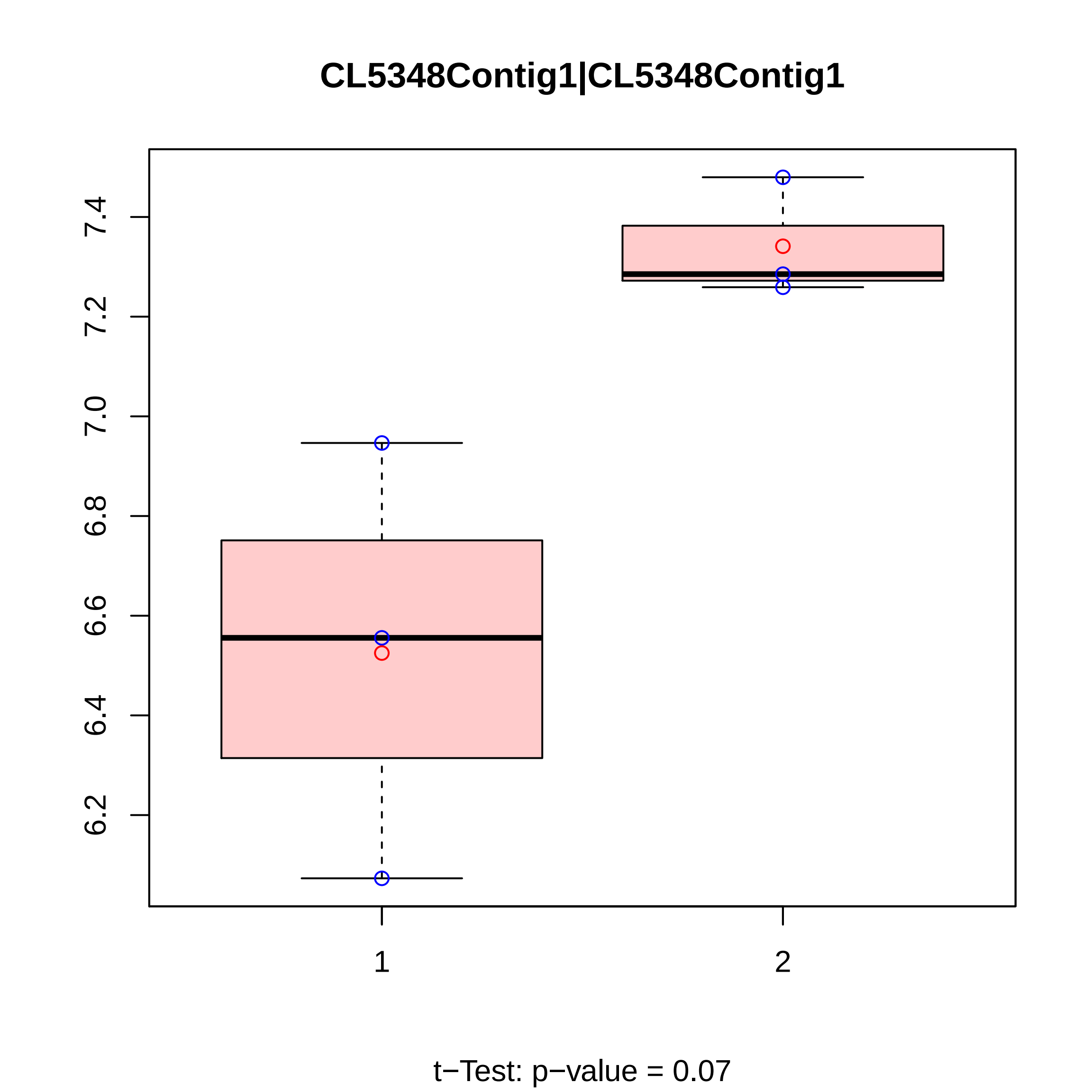
CL5348Contig1|CL5348Contig1
7.4
7.2
7.0
6.8
6.6
6.4
6.2
1
2
t−Test: p−value = 0.07

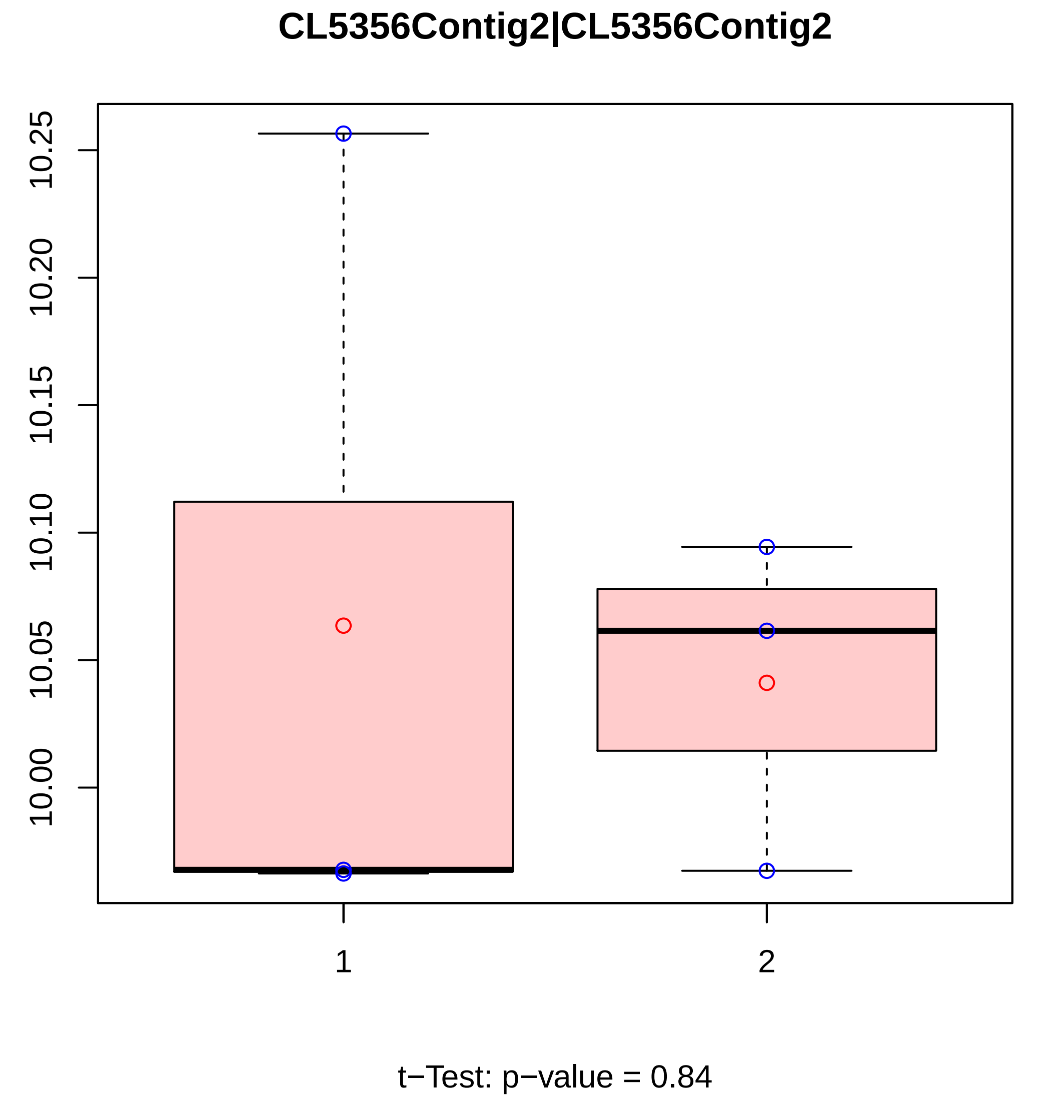
CL5356Contig2|CL5356Contig2
10.00
10.05
10.10
10.15
10.20
10.25
1
2
t−Test: p−value = 0.84

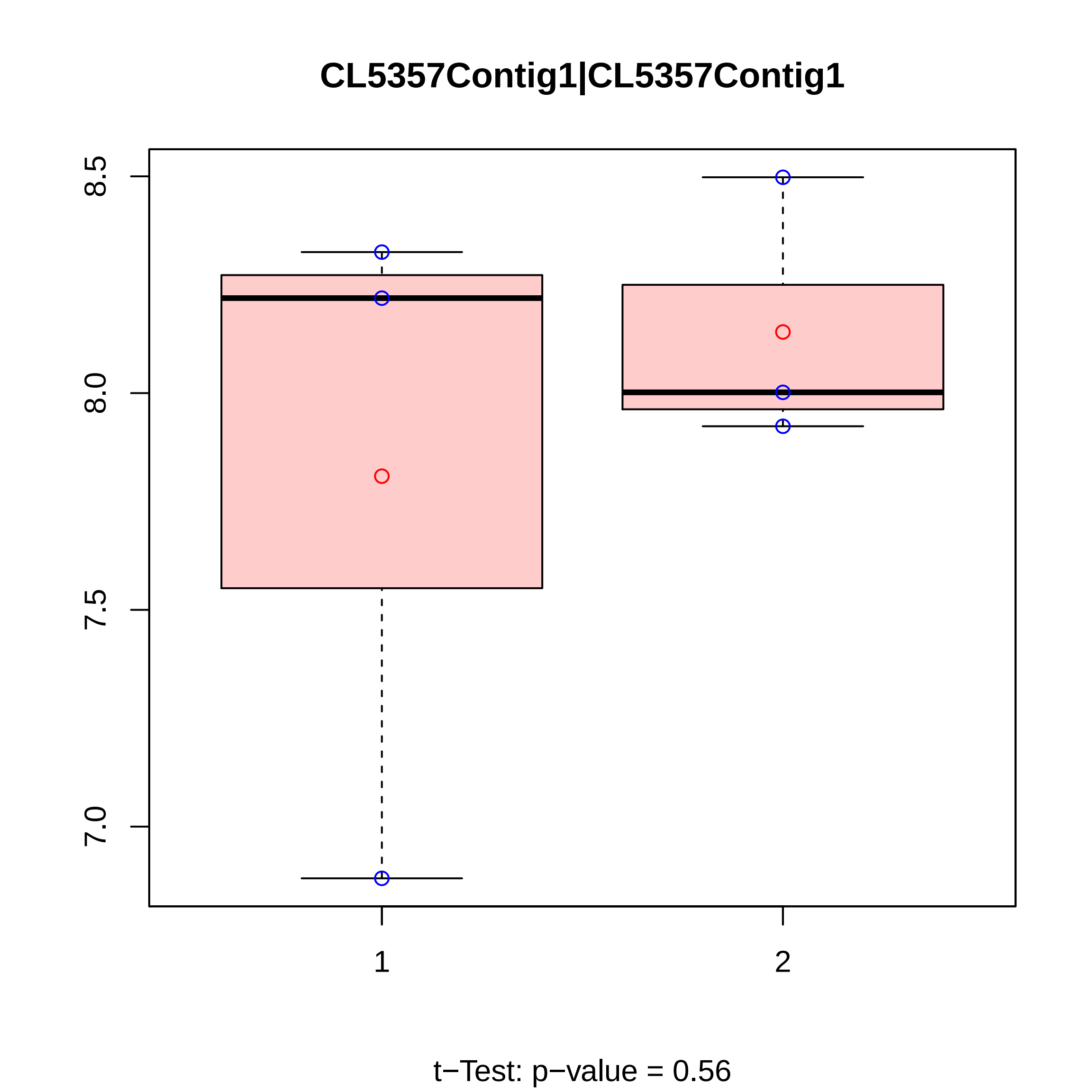

CL5357Contig1|CL5357Contig1
8.5
8.0
7.5
7.0
1
2
t−Test: p−value = 0.56

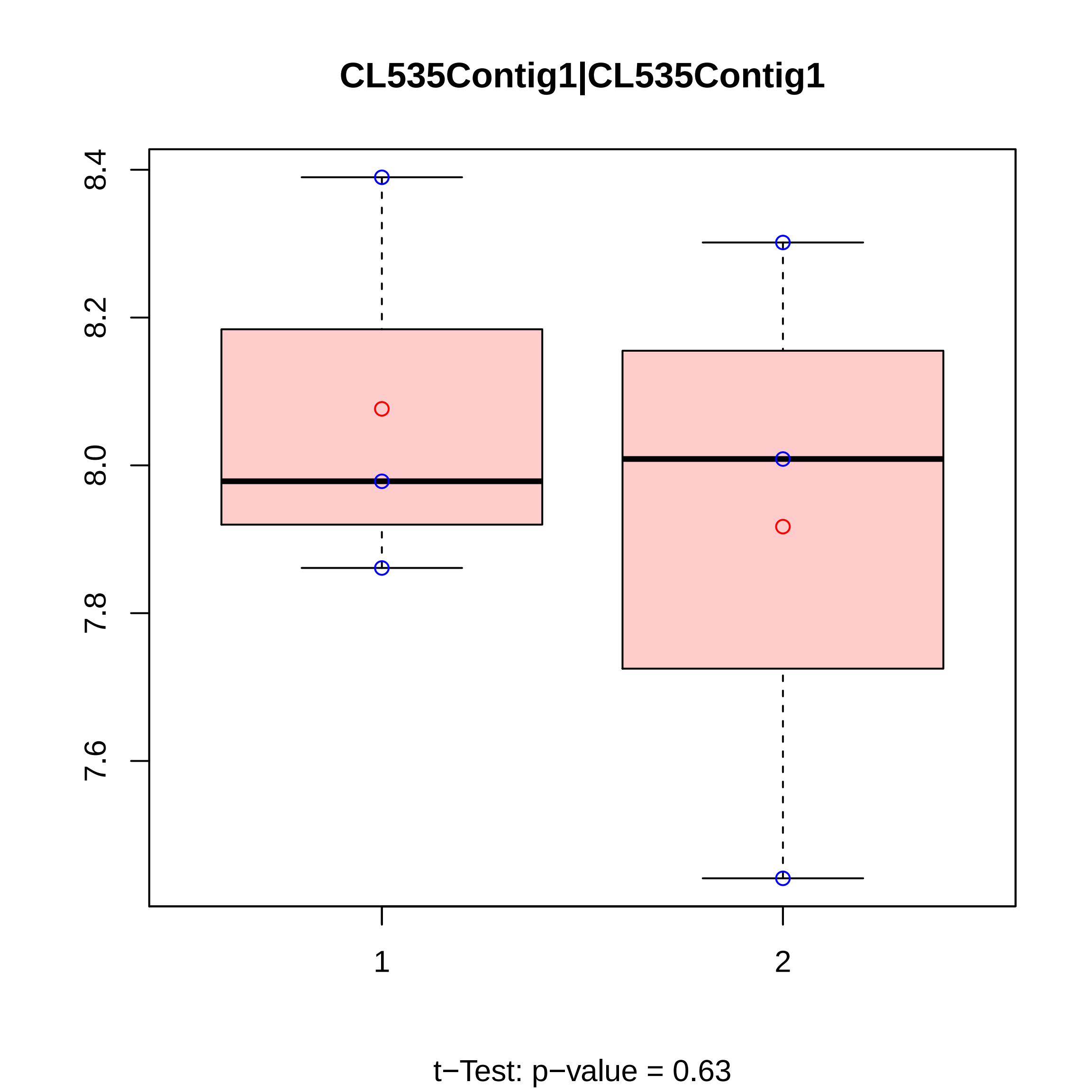
CL535Contig1|CL535Contig1
8.4
8.2
8.0
7.8
7.6
1
2
t−Test: p−value = 0.63

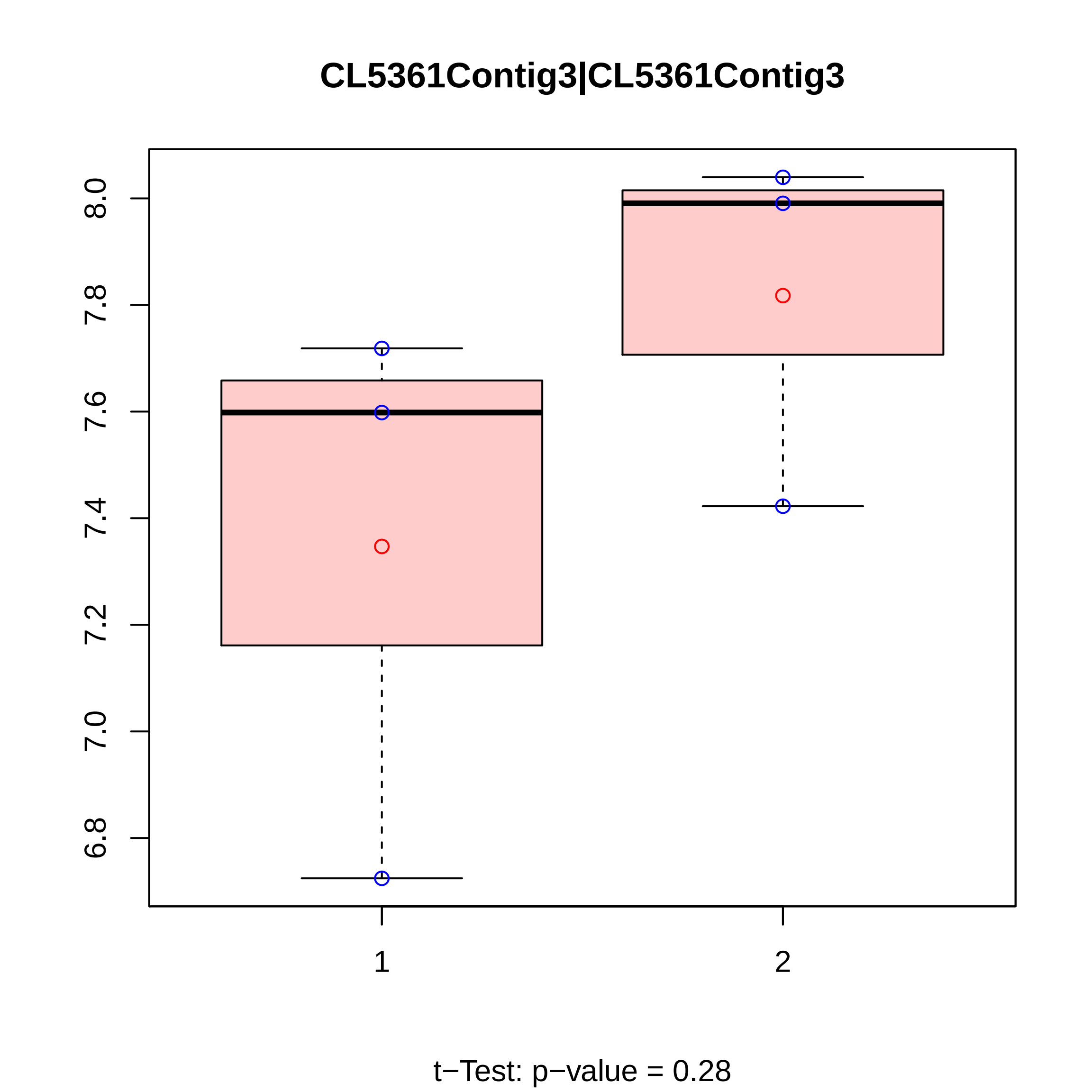

CL5361Contig3|CL5361Contig3
8.0
7.8
7.6
7.4
7.2
7.0
6.8
1
2
t−Test: p−value = 0.28

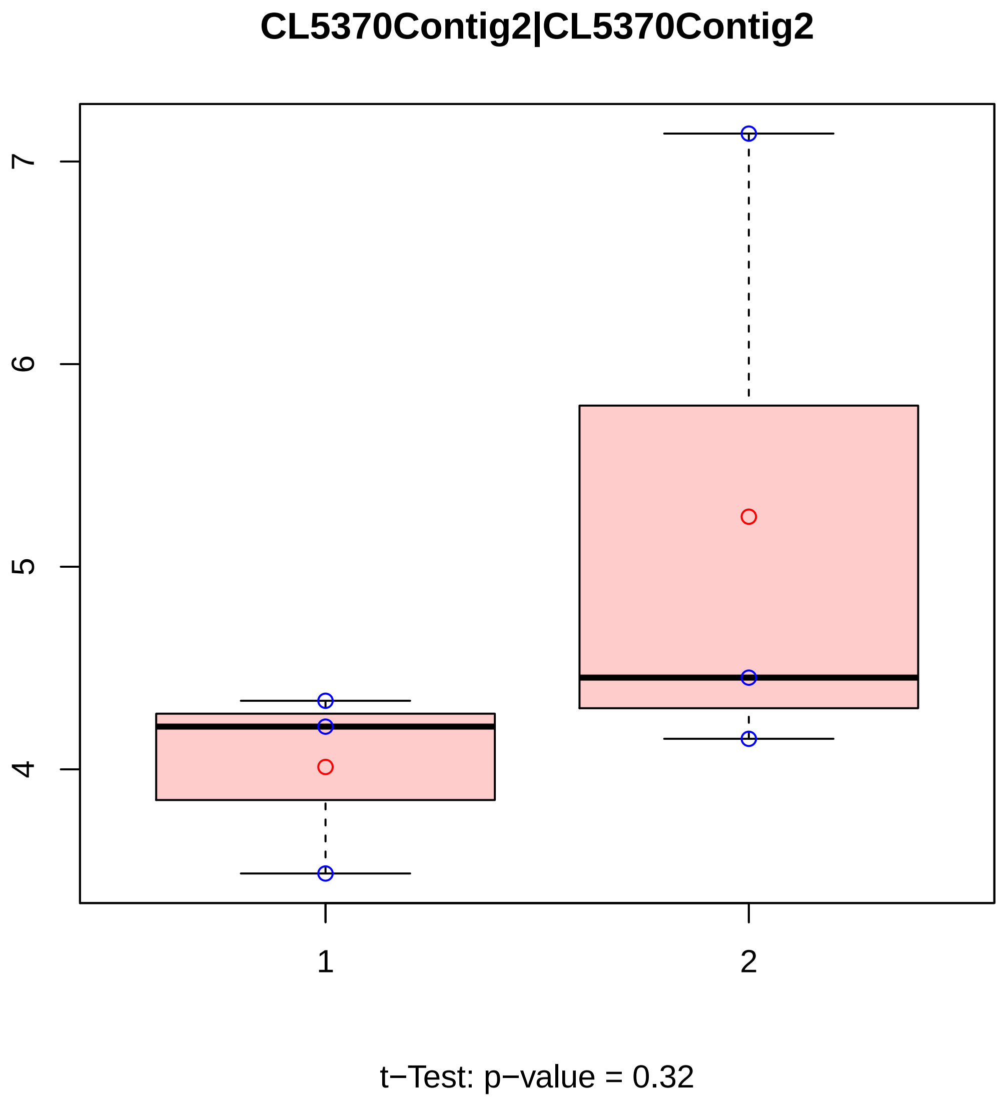

CL5370Contig2|CL5370Contig2
7
6
5
4
1
2
t−Test: p−value = 0.32

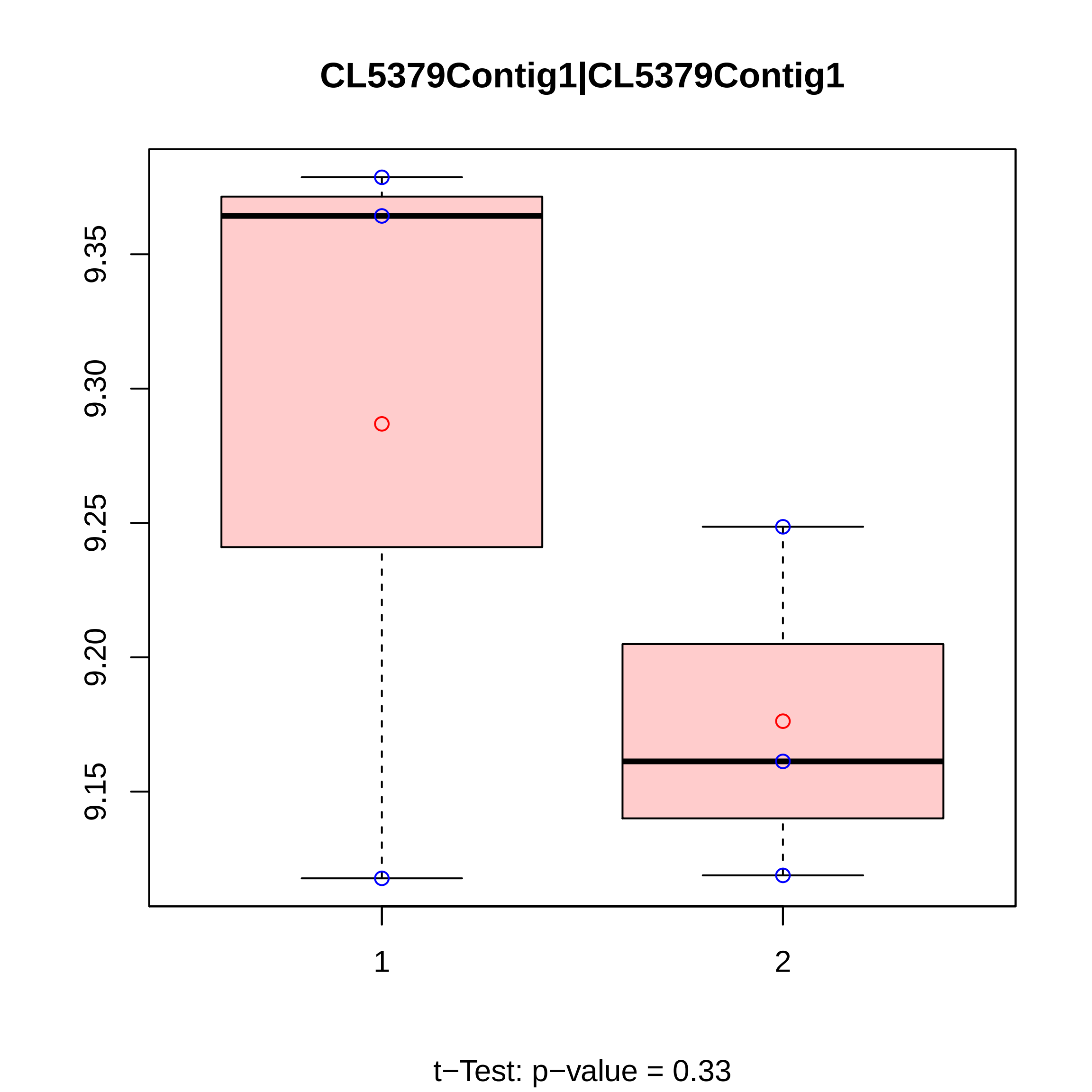

CL5379Contig1|CL5379Contig1
9.15
9.20
9.25
9.30
9.35
1
2
t−Test: p−value = 0.33

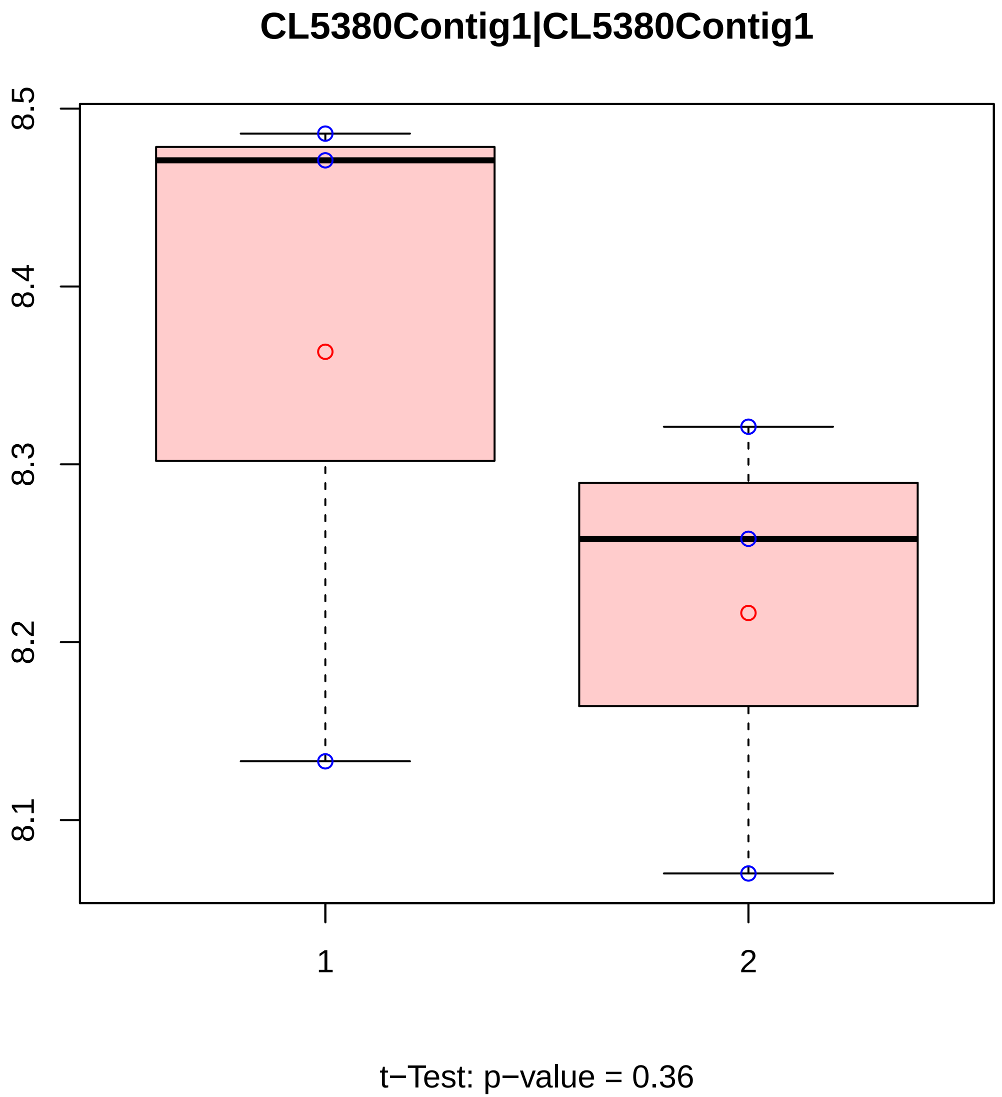
CL5380Contig1|CL5380Contig1
8.5
8.4
8.3
8.2
8.1
1
2
t−Test: p−value = 0.36

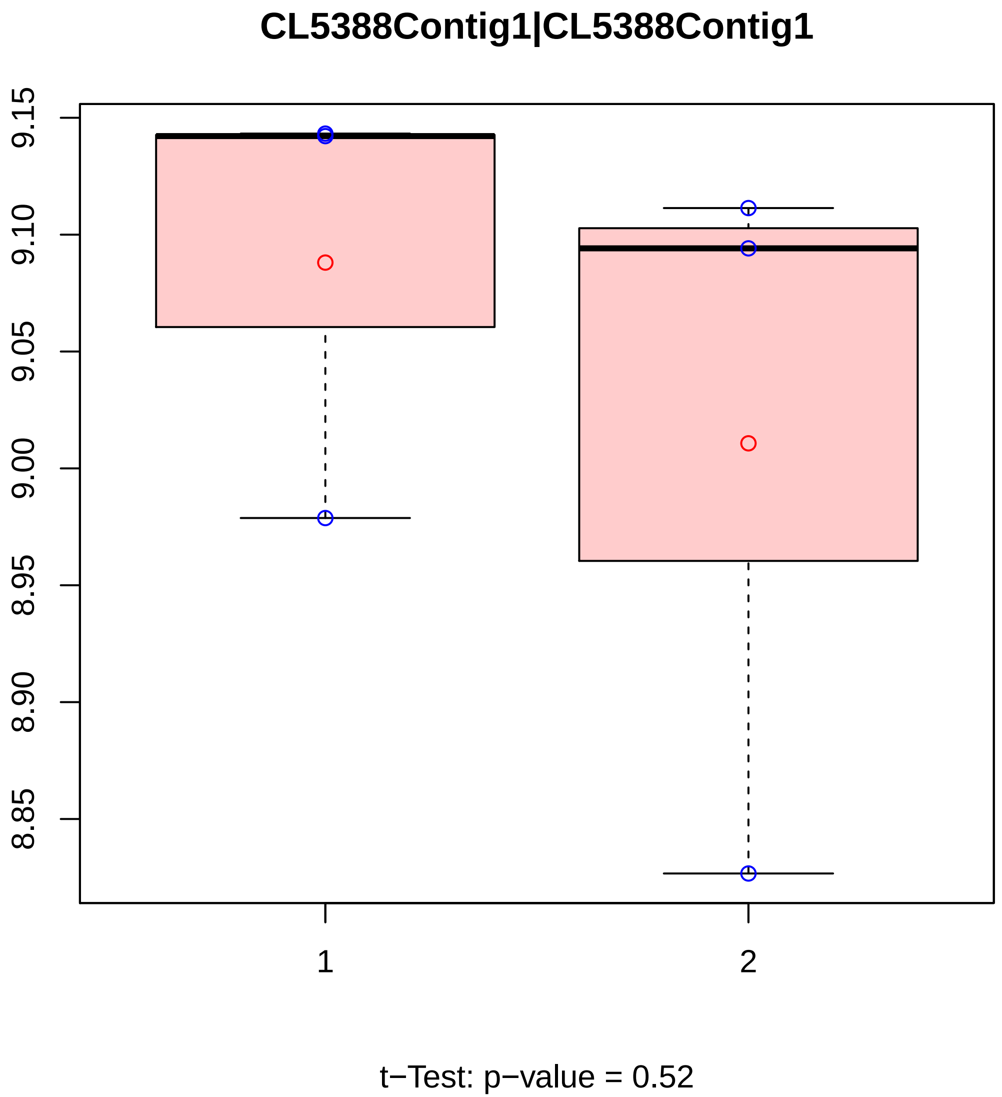
CL5388Contig1|CL5388Contig1
9.15
9.10
9.05
9.00
8.95
8.90
8.85
1
2
t−Test: p−value = 0.52

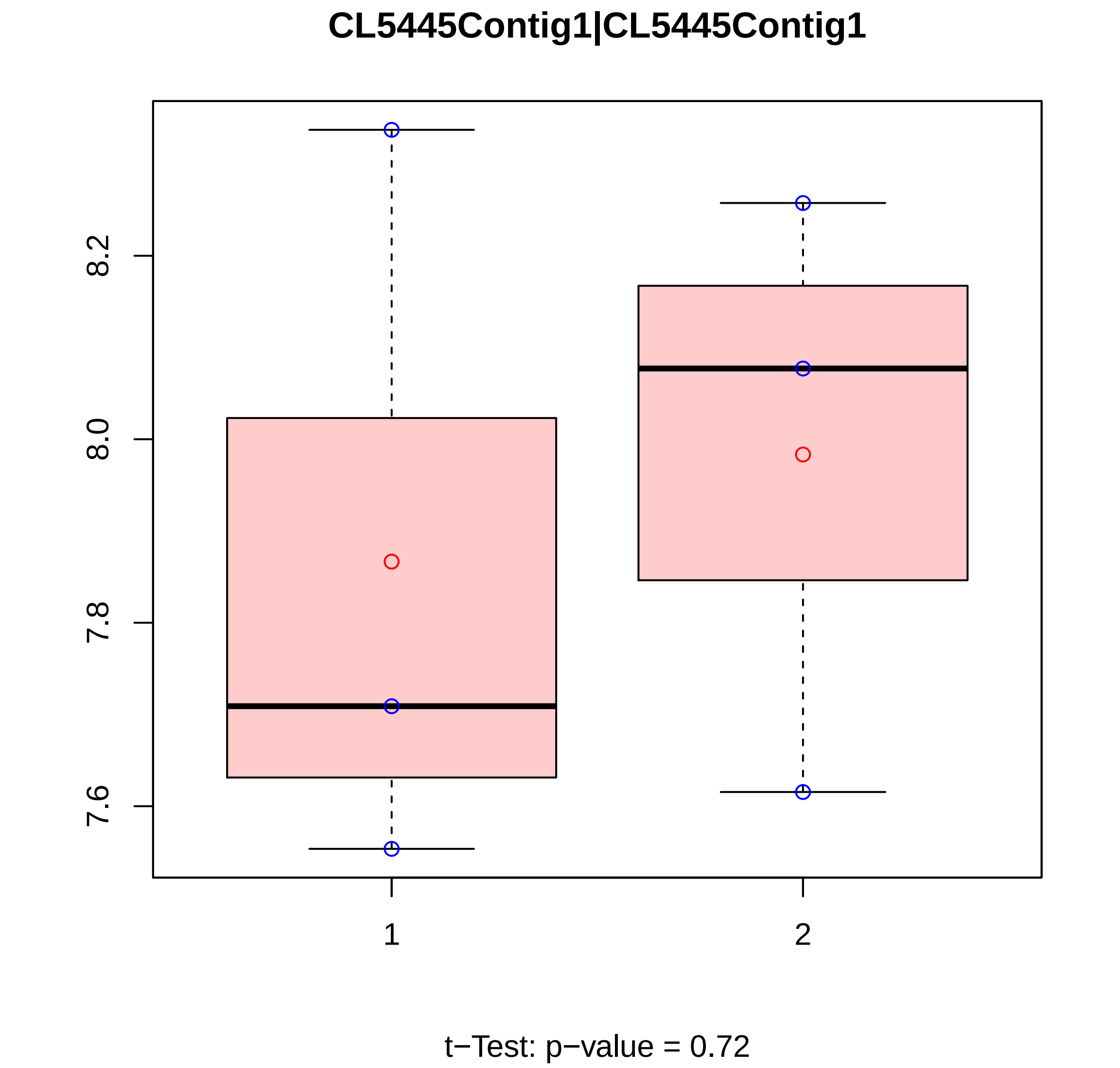
CL5445Contig1|CL5445Contig1
8.2
8.0
7.8
7.6
1
2
t−Test: p−value = 0.72

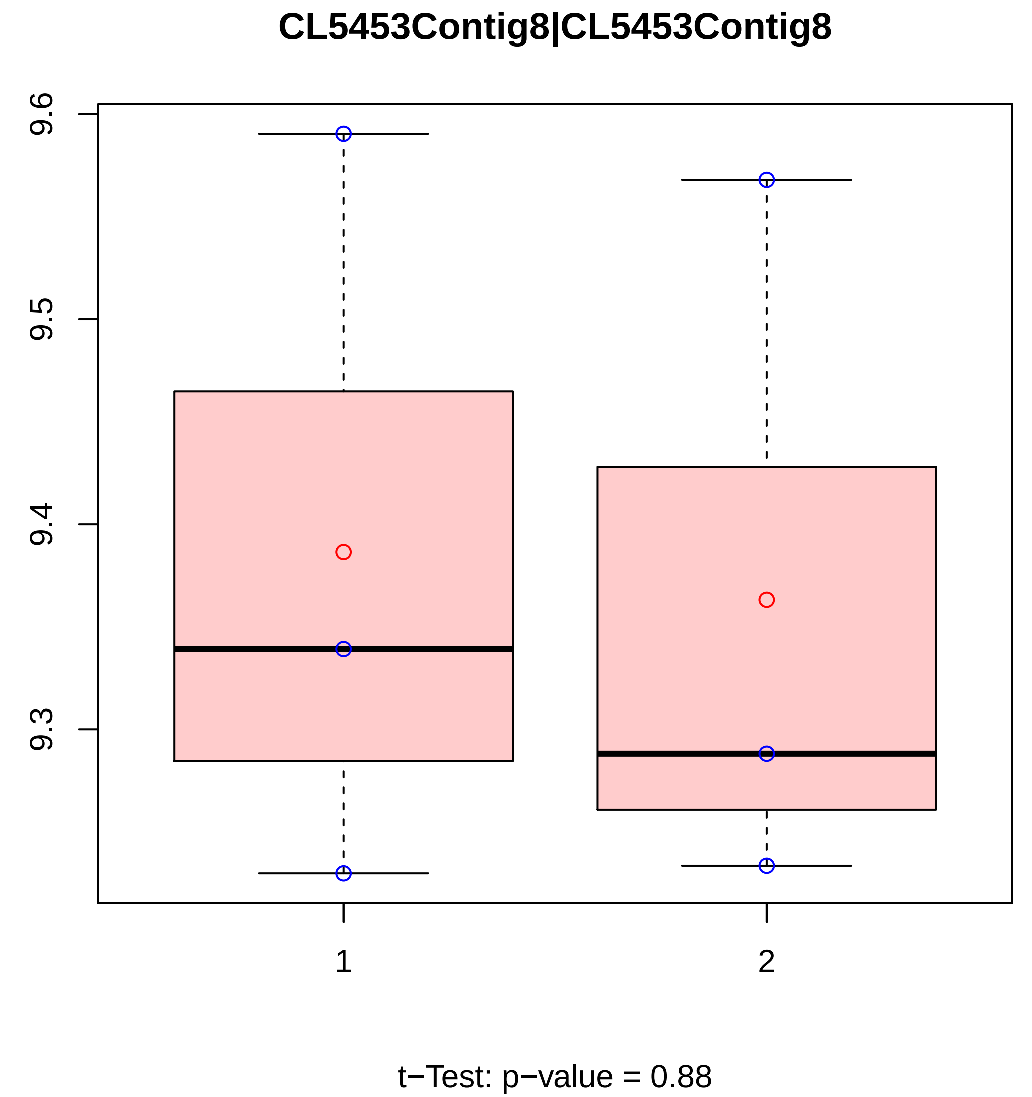
CL5453Contig8|CL5453Contig8
9.6
9.5
9.4
9.3
1
2
t−Test: p−value = 0.88

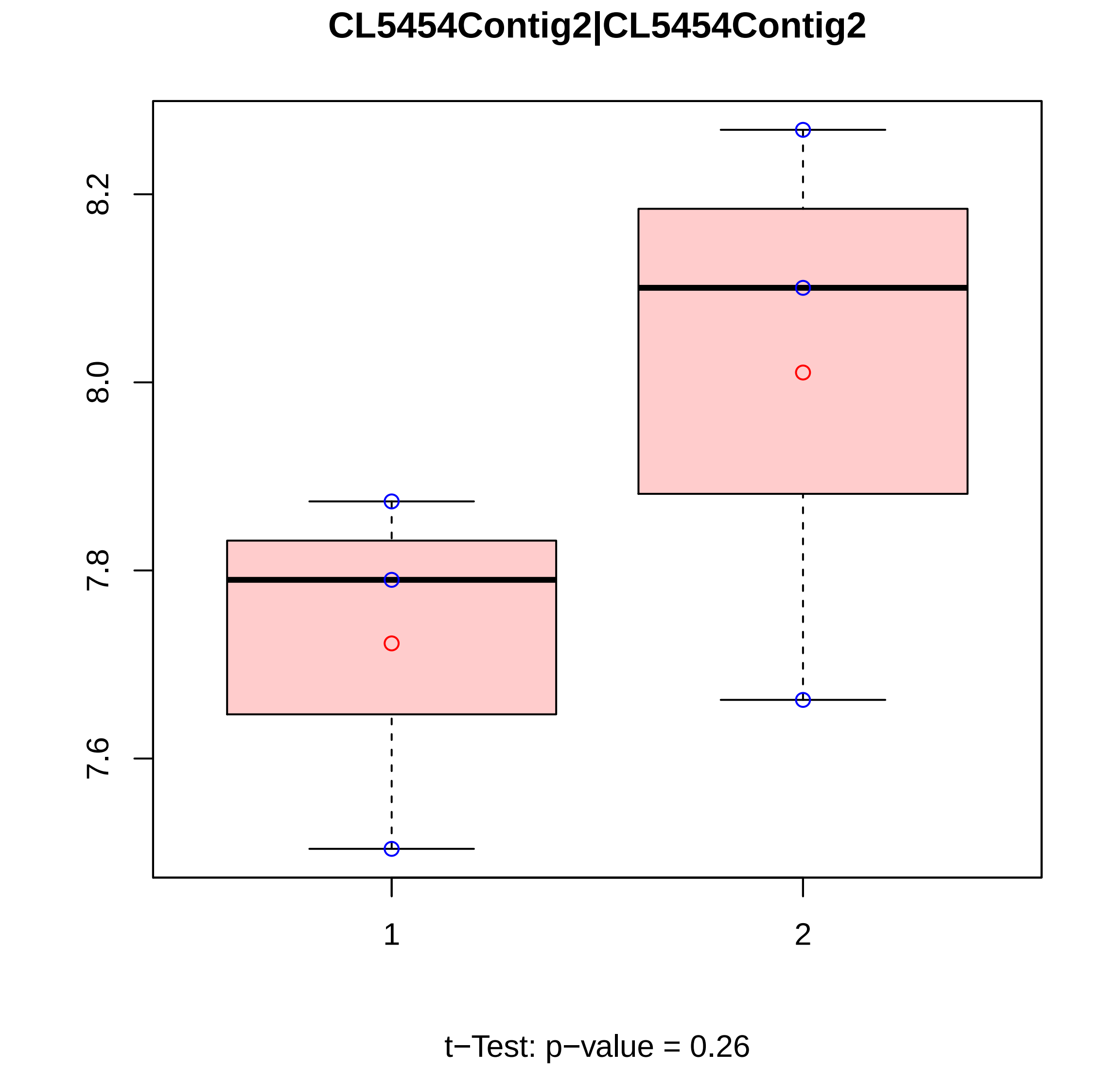

CL5454Contig2|CL5454Contig2
8.2
8.0
7.8
7.6
1
2
t−Test: p−value = 0.26

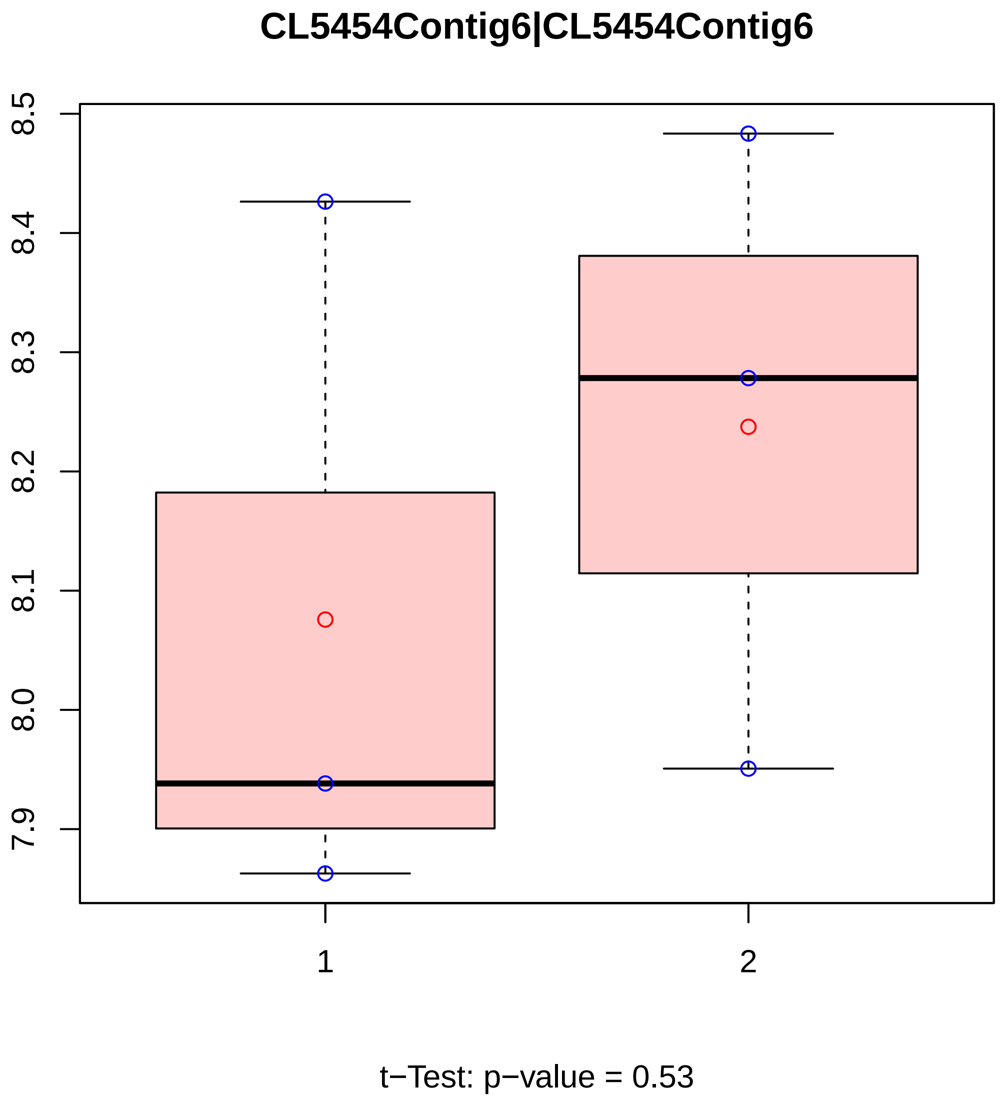
CL5454Contig6|CL5454Contig6
8.5
8.4
8.3
8.2
8.1
8.0
7.9
1
2
t−Test: p−value = 0.53

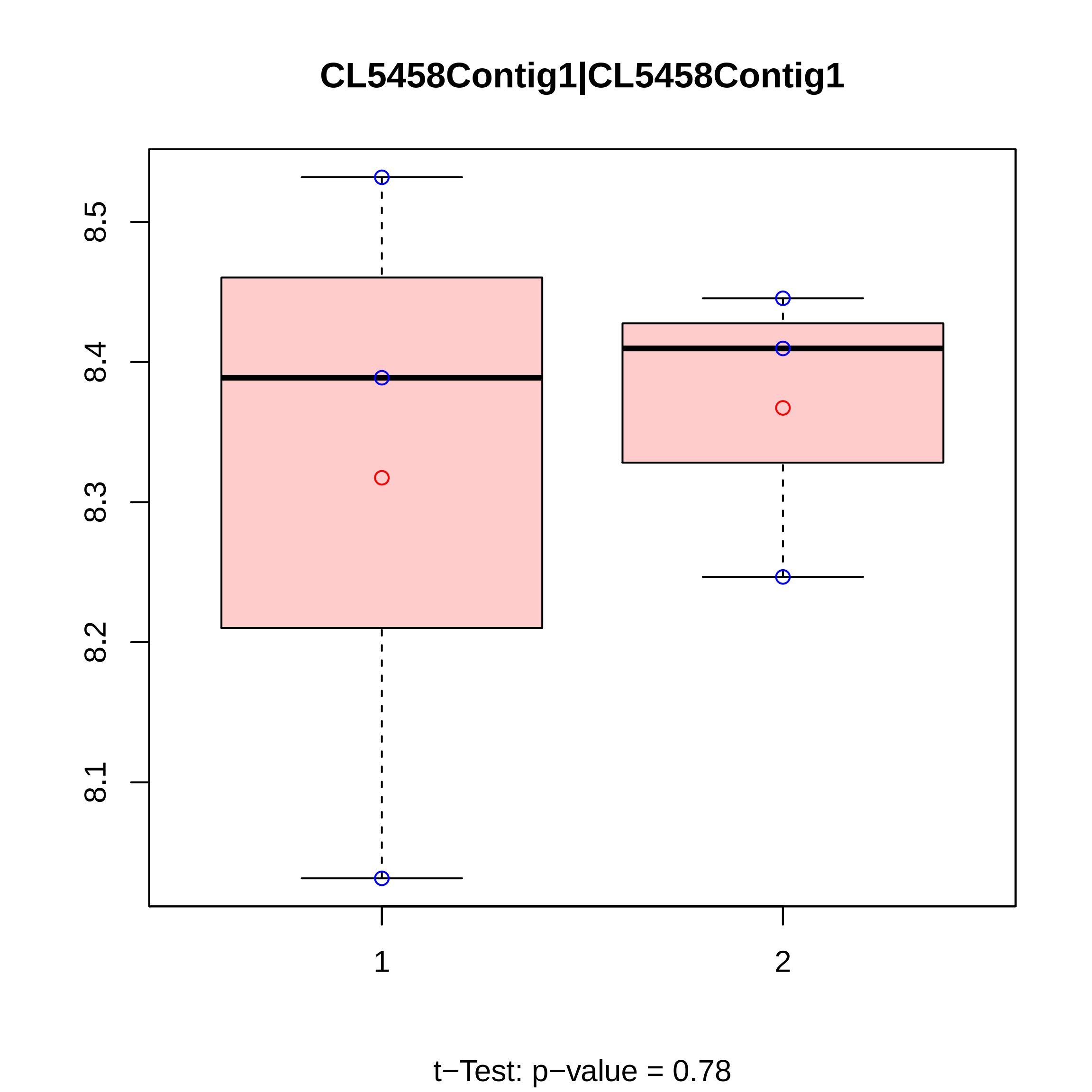
CL5458Contig1|CL5458Contig1
8.1
8.2
8.3
8.4
8.5
1
2
t−Test: p−value = 0.78

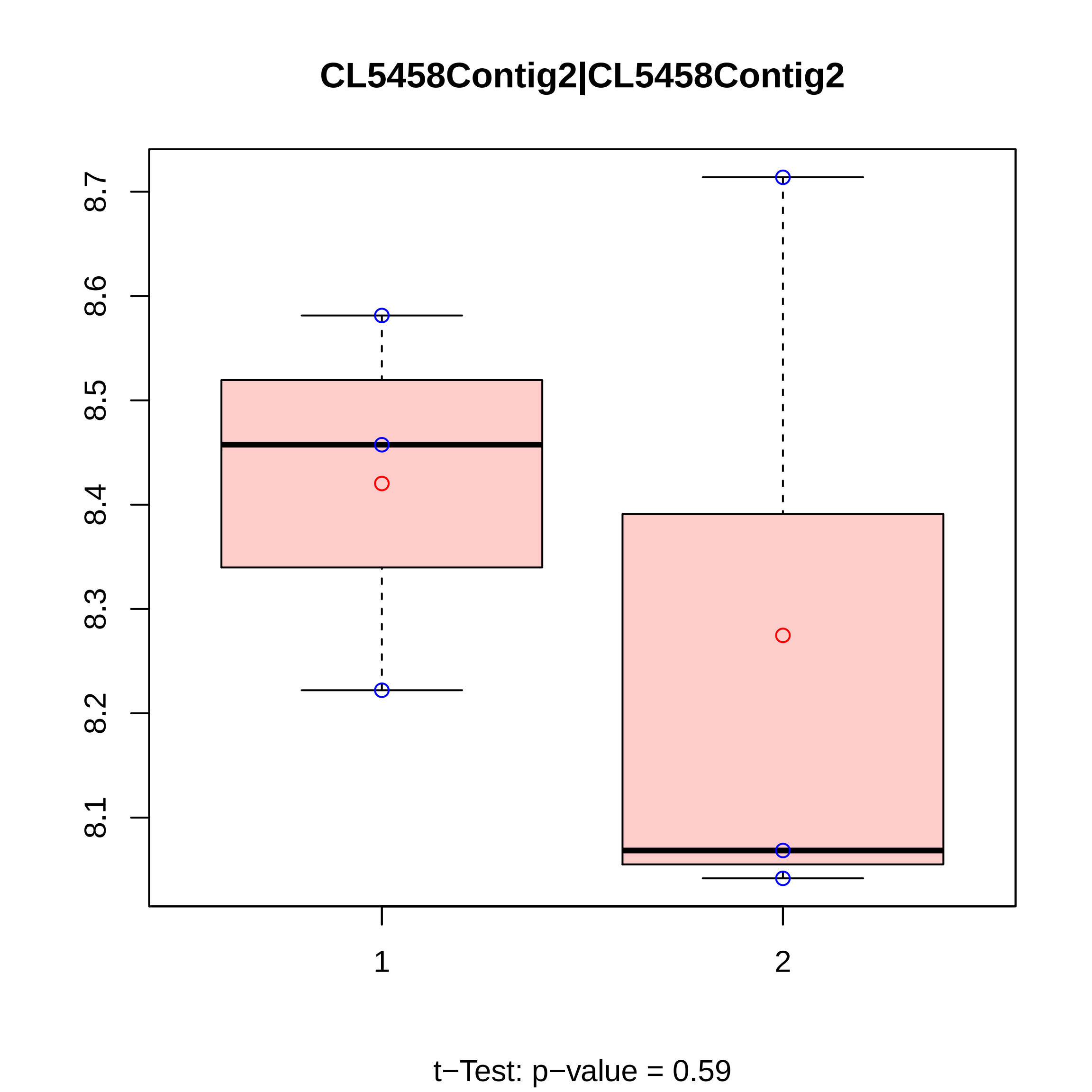
CL5458Contig2|CL5458Contig2
8.1
8.2
8.3
8.4
8.5
8.6
8.7
1
2
t−Test: p−value = 0.59

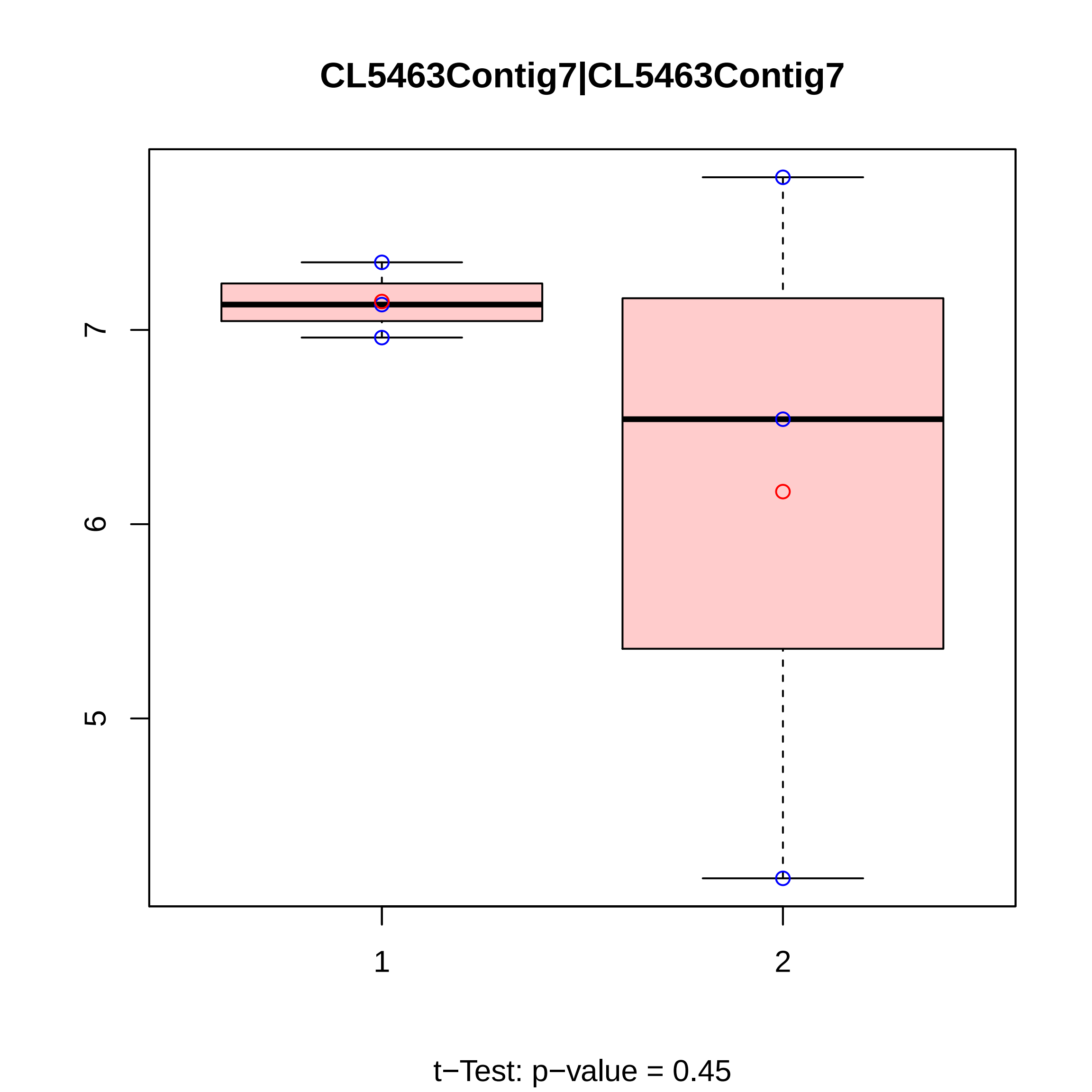

CL5463Contig7|CL5463Contig7
7
6
5
1
2
t−Test: p−value = 0.45

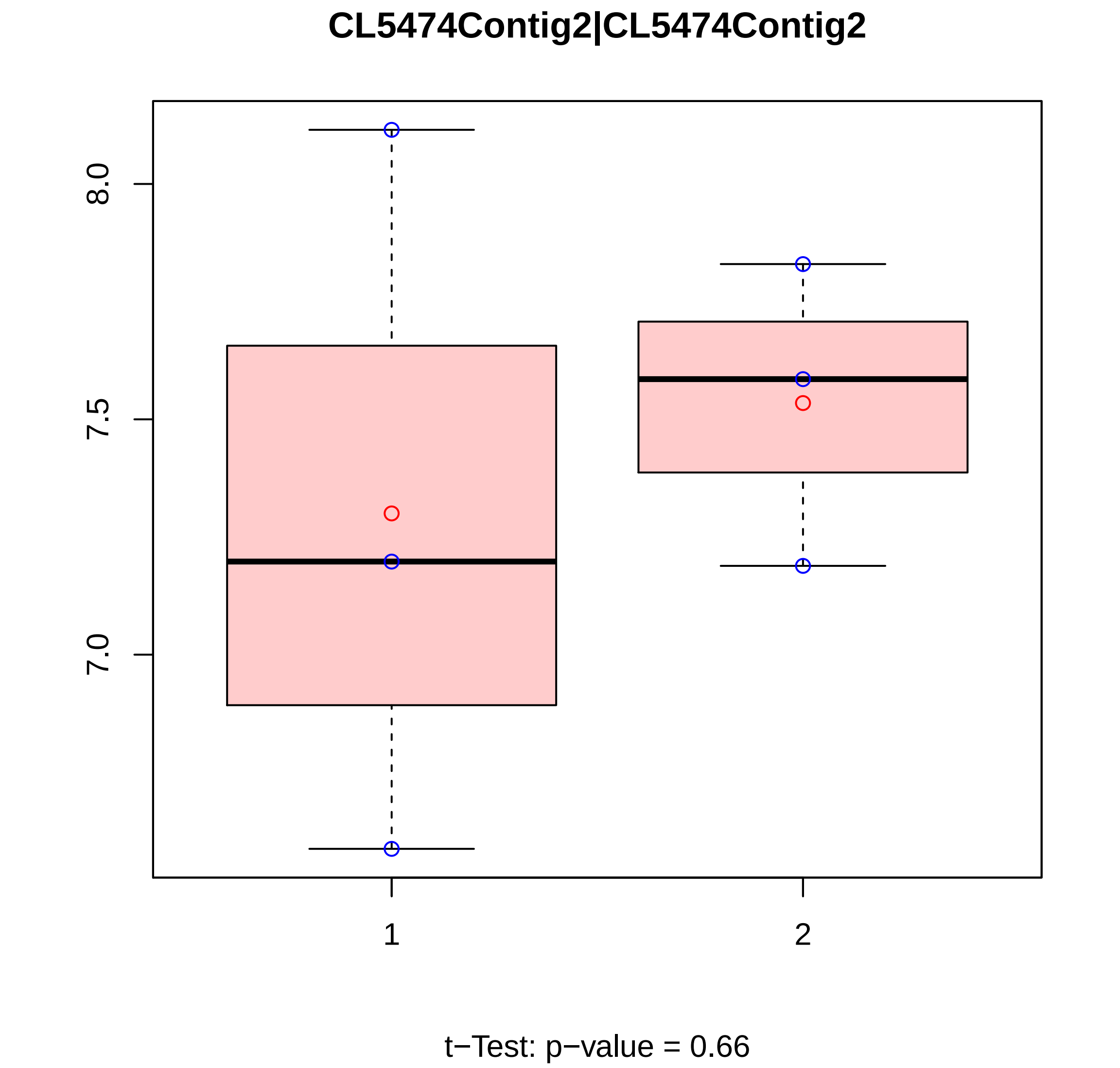
CL5474Contig2|CL5474Contig2
8.0
7.5
7.0
1
2
t−Test: p−value = 0.66

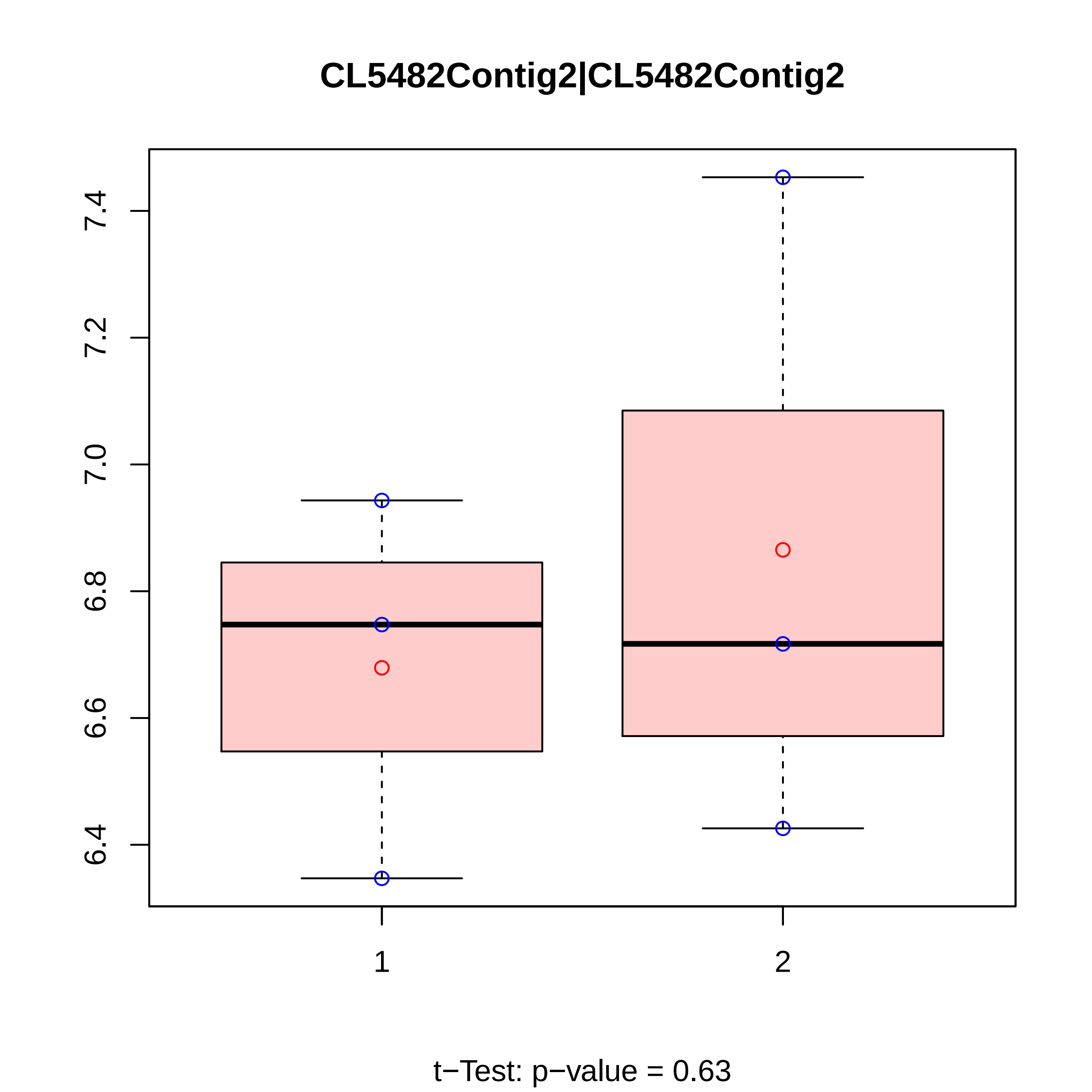

CL5482Contig2|CL5482Contig2
7.4
7.2
7.0
6.8
6.6
6.4
1
2
t−Test: p−value = 0.63

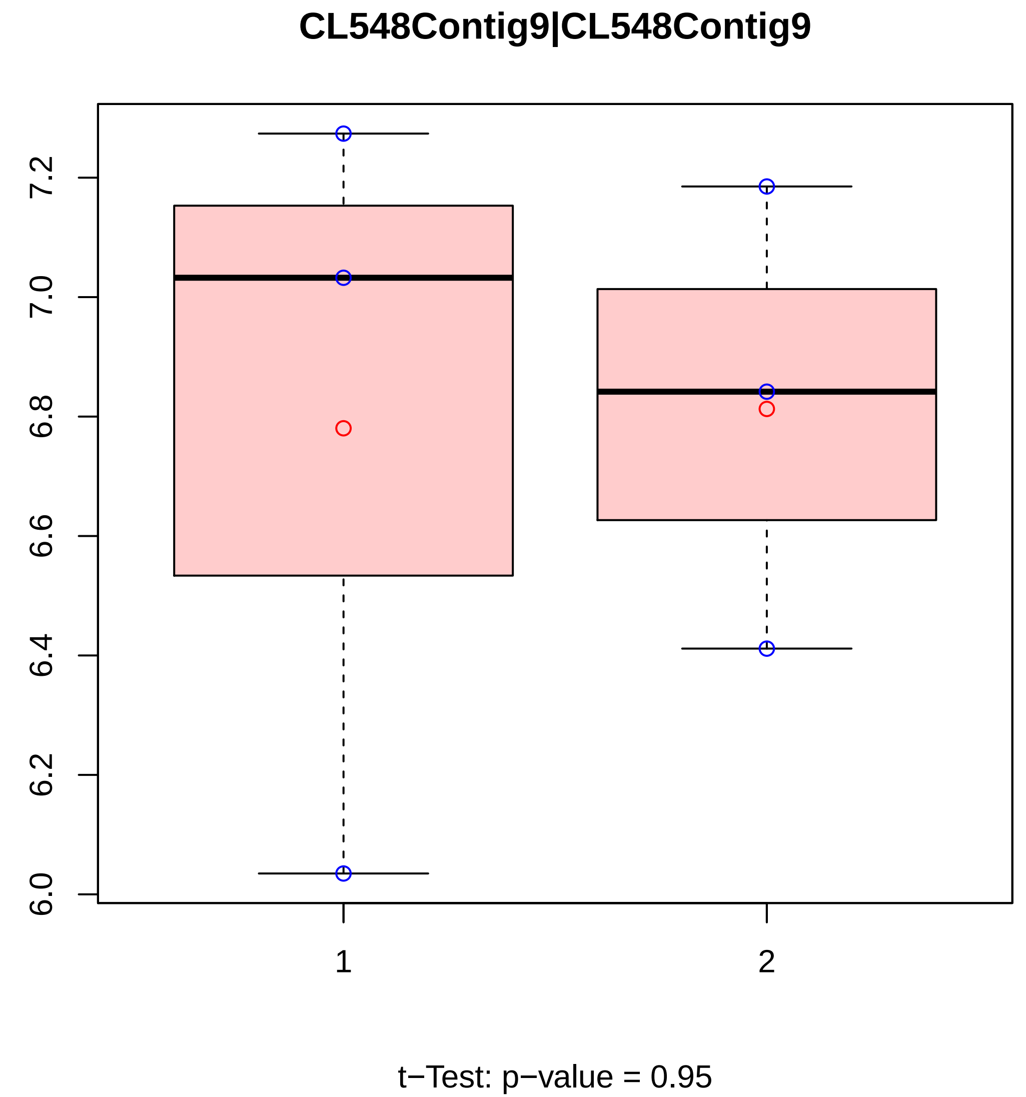
CL548Contig9|CL548Contig9
7.2
7.0
6.8
6.6
6.4
6.2
6.0
1
2
t−Test: p−value = 0.95

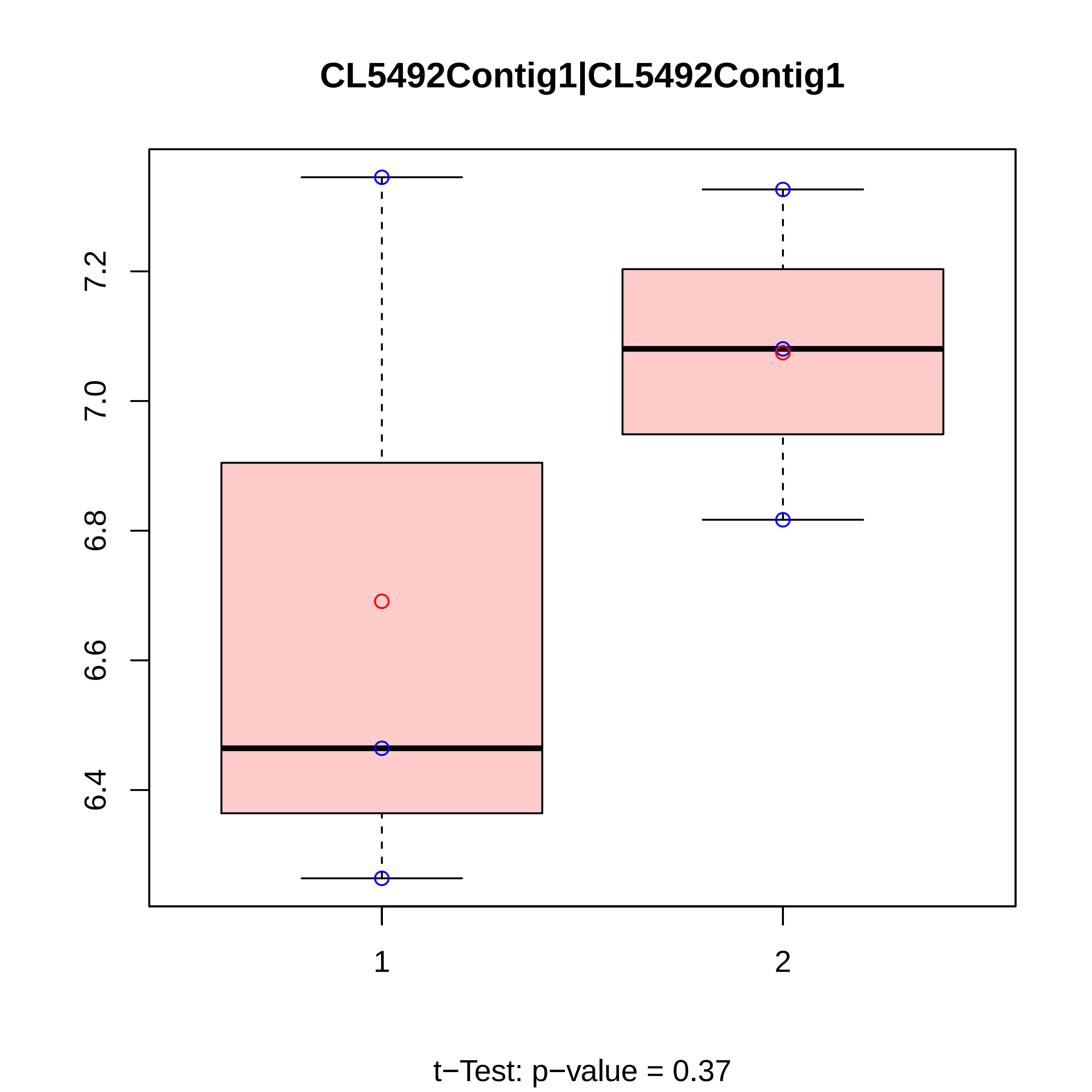
CL5492Contig1|CL5492Contig1
7.2
7.0
6.8
6.6
6.4
1
2
t−Test: p−value = 0.37

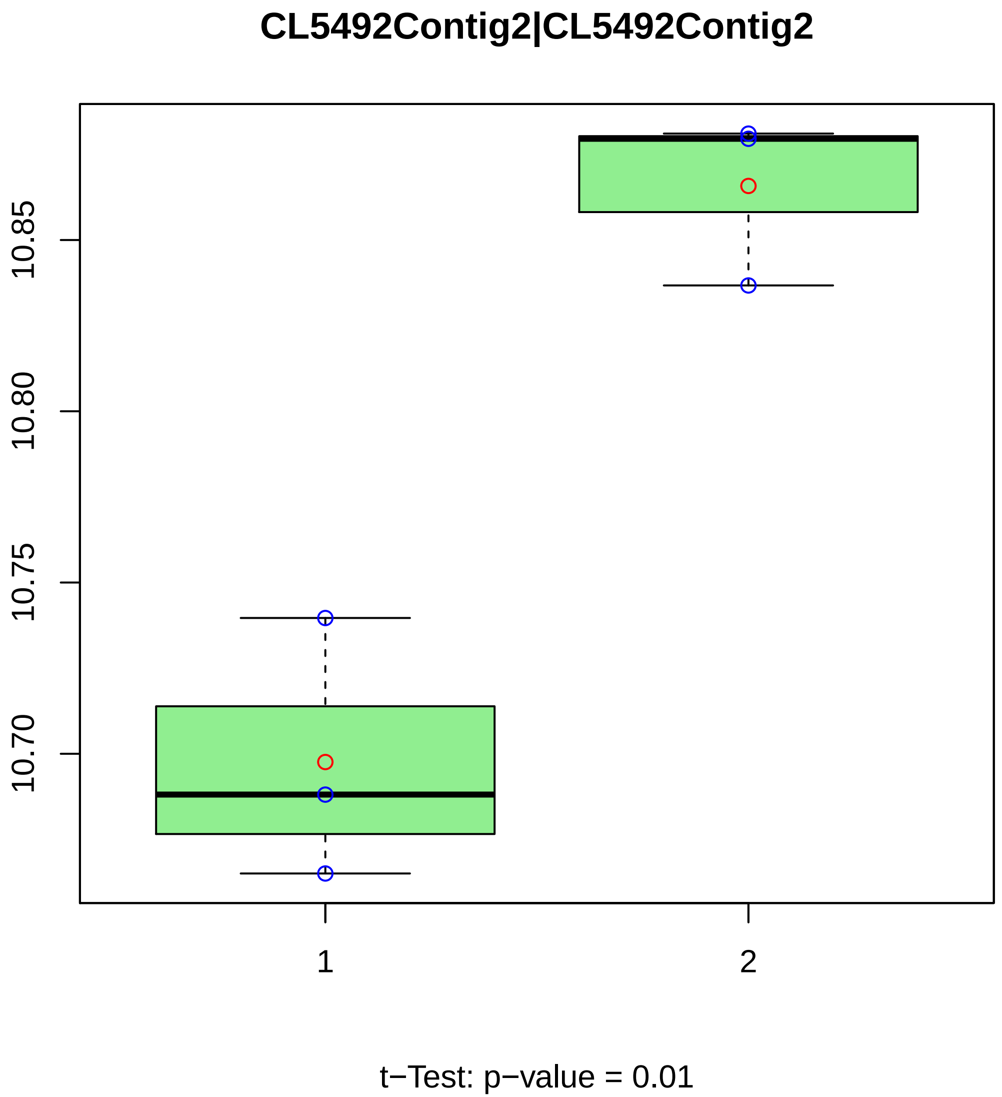
CL5492Contig2|CL5492Contig2
10.70
10.75
10.80
10.85
1
2
t−Test: p−value = 0.01

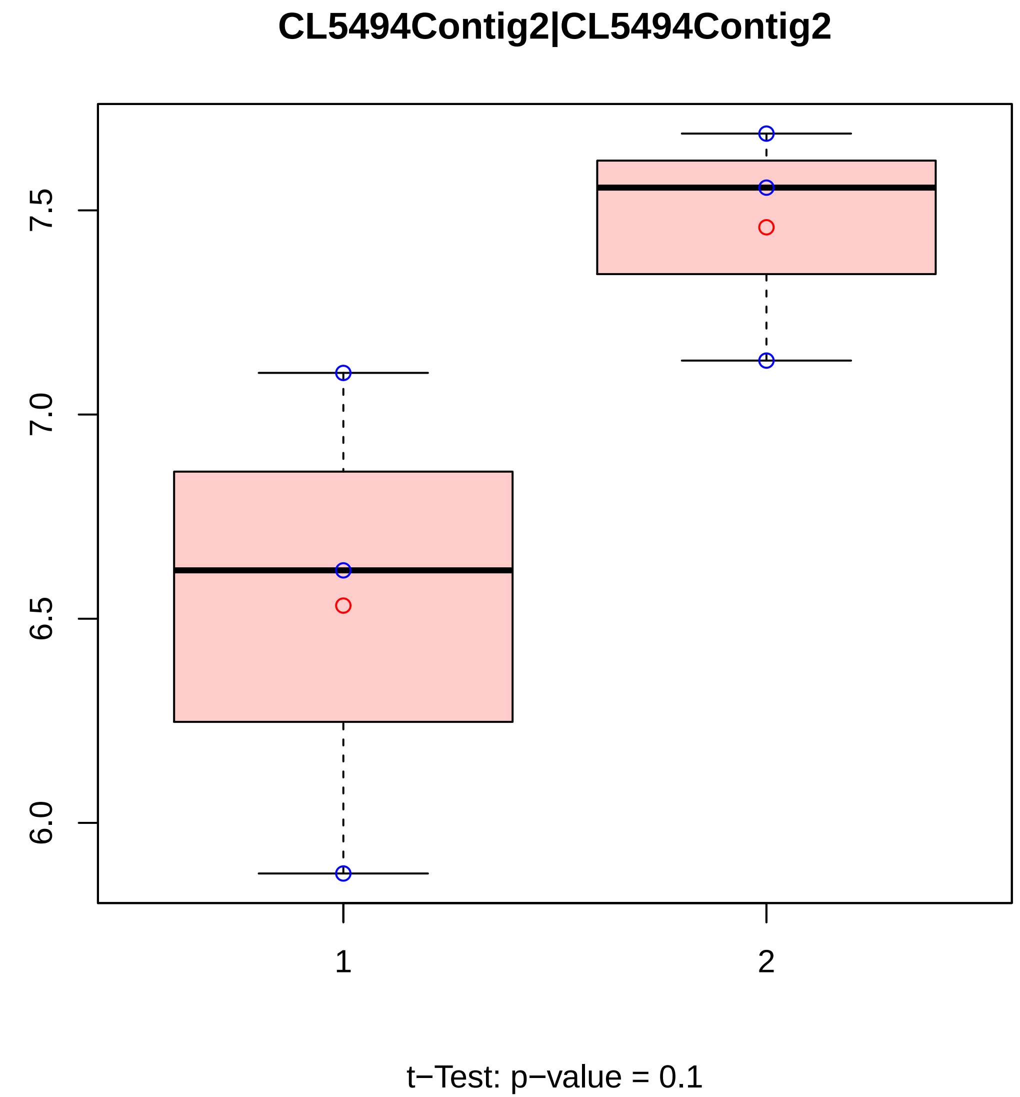
CL5494Contig2|CL5494Contig2
7.5
7.0
6.5
6.0
1
2
t−Test: p−value = 0.1

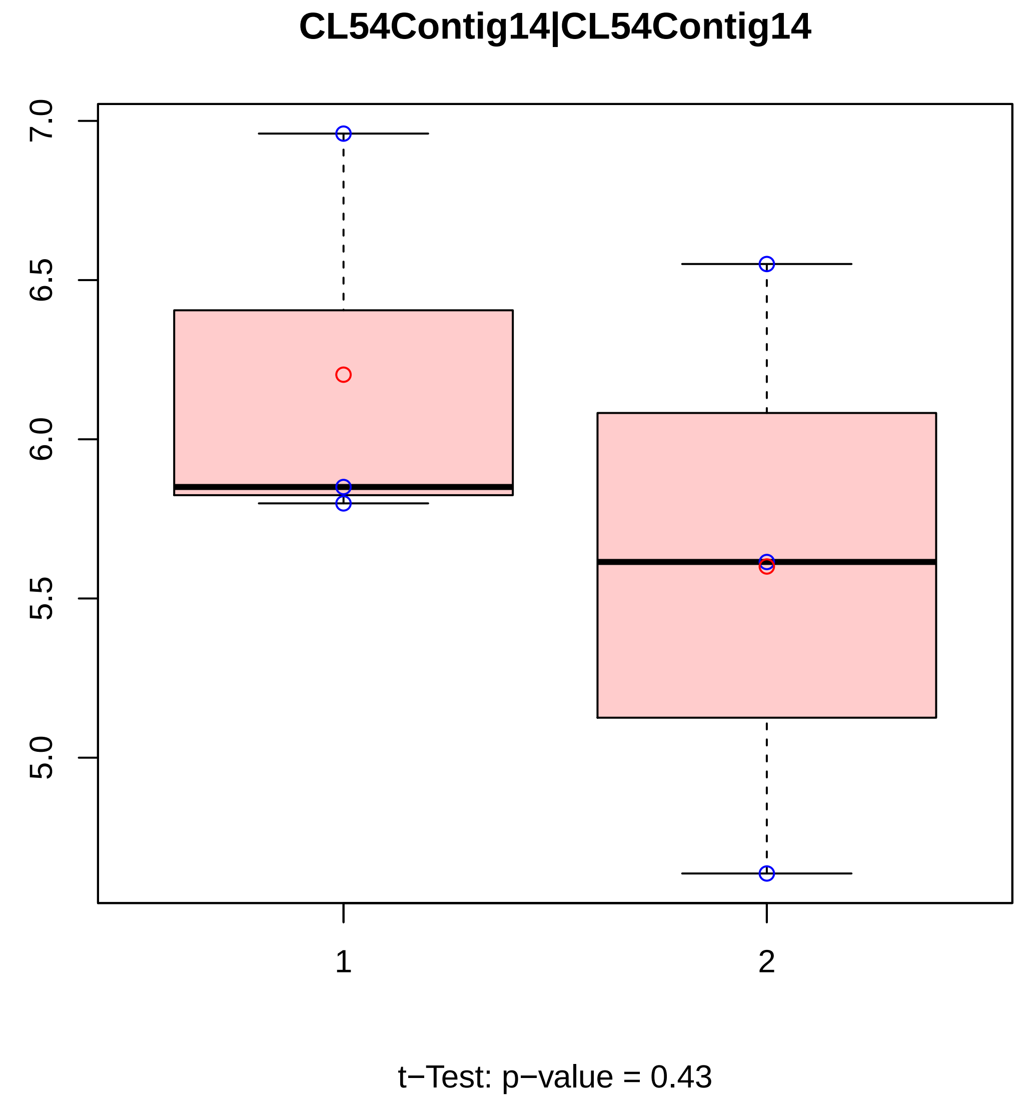
CL54Contig14|CL54Contig14
7.0
6.5
6.0
5.5
5.0
1
2
t−Test: p−value = 0.43

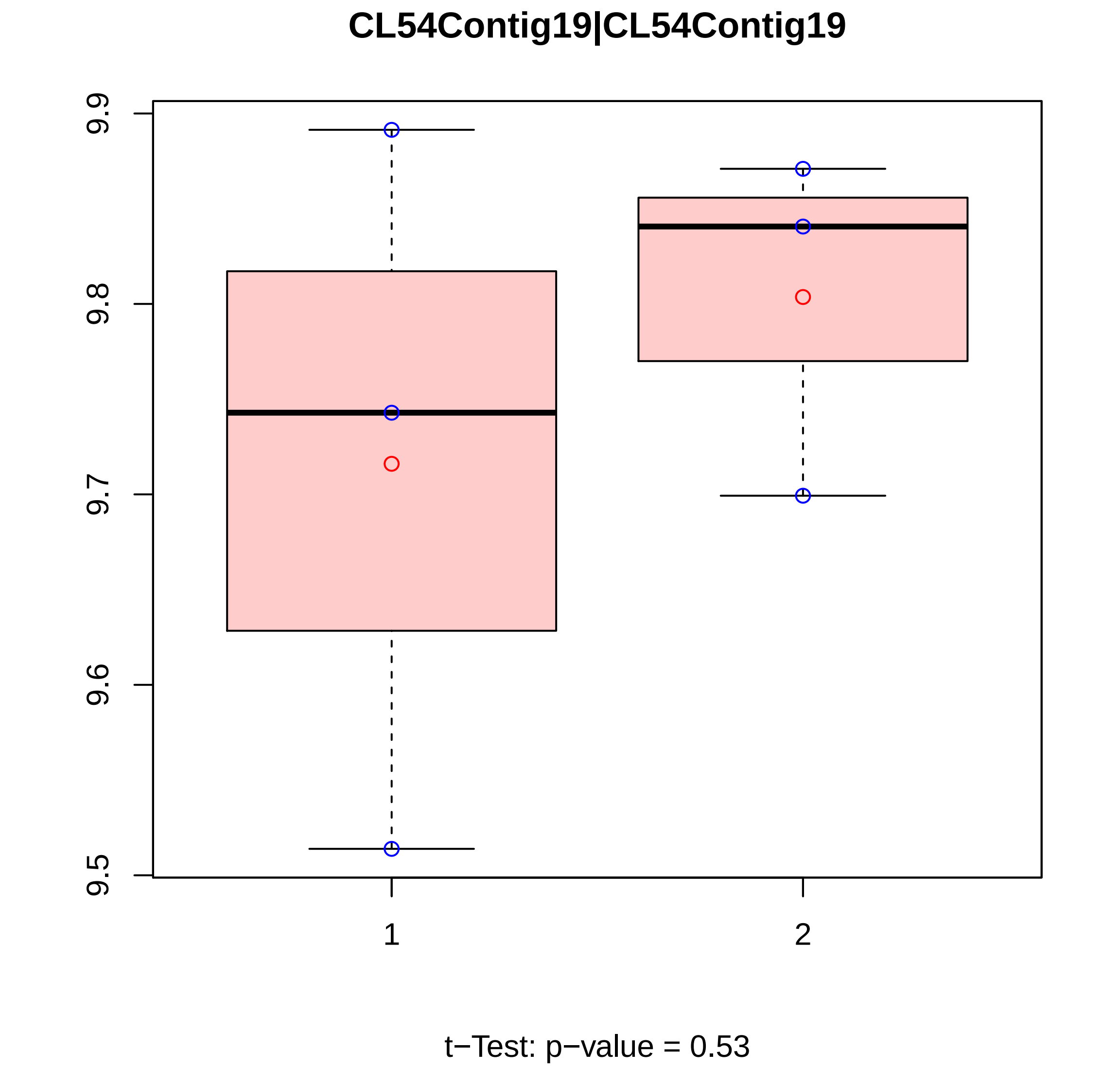
CL54Contig19|CL54Contig19
9.5
9.6
9.7
9.8
9.9
1
2
t−Test: p−value = 0.53

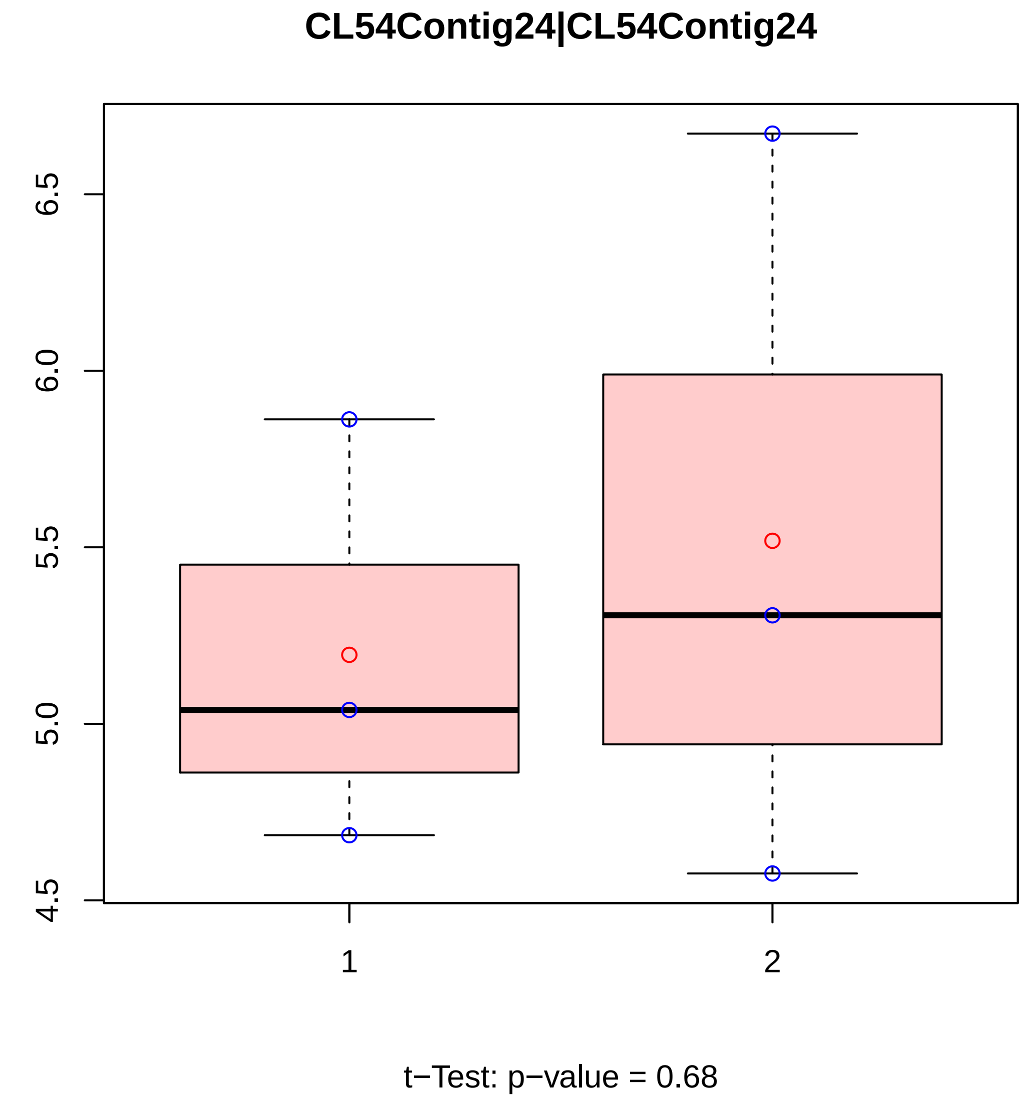

CL54Contig24|CL54Contig24
4.5
5.0
5.5
6.0
6.5
1
2
t−Test: p−value = 0.68

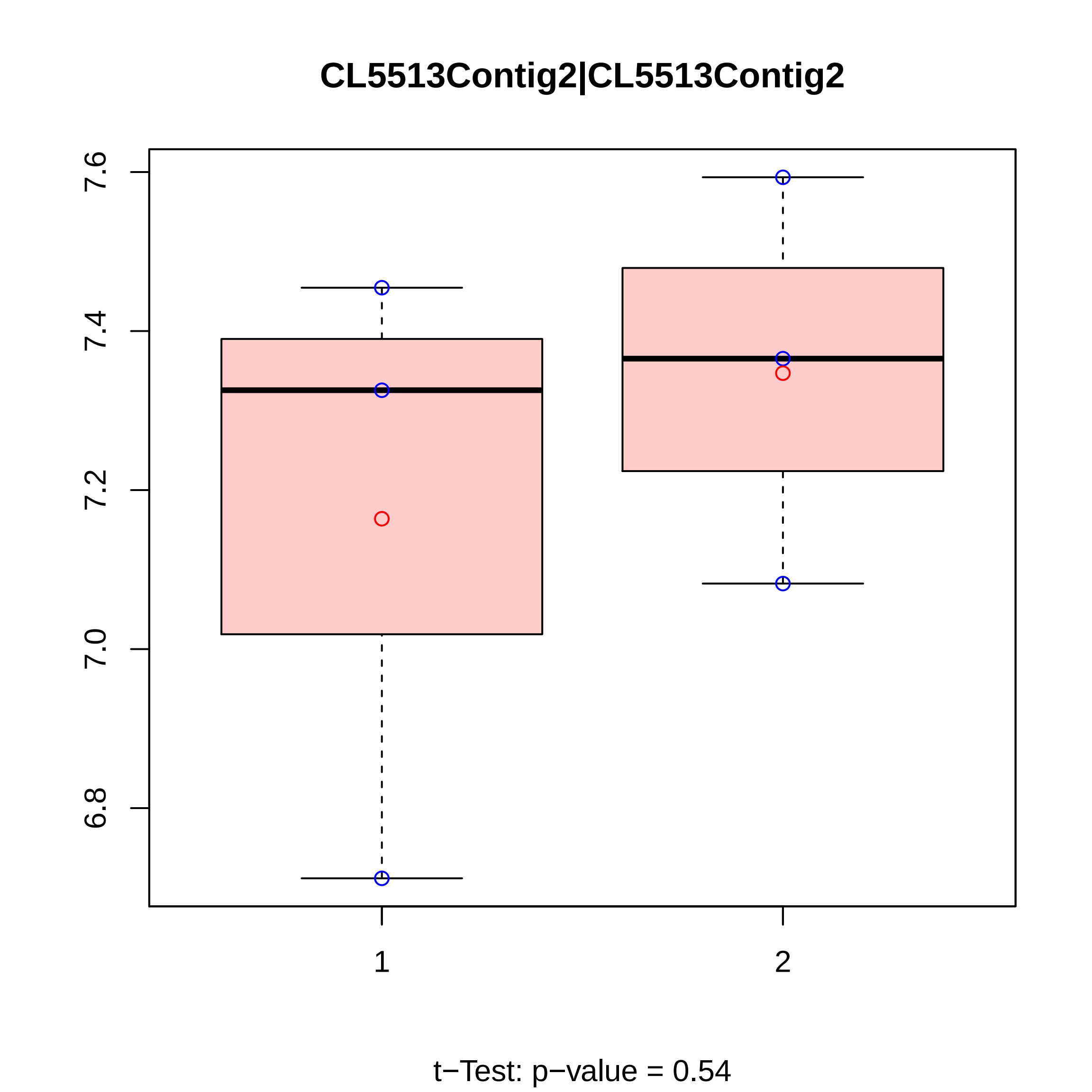

CL5513Contig2|CL5513Contig2
7.6
7.4
7.2
7.0
6.8
1
2
t−Test: p−value = 0.54

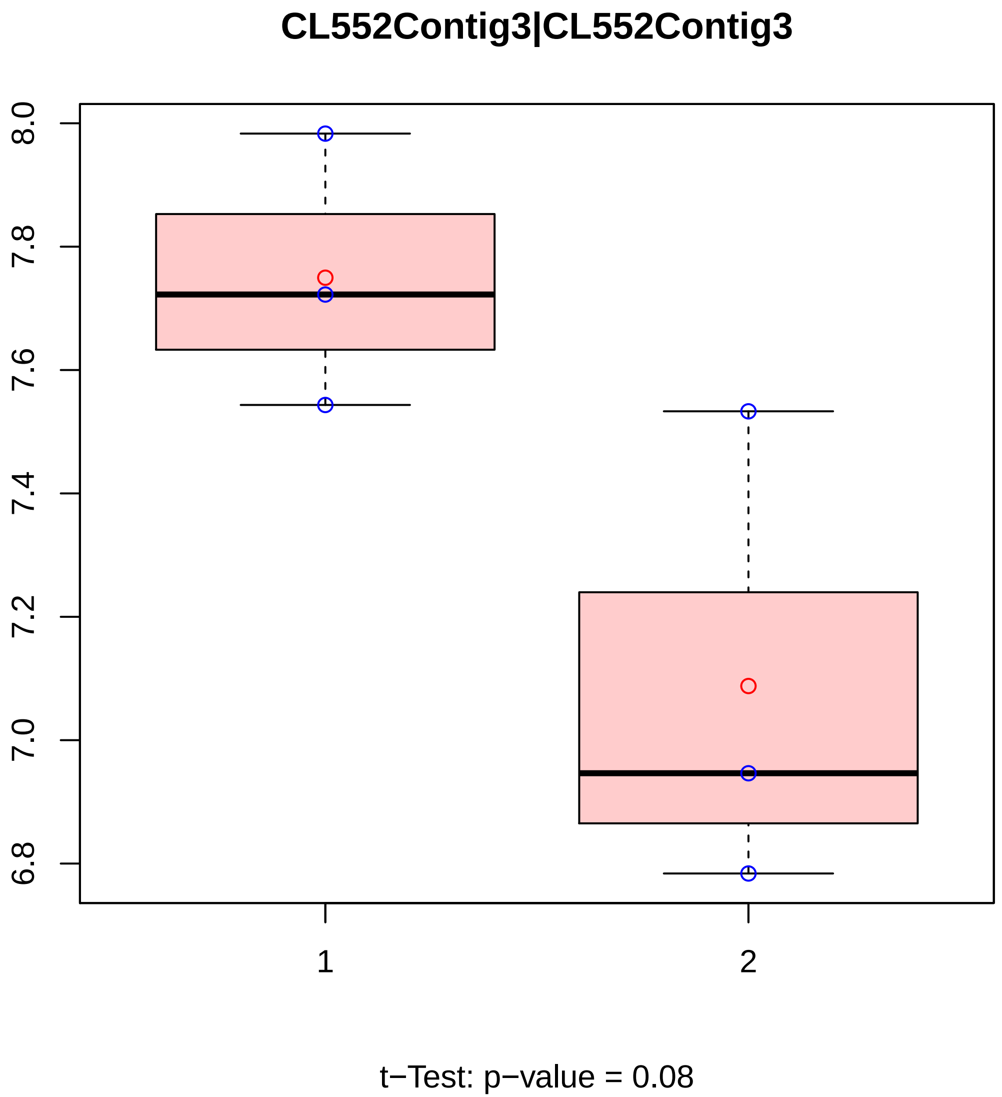
CL552Contig3|CL552Contig3
8.0
7.8
7.6
7.4
7.2
7.0
6.8
1
2
t−Test: p−value = 0.08

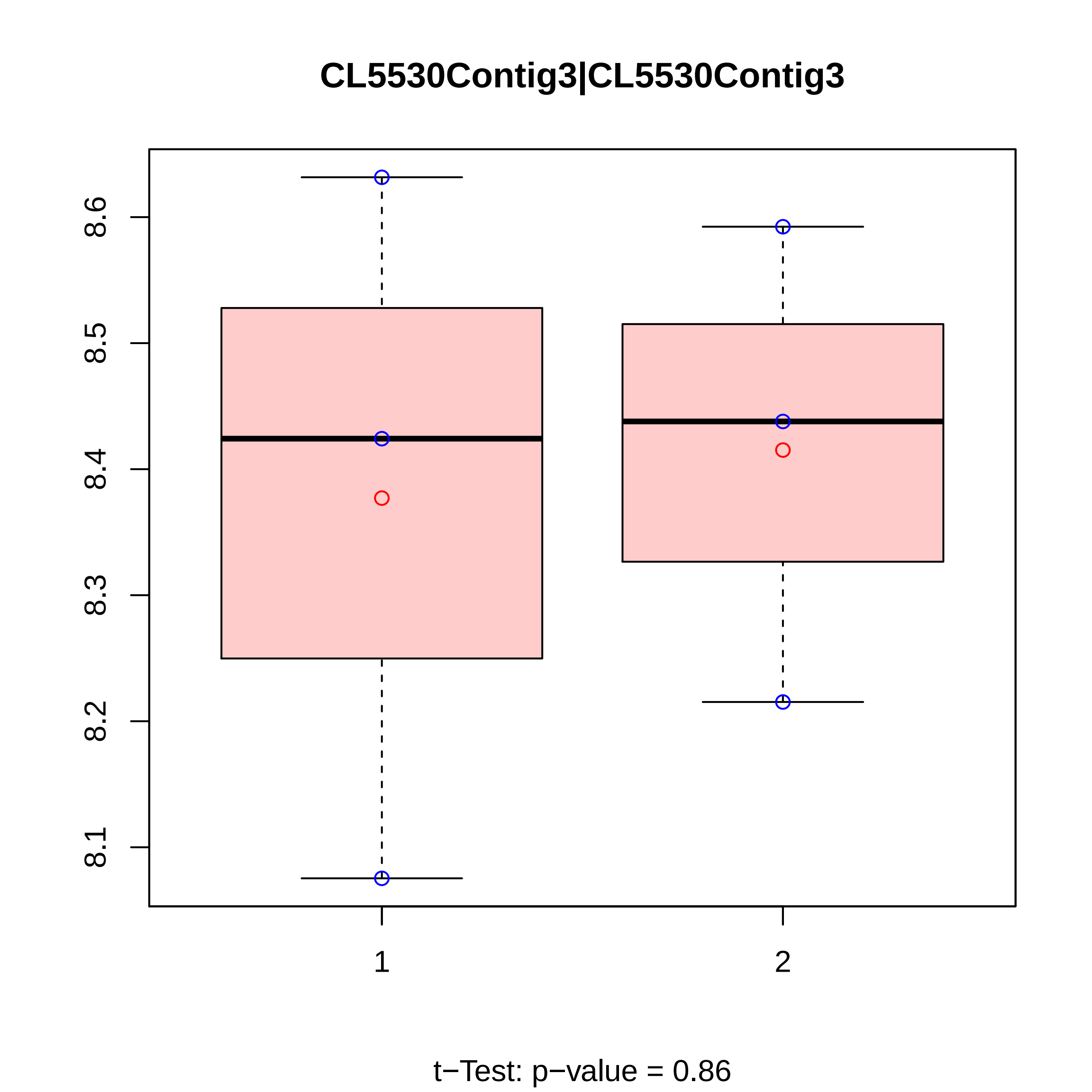

CL5530Contig3|CL5530Contig3
8.1
8.2
8.3
8.4
8.5
8.6
1
2
t−Test: p−value = 0.86

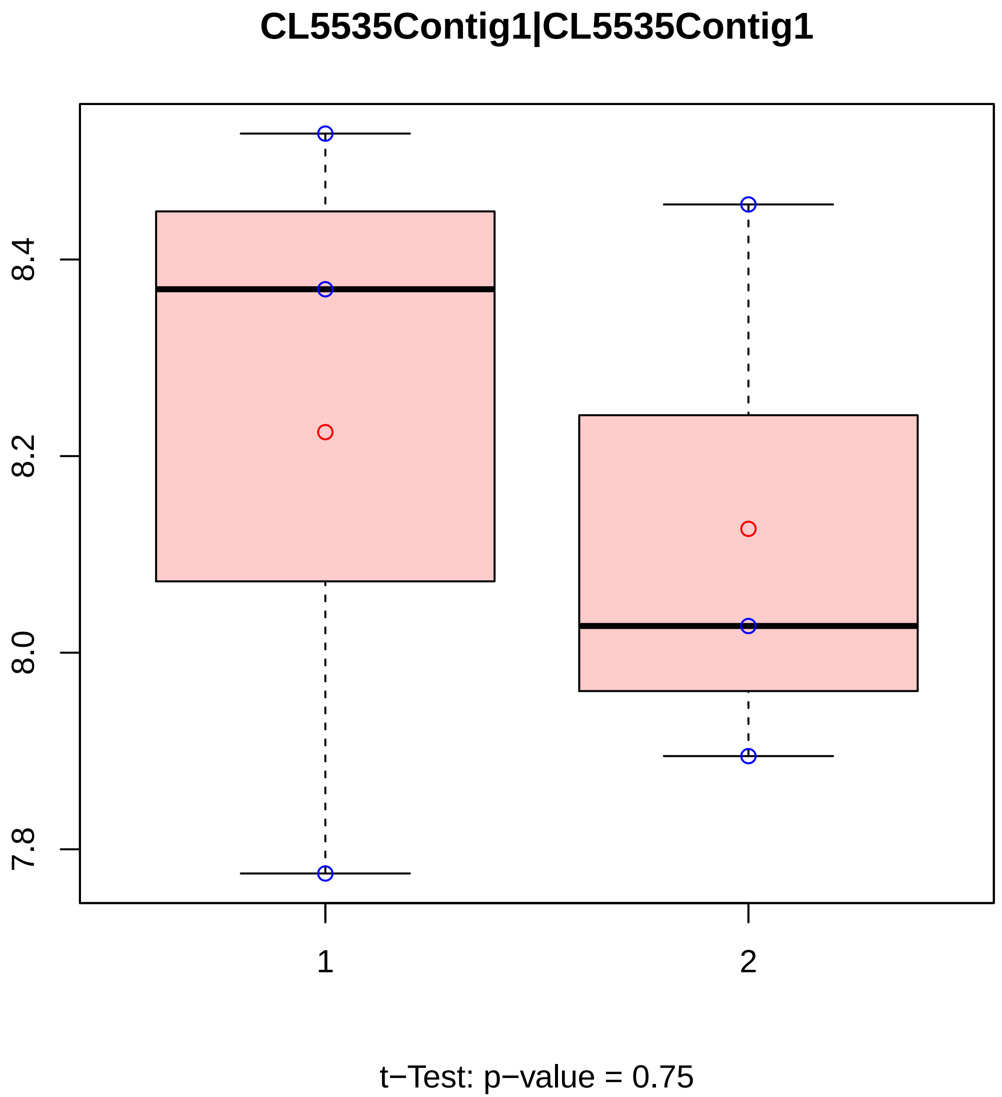
CL5535Contig1|CL5535Contig1
8.4
8.2
8.0
7.8
1
2
t−Test: p−value = 0.75

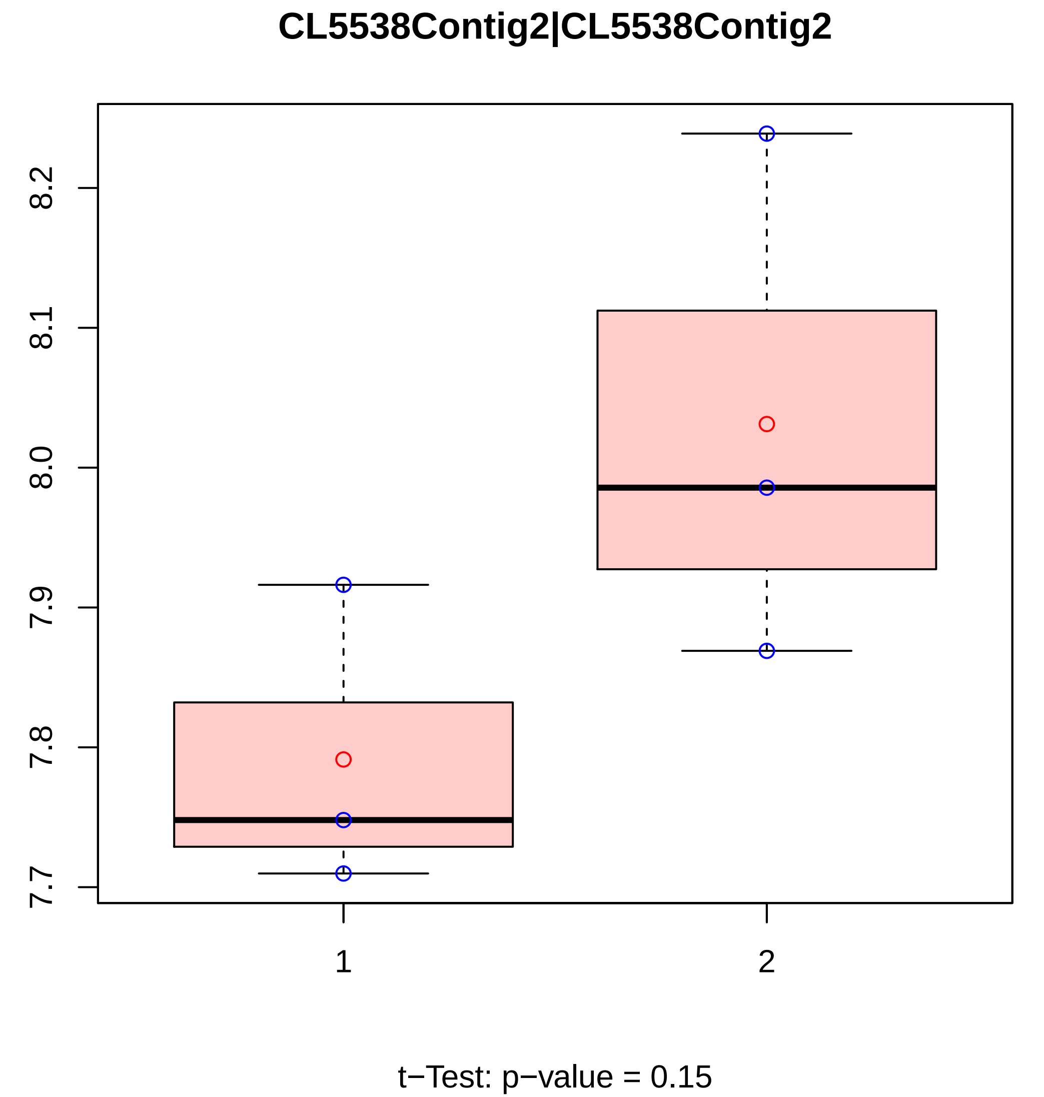
CL5538Contig2|CL5538Contig2
8.2
8.1
8.0
7.9
7.8
7.7
1
2
t−Test: p−value = 0.15

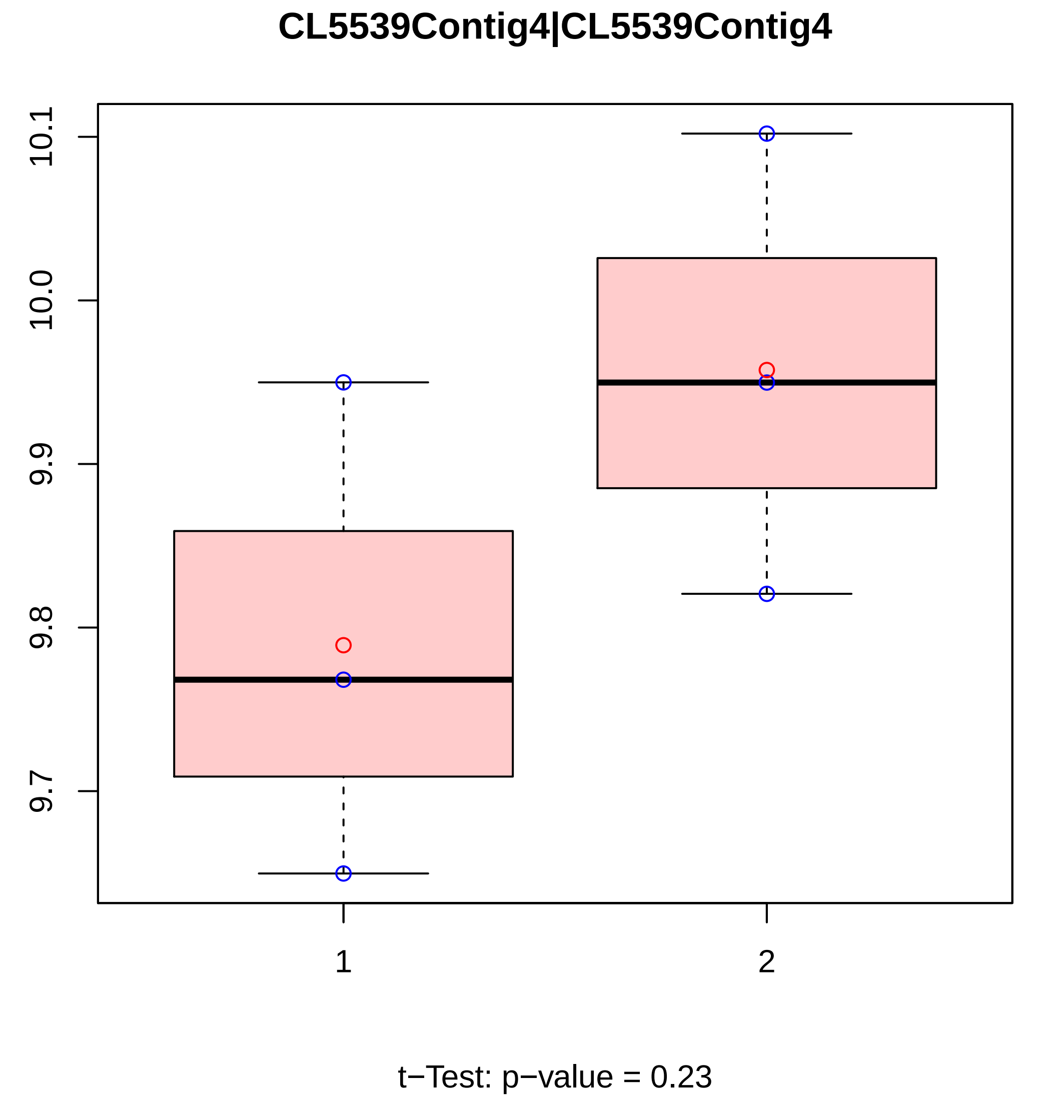

CL5539Contig4|CL5539Contig4
10.1
10.0
9.9
9.8
9.7
1
2
t−Test: p−value = 0.23

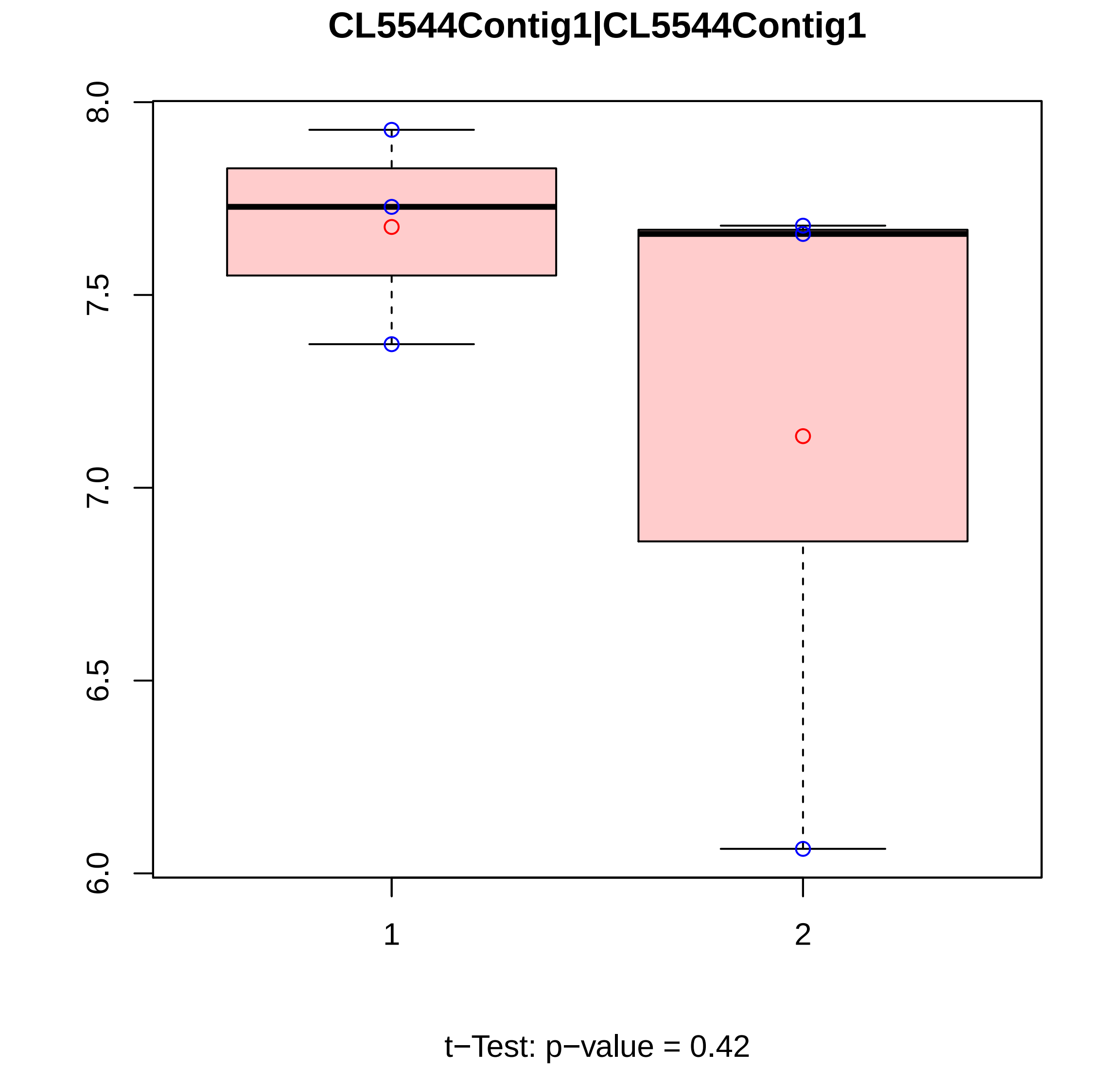
CL5544Contig1|CL5544Contig1
8.0
7.5
7.0
6.5
6.0
1
2
t−Test: p−value = 0.42

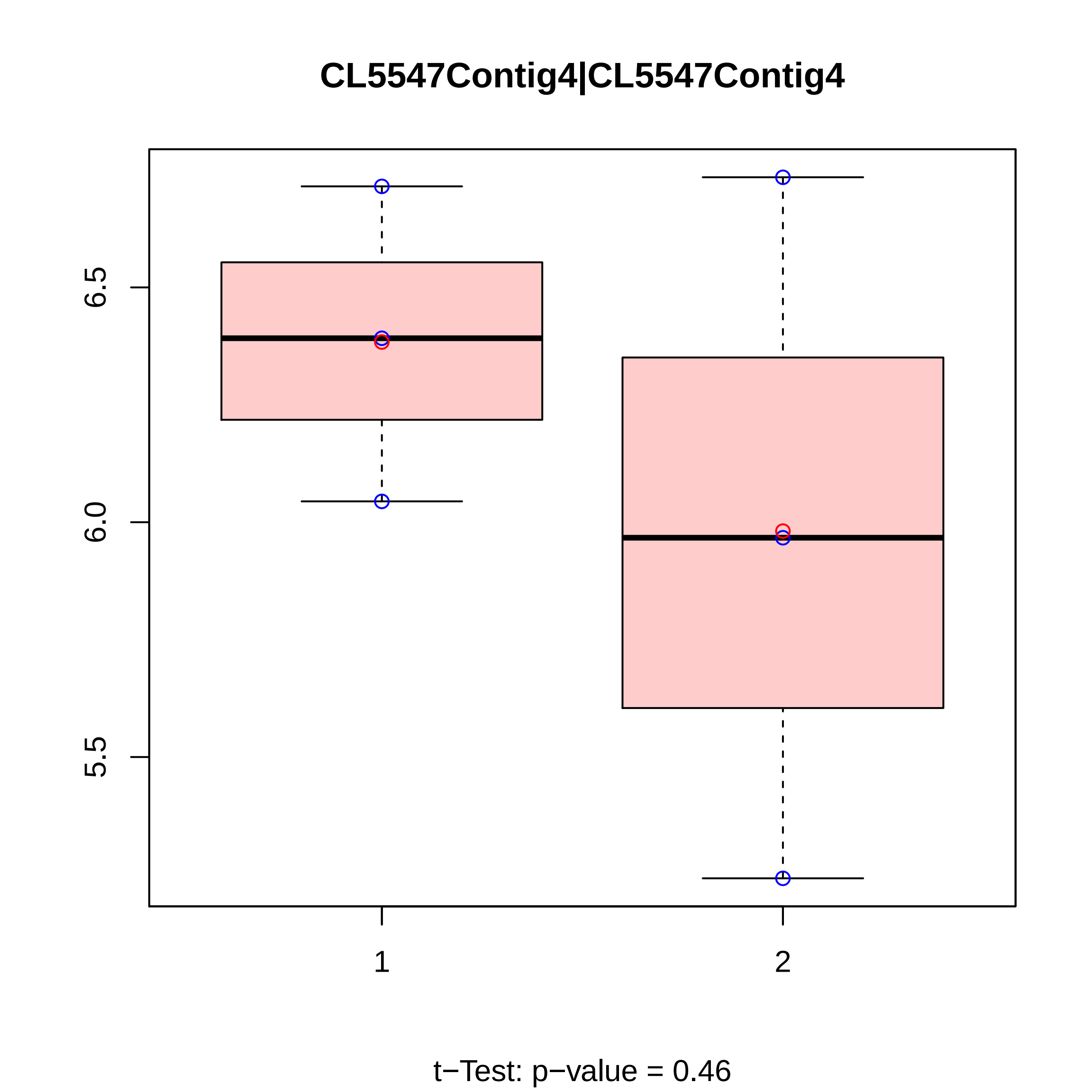

CL5547Contig4|CL5547Contig4
6.5
6.0
5.5
1
2
t−Test: p−value = 0.46

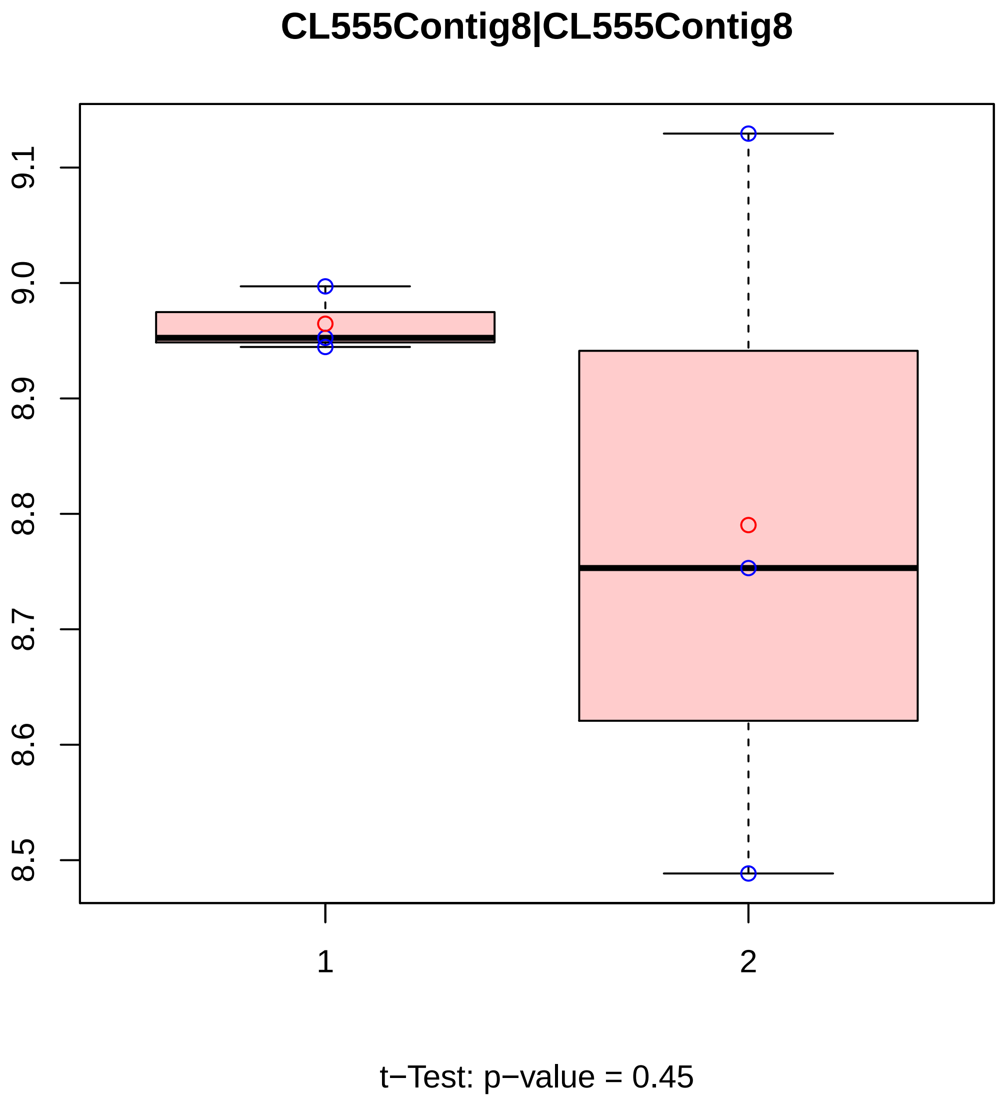
CL555Contig8|CL555Contig8
9.1
9.0
8.9
8.8
8.7
8.6
8.5
1
2
t−Test: p−value = 0.45

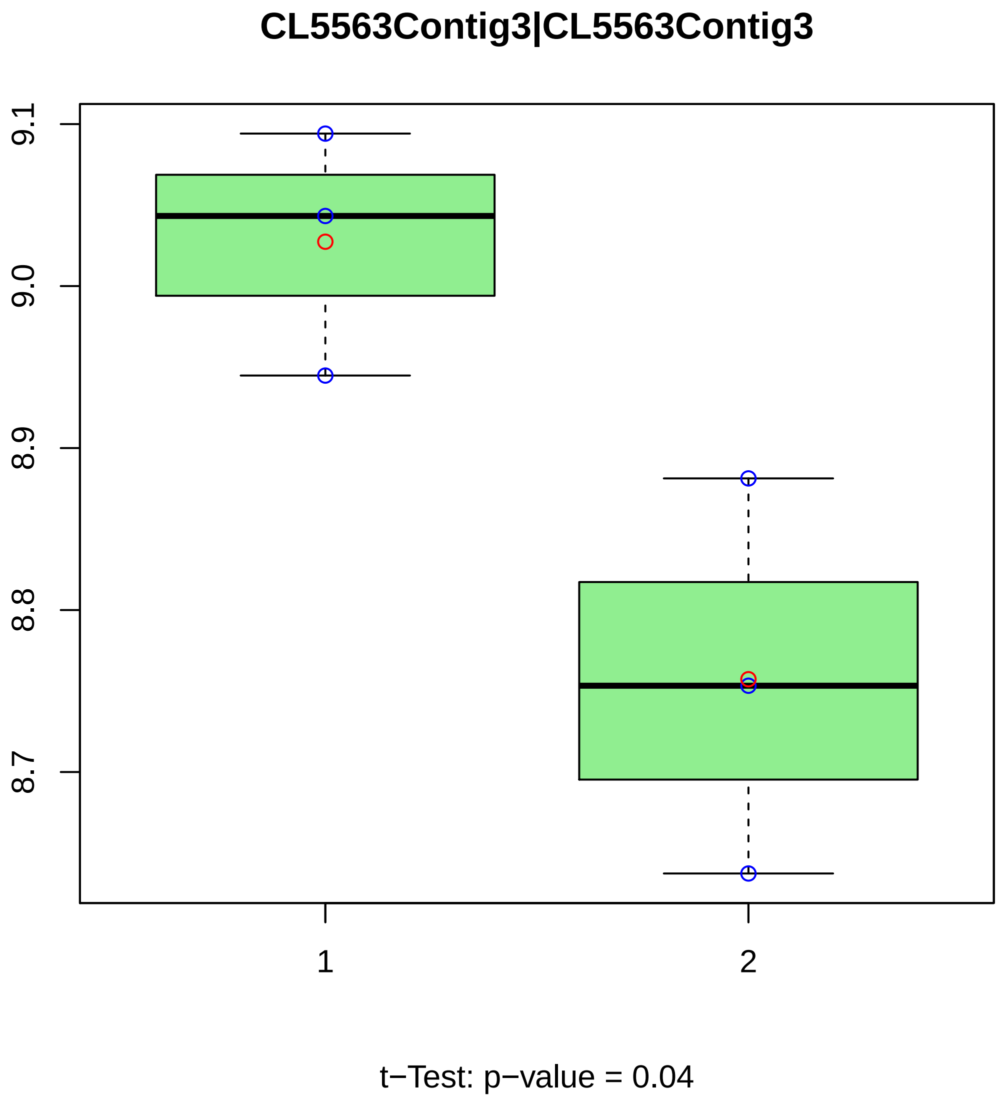

CL5563Contig3|CL5563Contig3
9.1
9.0
8.9
8.8
8.7
1
2
t−Test: p−value = 0.04

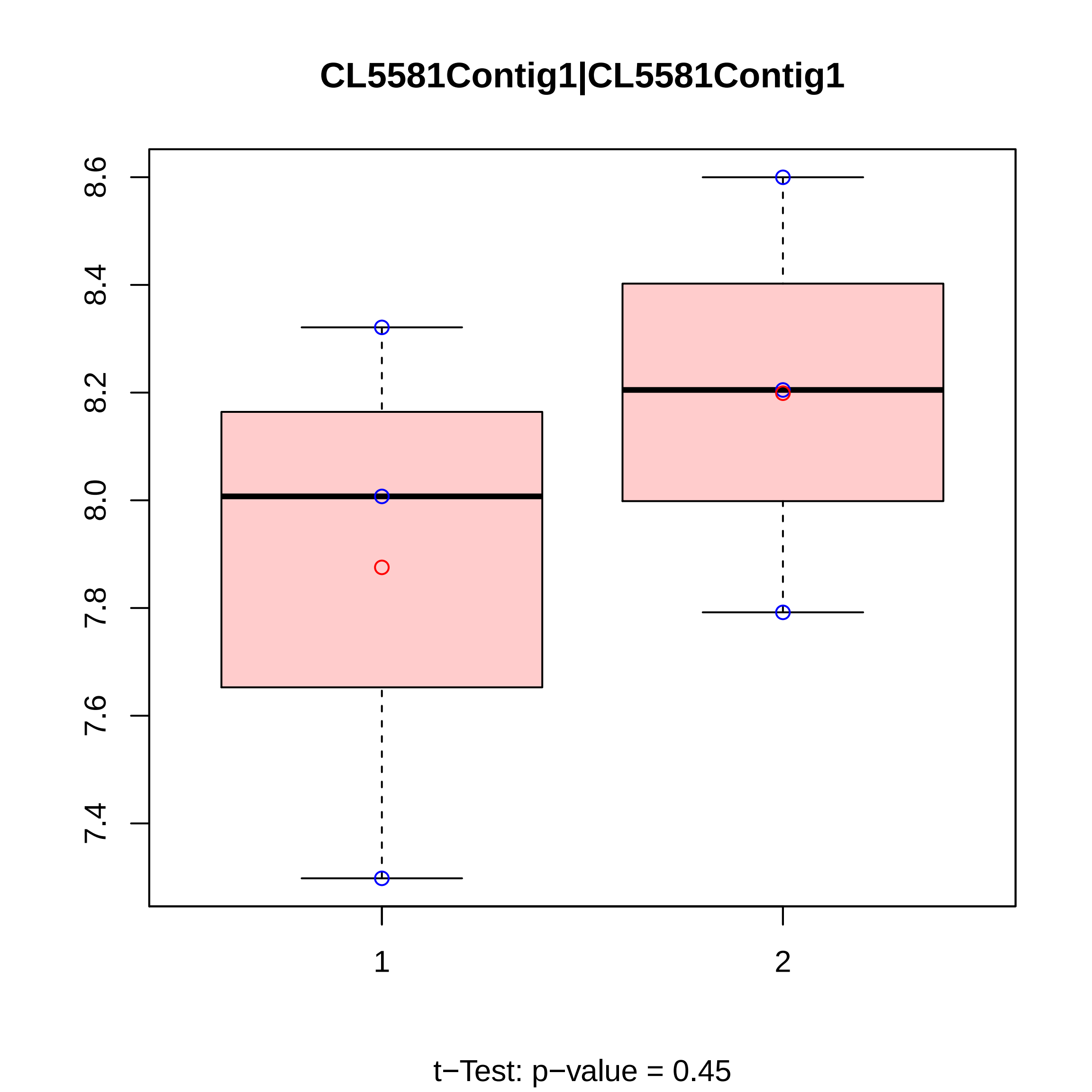
CL5581Contig1|CL5581Contig1
8.6
8.4
8.2
8.0
7.8
7.6
7.4
1
2
t−Test: p−value = 0.45

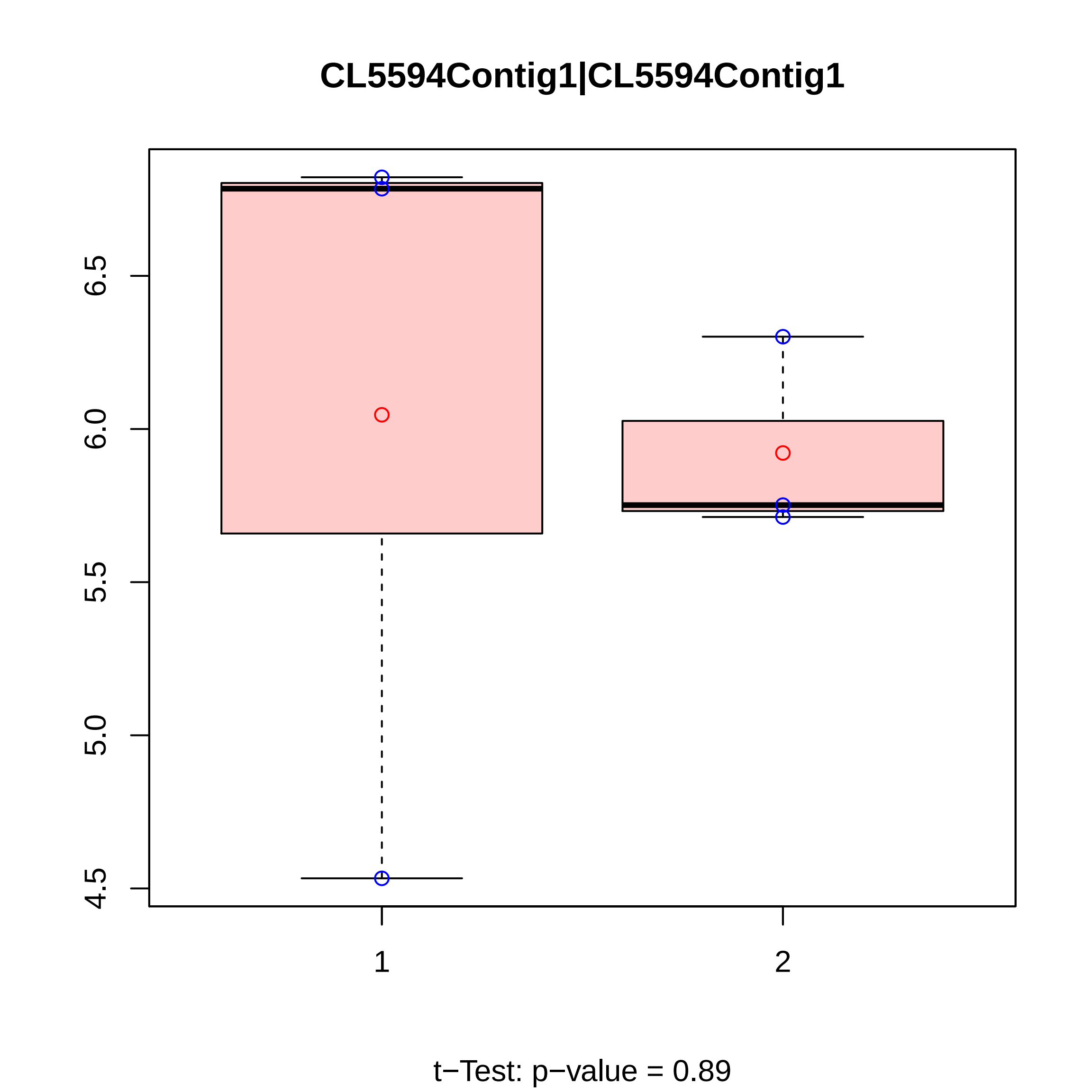

CL5594Contig1|CL5594Contig1
6.5
6.0
5.5
5.0
4.5
1
2
t−Test: p−value = 0.89

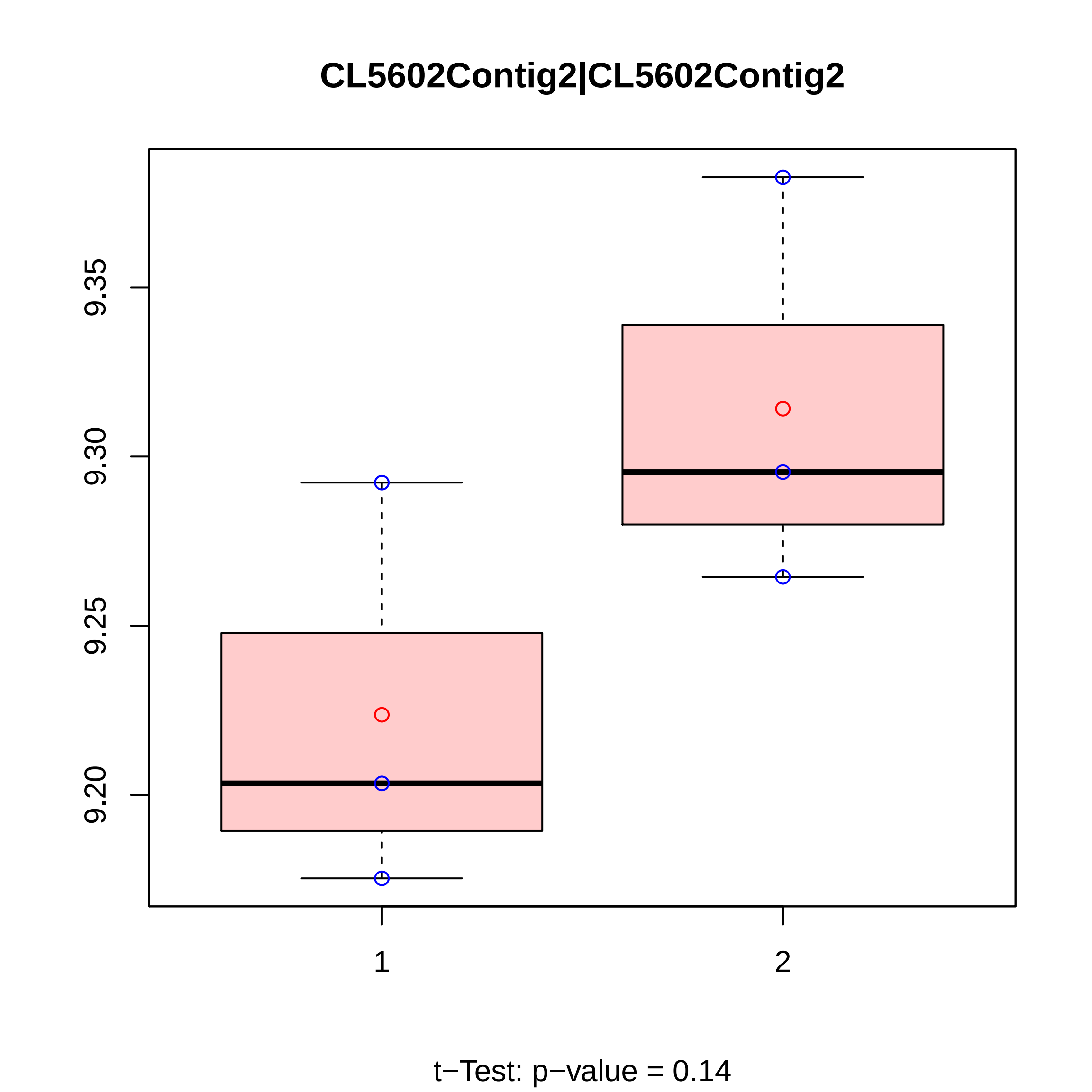
CL5602Contig2|CL5602Contig2
9.20
9.25
9.30
9.35
1
2
t−Test: p−value = 0.14

## CL5604Contig1|CL5604Contig1

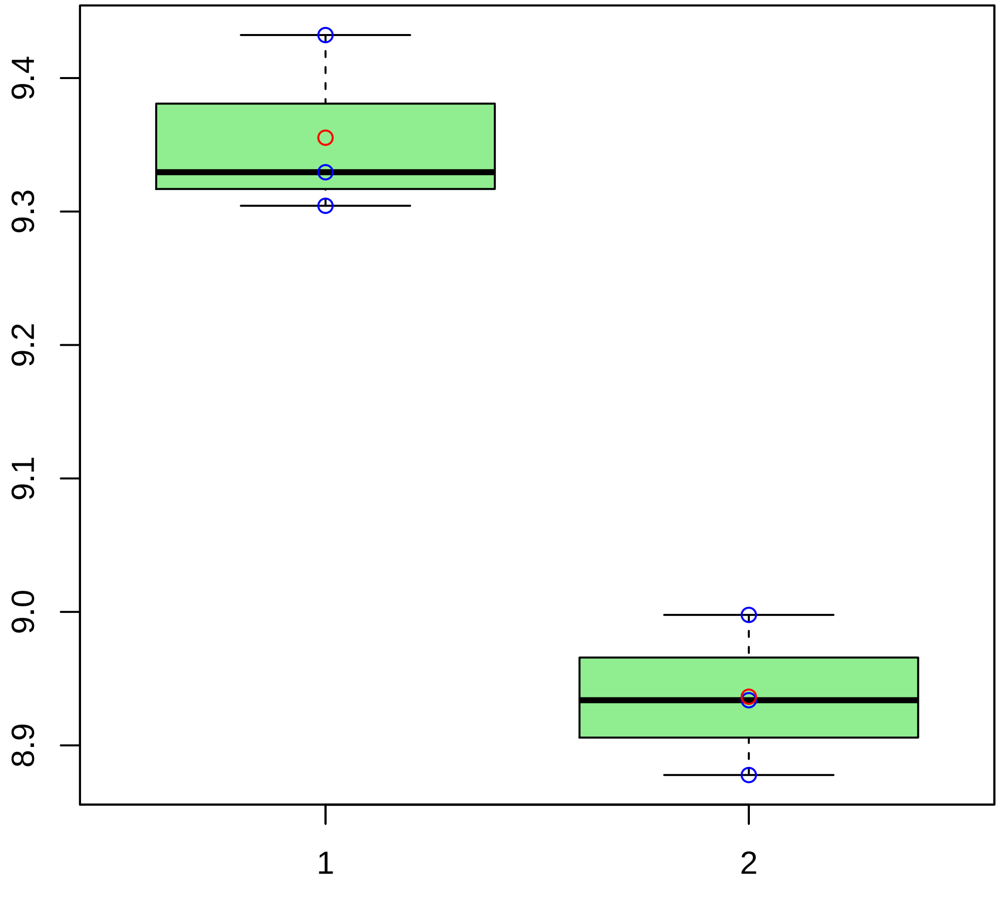


t−Test: p−value = 0

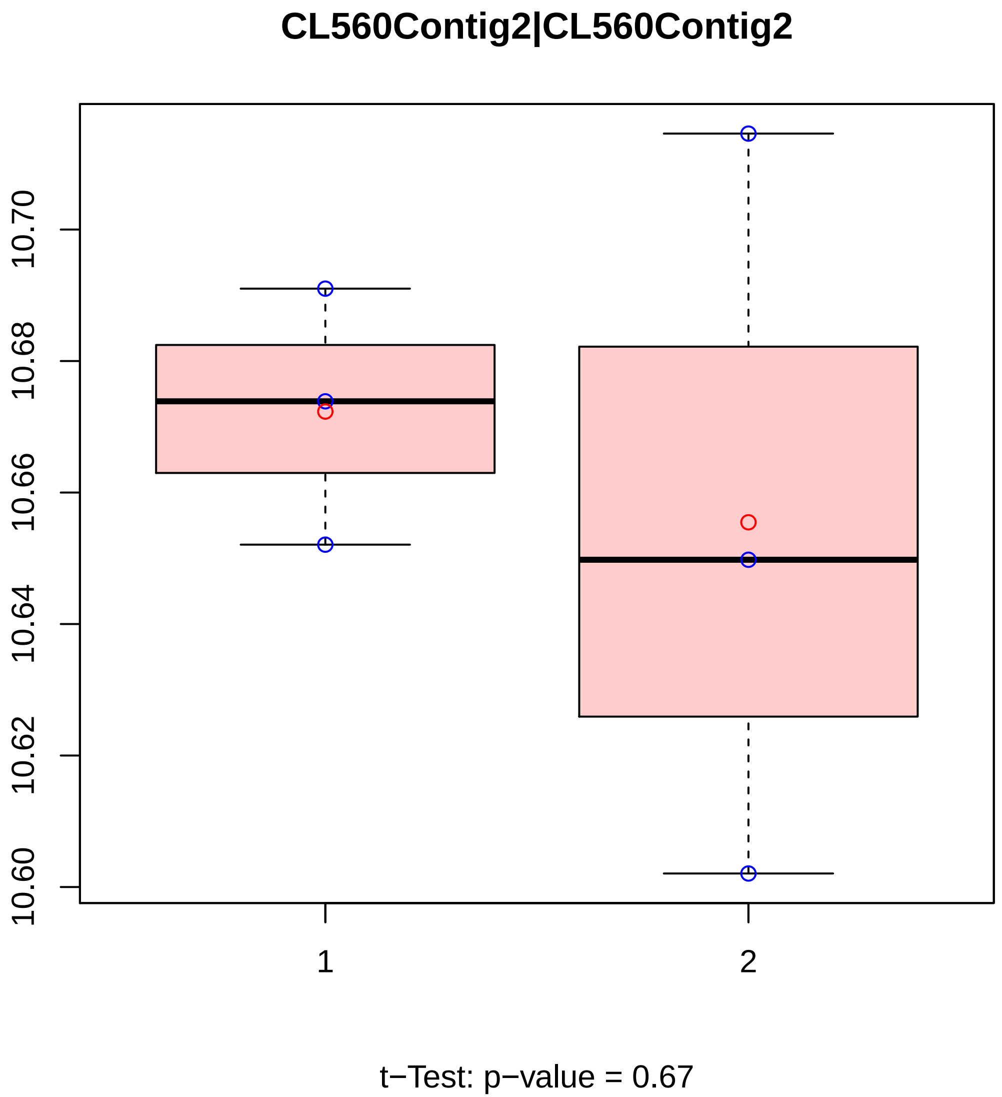
CL560Contig2|CL560Contig2
10.60
10.62
10.64
10.66
10.68
10.70
1
2
t−Test: p−value = 0.67

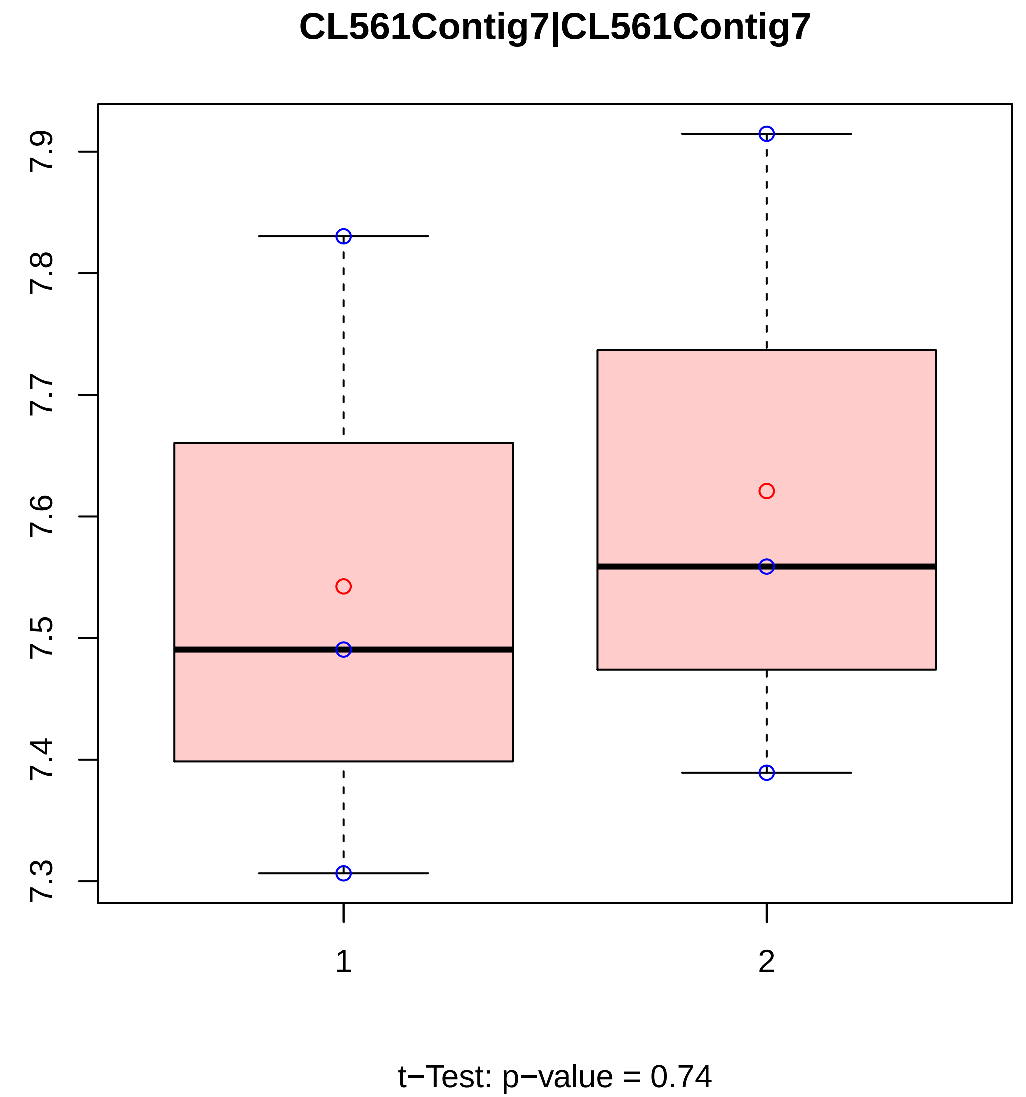
CL561Contig7|CL561Contig7
7.3
7.4
7.5
7.6
7.7
7.8
7.9
1
2
t−Test: p−value = 0.74

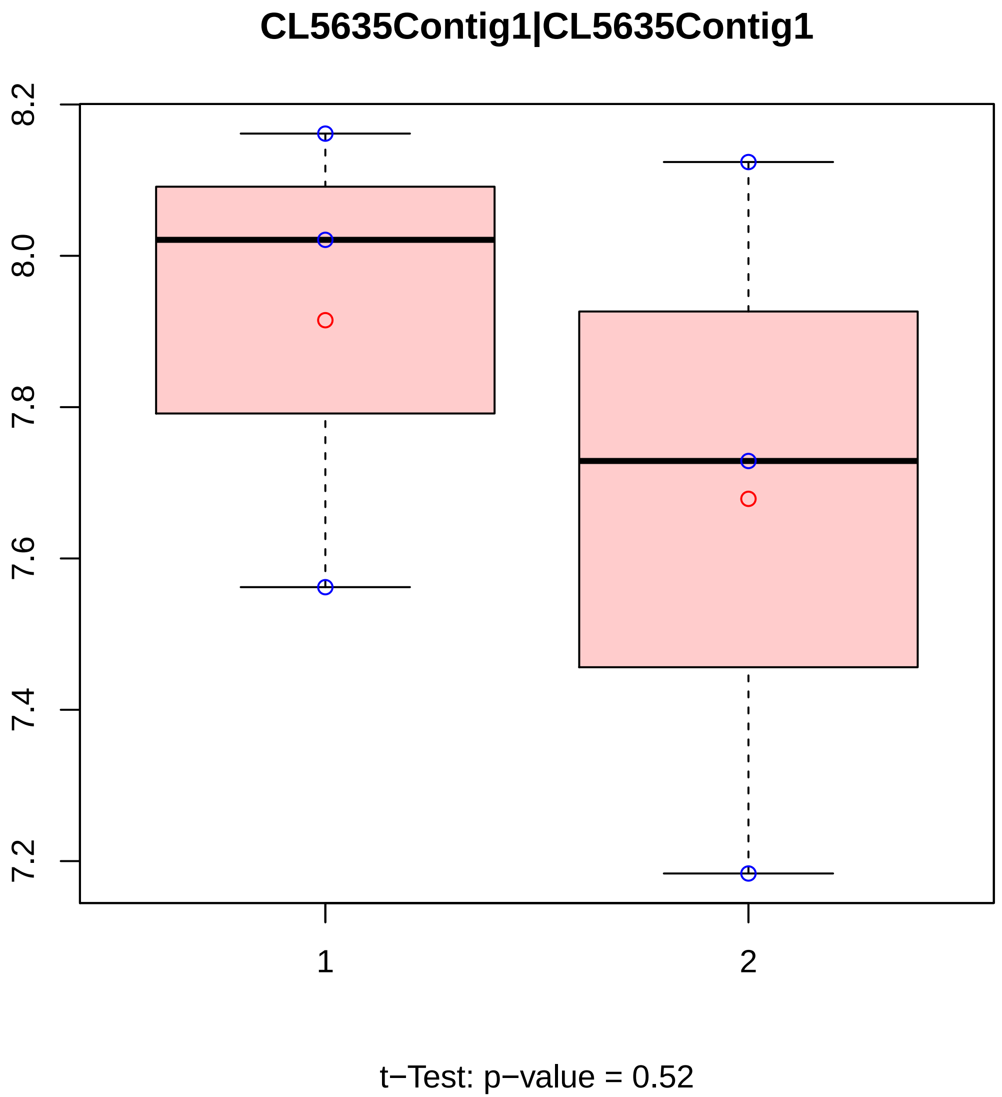

CL5635Contig1|CL5635Contig1
8.2
8.0
7.8
7.6
7.4
7.2
1
2
t−Test: p−value = 0.52

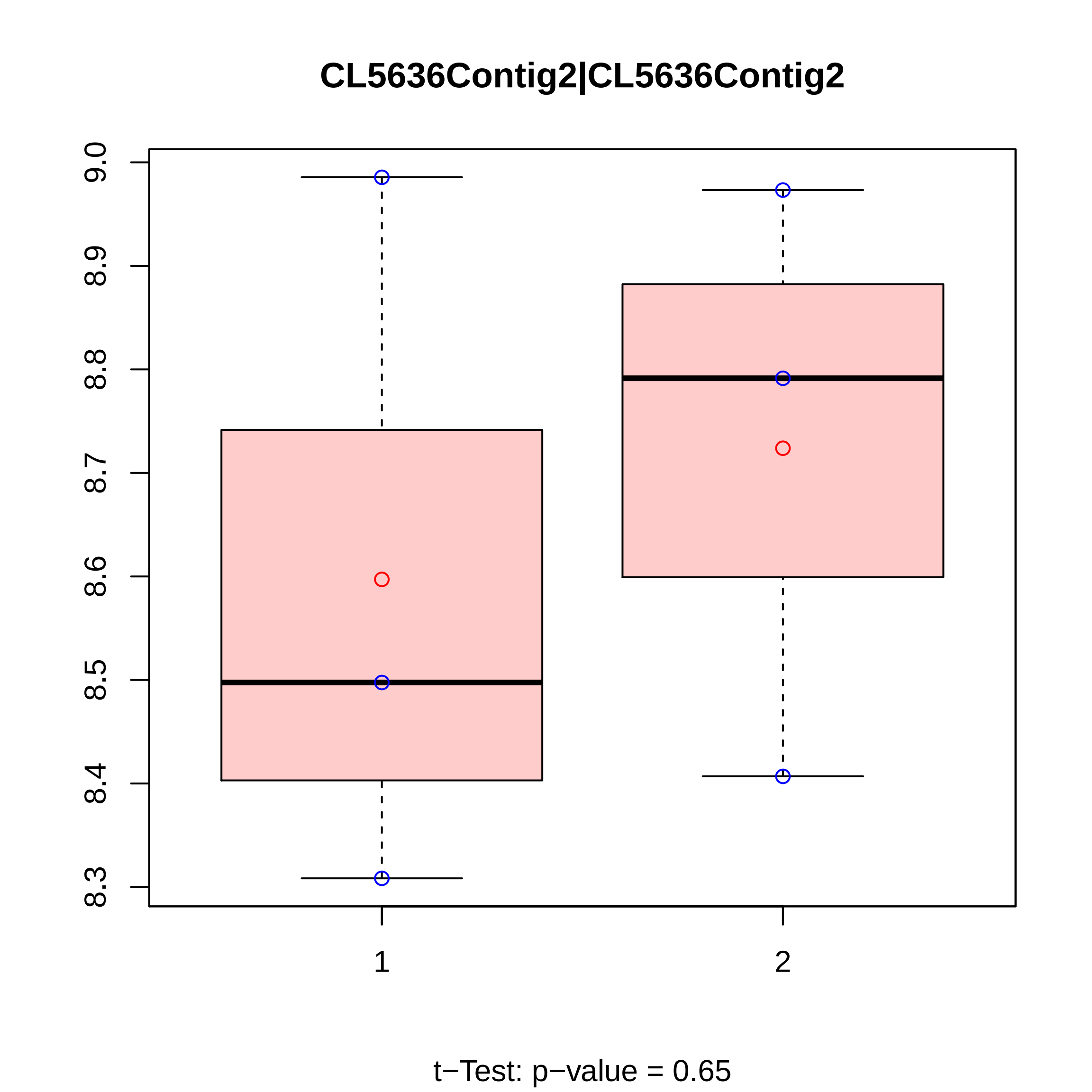
CL5636Contig2|CL5636Contig2
9.0
8.9
8.8
8.7
8.6
8.5
8.4
8.3
1
2
t−Test: p−value = 0.65

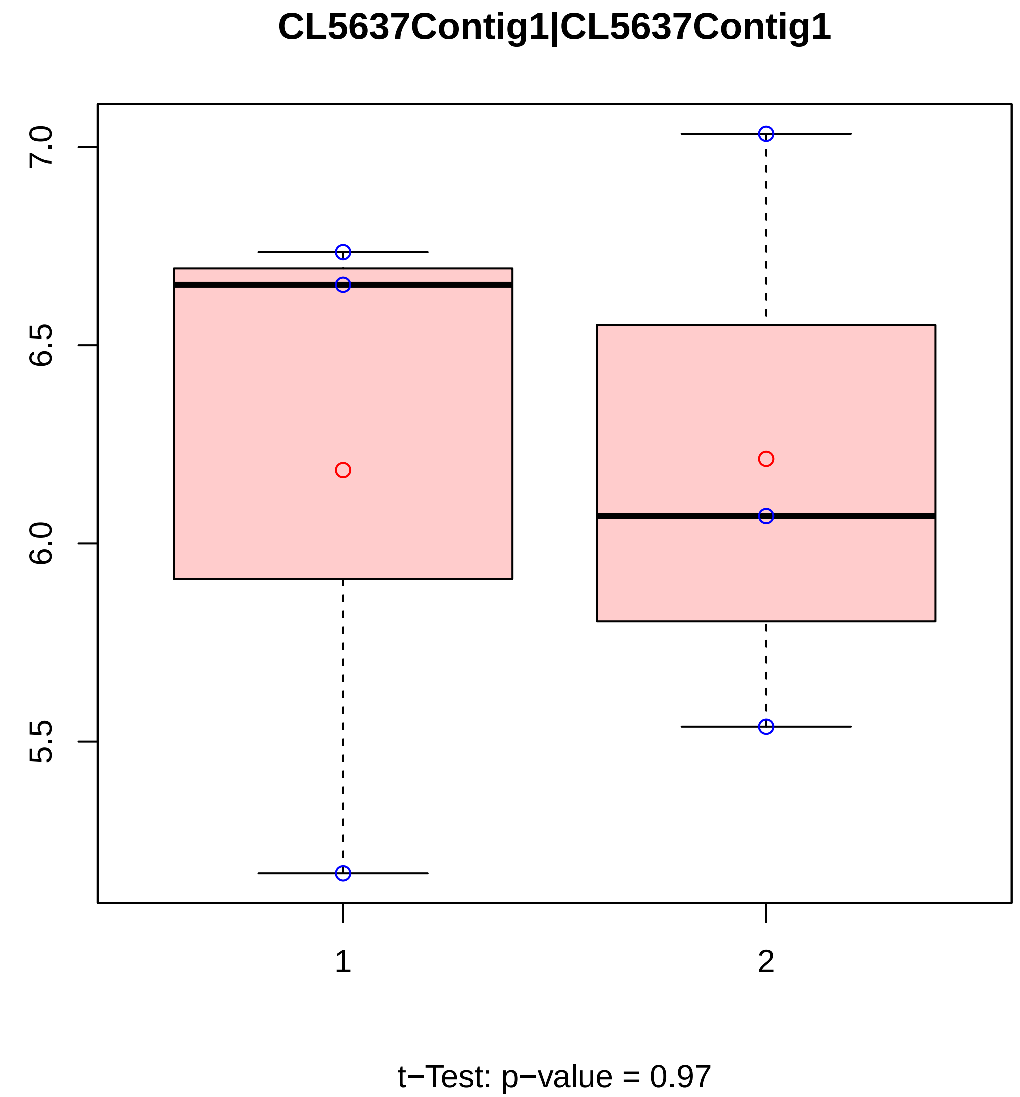

CL5637Contig1|CL5637Contig1
7.0
6.5
6.0
5.5
1
2
t−Test: p−value = 0.97

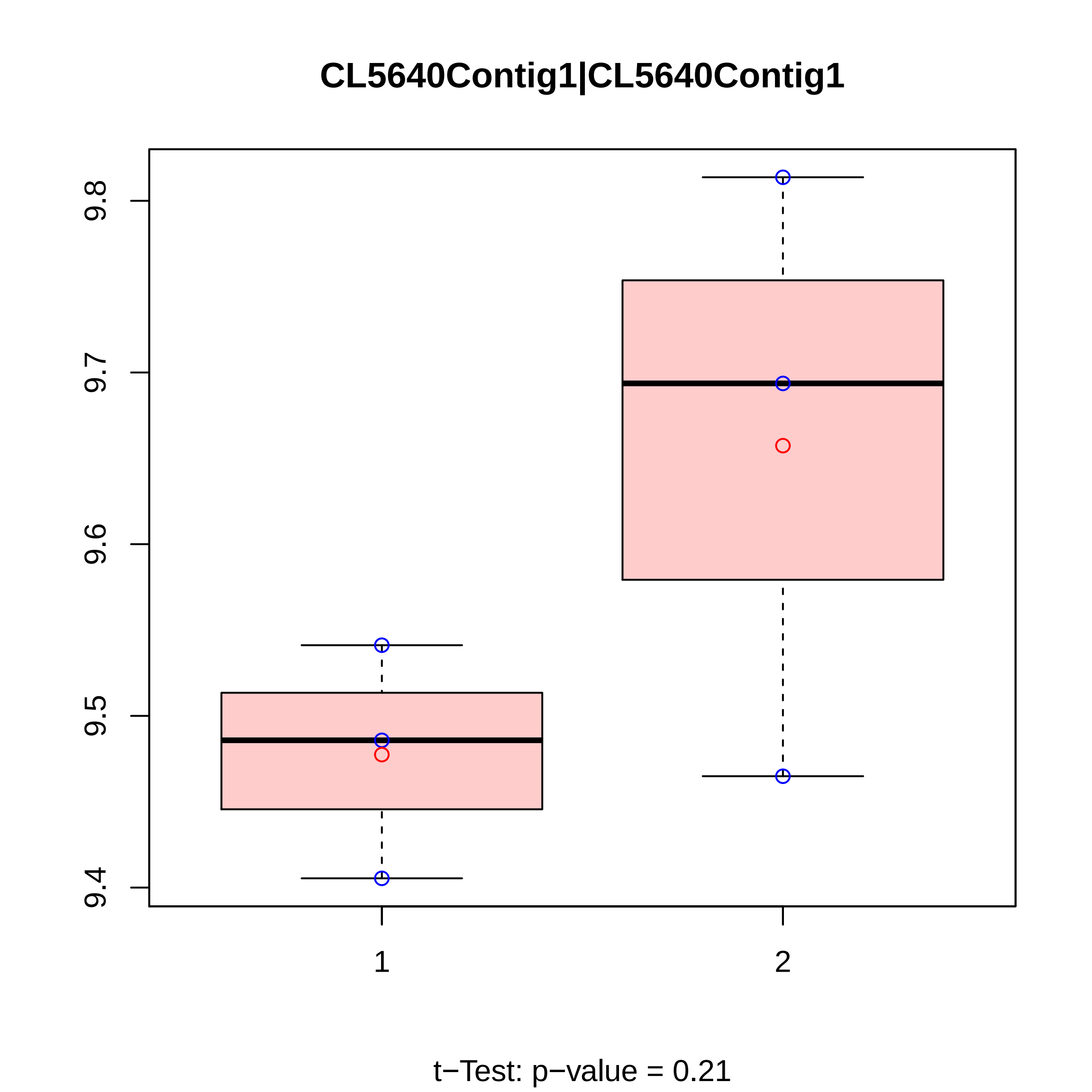
CL5640Contig1|CL5640Contig1
9.4
9.5
9.6
9.7
9.8
1
2
t−Test: p−value = 0.21

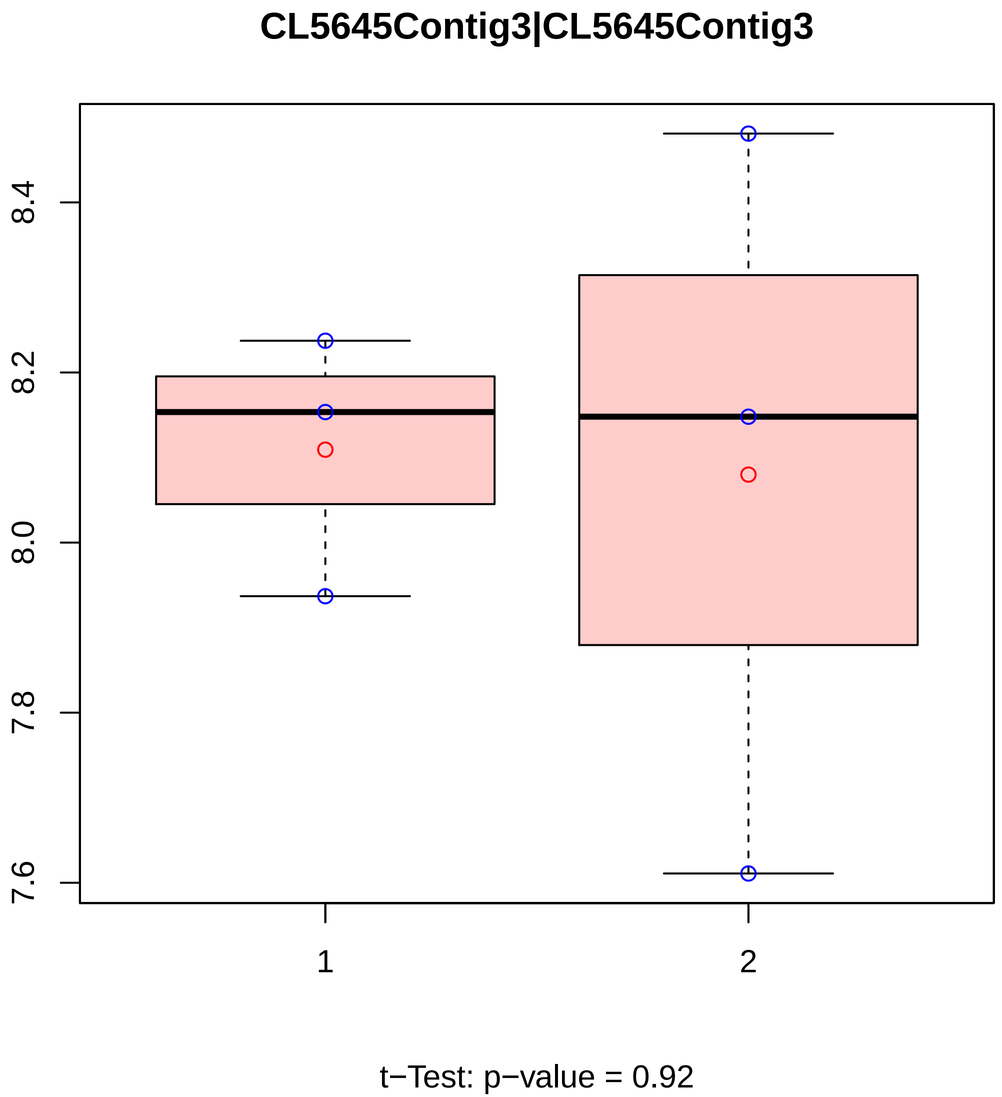
CL5645Contig3|CL5645Contig3
8.4
8.2
8.0
7.8
7.6
1
2
t−Test: p−value = 0.92

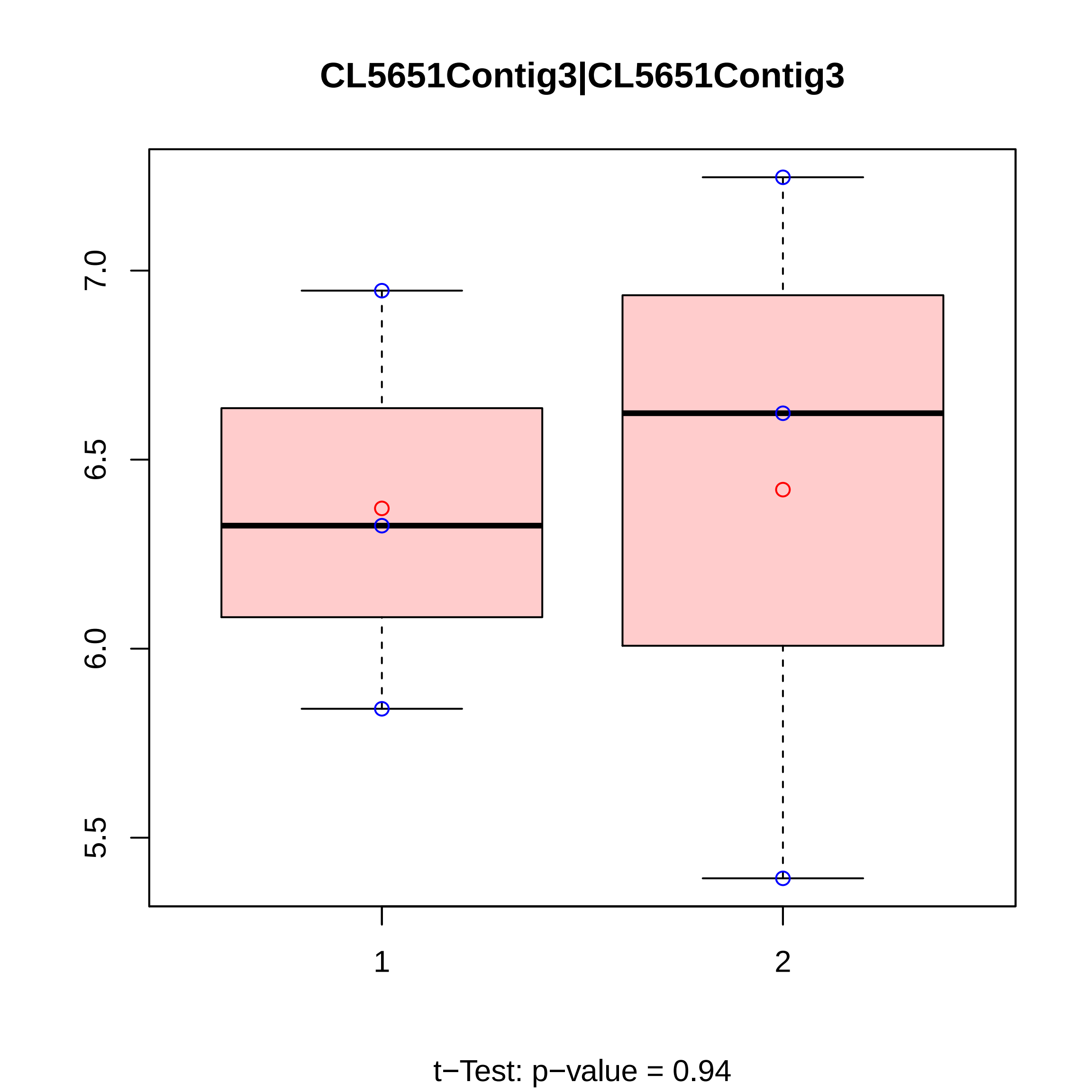
CL5651Contig3|CL5651Contig3
7.0
6.5
6.0
5.5
1
2
t−Test: p−value = 0.94

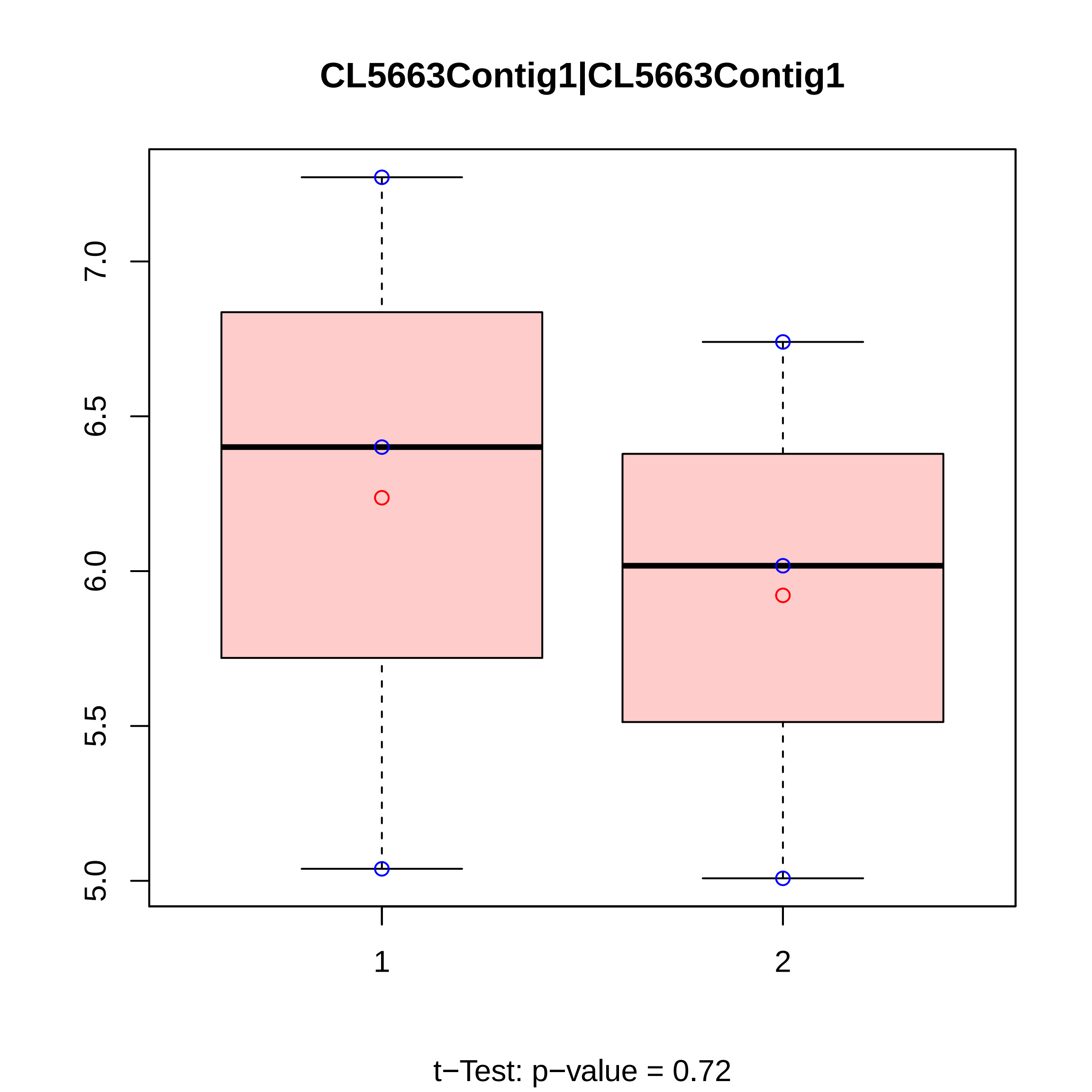

CL5663Contig1|CL5663Contig1
7.0
6.5
6.0
5.5
5.0
1
2
t−Test: p−value = 0.72

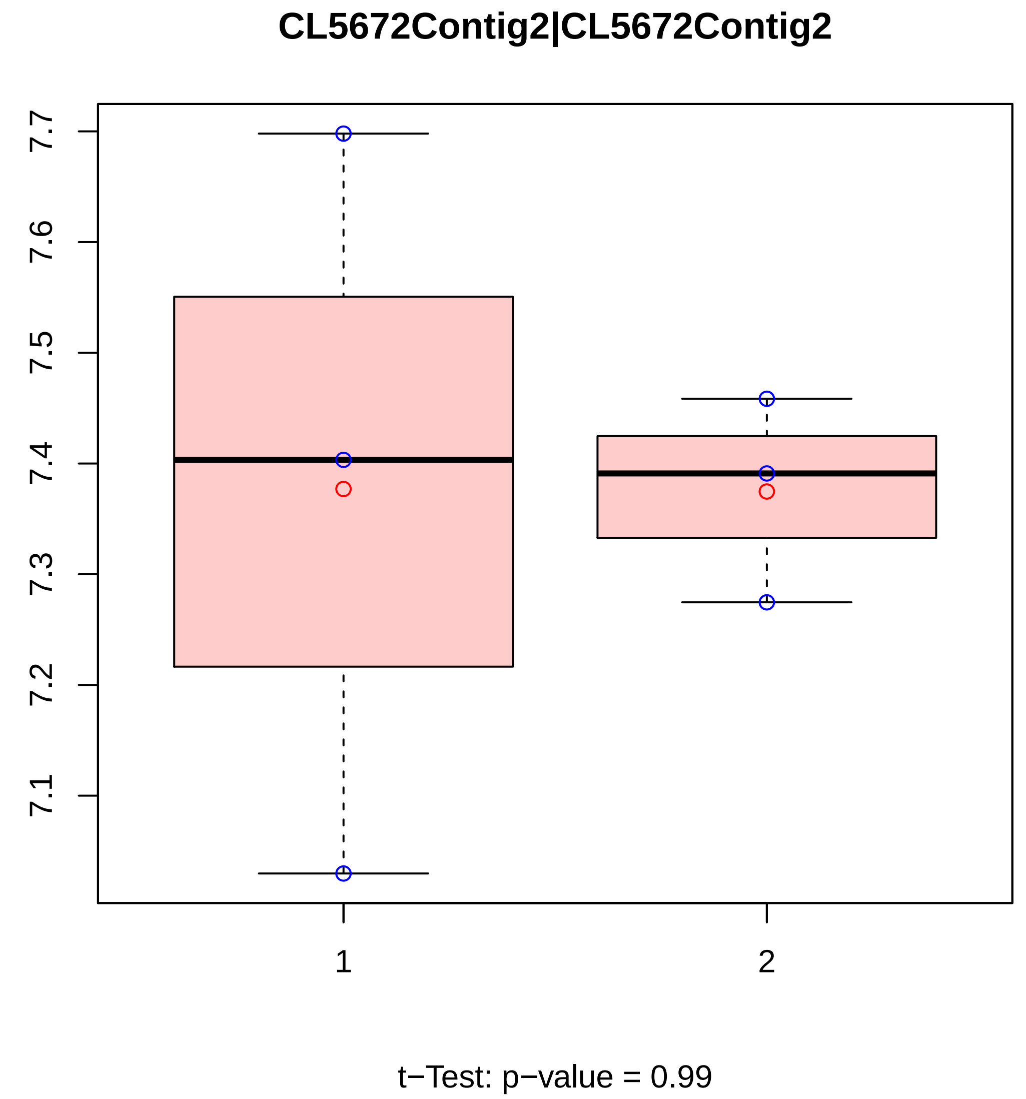

CL5672Contig2|CL5672Contig2
7.1
7.2
7.3
7.4
7.5
7.6
7.7
1
2
t−Test: p−value = 0.99

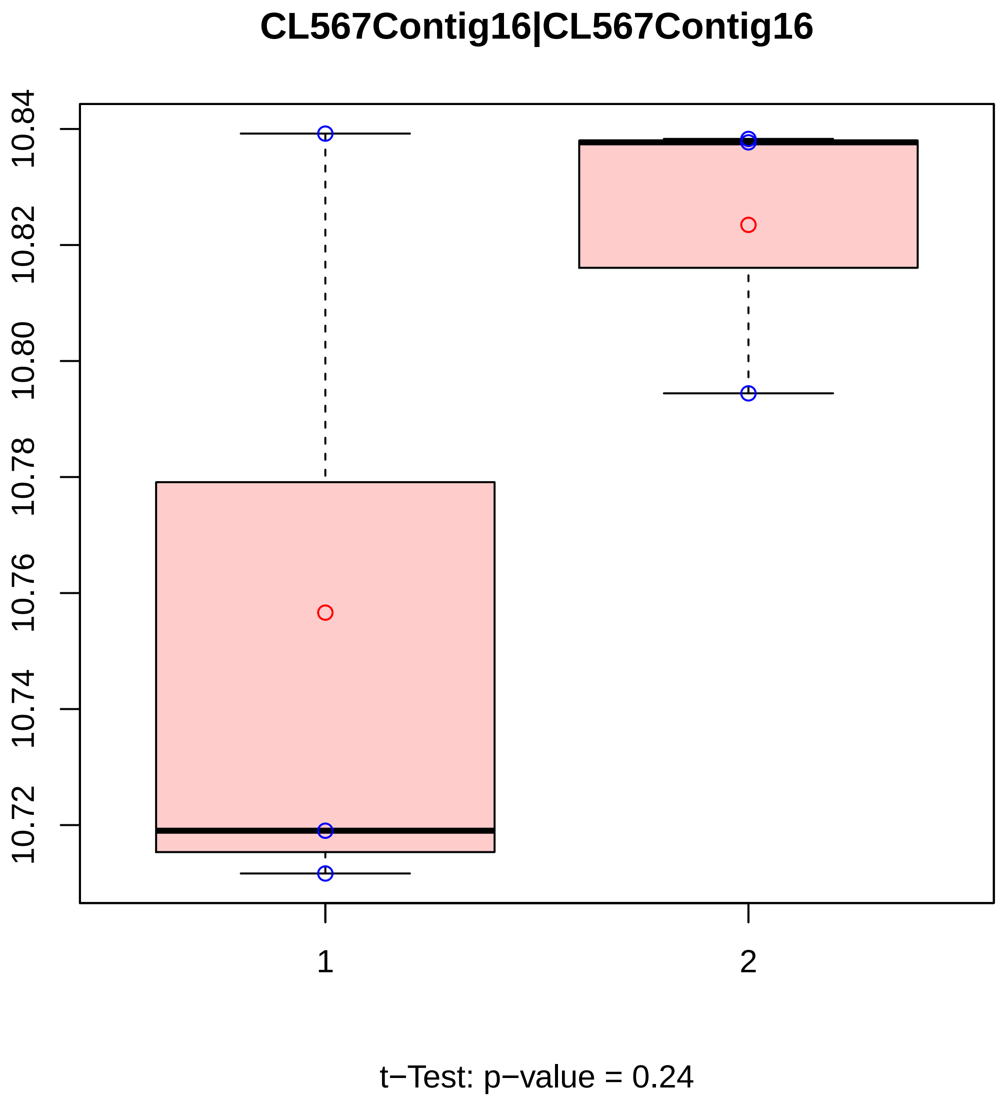

CL567Contig16|CL567Contig16
10.84
10.82
10.80
10.78
10.76
10.74
10.72
1
2
t−Test: p−value = 0.24

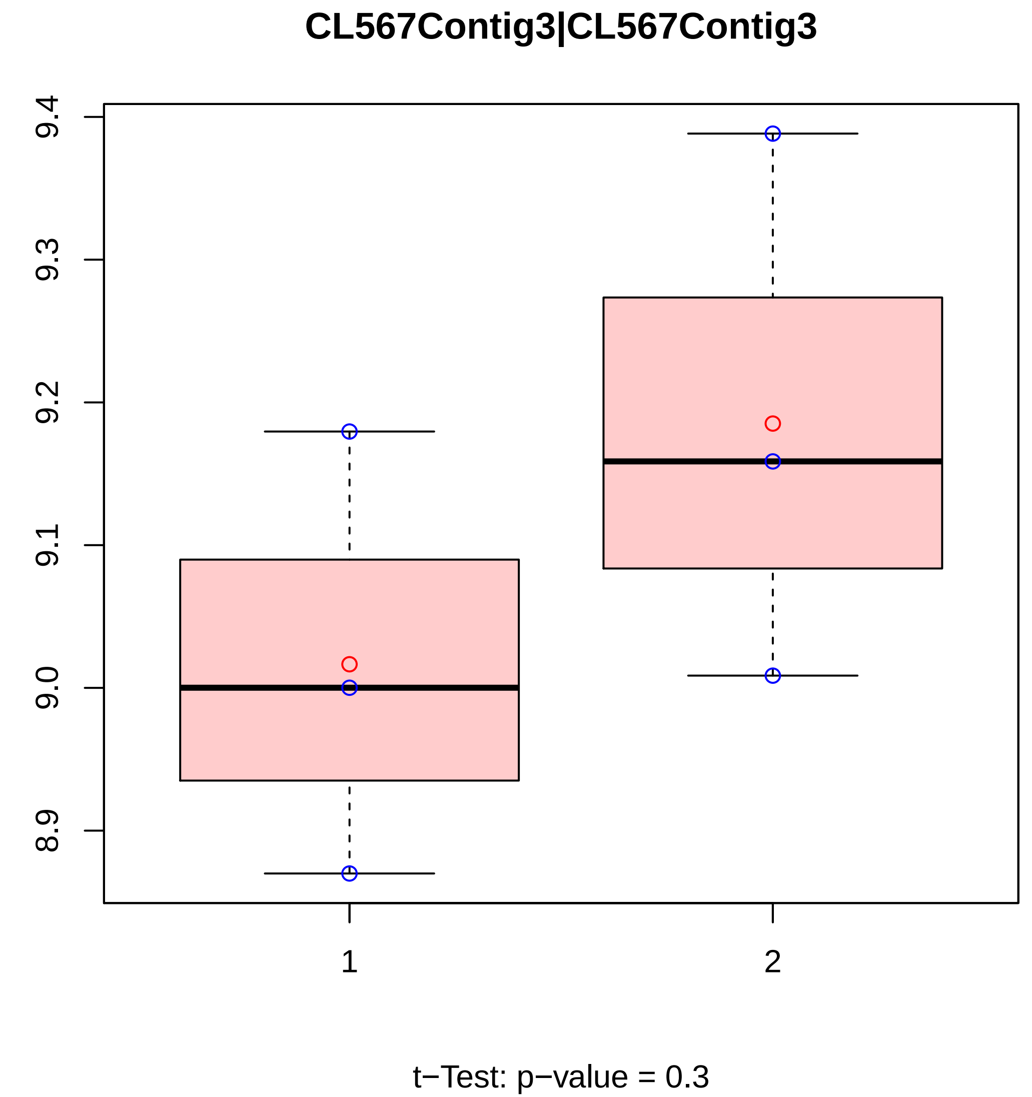
CL567Contig3|CL567Contig3
9.4
9.3
9.2
9.1
9.0
8.9
1
2
t−Test: p−value = 0.3

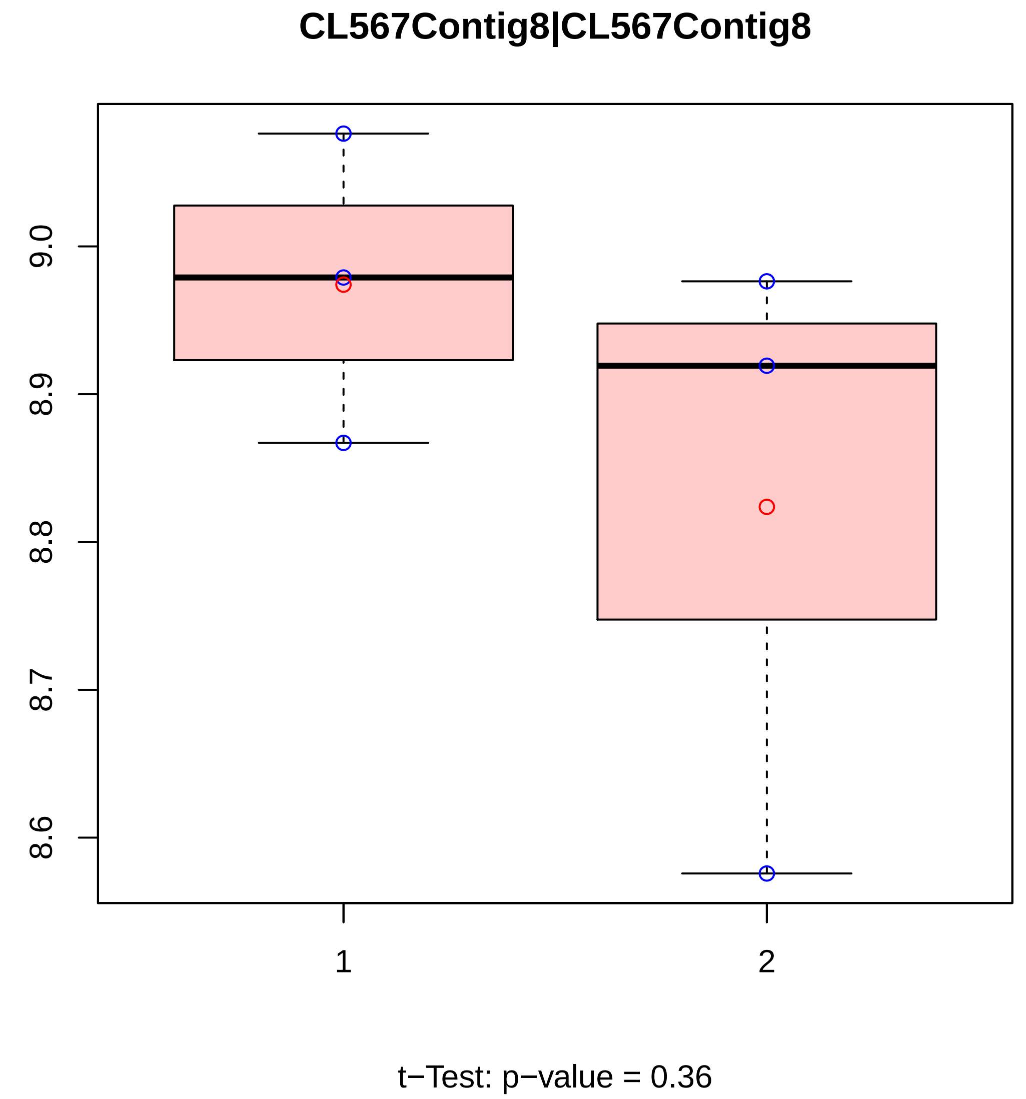
CL567Contig8|CL567Contig8
9.0
8.9
8.8
8.7
8.6
1
2
t−Test: p−value = 0.36

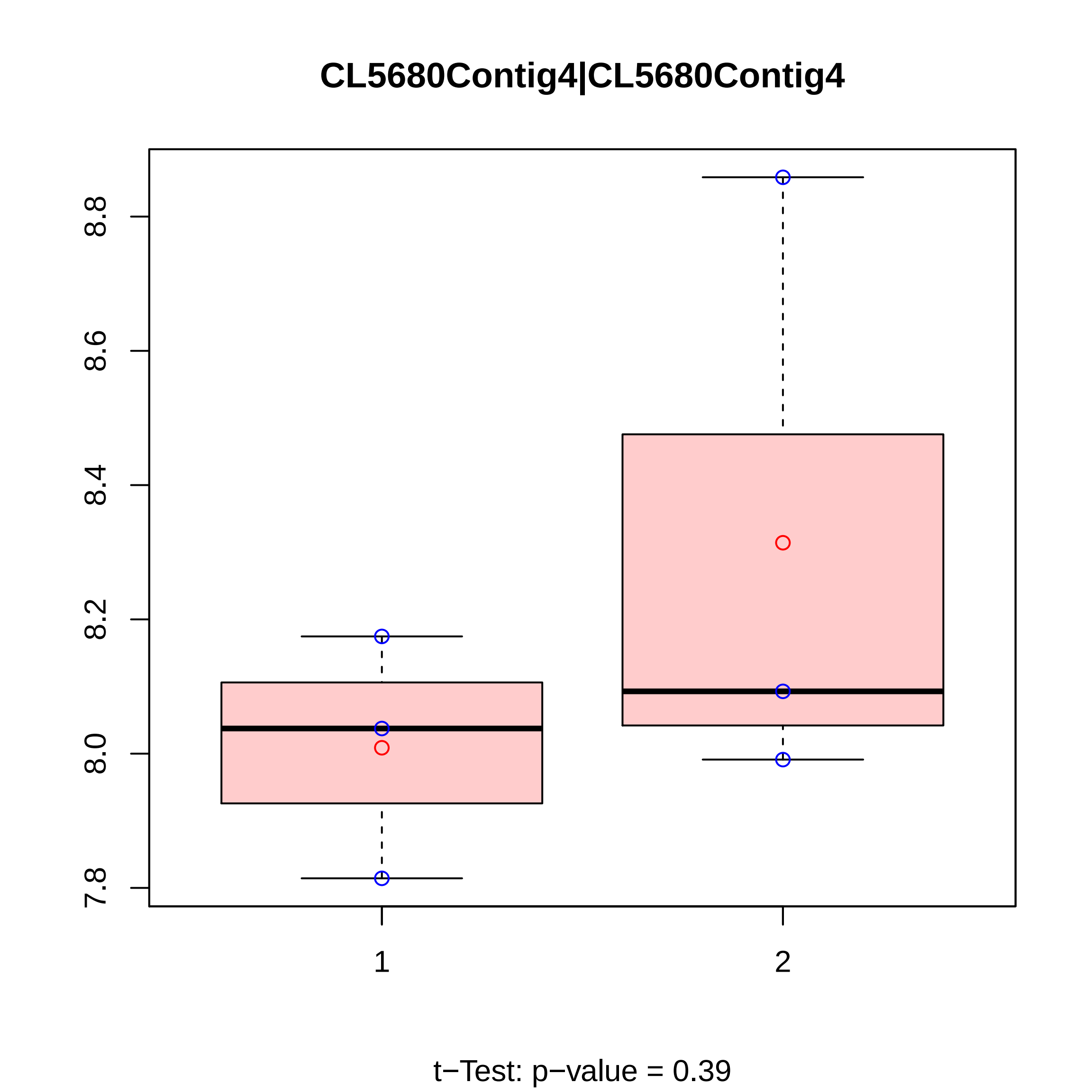
CL5680Contig4|CL5680Contig4
7.8
8.0
8.2
8.4
8.6
8.8
1
2
t−Test: p−value = 0.39

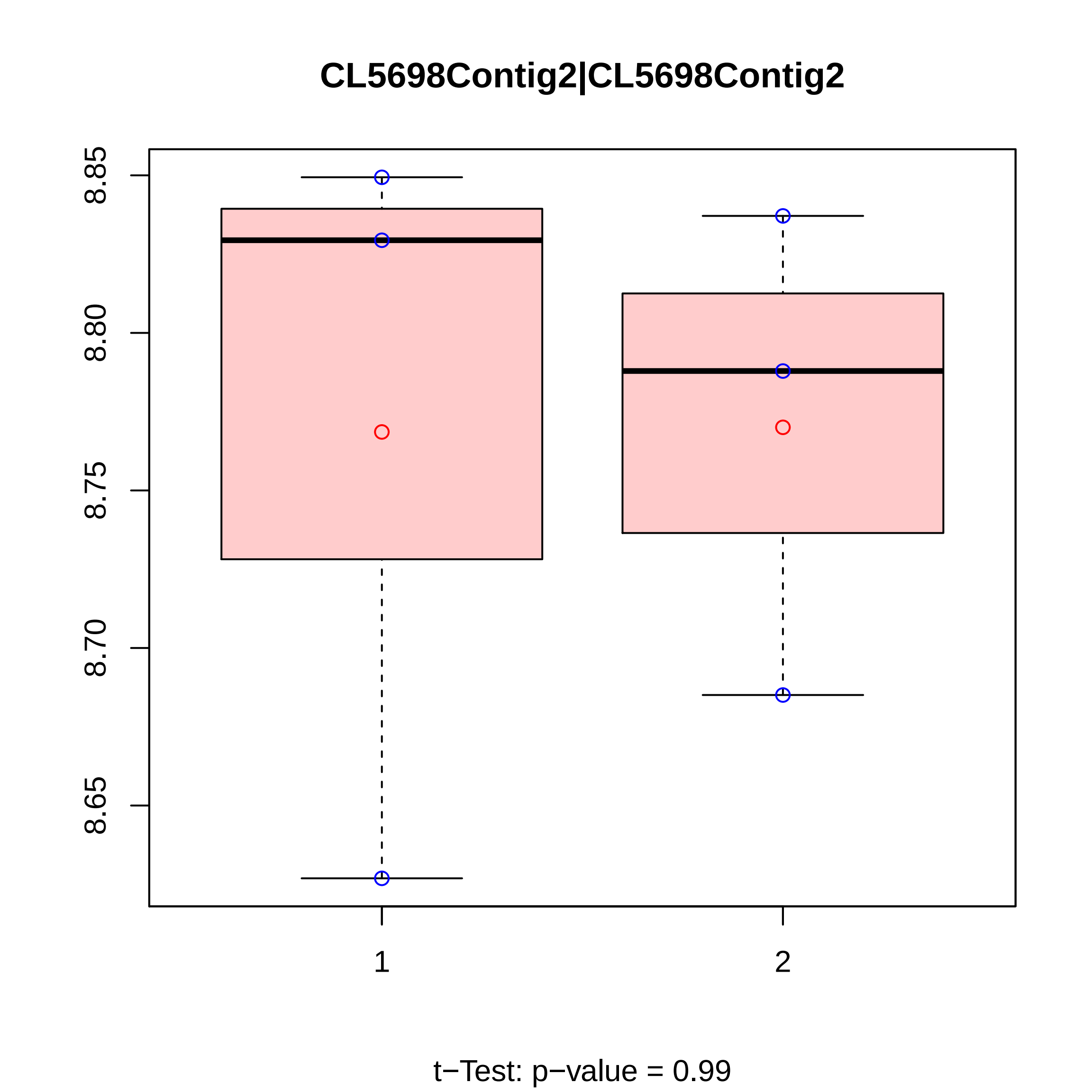

CL5698Contig2|CL5698Contig2
8.65
8.70
8.75
8.80
8.85
1
2
t−Test: p−value = 0.99

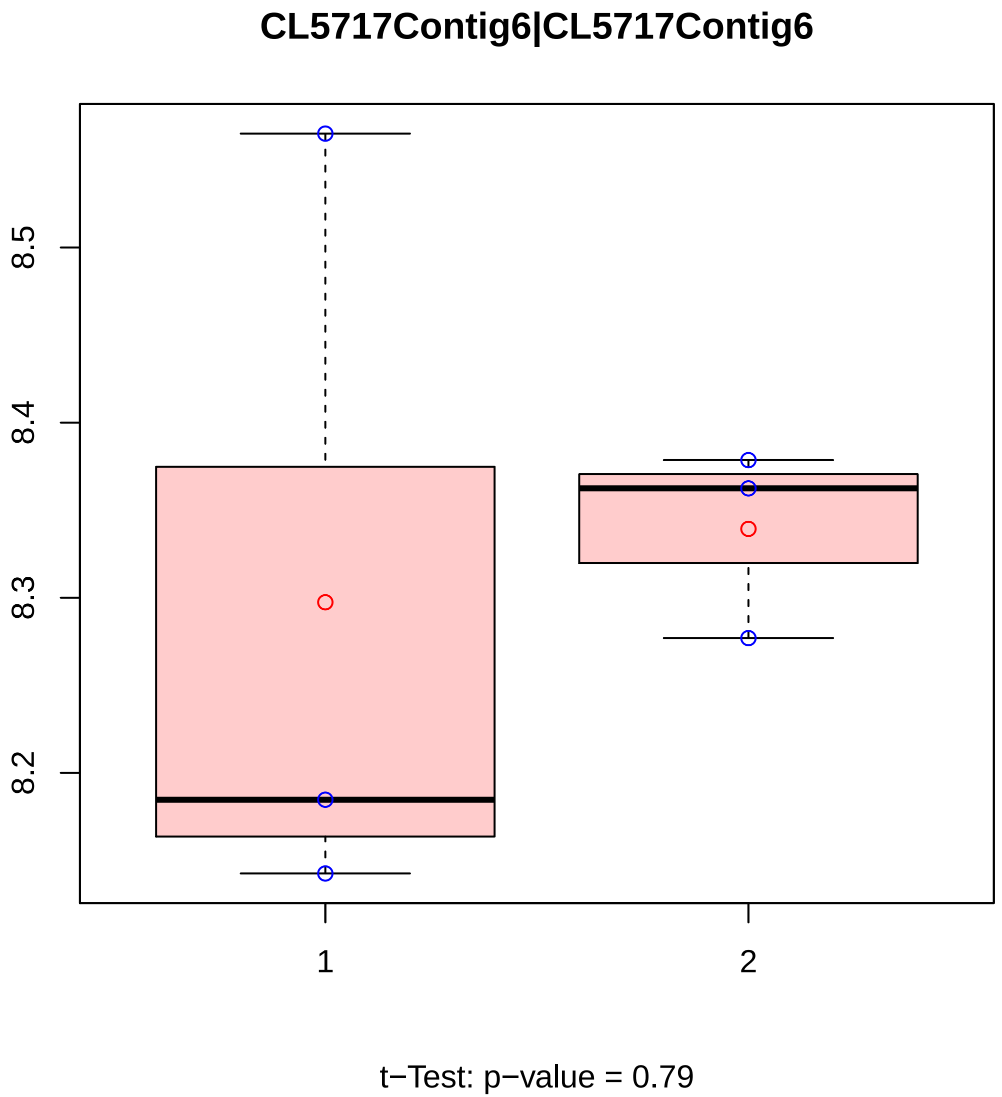
CL5717Contig6|CL5717Contig6
8.2
8.3
8.4
8.5
1
2
t−Test: p−value = 0.79

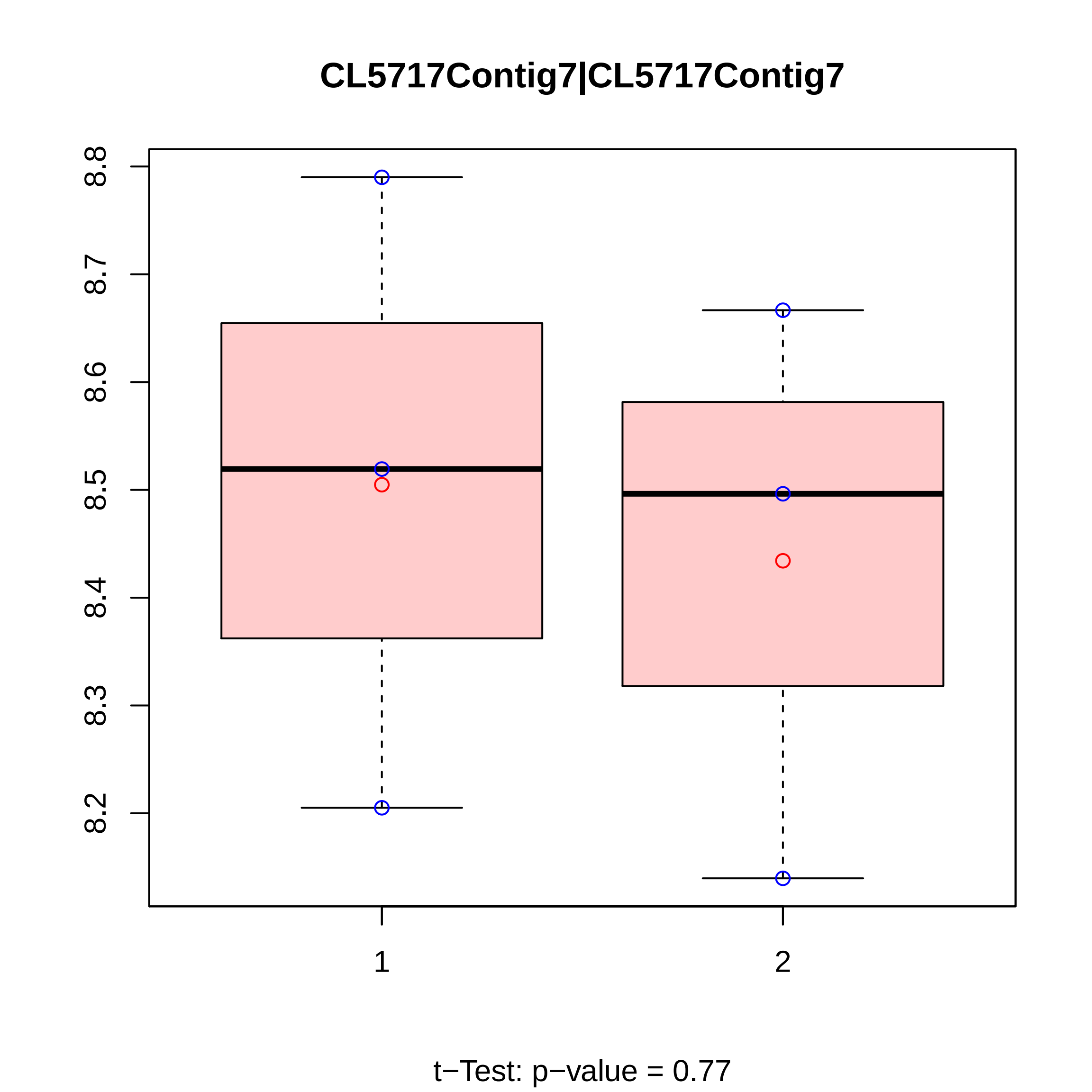

CL5717Contig7|CL5717Contig7
8.2
8.3
8.4
8.5
8.6
8.7
8.8
1
2
t−Test: p−value = 0.77

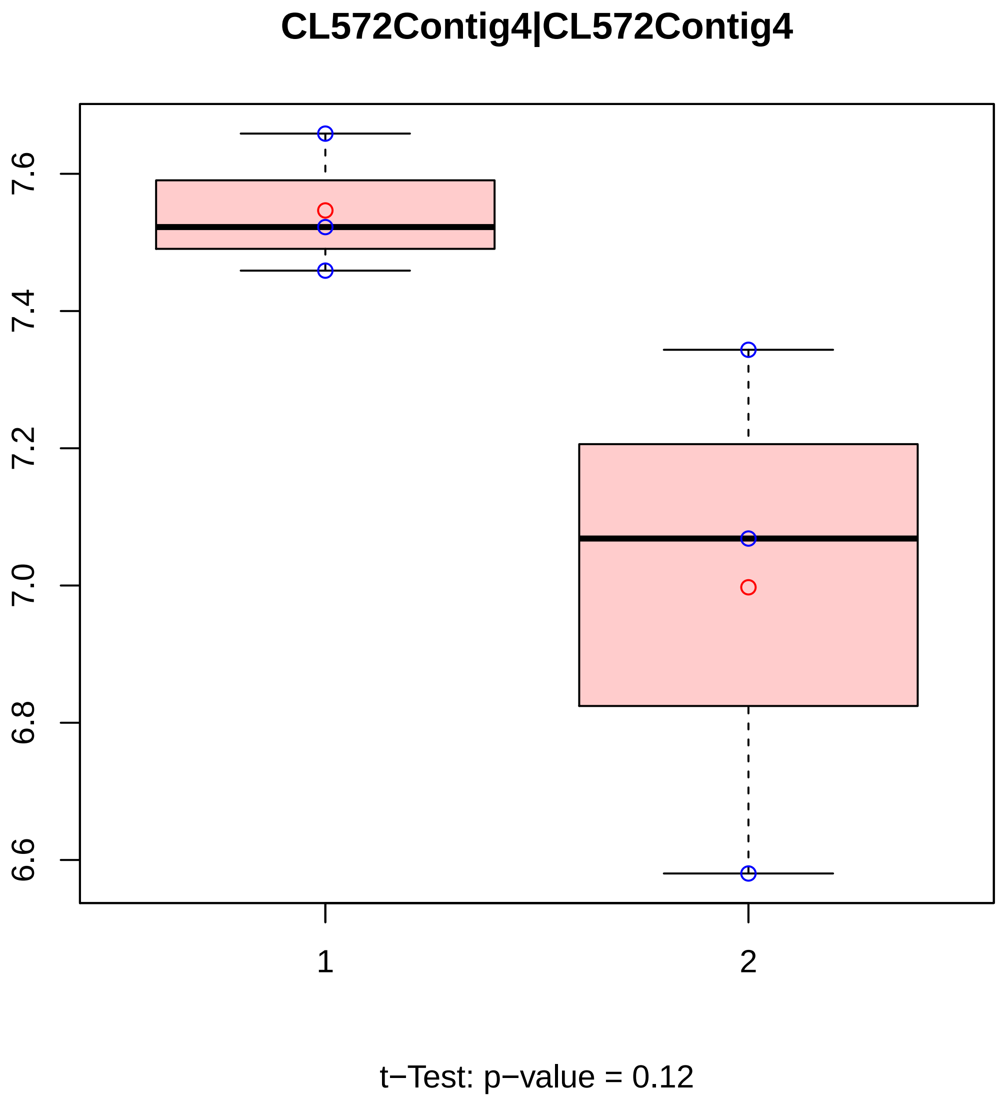
CL572Contig4|CL572Contig4
7.6
7.4
7.2
7.0
6.8
6.6
1
2
t−Test: p−value = 0.12

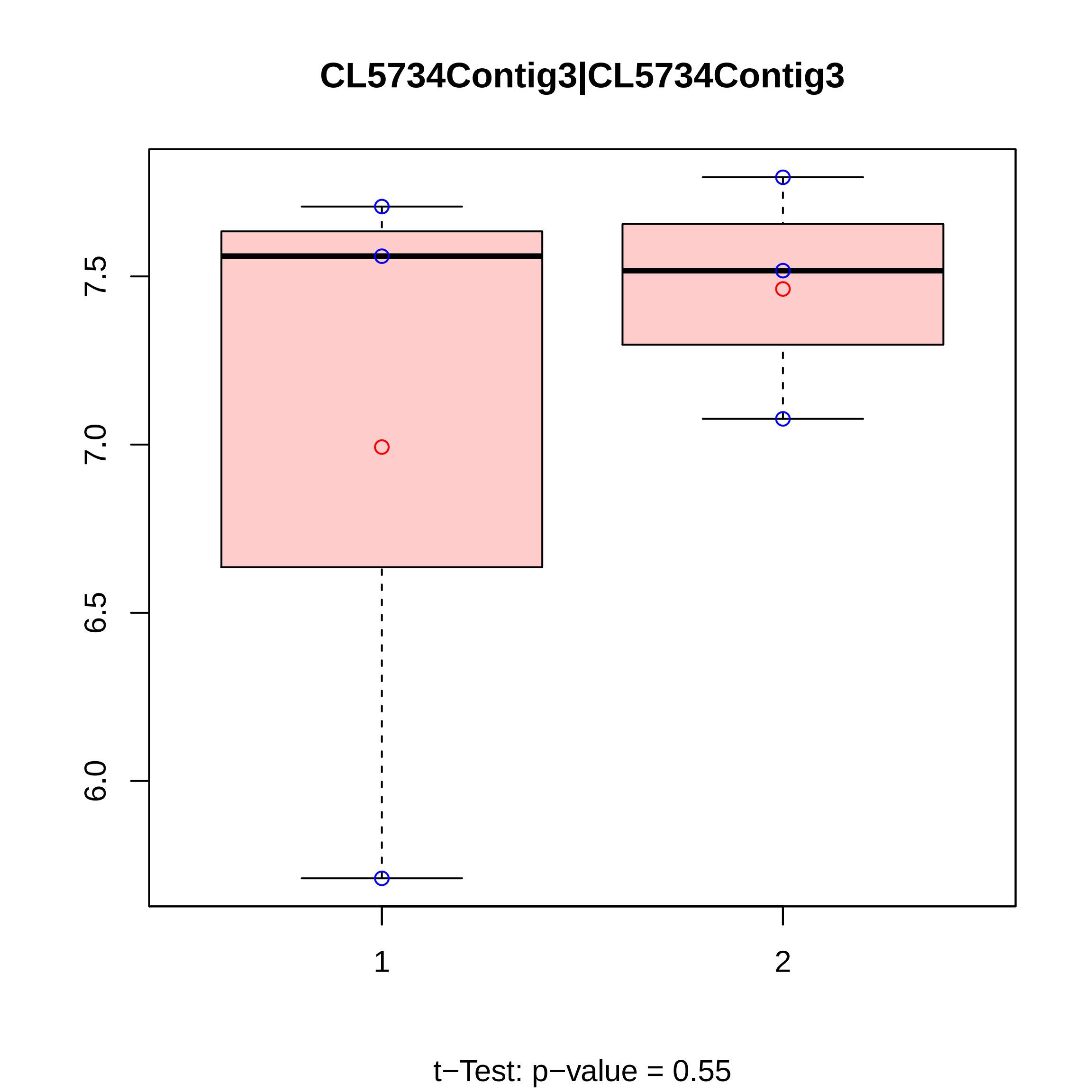

CL5734Contig3|CL5734Contig3
7.5
7.0
6.5
6.0
1
2
t−Test: p−value = 0.55

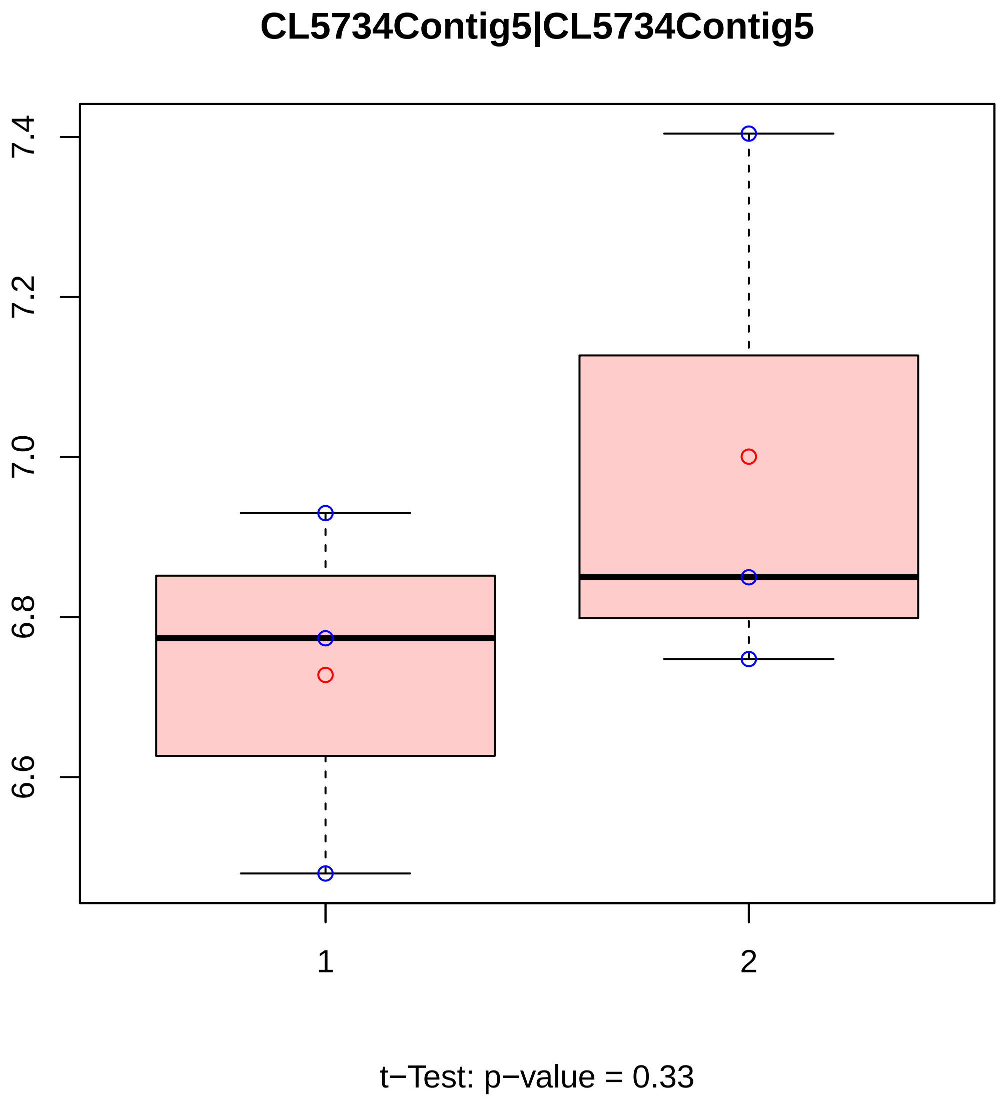

CL5734Contig5|CL5734Contig5
7.4
7.2
7.0
6.8
6.6
1
2
t−Test: p−value = 0.33

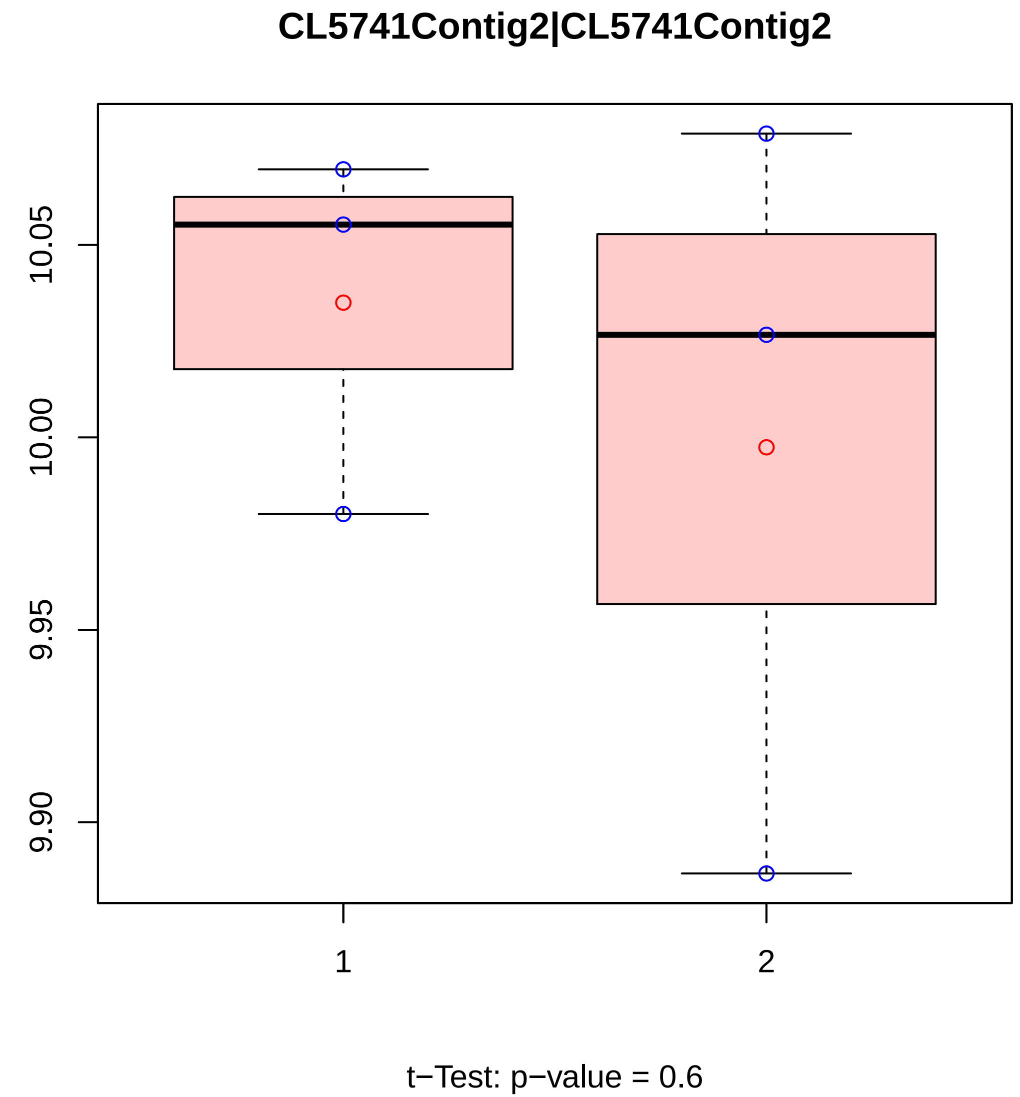
CL5741Contig2|CL5741Contig2
10.05
10.00
9.95
9.90
1
2
t−Test: p−value = 0.6

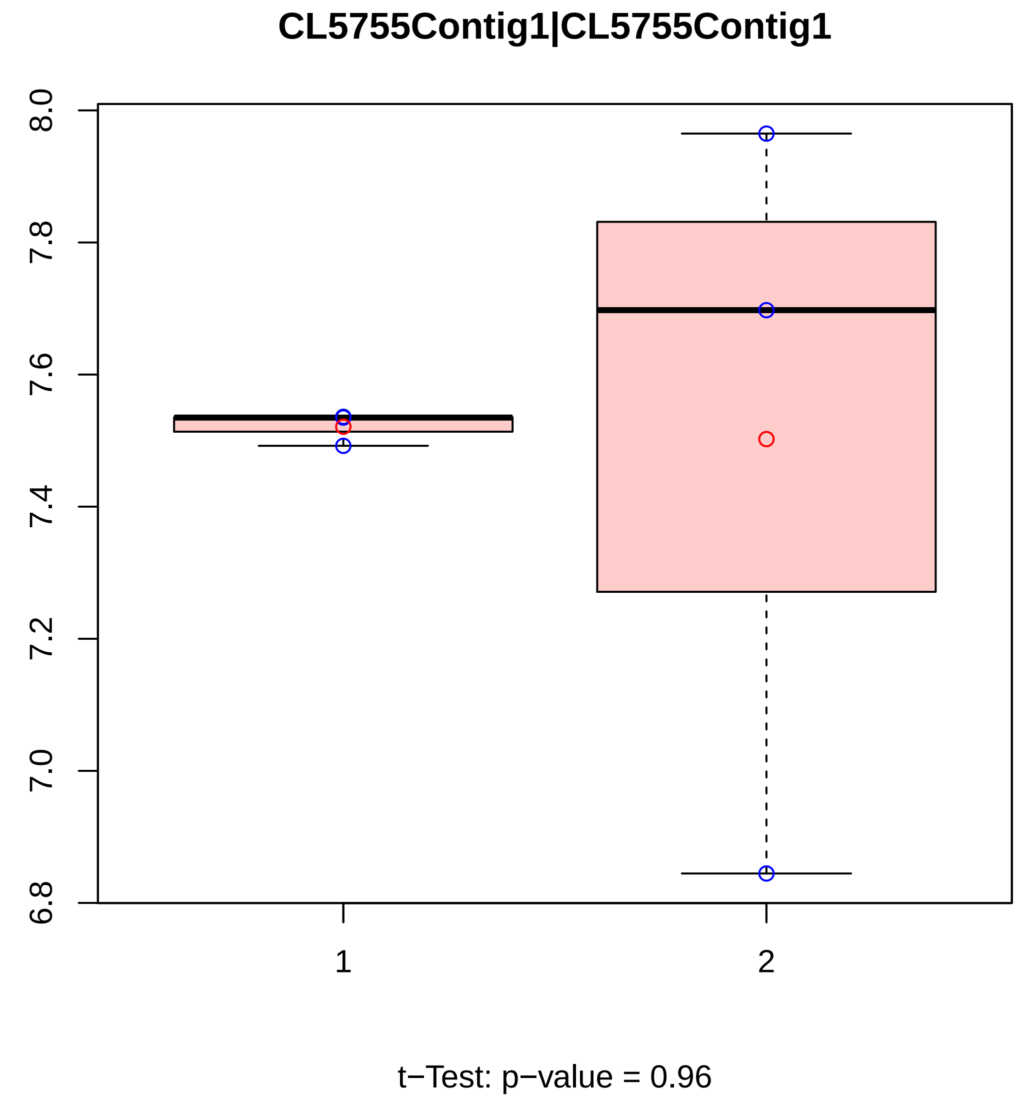
CL5755Contig1|CL5755Contig1
8.0
7.8
7.6
7.4
7.2
7.0
6.8
1
2
t−Test: p−value = 0.96

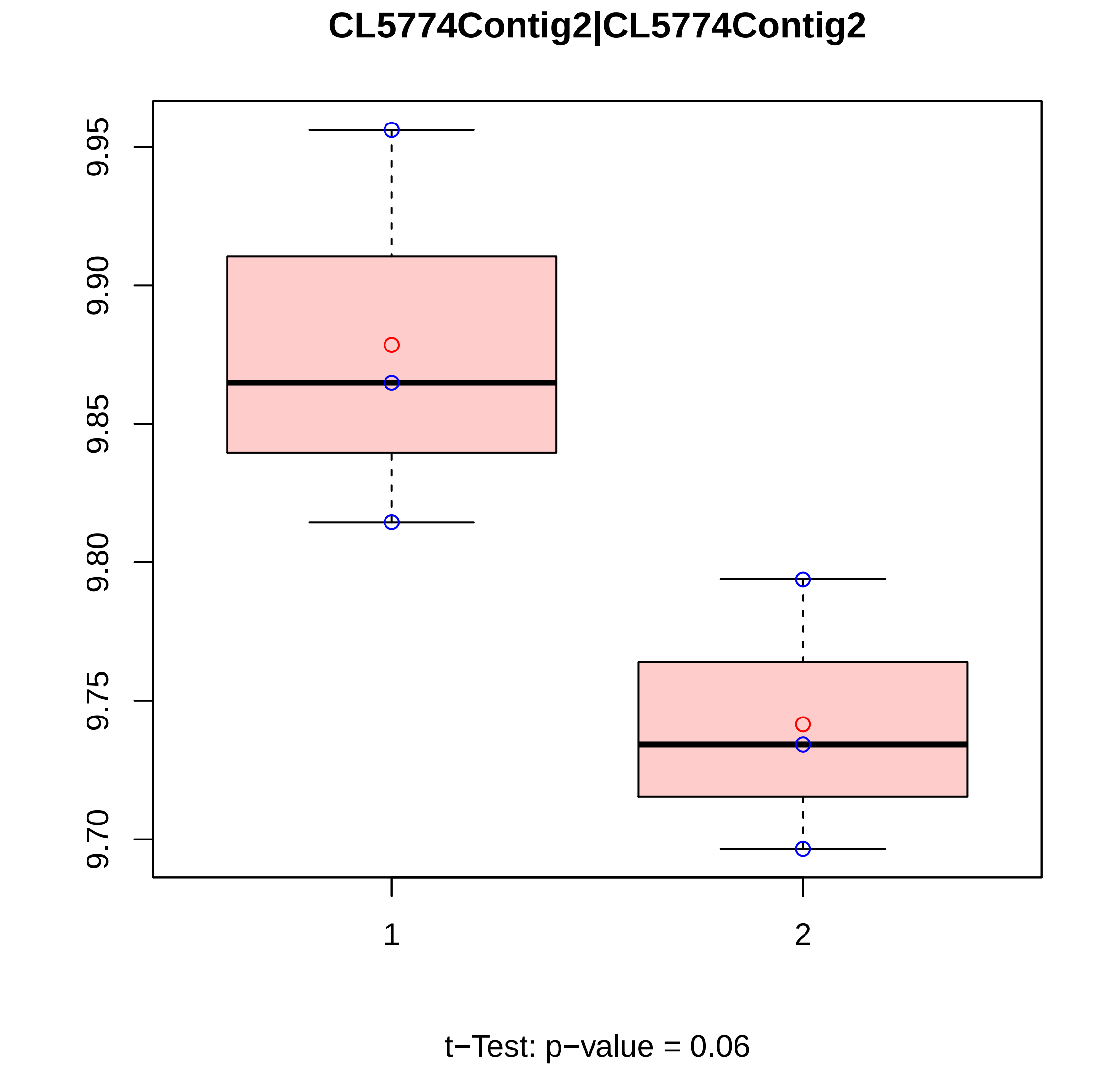

CL5774Contig2|CL5774Contig2
9.95
9.90
9.85
9.80
9.75
9.70
1
2
t−Test: p−value = 0.06

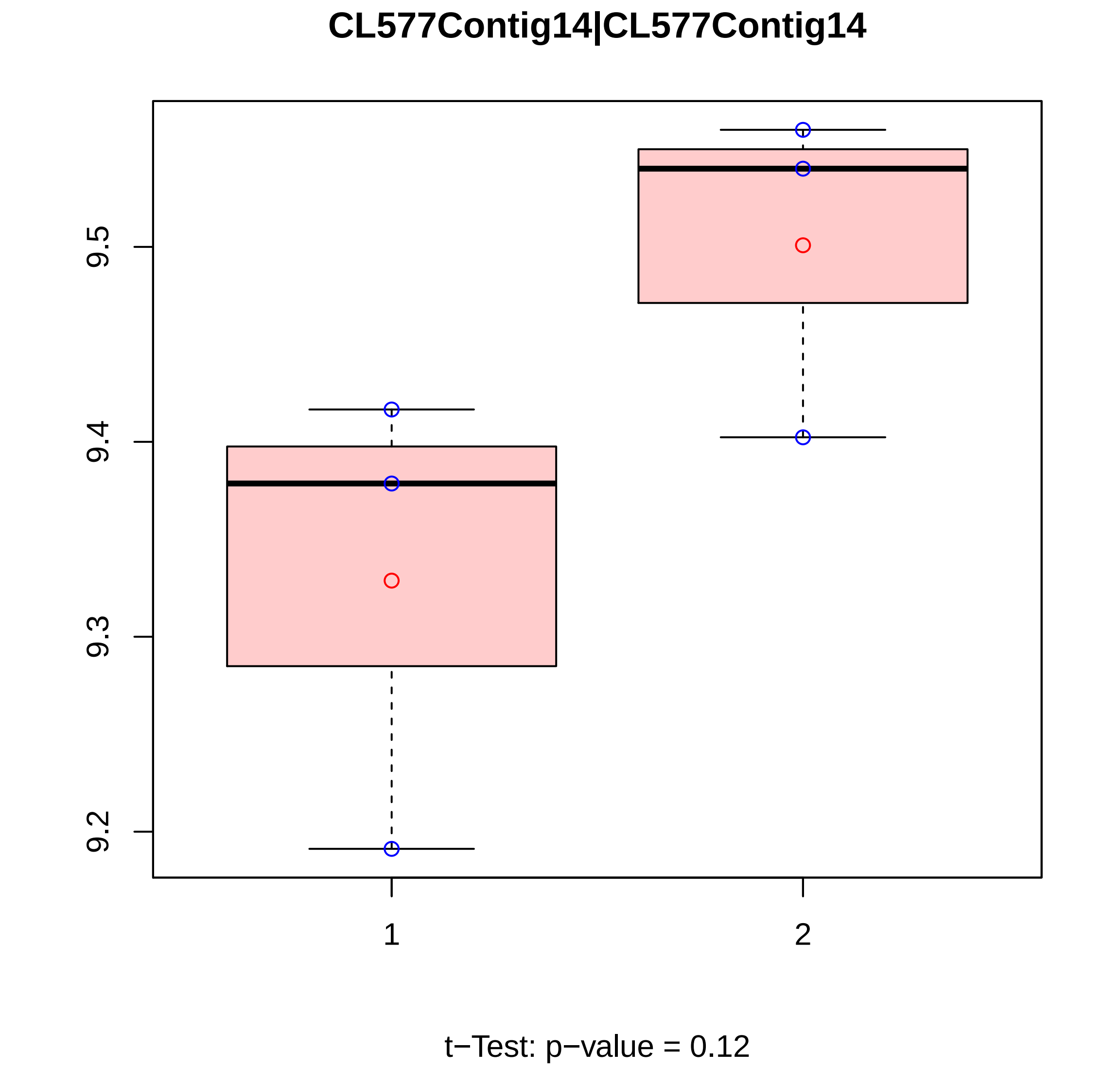
CL577Contig14|CL577Contig14
9.2
9.3
9.4
9.5
1
2
t−Test: p−value = 0.12

## CL5782Contig1|CL5782Contig1

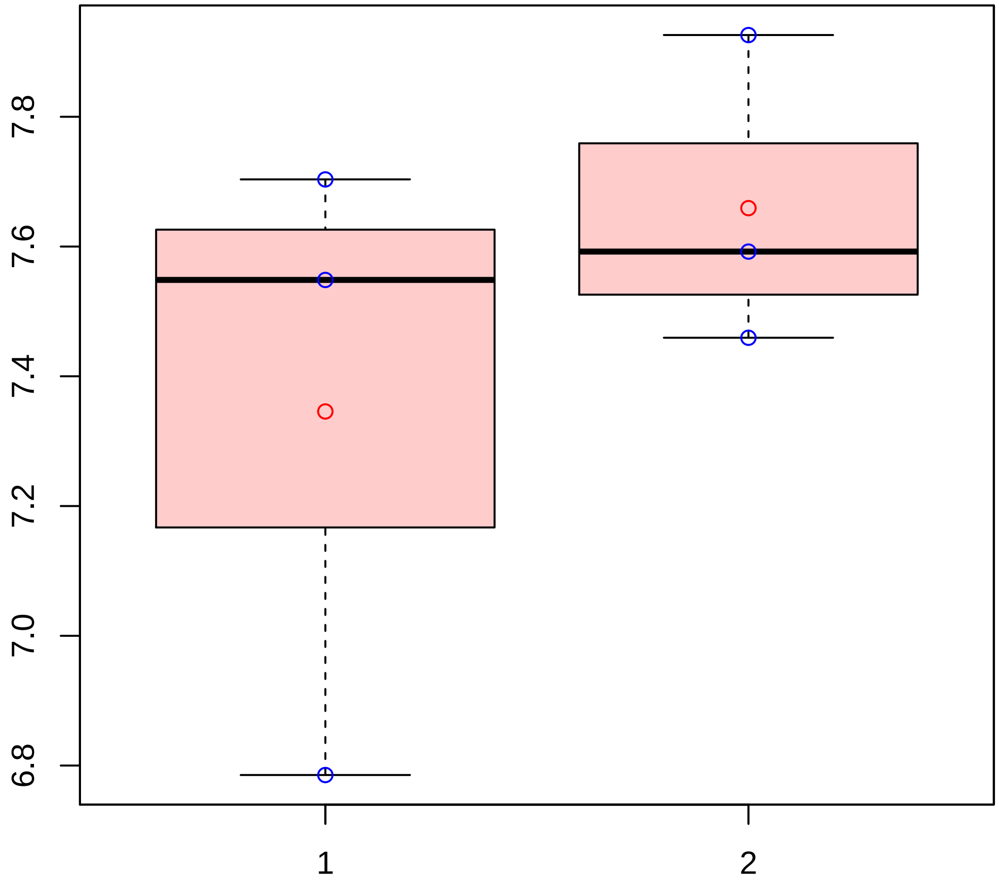


t−Test: p−value = 0.4

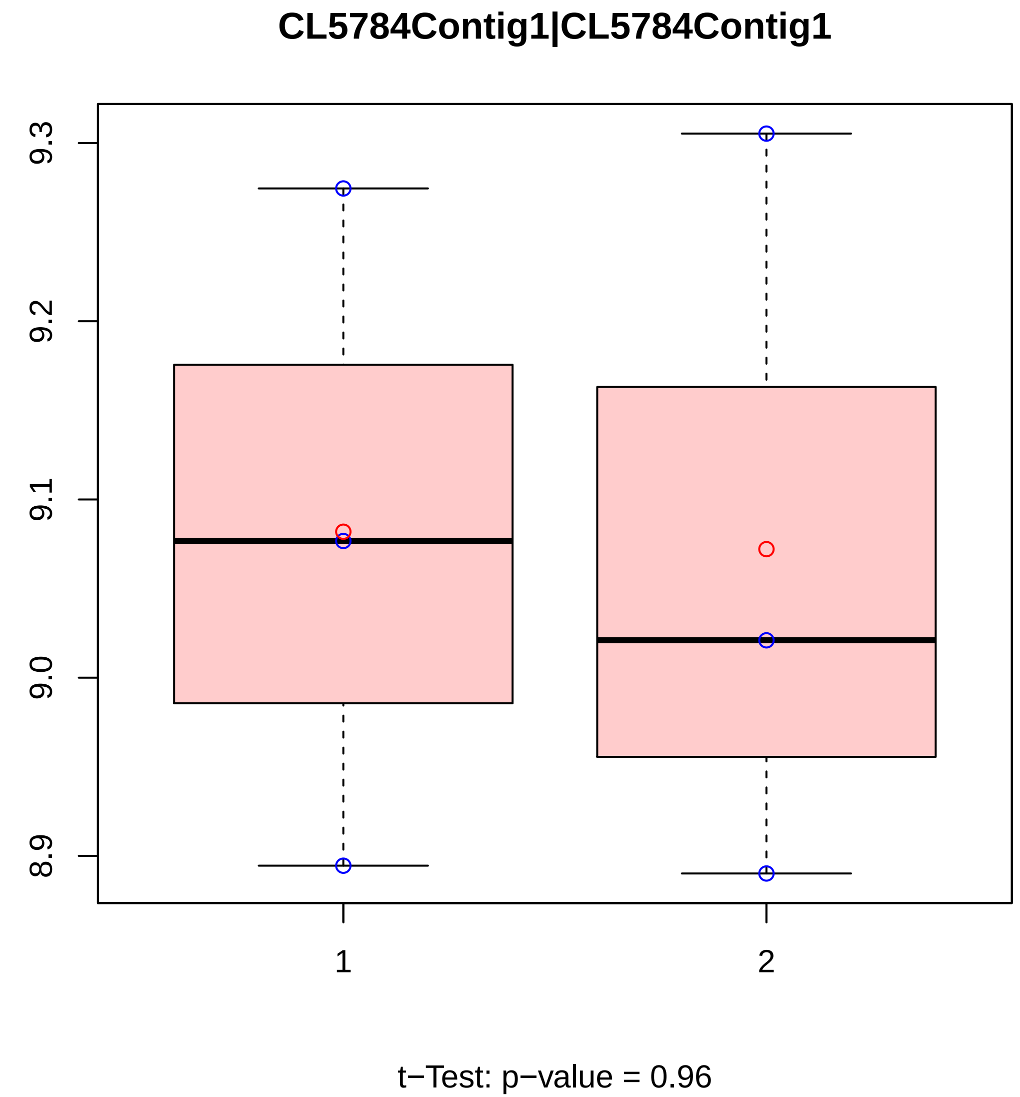
CL5784Contig1|CL5784Contig1
9.3
9.2
9.1
9.0
8.9
1
2
t−Test: p−value = 0.96

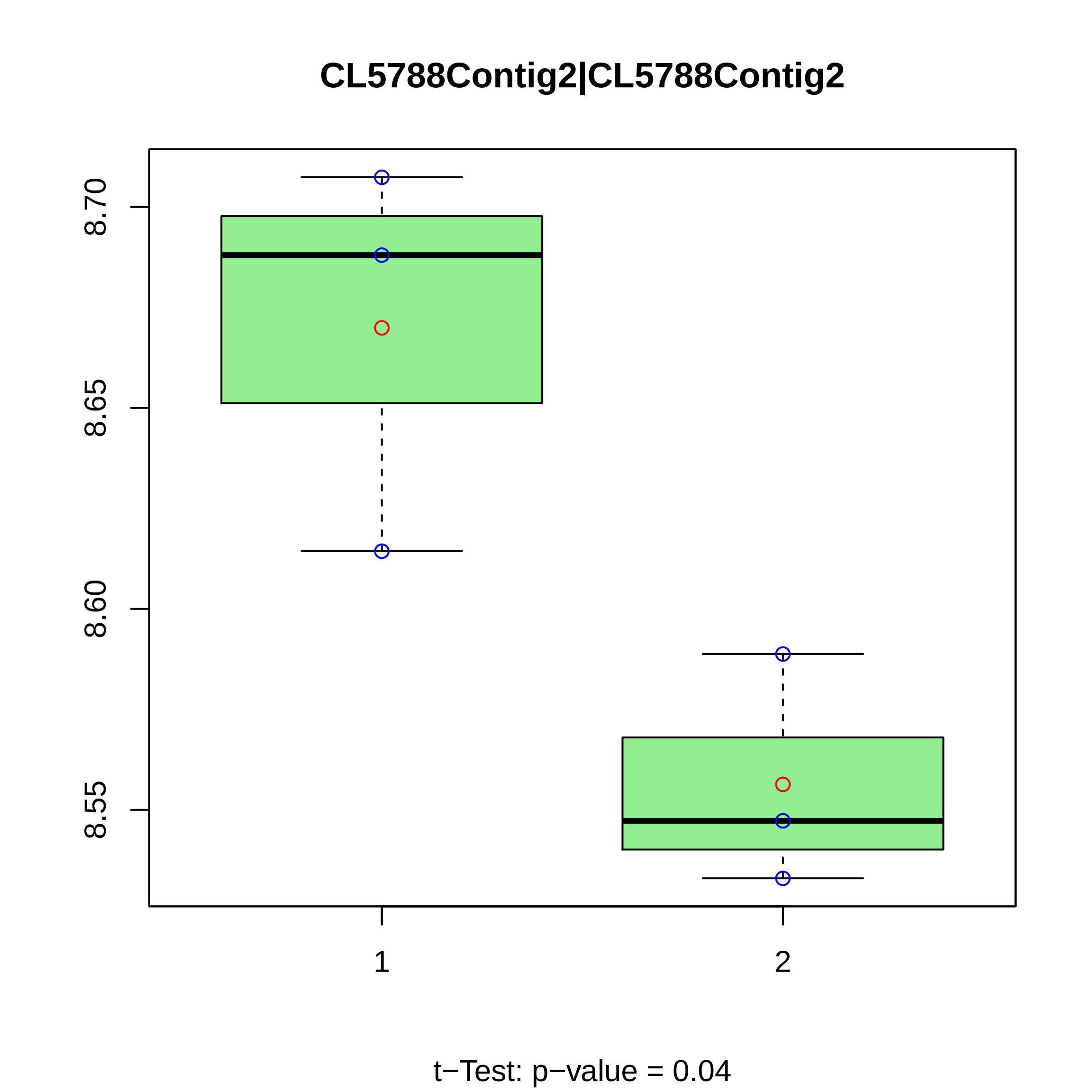
CL5788Contig2|CL5788Contig2
8.70
8.65
8.60
8.55
1
2
t−Test: p−value = 0.04

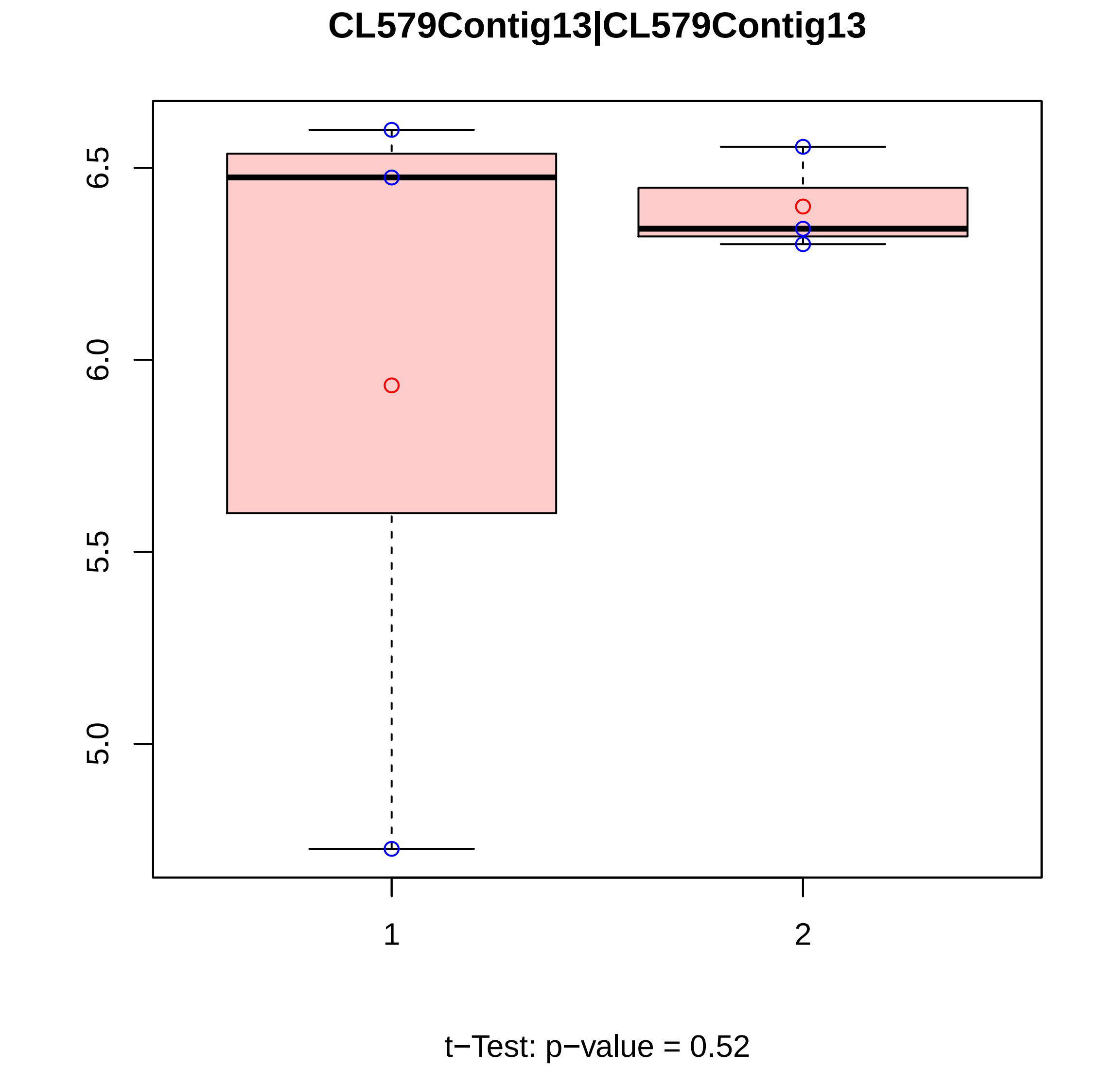
CL579Contig13|CL579Contig13
6.5
6.0
5.5
5.0
1
2
t−Test: p−value = 0.52

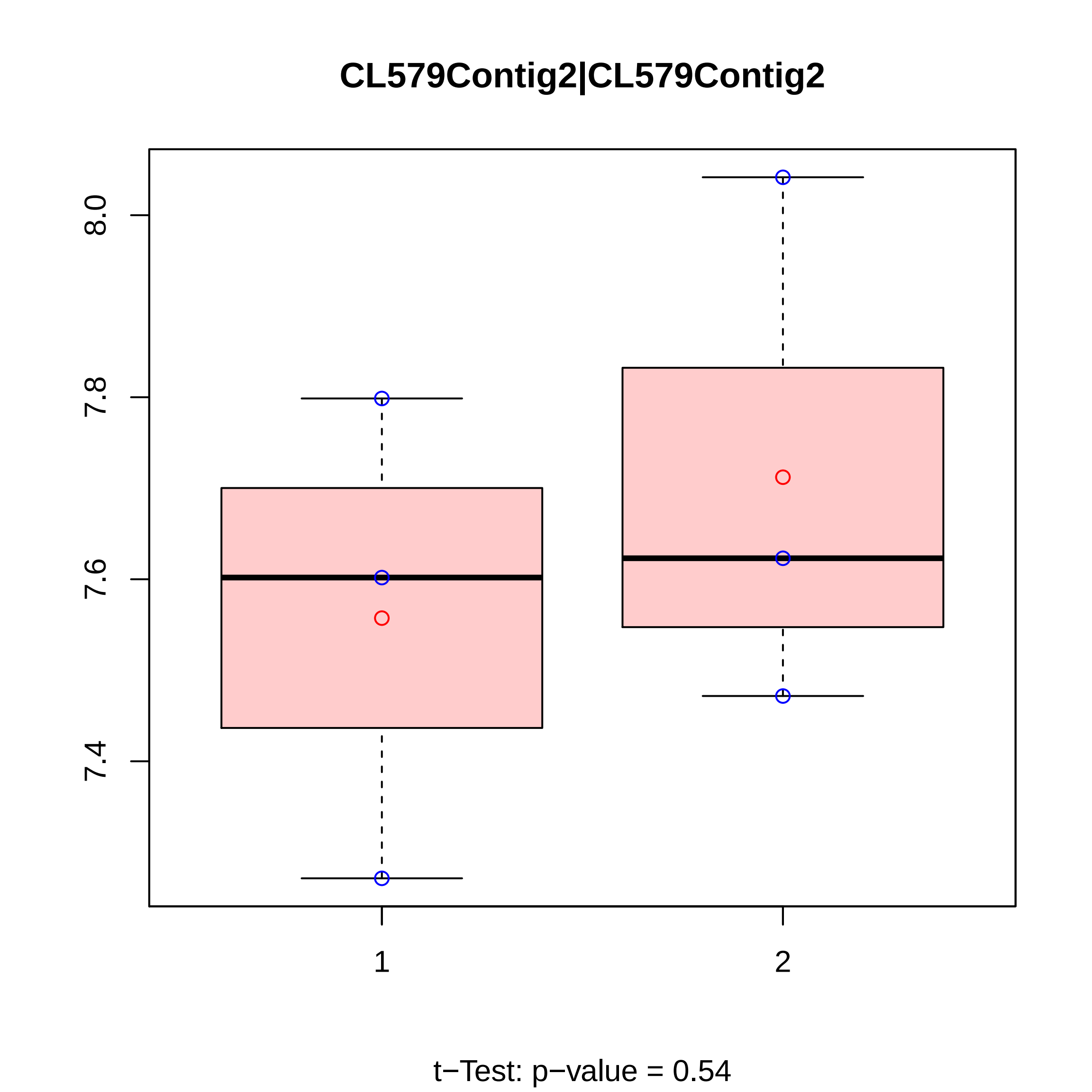
CL579Contig2|CL579Contig2
8.0
7.8
7.6
7.4
1
2
t−Test: p−value = 0.54

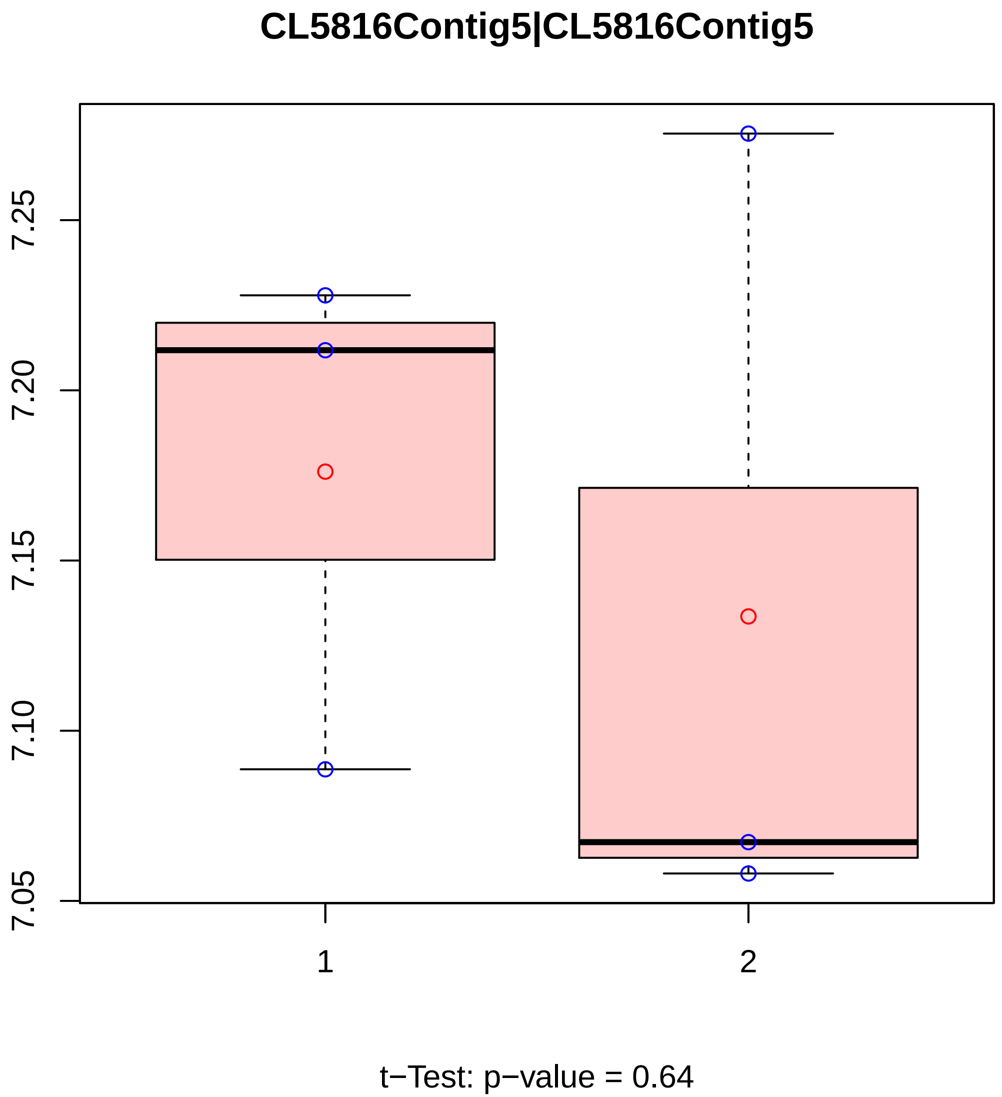

CL5816Contig5|CL5816Contig5
7.05
7.10
7.15
7.20
7.25
1
2
t−Test: p−value = 0.64

# CL5826Contig2|CL5826Contig2

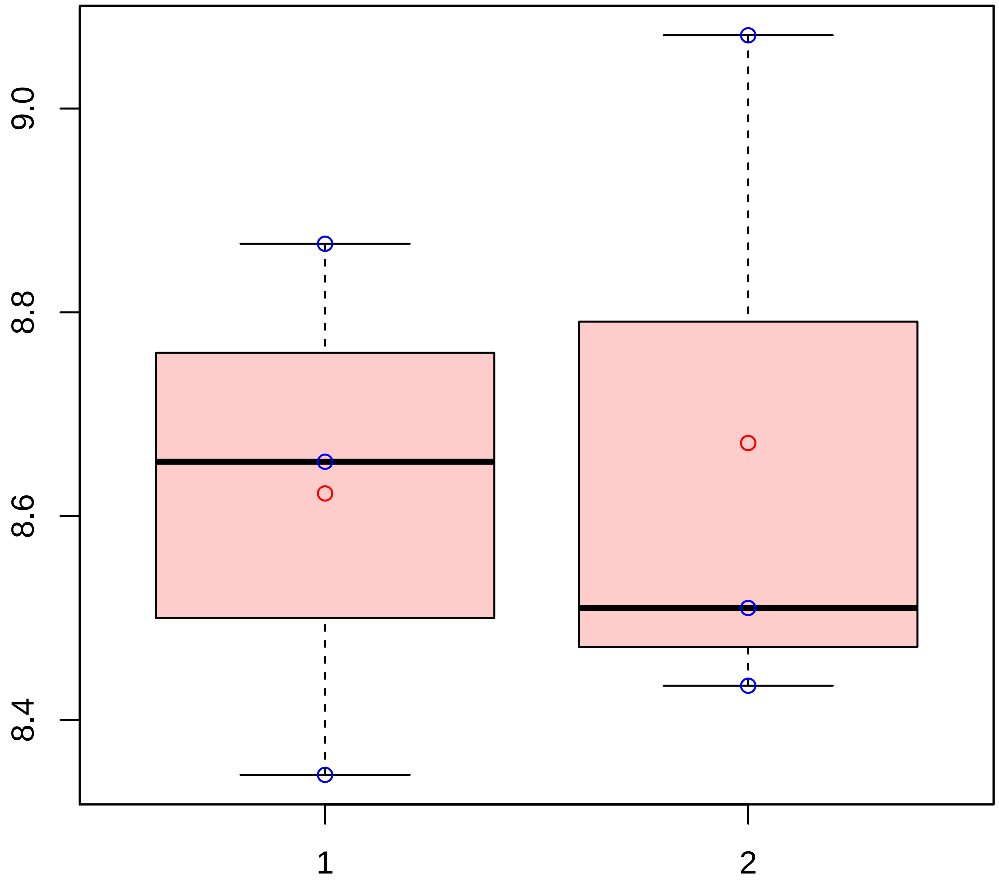


t−Test: p−value = 0.85

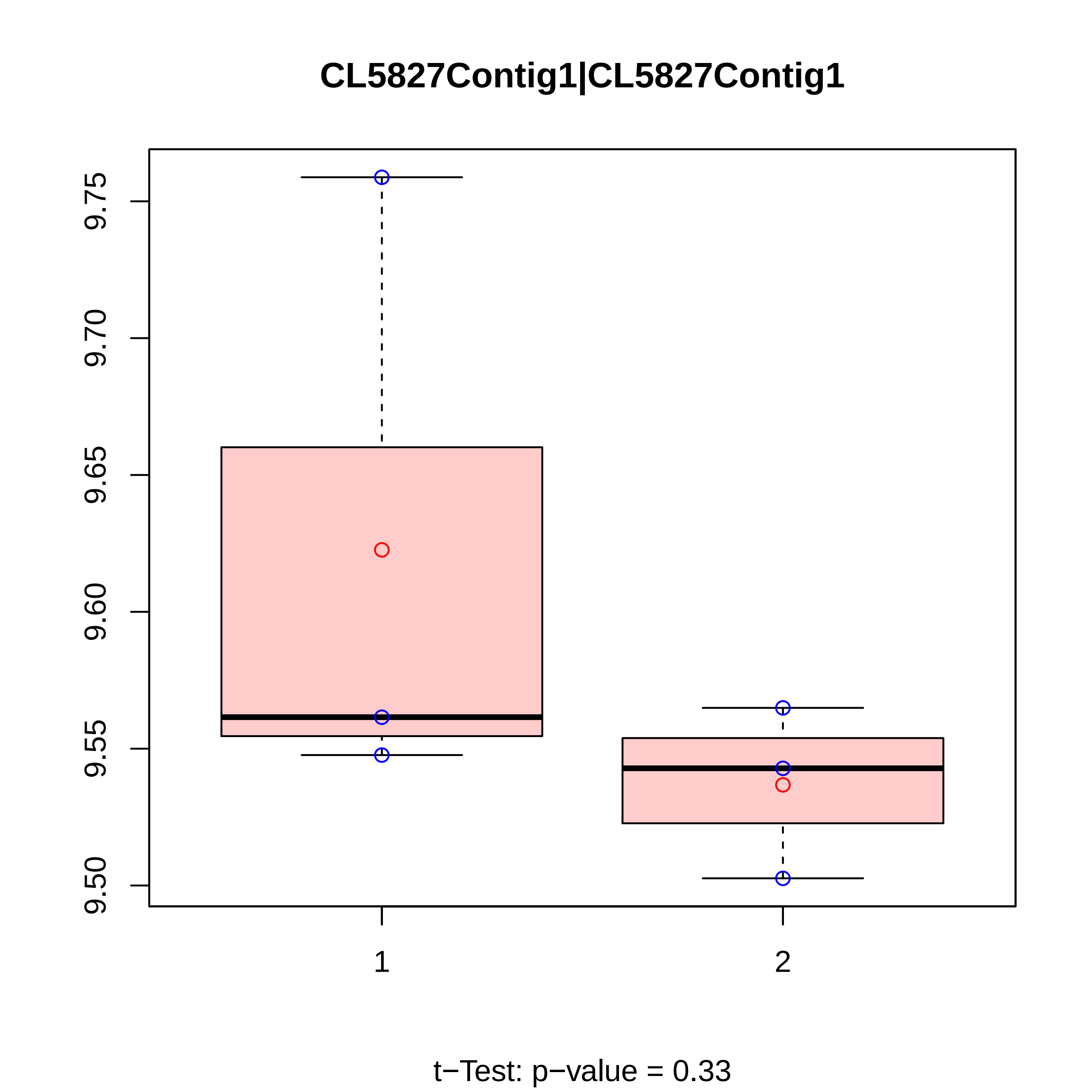

CL5827Contig1|CL5827Contig1
9.50
9.55
9.60
9.65
9.70
9.75
1
2
t−Test: p−value = 0.33

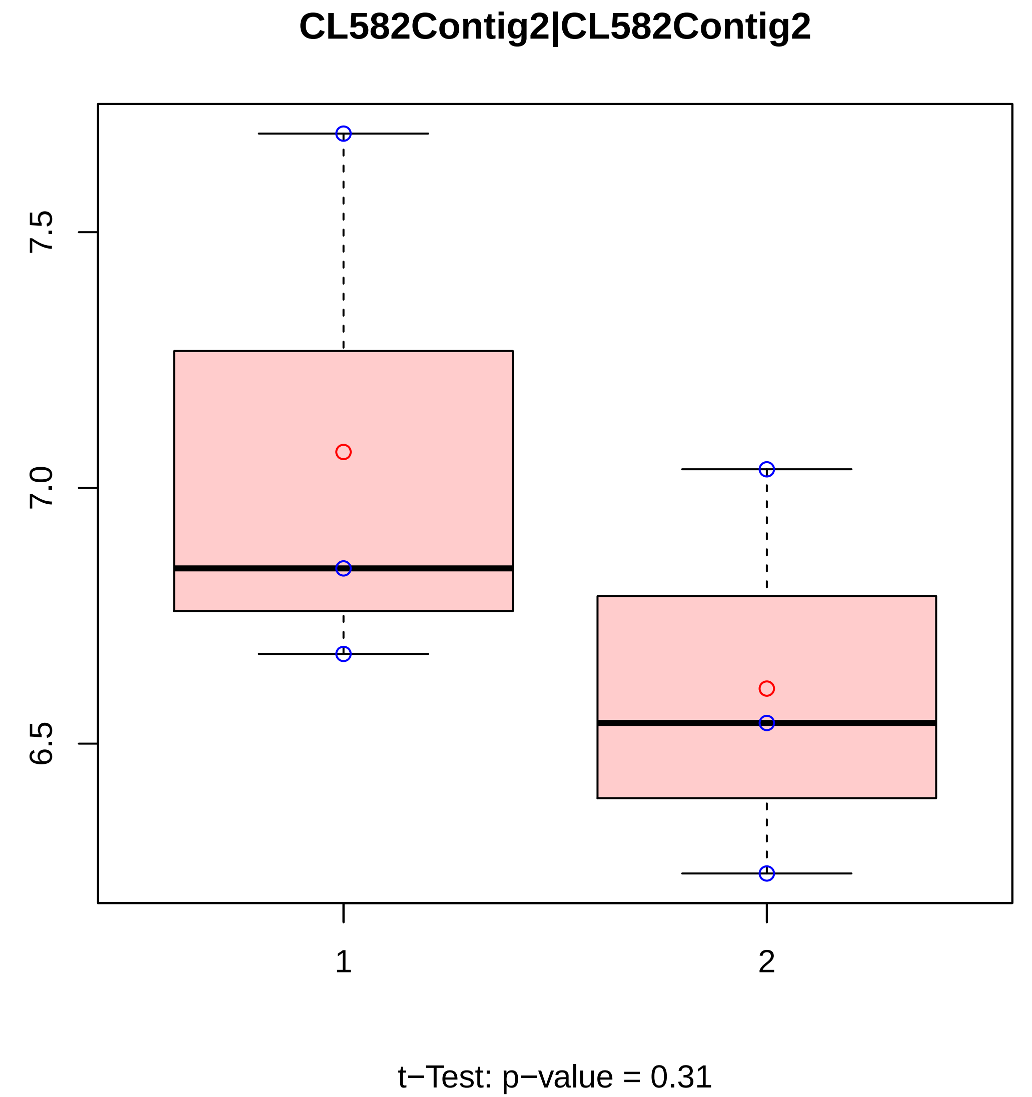
CL582Contig2|CL582Contig2
7.5
7.0
6.5
1
2
t−Test: p−value = 0.31

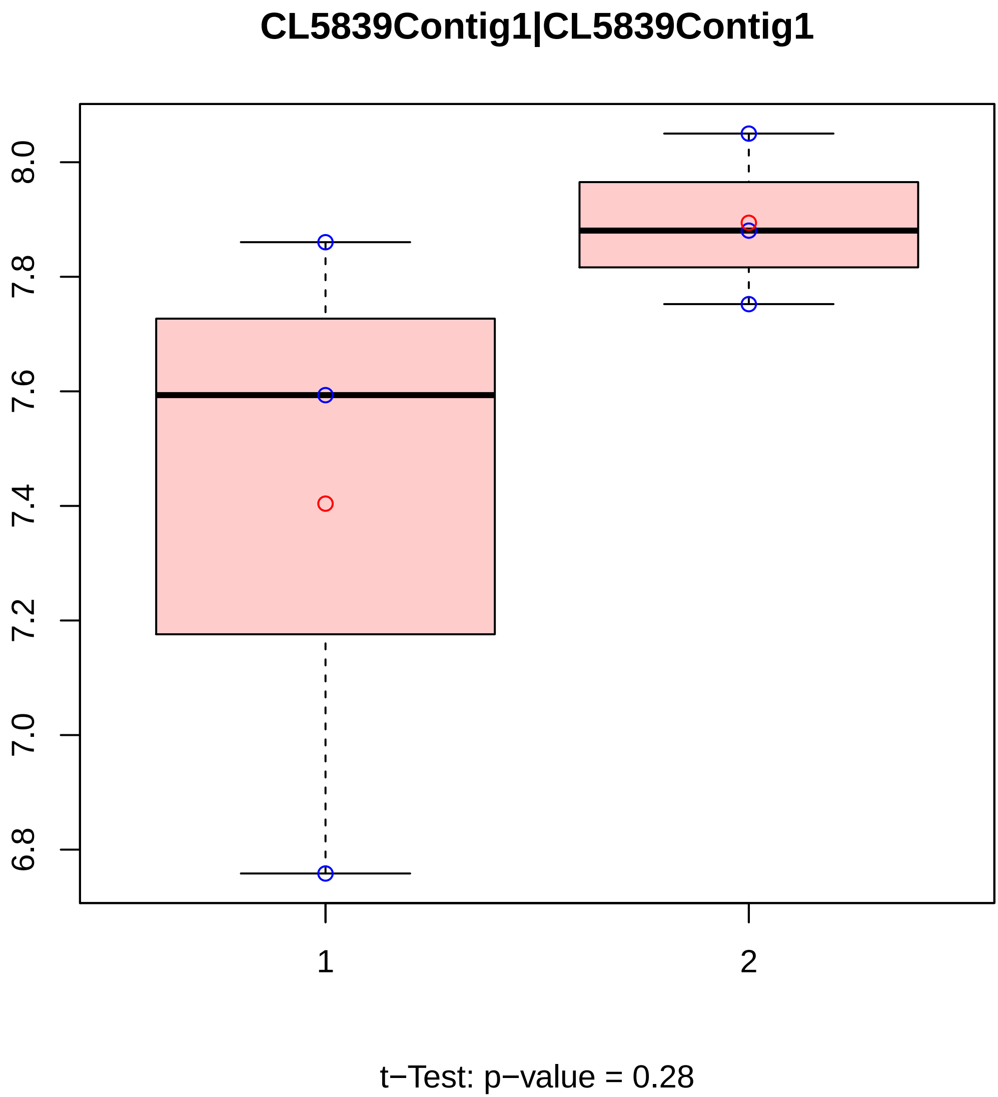
CL5839Contig1|CL5839Contig1
8.0
7.8
7.6
7.4
7.2
7.0
6.8
1
2
t−Test: p−value = 0.28

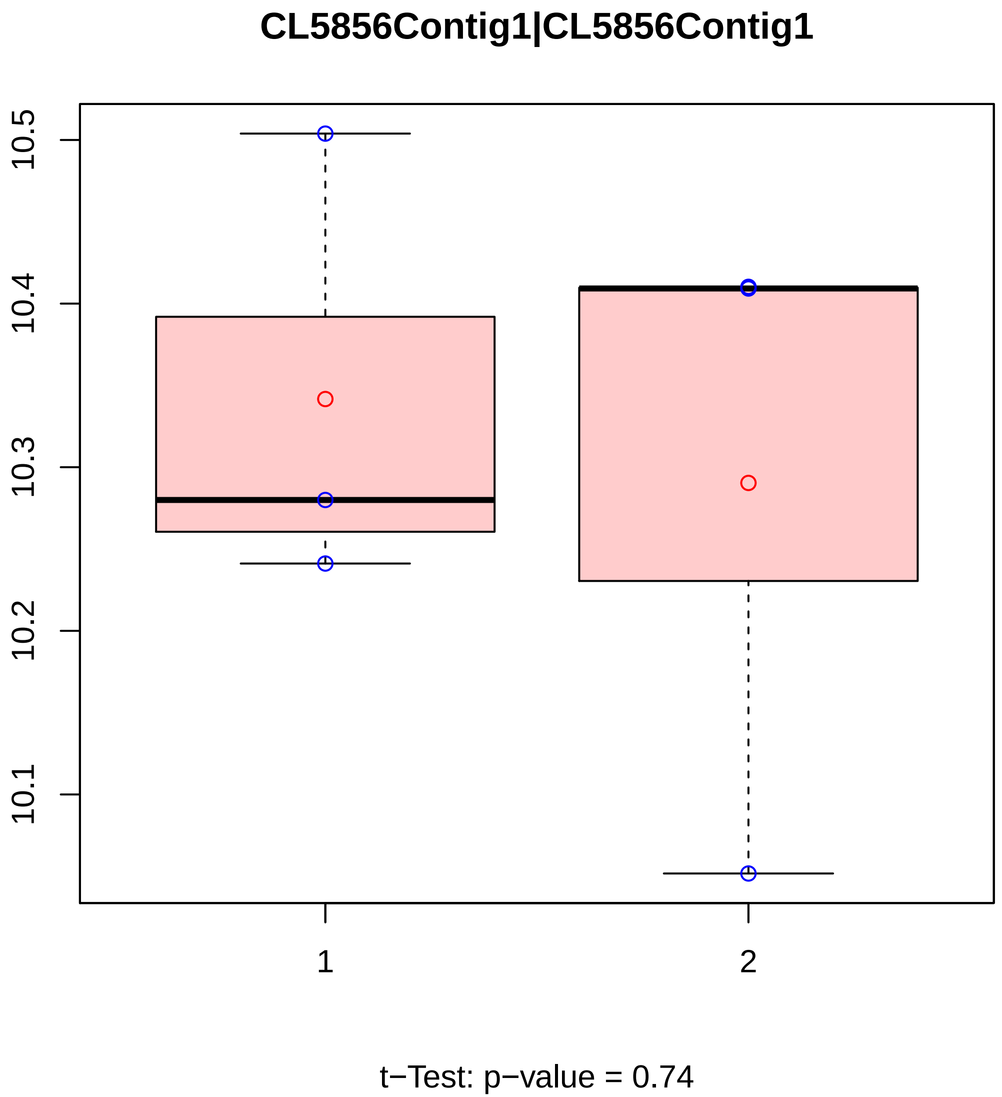
CL5856Contig1|CL5856Contig1
10.5
10.4
10.3
10.2
10.1
1
2
t−Test: p−value = 0.74

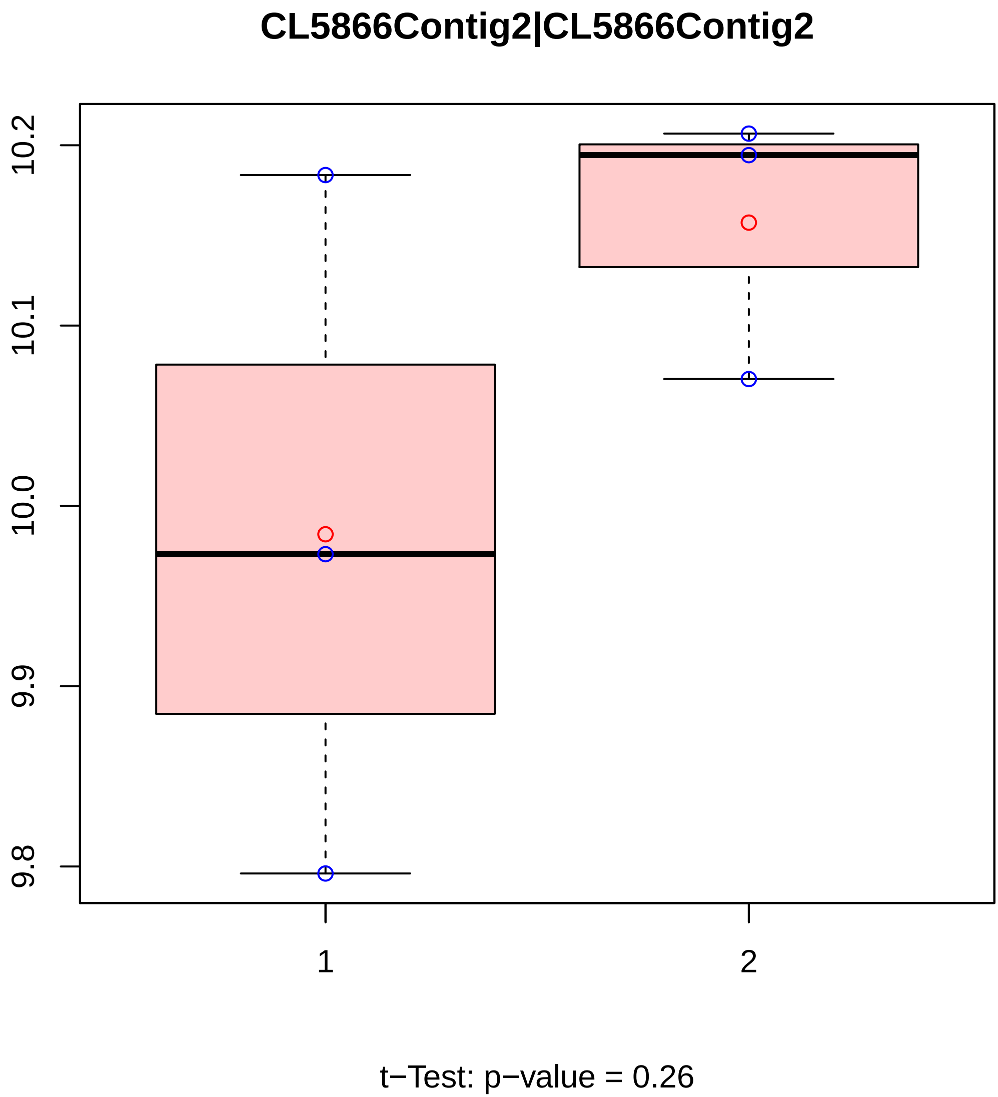
CL5866Contig2|CL5866Contig2
10.2
10.1
10.0
9.9
9.8
1
2
t−Test: p−value = 0.26

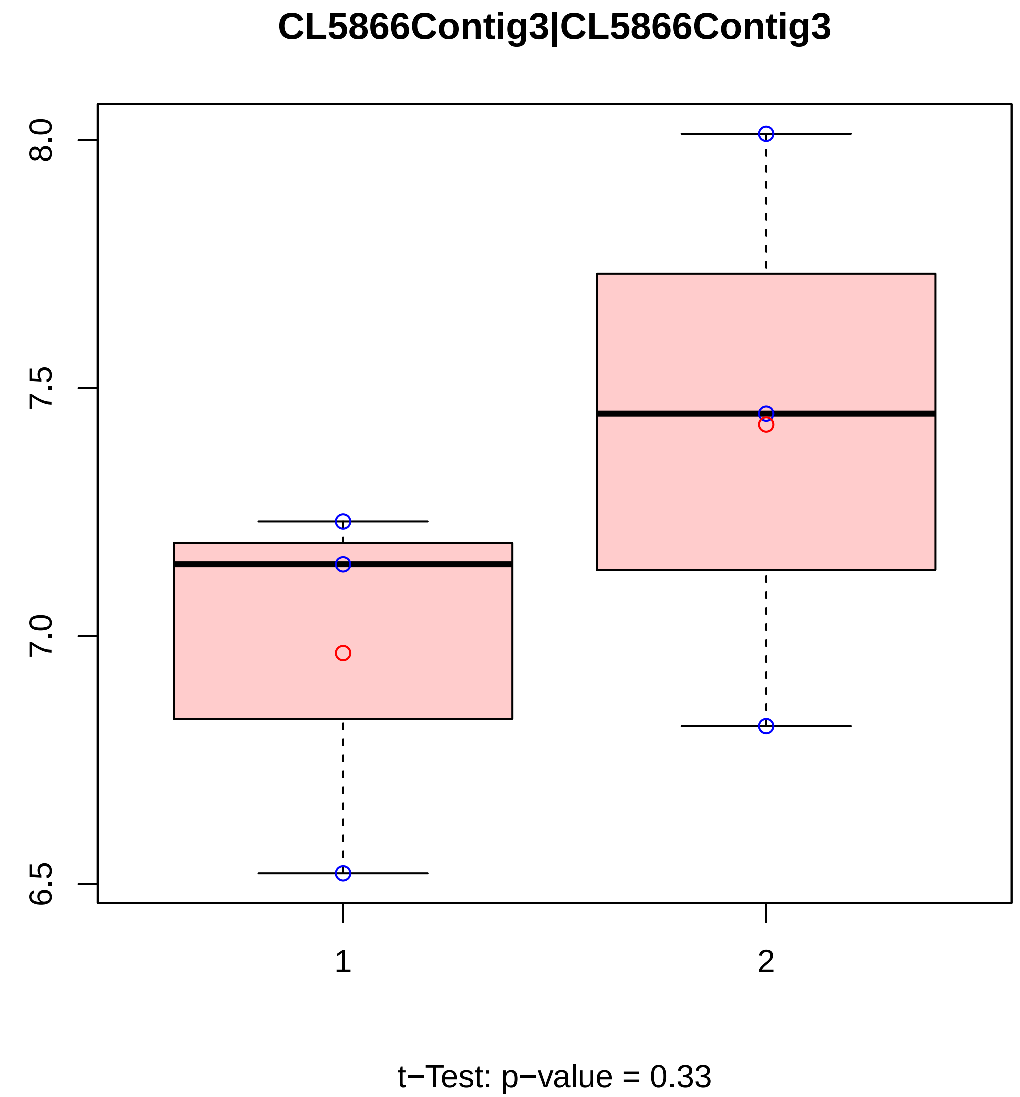
CL5866Contig3|CL5866Contig3
8.0
7.5
7.0
6.5
1
2
t−Test: p−value = 0.33

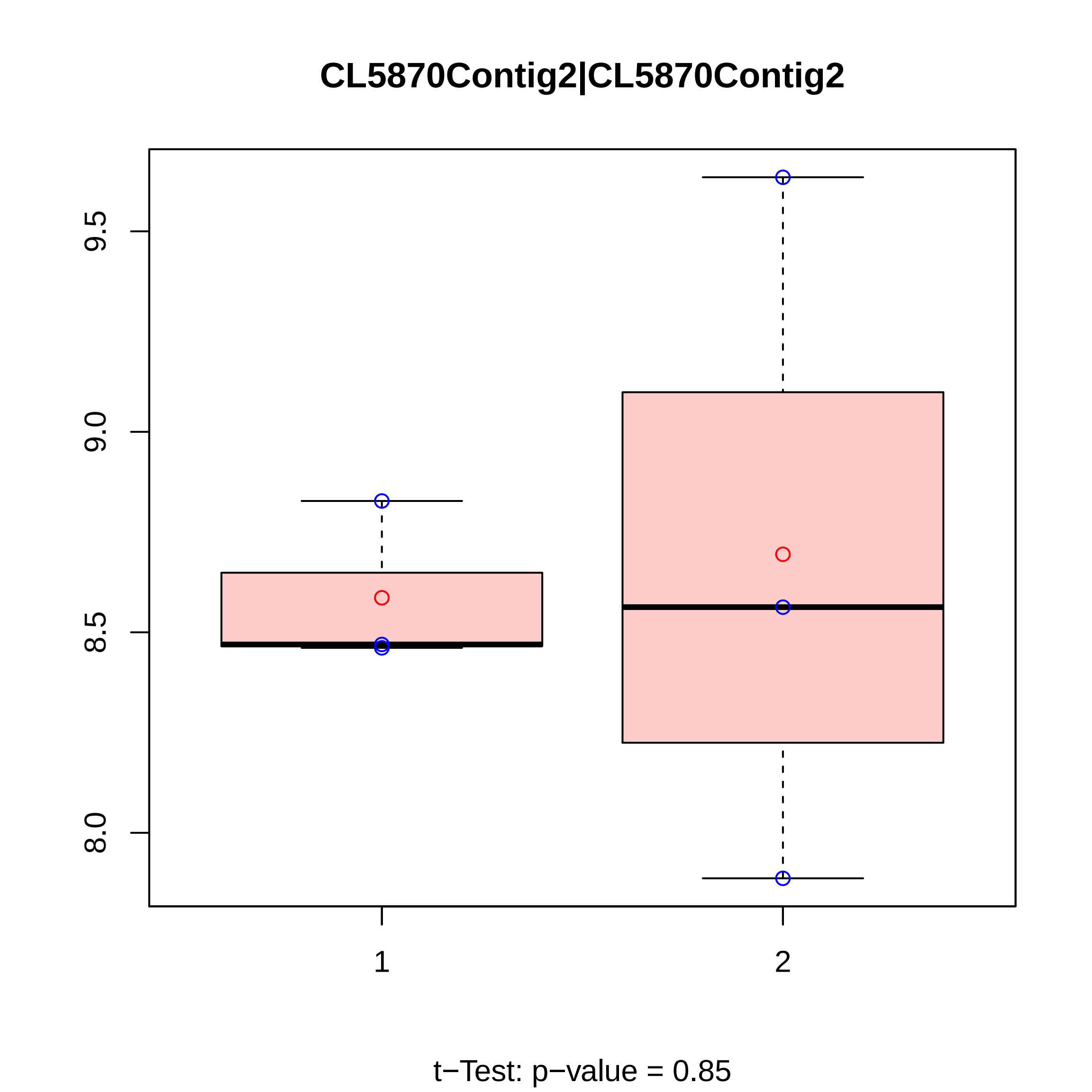

CL5870Contig2|CL5870Contig2
9.5
9.0
8.5
8.0
1
2
t−Test: p−value = 0.85

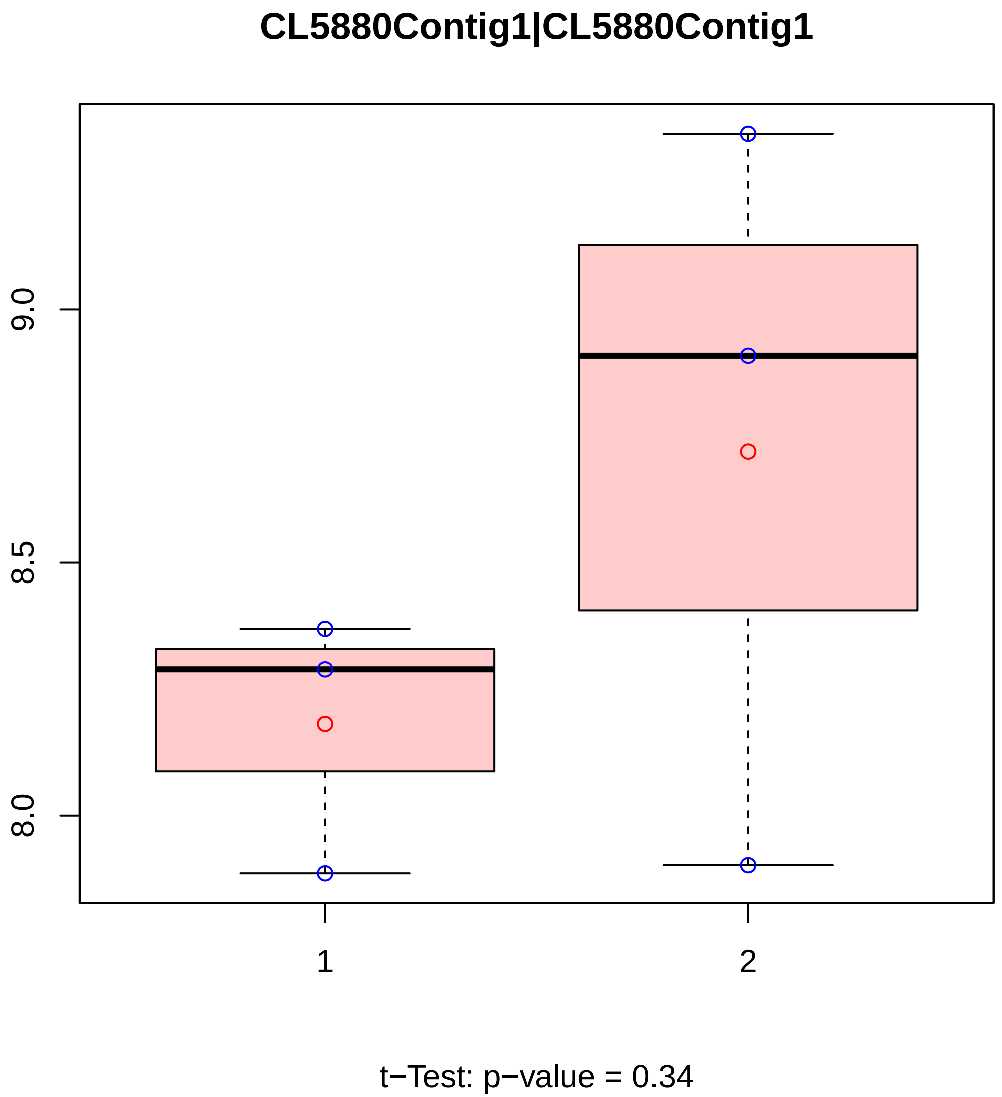

CL5880Contig1|CL5880Contig1
9.0
8.5
8.0
1
2
t−Test: p−value = 0.34

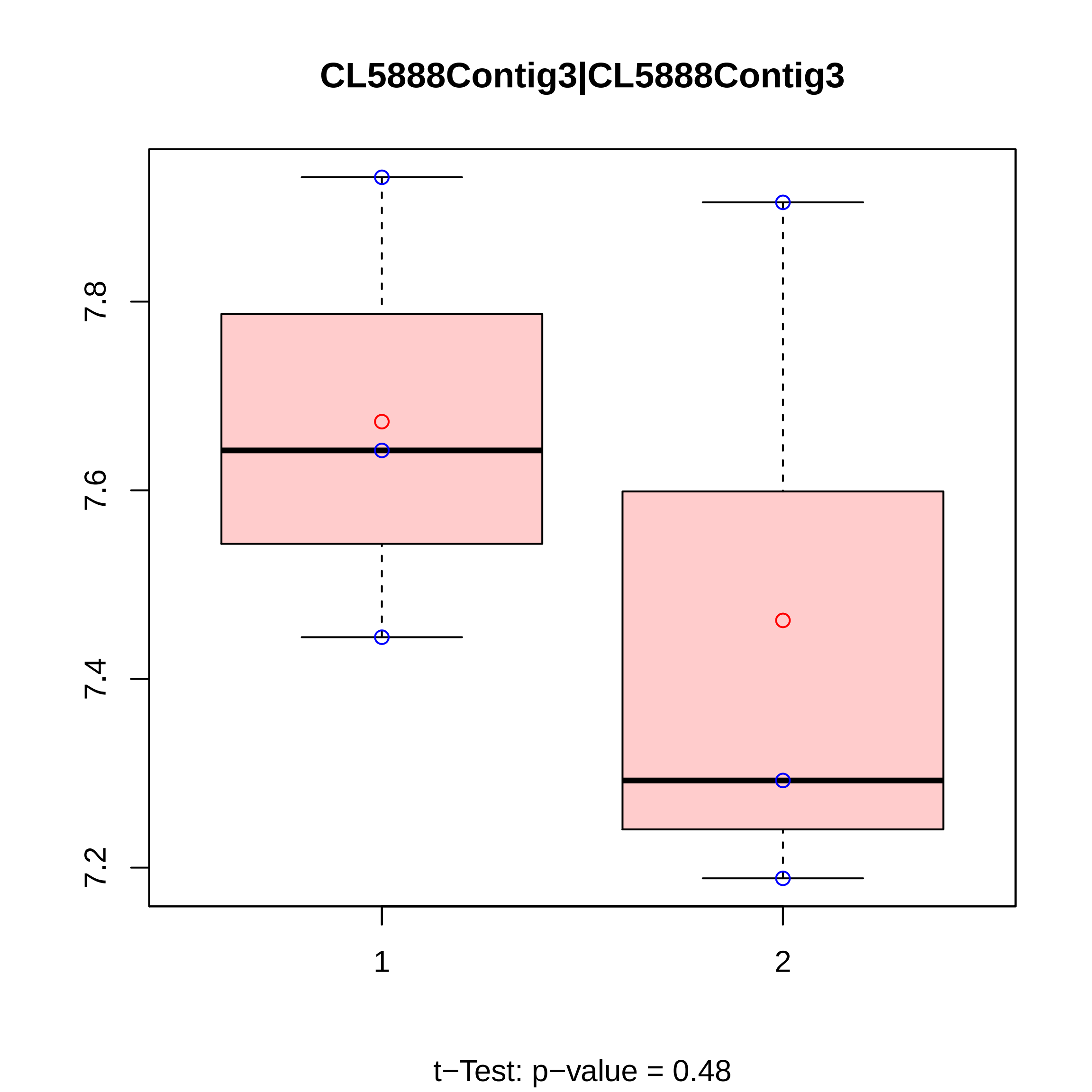
CL5888Contig3|CL5888Contig3
7.2
7.4
7.6
7.8
1
2
t−Test: p−value = 0.48

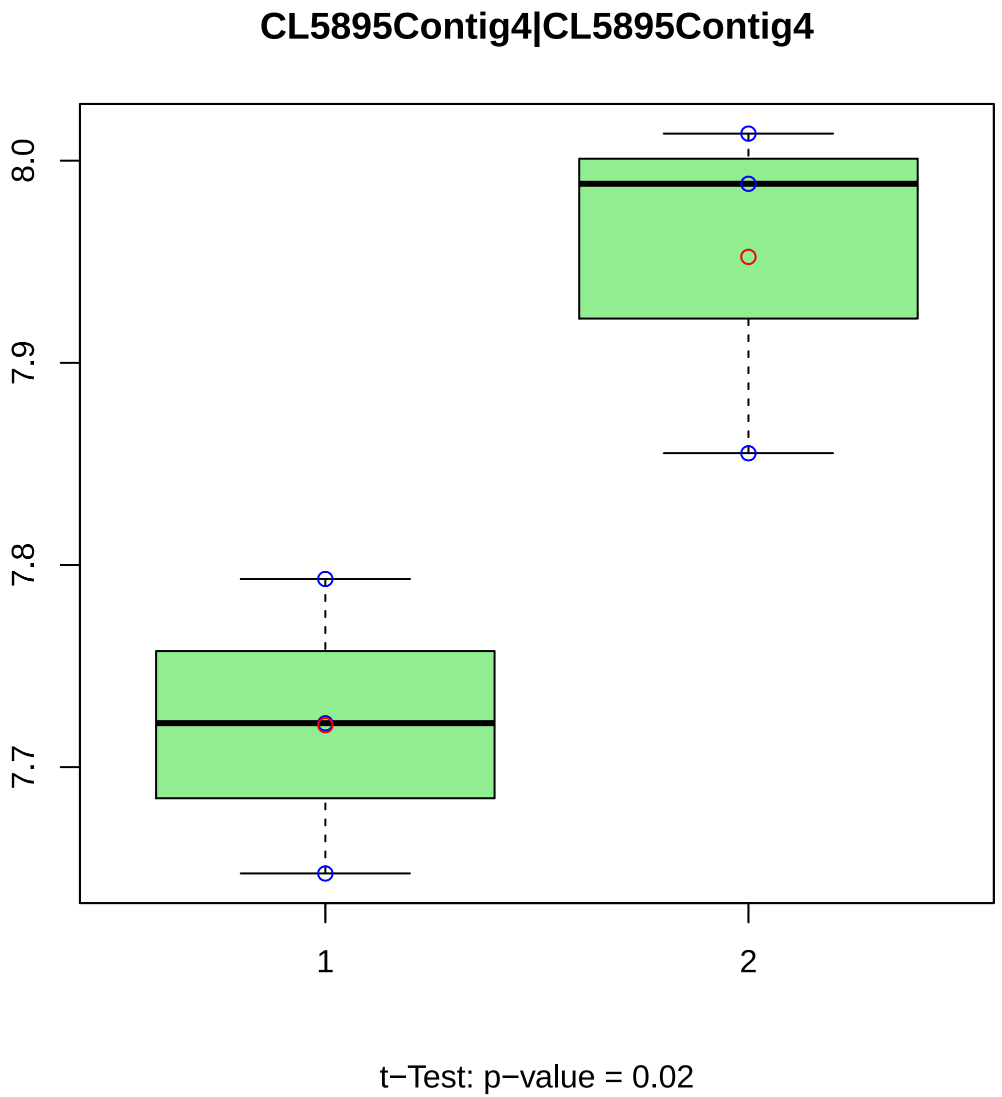

CL5895Contig4|CL5895Contig4
8.0
7.9
7.8
7.7
1
2
t−Test: p−value = 0.02

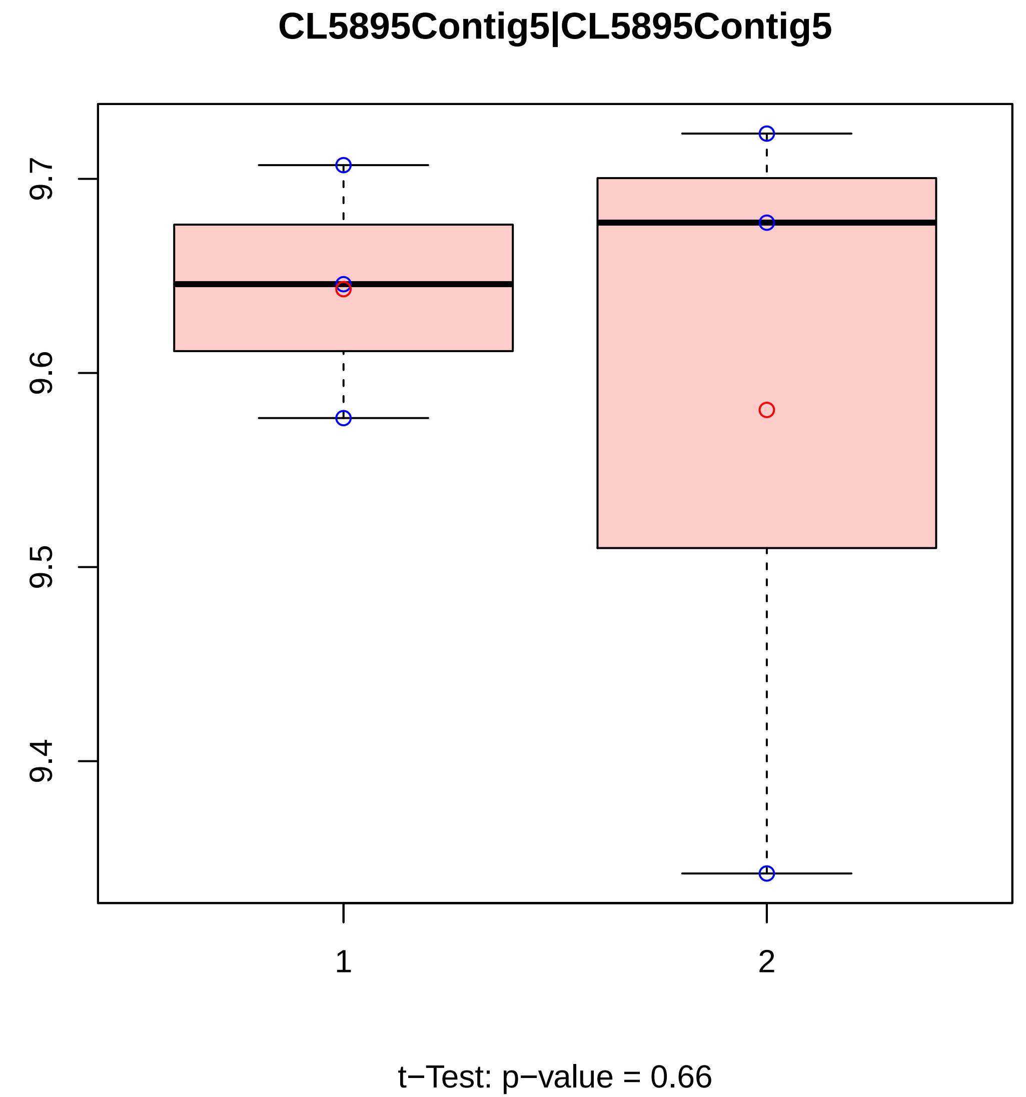
CL5895Contig5|CL5895Contig5
9.4
9.5
9.6
9.7
1
2
t−Test: p−value = 0.66

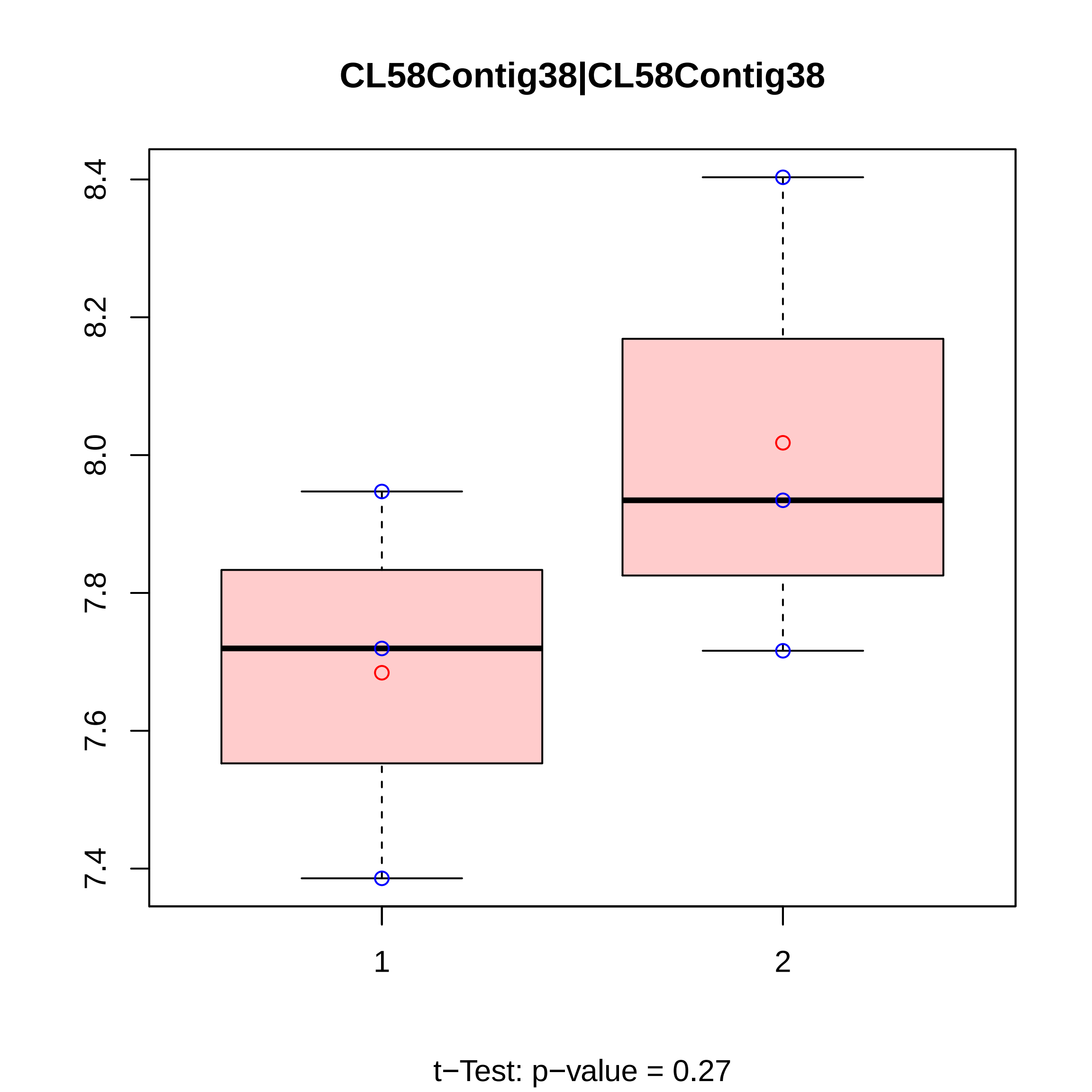
CL58Contig38|CL58Contig38
8.4
8.2
8.0
7.8
7.6
7.4
1
2
t−Test: p−value = 0.27

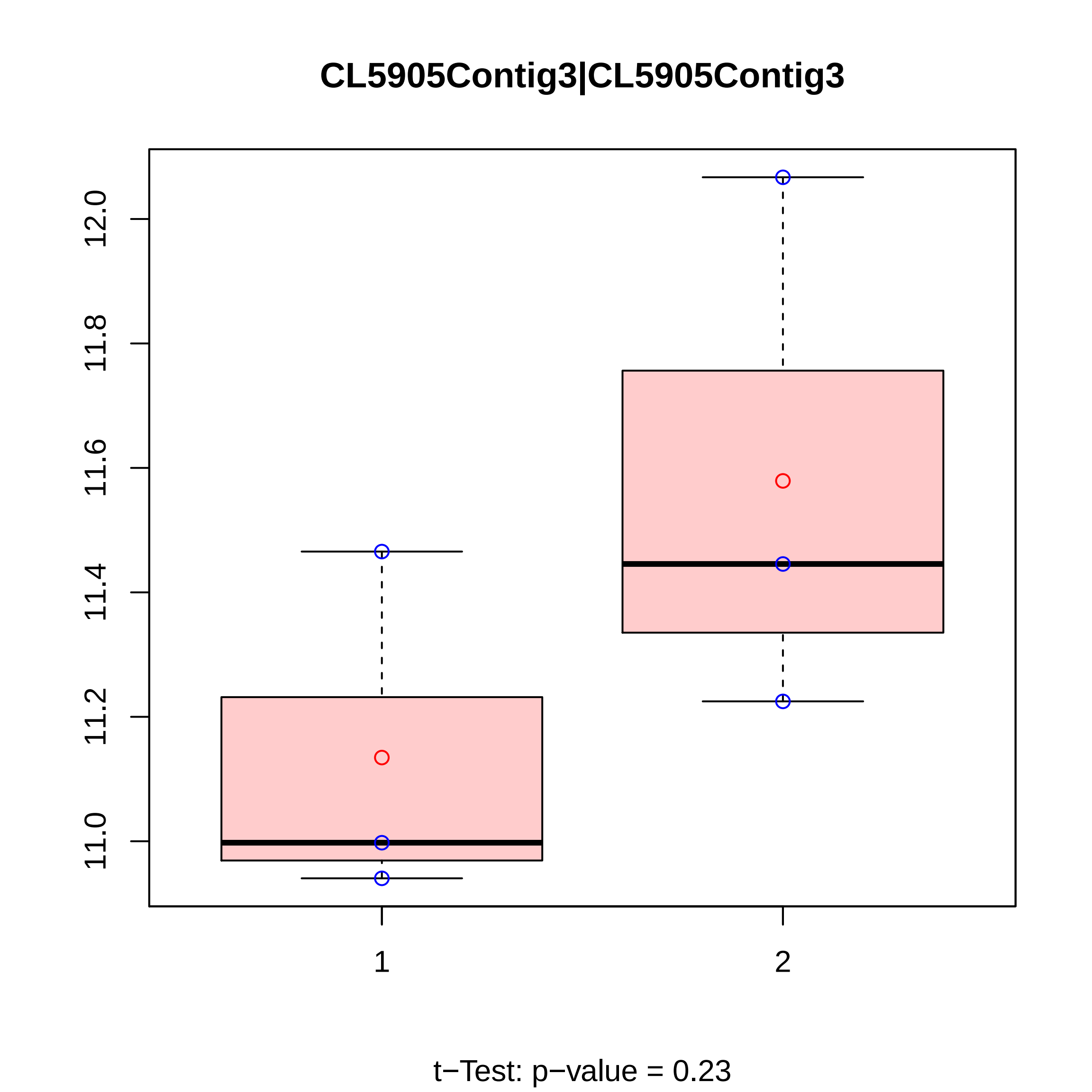
CL5905Contig3|CL5905Contig3
11.0
11.2
11.4
11.6
11.8
12.0
1
2
t−Test: p−value = 0.23

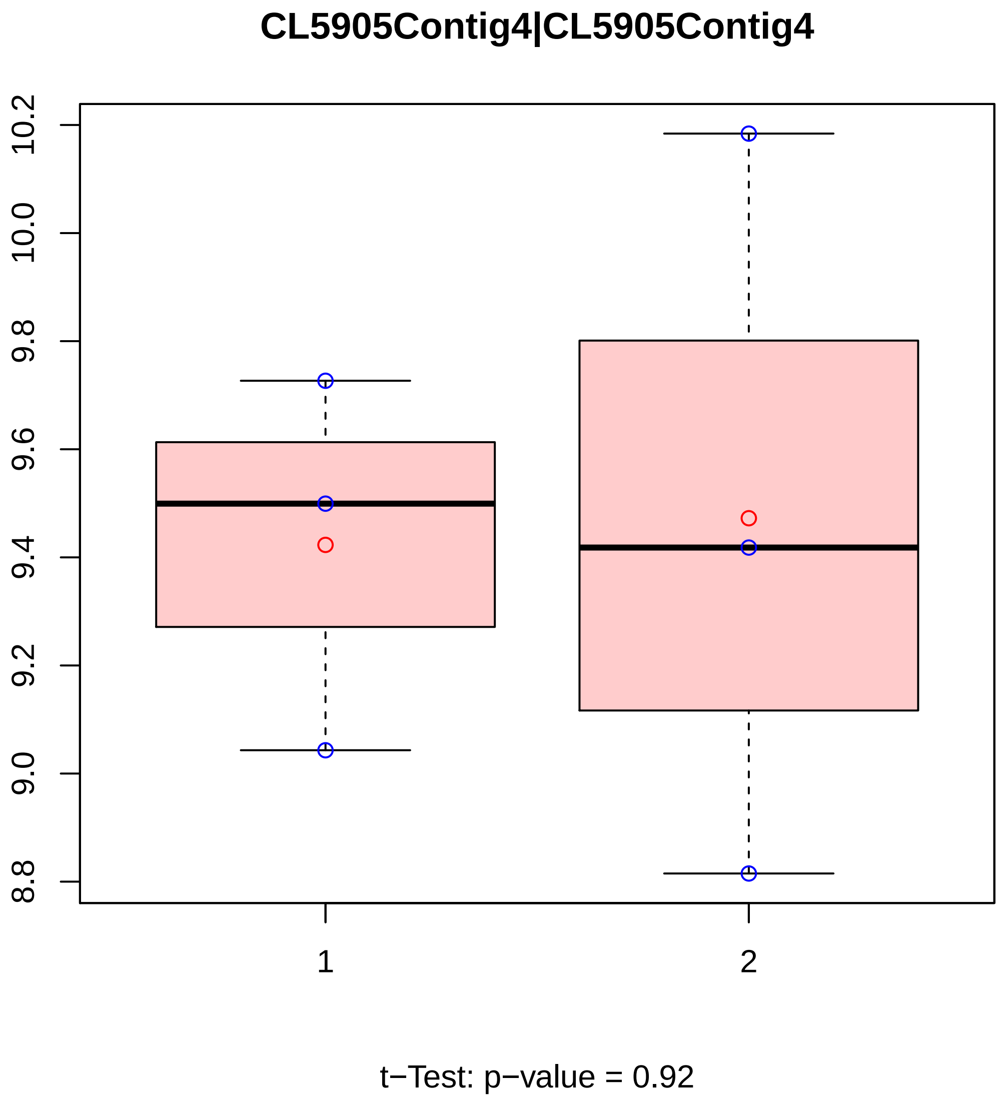
CL5905Contig4|CL5905Contig4
10.2
10.0
9.8
9.6
9.4
9.2
9.0
8.8
1
2
t−Test: p−value = 0.92

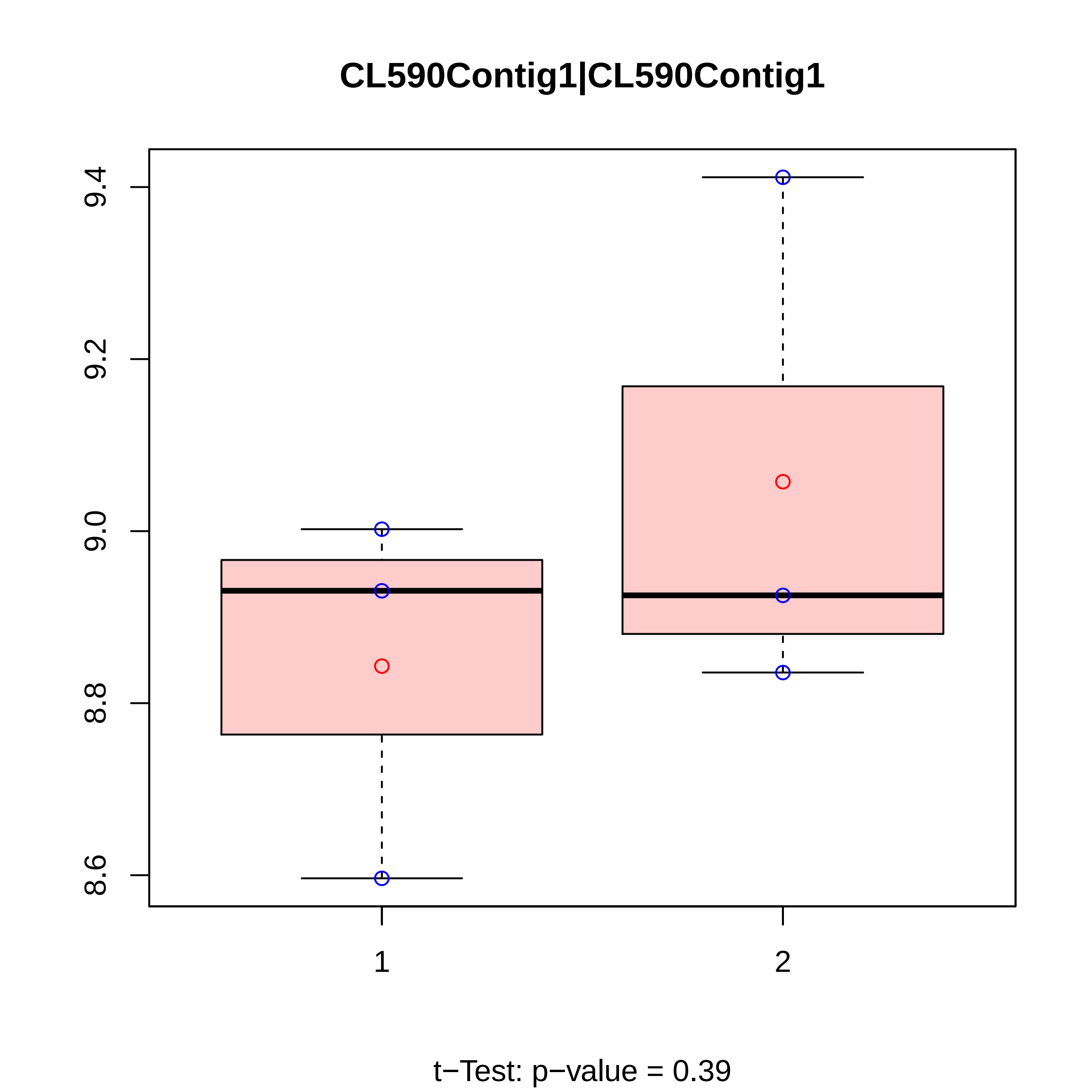

CL590Contig1|CL590Contig1
9.4
9.2
9.0
8.8
8.6
1
2
t−Test: p−value = 0.39

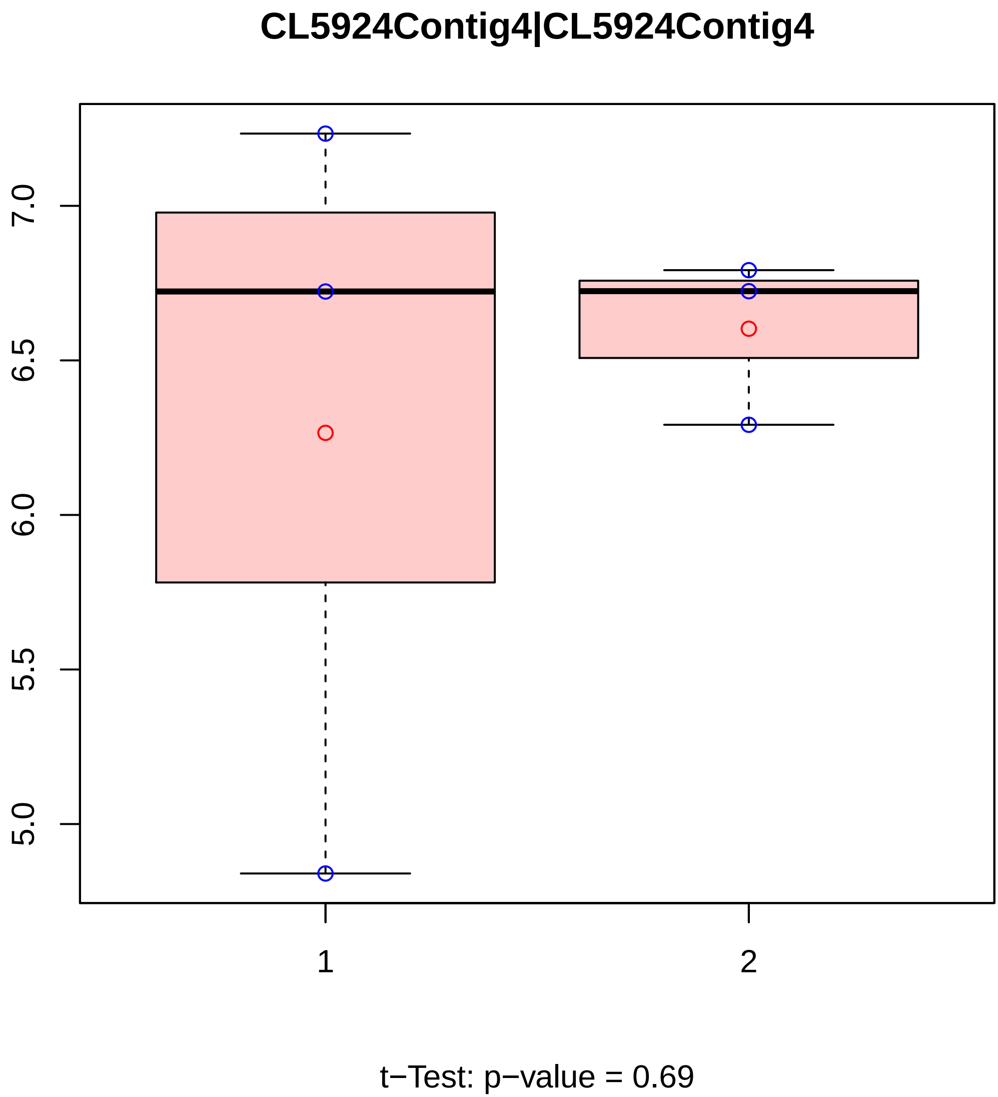

CL5924Contig4|CL5924Contig4
7.0
6.5
6.0
5.5
5.0
1
2
t−Test: p−value = 0.69

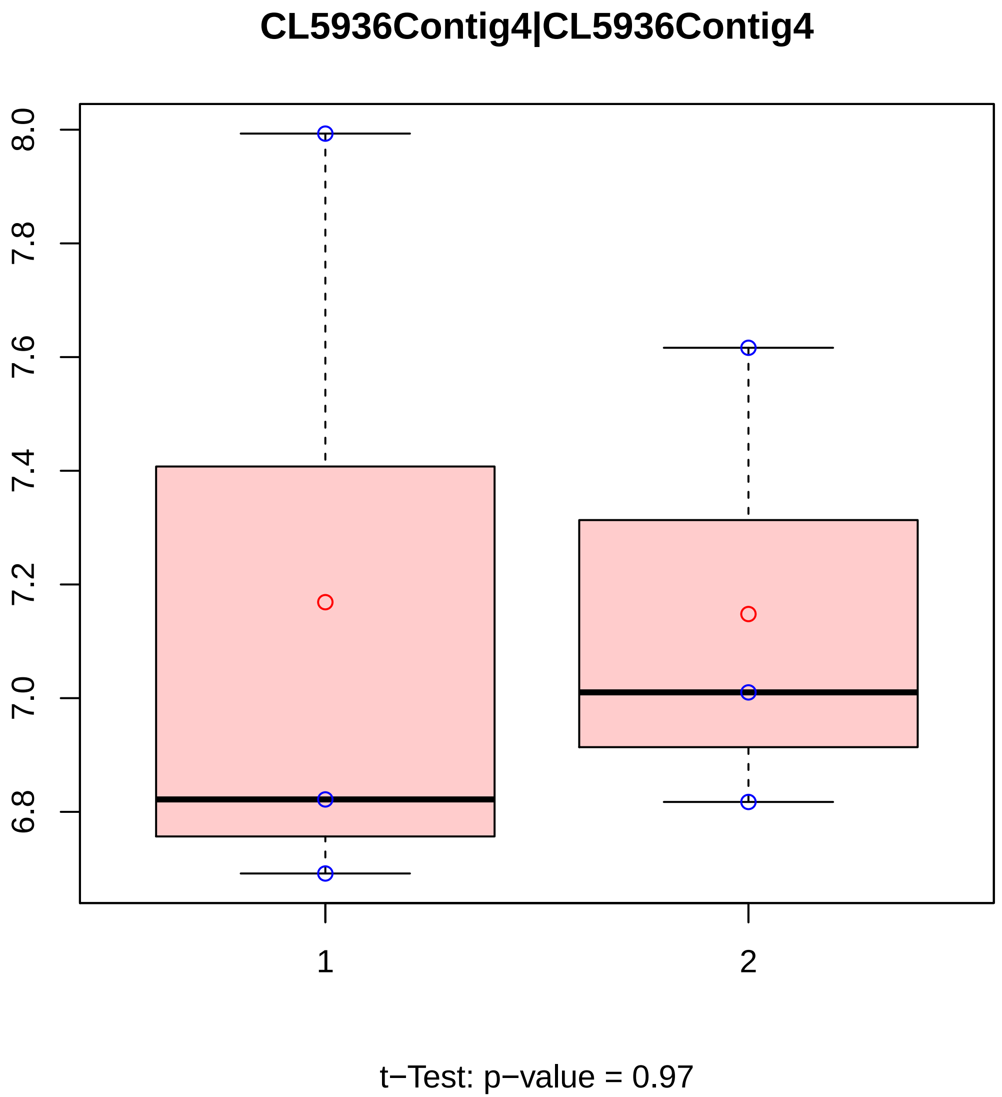
CL5936Contig4|CL5936Contig4
8.0
7.8
7.6
7.4
7.2
7.0
6.8
1
2
t−Test: p−value = 0.97

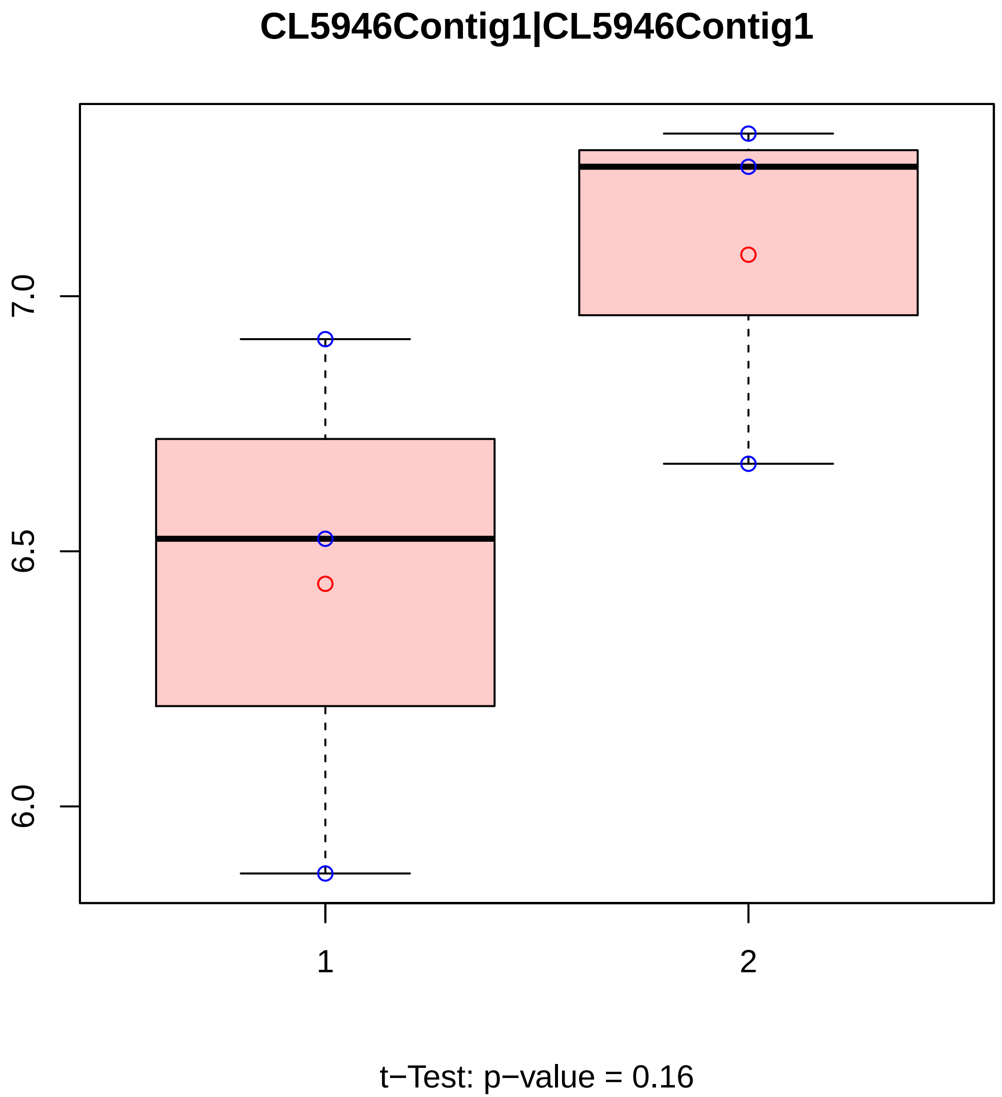
CL5946Contig1|CL5946Contig1
7.0
6.5
6.0
1
2
t−Test: p−value = 0.16

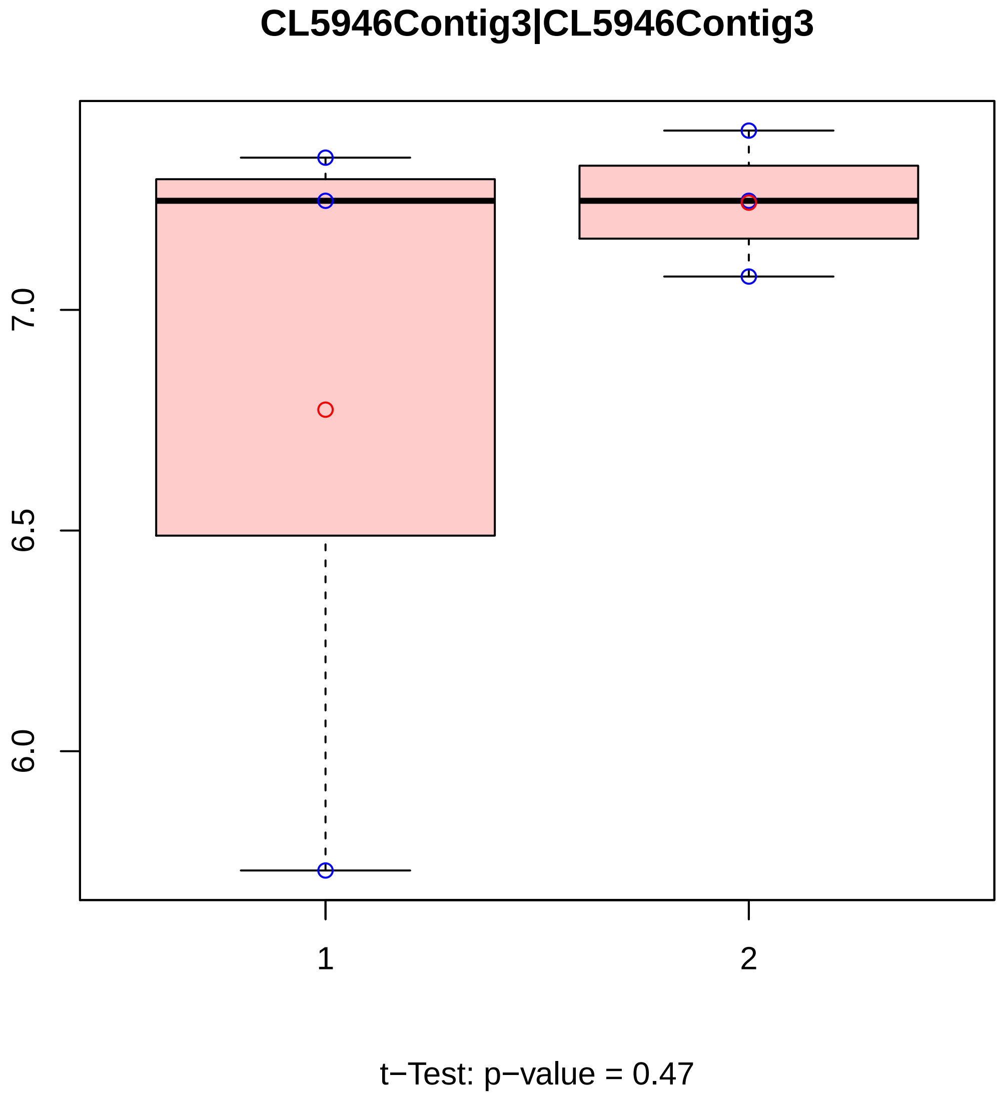

CL5946Contig3|CL5946Contig3
7.0
6.5
6.0
1
2
t−Test: p−value = 0.47

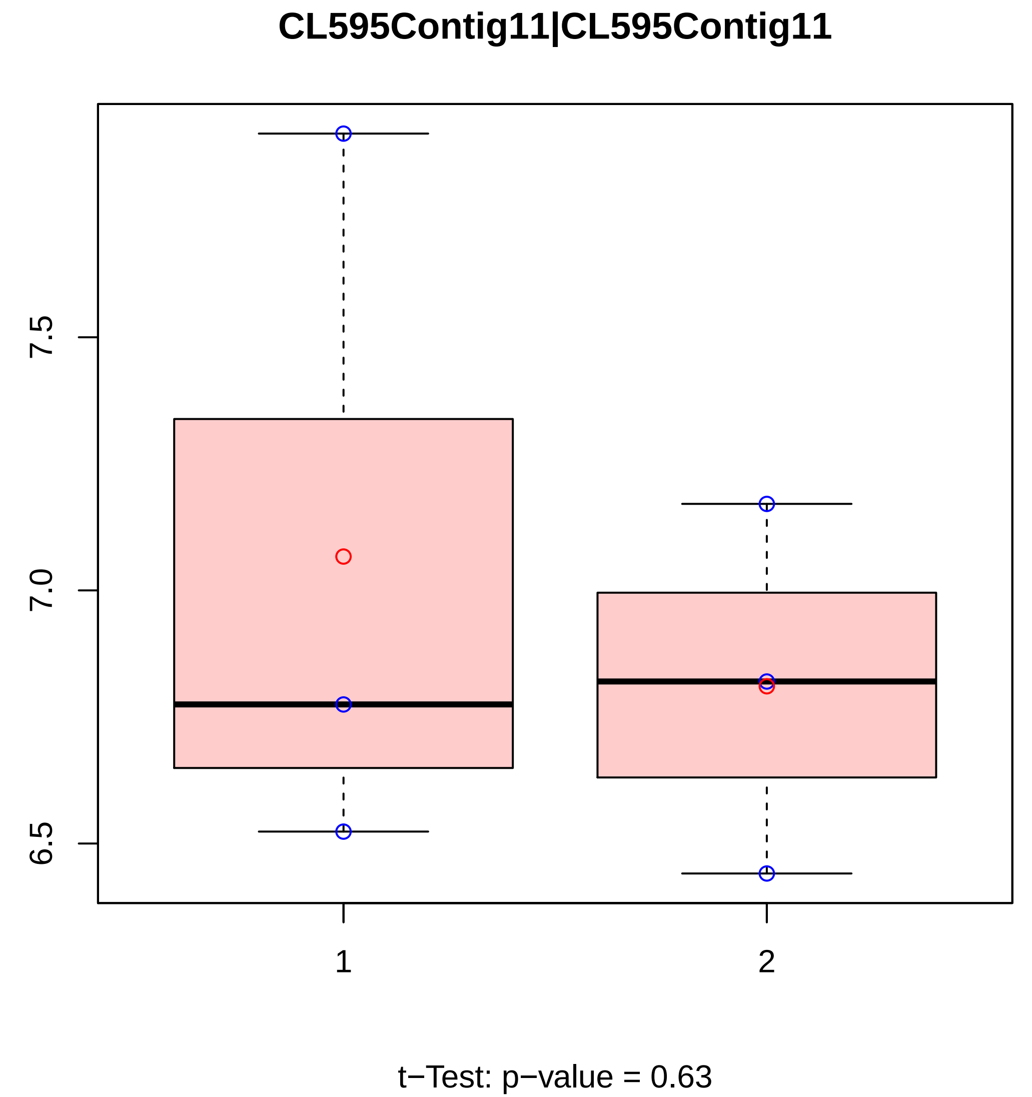
CL595Contig11|CL595Contig11
7.5
7.0
6.5
1
2
t−Test: p−value = 0.63

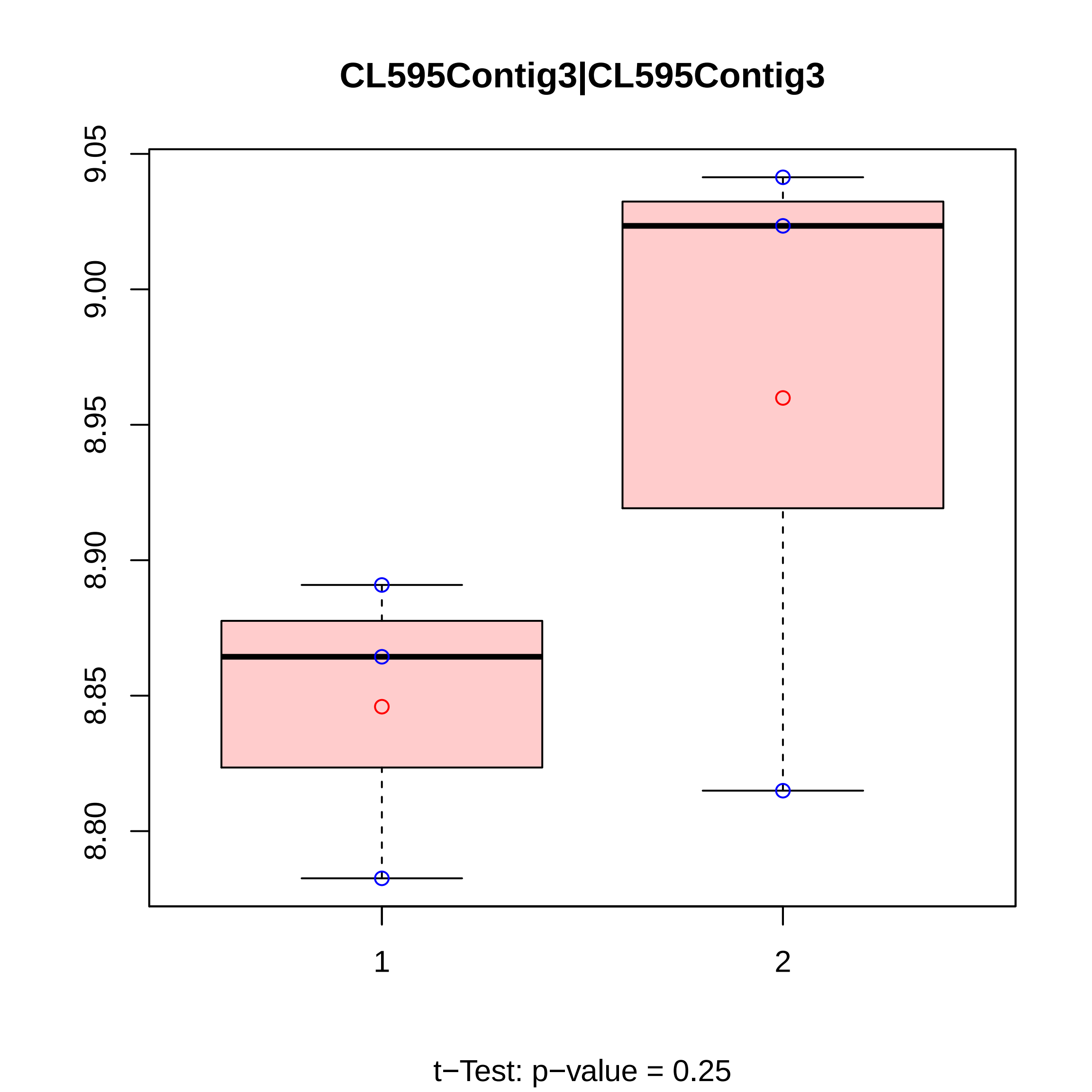

CL595Contig3|CL595Contig3
9.05
9.00
8.95
8.90
8.85
8.80
1
2
t−Test: p−value = 0.25

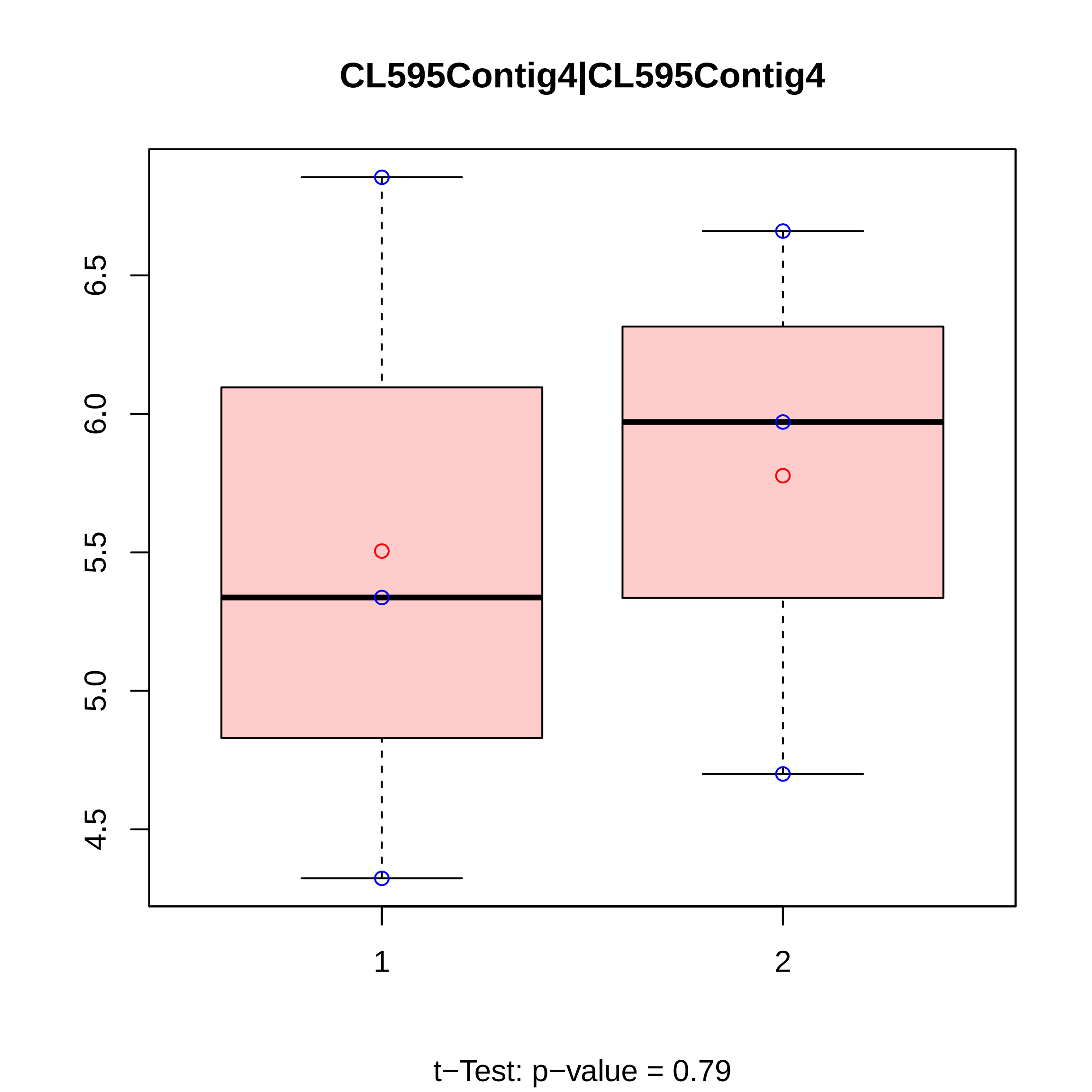
CL595Contig4|CL595Contig4
6.5
6.0
5.5
5.0
4.5
1
2
t−Test: p−value = 0.79

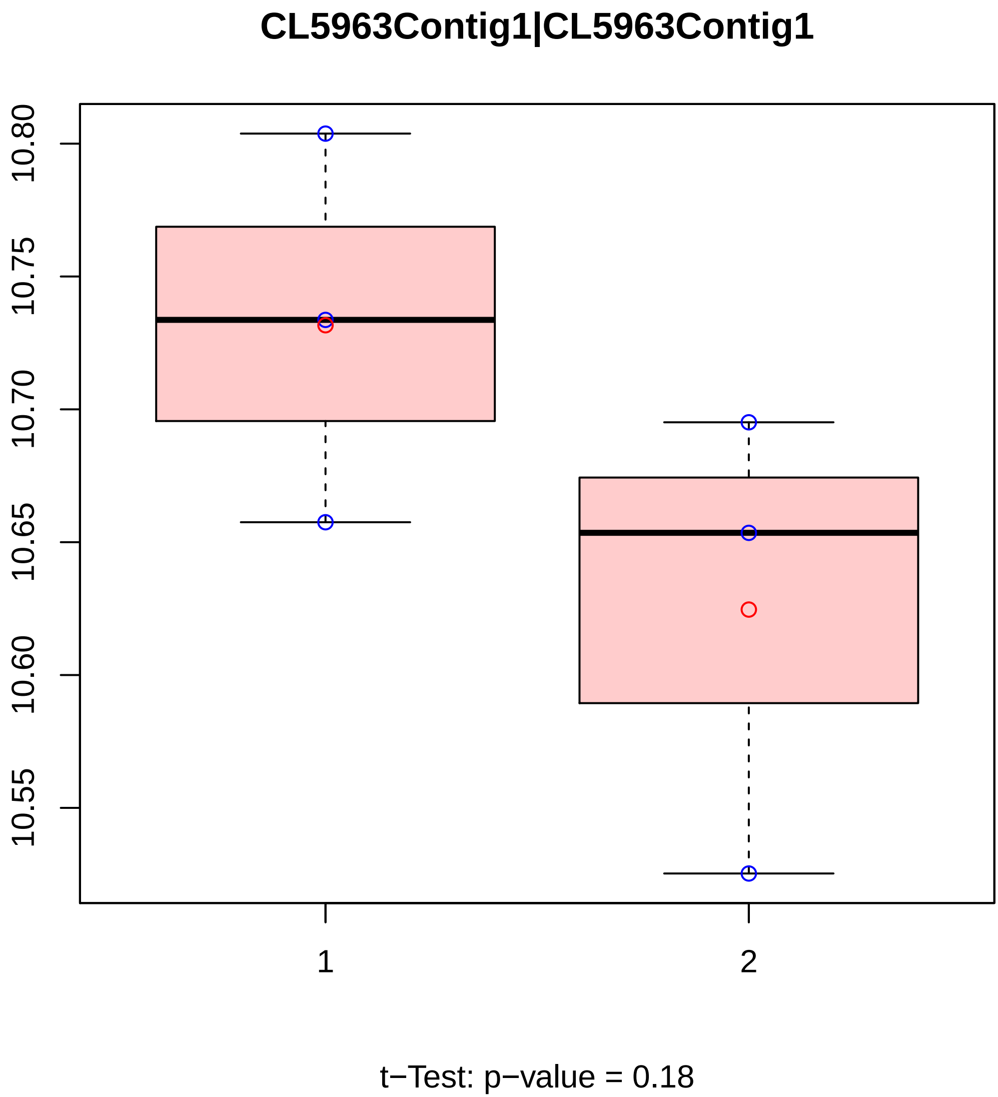
CL5963Contig1|CL5963Contig1
10.80
10.75
10.70
10.65
10.60
10.55
1
2
t−Test: p−value = 0.18

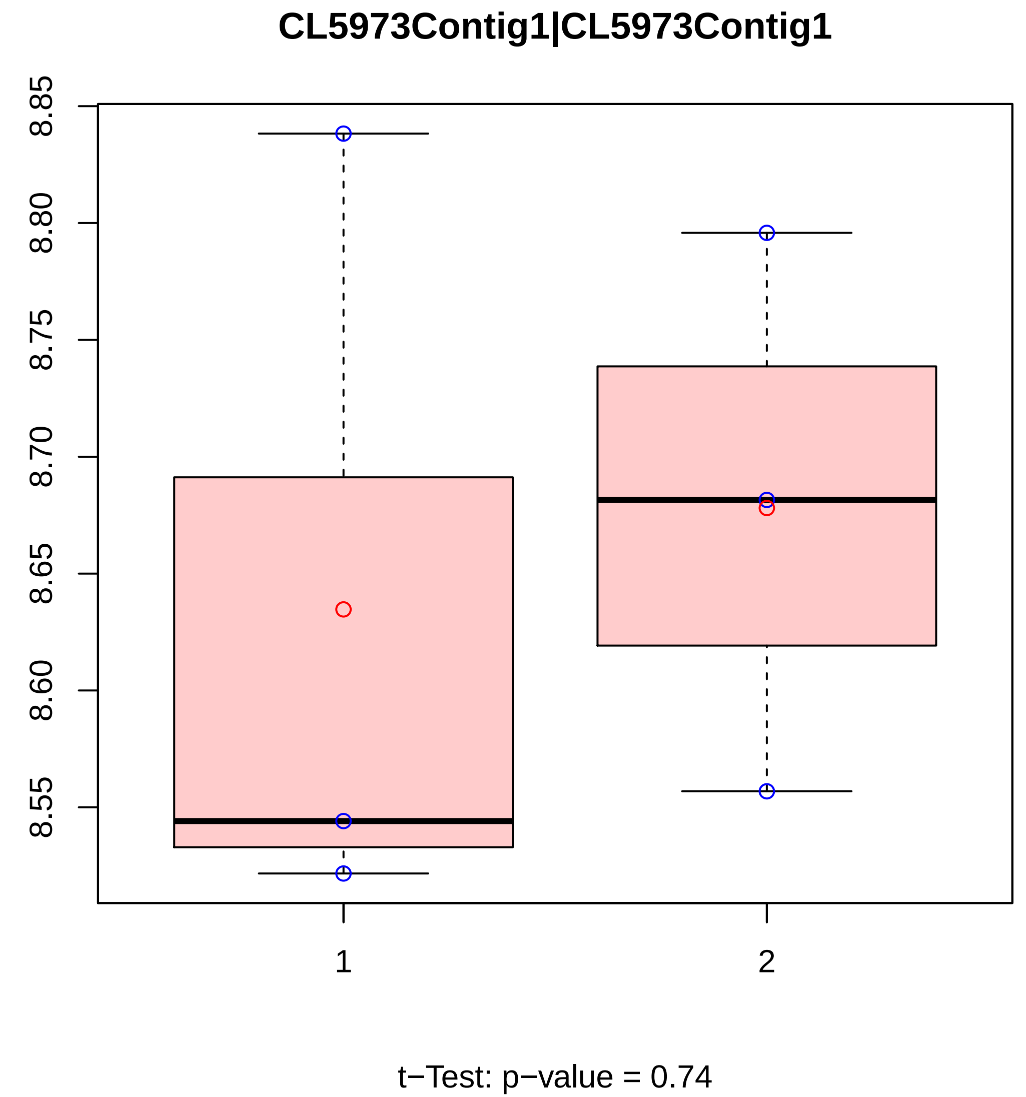

CL5973Contig1|CL5973Contig1
8.85
8.80
8.75
8.70
8.65
8.60
8.55
1
2
t−Test: p−value = 0.74

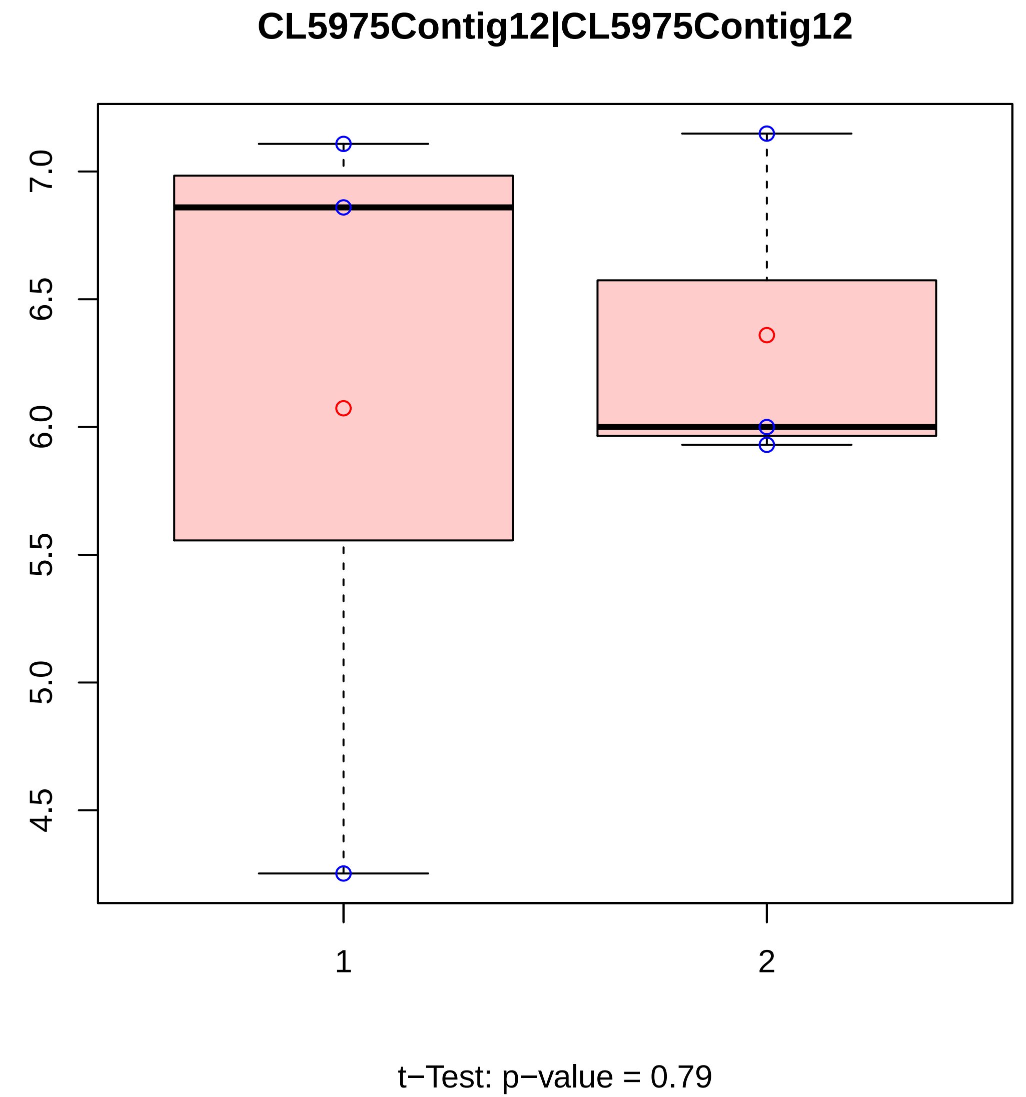
CL5975Contig12|CL5975Contig12
7.0
6.5
6.0
5.5
5.0
4.5
1
2
t−Test: p−value = 0.79

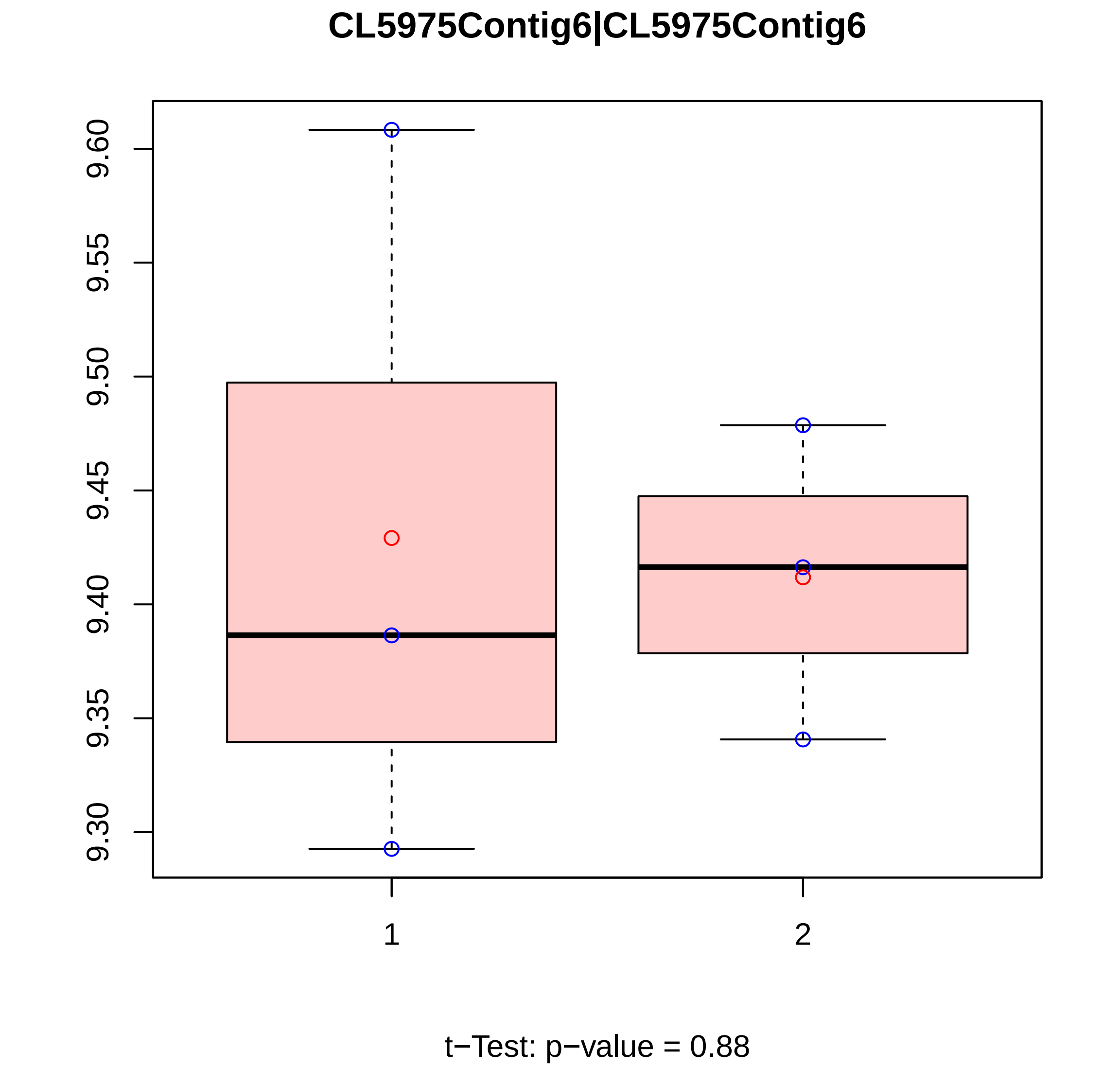

CL5975Contig6|CL5975Contig6
9.60
9.55
9.50
9.45
9.40
9.35
9.30
1
2
t−Test: p−value = 0.88

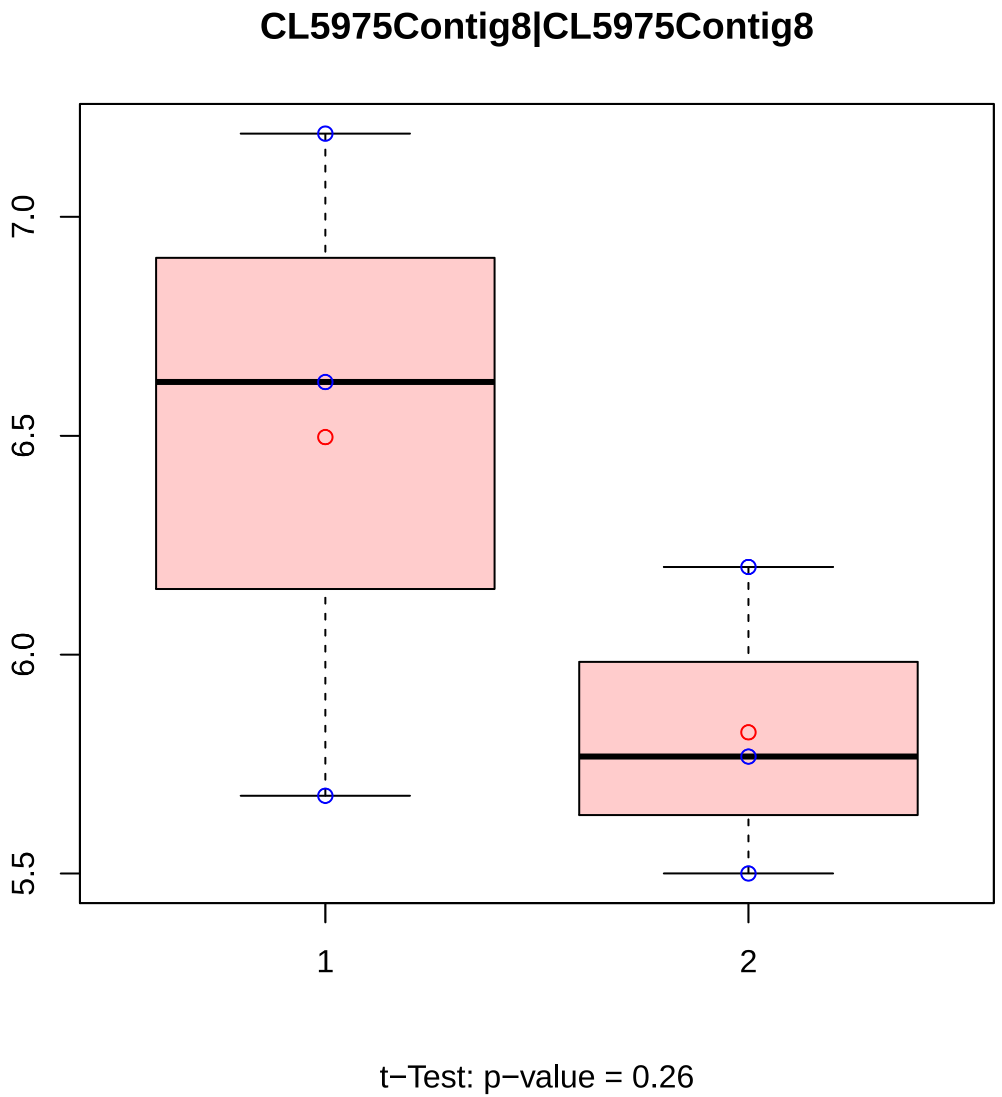

CL5975Contig8|CL5975Contig8
7.0
6.5
6.0
5.5
1
2
t−Test: p−value = 0.26

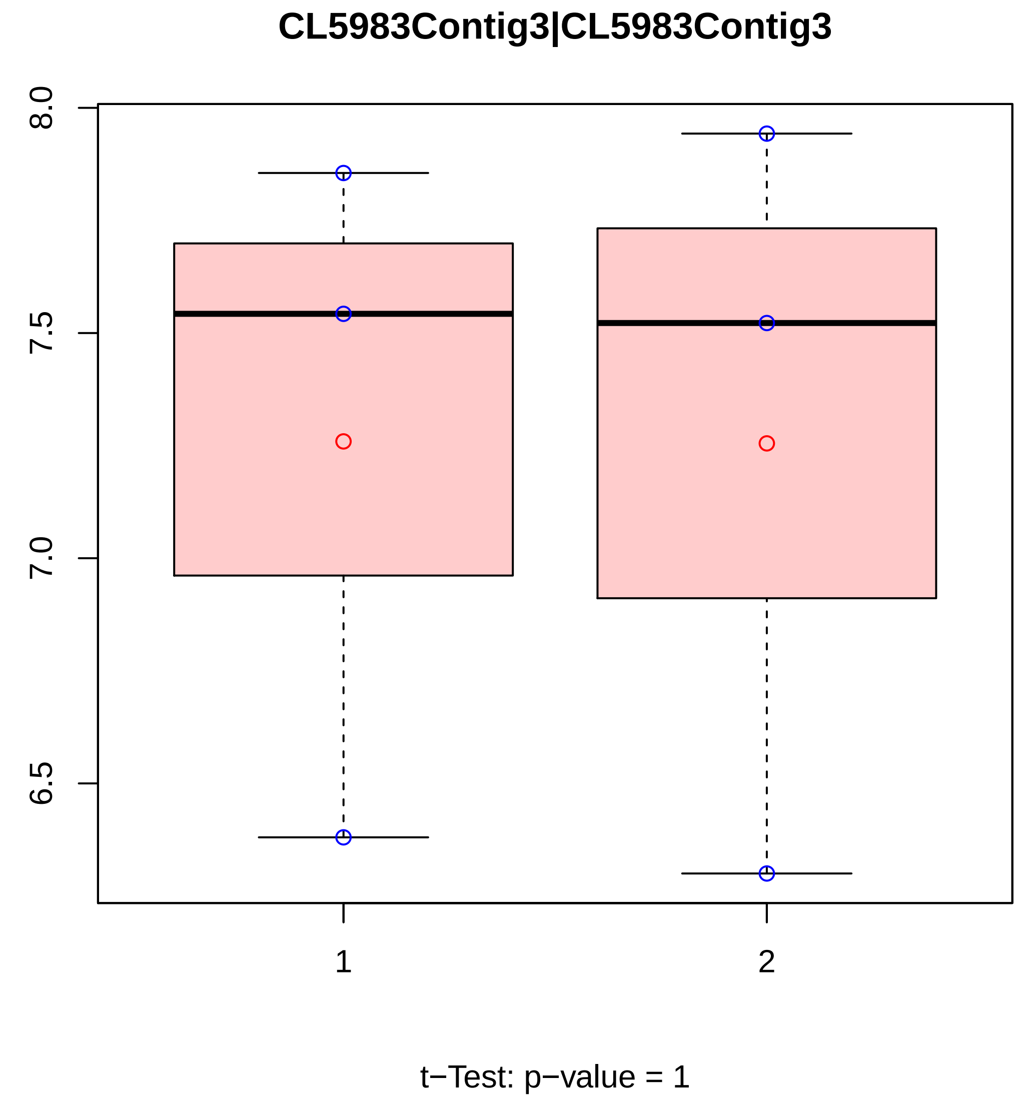

CL5983Contig3|CL5983Contig3
8.0
7.5
7.0
6.5
1
2
t−Test: p−value = 1

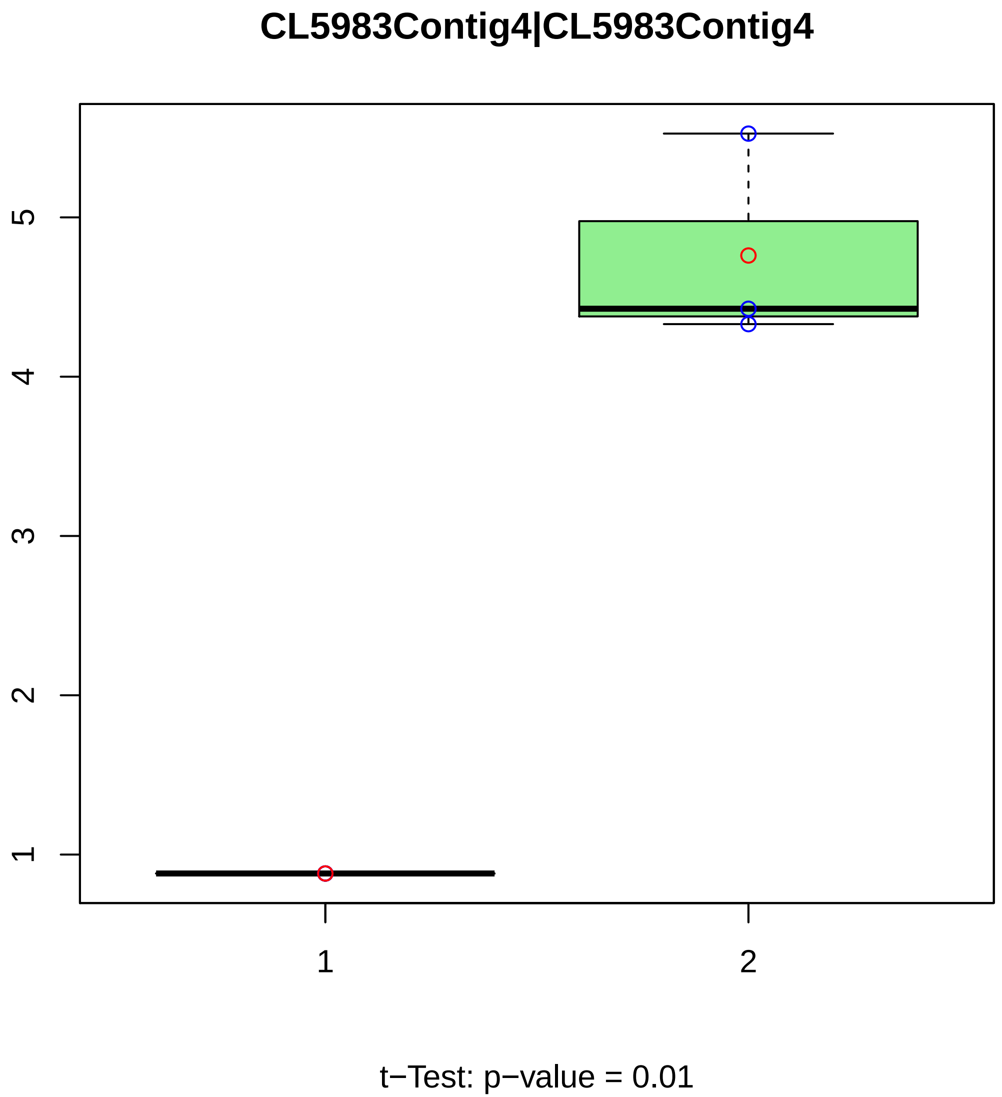

CL5983Contig4|CL5983Contig4
1
2
3
4
5
1
2
t−Test: p−value = 0.01

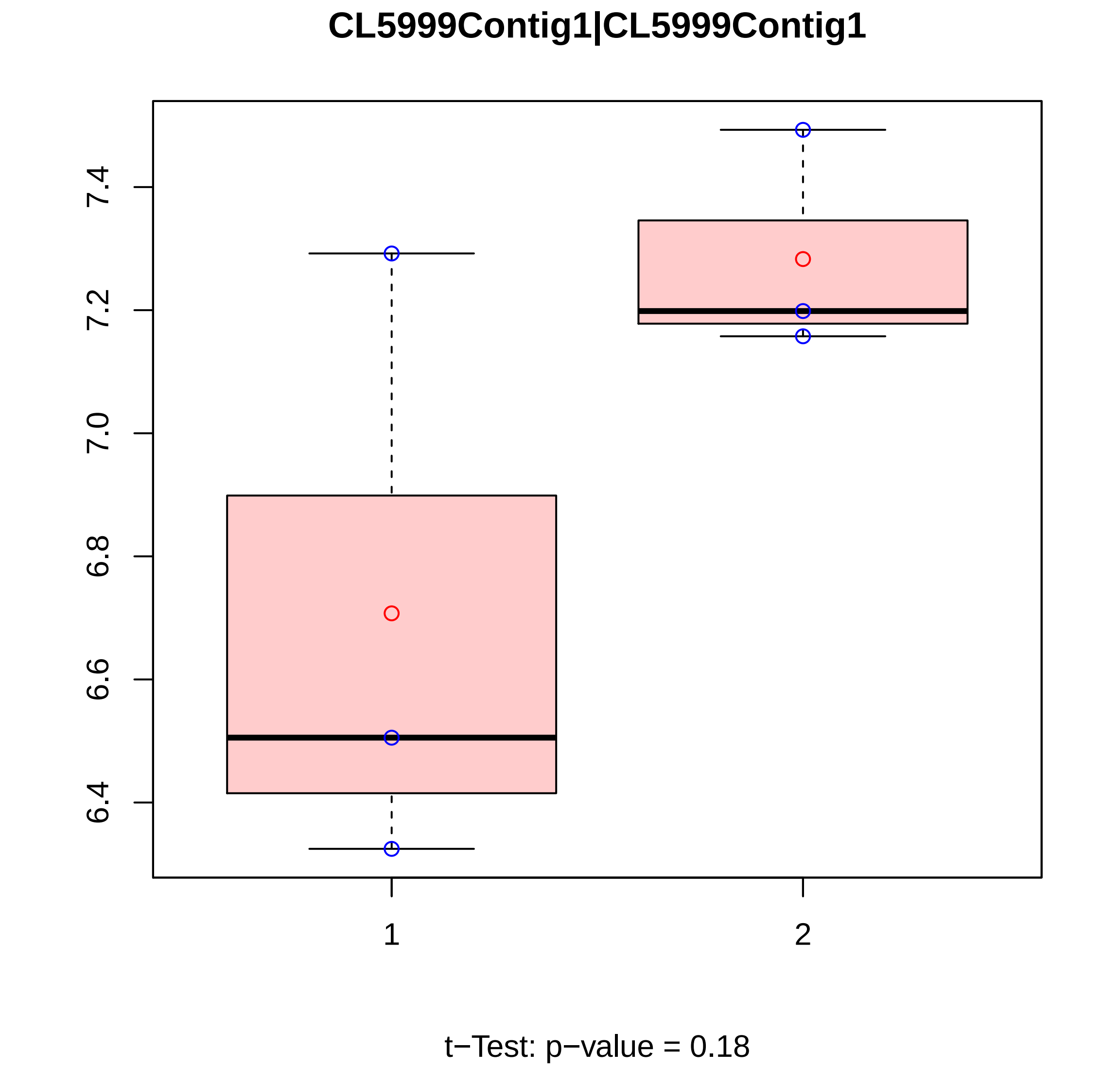
CL5999Contig1|CL5999Contig1
7.4
7.2
7.0
6.8
6.6
6.4
1
2
t−Test: p−value = 0.18

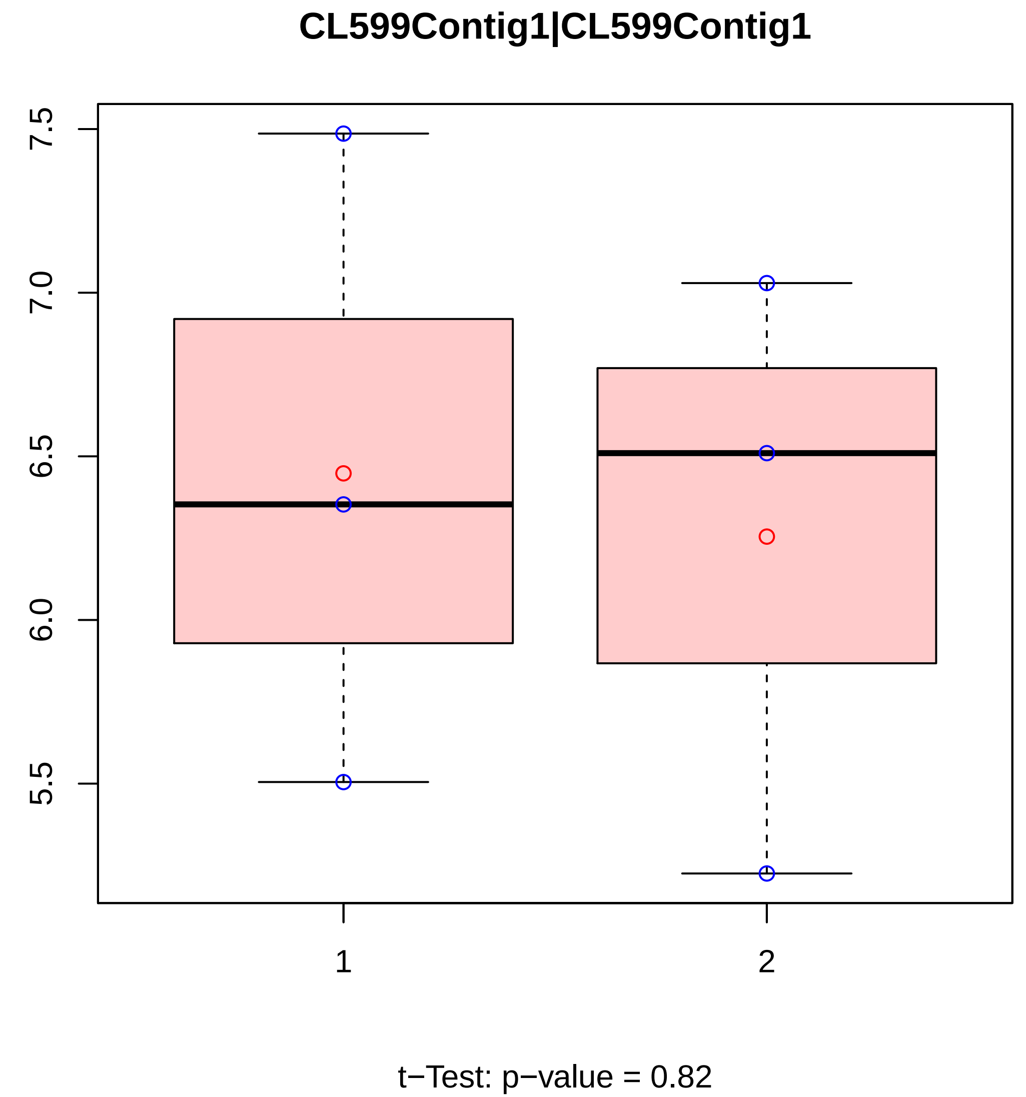
CL599Contig1|CL599Contig1
7.5
7.0
6.5
6.0
5.5
1
2
t−Test: p−value = 0.82

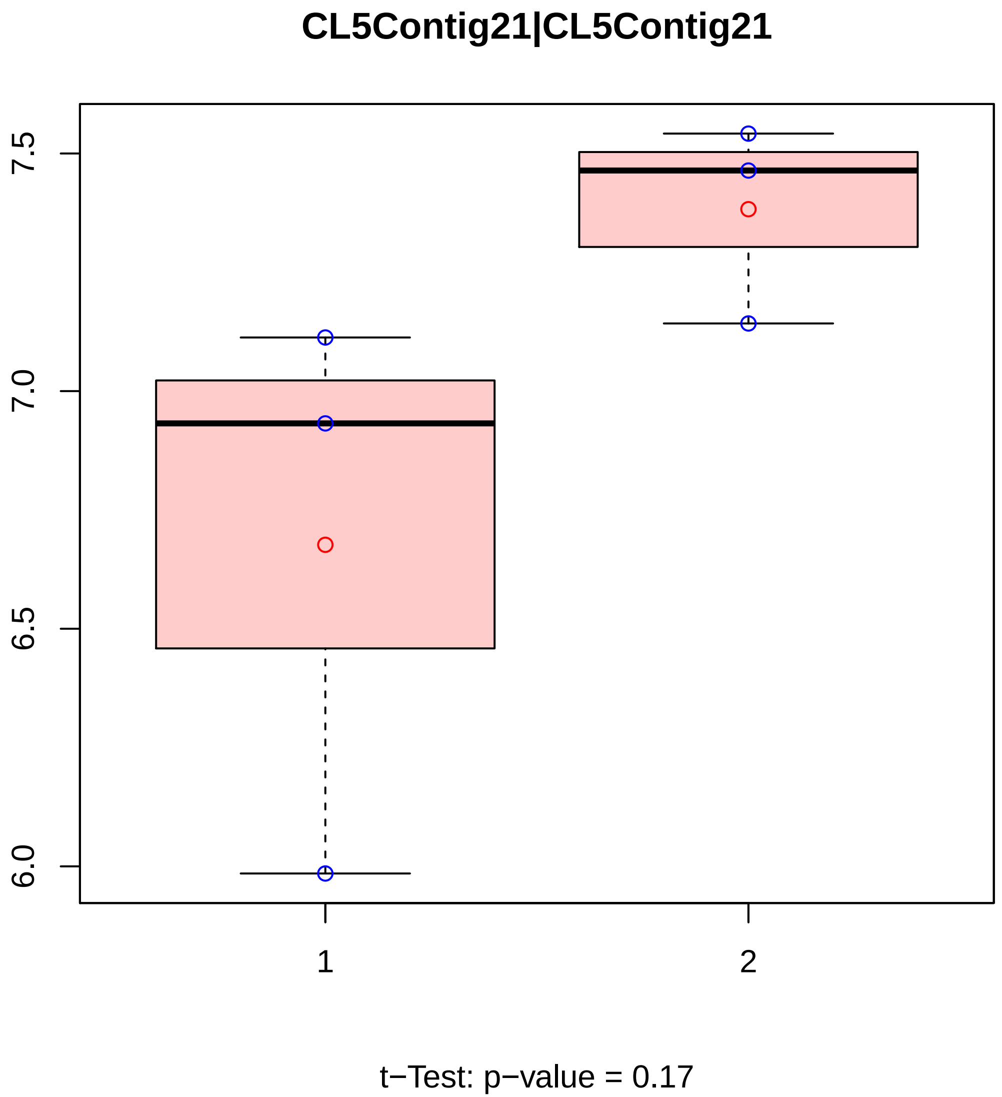

CL5Contig21|CL5Contig21
7.5
7.0
6.5
6.0
1
2
t−Test: p−value = 0.17

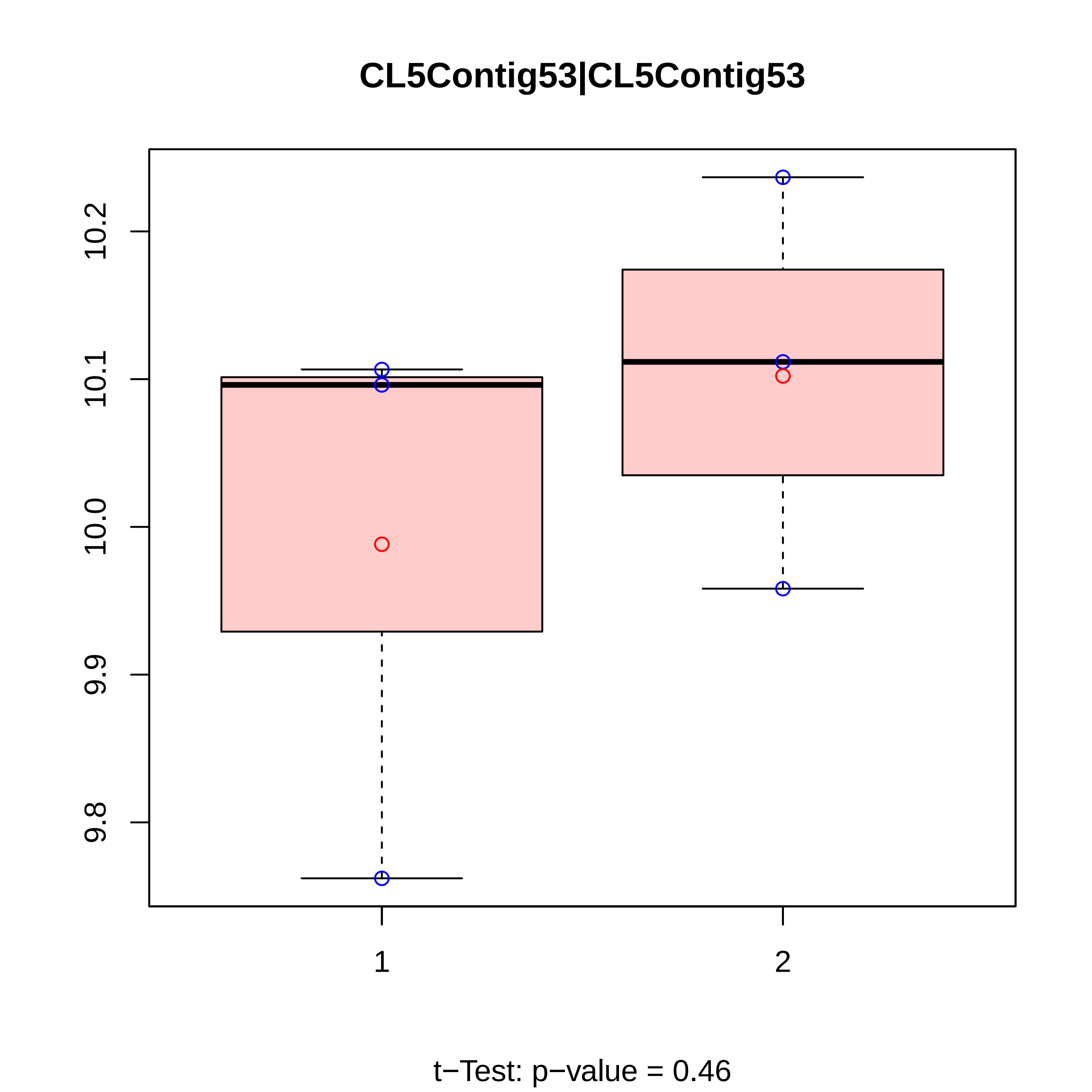
CL5Contig53|CL5Contig53
10.2
10.1
10.0
9.9
9.8
1
2
t−Test: p−value = 0.46

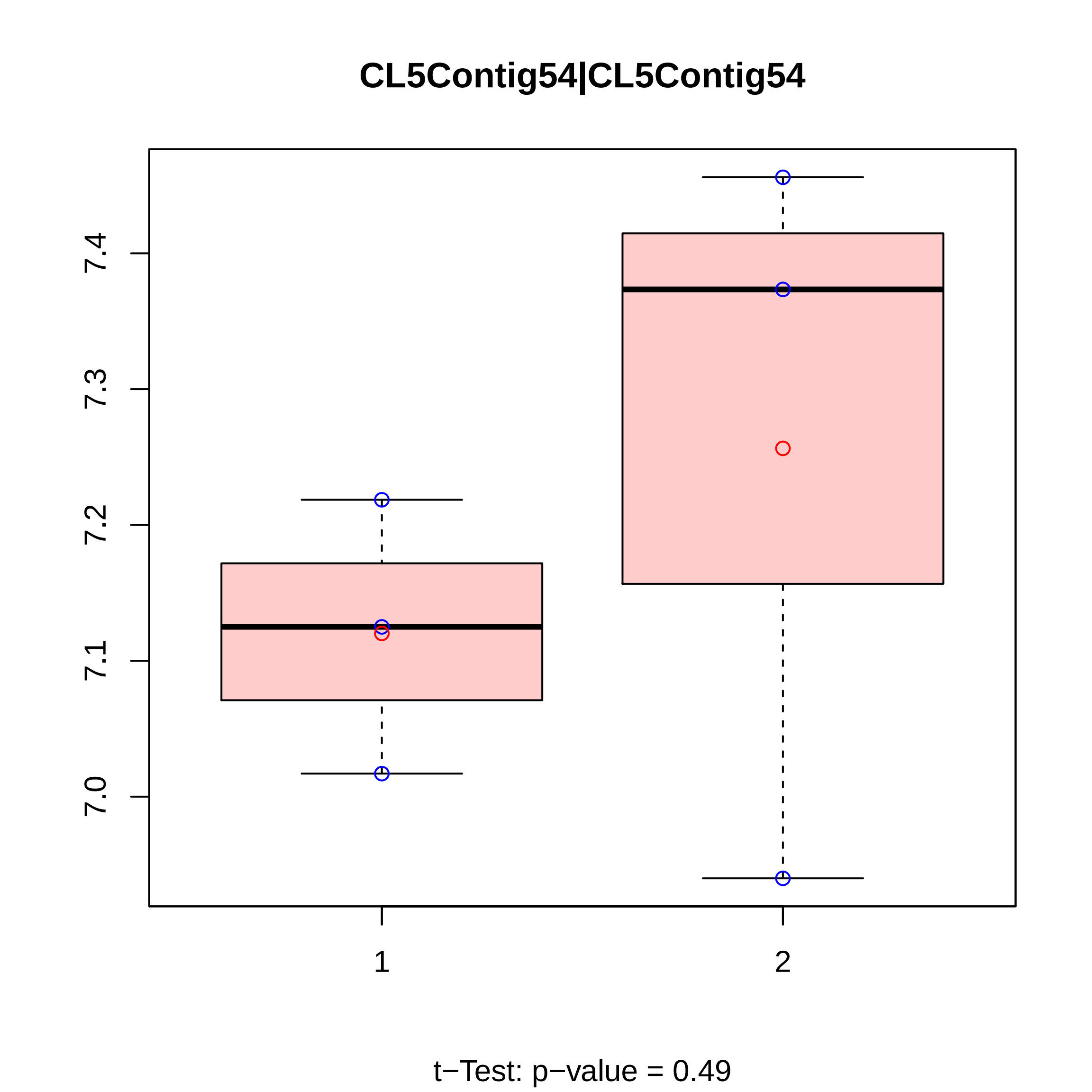
CL5Contig54|CL5Contig54
7.0
7.1
7.2
7.3
7.4
1
2
t−Test: p−value = 0.49

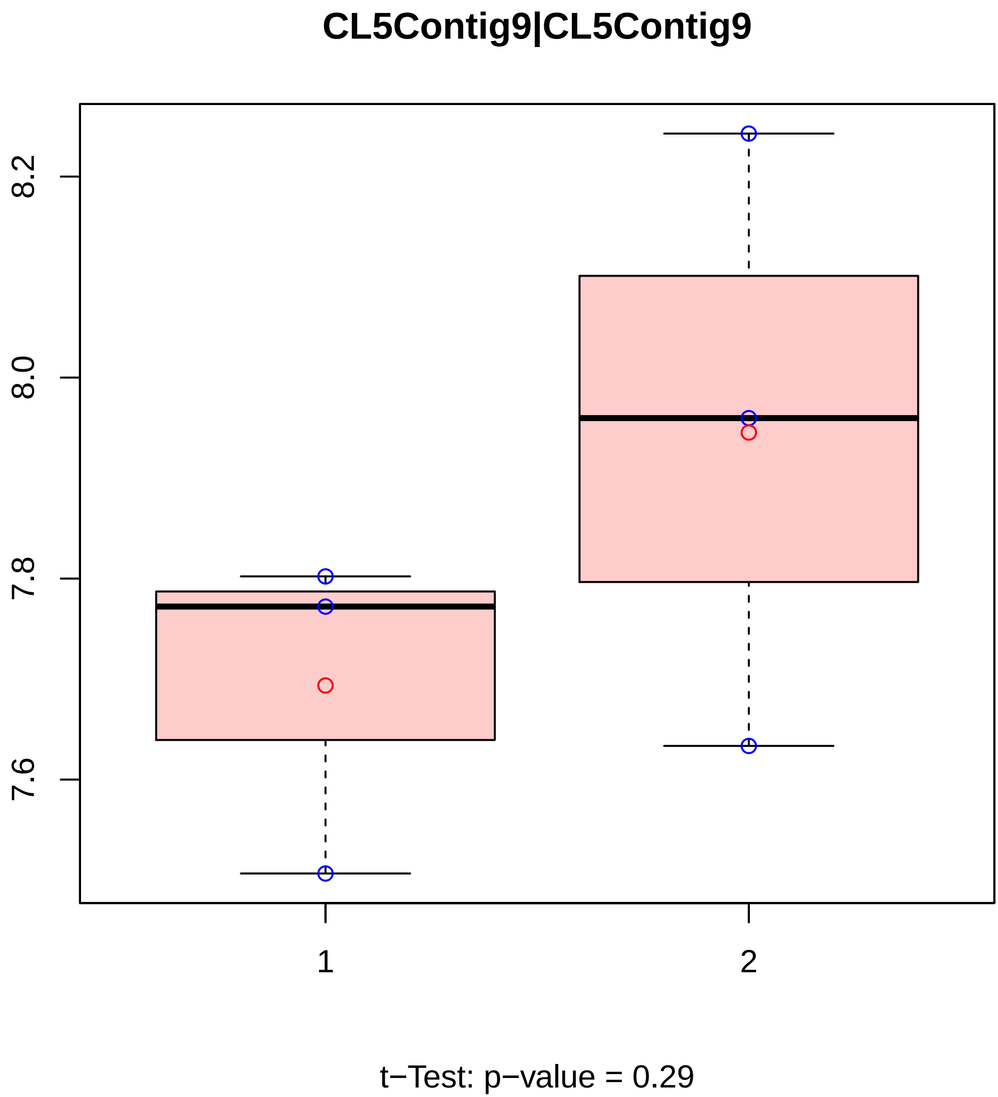

CL5Contig9|CL5Contig9
8.2
8.0
7.8
7.6
1
2
t−Test: p−value = 0.29

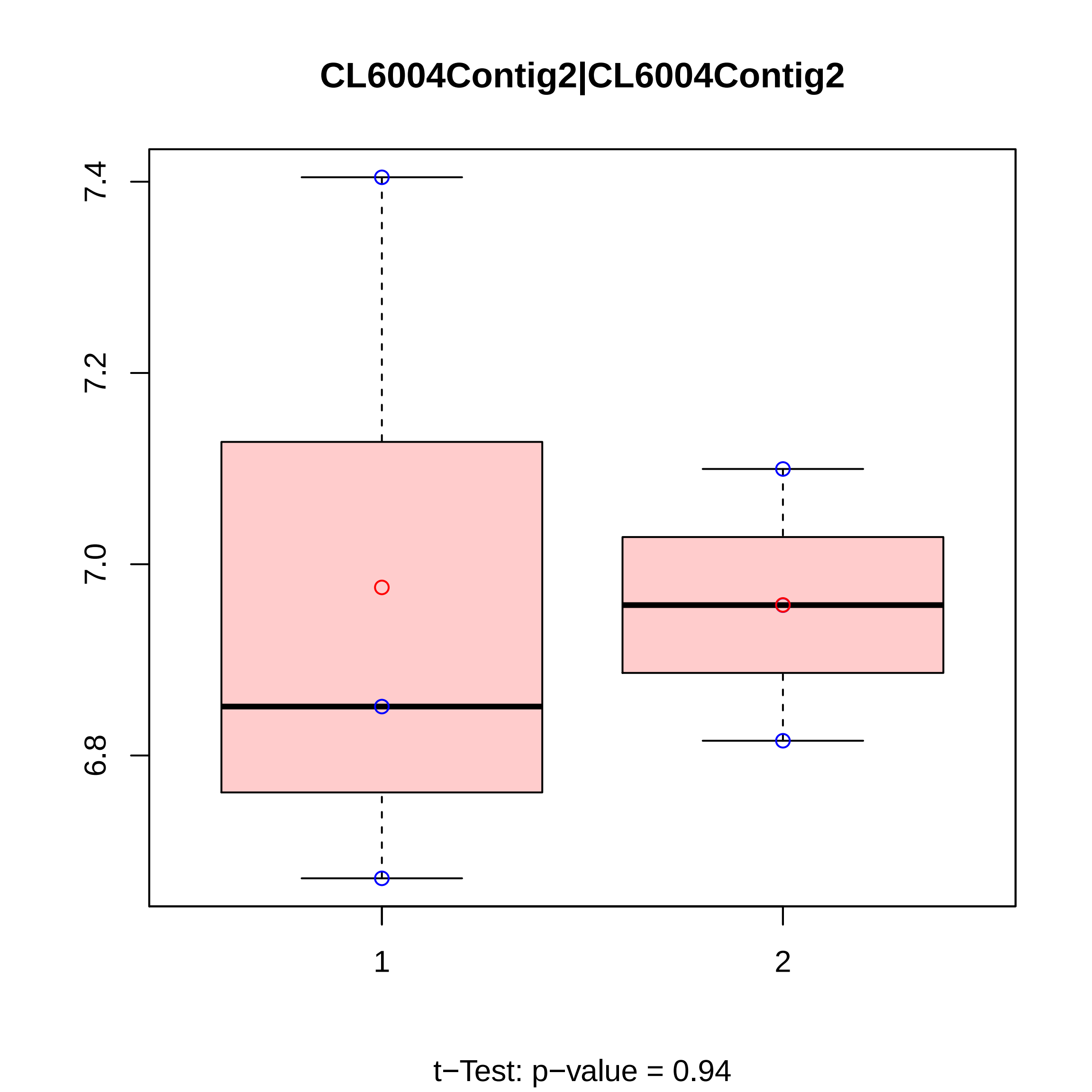
CL6004Contig2|CL6004Contig2
7.4
7.2
7.0
6.8
1
2
t−Test: p−value = 0.94

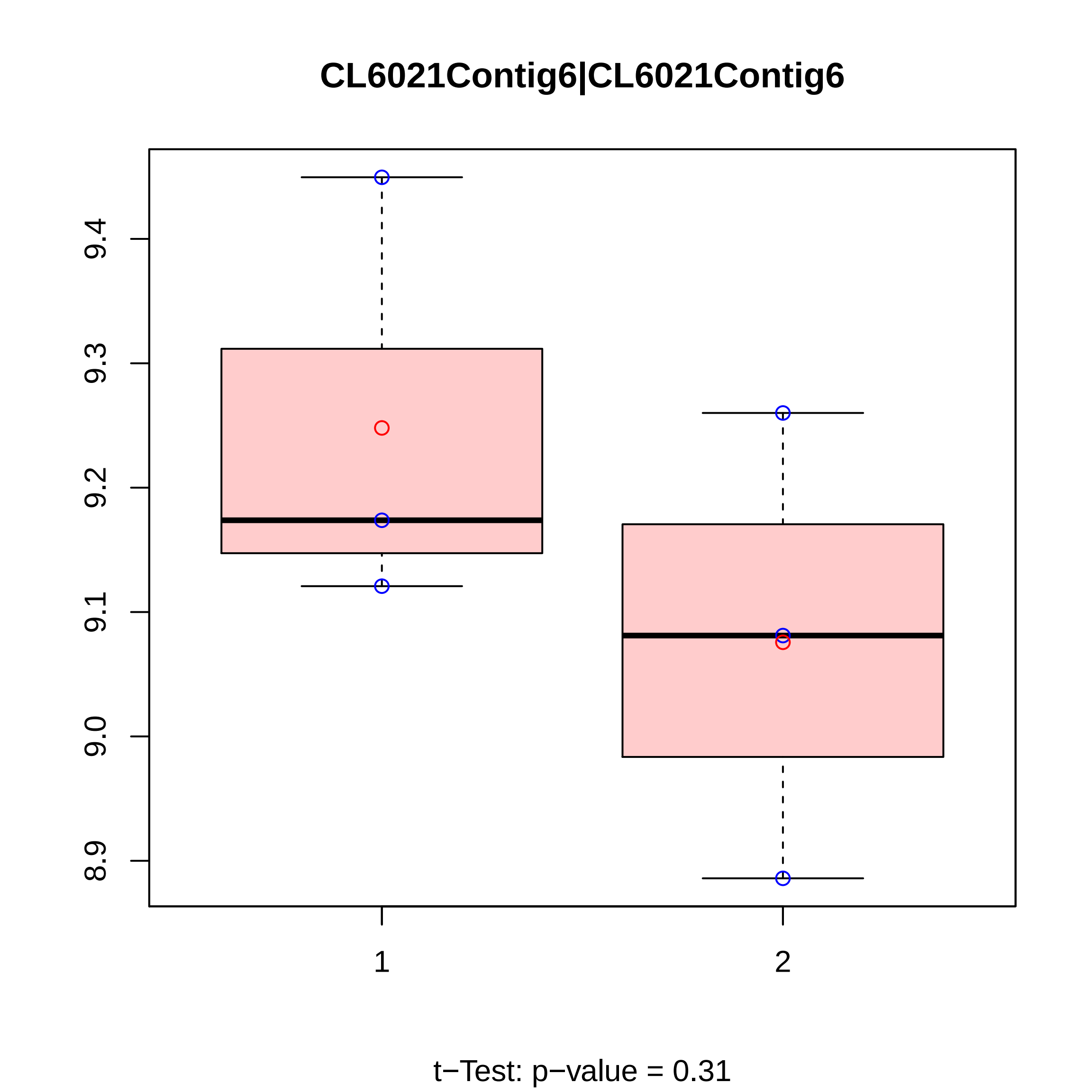
CL6021Contig6|CL6021Contig6
9.4
9.3
9.2
9.1
9.0
8.9
1
2
t−Test: p−value = 0.31

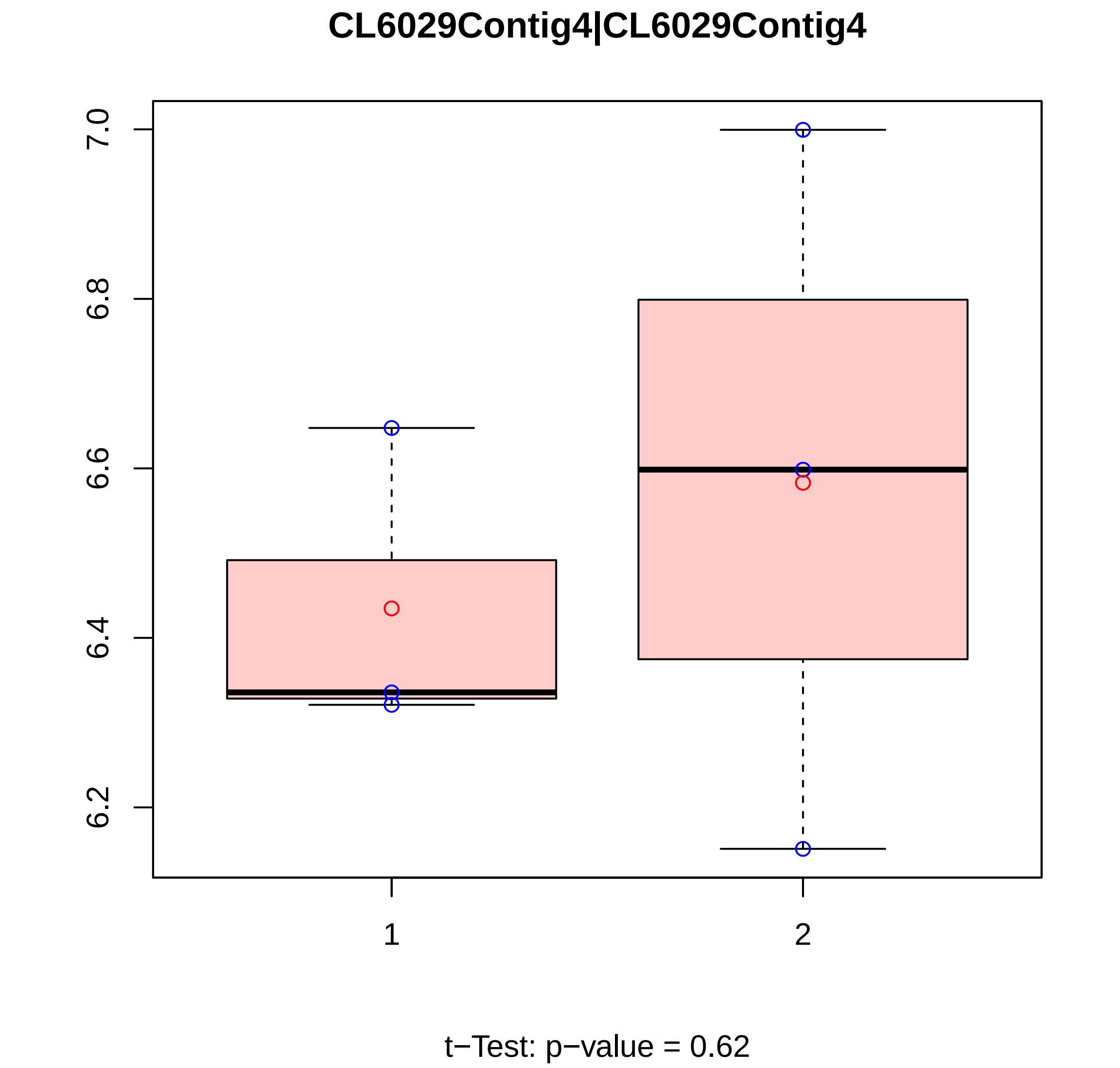
CL6029Contig4|CL6029Contig4
7.0
6.8
6.6
6.4
6.2
1
2
t−Test: p−value = 0.62

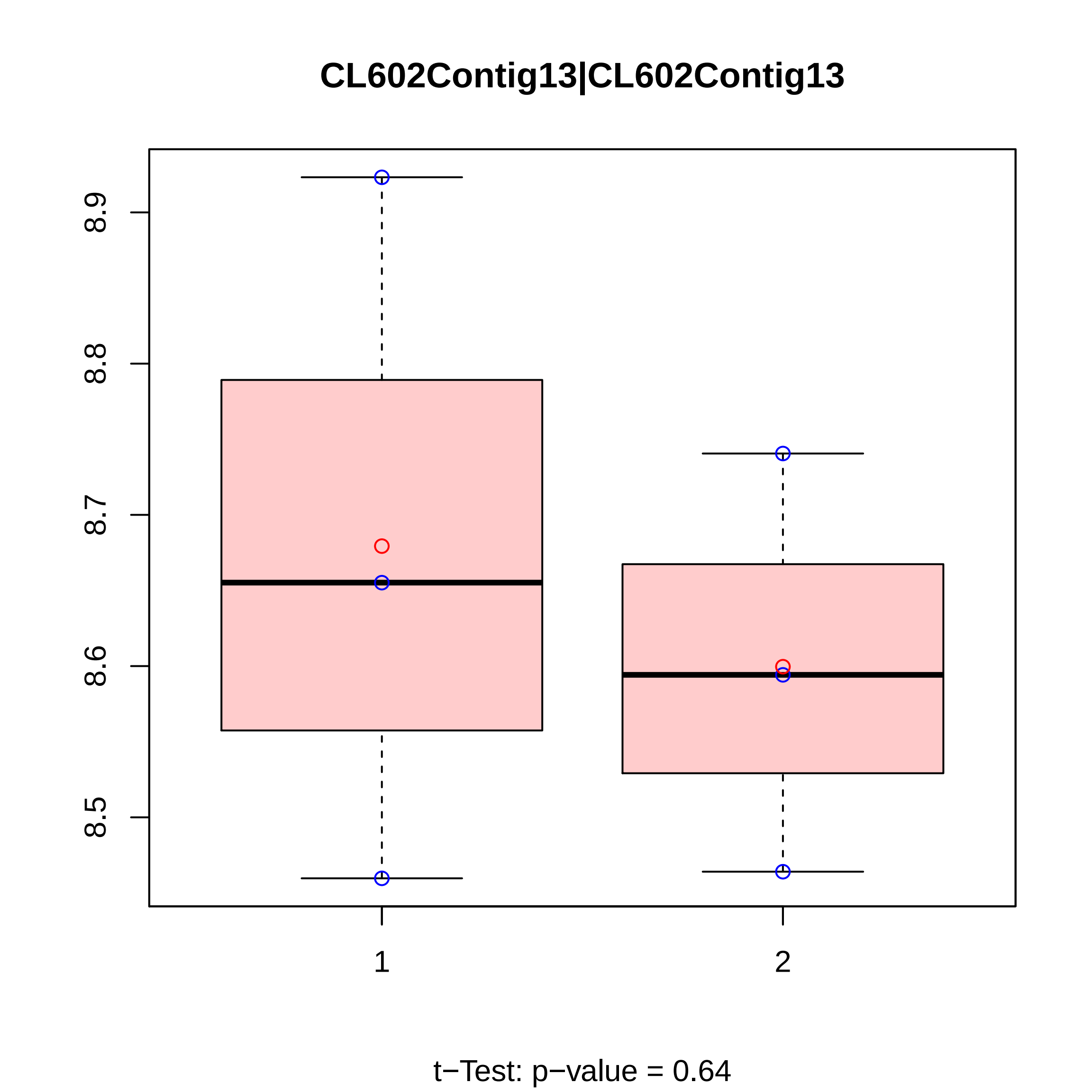

CL602Contig13|CL602Contig13
8.9
8.8
8.7
8.6
8.5
1
2
t−Test: p−value = 0.64

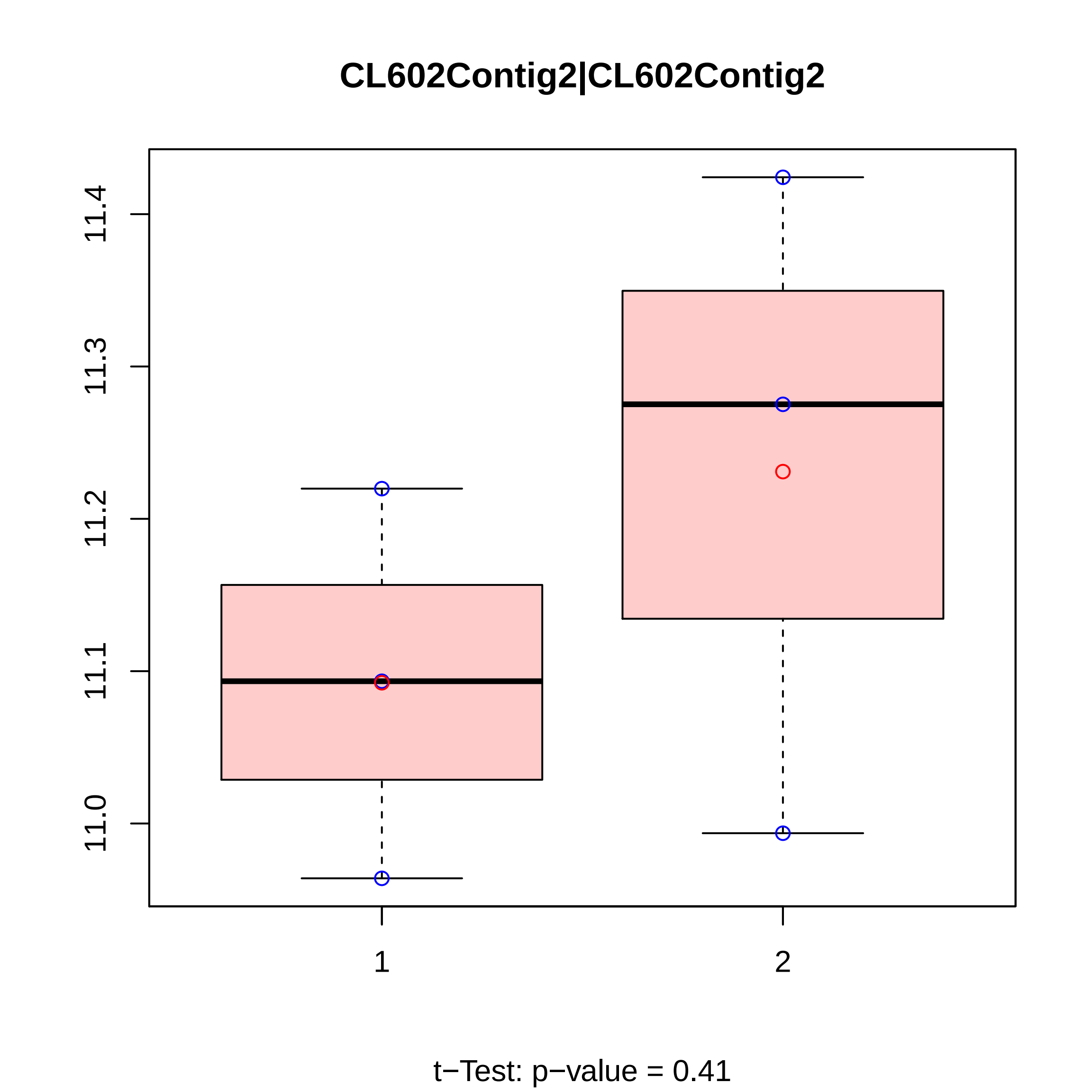
CL602Contig2|CL602Contig2
11.0
11.1
11.2
11.3
11.4
1
2
t−Test: p−value = 0.41

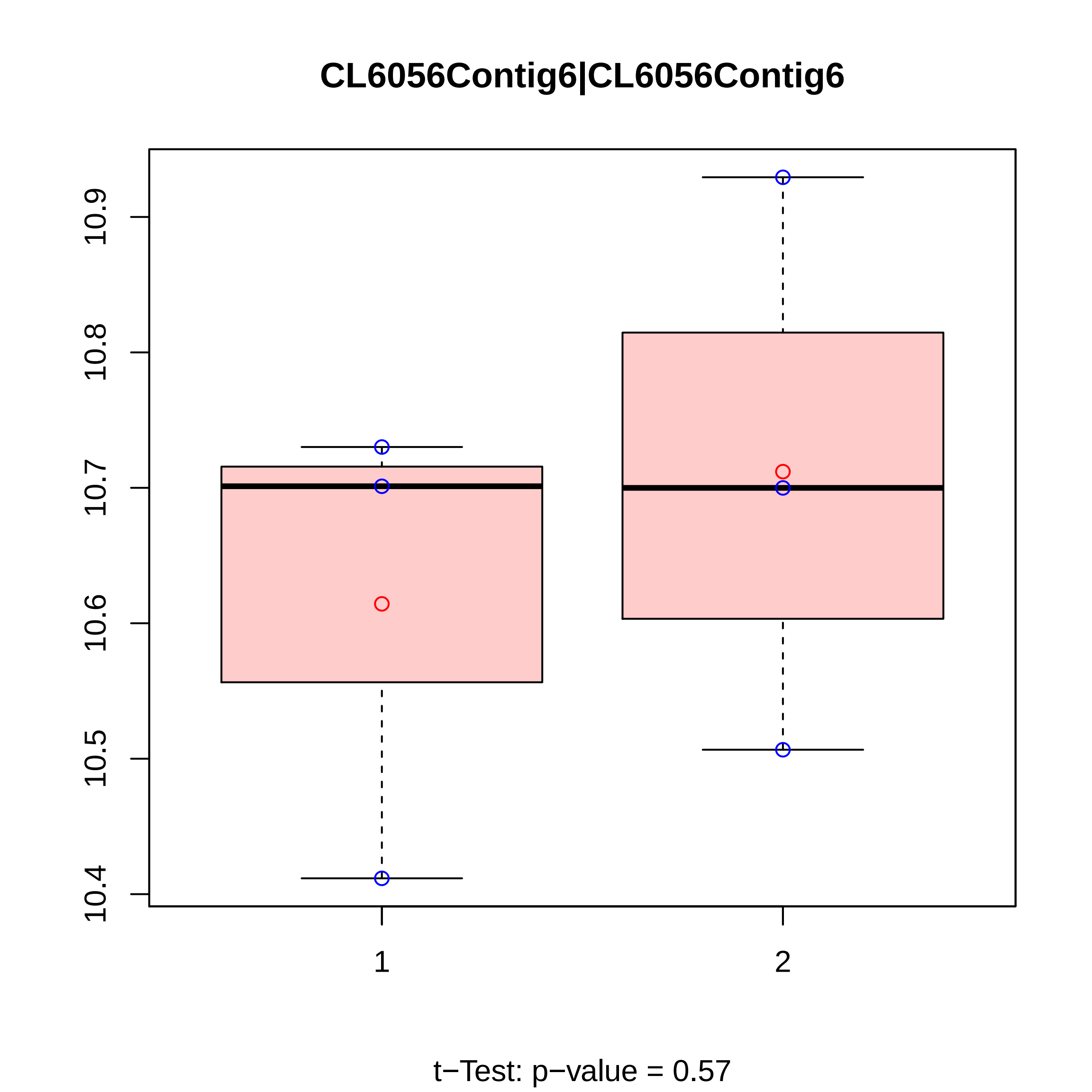

CL6056Contig6|CL6056Contig6
10.4
10.5
10.6
10.7
10.8
10.9
1
2
t−Test: p−value = 0.57

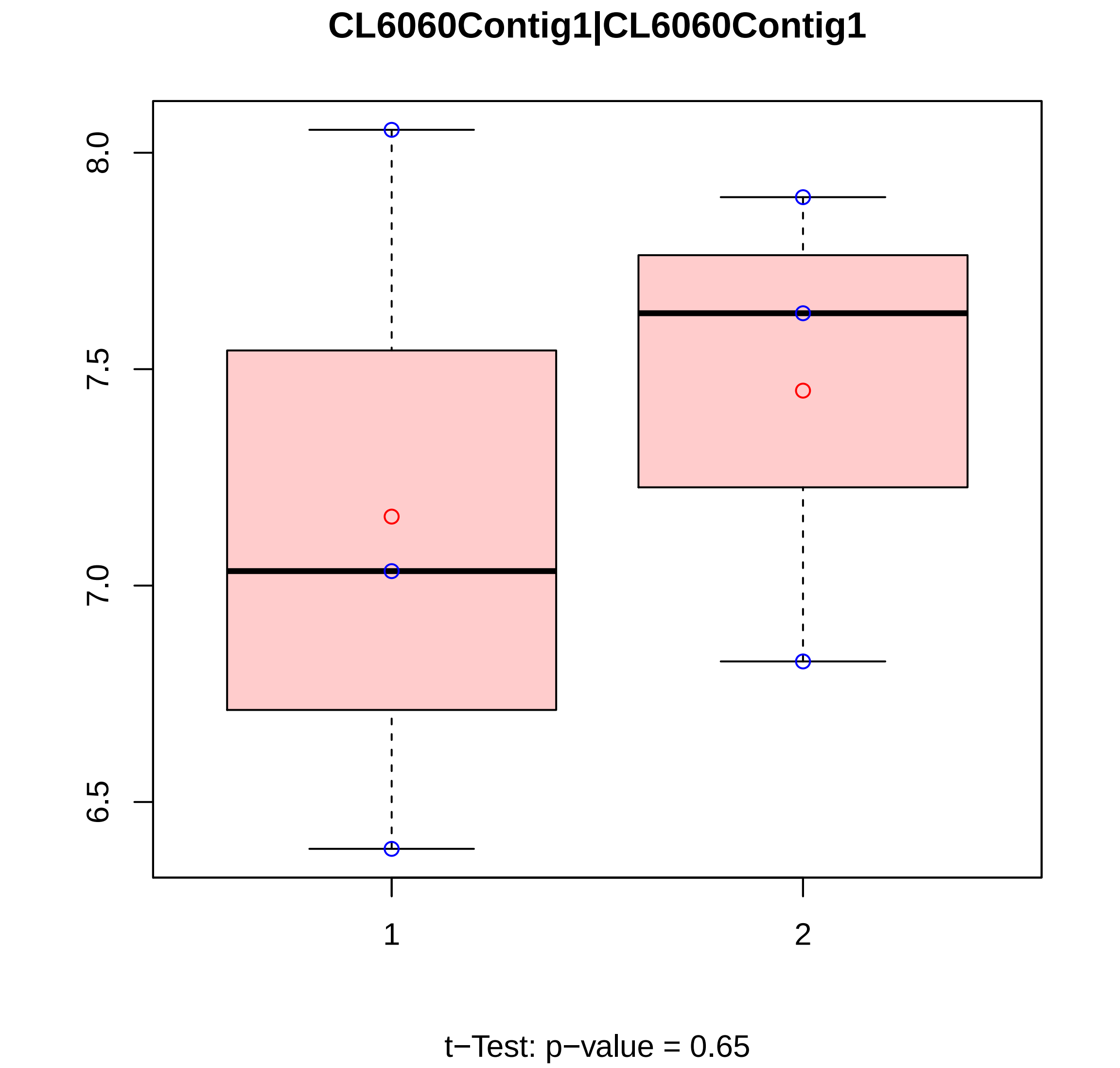
CL6060Contig1|CL6060Contig1
8.0
7.5
7.0
6.5
1
2
t−Test: p−value = 0.65

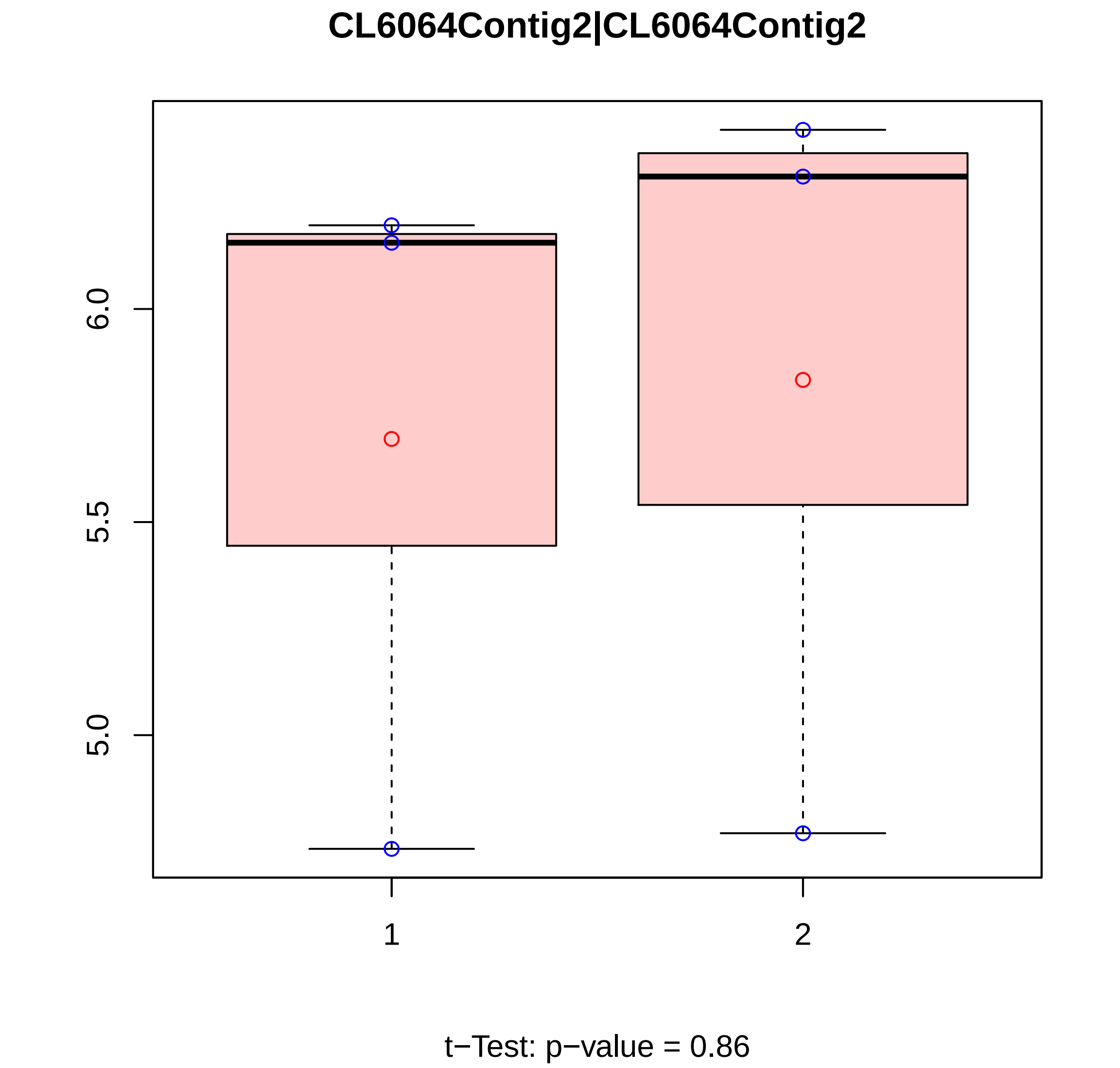

CL6064Contig2|CL6064Contig2
6.0
5.5
5.0
1
2
t−Test: p−value = 0.86

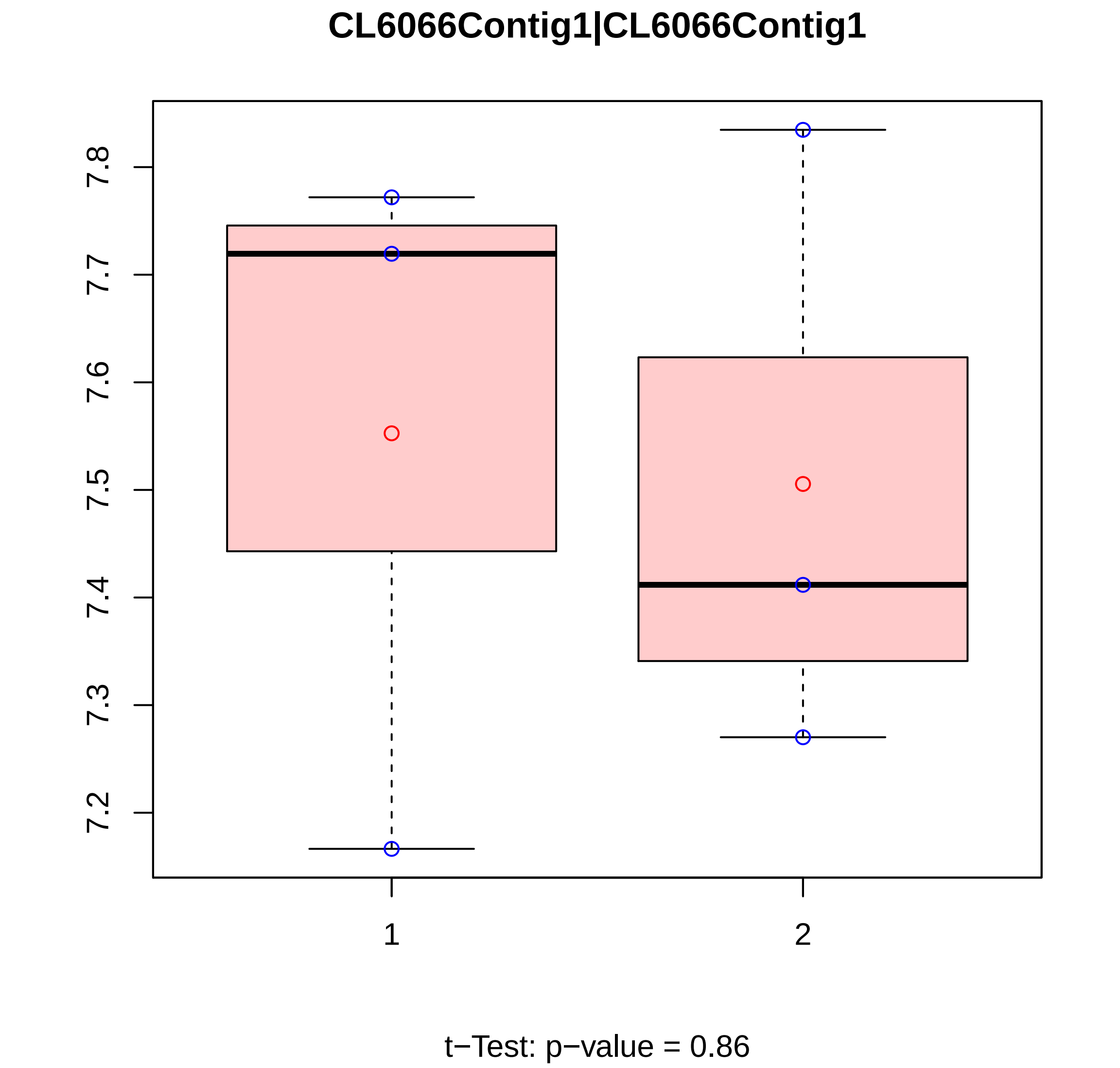

CL6066Contig1|CL6066Contig1
7.2
7.3
7.4
7.5
7.6
7.7
7.8
1
2
t−Test: p−value = 0.86

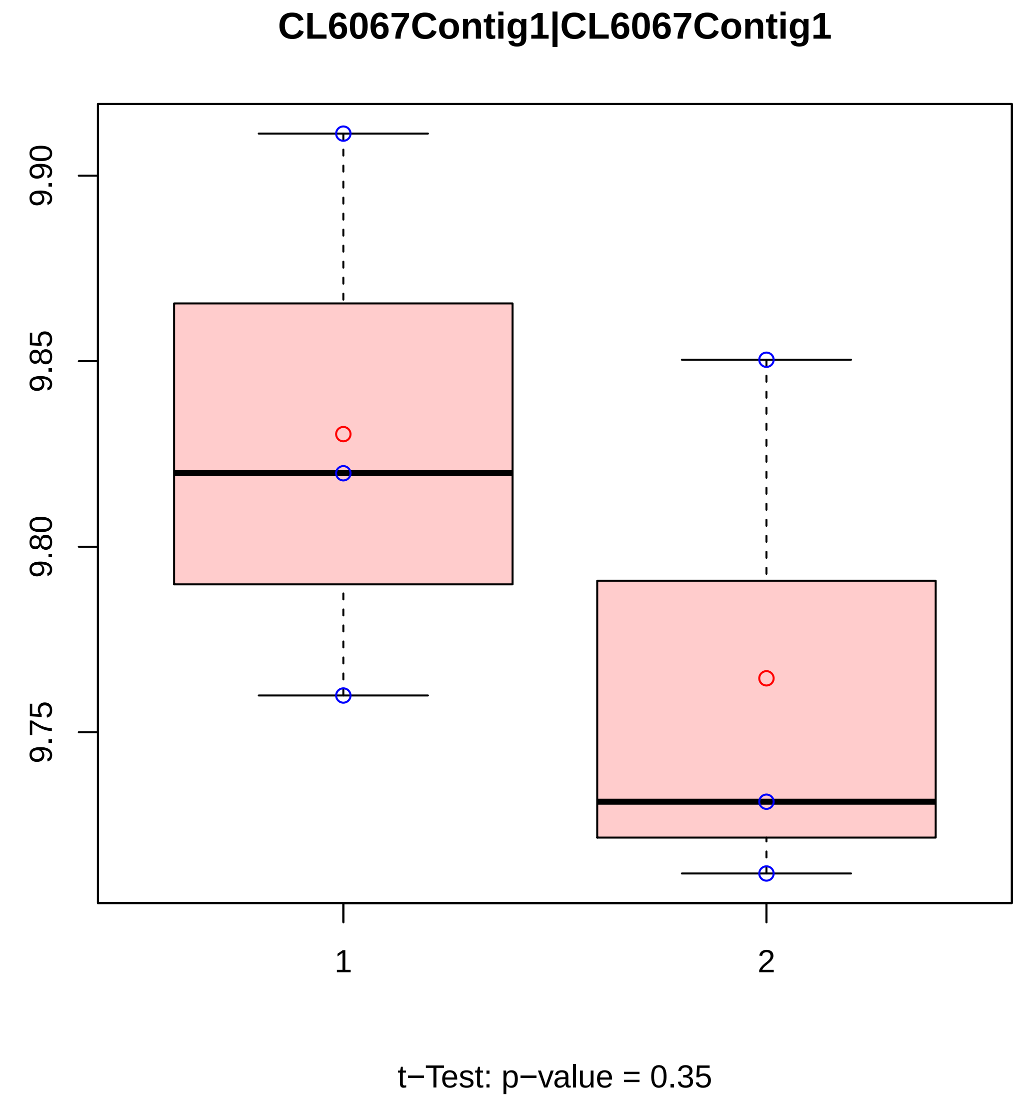
CL6067Contig1|CL6067Contig1
9.75
9.80
9.85
9.90
1
2
t−Test: p−value = 0.35

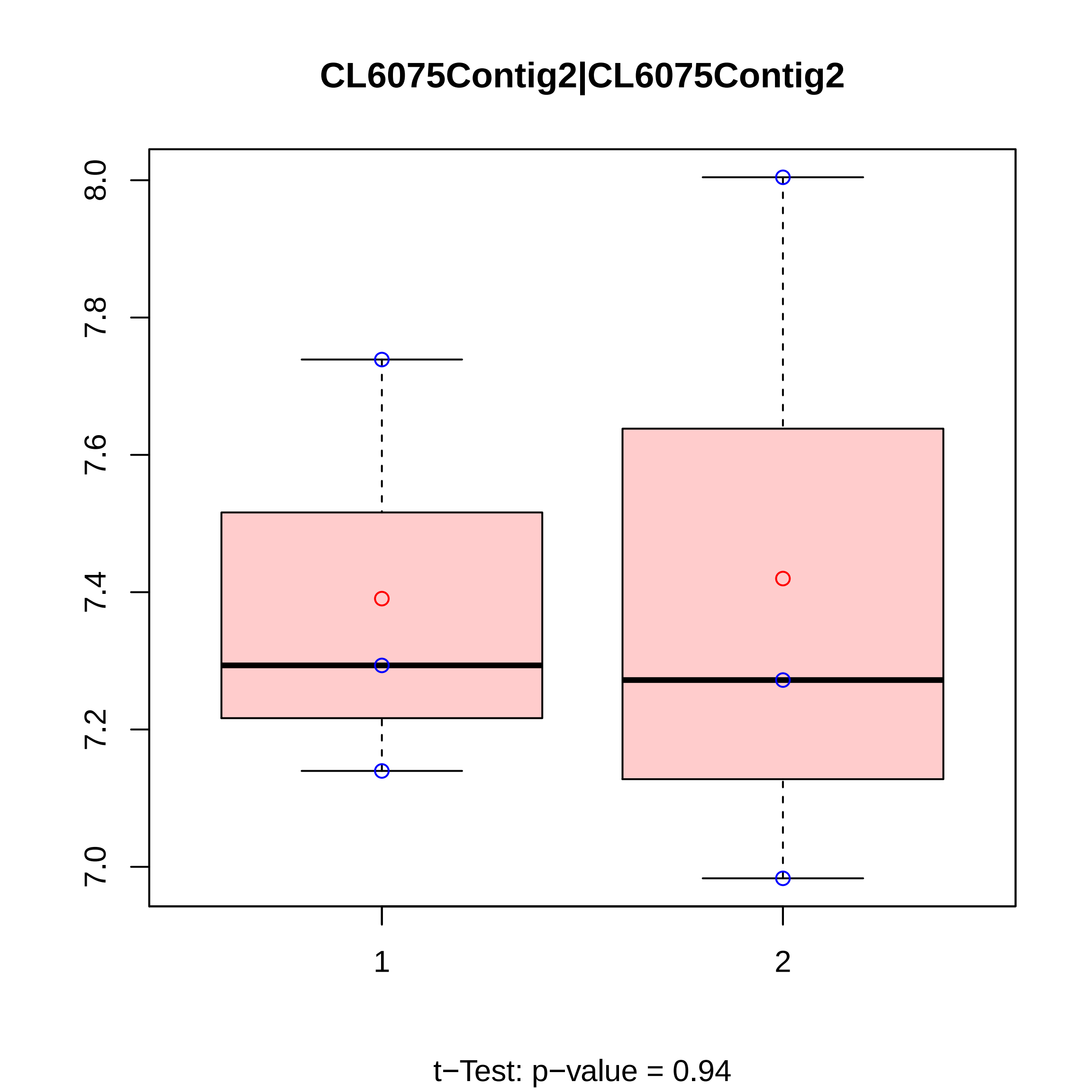

CL6075Contig2|CL6075Contig2
8.0
7.8
7.6
7.4
7.2
7.0
1
2
t−Test: p−value = 0.94

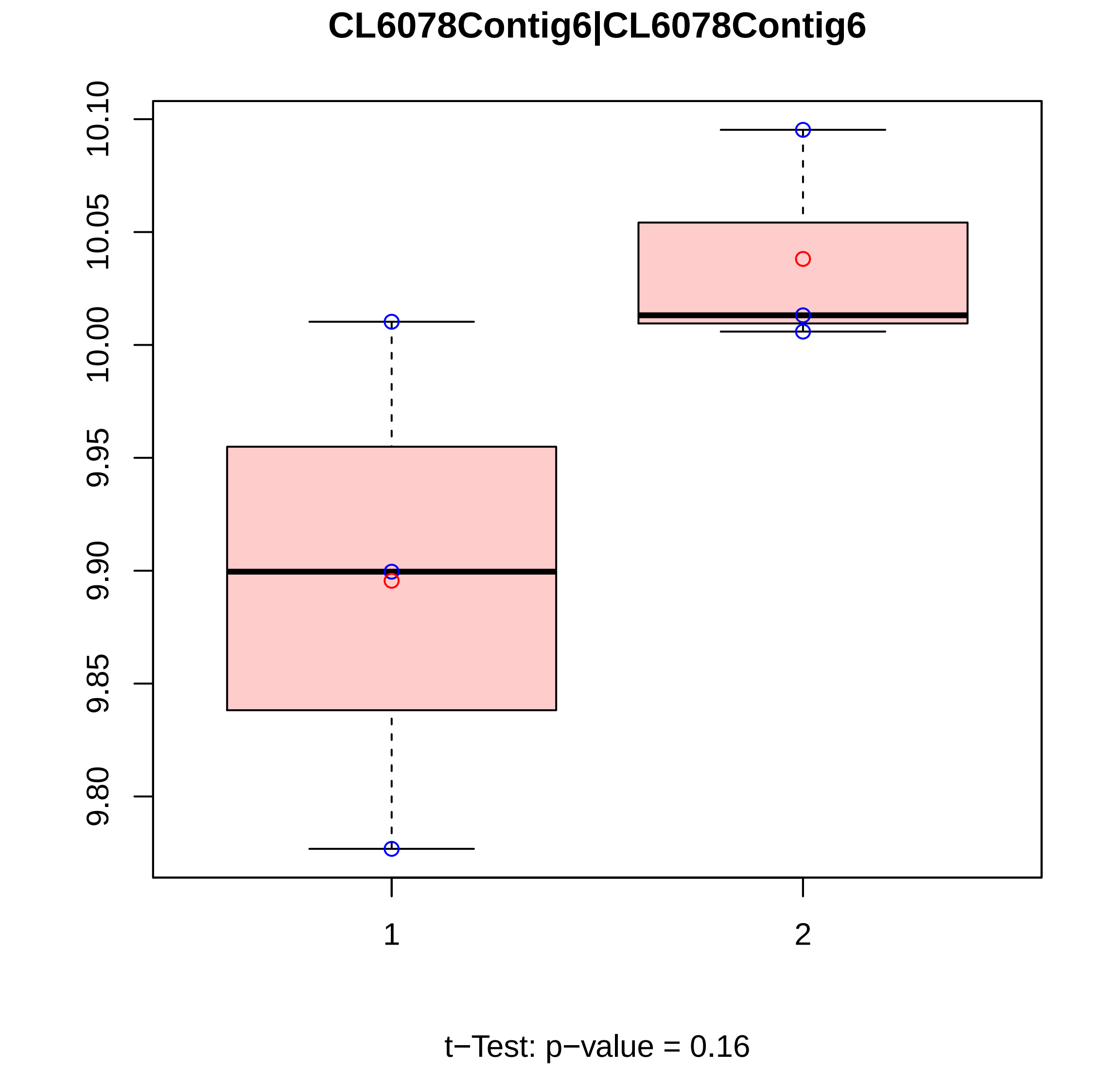
CL6078Contig6|CL6078Contig6
10.10
10.05
10.00
9.95
9.90
9.85
9.80
1
2
t−Test: p−value = 0.16

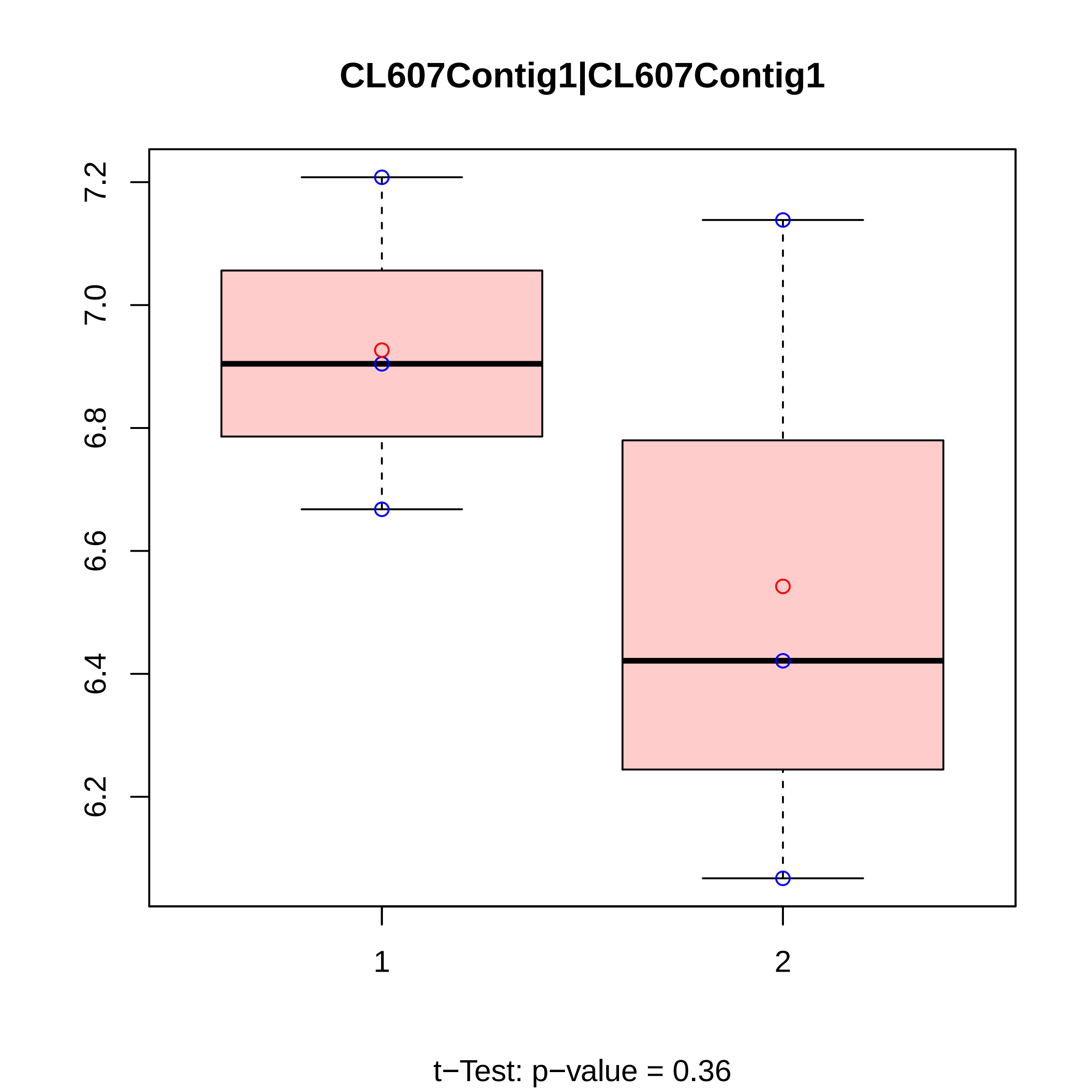

CL607Contig1|CL607Contig1
7.2
7.0
6.8
6.6
6.4
6.2
1
2
t−Test: p−value = 0.36

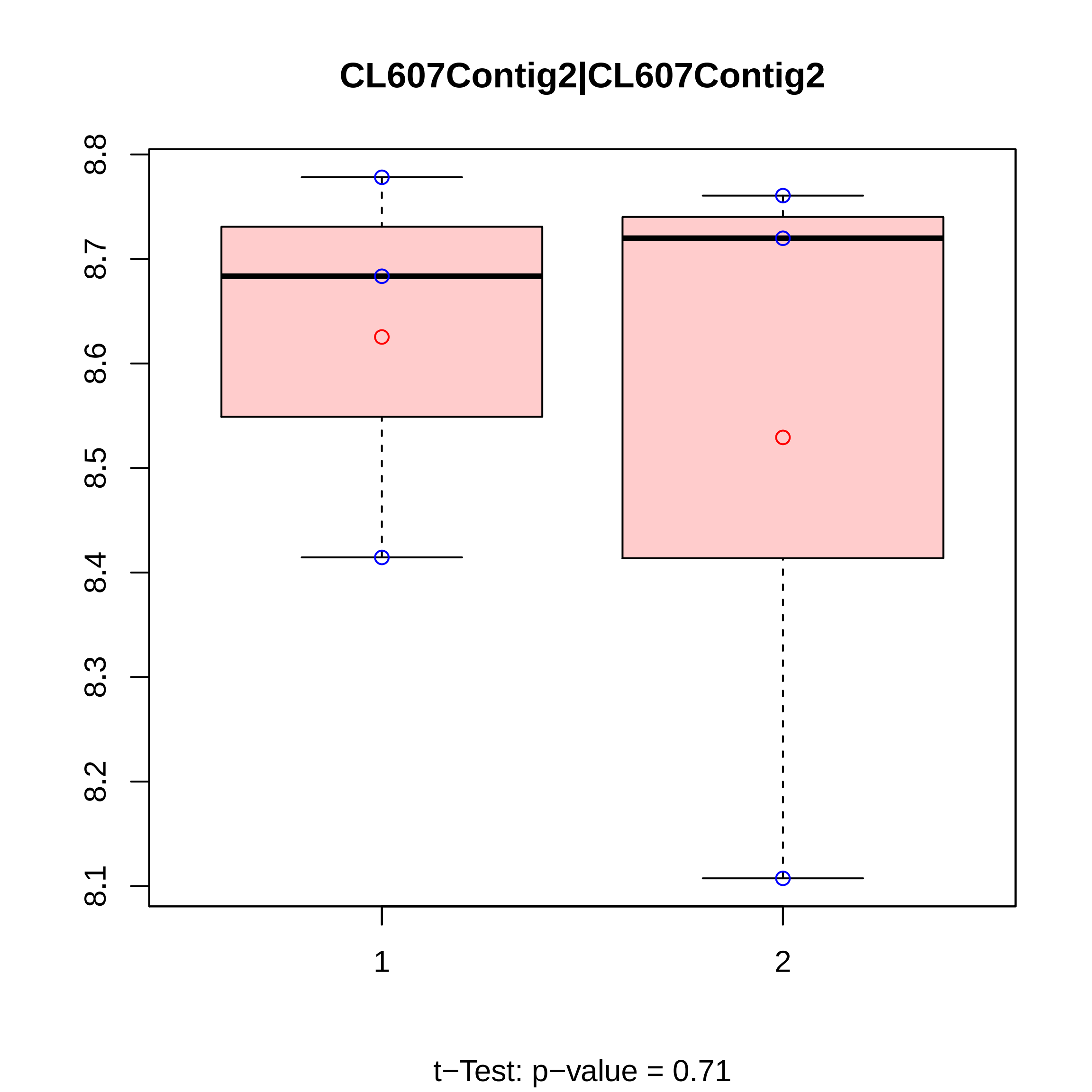

CL607Contig2|CL607Contig2
8.8
8.7
8.6
8.5
8.4
8.3
8.2
8.1
1
2
t−Test: p−value = 0.71

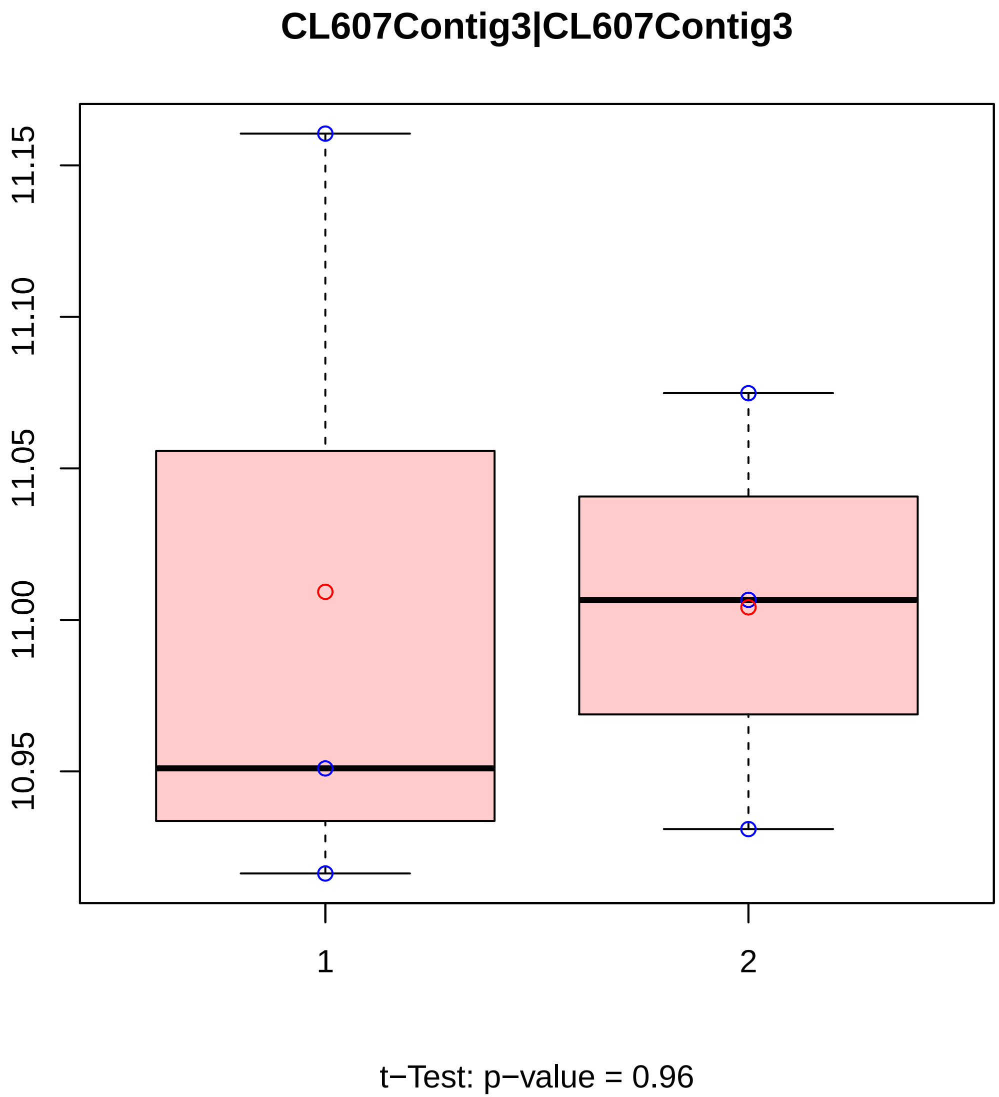

CL607Contig3|CL607Contig3
11.15
11.10
11.05
11.00
10.95
1
2
t−Test: p−value = 0.96

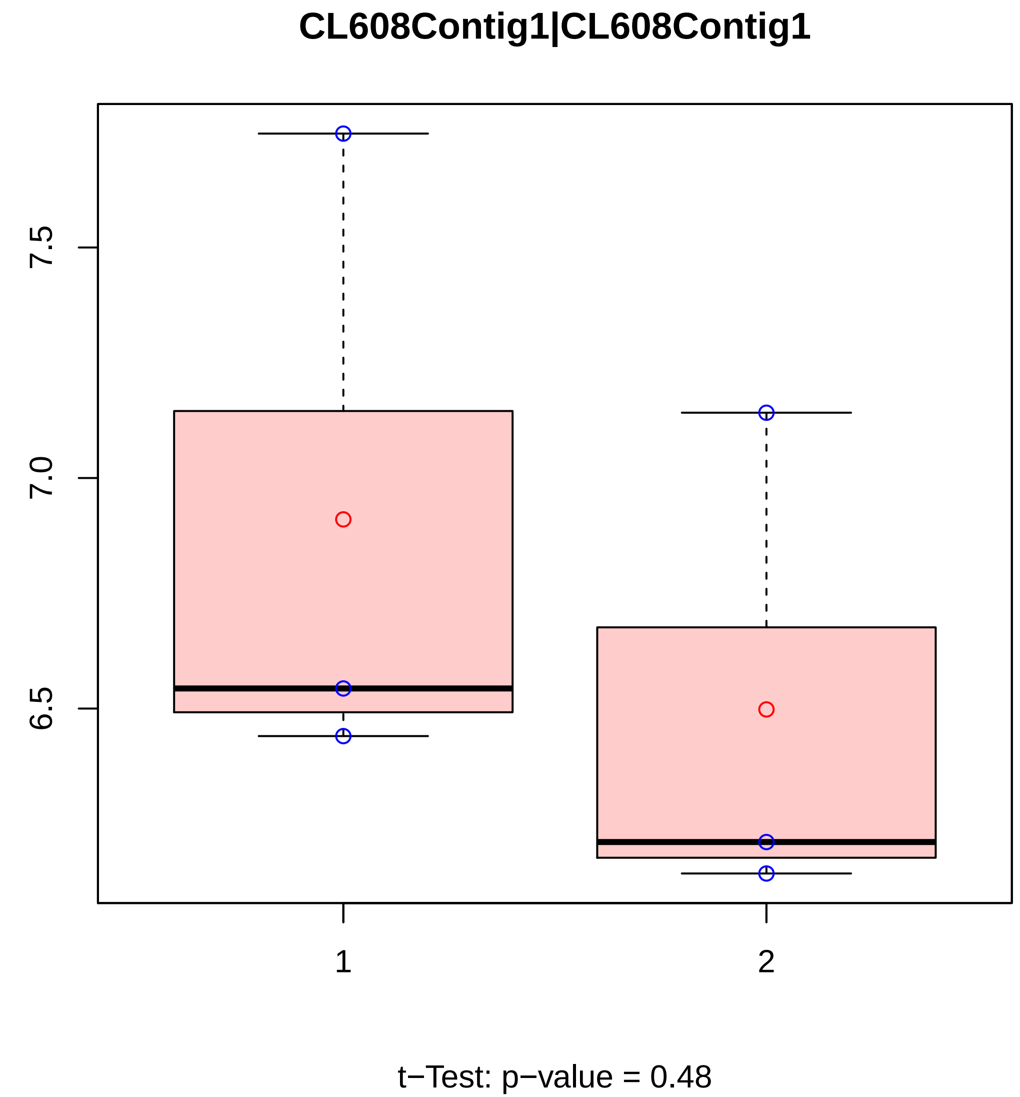
CL608Contig1|CL608Contig1
7.5
7.0
6.5
1
2
t−Test: p−value = 0.48

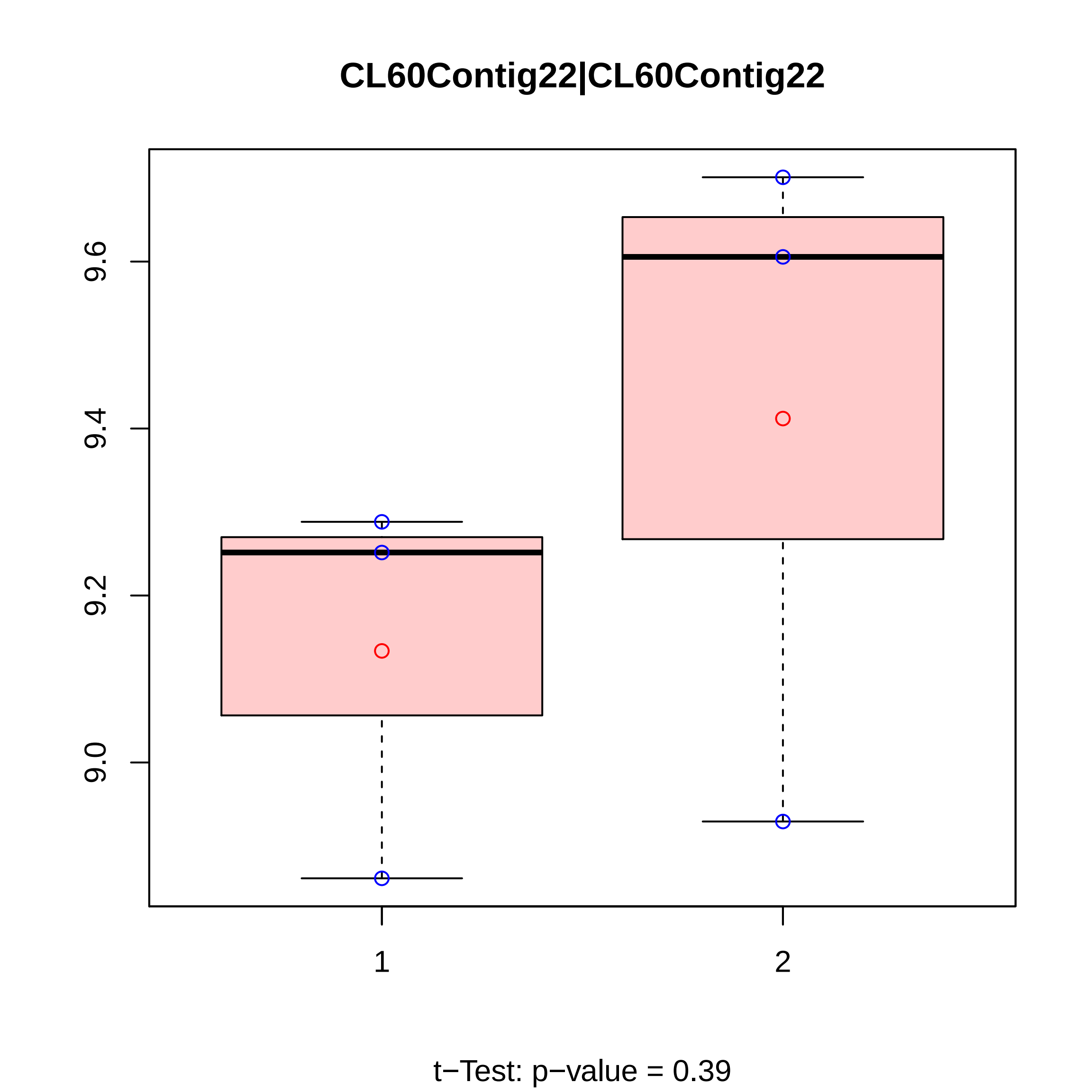

CL60Contig22|CL60Contig22
9.0
9.2
9.4
9.6
1
2
t−Test: p−value = 0.39

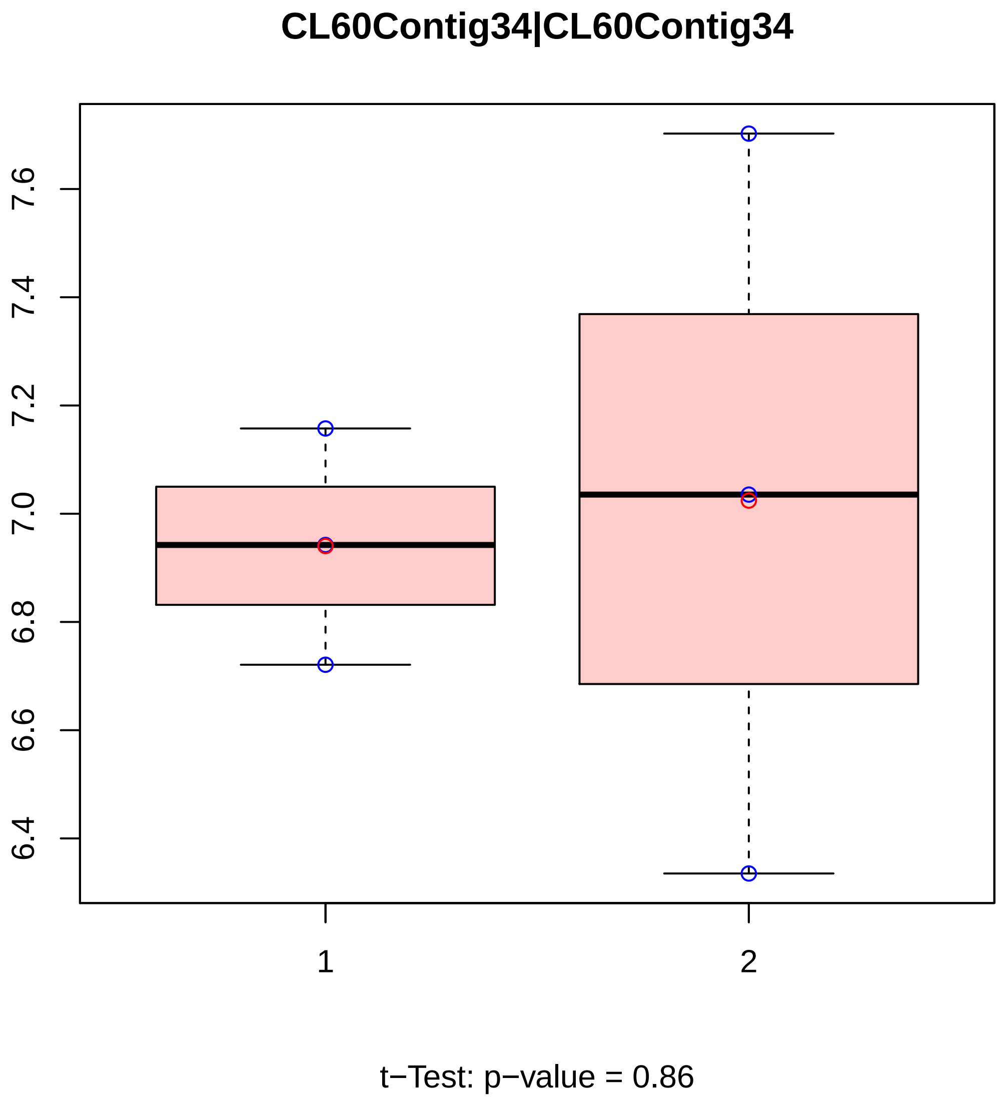
CL60Contig34|CL60Contig34
7.6
7.4
7.2
7.0
6.8
6.6
6.4
1
2
t−Test: p−value = 0.86

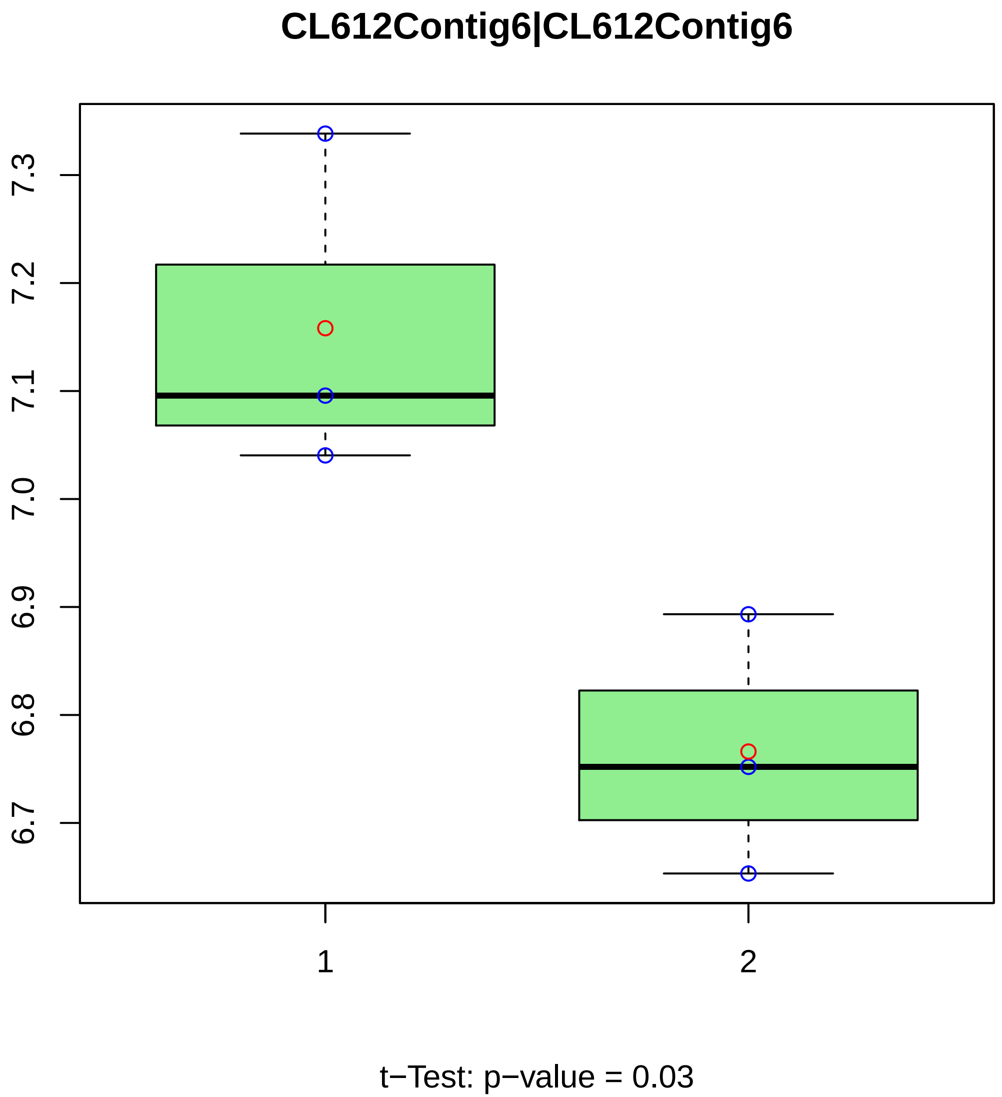
CL612Contig6|CL612Contig6
7.3
7.2
7.1
7.0
6.9
6.8
6.7
1
2
t−Test: p−value = 0.03

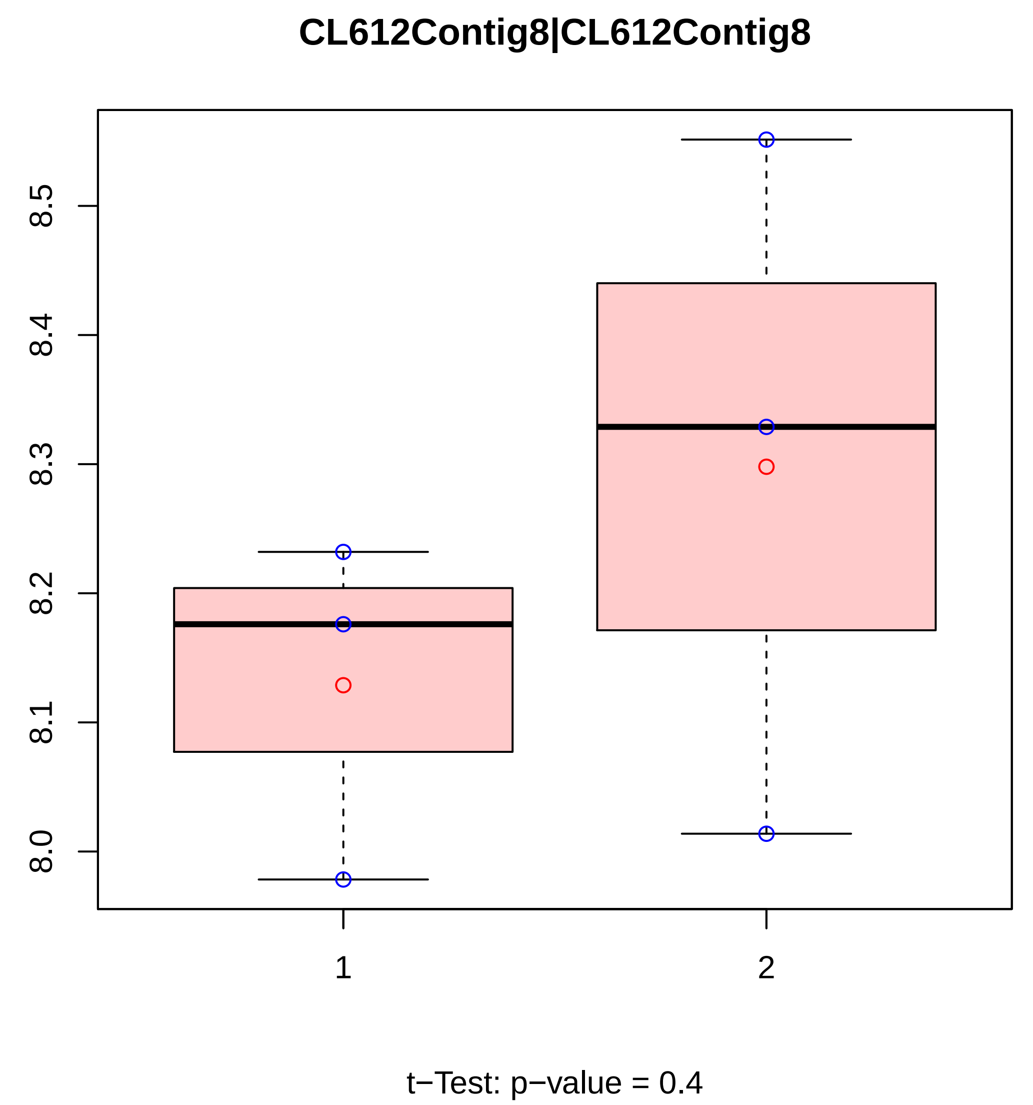

CL612Contig8|CL612Contig8
8.0
8.1
8.2
8.3
8.4
8.5
1
2
t−Test: p−value = 0.4

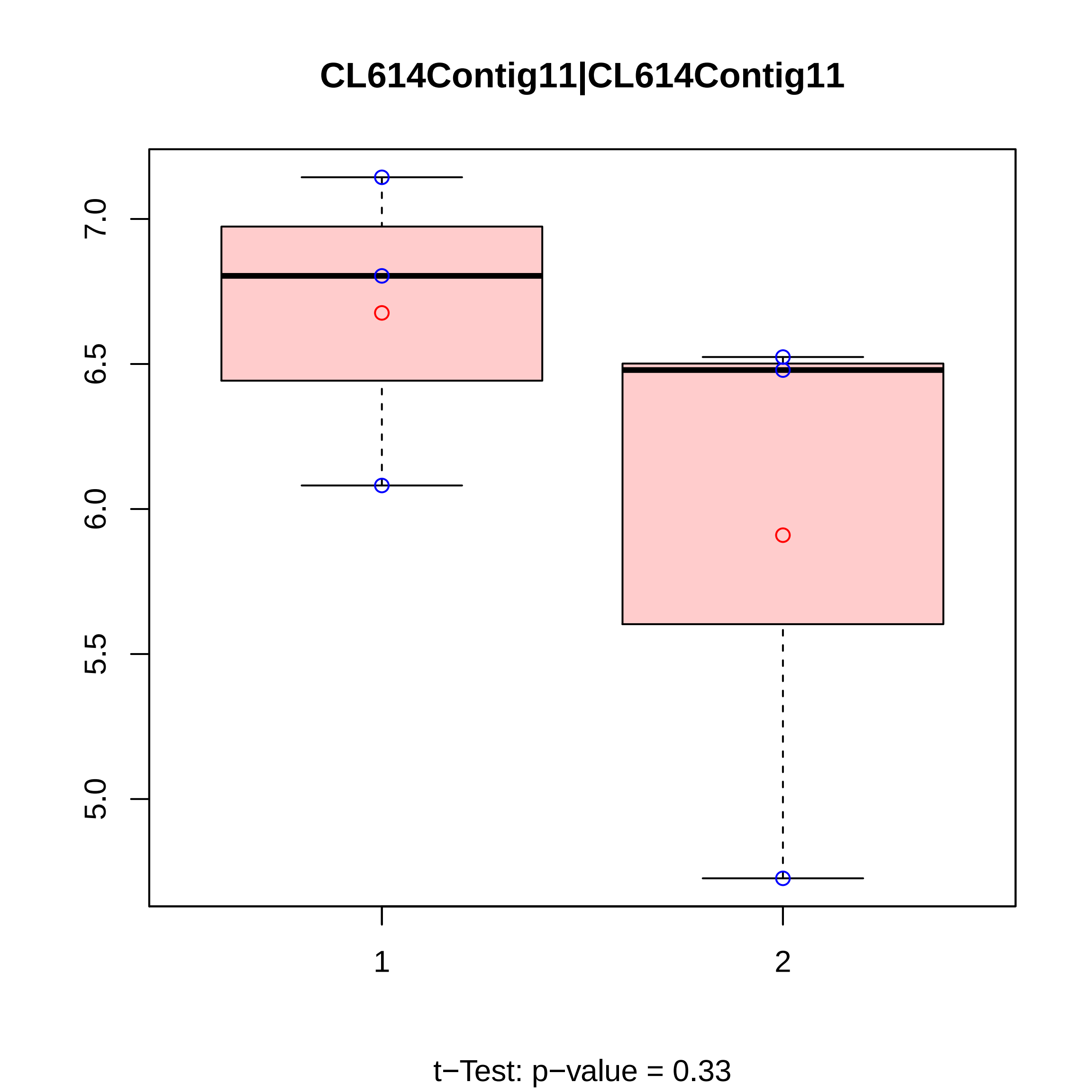
CL614Contig11|CL614Contig11
7.0
6.5
6.0
5.5
5.0
1
2
t−Test: p−value = 0.33

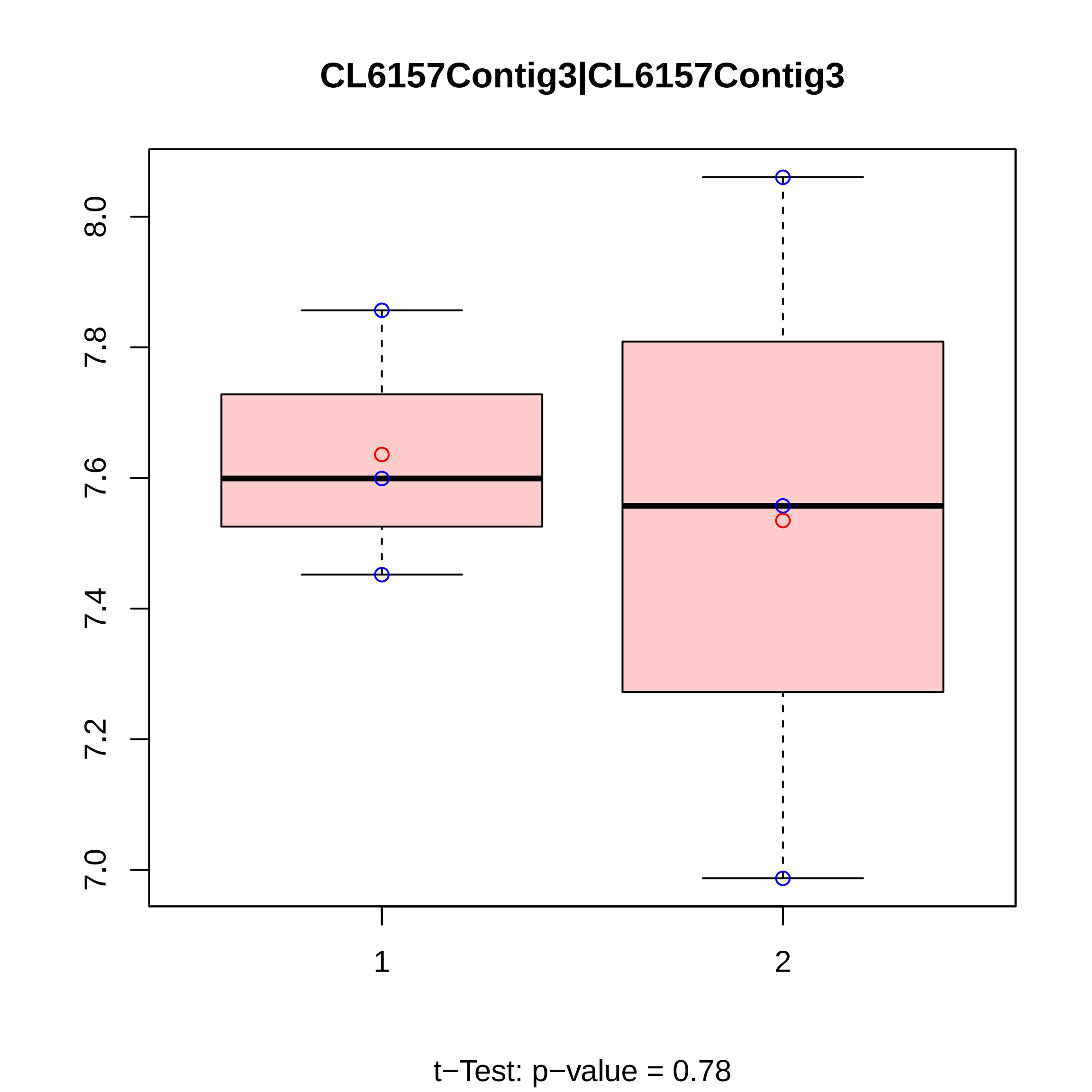
CL6157Contig3|CL6157Contig3
8.0
7.8
7.6
7.4
7.2
7.0
1
2
t−Test: p−value = 0.78

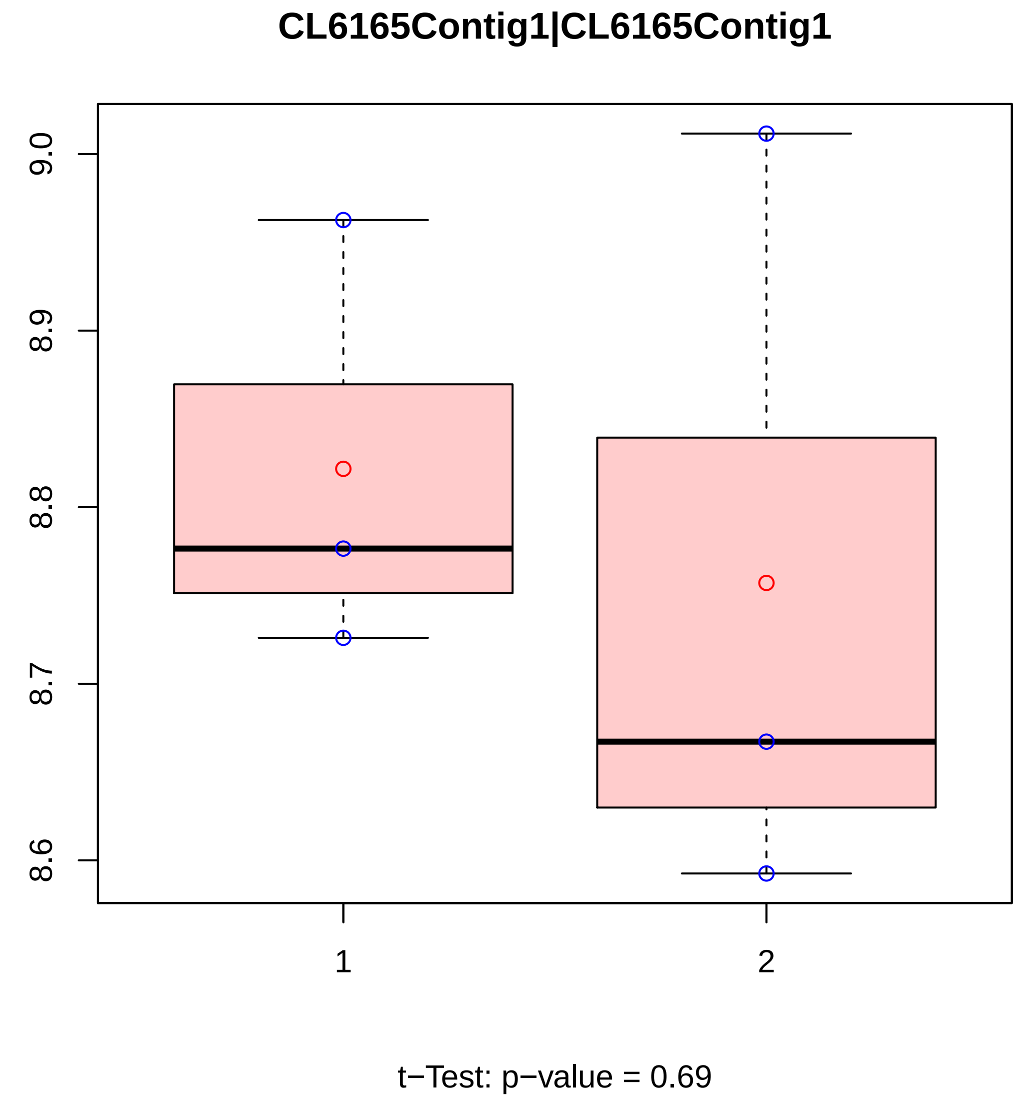
CL6165Contig1|CL6165Contig1
9.0
8.9
8.8
8.7
8.6
1
2
t−Test: p−value = 0.69

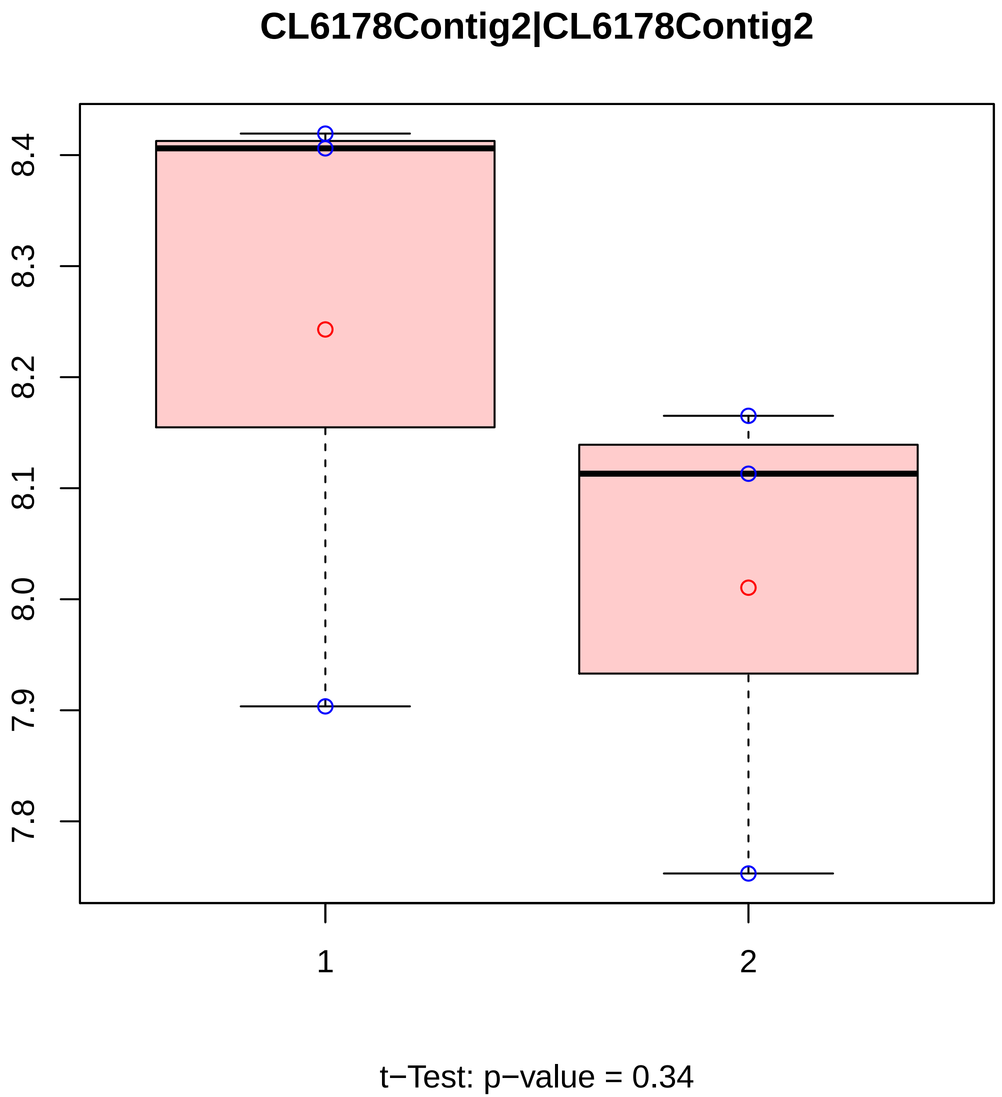

CL6178Contig2|CL6178Contig2
8.4
8.3
8.2
8.1
8.0
7.9
7.8
1
2
t−Test: p−value = 0.34

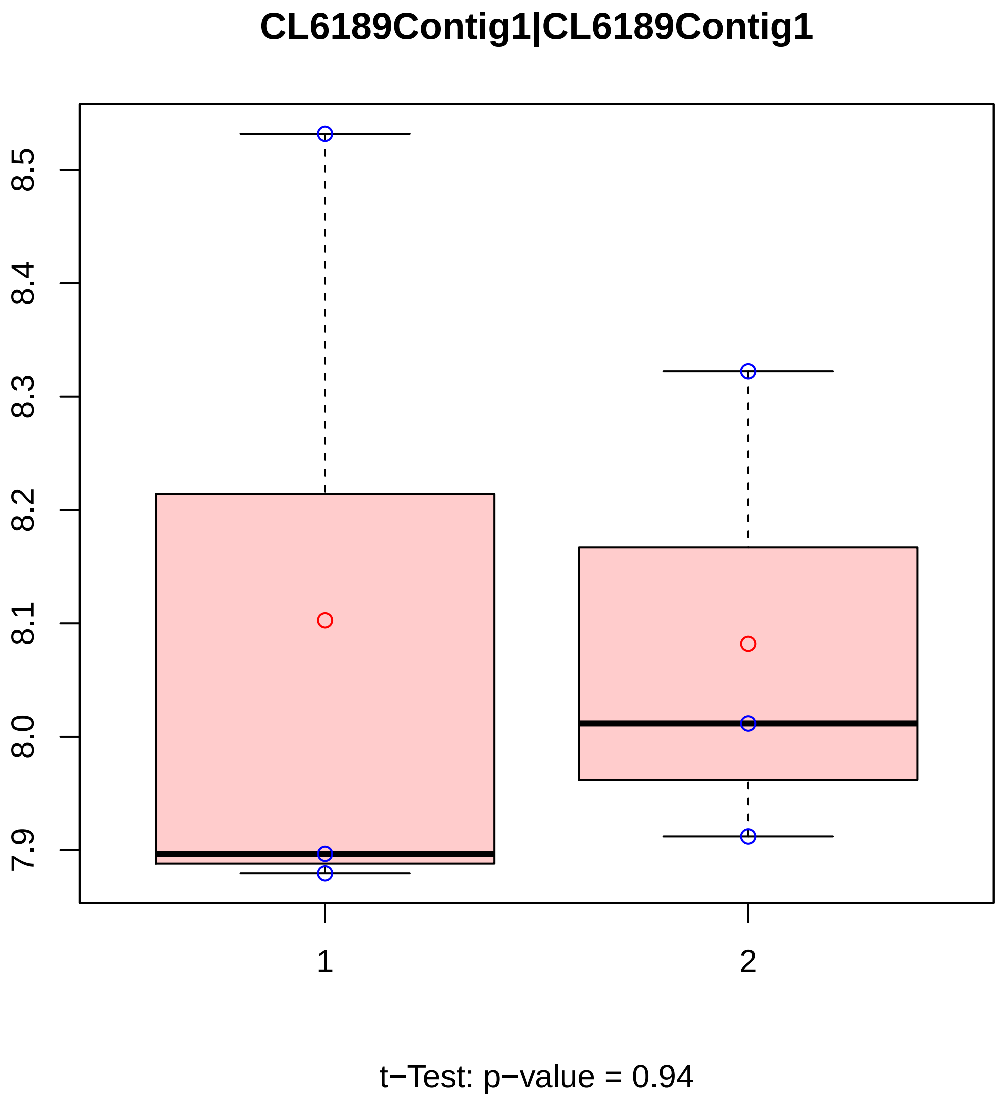

CL6189Contig1|CL6189Contig1
8.5
8.4
8.3
8.2
8.1
8.0
7.9
1
2
t−Test: p−value = 0.94

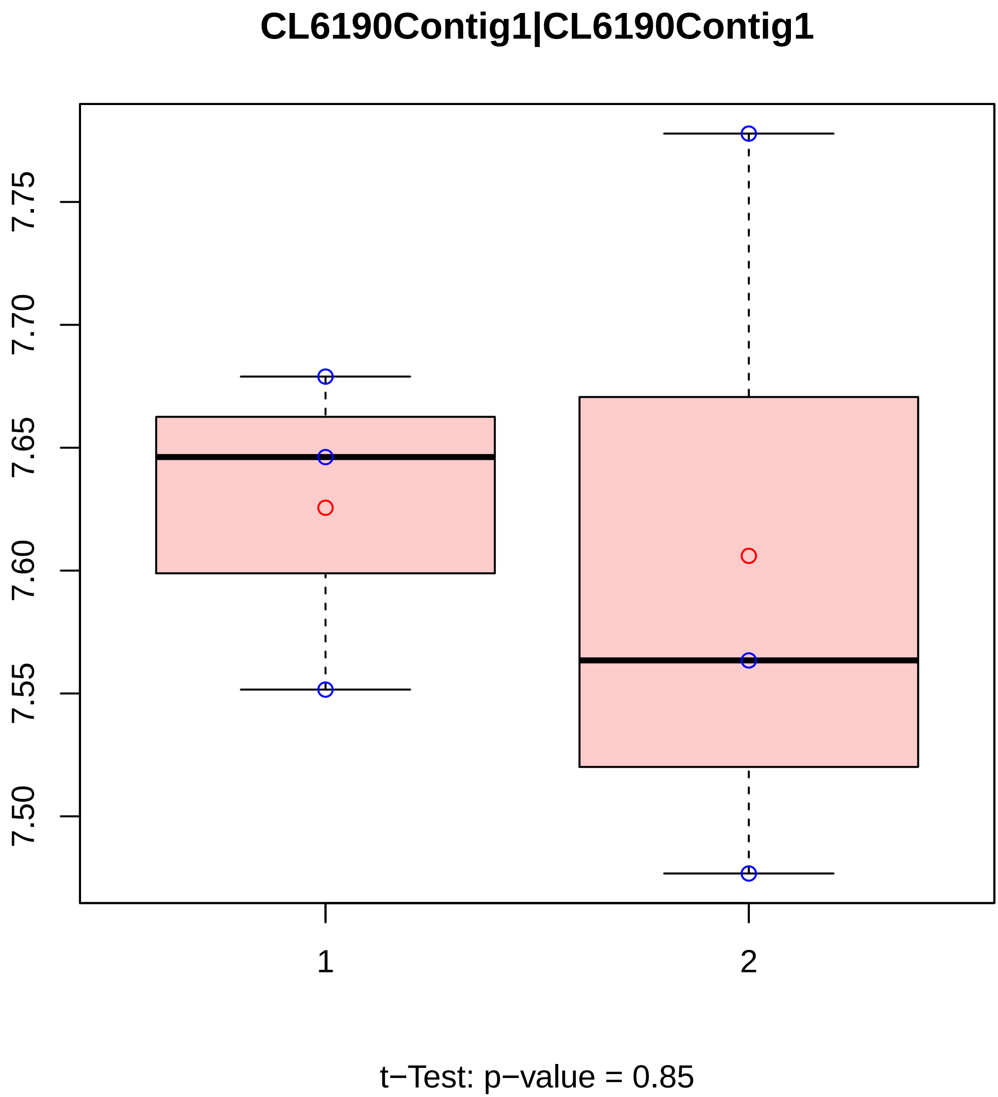
CL6190Contig1|CL6190Contig1
7.50
7.55
7.60
7.65
7.70
7.75
1
2
t−Test: p−value = 0.85

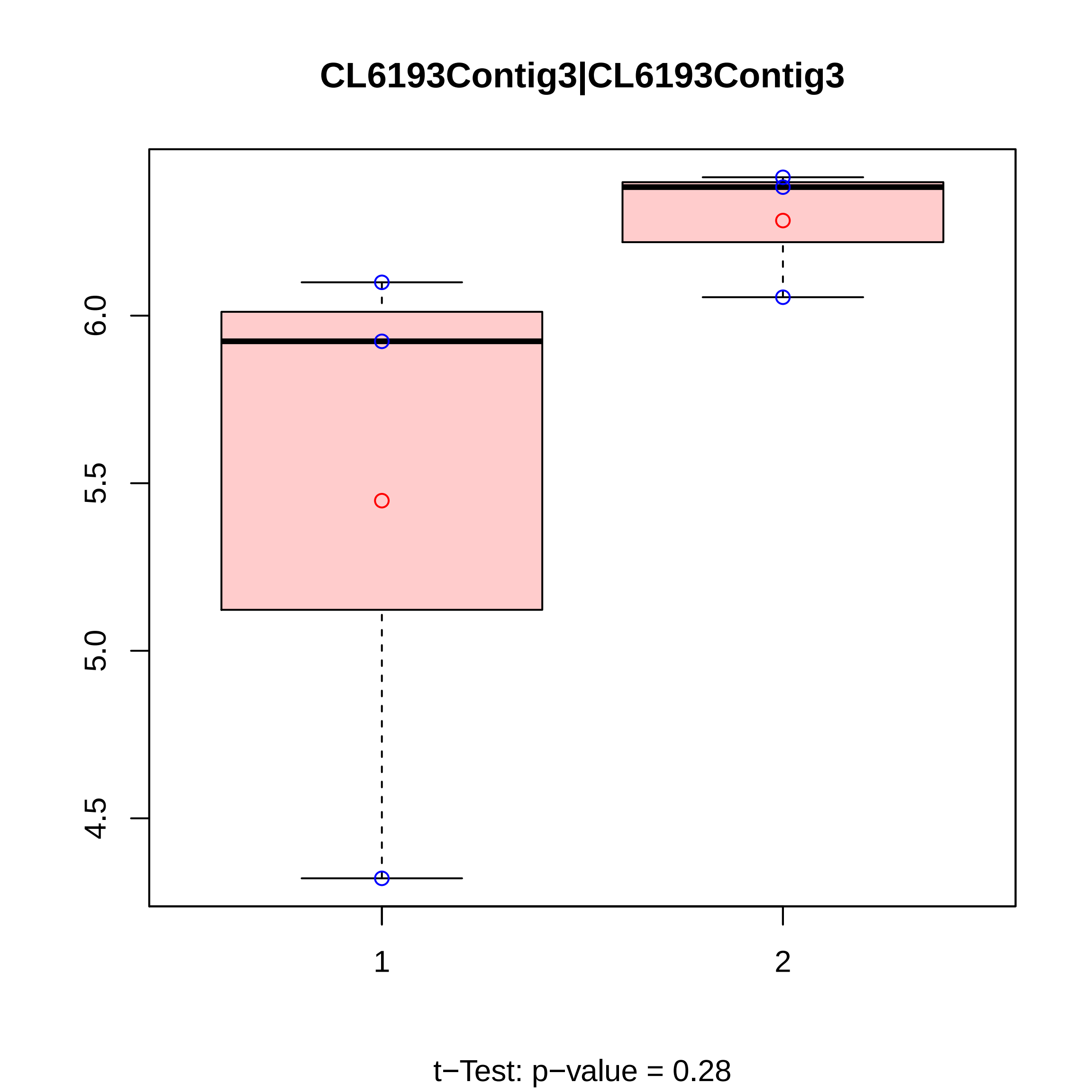

CL6193Contig3|CL6193Contig3
6.0
5.5
5.0
4.5
1
2
t−Test: p−value = 0.28

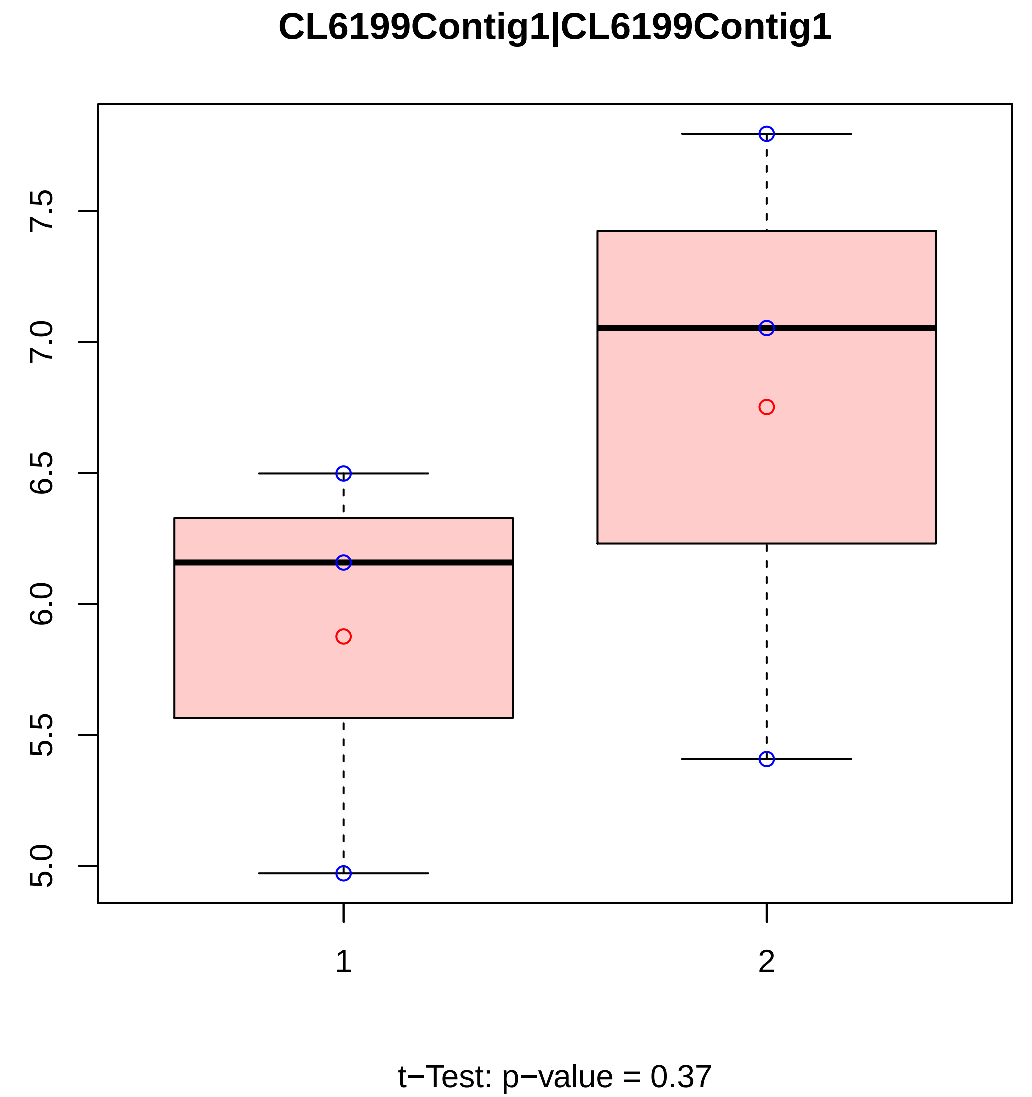
CL6199Contig1|CL6199Contig1
7.5
7.0
6.5
6.0
5.5
5.0
1
2
t−Test: p−value = 0.37

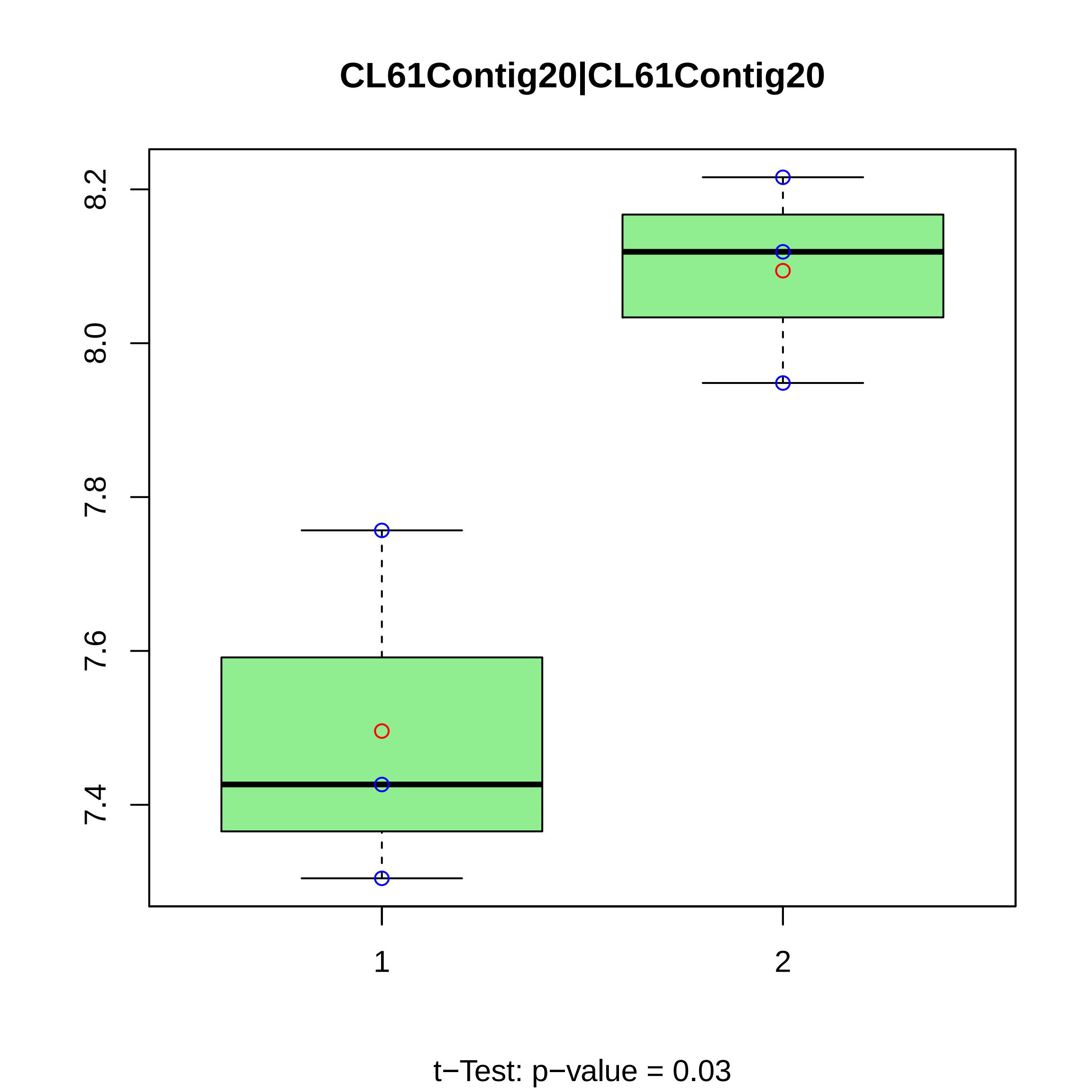

CL61Contig20|CL61Contig20
8.2
8.0
7.8
7.6
7.4
1
2
t−Test: p−value = 0.03

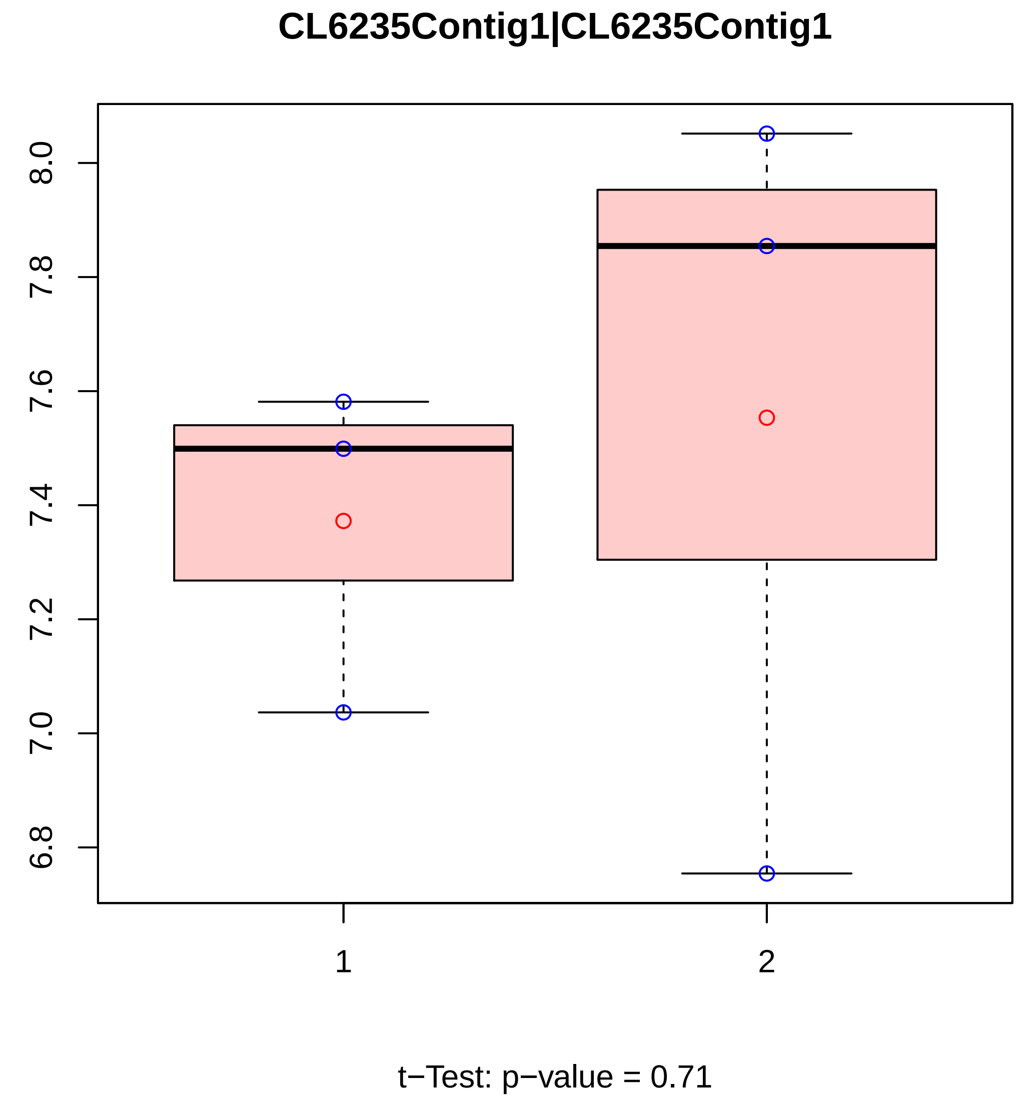

CL6235Contig1|CL6235Contig1
8.0
7.8
7.6
7.4
7.2
7.0
6.8
1
2
t−Test: p−value = 0.71

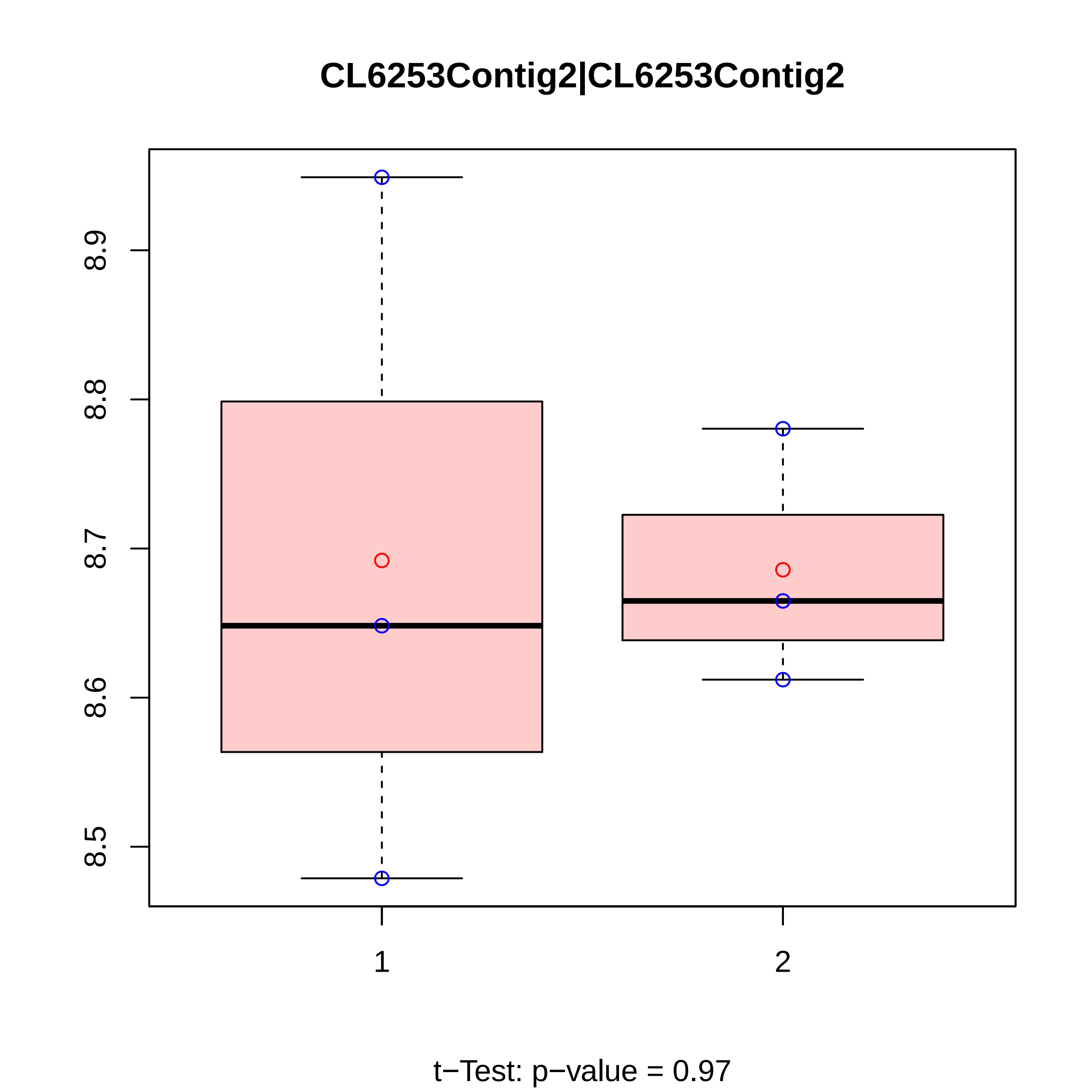

CL6253Contig2|CL6253Contig2
8.5
8.6
8.7
8.8
8.9
1
2
t−Test: p−value = 0.97

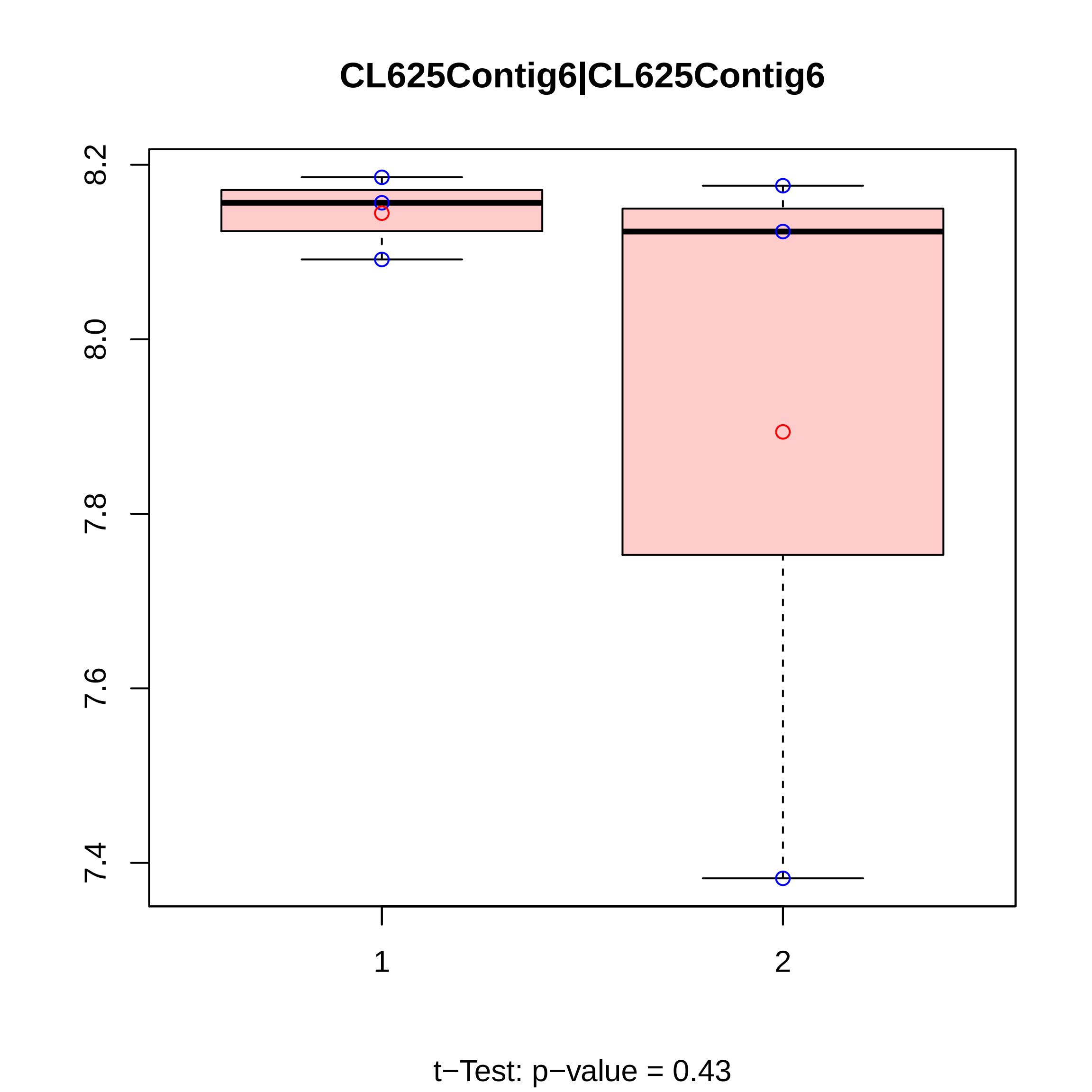
CL625Contig6|CL625Contig6
8.2
8.0
7.8
7.6
7.4
1
2
t−Test: p−value = 0.43

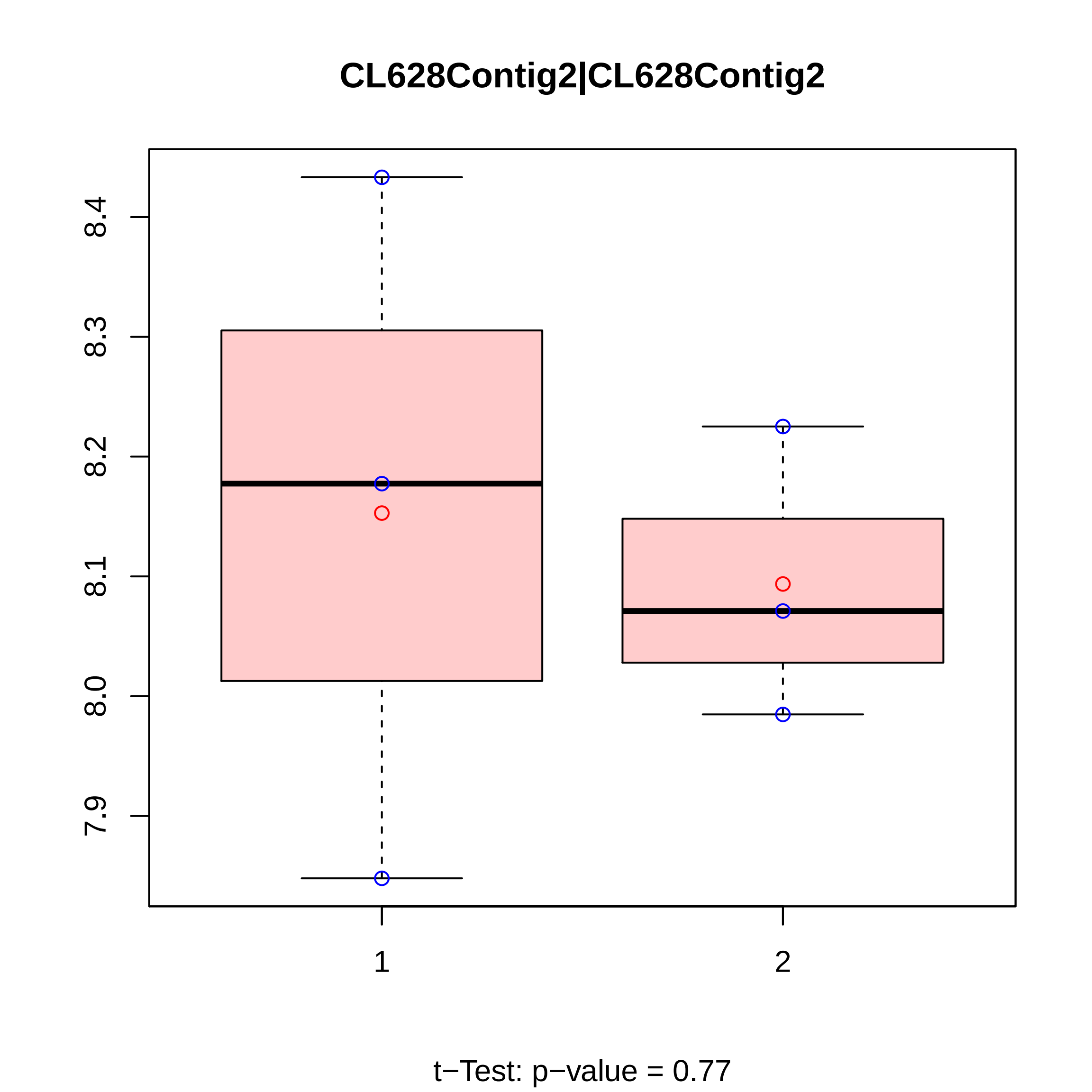

CL628Contig2|CL628Contig2
8.4
8.3
8.2
8.1
8.0
7.9
1
2
t−Test: p−value = 0.77

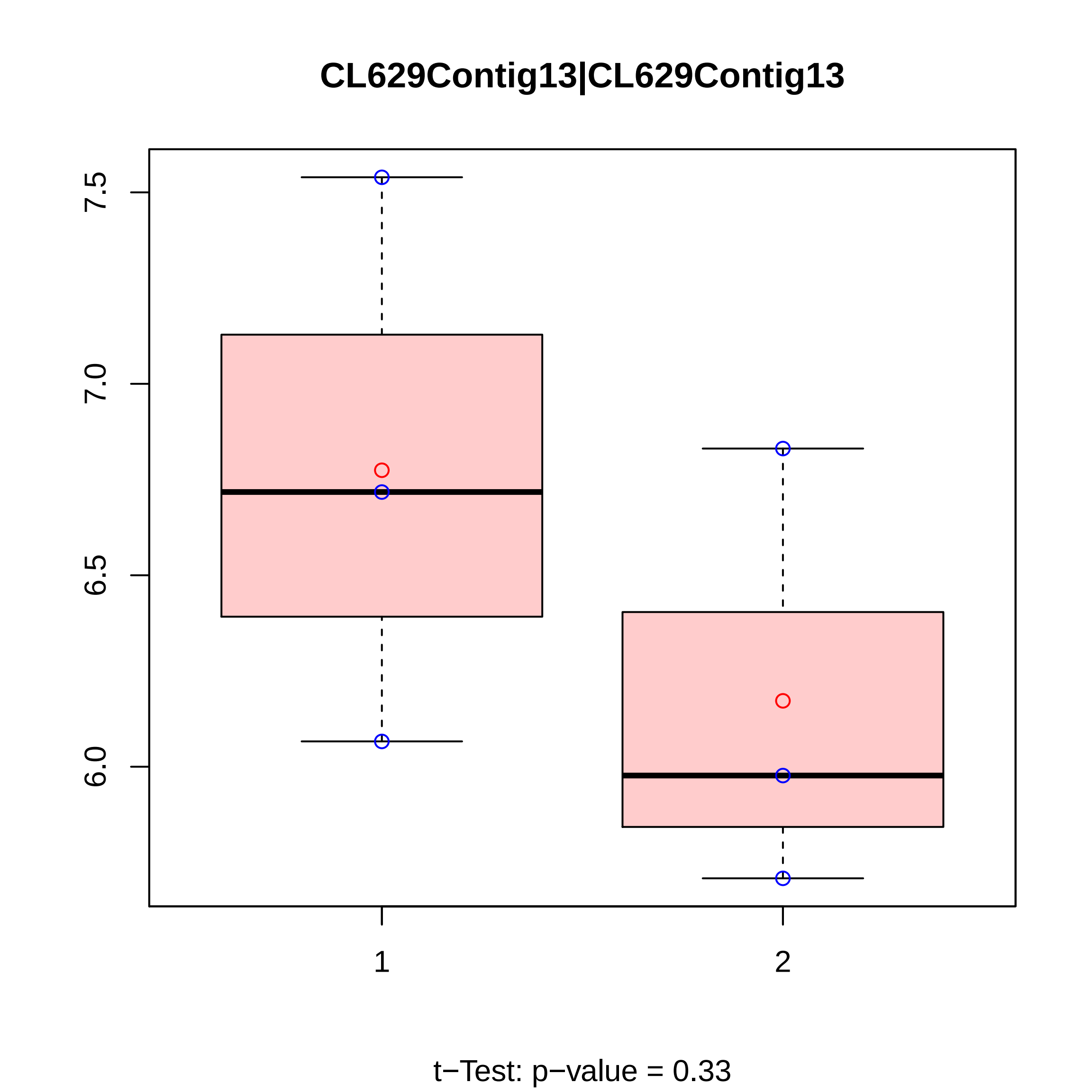
CL629Contig13|CL629Contig13
7.5
7.0
6.5
6.0
1
2
t−Test: p−value = 0.33

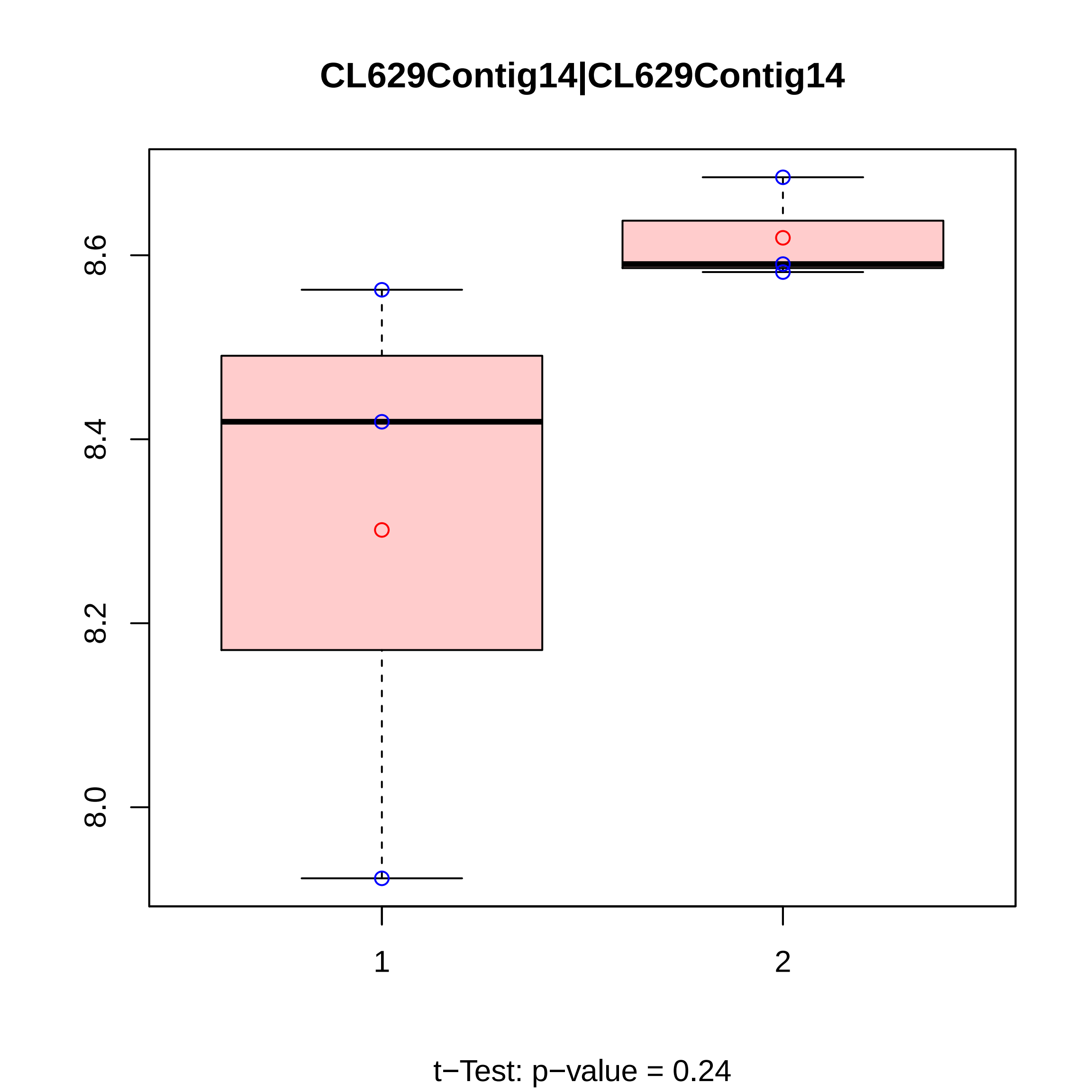
CL629Contig14|CL629Contig14
8.0
8.2
8.4
8.6
1
2
t−Test: p−value = 0.24

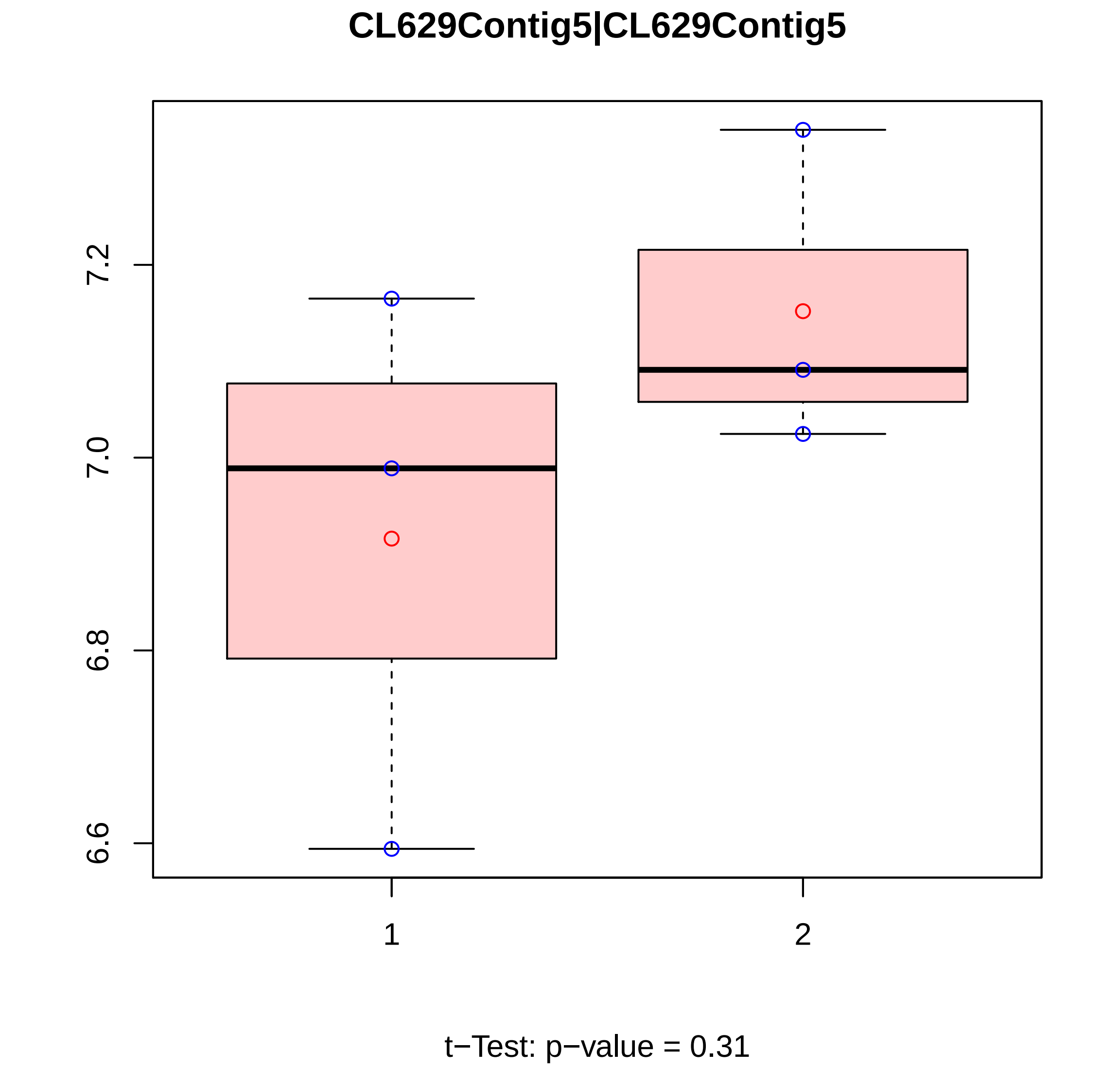
CL629Contig5|CL629Contig5
7.2
7.0
6.8
6.6
1
2
t−Test: p−value = 0.31

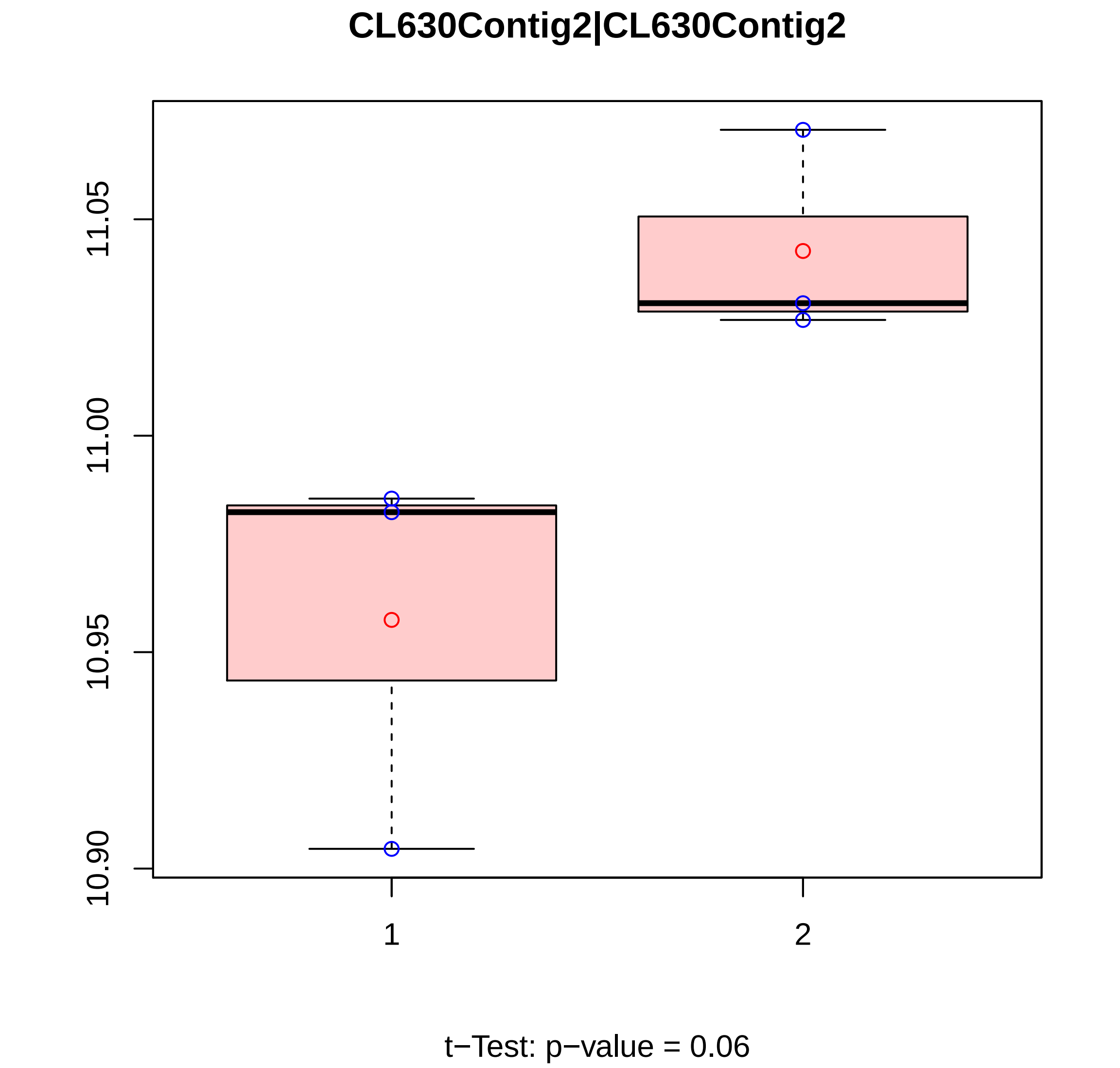
CL630Contig2|CL630Contig2
11.05
11.00
10.95
10.90
1
2
t−Test: p−value = 0.06

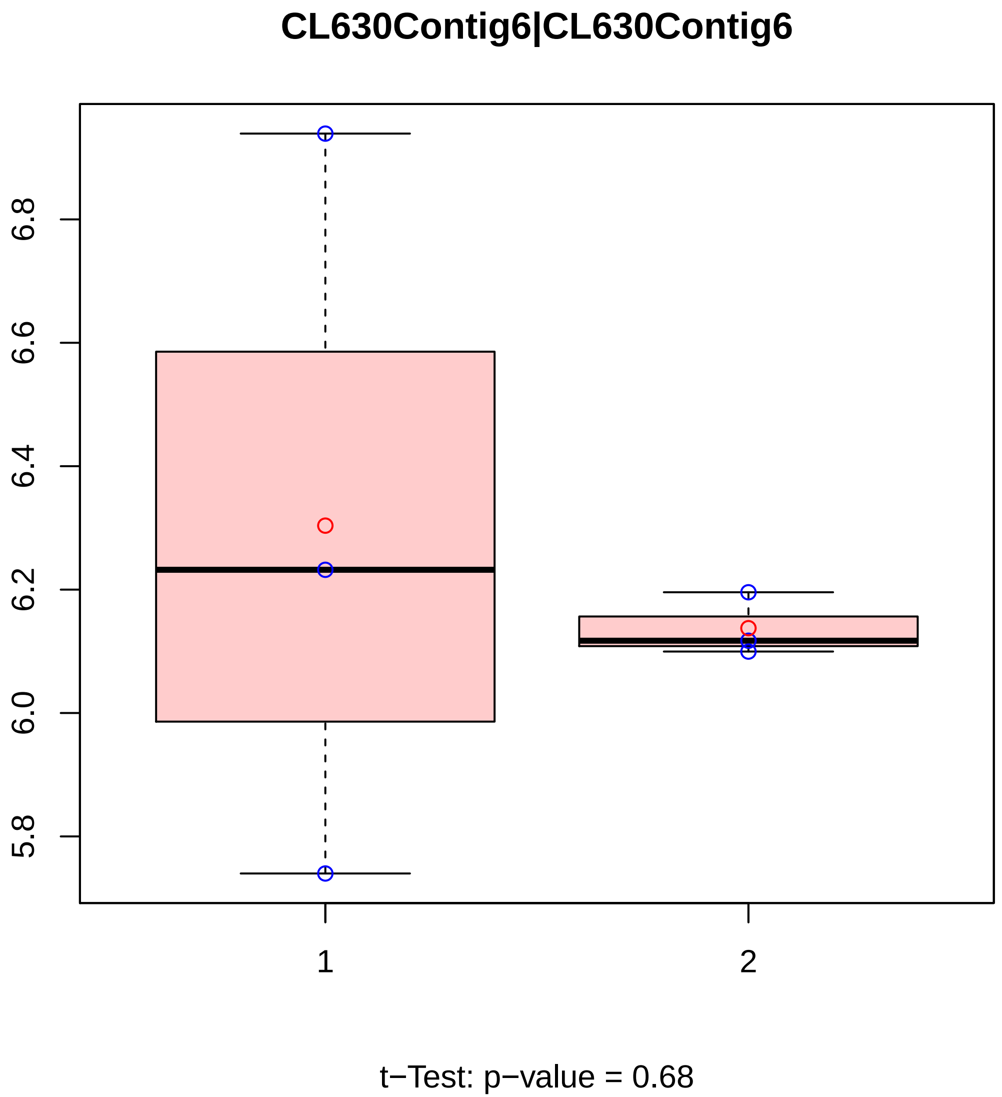
CL630Contig6|CL630Contig6
6.8
6.6
6.4
6.2
6.0
5.8
1
2
t−Test: p−value = 0.68

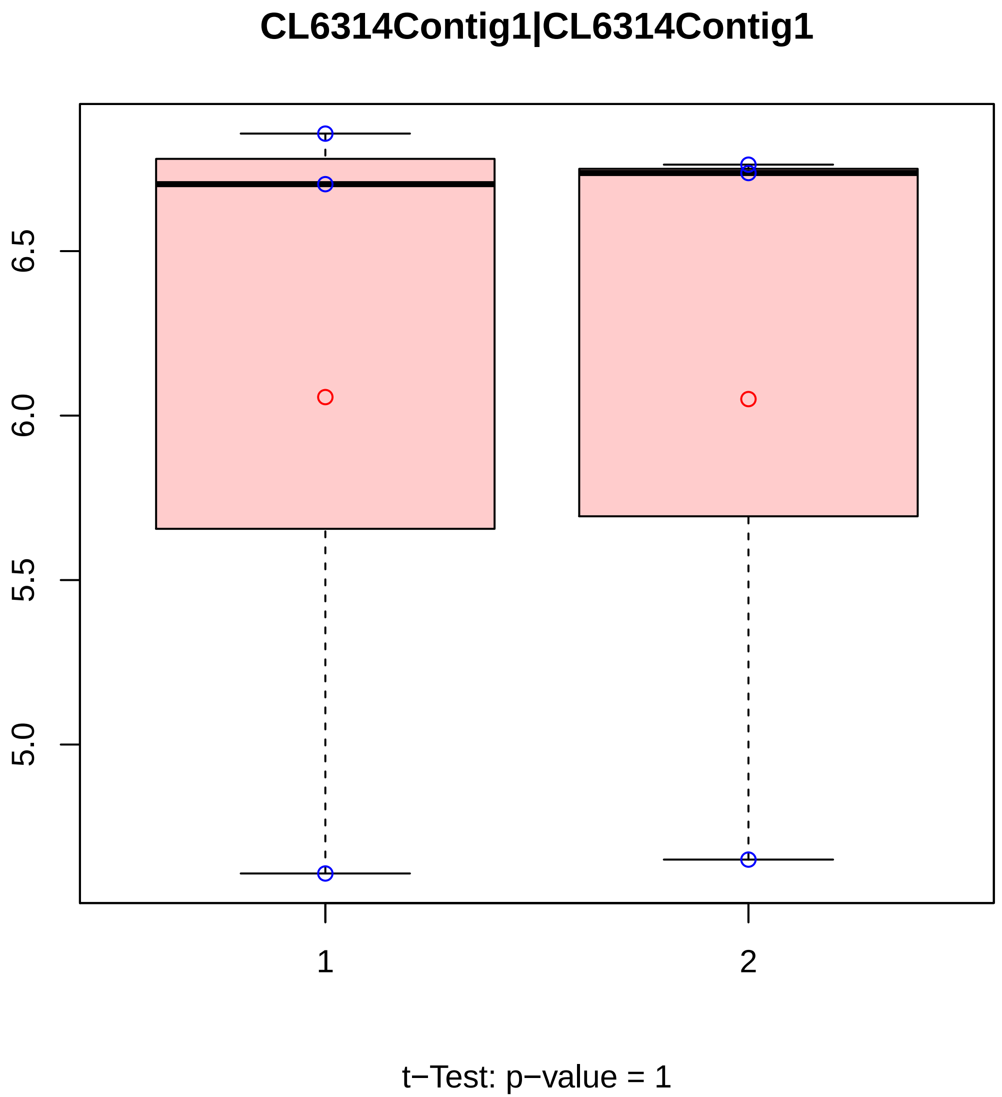
CL6314Contig1|CL6314Contig1
5.0
5.5
6.0
6.5
1
2
t−Test: p−value = 1

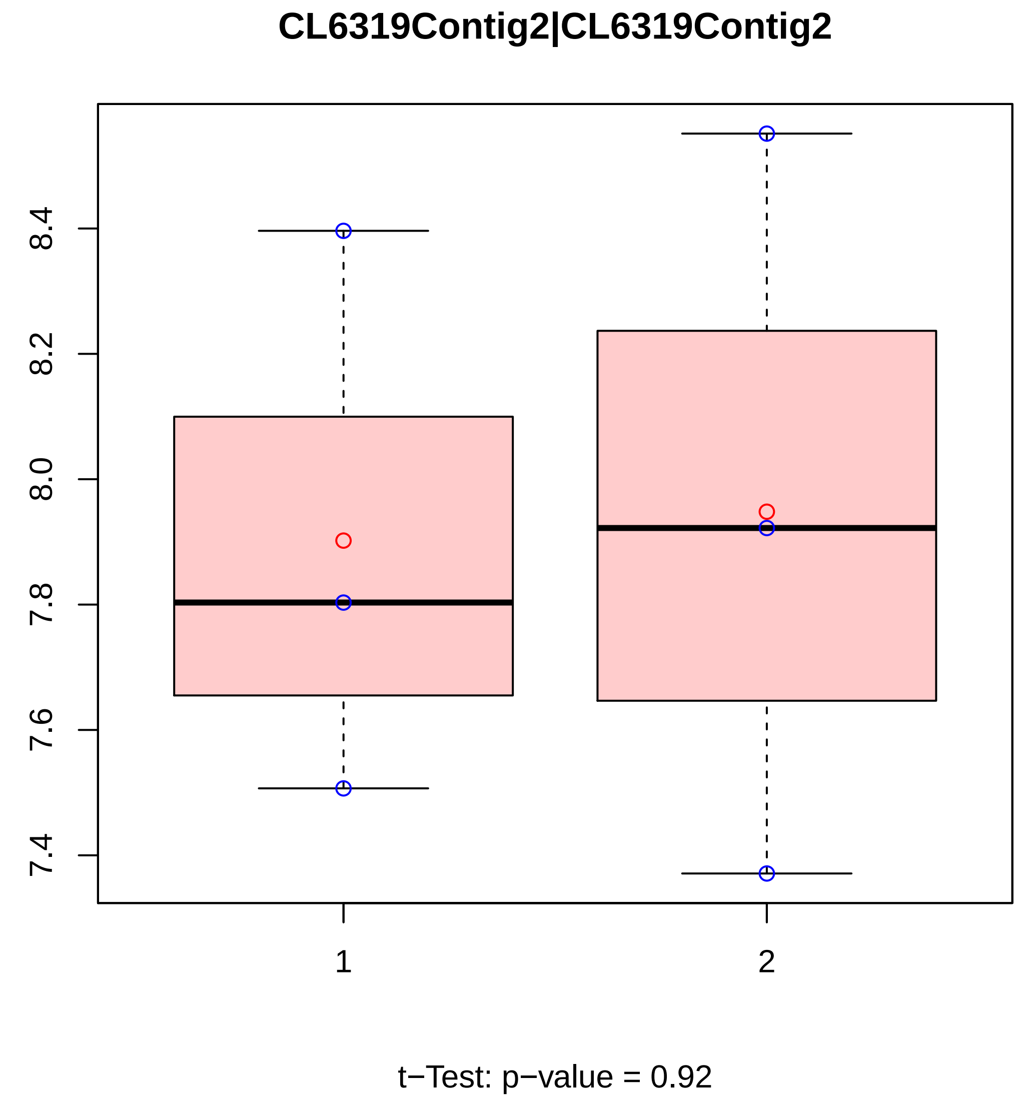
CL6319Contig2|CL6319Contig2
8.4
8.2
8.0
7.8
7.6
7.4
1
2
t−Test: p−value = 0.92

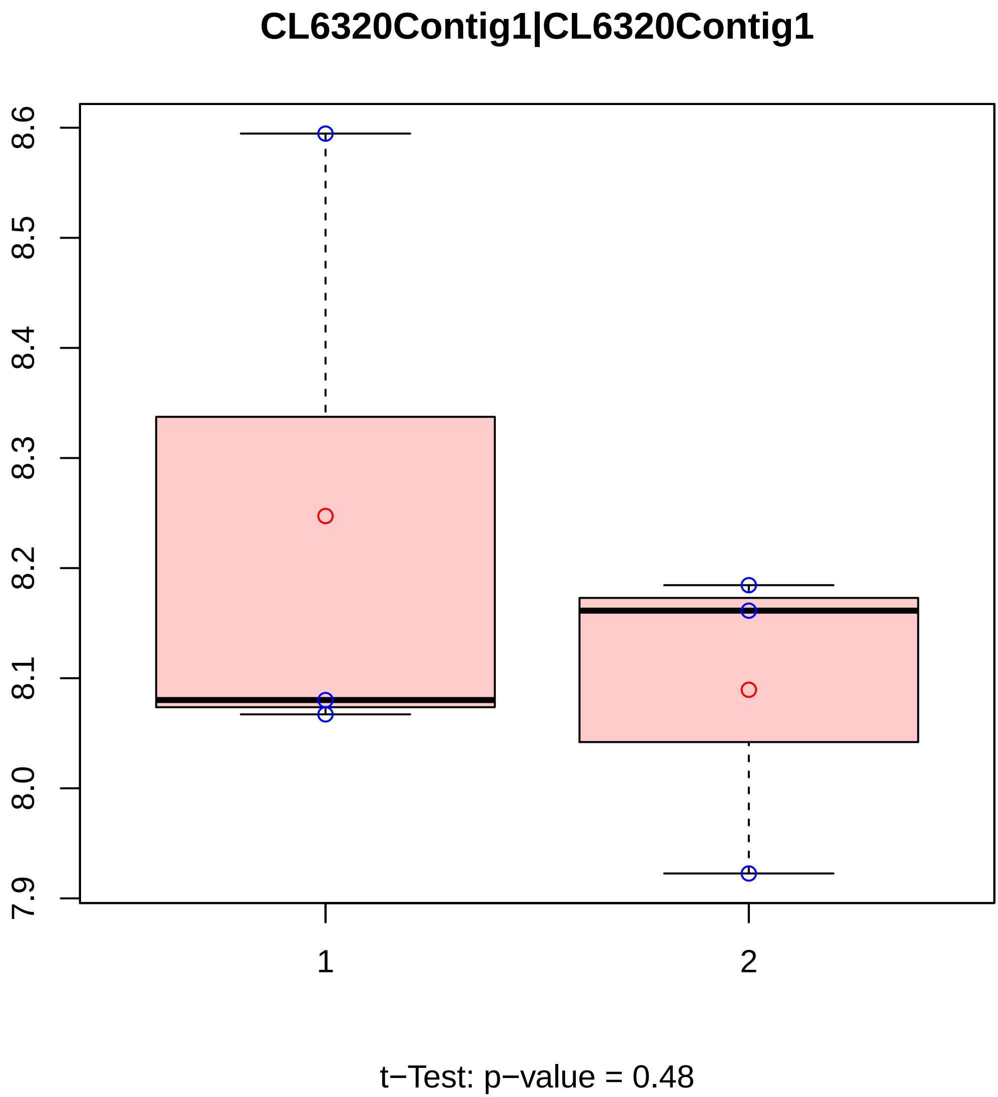
CL6320Contig1|CL6320Contig1
8.6
8.5
8.4
8.3
8.2
8.1
8.0
7.9
1
2
t−Test: p−value = 0.48

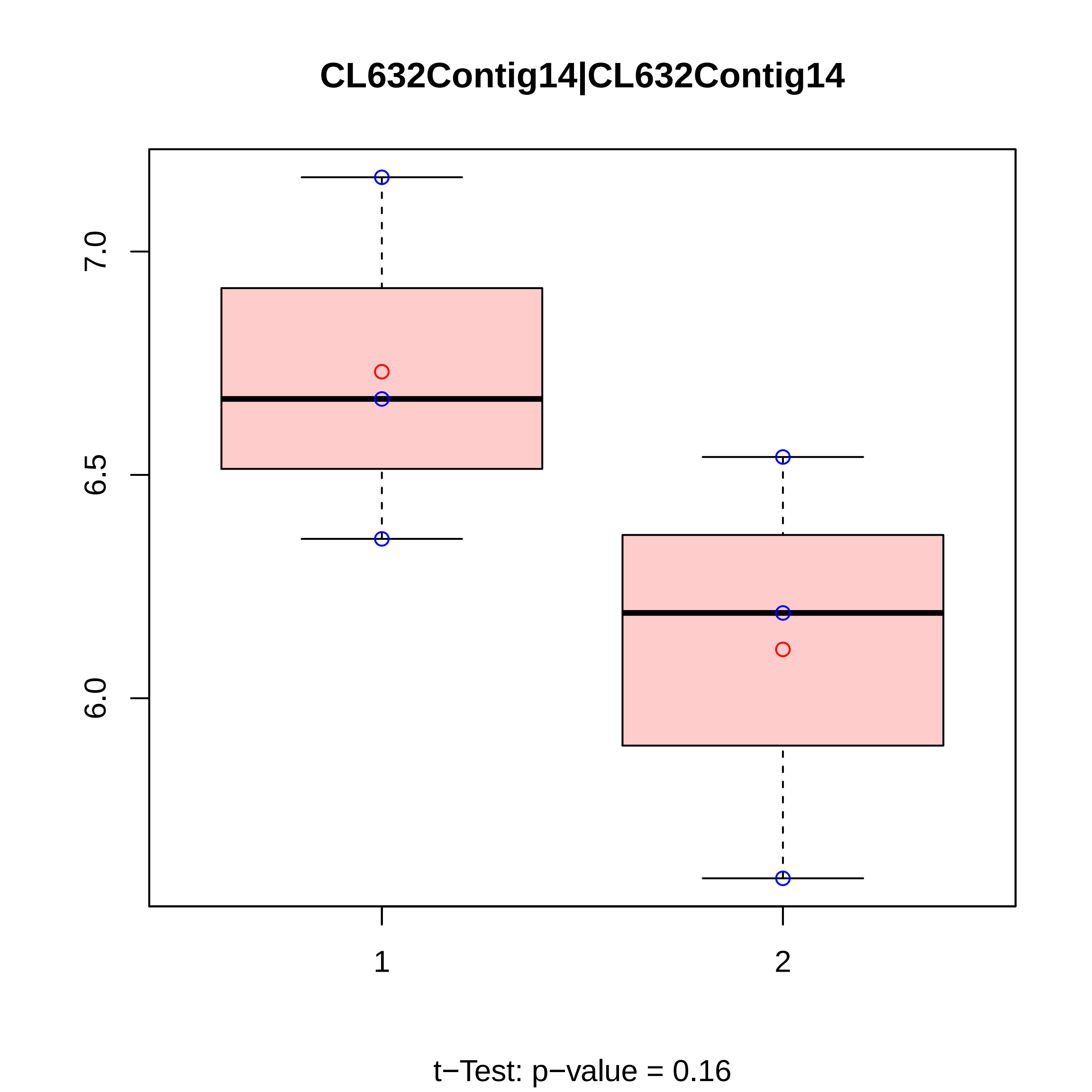

CL632Contig14|CL632Contig14
7.0
6.5
6.0
1
2
t−Test: p−value = 0.16

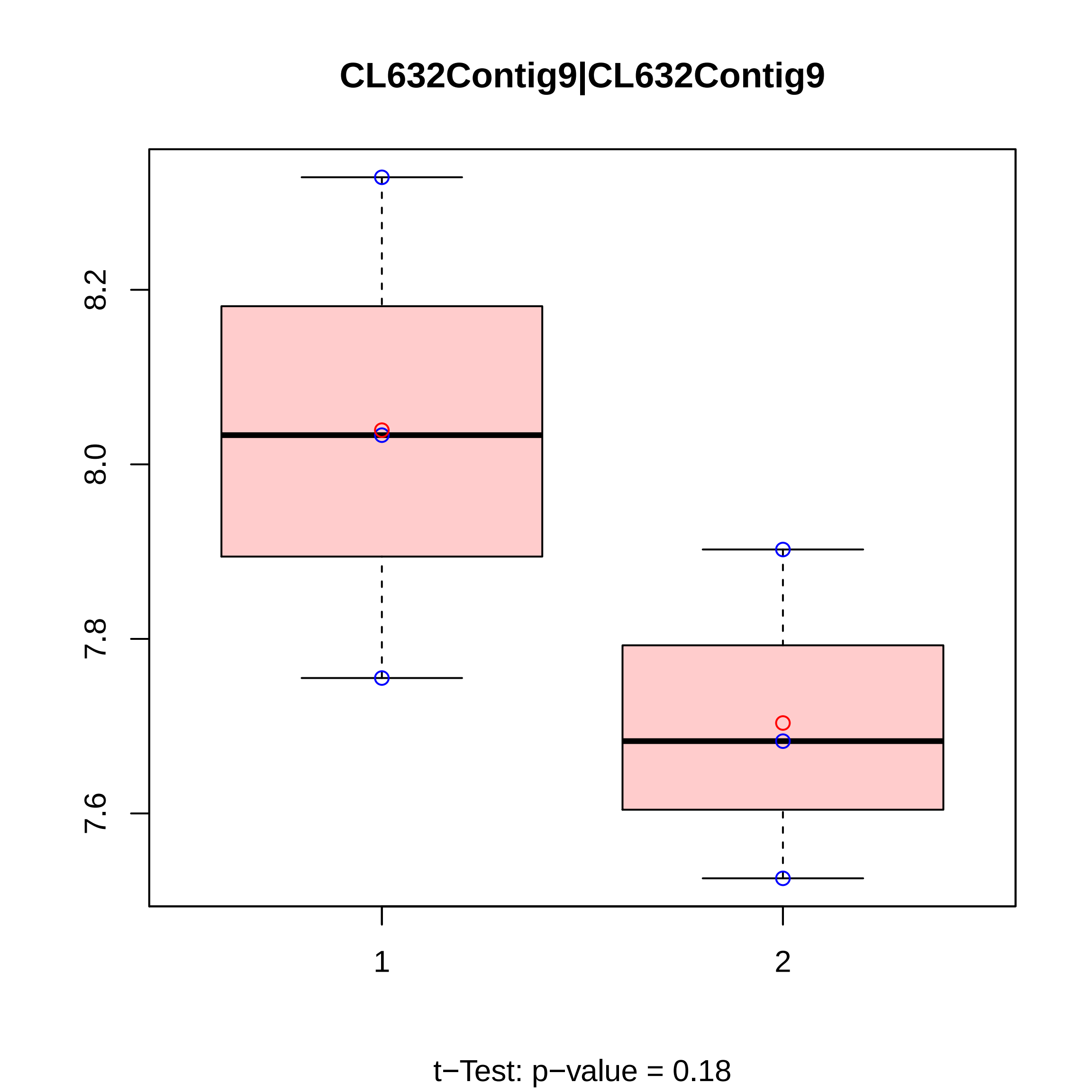
CL632Contig9|CL632Contig9
8.2
8.0
7.8
7.6
1
2
t−Test: p−value = 0.18

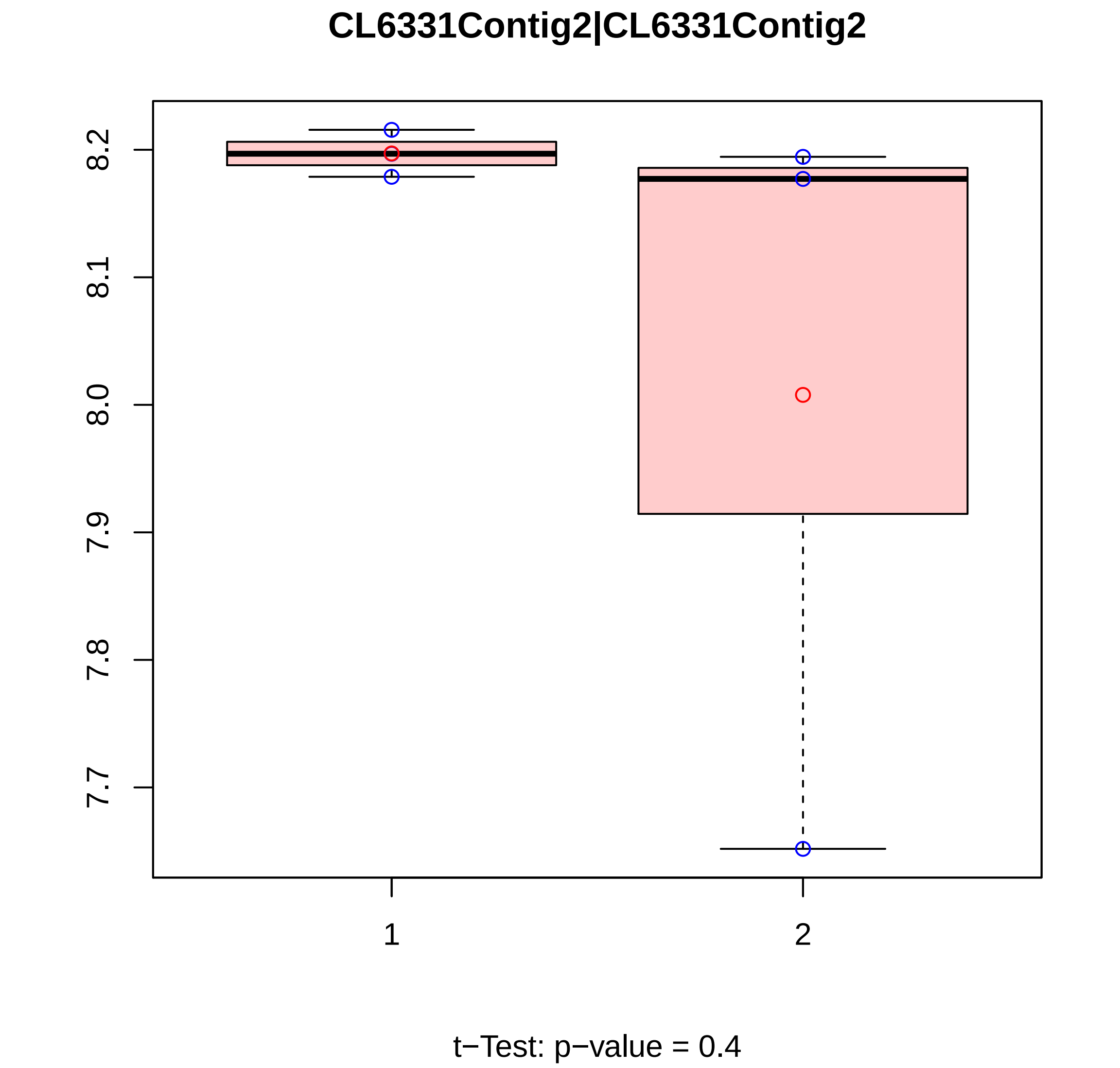
CL6331Contig2|CL6331Contig2
8.2
8.1
8.0
7.9
7.8
7.7
1
2
t−Test: p−value = 0.4

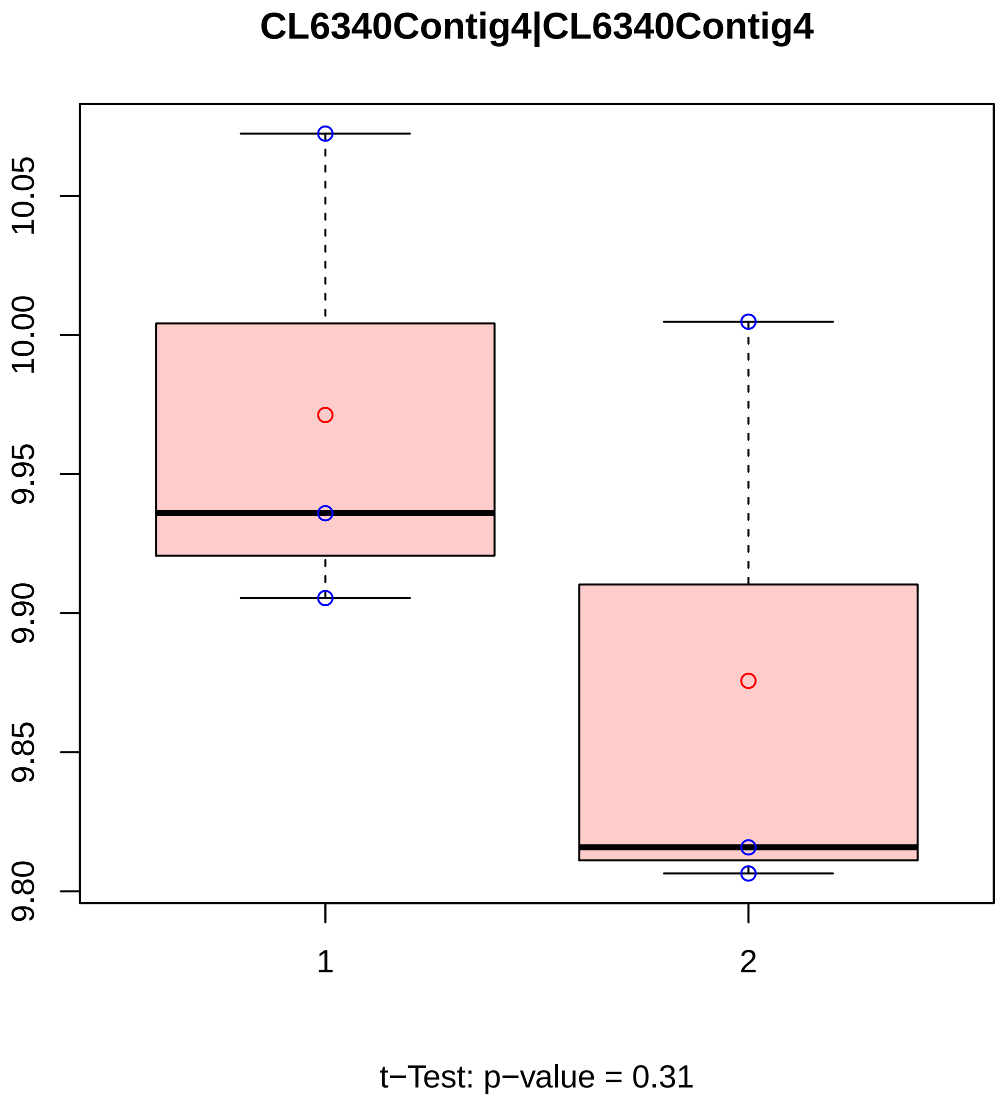

CL6340Contig4|CL6340Contig4
10.05
10.00
9.95
9.90
9.85
9.80
1
2
t−Test: p−value = 0.31

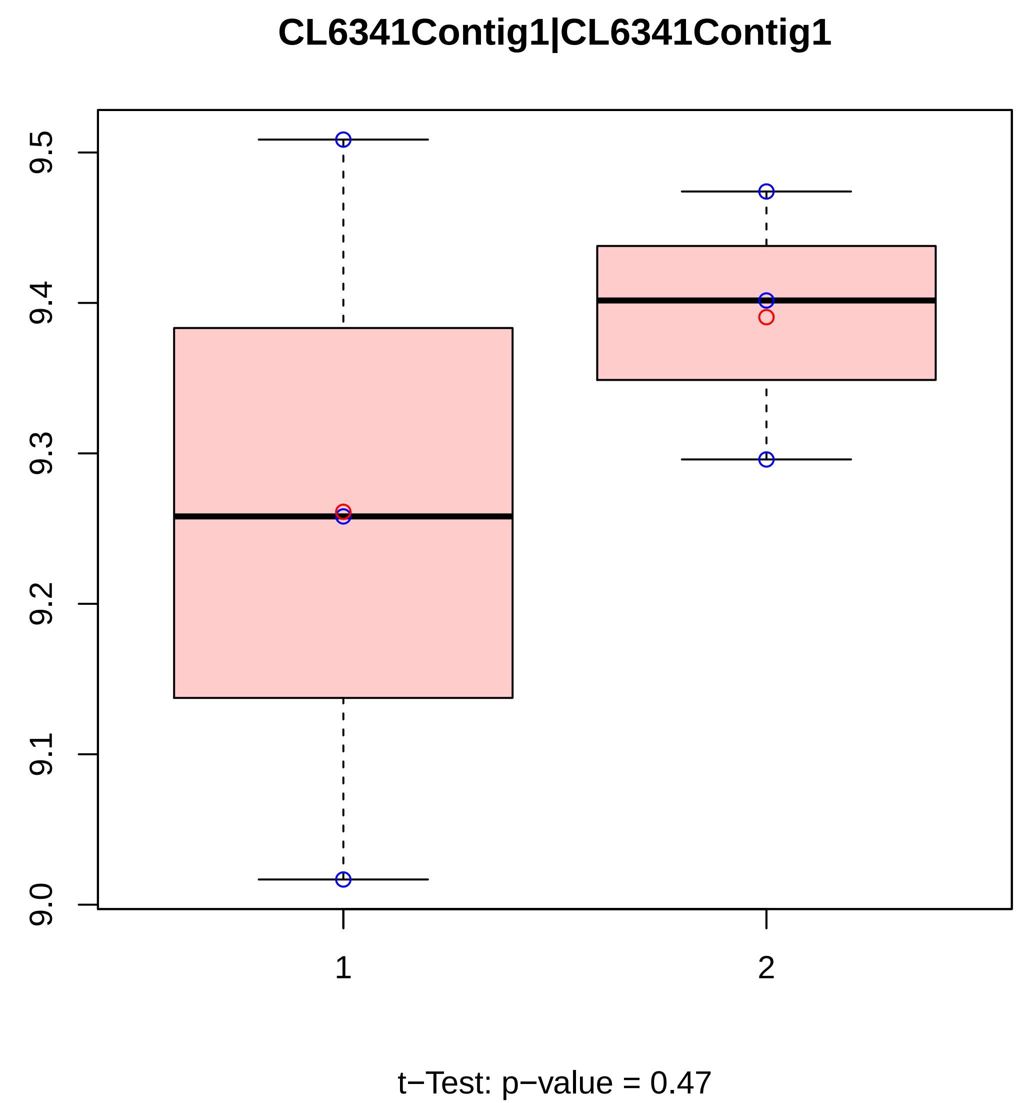
CL6341Contig1|CL6341Contig1
9.0
9.1
9.2
9.3
9.4
9.5
1
2
t−Test: p−value = 0.47

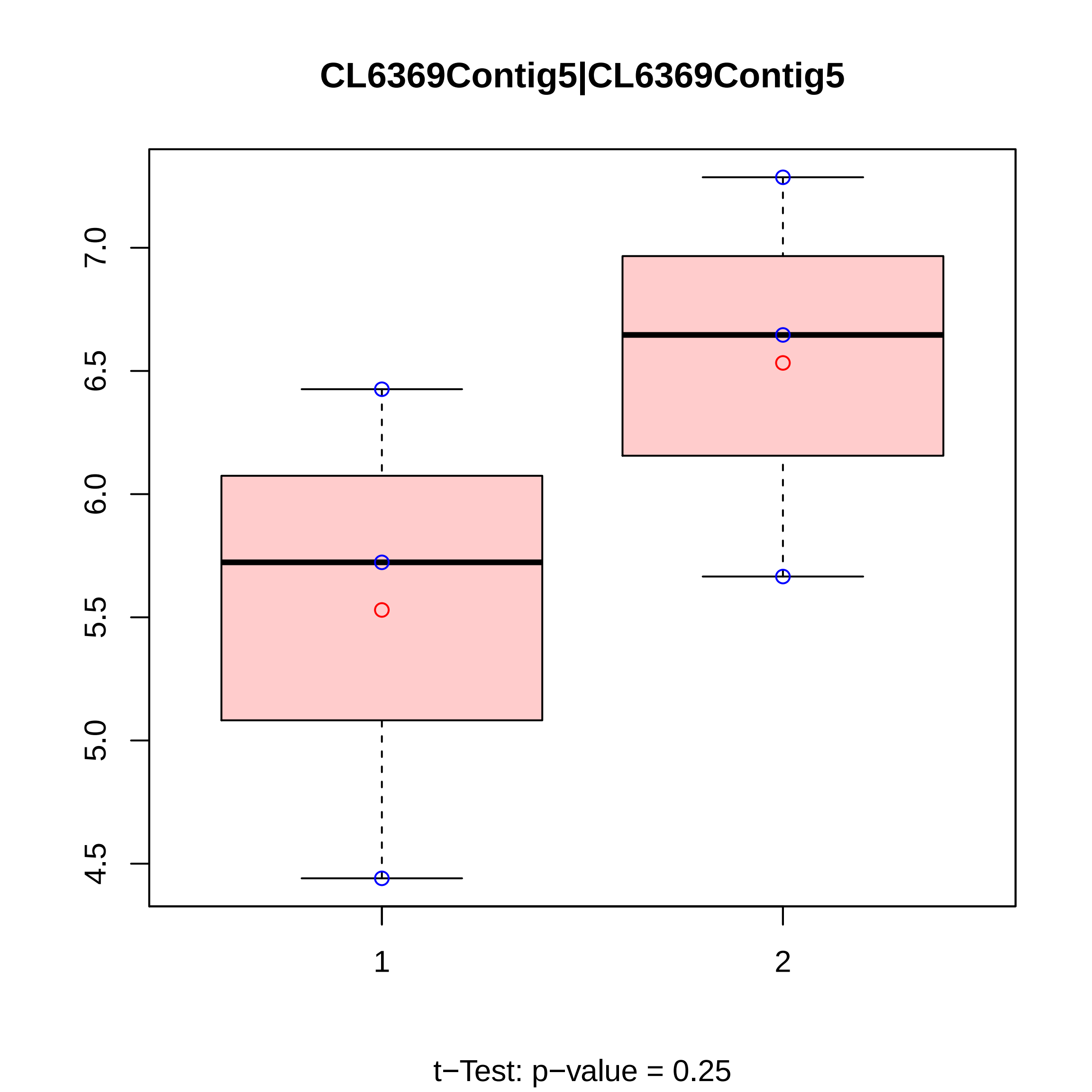
CL6369Contig5|CL6369Contig5
7.0
6.5
6.0
5.5
5.0
4.5
1
2
t−Test: p−value = 0.25

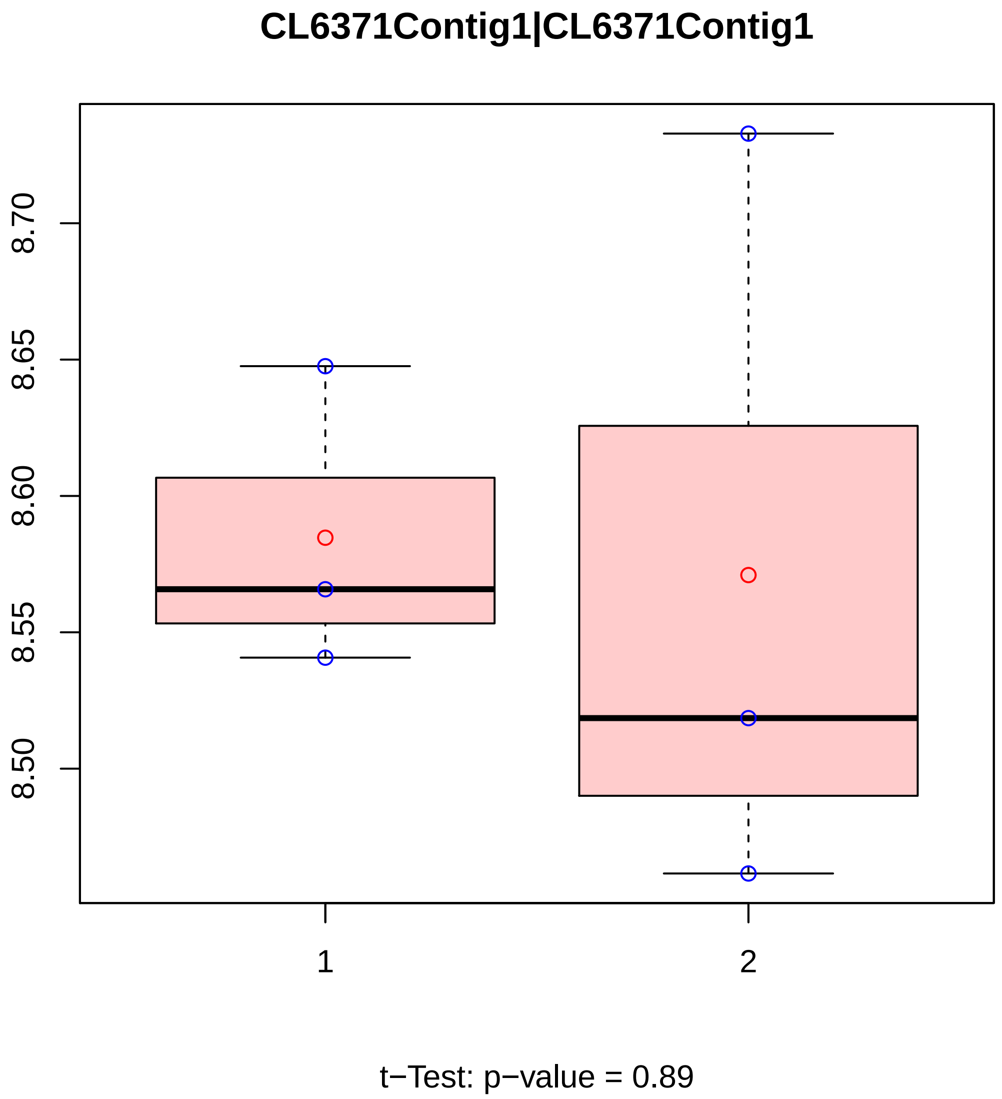
CL6371Contig1|CL6371Contig1
8.50
8.55
8.60
8.65
8.70
1
2
t−Test: p−value = 0.89

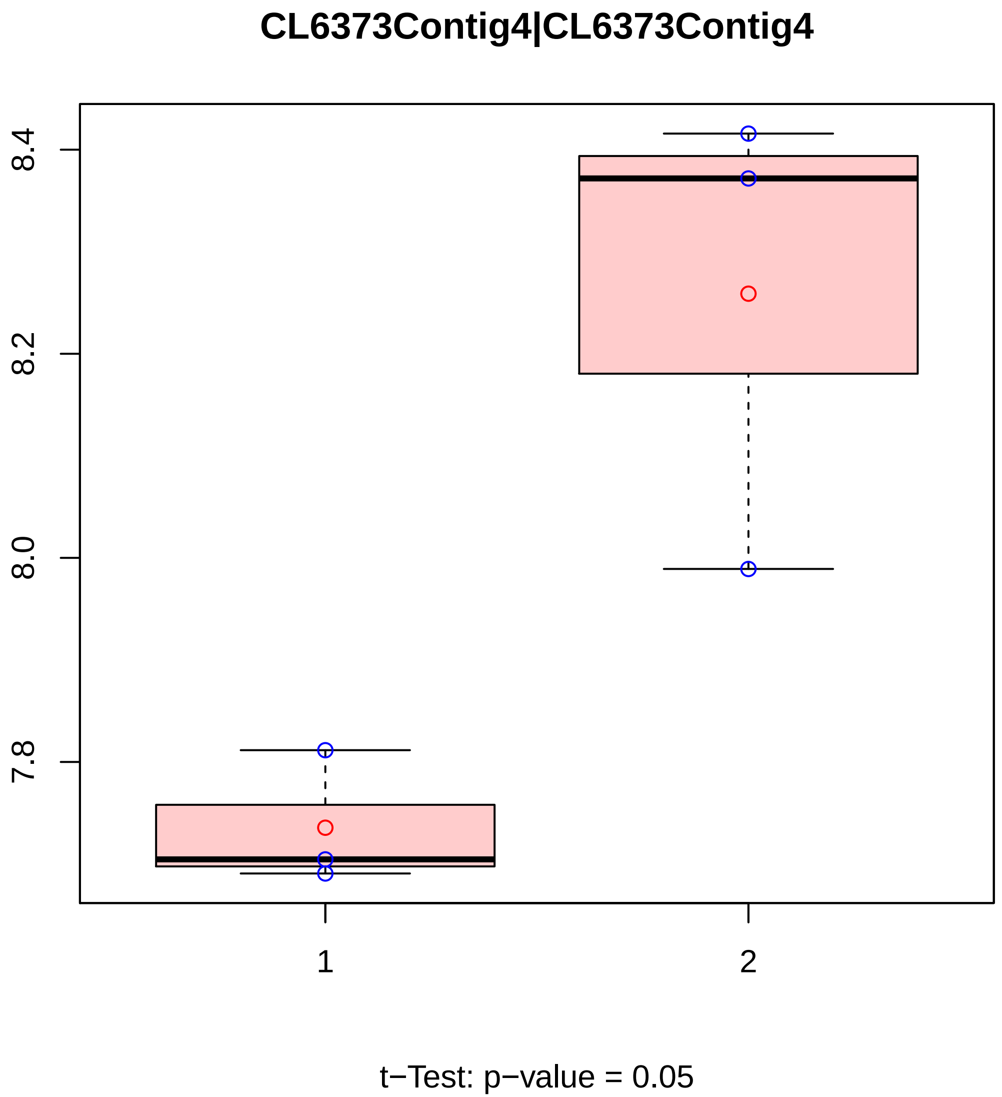

CL6373Contig4|CL6373Contig4
8.4
8.2
8.0
7.8
1
2
t−Test: p−value = 0.05

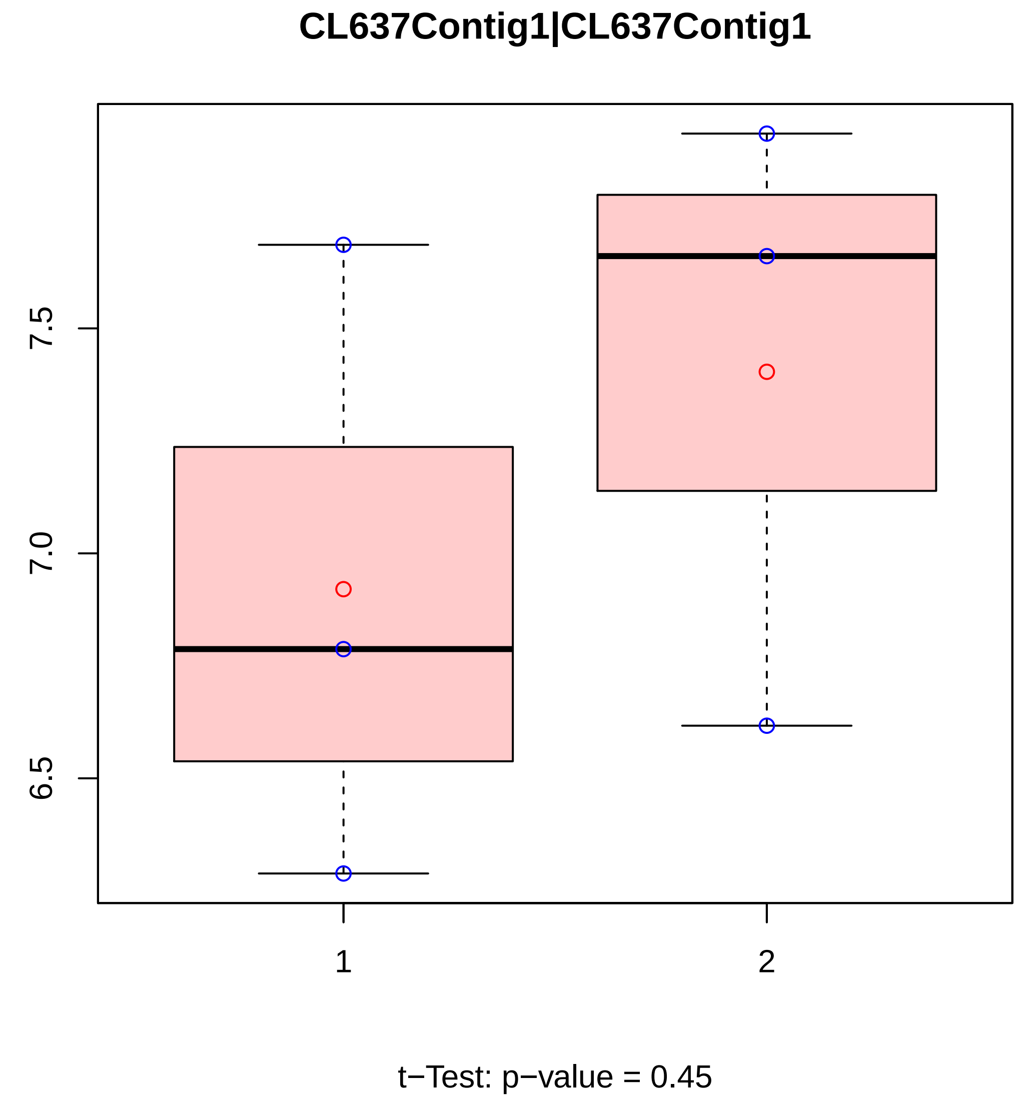
CL637Contig1|CL637Contig1
7.5
7.0
6.5
1
2
t−Test: p−value = 0.45

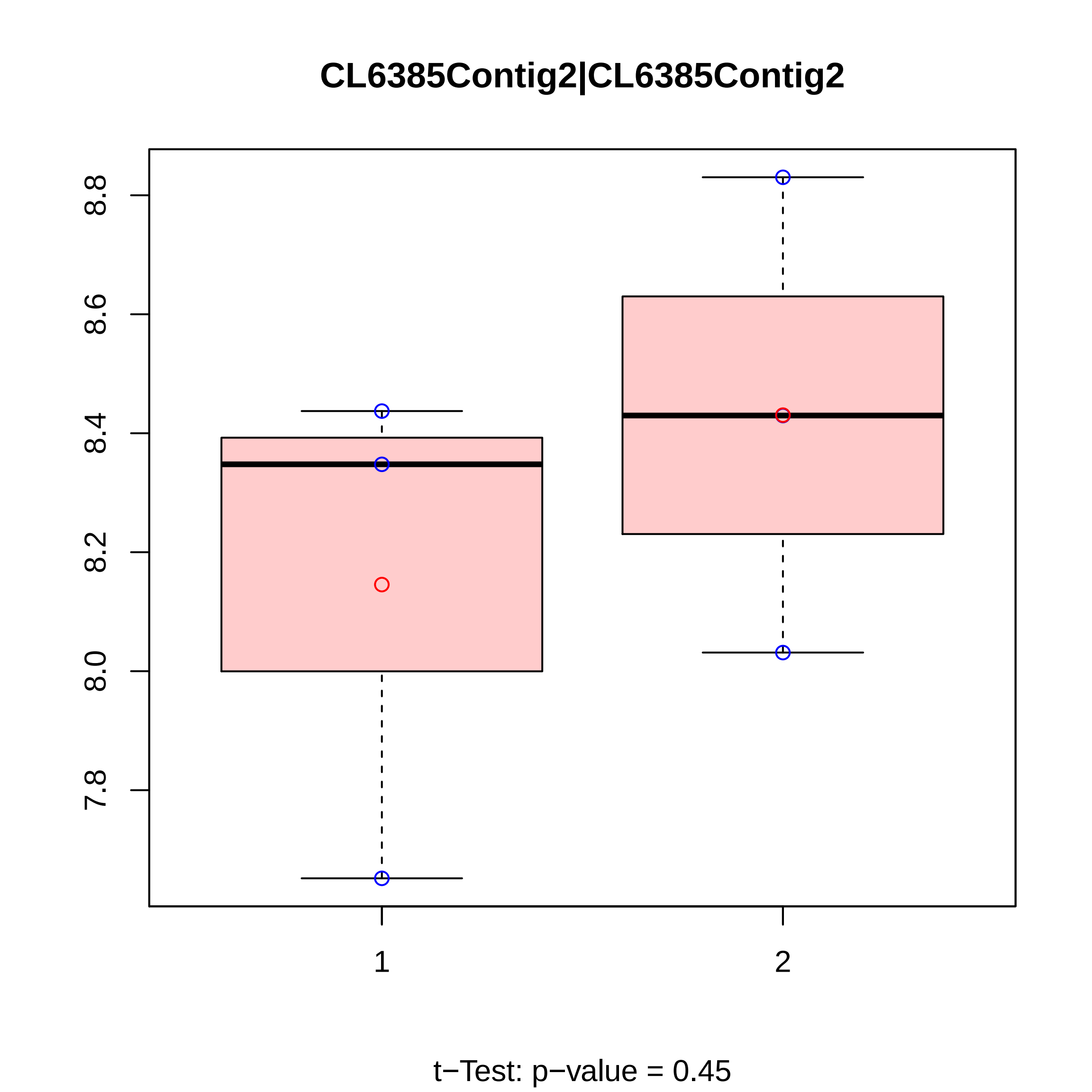

CL6385Contig2|CL6385Contig2
8.8
8.6
8.4
8.2
8.0
7.8
1
2
t−Test: p−value = 0.45

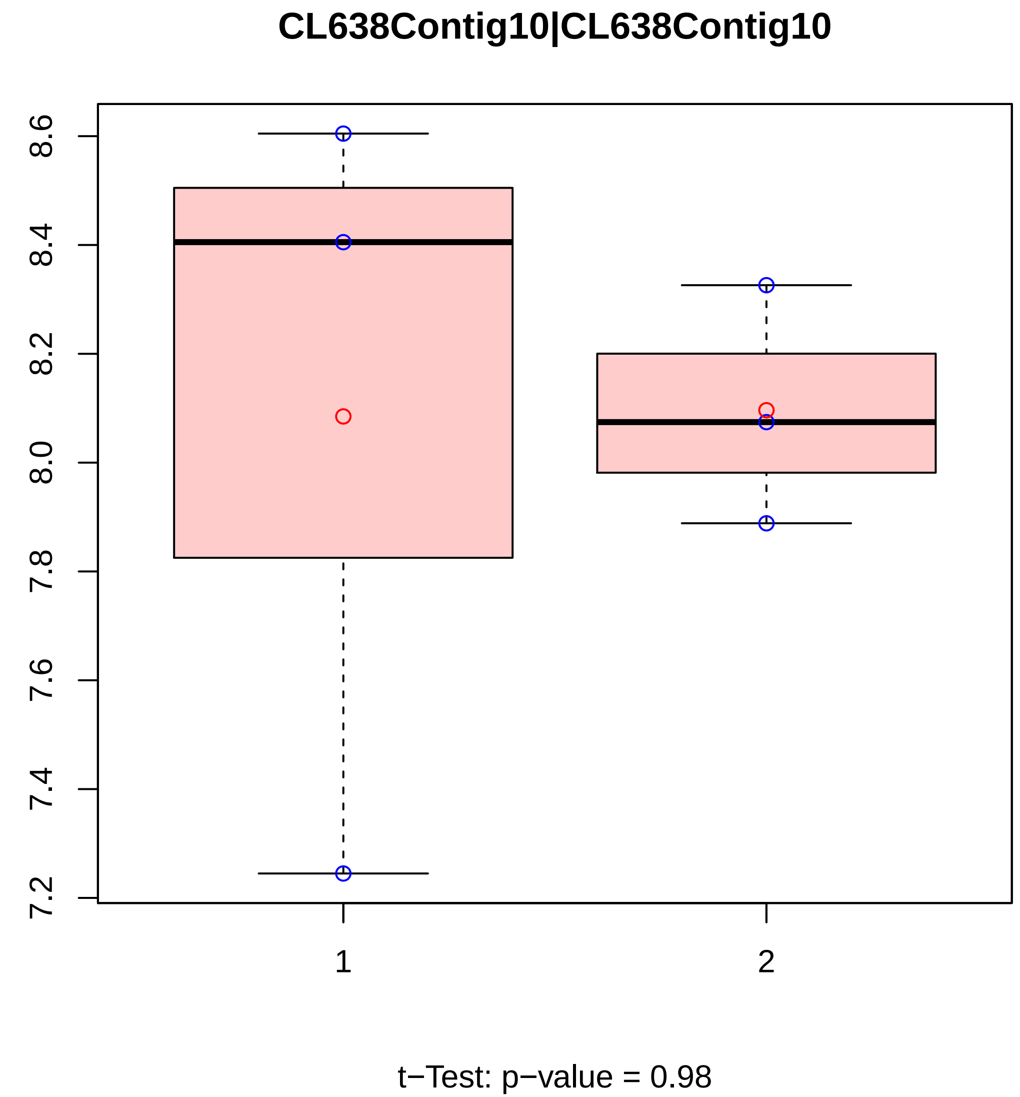
CL638Contig10|CL638Contig10
8.6
8.4
8.2
8.0
7.8
7.6
7.4
7.2
1
2
t−Test: p−value = 0.98

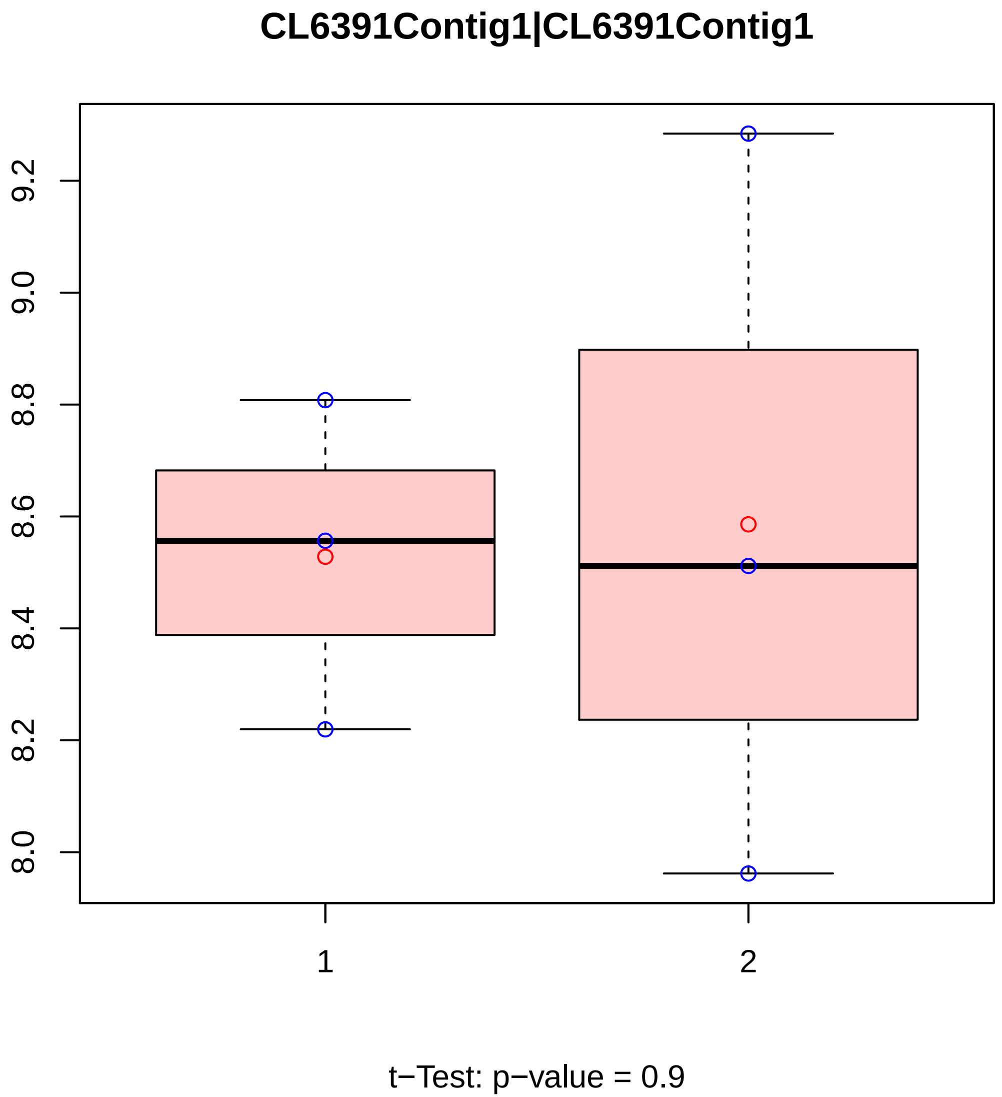

CL6391Contig1|CL6391Contig1
9.2
9.0
8.8
8.6
8.4
8.2
8.0
1
2
t−Test: p−value = 0.9

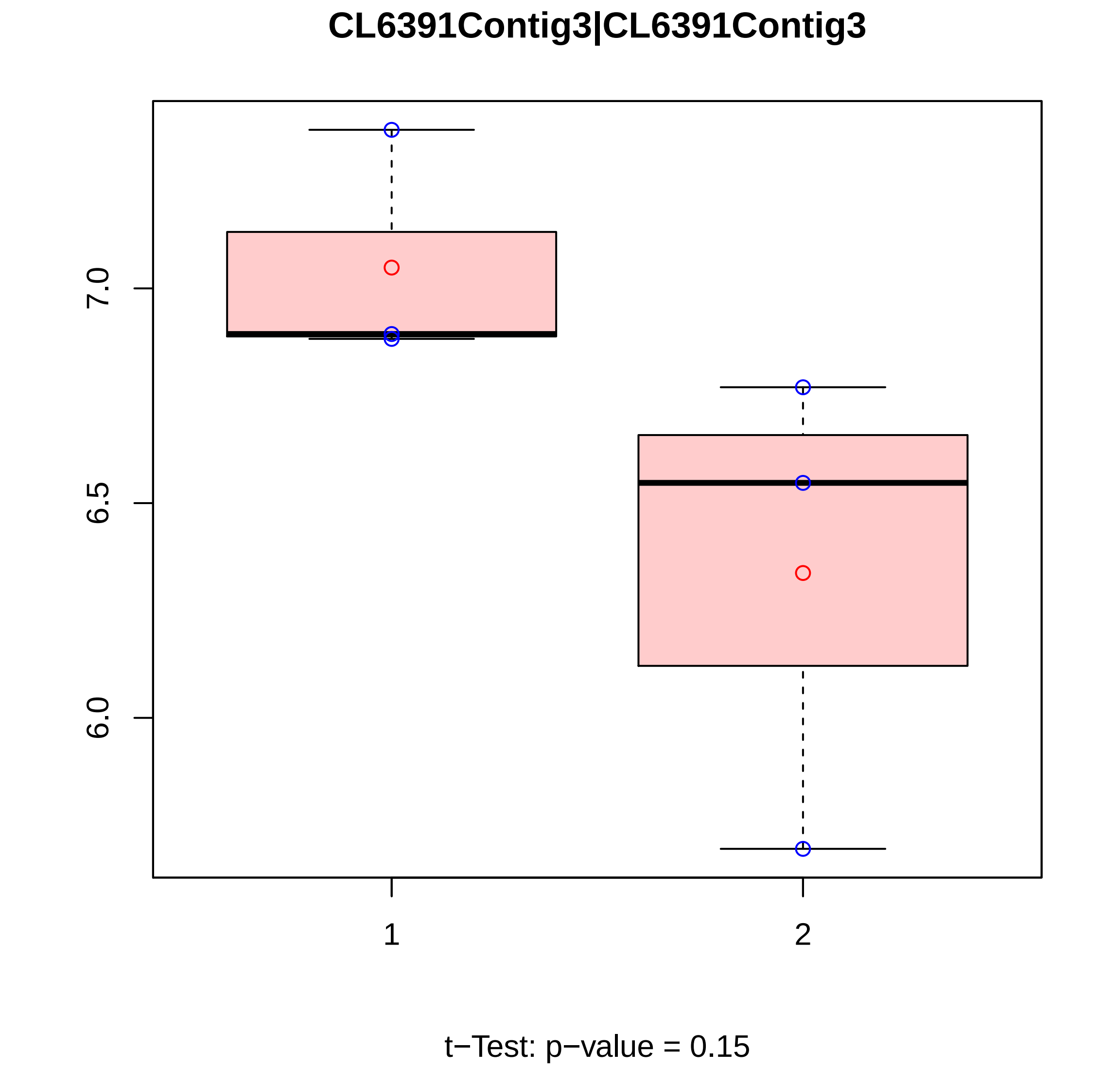
CL6391Contig3|CL6391Contig3
7.0
6.5
6.0
1
2
t−Test: p−value = 0.15

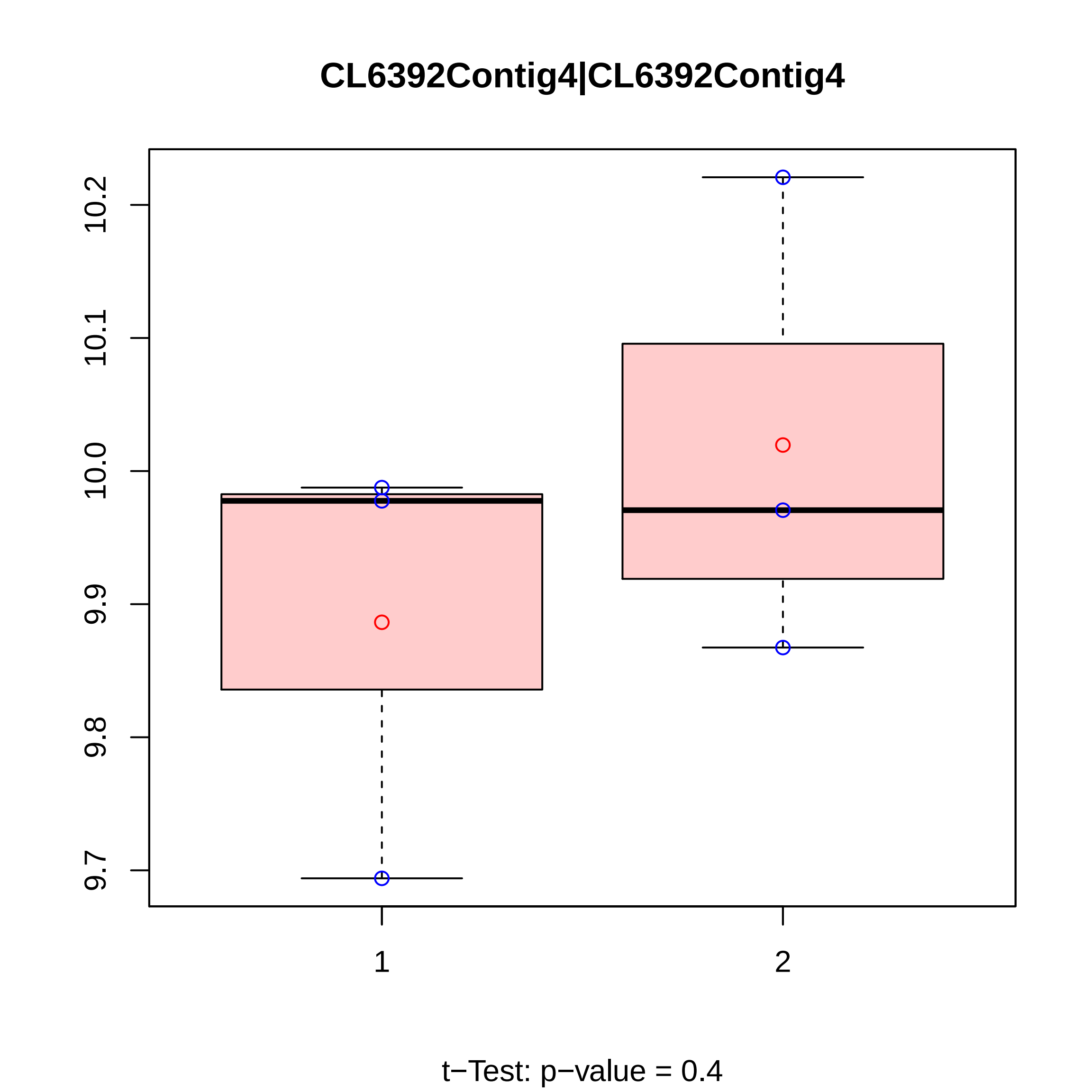
CL6392Contig4|CL6392Contig4
10.2
10.1
10.0
9.9
9.8
9.7
1
2
t−Test: p−value = 0.4

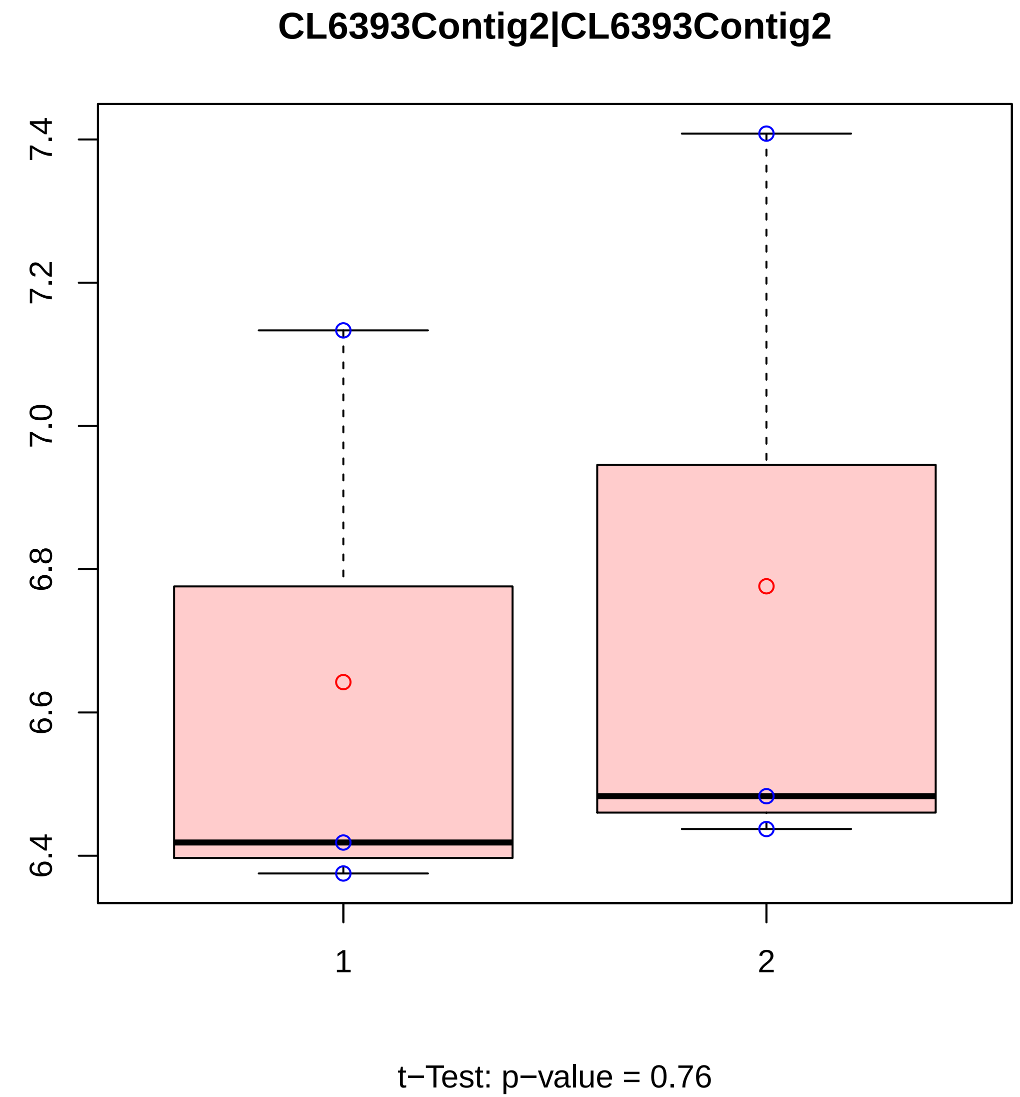

CL6393Contig2|CL6393Contig2
7.4
7.2
7.0
6.8
6.6
6.4
1
2
t−Test: p−value = 0.76

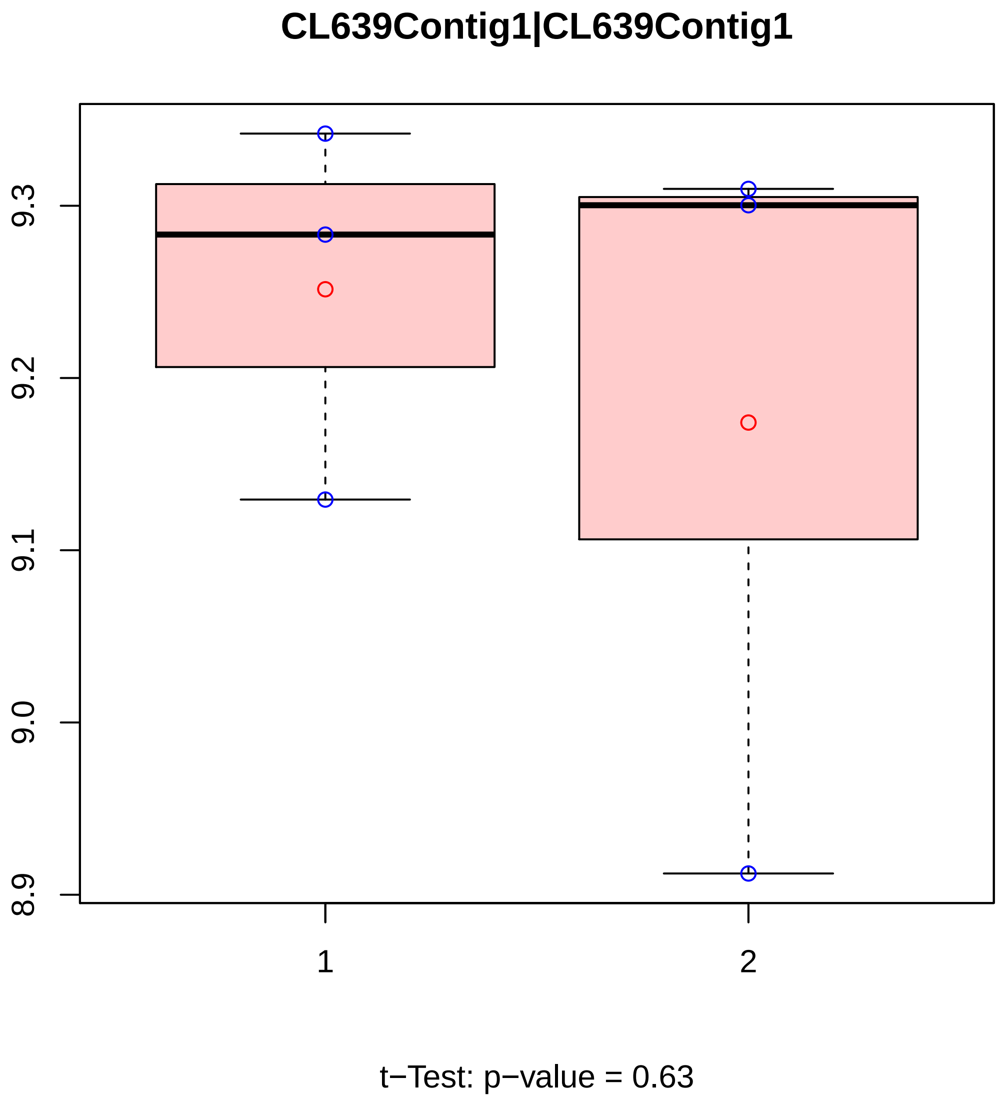
CL639Contig1|CL639Contig1
9.3
9.2
9.1
9.0
8.9
1
2
t−Test: p−value = 0.63

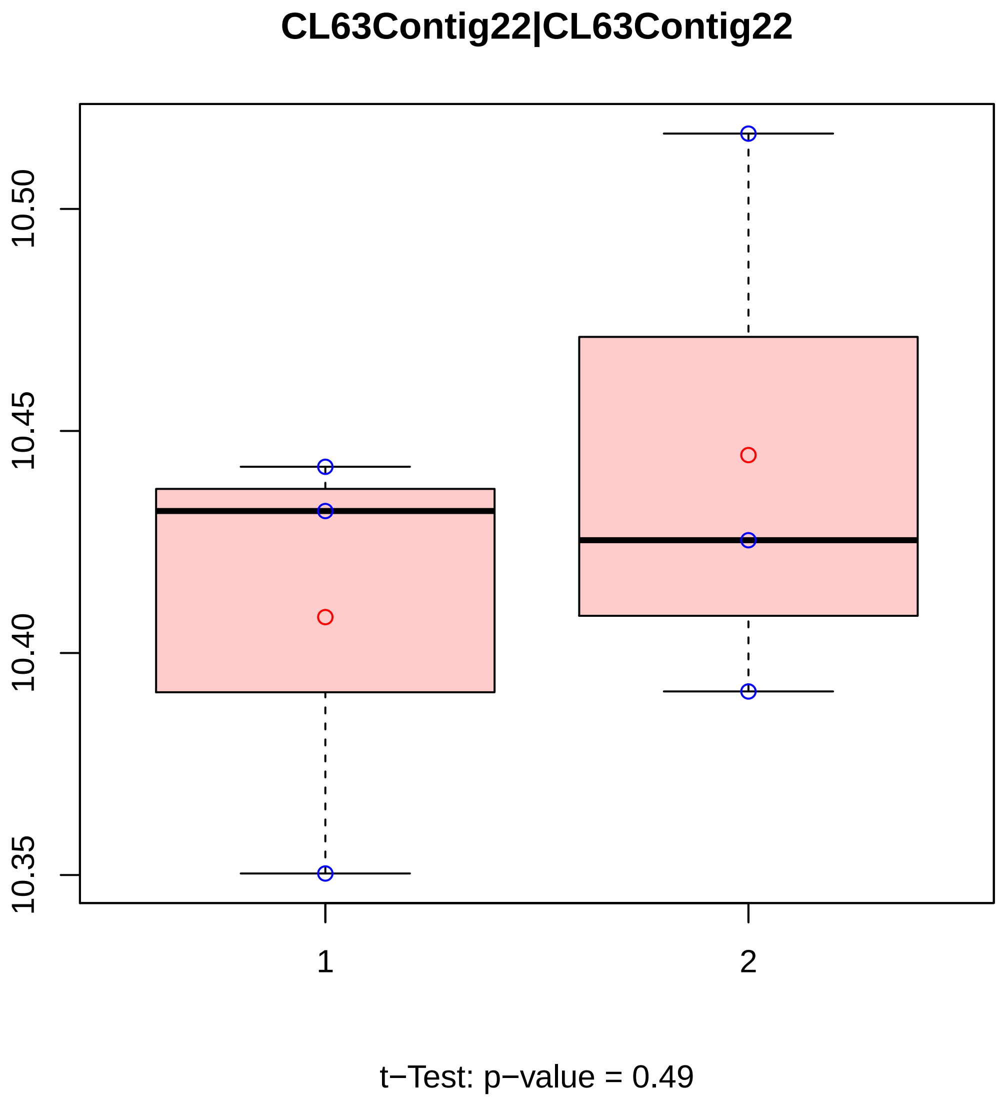
CL63Contig22|CL63Contig22
10.35
10.40
10.45
10.50
1
2
t−Test: p−value = 0.49

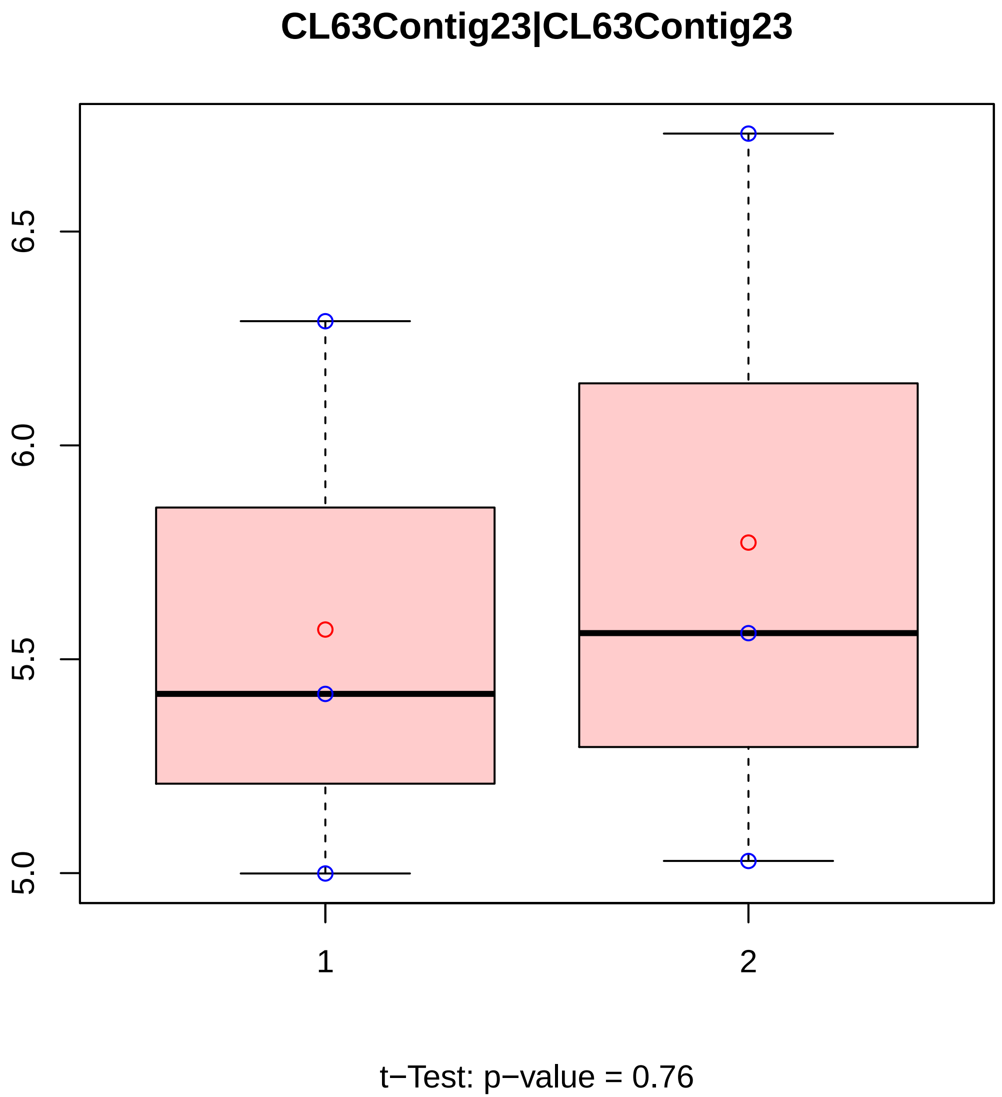

CL63Contig23|CL63Contig23
6.5
6.0
5.5
5.0
1
2
t−Test: p−value = 0.76

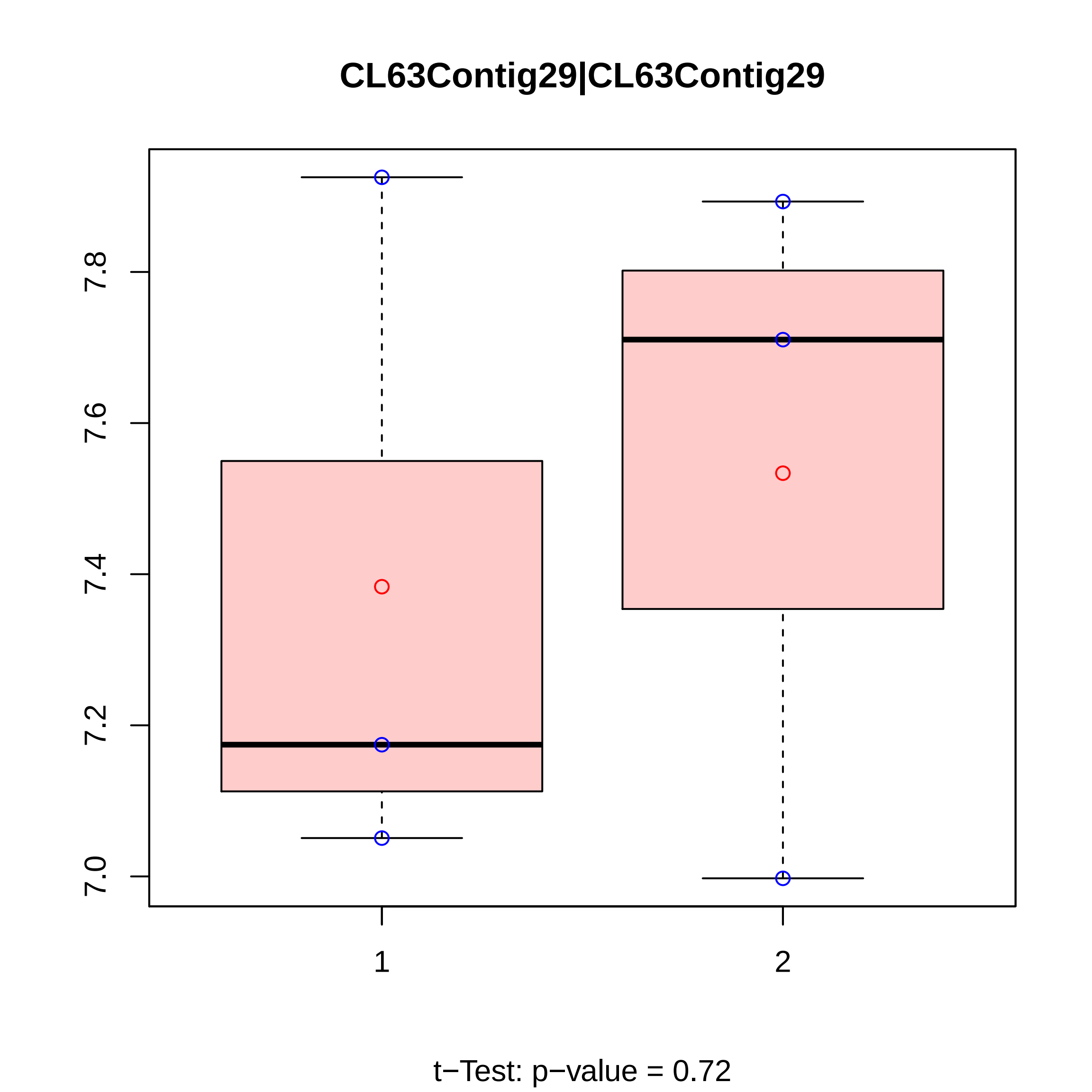
CL63Contig29|CL63Contig29
7.0
7.2
7.4
7.6
7.8
1
2
t−Test: p−value = 0.72

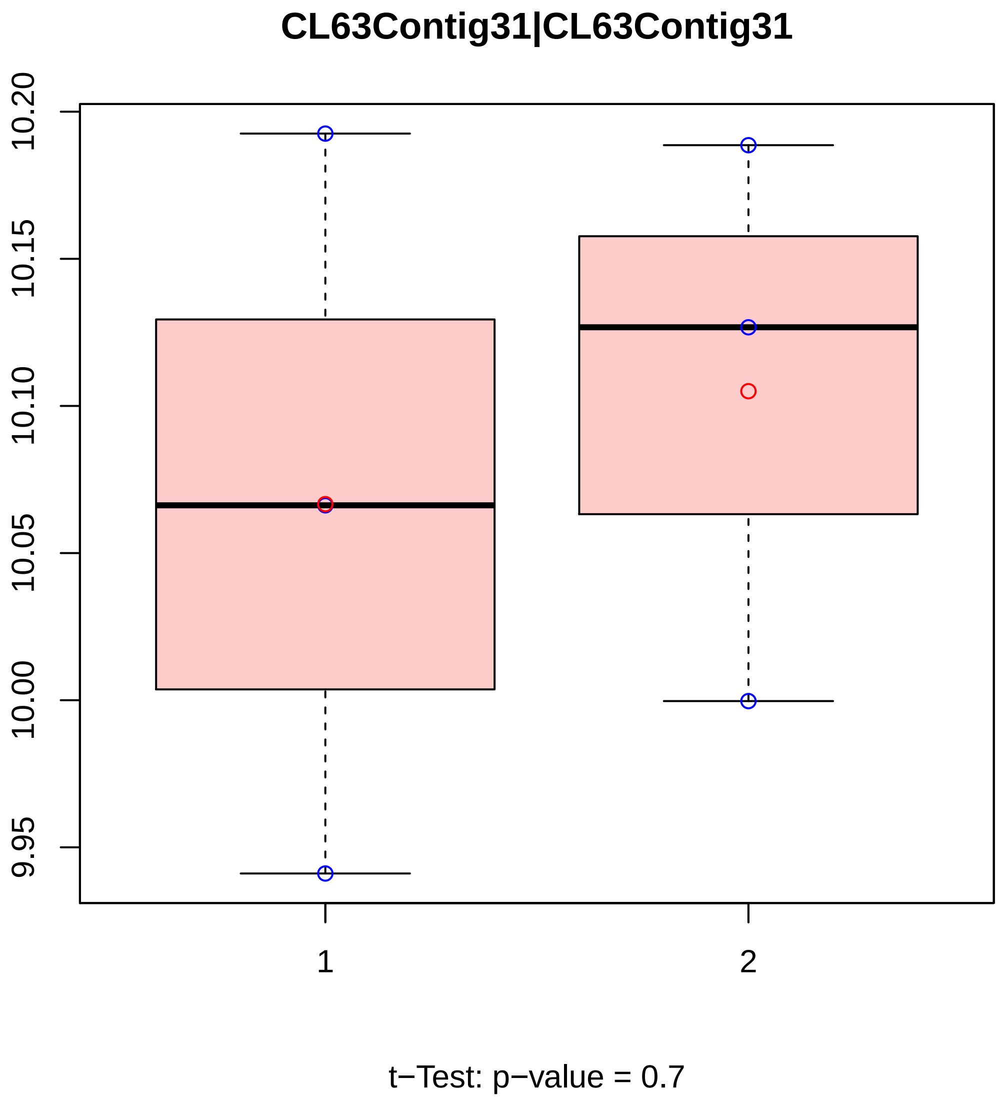

CL63Contig31|CL63Contig31
10.20
10.15
10.10
10.05
10.00
9.95
1
2
t−Test: p−value = 0.7

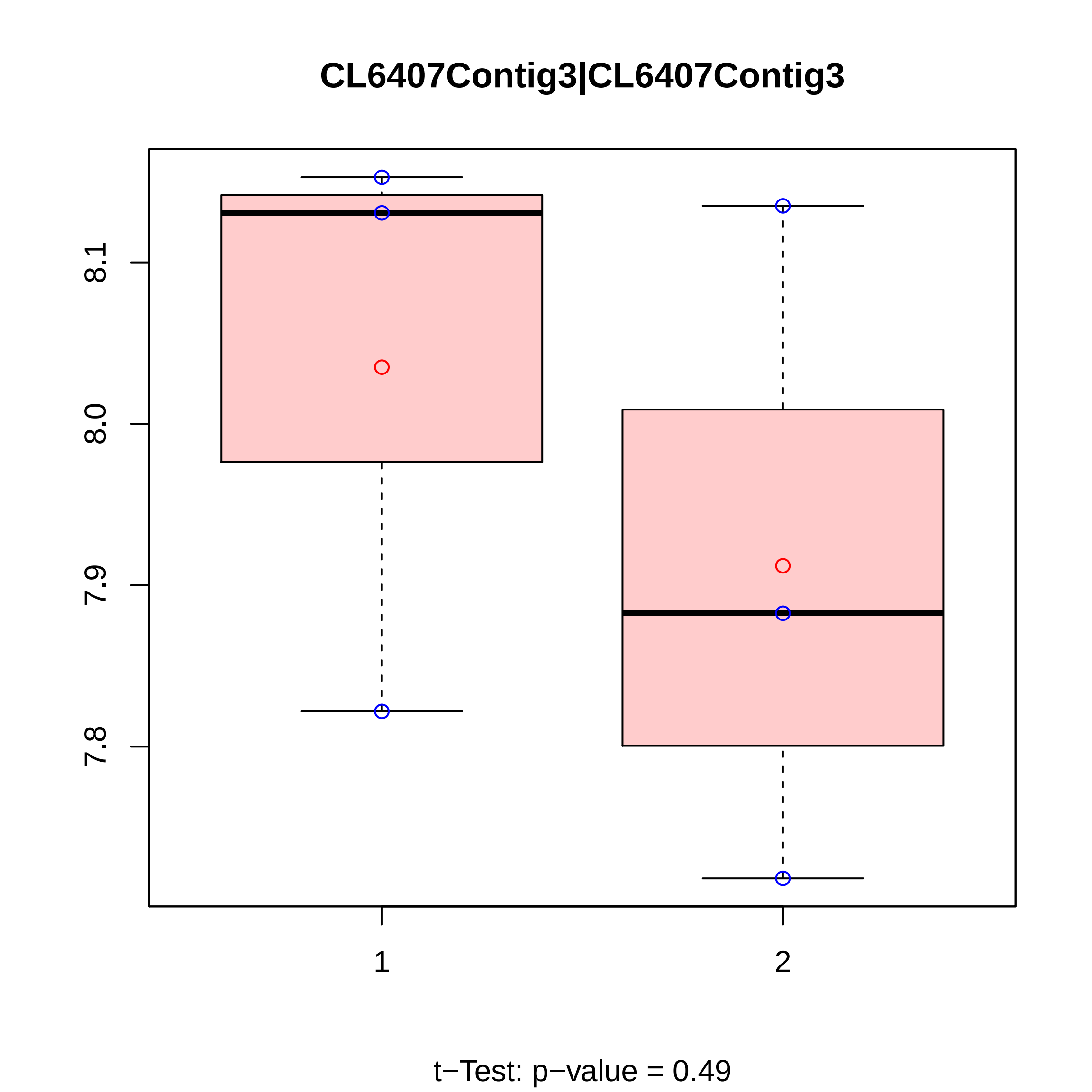

CL6407Contig3|CL6407Contig3
8.1
8.0
7.9
7.8
1
2
t−Test: p−value = 0.49

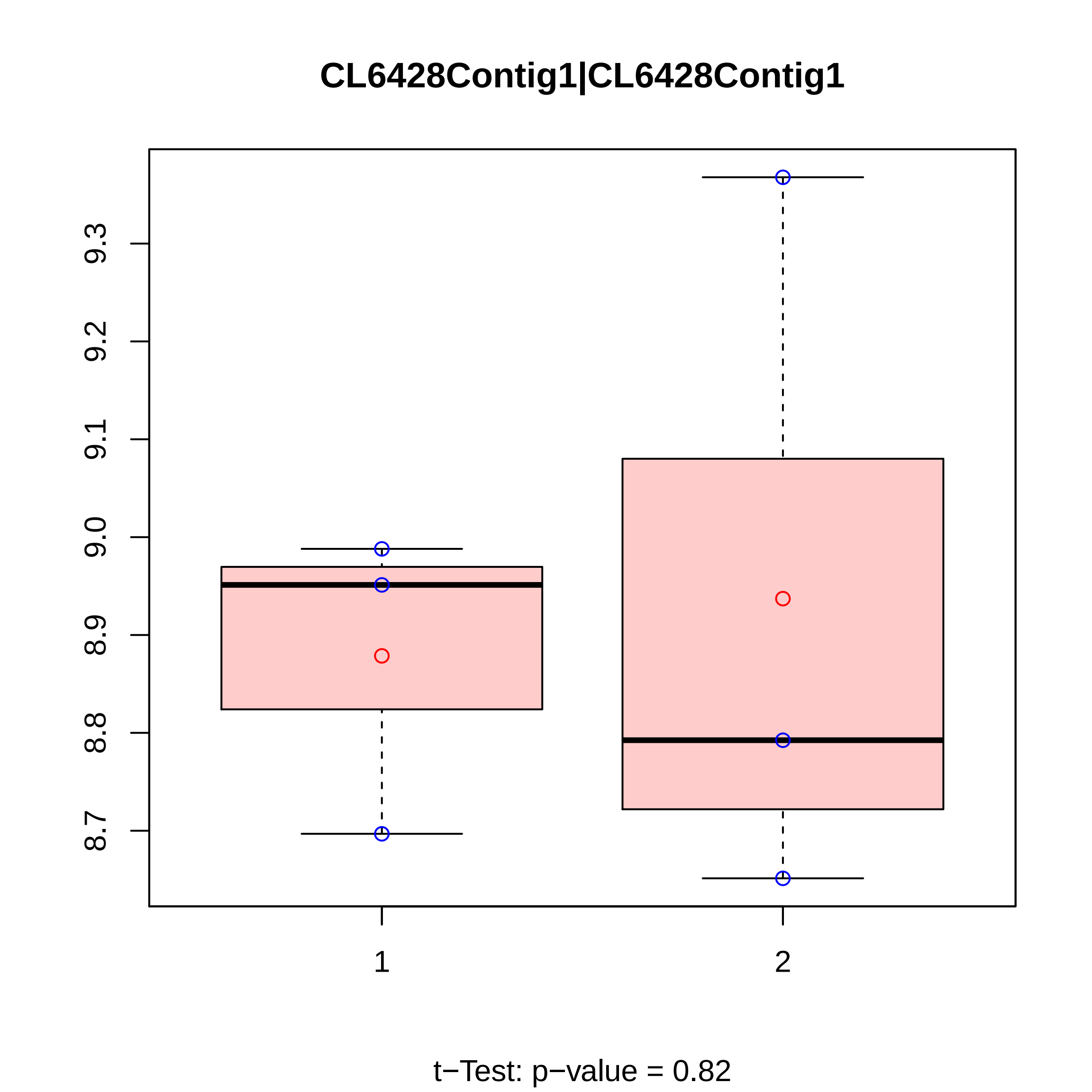
CL6428Contig1|CL6428Contig1
9.3
9.2
9.1
9.0
8.9
8.8
8.7
1
2
t−Test: p−value = 0.82

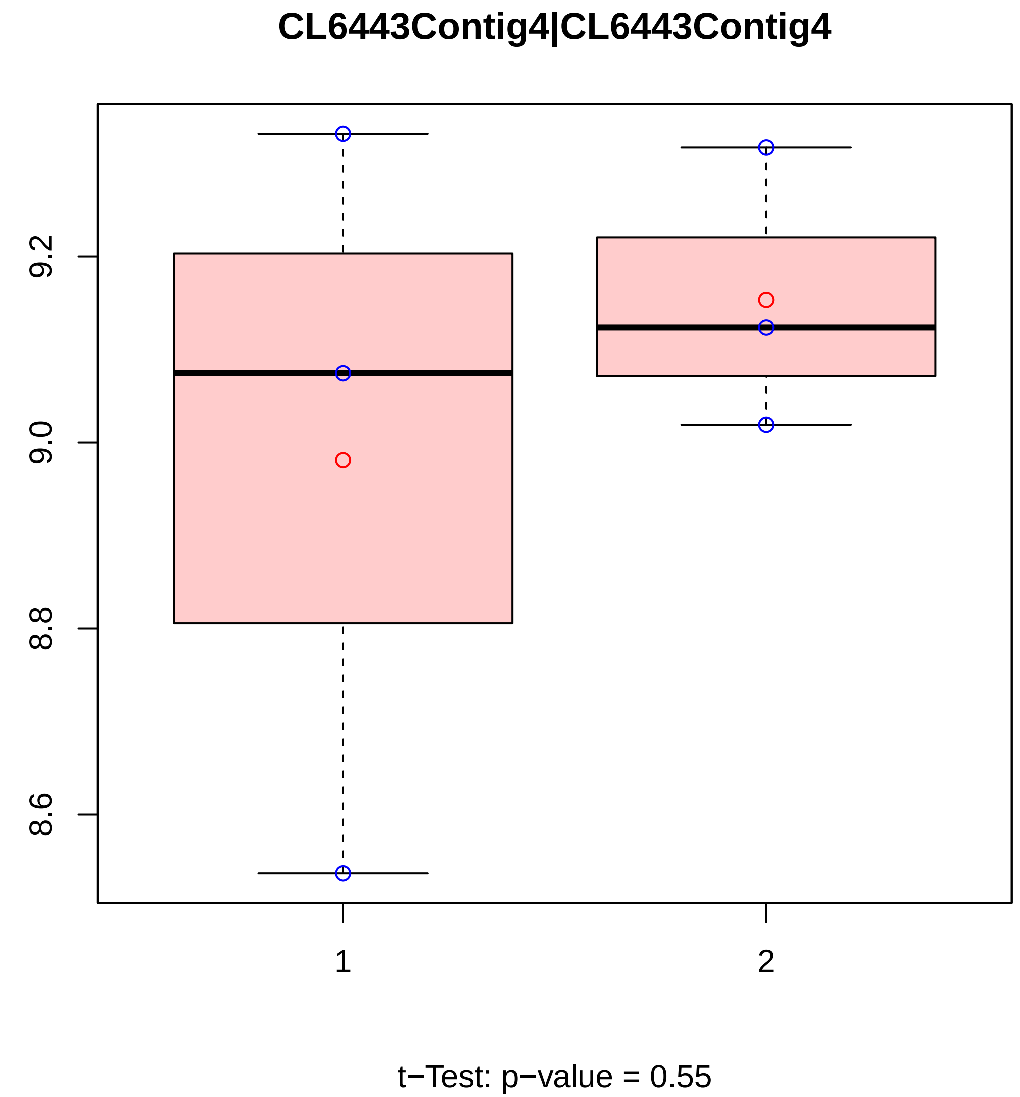

CL6443Contig4|CL6443Contig4
9.2
9.0
8.8
8.6
1
2
t−Test: p−value = 0.55

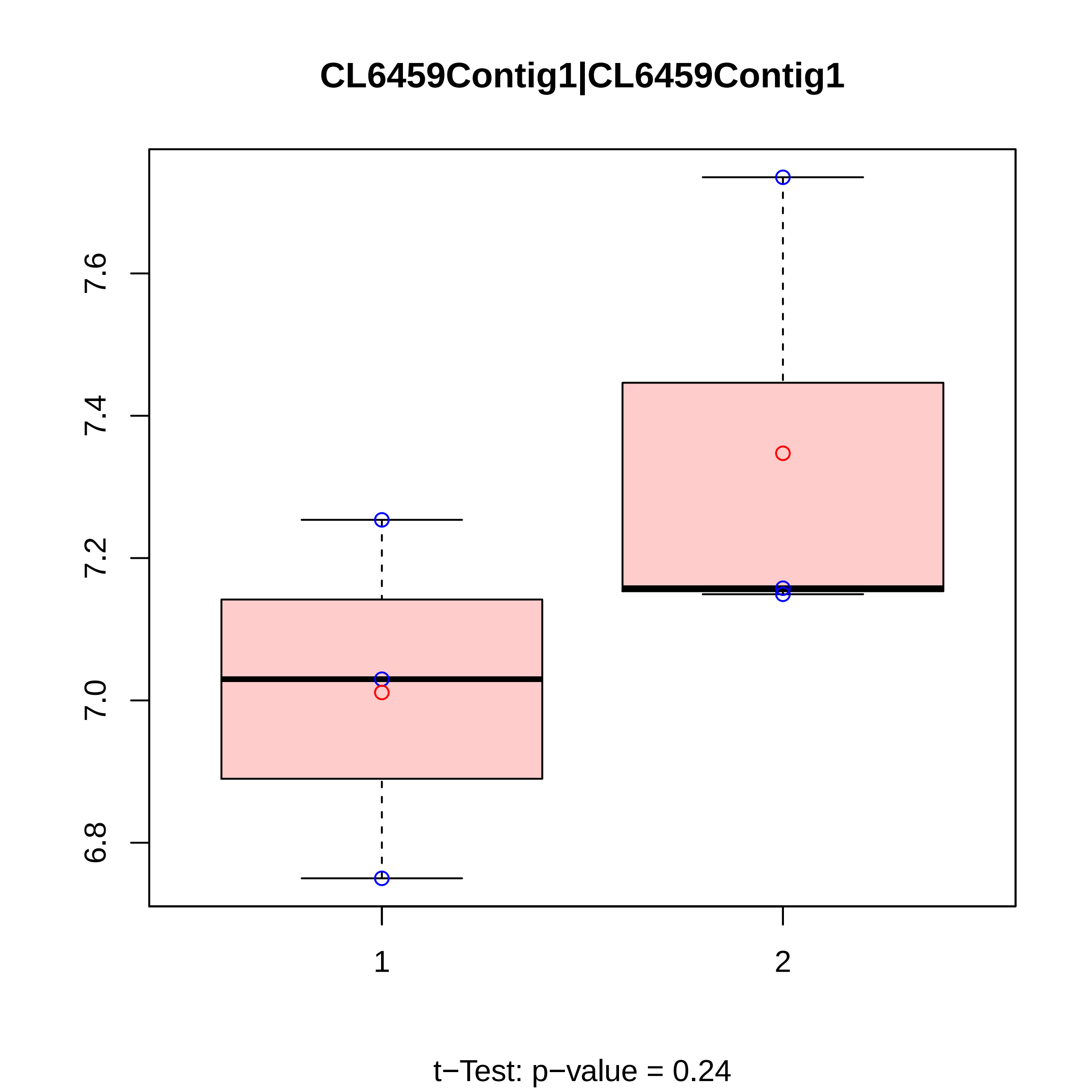
CL6459Contig1|CL6459Contig1
7.6
7.4
7.2
7.0
6.8
1
2
t−Test: p−value = 0.24

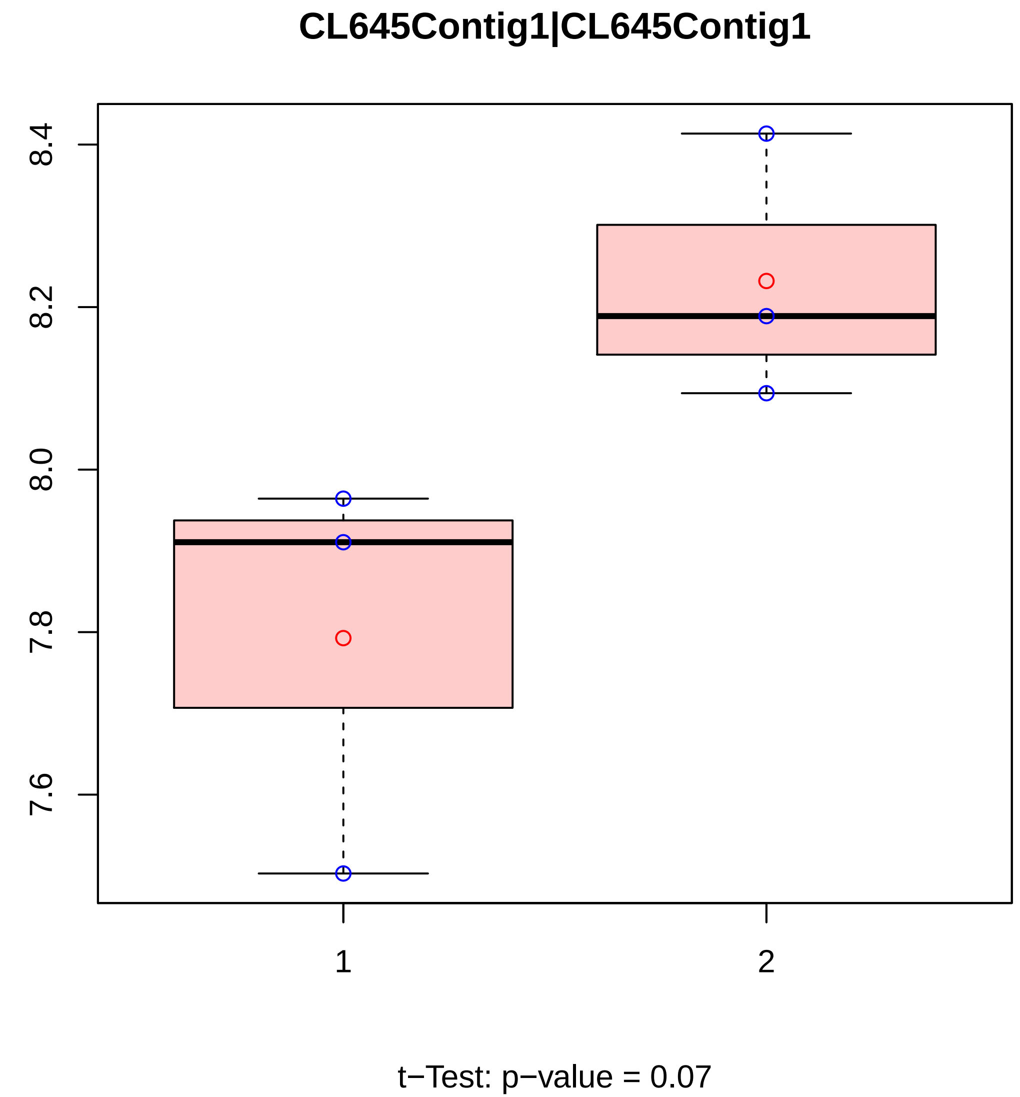

CL645Contig1|CL645Contig1
8.4
8.2
8.0
7.8
7.6
1
2
t−Test: p−value = 0.07

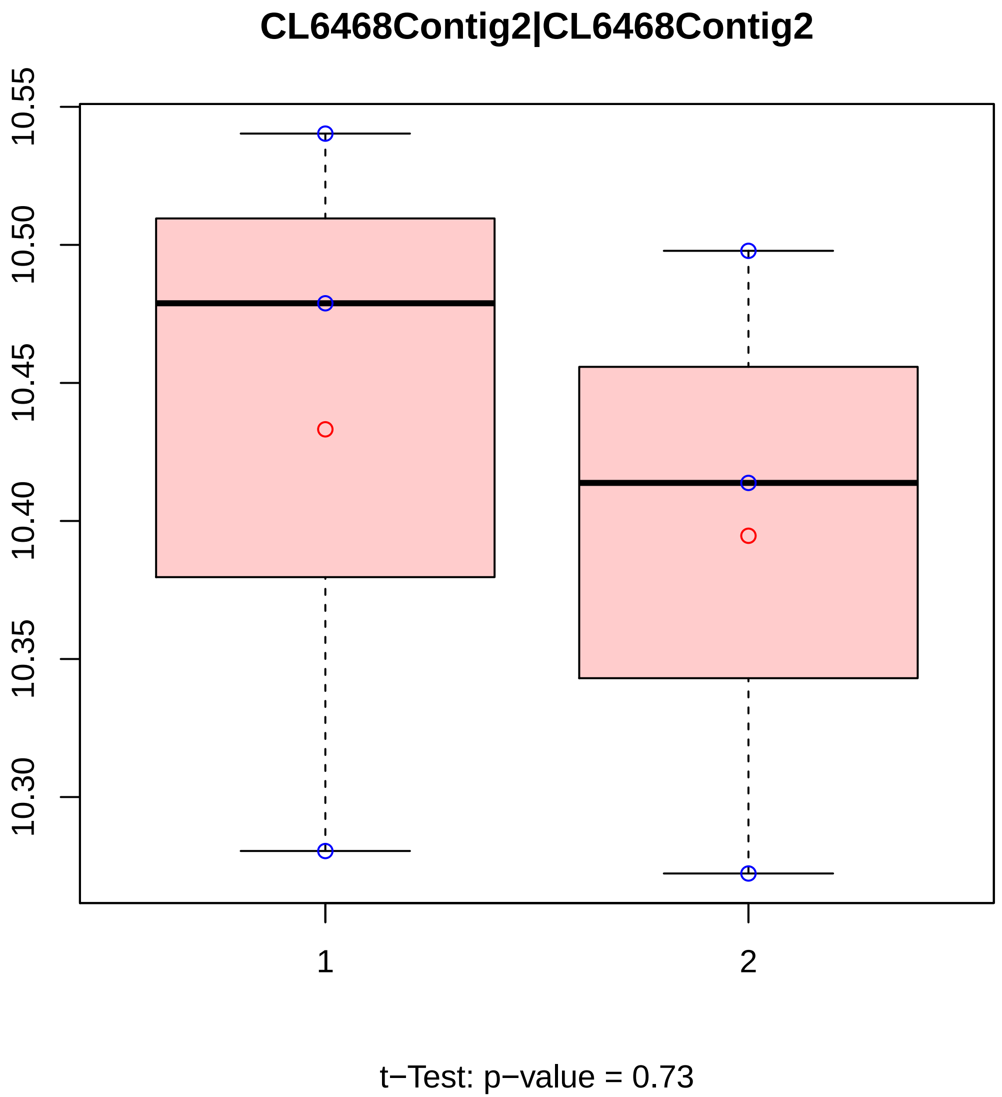

CL6468Contig2|CL6468Contig2
10.55
10.50
10.45
10.40
10.35
10.30
1
2
t−Test: p−value = 0.73

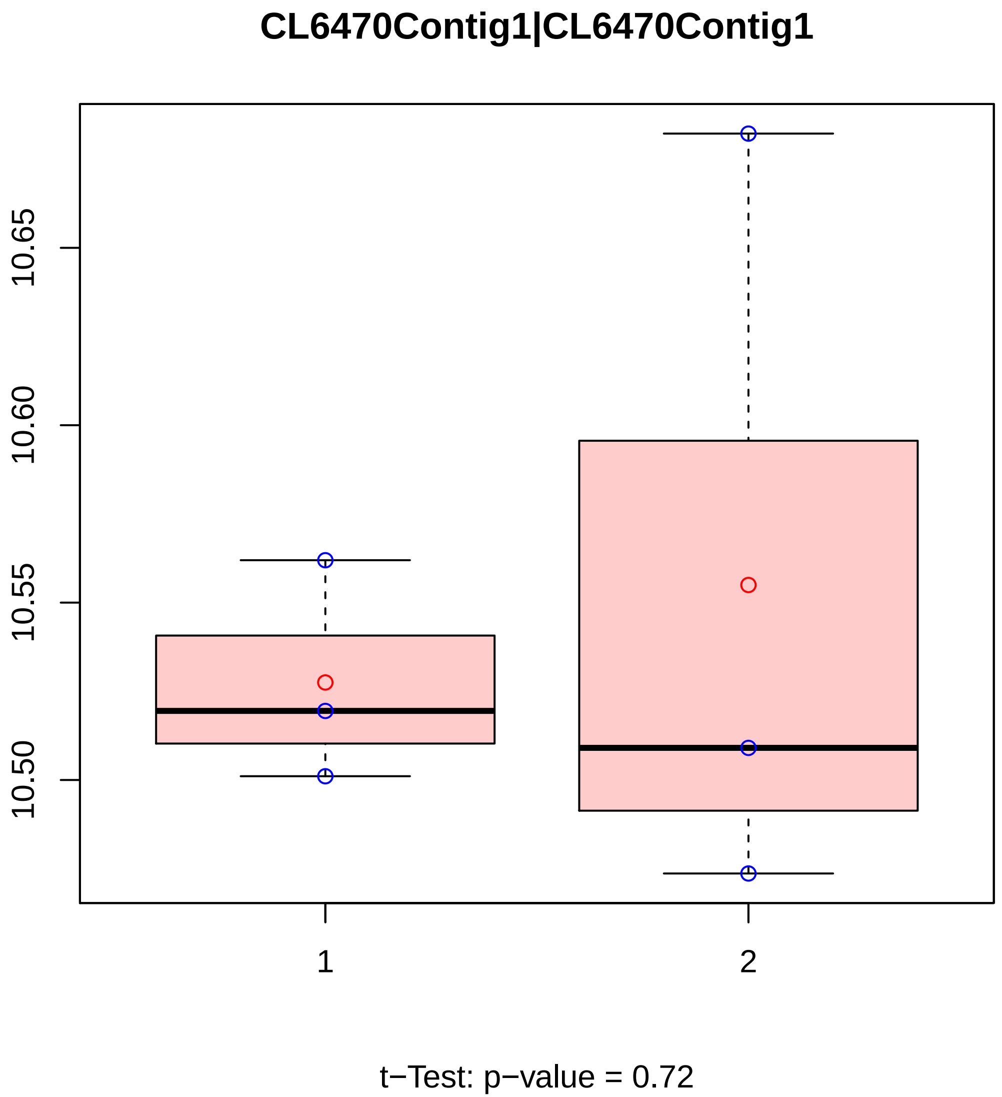

CL6470Contig1|CL6470Contig1
10.50
10.55
10.60
10.65
1
2
t−Test: p−value = 0.72

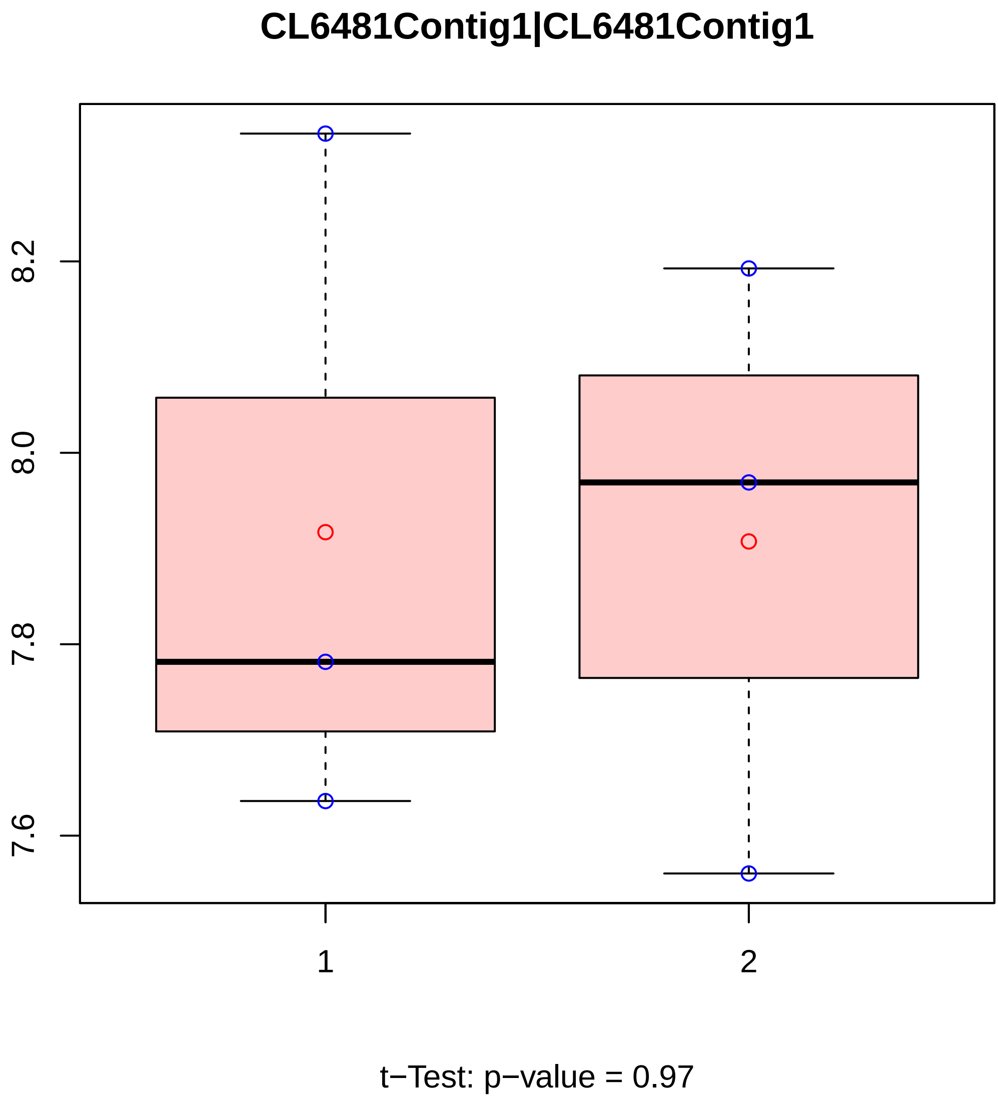
CL6481Contig1|CL6481Contig1
8.2
8.0
7.8
7.6
1
2
t−Test: p−value = 0.97

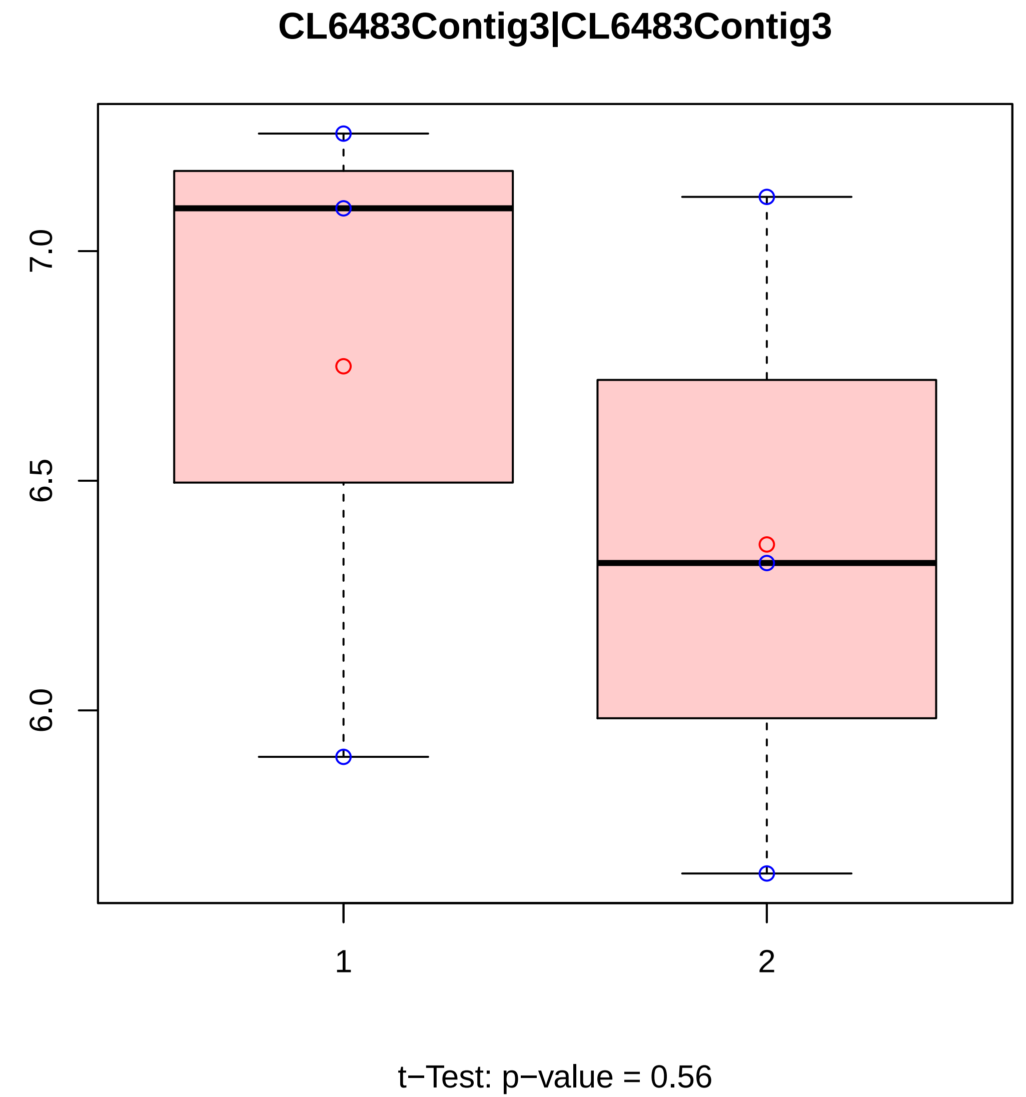

CL6483Contig3|CL6483Contig3
7.0
6.5
6.0
1
2
t−Test: p−value = 0.56

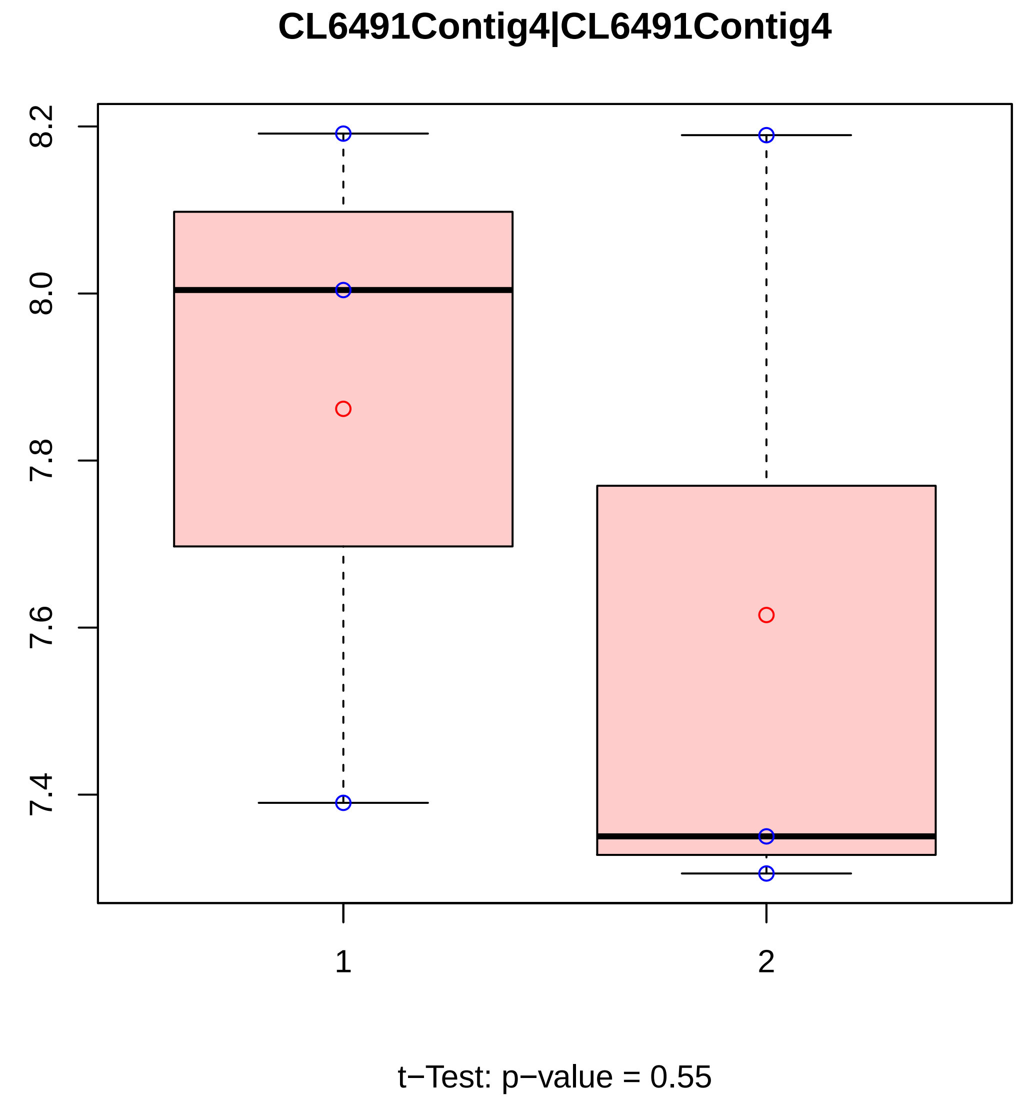

CL6491Contig4|CL6491Contig4
8.2
8.0
7.8
7.6
7.4
1
2
t−Test: p−value = 0.55

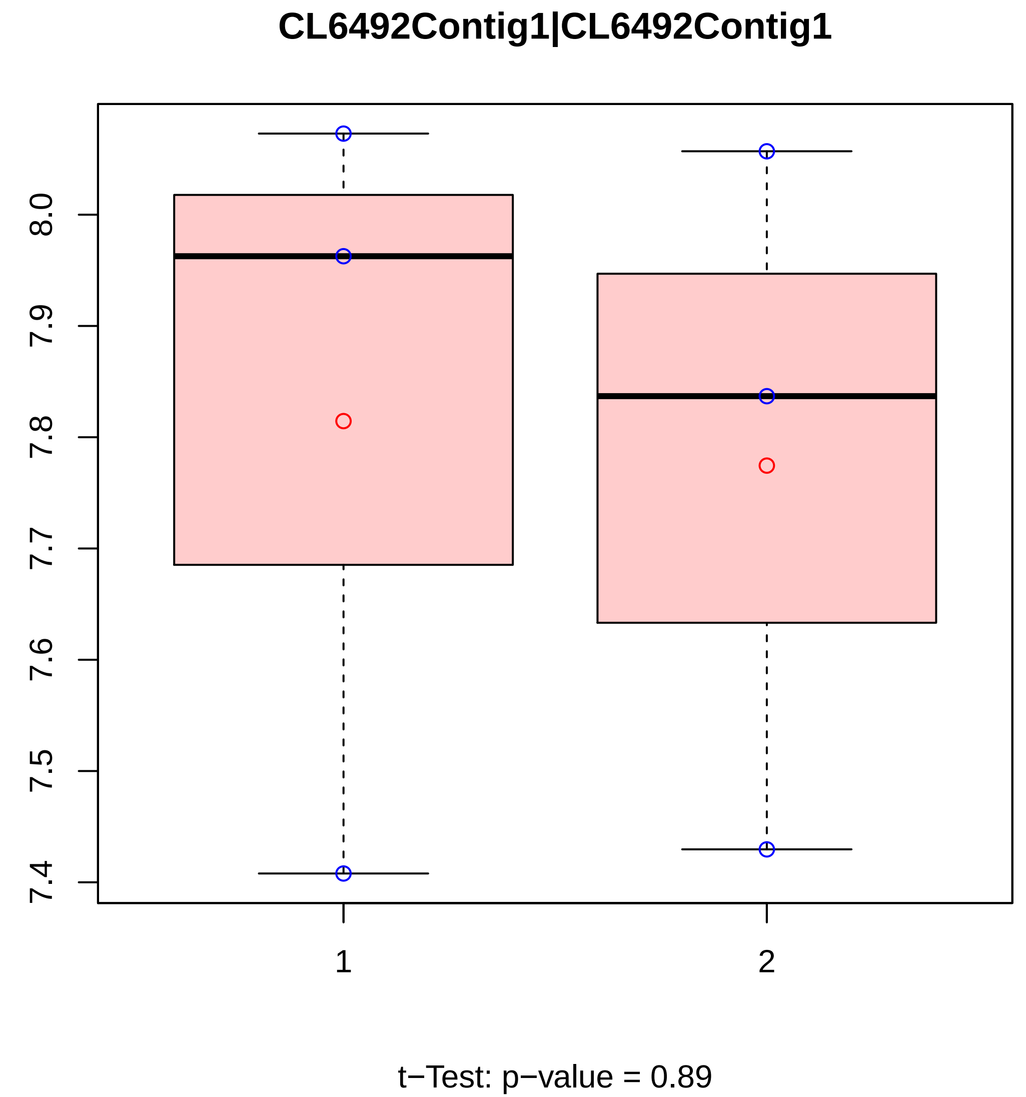

CL6492Contig1|CL6492Contig1
8.0
7.9
7.8
7.7
7.6
7.5
7.4
1
2
t−Test: p−value = 0.89

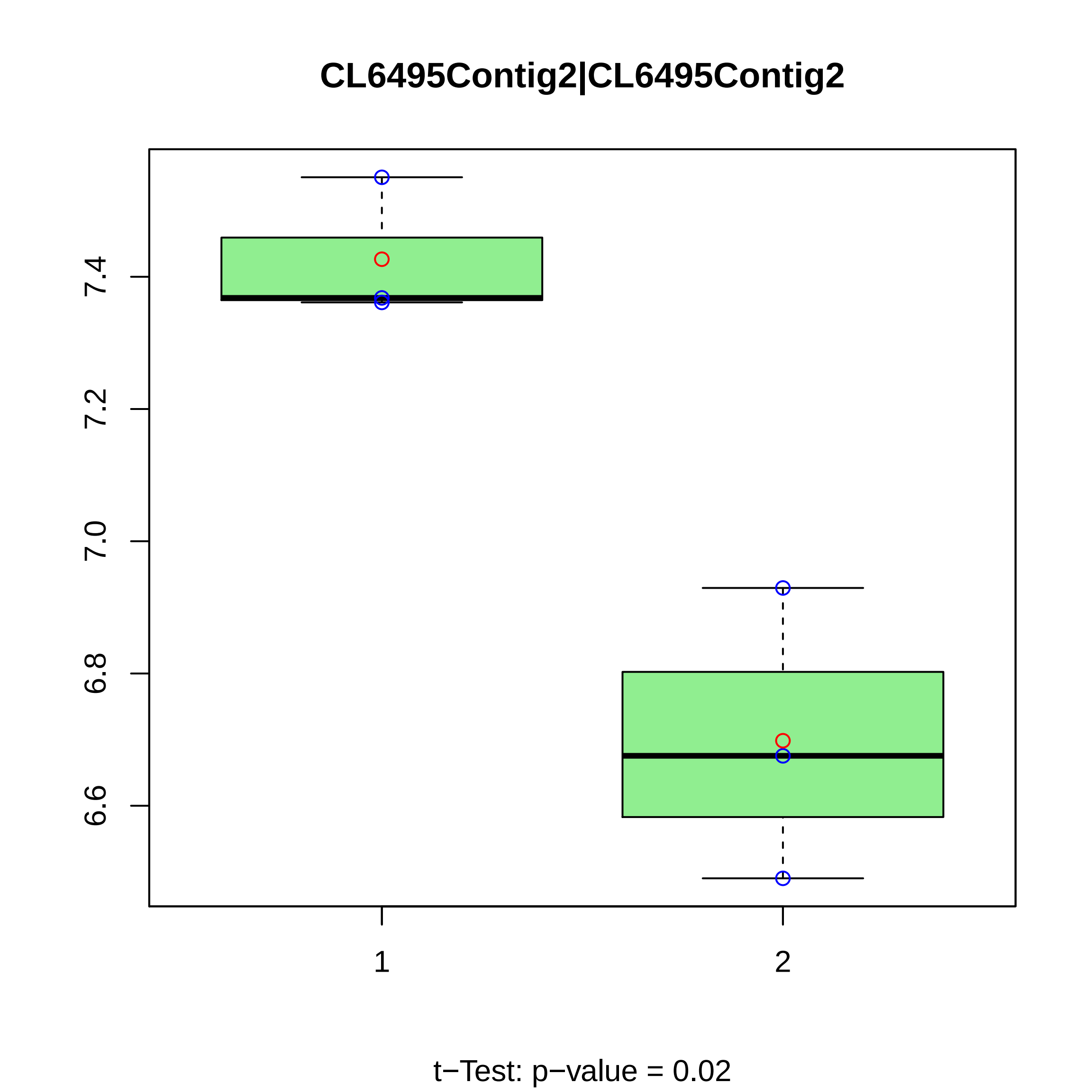
CL6495Contig2|CL6495Contig2
7.4
7.2
7.0
6.8
6.6
1
2
t−Test: p−value = 0.02

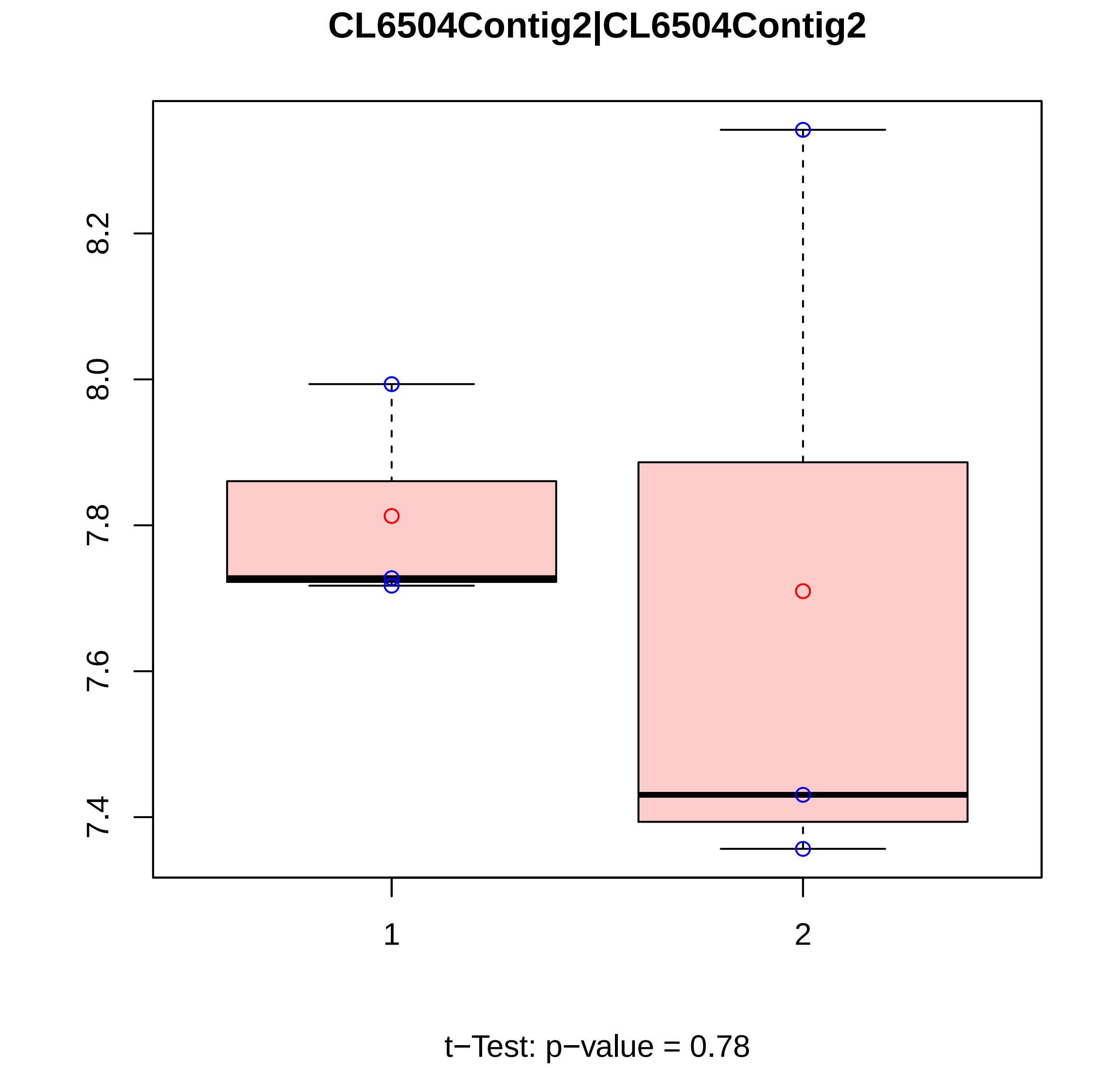

CL6504Contig2|CL6504Contig2
8.2
8.0
7.8
7.6
7.4
1
2
t−Test: p−value = 0.78

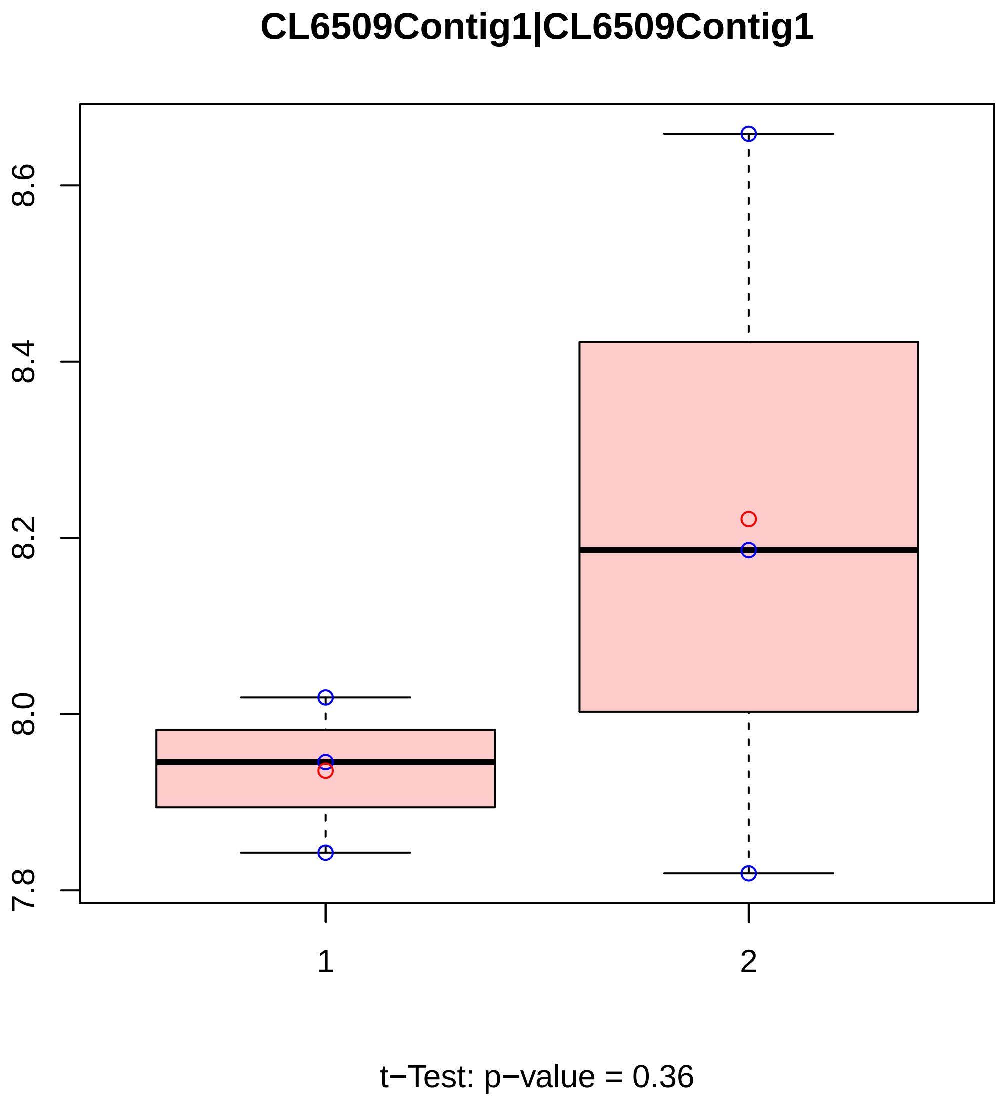
CL6509Contig1|CL6509Contig1
8.6
8.4
8.2
8.0
7.8
1
2
t−Test: p−value = 0.36

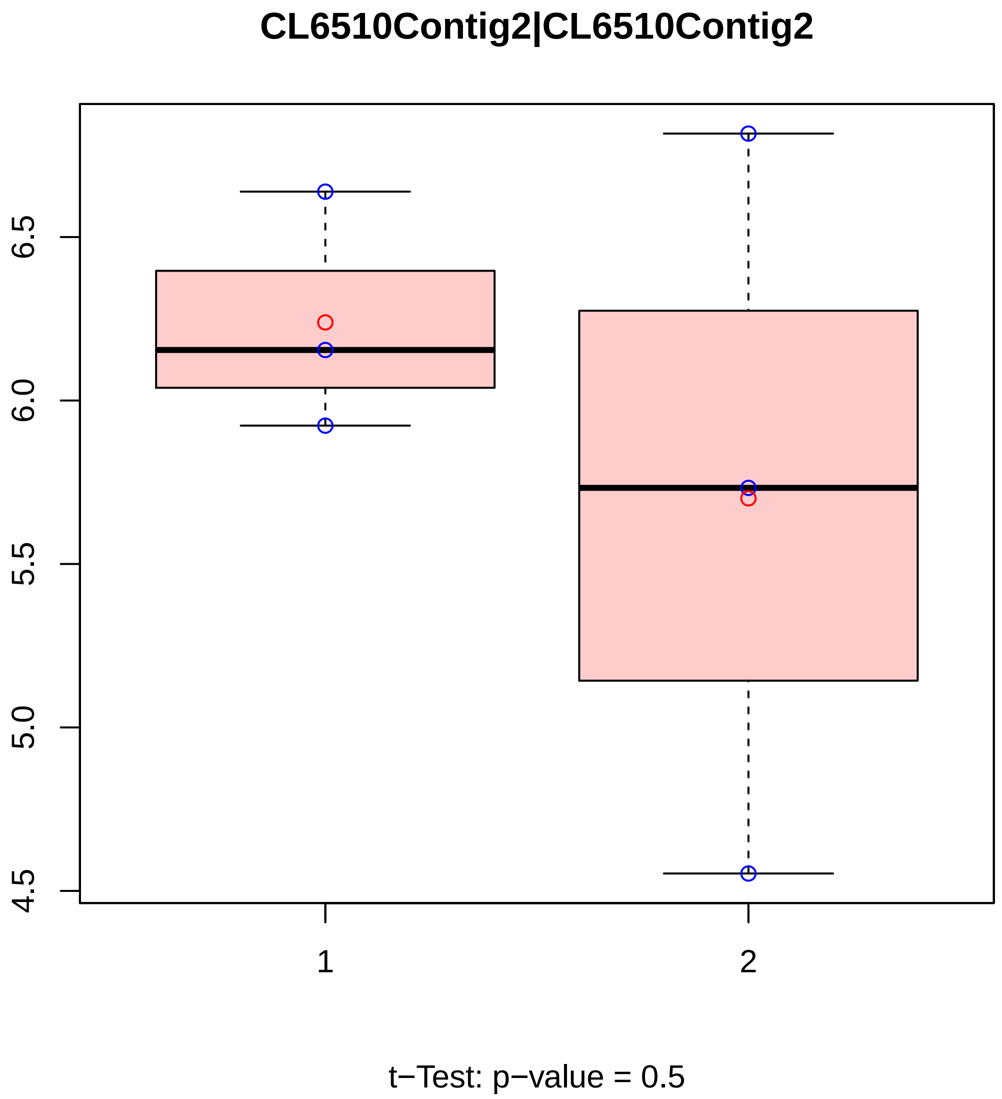

CL6510Contig2|CL6510Contig2
6.5
6.0
5.5
5.0
4.5
1
2
t−Test: p−value = 0.5

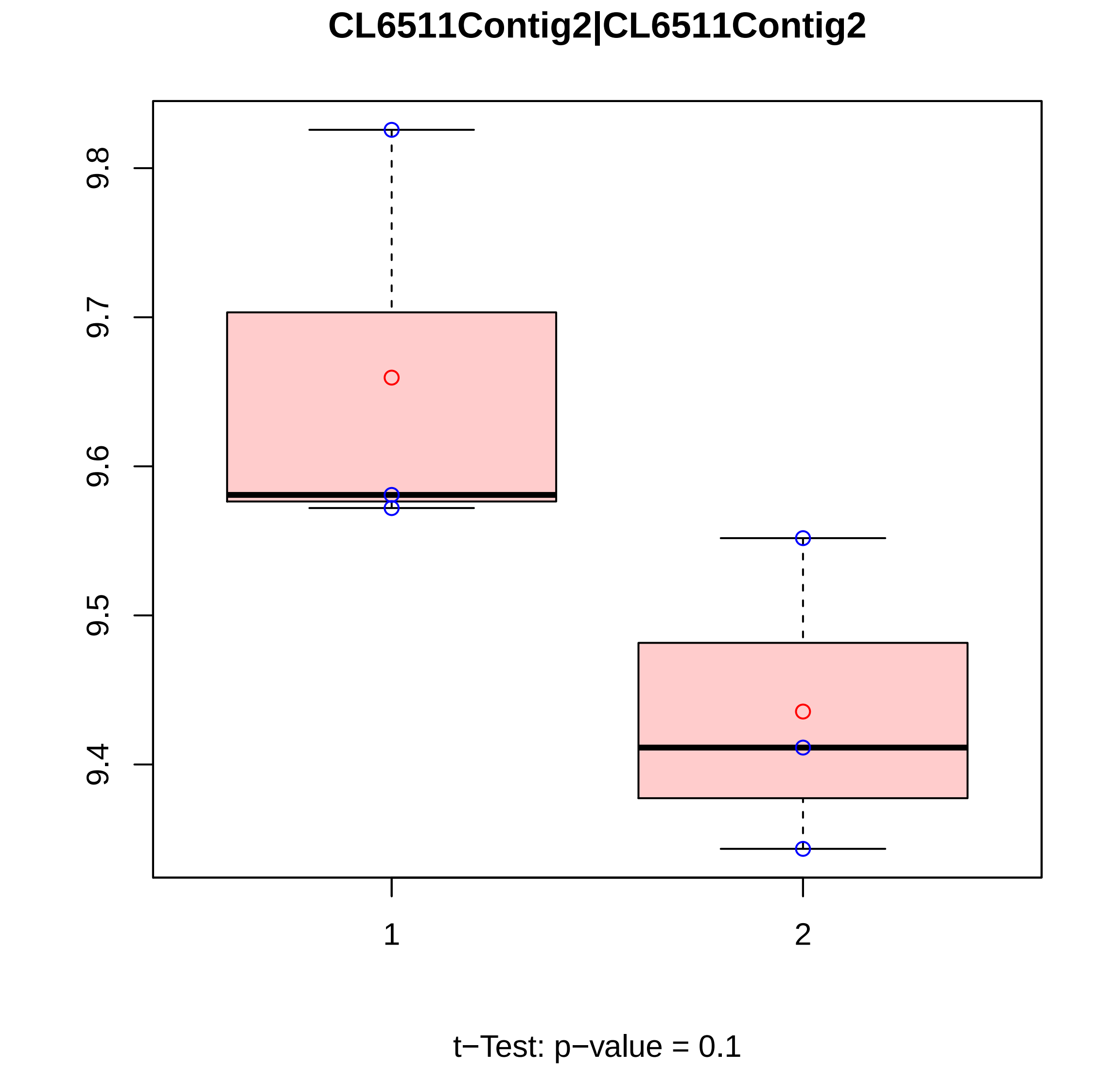
CL6511Contig2|CL6511Contig2
9.8
9.7
9.6
9.5
9.4
1
2
t−Test: p−value = 0.1

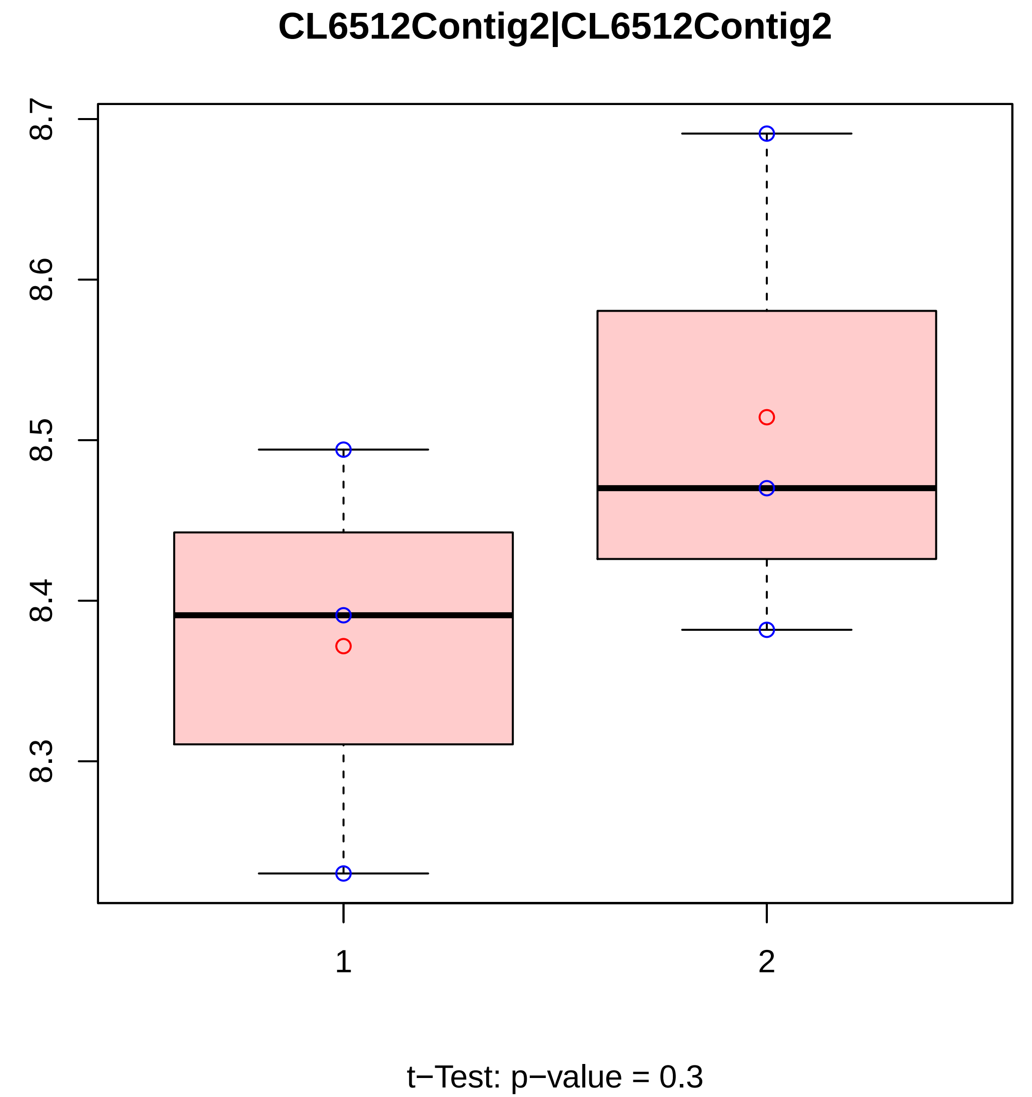
CL6512Contig2|CL6512Contig2
8.7
8.6
8.5
8.4
8.3
1
2
t−Test: p−value = 0.3

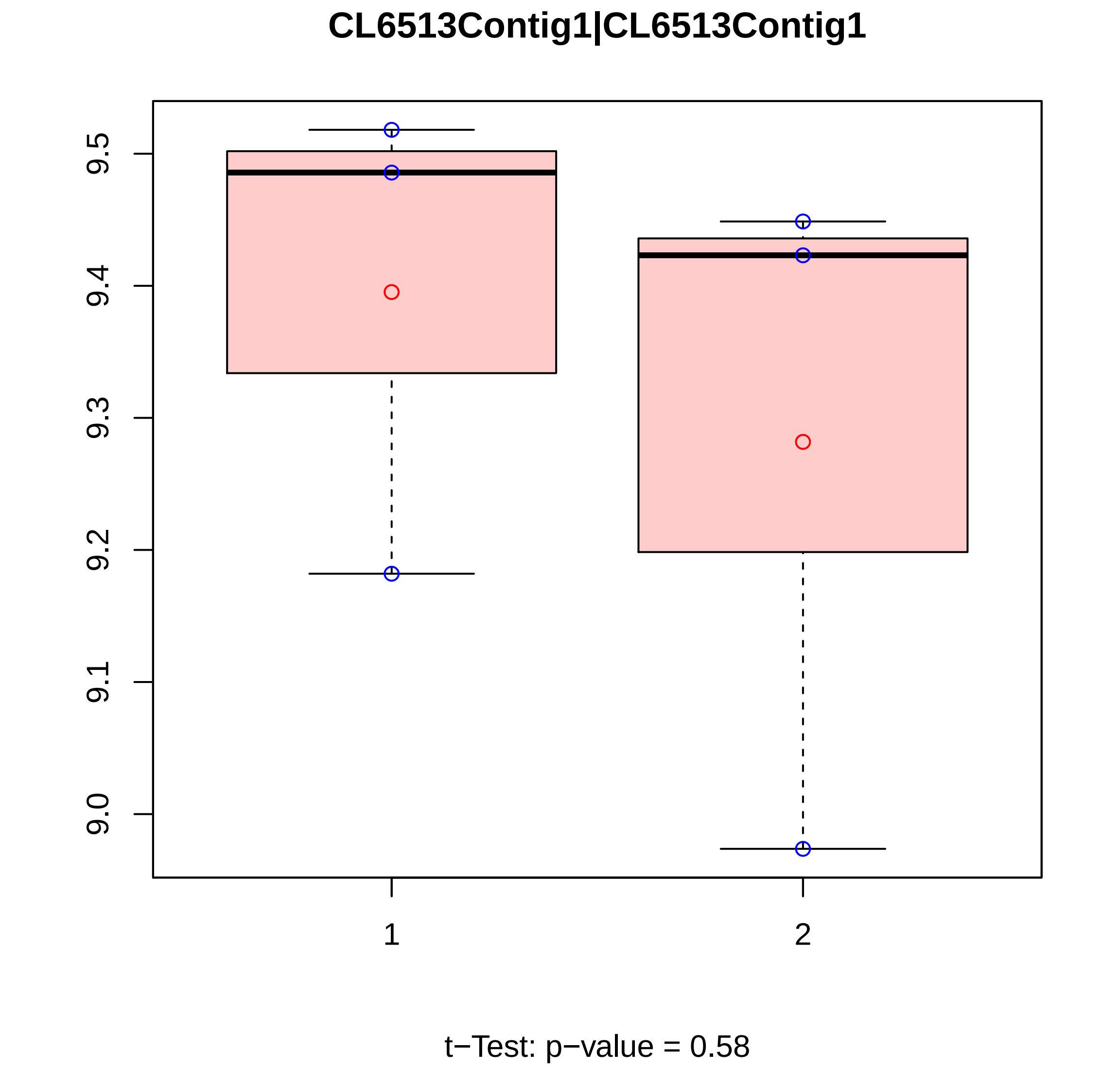
CL6513Contig1|CL6513Contig1
9.5
9.4
9.3
9.2
9.1
9.0
1
2
t−Test: p−value = 0.58

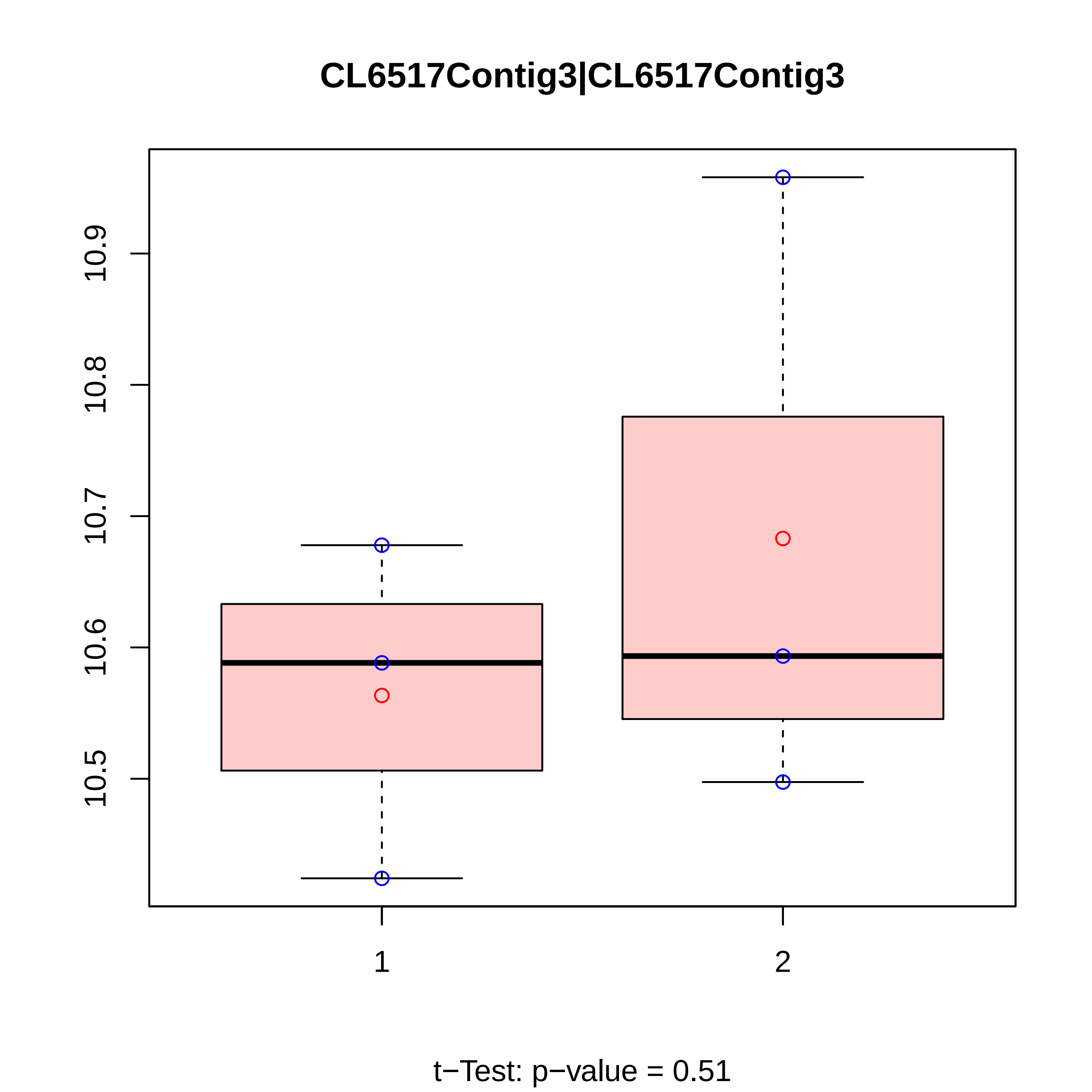

CL6517Contig3|CL6517Contig3
10.5
10.6
10.7
10.8
10.9
1
2
t−Test: p−value = 0.51

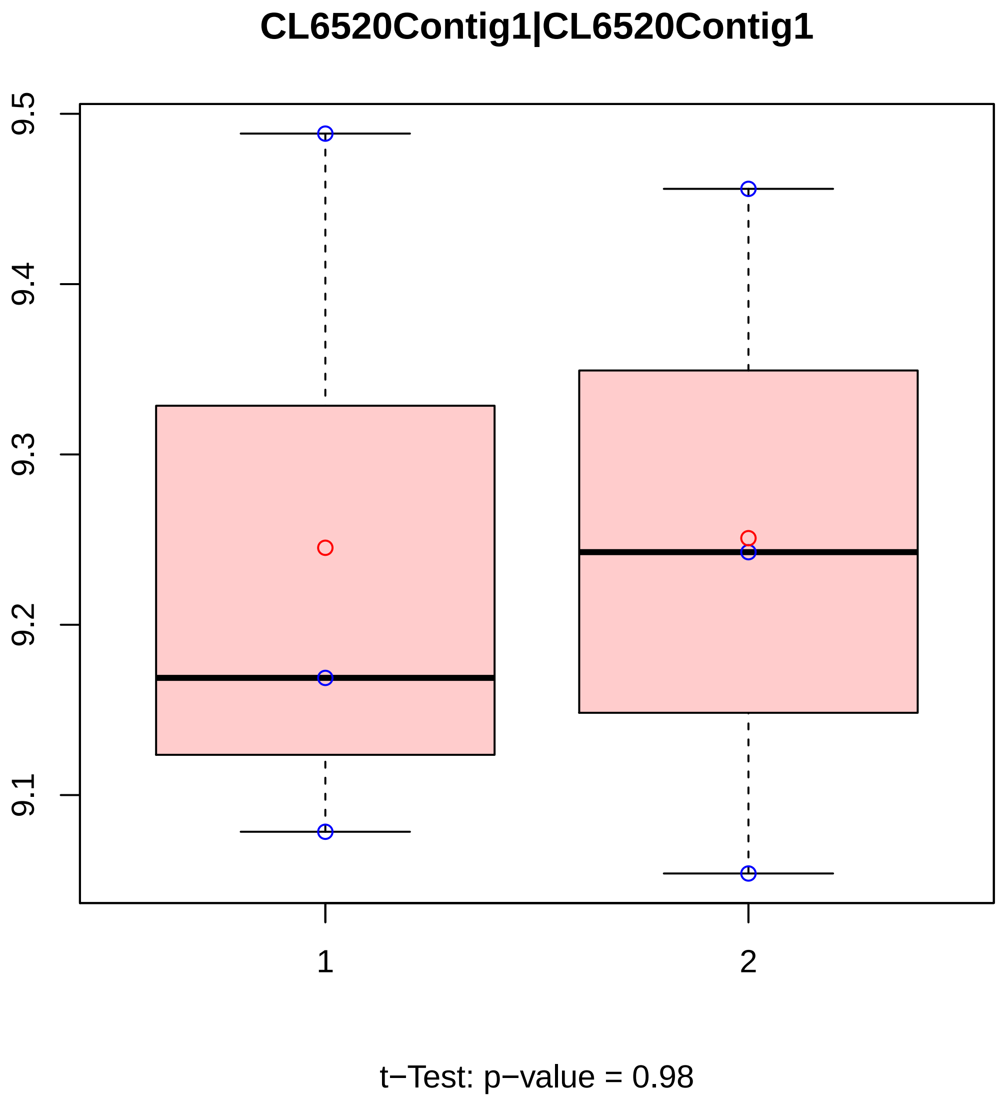

CL6520Contig1|CL6520Contig1
9.1
9.2
9.3
9.4
9.5
1
2
t−Test: p−value = 0.98

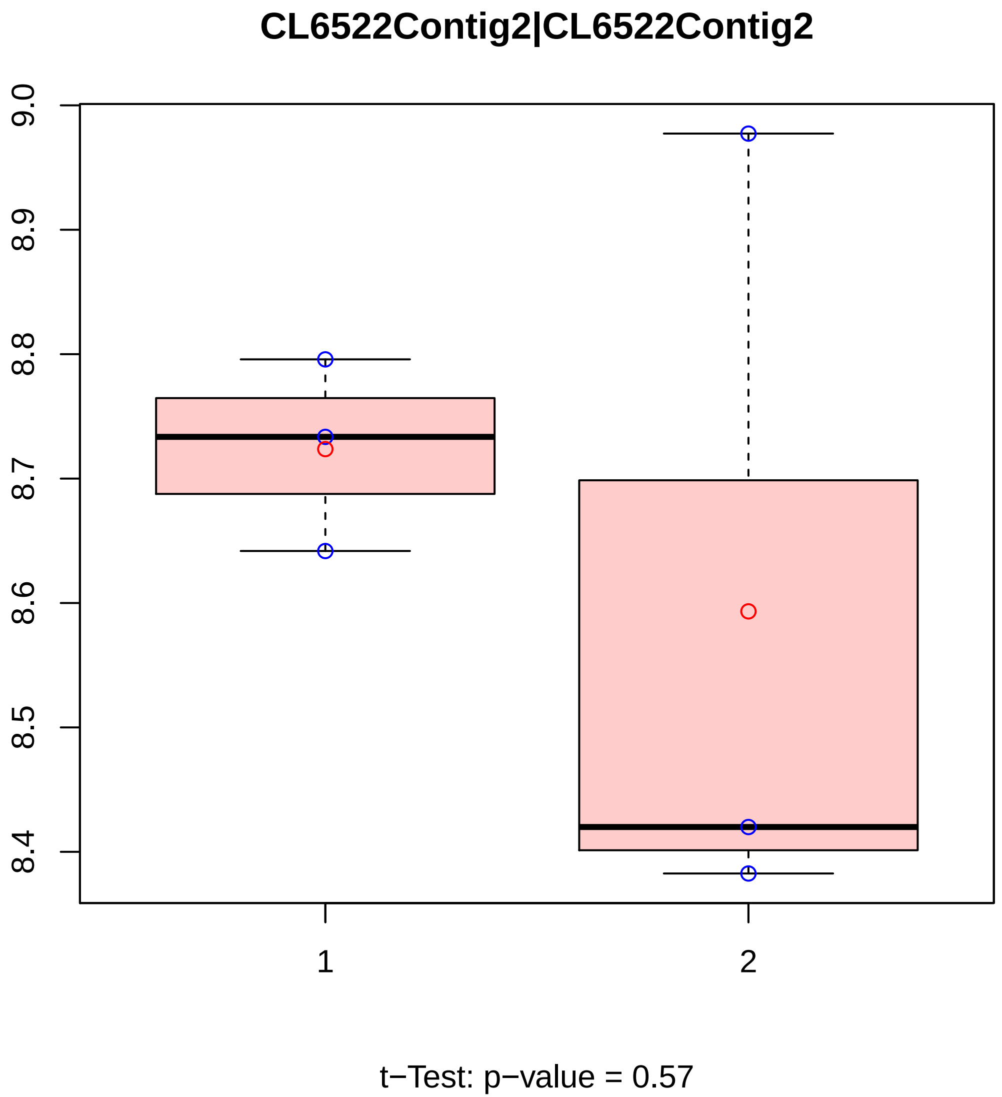
CL6522Contig2|CL6522Contig2
9.0
8.9
8.8
8.7
8.6
8.5
8.4
1
2
t−Test: p−value = 0.57

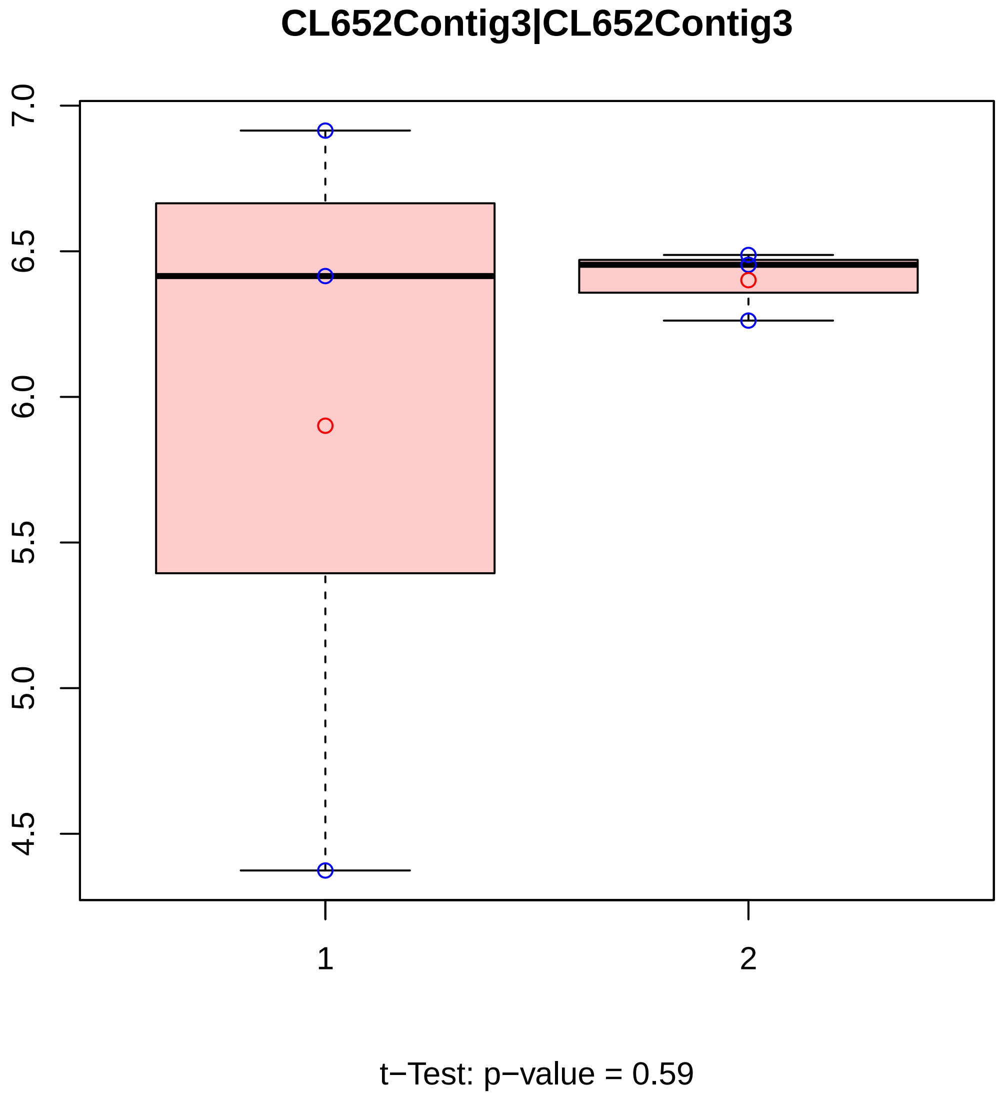

CL652Contig3|CL652Contig3
7.0
6.5
6.0
5.5
5.0
4.5
1
2
t−Test: p−value = 0.59

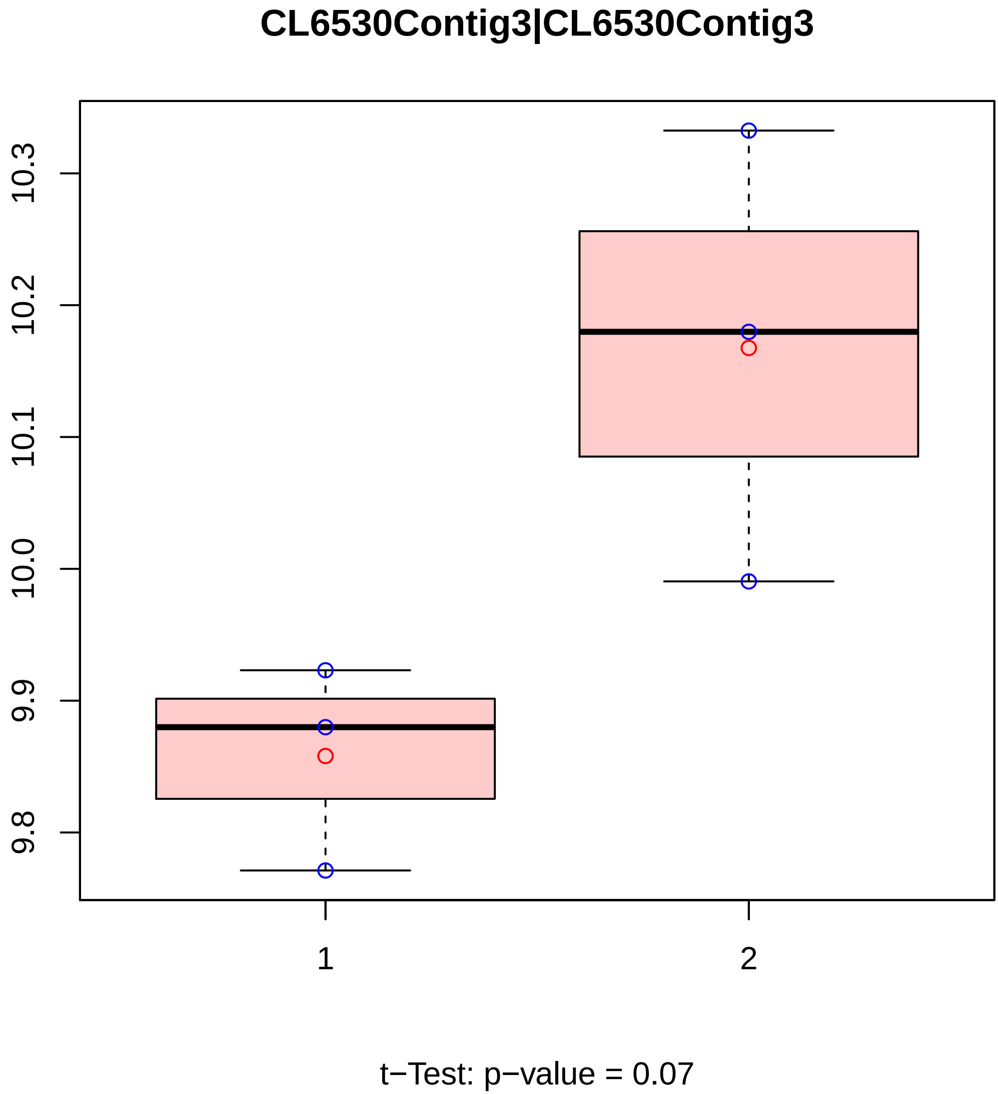

CL6530Contig3|CL6530Contig3
10.3
10.2
10.1
10.0
9.9
9.8
1
2
t−Test: p−value = 0.07

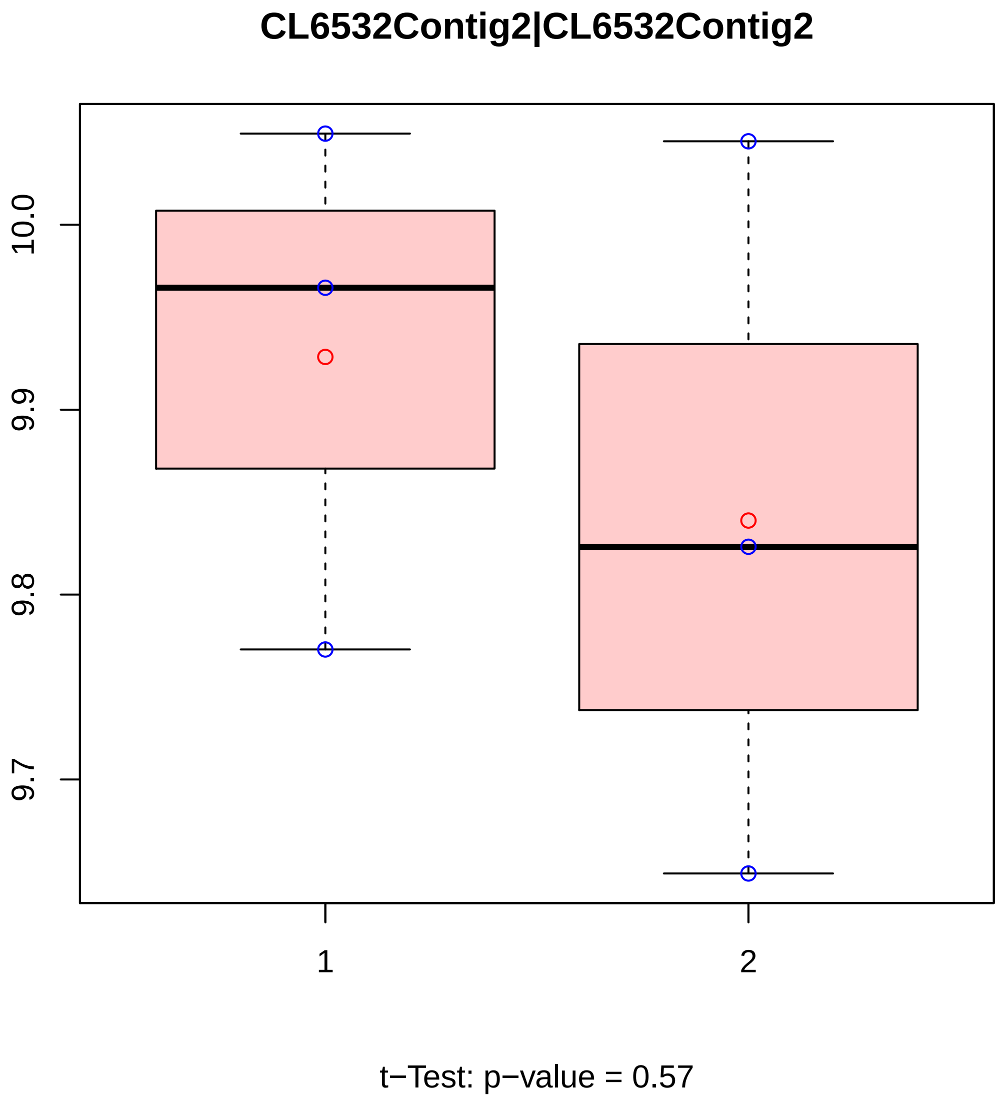
CL6532Contig2|CL6532Contig2
10.0
9.9
9.8
9.7
1
2
t−Test: p−value = 0.57

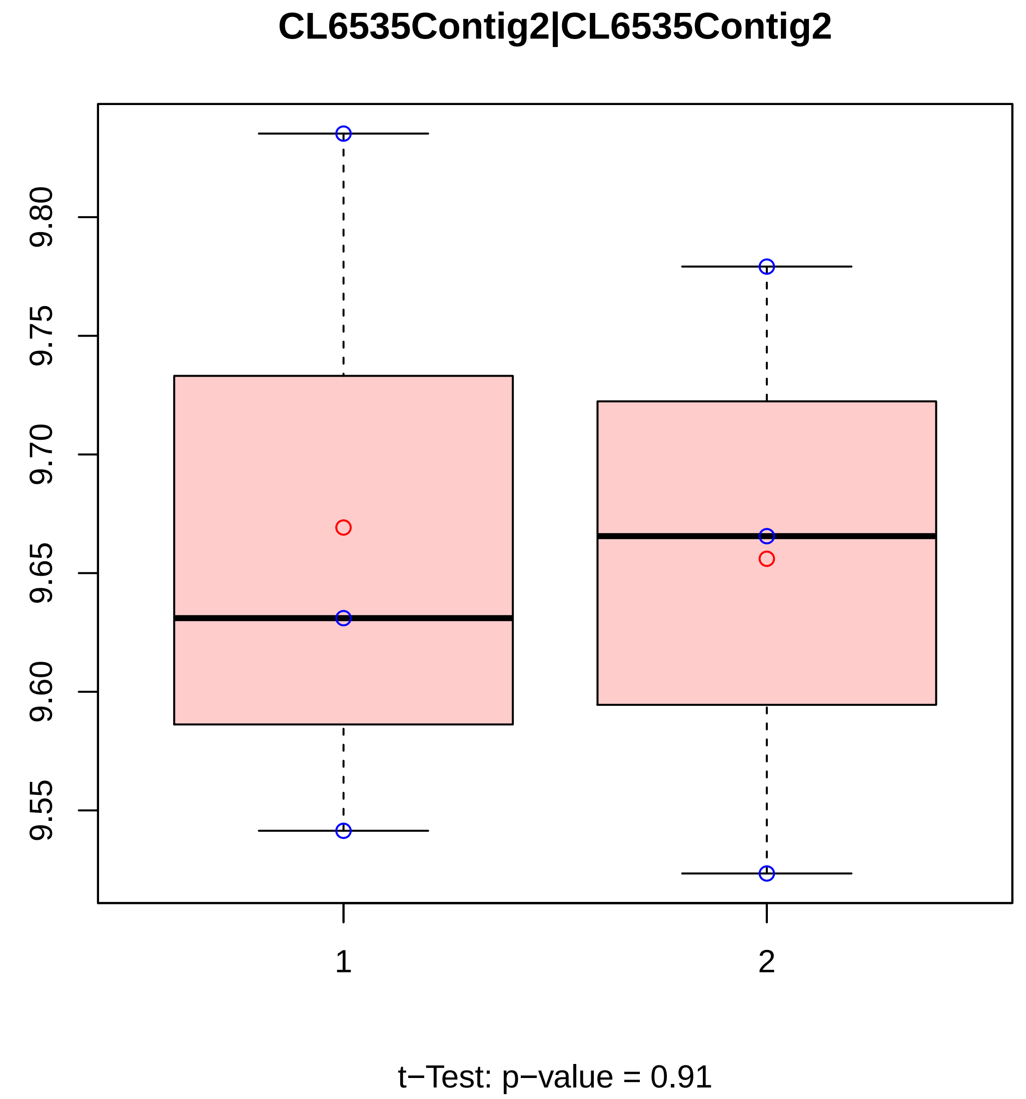
CL6535Contig2|CL6535Contig2
9.55
9.60
9.65
9.70
9.75
9.80
1
2
t−Test: p−value = 0.91

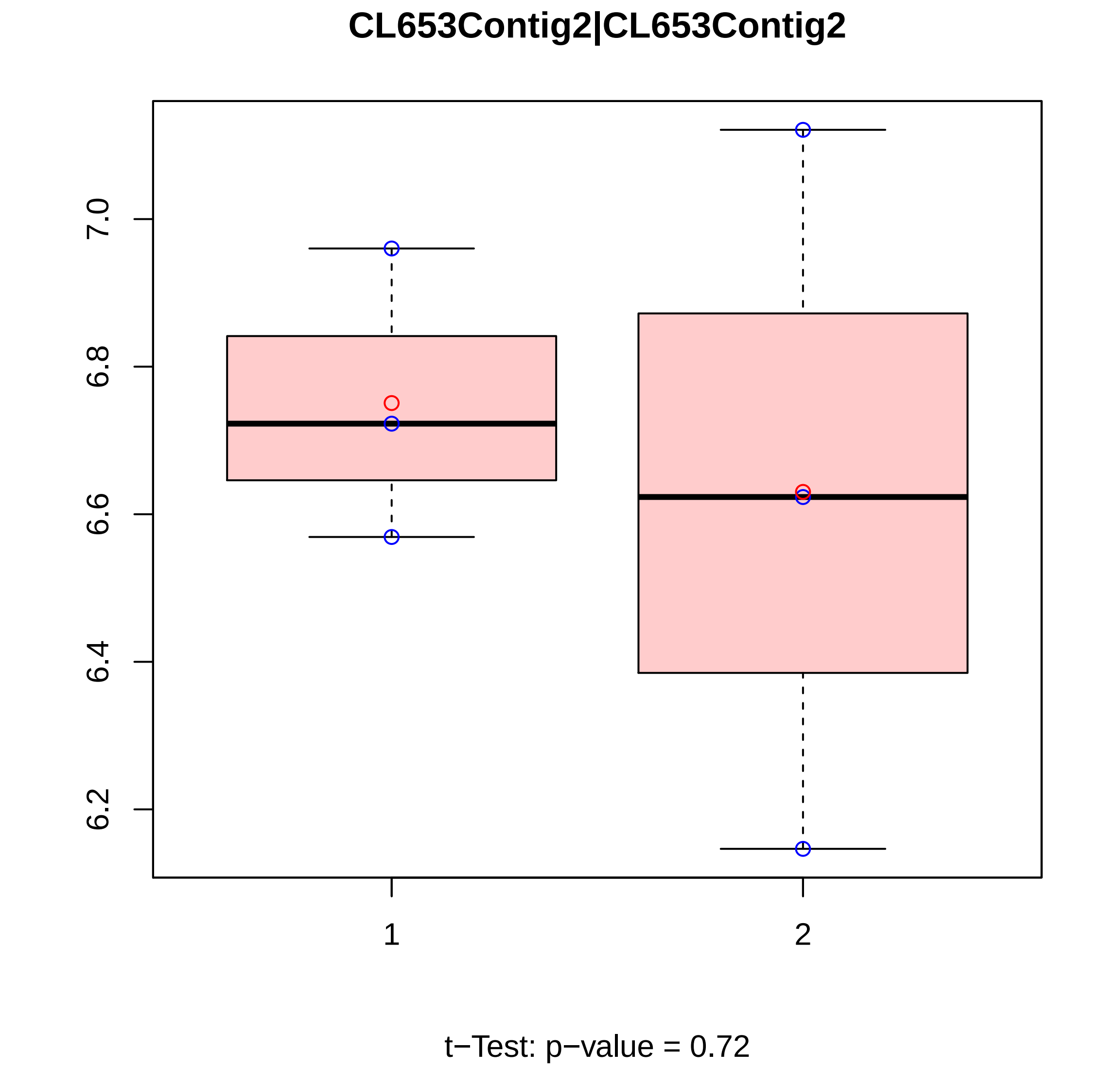
CL653Contig2|CL653Contig2
7.0
6.8
6.6
6.4
6.2
1
2
t−Test: p−value = 0.72

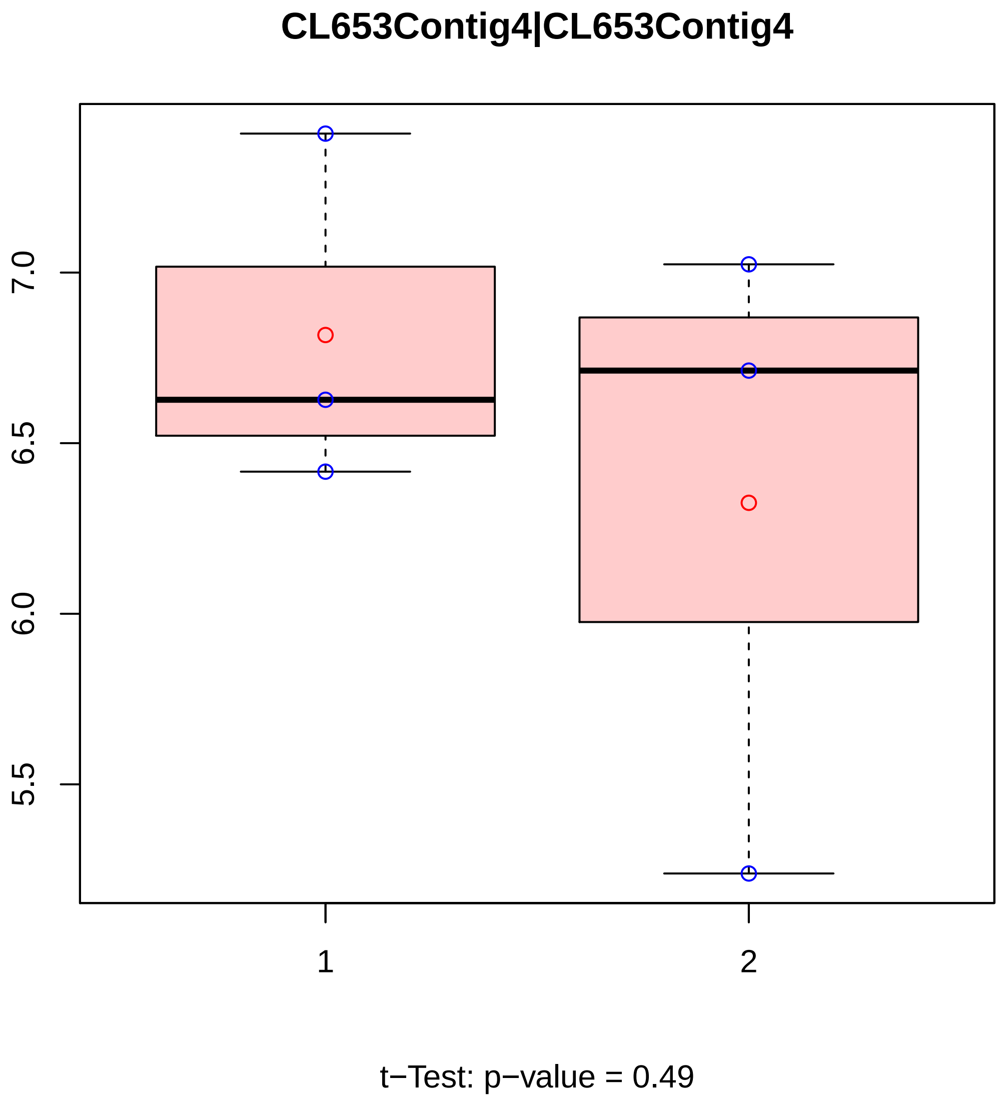
CL653Contig4|CL653Contig4
7.0
6.5
6.0
5.5
1
2
t−Test: p−value = 0.49

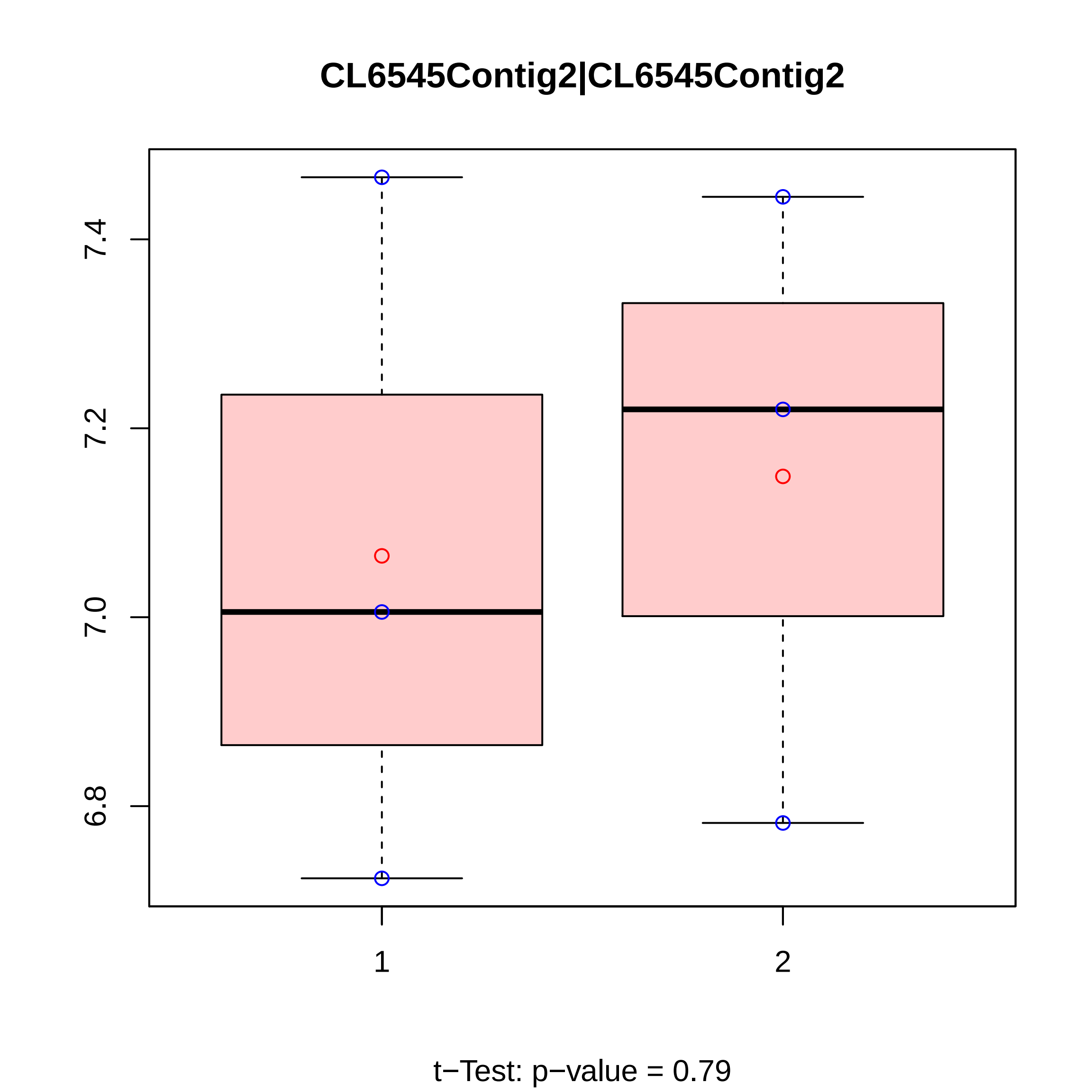
CL6545Contig2|CL6545Contig2
7.4
7.2
7.0
6.8
1
2
t−Test: p−value = 0.79

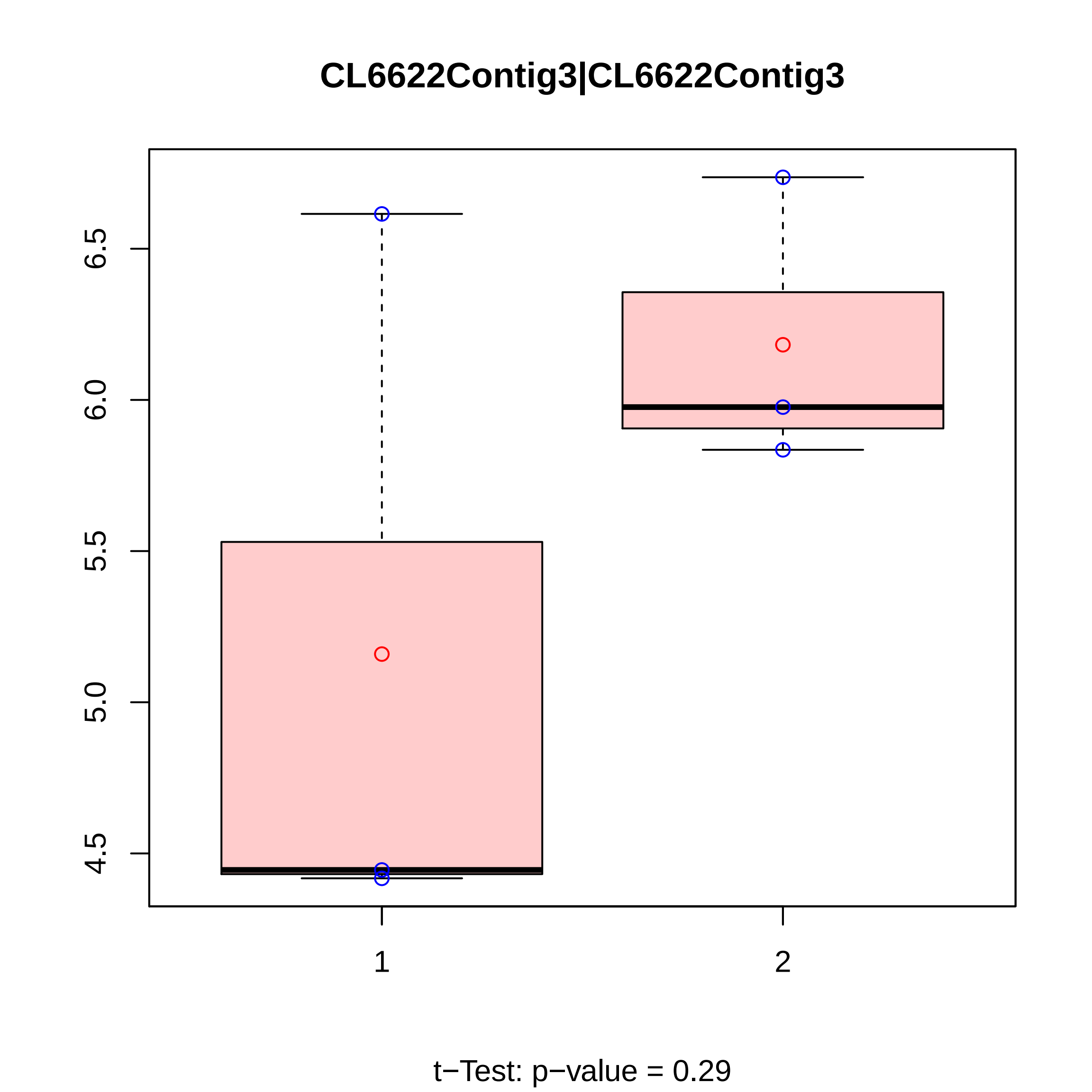

CL6622Contig3|CL6622Contig3
4.5
5.0
5.5
6.0
6.5
1
2
t−Test: p−value = 0.29

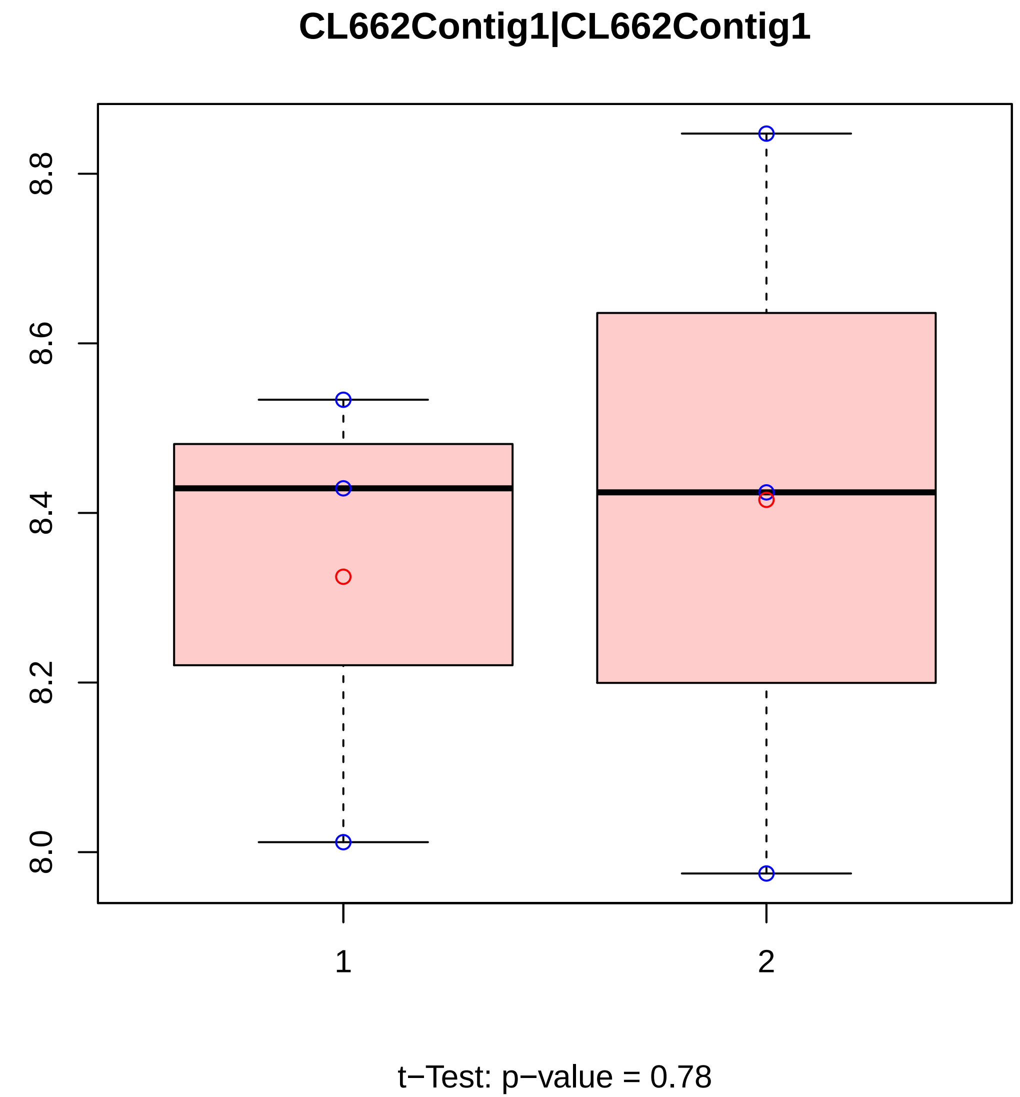
CL662Contig1|CL662Contig1
8.0
8.2
8.4
8.6
8.8
1
2
t−Test: p−value = 0.78

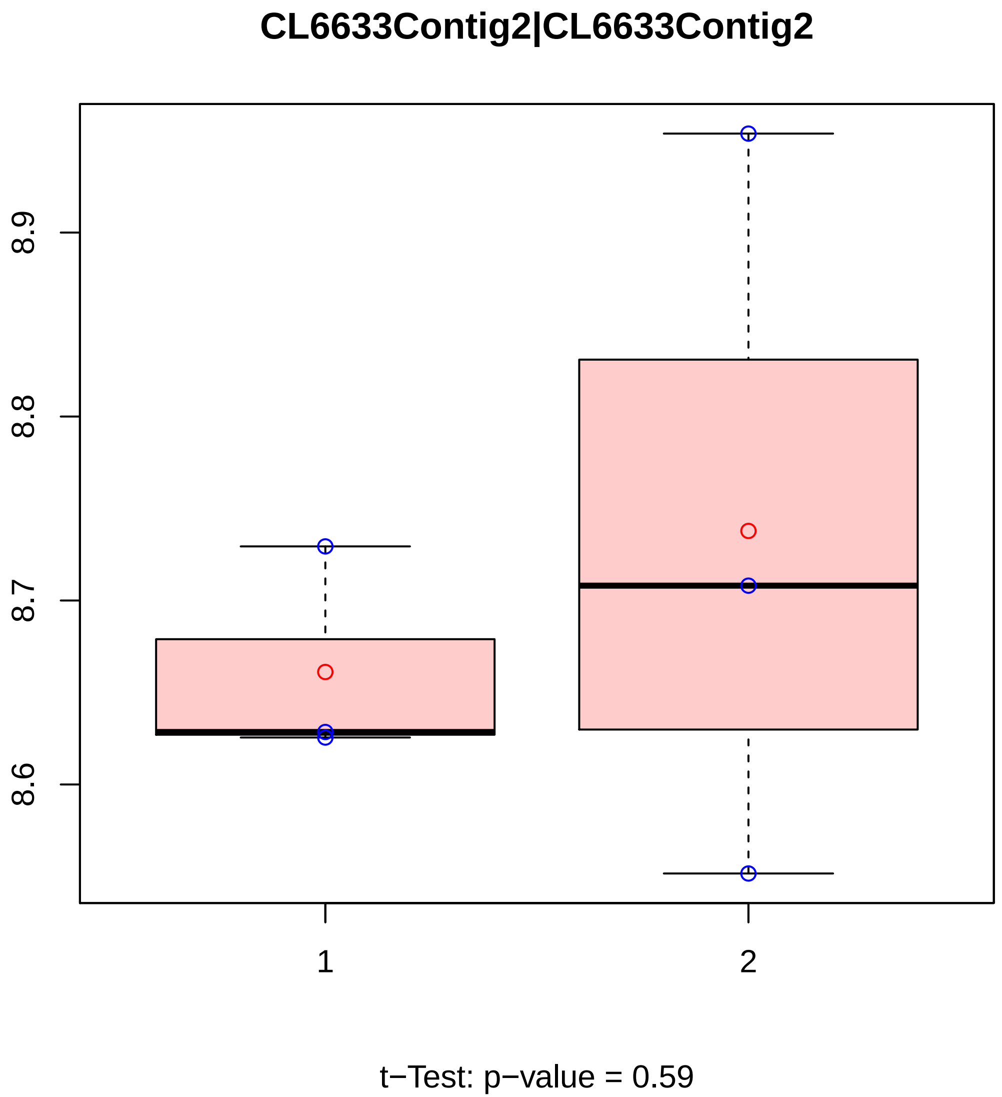
CL6633Contig2|CL6633Contig2
8.6
8.7
8.8
8.9
1
2
t−Test: p−value = 0.59

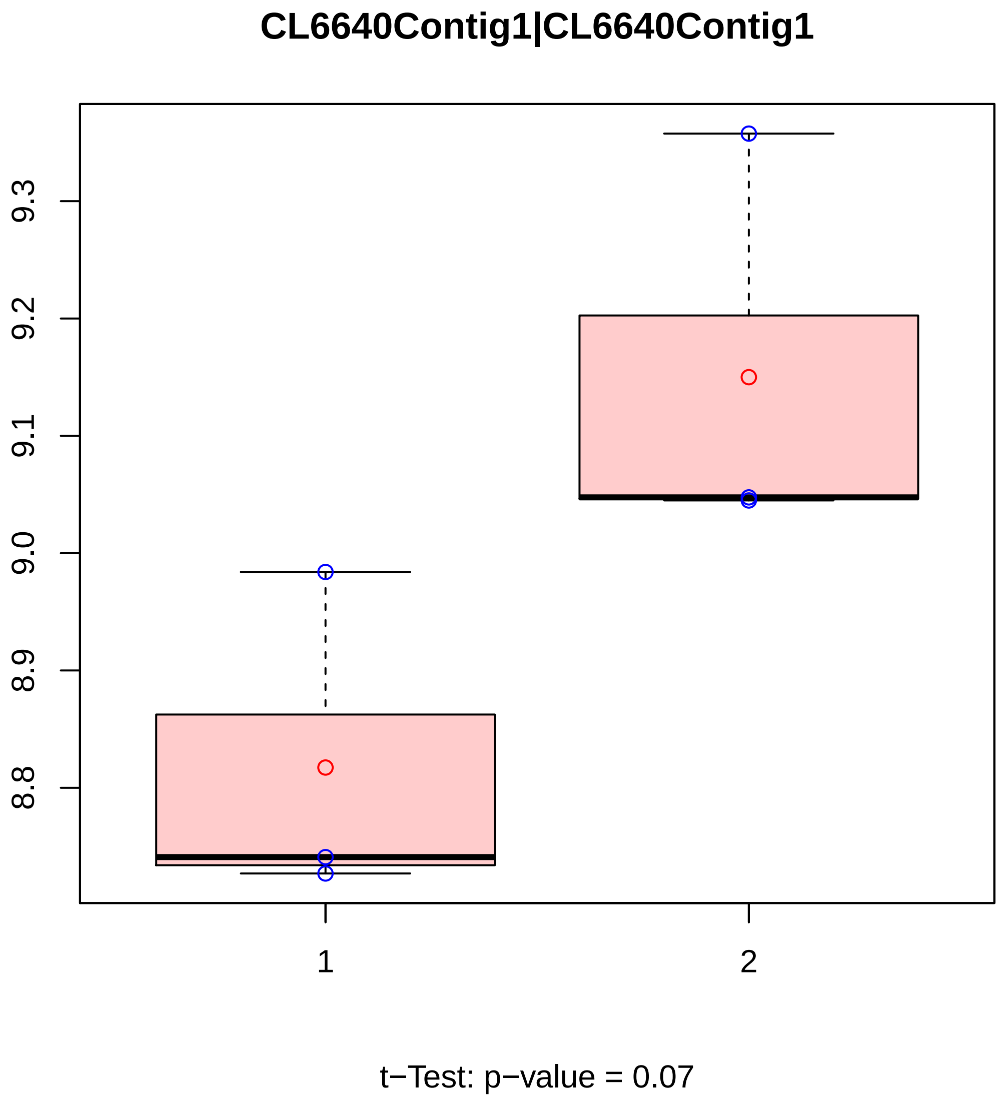
CL6640Contig1|CL6640Contig1
9.3
9.2
9.1
9.0
8.9
8.8
1
2
t−Test: p−value = 0.07

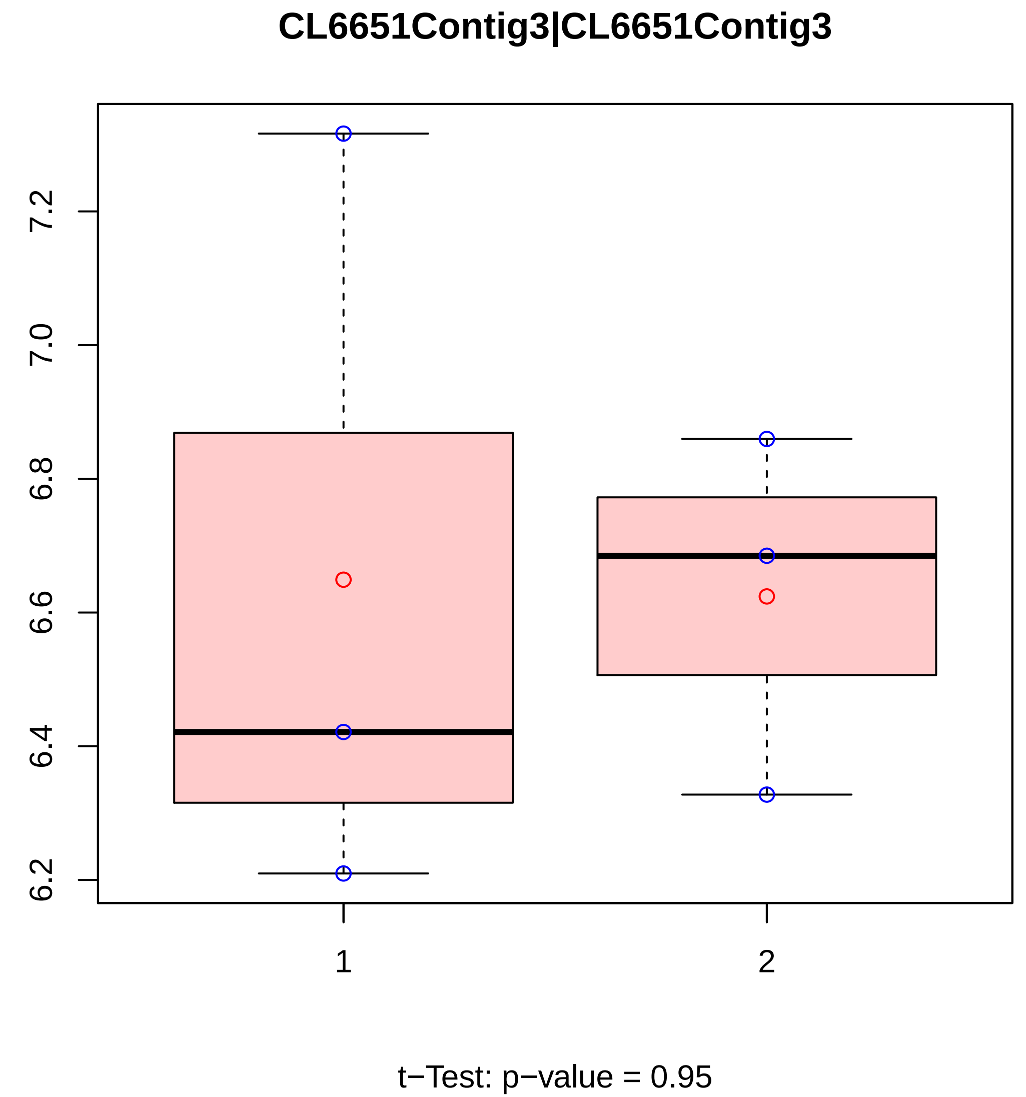
CL6651Contig3|CL6651Contig3
7.2
7.0
6.8
6.6
6.4
6.2
1
2
t−Test: p−value = 0.95

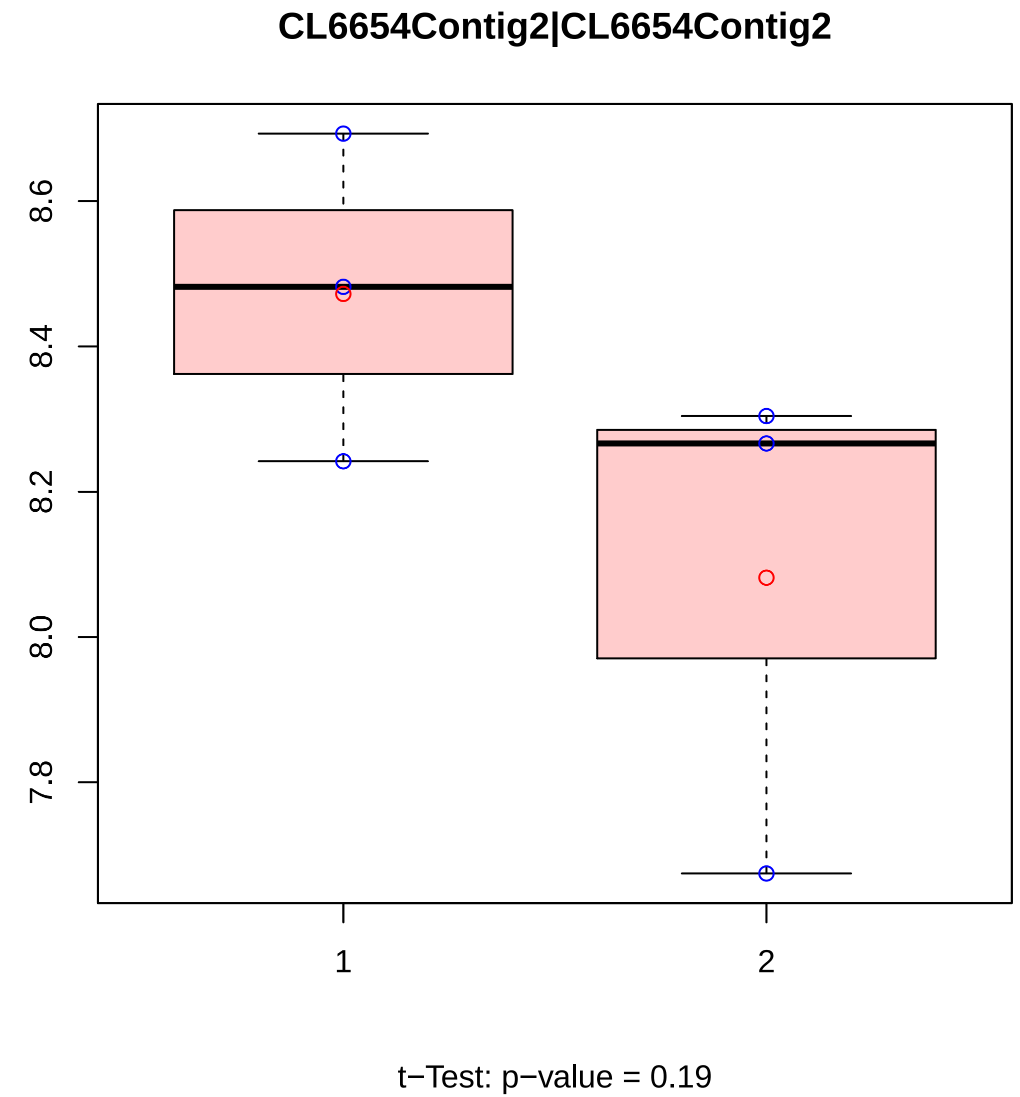

CL6654Contig2|CL6654Contig2
8.6
8.4
8.2
8.0
7.8
1
2
t−Test: p−value = 0.19

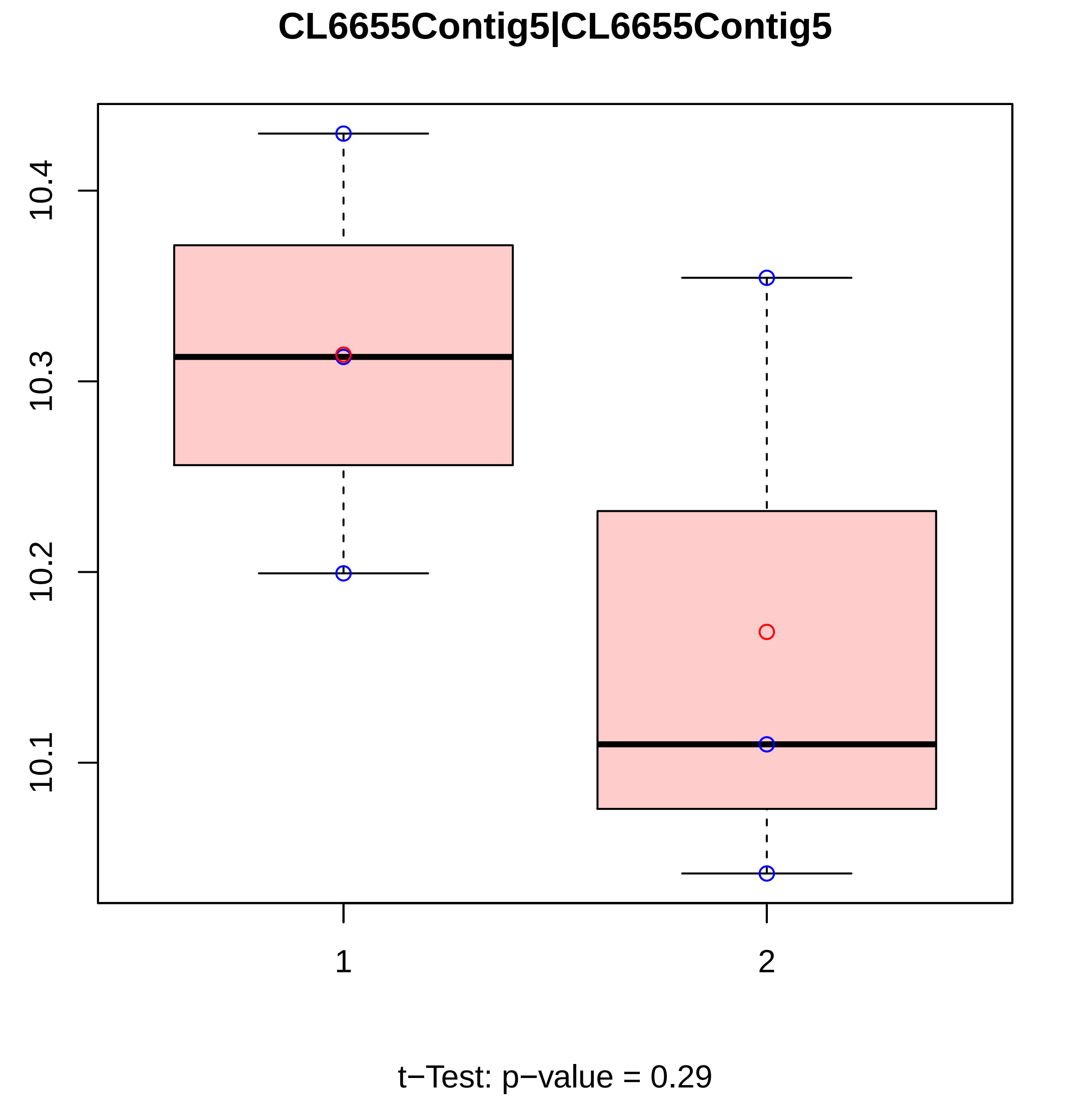
CL6655Contig5|CL6655Contig5
10.1
10.2
10.3
10.4
1
2
t−Test: p−value = 0.29

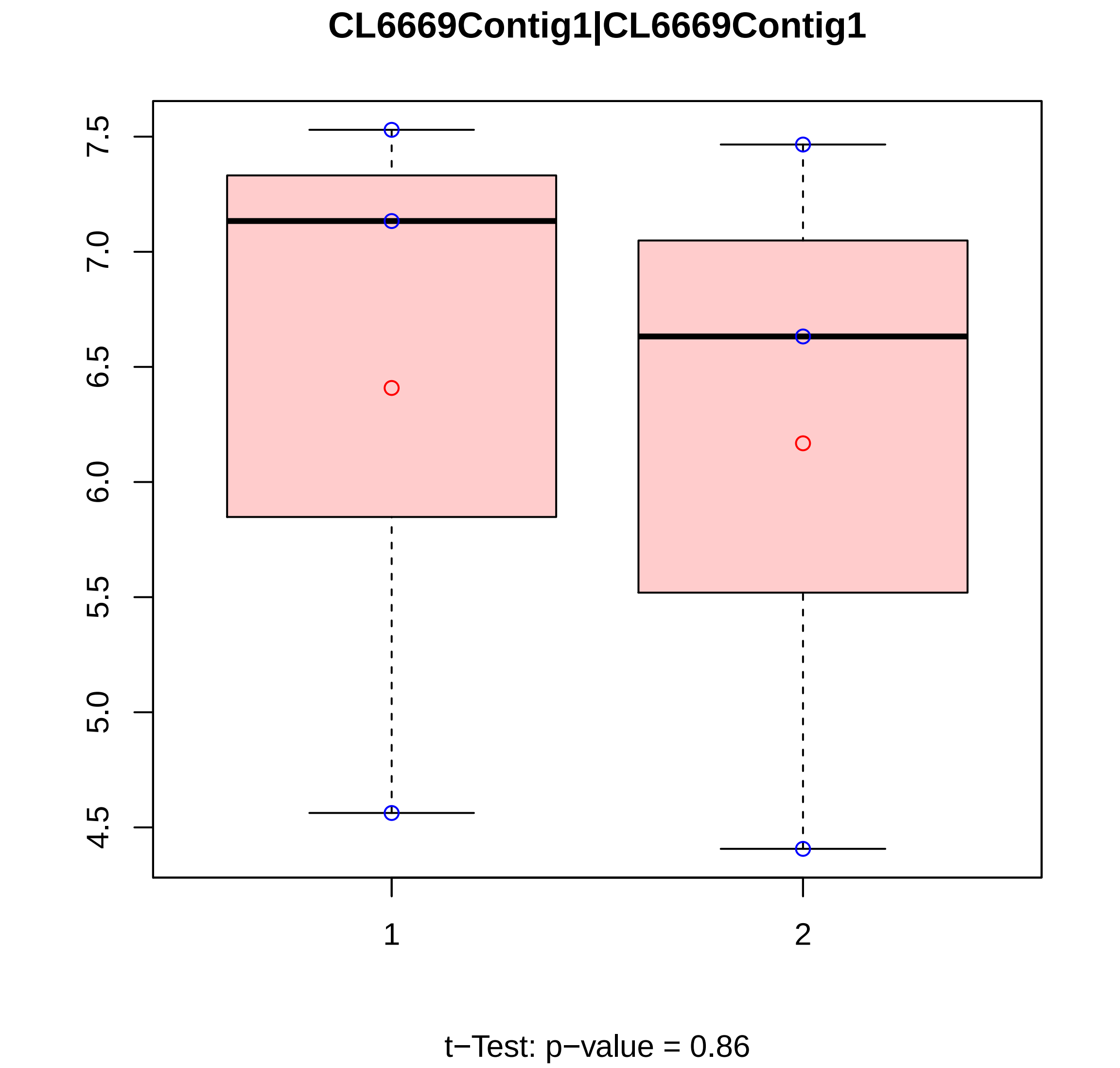
CL6669Contig1|CL6669Contig1
7.5
7.0
6.5
6.0
5.5
5.0
4.5
1
2
t−Test: p−value = 0.86

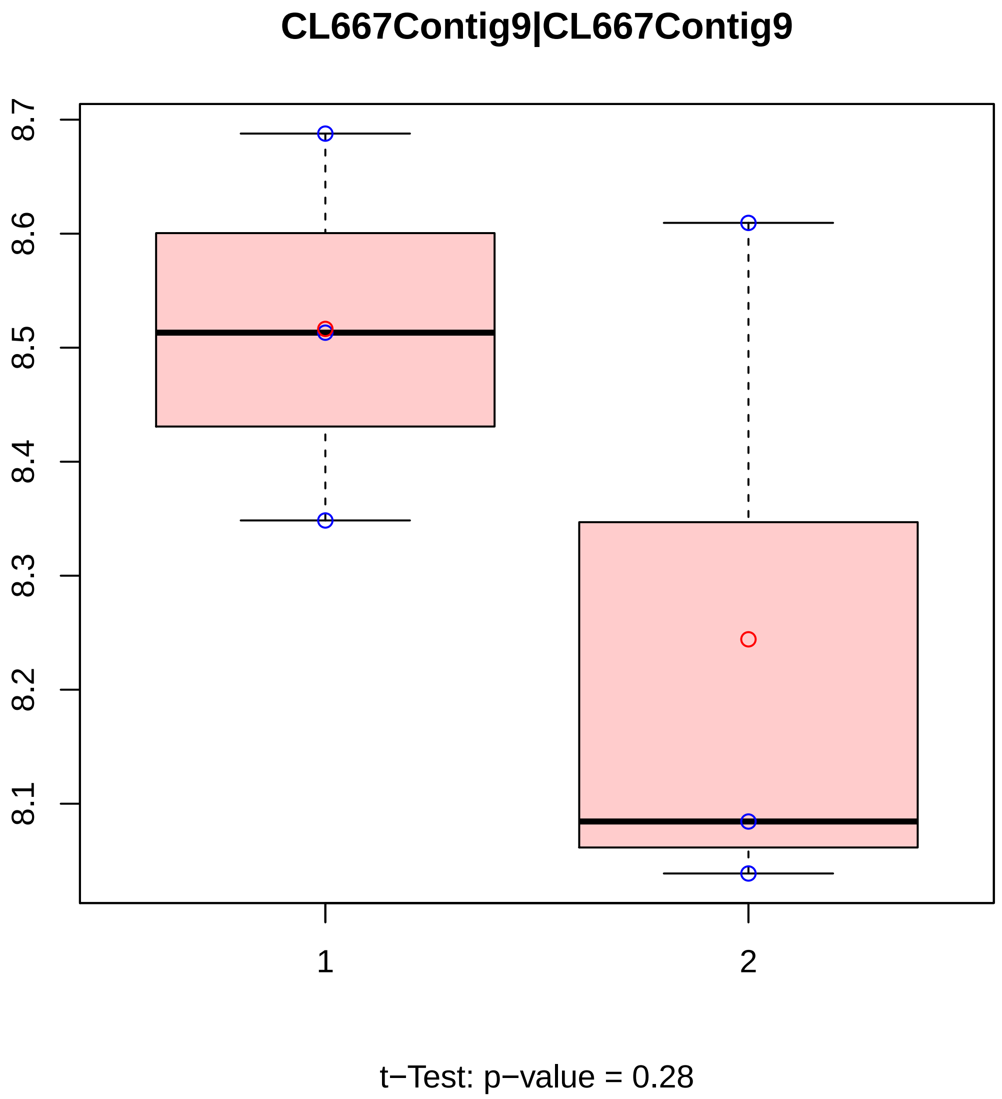
CL667Contig9|CL667Contig9
8.1
8.2
8.3
8.4
8.5
8.6
8.7
1
2
t−Test: p−value = 0.28

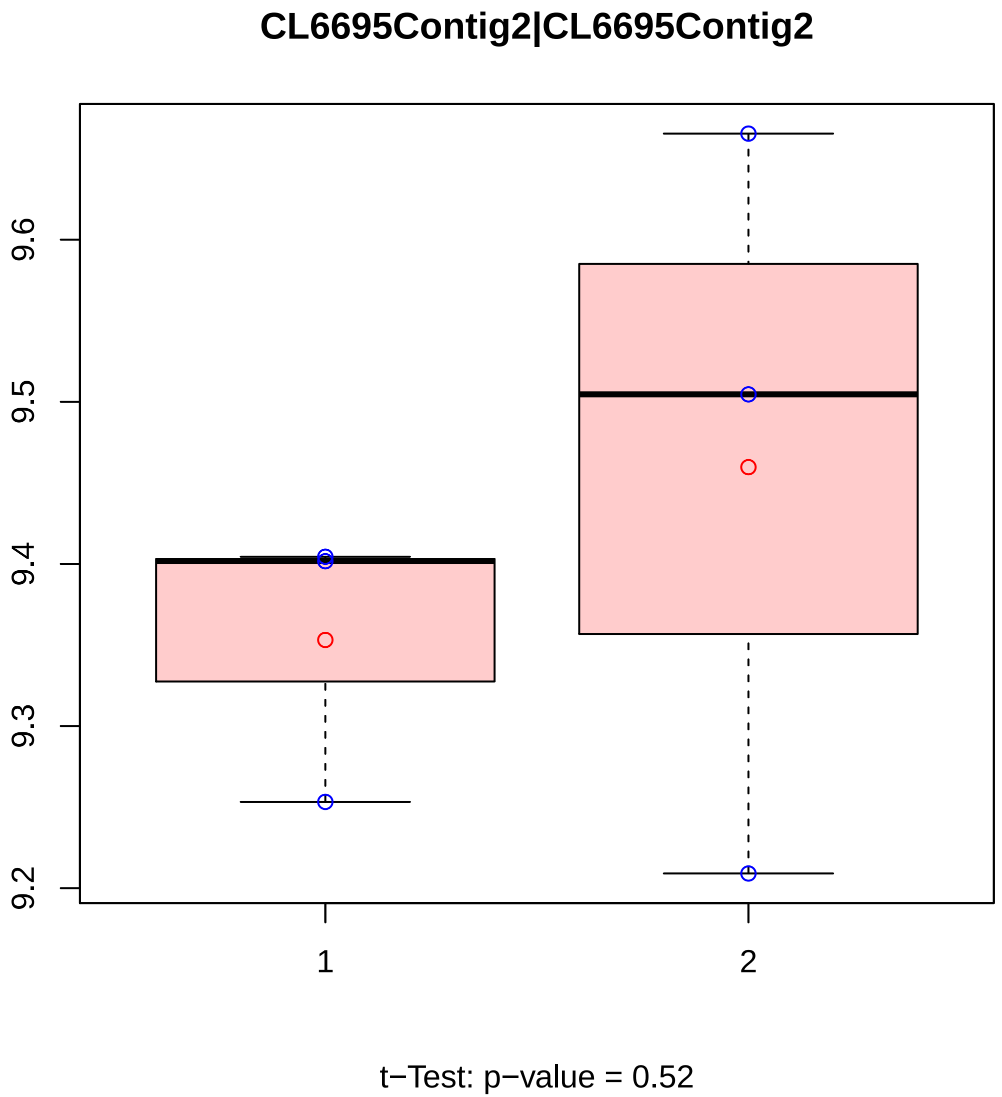

CL6695Contig2|CL6695Contig2
9.2
9.3
9.4
9.5
9.6
1
2
t−Test: p−value = 0.52

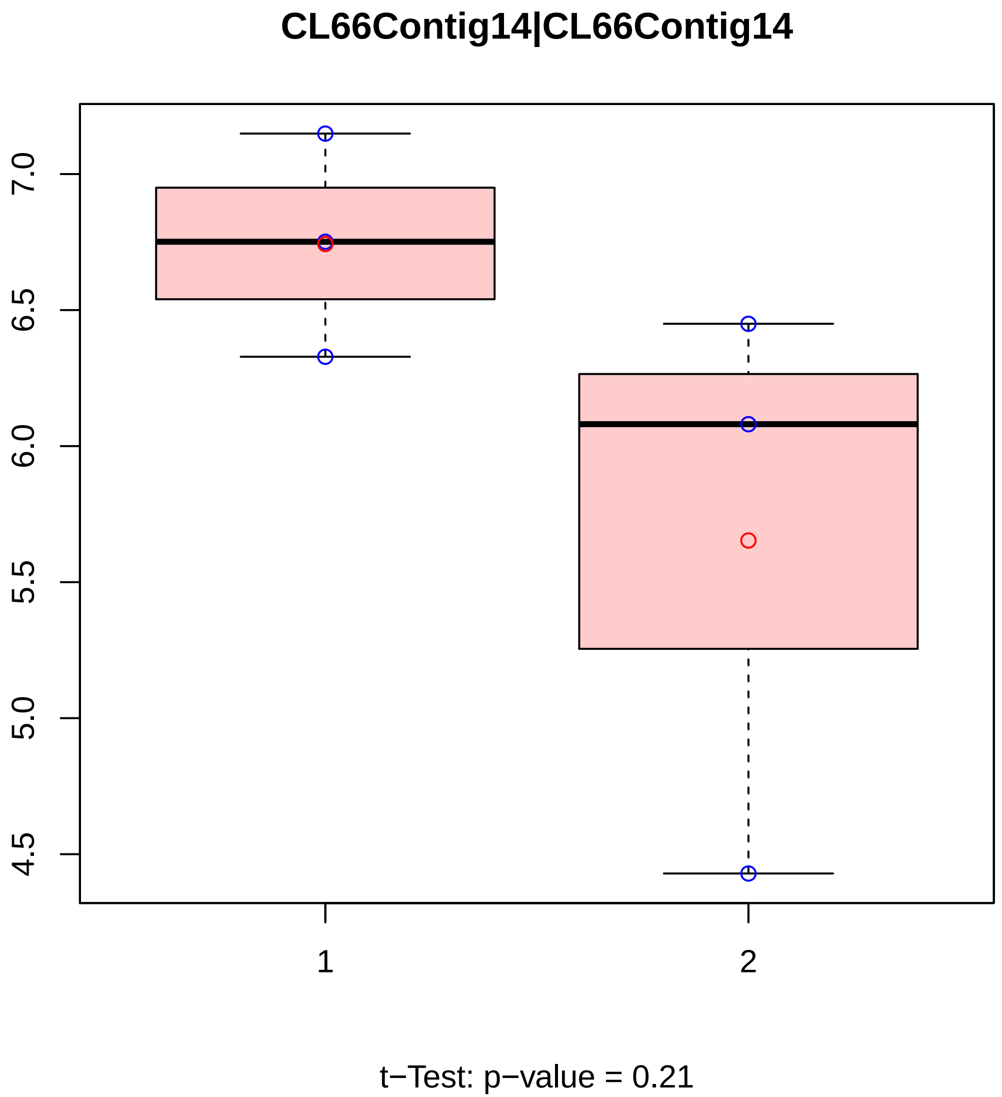

CL66Contig14|CL66Contig14
7.0
6.5
6.0
5.5
5.0
4.5
1
2
t−Test: p−value = 0.21

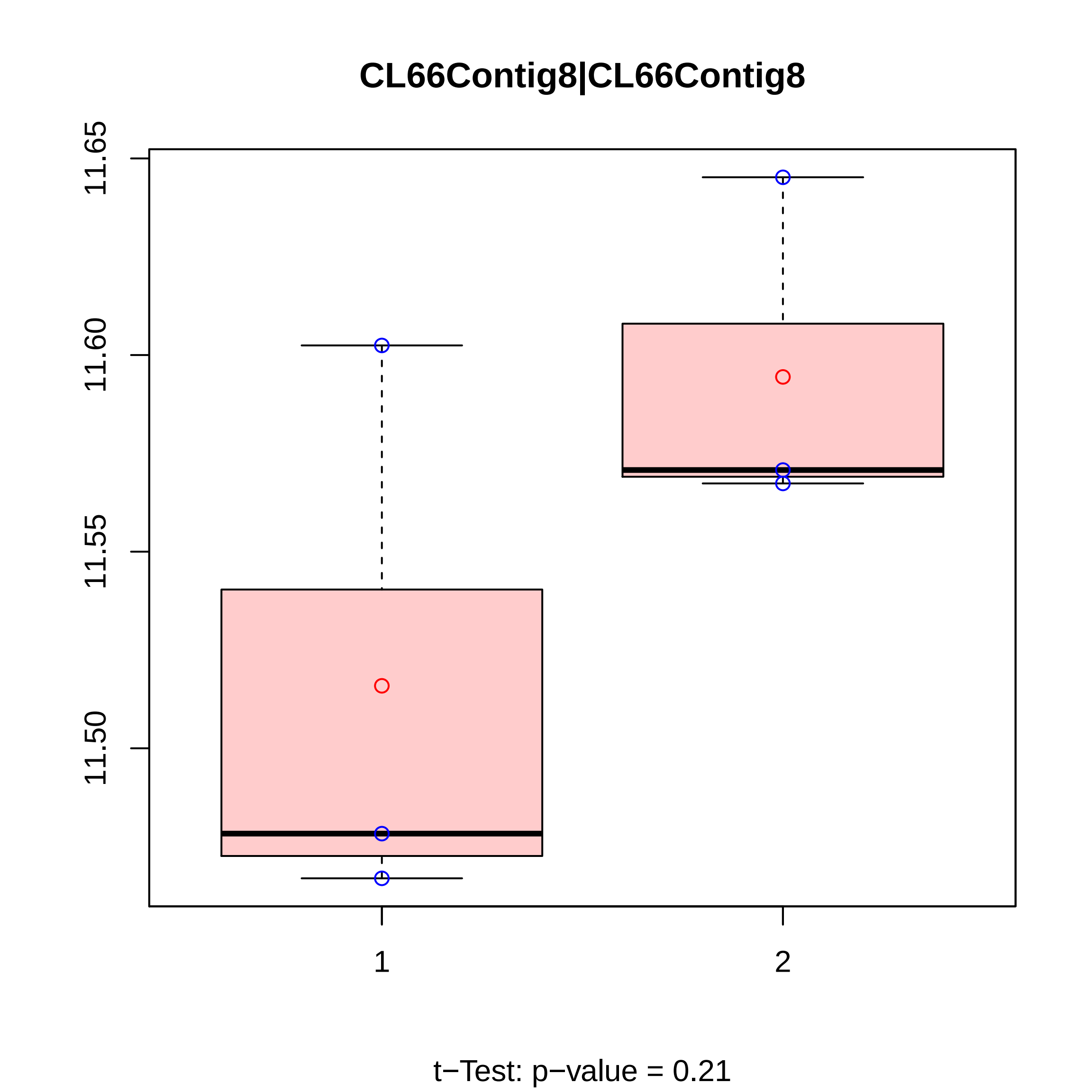

CL66Contig8|CL66Contig8
11.65
11.60
11.55
11.50
1
2
t−Test: p−value = 0.21

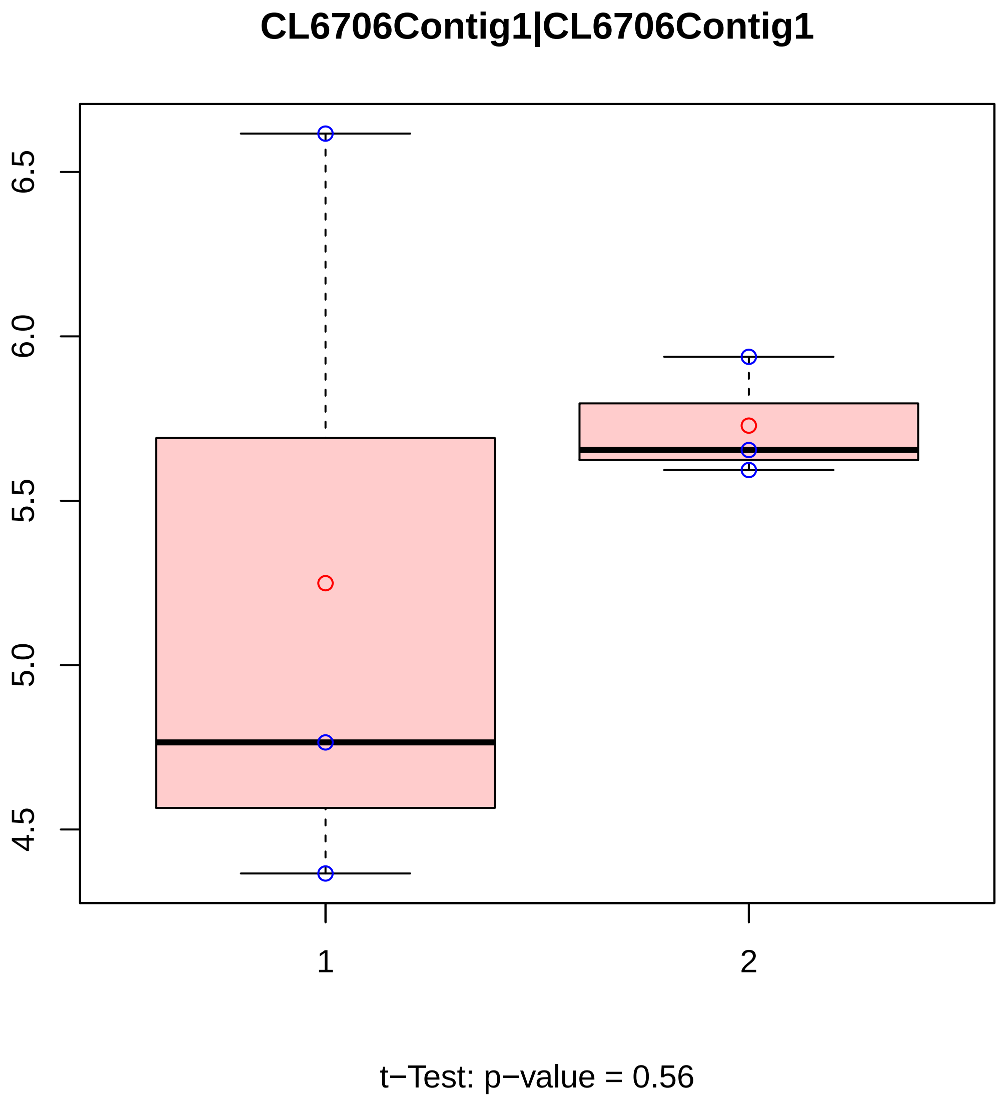
CL6706Contig1|CL6706Contig1
6.5
6.0
5.5
5.0
4.5
1
2
t−Test: p−value = 0.56

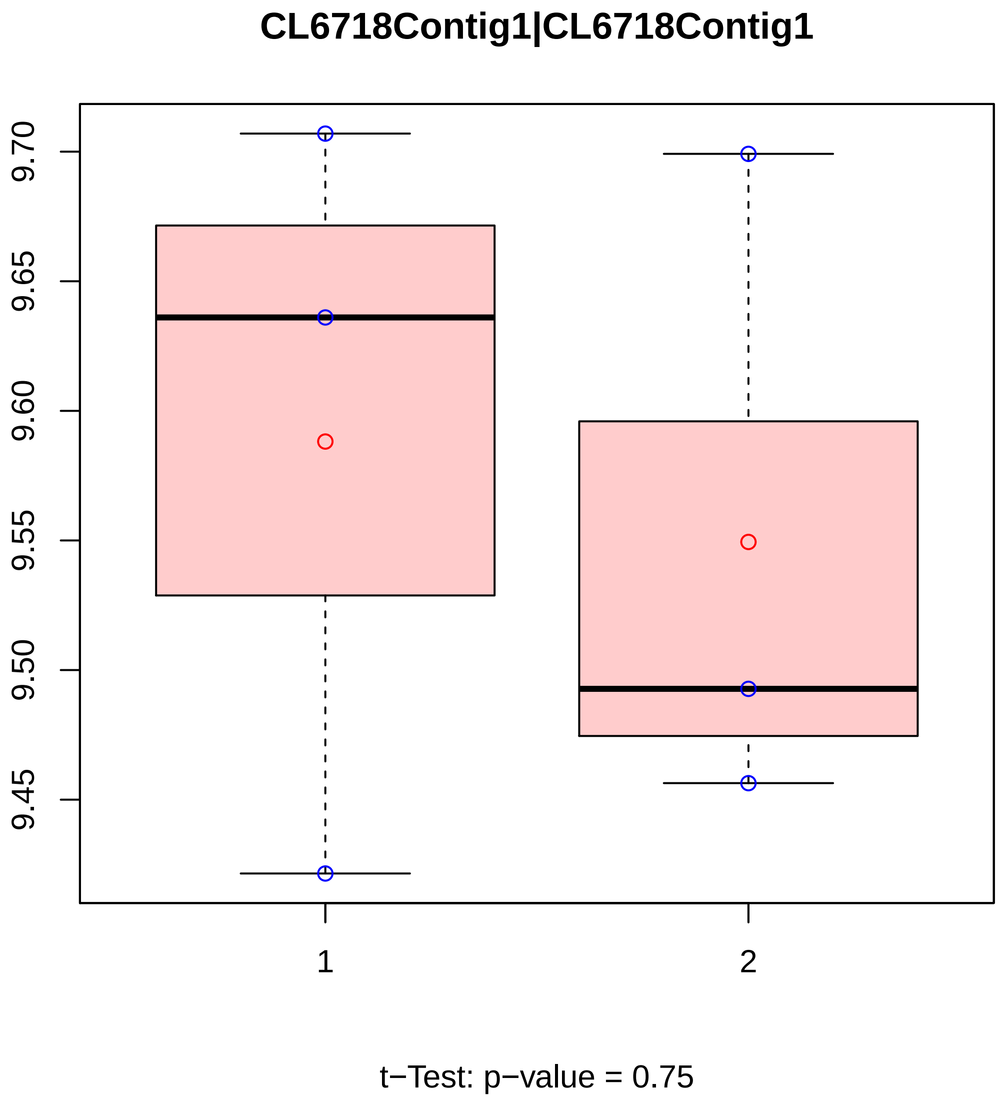

CL6718Contig1|CL6718Contig1
9.70
9.65
9.60
9.55
9.50
9.45
1
2
t−Test: p−value = 0.75

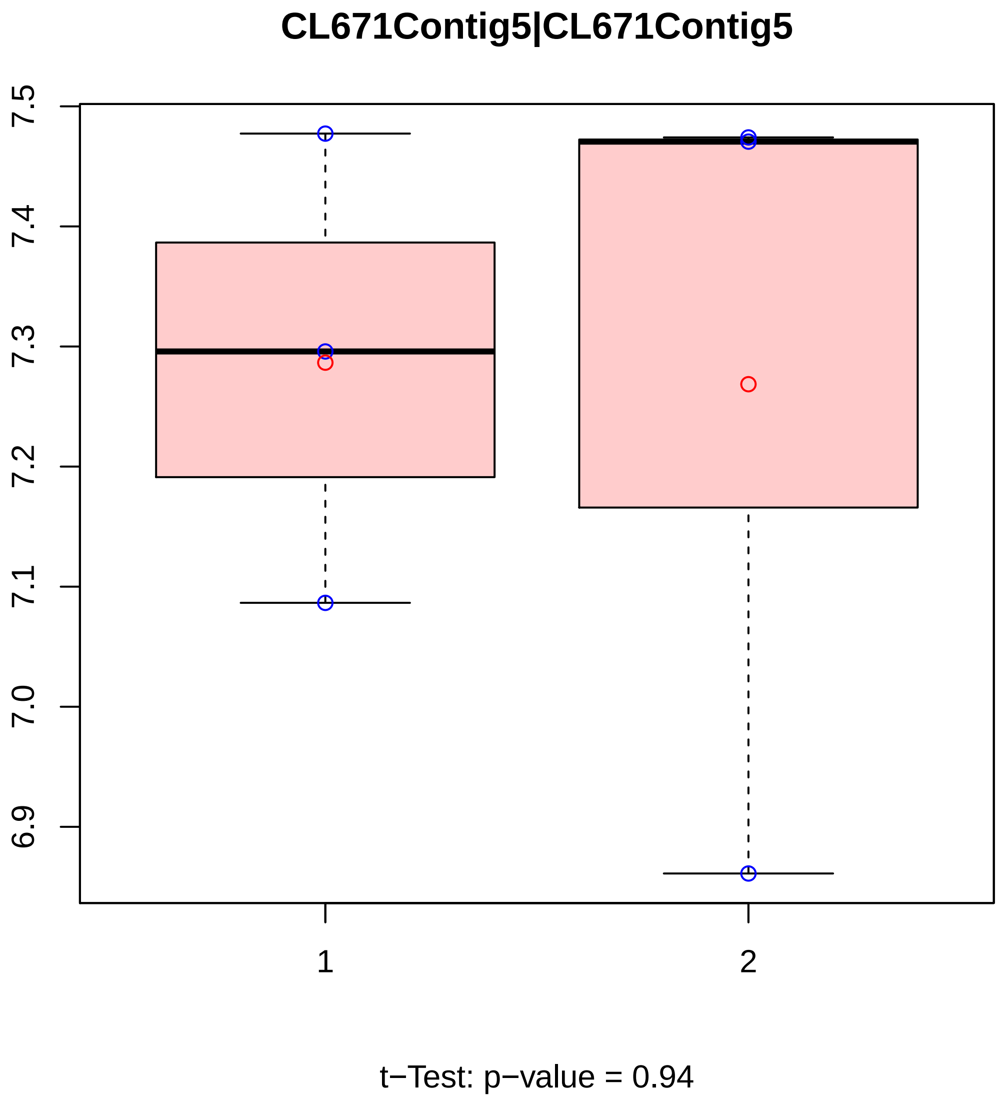
CL671Contig5|CL671Contig5
7.5
7.4
7.3
7.2
7.1
7.0
6.9
1
2
t−Test: p−value = 0.94

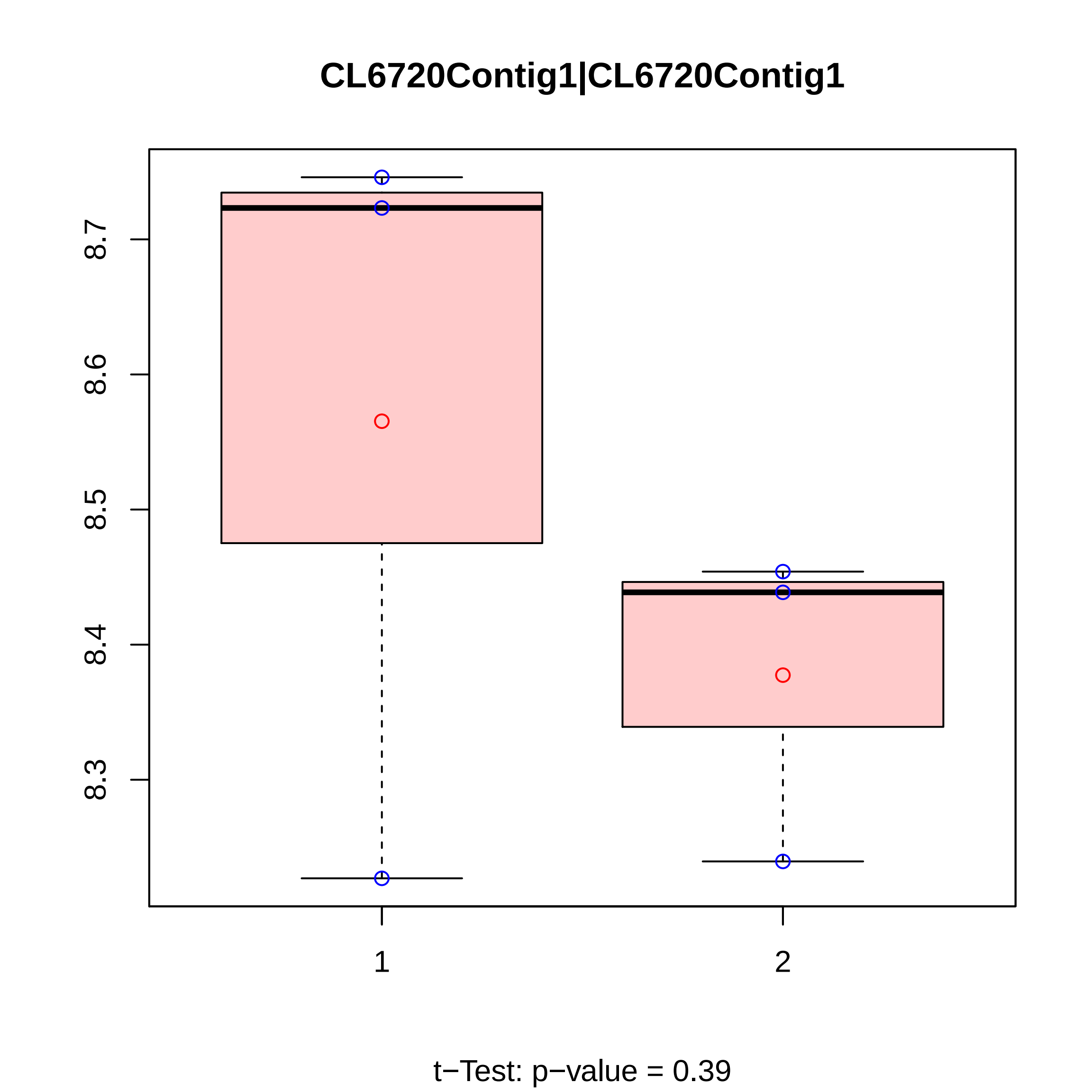

CL6720Contig1|CL6720Contig1
8.3
8.4
8.5
8.6
8.7
1
2
t−Test: p−value = 0.39

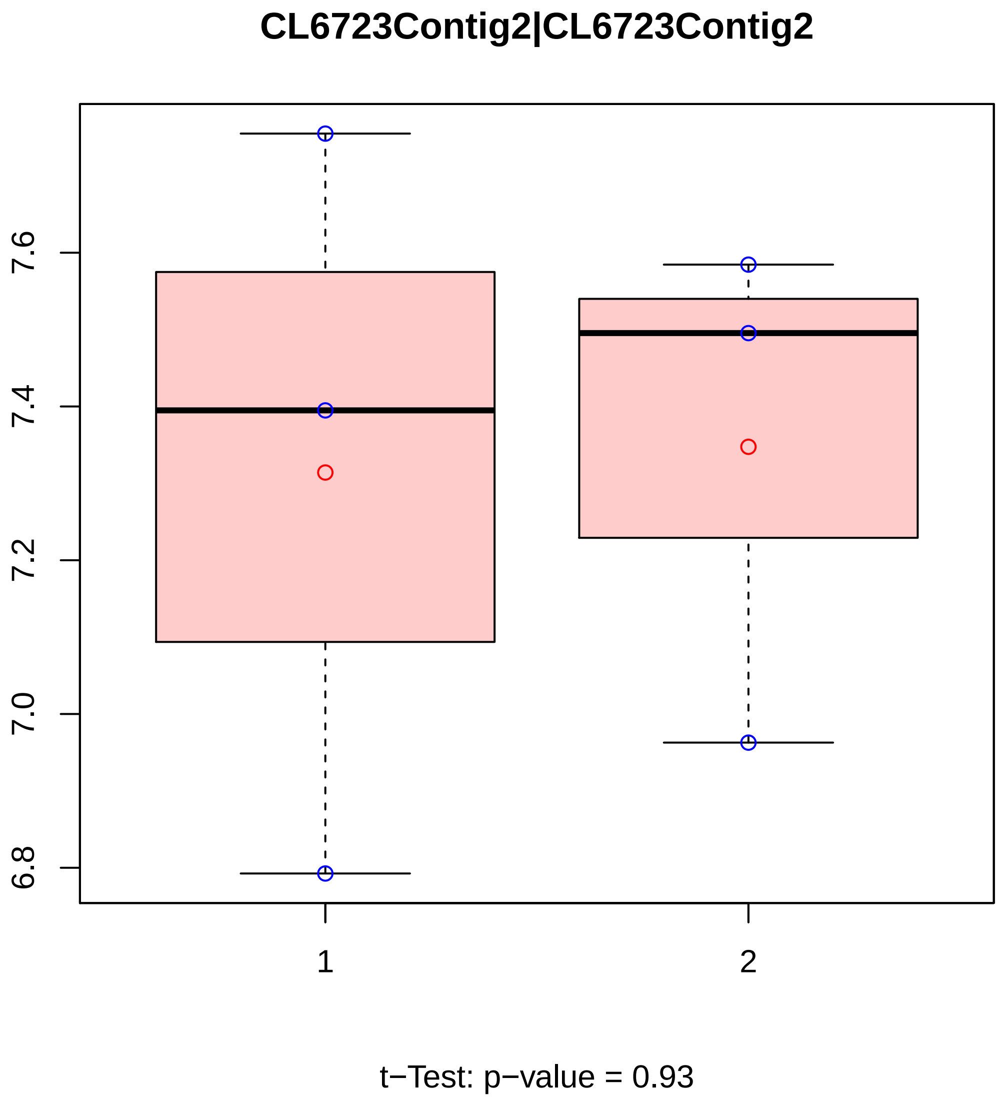

CL6723Contig2|CL6723Contig2
6.8
7.0
7.2
7.4
7.6
1
2
t−Test: p−value = 0.93

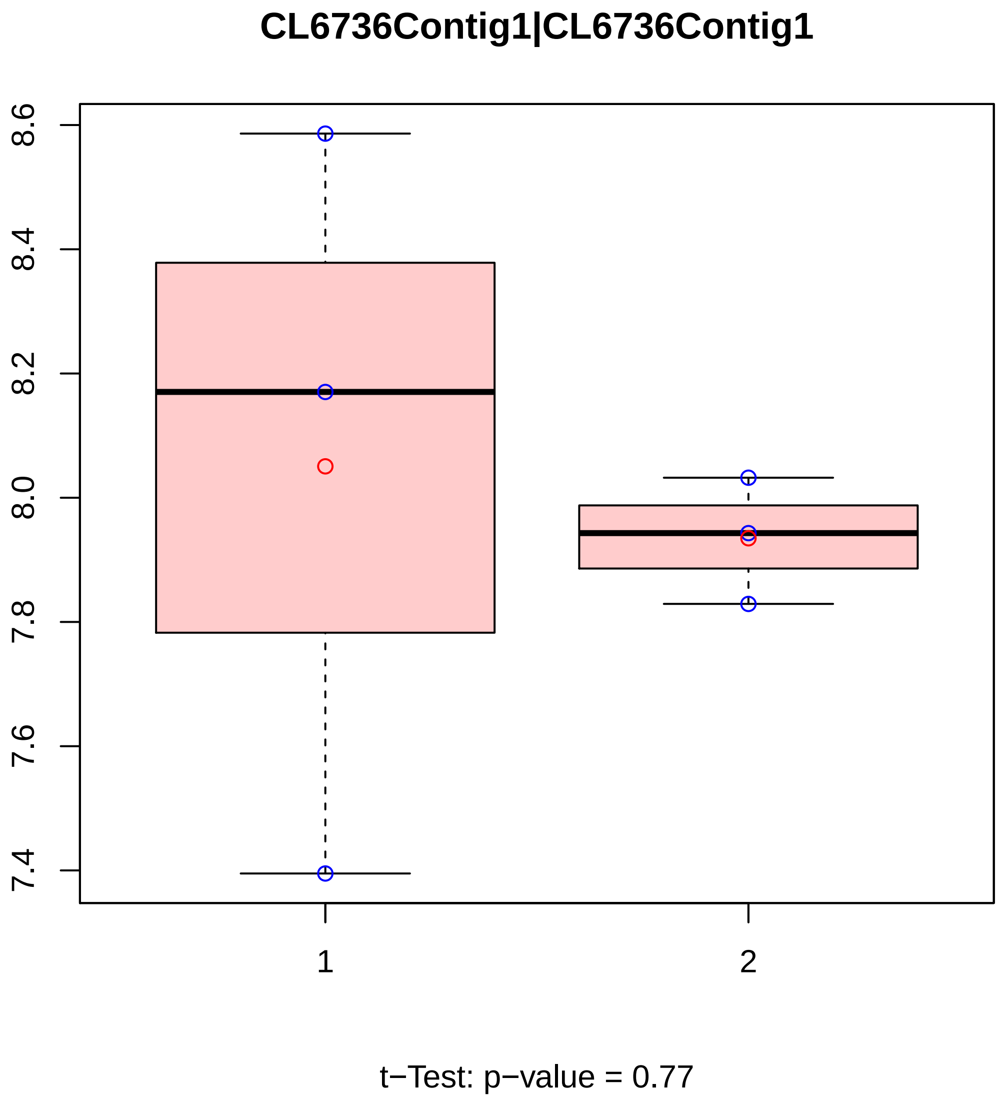

CL6736Contig1|CL6736Contig1
8.6
8.4
8.2
8.0
7.8
7.6
7.4
1
2
t−Test: p−value = 0.77

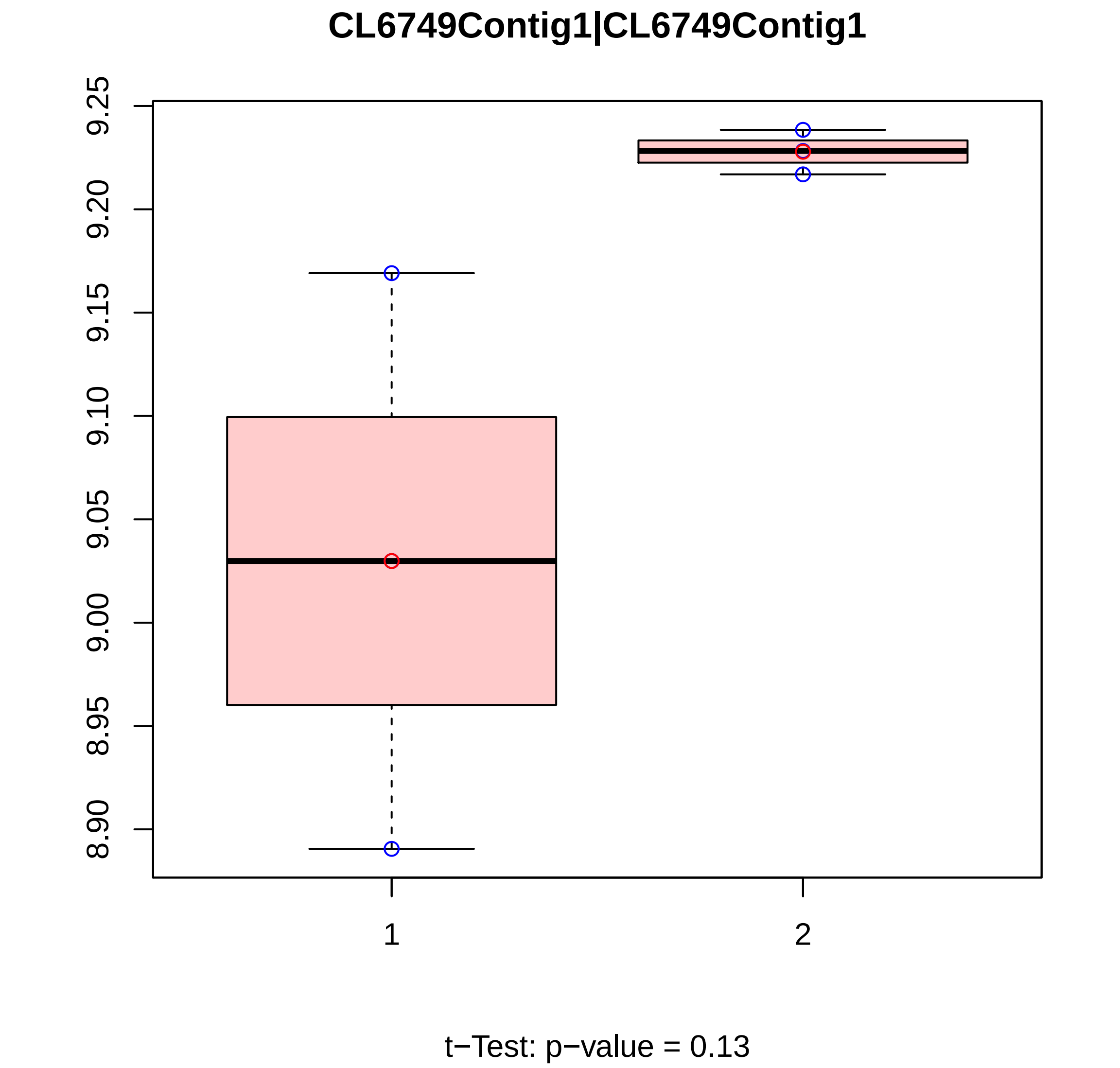

CL6749Contig1|CL6749Contig1
9.25
9.20
9.15
9.10
9.05
9.00
8.95
8.90
1
2
t−Test: p−value = 0.13

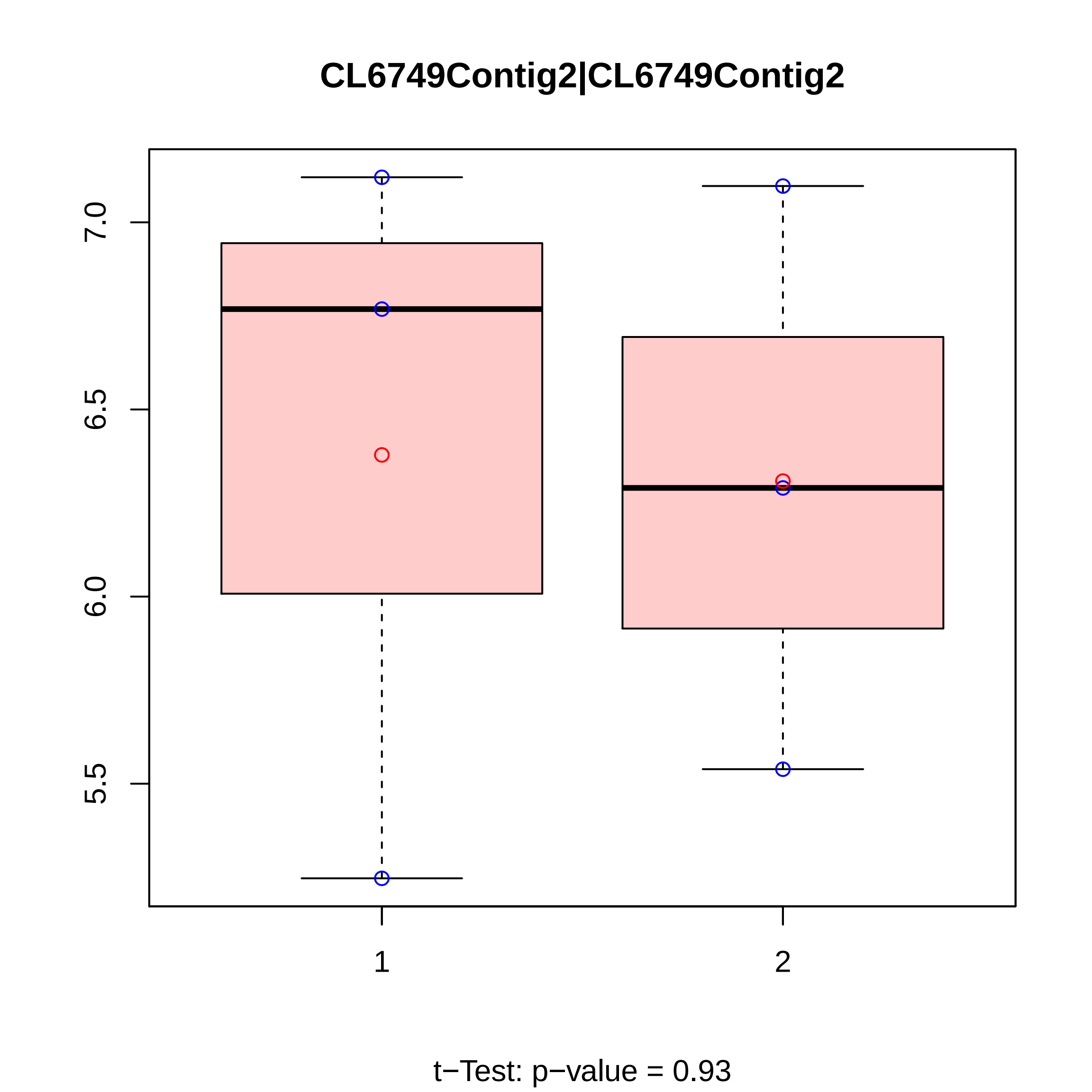

CL6749Contig2|CL6749Contig2
7.0
6.5
6.0
5.5
1
2
t−Test: p−value = 0.93

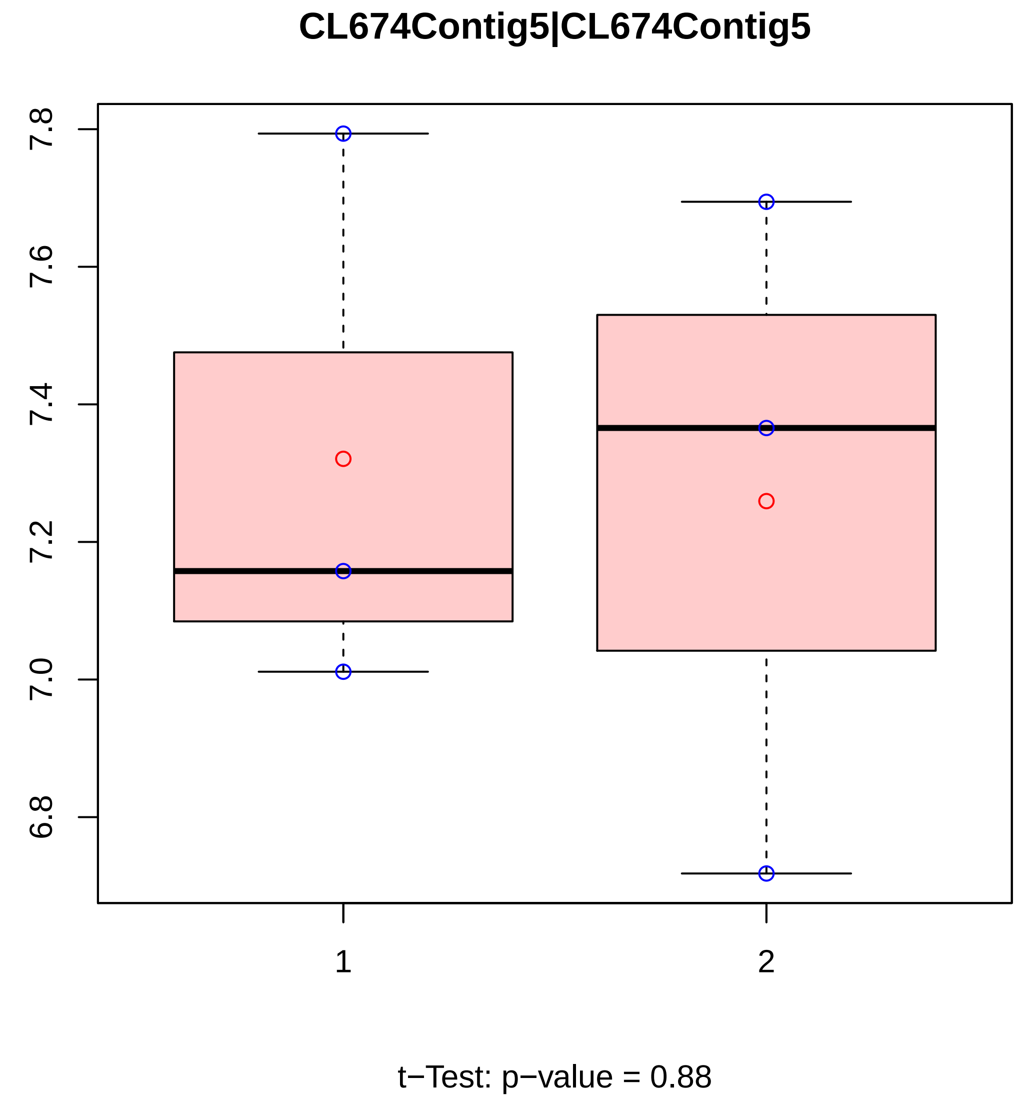
CL674Contig5|CL674Contig5
7.8
7.6
7.4
7.2
7.0
6.8
1
2
t−Test: p−value = 0.88

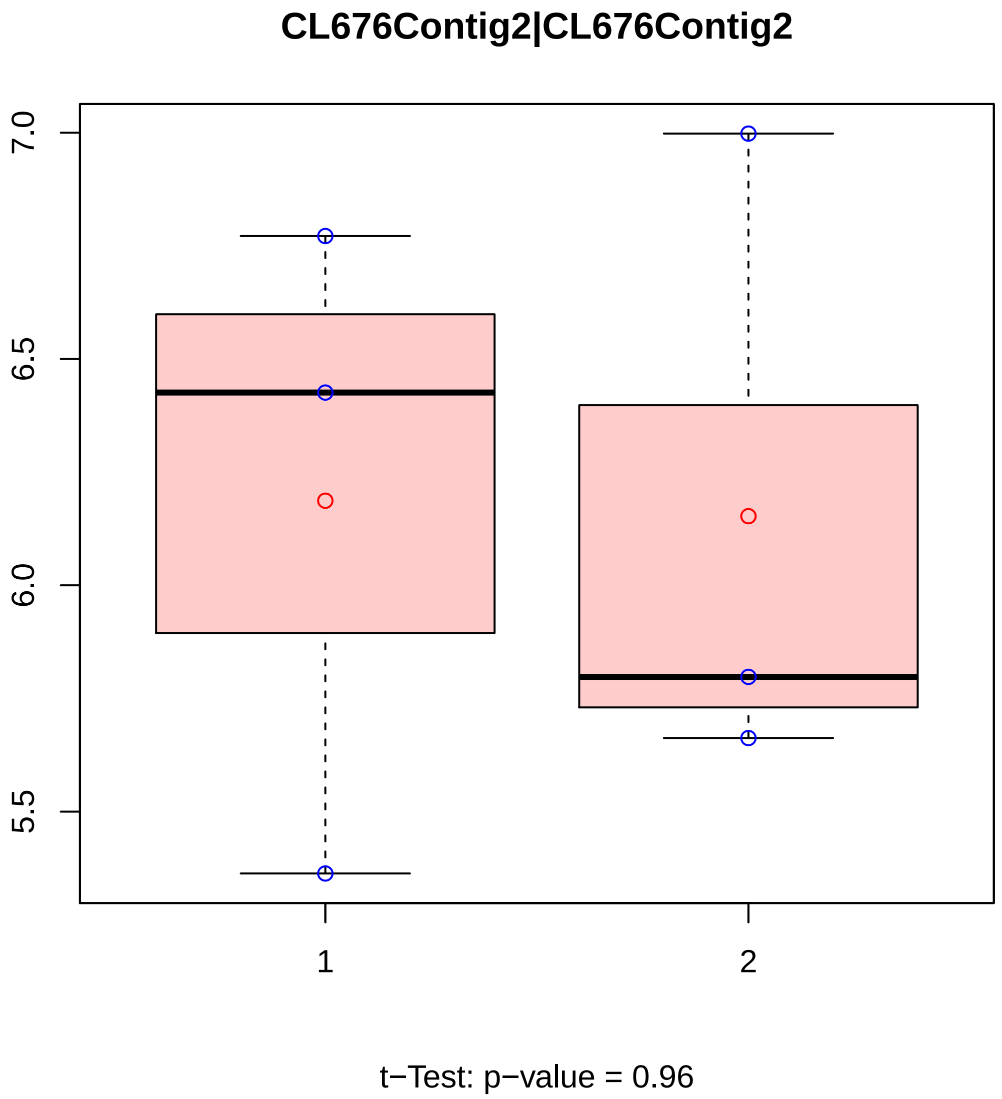
CL676Contig2|CL676Contig2
7.0
6.5
6.0
5.5
1
2
t−Test: p−value = 0.96

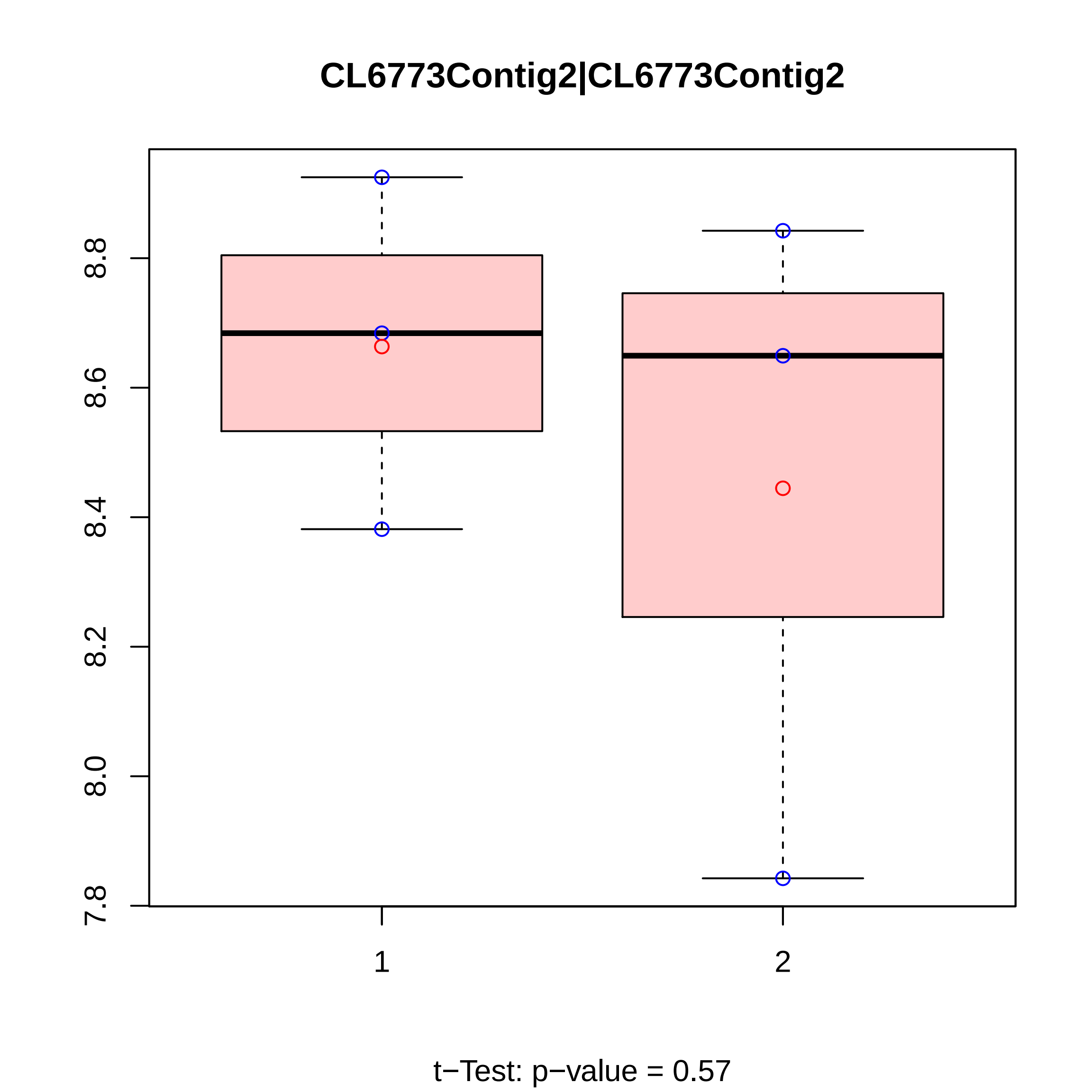
CL6773Contig2|CL6773Contig2
7.8
8.0
8.2
8.4
8.6
8.8
1
2
t−Test: p−value = 0.57

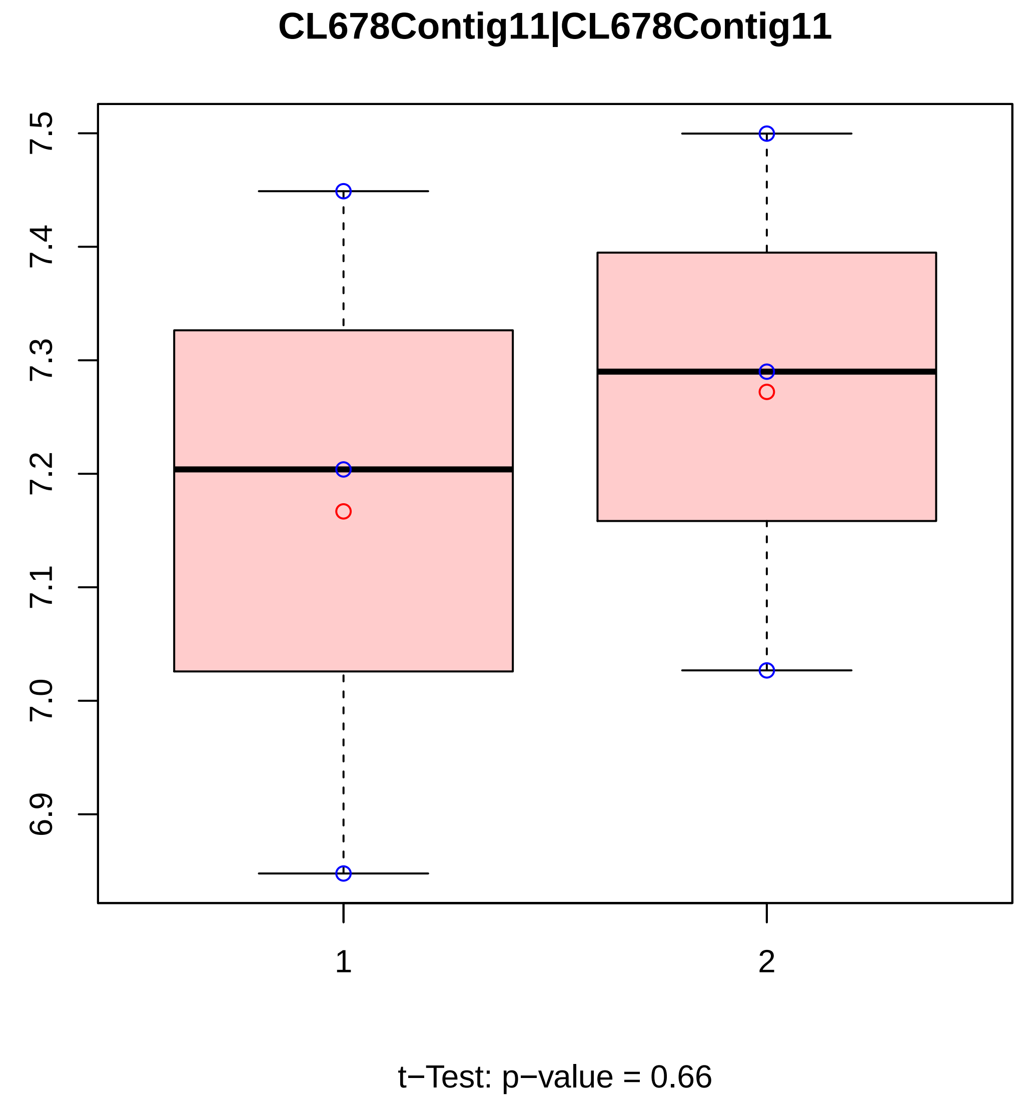

CL678Contig11|CL678Contig11
7.5
7.4
7.3
7.2
7.1
7.0
6.9
1
2
t−Test: p−value = 0.66

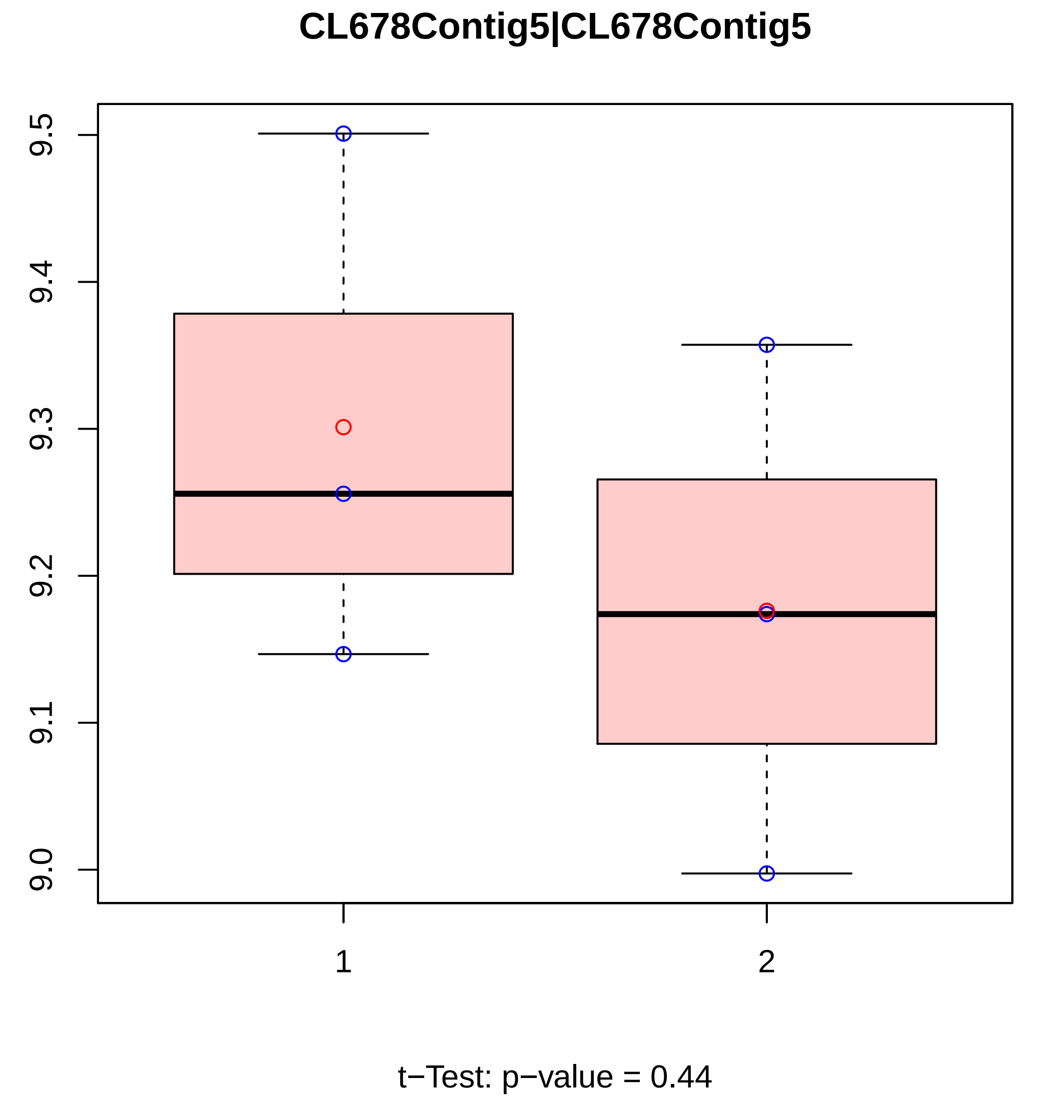
CL678Contig5|CL678Contig5
9.0
9.1
9.2
9.3
9.4
9.5
1
2
t−Test: p−value = 0.44

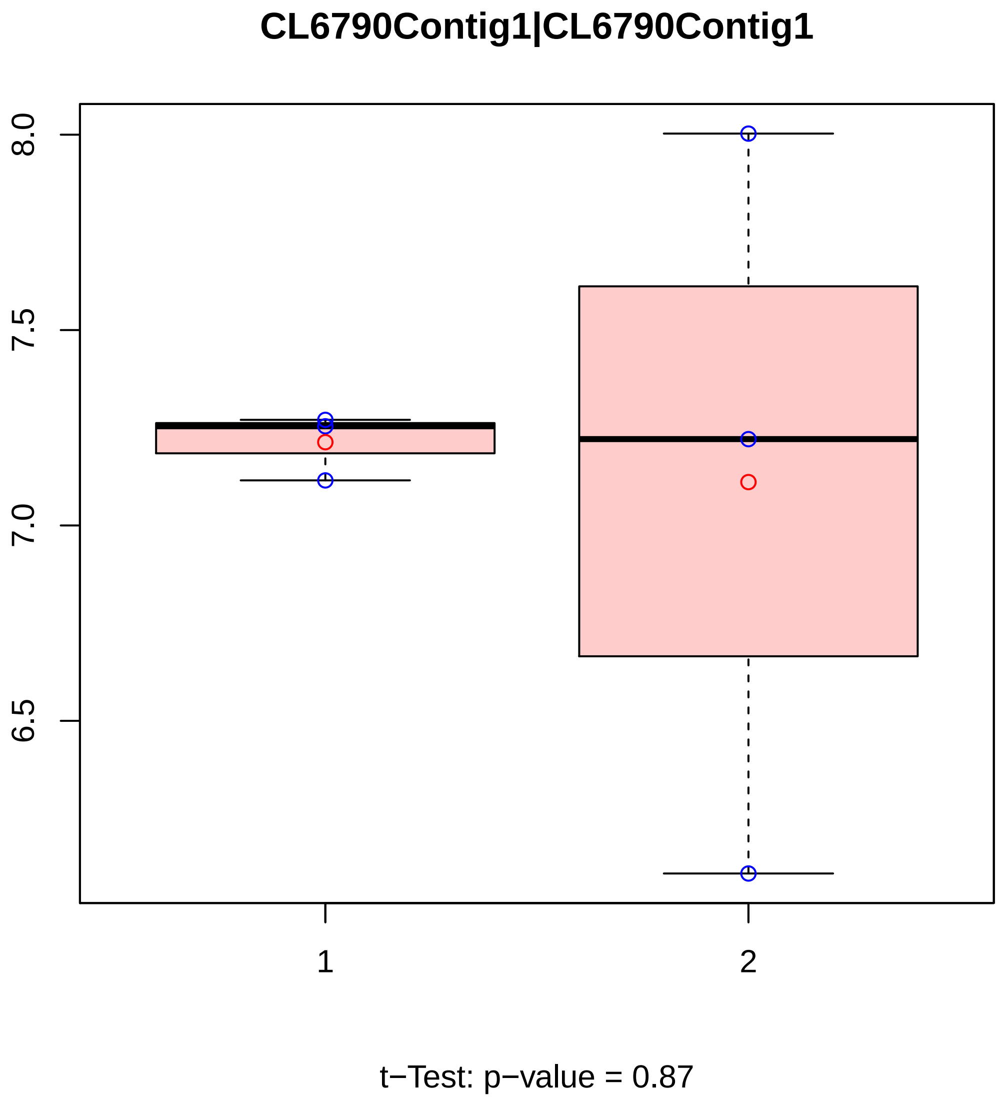
CL6790Contig1|CL6790Contig1
8.0
7.5
7.0
6.5
1
2
t−Test: p−value = 0.87

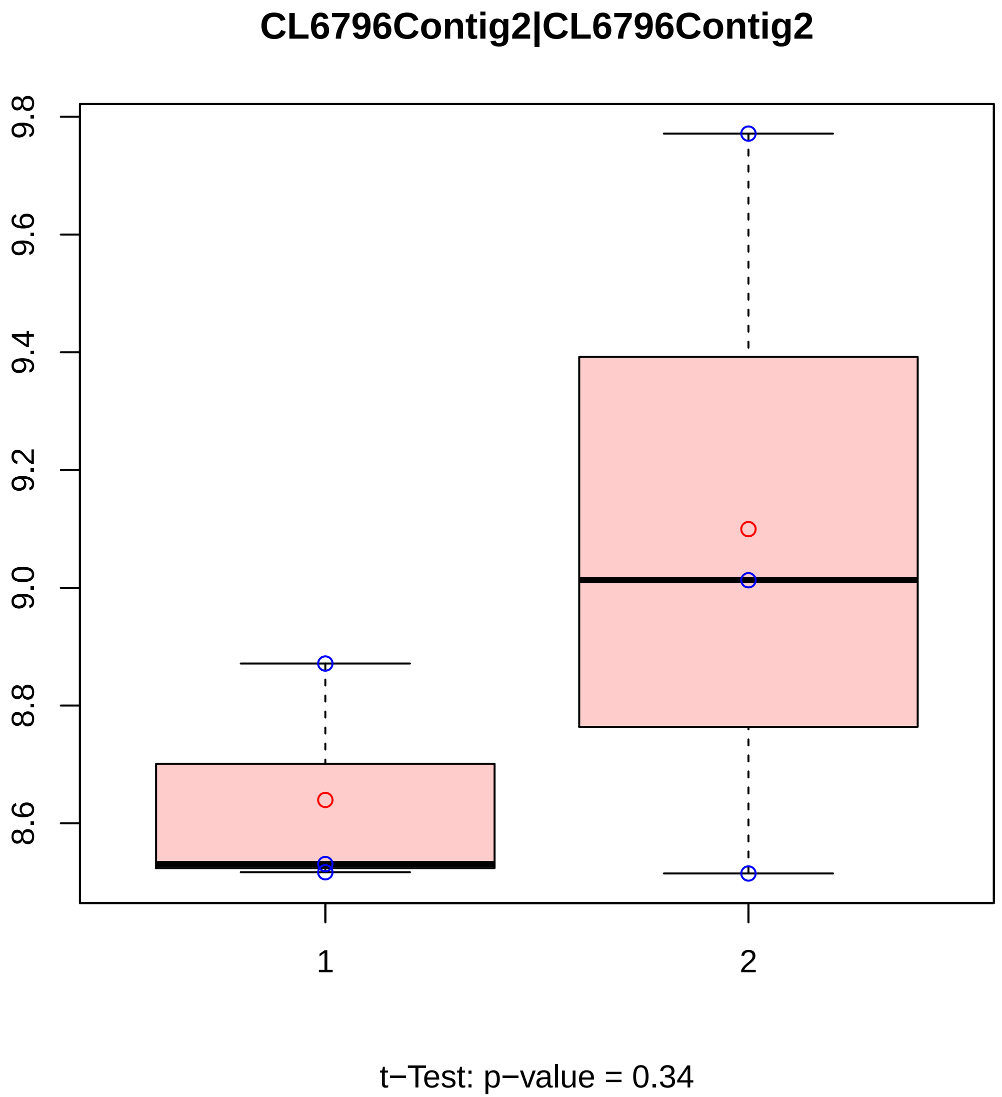
CL6796Contig2|CL6796Contig2
9.8
9.6
9.4
9.2
9.0
8.8
8.6
1
2
t−Test: p−value = 0.34

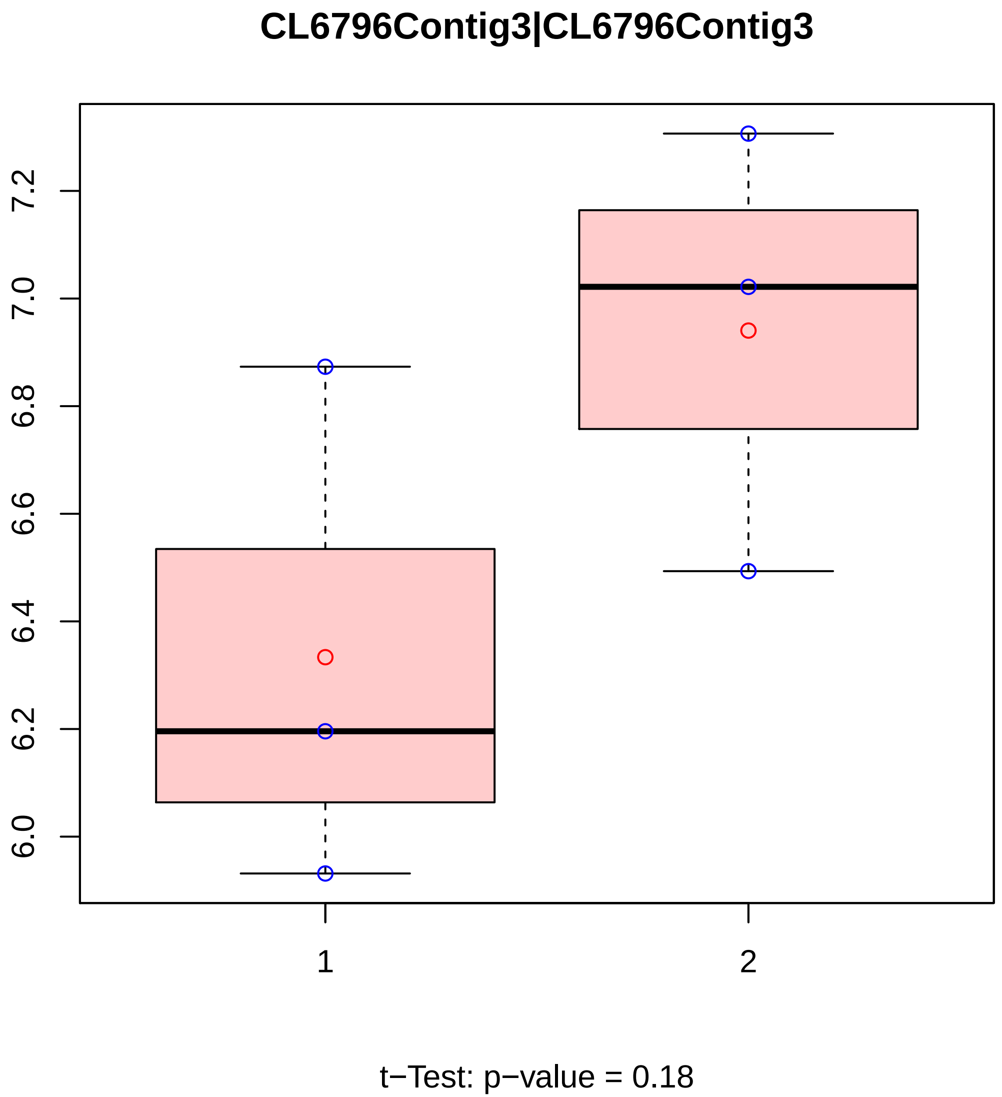

CL6796Contig3|CL6796Contig3
7.2
7.0
6.8
6.6
6.4
6.2
6.0
1
2
t−Test: p−value = 0.18

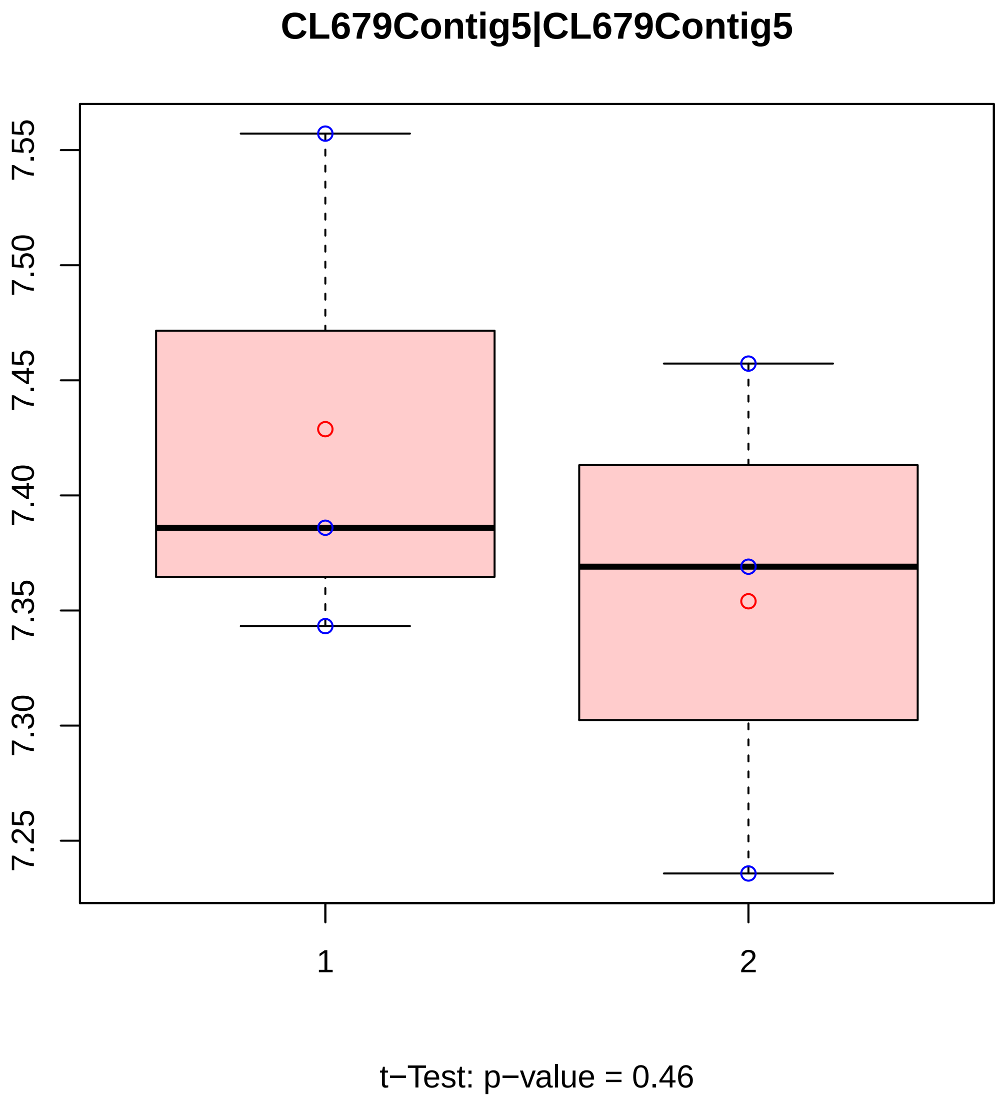
CL679Contig5|CL679Contig5
7.55
7.50
7.45
7.40
7.35
7.30
7.25
1
2
t−Test: p−value = 0.46

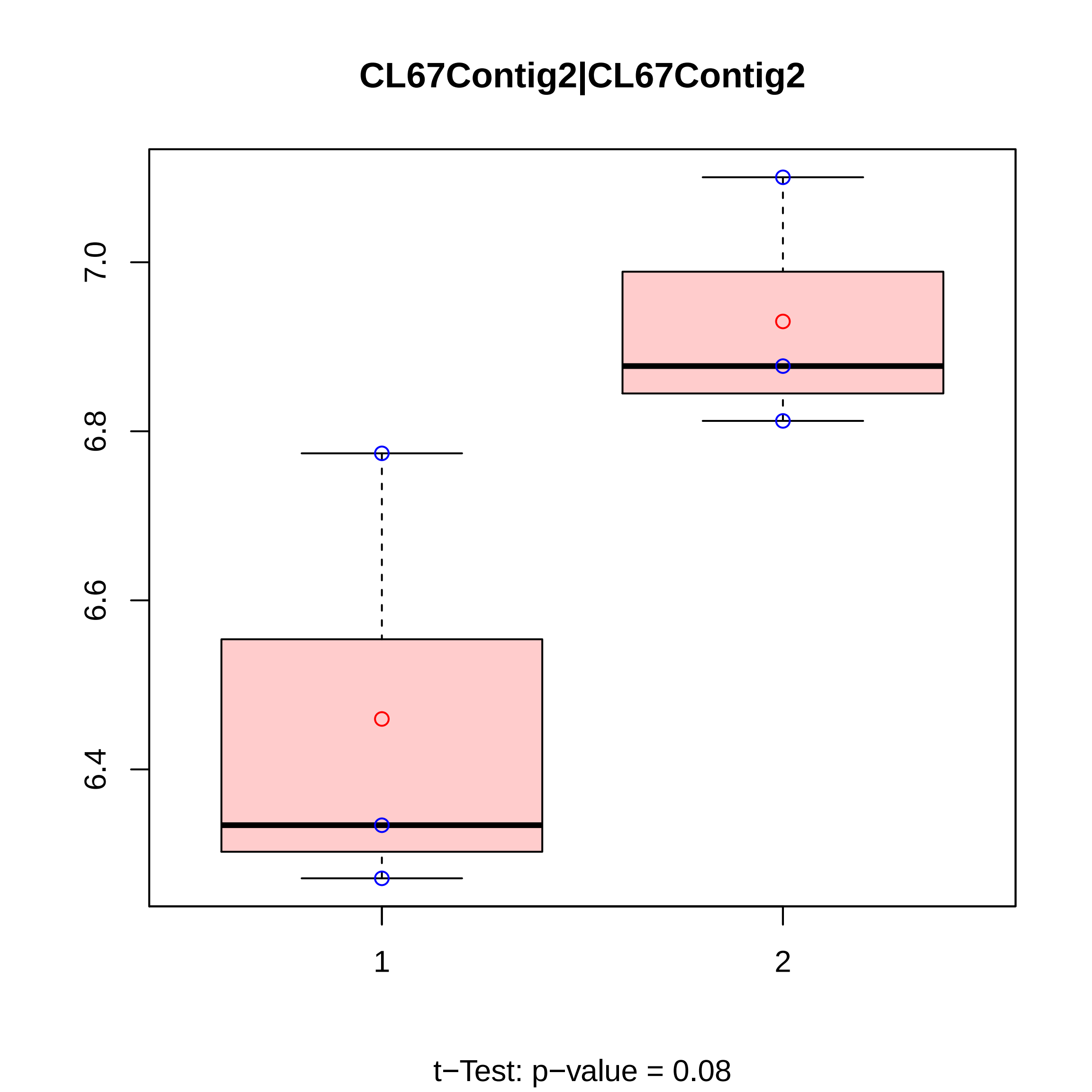

CL67Contig2|CL67Contig2
7.0
6.8
6.6
6.4
1
2
t−Test: p−value = 0.08

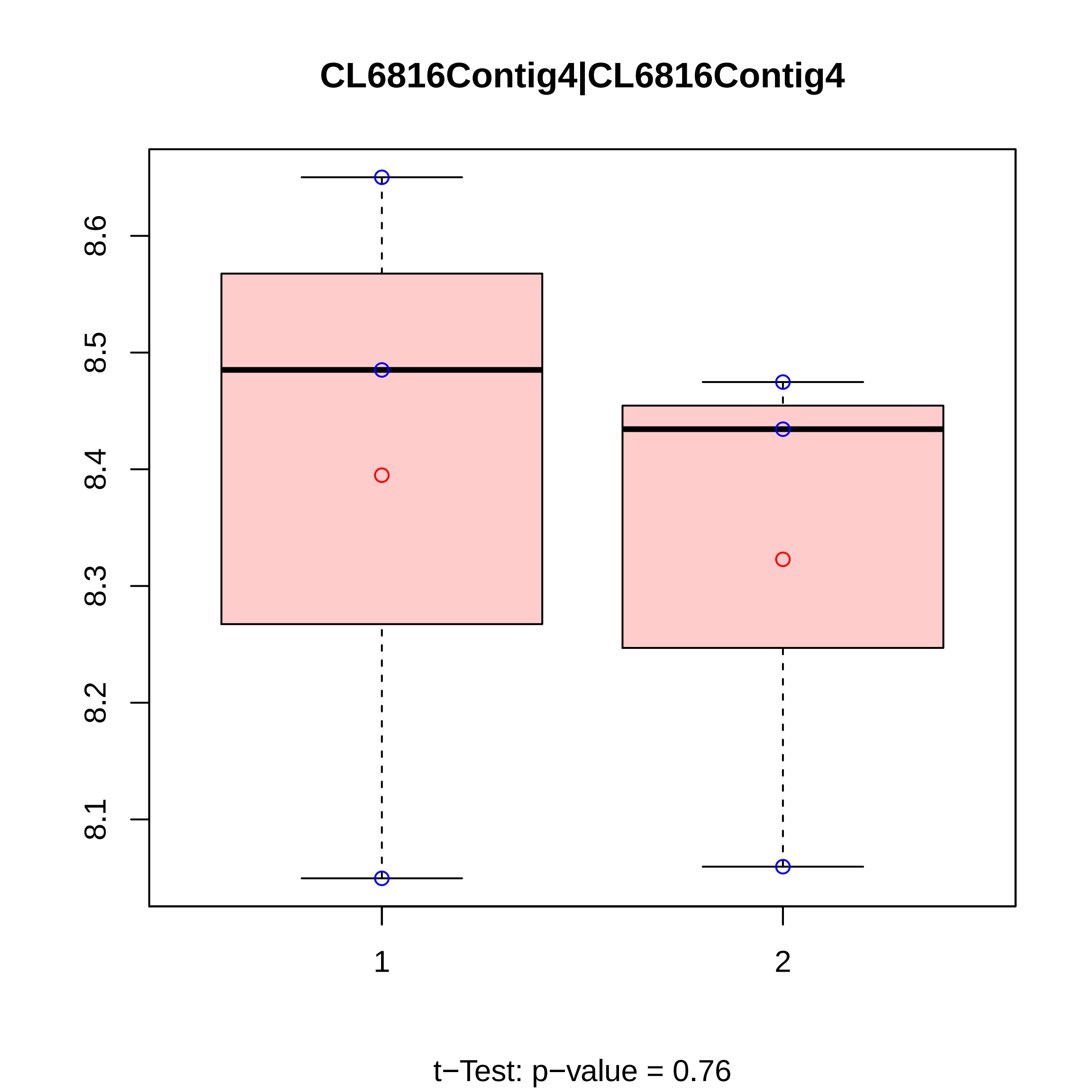
CL6816Contig4|CL6816Contig4
8.1
8.2
8.3
8.4
8.5
8.6
1
2
t−Test: p−value = 0.76

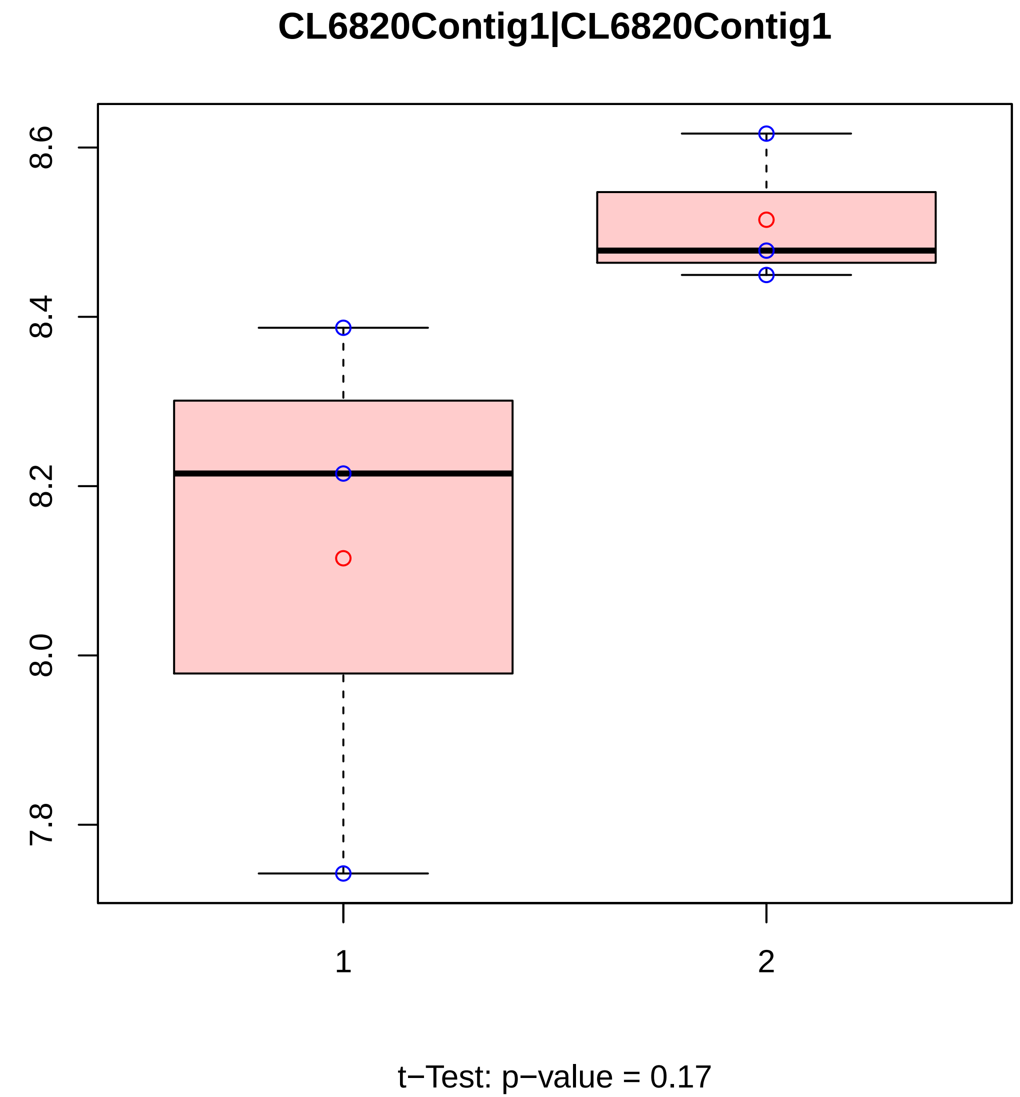

CL6820Contig1|CL6820Contig1
8.6
8.4
8.2
8.0
7.8
1
2
t−Test: p−value = 0.17

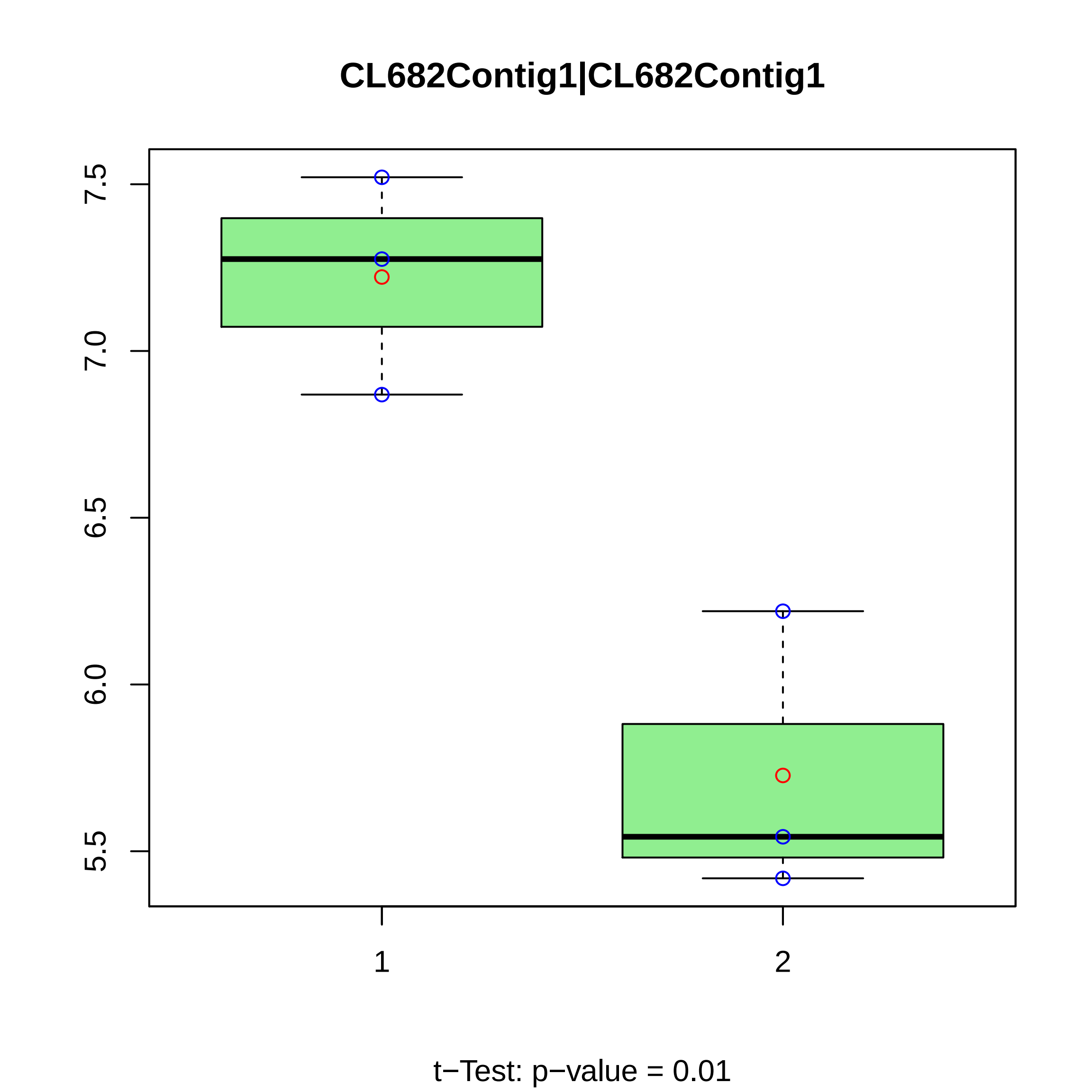

CL682Contig1|CL682Contig1
7.5
7.0
6.5
6.0
5.5
1
2
t−Test: p−value = 0.01

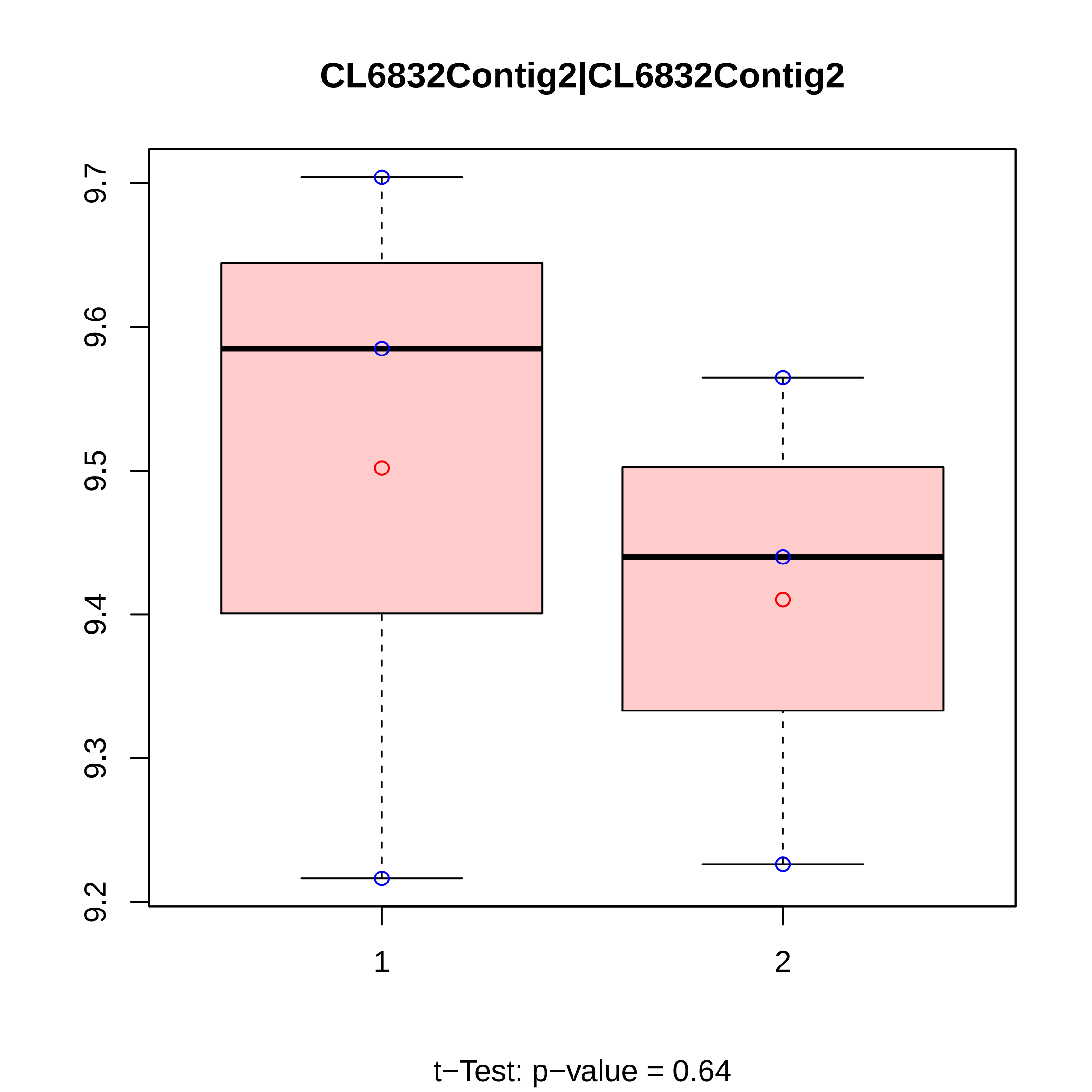

CL6832Contig2|CL6832Contig2
9.2
9.3
9.4
9.5
9.6
9.7
1
2
t−Test: p−value = 0.64

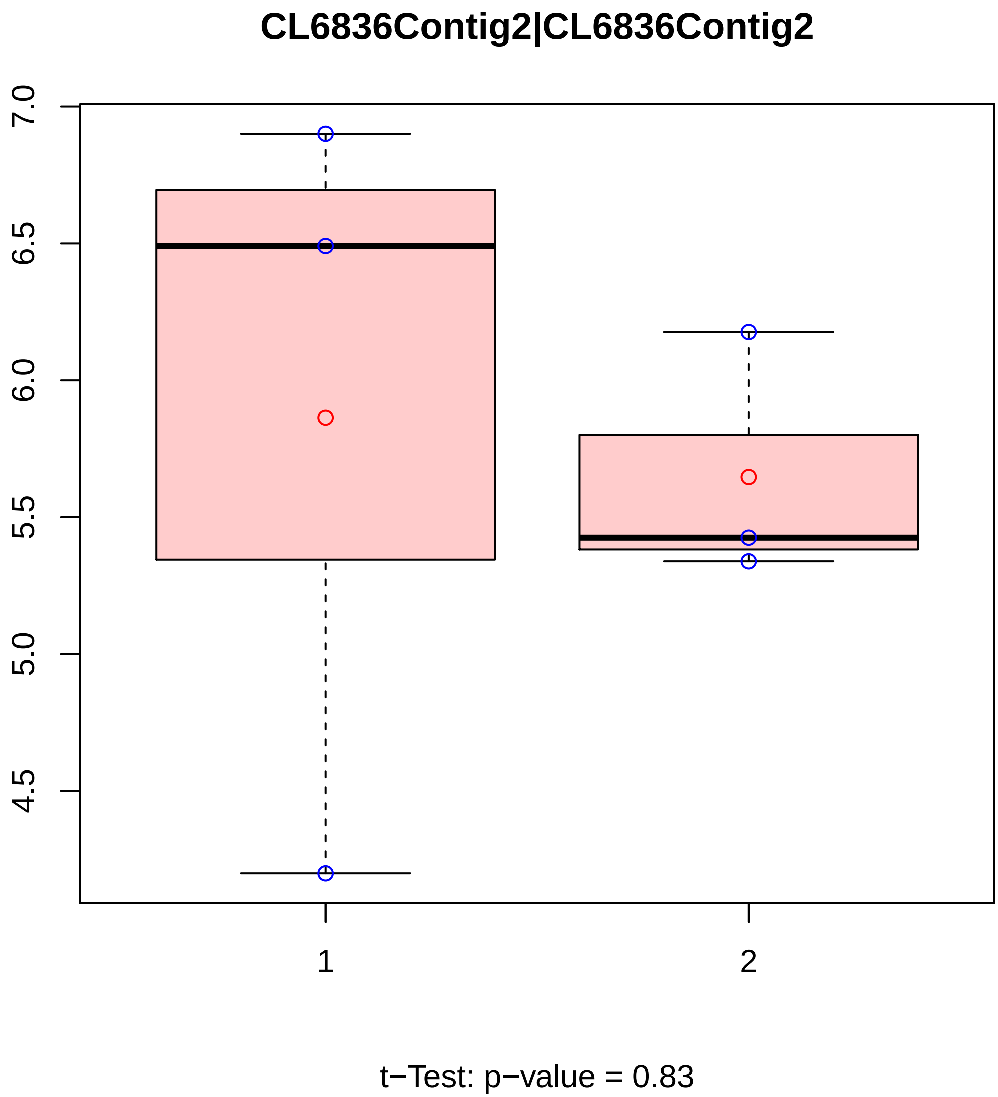

CL6836Contig2|CL6836Contig2
7.0
6.5
6.0
5.5
5.0
4.5
1
2
t−Test: p−value = 0.83

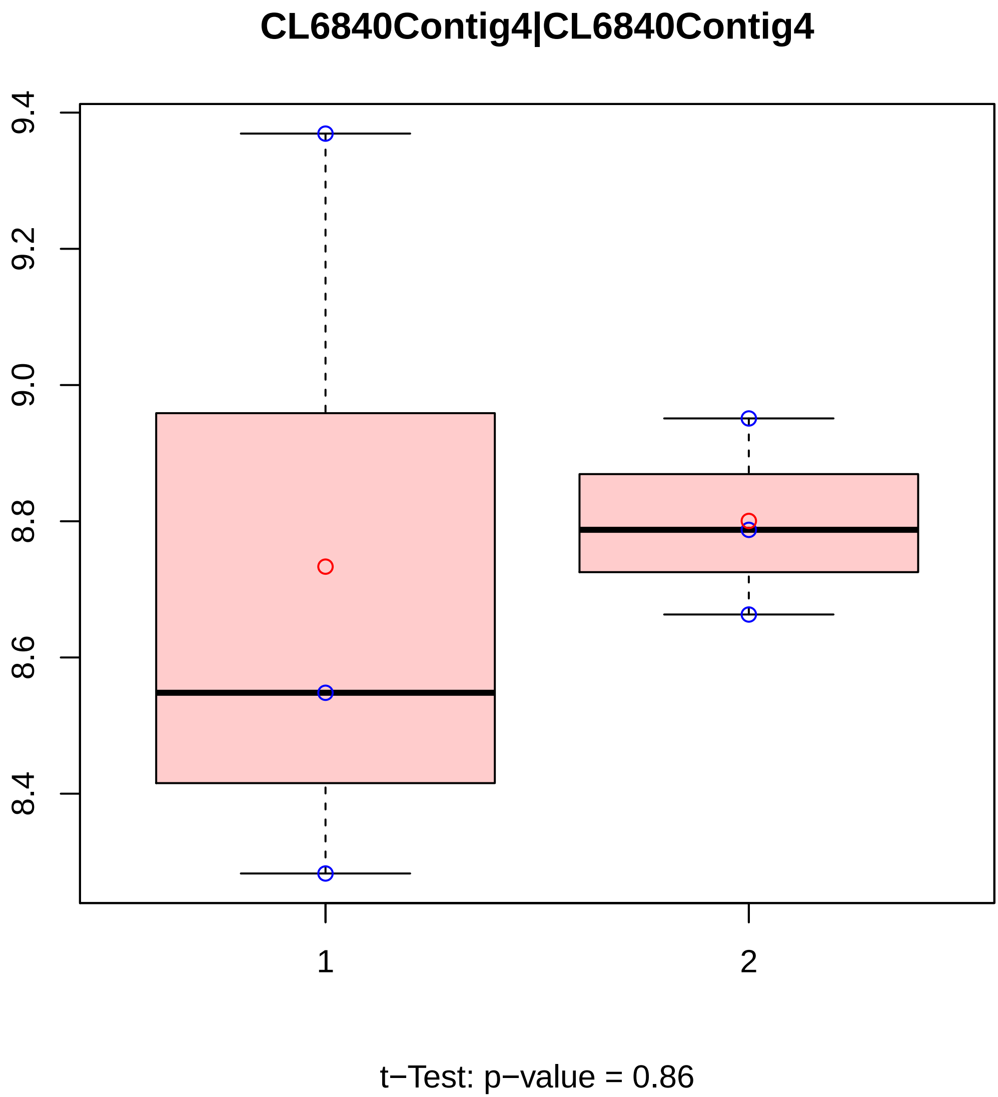

CL6840Contig4|CL6840Contig4
9.4
9.2
9.0
8.8
8.6
8.4
1
2
t−Test: p−value = 0.86

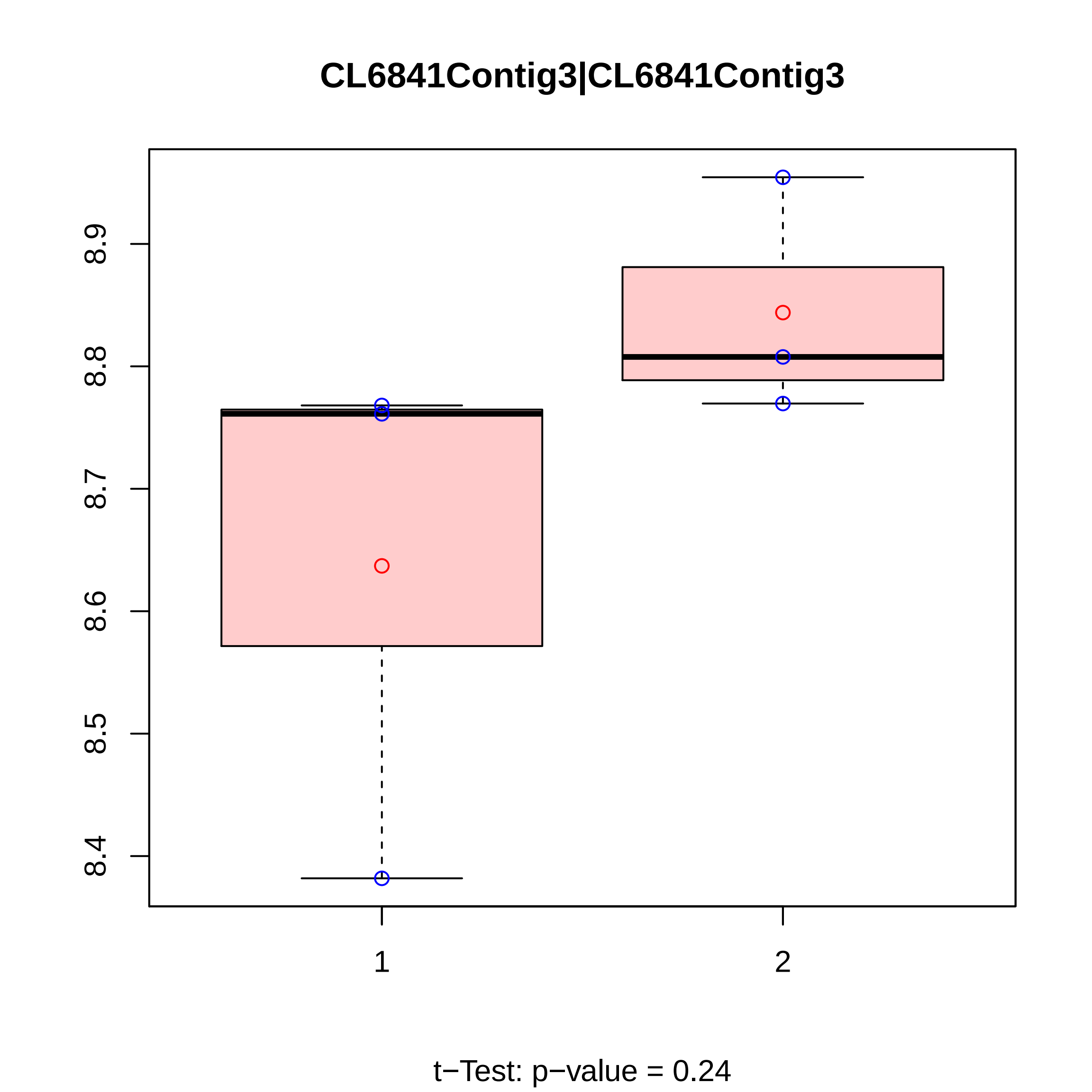

CL6841Contig3|CL6841Contig3
8.4
8.5
8.6
8.7
8.8
8.9
1
2
t−Test: p−value = 0.24

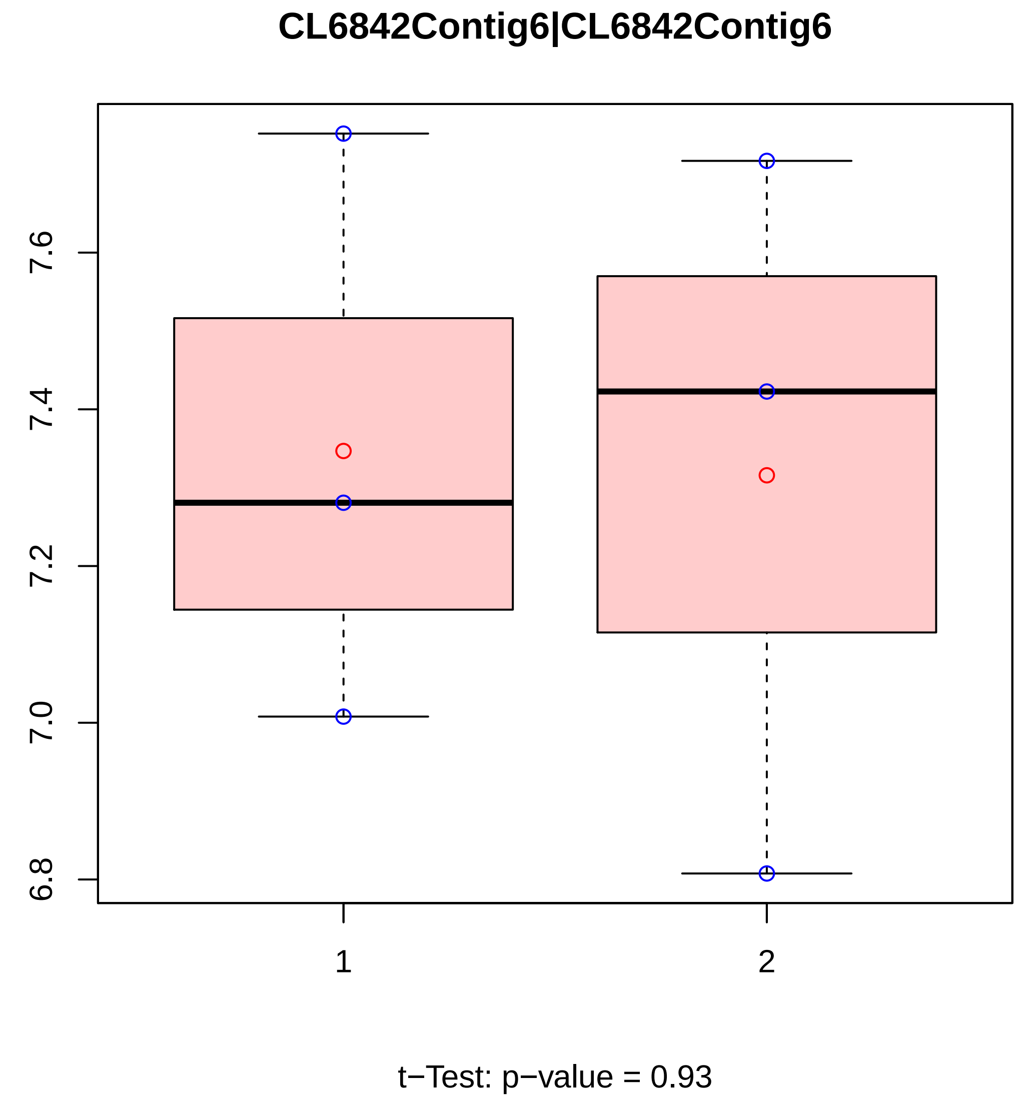

CL6842Contig6|CL6842Contig6
7.6
7.4
7.2
7.0
6.8
1
2
t−Test: p−value = 0.93

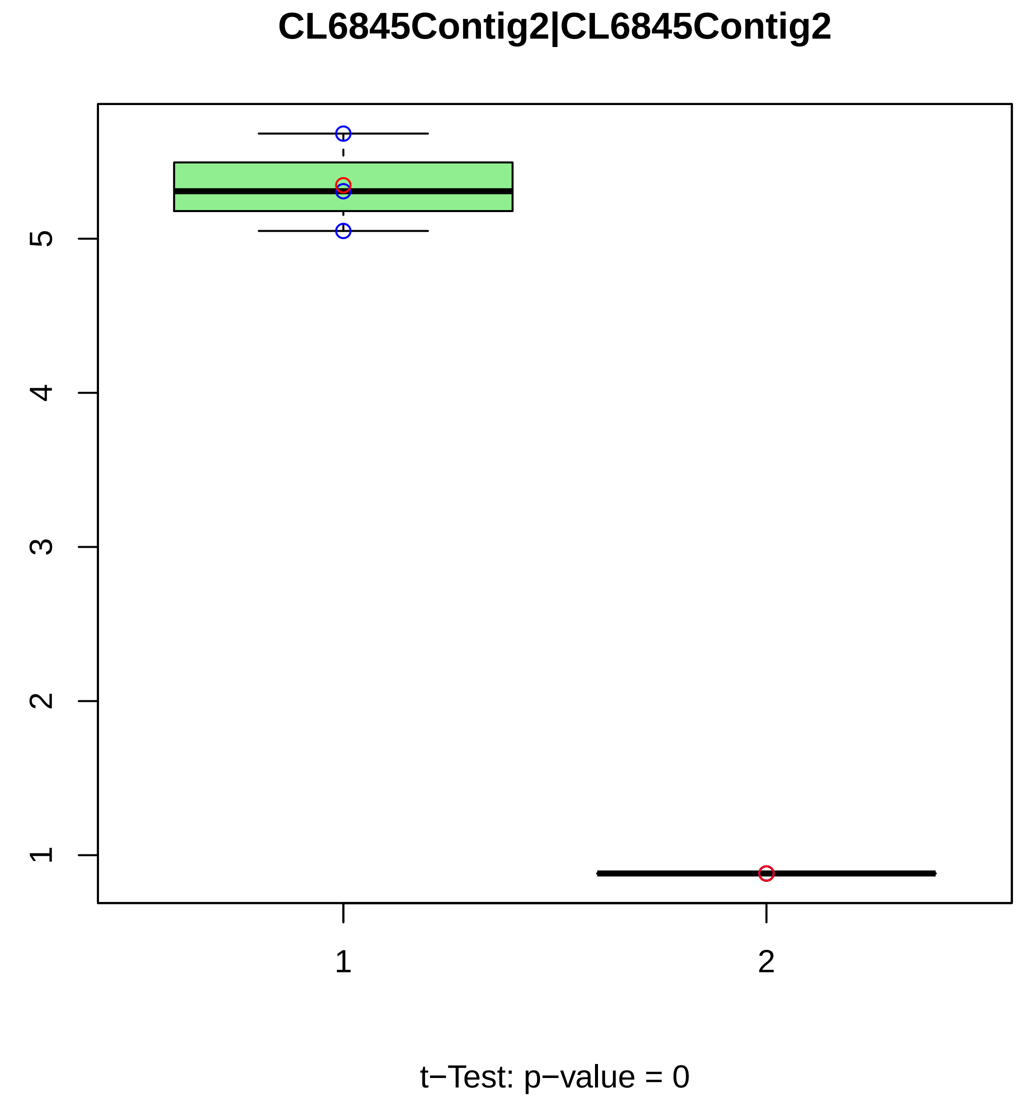
CL6845Contig2|CL6845Contig2
5
4
3
2
1
1
2
t−Test: p−value = 0

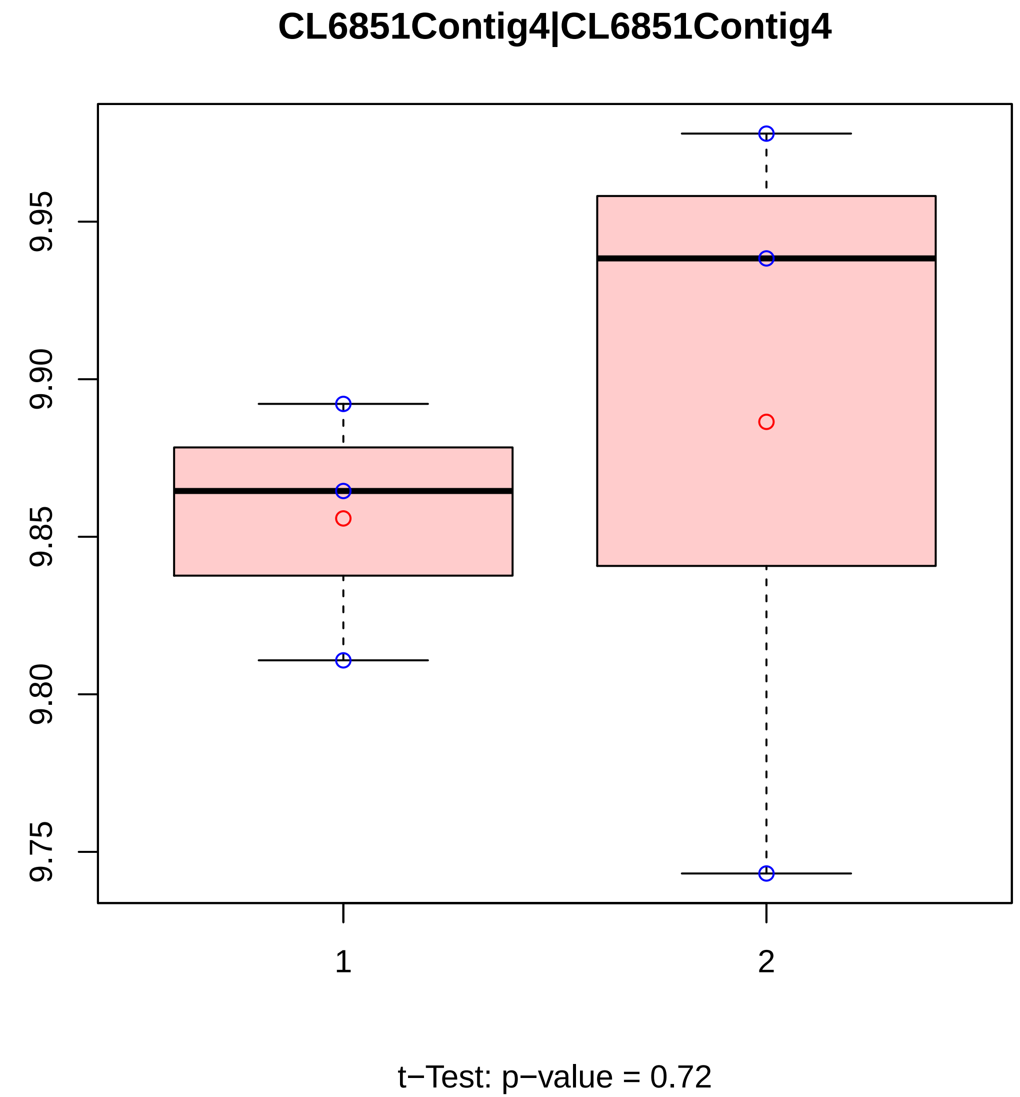

CL6851Contig4|CL6851Contig4
9.75
9.80
9.85
9.90
9.95
1
2
t−Test: p−value = 0.72

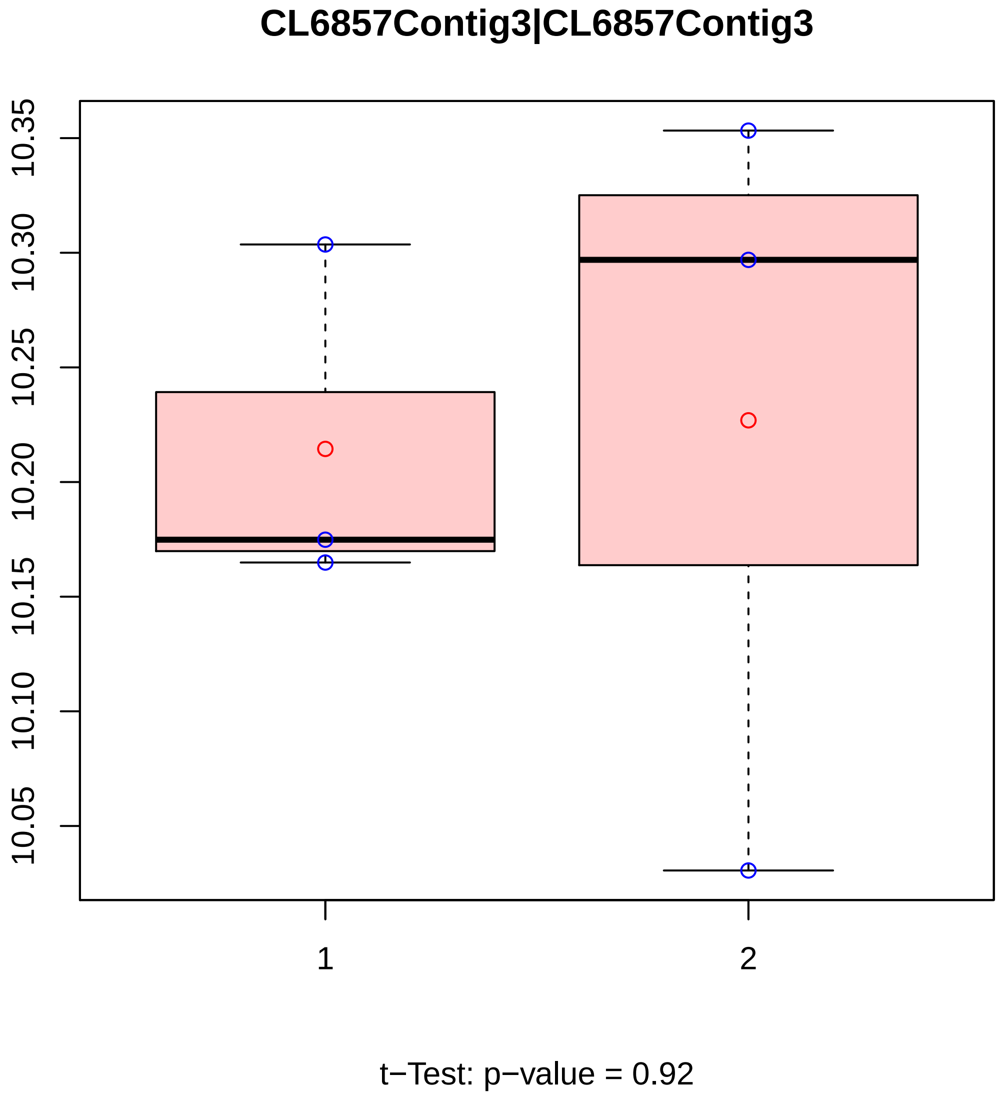

CL6857Contig3|CL6857Contig3
10.35
10.30
10.25
10.20
10.15
10.10
10.05
1
2
t−Test: p−value = 0.92

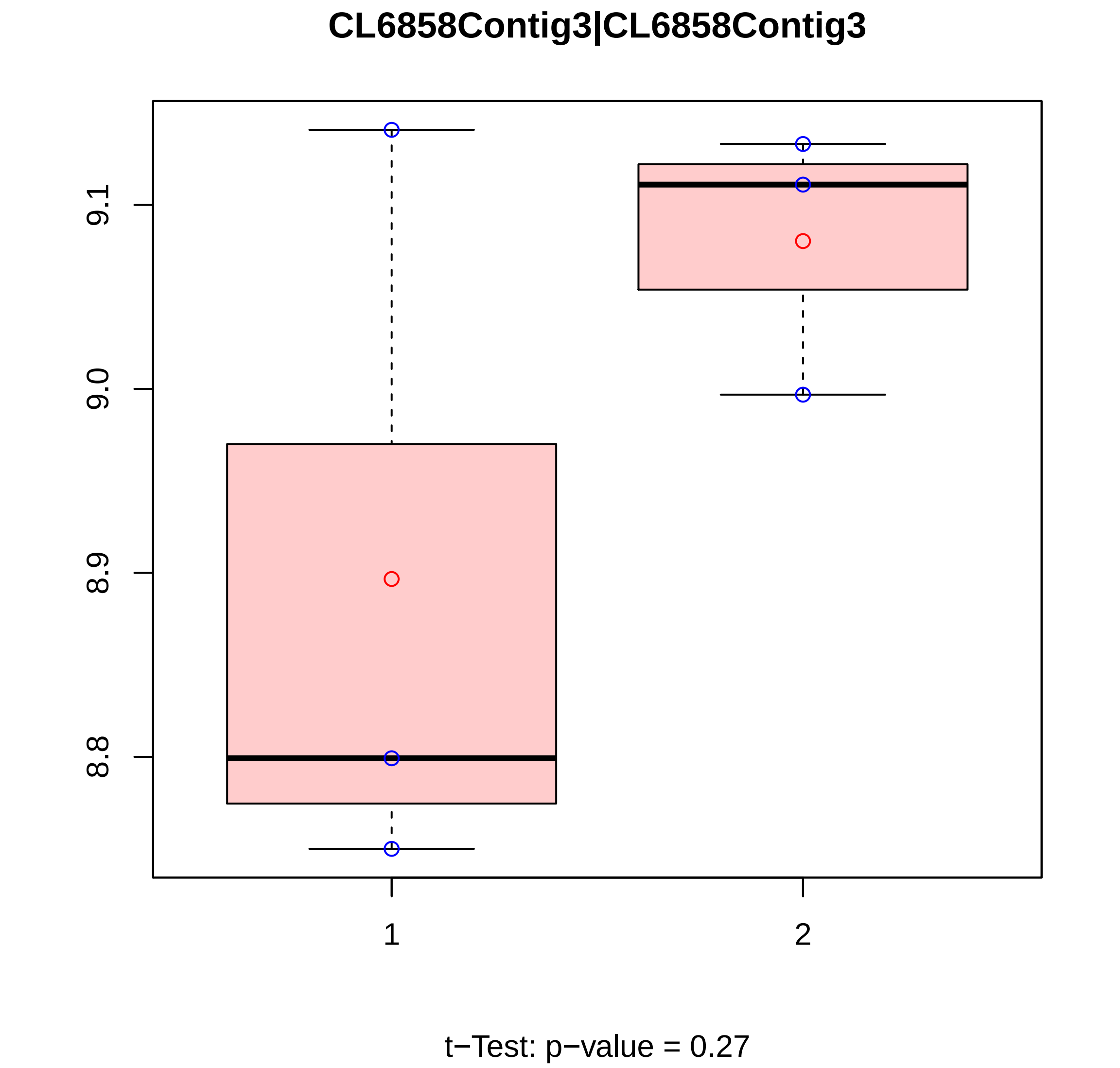
CL6858Contig3|CL6858Contig3
9.1
9.0
8.9
8.8
1
2
t−Test: p−value = 0.27

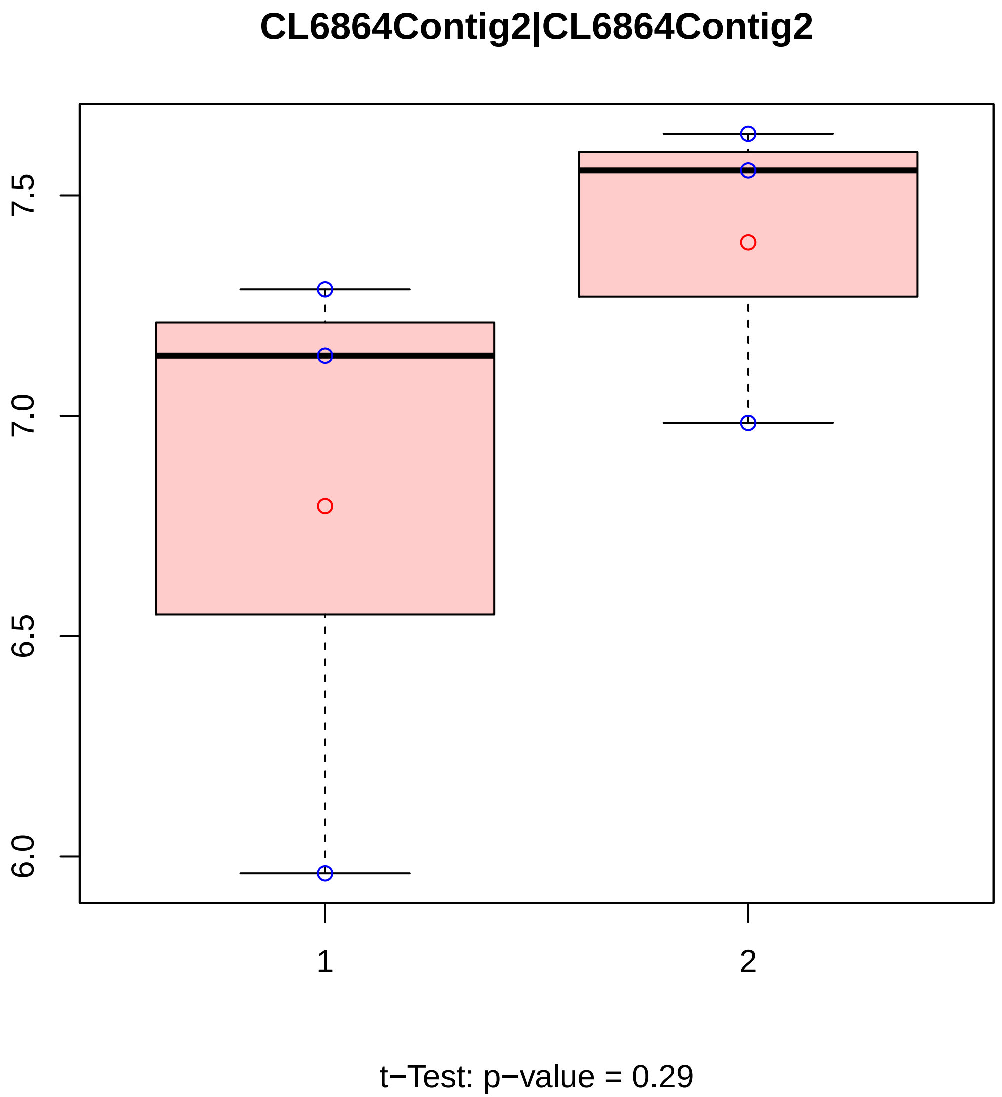

CL6864Contig2|CL6864Contig2
7.5
7.0
6.5
6.0
1
2
t−Test: p−value = 0.29

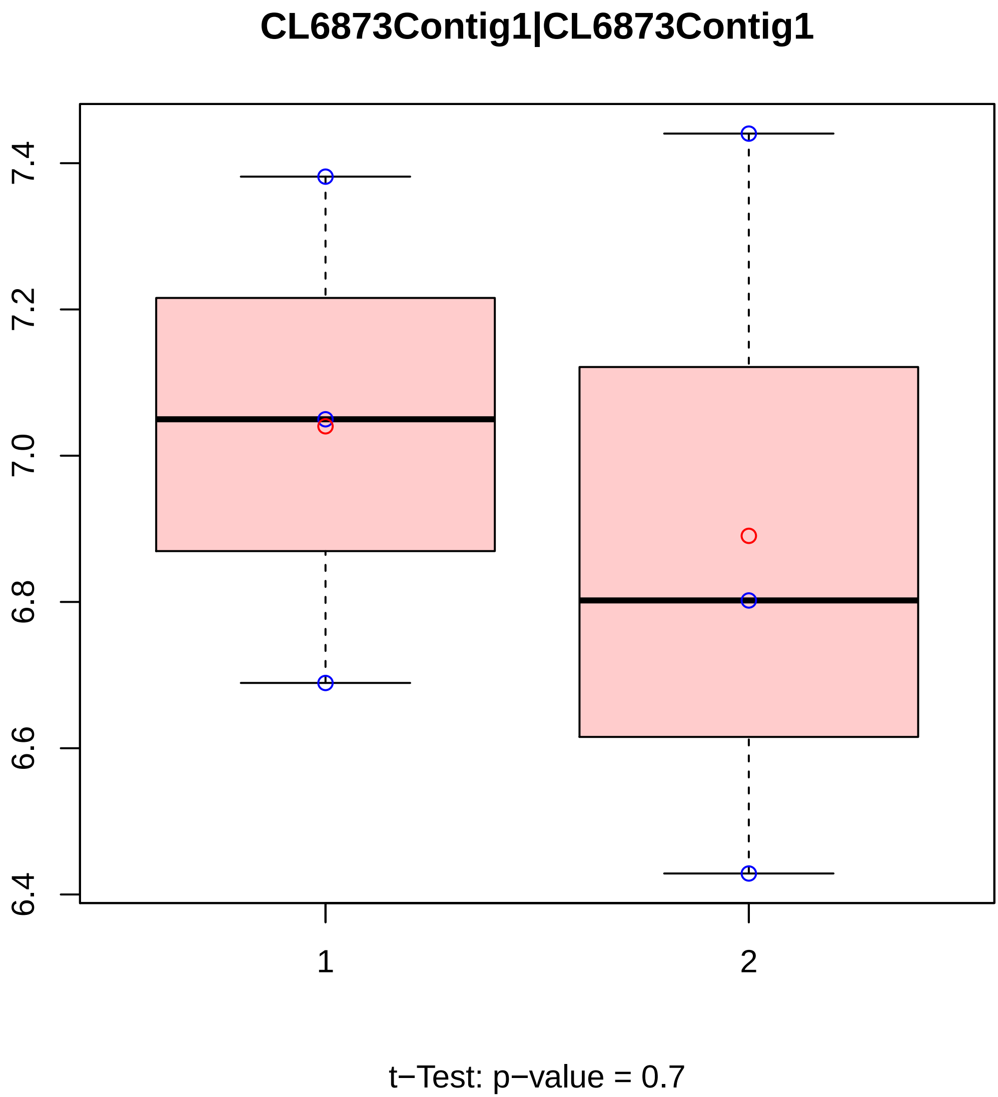
CL6873Contig1|CL6873Contig1
7.4
7.2
7.0
6.8
6.6
6.4
1
2
t−Test: p−value = 0.7

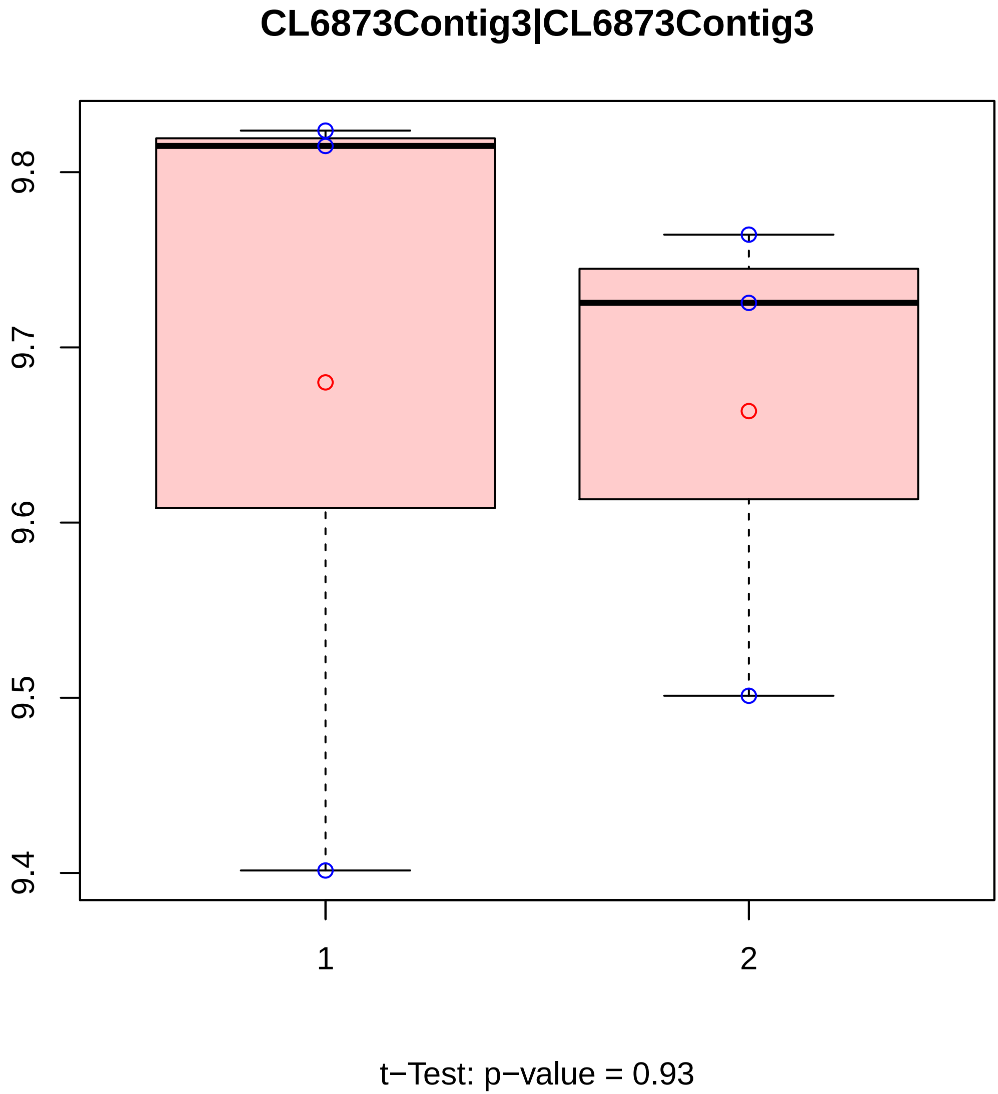

CL6873Contig3|CL6873Contig3
9.4
9.5
9.6
9.7
9.8
1
2
t−Test: p−value = 0.93

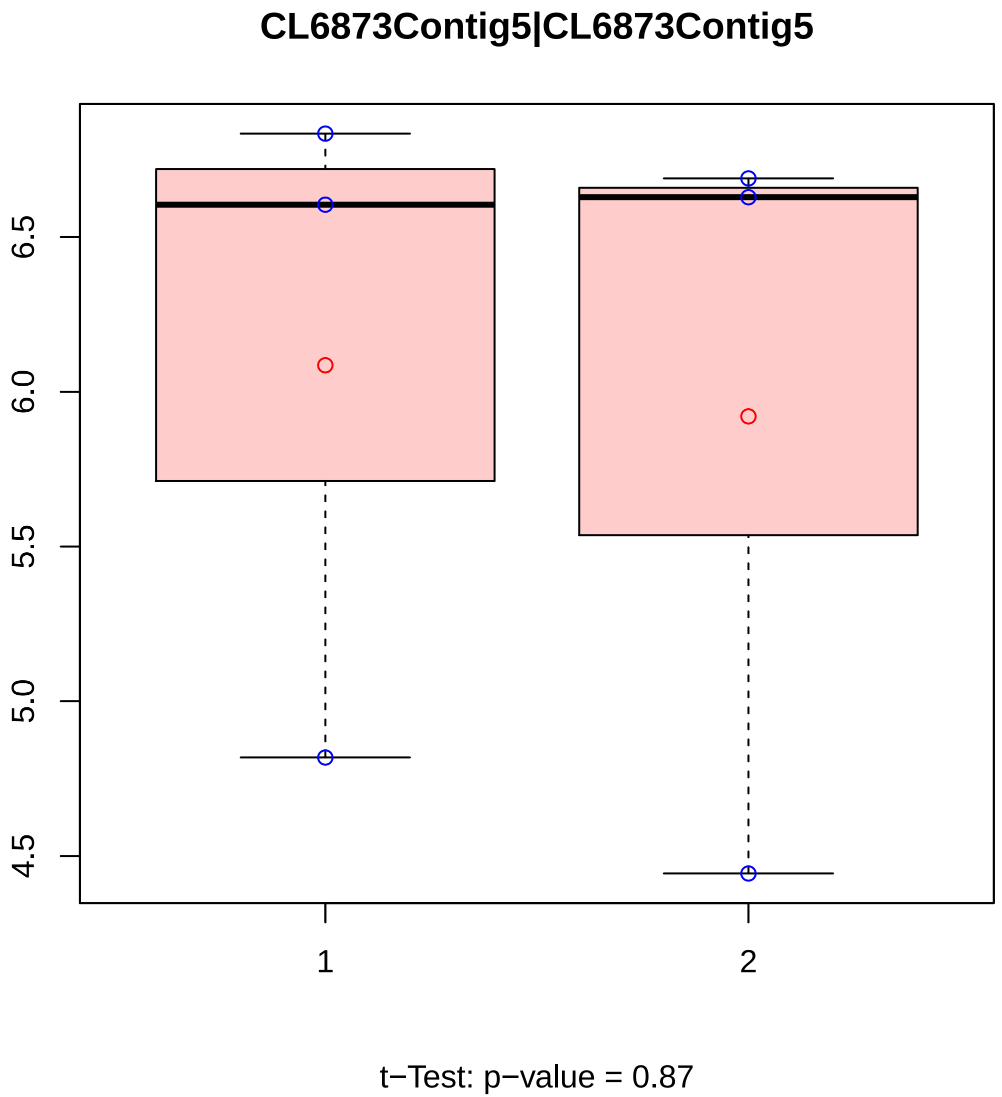

CL6873Contig5|CL6873Contig5
6.5
6.0
5.5
5.0
4.5
1
2
t−Test: p−value = 0.87

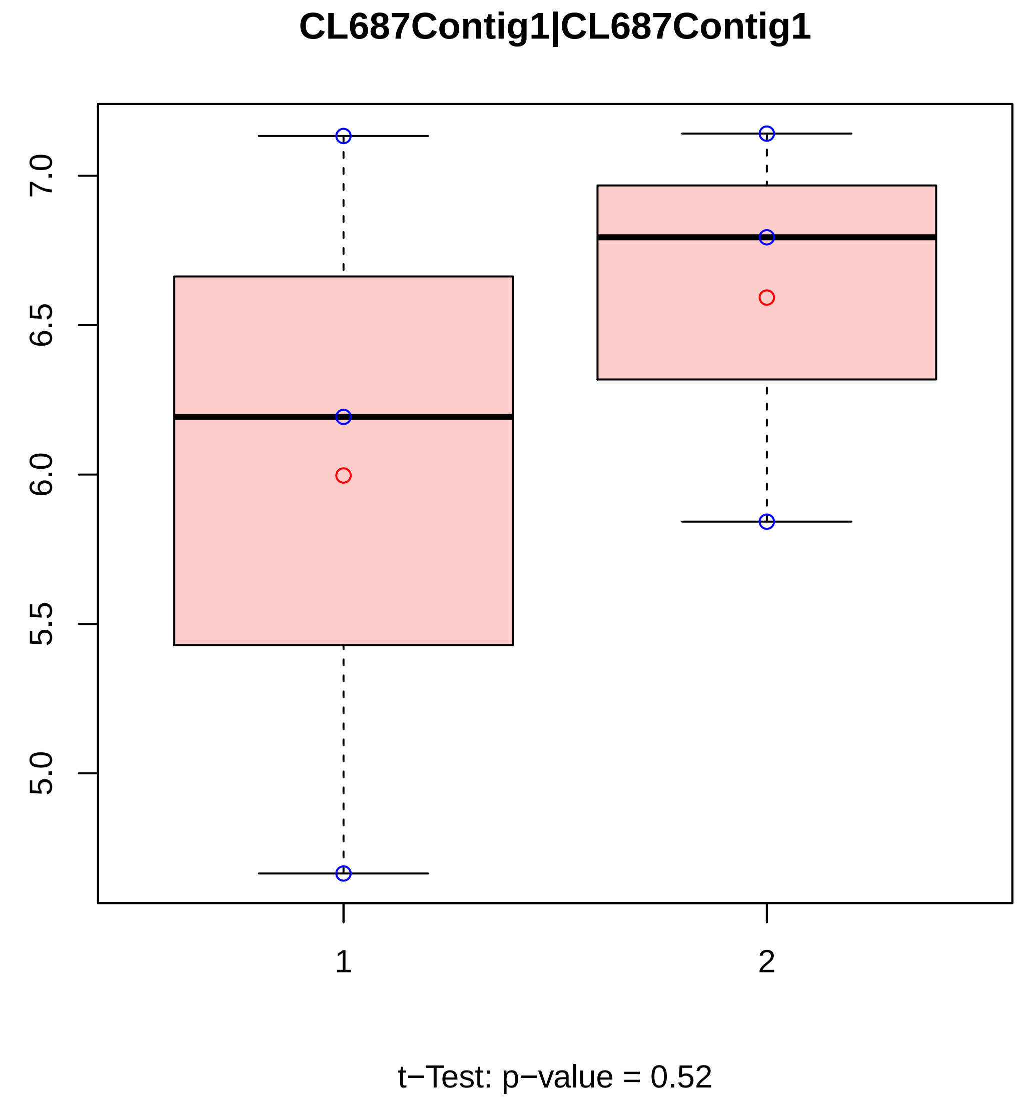
CL687Contig1|CL687Contig1
7.0
6.5
6.0
5.5
5.0
1
2
t−Test: p−value = 0.52

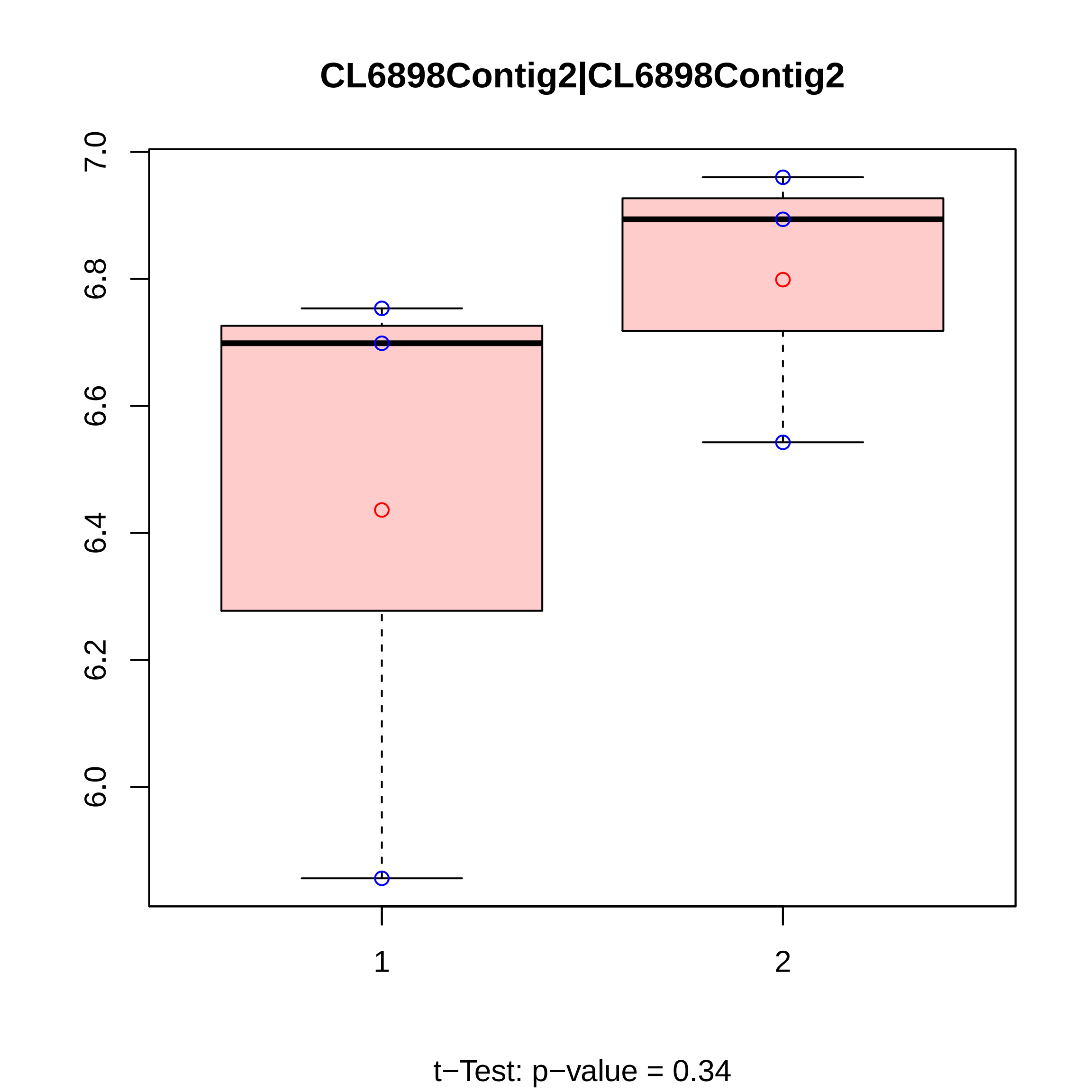
CL6898Contig2|CL6898Contig2
7.0
6.8
6.6
6.4
6.2
6.0
1
2
t−Test: p−value = 0.34

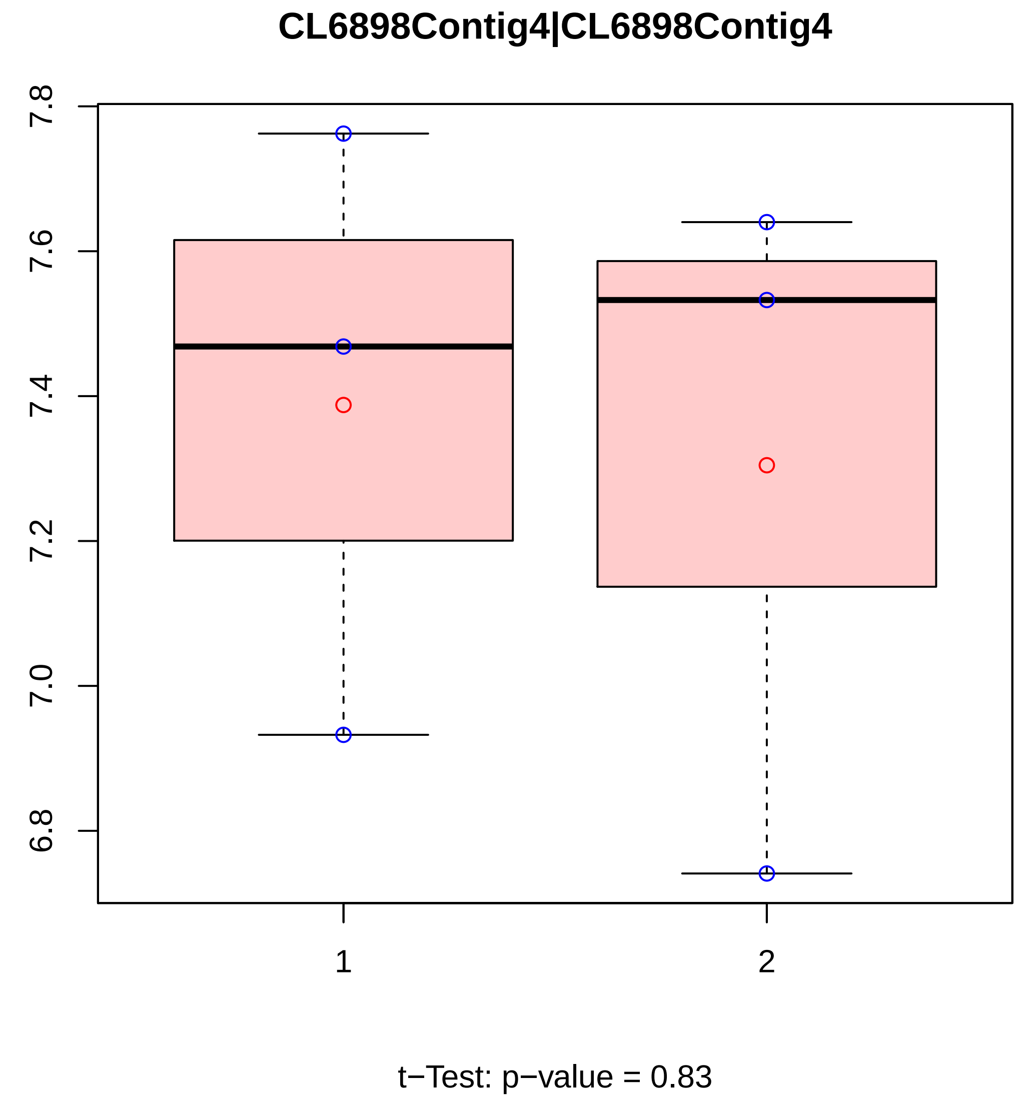
CL6898Contig4|CL6898Contig4
7.8
7.6
7.4
7.2
7.0
6.8
1
2
t−Test: p−value = 0.83

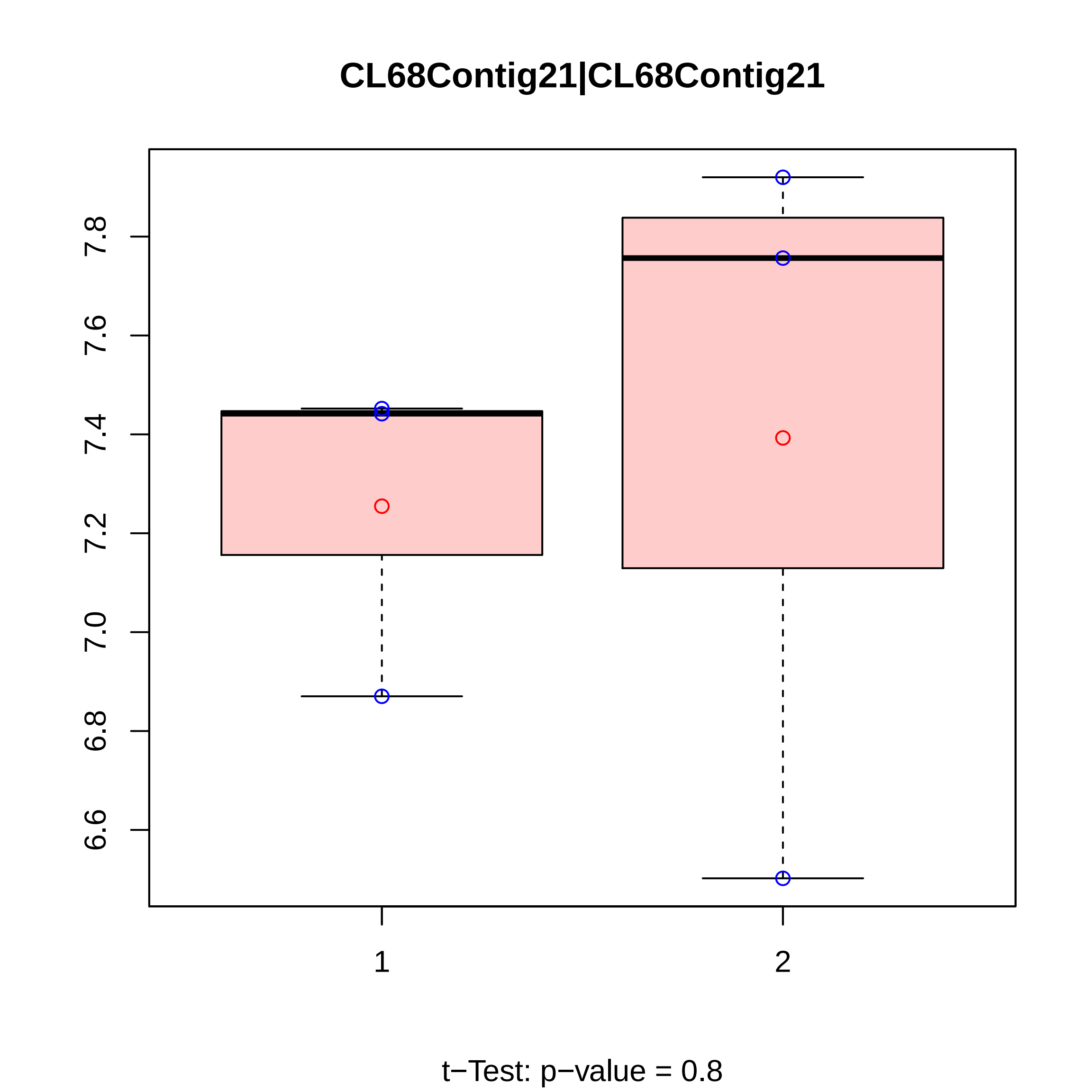

CL68Contig21|CL68Contig21
6.6
6.8
7.0
7.2
7.4
7.6
7.8
1
2
t−Test: p−value = 0.8

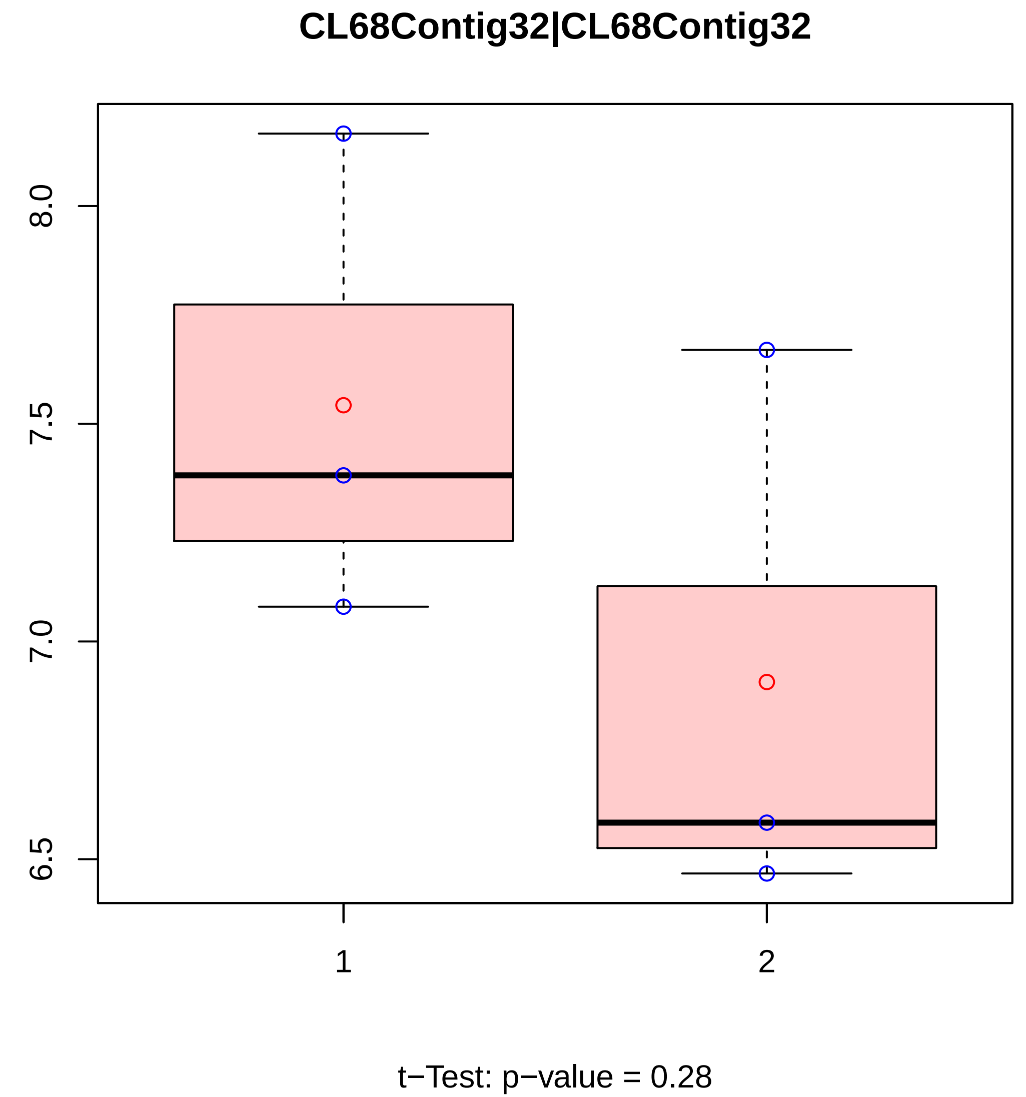
CL68Contig32|CL68Contig32
8.0
7.5
7.0
6.5
1
2
t−Test: p−value = 0.28

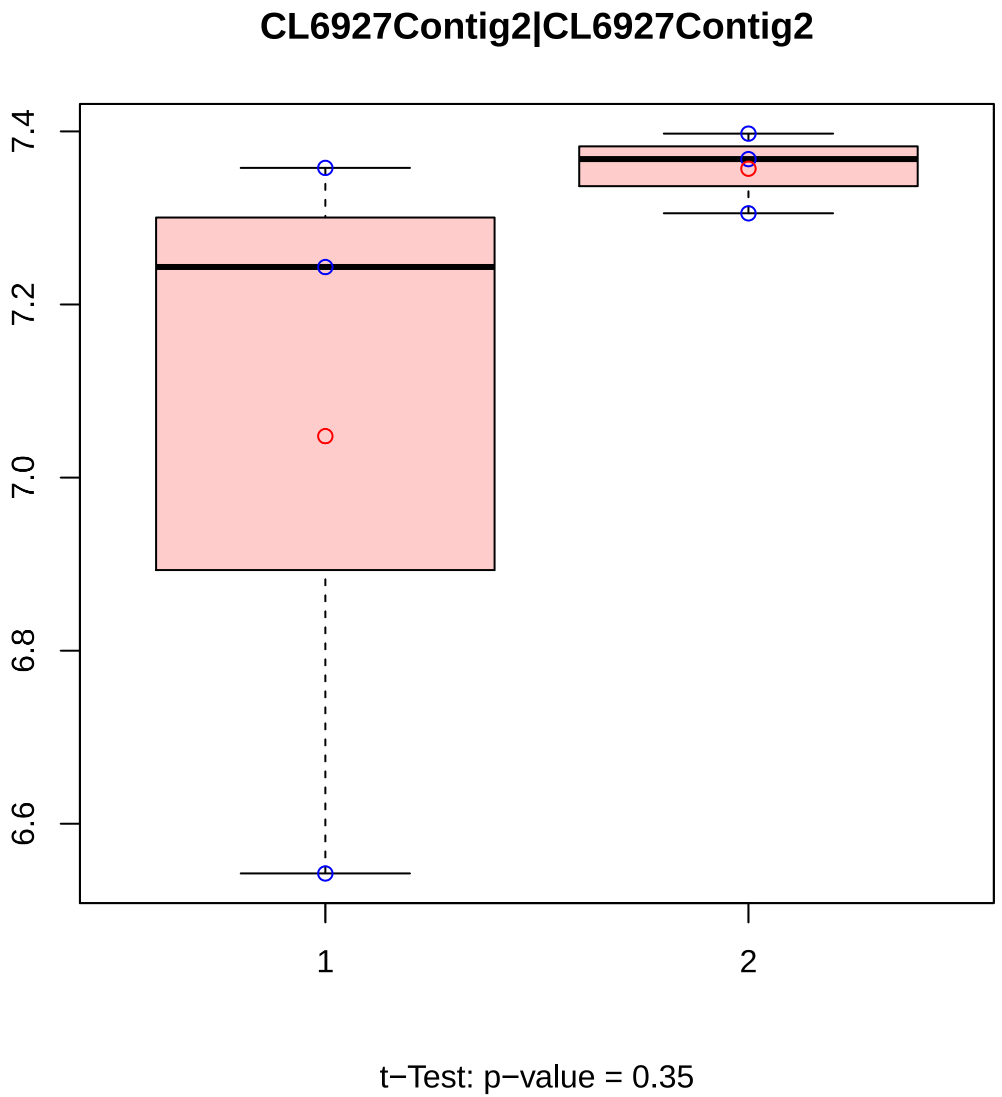

CL6927Contig2|CL6927Contig2
7.4
7.2
7.0
6.8
6.6
1
2
t−Test: p−value = 0.35

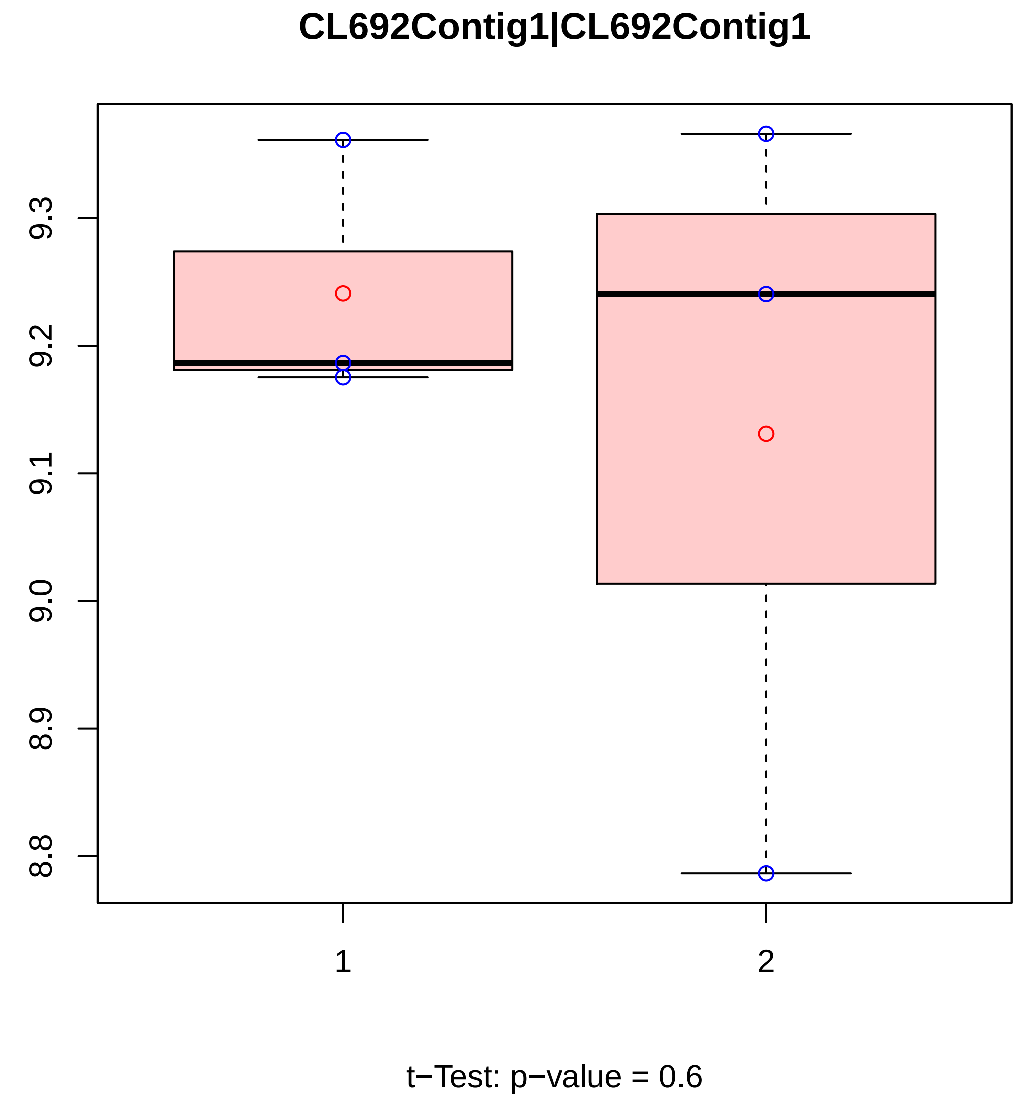

CL692Contig1|CL692Contig1
9.3
9.2
9.1
9.0
8.9
8.8
1
2
t−Test: p−value = 0.6

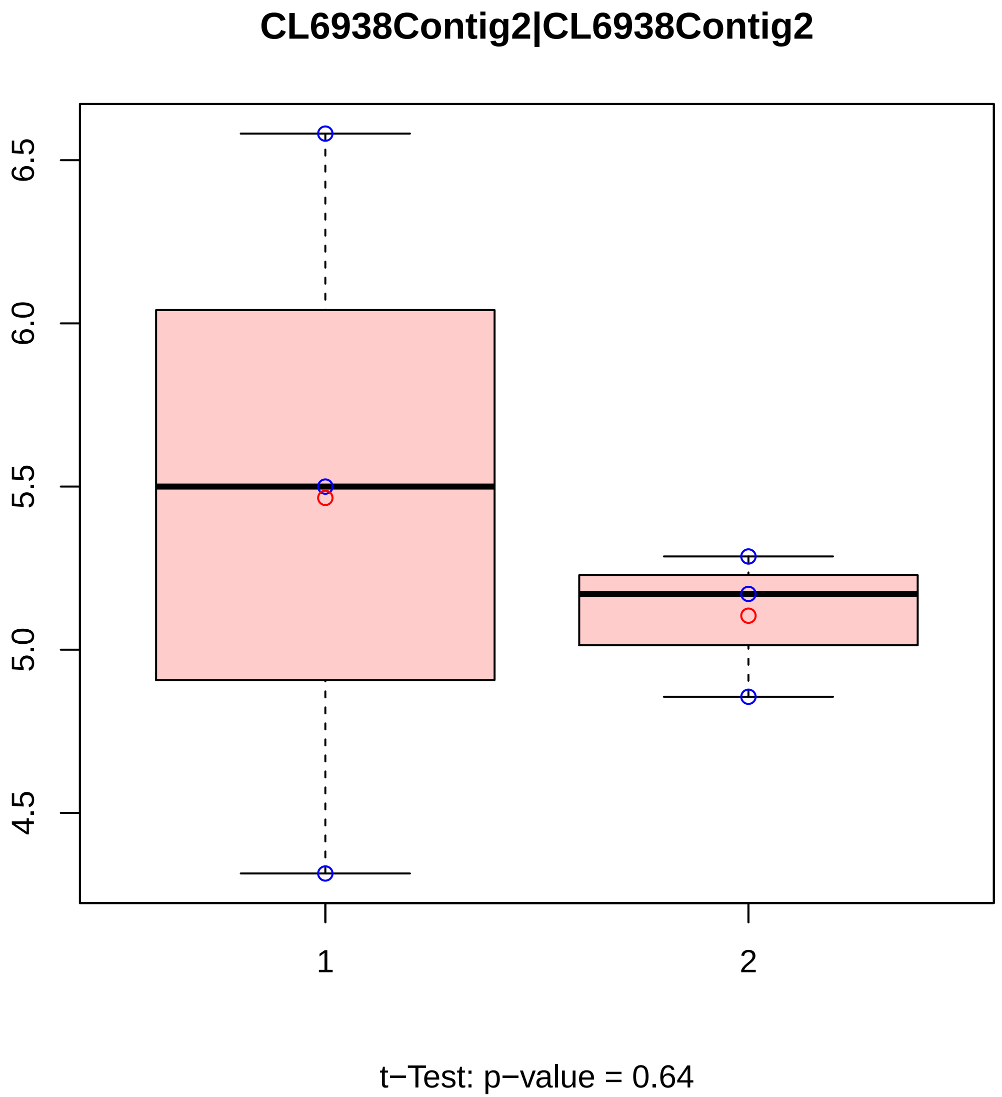
CL6938Contig2|CL6938Contig2
6.5
6.0
5.5
5.0
4.5
1
2
t−Test: p−value = 0.64

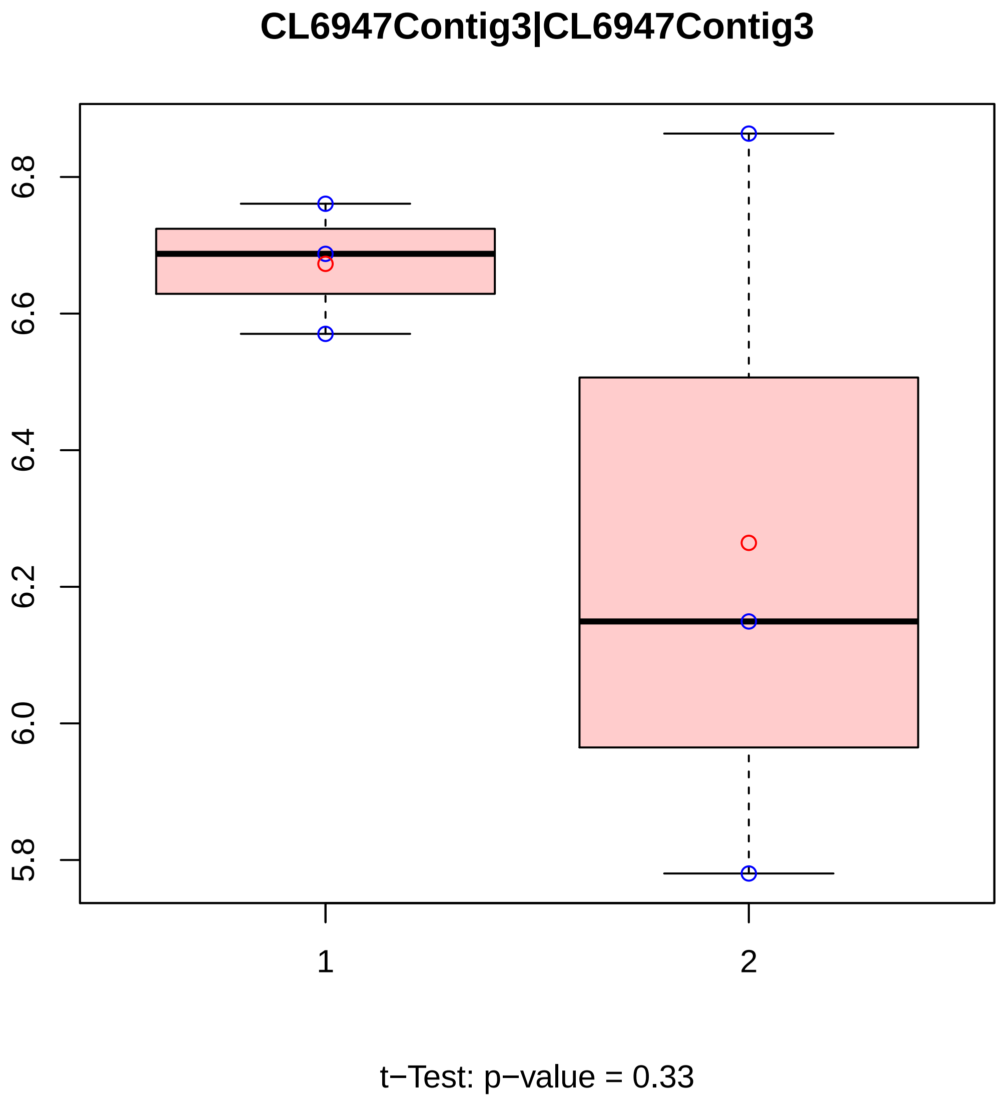
CL6947Contig3|CL6947Contig3
6.8
6.6
6.4
6.2
6.0
5.8
1
2
t−Test: p−value = 0.33

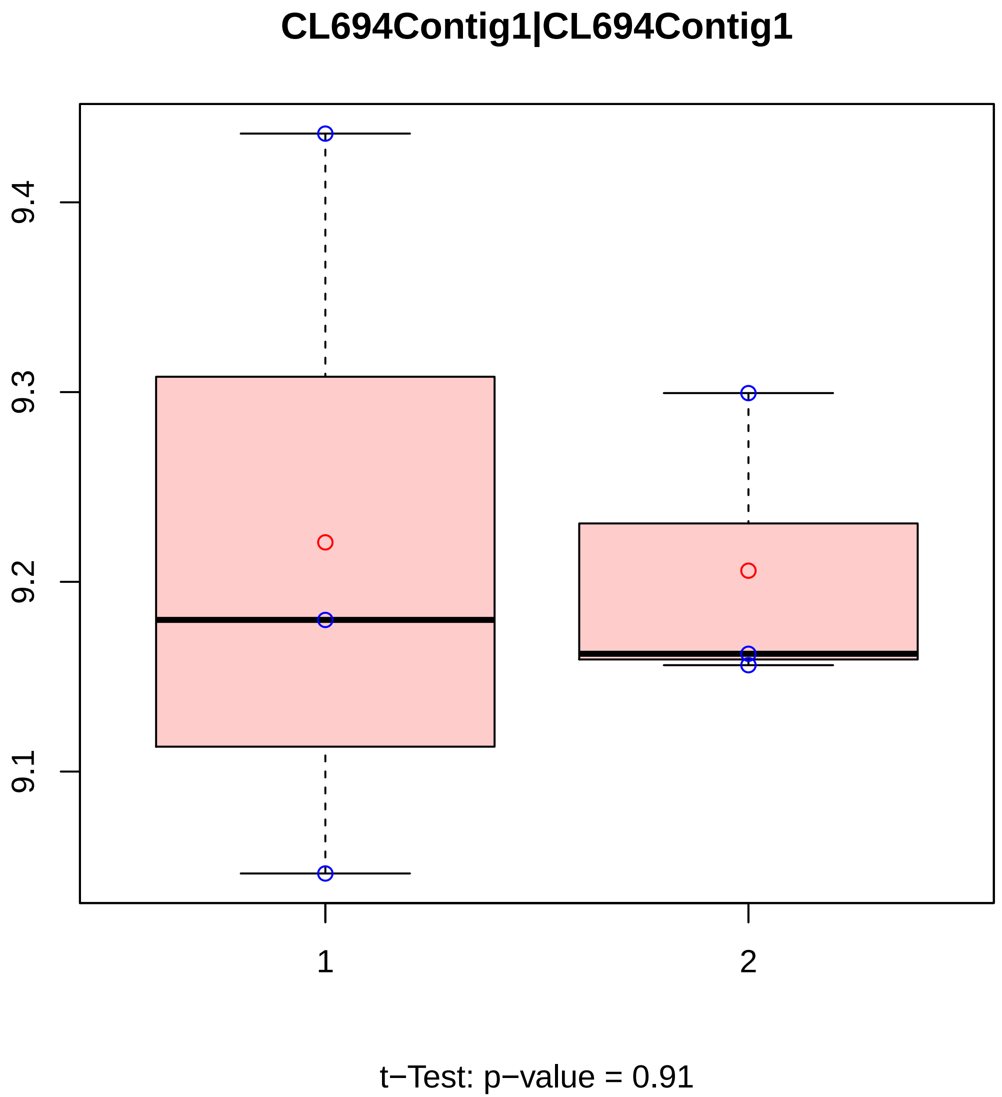
CL694Contig1|CL694Contig1
9.1
9.2
9.3
9.4
1
2
t−Test: p−value = 0.91

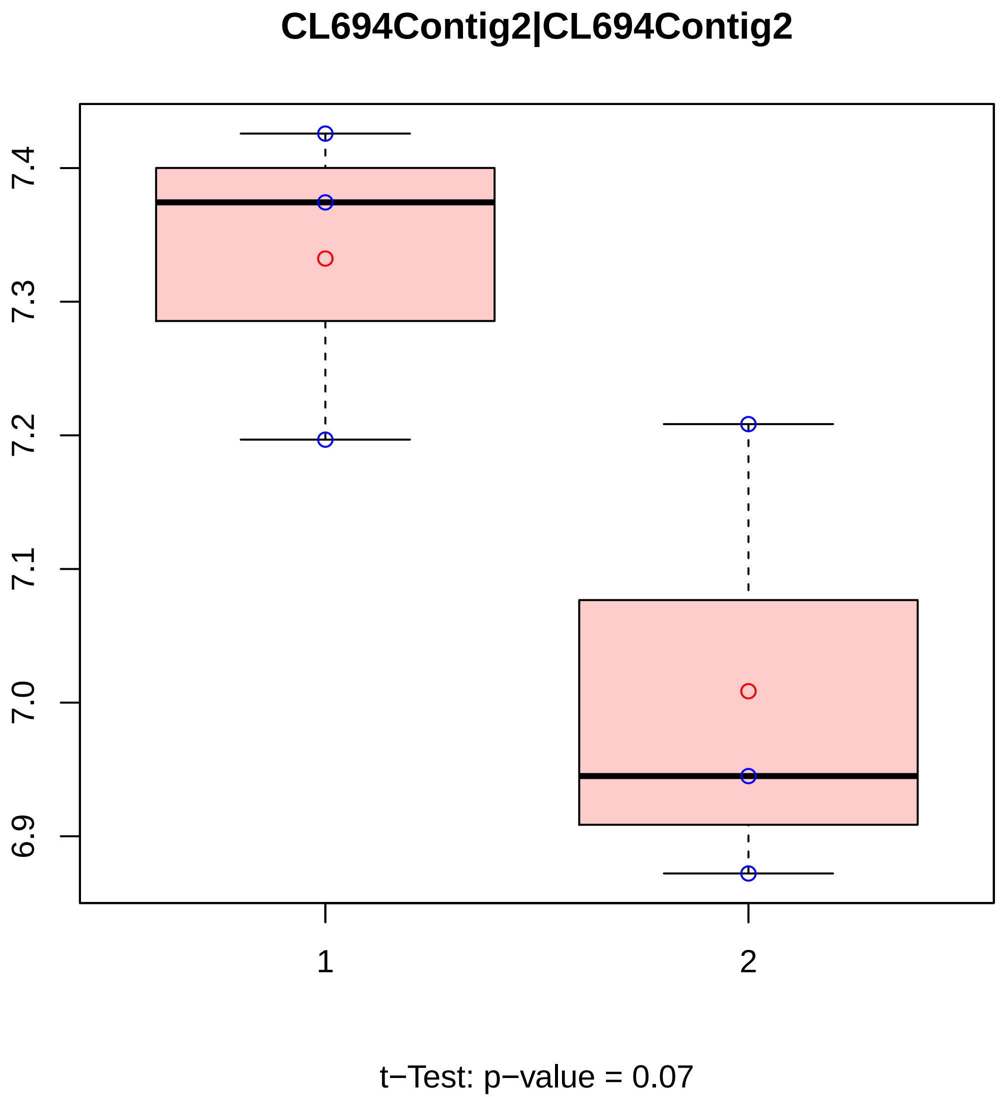
CL694Contig2|CL694Contig2
7.4
7.3
7.2
7.1
7.0
6.9
1
2
t−Test: p−value = 0.07

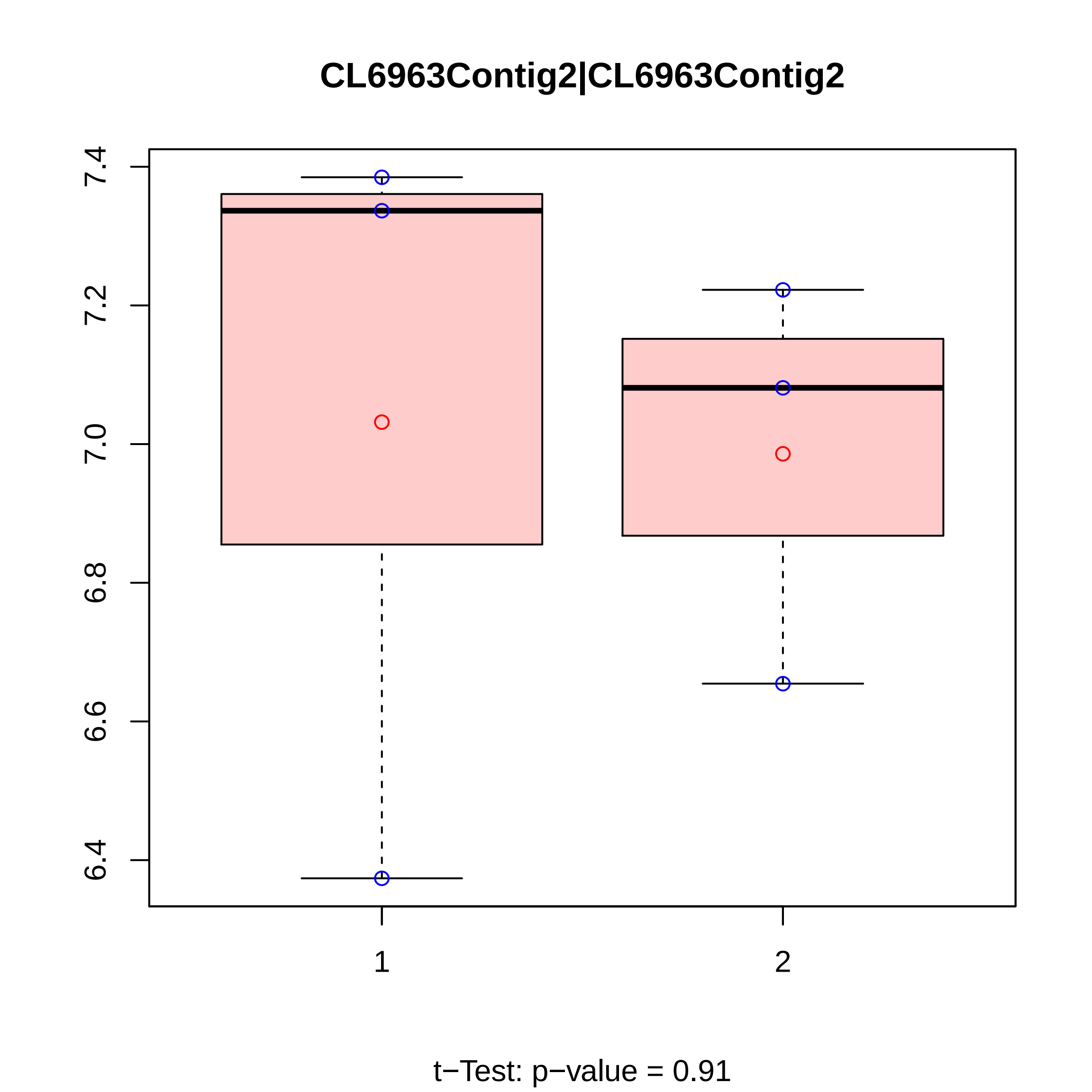

CL6963Contig2|CL6963Contig2
7.4
7.2
7.0
6.8
6.6
6.4
1
2
t−Test: p−value = 0.91

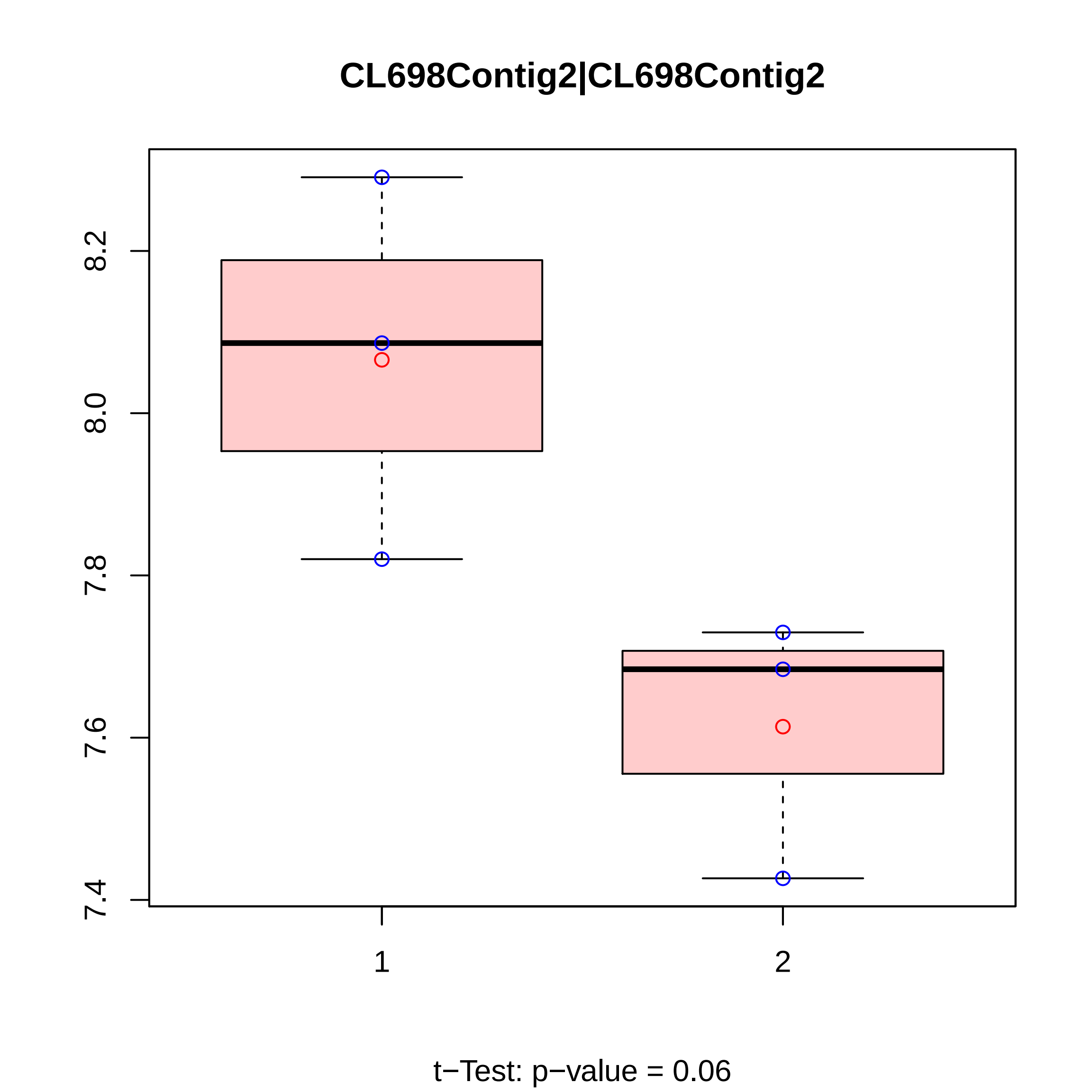

CL698Contig2|CL698Contig2
8.2
8.0
7.8
7.6
7.4
1
2
t−Test: p−value = 0.06

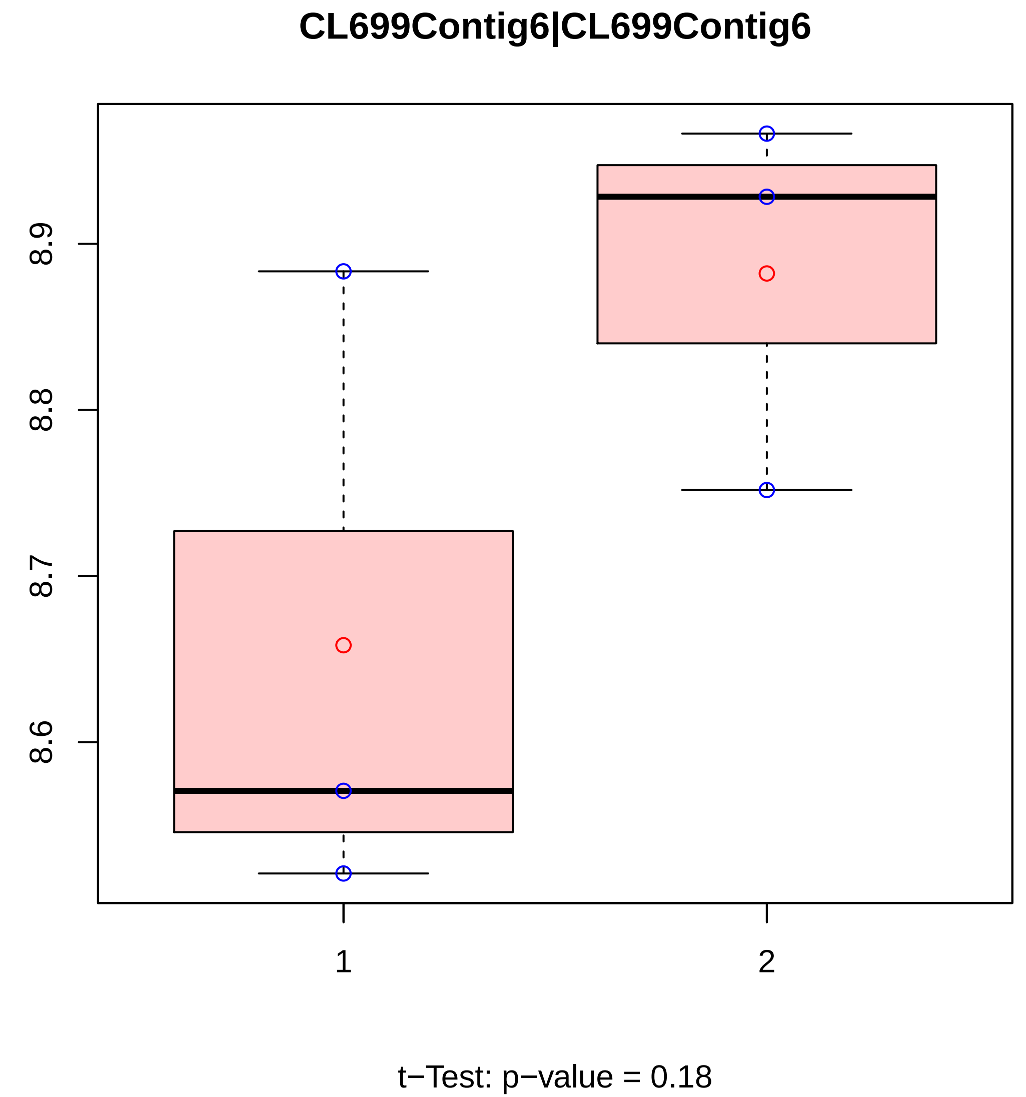

CL699Contig6|CL699Contig6
8.6
8.7
8.8
8.9
1
2
t−Test: p−value = 0.18

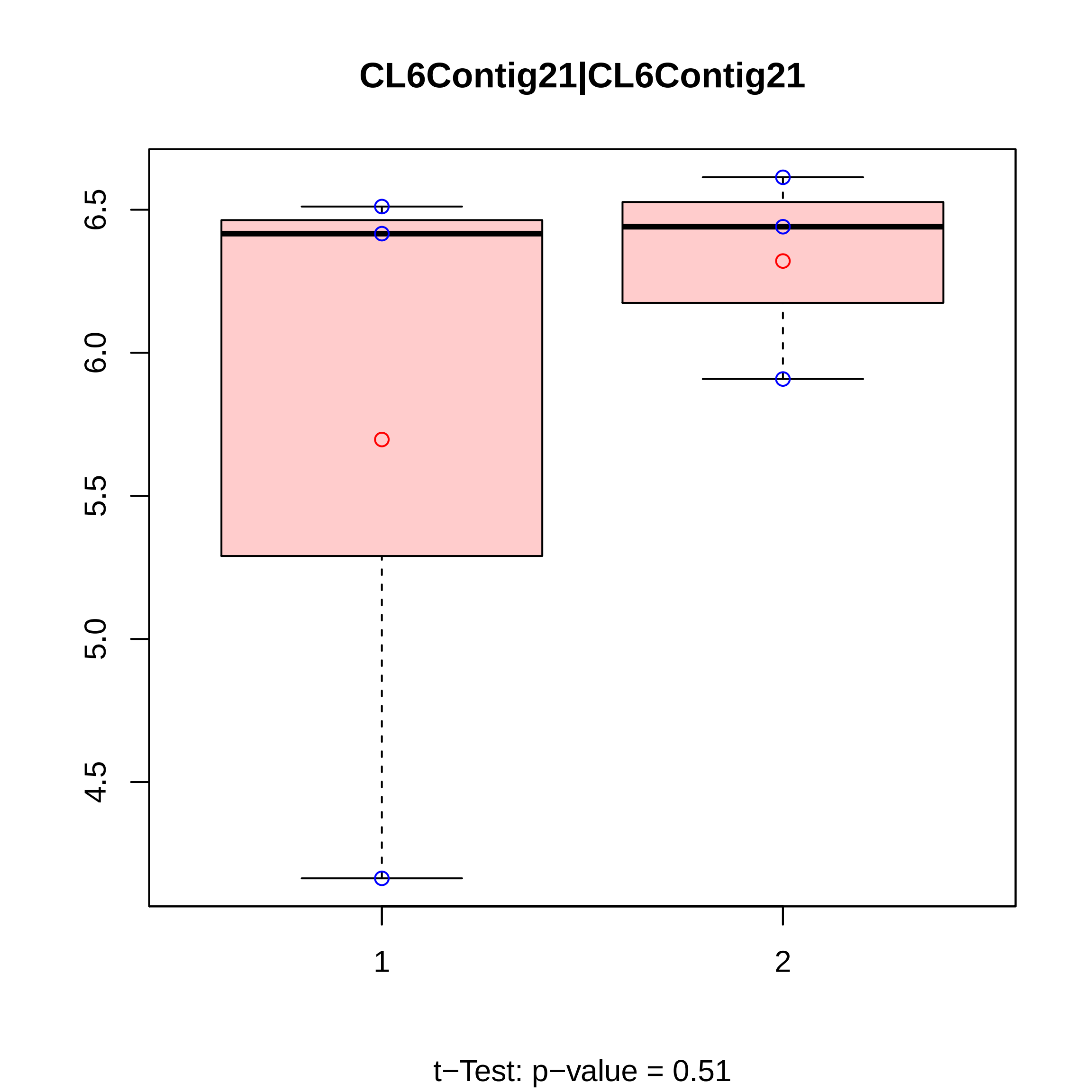

CL6Contig21|CL6Contig21
6.5
6.0
5.5
5.0
4.5
1
2
t−Test: p−value = 0.51

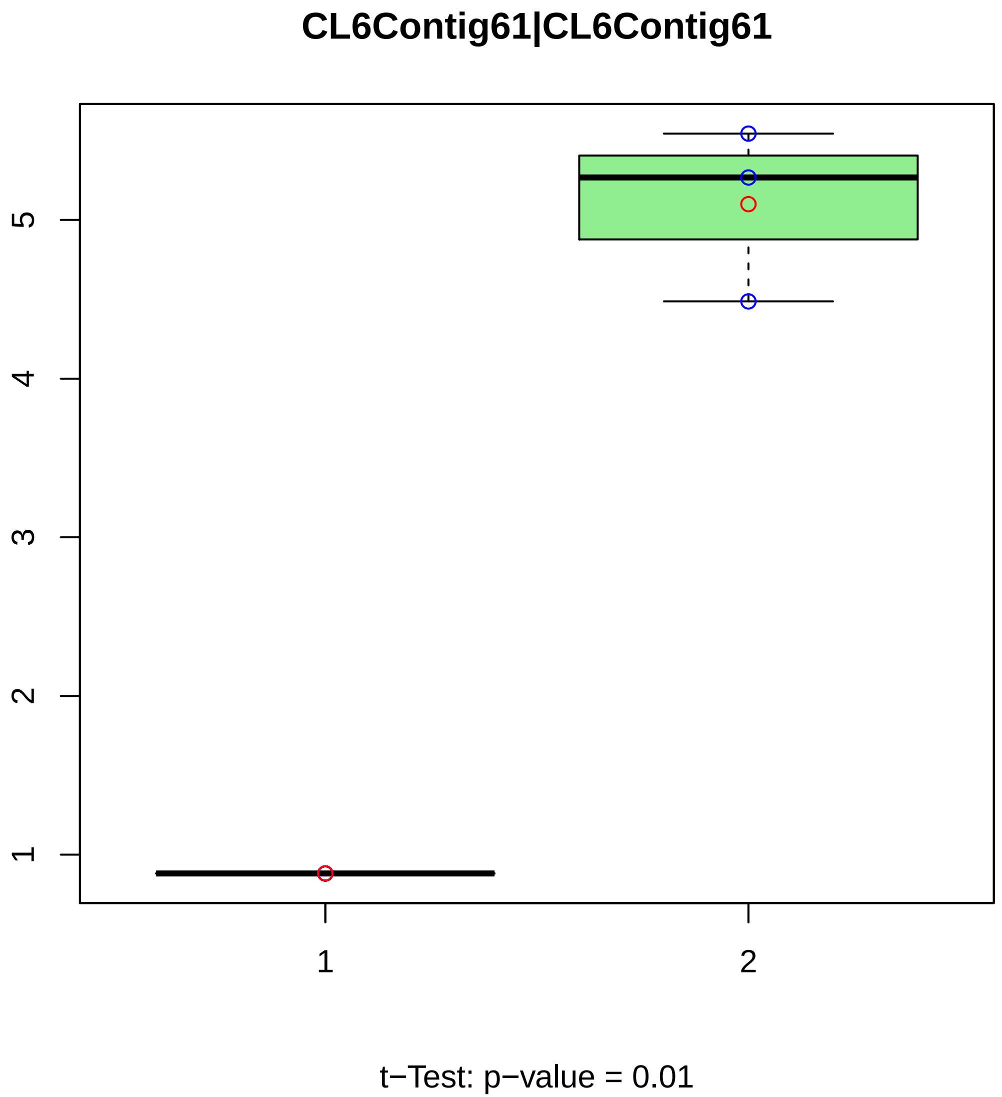

CL6Contig61|CL6Contig61
1
2
3
4
5
1
2
t−Test: p−value = 0.01

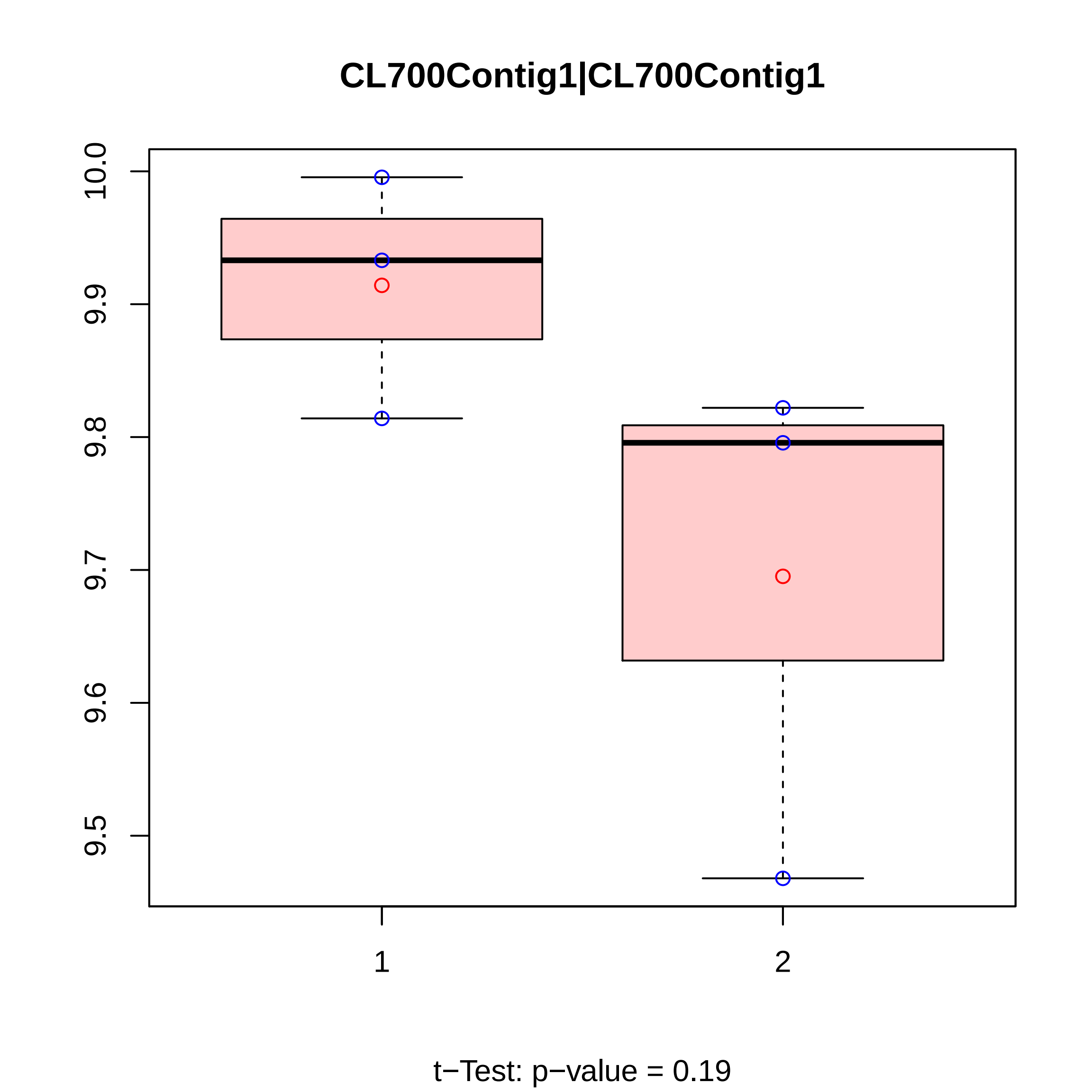
CL700Contig1|CL700Contig1
10.0
9.9
9.8
9.7
9.6
9.5
1
2
t−Test: p−value = 0.19

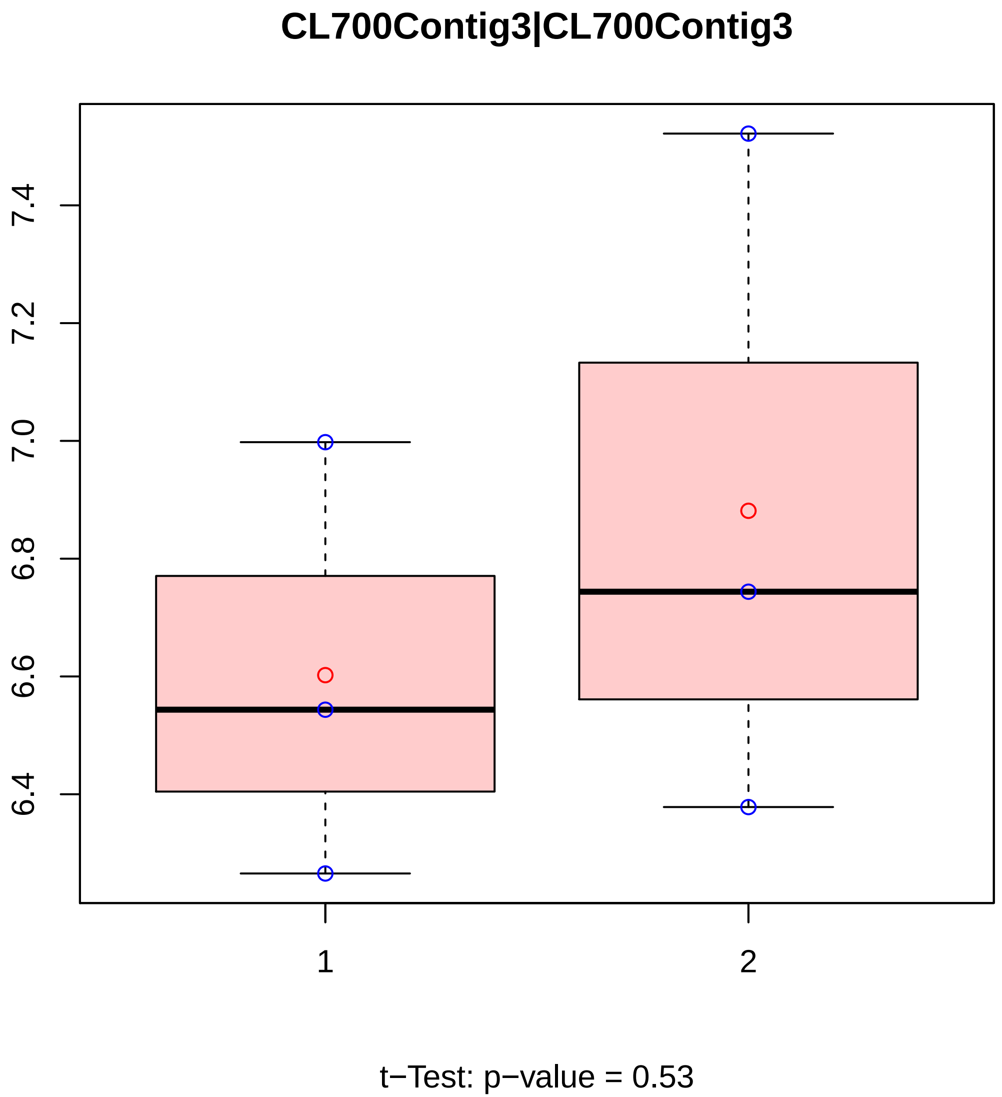
CL700Contig3|CL700Contig3
7.4
7.2
7.0
6.8
6.6
6.4
1
2
t−Test: p−value = 0.53

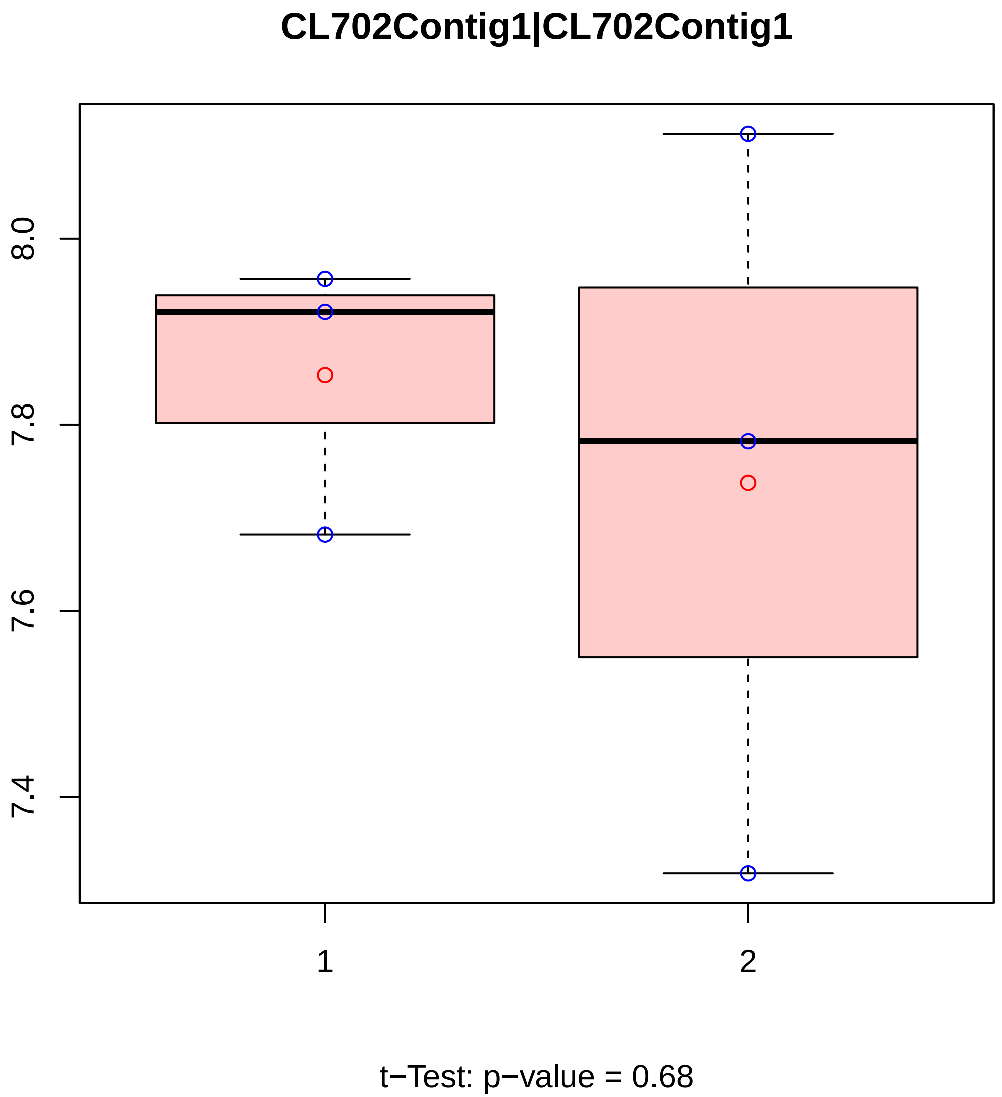

CL702Contig1|CL702Contig1
8.0
7.8
7.6
7.4
1
2
t−Test: p−value = 0.68

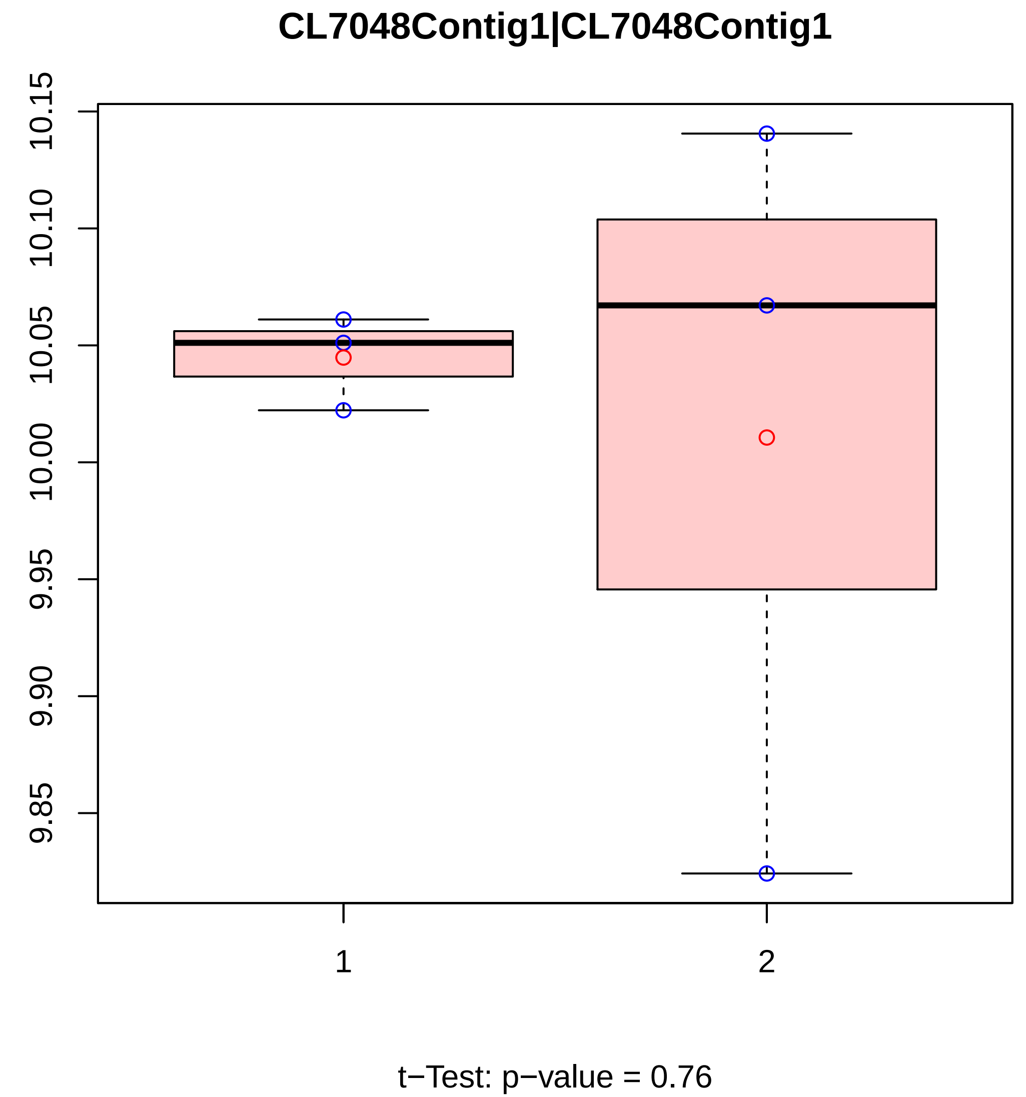

CL7048Contig1|CL7048Contig1
10.15
10.10
10.05
10.00
9.95
9.90
9.85
1
2
t−Test: p−value = 0.76

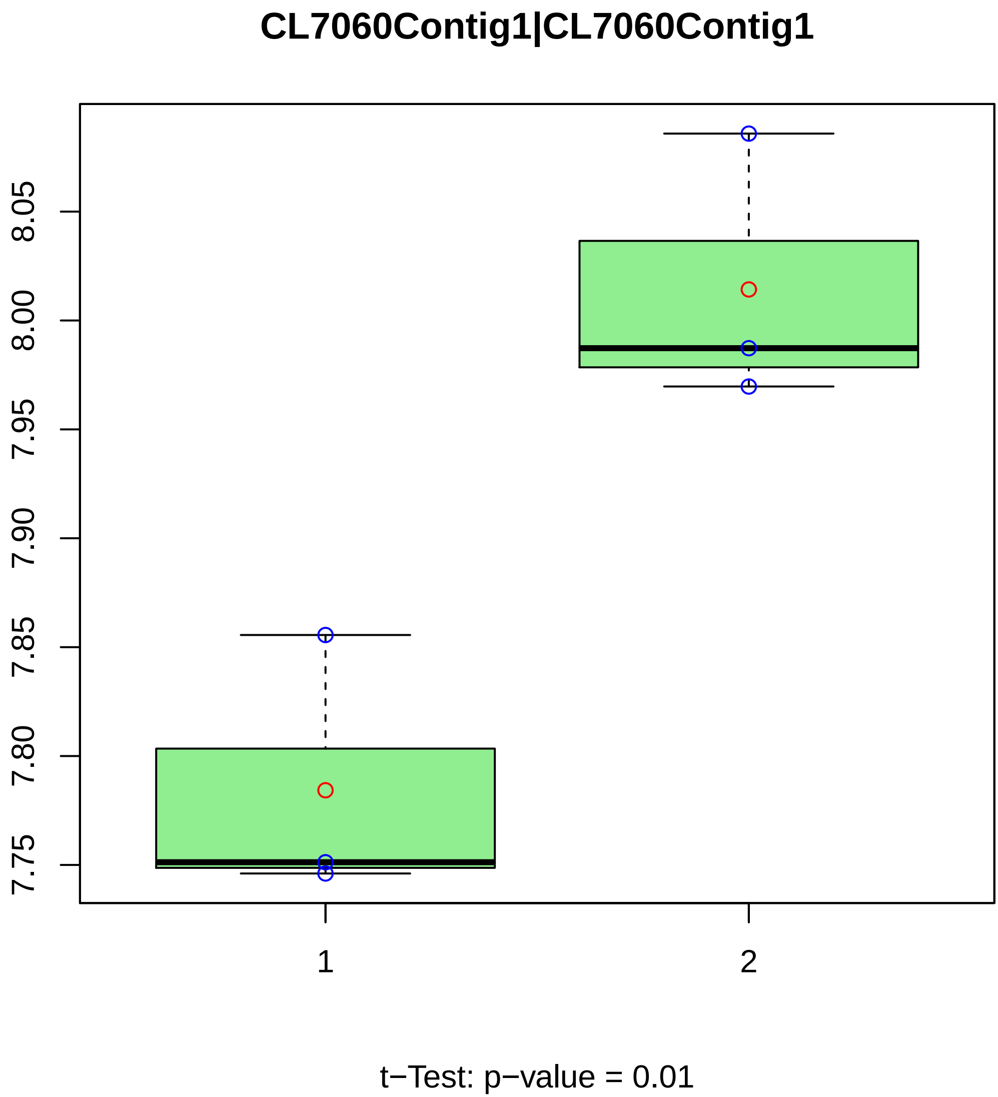

CL7060Contig1|CL7060Contig1
8.05
8.00
7.95
7.90
7.85
7.80
7.75
1
2
t−Test: p−value = 0.01

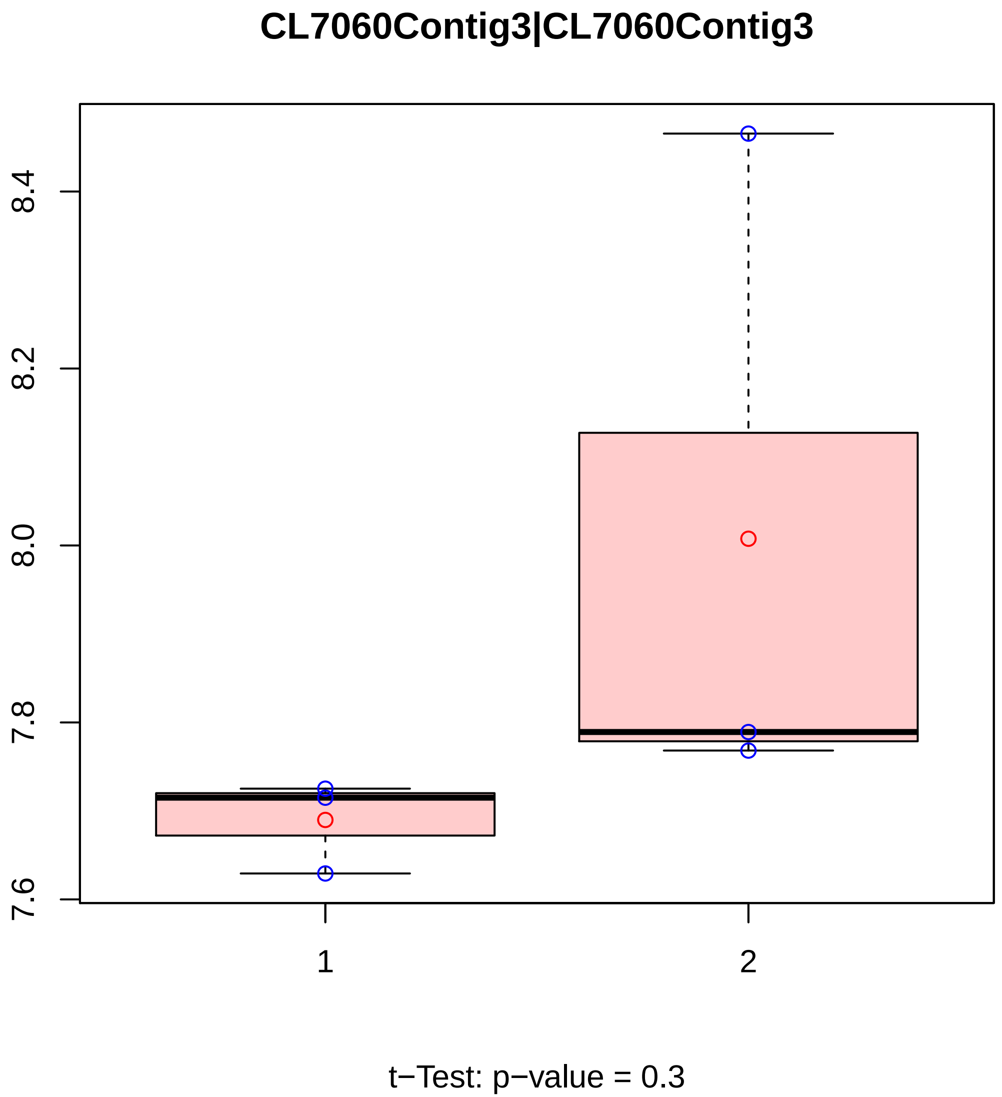
CL7060Contig3|CL7060Contig3
7.6
7.8
8.0
8.2
8.4
1
2
t−Test: p−value = 0.3

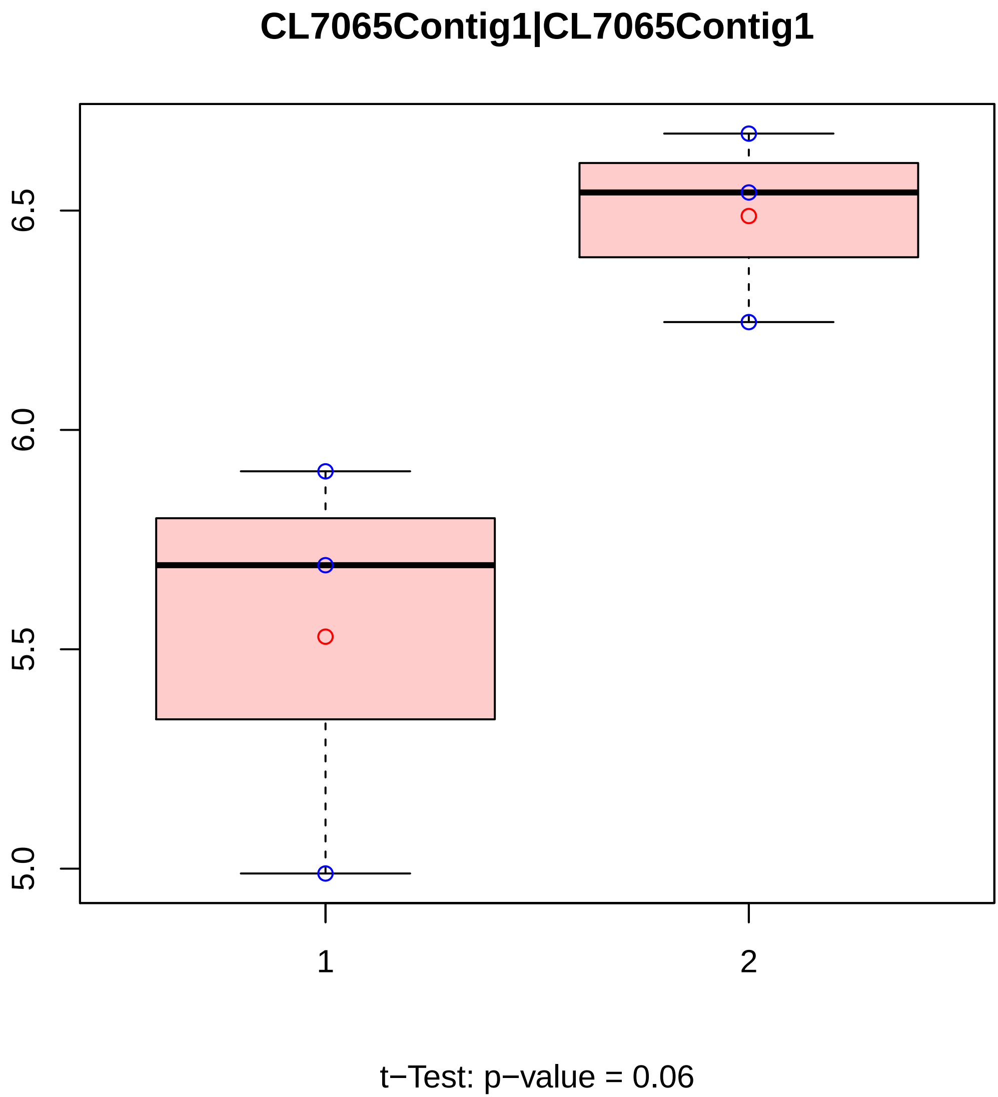
CL7065Contig1|CL7065Contig1
6.5
6.0
5.5
5.0
1
2
t−Test: p−value = 0.06

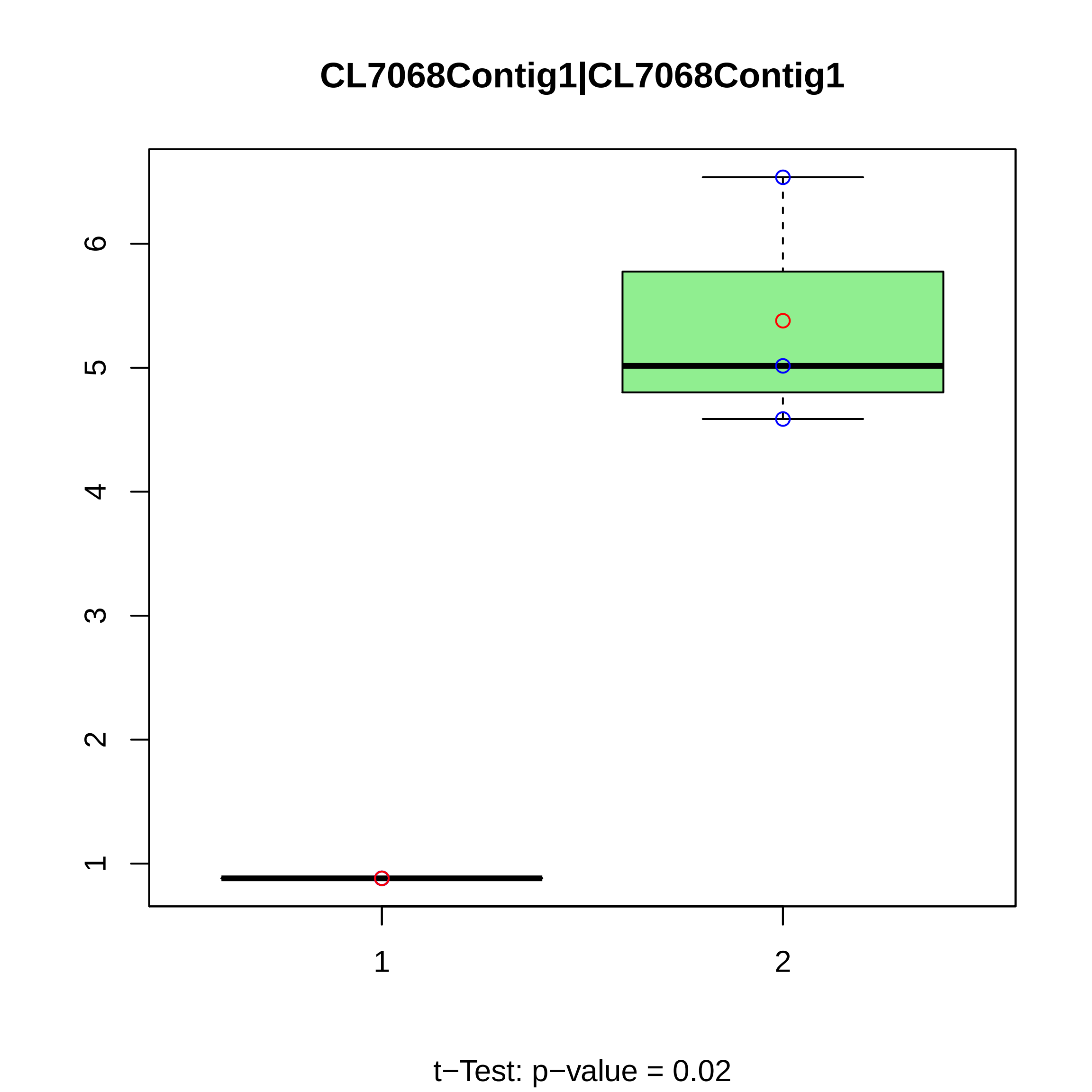

CL7068Contig1|CL7068Contig1
1
2
3
4
5
6
1
2
t−Test: p−value = 0.02

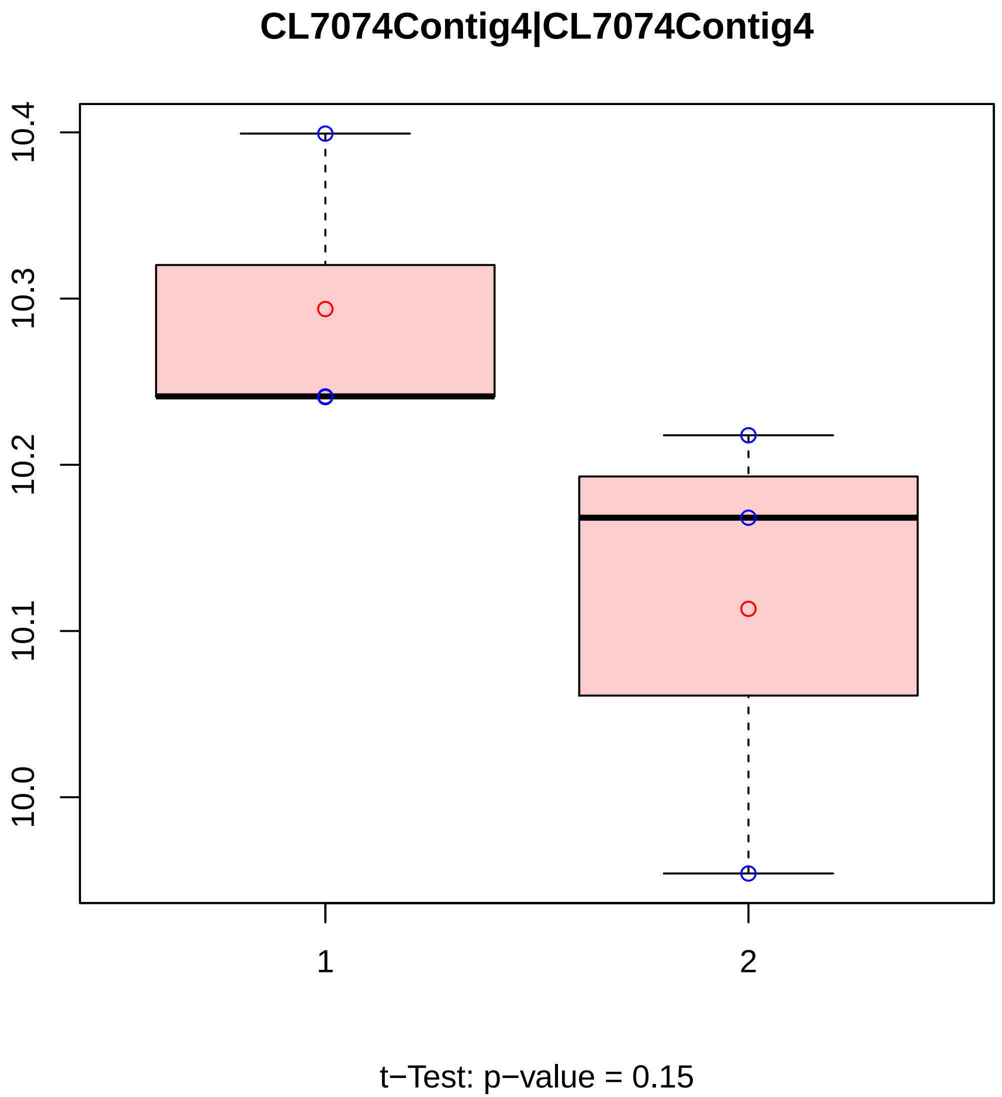

CL7074Contig4|CL7074Contig4
10.0
10.1
10.2
10.3
10.4
1
2
t−Test: p−value = 0.15

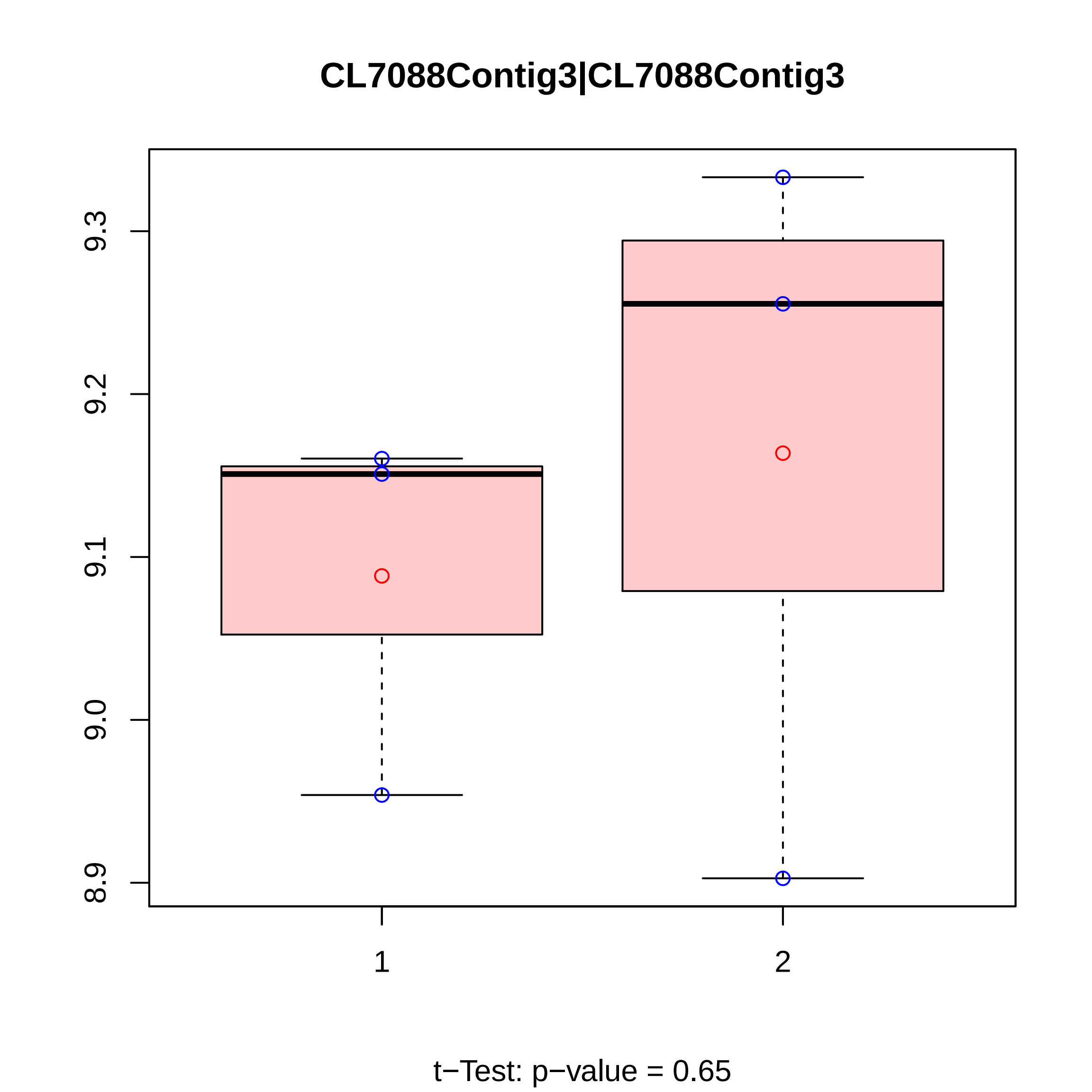
CL7088Contig3|CL7088Contig3
9.3
9.2
9.1
9.0
8.9
1
2
t−Test: p−value = 0.65

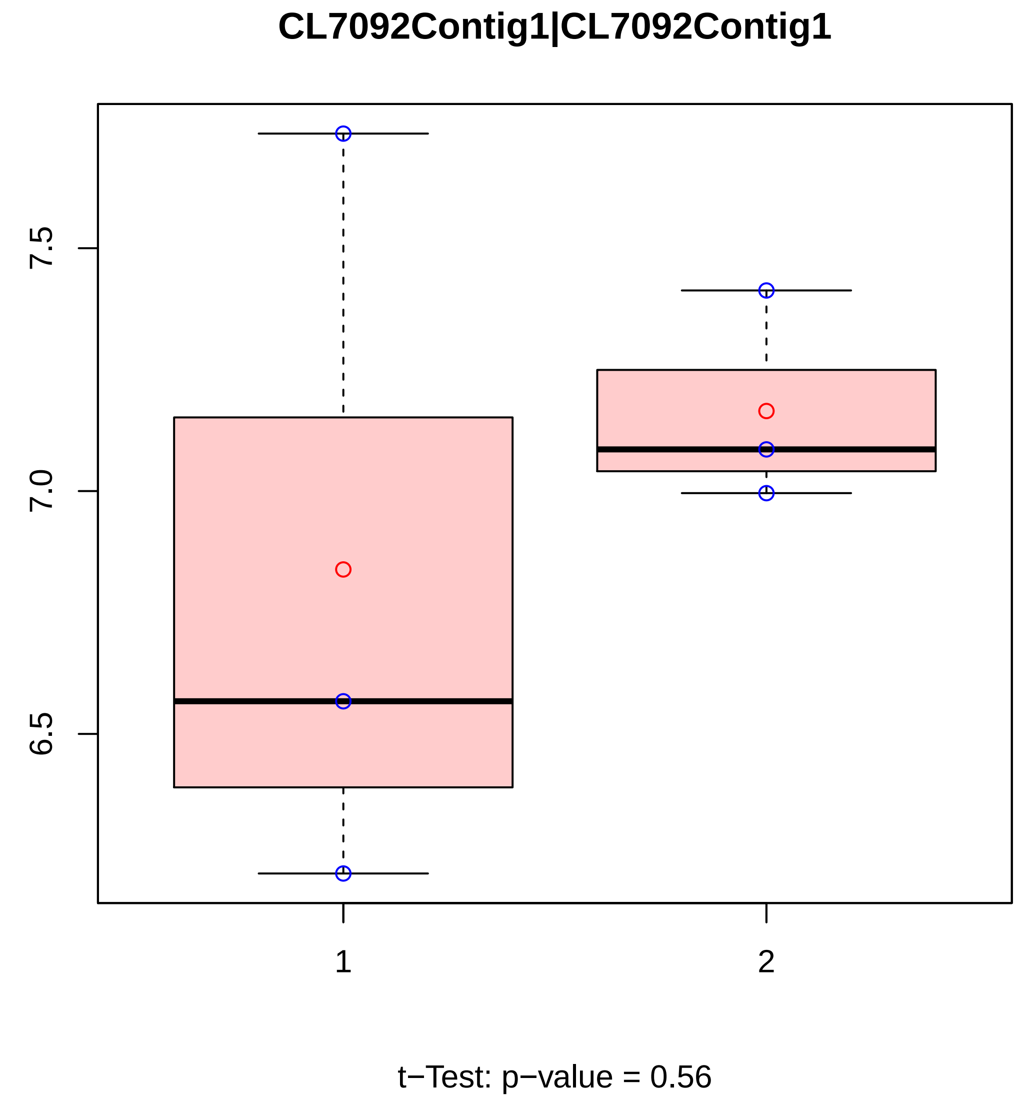

CL7092Contig1|CL7092Contig1
7.5
7.0
6.5
1
2
t−Test: p−value = 0.56

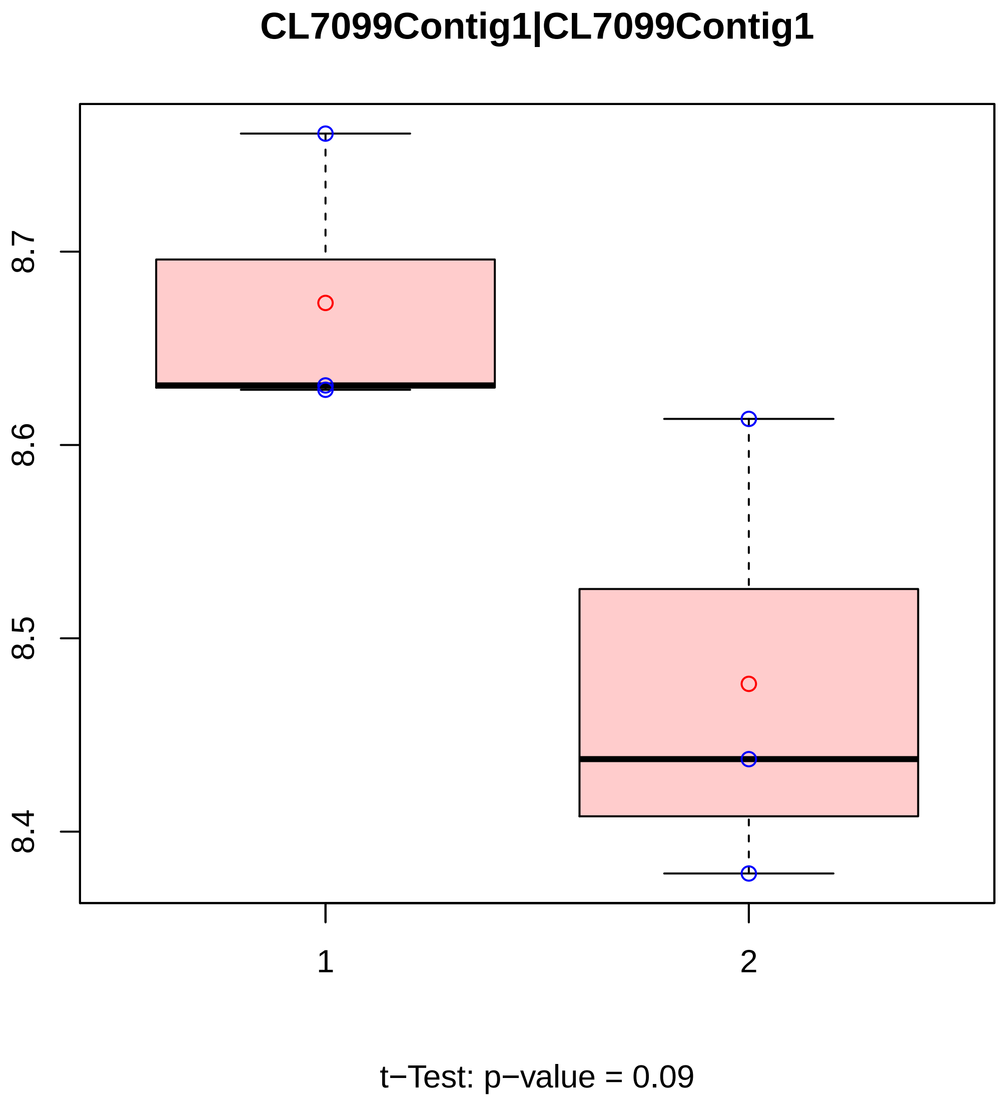

CL7099Contig1|CL7099Contig1
8.4
8.5
8.6
8.7
1
2
t−Test: p−value = 0.09

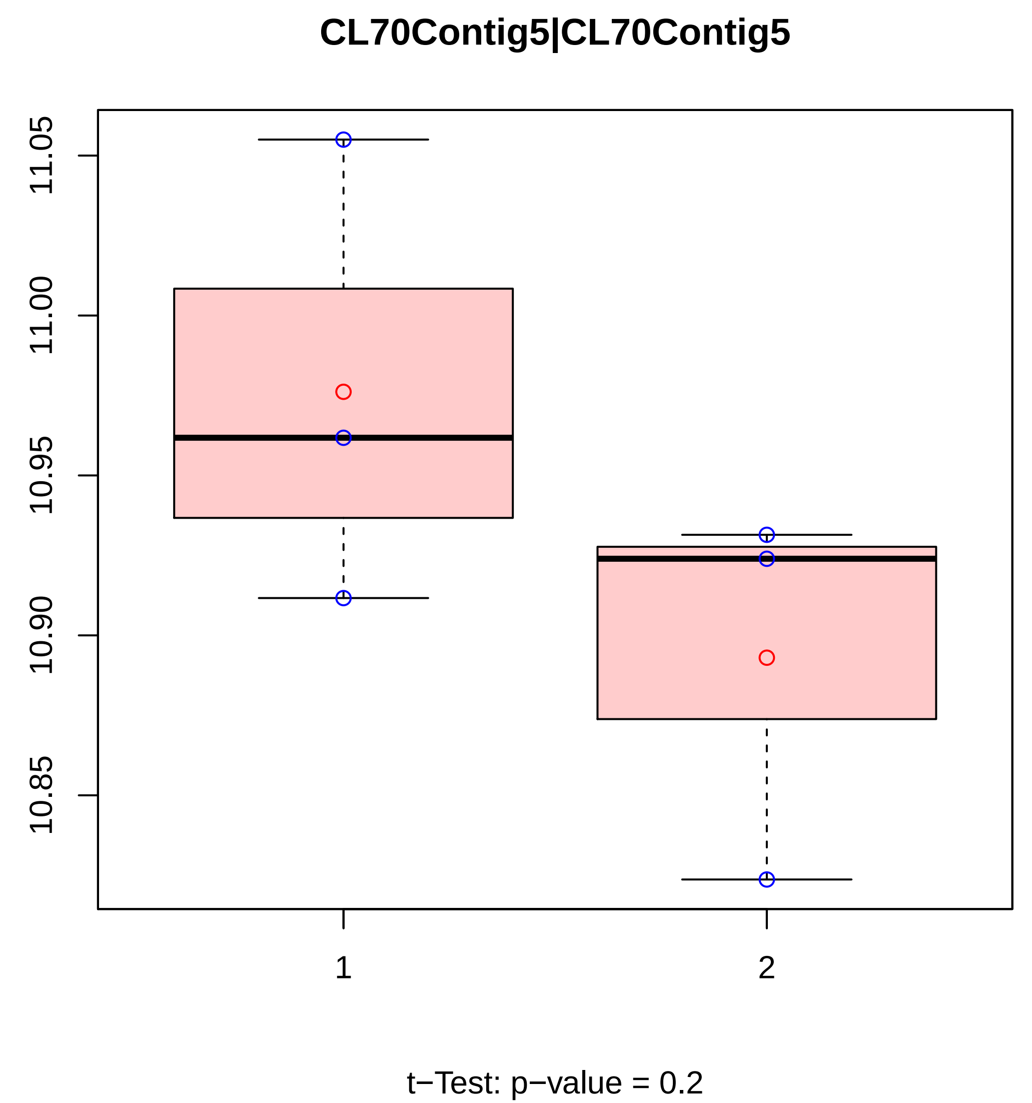
CL70Contig5|CL70Contig5
11.05
11.00
10.95
10.90
10.85
1
2
t−Test: p−value = 0.2

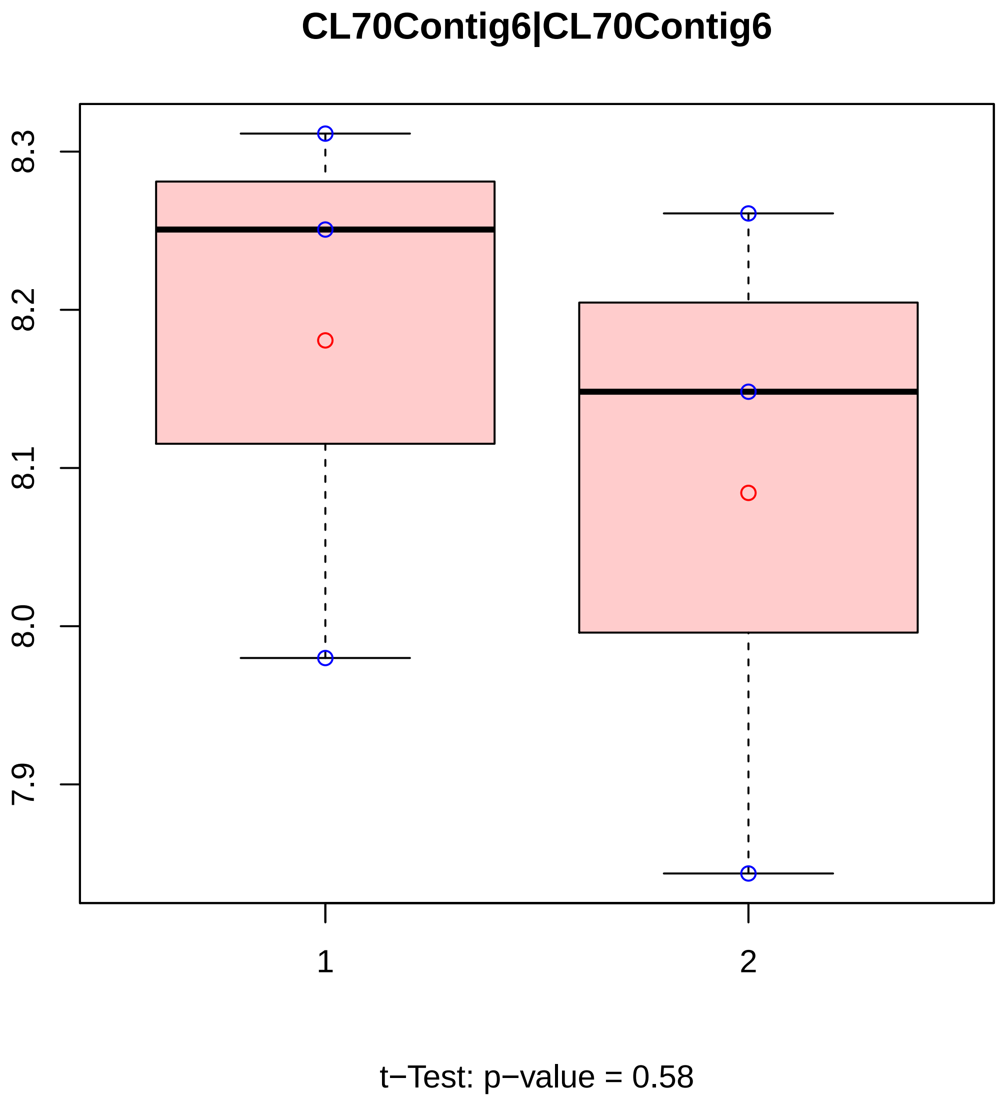

CL70Contig6|CL70Contig6
8.3
8.2
8.1
8.0
7.9
1
2
t−Test: p−value = 0.58

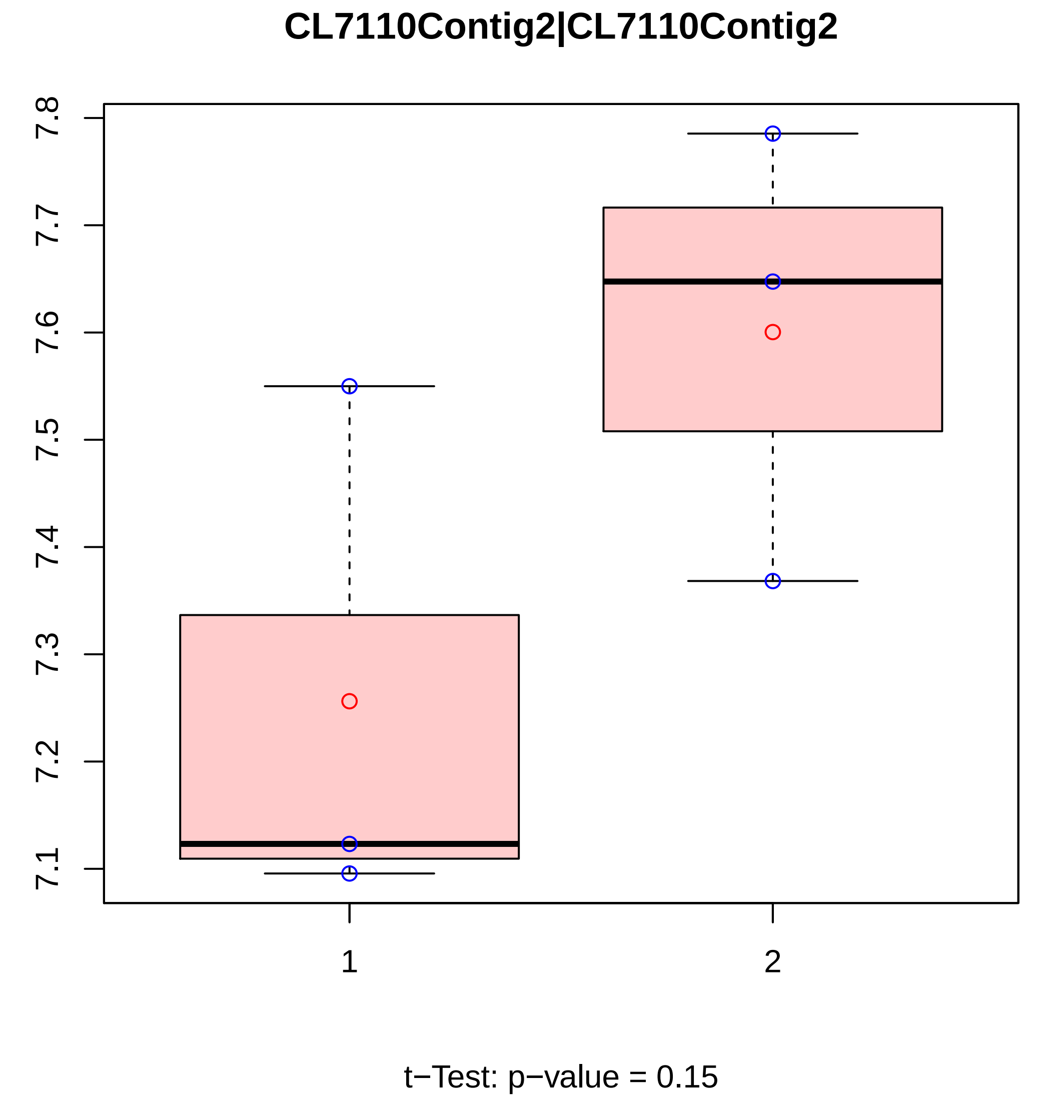
CL7110Contig2|CL7110Contig2
7.1
7.2
7.3
7.4
7.5
7.6
7.7
7.8
1
2
t−Test: p−value = 0.15

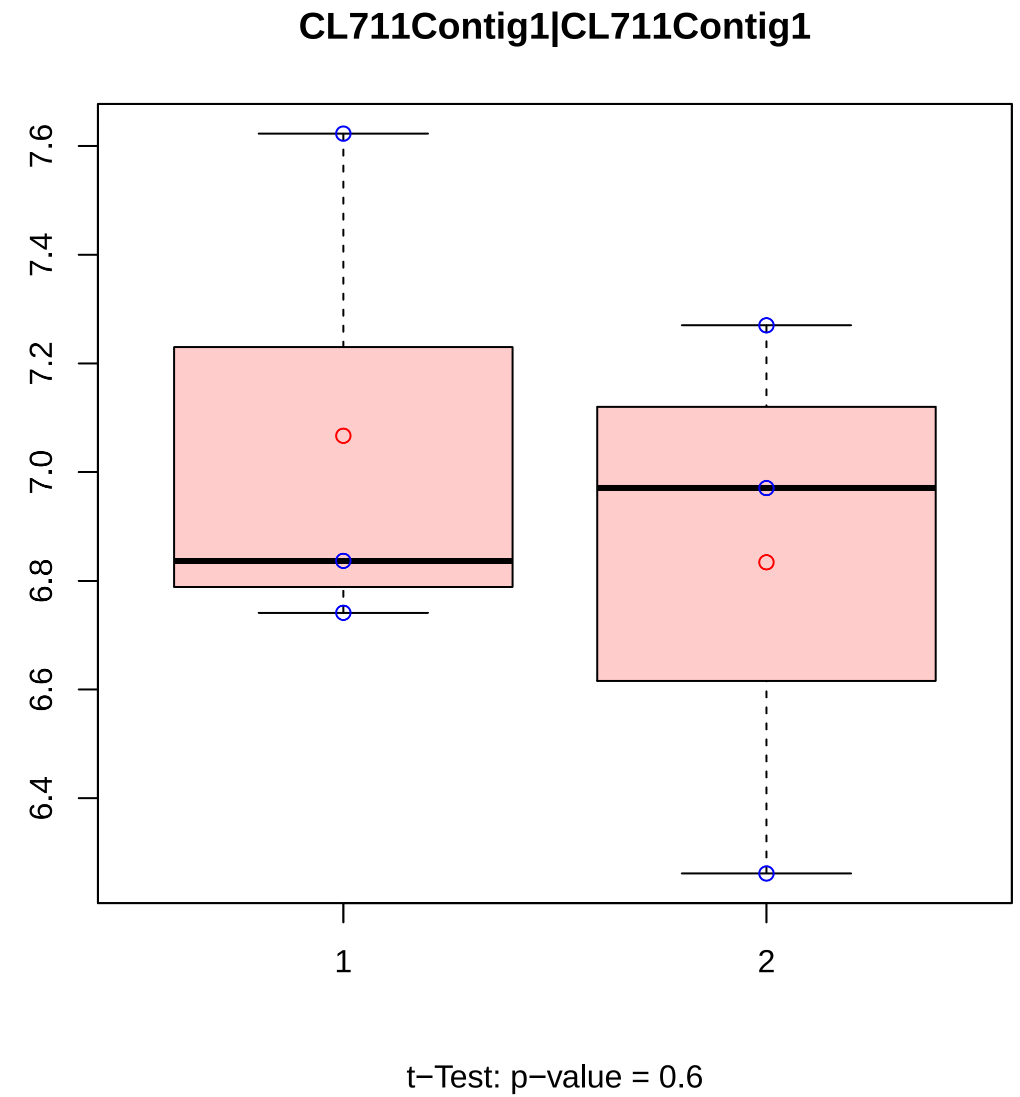

CL711Contig1|CL711Contig1
7.6
7.4
7.2
7.0
6.8
6.6
6.4
1
2
t−Test: p−value = 0.6

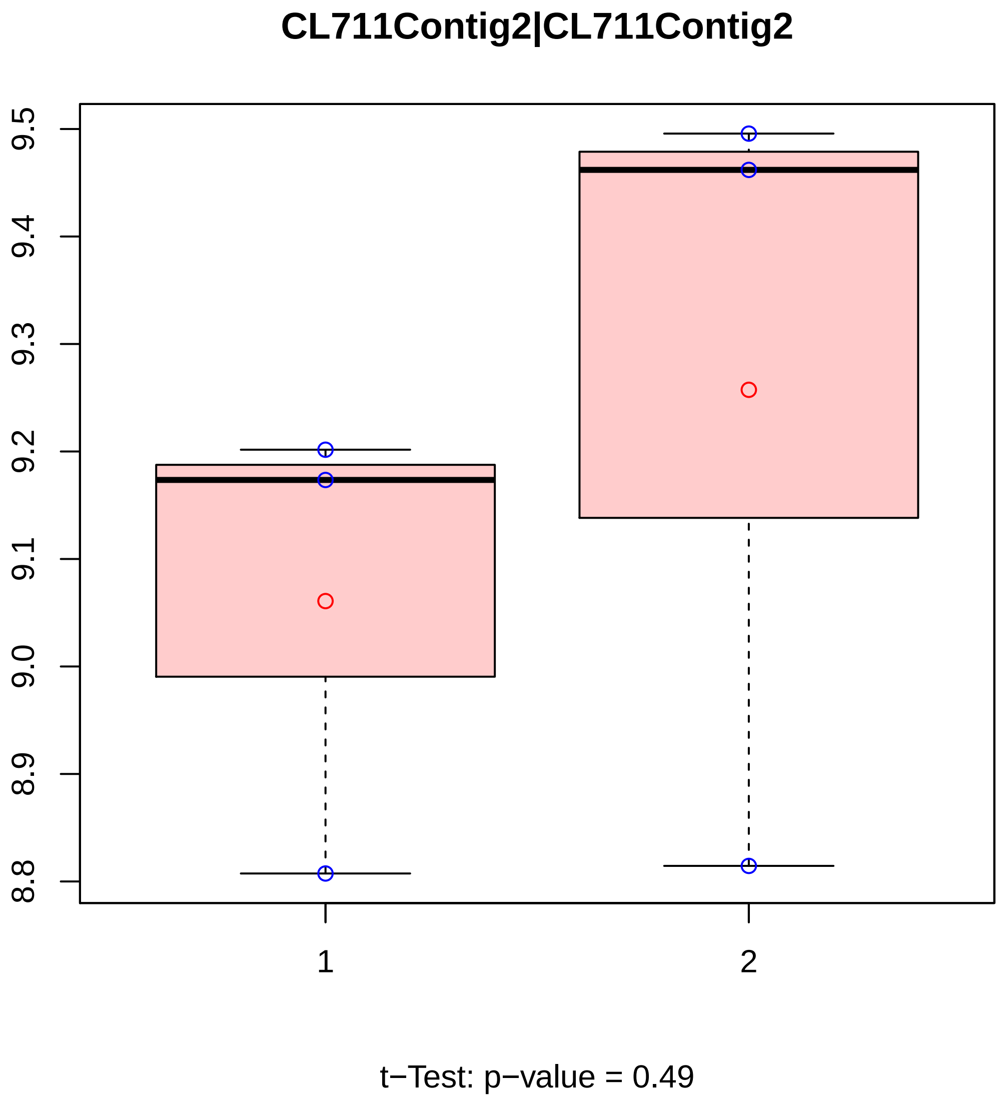

CL711Contig2|CL711Contig2
9.5
9.4
9.3
9.2
9.1
9.0
8.9
8.8
1
2
t−Test: p−value = 0.49

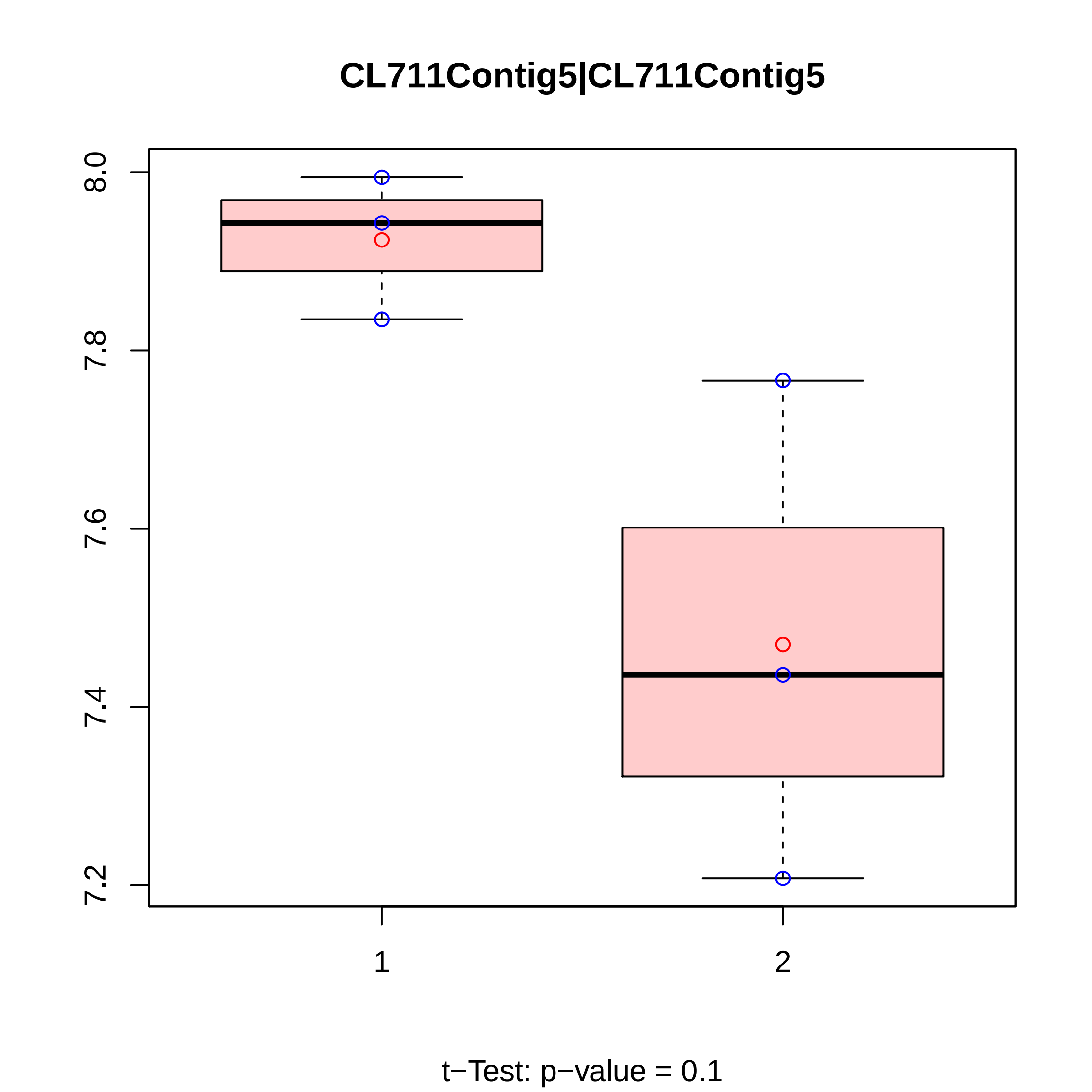

CL711Contig5|CL711Contig5
8.0
7.8
7.6
7.4
7.2
1
2
t−Test: p−value = 0.1

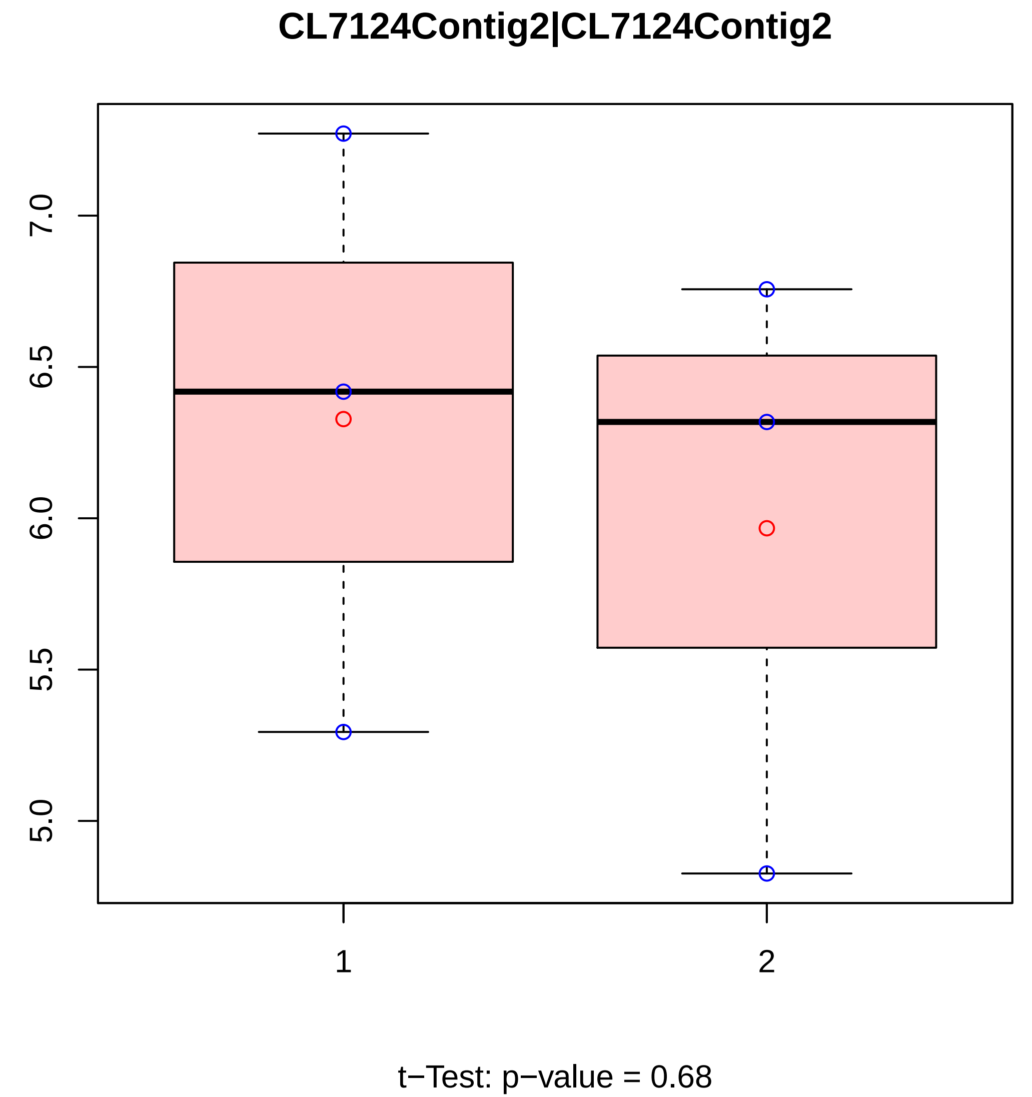

CL7124Contig2|CL7124Contig2
7.0
6.5
6.0
5.5
5.0
1
2
t−Test: p−value = 0.68

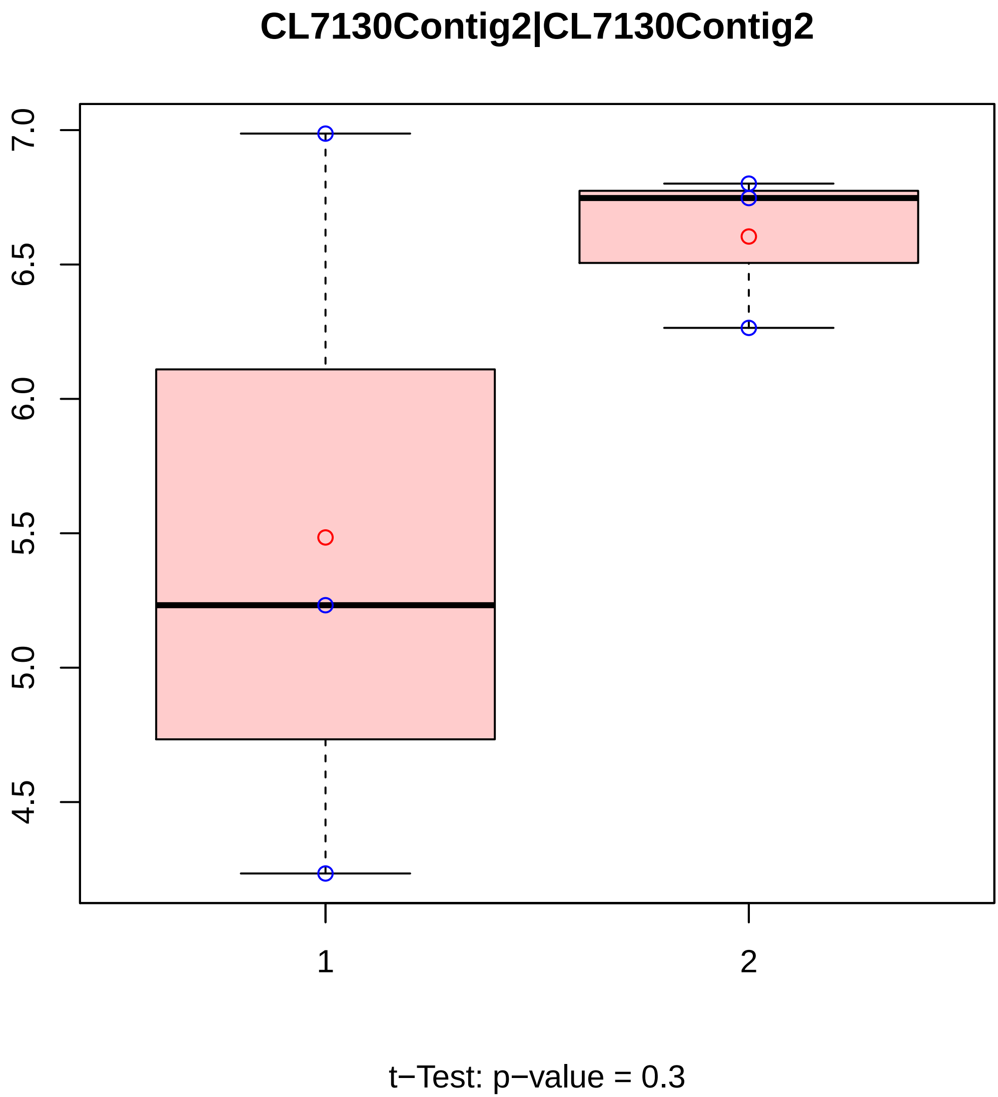
CL7130Contig2|CL7130Contig2
7.0
6.5
6.0
5.5
5.0
4.5
1
2
t−Test: p−value = 0.3

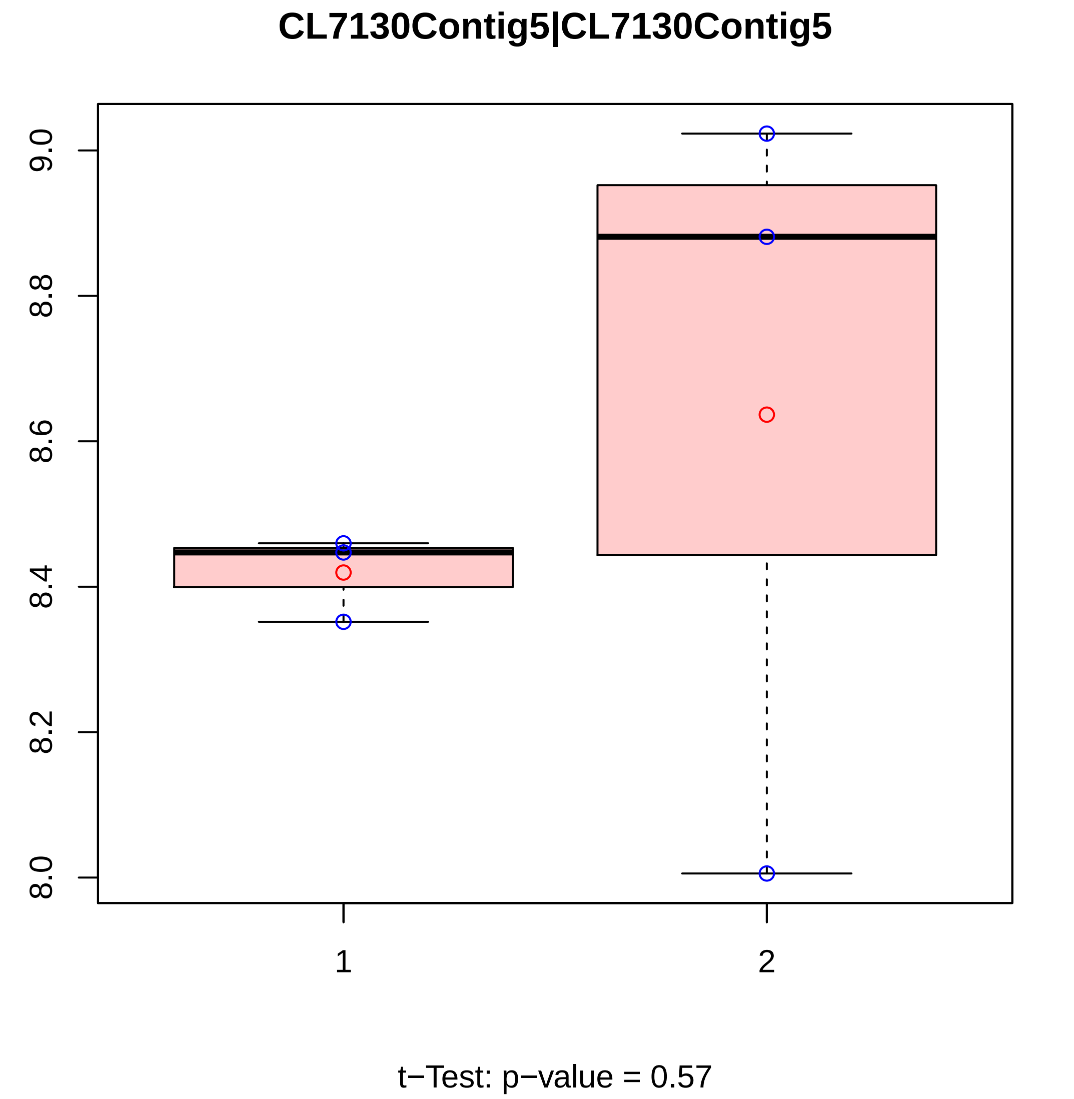
CL7130Contig5|CL7130Contig5
9.0
8.8
8.6
8.4
8.2
8.0
1
2
t−Test: p−value = 0.57

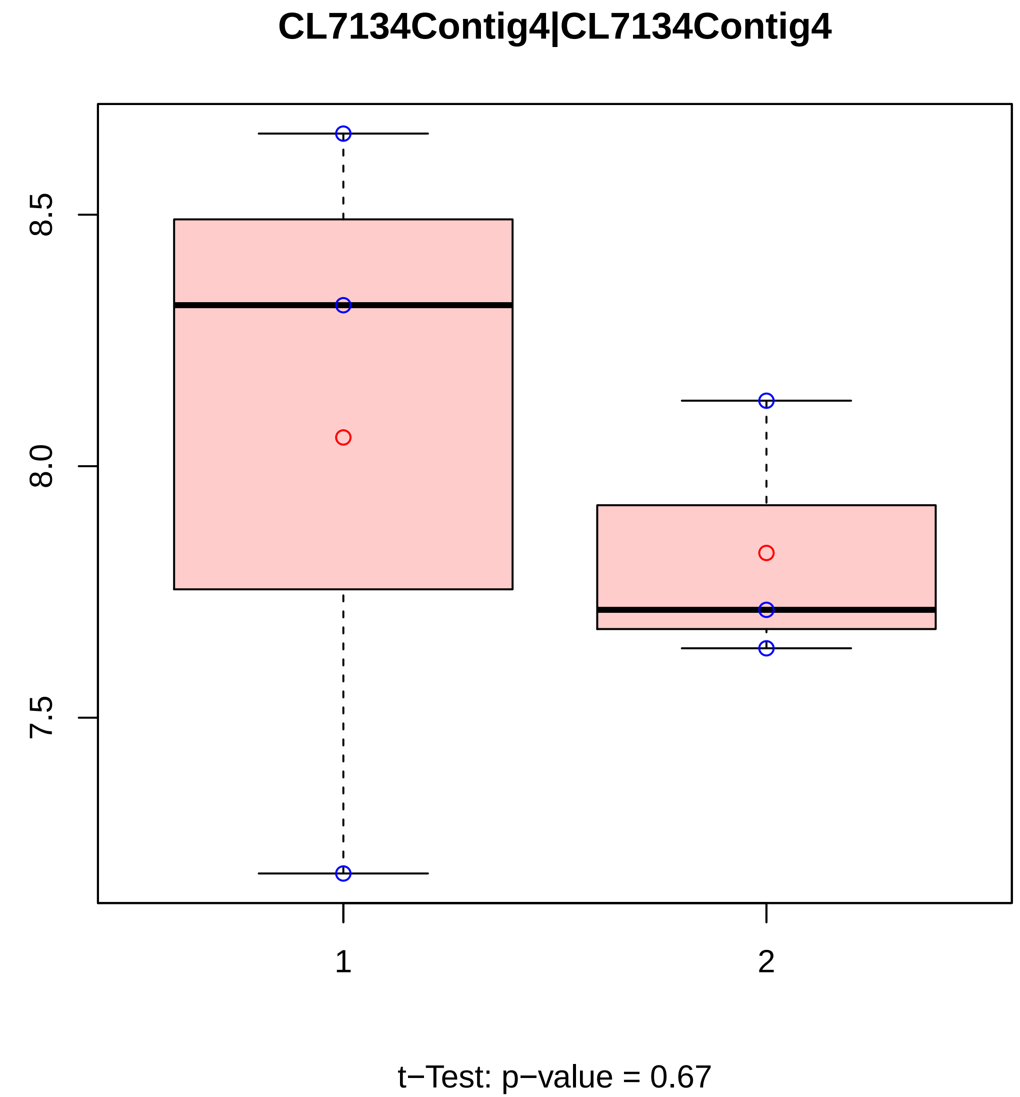
CL7134Contig4|CL7134Contig4
8.5
8.0
7.5
1
2
t−Test: p−value = 0.67

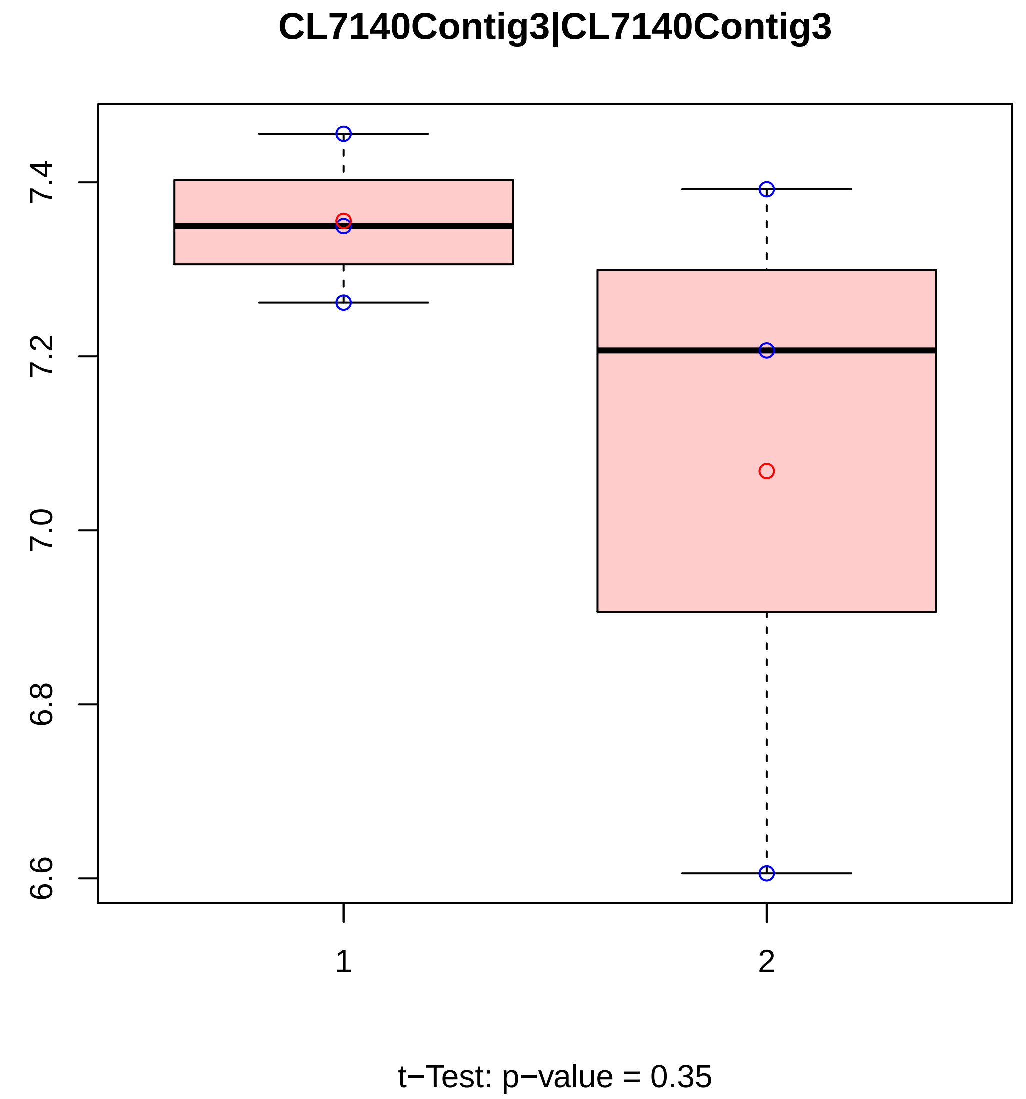
CL7140Contig3|CL7140Contig3
7.4
7.2
7.0
6.8
6.6
1
2
t−Test: p−value = 0.35

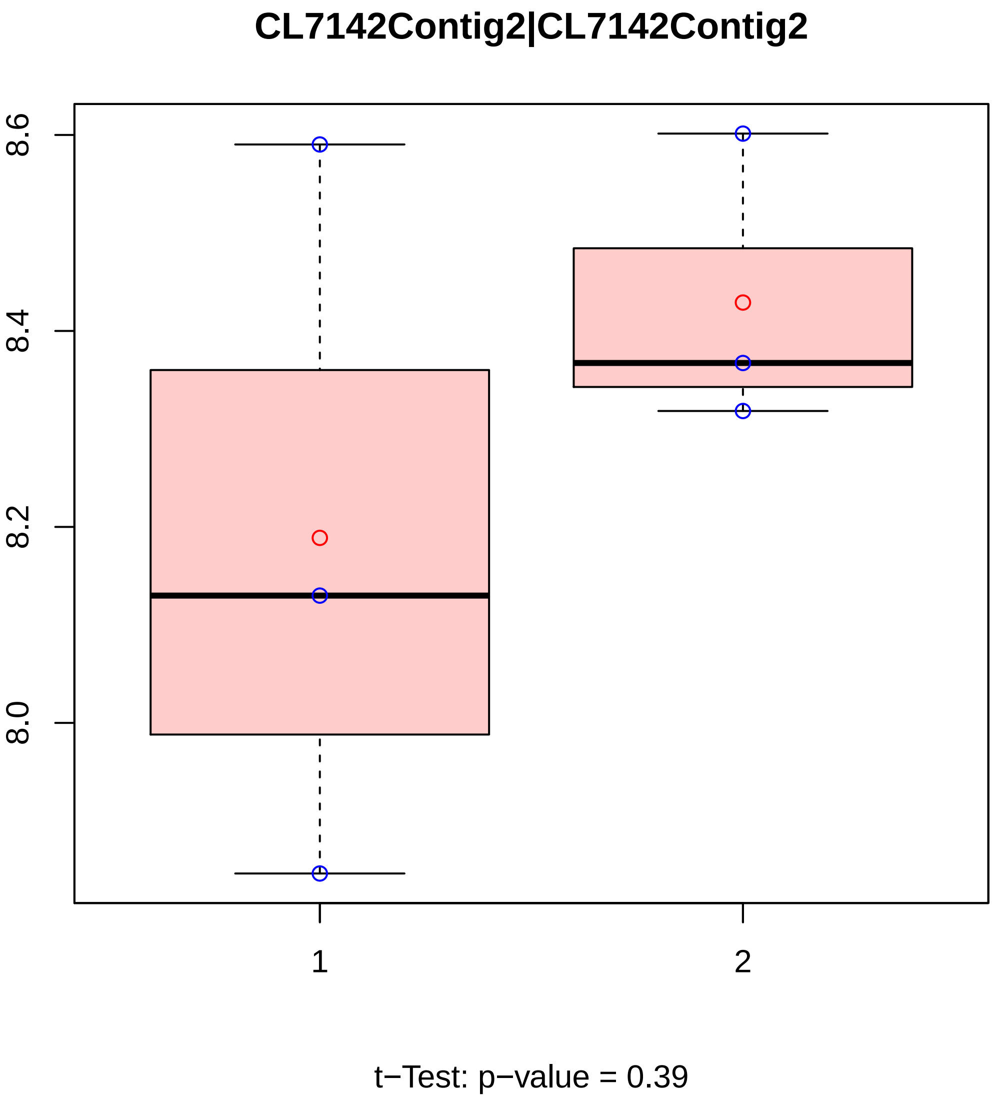

CL7142Contig2|CL7142Contig2
8.6
8.4
8.2
8.0
1
2
t−Test: p−value = 0.39

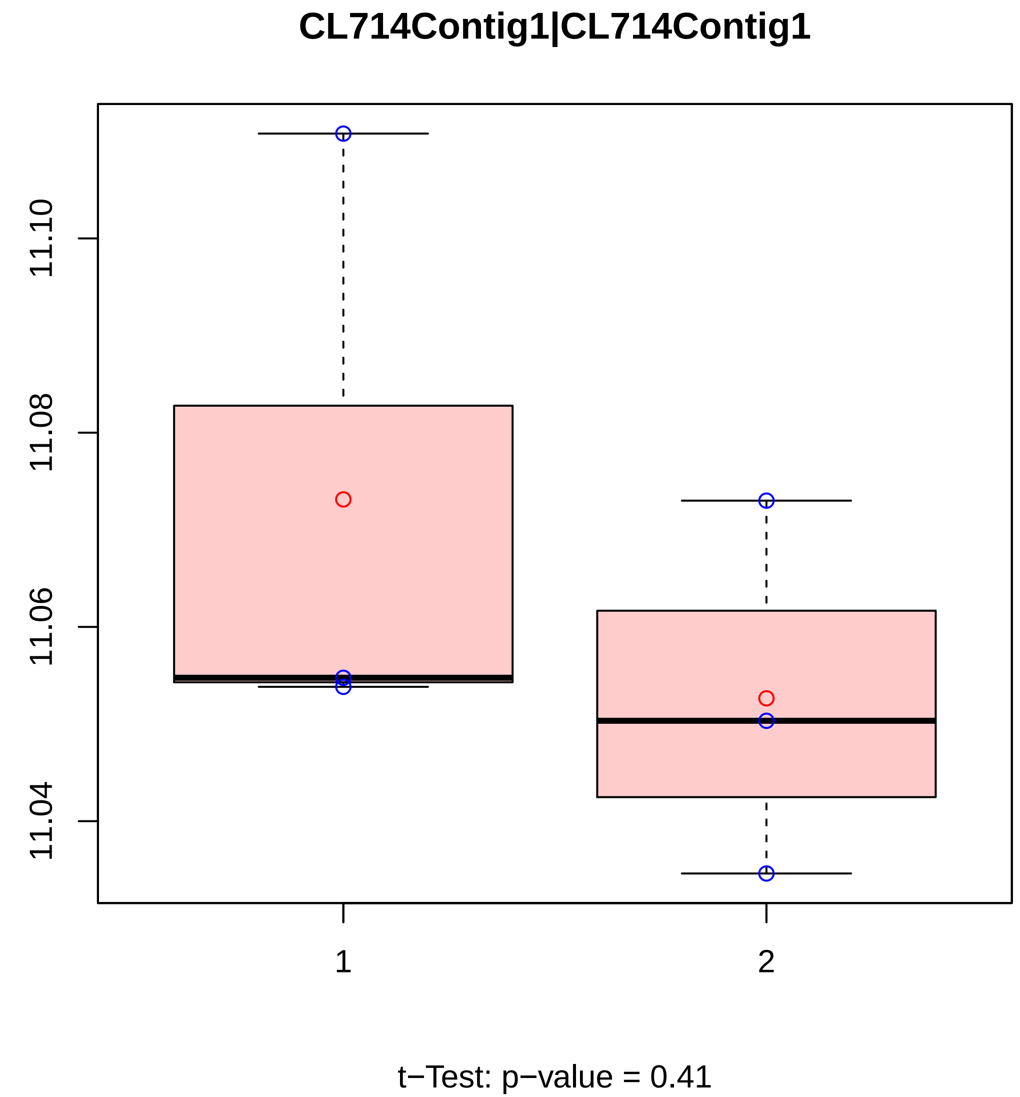
CL714Contig1|CL714Contig1
11.10
11.08
11.06
11.04
1
2
t−Test: p−value = 0.41

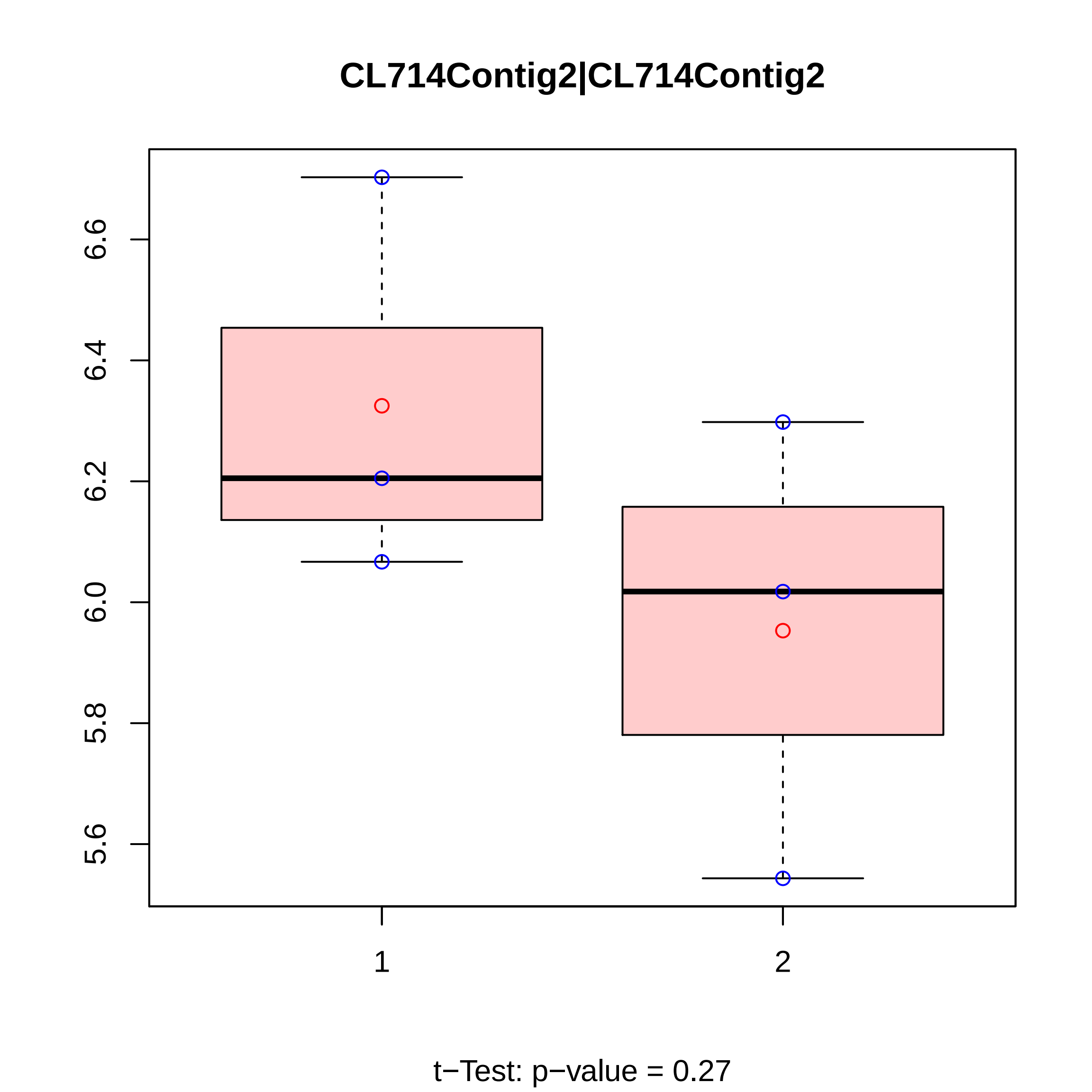

CL714Contig2|CL714Contig2
6.6
6.4
6.2
6.0
5.8
5.6
1
2
t−Test: p−value = 0.27

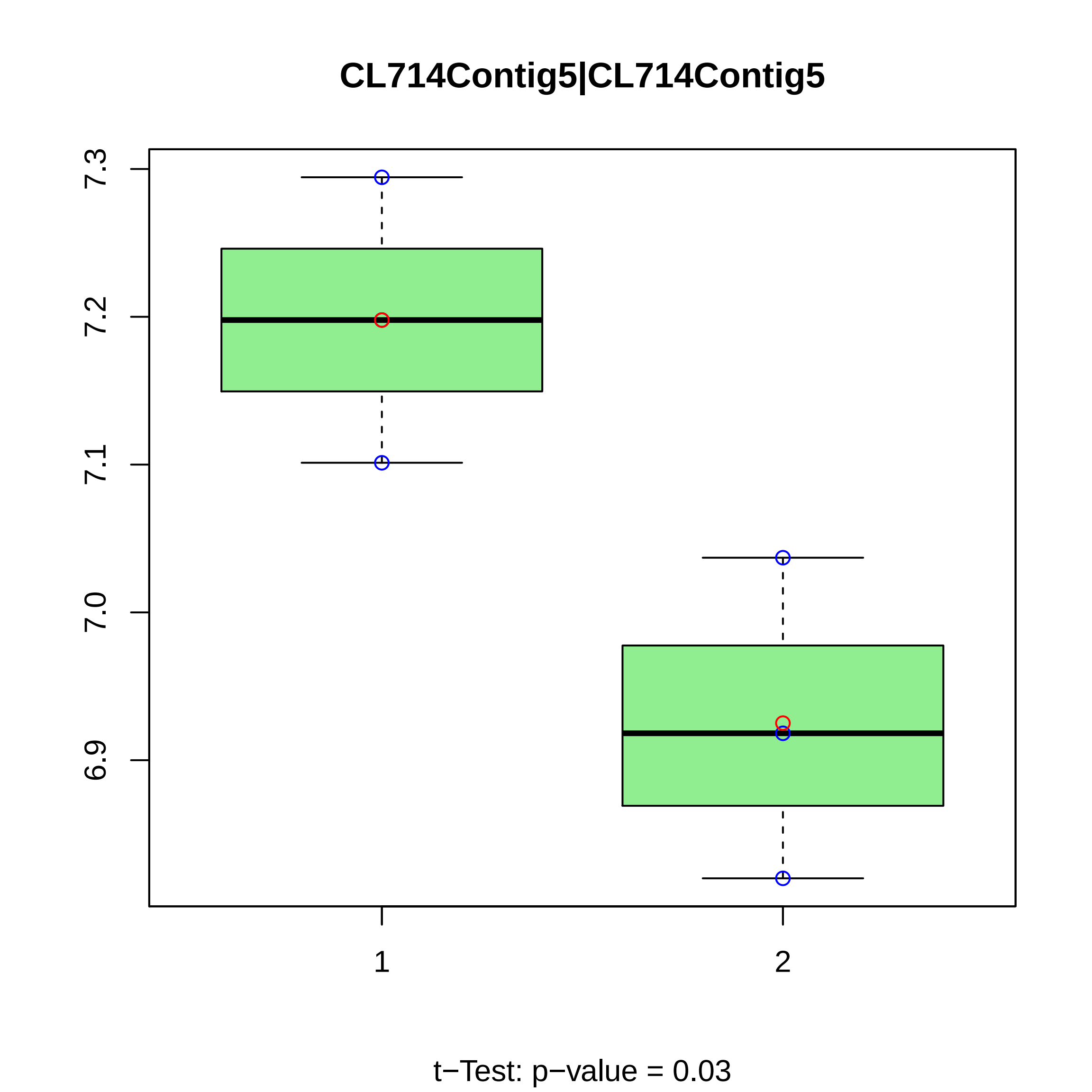
CL714Contig5|CL714Contig5
7.3
7.2
7.1
7.0
6.9
1
2
t−Test: p−value = 0.03

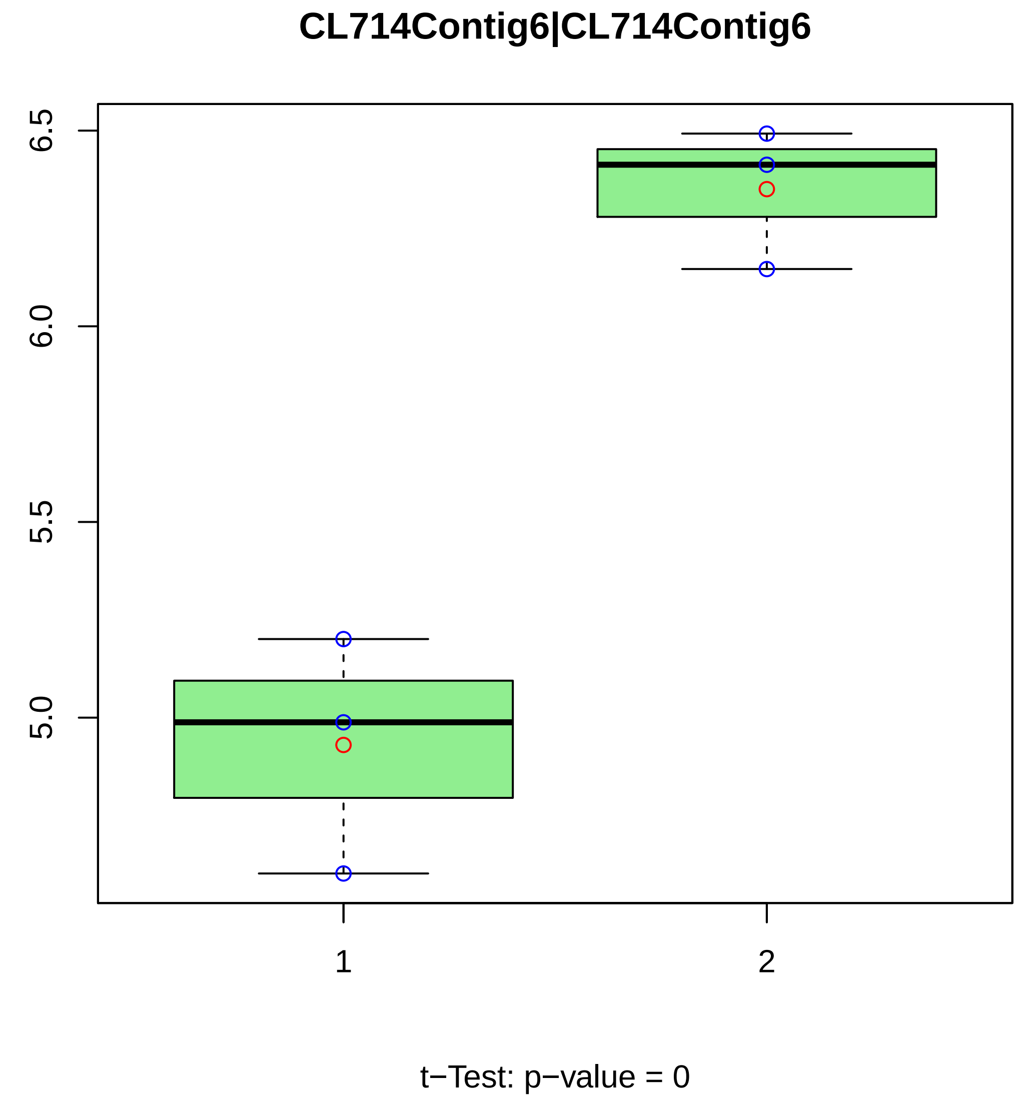

CL714Contig6|CL714Contig6
6.5
6.0
5.5
5.0
1
2
t−Test: p−value = 0

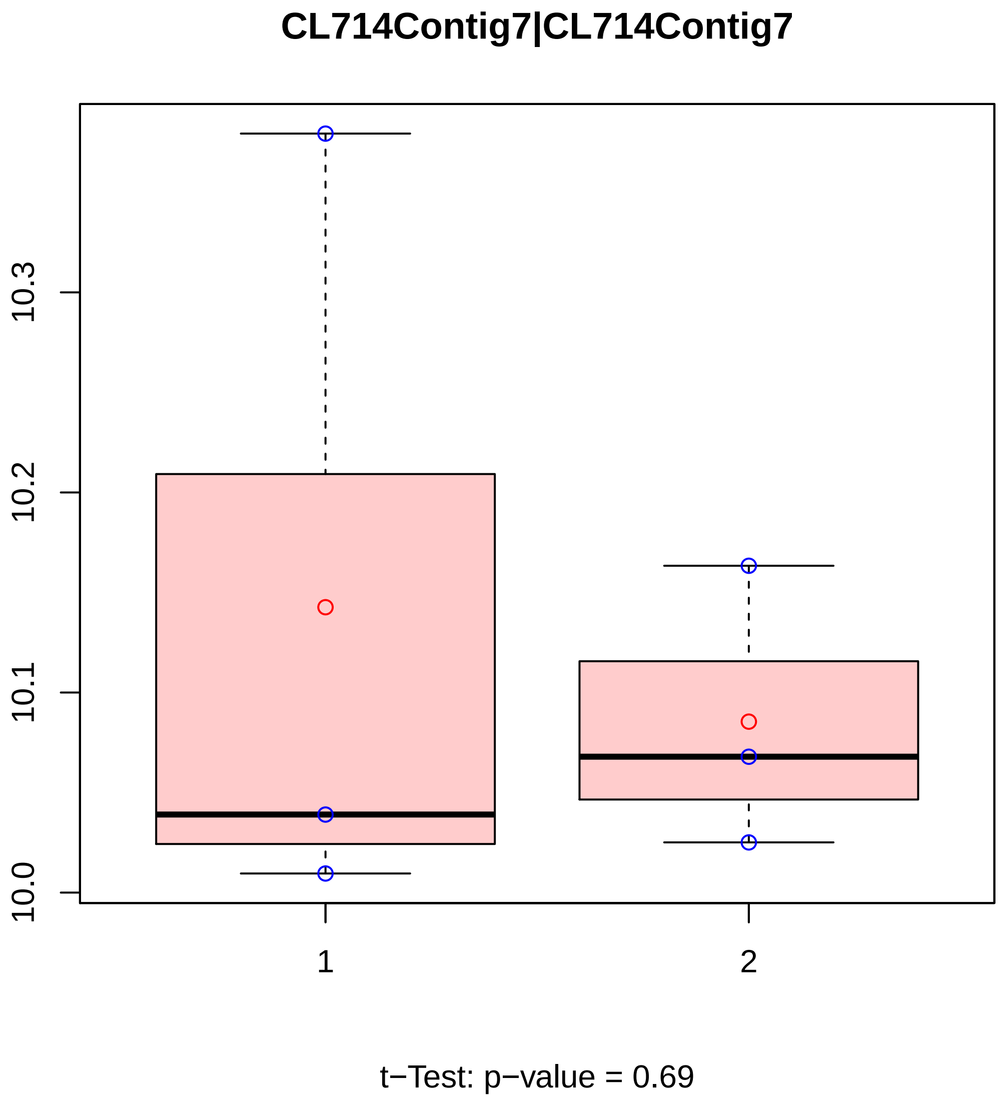

CL714Contig7|CL714Contig7
10.0
10.1
10.2
10.3
1
2
t−Test: p−value = 0.69

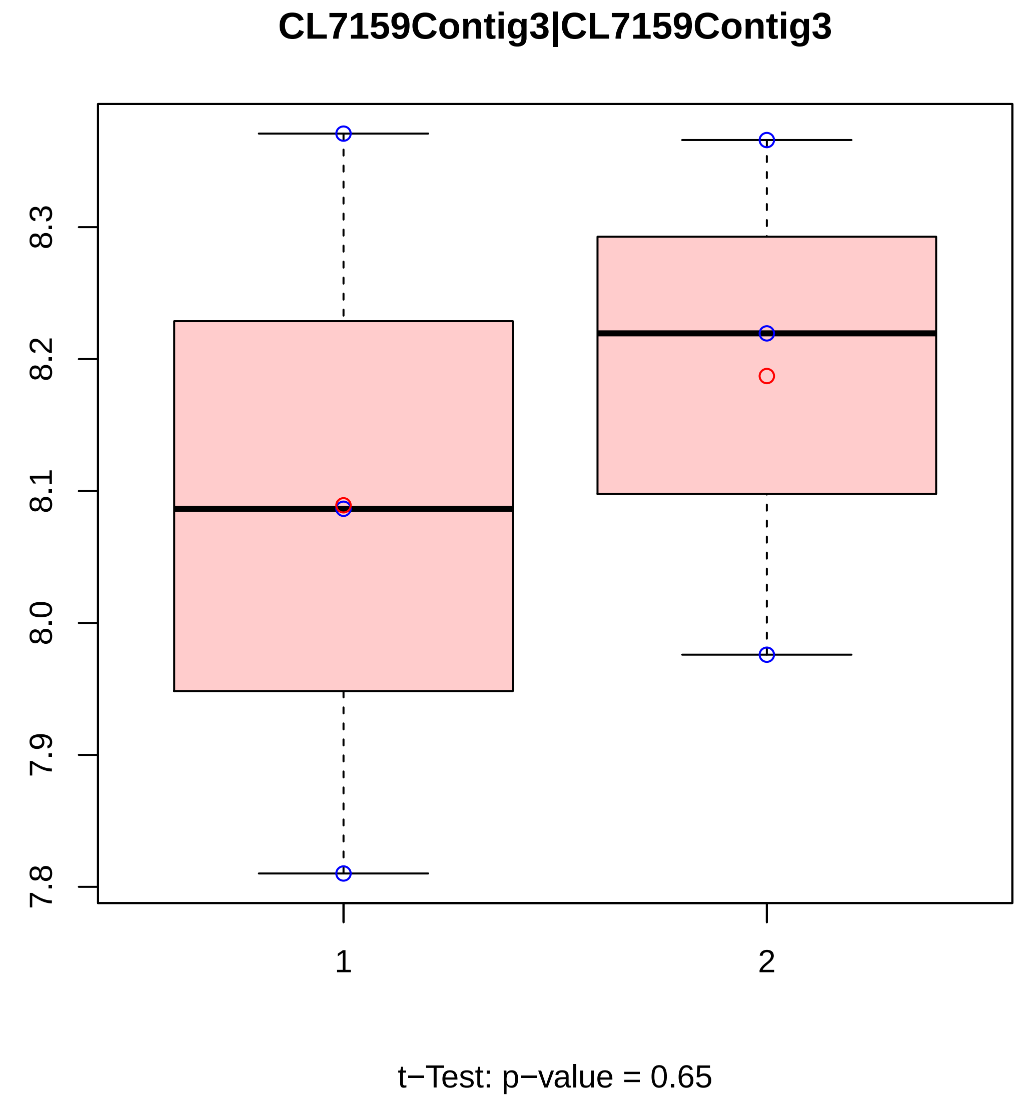
CL7159Contig3|CL7159Contig3
8.3
8.2
8.1
8.0
7.9
7.8
1
2
t−Test: p−value = 0.65

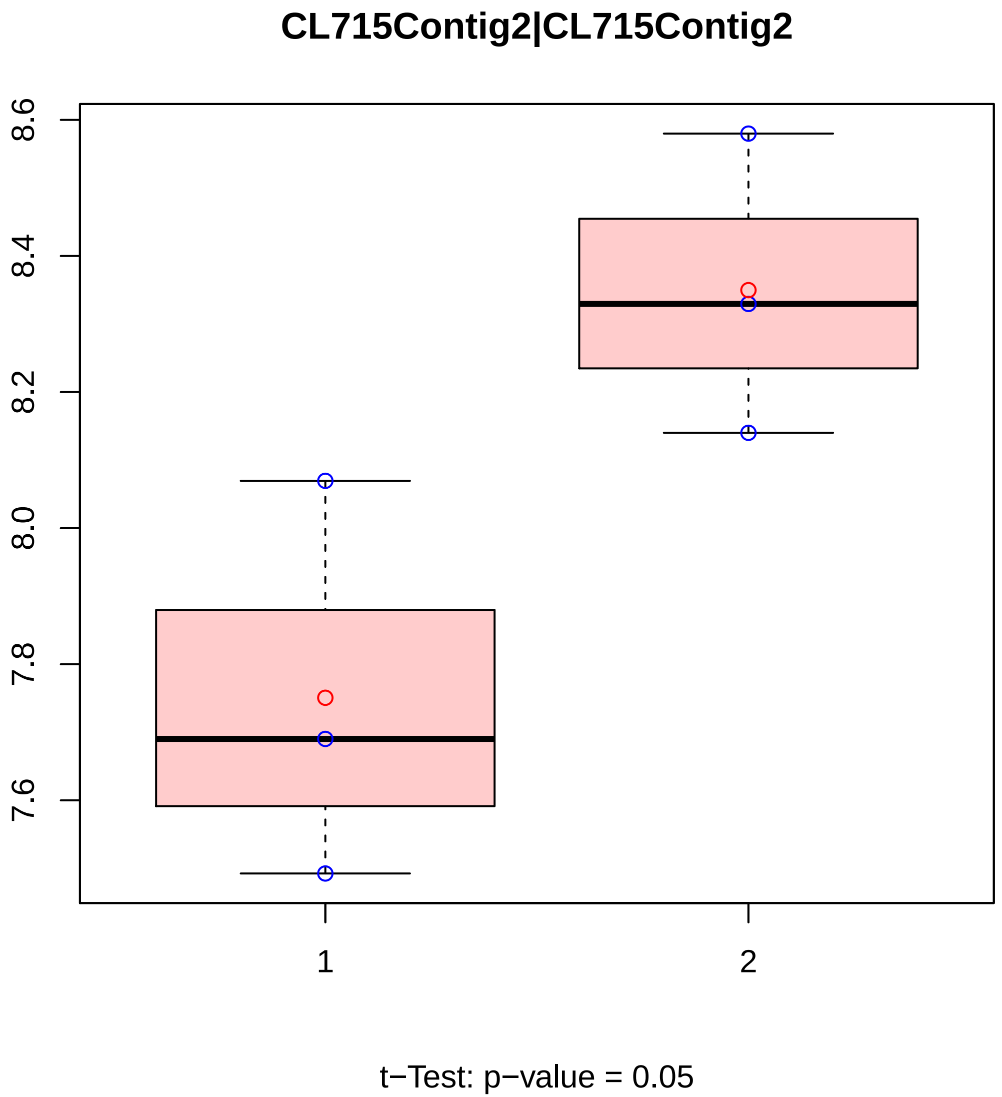
CL715Contig2|CL715Contig2
8.6
8.4
8.2
8.0
7.8
7.6
1
2
t−Test: p−value = 0.05

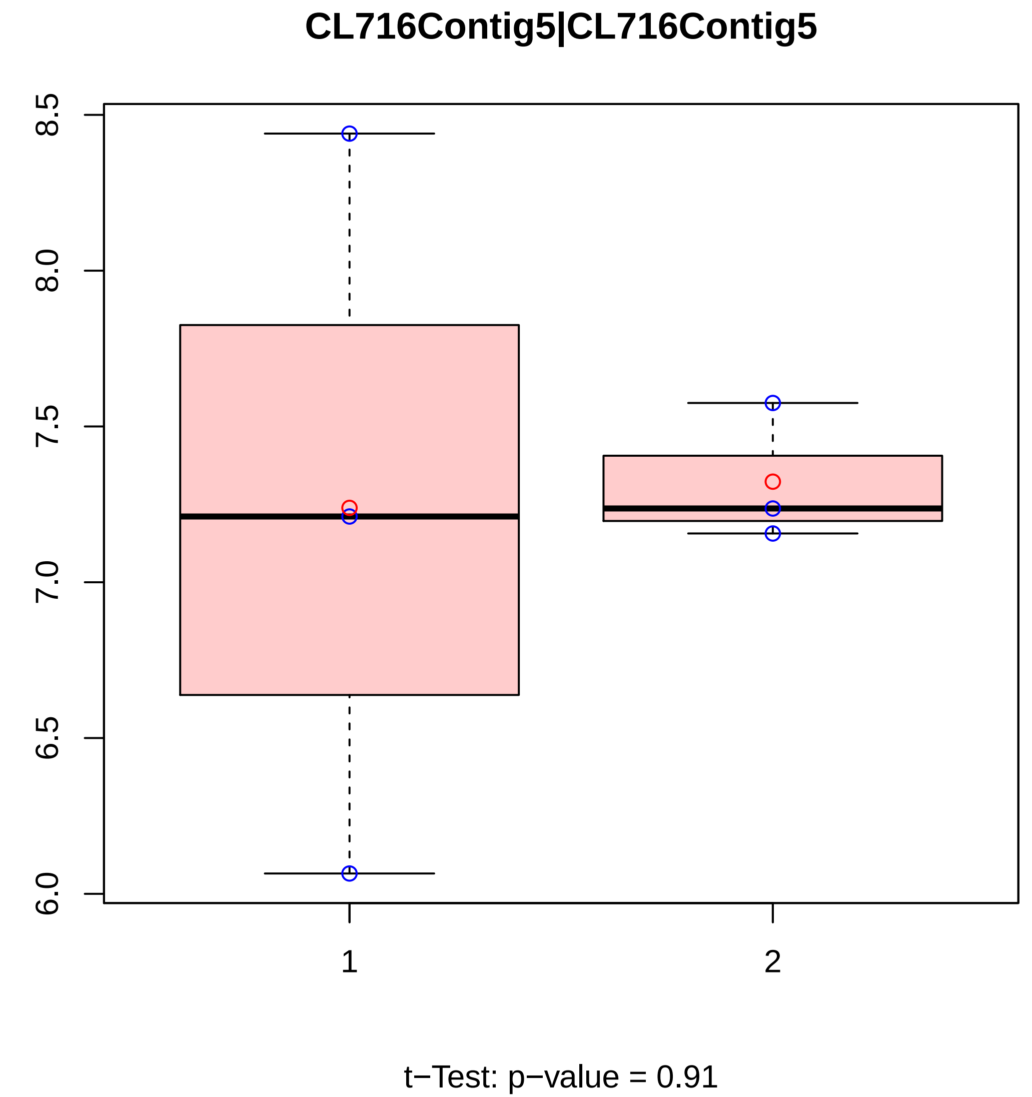

CL716Contig5|CL716Contig5
8.5
8.0
7.5
7.0
6.5
6.0
1
2
t−Test: p−value = 0.91

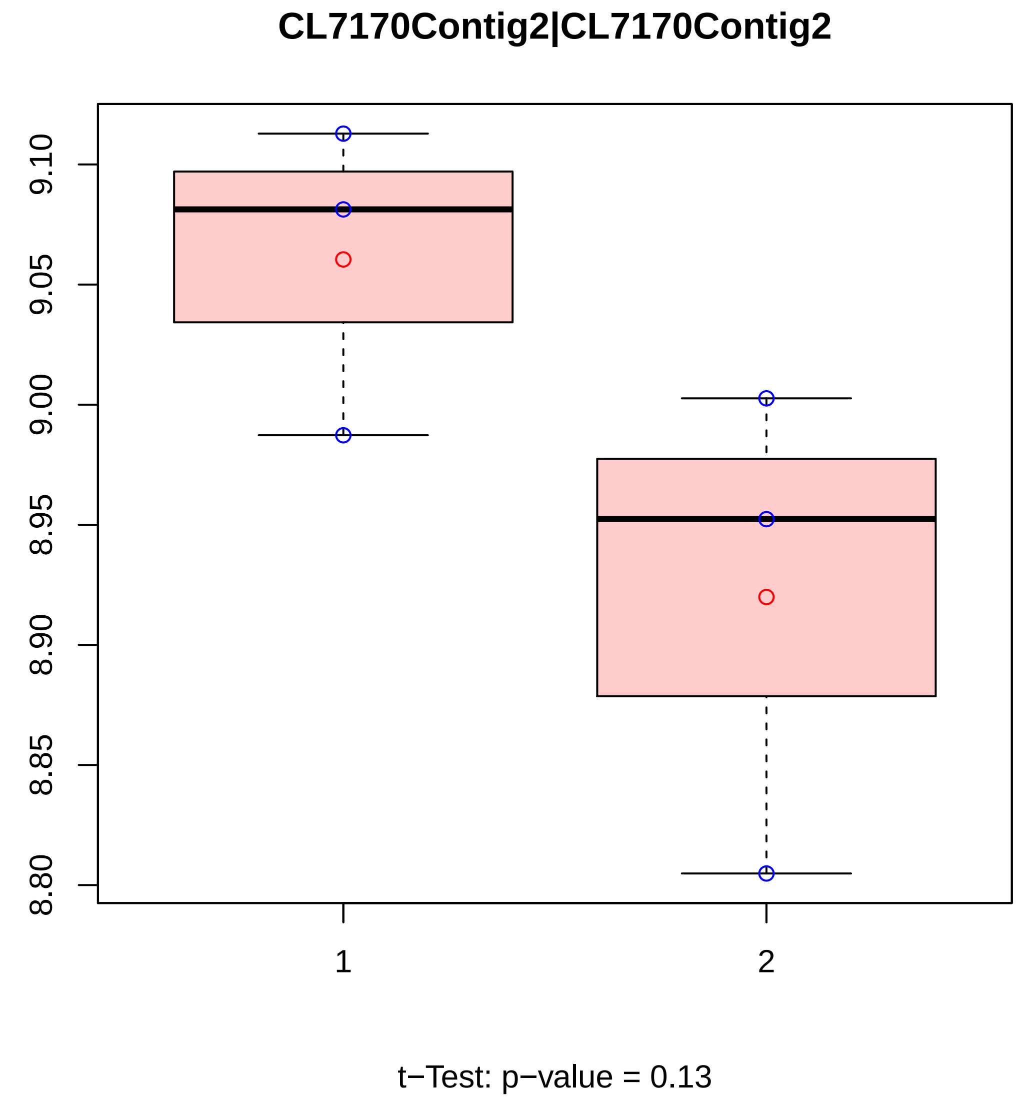

CL7170Contig2|CL7170Contig2
9.10
9.05
9.00
8.95
8.90
8.85
8.80
1
2
t−Test: p−value = 0.13

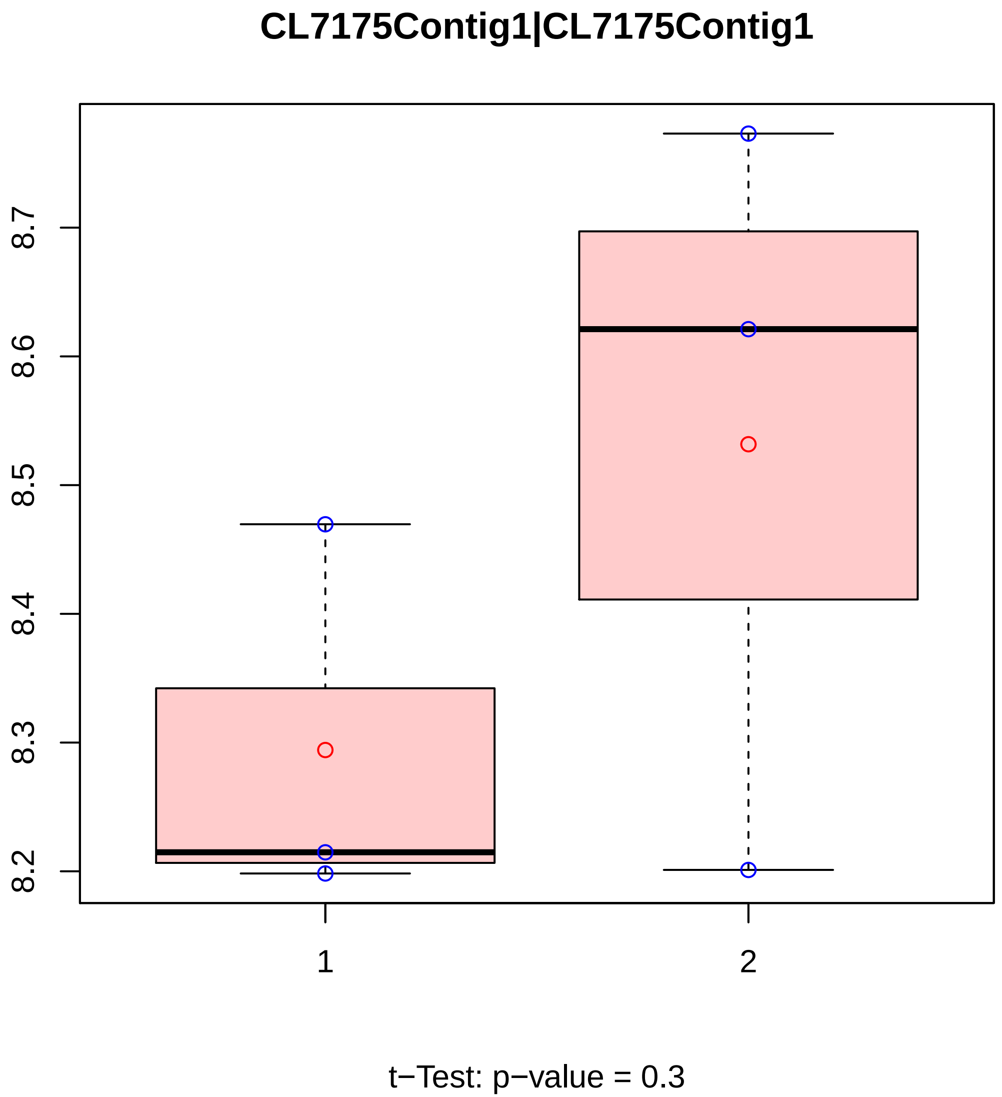
CL7175Contig1|CL7175Contig1
8.2
8.3
8.4
8.5
8.6
8.7
1
2
t−Test: p−value = 0.3

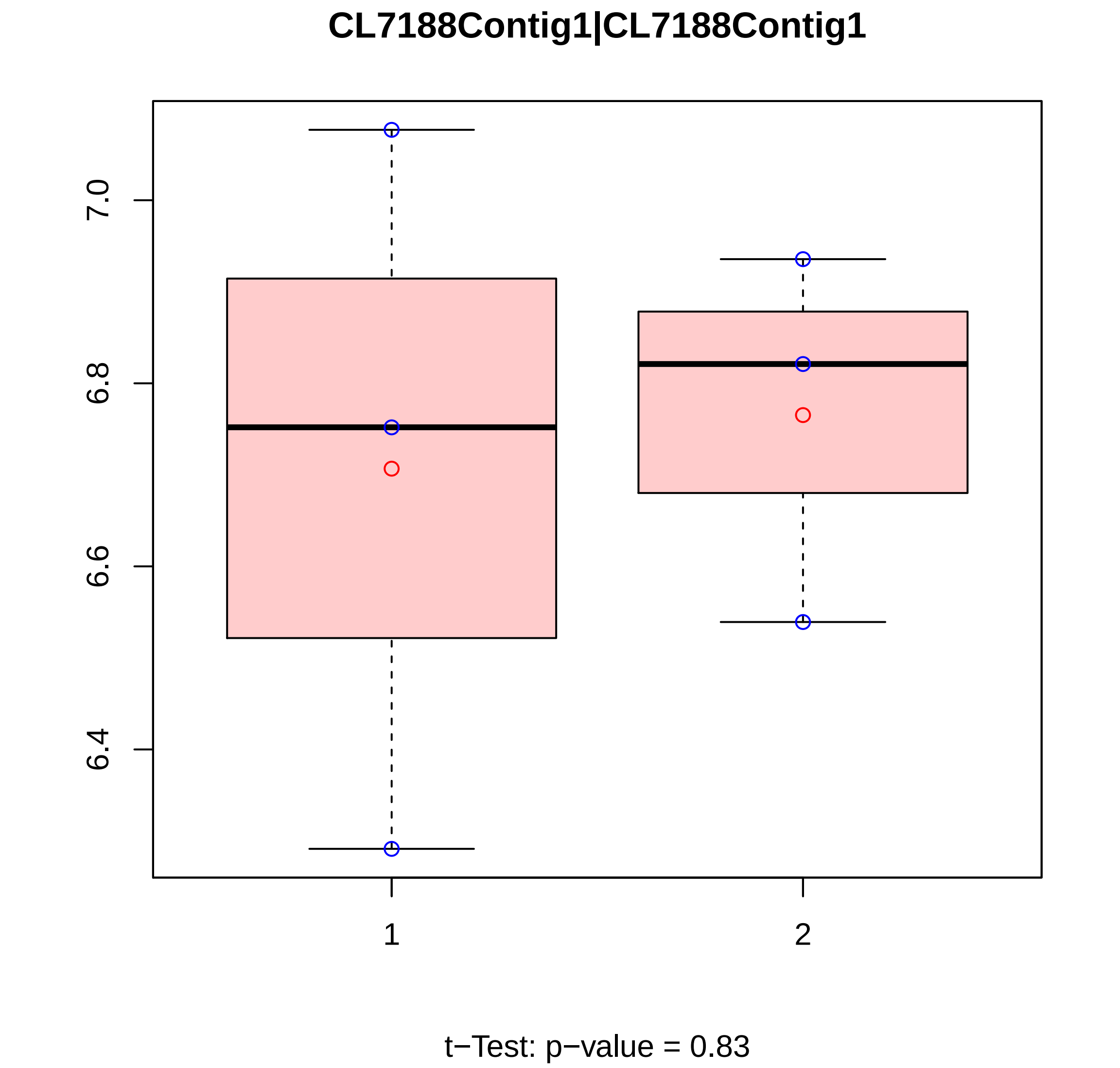

CL7188Contig1|CL7188Contig1
7.0
6.8
6.6
6.4
1
2
t−Test: p−value = 0.83

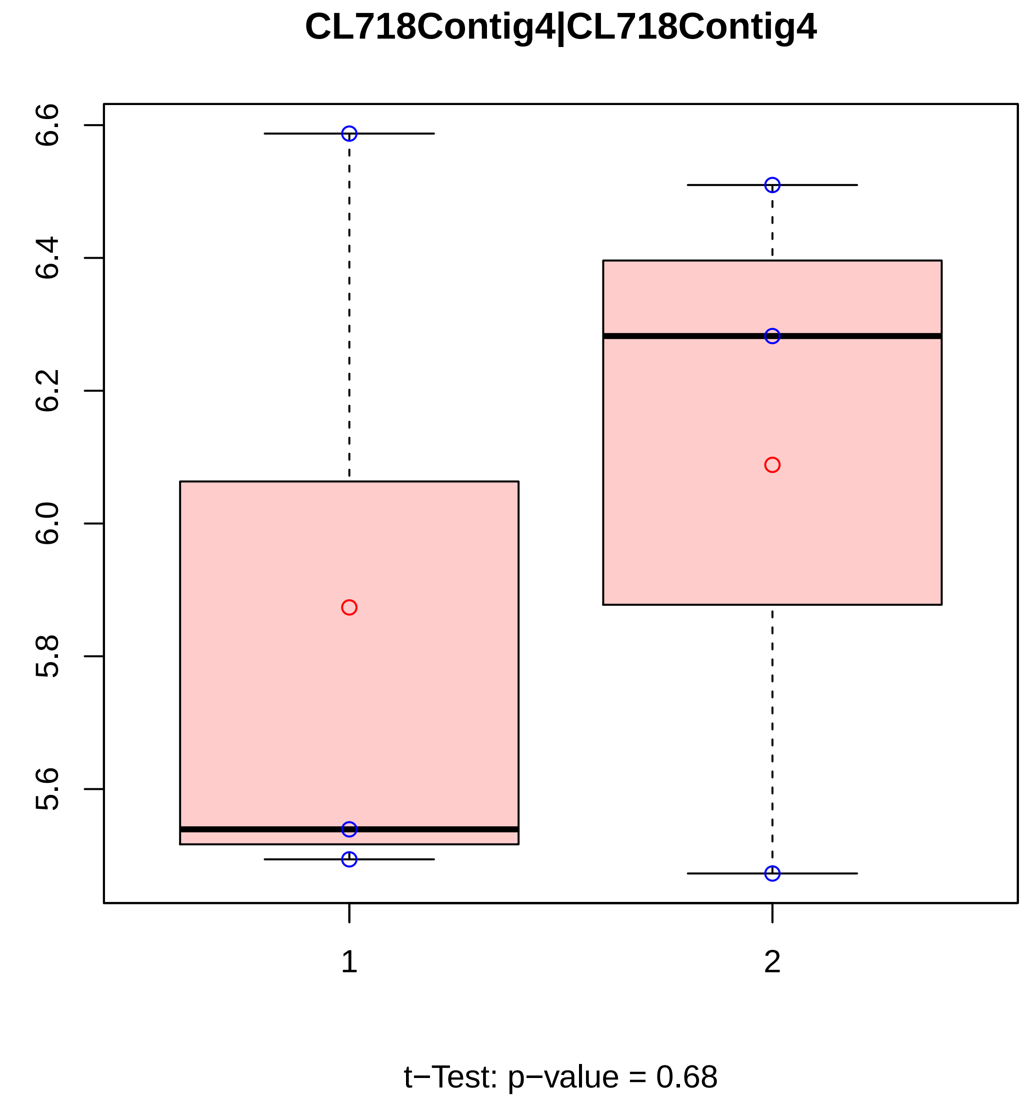

CL718Contig4|CL718Contig4
6.6
6.4
6.2
6.0
5.8
5.6
1
2
t−Test: p−value = 0.68

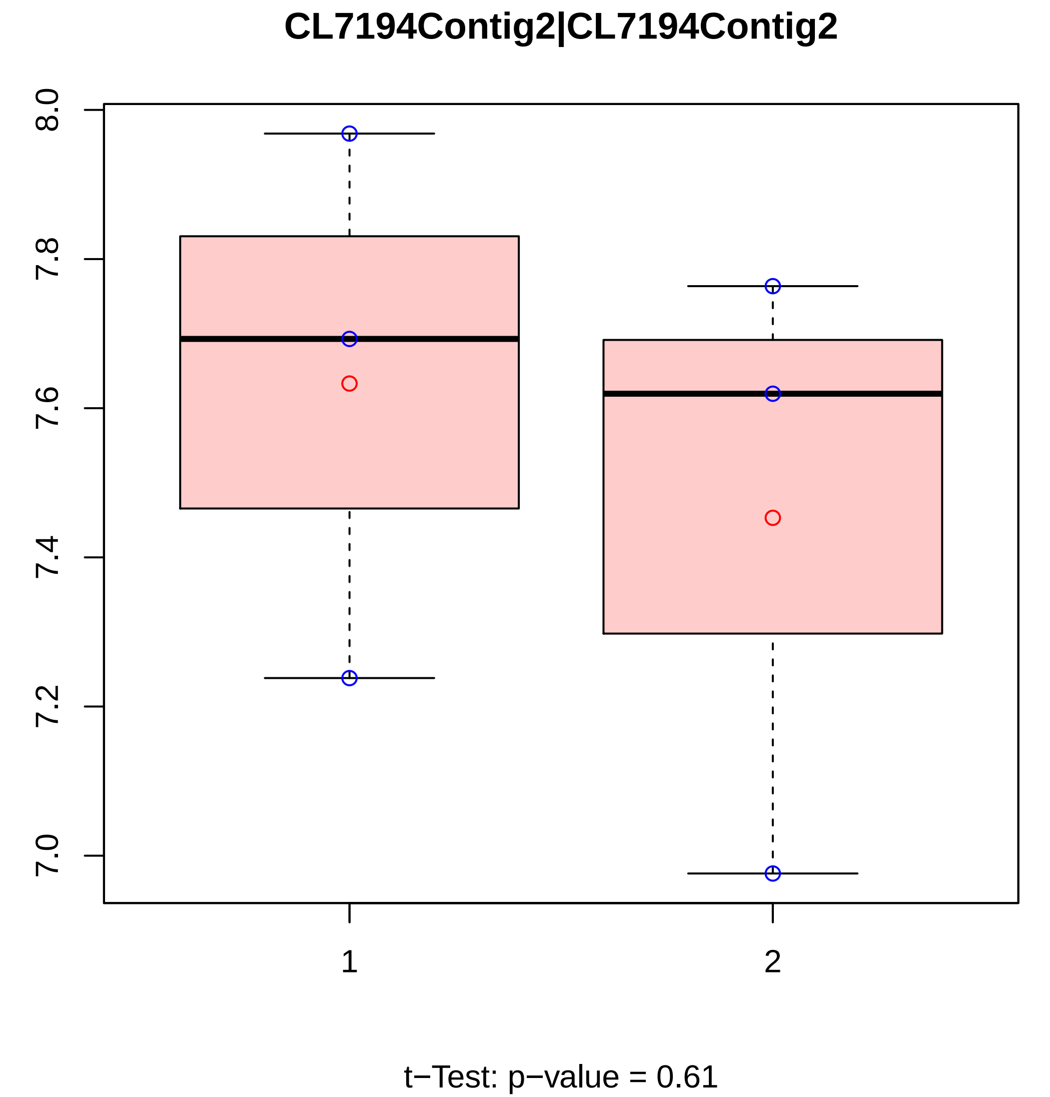
CL7194Contig2|CL7194Contig2
8.0
7.8
7.6
7.4
7.2
7.0
1
2
t−Test: p−value = 0.61

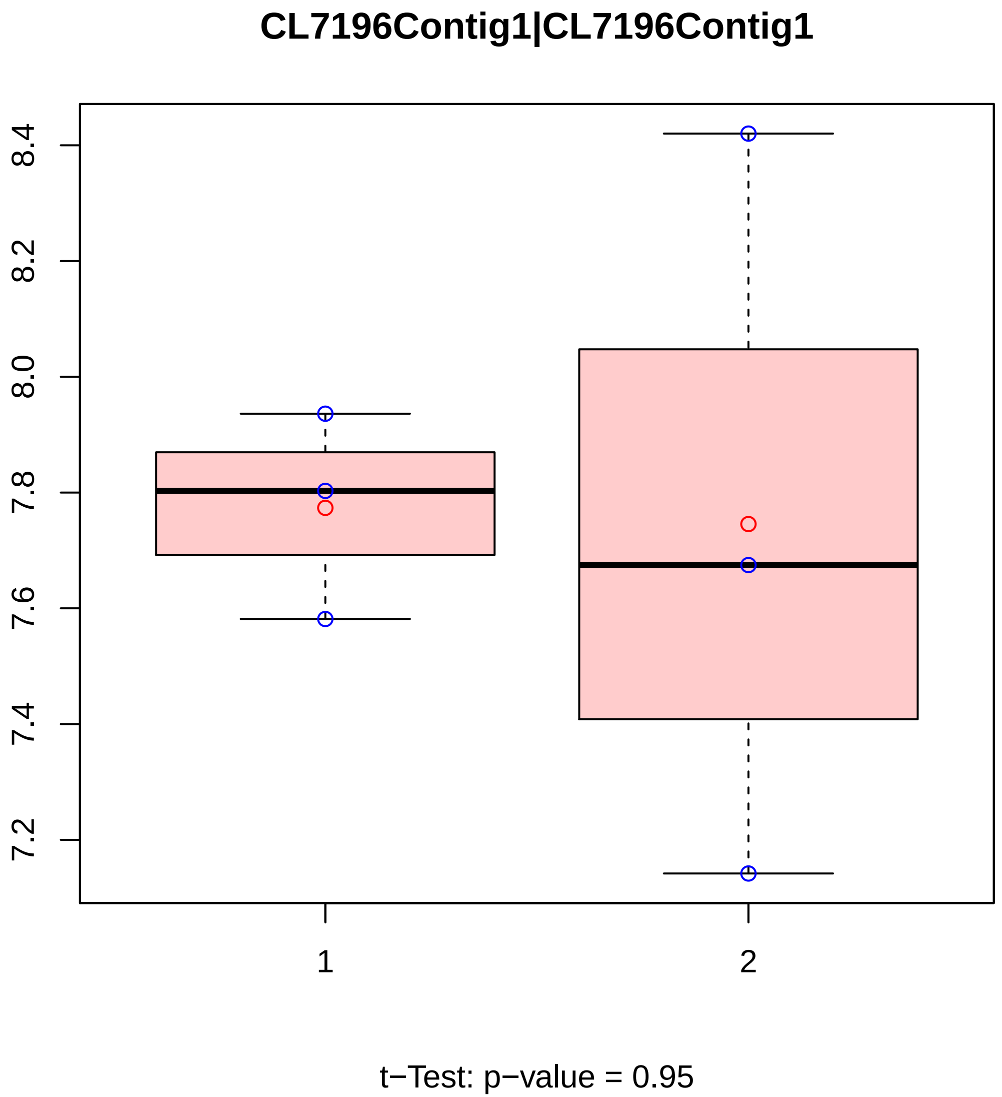
CL7196Contig1|CL7196Contig1
8.4
8.2
8.0
7.8
7.6
7.4
7.2
1
2
t−Test: p−value = 0.95

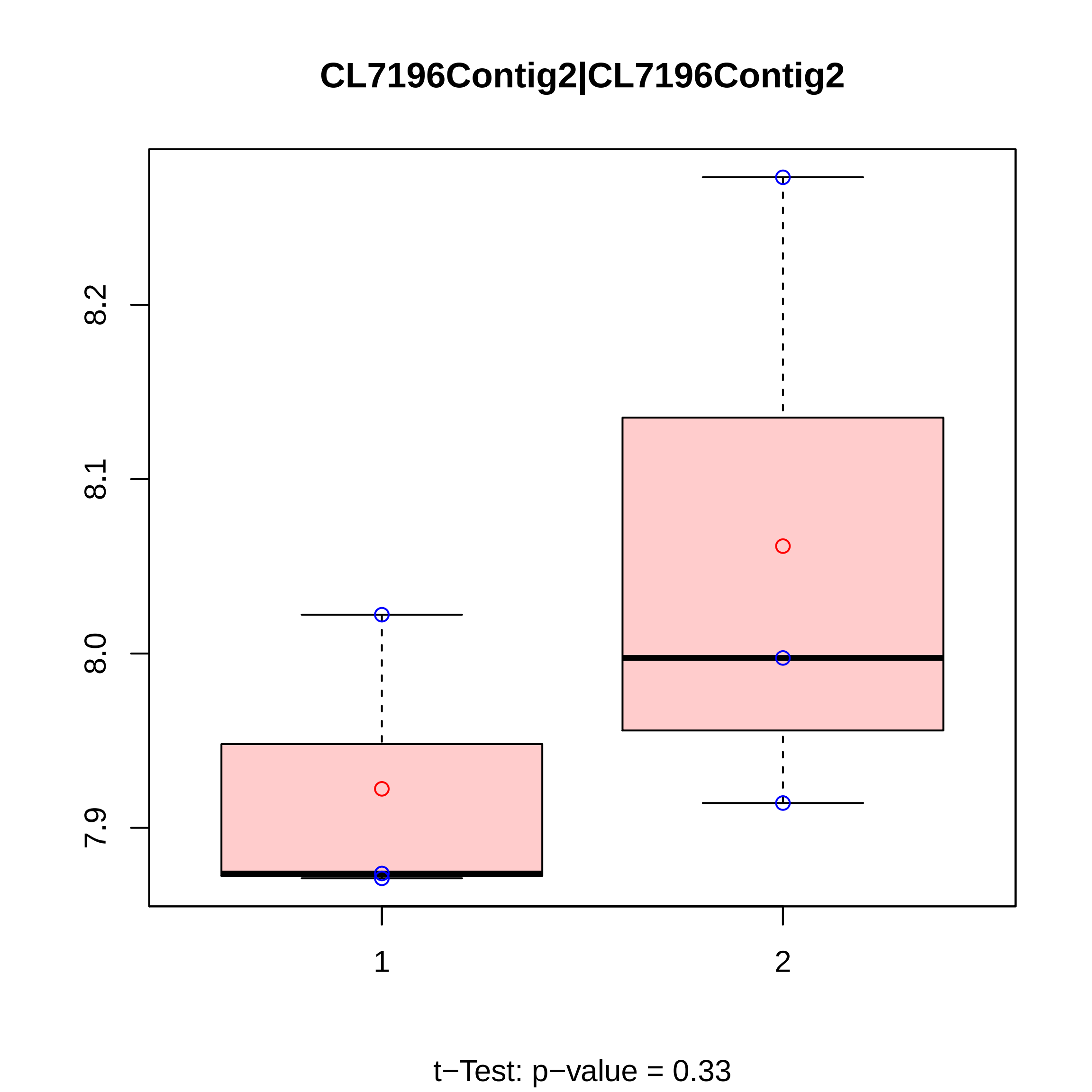

CL7196Contig2|CL7196Contig2
8.2
8.1
8.0
7.9
1
2
t−Test: p−value = 0.33

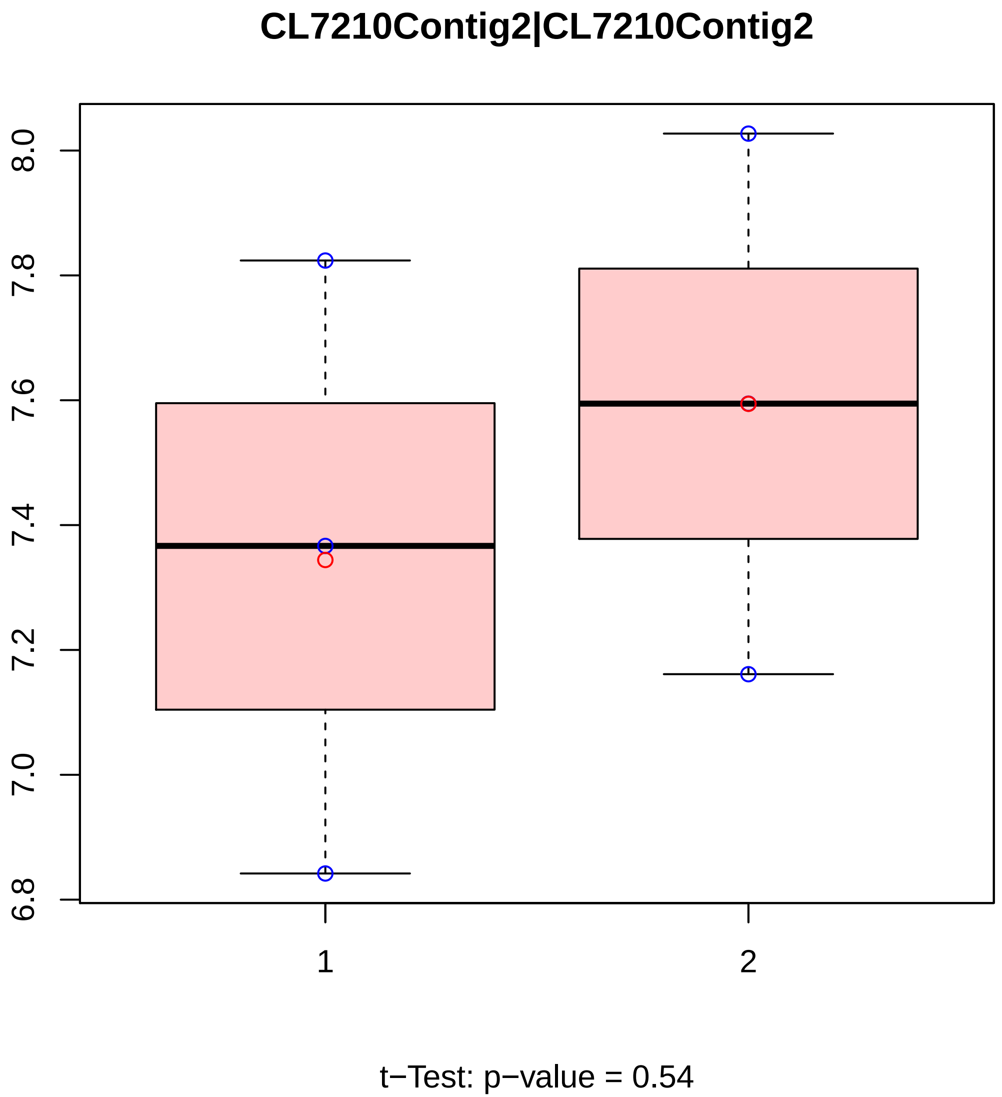

CL7210Contig2|CL7210Contig2
8.0
7.8
7.6
7.4
7.2
7.0
6.8
1
2
t−Test: p−value = 0.54

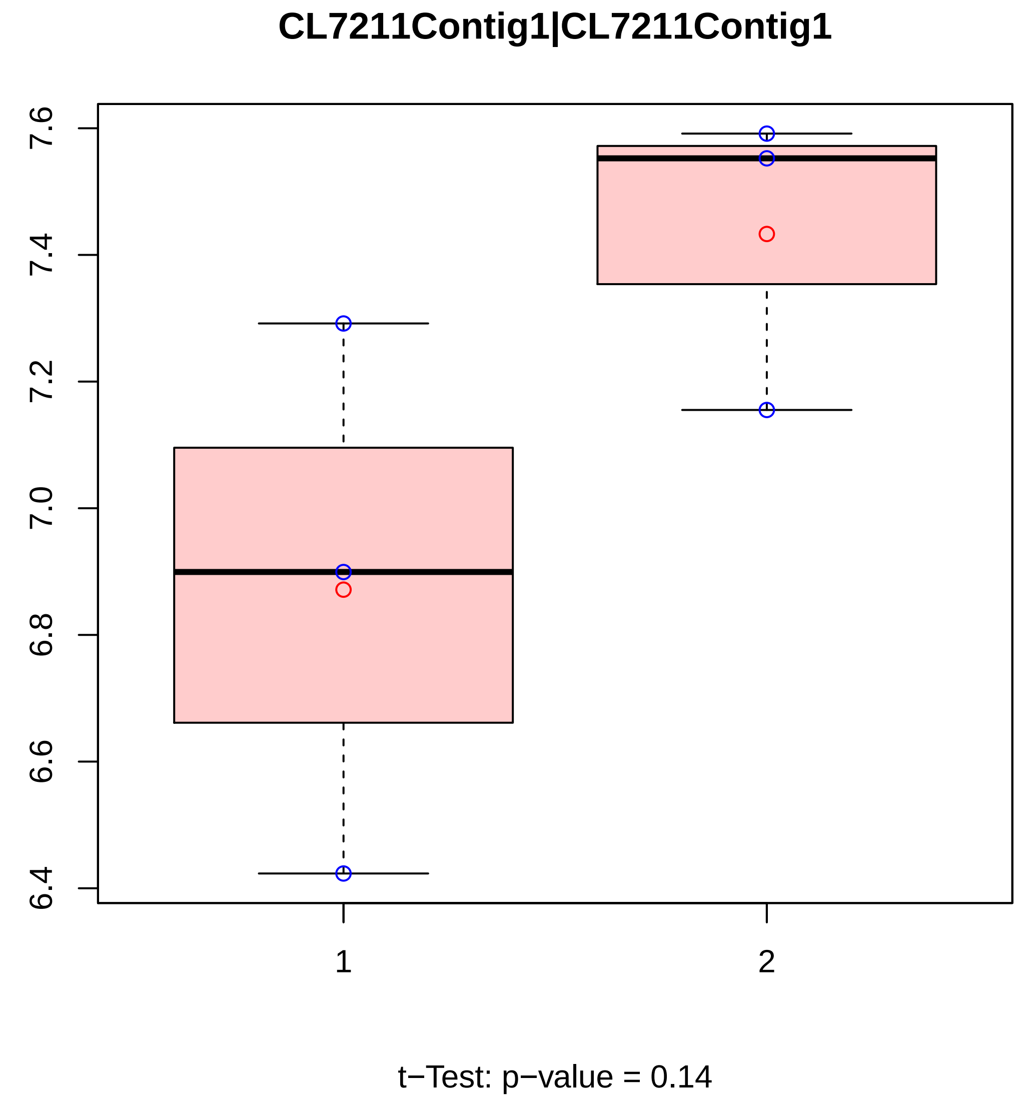

CL7211Contig1|CL7211Contig1
7.6
7.4
7.2
7.0
6.8
6.6
6.4
1
2
t−Test: p−value = 0.14

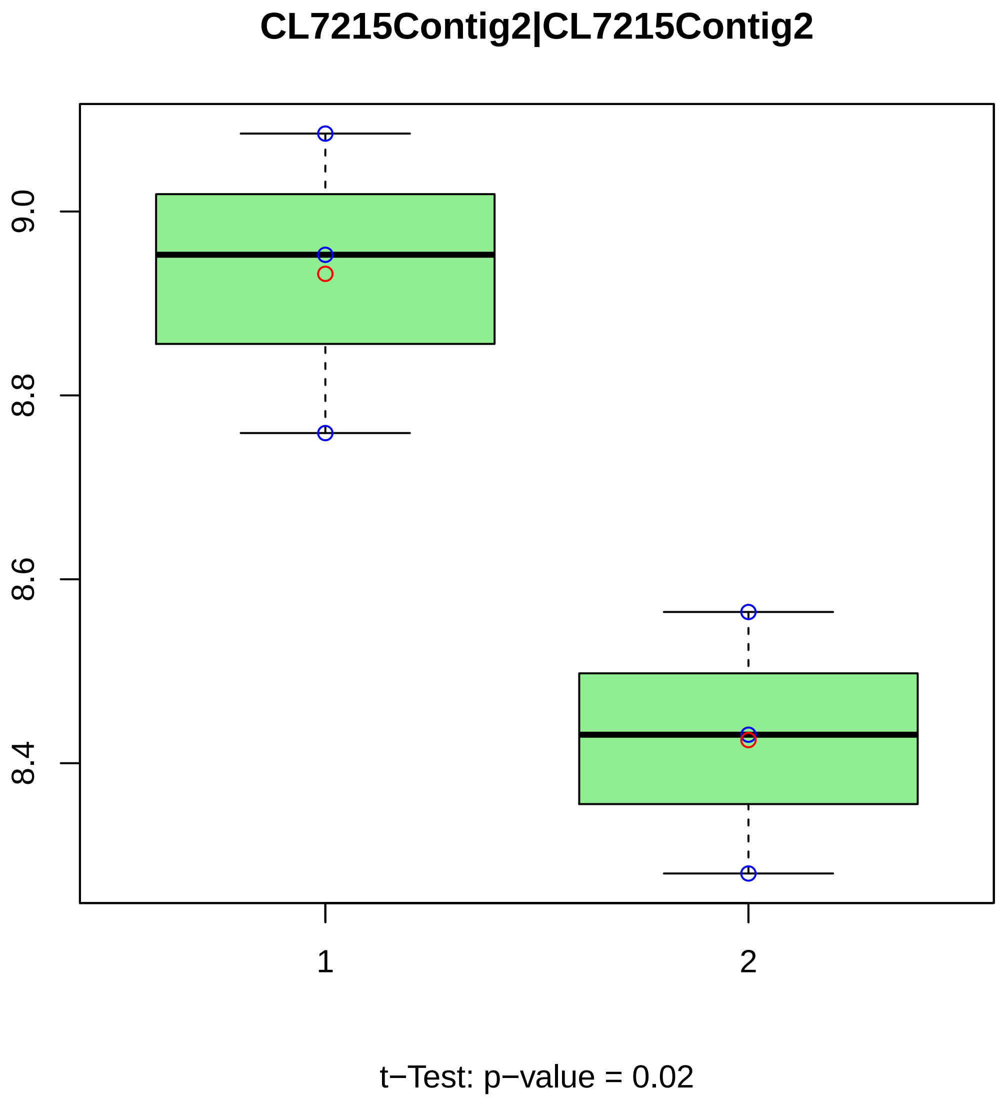
CL7215Contig2|CL7215Contig2
9.0
8.8
8.6
8.4
1
2
t−Test: p−value = 0.02

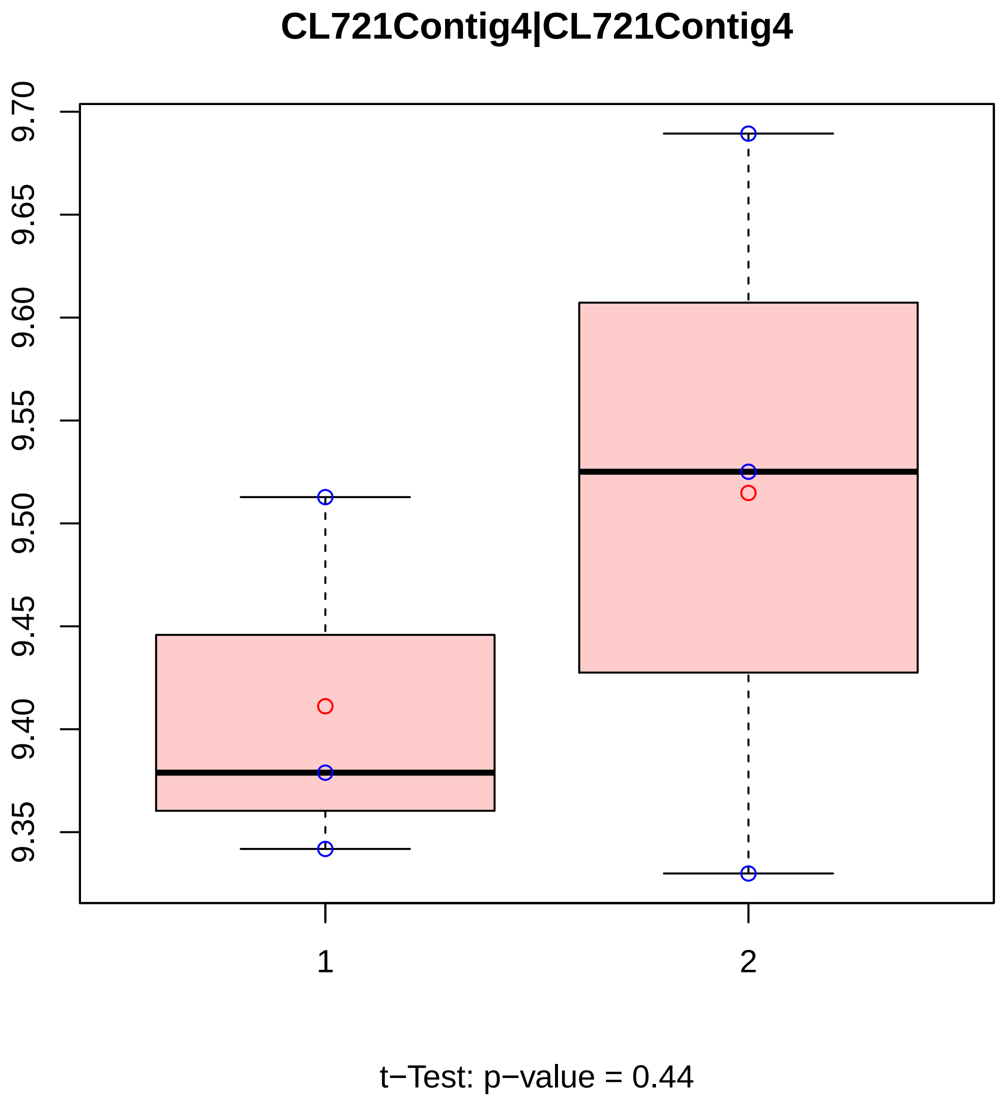

CL721Contig4|CL721Contig4
9.35
9.40
9.45
9.50
9.55
9.60
9.65
9.70
1
2
t−Test: p−value = 0.44

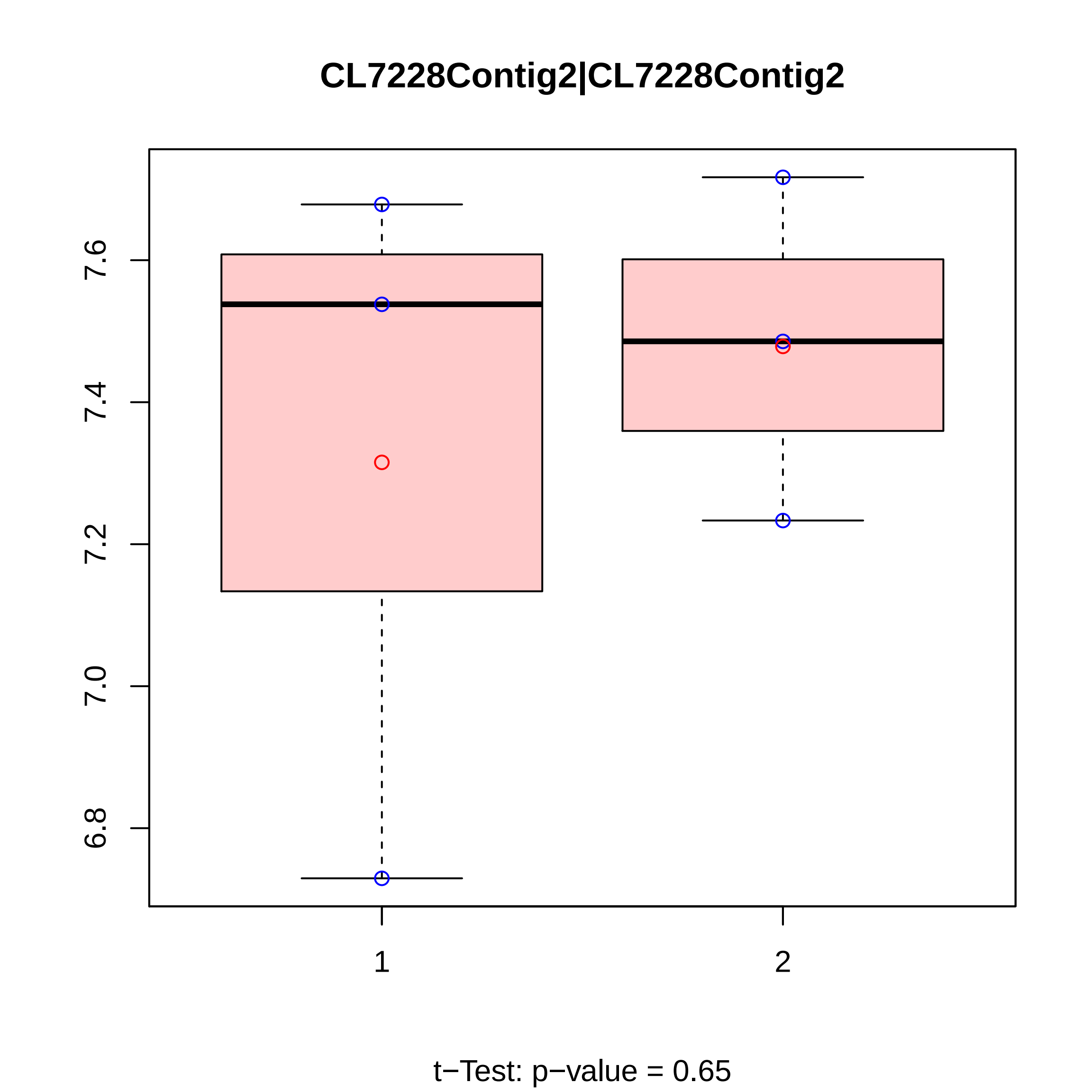

CL7228Contig2|CL7228Contig2
7.6
7.4
7.2
7.0
6.8
1
2
t−Test: p−value = 0.65

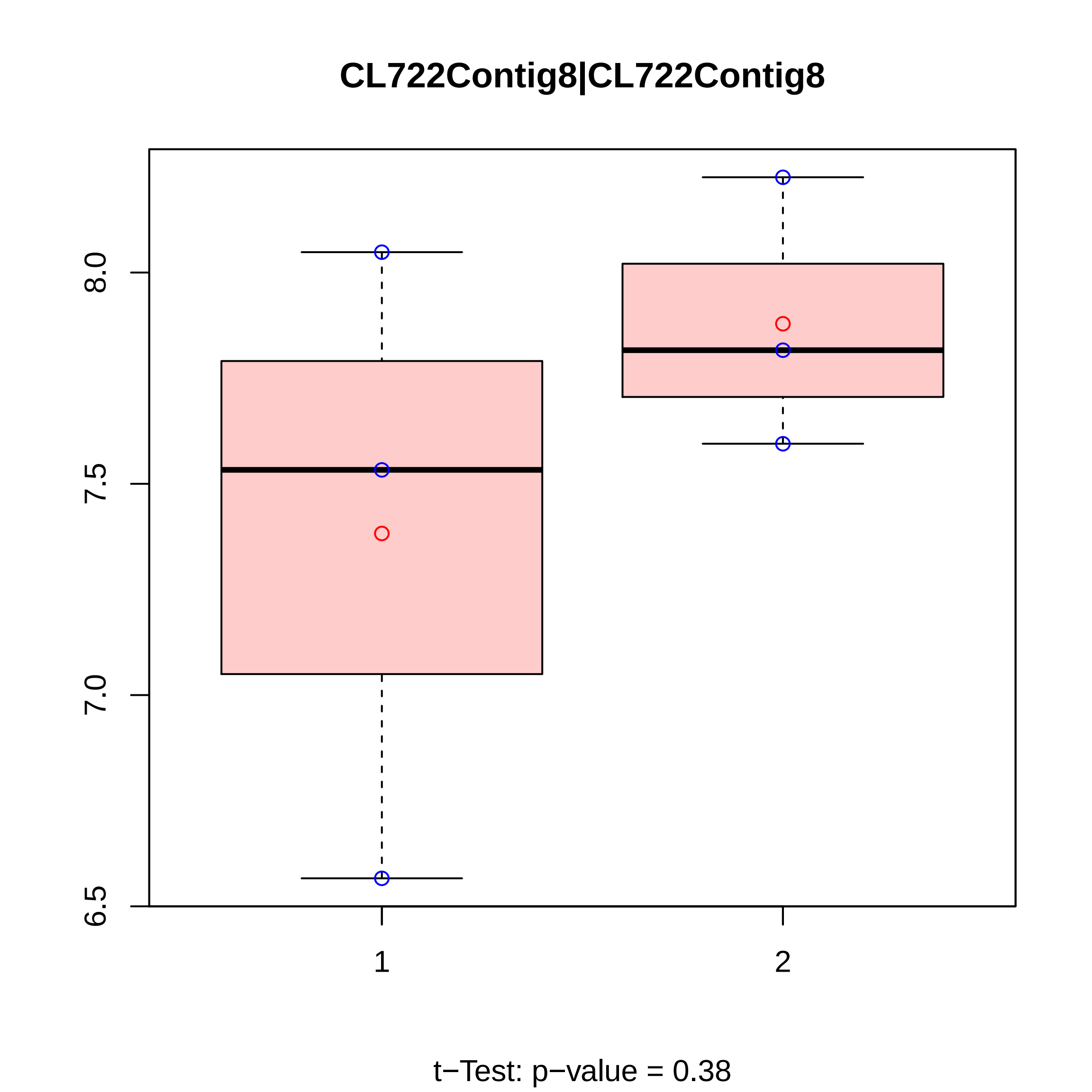

CL722Contig8|CL722Contig8
8.0
7.5
7.0
6.5
1
2
t−Test: p−value = 0.38

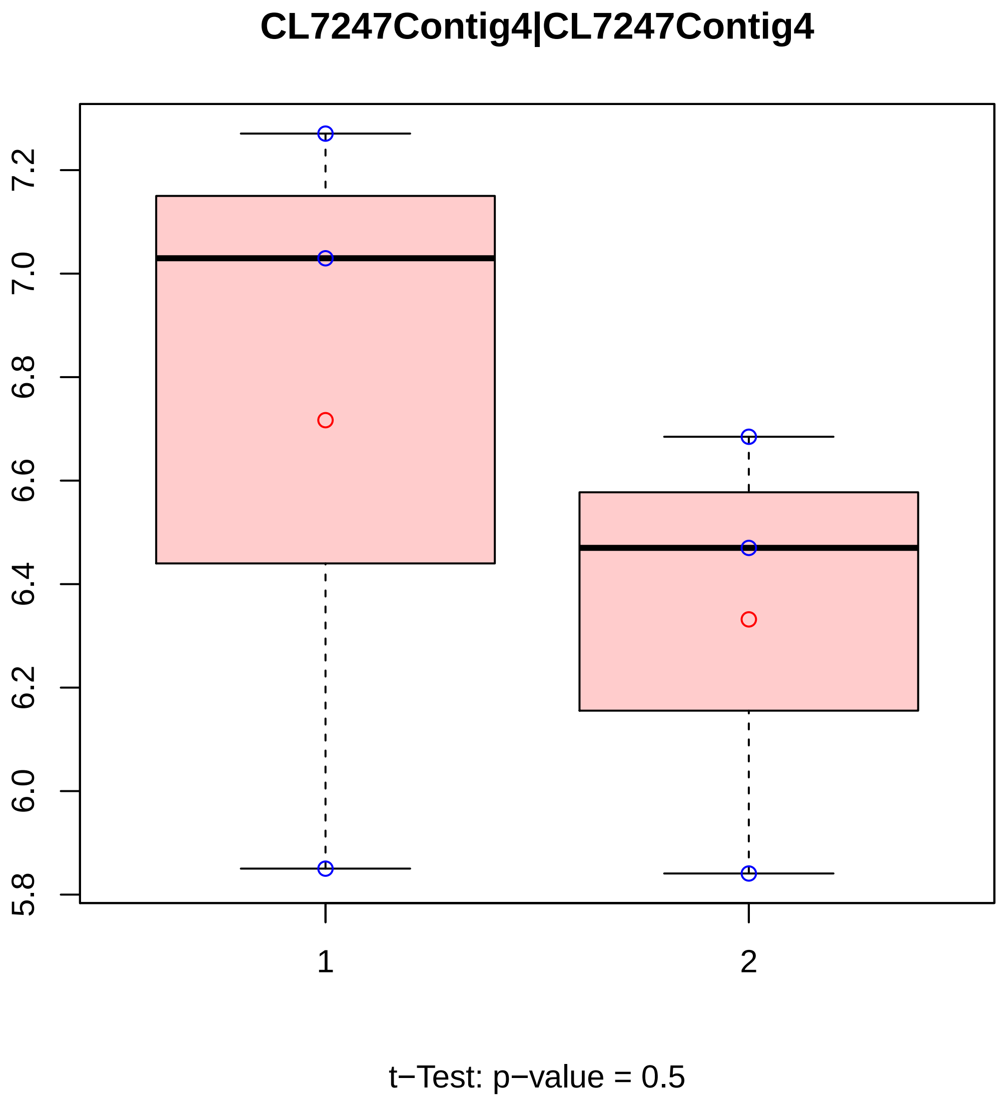

CL7247Contig4|CL7247Contig4
7.2
7.0
6.8
6.6
6.4
6.2
6.0
5.8
1
2
t−Test: p−value = 0.5

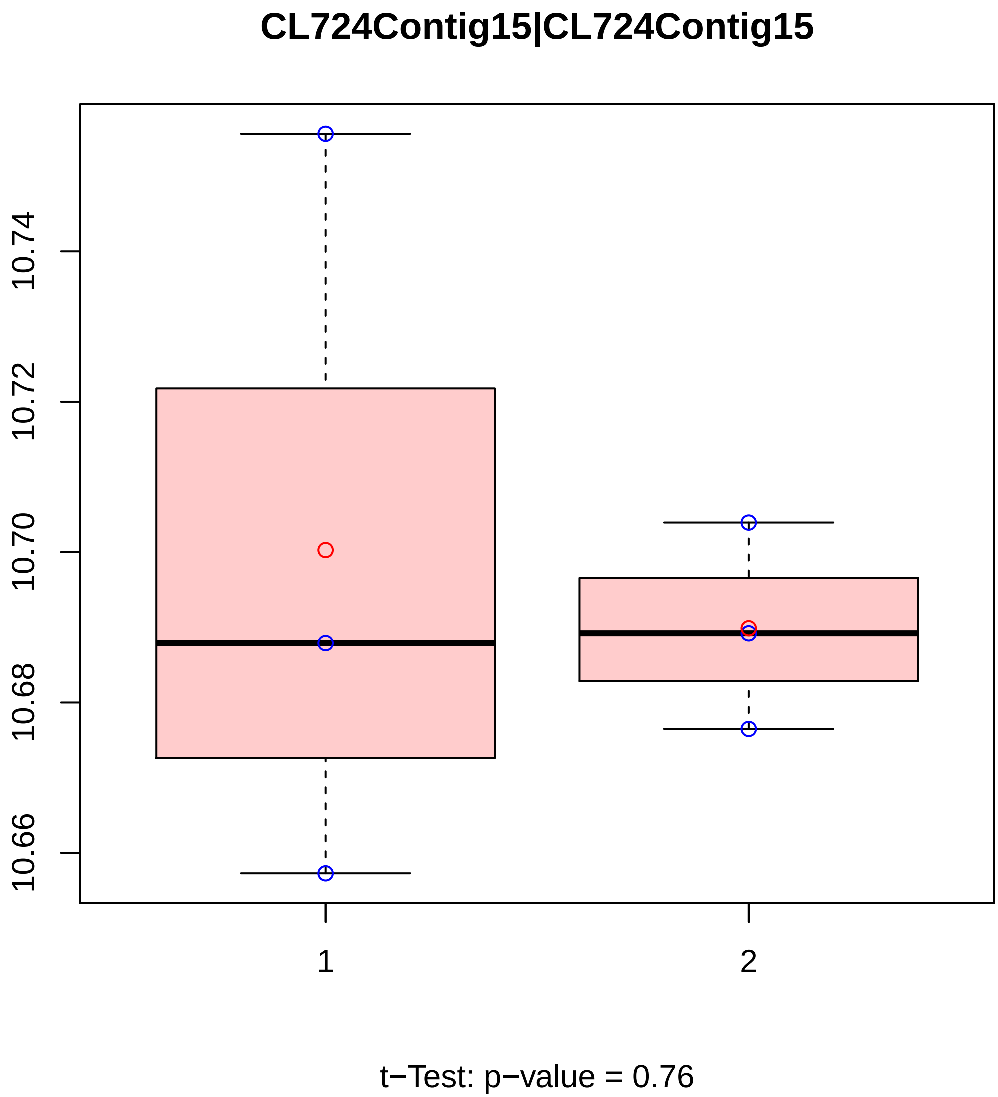

CL724Contig15|CL724Contig15
10.66
10.68
10.70
10.72
10.74
1
2
t−Test: p−value = 0.76

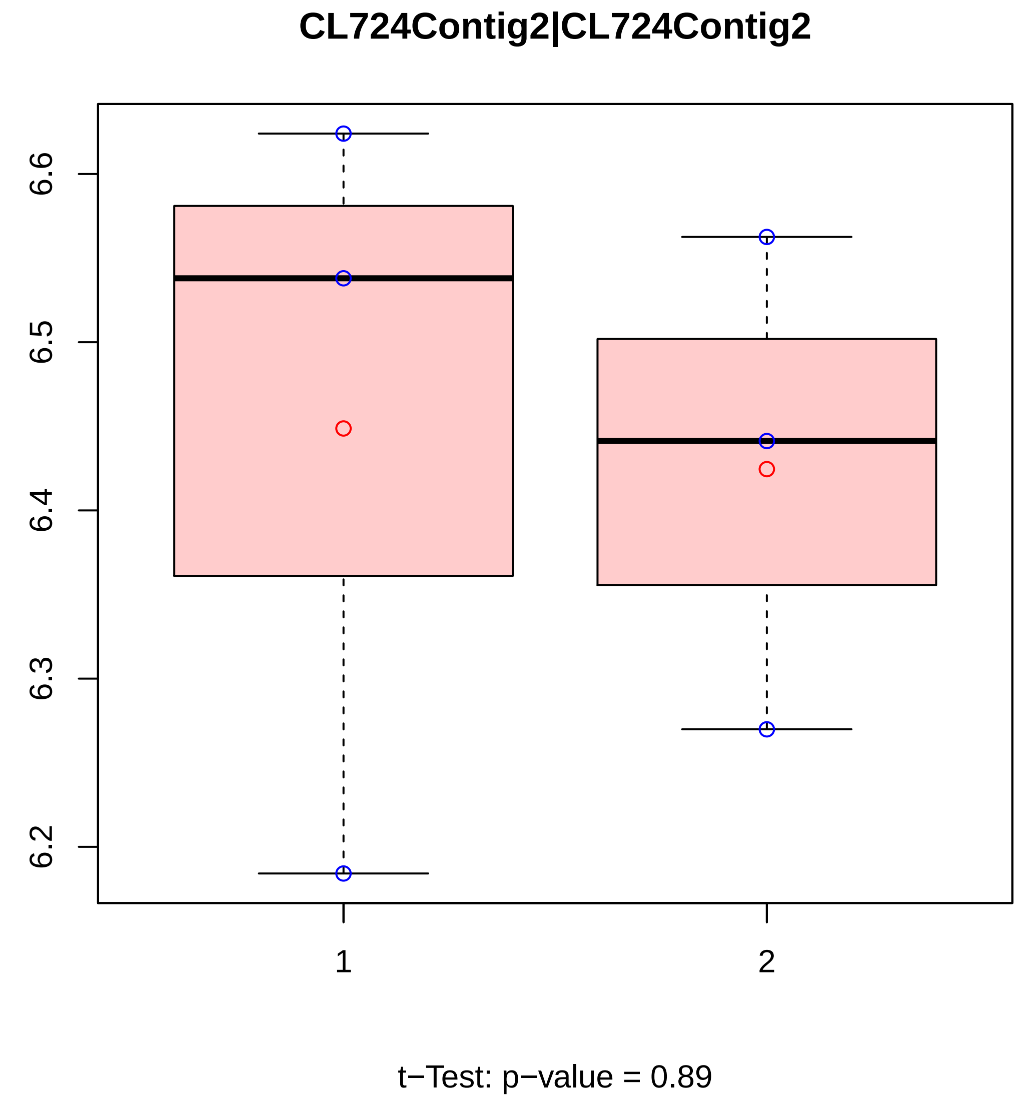
CL724Contig2|CL724Contig2
6.2
6.3
6.4
6.5
6.6
1
2
t−Test: p−value = 0.89

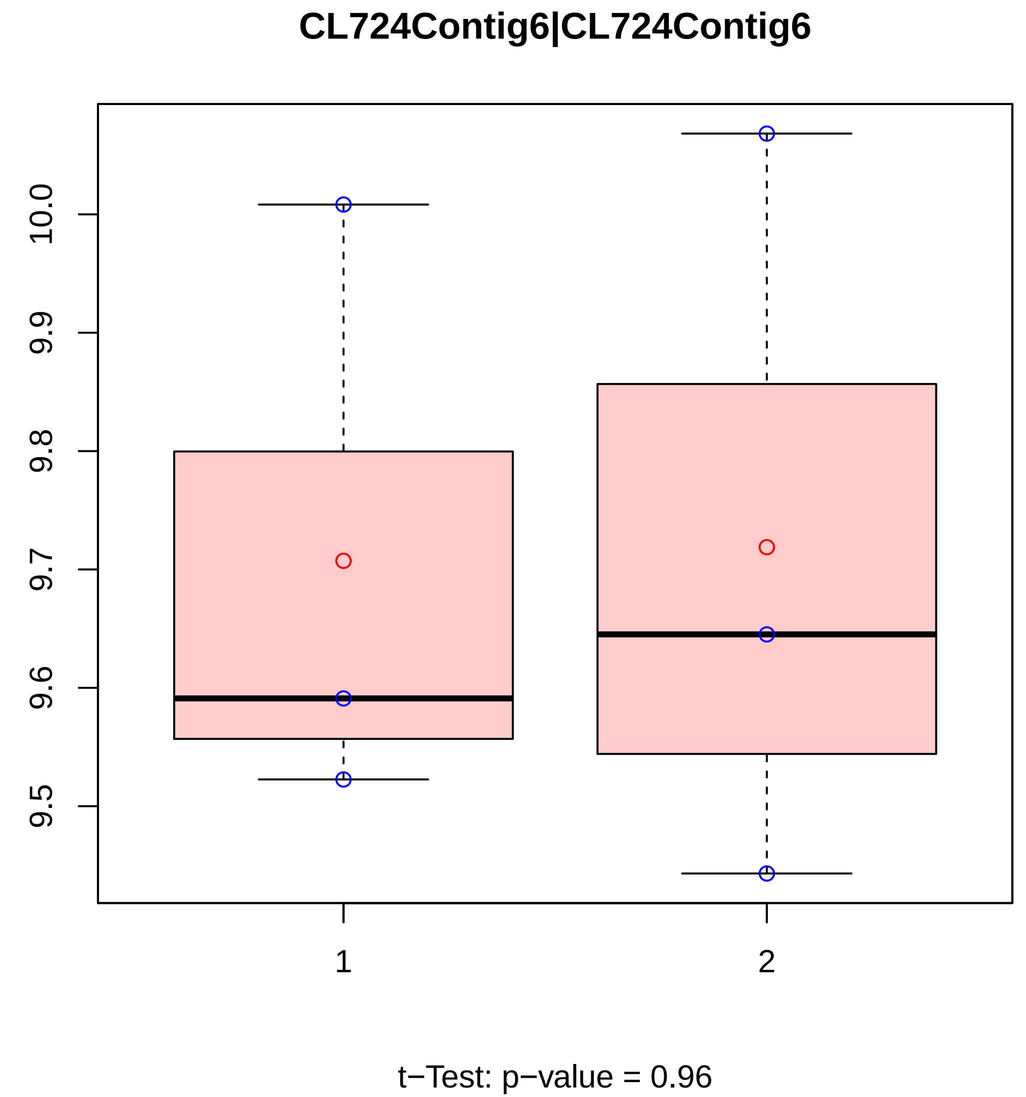
CL724Contig6|CL724Contig6
10.0
9.9
9.8
9.7
9.6
9.5
1
2
t−Test: p−value = 0.96

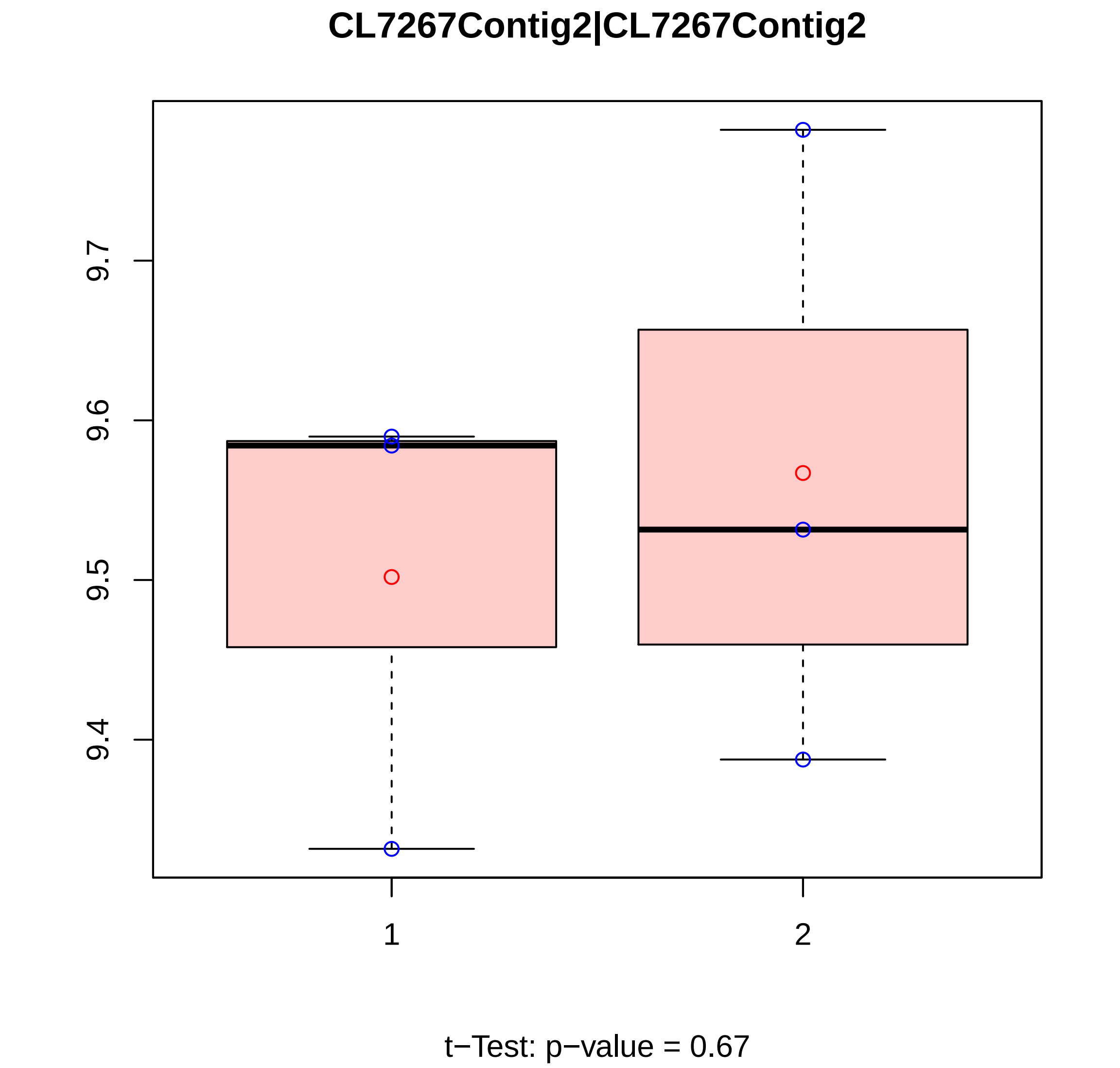
CL7267Contig2|CL7267Contig2
9.4
9.5
9.6
9.7
1
2
t−Test: p−value = 0.67

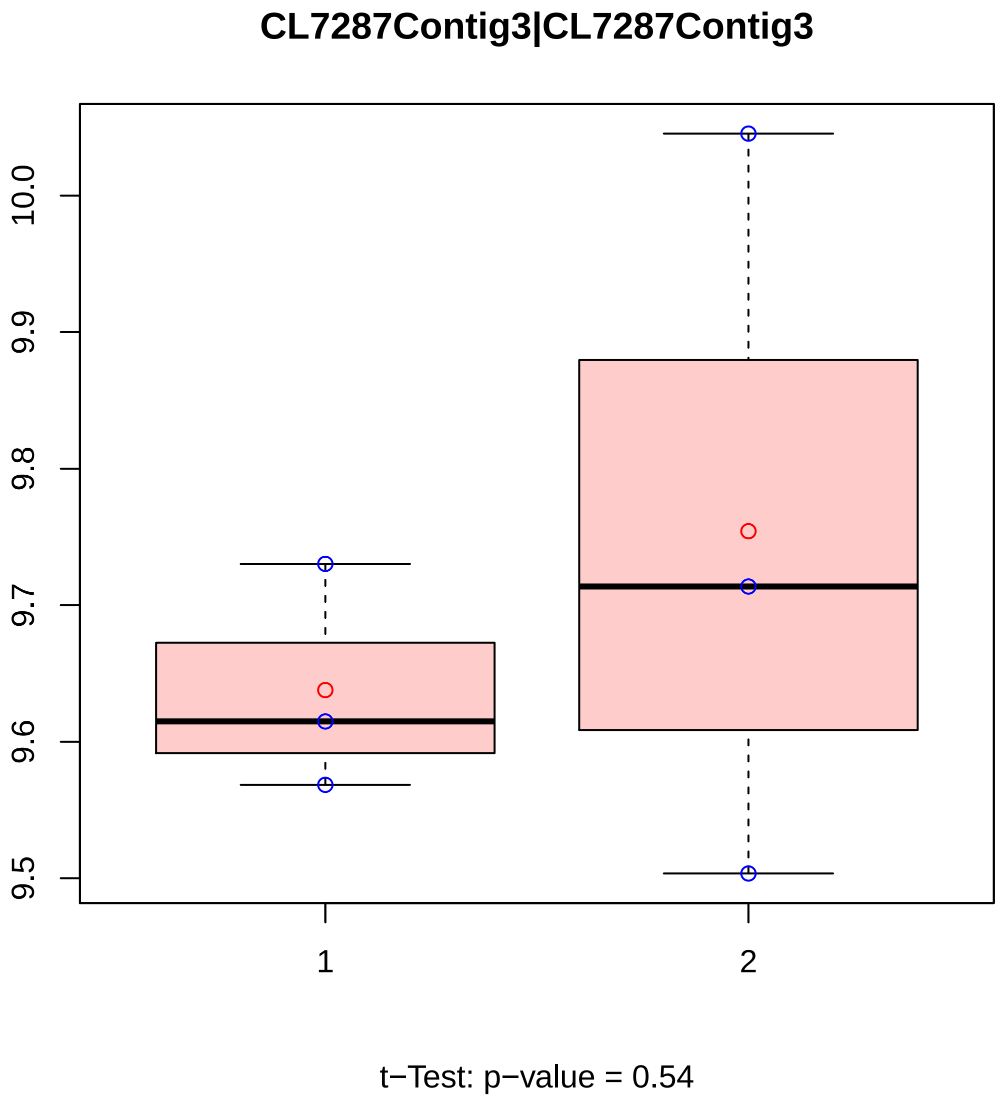

CL7287Contig3|CL7287Contig3
10.0
9.9
9.8
9.7
9.6
9.5
1
2
t−Test: p−value = 0.54

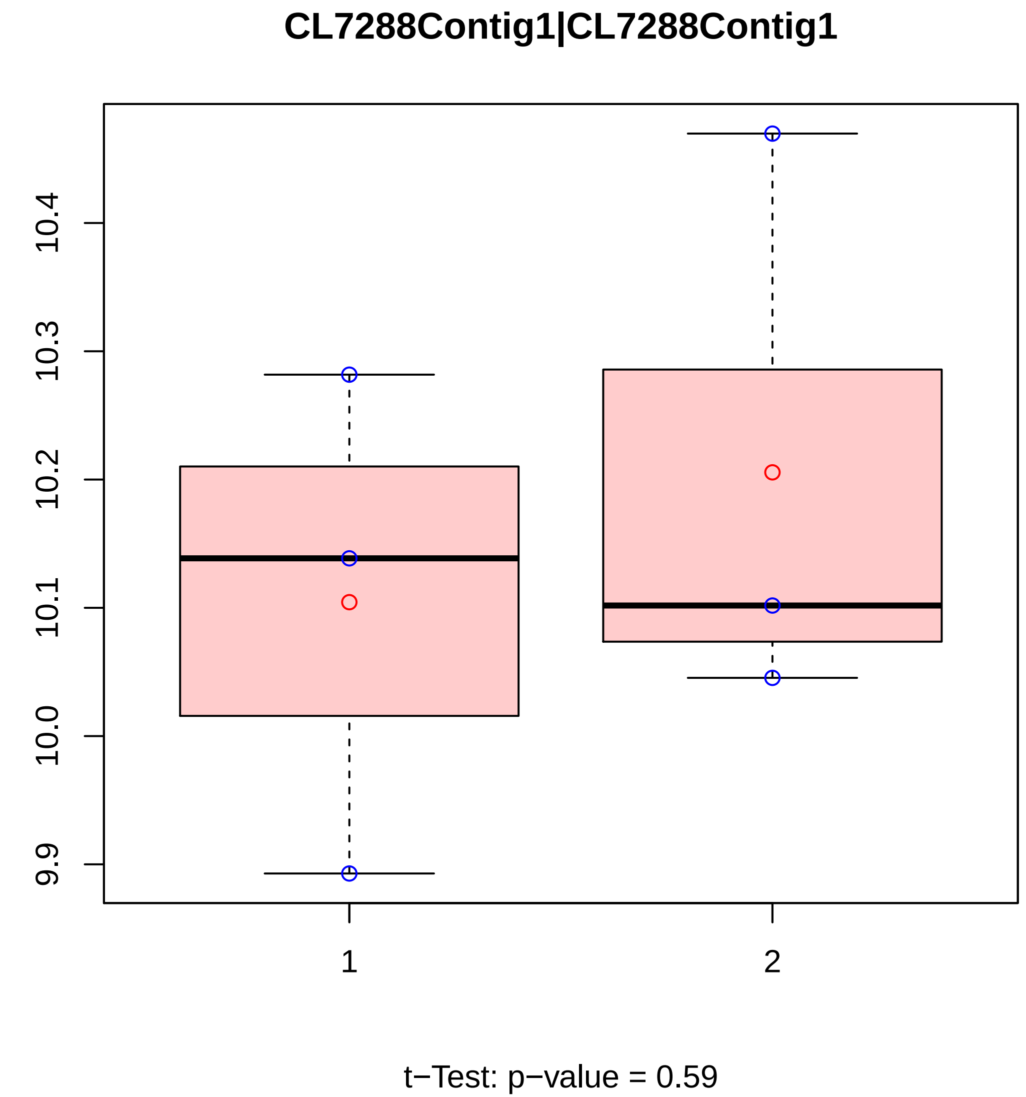
CL7288Contig1|CL7288Contig1
10.4
10.3
10.2
10.1
10.0
9.9
1
2
t−Test: p−value = 0.59

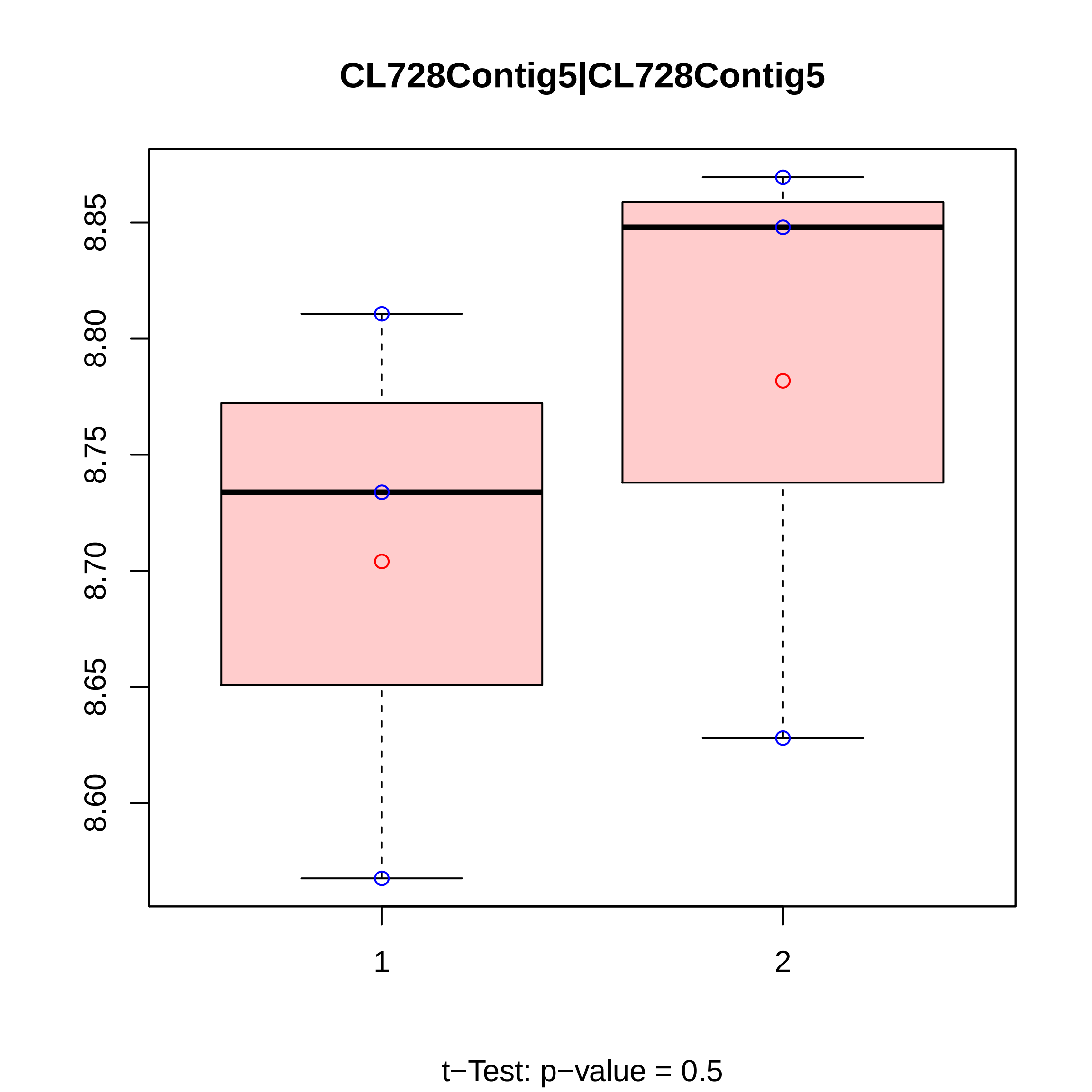
CL728Contig5|CL728Contig5
8.60
8.65
8.70
8.75
8.80
8.85
1
2
t−Test: p−value = 0.5

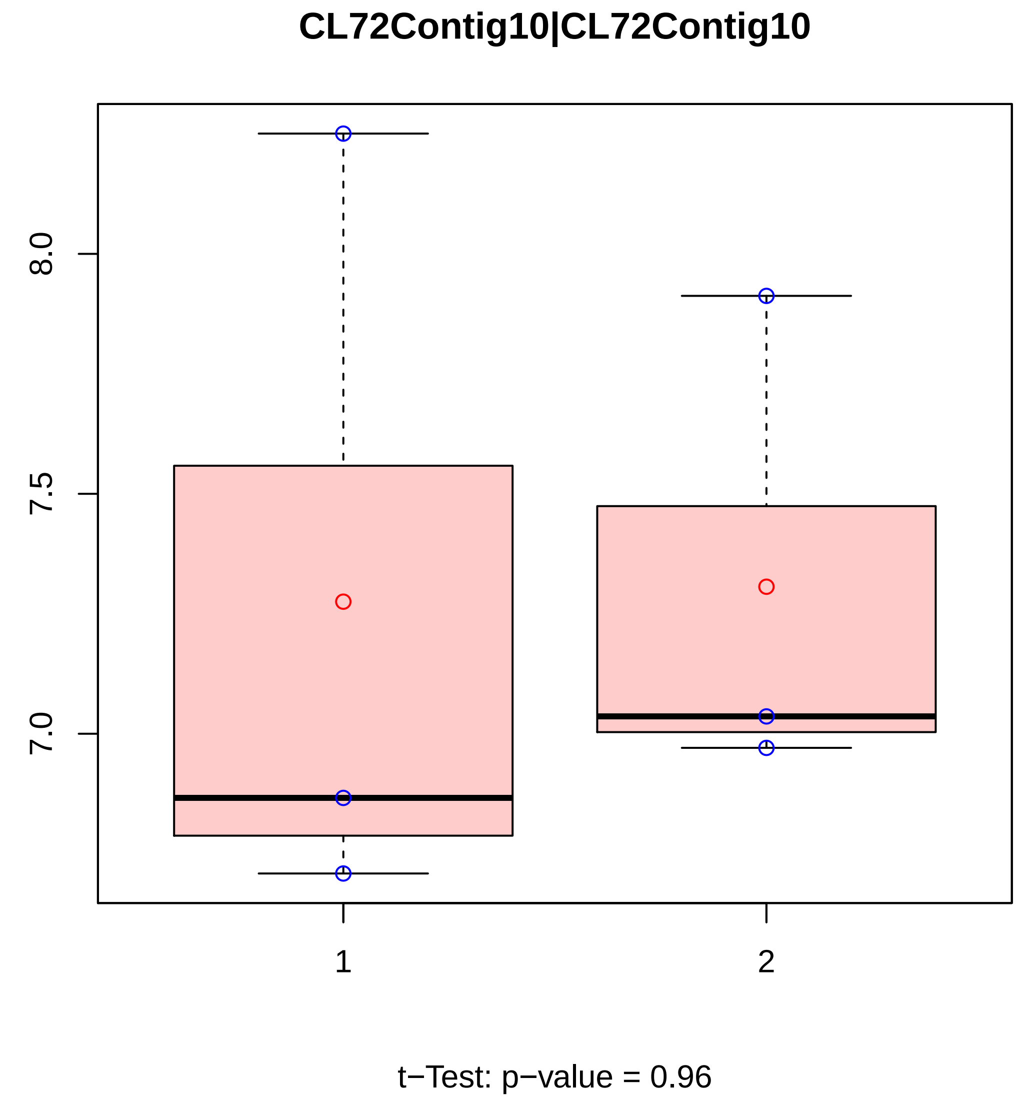
CL72Contig10|CL72Contig10
8.0
7.5
7.0
1
2
t−Test: p−value = 0.96

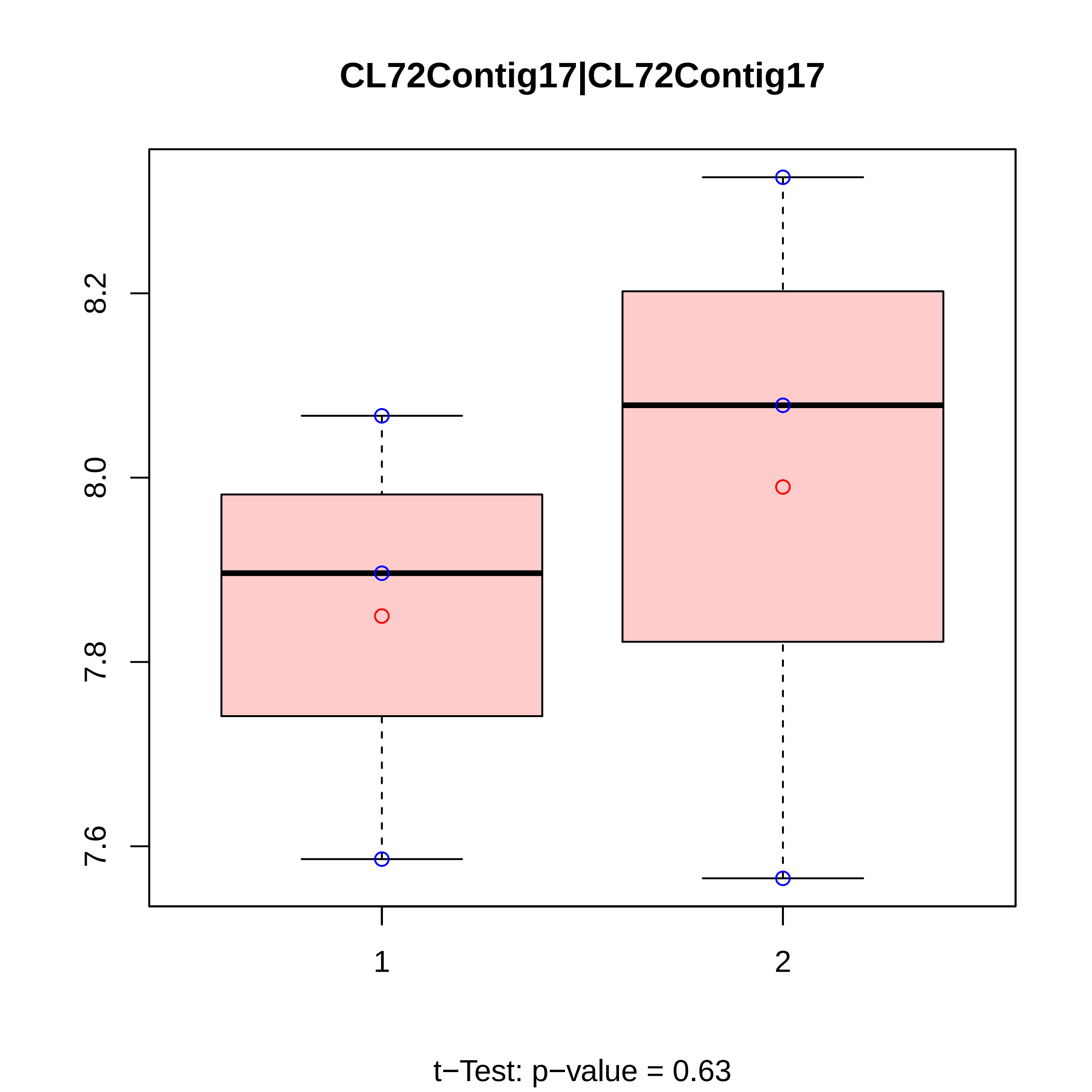
CL72Contig17|CL72Contig17
8.2
8.0
7.8
7.6
1
2
t−Test: p−value = 0.63

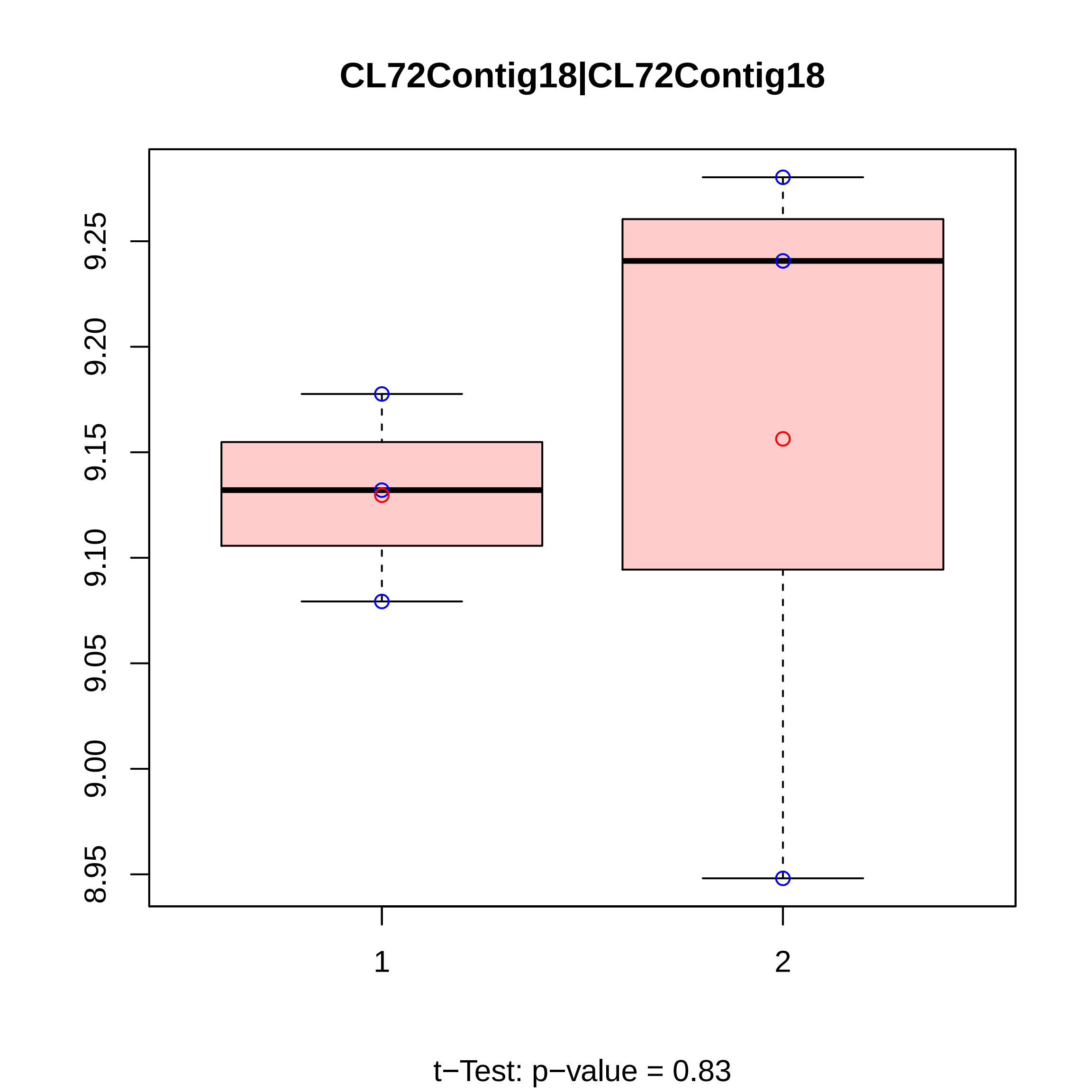

CL72Contig18|CL72Contig18
9.25
9.20
9.15
9.10
9.05
9.00
8.95
1
2
t−Test: p−value = 0.83

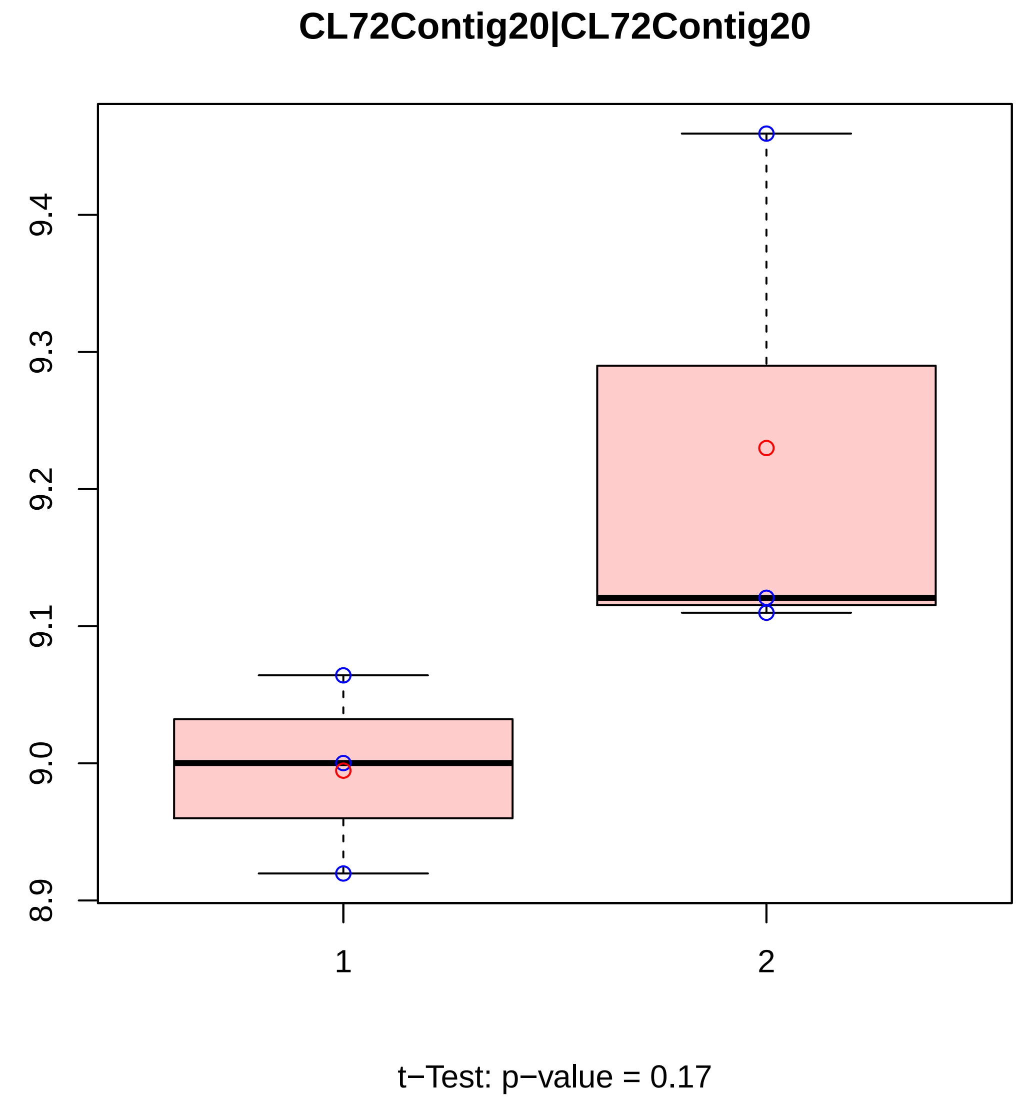
CL72Contig20|CL72Contig20
9.4
9.3
9.2
9.1
9.0
8.9
1
2
t−Test: p−value = 0.17

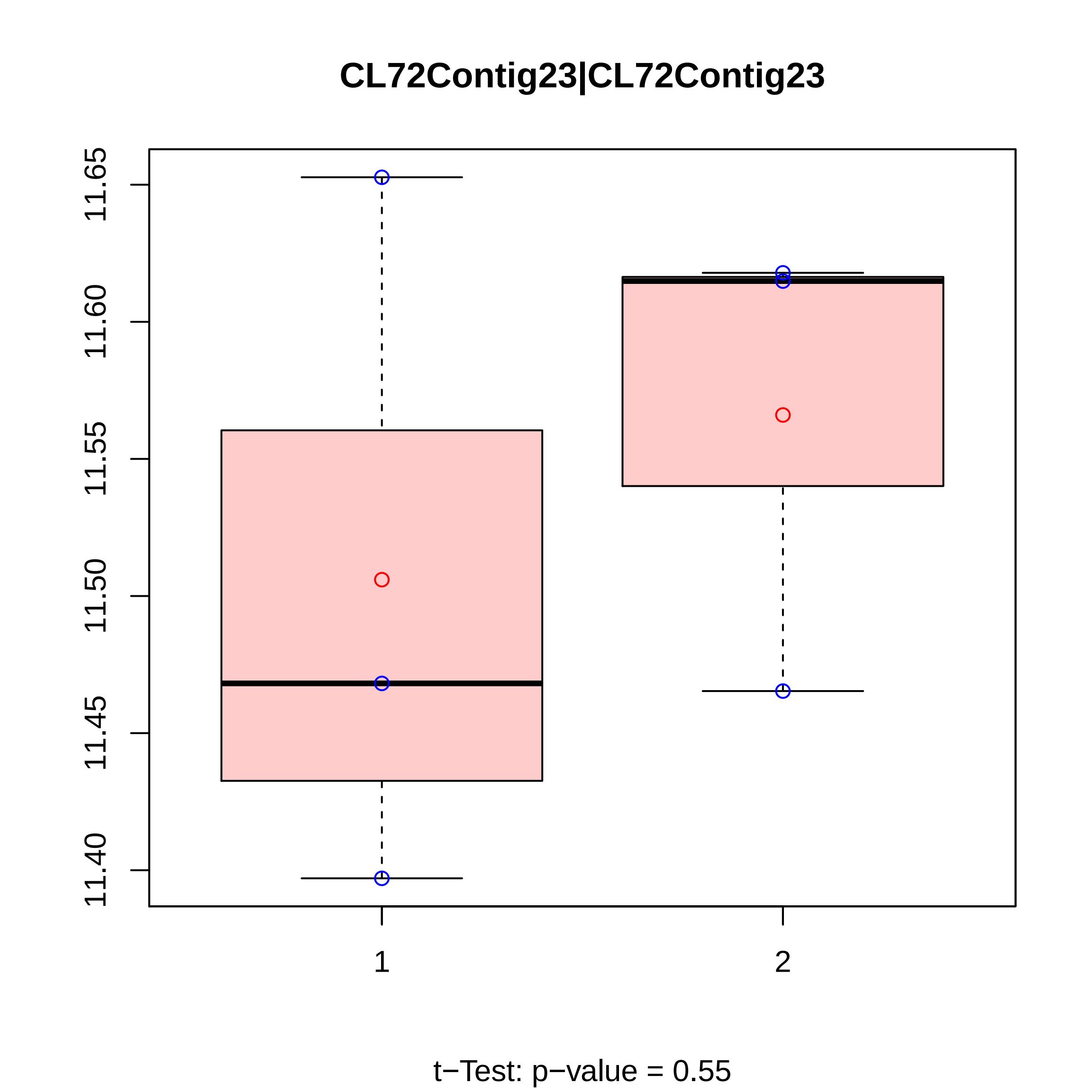

CL72Contig23|CL72Contig23
11.40
11.45
11.50
11.55
11.60
11.65
1
2
t−Test: p−value = 0.55

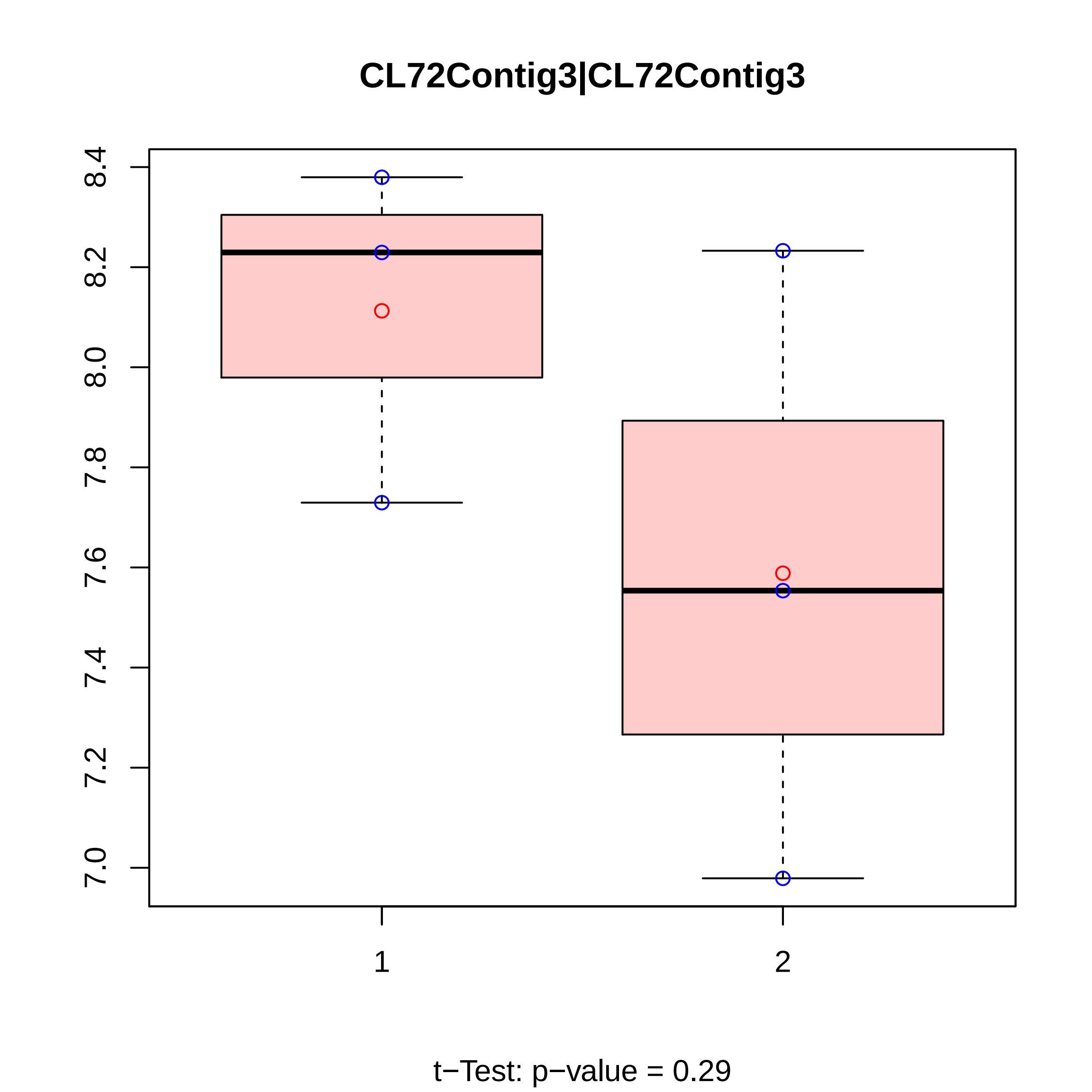
CL72Contig3|CL72Contig3
8.4
8.2
8.0
7.8
7.6
7.4
7.2
7.0
1
2
t−Test: p−value = 0.29

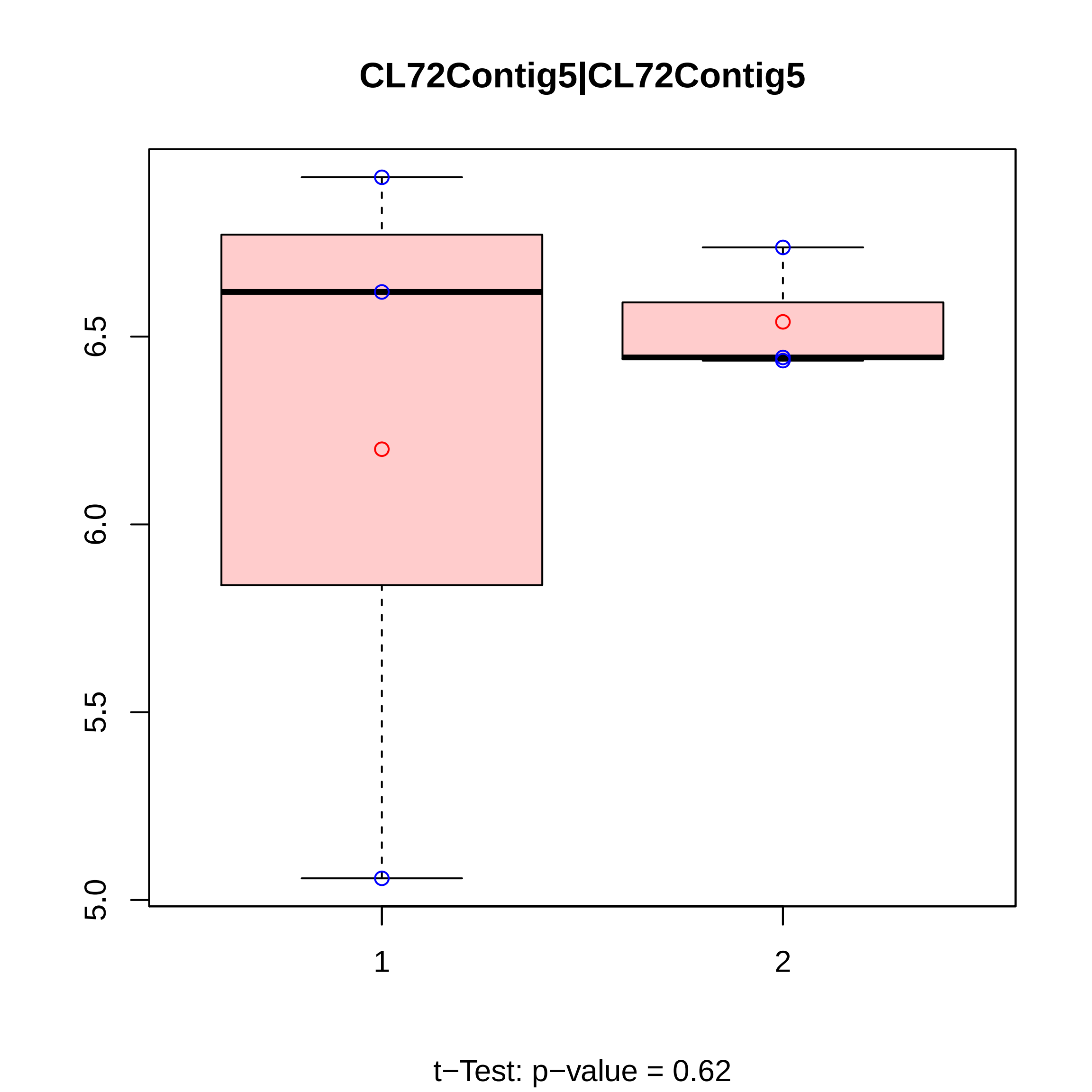

CL72Contig5|CL72Contig5
6.5
6.0
5.5
5.0
1
2
t−Test: p−value = 0.62

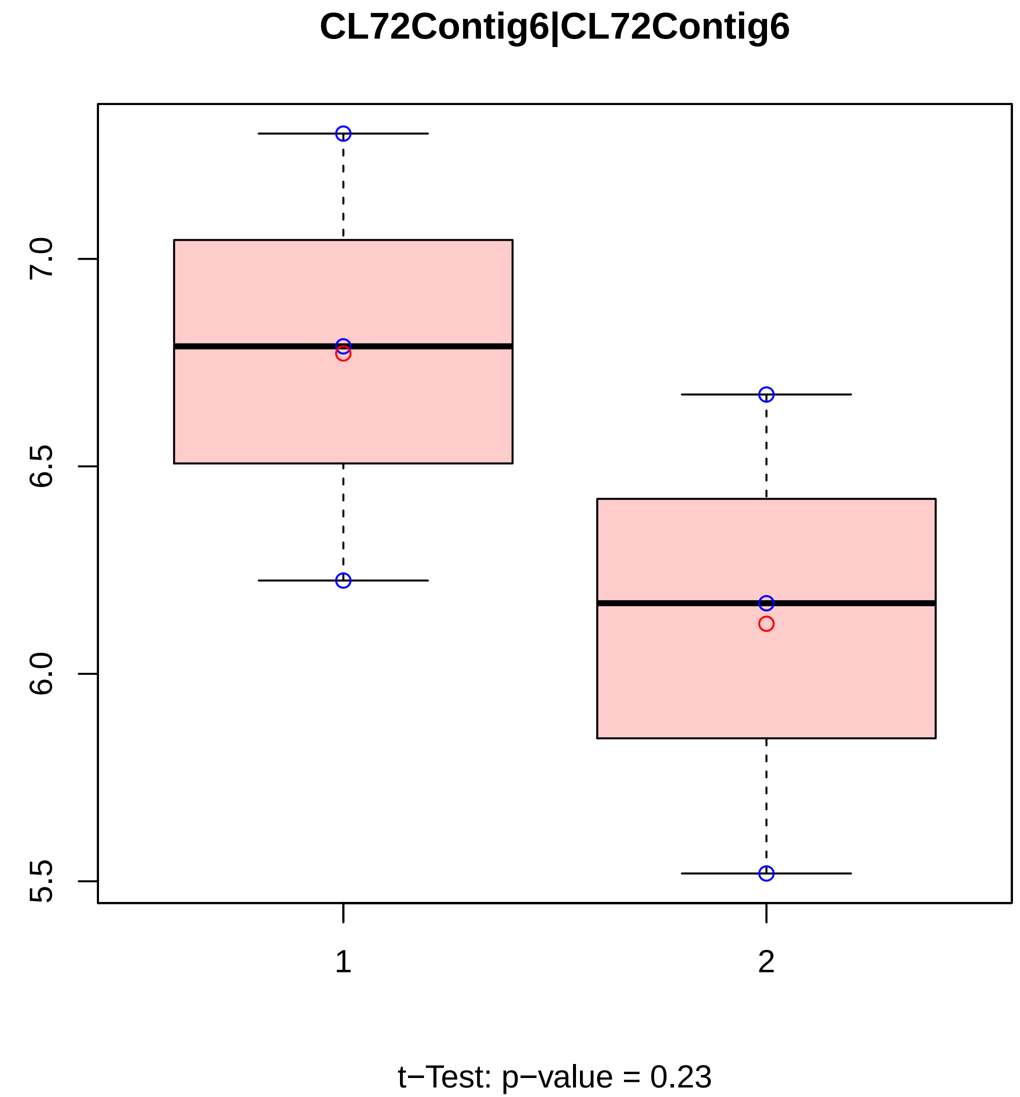

CL72Contig6|CL72Contig6
7.0
6.5
6.0
5.5
1
2
t−Test: p−value = 0.23

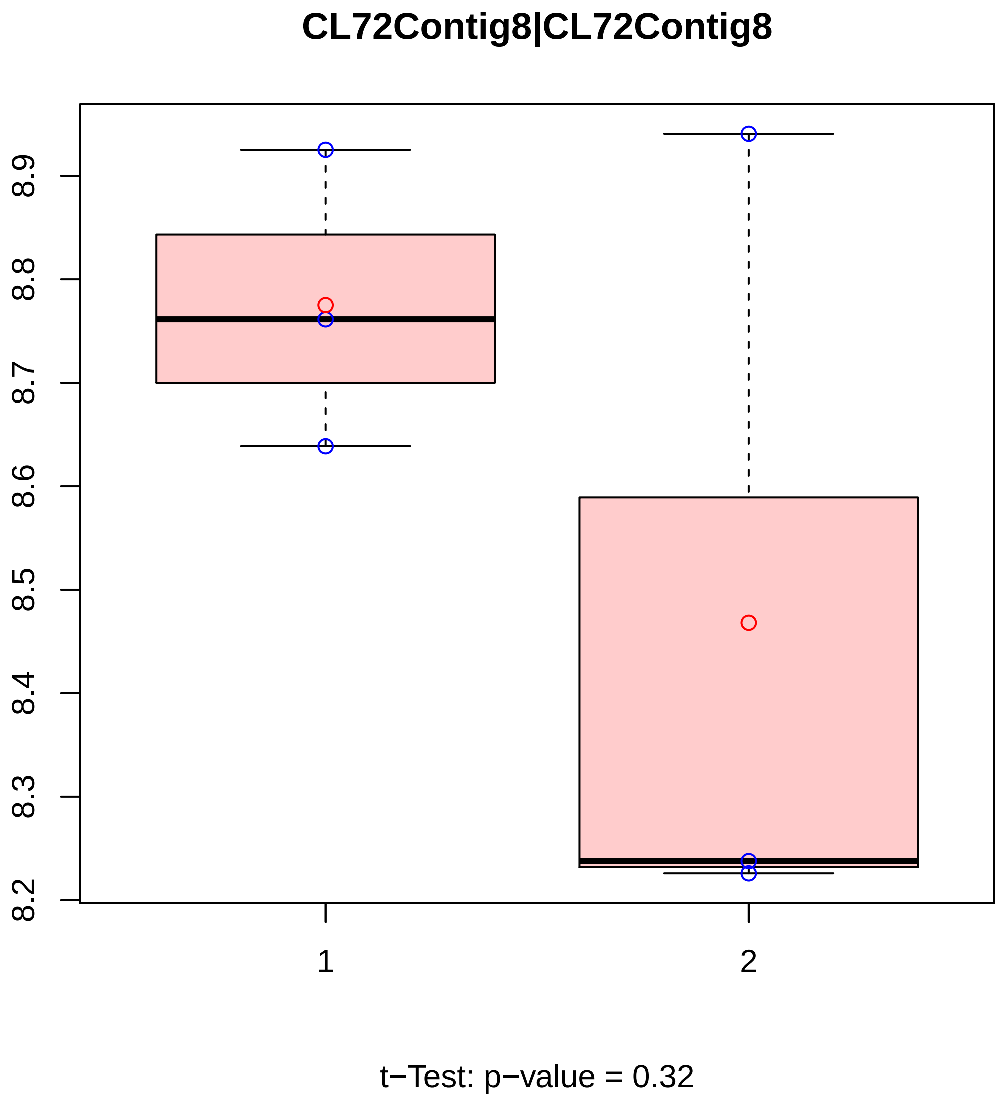

CL72Contig8|CL72Contig8
8.2
8.3
8.4
8.5
8.6
8.7
8.8
8.9
1
2
t−Test: p−value = 0.32

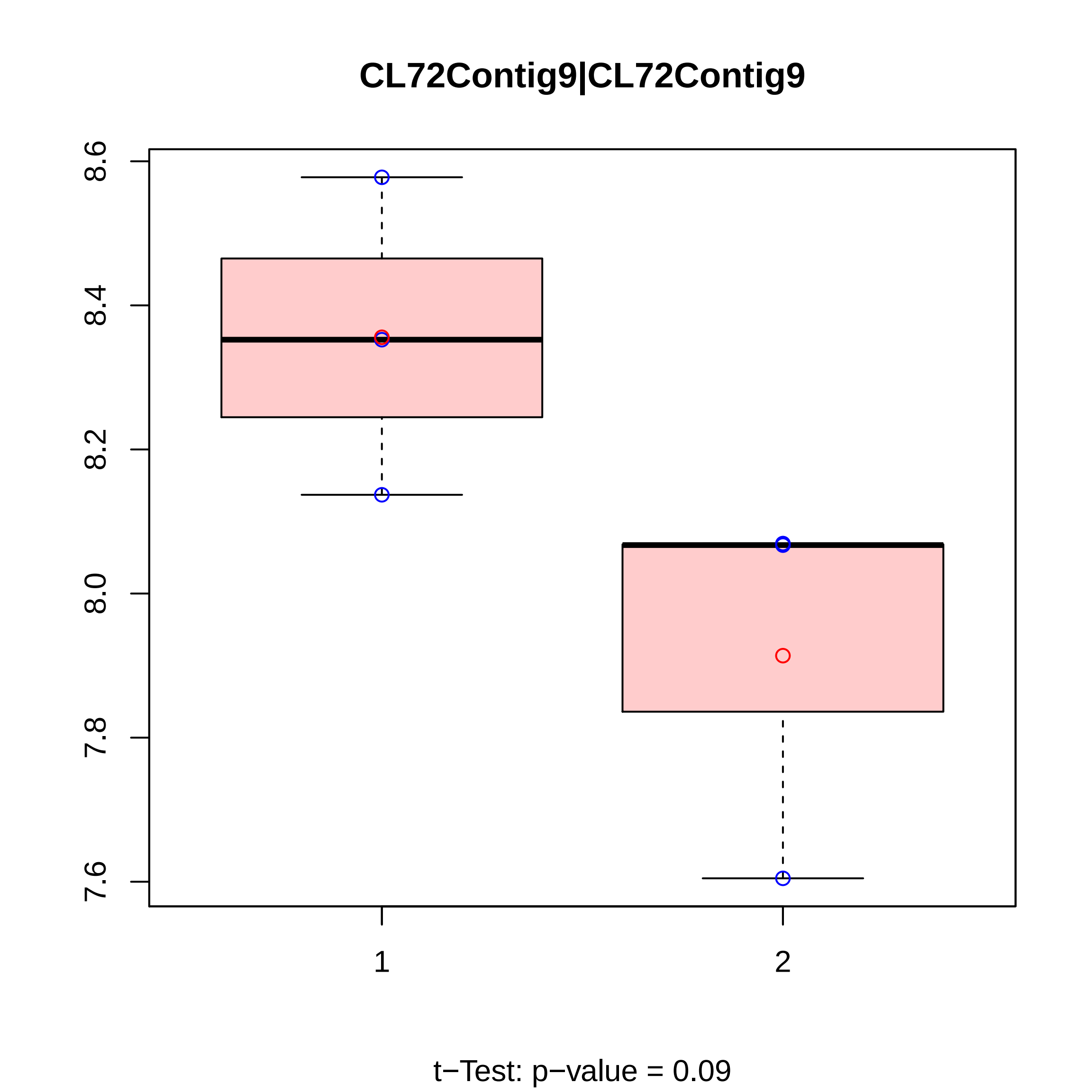
CL72Contig9|CL72Contig9
8.6
8.4
8.2
8.0
7.8
7.6
1
2
t−Test: p−value = 0.09

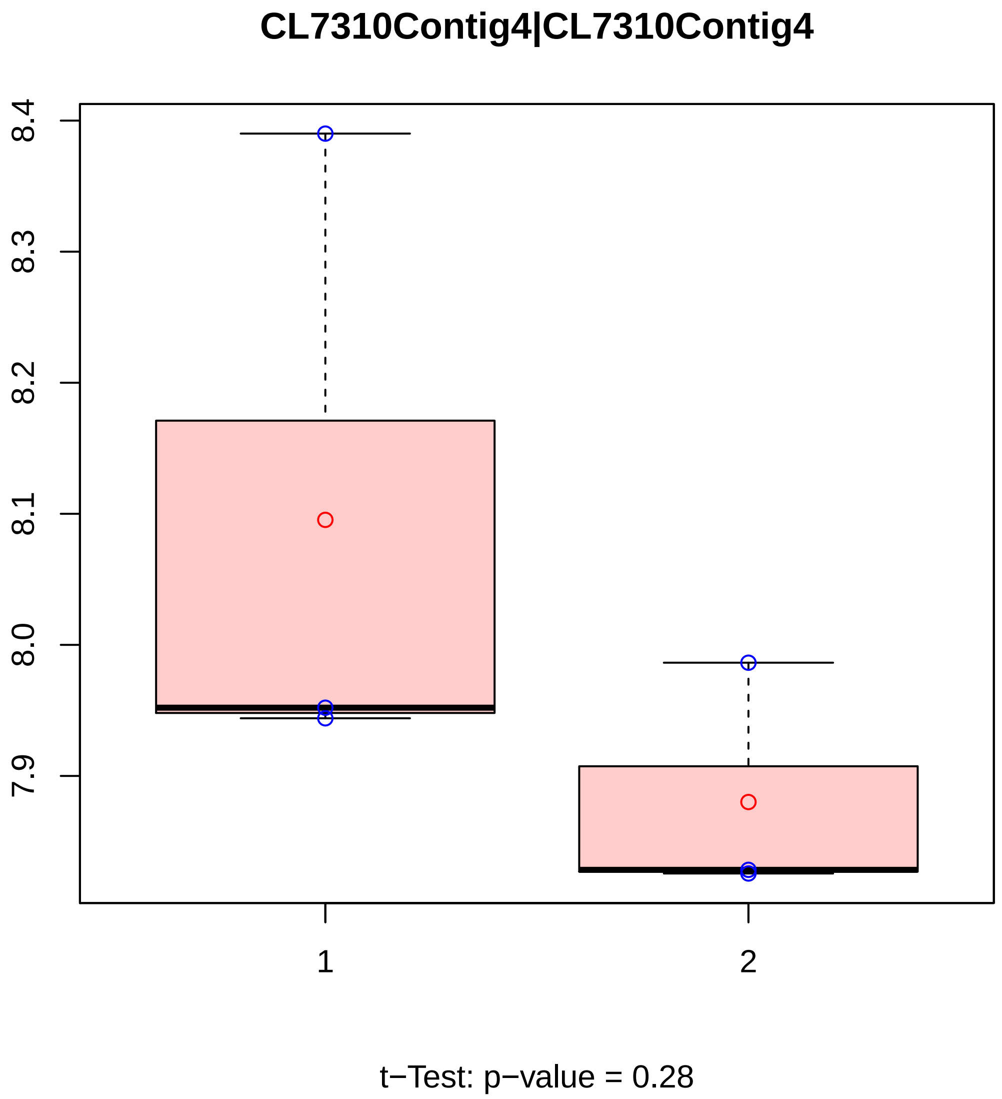

CL7310Contig4|CL7310Contig4
8.4
8.3
8.2
8.1
8.0
7.9
1
2
t−Test: p−value = 0.28

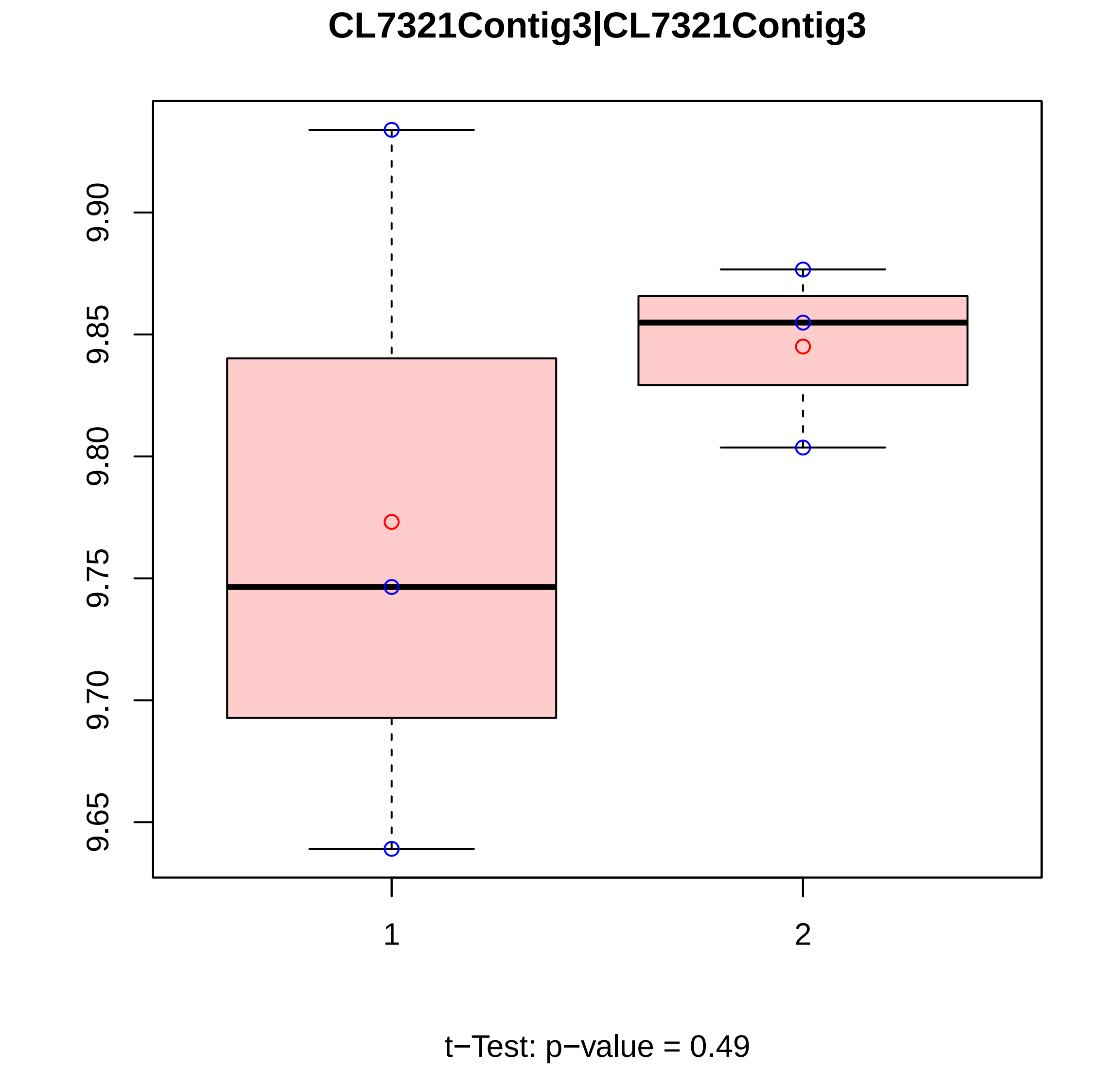

CL7321Contig3|CL7321Contig3
9.65
9.70
9.75
9.80
9.85
9.90
1
2
t−Test: p−value = 0.49

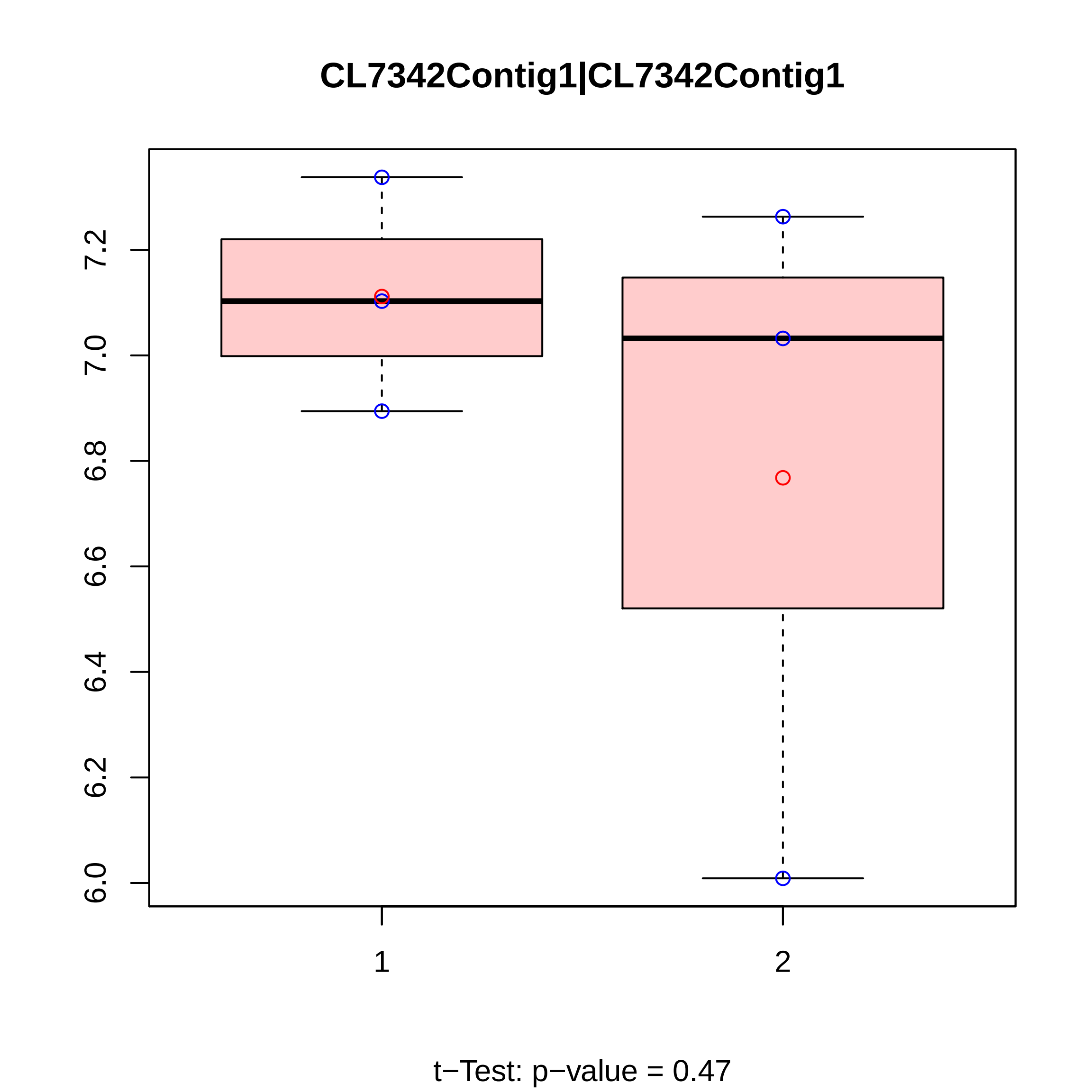
CL7342Contig1|CL7342Contig1
7.2
7.0
6.8
6.6
6.4
6.2
6.0
1
2
t−Test: p−value = 0.47

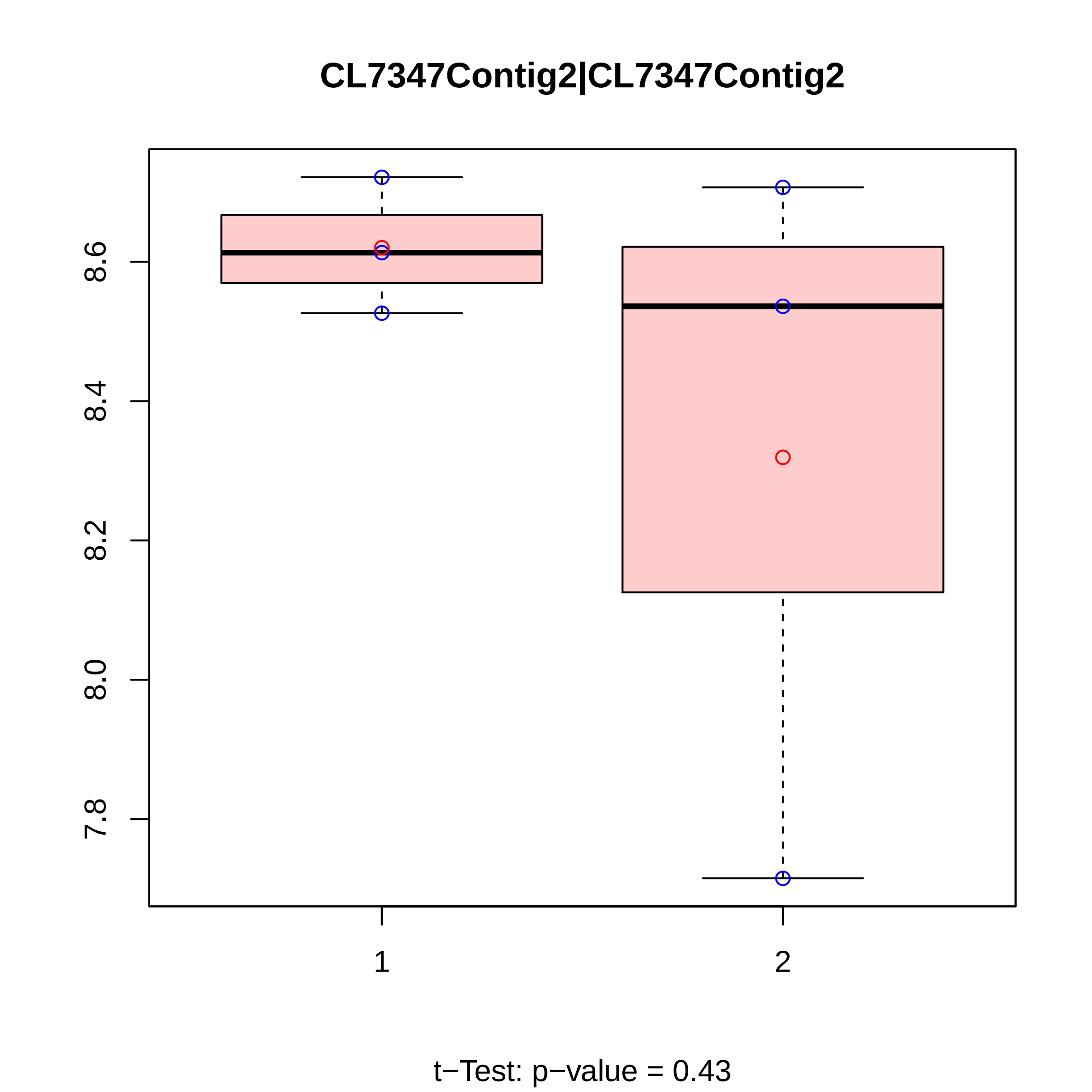

CL7347Contig2|CL7347Contig2
7.8
8.0
8.2
8.4
8.6
1
2
t−Test: p−value = 0.43

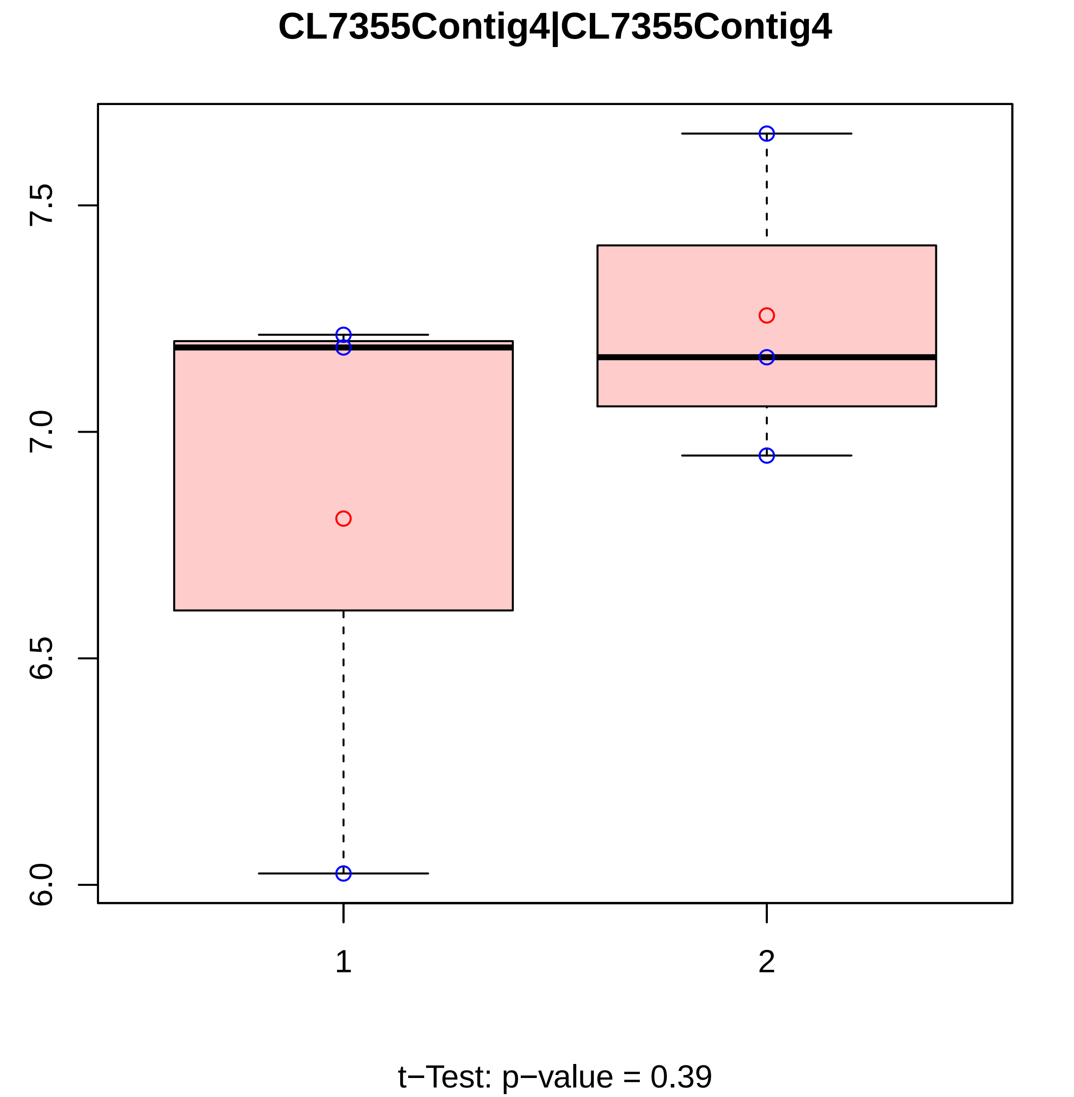
CL7355Contig4|CL7355Contig4
7.5
7.0
6.5
6.0
1
2
t−Test: p−value = 0.39

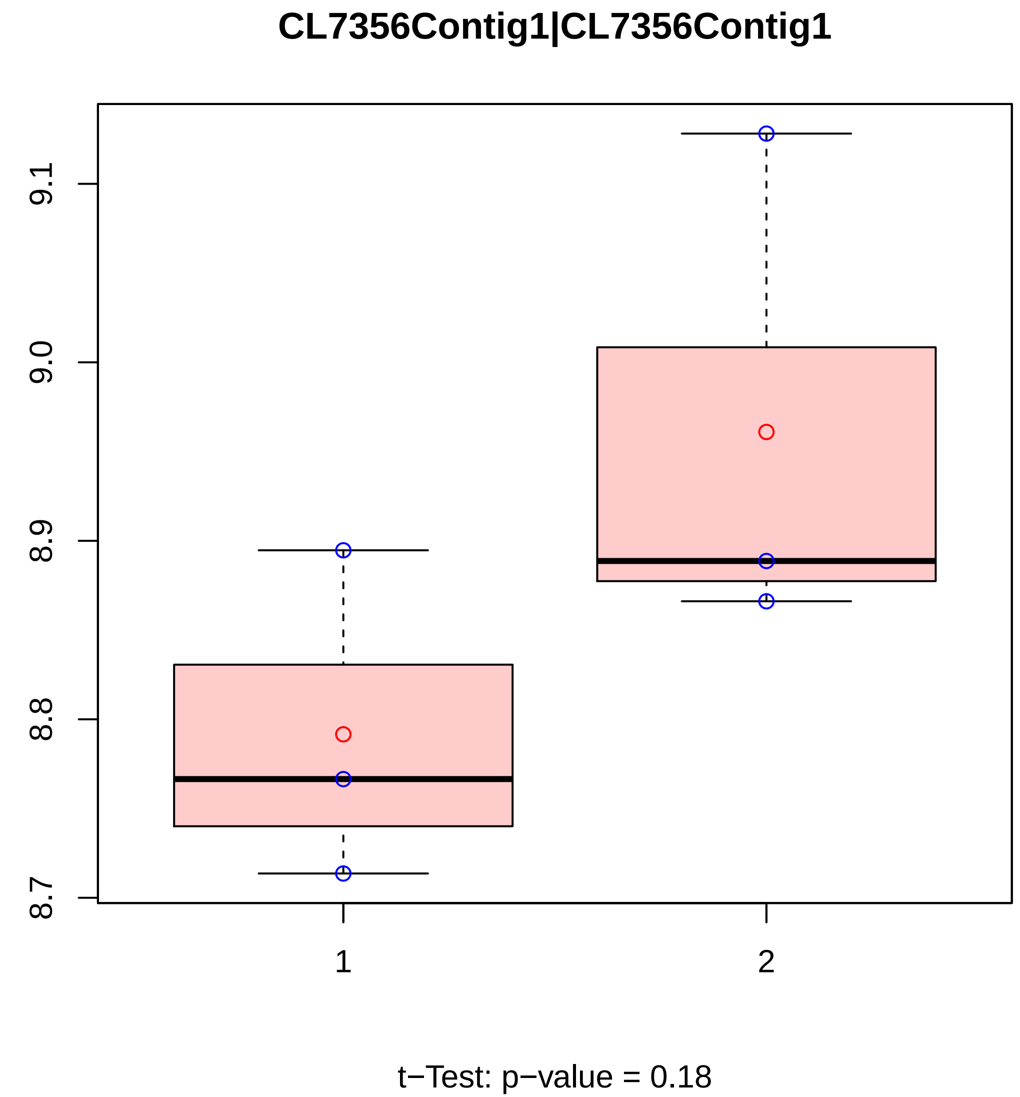
CL7356Contig1|CL7356Contig1
9.1
9.0
8.9
8.8
8.7
1
2
t−Test: p−value = 0.18

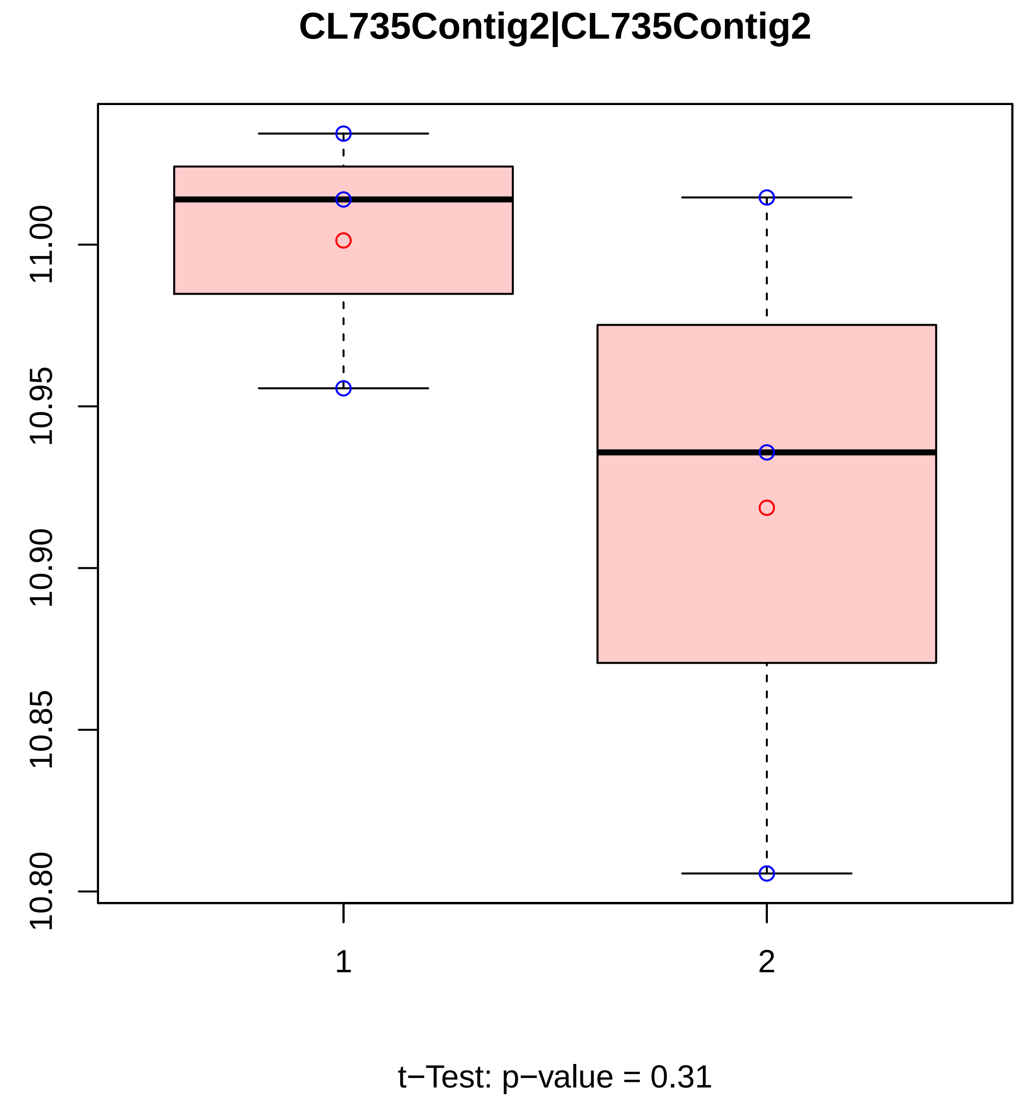

CL735Contig2|CL735Contig2
11.00
10.95
10.90
10.85
10.80
1
2
t−Test: p−value = 0.31

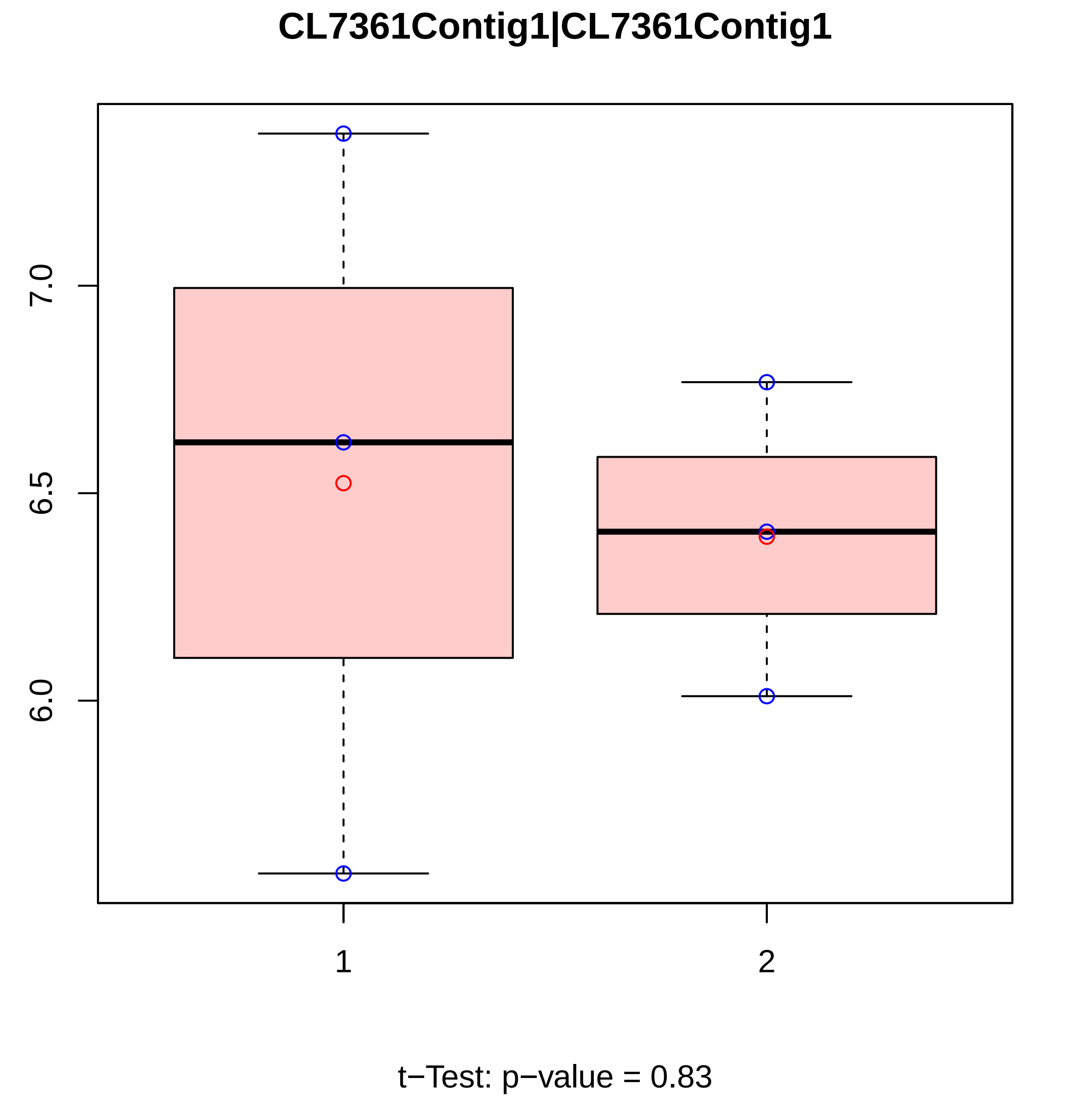
CL7361Contig1|CL7361Contig1
7.0
6.5
6.0
1
2
t−Test: p−value = 0.83

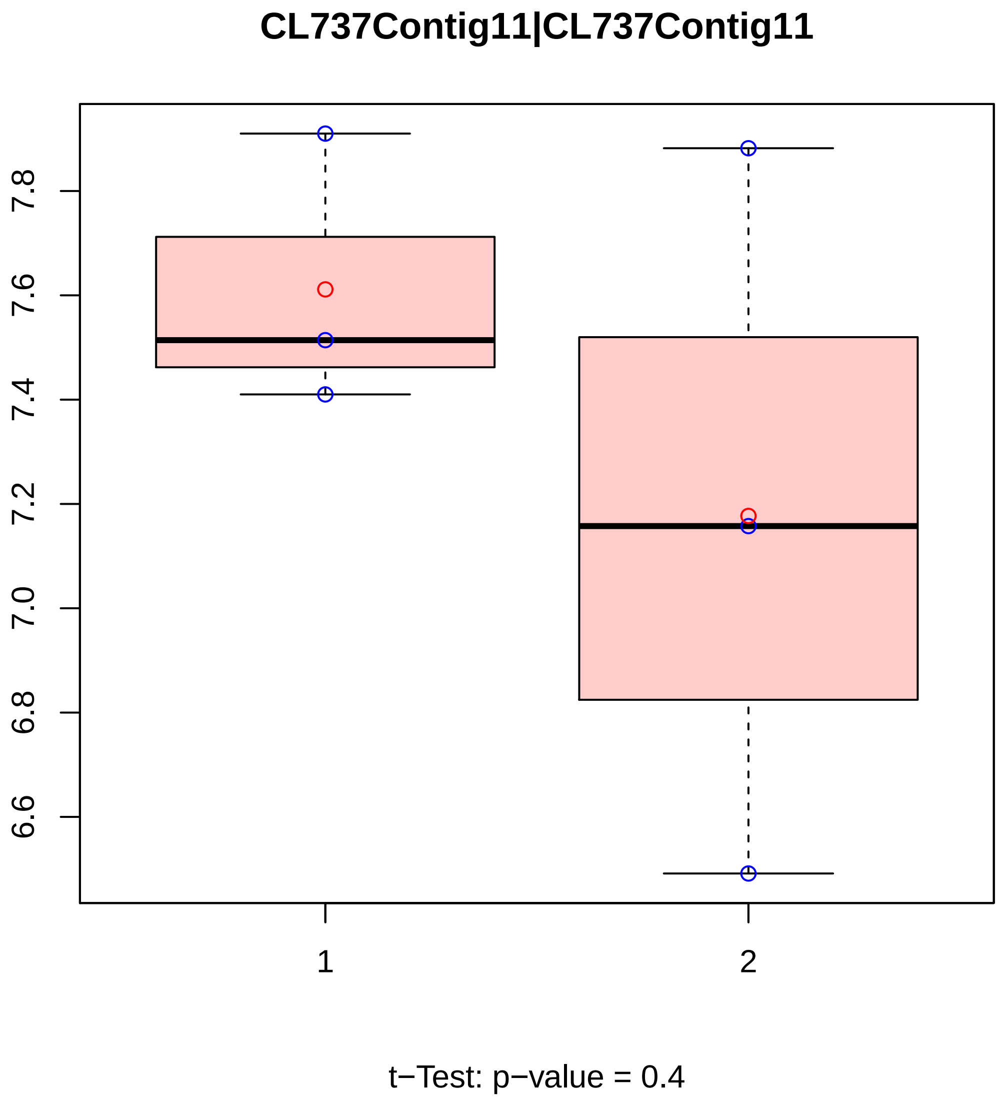
CL737Contig11|CL737Contig11
7.8
7.6
7.4
7.2
7.0
6.8
6.6
1
2
t−Test: p−value = 0.4

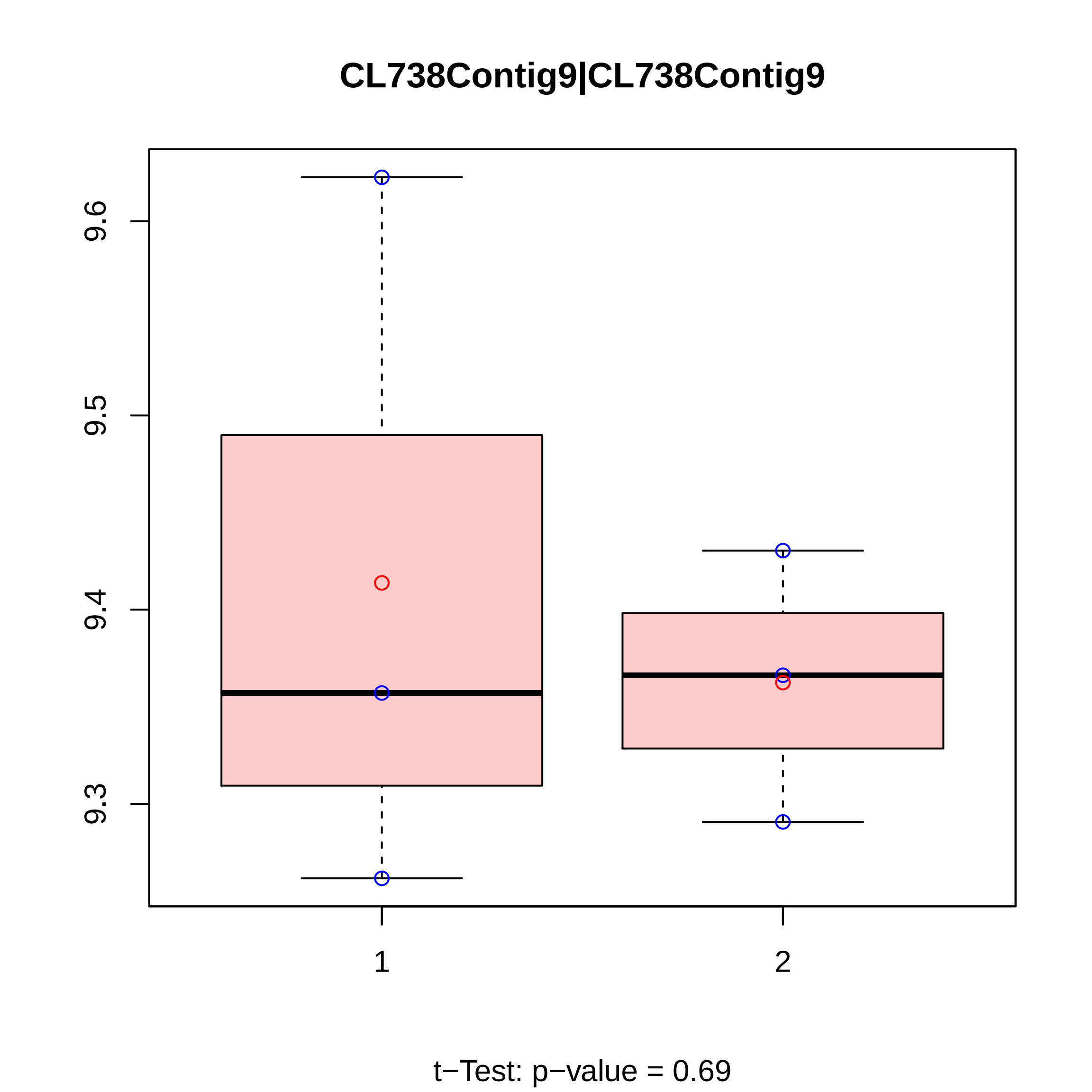

CL738Contig9|CL738Contig9
9.3
9.4
9.5
9.6
1
2
t−Test: p−value = 0.69

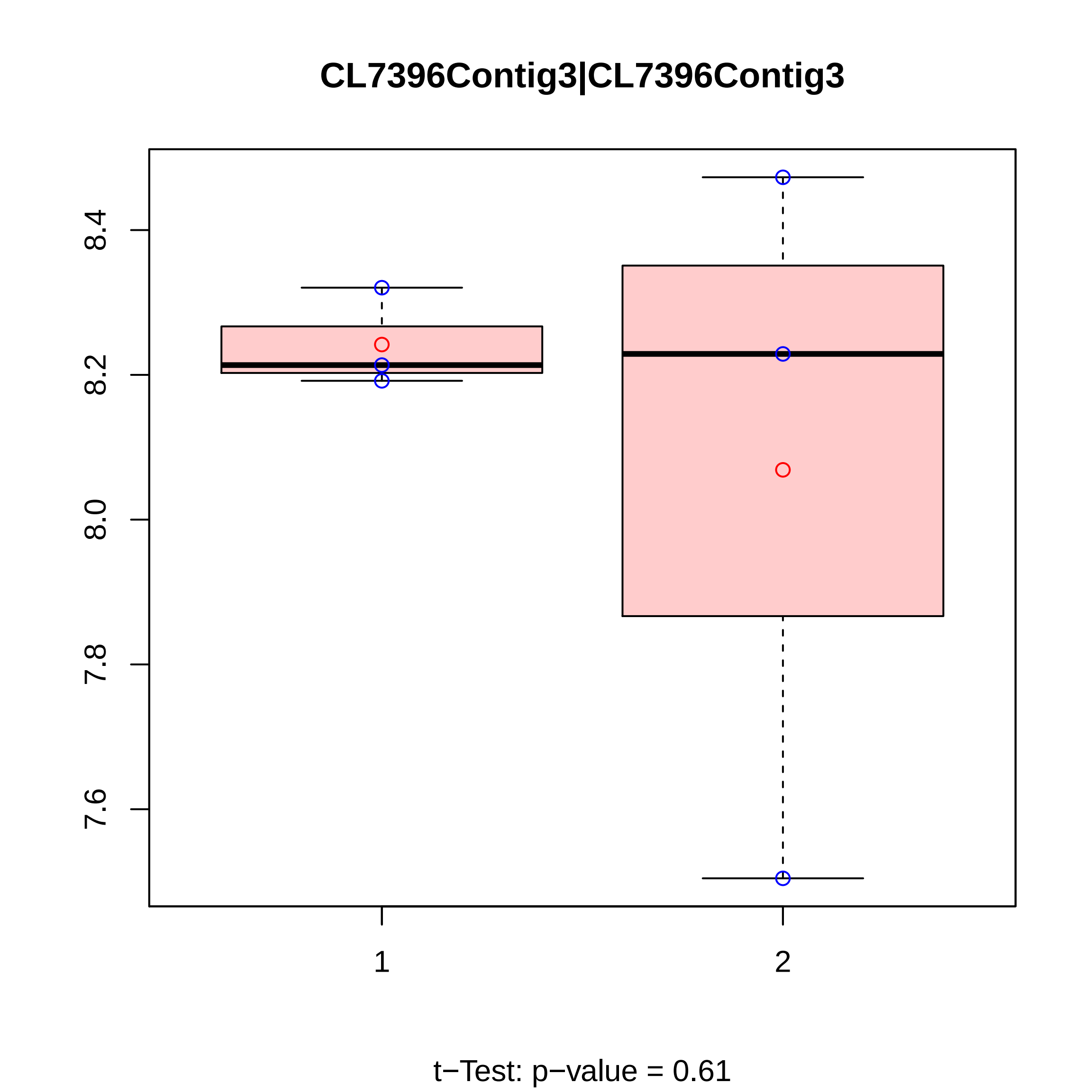

CL7396Contig3|CL7396Contig3
8.4
8.2
8.0
7.8
7.6
1
2
t−Test: p−value = 0.61

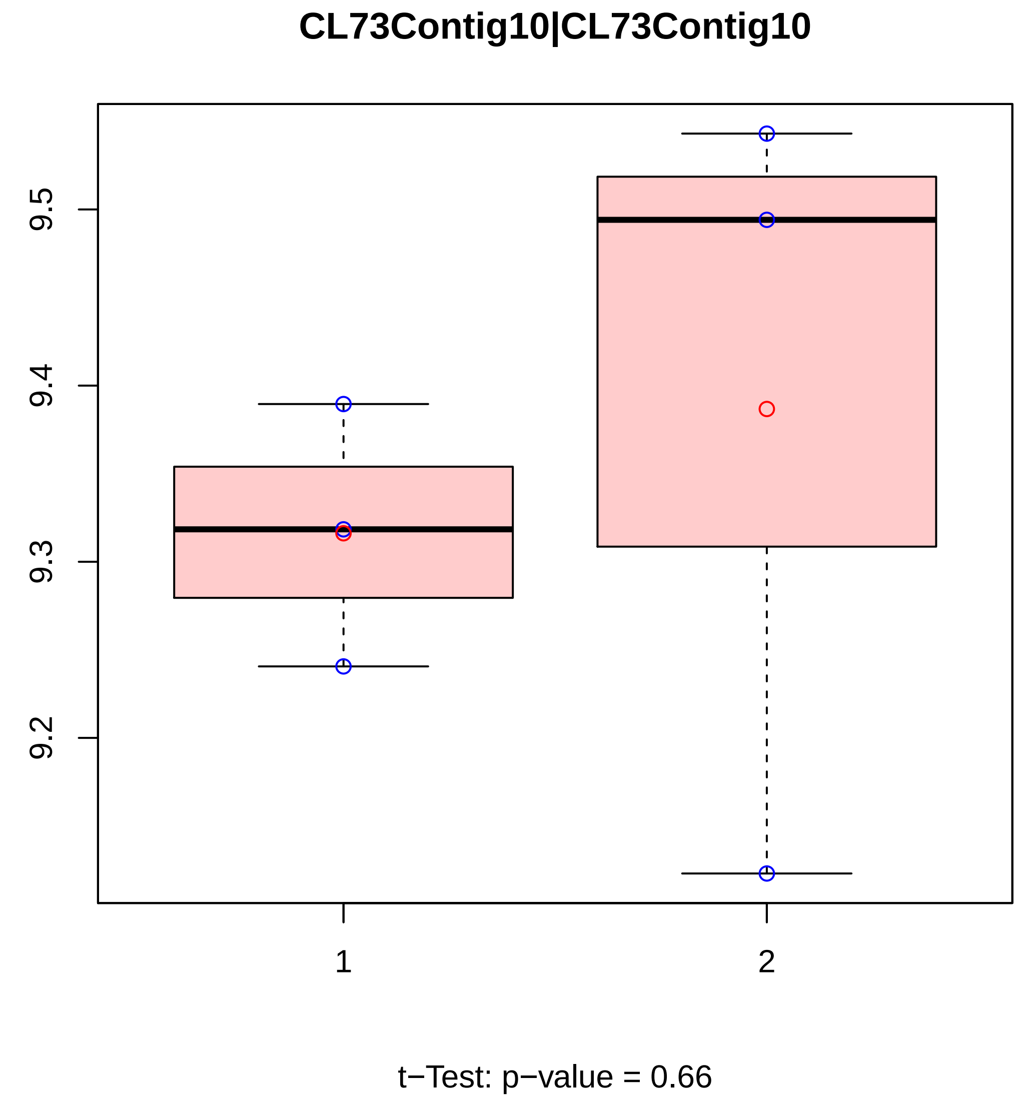
CL73Contig10|CL73Contig10
9.2
9.3
9.4
9.5
1
2
t−Test: p−value = 0.66

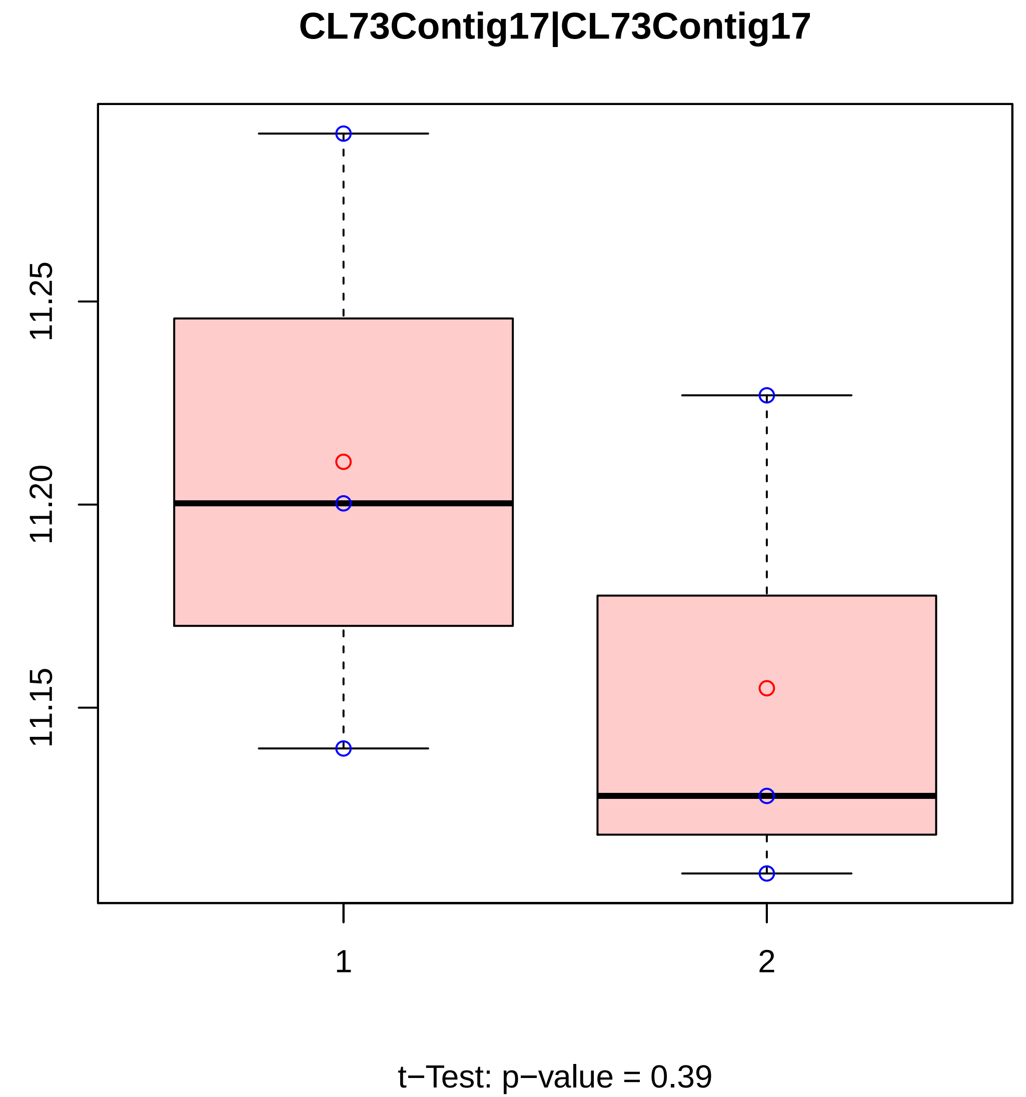
CL73Contig17|CL73Contig17
11.15
11.20
11.25
1
2
t−Test: p−value = 0.39

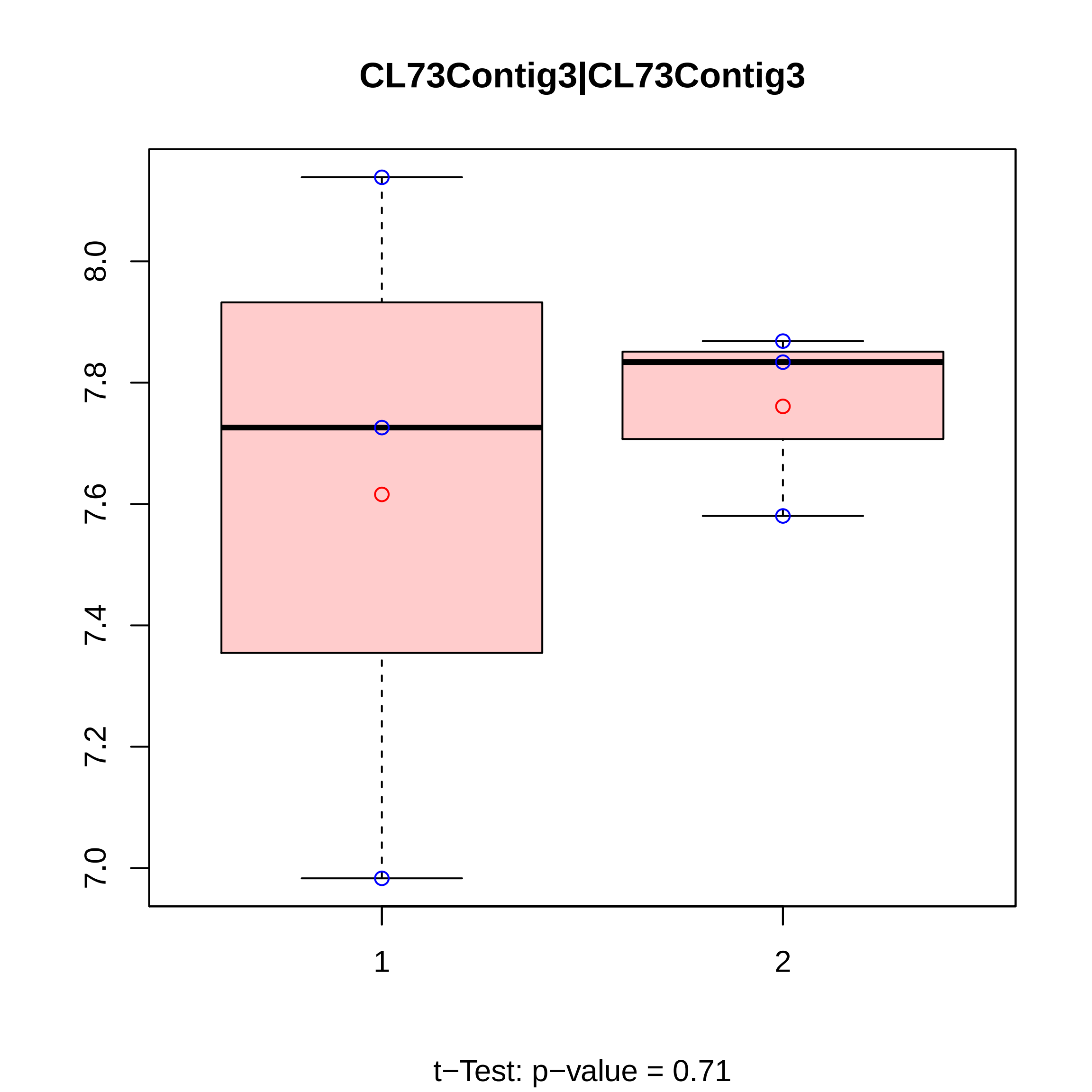
CL73Contig3|CL73Contig3
8.0
7.8
7.6
7.4
7.2
7.0
1
2
t−Test: p−value = 0.71

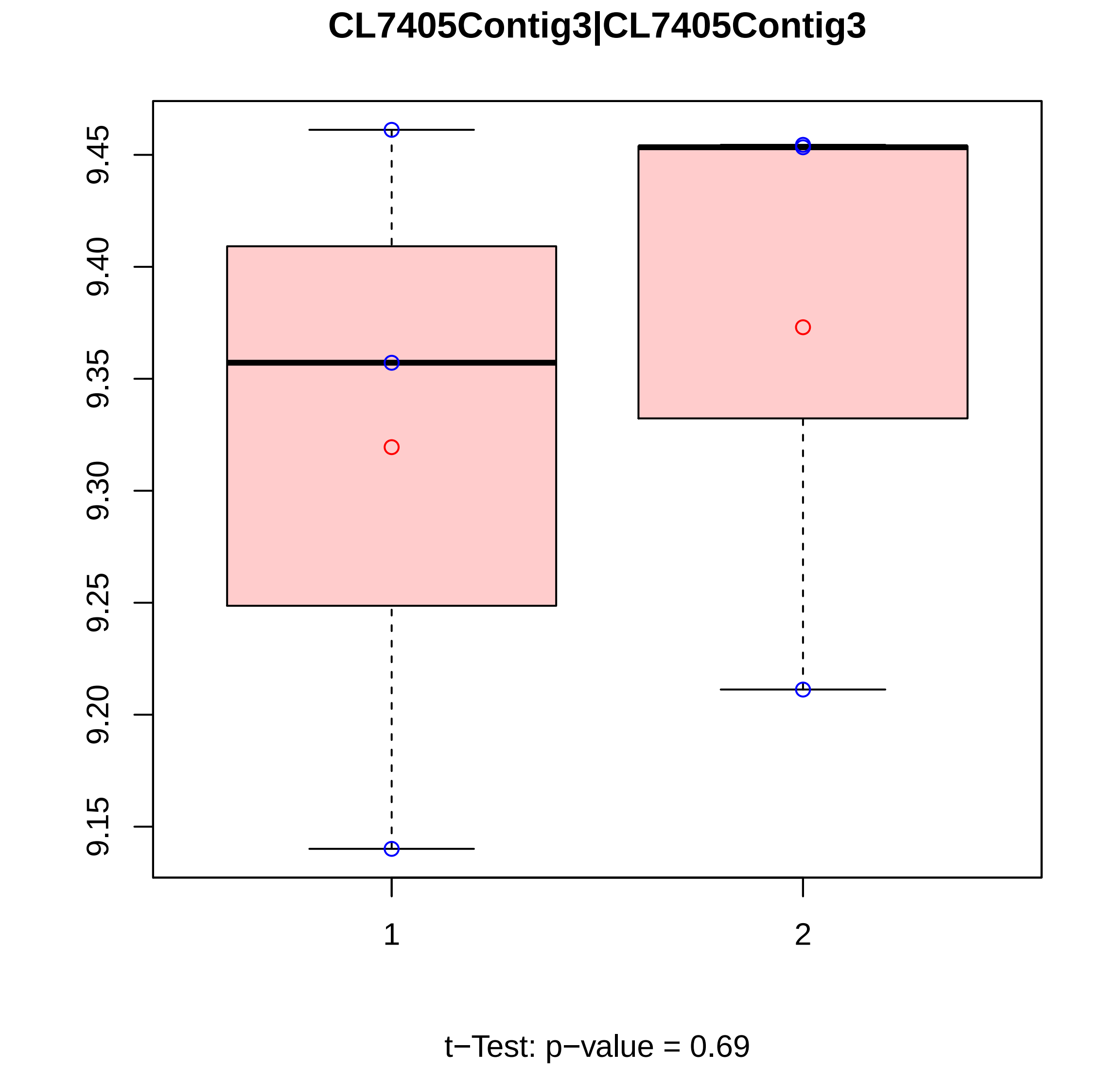
CL7405Contig3|CL7405Contig3
9.15
9.20
9.25
9.30
9.35
9.40
9.45
1
2
t−Test: p−value = 0.69

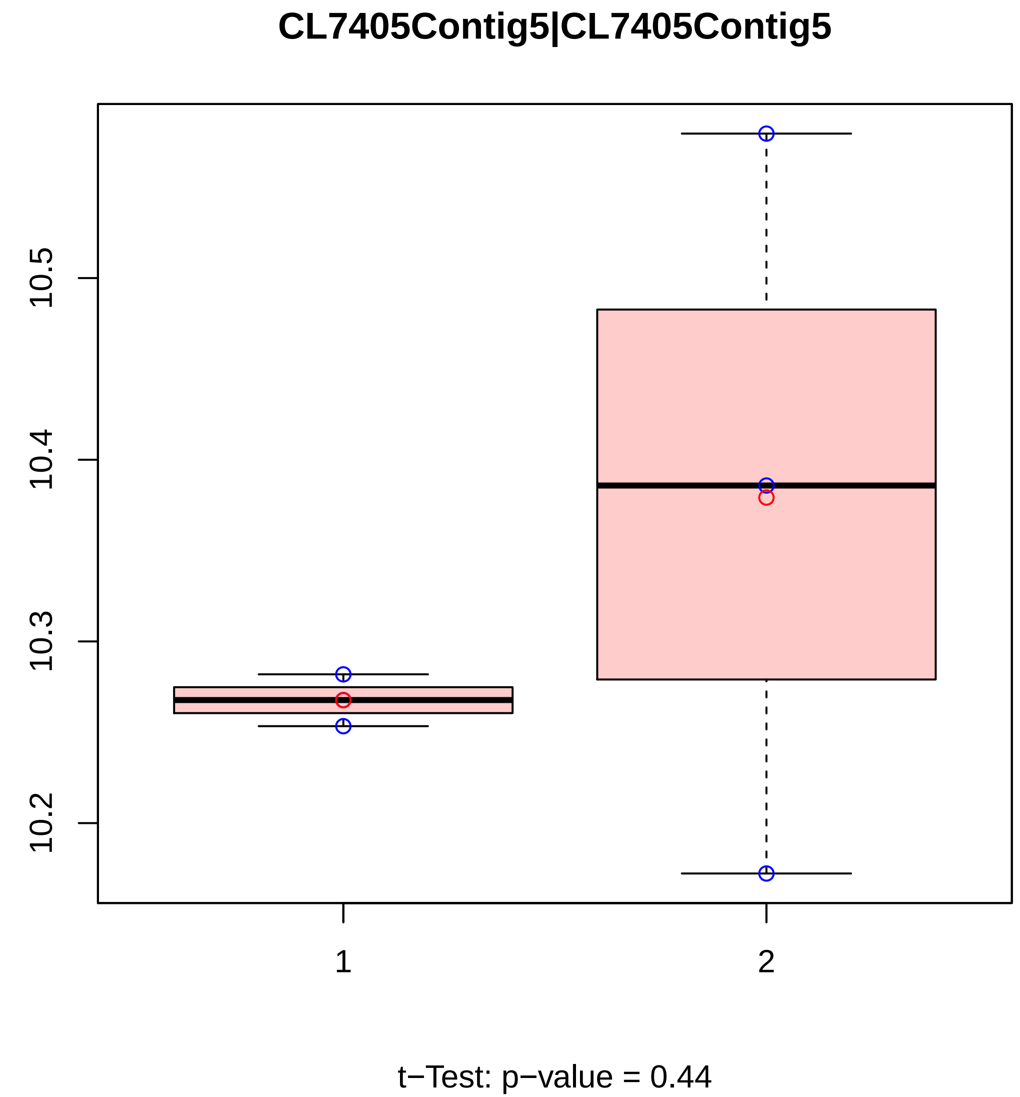

CL7405Contig5|CL7405Contig5
10.2
10.3
10.4
10.5
1
2
t−Test: p−value = 0.44

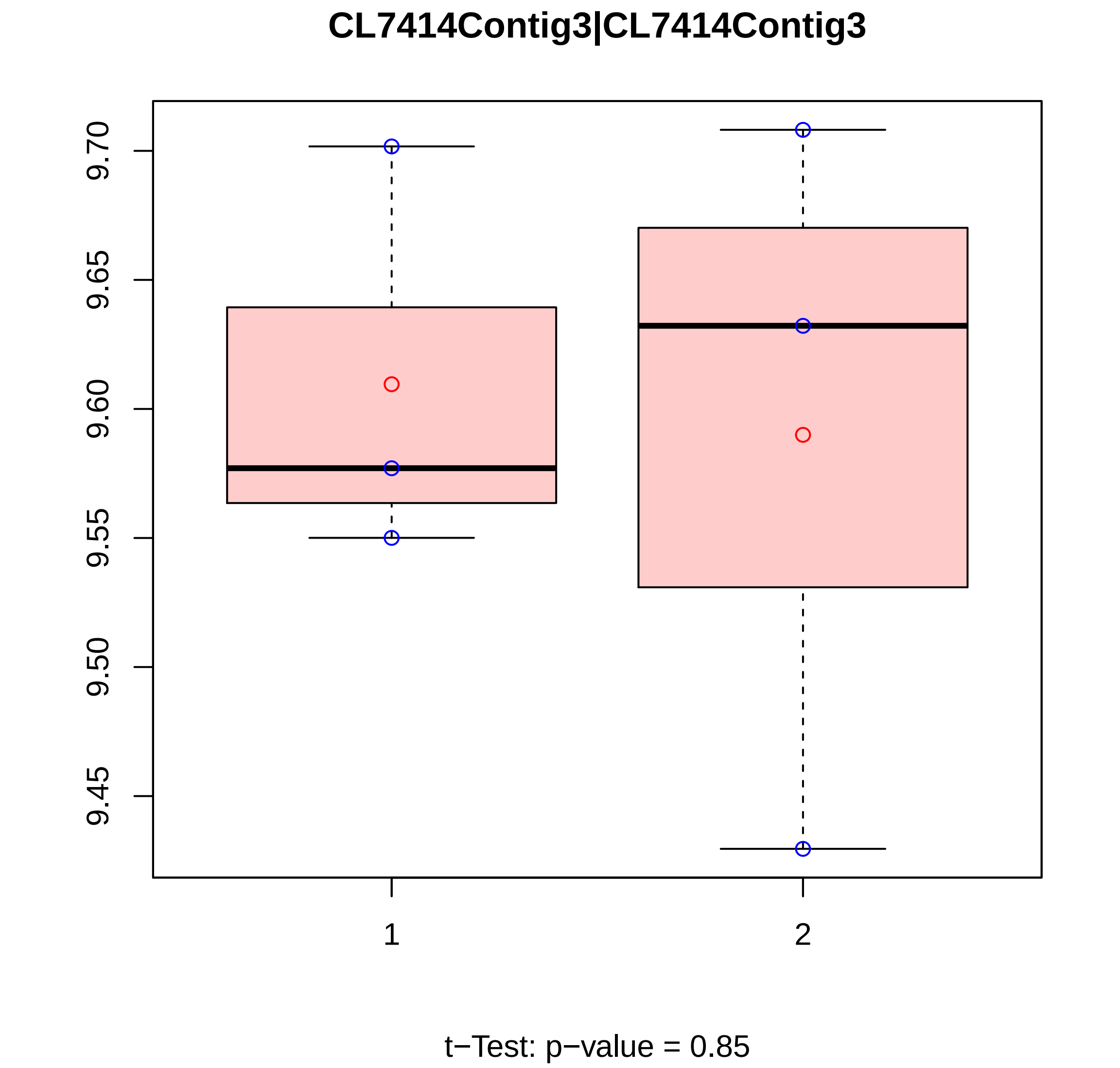
CL7414Contig3|CL7414Contig3
9.70
9.65
9.60
9.55
9.50
9.45
1
2
t−Test: p−value = 0.85

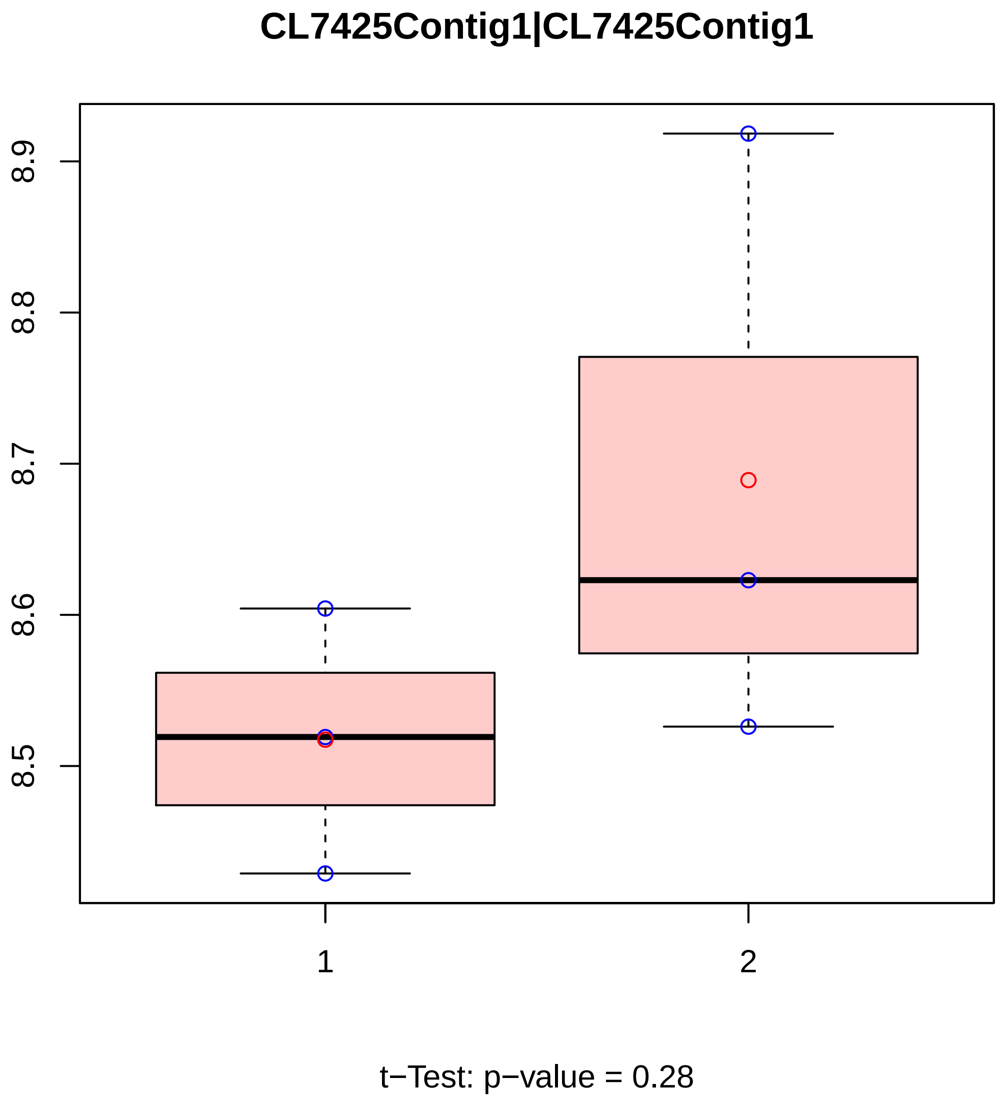

CL7425Contig1|CL7425Contig1
8.5
8.6
8.7
8.8
8.9
1
2
t−Test: p−value = 0.28

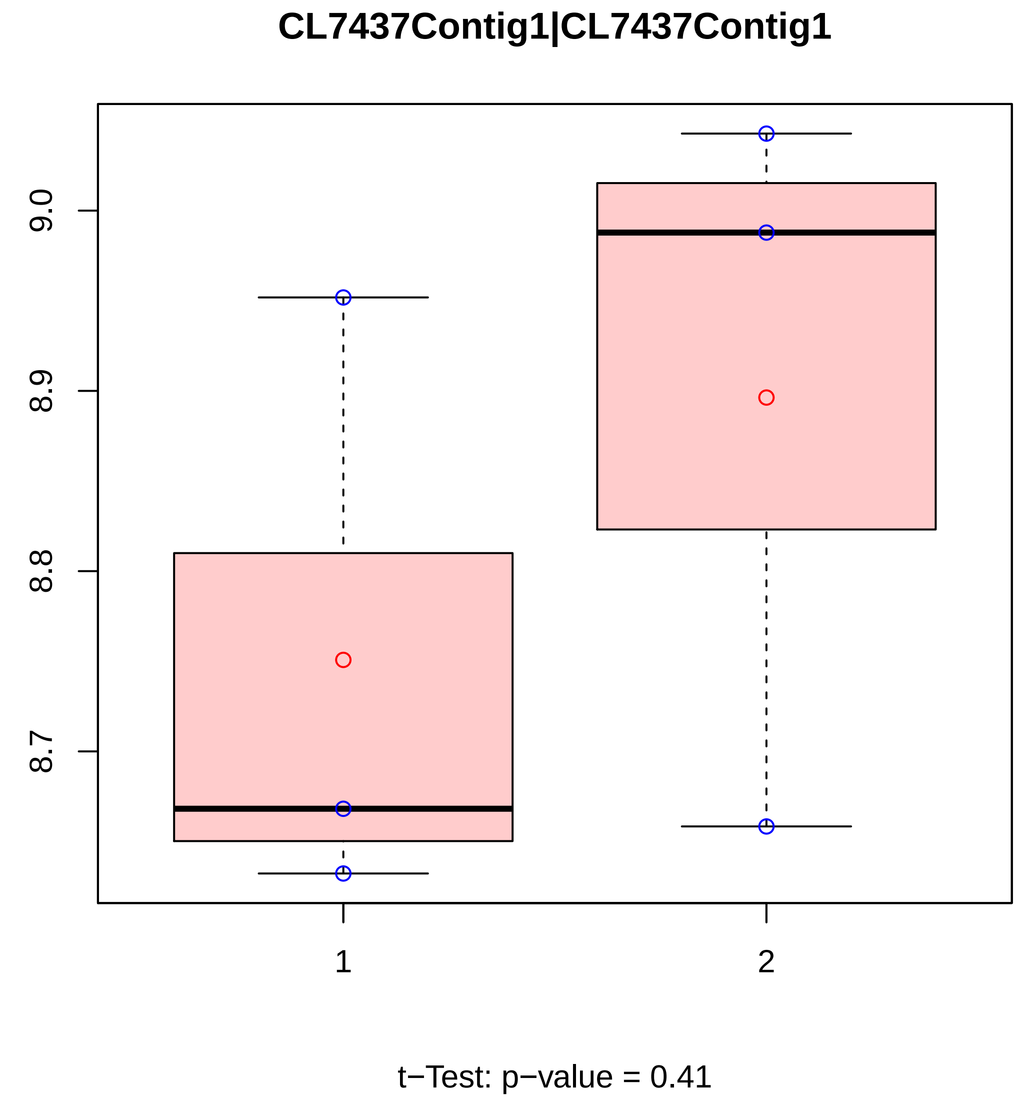
CL7437Contig1|CL7437Contig1
9.0
8.9
8.8
8.7
1
2
t−Test: p−value = 0.41

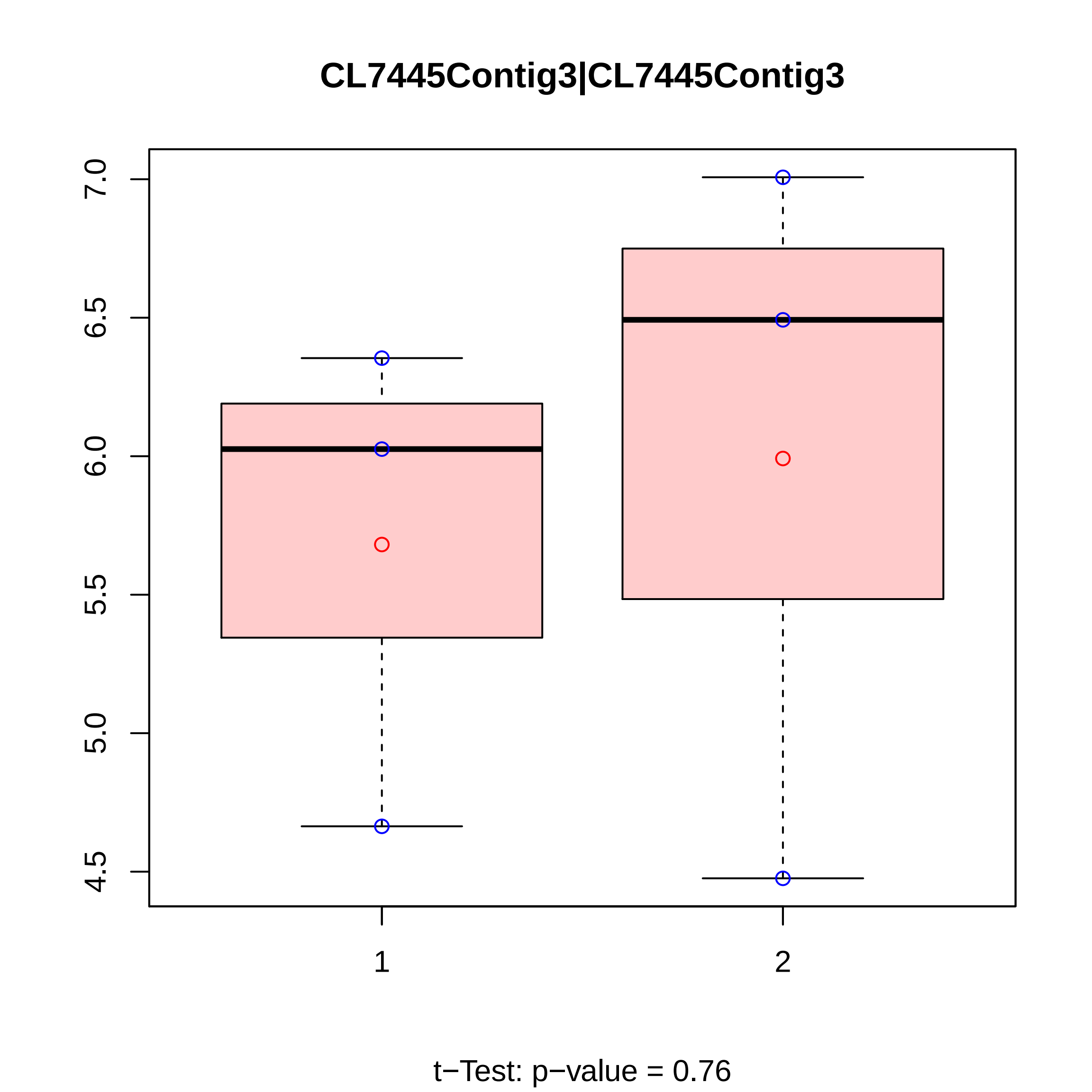

CL7445Contig3|CL7445Contig3
7.0
6.5
6.0
5.5
5.0
4.5
1
2
t−Test: p−value = 0.76

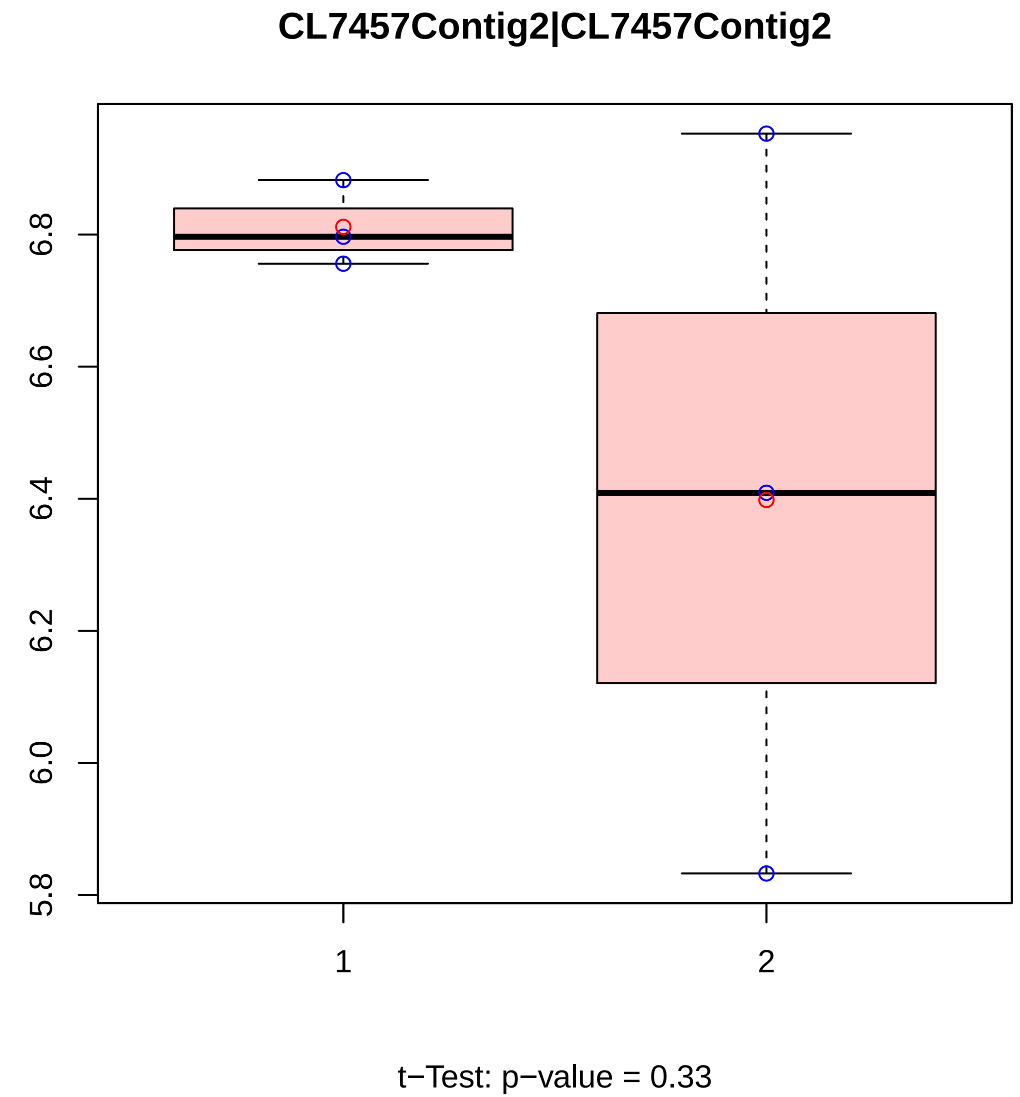
CL7457Contig2|CL7457Contig2
6.8
6.6
6.4
6.2
6.0
5.8
1
2
t−Test: p−value = 0.33

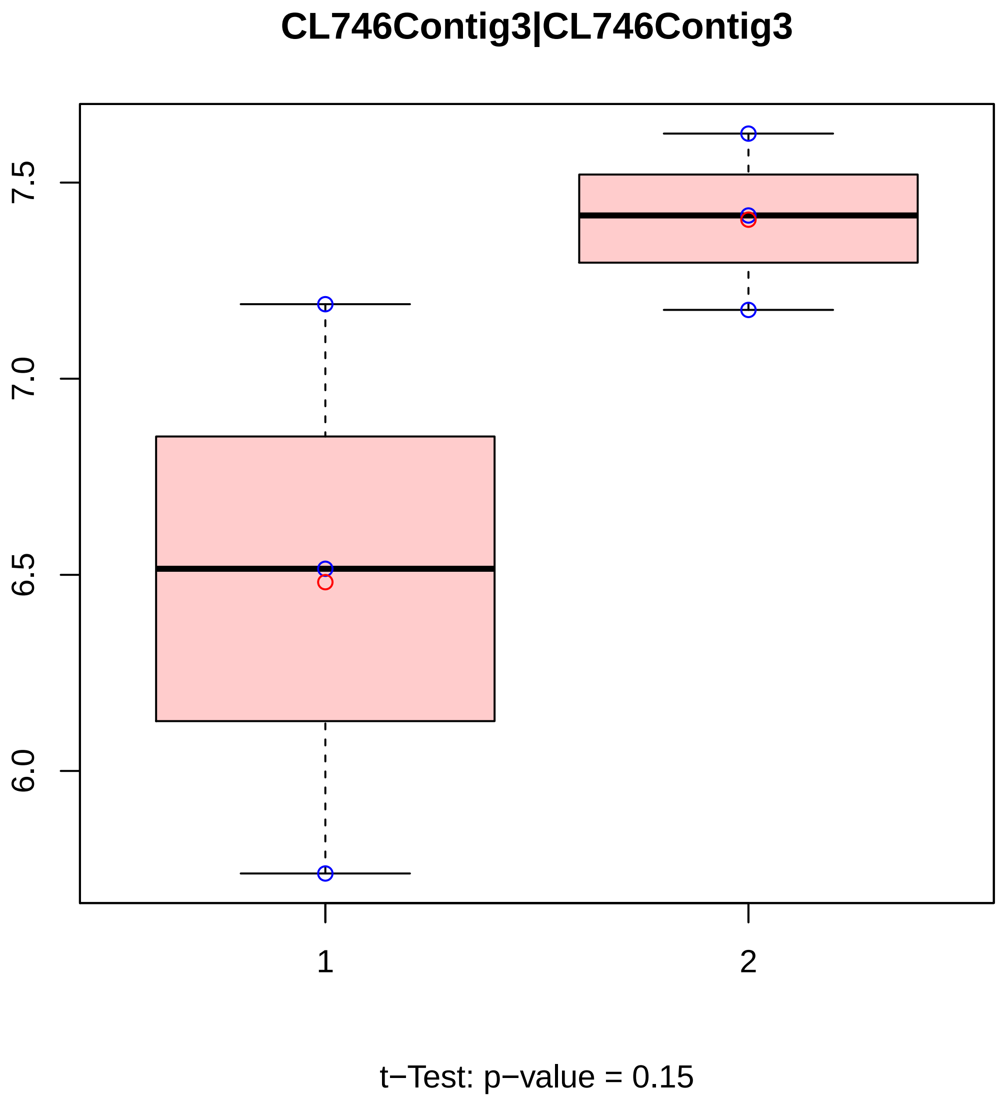

CL746Contig3|CL746Contig3
7.5
7.0
6.5
6.0
1
2
t−Test: p−value = 0.15

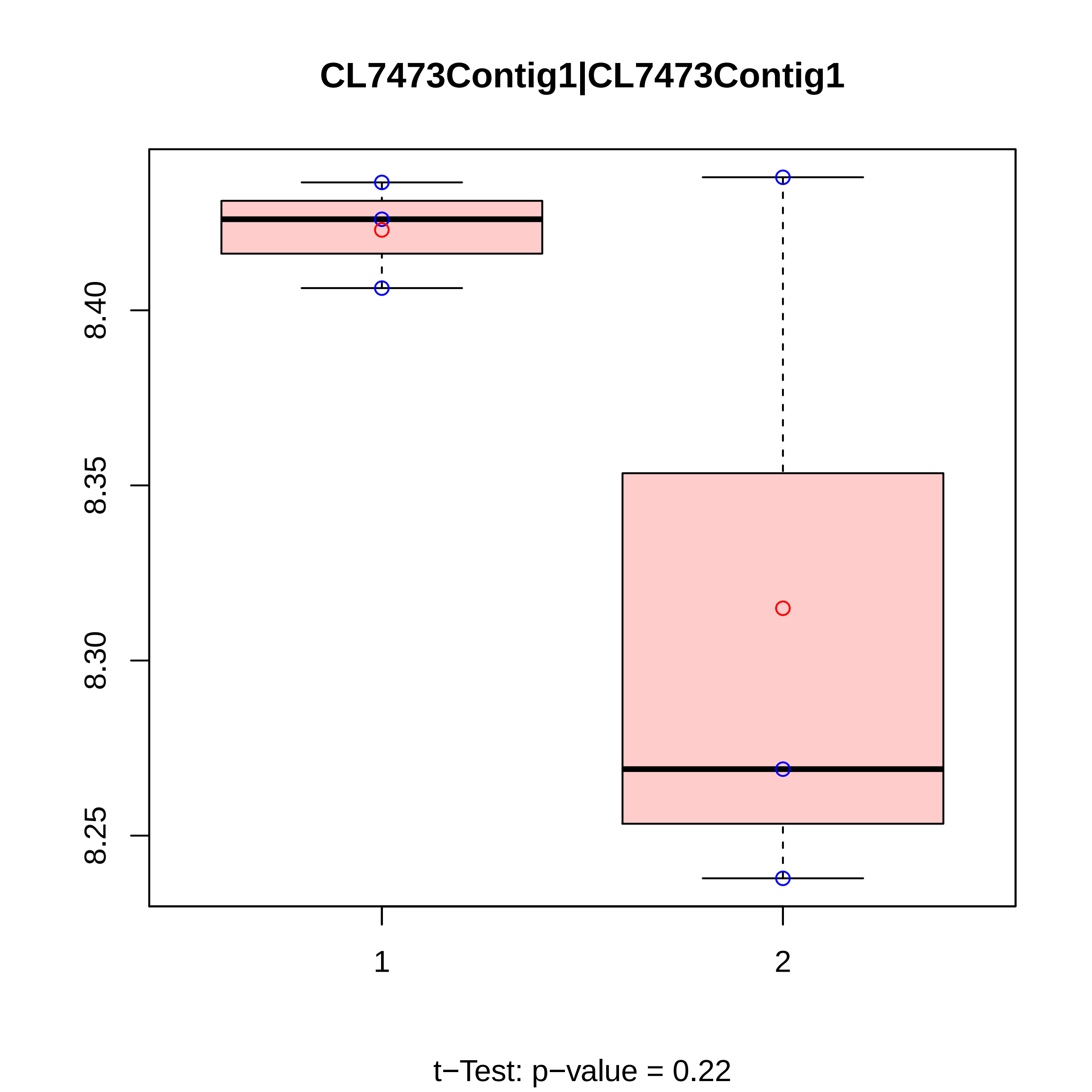
CL7473Contig1|CL7473Contig1
8.25
8.30
8.35
8.40
1
2
t−Test: p−value = 0.22

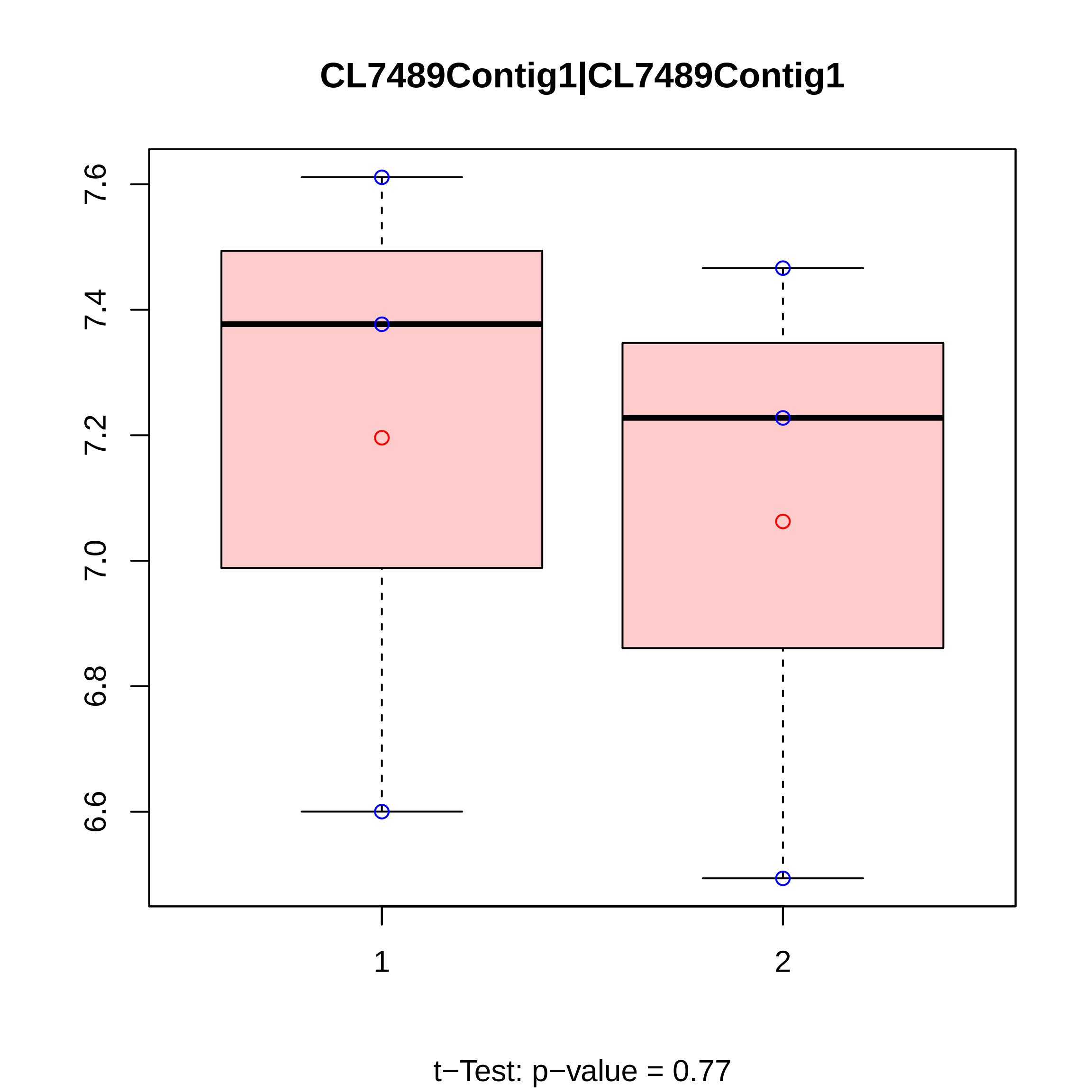
CL7489Contig1|CL7489Contig1
7.6
7.4
7.2
7.0
6.8
6.6
1
2
t−Test: p−value = 0.77

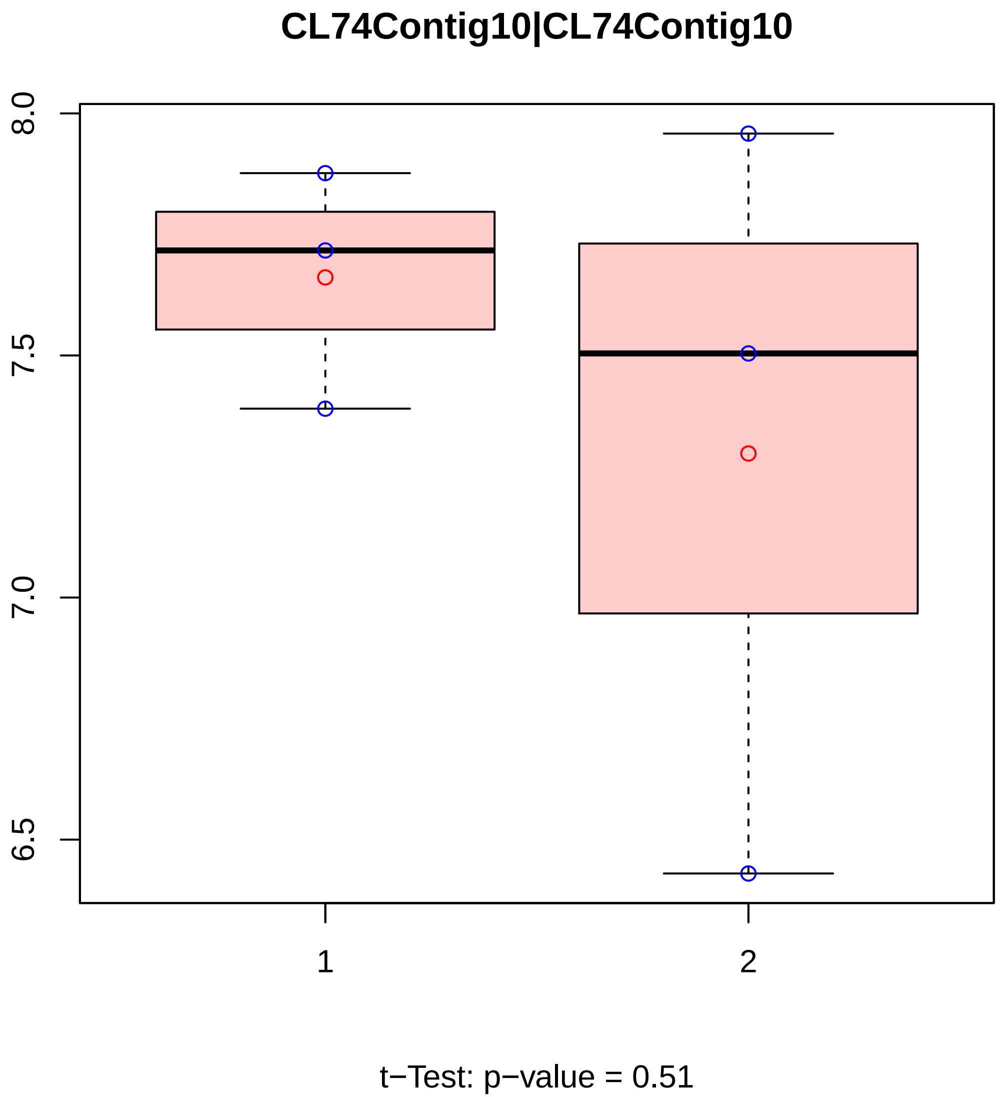
CL74Contig10|CL74Contig10
8.0
7.5
7.0
6.5
1
2
t−Test: p−value = 0.51

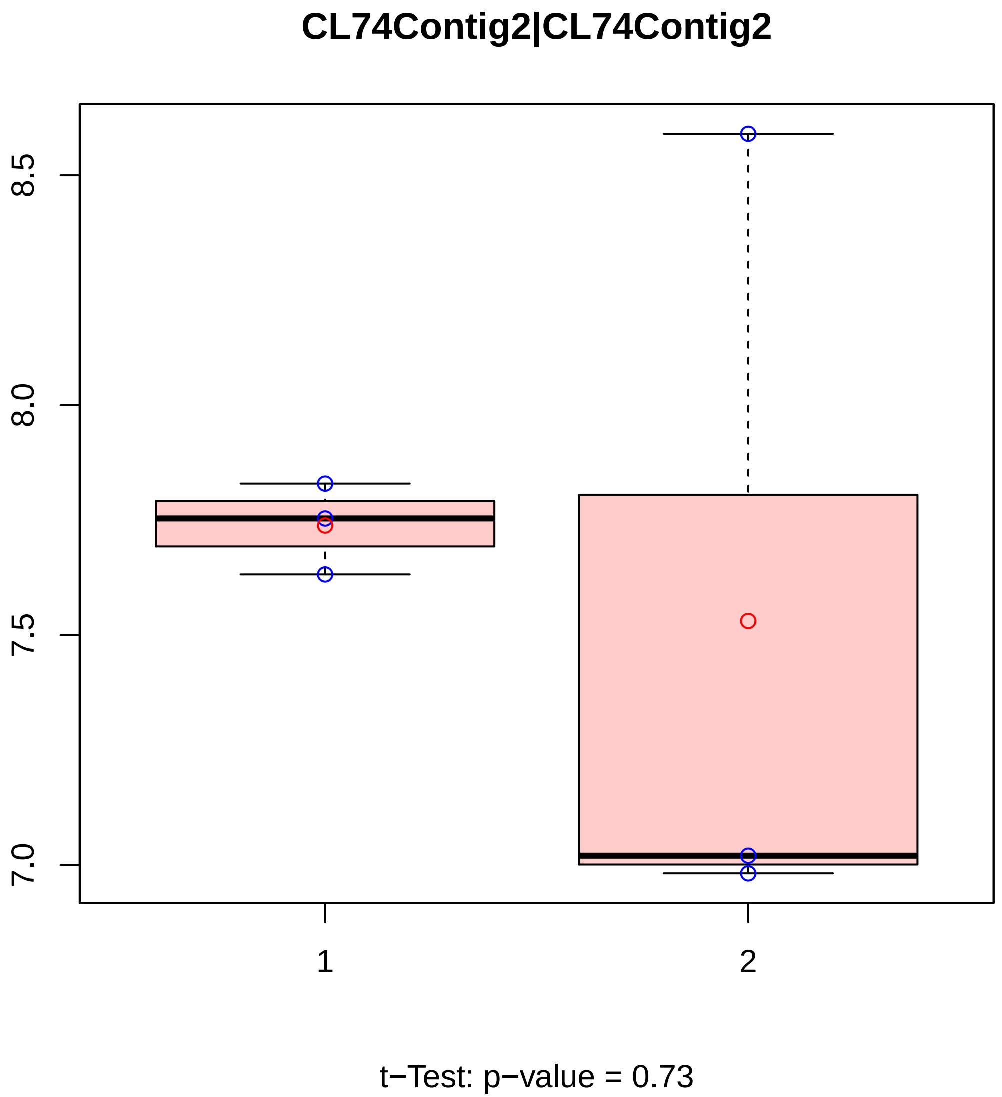

CL74Contig2|CL74Contig2
8.5
8.0
7.5
7.0
1
2
t−Test: p−value = 0.73

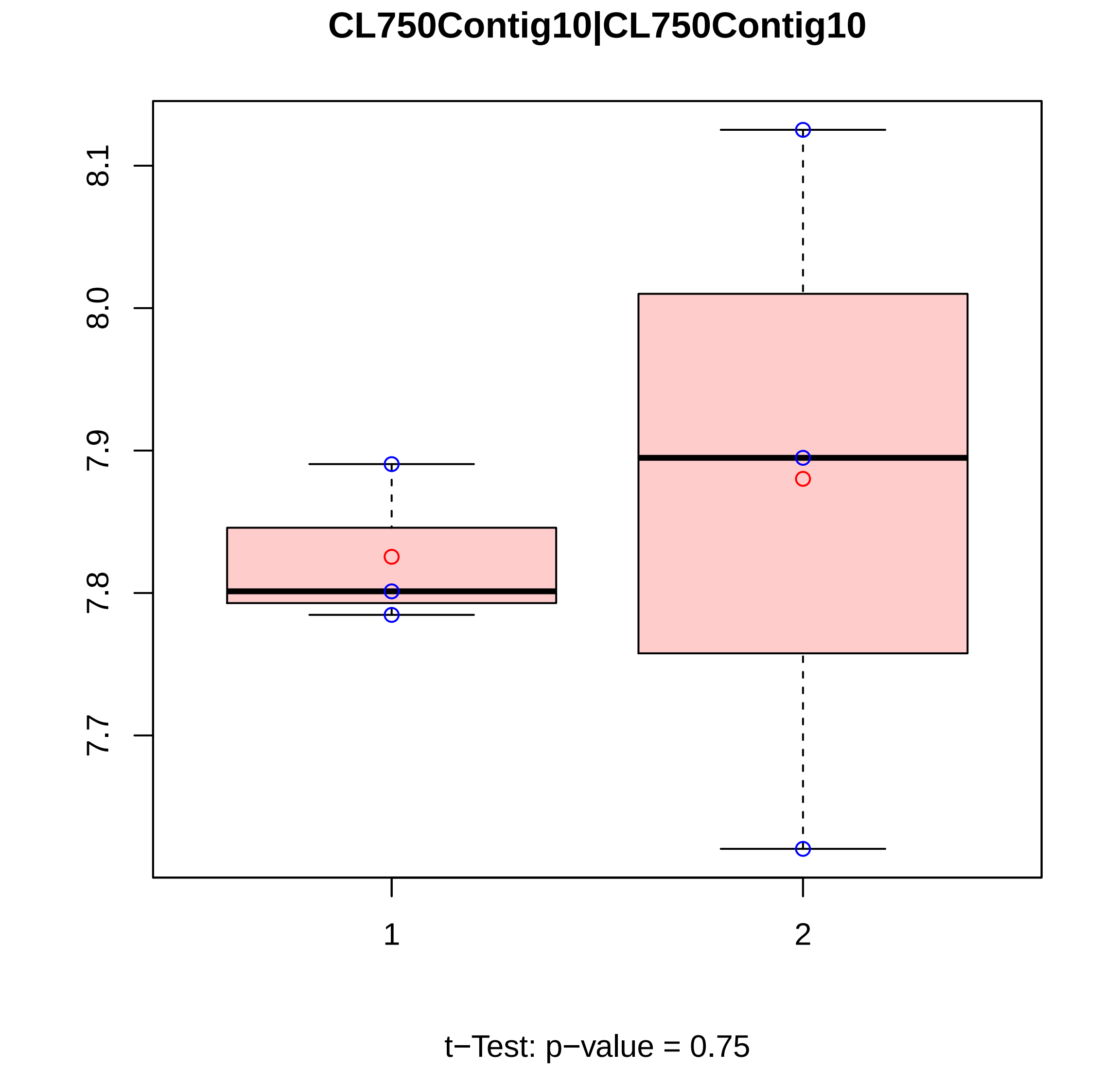
CL750Contig10|CL750Contig10
8.1
8.0
7.9
7.8
7.7
1
2
t−Test: p−value = 0.75

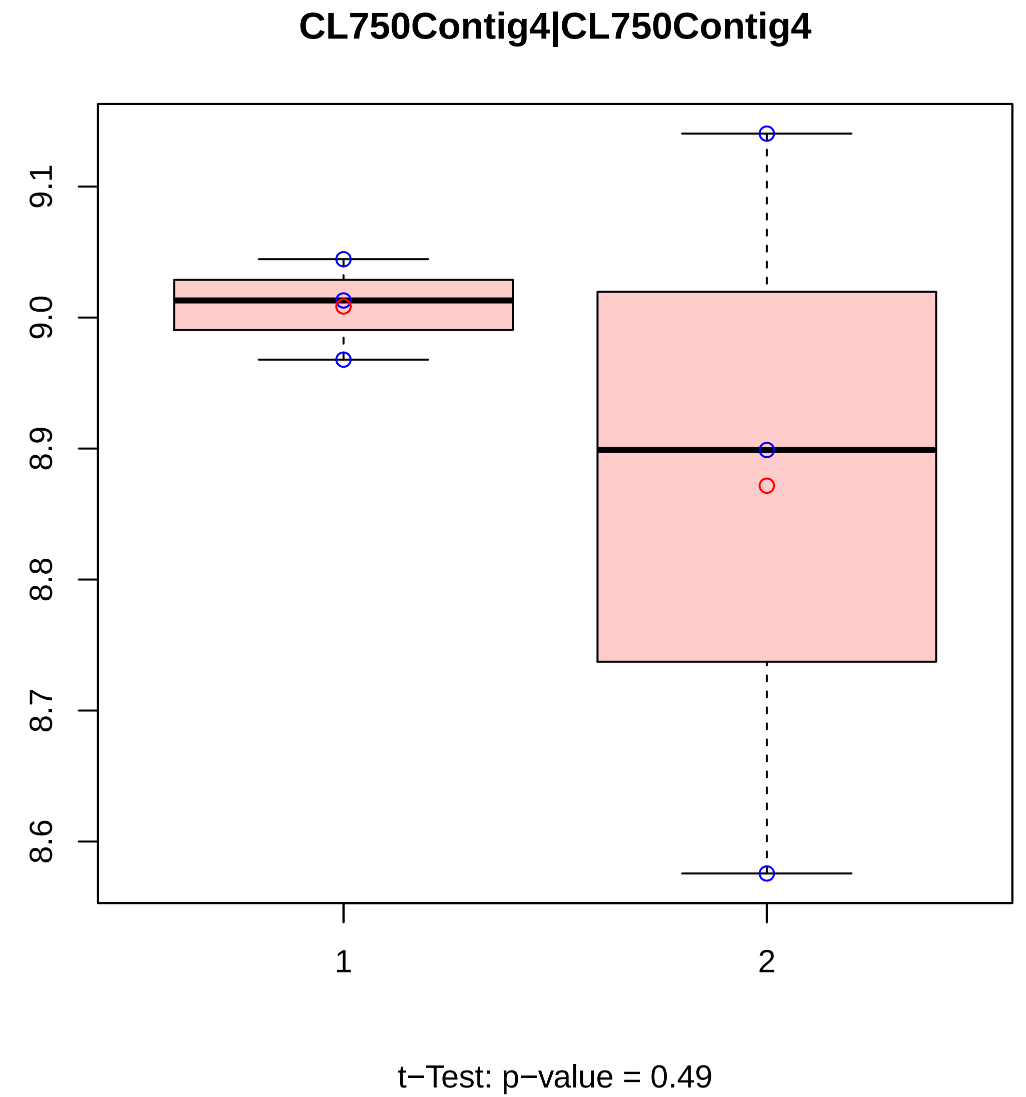

CL750Contig4|CL750Contig4
9.1
9.0
8.9
8.8
8.7
8.6
1
2
t−Test: p−value = 0.49

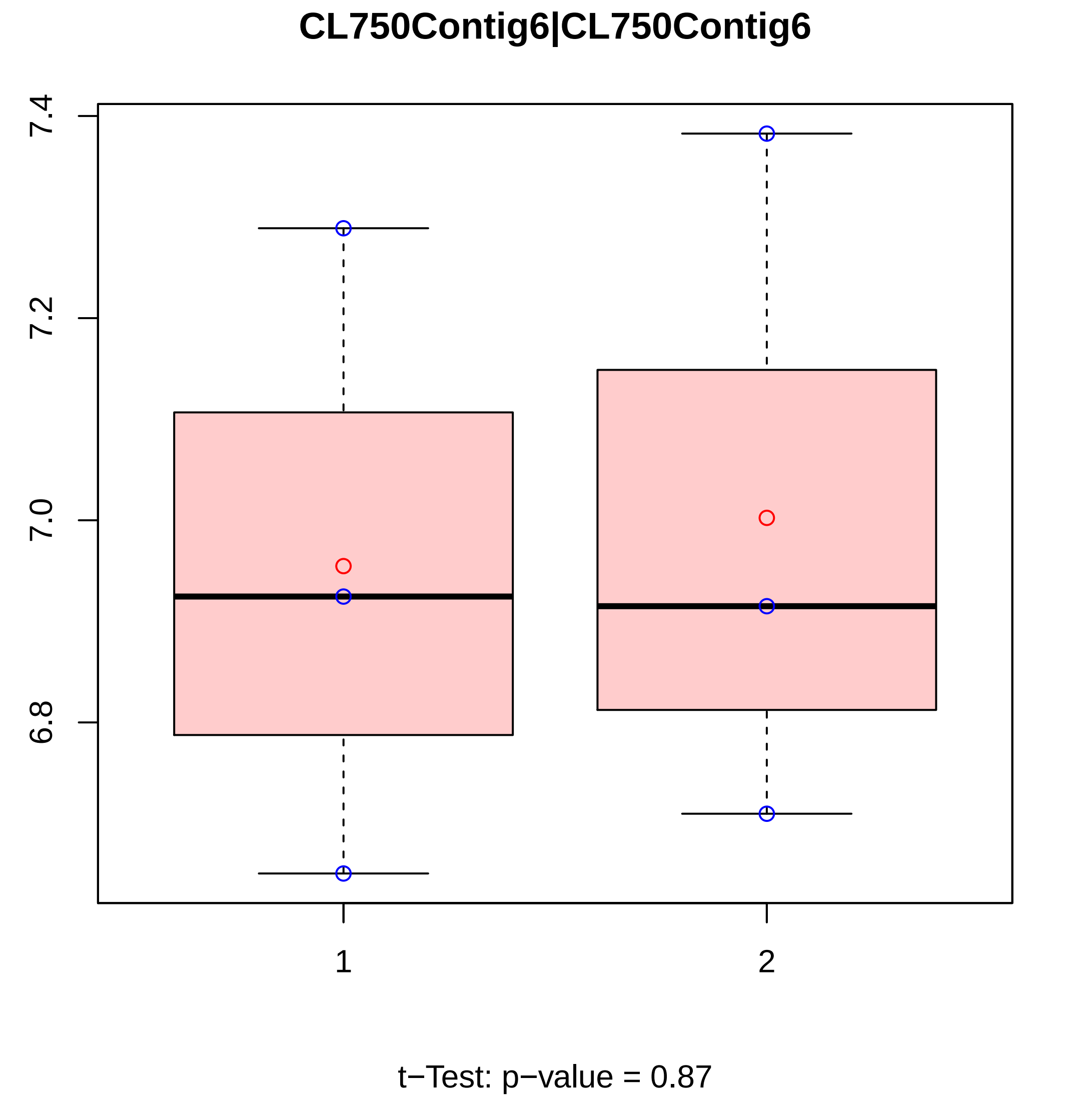
CL750Contig6|CL750Contig6
7.4
7.2
7.0
6.8
1
2
t−Test: p−value = 0.87

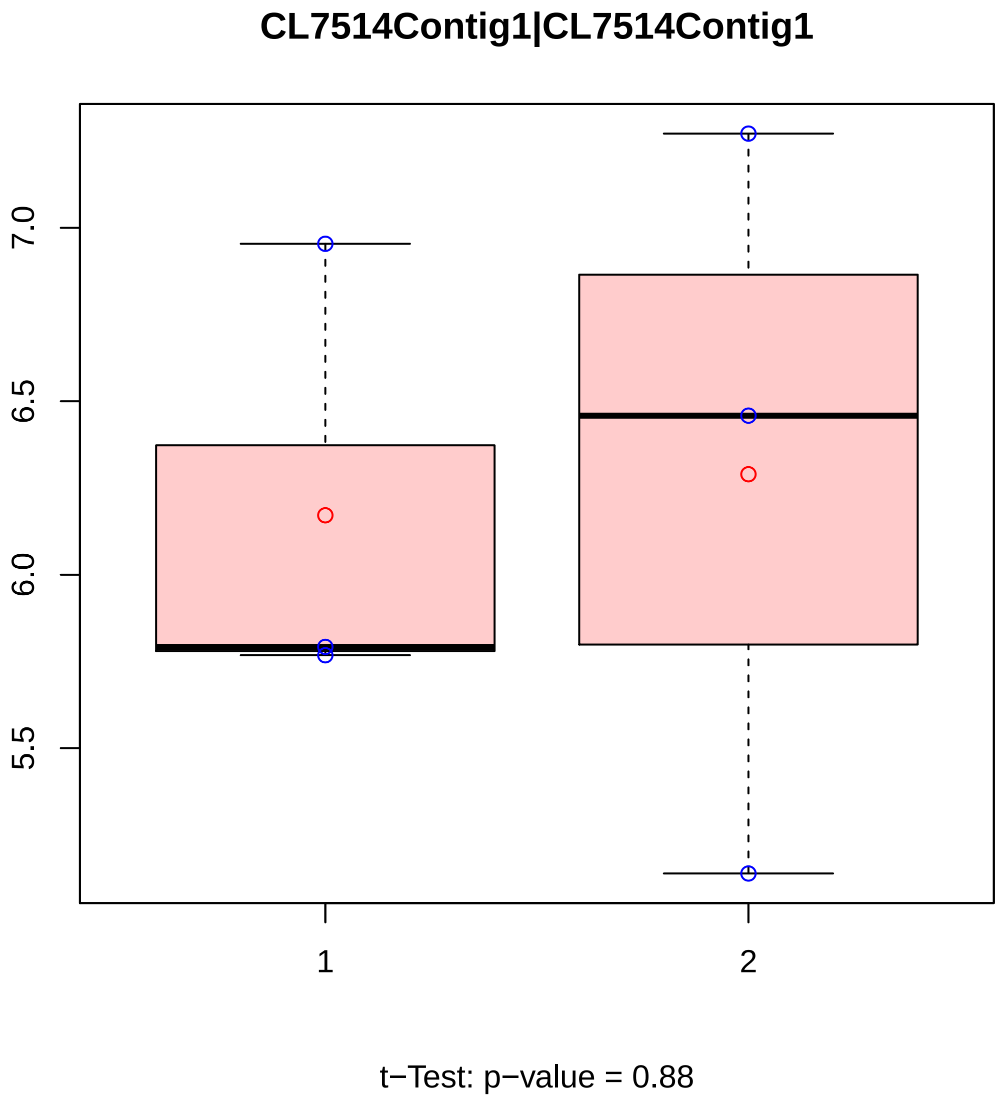

CL7514Contig1|CL7514Contig1
7.0
6.5
6.0
5.5
1
2
t−Test: p−value = 0.88

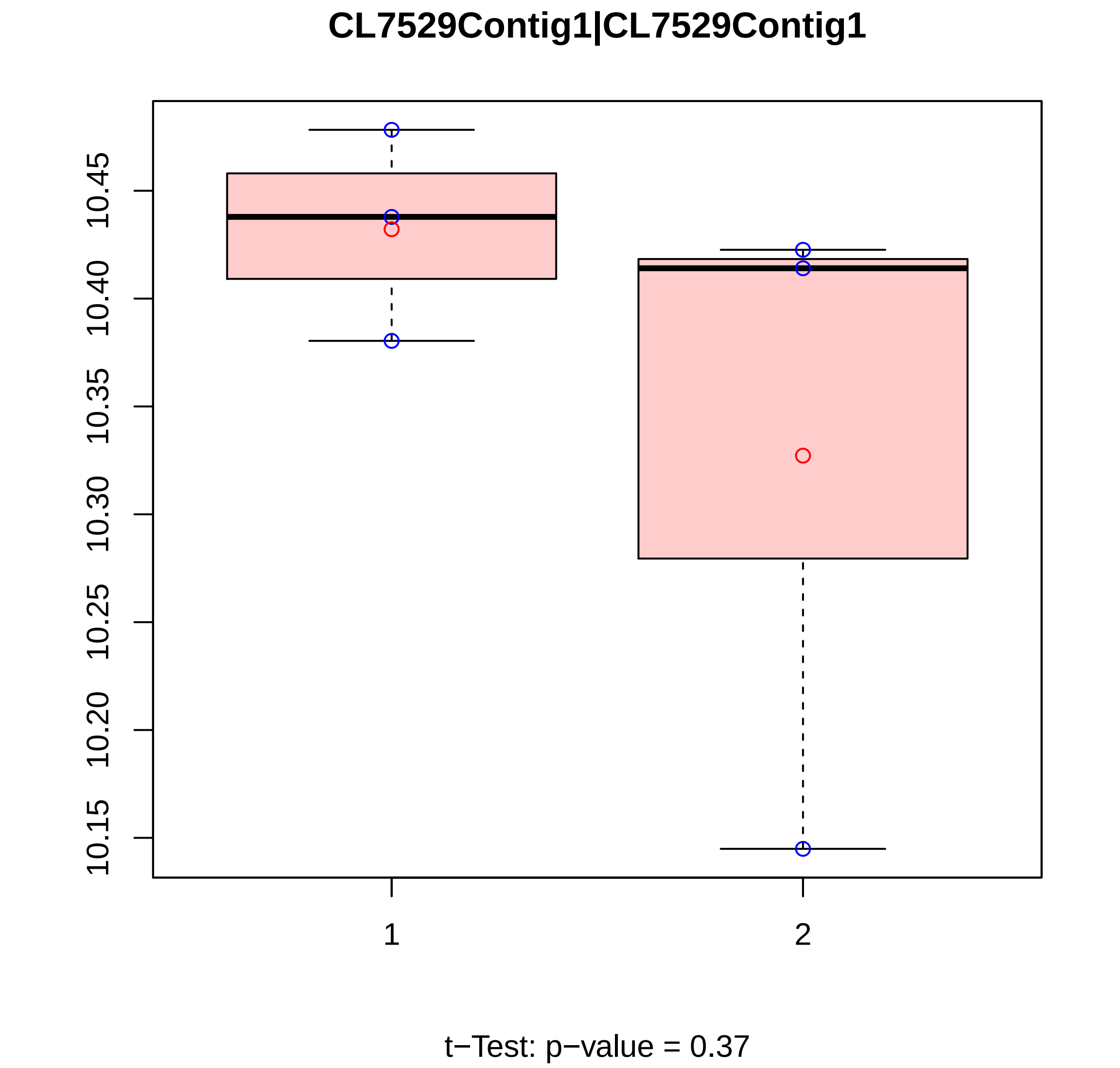
CL7529Contig1|CL7529Contig1
10.15
10.20
10.25
10.30
10.35
10.40
10.45
1
2
t−Test: p−value = 0.37

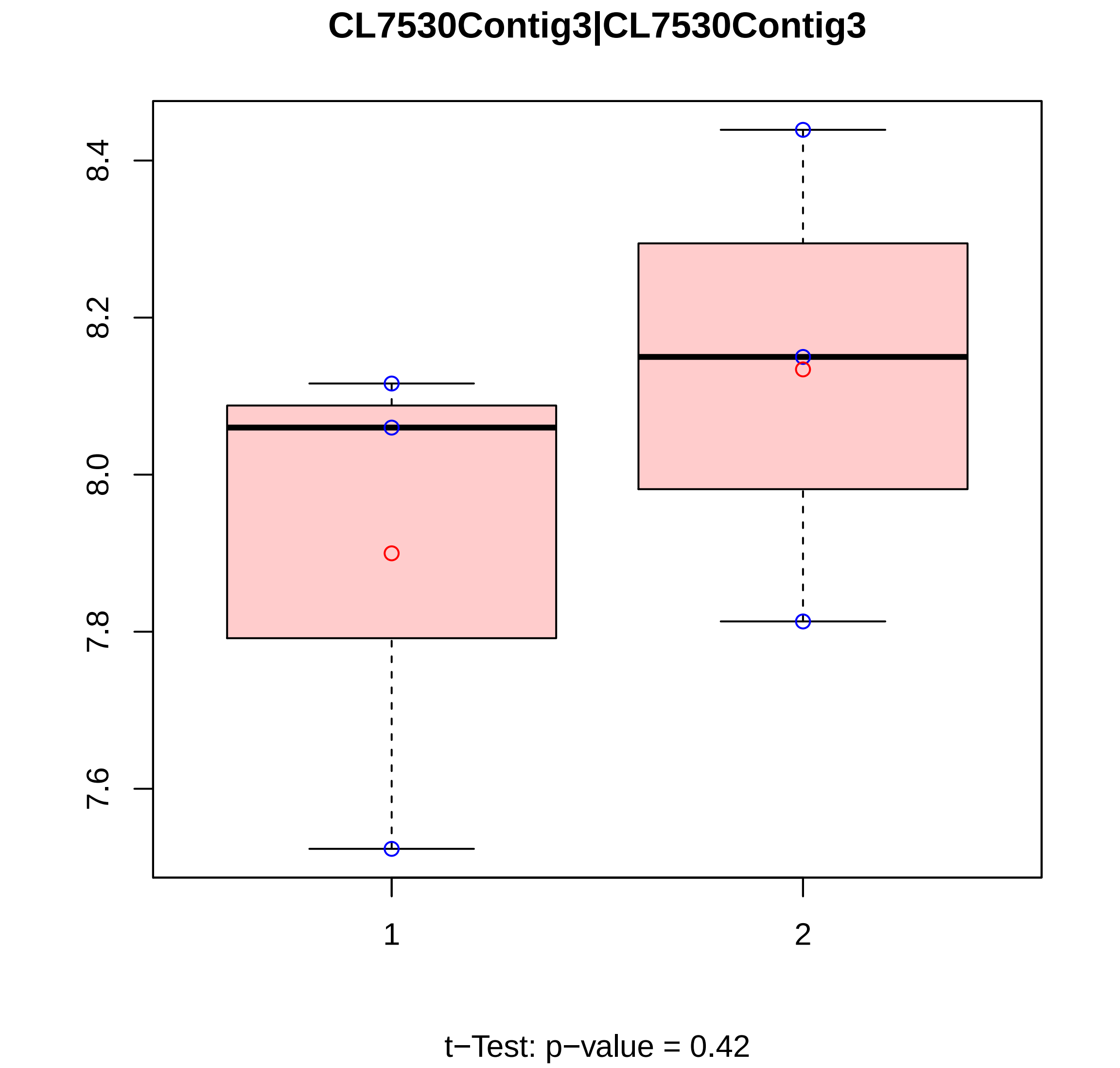
CL7530Contig3|CL7530Contig3
8.4
8.2
8.0
7.8
7.6
1
2
t−Test: p−value = 0.42

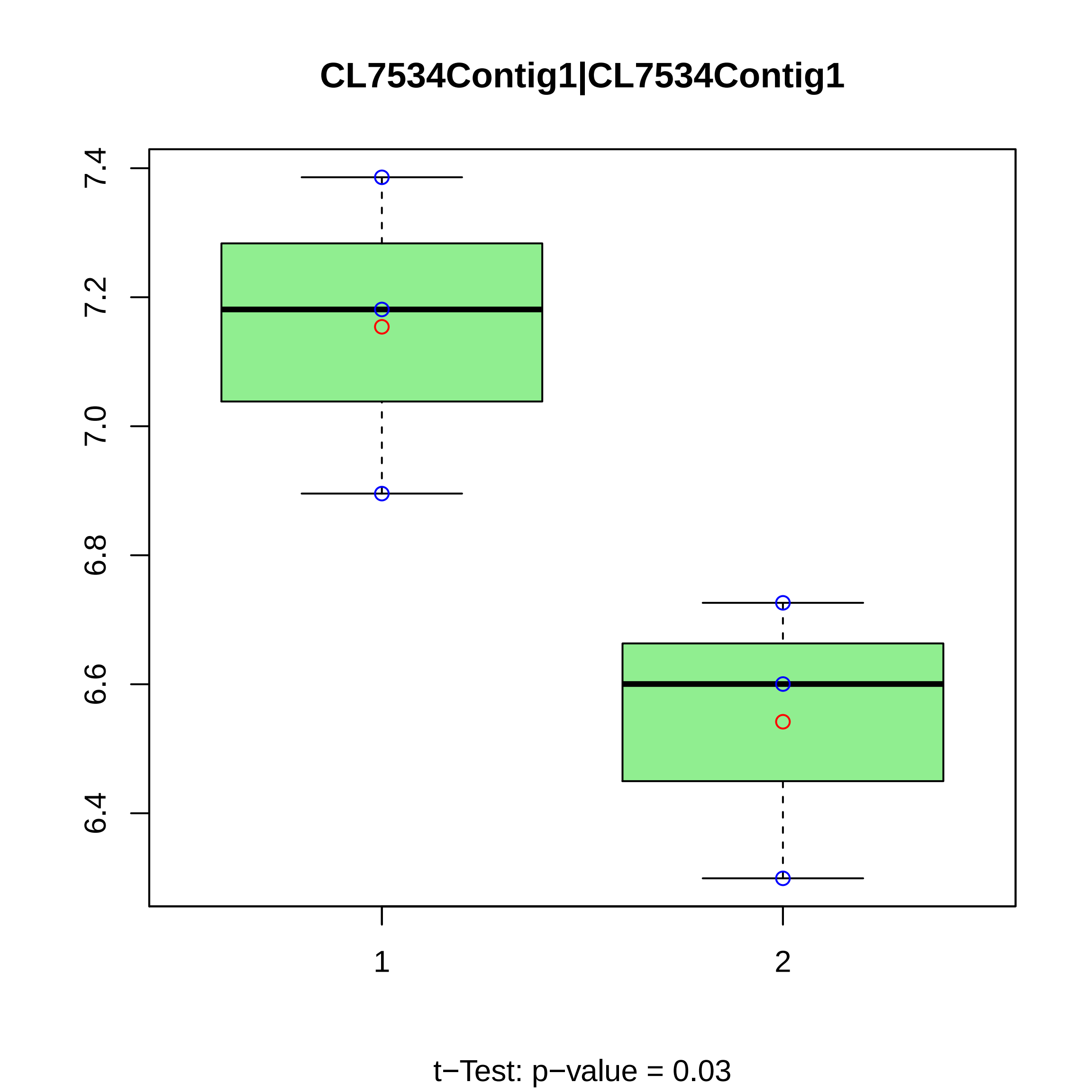

CL7534Contig1|CL7534Contig1
7.4
7.2
7.0
6.8
6.6
6.4
1
2
t−Test: p−value = 0.03

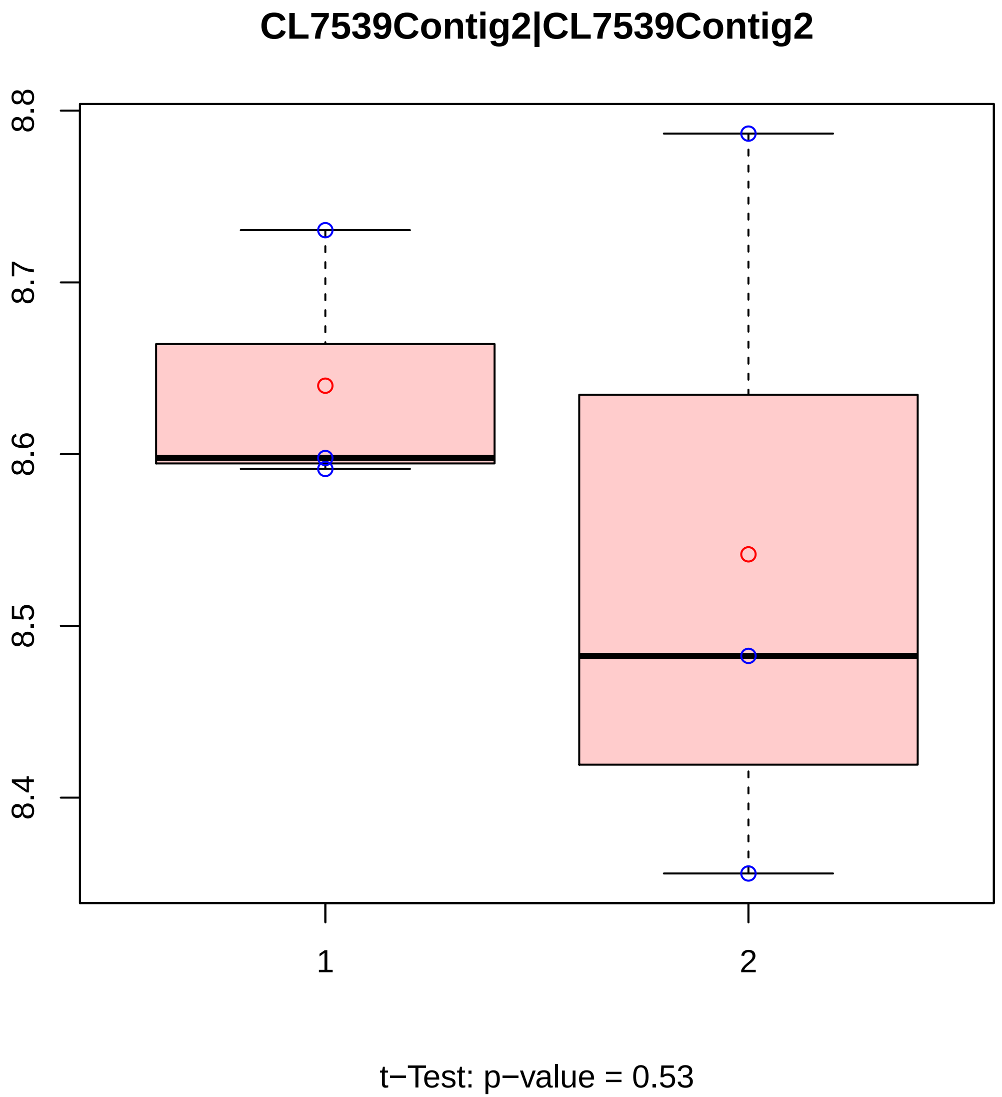

CL7539Contig2|CL7539Contig2
8.8
8.7
8.6
8.5
8.4
1
2
t−Test: p−value = 0.53

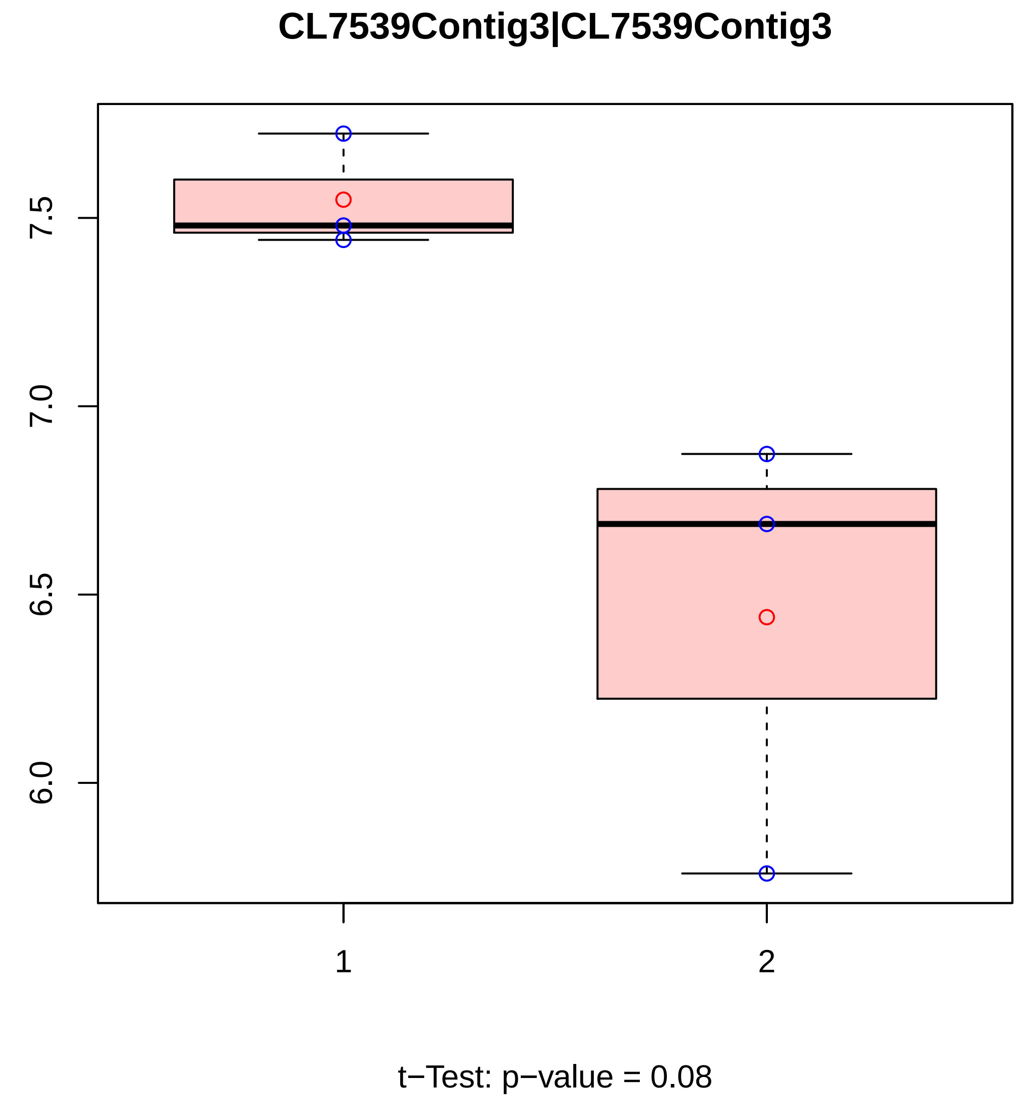
CL7539Contig3|CL7539Contig3
7.5
7.0
6.5
6.0
1
2
t−Test: p−value = 0.08

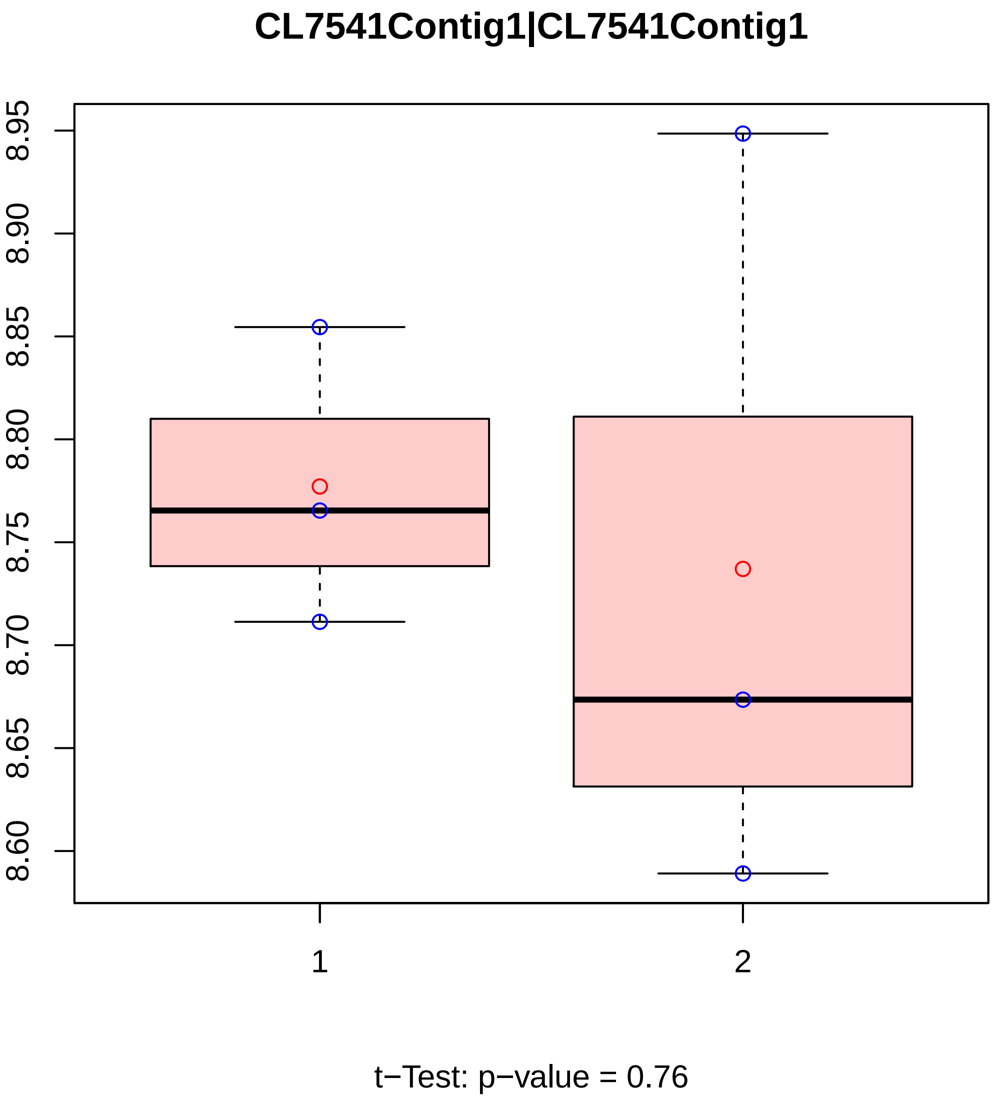
CL7541Contig1|CL7541Contig1
8.60
8.65
8.70
8.75
8.80
8.85
8.90
8.95
1
2
t−Test: p−value = 0.76

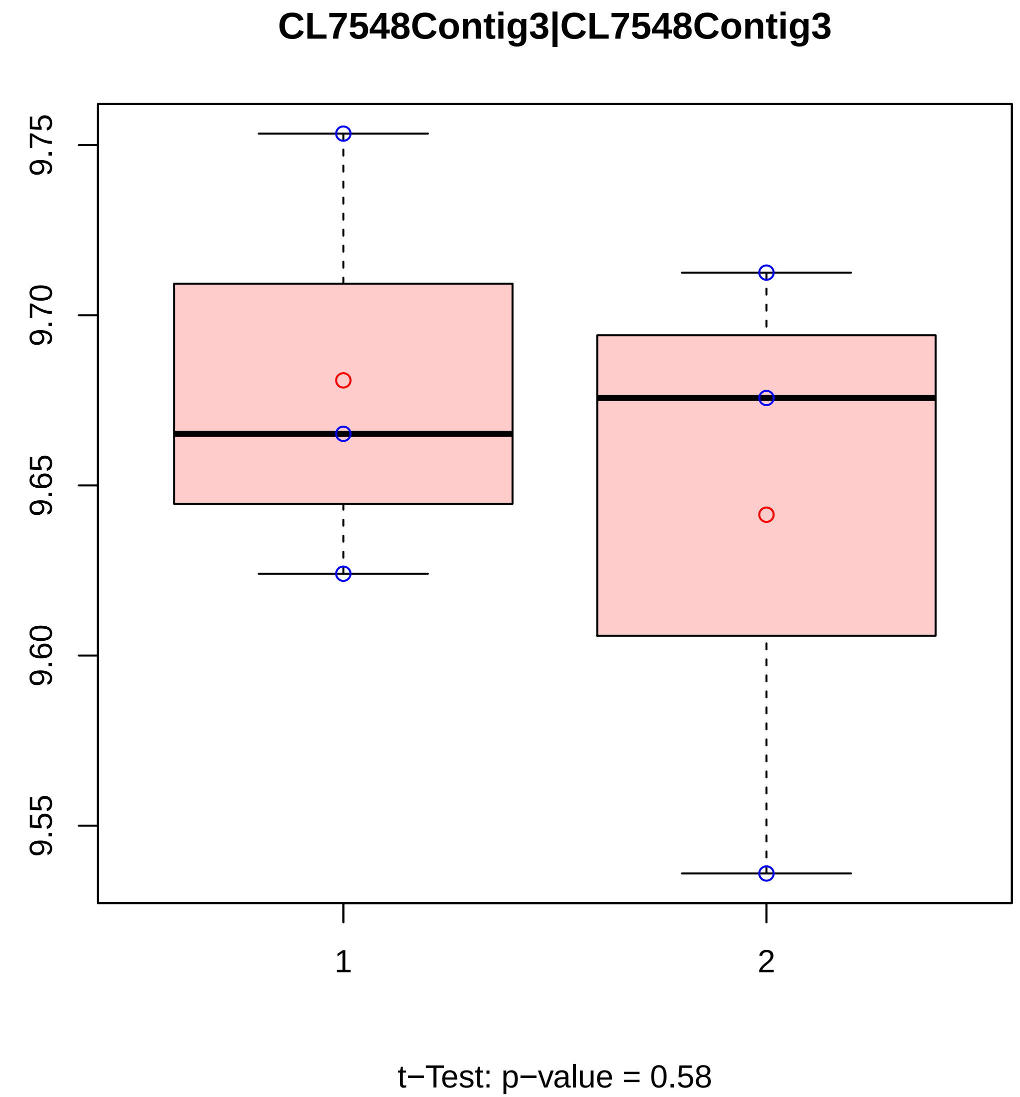
CL7548Contig3|CL7548Contig3
9.75
9.70
9.65
9.60
9.55
1
2
t−Test: p−value = 0.58

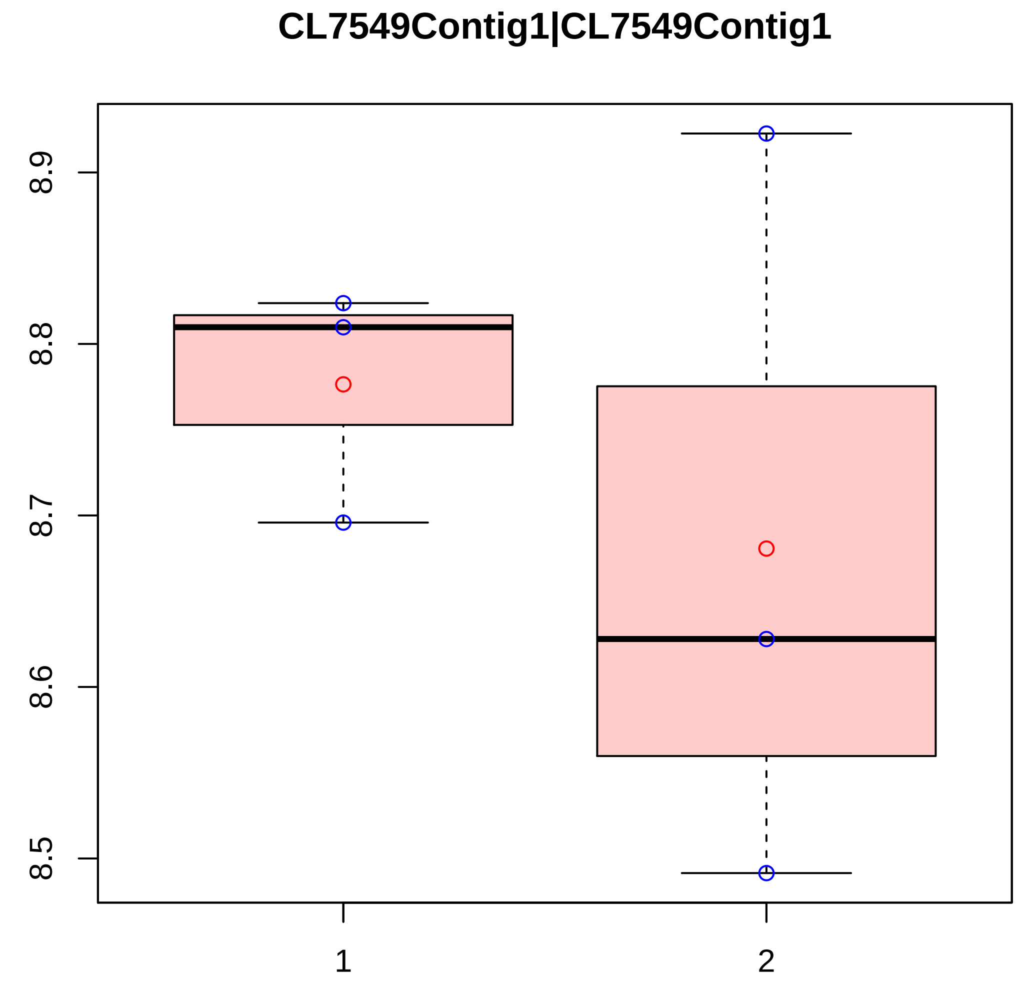


t−Test: p−value = 0.54

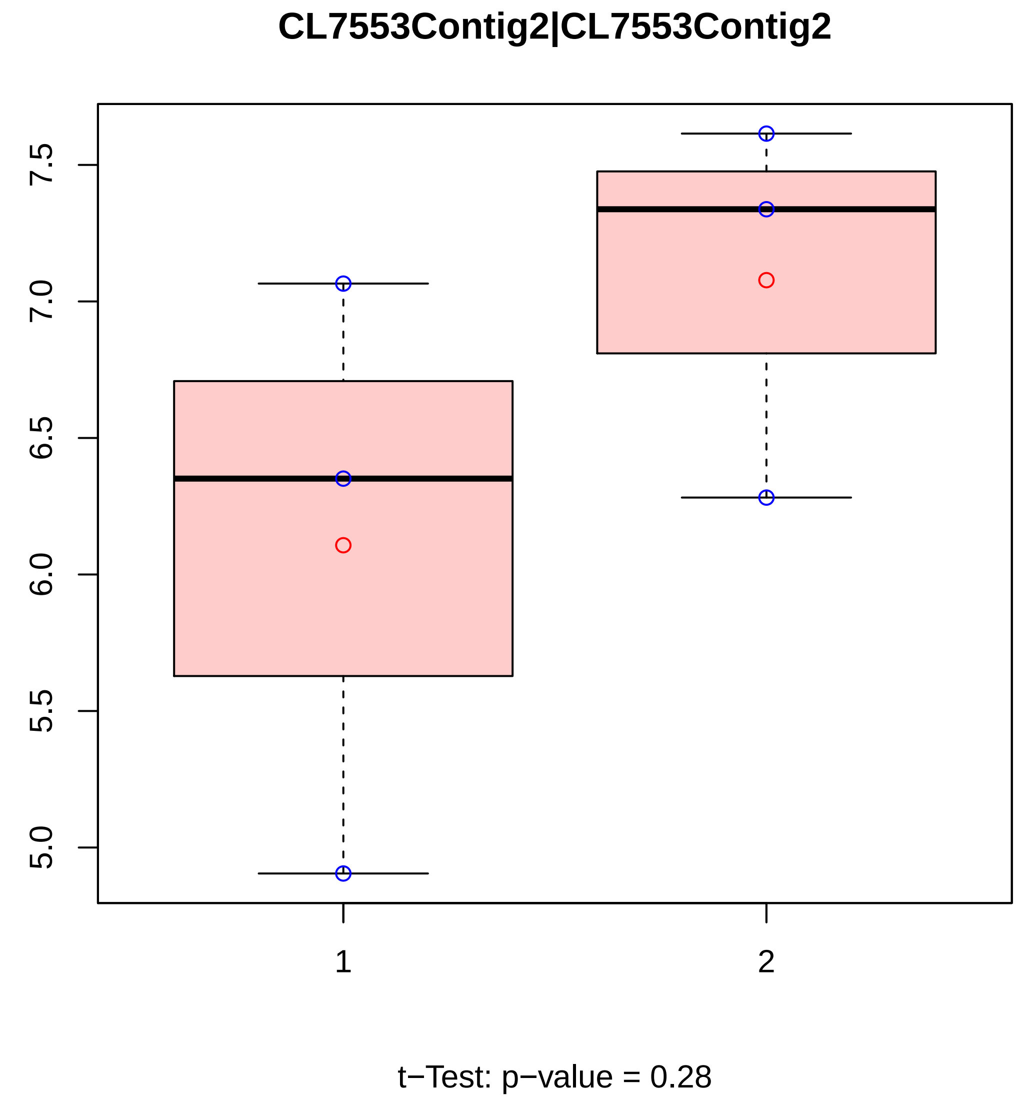
CL7553Contig2|CL7553Contig2
7.5
7.0
6.5
6.0
5.5
5.0
1
2
t−Test: p−value = 0.28

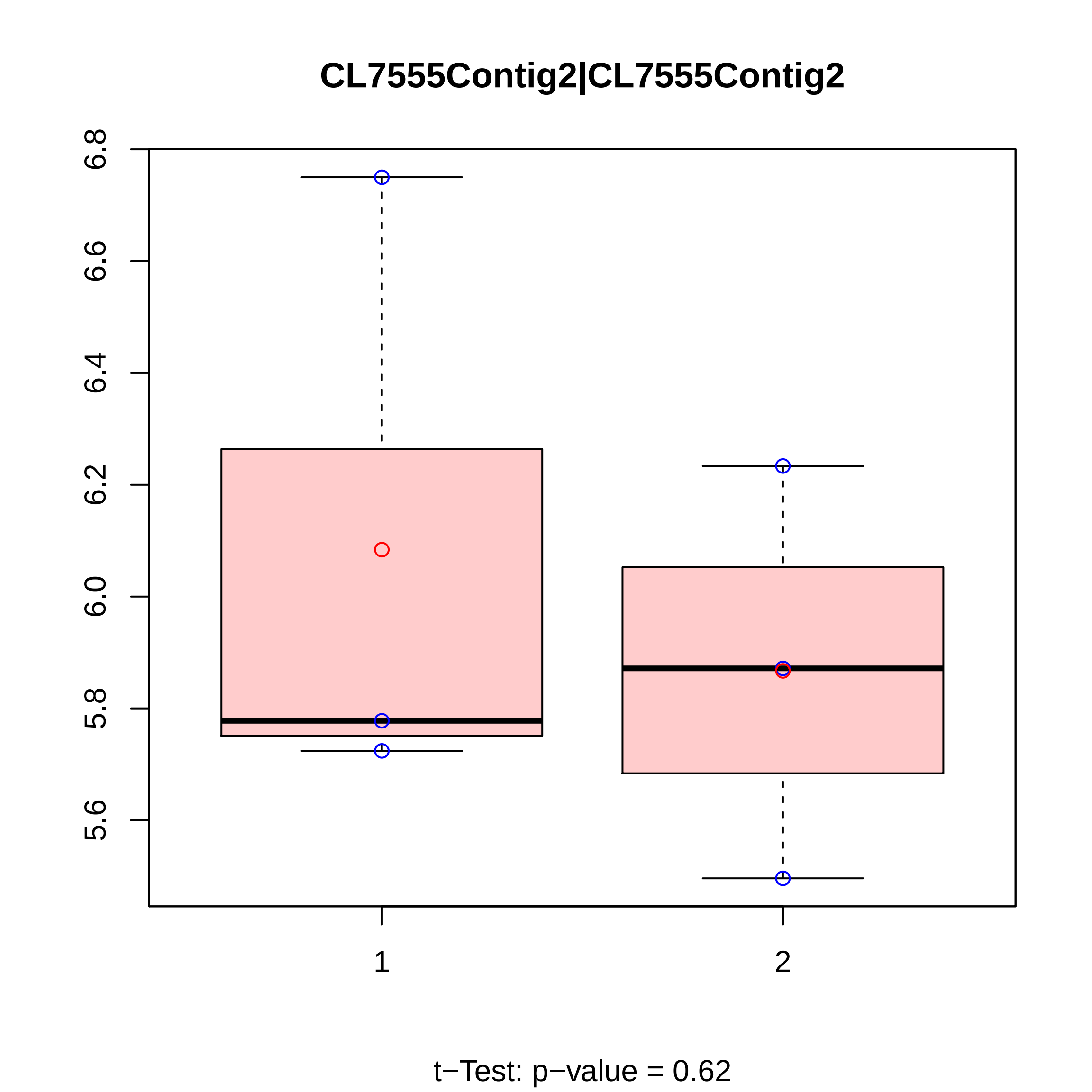

CL7555Contig2|CL7555Contig2
6.8
6.6
6.4
6.2
6.0
5.8
5.6
1
2
t−Test: p−value = 0.62

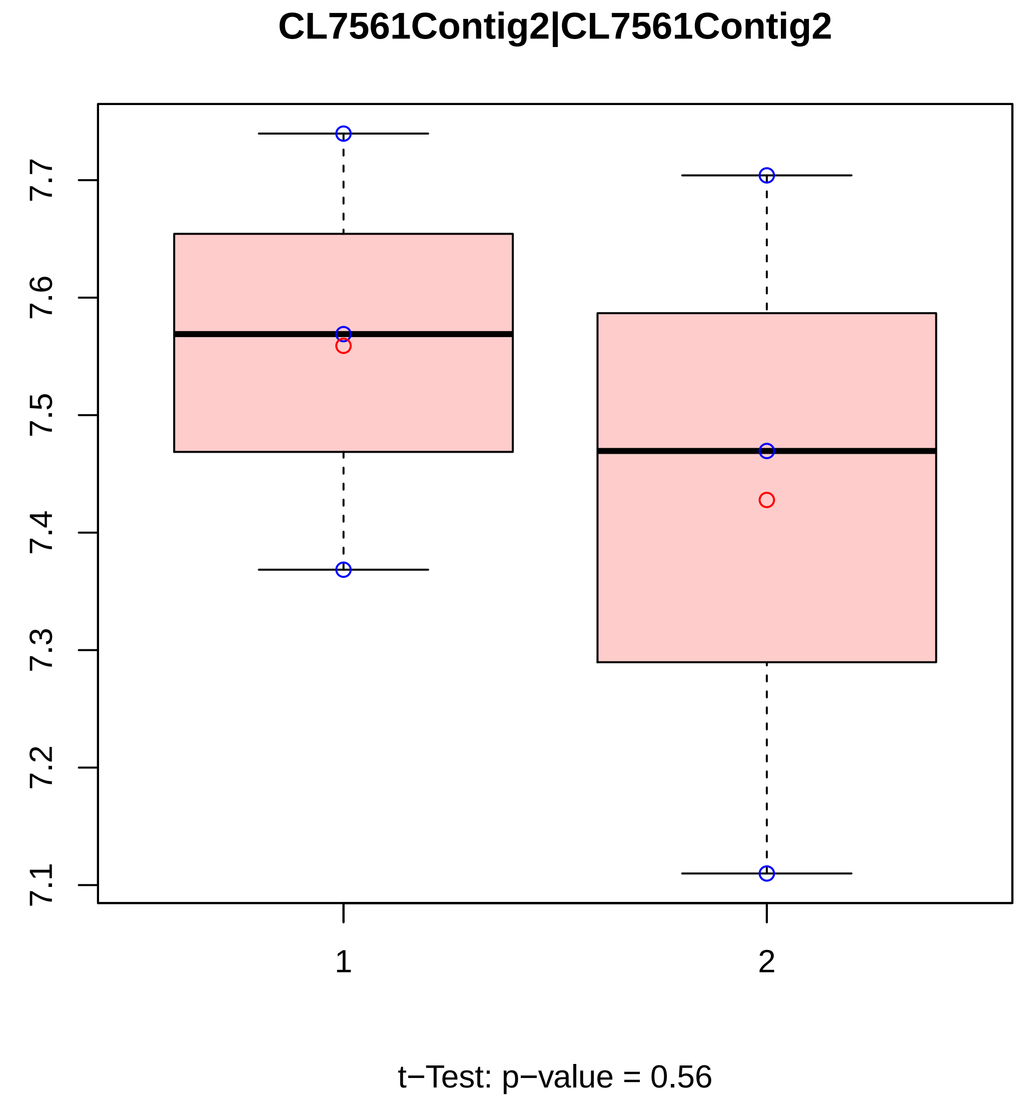
CL7561Contig2|CL7561Contig2
7.1
7.2
7.3
7.4
7.5
7.6
7.7
1
2
t−Test: p−value = 0.56

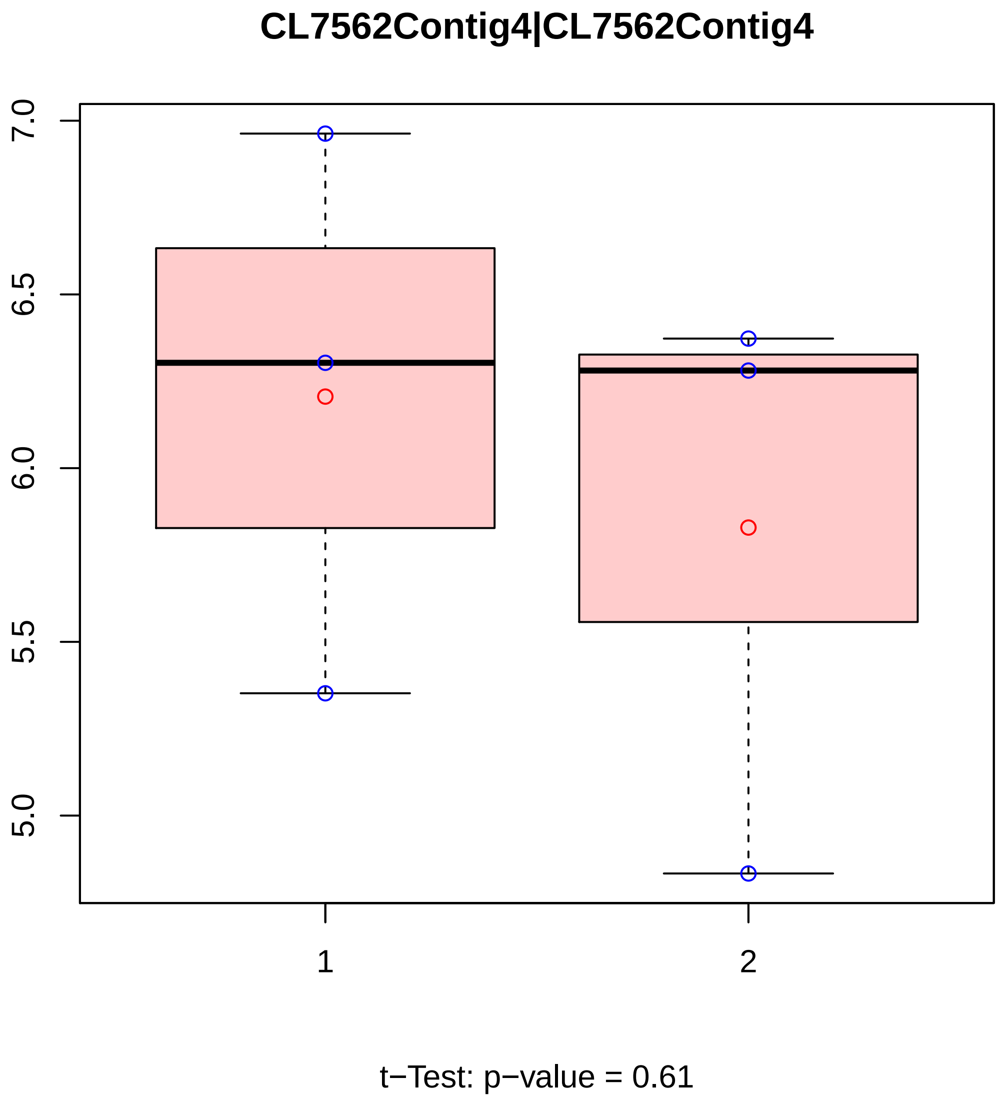

CL7562Contig4|CL7562Contig4
7.0
6.5
6.0
5.5
5.0
1
2
t−Test: p−value = 0.61

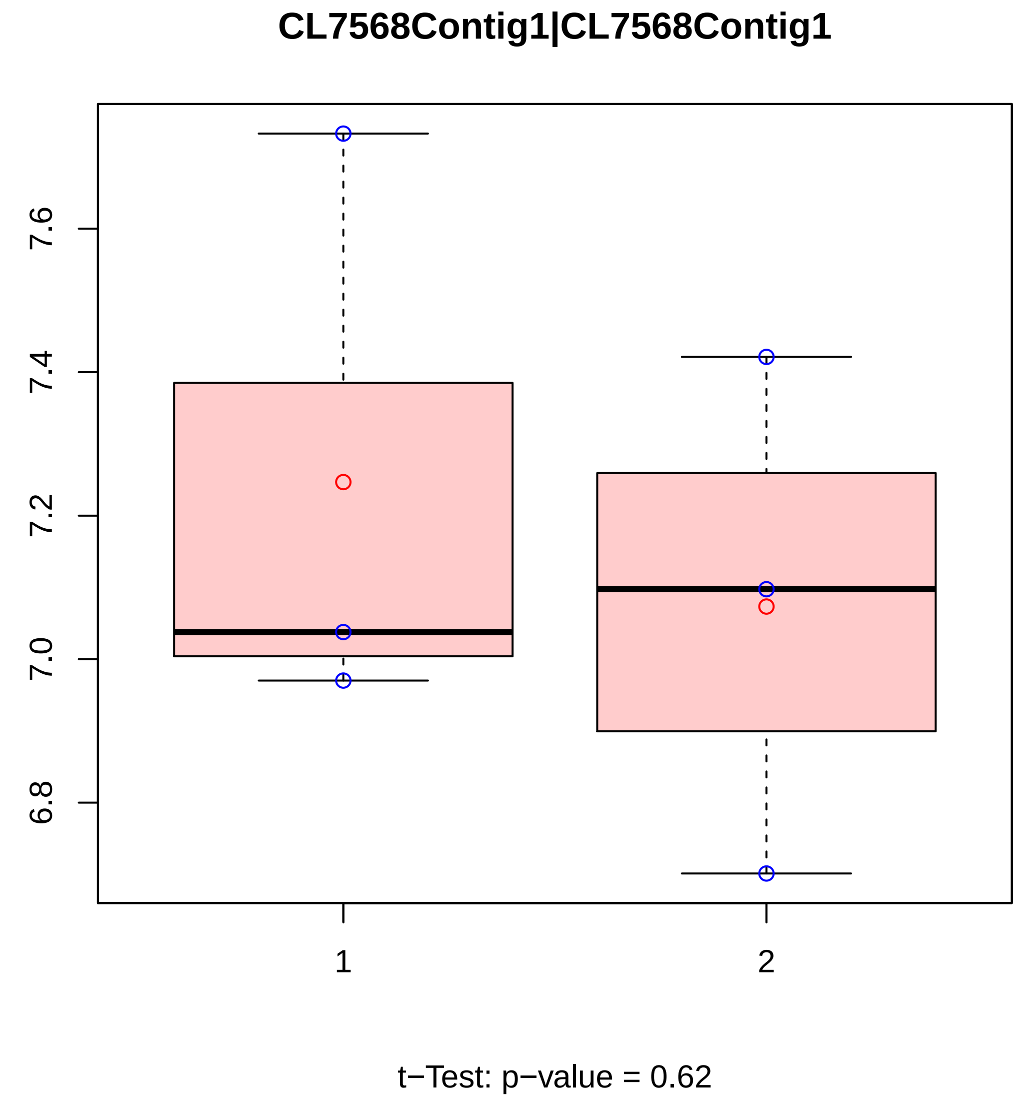
CL7568Contig1|CL7568Contig1
7.6
7.4
7.2
7.0
6.8
1
2
t−Test: p−value = 0.62

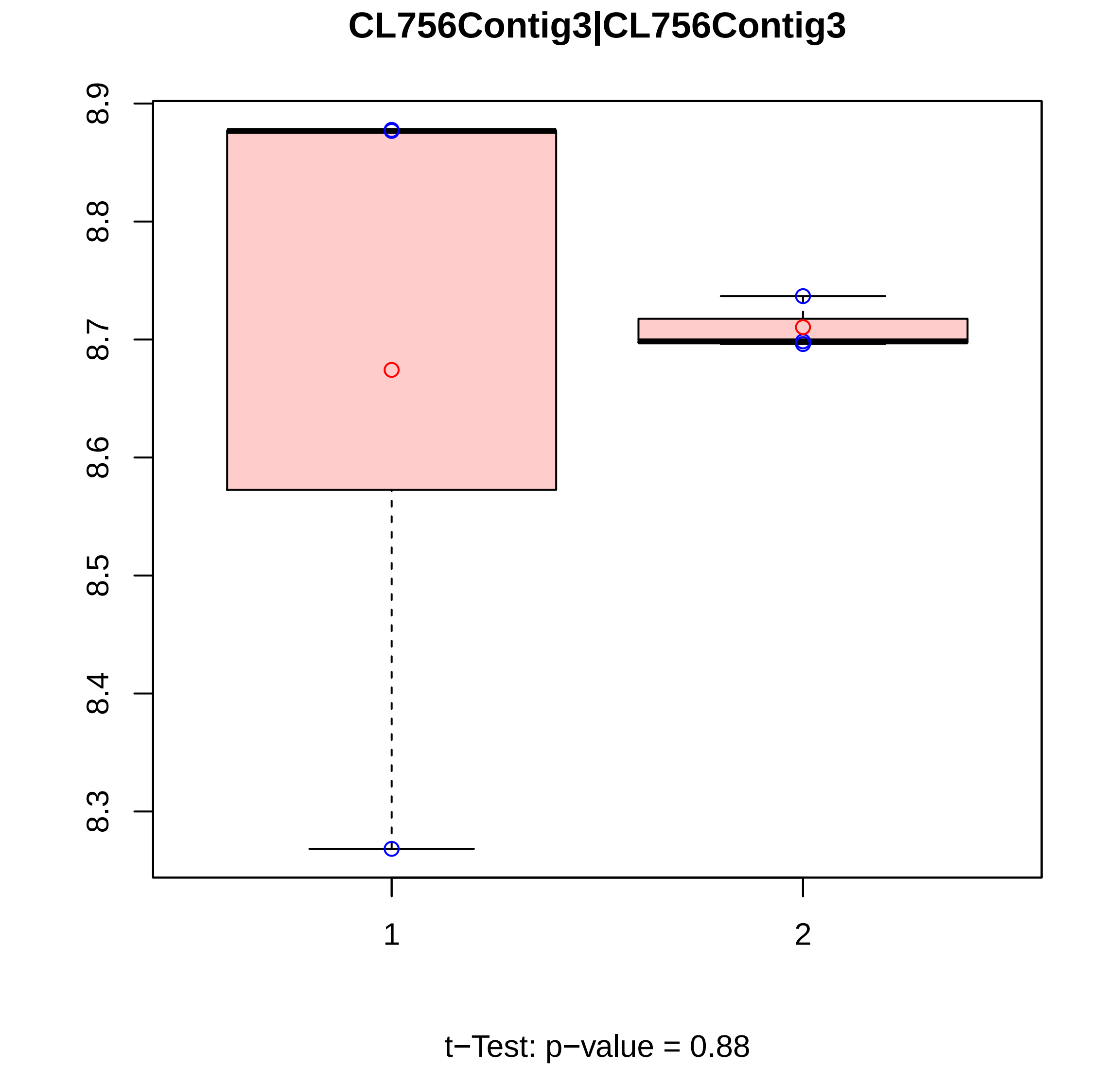
CL756Contig3|CL756Contig3
8.9
8.8
8.7
8.6
8.5
8.4
8.3
1
2
t−Test: p−value = 0.88

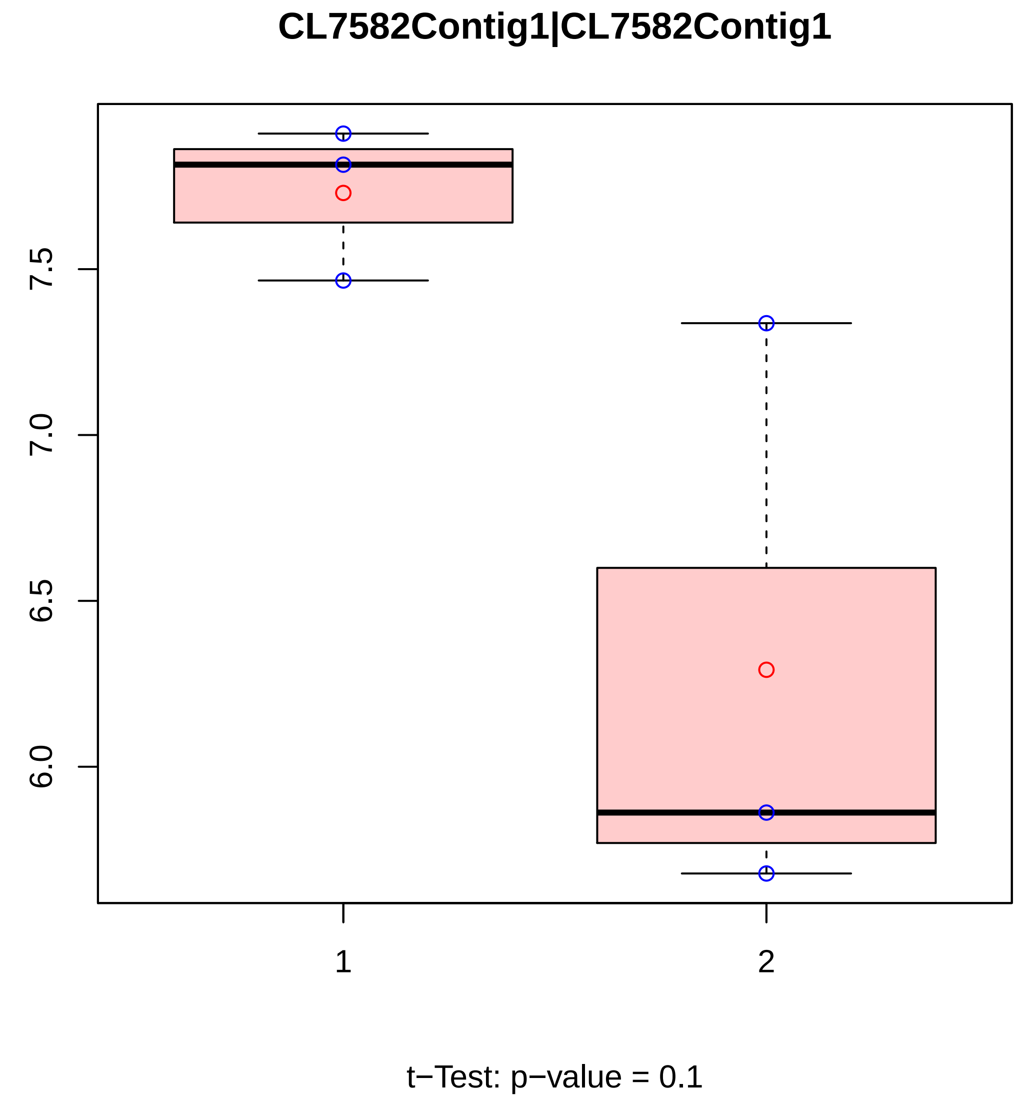

CL7582Contig1|CL7582Contig1
7.5
7.0
6.5
6.0
1
2
t−Test: p−value = 0.1

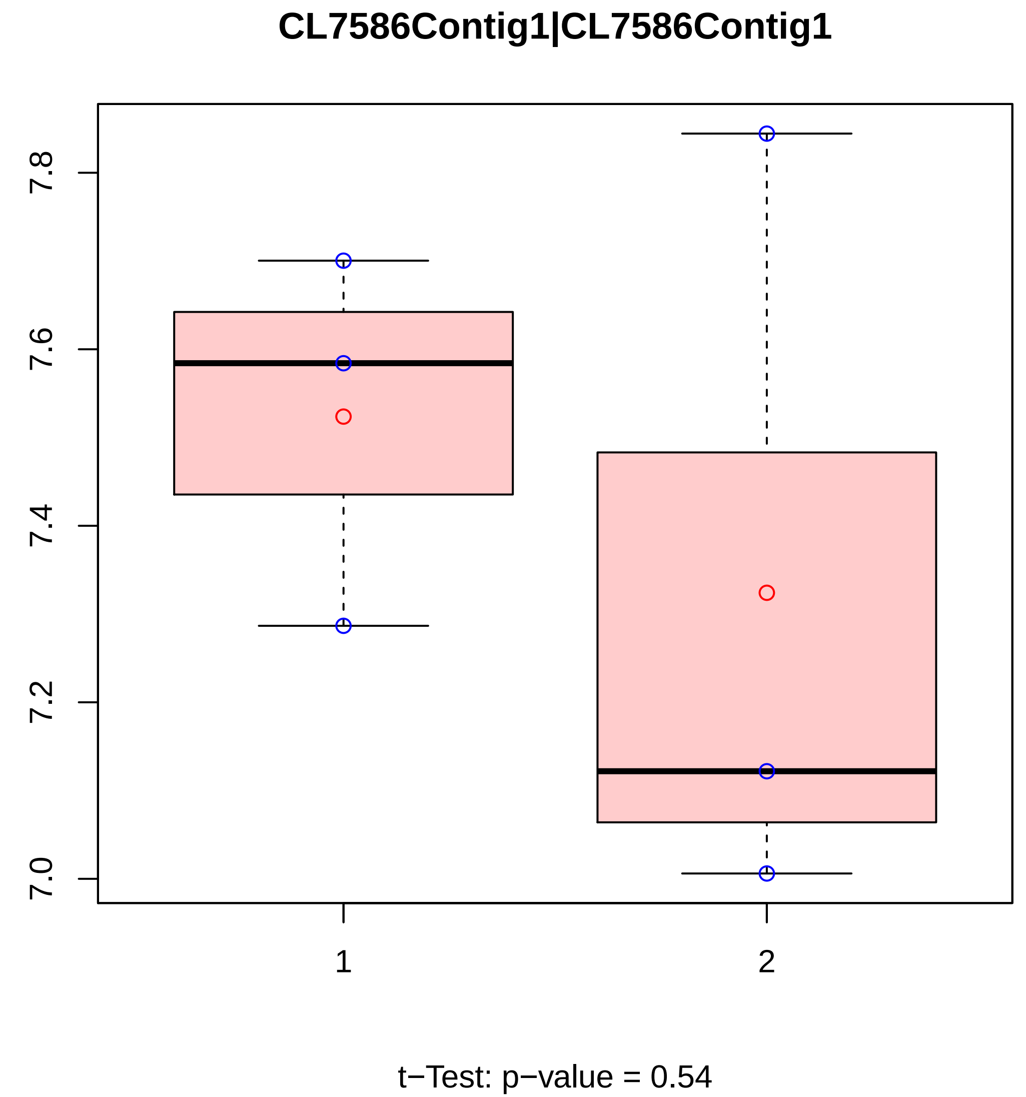
CL7586Contig1|CL7586Contig1
7.0
7.2
7.4
7.6
7.8
1
2
t−Test: p−value = 0.54

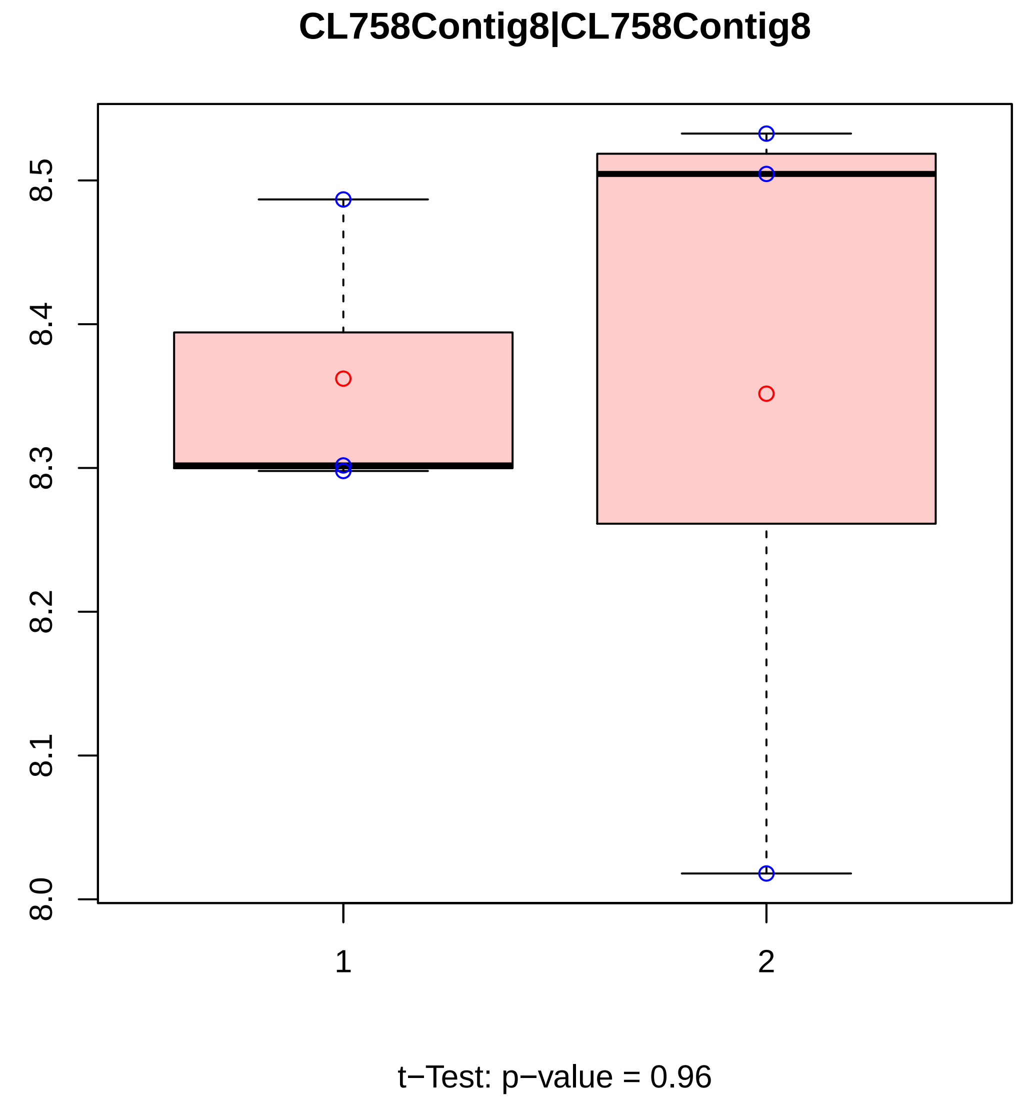
CL758Contig8|CL758Contig8
8.0
8.1
8.2
8.3
8.4
8.5
1
2
t−Test: p−value = 0.96

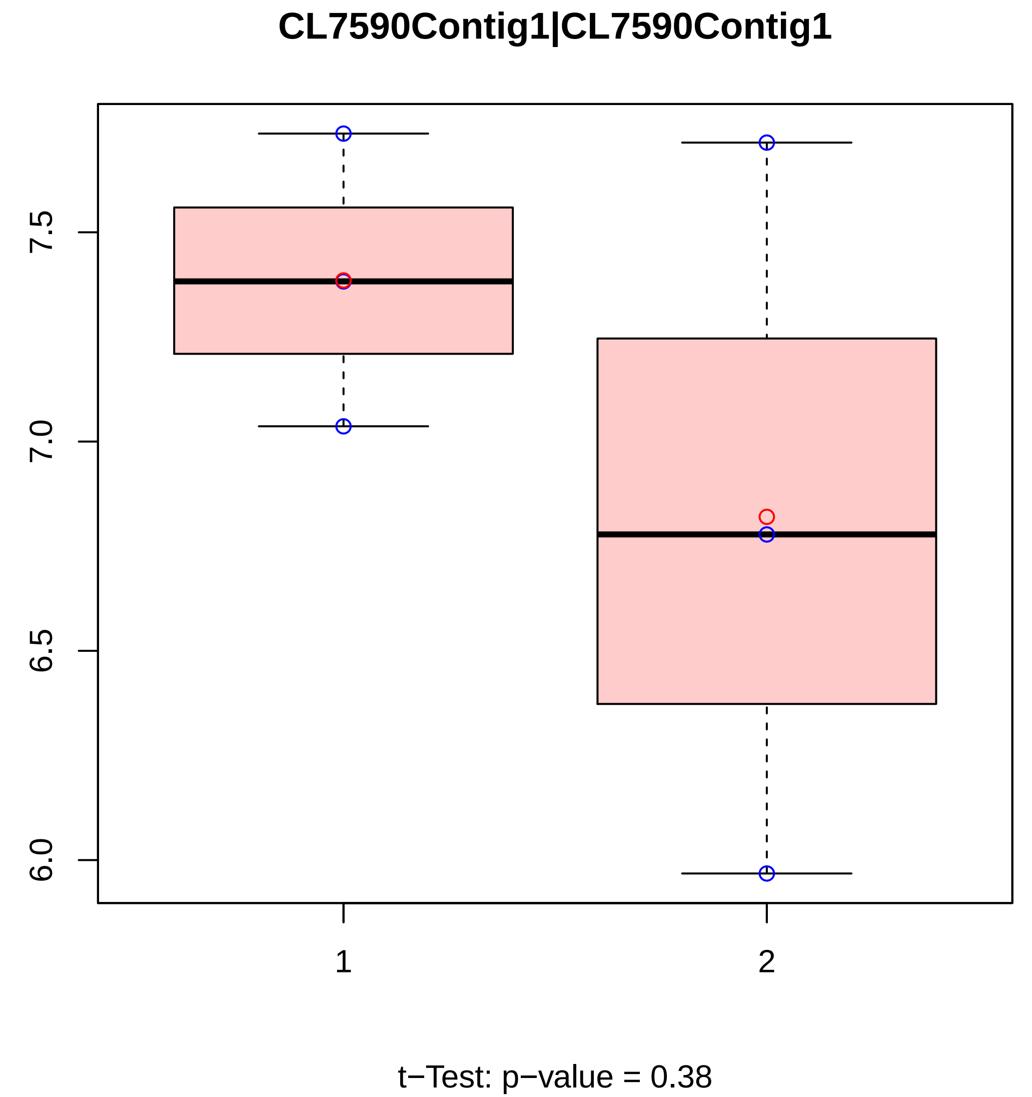
CL7590Contig1|CL7590Contig1
7.5
7.0
6.5
6.0
1
2
t−Test: p−value = 0.38

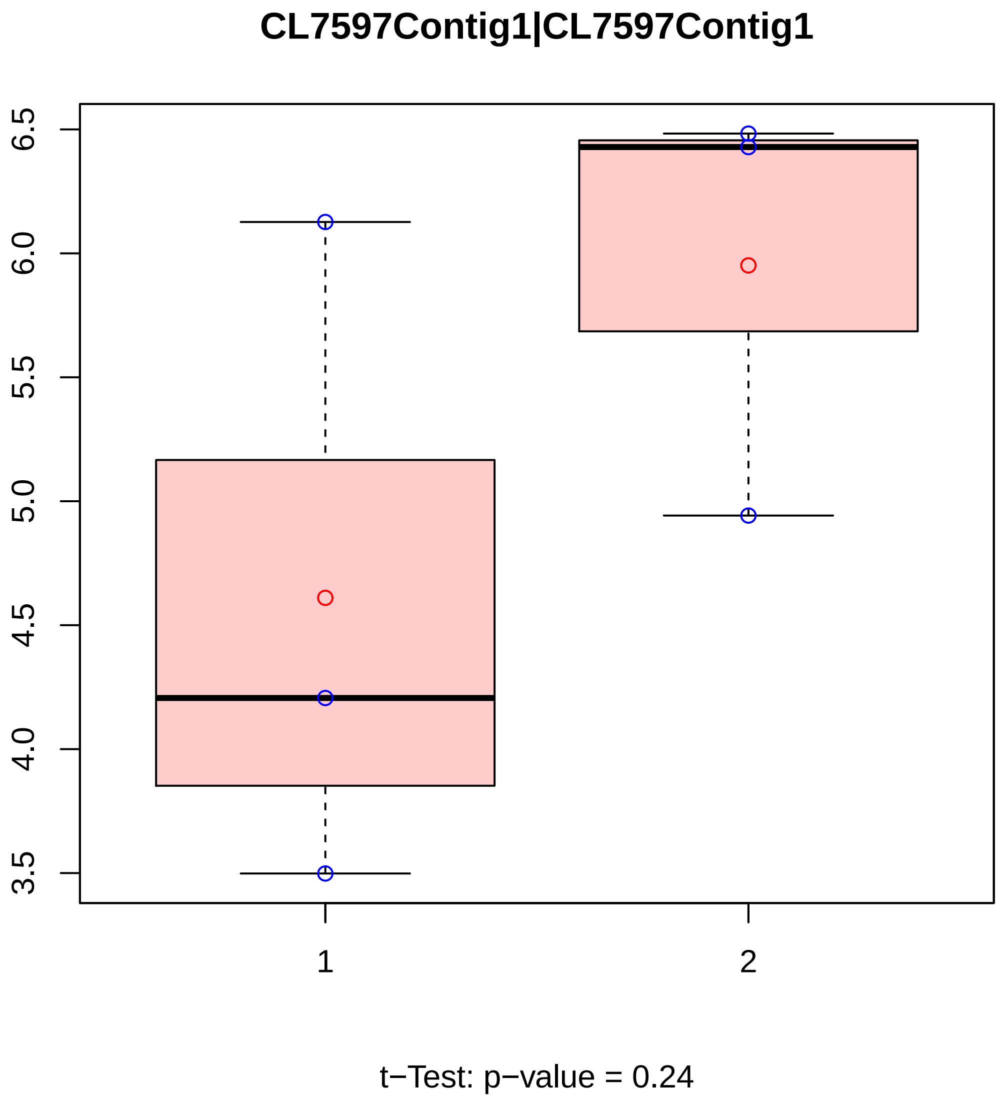
CL7597Contig1|CL7597Contig1
6.5
6.0
5.5
5.0
4.5
4.0
3.5
1
2
t−Test: p−value = 0.24

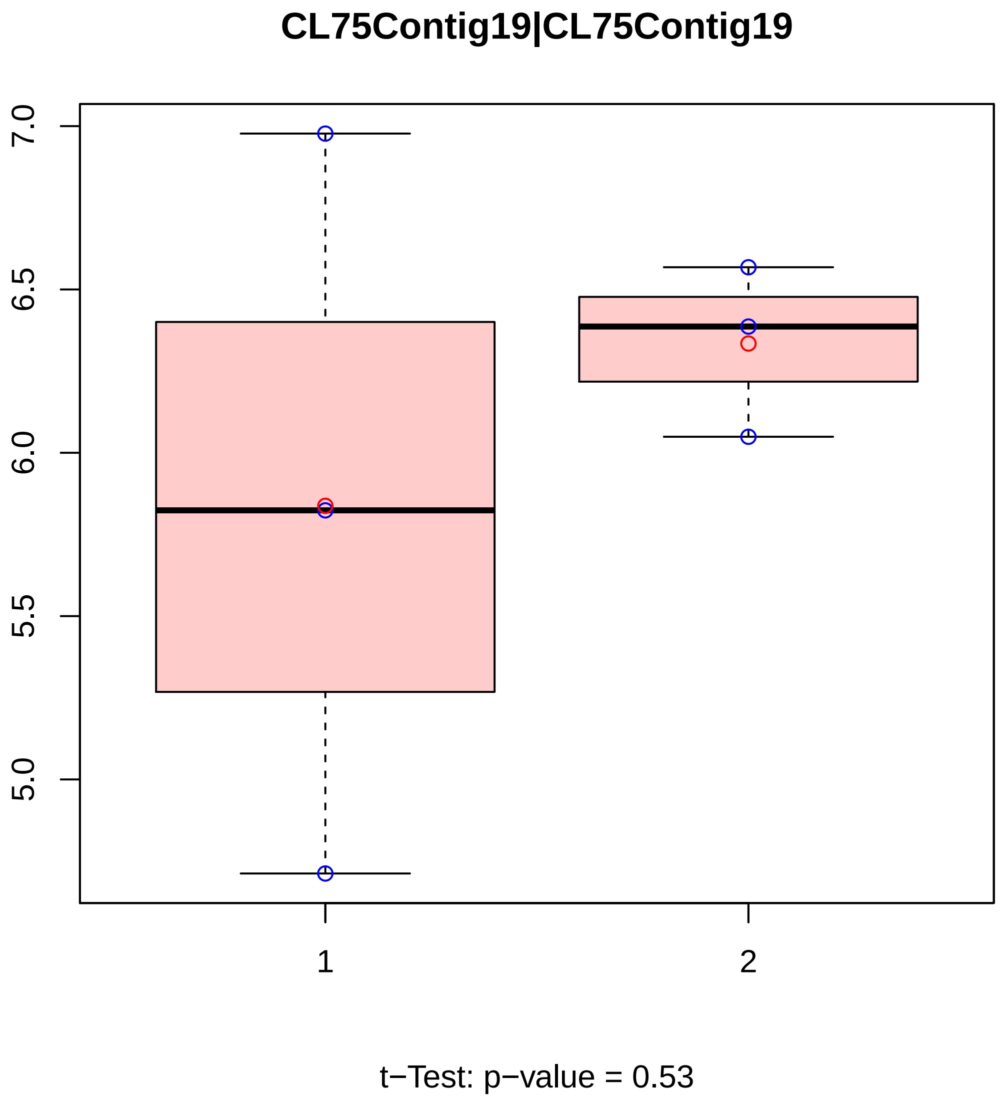

CL75Contig19|CL75Contig19
7.0
6.5
6.0
5.5
5.0
1
2
t−Test: p−value = 0.53

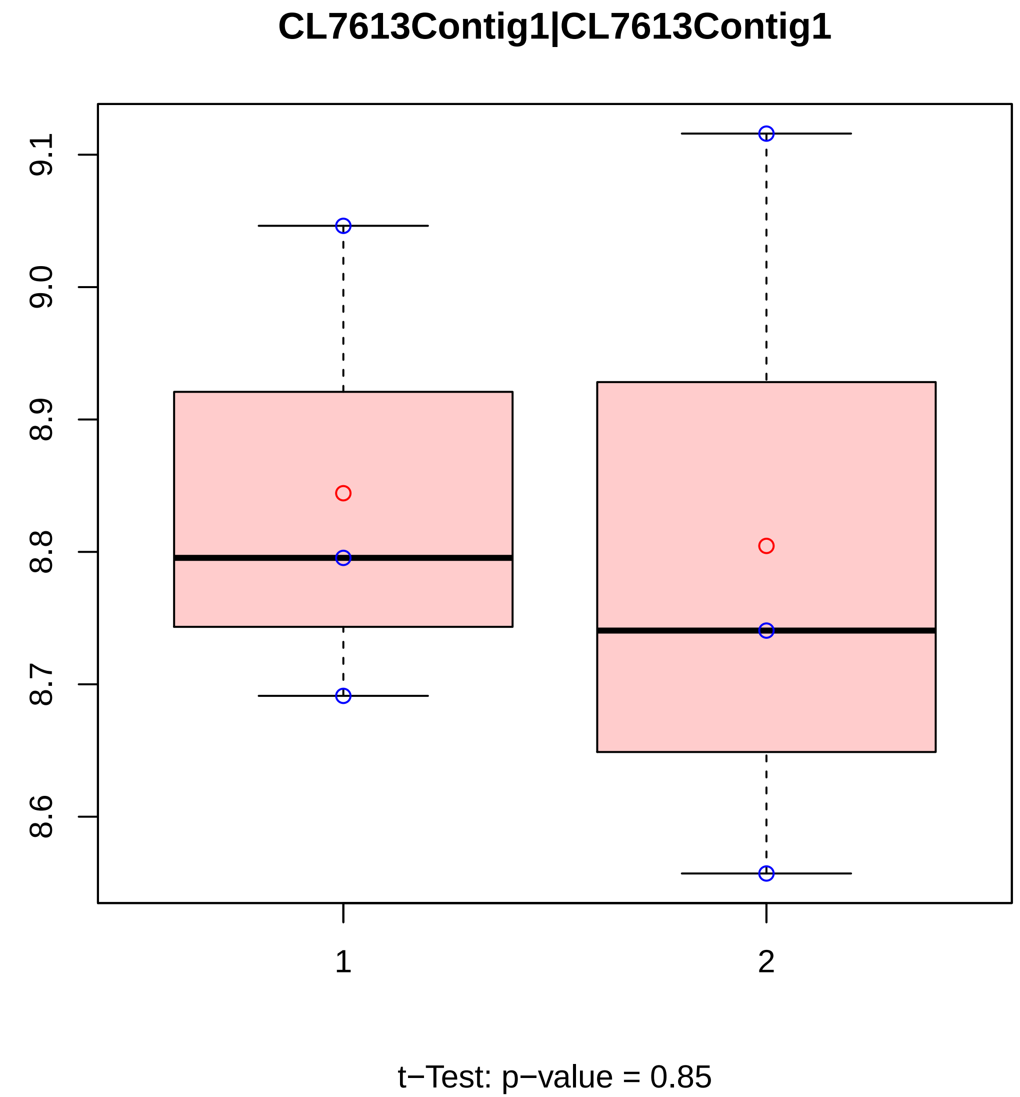
CL7613Contig1|CL7613Contig1
9.1
9.0
8.9
8.8
8.7
8.6
1
2
t−Test: p−value = 0.85

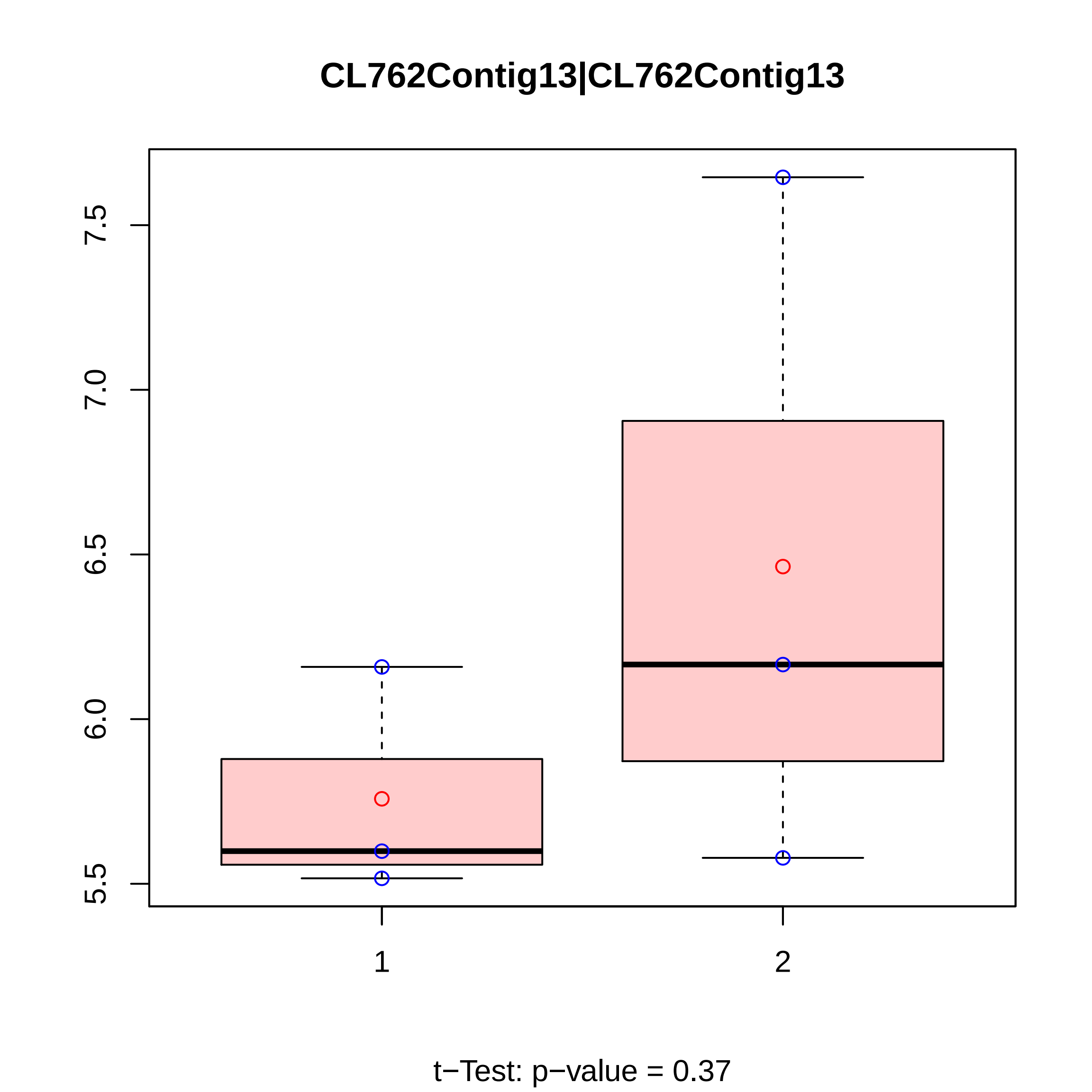
CL762Contig13|CL762Contig13
7.5
7.0
6.5
6.0
5.5
1
2
t−Test: p−value = 0.37

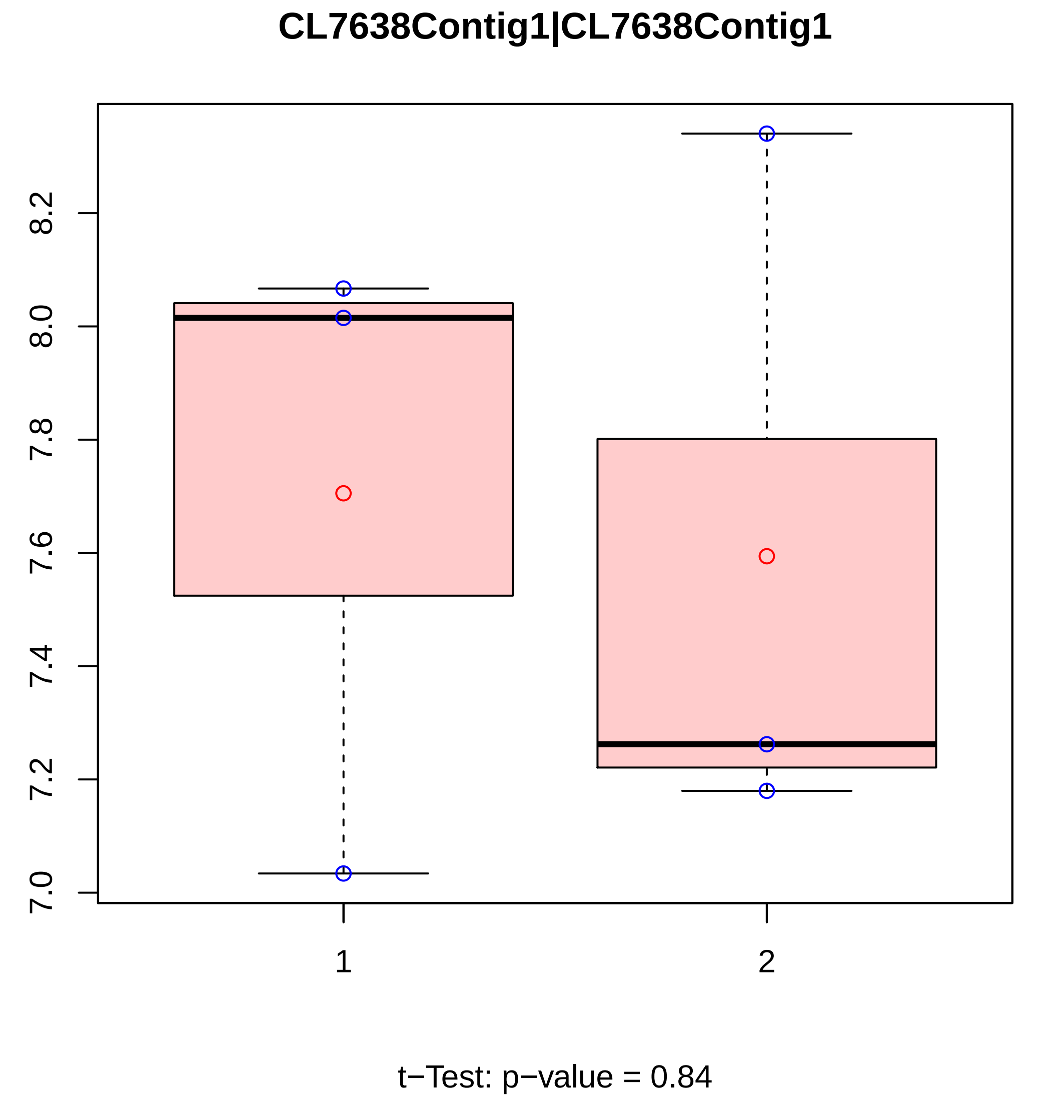
CL7638Contig1|CL7638Contig1
7.0
7.2
7.4
7.6
7.8
8.0
8.2
1
2
t−Test: p−value = 0.84

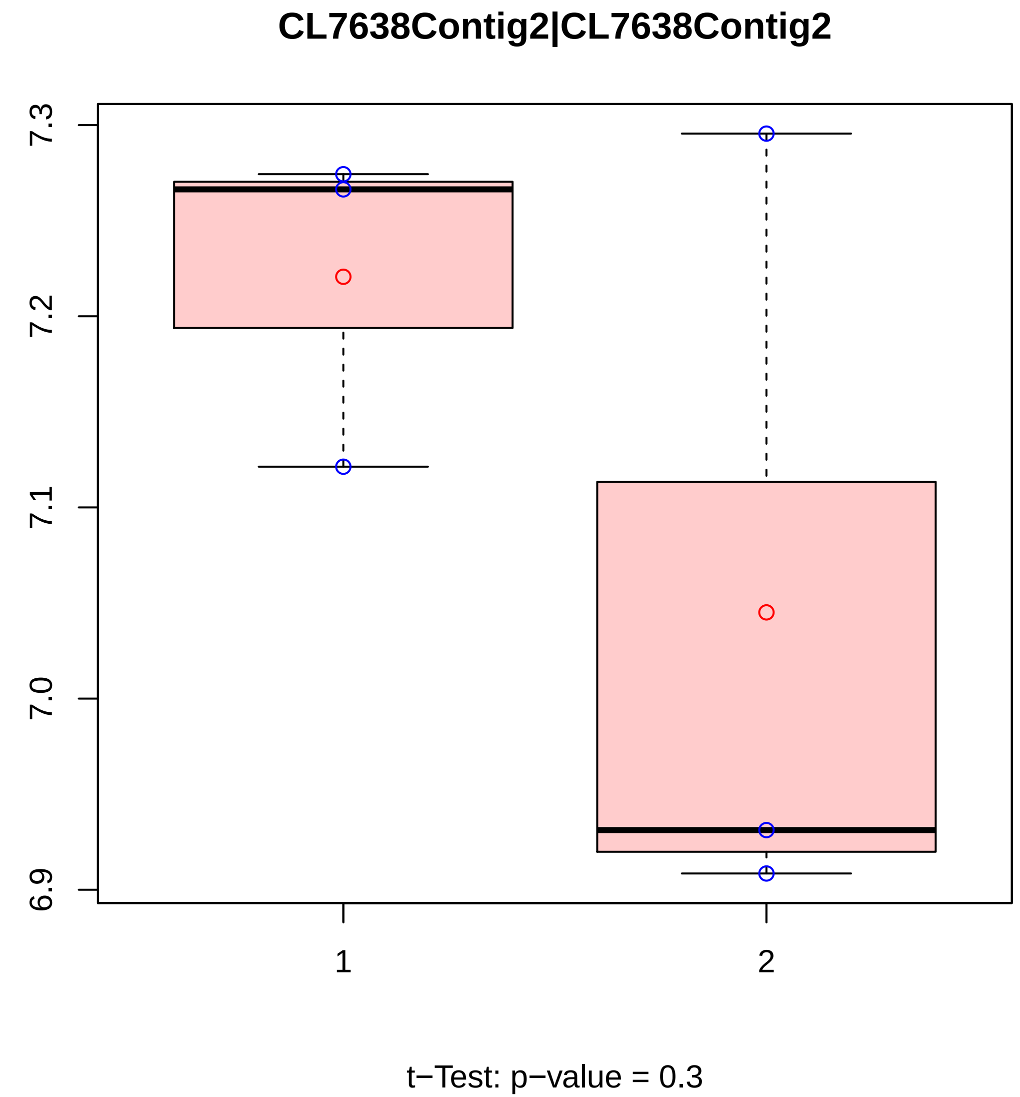

CL7638Contig2|CL7638Contig2
7.3
7.2
7.1
7.0
6.9
1
2
t−Test: p−value = 0.3

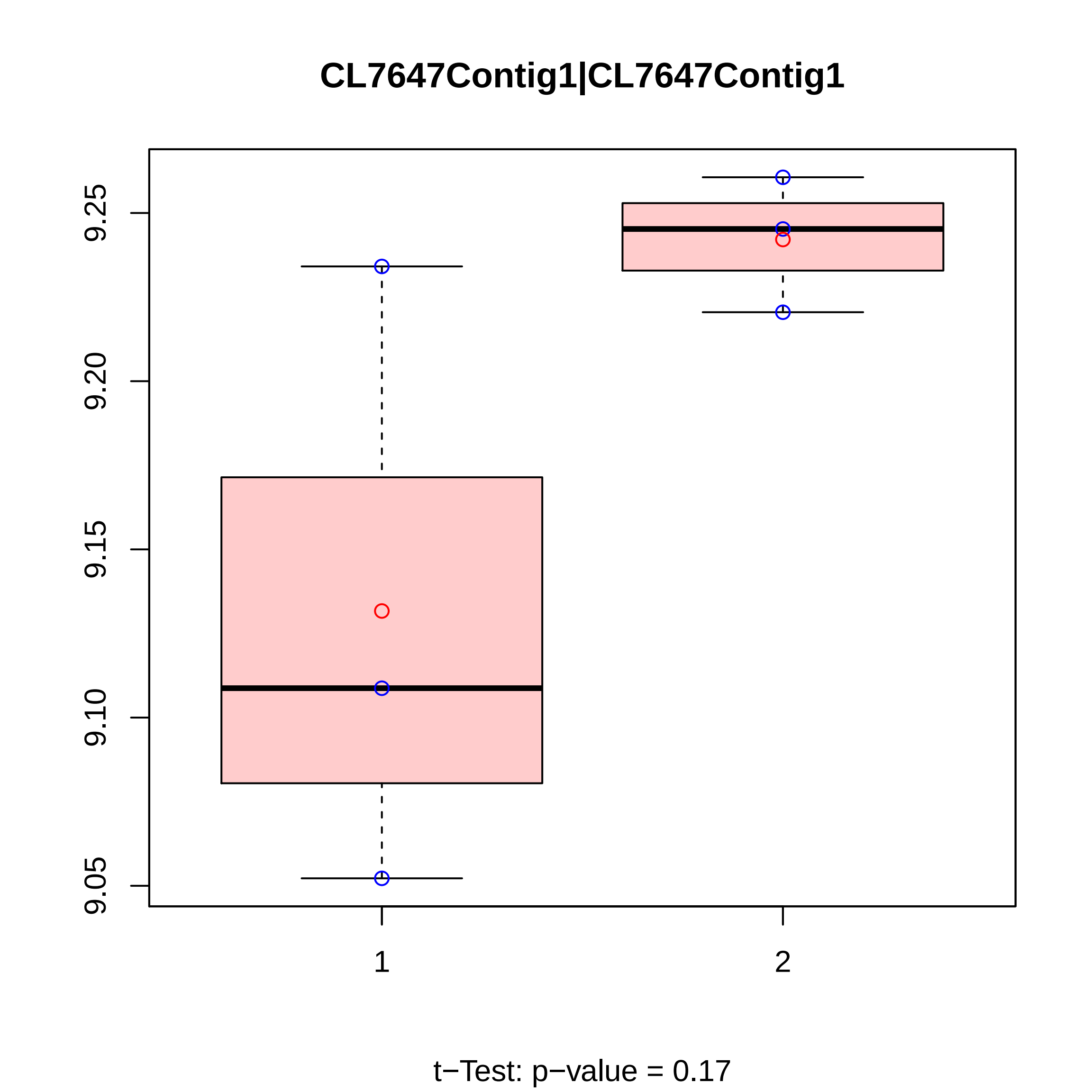
CL7647Contig1|CL7647Contig1
9.05
9.10
9.15
9.20
9.25
1
2
t−Test: p−value = 0.17

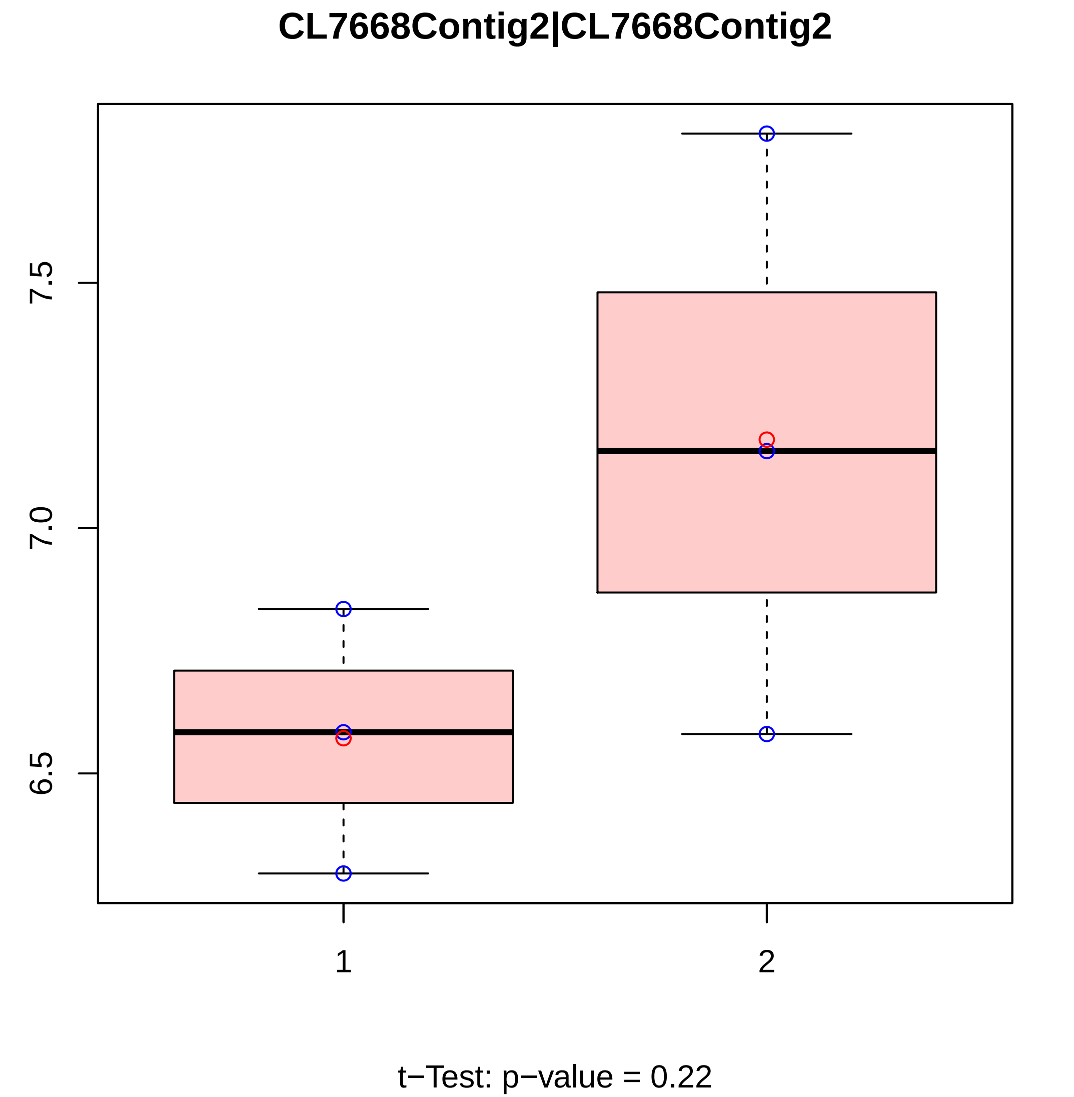

CL7668Contig2|CL7668Contig2
7.5
7.0
6.5
1
2
t−Test: p−value = 0.22

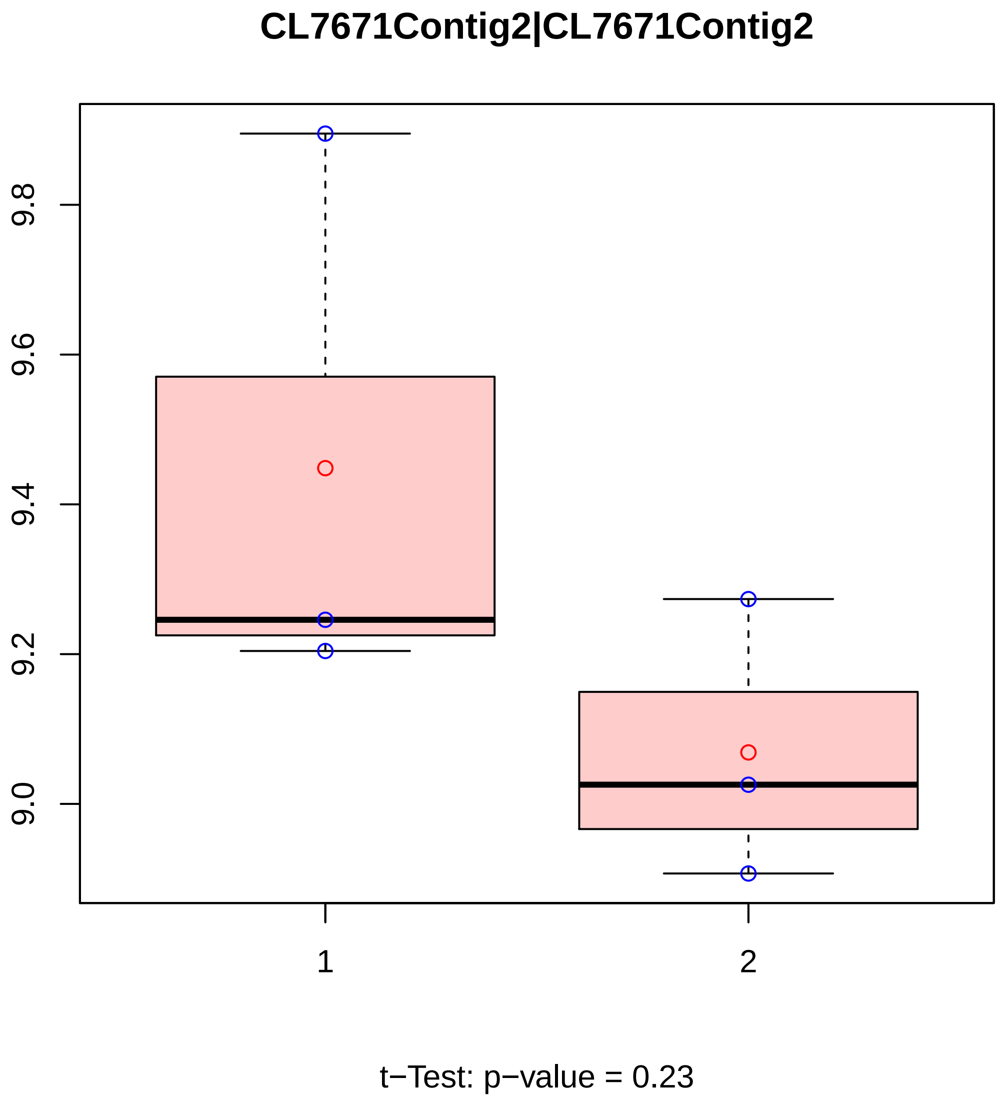
CL7671Contig2|CL7671Contig2
9.0
9.2
9.4
9.6
9.8
1
2
t−Test: p−value = 0.23

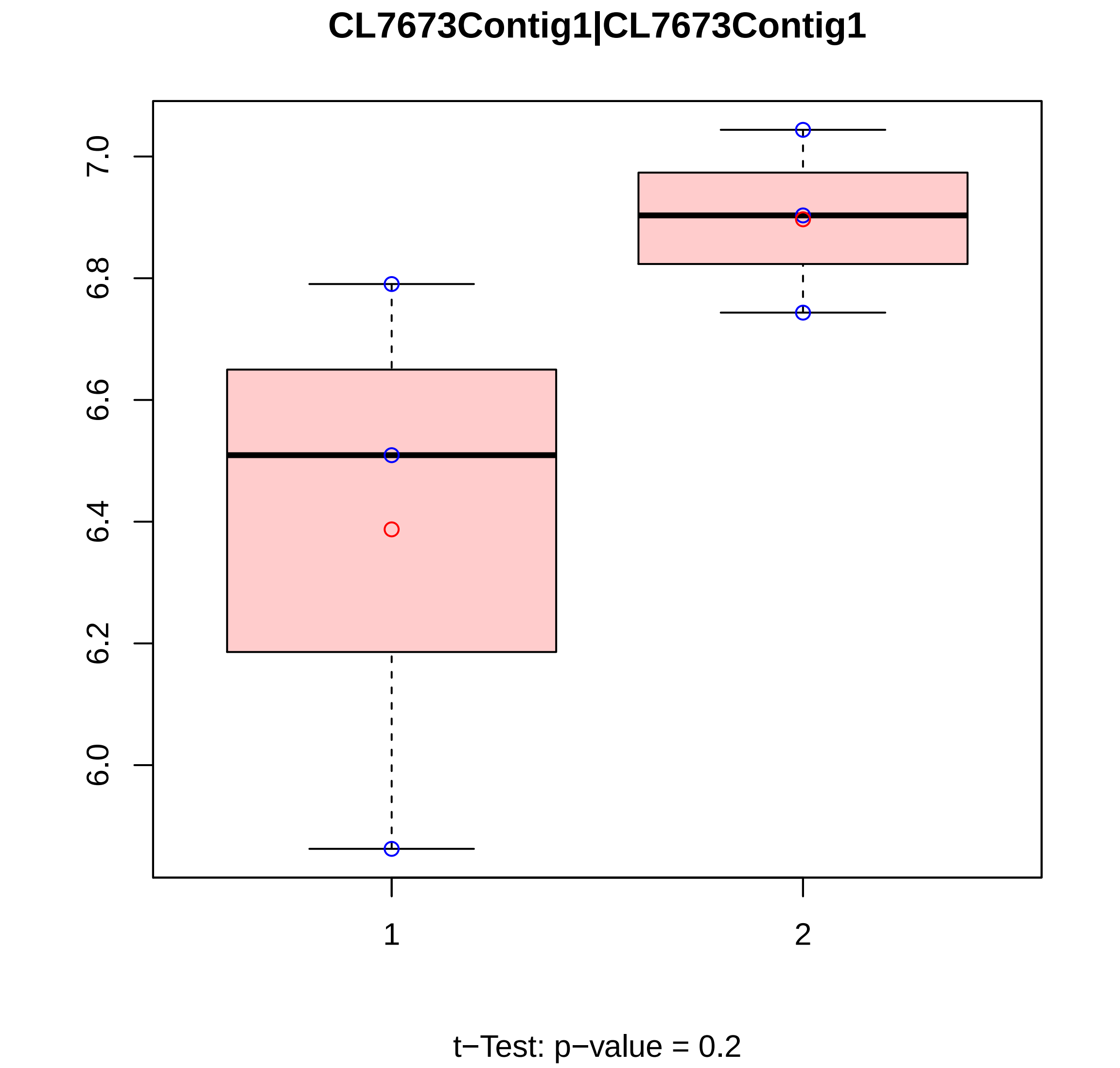
CL7673Contig1|CL7673Contig1
7.0
6.8
6.6
6.4
6.2
6.0
1
2
t−Test: p−value = 0.2

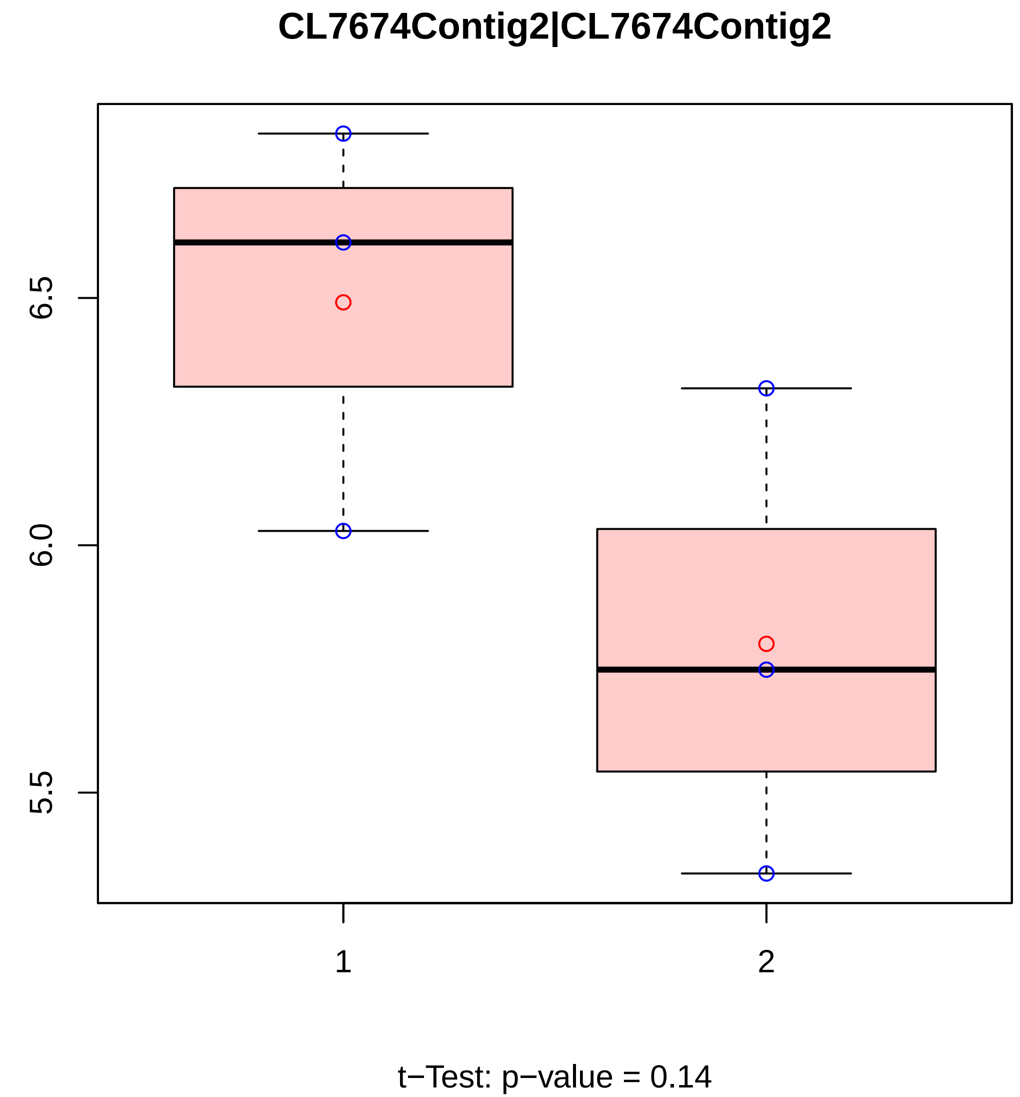

CL7674Contig2|CL7674Contig2
6.5
6.0
5.5
1
2
t−Test: p−value = 0.14

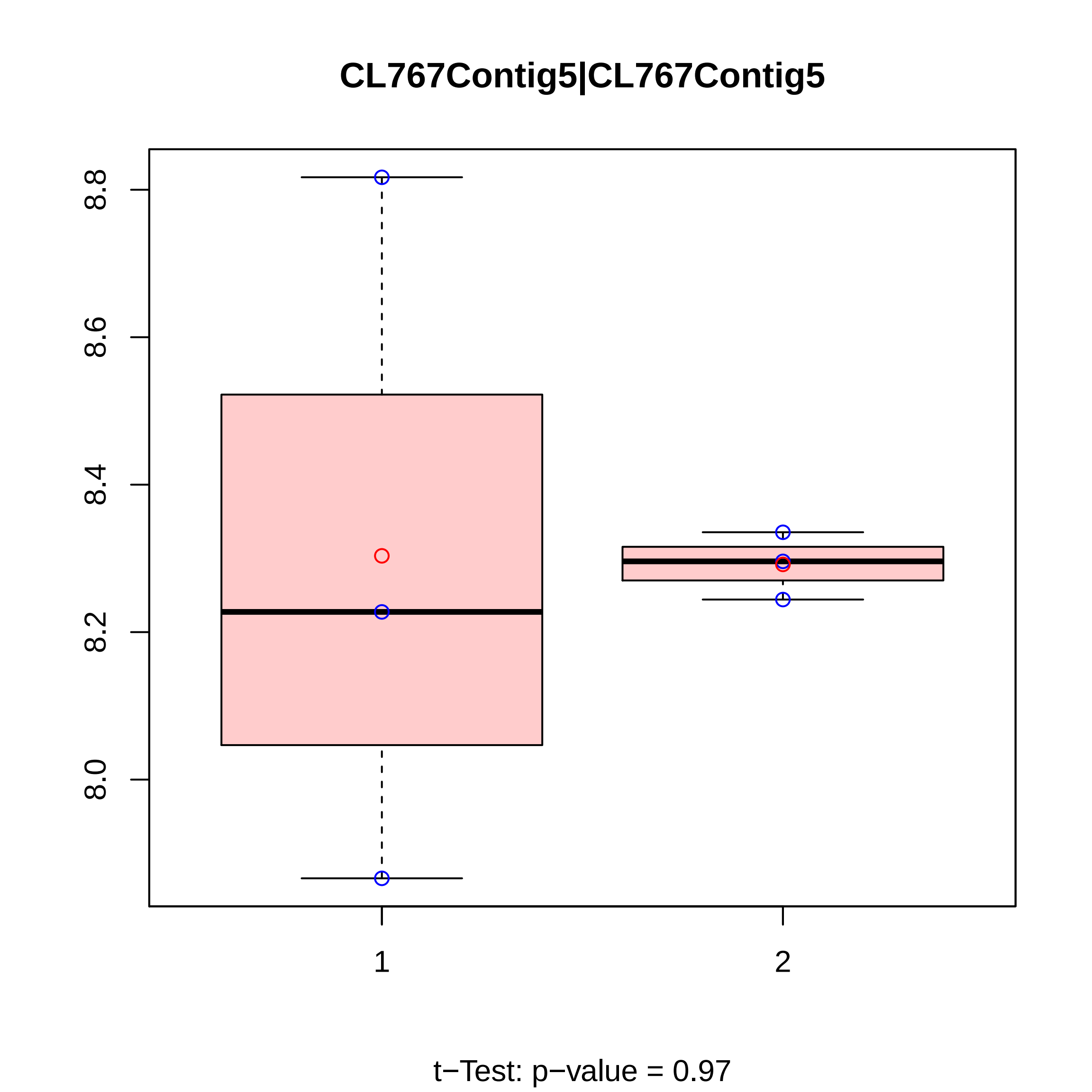

CL767Contig5|CL767Contig5
8.0
8.2
8.4
8.6
8.8
1
2
t−Test: p−value = 0.97

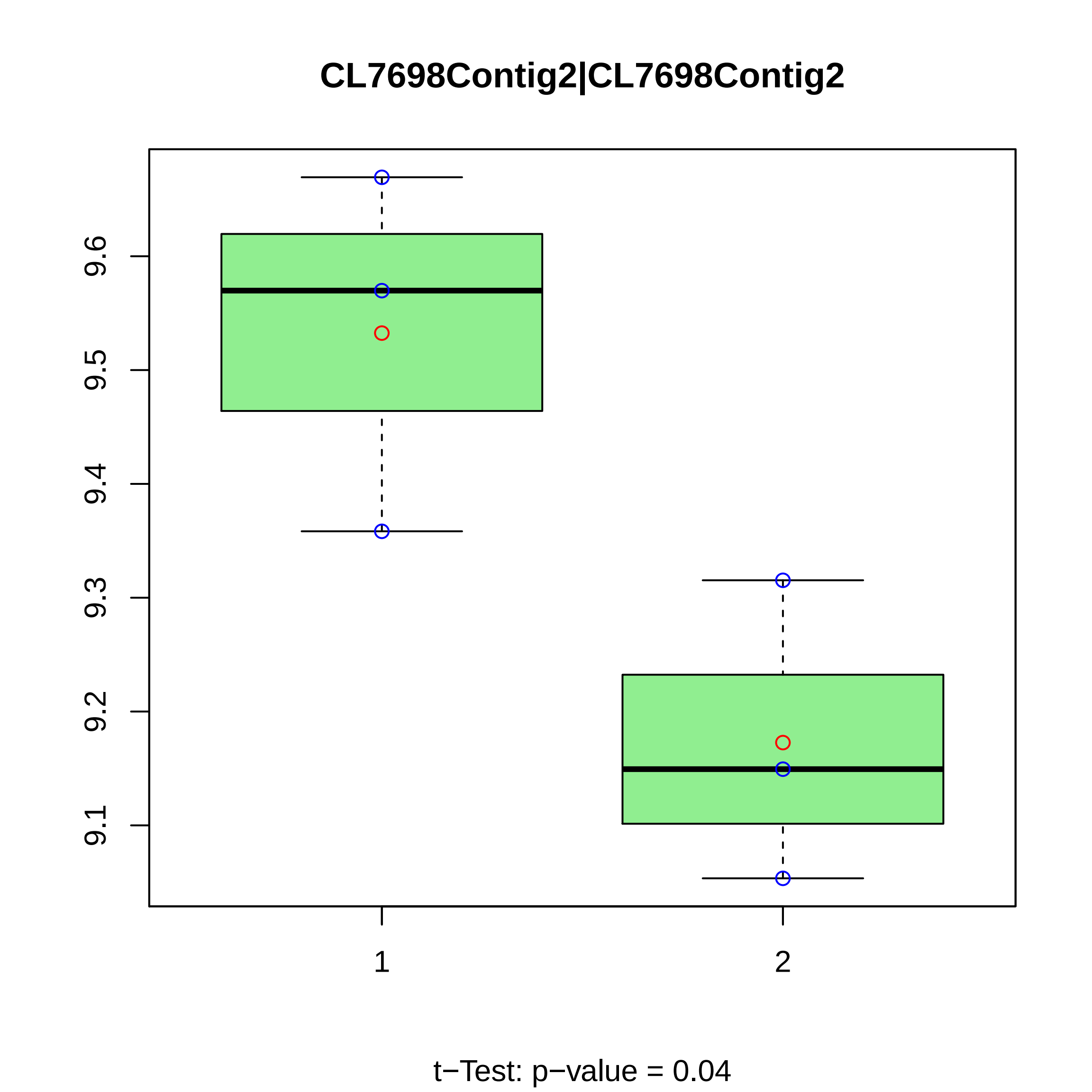

CL7698Contig2|CL7698Contig2
9.1
9.2
9.3
9.4
9.5
9.6
1
2
t−Test: p−value = 0.04

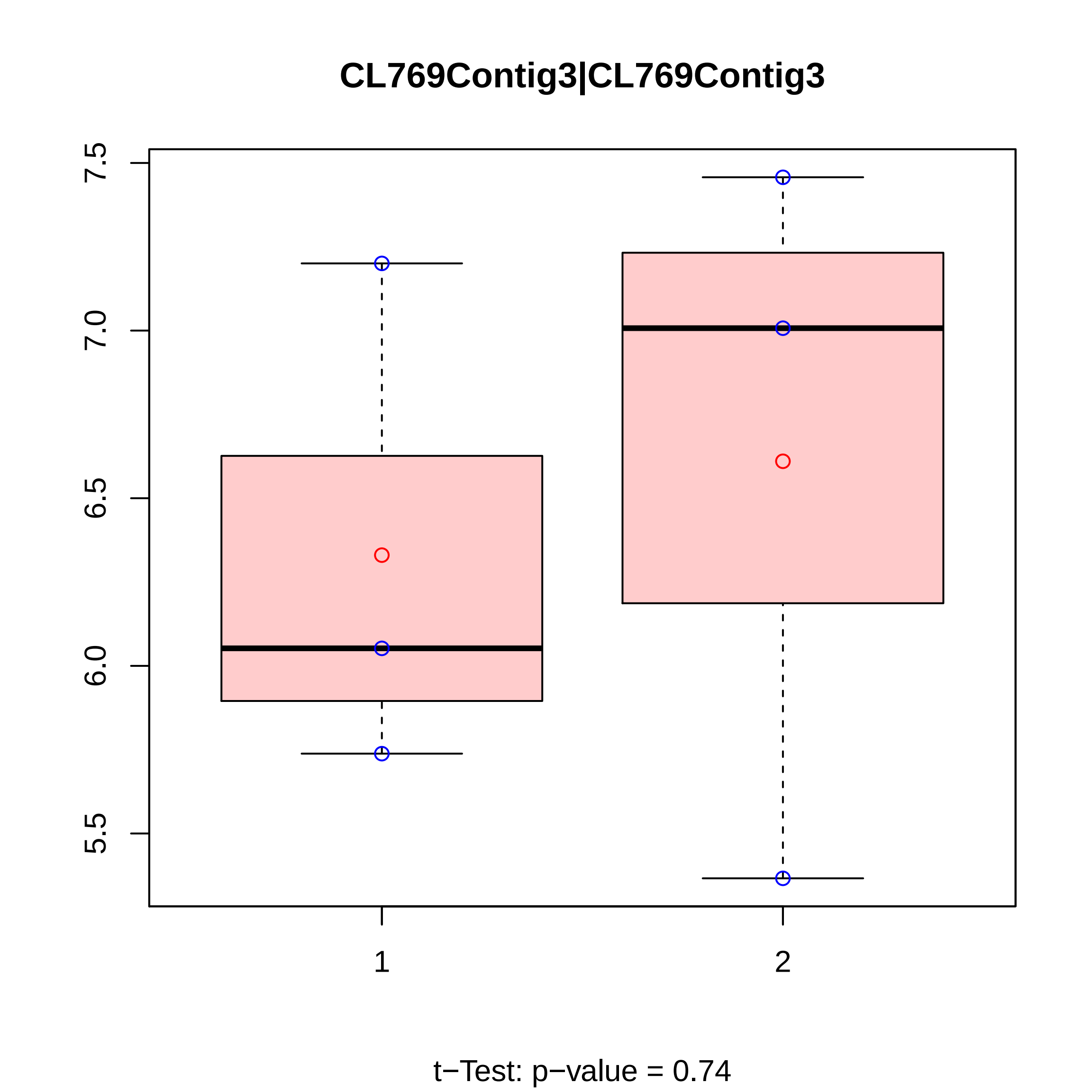

CL769Contig3|CL769Contig3
7.5
7.0
6.5
6.0
5.5
1
2
t−Test: p−value = 0.74

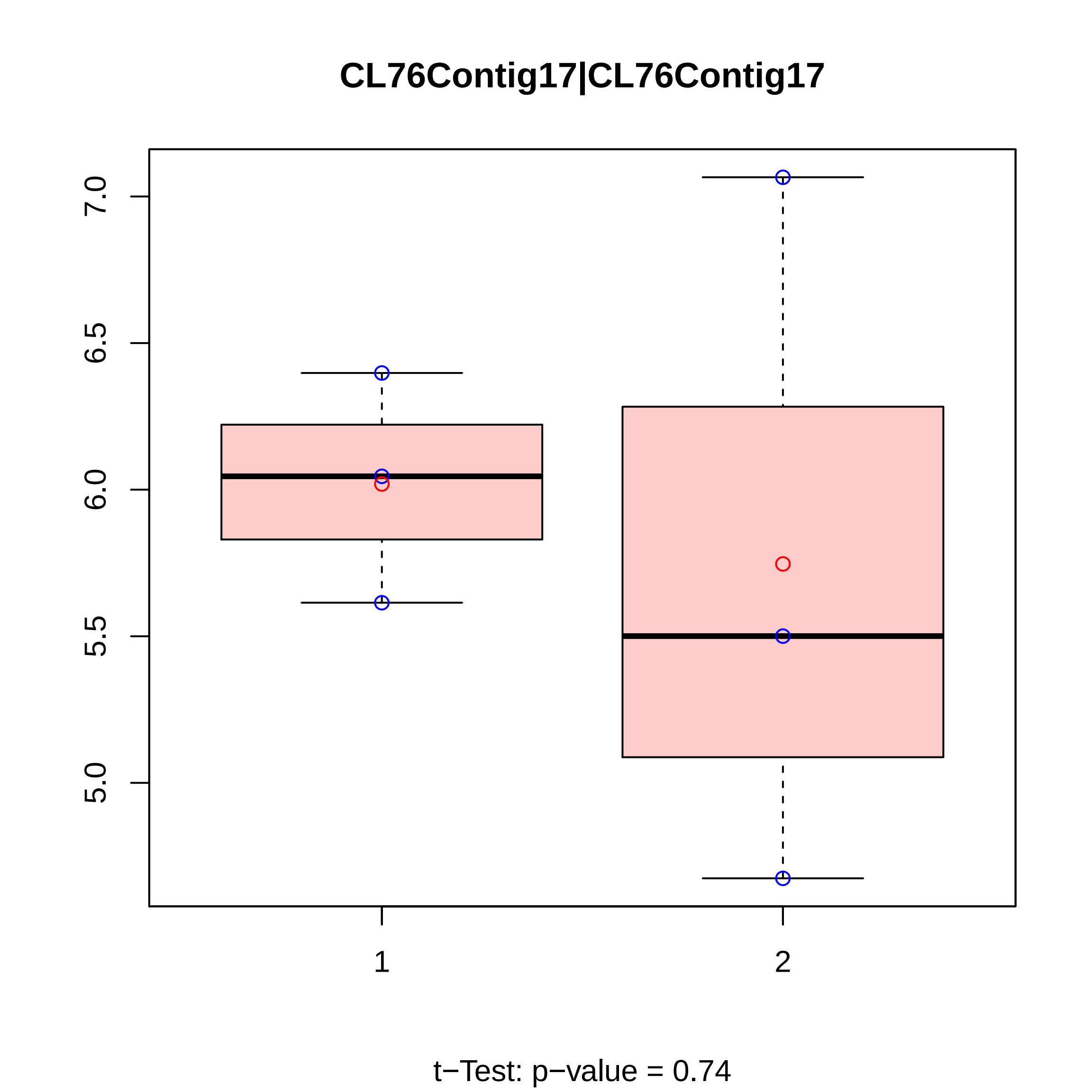

CL76Contig17|CL76Contig17
7.0
6.5
6.0
5.5
5.0
1
2
t−Test: p−value = 0.74

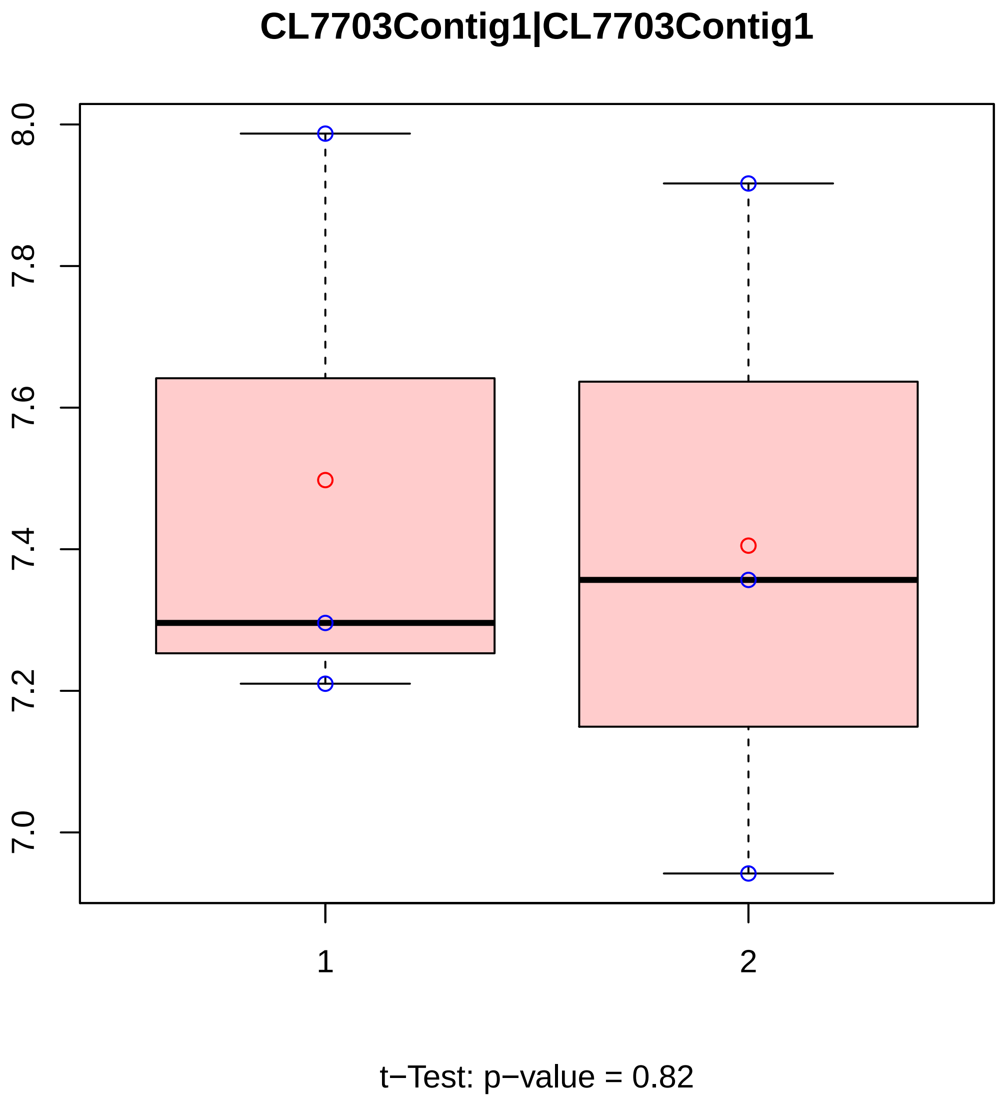

CL7703Contig1|CL7703Contig1
8.0
7.8
7.6
7.4
7.2
7.0
1
2
t−Test: p−value = 0.82

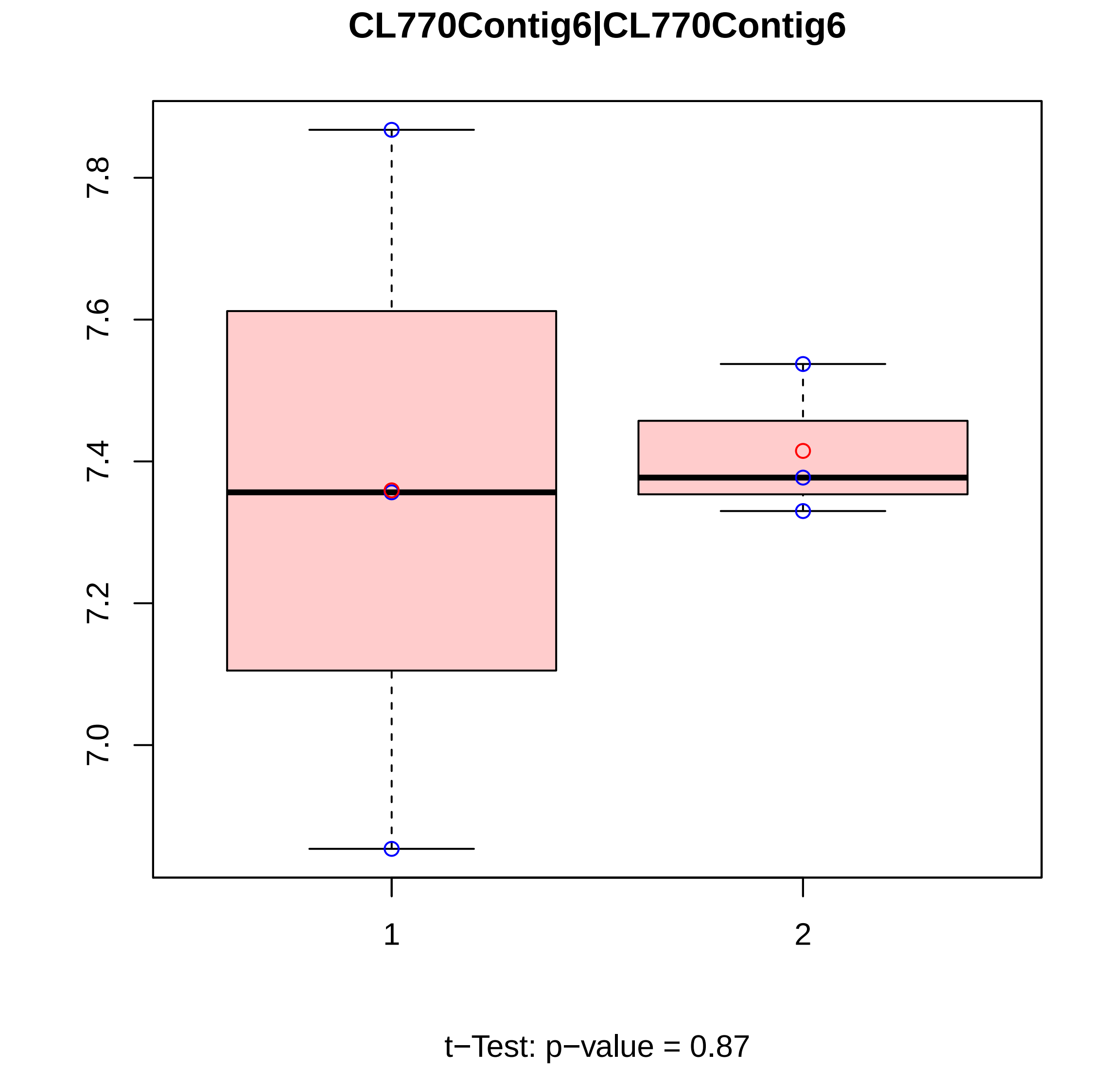

CL770Contig6|CL770Contig6
7.0
7.2
7.4
7.6
7.8
1
2
t−Test: p−value = 0.87

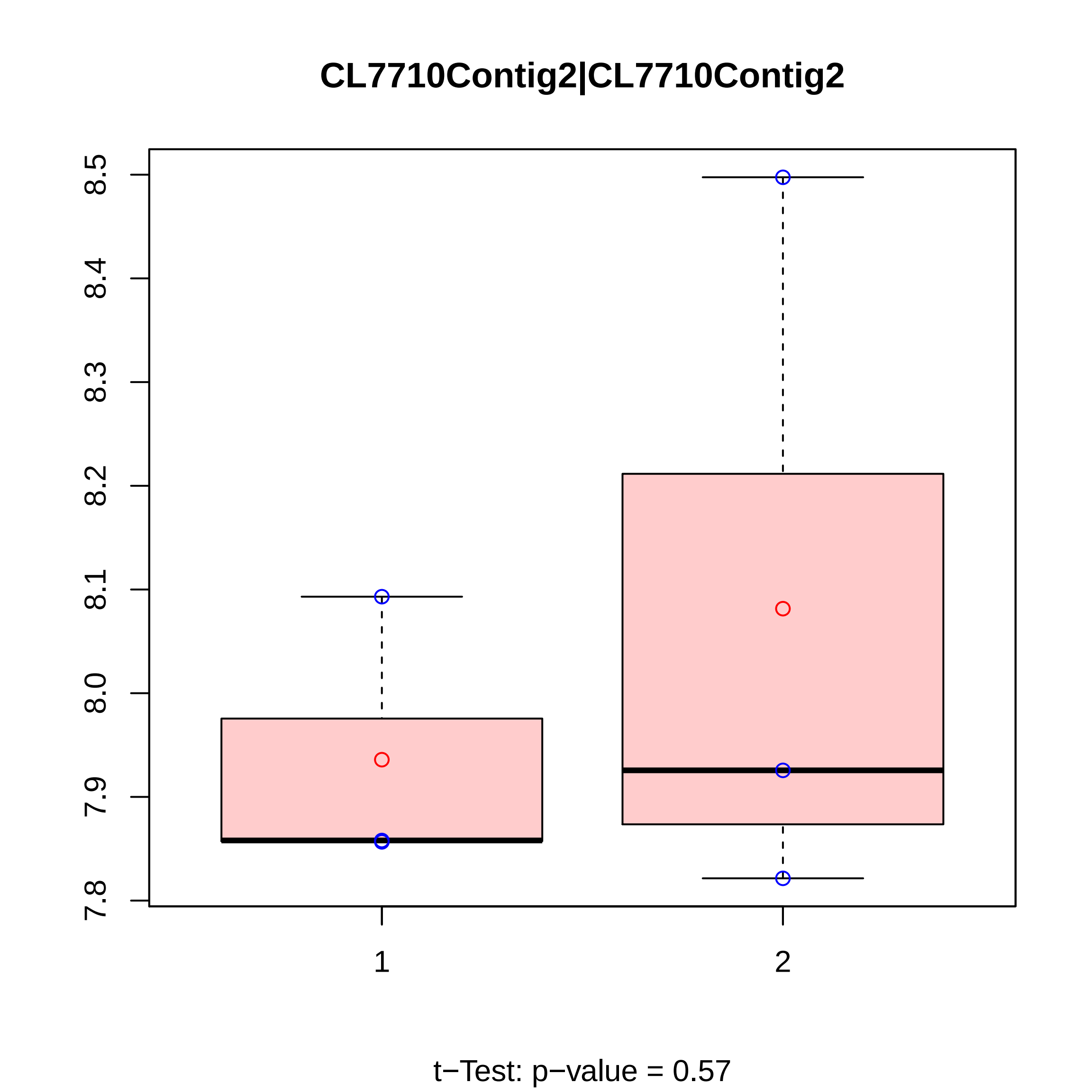

CL7710Contig2|CL7710Contig2
8.5
8.4
8.3
8.2
8.1
8.0
7.9
7.8
1
2
t−Test: p−value = 0.57

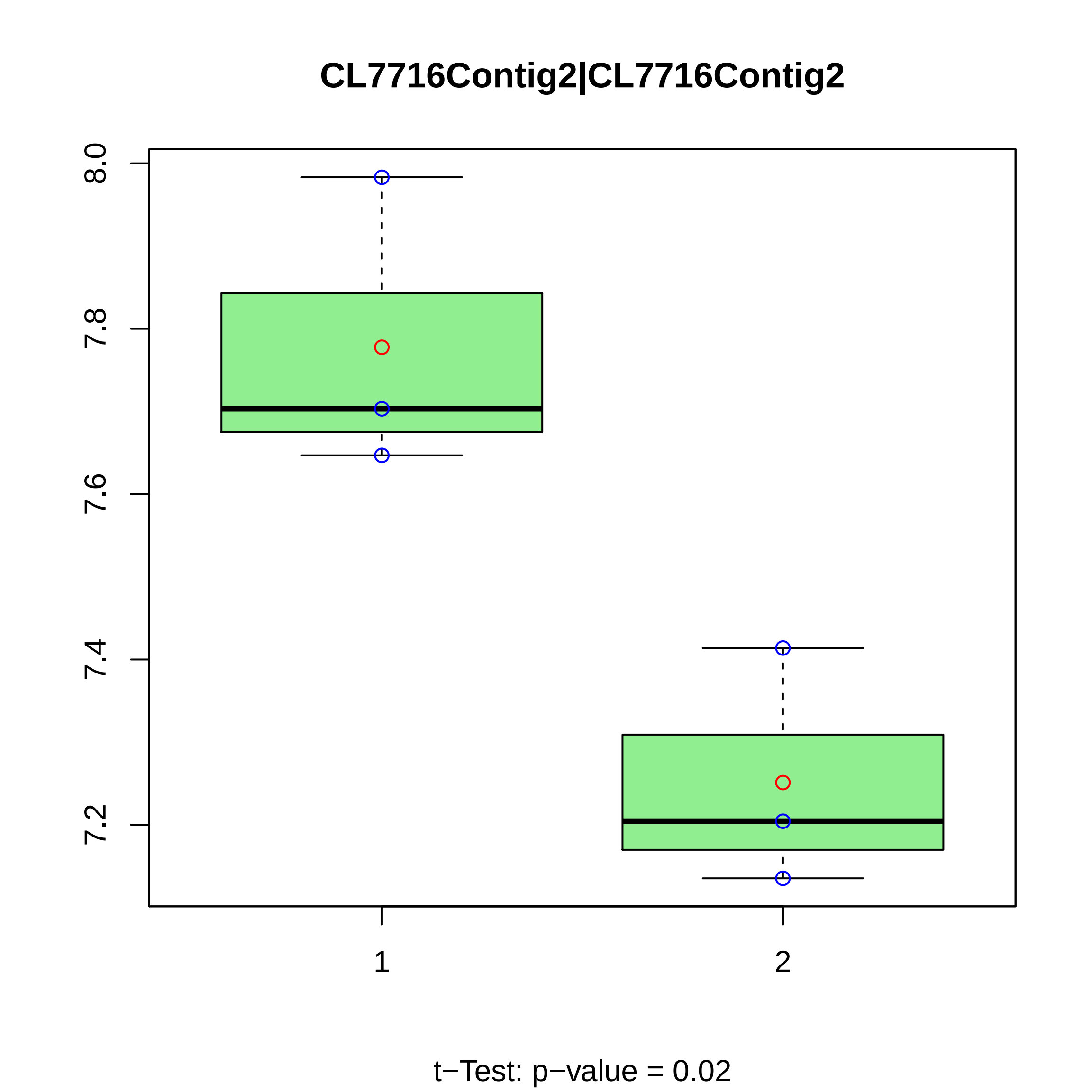
CL7716Contig2|CL7716Contig2
8.0
7.8
7.6
7.4
7.2
1
2
t−Test: p−value = 0.02

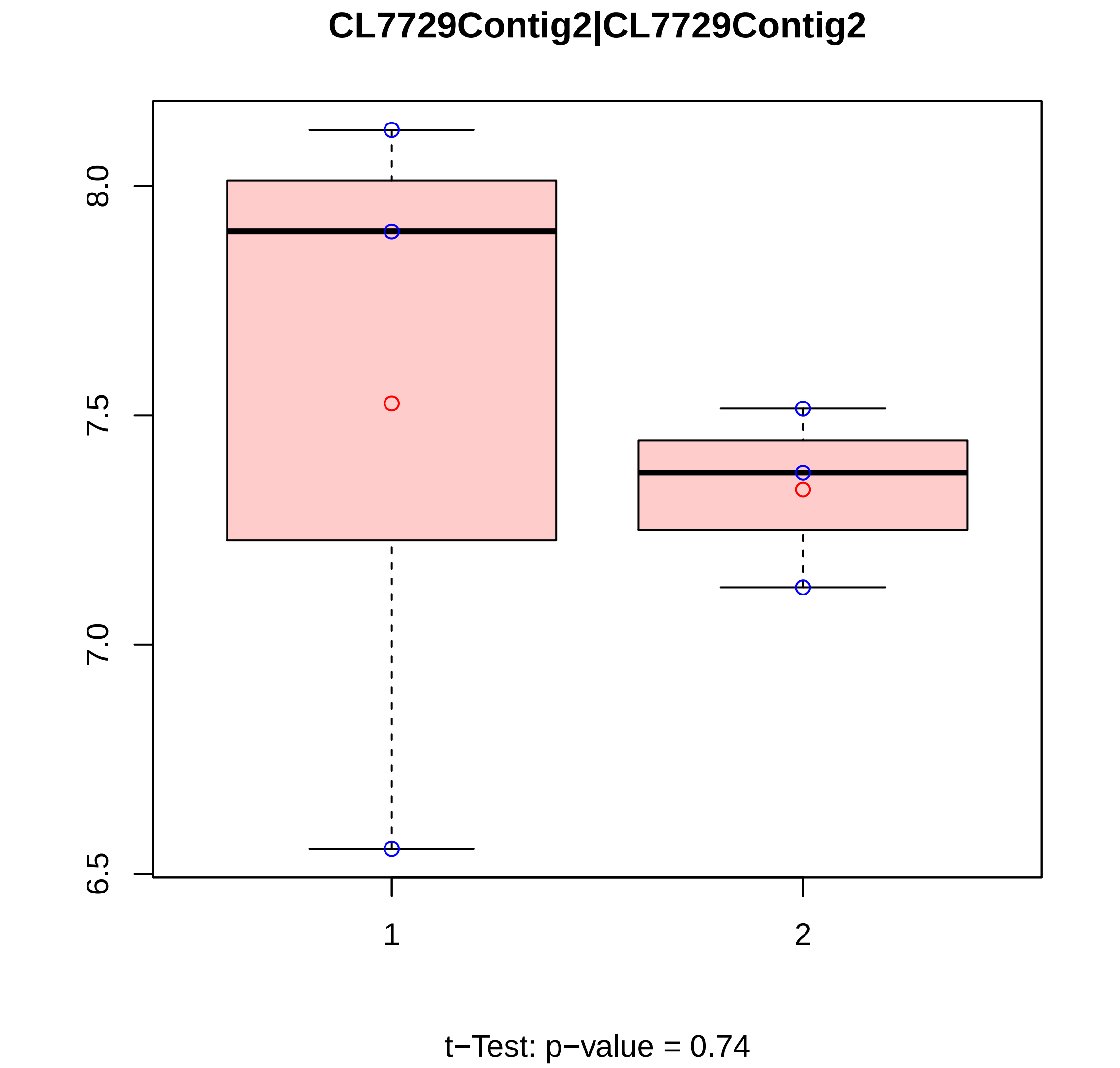
CL7729Contig2|CL7729Contig2
8.0
7.5
7.0
6.5
1
2
t−Test: p−value = 0.74

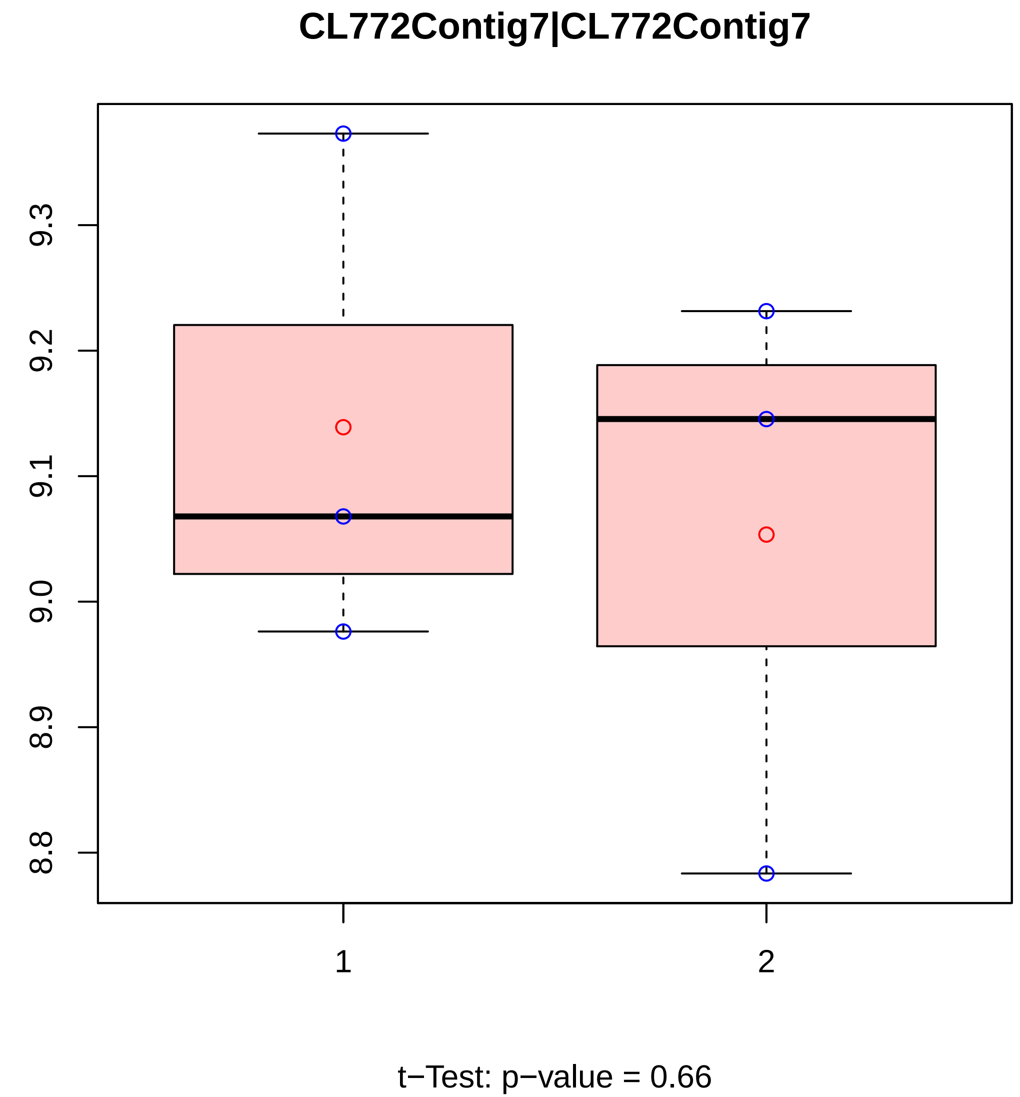

CL772Contig7|CL772Contig7
9.3
9.2
9.1
9.0
8.9
8.8
1
2
t−Test: p−value = 0.66

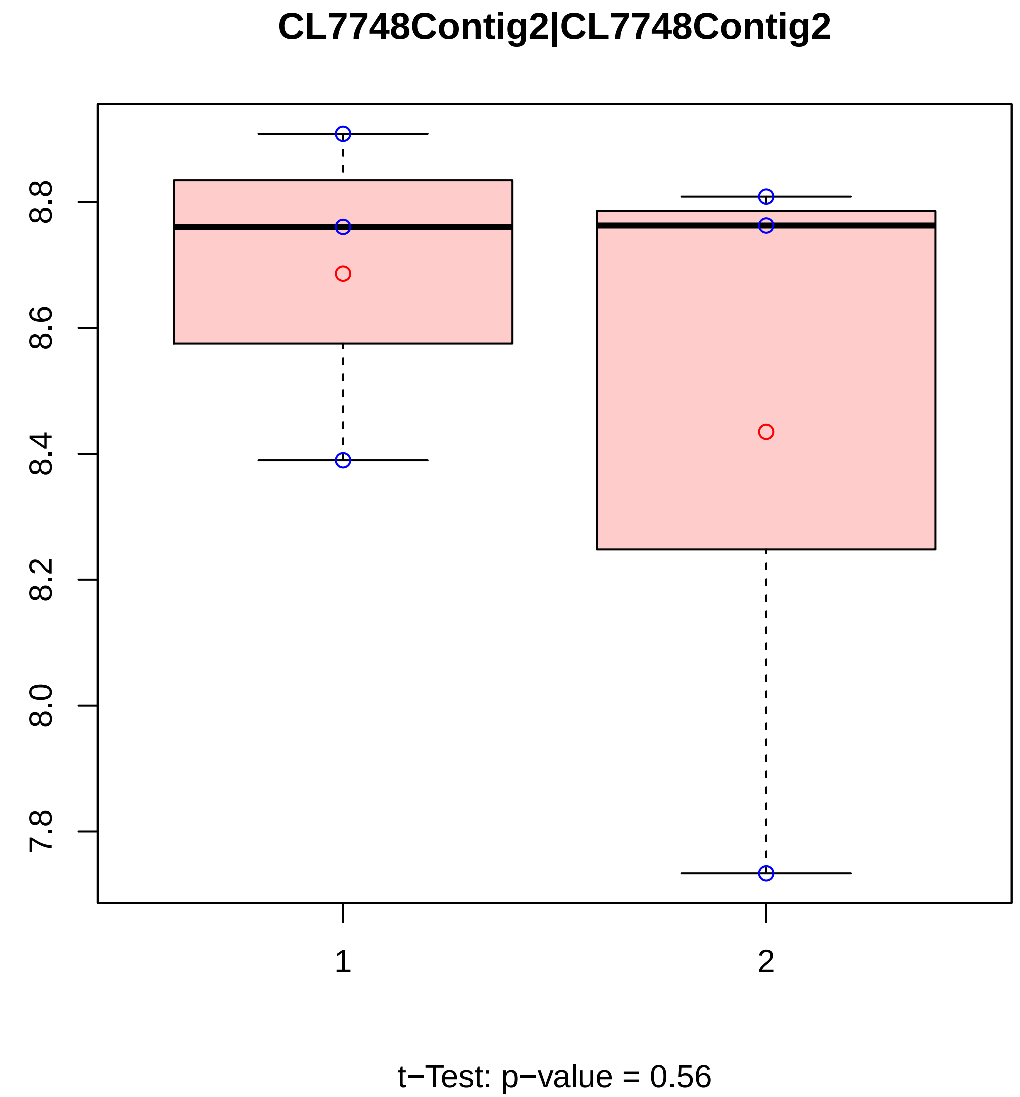
CL7748Contig2|CL7748Contig2
8.8
8.6
8.4
8.2
8.0
7.8
1
2
t−Test: p−value = 0.56

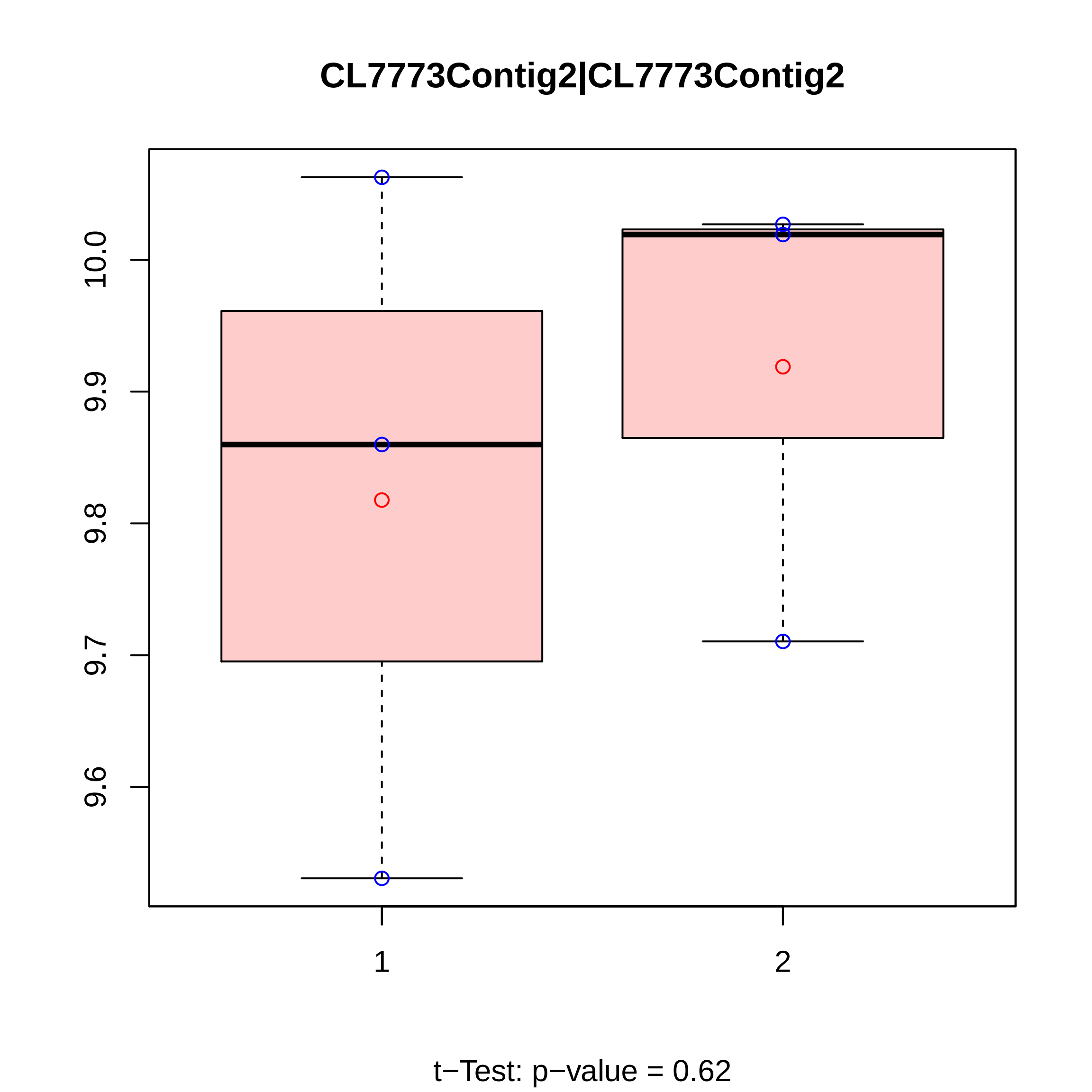

CL7773Contig2|CL7773Contig2
10.0
9.9
9.8
9.7
9.6
1
2
t−Test: p−value = 0.62

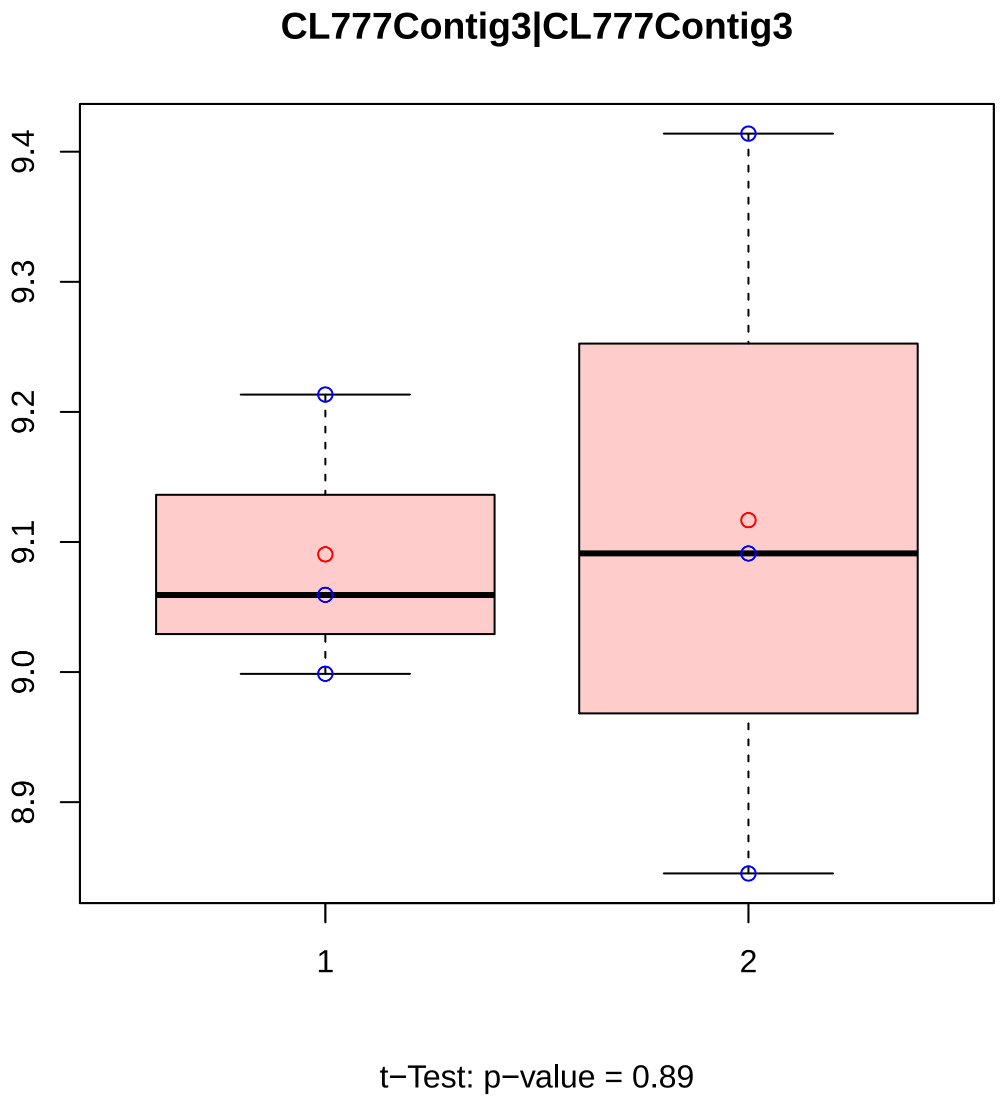
CL777Contig3|CL777Contig3
9.4
9.3
9.2
9.1
9.0
8.9
1
2
t−Test: p−value = 0.89

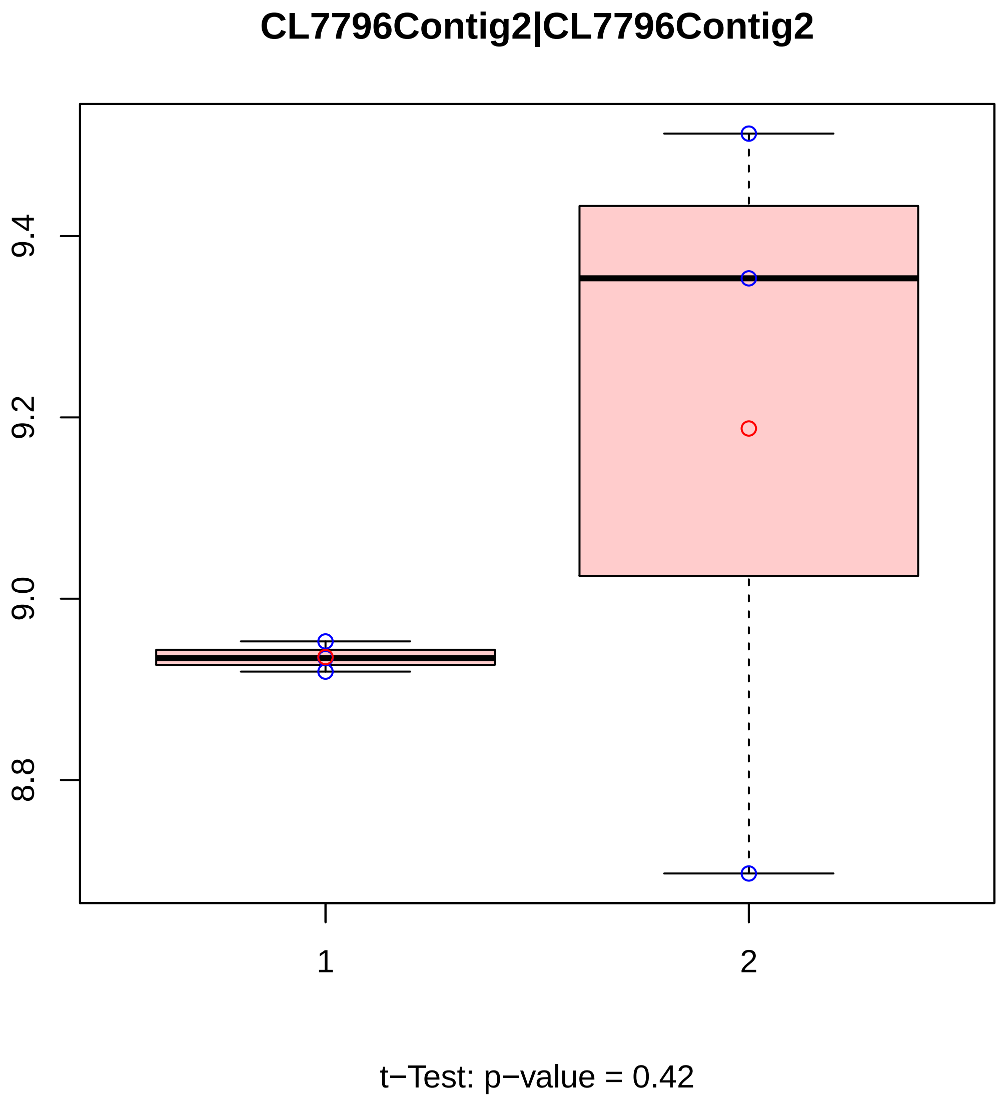

CL7796Contig2|CL7796Contig2
9.4
9.2
9.0
8.8
1
2
t−Test: p−value = 0.42

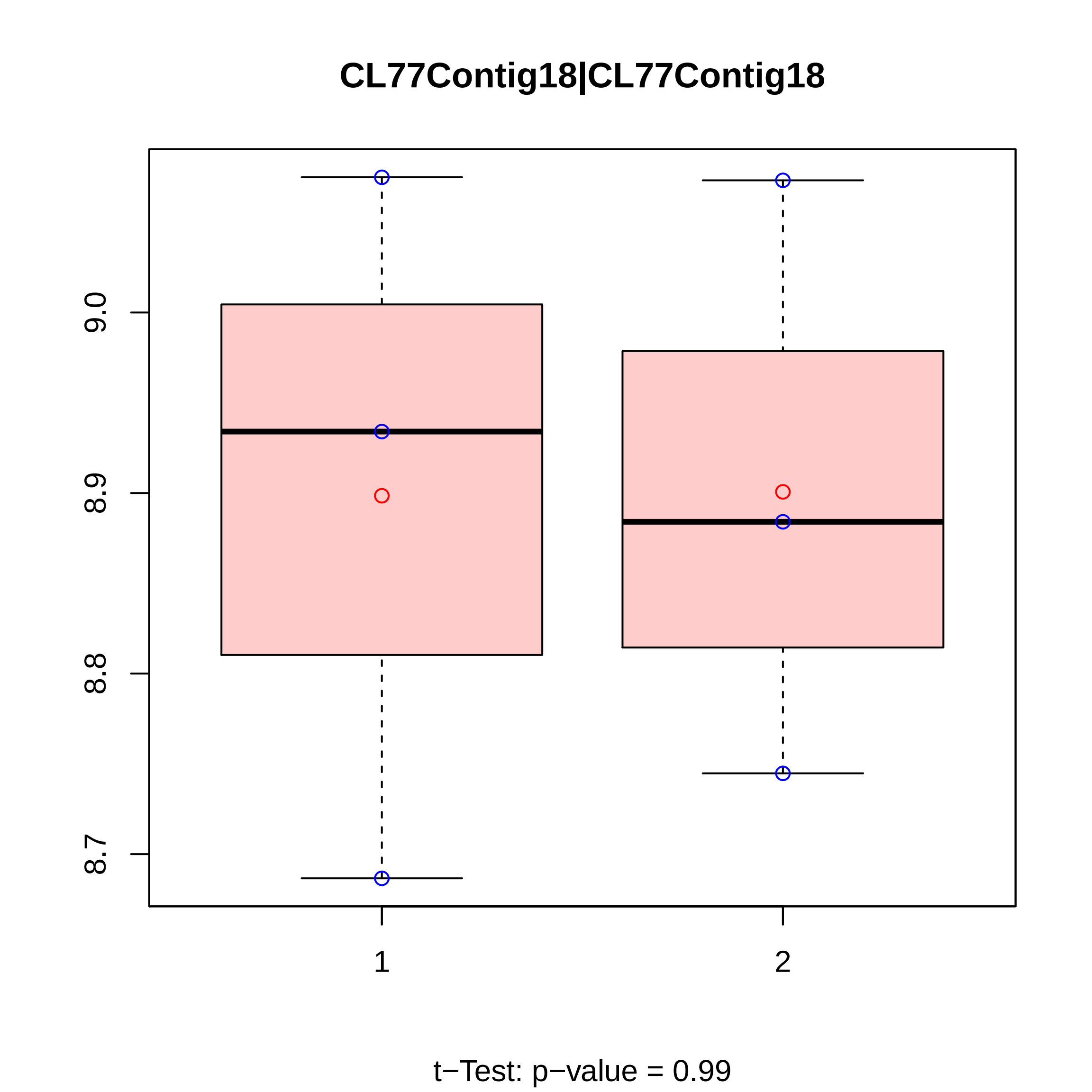

CL77Contig18|CL77Contig18
9.0
8.9
8.8
8.7
1
2
t−Test: p−value = 0.99

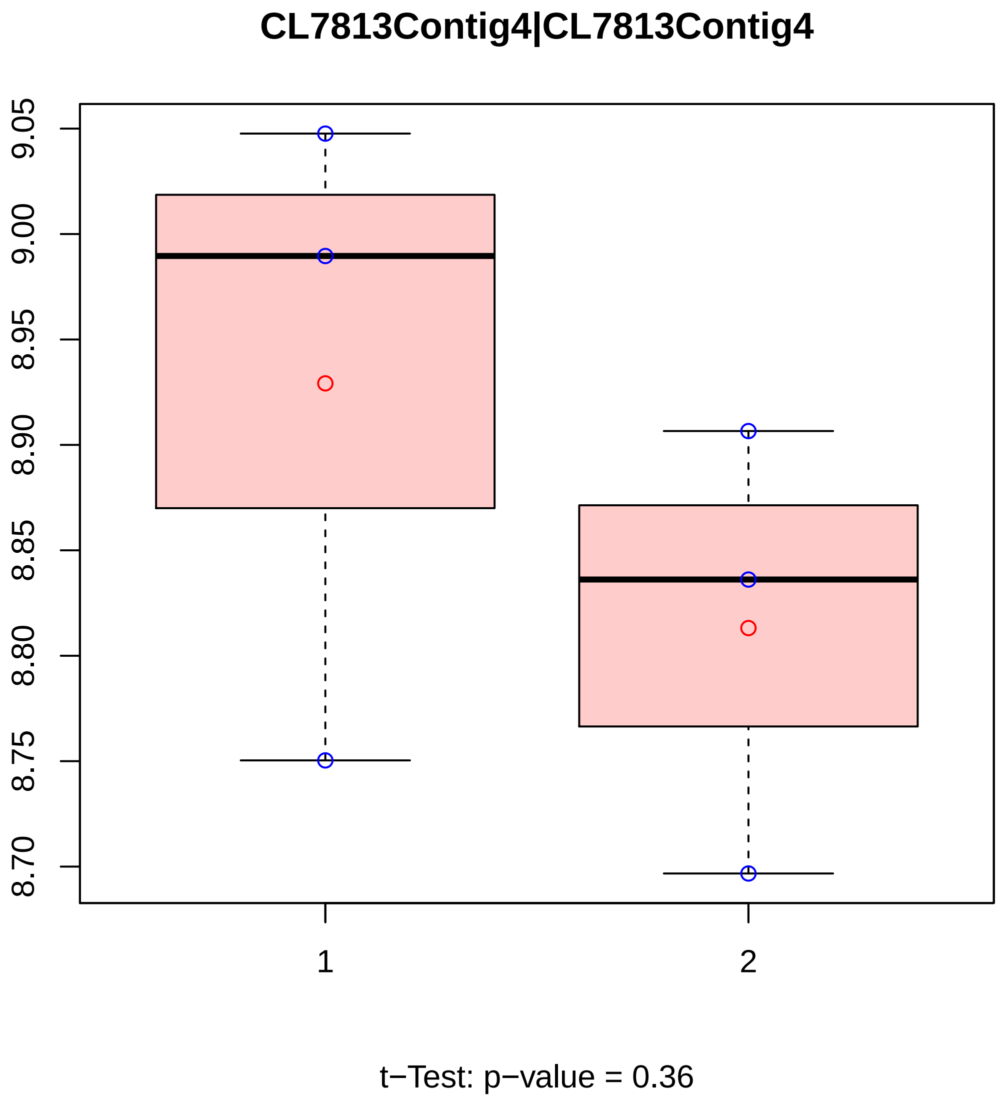
CL7813Contig4|CL7813Contig4
9.05
9.00
8.95
8.90
8.85
8.80
8.75
8.70
1
2
t−Test: p−value = 0.36

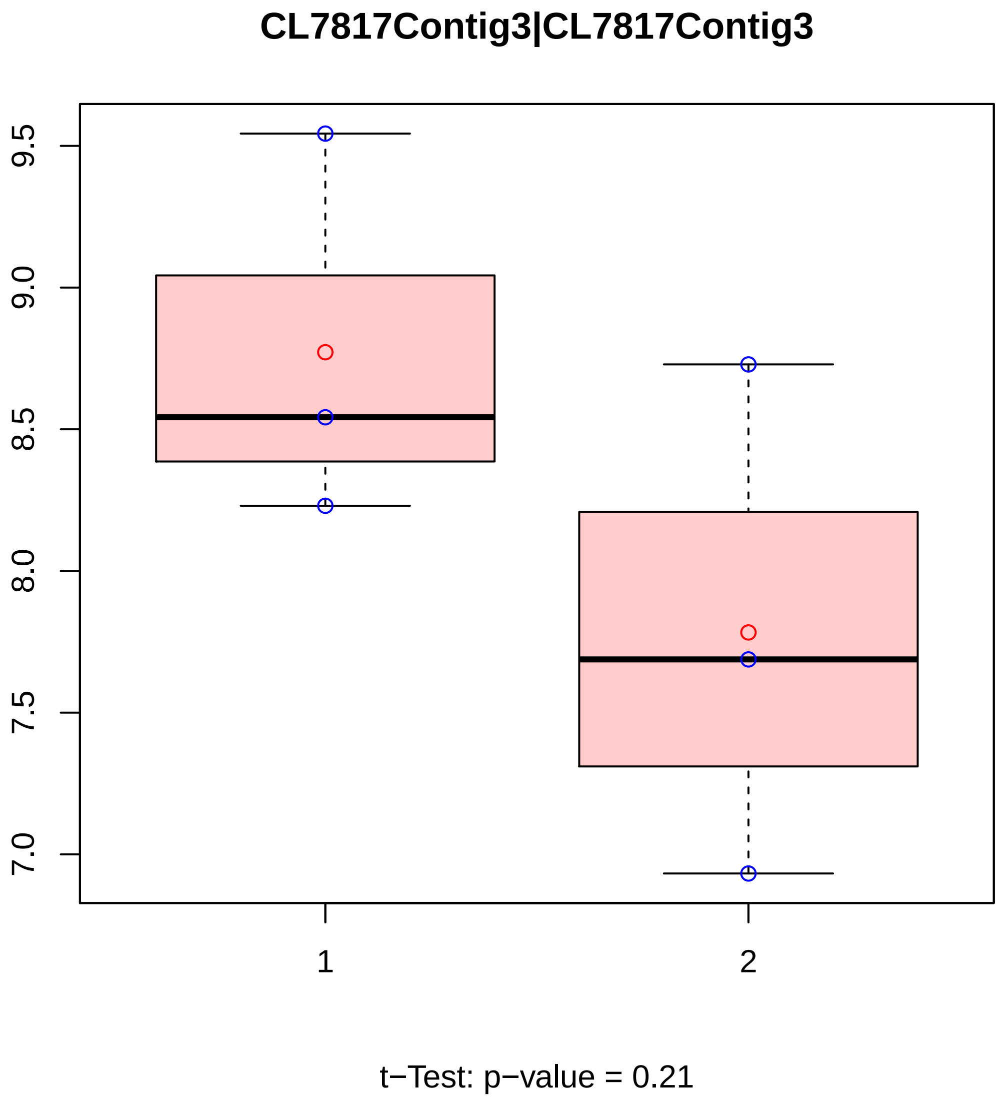

CL7817Contig3|CL7817Contig3
9.5
9.0
8.5
8.0
7.5
7.0
1
2
t−Test: p−value = 0.21

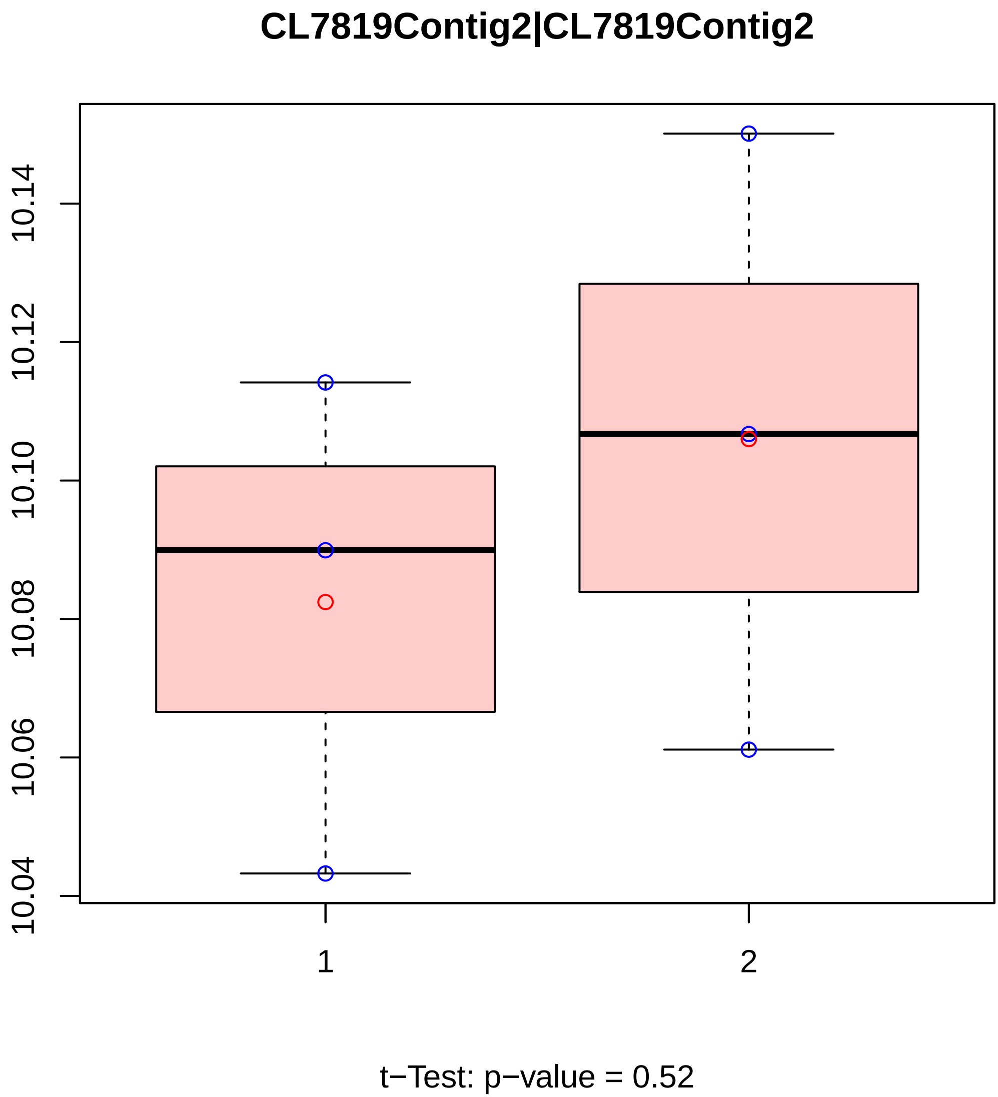

CL7819Contig2|CL7819Contig2
10.04
10.06
10.08
10.10
10.12
10.14
1
2
t−Test: p−value = 0.52

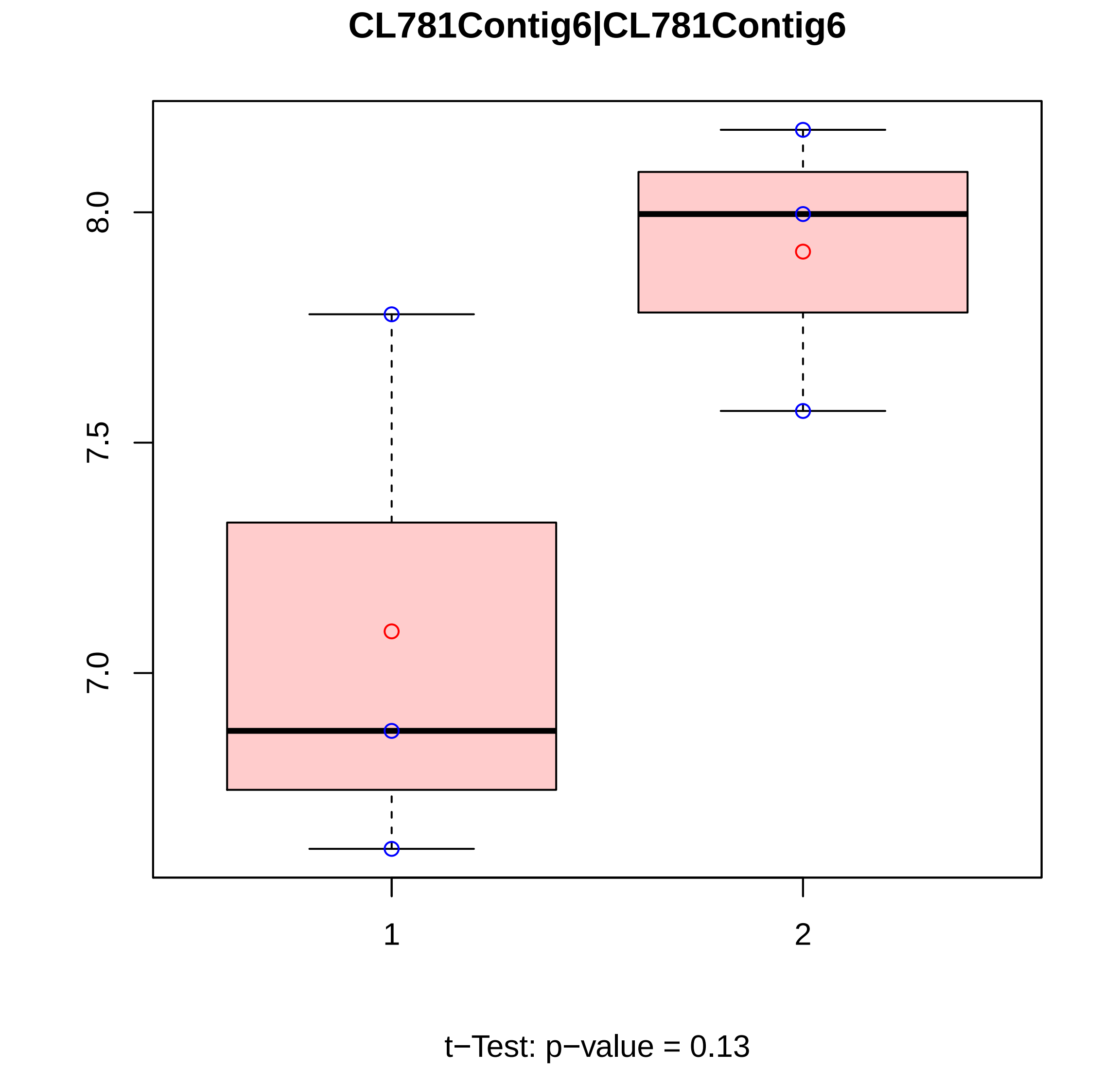
CL781Contig6|CL781Contig6
8.0
7.5
7.0
1
2
t−Test: p−value = 0.13

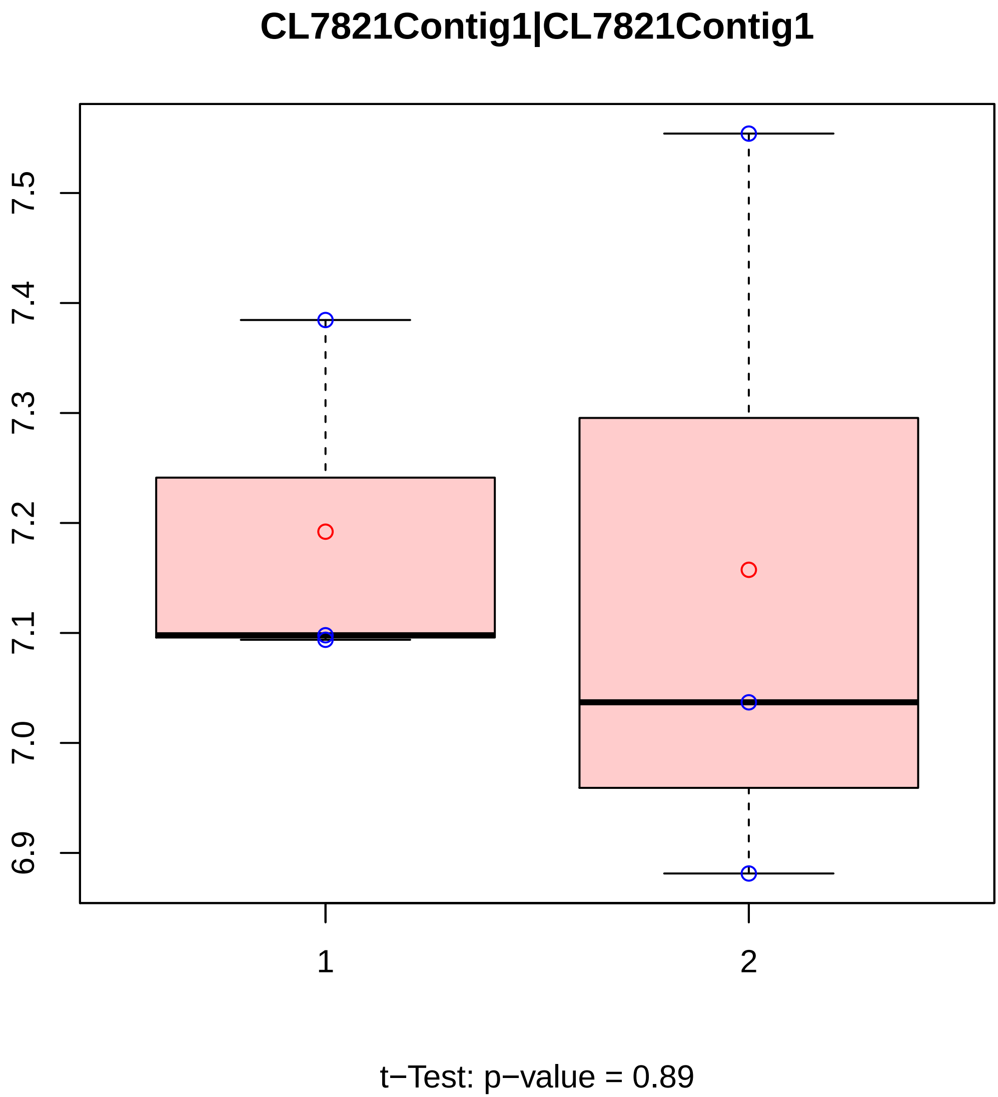
CL7821Contig1|CL7821Contig1
7.5
7.4
7.3
7.2
7.1
7.0
6.9
1
2
t−Test: p−value = 0.89

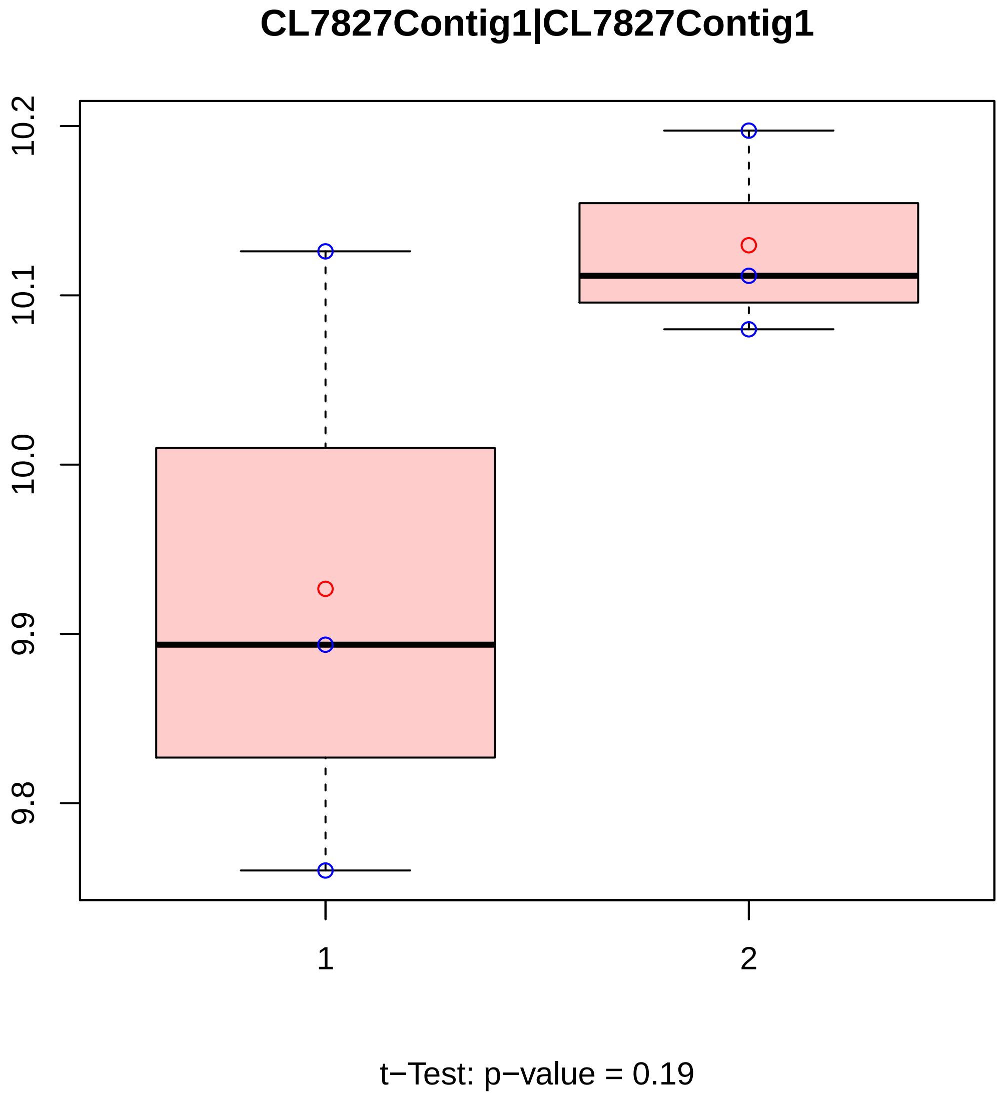
CL7827Contig1|CL7827Contig1
10.2
10.1
10.0
9.9
9.8
1
2
t−Test: p−value = 0.19

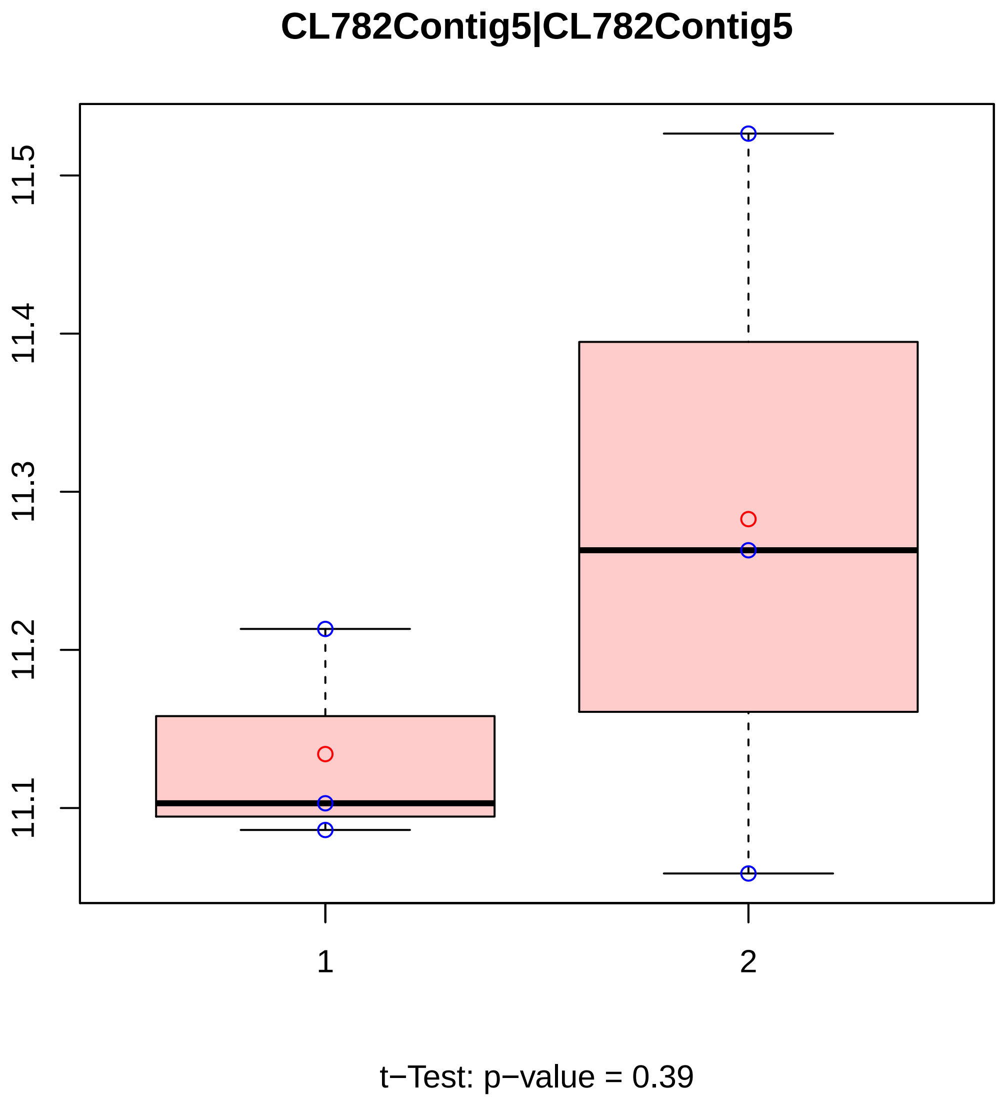

CL782Contig5|CL782Contig5
11.1
11.2
11.3
11.4
11.5
1
2
t−Test: p−value = 0.39

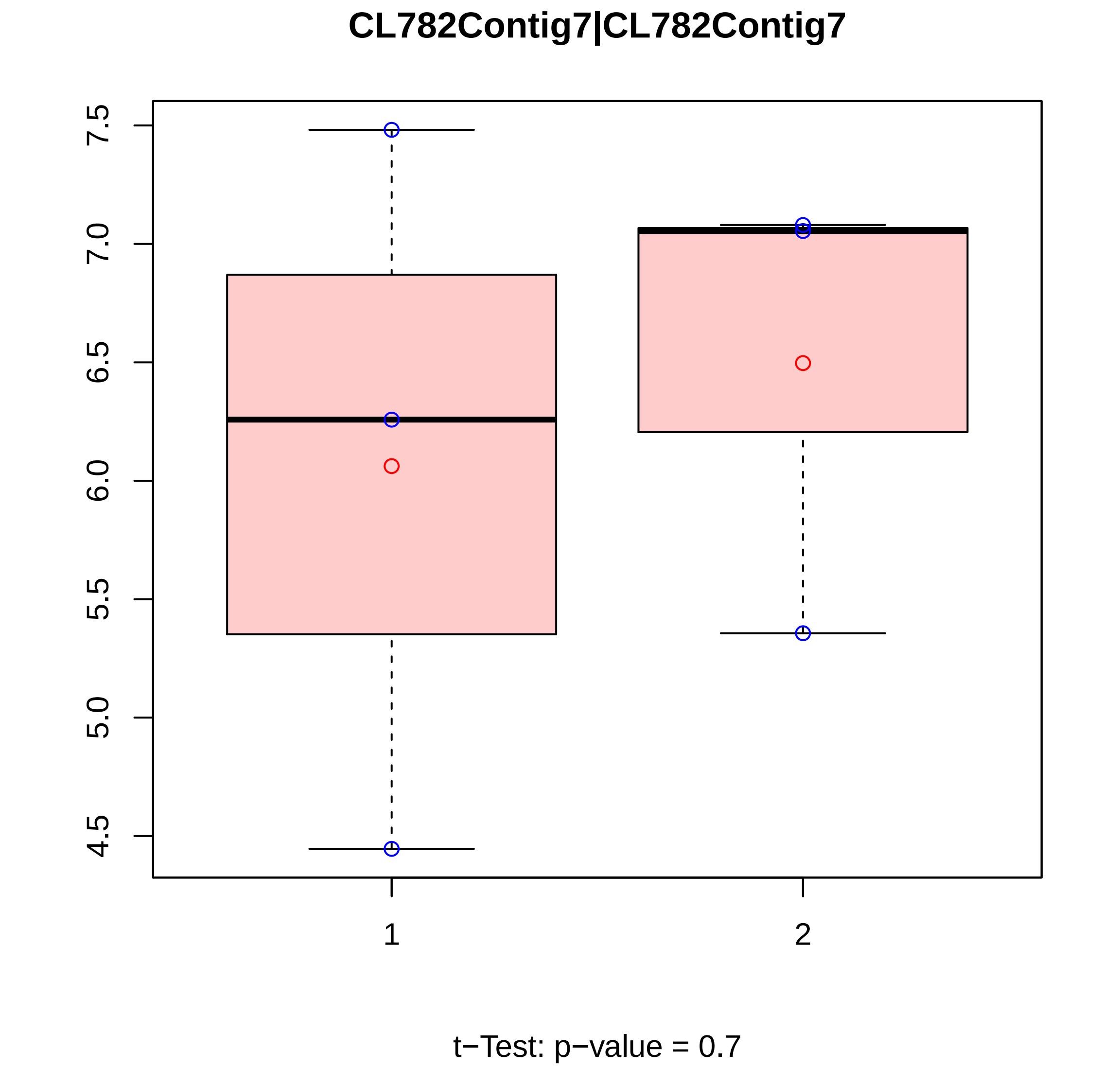

CL782Contig7|CL782Contig7
7.5
7.0
6.5
6.0
5.5
5.0
4.5
1
2
t−Test: p−value = 0.7

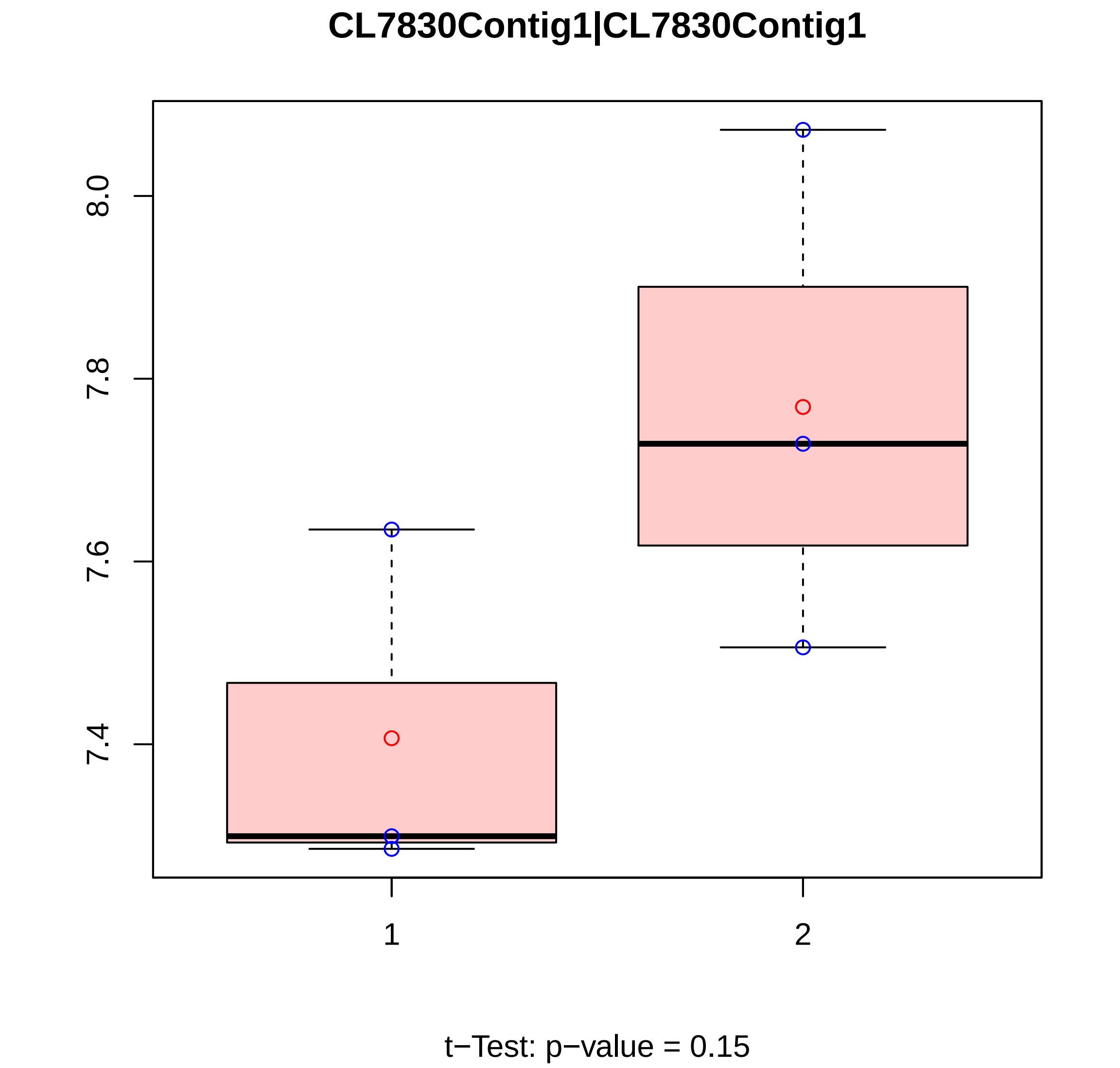
CL7830Contig1|CL7830Contig1
8.0
7.8
7.6
7.4
1
2
t−Test: p−value = 0.15

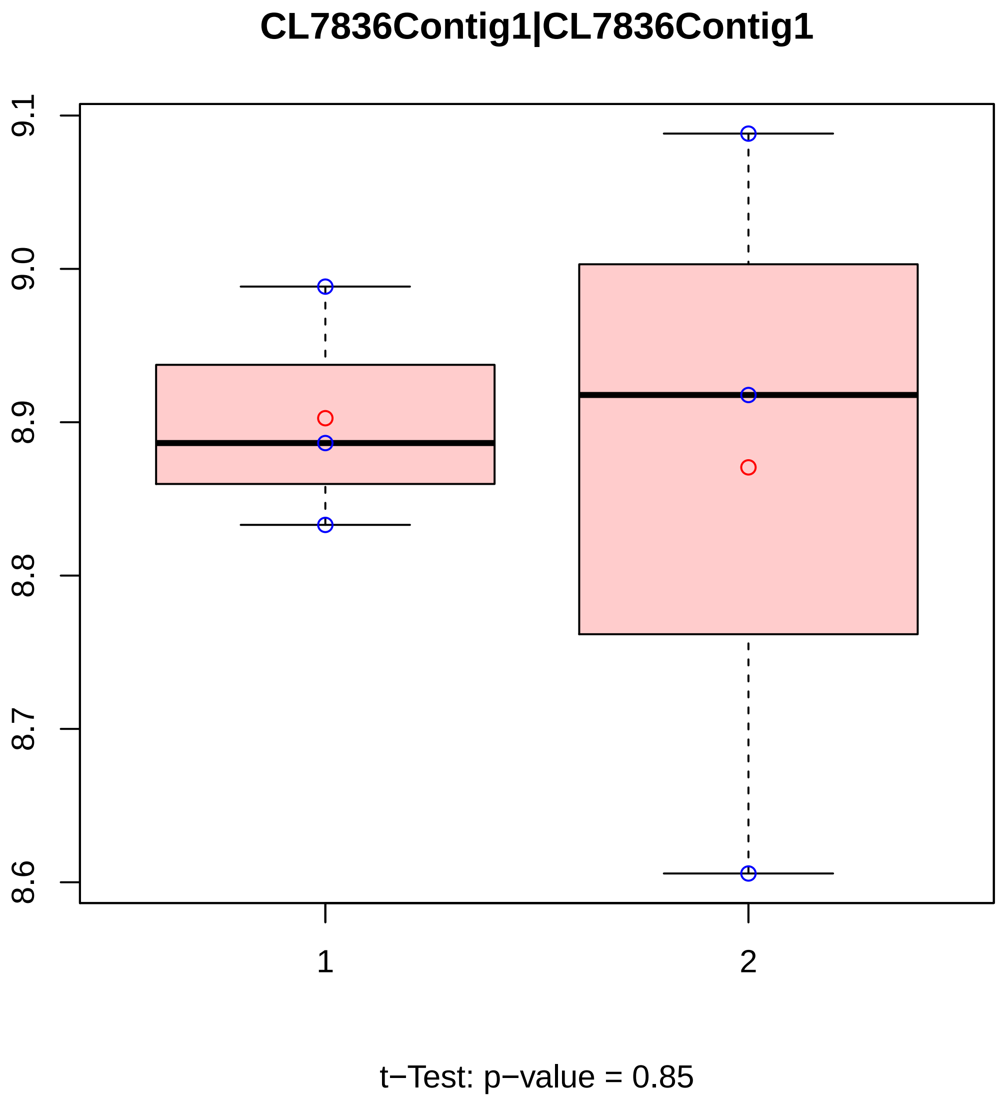
CL7836Contig1|CL7836Contig1
9.1
9.0
8.9
8.8
8.7
8.6
1
2
t−Test: p−value = 0.85

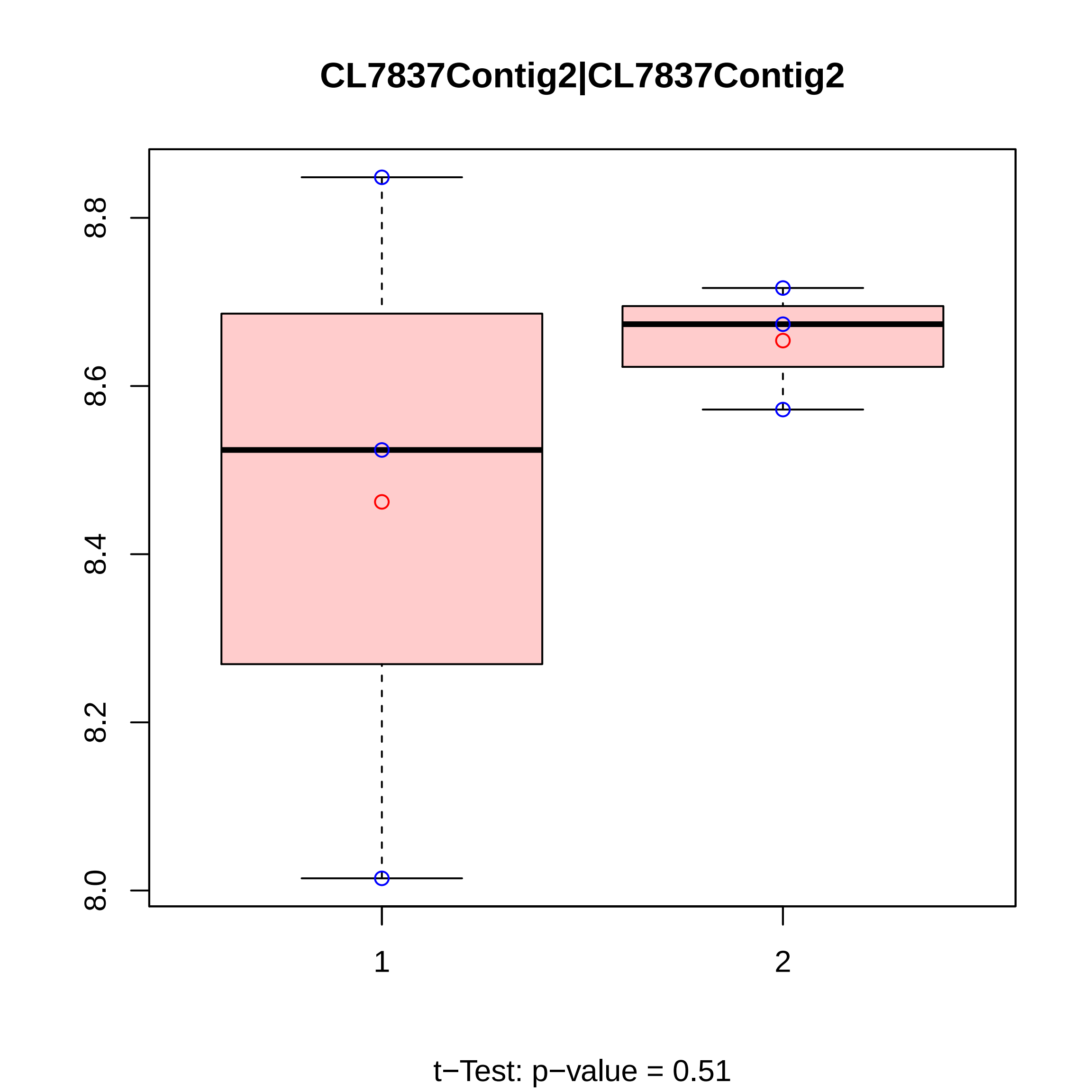
CL7837Contig2|CL7837Contig2
8.0
8.2
8.4
8.6
8.8
1
2
t−Test: p−value = 0.51

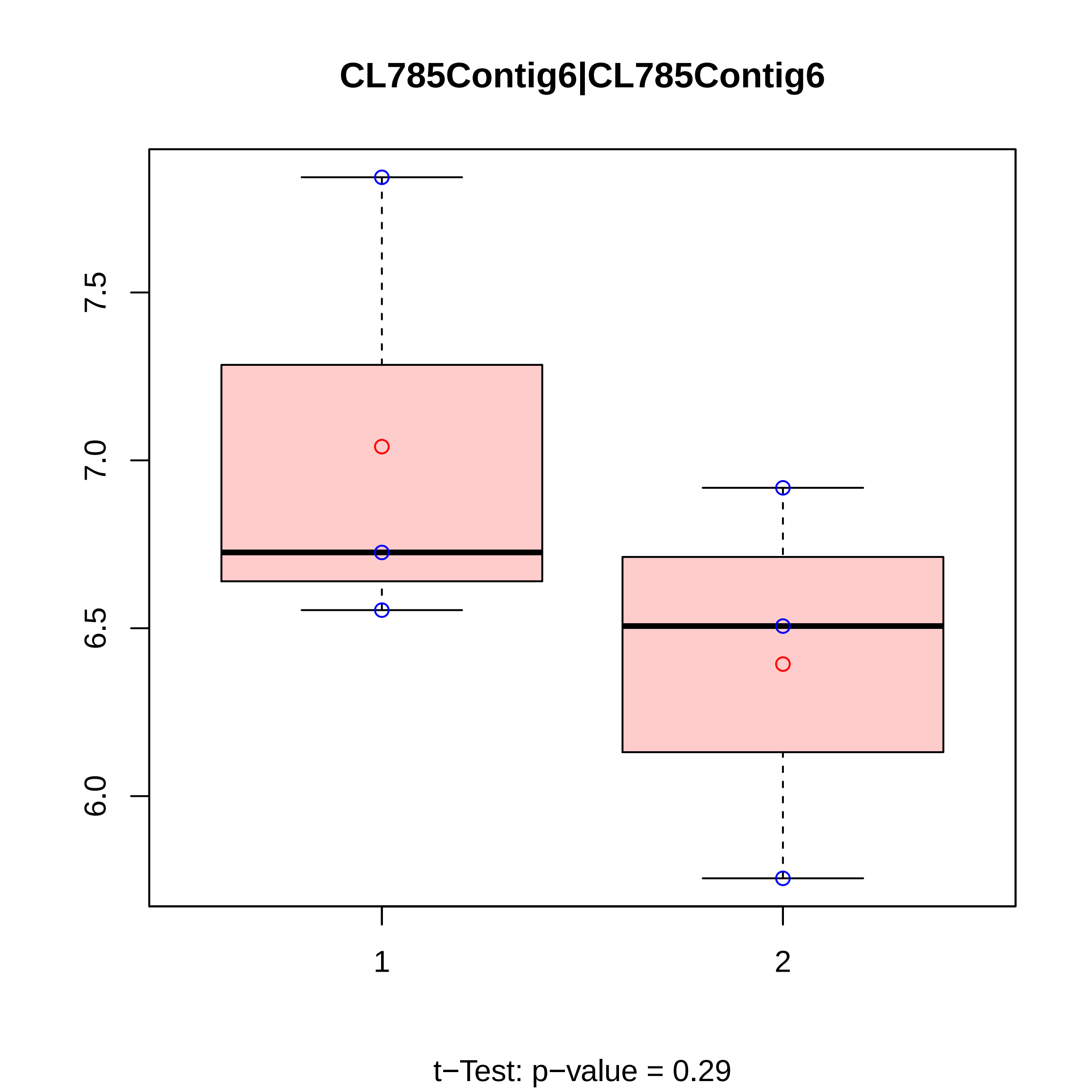

CL785Contig6|CL785Contig6
7.5
7.0
6.5
6.0
1
2
t−Test: p−value = 0.29

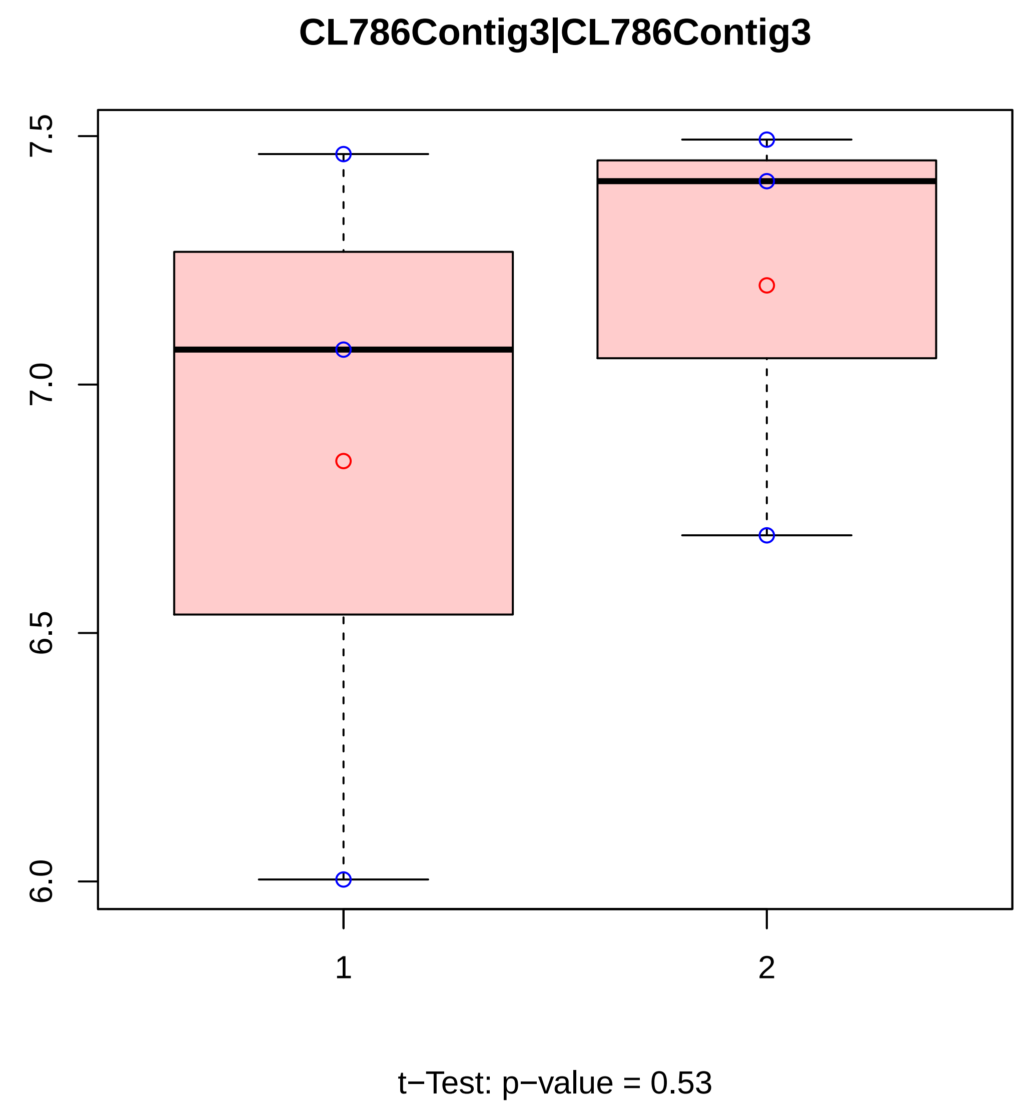

CL786Contig3|CL786Contig3
7.5
7.0
6.5
6.0
1
2
t−Test: p−value = 0.53

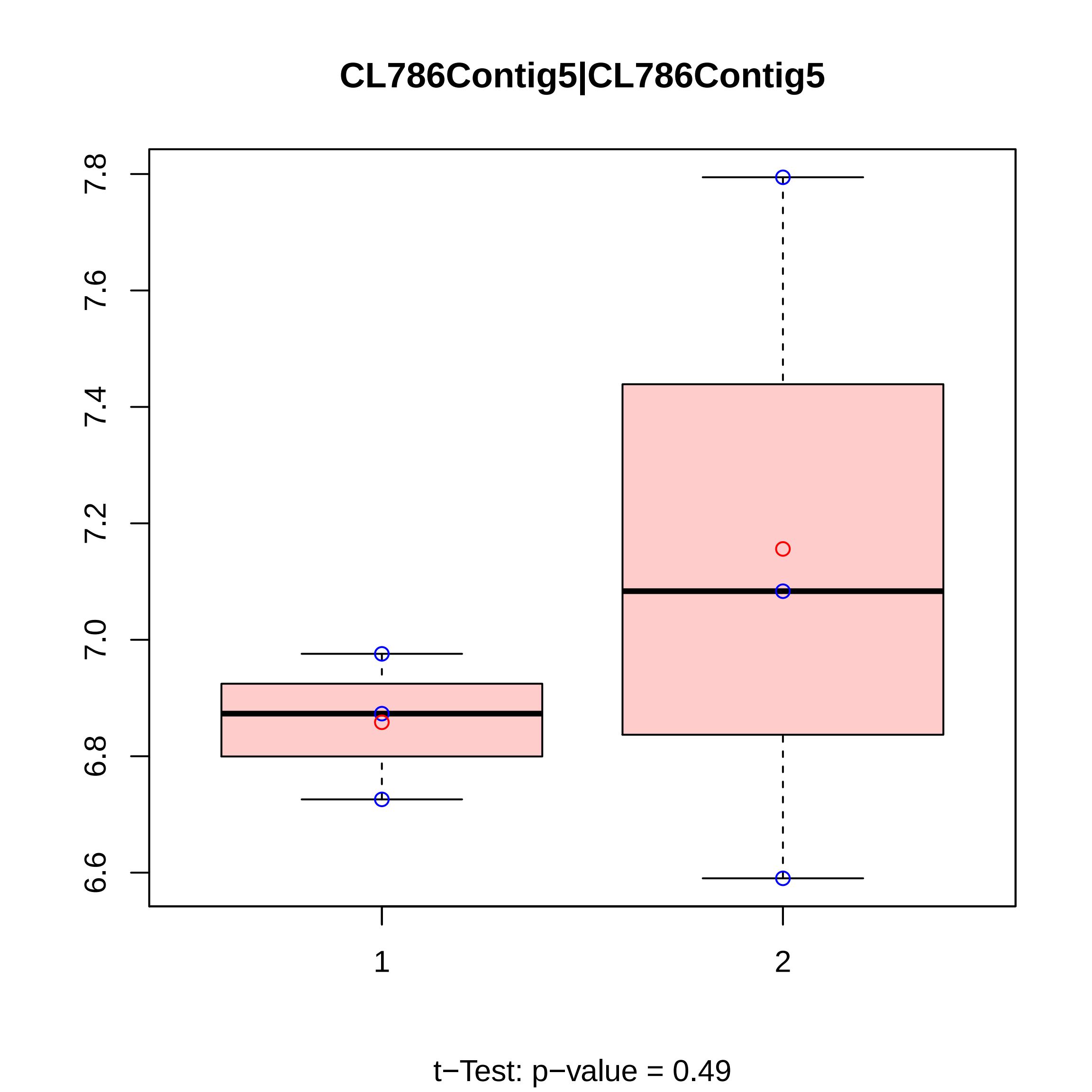

CL786Contig5|CL786Contig5
7.8
7.6
7.4
7.2
7.0
6.8
6.6
1
2
t−Test: p−value = 0.49

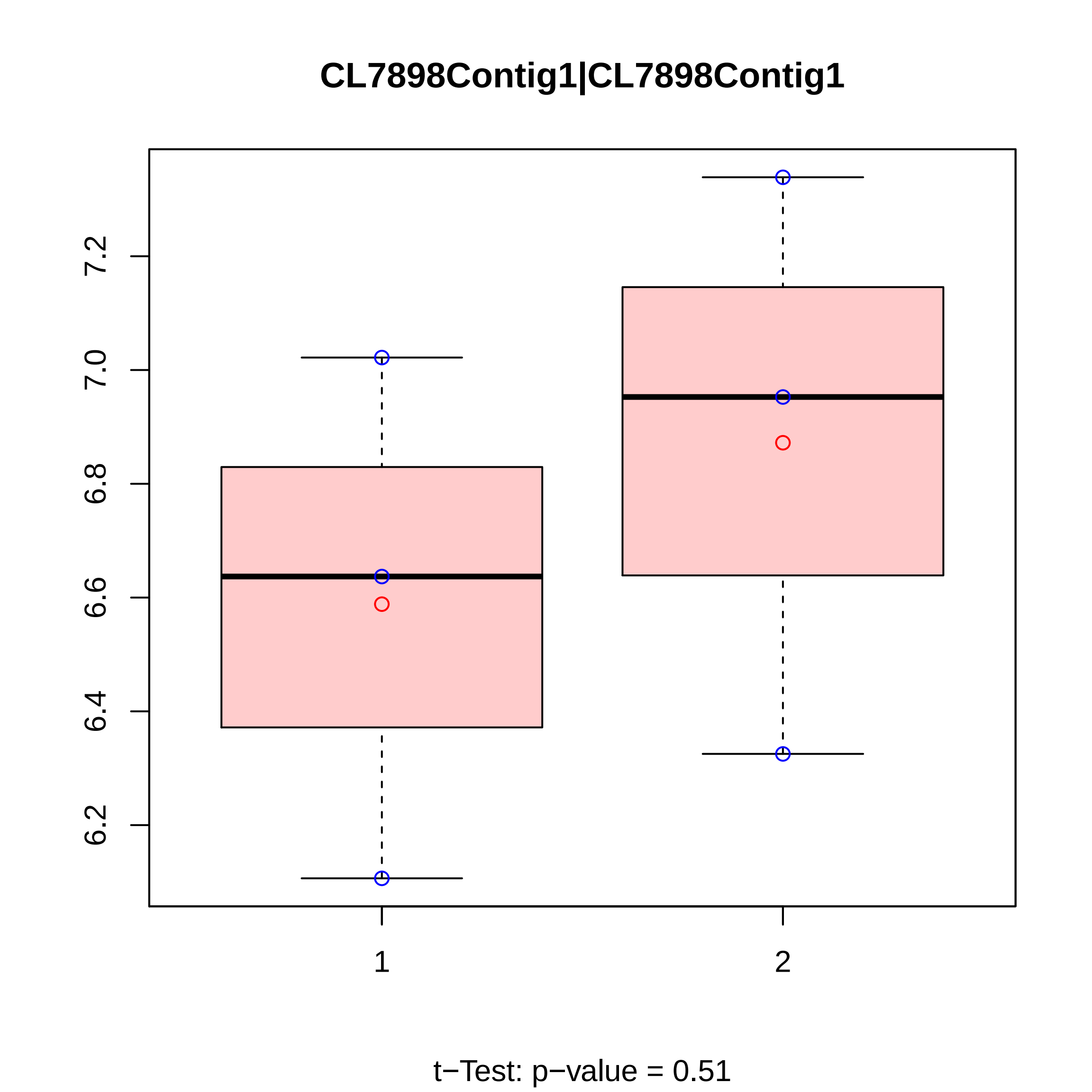

CL7898Contig1|CL7898Contig1
7.2
7.0
6.8
6.6
6.4
6.2
1
2
t−Test: p−value = 0.51

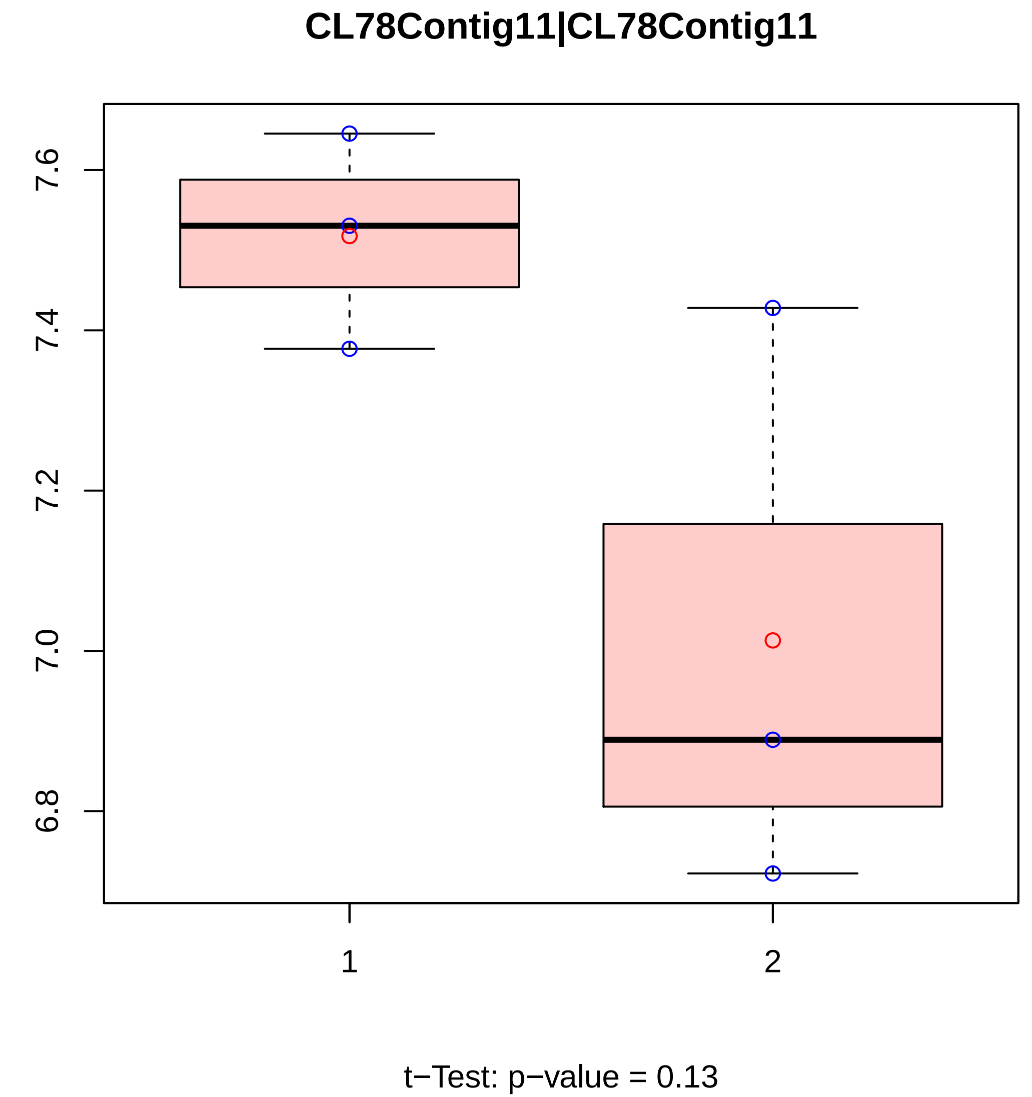
CL78Contig11|CL78Contig11
7.6
7.4
7.2
7.0
6.8
1
2
t−Test: p−value = 0.13

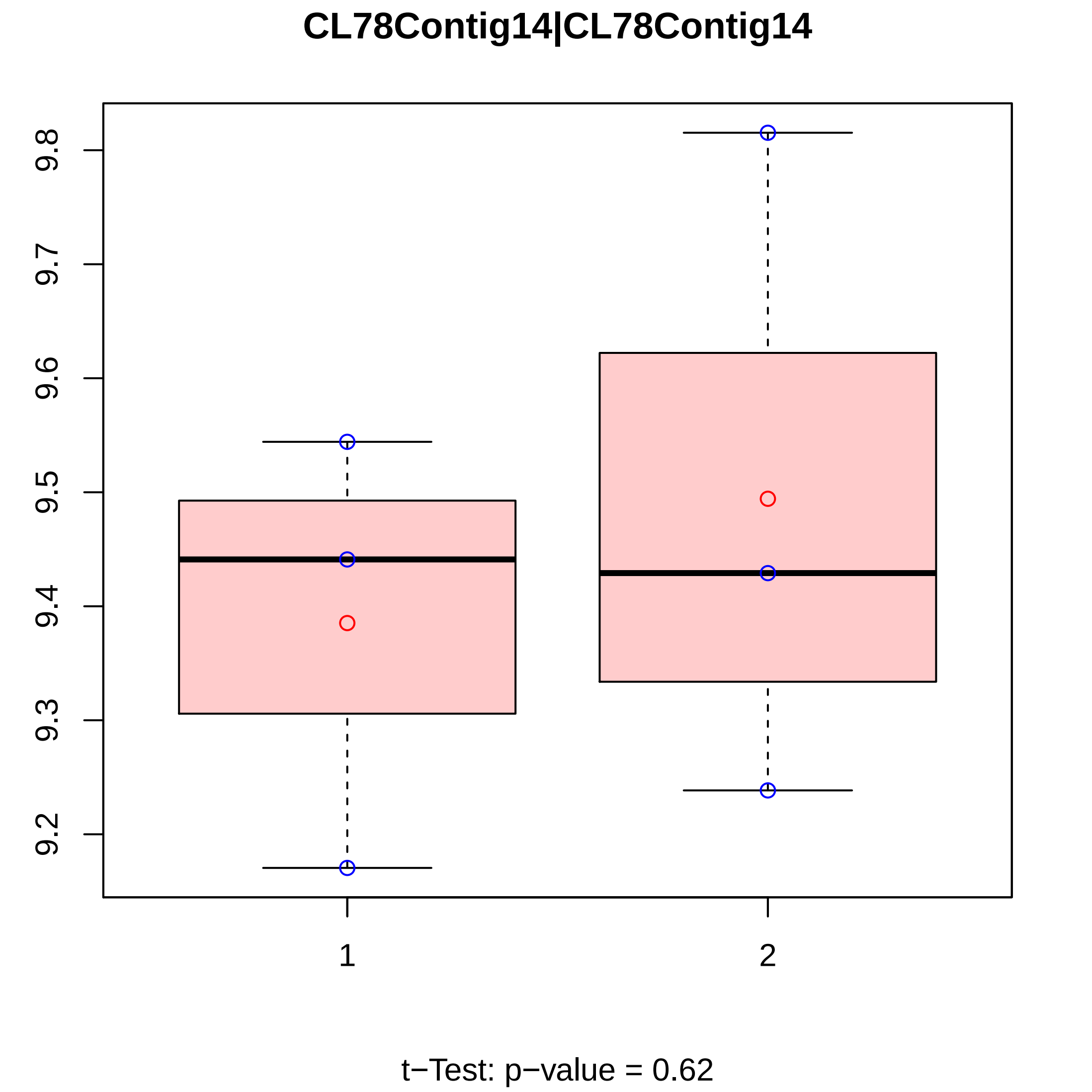
CL78Contig14|CL78Contig14
9.2
9.3
9.4
9.5
9.6
9.7
9.8
1
2
t−Test: p−value = 0.62

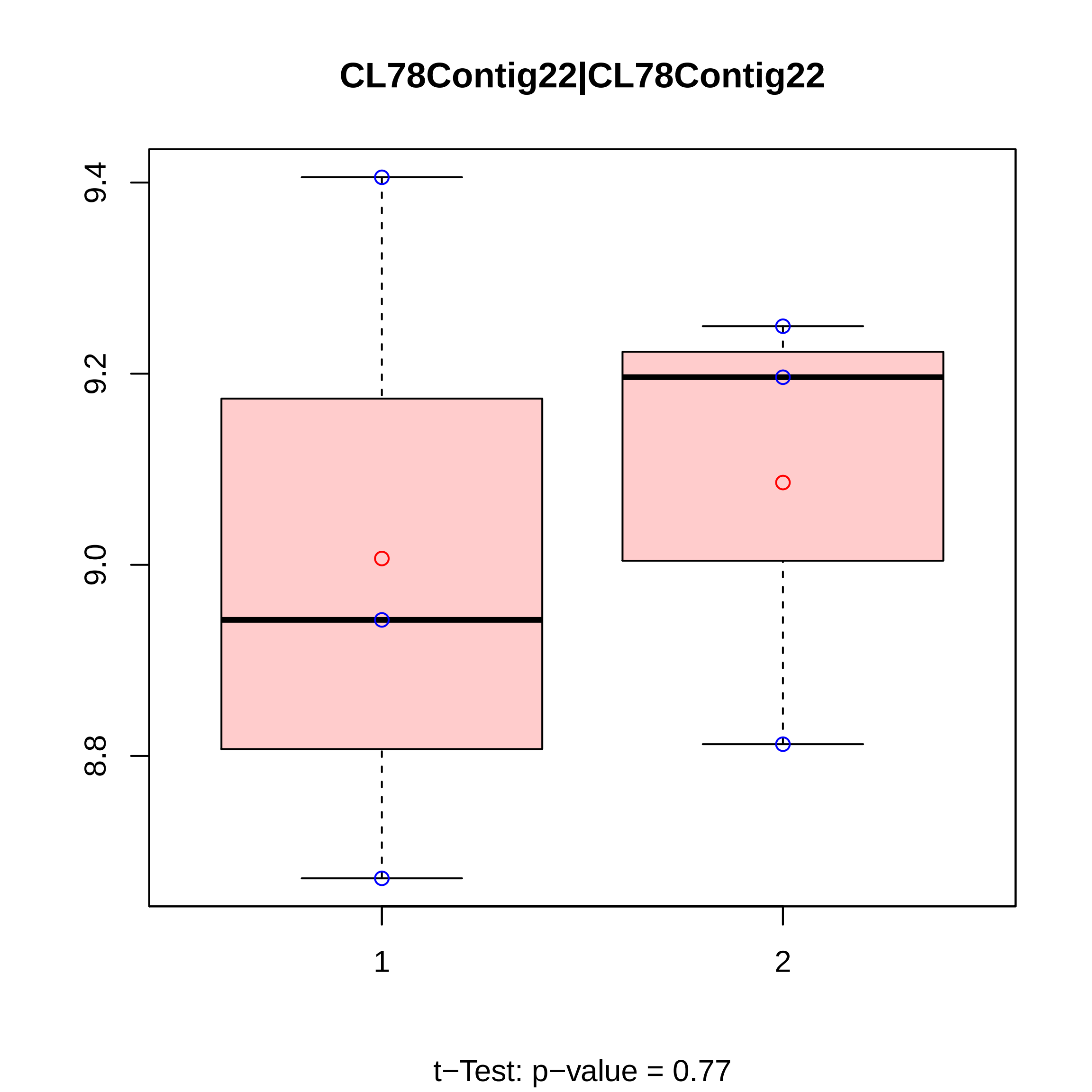
CL78Contig22|CL78Contig22
9.4
9.2
9.0
8.8
1
2
t−Test: p−value = 0.77

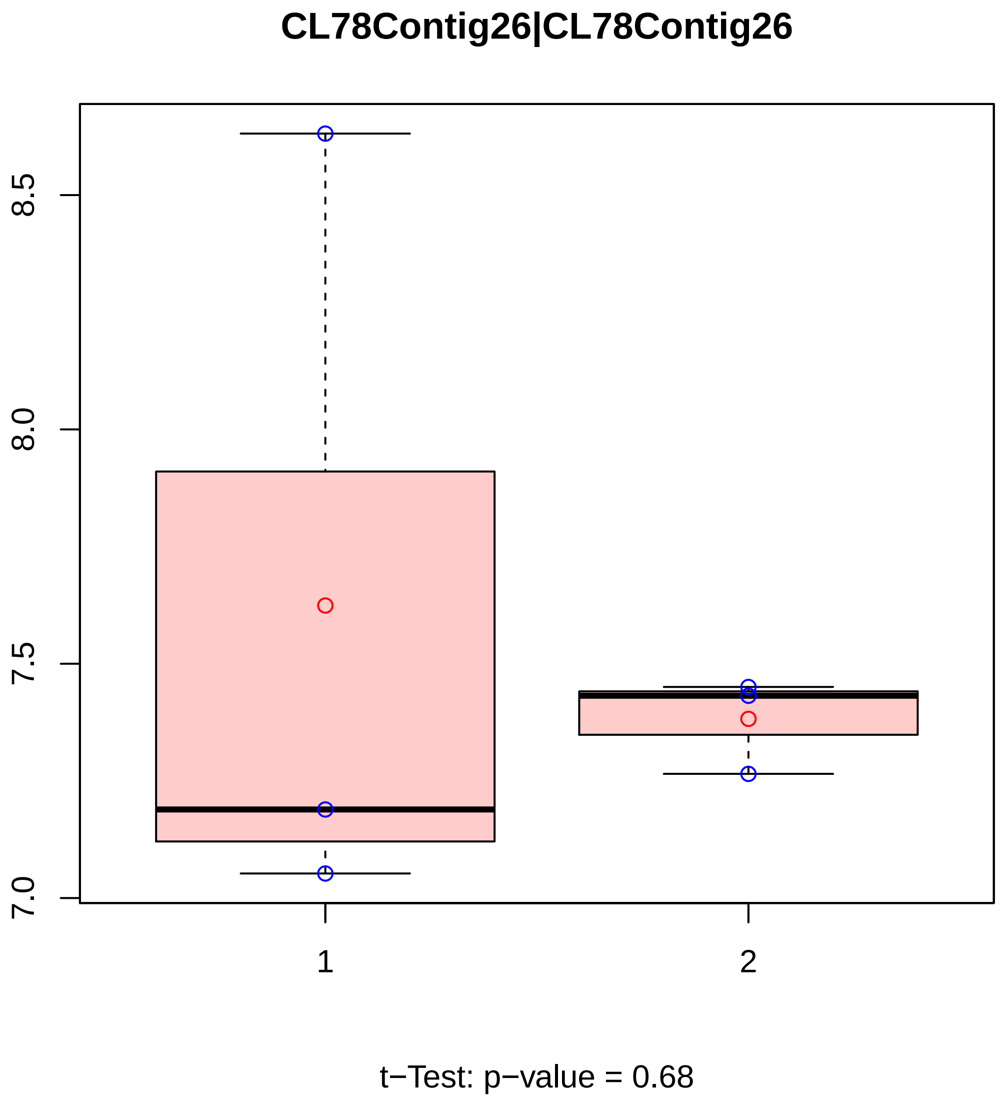
CL78Contig26|CL78Contig26
8.5
8.0
7.5
7.0
1
2
t−Test: p−value = 0.68

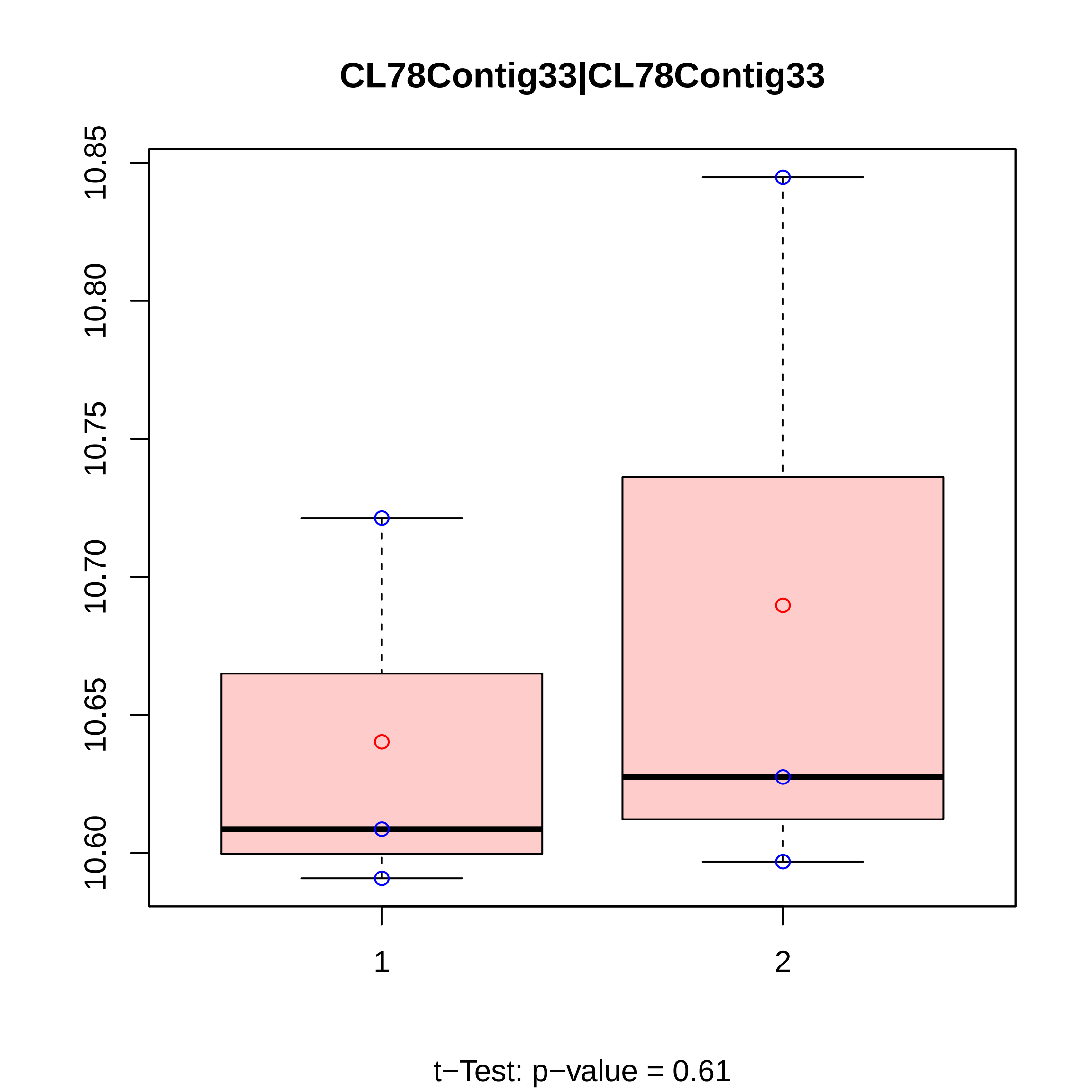

CL78Contig33|CL78Contig33
10.60
10.65
10.70
10.75
10.80
10.85
1
2
t−Test: p−value = 0.61

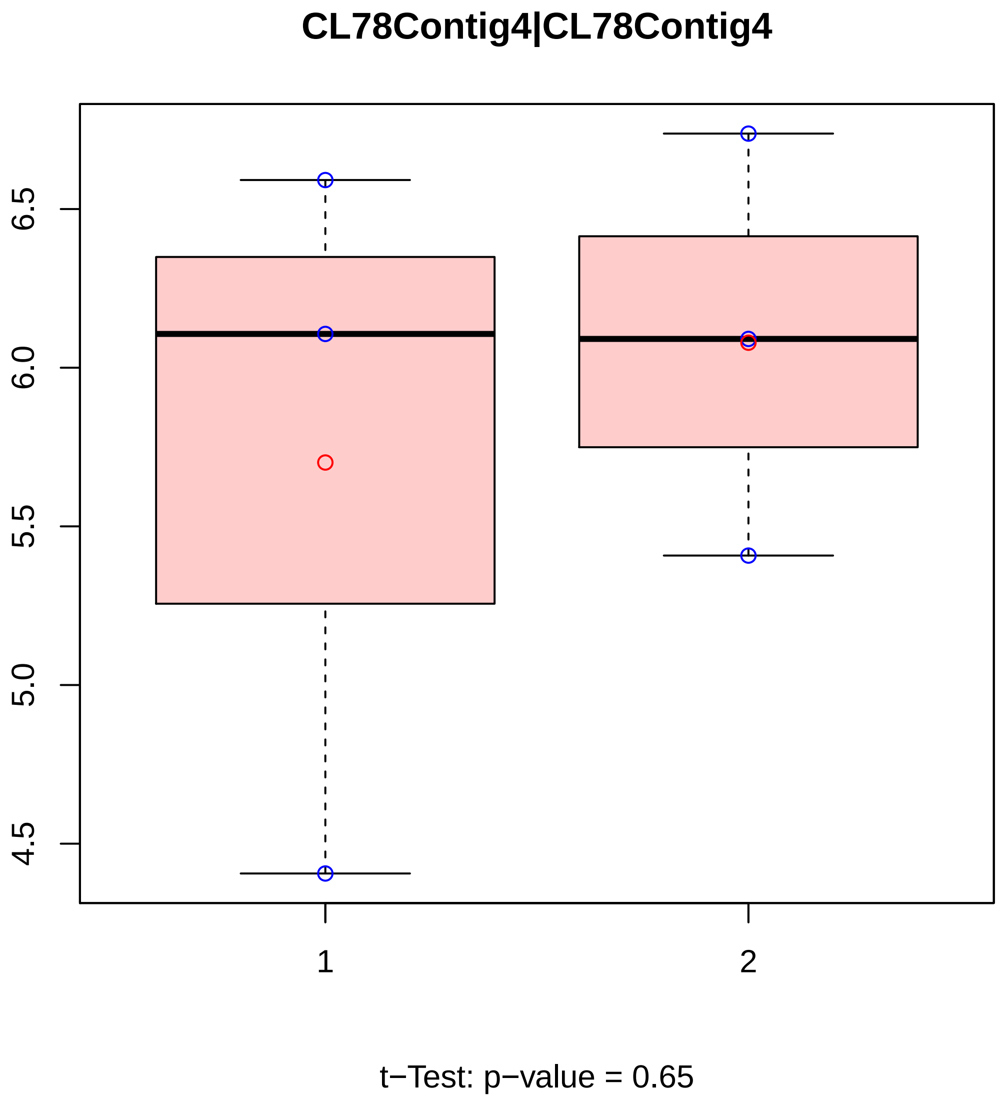
CL78Contig4|CL78Contig4
6.5
6.0
5.5
5.0
4.5
1
2
t−Test: p−value = 0.65

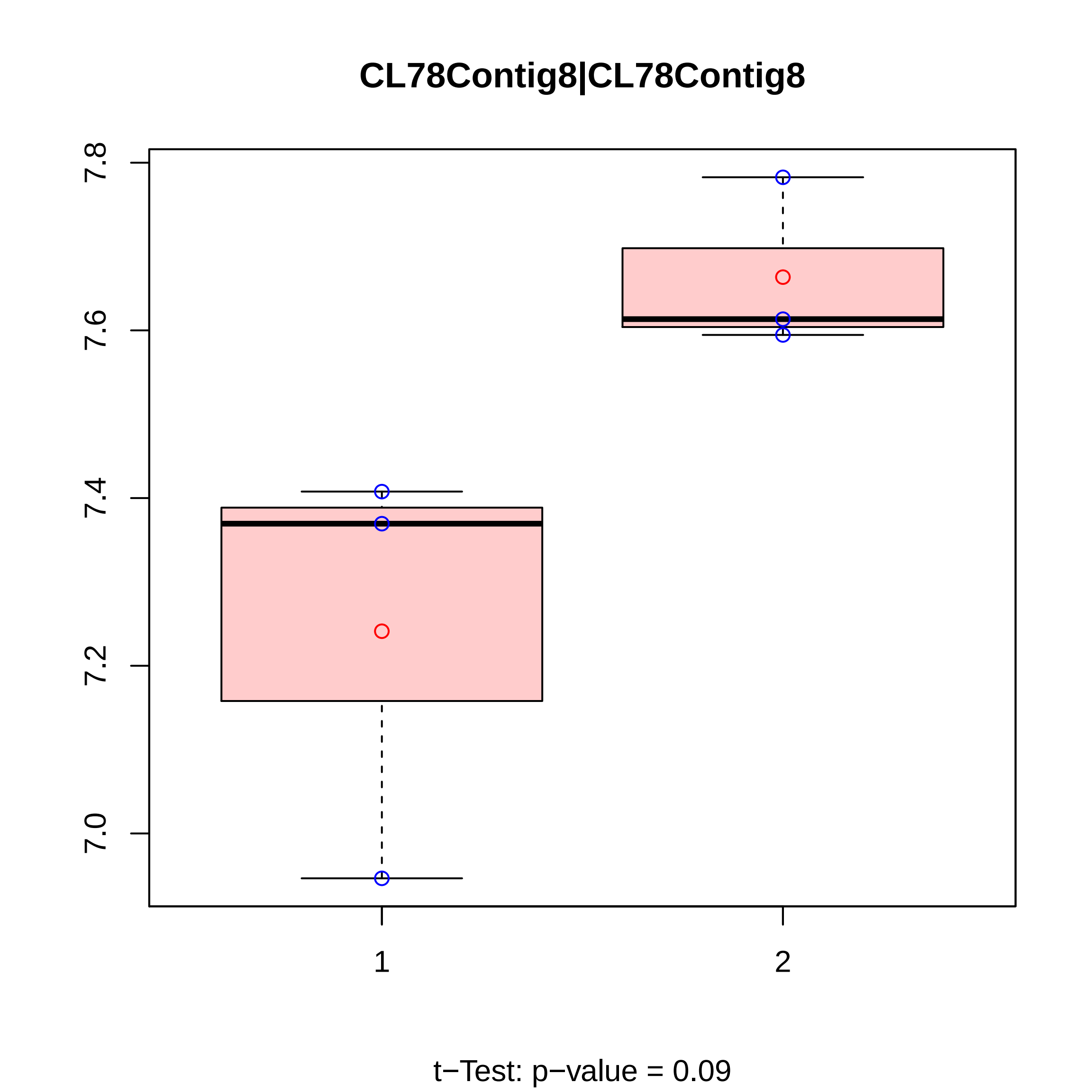

CL78Contig8|CL78Contig8
7.0
7.2
7.4
7.6
7.8
1
2
t−Test: p−value = 0.09

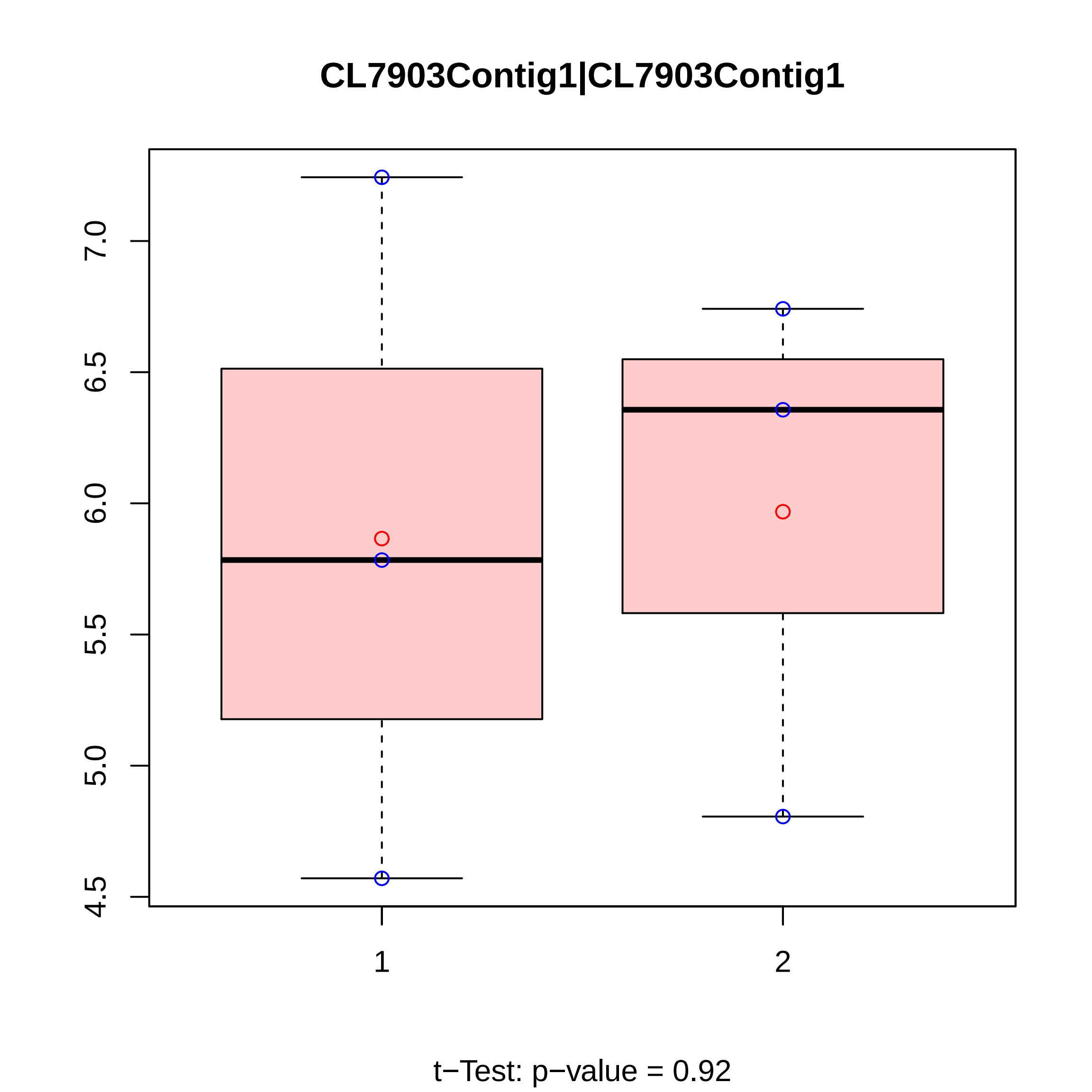

CL7903Contig1|CL7903Contig1
7.0
6.5
6.0
5.5
5.0
4.5
1
2
t−Test: p−value = 0.92

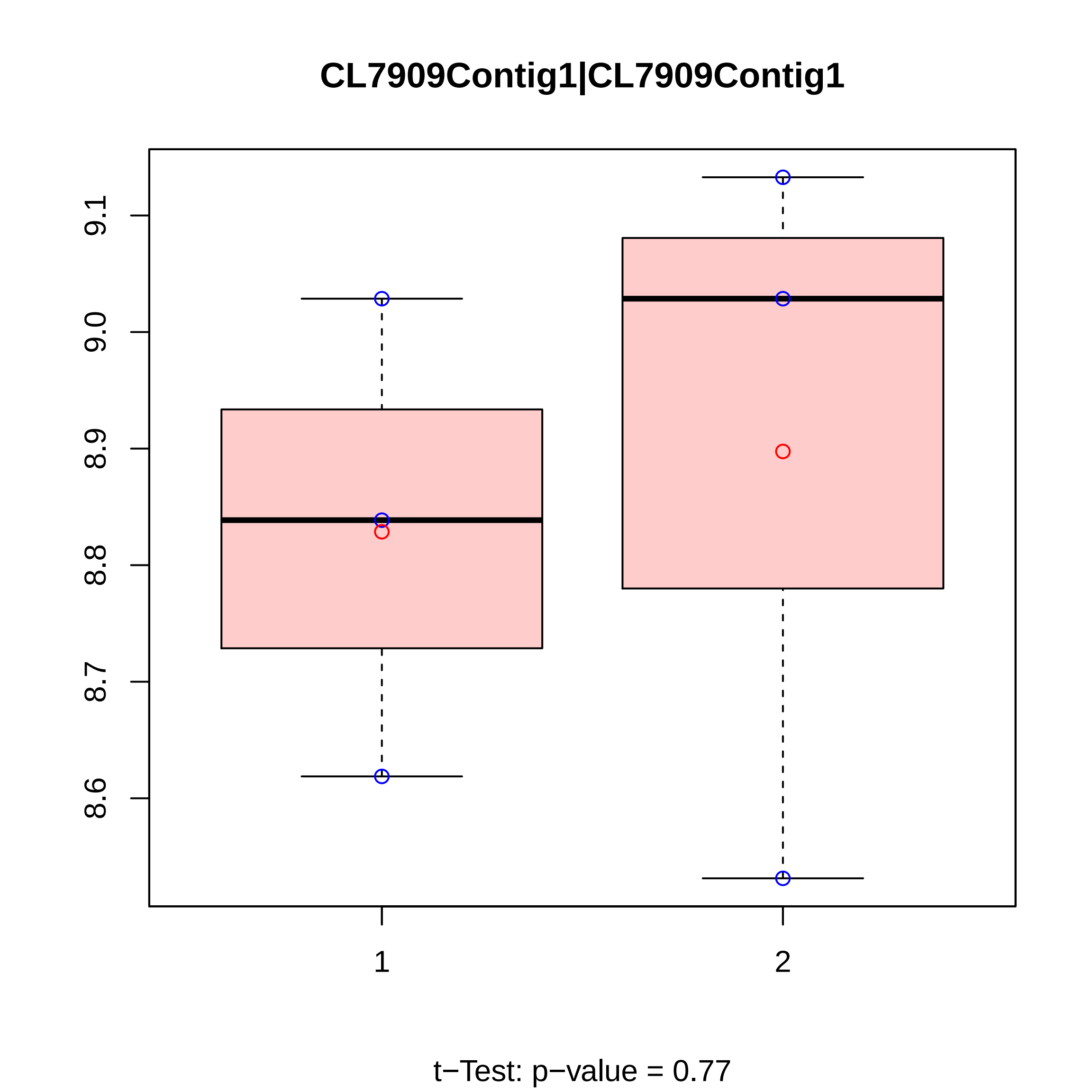

CL7909Contig1|CL7909Contig1
9.1
9.0
8.9
8.8
8.7
8.6
1
2
t−Test: p−value = 0.77

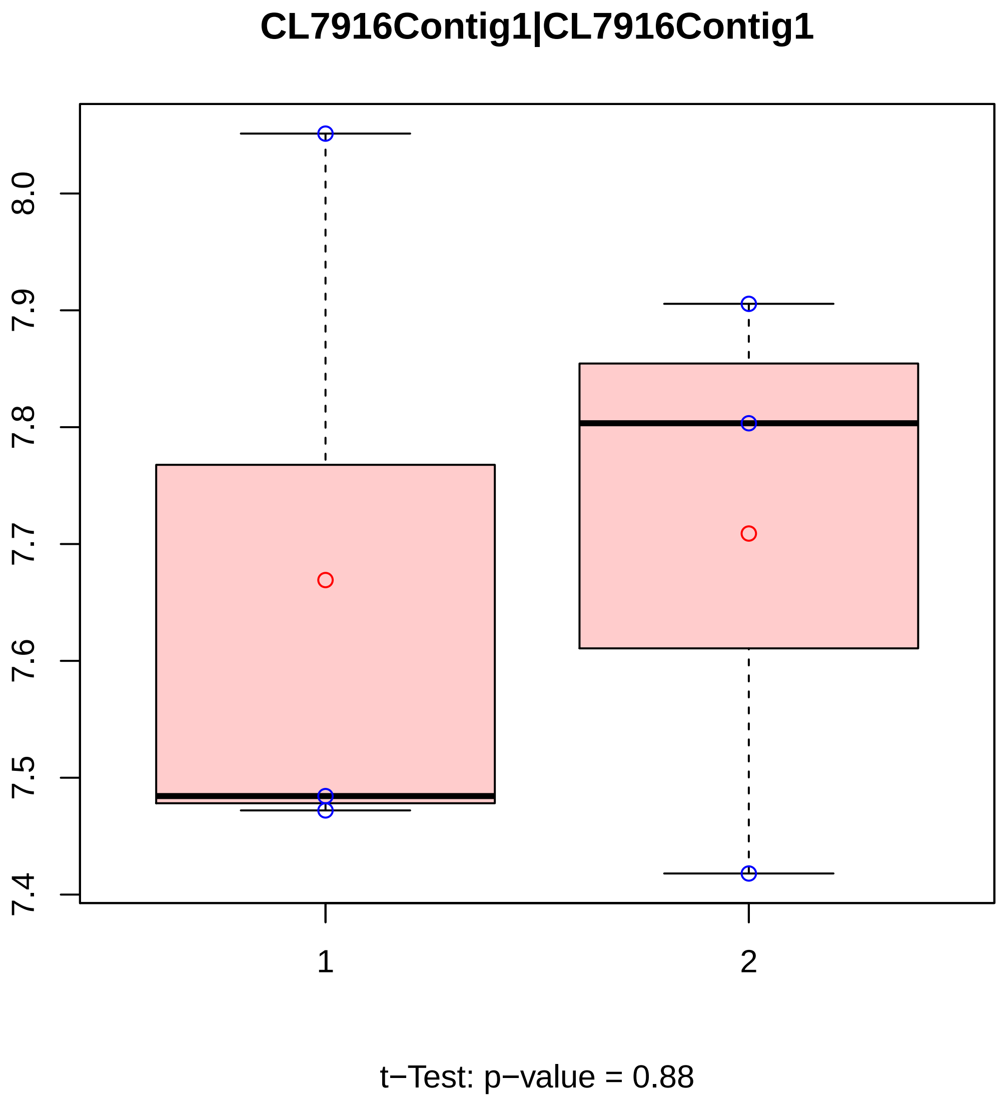
CL7916Contig1|CL7916Contig1
8.0
7.9
7.8
7.7
7.6
7.5
7.4
1
2
t−Test: p−value = 0.88

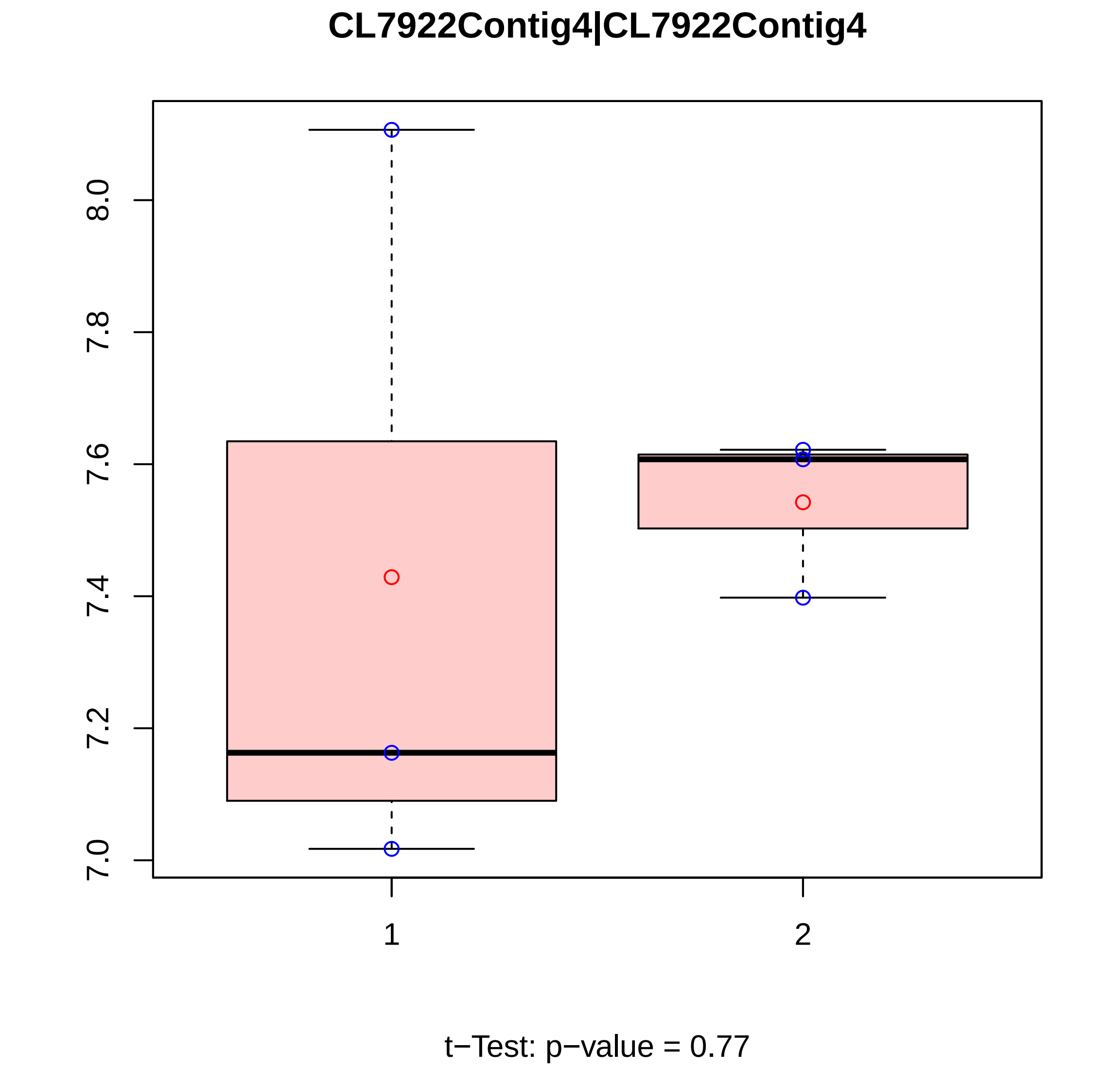
CL7922Contig4|CL7922Contig4
8.0
7.8
7.6
7.4
7.2
7.0
1
2
t−Test: p−value = 0.77

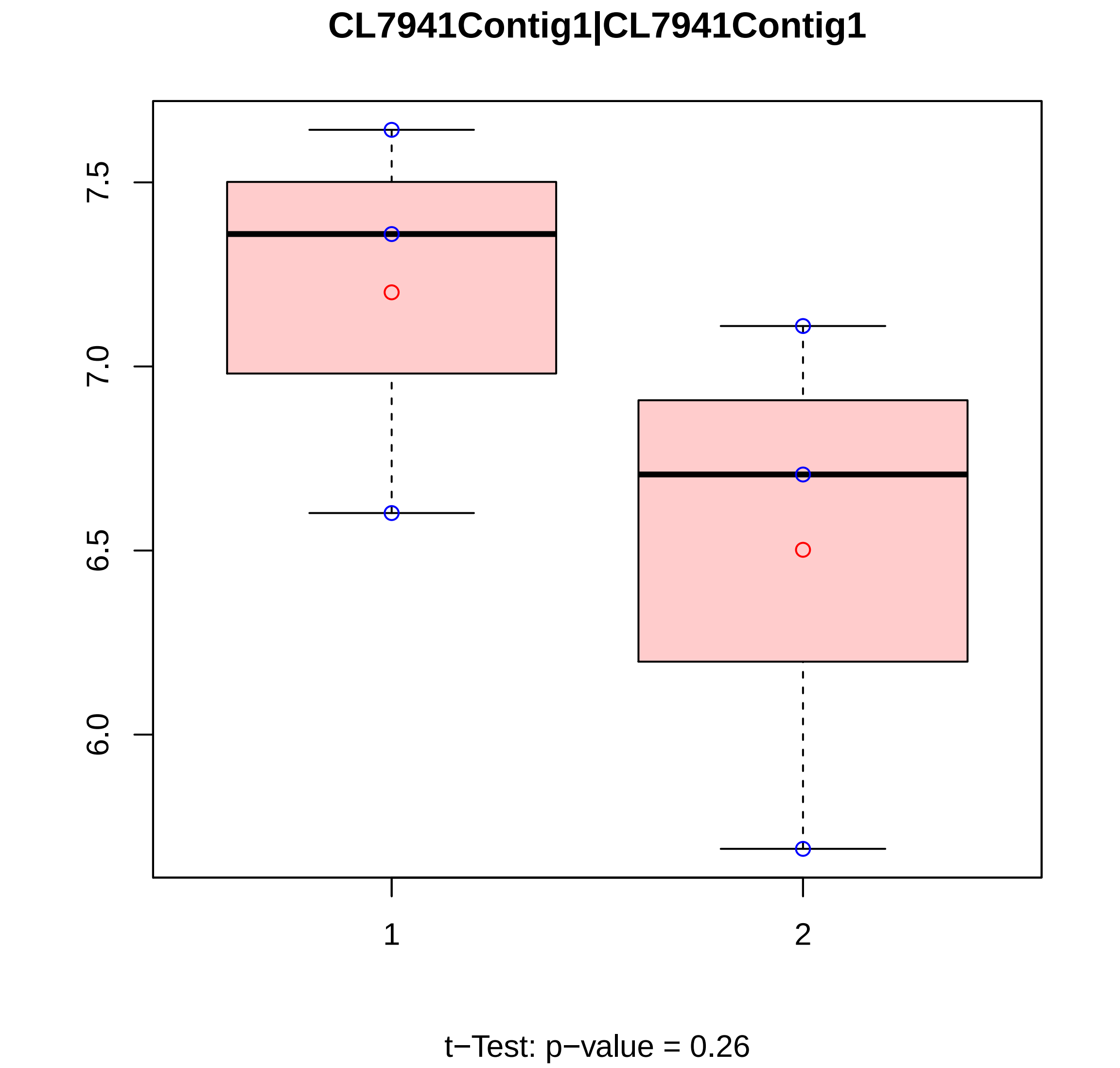

CL7941Contig1|CL7941Contig1
7.5
7.0
6.5
6.0
1
2
t−Test: p−value = 0.26

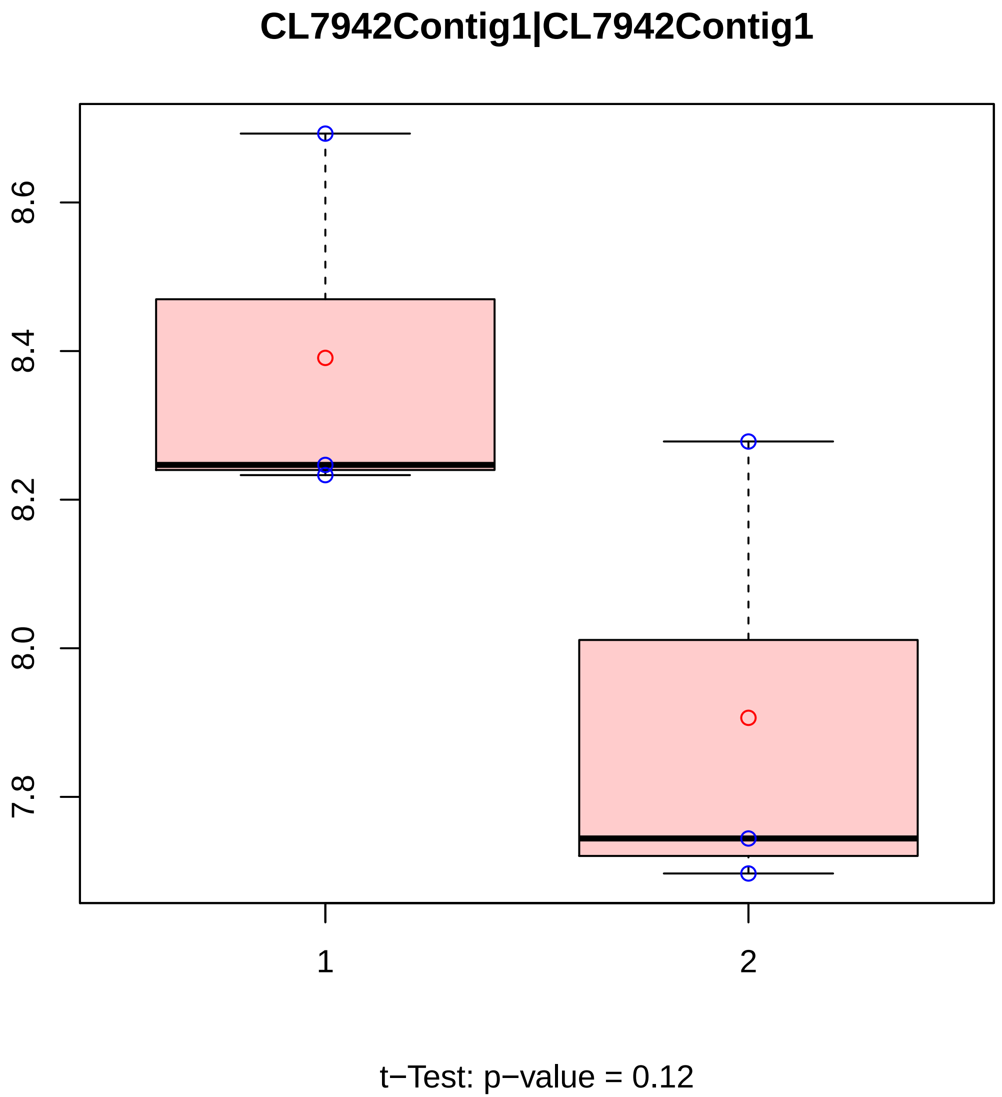
CL7942Contig1|CL7942Contig1
8.6
8.4
8.2
8.0
7.8
1
2
t−Test: p−value = 0.12

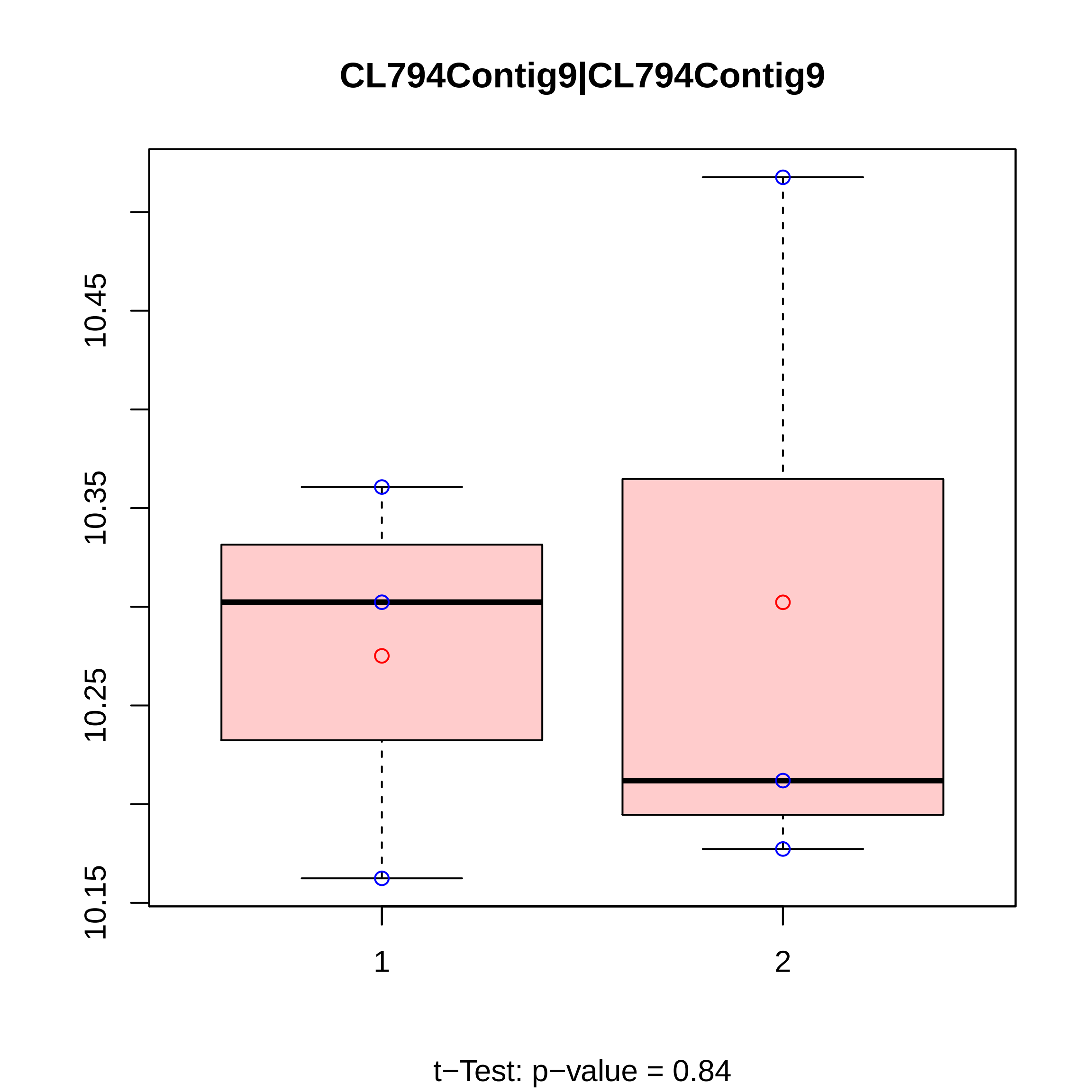

CL794Contig9|CL794Contig9
10.15
10.25
10.35
10.45
1
2
t−Test: p−value = 0.84

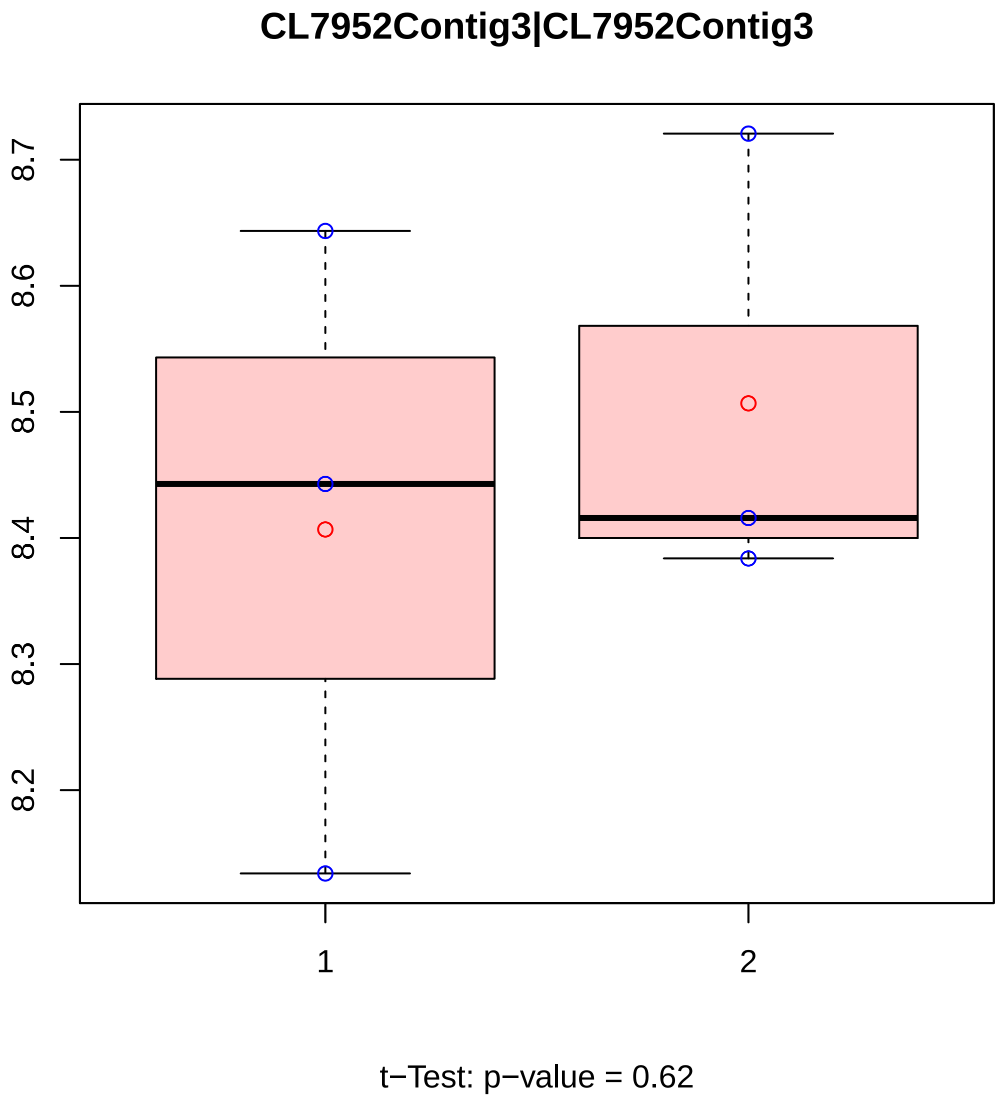

CL7952Contig3|CL7952Contig3
8.7
8.6
8.5
8.4
8.3
8.2
1
2
t−Test: p−value = 0.62

# CL7965Contig3|CL7965Contig3

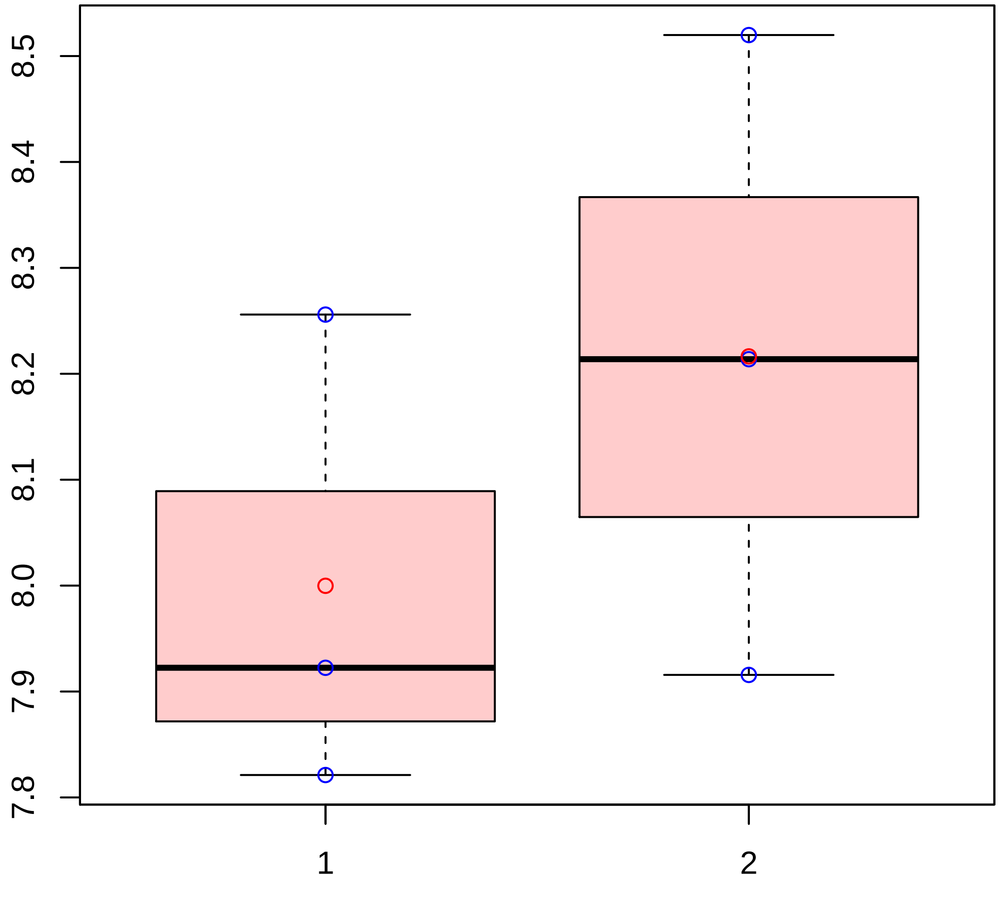


t−Test: p−value = 0.38

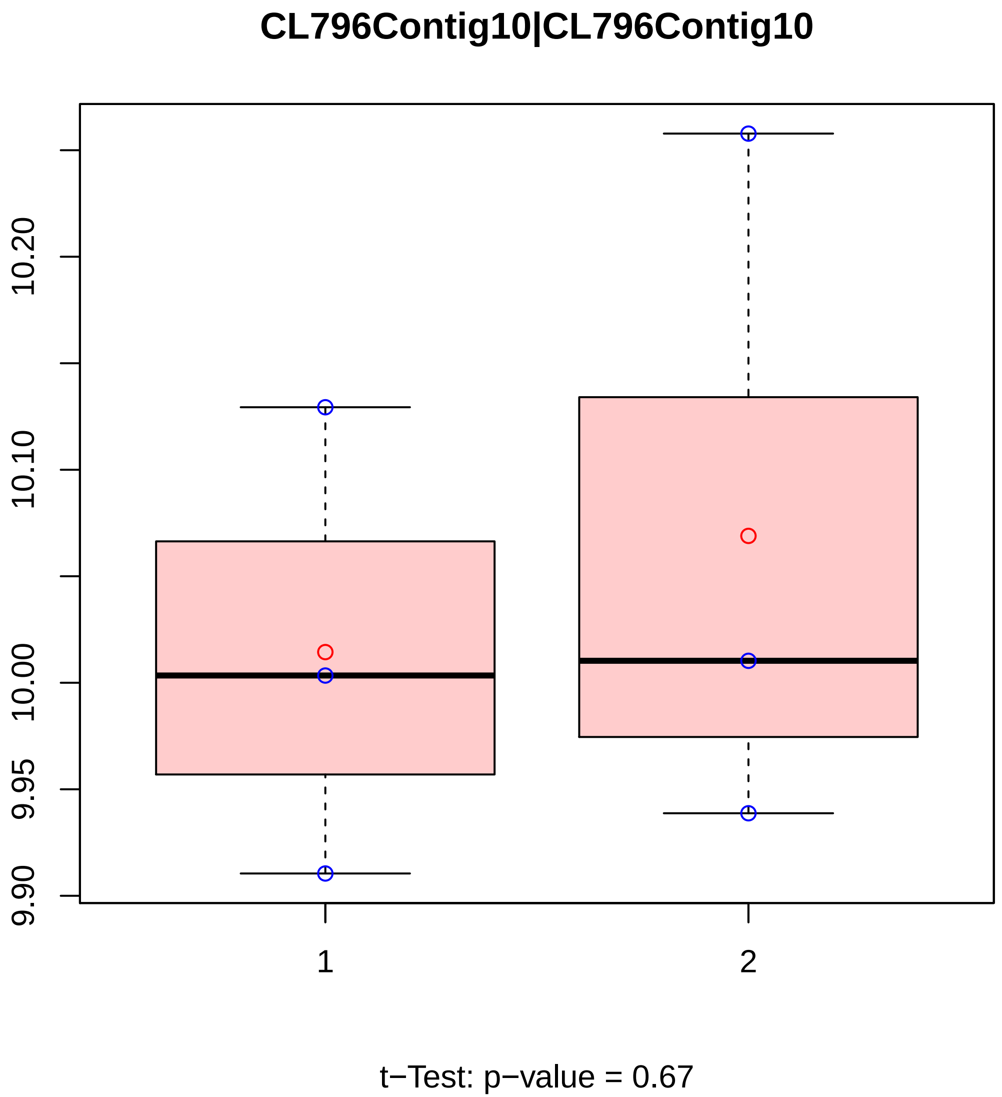
CL796Contig10|CL796Contig10
9.90
9.95
10.00
10.10
10.20
1
2
t−Test: p−value = 0.67

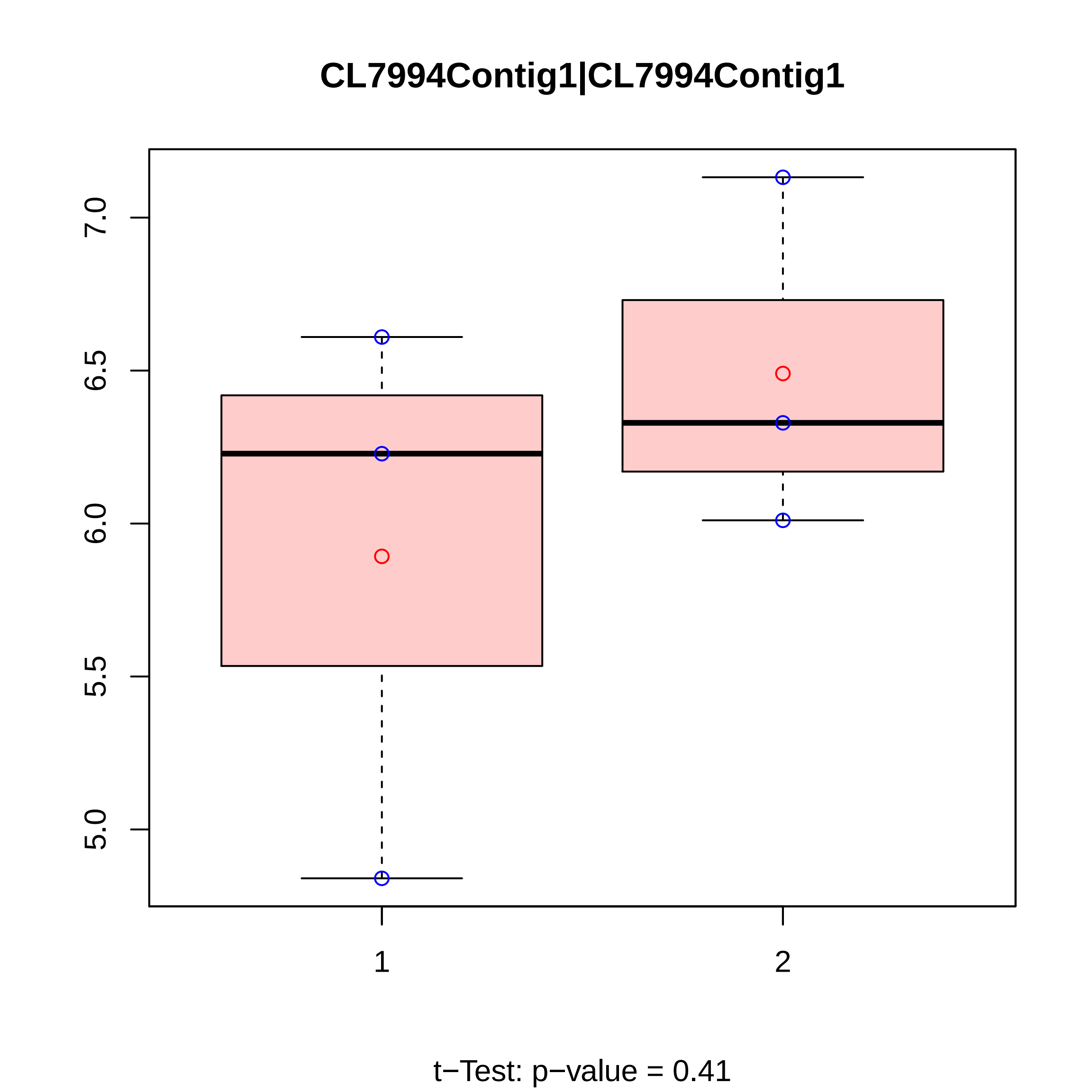

CL7994Contig1|CL7994Contig1
7.0
6.5
6.0
5.5
5.0
1
2
t−Test: p−value = 0.41

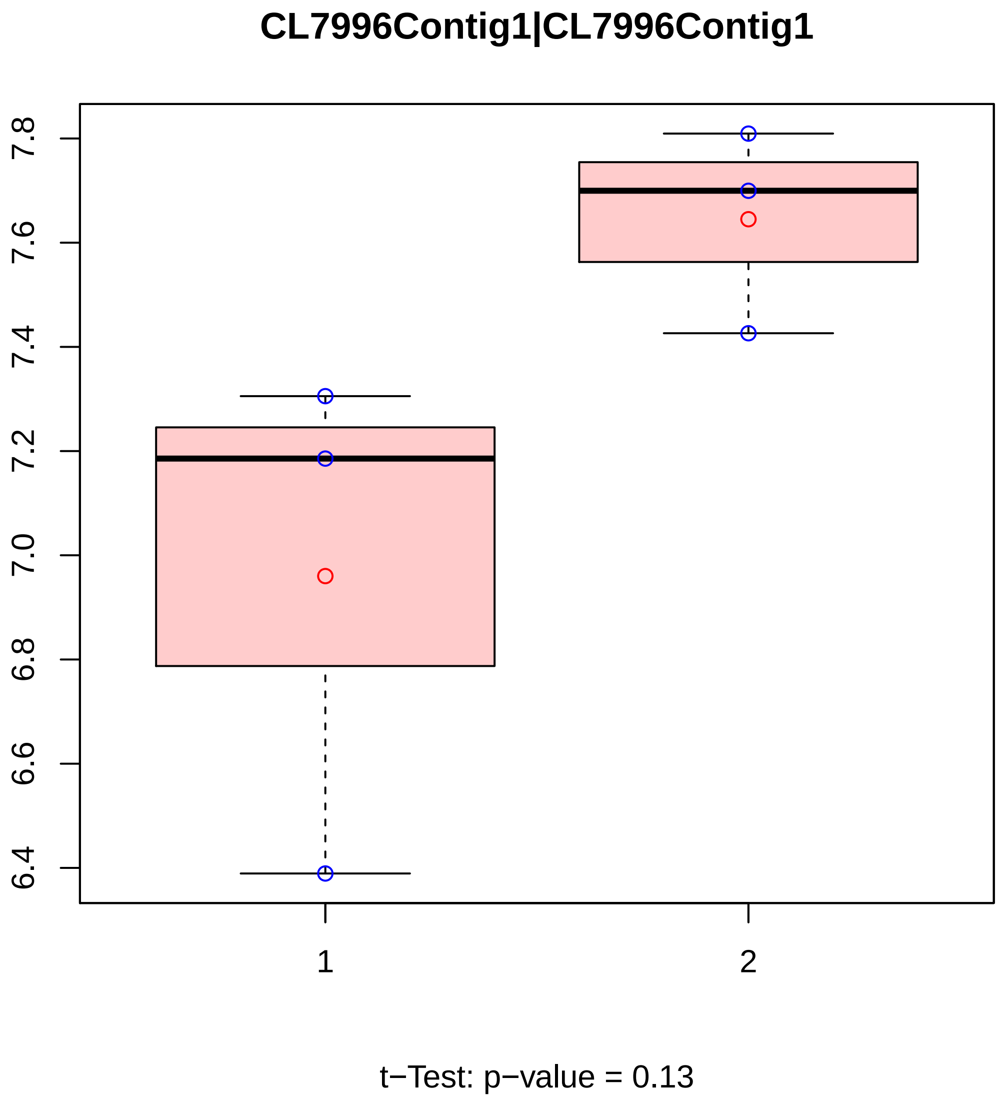

CL7996Contig1|CL7996Contig1
7.8
7.6
7.4
7.2
7.0
6.8
6.6
6.4
1
2
t−Test: p−value = 0.13

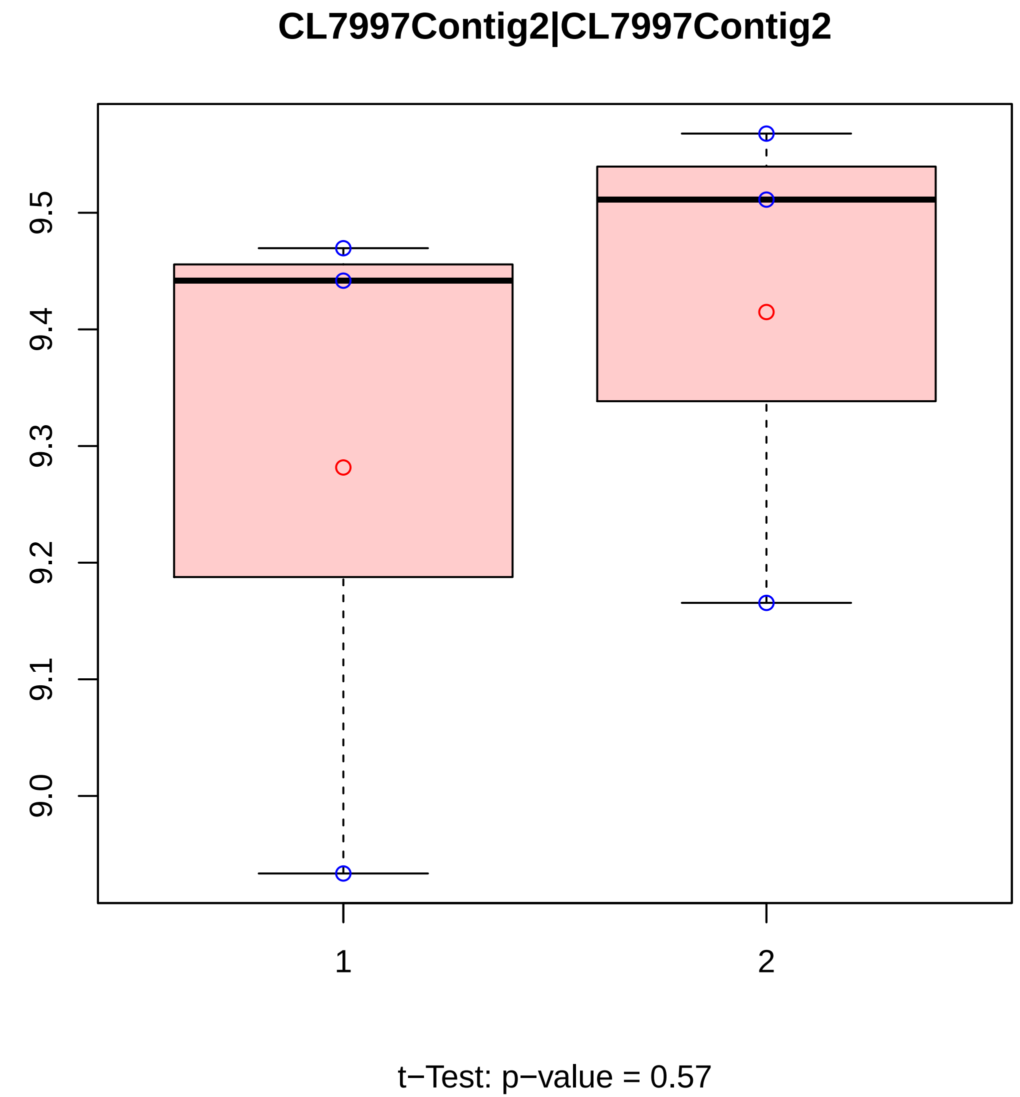
CL7997Contig2|CL7997Contig2
9.0
9.1
9.2
9.3
9.4
9.5
1
2
t−Test: p−value = 0.57

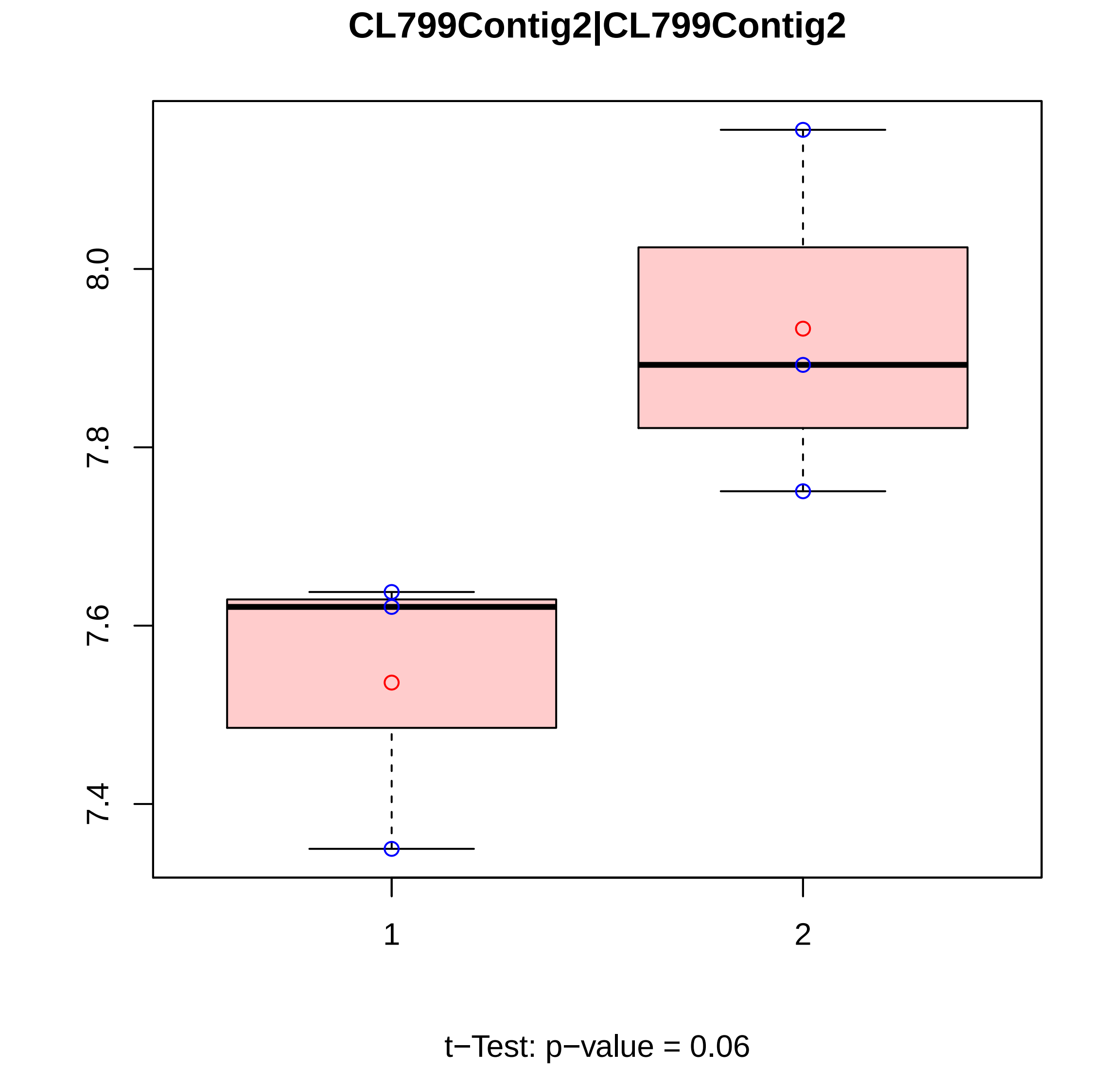
CL799Contig2|CL799Contig2
7.4
7.6
7.8
8.0
1
2
t−Test: p−value = 0.06

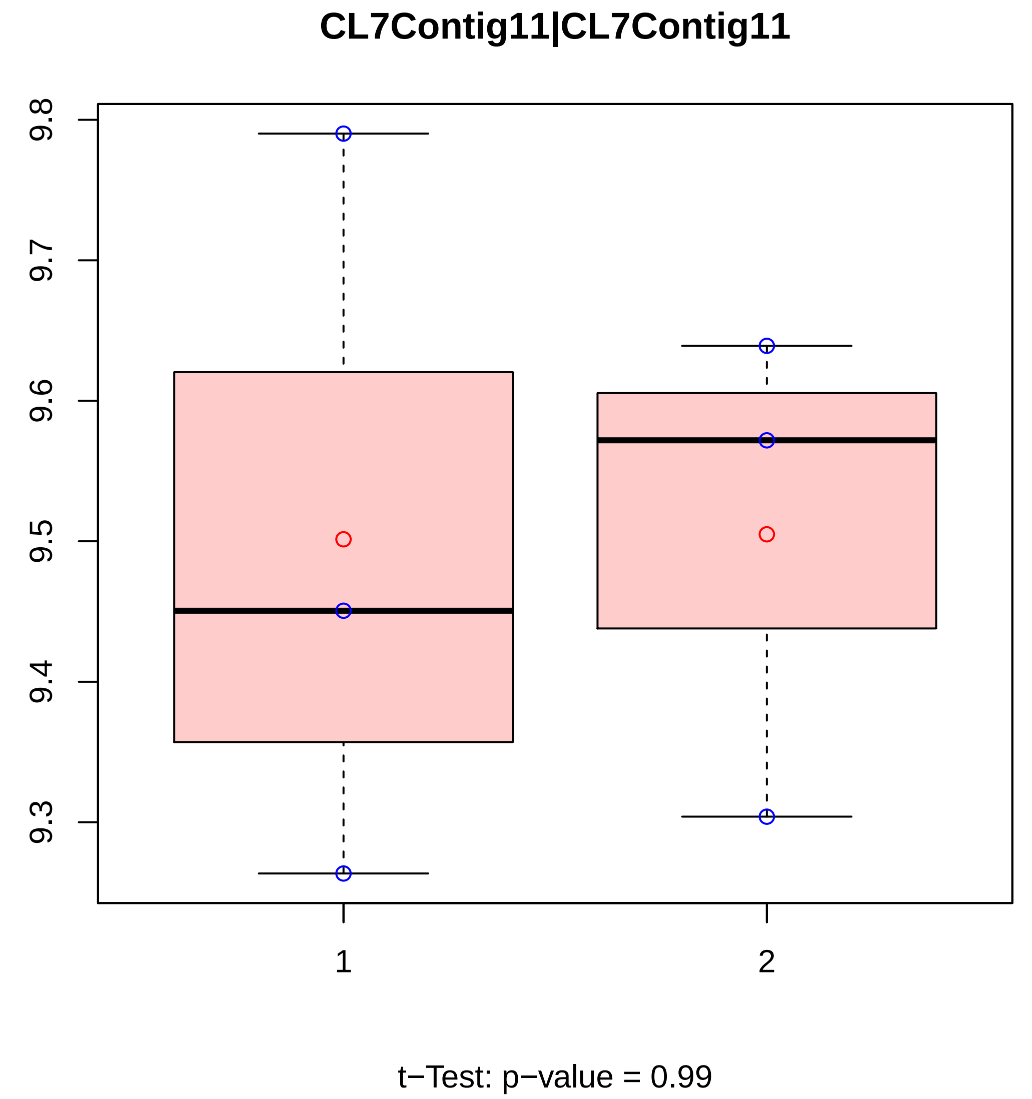
CL7Contig11|CL7Contig11
9.3
9.4
9.5
9.6
9.7
9.8
1
2
t−Test: p−value = 0.99

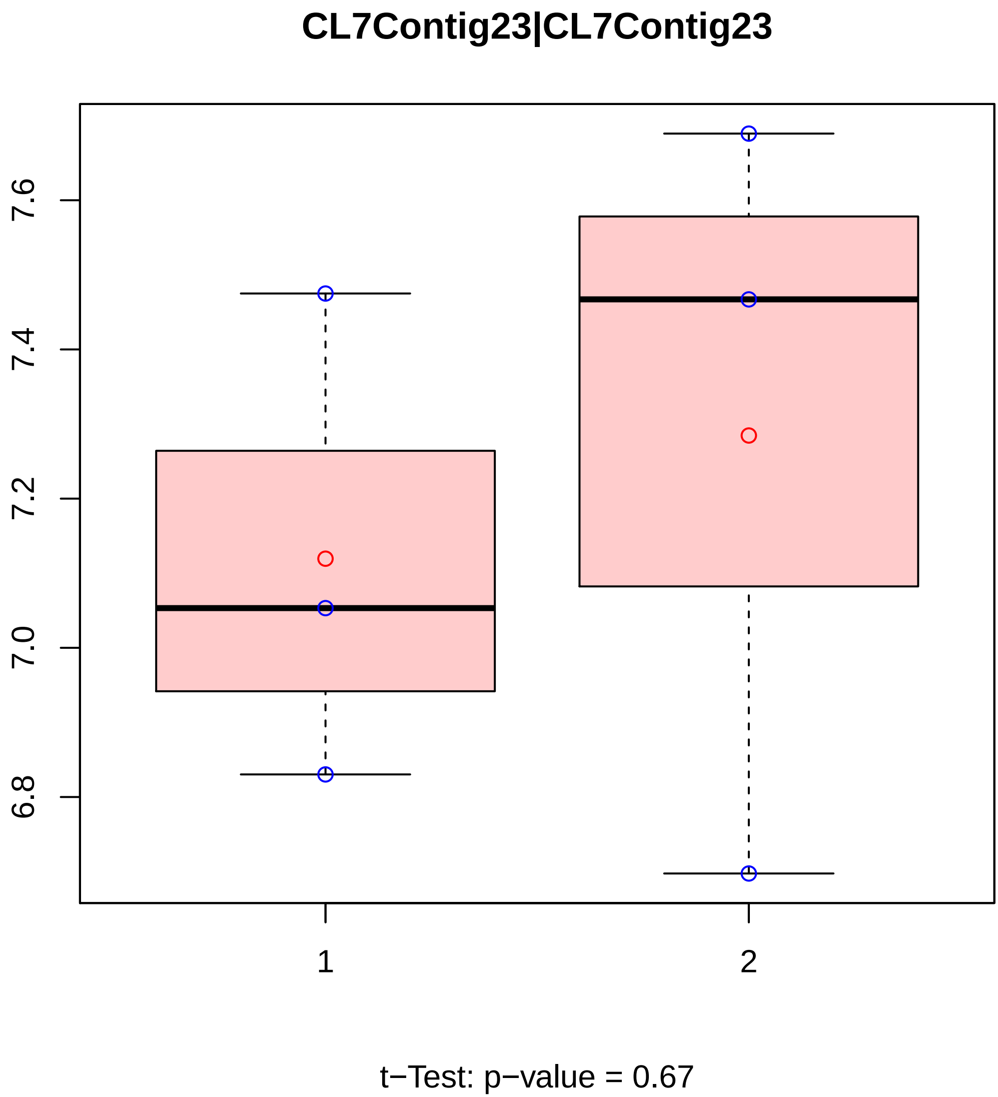

CL7Contig23|CL7Contig23
7.6
7.4
7.2
7.0
6.8
1
2
t−Test: p−value = 0.67

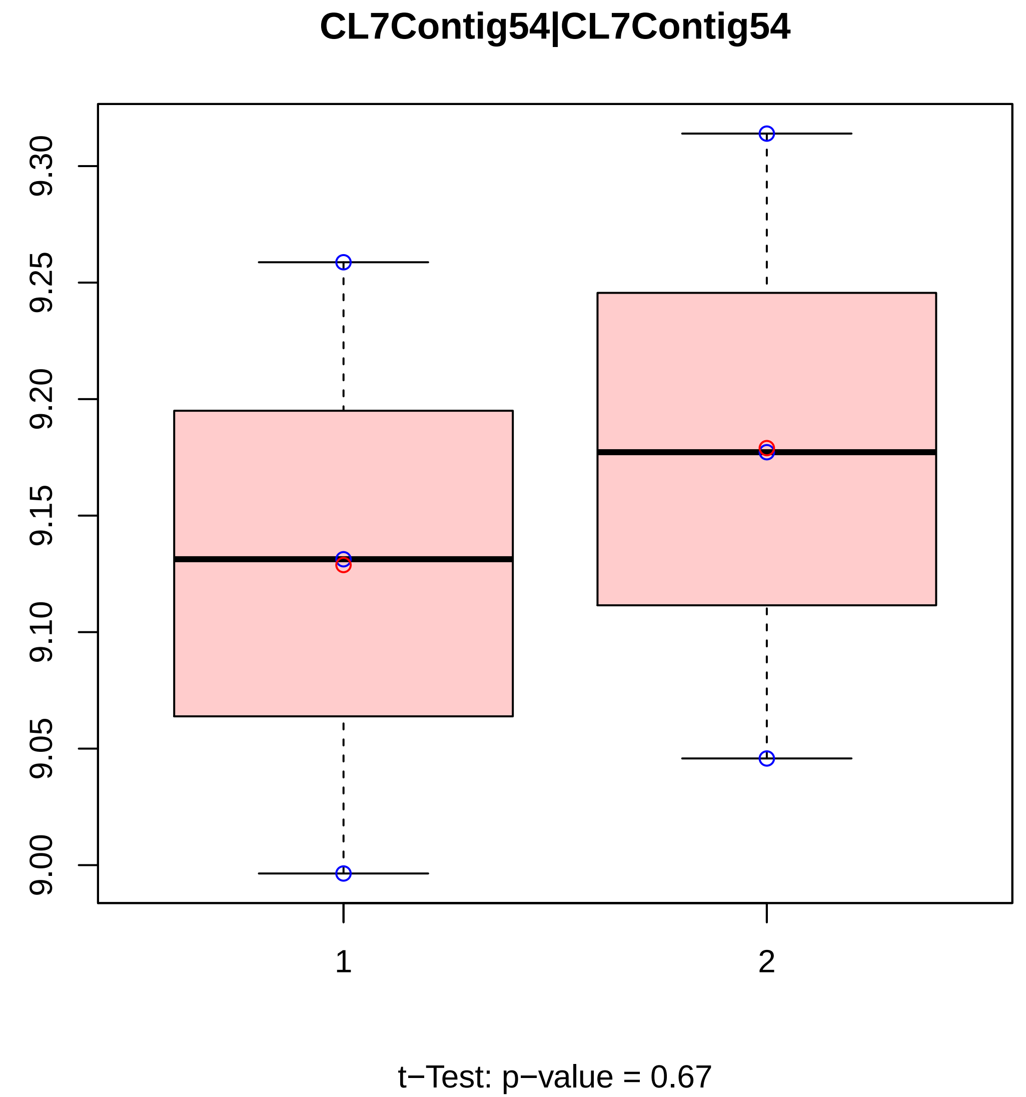
CL7Contig54|CL7Contig54
9.30
9.25
9.20
9.15
9.10
9.05
9.00
1
2
t−Test: p−value = 0.67

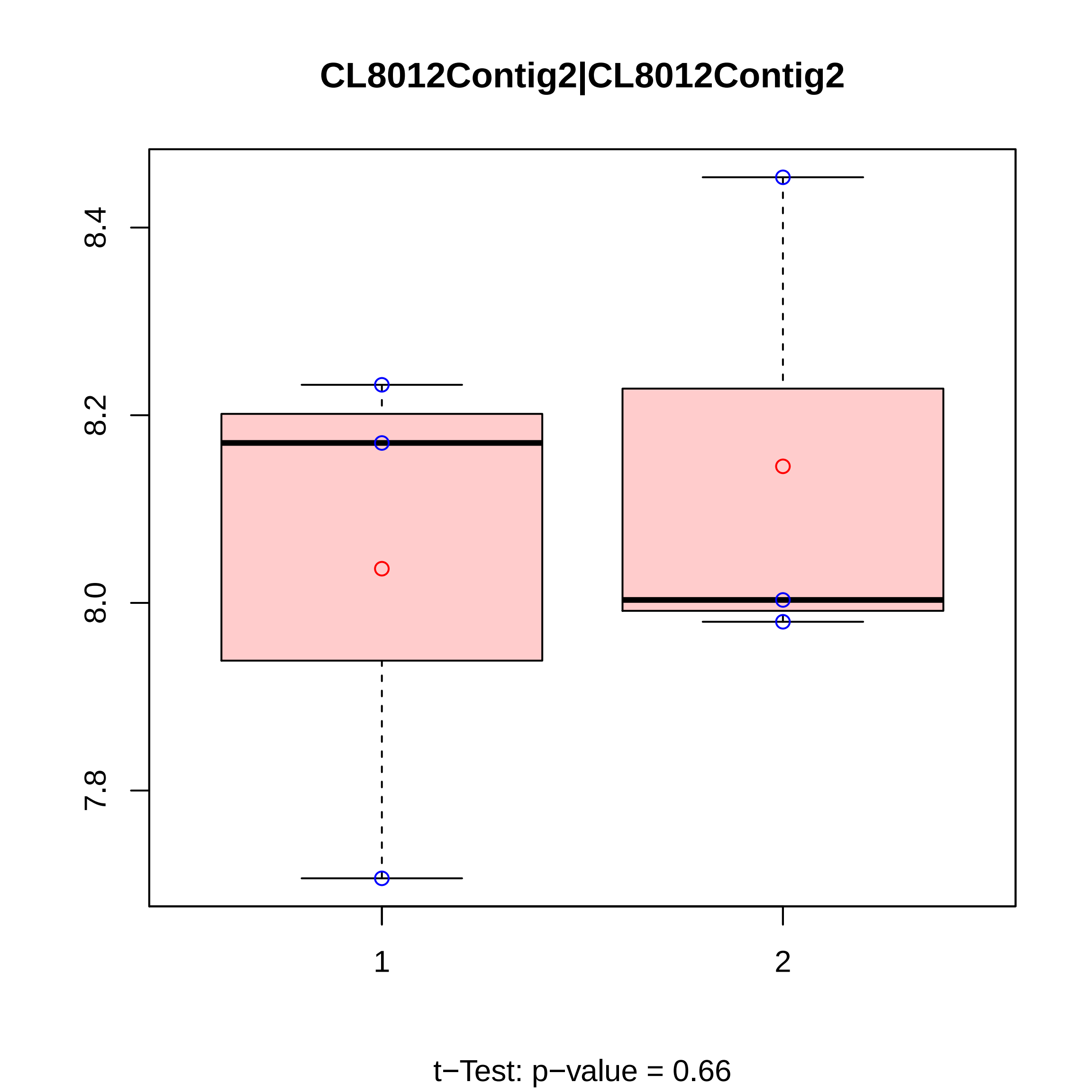

CL8012Contig2|CL8012Contig2
8.4
8.2
8.0
7.8
1
2
t−Test: p−value = 0.66

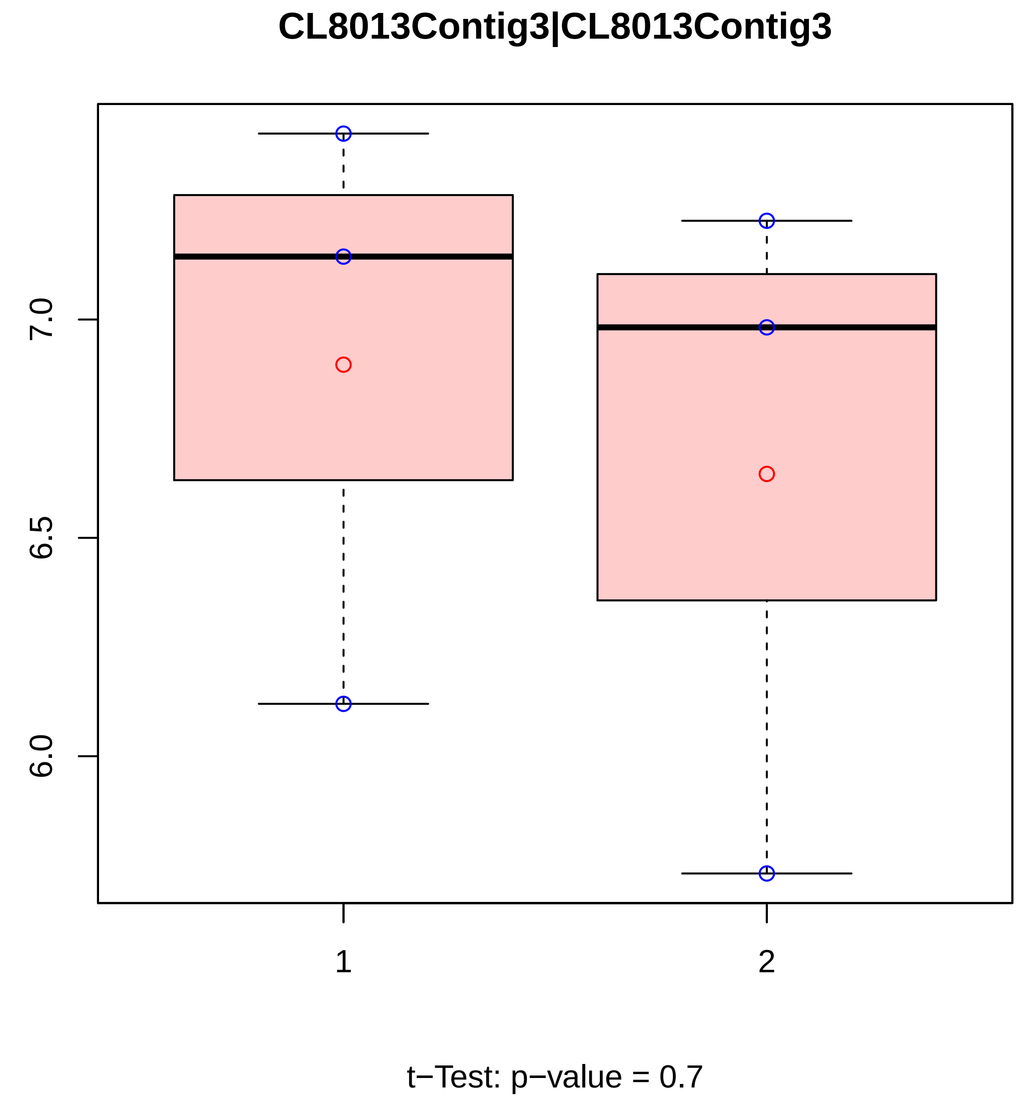
CL8013Contig3|CL8013Contig3
7.0
6.5
6.0
1
2
t−Test: p−value = 0.7

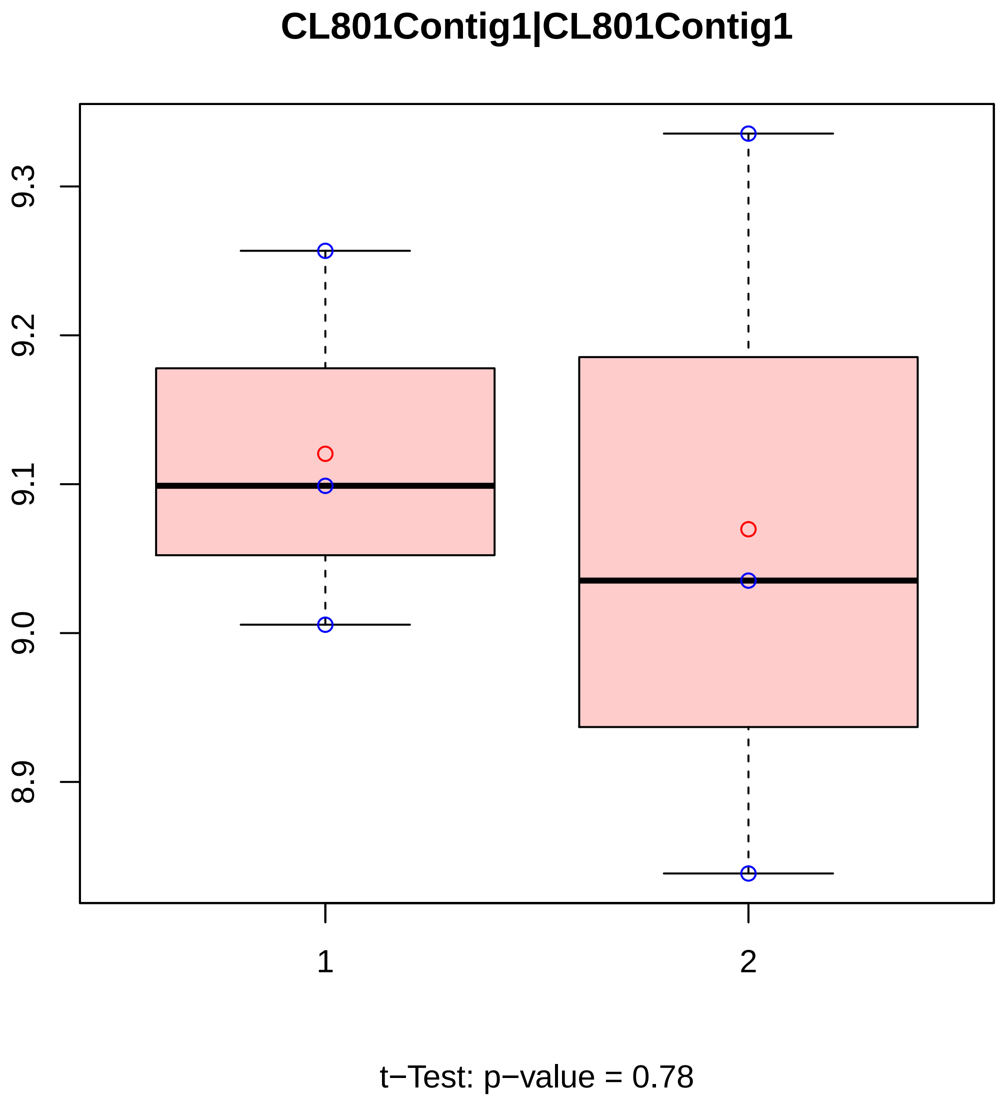

CL801Contig1|CL801Contig1
9.3
9.2
9.1
9.0
8.9
1
2
t−Test: p−value = 0.78

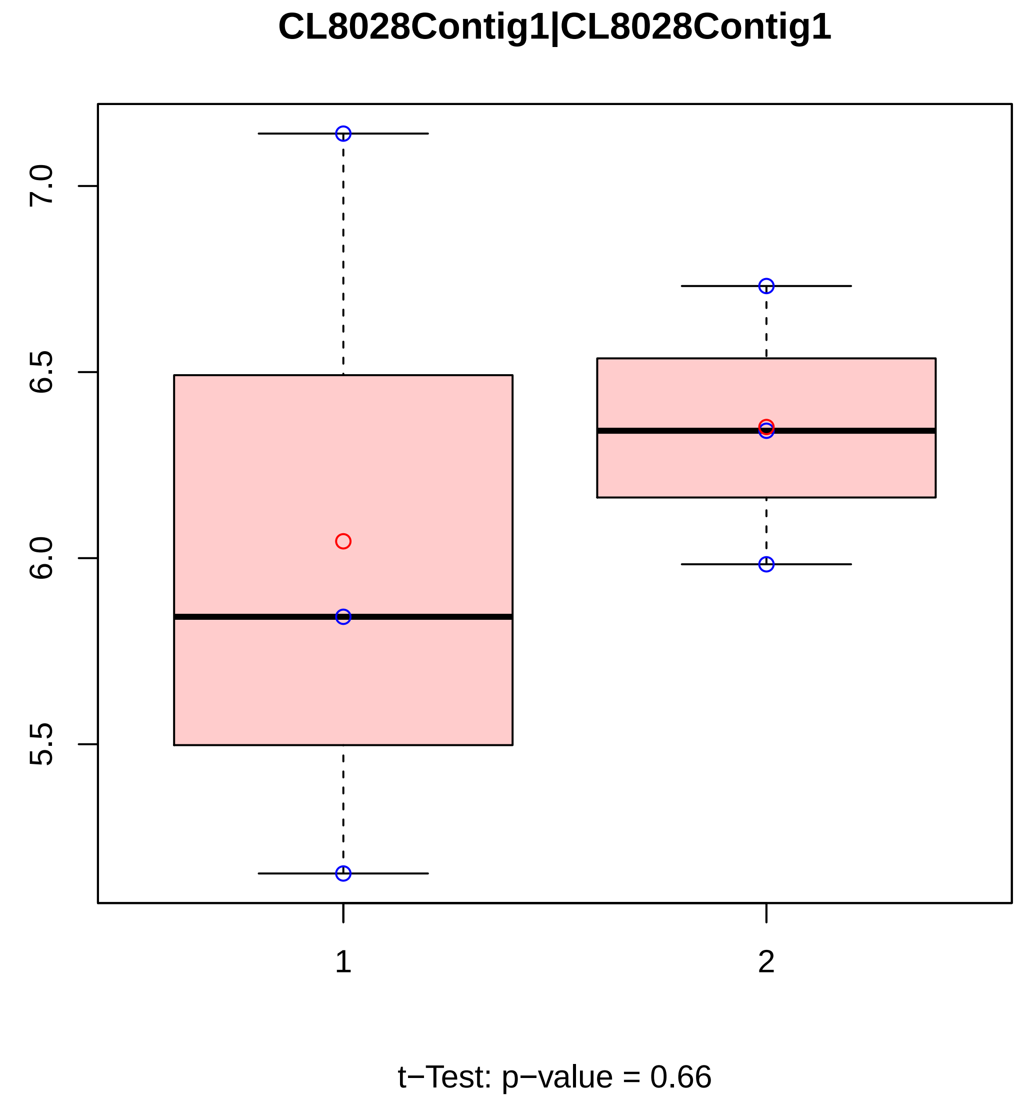
CL8028Contig1|CL8028Contig1
7.0
6.5
6.0
5.5
1
2
t−Test: p−value = 0.66

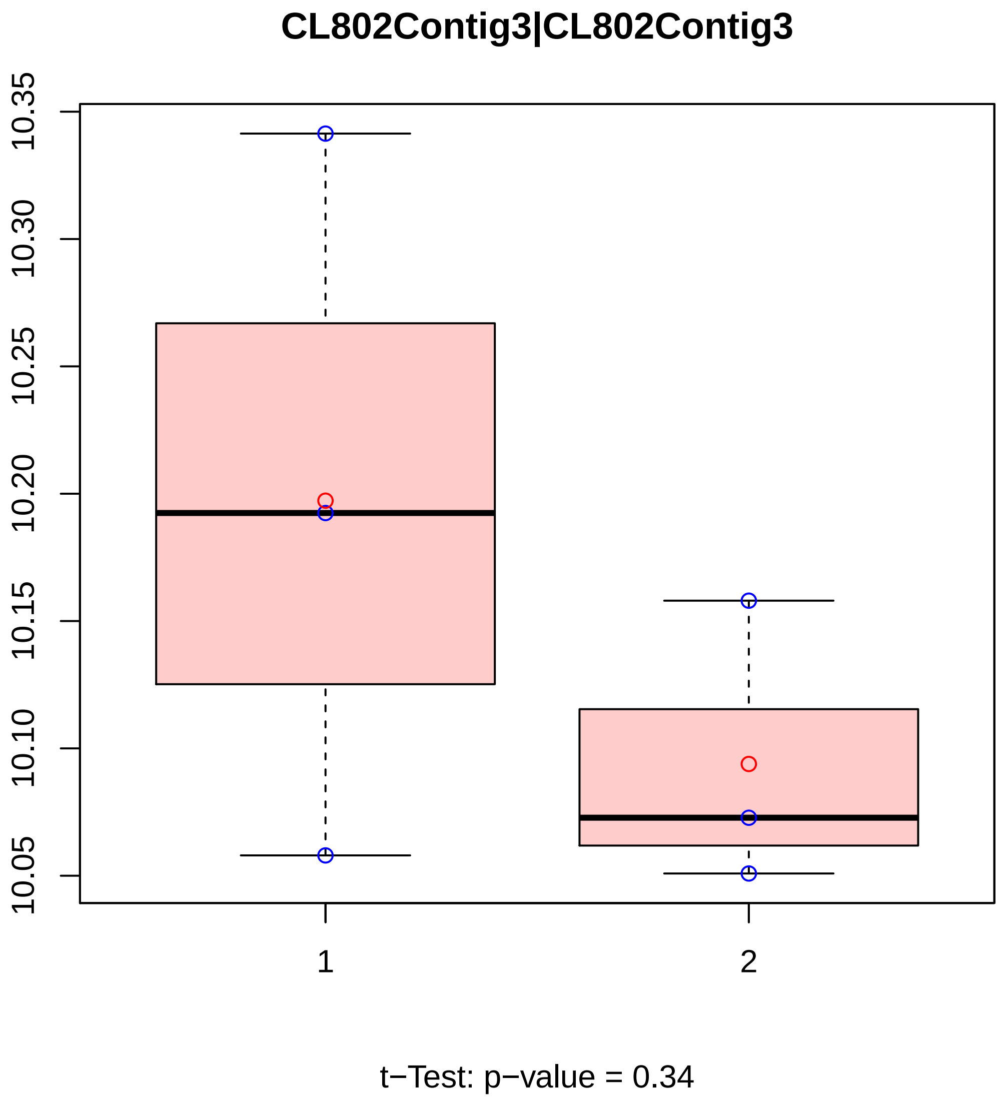

CL802Contig3|CL802Contig3
10.05
10.10
10.15
10.20
10.25
10.30
10.35
1
2
t−Test: p−value = 0.34

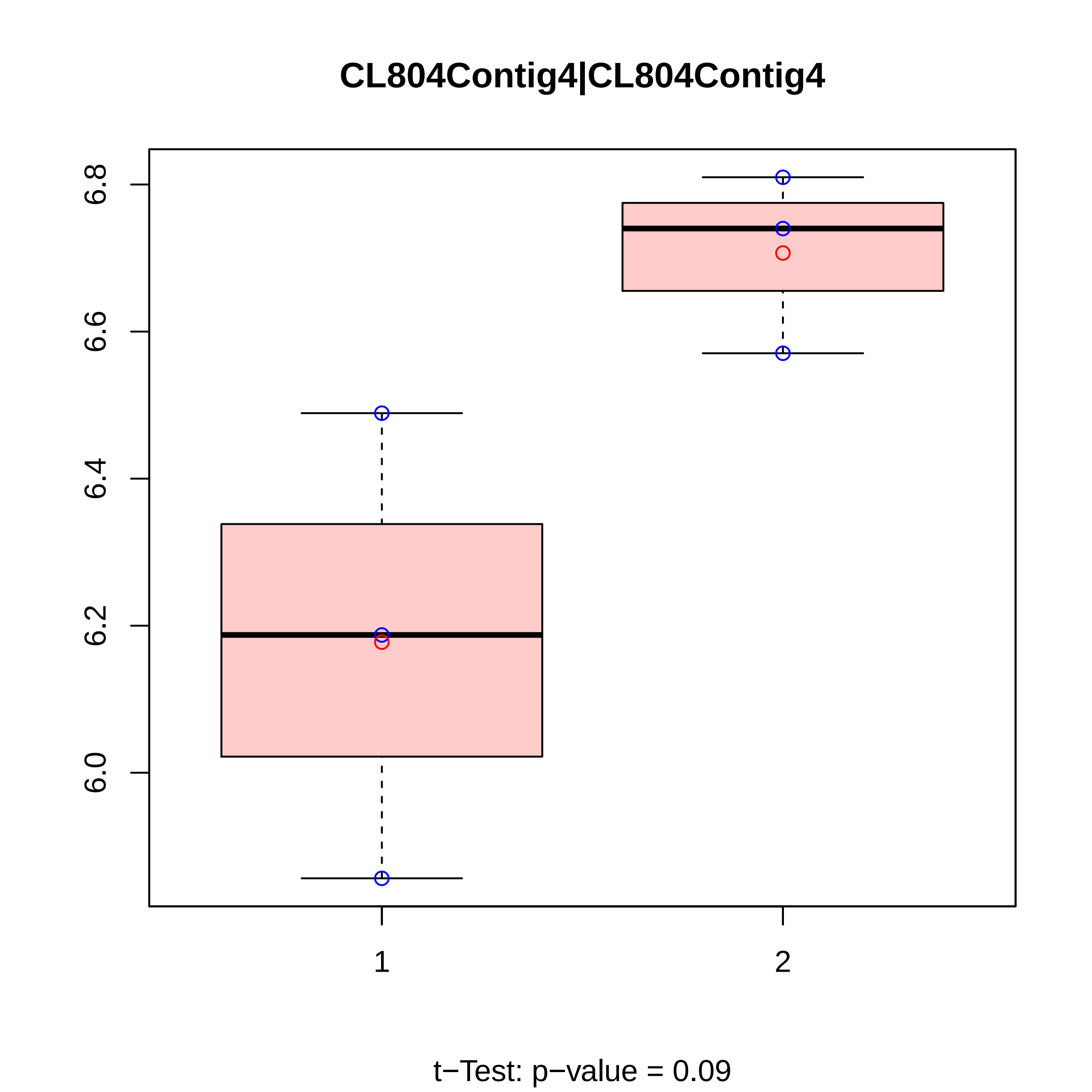

CL804Contig4|CL804Contig4
6.0
6.2
6.4
6.6
6.8
1
2
t−Test: p−value = 0.09

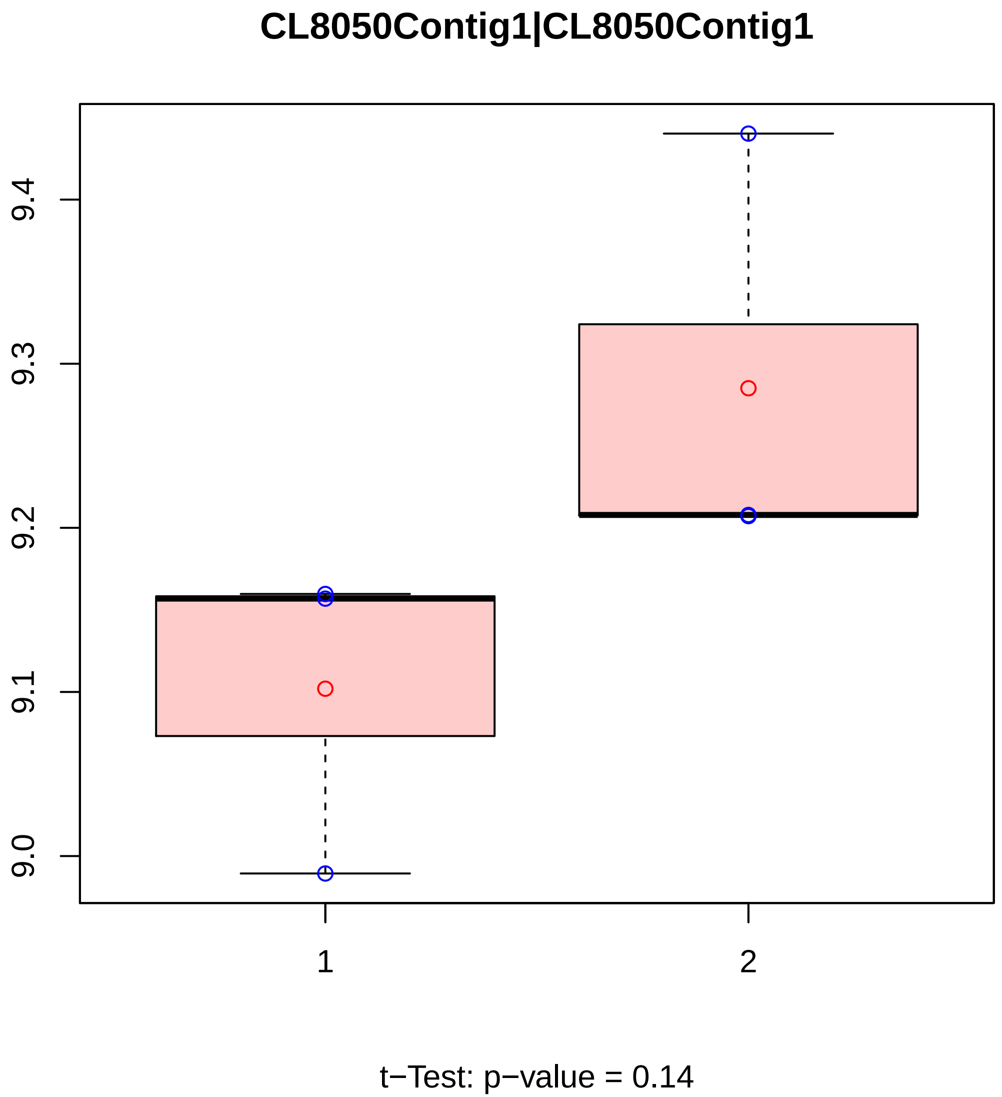

CL8050Contig1|CL8050Contig1
9.0
9.1
9.2
9.3
9.4
1
2
t−Test: p−value = 0.14

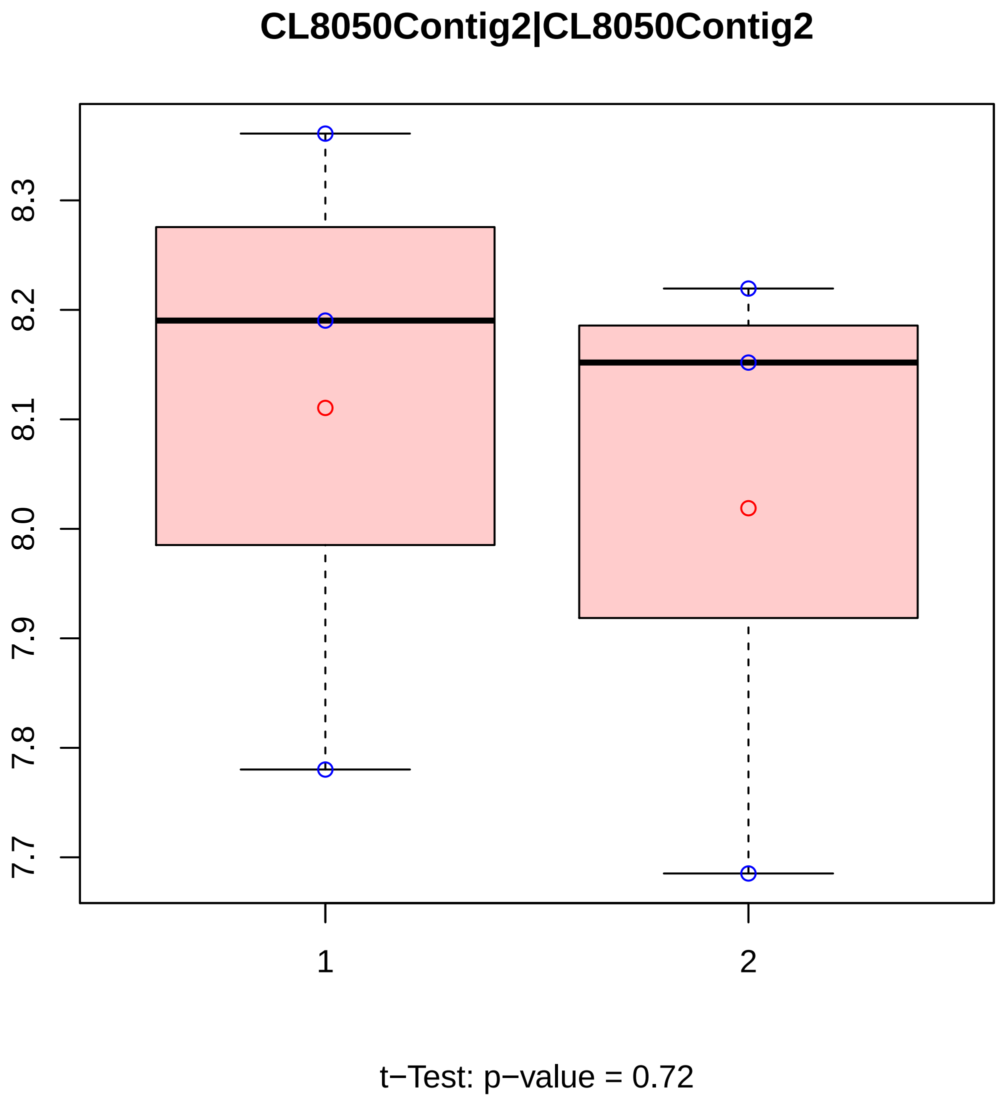
CL8050Contig2|CL8050Contig2
8.3
8.2
8.1
8.0
7.9
7.8
7.7
1
2
t−Test: p−value = 0.72

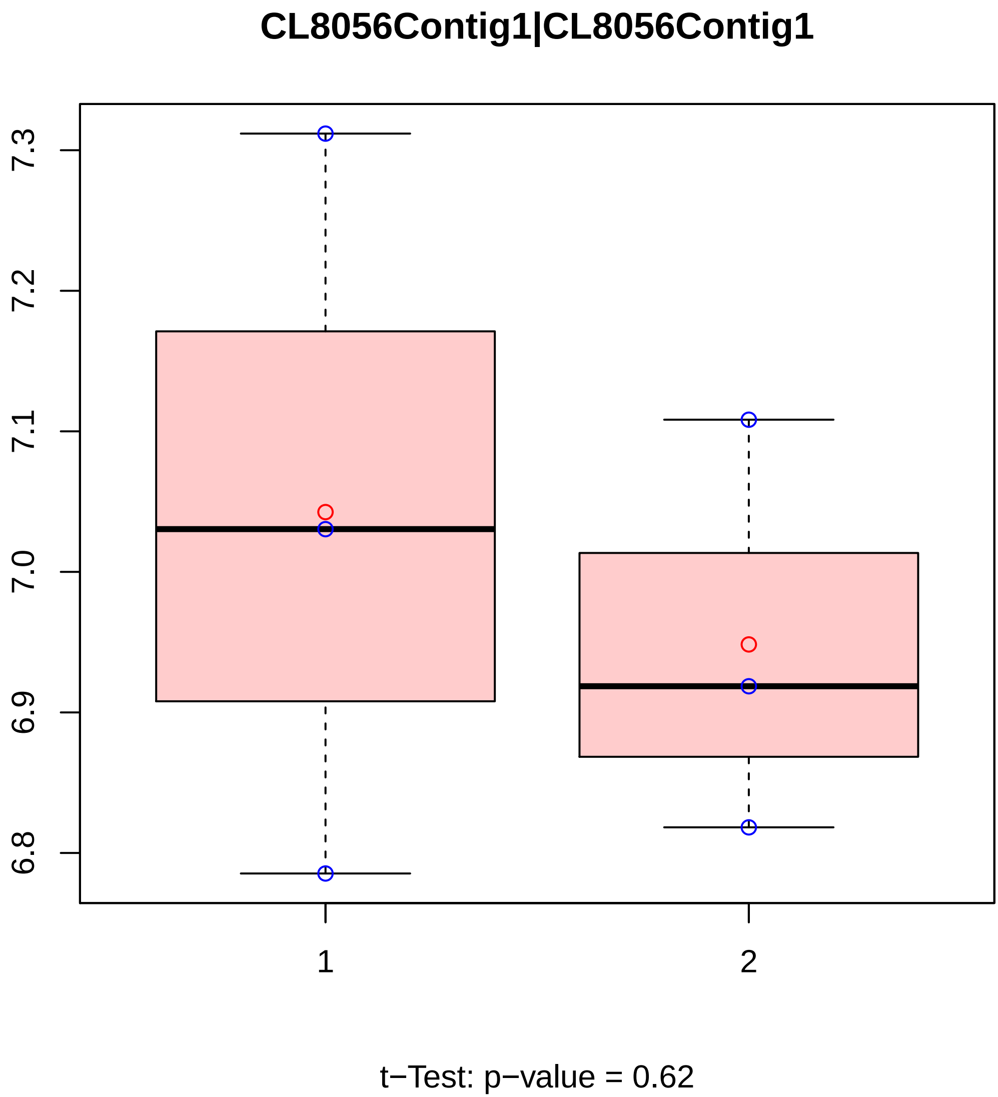

CL8056Contig1|CL8056Contig1
7.3
7.2
7.1
7.0
6.9
6.8
1
2
t−Test: p−value = 0.62

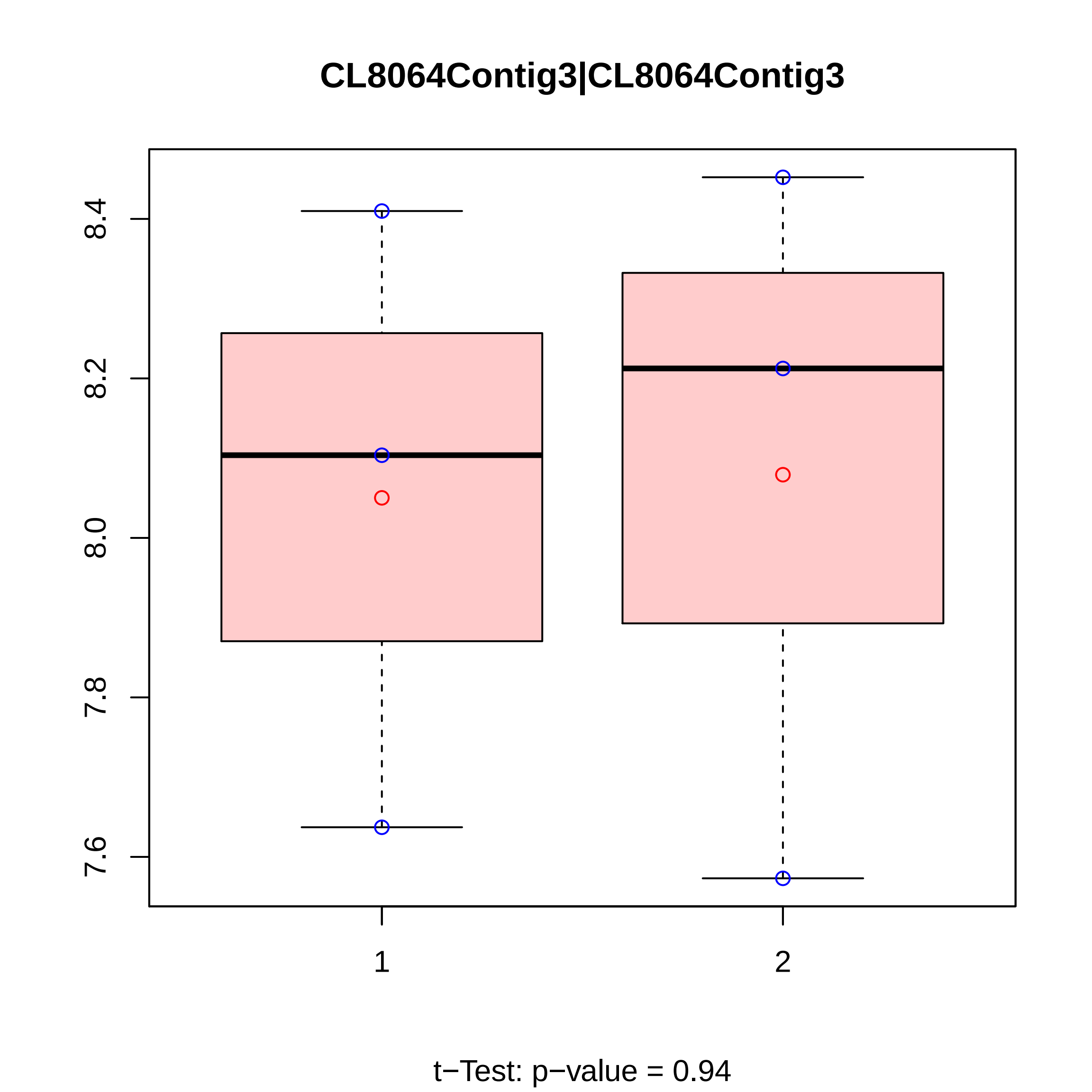
CL8064Contig3|CL8064Contig3
8.4
8.2
8.0
7.8
7.6
1
2
t−Test: p−value = 0.94

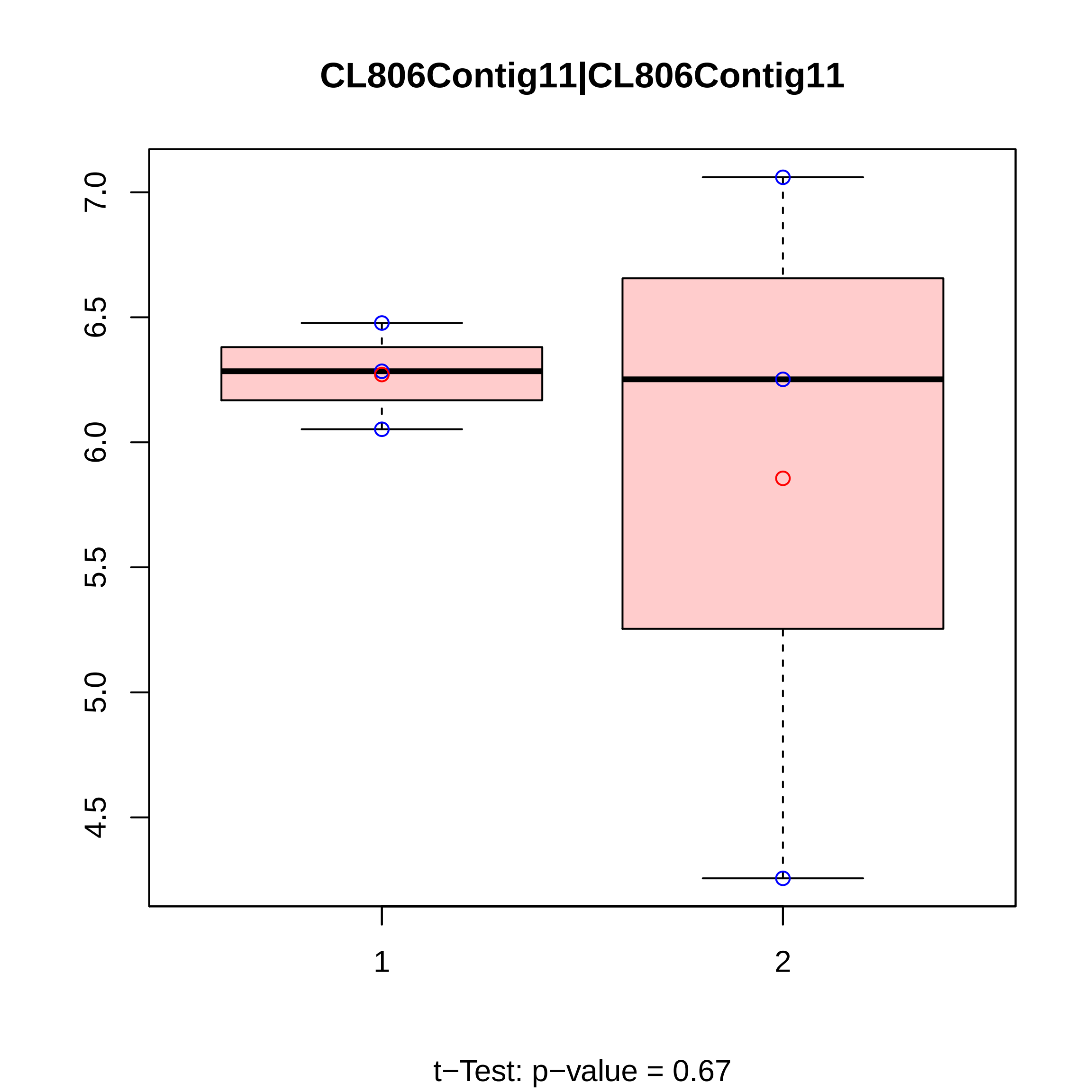
CL806Contig11|CL806Contig11
7.0
6.5
6.0
5.5
5.0
4.5
1
2
t−Test: p−value = 0.67

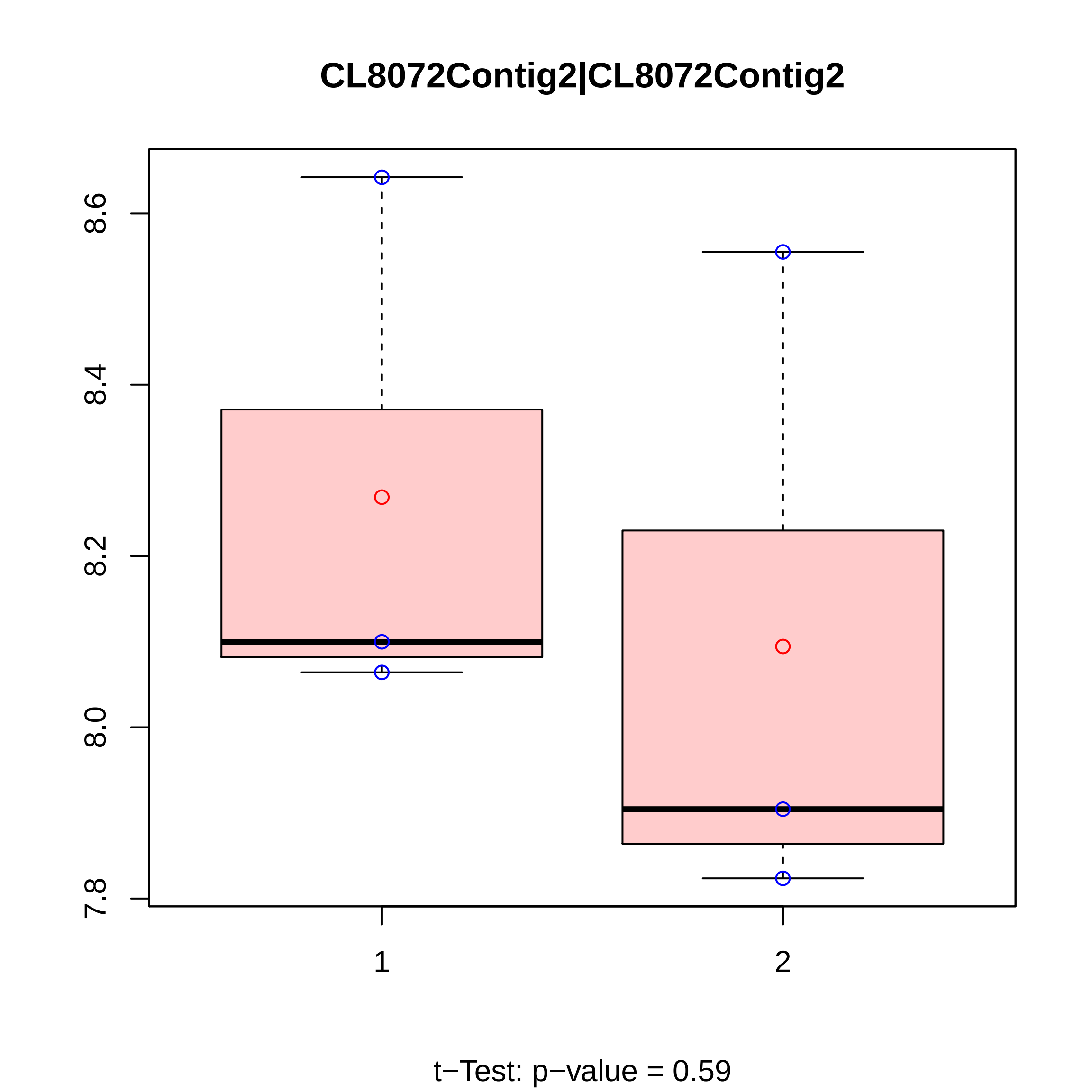

CL8072Contig2|CL8072Contig2
8.6
8.4
8.2
8.0
7.8
1
2
t−Test: p−value = 0.59

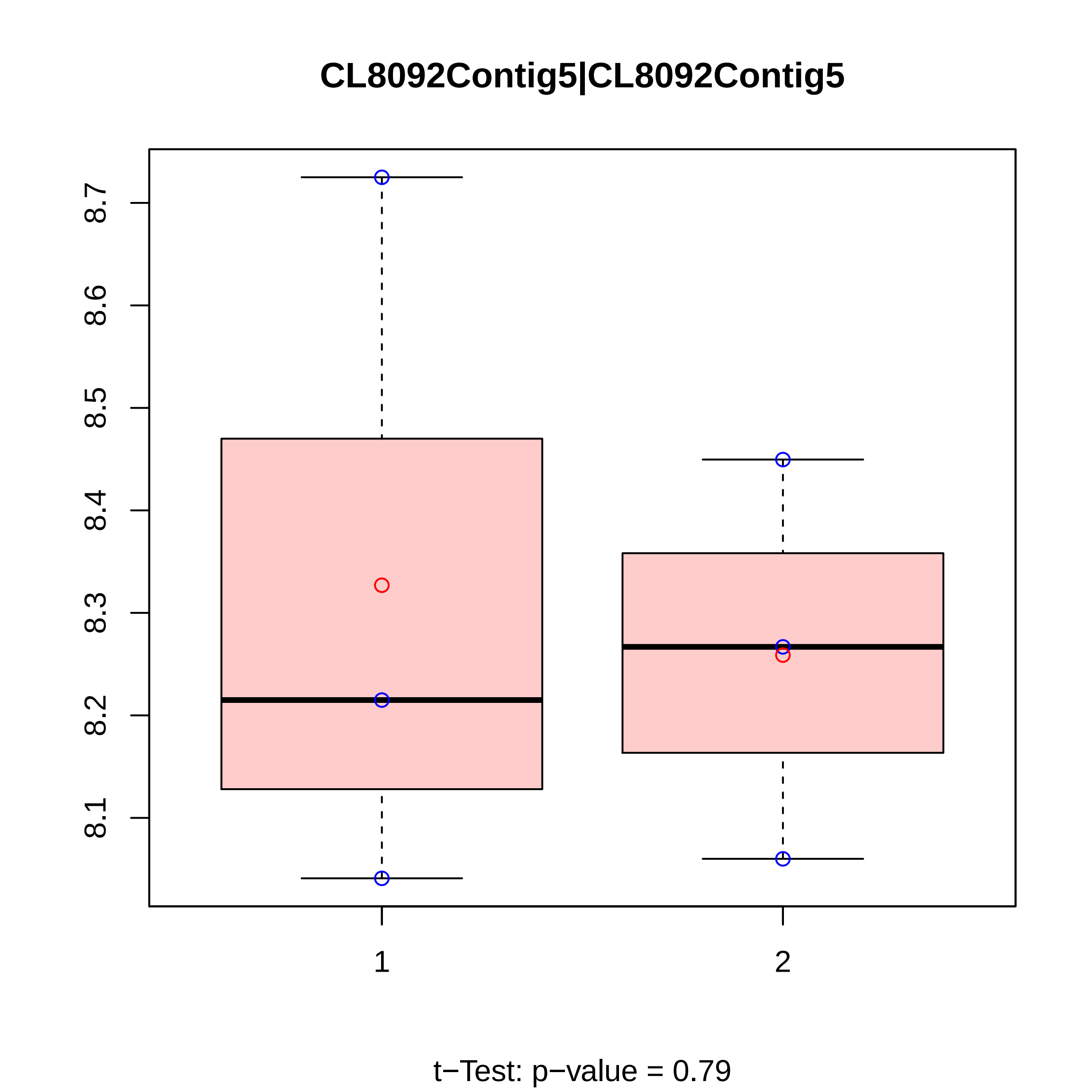
CL8092Contig5|CL8092Contig5
8.1
8.2
8.3
8.4
8.5
8.6
8.7
1
2
t−Test: p−value = 0.79

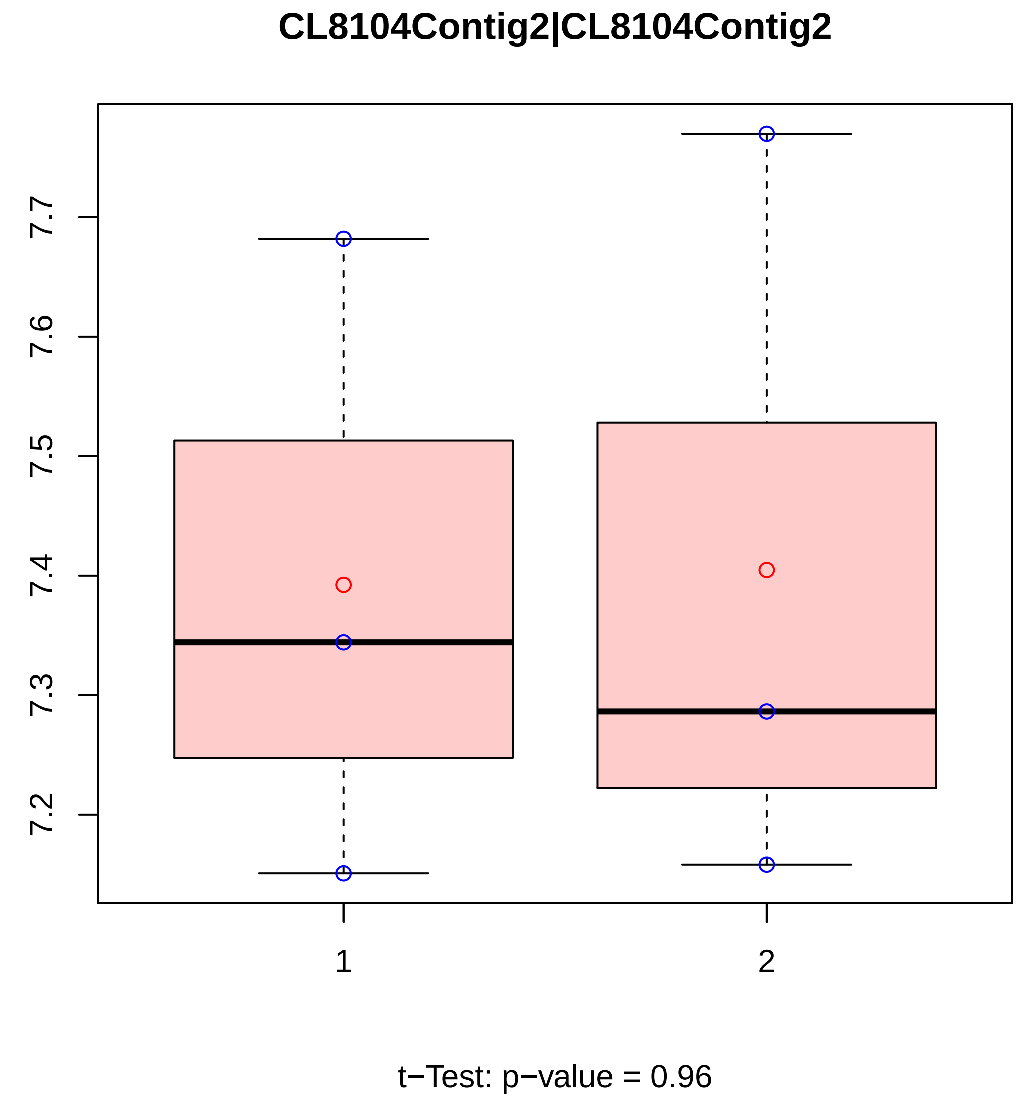
CL8104Contig2|CL8104Contig2
7.2
7.3
7.4
7.5
7.6
7.7
1
2
t−Test: p−value = 0.96

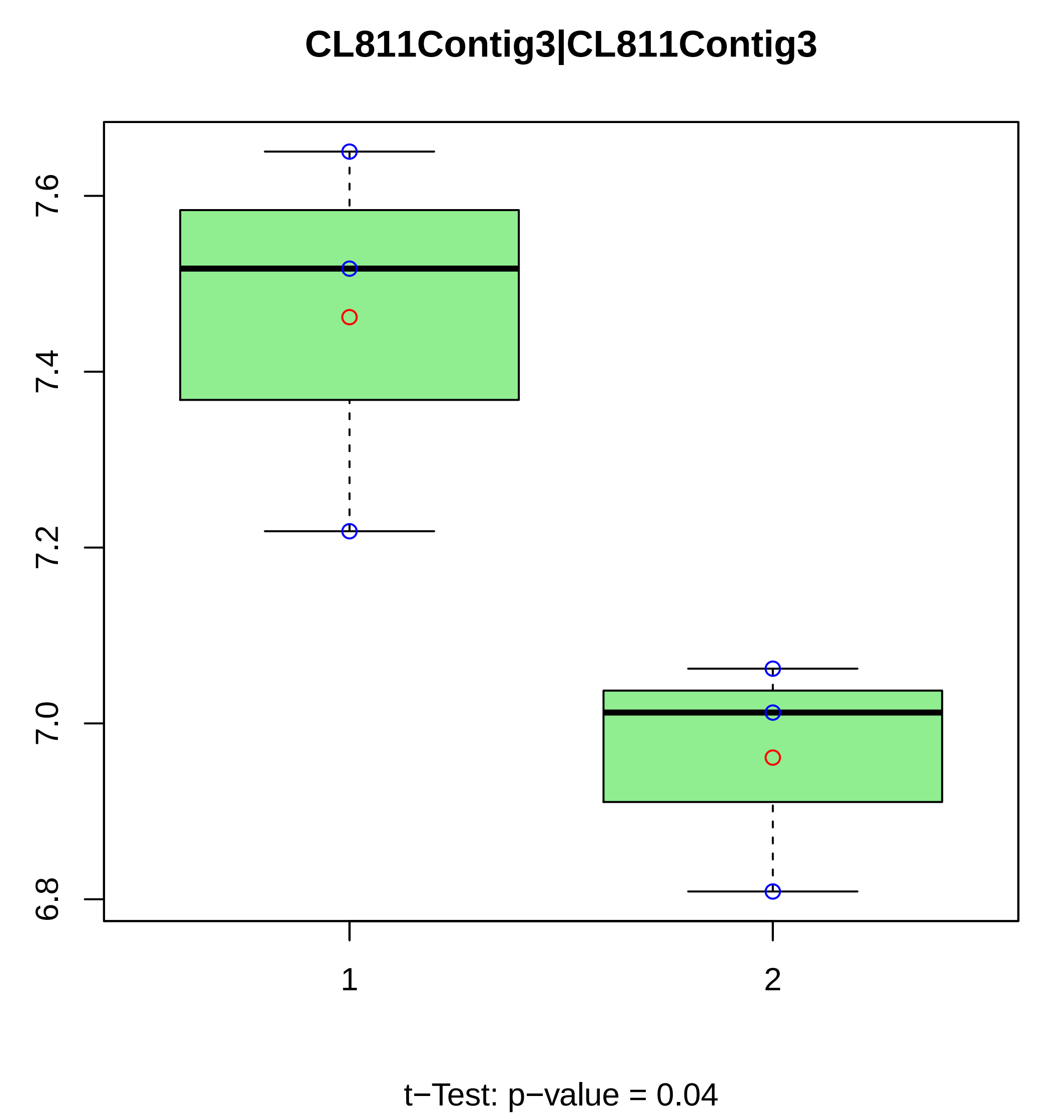

CL811Contig3|CL811Contig3
7.6
7.4
7.2
7.0
6.8
1
2
t−Test: p−value = 0.04

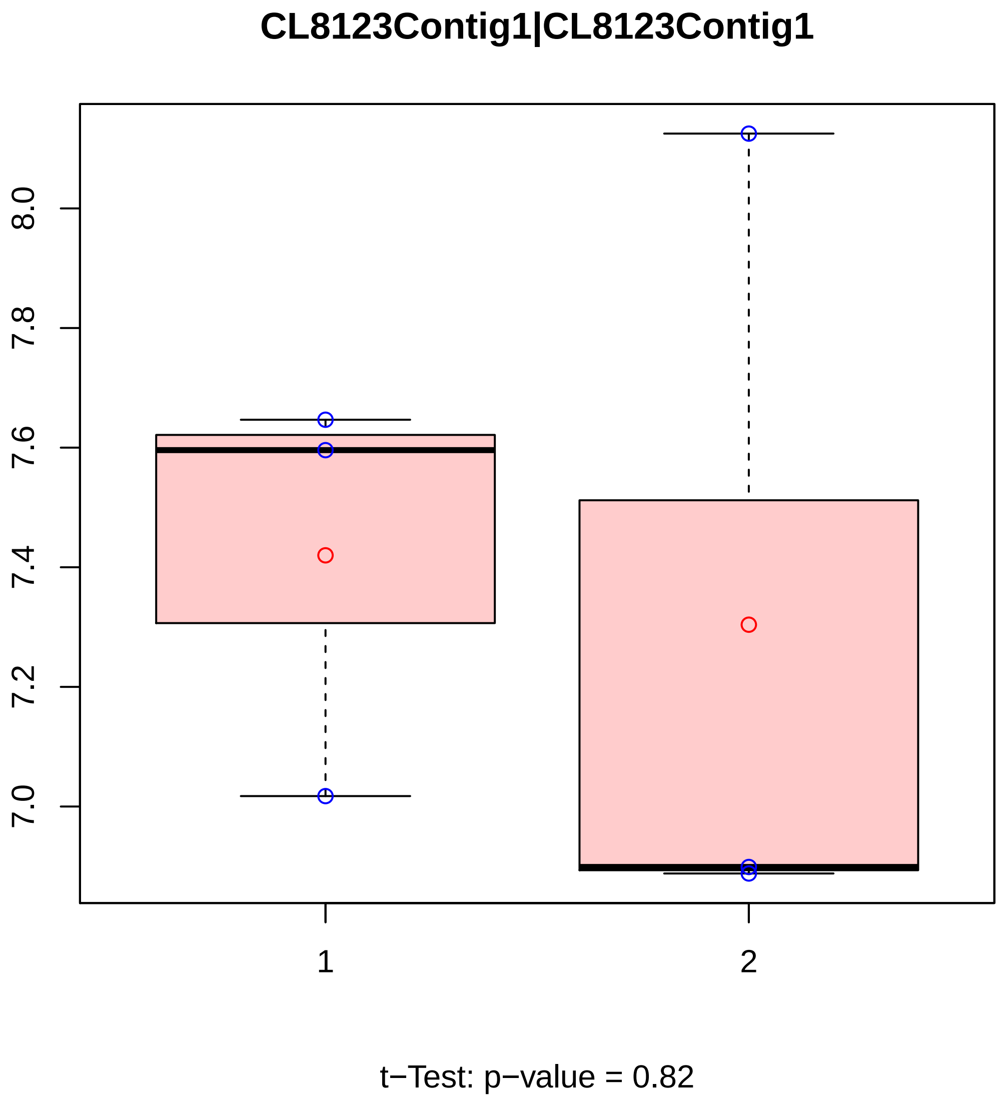
CL8123Contig1|CL8123Contig1
8.0
7.8
7.6
7.4
7.2
7.0
1
2
t−Test: p−value = 0.82

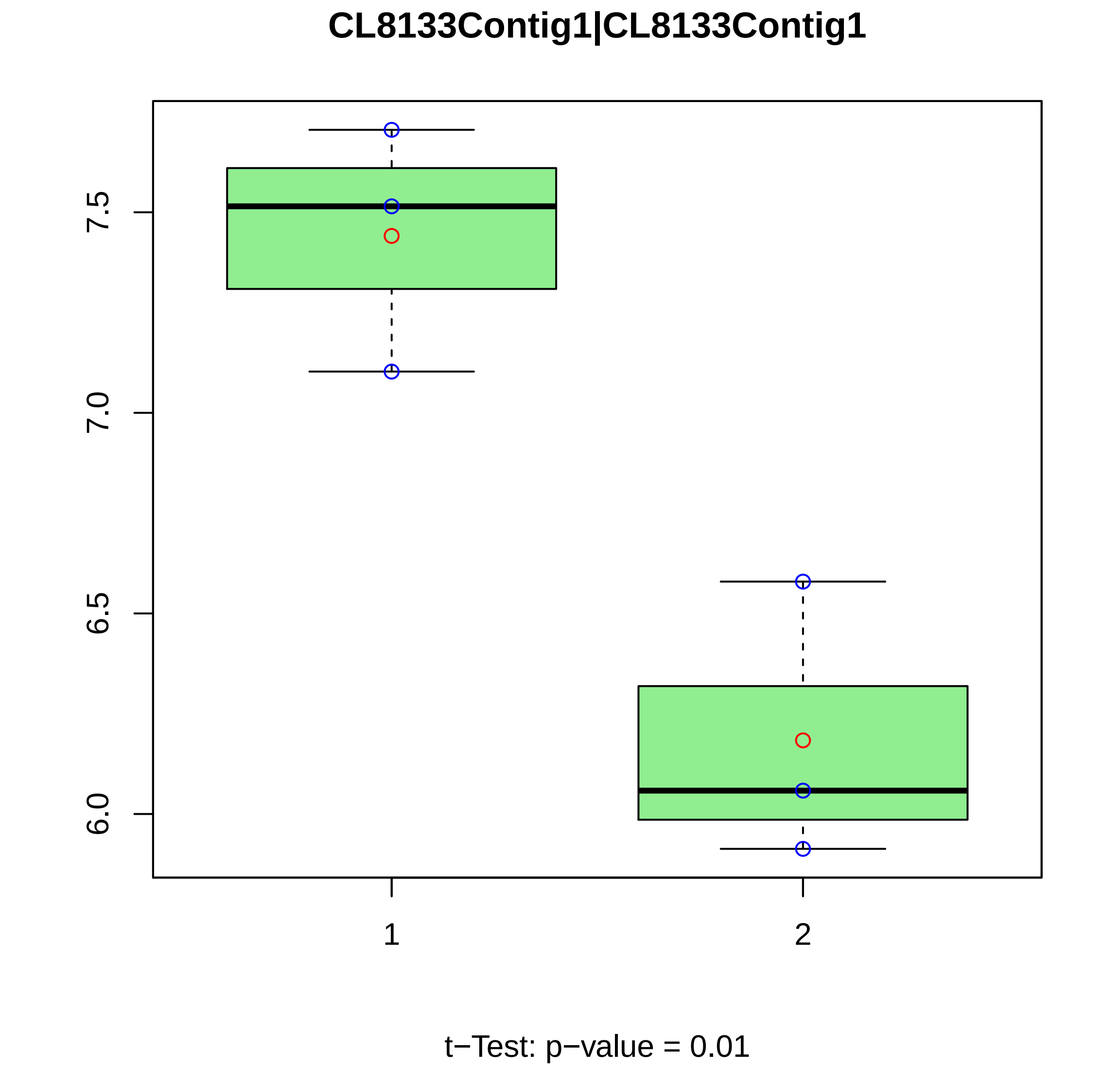
CL8133Contig1|CL8133Contig1
7.5
7.0
6.5
6.0
1
2
t−Test: p−value = 0.01

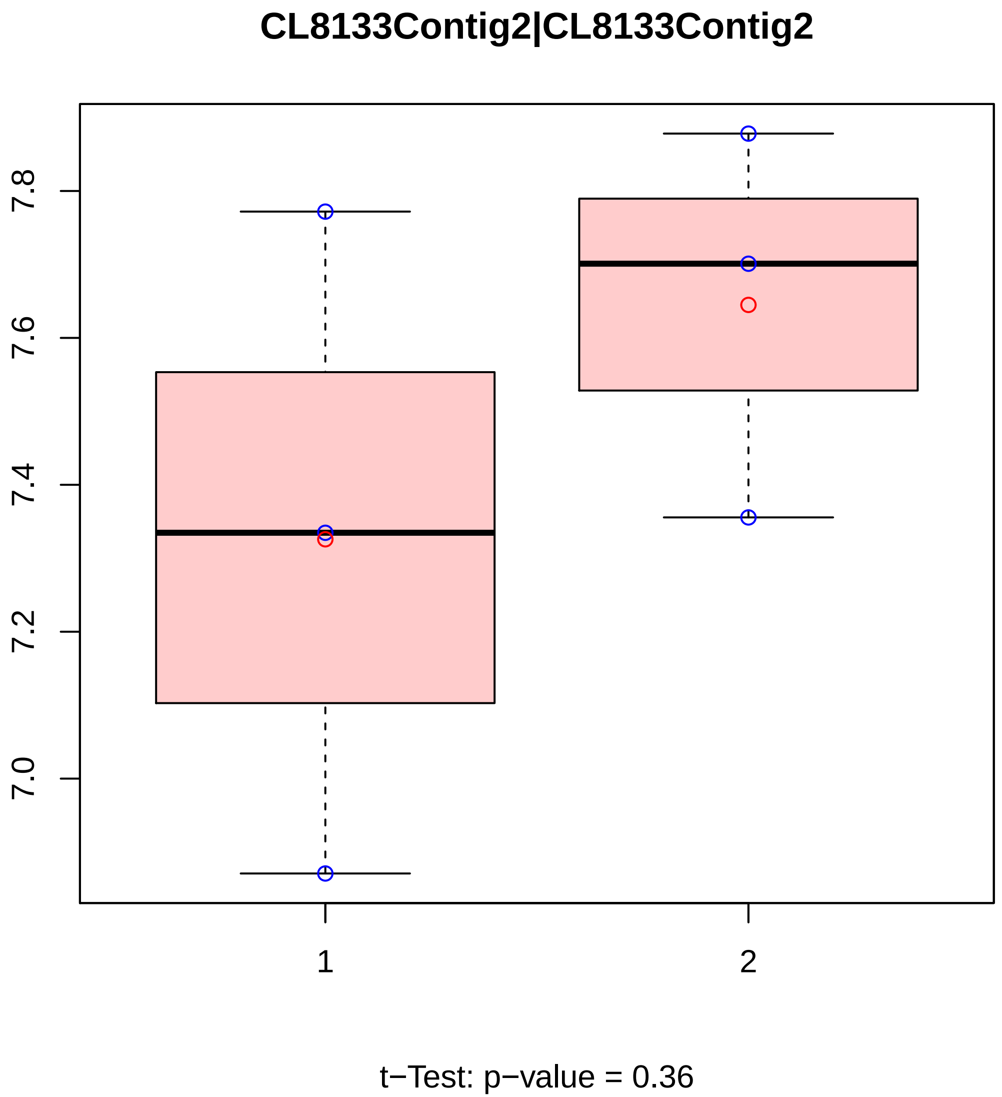
CL8133Contig2|CL8133Contig2
7.0
7.2
7.4
7.6
7.8
1
2
t−Test: p−value = 0.36

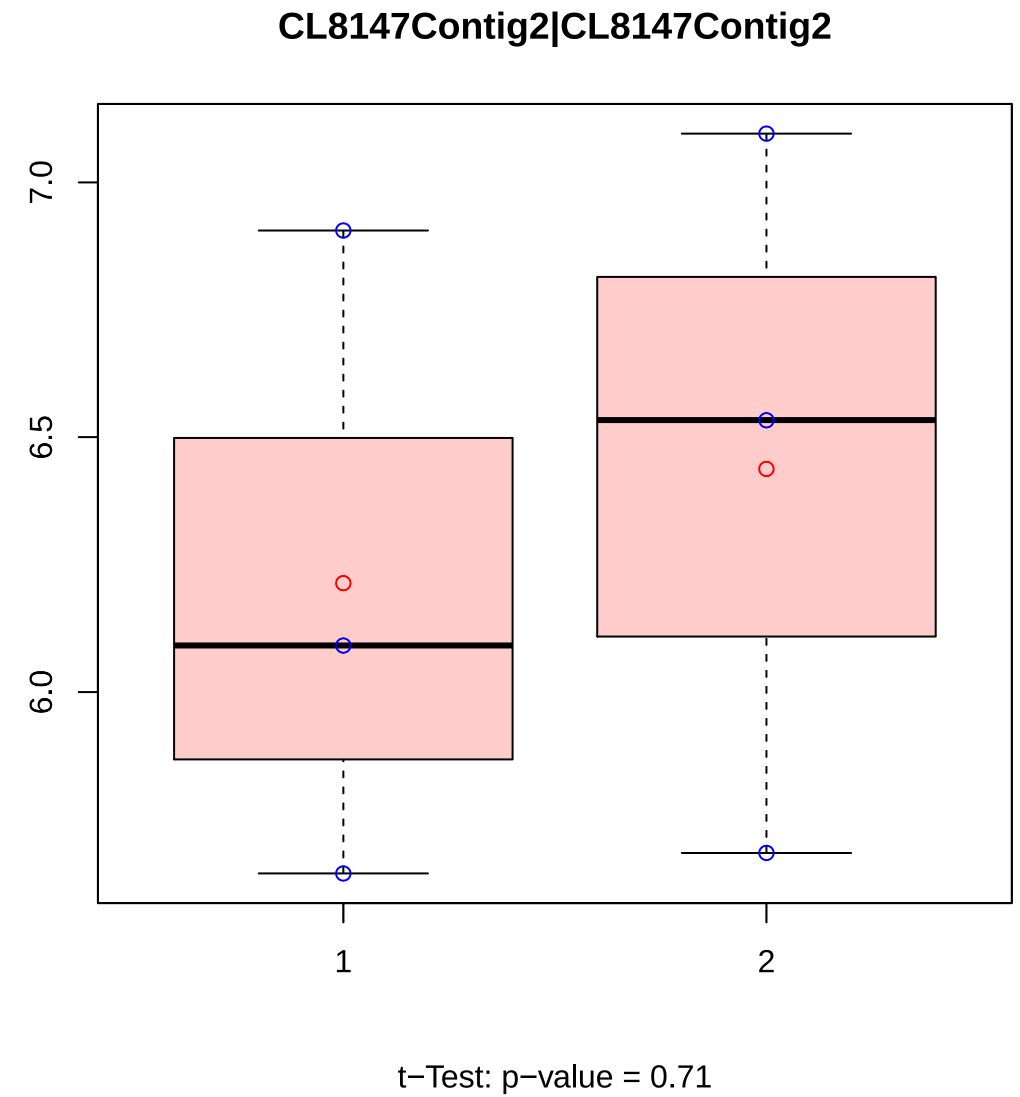
CL8147Contig2|CL8147Contig2
7.0
6.5
6.0
1
2
t−Test: p−value = 0.71

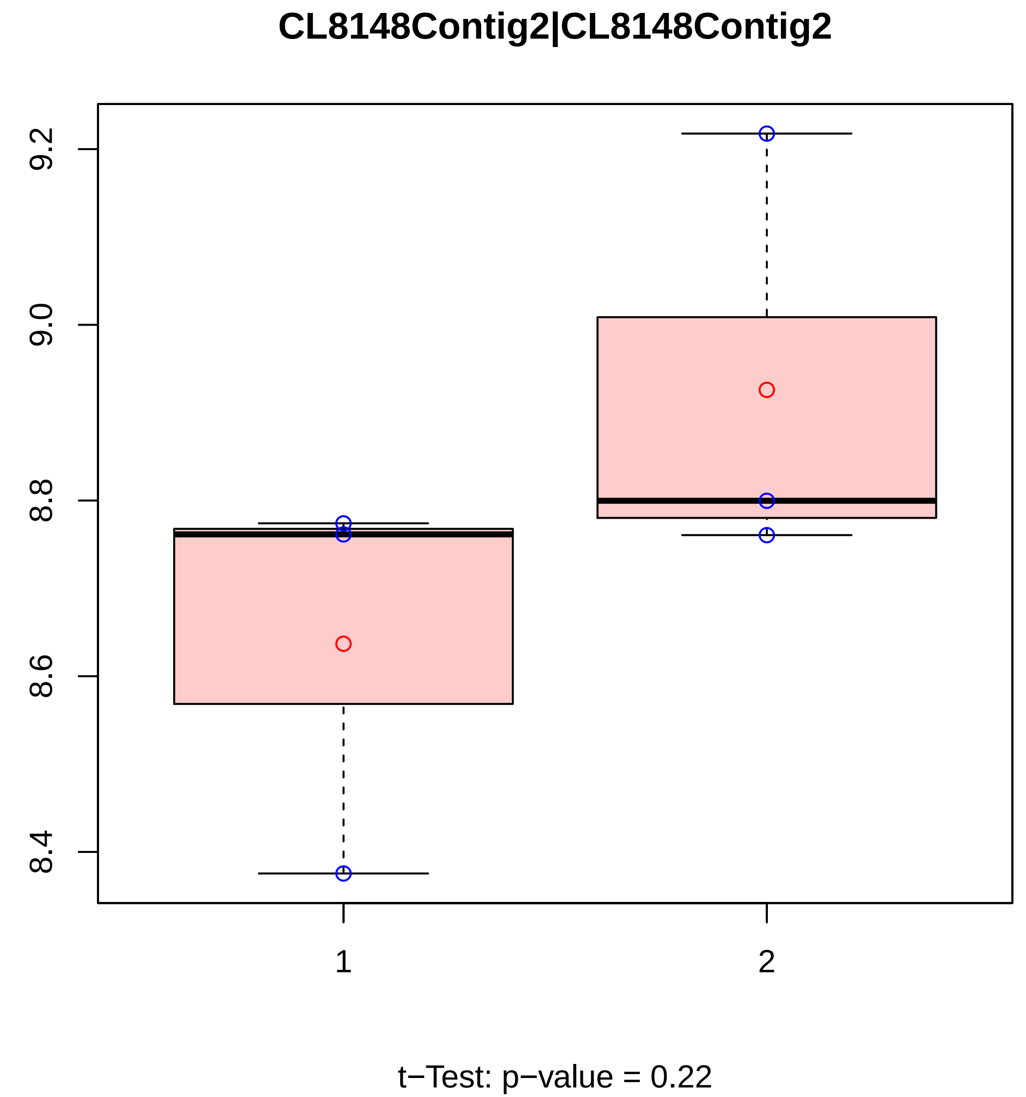
CL8148Contig2|CL8148Contig2
9.2
9.0
8.8
8.6
8.4
1
2
t−Test: p−value = 0.22

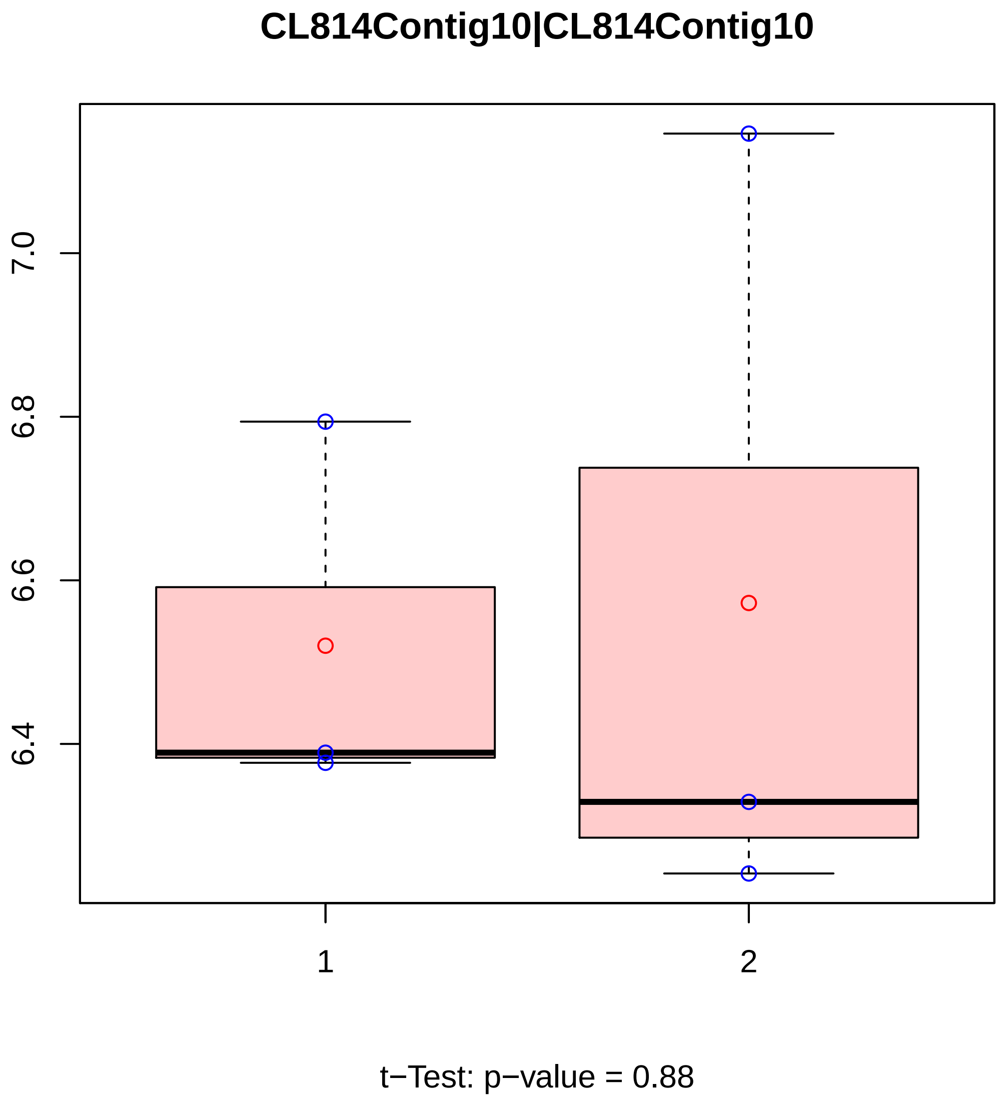

CL814Contig10|CL814Contig10
7.0
6.8
6.6
6.4
1
2
t−Test: p−value = 0.88

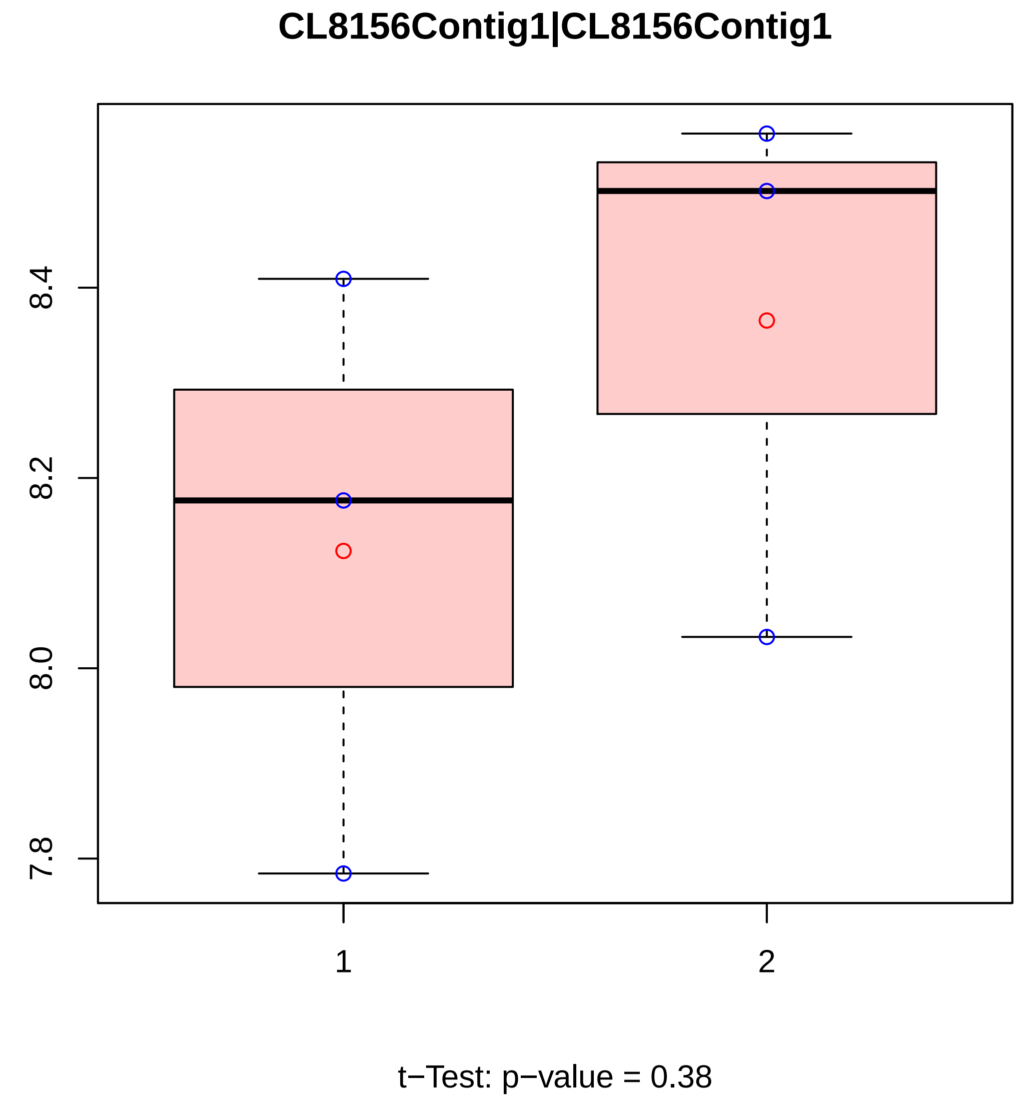
CL8156Contig1|CL8156Contig1
8.4
8.2
8.0
7.8
1
2
t−Test: p−value = 0.38

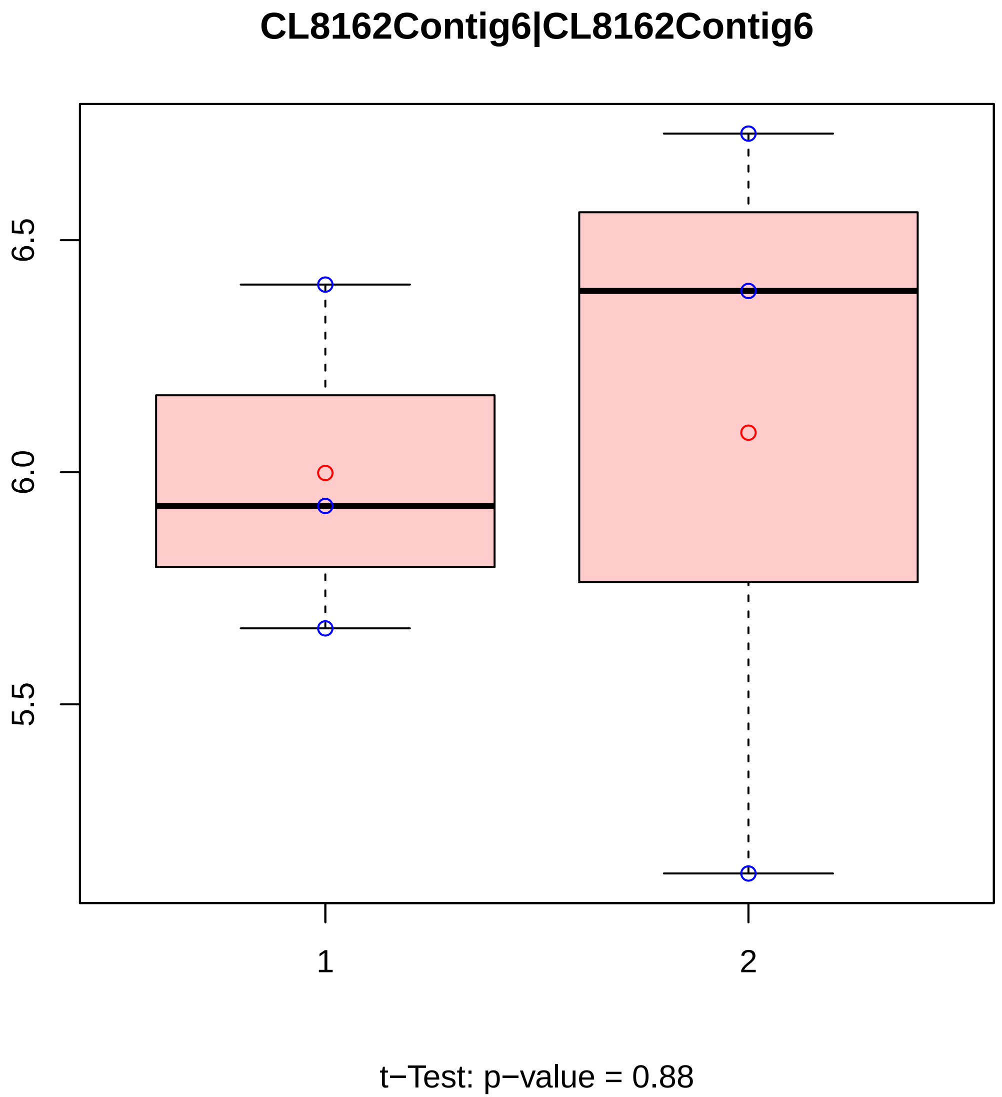
CL8162Contig6|CL8162Contig6
6.5
6.0
5.5
1
2
t−Test: p−value = 0.88

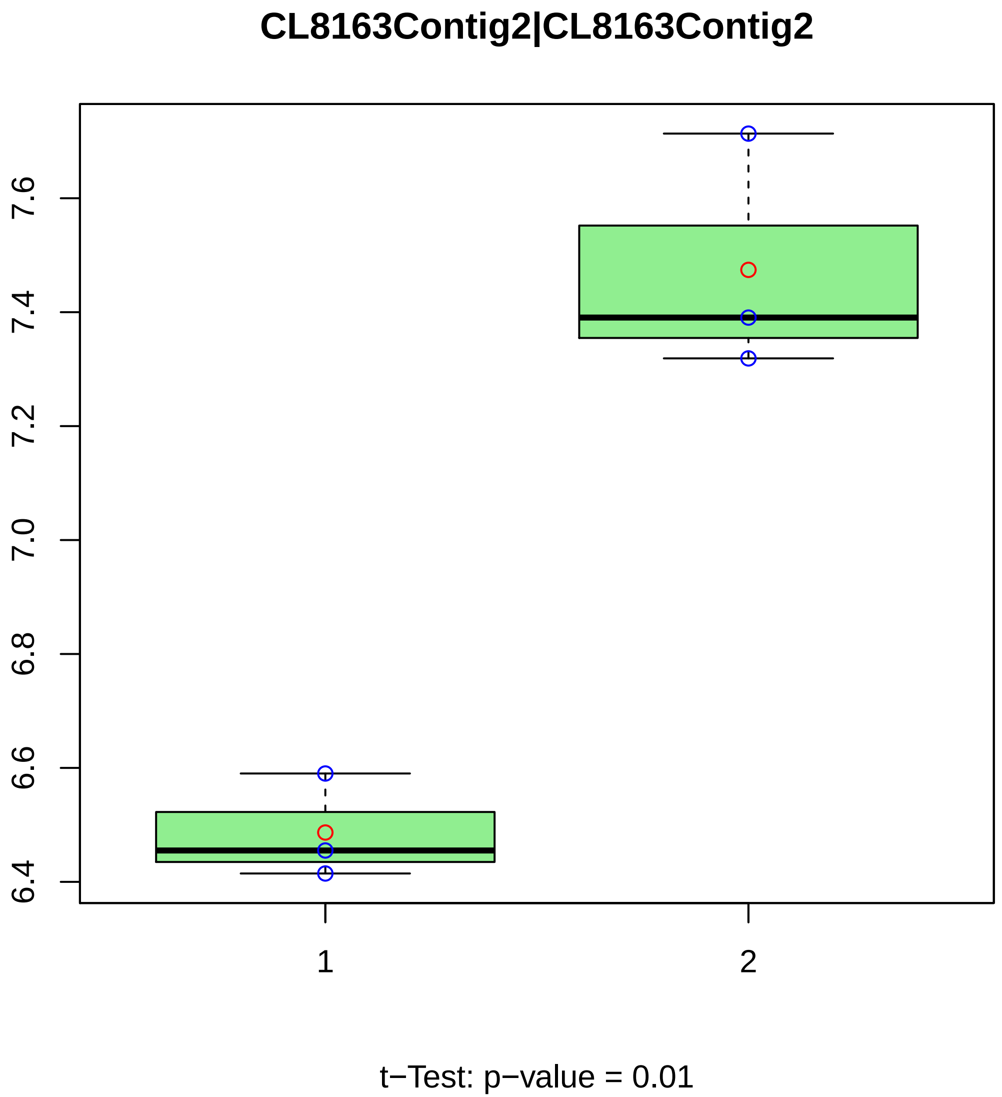
CL8163Contig2|CL8163Contig2
7.6
7.4
7.2
7.0
6.8
6.6
6.4
1
2
t−Test: p−value = 0.01

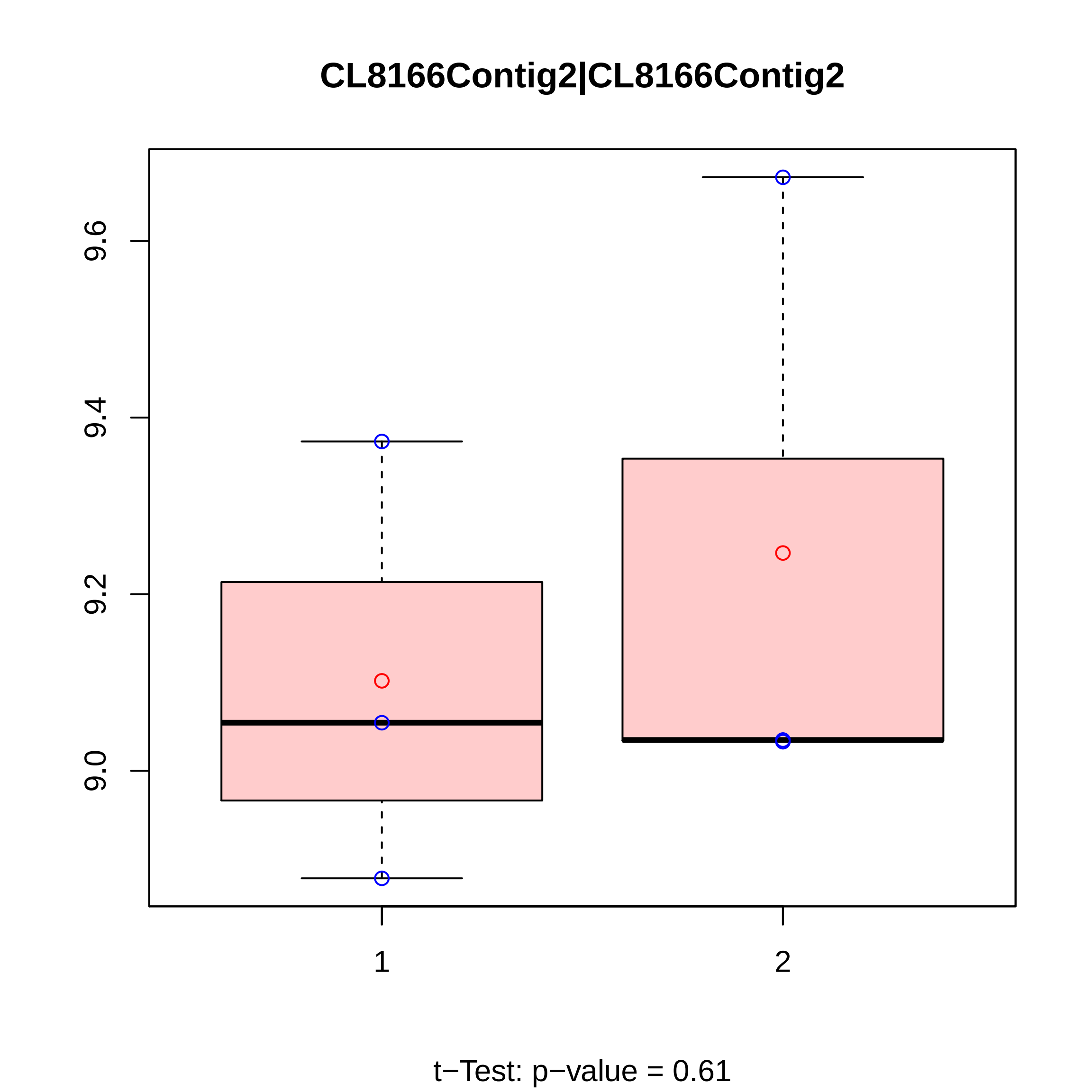

CL8166Contig2|CL8166Contig2
9.0
9.2
9.4
9.6
1
2
t−Test: p−value = 0.61

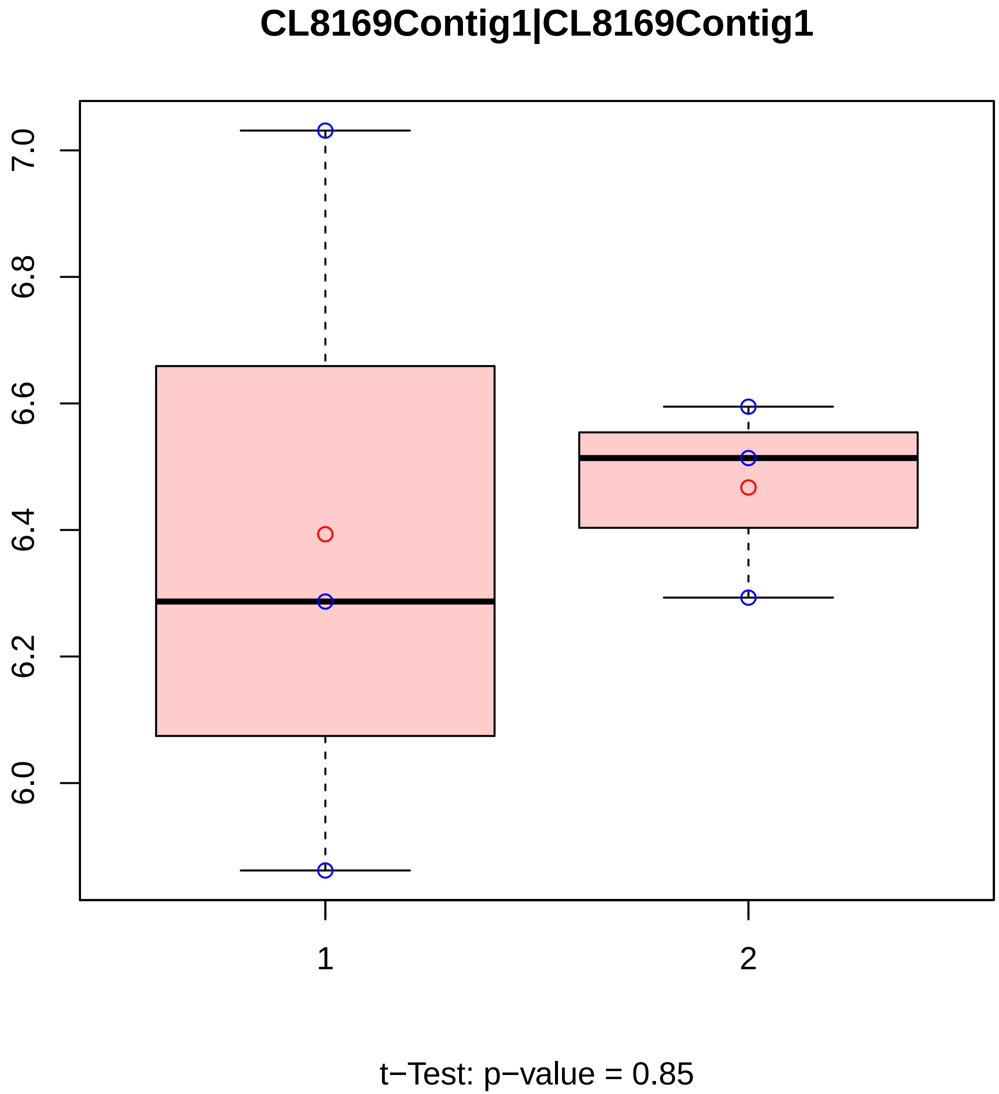
CL8169Contig1|CL8169Contig1
7.0
6.8
6.6
6.4
6.2
6.0
1
2
t−Test: p−value = 0.85

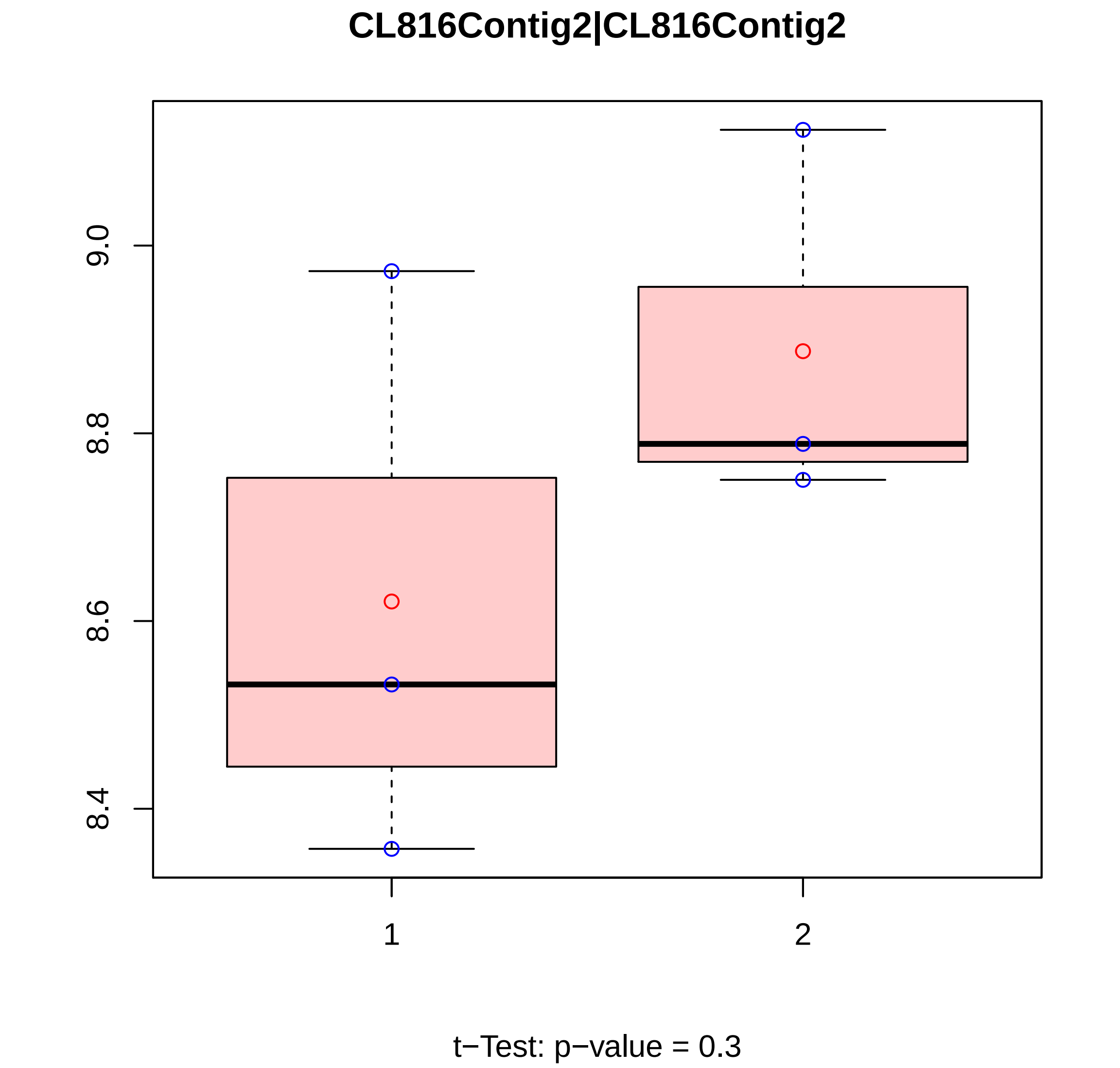
CL816Contig2|CL816Contig2
9.0
8.8
8.6
8.4
1
2
t−Test: p−value = 0.3

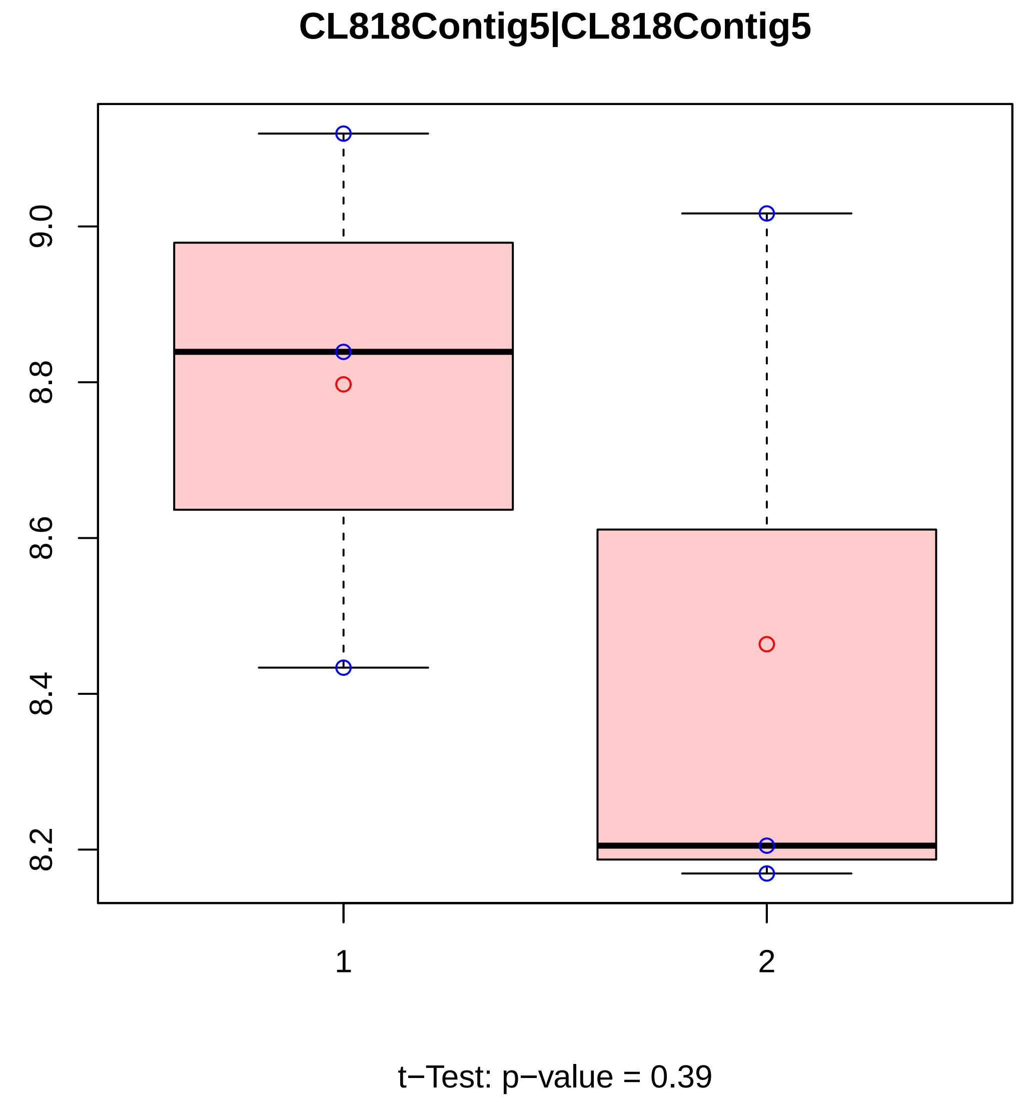
CL818Contig5|CL818Contig5
9.0
8.8
8.6
8.4
8.2
1
2
t−Test: p−value = 0.39

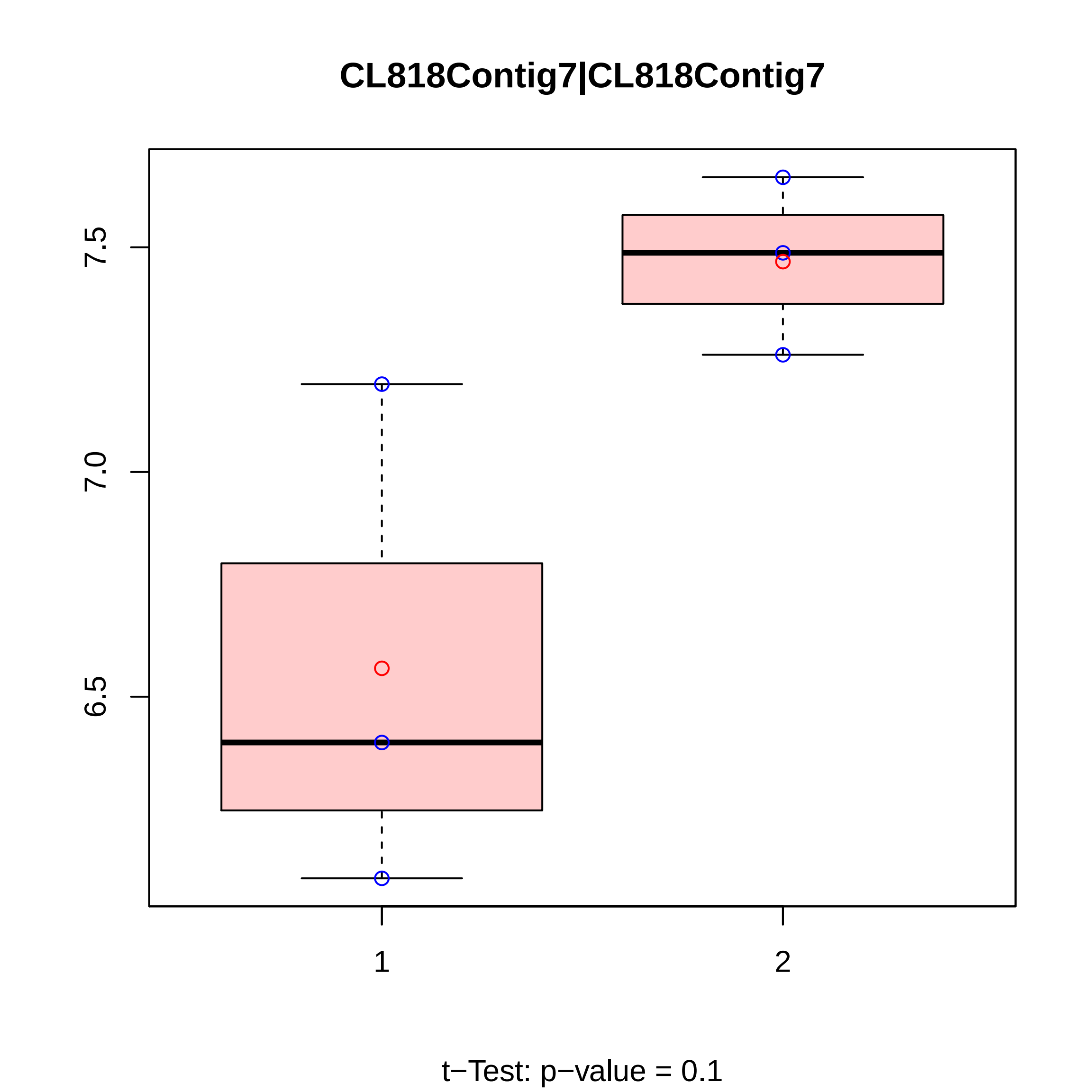

CL818Contig7|CL818Contig7
7.5
7.0
6.5
1
2
t−Test: p−value = 0.1

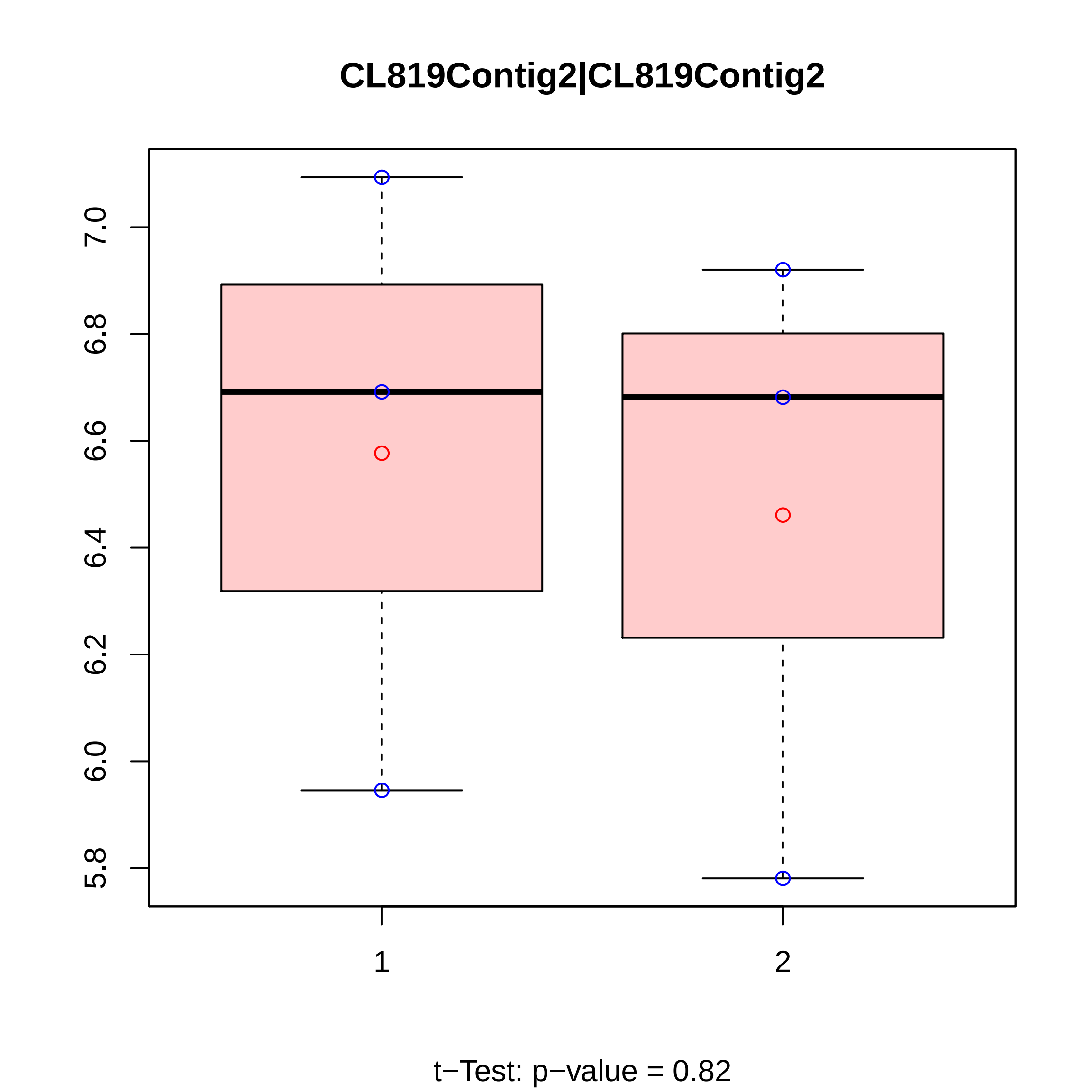
CL819Contig2|CL819Contig2
7.0
6.8
6.6
6.4
6.2
6.0
5.8
1
2
t−Test: p−value = 0.82

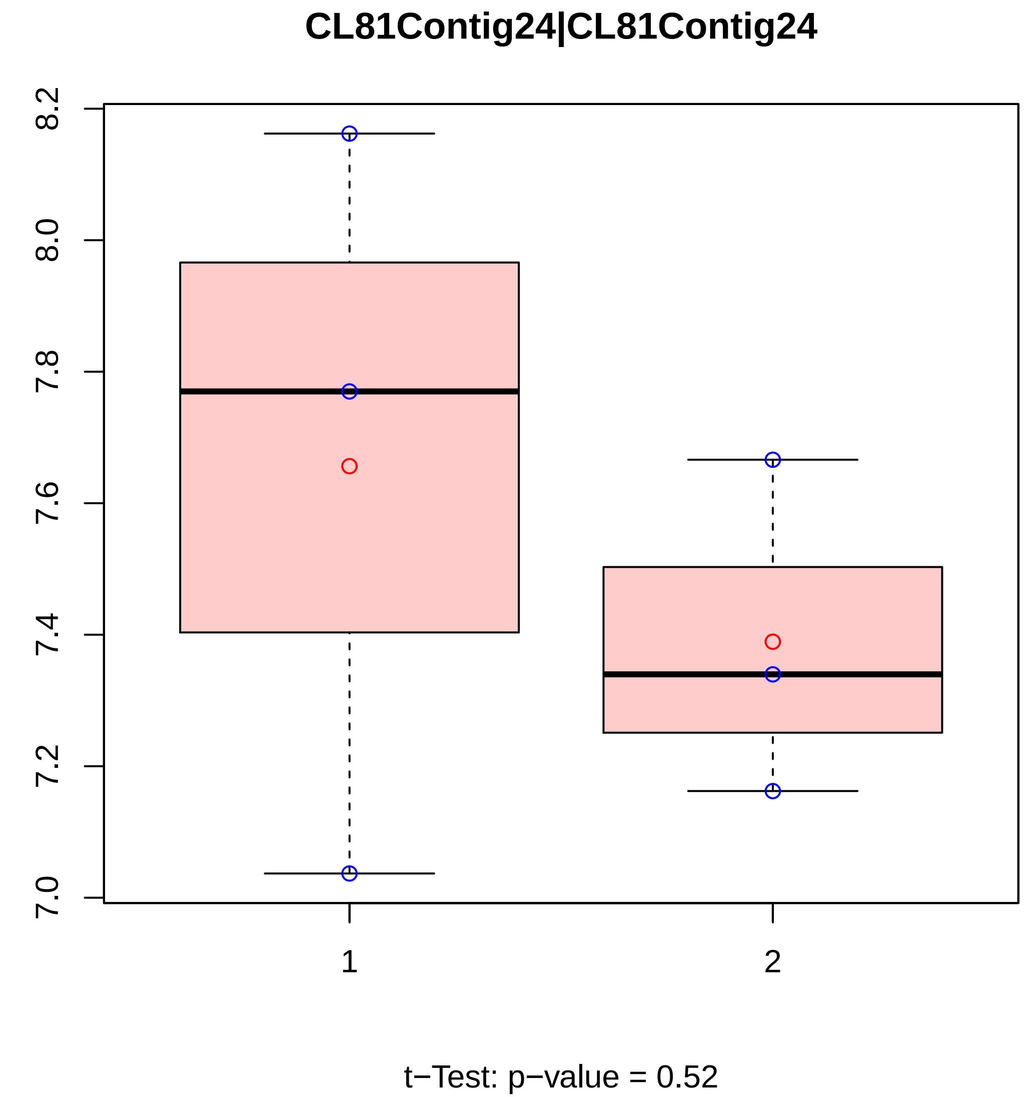
CL81Contig24|CL81Contig24
8.2
8.0
7.8
7.6
7.4
7.2
7.0
1
2
t−Test: p−value = 0.52

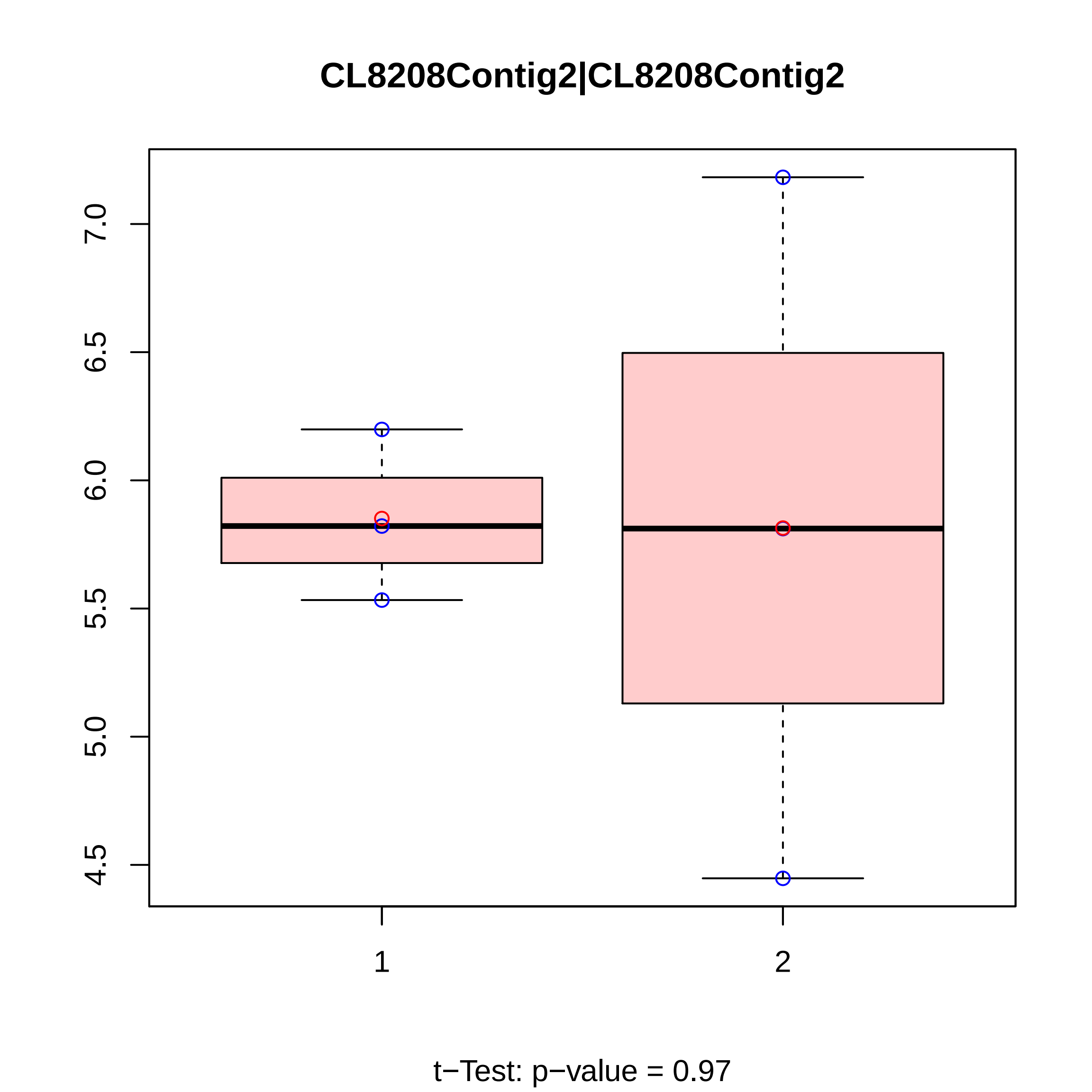

CL8208Contig2|CL8208Contig2
7.0
6.5
6.0
5.5
5.0
4.5
1
2
t−Test: p−value = 0.97

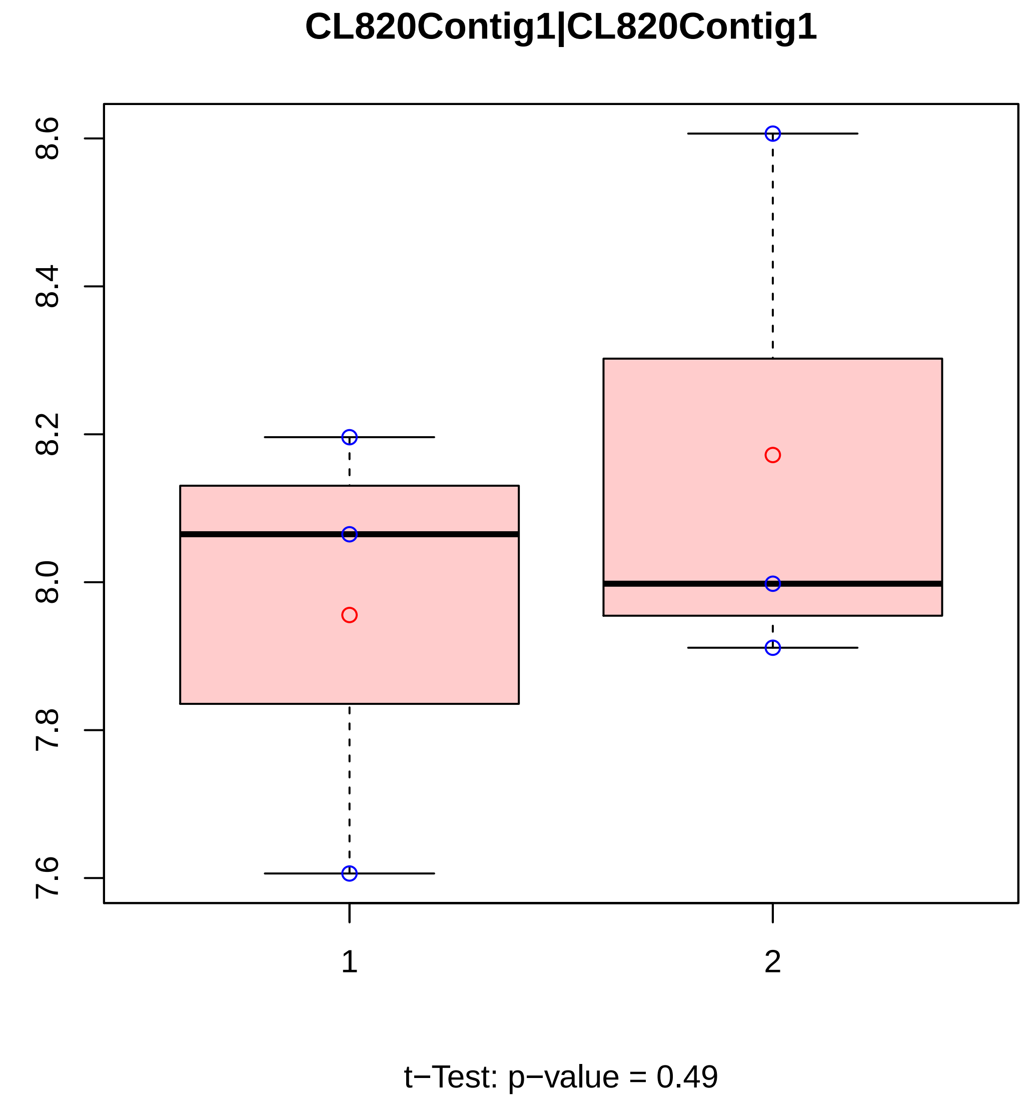

CL820Contig1|CL820Contig1
8.6
8.4
8.2
8.0
7.8
7.6
1
2
t−Test: p−value = 0.49

## CL820Contig8|CL820Contig8

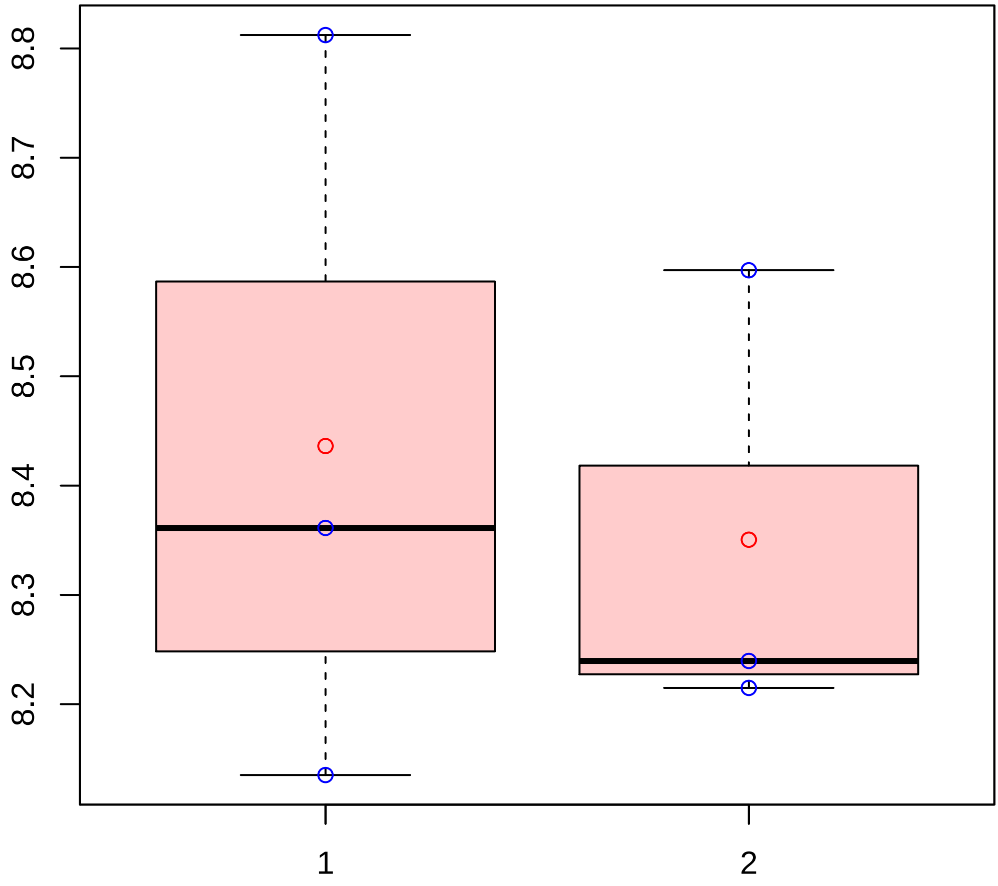


t−Test: p−value = 0.74

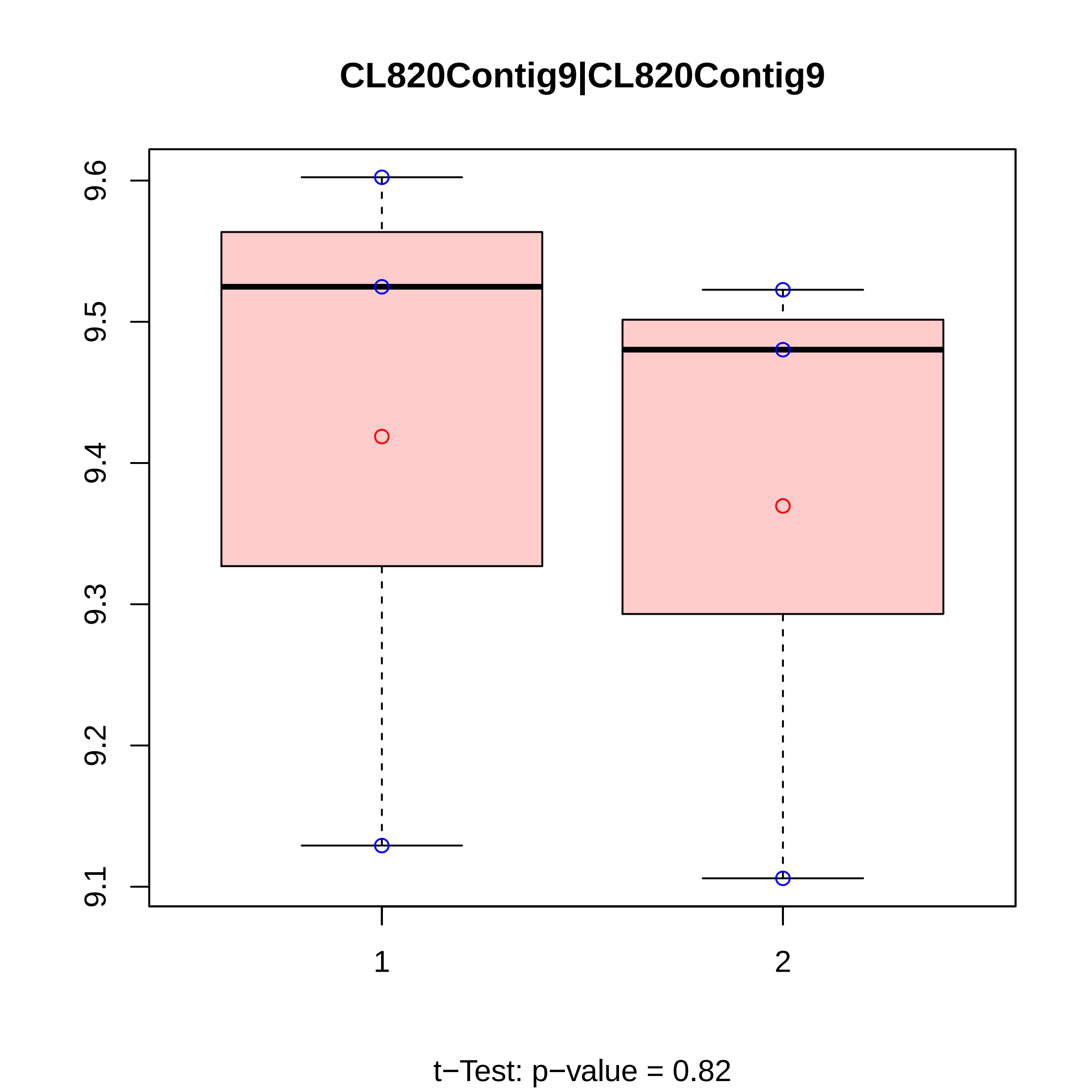

CL820Contig9|CL820Contig9
9.1
9.2
9.3
9.4
9.5
9.6
1
2
t−Test: p−value = 0.82

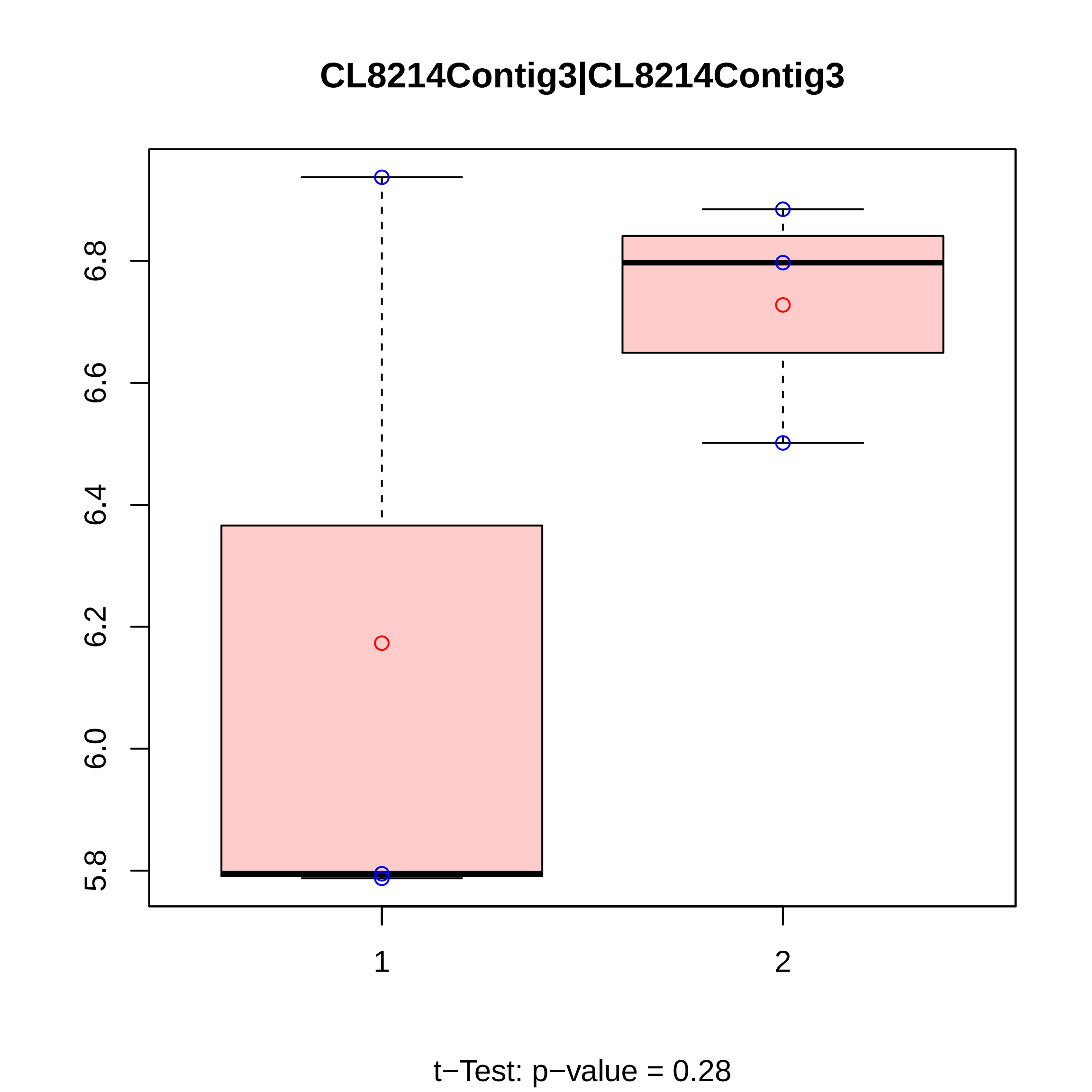
CL8214Contig3|CL8214Contig3
6.8
6.6
6.4
6.2
6.0
5.8
1
2
t−Test: p−value = 0.28

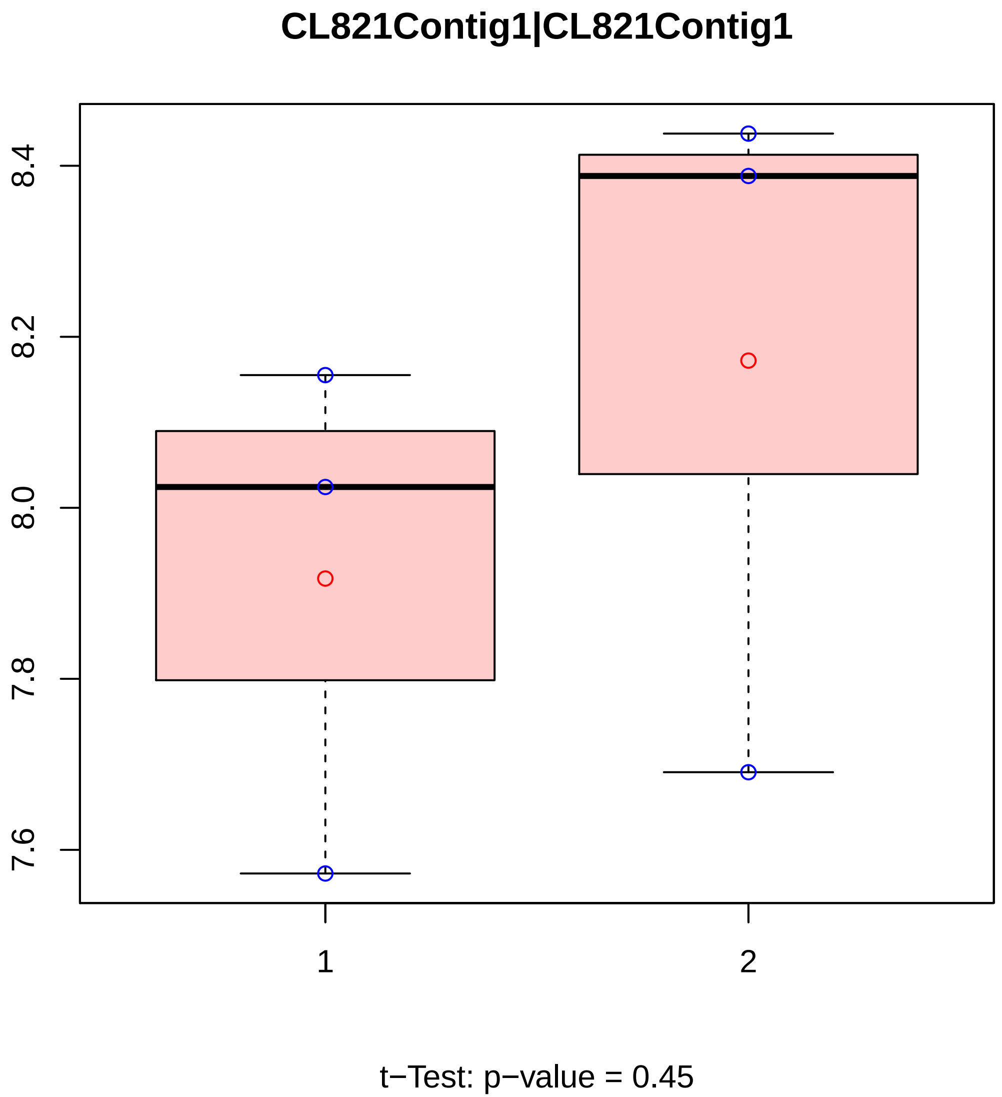
CL821Contig1|CL821Contig1
8.4
8.2
8.0
7.8
7.6
1
2
t−Test: p−value = 0.45

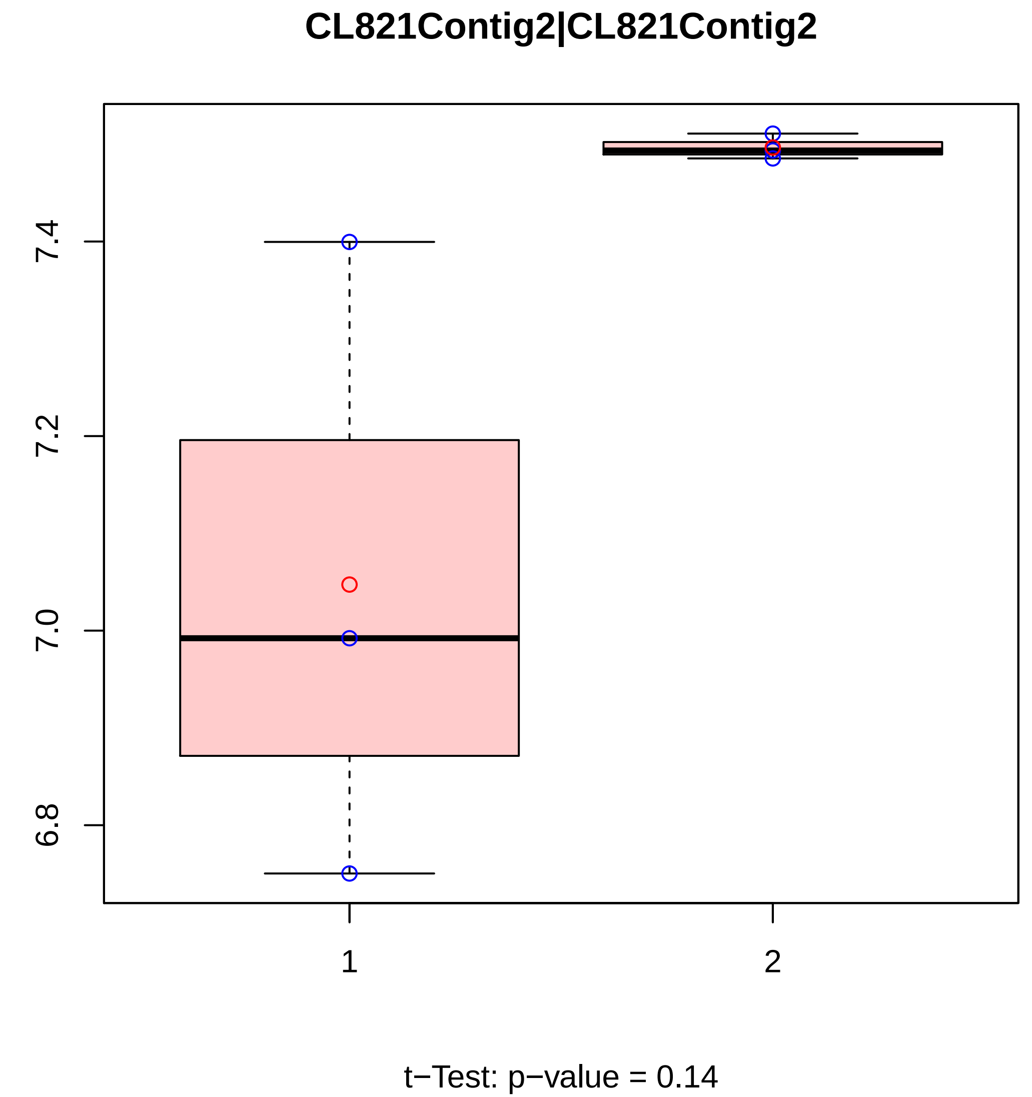
CL821Contig2|CL821Contig2
7.4
7.2
7.0
6.8
1
2
t−Test: p−value = 0.14

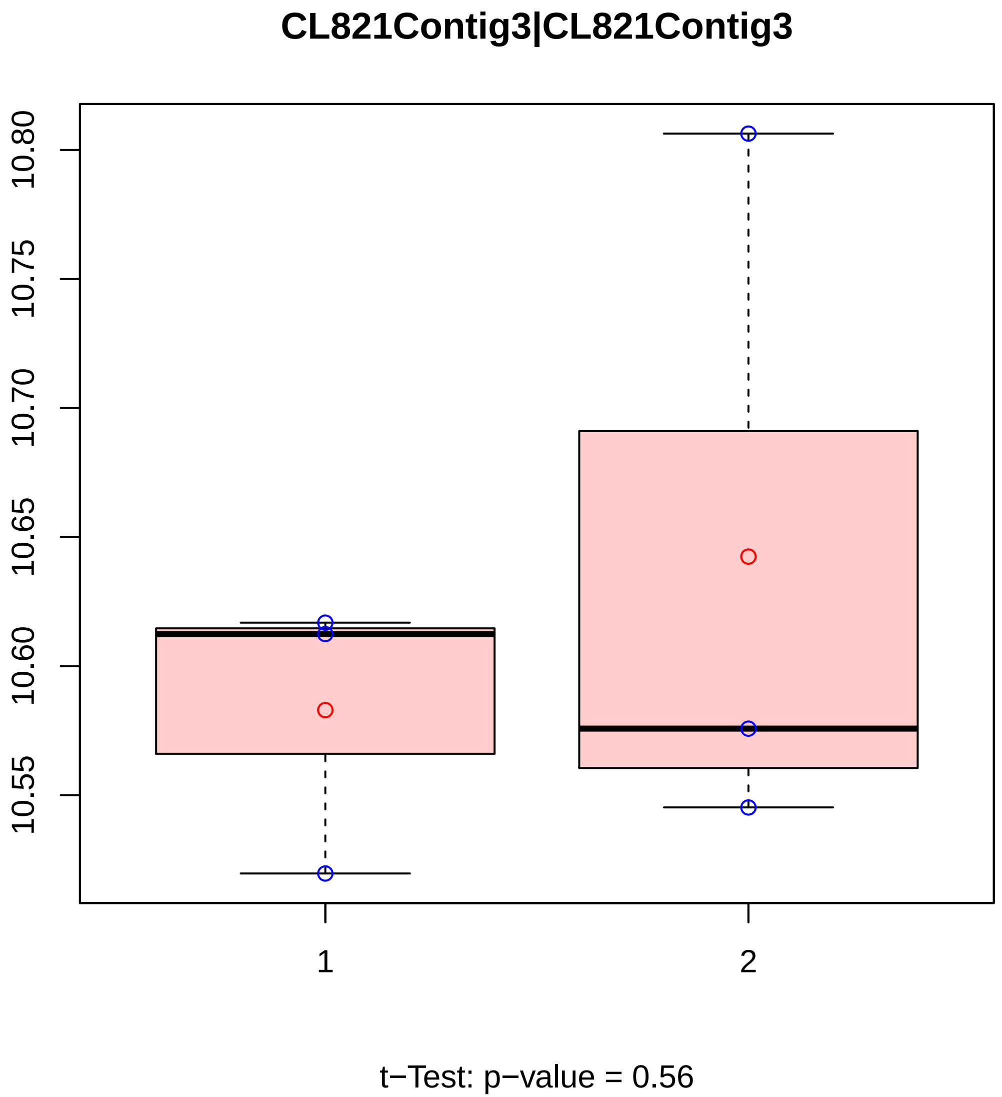
CL821Contig3|CL821Contig3
10.55
10.60
10.65
10.70
10.75
10.80
1
2
t−Test: p−value = 0.56

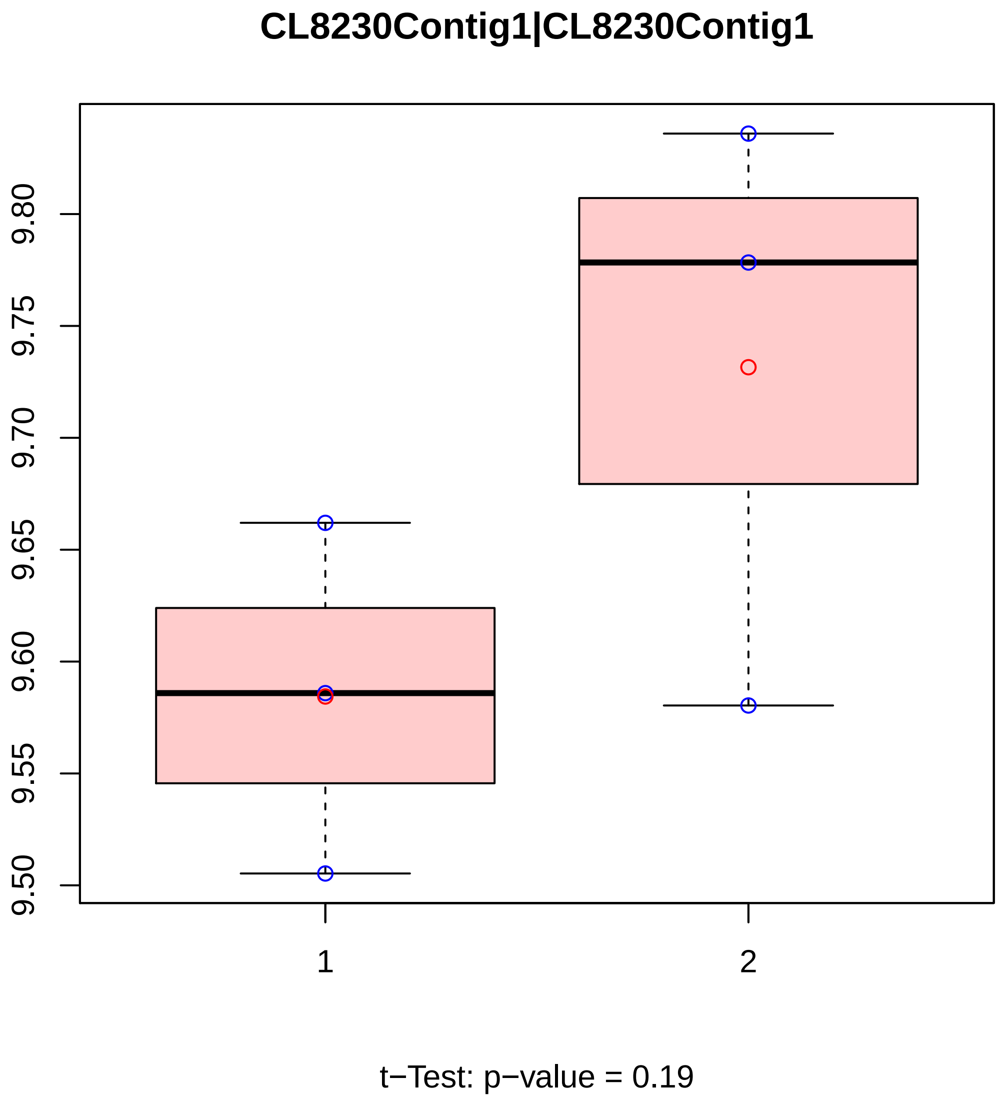
CL8230Contig1|CL8230Contig1
9.50
9.55
9.60
9.65
9.70
9.75
9.80
1
2
t−Test: p−value = 0.19

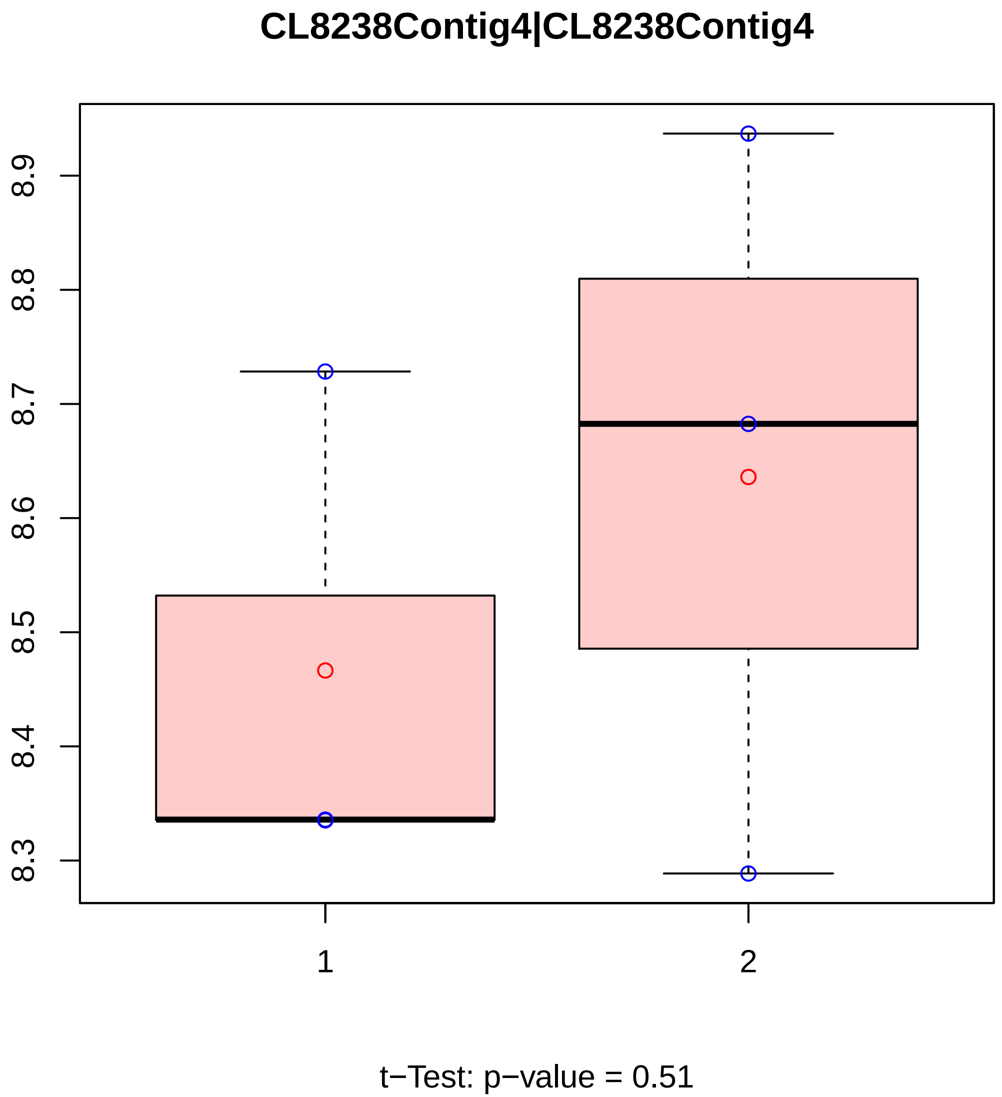
CL8238Contig4|CL8238Contig4
8.3
8.4
8.5
8.6
8.7
8.8
8.9
1
2
t−Test: p−value = 0.51

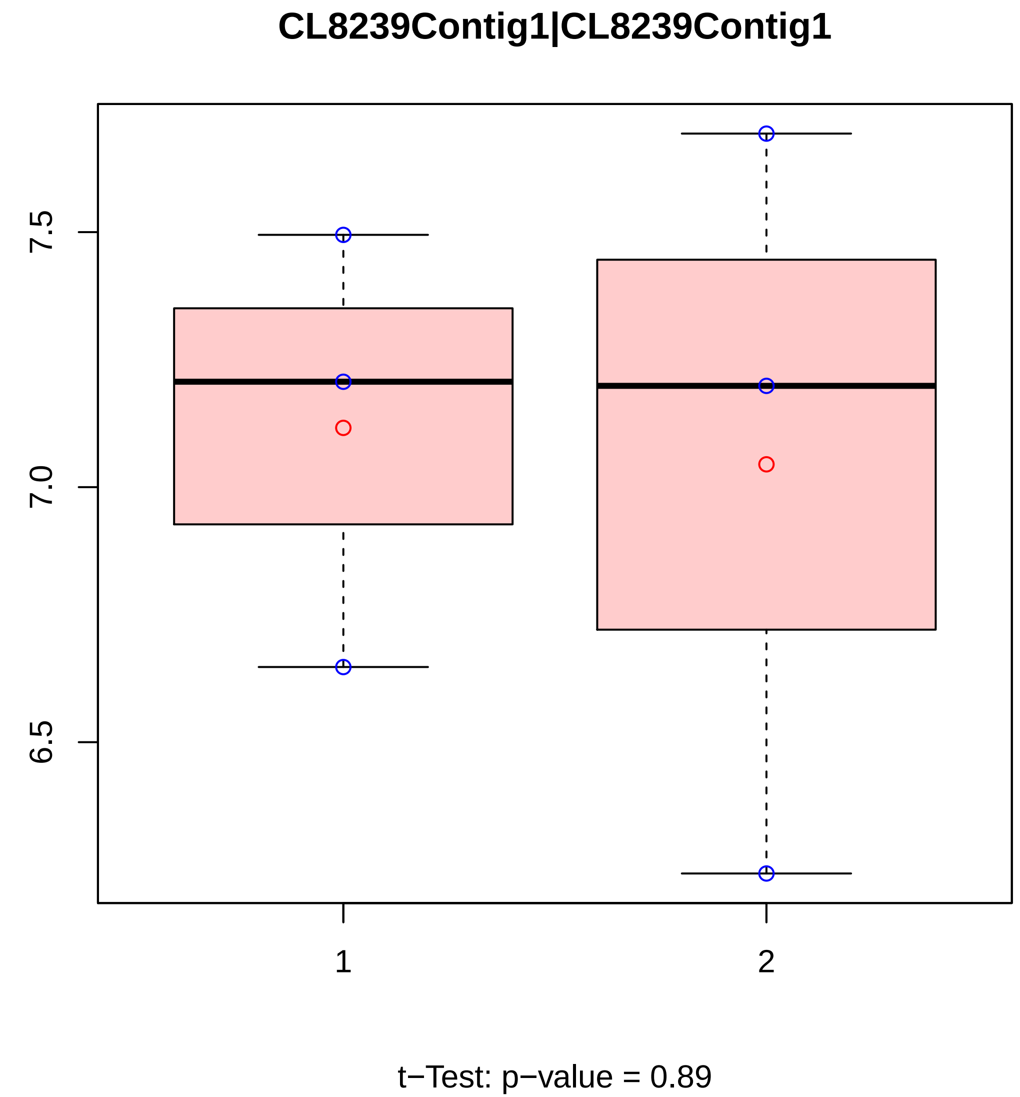

CL8239Contig1|CL8239Contig1
7.5
7.0
6.5
1
2
t−Test: p−value = 0.89

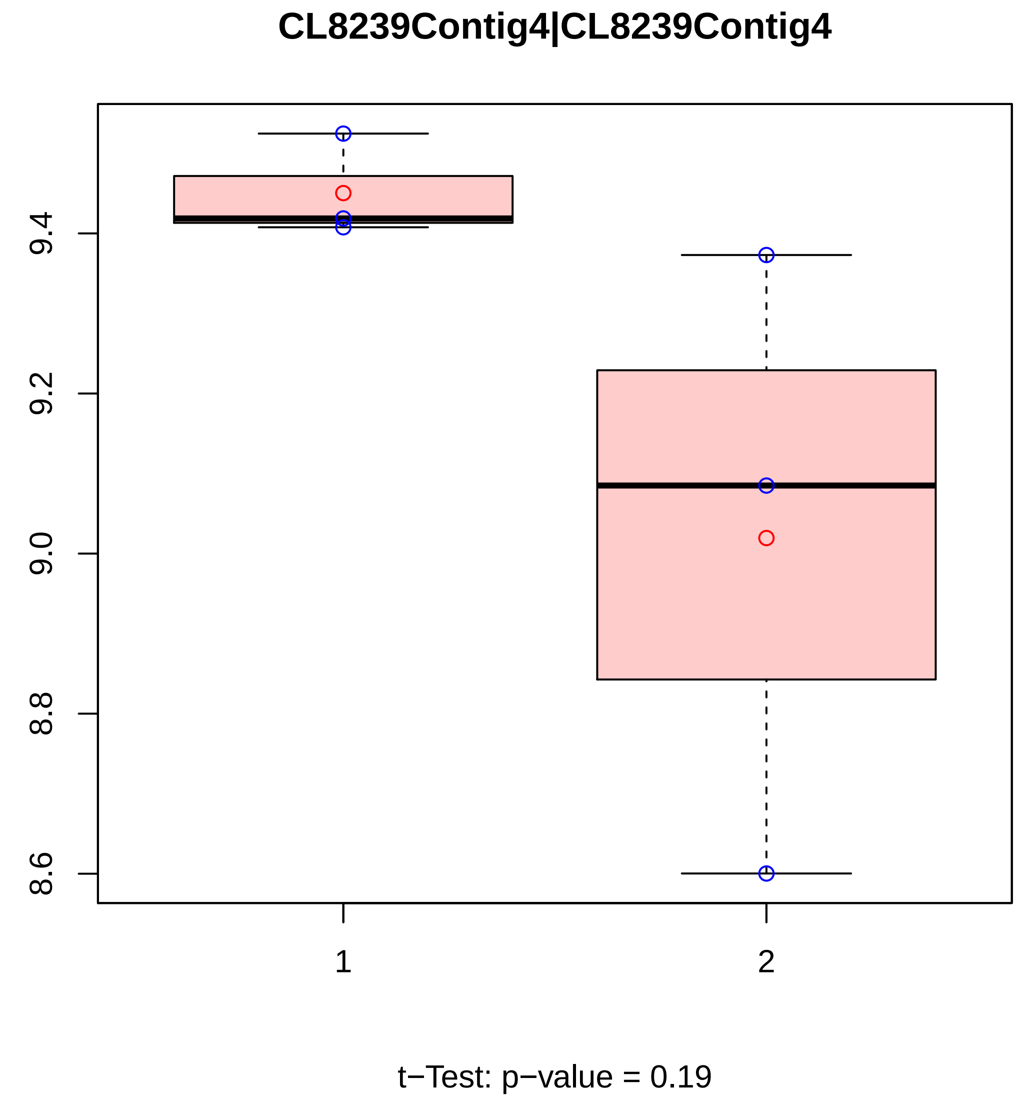
CL8239Contig4|CL8239Contig4
9.4
9.2
9.0
8.8
8.6
1
2
t−Test: p−value = 0.19

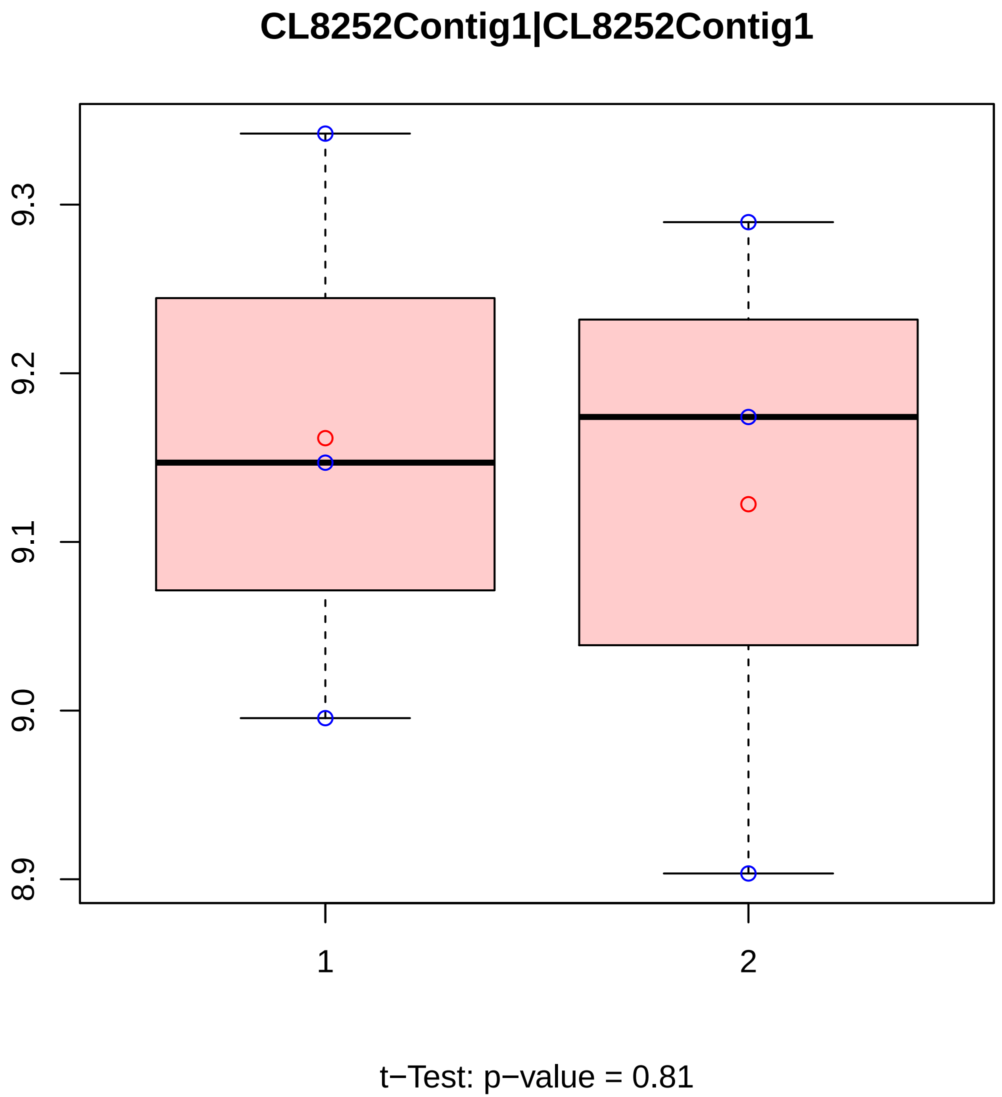
CL8252Contig1|CL8252Contig1
9.3
9.2
9.1
9.0
8.9
1
2
t−Test: p−value = 0.81

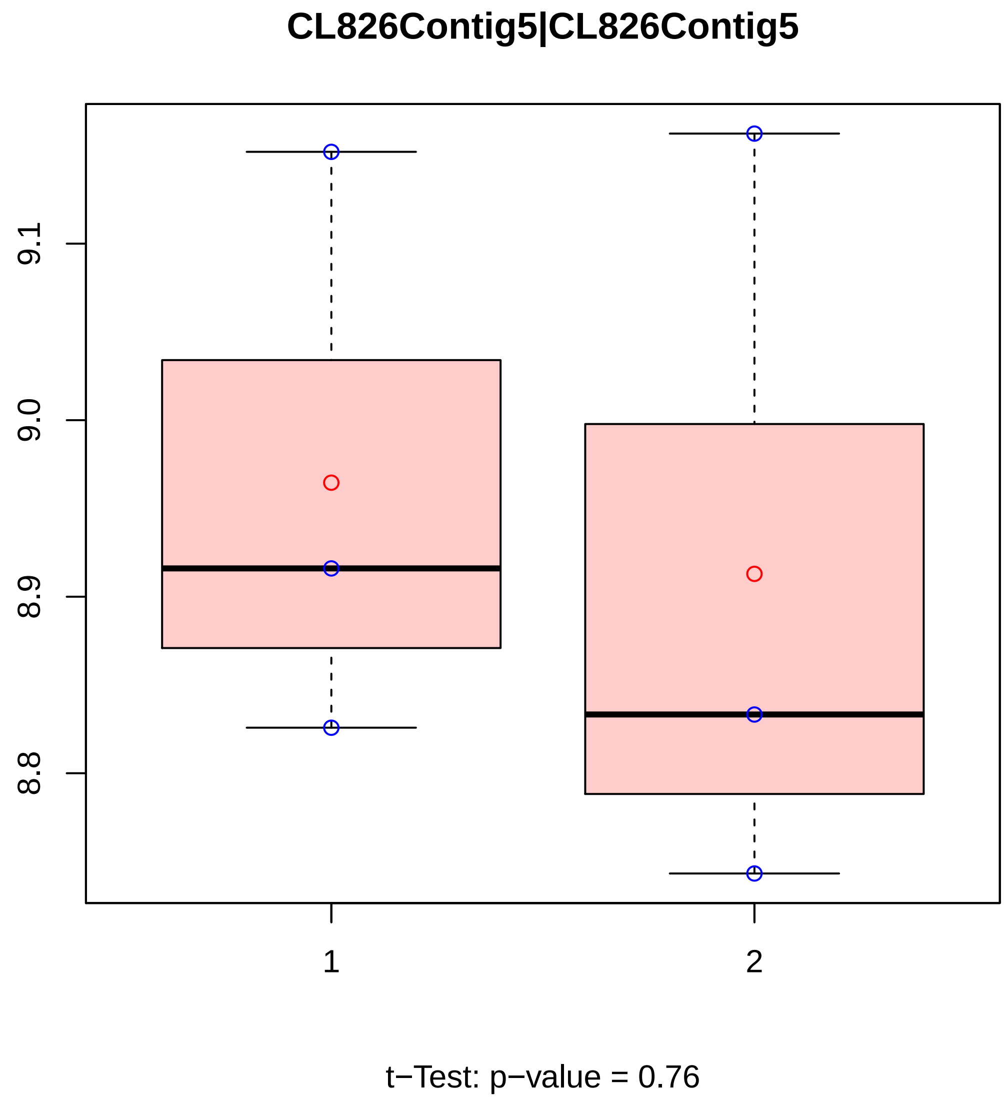
CL826Contig5|CL826Contig5
9.1
9.0
8.9
8.8
1
2
t−Test: p−value = 0.76

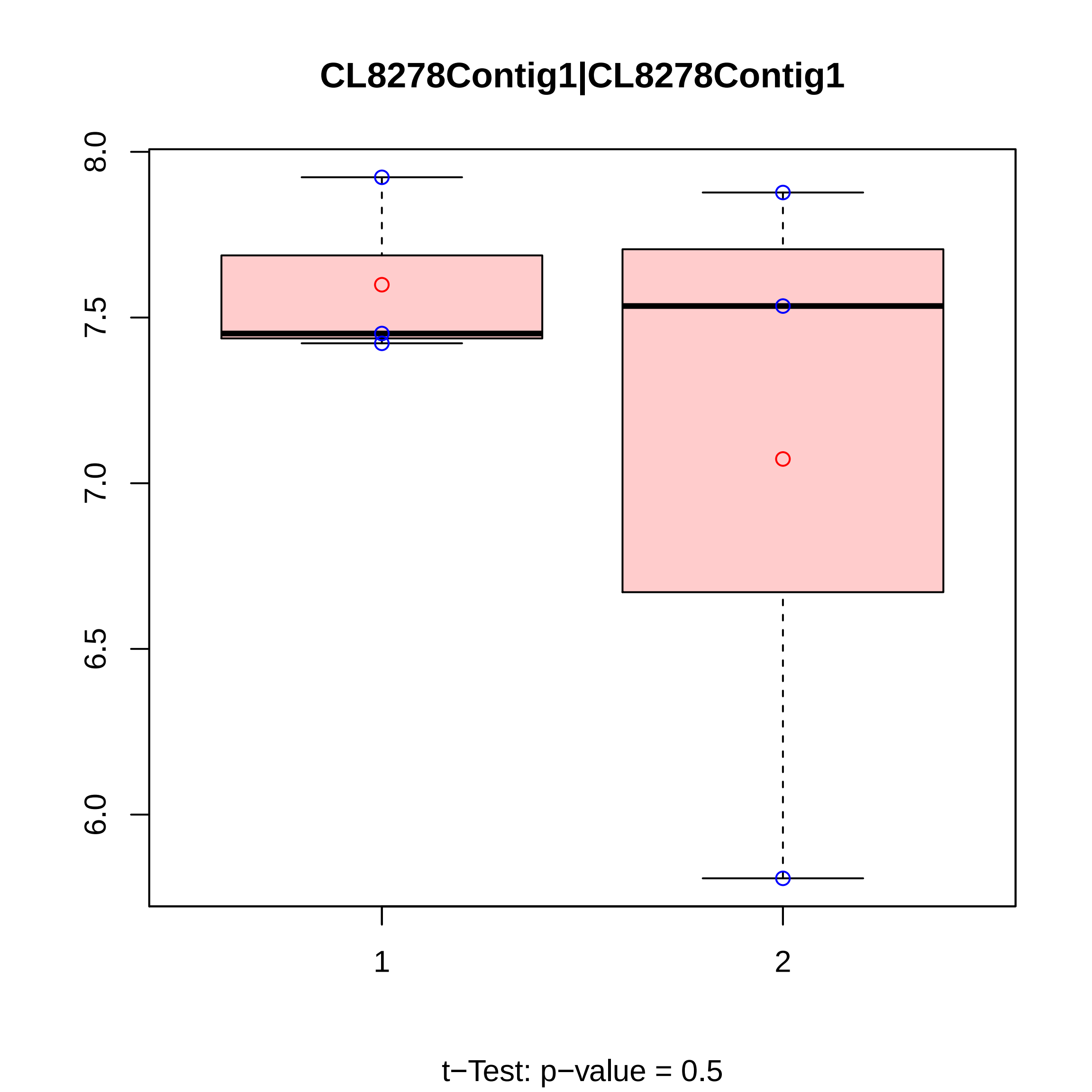

CL8278Contig1|CL8278Contig1
8.0
7.5
7.0
6.5
6.0
1
2
t−Test: p−value = 0.5

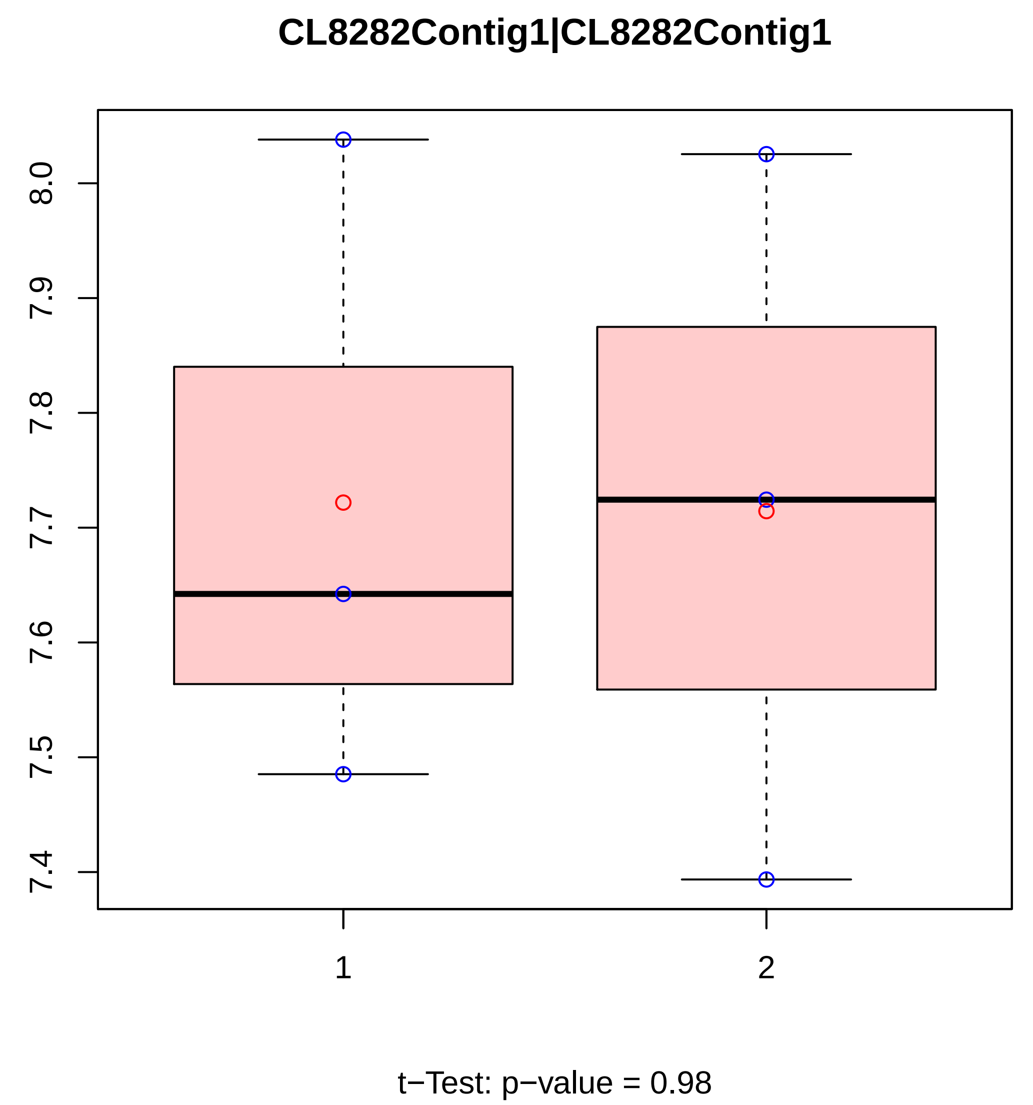
CL8282Contig1|CL8282Contig1
8.0
7.9
7.8
7.7
7.6
7.5
7.4
1
2
t−Test: p−value = 0.98

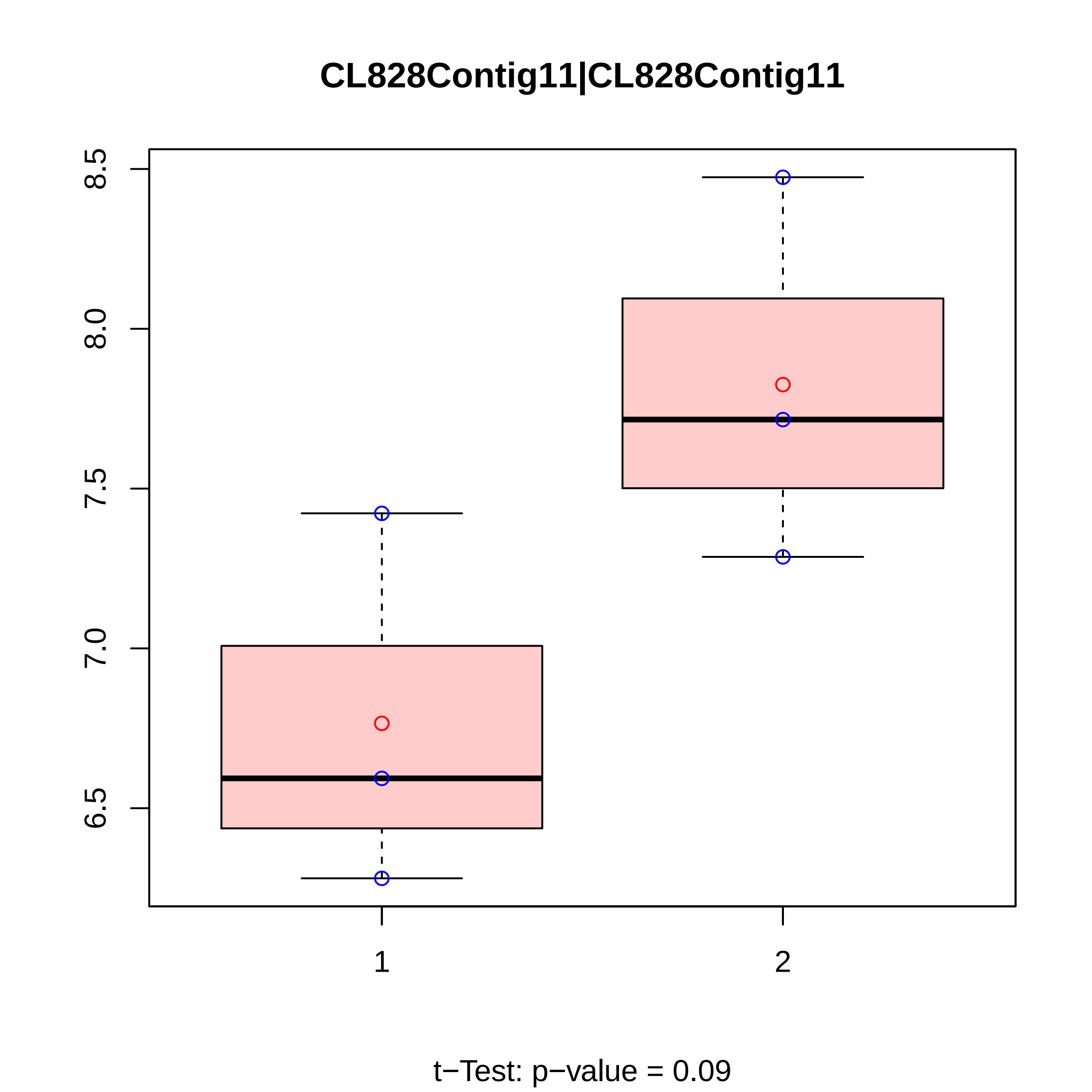

CL828Contig11|CL828Contig11
6.5
7.0
7.5
8.0
8.5
1
2
t−Test: p−value = 0.09

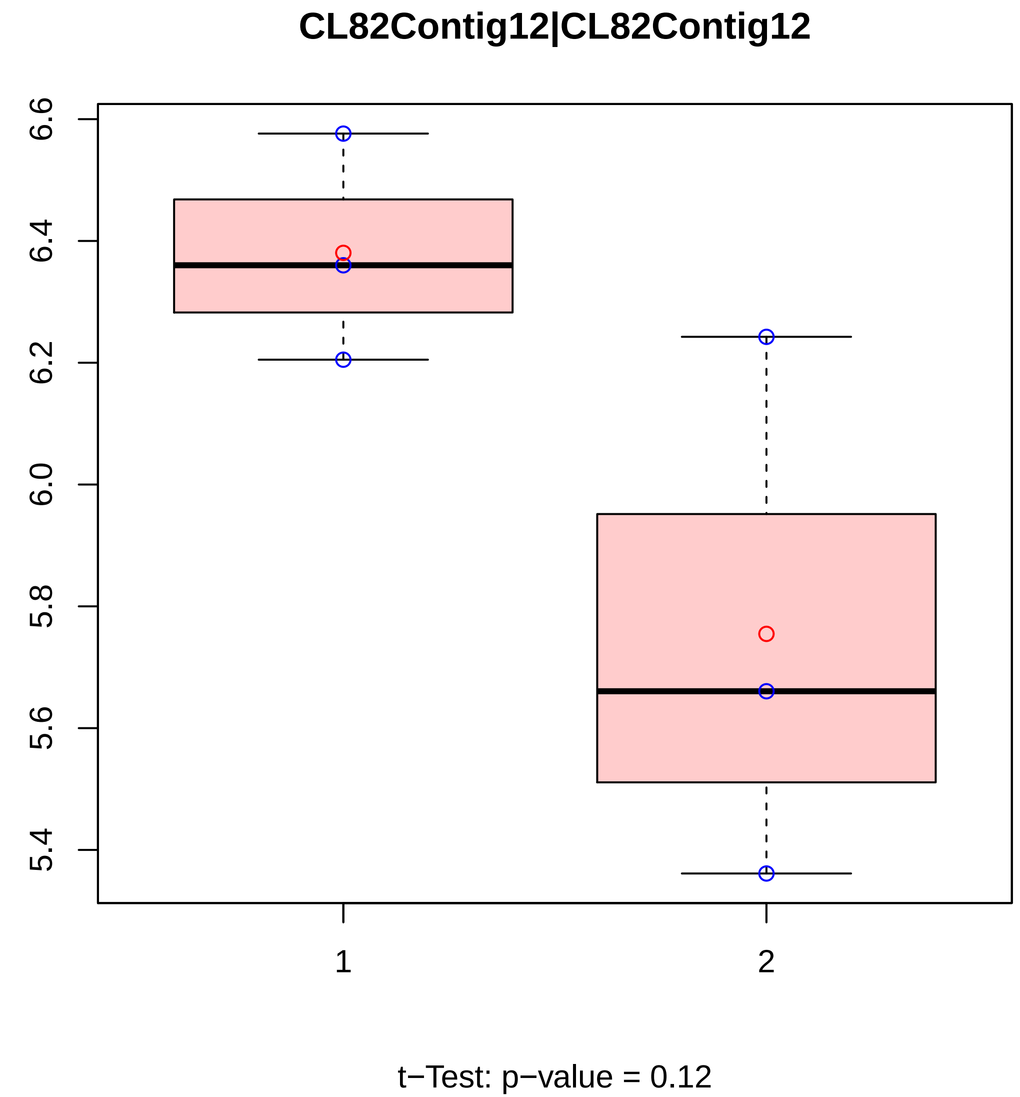
CL82Contig12|CL82Contig12
6.6
6.4
6.2
6.0
5.8
5.6
5.4
1
2
t−Test: p−value = 0.12

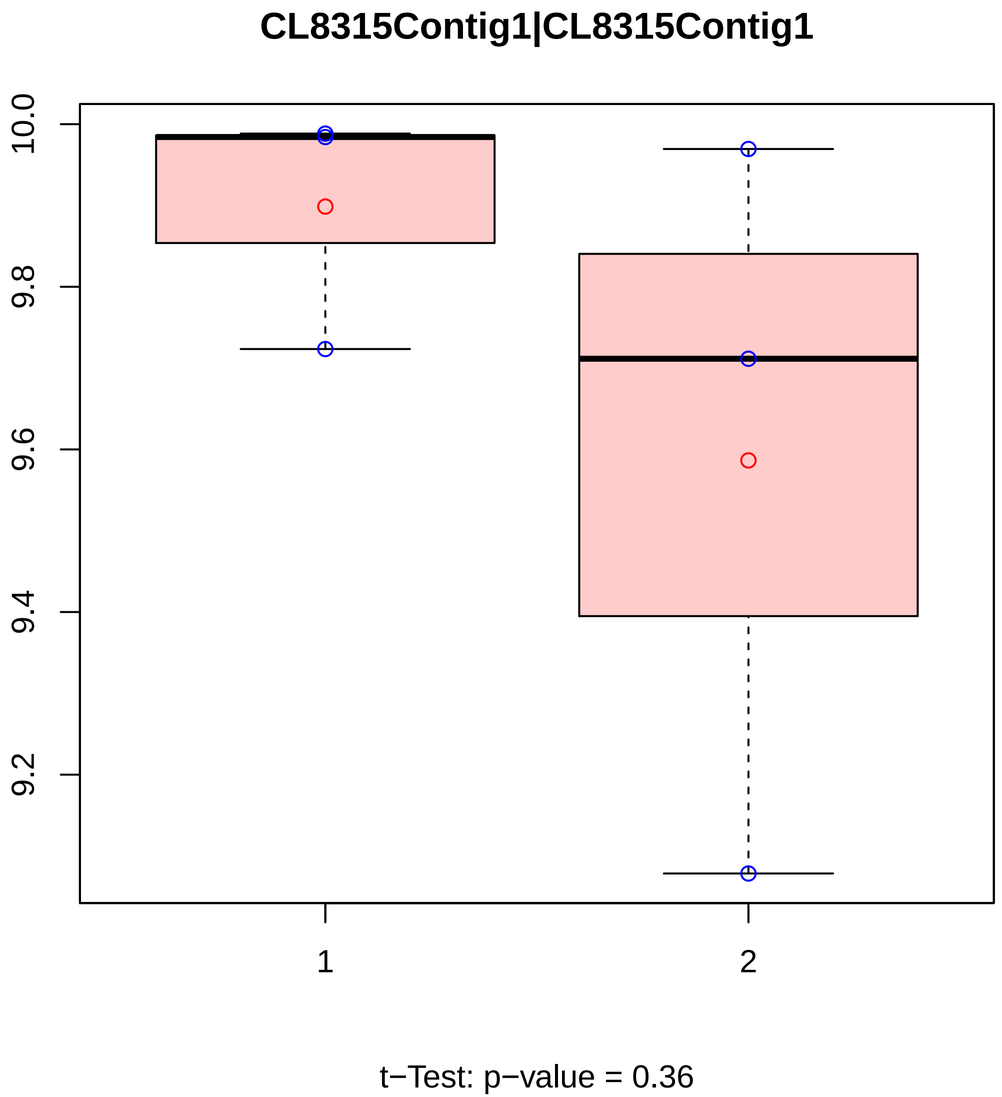
CL8315Contig1|CL8315Contig1
10.0
9.8
9.6
9.4
9.2
1
2
t−Test: p−value = 0.36

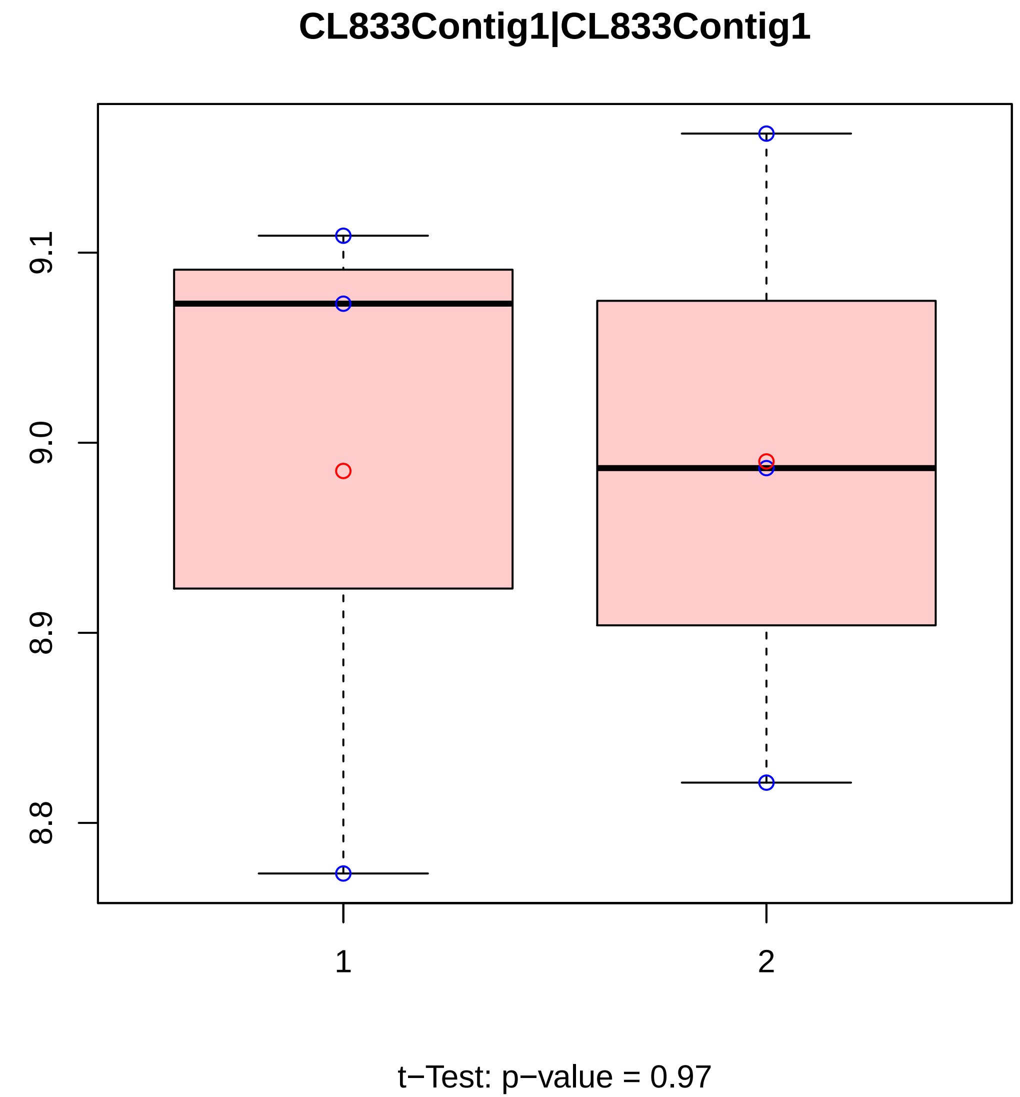
CL833Contig1|CL833Contig1
9.1
9.0
8.9
8.8
1
2
t−Test: p−value = 0.97

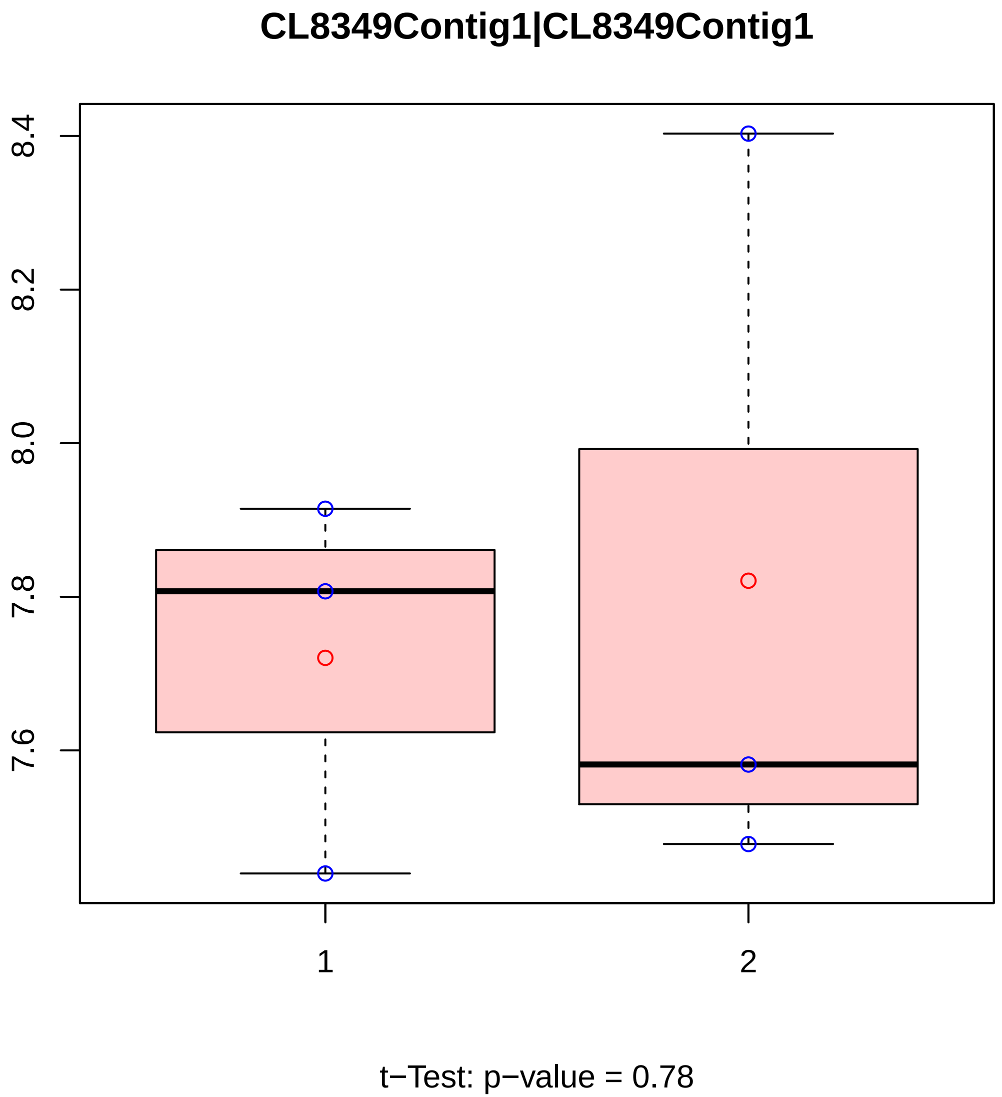

CL8349Contig1|CL8349Contig1
8.4
8.2
8.0
7.8
7.6
1
2
t−Test: p−value = 0.78

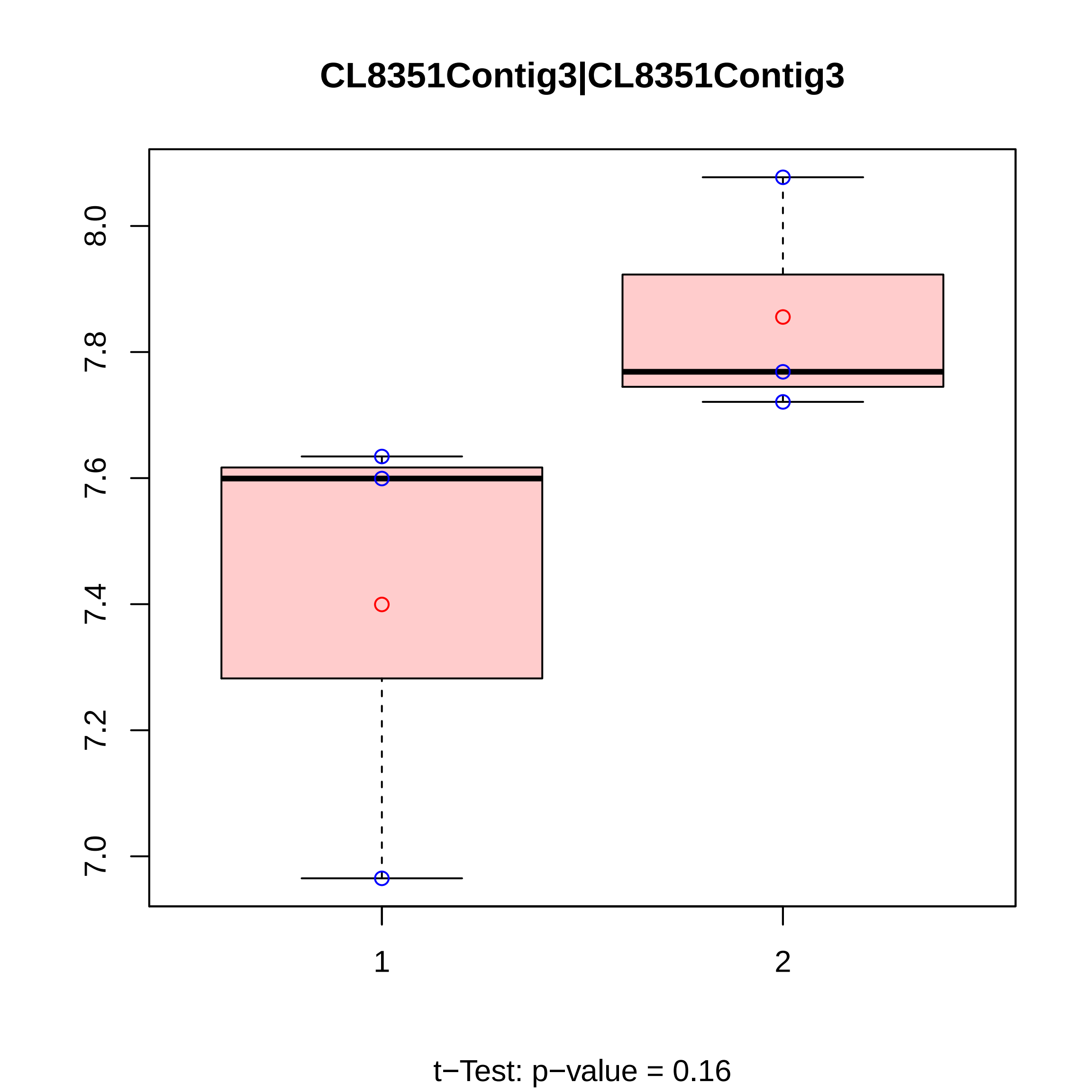
CL8351Contig3|CL8351Contig3
8.0
7.8
7.6
7.4
7.2
7.0
1
2
t−Test: p−value = 0.16

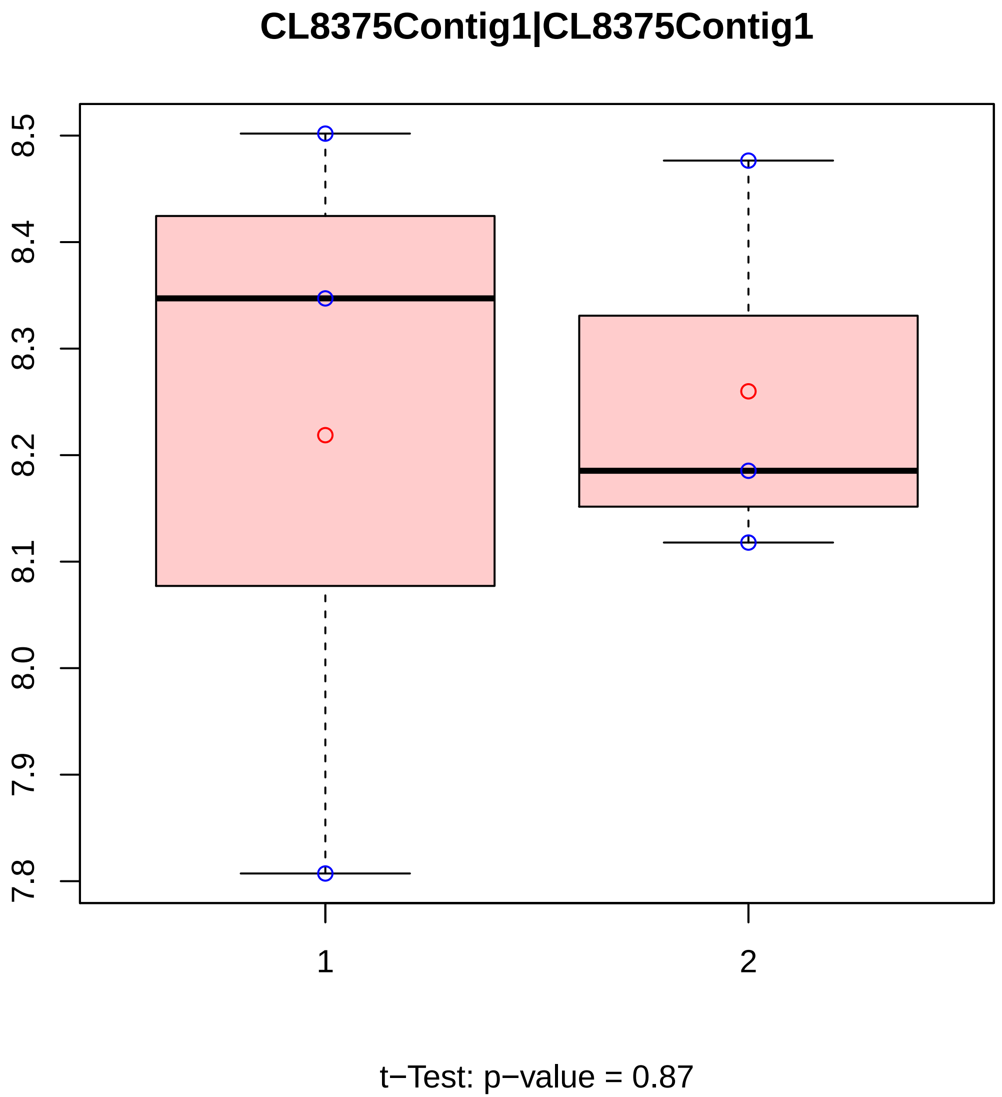
CL8375Contig1|CL8375Contig1
8.5
8.4
8.3
8.2
8.1
8.0
7.9
7.8
1
2
t−Test: p−value = 0.87

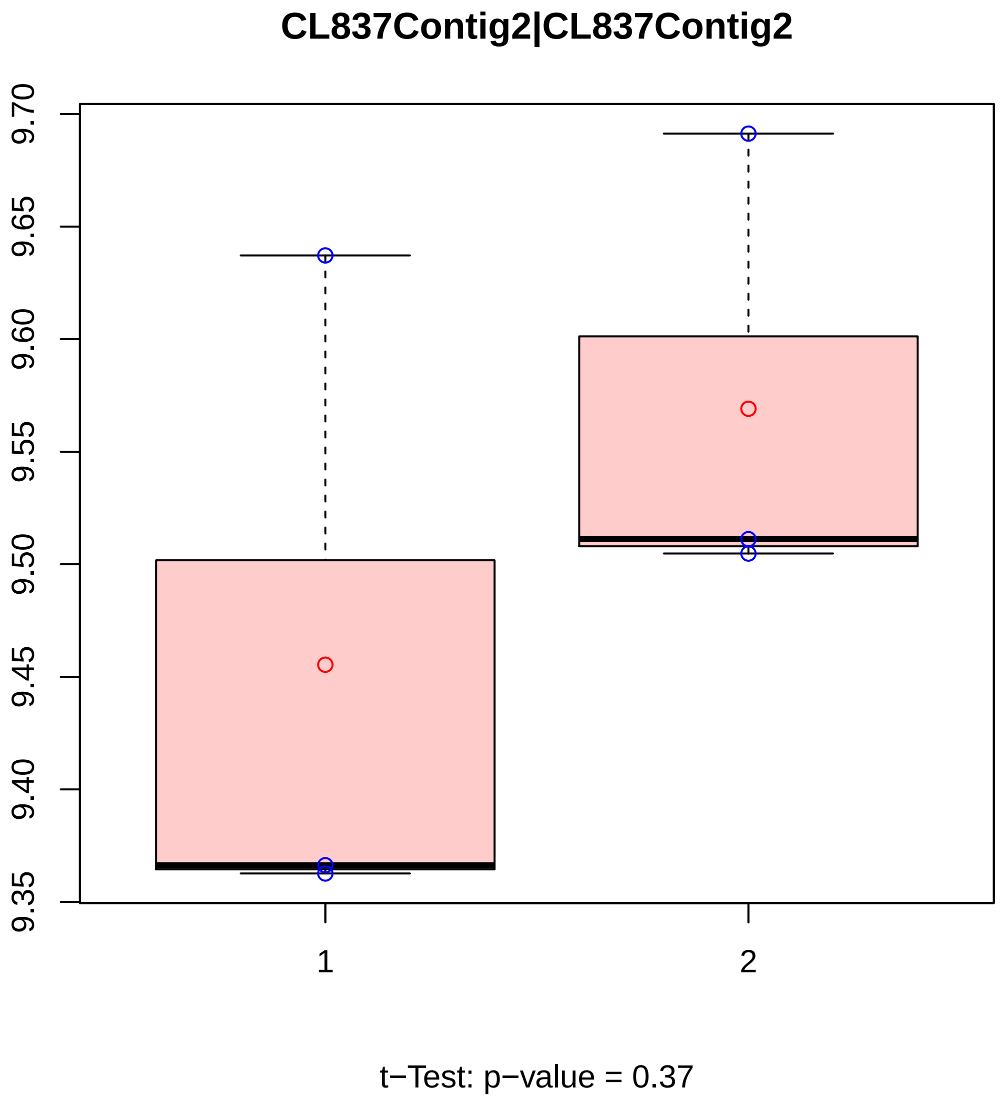
CL837Contig2|CL837Contig2
9.35
9.40
9.45
9.50
9.55
9.60
9.65
9.70
1
2
t−Test: p−value = 0.37

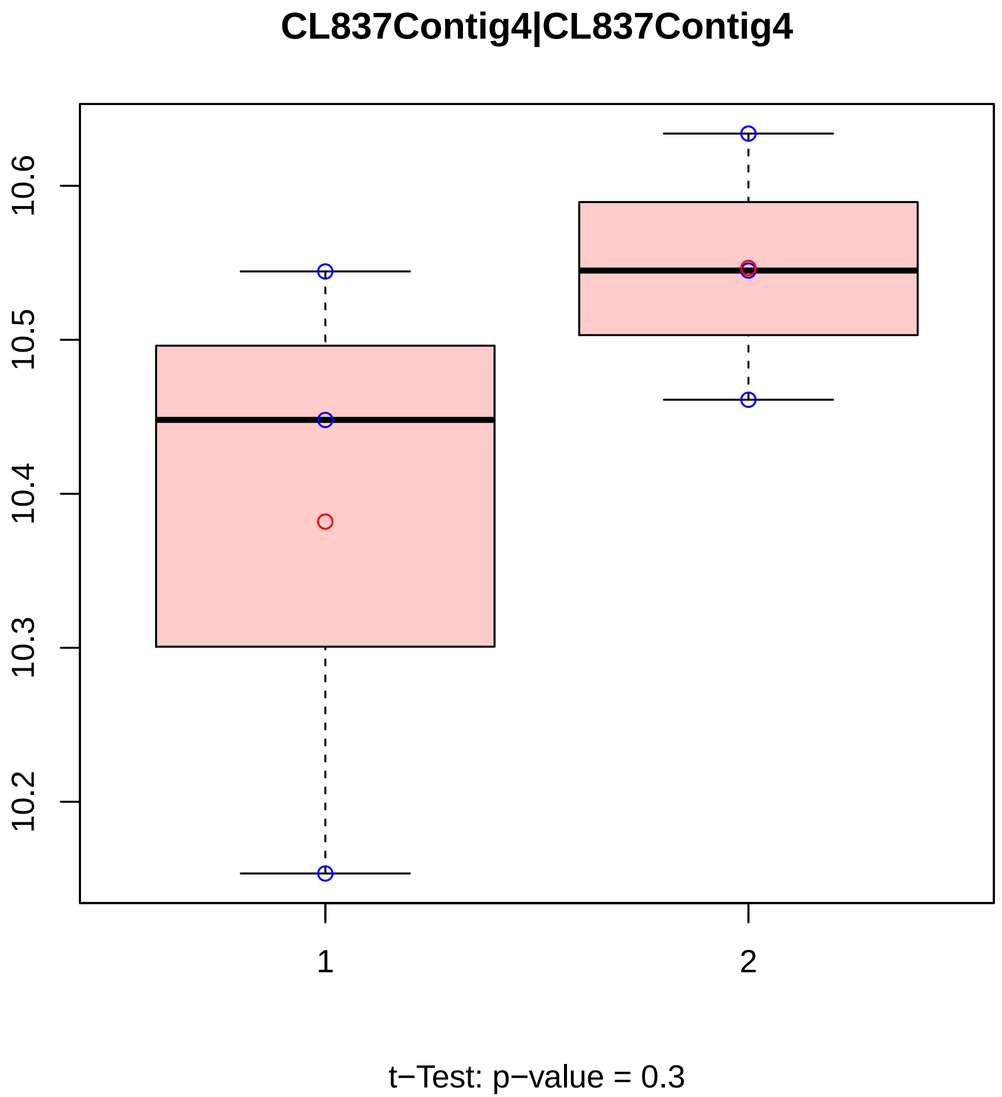
CL837Contig4|CL837Contig4
10.2
10.3
10.4
10.5
10.6
1
2
t−Test: p−value = 0.3

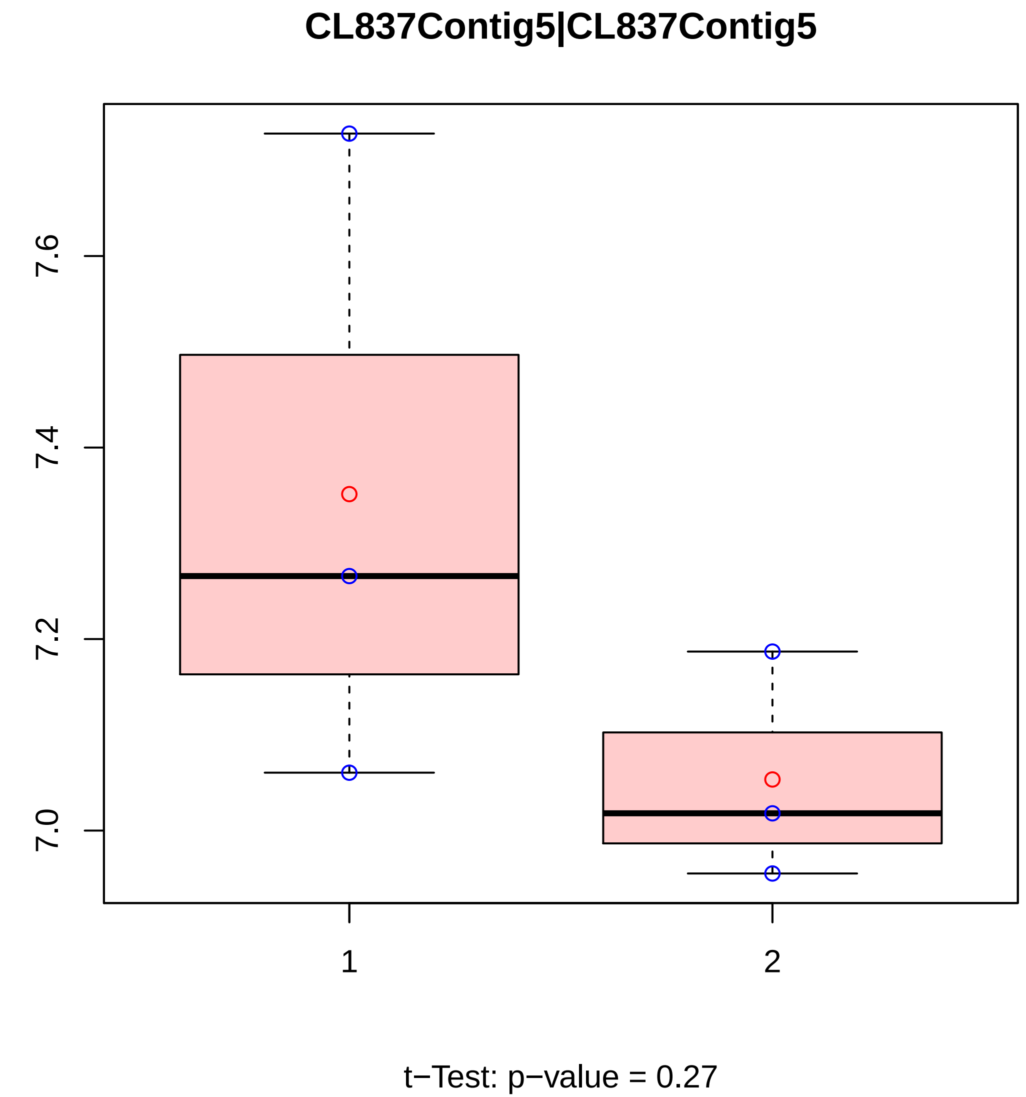
CL837Contig5|CL837Contig5
7.0
7.2
7.4
7.6
1
2
t−Test: p−value = 0.27

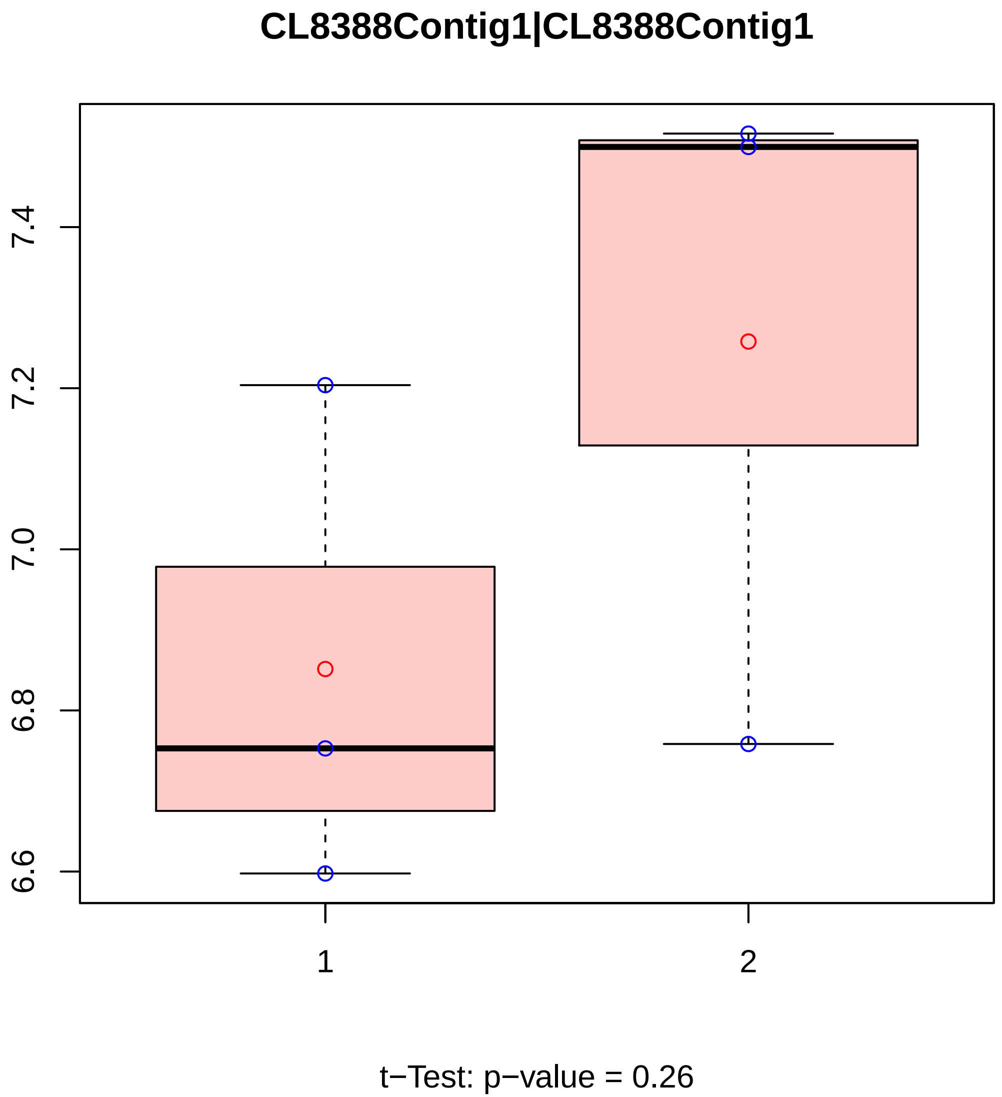
CL8388Contig1|CL8388Contig1
7.4
7.2
7.0
6.8
6.6
1
2
t−Test: p−value = 0.26

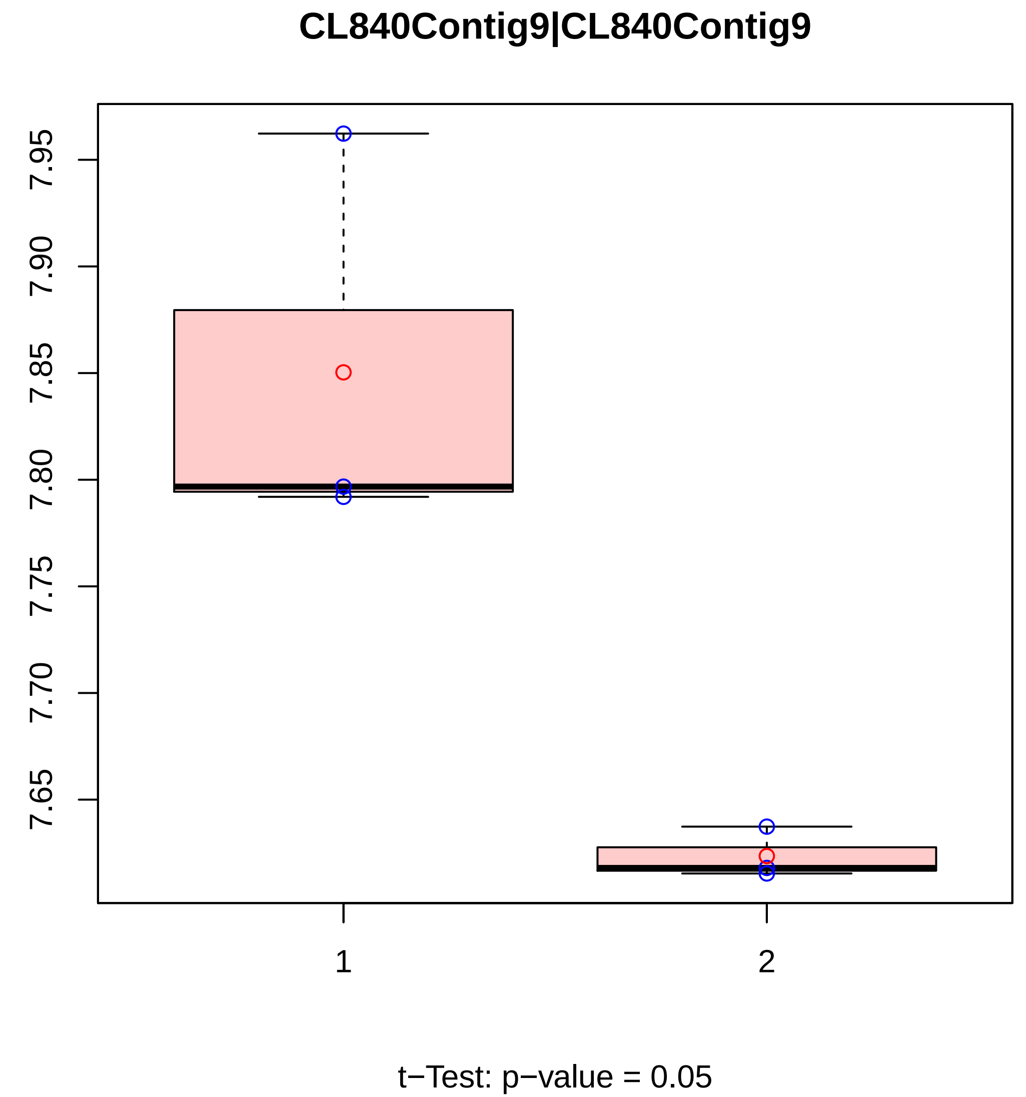
CL840Contig9|CL840Contig9
7.65
7.70
7.75
7.80
7.85
7.90
7.95
1
2
t−Test: p−value = 0.05

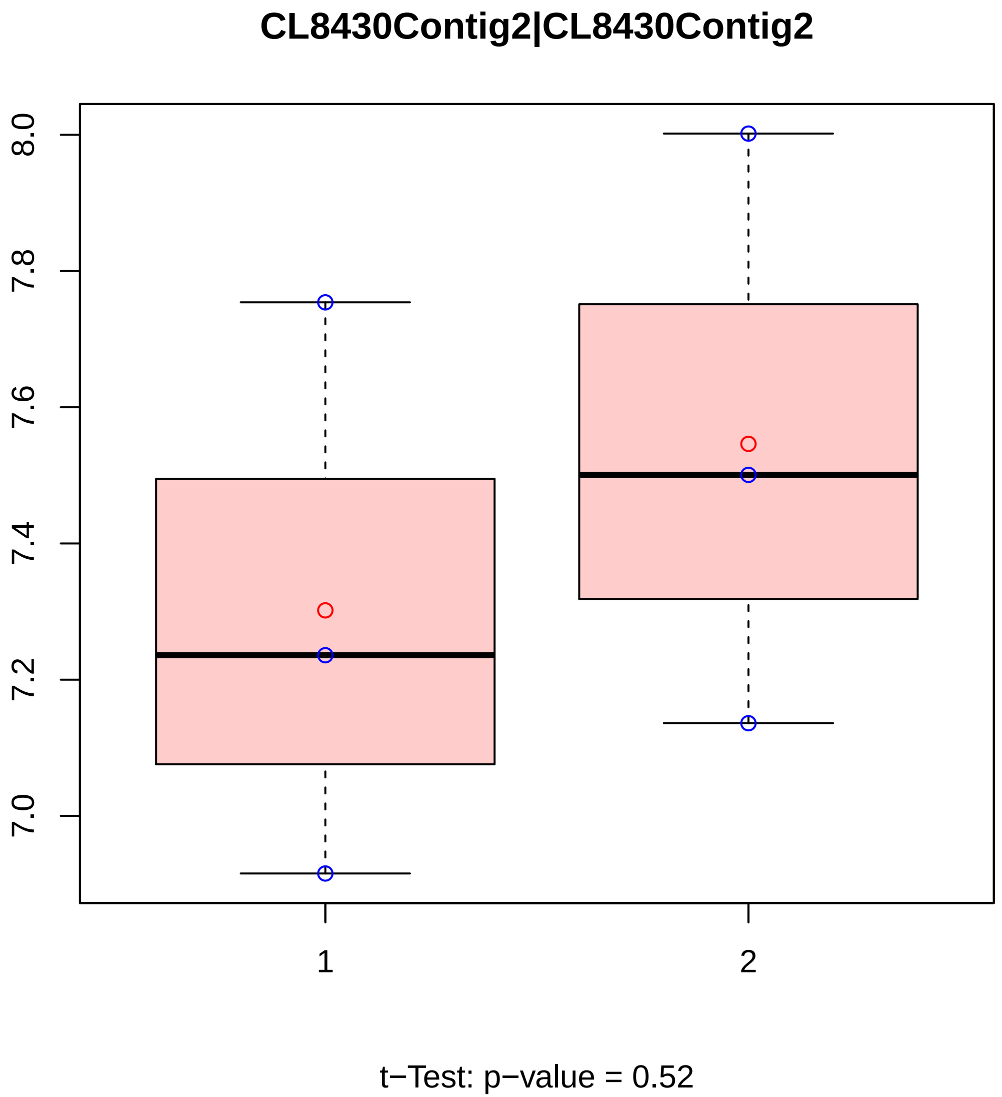

CL8430Contig2|CL8430Contig2
8.0
7.8
7.6
7.4
7.2
7.0
1
2
t−Test: p−value = 0.52

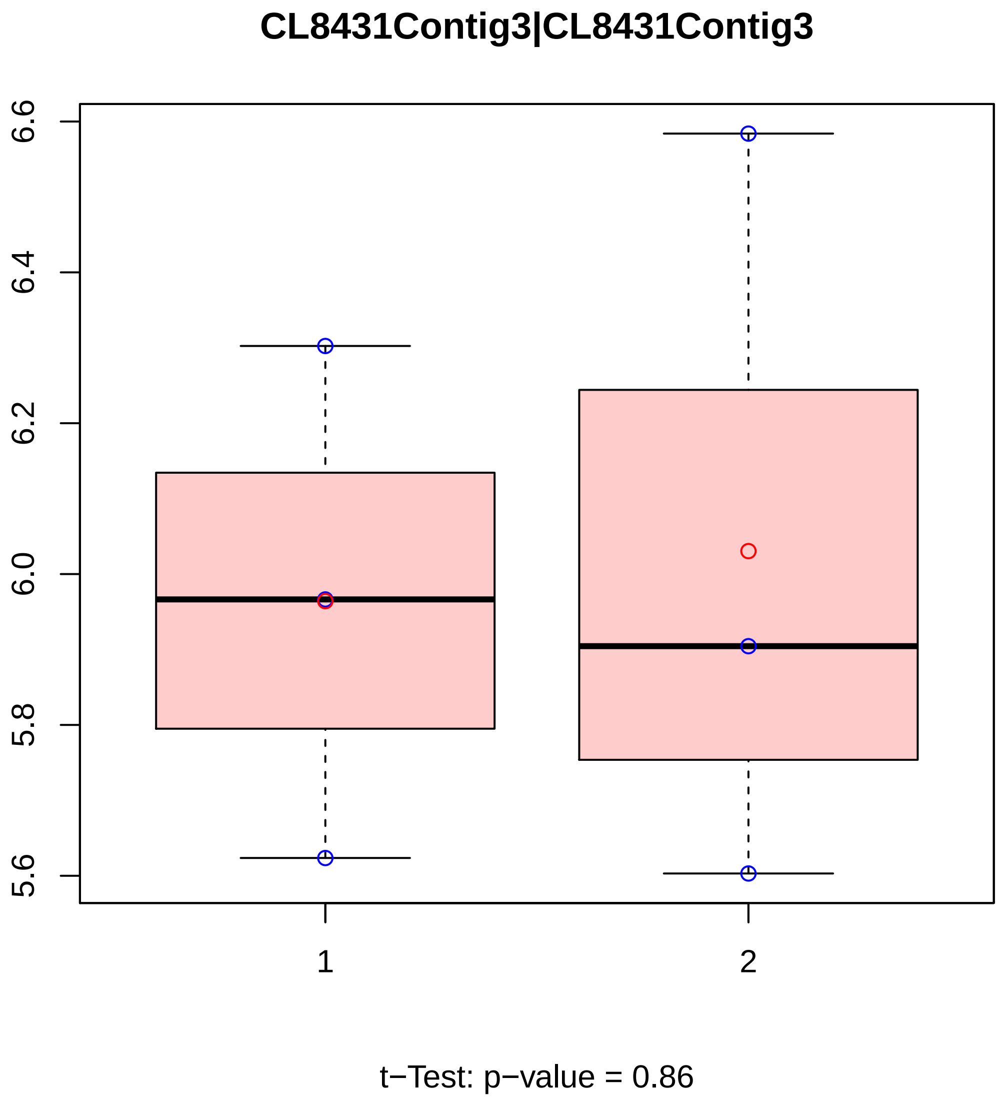
CL8431Contig3|CL8431Contig3
6.6
6.4
6.2
6.0
5.8
5.6
1
2
t−Test: p−value = 0.86

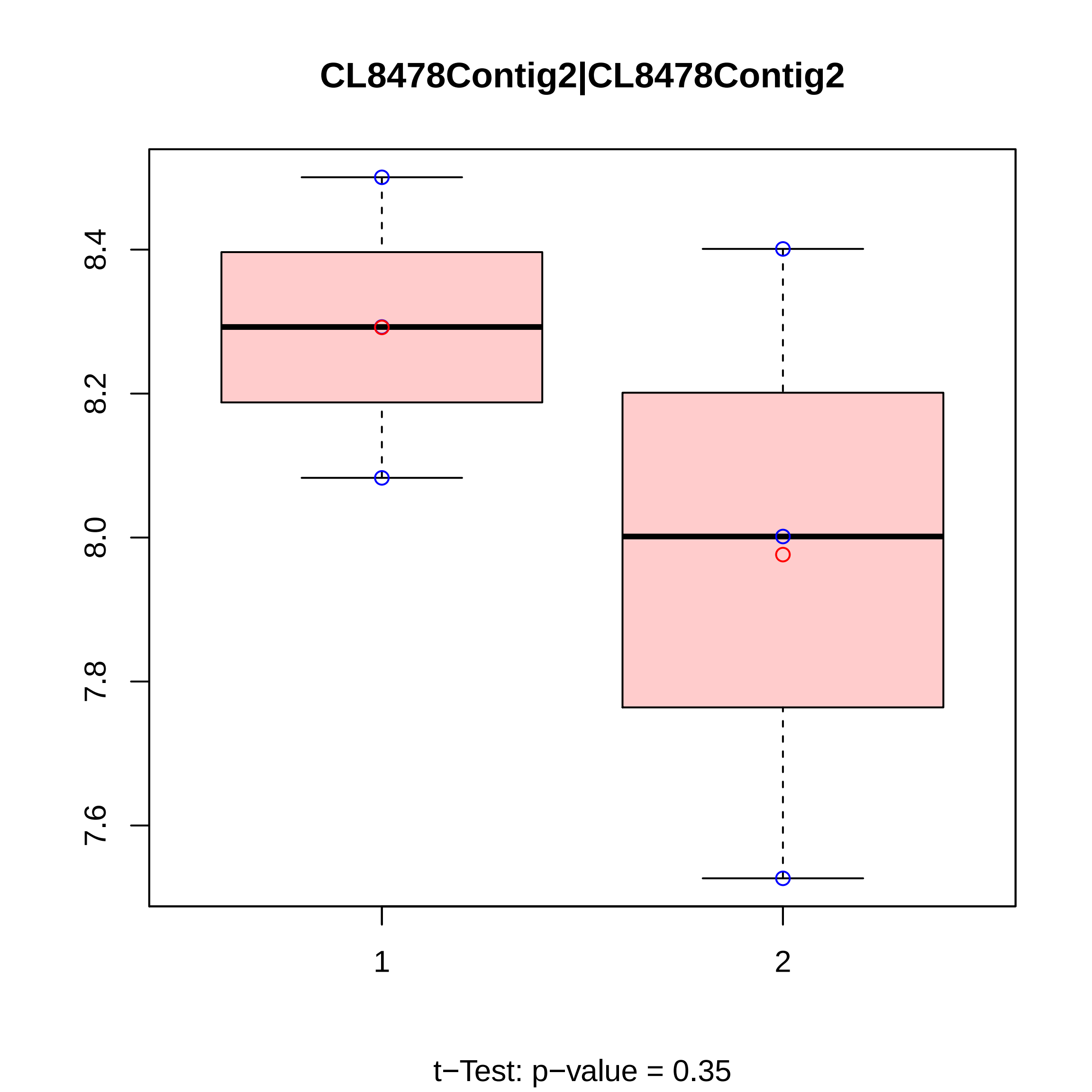
CL8478Contig2|CL8478Contig2
8.4
8.2
8.0
7.8
7.6
1
2
t−Test: p−value = 0.35

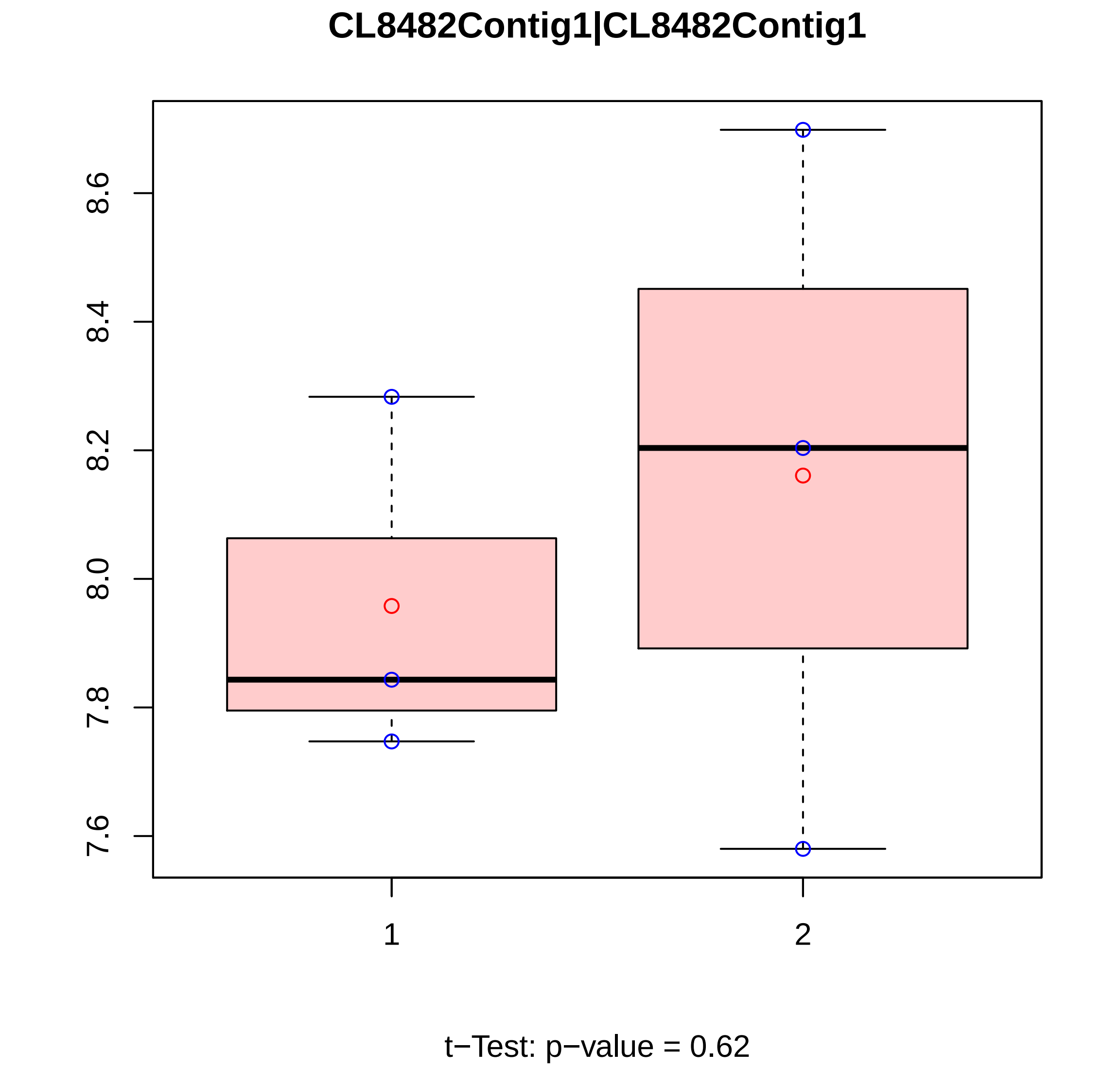
CL8482Contig1|CL8482Contig1
8.6
8.4
8.2
8.0
7.8
7.6
1
2
t−Test: p−value = 0.62

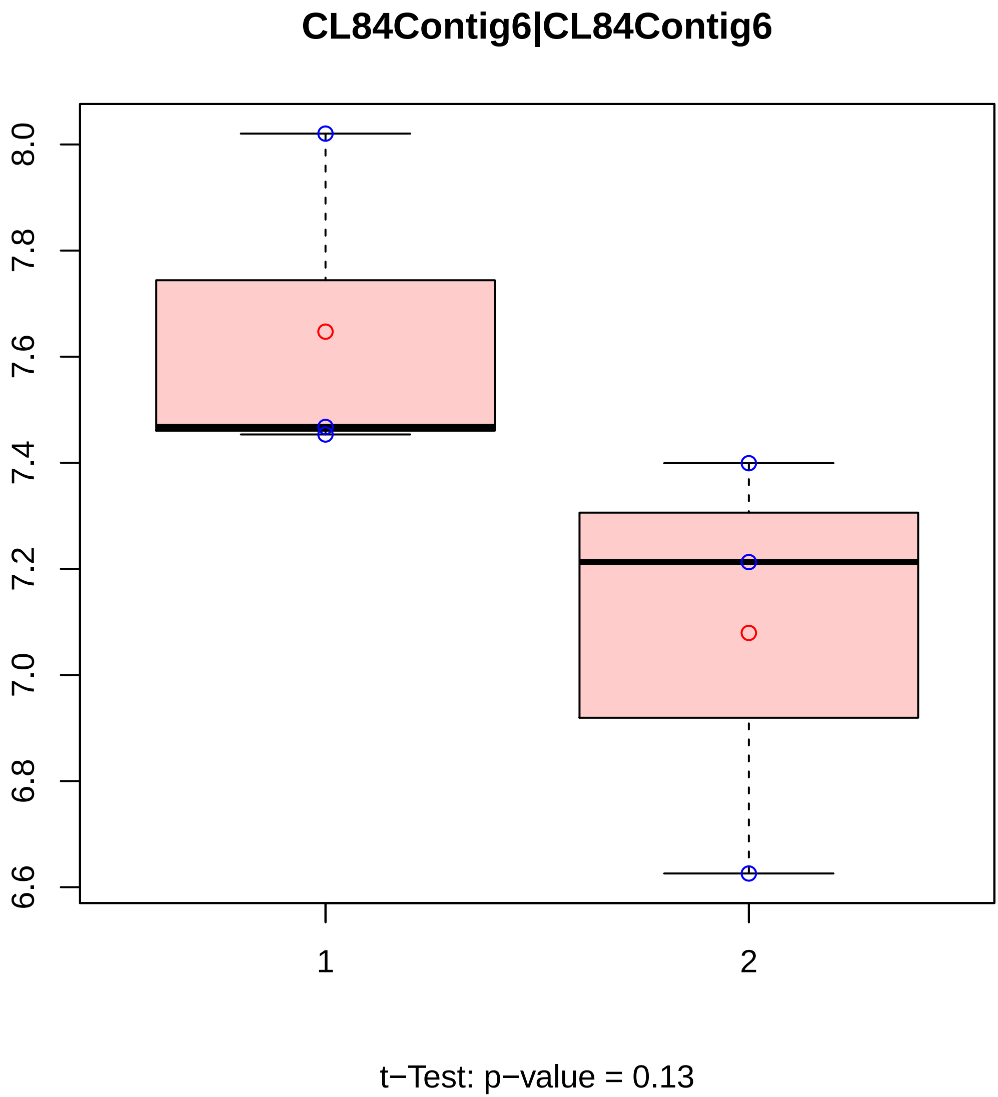

CL84Contig6|CL84Contig6
8.0
7.8
7.6
7.4
7.2
7.0
6.8
6.6
1
2
t−Test: p−value = 0.13

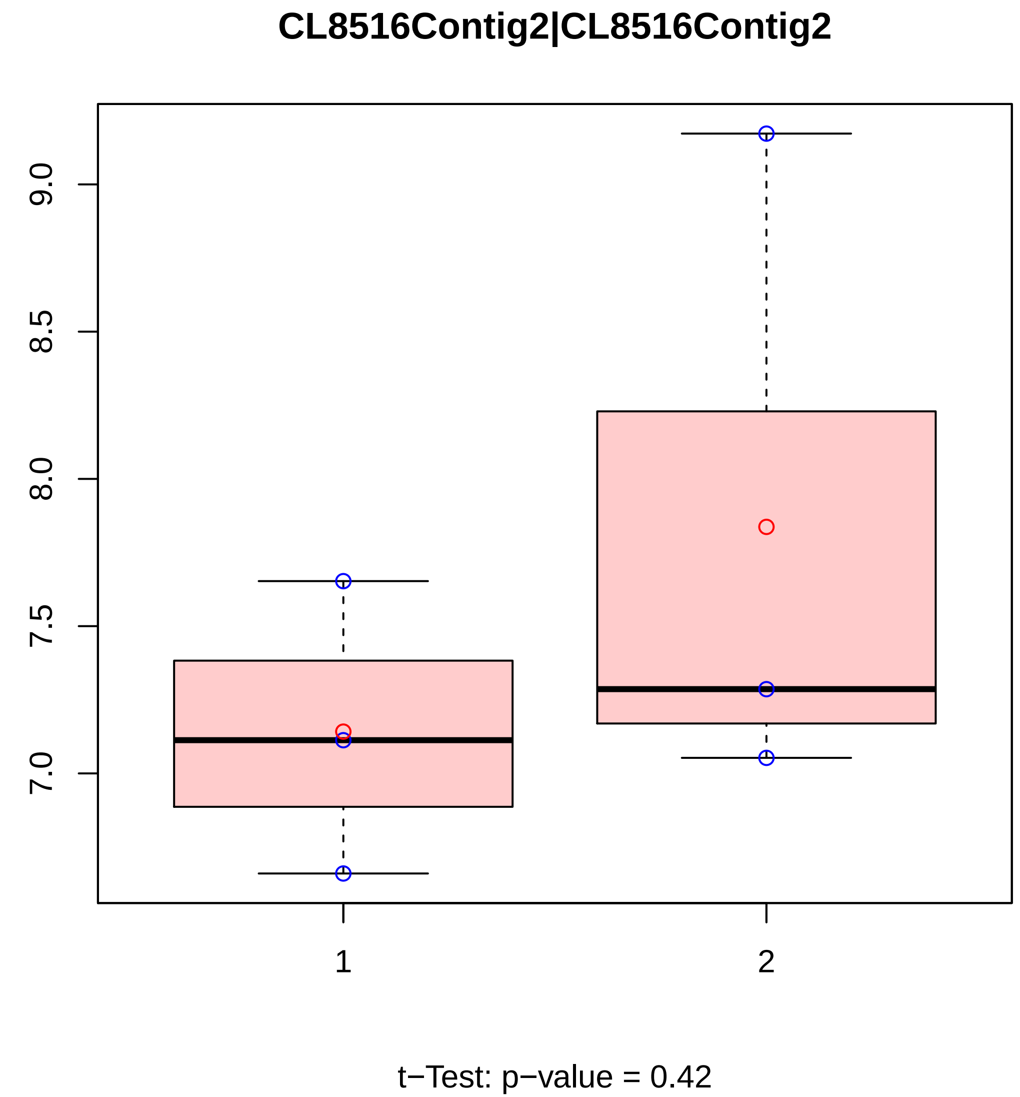
CL8516Contig2|CL8516Contig2
9.0
8.5
8.0
7.5
7.0
1
2
t−Test: p−value = 0.42

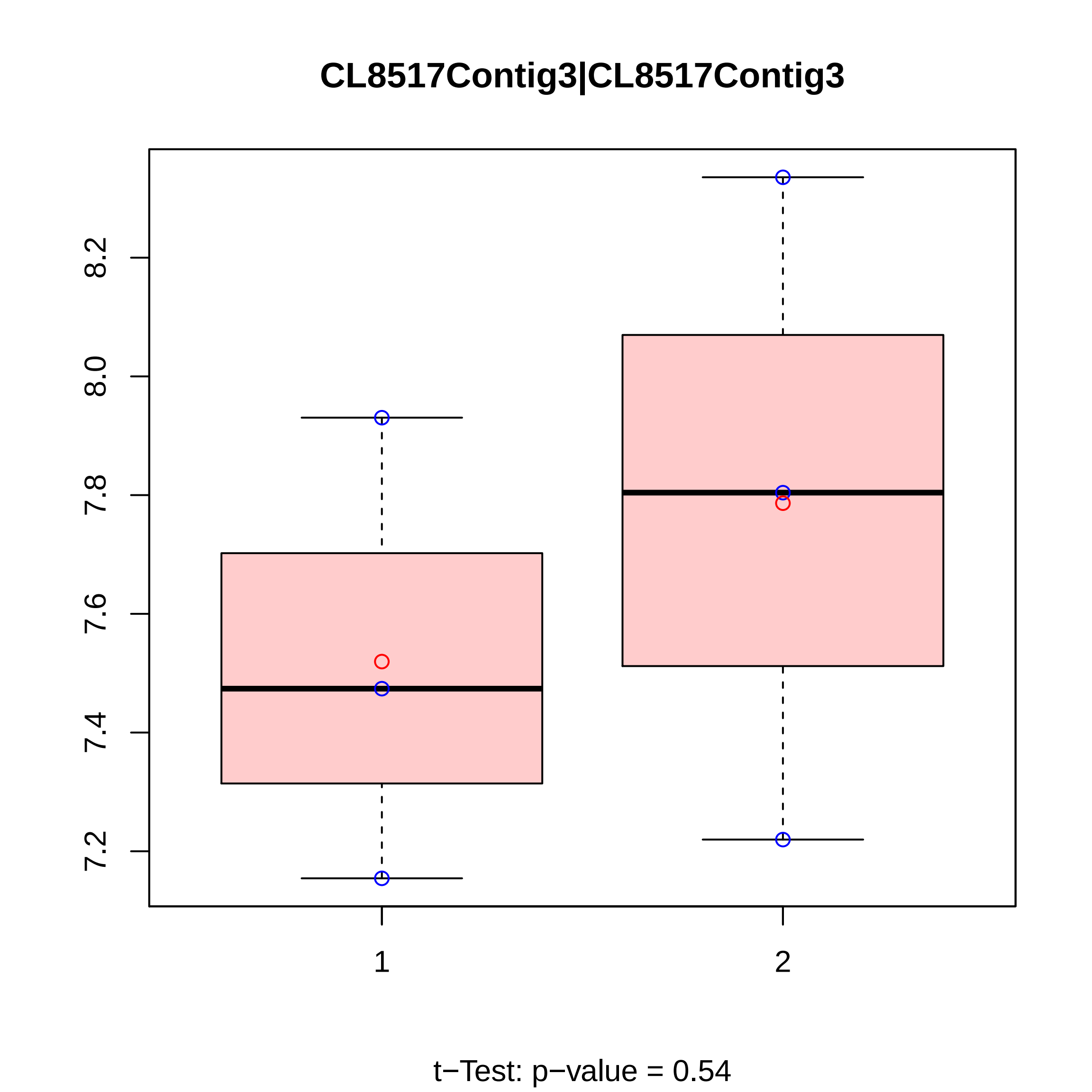

CL8517Contig3|CL8517Contig3
8.2
8.0
7.8
7.6
7.4
7.2
1
2
t−Test: p−value = 0.54

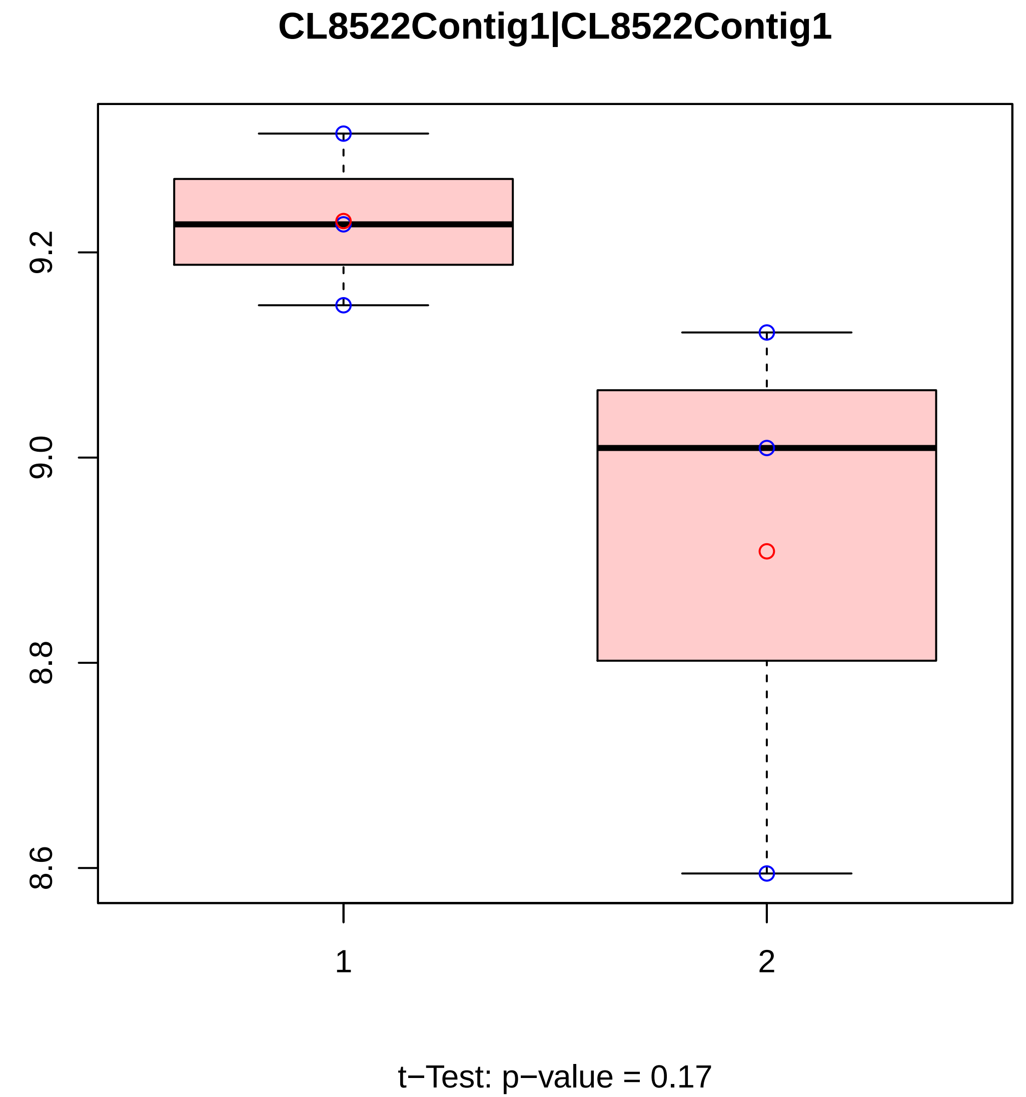
CL8522Contig1|CL8522Contig1
9.2
9.0
8.8
8.6
1
2
t−Test: p−value = 0.17

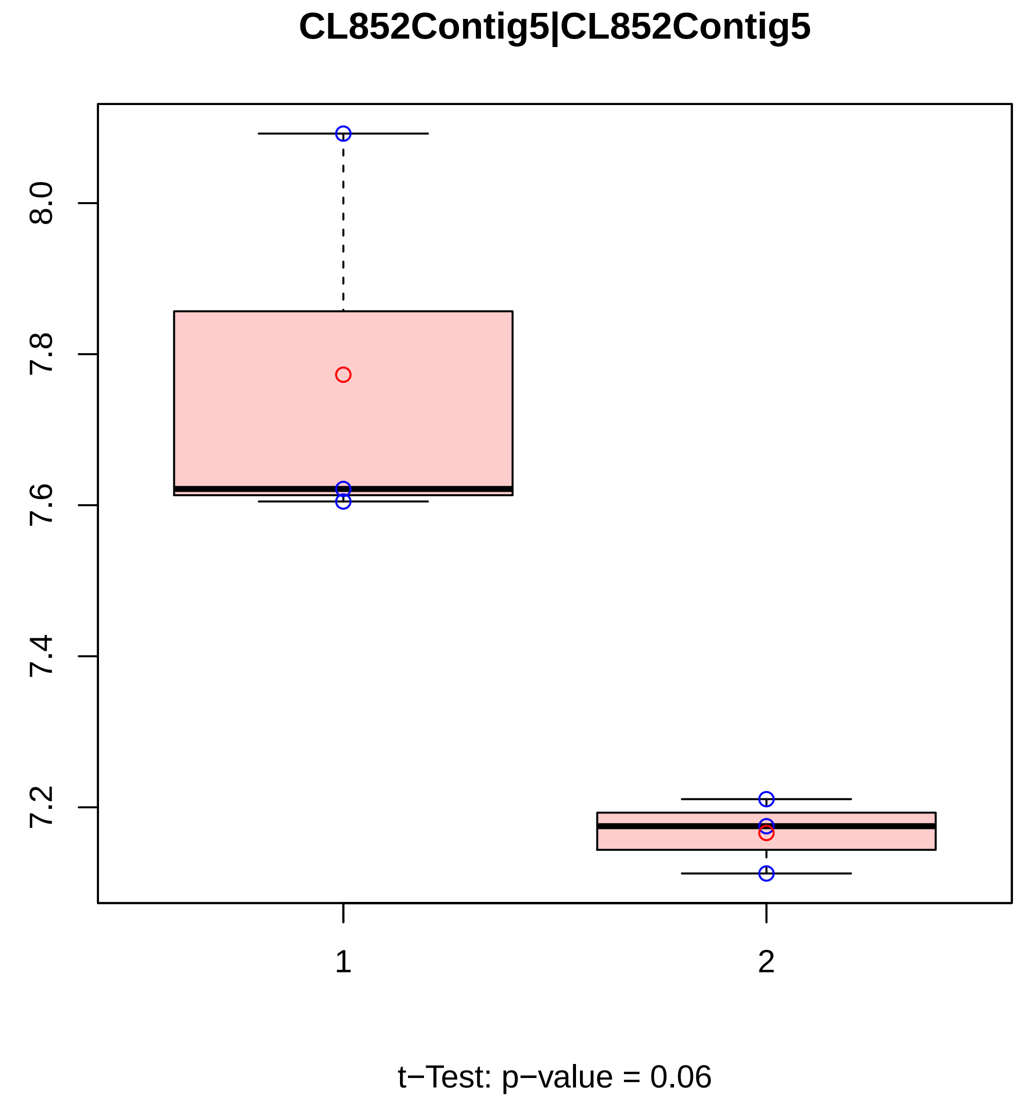

CL852Contig5|CL852Contig5
8.0
7.8
7.6
7.4
7.2
1
2
t−Test: p−value = 0.06

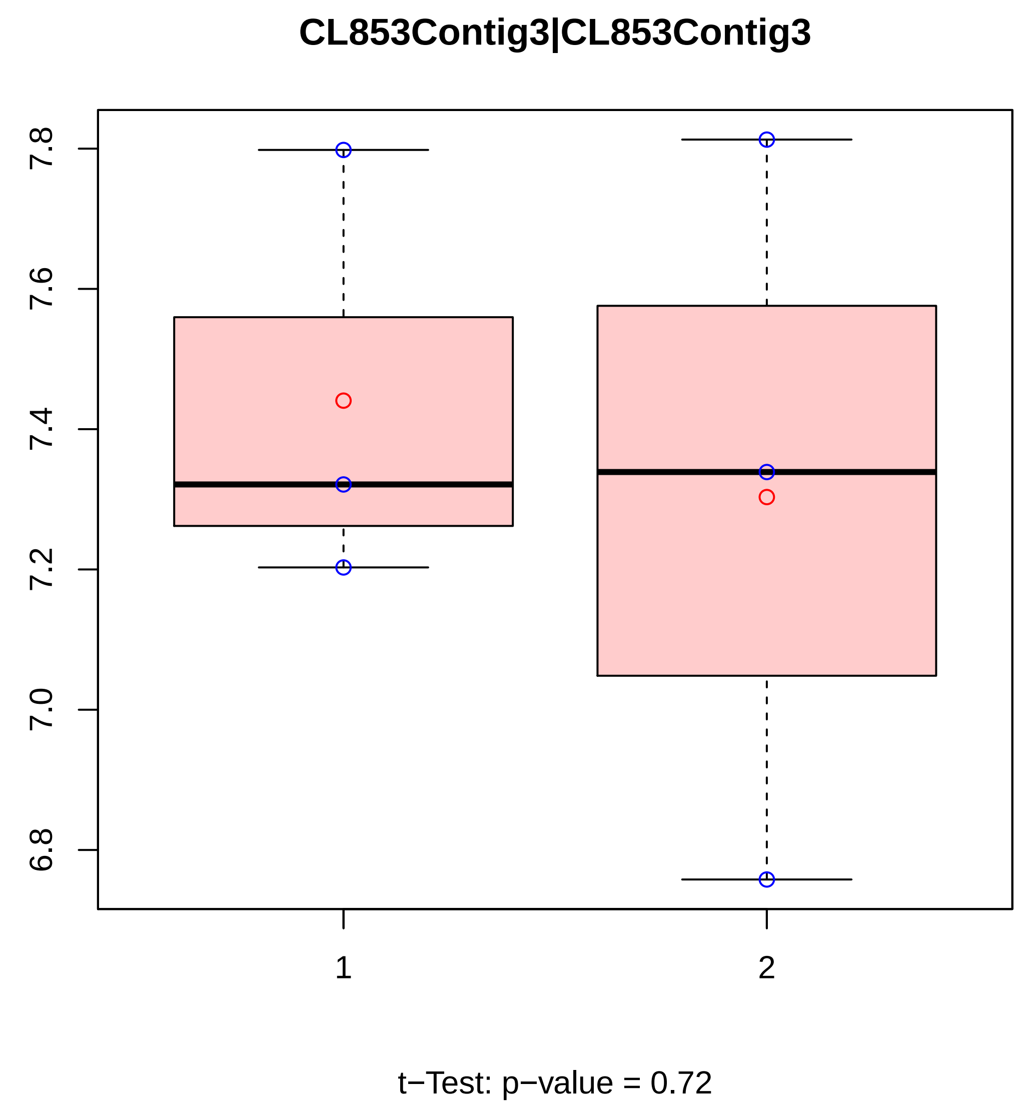
CL853Contig3|CL853Contig3
7.8
7.6
7.4
7.2
7.0
6.8
1
2
t−Test: p−value = 0.72

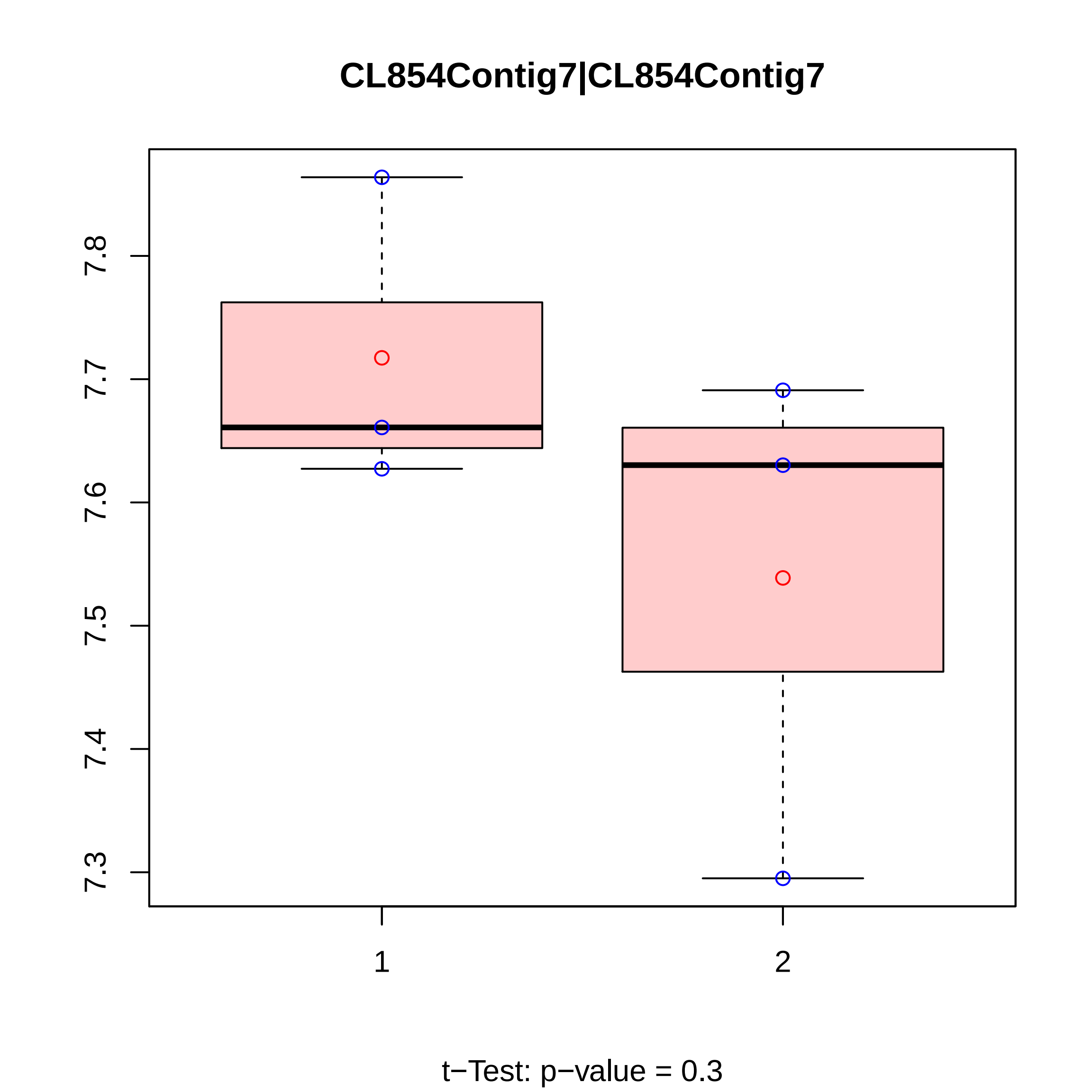

CL854Contig7|CL854Contig7
7.3
7.4
7.5
7.6
7.7
7.8
1
2
t−Test: p−value = 0.3

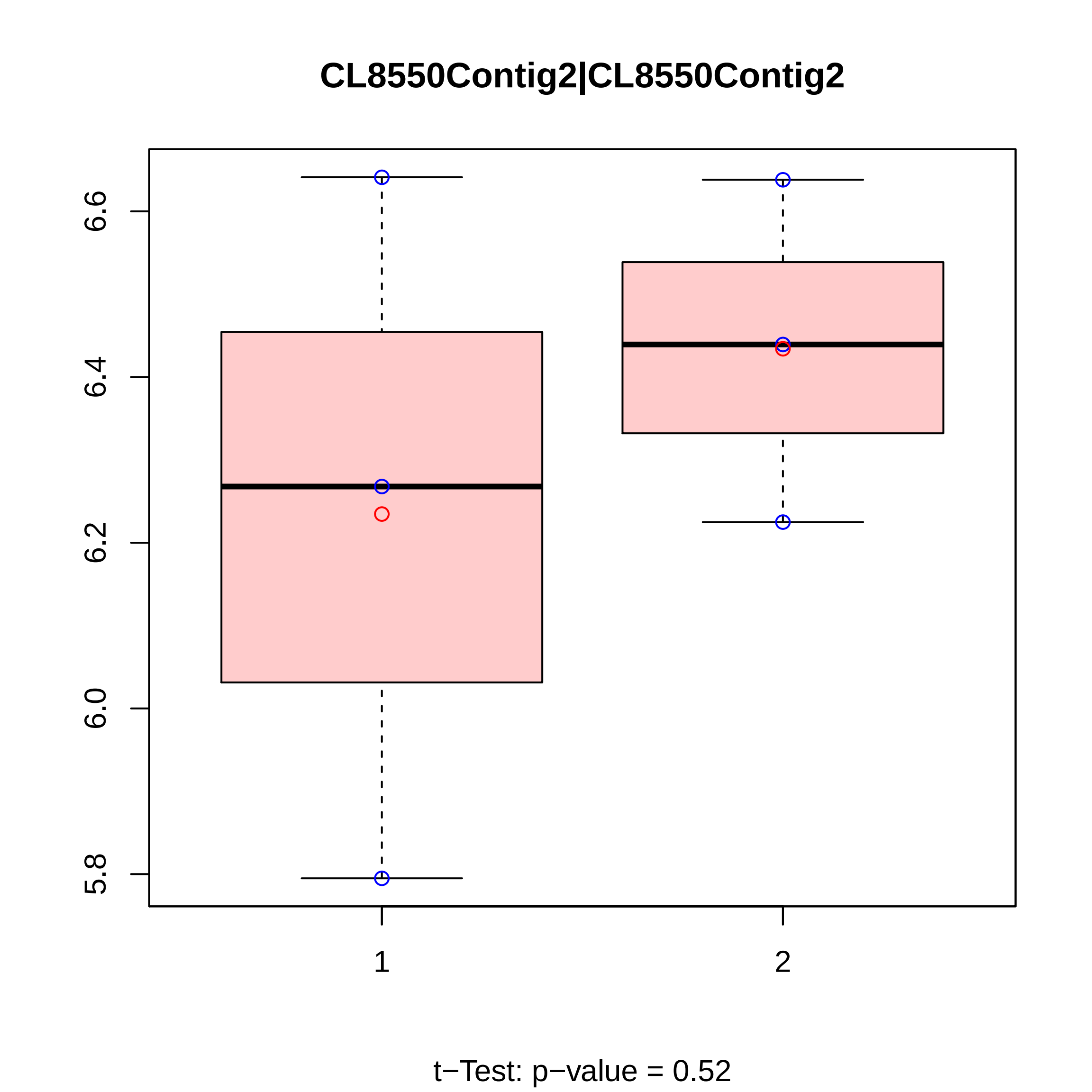
CL8550Contig2|CL8550Contig2
6.6
6.4
6.2
6.0
5.8
1
2
t−Test: p−value = 0.52

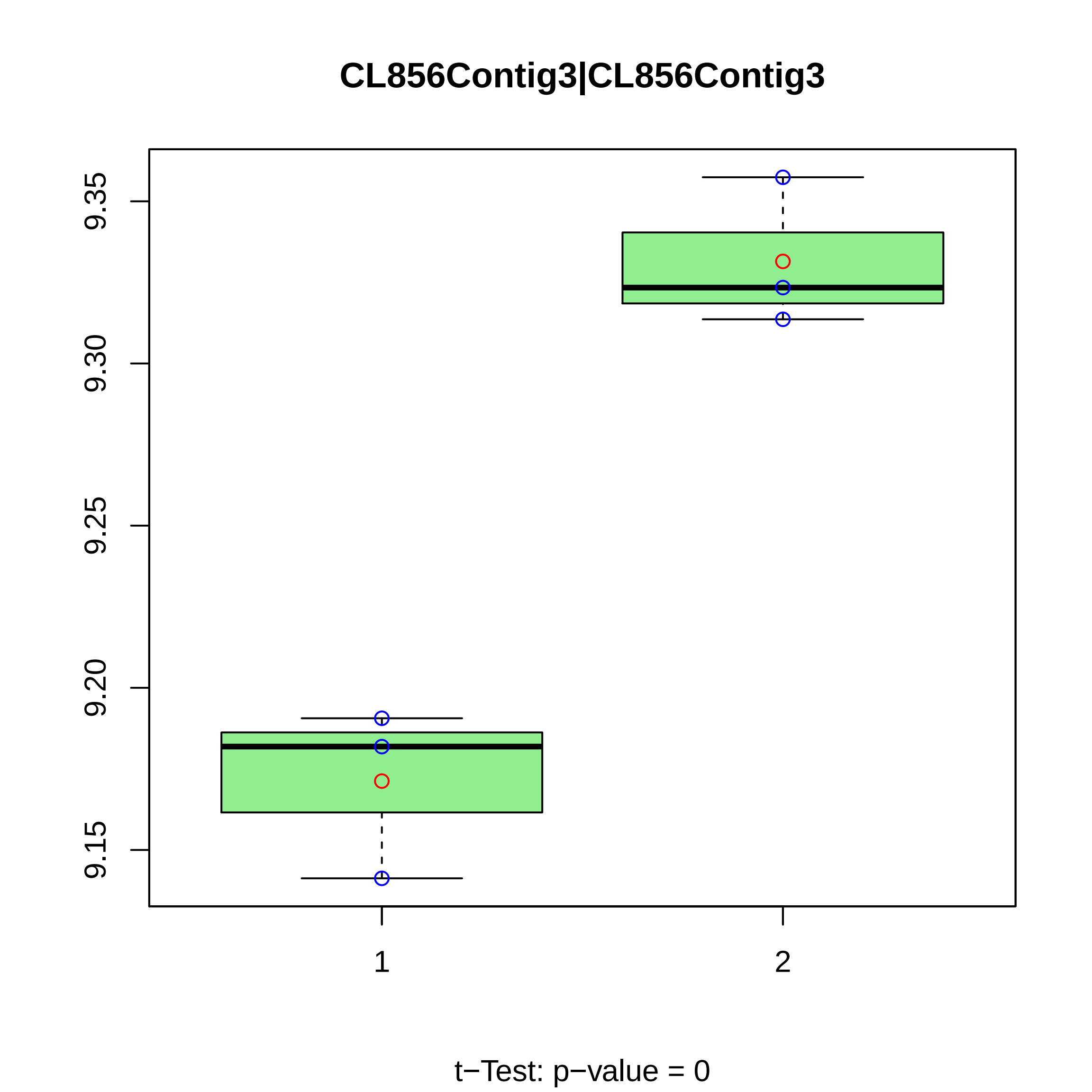
CL856Contig3|CL856Contig3
9.15
9.20
9.25
9.30
9.35
1
2
t−Test: p−value = 0

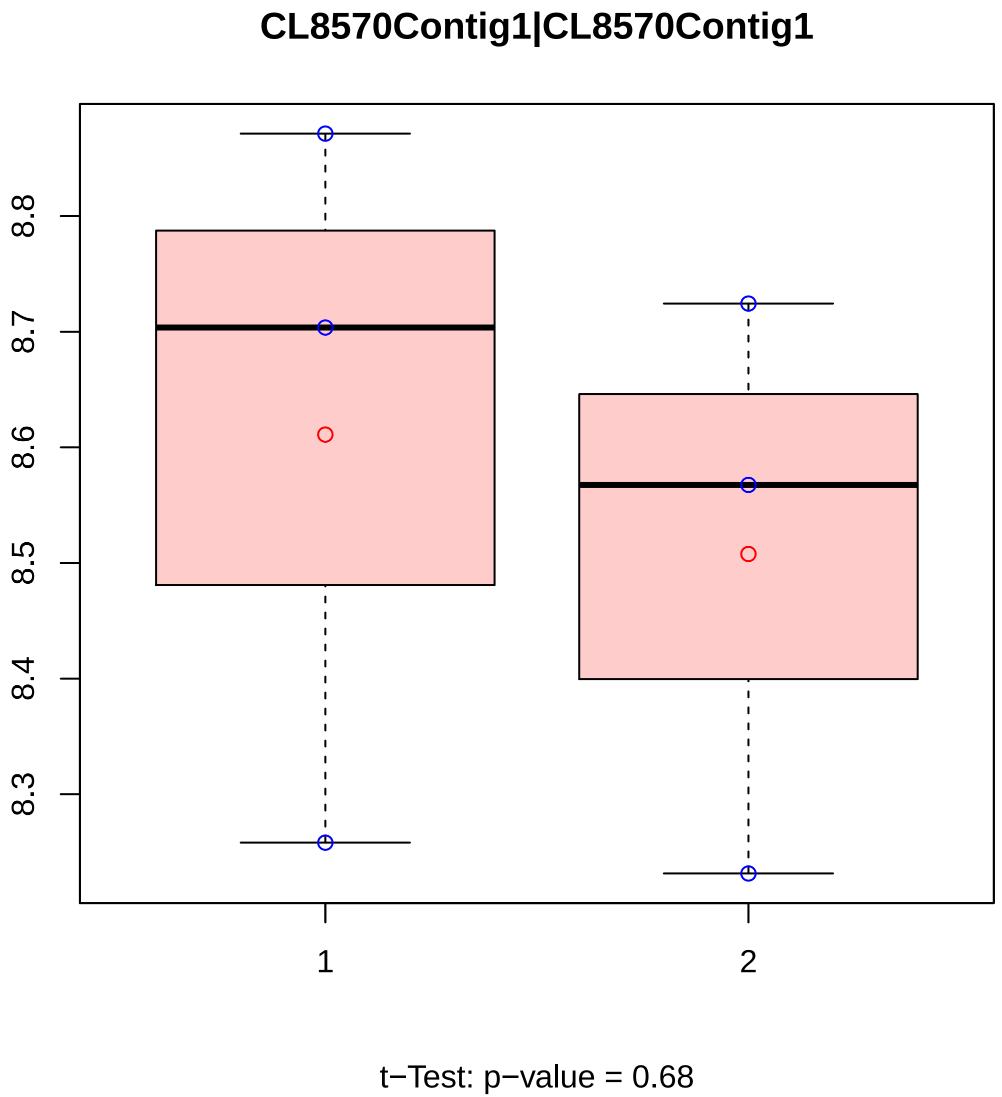
CL8570Contig1|CL8570Contig1
8.3
8.4
8.5
8.6
8.7
8.8
1
2
t−Test: p−value = 0.68

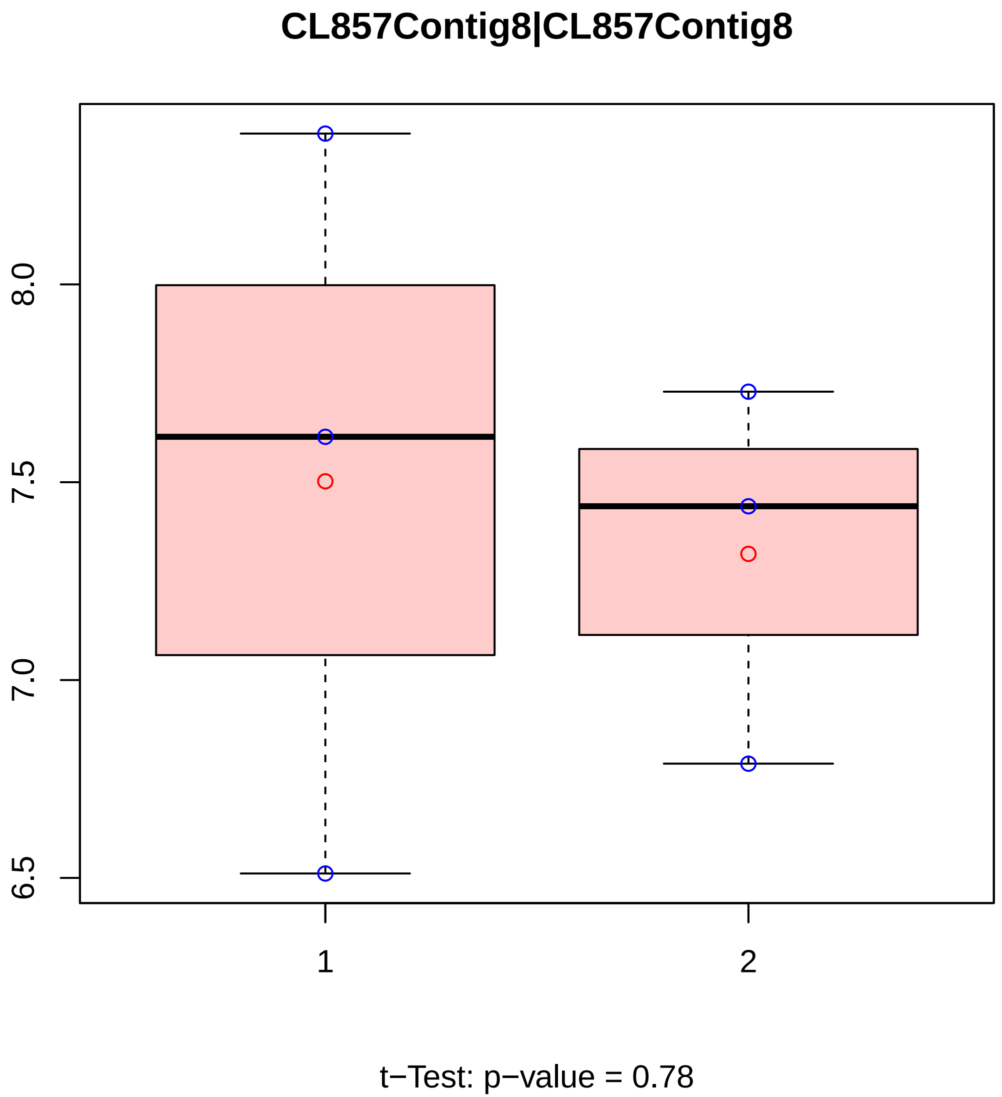
CL857Contig8|CL857Contig8
8.0
7.5
7.0
6.5
1
2
t−Test: p−value = 0.78

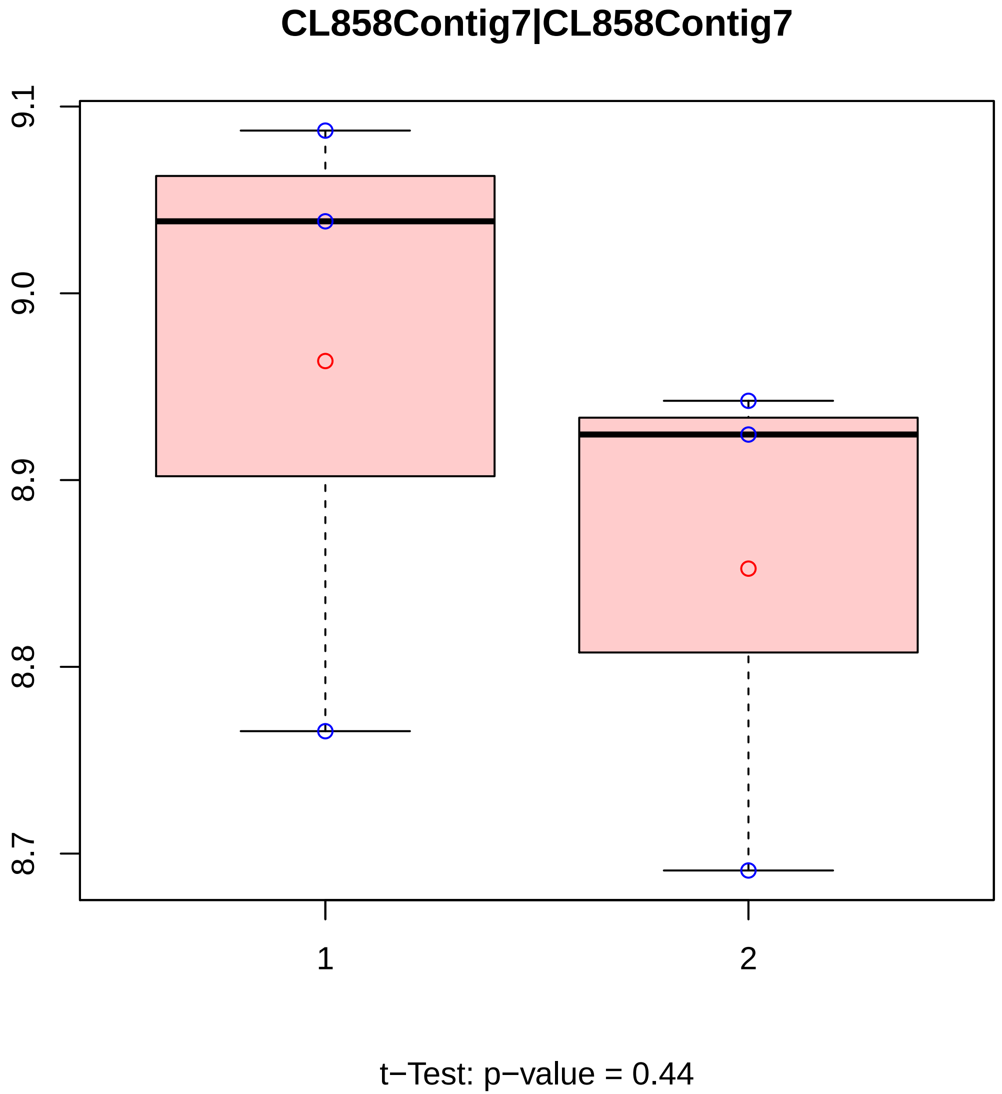

CL858Contig7|CL858Contig7
9.1
9.0
8.9
8.8
8.7
1
2
t−Test: p−value = 0.44

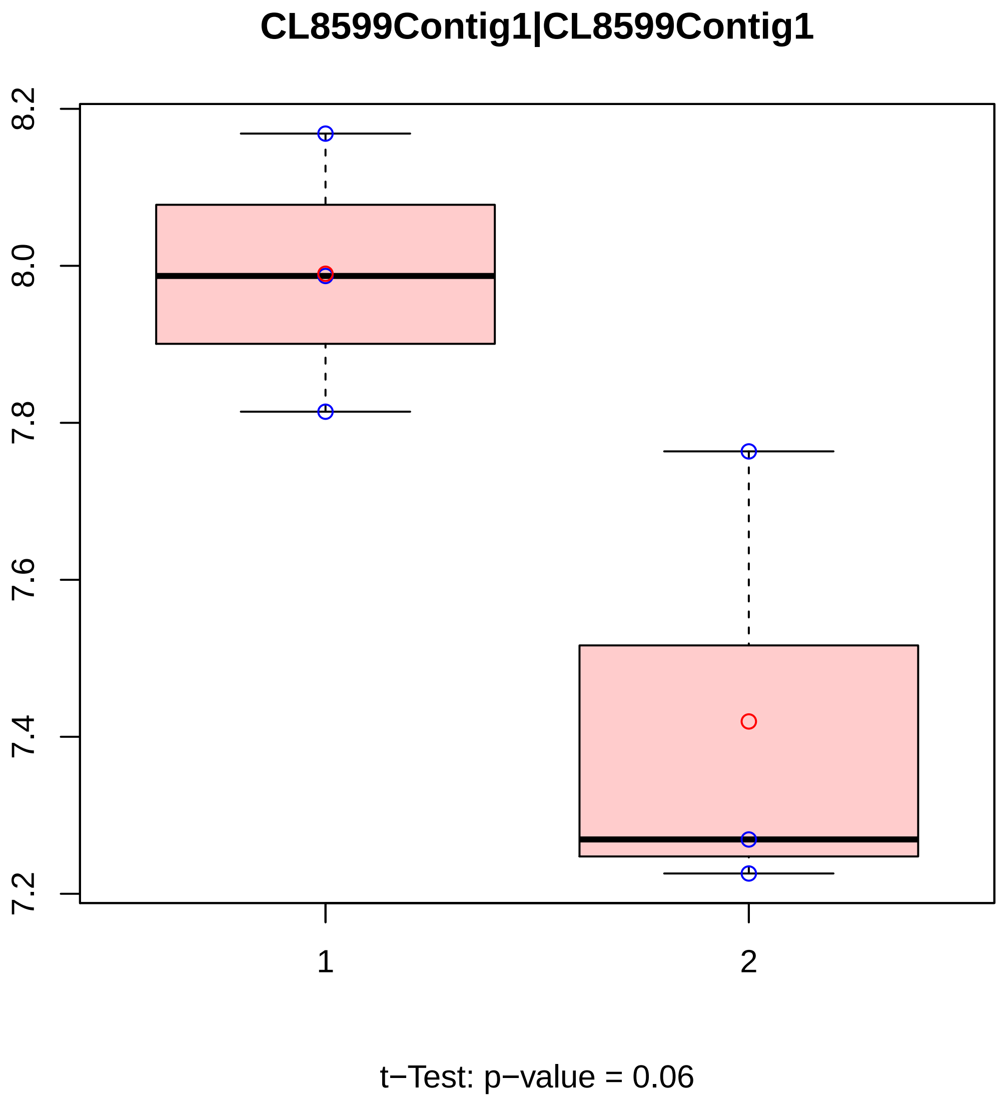

CL8599Contig1|CL8599Contig1
8.2
8.0
7.8
7.6
7.4
7.2
1
2
t−Test: p−value = 0.06

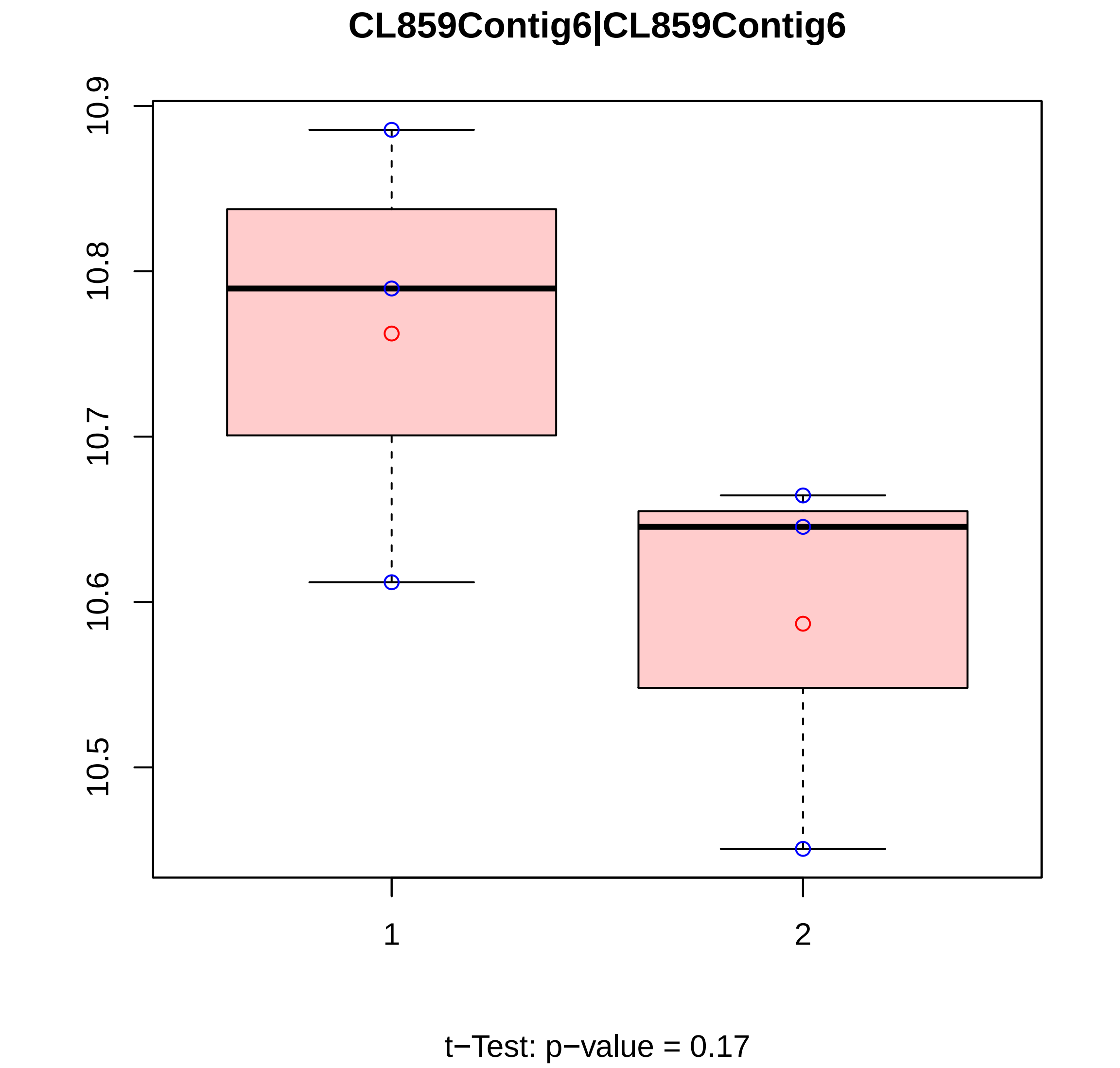
CL859Contig6|CL859Contig6
10.9
10.8
10.7
10.6
10.5
1
2
t−Test: p−value = 0.17

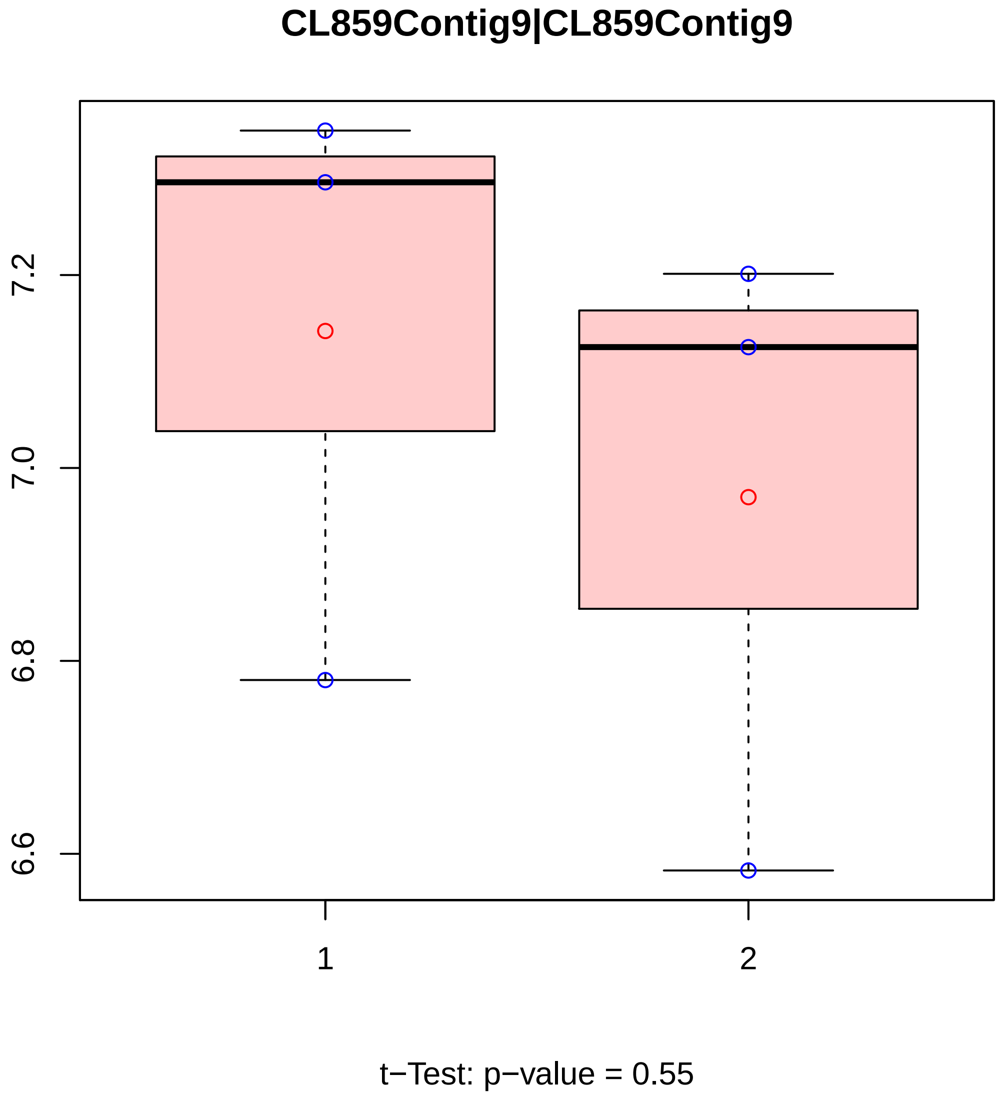

CL859Contig9|CL859Contig9
7.2
7.0
6.8
6.6
1
2
t−Test: p−value = 0.55

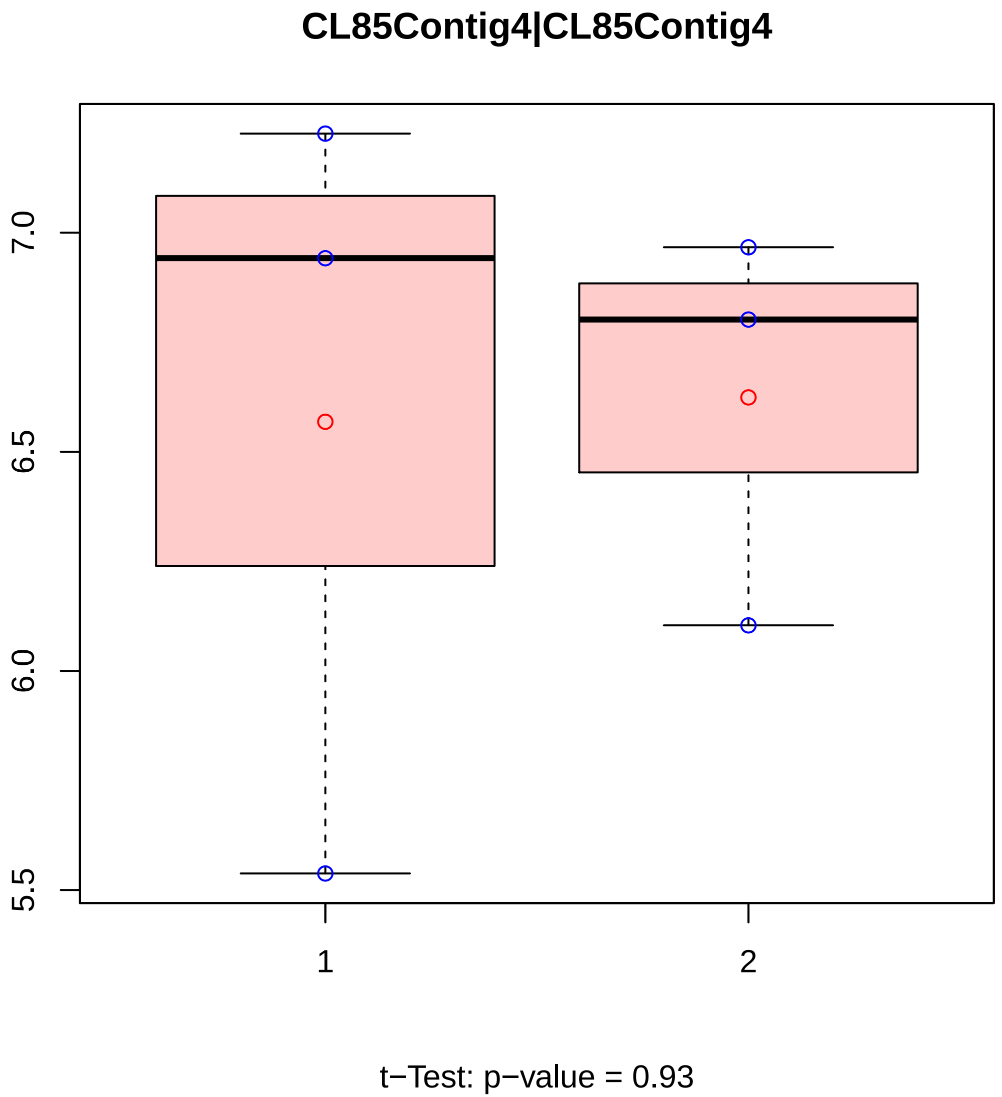
CL85Contig4|CL85Contig4
7.0
6.5
6.0
5.5
1
2
t−Test: p−value = 0.93

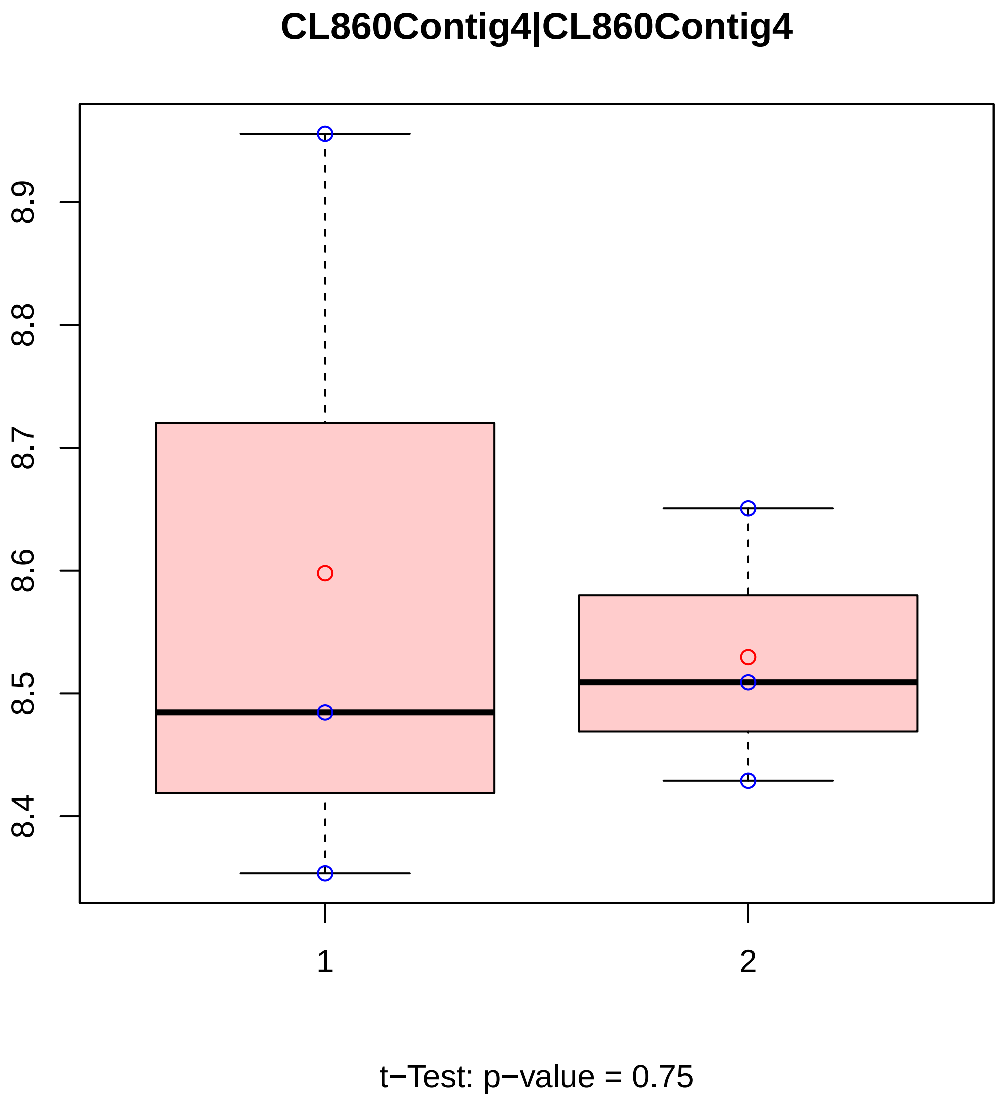
CL860Contig4|CL860Contig4
8.4
8.5
8.6
8.7
8.8
8.9
1
2
t−Test: p−value = 0.75

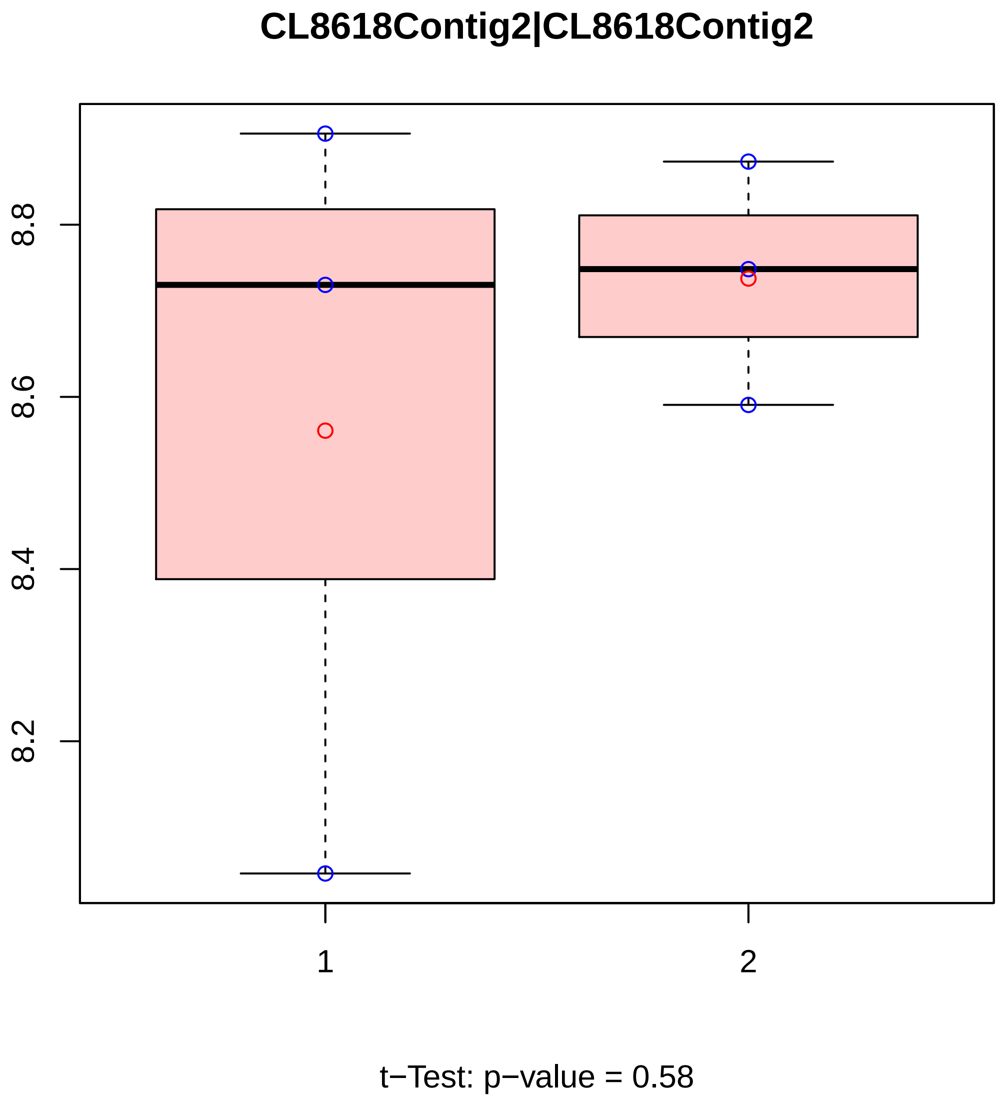
CL8618Contig2|CL8618Contig2
8.2
8.4
8.6
8.8
1
2
t−Test: p−value = 0.58

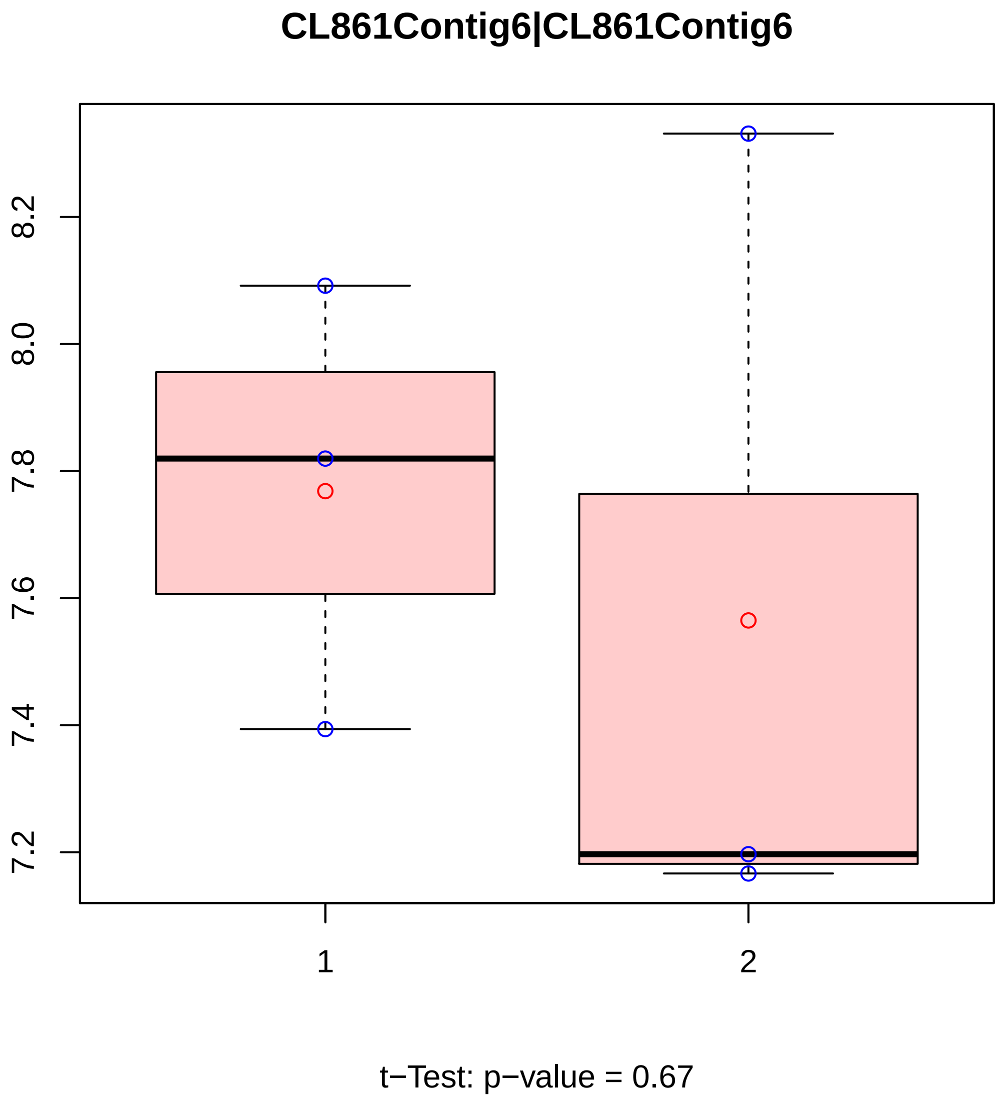
CL861Contig6|CL861Contig6
8.2
8.0
7.8
7.6
7.4
7.2
1
2
t−Test: p−value = 0.67

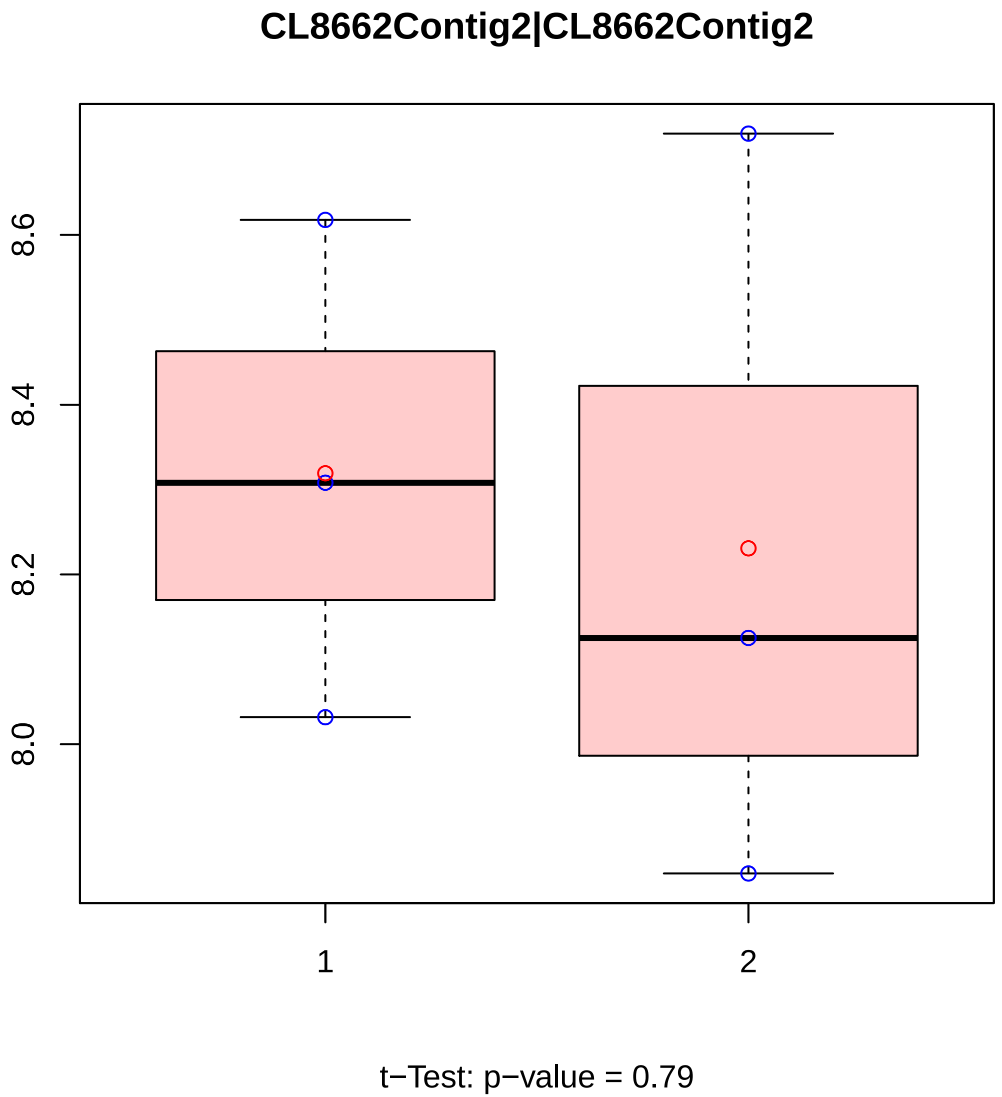

CL8662Contig2|CL8662Contig2
8.0
8.2
8.4
8.6
1
2
t−Test: p−value = 0.79

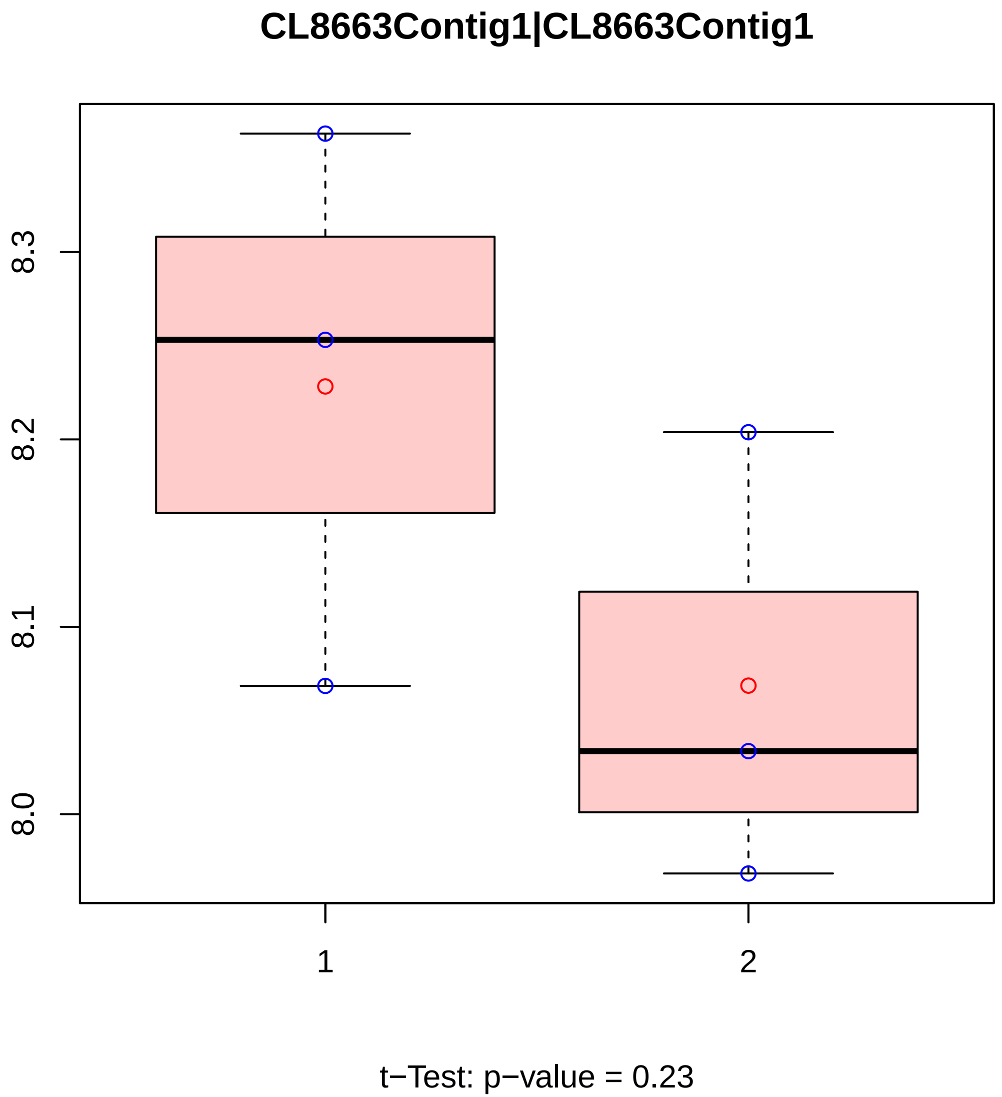
CL8663Contig1|CL8663Contig1
8.0
8.1
8.2
8.3
1
2
t−Test: p−value = 0.23

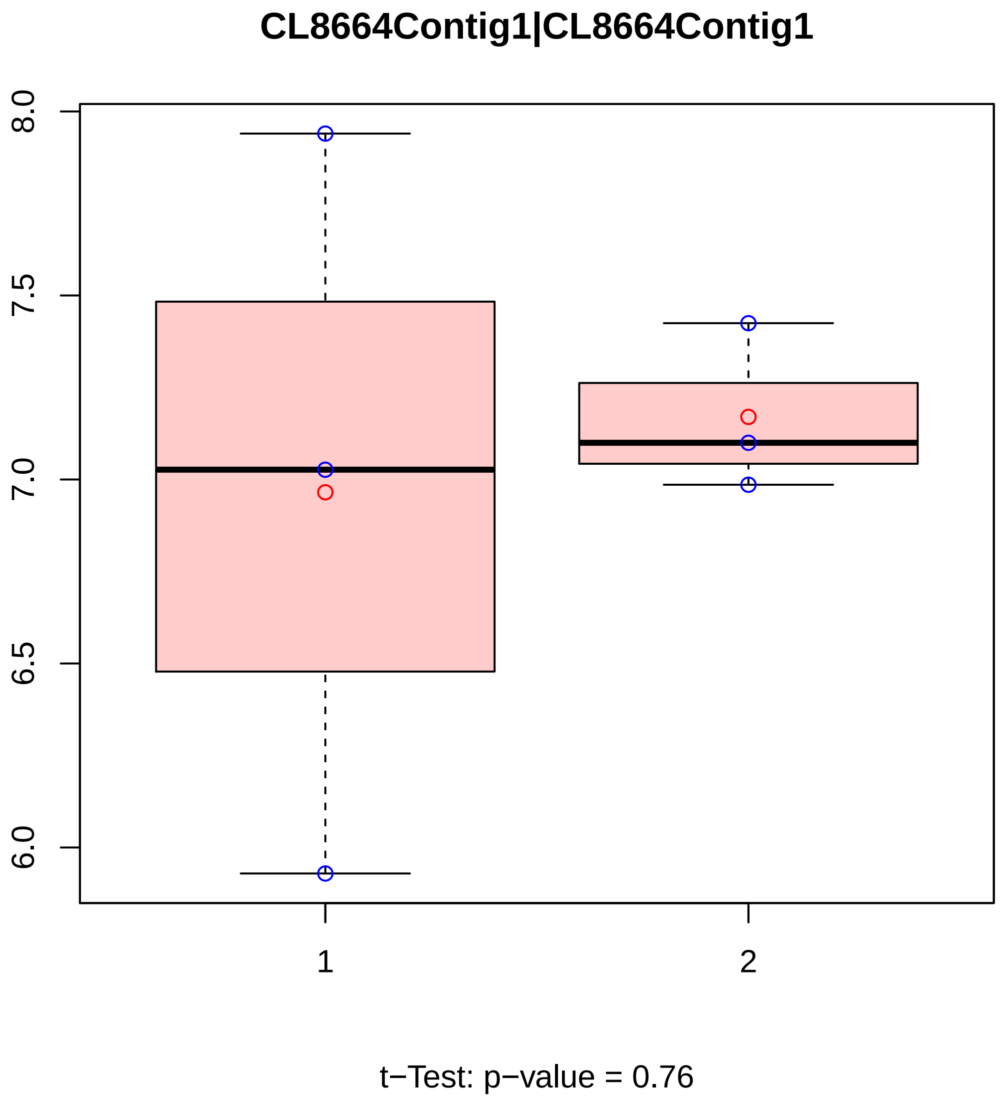

CL8664Contig1|CL8664Contig1
8.0
7.5
7.0
6.5
6.0
1
2
t−Test: p−value = 0.76

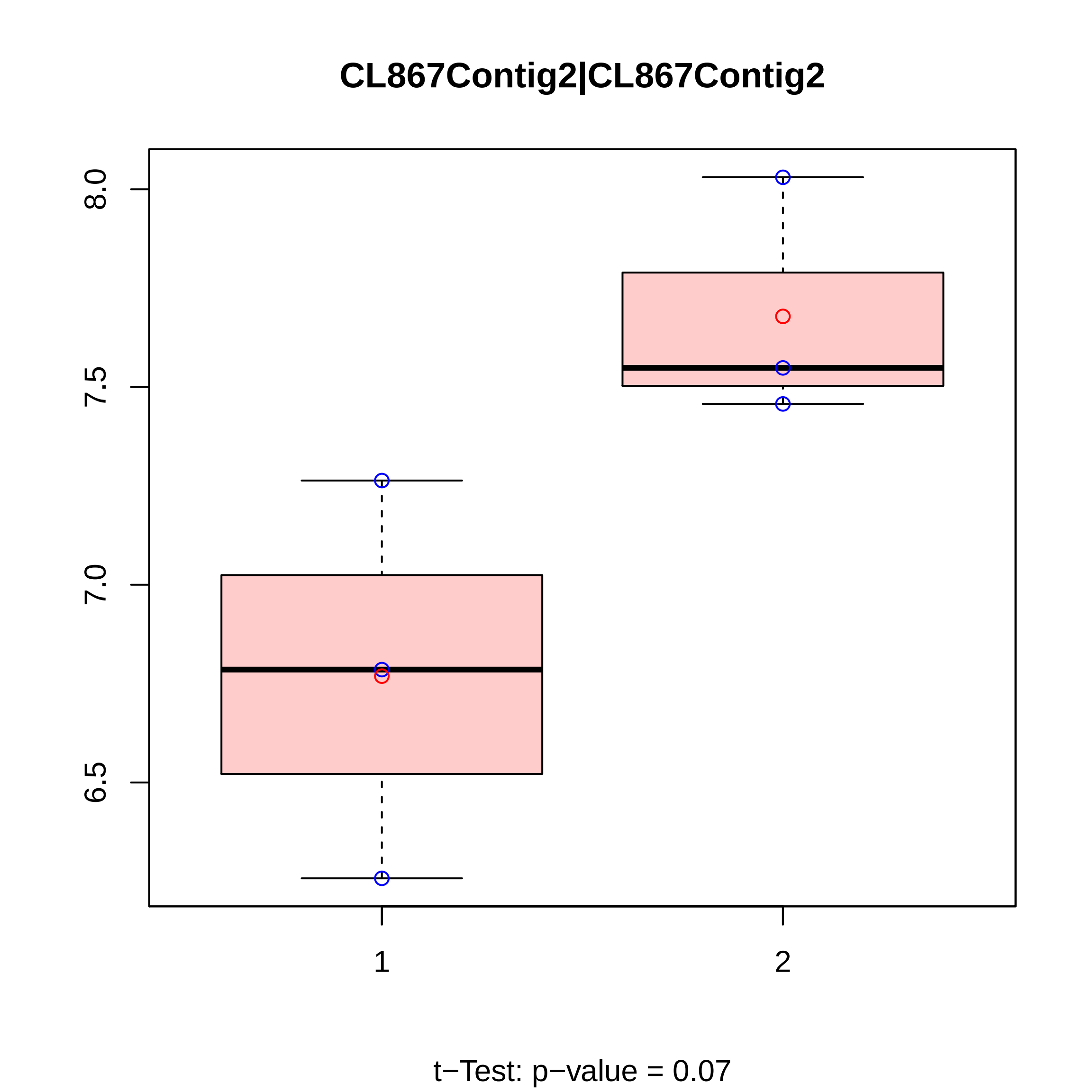
CL867Contig2|CL867Contig2
8.0
7.5
7.0
6.5
1
2
t−Test: p−value = 0.07

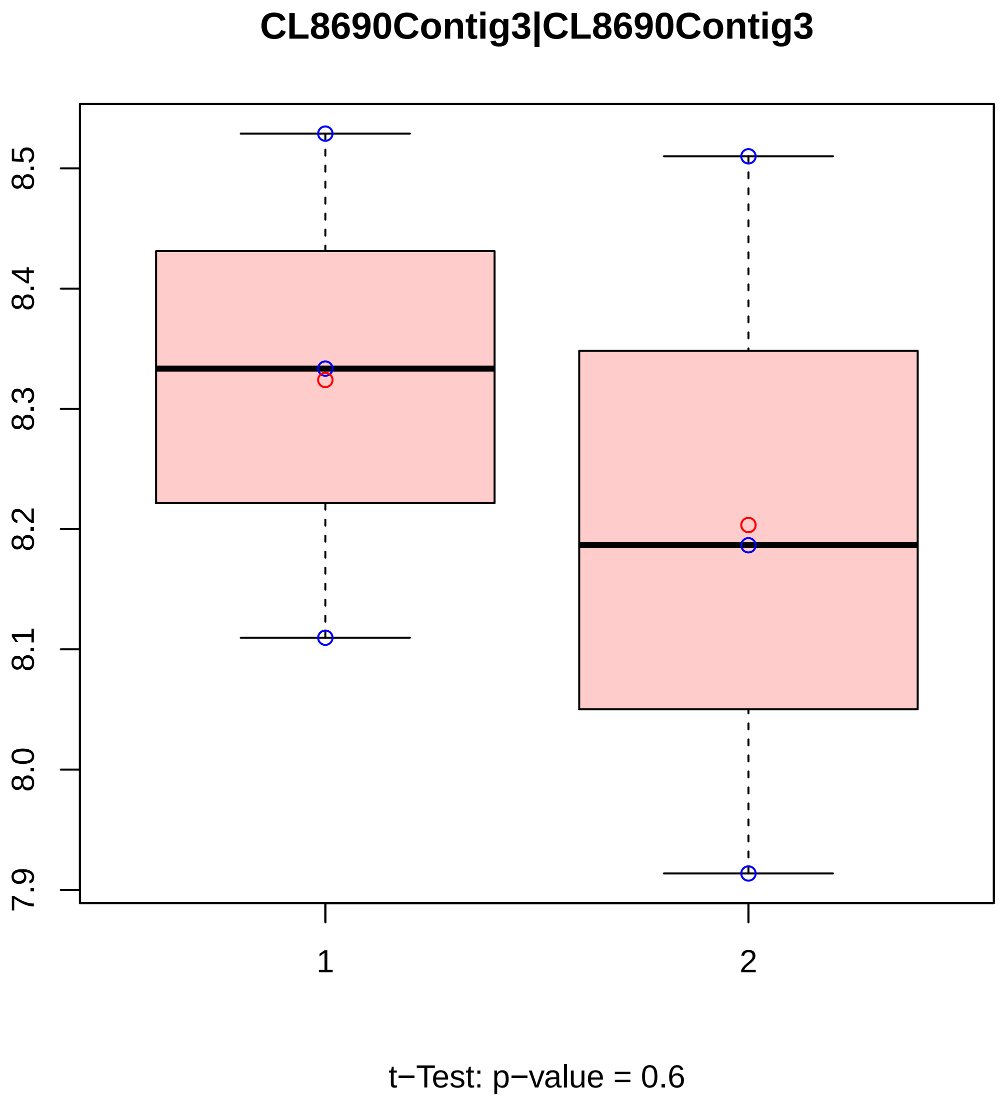
CL8690Contig3|CL8690Contig3
8.5
8.4
8.3
8.2
8.1
8.0
7.9
1
2
t−Test: p−value = 0.6

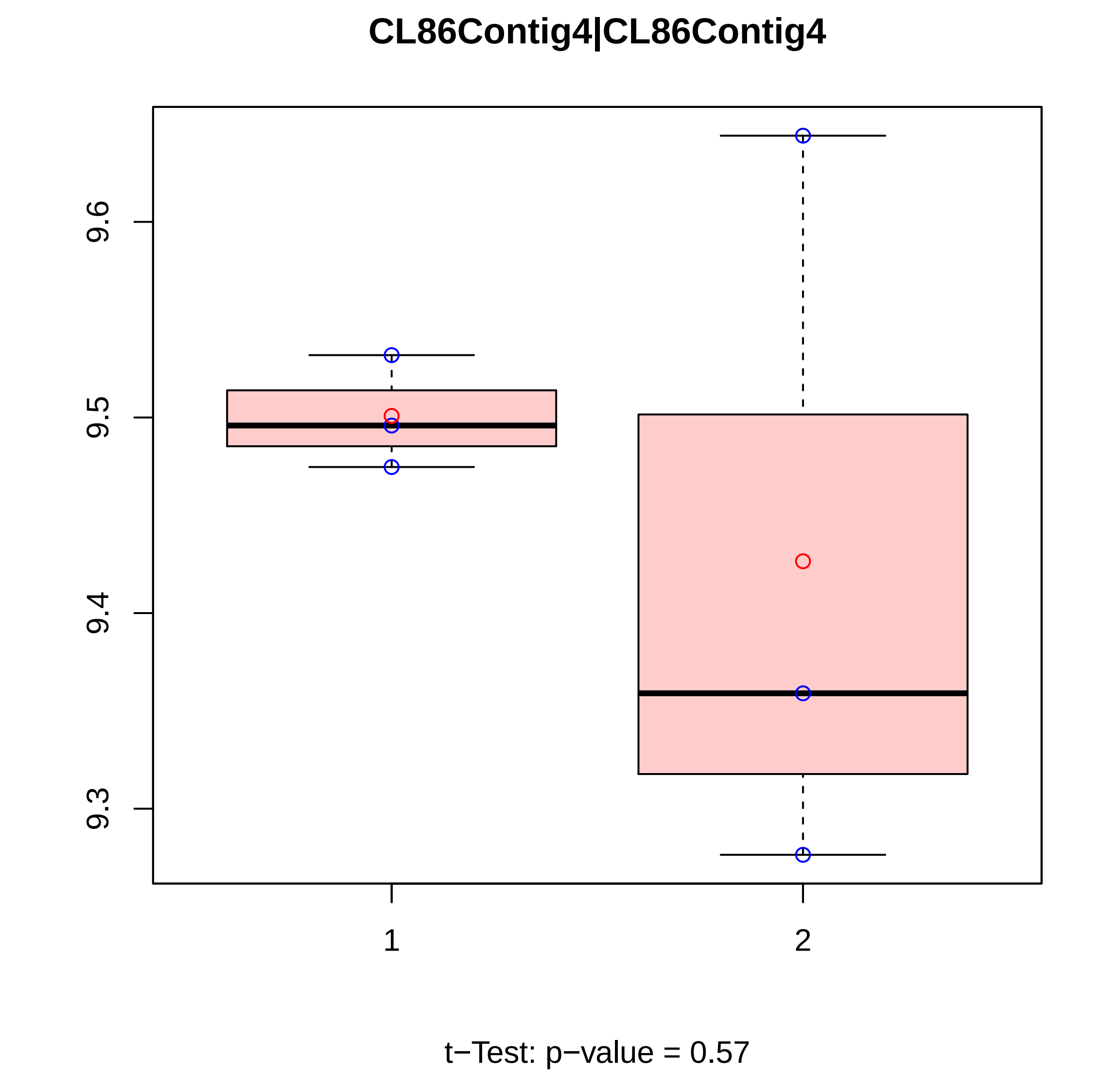
CL86Contig4|CL86Contig4
9.3
9.4
9.5
9.6
1
2
t−Test: p−value = 0.57

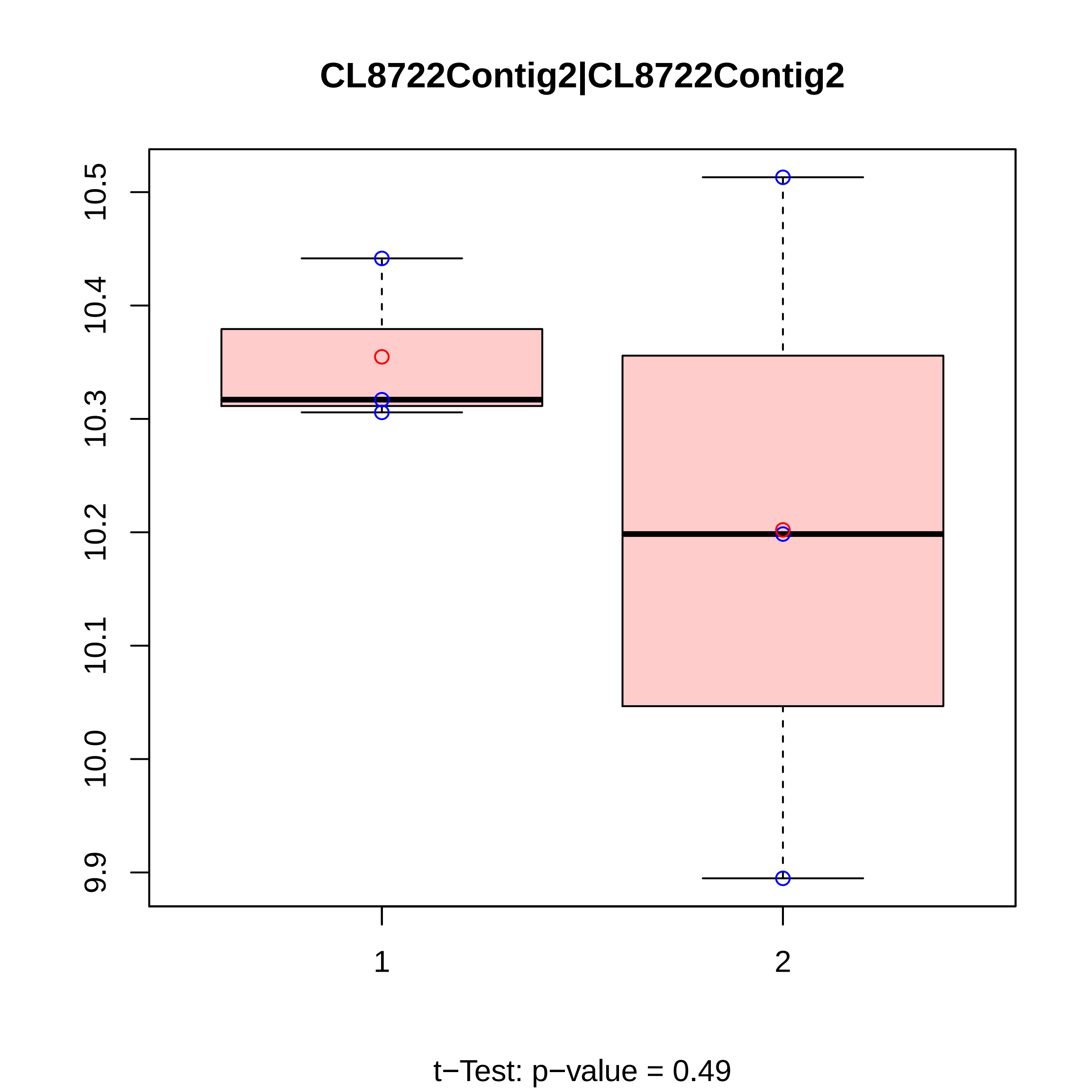

CL8722Contig2|CL8722Contig2
10.5
10.4
10.3
10.2
10.1
10.0
9.9
1
2
t−Test: p−value = 0.49

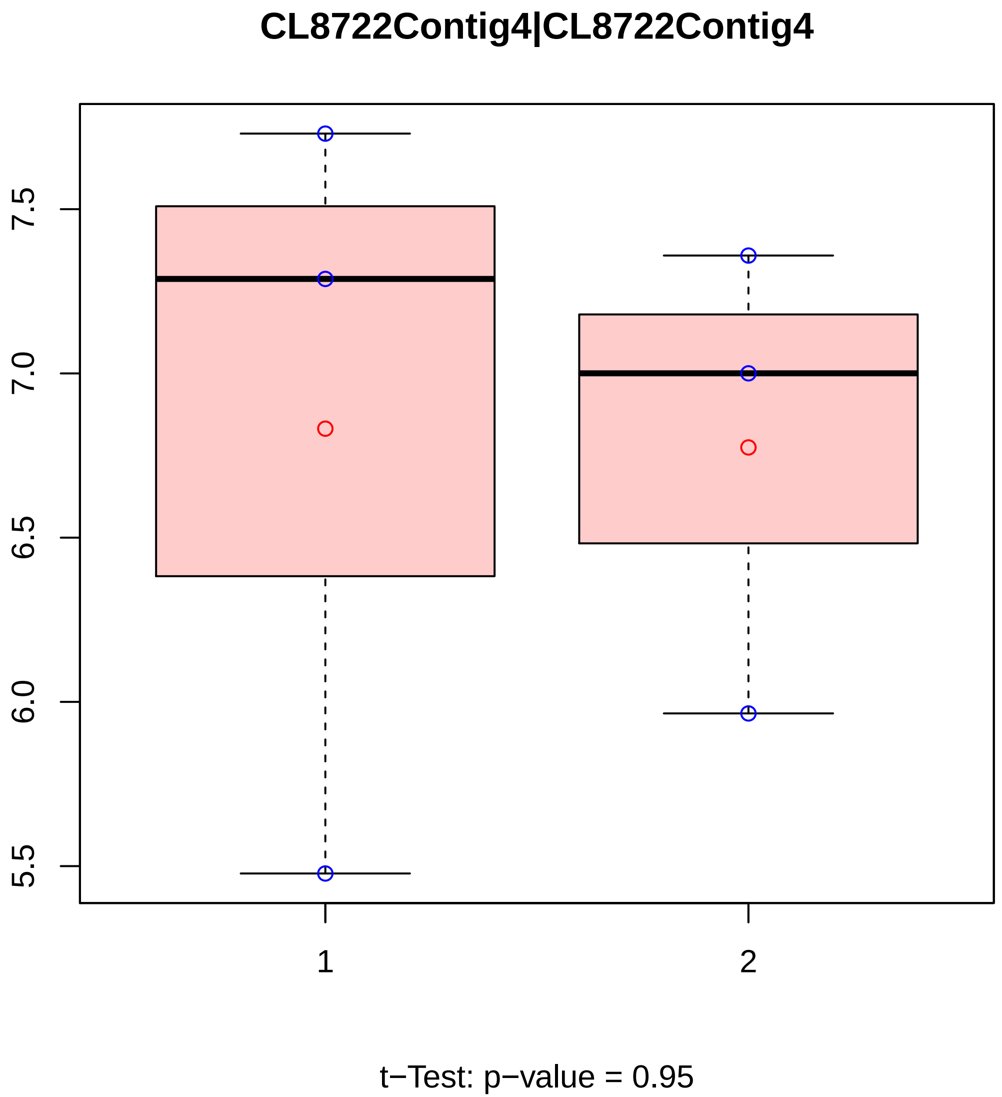
CL8722Contig4|CL8722Contig4
7.5
7.0
6.5
6.0
5.5
1
2
t−Test: p−value = 0.95

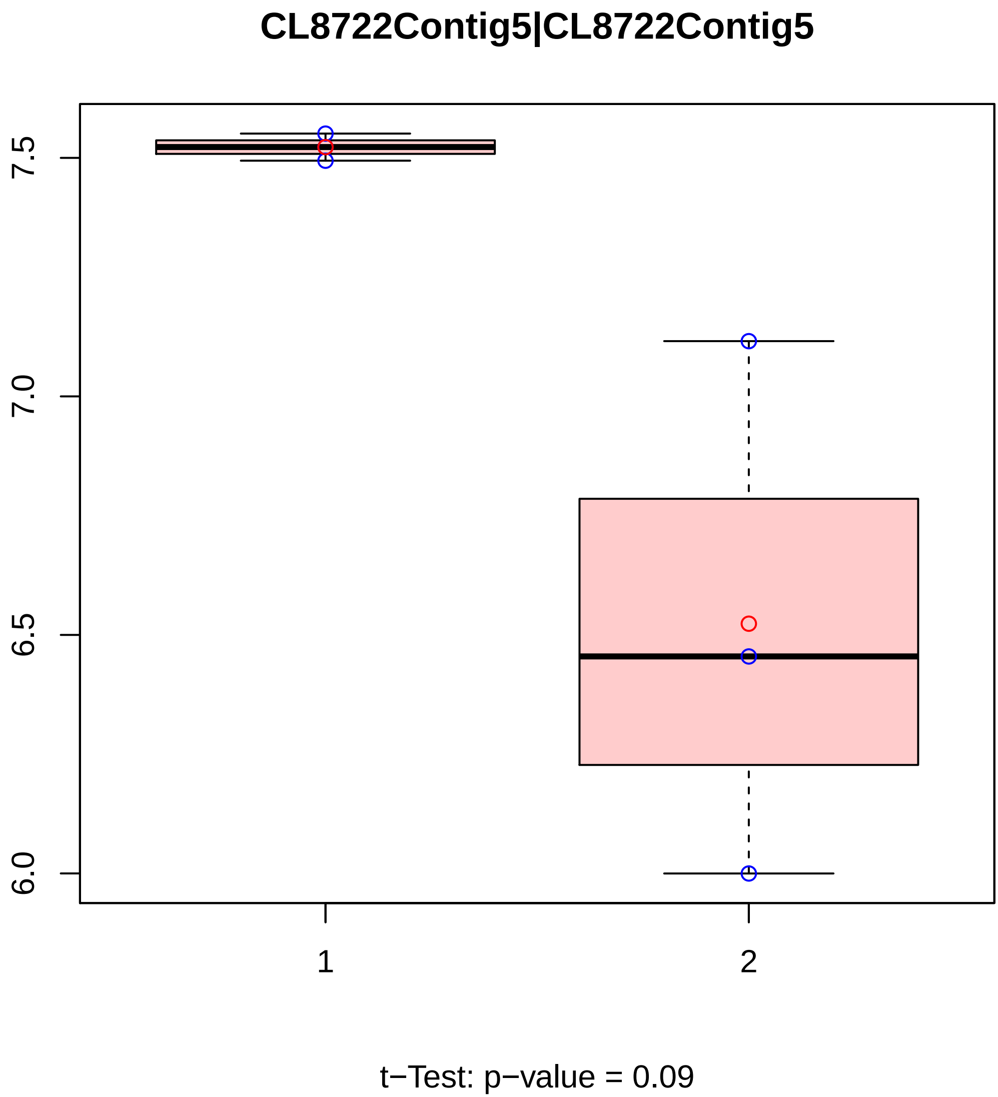
CL8722Contig5|CL8722Contig5
7.5
7.0
6.5
6.0
1
2
t−Test: p−value = 0.09

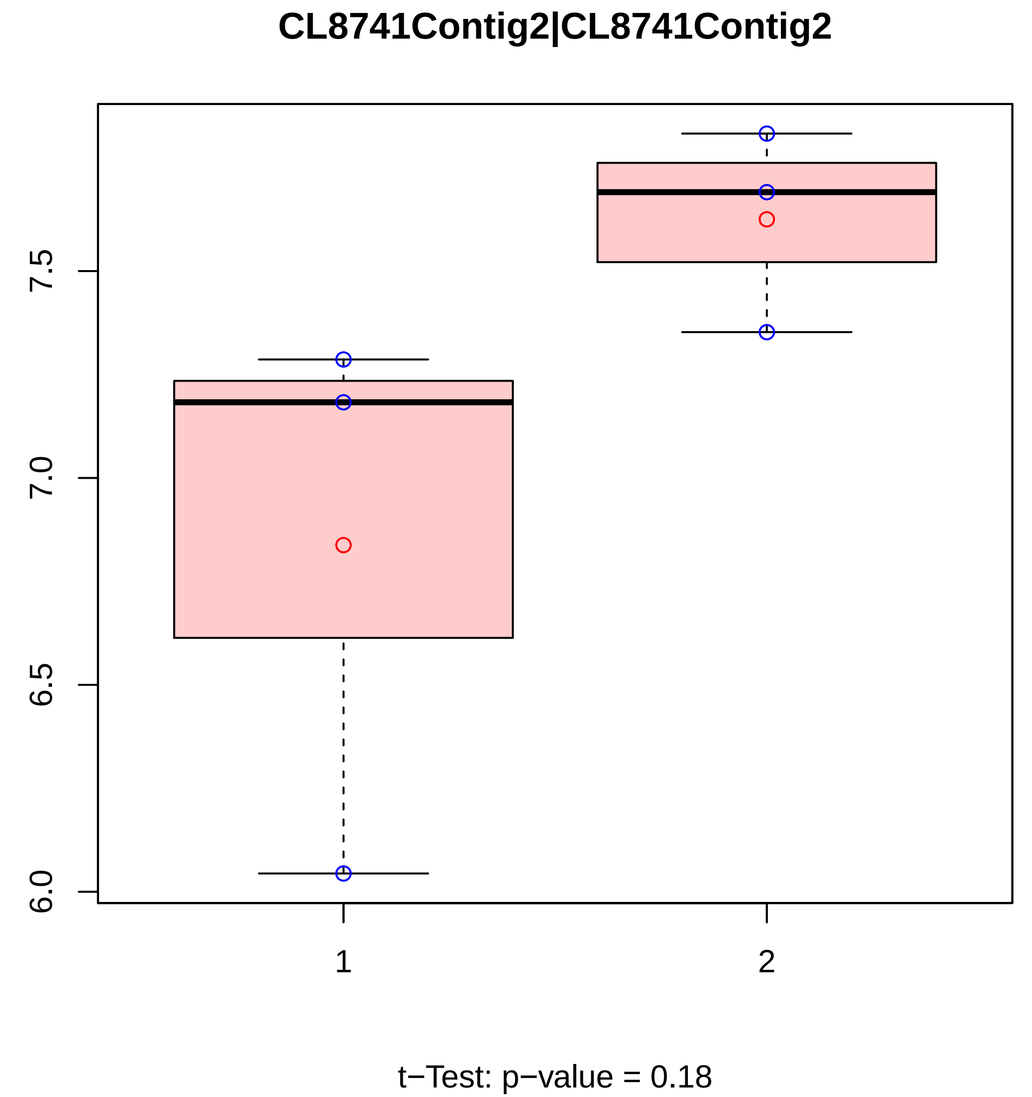
CL8741Contig2|CL8741Contig2
7.5
7.0
6.5
6.0
1
2
t−Test: p−value = 0.18

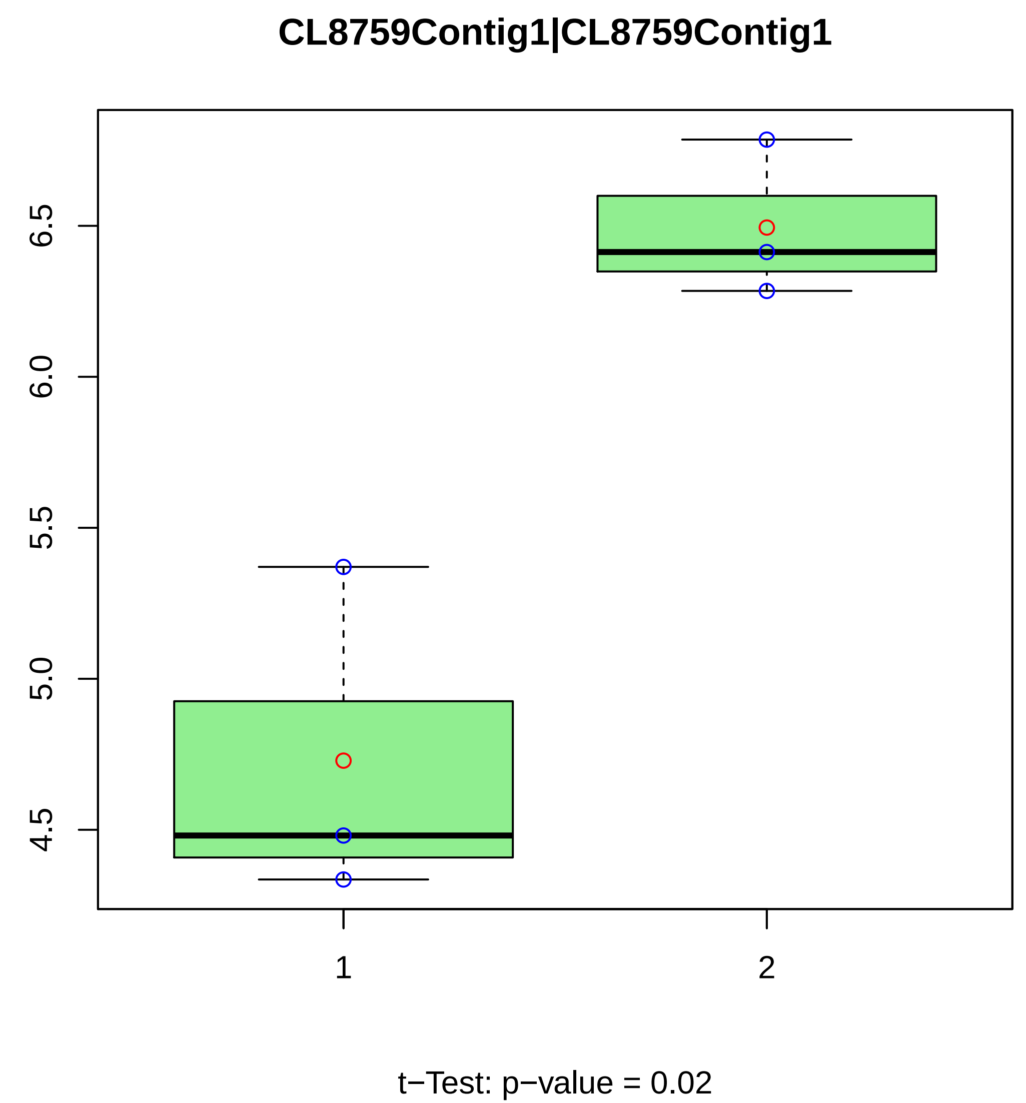
CL8759Contig1|CL8759Contig1
6.5
6.0
5.5
5.0
4.5
1
2
t−Test: p−value = 0.02

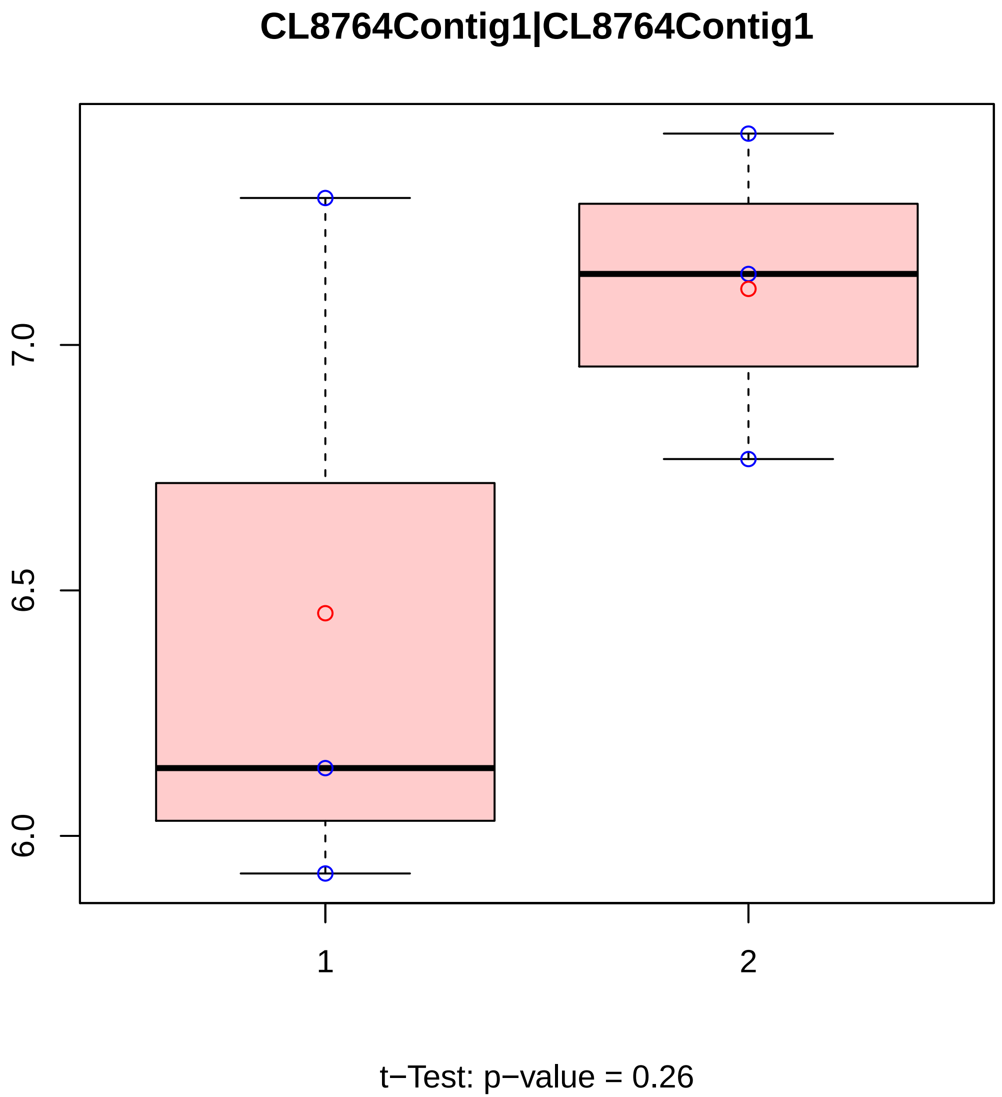
CL8764Contig1|CL8764Contig1
7.0
6.5
6.0
1
2
t−Test: p−value = 0.26

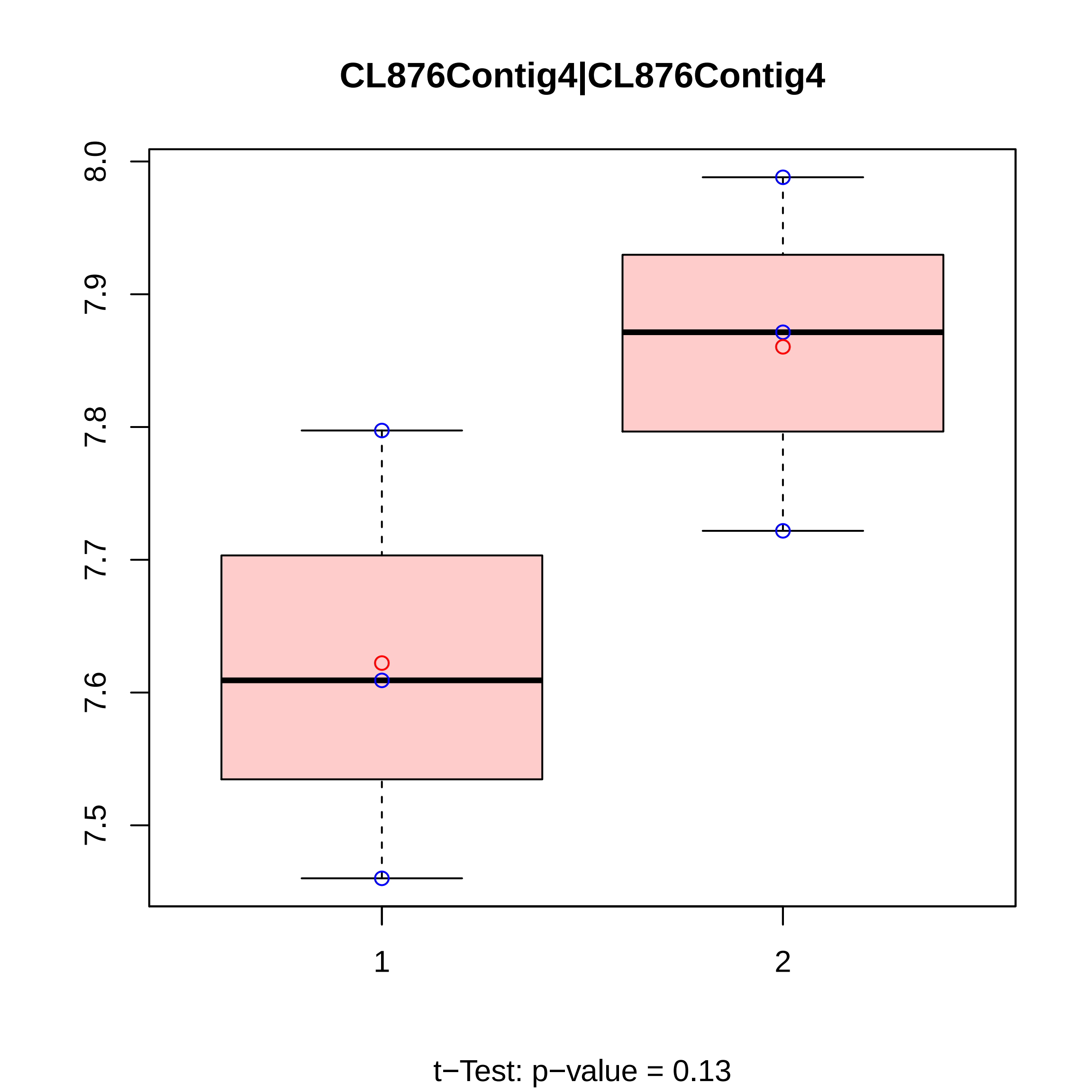

CL876Contig4|CL876Contig4
8.0
7.9
7.8
7.7
7.6
7.5
1
2
t−Test: p−value = 0.13

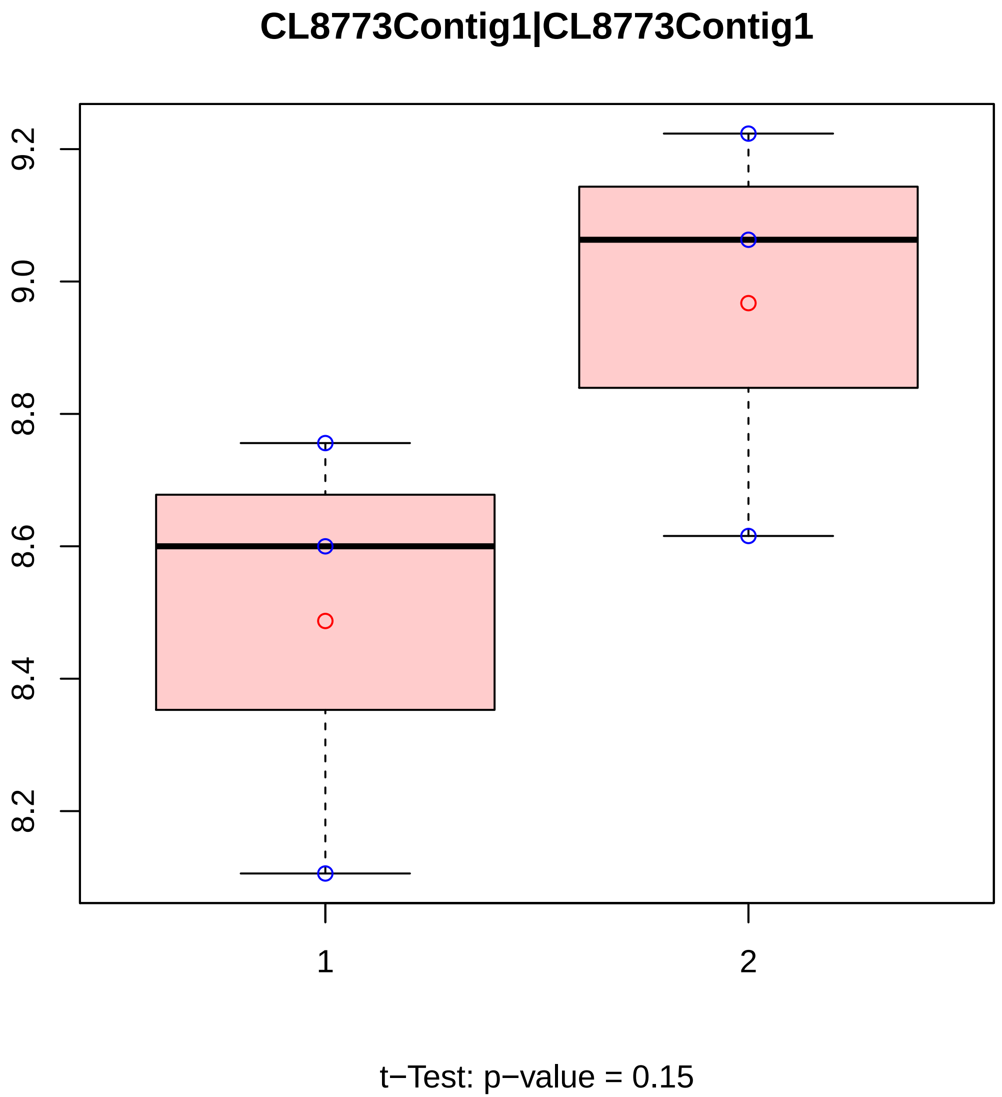
CL8773Contig1|CL8773Contig1
9.2
9.0
8.8
8.6
8.4
8.2
1
2
t−Test: p−value = 0.15

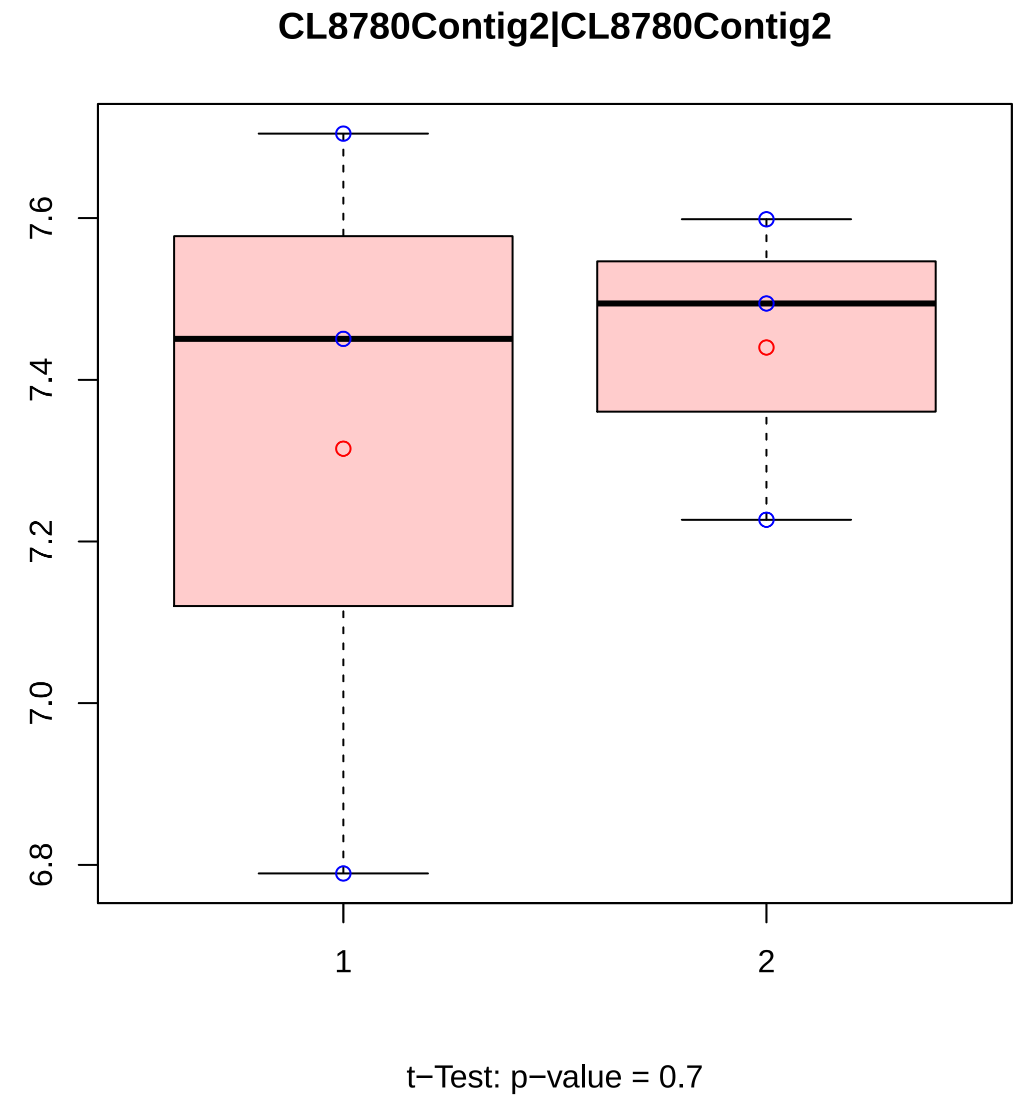

CL8780Contig2|CL8780Contig2
6.8
7.0
7.2
7.4
7.6
1
2
t−Test: p−value = 0.7

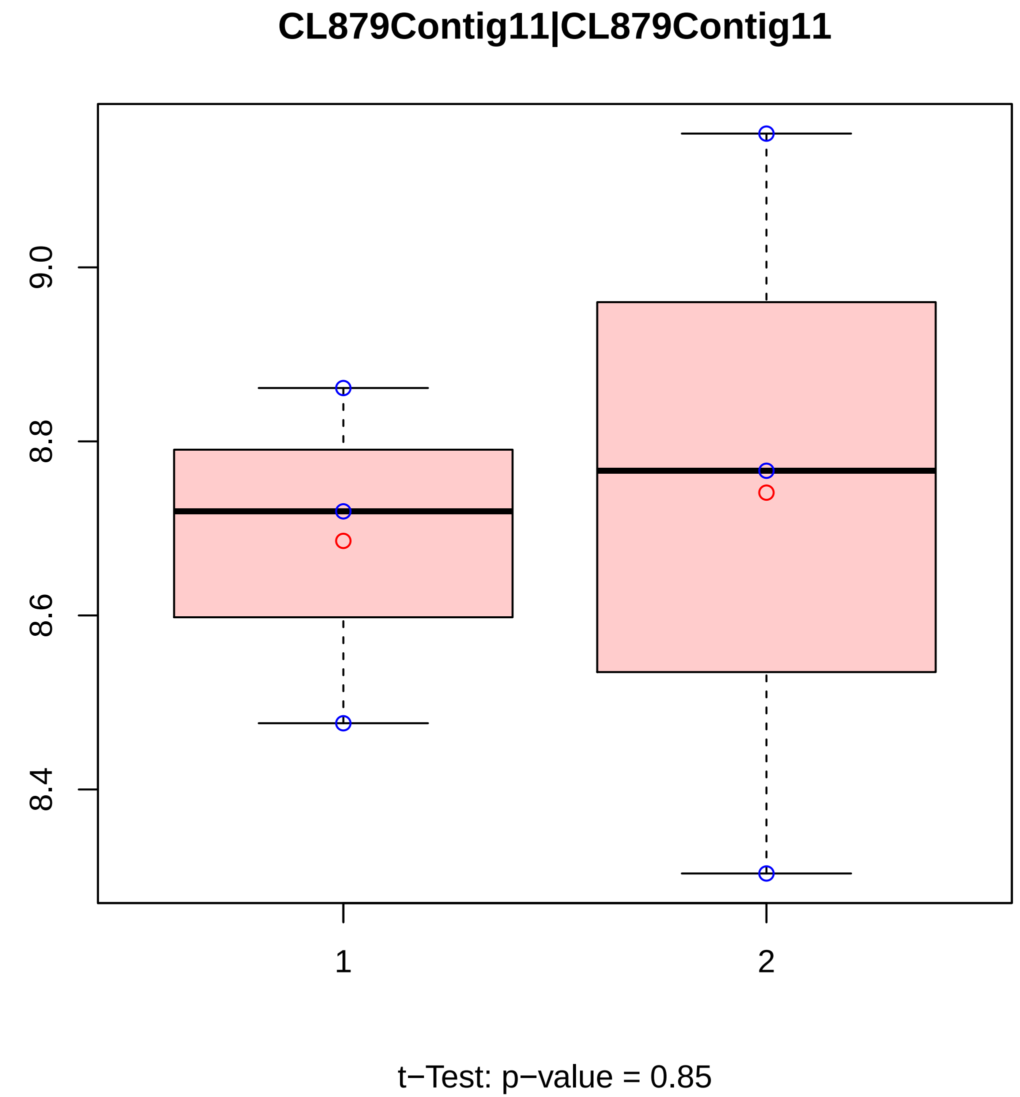

CL879Contig11|CL879Contig11
9.0
8.8
8.6
8.4
1
2
t−Test: p−value = 0.85

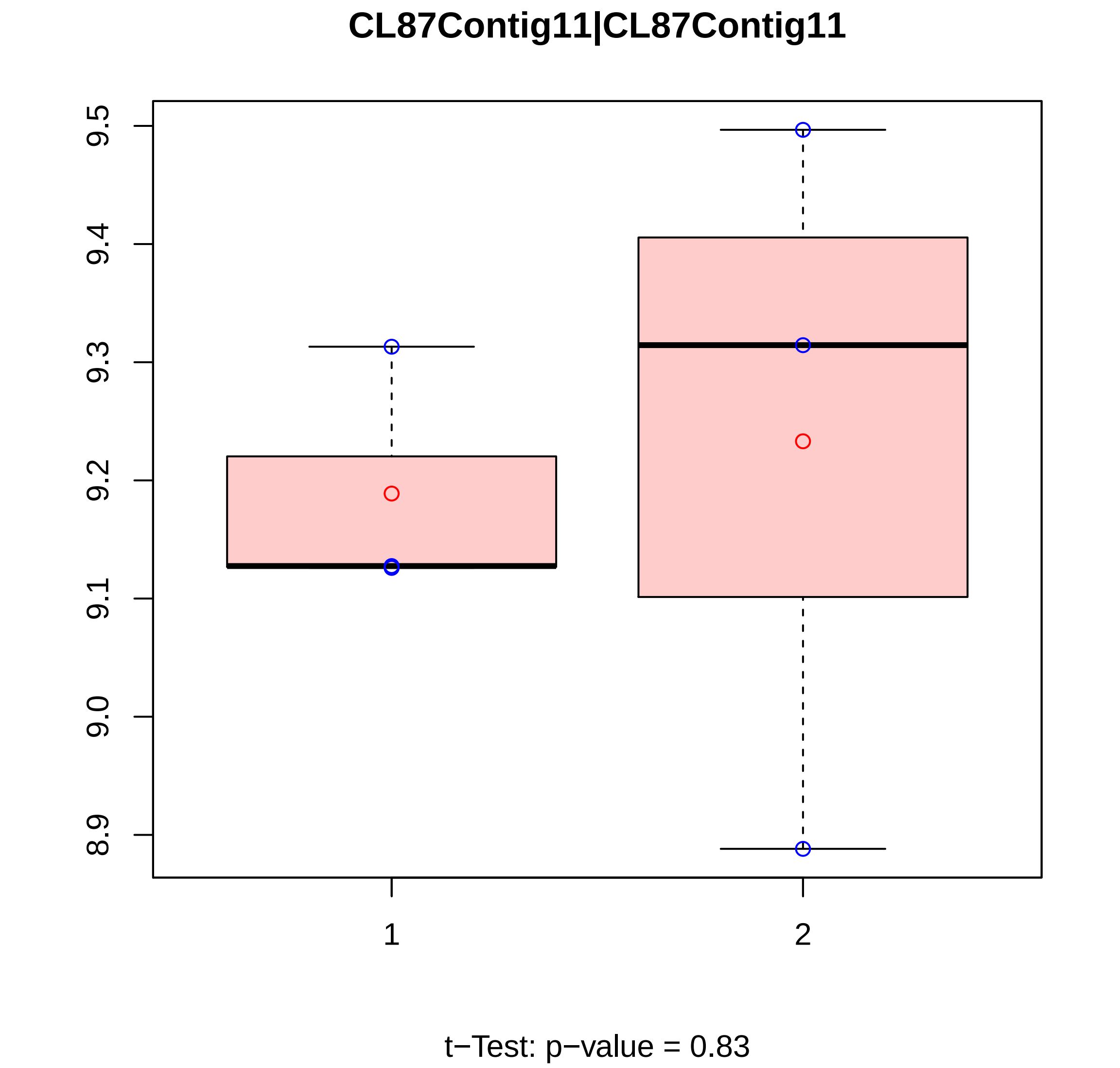
CL87Contig11|CL87Contig11
9.5
9.4
9.3
9.2
9.1
9.0
8.9
1
2
t−Test: p−value = 0.83

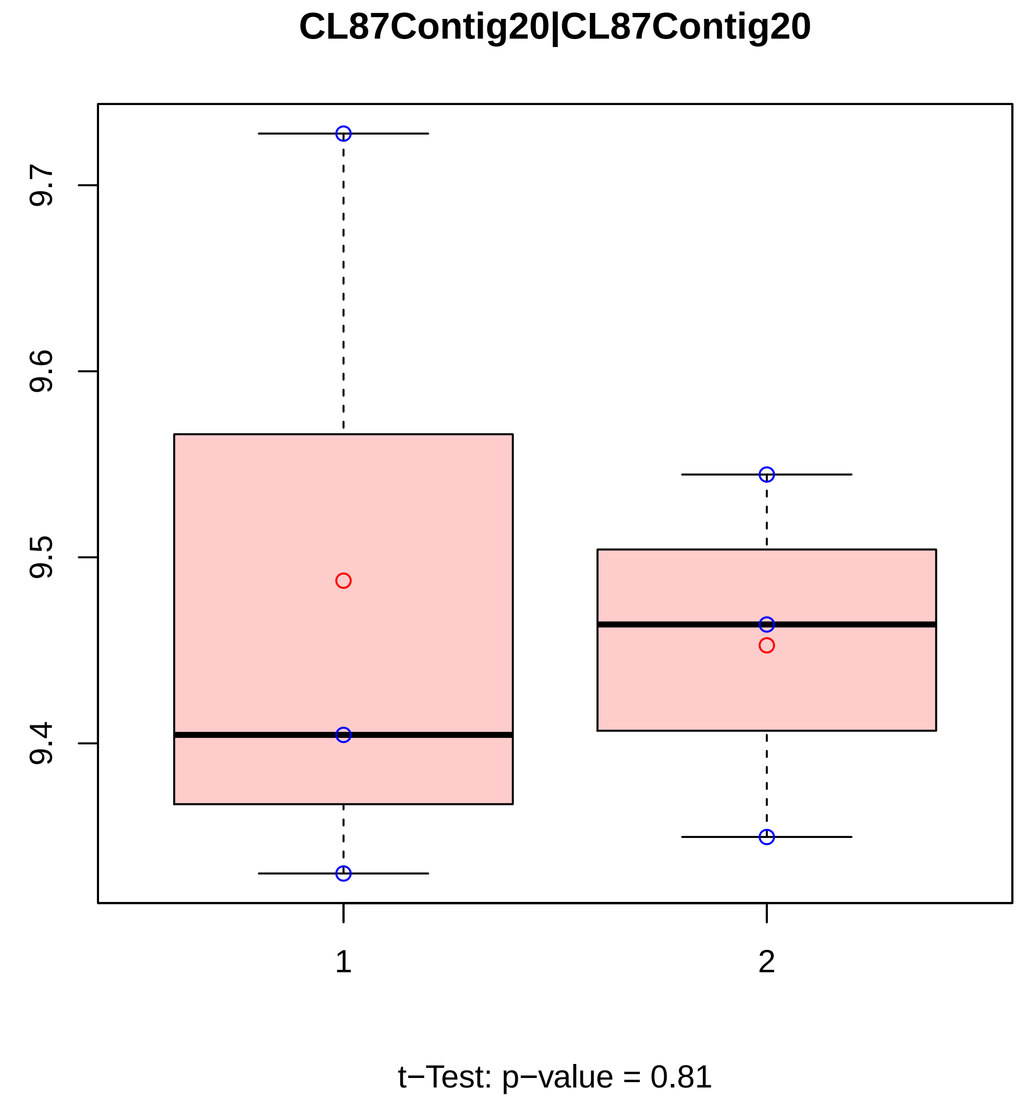

CL87Contig20|CL87Contig20
9.4
9.5
9.6
9.7
1
2
t−Test: p−value = 0.81

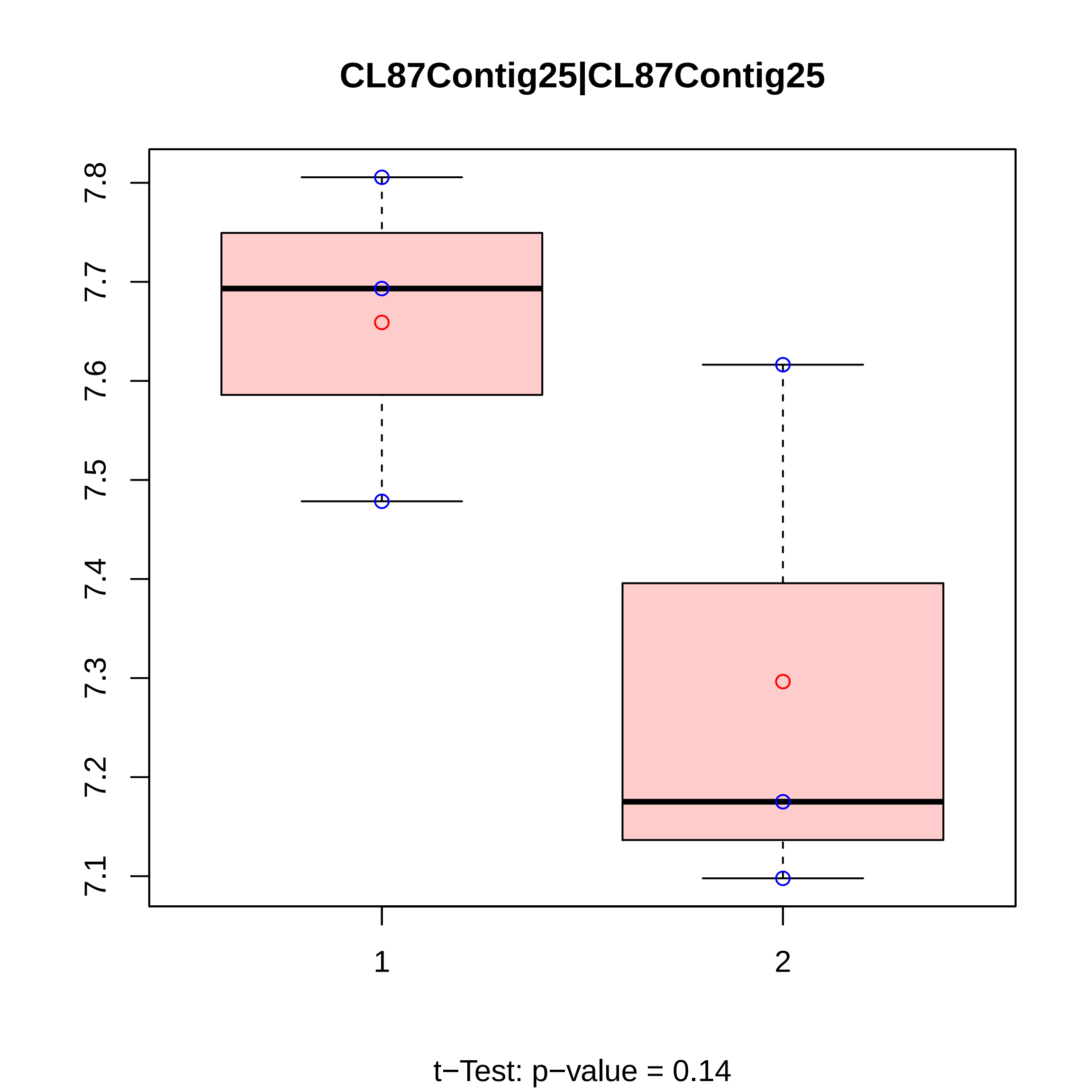

CL87Contig25|CL87Contig25
7.1
7.2
7.3
7.4
7.5
7.6
7.7
7.8
1
2
t−Test: p−value = 0.14

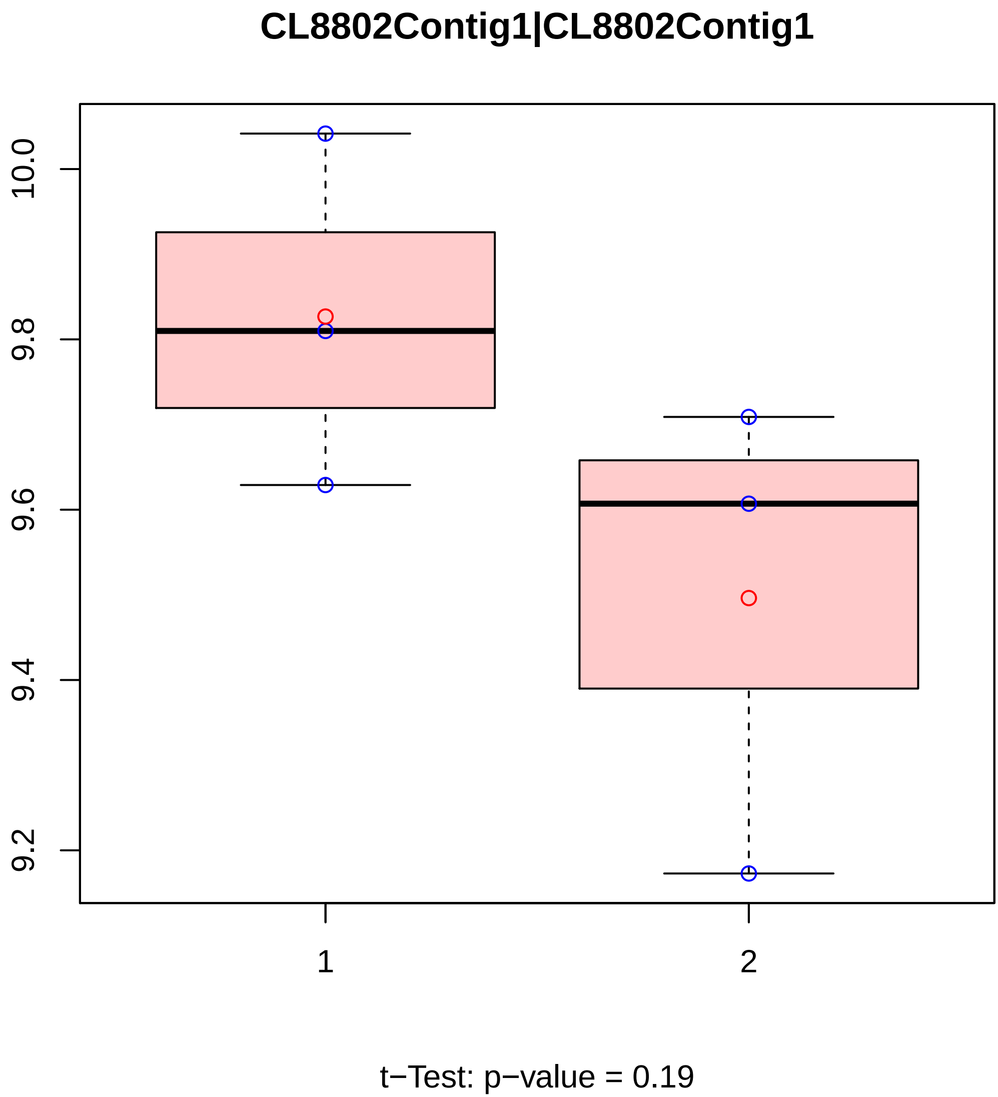
CL8802Contig1|CL8802Contig1
10.0
9.8
9.6
9.4
9.2
1
2
t−Test: p−value = 0.19

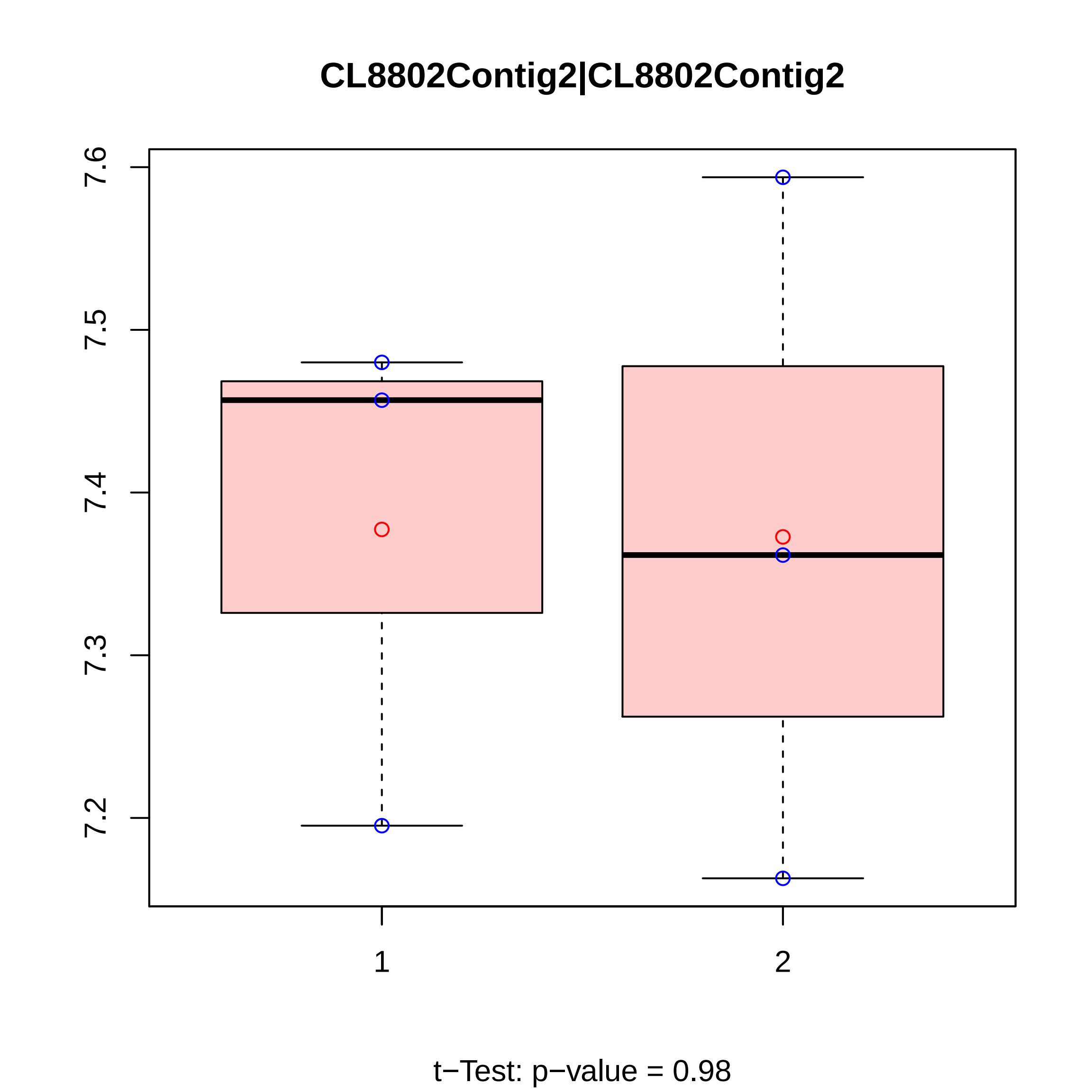
CL8802Contig2|CL8802Contig2
7.6
7.5
7.4
7.3
7.2
1
2
t−Test: p−value = 0.98

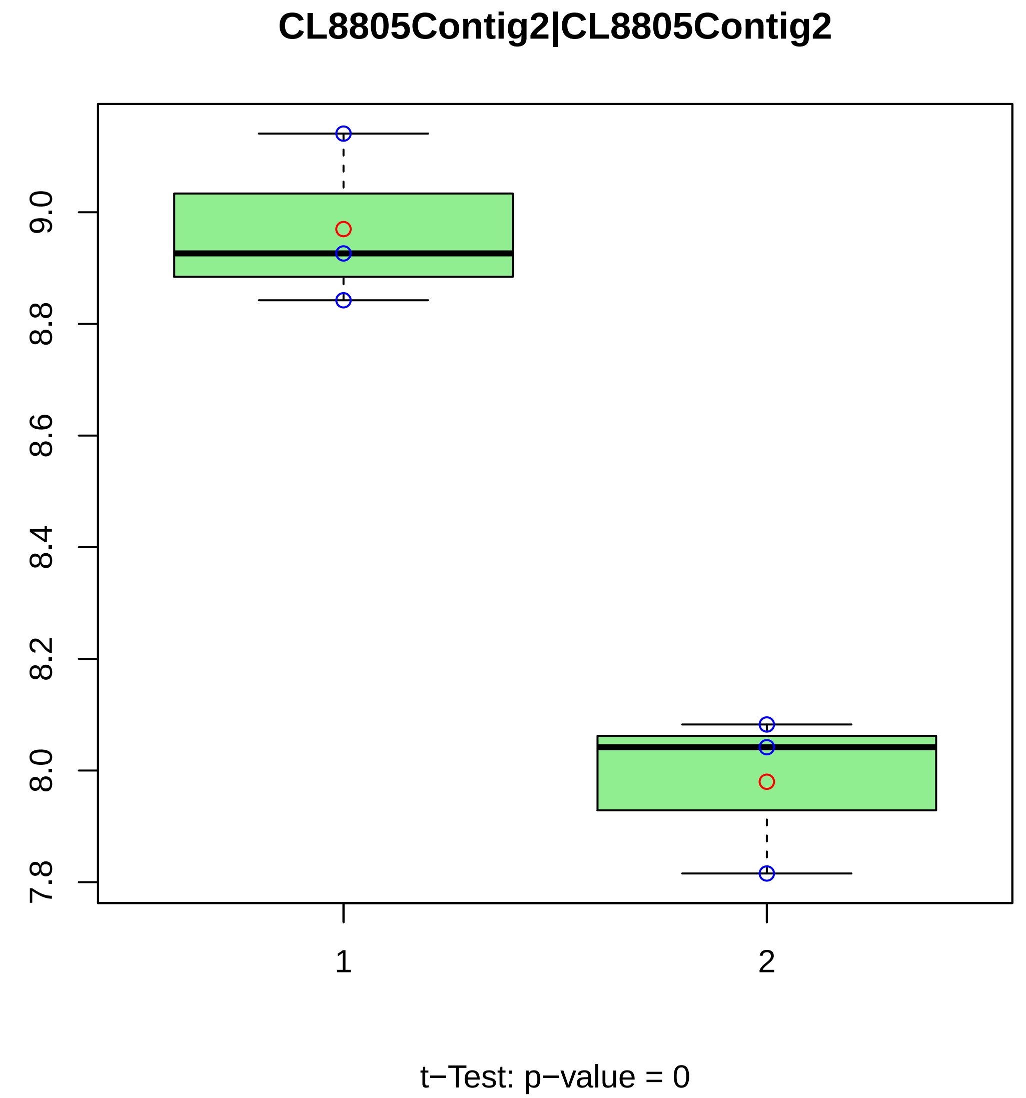

CL8805Contig2|CL8805Contig2
9.0
8.8
8.6
8.4
8.2
8.0
7.8
1
2
t−Test: p−value = 0

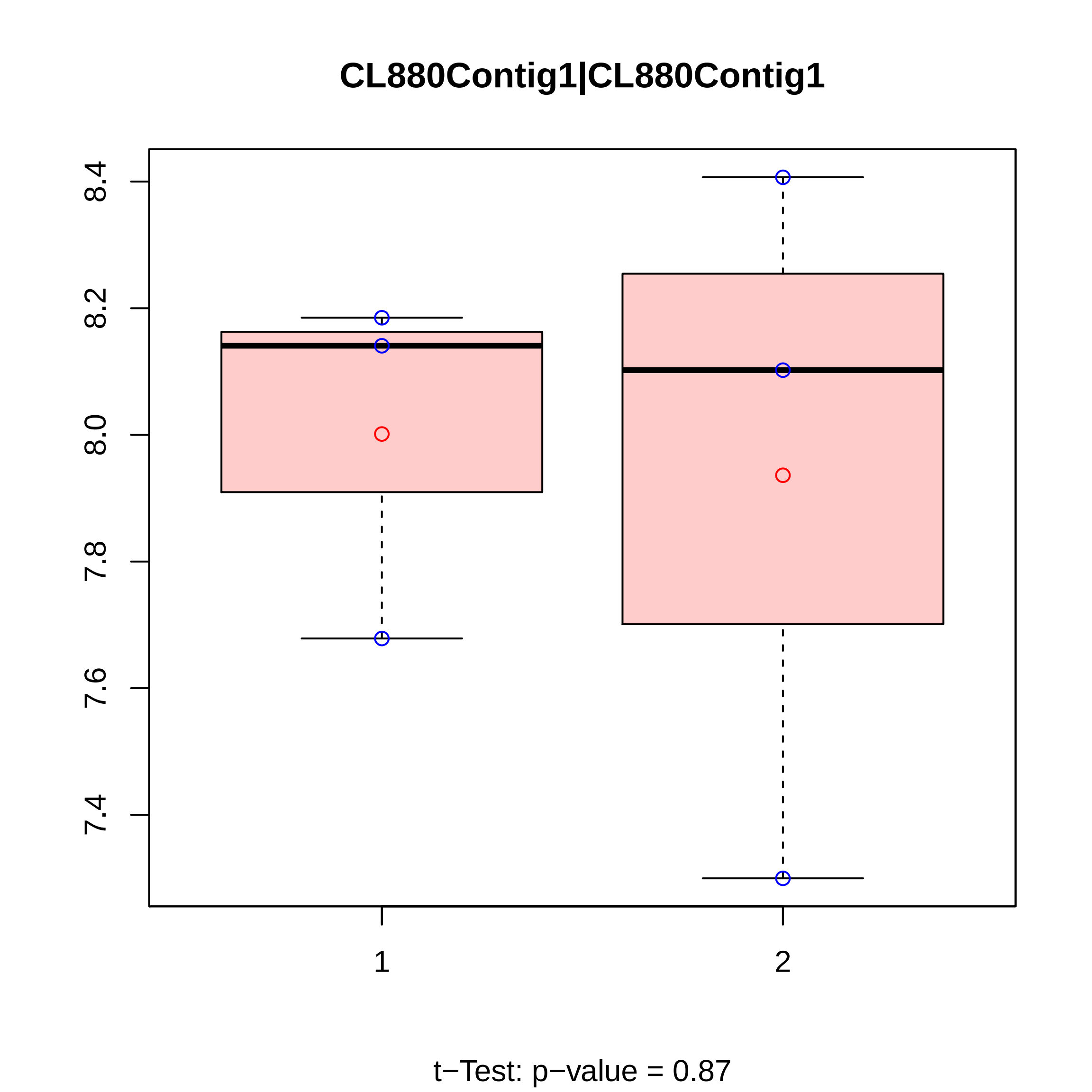

CL880Contig1|CL880Contig1
8.4
8.2
8.0
7.8
7.6
7.4
1
2
t−Test: p−value = 0.87

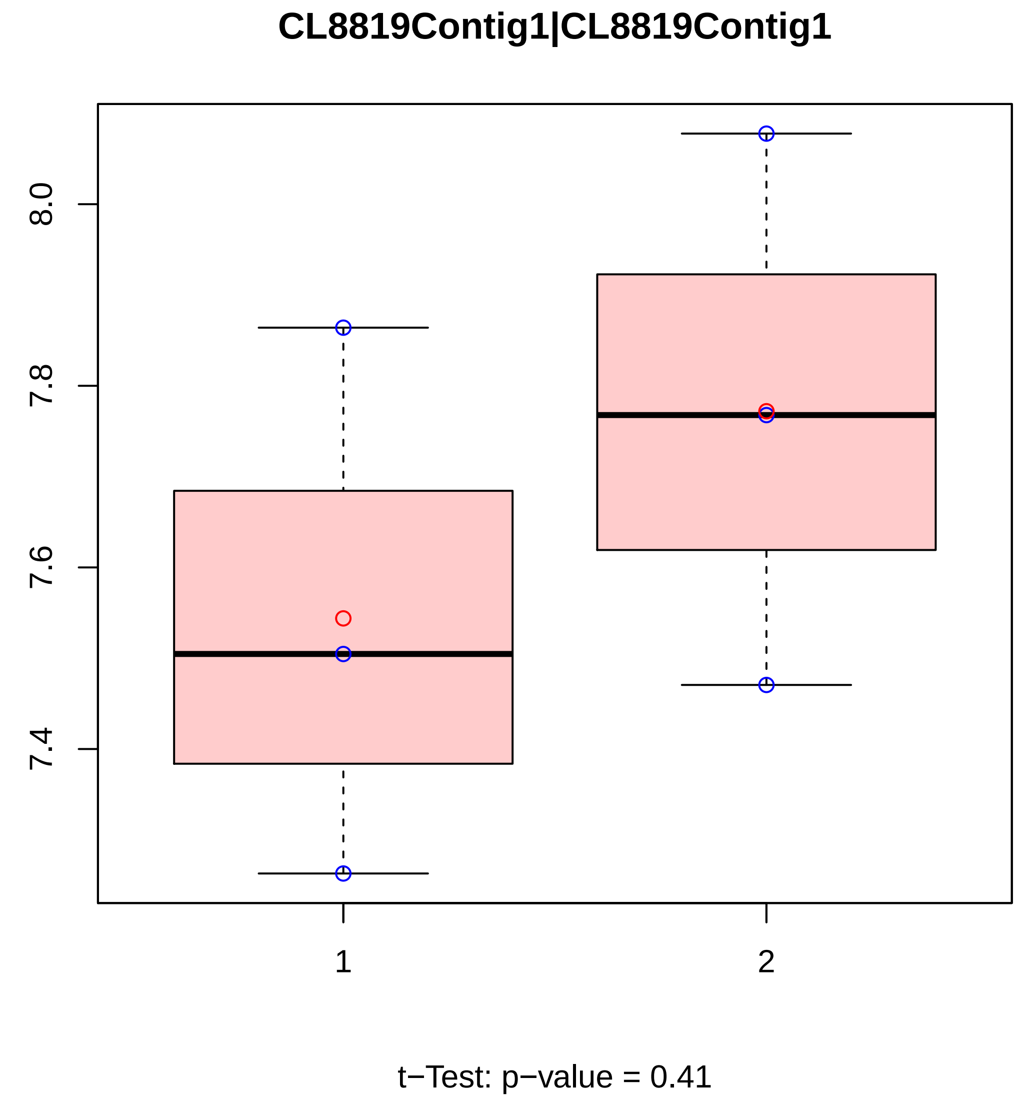

CL8819Contig1|CL8819Contig1
8.0
7.8
7.6
7.4
1
2
t−Test: p−value = 0.41

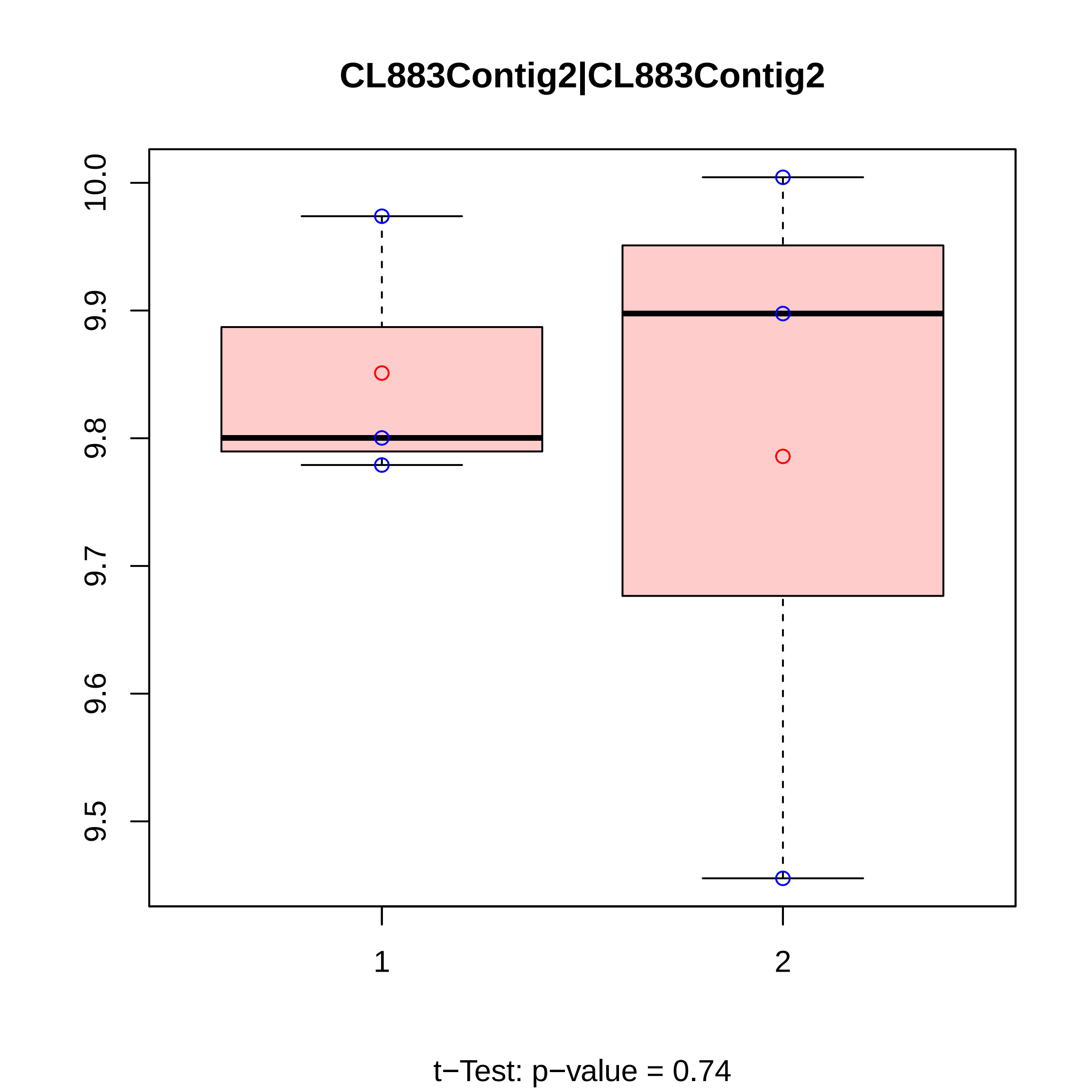
CL883Contig2|CL883Contig2
10.0
9.9
9.8
9.7
9.6
9.5
1
2
t−Test: p−value = 0.74

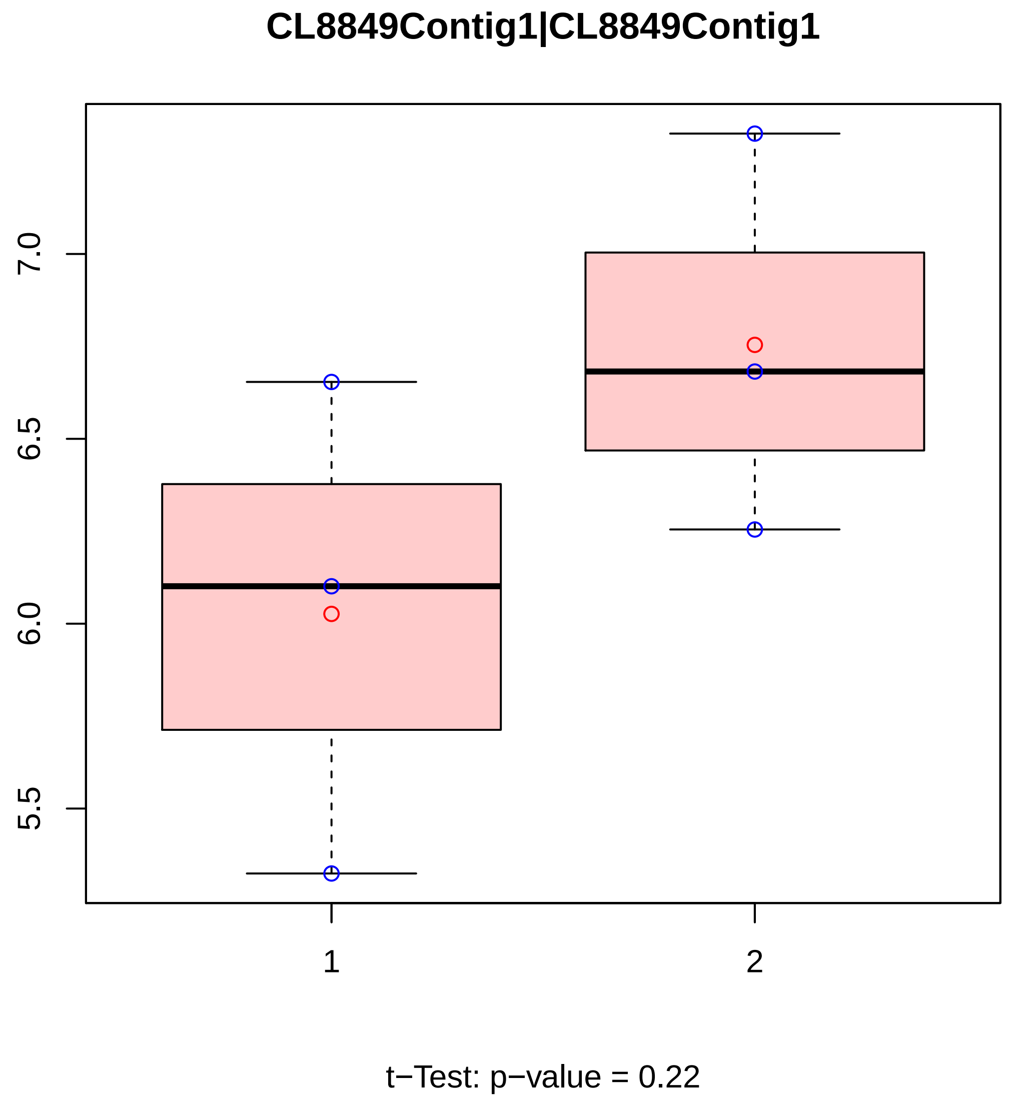
CL8849Contig1|CL8849Contig1
7.0
6.5
6.0
5.5
1
2
t−Test: p−value = 0.22

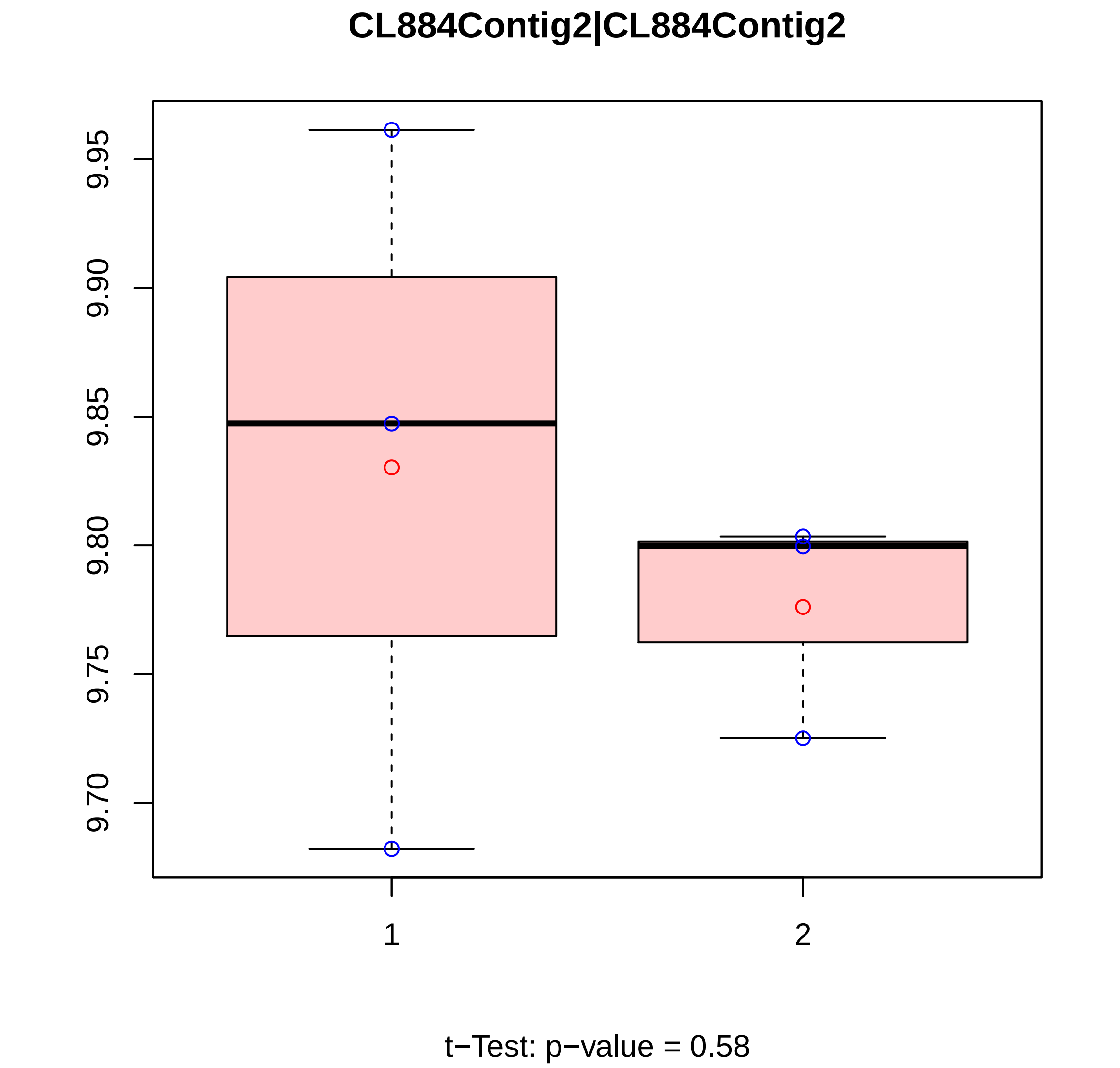
CL884Contig2|CL884Contig2
9.70
9.75
9.80
9.85
9.90
9.95
1
2
t−Test: p−value = 0.58

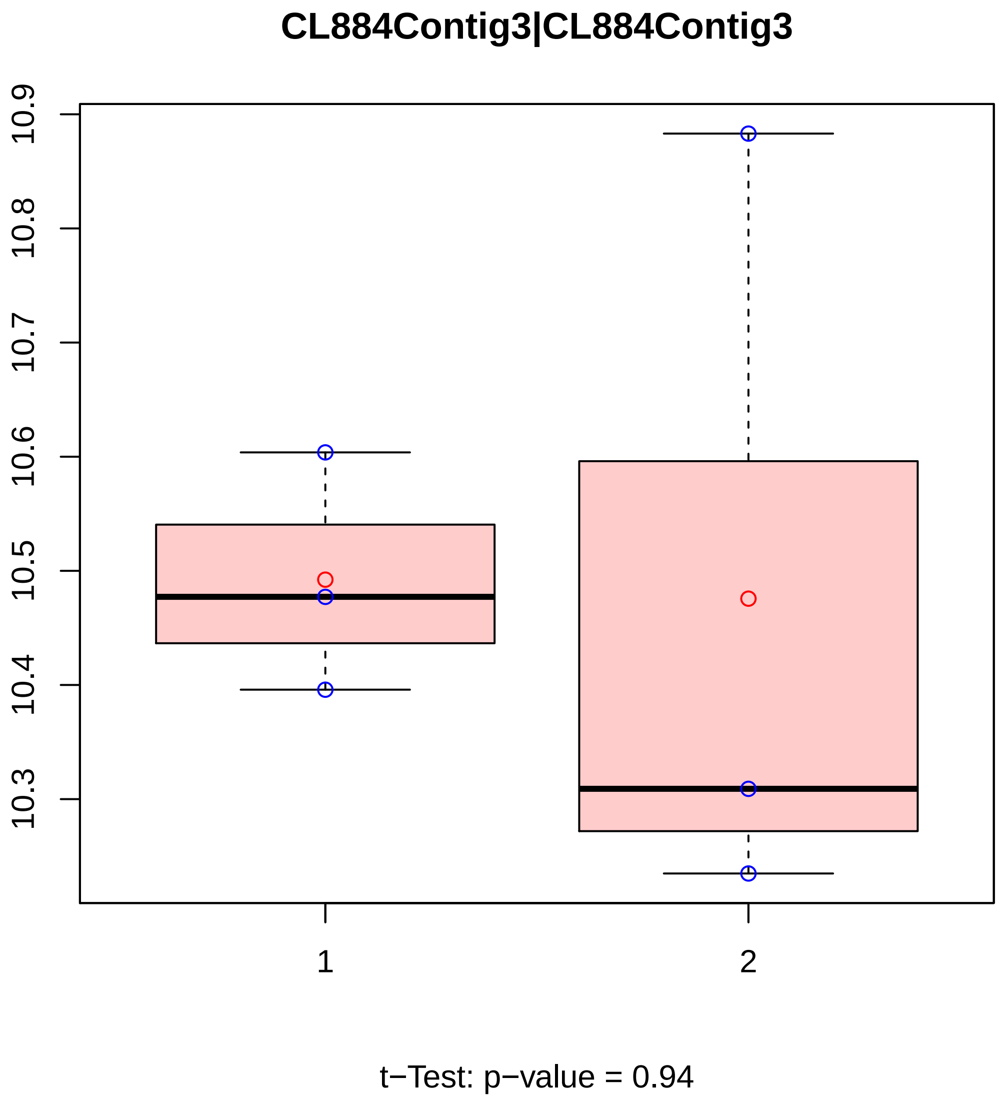

CL884Contig3|CL884Contig3
10.9
10.8
10.7
10.6
10.5
10.4
10.3
1
2
t−Test: p−value = 0.94

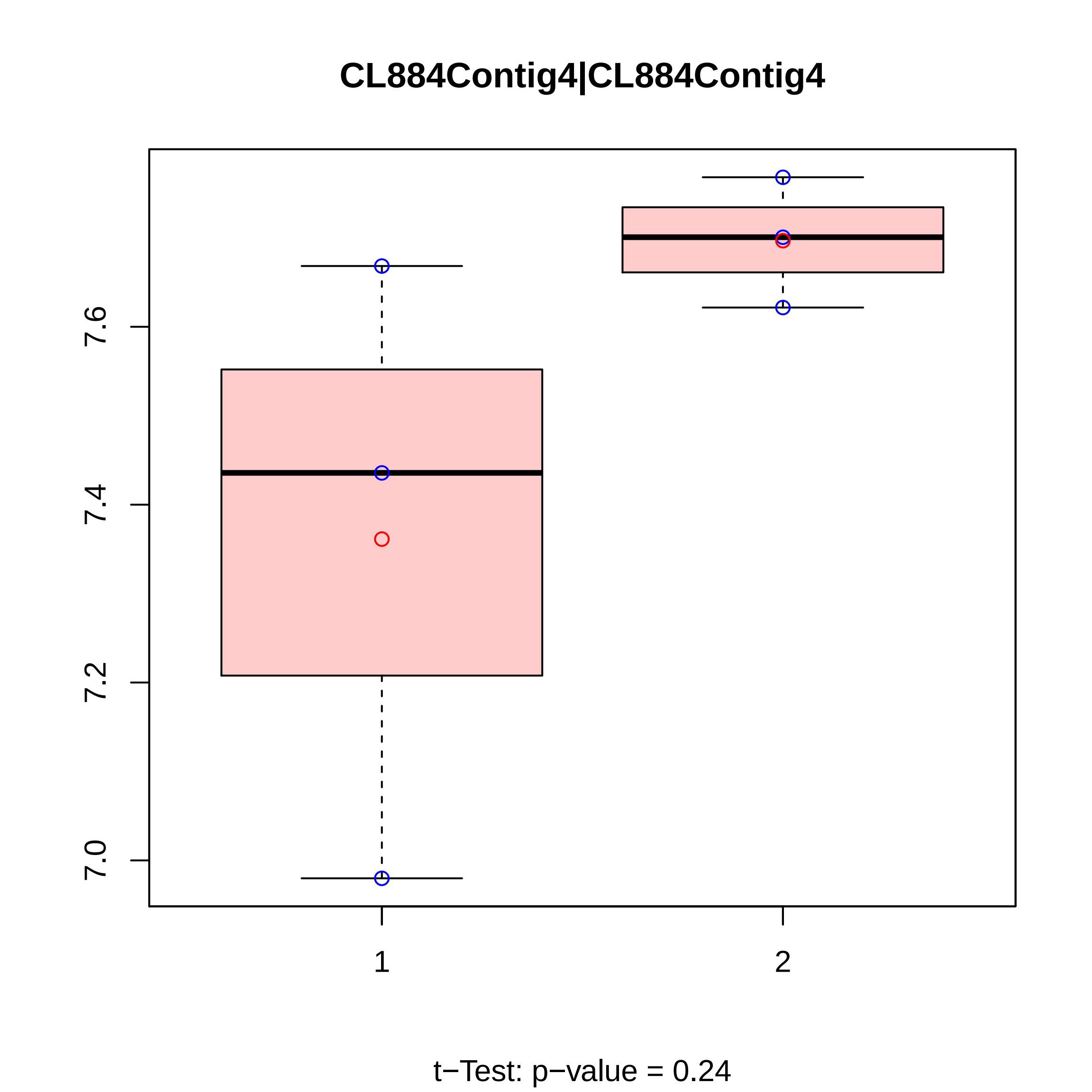
CL884Contig4|CL884Contig4
7.0
7.2
7.4
7.6
1
2
t−Test: p−value = 0.24

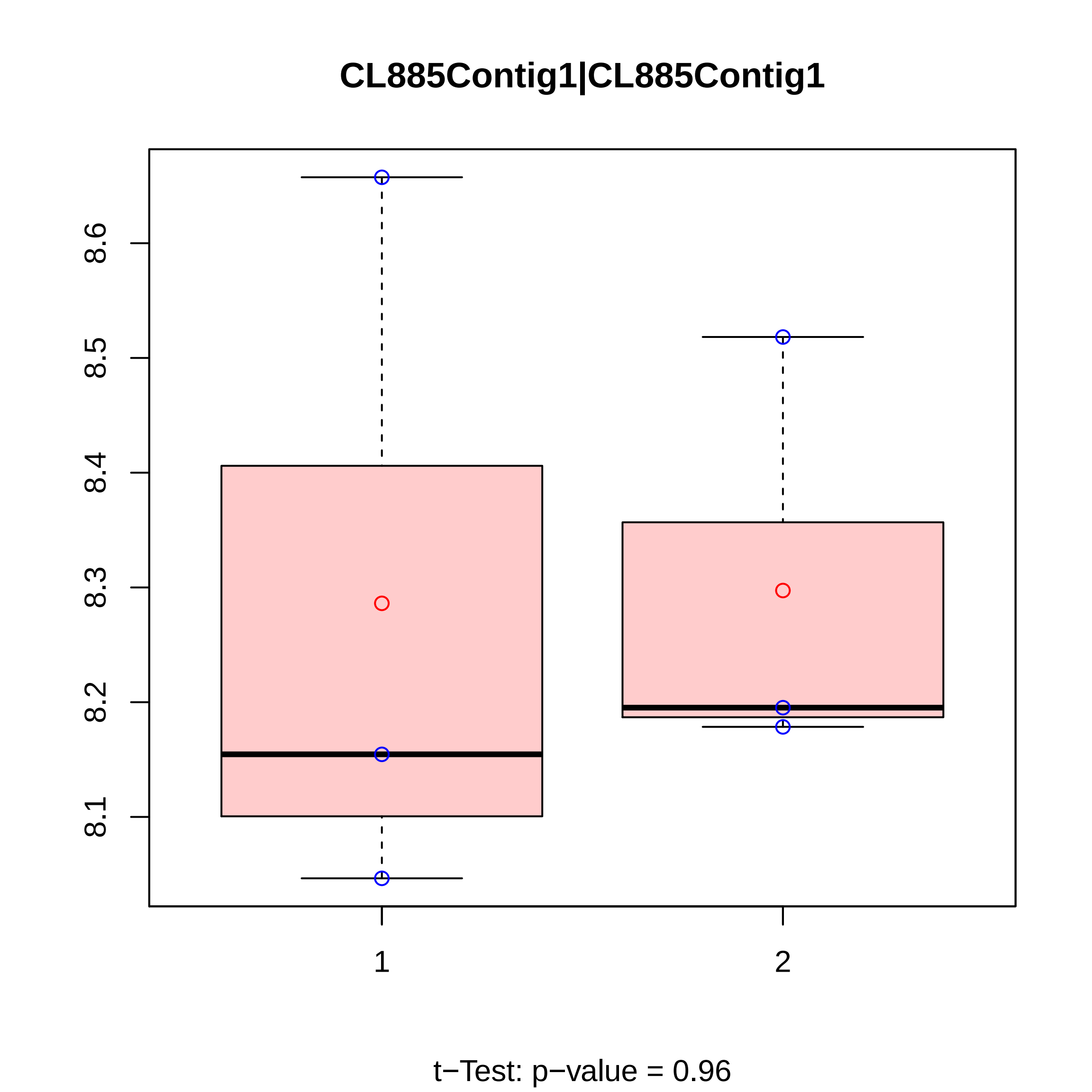

CL885Contig1|CL885Contig1
8.1
8.2
8.3
8.4
8.5
8.6
1
2
t−Test: p−value = 0.96

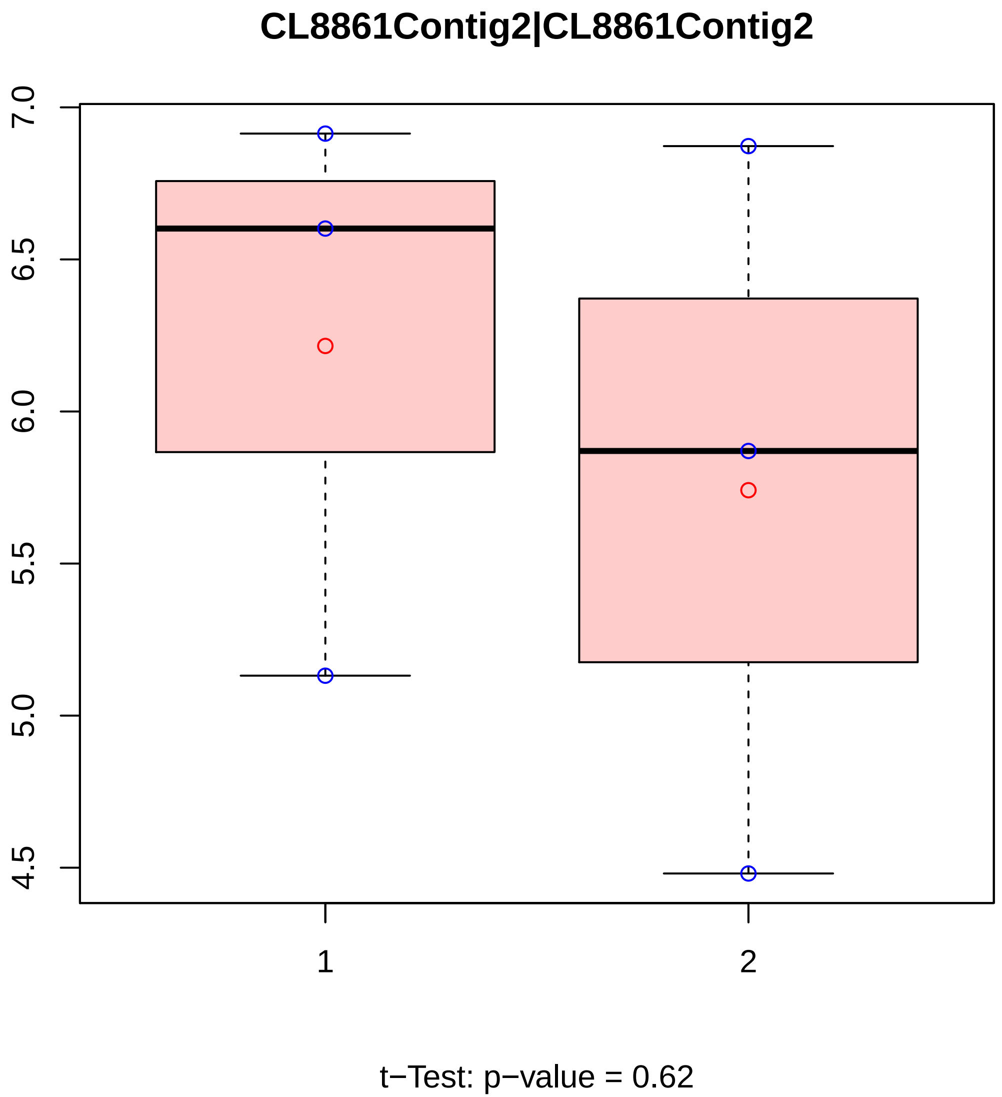
CL8861Contig2|CL8861Contig2
7.0
6.5
6.0
5.5
5.0
4.5
1
2
t−Test: p−value = 0.62

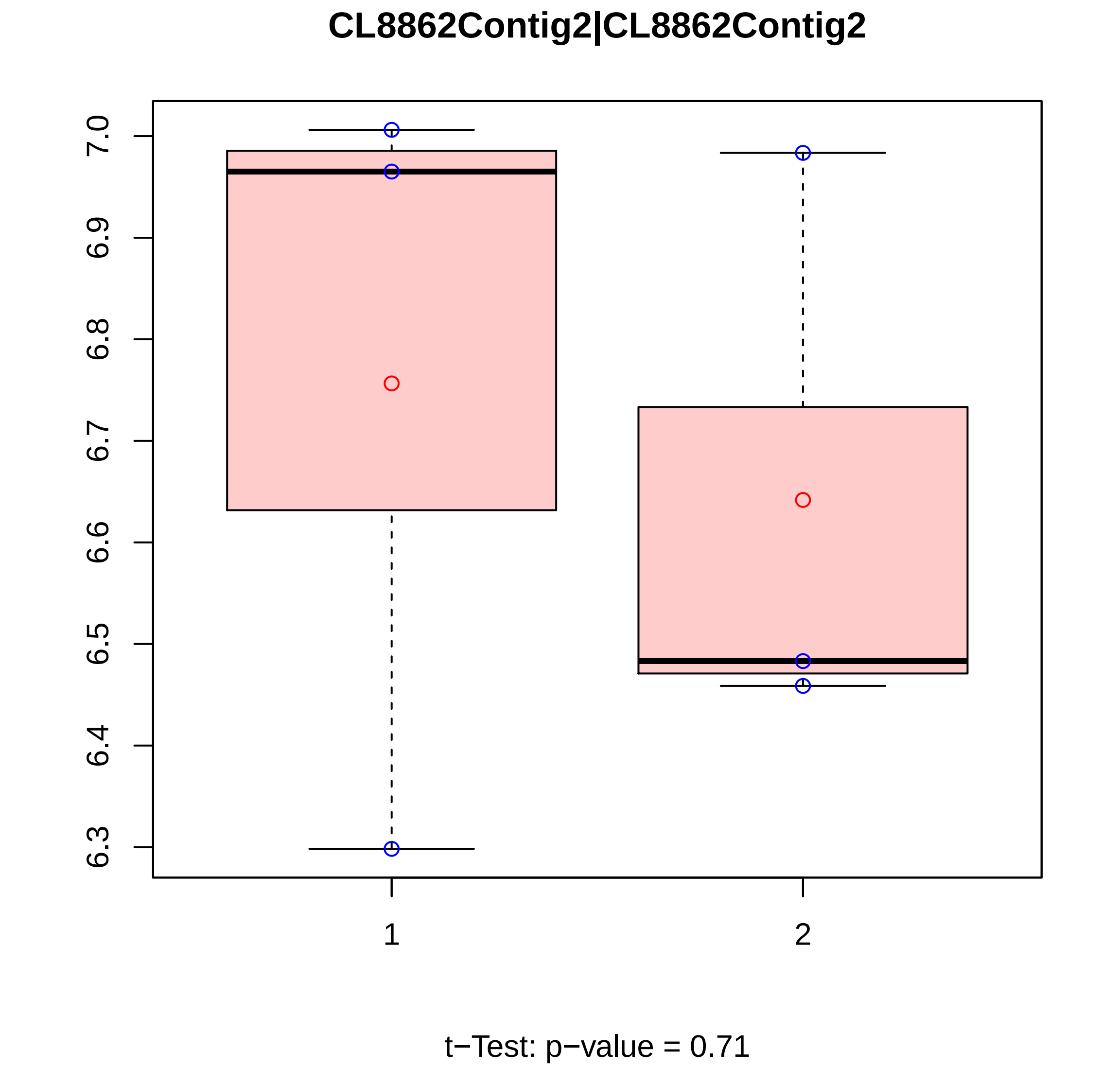
CL8862Contig2|CL8862Contig2
7.0
6.9
6.8
6.7
6.6
6.5
6.4
6.3
1
2
t−Test: p−value = 0.71

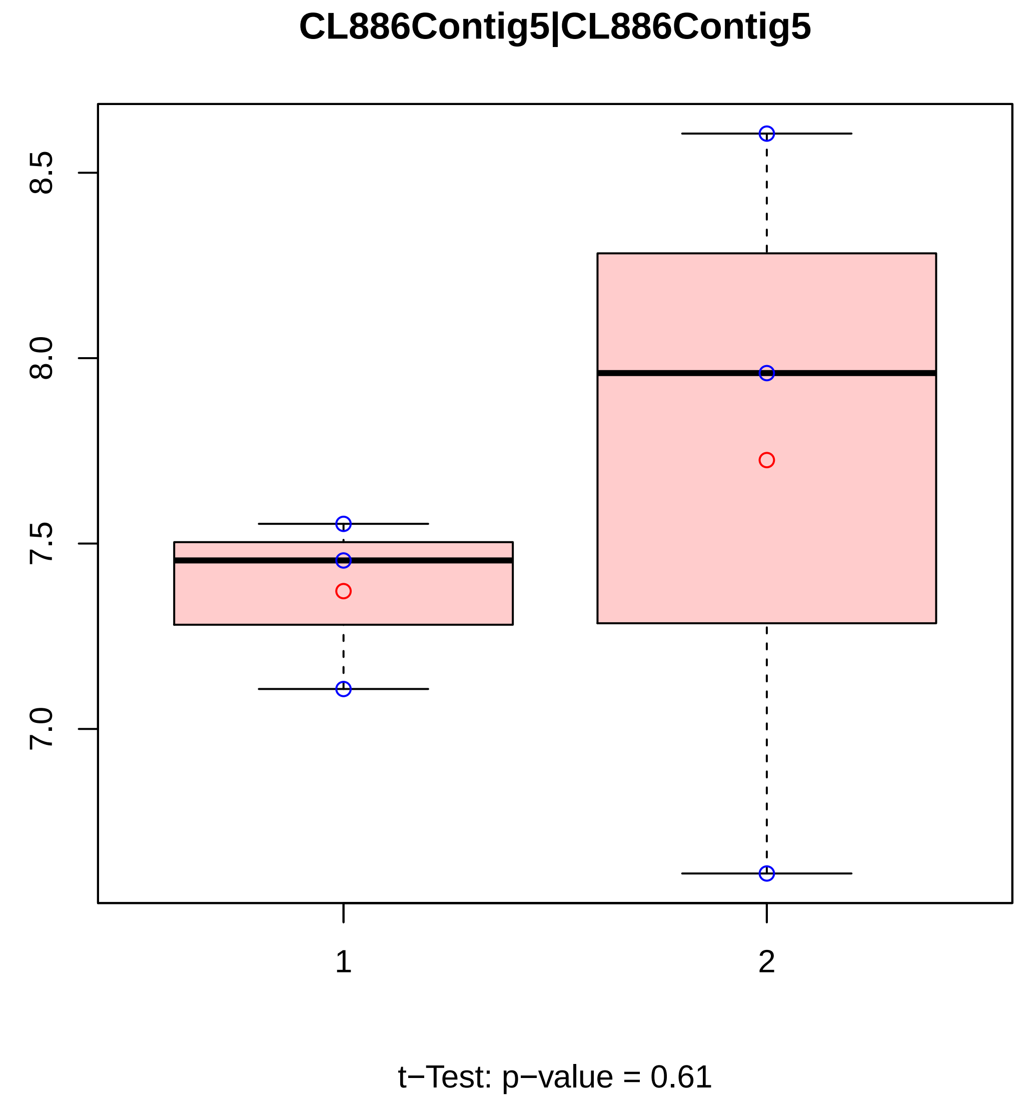
CL886Contig5|CL886Contig5
8.5
8.0
7.5
7.0
1
2
t−Test: p−value = 0.61

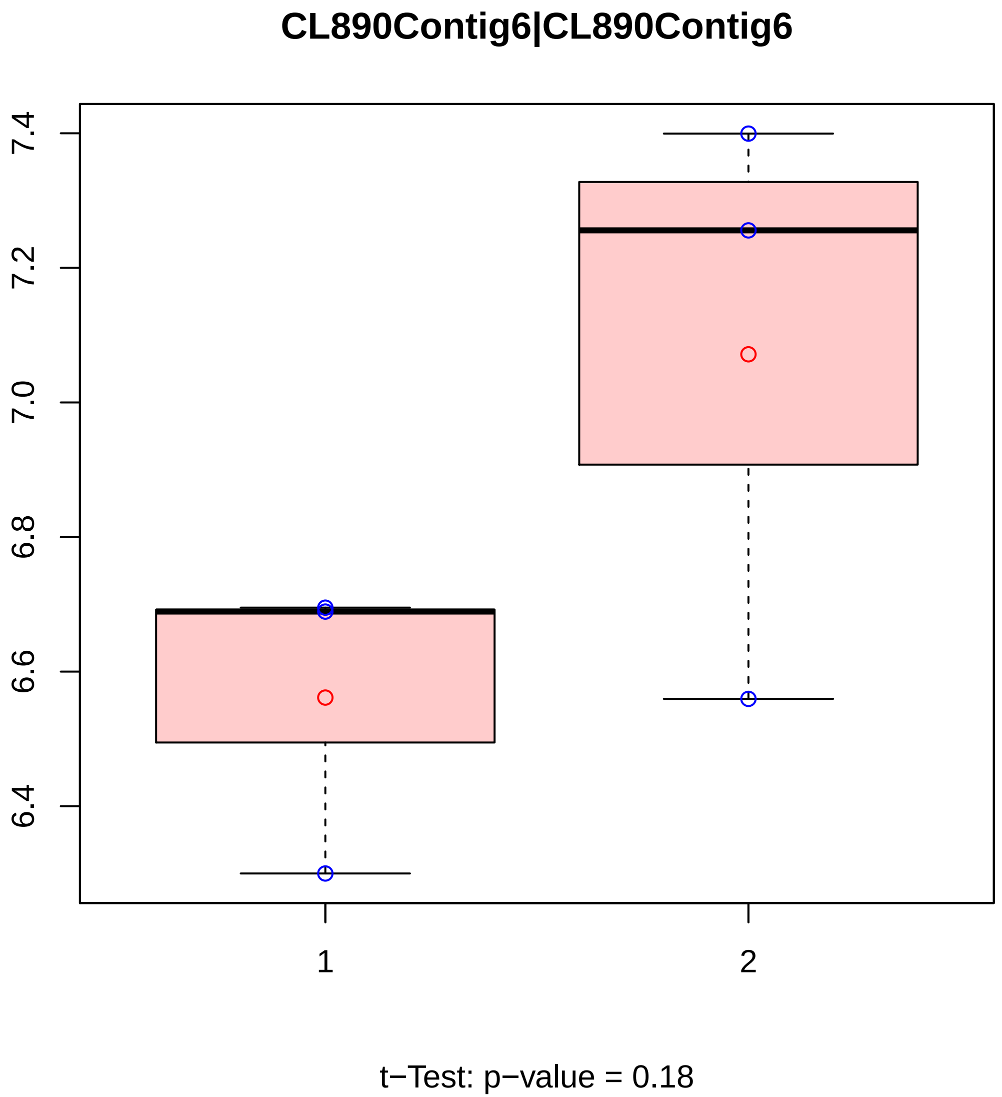
CL890Contig6|CL890Contig6
7.4
7.2
7.0
6.8
6.6
6.4
1
2
t−Test: p−value = 0.18

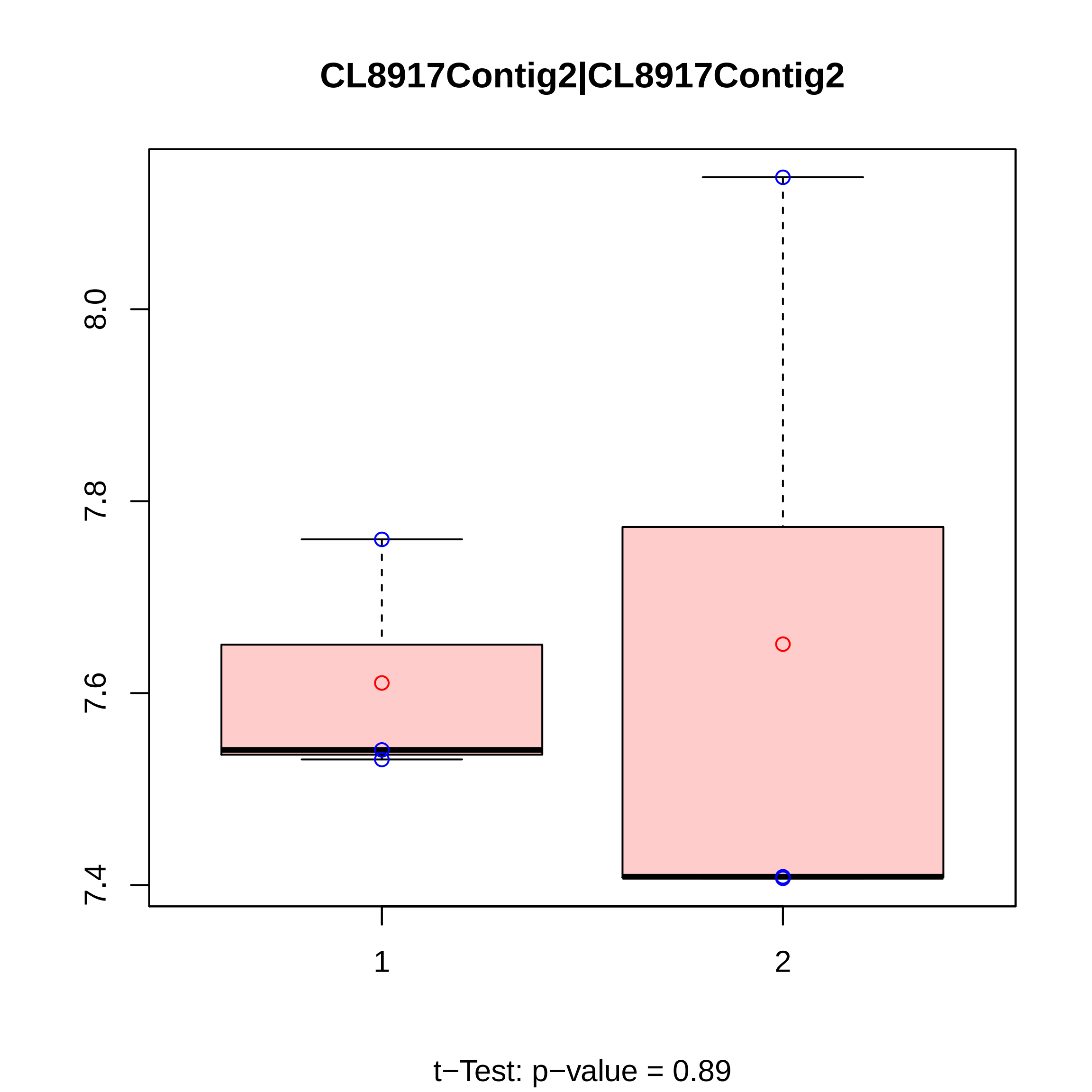
CL8917Contig2|CL8917Contig2
8.0
7.8
7.6
7.4
1
2
t−Test: p−value = 0.89

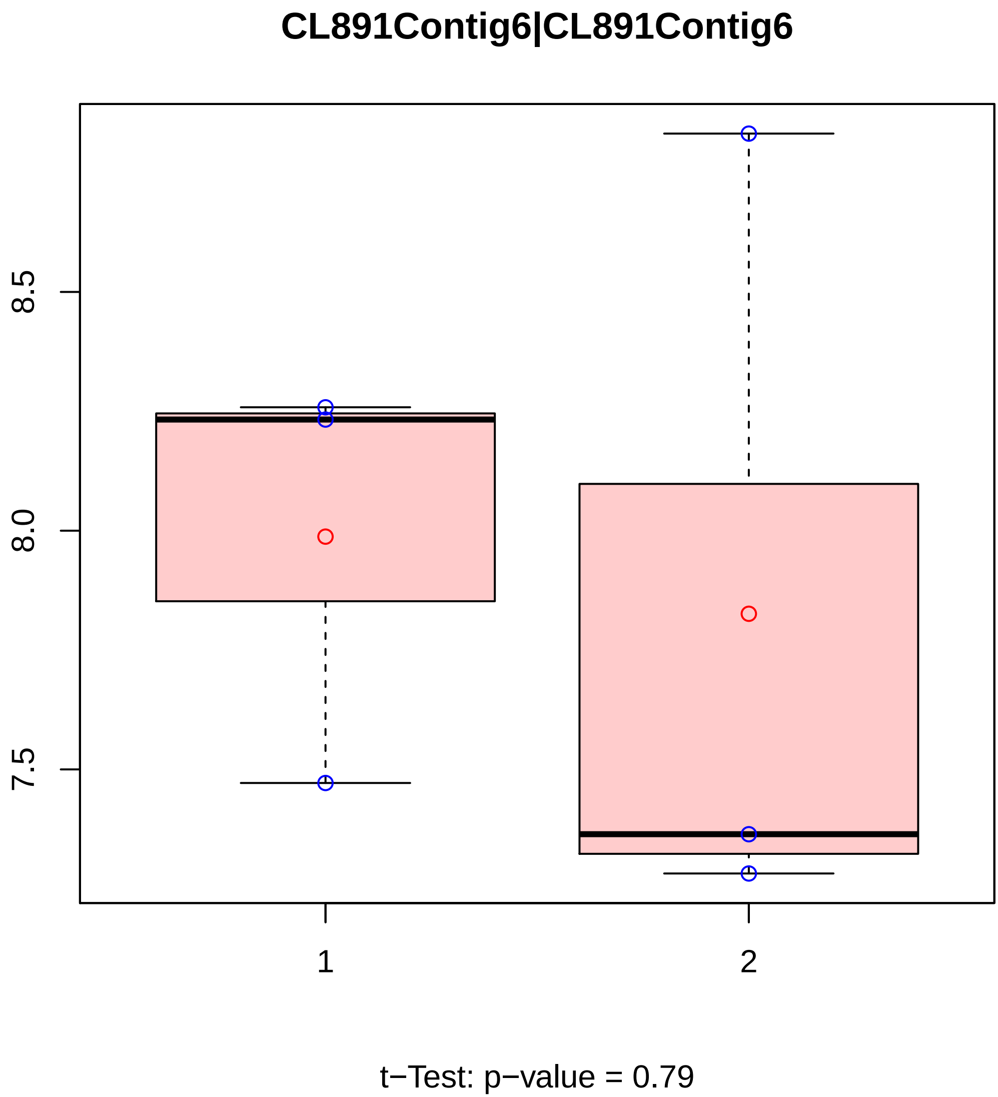

CL891Contig6|CL891Contig6
8.5
8.0
7.5
1
2
t−Test: p−value = 0.79

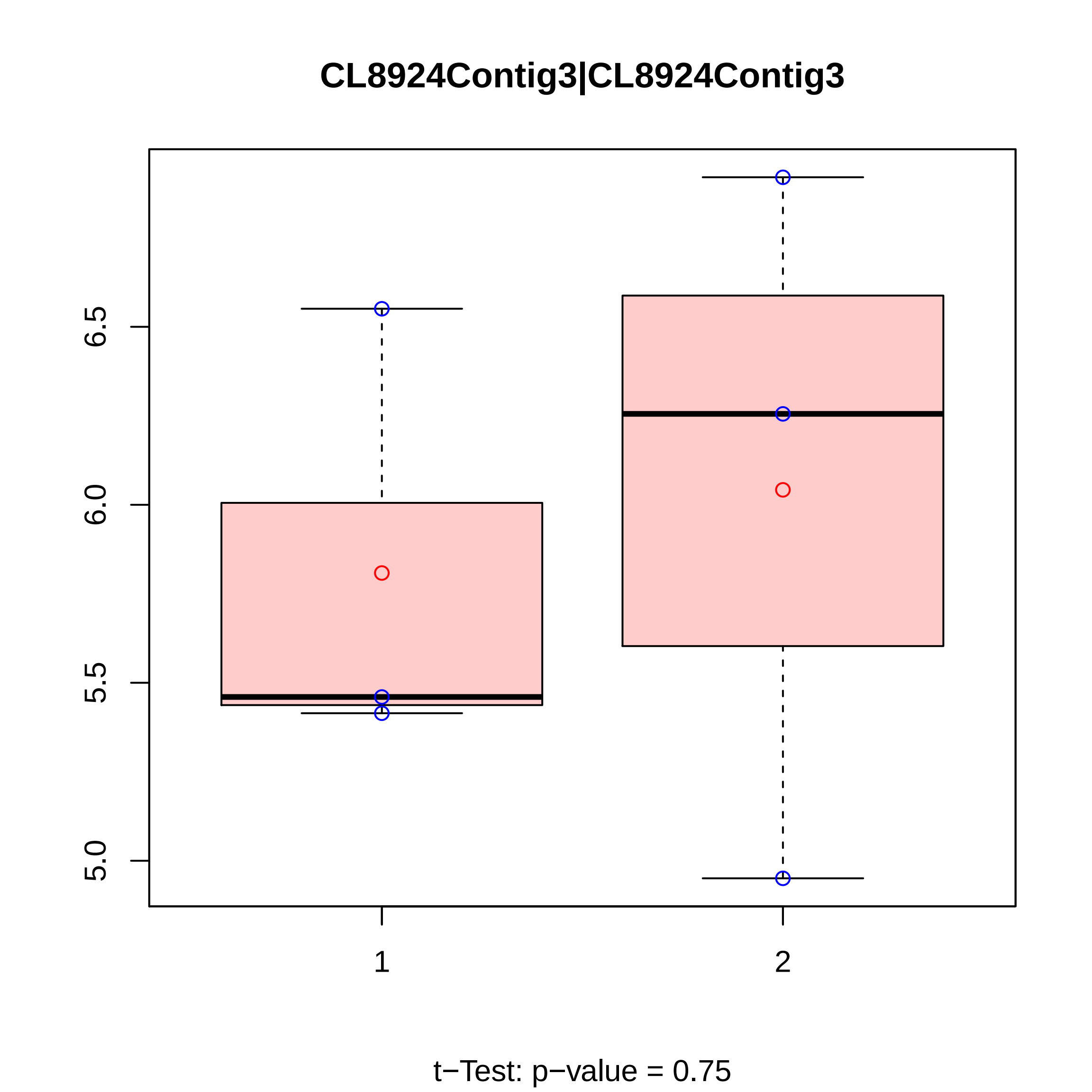
CL8924Contig3|CL8924Contig3
6.5
6.0
5.5
5.0
1
2
t−Test: p−value = 0.75

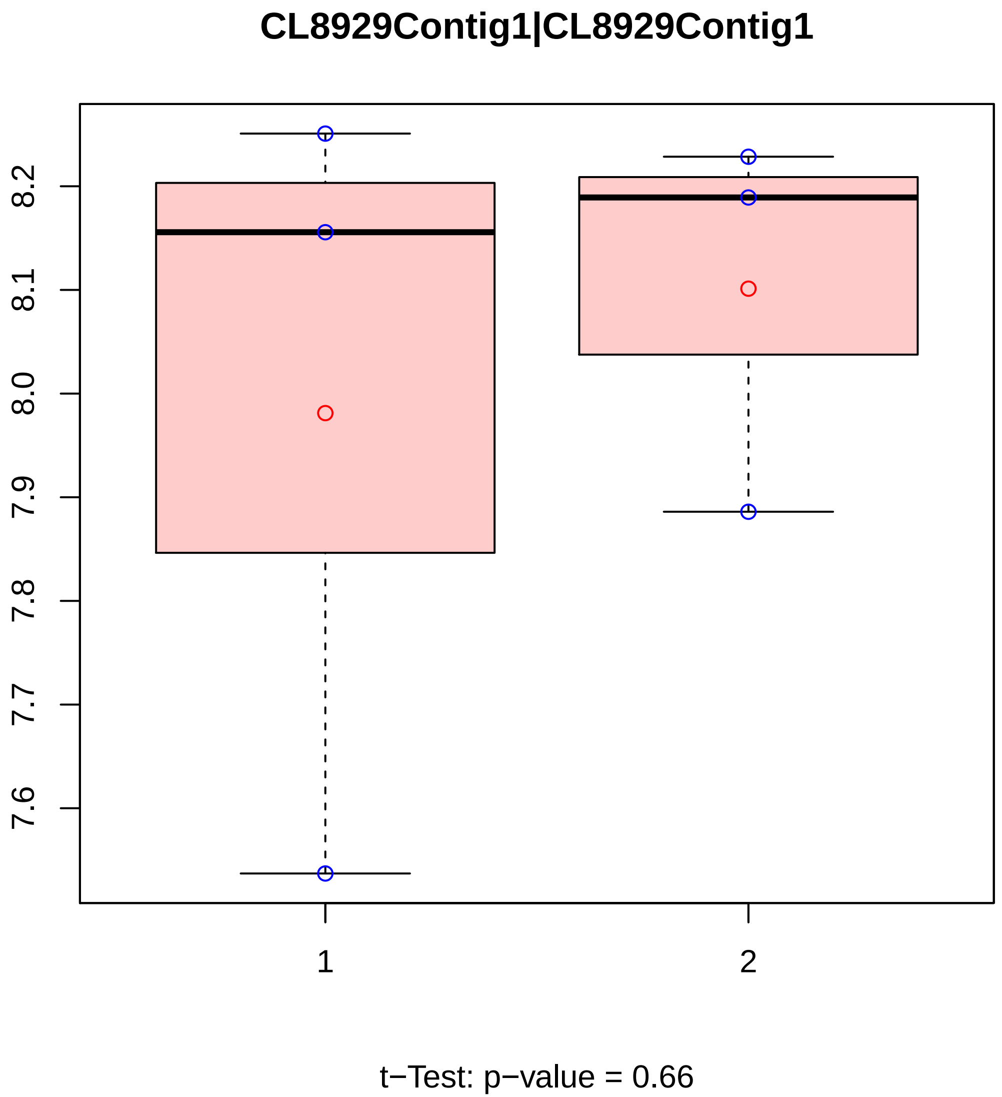
CL8929Contig1|CL8929Contig1
8.2
8.1
8.0
7.9
7.8
7.7
7.6
1
2
t−Test: p−value = 0.66

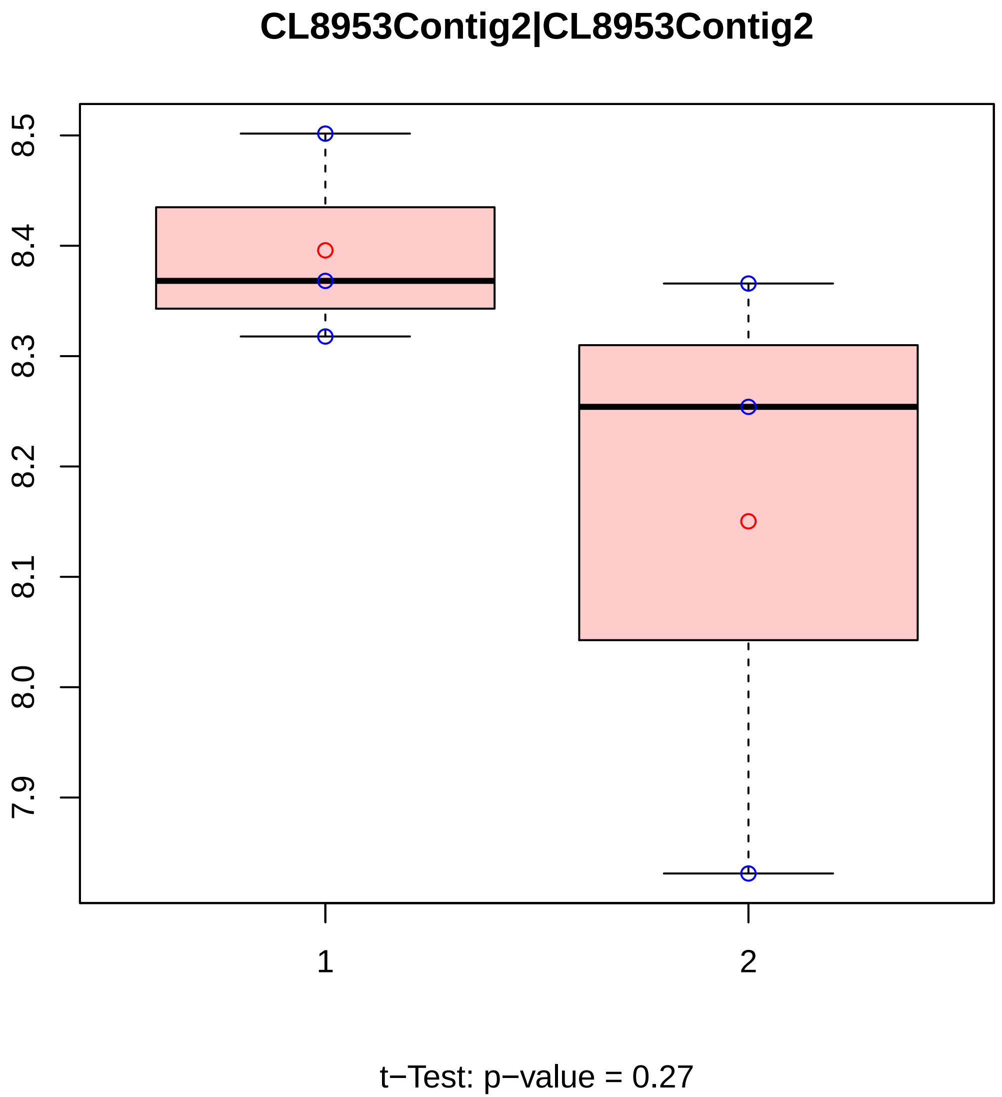

CL8953Contig2|CL8953Contig2
8.5
8.4
8.3
8.2
8.1
8.0
7.9
1
2
t−Test: p−value = 0.27

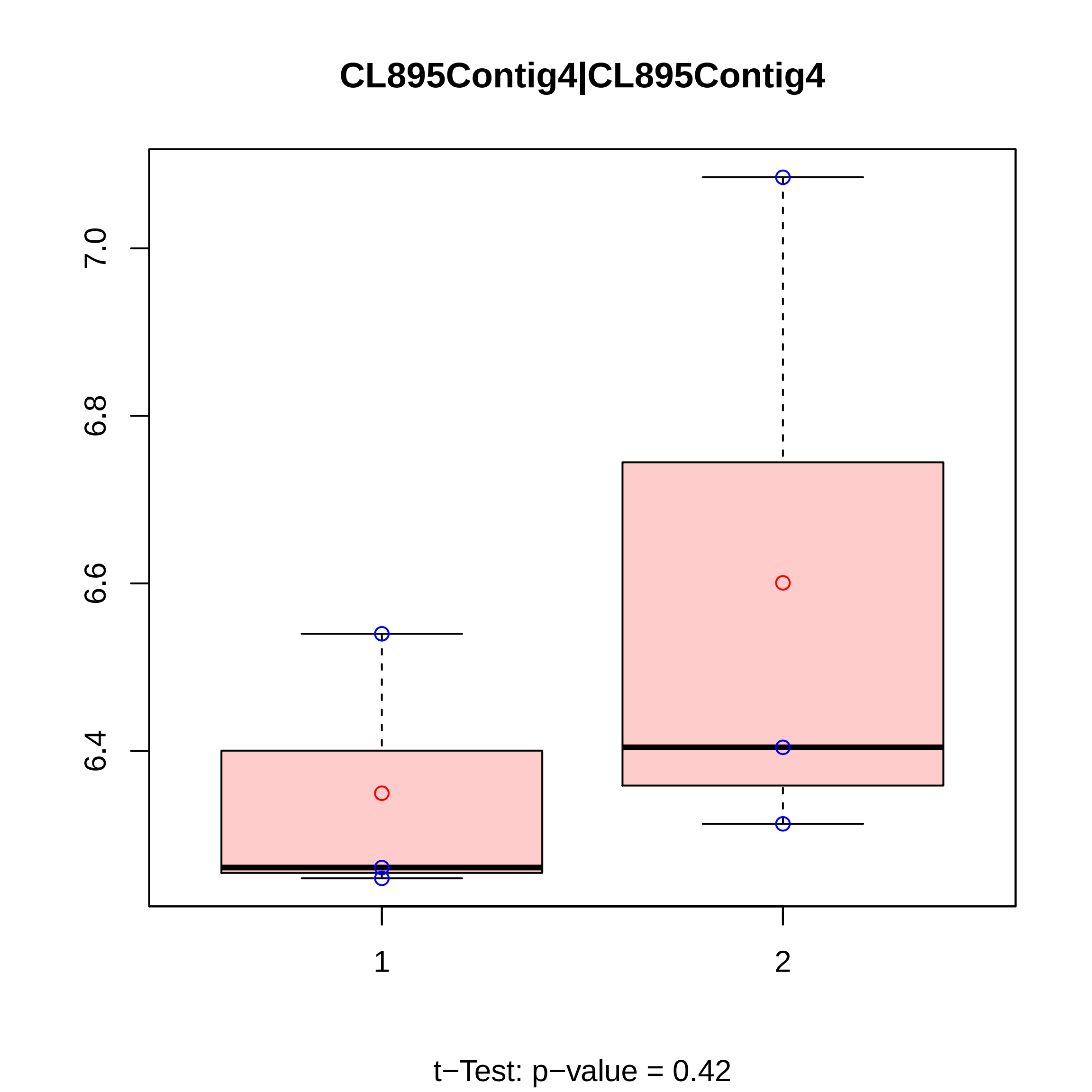
CL895Contig4|CL895Contig4
7.0
6.8
6.6
6.4
1
2
t−Test: p−value = 0.42

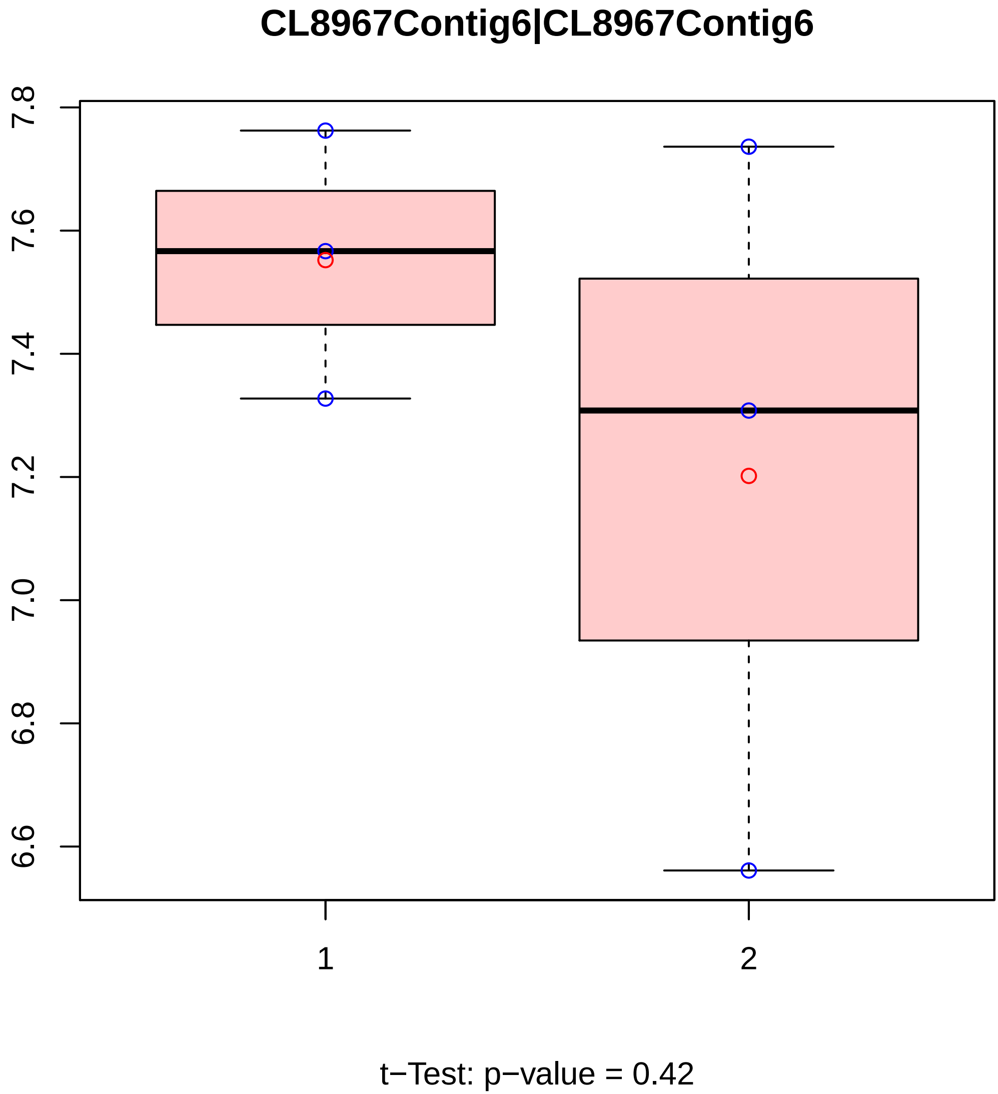
CL8967Contig6|CL8967Contig6
7.8
7.6
7.4
7.2
7.0
6.8
6.6
1
2
t−Test: p−value = 0.42

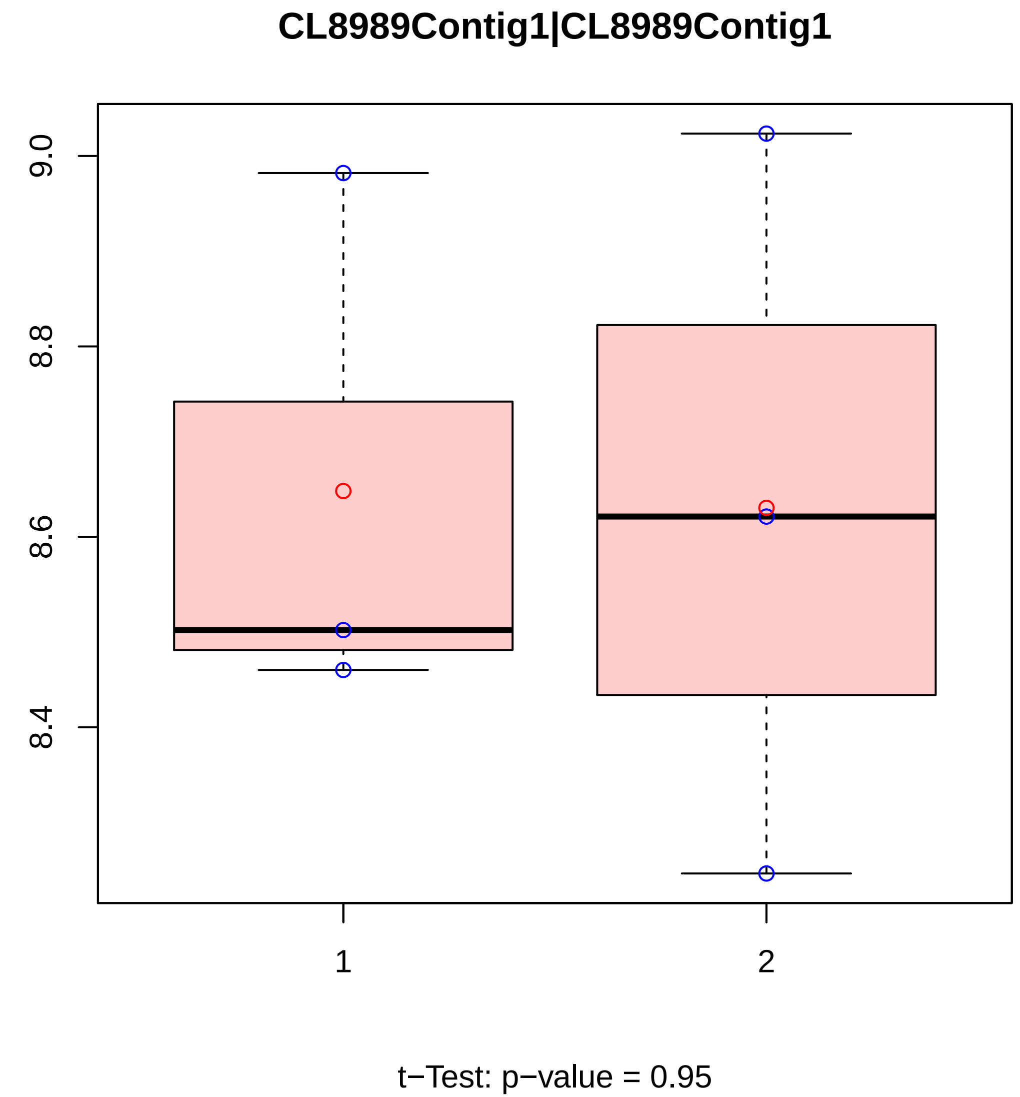
CL8989Contig1|CL8989Contig1
9.0
8.8
8.6
8.4
1
2
t−Test: p−value = 0.95

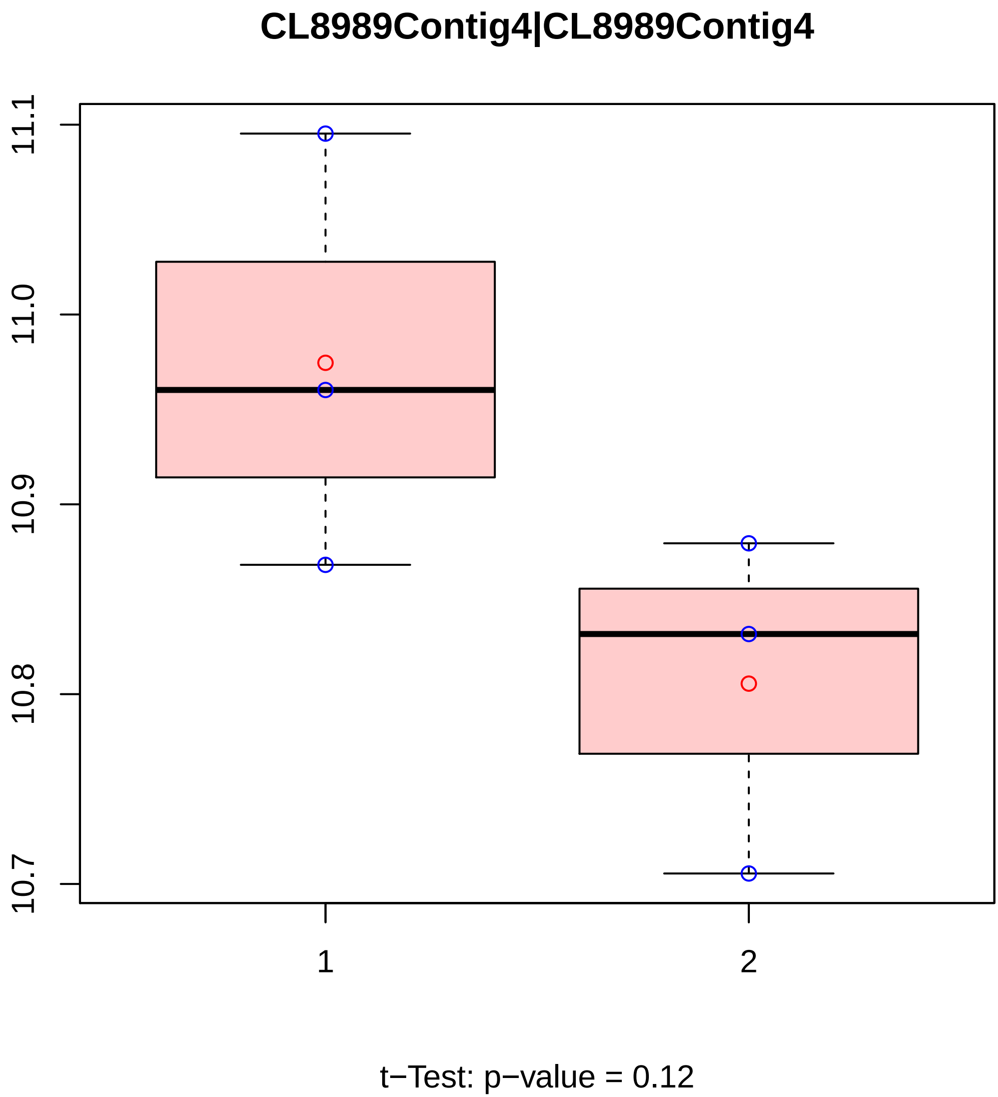

CL8989Contig4|CL8989Contig4
11.1
11.0
10.9
10.8
10.7
1
2
t−Test: p−value = 0.12

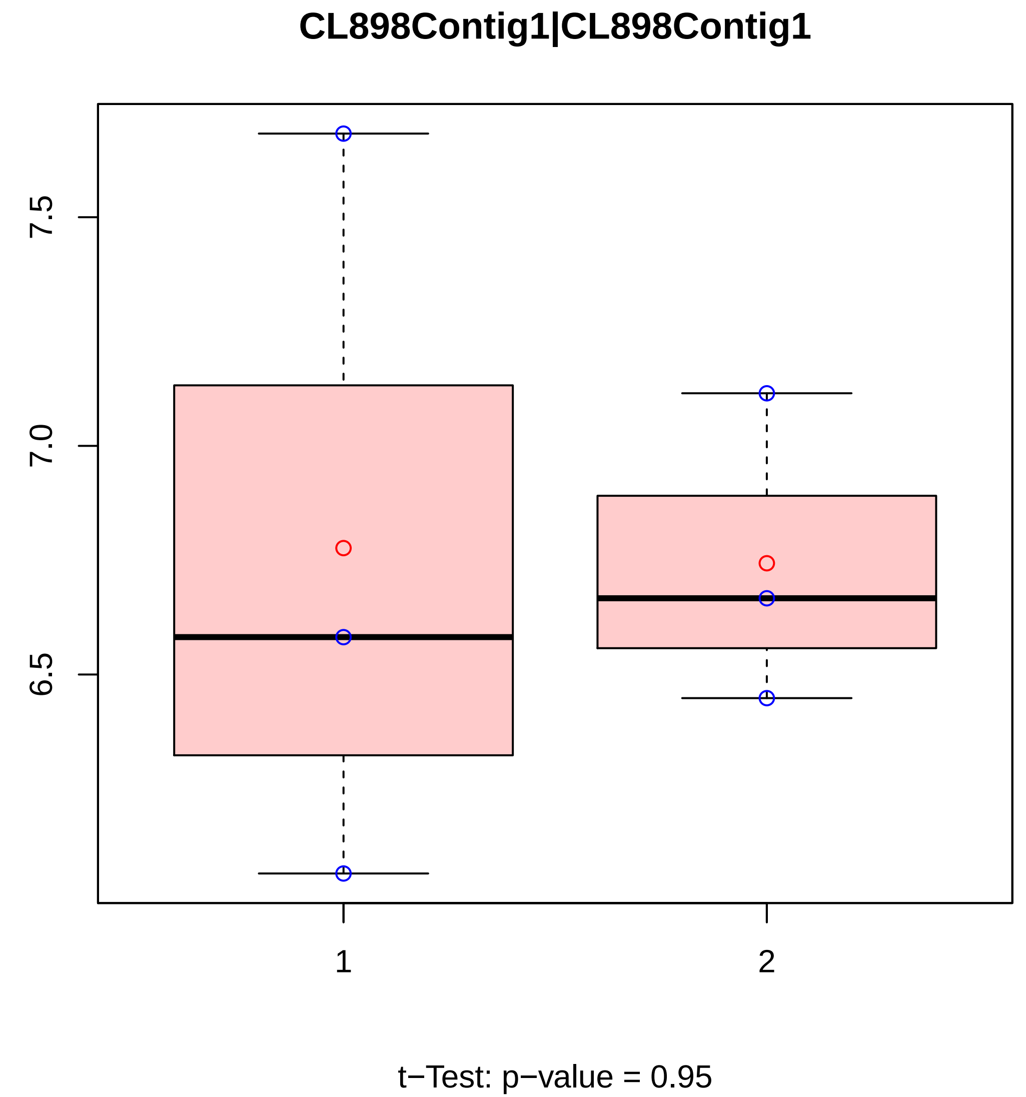
CL898Contig1|CL898Contig1
7.5
7.0
6.5
1
2
t−Test: p−value = 0.95

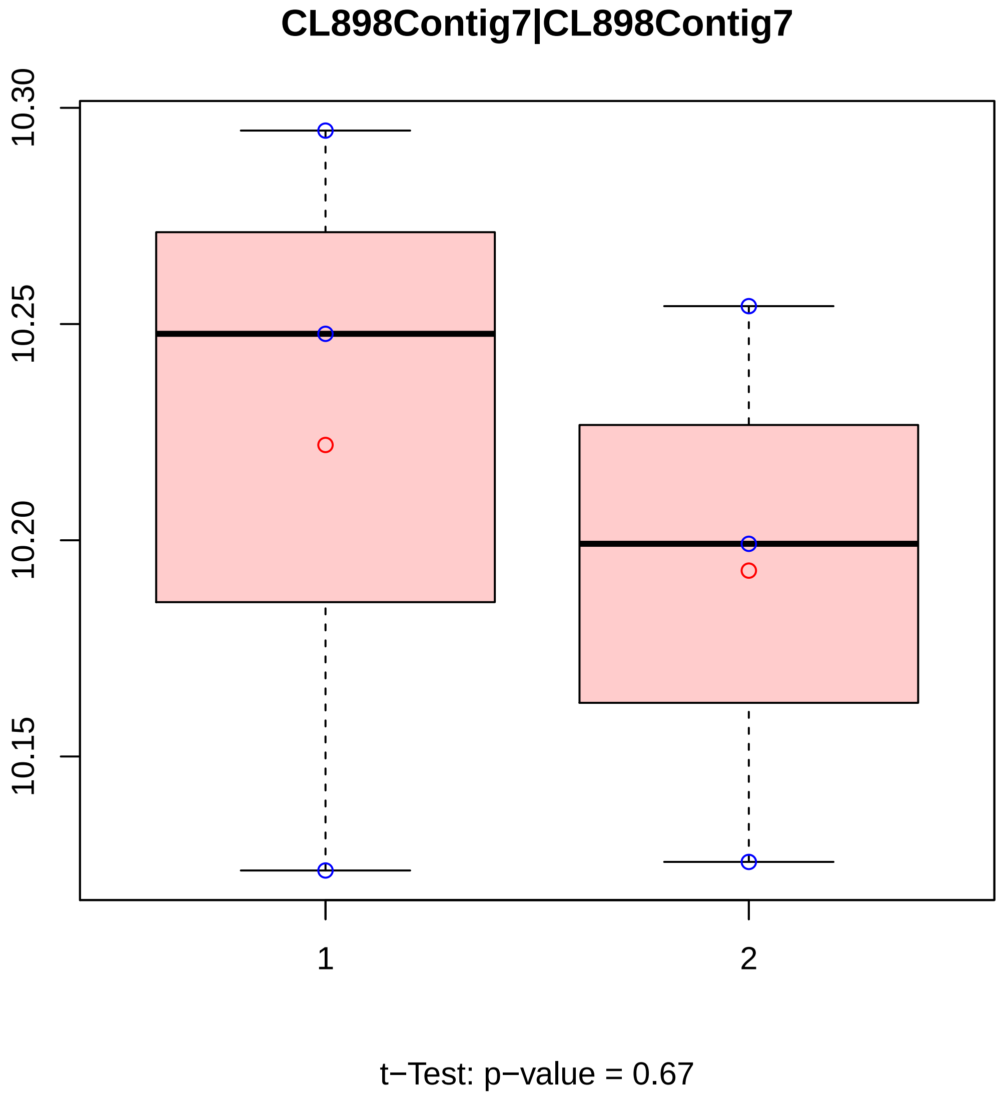
CL898Contig7|CL898Contig7
10.30
10.25
10.20
10.15
1
2
t−Test: p−value = 0.67

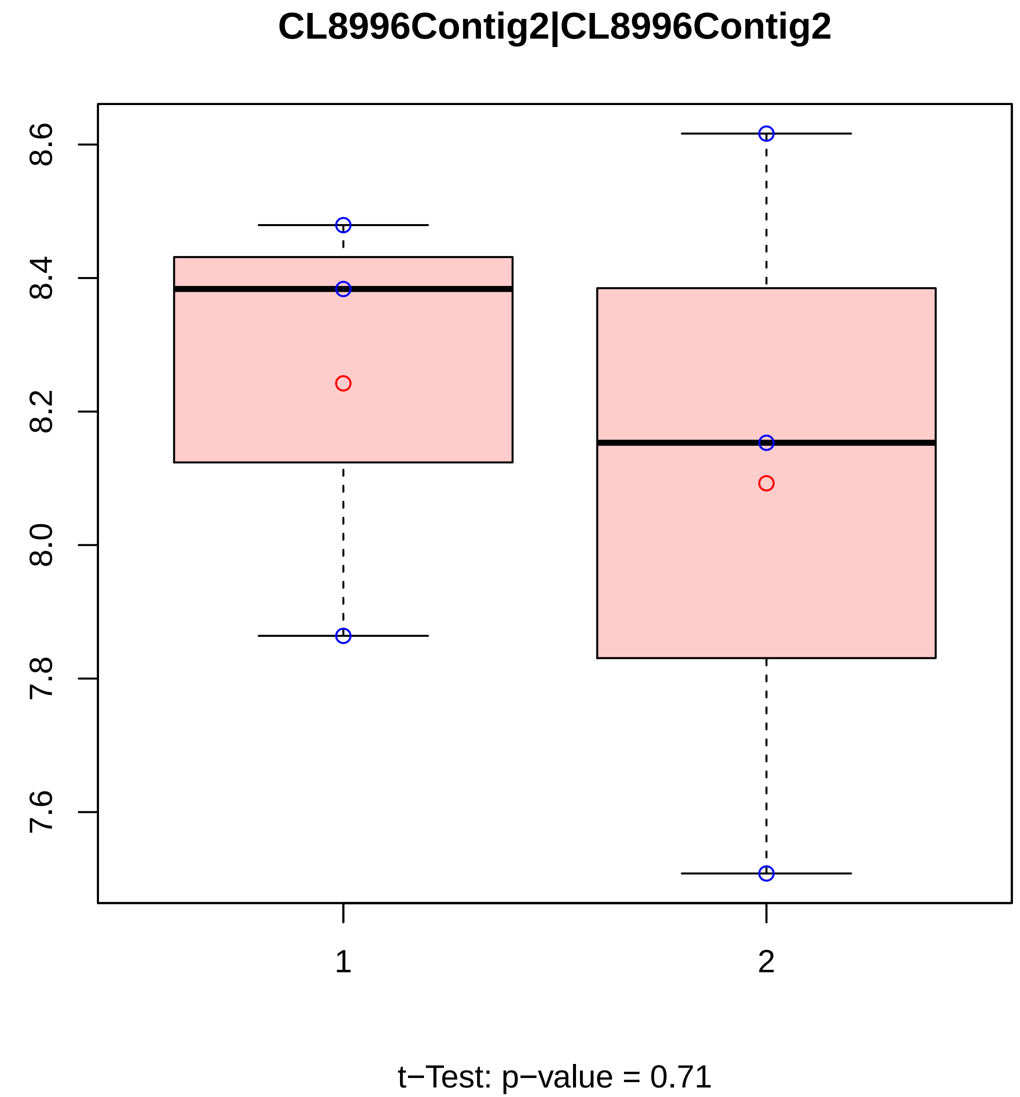
CL8996Contig2|CL8996Contig2
8.6
8.4
8.2
8.0
7.8
7.6
1
2
t−Test: p−value = 0.71

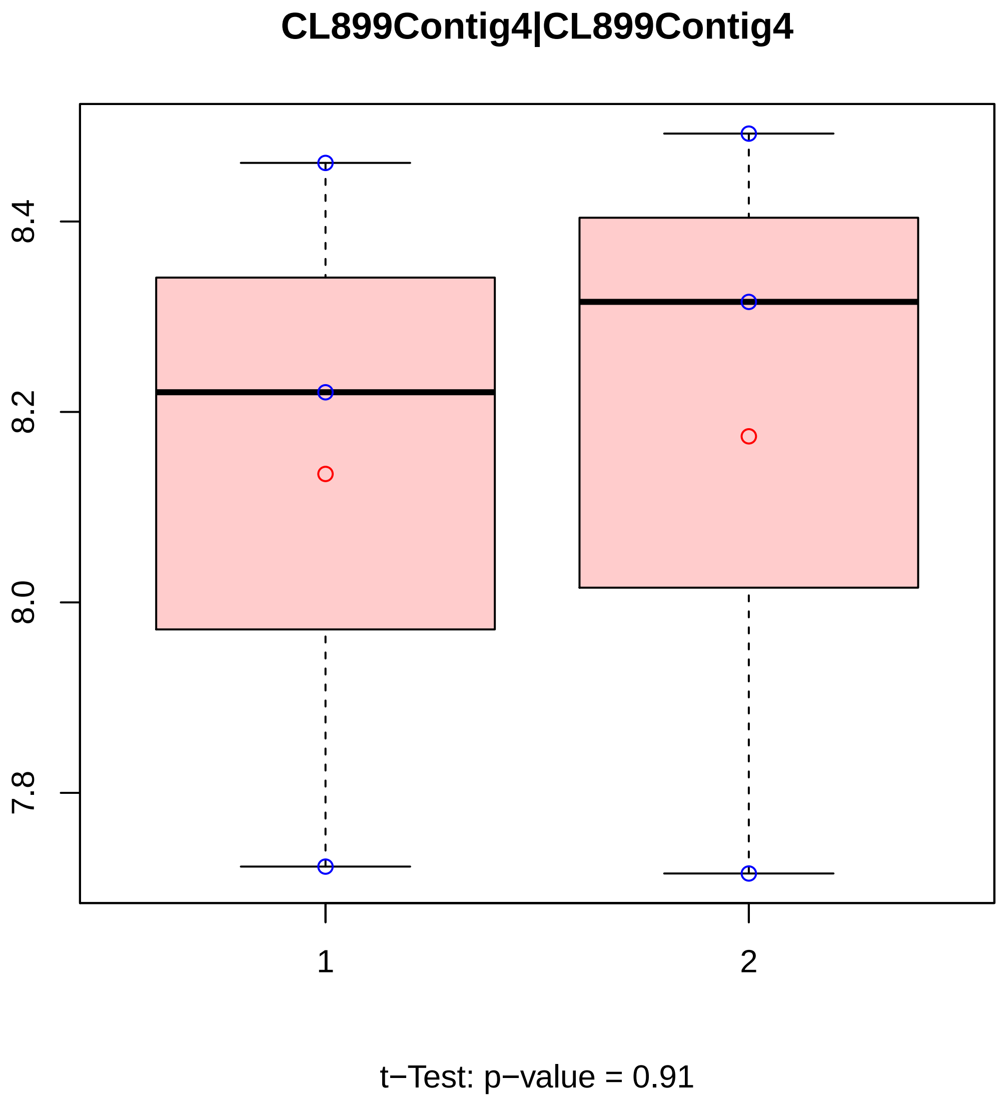
CL899Contig4|CL899Contig4
8.4
8.2
8.0
7.8
1
2
t−Test: p−value = 0.91

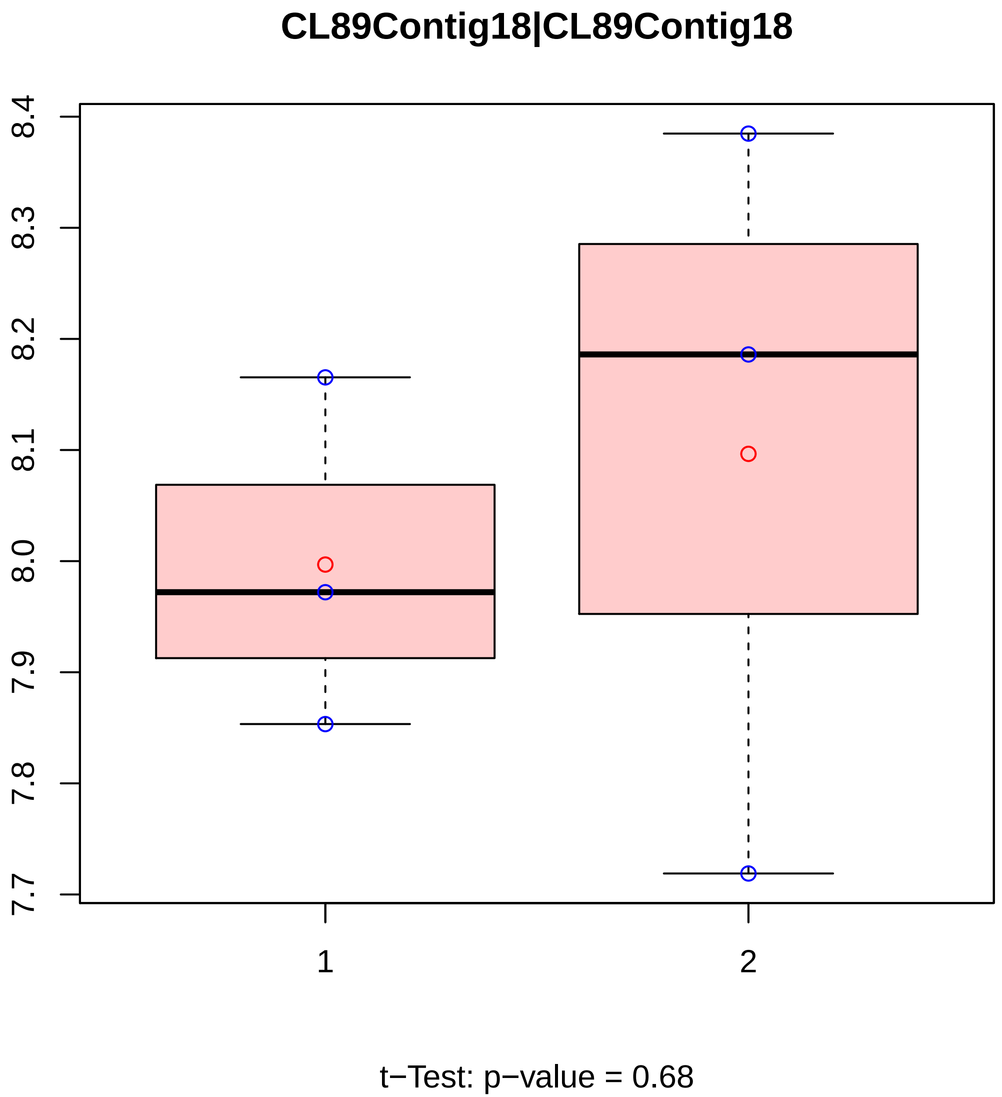
CL89Contig18|CL89Contig18
8.4
8.3
8.2
8.1
8.0
7.9
7.8
7.7
1
2
t−Test: p−value = 0.68

**CL9002Contig5|CL9002Contig5**

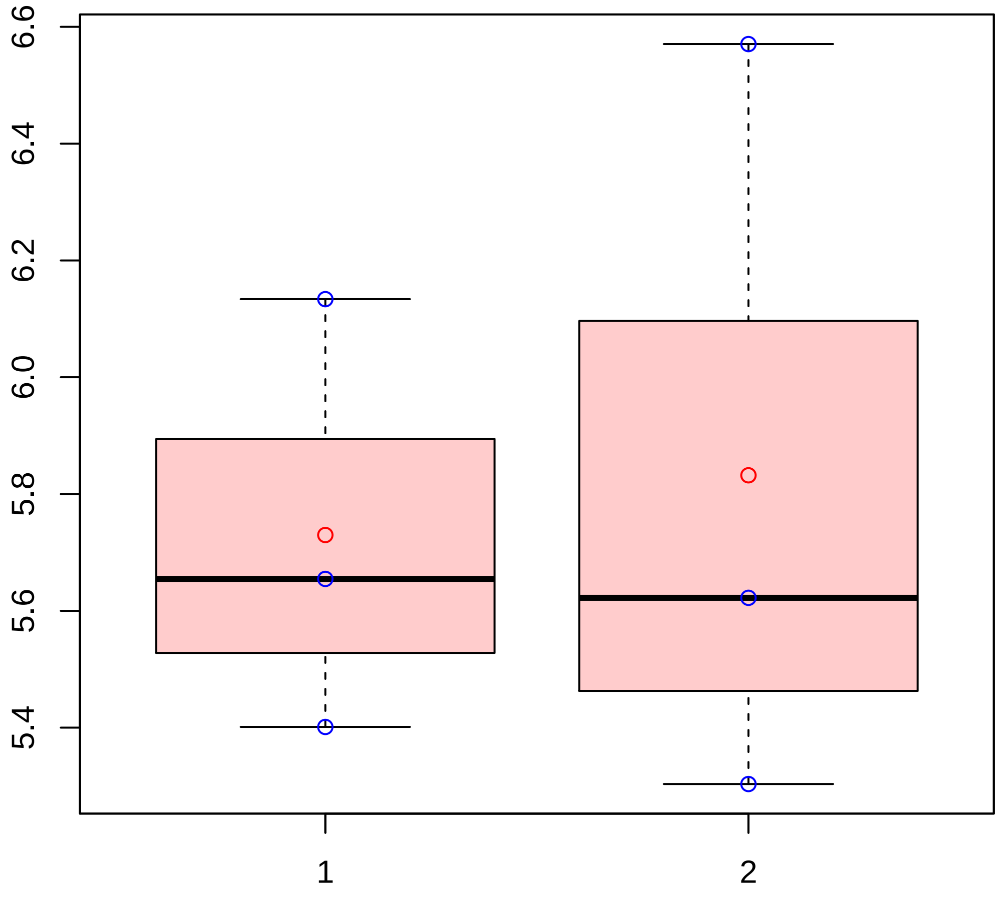


t−Test: p−value = 0.83

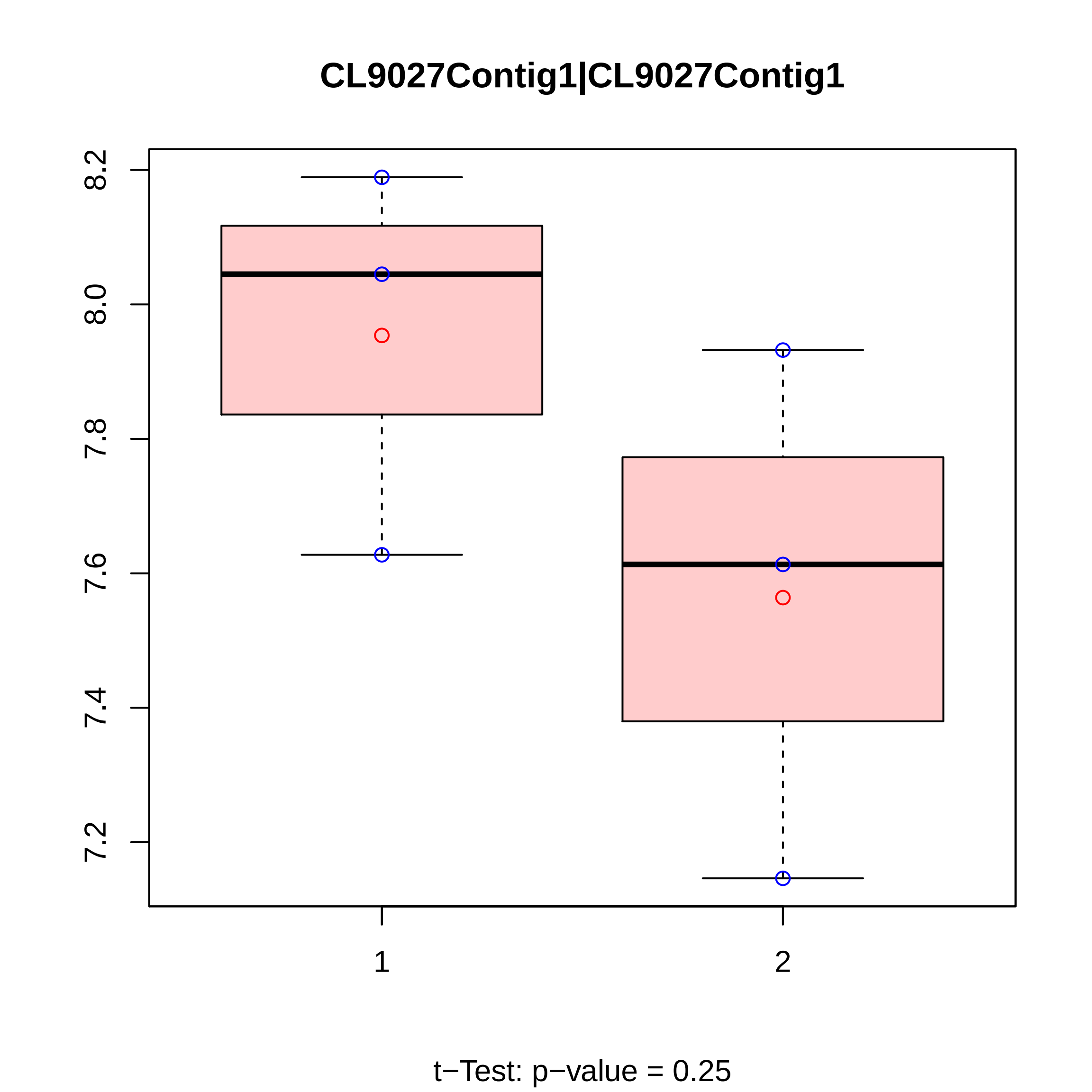
CL9027Contig1|CL9027Contig1
8.2
8.0
7.8
7.6
7.4
7.2
1
2
t−Test: p−value = 0.25

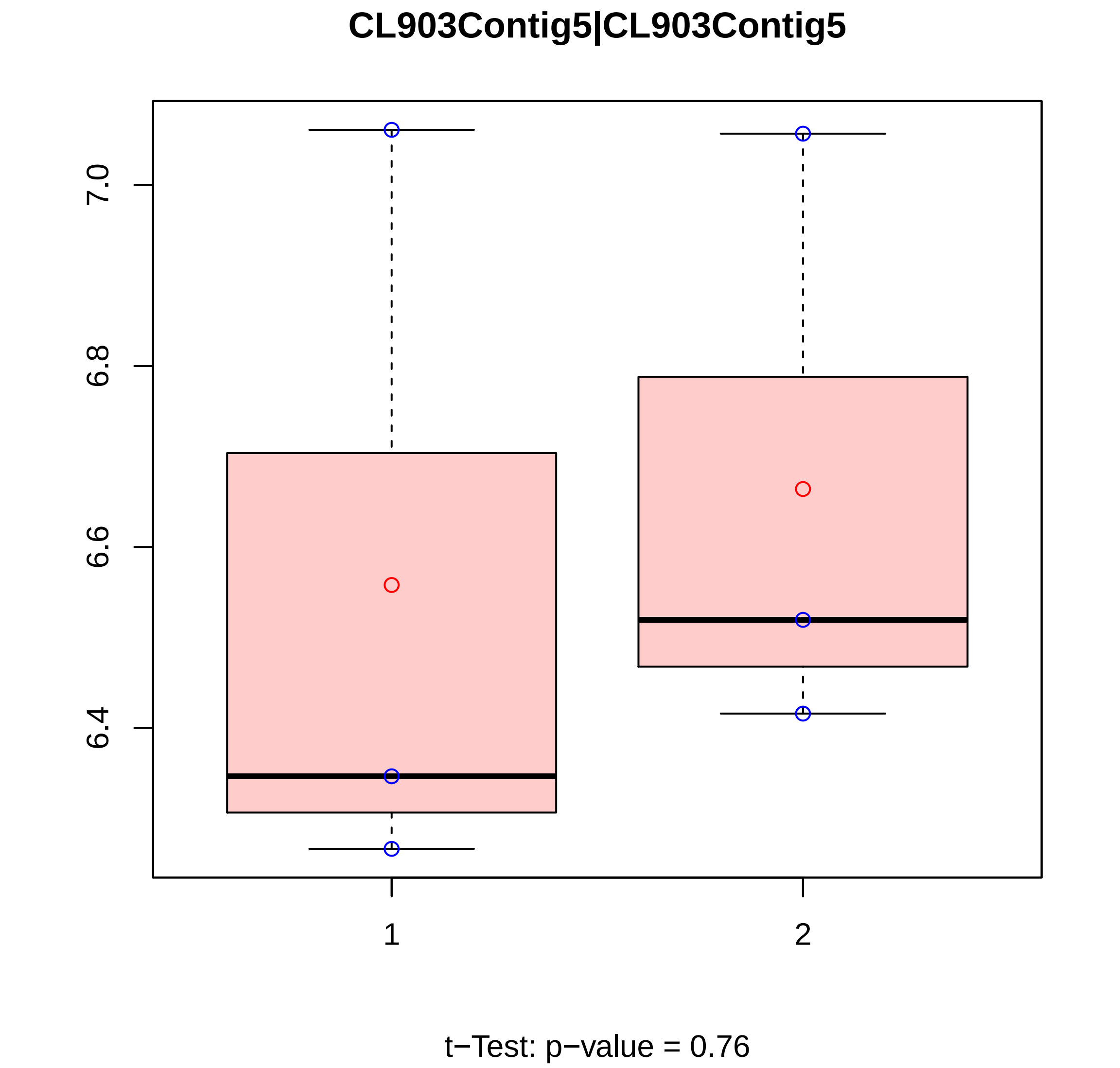
CL903Contig5|CL903Contig5
7.0
6.8
6.6
6.4
1
2
t−Test: p−value = 0.76

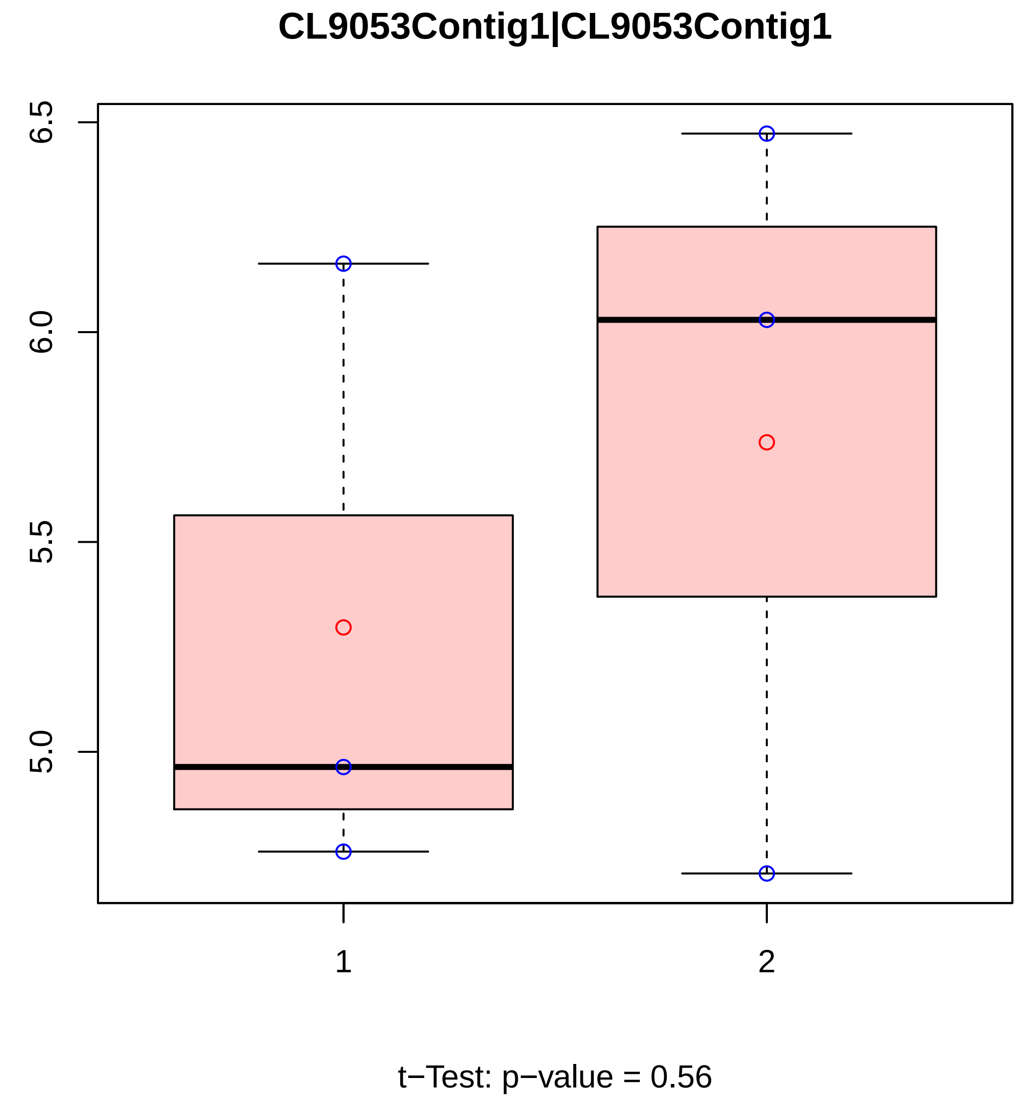

CL9053Contig1|CL9053Contig1
6.5
6.0
5.5
5.0
1
2
t−Test: p−value = 0.56

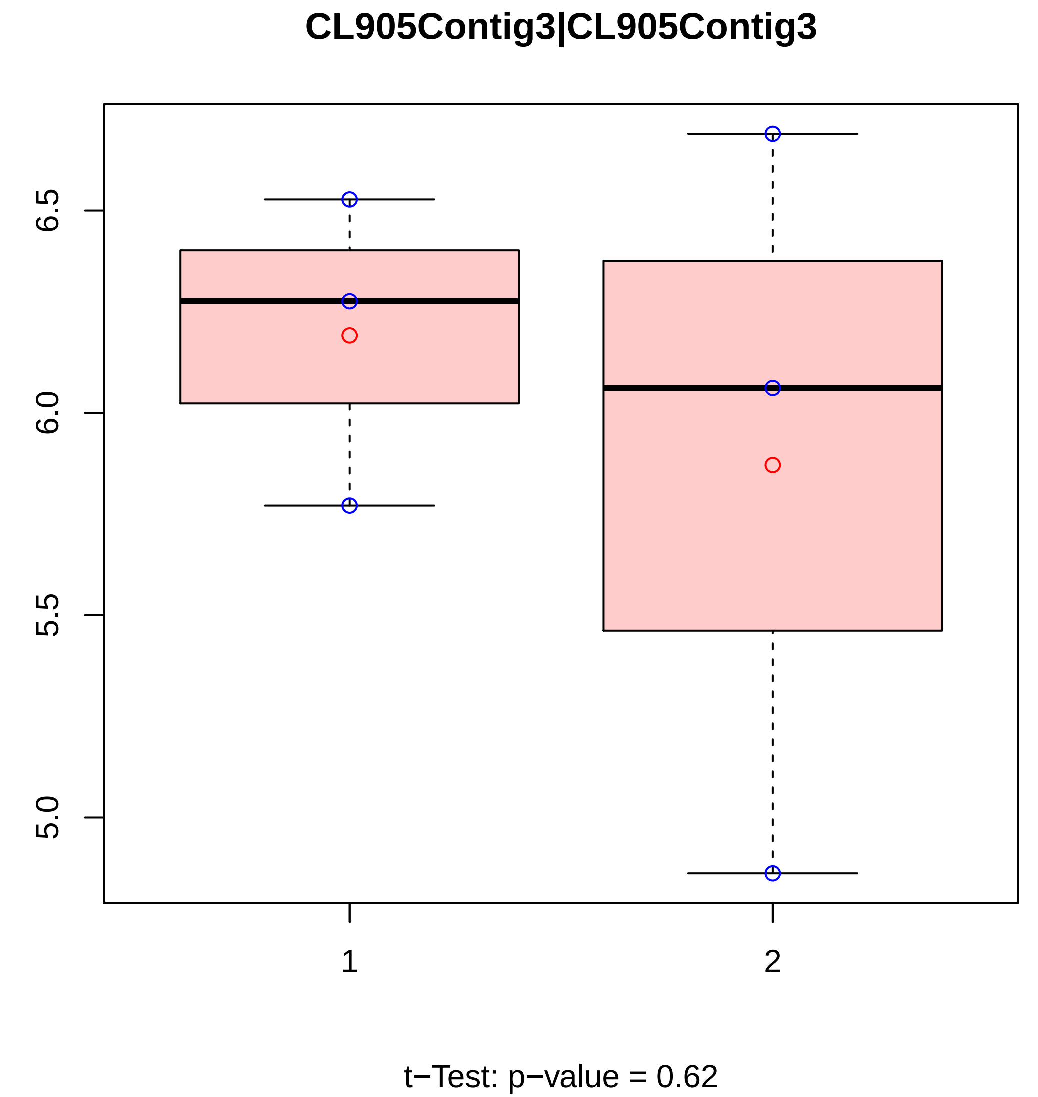

CL905Contig3|CL905Contig3
6.5
6.0
5.5
5.0
1
2
t−Test: p−value = 0.62

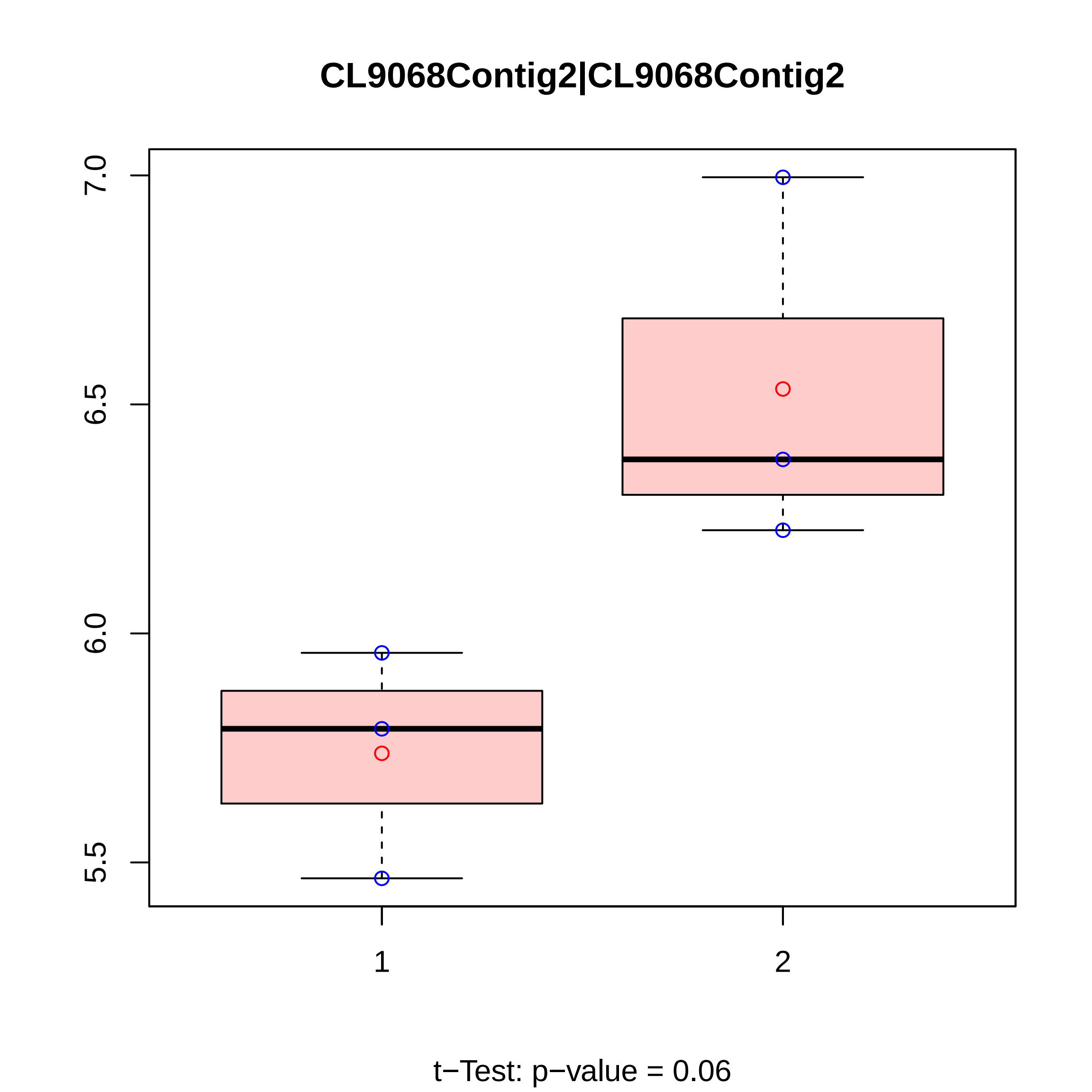

CL9068Contig2|CL9068Contig2
7.0
6.5
6.0
5.5
1
2
t−Test: p−value = 0.06

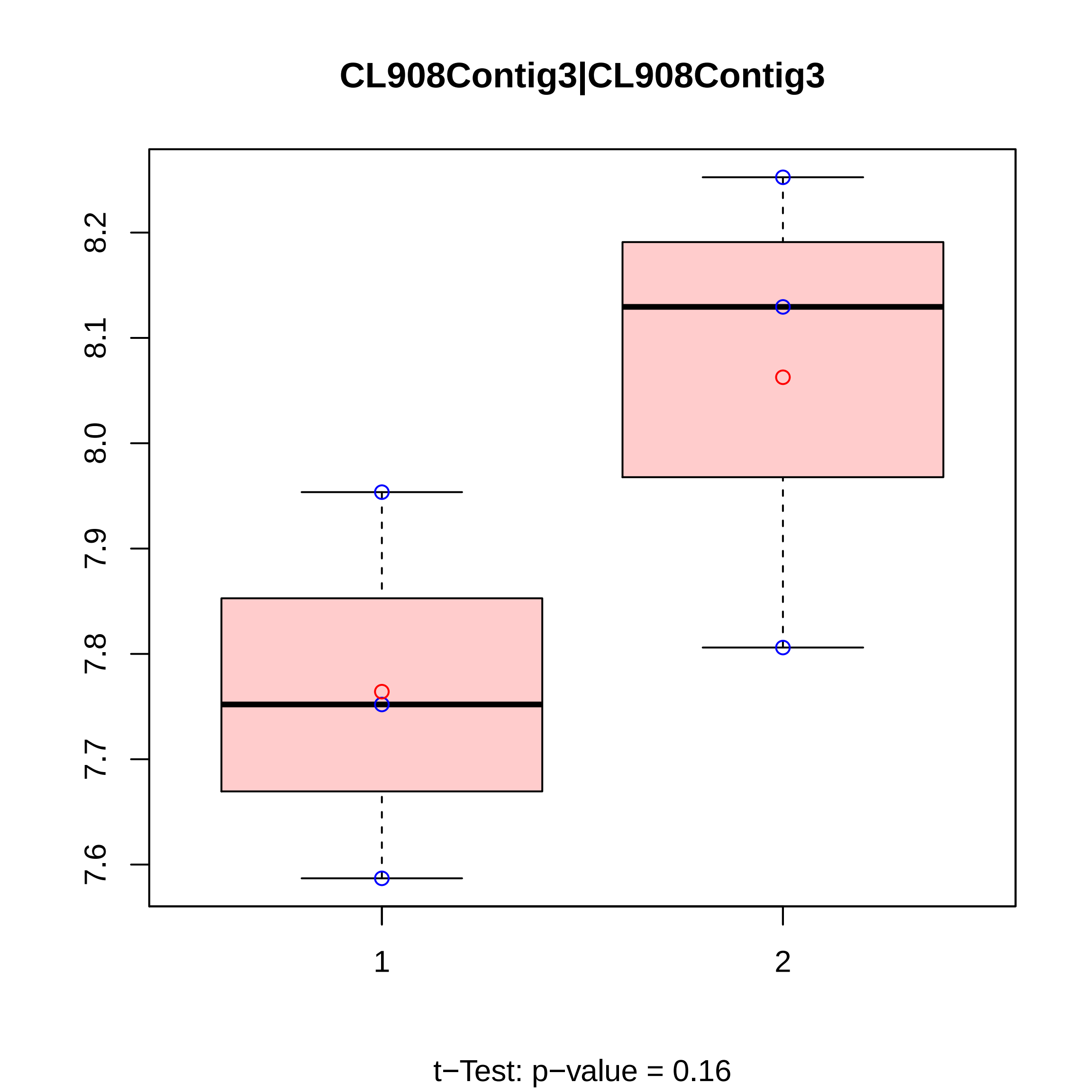

CL908Contig3|CL908Contig3
8.2
8.1
8.0
7.9
7.8
7.7
7.6
1
2
t−Test: p−value = 0.16

# CL909Contig5|CL909Contig5

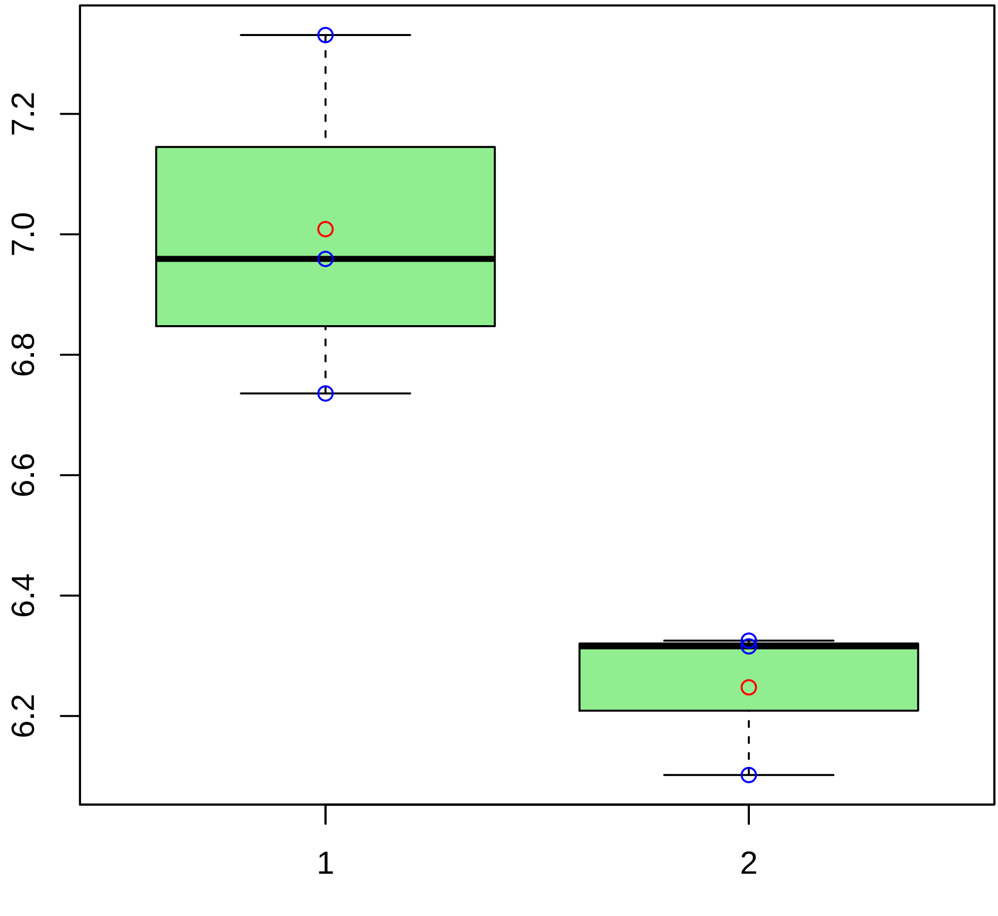

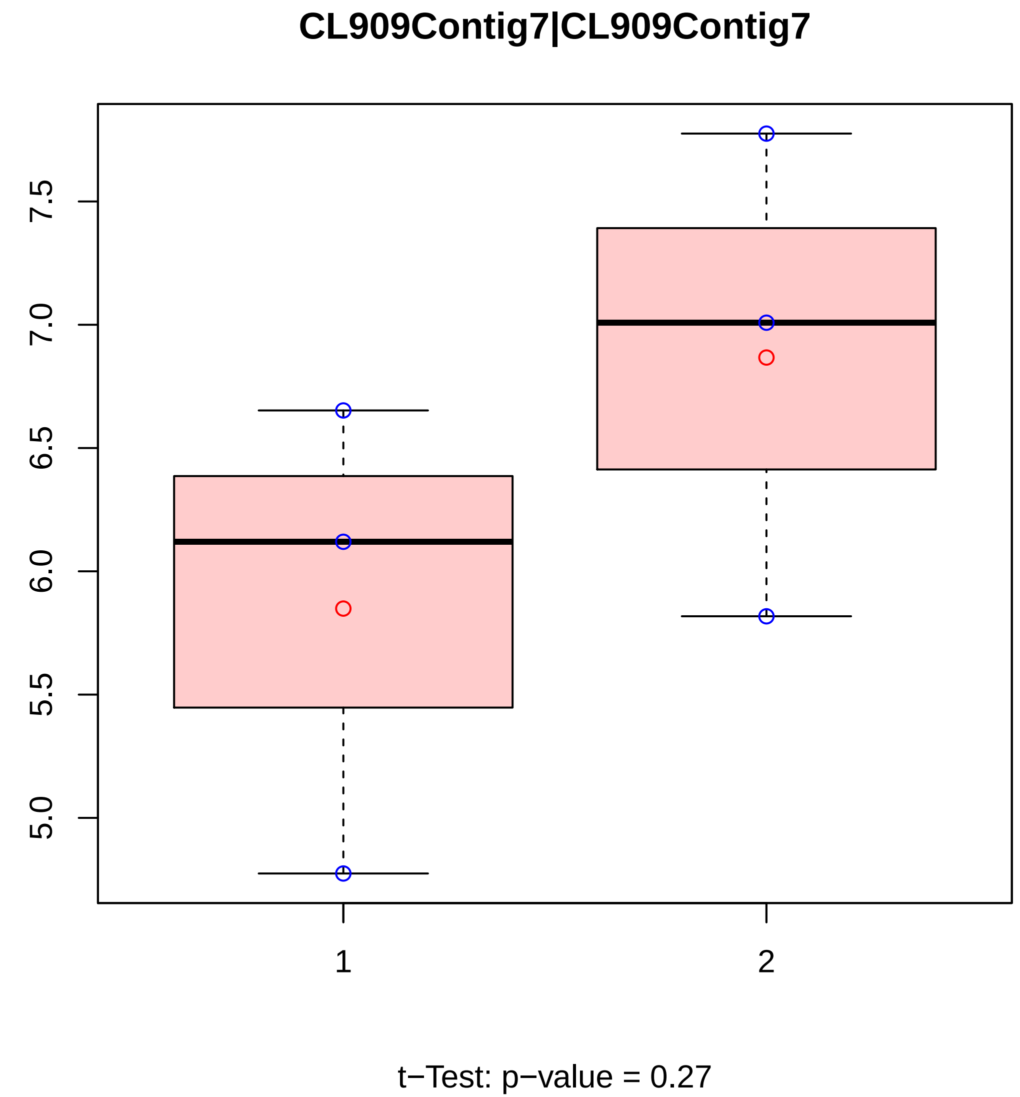
CL909Contig7|CL909Contig7
7.5
7.0
6.5
6.0
5.5
5.0
1
2
t−Test: p−value = 0.27

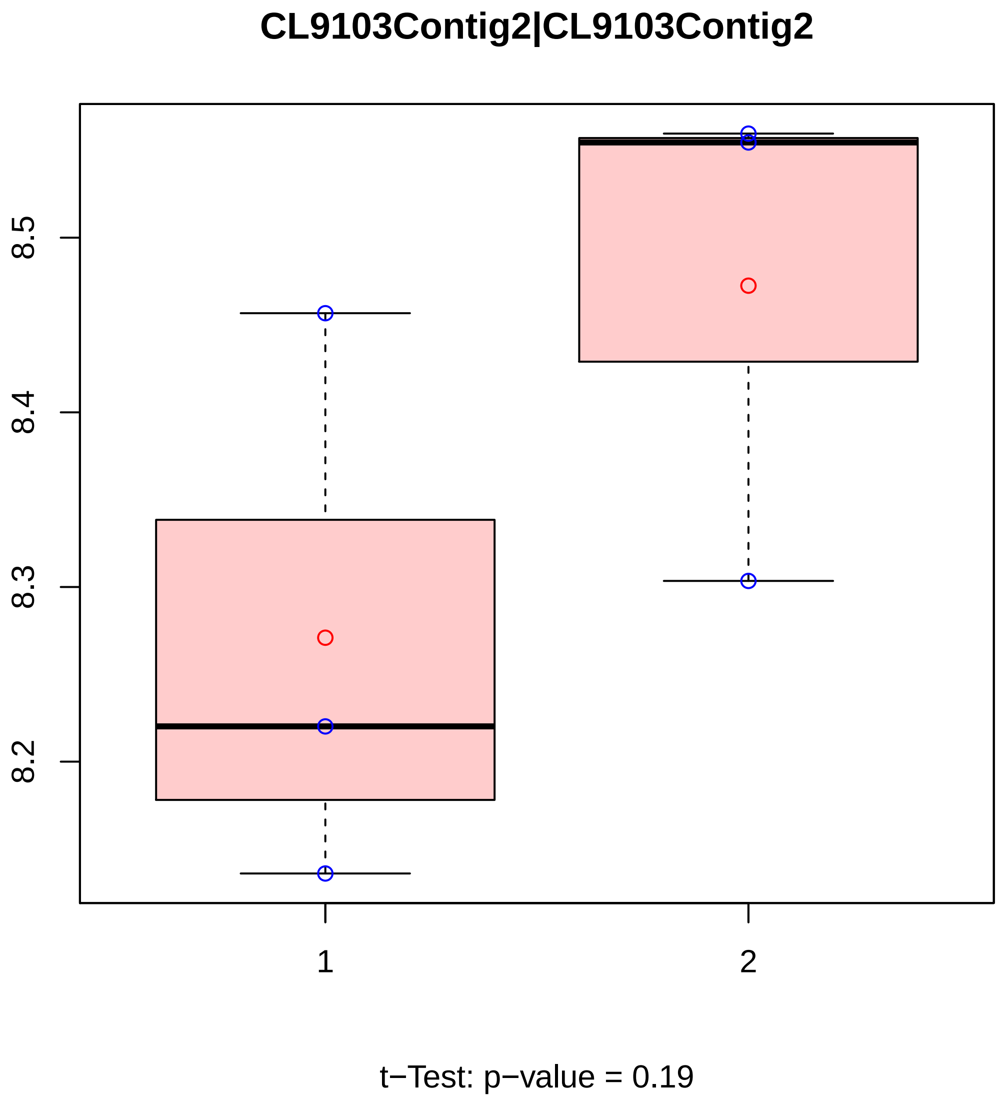
CL9103Contig2|CL9103Contig2
8.2
8.3
8.4
8.5
1
2
t−Test: p−value = 0.19

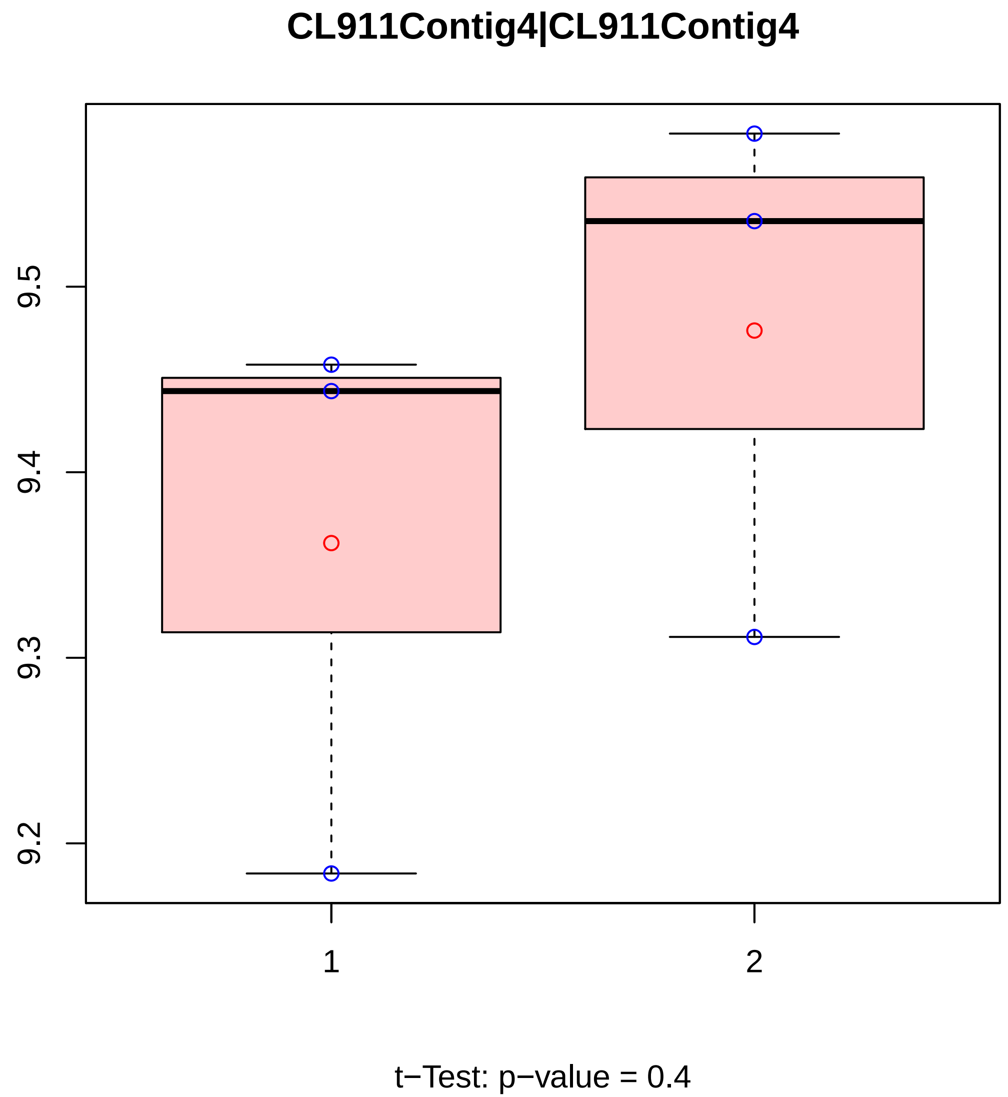
CL911Contig4|CL911Contig4
9.2
9.3
9.4
9.5
1
2
t−Test: p−value = 0.4

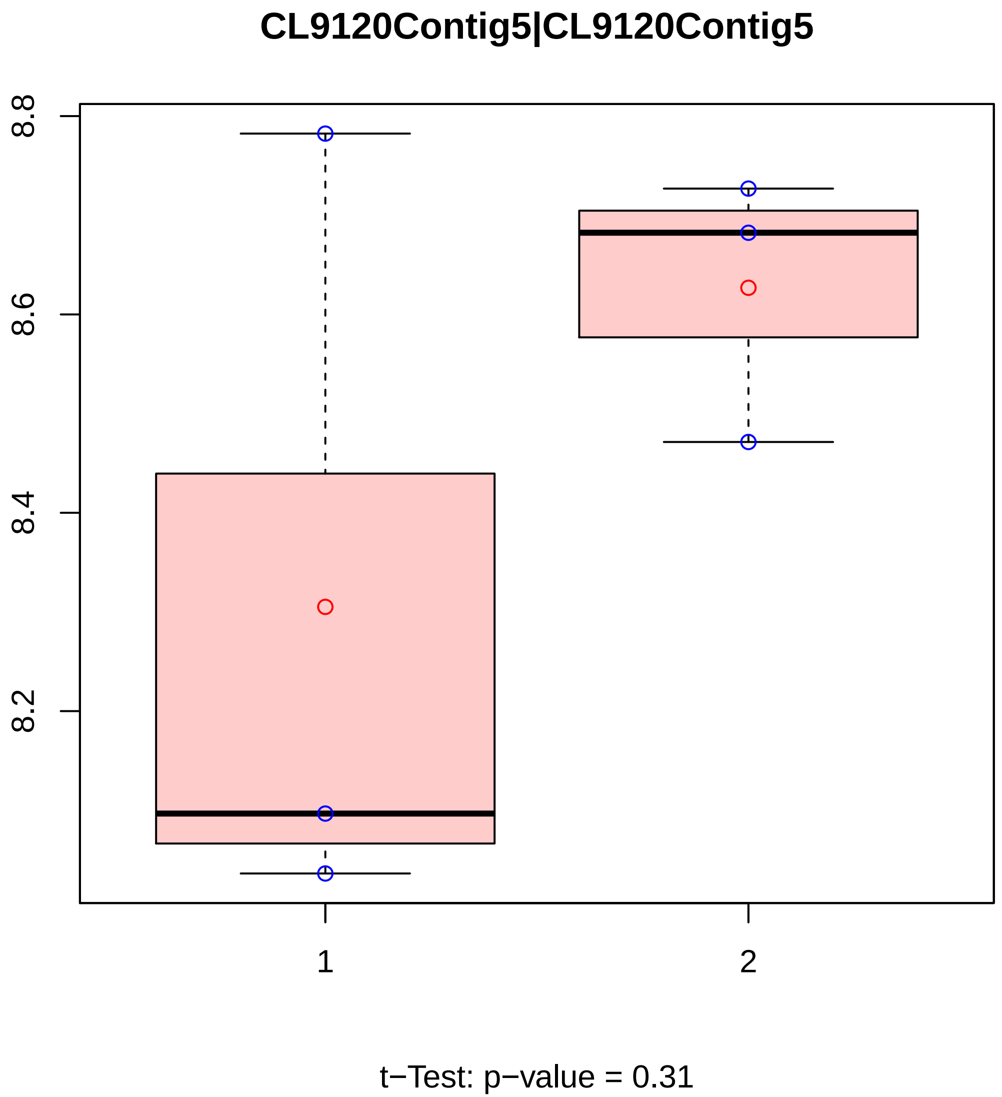

CL9120Contig5|CL9120Contig5
8.8
8.6
8.4
8.2
1
2
t−Test: p−value = 0.31

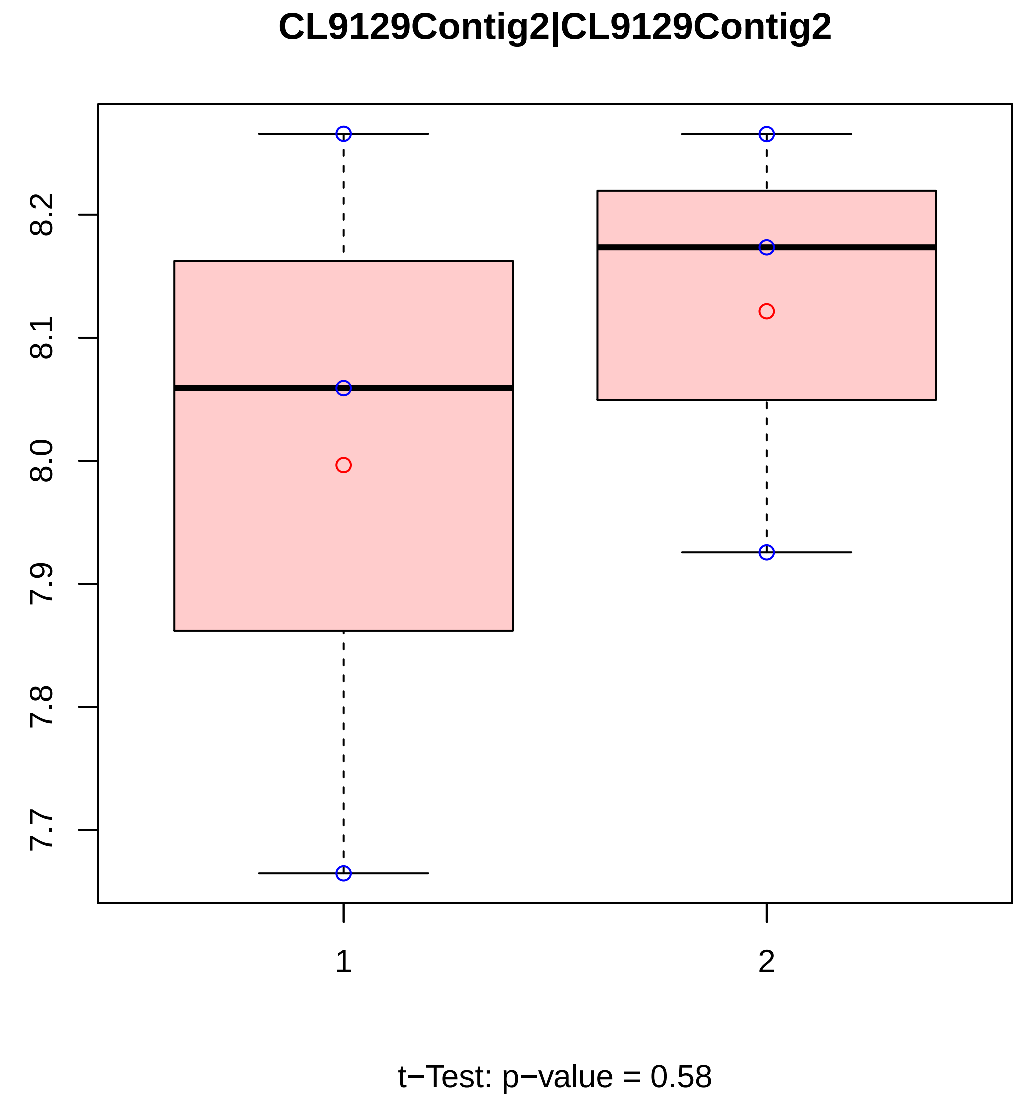
CL9129Contig2|CL9129Contig2
8.2
8.1
8.0
7.9
7.8
7.7
1
2
t−Test: p−value = 0.58

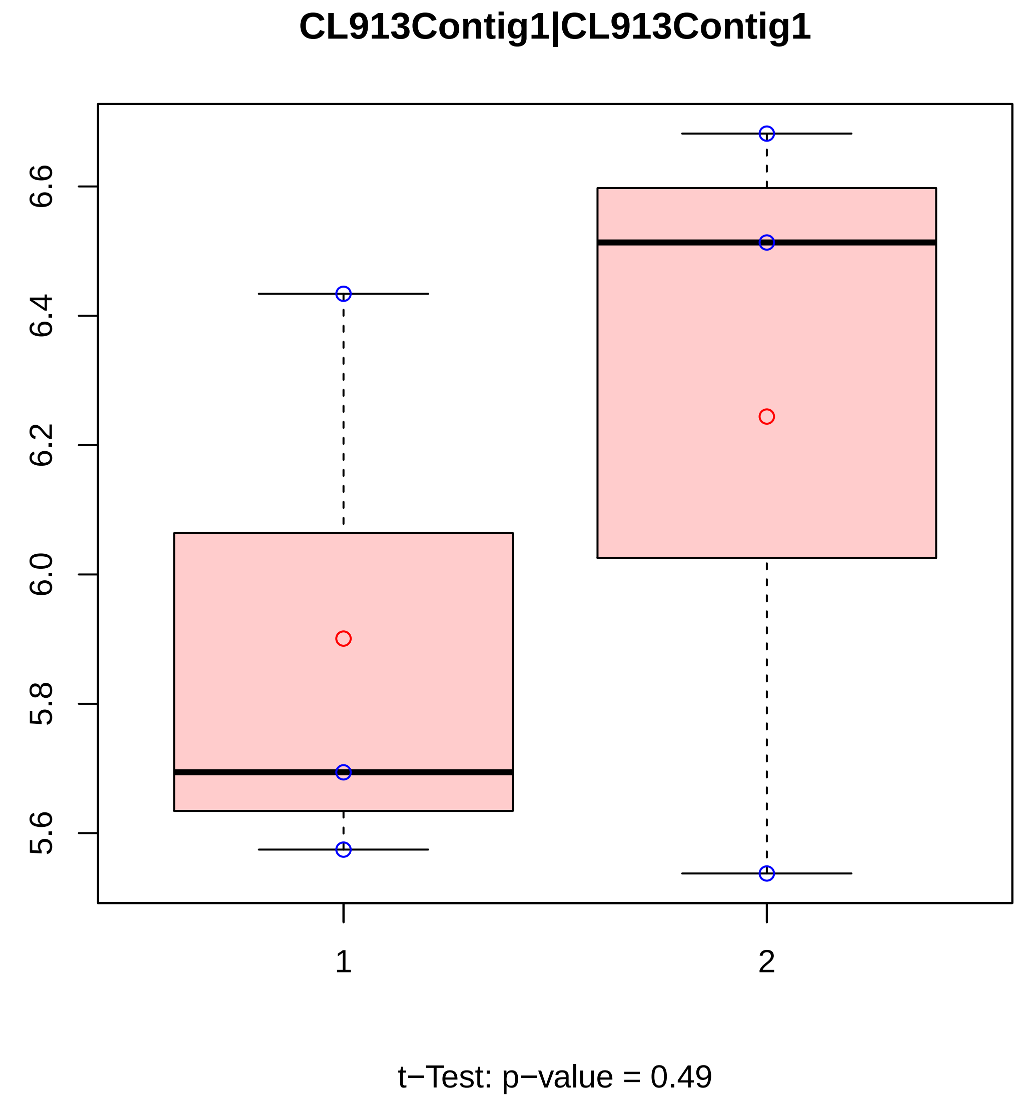
CL913Contig1|CL913Contig1
6.6
6.4
6.2
6.0
5.8
5.6
1
2
t−Test: p−value = 0.49

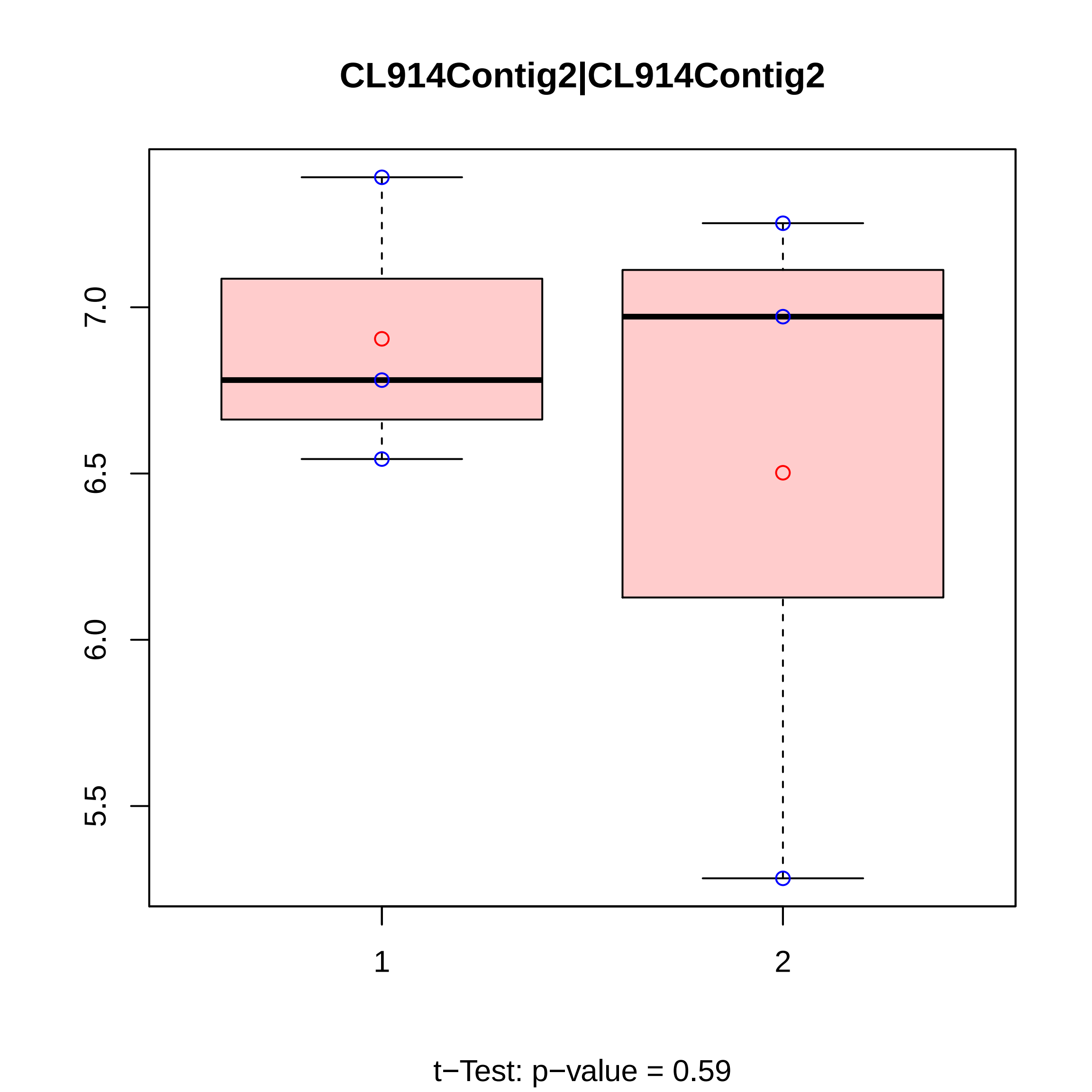
CL914Contig2|CL914Contig2
7.0
6.5
6.0
5.5
1
2
t−Test: p−value = 0.59

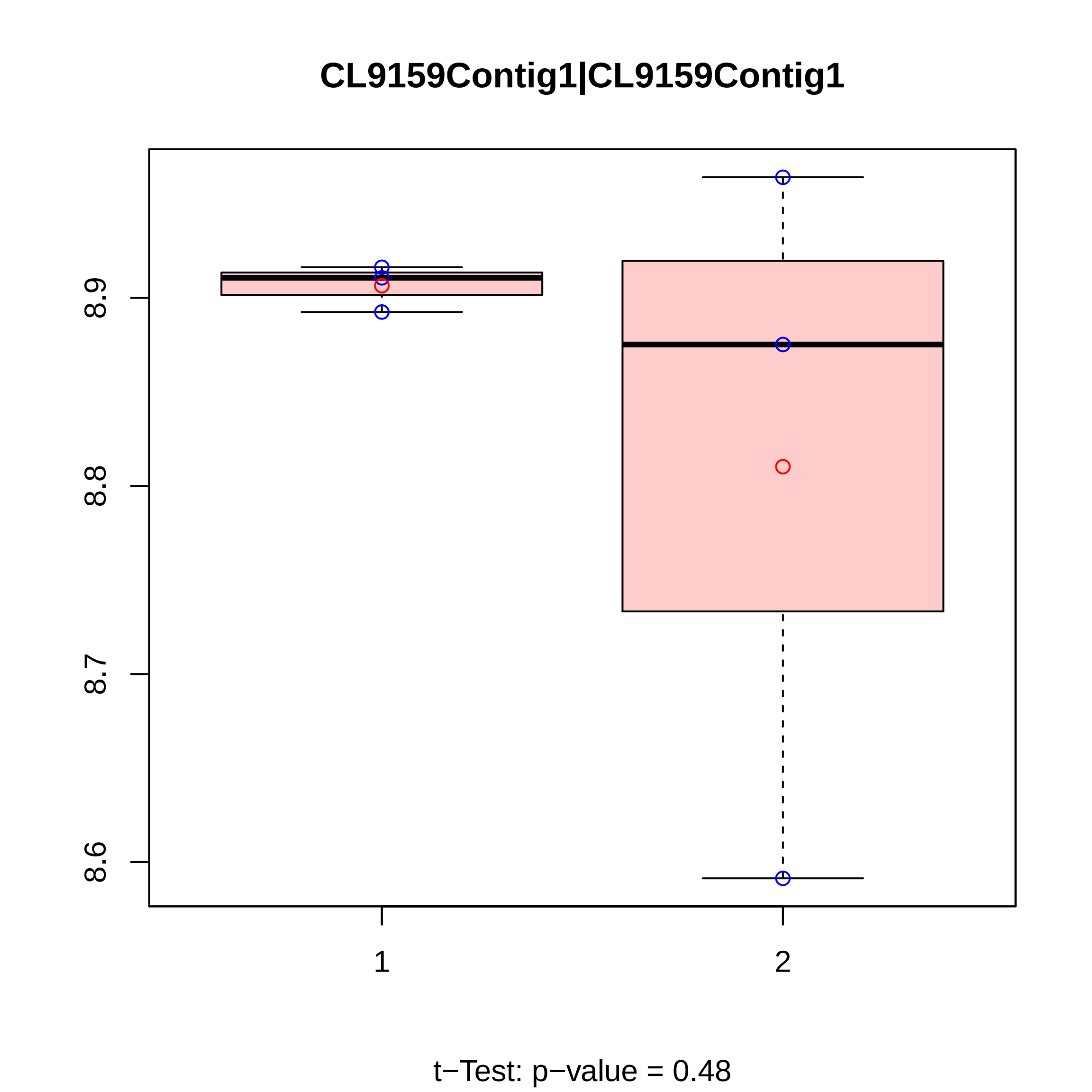
CL9159Contig1|CL9159Contig1
8.6
8.7
8.8
8.9
1
2
t−Test: p−value = 0.48

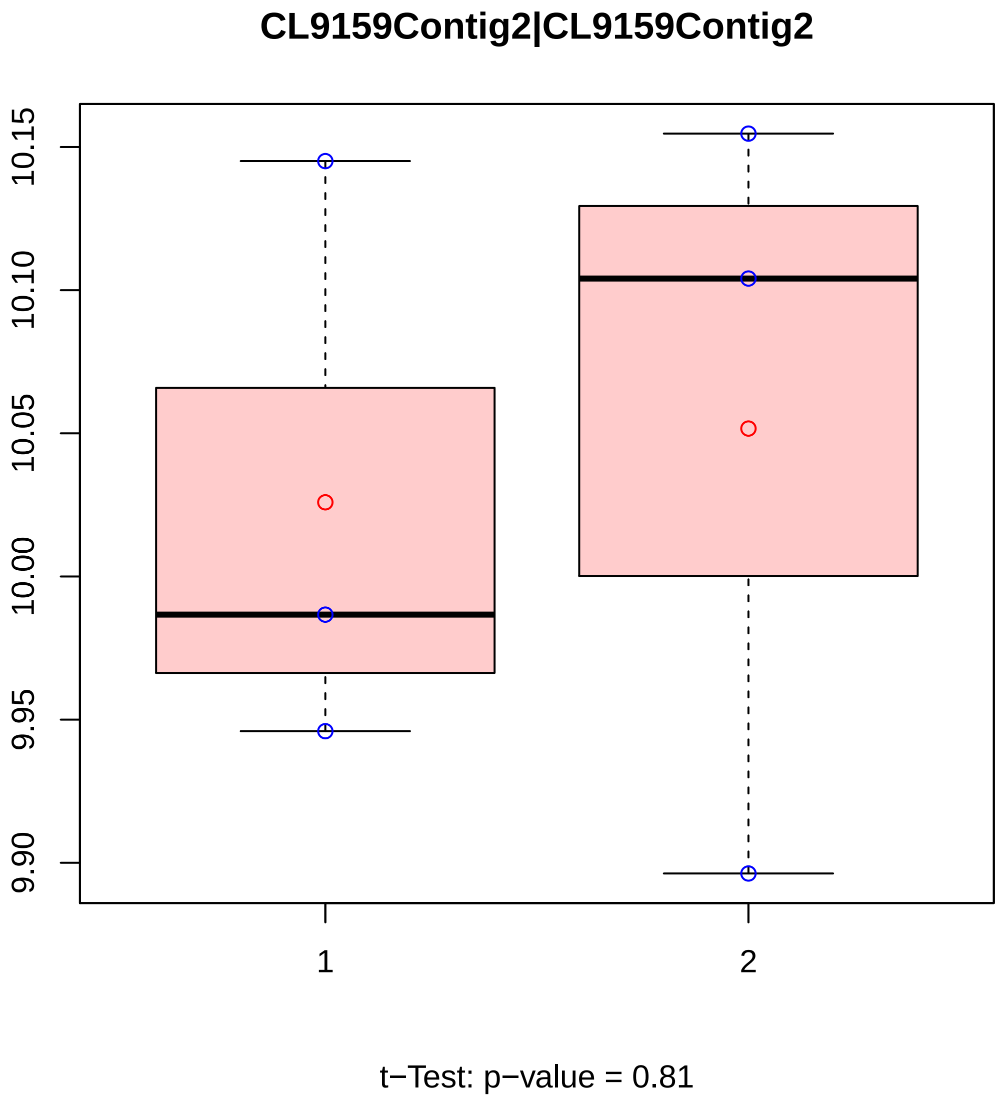
CL9159Contig2|CL9159Contig2
10.15
10.10
10.05
10.00
9.95
9.90
1
2
t−Test: p−value = 0.81

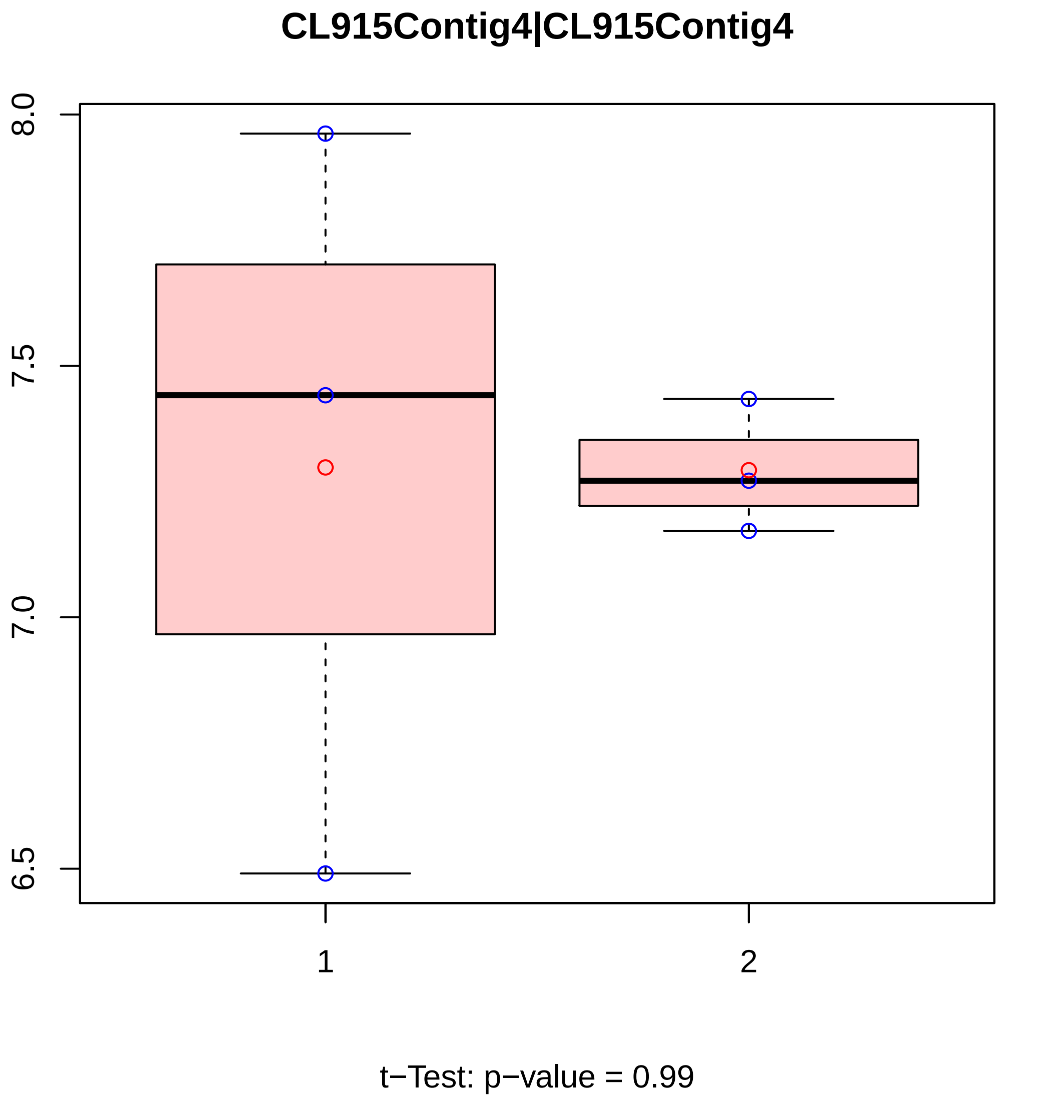
CL915Contig4|CL915Contig4
8.0
7.5
7.0
6.5
1
2
t−Test: p−value = 0.99

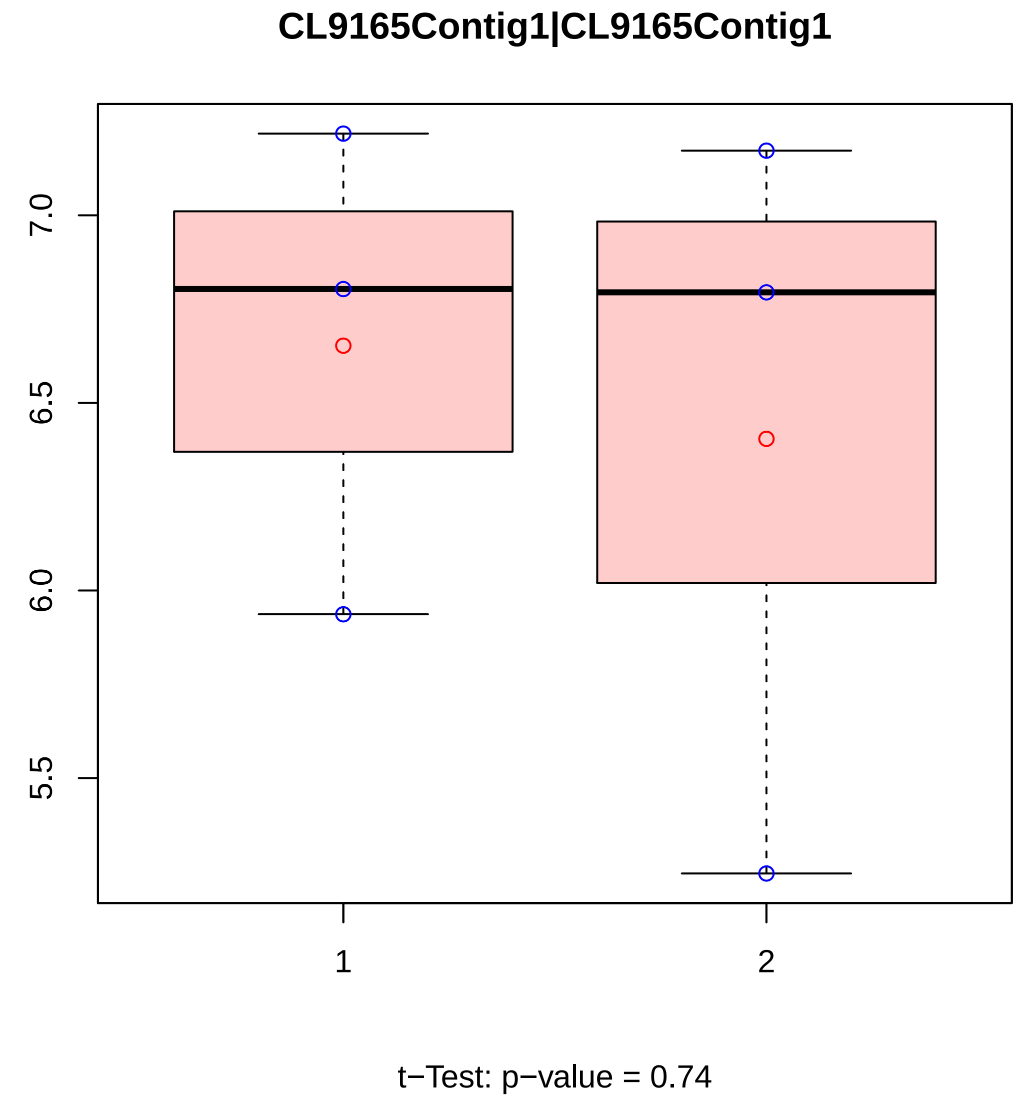

CL9165Contig1|CL9165Contig1
7.0
6.5
6.0
5.5
1
2
t−Test: p−value = 0.74

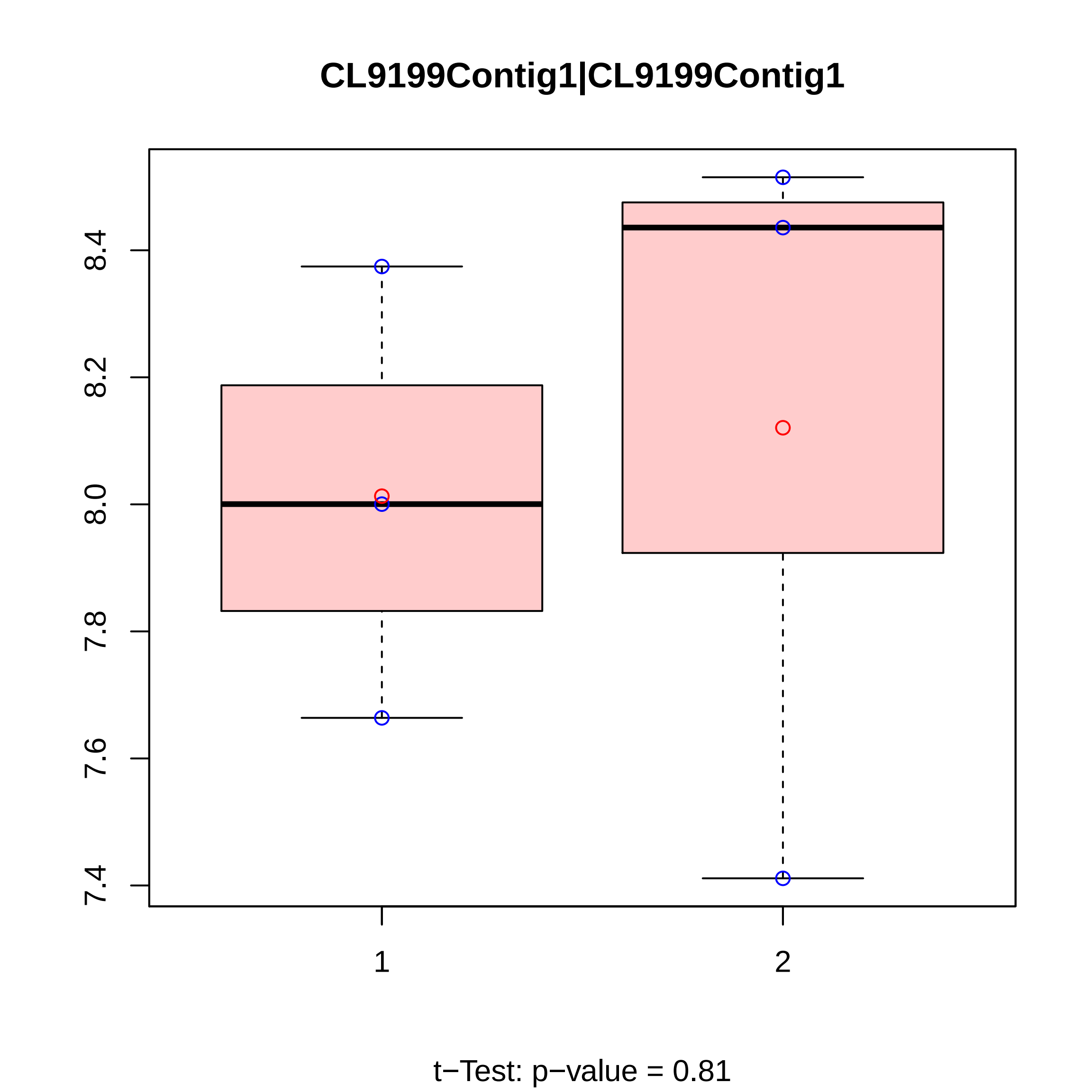
CL9199Contig1|CL9199Contig1
8.4
8.2
8.0
7.8
7.6
7.4
1
2
t−Test: p−value = 0.81

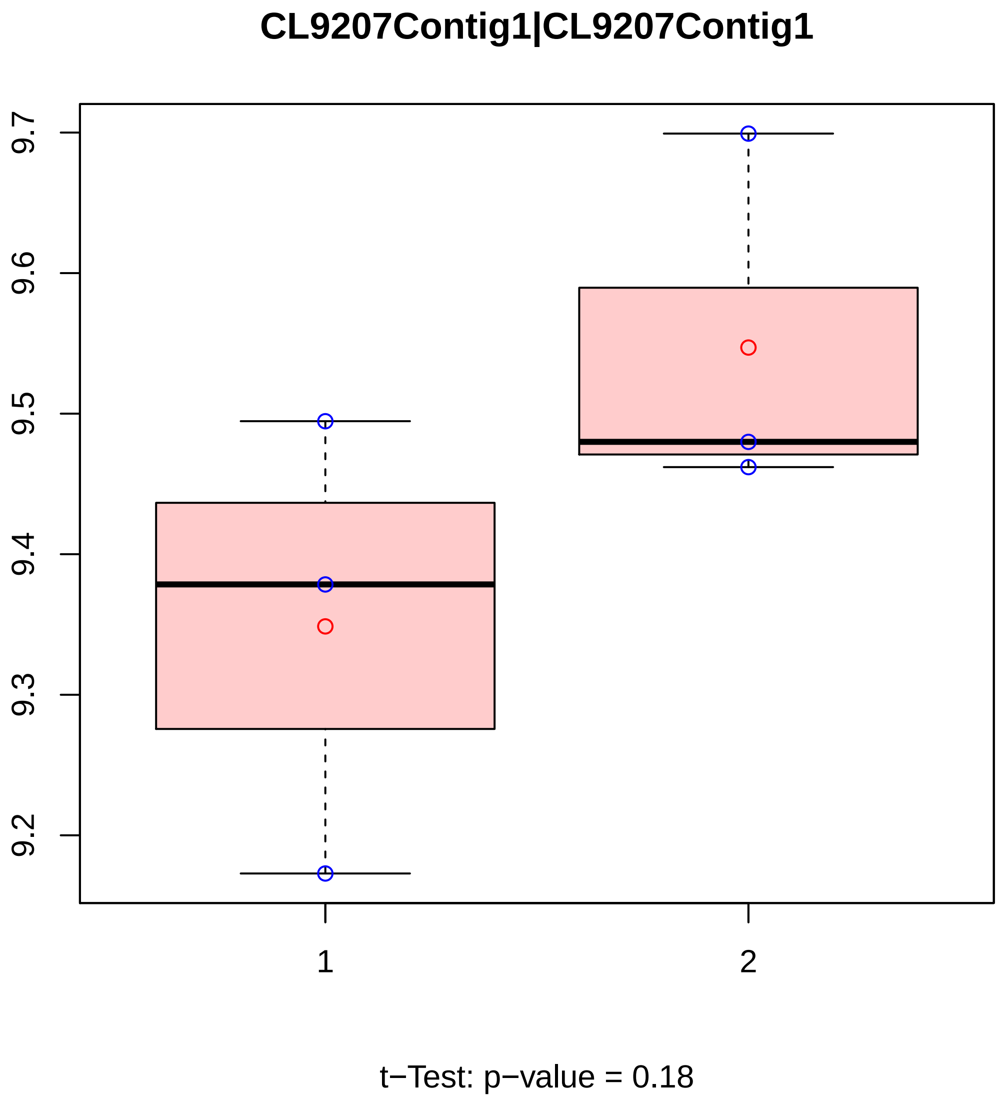
CL9207Contig1|CL9207Contig1
9.2
9.3
9.4
9.5
9.6
9.7
1
2
t−Test: p−value = 0.18

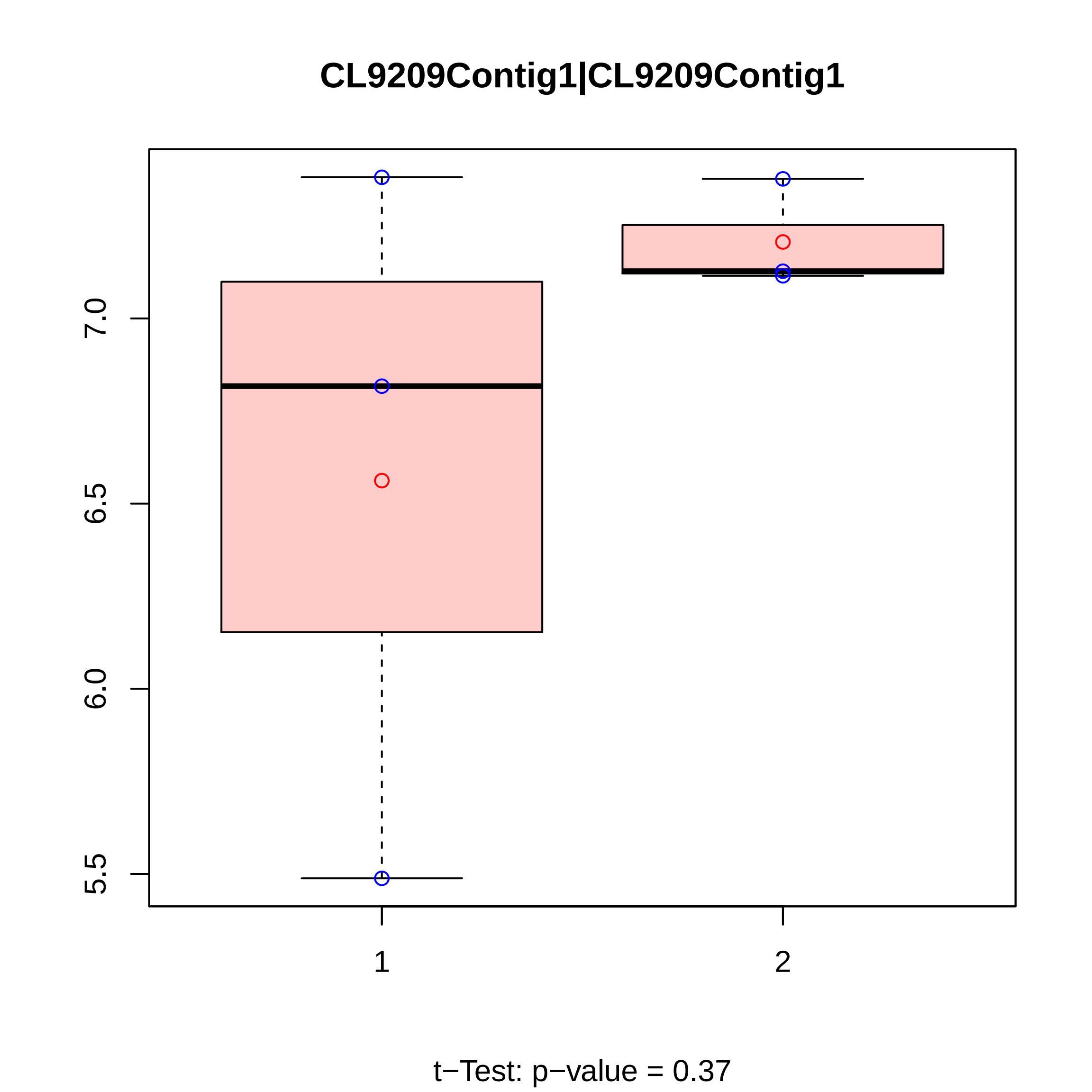
CL9209Contig1|CL9209Contig1
7.0
6.5
6.0
5.5
1
2
t−Test: p−value = 0.37

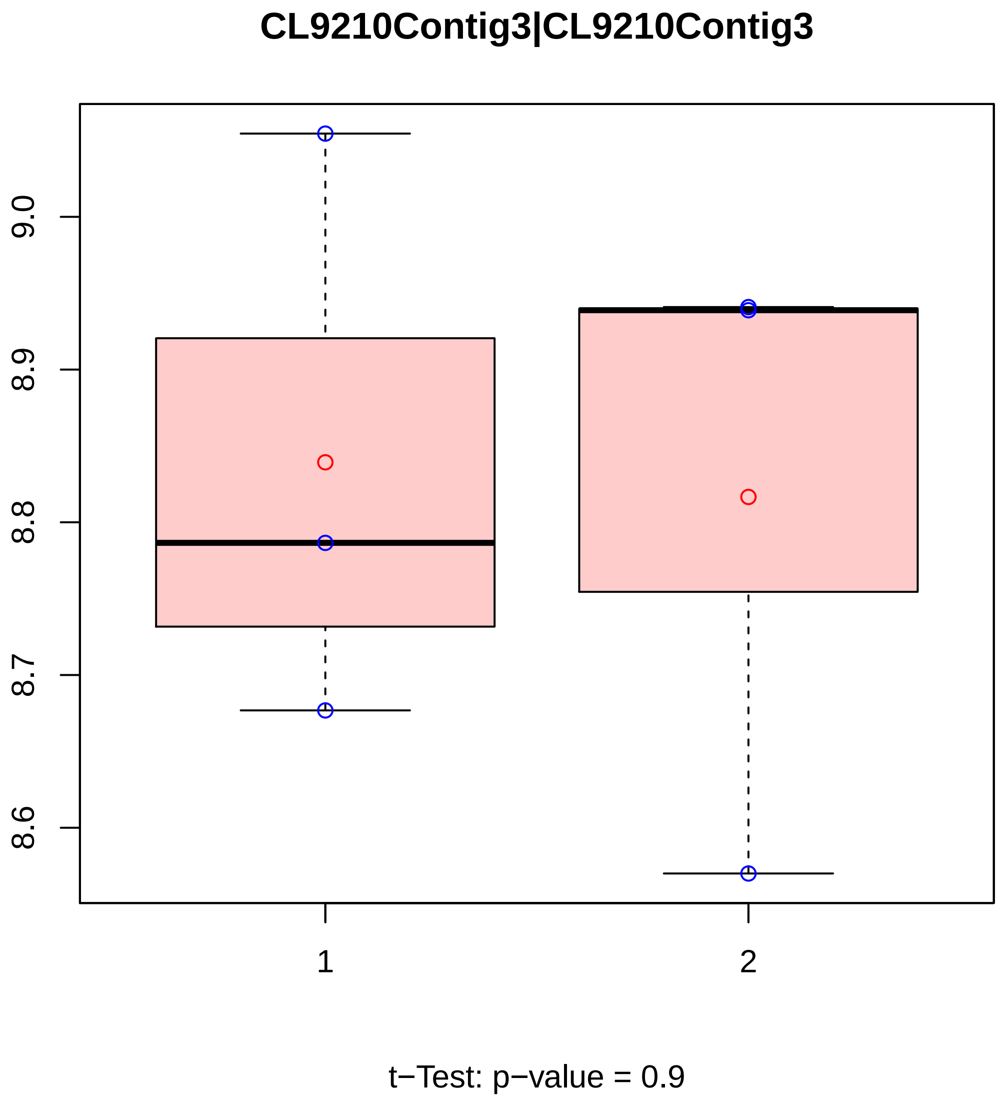
CL9210Contig3|CL9210Contig3
9.0
8.9
8.8
8.7
8.6
1
2
t−Test: p−value = 0.9

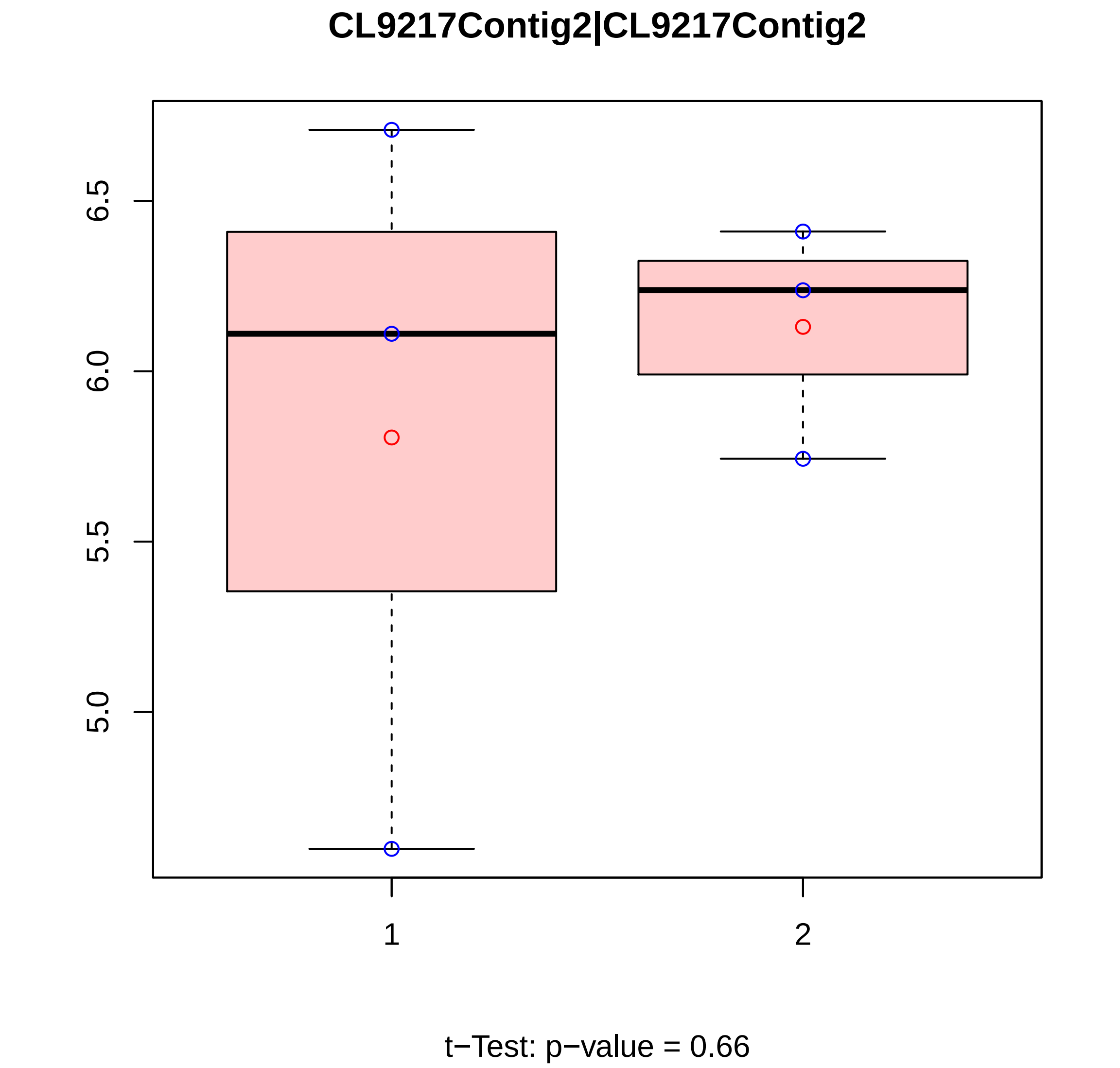

CL9217Contig2|CL9217Contig2
6.5
6.0
5.5
5.0
1
2
t−Test: p−value = 0.66

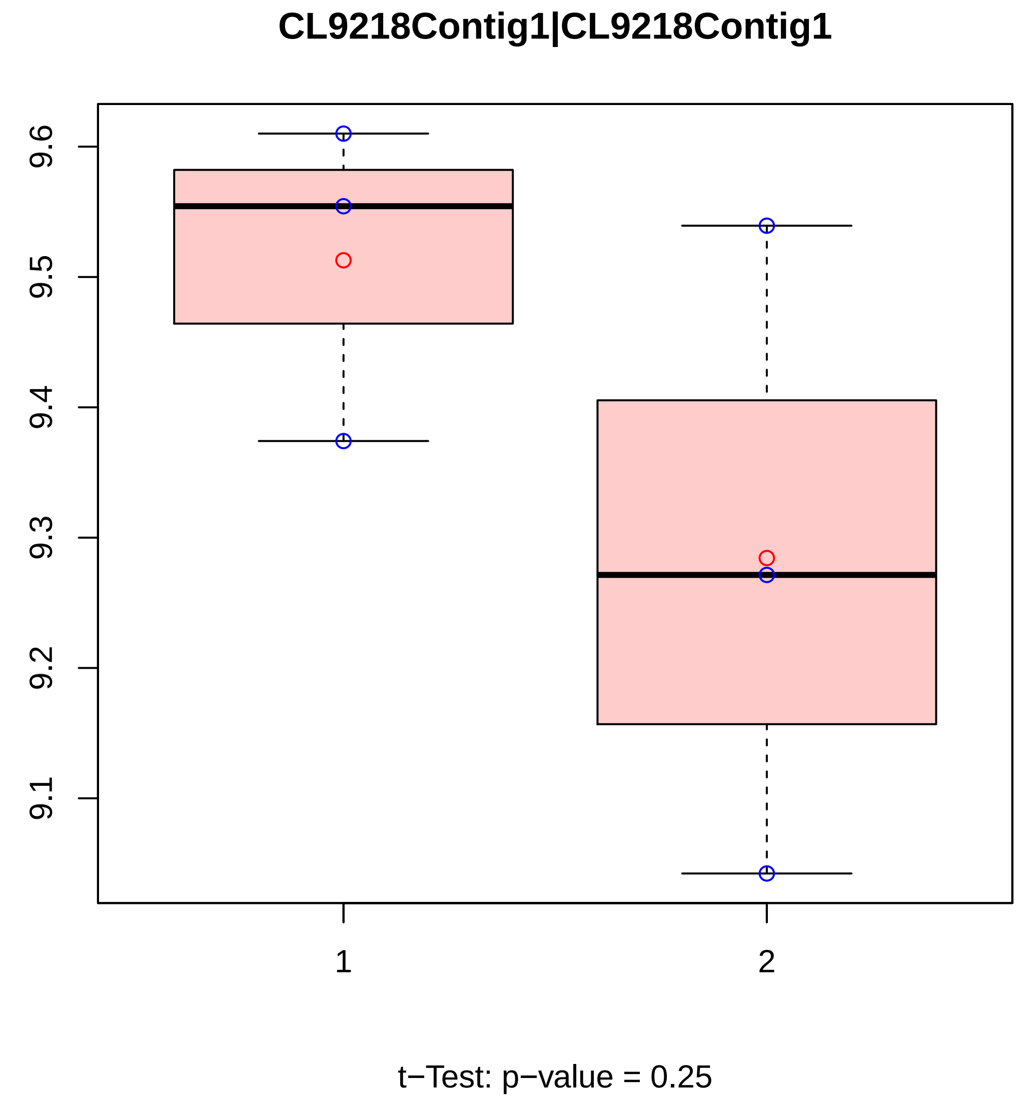
CL9218Contig1|CL9218Contig1
9.1
9.2
9.3
9.4
9.5
9.6
1
2
t−Test: p−value = 0.25

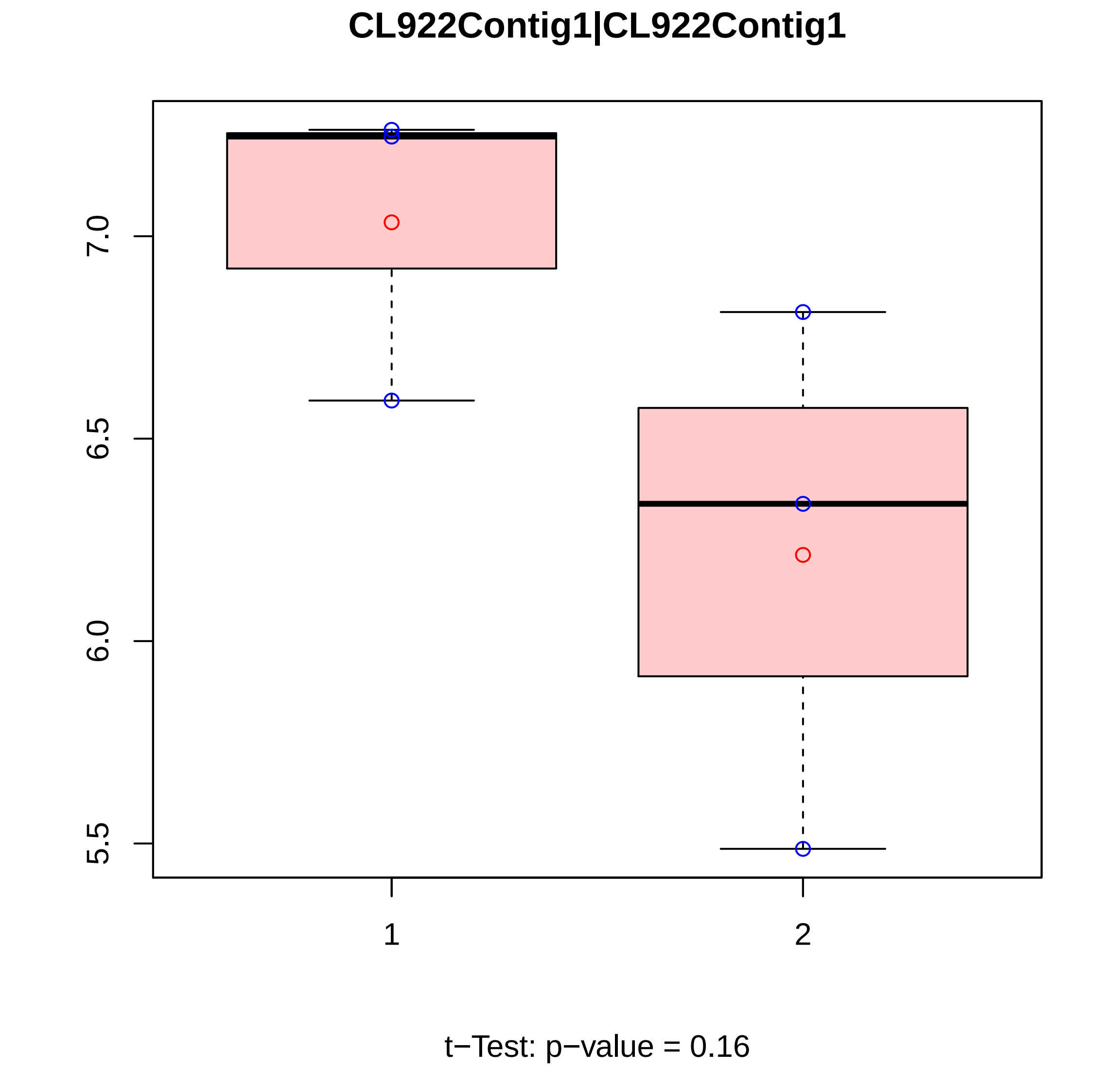

CL922Contig1|CL922Contig1
7.0
6.5
6.0
5.5
1
2
t−Test: p−value = 0.16

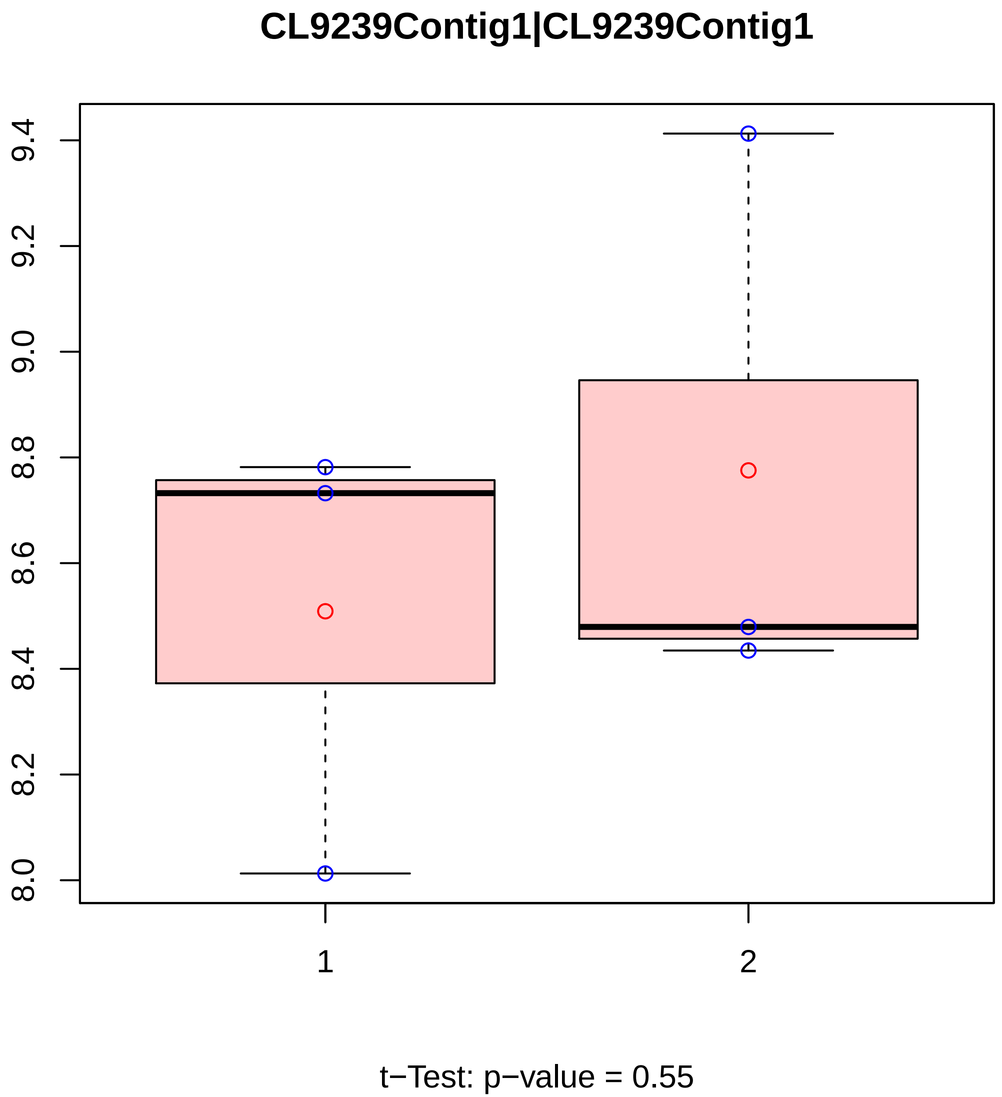

CL9239Contig1|CL9239Contig1
8.0
8.2
8.4
8.6
8.8
9.0
9.2
9.4
1
2
t−Test: p−value = 0.55

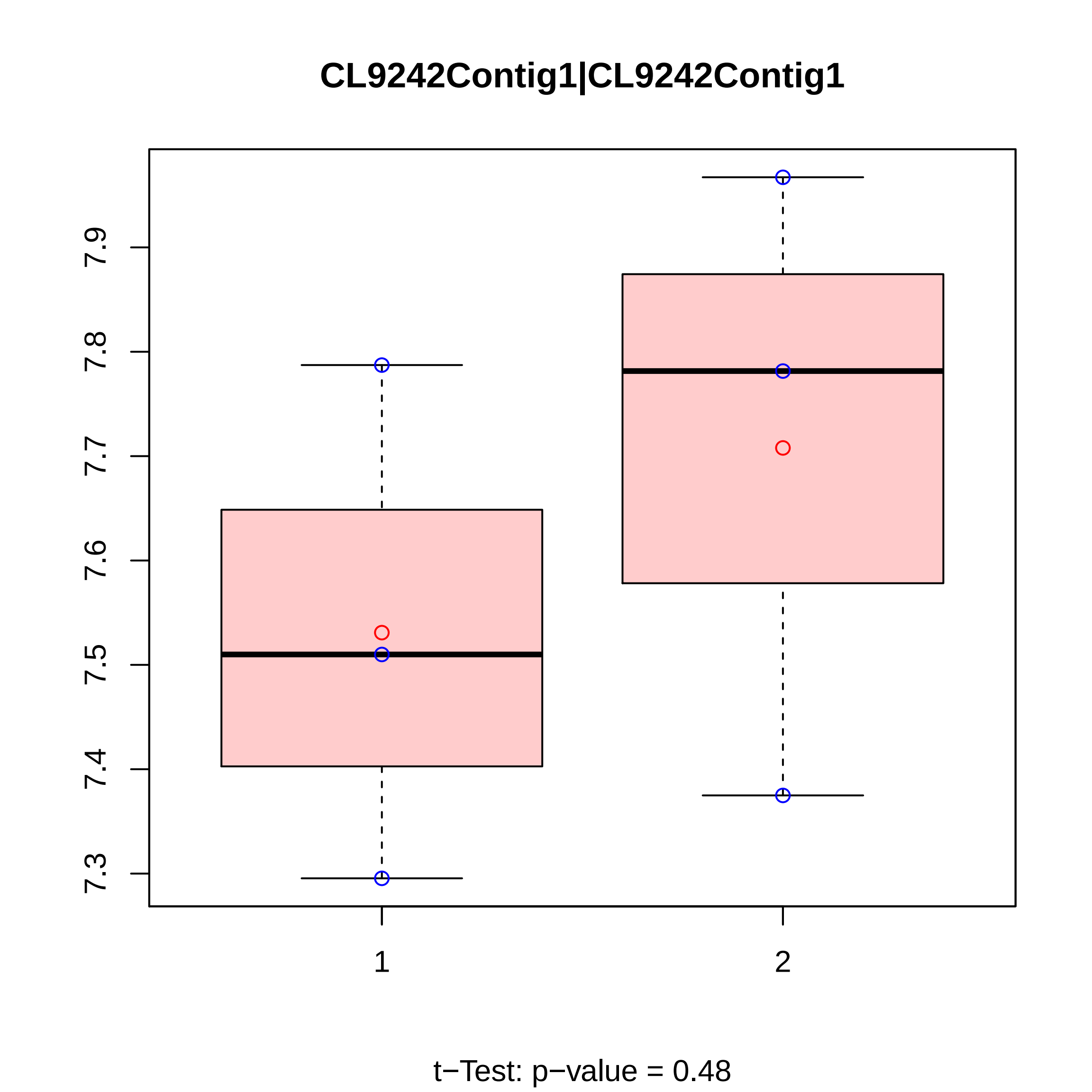
CL9242Contig1|CL9242Contig1
7.3
7.4
7.5
7.6
7.7
7.8
7.9
1
2
t−Test: p−value = 0.48

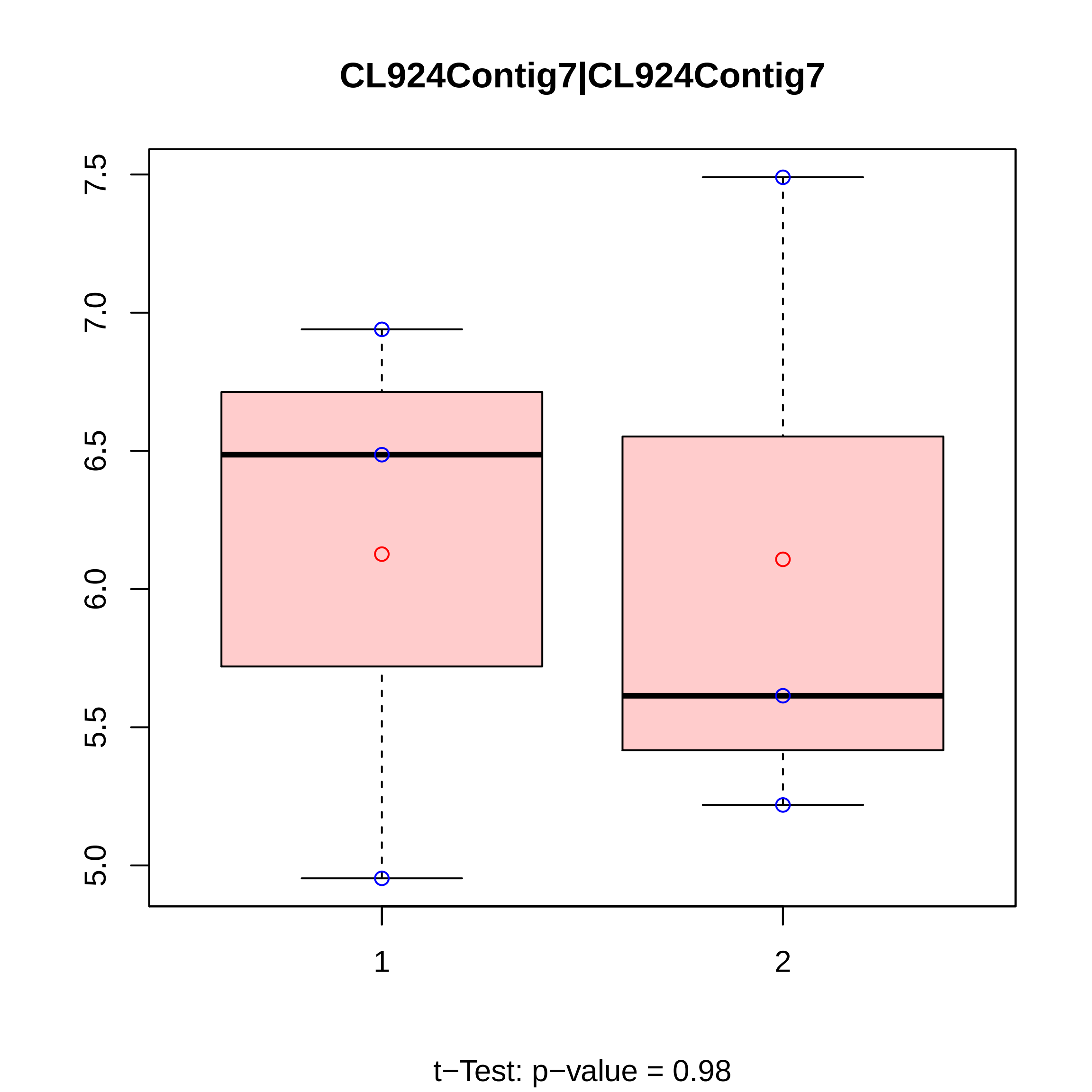
CL924Contig7|CL924Contig7
7.5
7.0
6.5
6.0
5.5
5.0
1
2
t−Test: p−value = 0.98

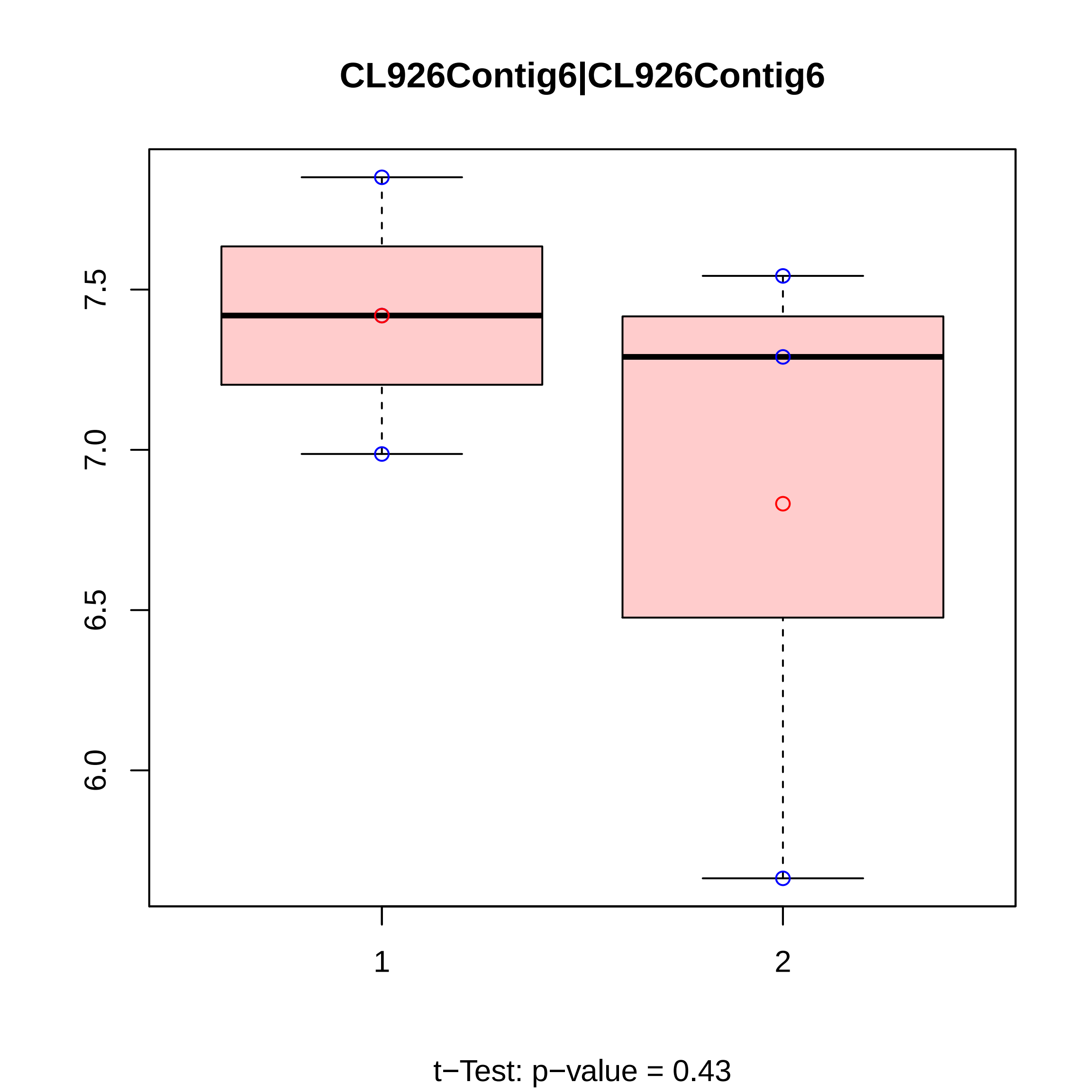

CL926Contig6|CL926Contig6
7.5
7.0
6.5
6.0
1
2
t−Test: p−value = 0.43

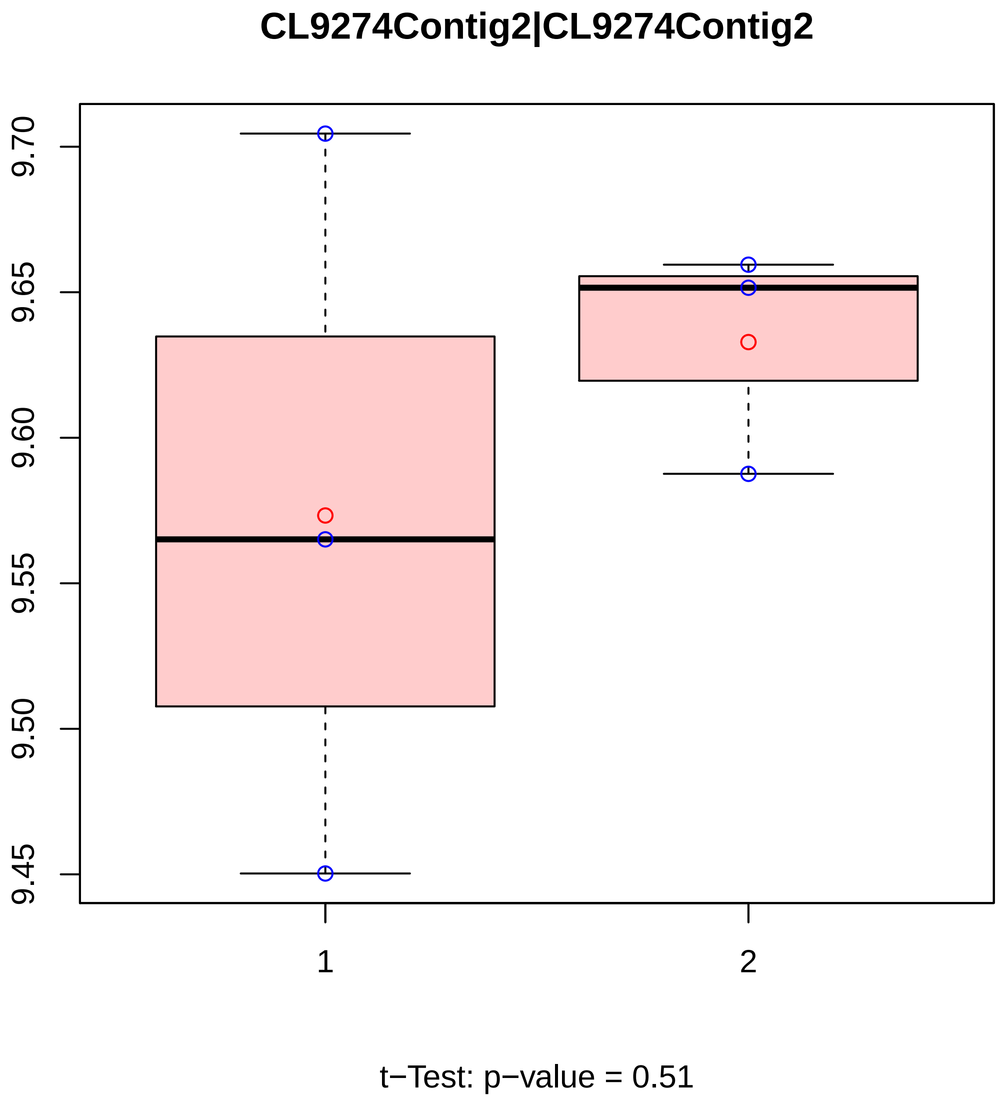

CL9274Contig2|CL9274Contig2
9.45
9.50
9.55
9.60
9.65
9.70
1
2
t−Test: p−value = 0.51

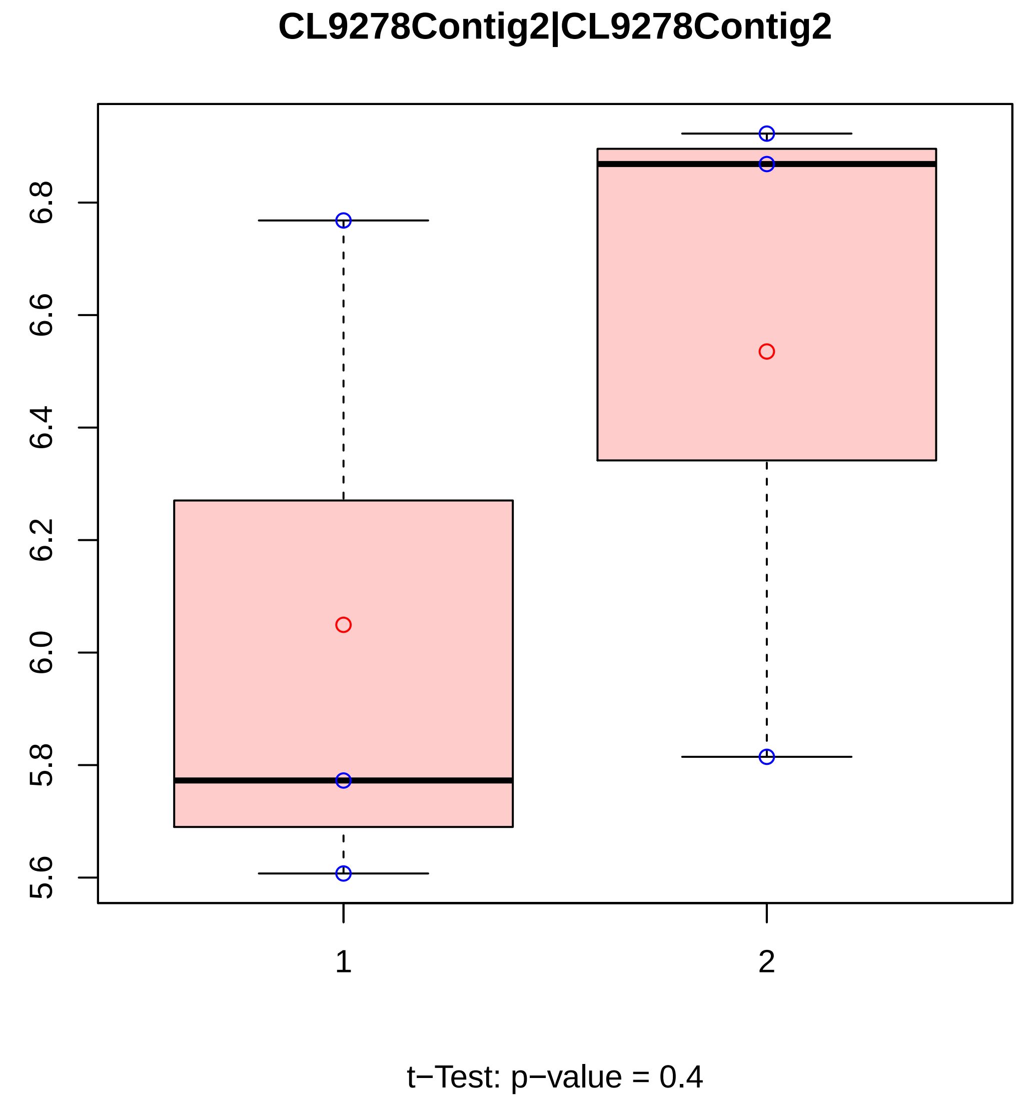
CL9278Contig2|CL9278Contig2
5.6
5.8
6.0
6.2
6.4
6.6
6.8
1
2
t−Test: p−value = 0.4

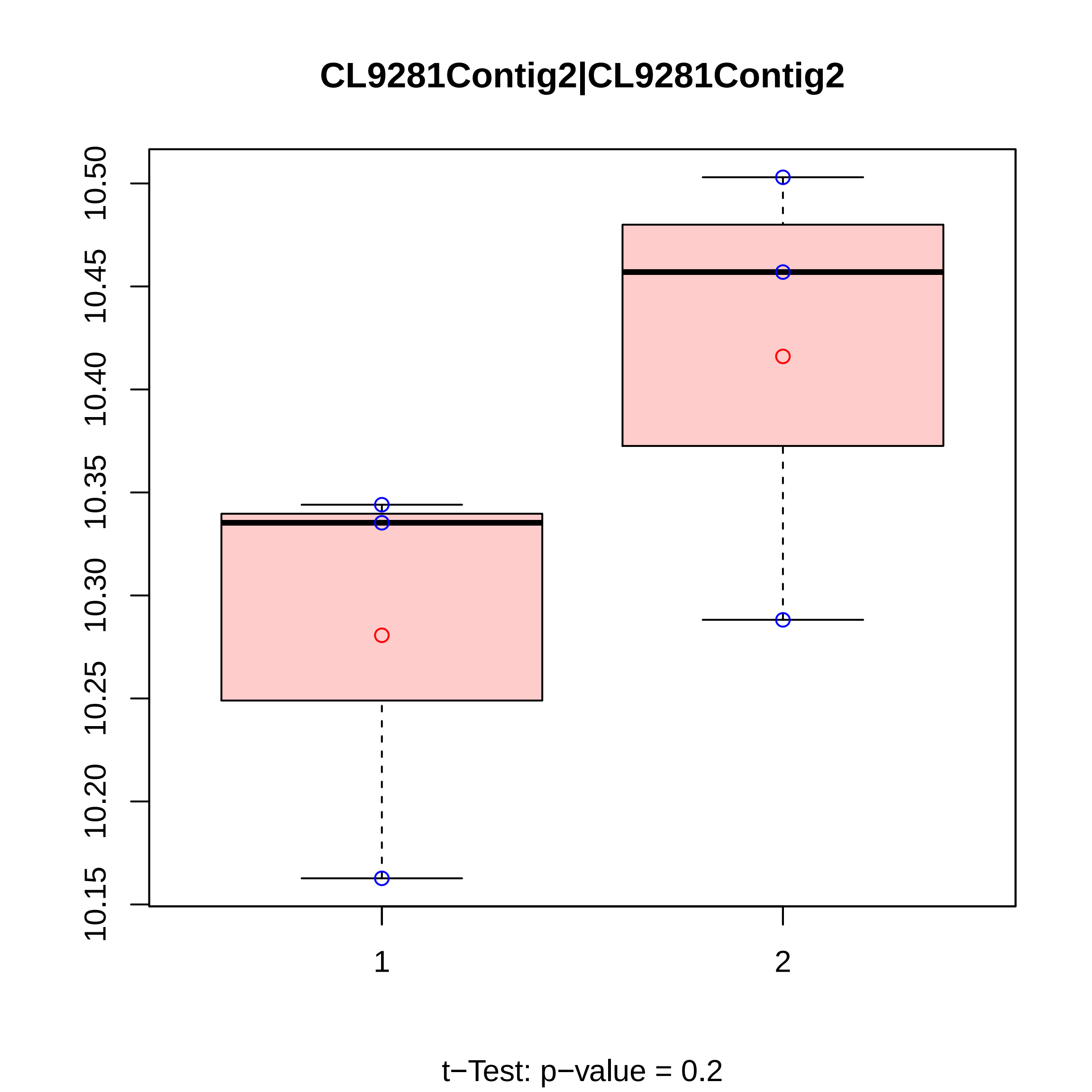

CL9281Contig2|CL9281Contig2
10.15
10.20
10.25
10.30
10.35
10.40
10.45
10.50
1
2
t−Test: p−value = 0.2

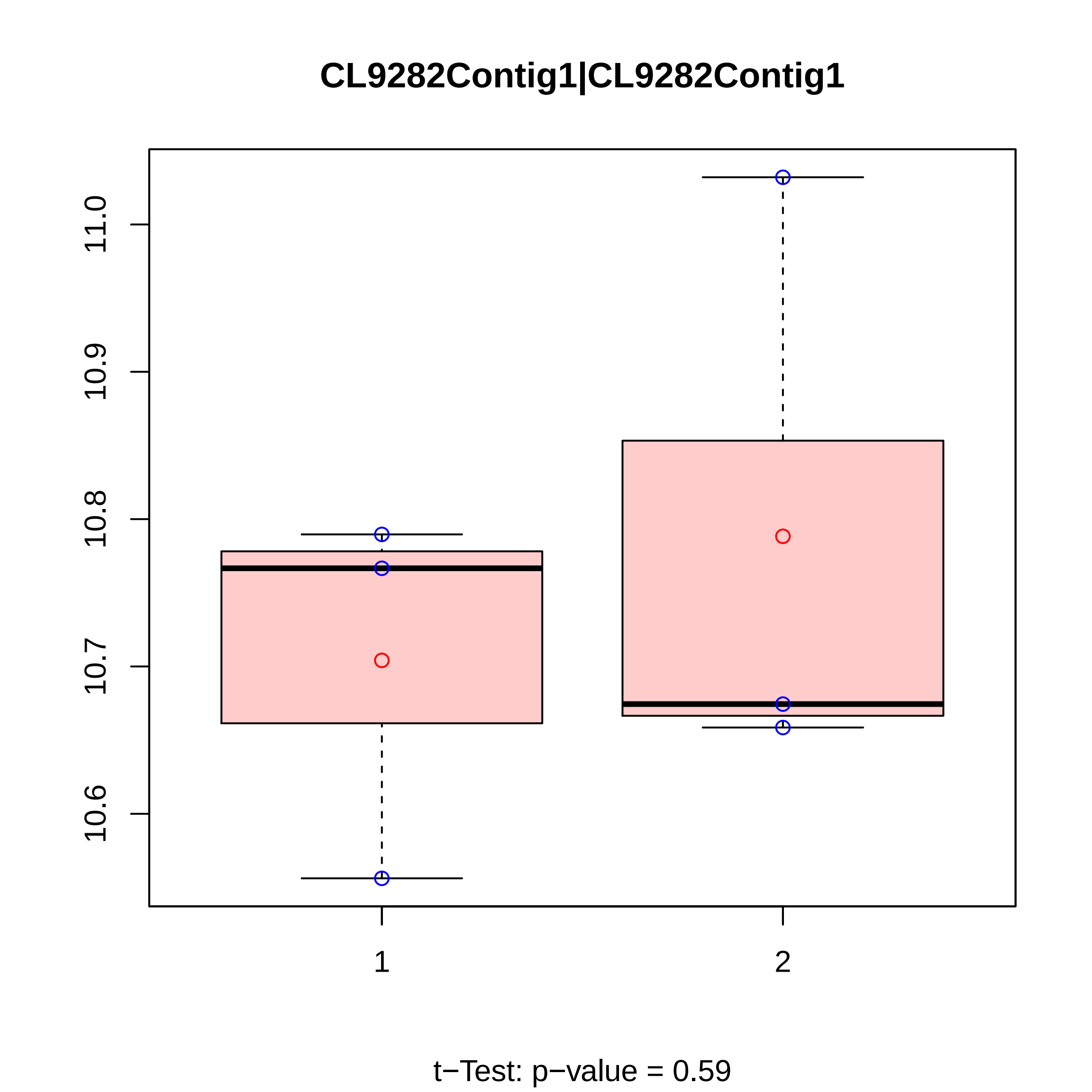
CL9282Contig1|CL9282Contig1
10.6
10.7
10.8
10.9
11.0
1
2
t−Test: p−value = 0.59

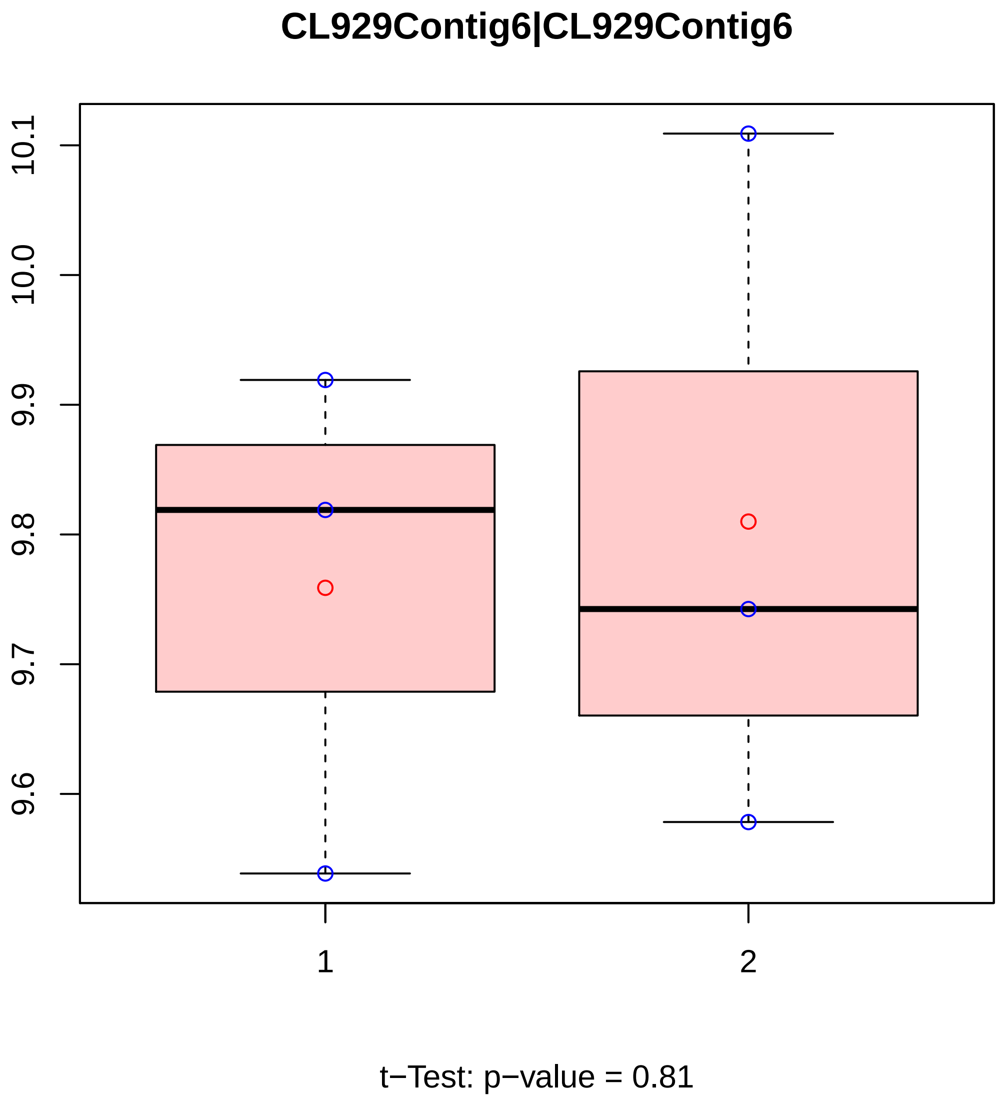

CL929Contig6|CL929Contig6
10.1
10.0
9.9
9.8
9.7
9.6
1
2
t−Test: p−value = 0.81

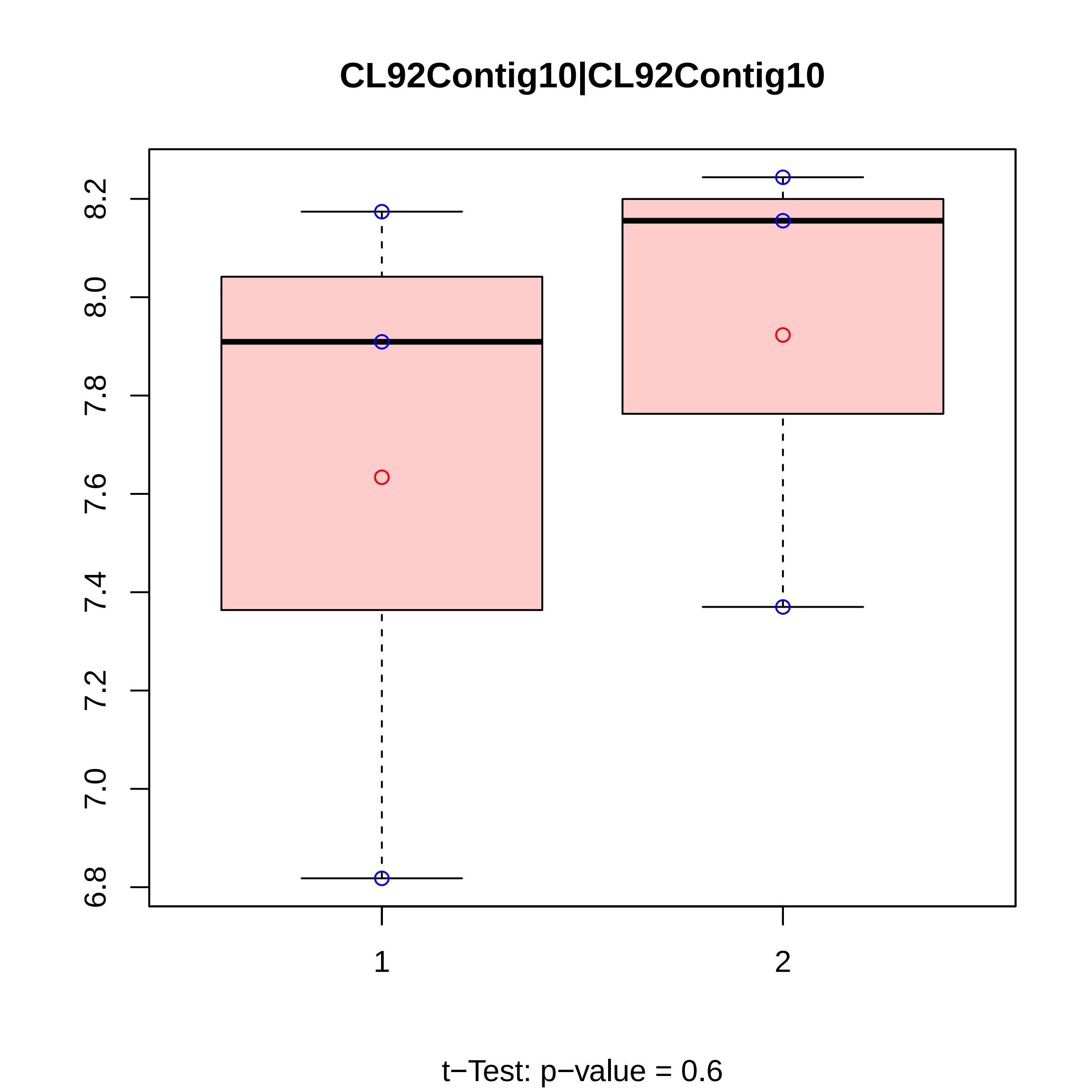
CL92Contig10|CL92Contig10
8.2
8.0
7.8
7.6
7.4
7.2
7.0
6.8
1
2
t−Test: p−value = 0.6

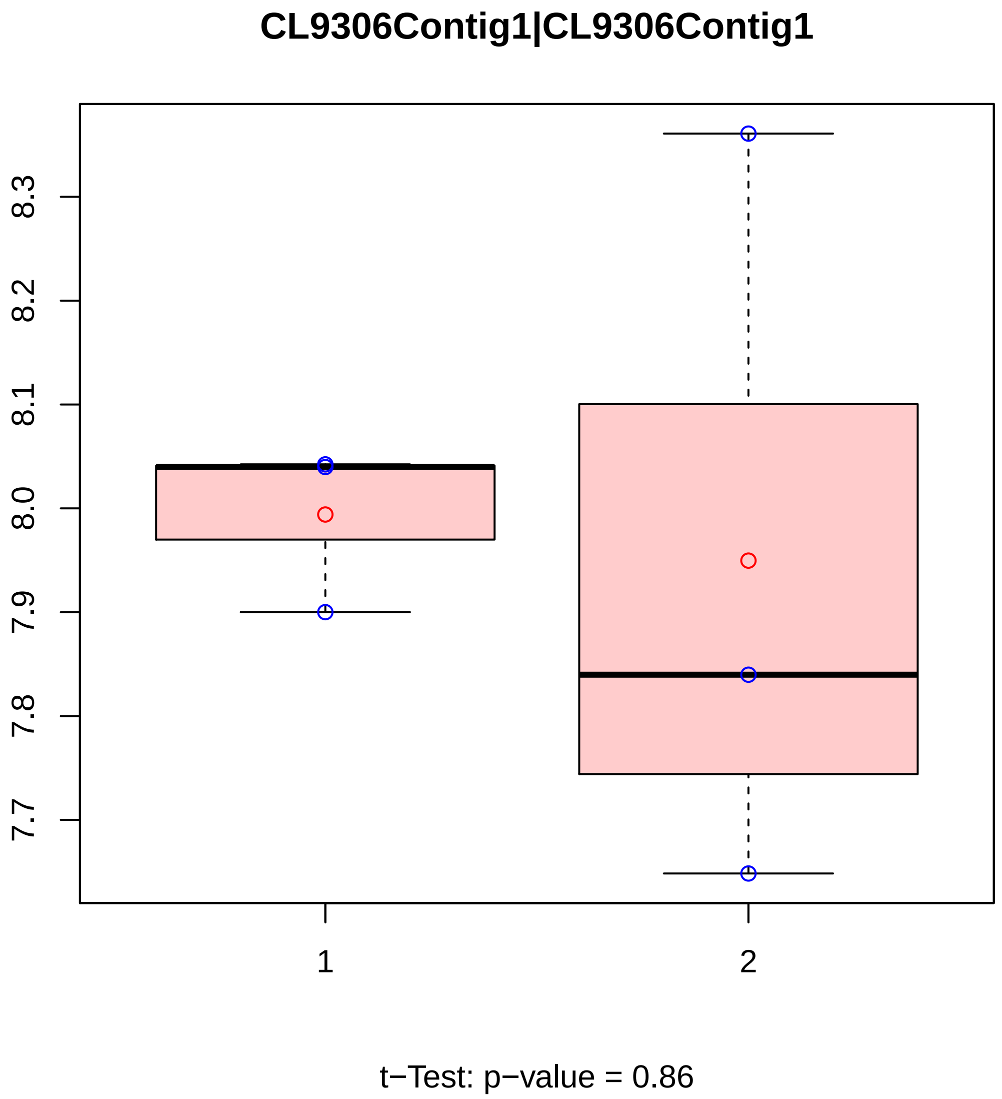
CL9306Contig1|CL9306Contig1
8.3
8.2
8.1
8.0
7.9
7.8
7.7
1
2
t−Test: p−value = 0.86

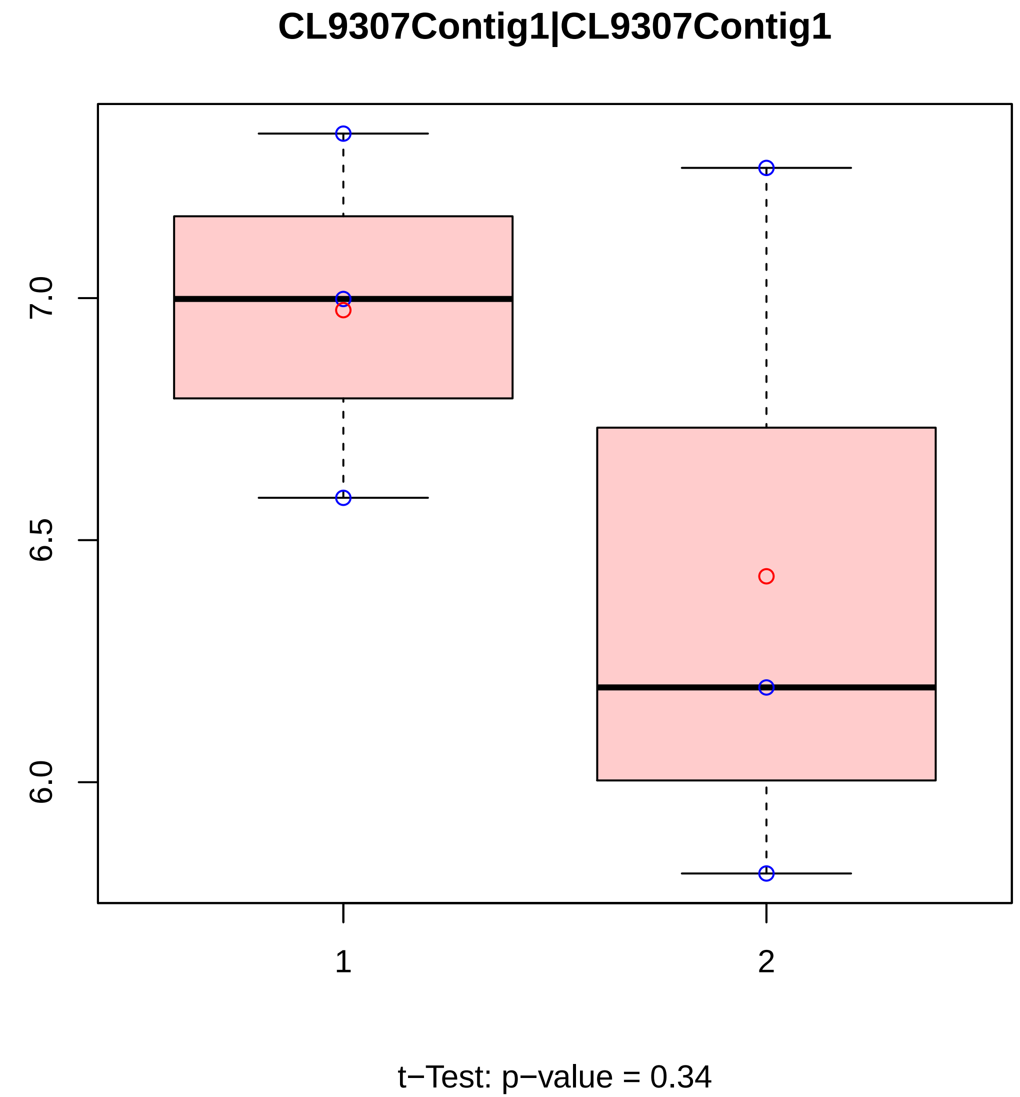
CL9307Contig1|CL9307Contig1
7.0
6.5
6.0
1
2
t−Test: p−value = 0.34

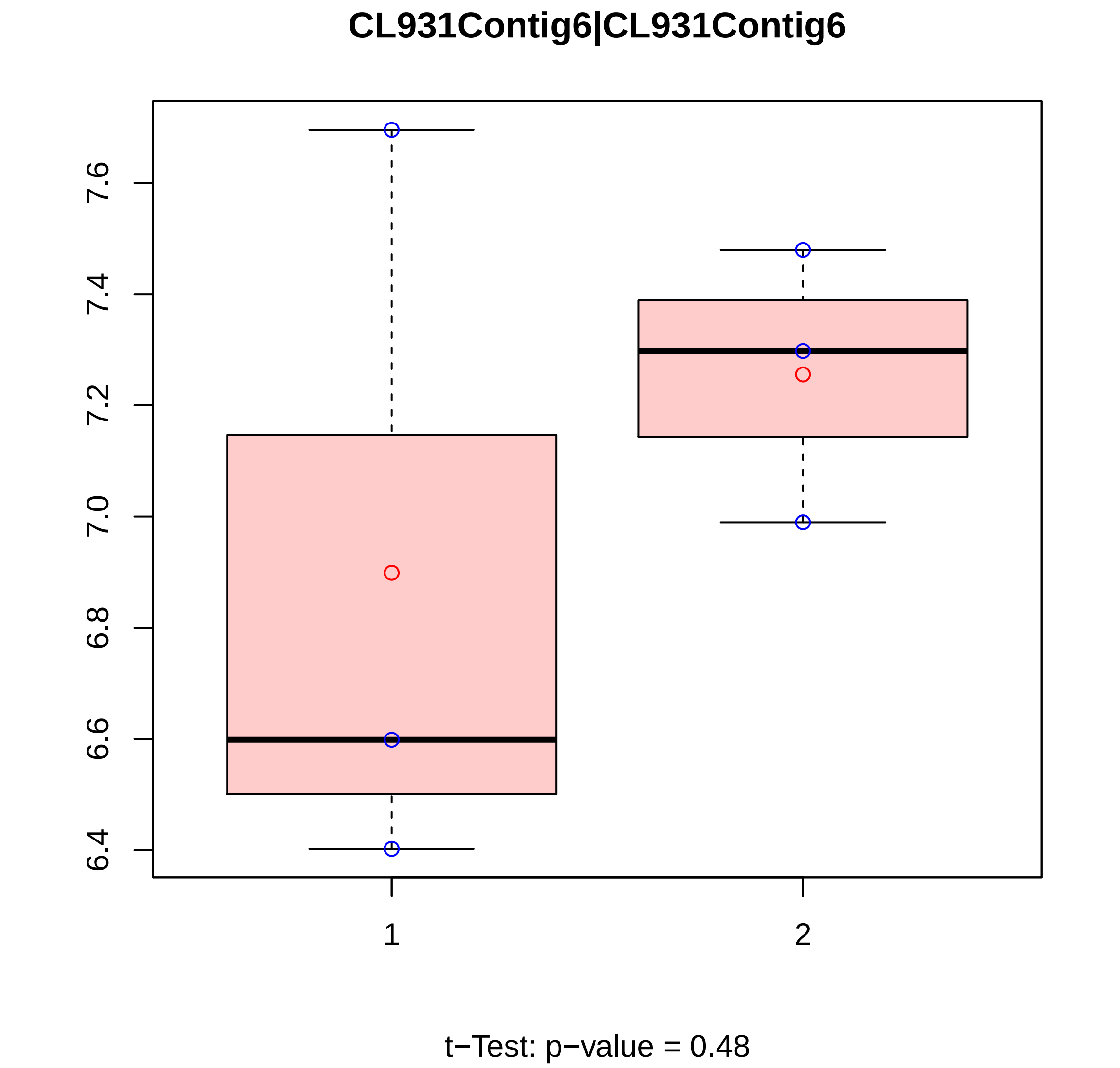
CL931Contig6|CL931Contig6
7.6
7.4
7.2
7.0
6.8
6.6
6.4
1
2
t−Test: p−value = 0.48

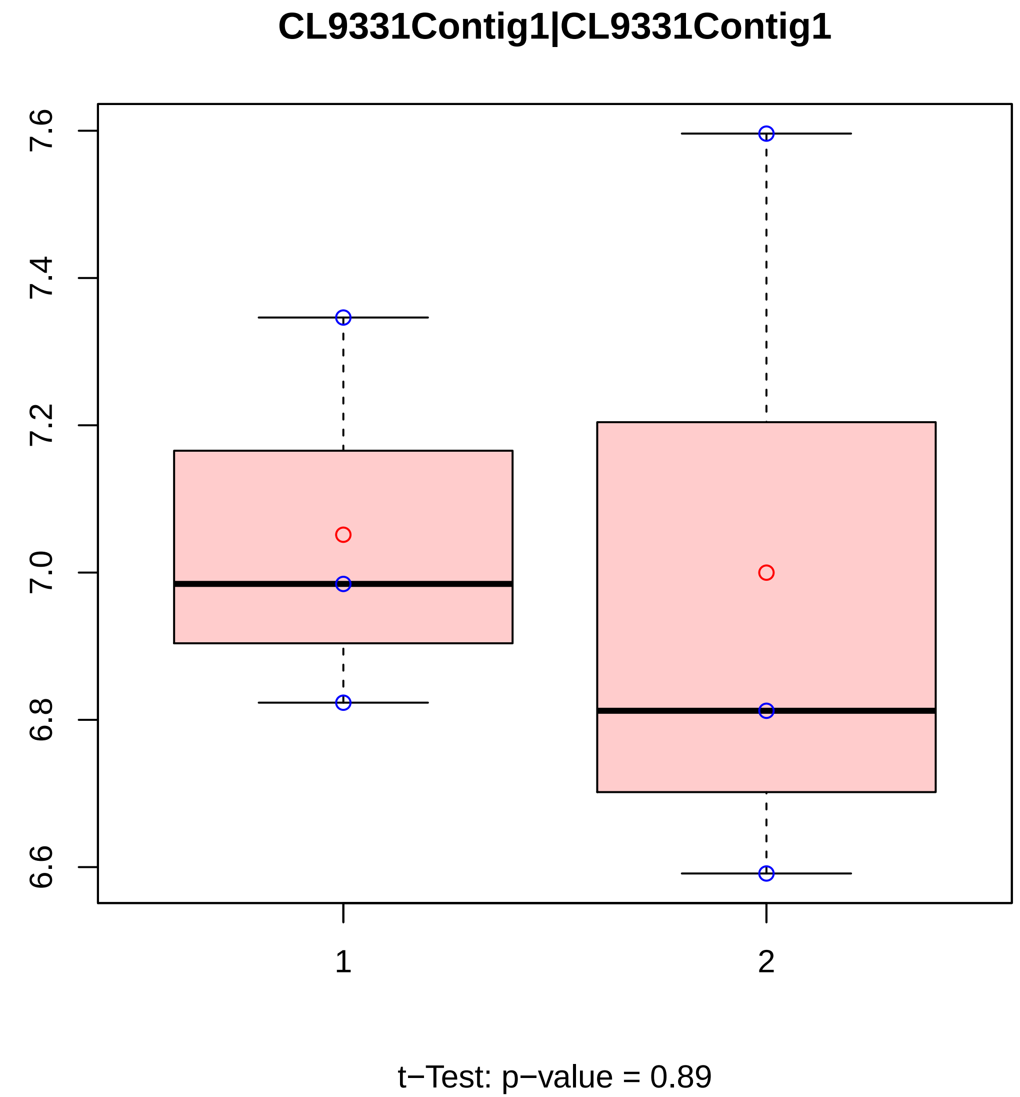
CL9331Contig1|CL9331Contig1
7.6
7.4
7.2
7.0
6.8
6.6
1
2
t−Test: p−value = 0.89

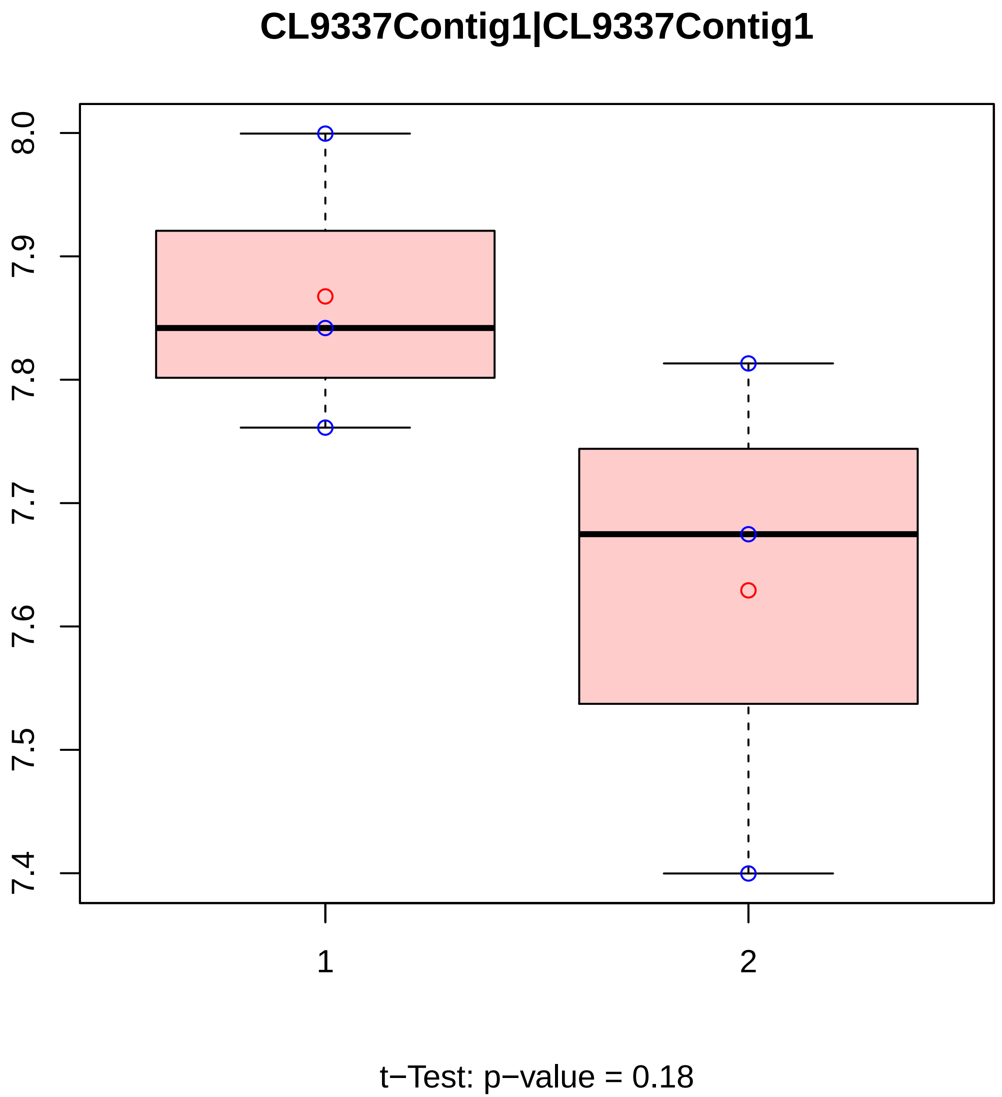
CL9337Contig1|CL9337Contig1
8.0
7.9
7.8
7.7
7.6
7.5
7.4
1
2
t−Test: p−value = 0.18

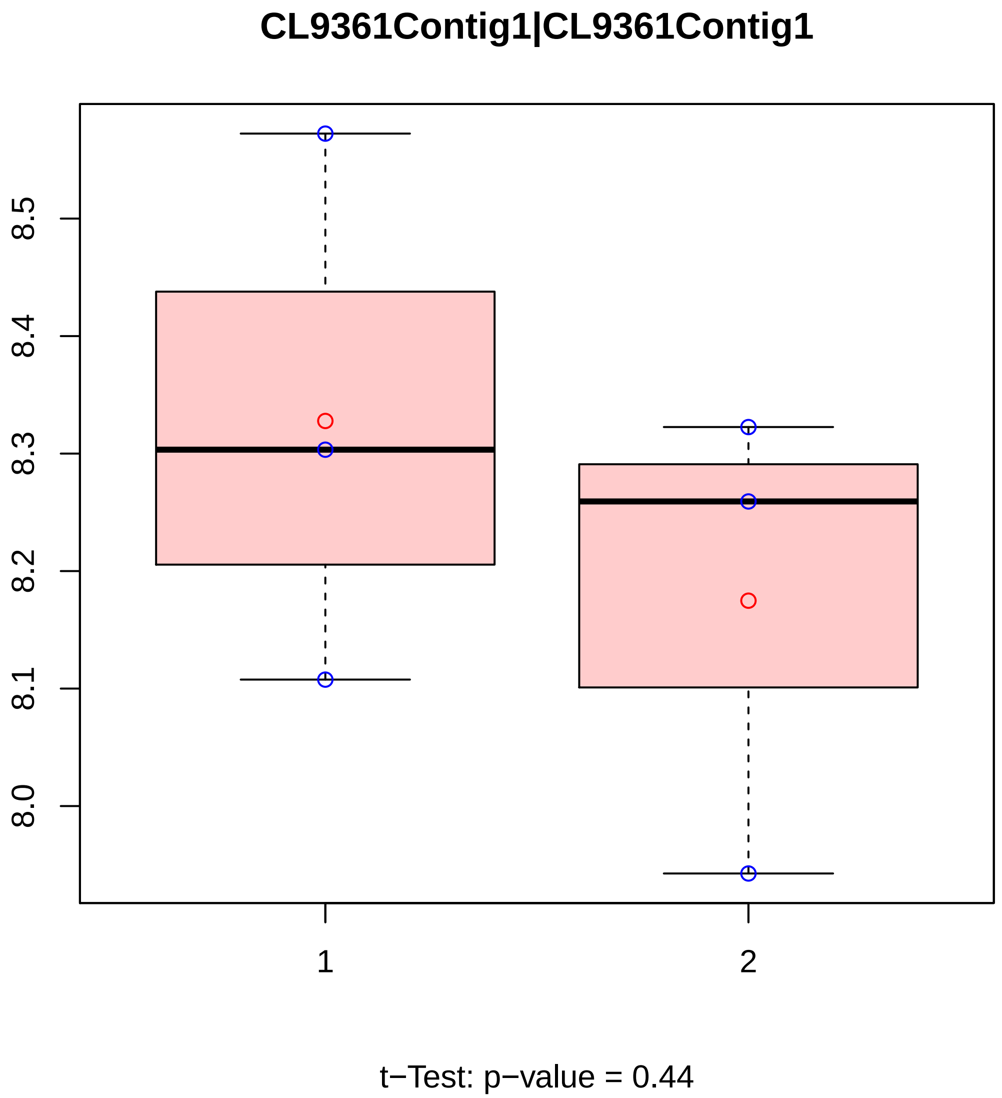

CL9361Contig1|CL9361Contig1
8.0
8.1
8.2
8.3
8.4
8.5
1
2
t−Test: p−value = 0.44

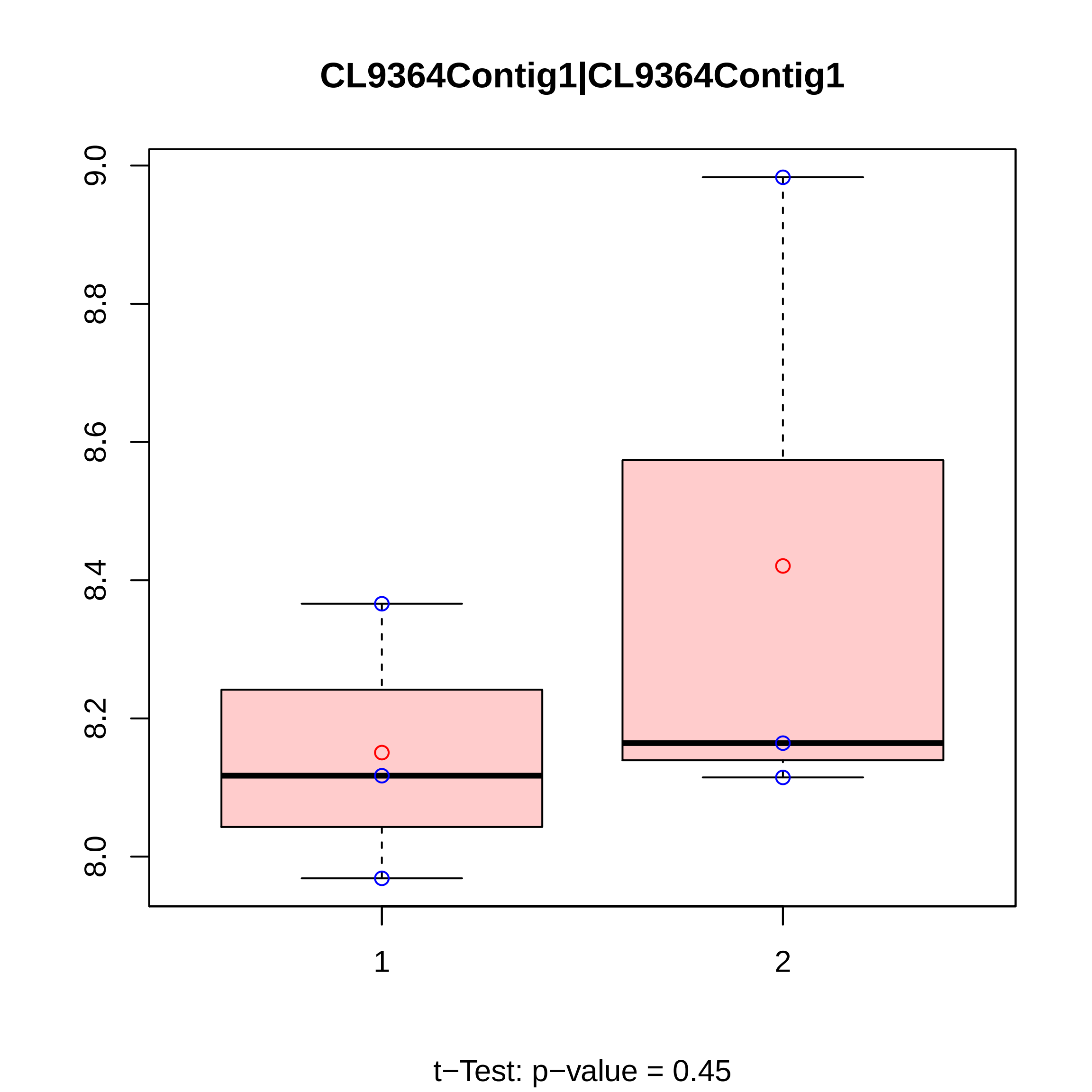
CL9364Contig1|CL9364Contig1
9.0
8.8
8.6
8.4
8.2
8.0
1
2
t−Test: p−value = 0.45

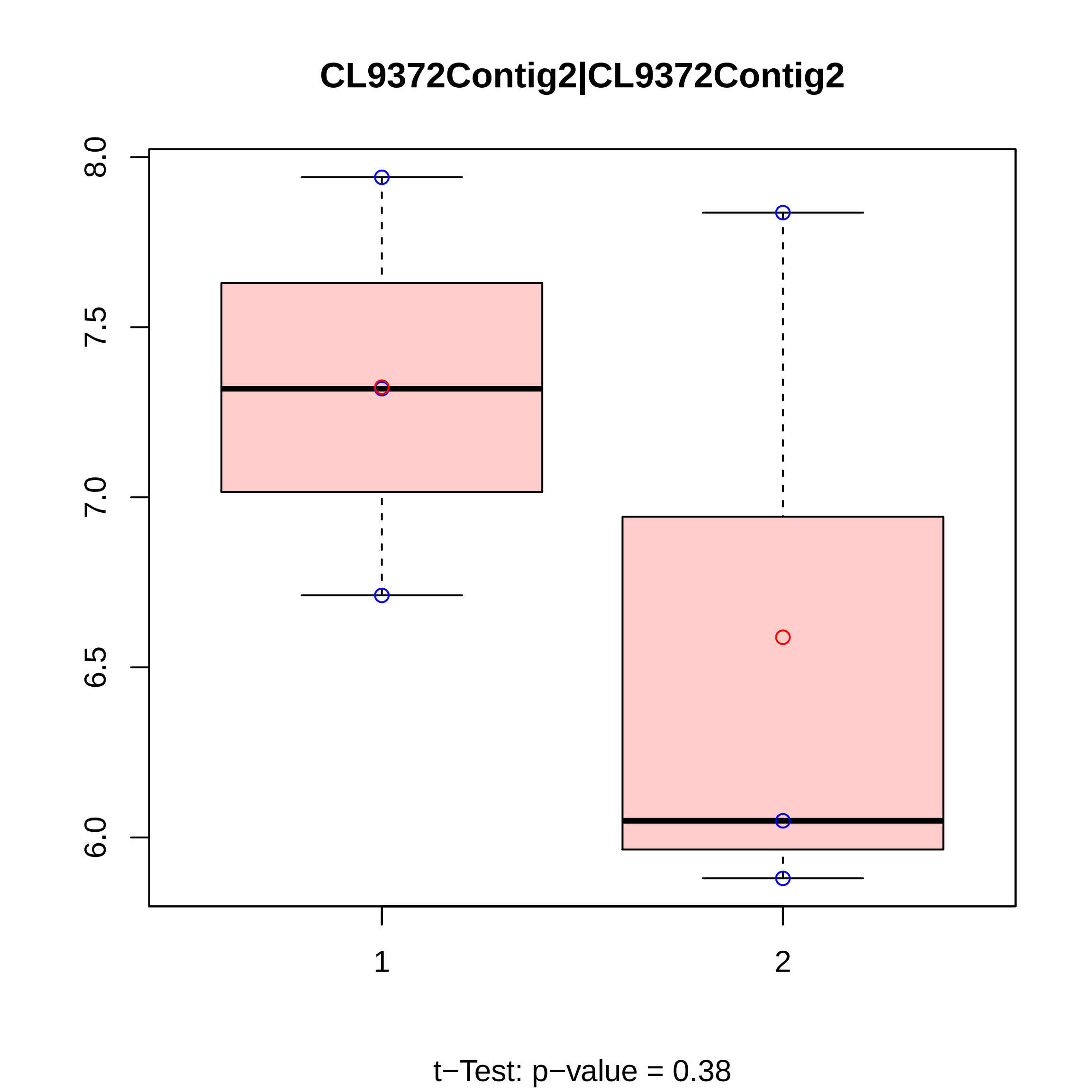

CL9372Contig2|CL9372Contig2
8.0
7.5
7.0
6.5
6.0
1
2
t−Test: p−value = 0.38

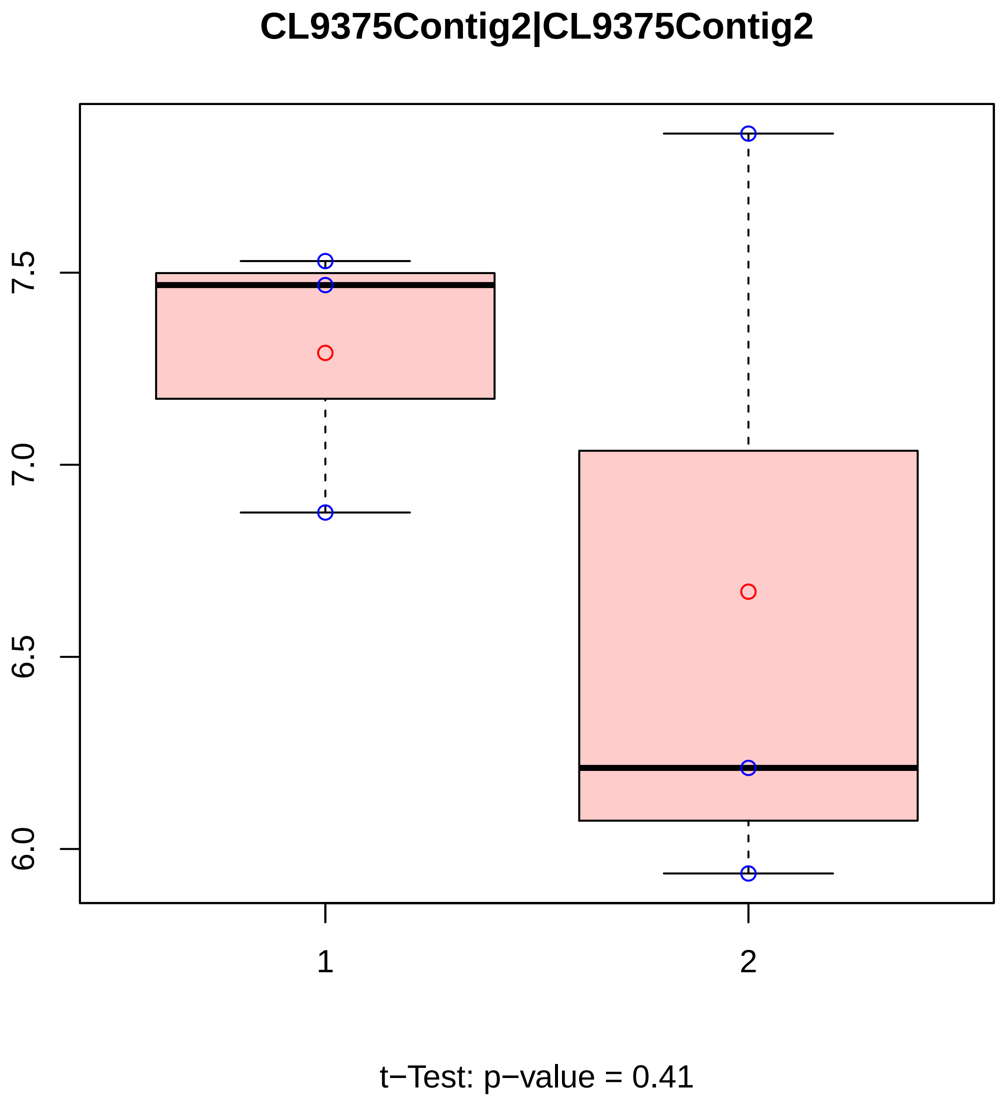
CL9375Contig2|CL9375Contig2
7.5
7.0
6.5
6.0
1
2
t−Test: p−value = 0.41

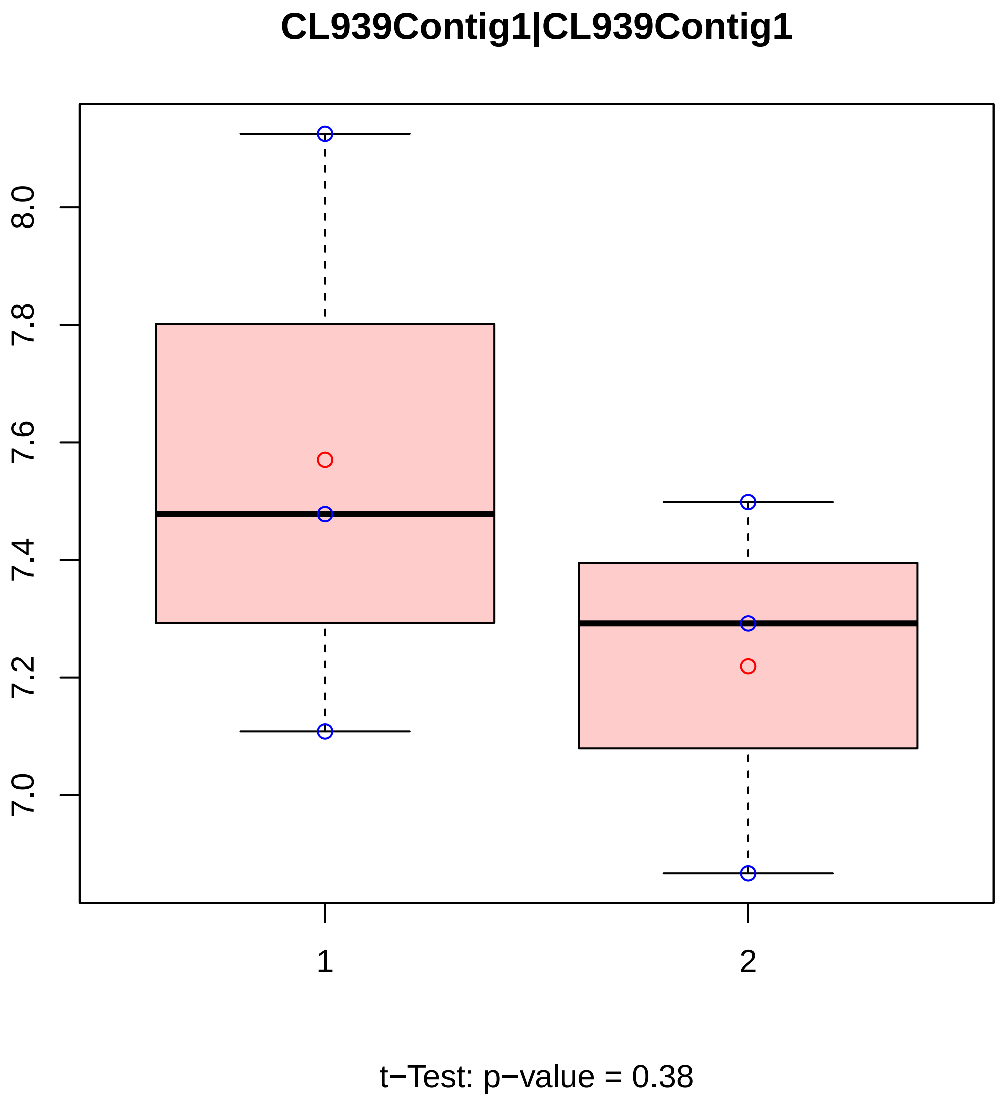
CL939Contig1|CL939Contig1
8.0
7.8
7.6
7.4
7.2
7.0
1
2
t−Test: p−value = 0.38

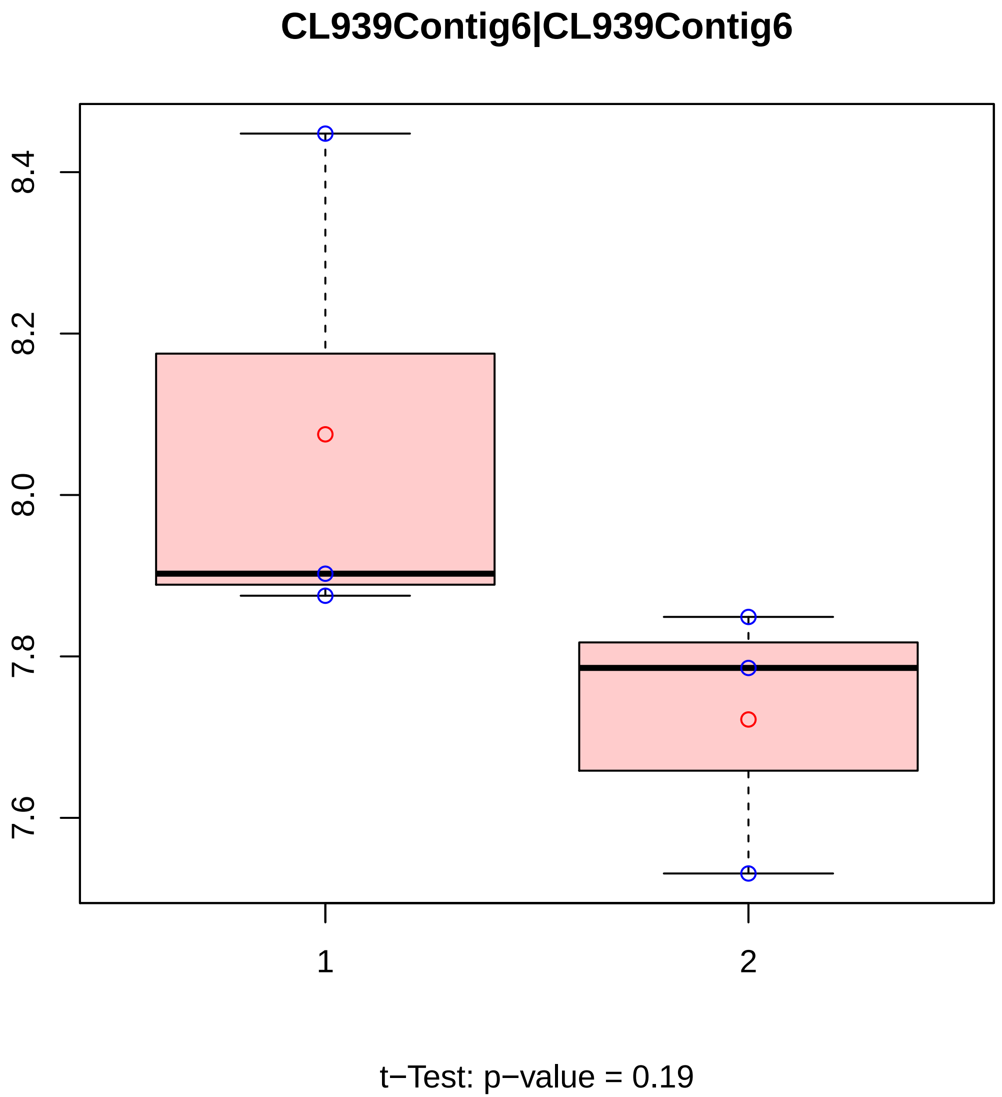

CL939Contig6|CL939Contig6
8.4
8.2
8.0
7.8
7.6
1
2
t−Test: p−value = 0.19

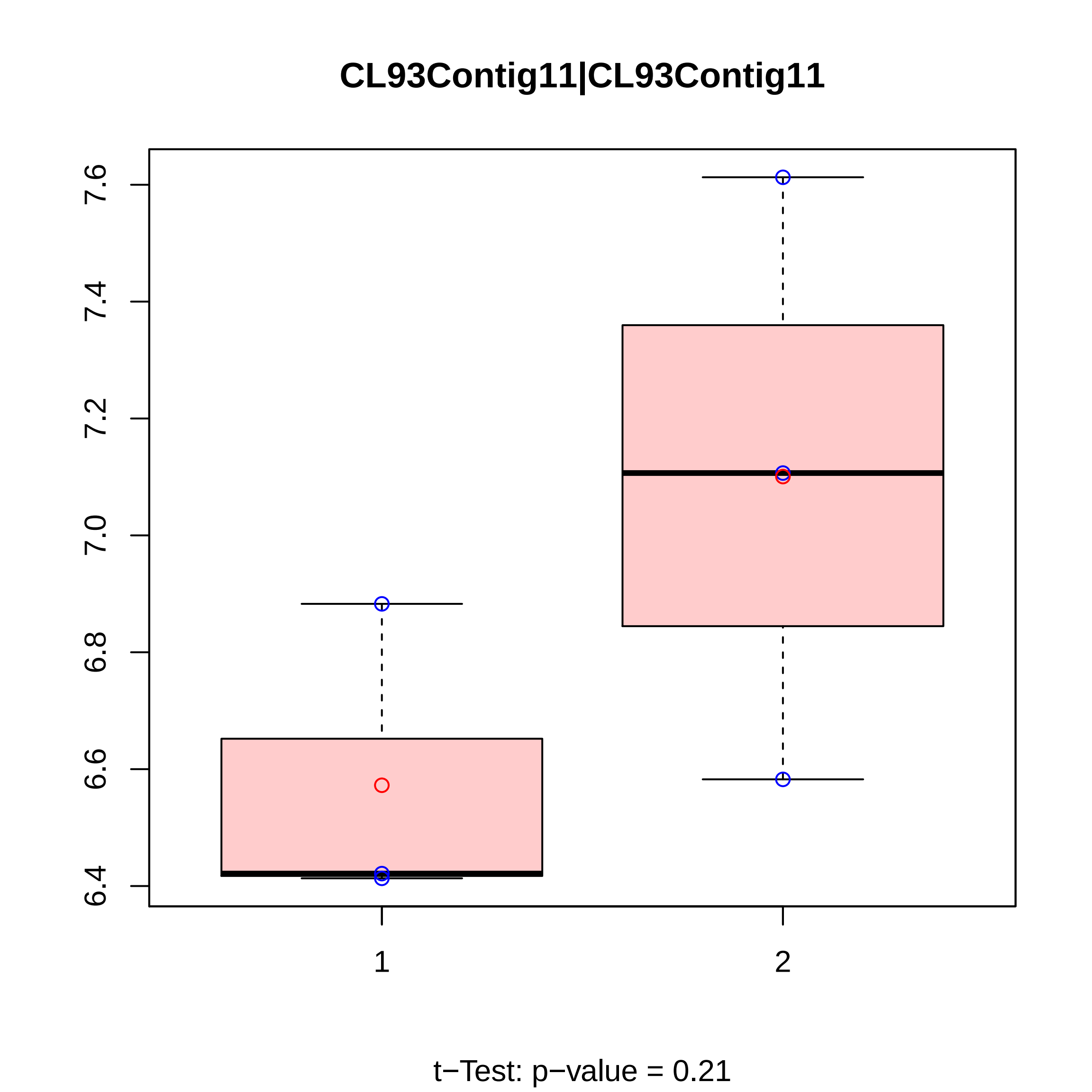

CL93Contig11|CL93Contig11
7.6
7.4
7.2
7.0
6.8
6.6
6.4
1
2
t−Test: p−value = 0.21

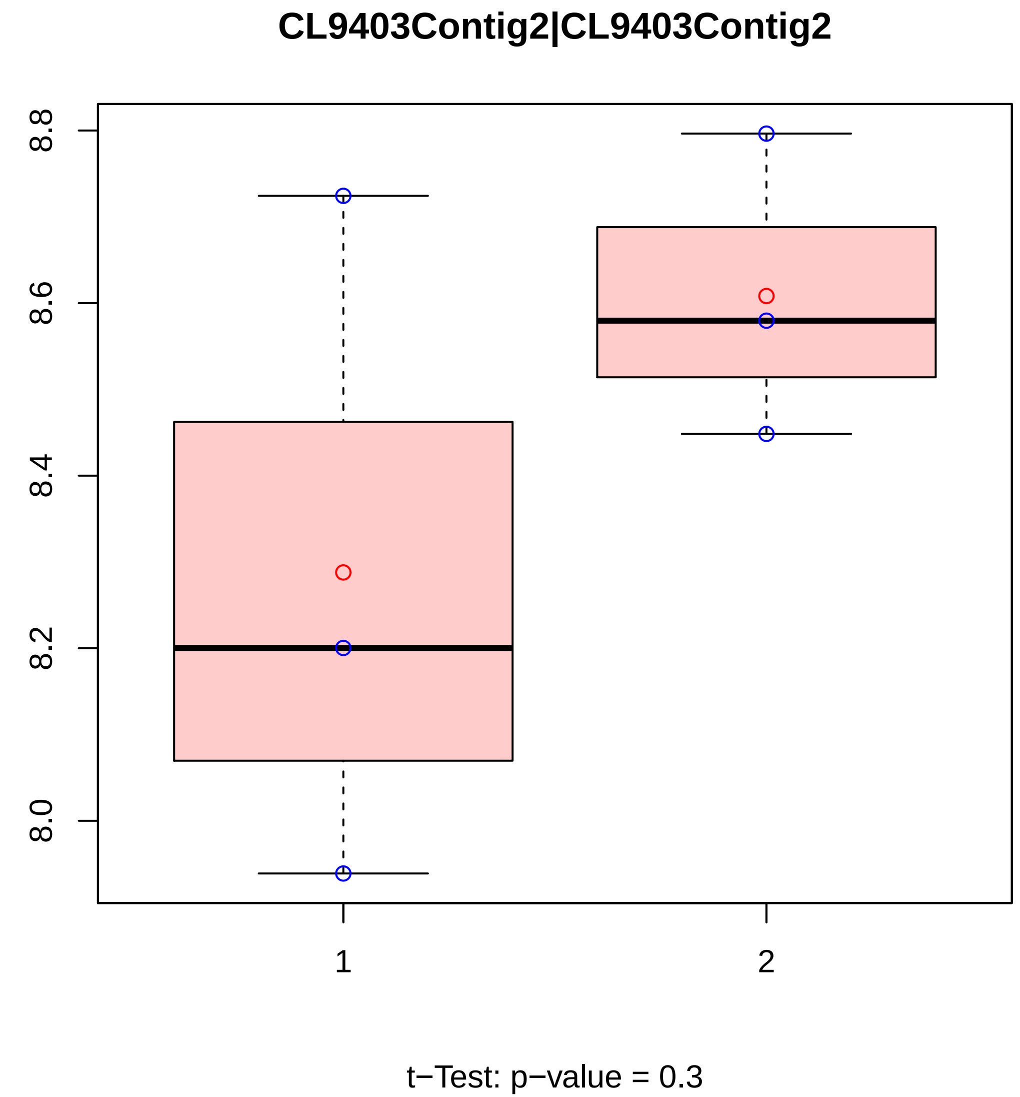

CL9403Contig2|CL9403Contig2
8.0
8.2
8.4
8.6
8.8
1
2
t−Test: p−value = 0.3

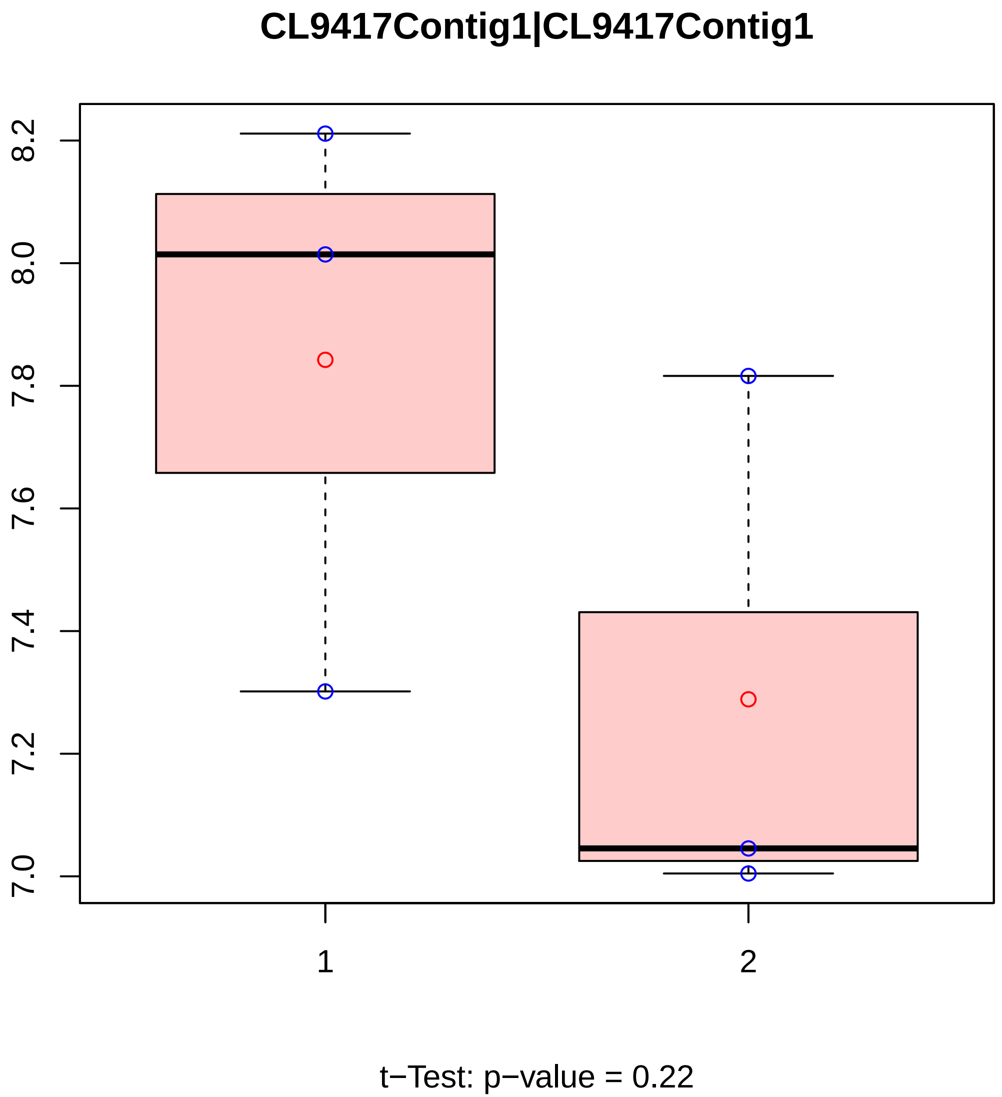

CL9417Contig1|CL9417Contig1
8.2
8.0
7.8
7.6
7.4
7.2
7.0
1
2
t−Test: p−value = 0.22

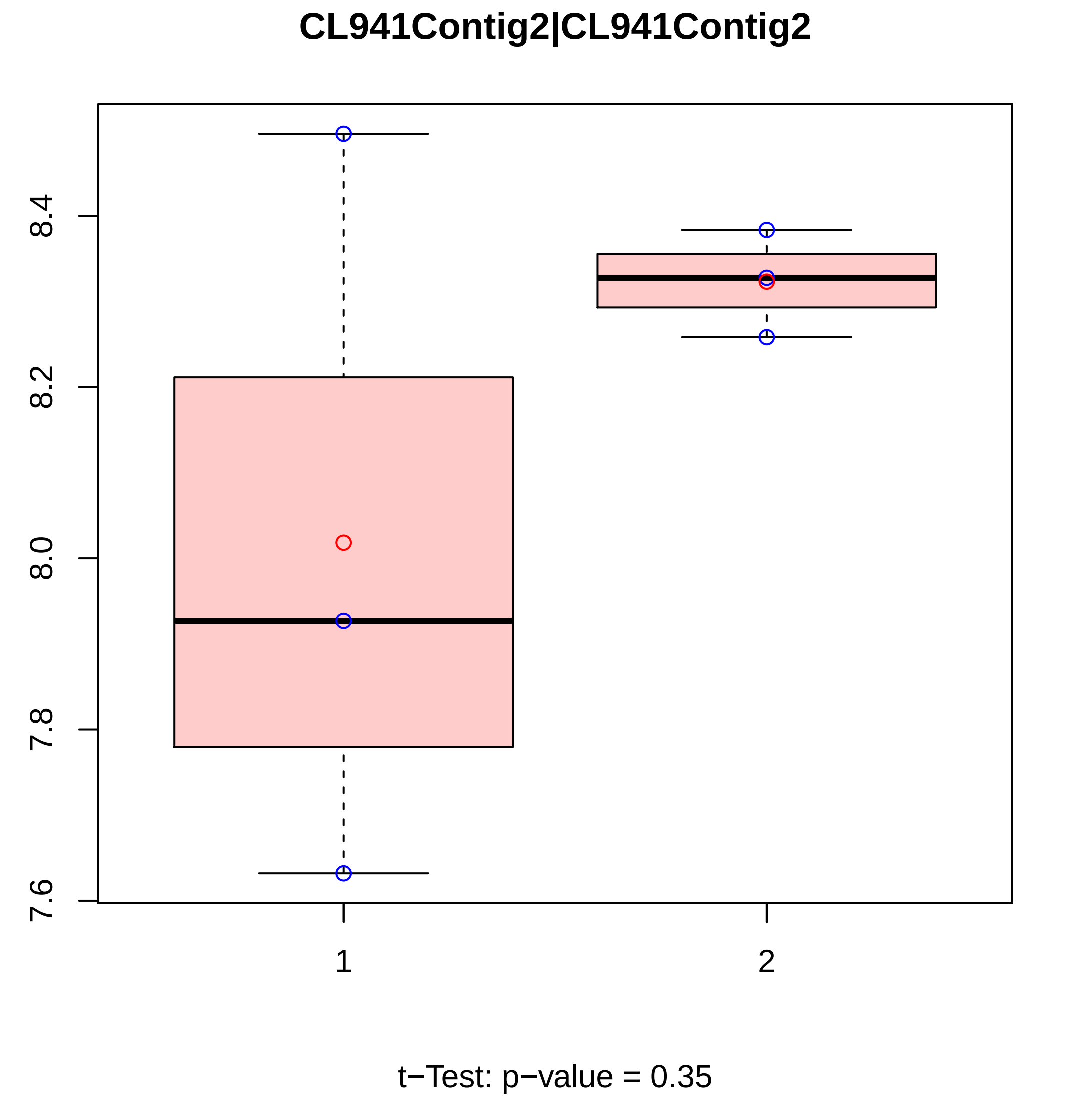
CL941Contig2|CL941Contig2
8.4
8.2
8.0
7.8
7.6
1
2
t−Test: p−value = 0.35

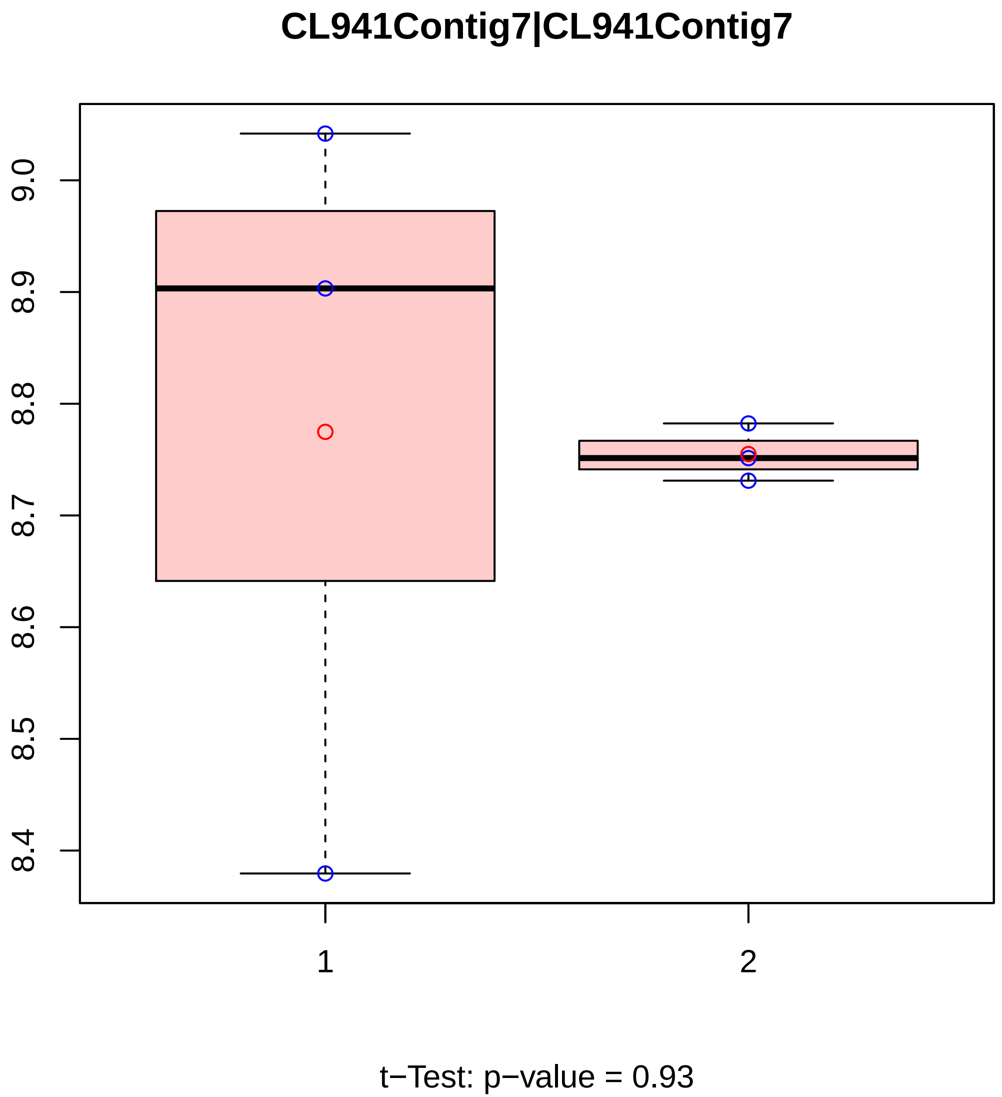
CL941Contig7|CL941Contig7
9.0
8.9
8.8
8.7
8.6
8.5
8.4
1
2
t−Test: p−value = 0.93

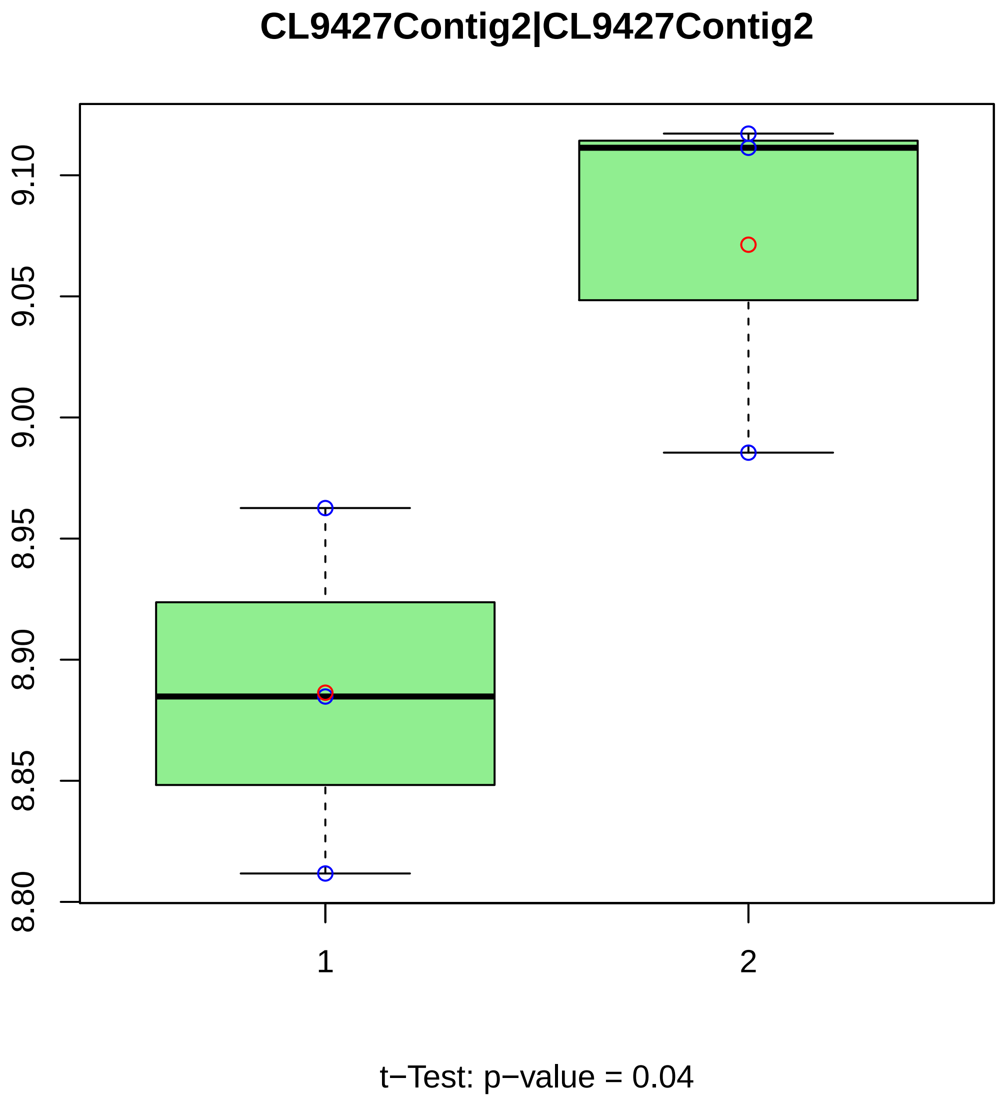
CL9427Contig2|CL9427Contig2
9.10
9.05
9.00
8.95
8.90
8.85
8.80
1
2
t−Test: p−value = 0.04

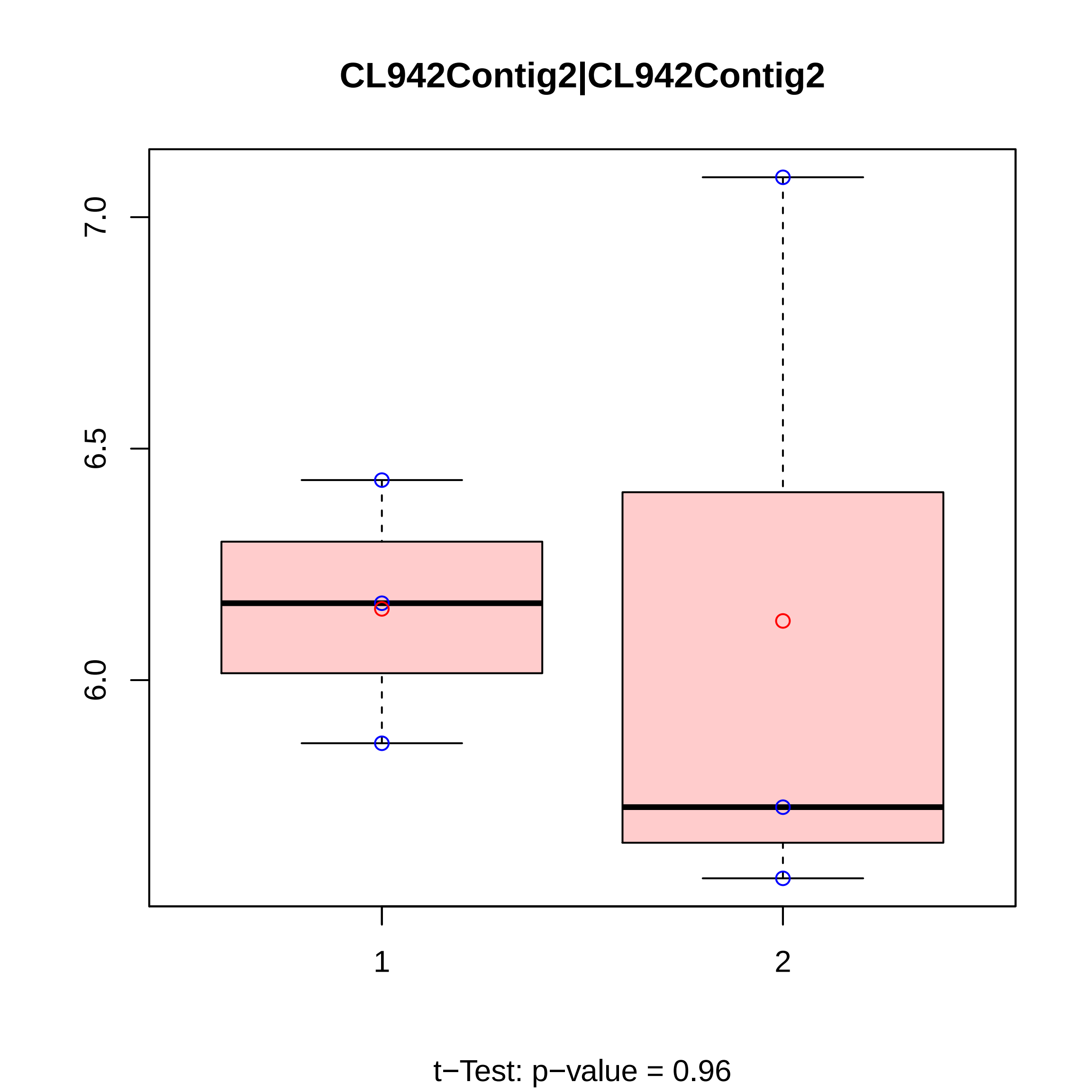

CL942Contig2|CL942Contig2
7.0
6.5
6.0
1
2
t−Test: p−value = 0.96

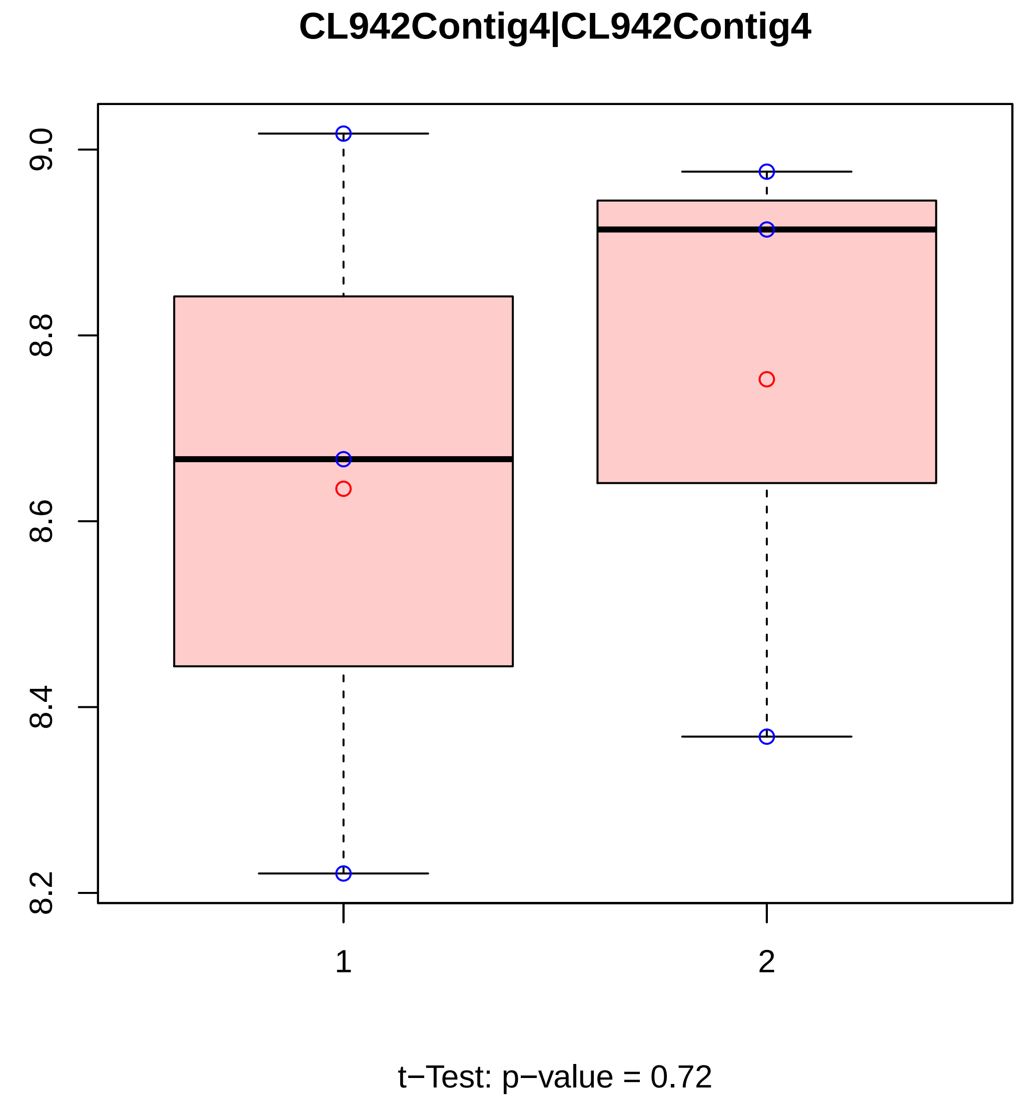

CL942Contig4|CL942Contig4
9.0
8.8
8.6
8.4
8.2
1
2
t−Test: p−value = 0.72

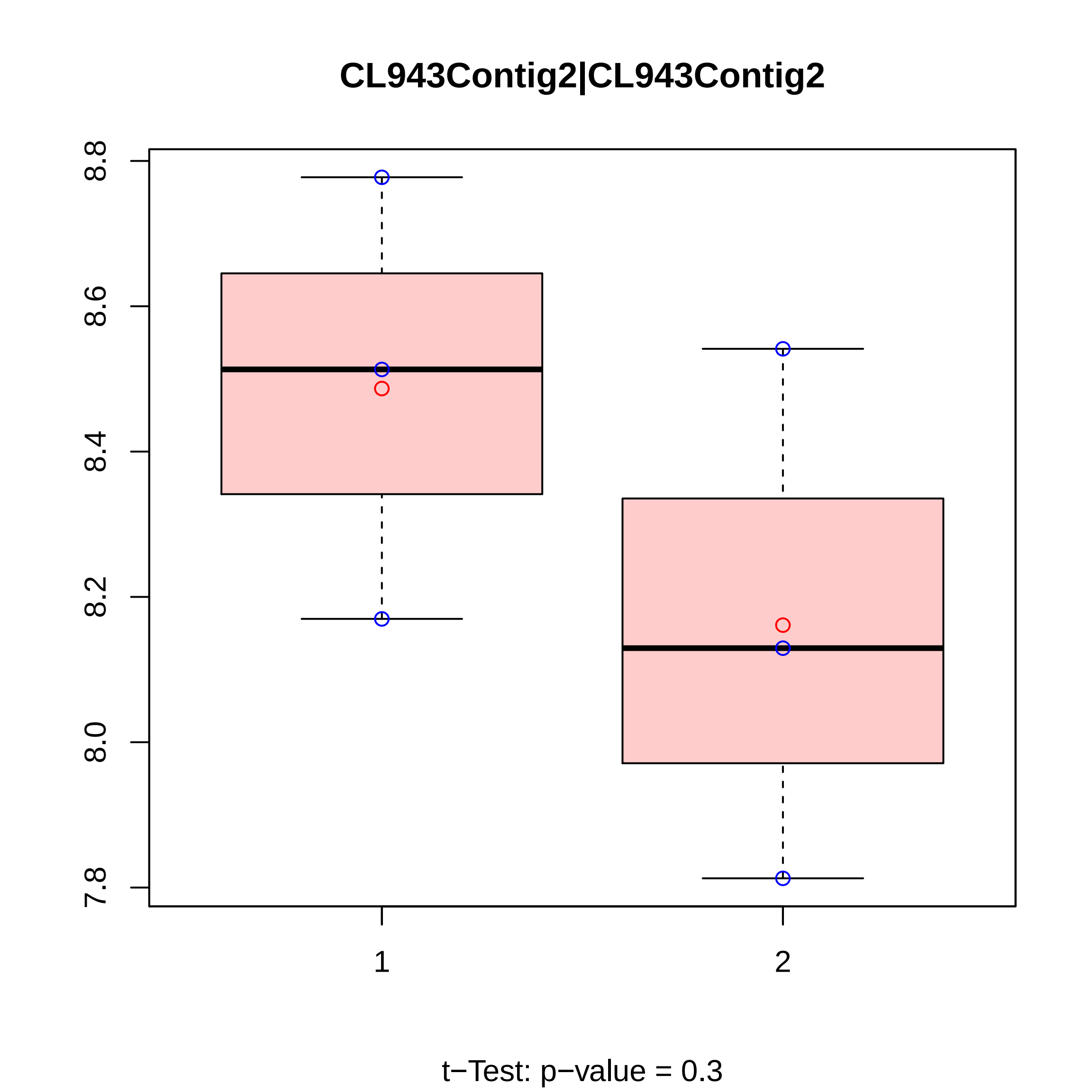

CL943Contig2|CL943Contig2
8.8
8.6
8.4
8.2
8.0
7.8
1
2
t−Test: p−value = 0.3

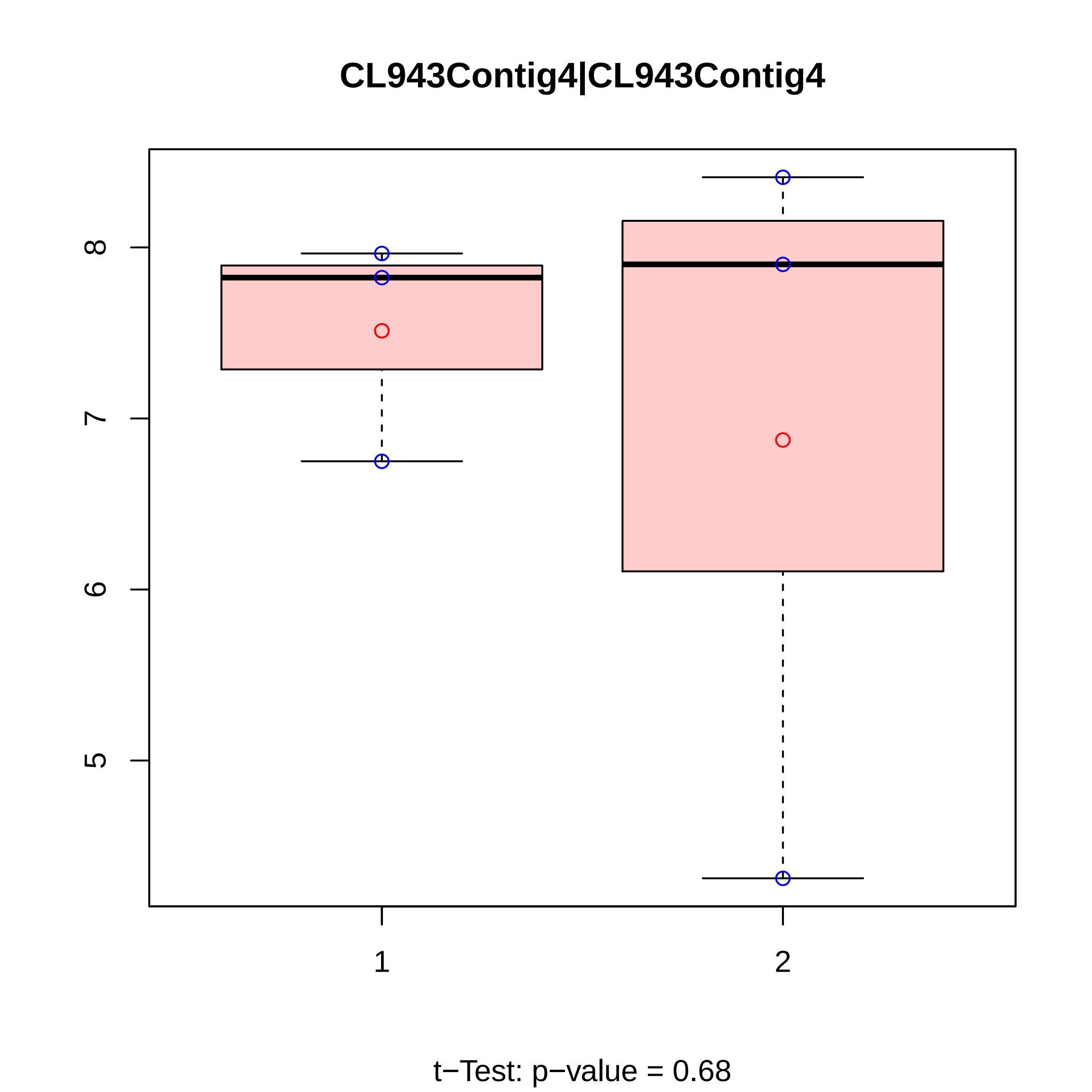
CL943Contig4|CL943Contig4
8
7
6
5
1
2
t−Test: p−value = 0.68

# CL9459Contig2|CL9459Contig2

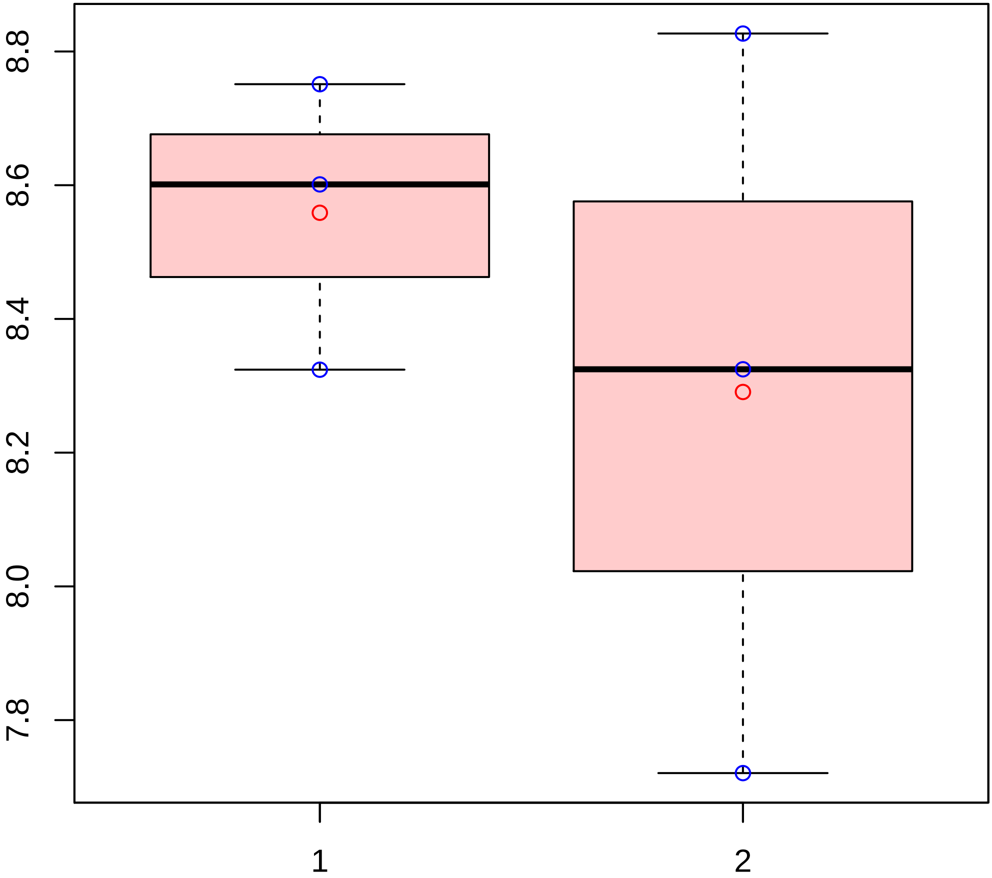


t−Test: p−value = 0.5

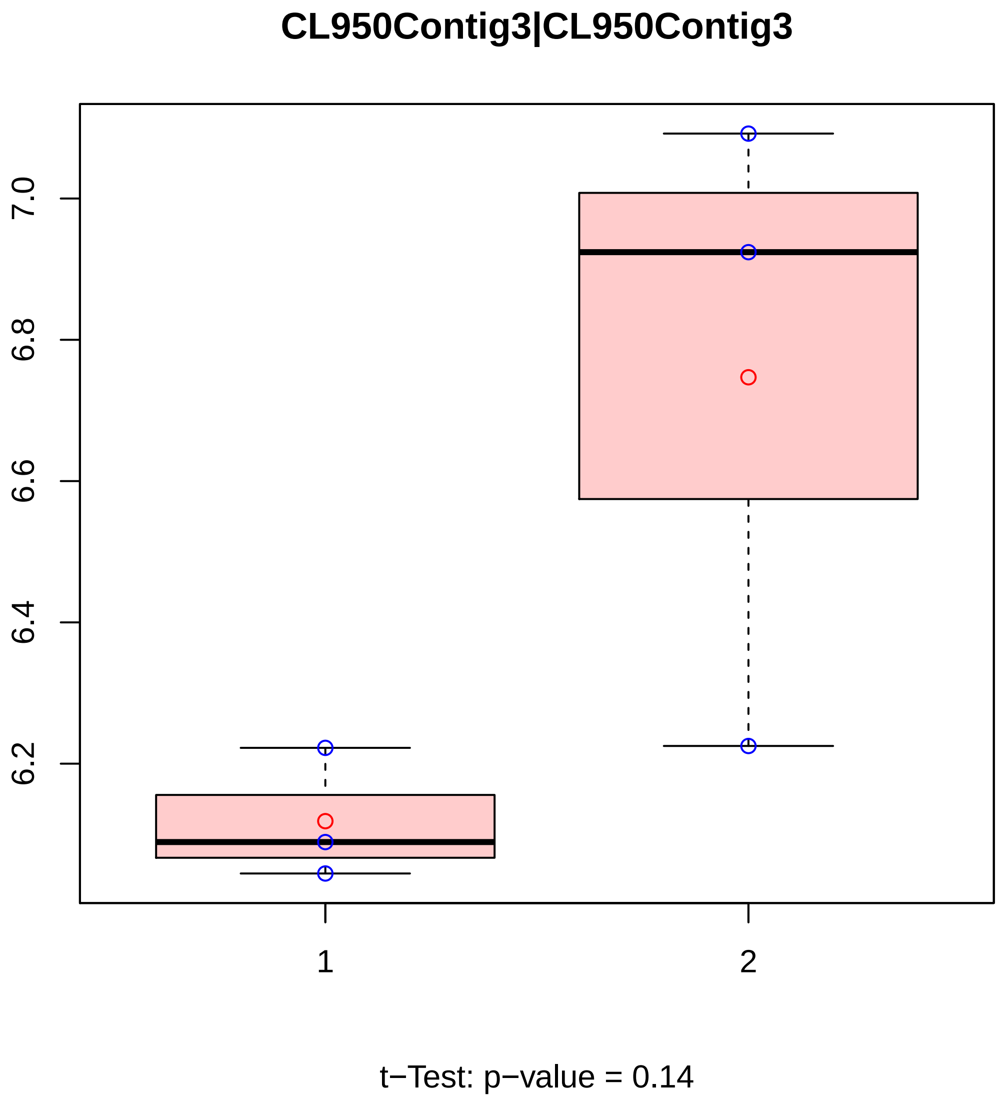
CL950Contig3|CL950Contig3
7.0
6.8
6.6
6.4
6.2
1
2
t−Test: p−value = 0.14

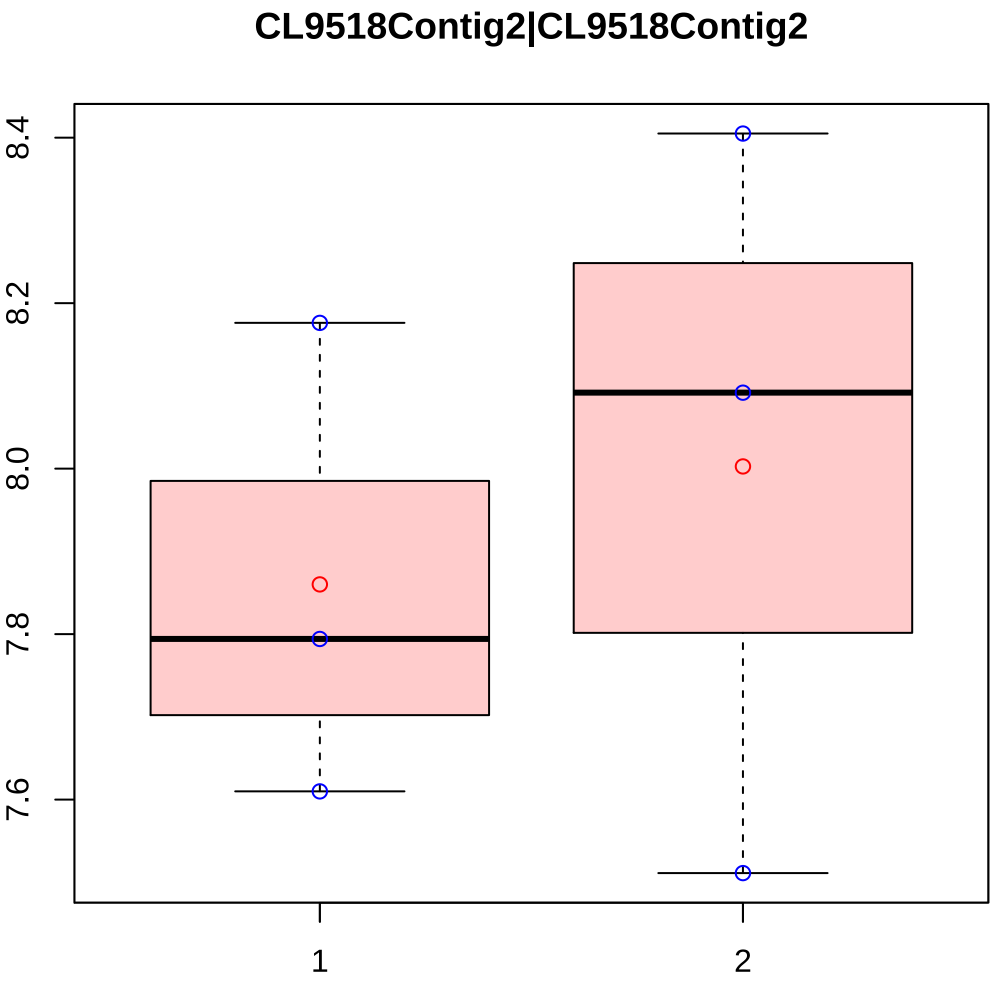


t−Test: p−value = 0.67

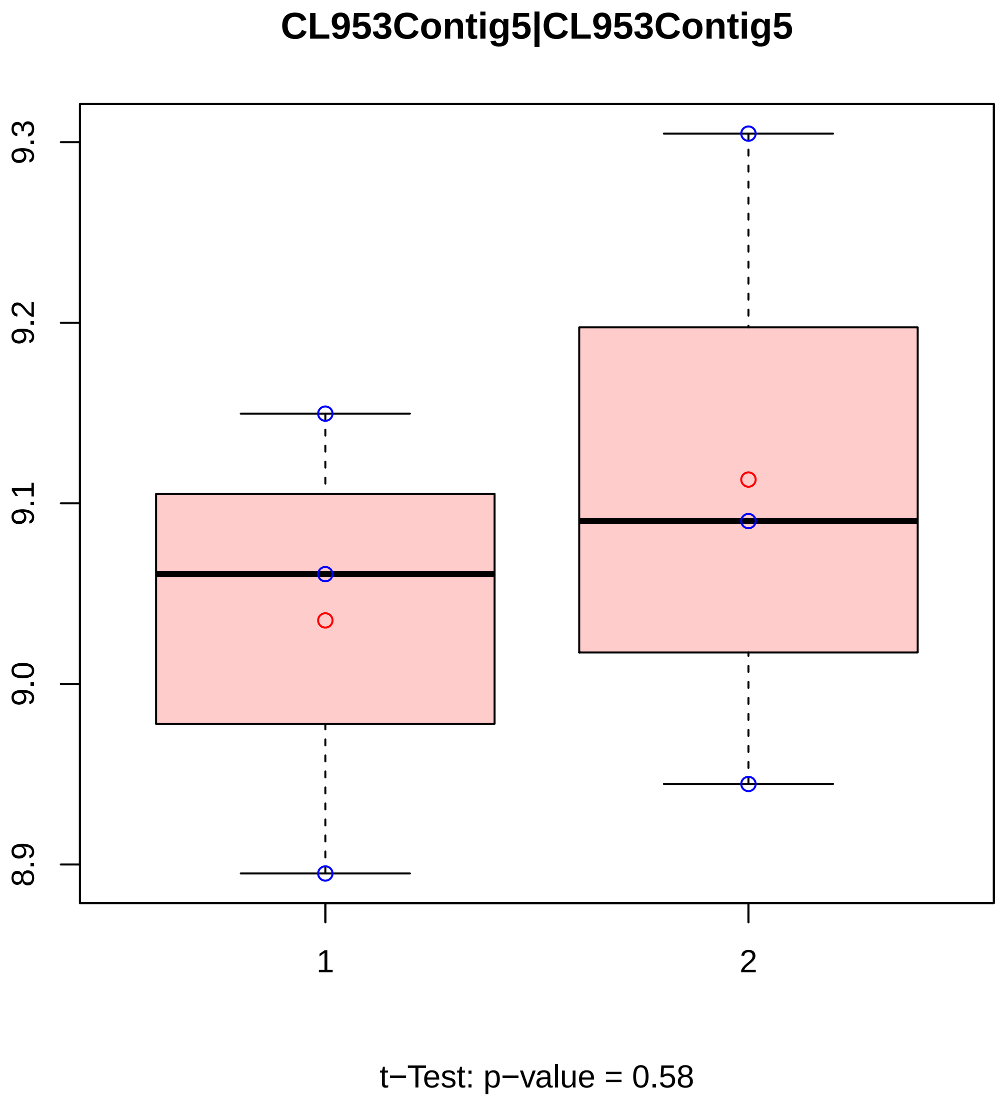

CL953Contig5|CL953Contig5
9.3
9.2
9.1
9.0
8.9
1
2
t−Test: p−value = 0.58

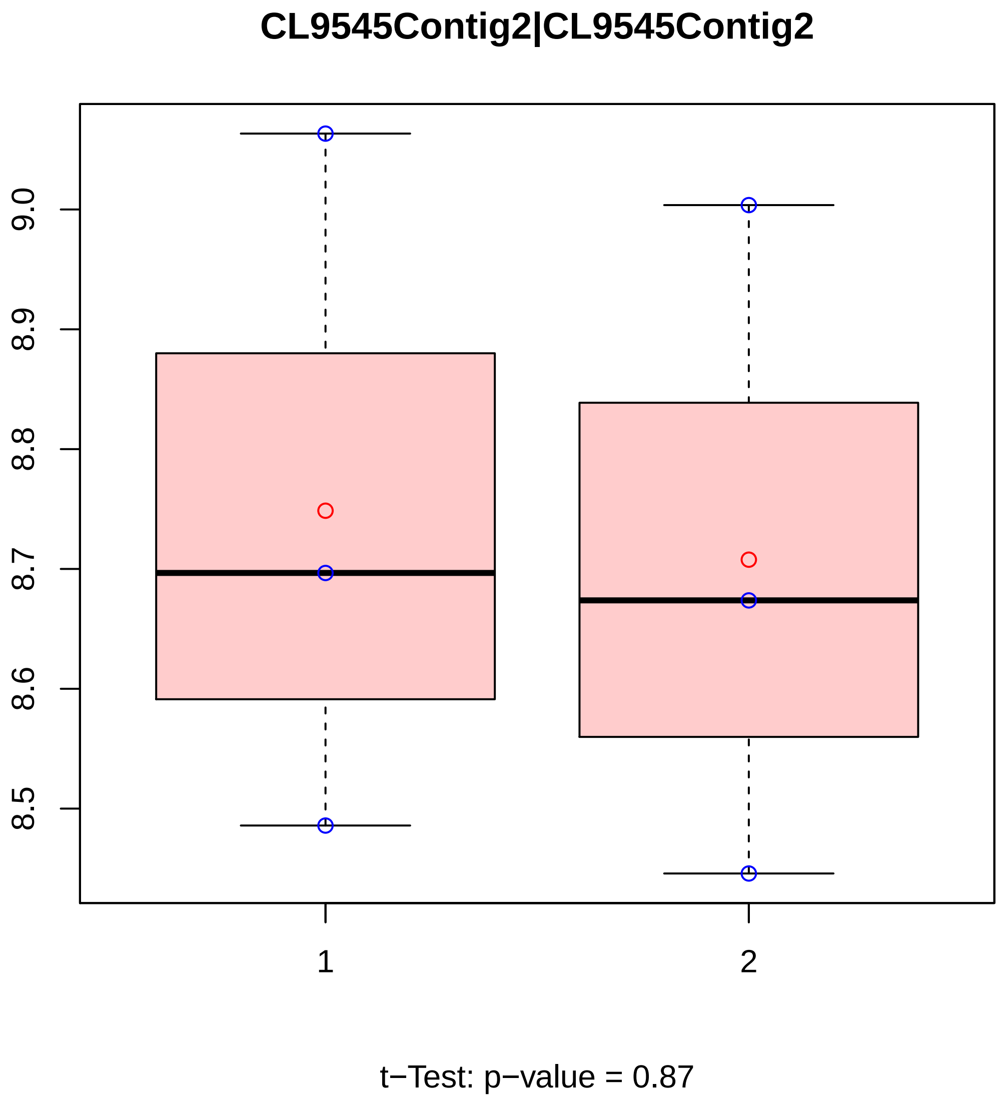
CL9545Contig2|CL9545Contig2
9.0
8.9
8.8
8.7
8.6
8.5
1
2
t−Test: p−value = 0.87

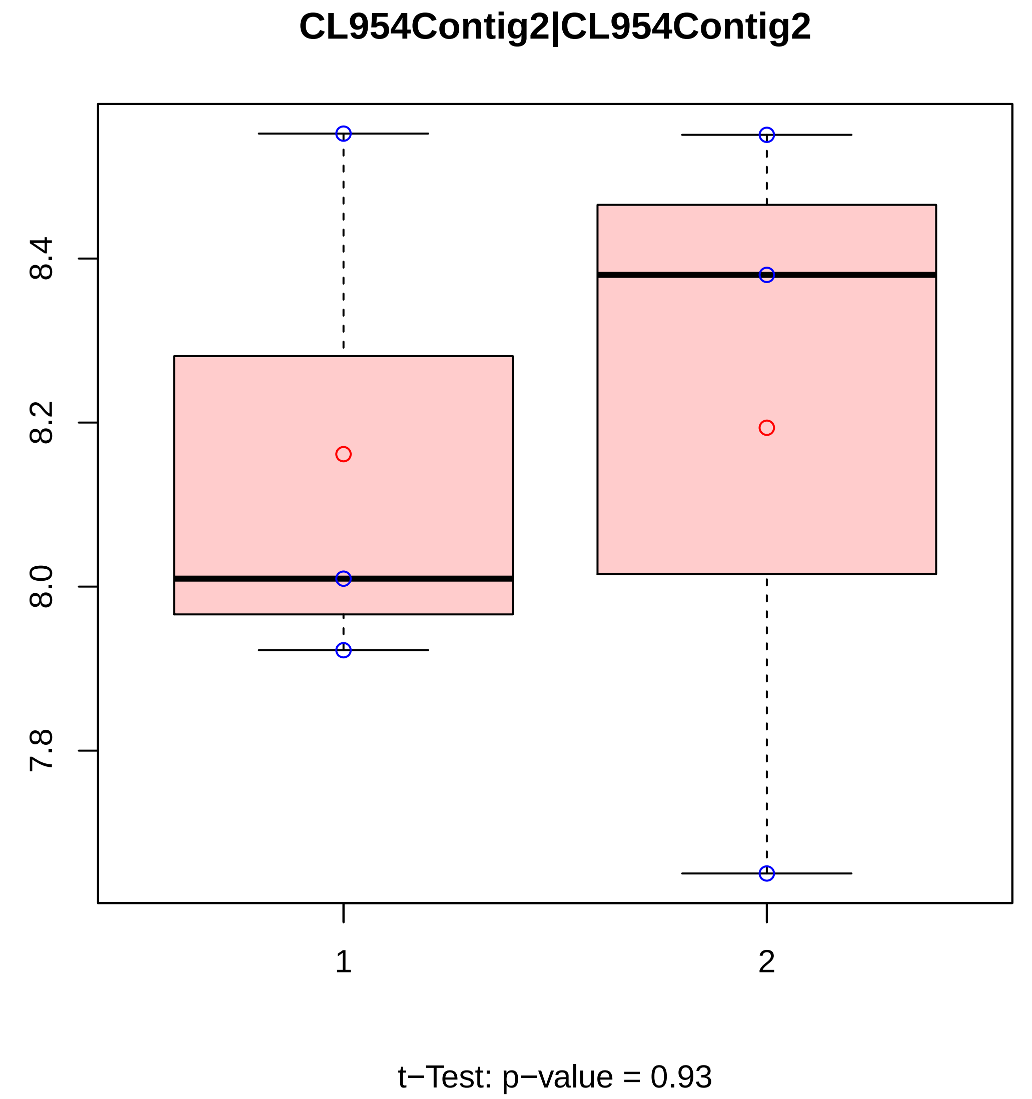
CL954Contig2|CL954Contig2
8.4
8.2
8.0
7.8
1
2
t−Test: p−value = 0.93

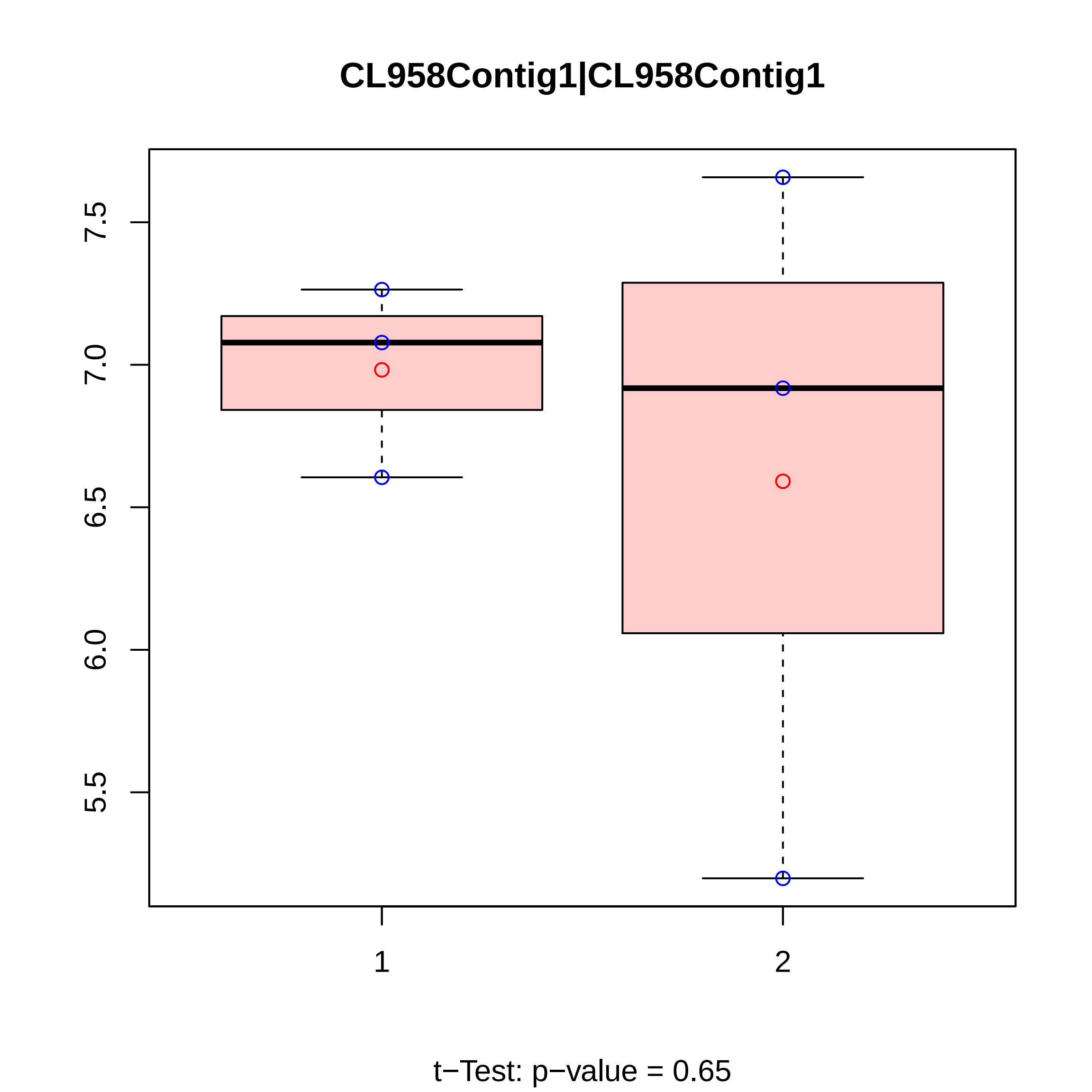
CL958Contig1|CL958Contig1
7.5
7.0
6.5
6.0
5.5
1
2
t−Test: p−value = 0.65

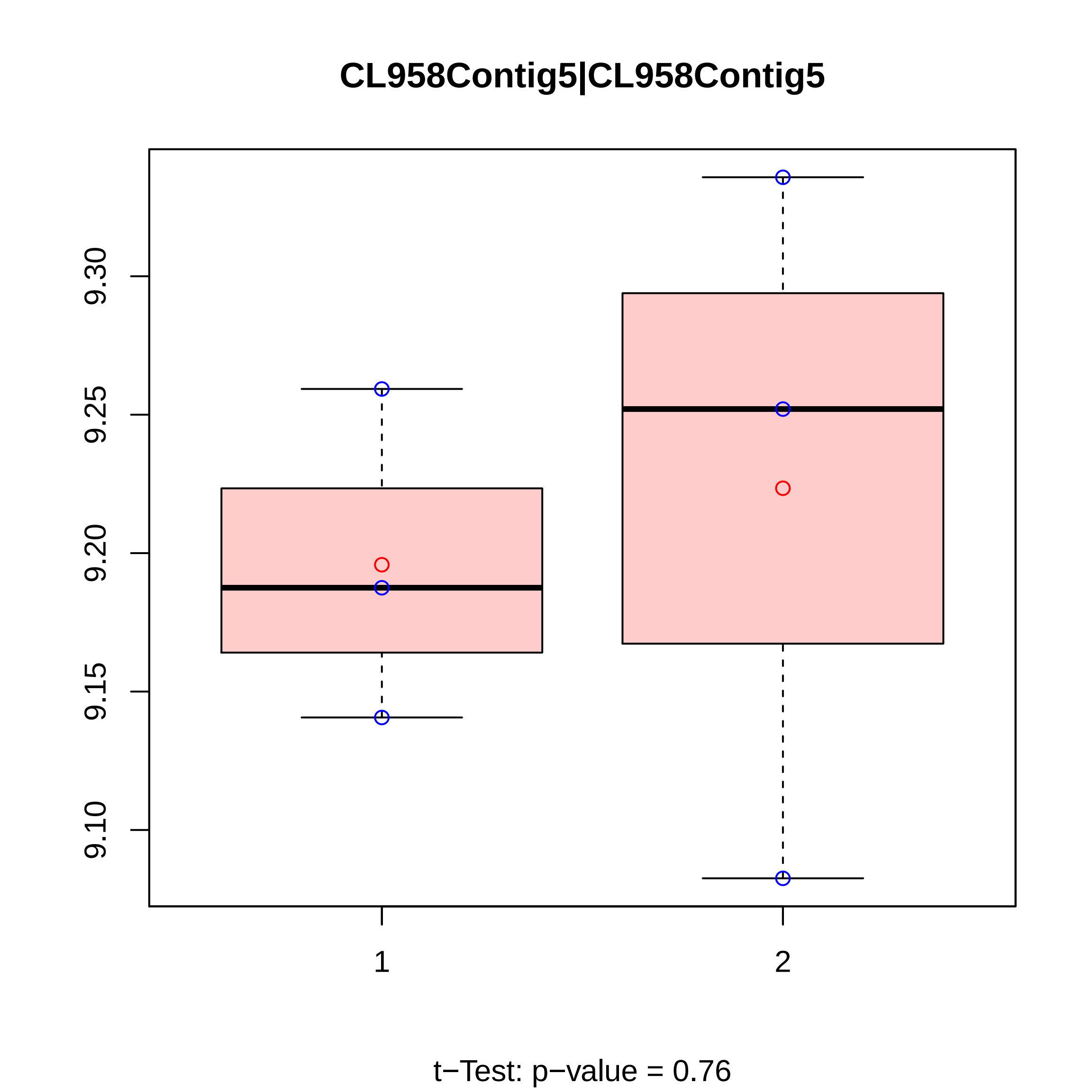

CL958Contig5|CL958Contig5
9.30
9.25
9.20
9.15
9.10
1
2
t−Test: p−value = 0.76

# CL959Contig12|CL959Contig12

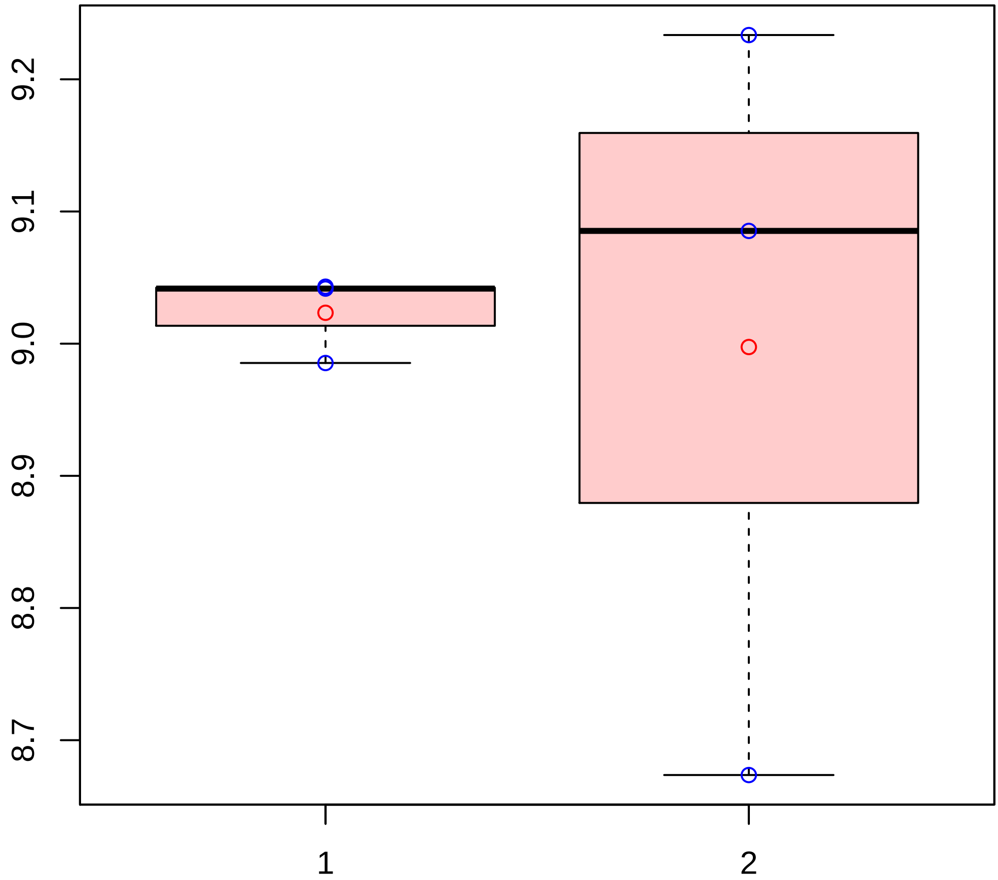


t−Test: p−value = 0.89

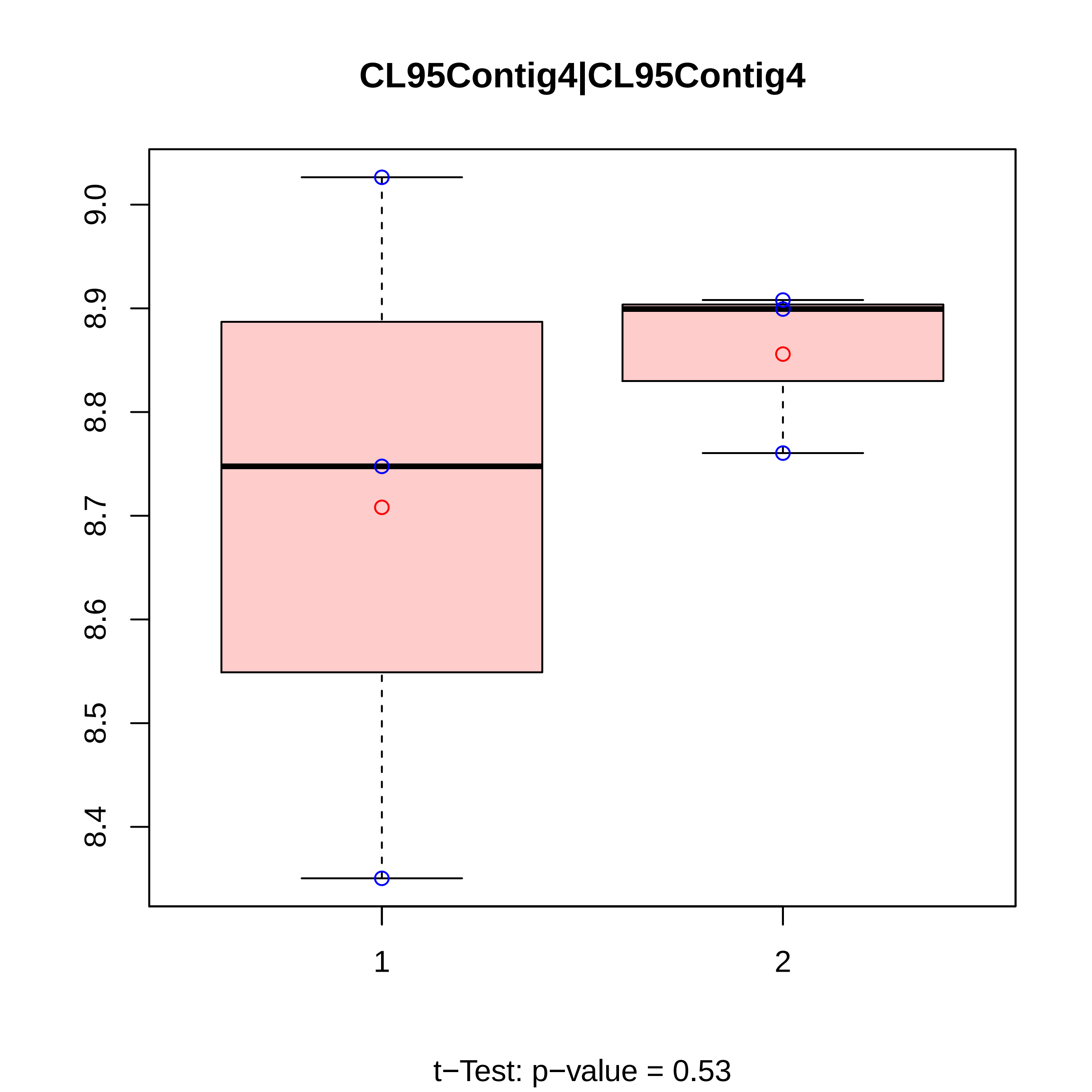
CL95Contig4|CL95Contig4
9.0
8.9
8.8
8.7
8.6
8.5
8.4
1
2
t−Test: p−value = 0.53

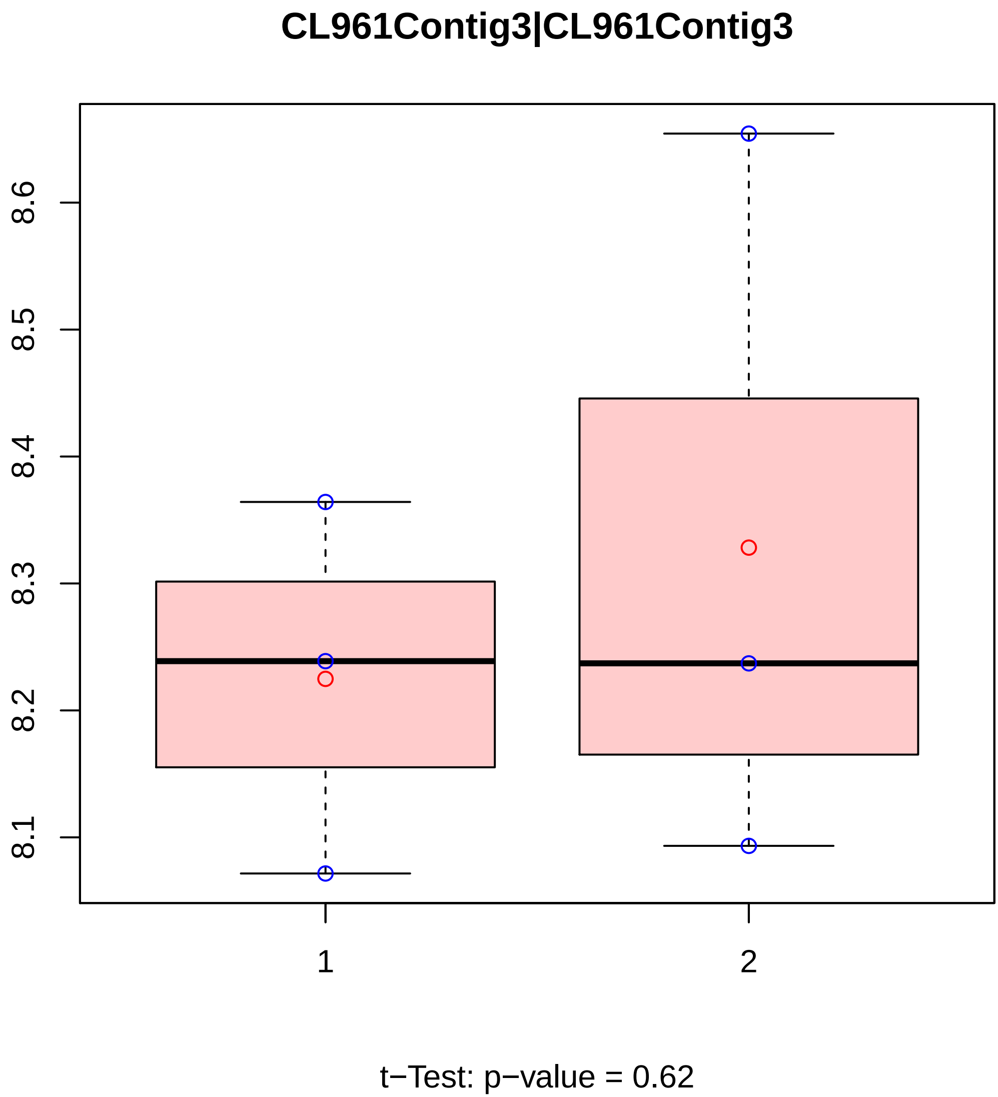

CL961Contig3|CL961Contig3
8.1
8.2
8.3
8.4
8.5
8.6
1
2
t−Test: p−value = 0.62

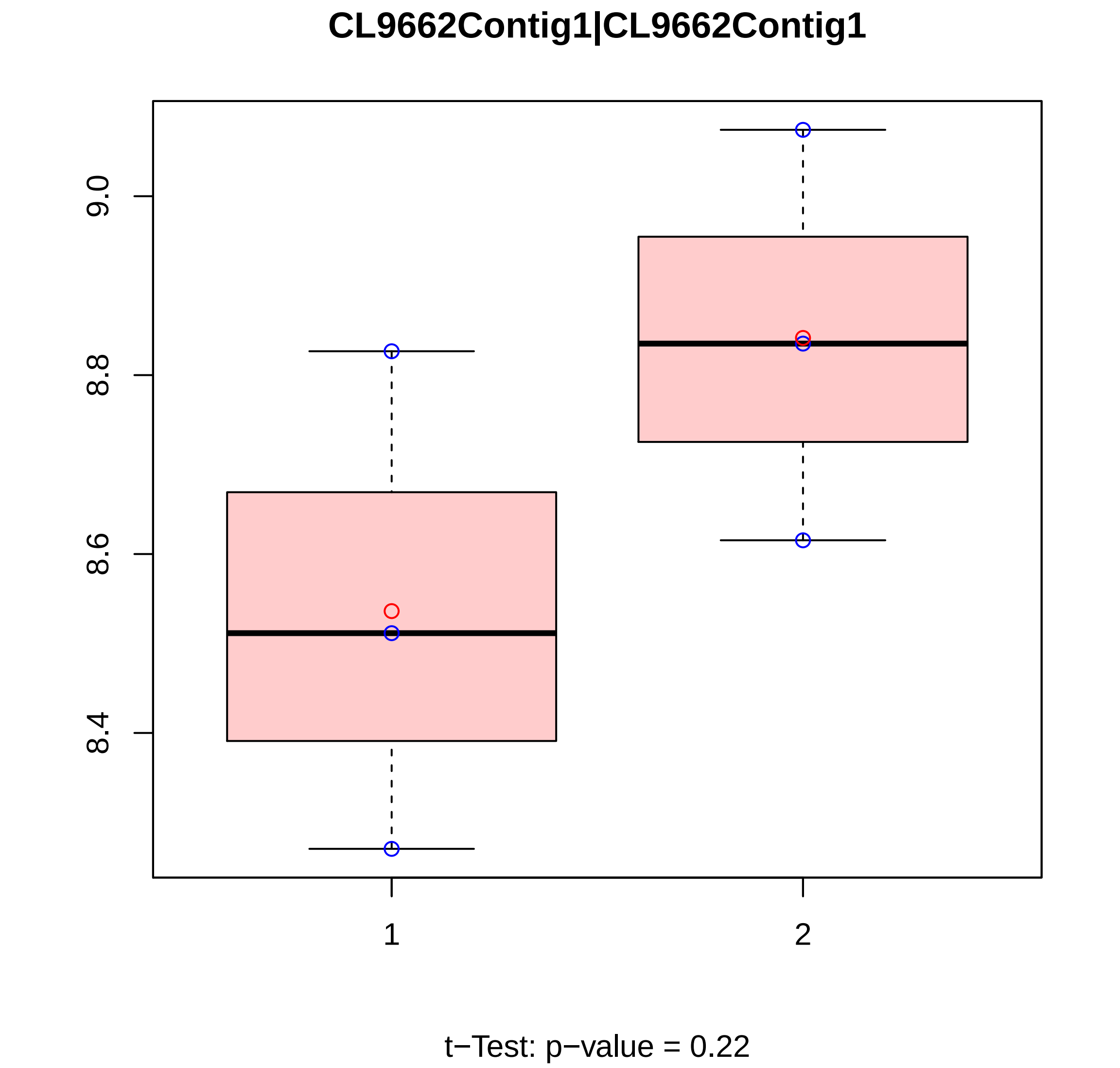

CL9662Contig1|CL9662Contig1
9.0
8.8
8.6
8.4
1
2
t−Test: p−value = 0.22

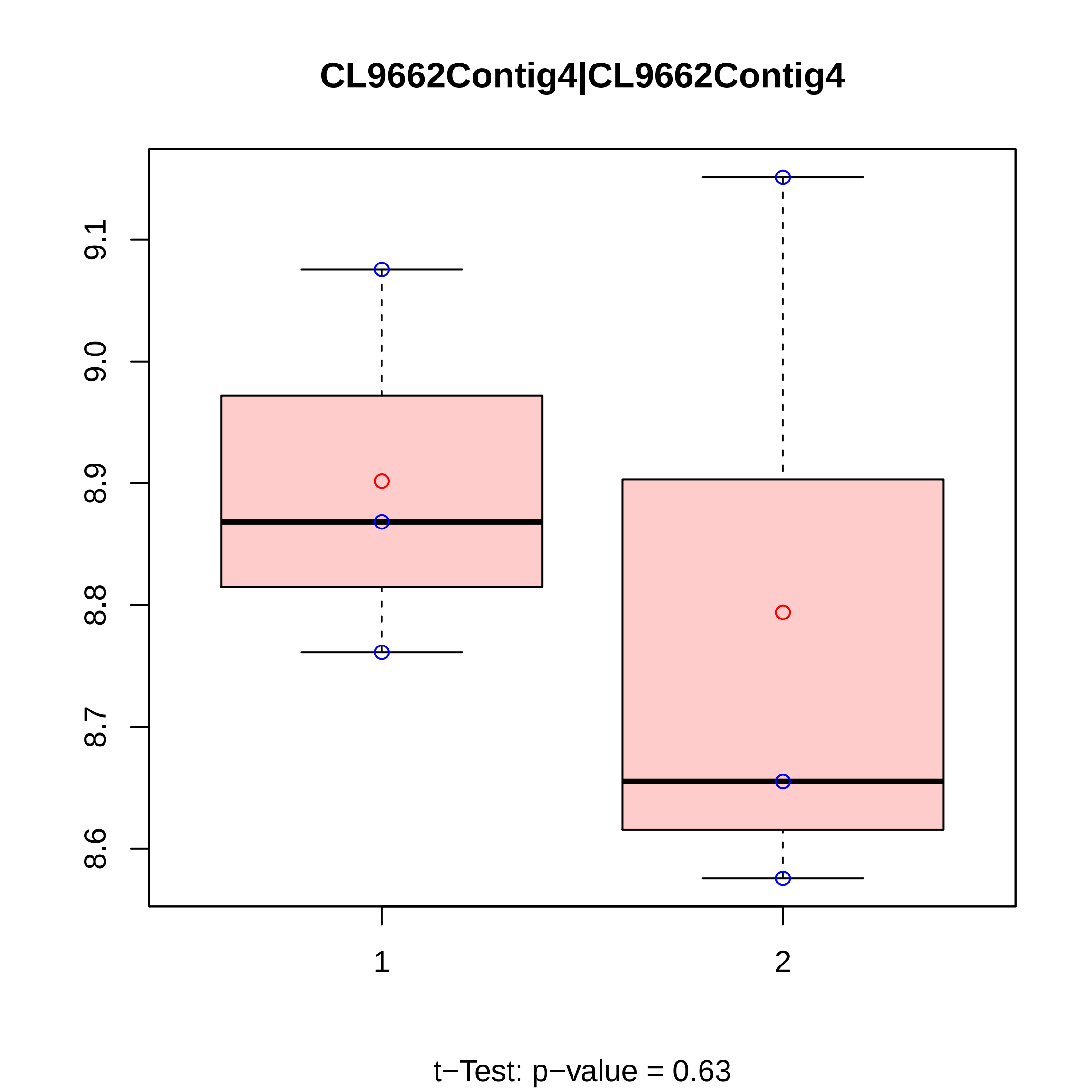
CL9662Contig4|CL9662Contig4
9.1
9.0
8.9
8.8
8.7
8.6
1
2
t−Test: p−value = 0.63

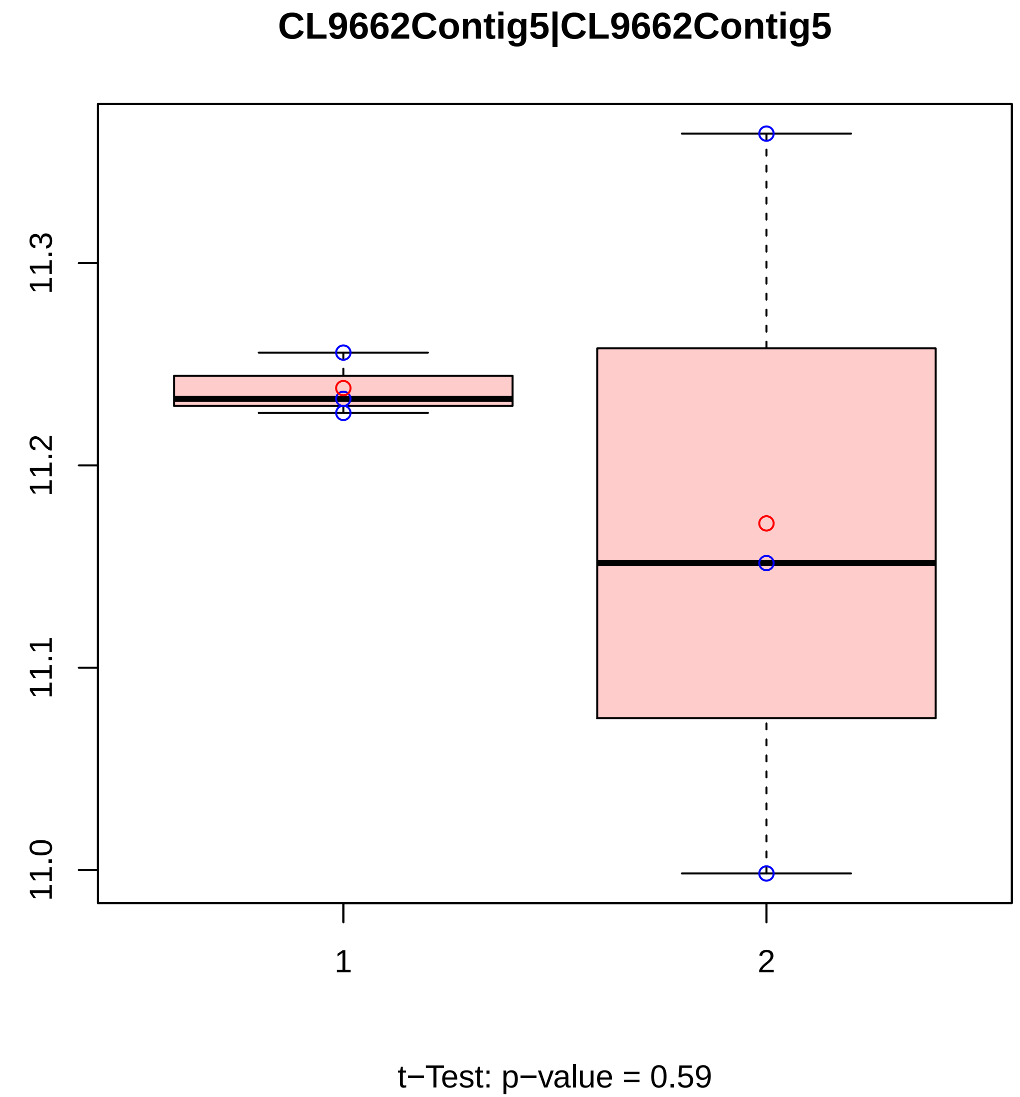

CL9662Contig5|CL9662Contig5
11.0
11.1
11.2
11.3
1
2
t−Test: p−value = 0.59

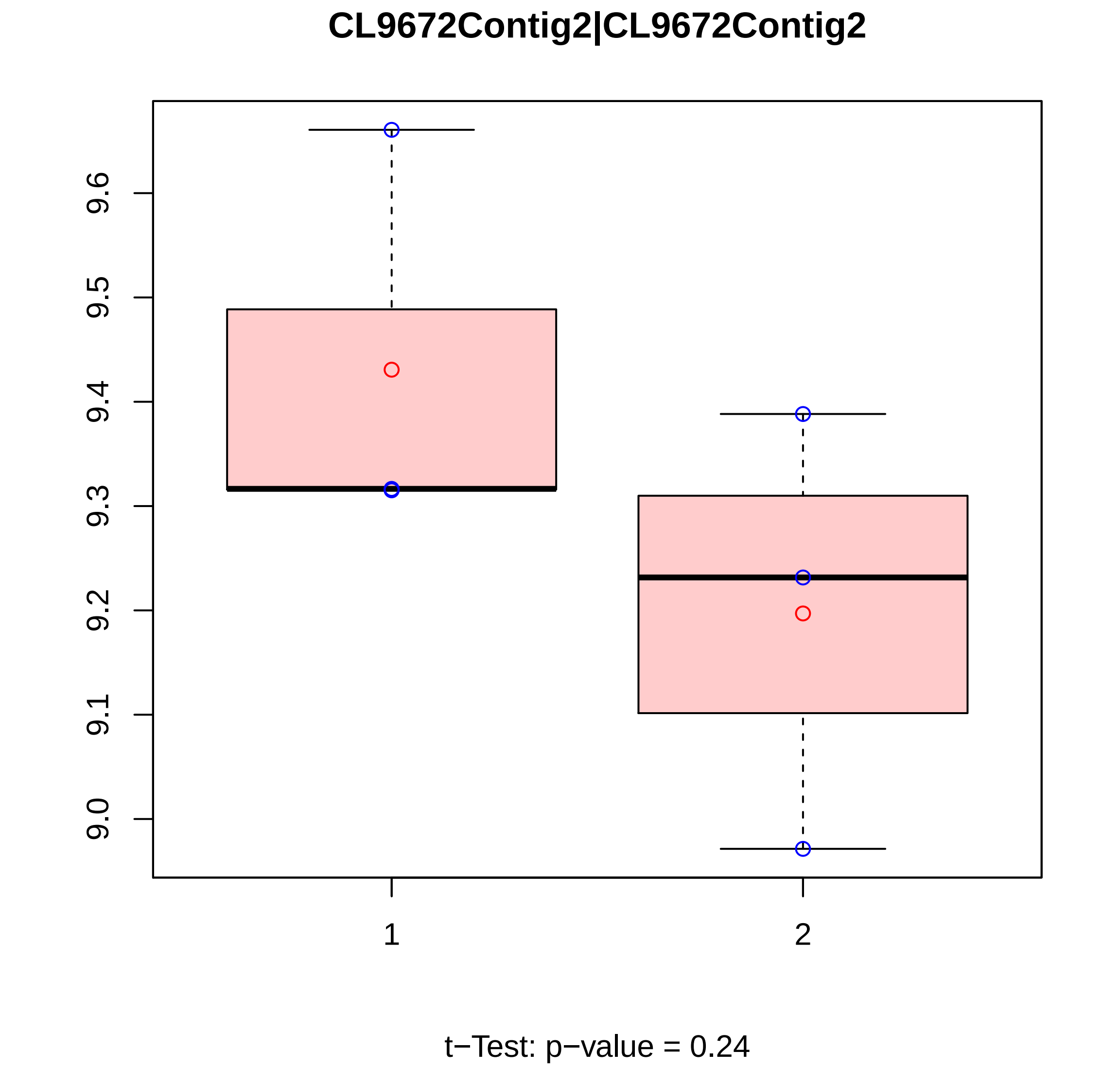
CL9672Contig2|CL9672Contig2
9.0
9.1
9.2
9.3
9.4
9.5
9.6
1
2
t−Test: p−value = 0.24

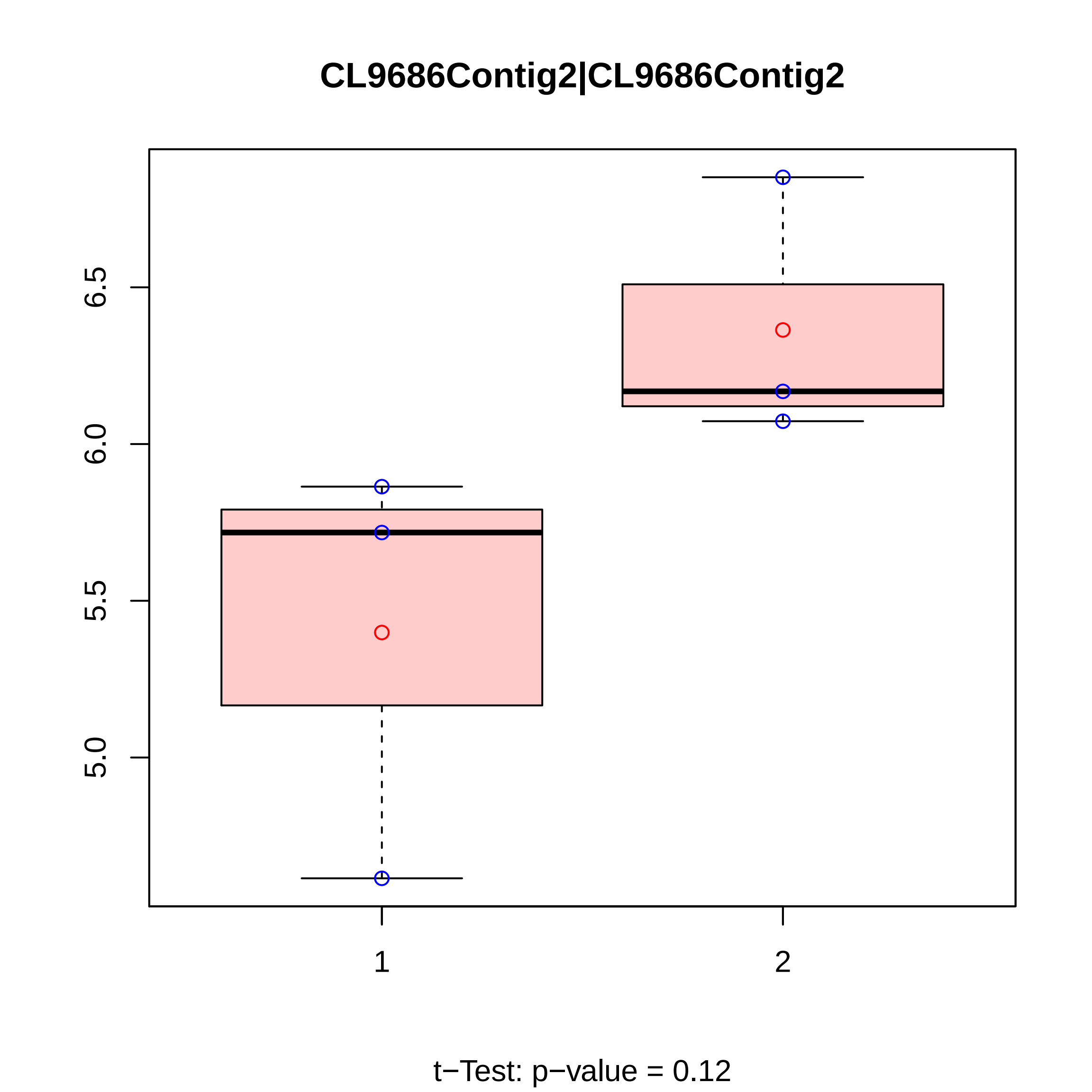
CL9686Contig2|CL9686Contig2
6.5
6.0
5.5
5.0
1
2
t−Test: p−value = 0.12

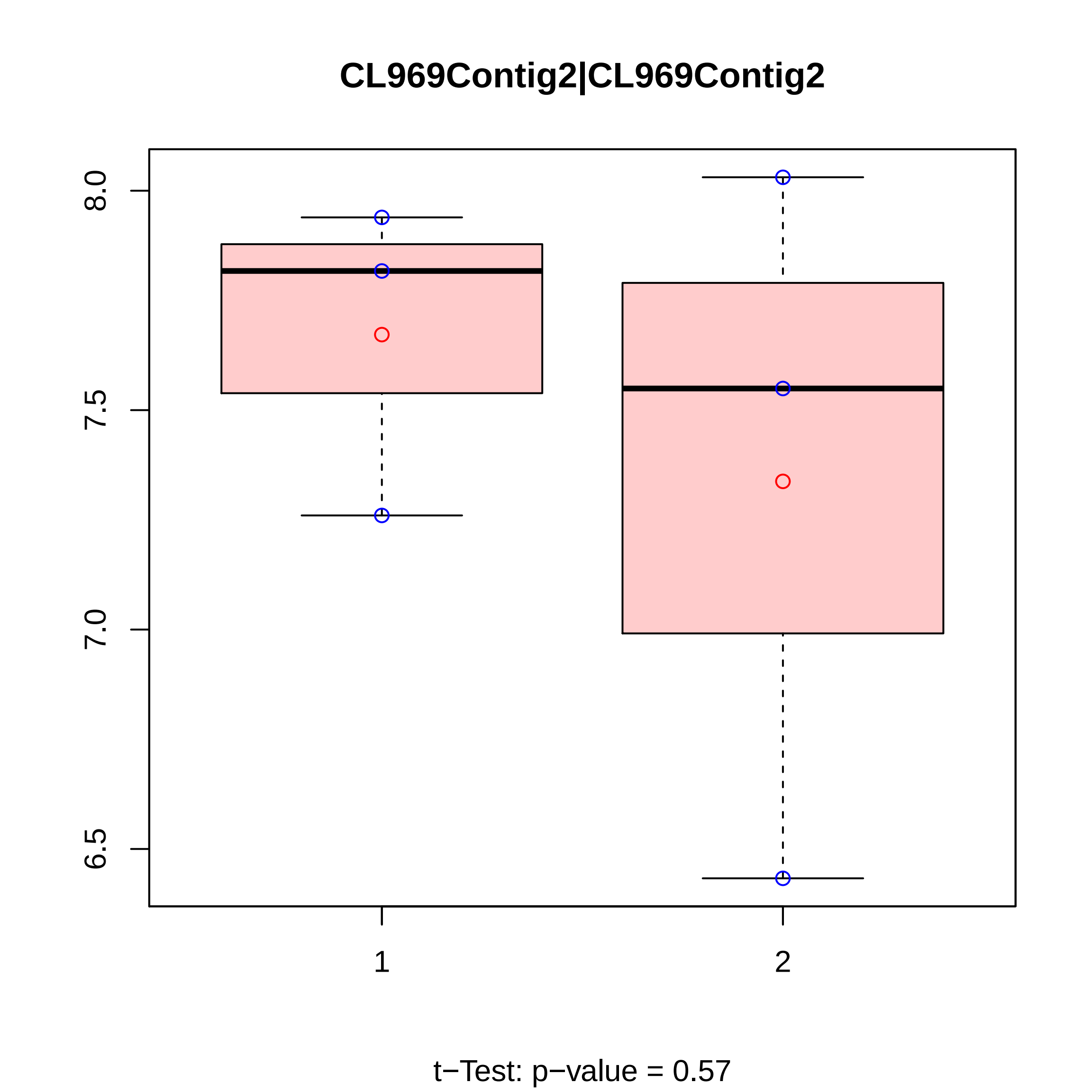
CL969Contig2|CL969Contig2
8.0
7.5
7.0
6.5
1
2
t−Test: p−value = 0.57

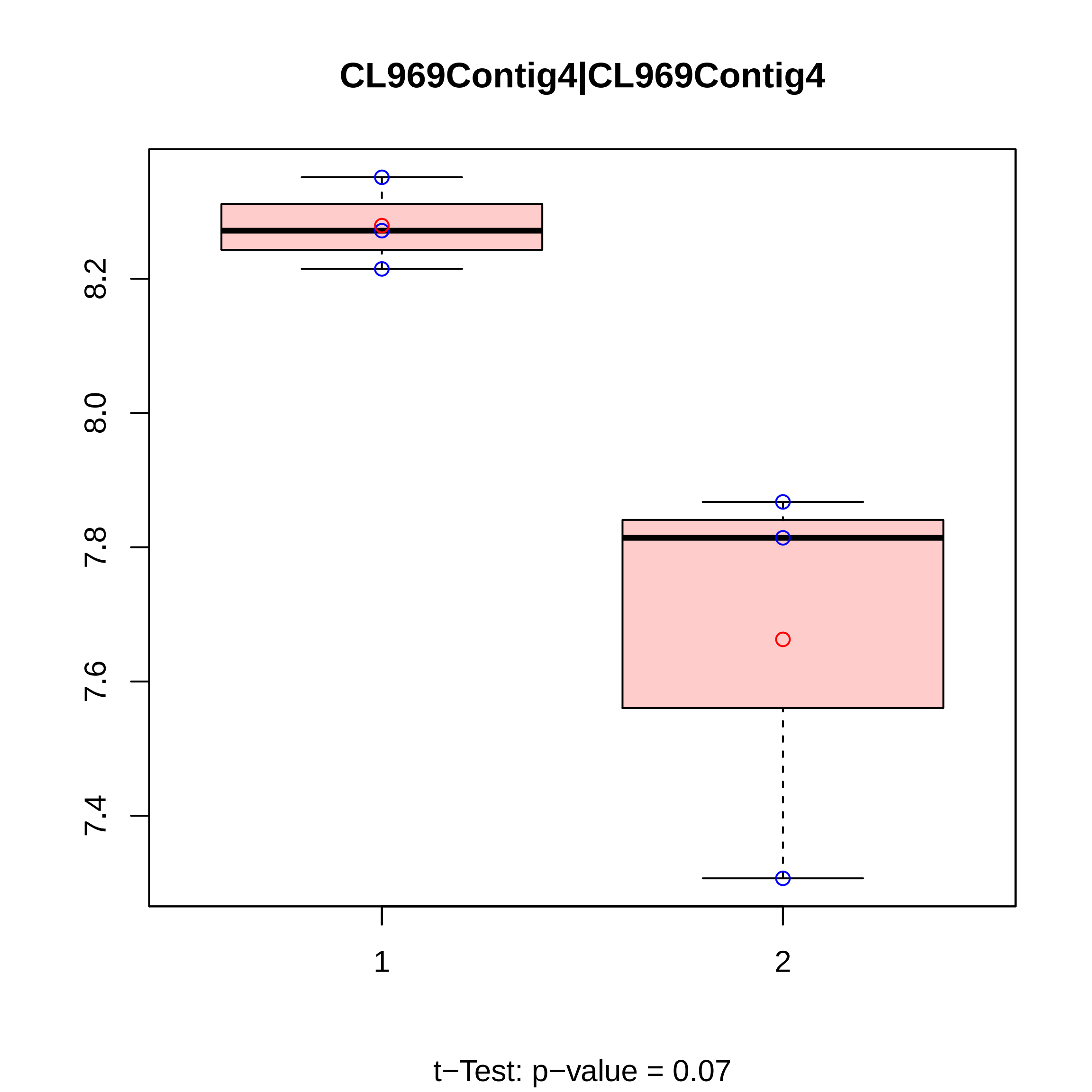
CL969Contig4|CL969Contig4
8.2
8.0
7.8
7.6
7.4
1
2
t−Test: p−value = 0.07

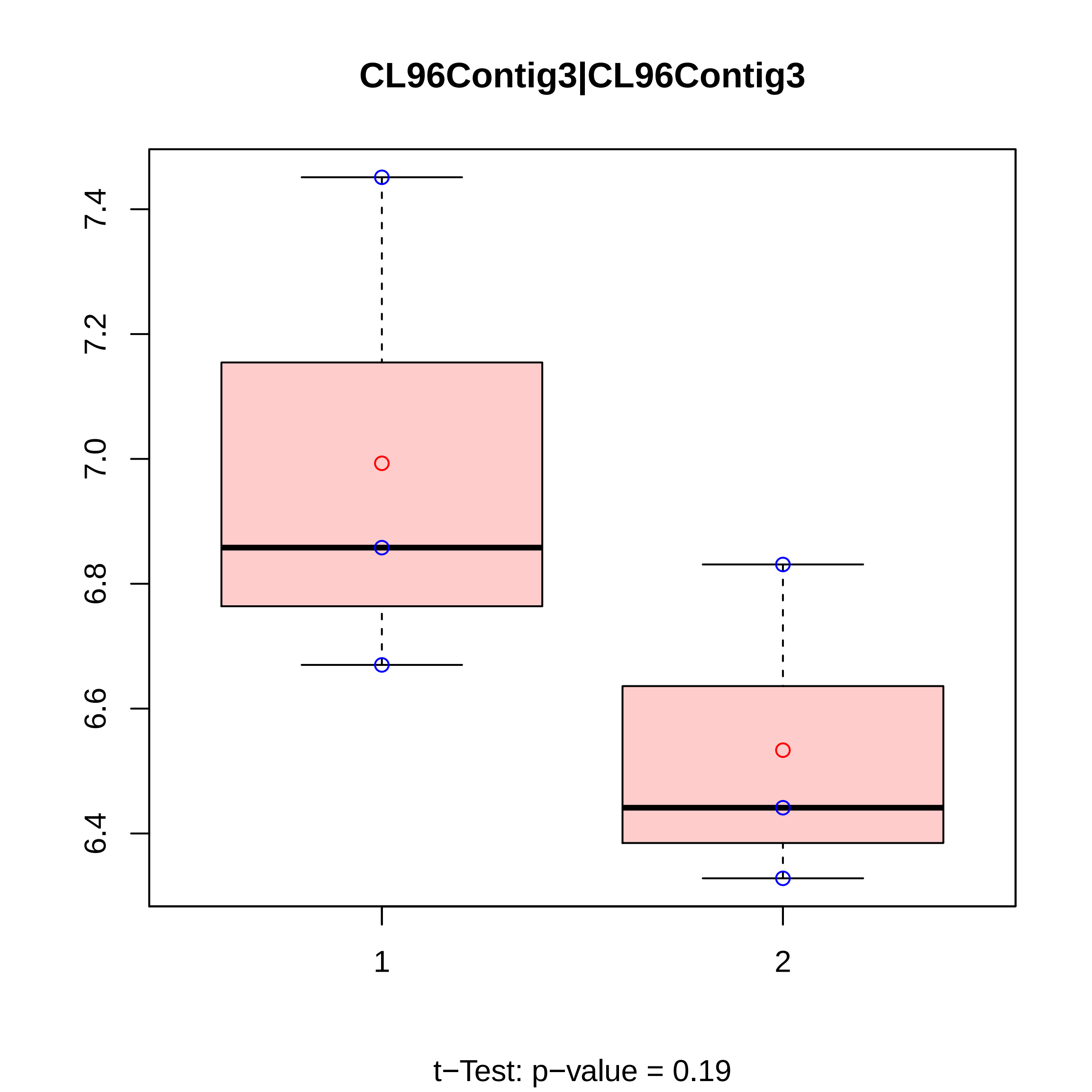

CL96Contig3|CL96Contig3
7.4
7.2
7.0
6.8
6.6
6.4
1
2
t−Test: p−value = 0.19

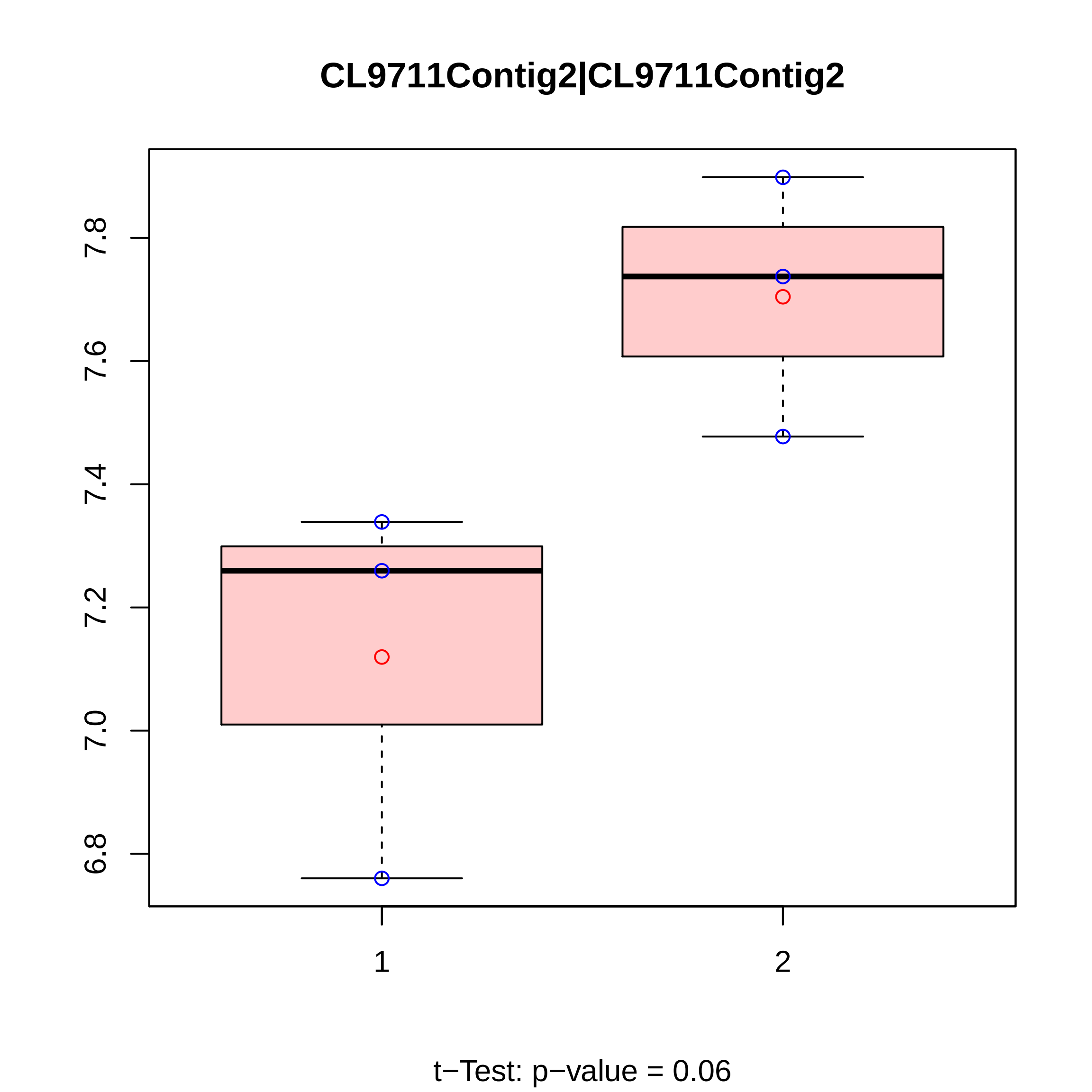
CL9711Contig2|CL9711Contig2
7.8
7.6
7.4
7.2
7.0
6.8
1
2
t−Test: p−value = 0.06

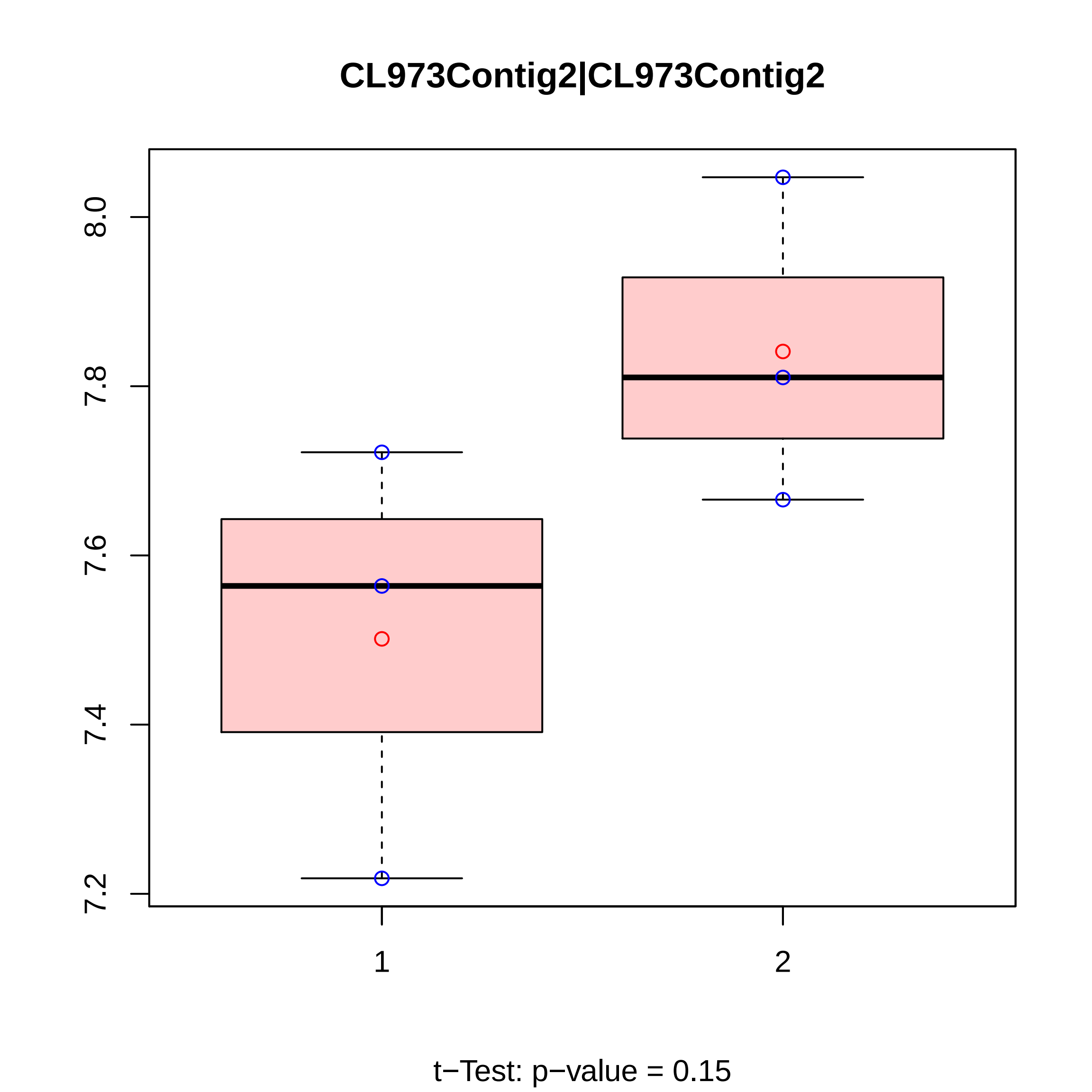
CL973Contig2|CL973Contig2
8.0
7.8
7.6
7.4
7.2
1
2
t−Test: p−value = 0.15

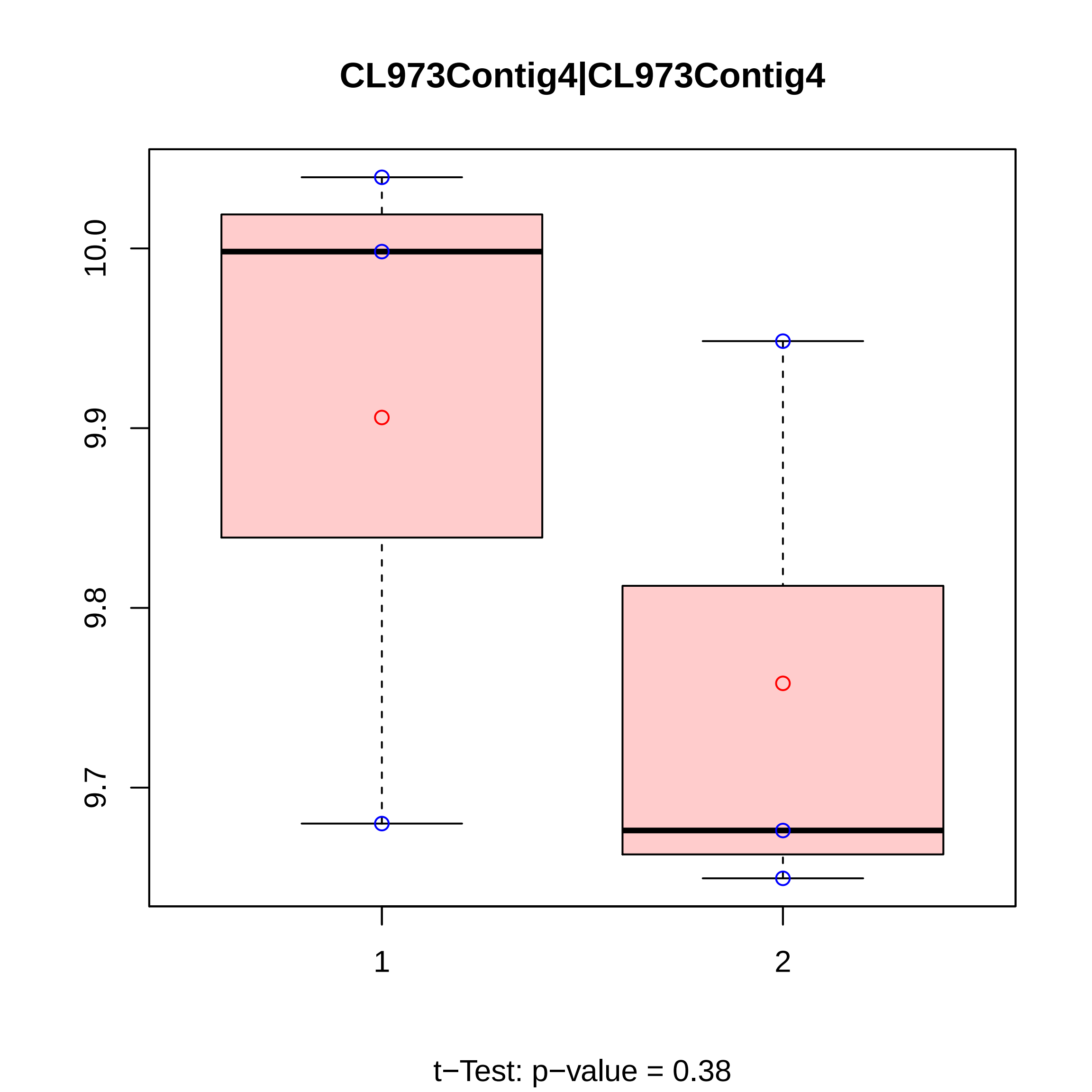

CL973Contig4|CL973Contig4
10.0
9.9
9.8
9.7
1
2
t−Test: p−value = 0.38

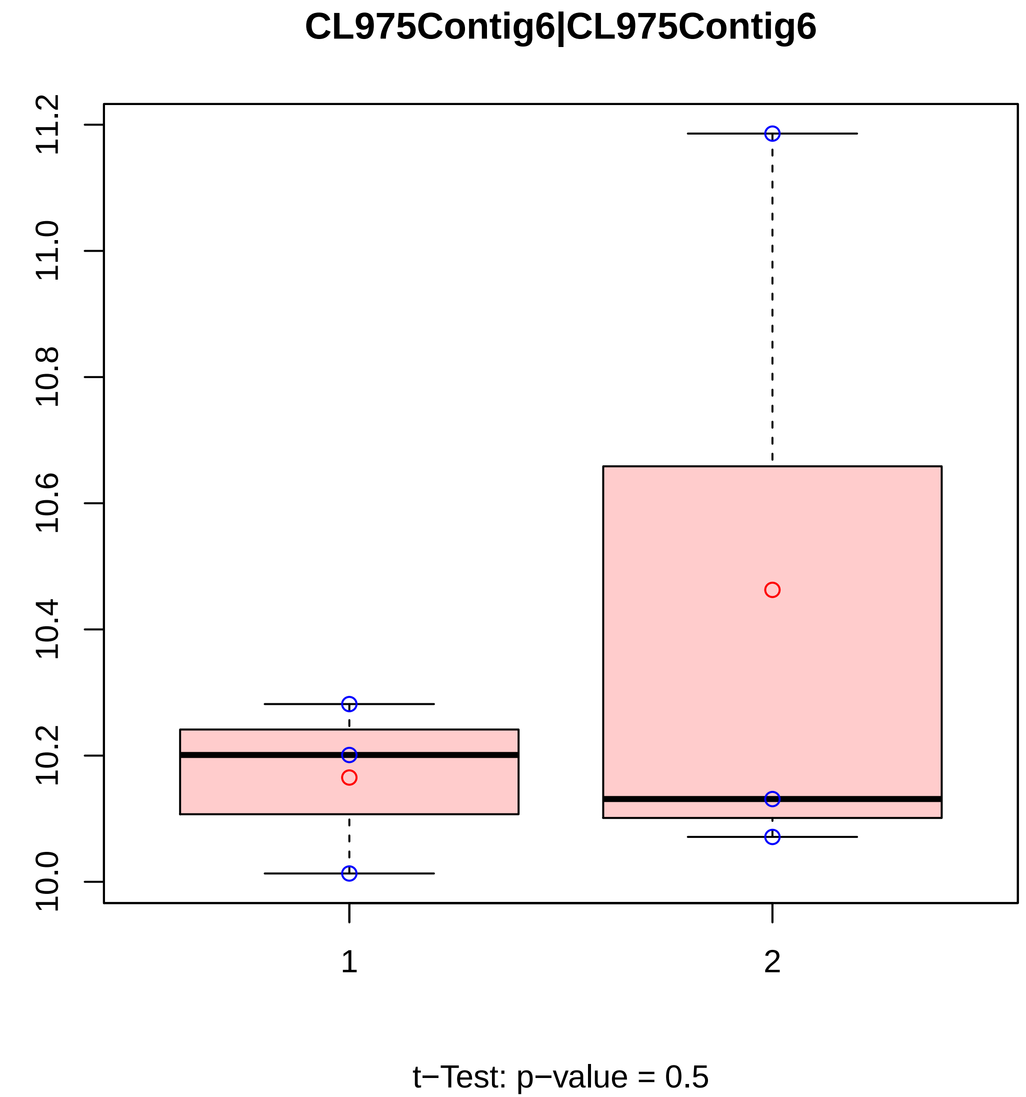
CL975Contig6|CL975Contig6
11.2
11.0
10.8
10.6
10.4
10.2
10.0
1
2
t−Test: p−value = 0.5

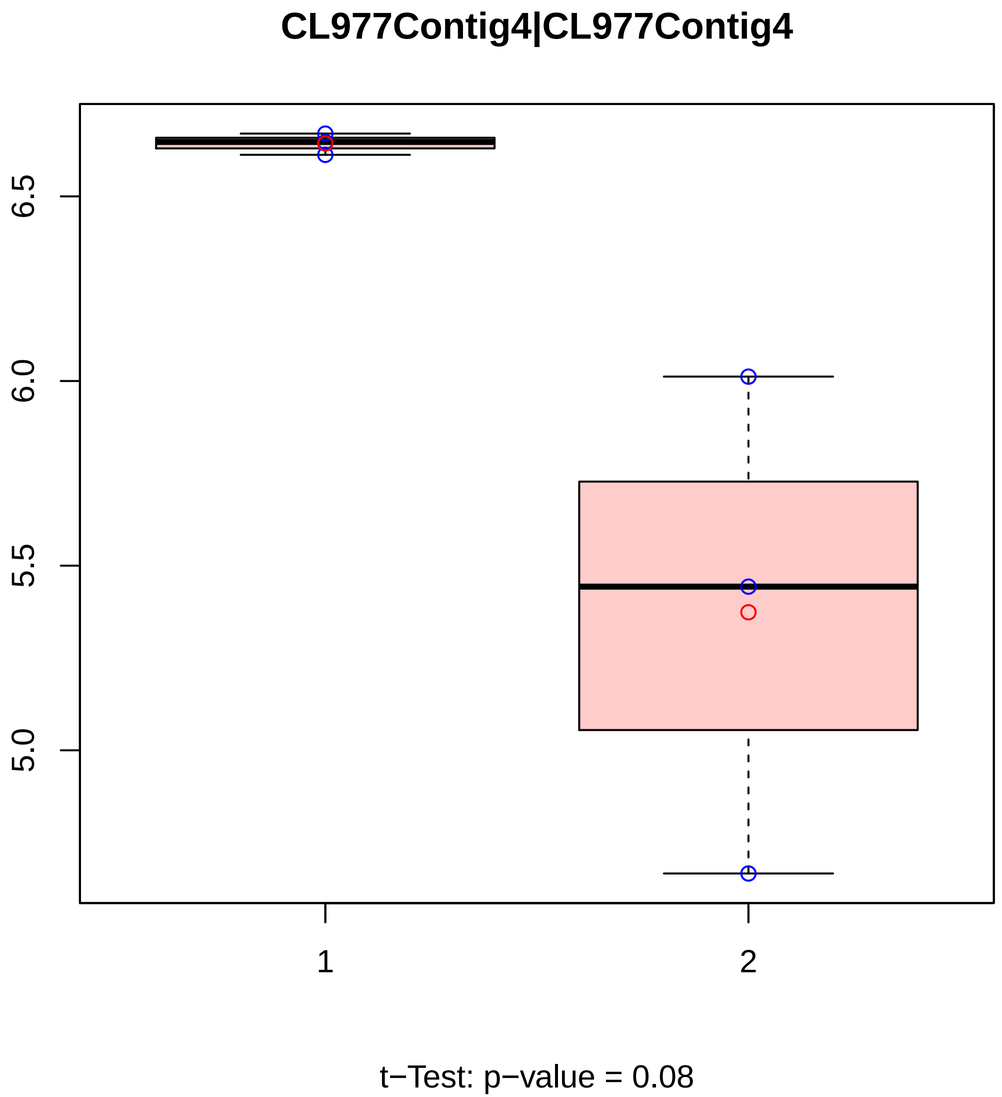

CL977Contig4|CL977Contig4
6.5
6.0
5.5
5.0
1
2
t−Test: p−value = 0.08

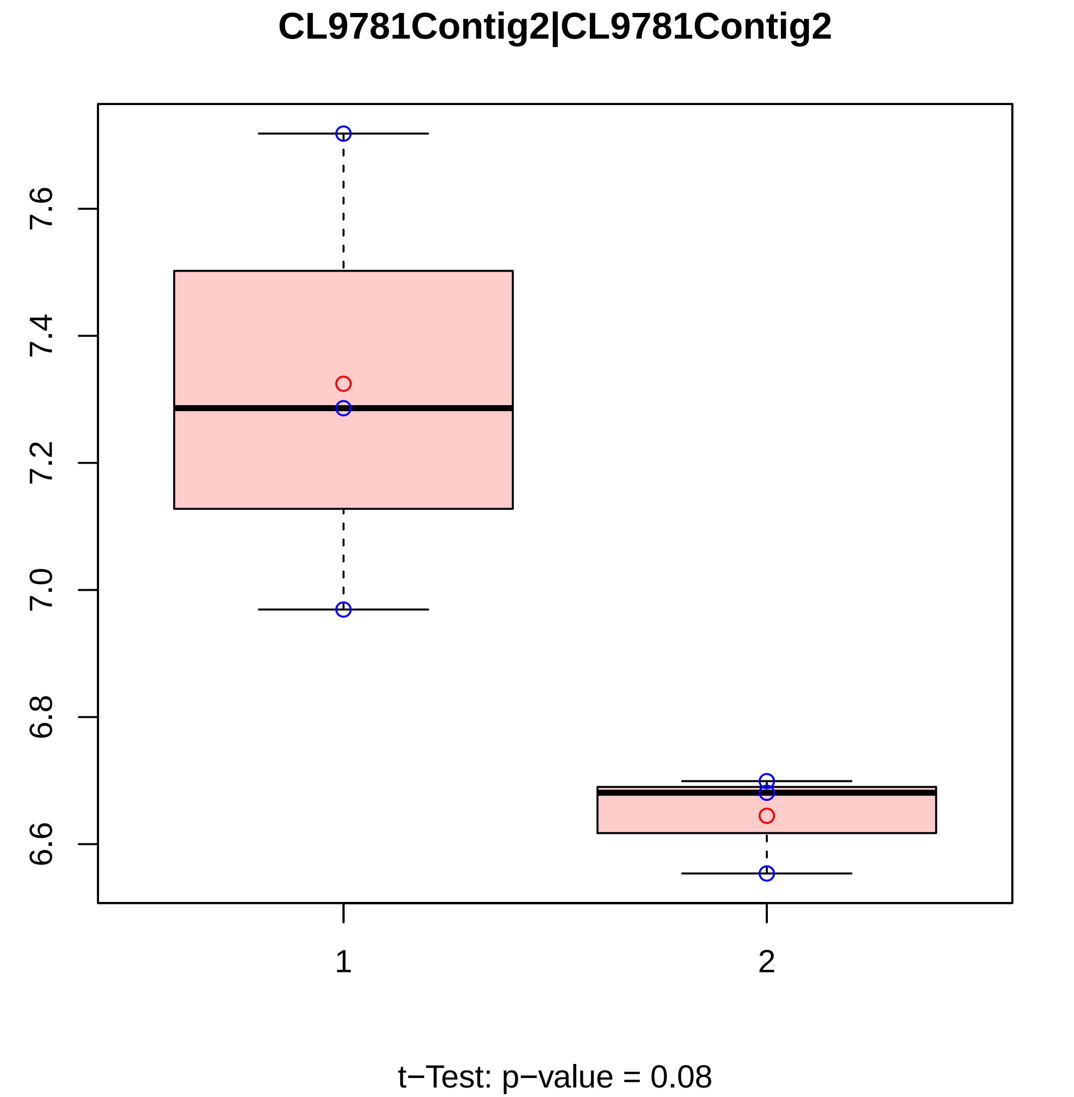
CL9781Contig2|CL9781Contig2
7.6
7.4
7.2
7.0
6.8
6.6
1
2
t−Test: p−value = 0.08

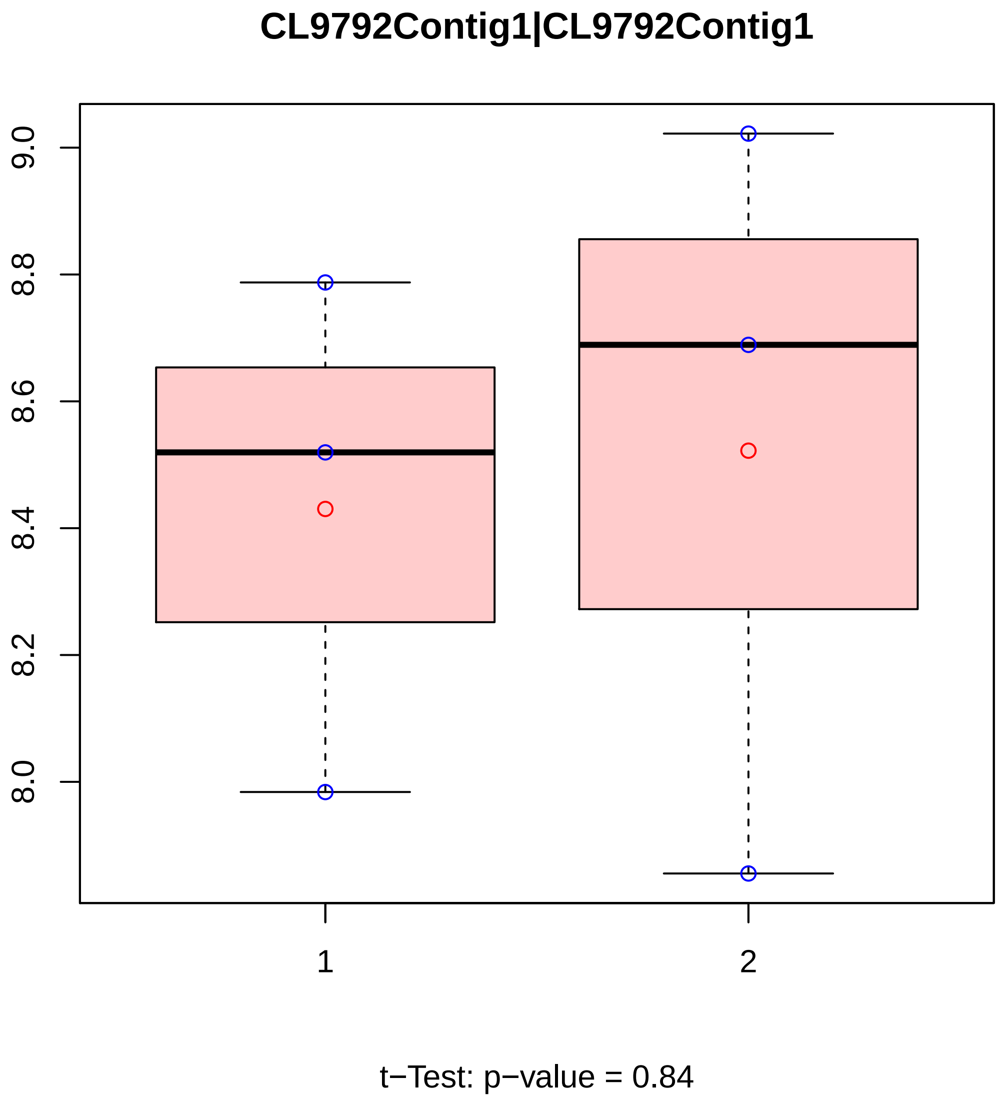
CL9792Contig1|CL9792Contig1
9.0
8.8
8.6
8.4
8.2
8.0
1
2
t−Test: p−value = 0.84

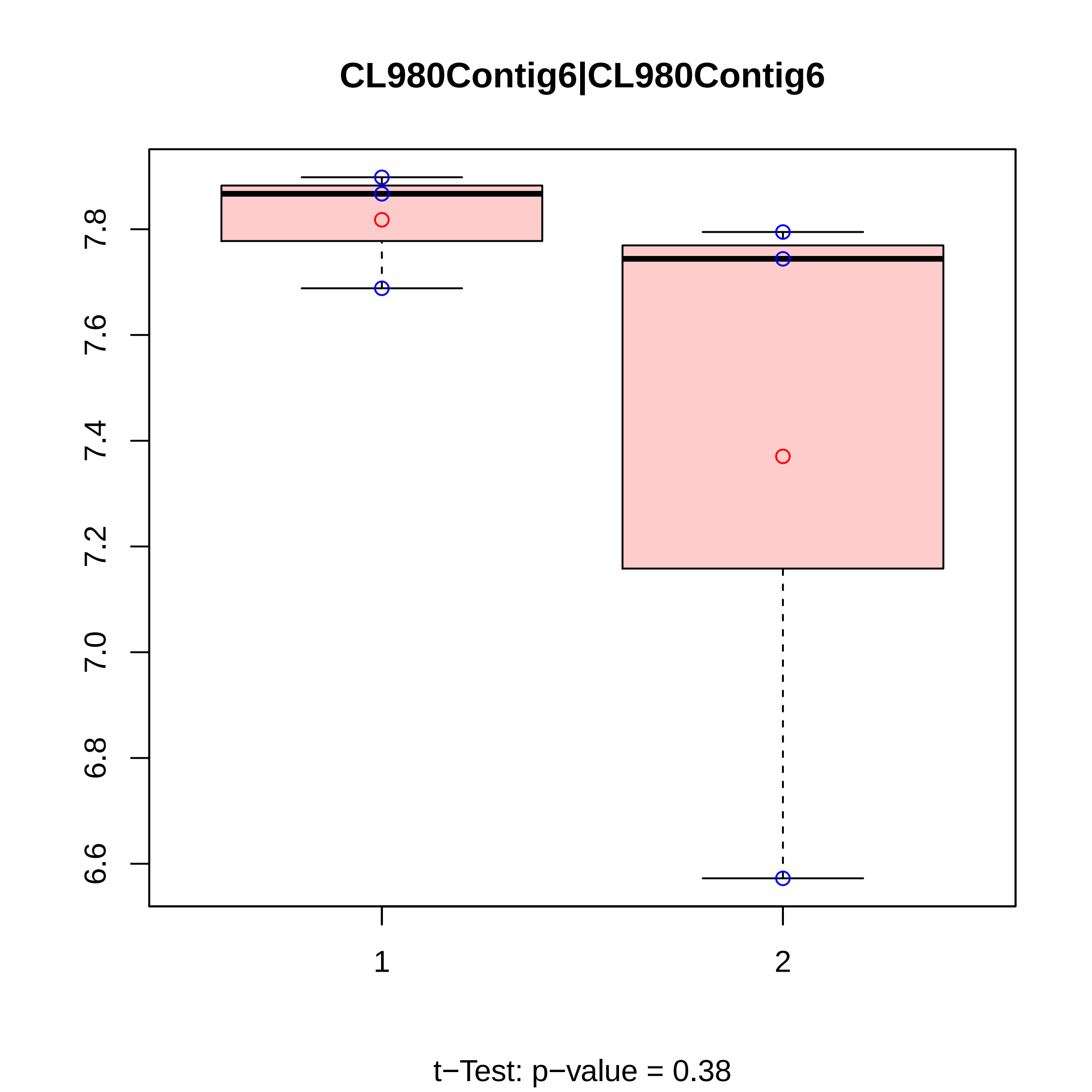
CL980Contig6|CL980Contig6
7.8
7.6
7.4
7.2
7.0
6.8
6.6
1
2
t−Test: p−value = 0.38

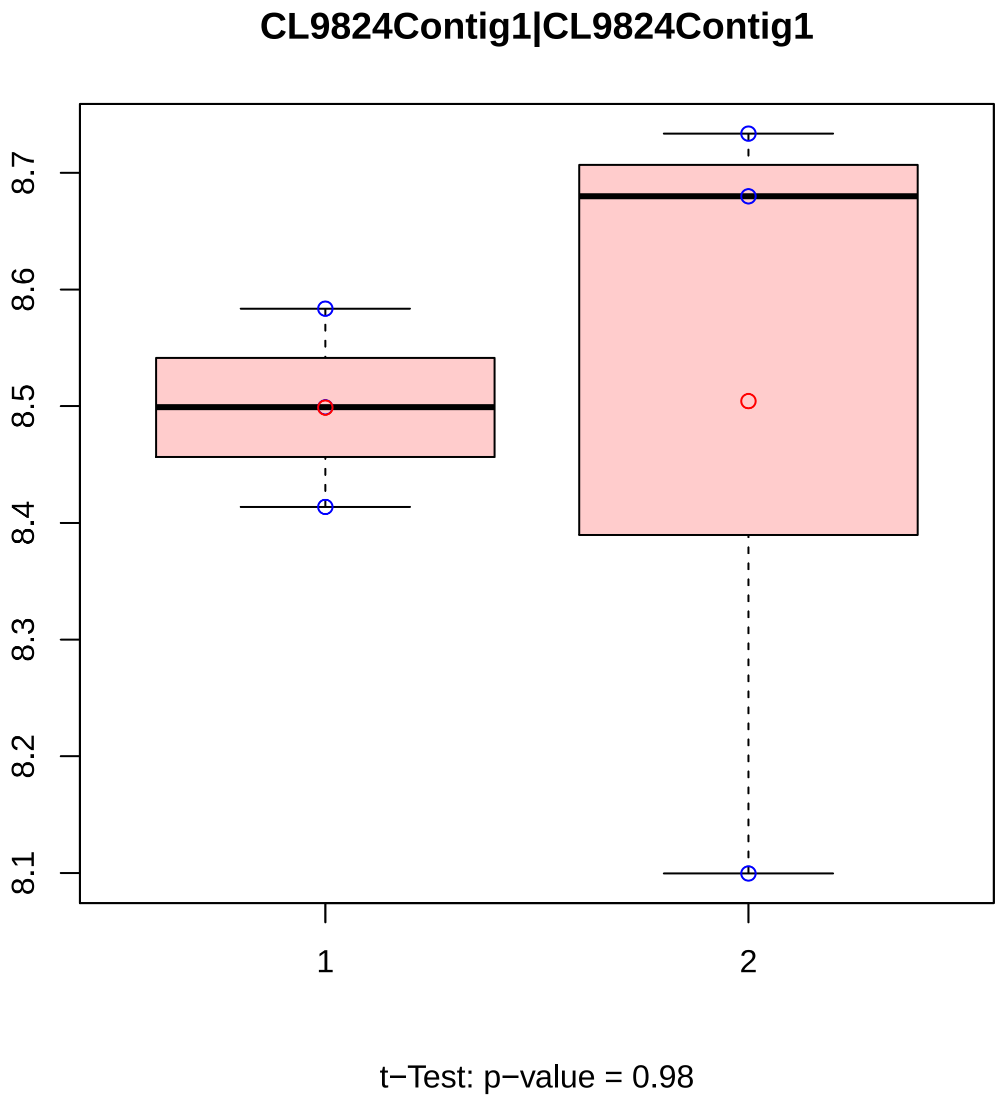
CL9824Contig1|CL9824Contig1
8.1
8.2
8.3
8.4
8.5
8.6
8.7
1
2
t−Test: p−value = 0.98

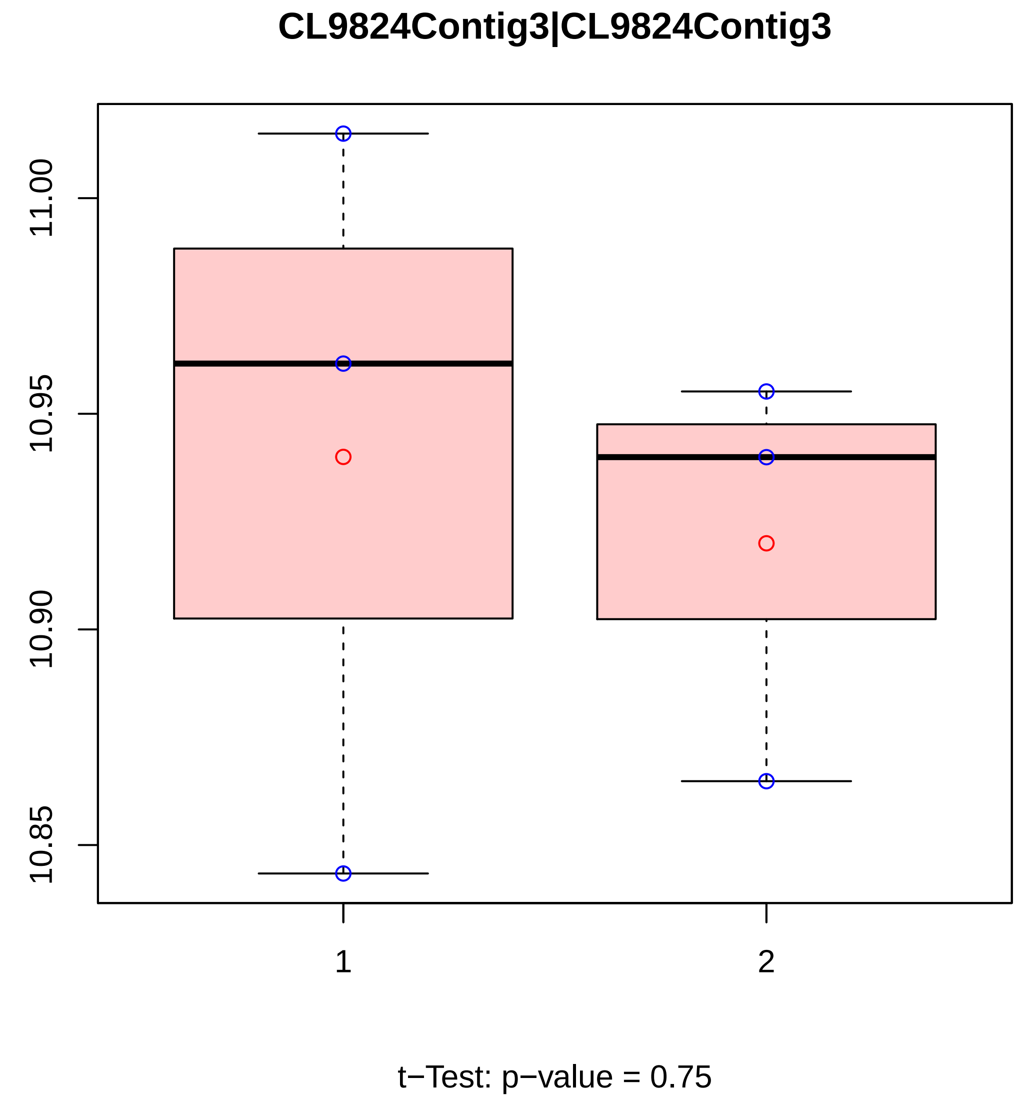
CL9824Contig3|CL9824Contig3
11.00
10.95
10.90
10.85
1
2
t−Test: p−value = 0.75

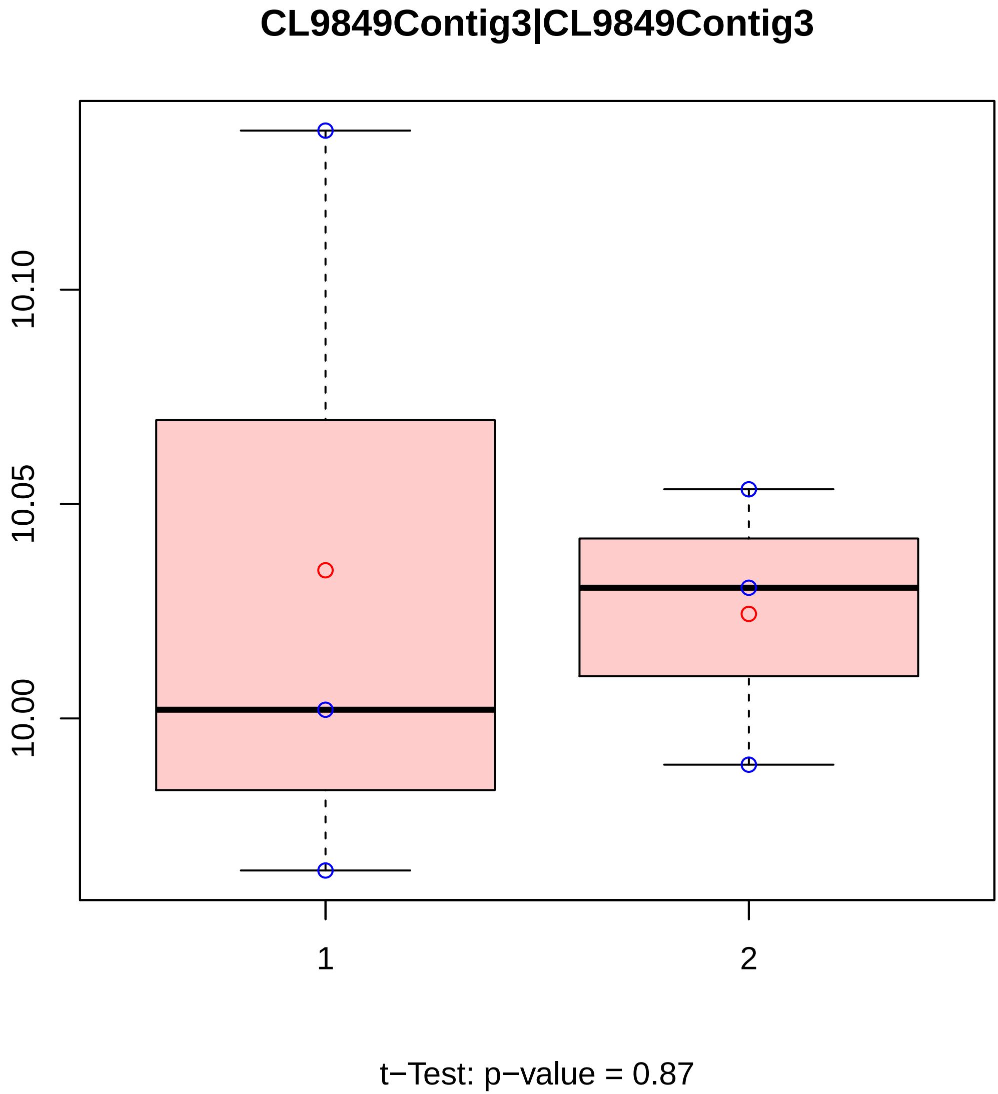
CL9849Contig3|CL9849Contig3
10.10
10.05
10.00
1
2
t−Test: p−value = 0.87

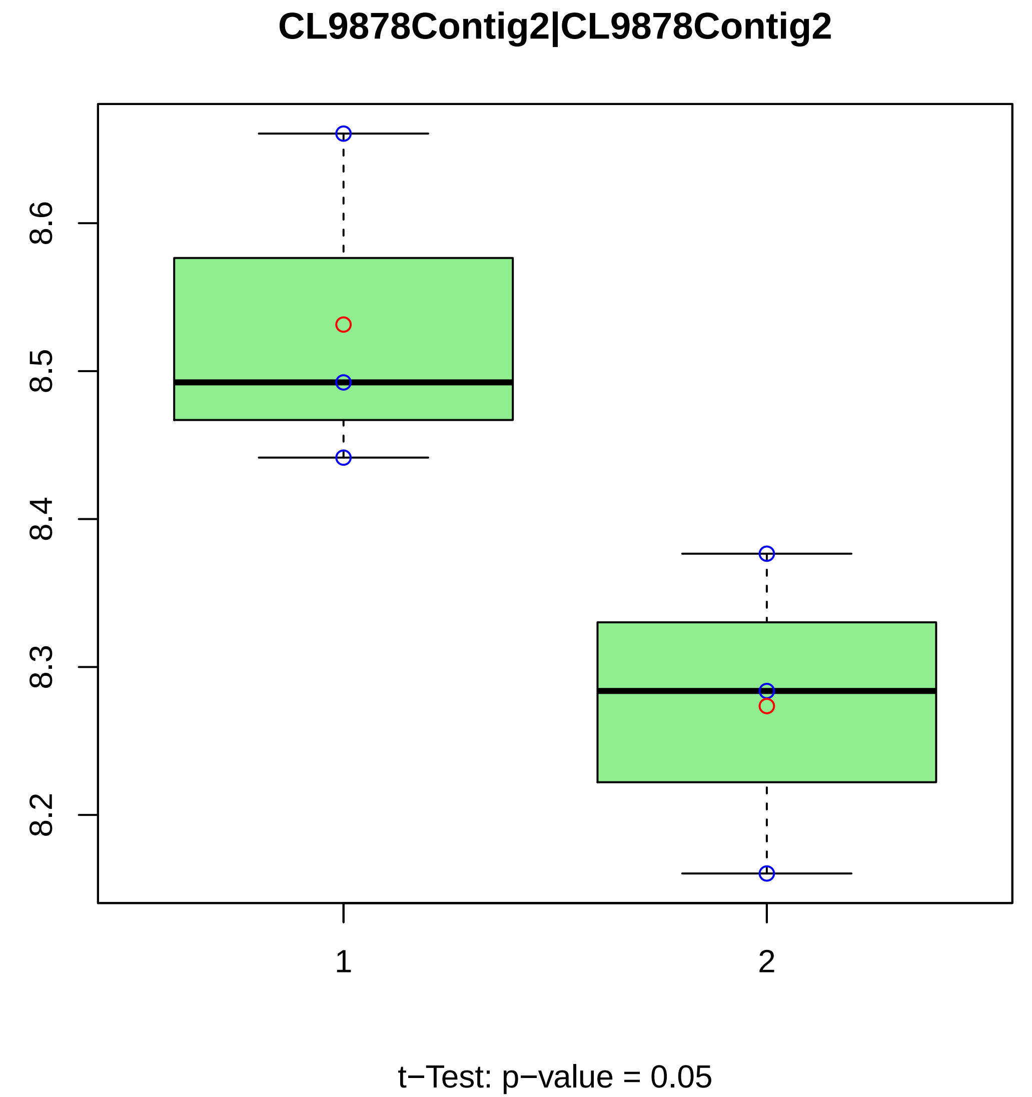

CL9878Contig2|CL9878Contig2
8.2
8.3
8.4
8.5
8.6
1
2
t−Test: p−value = 0.05

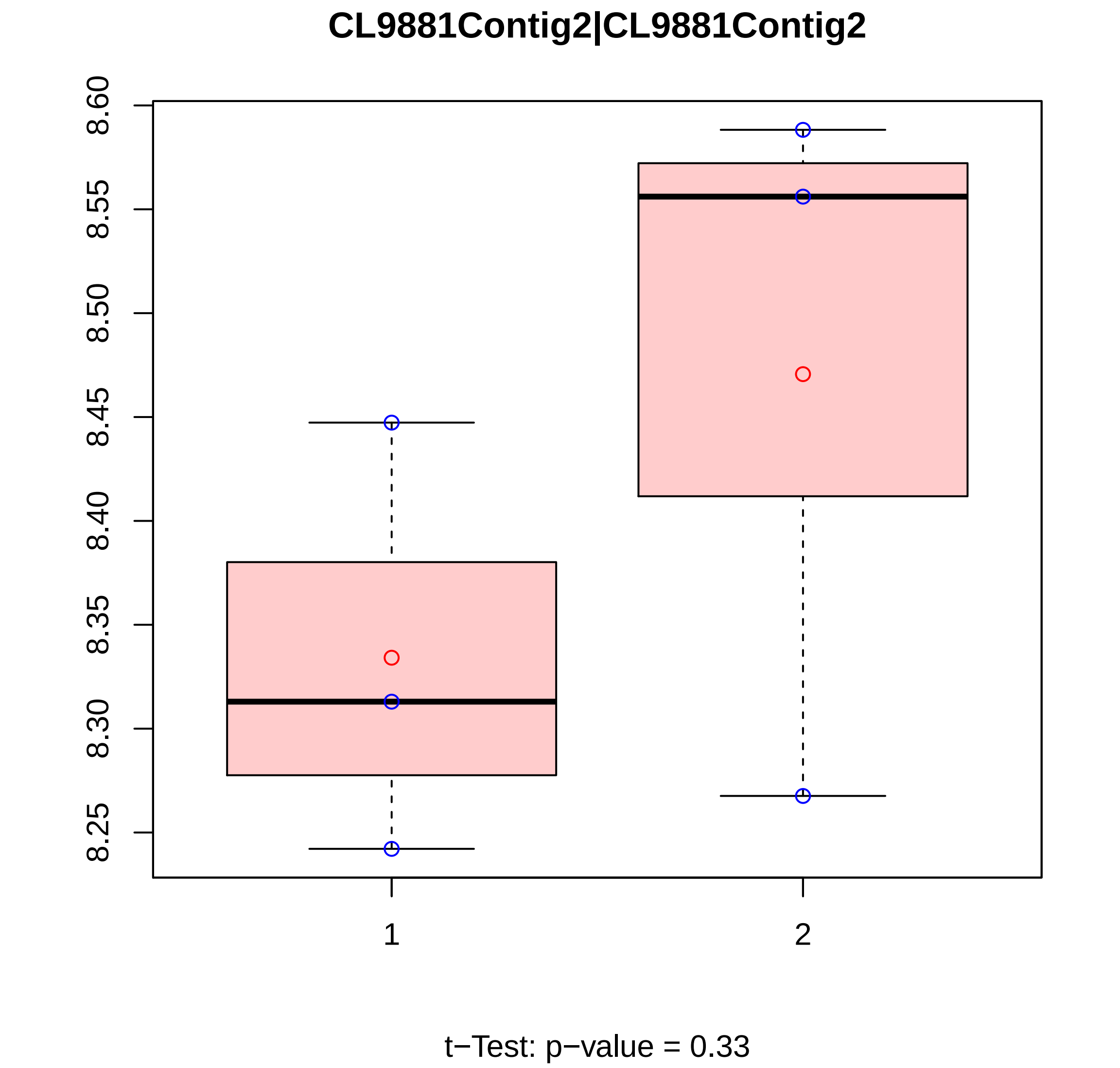
CL9881Contig2|CL9881Contig2
8.60
8.55
8.50
8.45
8.40
8.35
8.30
8.25
1
2
t−Test: p−value = 0.33

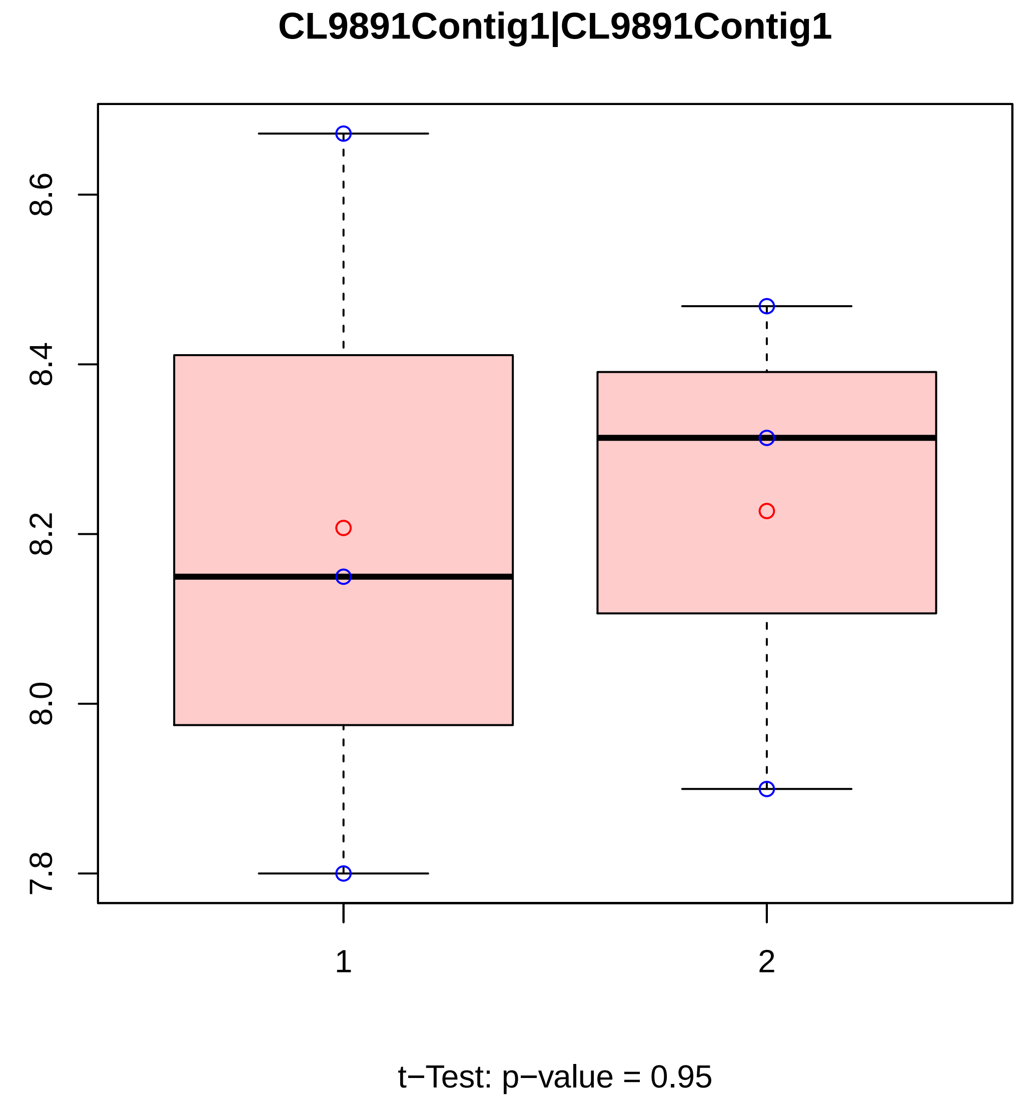
CL9891Contig1|CL9891Contig1
8.6
8.4
8.2
8.0
7.8
1
2
t−Test: p−value = 0.95

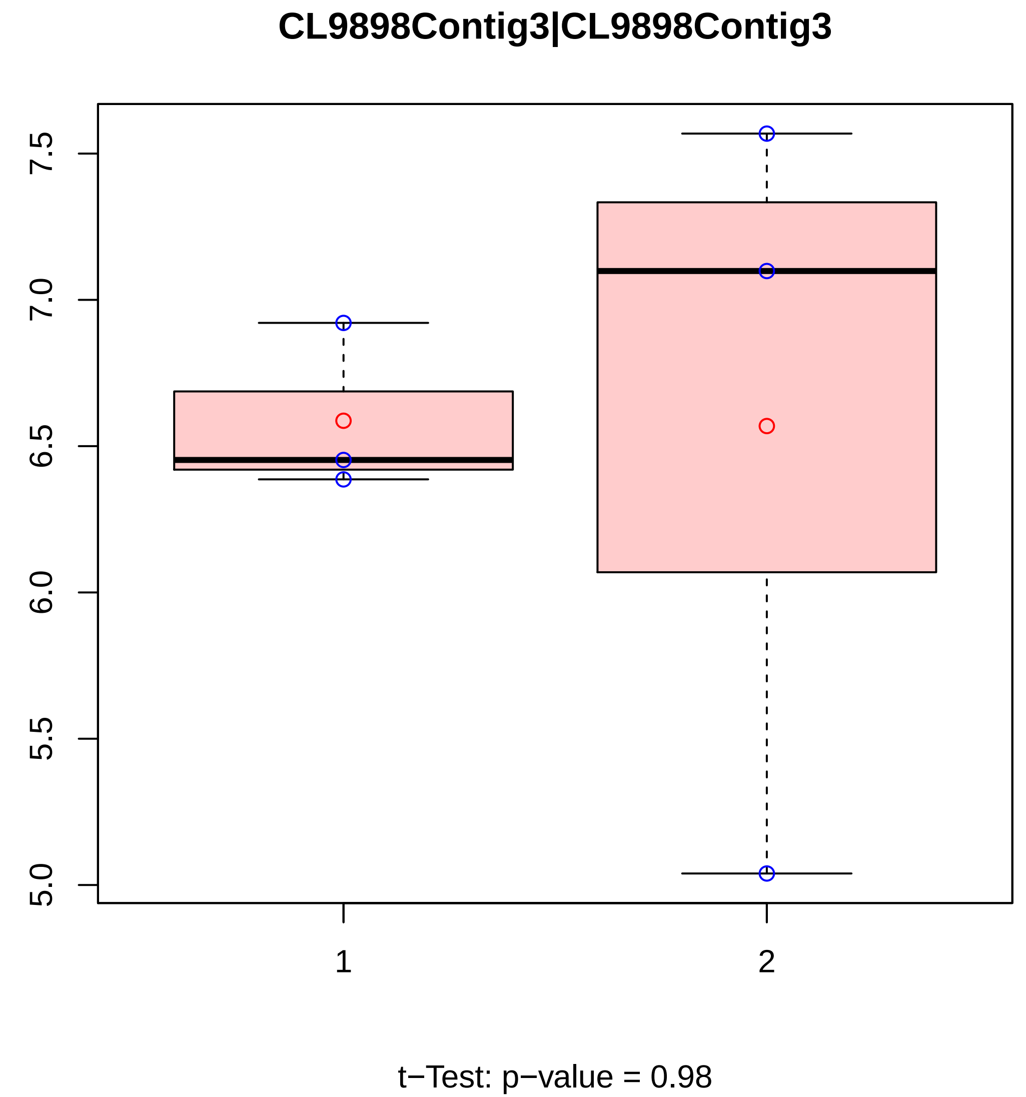
CL9898Contig3|CL9898Contig3
7.5
7.0
6.5
6.0
5.5
5.0
1
2
t−Test: p−value = 0.98

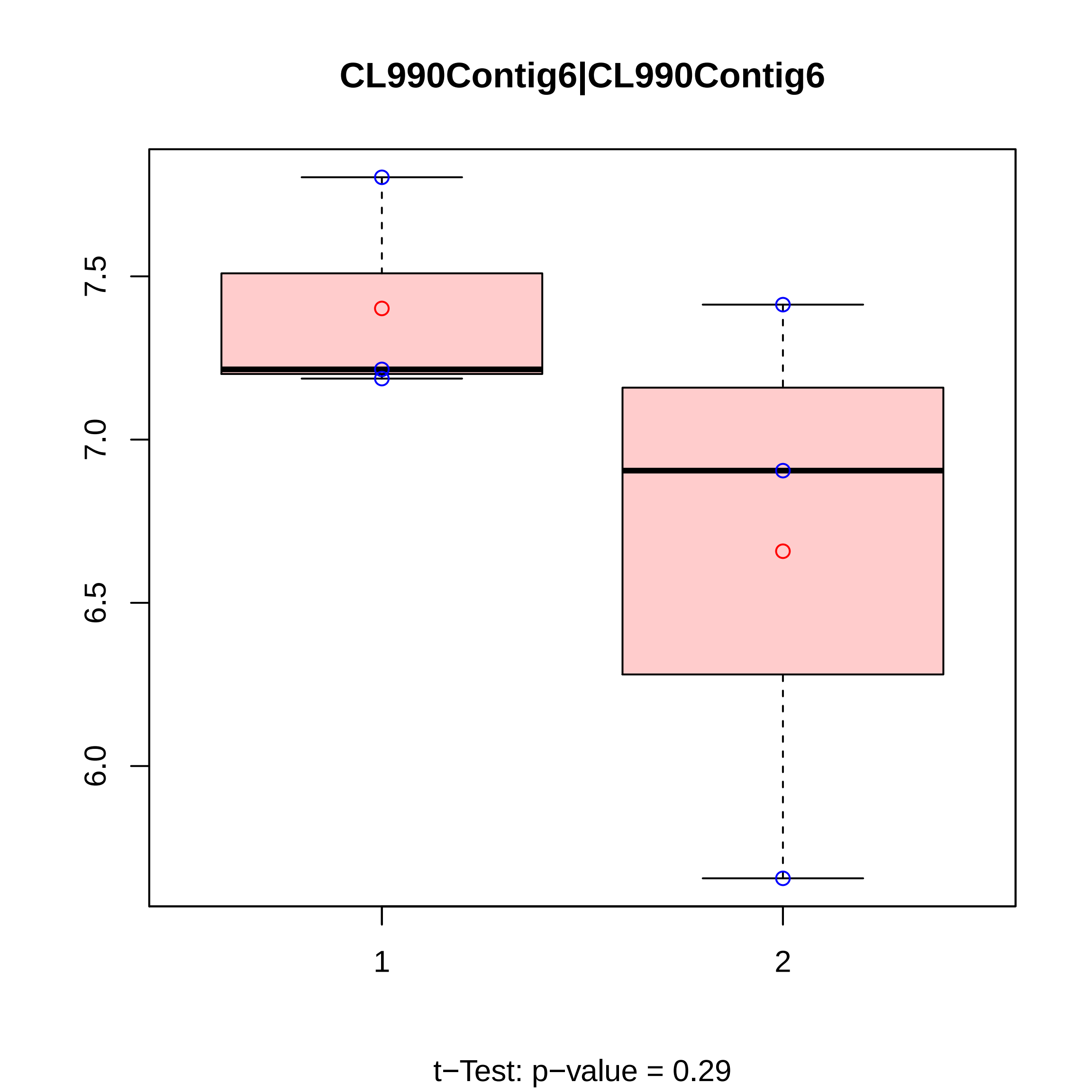
CL990Contig6|CL990Contig6
7.5
7.0
6.5
6.0
1
2
t−Test: p−value = 0.29

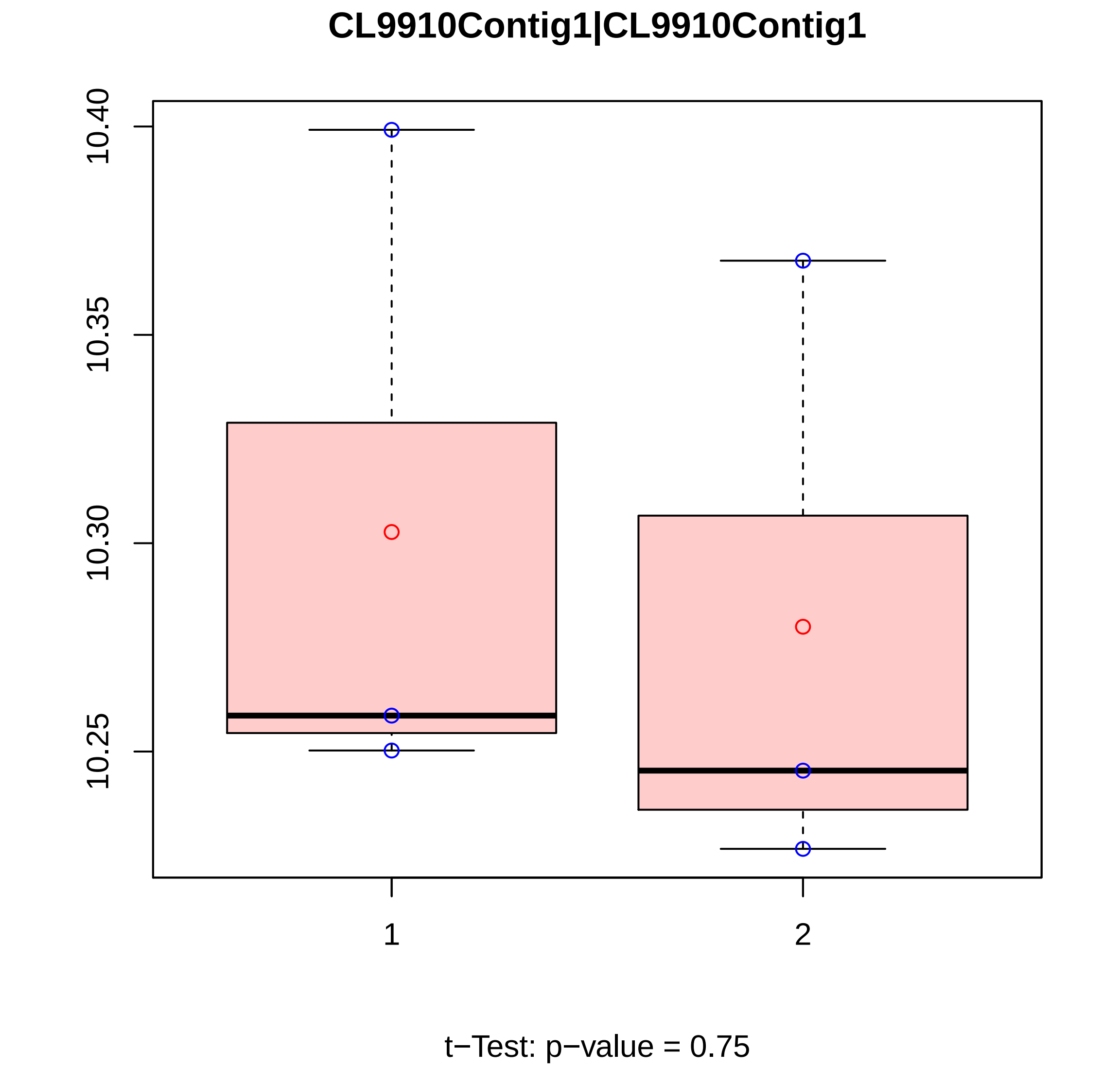
CL9910Contig1|CL9910Contig1
10.40
10.35
10.30
10.25
1
2
t−Test: p−value = 0.75

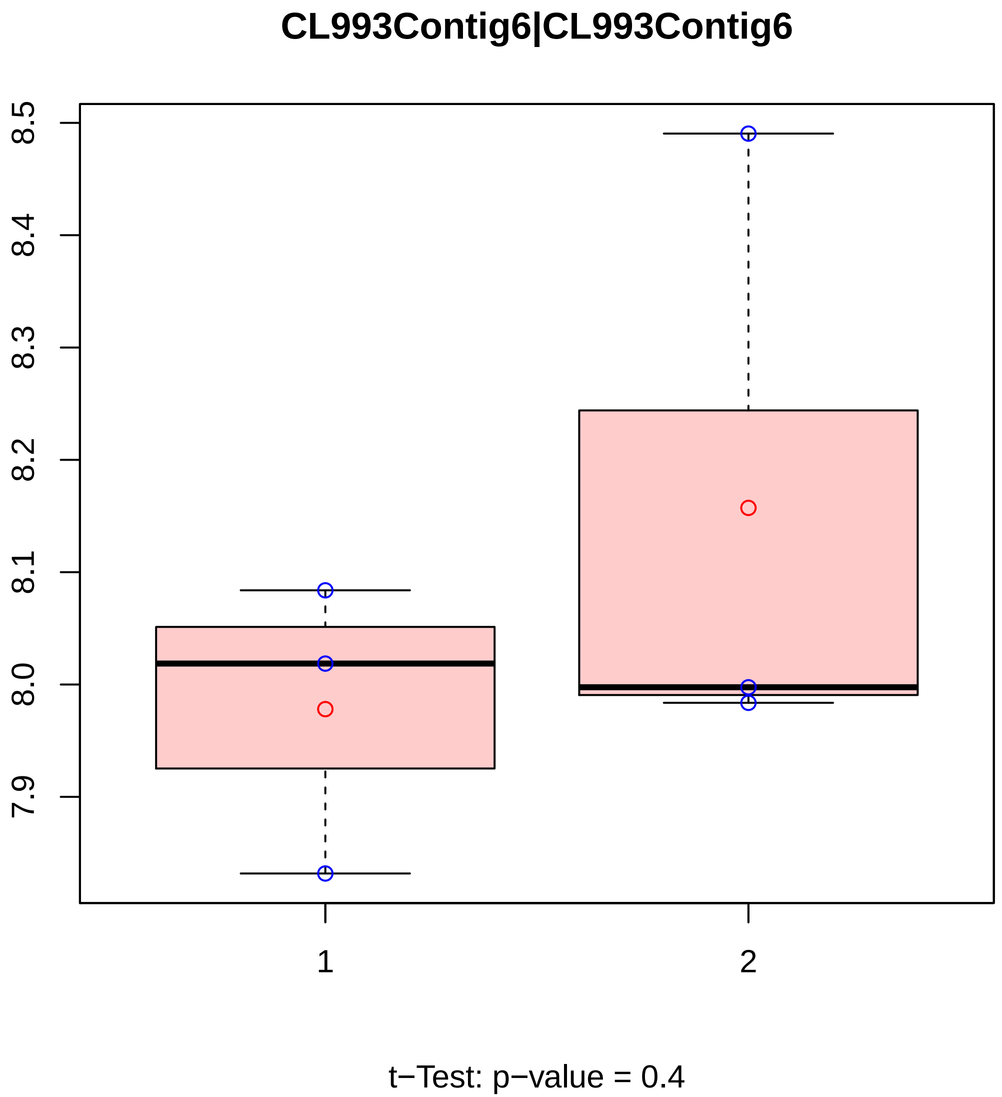
CL993Contig6|CL993Contig6
8.5
8.4
8.3
8.2
8.1
8.0
7.9
1
2
t−Test: p−value = 0.4

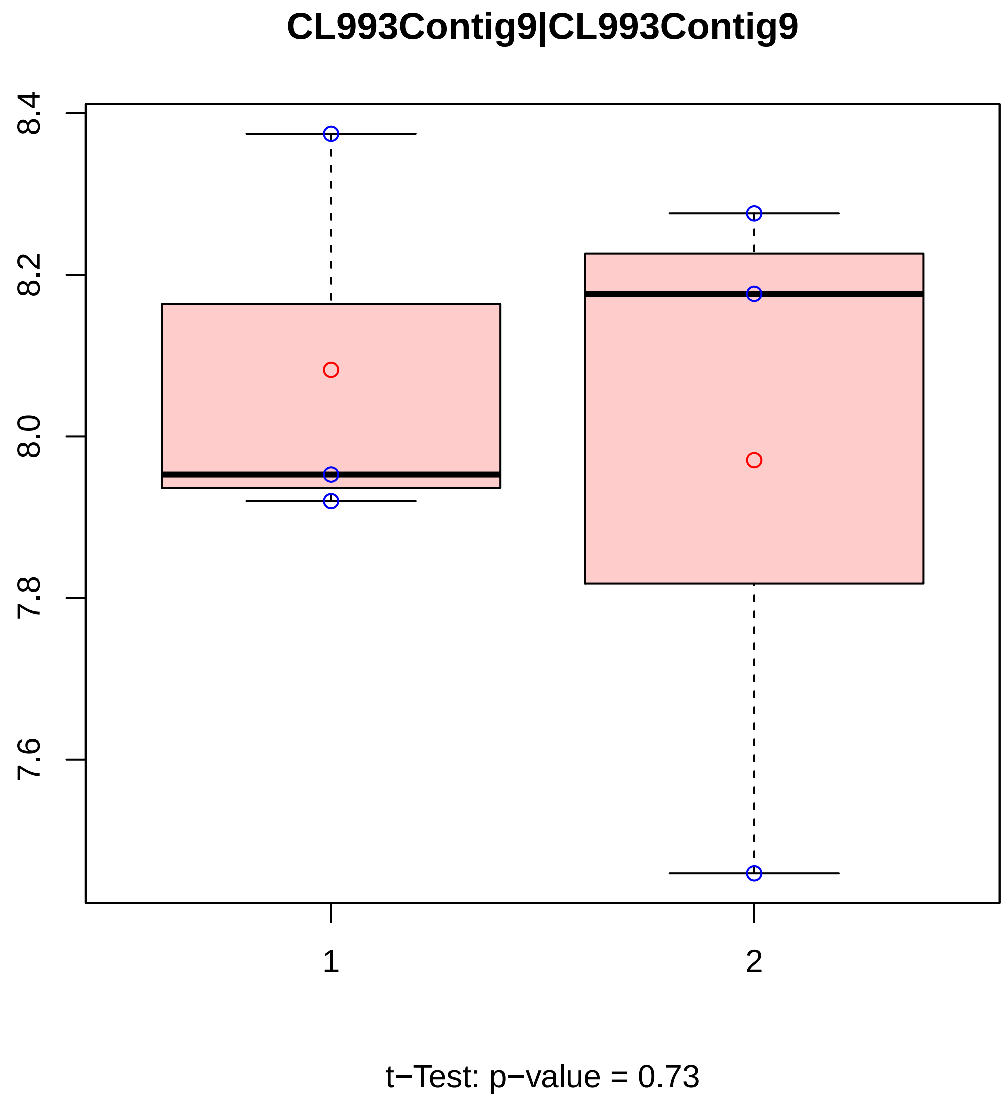

CL993Contig9|CL993Contig9
8.4
8.2
8.0
7.8
7.6
1
2
t−Test: p−value = 0.73

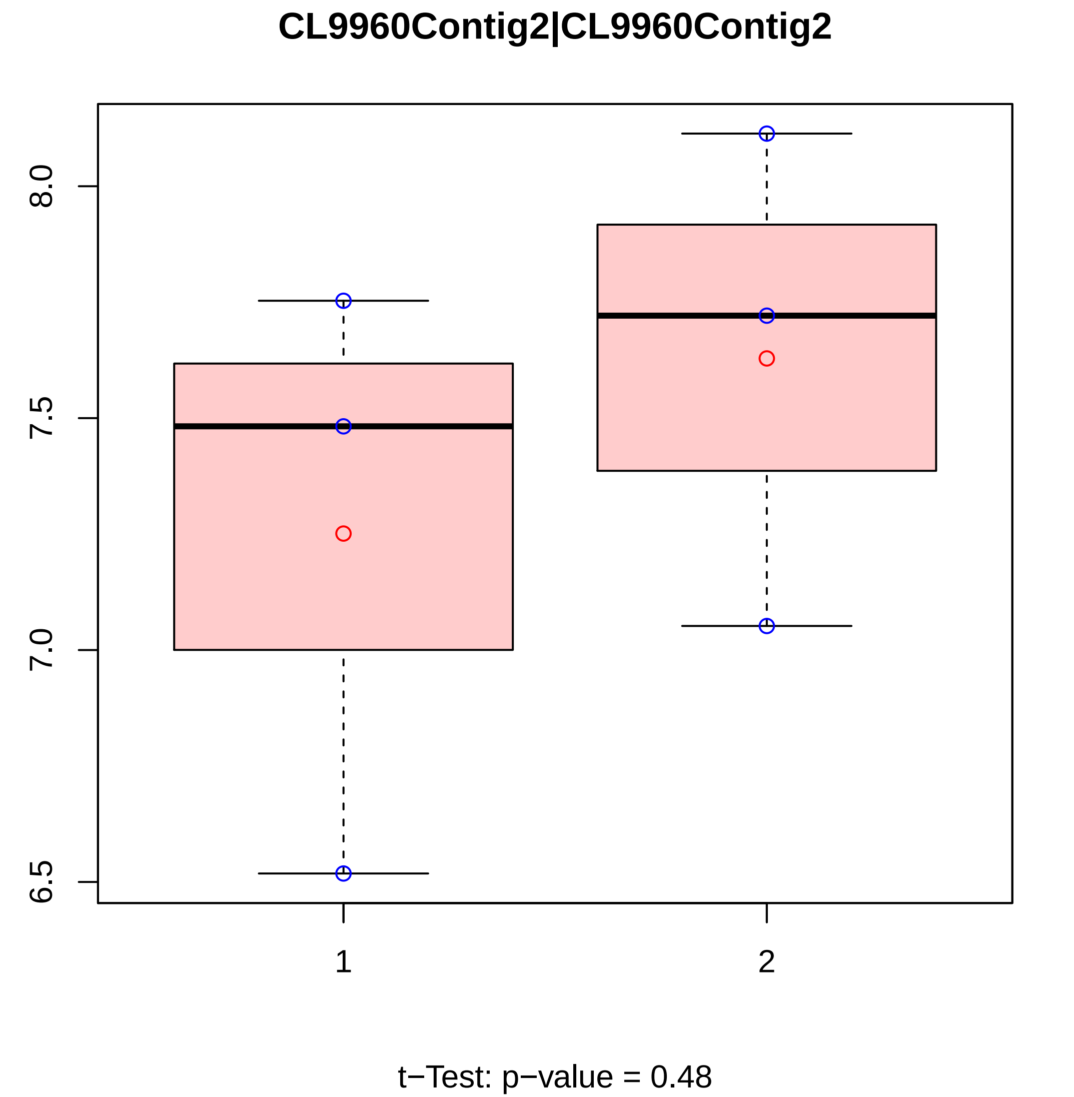
CL9960Contig2|CL9960Contig2
8.0
7.5
7.0
6.5
1
2
t−Test: p−value = 0.48

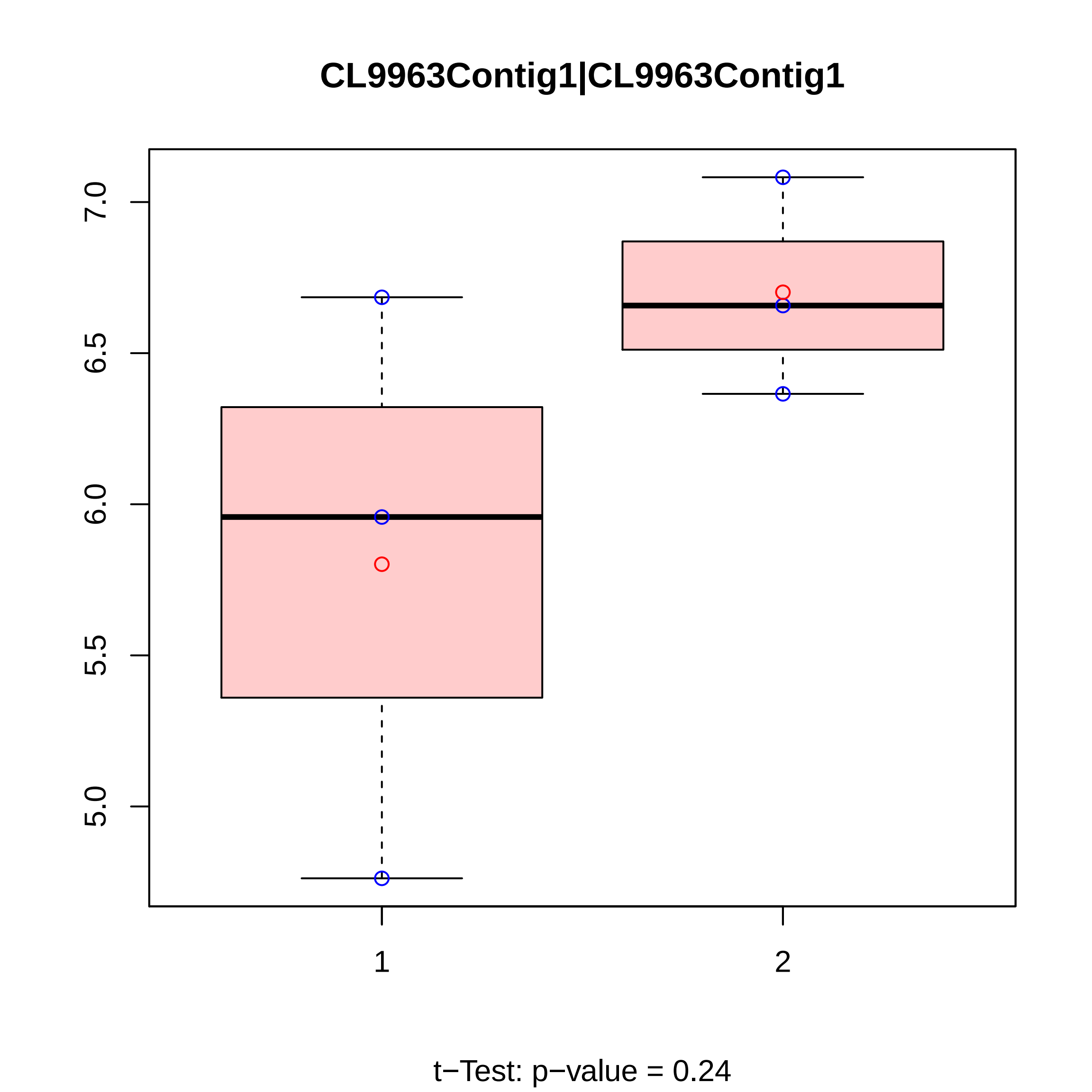

CL9963Contig1|CL9963Contig1
7.0
6.5
6.0
5.5
5.0
1
2
t−Test: p−value = 0.24

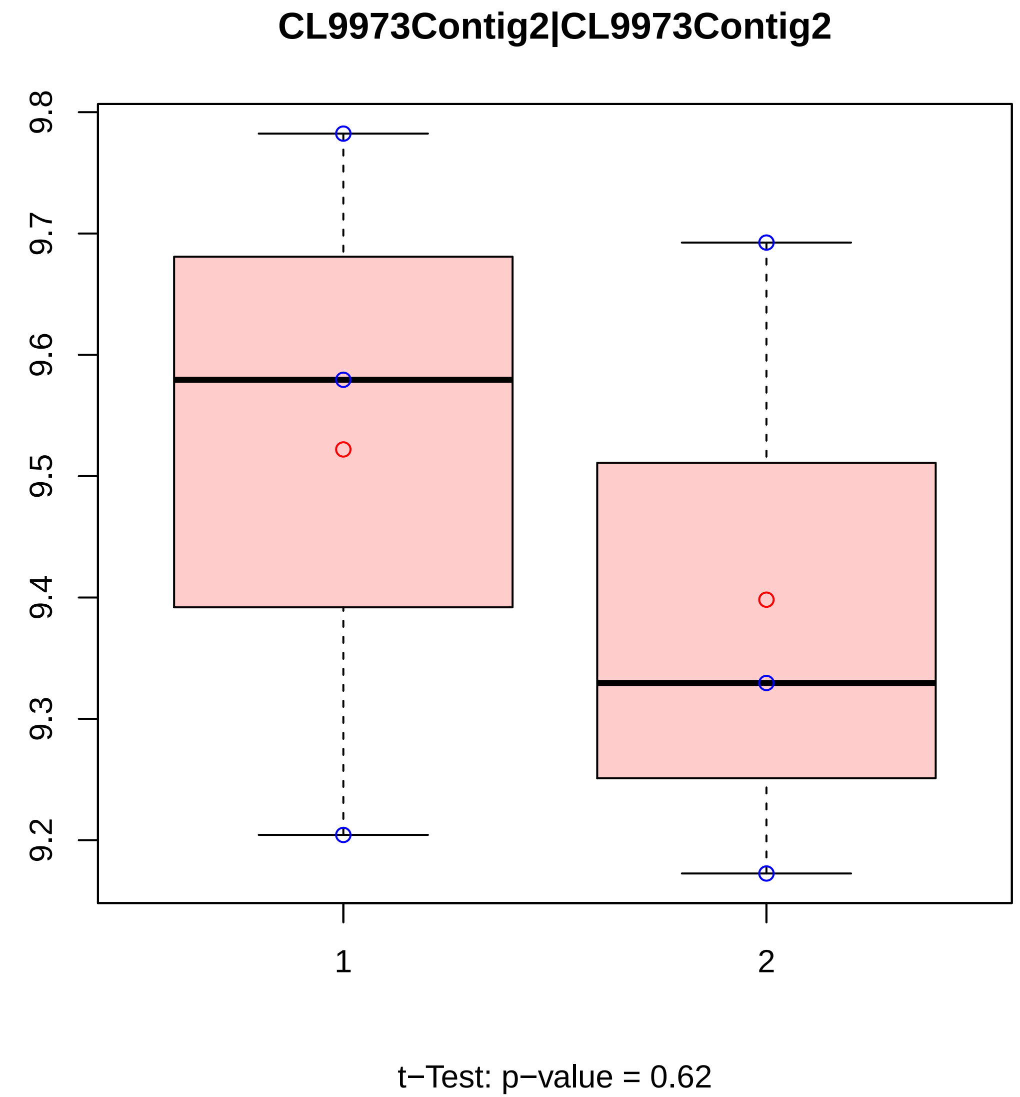

CL9973Contig2|CL9973Contig2
9.2
9.3
9.4
9.5
9.6
9.7
9.8
1
2
t−Test: p−value = 0.62

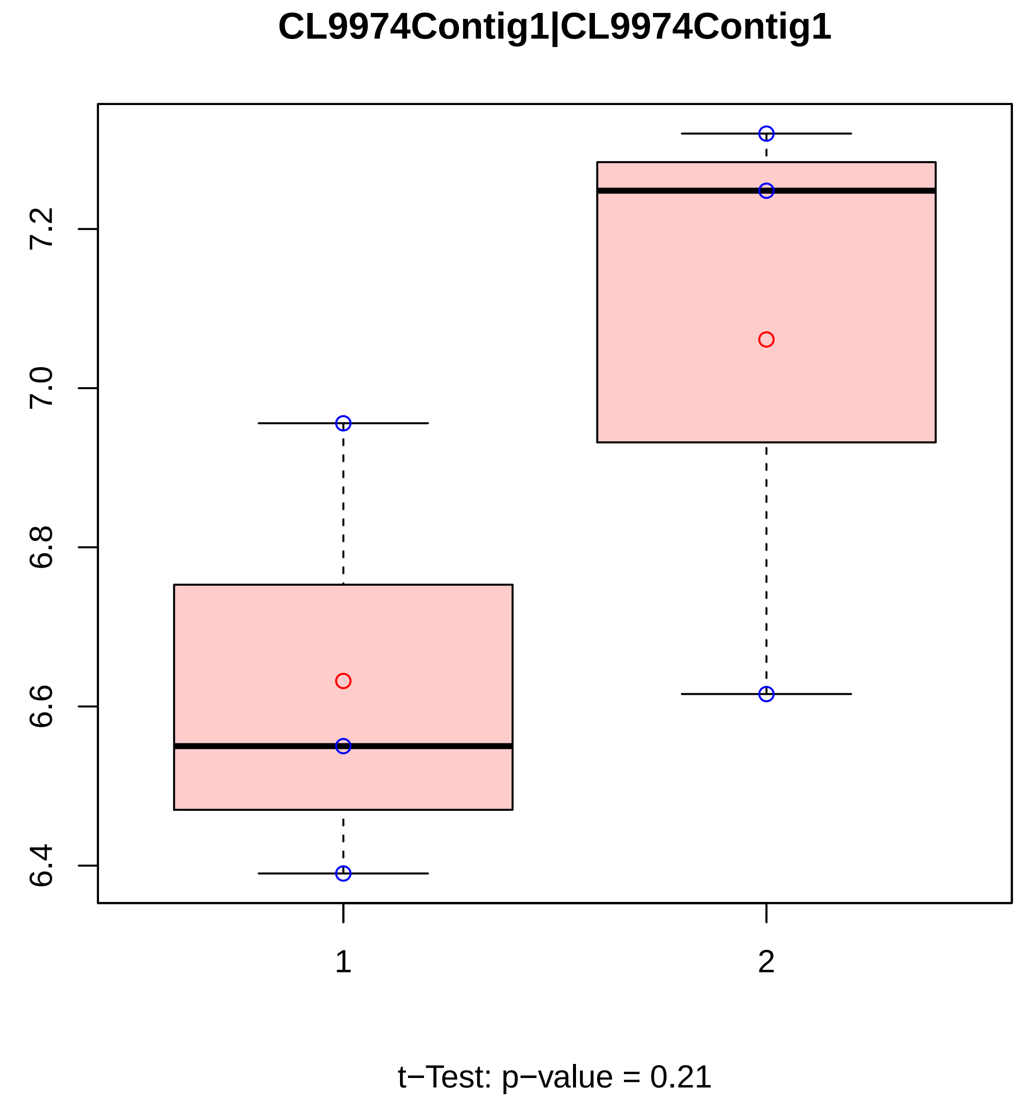
CL9974Contig1|CL9974Contig1
6.4
6.6
6.8
7.0
7.2
1
2
t−Test: p−value = 0.21

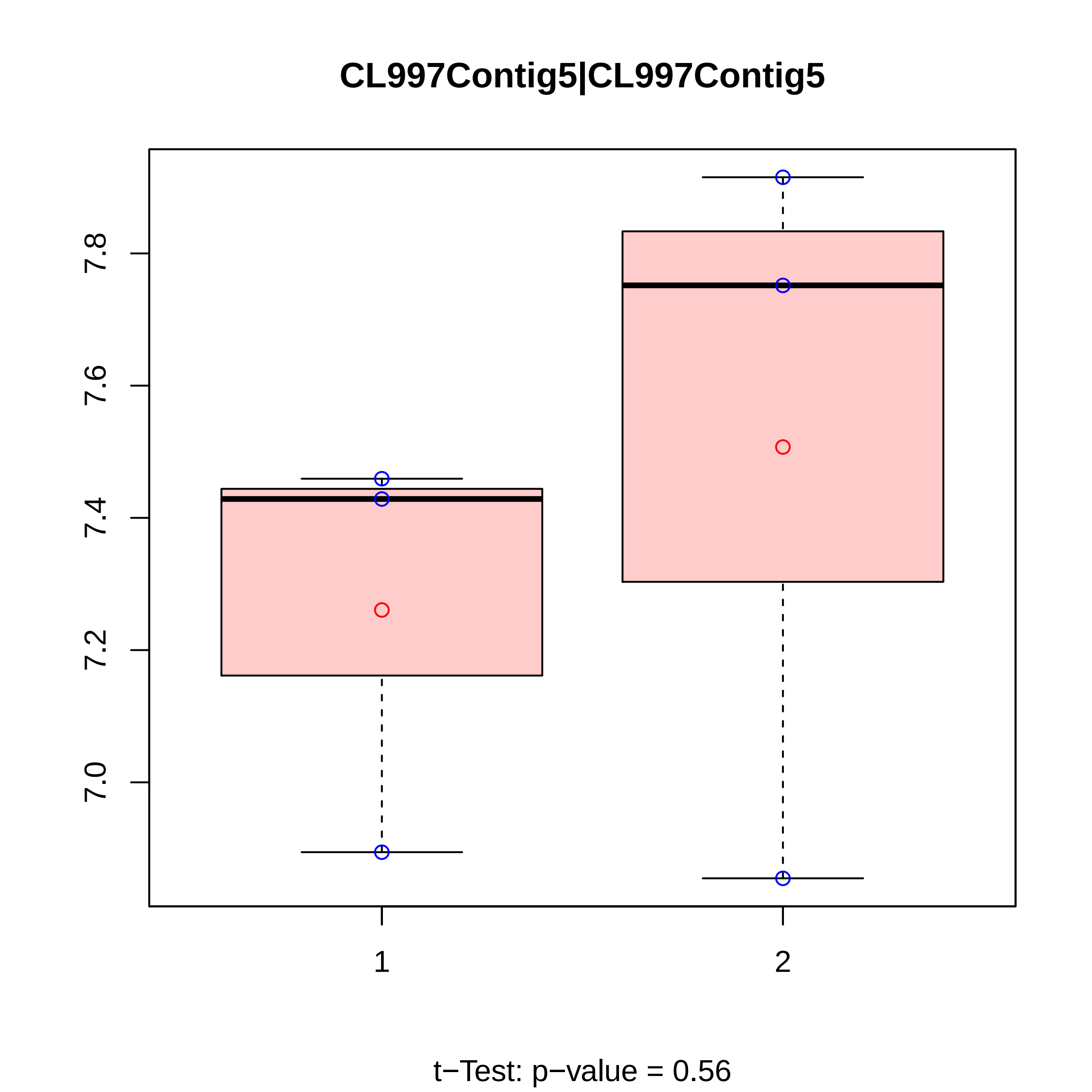

CL997Contig5|CL997Contig5
7.0
7.2
7.4
7.6
7.8
1
2
t−Test: p−value = 0.56

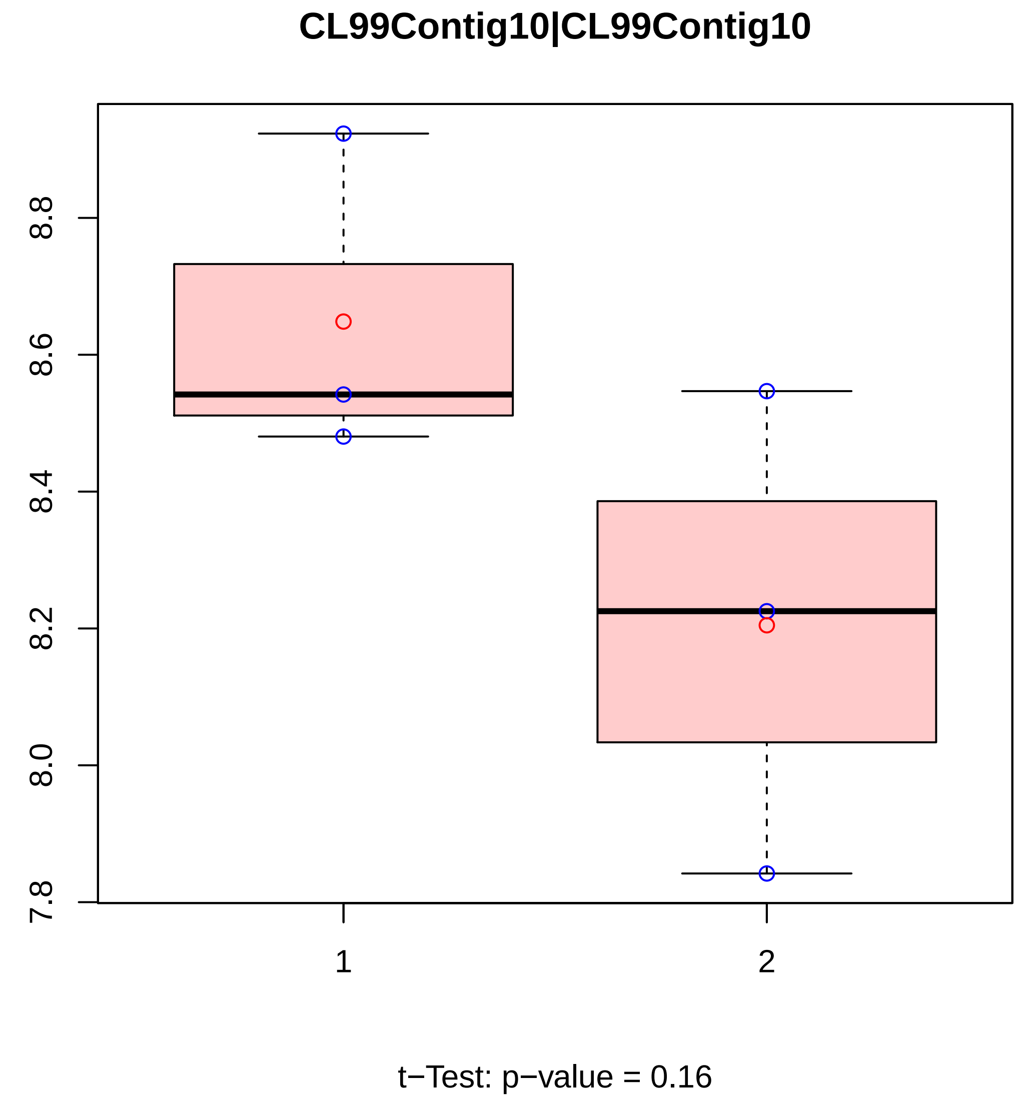
CL99Contig10|CL99Contig10
8.8
8.6
8.4
8.2
8.0
7.8
1
2
t−Test: p−value = 0.16

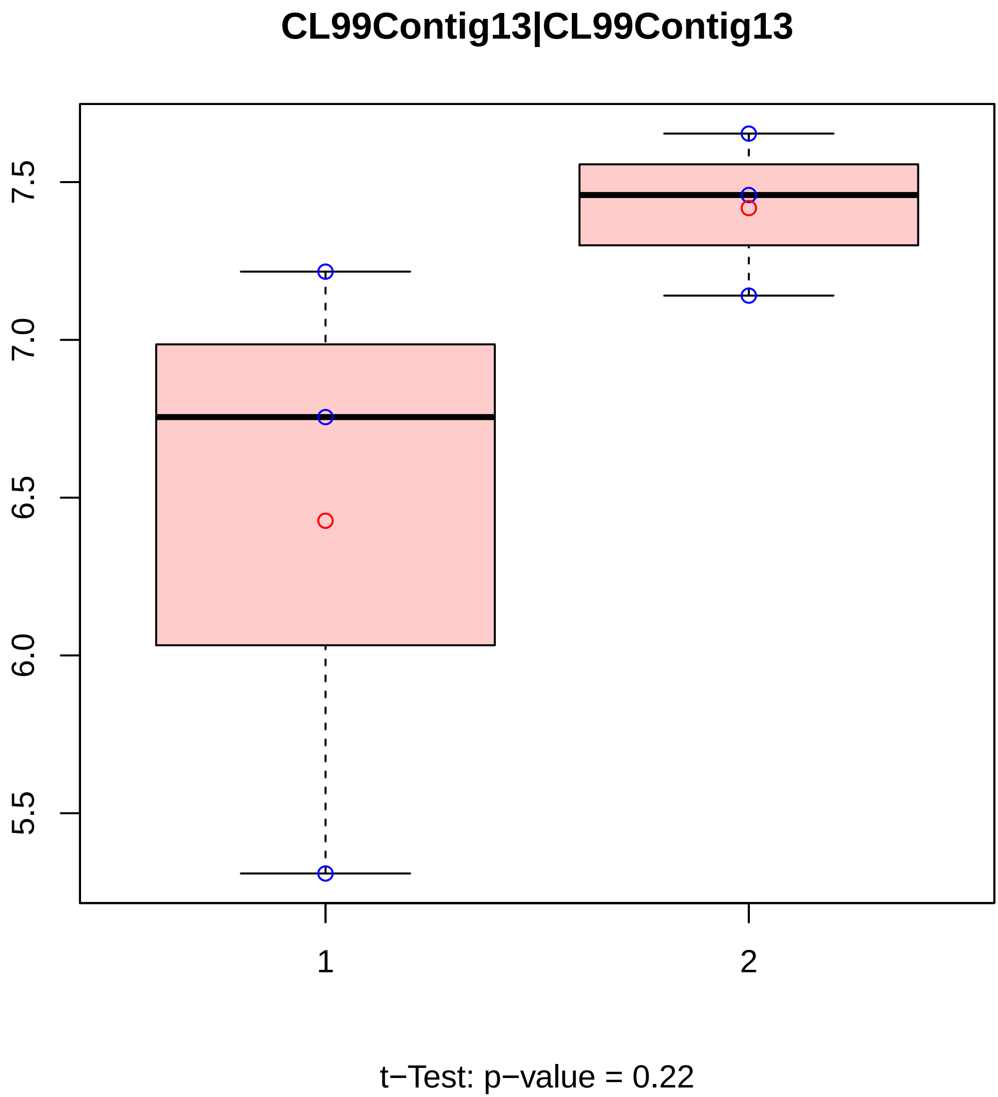

CL99Contig13|CL99Contig13
7.5
7.0
6.5
6.0
5.5
1
2
t−Test: p−value = 0.22

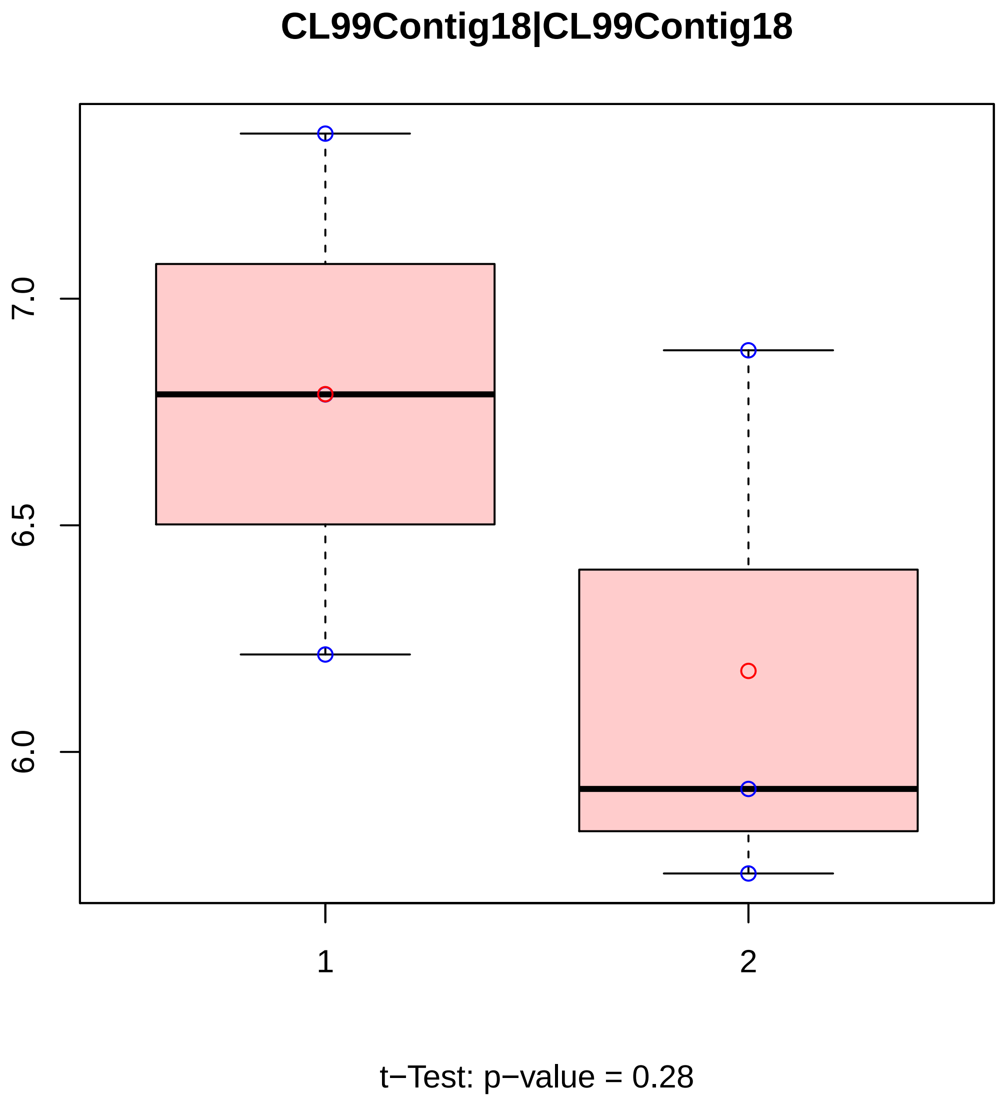
CL99Contig18|CL99Contig18
7.0
6.5
6.0
1
2
t−Test: p−value = 0.28

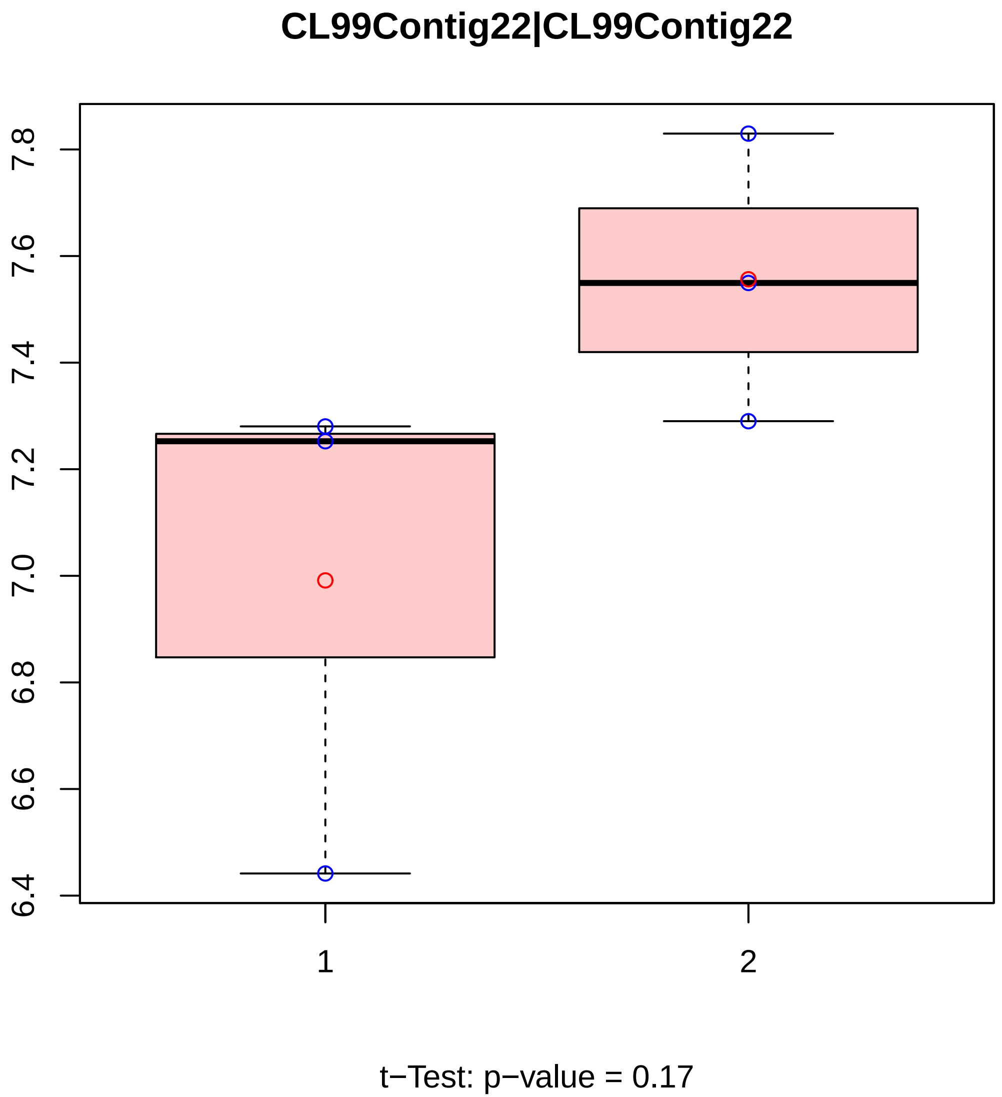
CL99Contig22|CL99Contig22
7.8
7.6
7.4
7.2
7.0
6.8
6.6
6.4
1
2
t−Test: p−value = 0.17

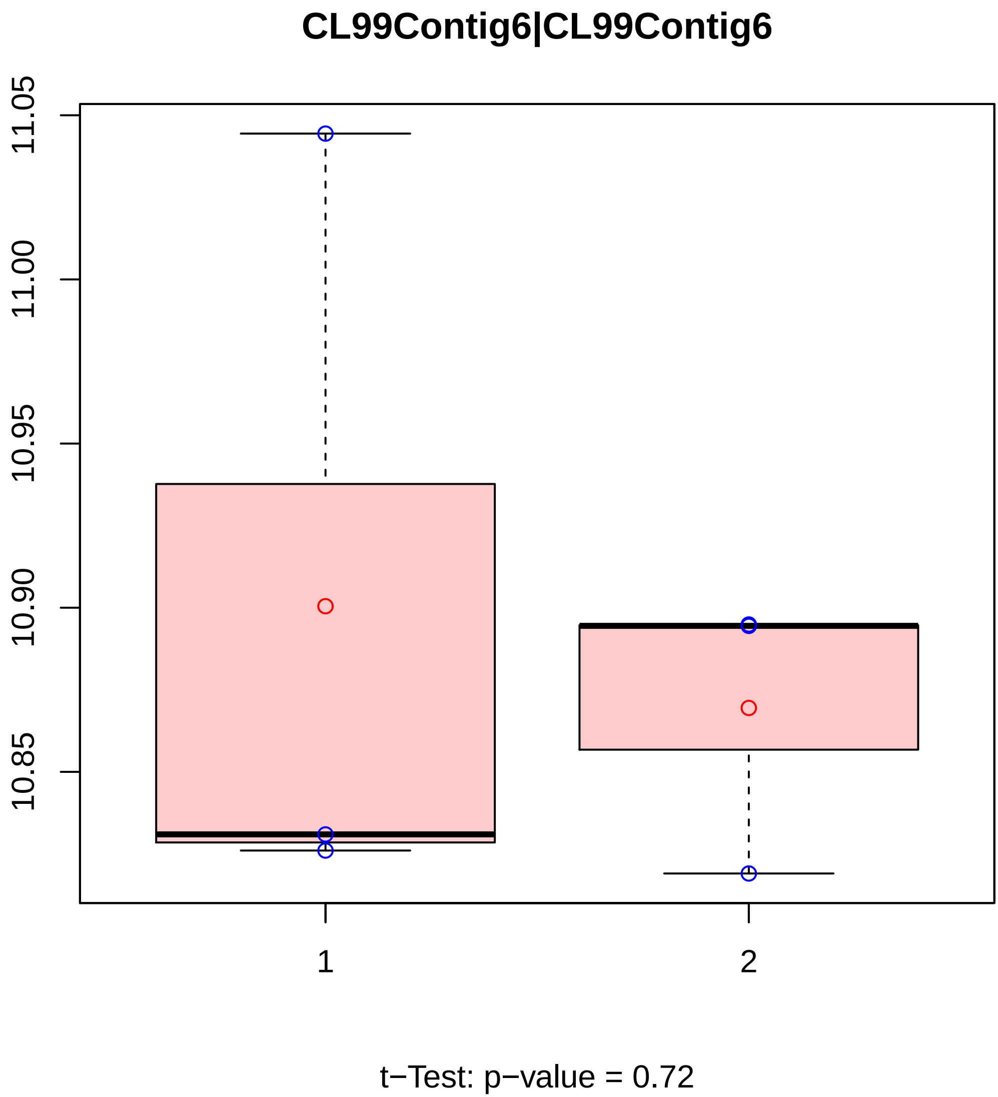
CL99Contig6|CL99Contig6
11.05
11.00
10.95
10.90
10.85
1
2
t−Test: p−value = 0.72

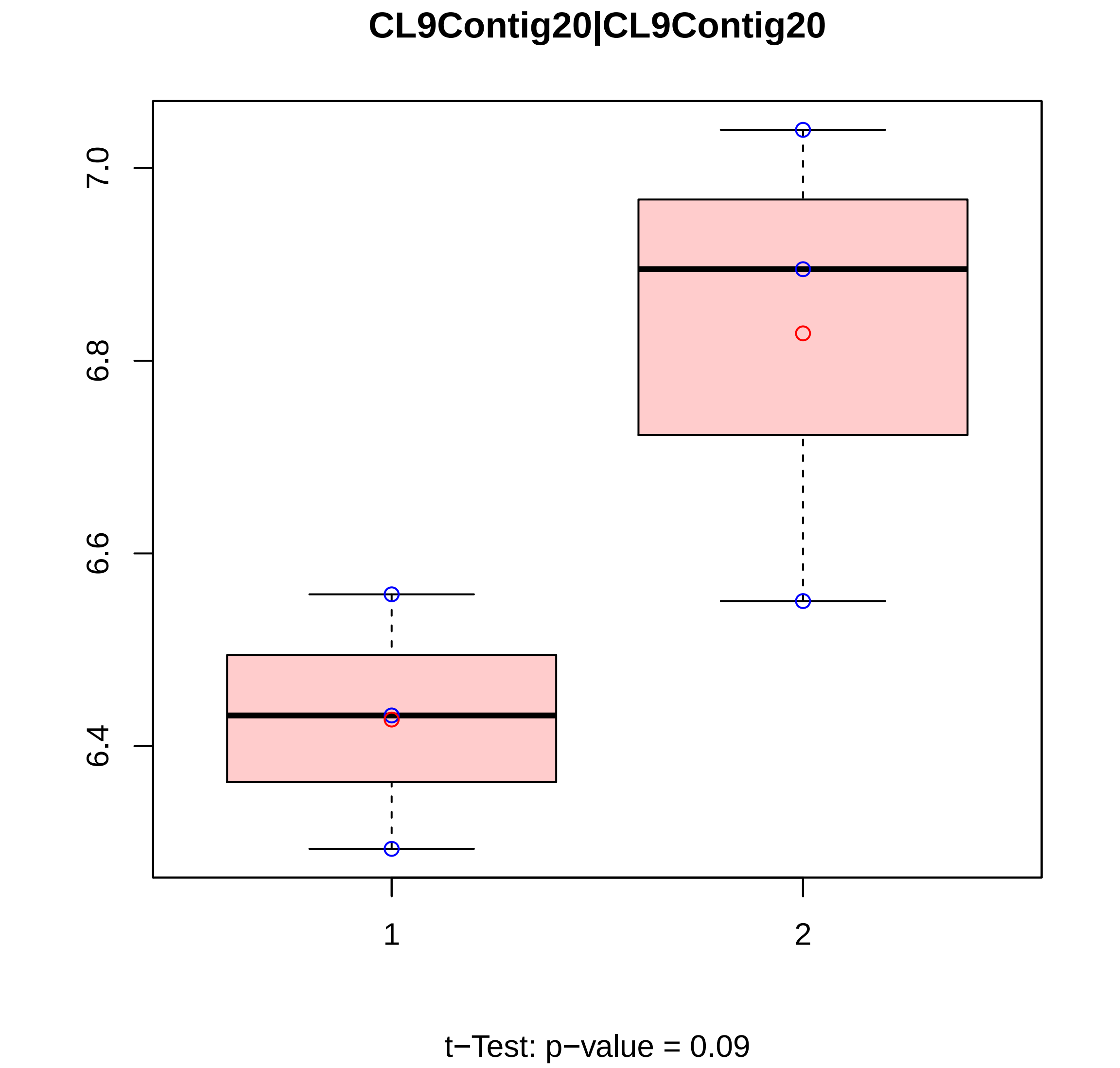
CL9Contig20|CL9Contig20
7.0
6.8
6.6
6.4
1
2
t−Test: p−value = 0.09

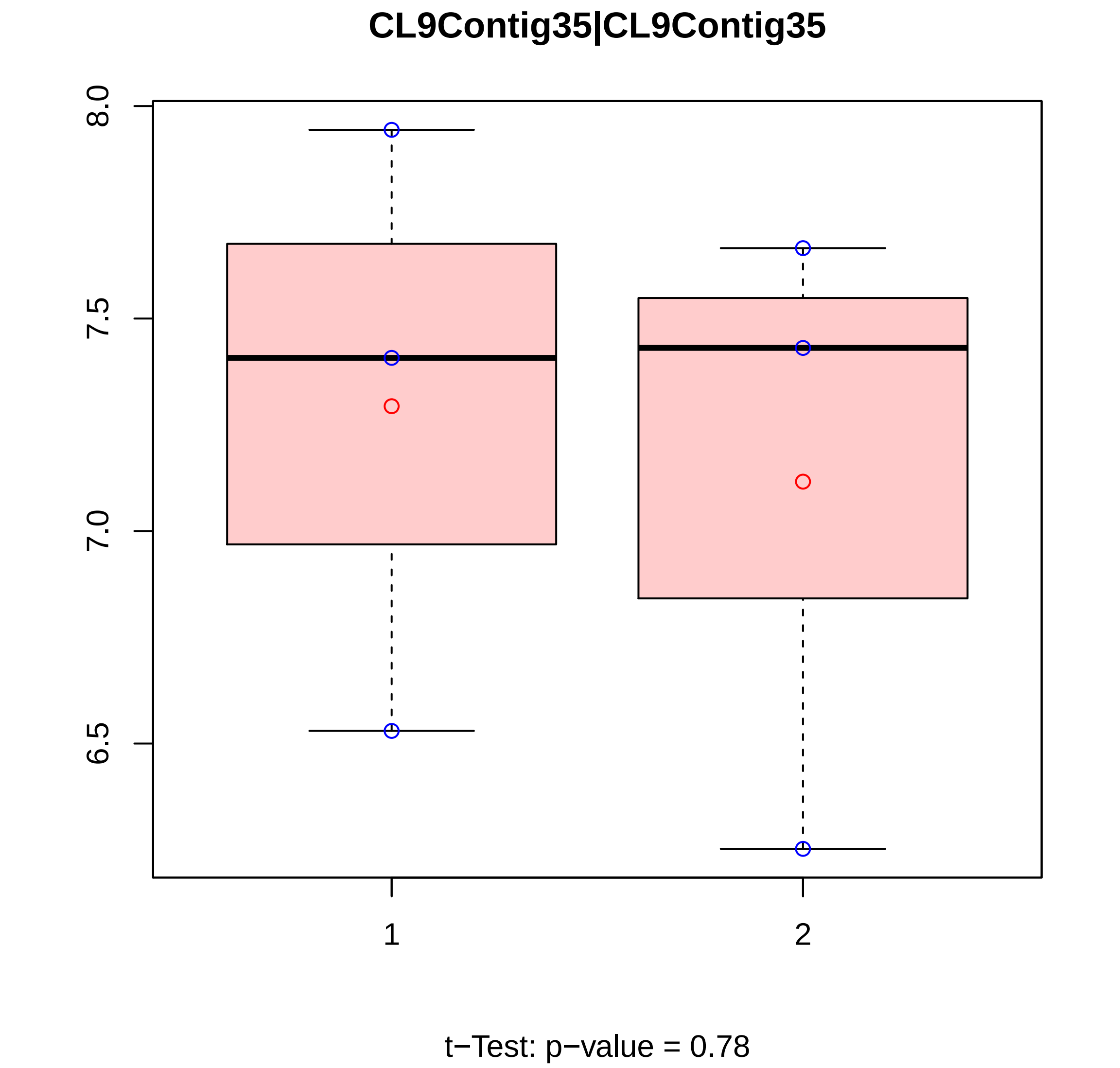
CL9Contig35|CL9Contig35
8.0
7.5
7.0
6.5
1
2
t−Test: p−value = 0.78

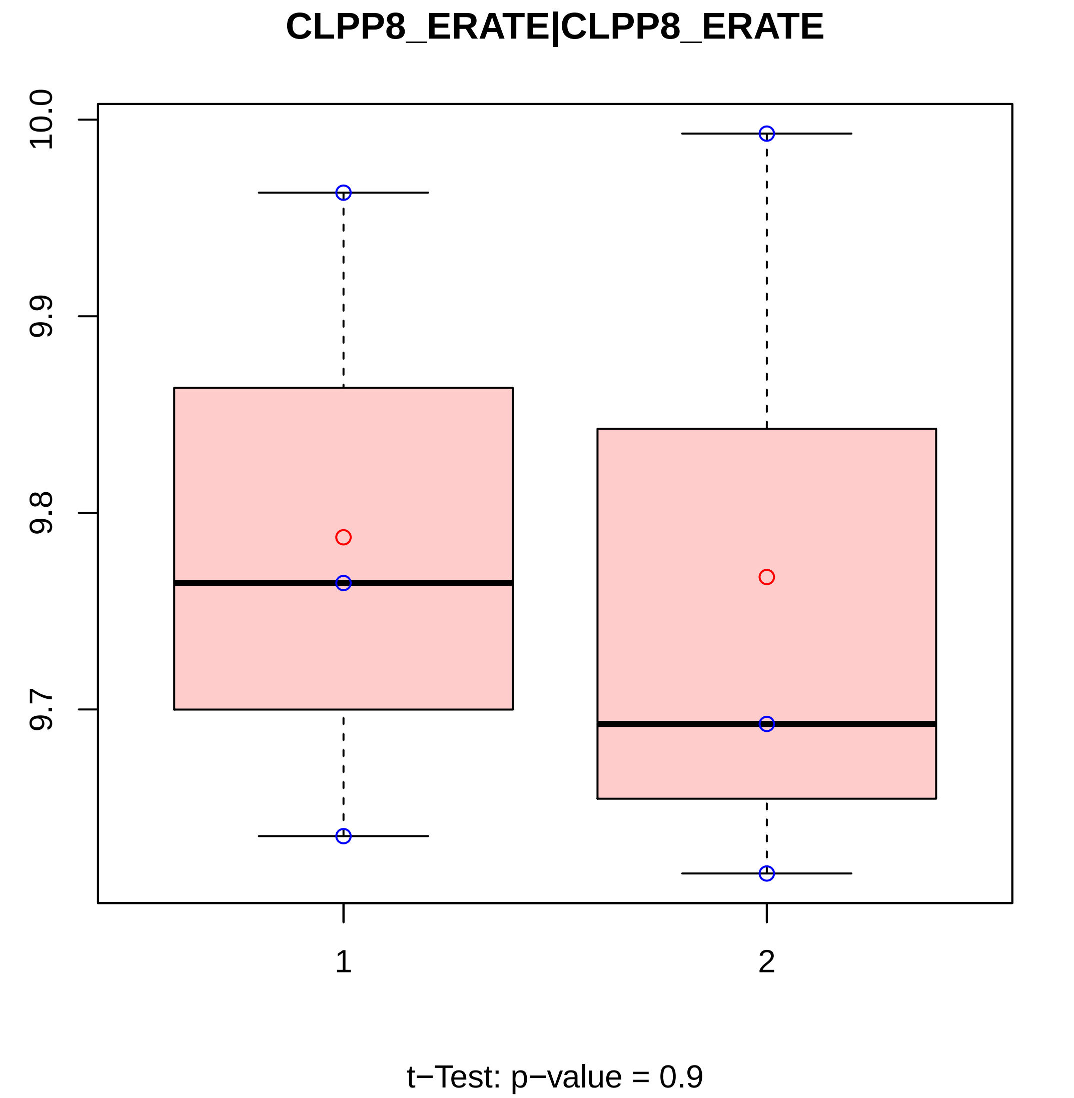
CLPP8_ERATE|CLPP8_ERATE
10.0
9.9
9.8
9.7
1
2
t−Test: p−value = 0.9

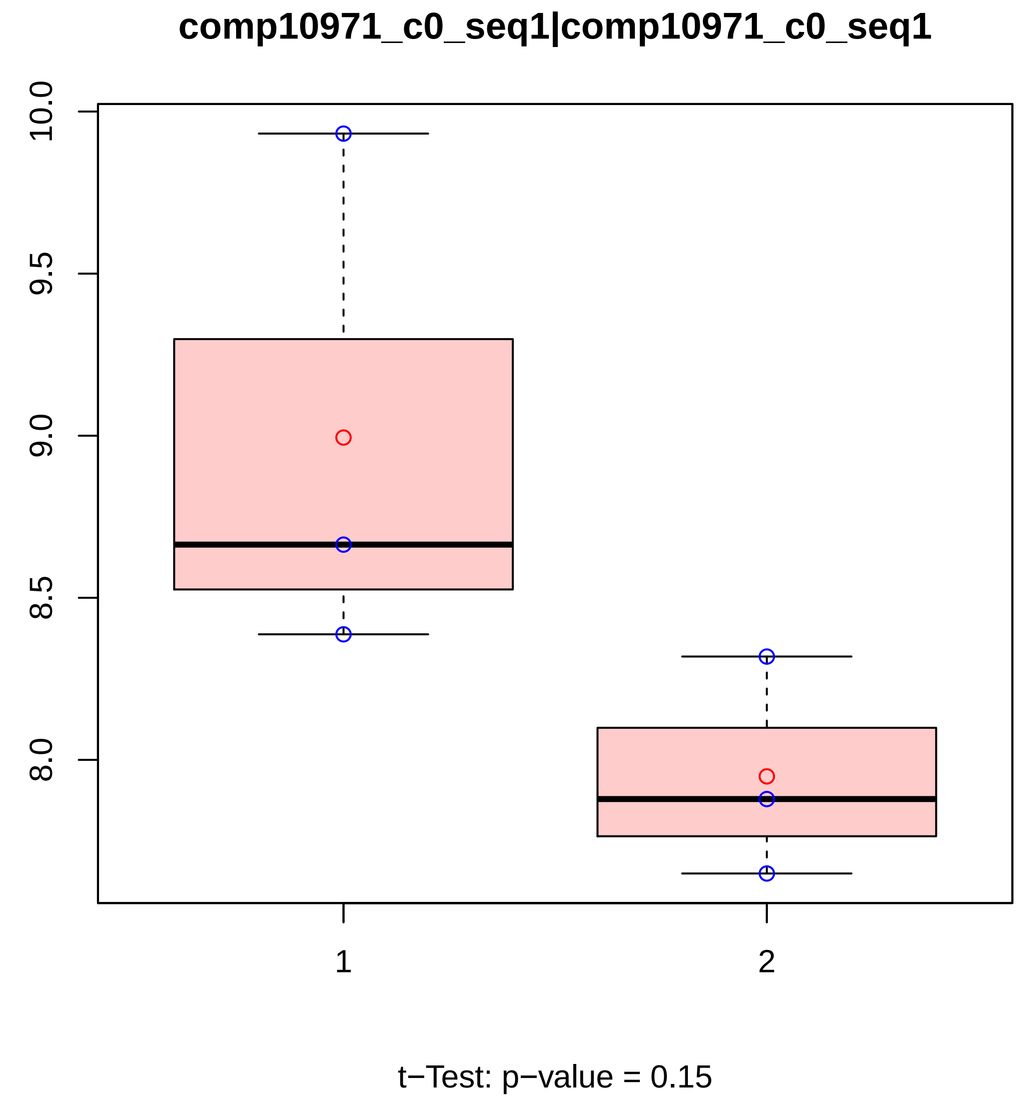
comp10971_c0_seq1|comp10971_c0_seq1
10.0
9.5
9.0
8.5
8.0
1
2
t−Test: p−value = 0.15

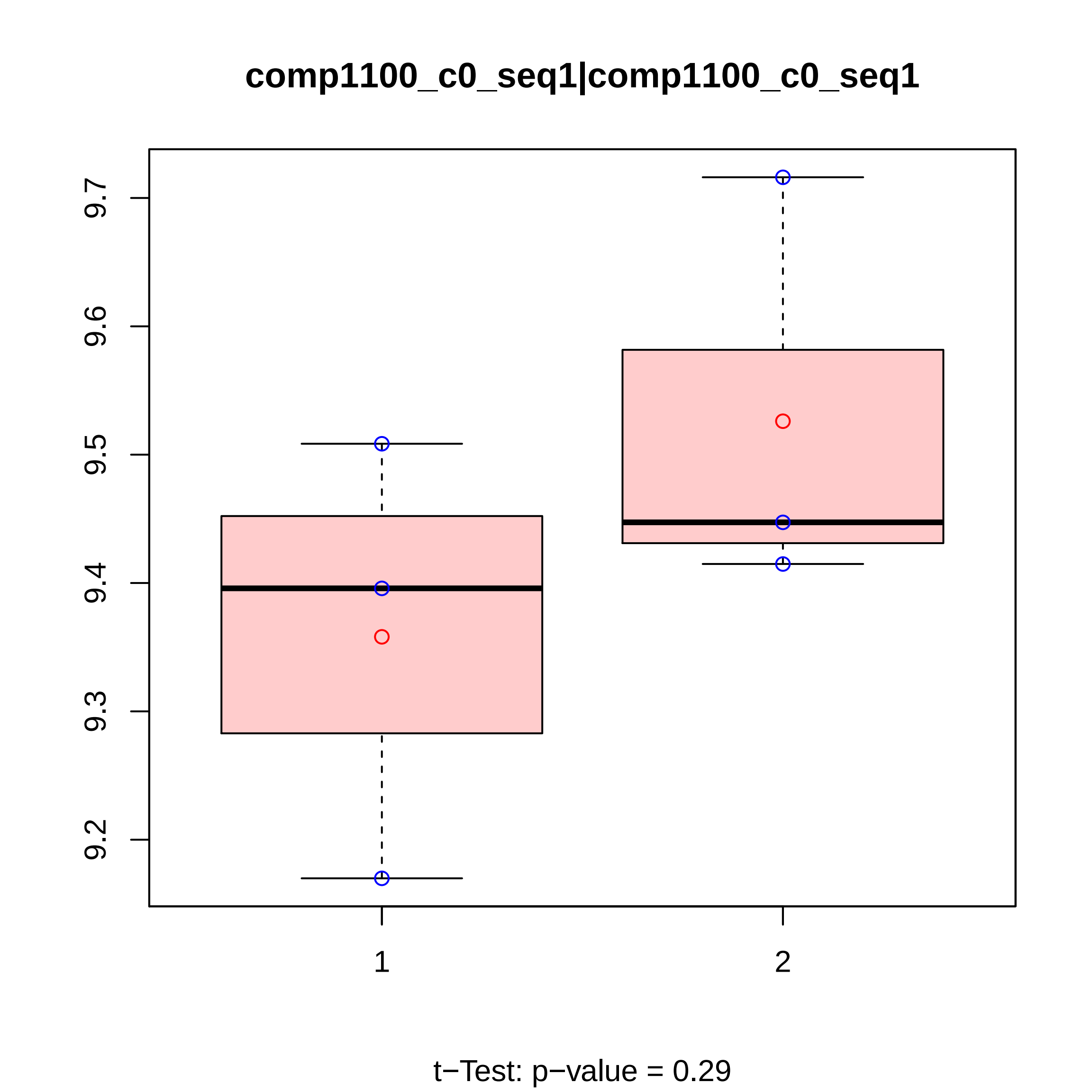

comp1100_c0_seq1|comp1100_c0_seq1
9.2
9.3
9.4
9.5
9.6
9.7
1
2
t−Test: p−value = 0.29

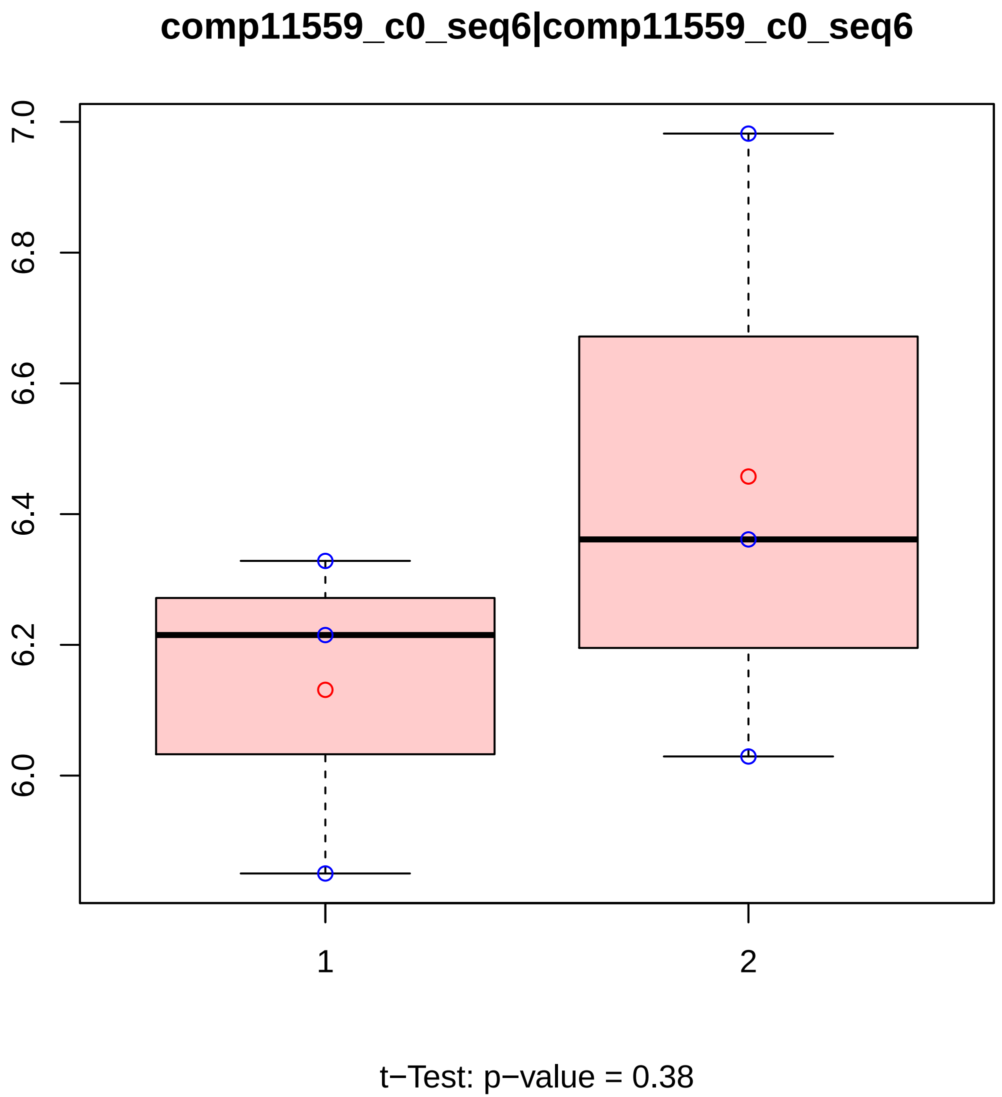
comp11559_c0_seq6|comp11559_c0_seq6
7.0
6.8
6.6
6.4
6.2
6.0
1
2
t−Test: p−value = 0.38

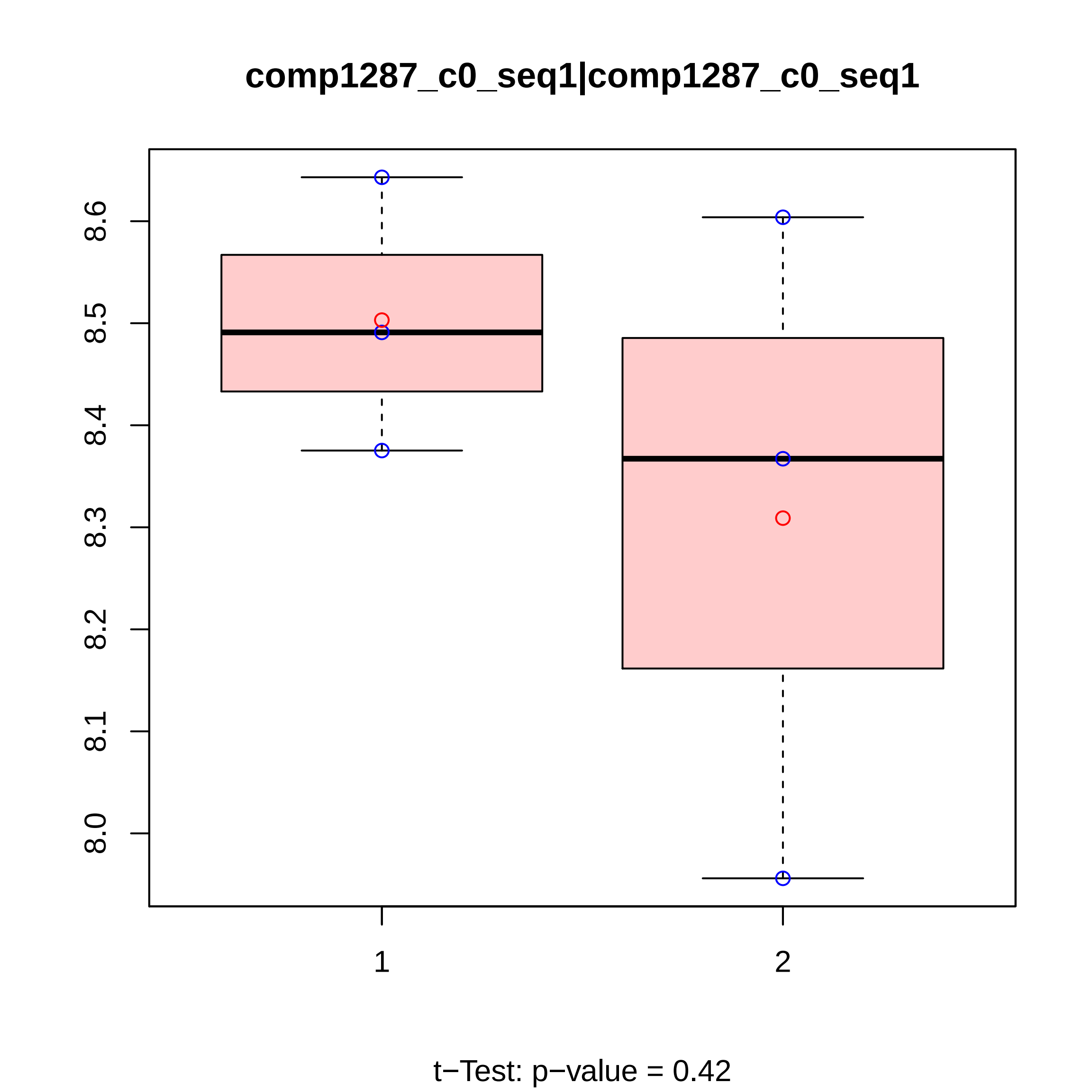
comp1287_c0_seq1|comp1287_c0_seq1
8.0
8.1
8.2
8.3
8.4
8.5
8.6
1
2
t−Test: p−value = 0.42

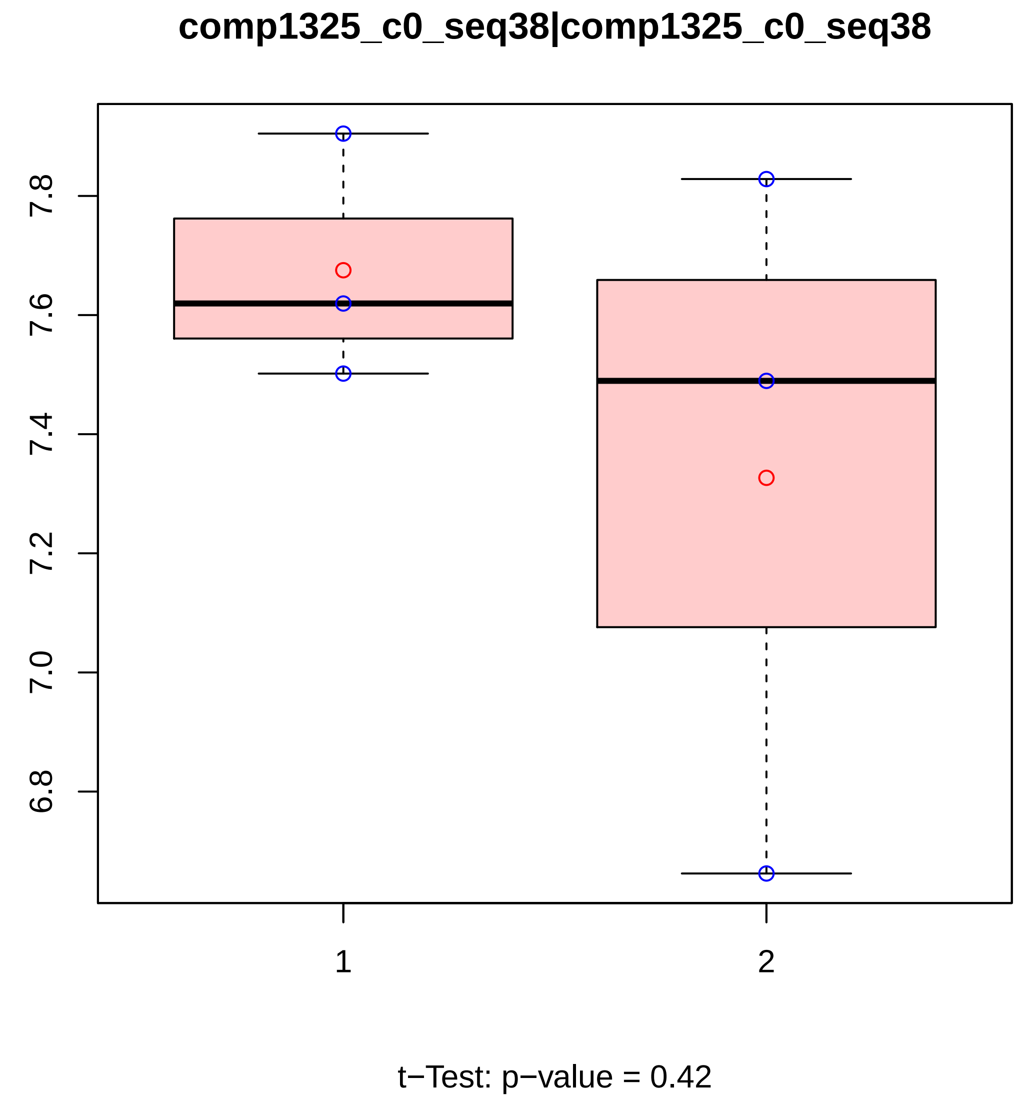
comp1325_c0_seq38|comp1325_c0_seq38
7.8
7.6
7.4
7.2
7.0
6.8
1
2
t−Test: p−value = 0.42

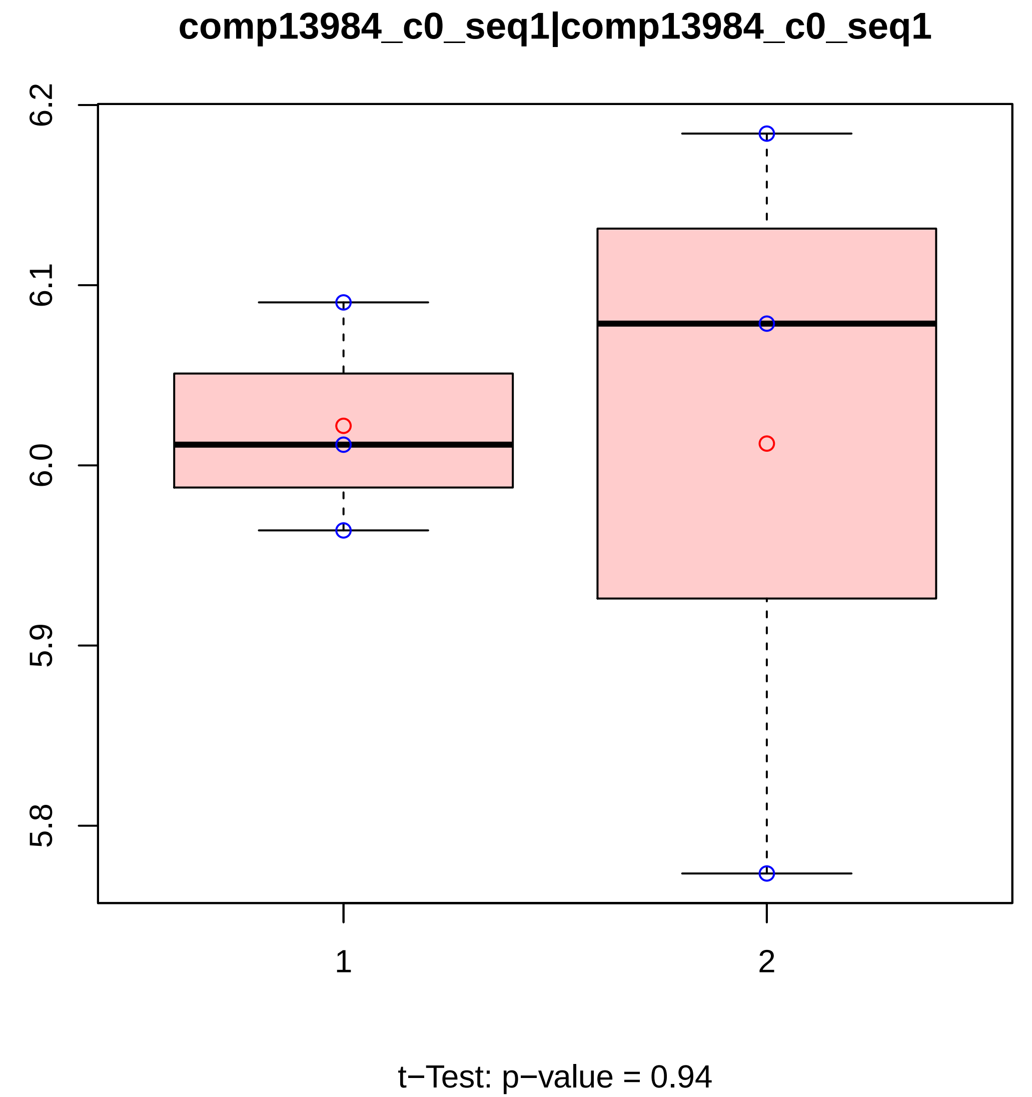
comp13984_c0_seq1|comp13984_c0_seq1
6.2
6.1
6.0
5.9
5.8
1
2
t−Test: p−value = 0.94

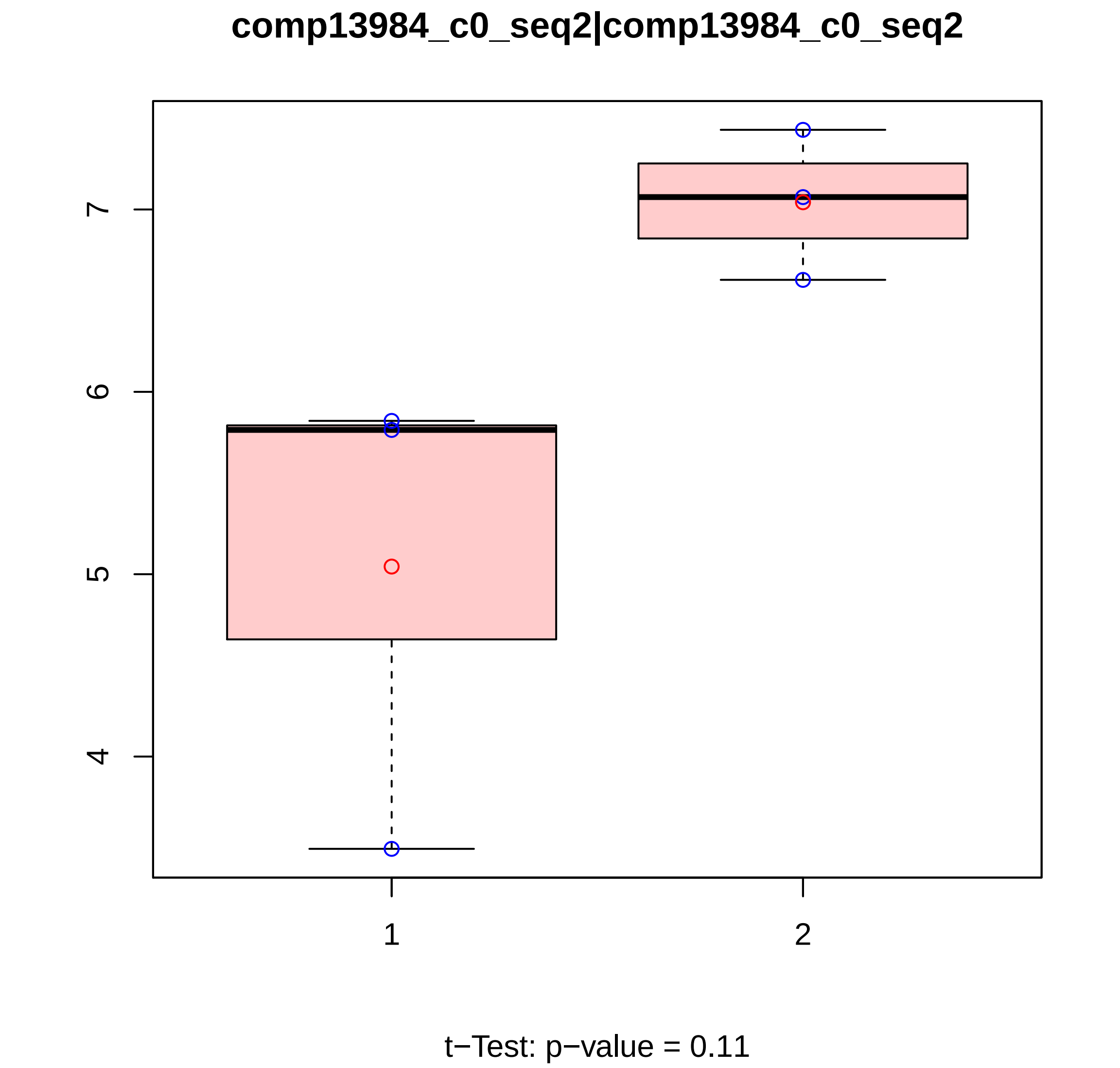
comp13984_c0_seq2|comp13984_c0_seq2
4
5
6
7
1
2
t−Test: p−value = 0.11

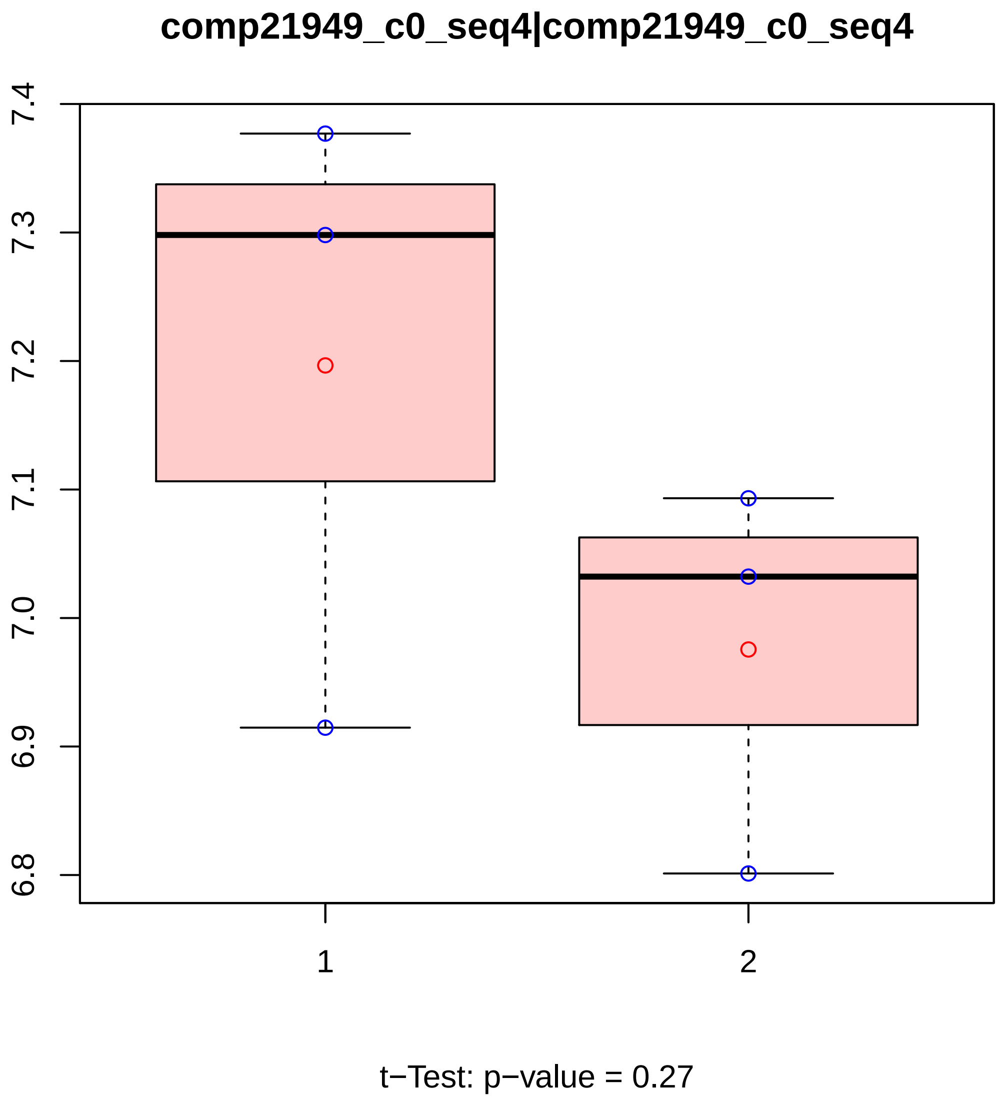
comp21949_c0_seq4|comp21949_c0_seq4
7.4
7.3
7.2
7.1
7.0
6.9
6.8
1
2
t−Test: p−value = 0.27

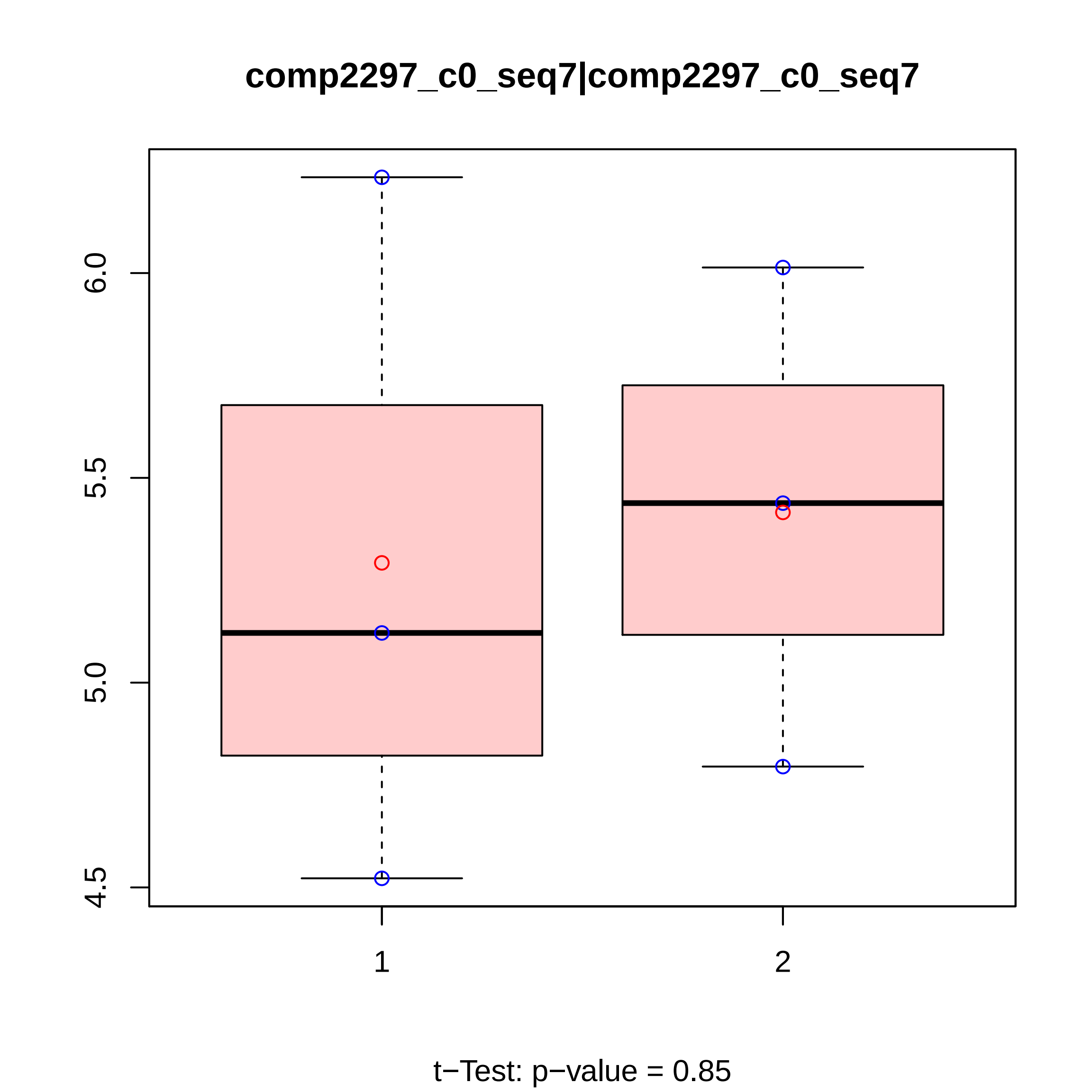
comp2297_c0_seq7|comp2297_c0_seq7
6.0
5.5
5.0
4.5
1
2
t−Test: p−value = 0.85

## comp2740_c0_seq1|comp2740_c0_seq1

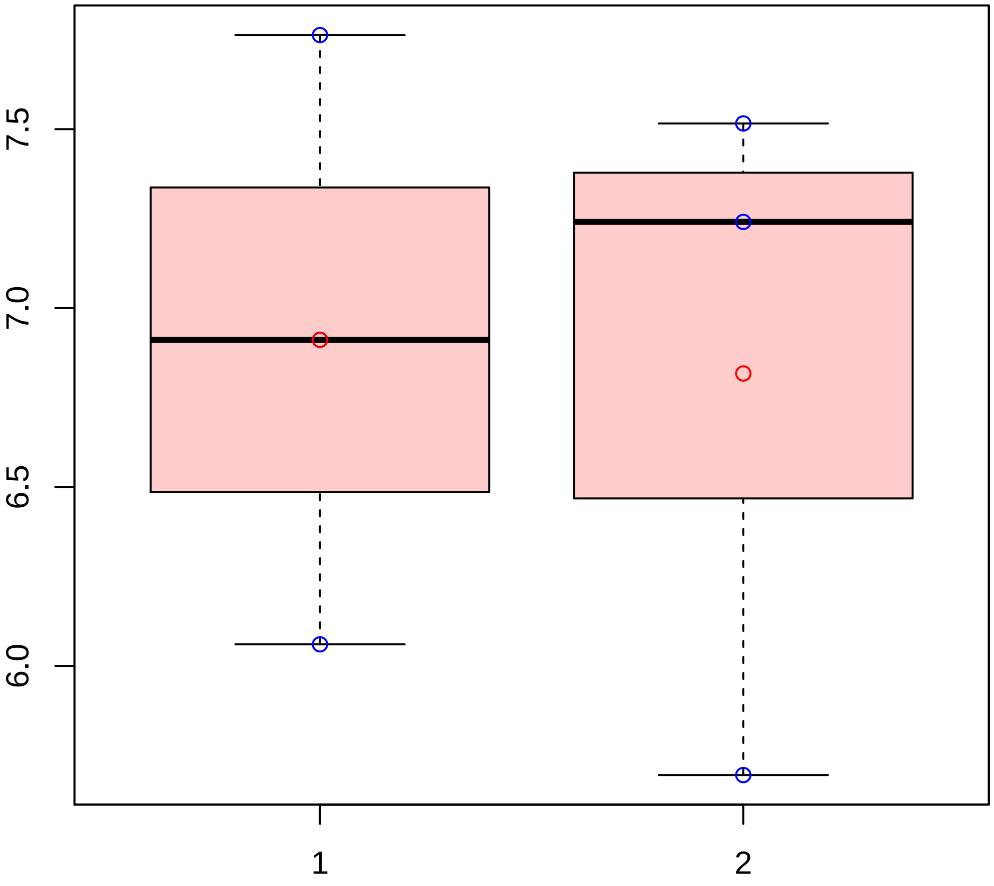


t−Test: p−value = 0.91

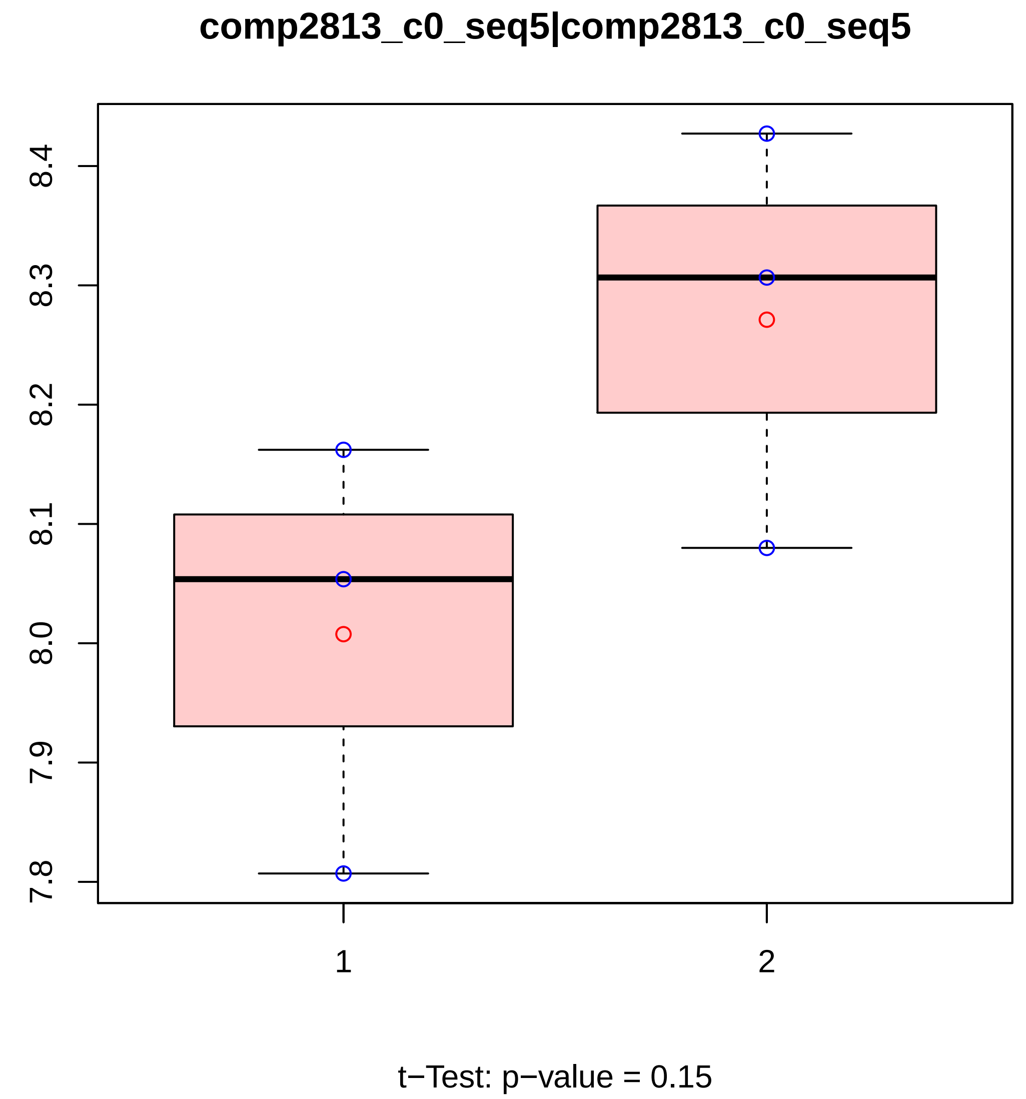
comp2813_c0_seq5|comp2813_c0_seq5
8.4
8.3
8.2
8.1
8.0
7.9
7.8
1
2
t−Test: p−value = 0.15

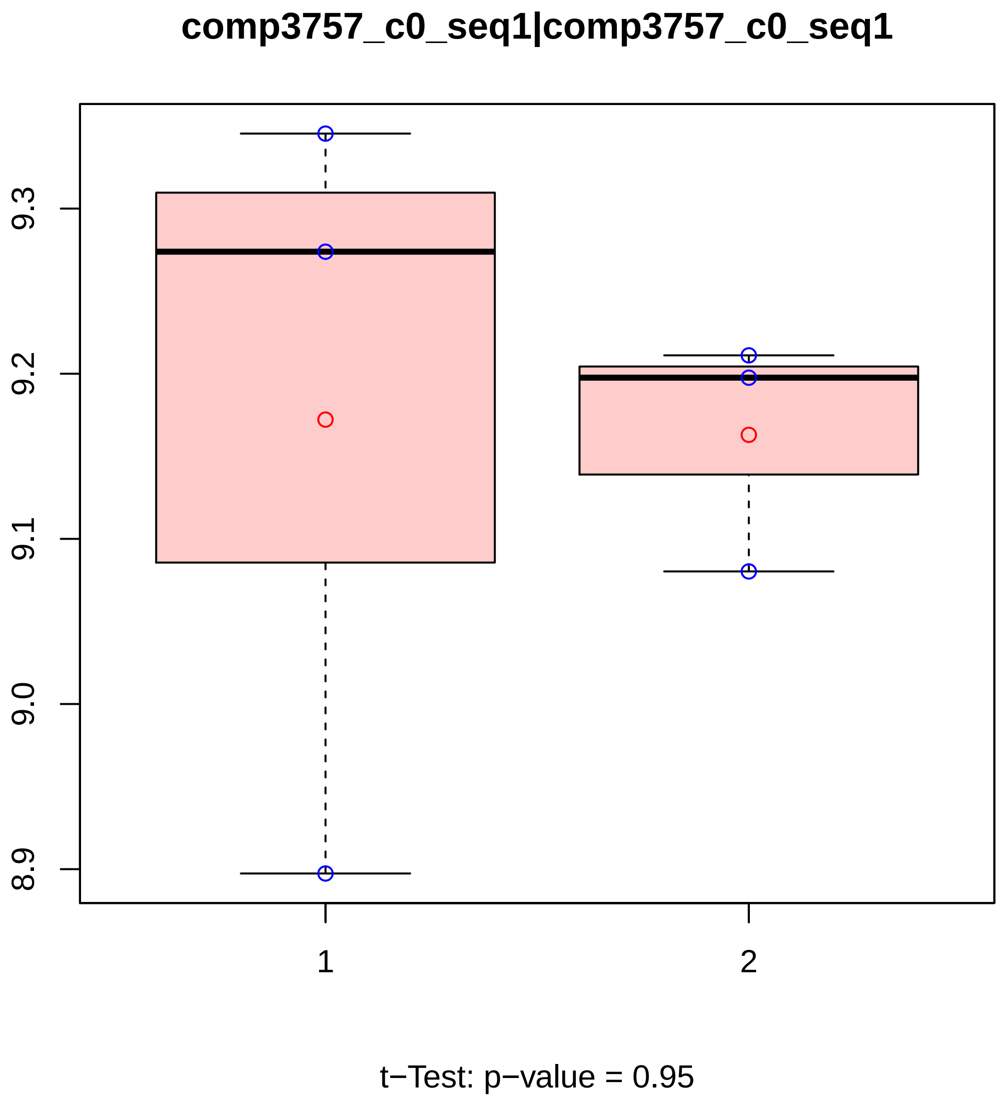
comp3757_c0_seq1|comp3757_c0_seq1
9.3
9.2
9.1
9.0
8.9
1
2
t−Test: p−value = 0.95

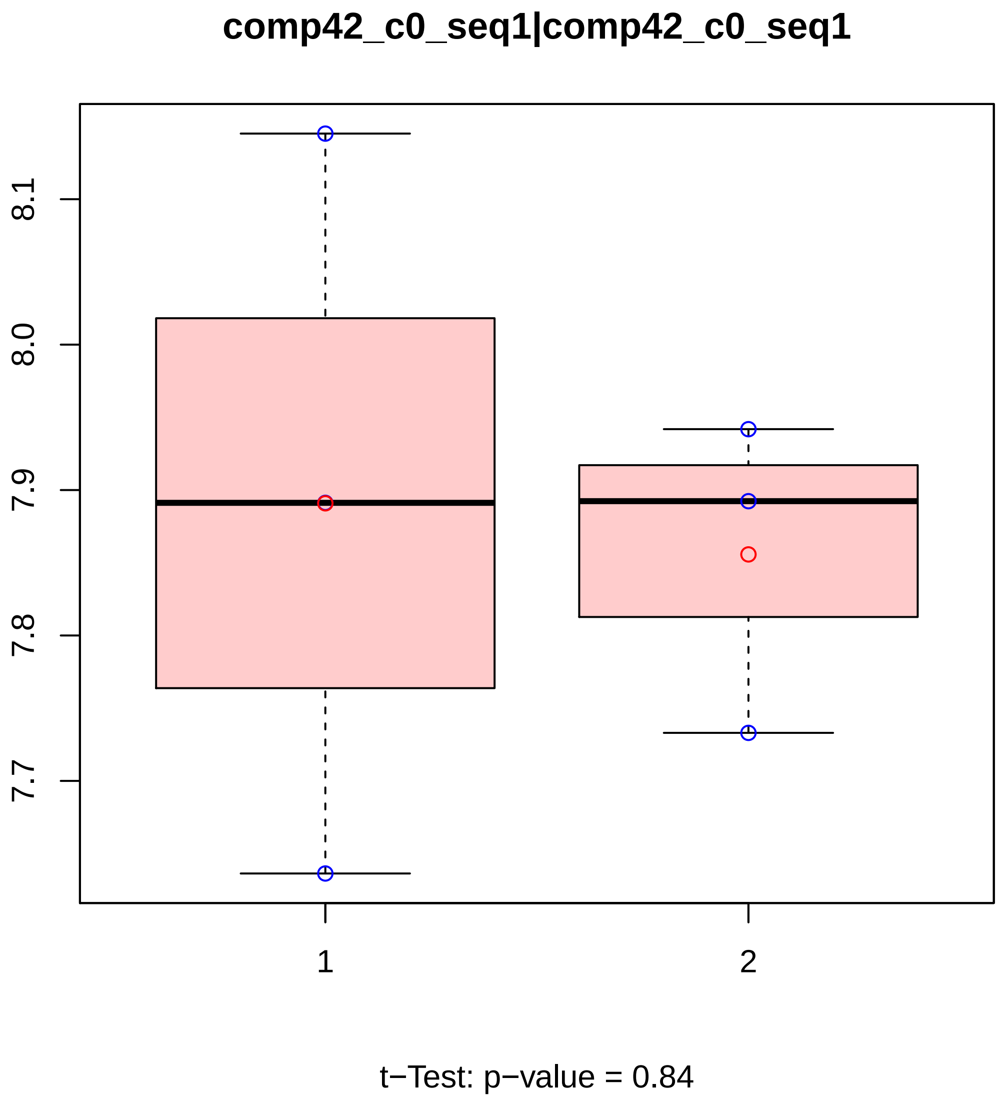
comp42_c0_seq1|comp42_c0_seq1
8.1
8.0
7.9
7.8
7.7
1
2
t−Test: p−value = 0.84

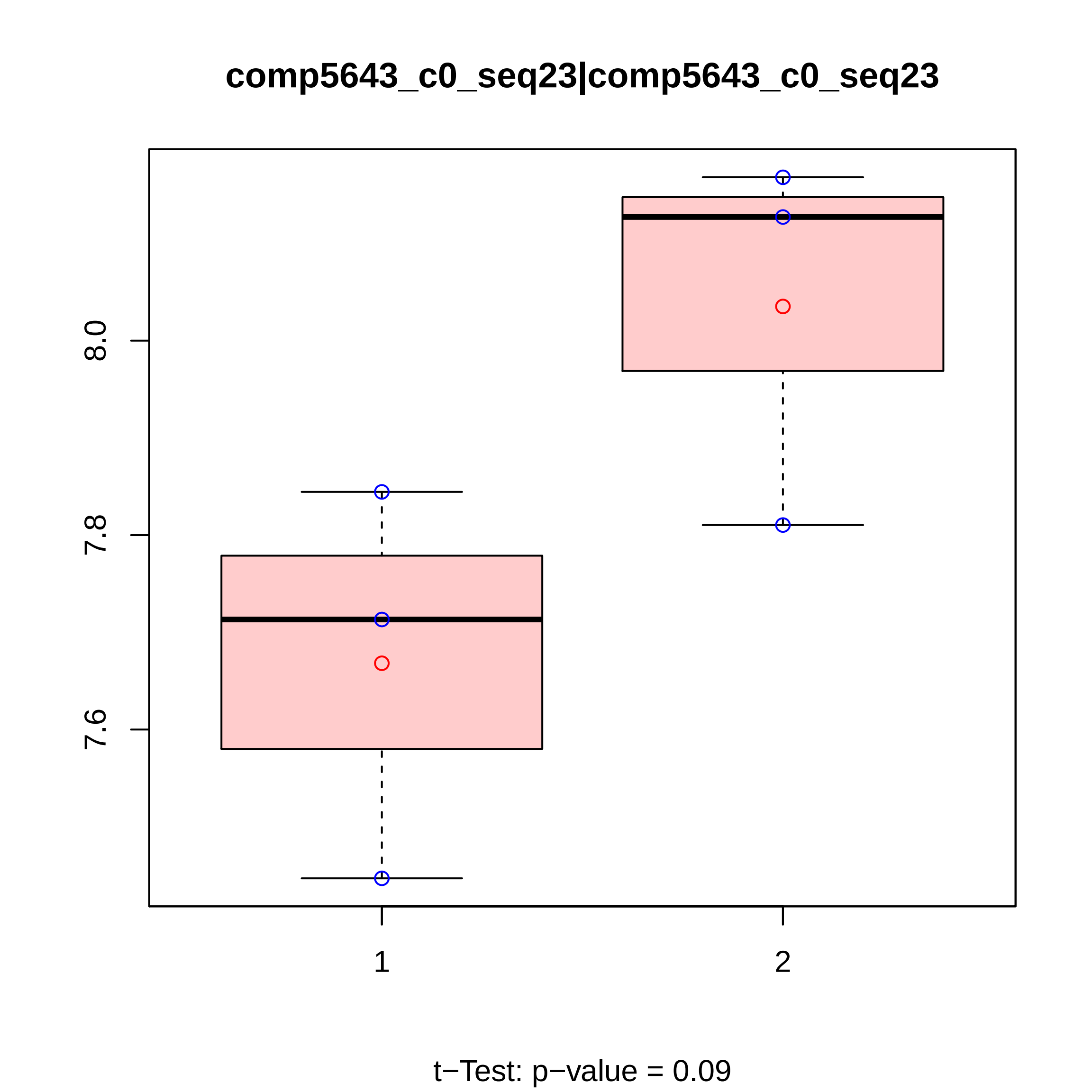
comp5643_c0_seq23|comp5643_c0_seq23
8.0
7.8
7.6
1
2
t−Test: p−value = 0.09

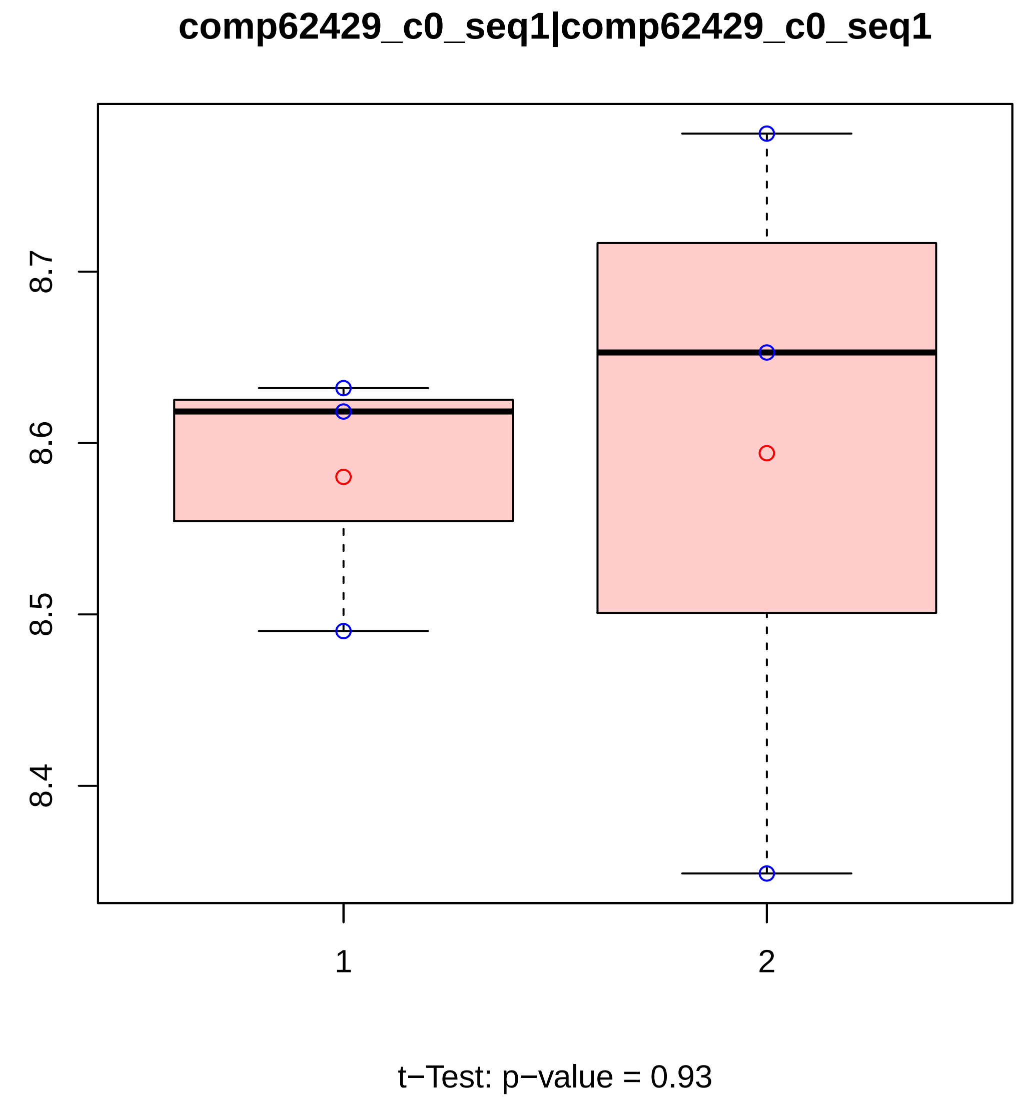
comp62429_c0_seq1|comp62429_c0_seq1
8.4
8.5
8.6
8.7
1
2
t−Test: p−value = 0.93

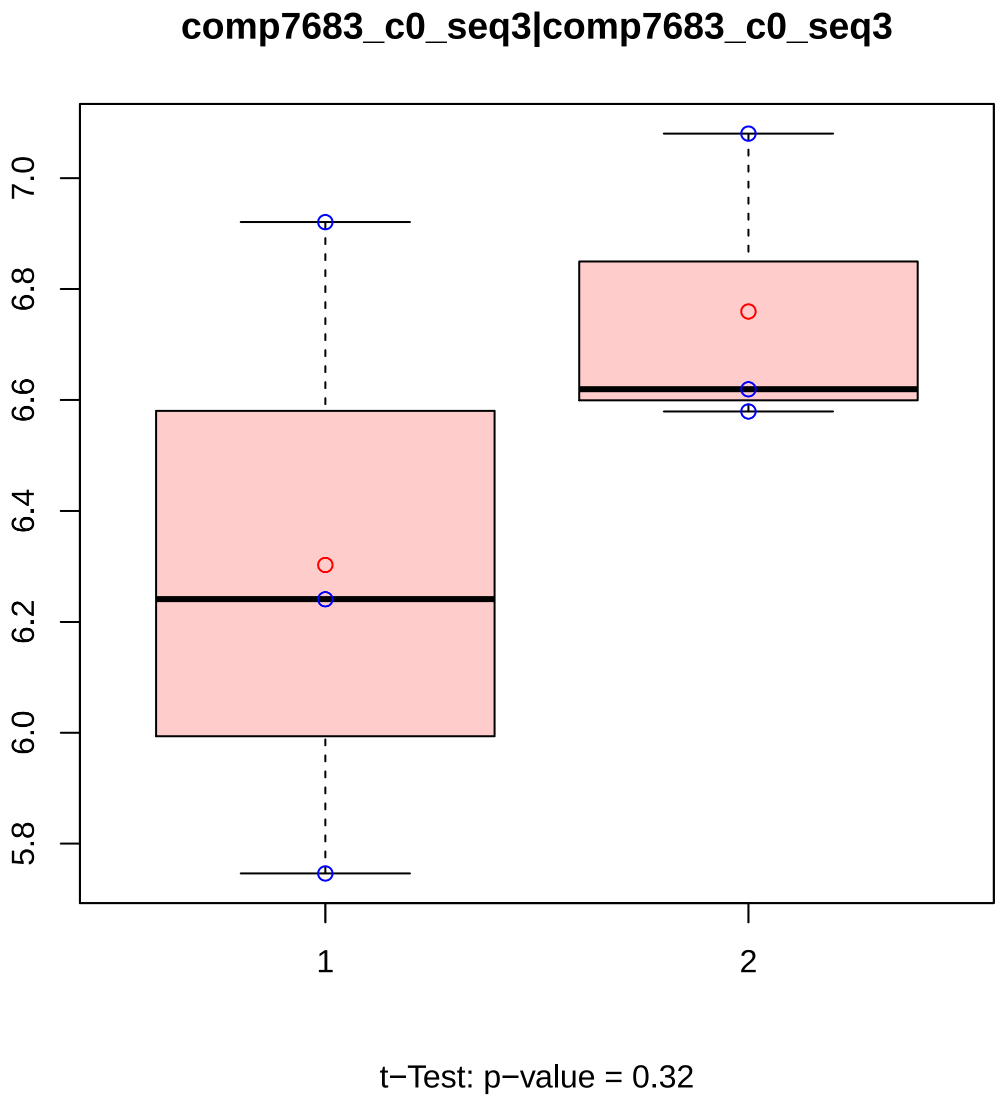

comp7683_c0_seq3|comp7683_c0_seq3
7.0
6.8
6.6
6.4
6.2
6.0
5.8
1
2
t−Test: p−value = 0.32

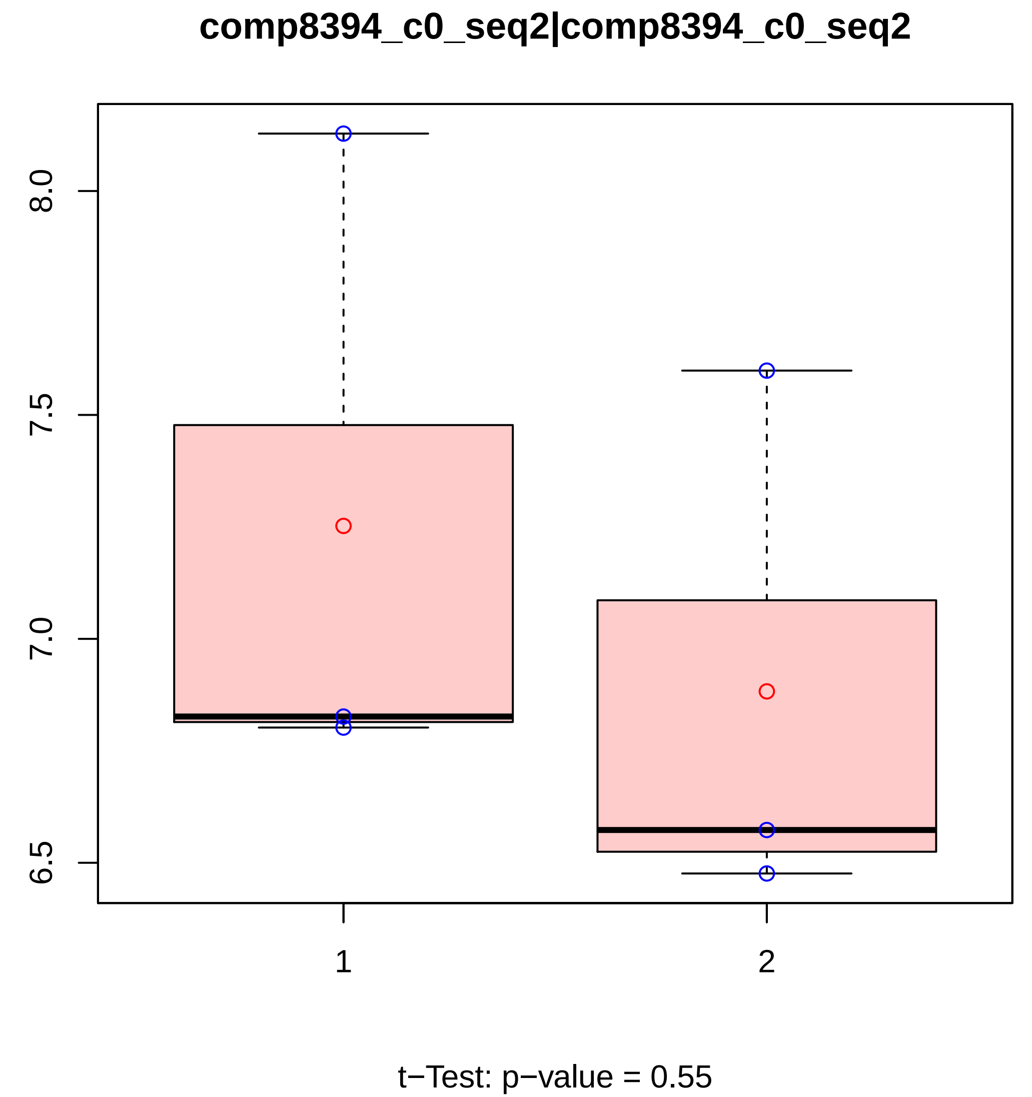
comp8394_c0_seq2|comp8394_c0_seq2
8.0
7.5
7.0
6.5
1
2
t−Test: p−value = 0.55

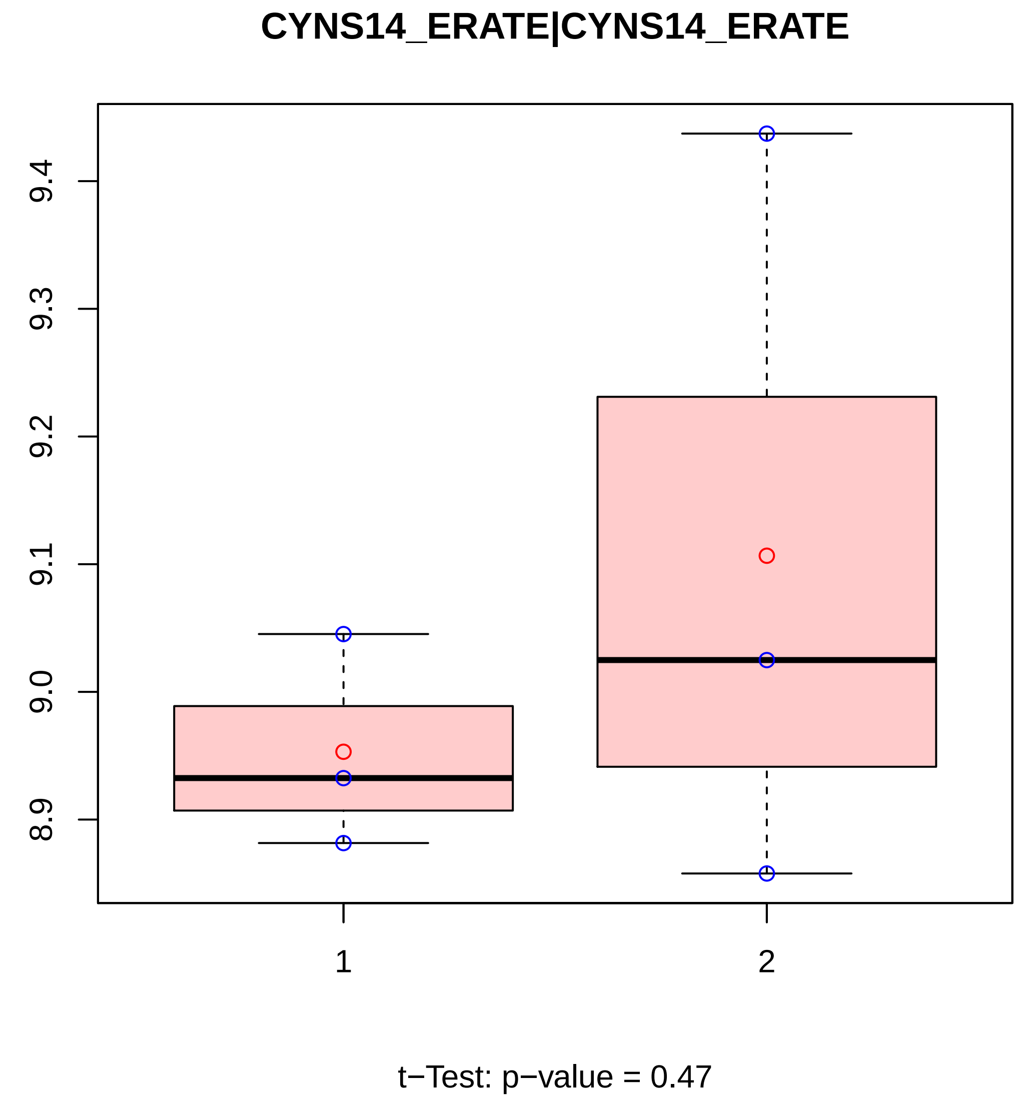
CYNS14_ERATE|CYNS14_ERATE
9.4
9.3
9.2
9.1
9.0
8.9
1
2
t−Test: p−value = 0.47

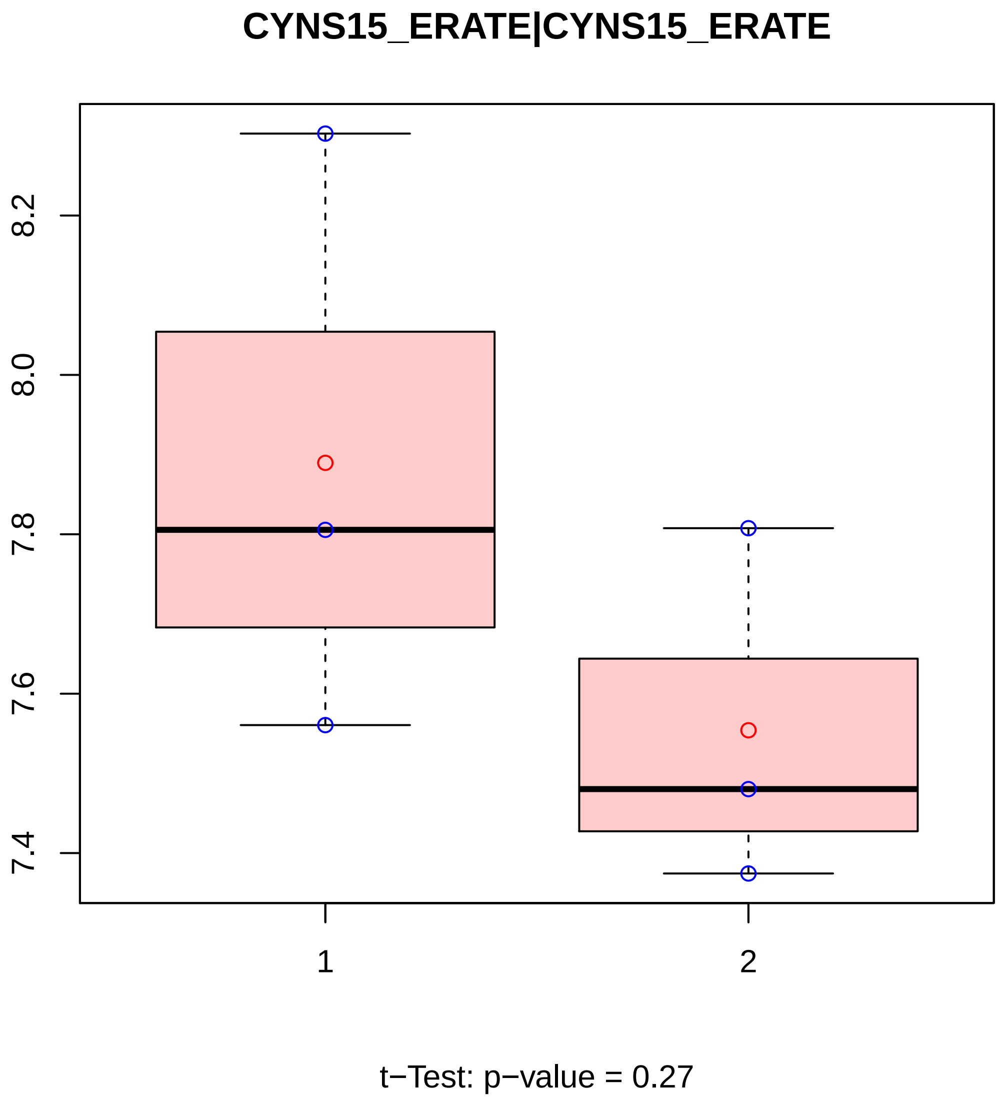
CYNS15_ERATE|CYNS15_ERATE
8.2
8.0
7.8
7.6
7.4
1
2
t−Test: p−value = 0.27

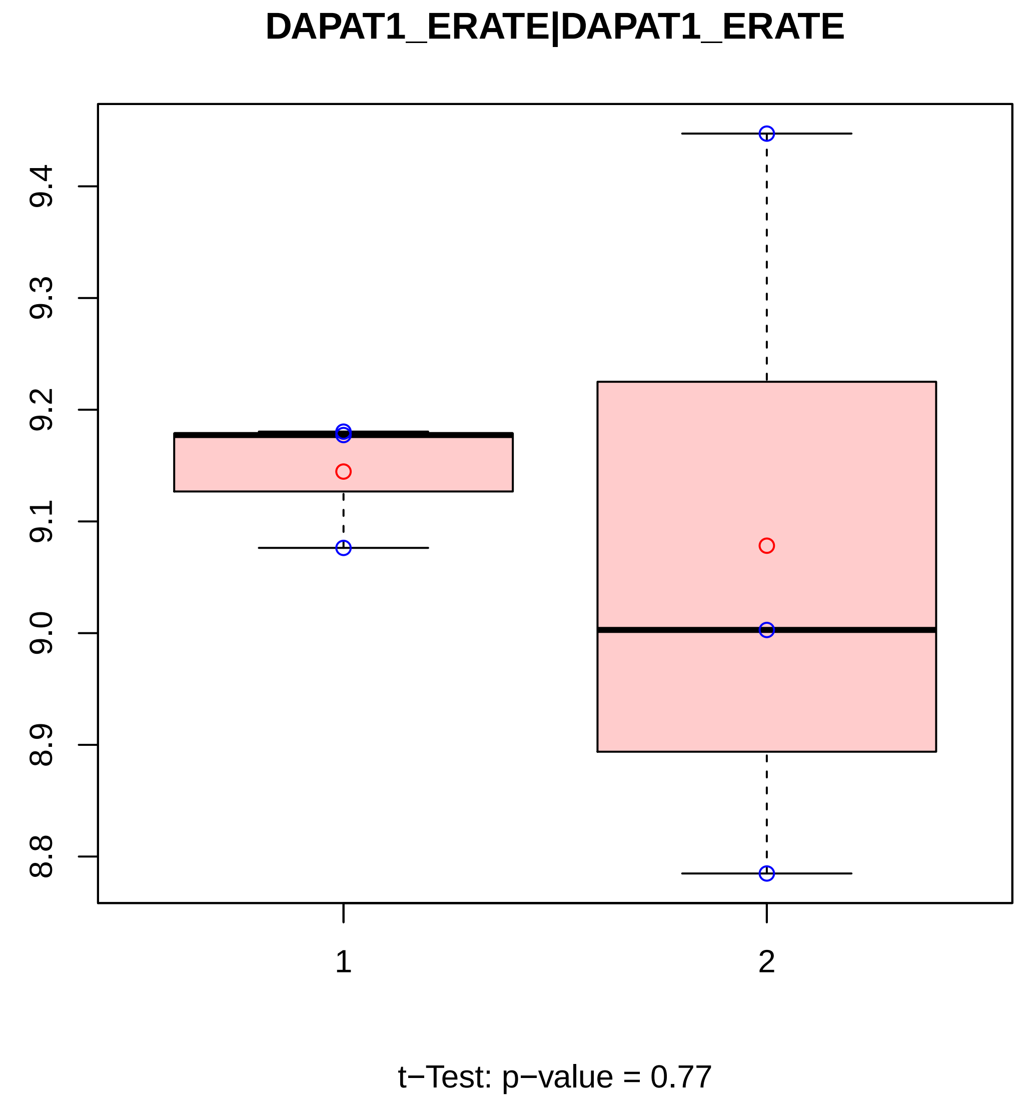
DAPAT1_ERATE|DAPAT1_ERATE
9.4
9.3
9.2
9.1
9.0
8.9
8.8
1
2
t−Test: p−value = 0.77

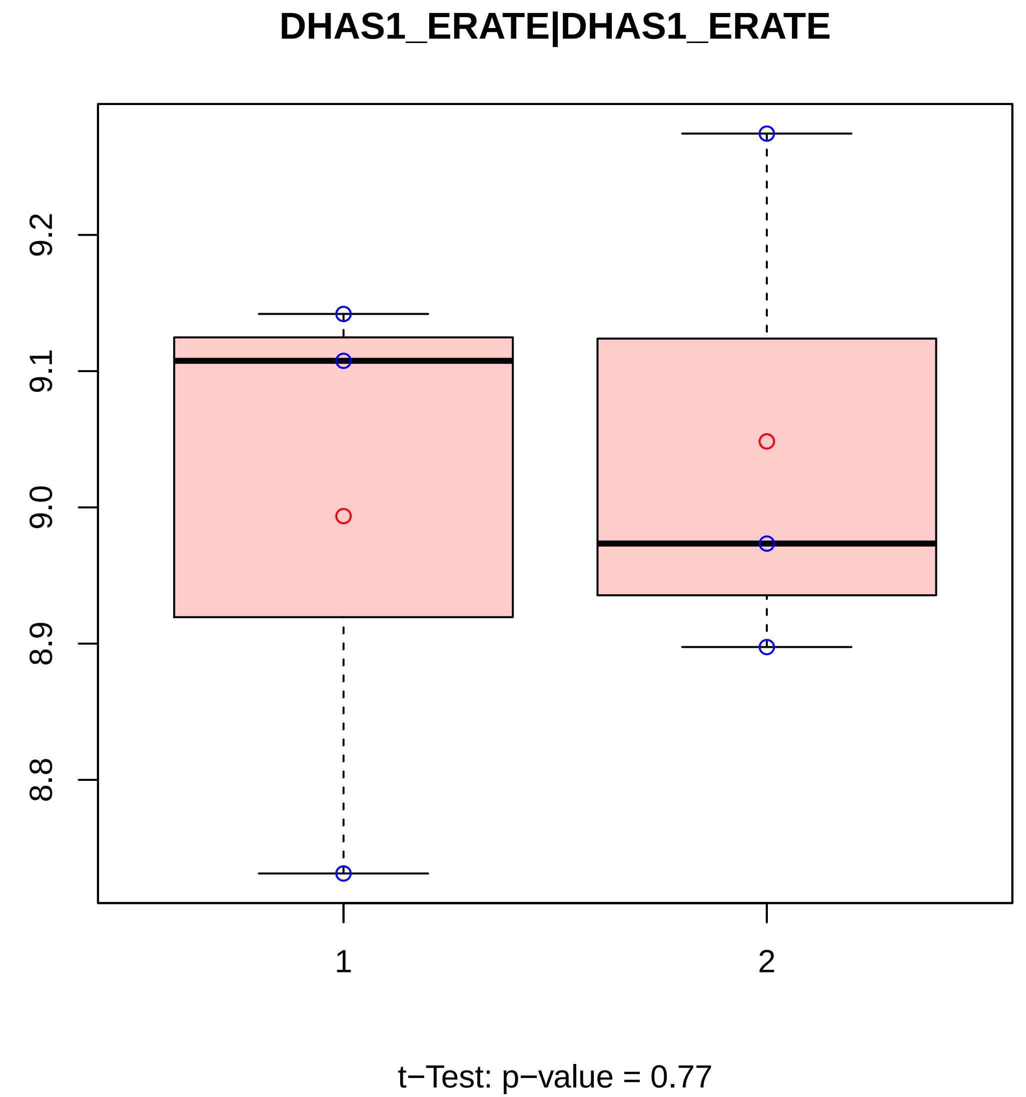

DHAS1_ERATE|DHAS1_ERATE
9.2
9.1
9.0
8.9
8.8
1
2
t−Test: p−value = 0.77

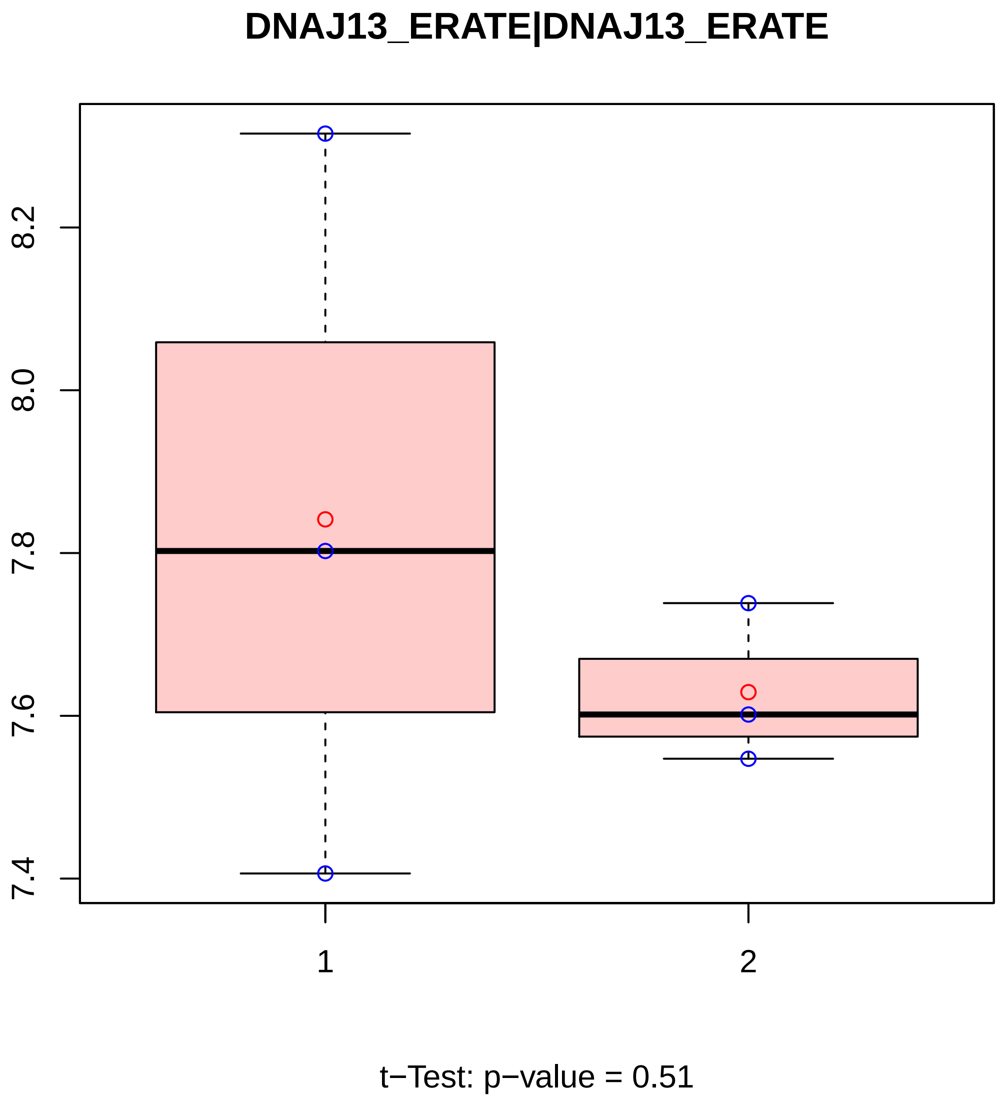
DNAJ13_ERATE|DNAJ13_ERATE
8.2
8.0
7.8
7.6
7.4
1
2
t−Test: p−value = 0.51

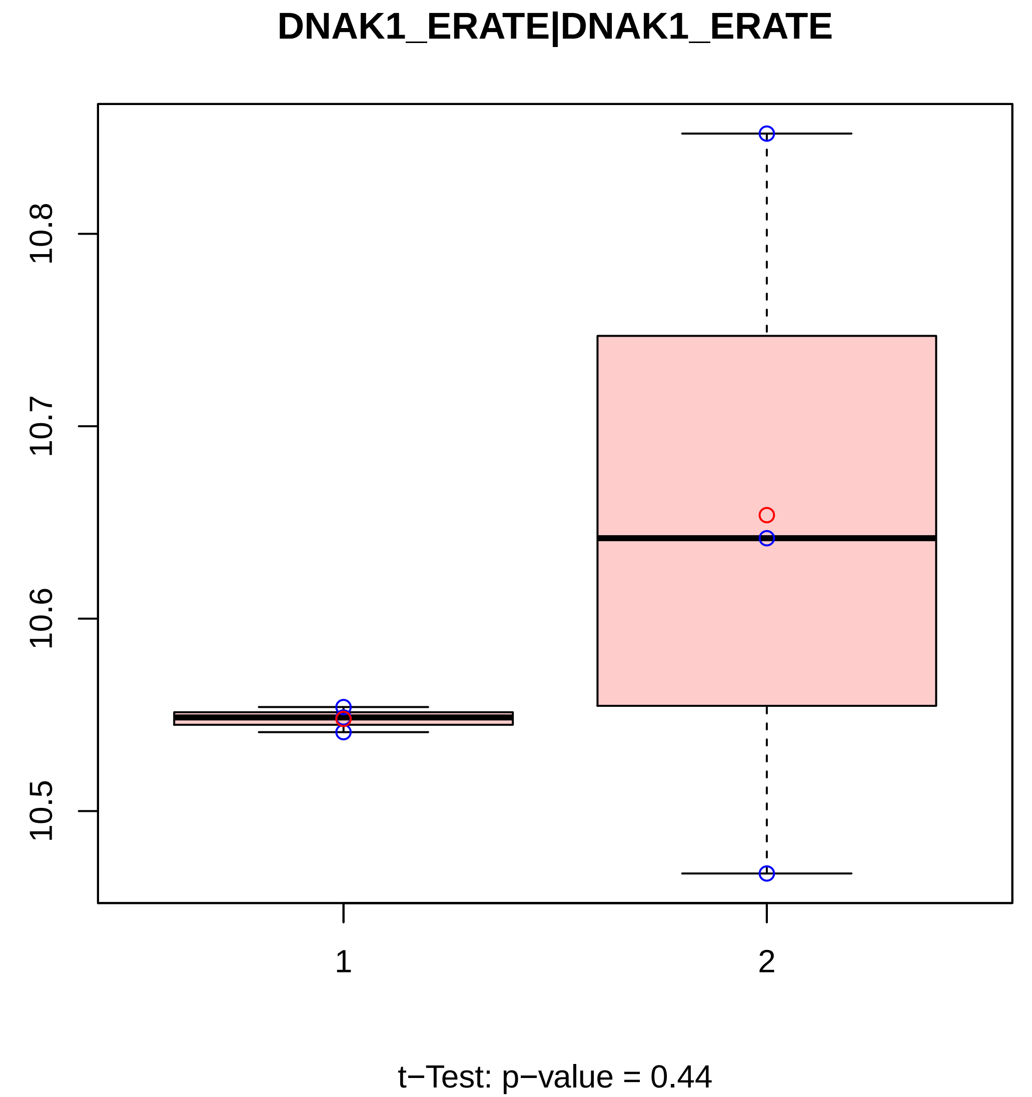
DNAK1_ERATE|DNAK1_ERATE
10.5
10.6
10.7
10.8
1
2
t−Test: p−value = 0.44

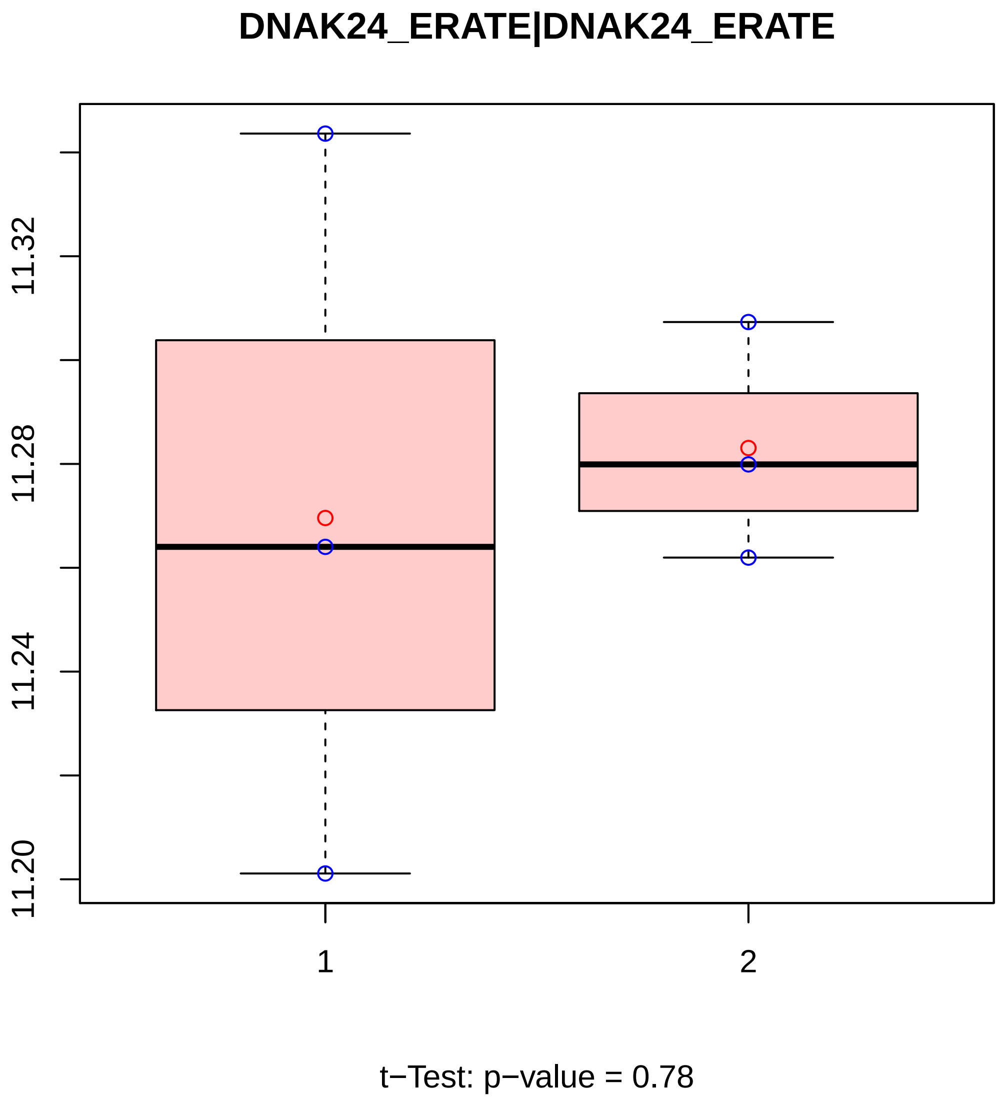

DNAK24_ERATE|DNAK24_ERATE
11.20
11.24
11.28
11.32
1
2
t−Test: p−value = 0.78

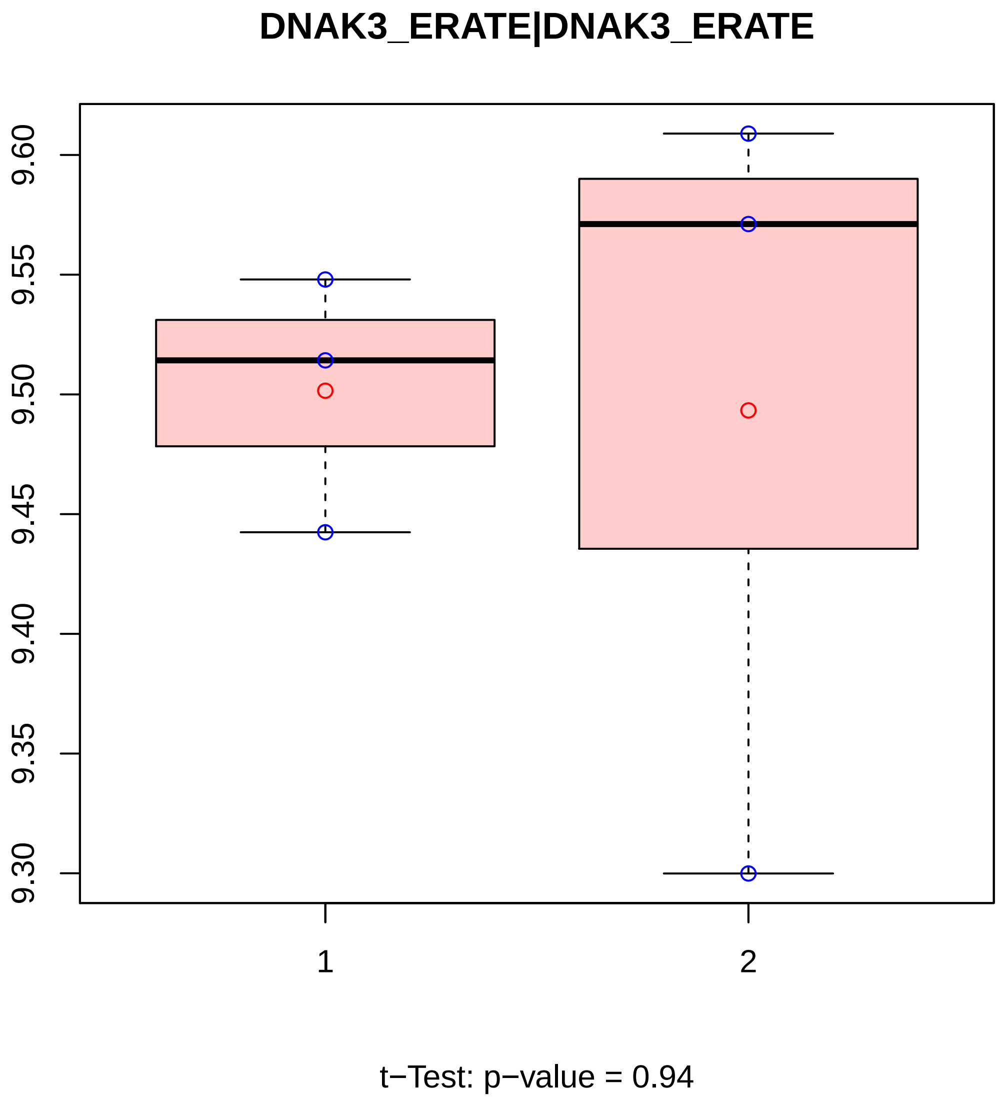

DNAK3_ERATE|DNAK3_ERATE
9.60
9.55
9.50
9.45
9.40
9.35
9.30
1
2
t−Test: p−value = 0.94

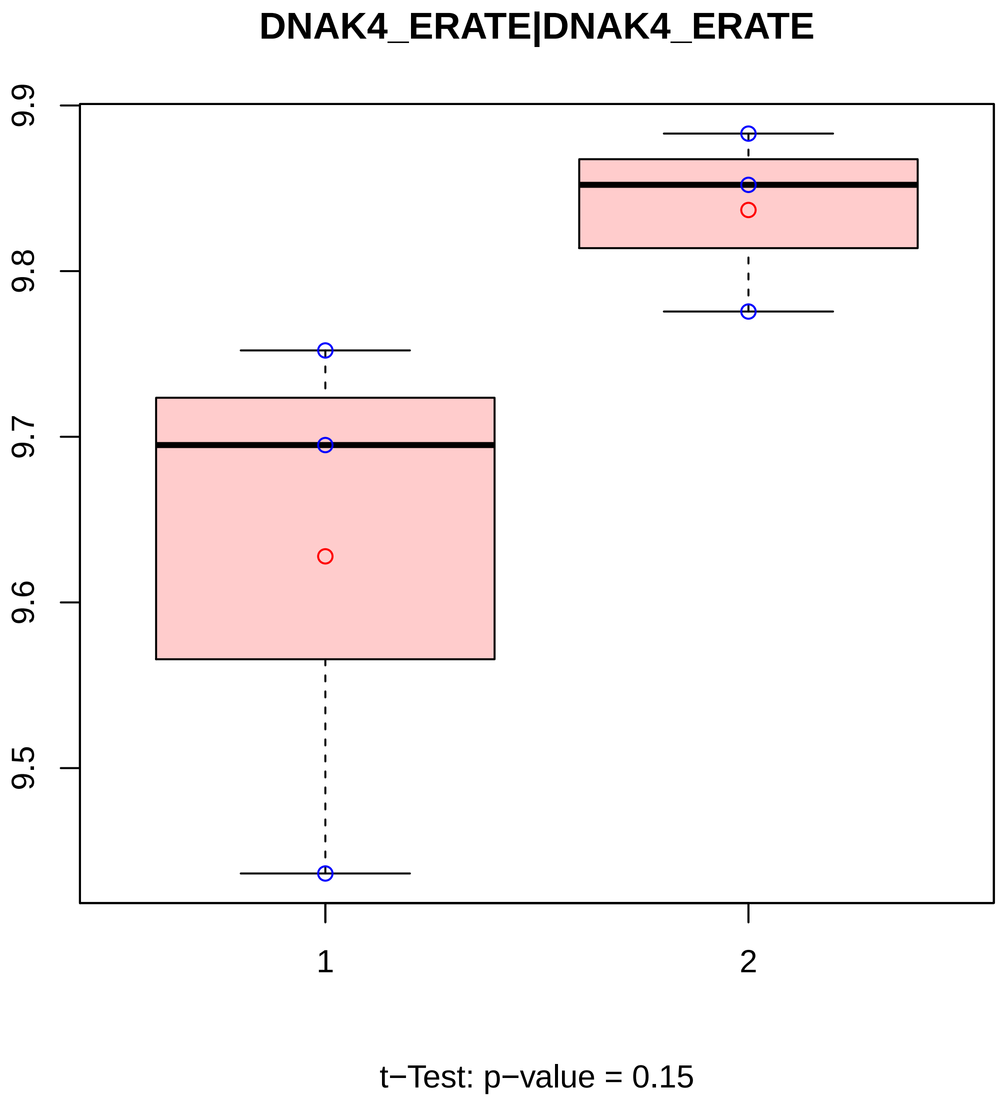

DNAK4_ERATE|DNAK4_ERATE
9.9
9.8
9.7
9.6
9.5
1
2
t−Test: p−value = 0.15

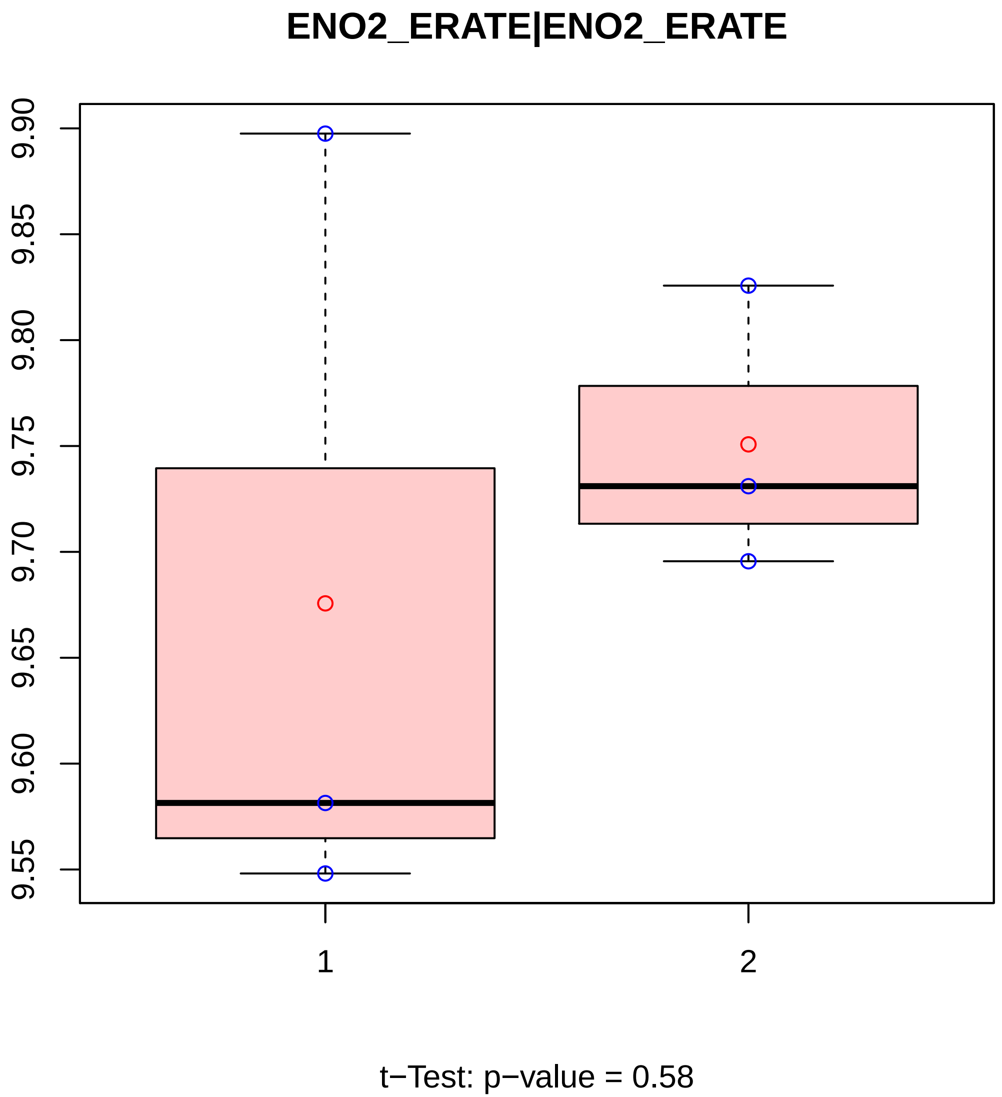
ENO2_ERATE|ENO2_ERATE
9.90
9.85
9.80
9.75
9.70
9.65
9.60
9.55
1
2
t−Test: p−value = 0.58

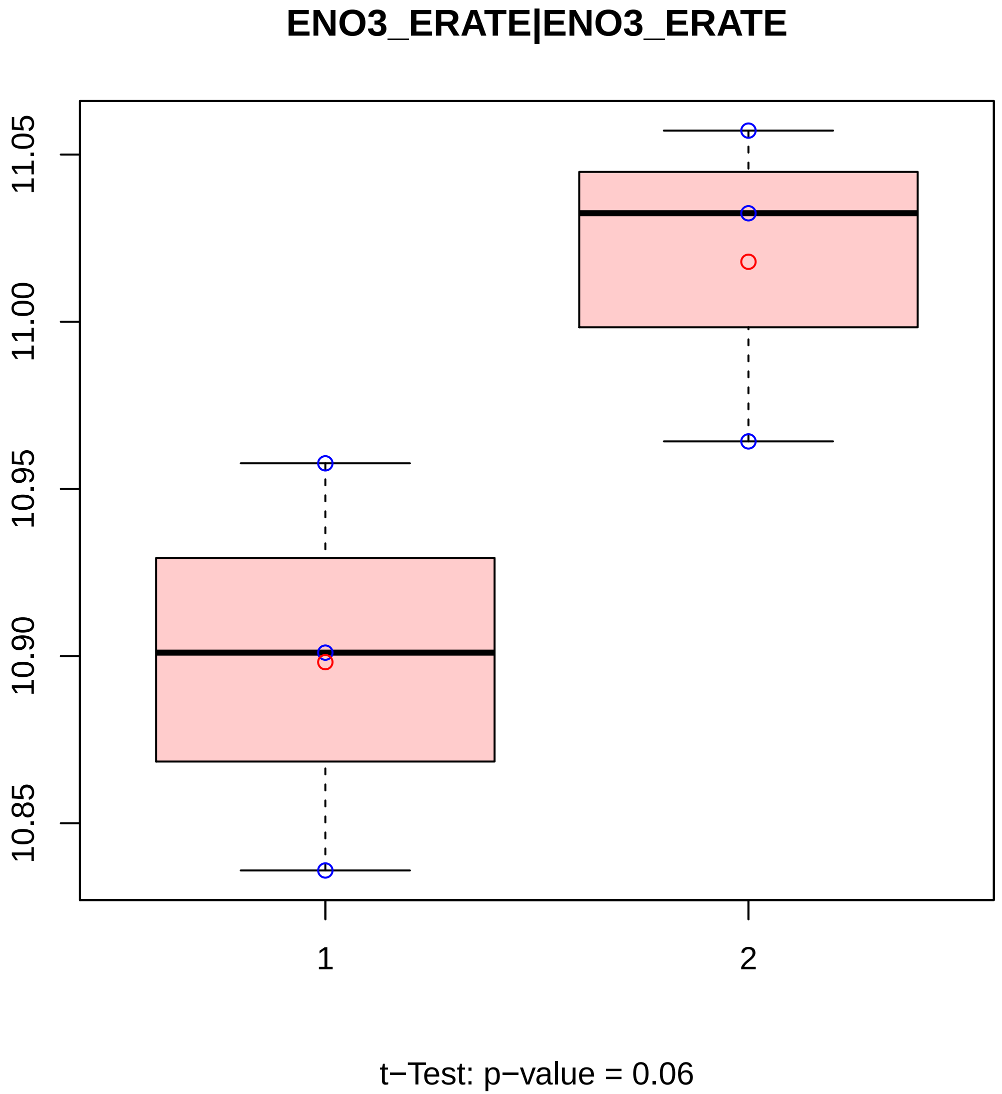
ENO3_ERATE|ENO3_ERATE
11.05
11.00
10.95
10.90
10.85
1
2
t−Test: p−value = 0.06

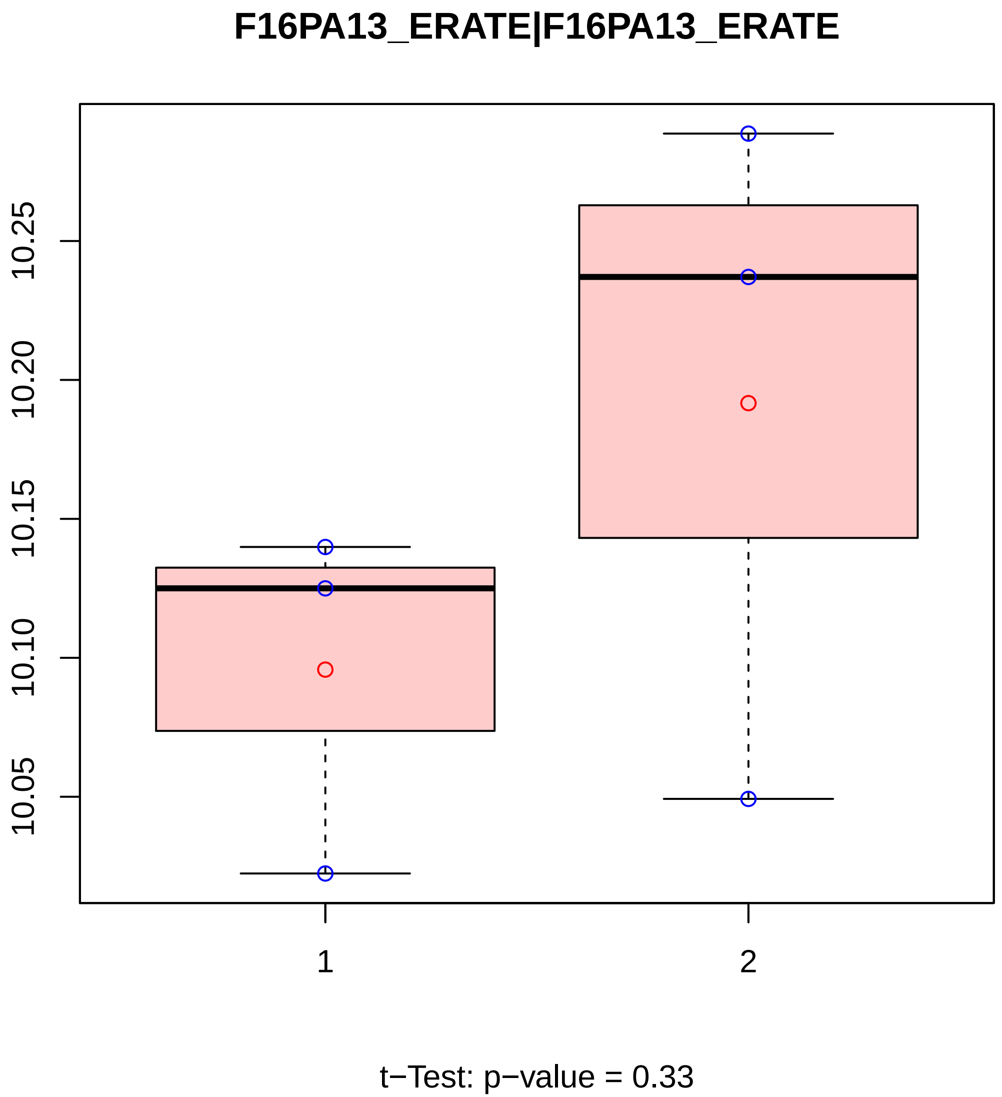
F16PA13_ERATE|F16PA13_ERATE
10.05
10.10
10.15
10.20
10.25
1
2
t−Test: p−value = 0.33

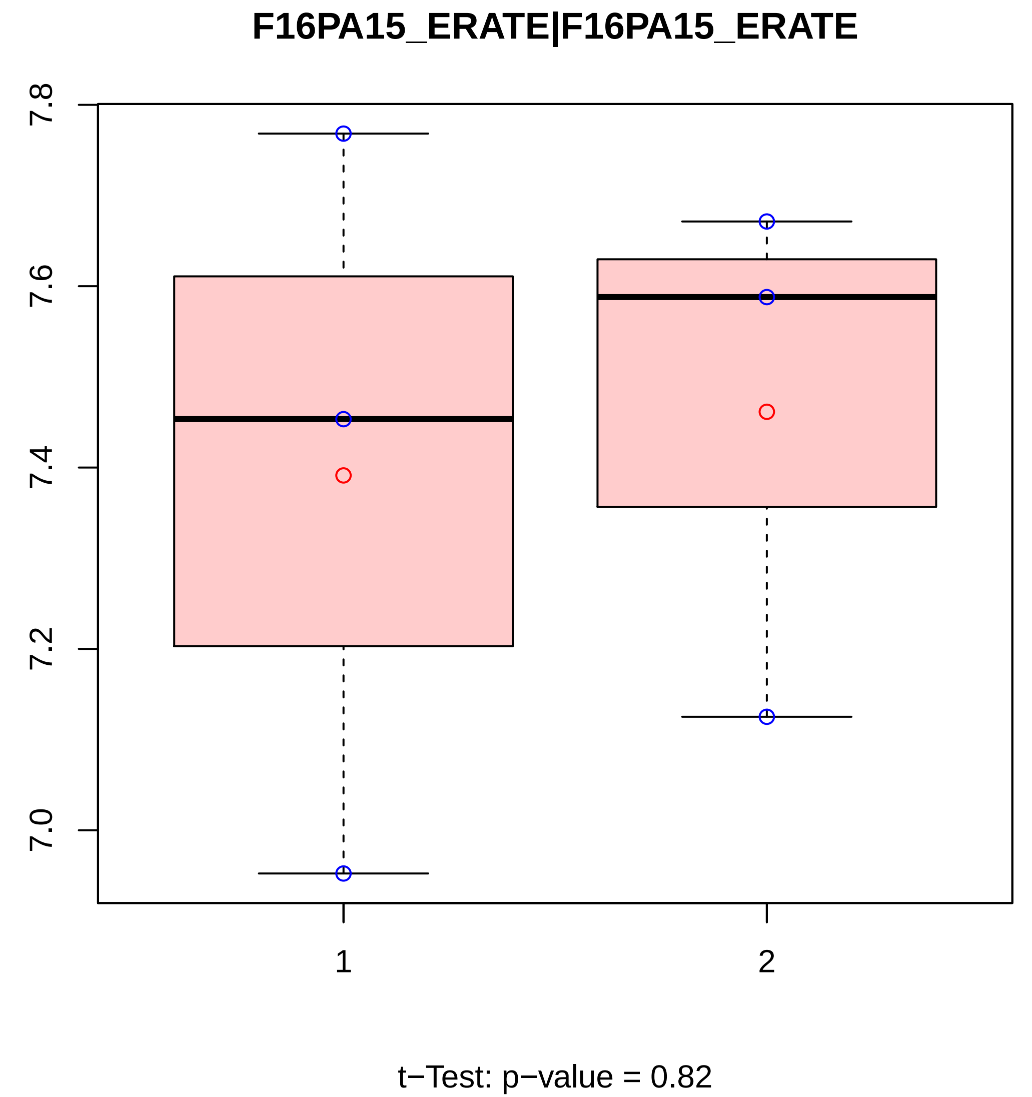
F16PA15_ERATE|F16PA15_ERATE
7.8
7.6
7.4
7.2
7.0
1
2
t−Test: p−value = 0.82

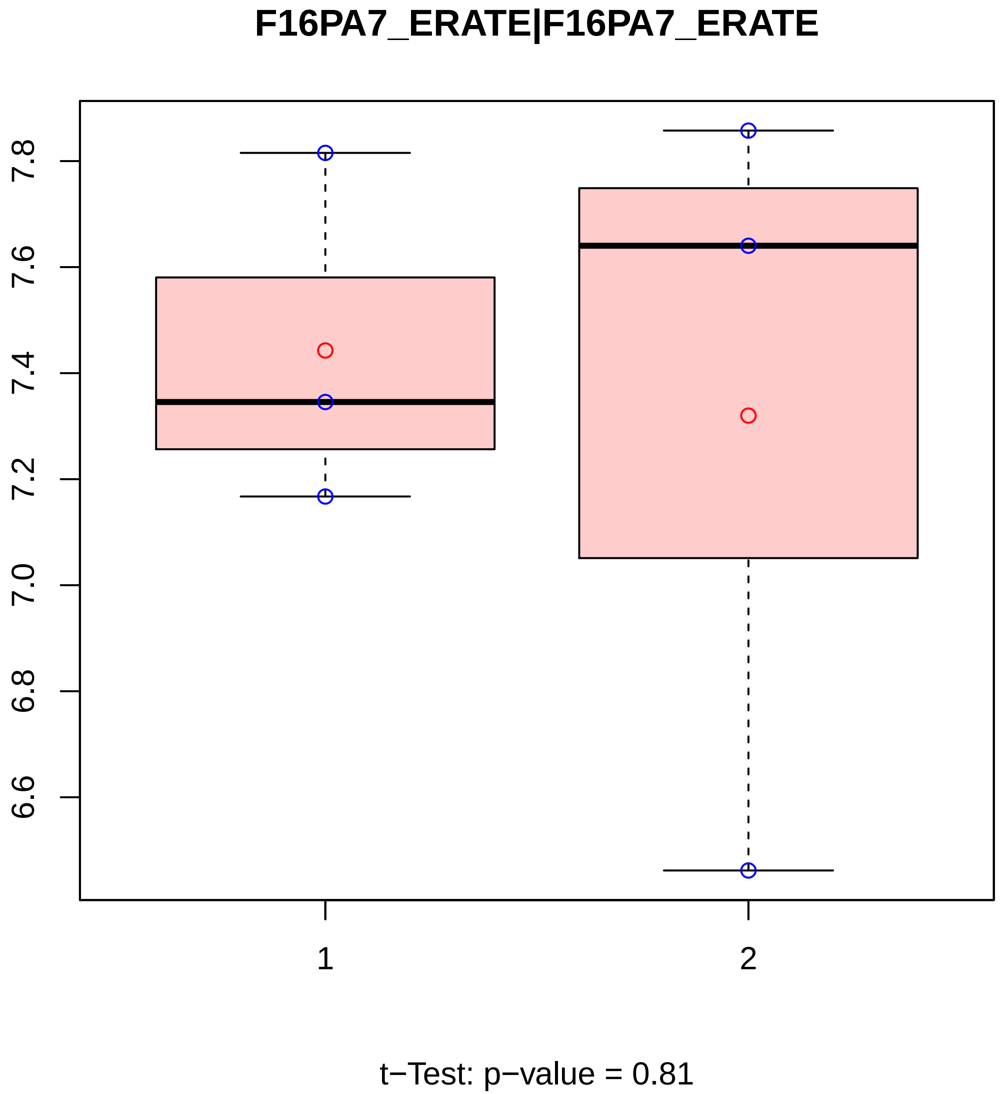
F16PA7_ERATE|F16PA7_ERATE
7.8
7.6
7.4
7.2
7.0
6.8
6.6
1
2
t−Test: p−value = 0.81

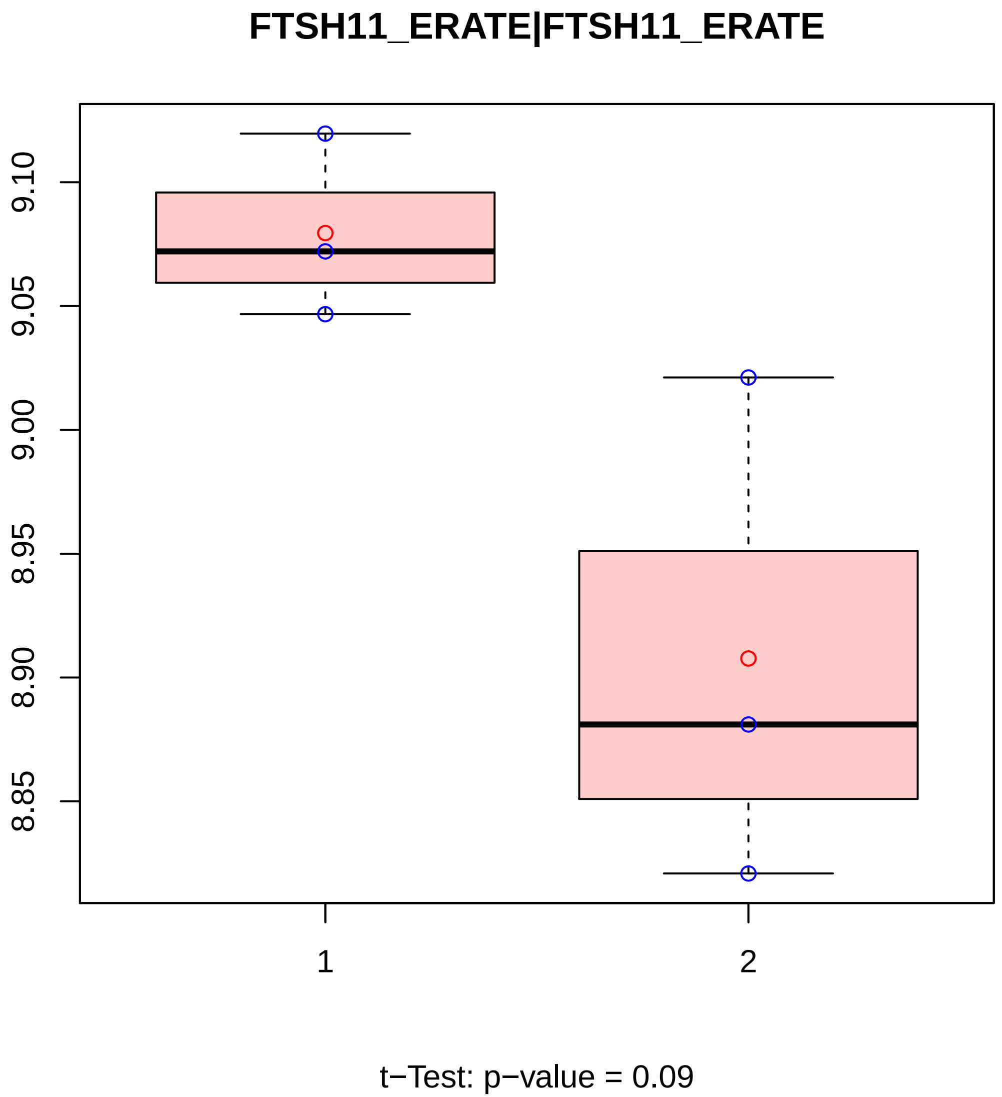
FTSH11_ERATE|FTSH11_ERATE
9.10
9.05
9.00
8.95
8.90
8.85
1
2
t−Test: p−value = 0.09

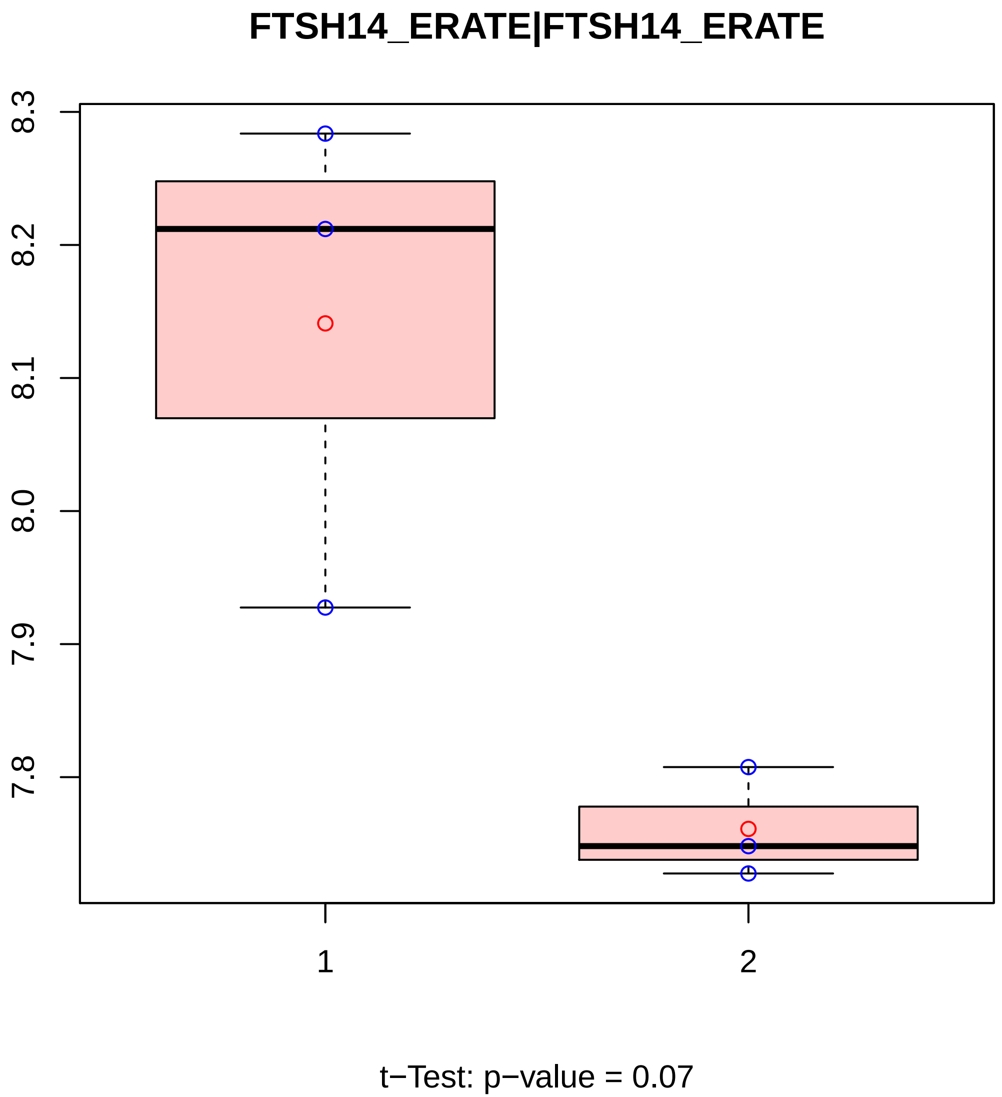
FTSH14_ERATE|FTSH14_ERATE
8.3
8.2
8.1
8.0
7.9
7.8
1
2
t−Test: p−value = 0.07

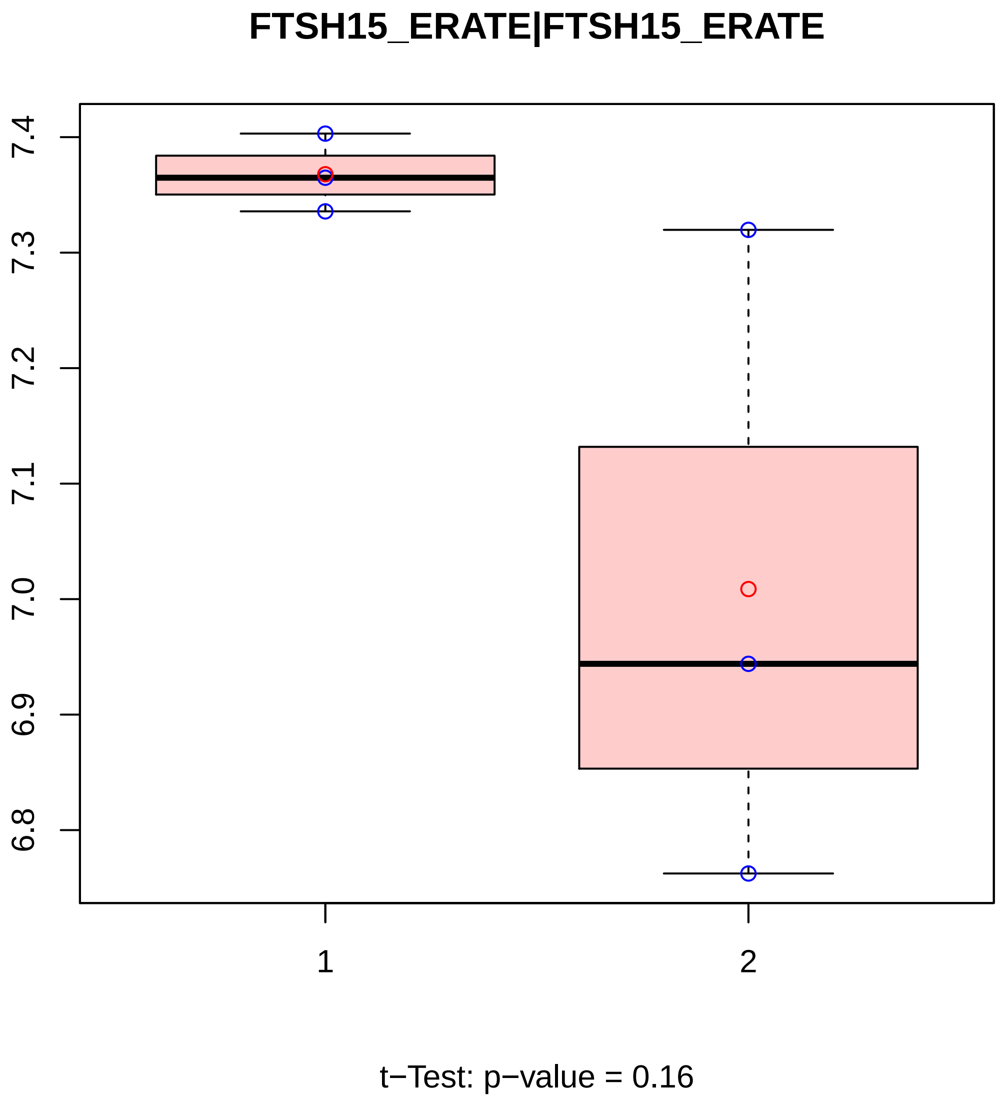

FTSH15_ERATE|FTSH15_ERATE
7.4
7.3
7.2
7.1
7.0
6.9
6.8
1
2
t−Test: p−value = 0.16

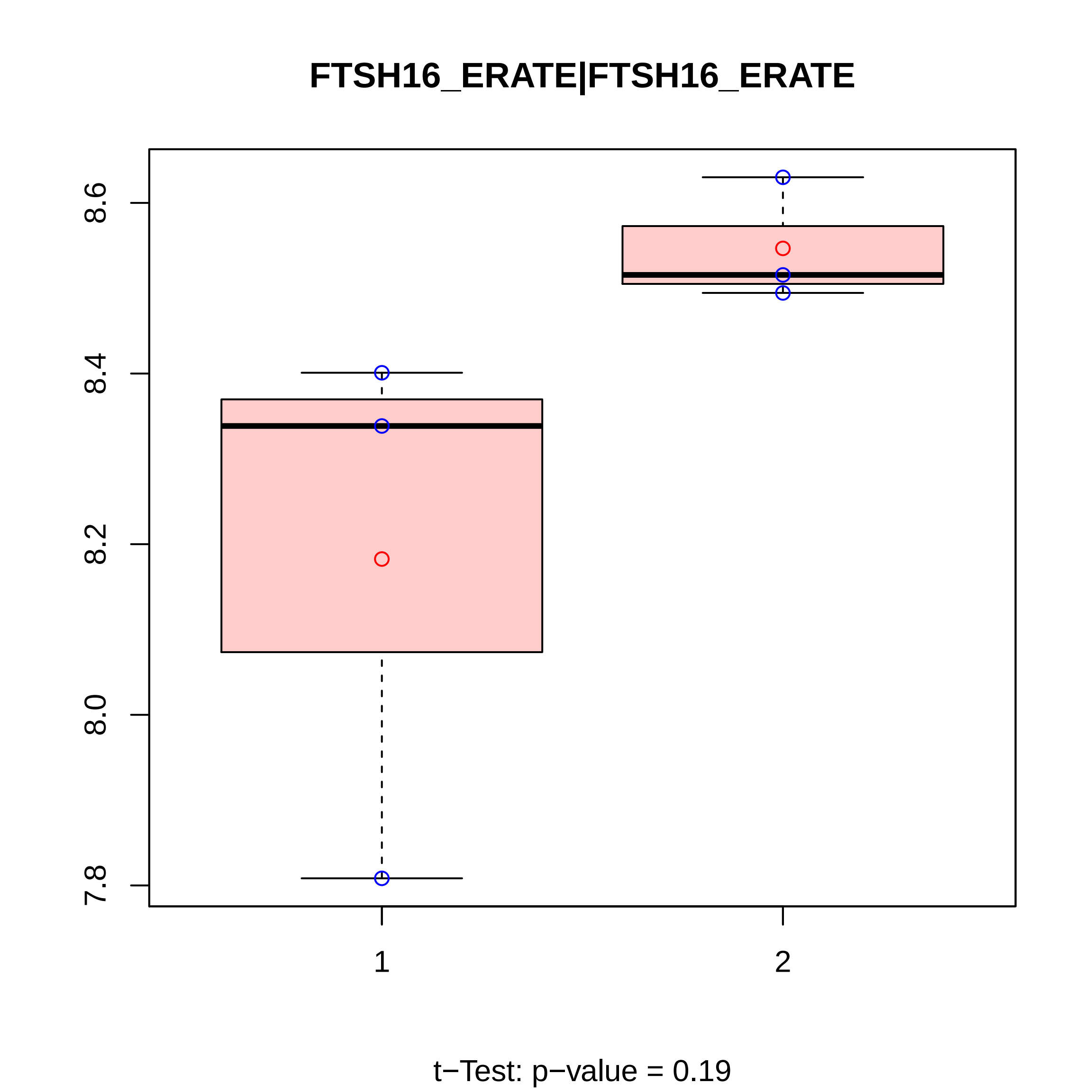
FTSH16_ERATE|FTSH16_ERATE
8.6
8.4
8.2
8.0
7.8
1
2
t−Test: p−value = 0.19

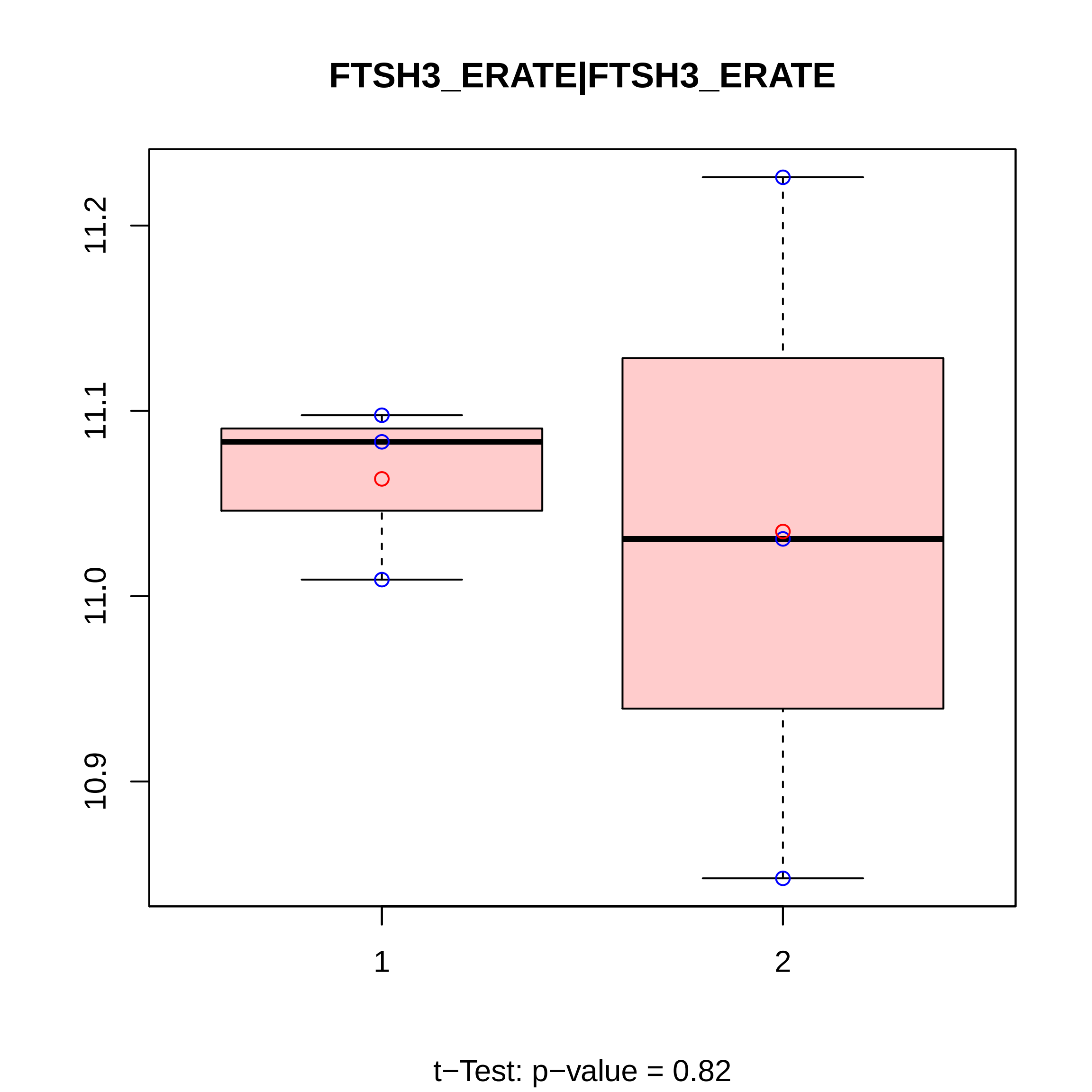
FTSH3_ERATE|FTSH3_ERATE
11.2
11.1
11.0
10.9
1
2
t−Test: p−value = 0.82

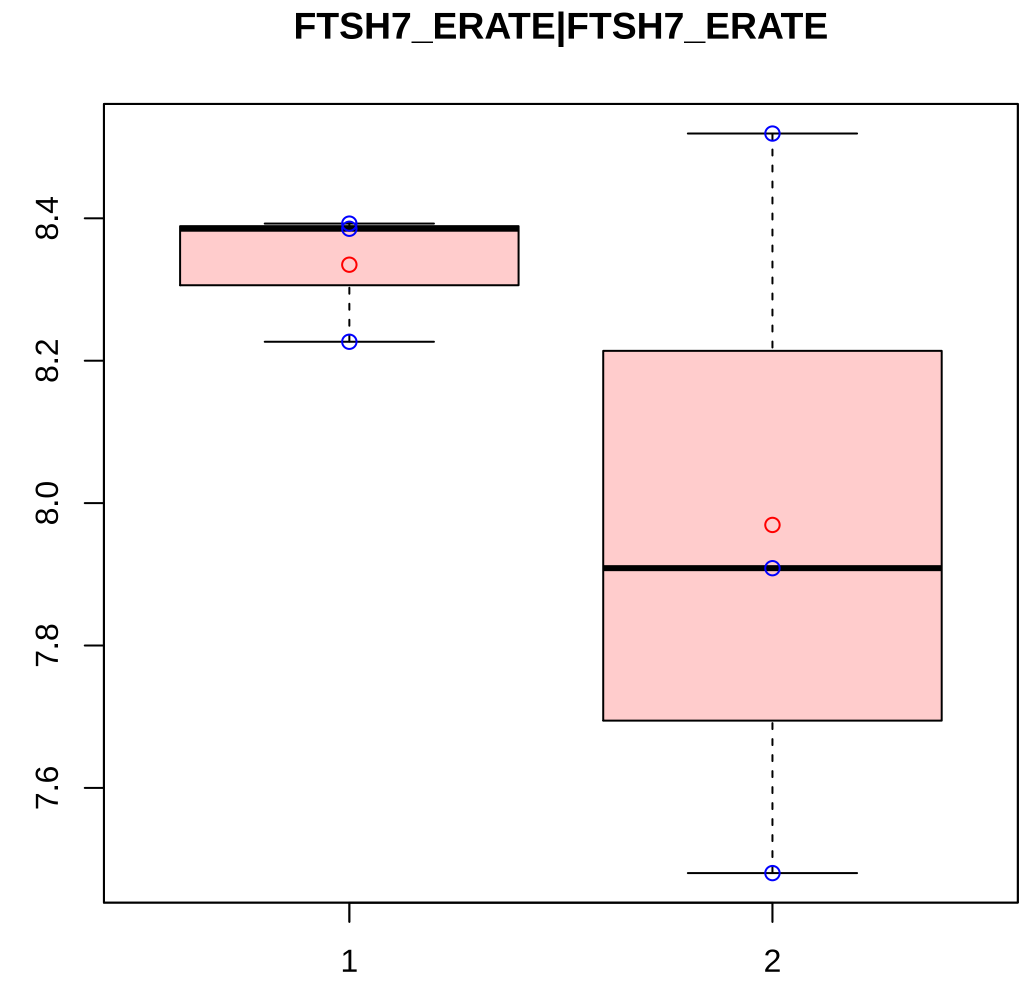


t−Test: p−value = 0.35

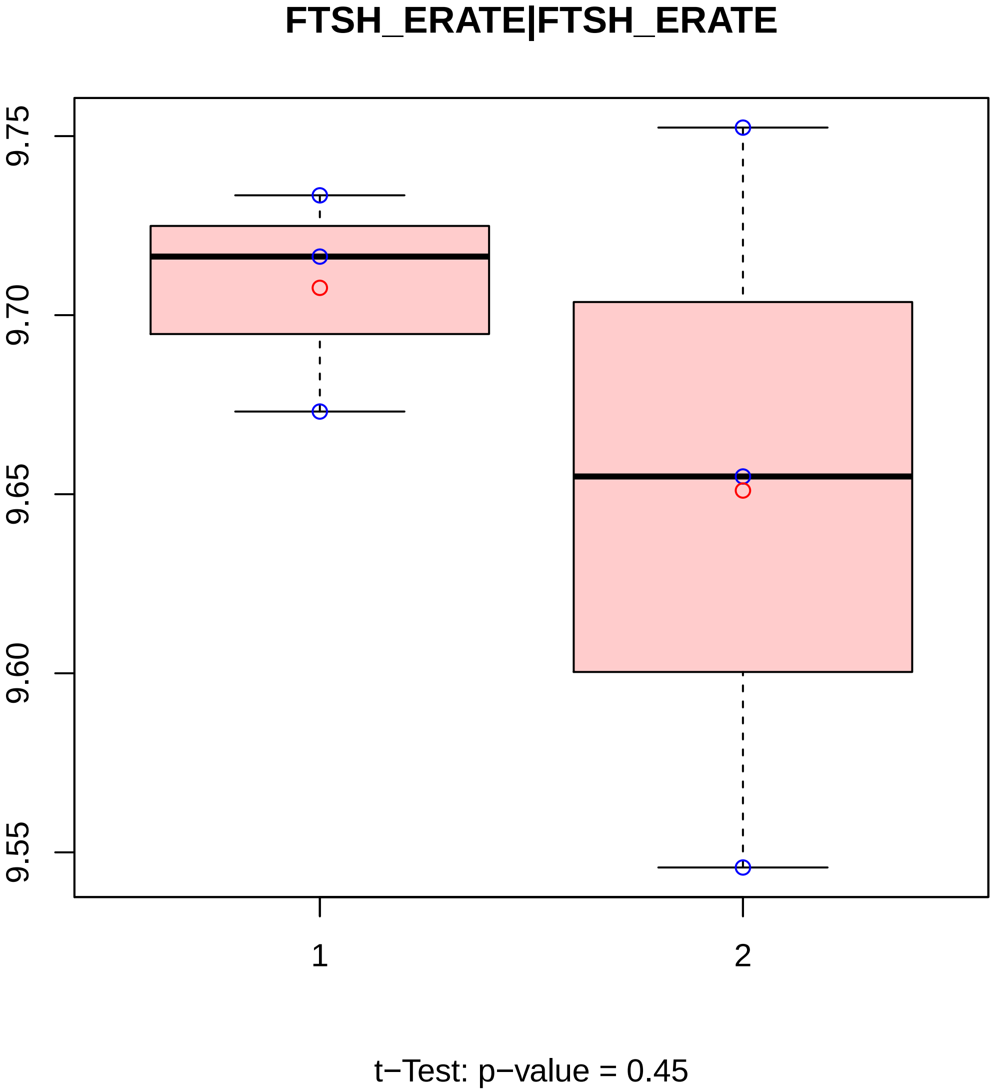
FTSH_ERATE|FTSH_ERATE
9.75
9.70
9.65
9.60
9.55
1
2
t−Test: p−value = 0.45

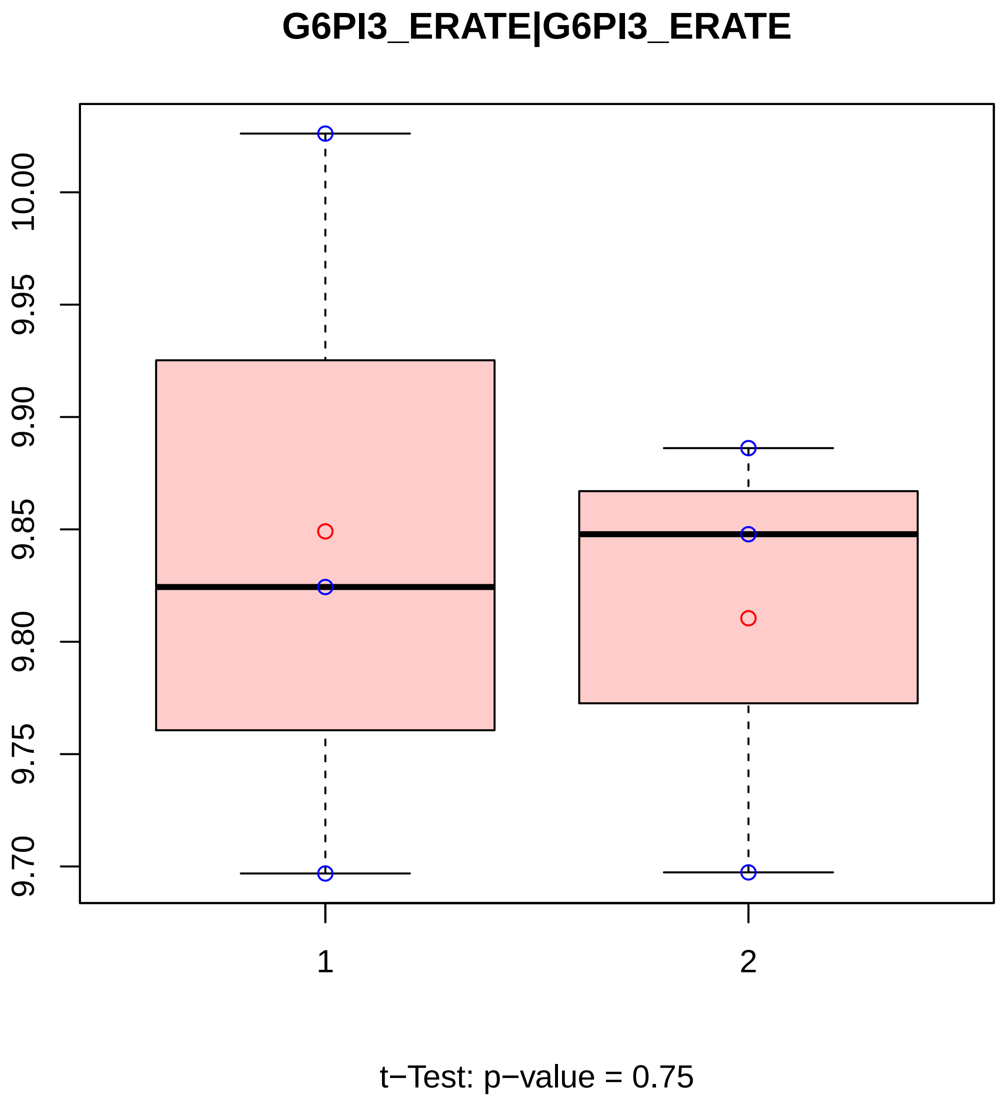
G6PI3_ERATE|G6PI3_ERATE
10.00
9.95
9.90
9.85
9.80
9.75
9.70
1
2
t−Test: p−value = 0.75

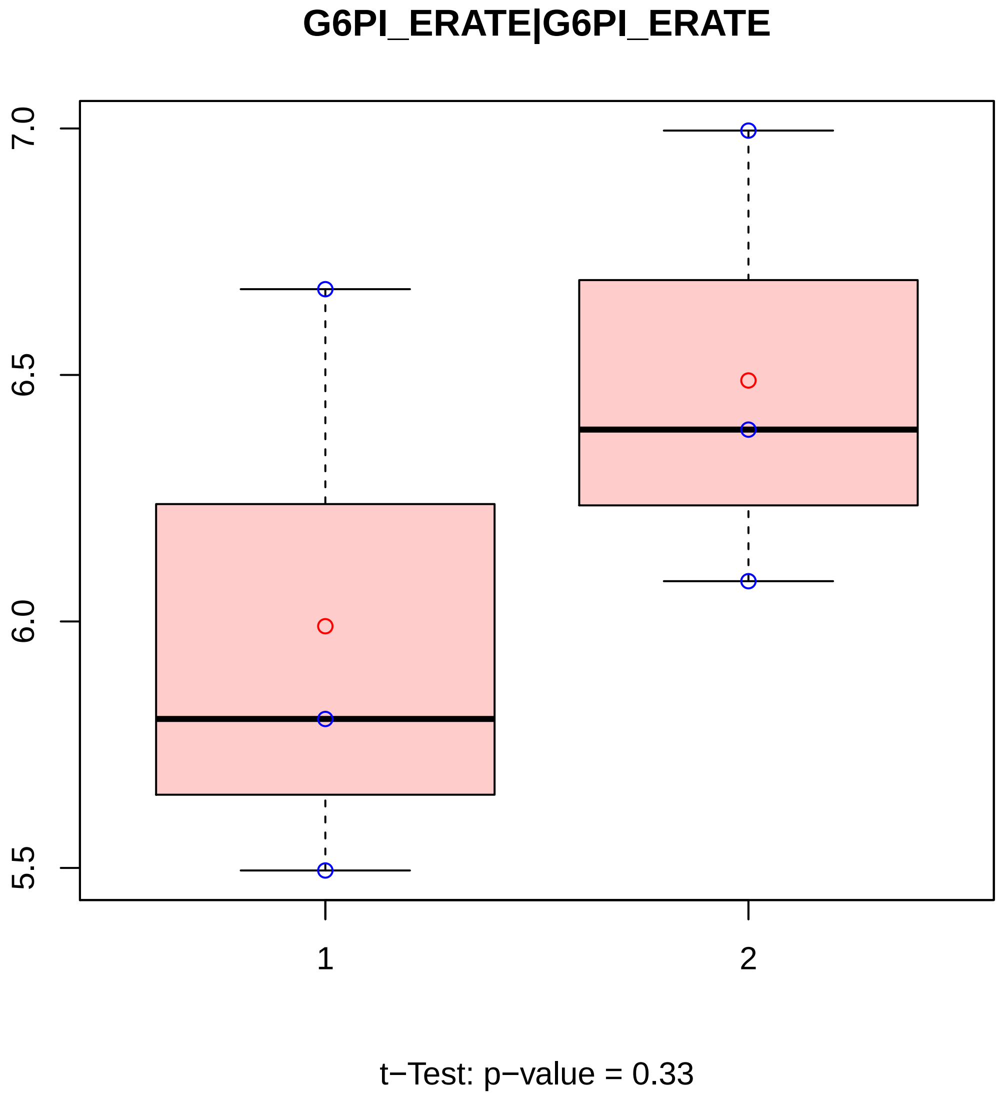
G6PI_ERATE|G6PI_ERATE
7.0
6.5
6.0
5.5
1
2
t−Test: p−value = 0.33

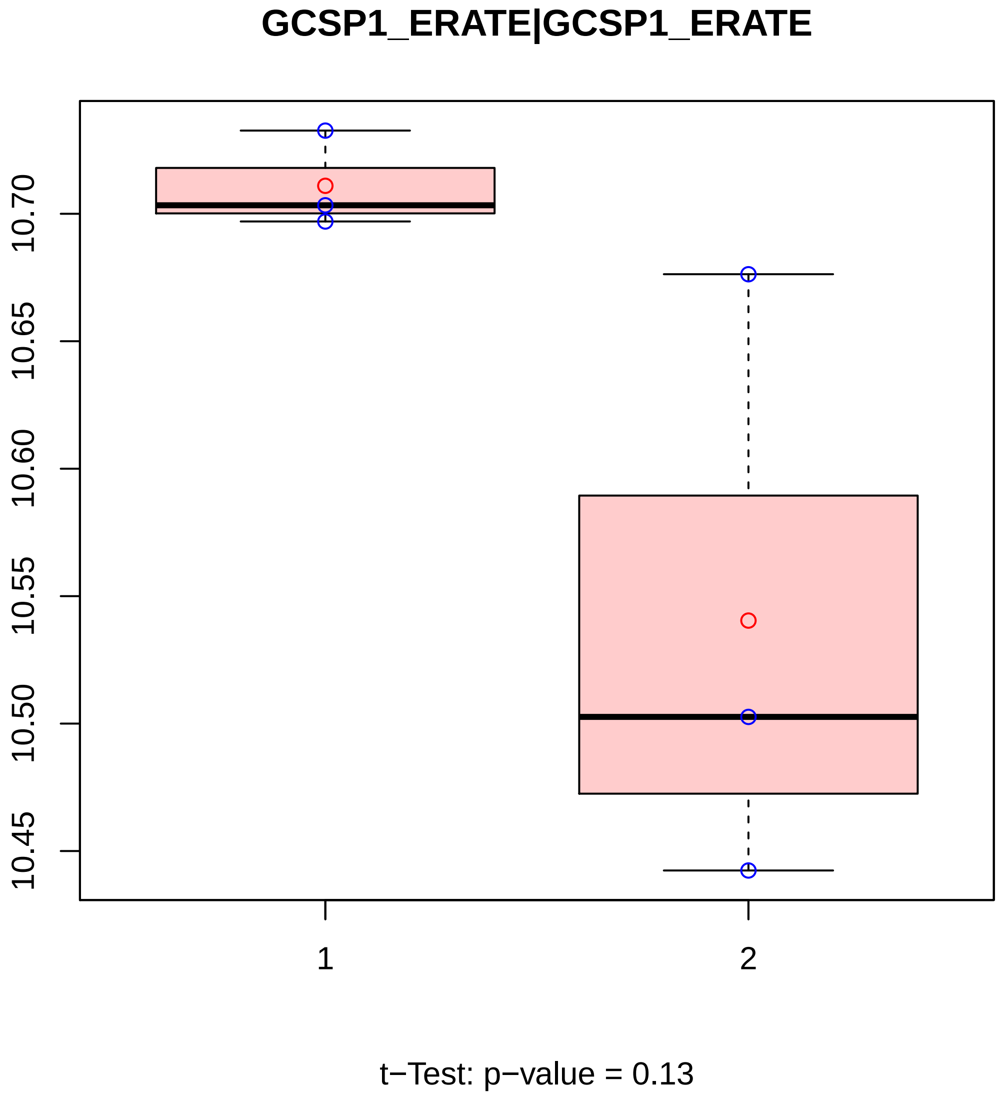
GCSP1_ERATE|GCSP1_ERATE
10.45
10.50
10.55
10.60
10.65
10.70
1
2
t−Test: p−value = 0.13

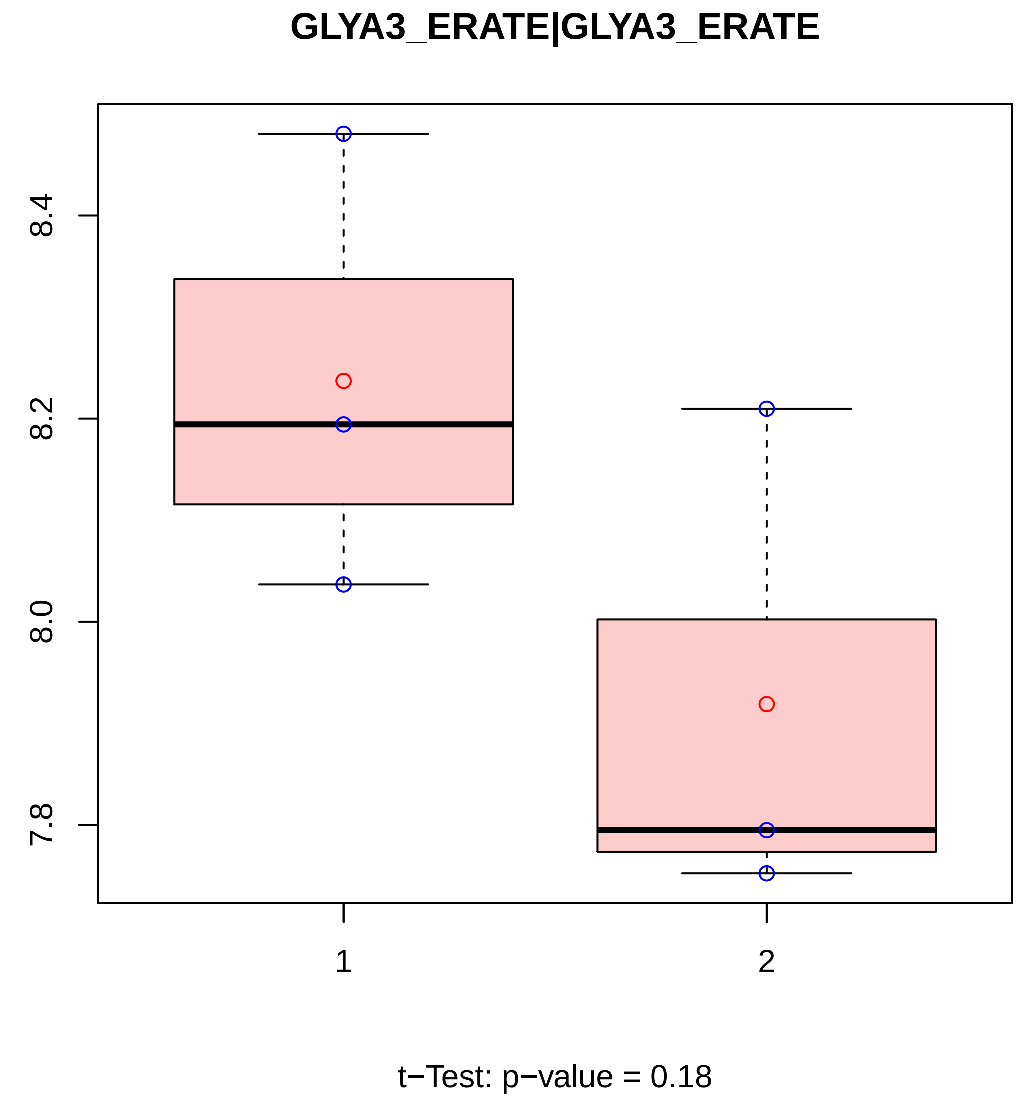
GLYA3_ERATE|GLYA3_ERATE
8.4
8.2
8.0
7.8
1
2
t−Test: p−value = 0.18

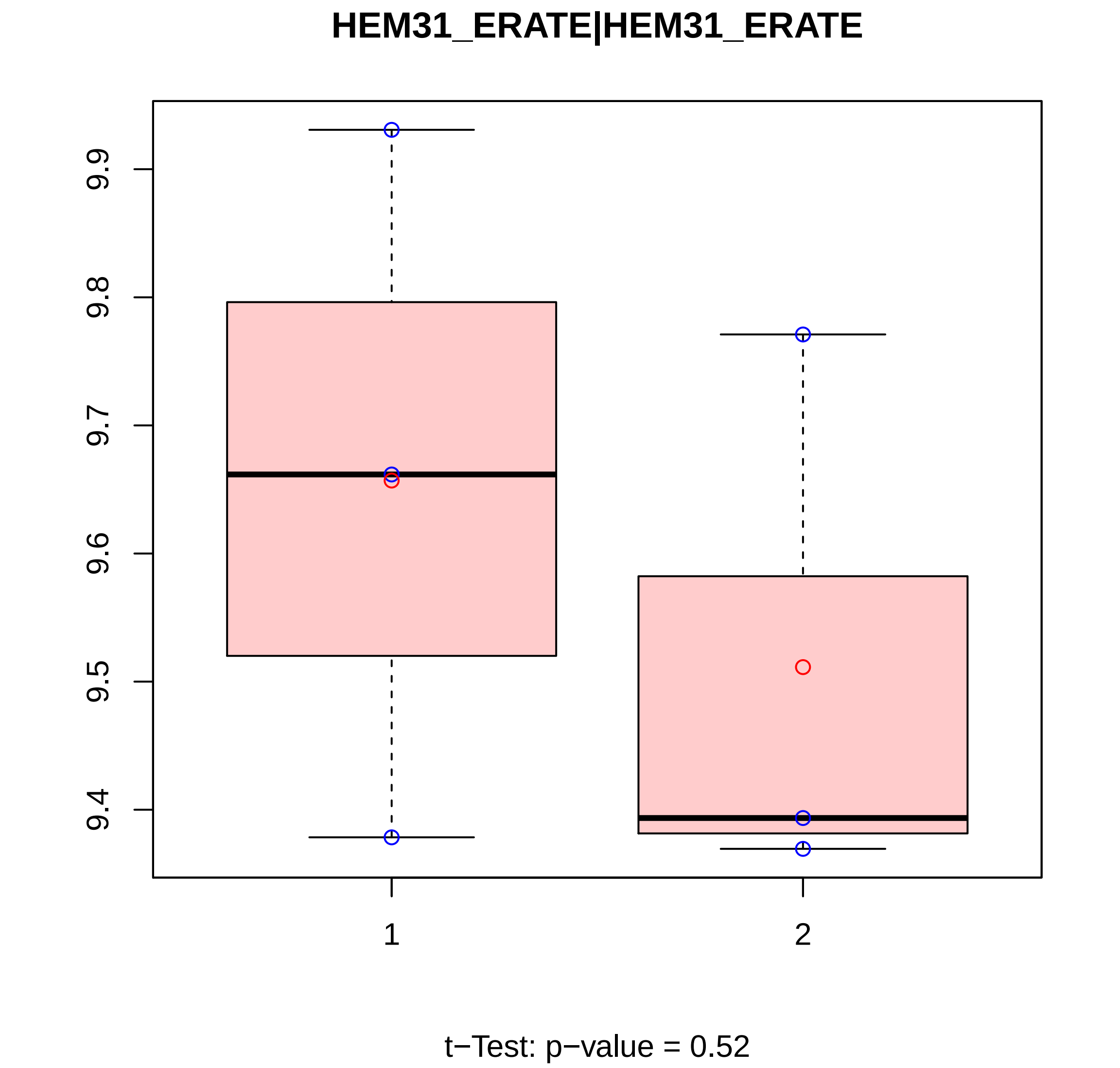

HEM31_ERATE|HEM31_ERATE
9.4
9.5
9.6
9.7
9.8
9.9
1
2
t−Test: p−value = 0.52

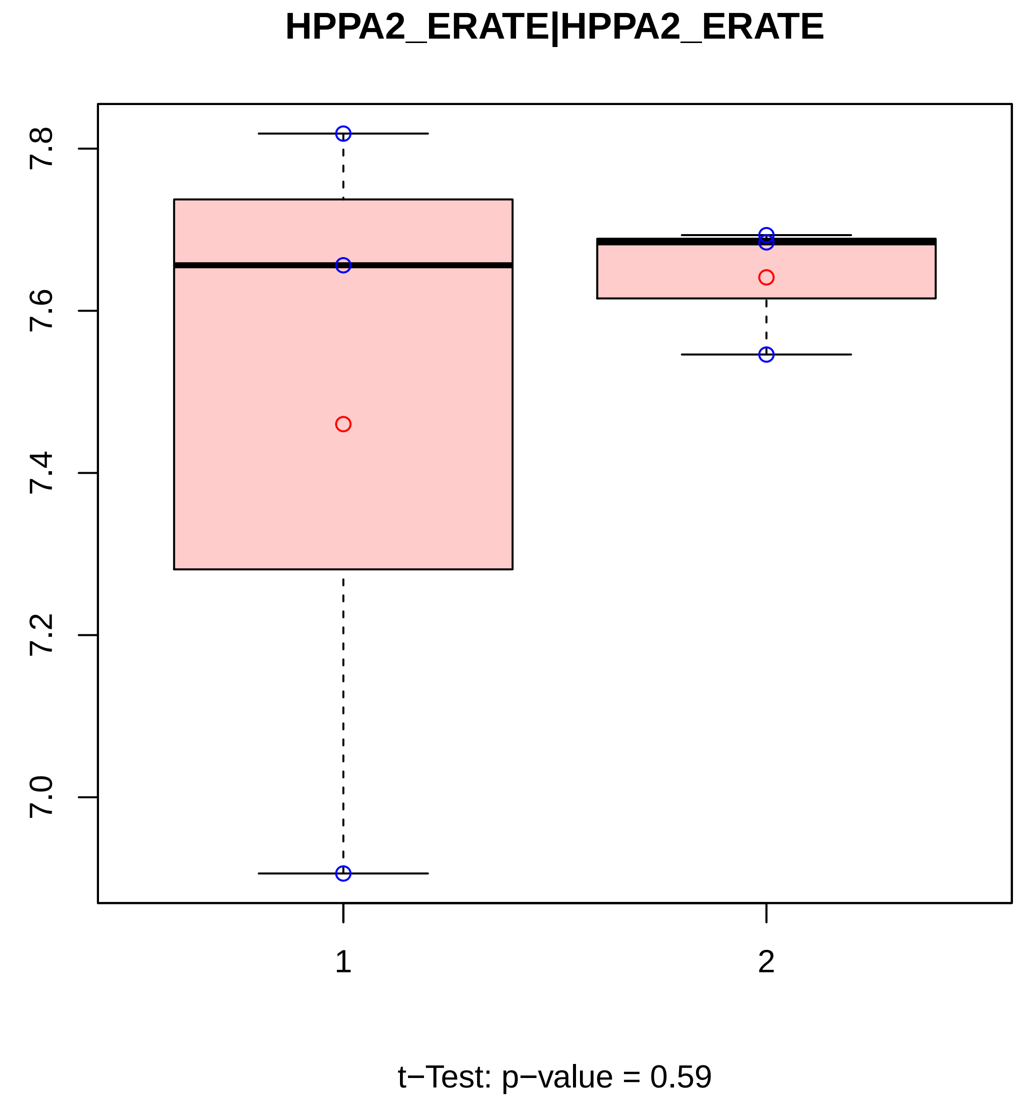
HPPA2_ERATE|HPPA2_ERATE
7.8
7.6
7.4
7.2
7.0
1
2
t−Test: p−value = 0.59

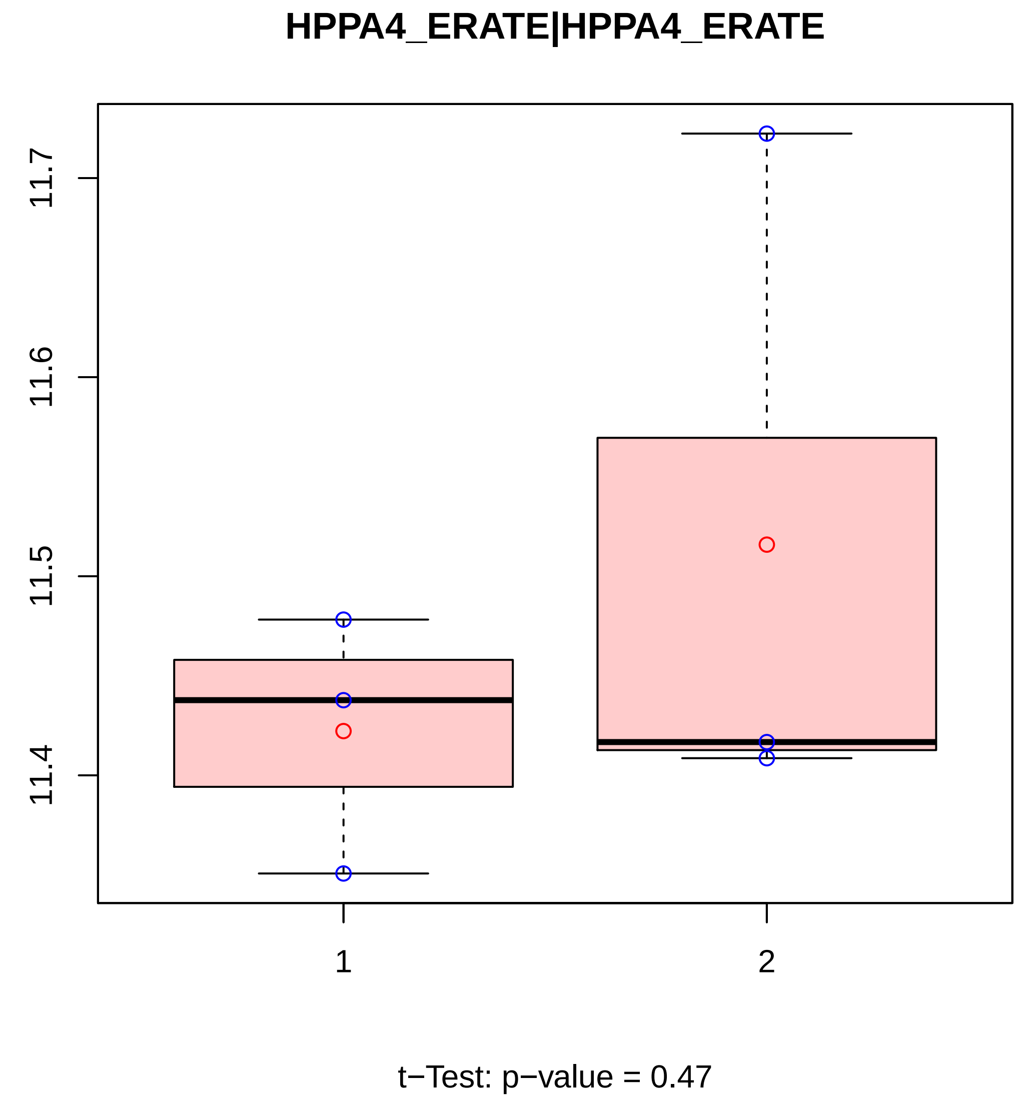
HPPA4_ERATE|HPPA4_ERATE
11.4
11.5
11.6
11.7
1
2
t−Test: p−value = 0.47

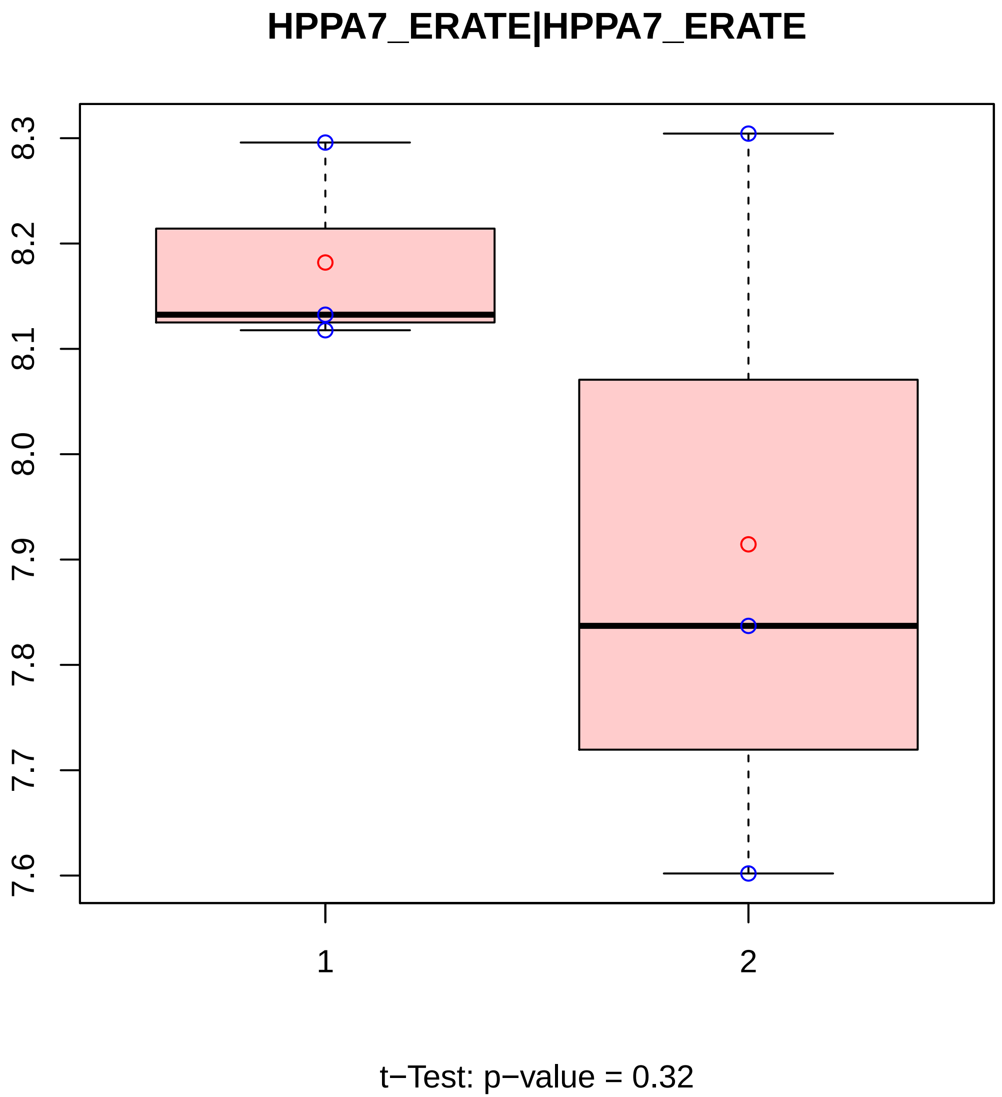

HPPA7_ERATE|HPPA7_ERATE
8.3
8.2
8.1
8.0
7.9
7.8
7.7
7.6
1
2
t−Test: p−value = 0.32

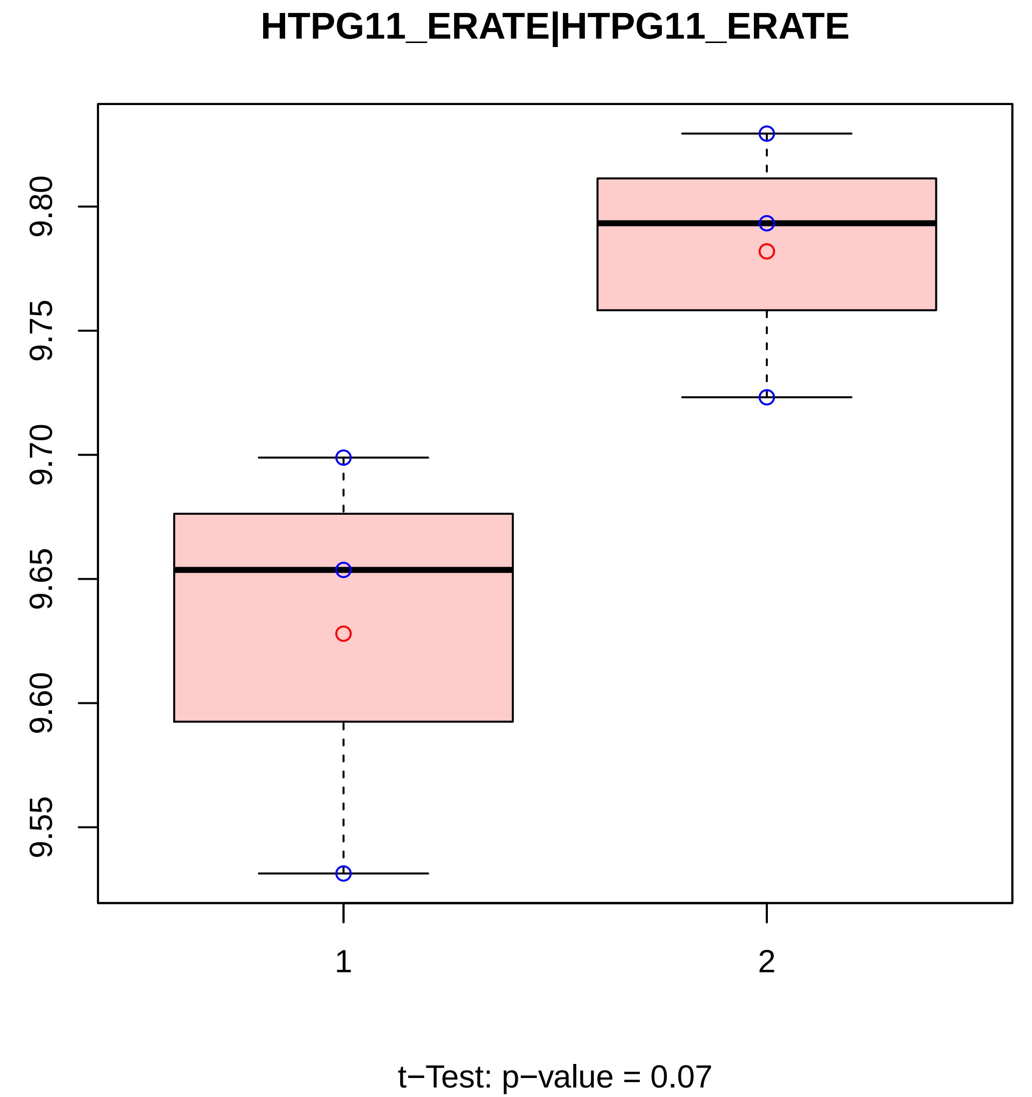
HTPG11_ERATE|HTPG11_ERATE
9.55
9.60
9.65
9.70
9.75
9.80
1
2
t−Test: p−value = 0.07

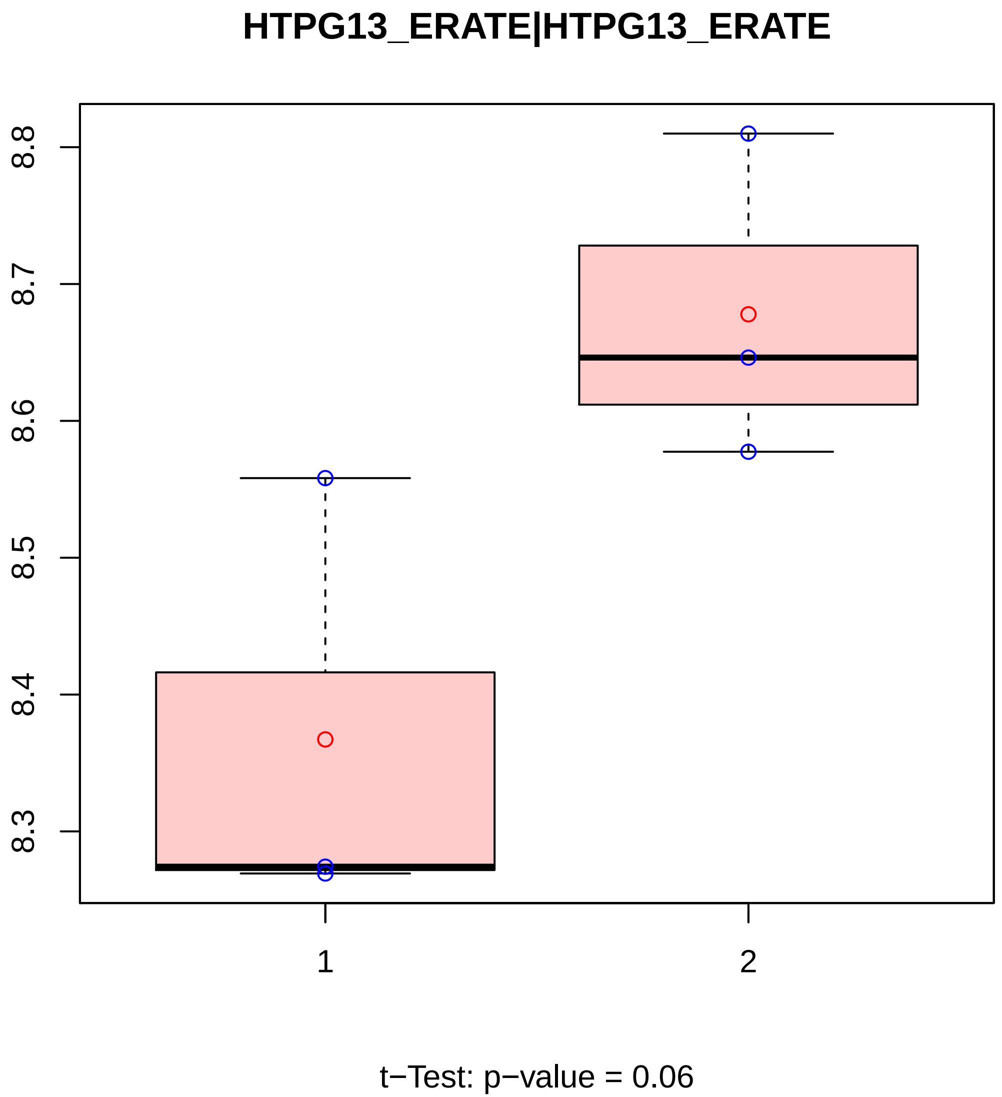

HTPG13_ERATE|HTPG13_ERATE
8.3
8.4
8.5
8.6
8.7
8.8
1
2
t−Test: p−value = 0.06

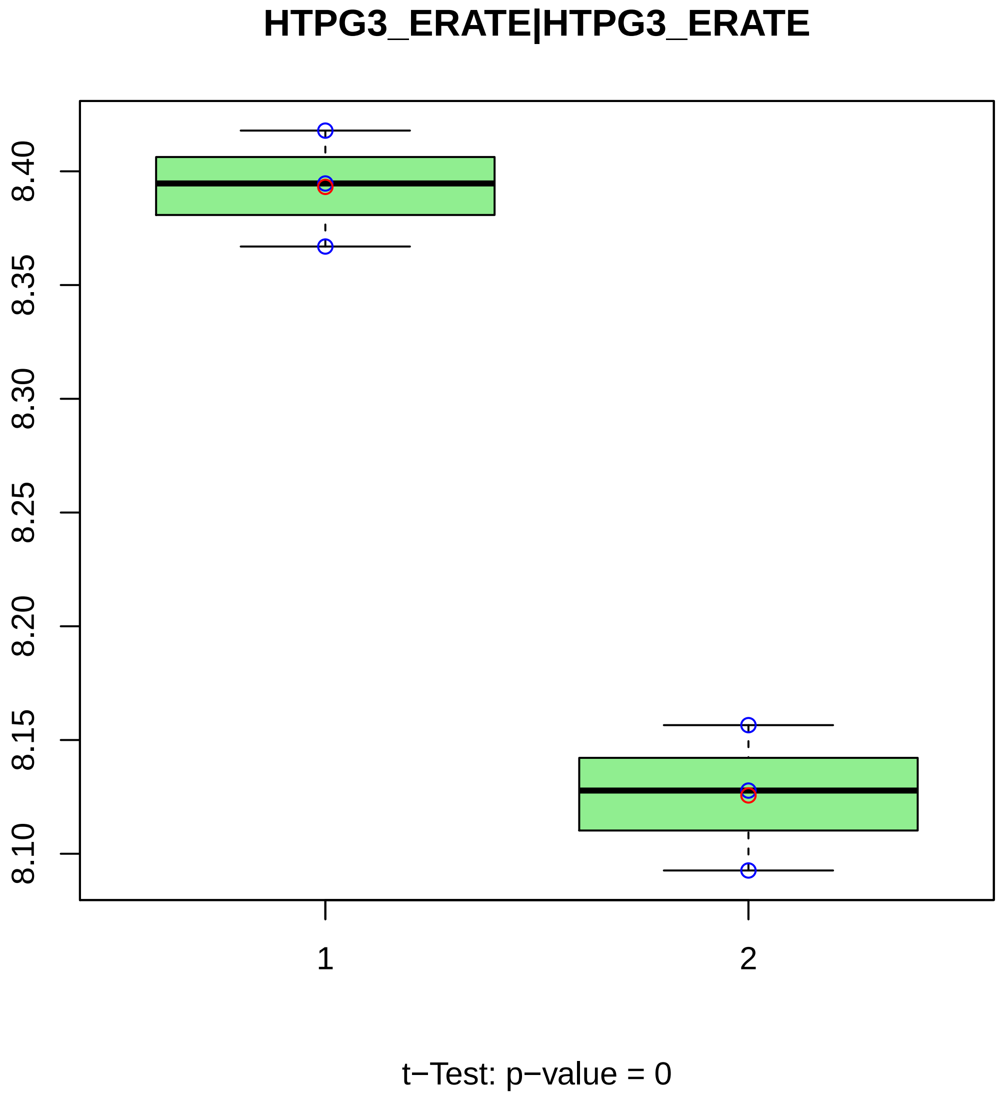
HTPG3_ERATE|HTPG3_ERATE
8.40
8.35
8.30
8.25
8.20
8.15
8.10
1
2
t−Test: p−value = 0

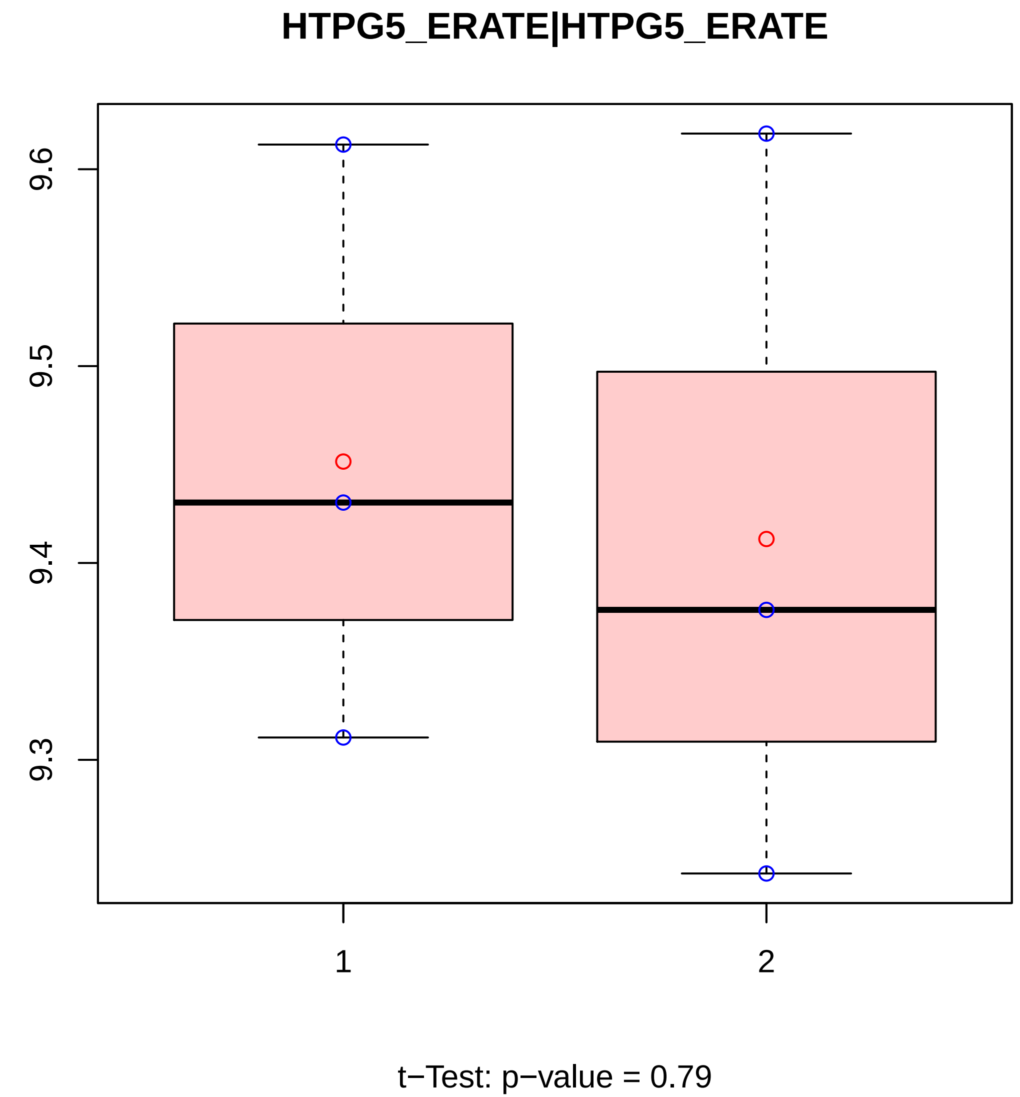
HTPG5_ERATE|HTPG5_ERATE
9.3
9.4
9.5
9.6
1
2
t−Test: p−value = 0.79

## HTPG8_ERATE|HTPG8_ERATE

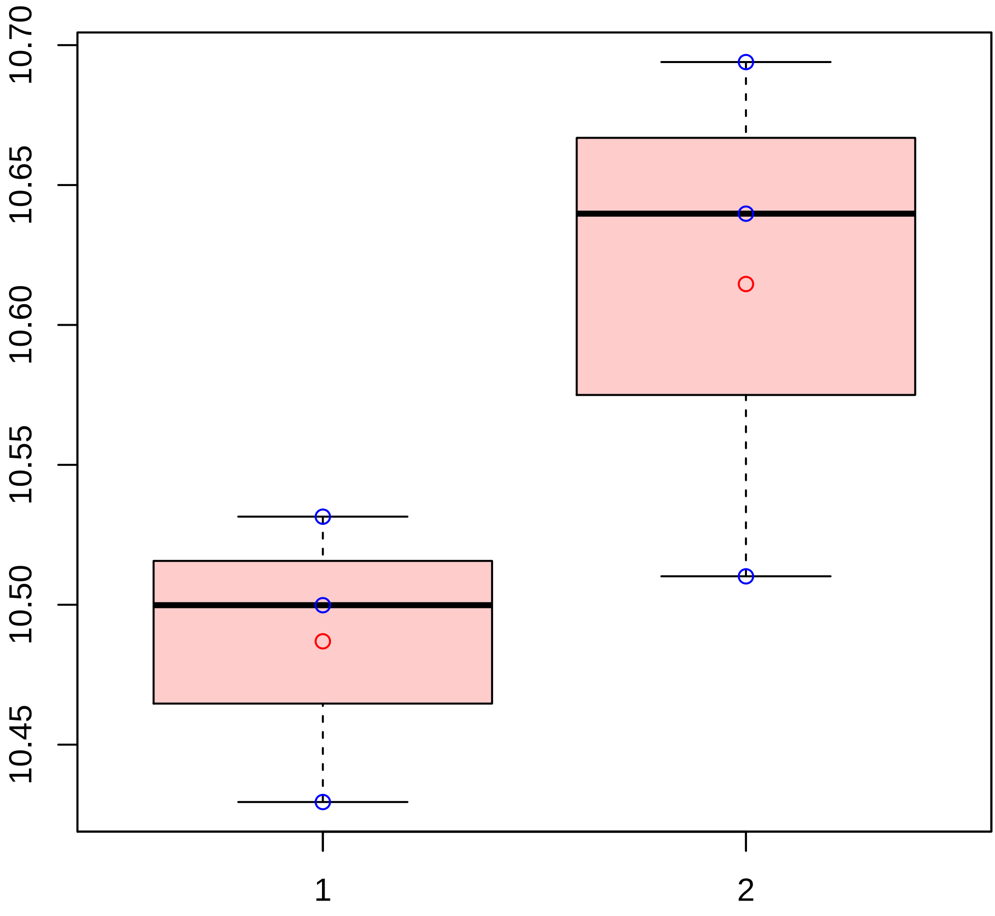


t−Test: p−value = 0.13

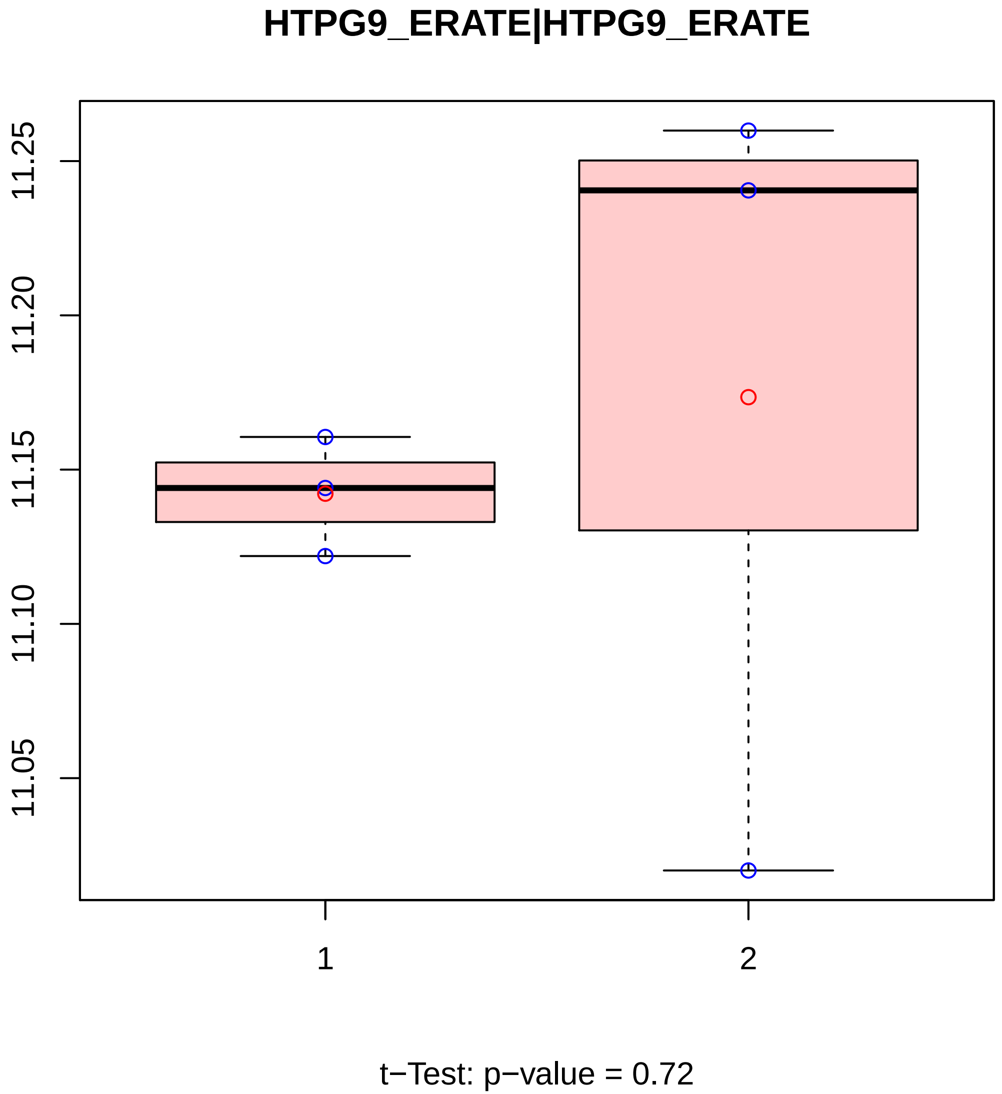

HTPG9_ERATE|HTPG9_ERATE
11.25
11.20
11.15
11.10
11.05
1
2
t−Test: p−value = 0.72

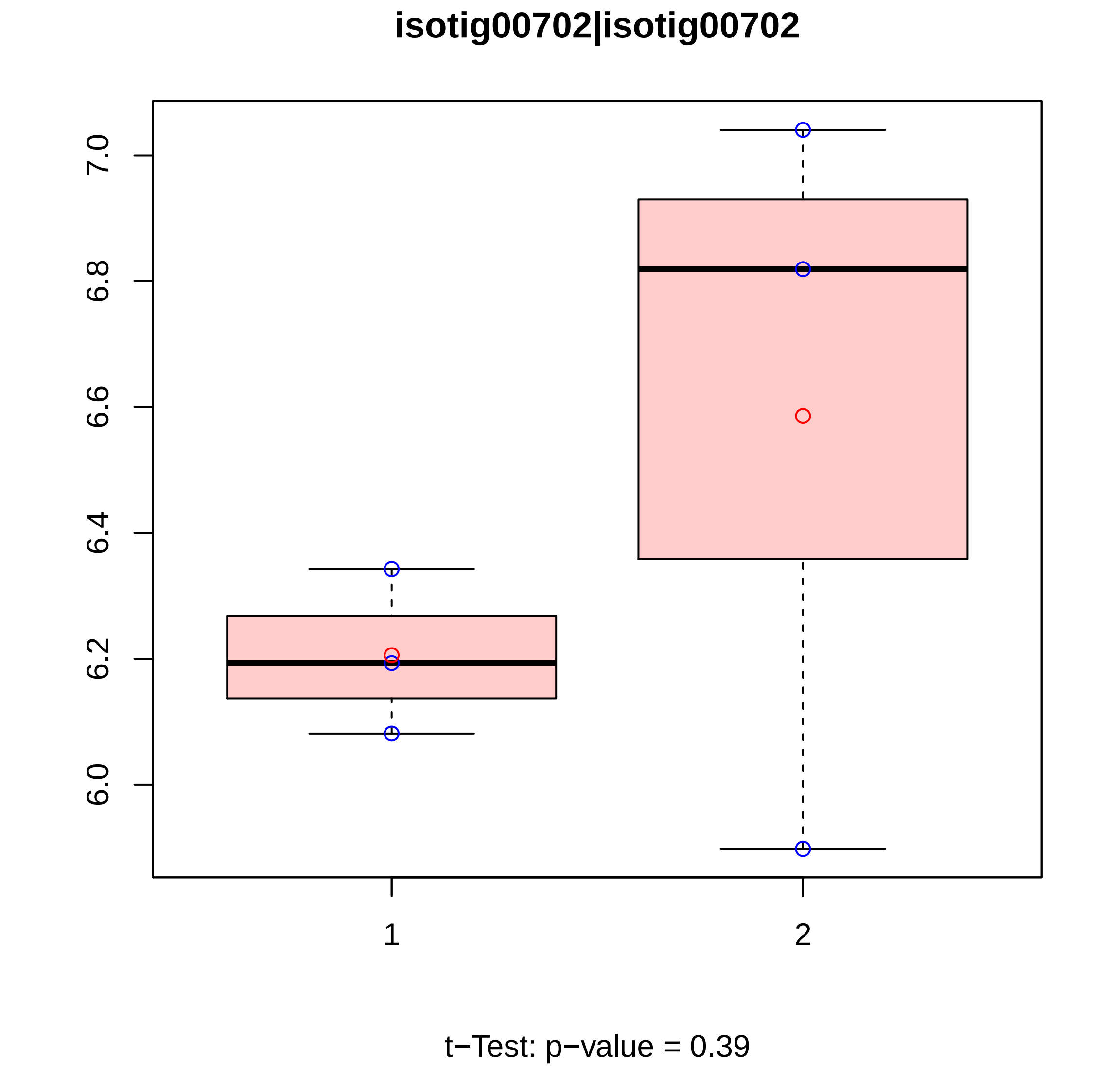
isotig00702|isotig00702
7.0
6.8
6.6
6.4
6.2
6.0
1
2
t−Test: p−value = 0.39

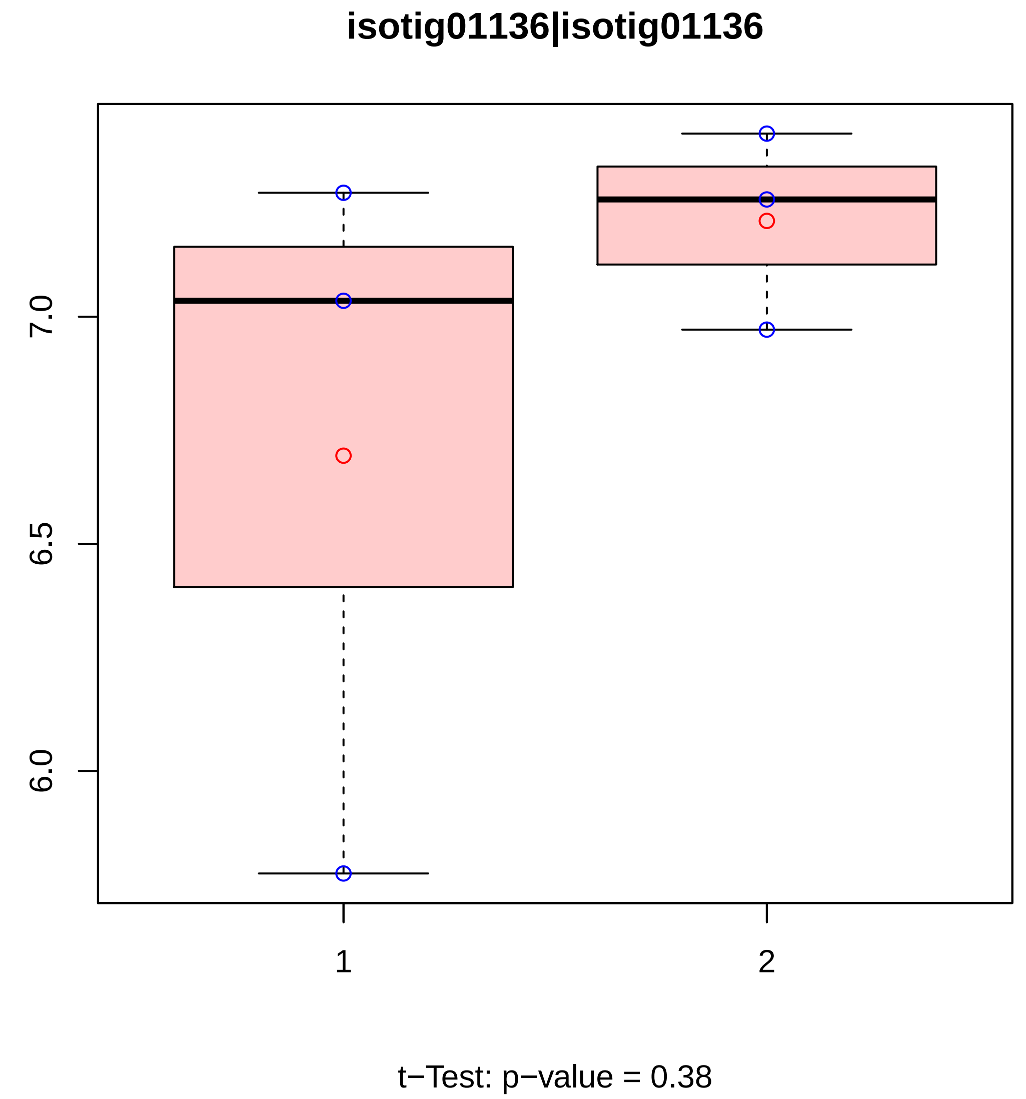
isotig01136|isotig01136
7.0
6.5
6.0
1
2
t−Test: p−value = 0.38

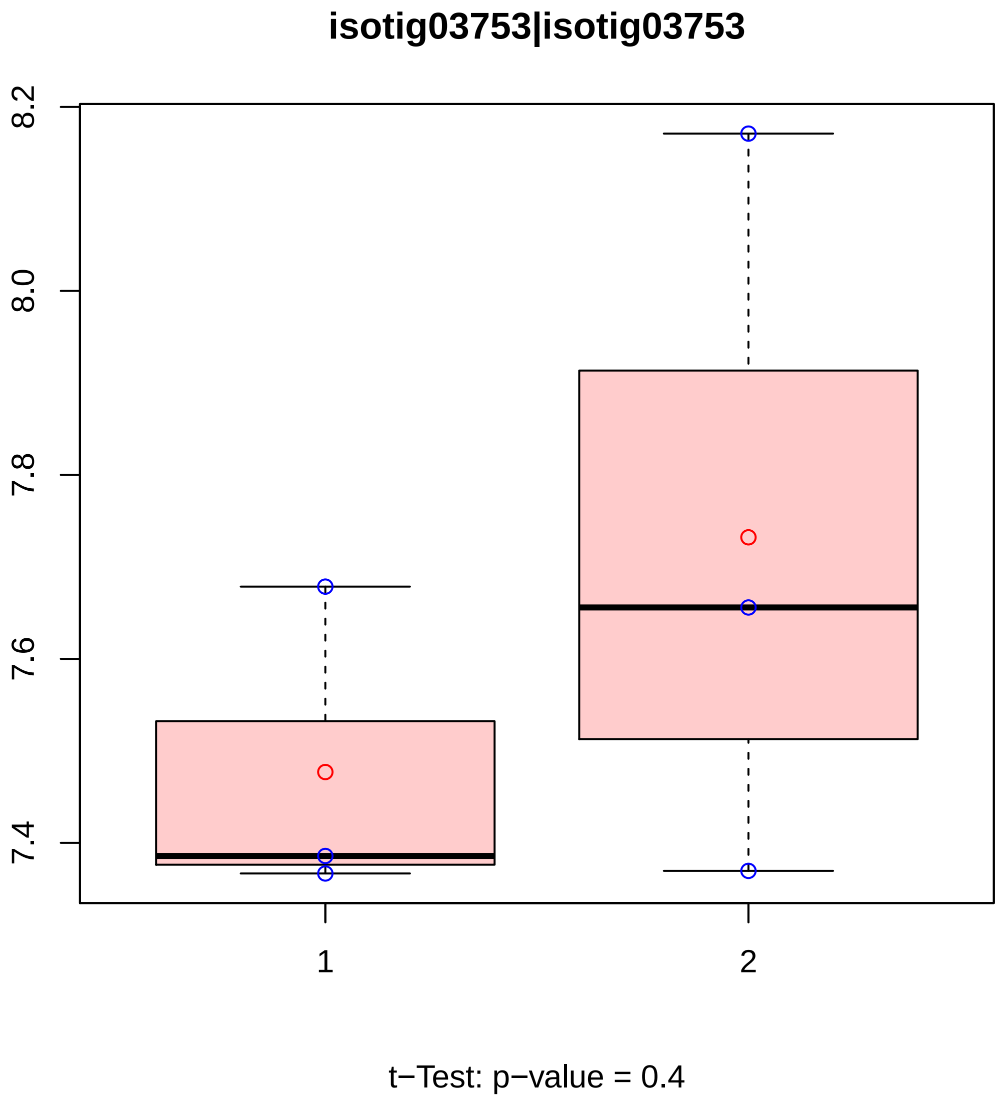
isotig03753|isotig03753
8.2
8.0
7.8
7.6
7.4
1
2
t−Test: p−value = 0.4

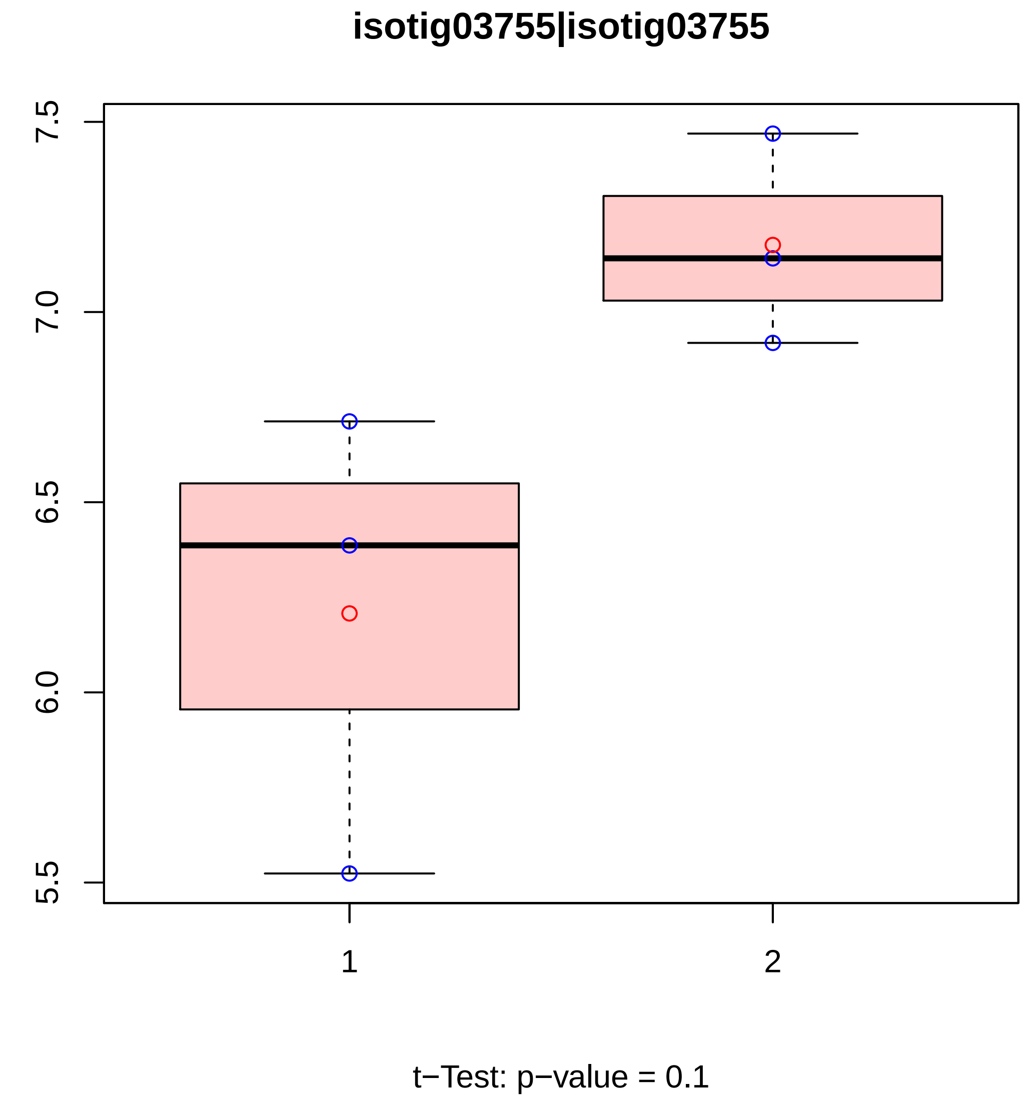
isotig03755|isotig03755
7.5
7.0
6.5
6.0
5.5
1
2
t−Test: p−value = 0.1

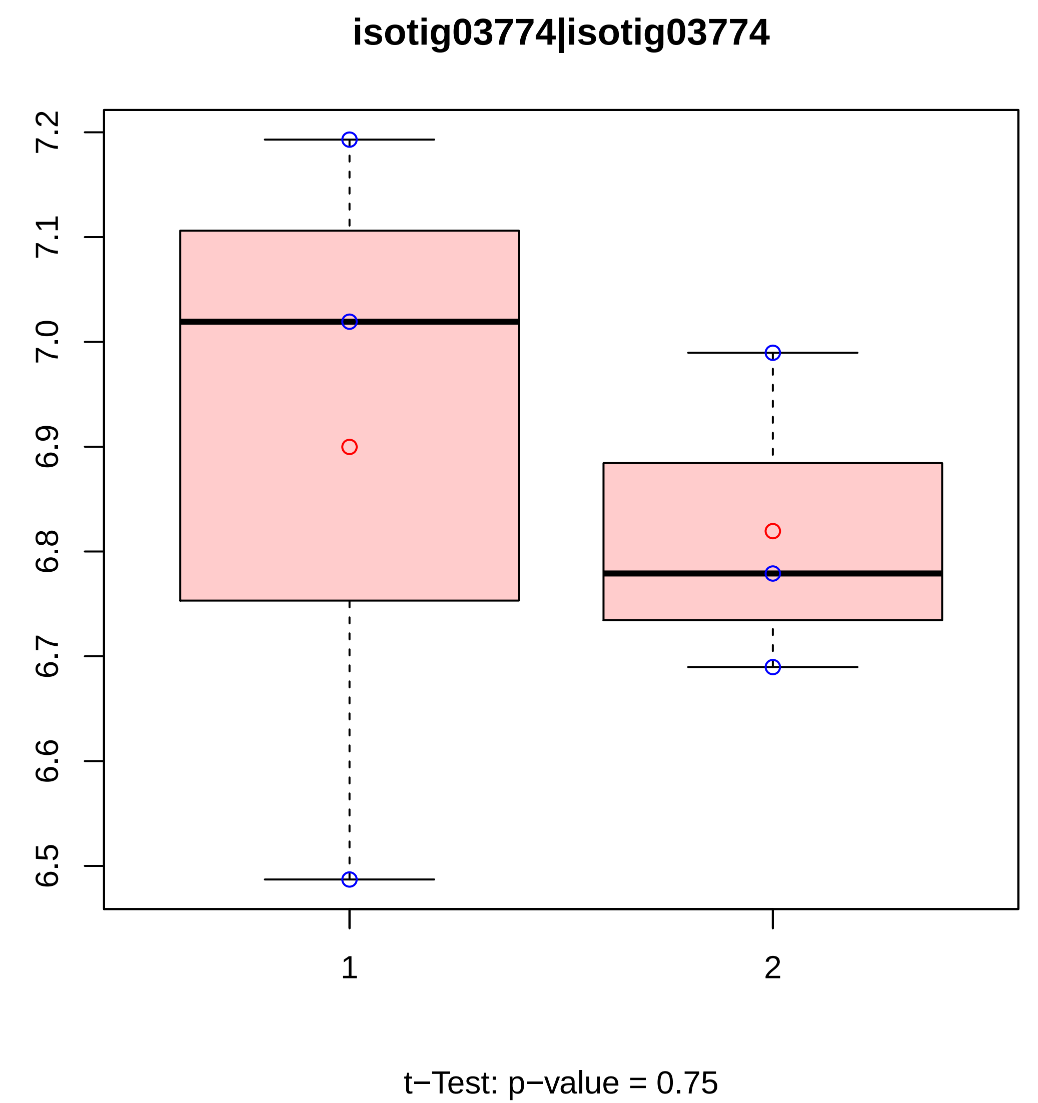
isotig03774|isotig03774
7.2
7.1
7.0
6.9
6.8
6.7
6.6
6.5
1
2
t−Test: p−value = 0.75

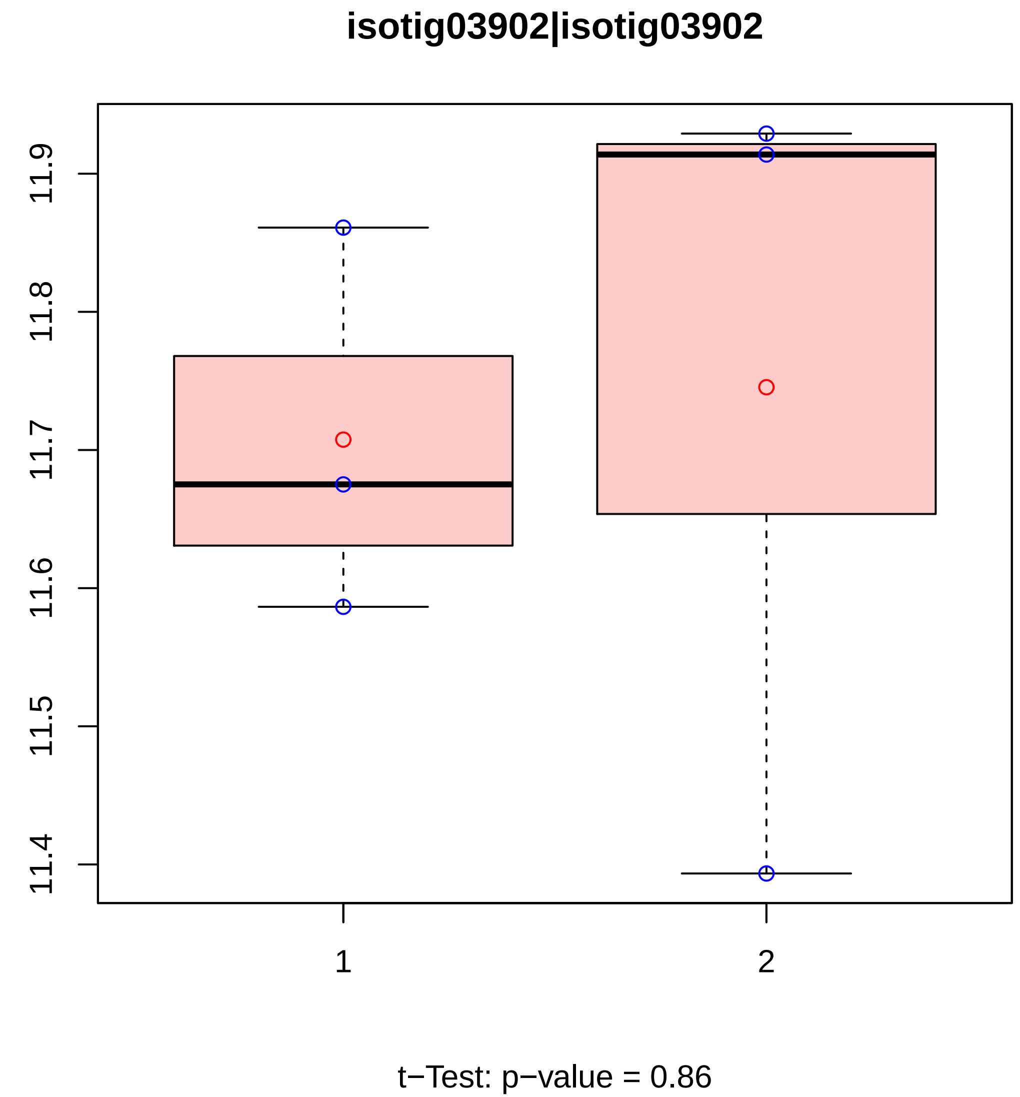
isotig03902|isotig03902
11.4
11.5
11.6
11.7
11.8
11.9
1
2
t−Test: p−value = 0.86

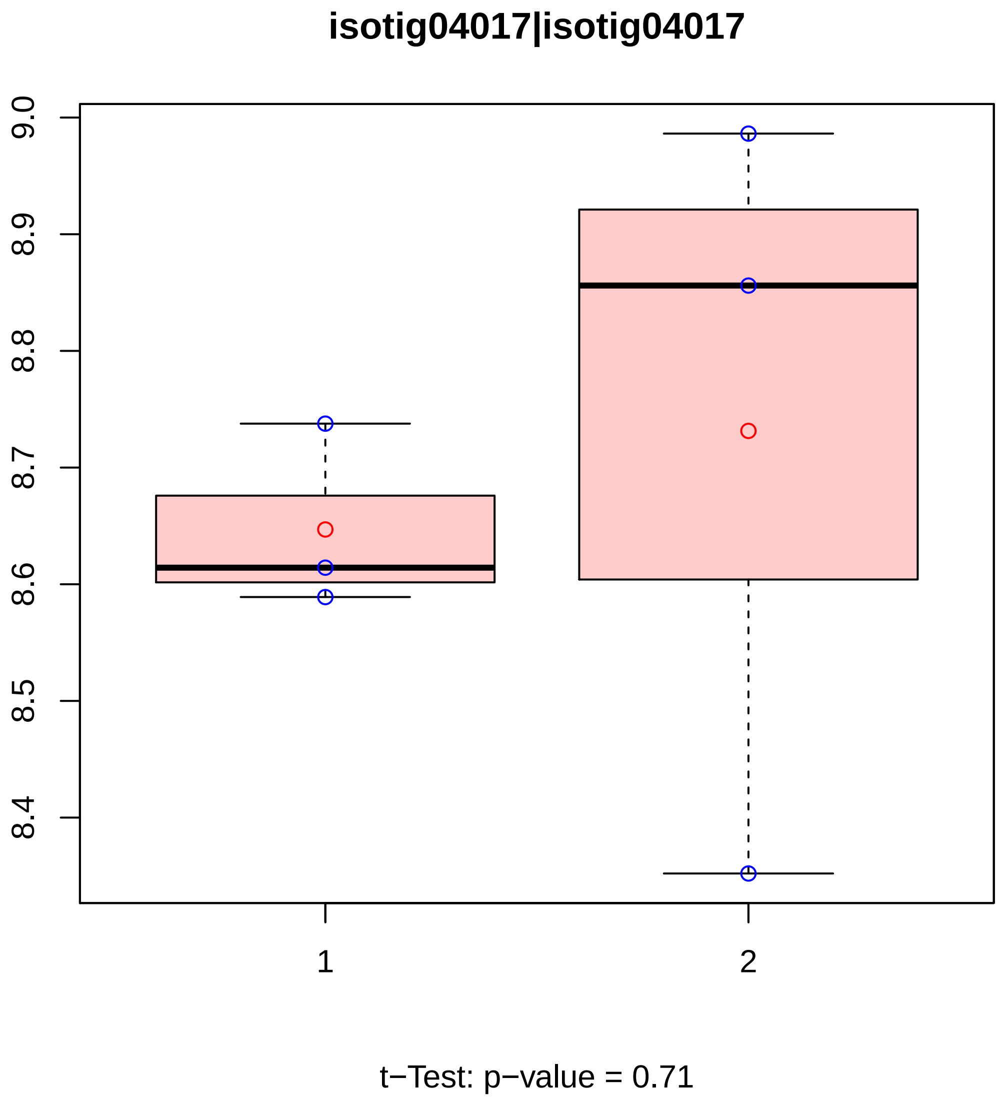
isotig04017|isotig04017
9.0
8.9
8.8
8.7
8.6
8.5
8.4
1
2
t−Test: p−value = 0.71

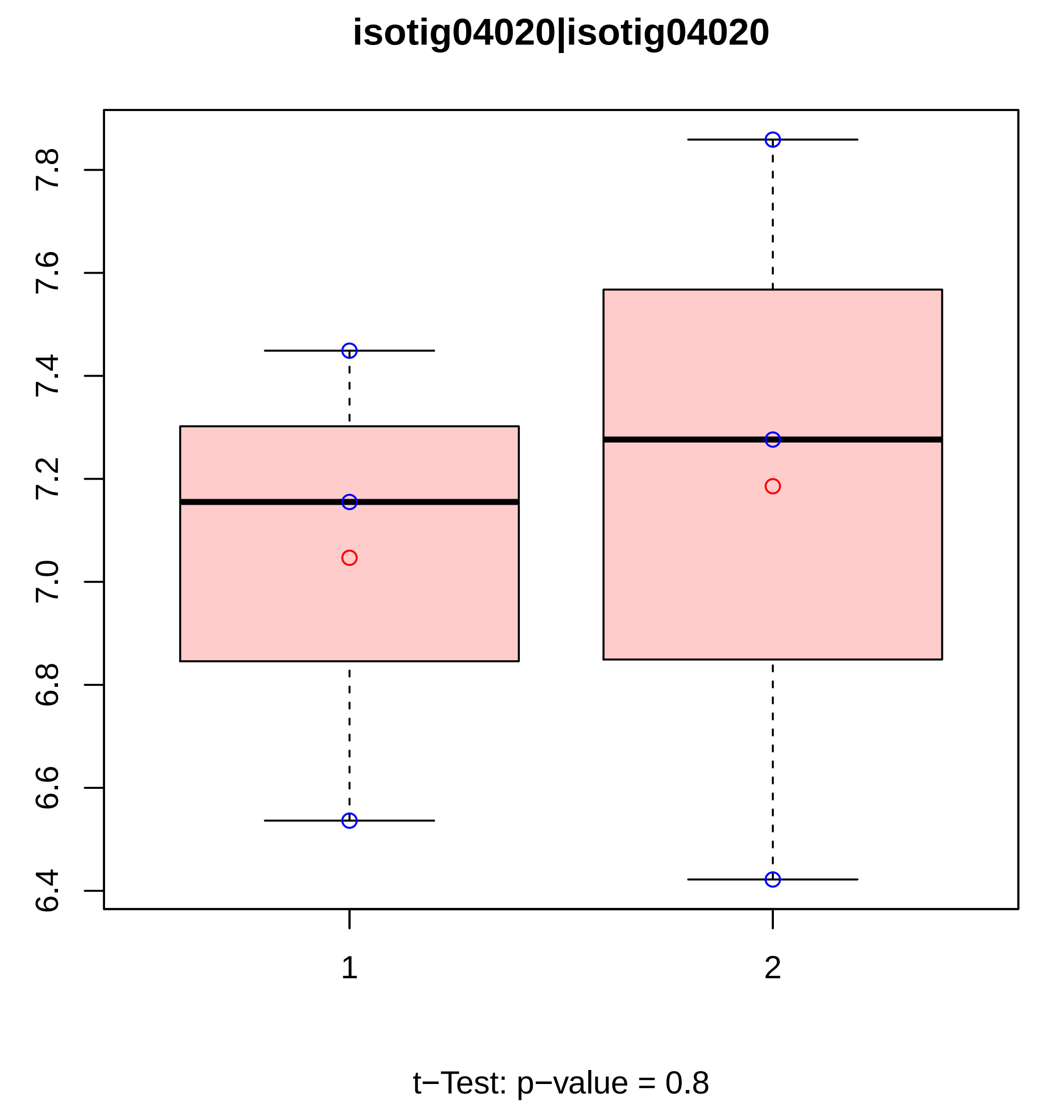

isotig04020|isotig04020
7.8
7.6
7.4
7.2
7.0
6.8
6.6
6.4
1
2
t−Test: p−value = 0.8

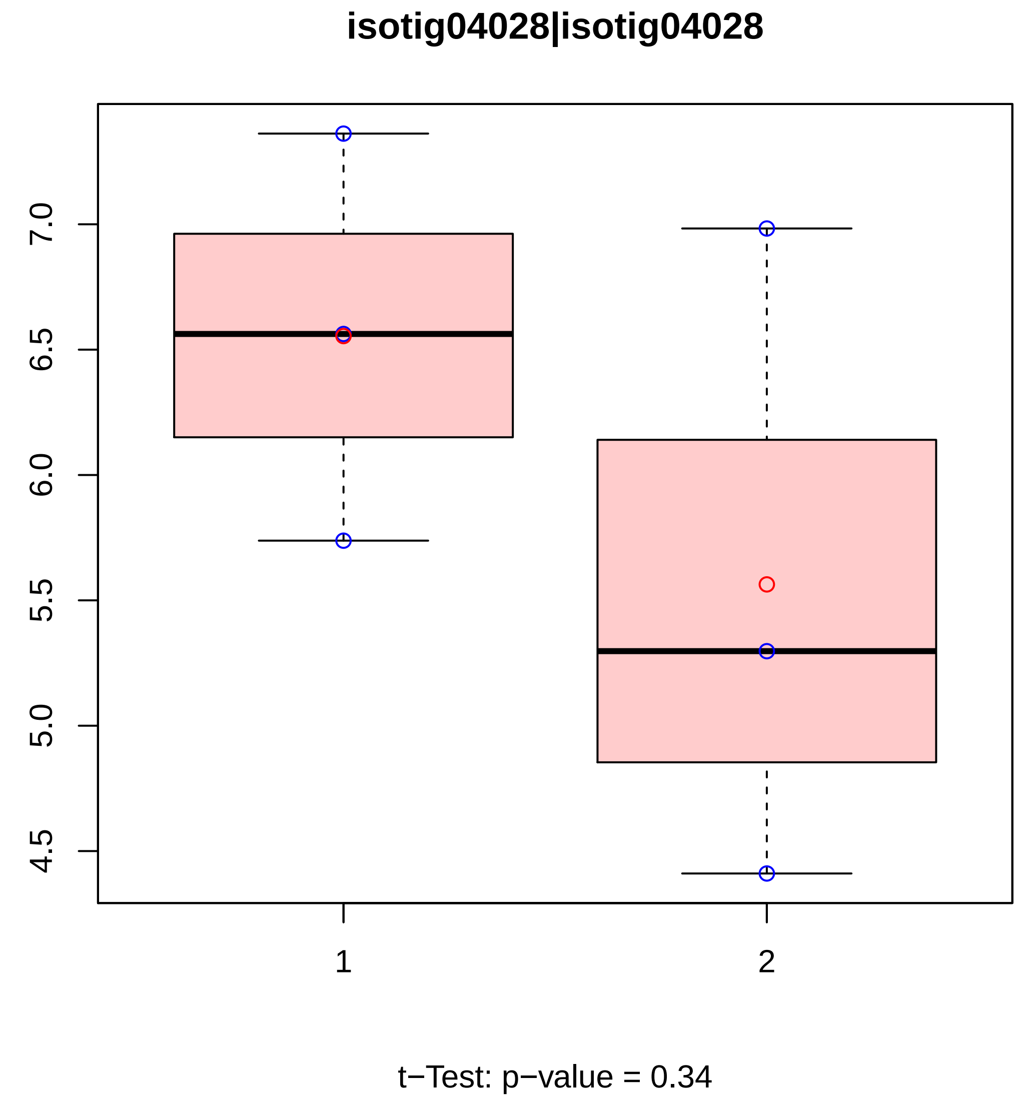
isotig04028|isotig04028
7.0
6.5
6.0
5.5
5.0
4.5
1
2
t−Test: p−value = 0.34

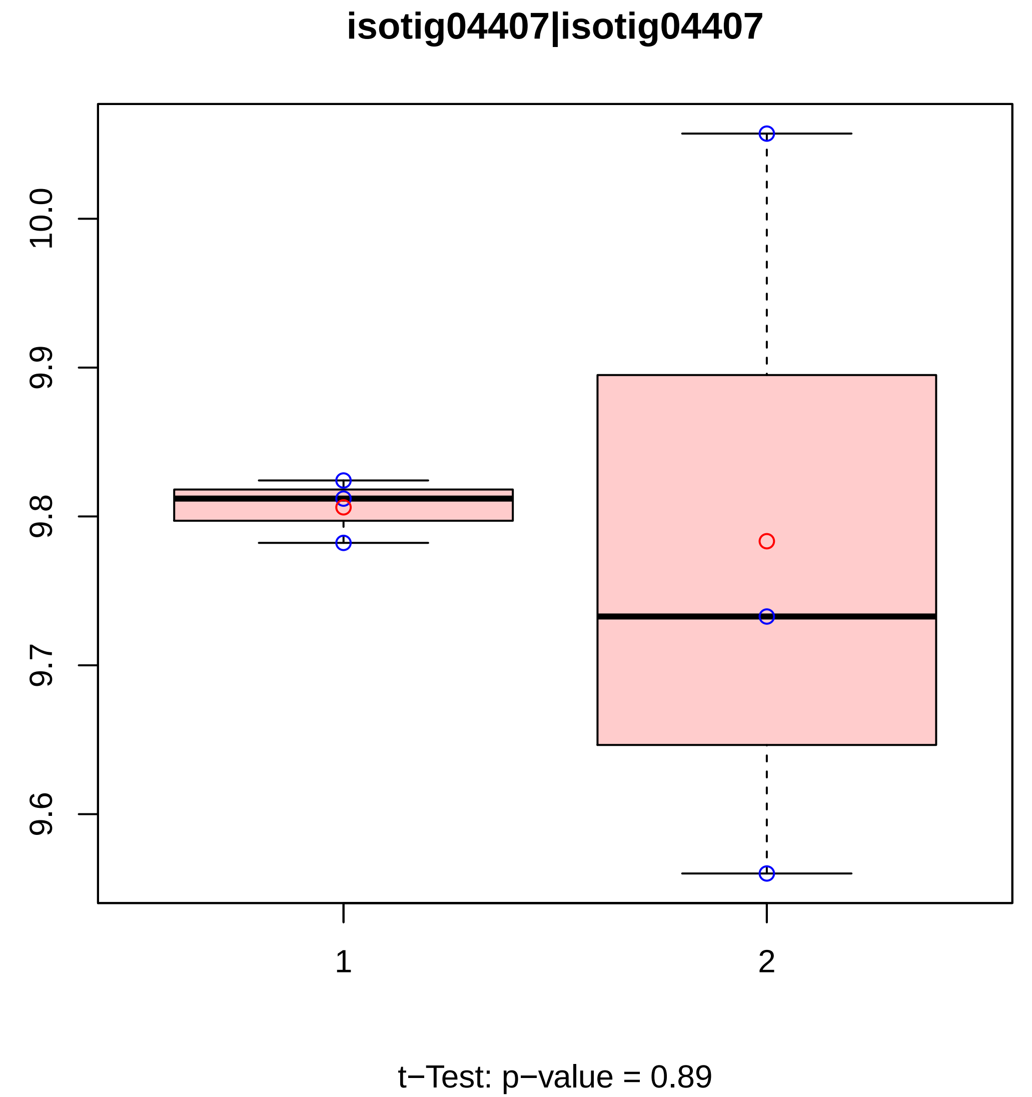
isotig04407|isotig04407
10.0
9.9
9.8
9.7
9.6
1
2
t−Test: p−value = 0.89

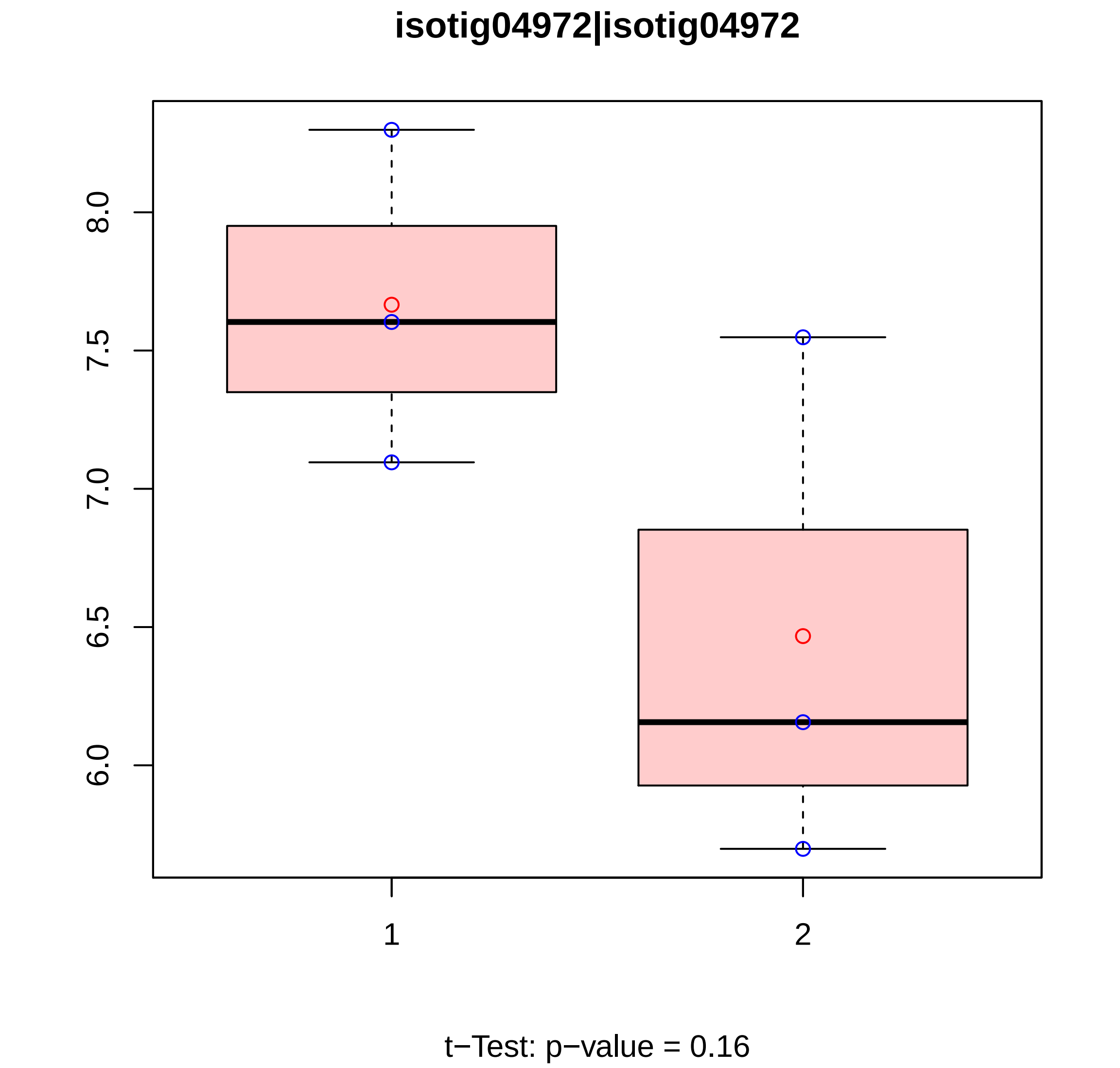
isotig04972|isotig04972
8.0
7.5
7.0
6.5
6.0
1
2
t−Test: p−value = 0.16

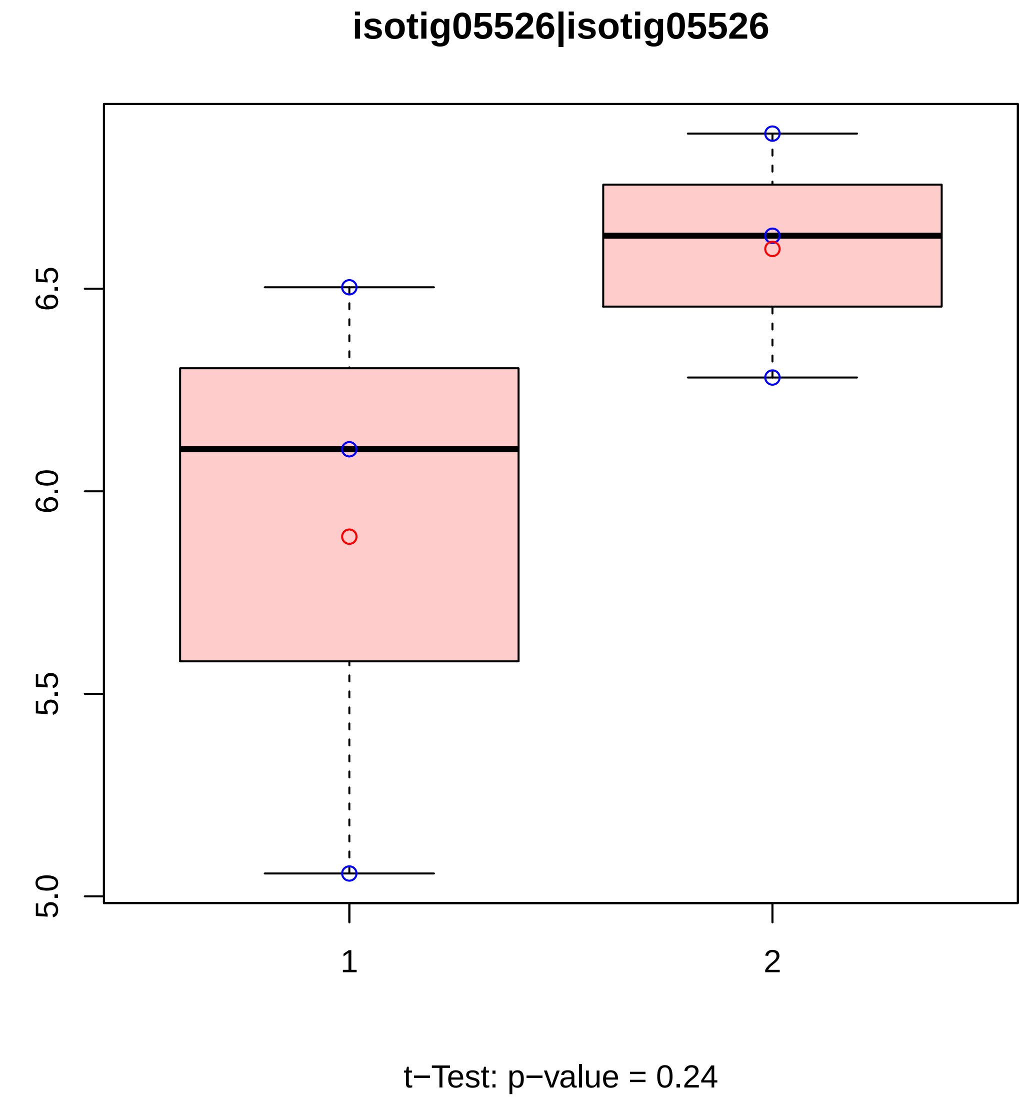

isotig05526|isotig05526
6.5
6.0
5.5
5.0
1
2
t−Test: p−value = 0.24

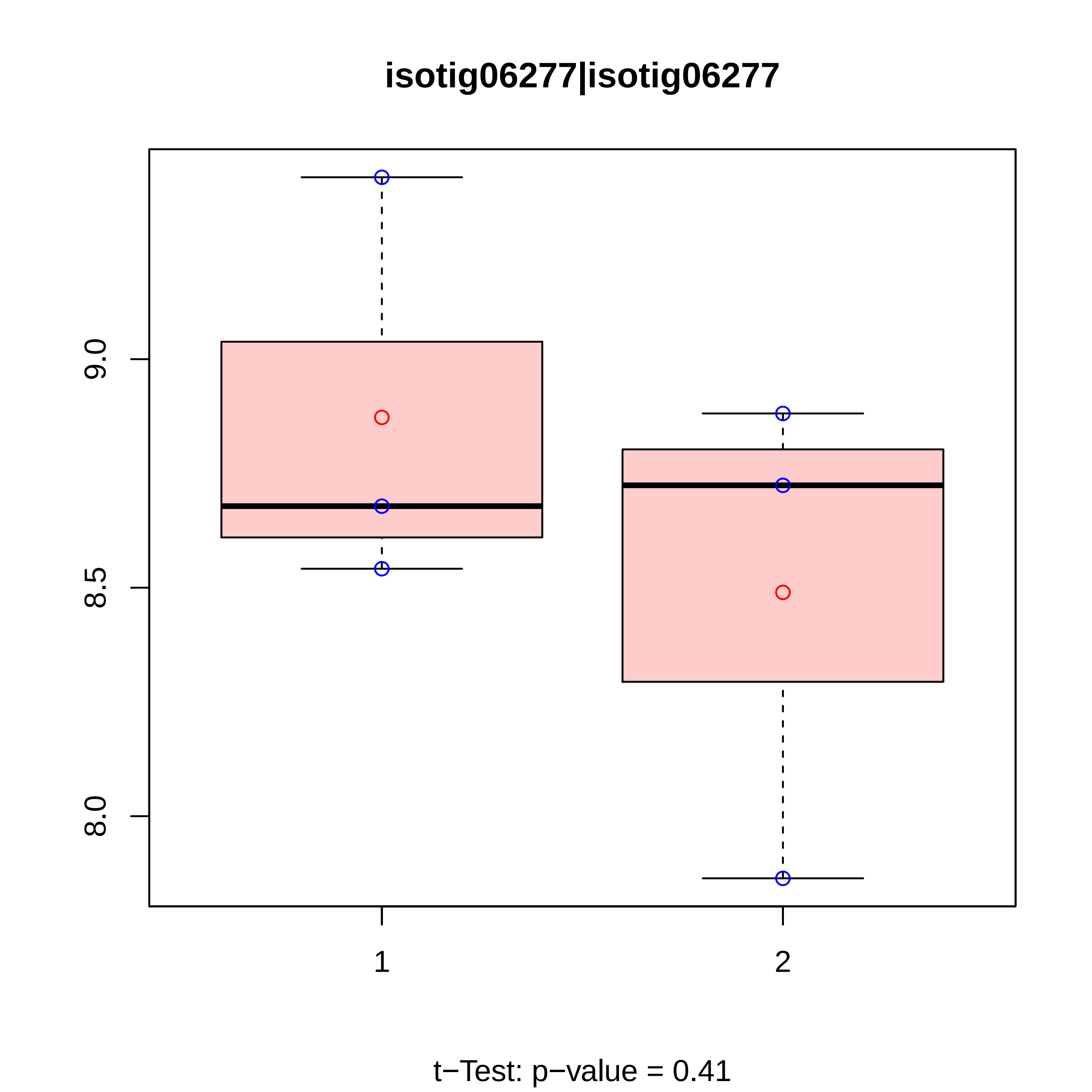
isotig06277|isotig06277
9.0
8.5
8.0
1
2
t−Test: p−value = 0.41

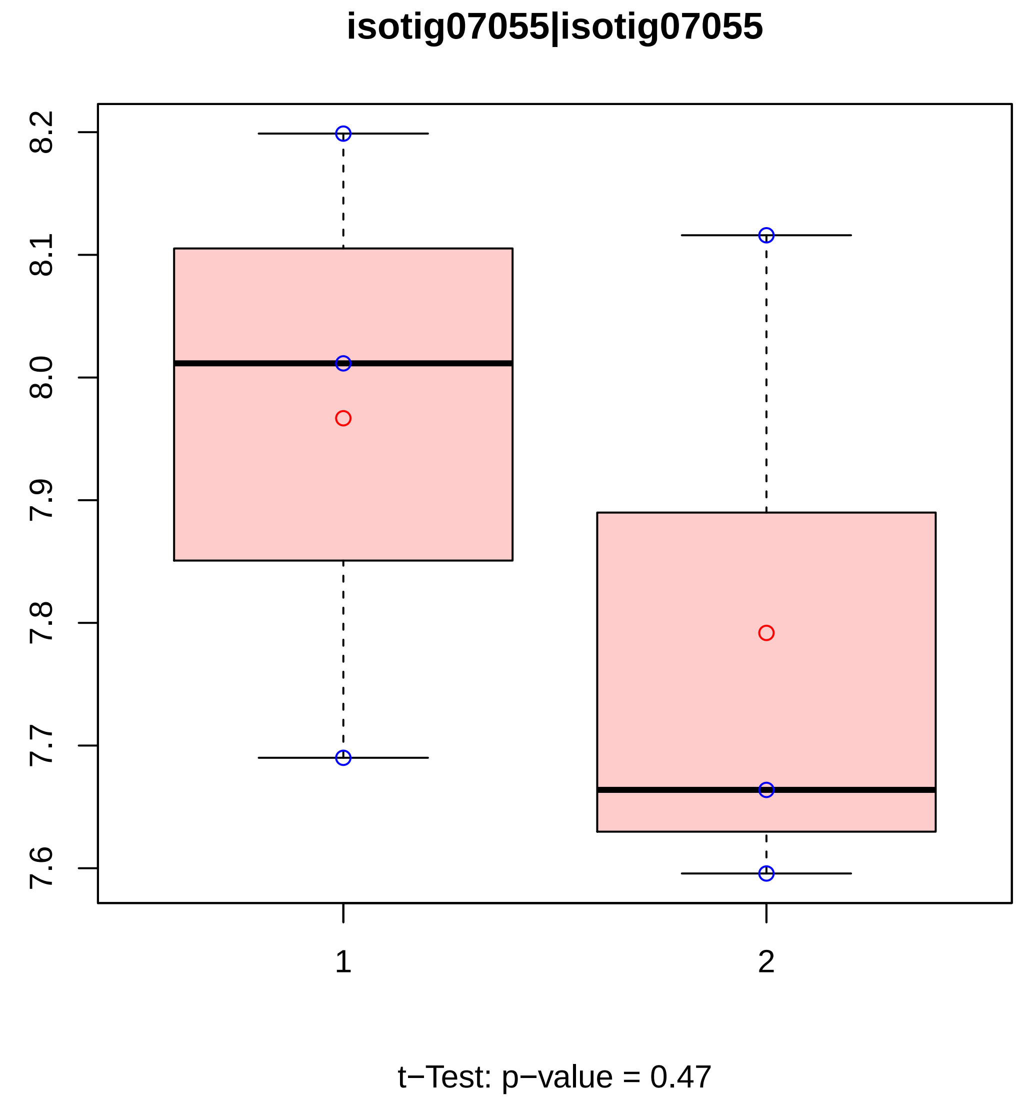
isotig07055|isotig07055
8.2
8.1
8.0
7.9
7.8
7.7
7.6
1
2
t−Test: p−value = 0.47

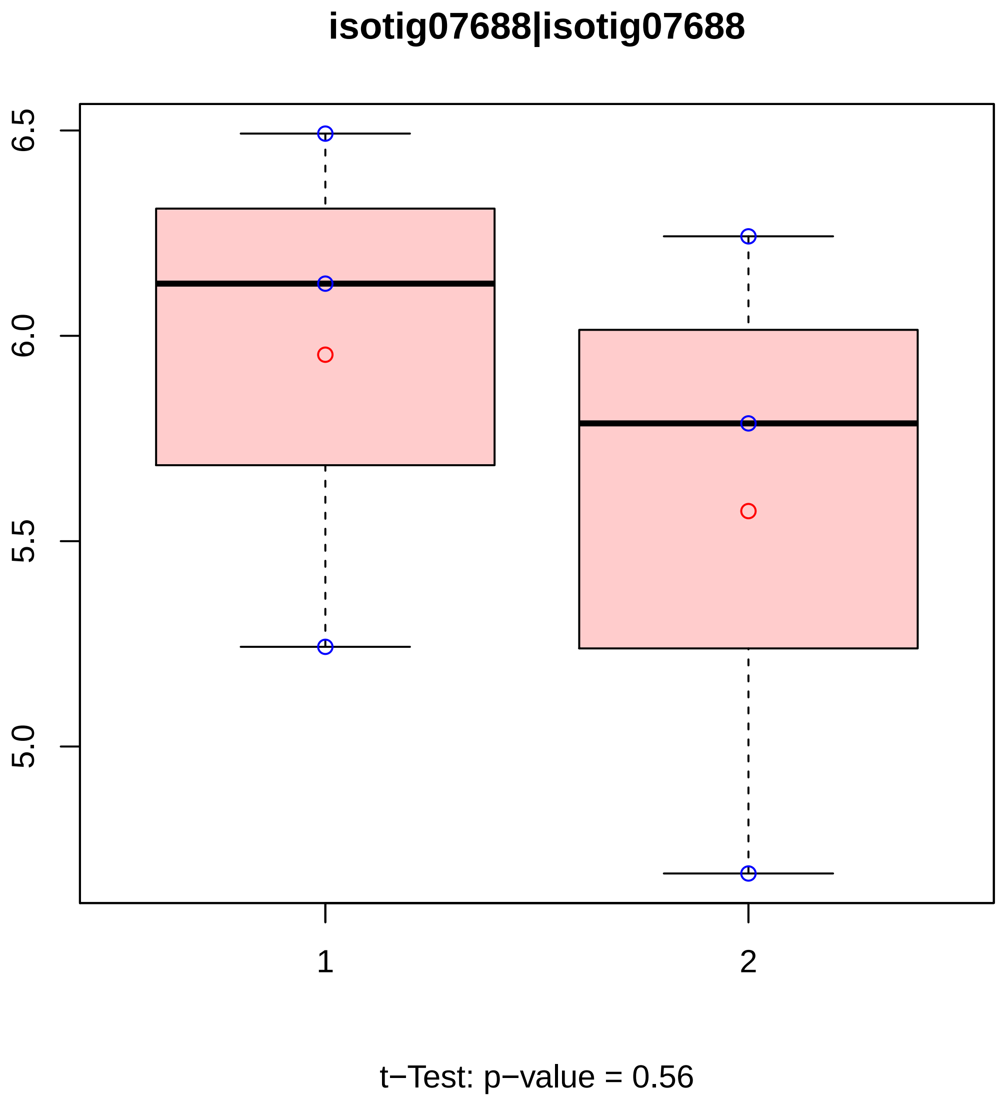
isotig07688|isotig07688
6.5
6.0
5.5
5.0
1
2
t−Test: p−value = 0.56

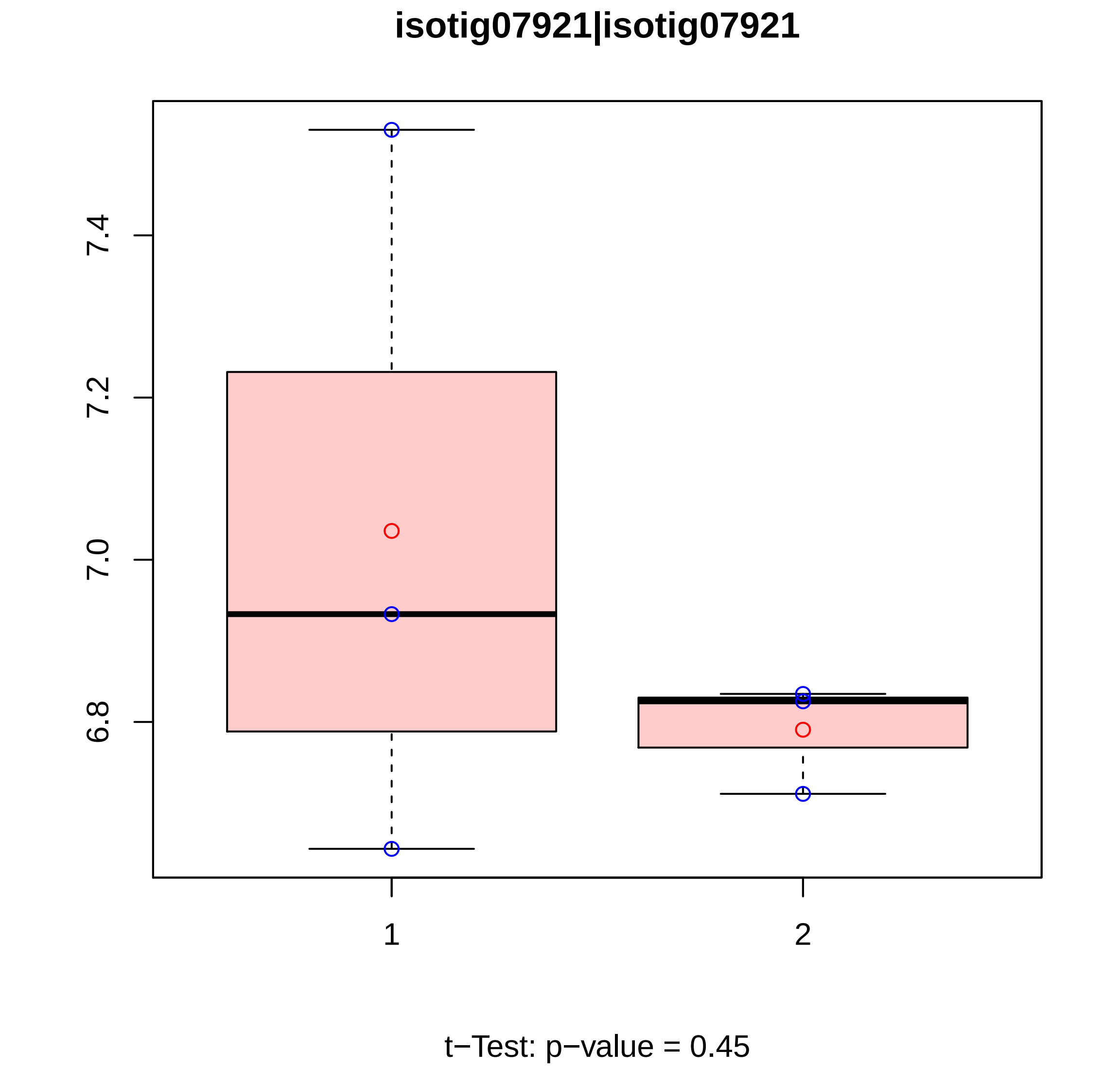
isotig07921|isotig07921
7.4
7.2
7.0
6.8
1
2
t−Test: p−value = 0.45

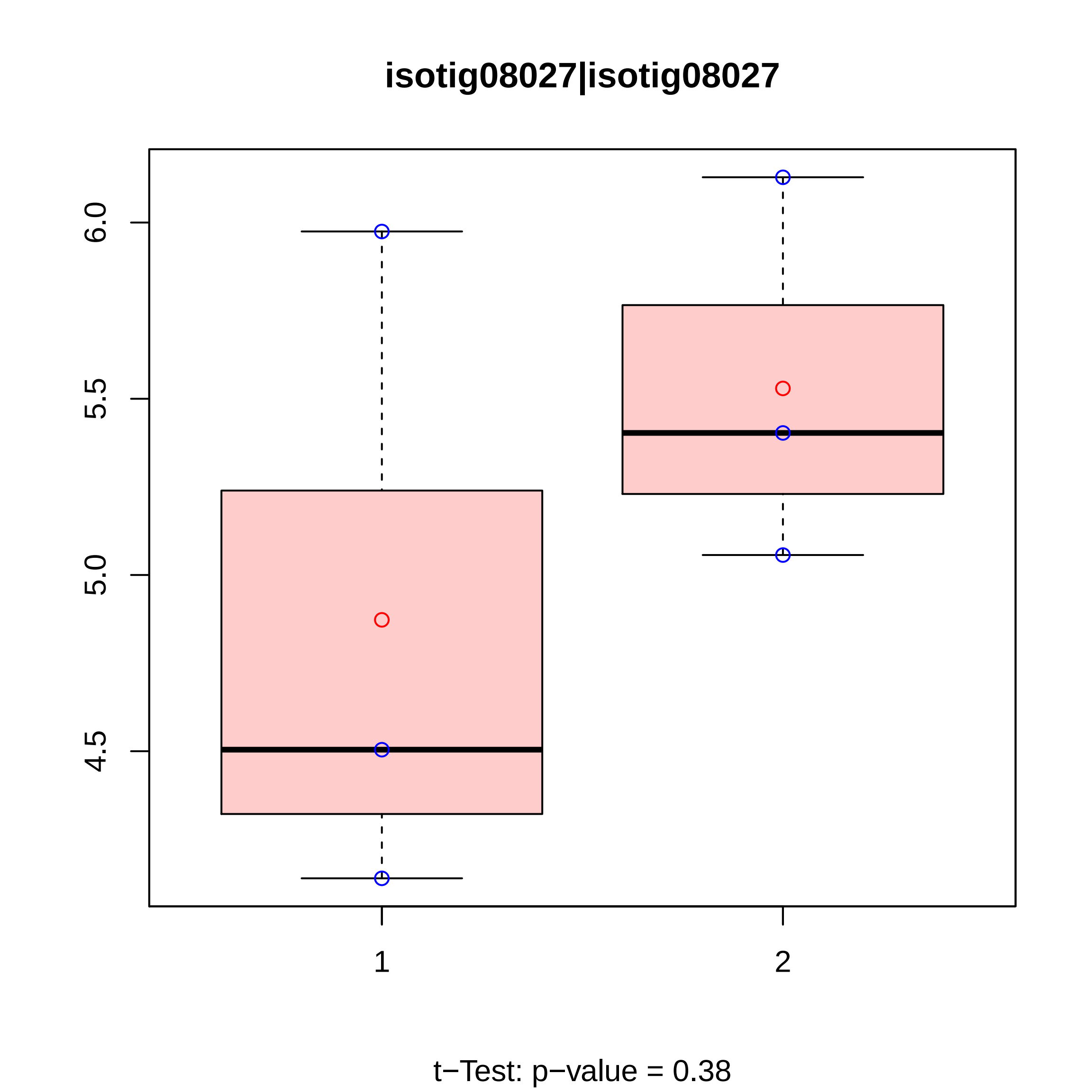
isotig08027|isotig08027
6.0
5.5
5.0
4.5
1
2
t−Test: p−value = 0.38

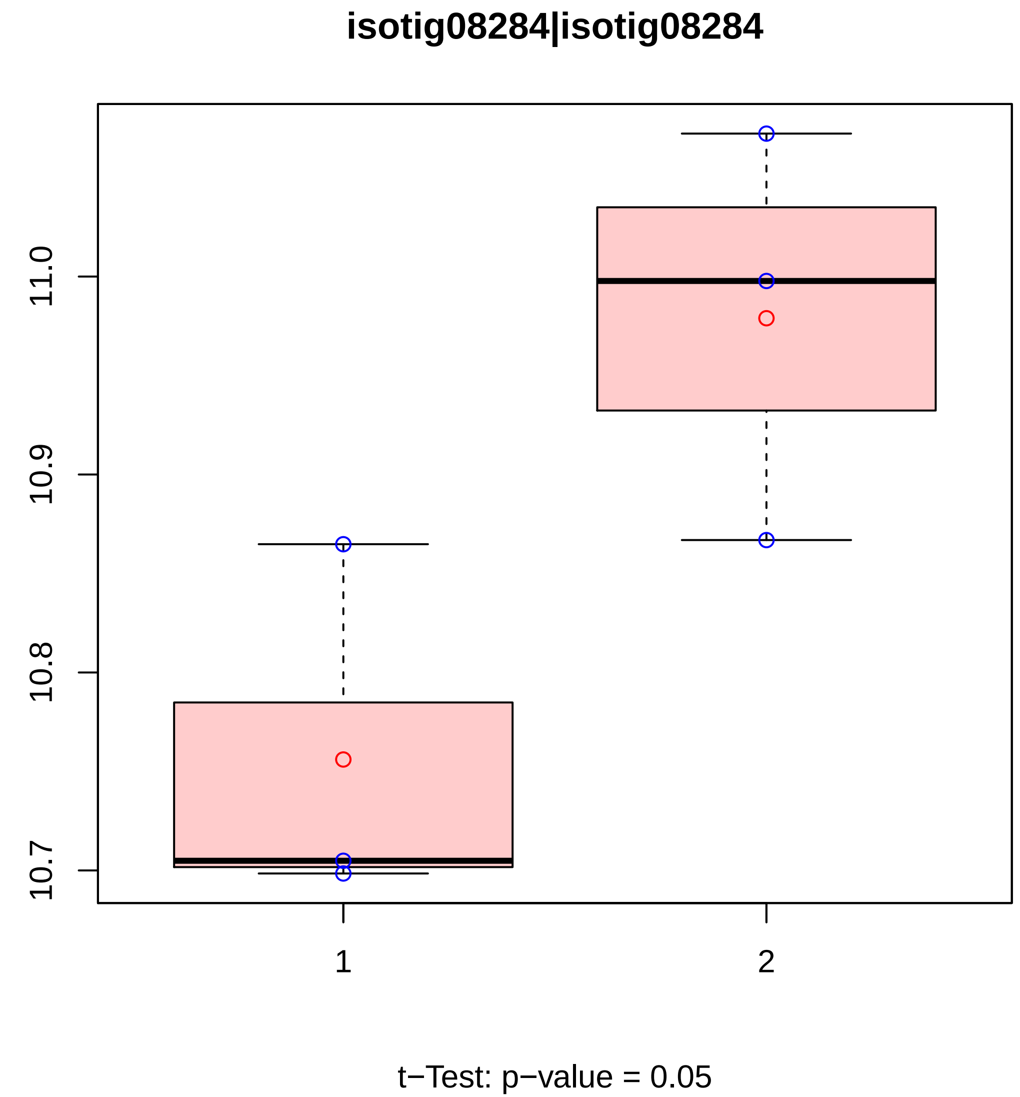
isotig08284|isotig08284
11.0
10.9
10.8
10.7
1
2
t−Test: p−value = 0.05

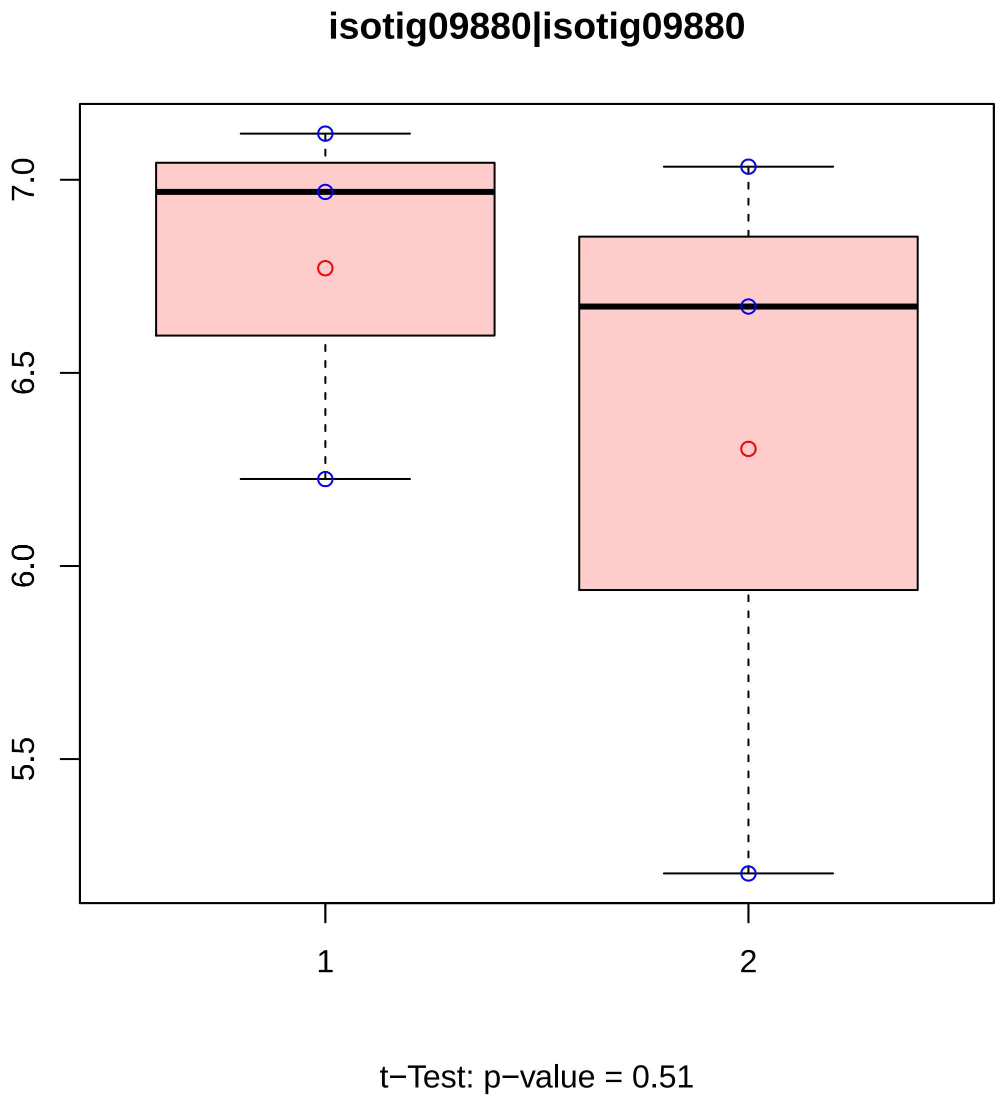
isotig09880|isotig09880
7.0
6.5
6.0
5.5
1
2
t−Test: p−value = 0.51

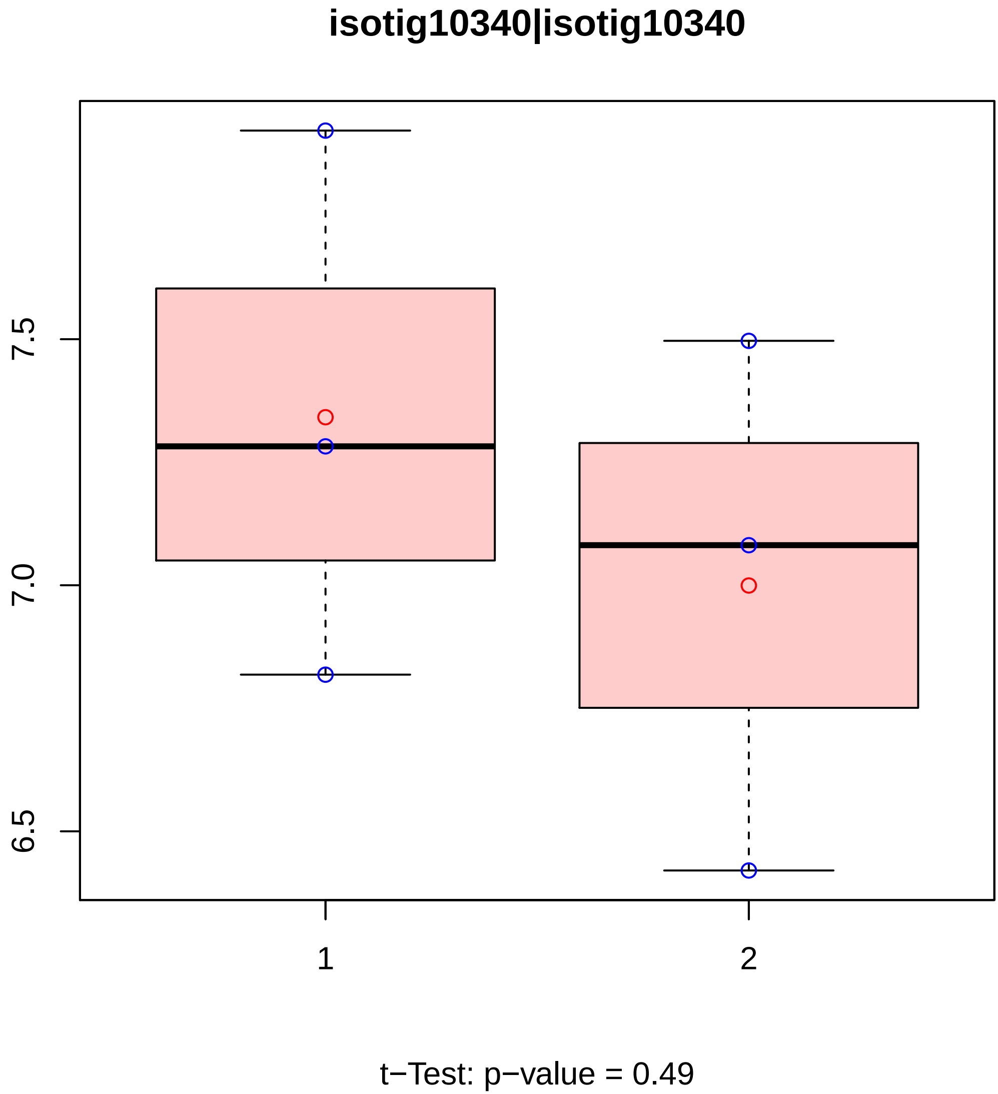
isotig10340|isotig10340
7.5
7.0
6.5
1
2
t−Test: p−value = 0.49

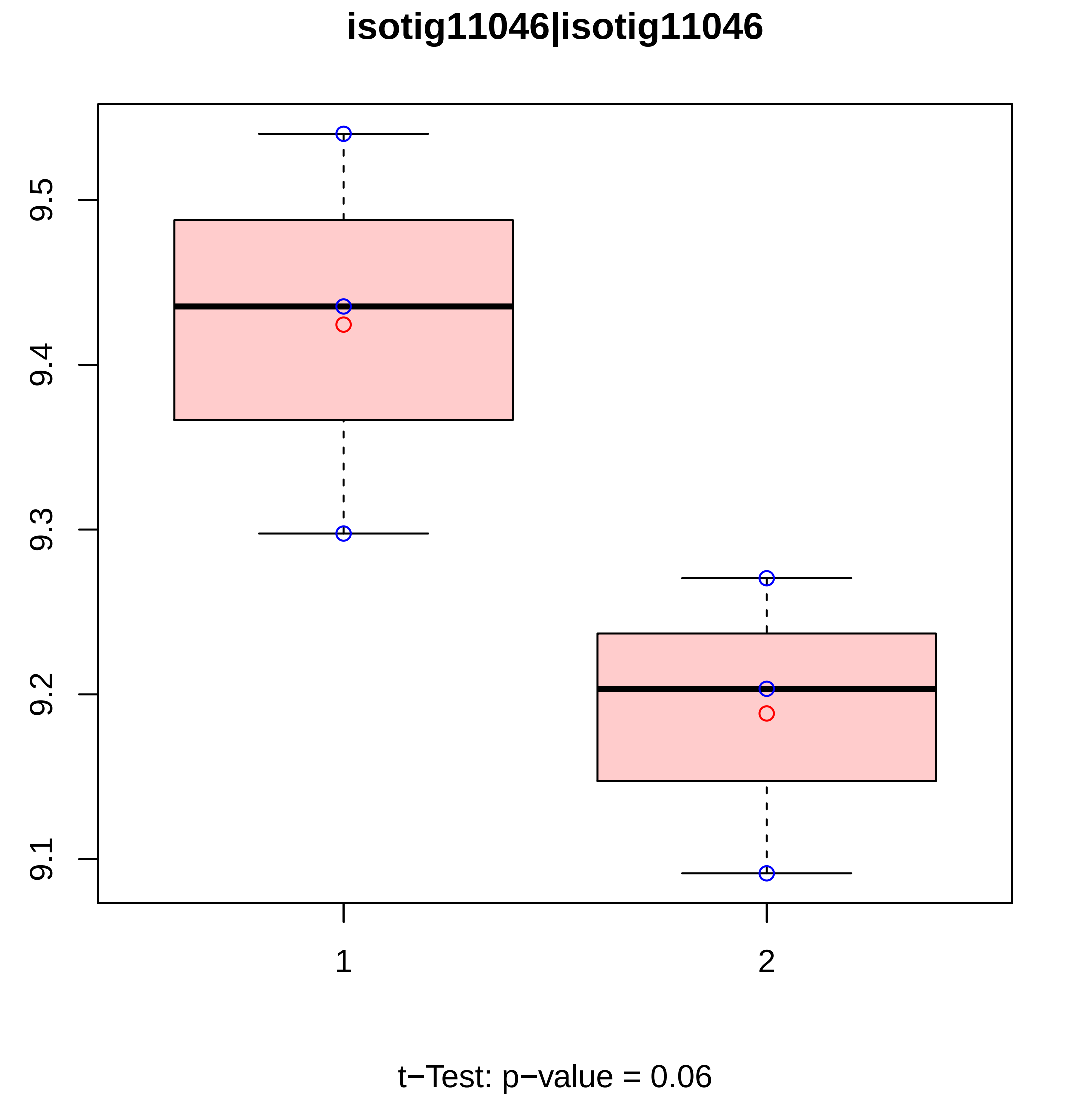

isotig11046|isotig11046
9.5
9.4
9.3
9.2
9.1
1
2
t−Test: p−value = 0.06

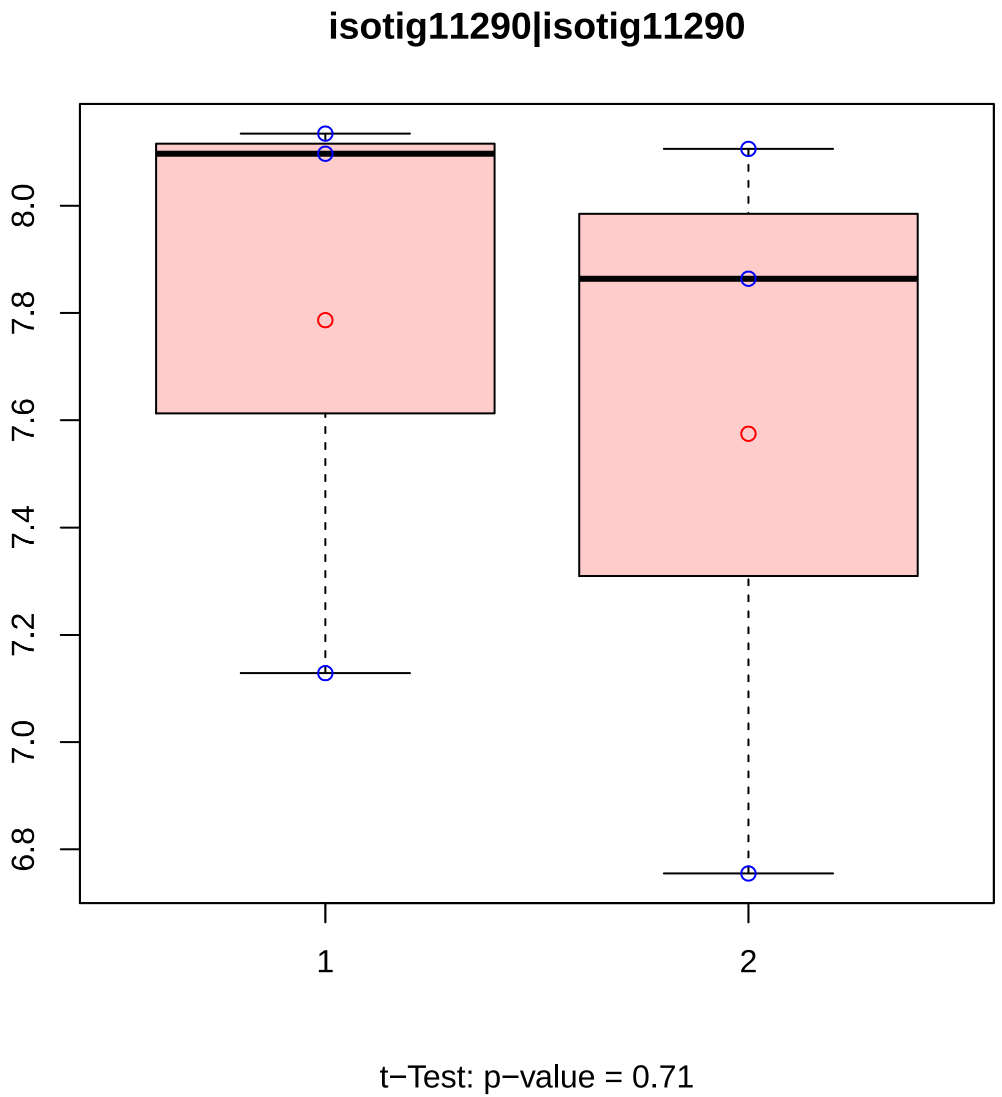
isotig11290|isotig11290
8.0
7.8
7.6
7.4
7.2
7.0
6.8
1
2
t−Test: p−value = 0.71

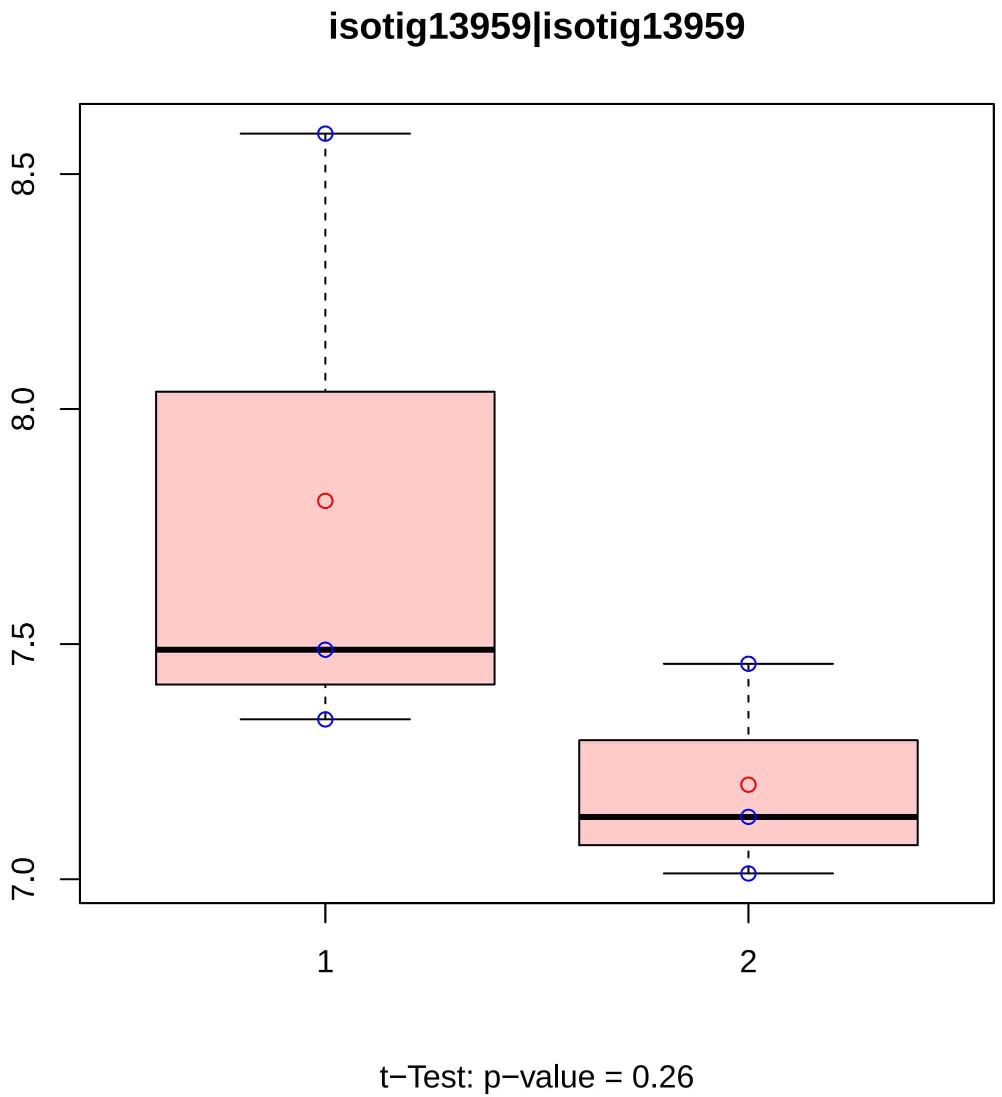
isotig13959|isotig13959
8.5
8.0
7.5
7.0
1
2
t−Test: p−value = 0.26

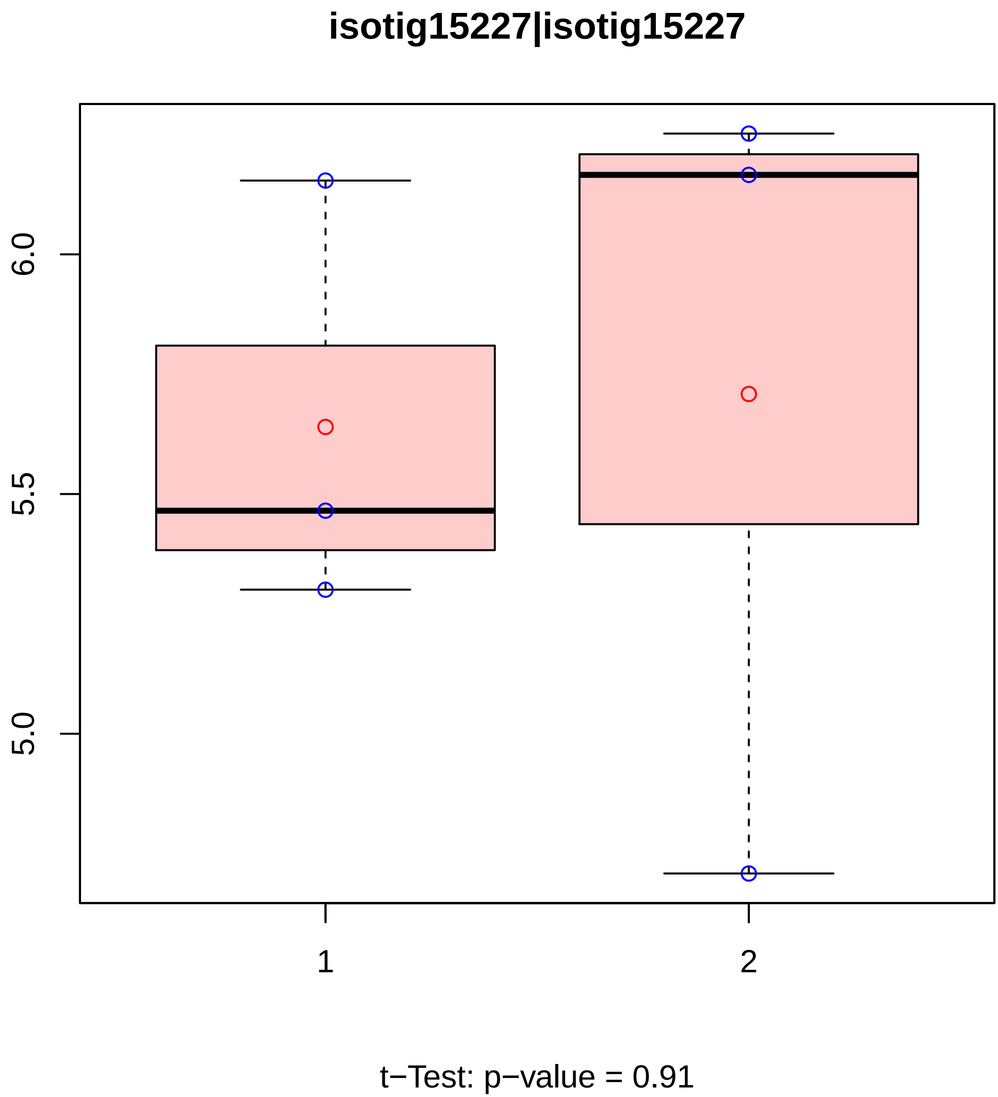

isotig15227|isotig15227
6.0
5.5
5.0
1
2
t−Test: p−value = 0.91

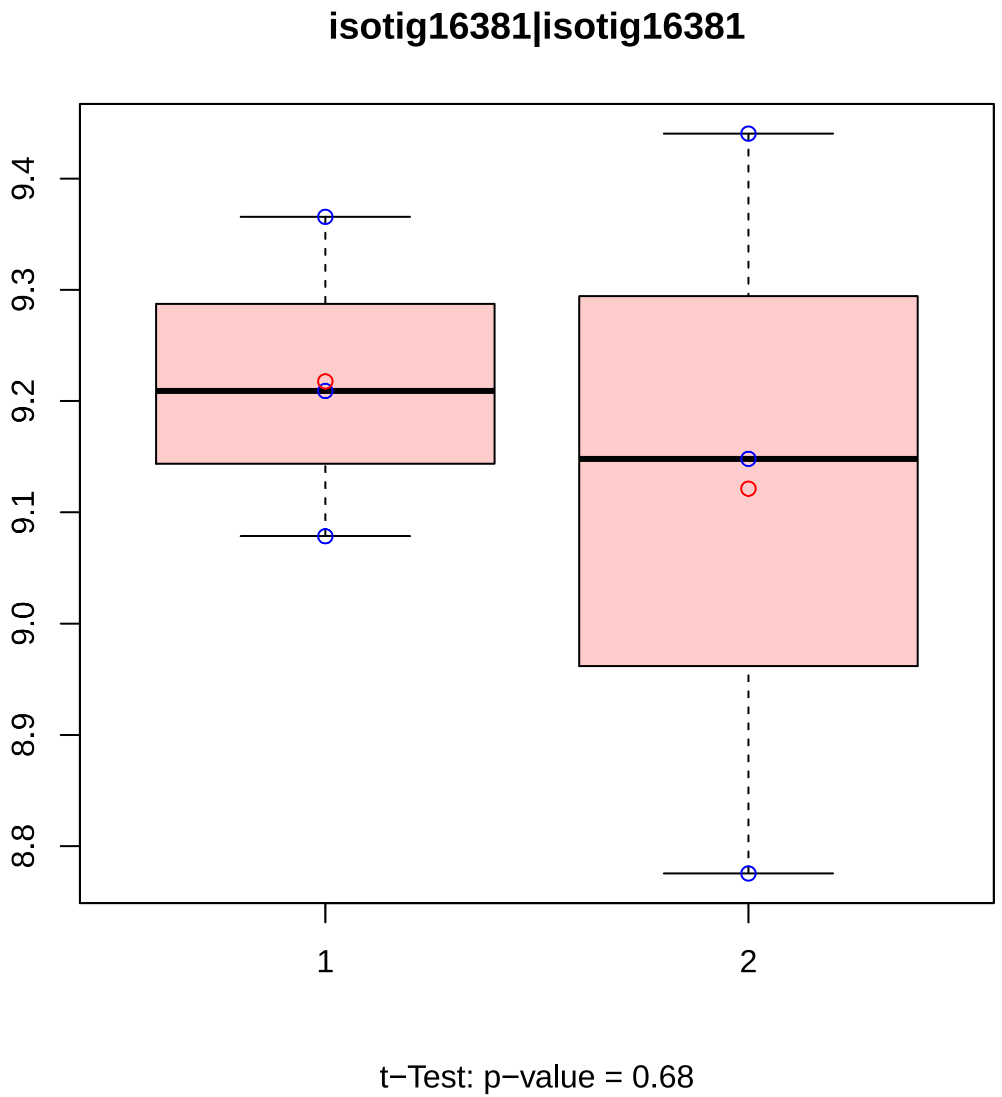
isotig16381|isotig16381
9.4
9.3
9.2
9.1
9.0
8.9
8.8
1
2
t−Test: p−value = 0.68

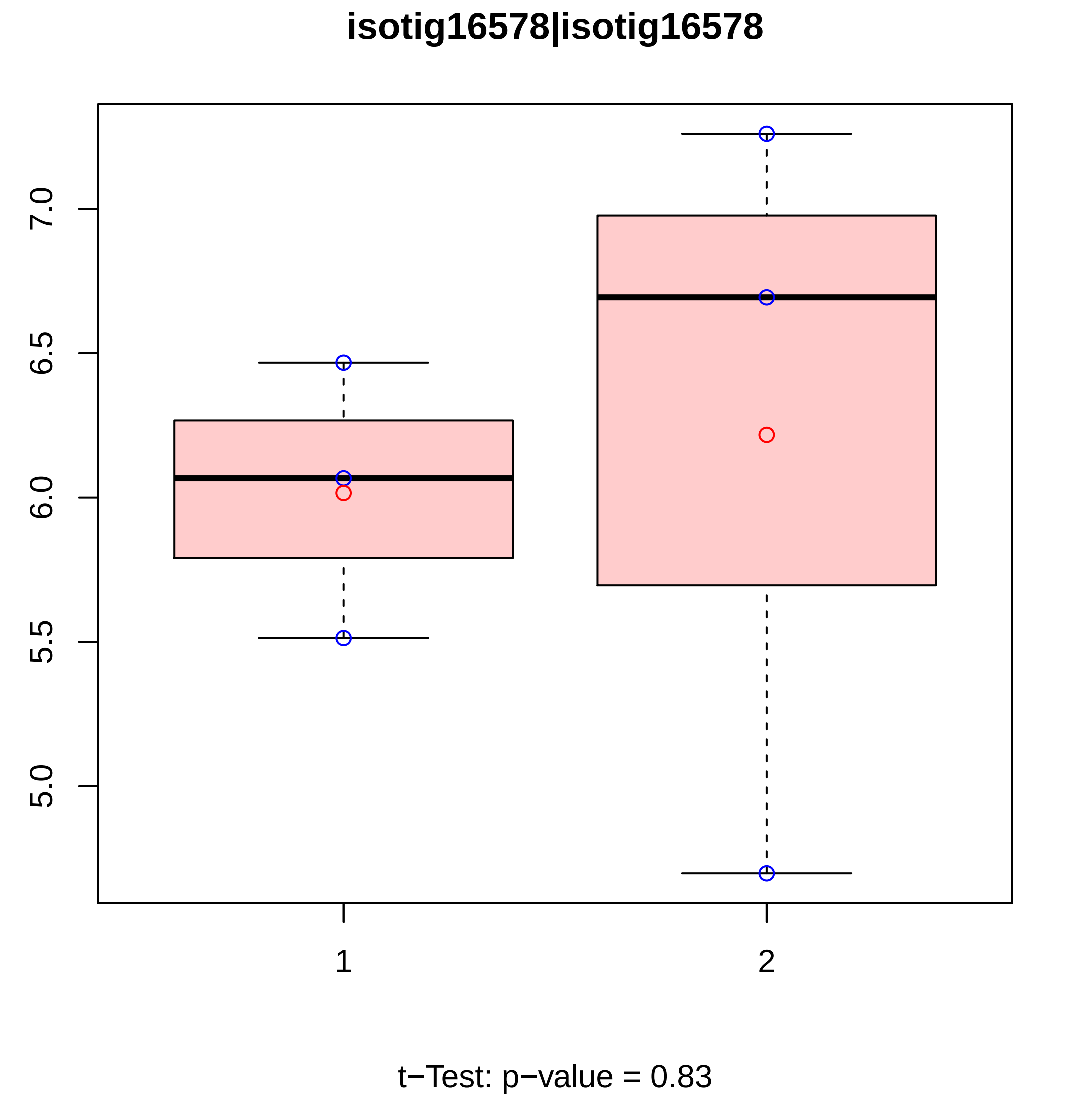
isotig16578|isotig16578
7.0
6.5
6.0
5.5
5.0
1
2
t−Test: p−value = 0.83

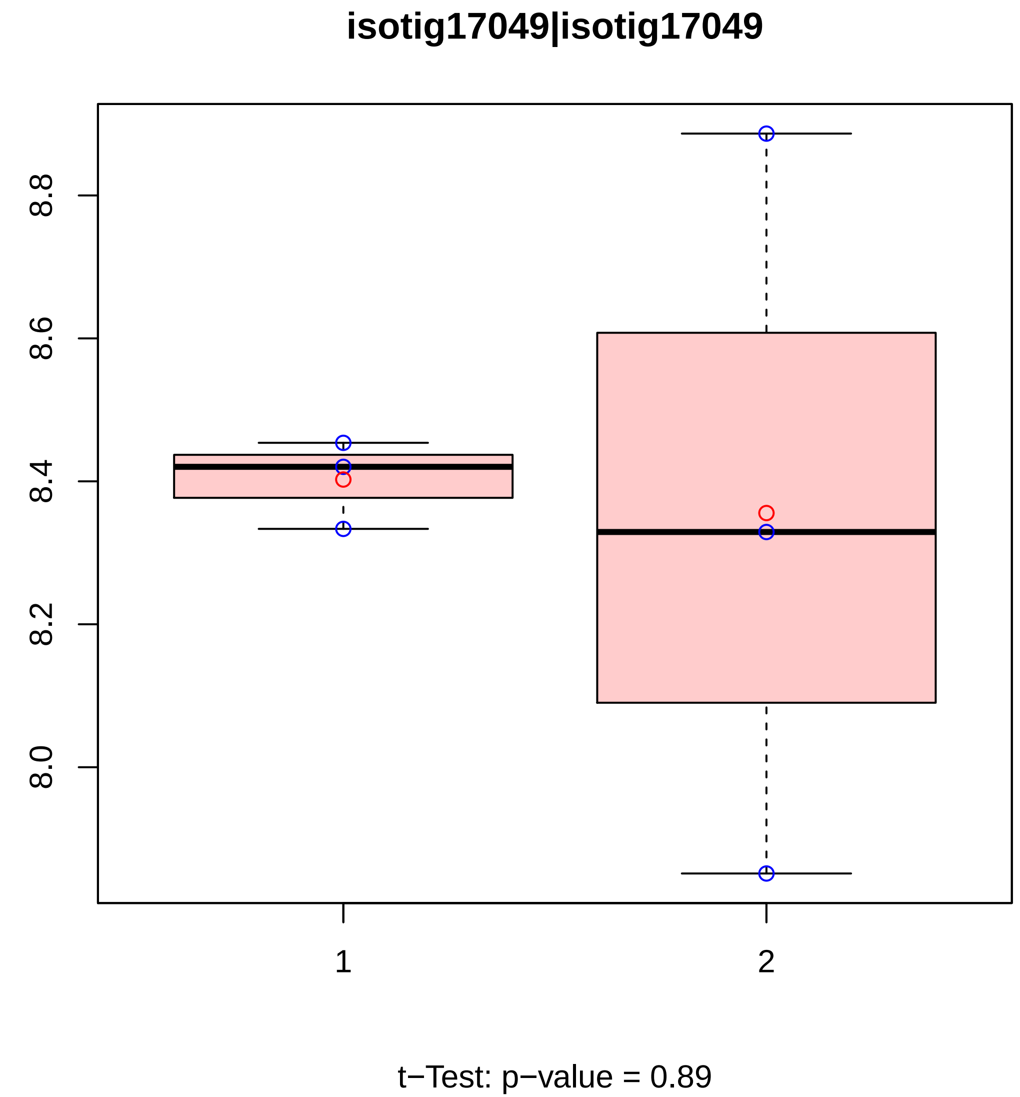
isotig17049|isotig17049
8.0
8.2
8.4
8.6
8.8
1
2
t−Test: p−value = 0.89

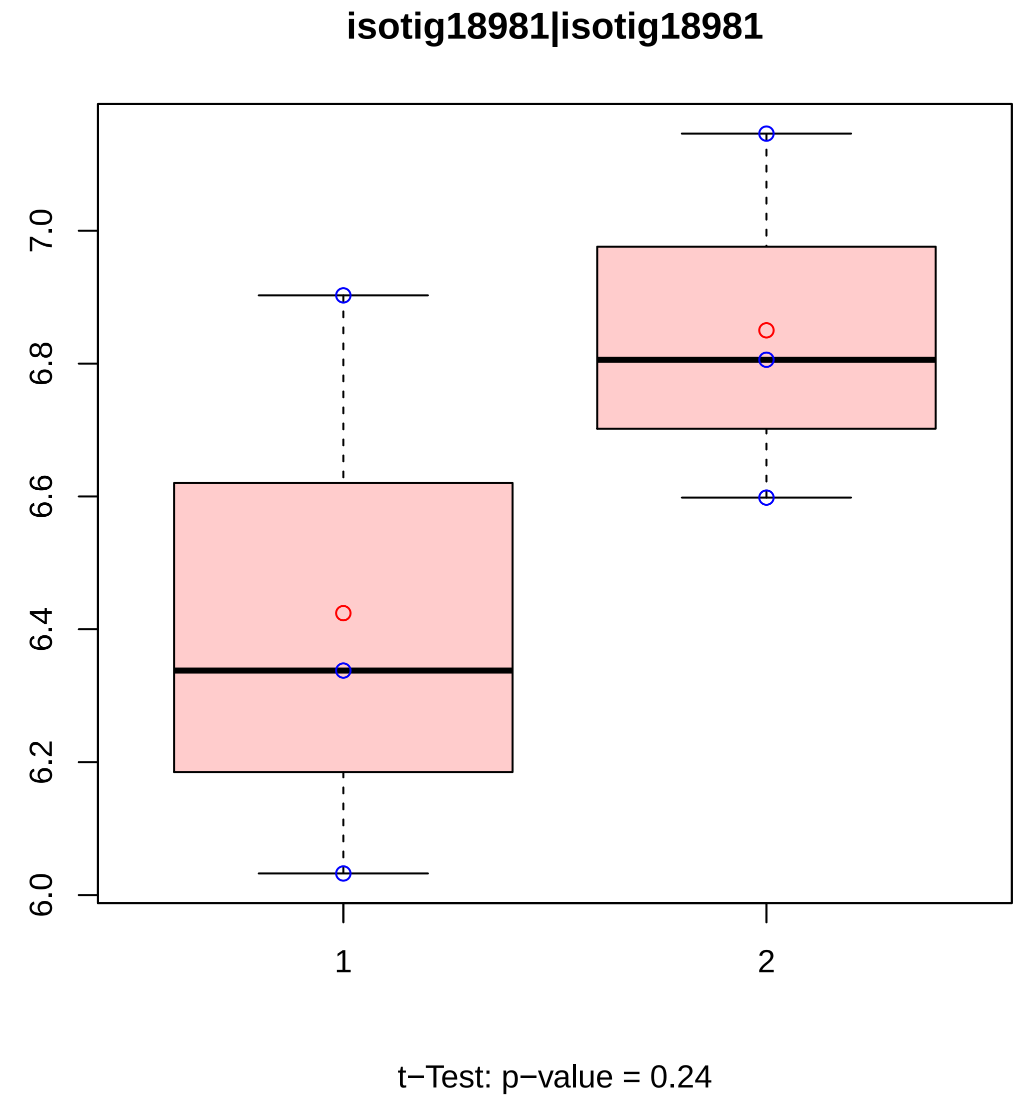

isotig18981|isotig18981
7.0
6.8
6.6
6.4
6.2
6.0
1
2
t−Test: p−value = 0.24

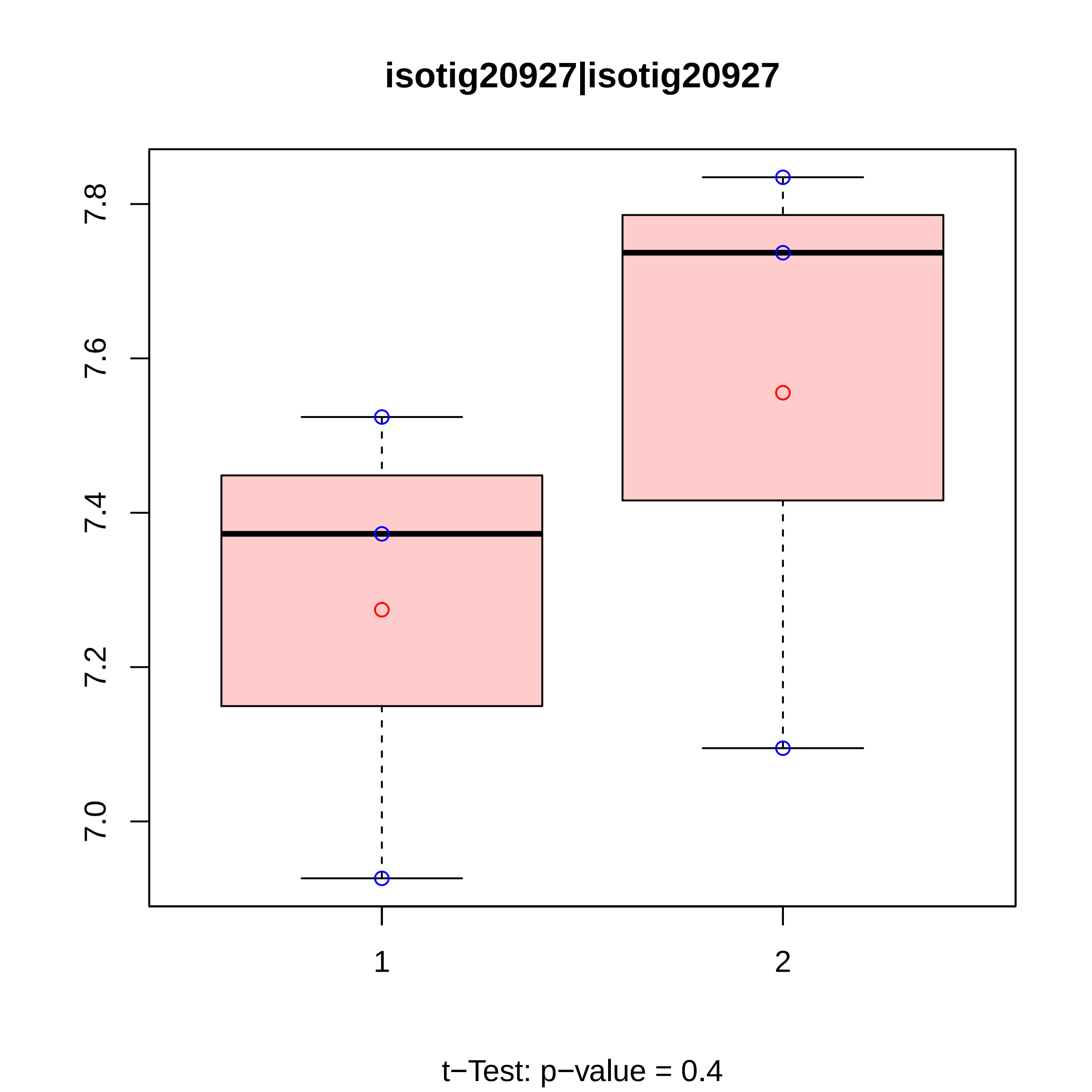
isotig20927|isotig20927
7.0
7.2
7.4
7.6
7.8
1
2
t−Test: p−value = 0.4

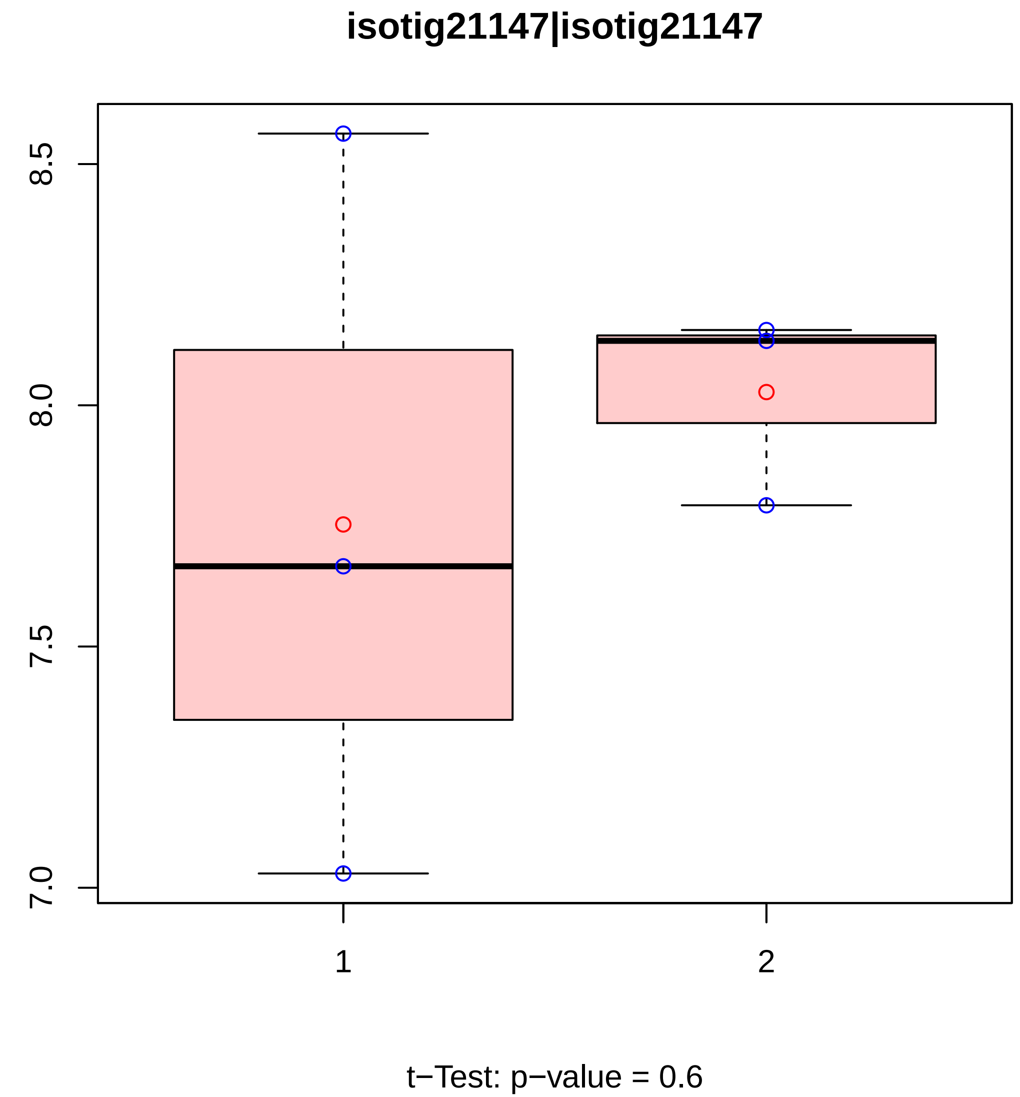
isotig21147|isotig21147
8.5
8.0
7.5
7.0
1
2
t−Test: p−value = 0.6

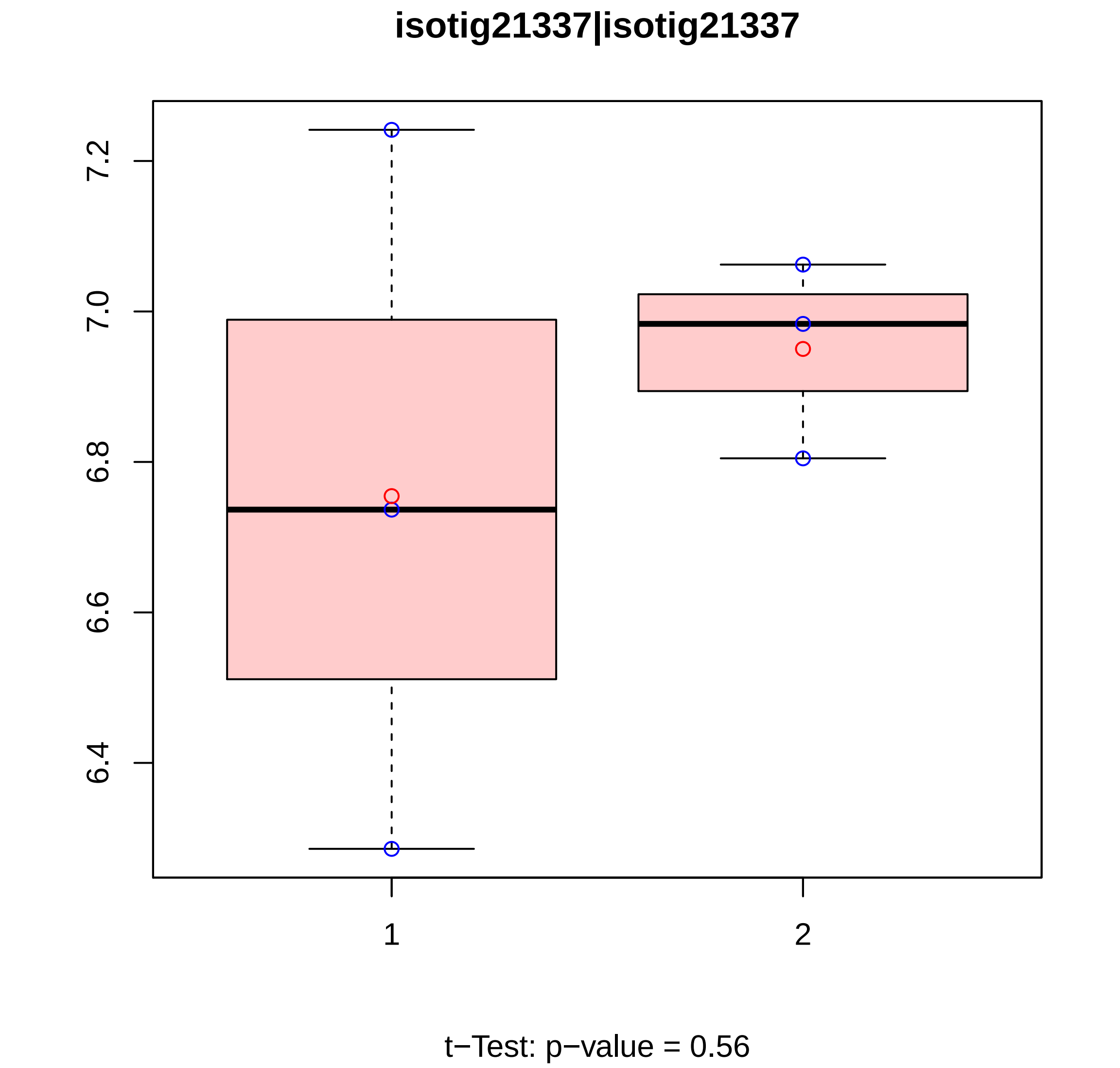
isotig21337|isotig21337
7.2
7.0
6.8
6.6
6.4
1
2
t−Test: p−value = 0.56

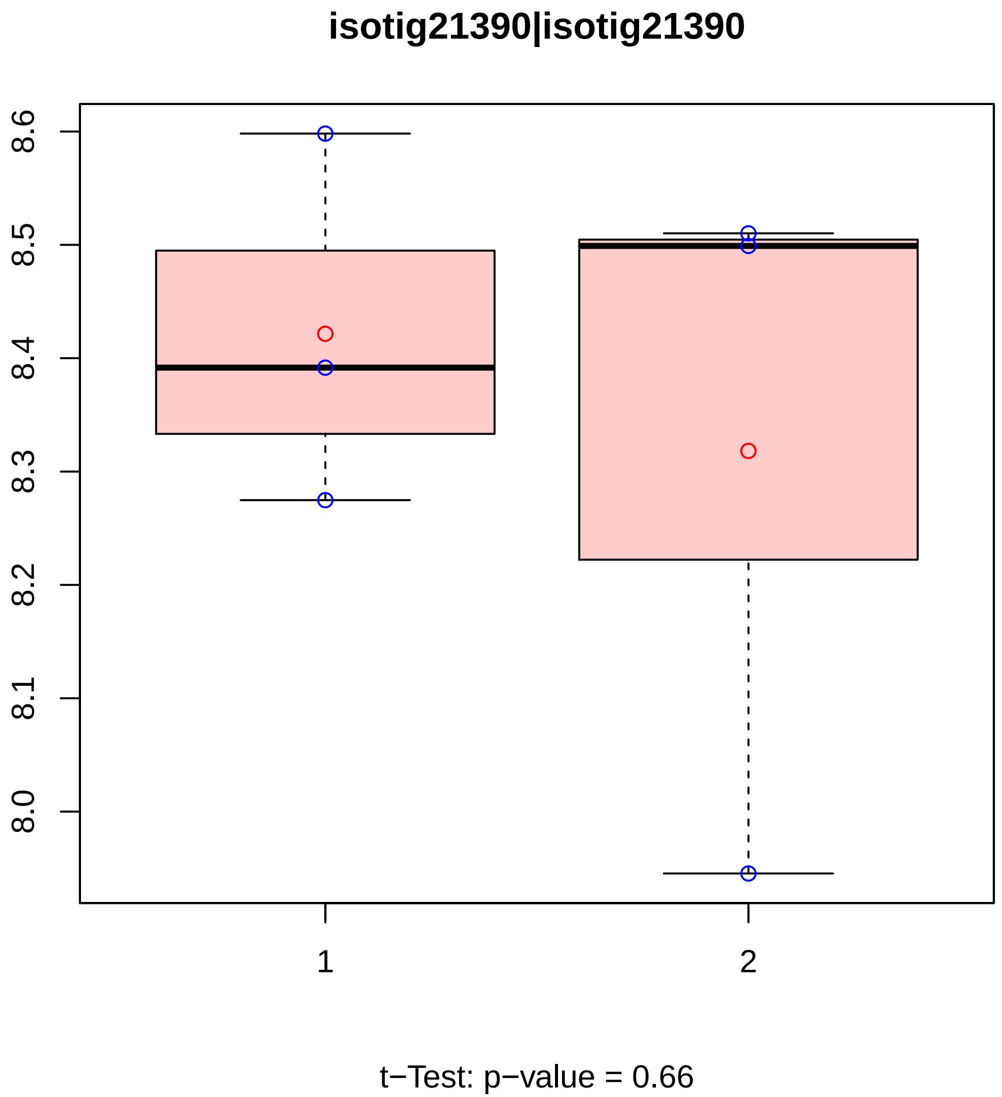
isotig21390|isotig21390
8.0
8.1
8.2
8.3
8.4
8.5
8.6
1
2
t−Test: p−value = 0.66

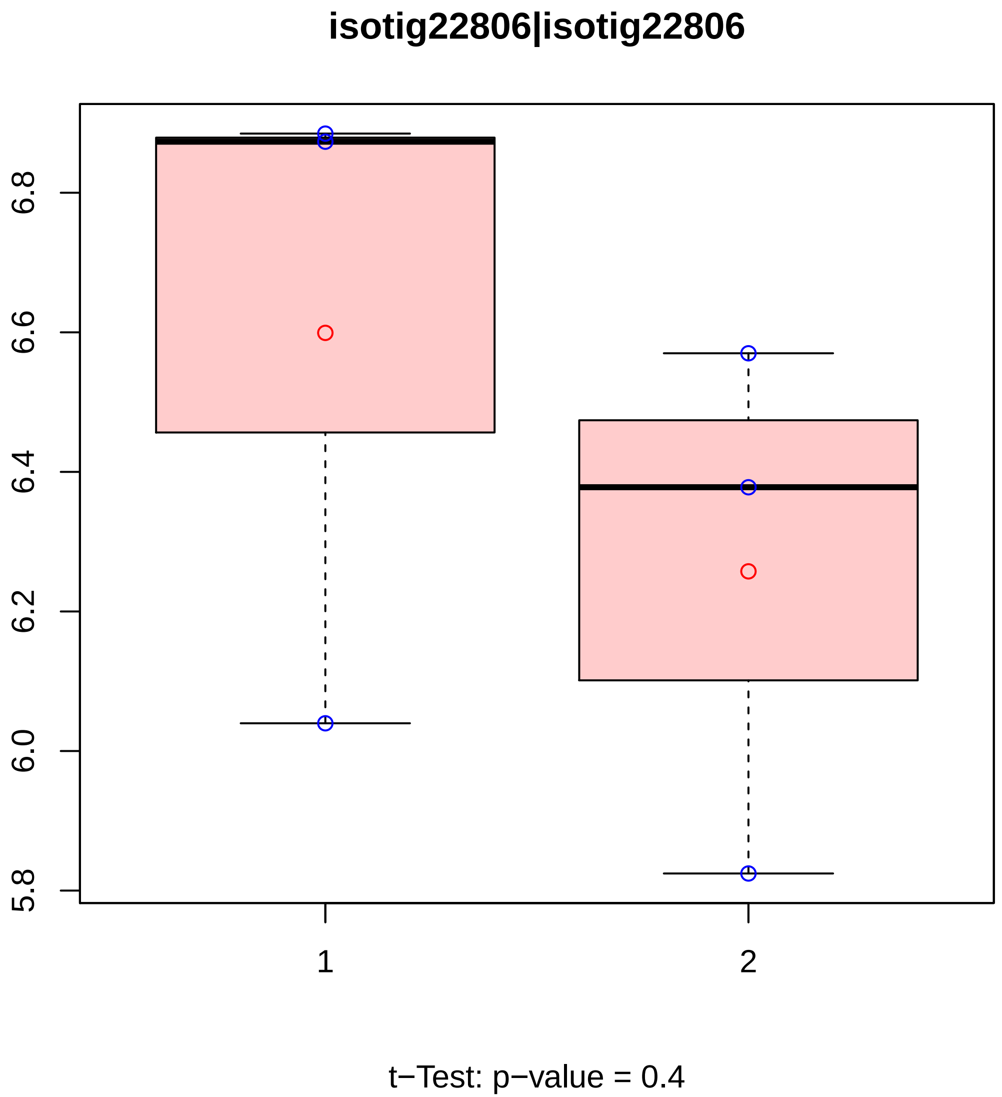

isotig22806|isotig22806
6.8
6.6
6.4
6.2
6.0
5.8
1
2
t−Test: p−value = 0.4

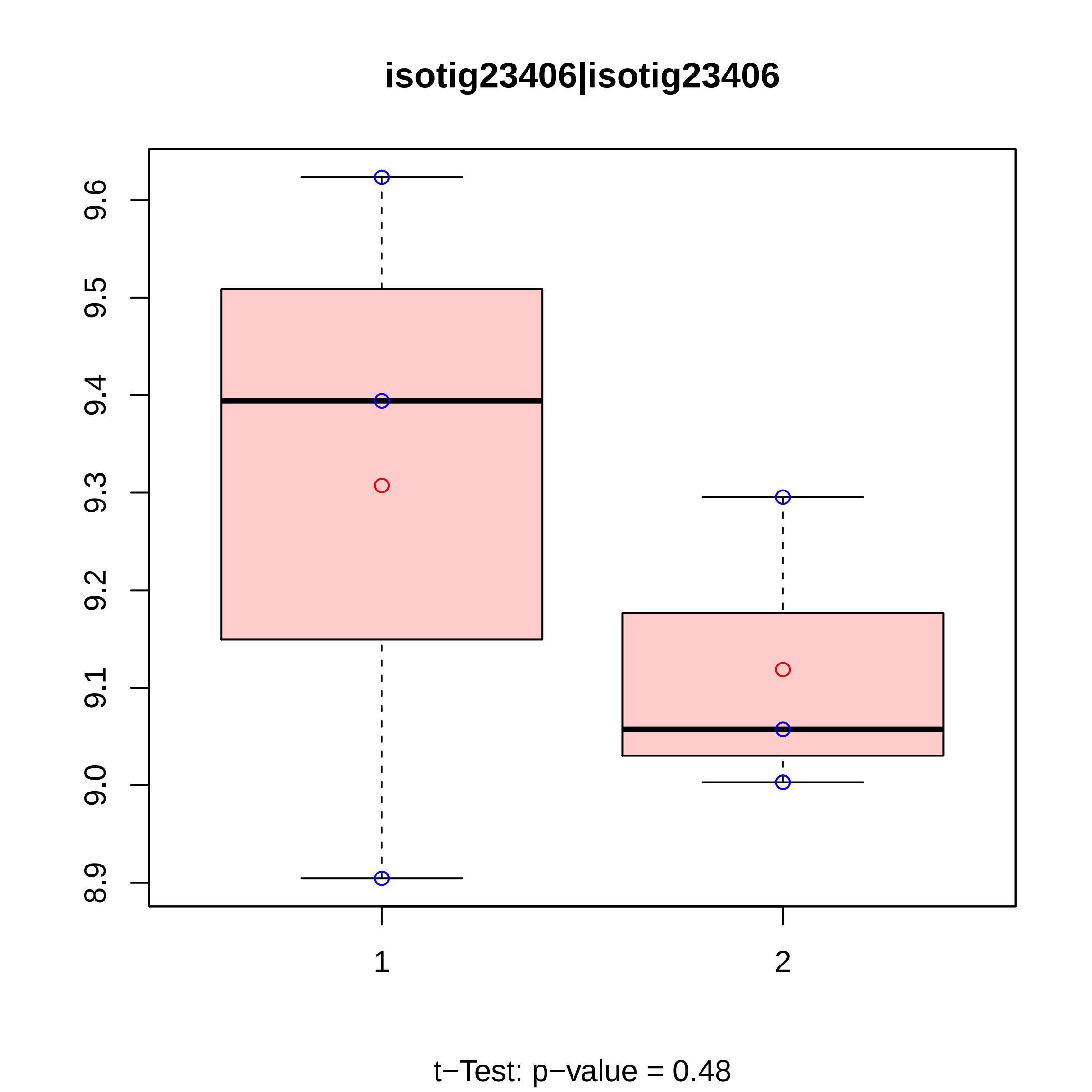

isotig23406|isotig23406
9.6
9.5
9.4
9.3
9.2
9.1
9.0
8.9
1
2
t−Test: p−value = 0.48

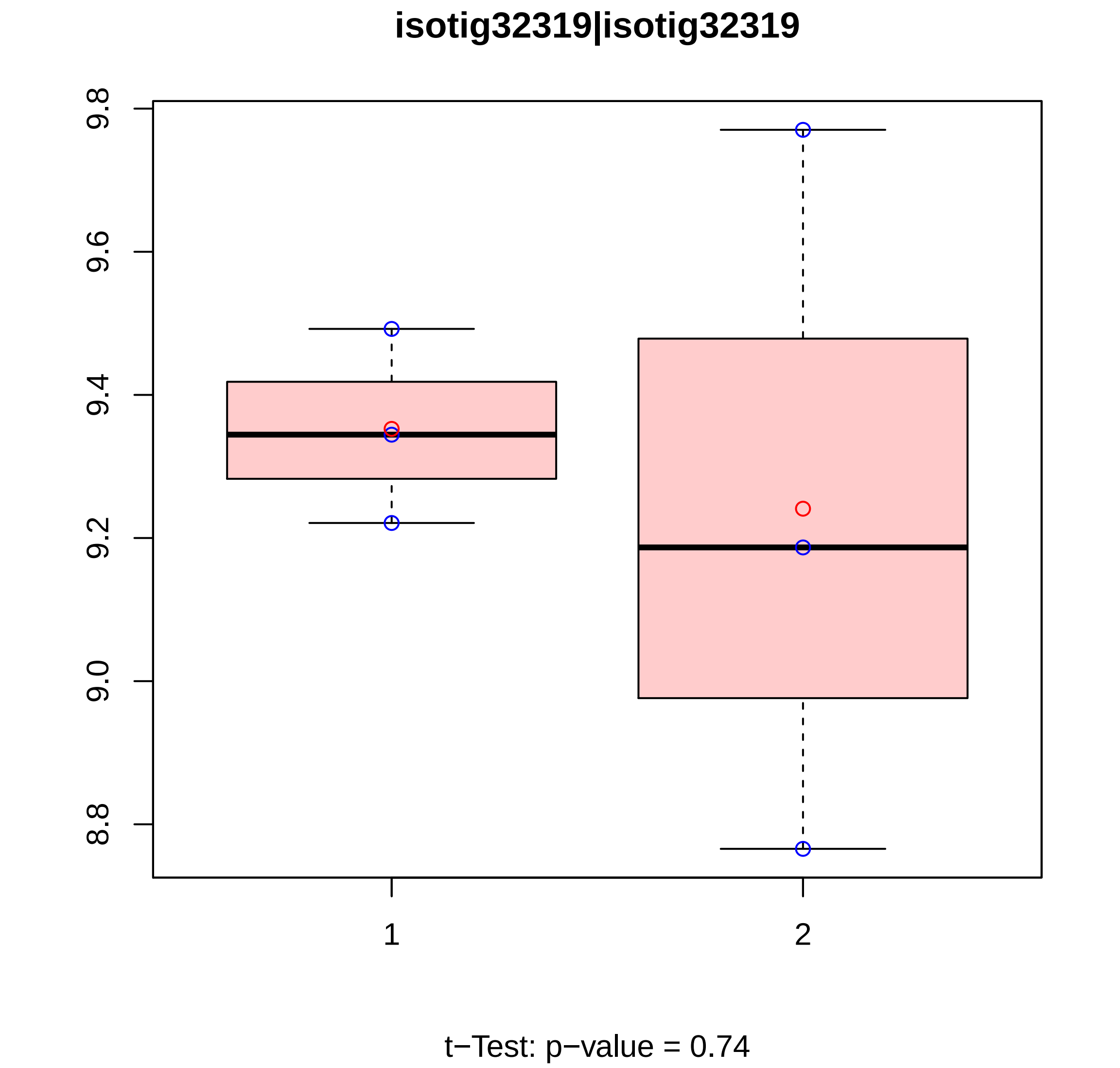
isotig32319|isotig32319
9.8
9.6
9.4
9.2
9.0
8.8
1
2
t−Test: p−value = 0.74

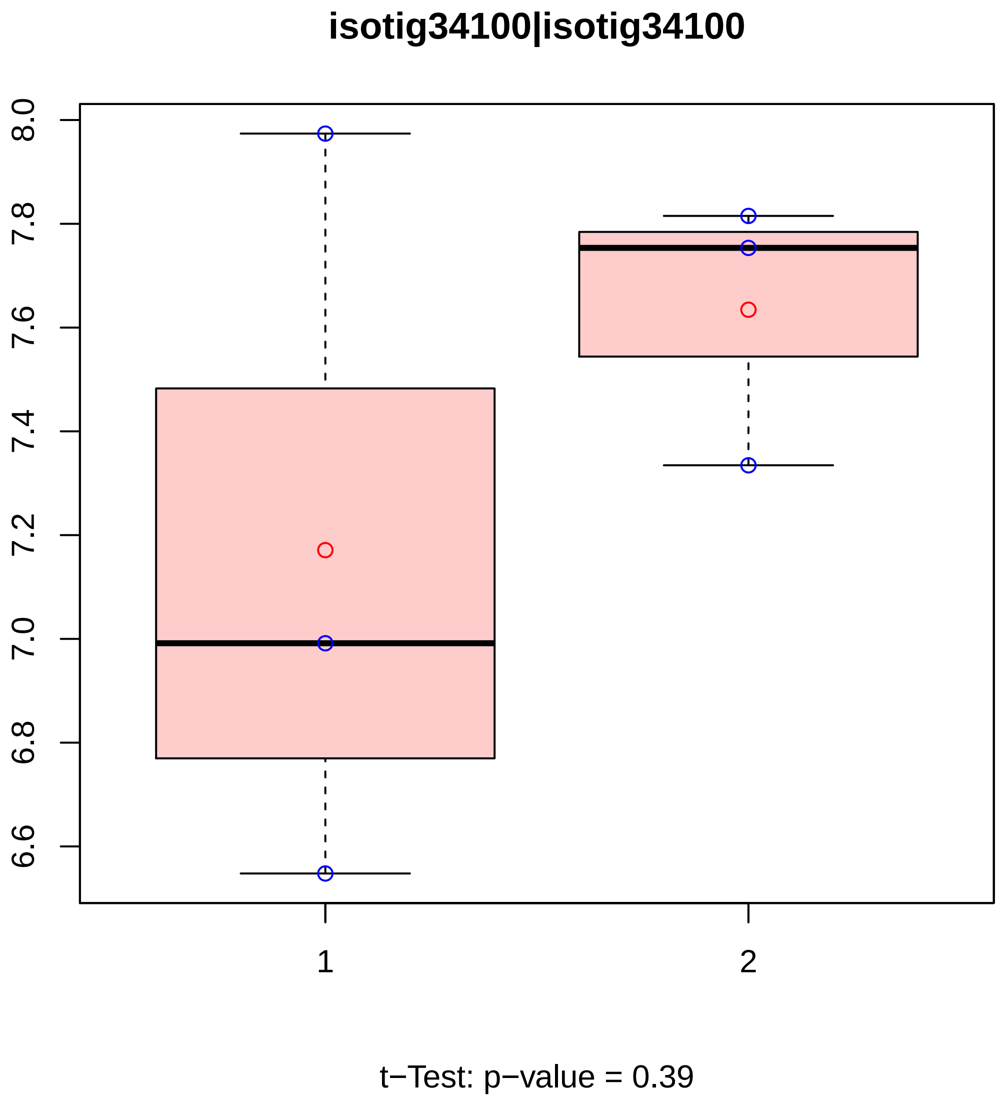

isotig34100|isotig34100
8.0
7.8
7.6
7.4
7.2
7.0
6.8
6.6
1
2
t−Test: p−value = 0.39

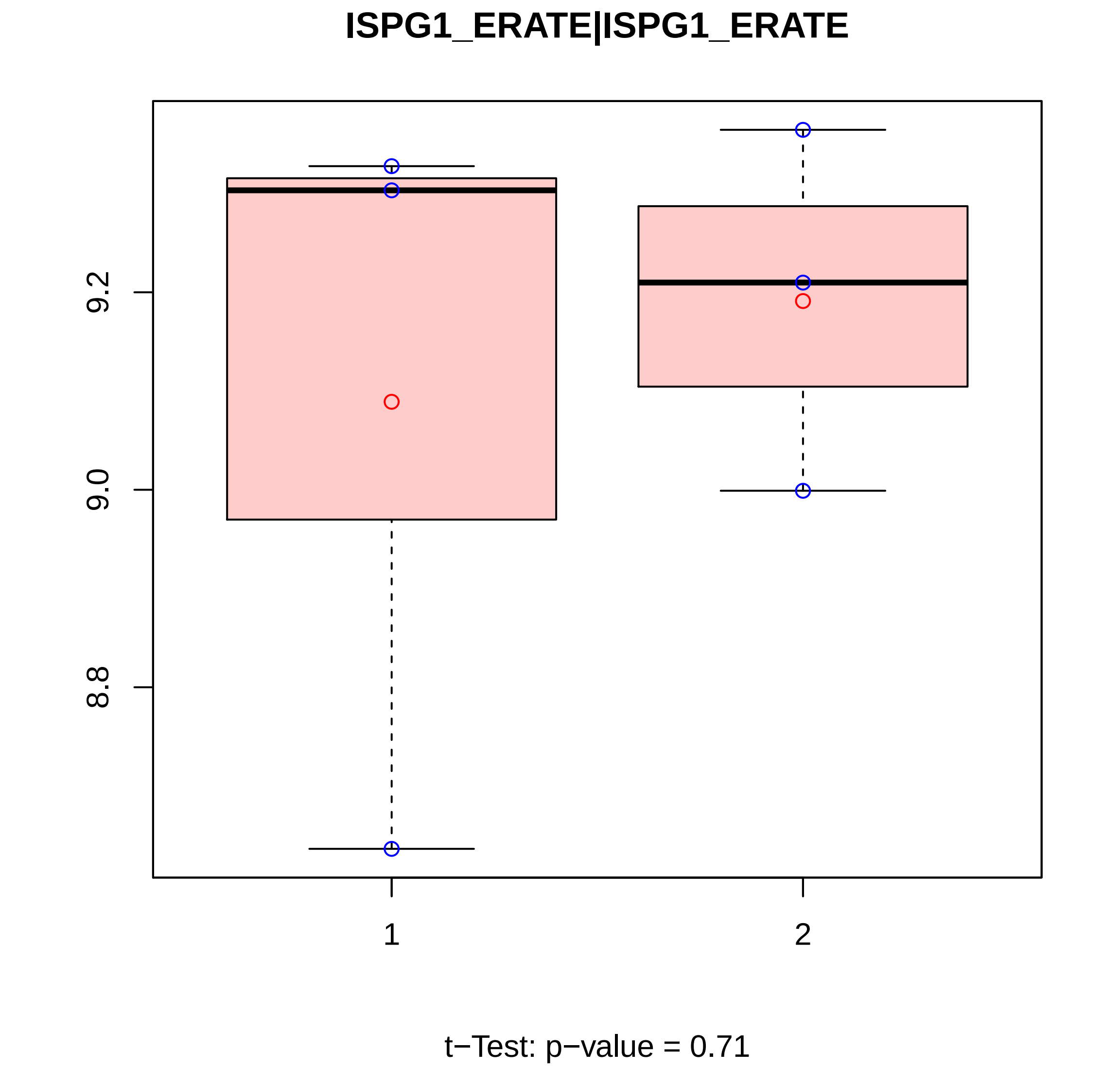
ISPG1_ERATE|ISPG1_ERATE
9.2
9.0
8.8
1
2
t−Test: p−value = 0.71

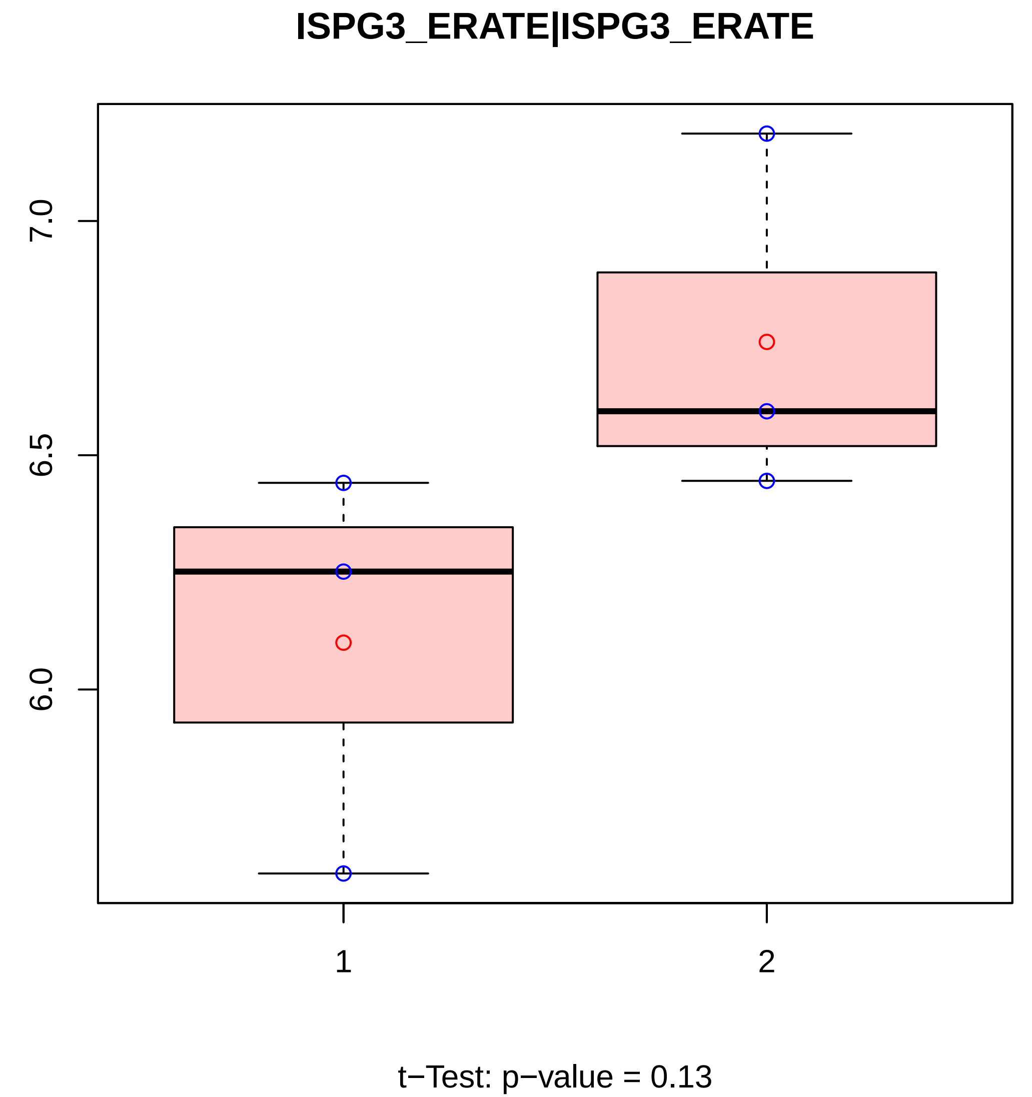

ISPG3_ERATE|ISPG3_ERATE
7.0
6.5
6.0
1
2
t−Test: p−value = 0.13

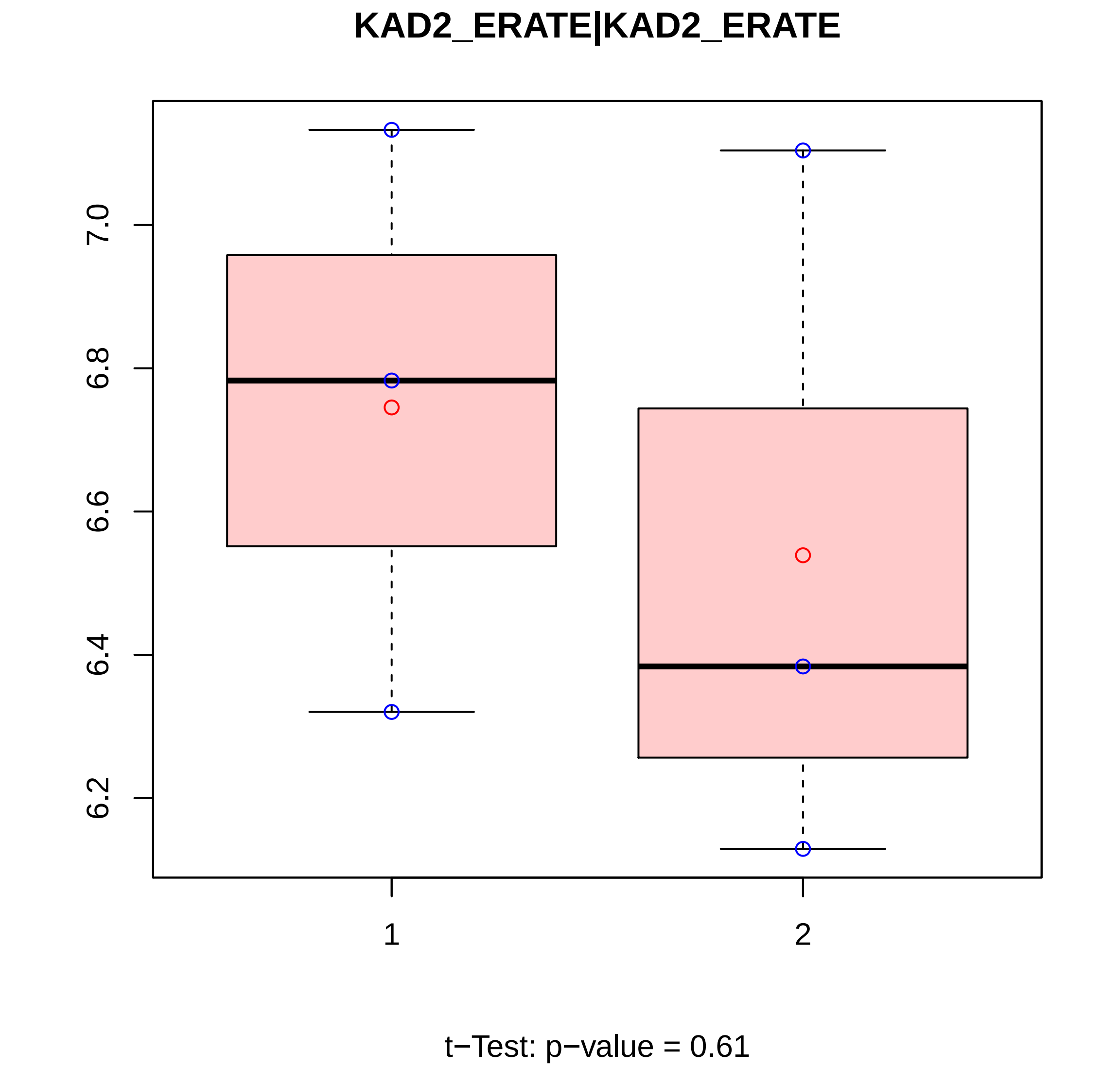

KAD2_ERATE|KAD2_ERATE
7.0
6.8
6.6
6.4
6.2
1
2
t−Test: p−value = 0.61

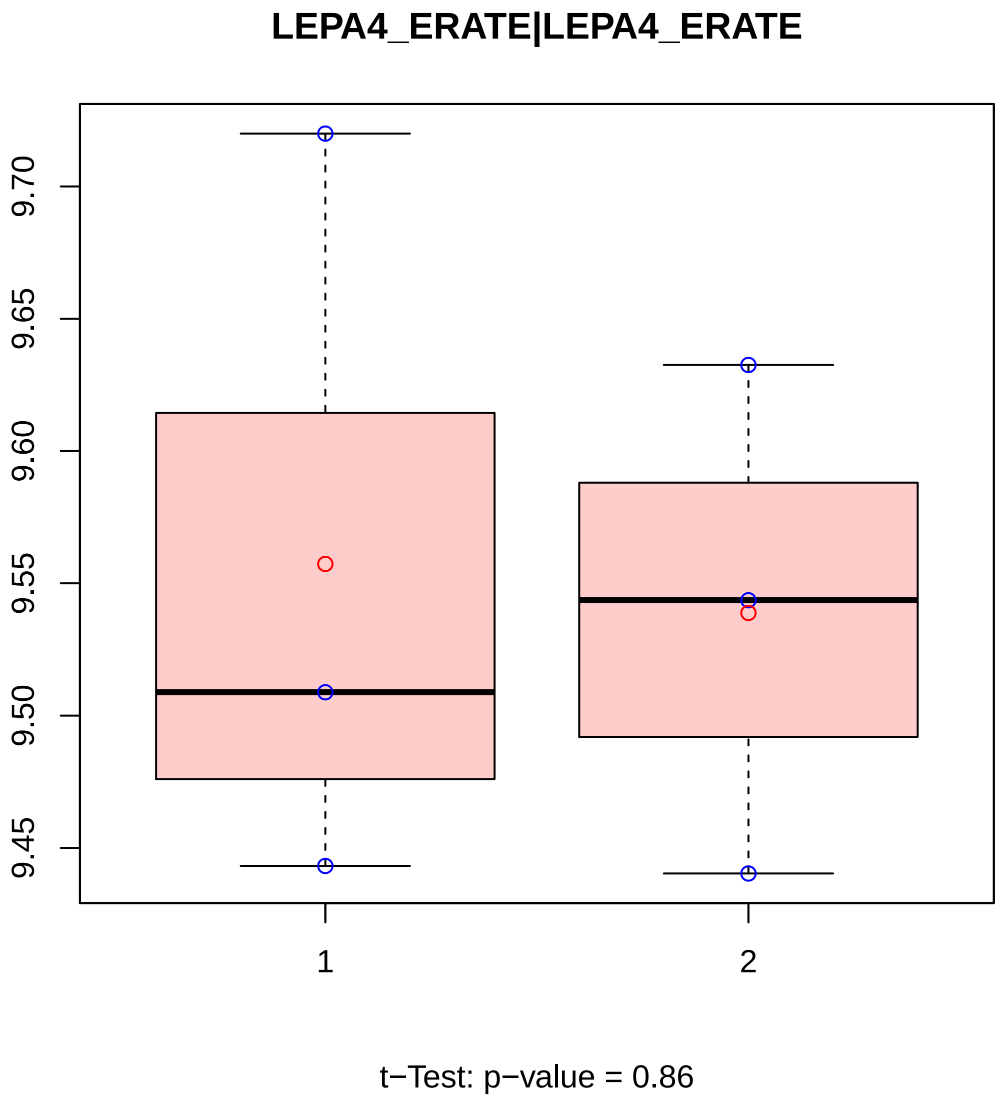
LEPA4_ERATE|LEPA4_ERATE
9.70
9.65
9.60
9.55
9.50
9.45
1
2
t−Test: p−value = 0.86

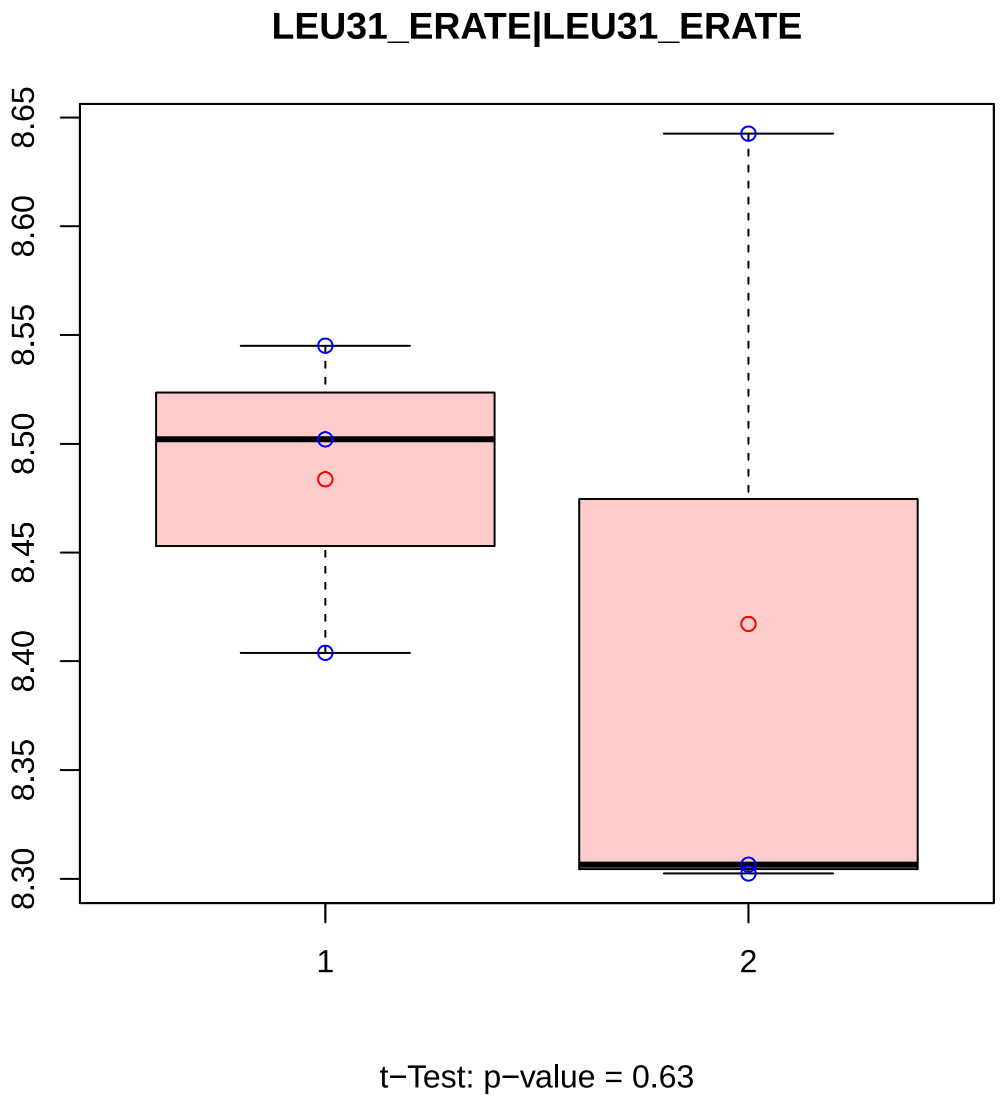

LEU31_ERATE|LEU31_ERATE
8.30
8.35
8.40
8.45
8.50
8.55
8.60
8.65
1
2
t−Test: p−value = 0.63

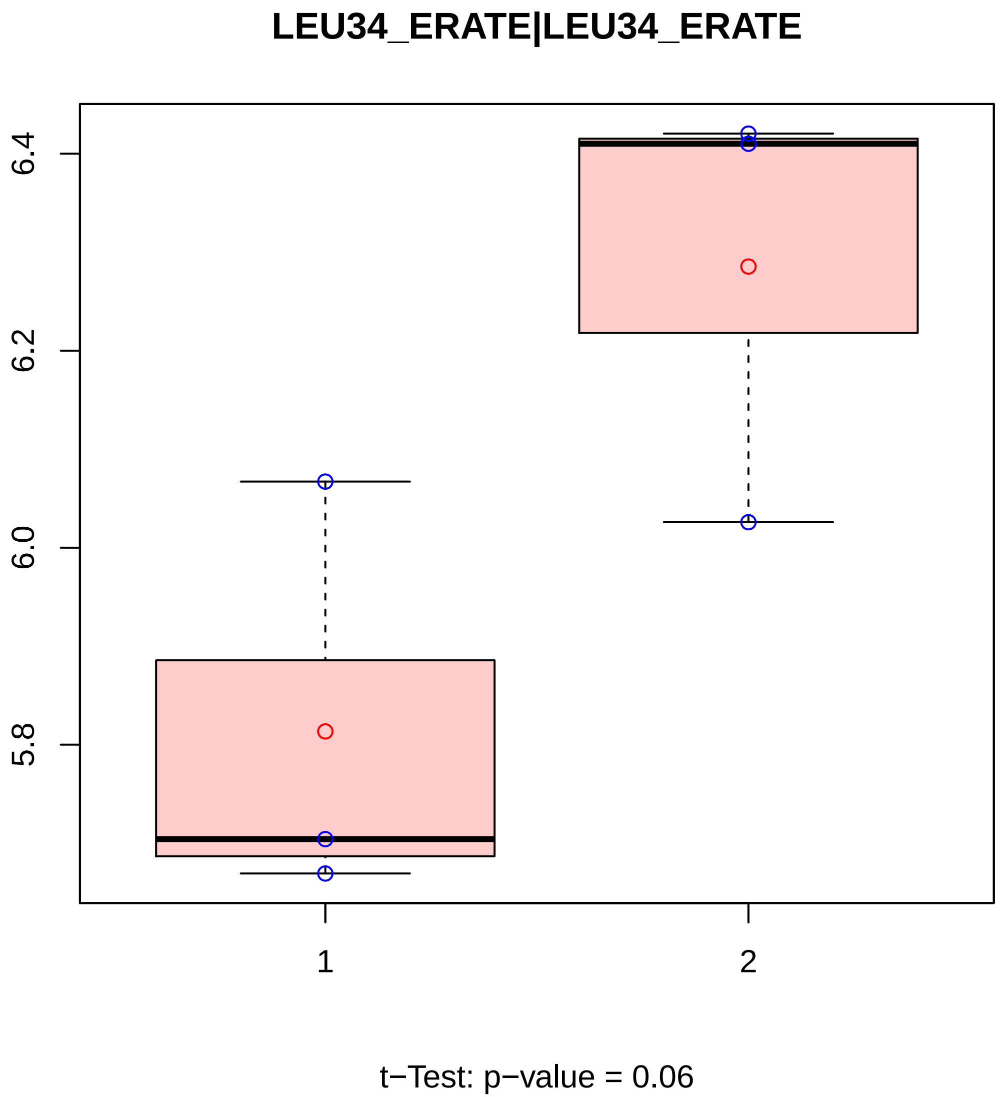

LEU34_ERATE|LEU34_ERATE
6.4
6.2
6.0
5.8
1
2
t−Test: p−value = 0.06

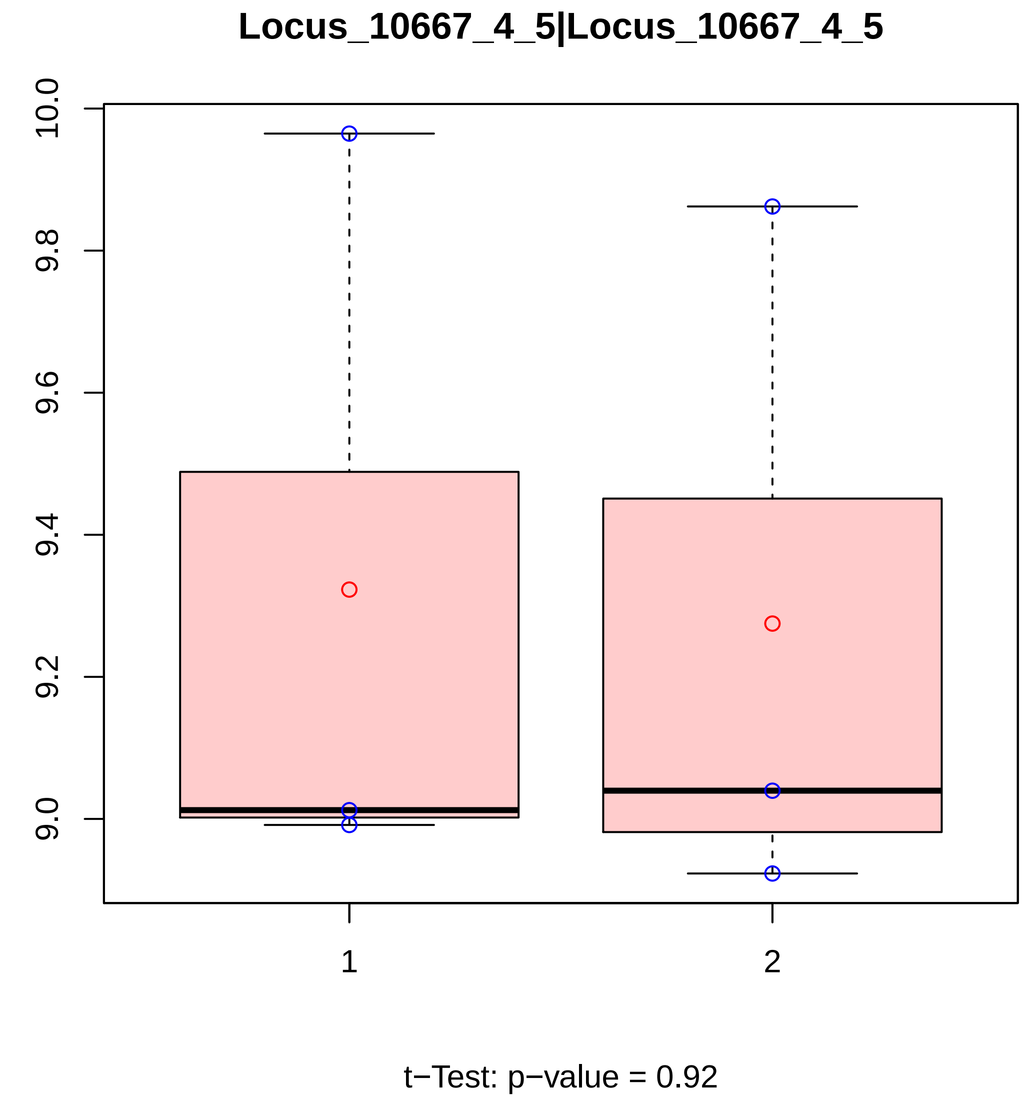
Locus_10667_4_5|Locus_10667_4_5
10.0
9.8
9.6
9.4
9.2
9.0
1
2
t−Test: p−value = 0.92

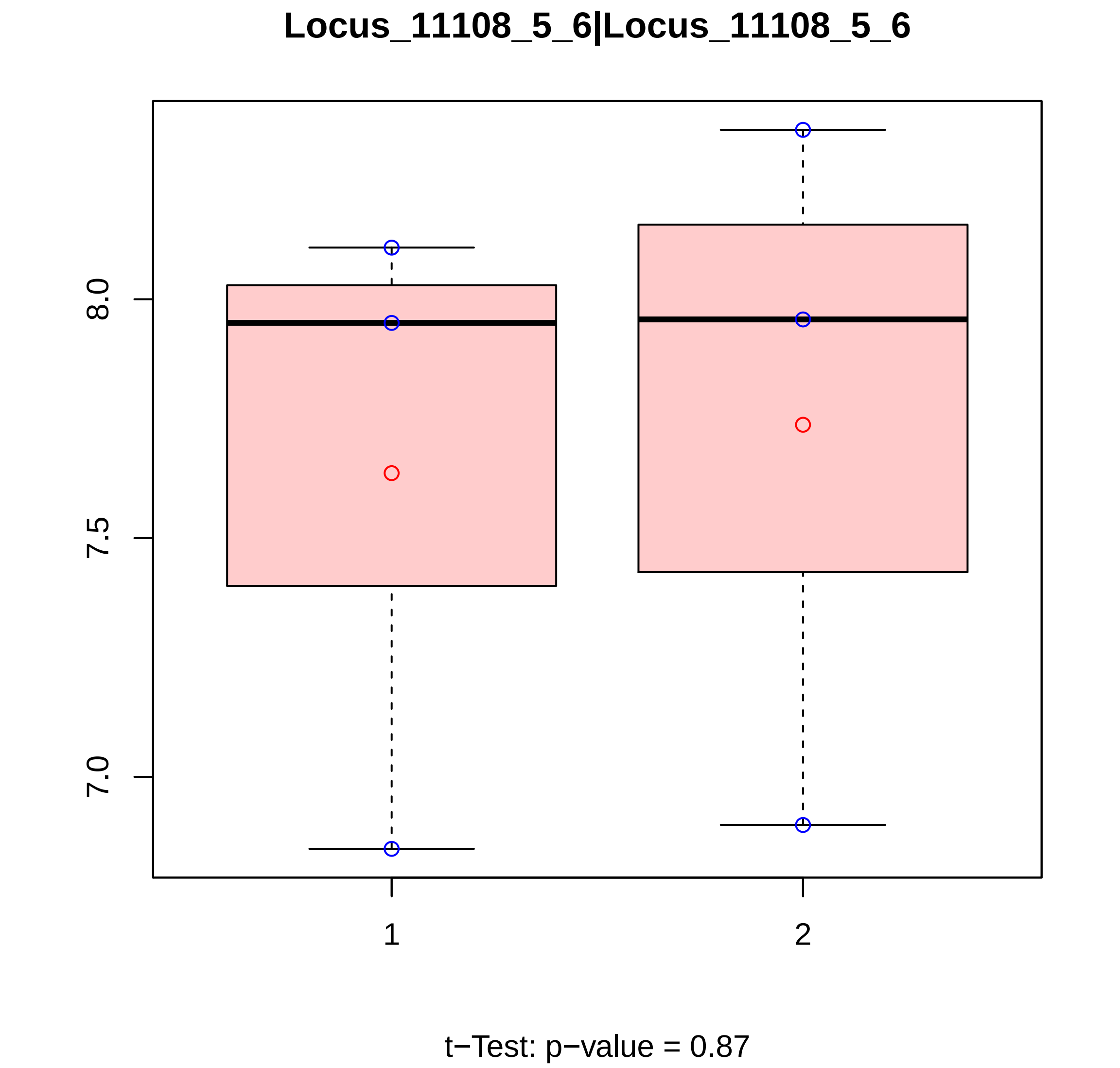
Locus_11108_5_6|Locus_11108_5_6
8.0
7.5
7.0
1
2
t−Test: p−value = 0.87

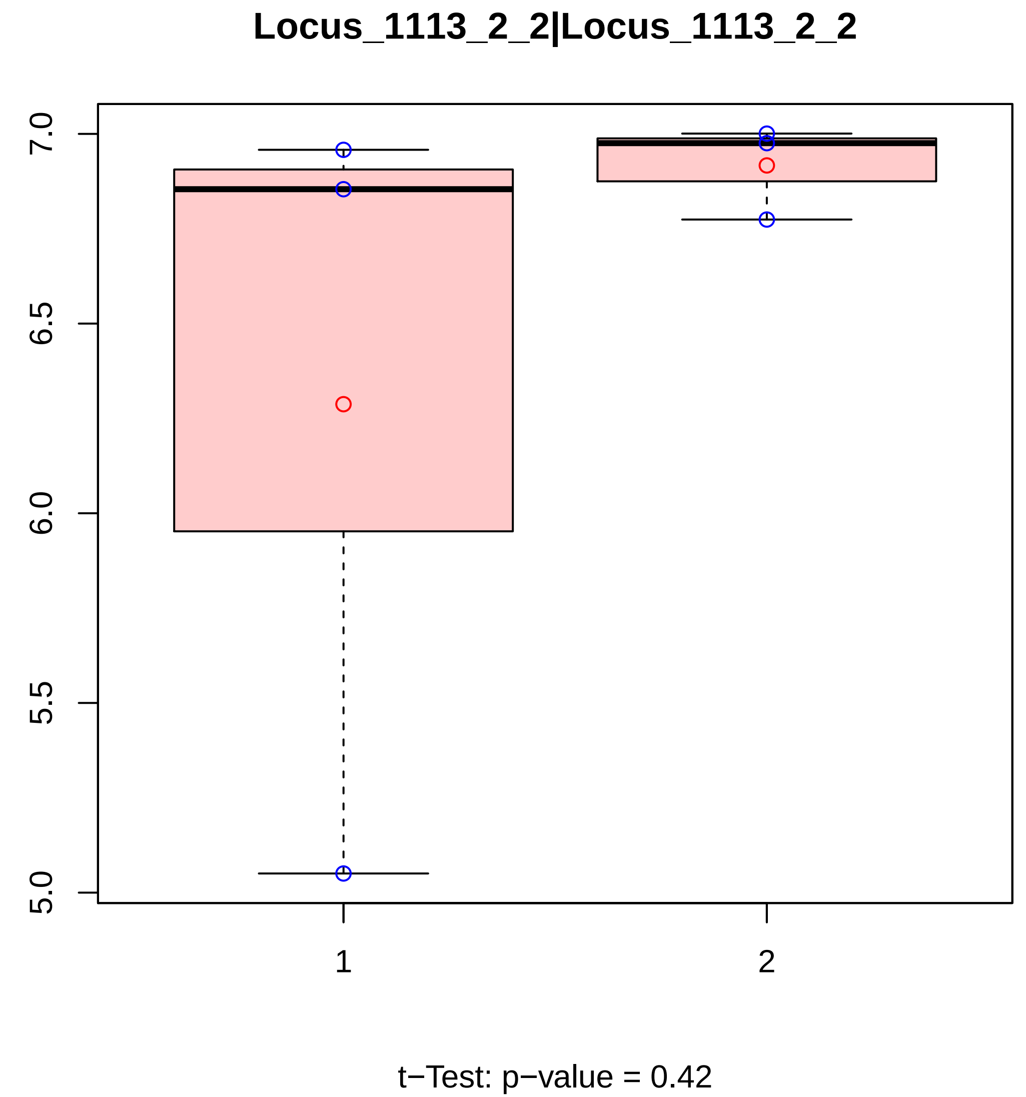
Locus_1113_2_2|Locus_1113_2_2
7.0
6.5
6.0
5.5
5.0
1
2
t−Test: p−value = 0.42

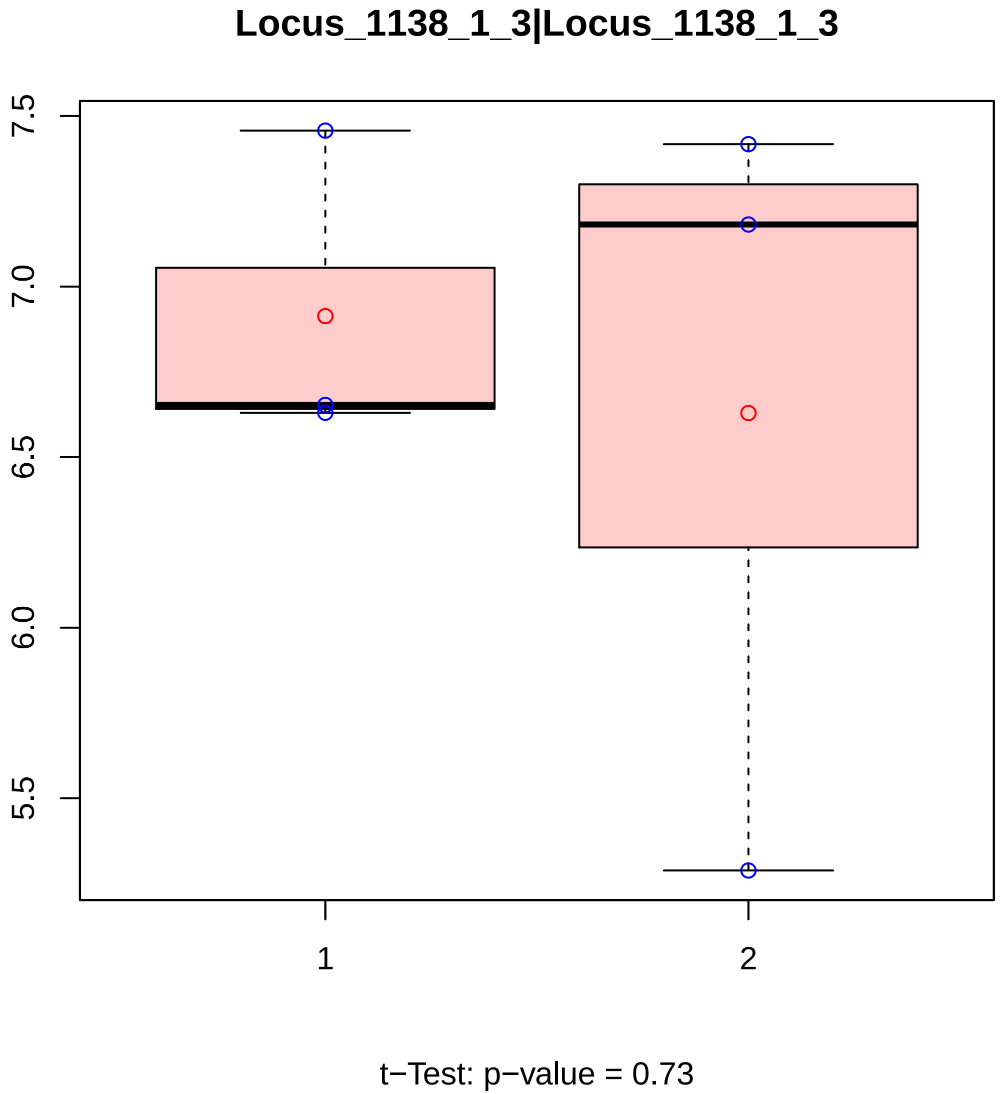

Locus_1138_1_3|Locus_1138_1_3
7.5
7.0
6.5
6.0
5.5
1
2
t−Test: p−value = 0.73

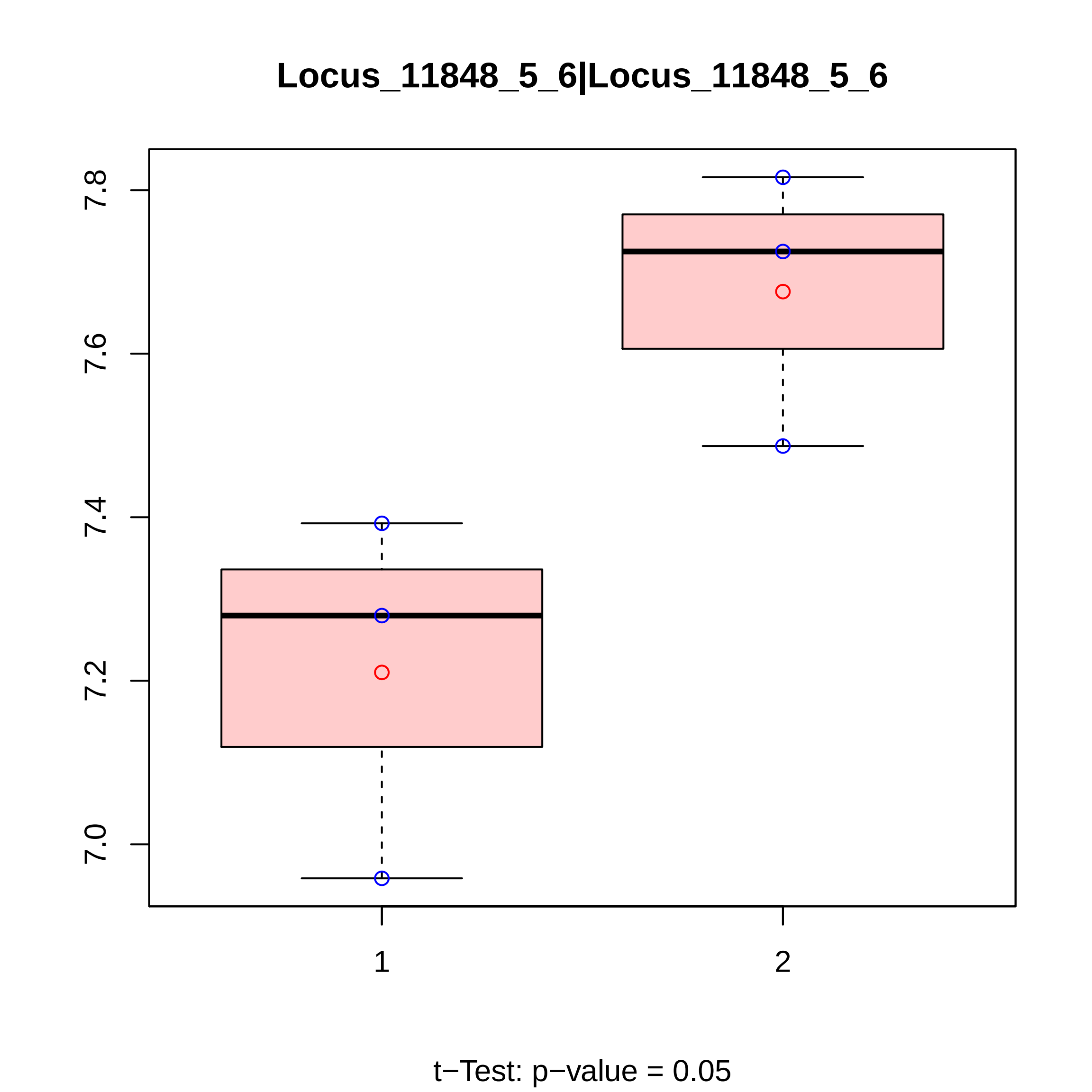
Locus_11848_5_6|Locus_11848_5_6
7.0
7.2
7.4
7.6
7.8
1
2
t−Test: p−value = 0.05

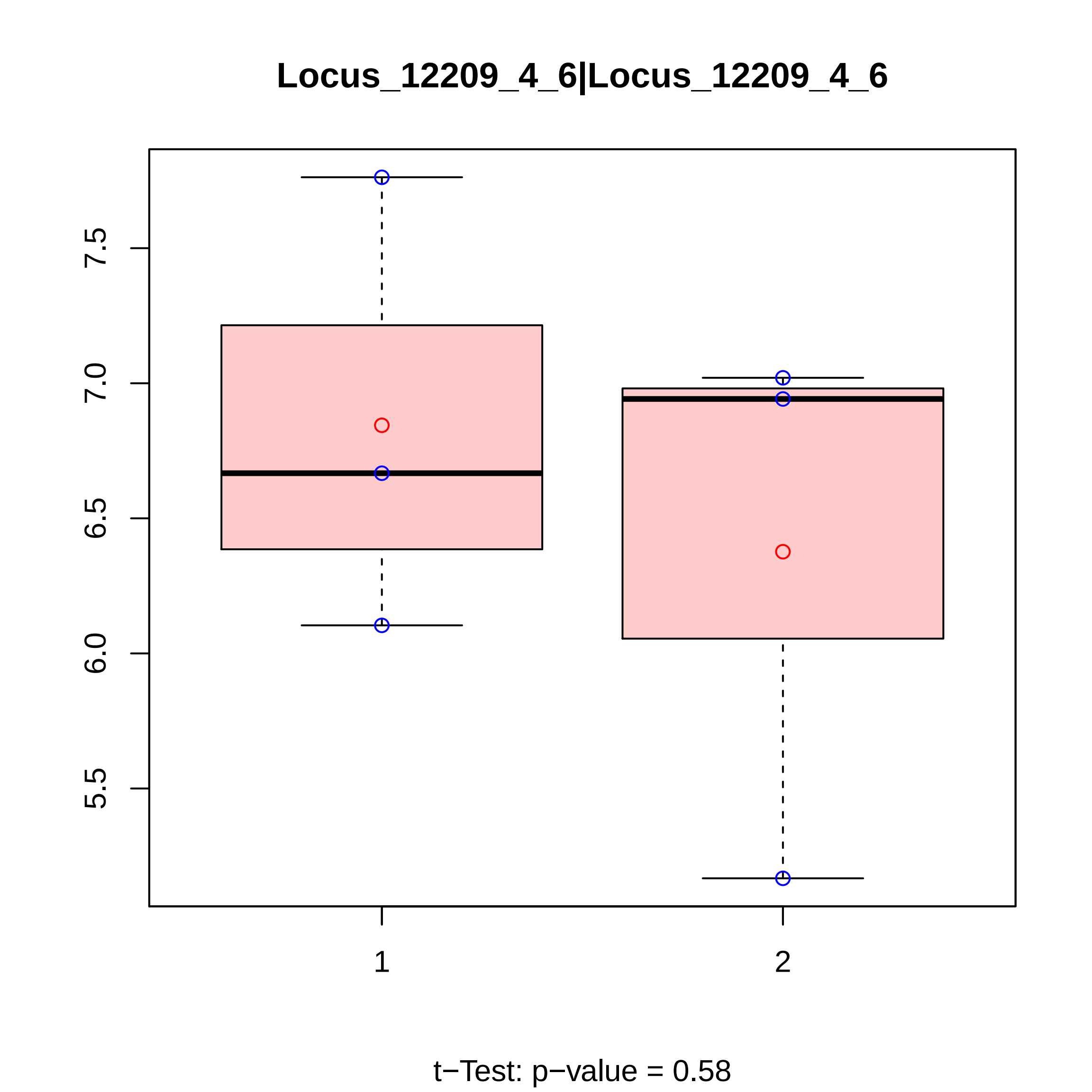
Locus_12209_4_6|Locus_12209_4_6
7.5
7.0
6.5
6.0
5.5
1
2
t−Test: p−value = 0.58

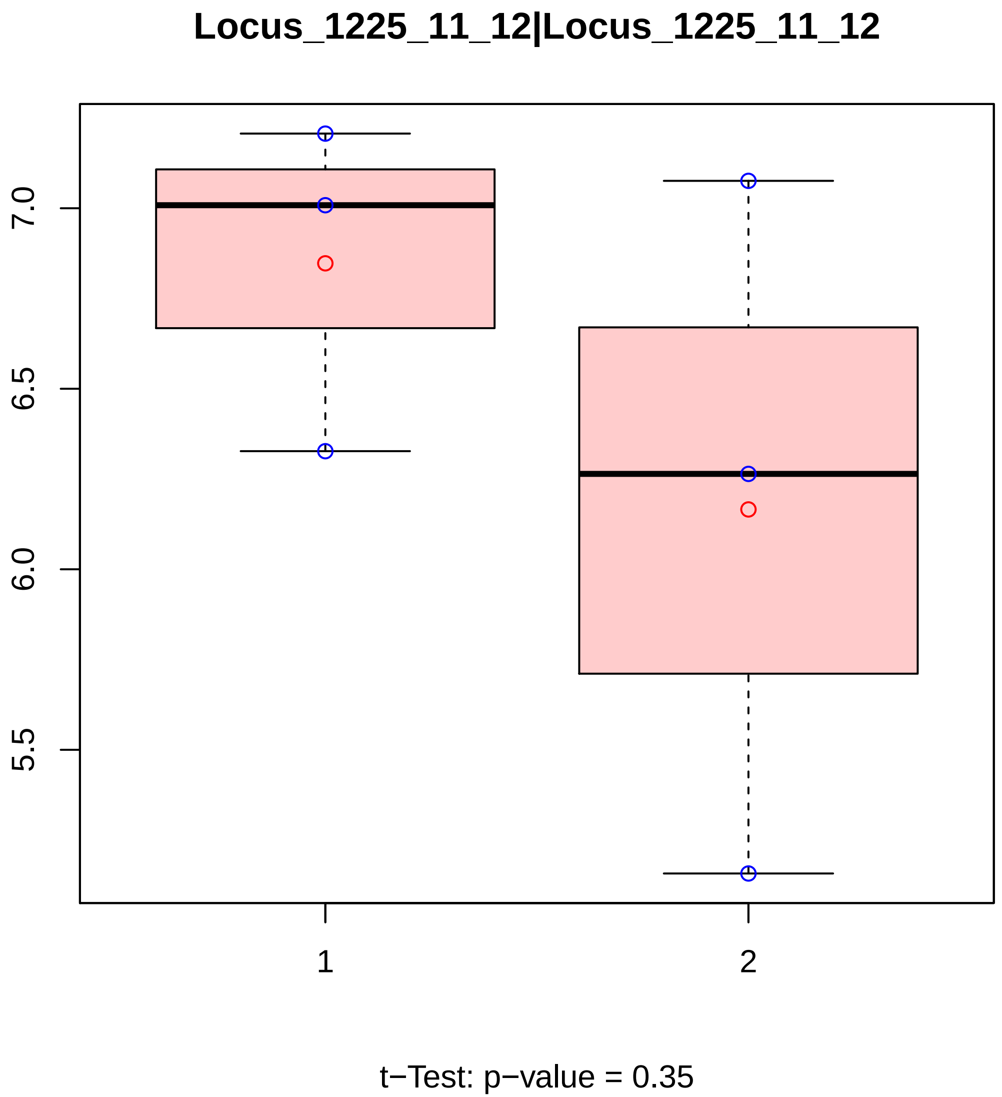

Locus_1225_11_12|Locus_1225_11_12
7.0
6.5
6.0
5.5
1
2
t−Test: p−value = 0.35

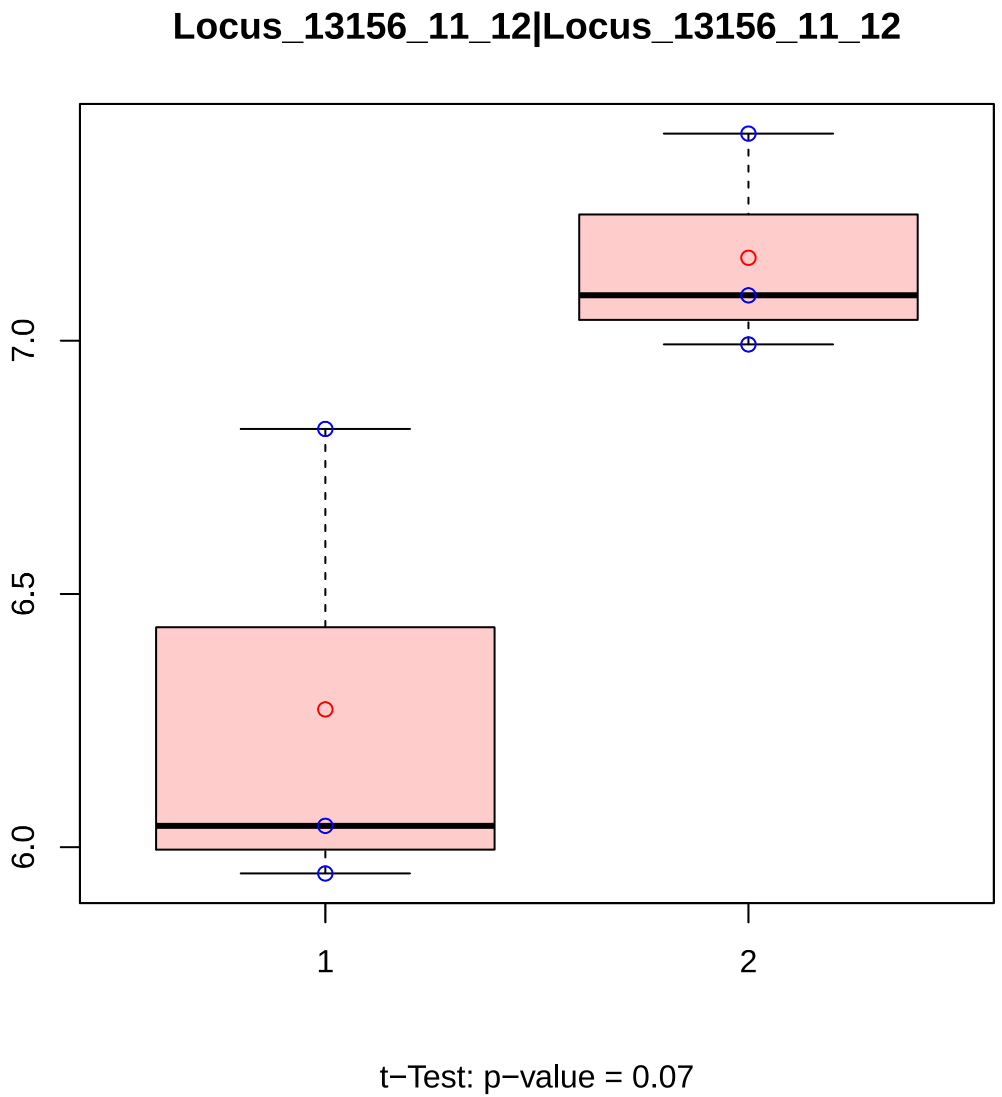
Locus_13156_11_12|Locus_13156_11_12
6.0
6.5
7.0
1
2
t−Test: p−value = 0.07

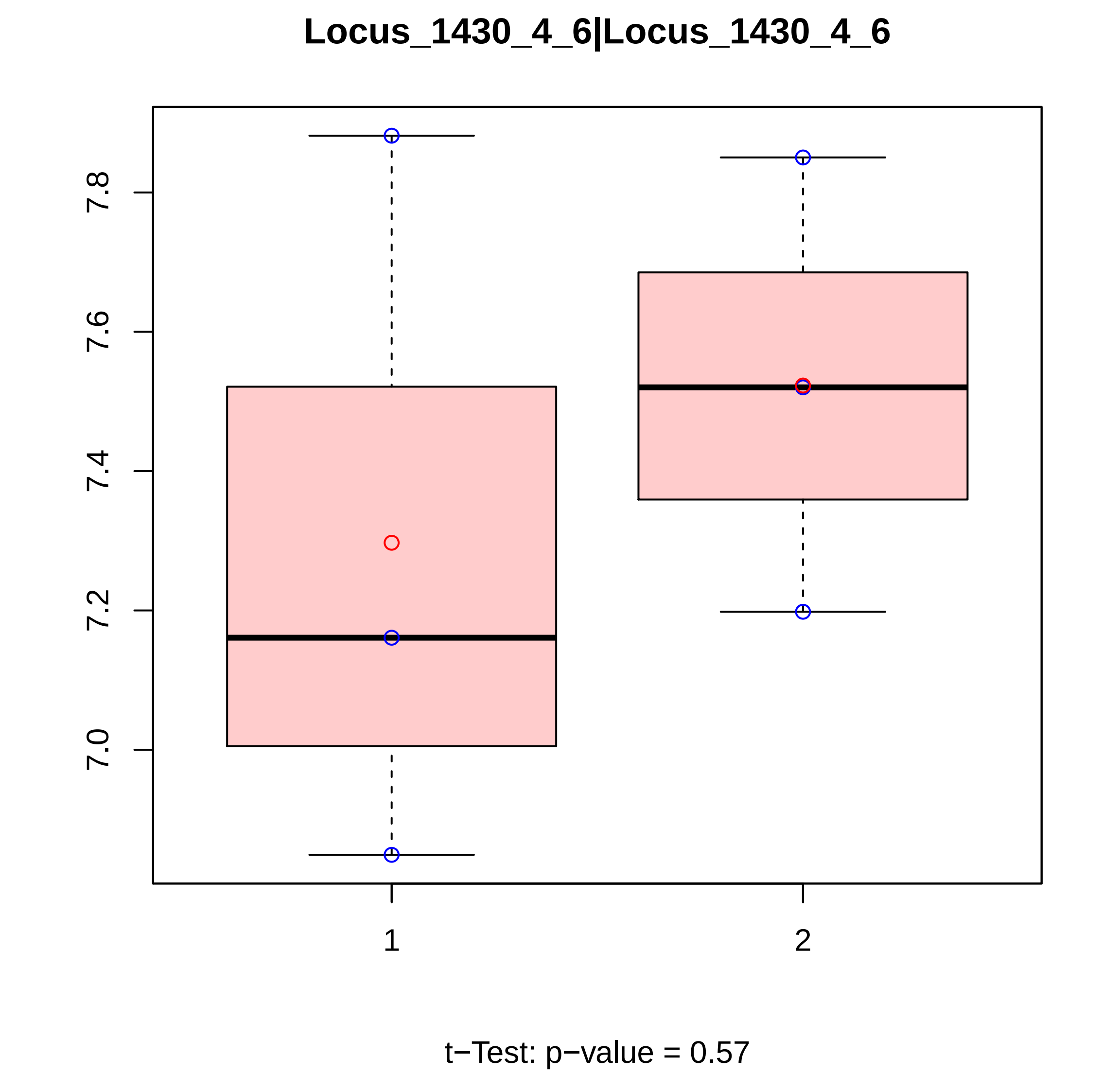

Locus_1430_4_6|Locus_1430_4_6
7.0
7.2
7.4
7.6
7.8
1
2
t−Test: p−value = 0.57

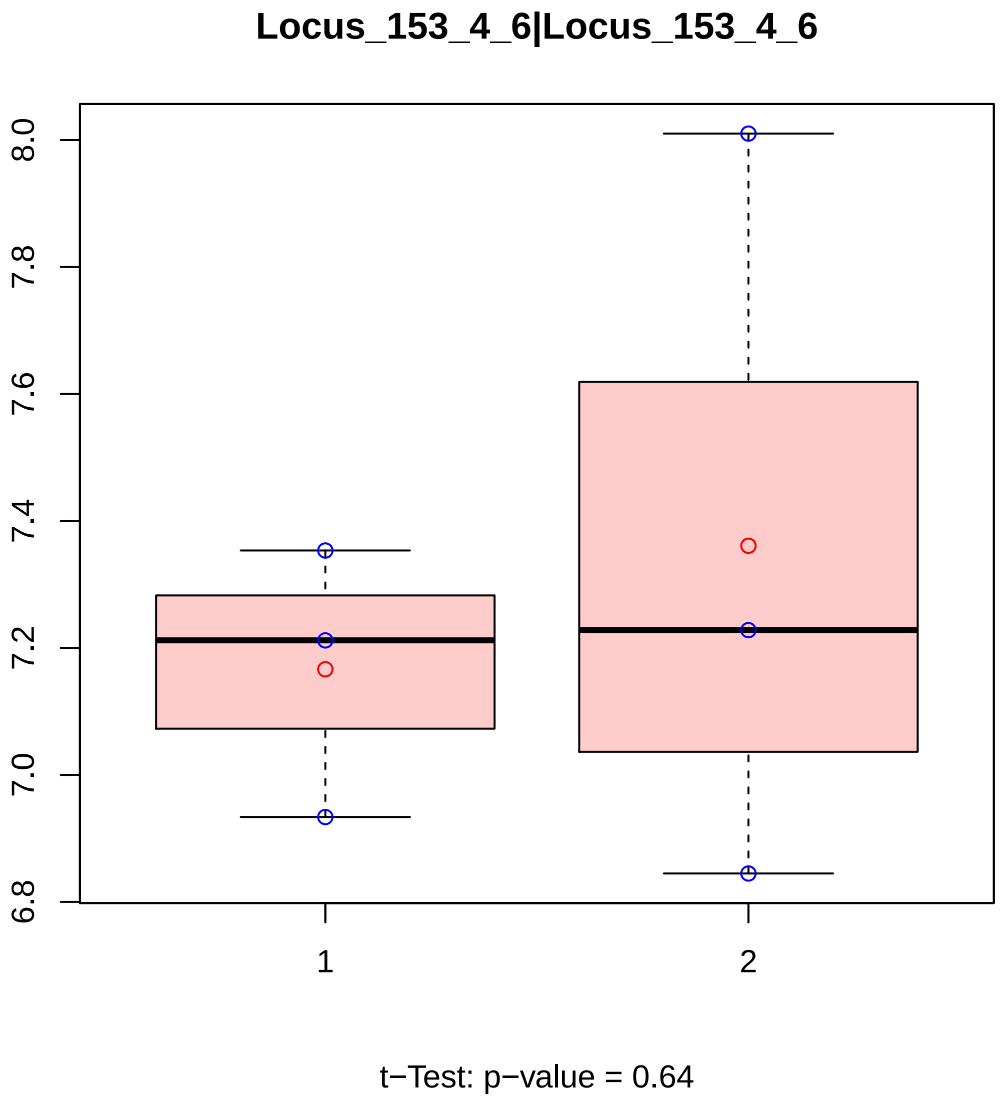
Locus_153_4_6|Locus_153_4_6
8.0
7.8
7.6
7.4
7.2
7.0
6.8
1
2
t−Test: p−value = 0.64

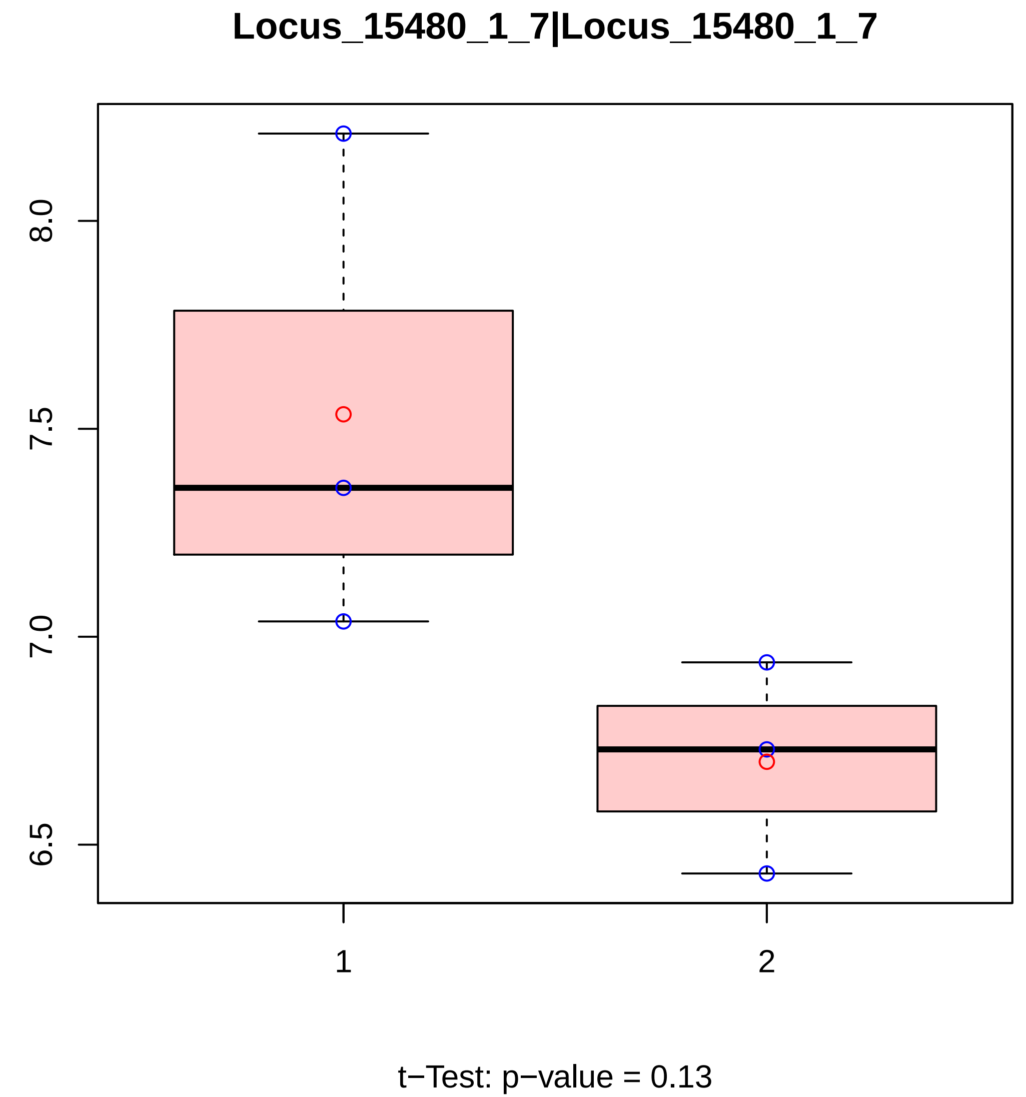
Locus_15480_1_7|Locus_15480_1_7
8.0
7.5
7.0
6.5
1
2
t−Test: p−value = 0.13

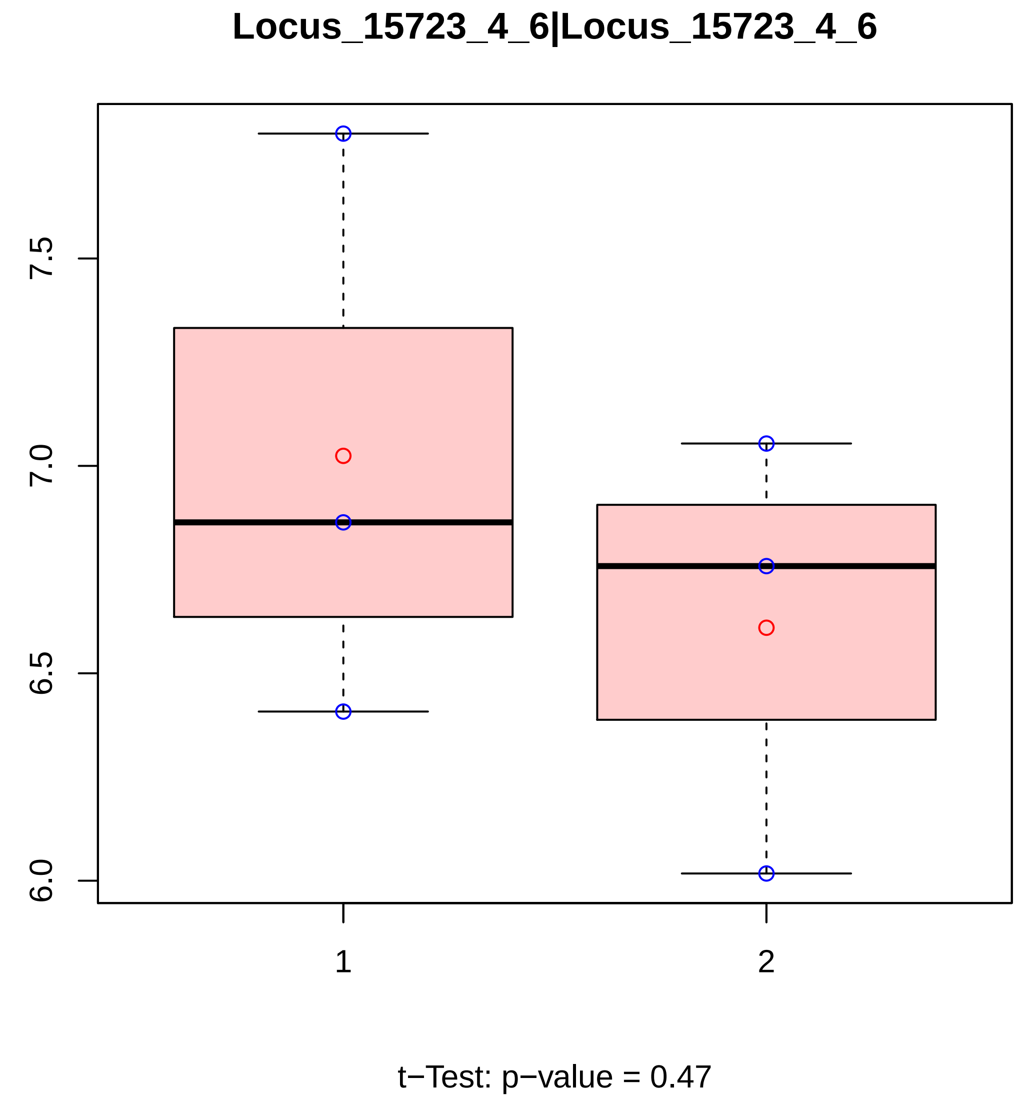
Locus_15723_4_6|Locus_15723_4_6
7.5
7.0
6.5
6.0
1
2
t−Test: p−value = 0.47

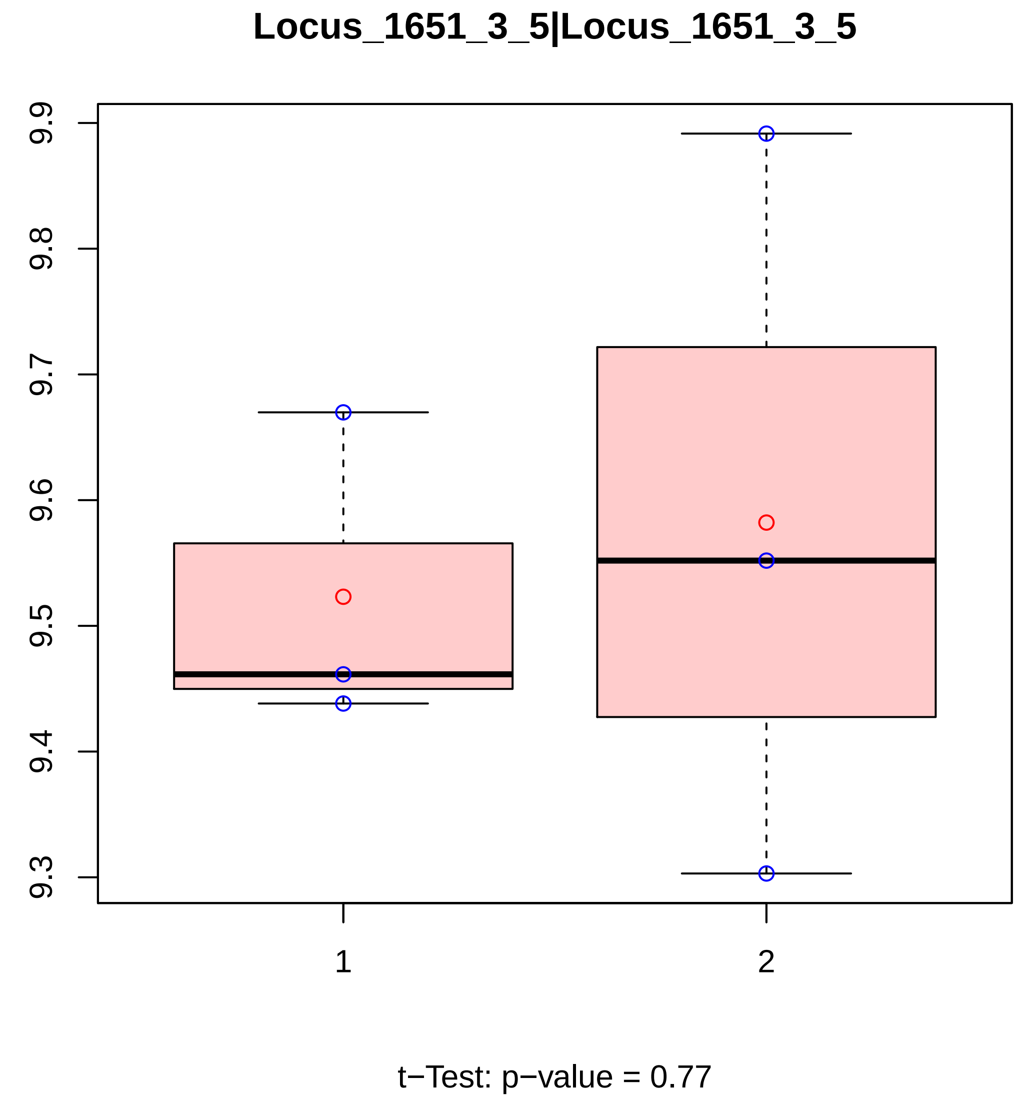

Locus_1651_3_5|Locus_1651_3_5
9.3
9.4
9.5
9.6
9.7
9.8
9.9
1
2
t−Test: p−value = 0.77

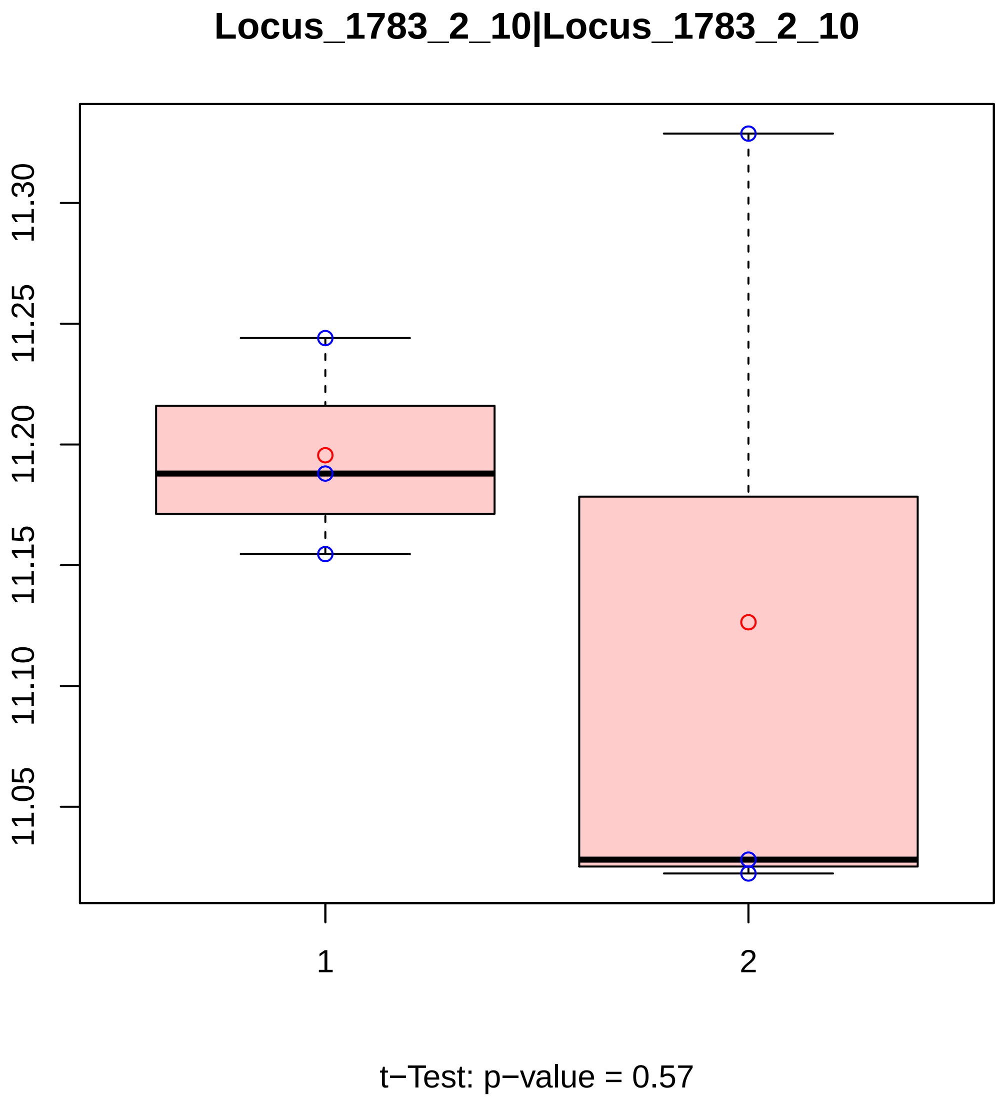
Locus_1783_2_10|Locus_1783_2_10
11.30
11.25
11.20
11.15
11.10
11.05
1
2
t−Test: p−value = 0.57

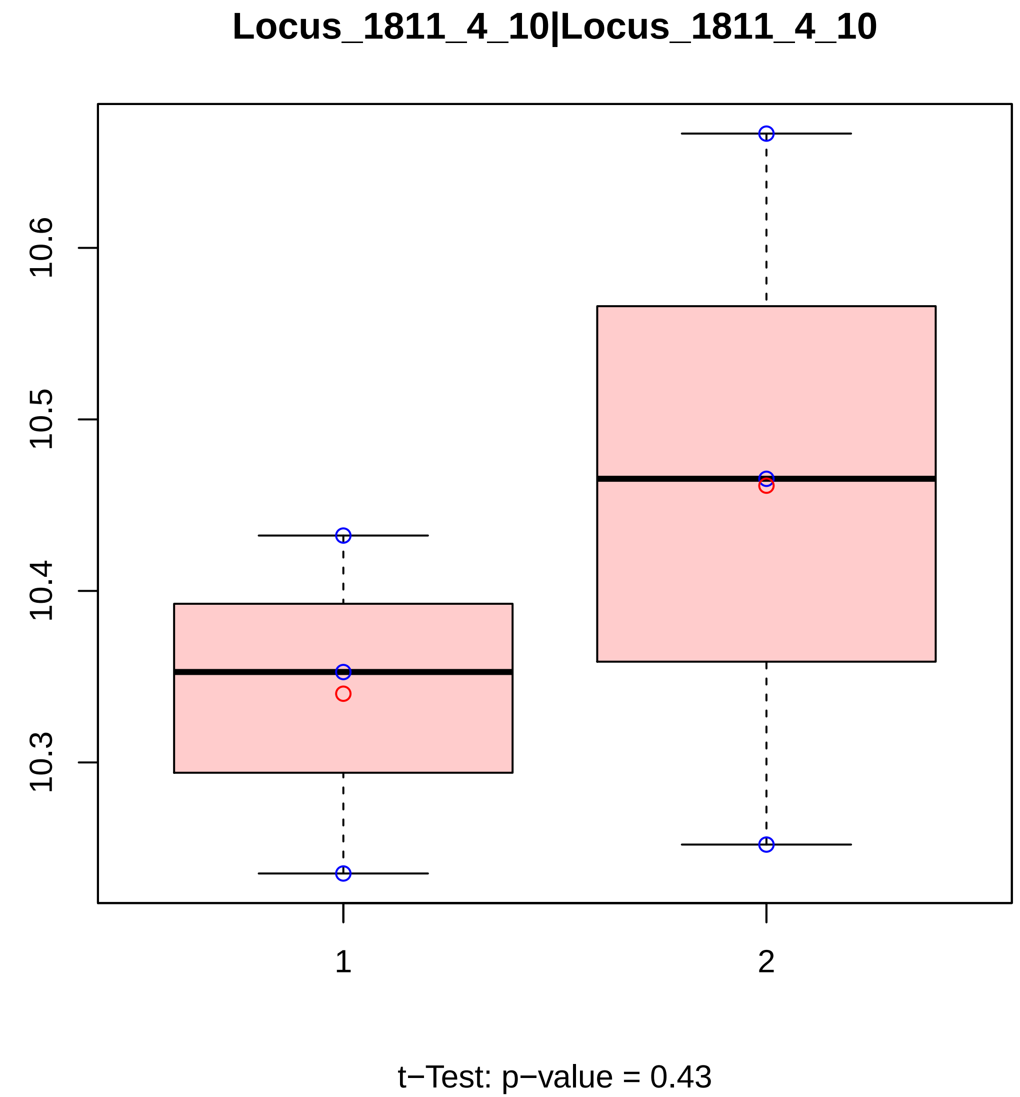

Locus_1811_4_10|Locus_1811_4_10
10.3
10.4
10.5
10.6
1
2
t−Test: p−value = 0.43

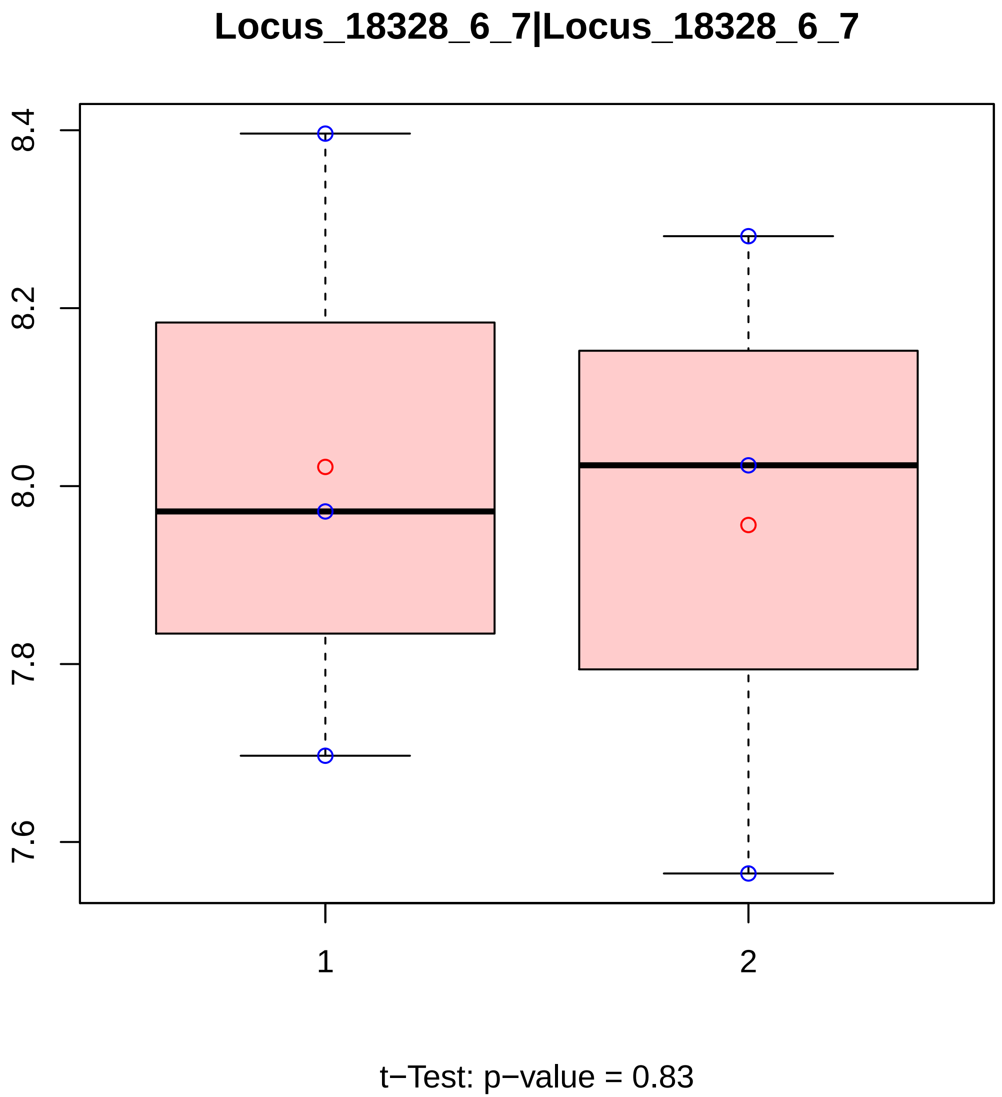

Locus_18328_6_7|Locus_18328_6_7
8.4
8.2
8.0
7.8
7.6
1
2
t−Test: p−value = 0.83

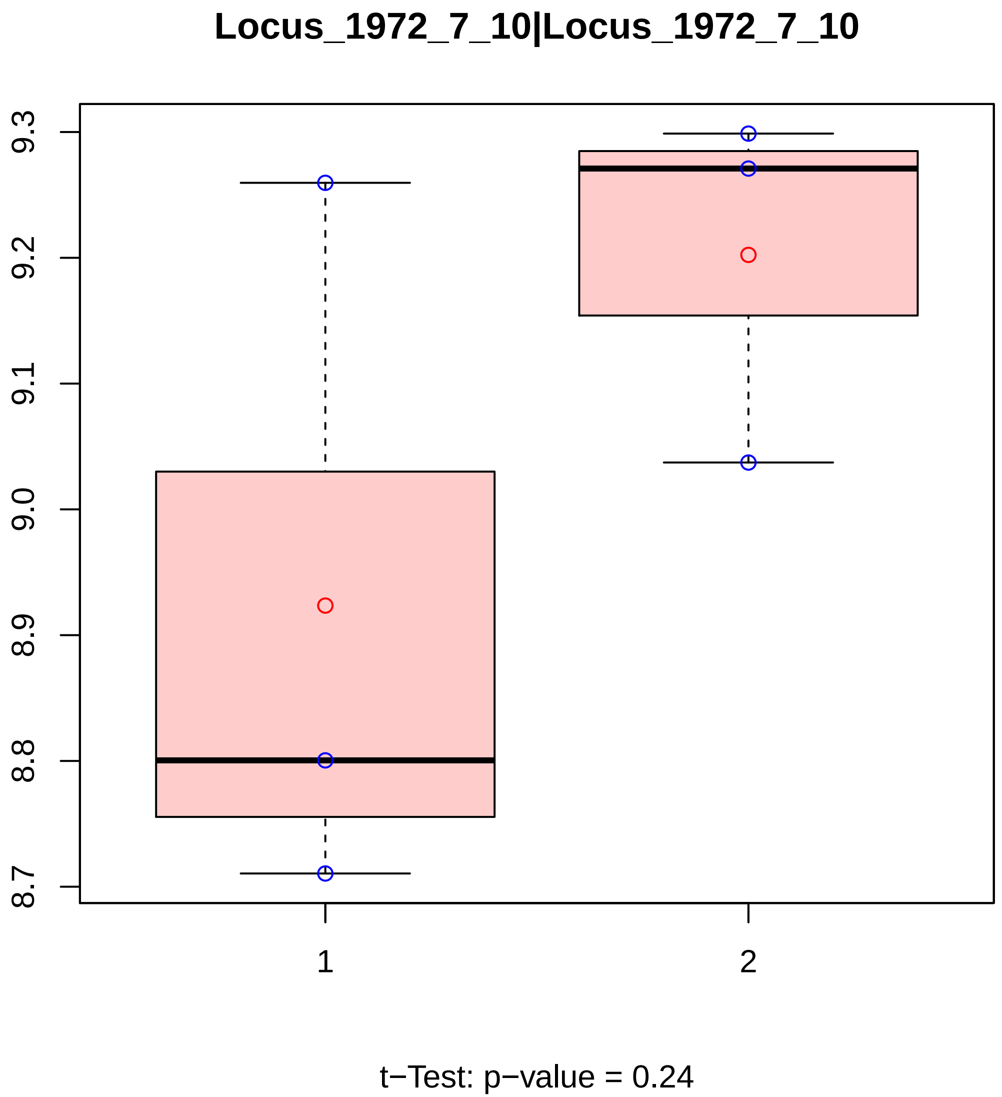
Locus_1972_7_10|Locus_1972_7_10
9.3
9.2
9.1
9.0
8.9
8.8
8.7
1
2
t−Test: p−value = 0.24

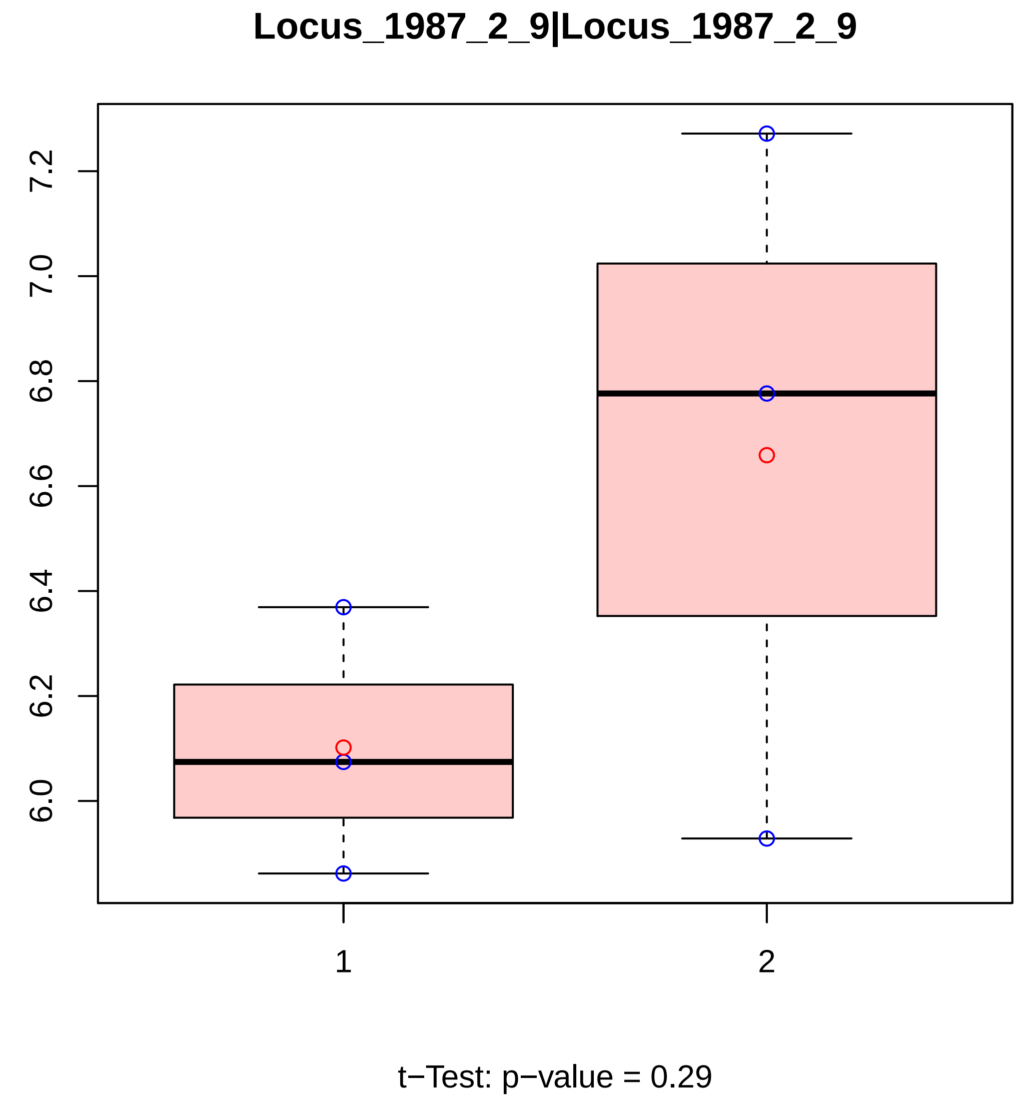
Locus_1987_2_9|Locus_1987_2_9
7.2
7.0
6.8
6.6
6.4
6.2
6.0
1
2
t−Test: p−value = 0.29

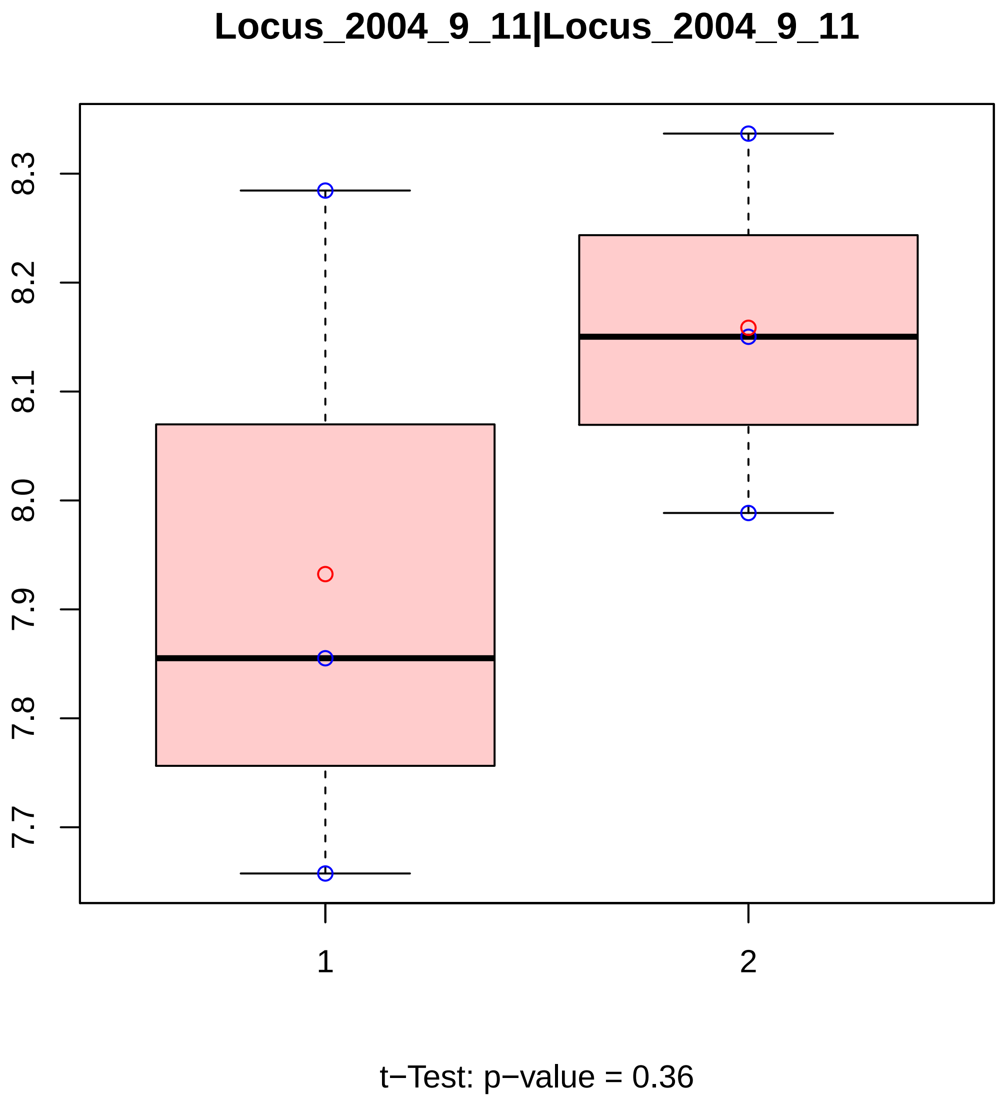
Locus_2004_9_11|Locus_2004_9_11
8.3
8.2
8.1
8.0
7.9
7.8
7.7
1
2
t−Test: p−value = 0.36

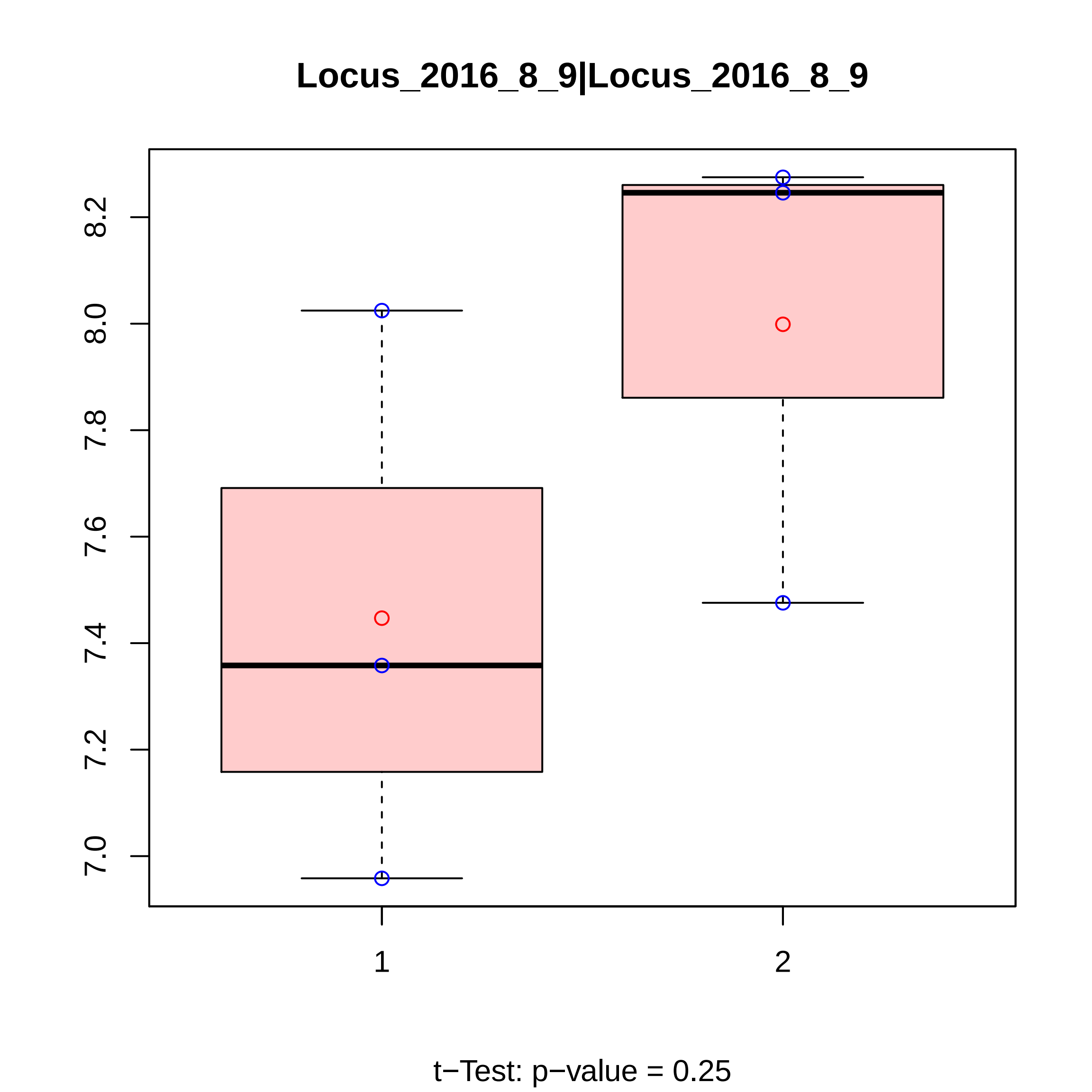
Locus_2016_8_9|Locus_2016_8_9
8.2
8.0
7.8
7.6
7.4
7.2
7.0
1
2
t−Test: p−value = 0.25

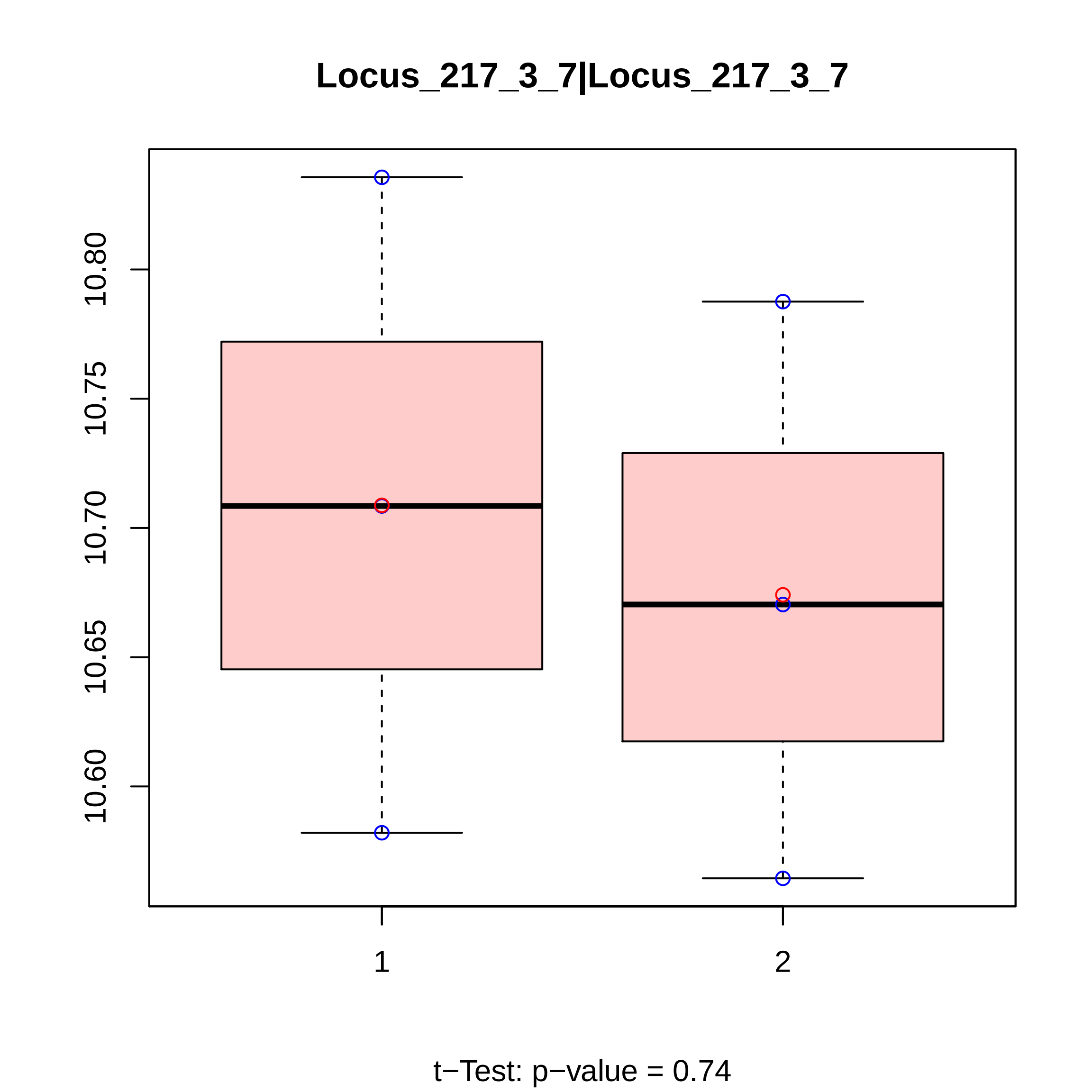
Locus_217_3_7|Locus_217_3_7
10.60
10.65
10.70
10.75
10.80
1
2
t−Test: p−value = 0.74

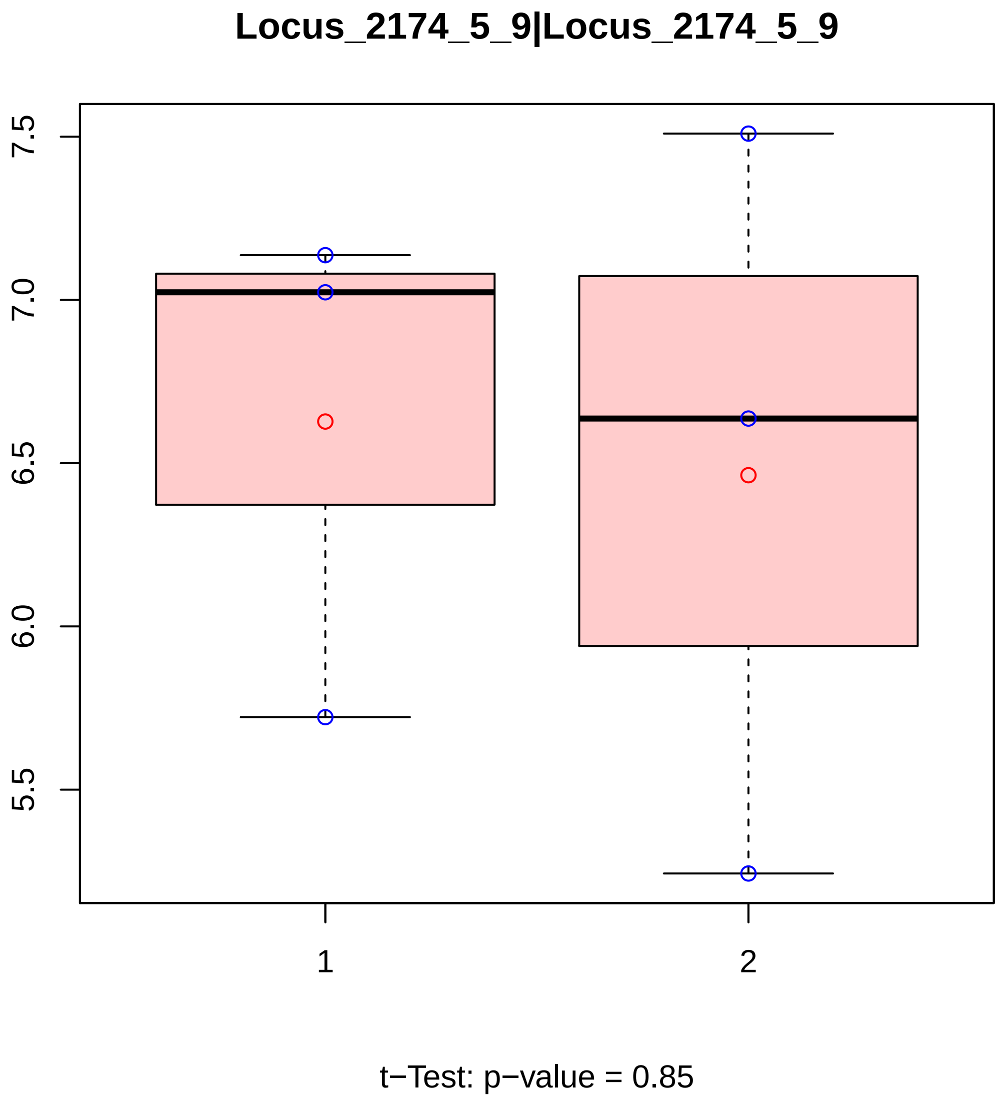
Locus_2174_5_9|Locus_2174_5_9
7.5
7.0
6.5
6.0
5.5
1
2
t−Test: p−value = 0.85

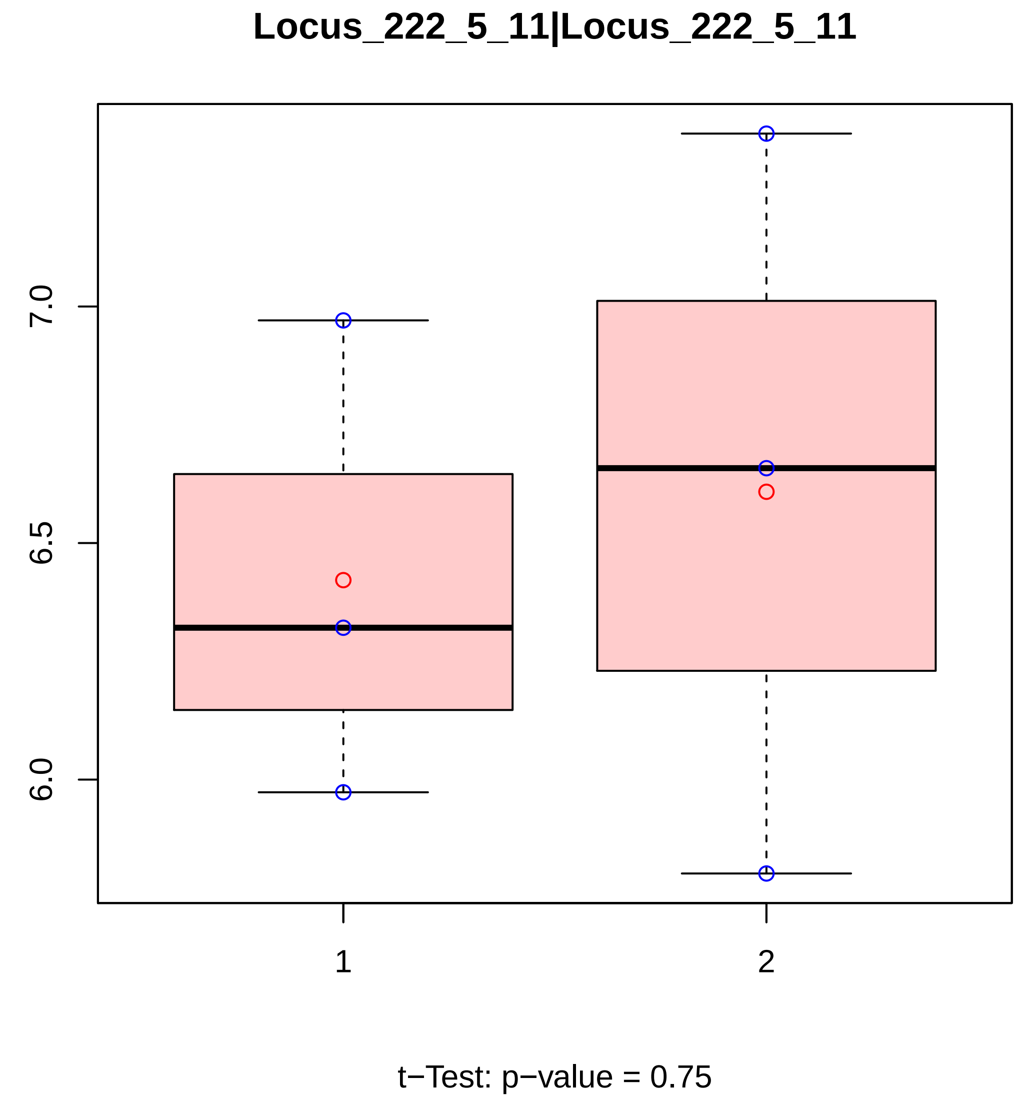
Locus_222_5_11|Locus_222_5_11
7.0
6.5
6.0
1
2
t−Test: p−value = 0.75

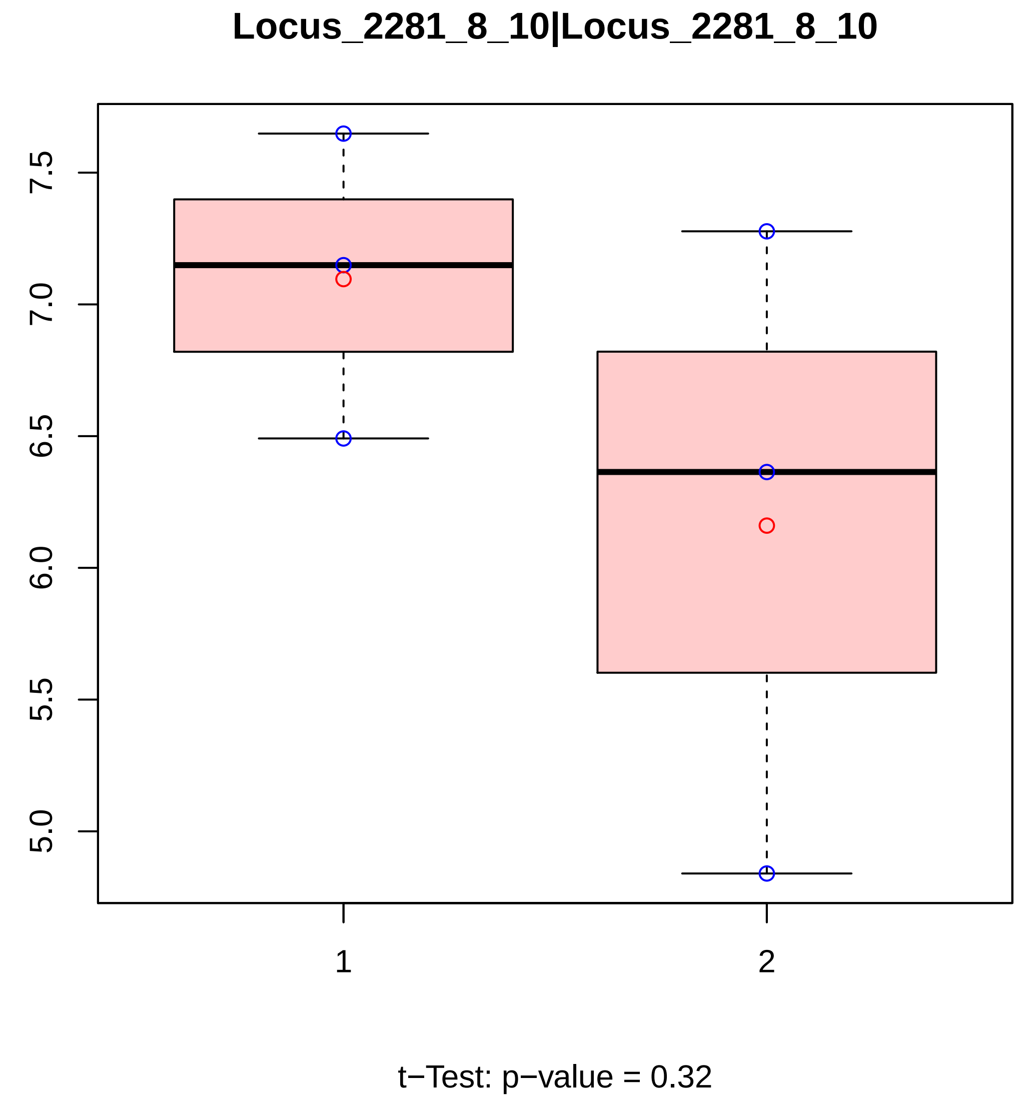
Locus_2281_8_10|Locus_2281_8_10
7.5
7.0
6.5
6.0
5.5
5.0
1
2
t−Test: p−value = 0.32

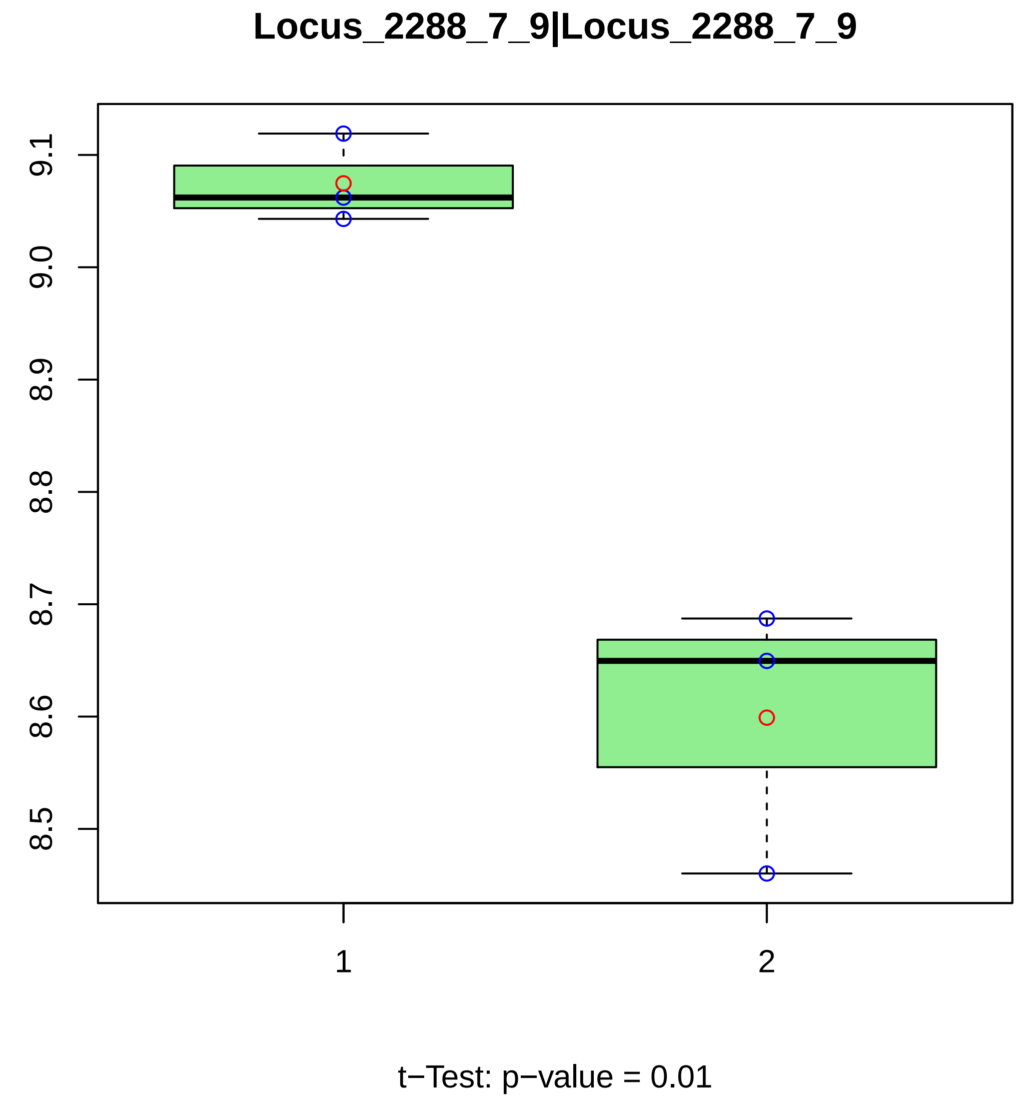
Locus_2288_7_9|Locus_2288_7_9
9.1
9.0
8.9
8.8
8.7
8.6
8.5
1
2
t−Test: p−value = 0.01

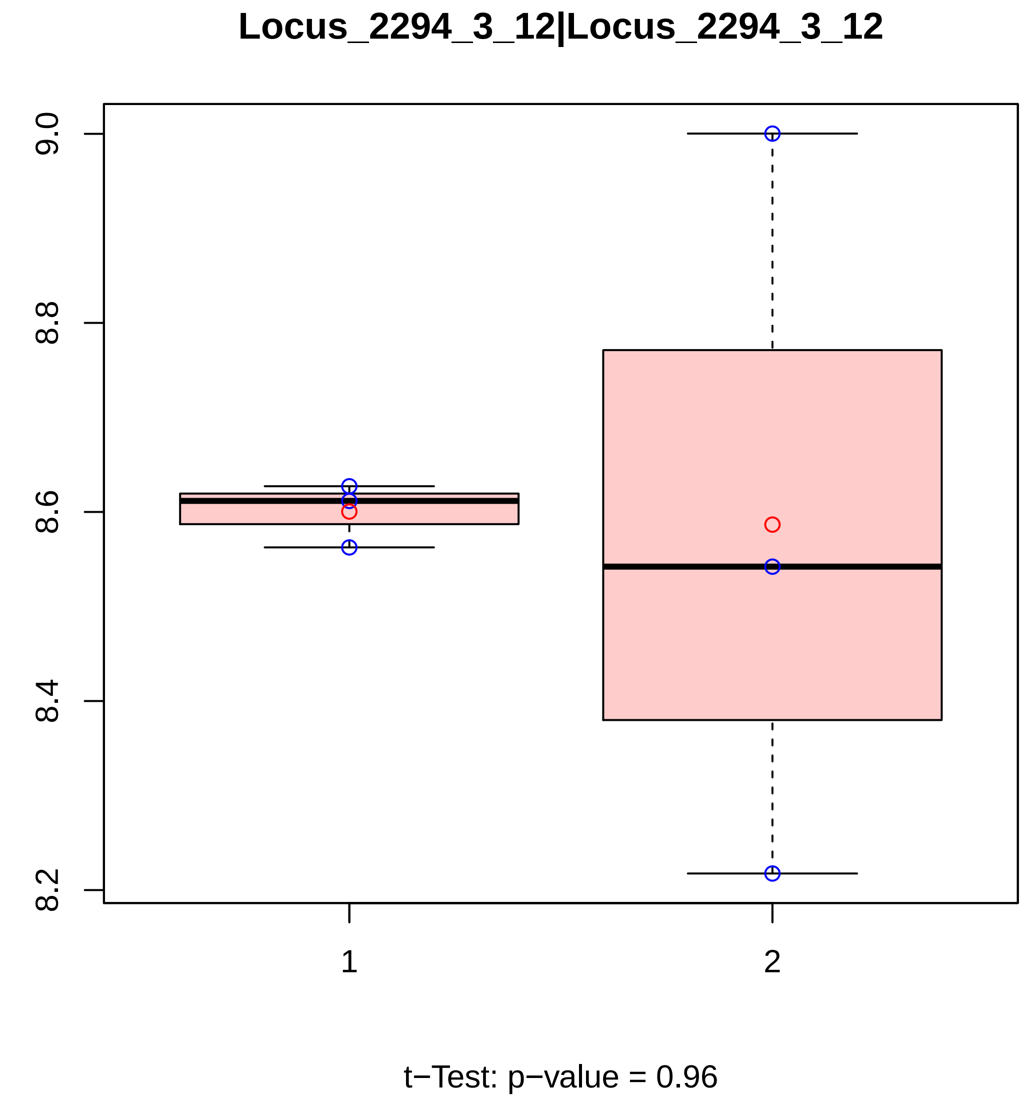
Locus_2294_3_12|Locus_2294_3_12
9.0
8.8
8.6
8.4
8.2
1
2
t−Test: p−value = 0.96

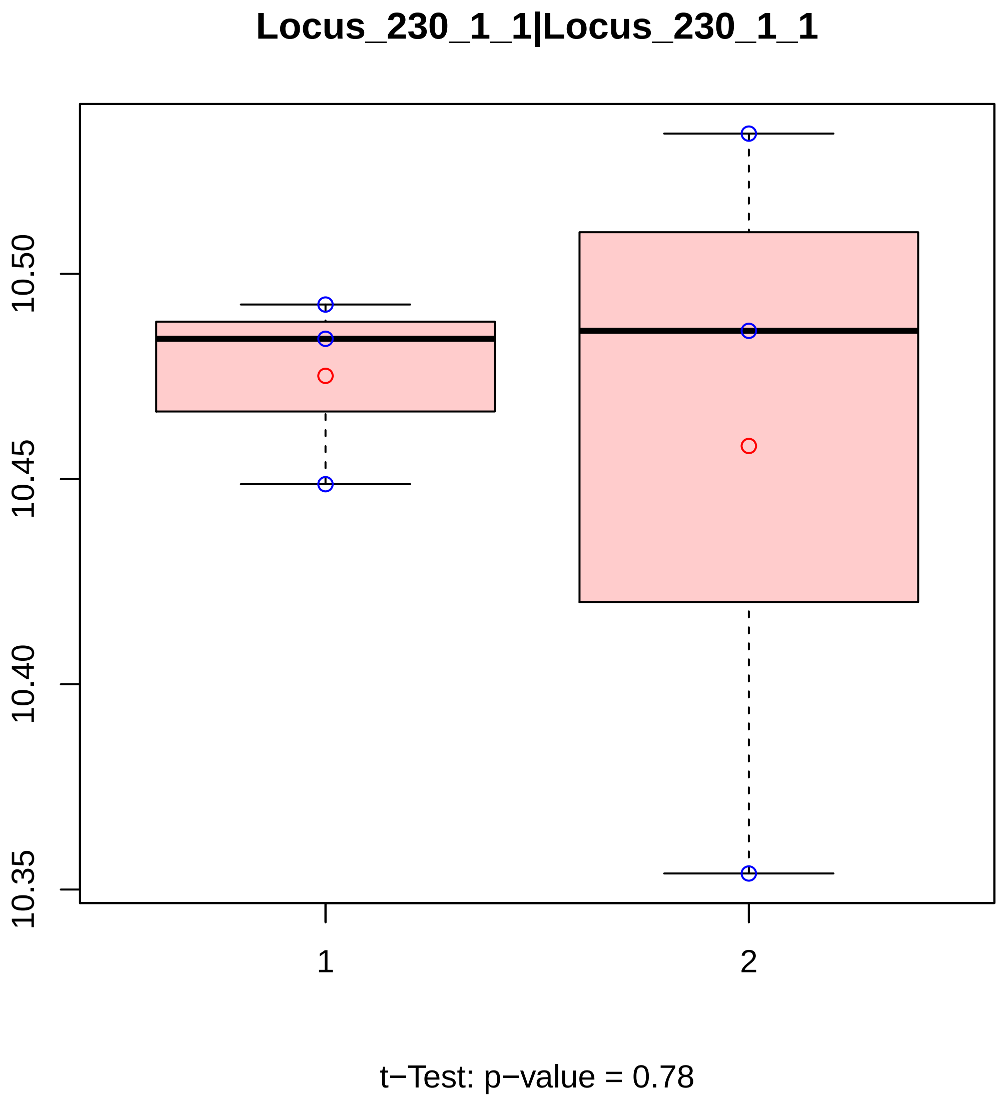

Locus_230_1_1|Locus_230_1_1
10.35
10.40
10.45
10.50
1
2
t−Test: p−value = 0.78

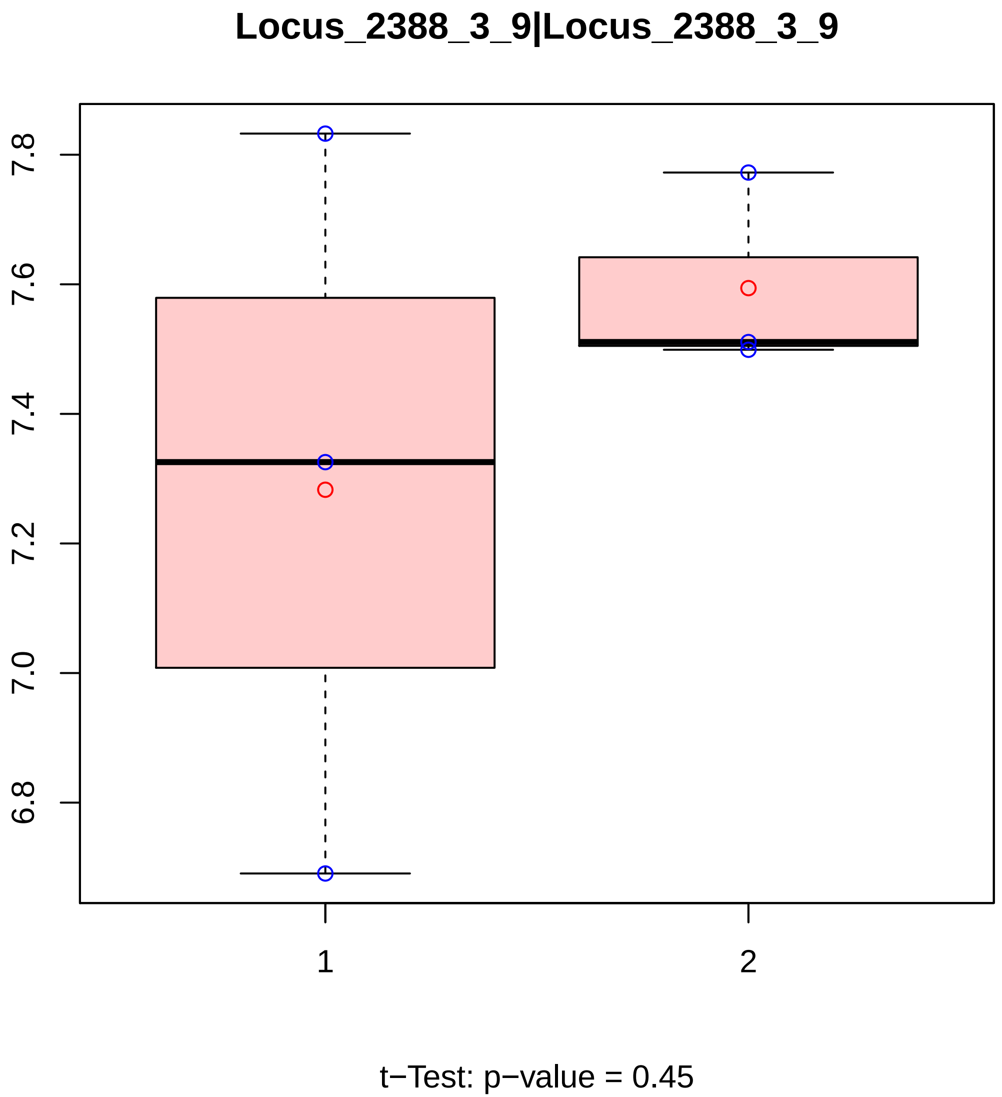

Locus_2388_3_9|Locus_2388_3_9
7.8
7.6
7.4
7.2
7.0
6.8
1
2
t−Test: p−value = 0.45

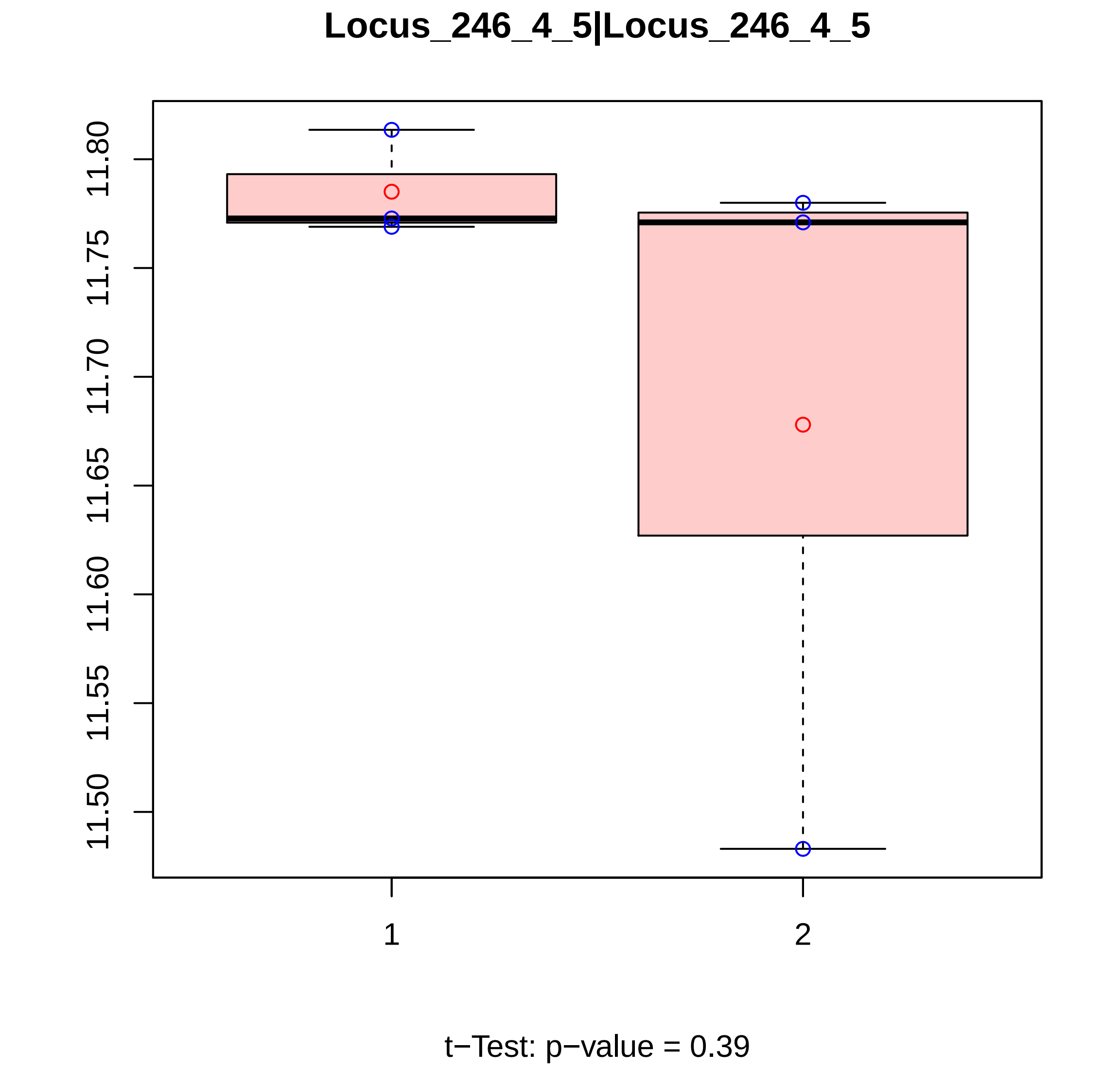

Locus_246_4_5|Locus_246_4_5
11.50
11.55
11.60
11.65
11.70
11.75
11.80
1
2
t−Test: p−value = 0.39

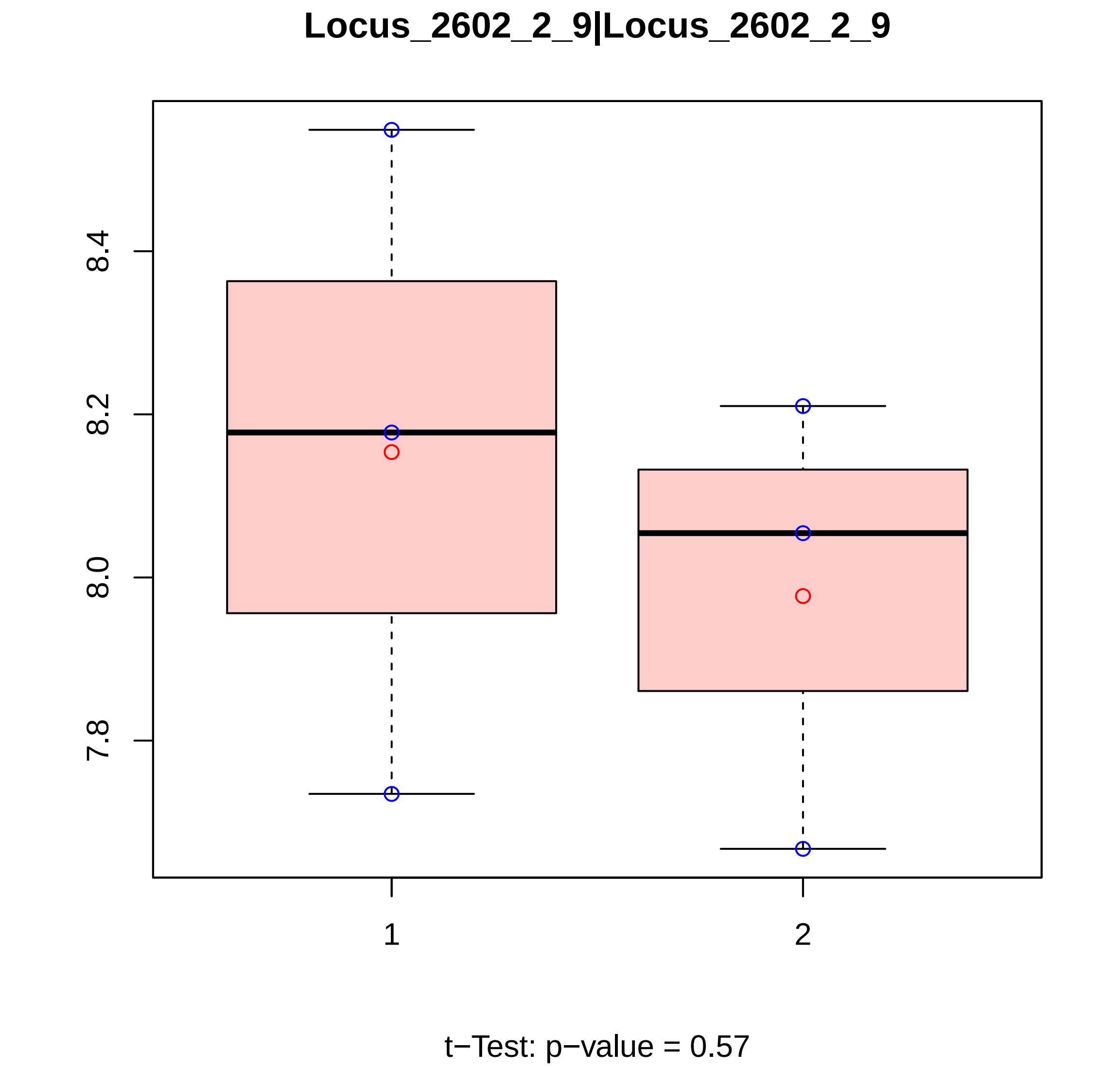
Locus_2602_2_9|Locus_2602_2_9
8.4
8.2
8.0
7.8
1
2
t−Test: p−value = 0.57

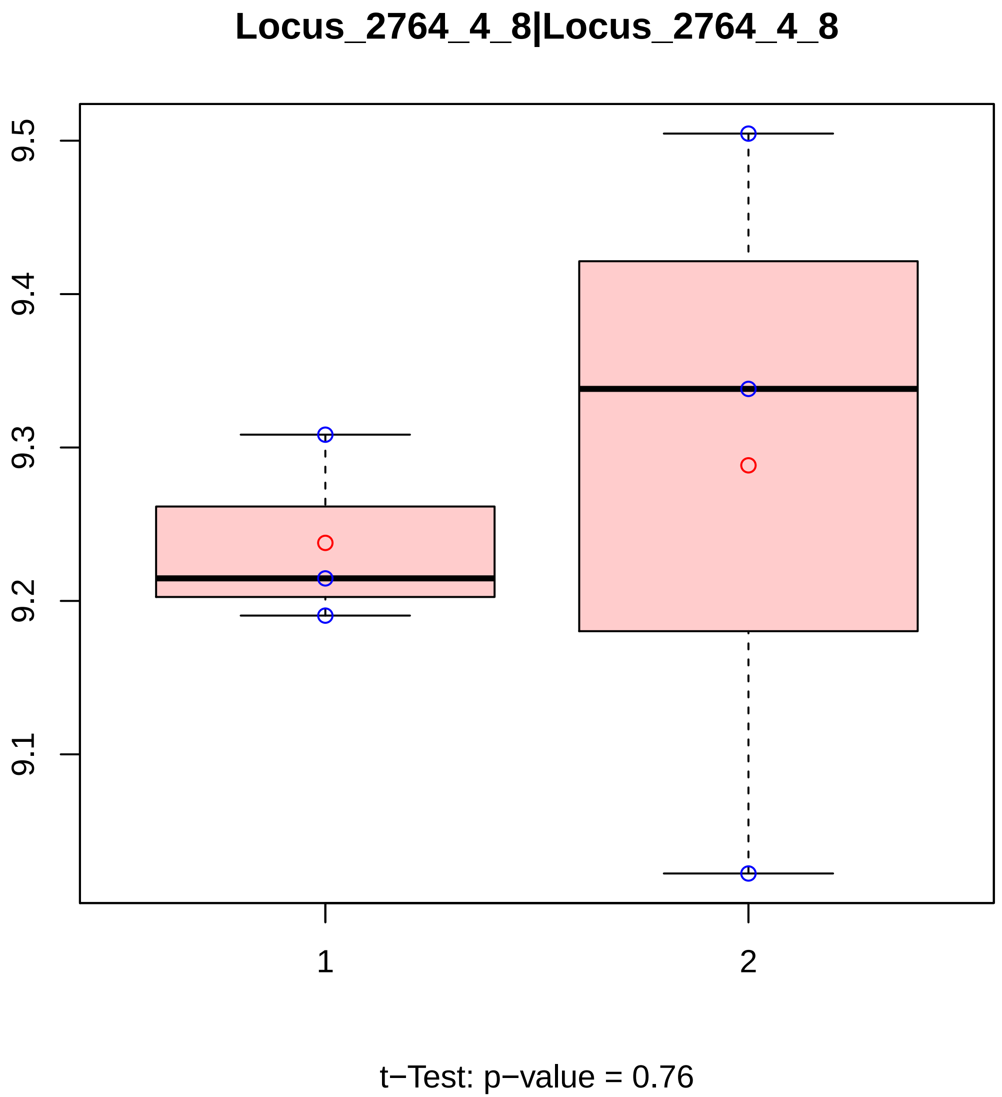

Locus_2764_4_8|Locus_2764_4_8
9.1
9.2
9.3
9.4
9.5
1
2
t−Test: p−value = 0.76

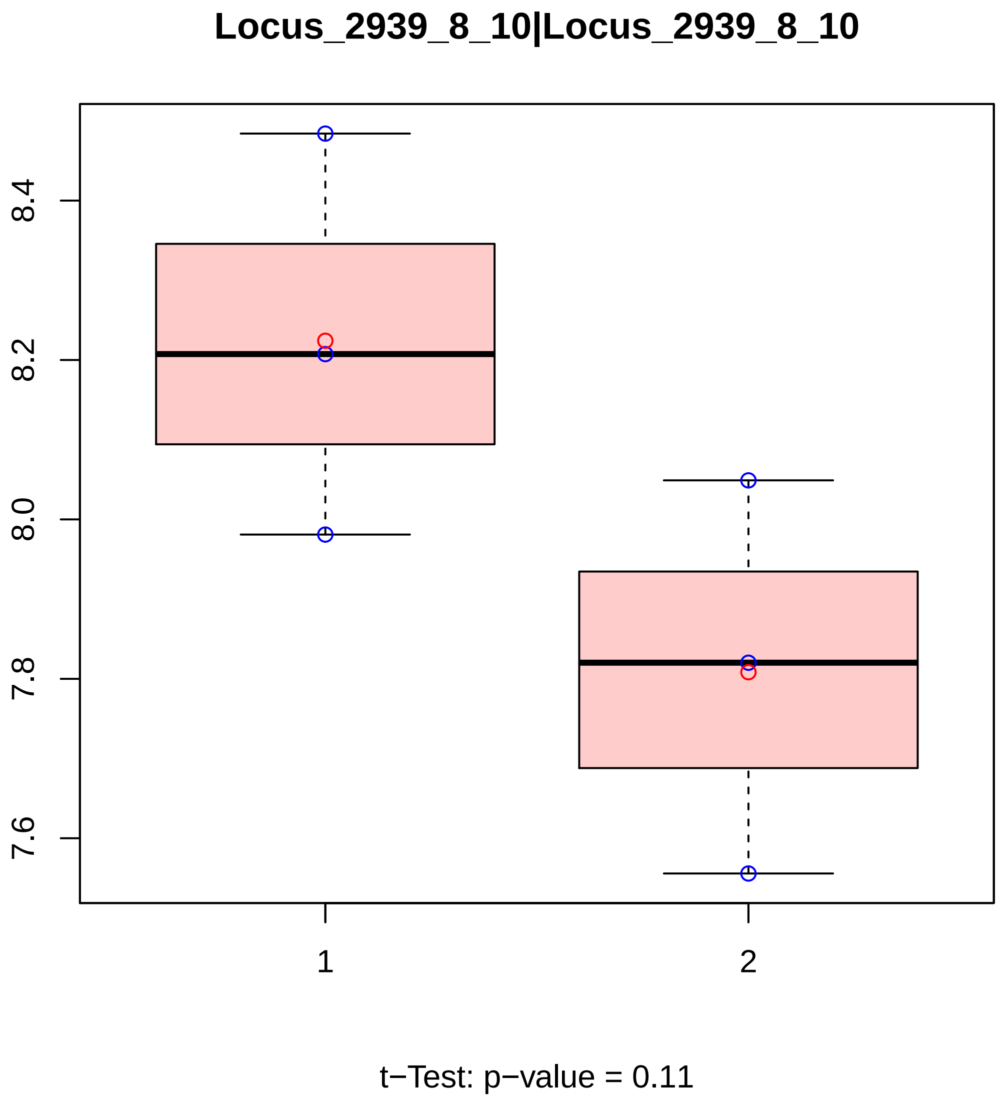

Locus_2939_8_10|Locus_2939_8_10
8.4
8.2
8.0
7.8
7.6
1
2
t−Test: p−value = 0.11

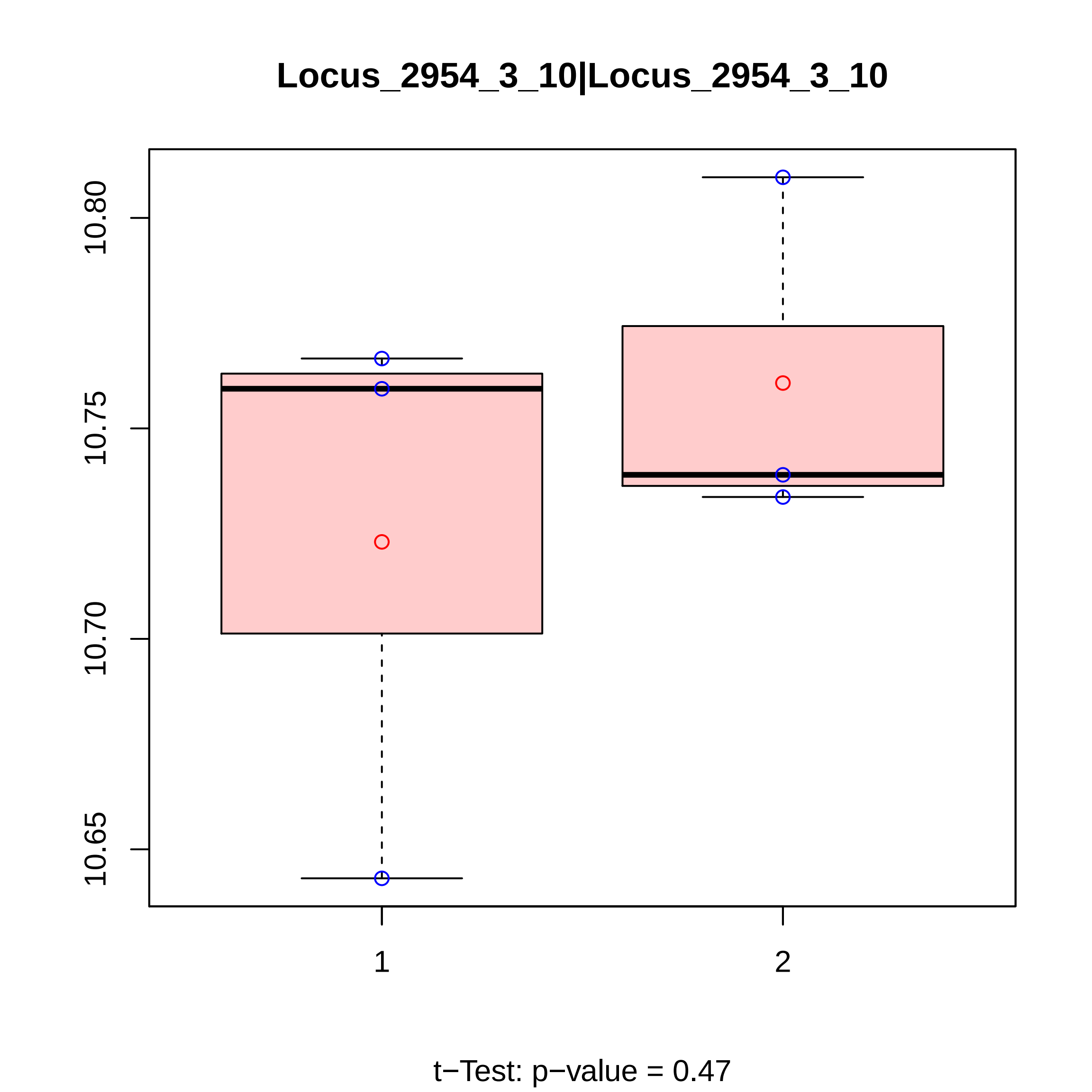

Locus_2954_3_10|Locus_2954_3_10
10.65
10.70
10.75
10.80
1
2
t−Test: p−value = 0.47

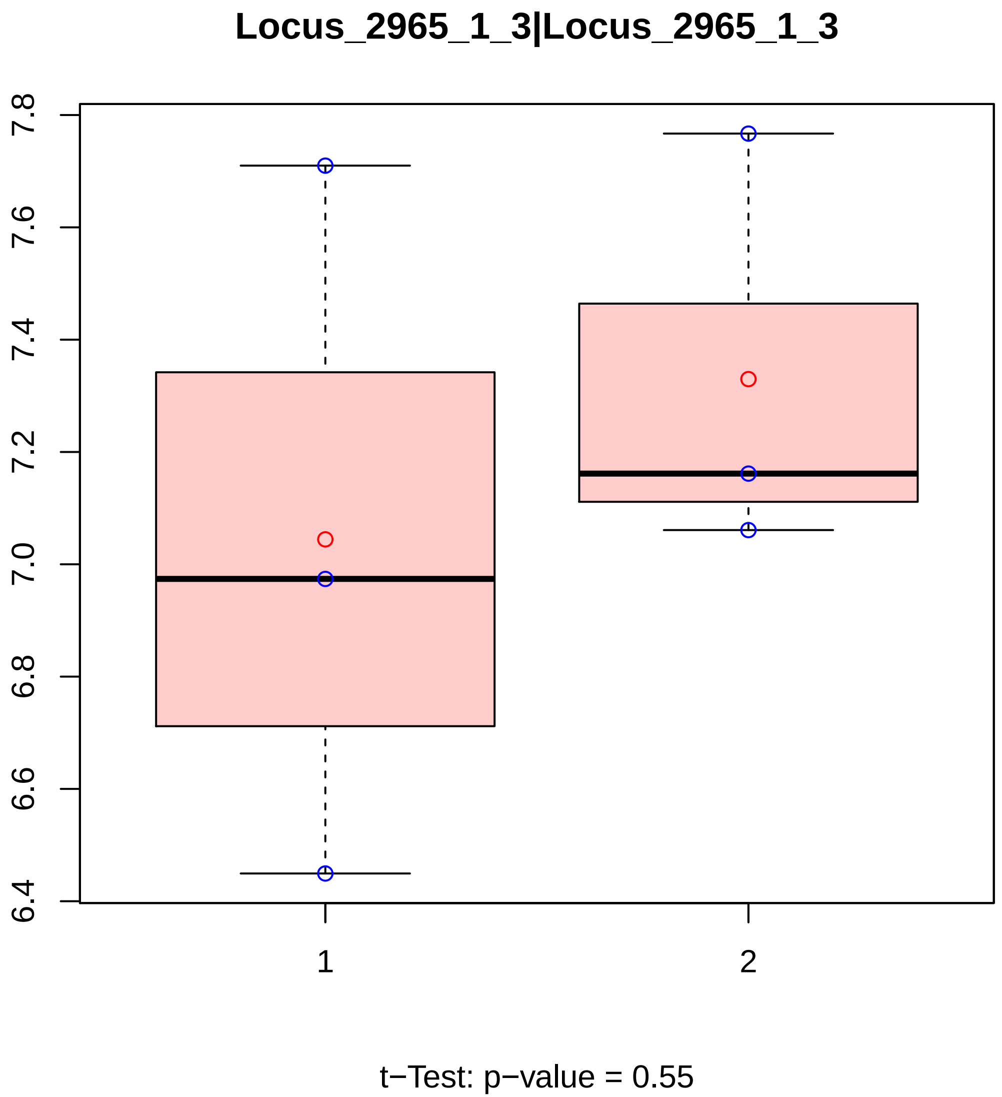
Locus_2965_1_3|Locus_2965_1_3
7.8
7.6
7.4
7.2
7.0
6.8
6.6
6.4
1
2
t−Test: p−value = 0.55

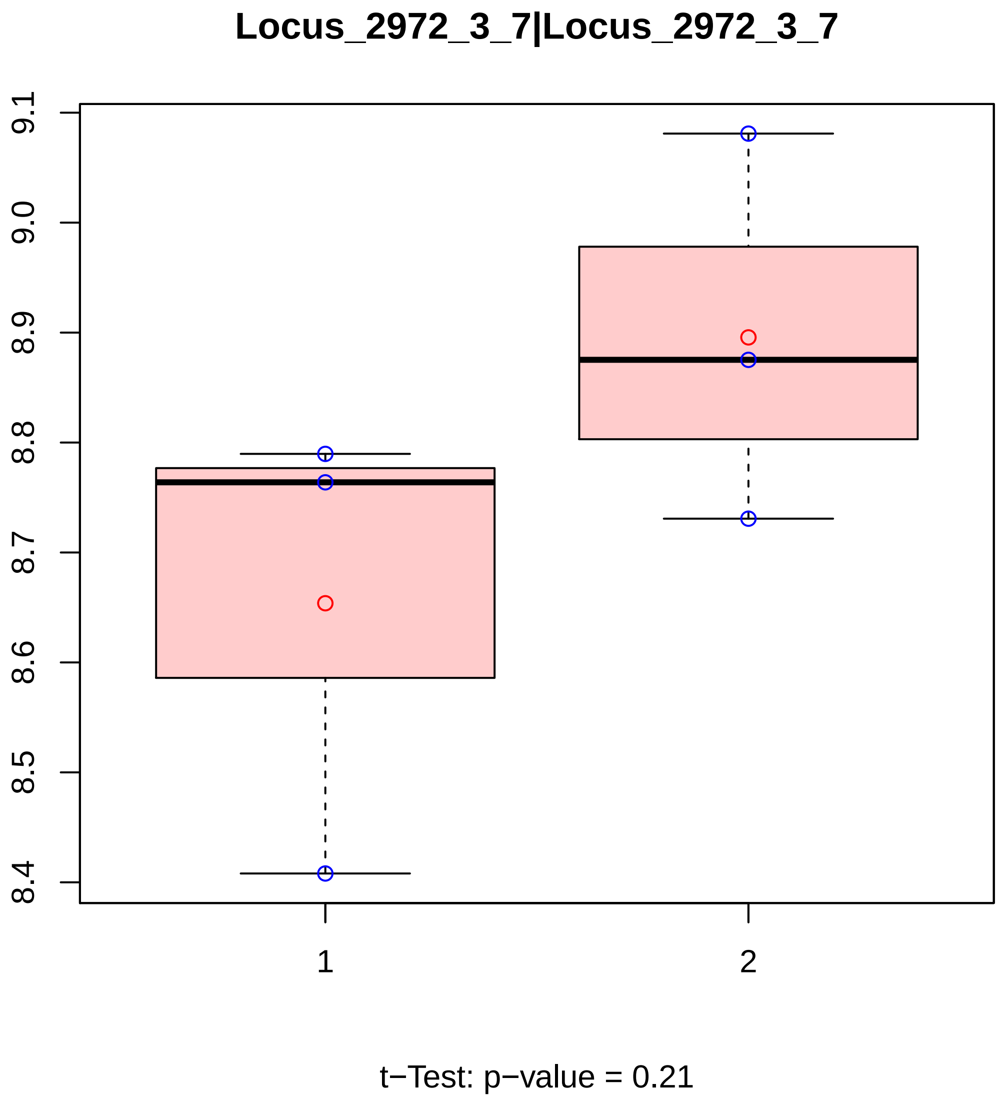

Locus_2972_3_7|Locus_2972_3_7
9.1
9.0
8.9
8.8
8.7
8.6
8.5
8.4
1
2
t−Test: p−value = 0.21

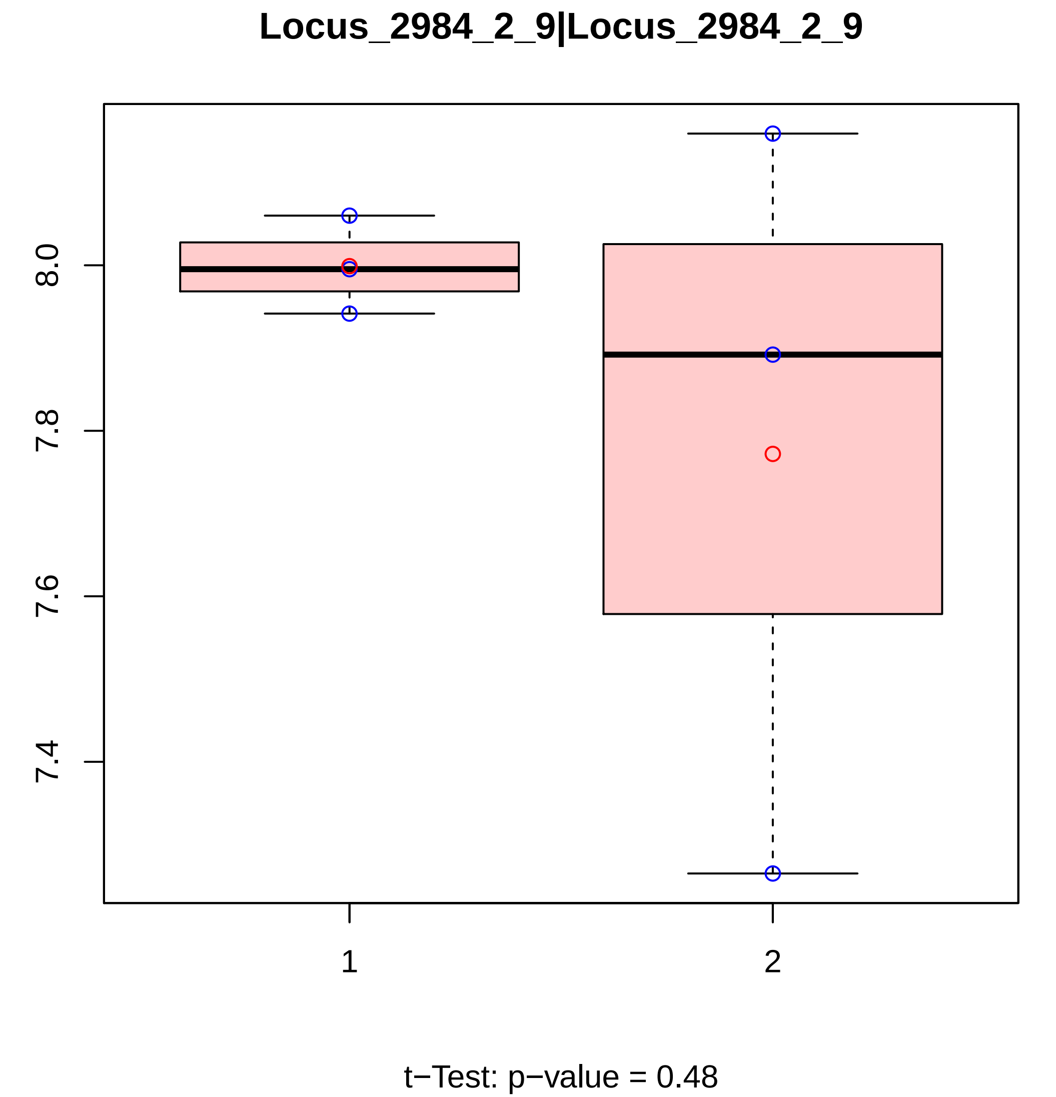
Locus_2984_2_9|Locus_2984_2_9
8.0
7.8
7.6
7.4
1
2
t−Test: p−value = 0.48

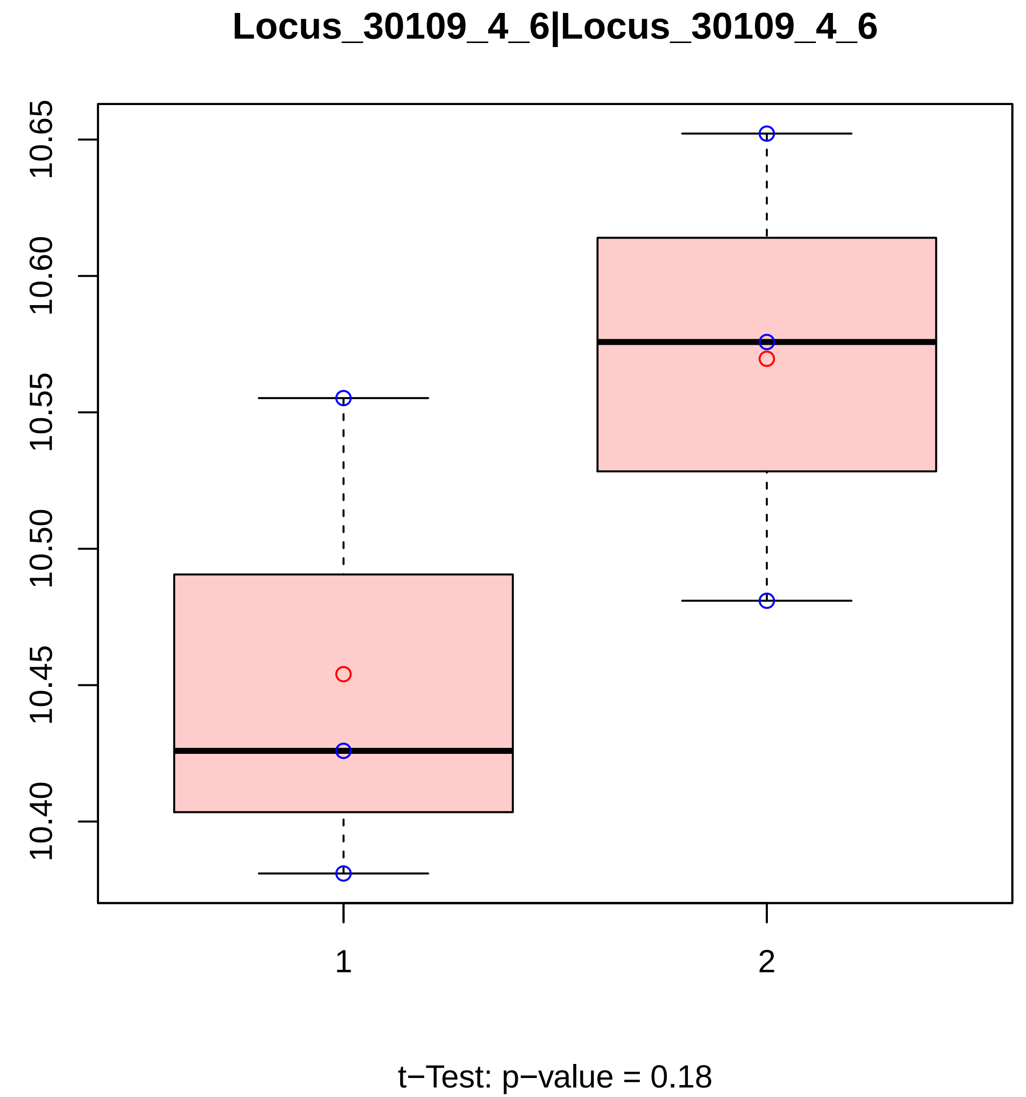

Locus_30109_4_6|Locus_30109_4_6
10.40
10.45
10.50
10.55
10.60
10.65
1
2
t−Test: p−value = 0.18

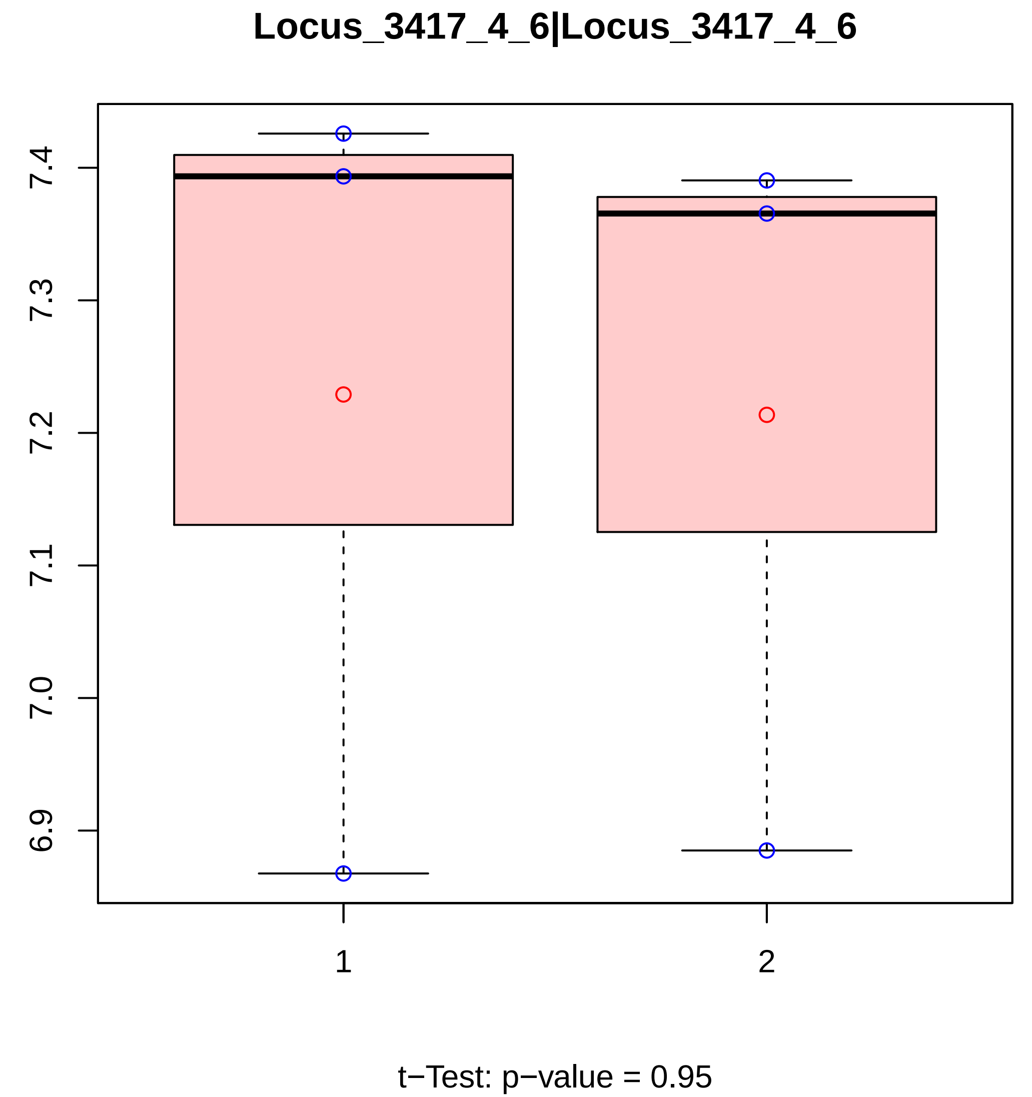

Locus_3417_4_6|Locus_3417_4_6
6.9
7.0
7.1
7.2
7.3
7.4
1
2
t−Test: p−value = 0.95

## Locus_3532_1_8|Locus_3532_1_8

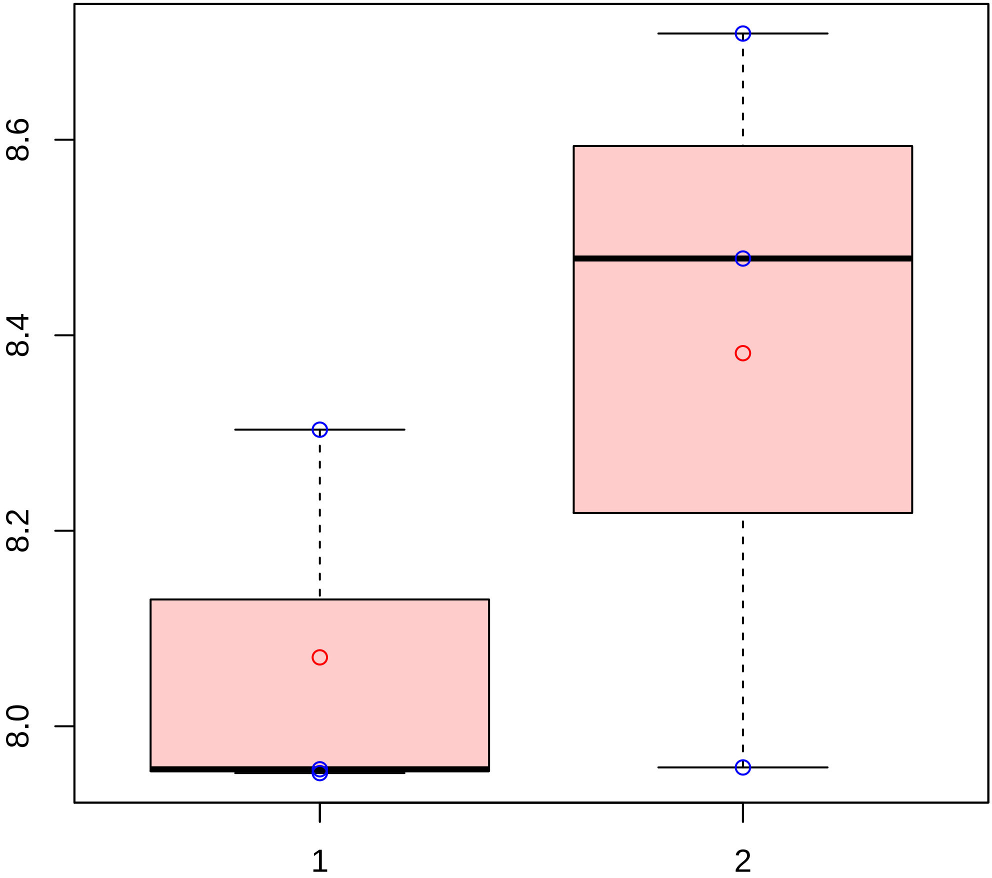


t−Test: p−value = 0.3

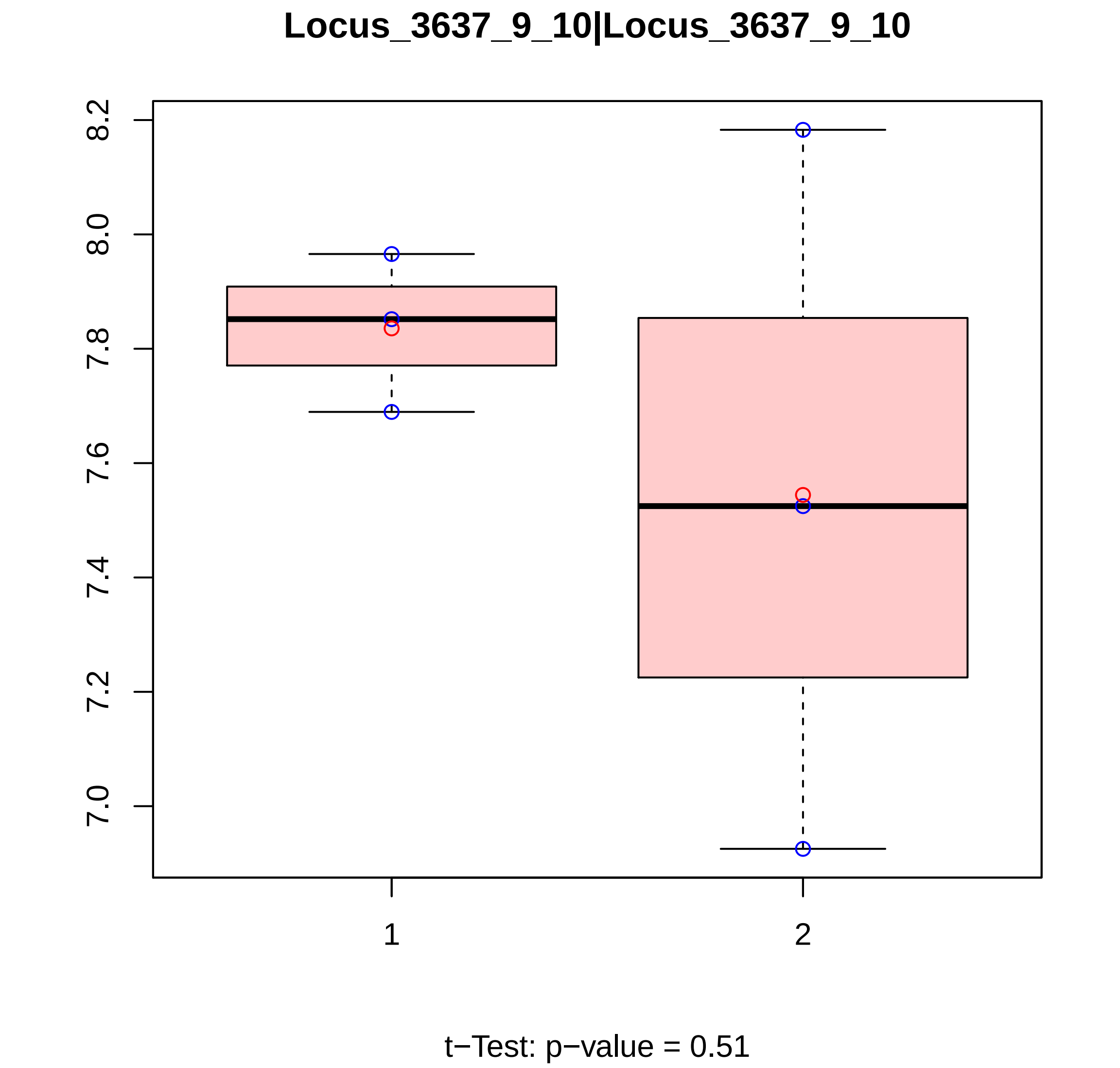
Locus_3637_9_10|Locus_3637_9_10
8.2
8.0
7.8
7.6
7.4
7.2
7.0
1
2
t−Test: p−value = 0.51

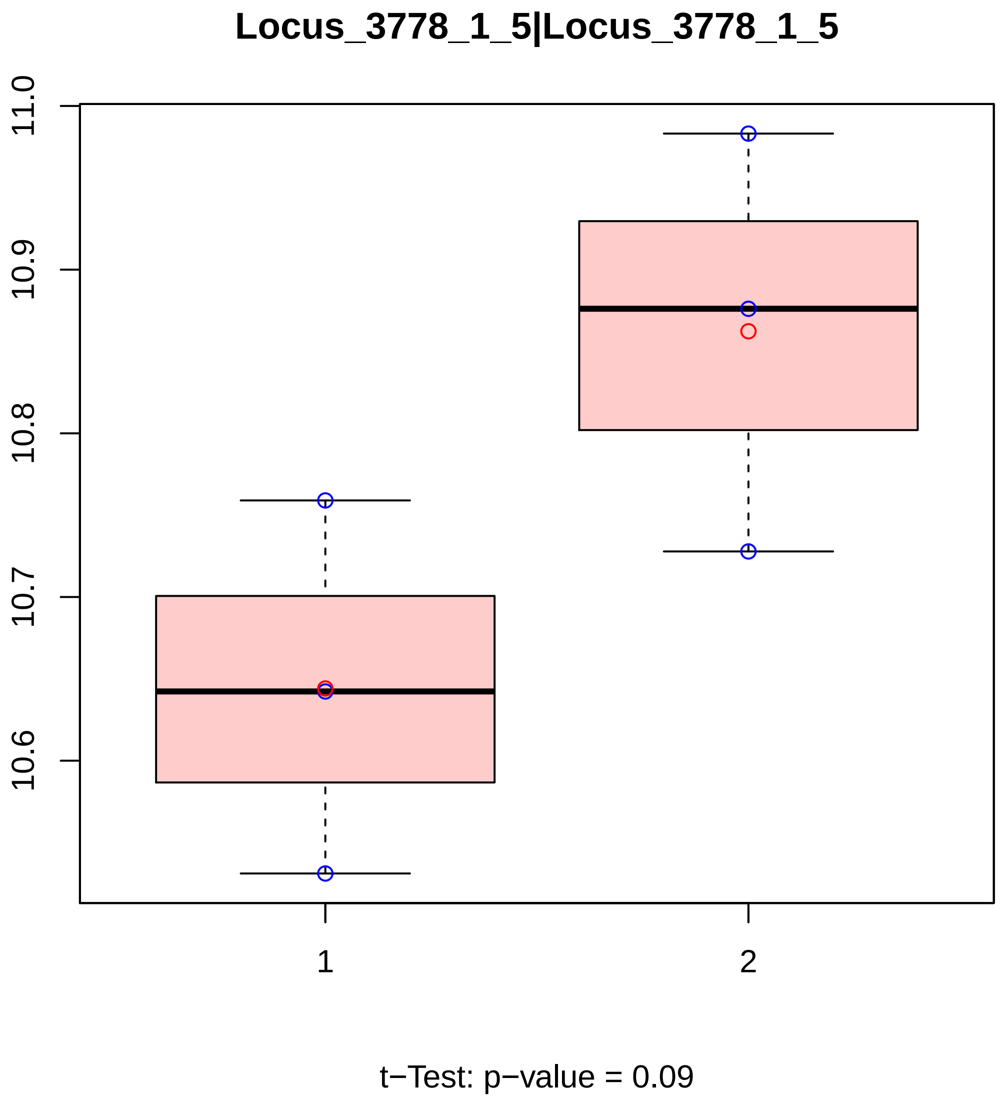

Locus_3778_1_5|Locus_3778_1_5
11.0
10.9
10.8
10.7
10.6
1
2
t−Test: p−value = 0.09

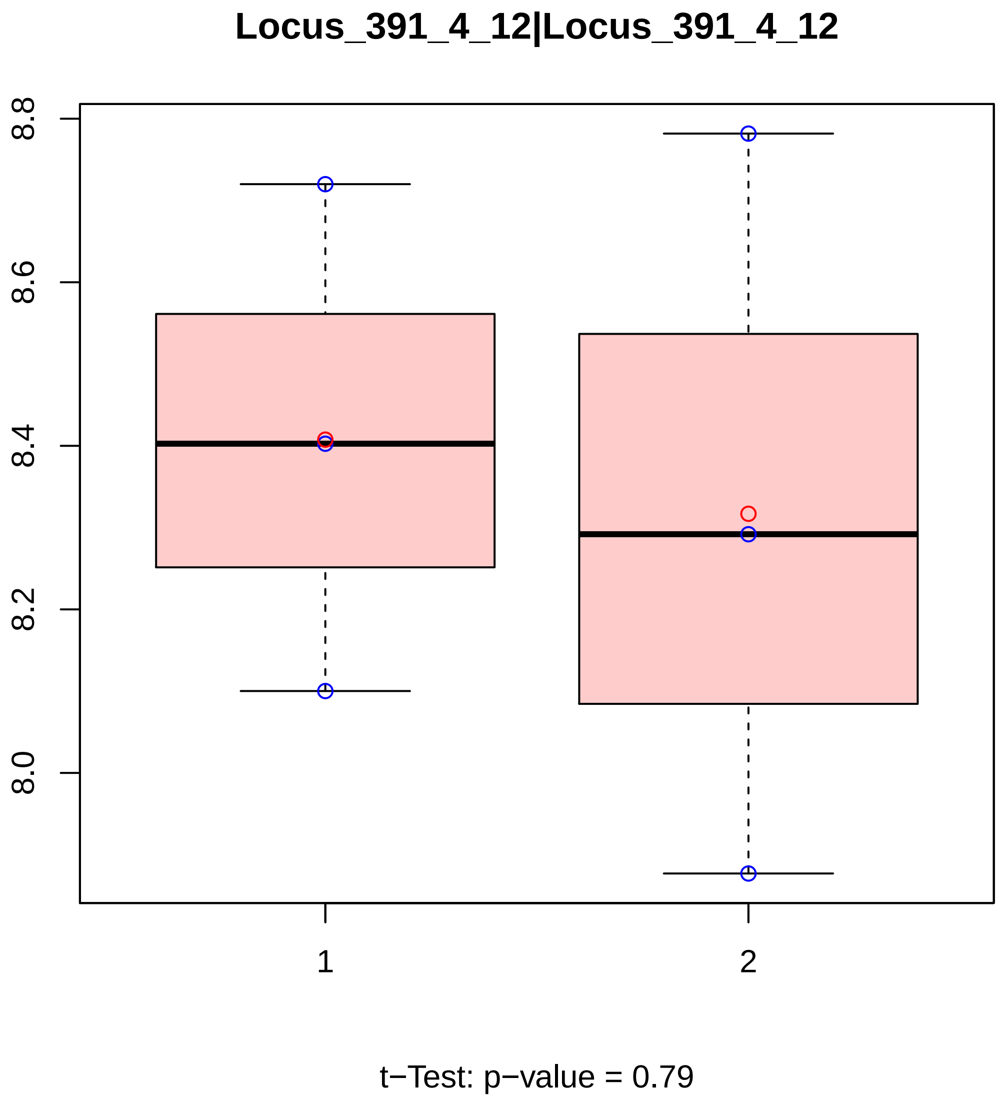

Locus_391_4_12|Locus_391_4_12
8.8
8.6
8.4
8.2
8.0
1
2
t−Test: p−value = 0.79

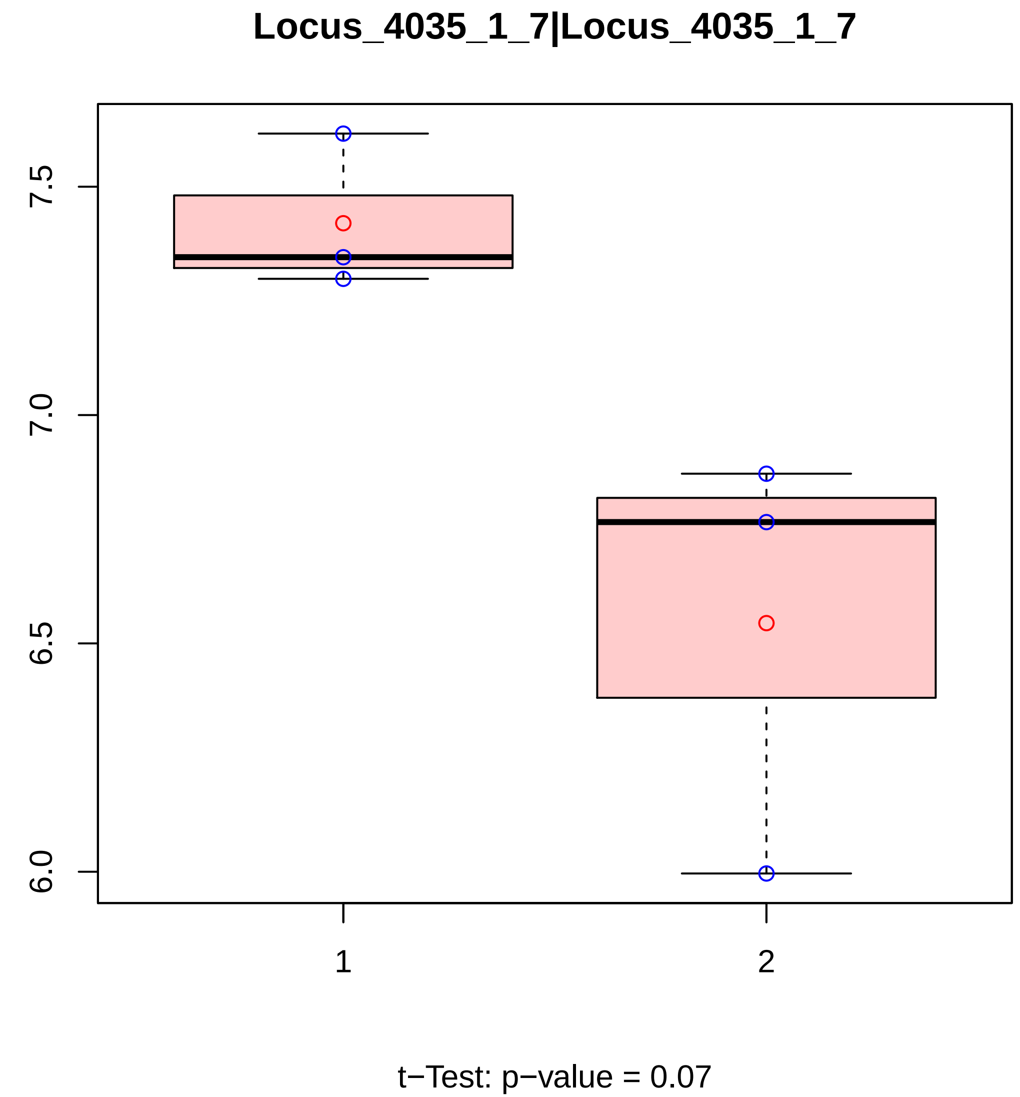
Locus_4035_1_7|Locus_4035_1_7
7.5
7.0
6.5
6.0
1
2
t−Test: p−value = 0.07

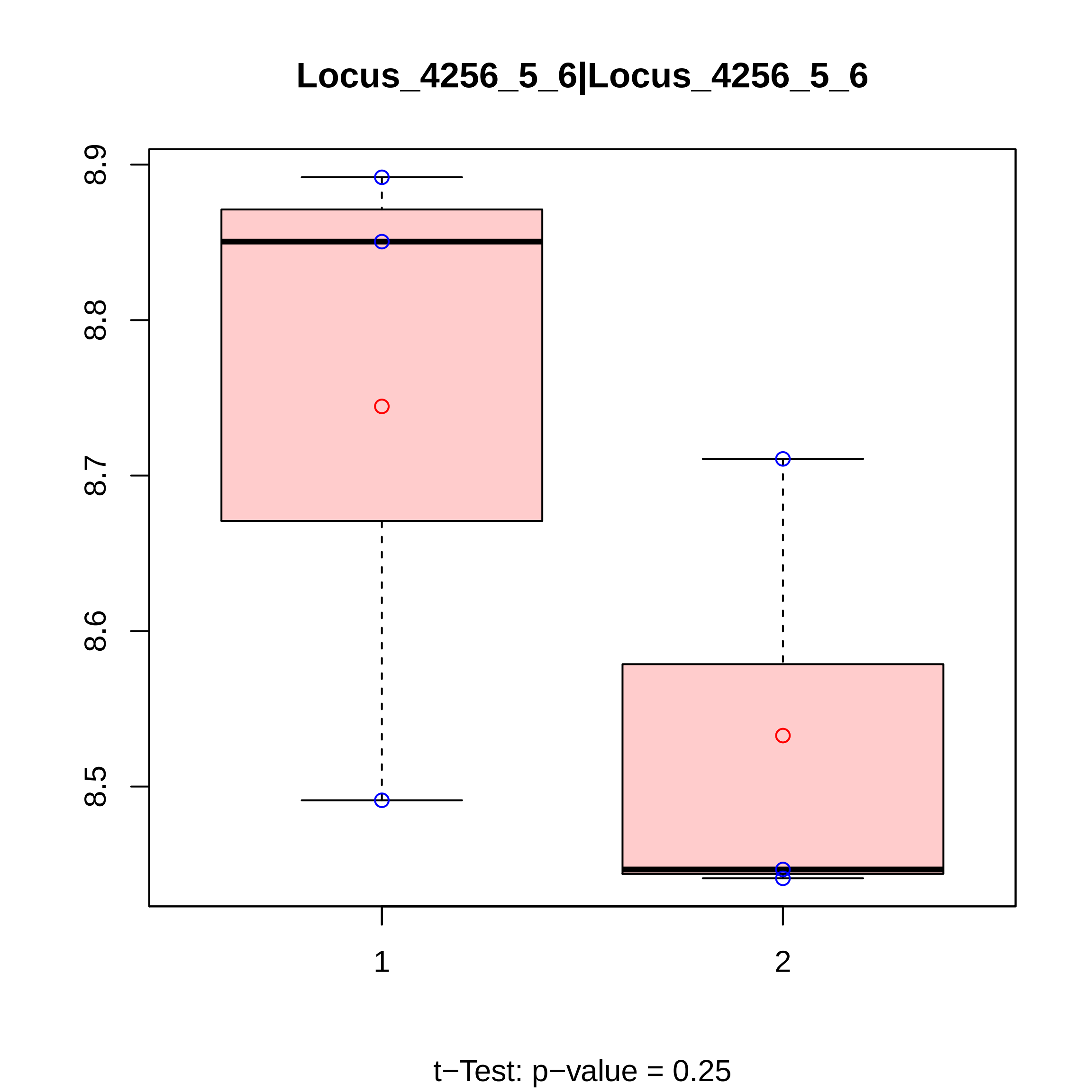

Locus_4256_5_6|Locus_4256_5_6
8.9
8.8
8.7
8.6
8.5
1
2
t−Test: p−value = 0.25

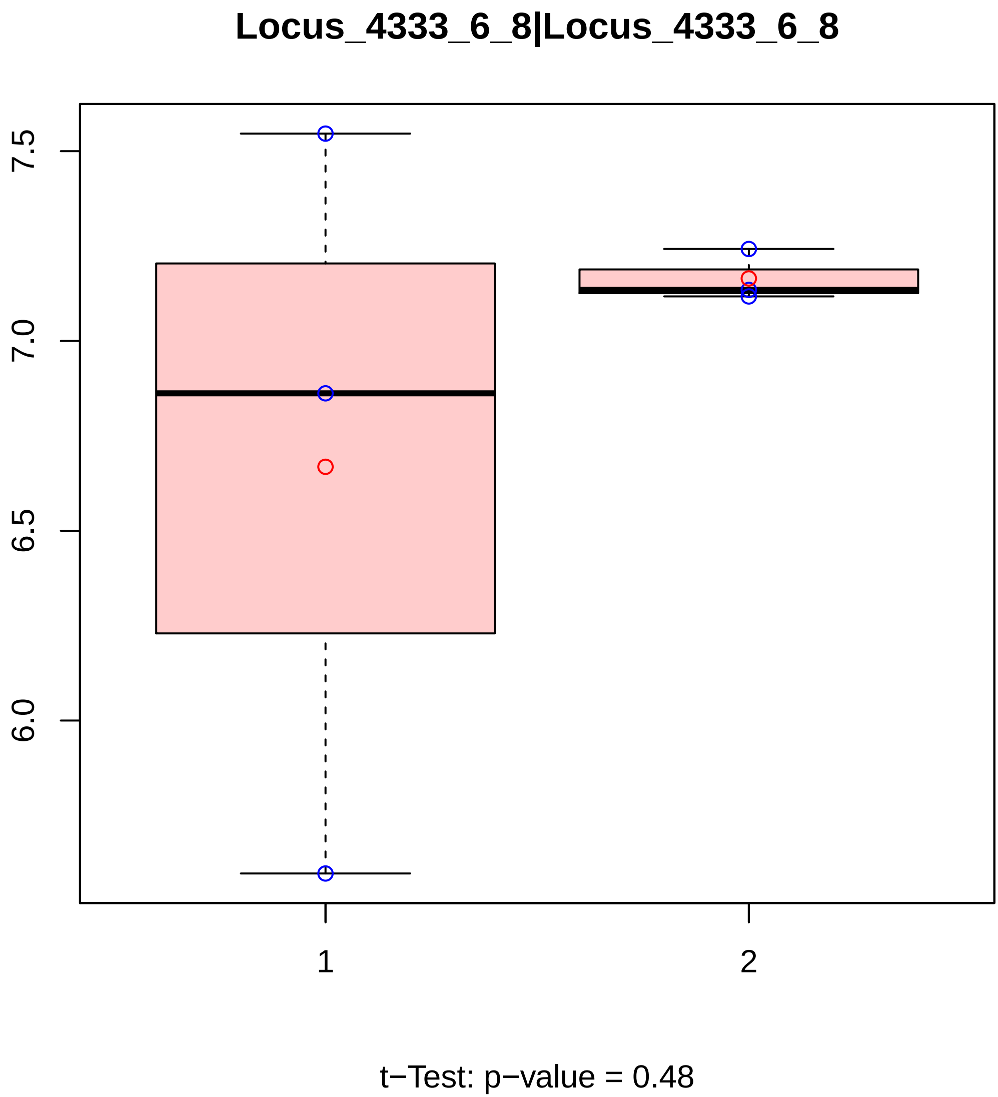

Locus_4333_6_8|Locus_4333_6_8
7.5
7.0
6.5
6.0
1
2
t−Test: p−value = 0.48

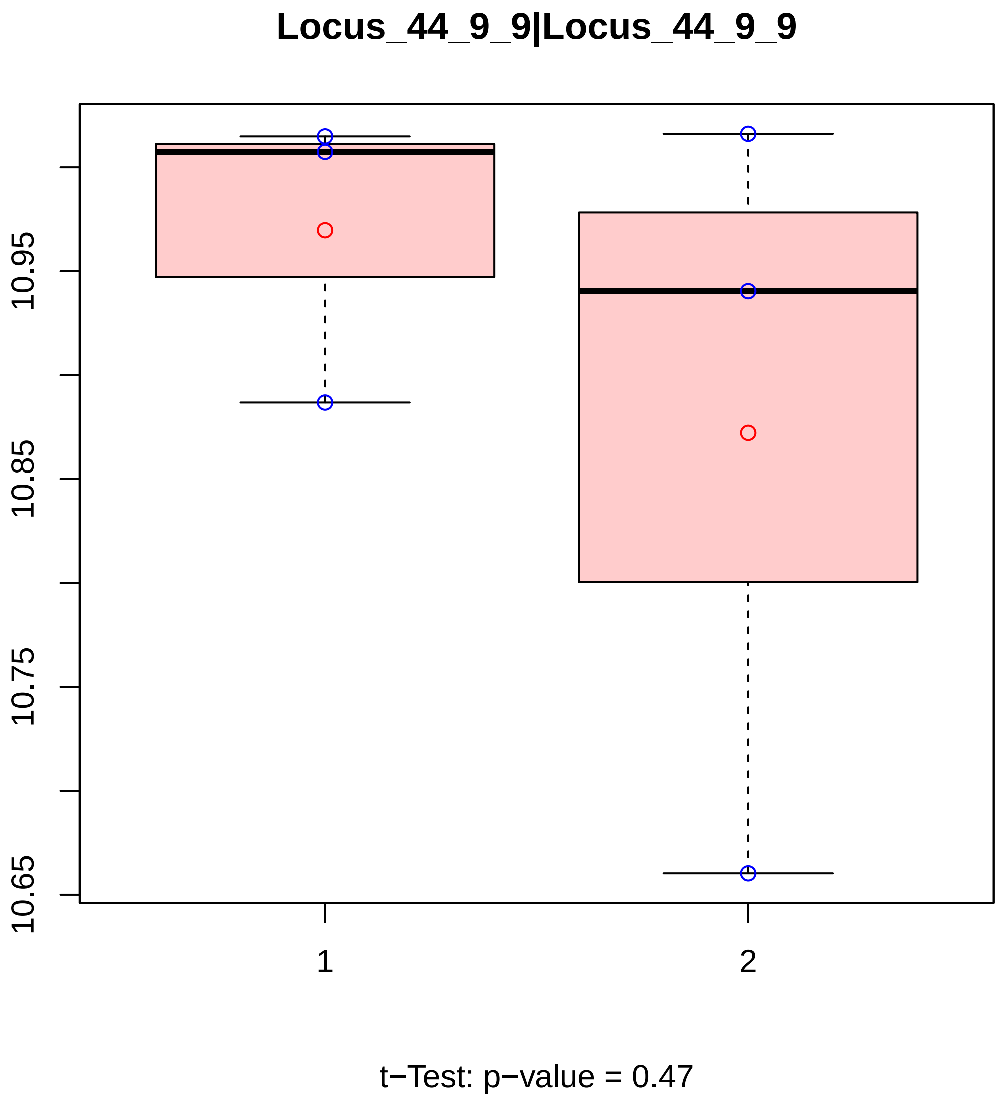
Locus_44_9_9|Locus_44_9_9
10.65
10.75
10.85
10.95
1
2
t−Test: p−value = 0.47

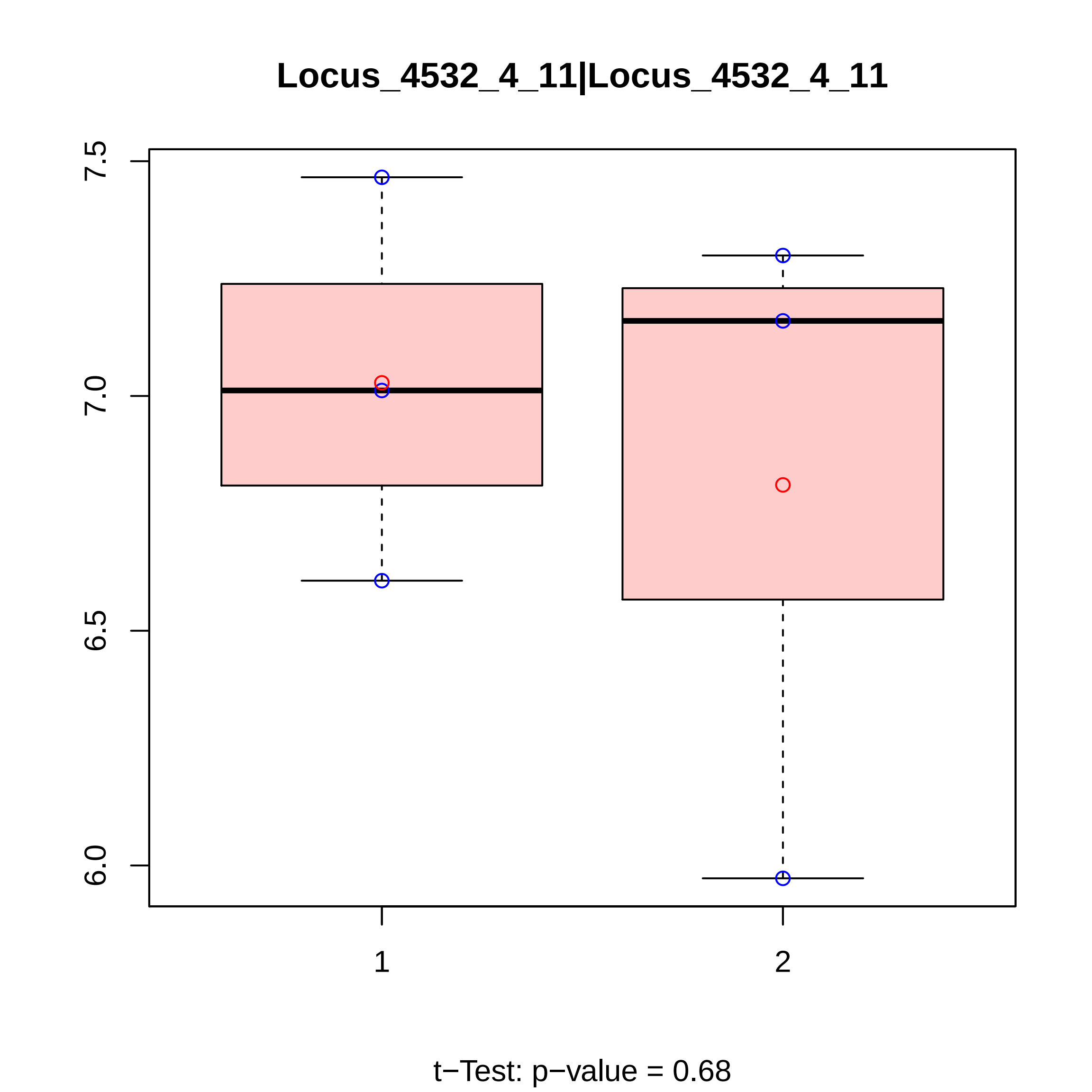

Locus_4532_4_11|Locus_4532_4_11
7.5
7.0
6.5
6.0
1
2
t−Test: p−value = 0.68

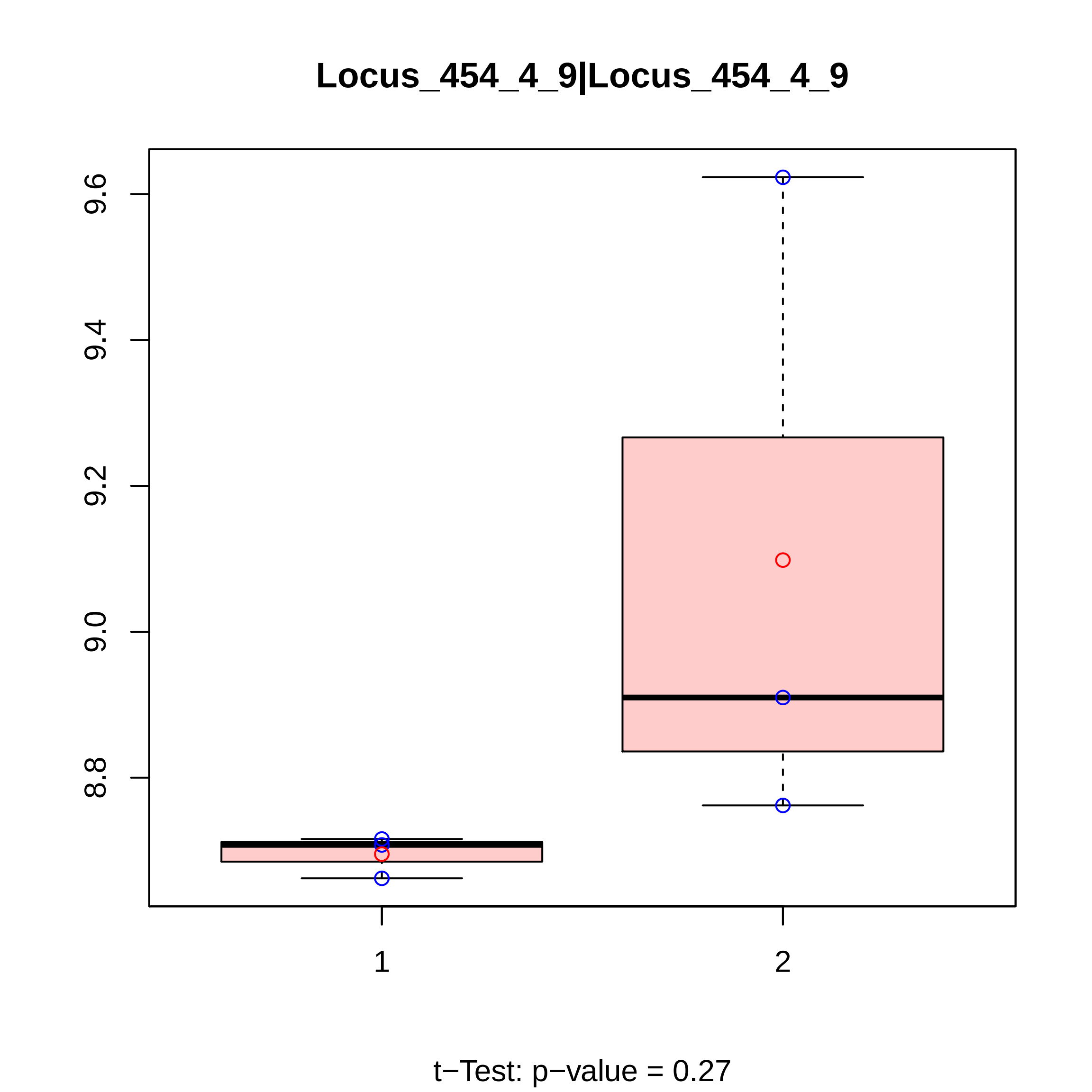

Locus_454_4_9|Locus_454_4_9
9.6
9.4
9.2
9.0
8.8
1
2
t−Test: p−value = 0.27

**Locus_4547_2_11|Locus_4547_2_11**

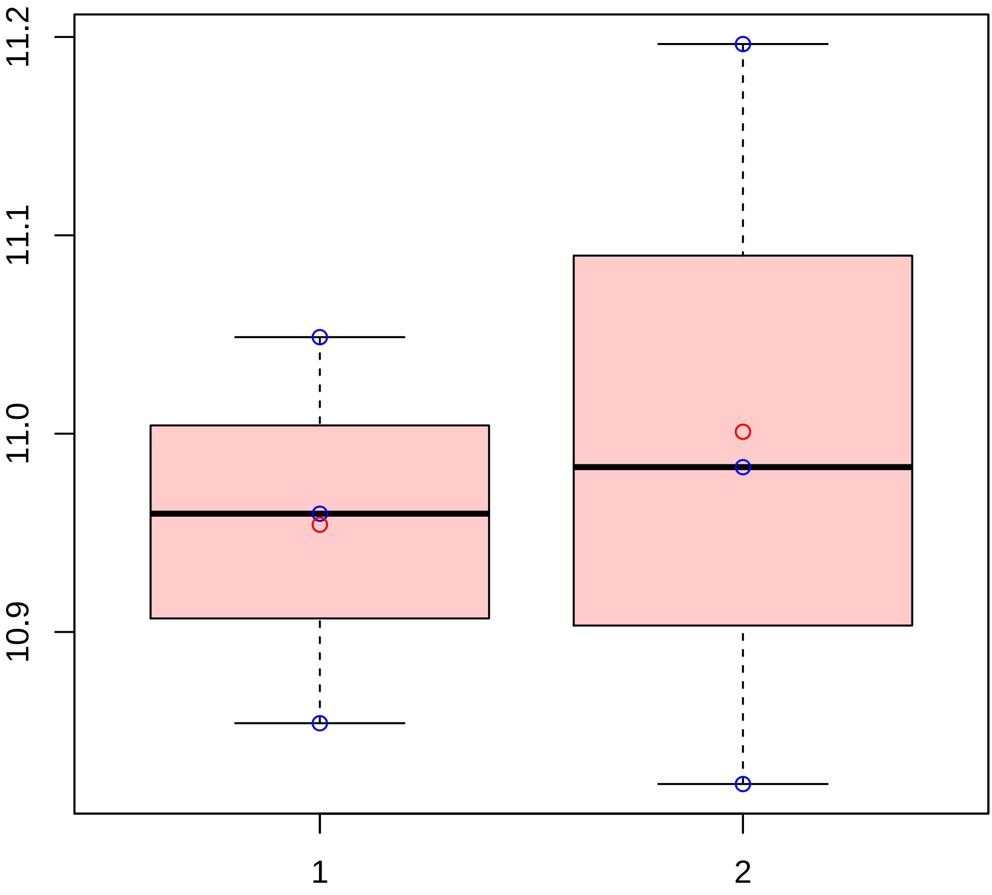


t−Test: p−value = 0.73

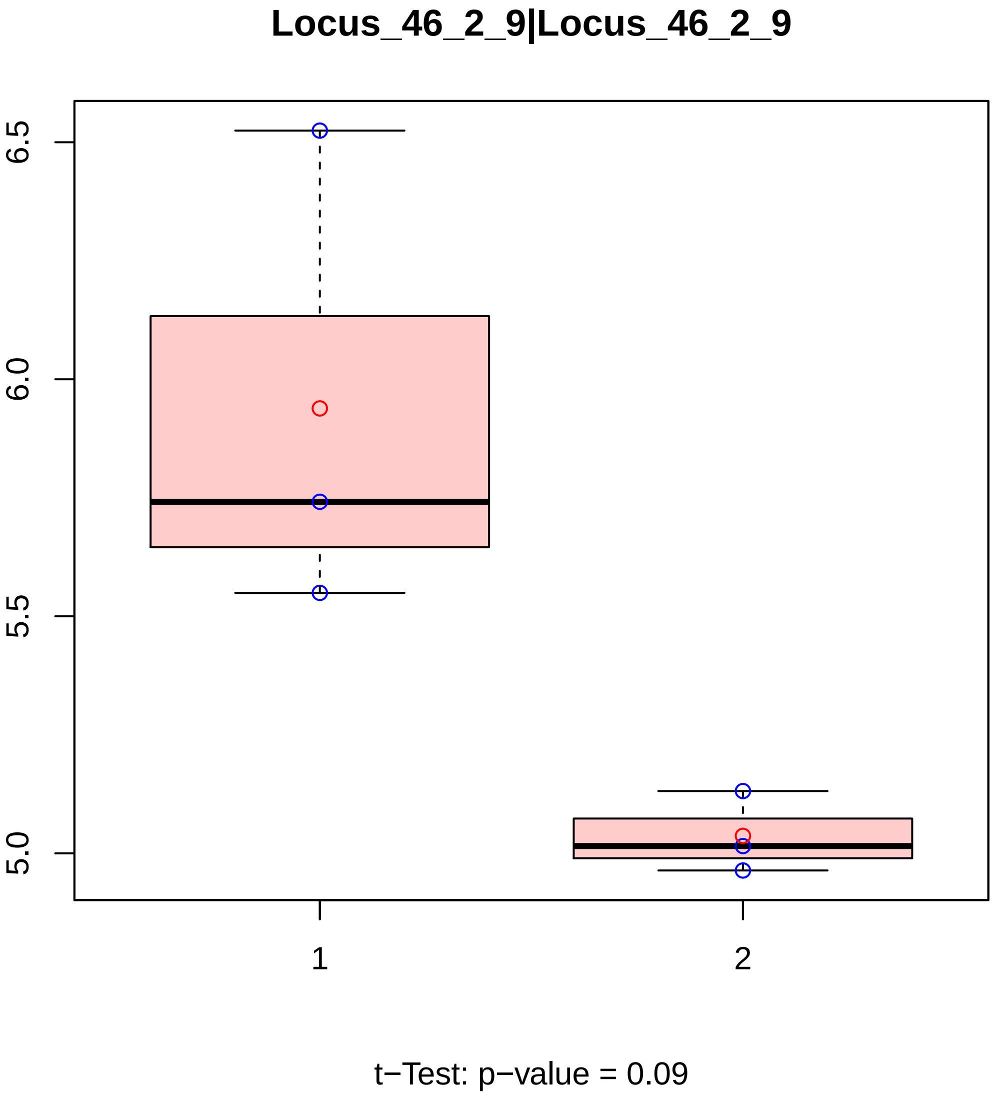

Locus_46_2_9|Locus_46_2_9
6.5
6.0
5.5
5.0
1
2
t−Test: p−value = 0.09

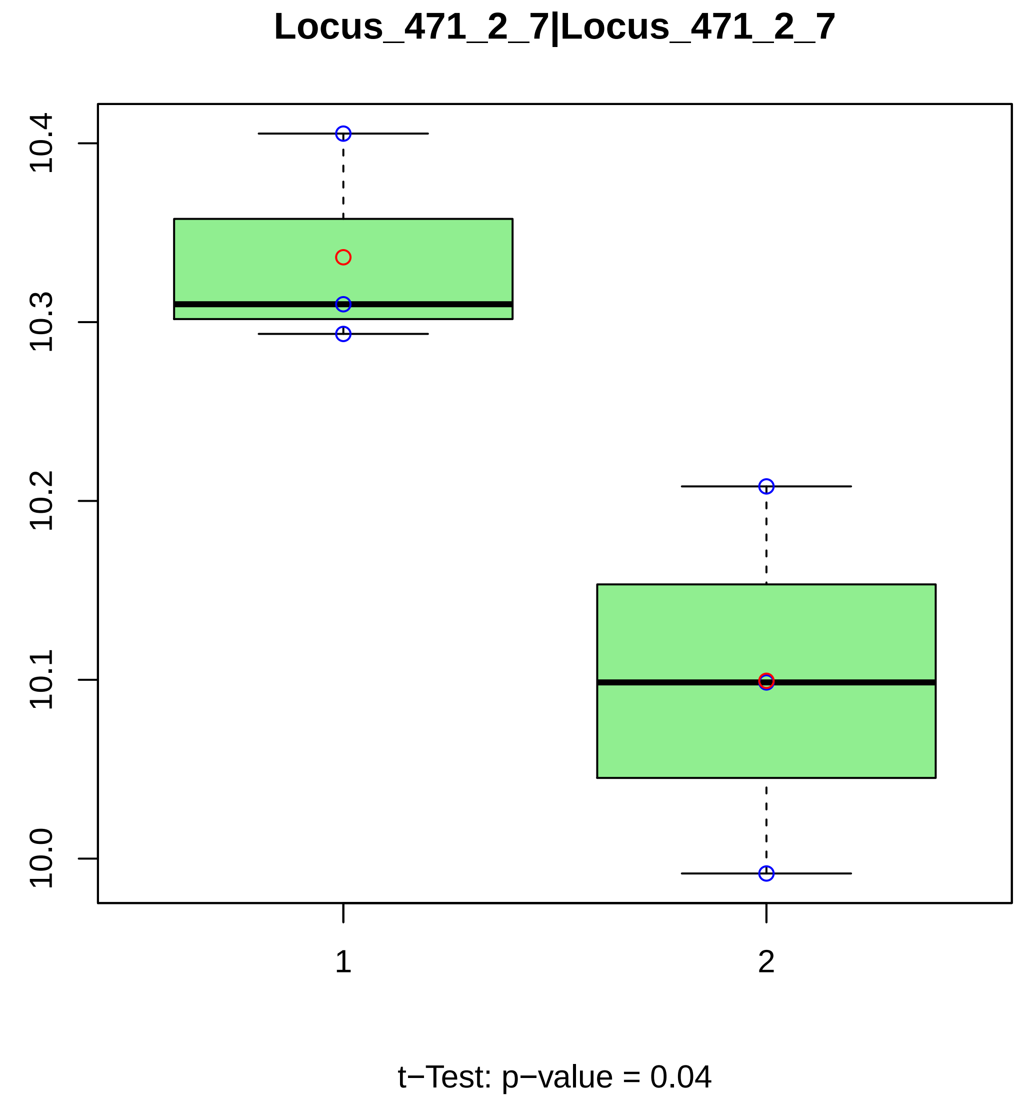

Locus_471_2_7|Locus_471_2_7
10.0
10.1
10.2
10.3
10.4
1
2
t−Test: p−value = 0.04

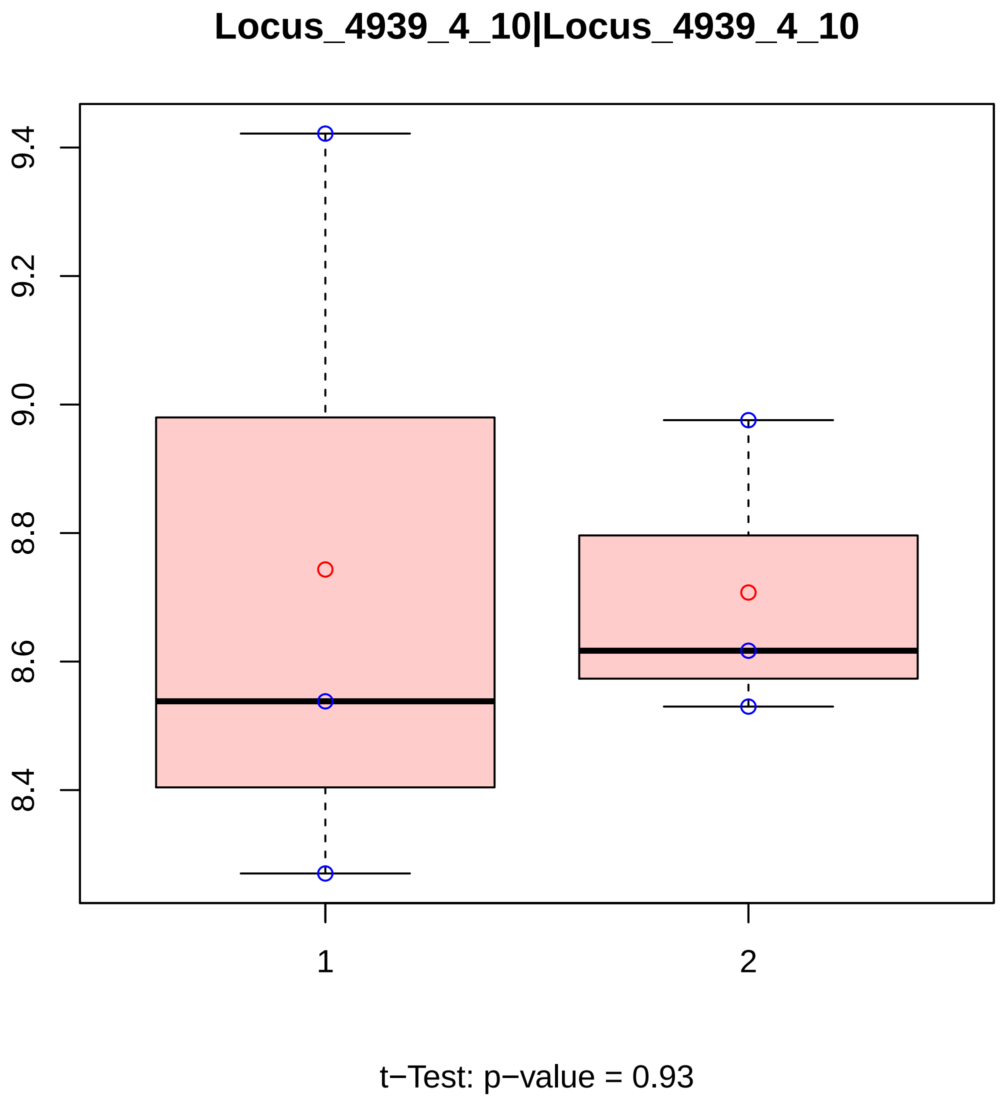

Locus_4939_4_10|Locus_4939_4_10
9.4
9.2
9.0
8.8
8.6
8.4
1
2
t−Test: p−value = 0.93

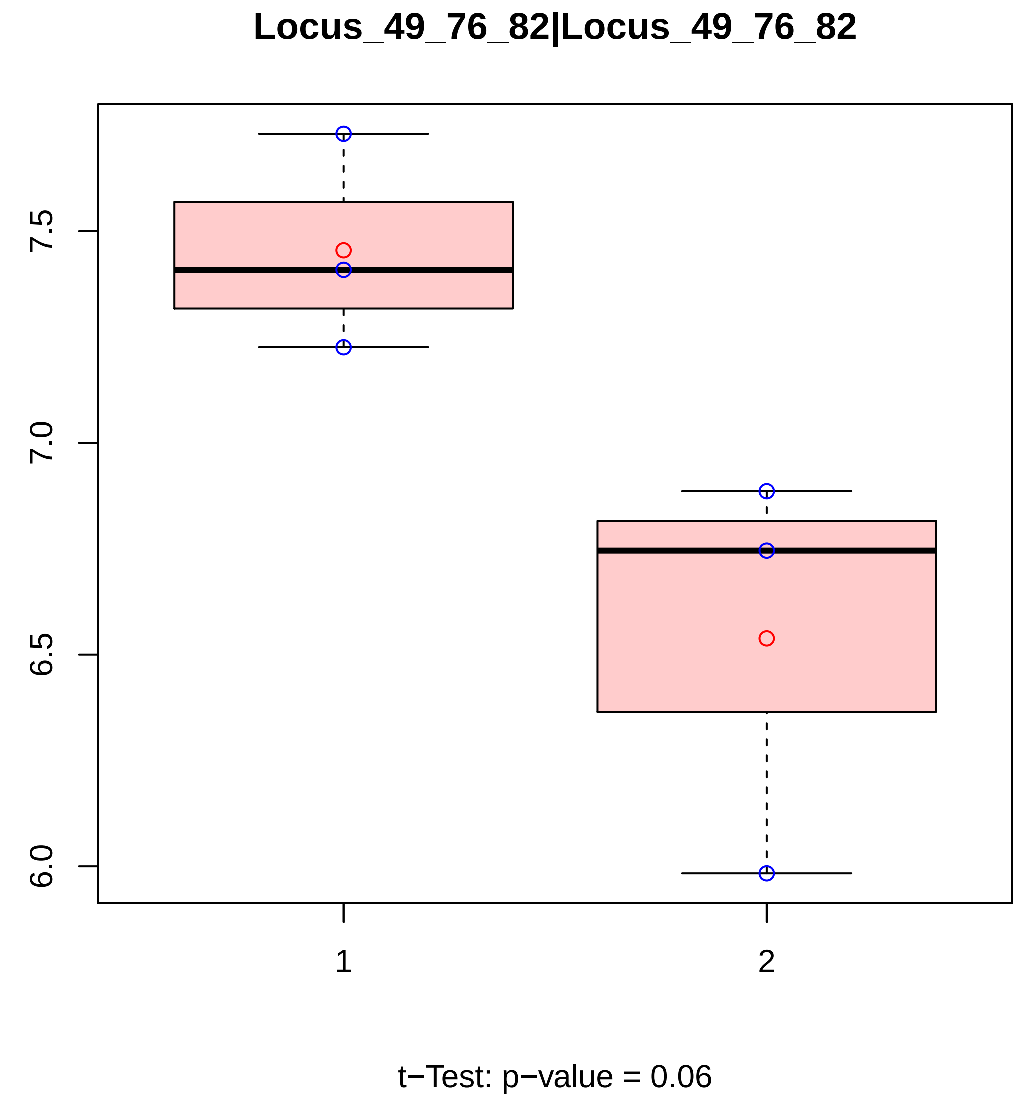
Locus_49_76_82|Locus_49_76_82
7.5
7.0
6.5
6.0
1
2
t−Test: p−value = 0.06

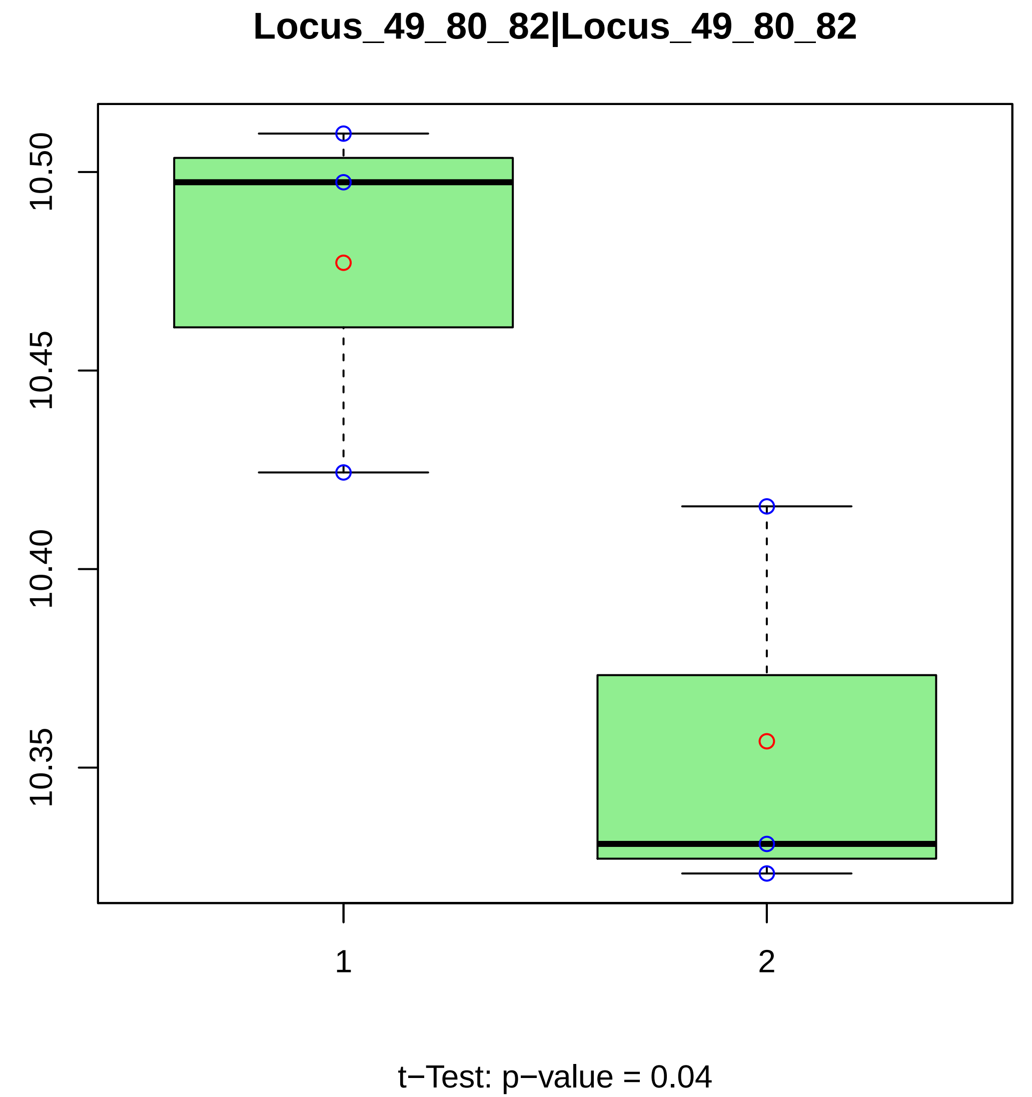
Locus_49_80_82|Locus_49_80_82
10.50
10.45
10.40
10.35
1
2
t−Test: p−value = 0.04

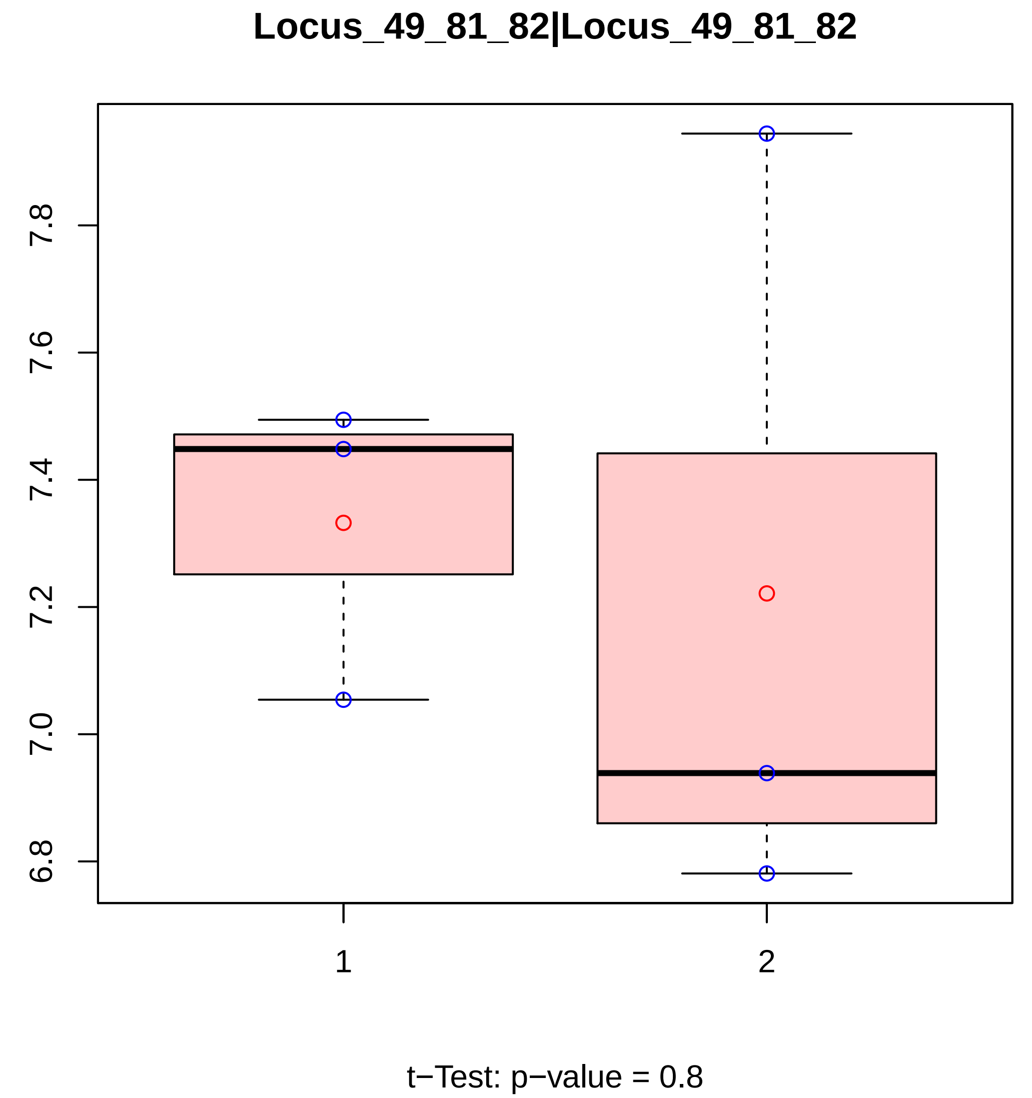

Locus_49_81_82|Locus_49_81_82
7.8
7.6
7.4
7.2
7.0
6.8
1
2
t−Test: p−value = 0.8

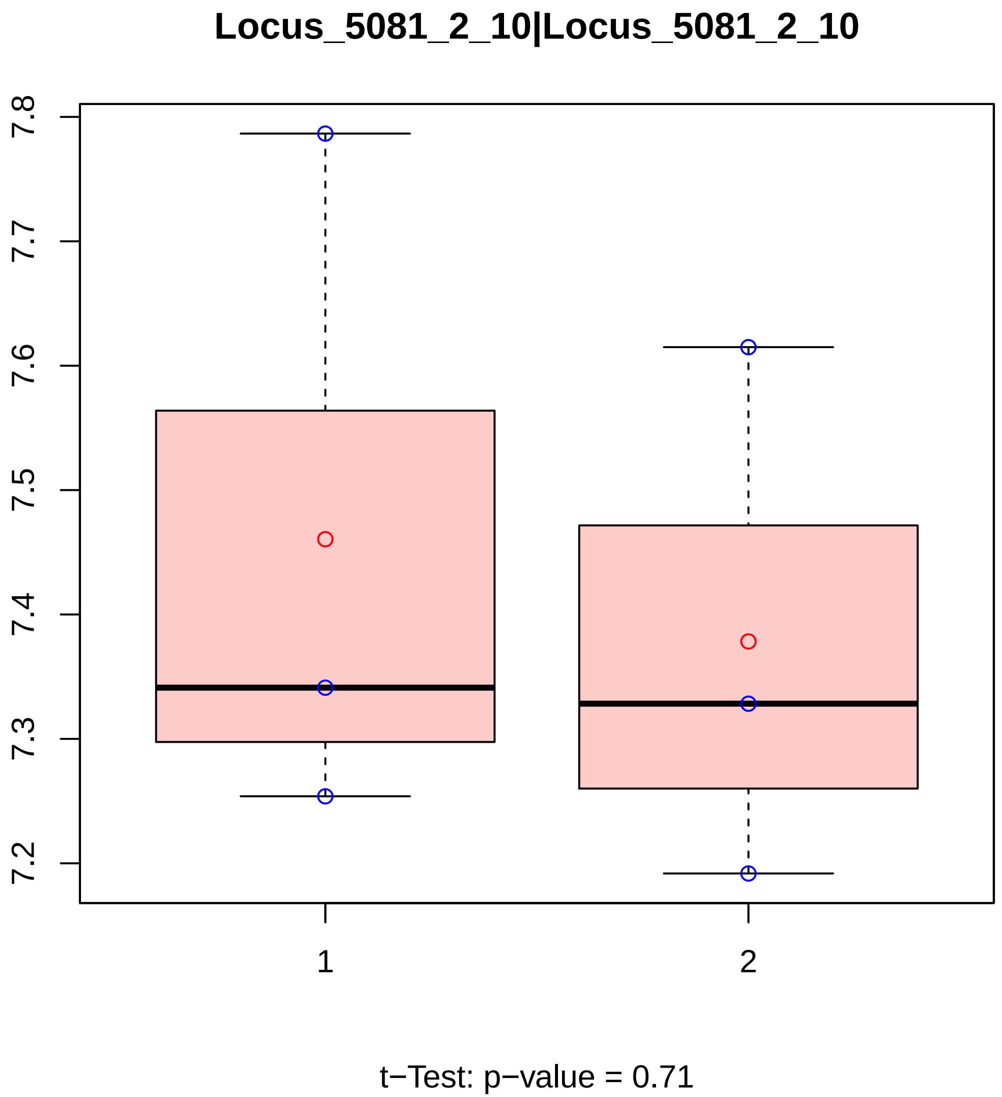

Locus_5081_2_10|Locus_5081_2_10
7.8
7.7
7.6
7.5
7.4
7.3
7.2
1
2
t−Test: p−value = 0.71

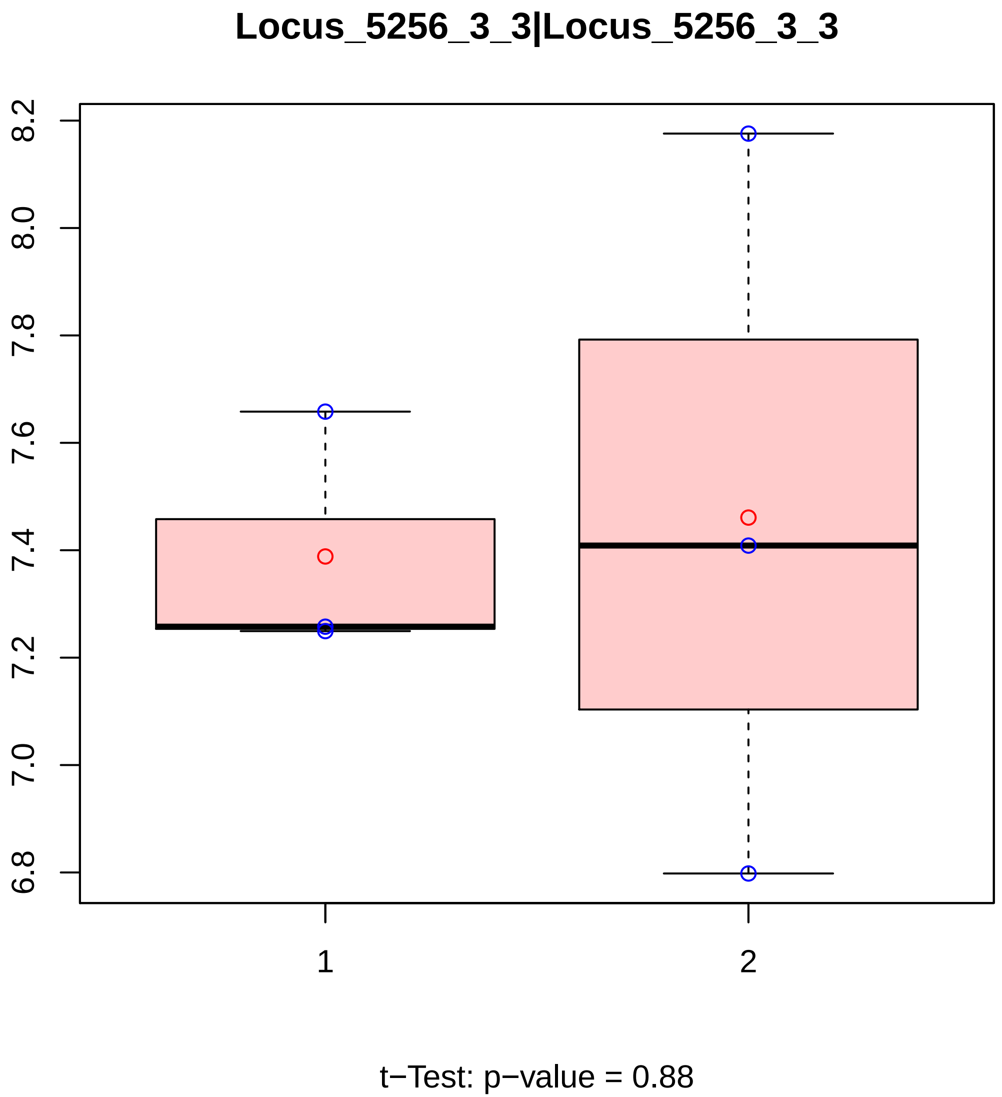

Locus_5256_3_3|Locus_5256_3_3
8.2
8.0
7.8
7.6
7.4
7.2
7.0
6.8
1
2
t−Test: p−value = 0.88

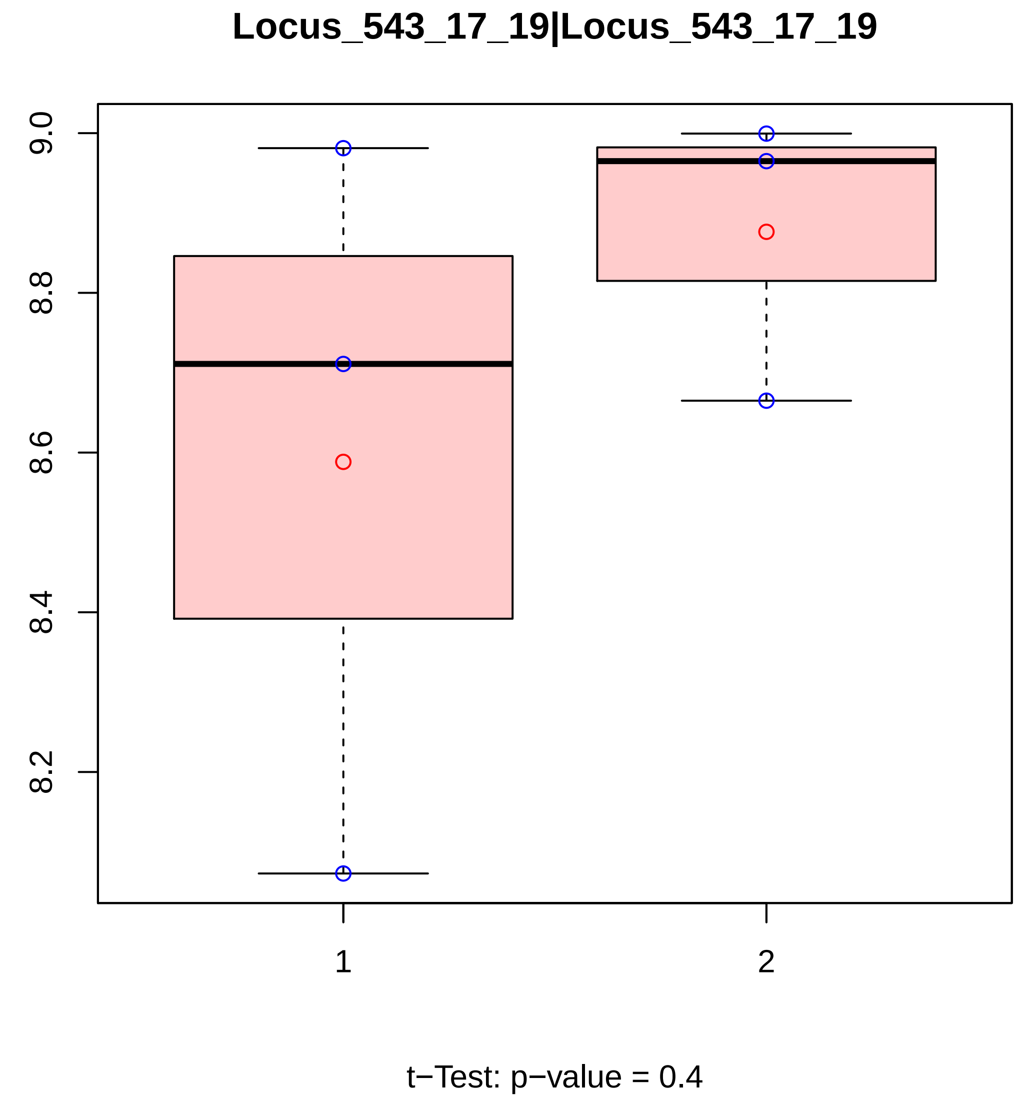

Locus_543_17_19|Locus_543_17_19
9.0
8.8
8.6
8.4
8.2
1
2
t−Test: p−value = 0.4

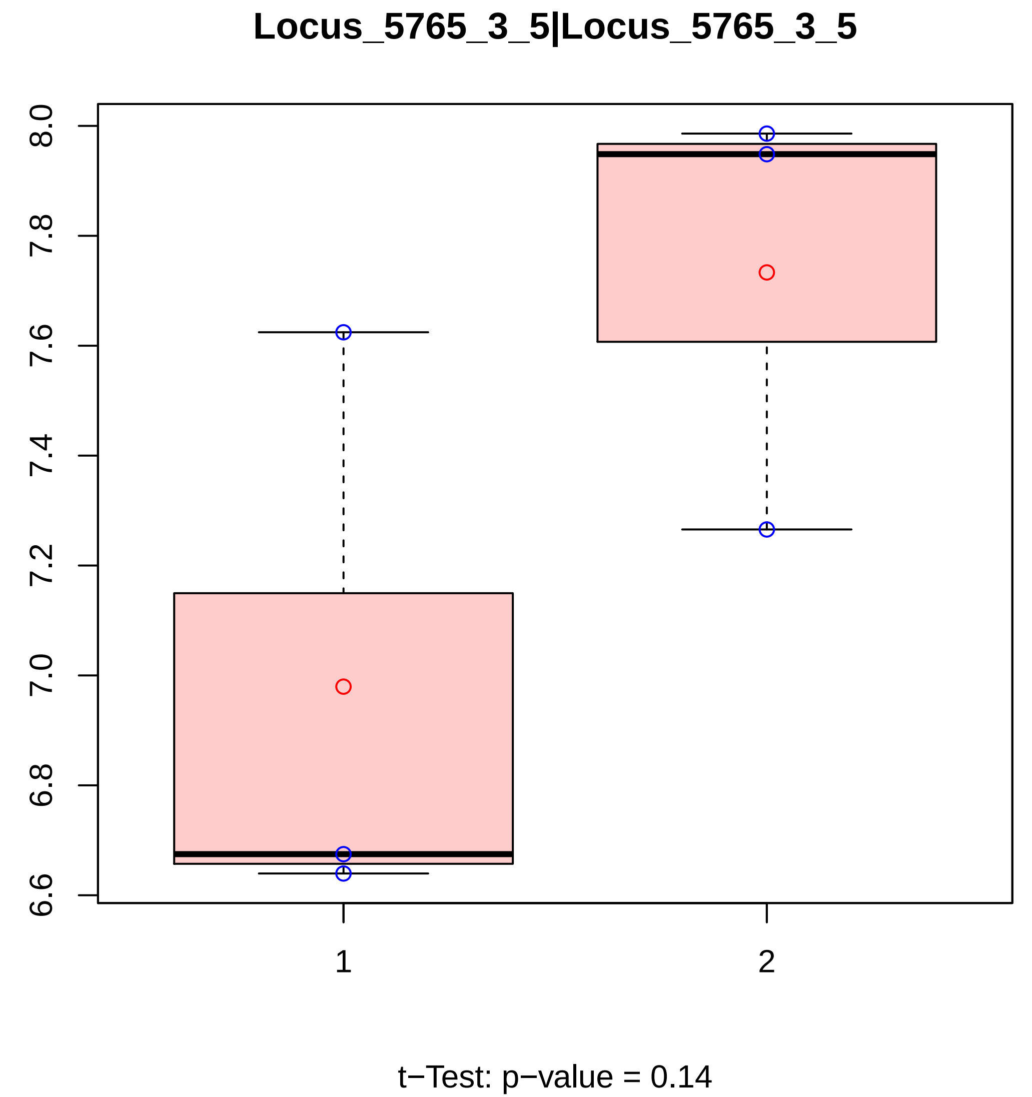
Locus_5765_3_5|Locus_5765_3_5
8.0
7.8
7.6
7.4
7.2
7.0
6.8
6.6
1
2
t−Test: p−value = 0.14

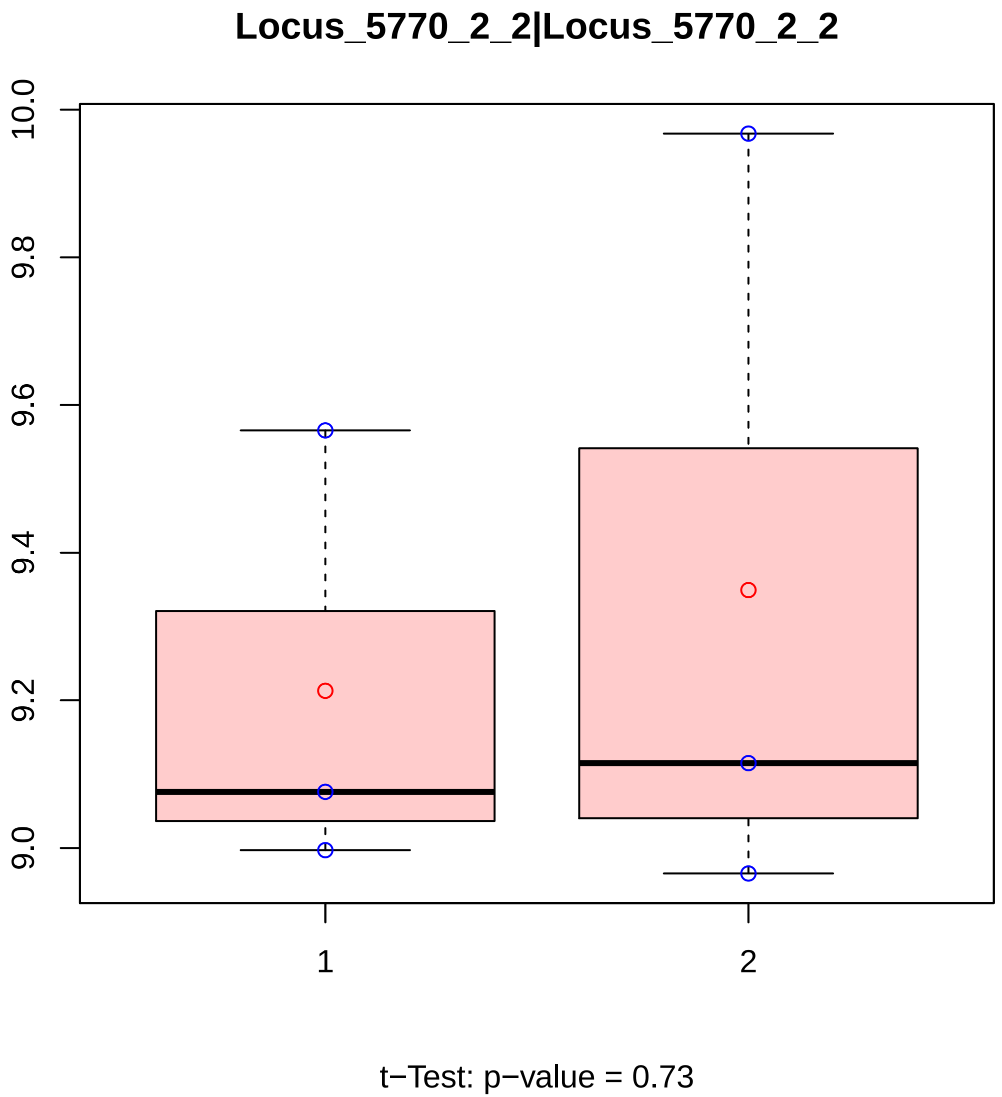
Locus_5770_2_2|Locus_5770_2_2
10.0
9.8
9.6
9.4
9.2
9.0
1
2
t−Test: p−value = 0.73

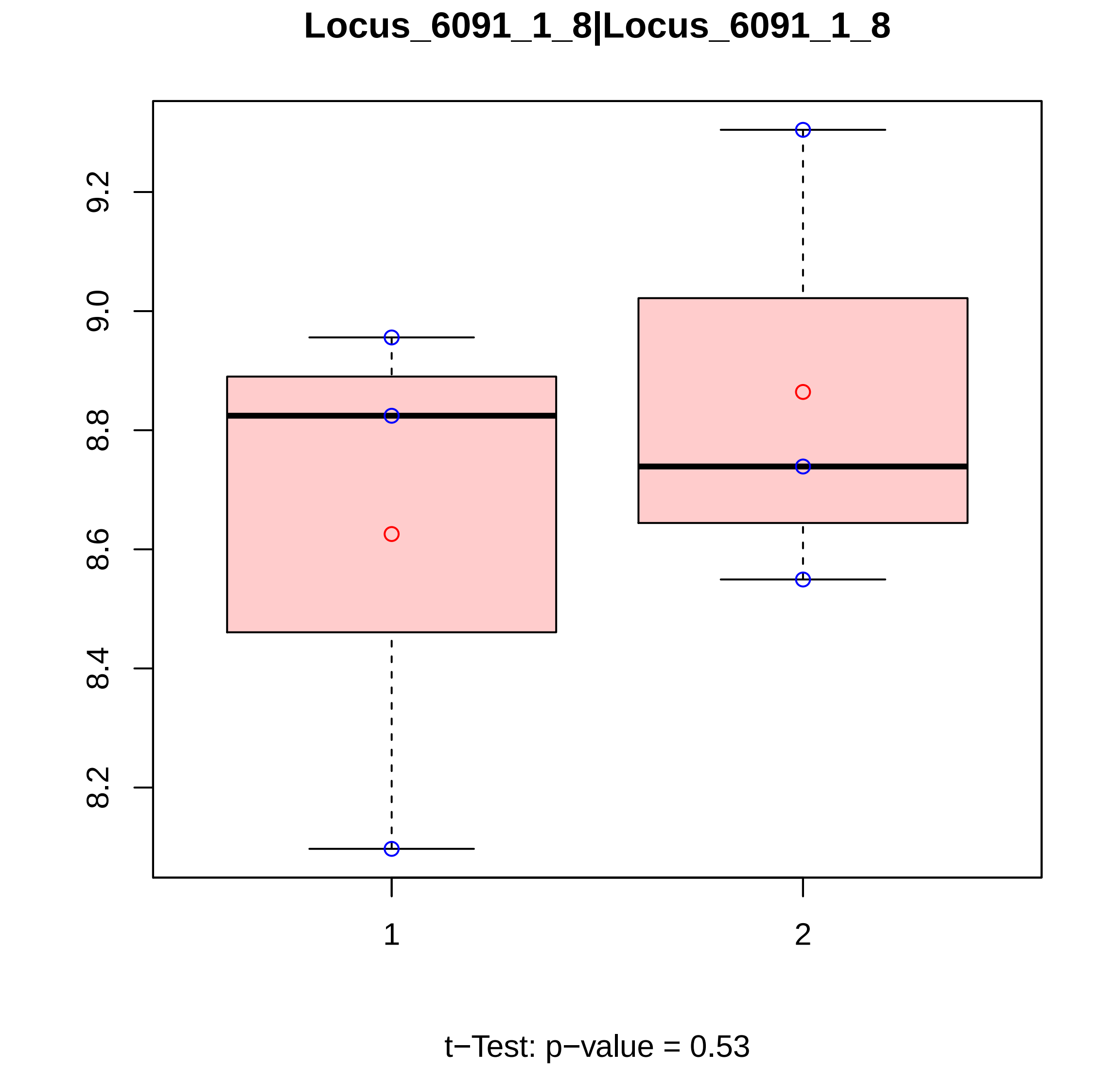

Locus_6091_1_8|Locus_6091_1_8
9.2
9.0
8.8
8.6
8.4
8.2
1
2
t−Test: p−value = 0.53

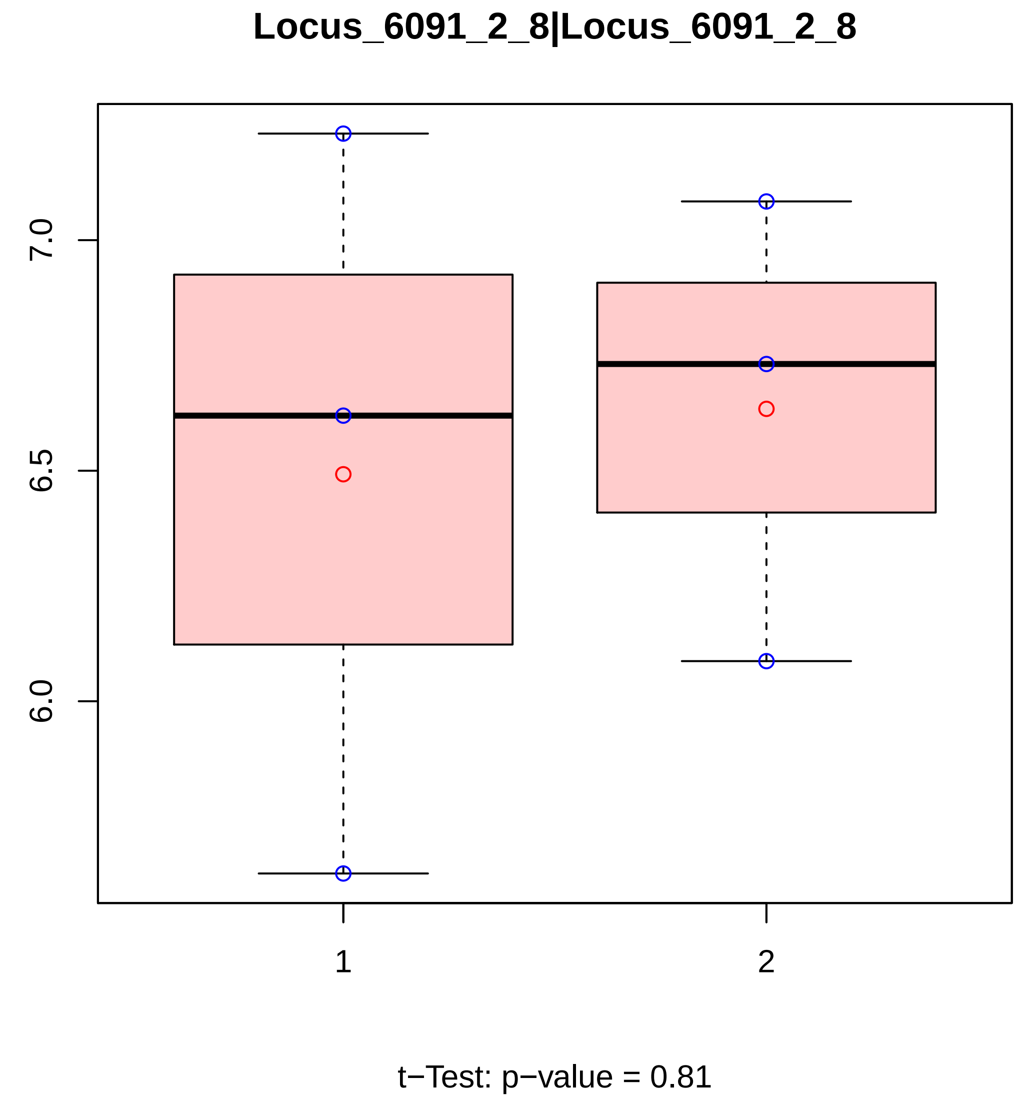
Locus_6091_2_8|Locus_6091_2_8
7.0
6.5
6.0
1
2
t−Test: p−value = 0.81

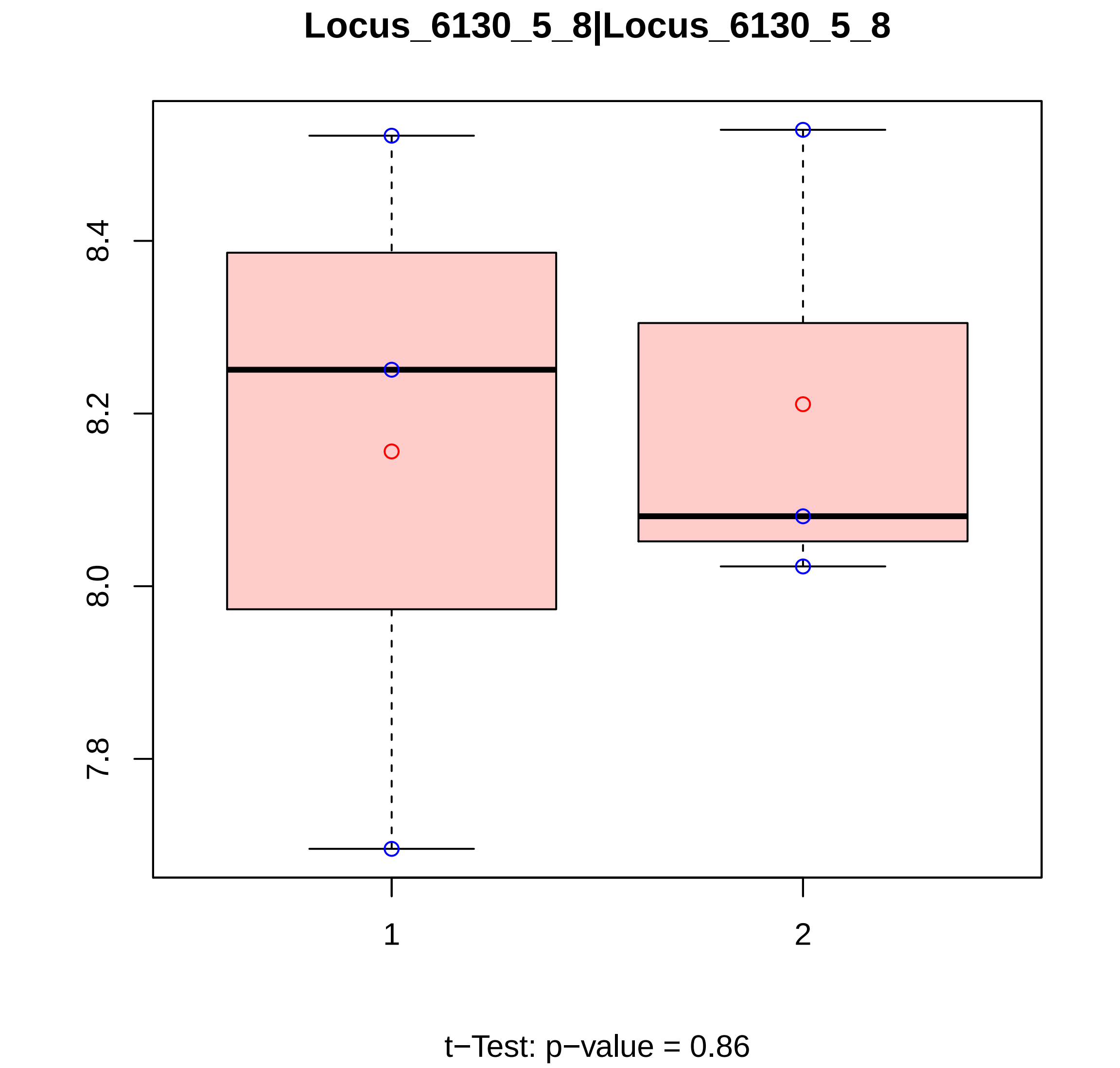

Locus_6130_5_8|Locus_6130_5_8
8.4
8.2
8.0
7.8
1
2
t−Test: p−value = 0.86

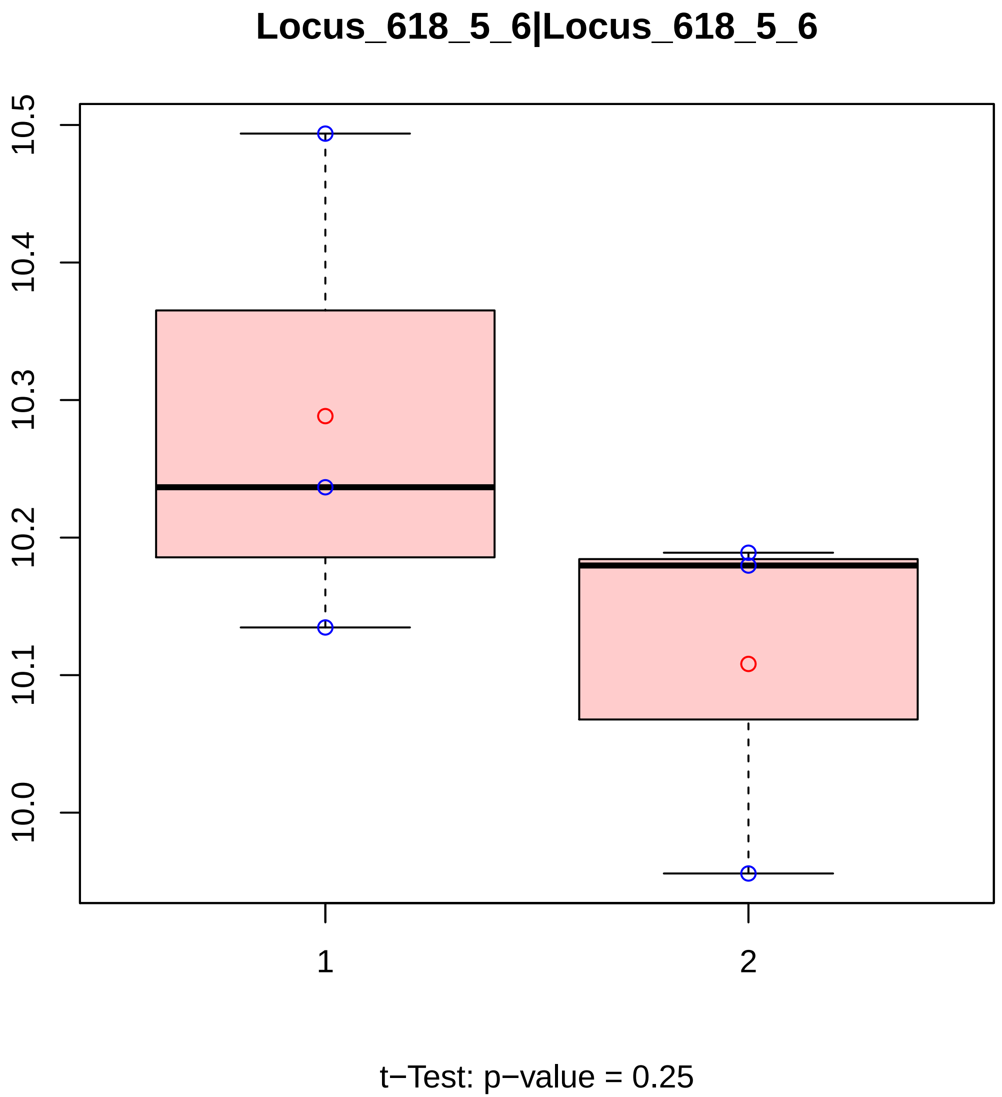

Locus_618_5_6|Locus_618_5_6
10.0
10.1
10.2
10.3
10.4
10.5
1
2
t−Test: p−value = 0.25

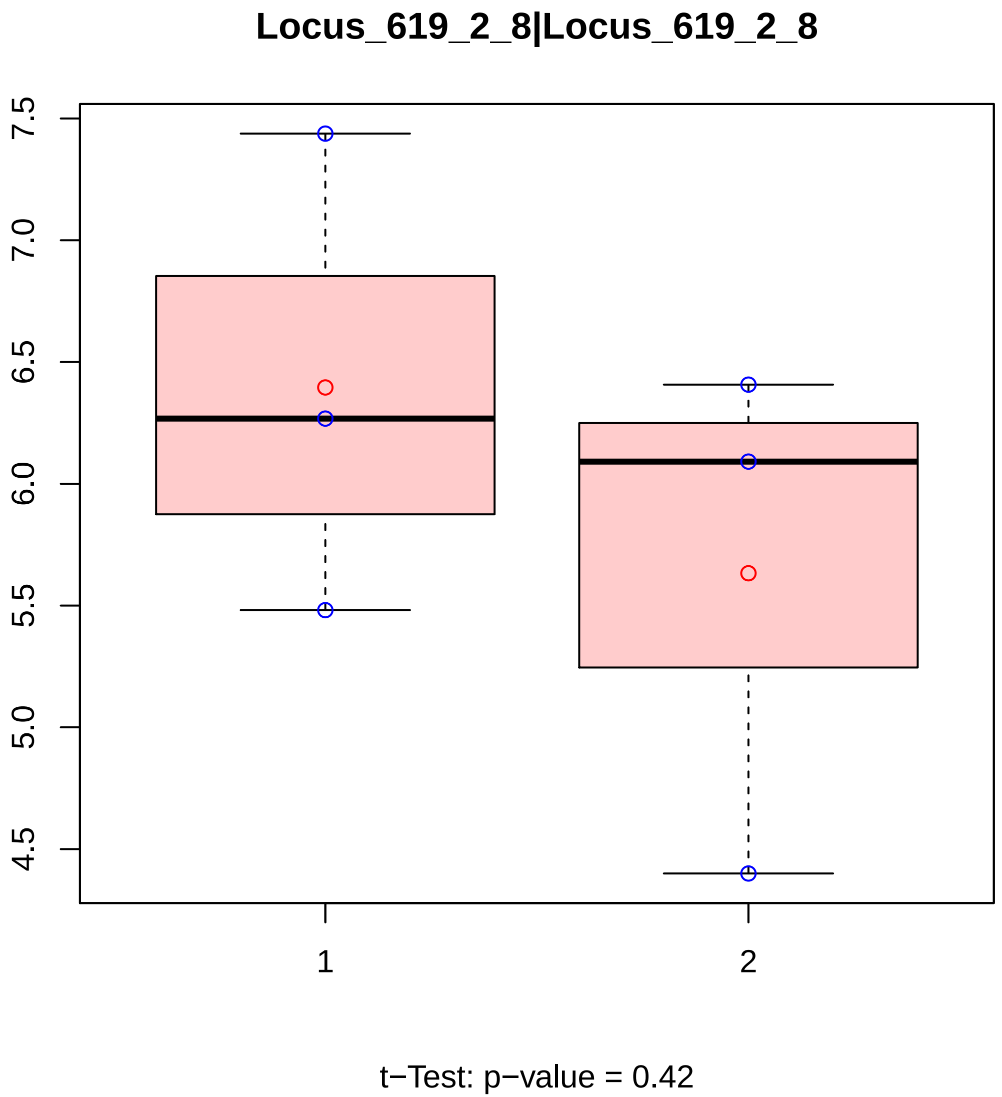

Locus_619_2_8|Locus_619_2_8
7.5
7.0
6.5
6.0
5.5
5.0
4.5
1
2
t−Test: p−value = 0.42

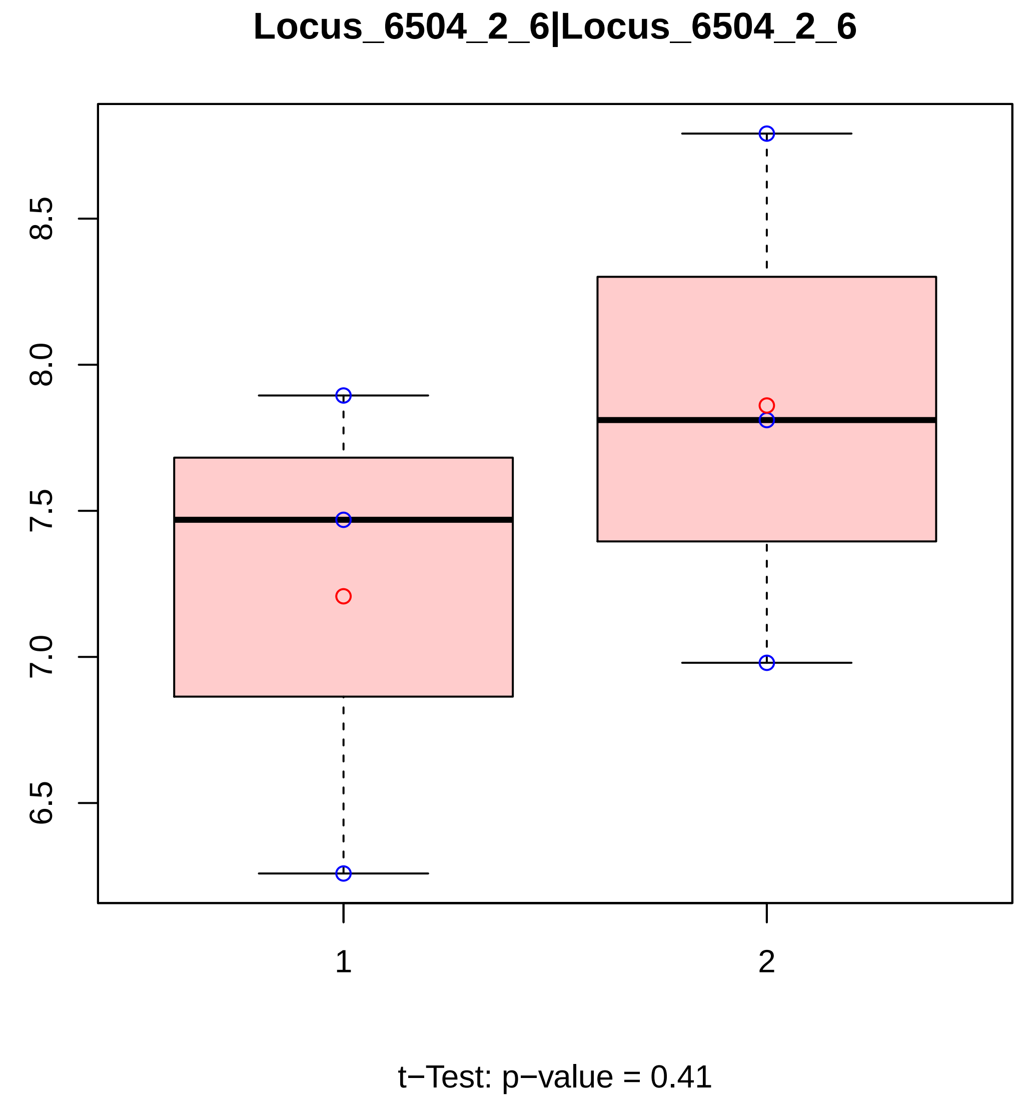

Locus_6504_2_6|Locus_6504_2_6
8.5
8.0
7.5
7.0
6.5
1
2
t−Test: p−value = 0.41

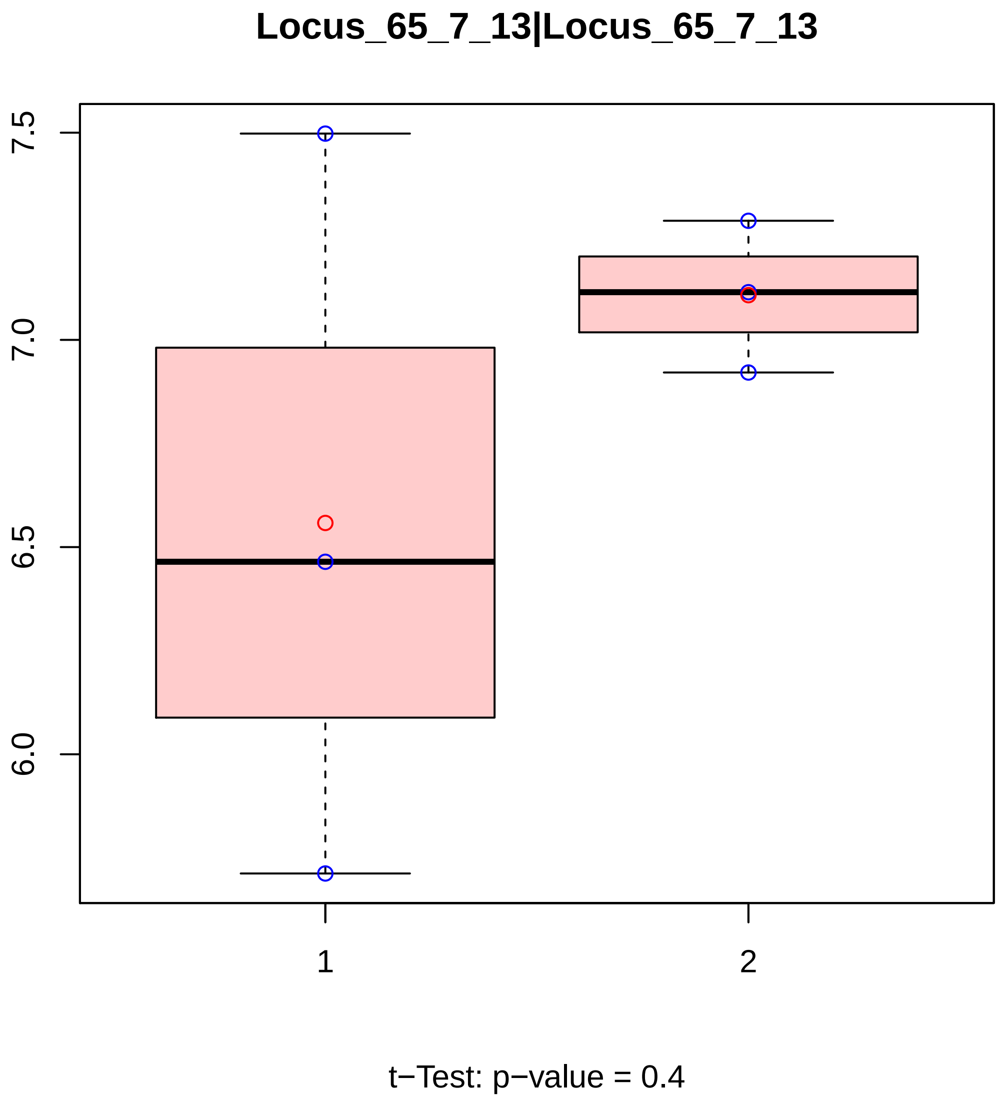
Locus_65_7_13|Locus_65_7_13
7.5
7.0
6.5
6.0
1
2
t−Test: p−value = 0.4

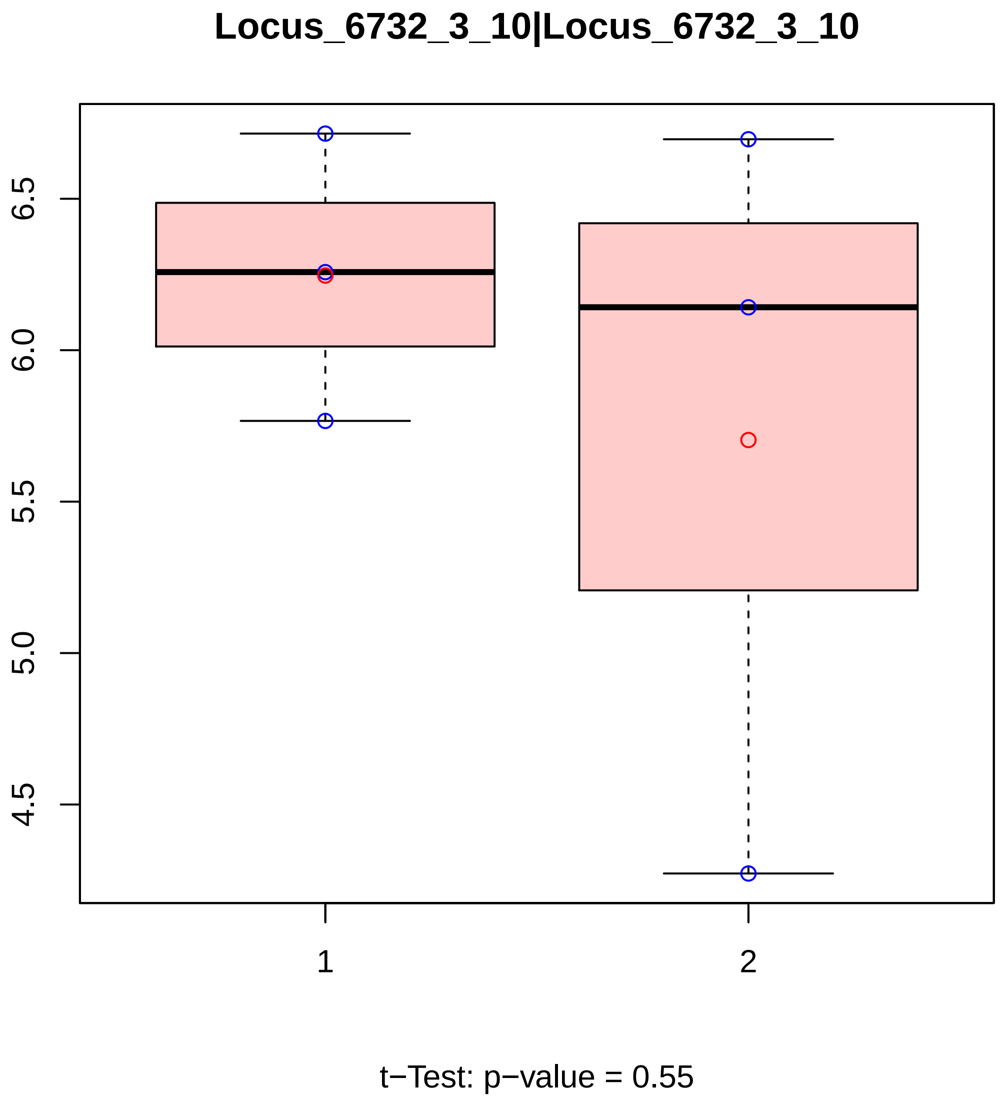
Locus_6732_3_10|Locus_6732_3_10
6.5
6.0
5.5
5.0
4.5
1
2
t−Test: p−value = 0.55

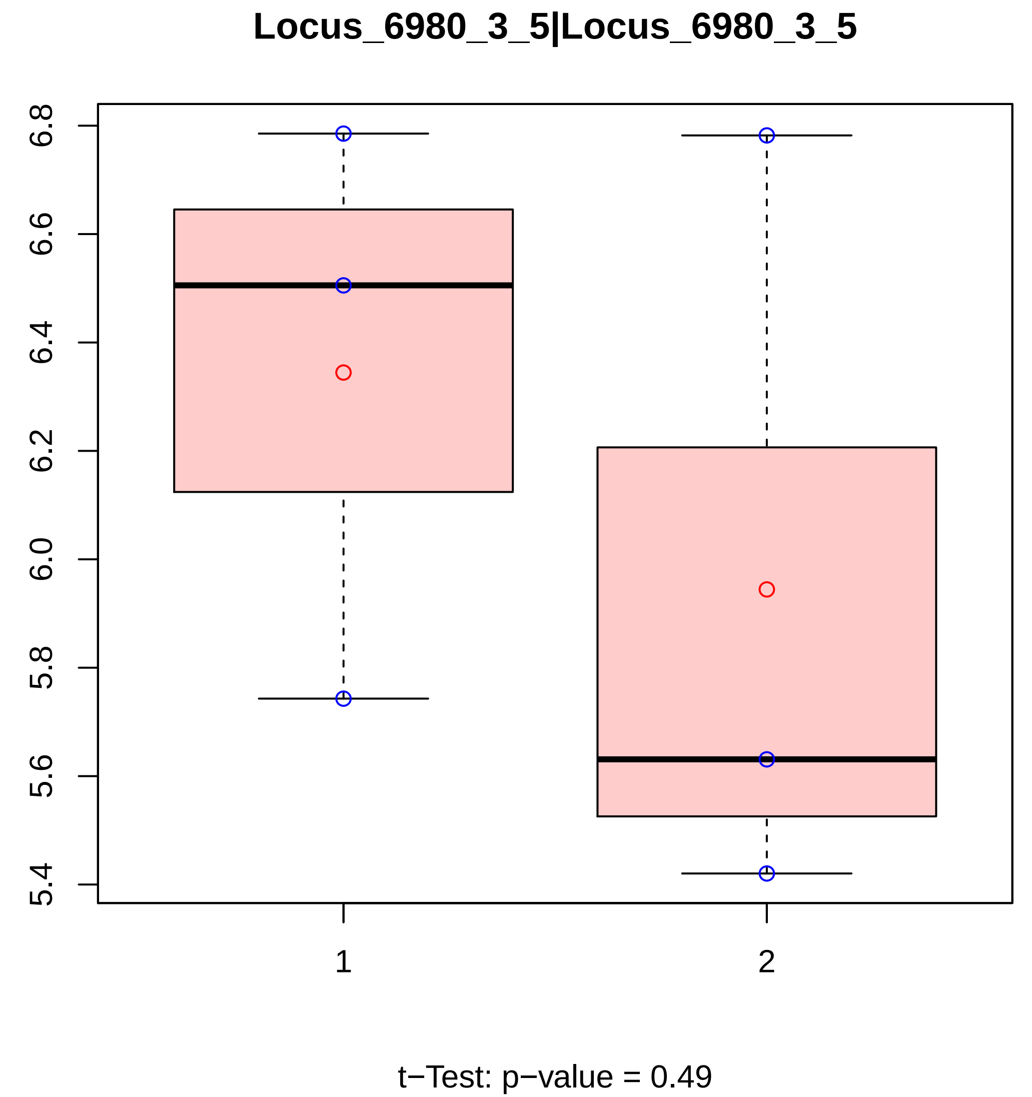

Locus_6980_3_5|Locus_6980_3_5
6.8
6.6
6.4
6.2
6.0
5.8
5.6
5.4
1
2
t−Test: p−value = 0.49

**Locus_7074_7_8|Locus_7074_7_8**

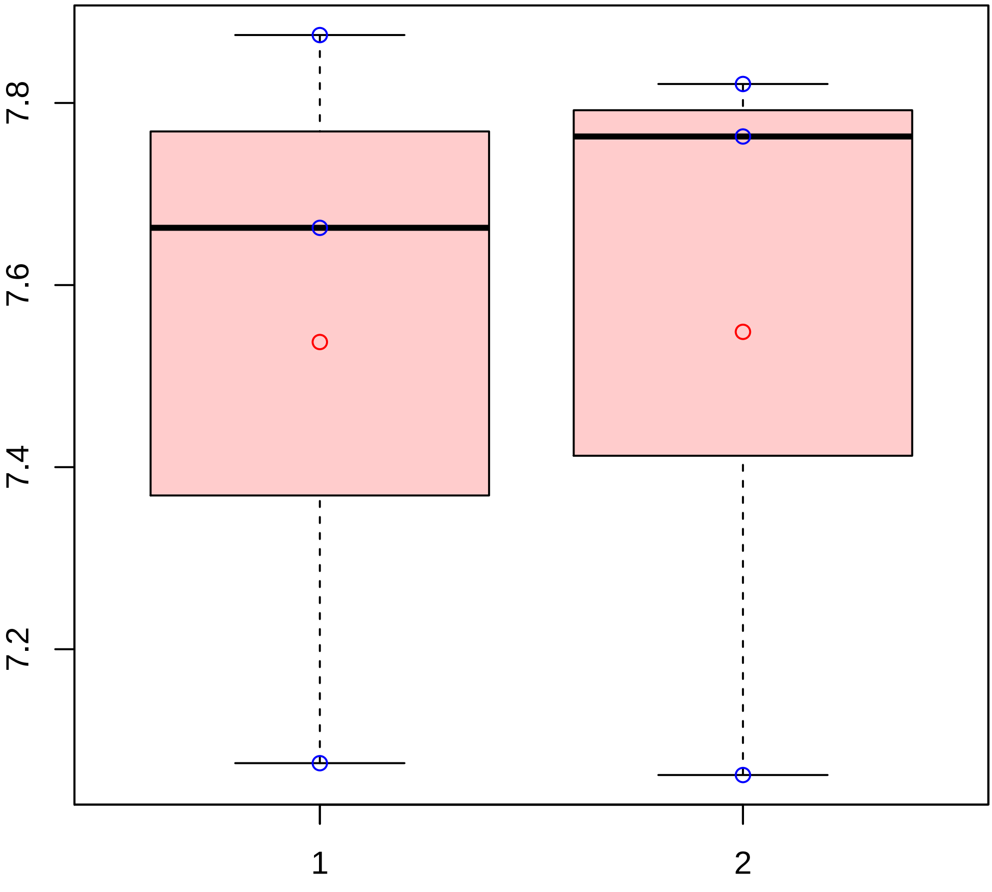


t−Test: p−value = 0.98

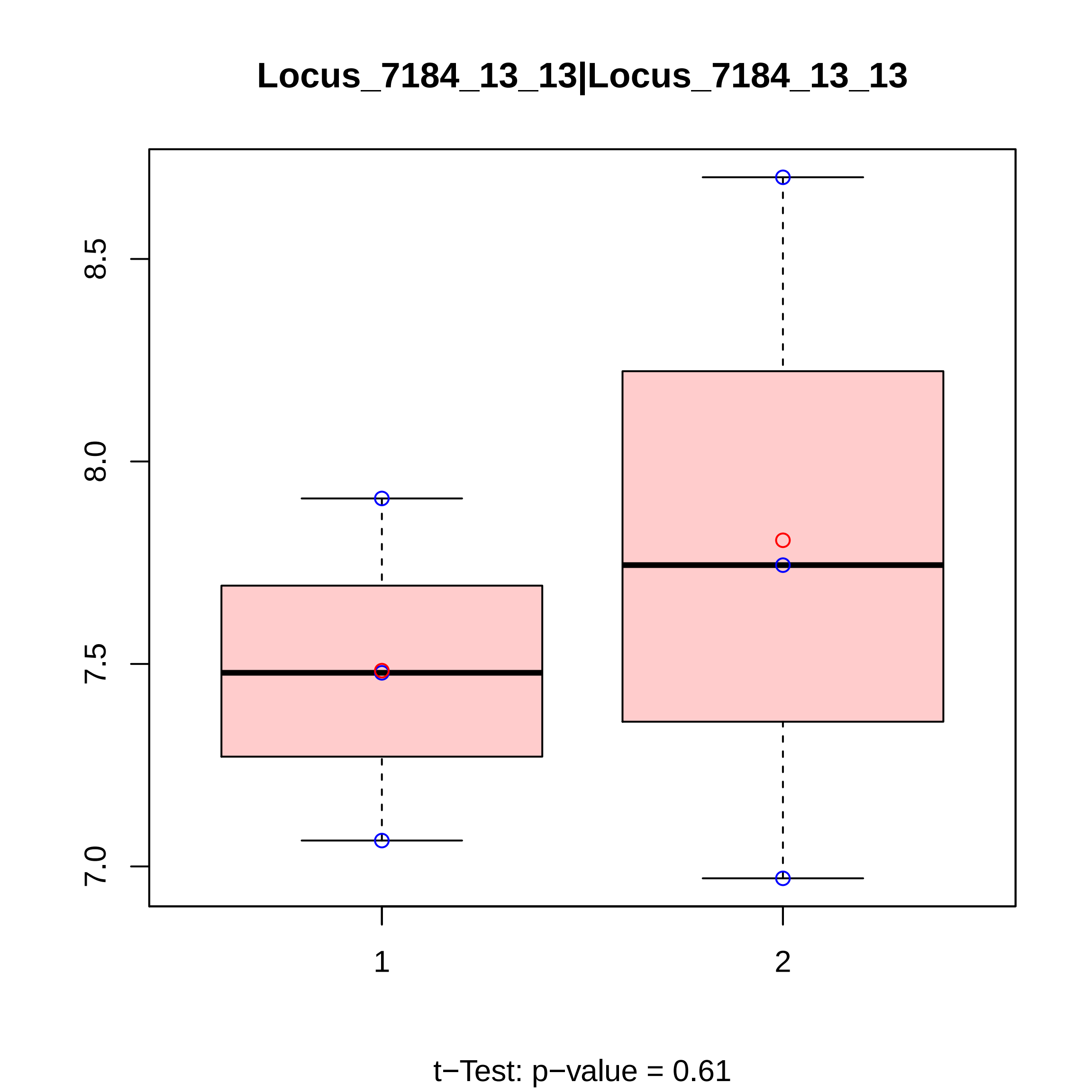

Locus_7184_13_13|Locus_7184_13_13
8.5
8.0
7.5
7.0
1
2
t−Test: p−value = 0.61

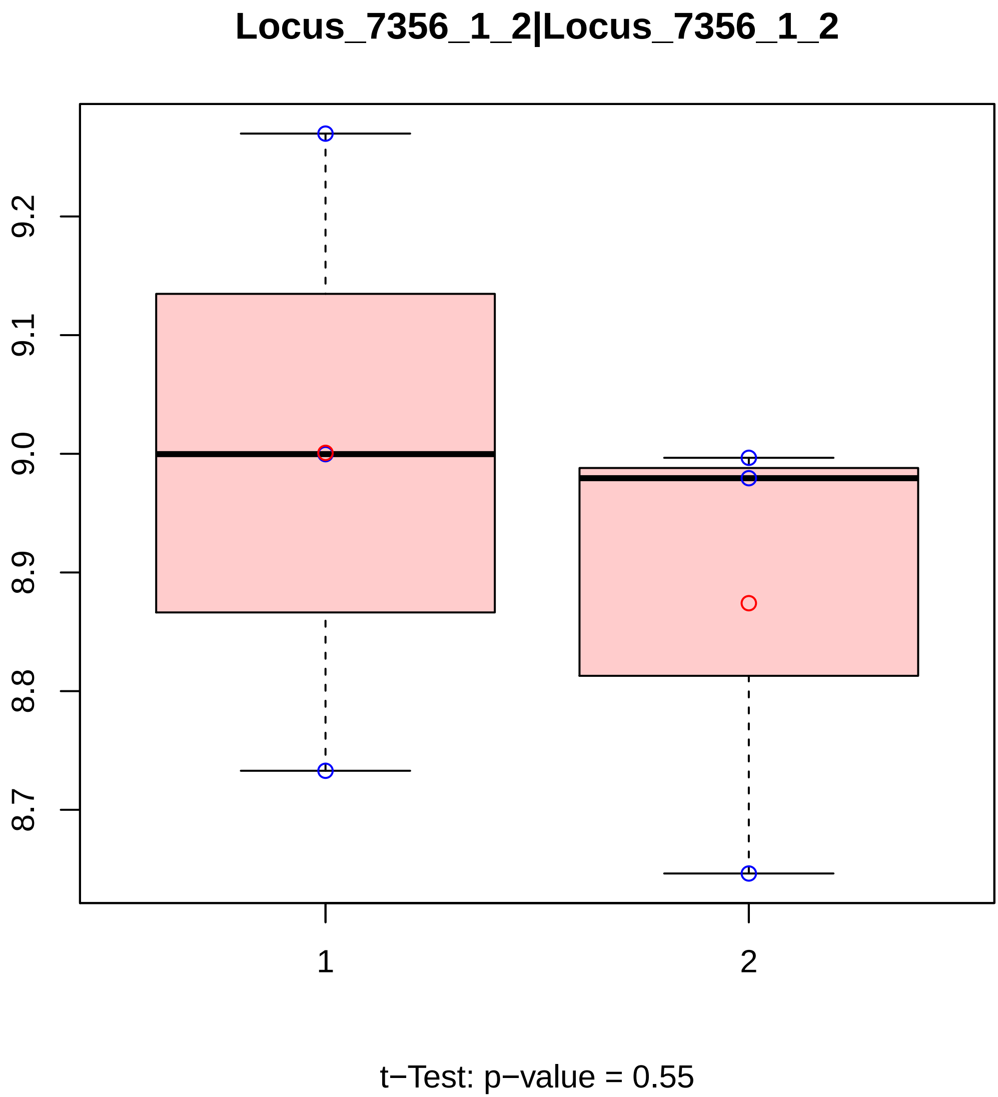
Locus_7356_1_2|Locus_7356_1_2
9.2
9.1
9.0
8.9
8.8
8.7
1
2
t−Test: p−value = 0.55

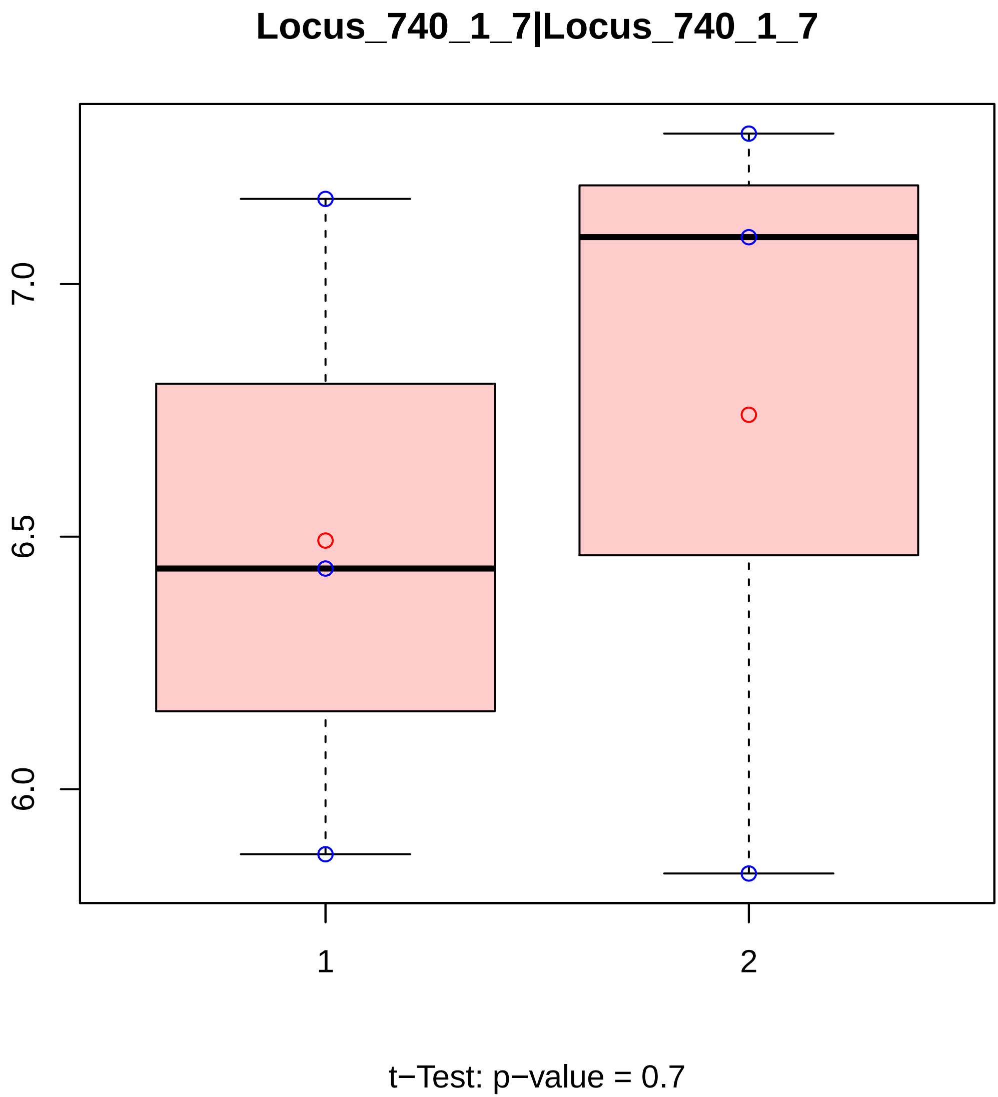

Locus_740_1_7|Locus_740_1_7
7.0
6.5
6.0
1
2
t−Test: p−value = 0.7

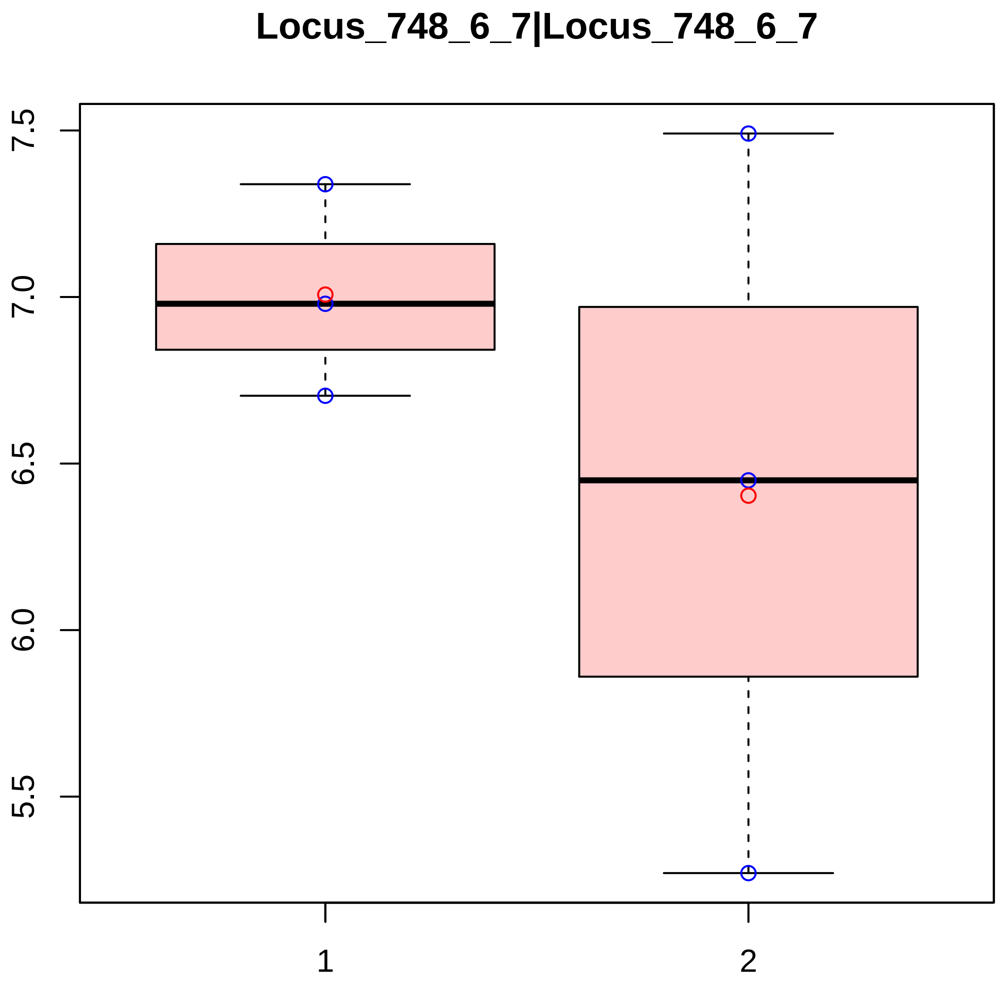

t−Test: p−value = 0.45

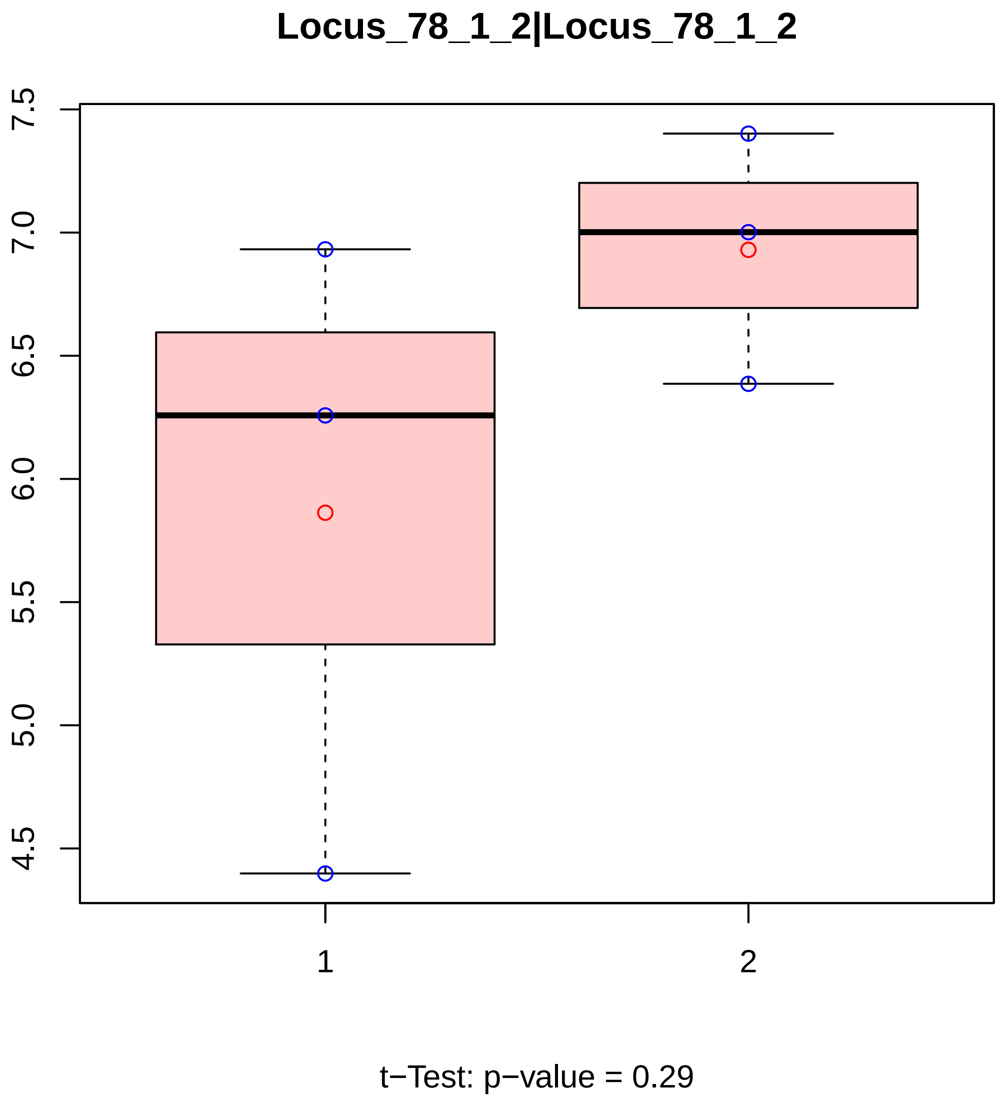
Locus_78_1_2|Locus_78_1_2
7.5
7.0
6.5
6.0
5.5
5.0
4.5
1
2
t−Test: p−value = 0.29

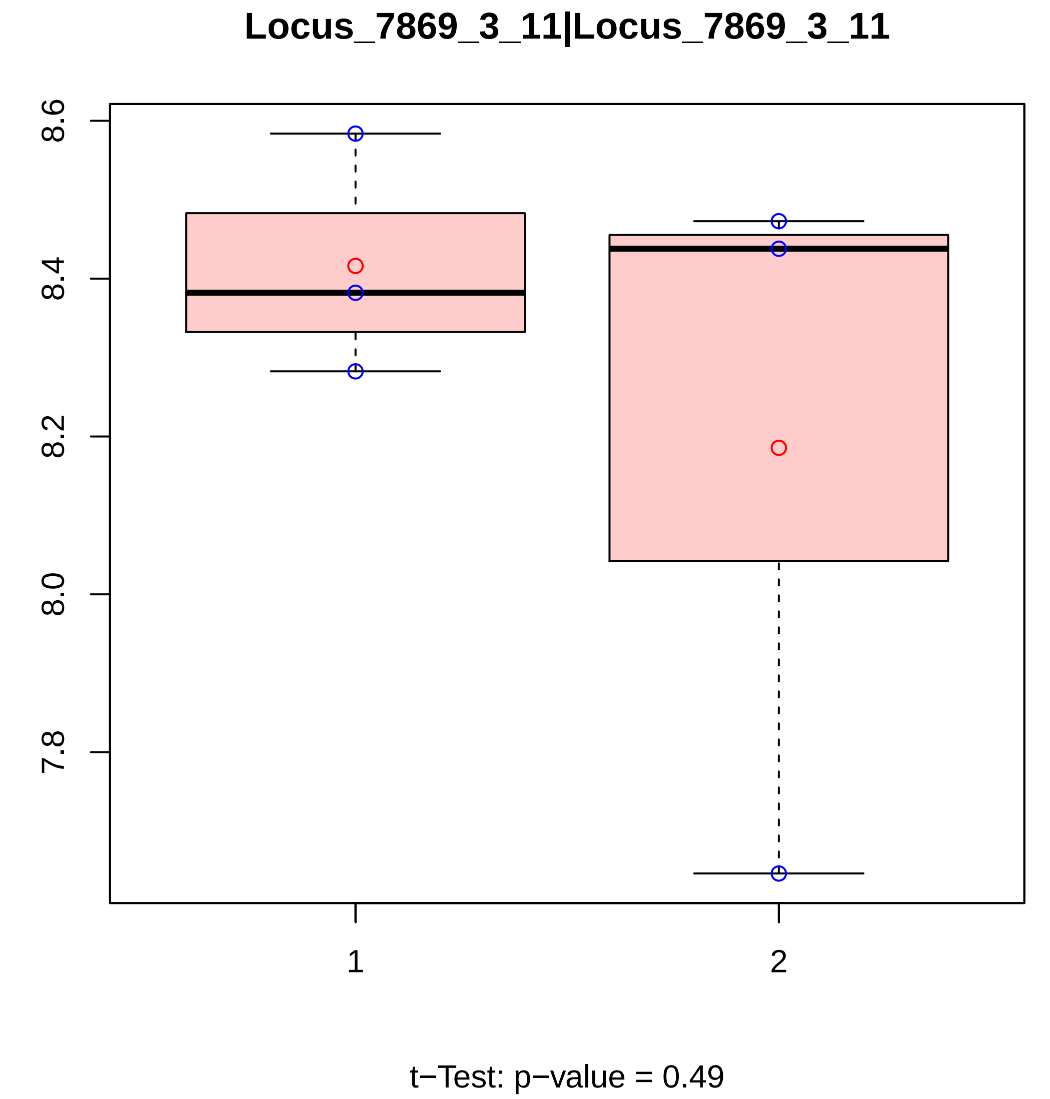

Locus_7869_3_11|Locus_7869_3_11
8.6
8.4
8.2
8.0
7.8
1
2
t−Test: p−value = 0.49

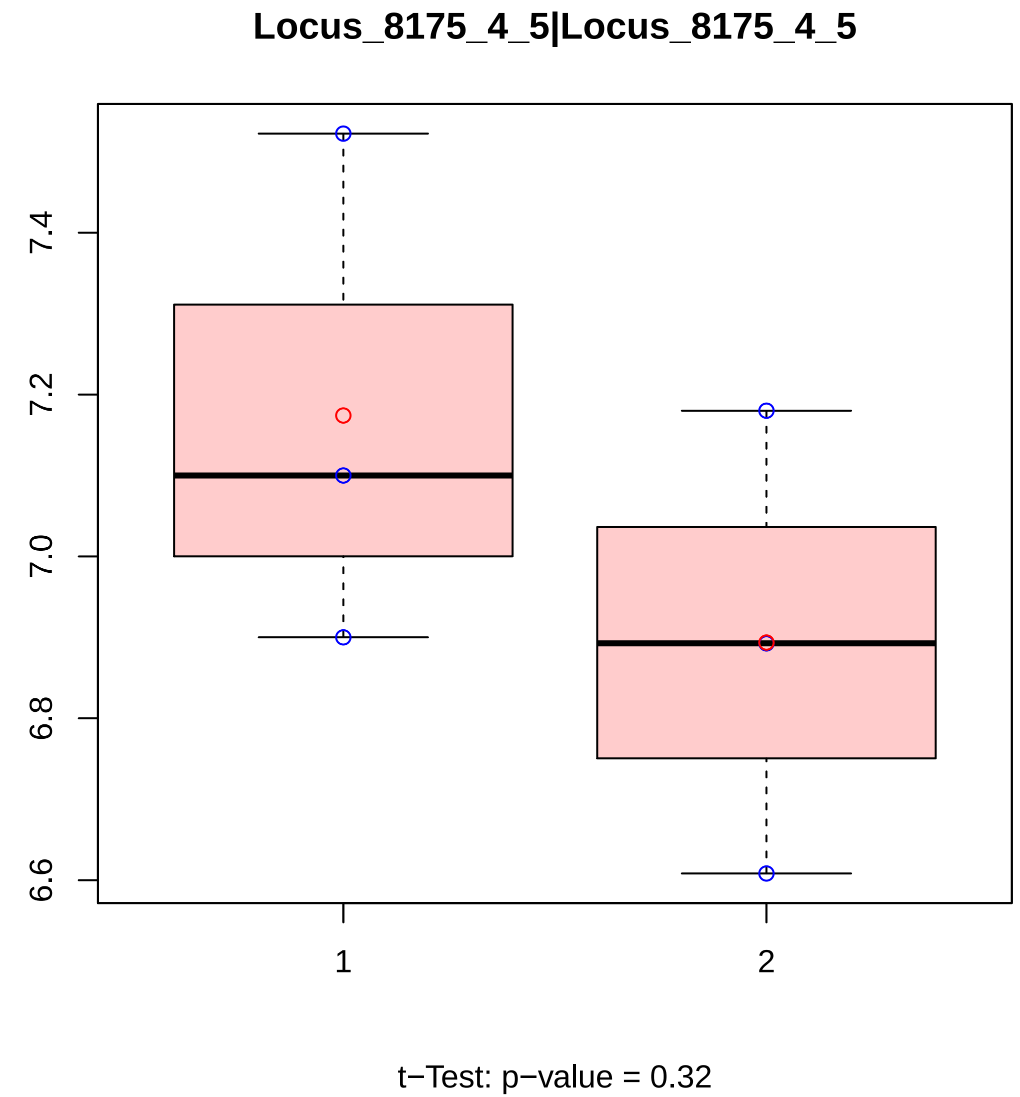

Locus_8175_4_5|Locus_8175_4_5
7.4
7.2
7.0
6.8
6.6
1
2
t−Test: p−value = 0.32

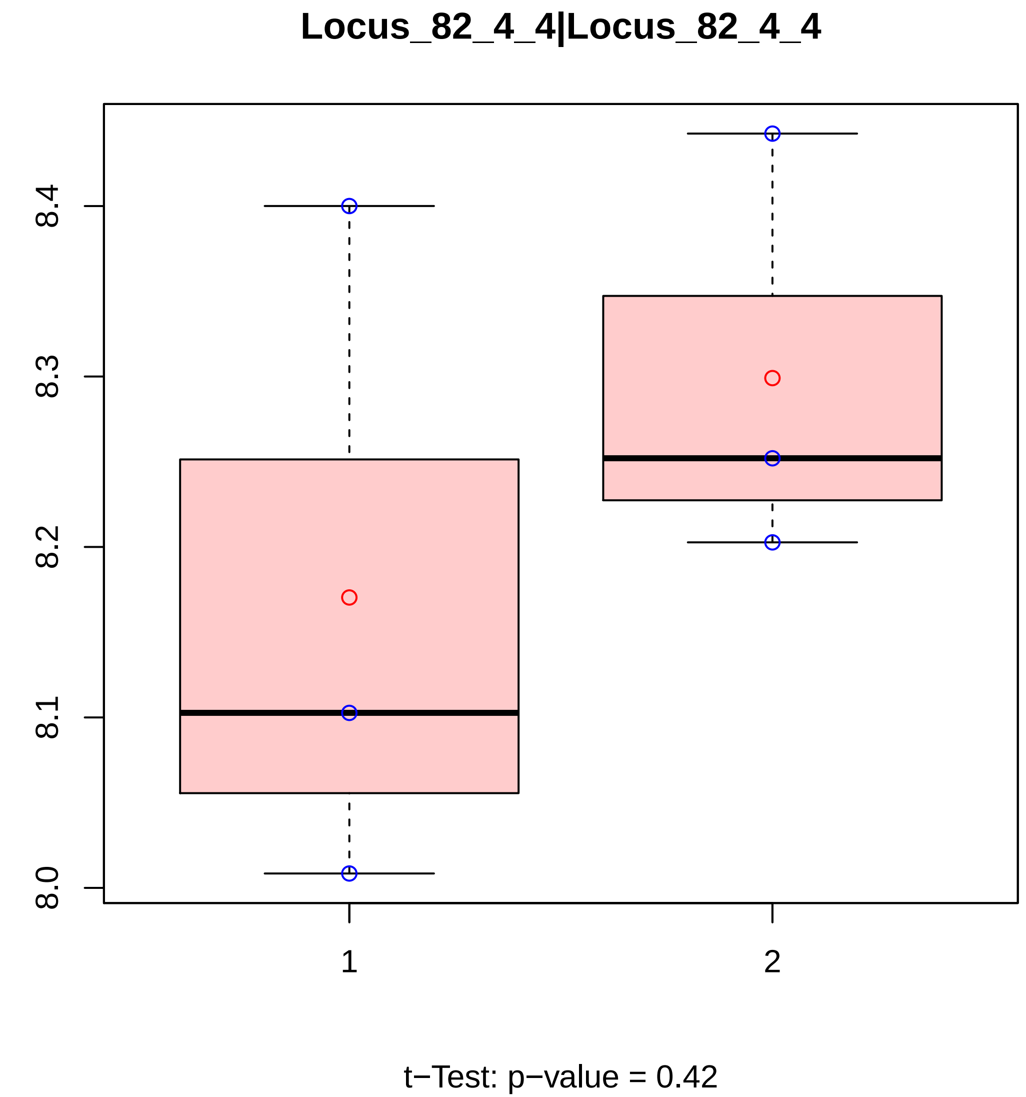
Locus_82_4_4|Locus_82_4_4
8.0
8.1
8.2
8.3
8.4
1
2
t−Test: p−value = 0.42

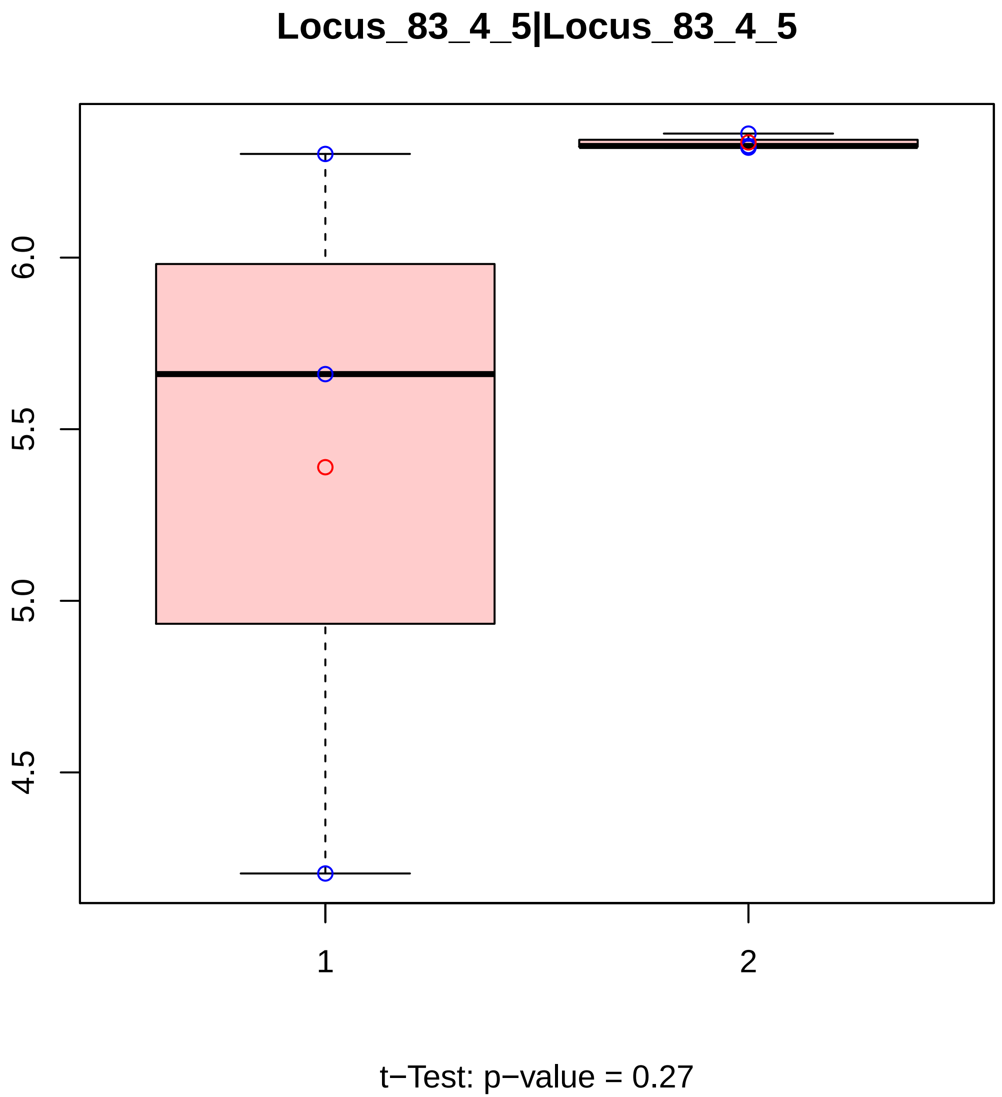

Locus_83_4_5|Locus_83_4_5
4.5
5.0
5.5
6.0
1
2
t−Test: p−value = 0.27

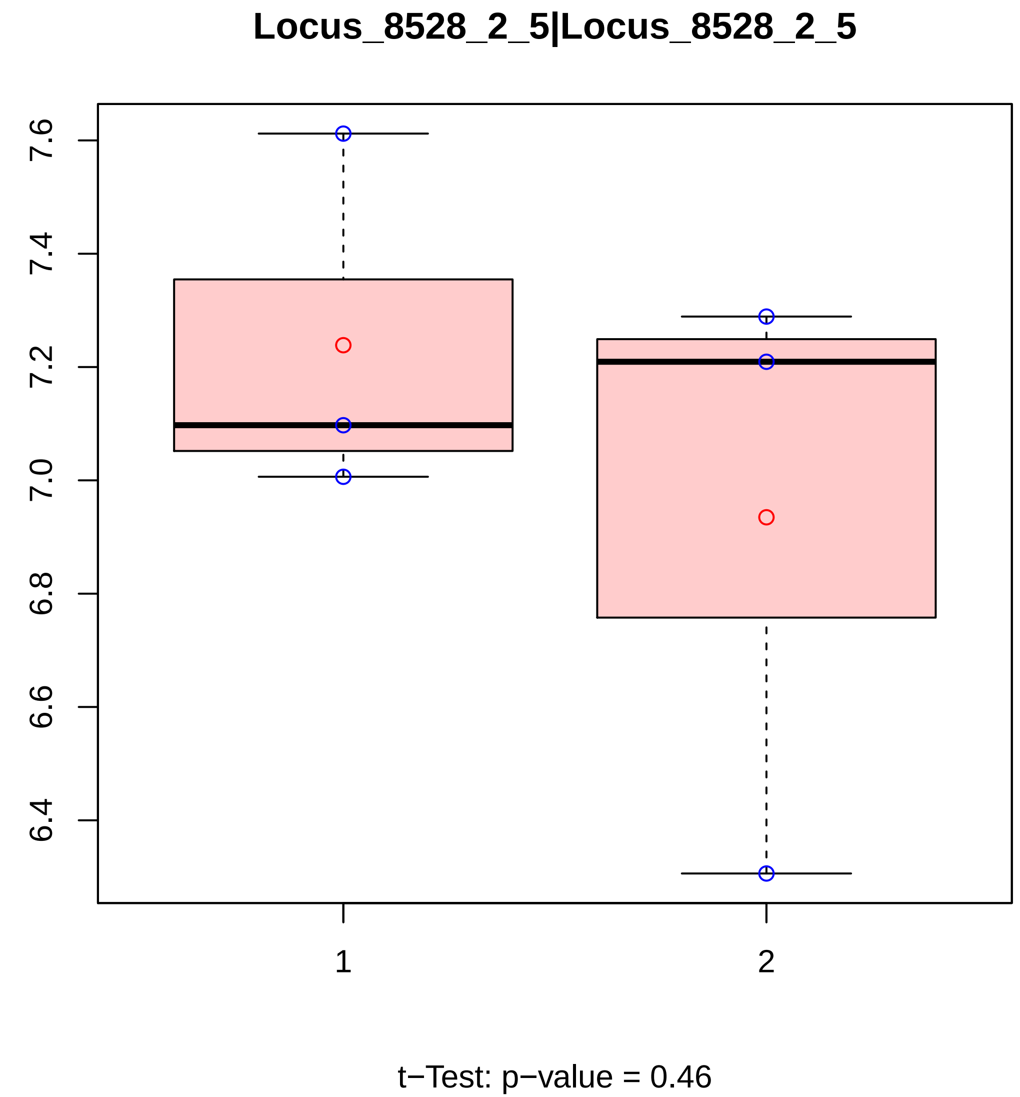

Locus_8528_2_5|Locus_8528_2_5
7.6
7.4
7.2
7.0
6.8
6.6
6.4
1
2
t−Test: p−value = 0.46

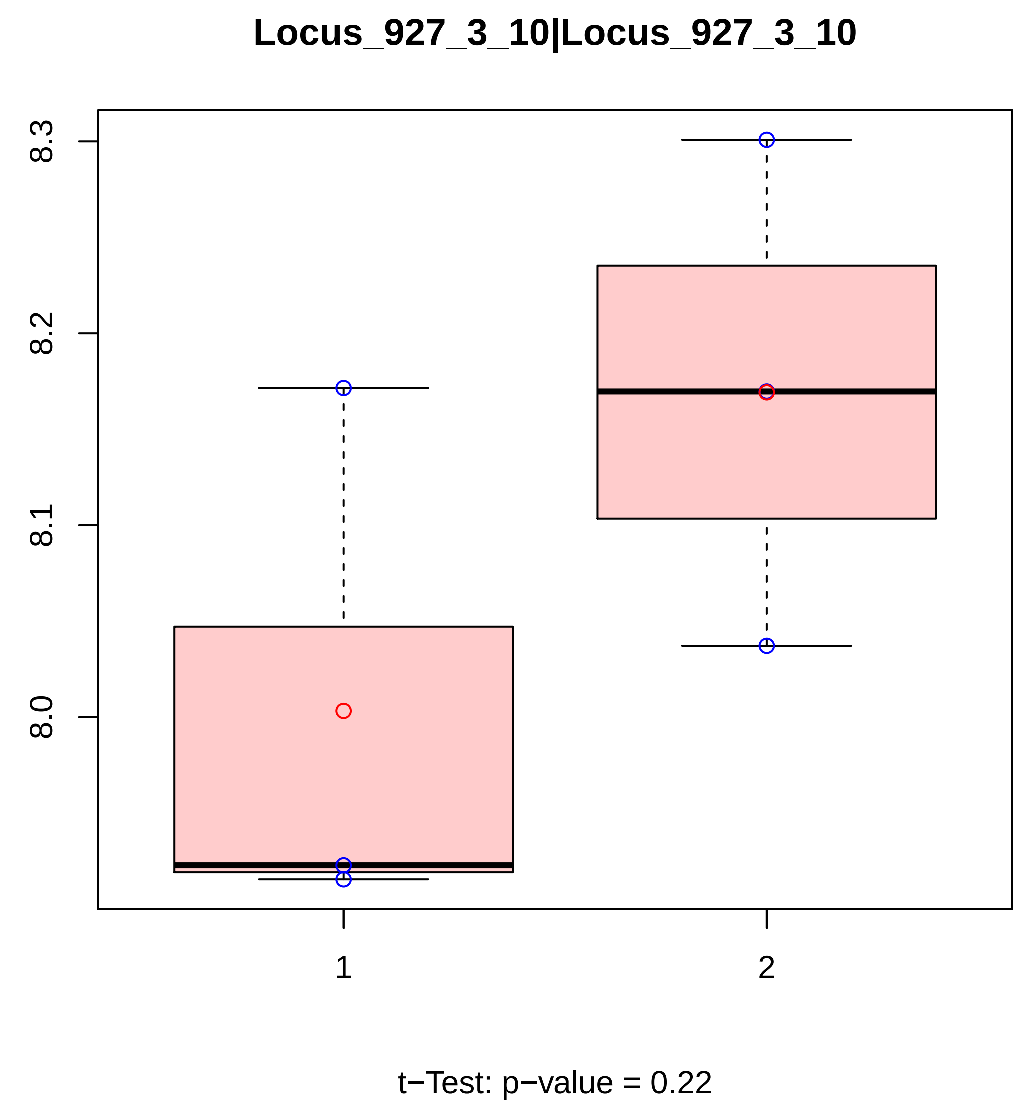
Locus_927_3_10|Locus_927_3_10
8.3
8.2
8.1
8.0
1
2
t−Test: p−value = 0.22

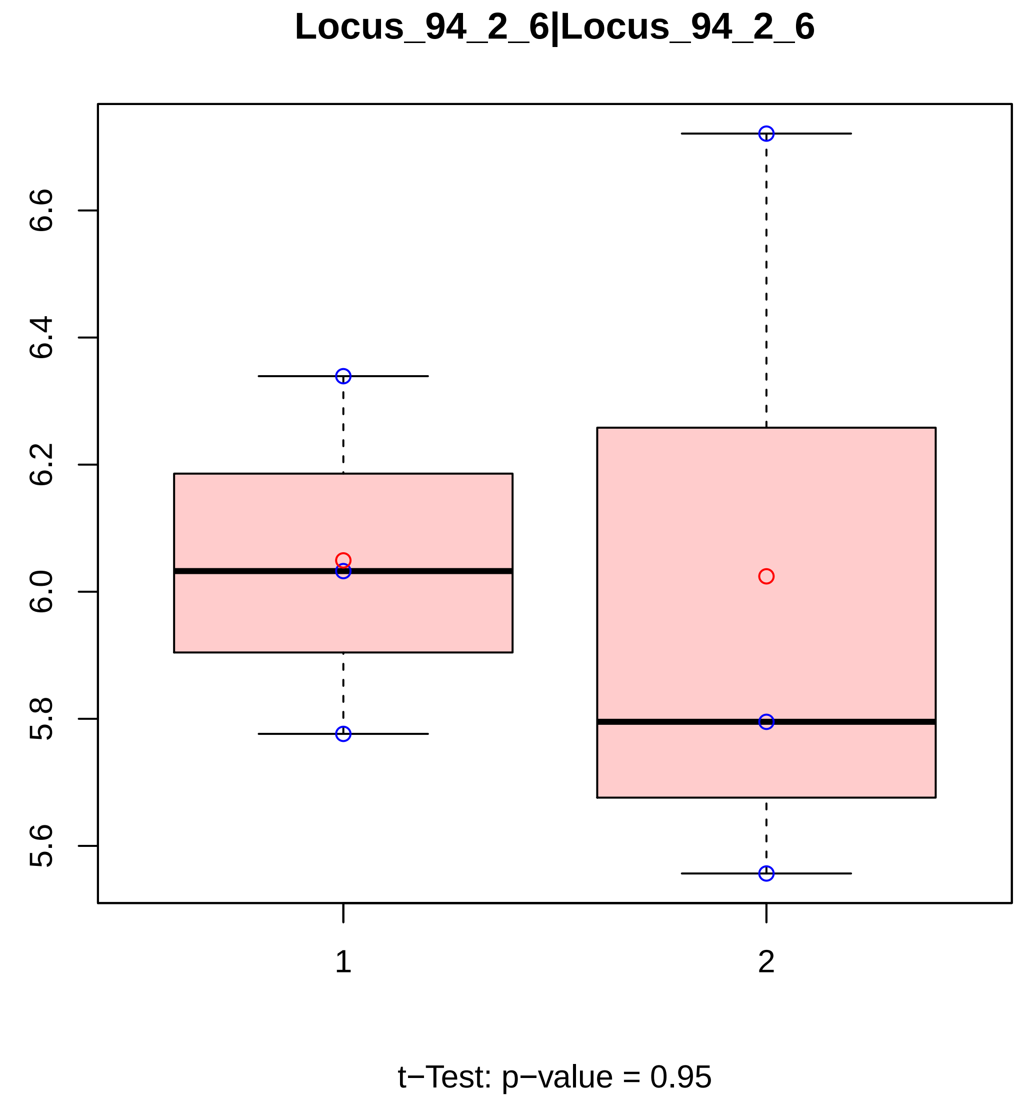

Locus_94_2_6|Locus_94_2_6
6.6
6.4
6.2
6.0
5.8
5.6
1
2
t−Test: p−value = 0.95

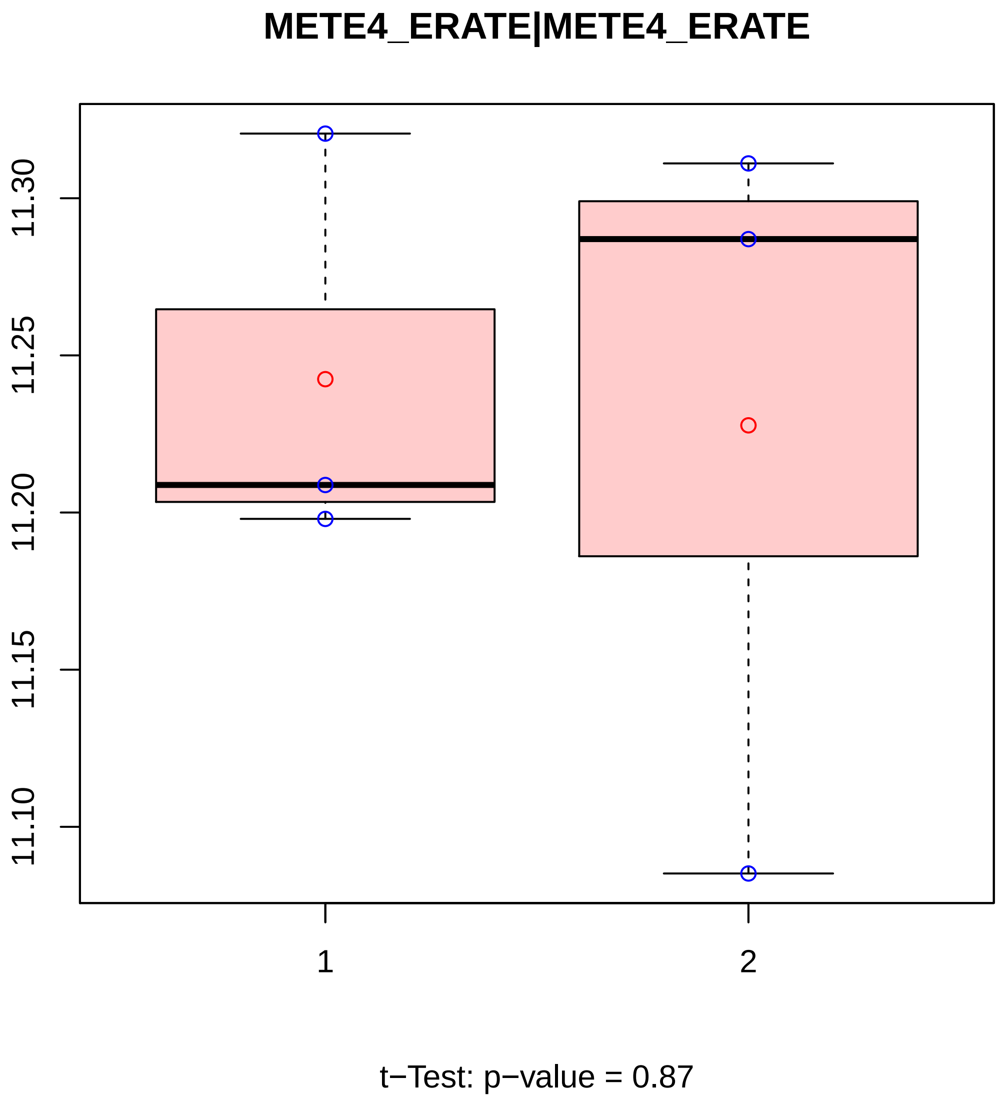
METE4_ERATE|METE4_ERATE
11.10
11.15
11.20
11.25
11.30
1
2
t−Test: p−value = 0.87

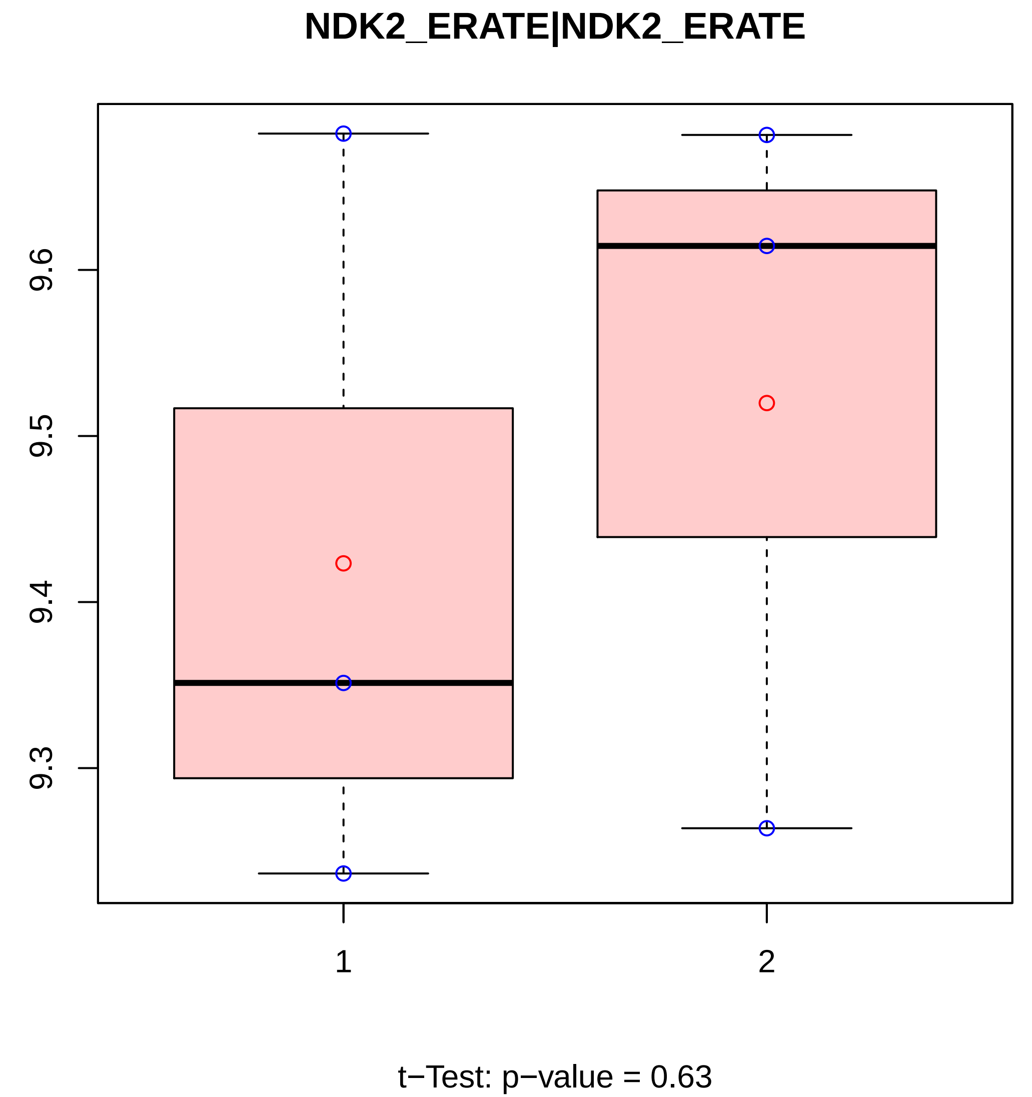

NDK2_ERATE|NDK2_ERATE
9.3
9.4
9.5
9.6
1
2
t−Test: p−value = 0.63

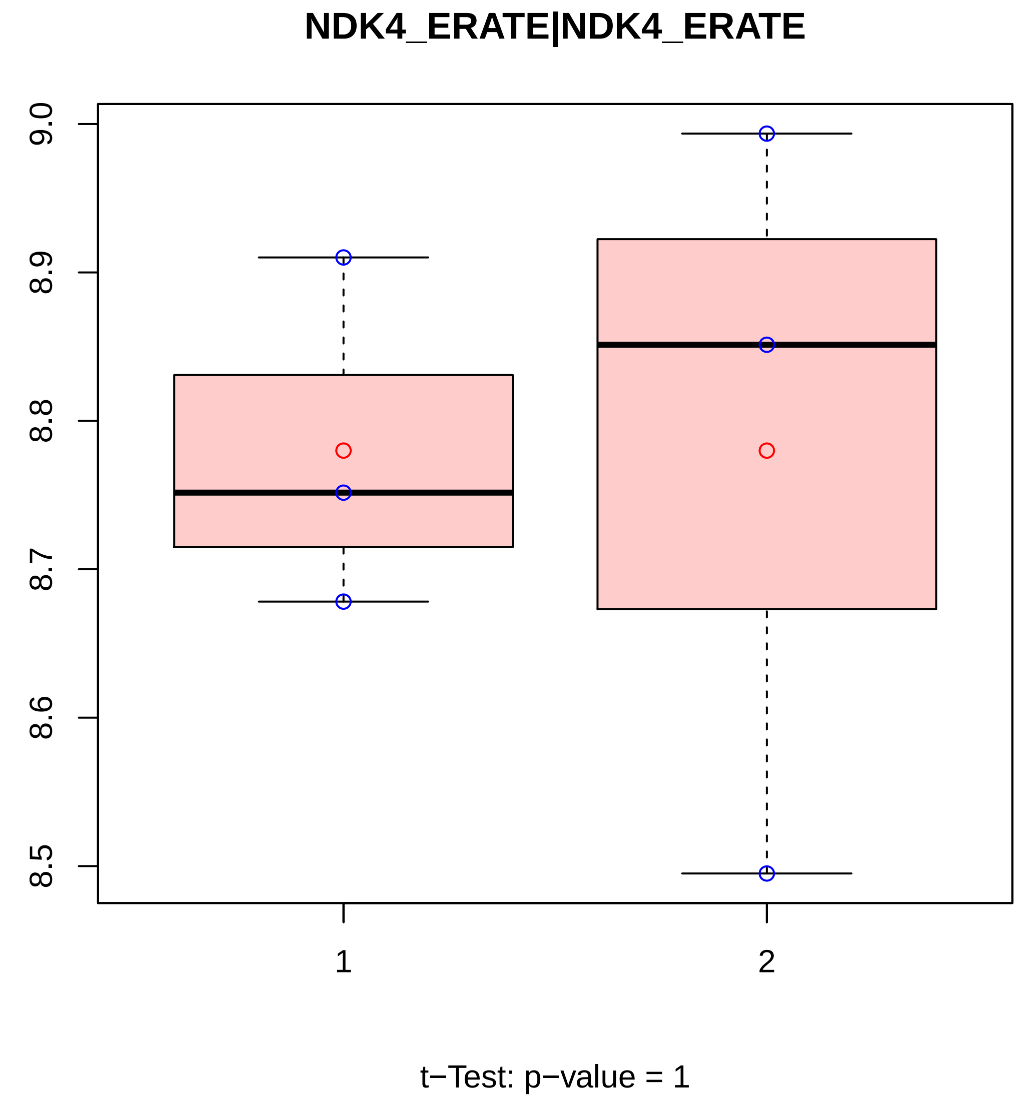

NDK4_ERATE|NDK4_ERATE
9.0
8.9
8.8
8.7
8.6
8.5
1
2
t−Test: p−value = 1

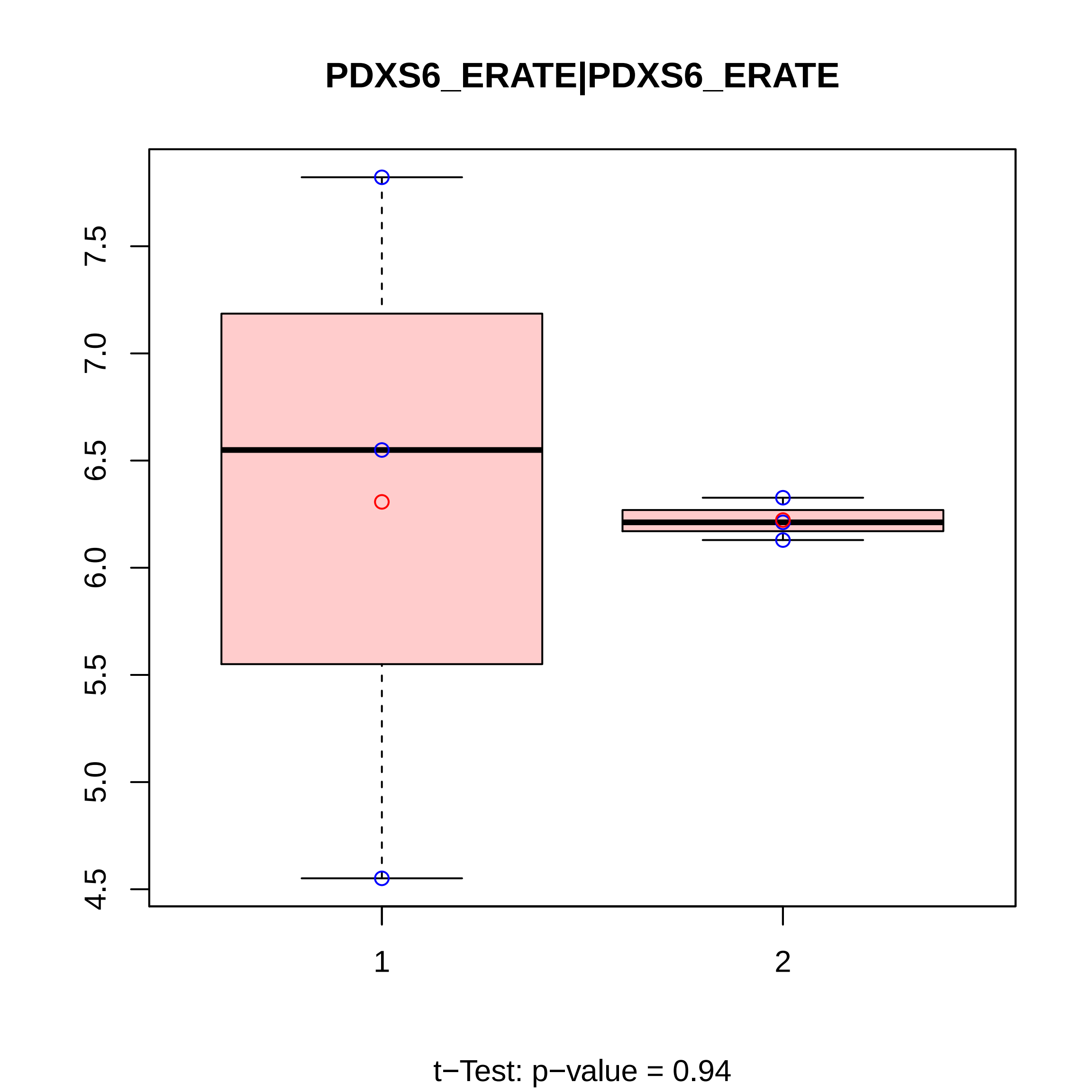
PDXS6_ERATE|PDXS6_ERATE
7.5
7.0
6.5
6.0
5.5
5.0
4.5
1
2
t−Test: p−value = 0.94

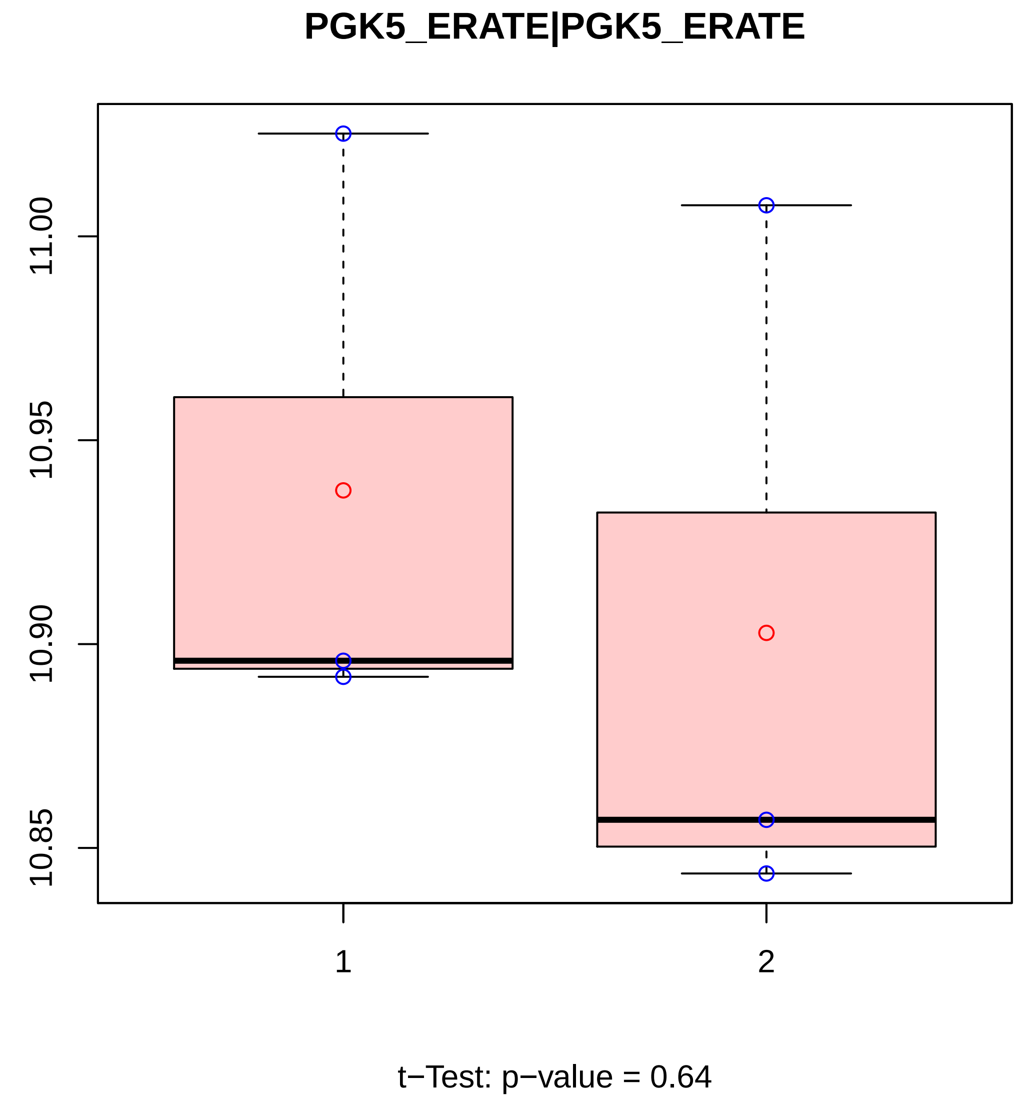
PGK5_ERATE|PGK5_ERATE
11.00
10.95
10.90
10.85
1
2
t−Test: p−value = 0.64

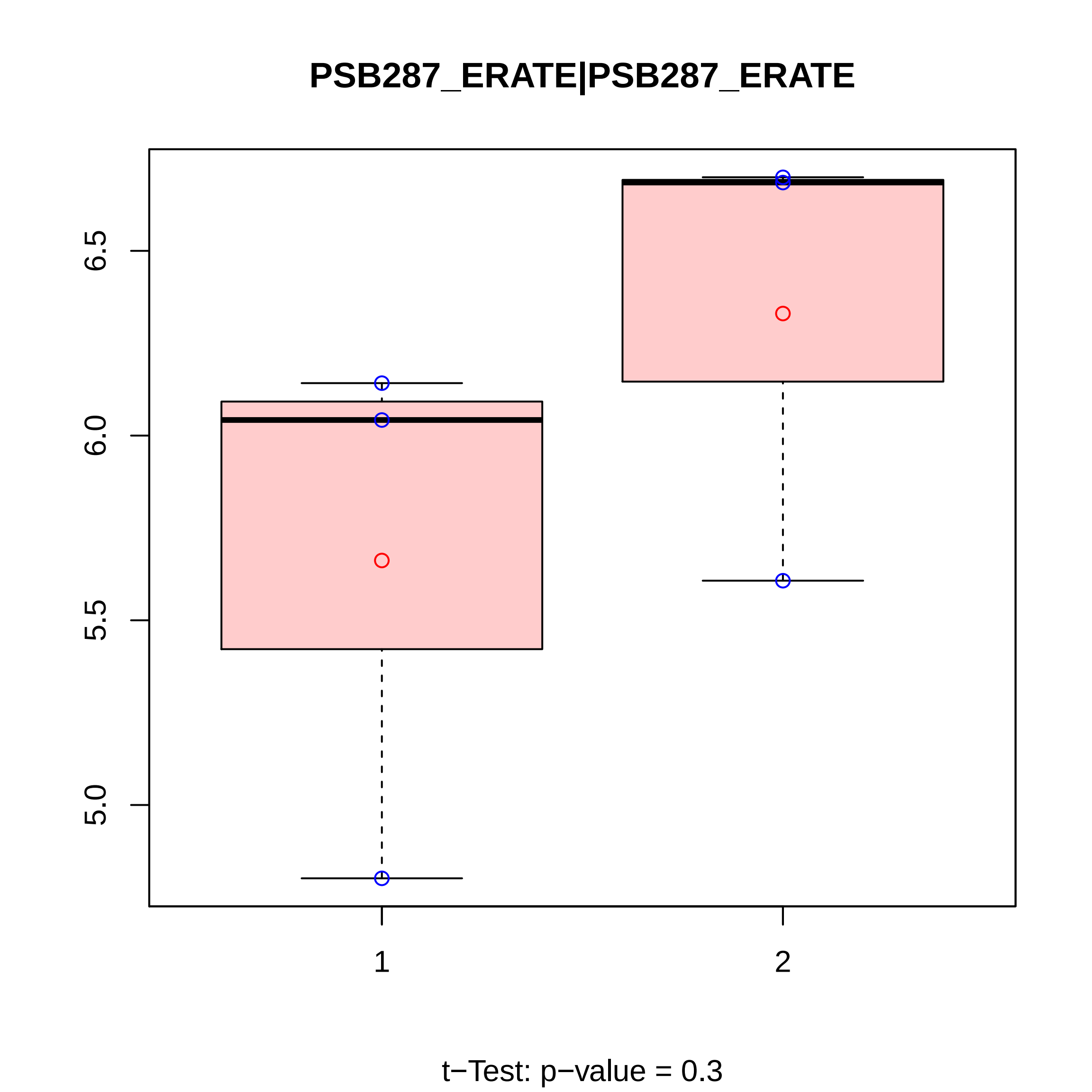
PSB287_ERATE|PSB287_ERATE
6.5
6.0
5.5
5.0
1
2
t−Test: p−value = 0.3

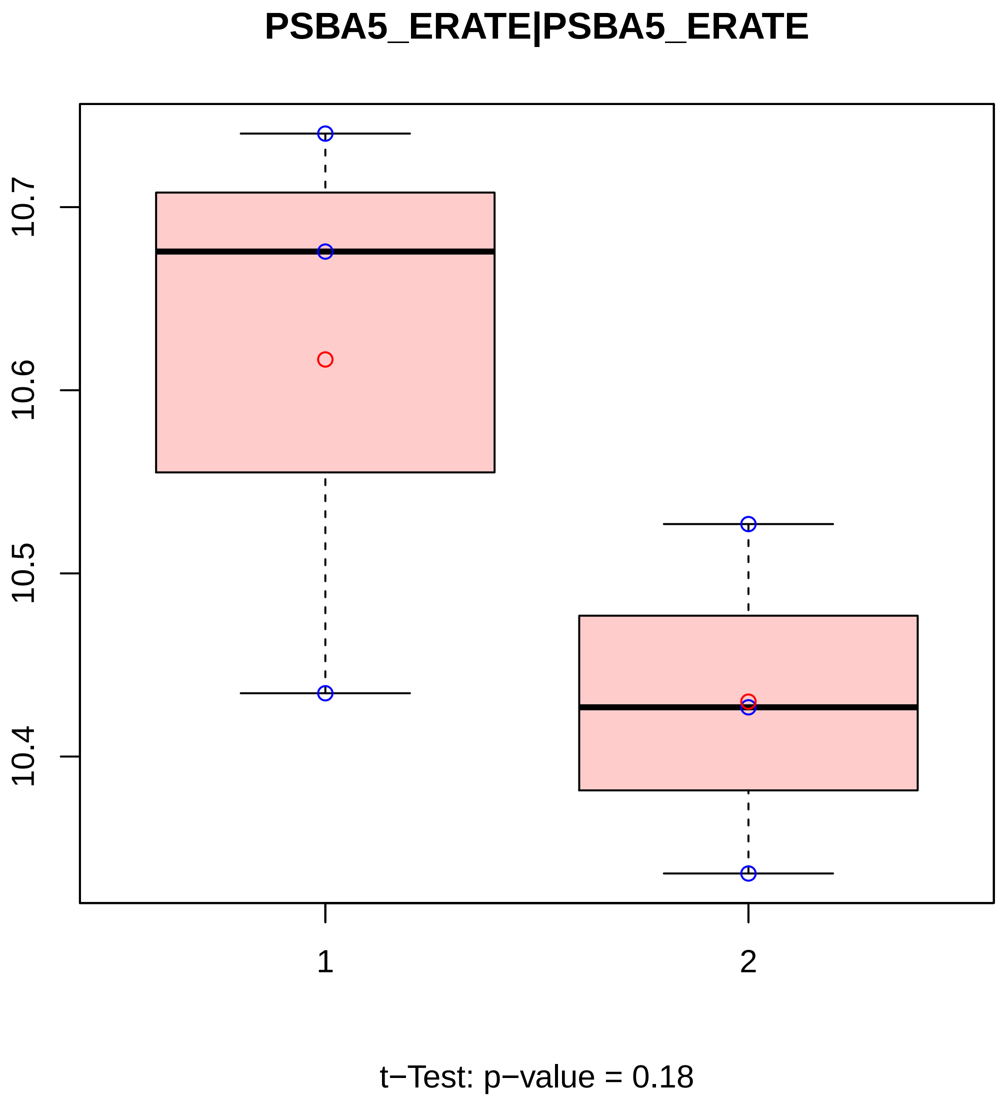
PSBA5_ERATE|PSBA5_ERATE
10.4
10.5
10.6
10.7
1
2
t−Test: p−value = 0.18

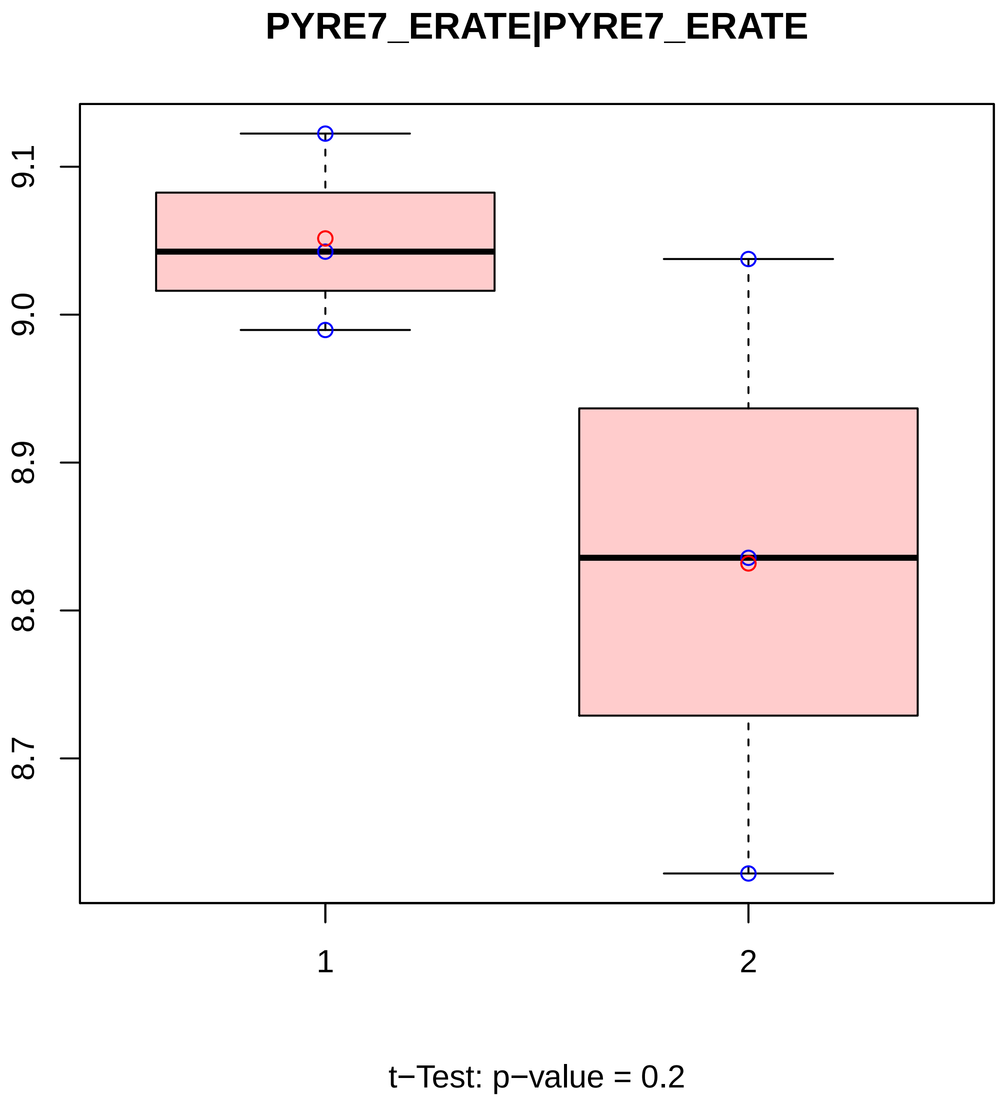

PYRE7_ERATE|PYRE7_ERATE
9.1
9.0
8.9
8.8
8.7
1
2
t−Test: p−value = 0.2

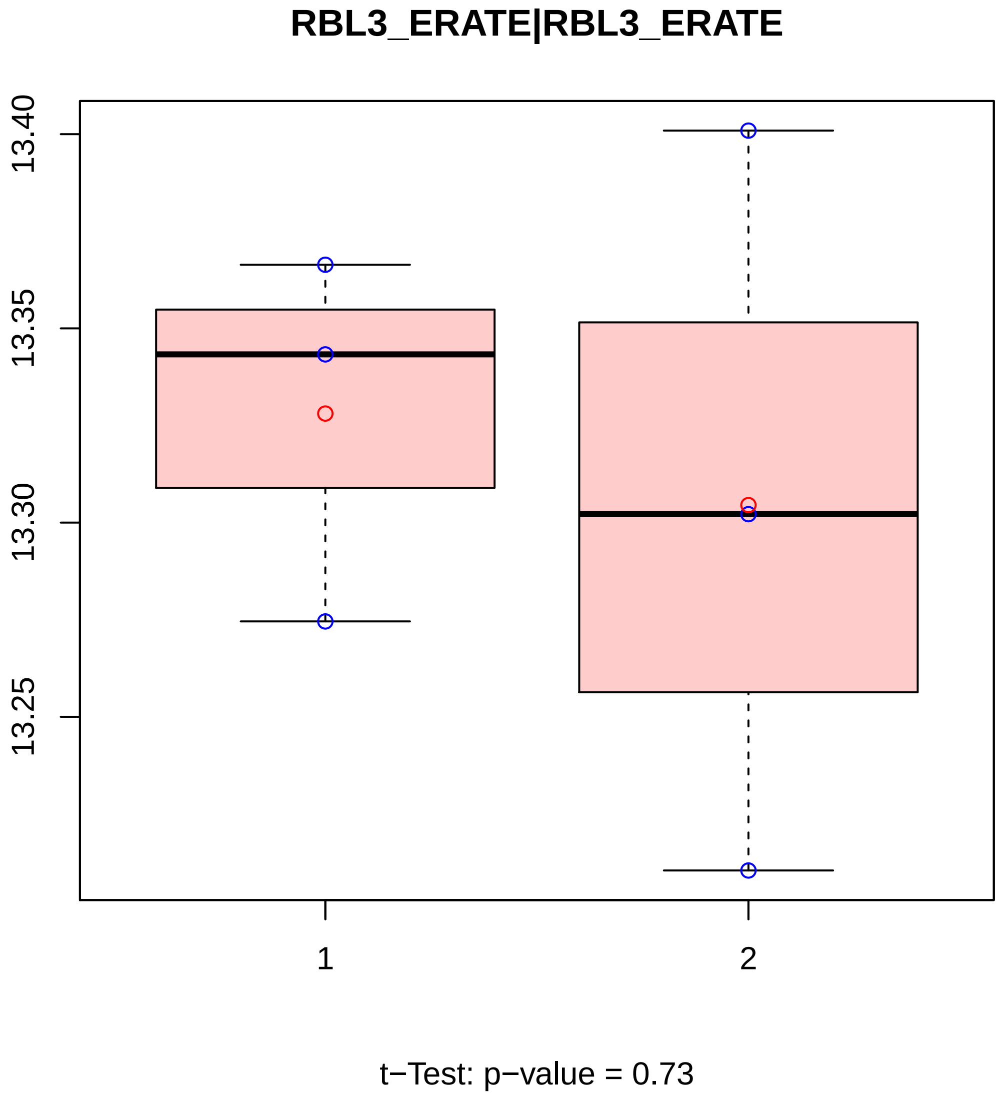
RBL3_ERATE|RBL3_ERATE
13.40
13.35
13.30
13.25
1
2
t−Test: p−value = 0.73

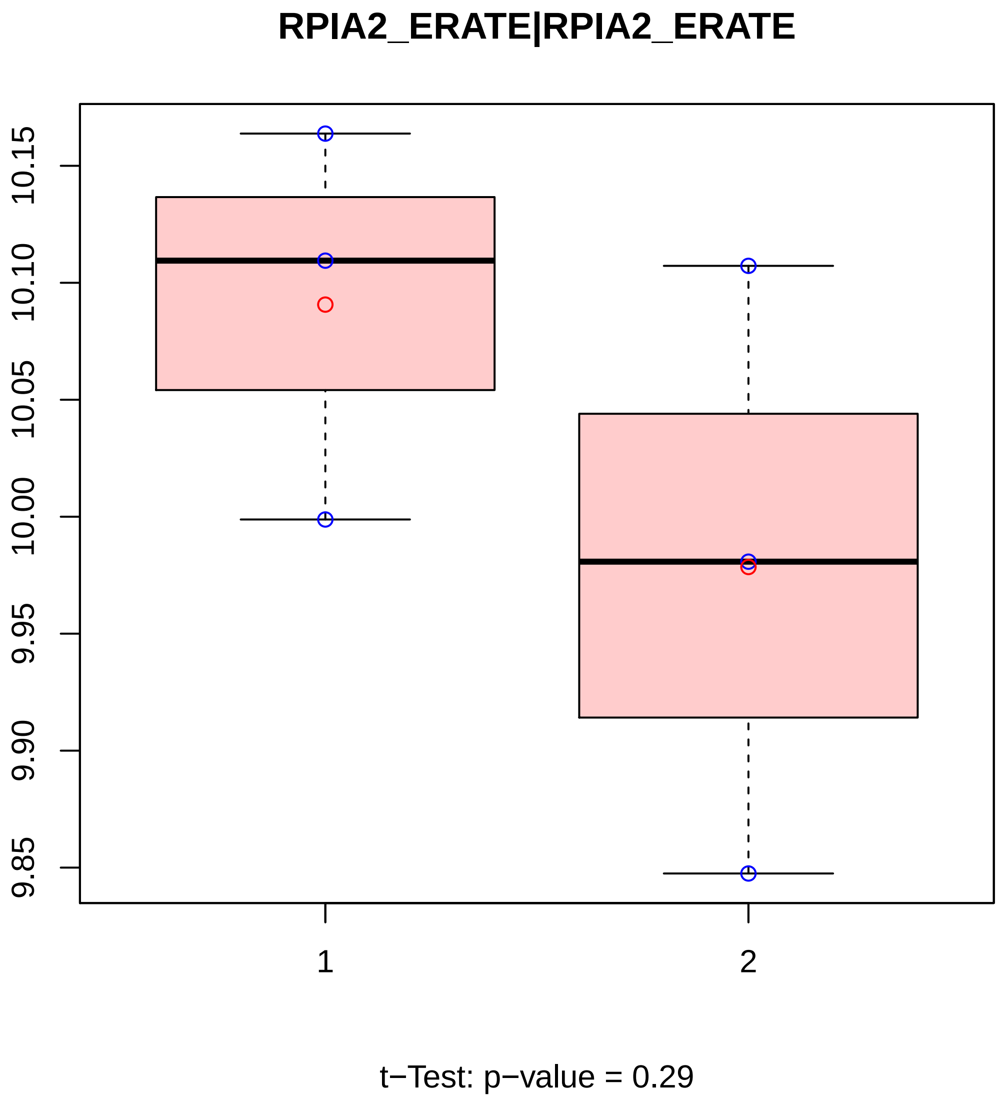
RPIA2_ERATE|RPIA2_ERATE
10.15
10.10
10.05
10.00
9.95
9.90
9.85
1
2
t−Test: p−value = 0.29

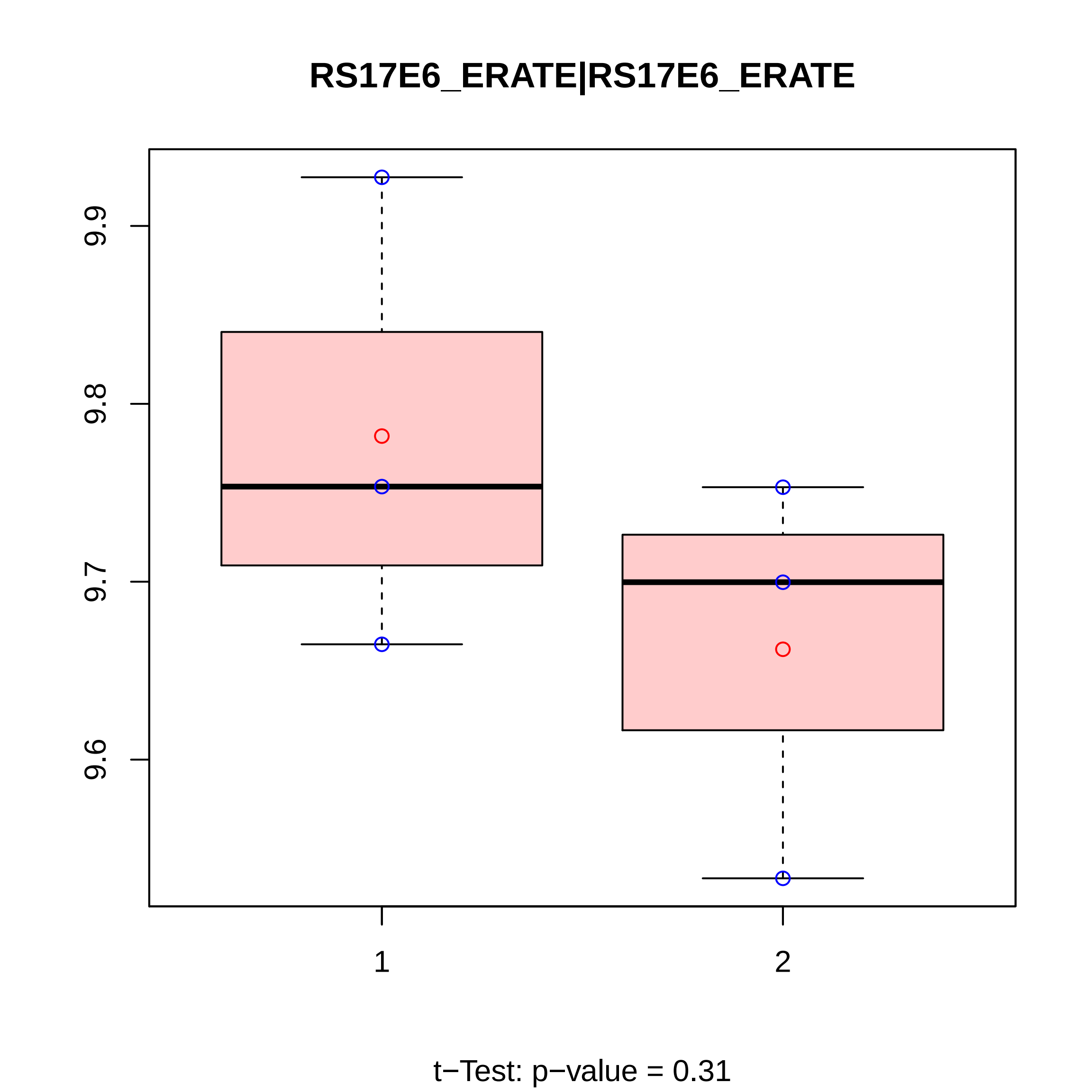

RS17E6_ERATE|RS17E6_ERATE
9.6
9.7
9.8
9.9
1
2
t−Test: p−value = 0.31

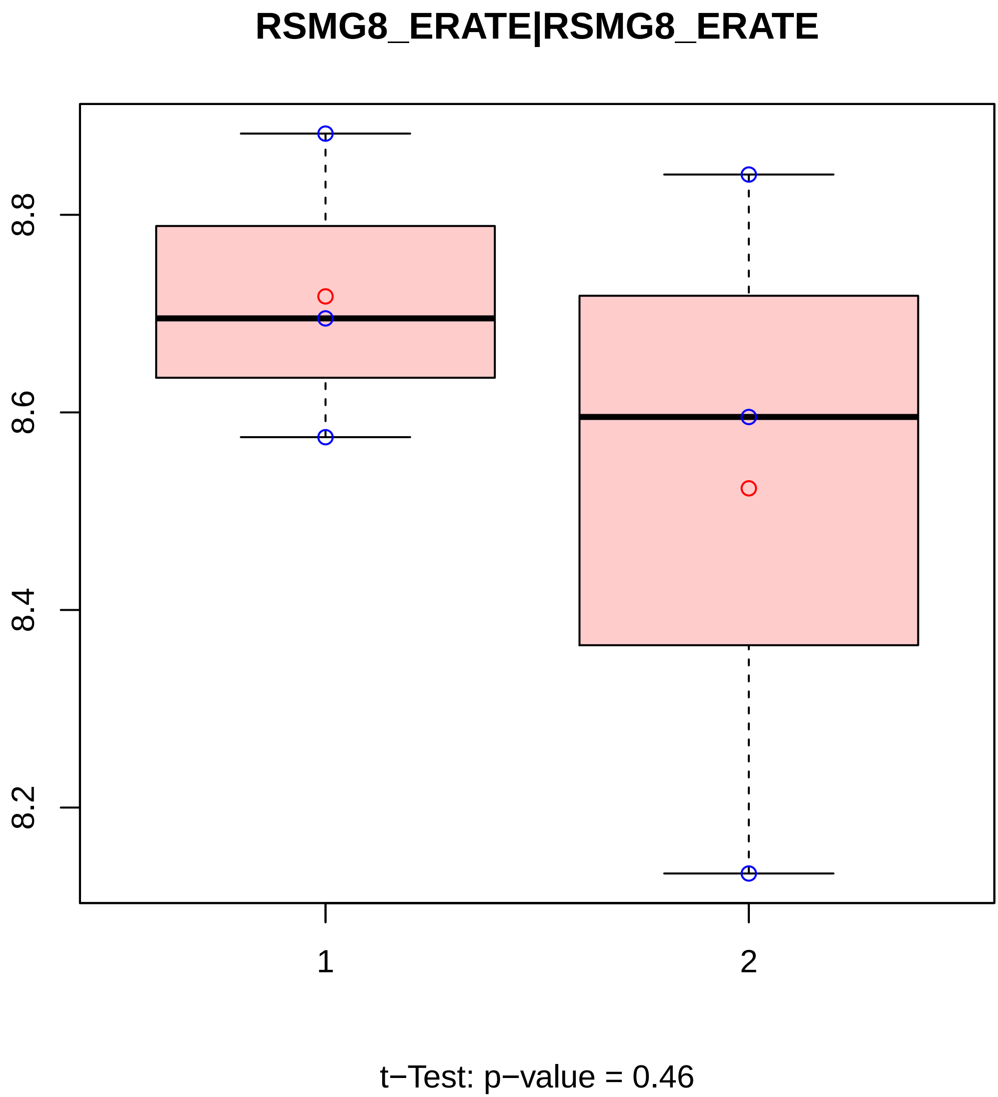
RSMG8_ERATE|RSMG8_ERATE
8.2
8.4
8.6
8.8
1
2
t−Test: p−value = 0.46

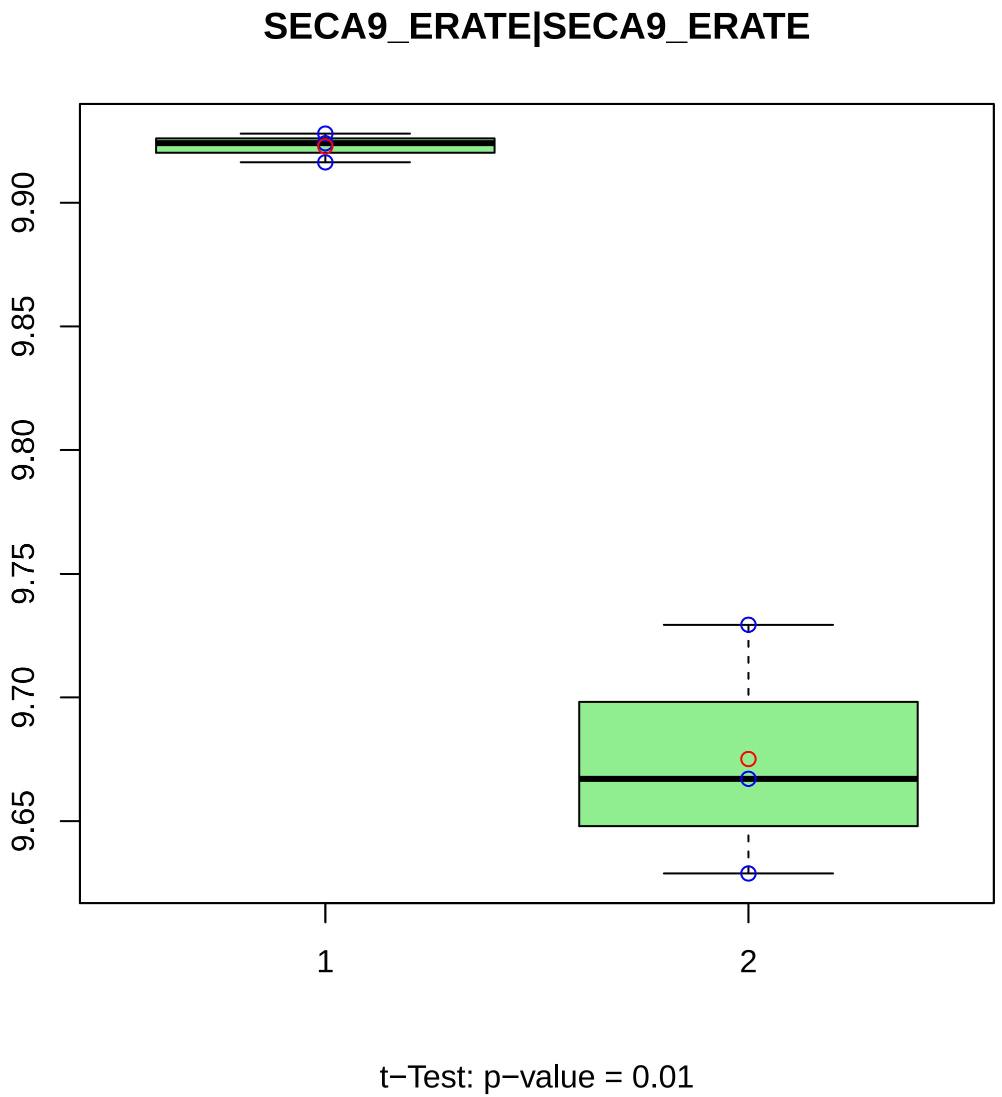

SECA9_ERATE|SECA9_ERATE
9.65
9.70
9.75
9.80
9.85
9.90
1
2
t−Test: p−value = 0.01

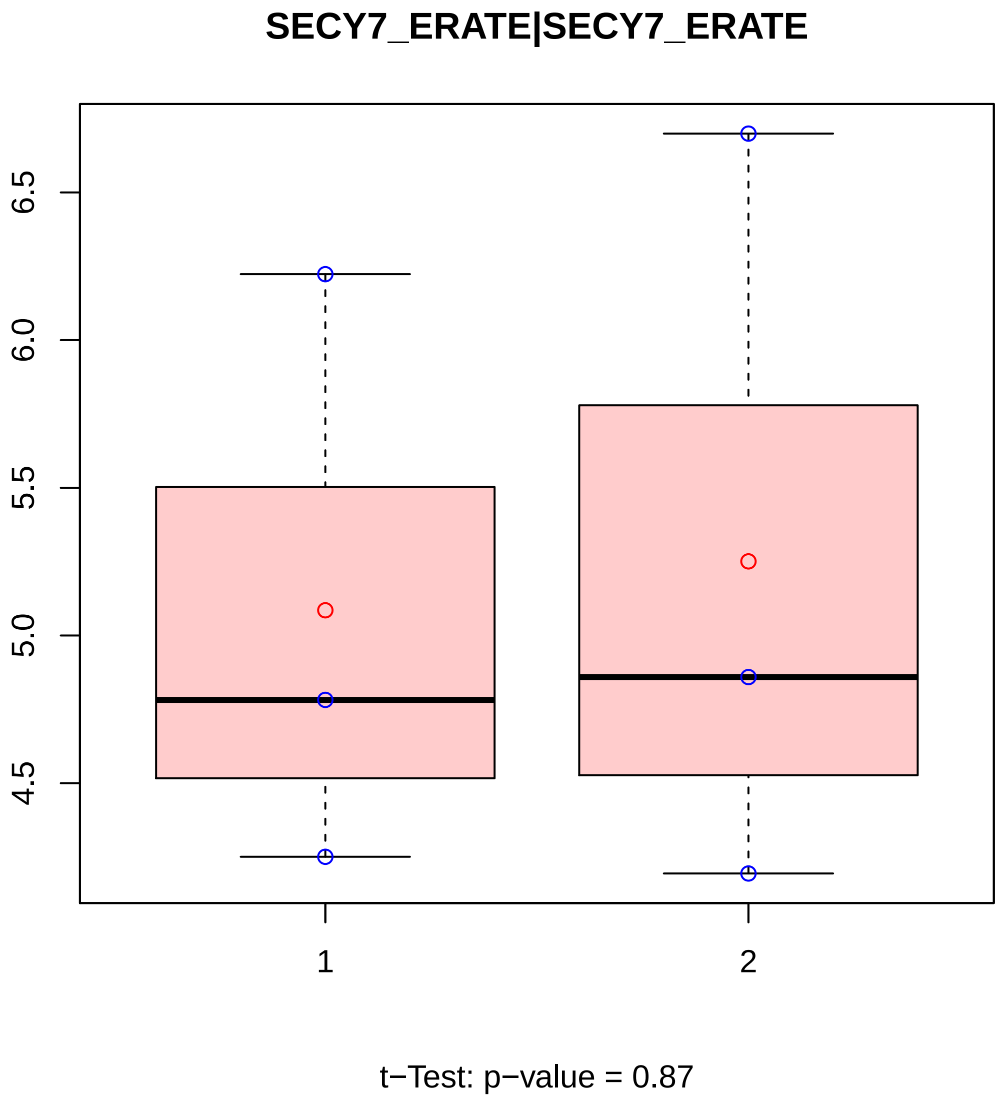

SECY7_ERATE|SECY7_ERATE
6.5
6.0
5.5
5.0
4.5
1
2
t−Test: p−value = 0.87

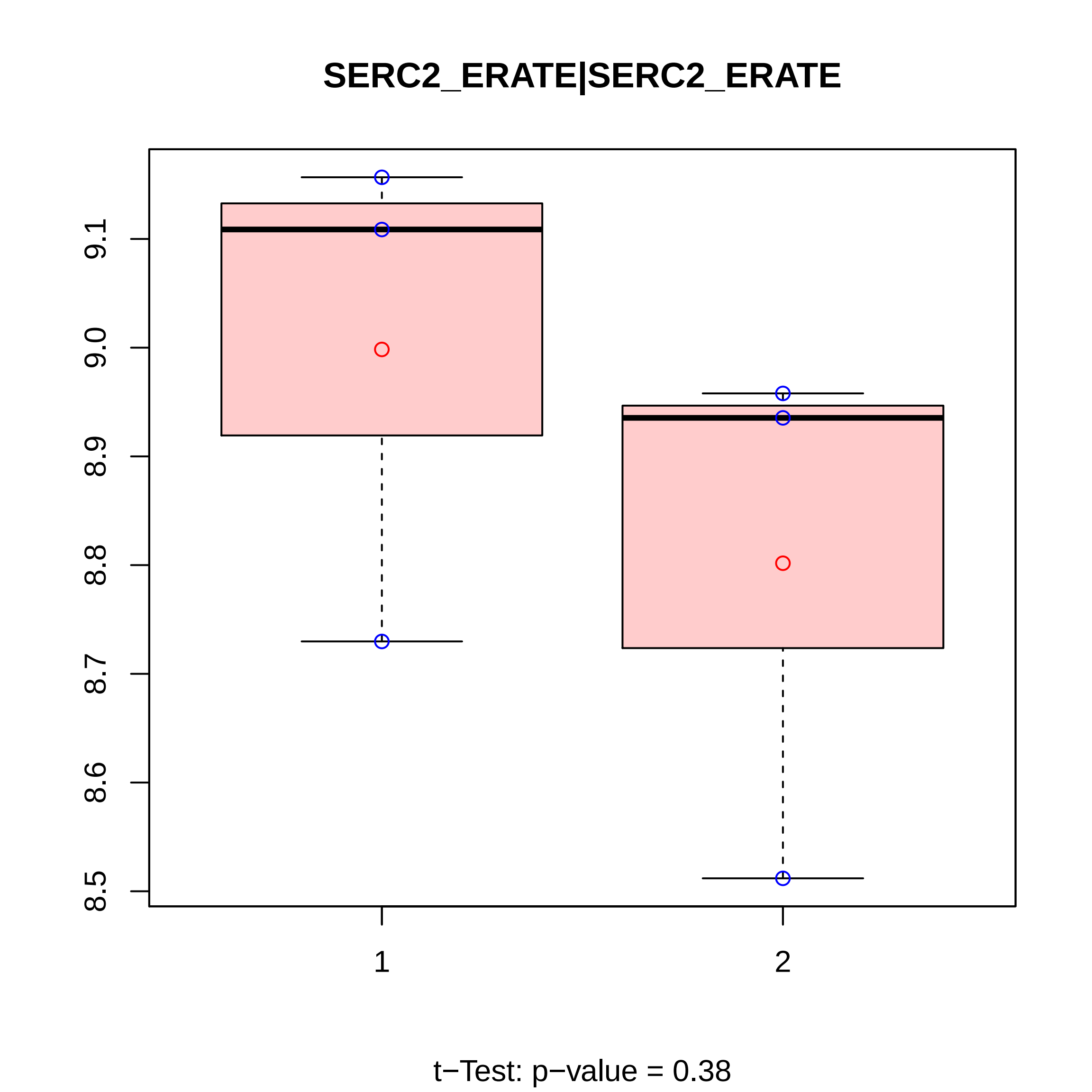
SERC2_ERATE|SERC2_ERATE
9.1
9.0
8.9
8.8
8.7
8.6
8.5
1
2
t−Test: p−value = 0.38

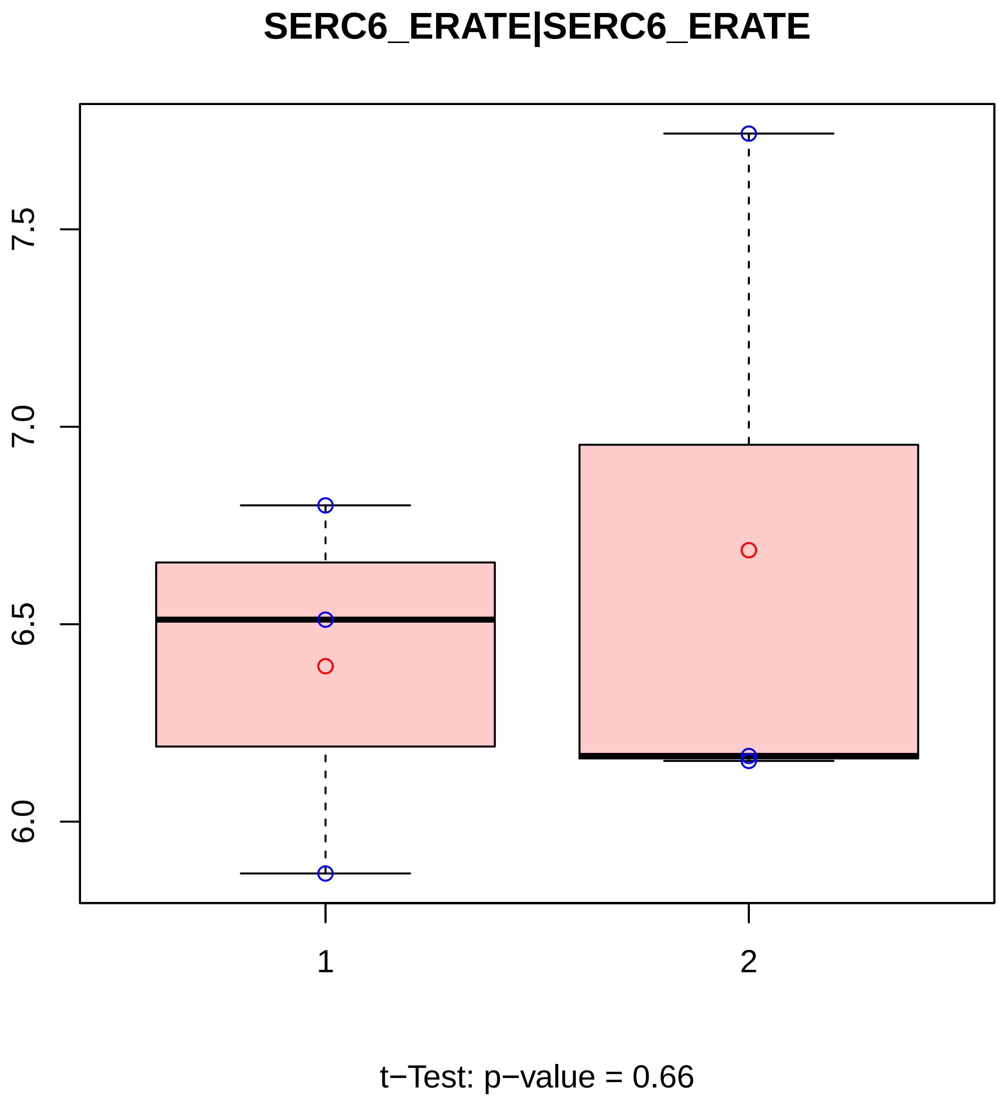

SERC6_ERATE|SERC6_ERATE
7.5
7.0
6.5
6.0
1
2
t−Test: p−value = 0.66

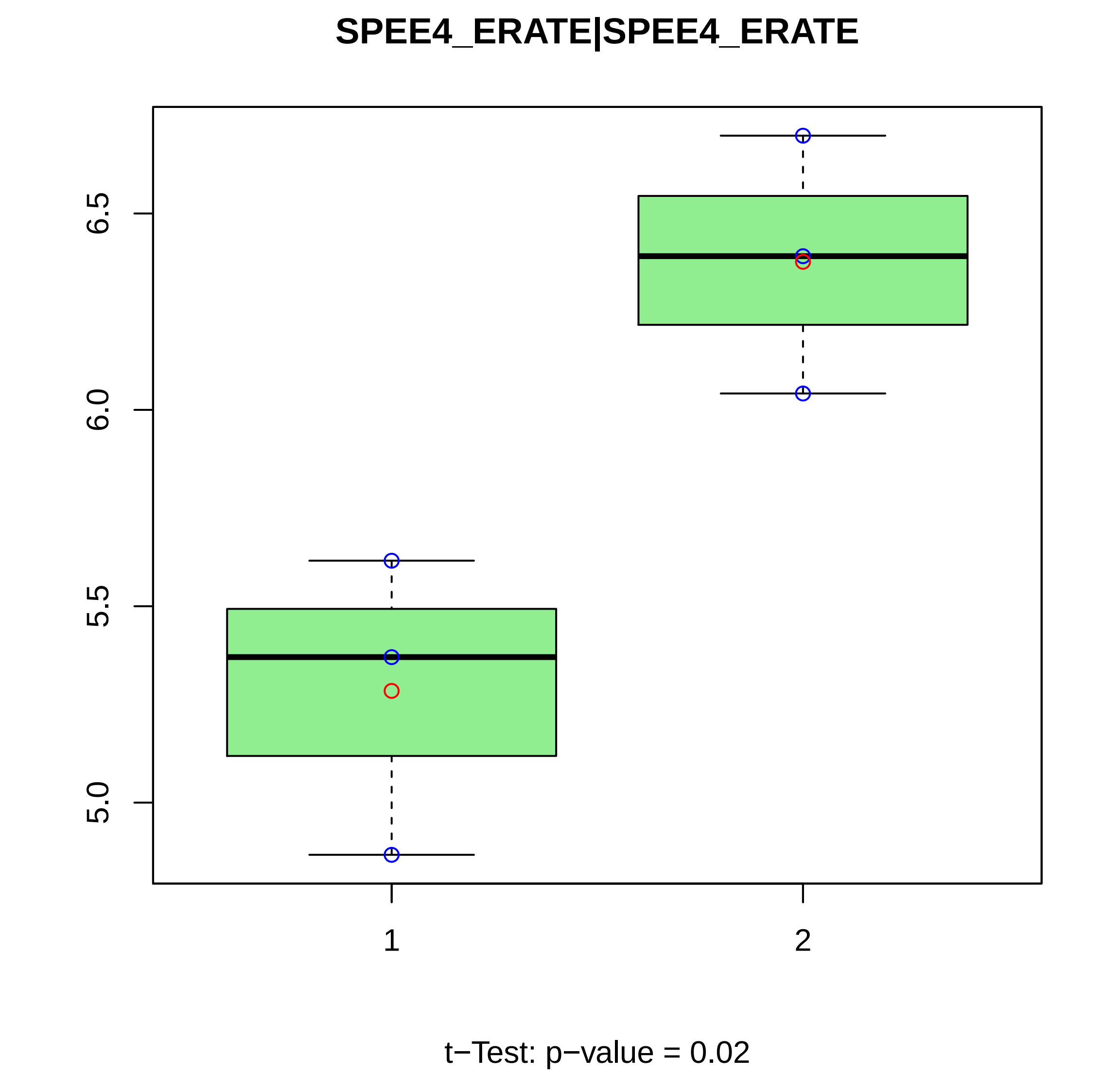
SPEE4_ERATE|SPEE4_ERATE
6.5
6.0
5.5
5.0
1
2
t−Test: p−value = 0.02

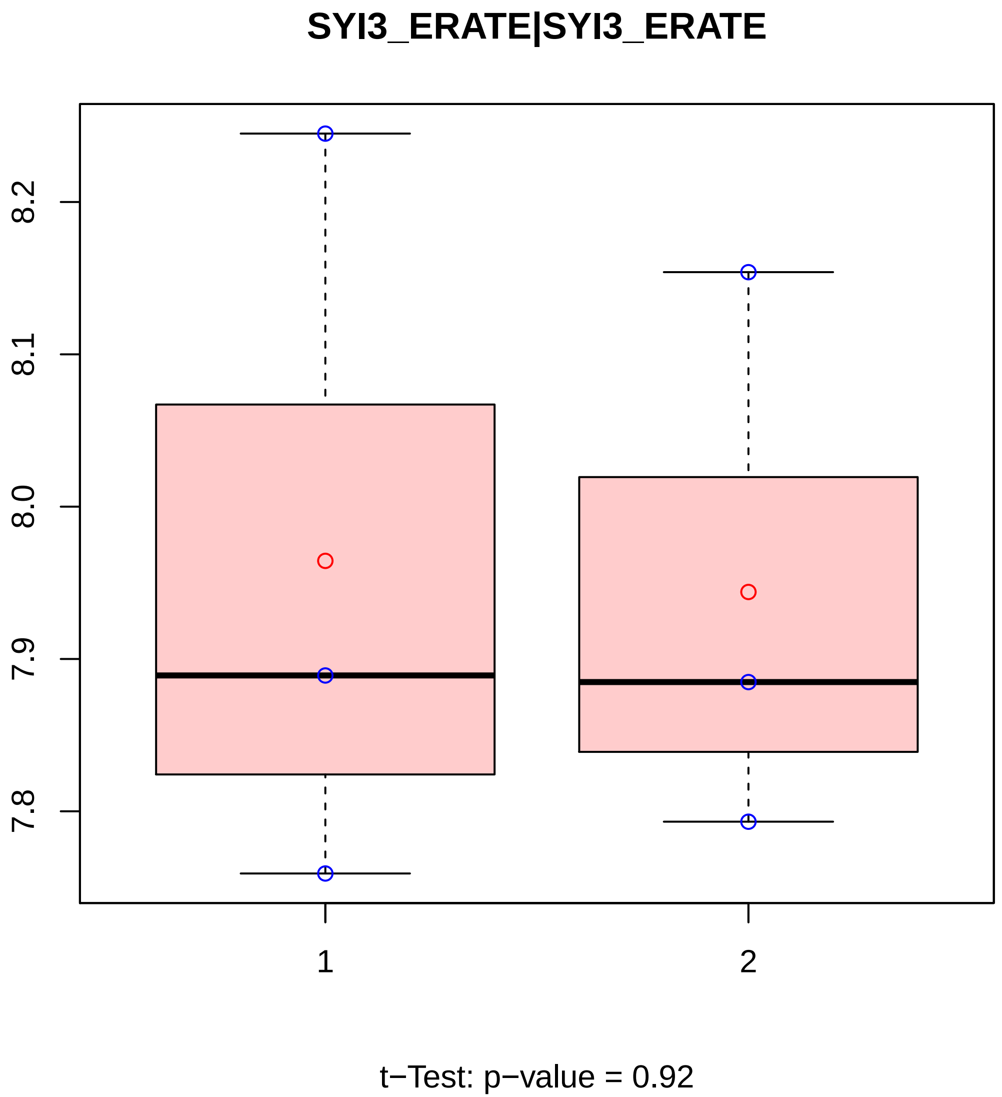

SYI3_ERATE|SYI3_ERATE
8.2
8.1
8.0
7.9
7.8
1
2
t−Test: p−value = 0.92

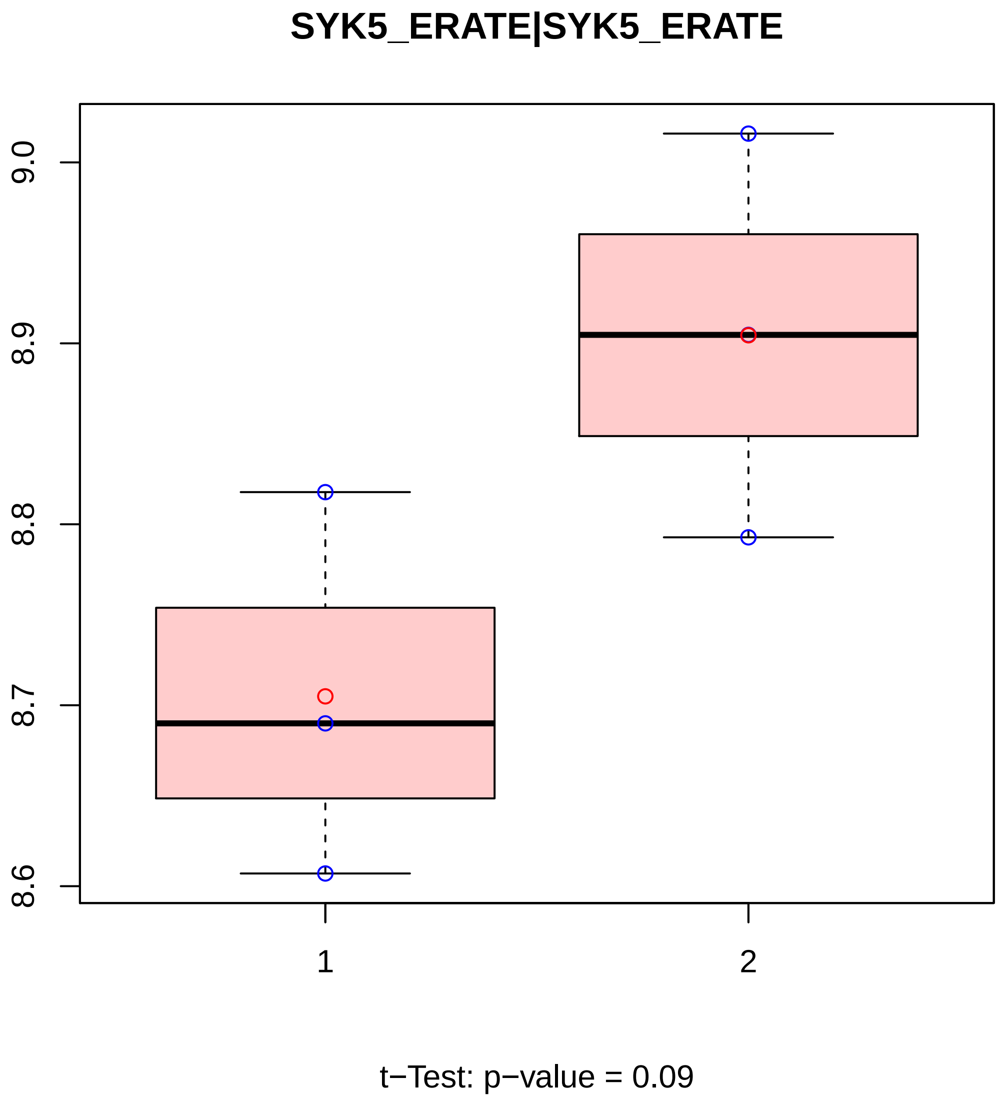
SYK5_ERATE|SYK5_ERATE
9.0
8.9
8.8
8.7
8.6
1
2
t−Test: p−value = 0.09

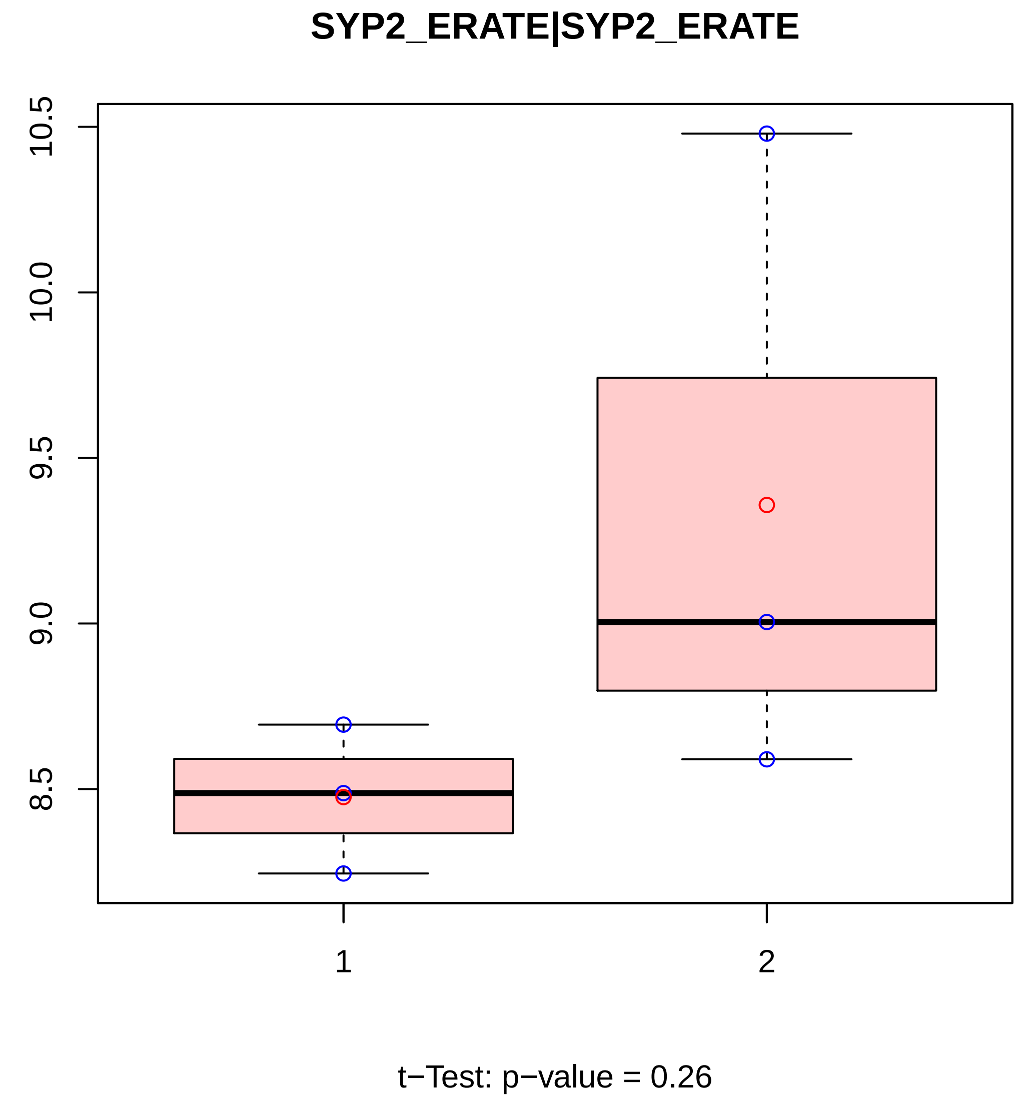
SYP2_ERATE|SYP2_ERATE
10.5
10.0
9.5
9.0
8.5
1
2
t−Test: p−value = 0.26

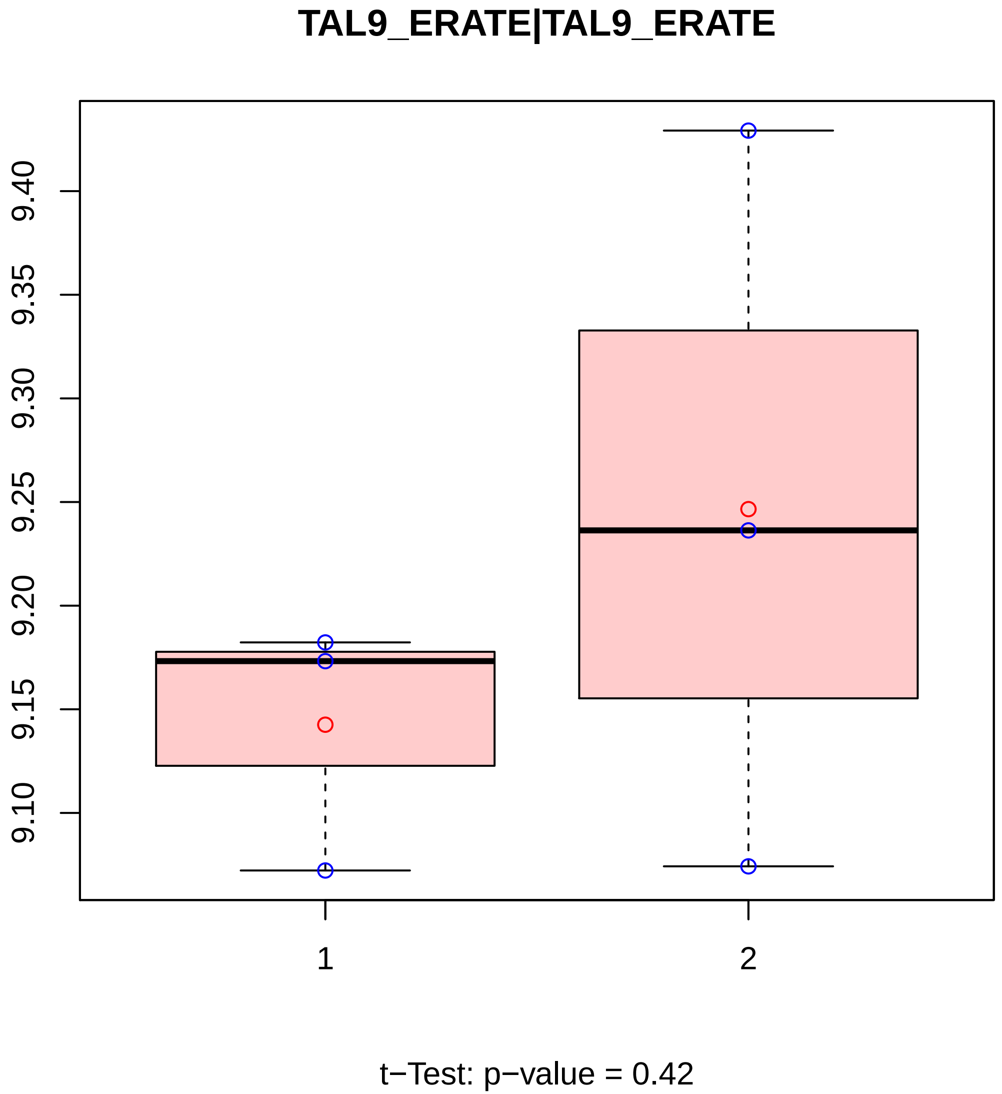
TAL9_ERATE|TAL9_ERATE
9.40
9.35
9.30
9.25
9.20
9.15
9.10
1
2
t−Test: p−value = 0.42

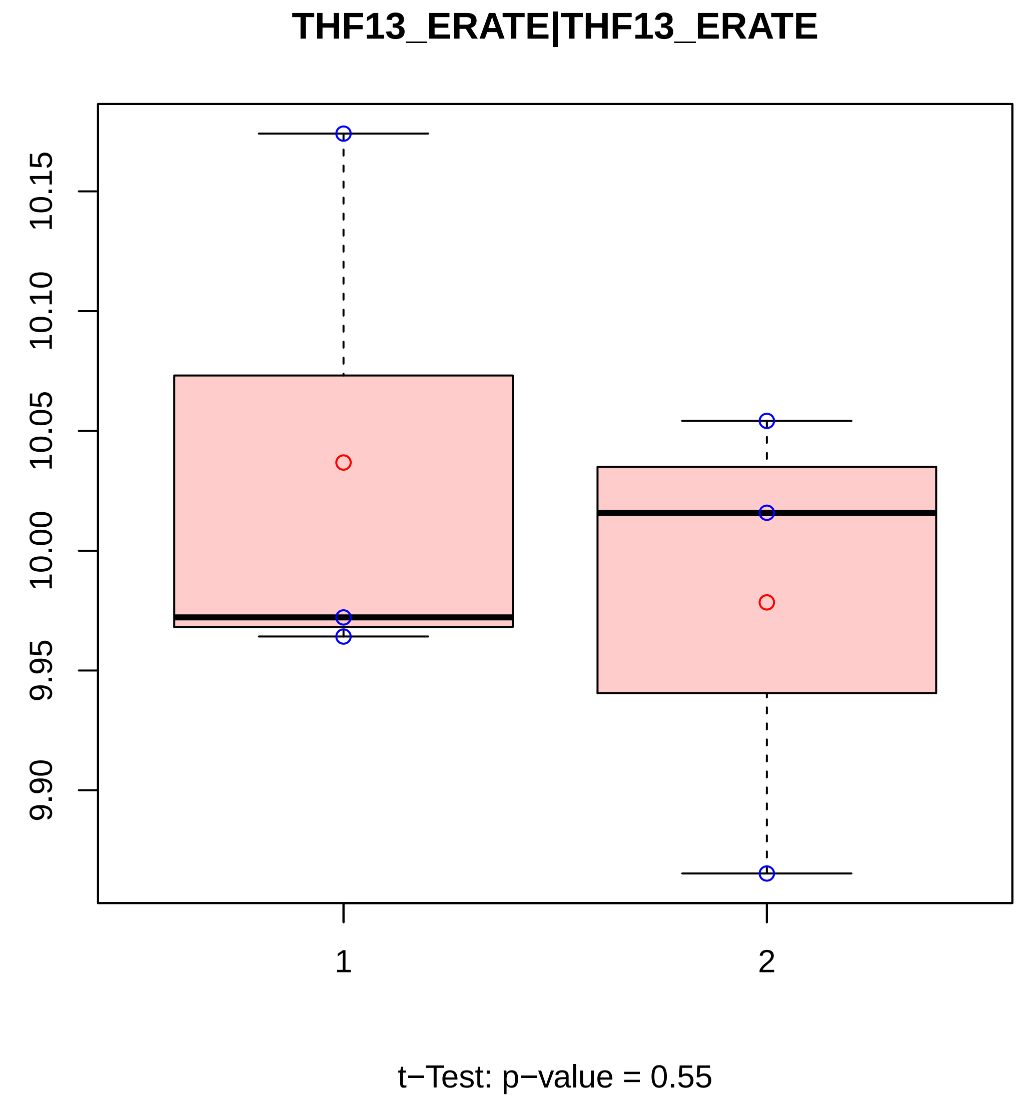
THF13_ERATE|THF13_ERATE
10.15
10.10
10.05
10.00
9.95
9.90
1
2
t−Test: p−value = 0.55

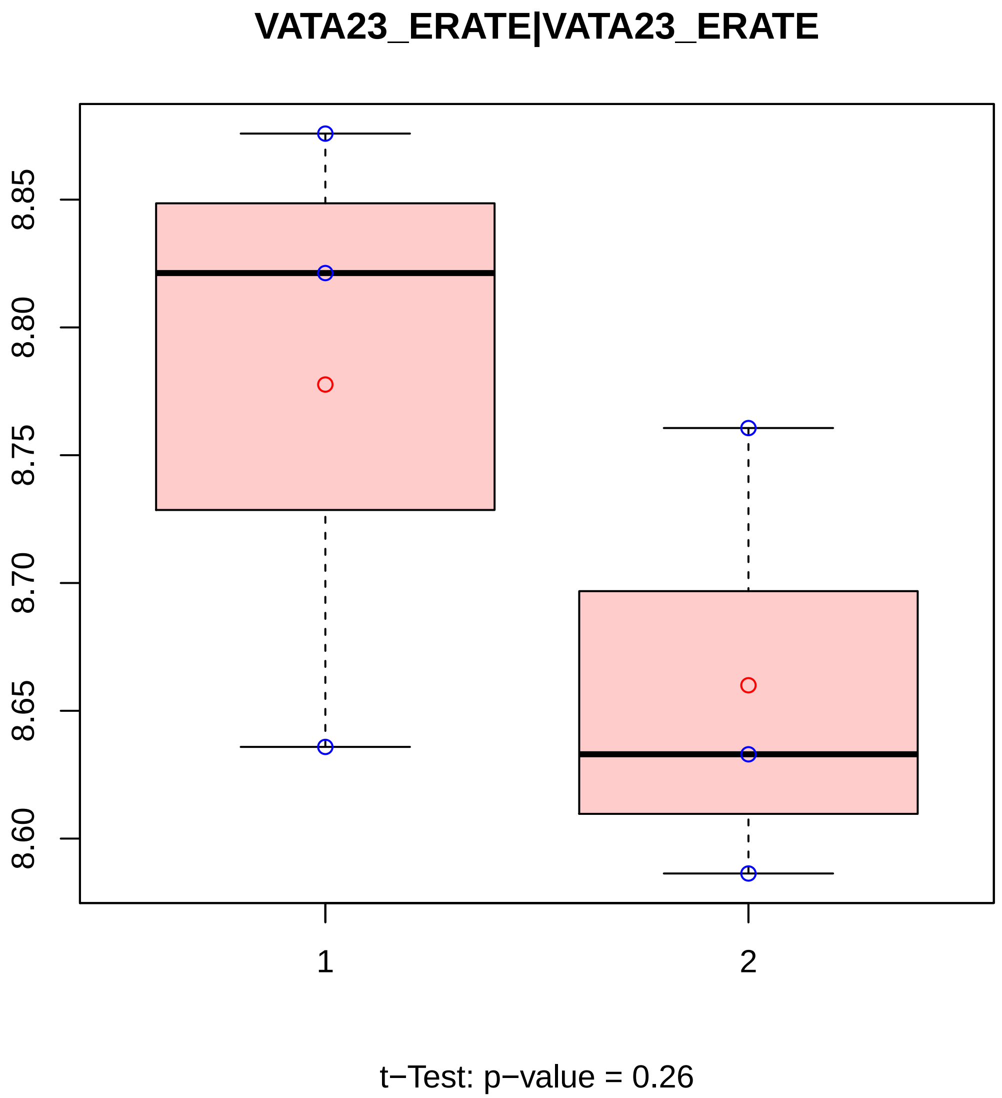
VATA23_ERATE|VATA23_ERATE
8.60
8.65
8.70
8.75
8.80
8.85
1
2
t−Test: p−value = 0.26

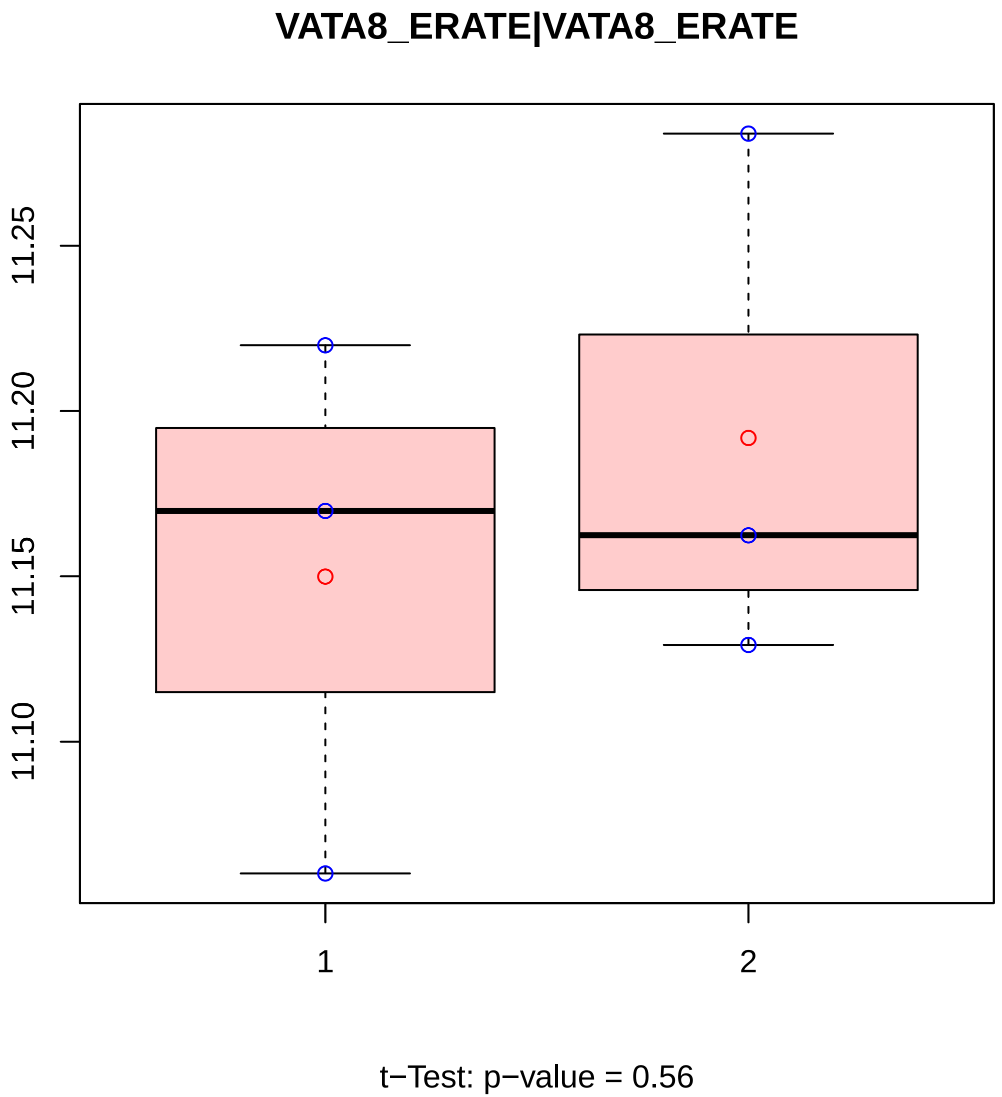

VATA8_ERATE|VATA8_ERATE
11.10
11.15
11.20
11.25
1
2
t−Test: p−value = 0.56

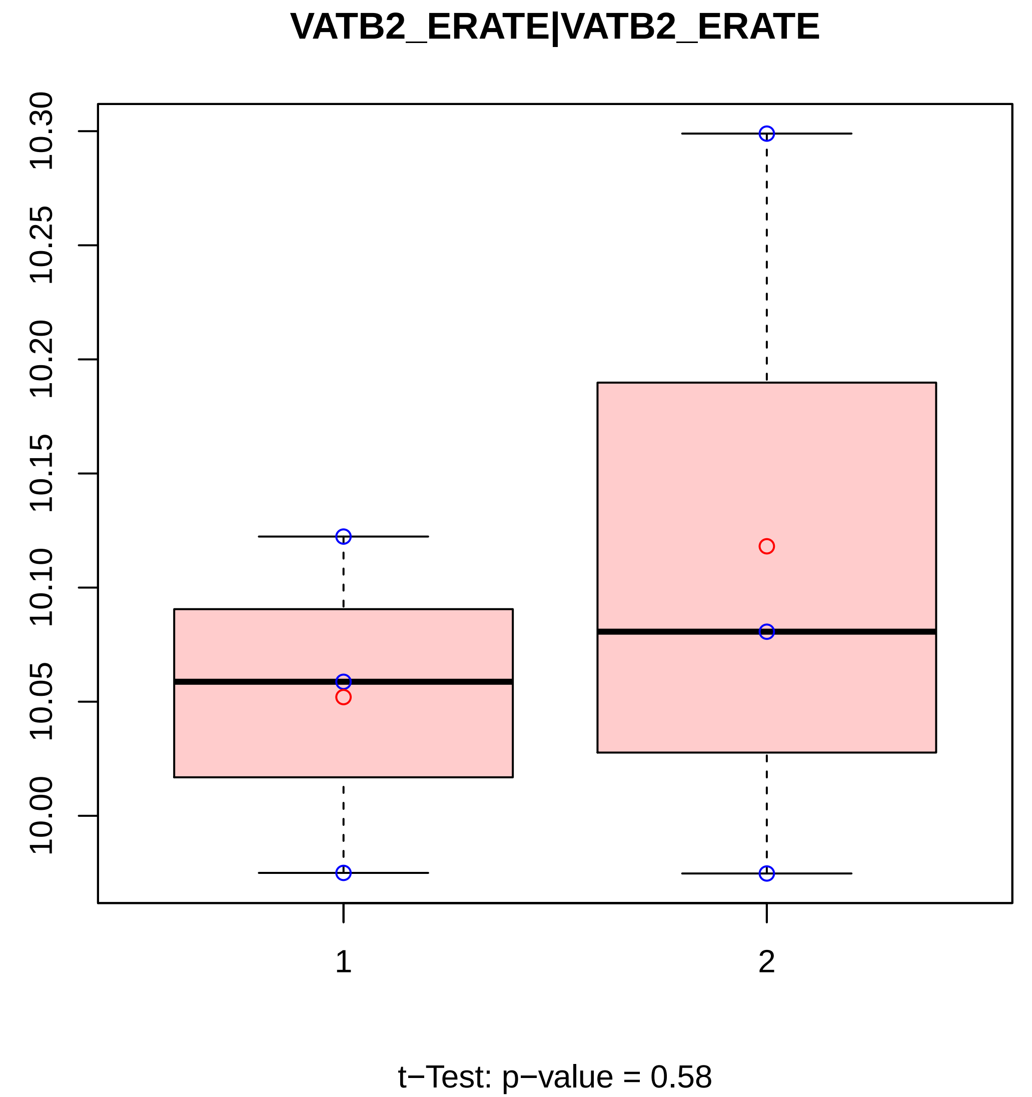
VATB2_ERATE|VATB2_ERATE
10.30
10.25
10.20
10.15
10.10
10.05
10.00
1
2
t−Test: p−value = 0.58

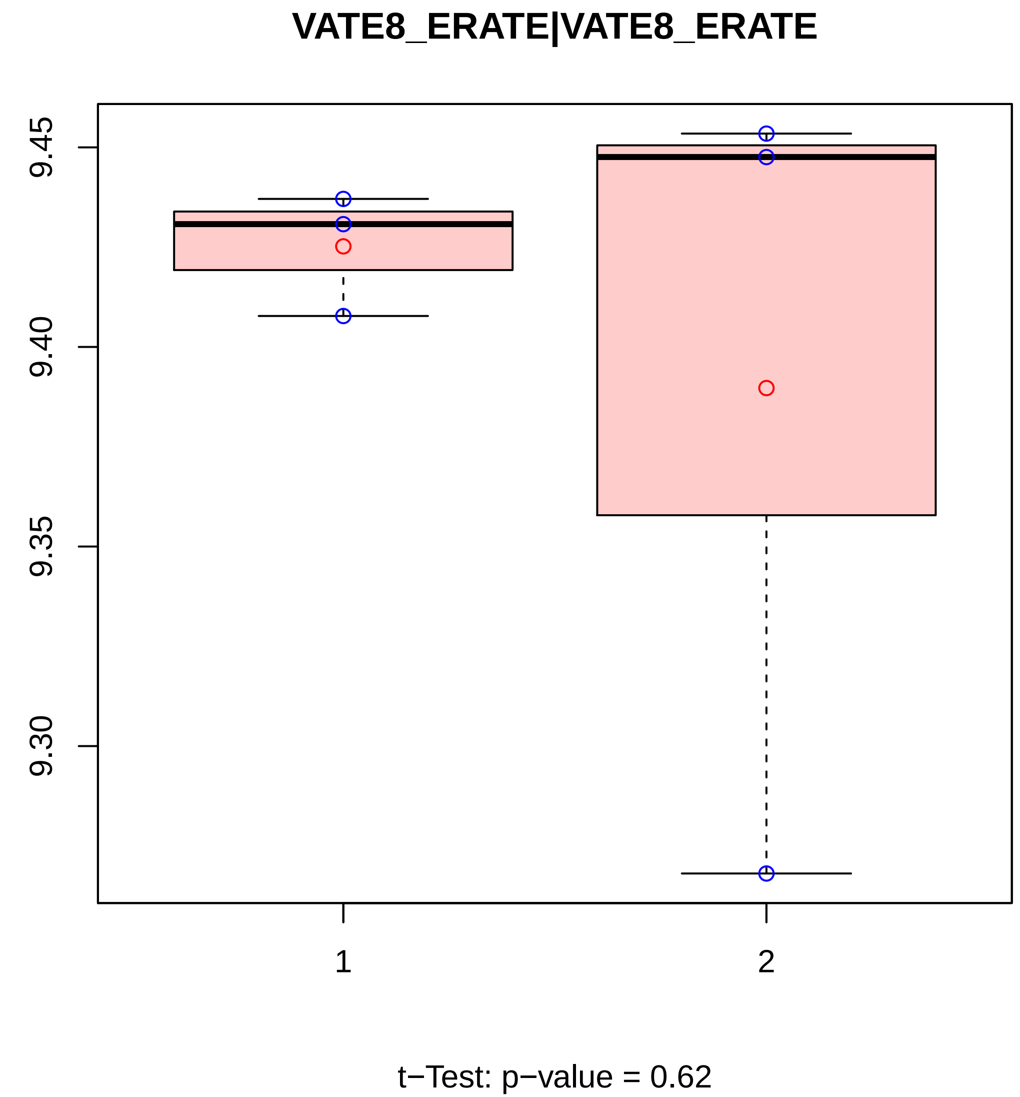
VATE8_ERATE|VATE8_ERATE
9.45
9.40
9.35
9.30
1
2
t−Test: p−value = 0.62
