# Supplementary material for: A Proteomic Approach to Investigate the Drought Response in the Orphan Crop Eragrostis tef
Source: Proteomes. 2017 Nov 15;5(4):32. doi: 10.3390/proteomes5040032 (PMC5748567; doi:10.3390/proteomes5040032)
Supplement: Supplementary file 1 [file S4.2 MU-two group analysis (boxplots).pdf › proteomes-208511 supplementary final/S4.2 MU-two group analysis (boxplots).pdf]

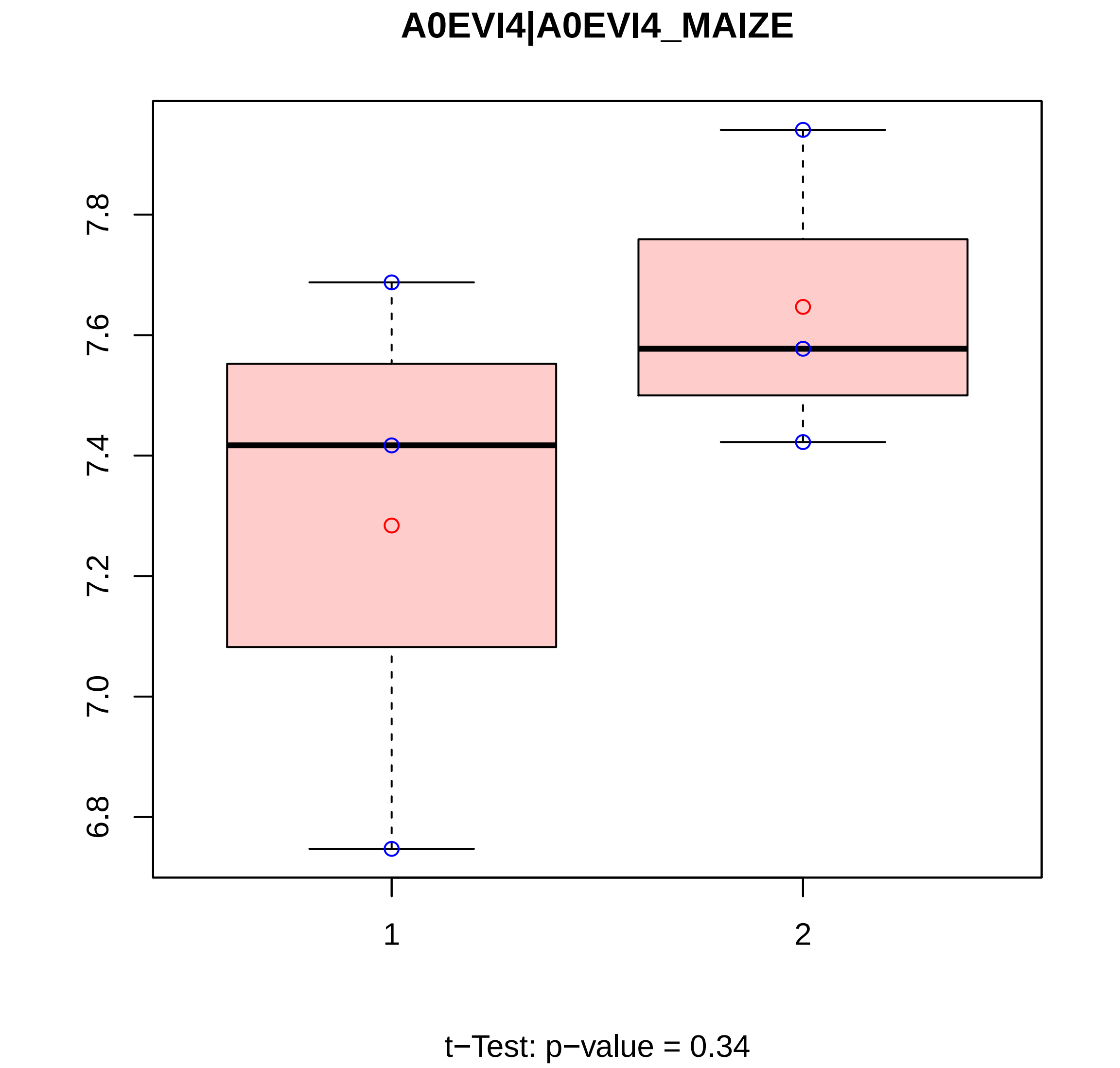
A0EVI4|A0EVI4_MAIZE
7.8
7.6
7.4
7.2
7.0
6.8
1
2
t−Test: p−value = 0.34

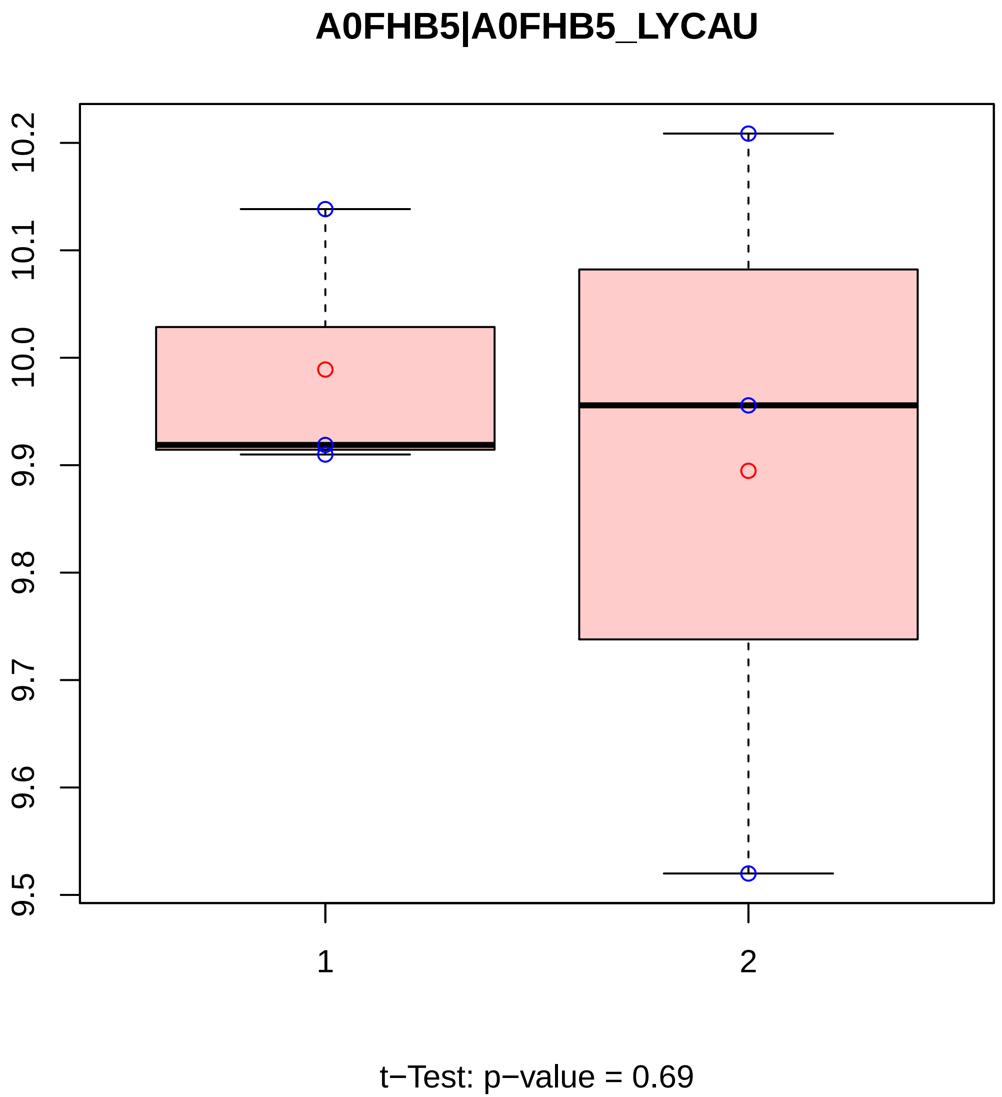

A0FHB5|A0FHB5_LYCAU
10.2
10.1
10.0
9.9
9.8
9.7
9.6
9.5
1
2
t−Test: p−value = 0.69

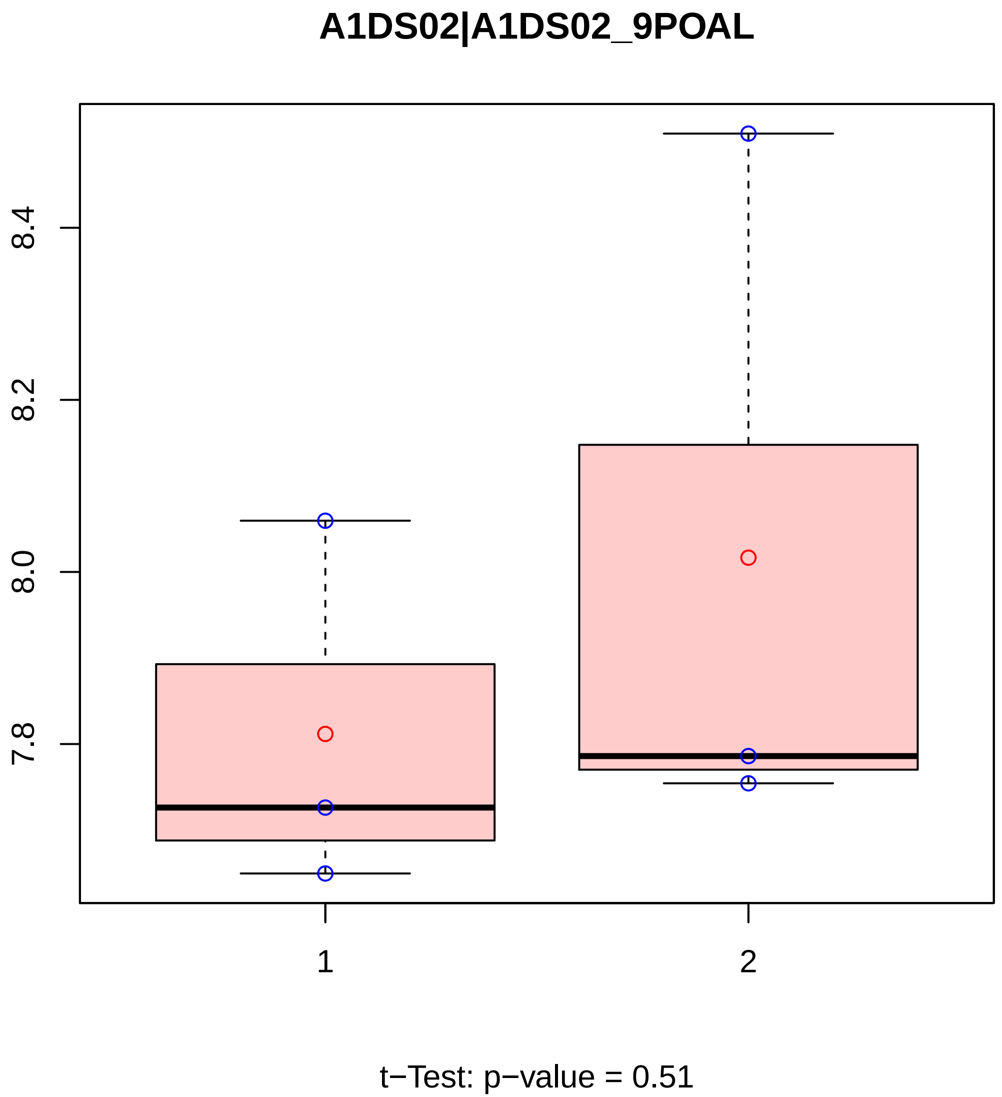
A1DS02|A1DS02_9POAL
8.4
8.2
8.0
7.8
1
2
t−Test: p−value = 0.51

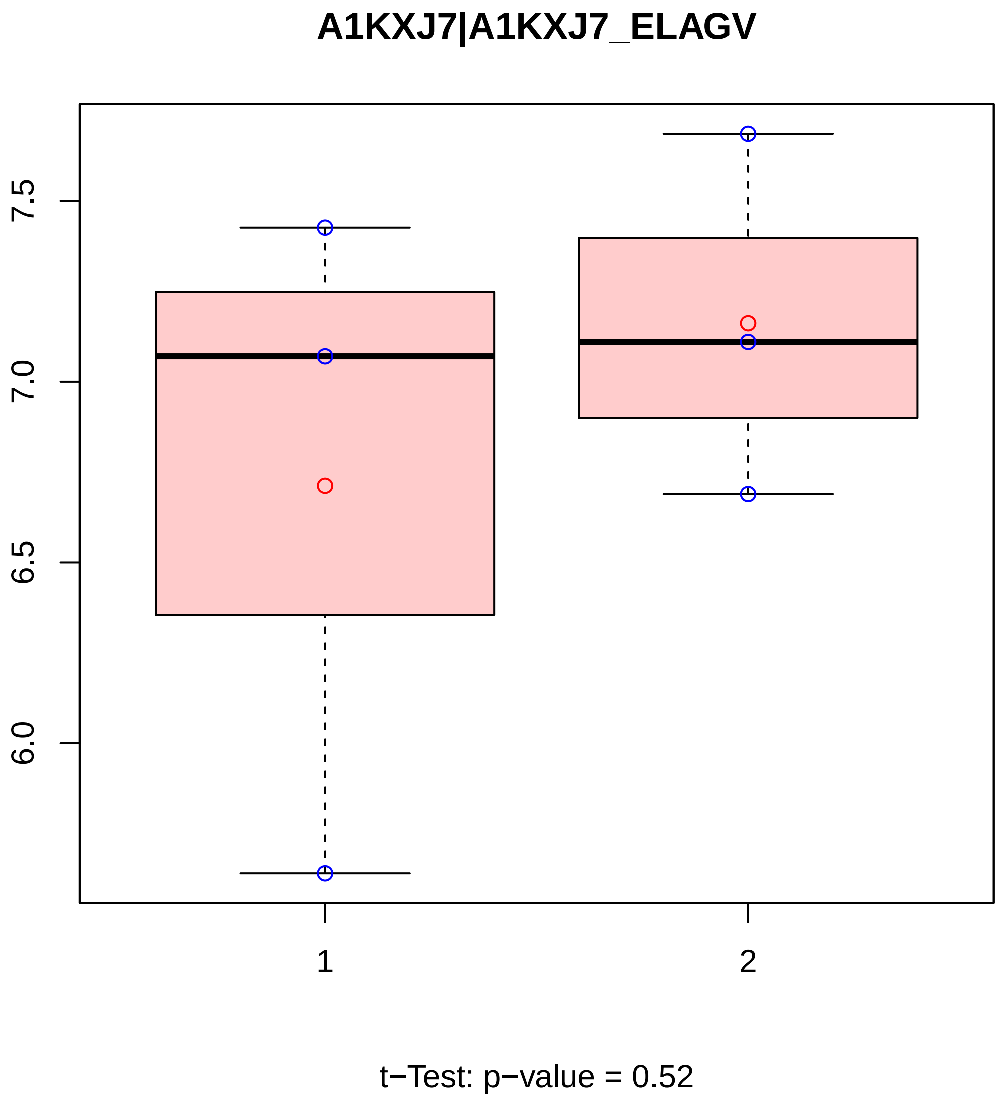
A1KXJ7|A1KXJ7_ELAGV
7.5
7.0
6.5
6.0
1
2
t−Test: p−value = 0.52

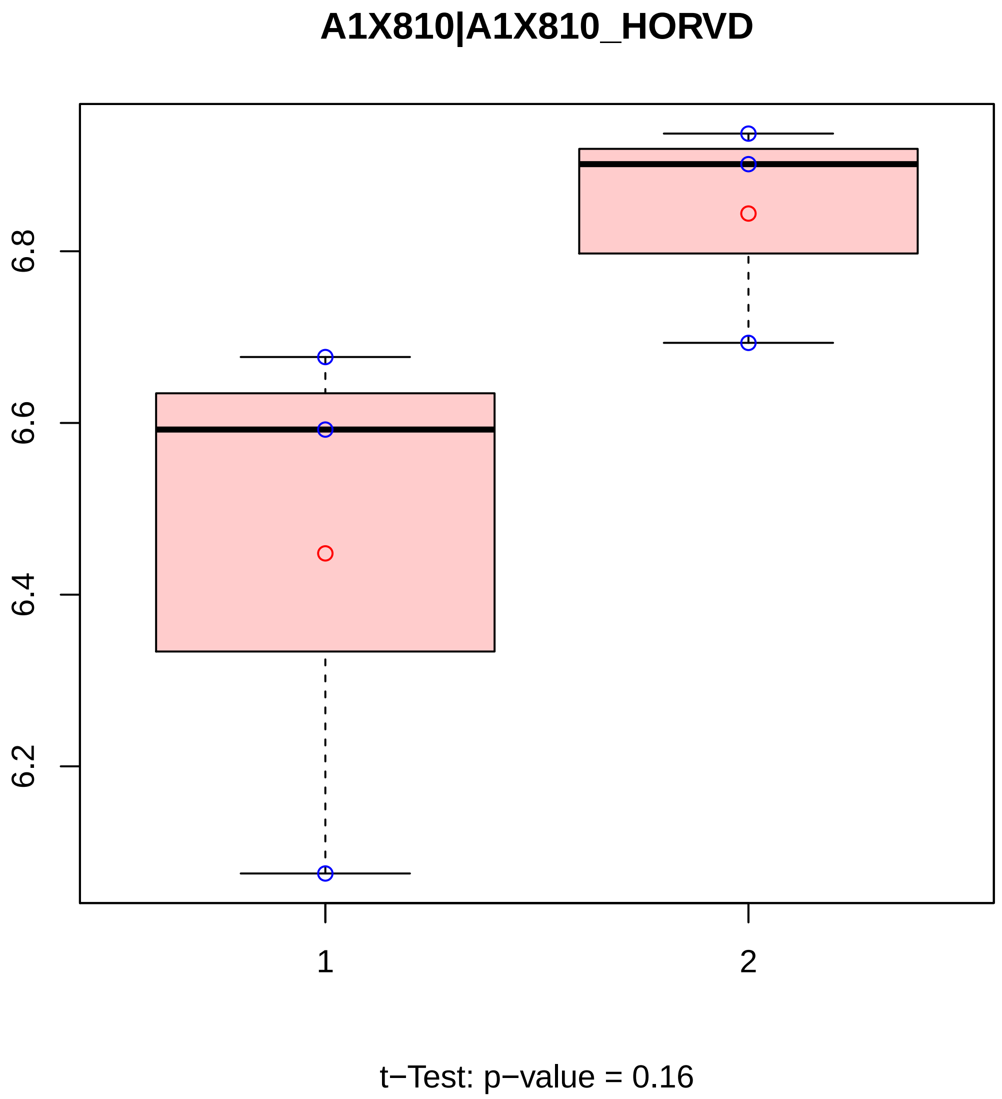
A1X810|A1X810_HORVD
6.2
6.4
6.6
6.8
1
2
t−Test: p−value = 0.16

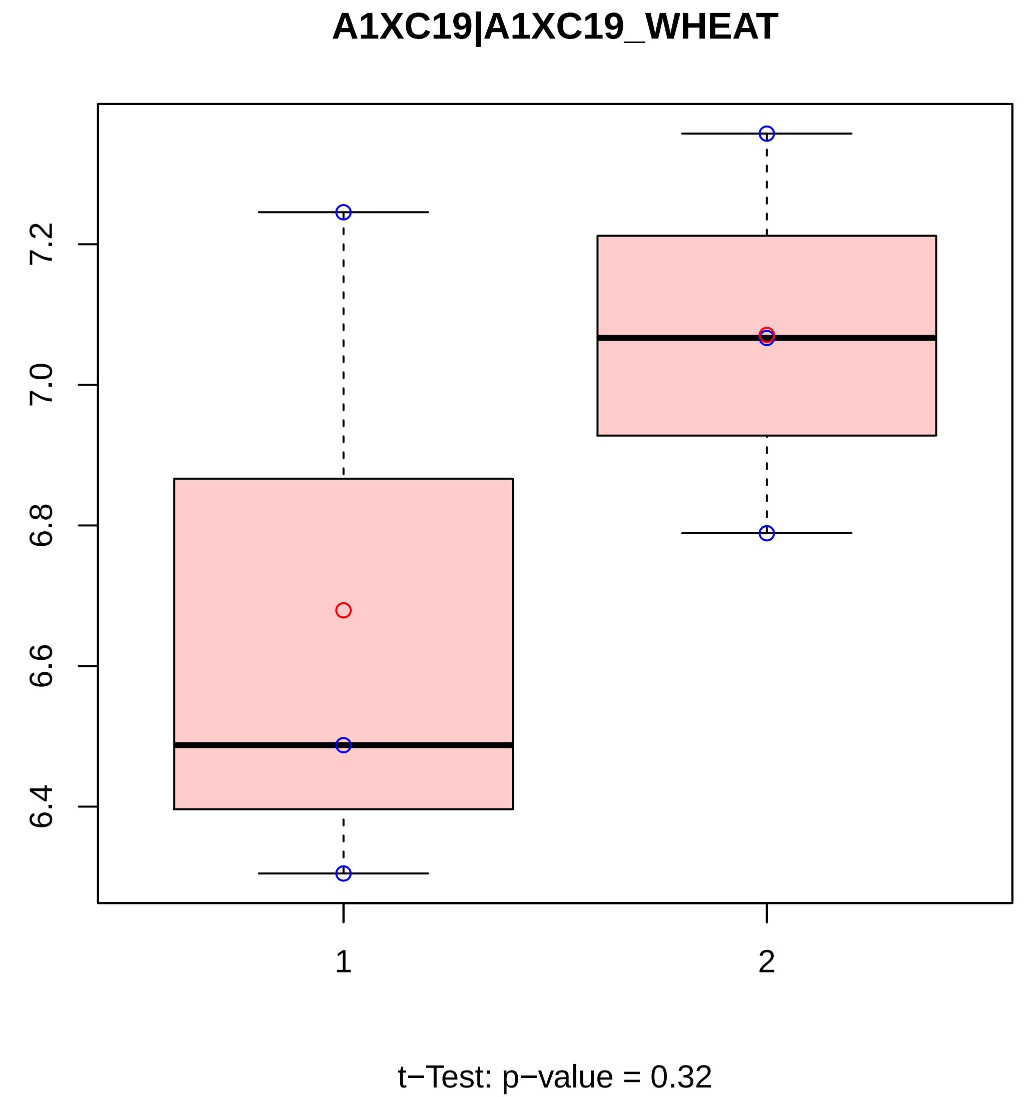
A1XC19|A1XC19_WHEAT
6.4
6.6
6.8
7.0
7.2
1
2
t−Test: p−value = 0.32

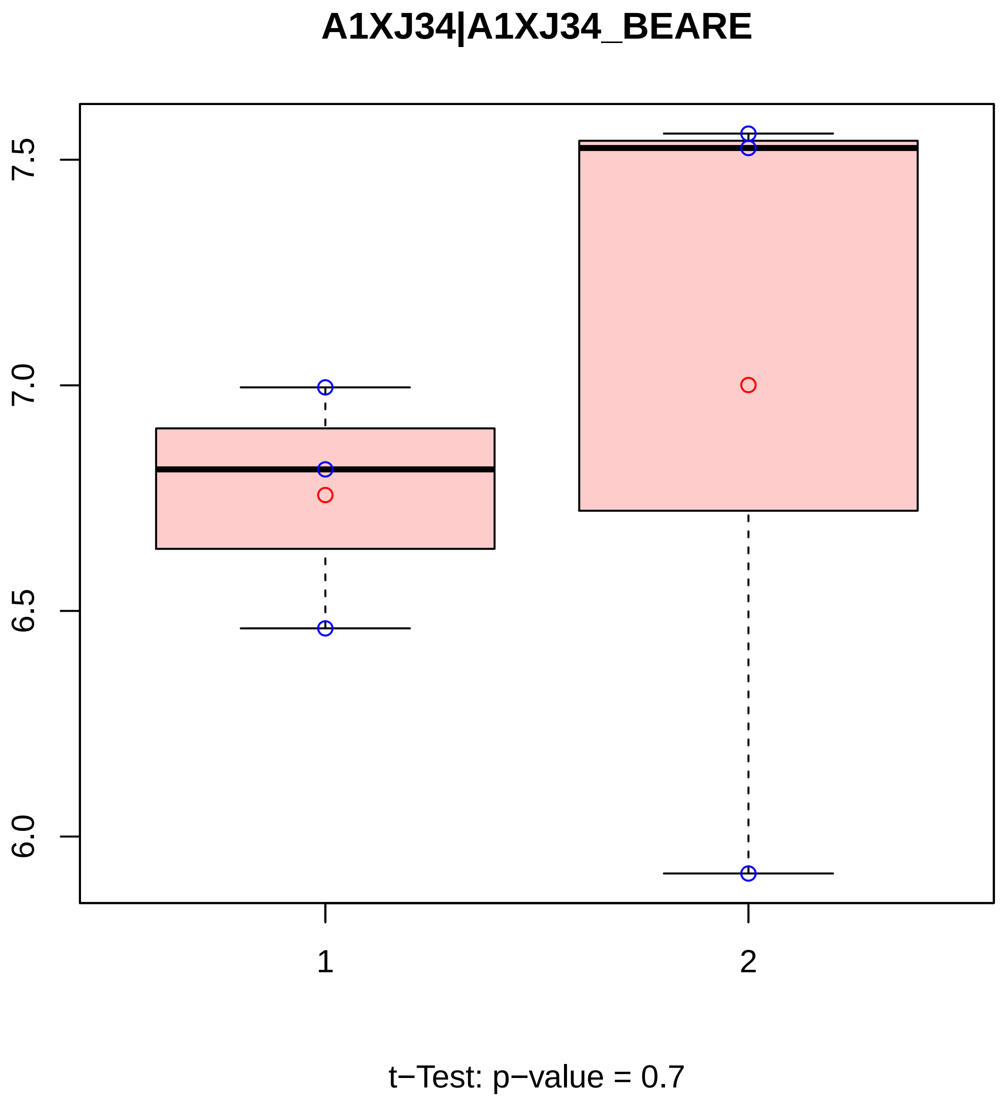

A1XJ34|A1XJ34_BEARE
7.5
7.0
6.5
6.0
1
2
t−Test: p−value = 0.7

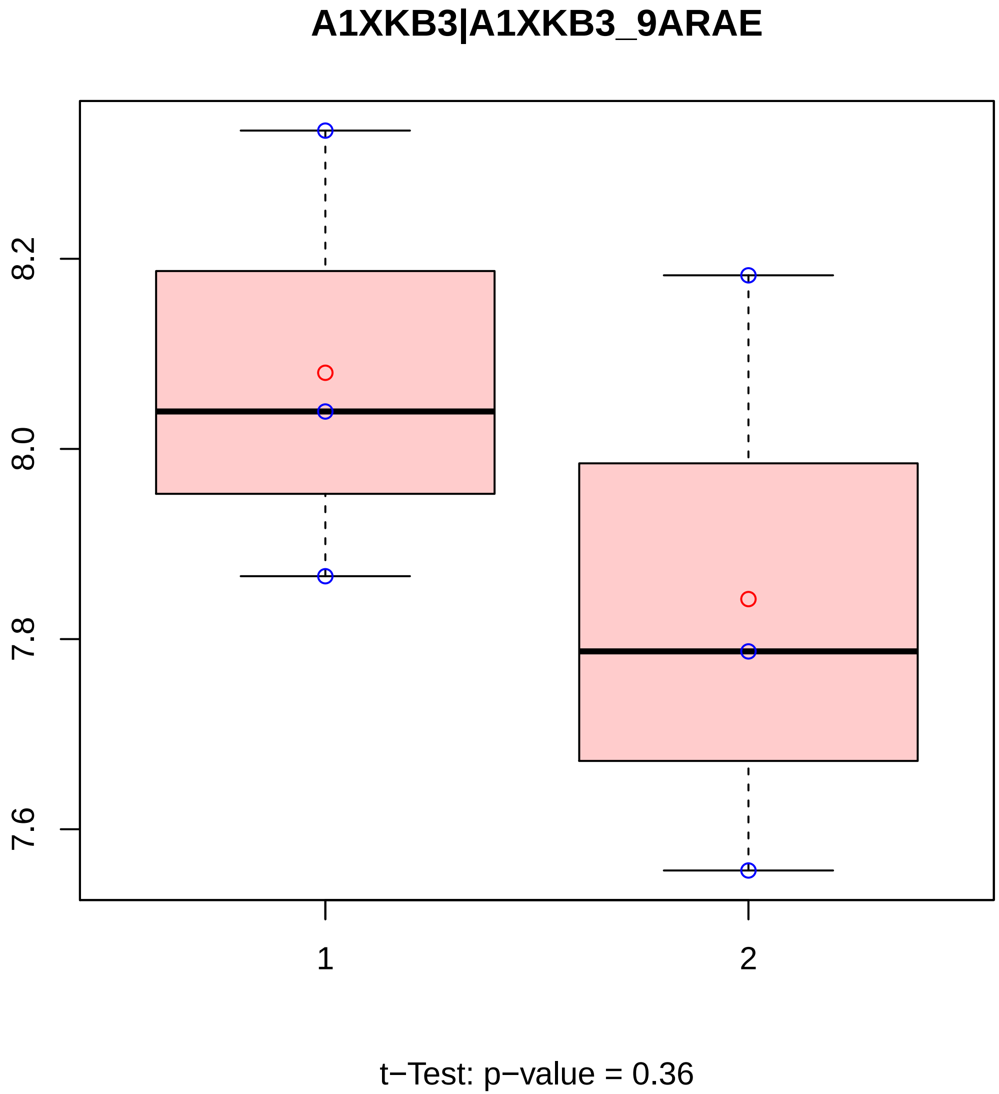
A1XKB3|A1XKB3_9ARAE
8.2
8.0
7.8
7.6
1
2
t−Test: p−value = 0.36

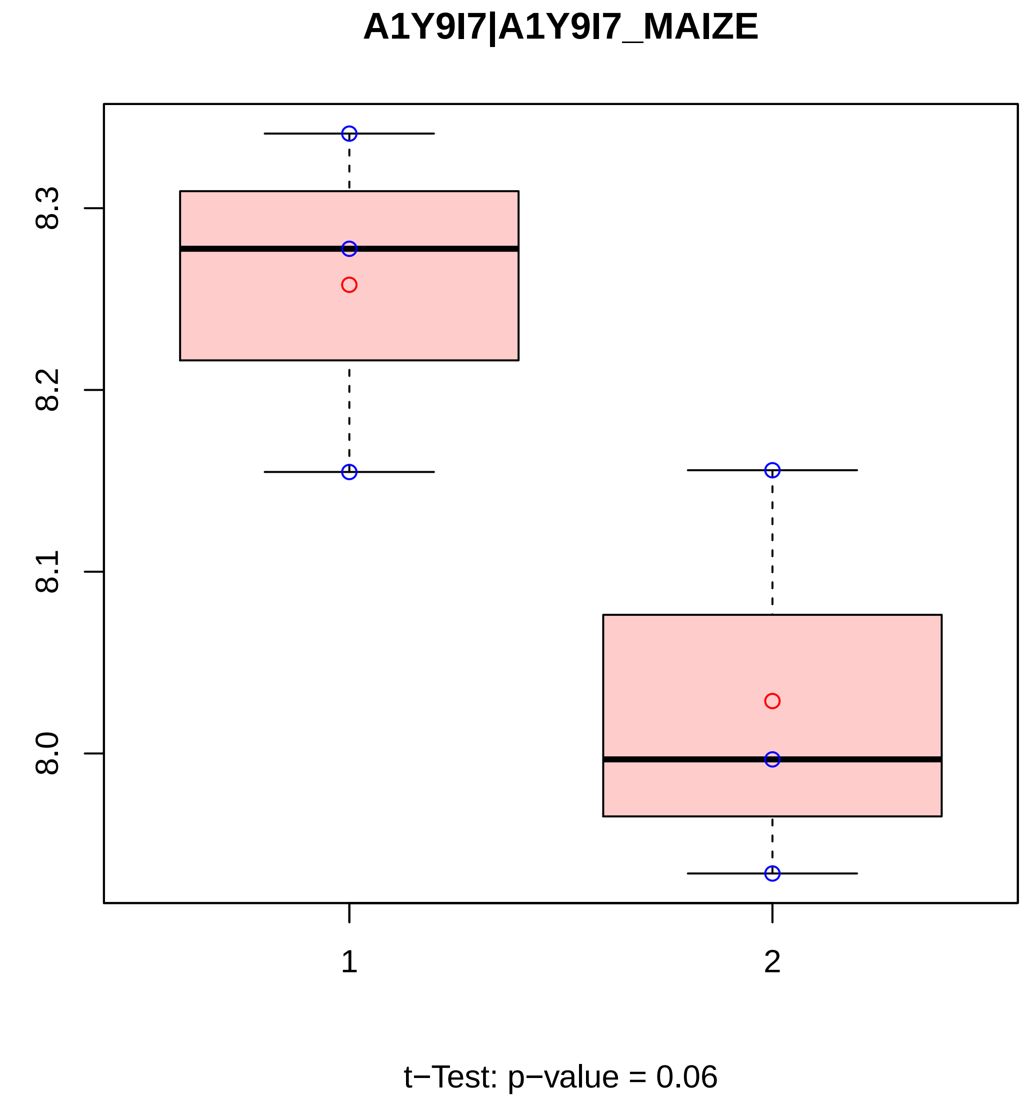
A1Y9I7|A1Y9I7_MAIZE
8.3
8.2
8.1
8.0
1
2
t−Test: p−value = 0.06

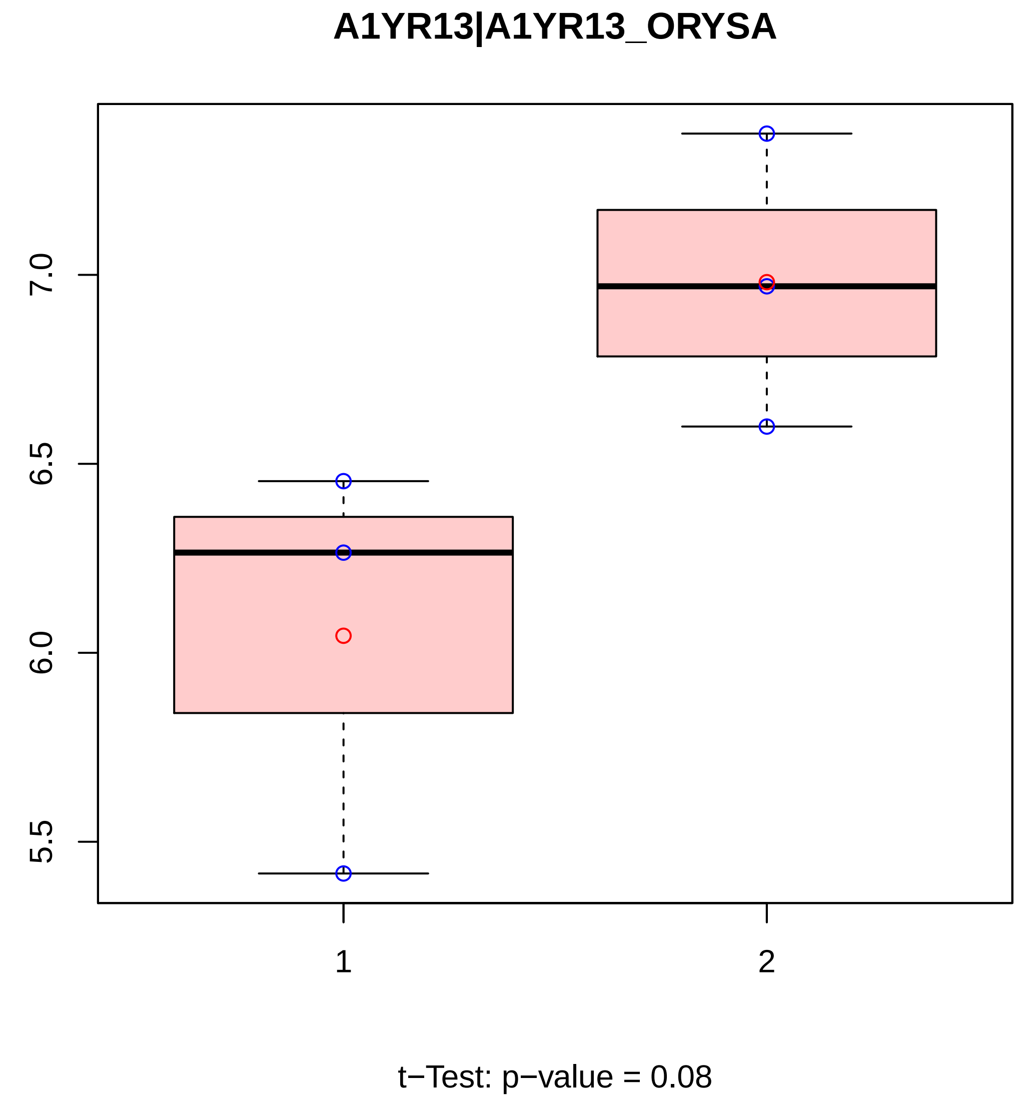

A1YR13|A1YR13_ORYSA
5.5
6.0
6.5
7.0
1
2
t−Test: p−value = 0.08

A2WKG1|A2WKG1_ORYSI

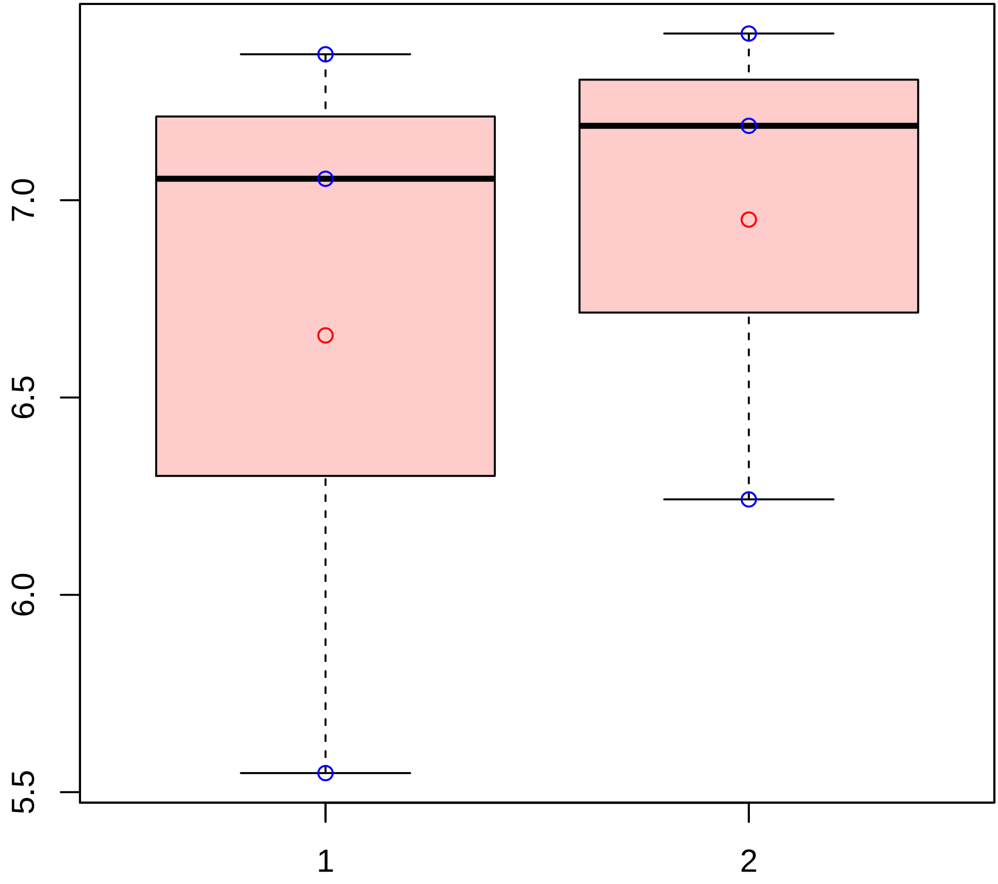

t−Test: p−value = 0.69

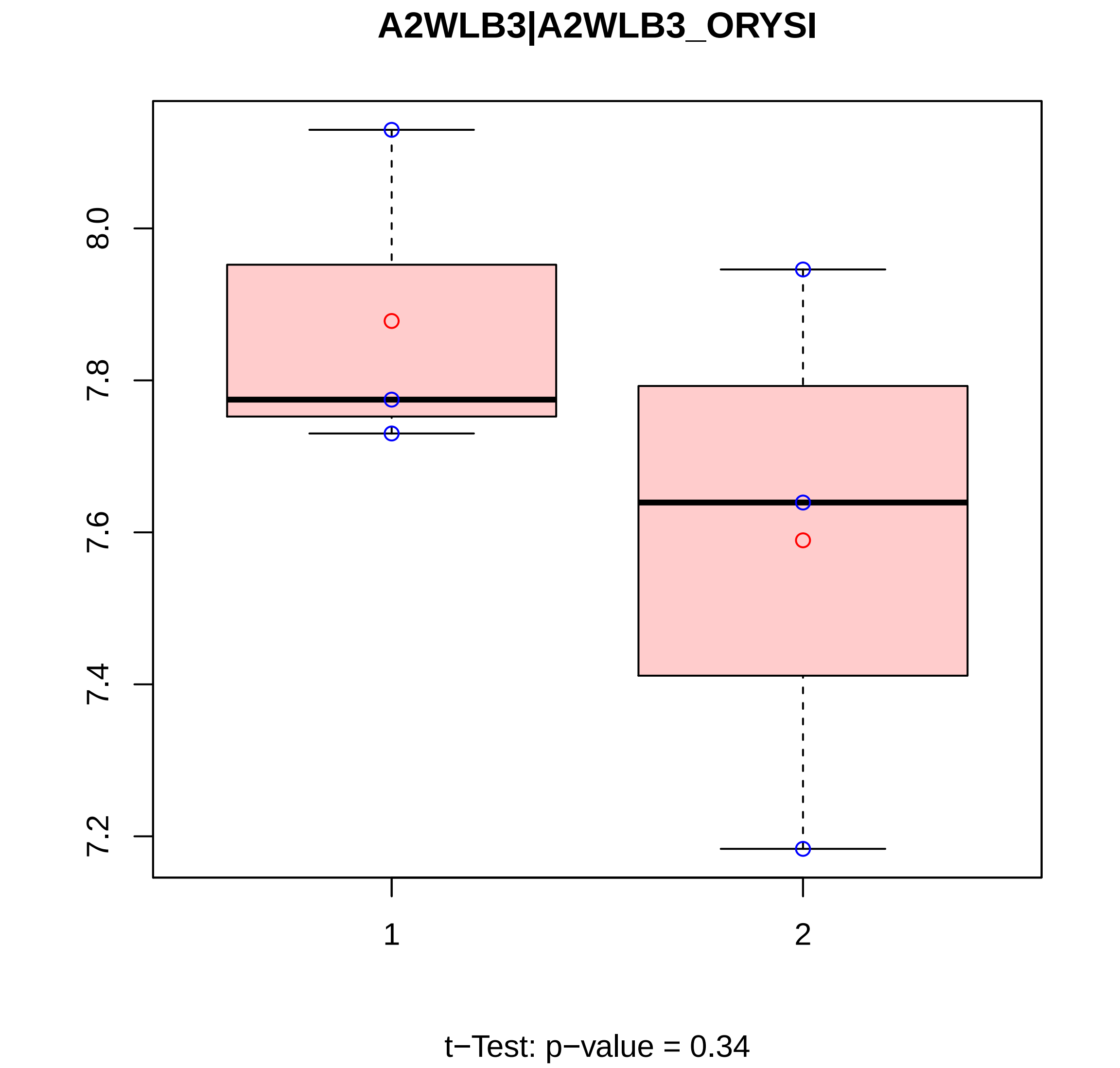
A2WLB3|A2WLB3_ORYSI
8.0
7.8
7.6
7.4
7.2
1
2
t−Test: p−value = 0.34

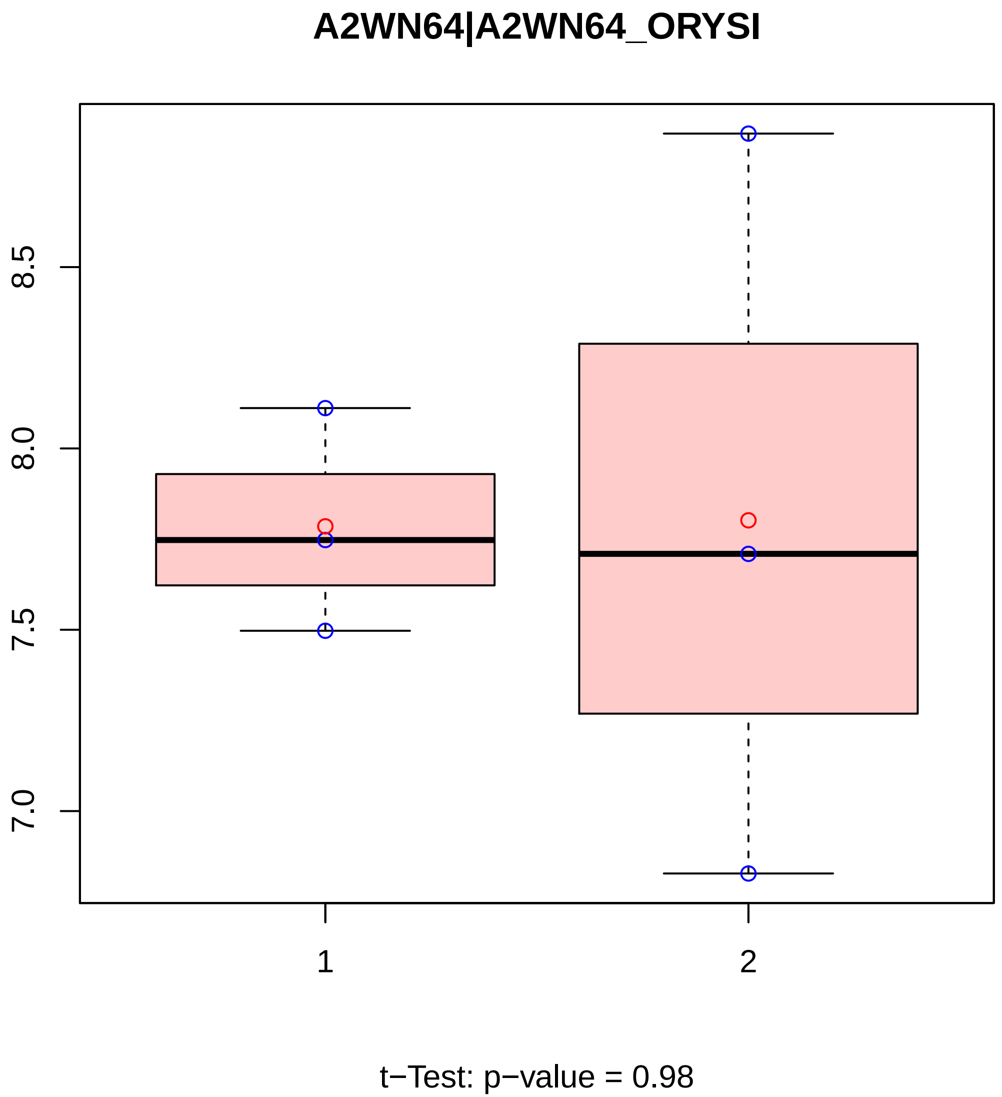
A2WN64|A2WN64_ORYSI
7.0
7.5
8.0
8.5
1
2
t−Test: p−value = 0.98

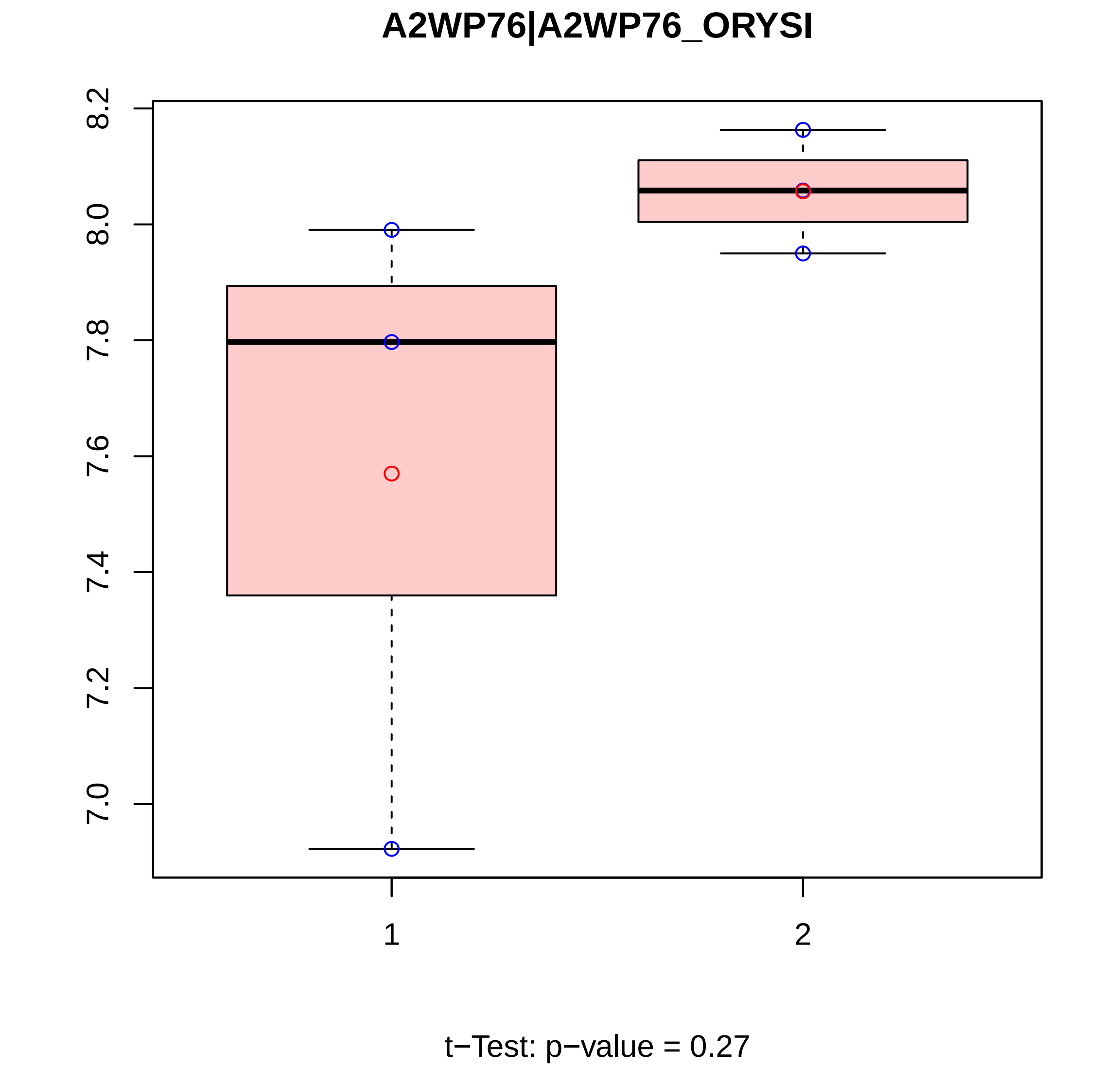
A2WP76|A2WP76_ORYSI
8.2
8.0
7.8
7.6
7.4
7.2
7.0
1
2
t−Test: p−value = 0.27

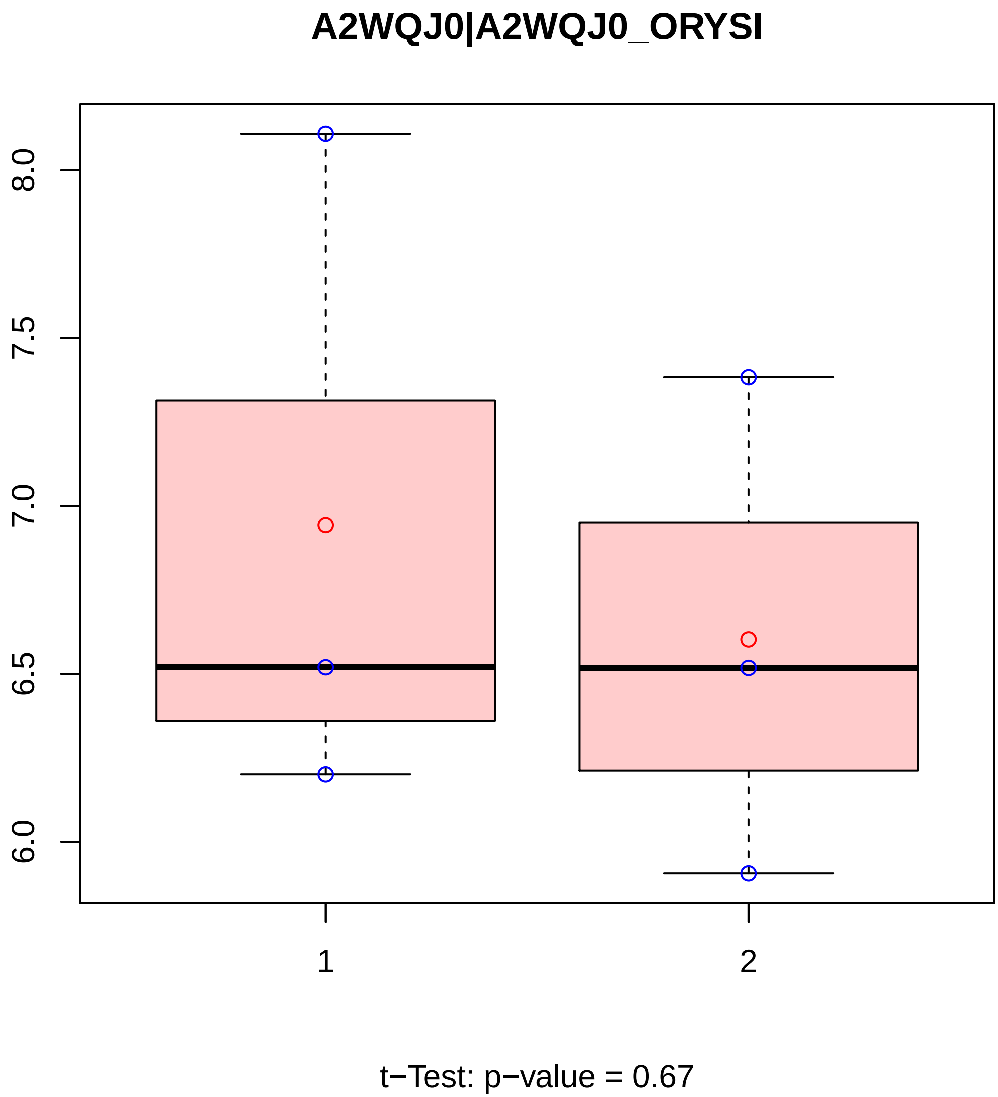

A2WQJ0|A2WQJ0_ORYSI
8.0
7.5
7.0
6.5
6.0
1
2
t−Test: p−value = 0.67

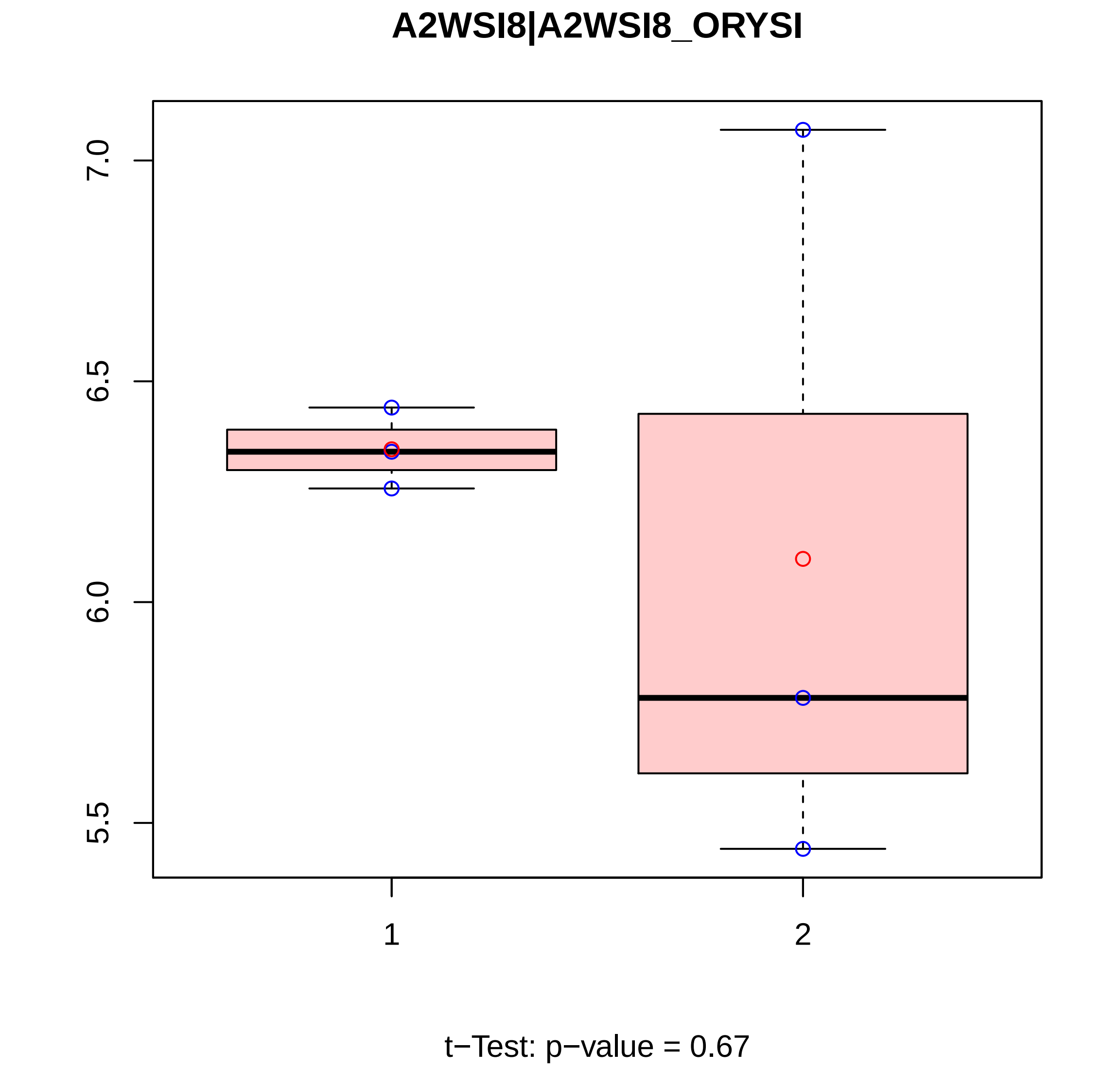
A2WSI8|A2WSI8_ORYSI
7.0
6.5
6.0
5.5
1
2
t−Test: p−value = 0.67

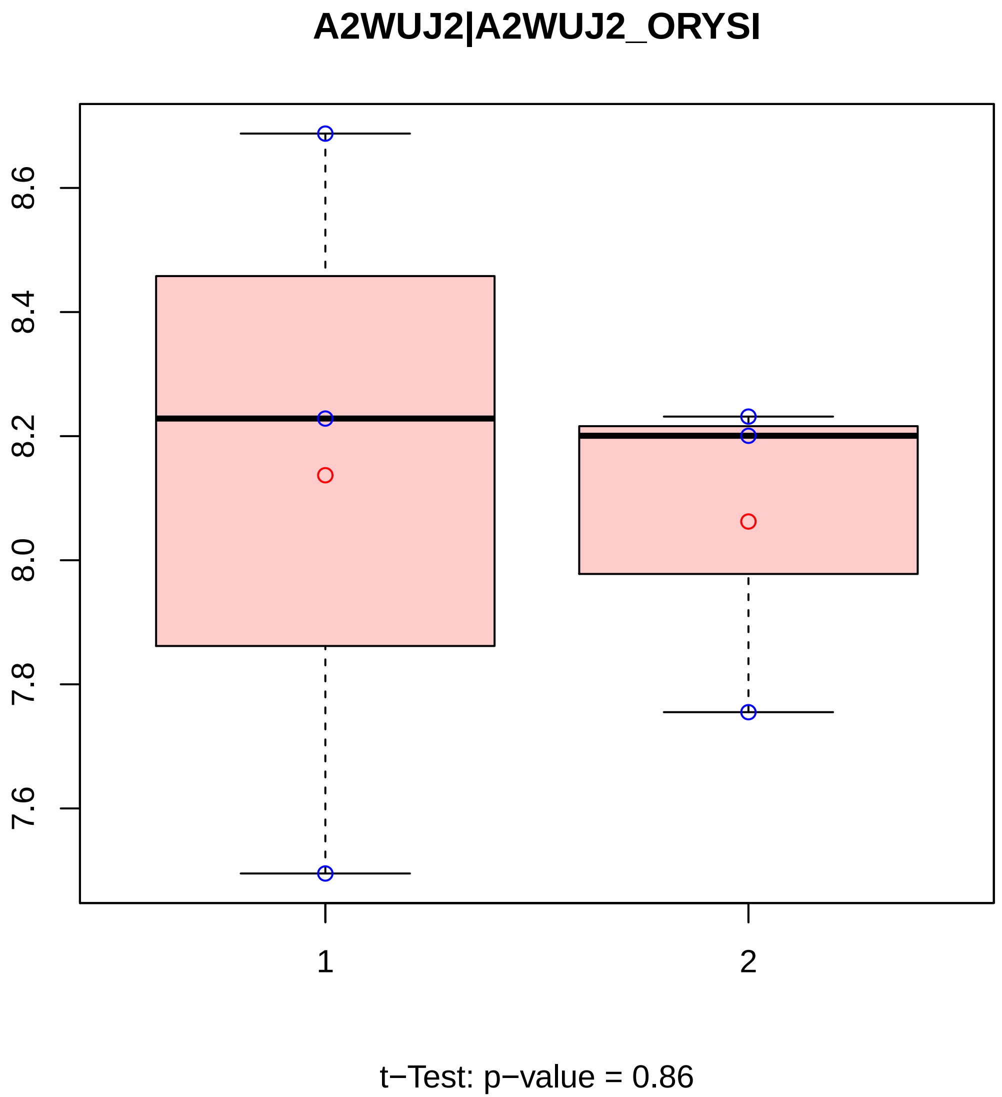
A2WUJ2|A2WUJ2_ORYSI
8.6
8.4
8.2
8.0
7.8
7.6
1
2
t−Test: p−value = 0.86

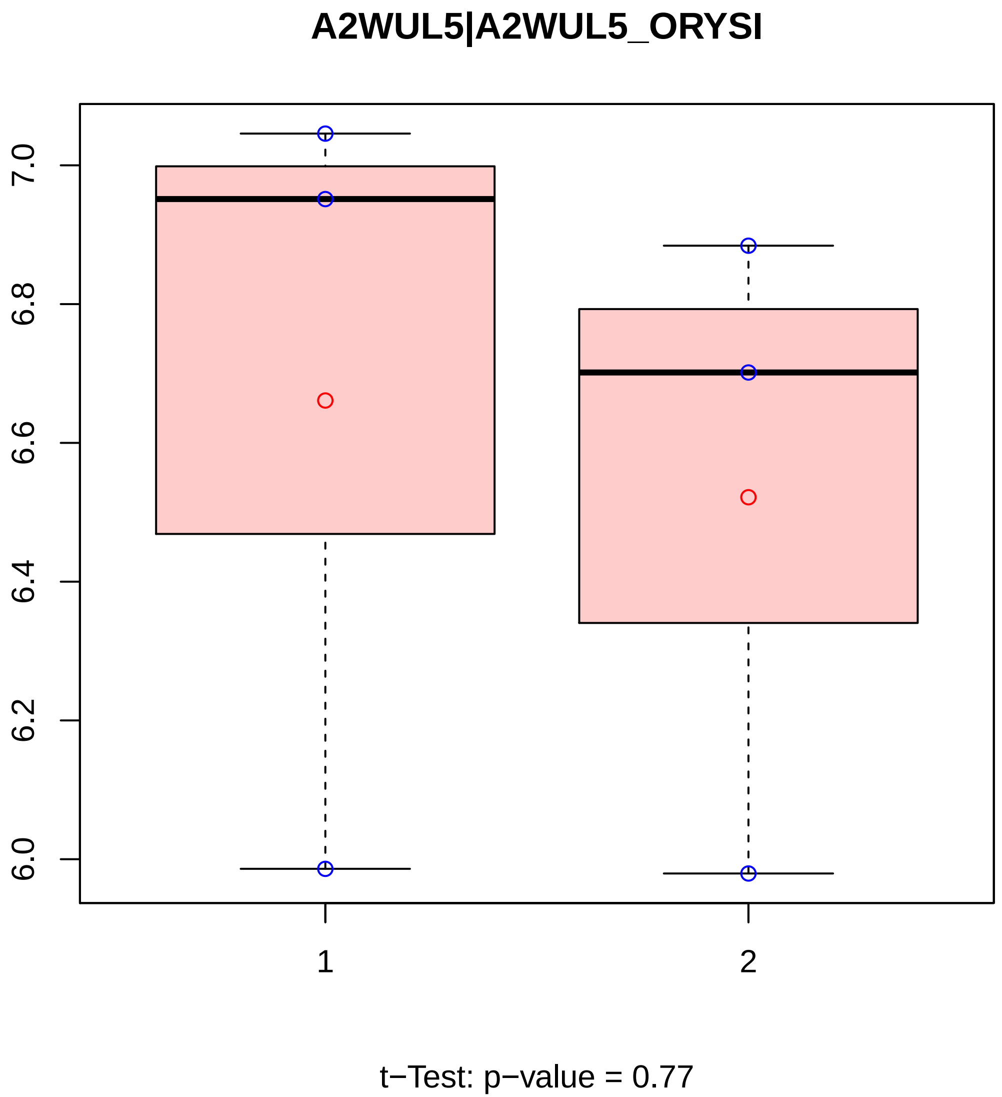
A2WUL5|A2WUL5_ORYSI
7.0
6.8
6.6
6.4
6.2
6.0
1
2
t−Test: p−value = 0.77

A2WWW5|A2WWW5_ORYSI

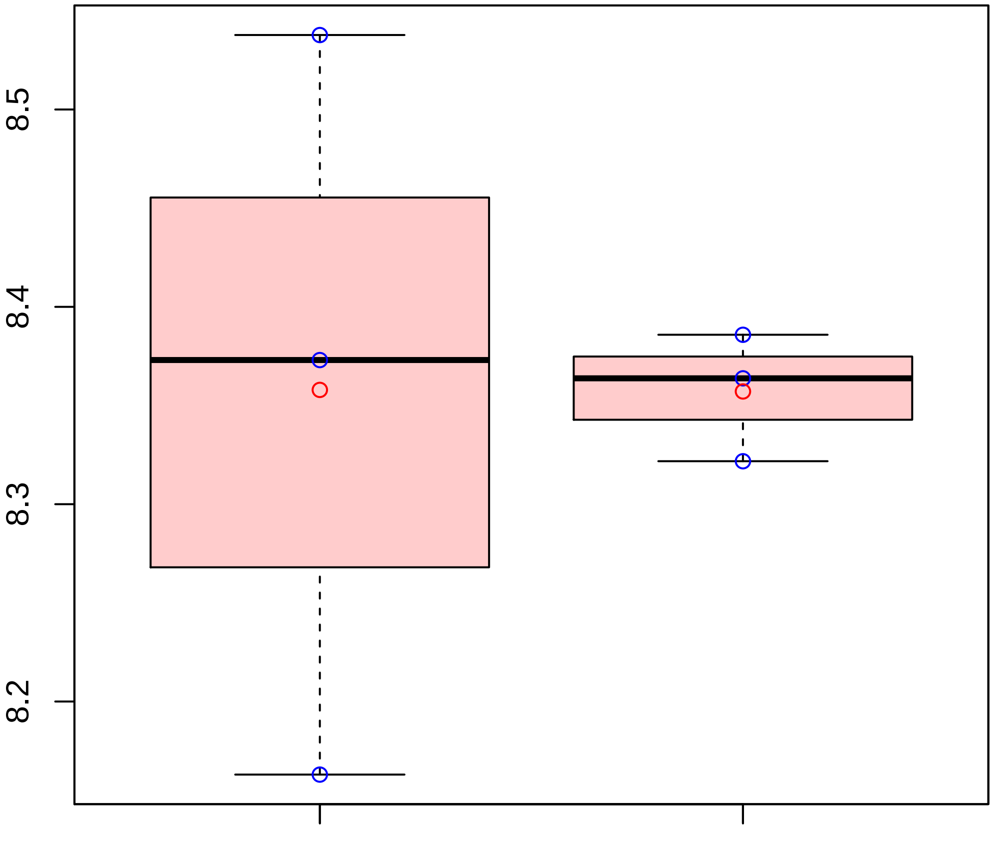

t−Test: p−value = 0.99

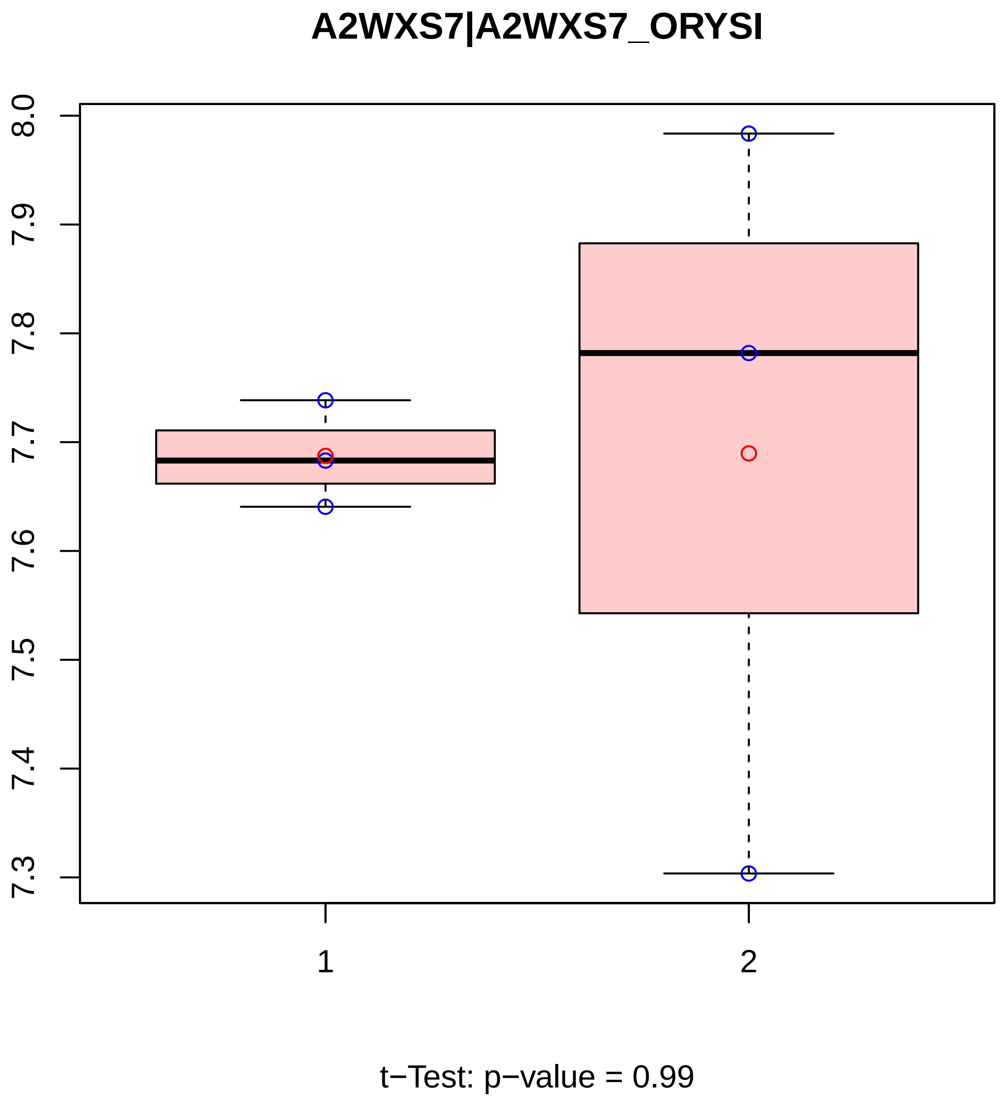

A2WXS7|A2WXS7_ORYSI
8.0
7.9
7.8
7.7
7.6
7.5
7.4
7.3
1
2
t−Test: p−value = 0.99

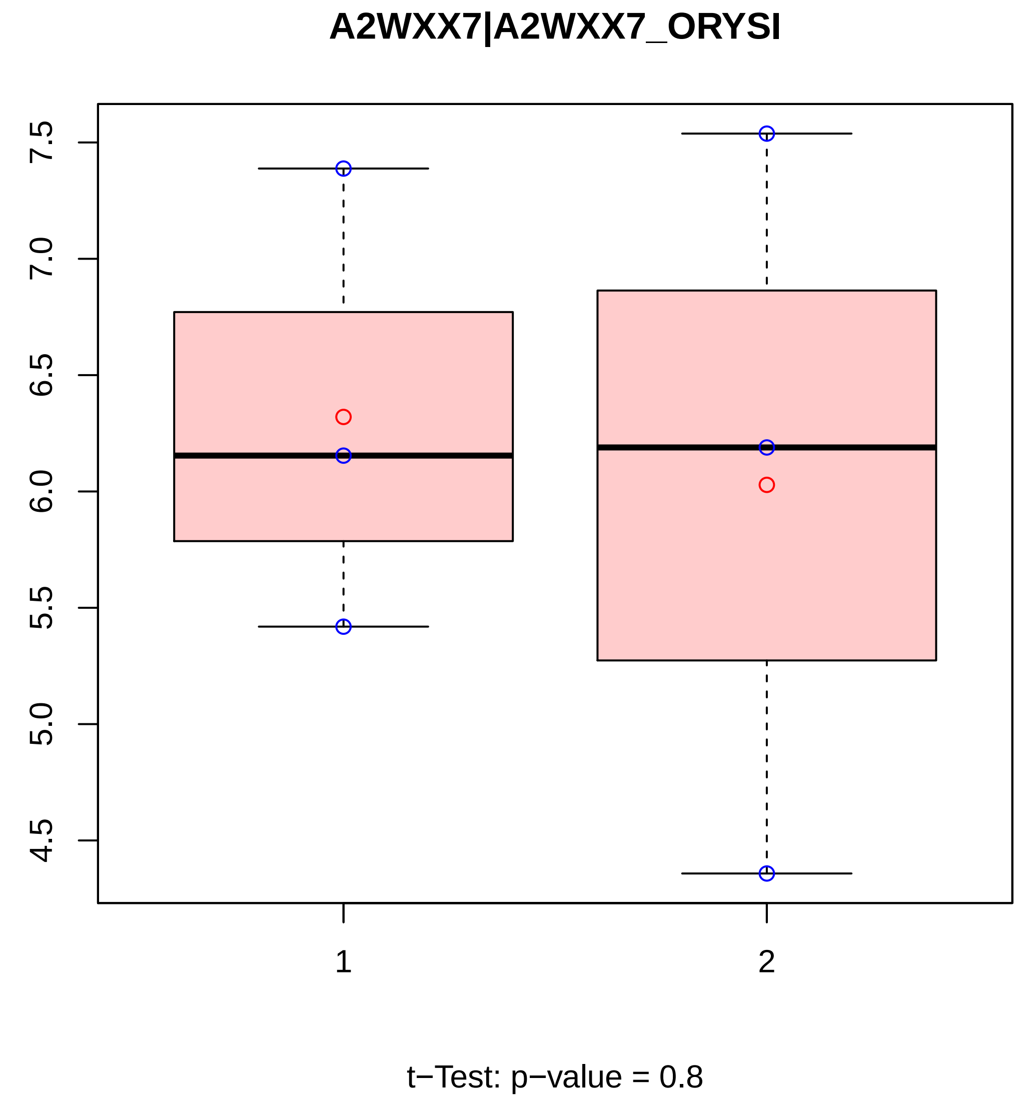

A2WXX7|A2WXX7_ORYSI
7.5
7.0
6.5
6.0
5.5
5.0
4.5
1
2
t−Test: p−value = 0.8

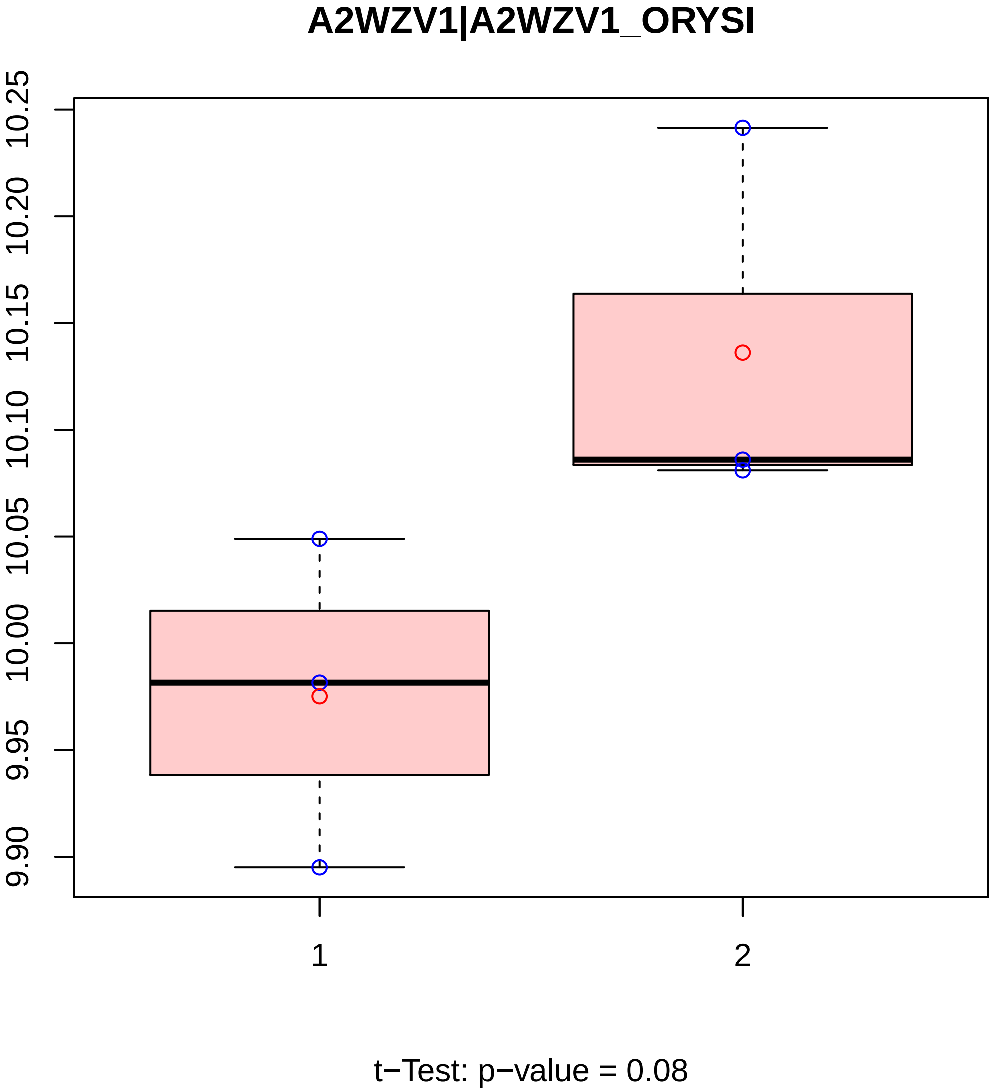
A2WZV1|A2WZV1_ORYSI
10.25
10.20
10.15
10.10
10.05
10.00
9.95
9.90
1
2
t−Test: p−value = 0.08

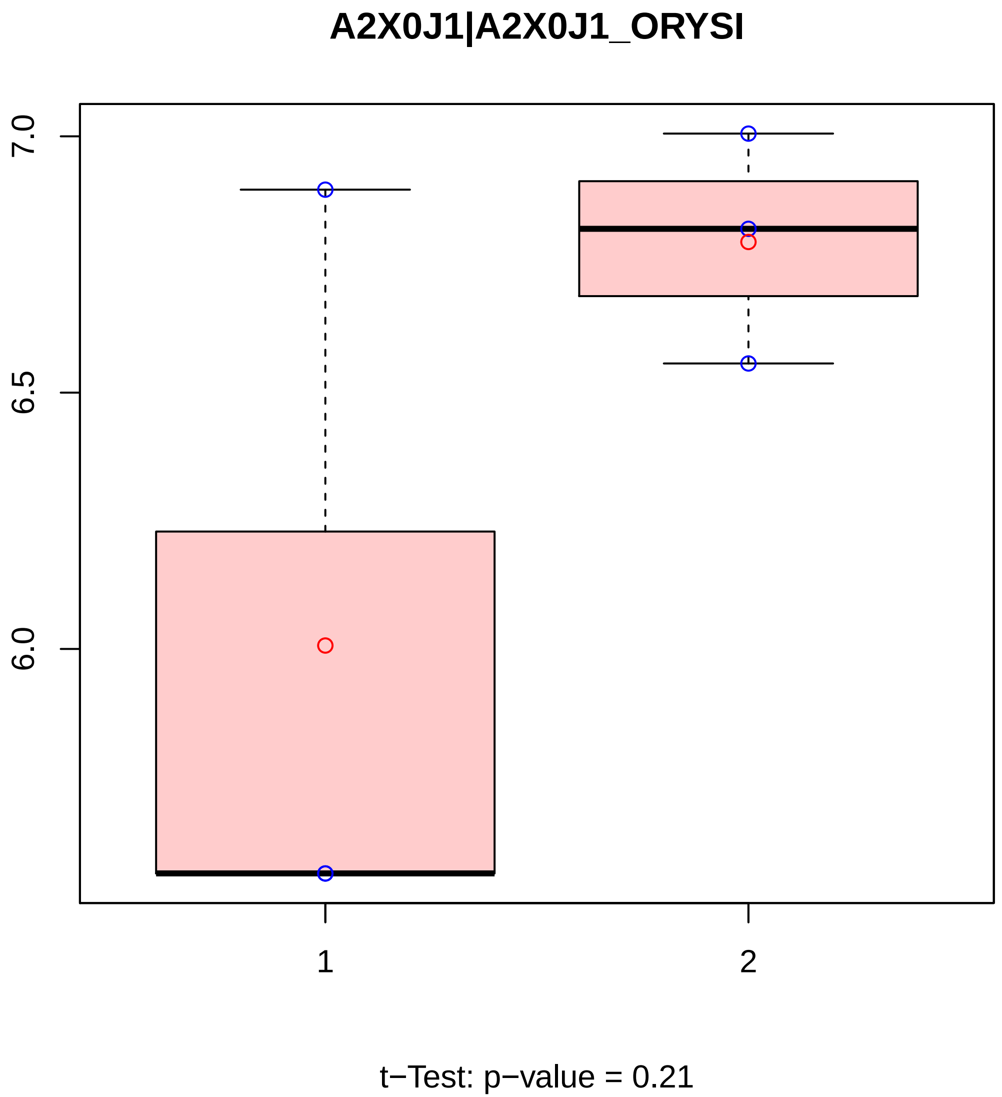
A2X0J1|A2X0J1_ORYSI
7.0
6.5
6.0
1
2
t−Test: p−value = 0.21

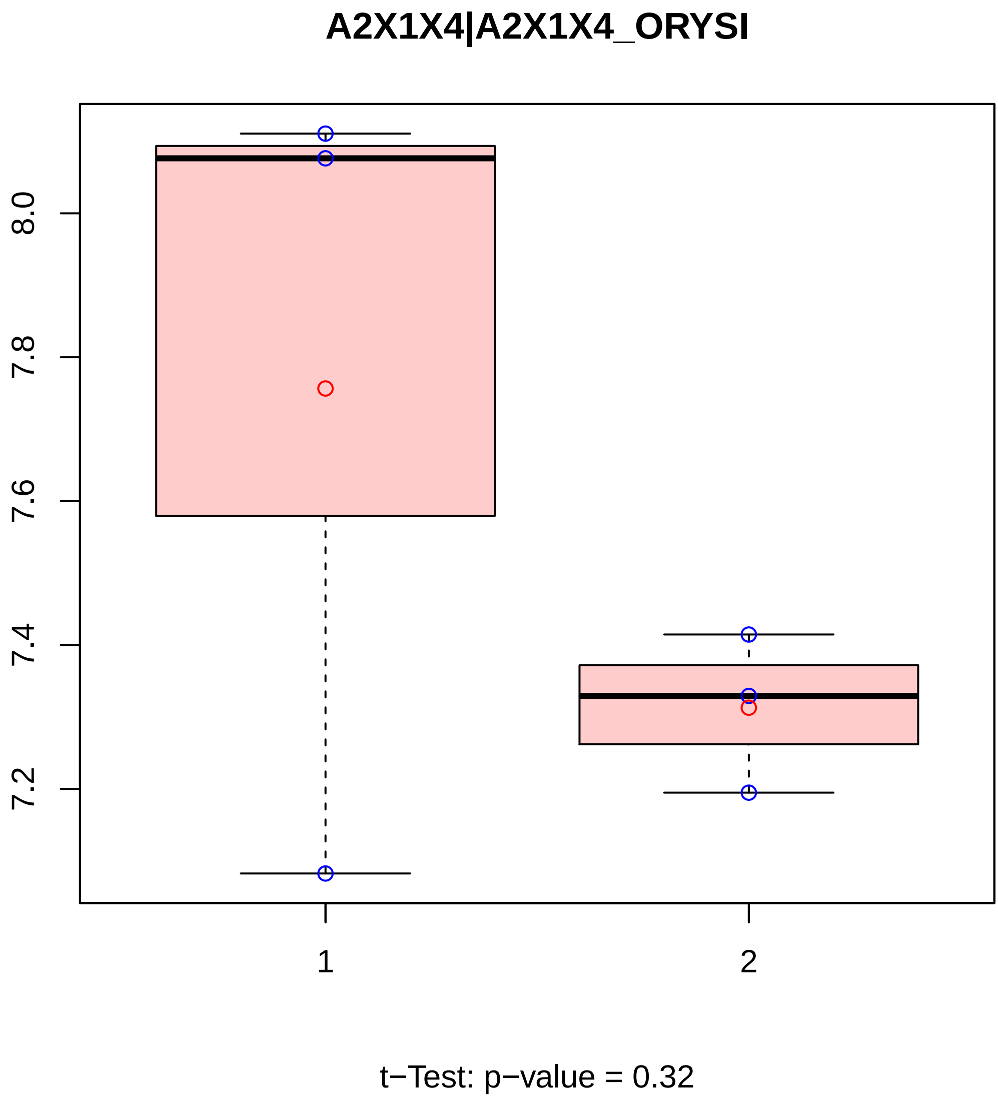

A2X1X4|A2X1X4_ORYSI
8.0
7.8
7.6
7.4
7.2
1
2
t−Test: p−value = 0.32

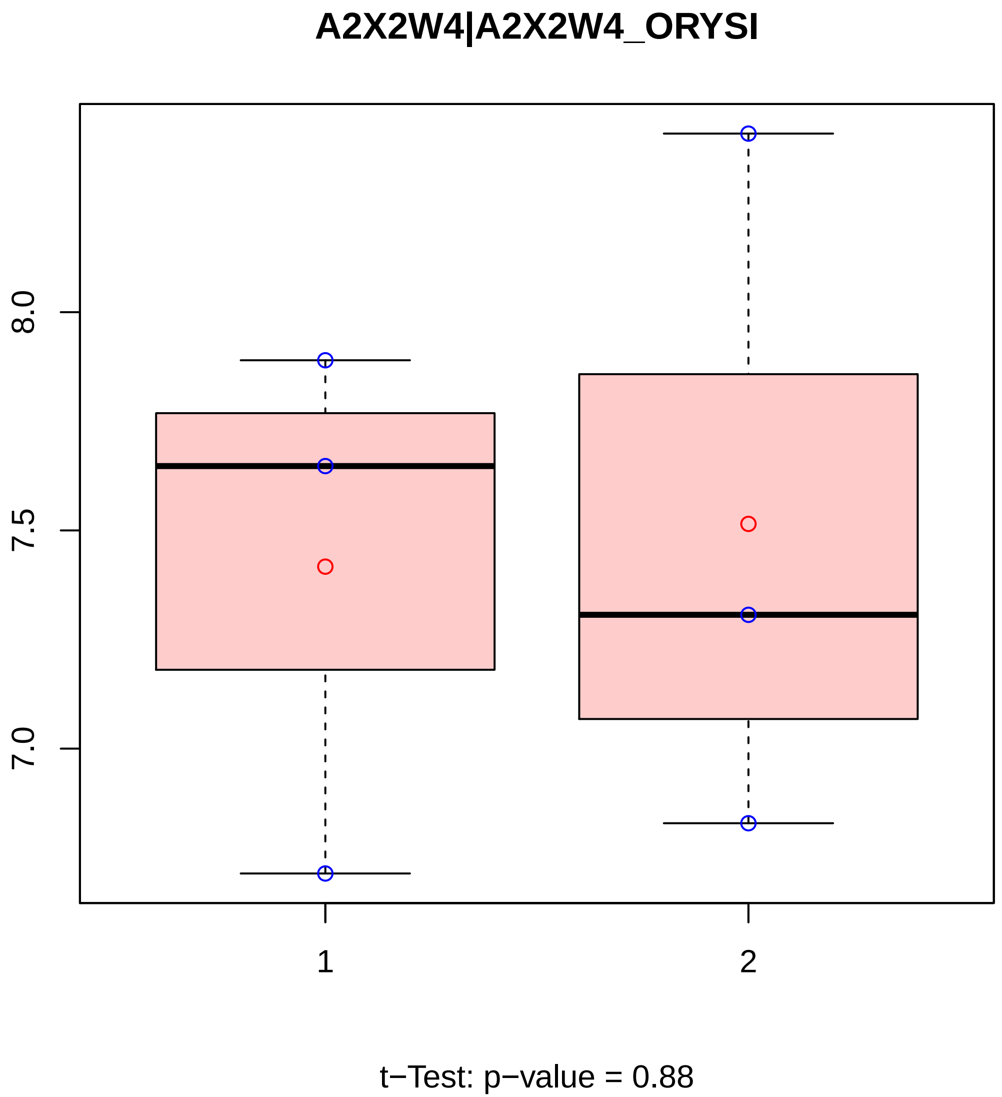

A2X2W4|A2X2W4_ORYSI
8.0
7.5
7.0
1
2
t−Test: p−value = 0.88

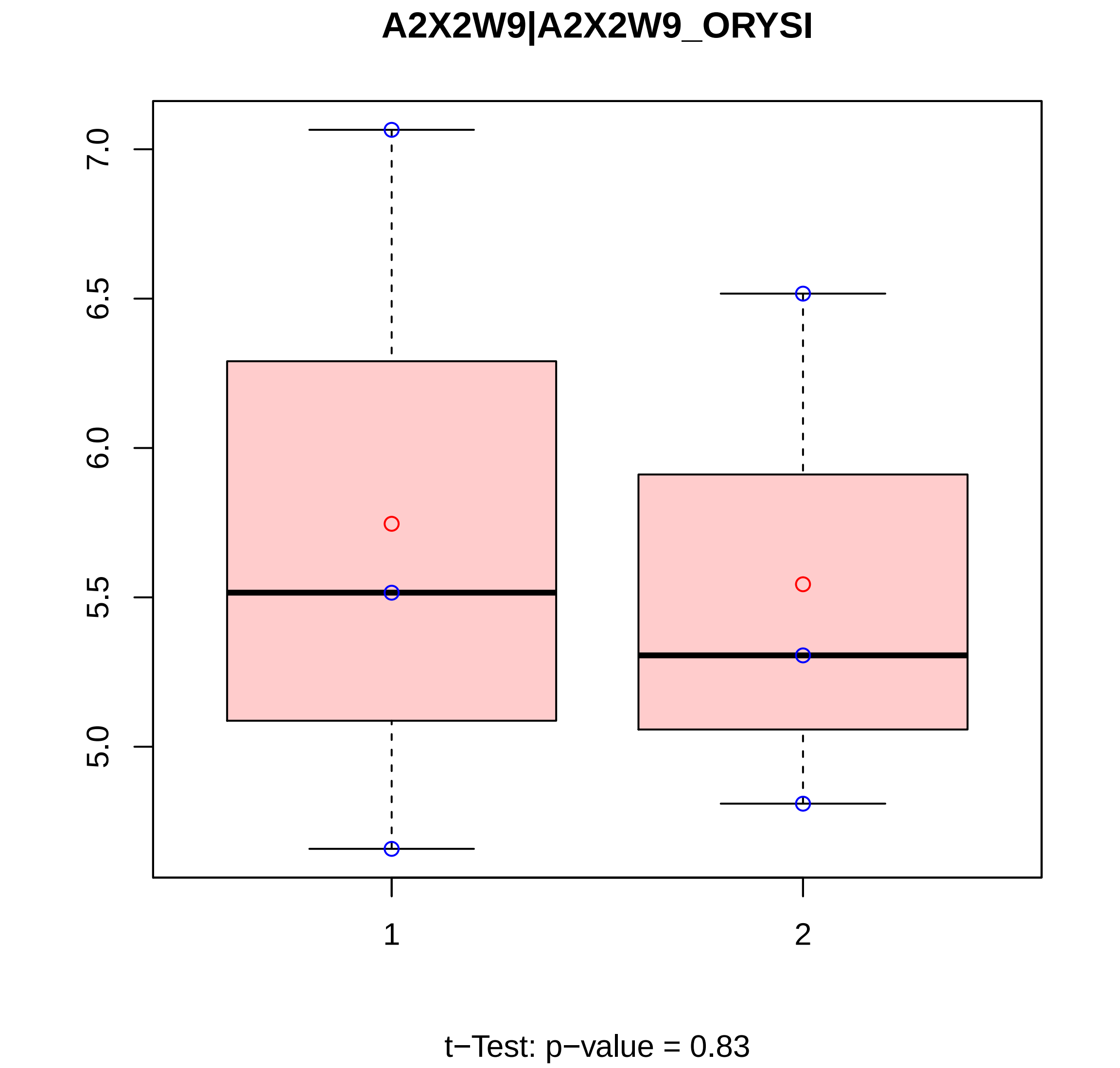
A2X2W9|A2X2W9_ORYSI
7.0
6.5
6.0
5.5
5.0
1
2
t−Test: p−value = 0.83

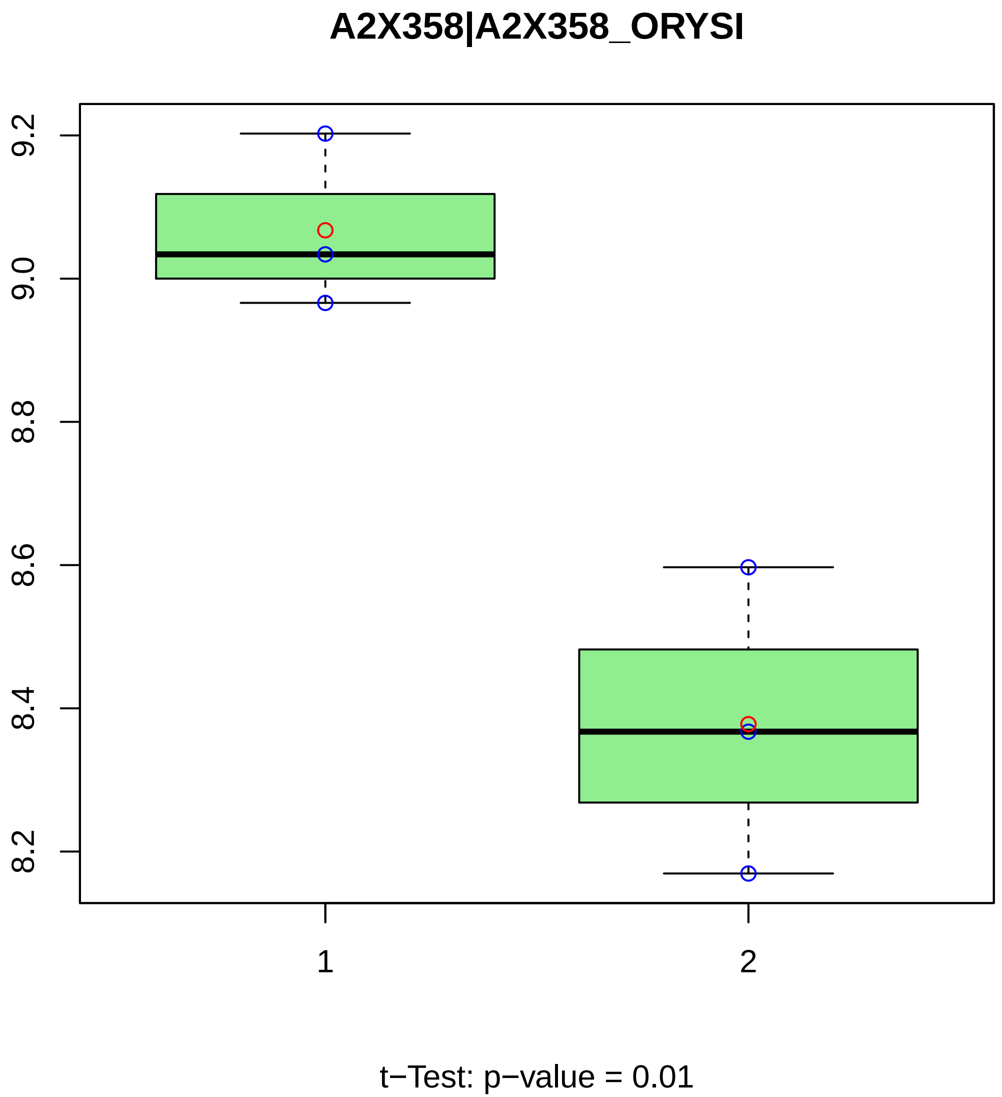
A2X358|A2X358_ORYSI
9.2
9.0
8.8
8.6
8.4
8.2
1
2
t−Test: p−value = 0.01

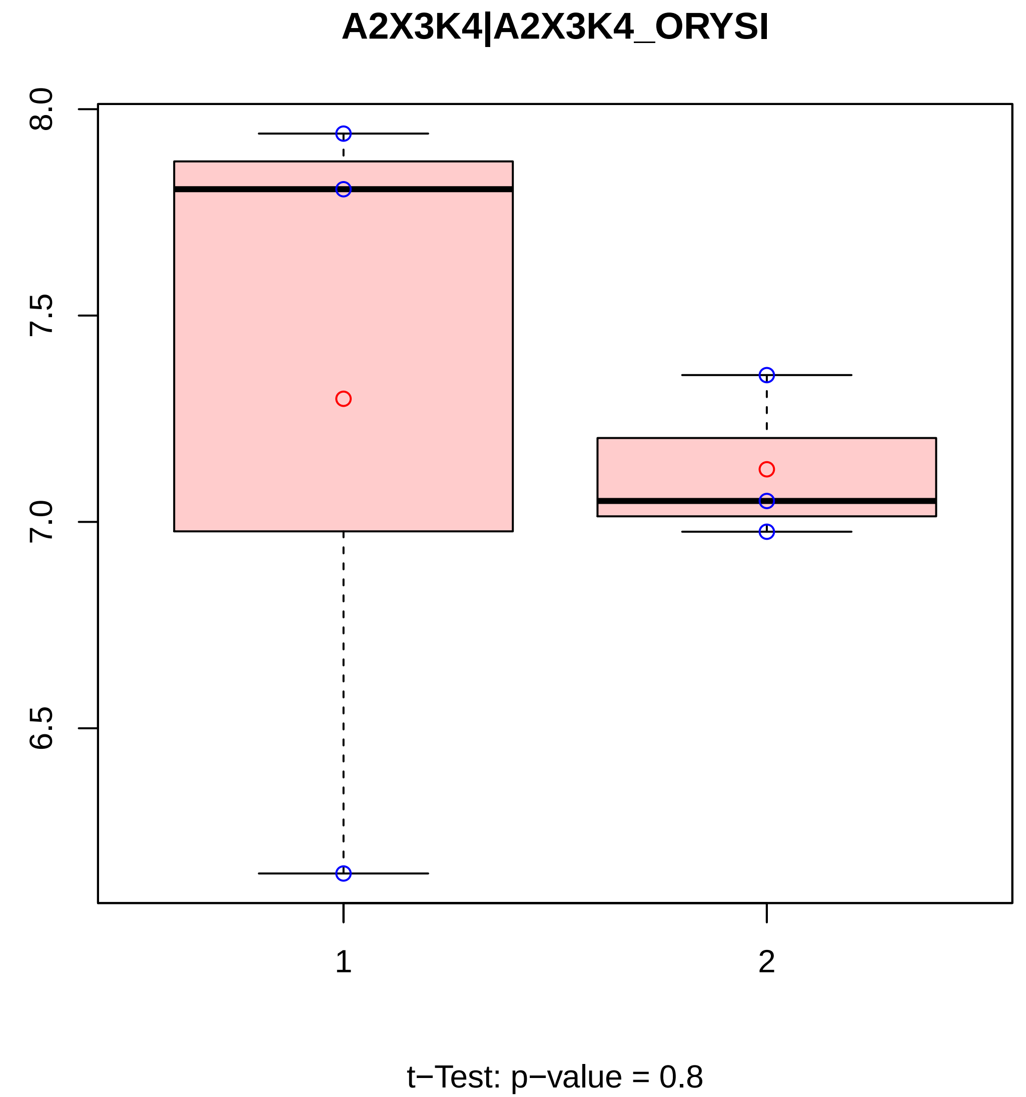
A2X3K4|A2X3K4_ORYSI
8.0
7.5
7.0
6.5
1
2
t−Test: p−value = 0.8

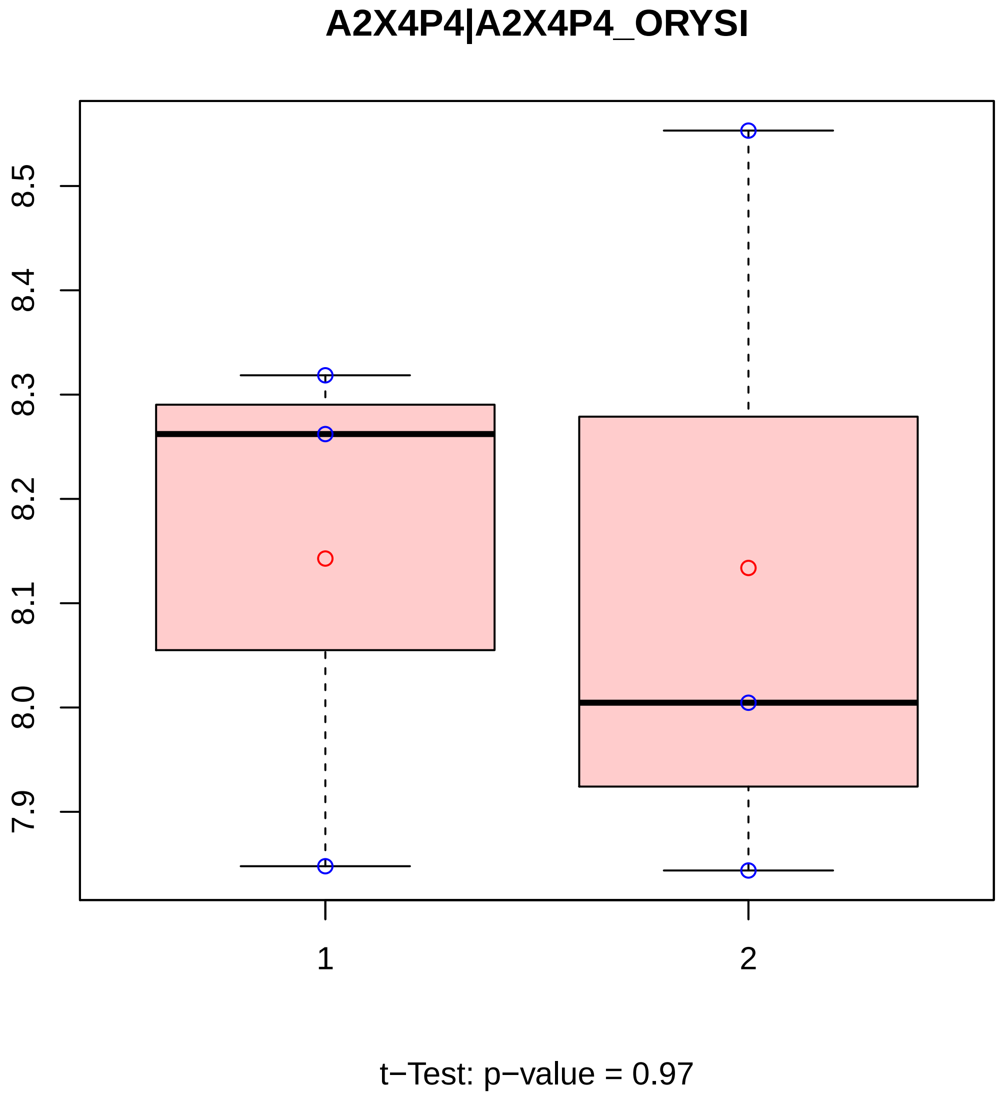
A2X4P4|A2X4P4_ORYSI
8.5
8.4
8.3
8.2
8.1
8.0
7.9
1
2
t−Test: p−value = 0.97

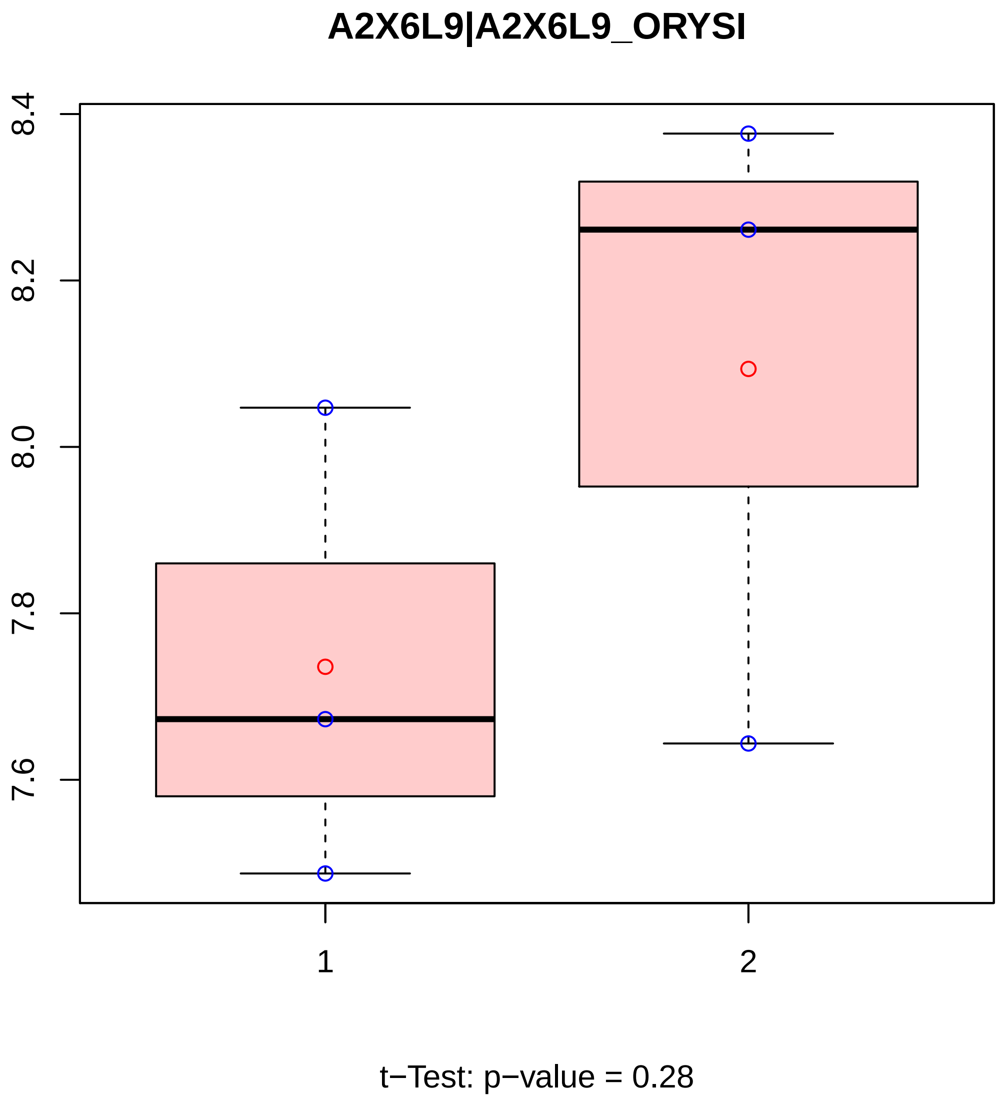

A2X6L9|A2X6L9_ORYSI
8.4
8.2
8.0
7.8
7.6
1
2
t−Test: p−value = 0.28

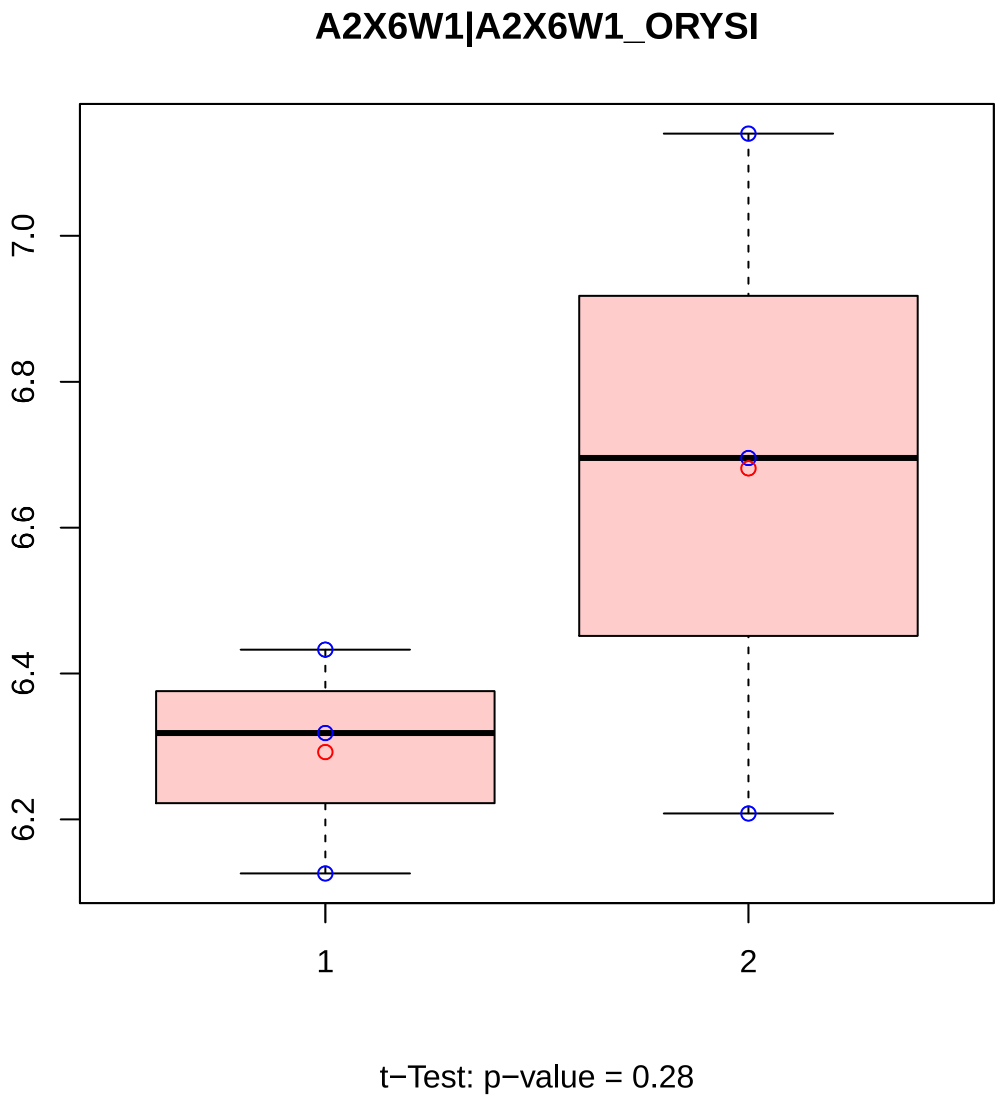
A2X6W1|A2X6W1_ORYSI
6.2
6.4
6.6
6.8
7.0
1
2
t−Test: p−value = 0.28

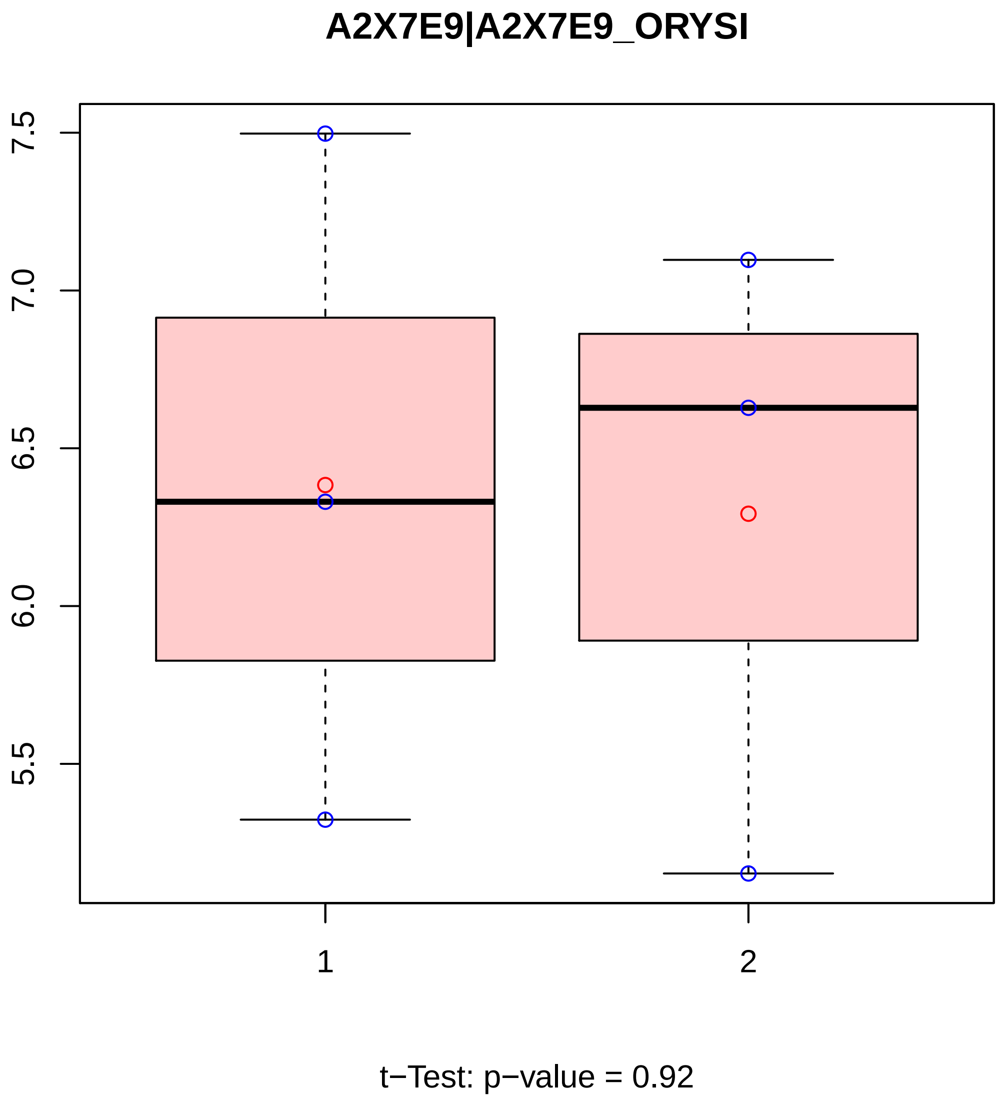
A2X7E9|A2X7E9_ORYSI
7.5
7.0
6.5
6.0
5.5
1
2
t−Test: p−value = 0.92

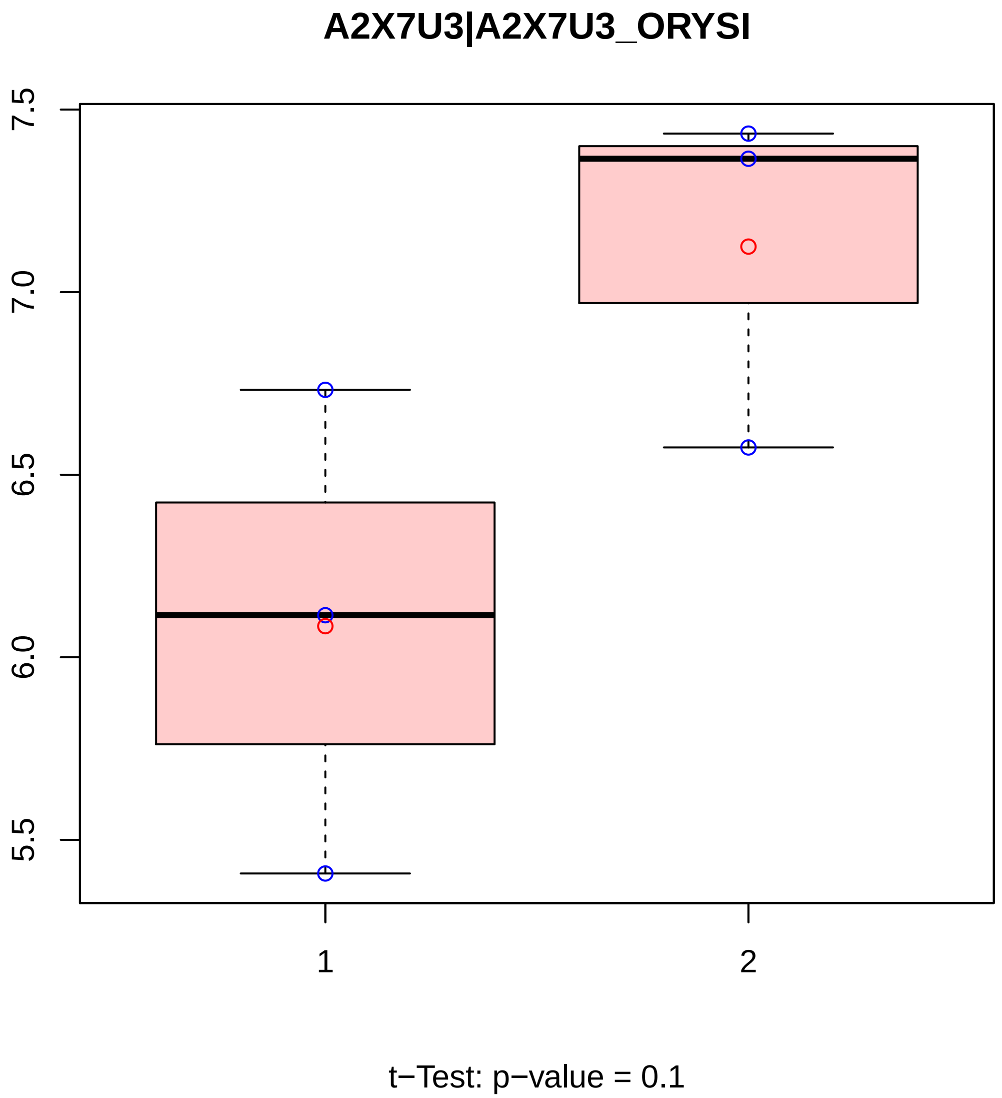

A2X7U3|A2X7U3_ORYSI
7.5
7.0
6.5
6.0
5.5
1
2
t−Test: p−value = 0.1

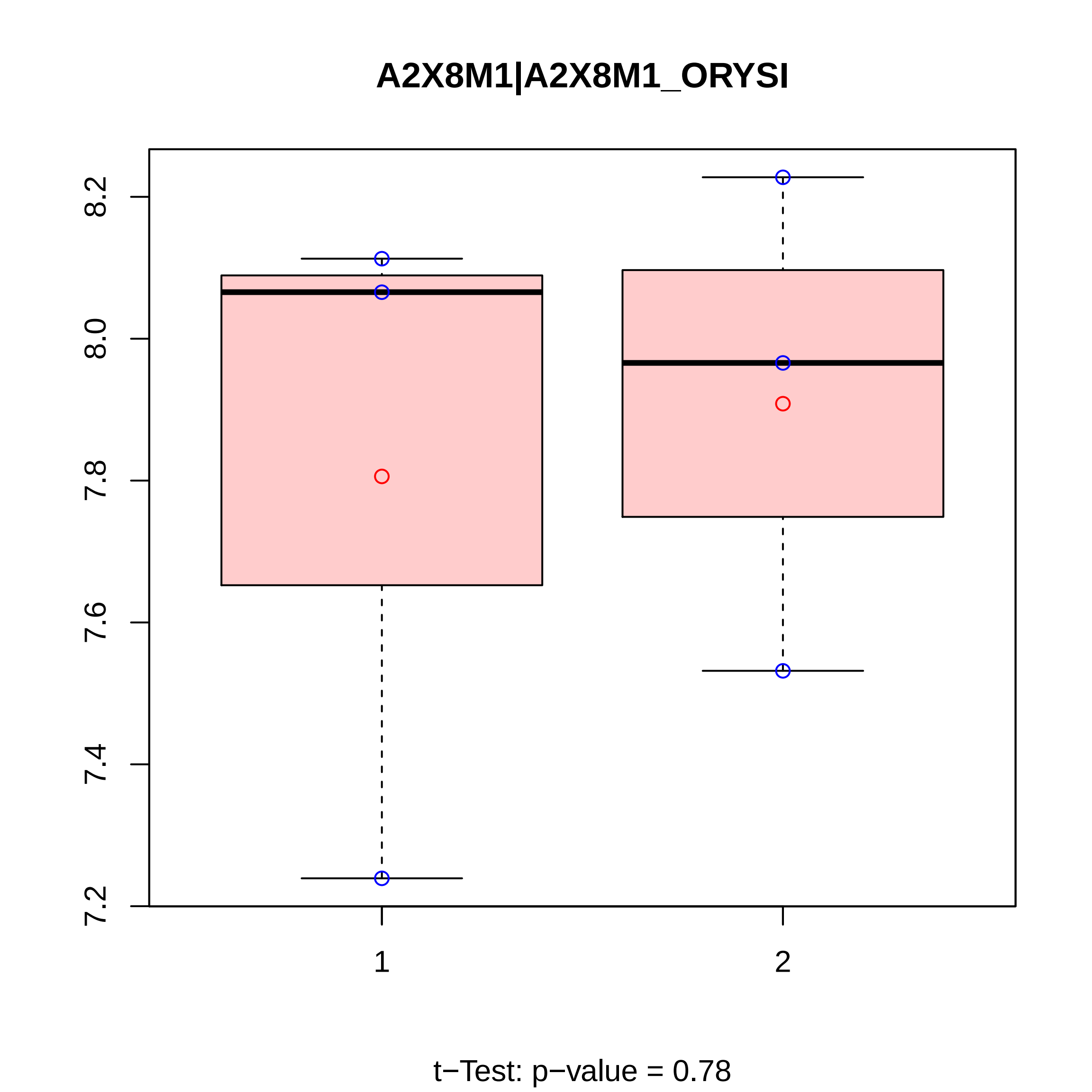
A2X8M1|A2X8M1_ORYSI
8.2
8.0
7.8
7.6
7.4
7.2
1
2
t−Test: p−value = 0.78

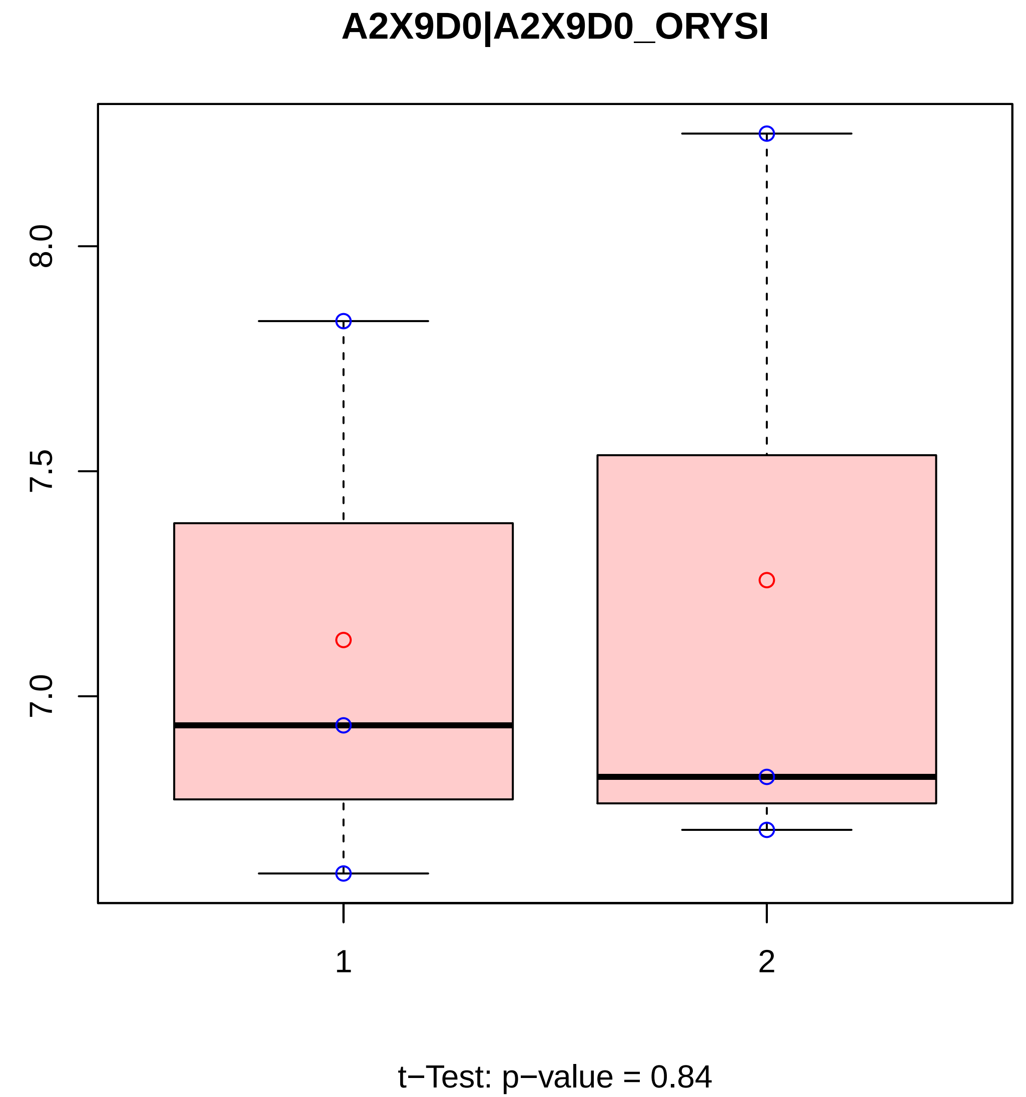
A2X9D0|A2X9D0_ORYSI
8.0
7.5
7.0
1
2
t−Test: p−value = 0.84

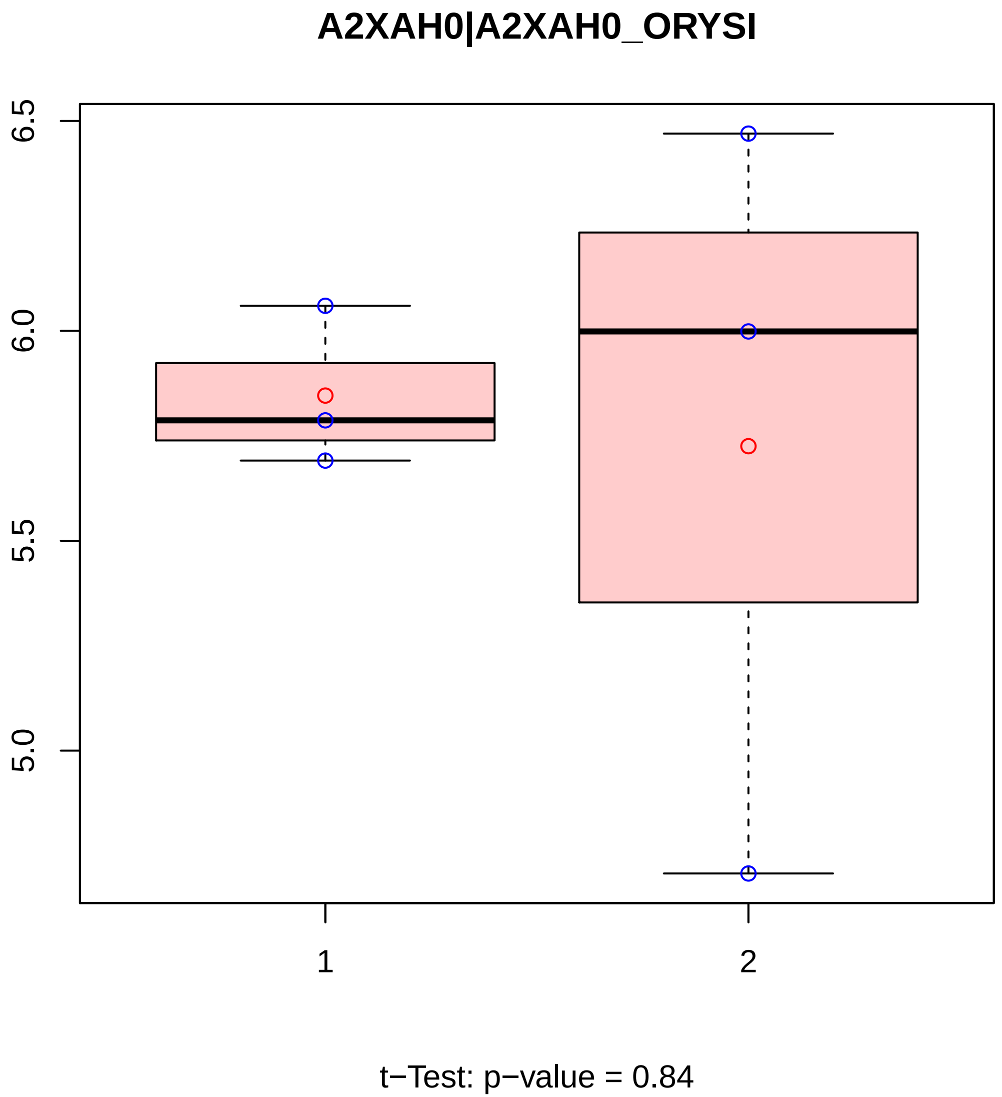
A2XAH0|A2XAH0_ORYSI
6.5
6.0
5.5
5.0
1
2
t−Test: p−value = 0.84

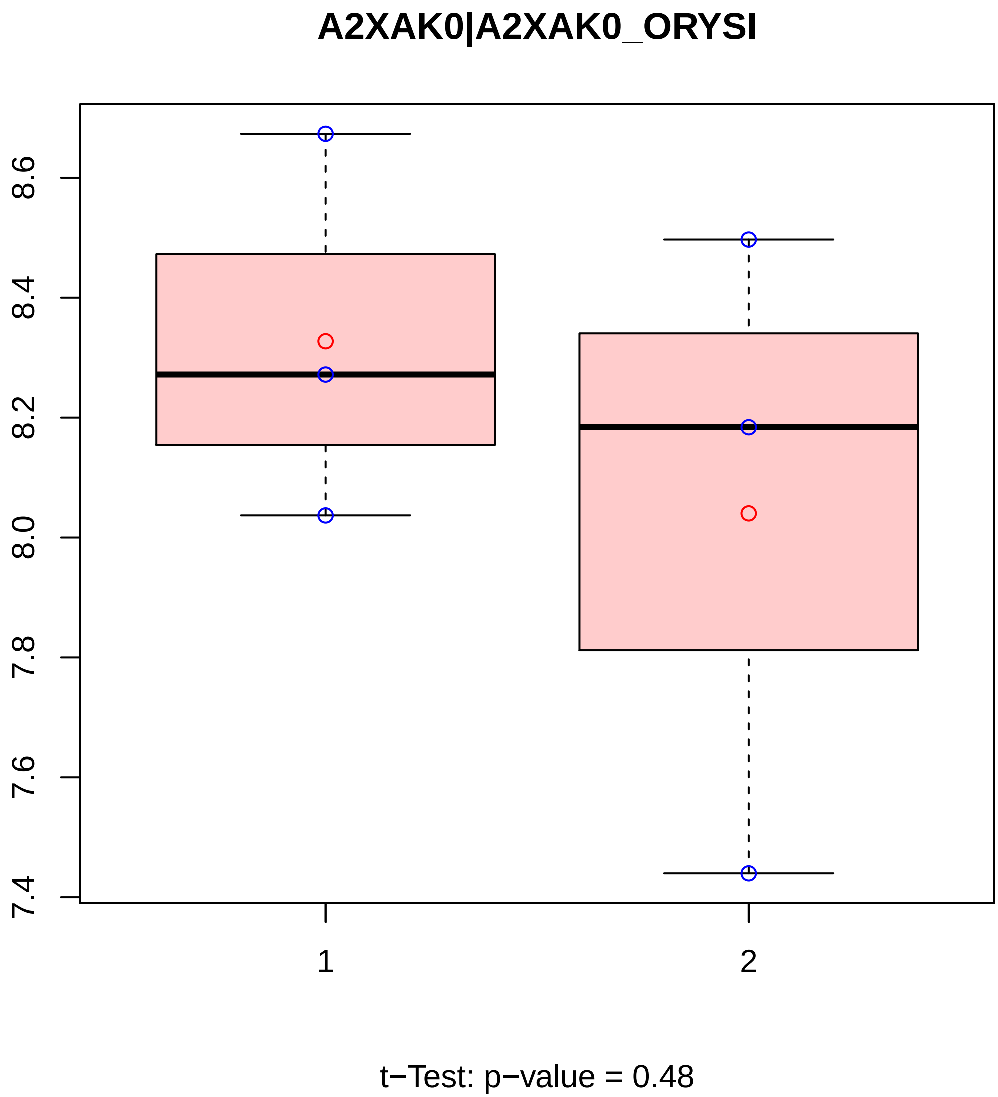
A2XAK0|A2XAK0_ORYSI
8.6
8.4
8.2
8.0
7.8
7.6
7.4
1
2
t−Test: p−value = 0.48

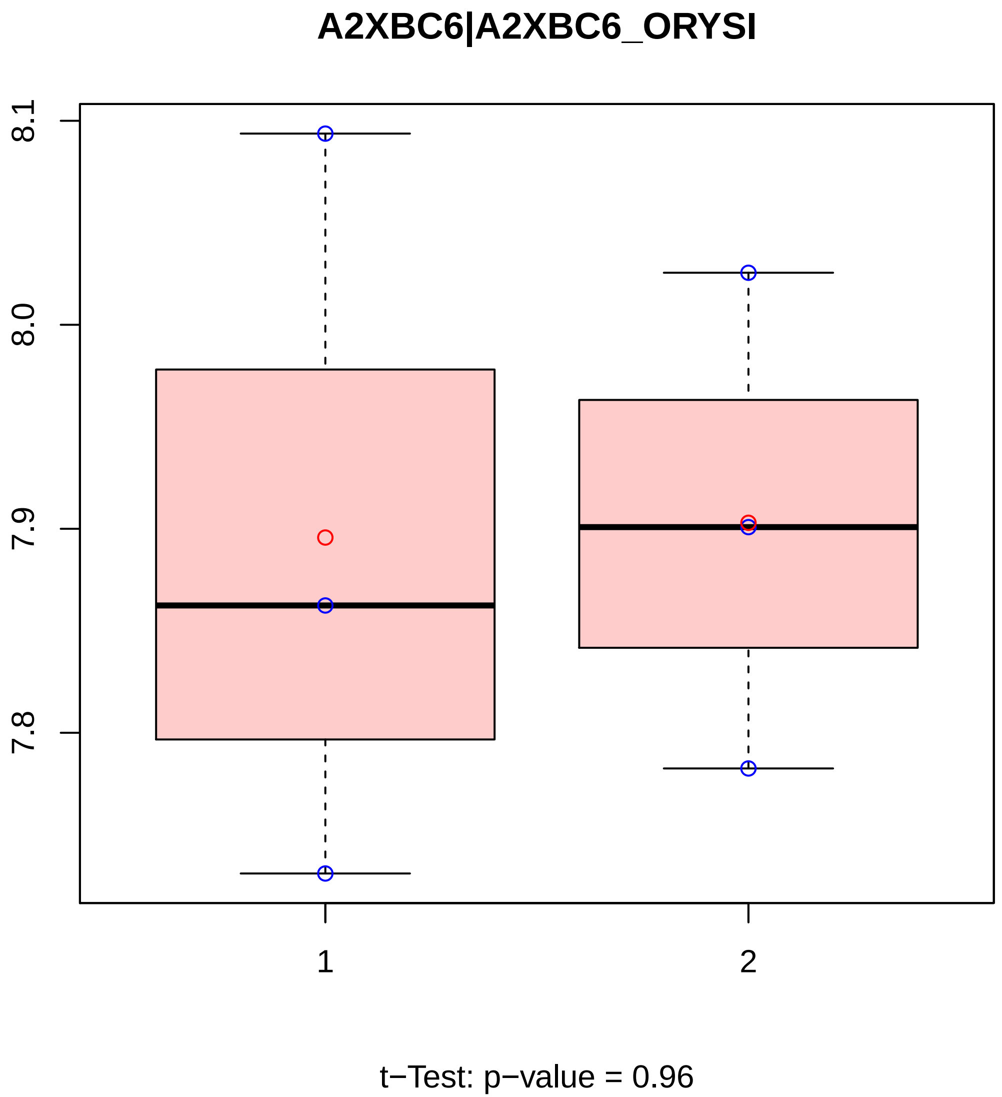
A2XBC6|A2XBC6_ORYSI
8.1
8.0
7.9
7.8
1
2
t−Test: p−value = 0.96

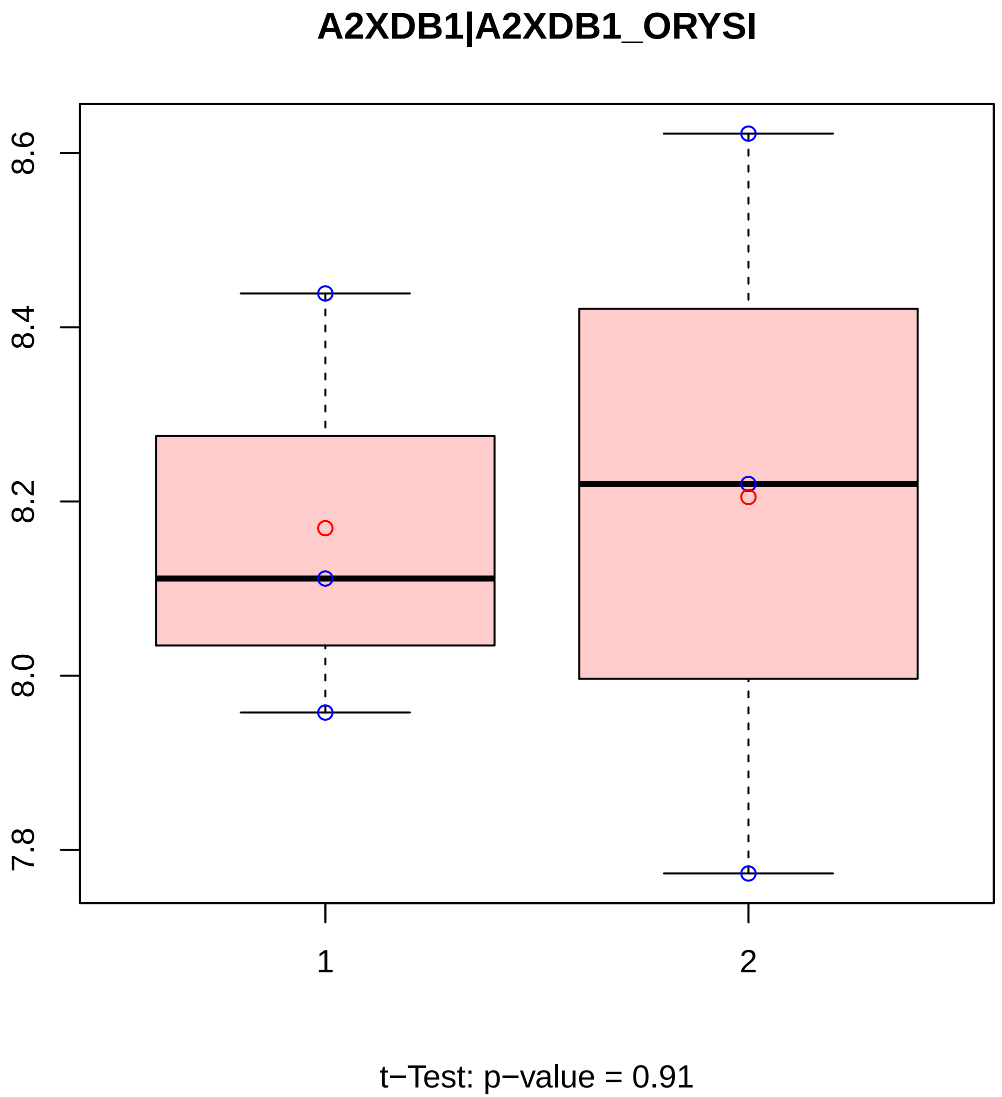
A2XDB1|A2XDB1_ORYSI
8.6
8.4
8.2
8.0
7.8
1
2
t−Test: p−value = 0.91

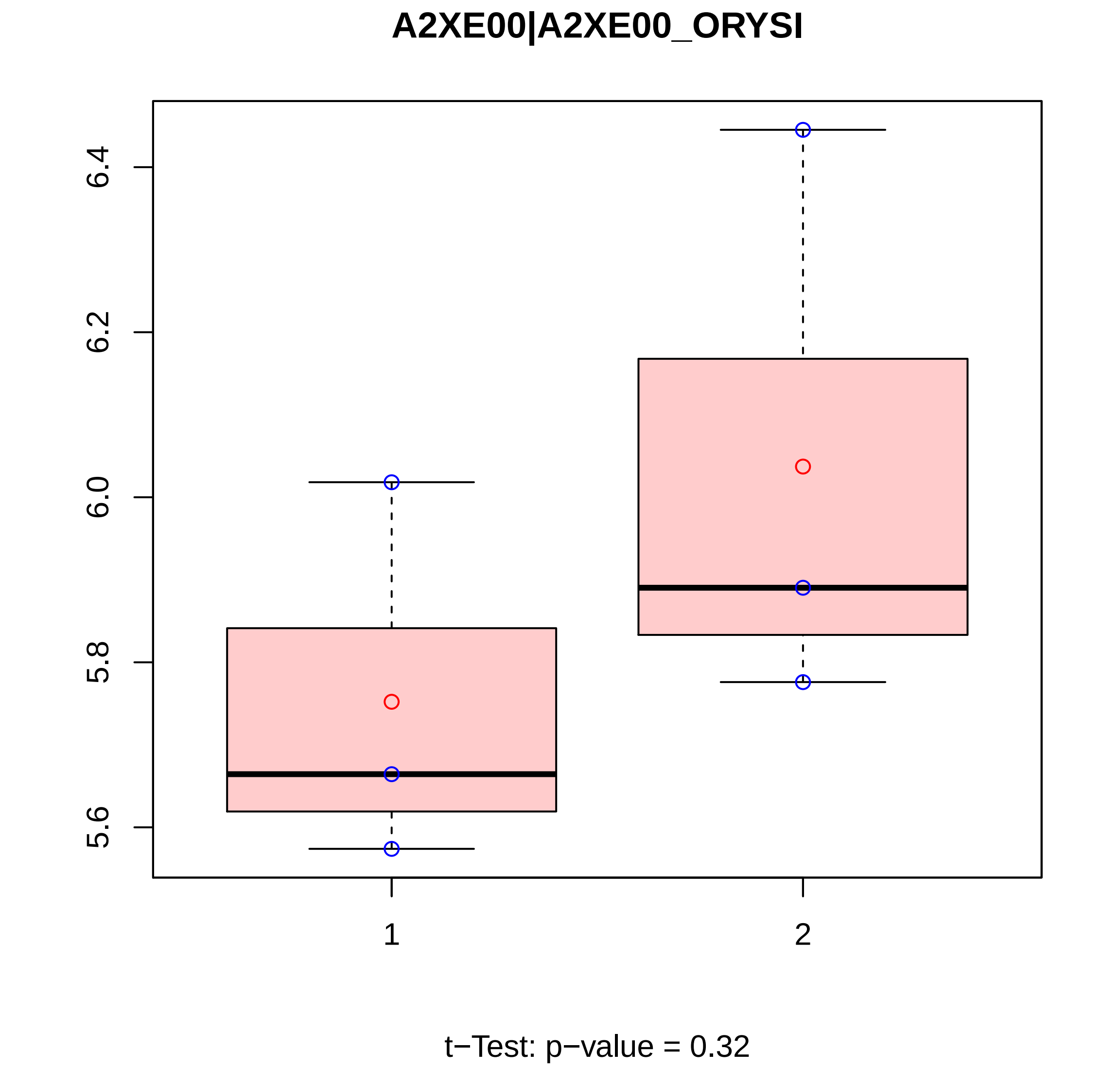
A2XE00|A2XE00_ORYSI
6.4
6.2
6.0
5.8
5.6
1
2
t−Test: p−value = 0.32

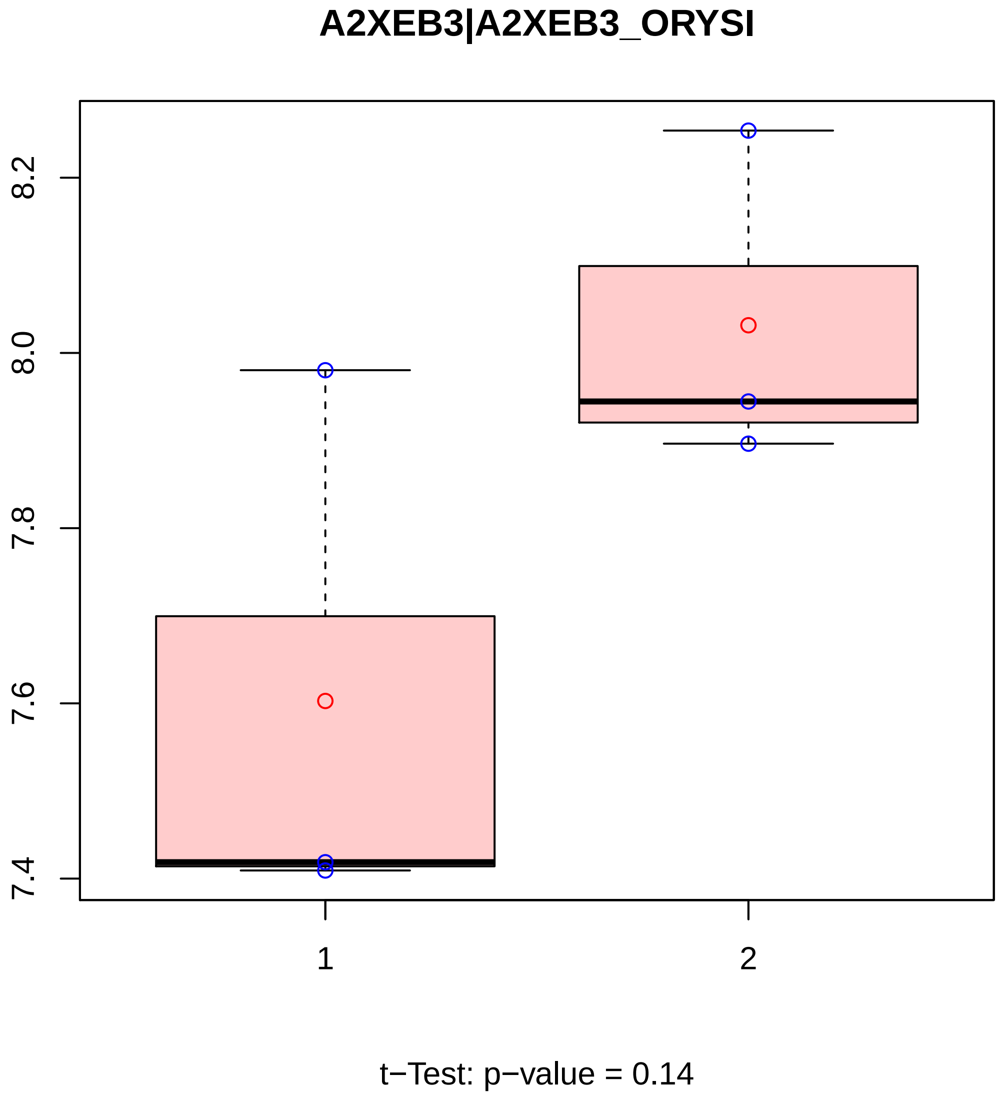
A2XEB3|A2XEB3_ORYSI
8.2
8.0
7.8
7.6
7.4
1
2
t−Test: p−value = 0.14

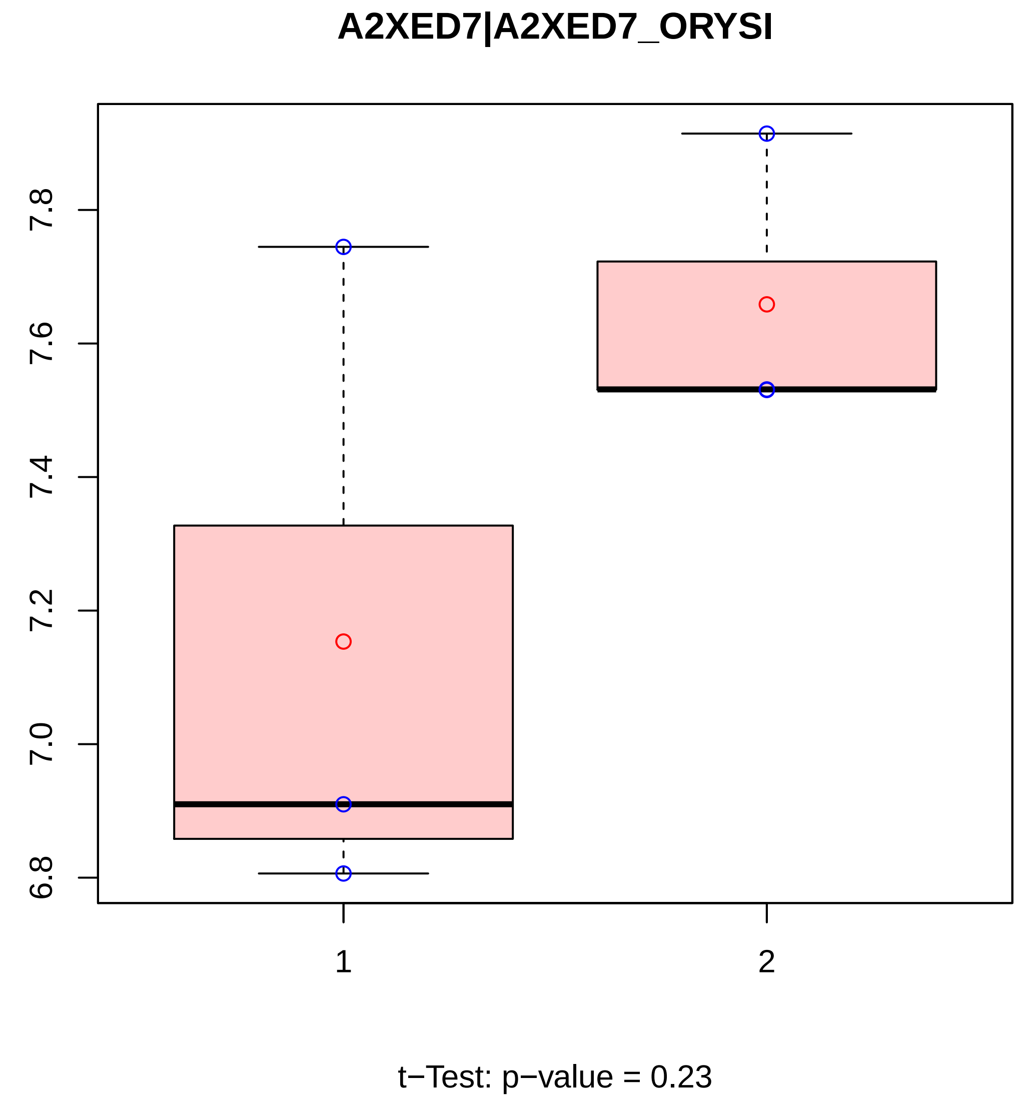
A2XED7|A2XED7_ORYSI
7.8
7.6
7.4
7.2
7.0
6.8
1
2
t−Test: p−value = 0.23

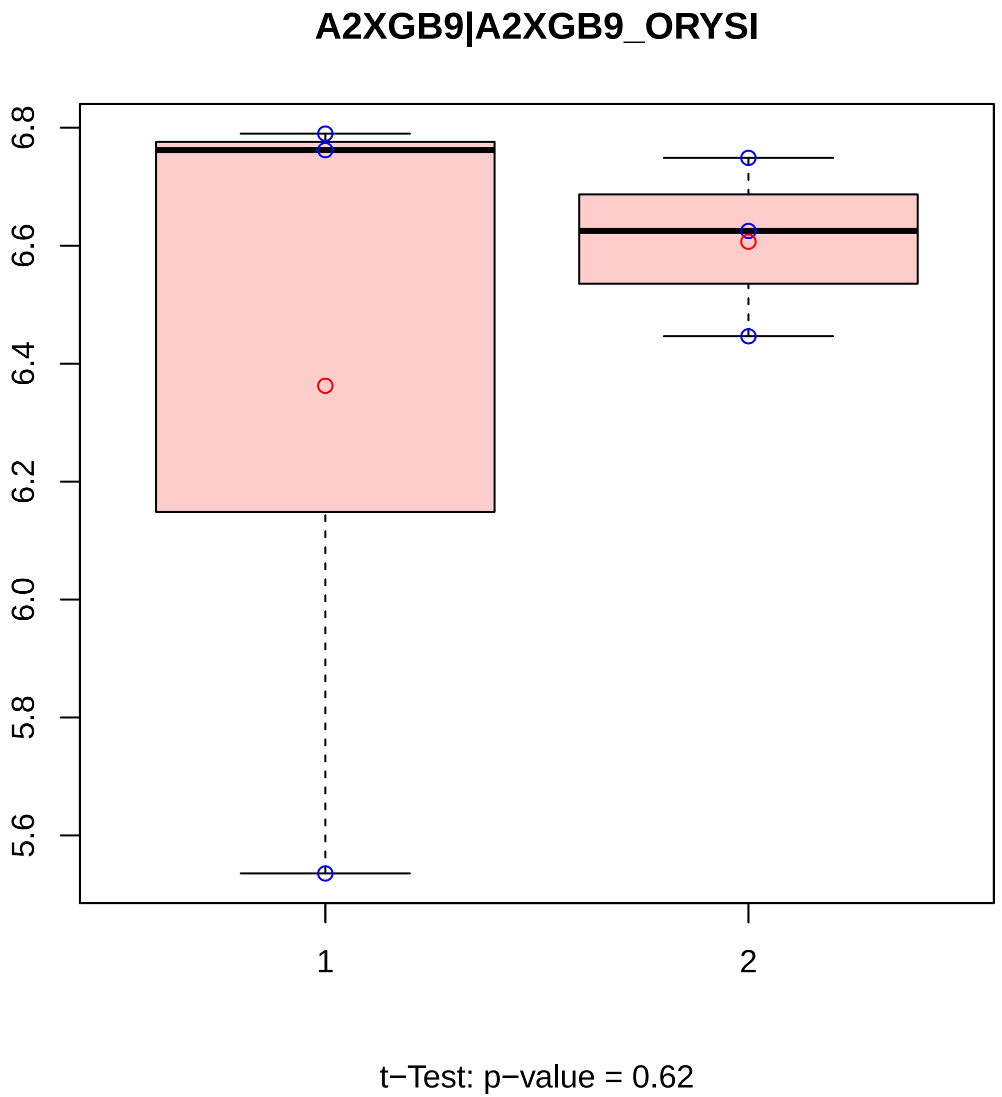
A2XGB9|A2XGB9_ORYSI
6.8
6.6
6.4
6.2
6.0
5.8
5.6
1
2
t−Test: p−value = 0.62

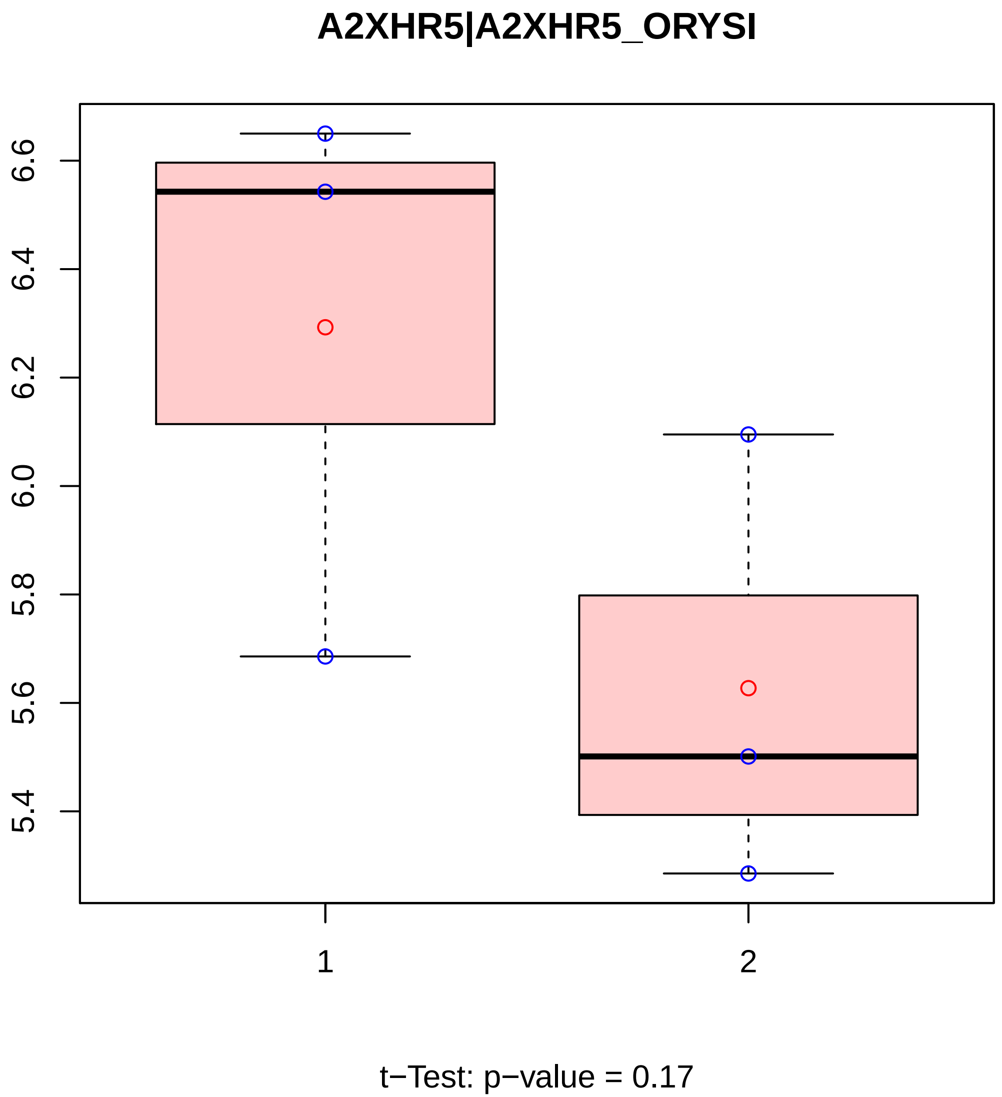
A2XHR5|A2XHR5_ORYSI
6.6
6.4
6.2
6.0
5.8
5.6
5.4
1
2
t−Test: p−value = 0.17

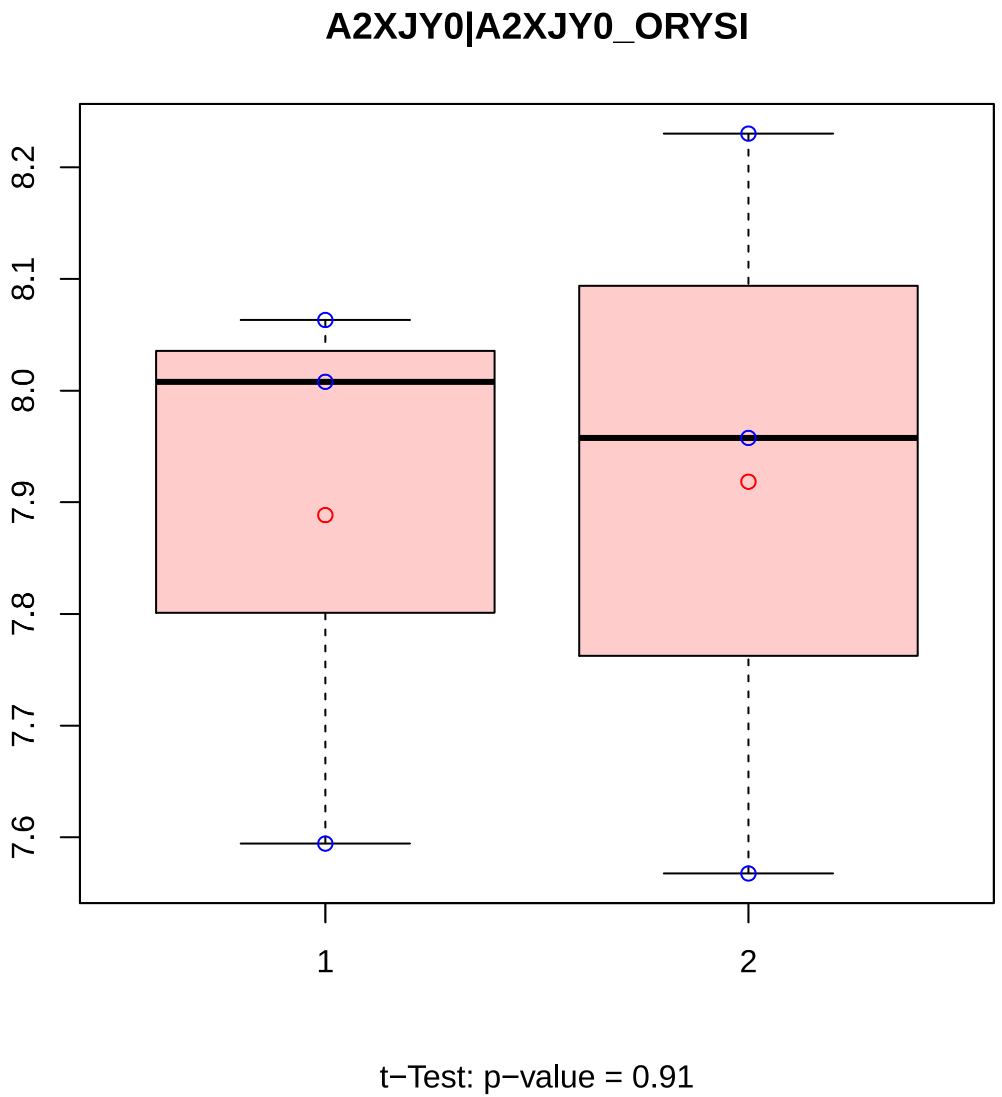
A2XJY0|A2XJY0_ORYSI
8.2
8.1
8.0
7.9
7.8
7.7
7.6
1
2
t−Test: p−value = 0.91

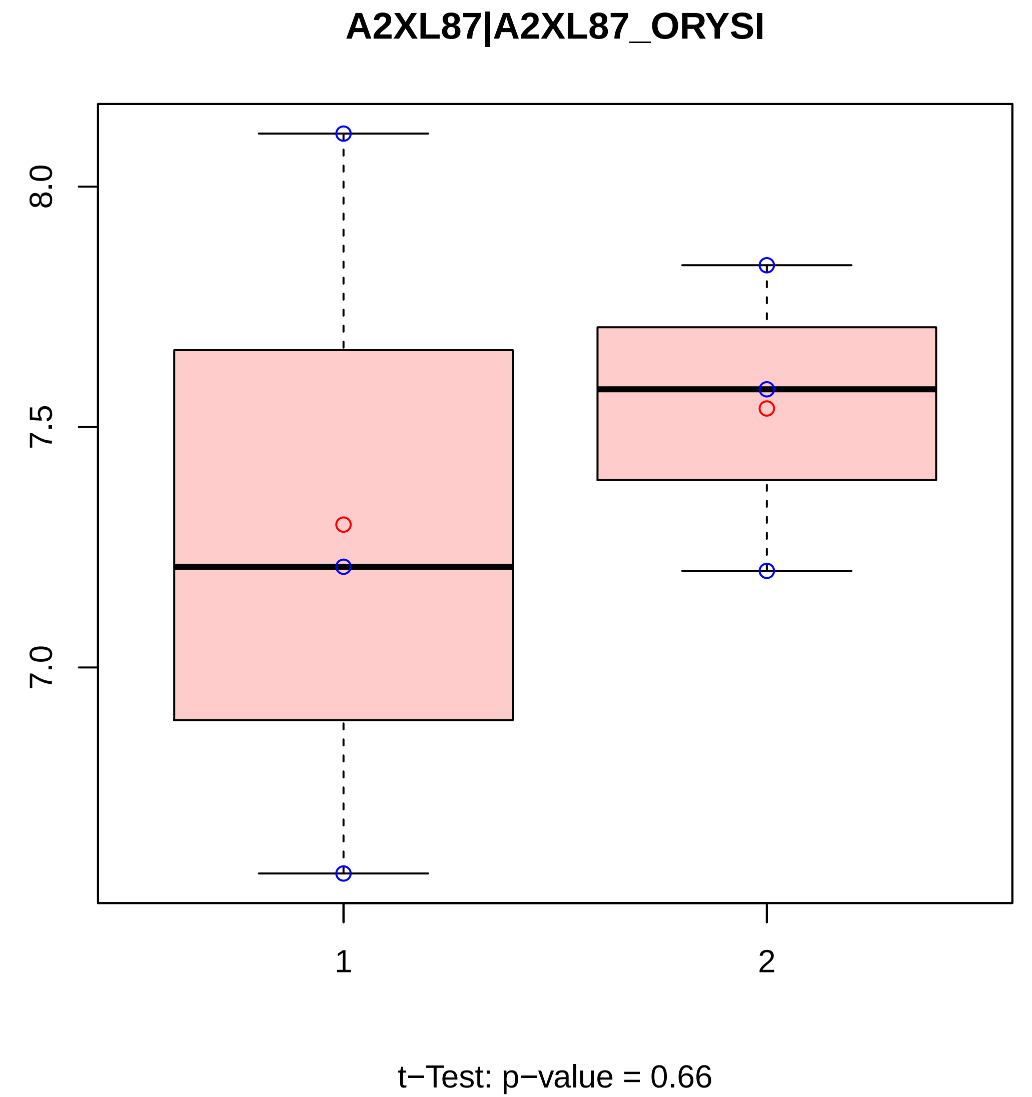
A2XL87|A2XL87_ORYSI
8.0
7.5
7.0
1
2
t−Test: p−value = 0.66

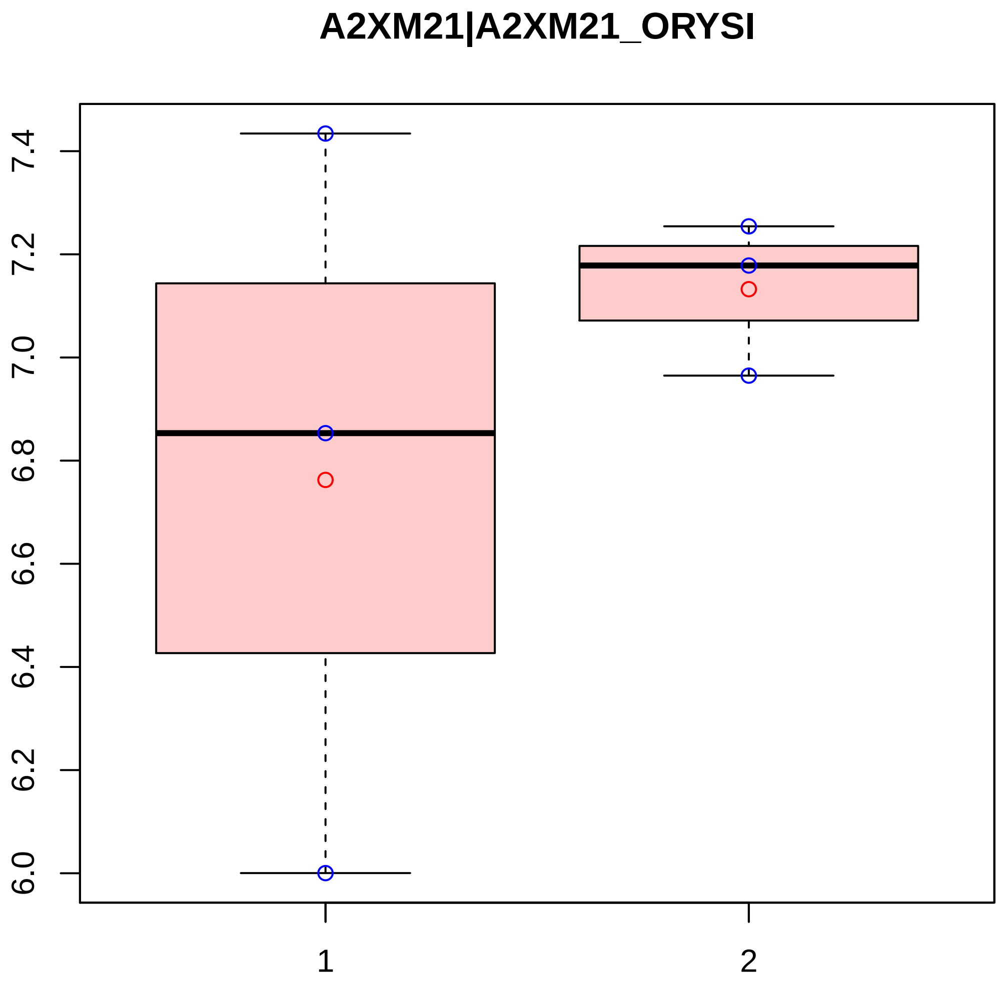

t−Test: p−value = 0.47

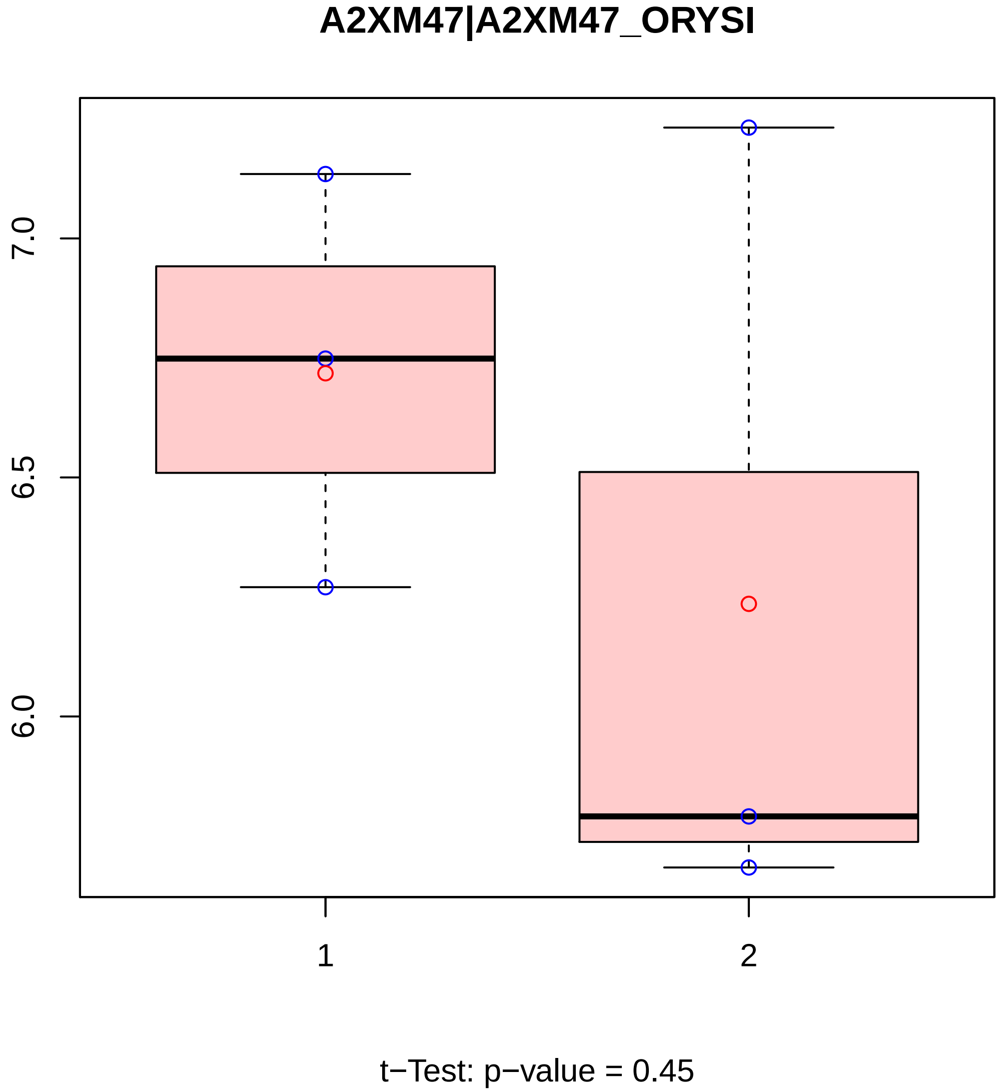
A2XM47|A2XM47_ORYSI
7.0
6.5
6.0
1
2
t−Test: p−value = 0.45

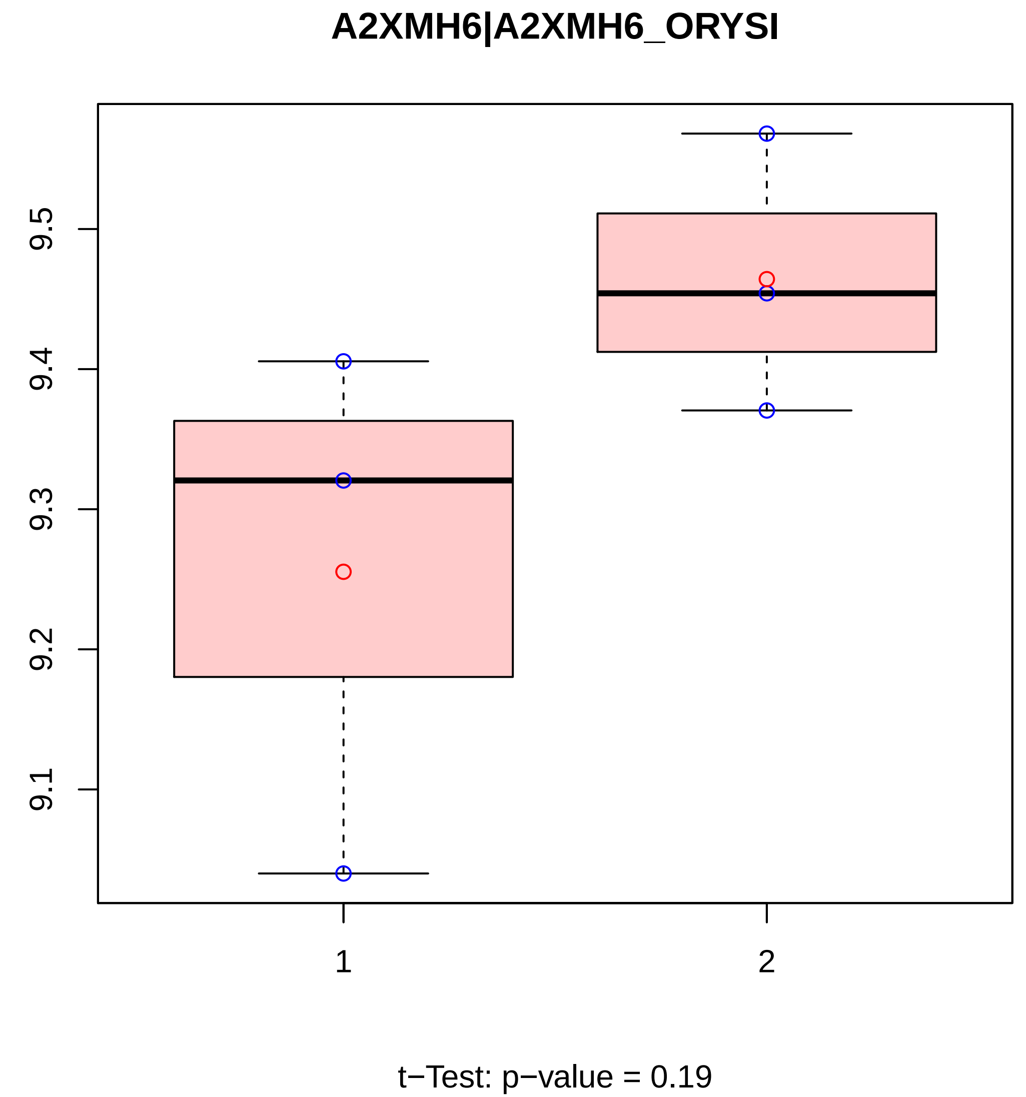

A2XMH6|A2XMH6_ORYSI
9.1
9.2
9.3
9.4
9.5
1
2
t−Test: p−value = 0.19

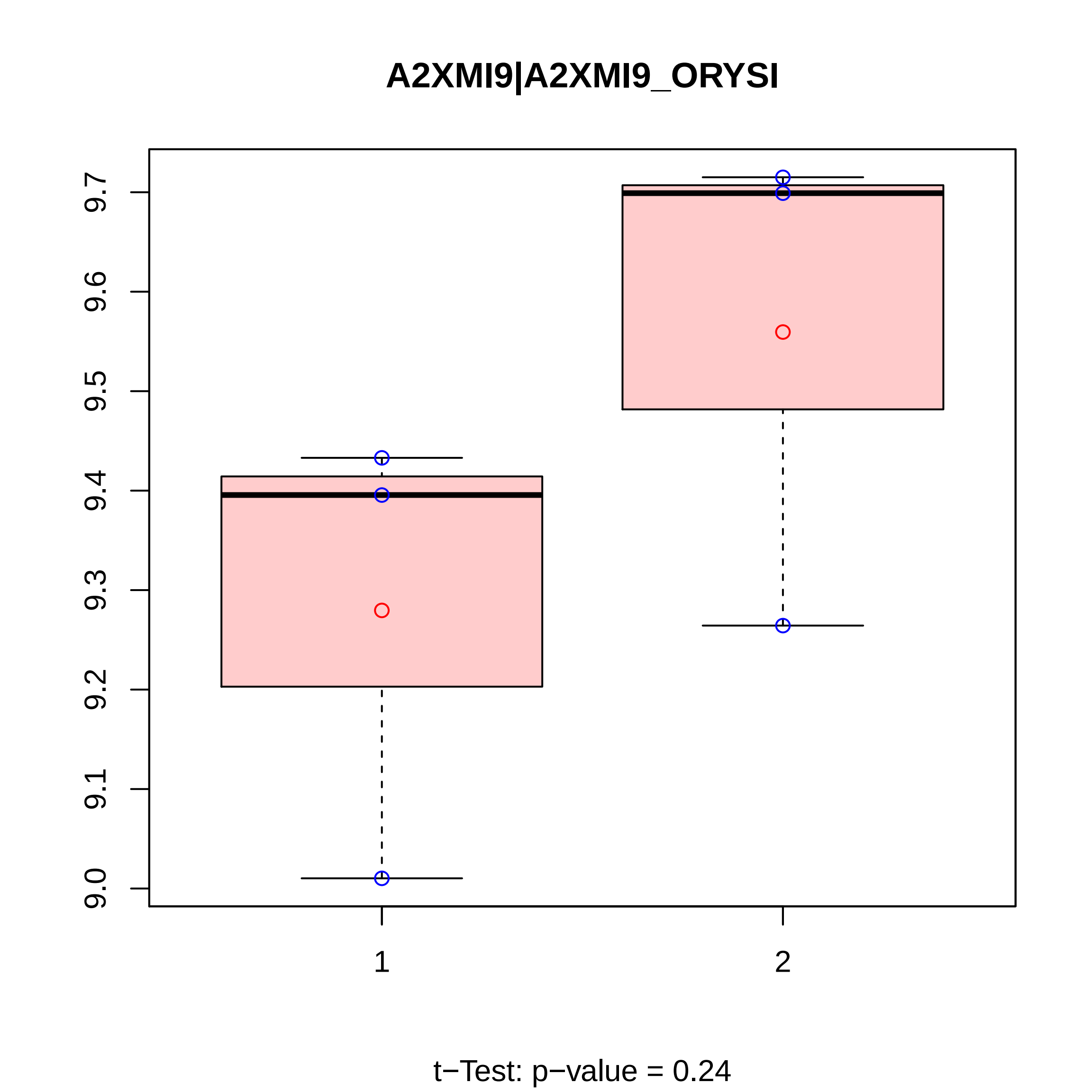
A2XMI9|A2XMI9_ORYSI
9.0
9.1
9.2
9.3
9.4
9.5
9.6
9.7
1
2
t−Test: p−value = 0.24

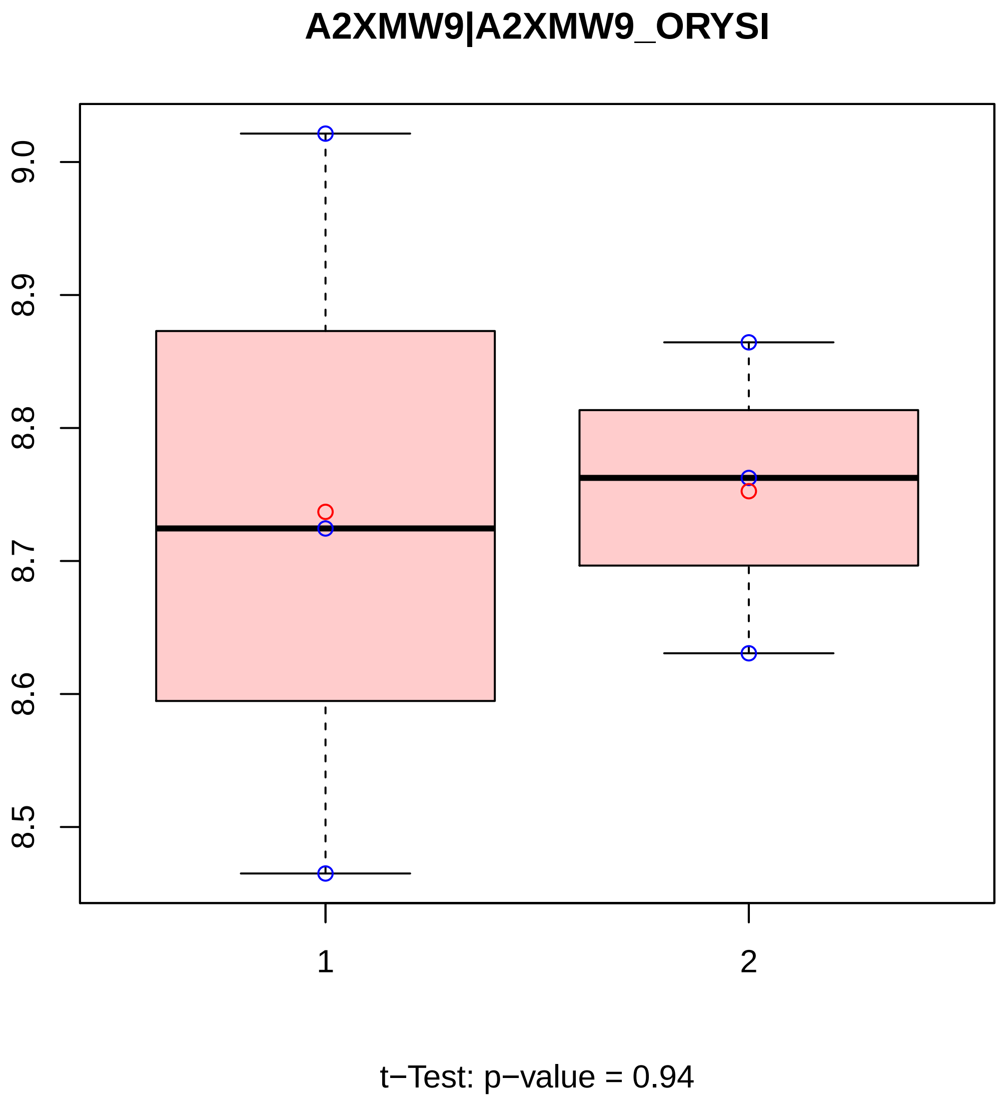
A2XMW9|A2XMW9_ORYSI
9.0
8.9
8.8
8.7
8.6
8.5
1
2
t−Test: p−value = 0.94

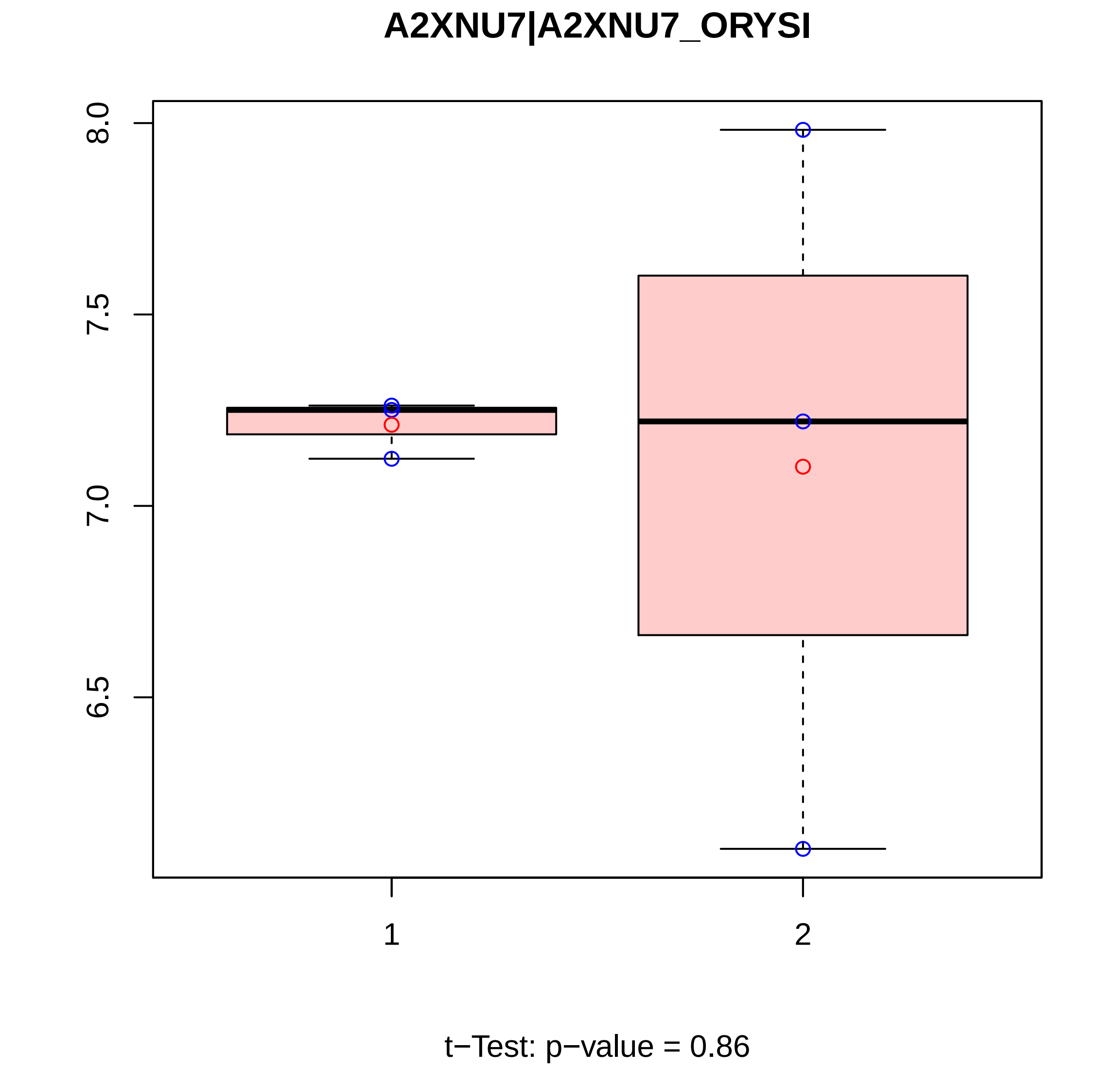

A2XNU7|A2XNU7_ORYSI
8.0
7.5
7.0
6.5
1
2
t−Test: p−value = 0.86

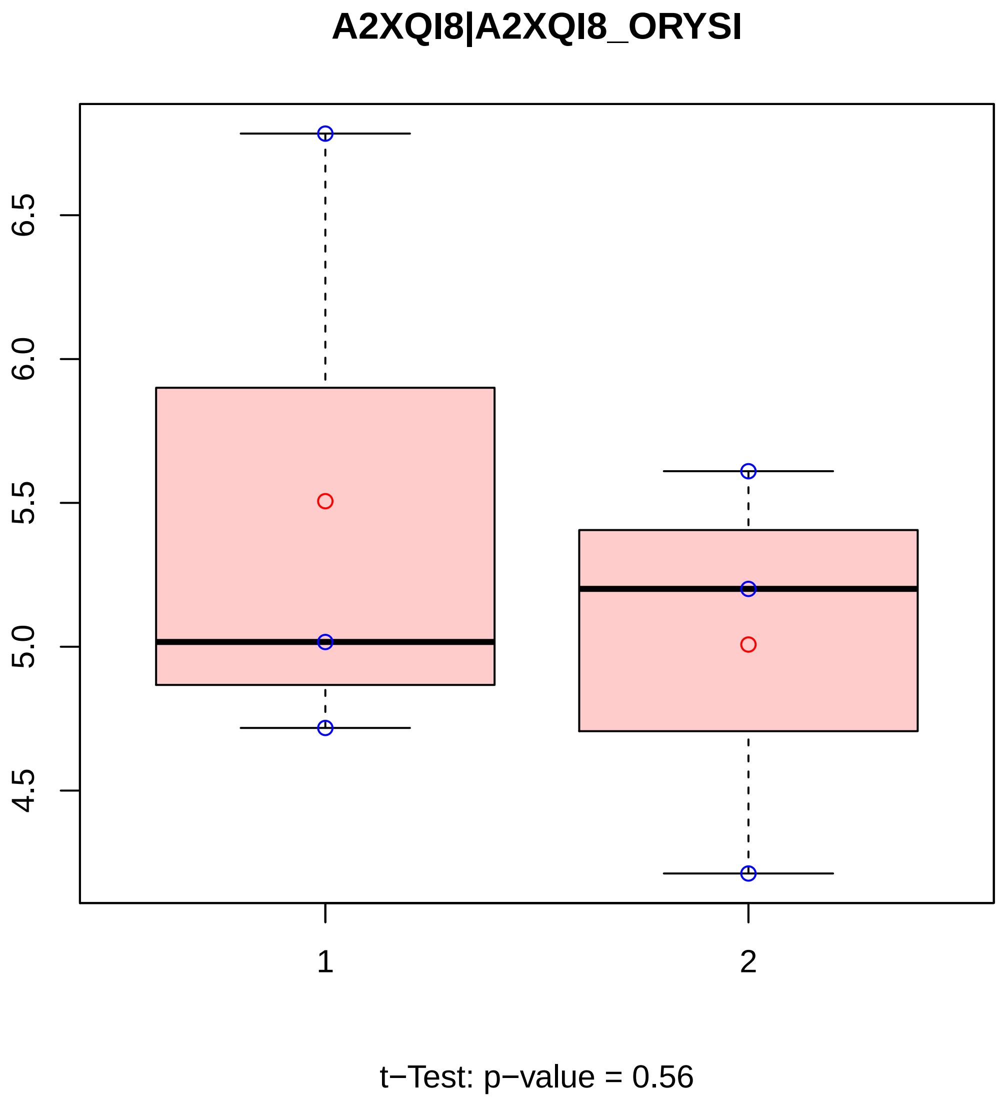
A2XQI8|A2XQI8_ORYSI
6.5
6.0
5.5
5.0
4.5
1
2
t−Test: p−value = 0.56

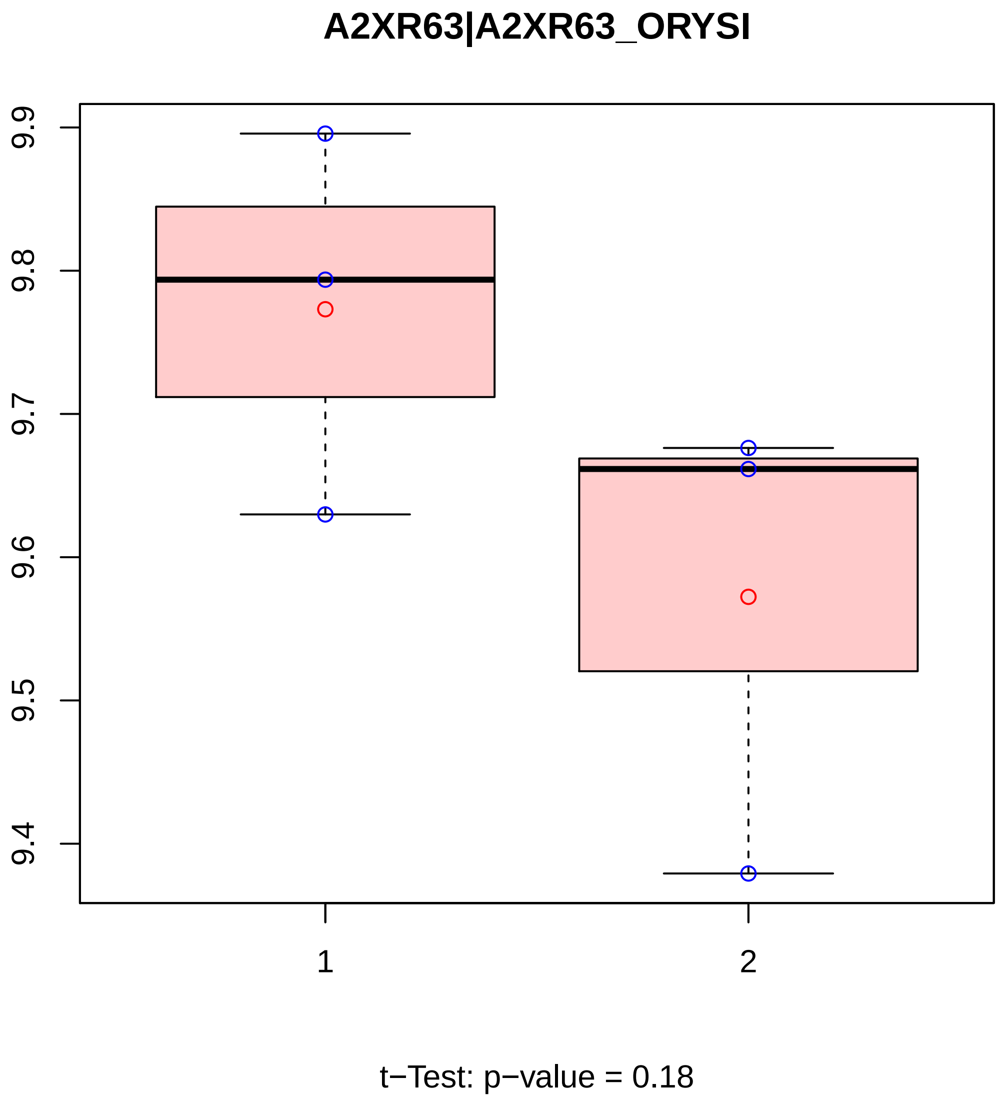
A2XR63|A2XR63_ORYSI
9.9
9.8
9.7
9.6
9.5
9.4
1
2
t−Test: p−value = 0.18

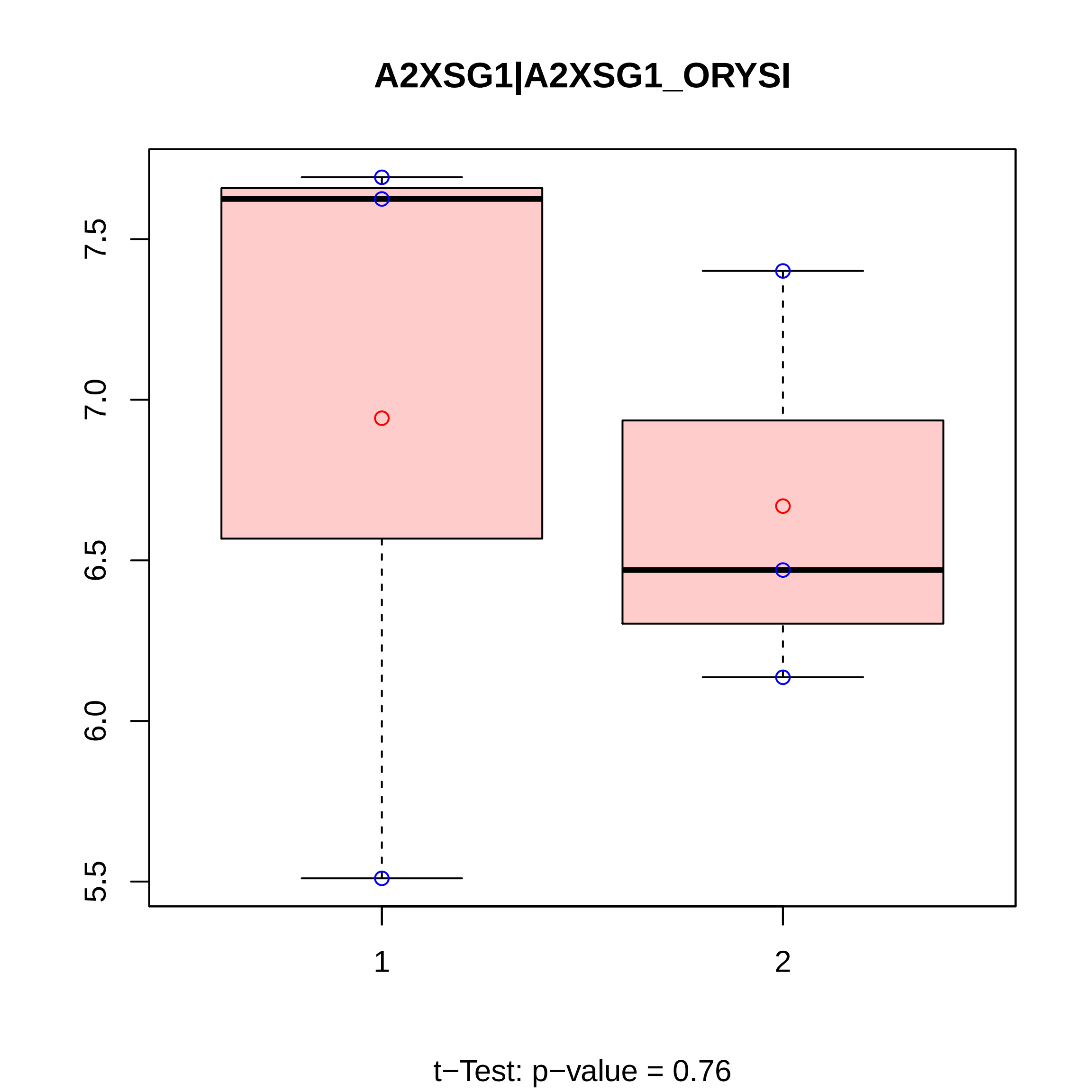
A2XSG1|A2XSG1_ORYSI
7.5
7.0
6.5
6.0
5.5
1
2
t−Test: p−value = 0.76

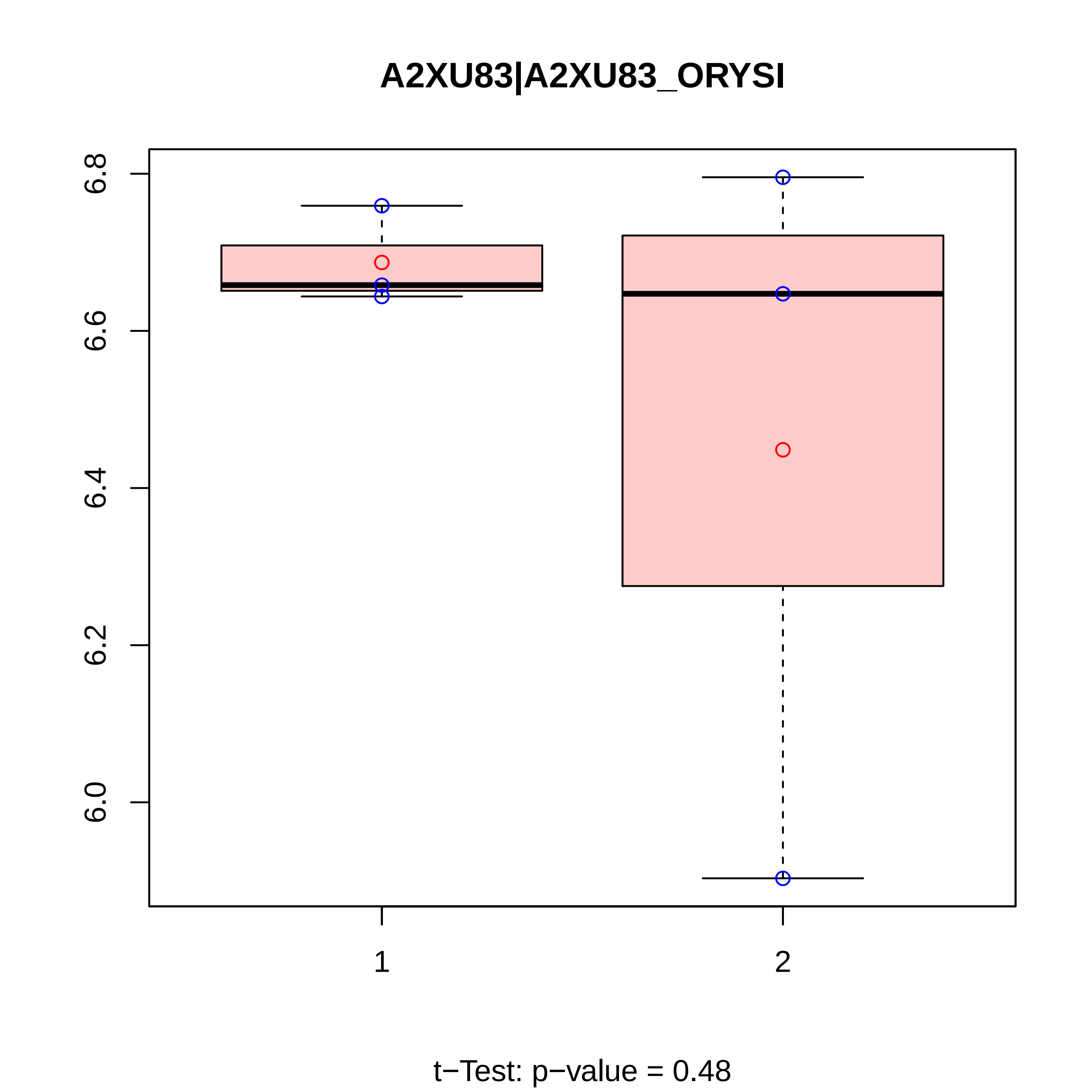
A2XU83|A2XU83_ORYSI
6.0
6.2
6.4
6.6
6.8
1
2
t−Test: p−value = 0.48

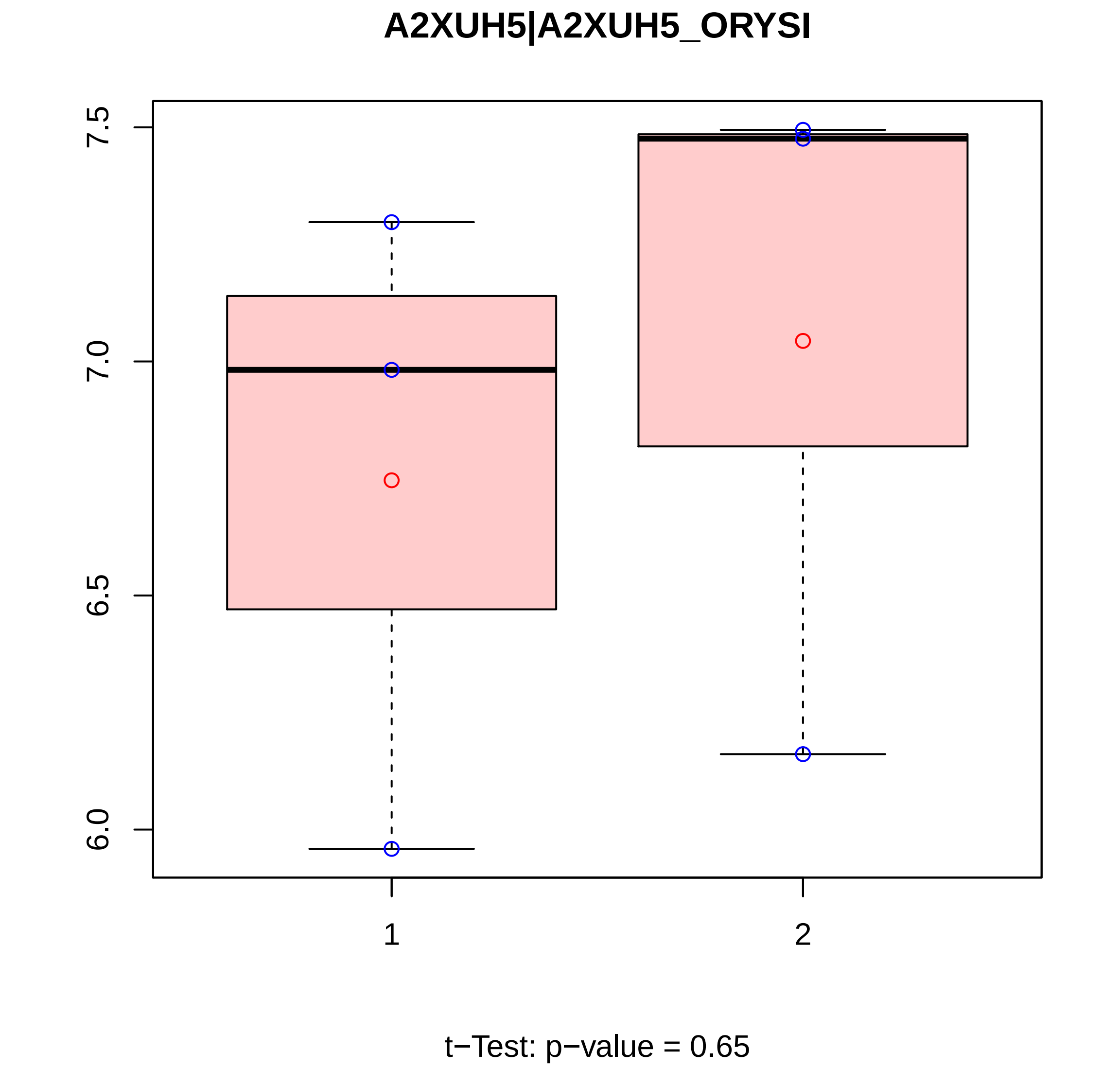
A2XUH5|A2XUH5_ORYSI
7.5
7.0
6.5
6.0
1
2
t−Test: p−value = 0.65

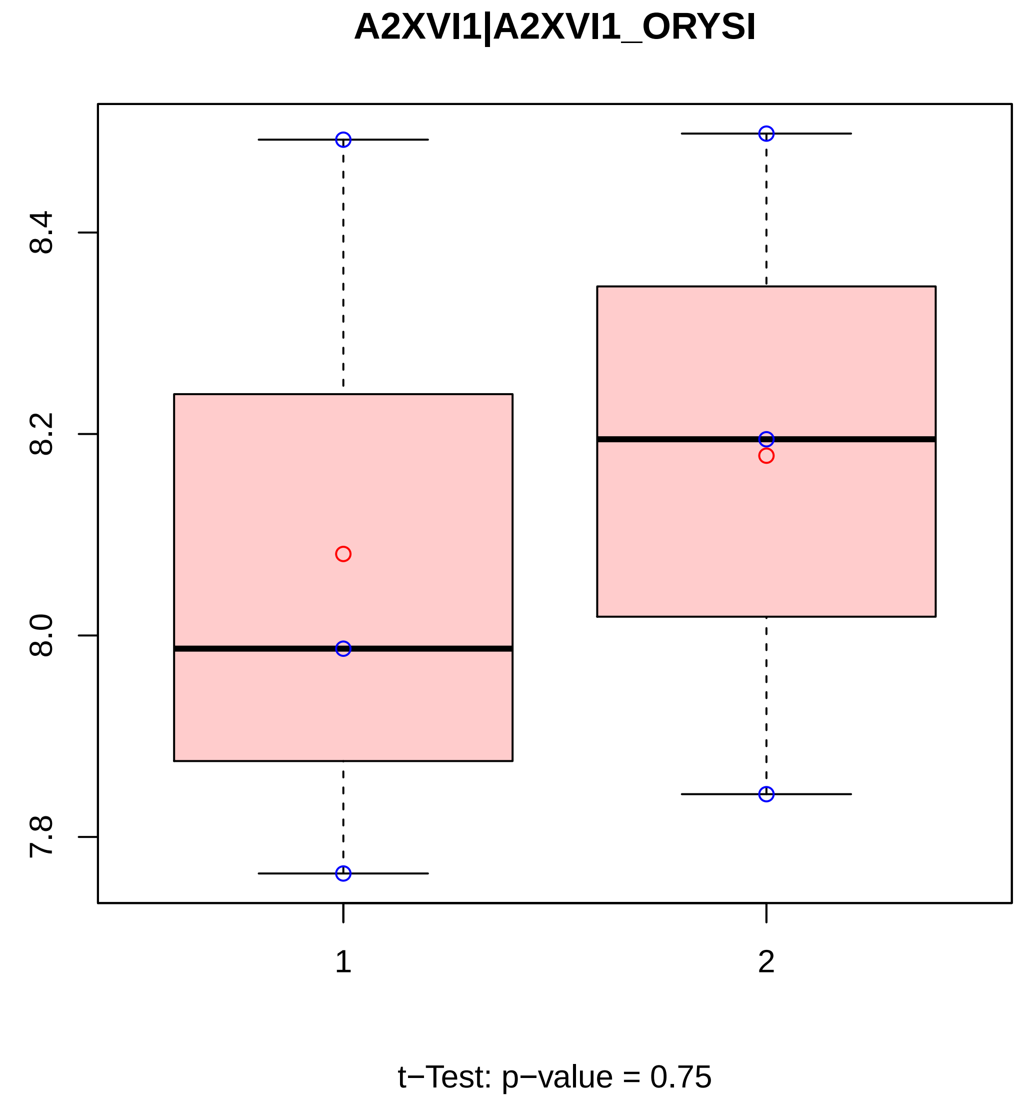
A2XVI1|A2XVI1_ORYSI
8.4
8.2
8.0
7.8
1
2
t−Test: p−value = 0.75

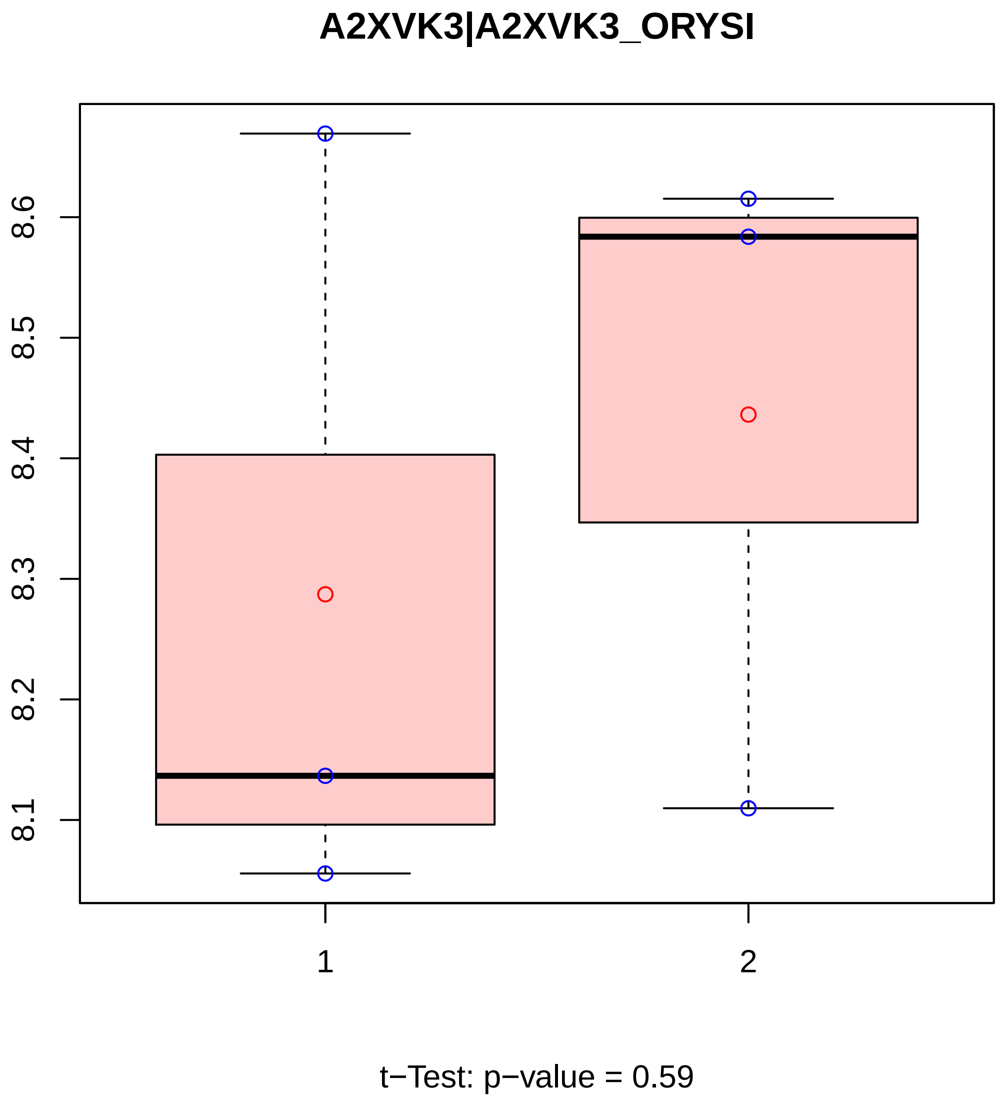

A2XVK3|A2XVK3_ORYSI
8.1
8.2
8.3
8.4
8.5
8.6
1
2
t−Test: p−value = 0.59

A2XVV0|A2XVV0_ORYSI

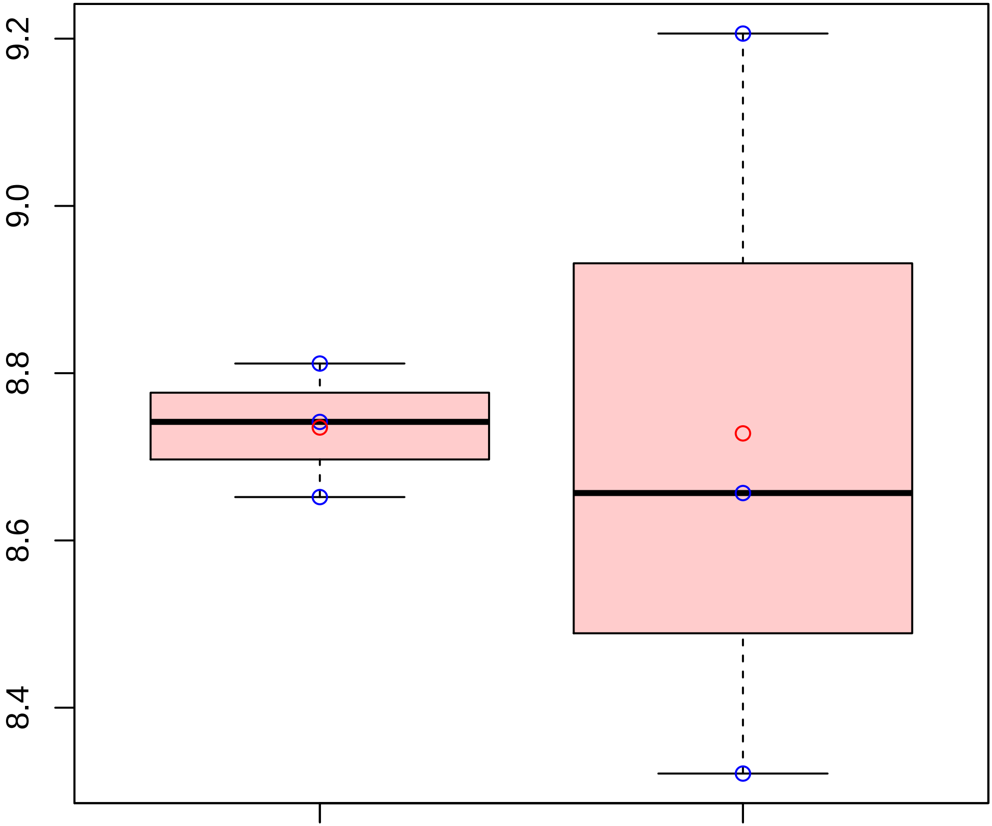

t−Test: p−value = 0.98

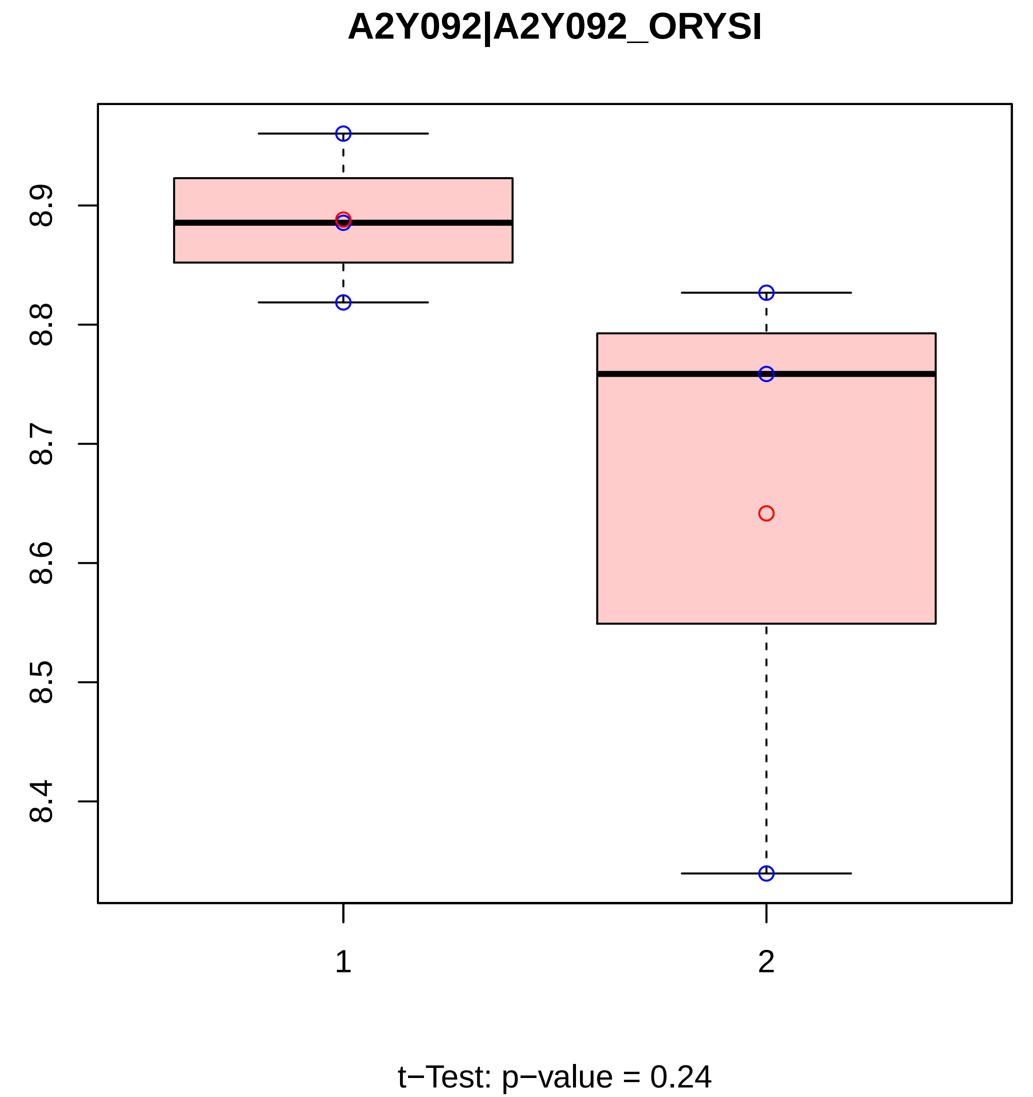
A2Y092|A2Y092_ORYSI
8.4
8.5
8.6
8.7
8.8
8.9
1
2
t−Test: p−value = 0.24

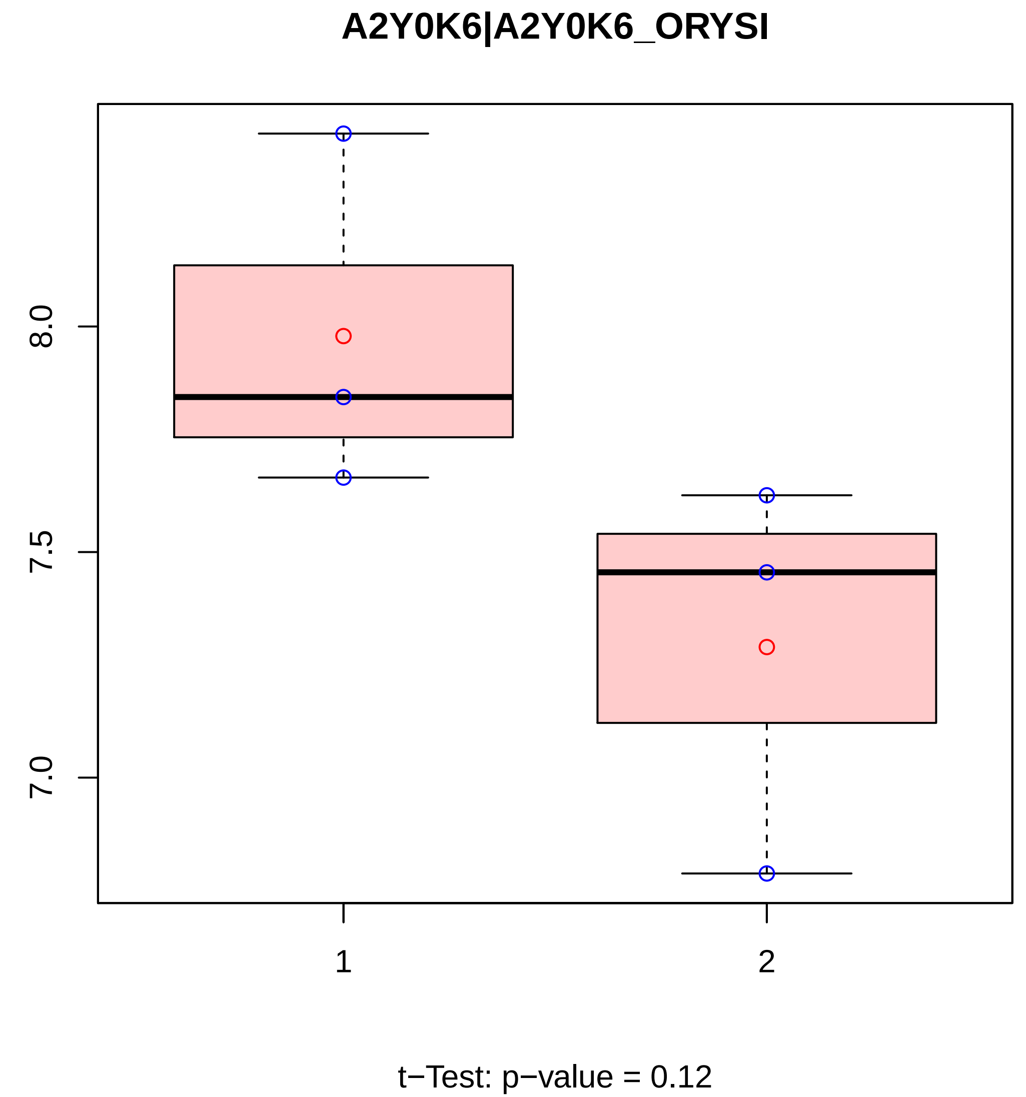
A2Y0K6|A2Y0K6_ORYSI
8.0
7.5
7.0
1
2
t−Test: p−value = 0.12

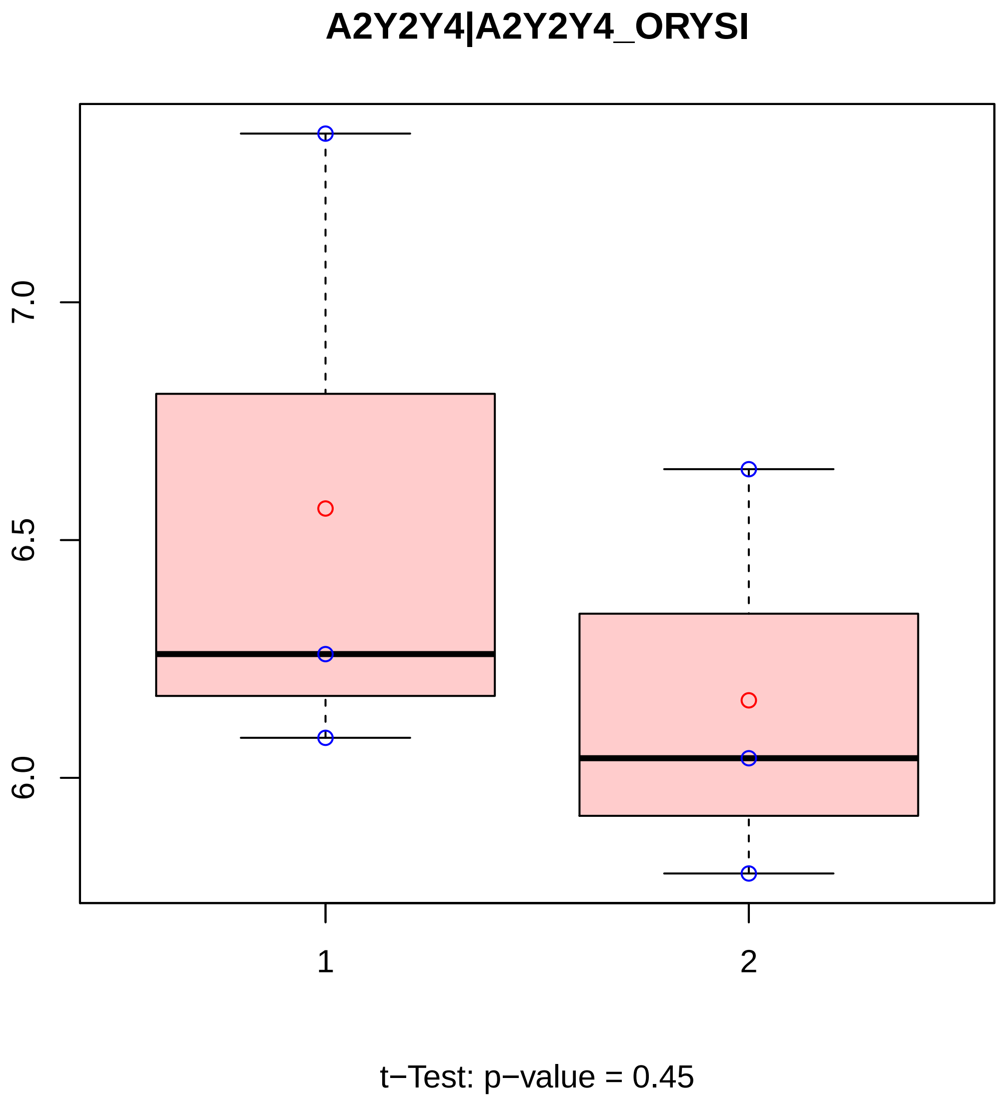
A2Y2Y4|A2Y2Y4_ORYSI
7.0
6.5
6.0
1
2
t−Test: p−value = 0.45

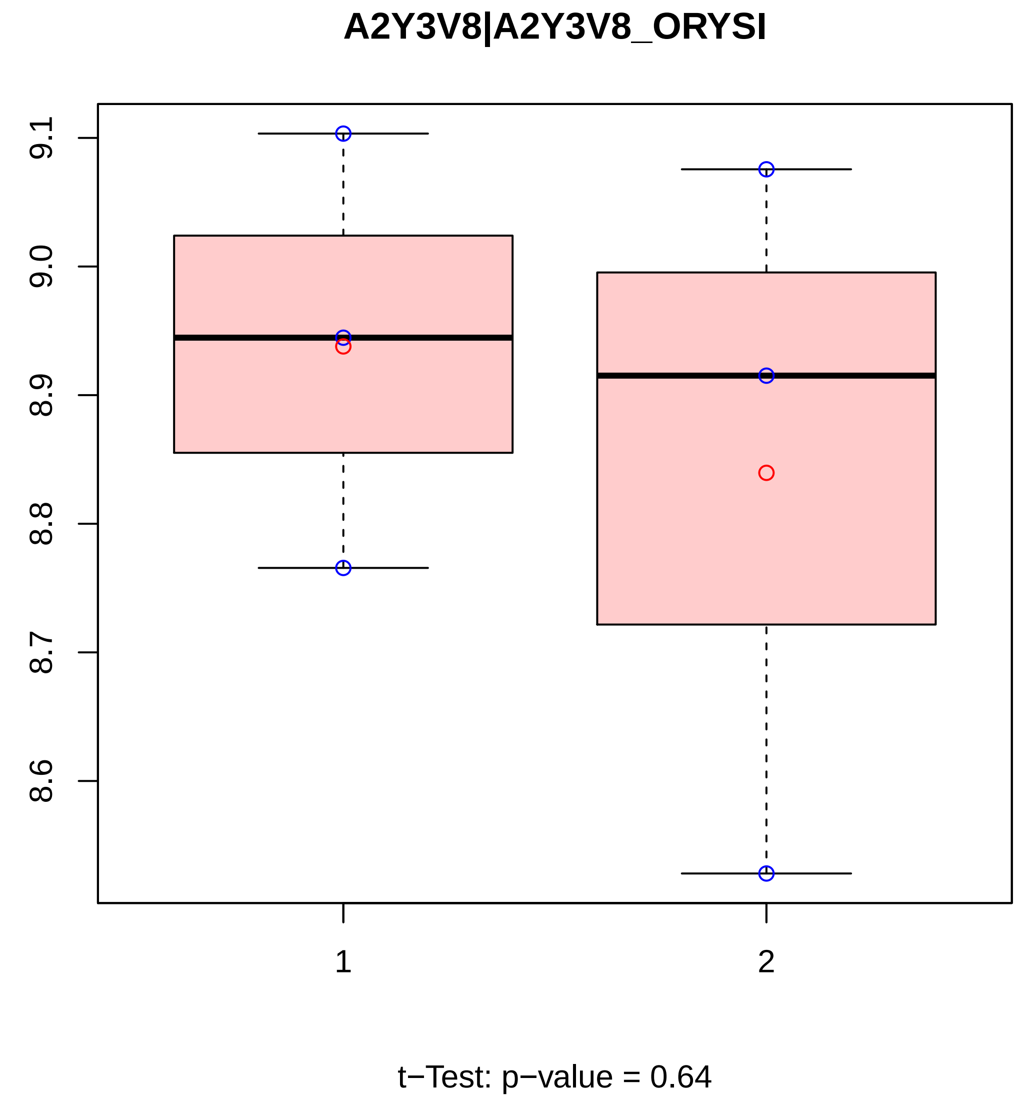

A2Y3V8|A2Y3V8_ORYSI
9.1
9.0
8.9
8.8
8.7
8.6
1
2
t−Test: p−value = 0.64

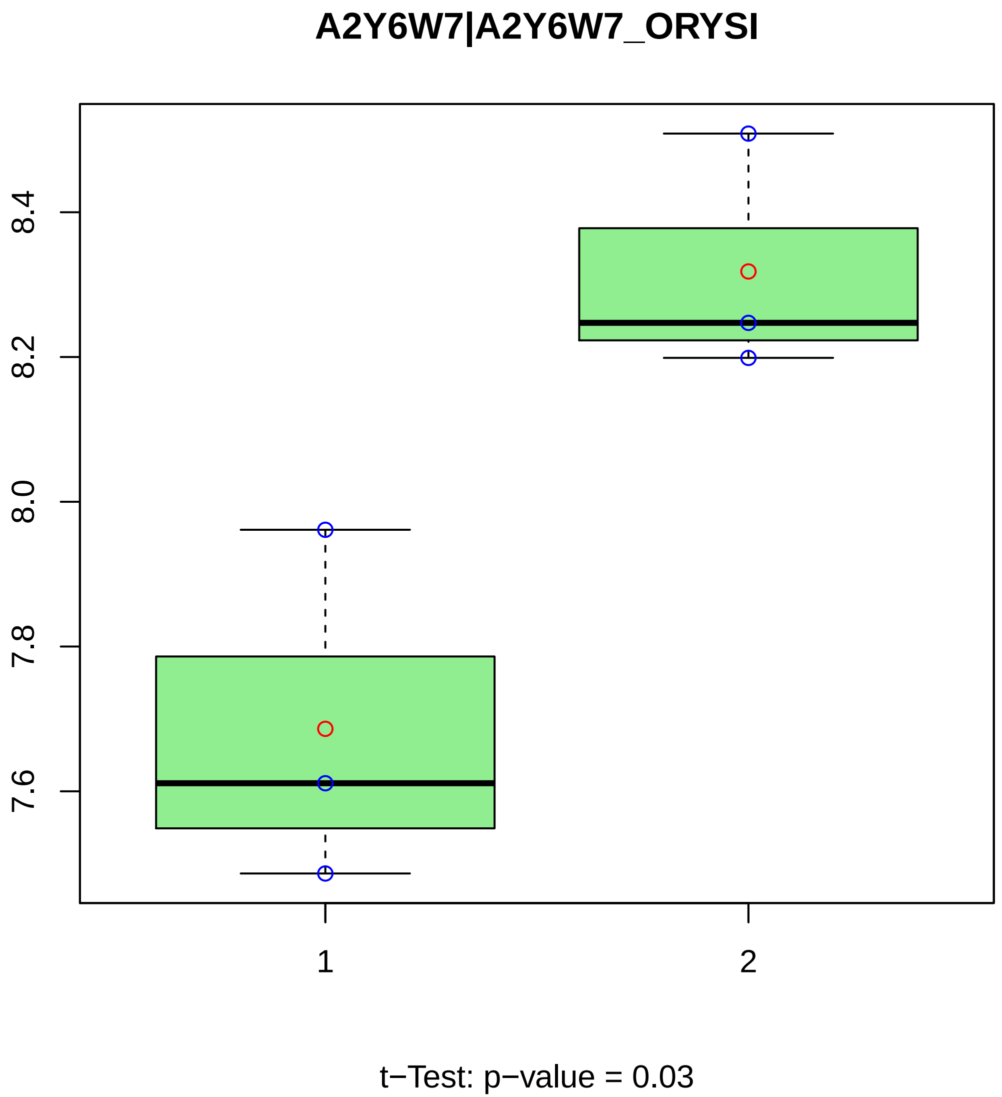
A2Y6W7|A2Y6W7_ORYSI
8.4
8.2
8.0
7.8
7.6
1
2
t−Test: p−value = 0.03

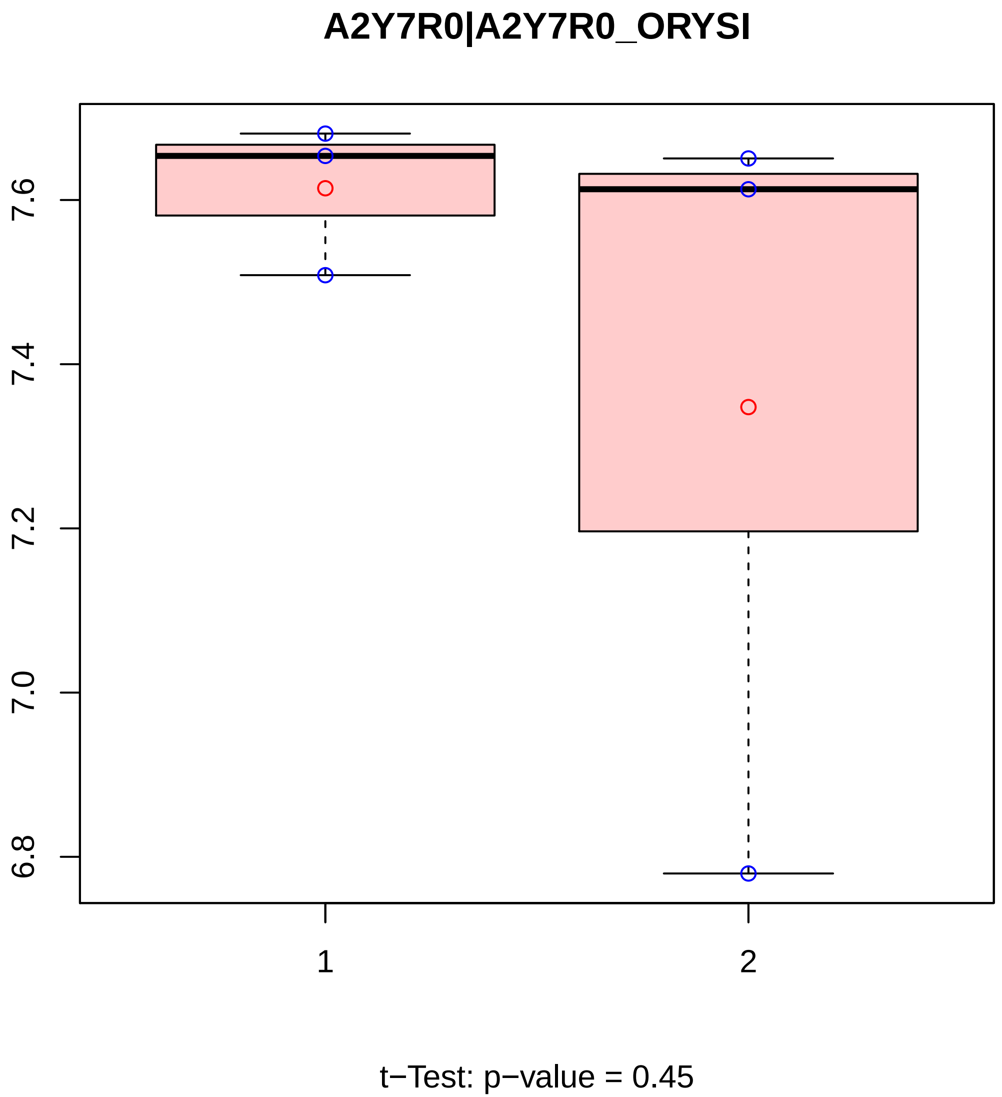

A2Y7R0|A2Y7R0_ORYSI
7.6
7.4
7.2
7.0
6.8
1
2
t−Test: p−value = 0.45

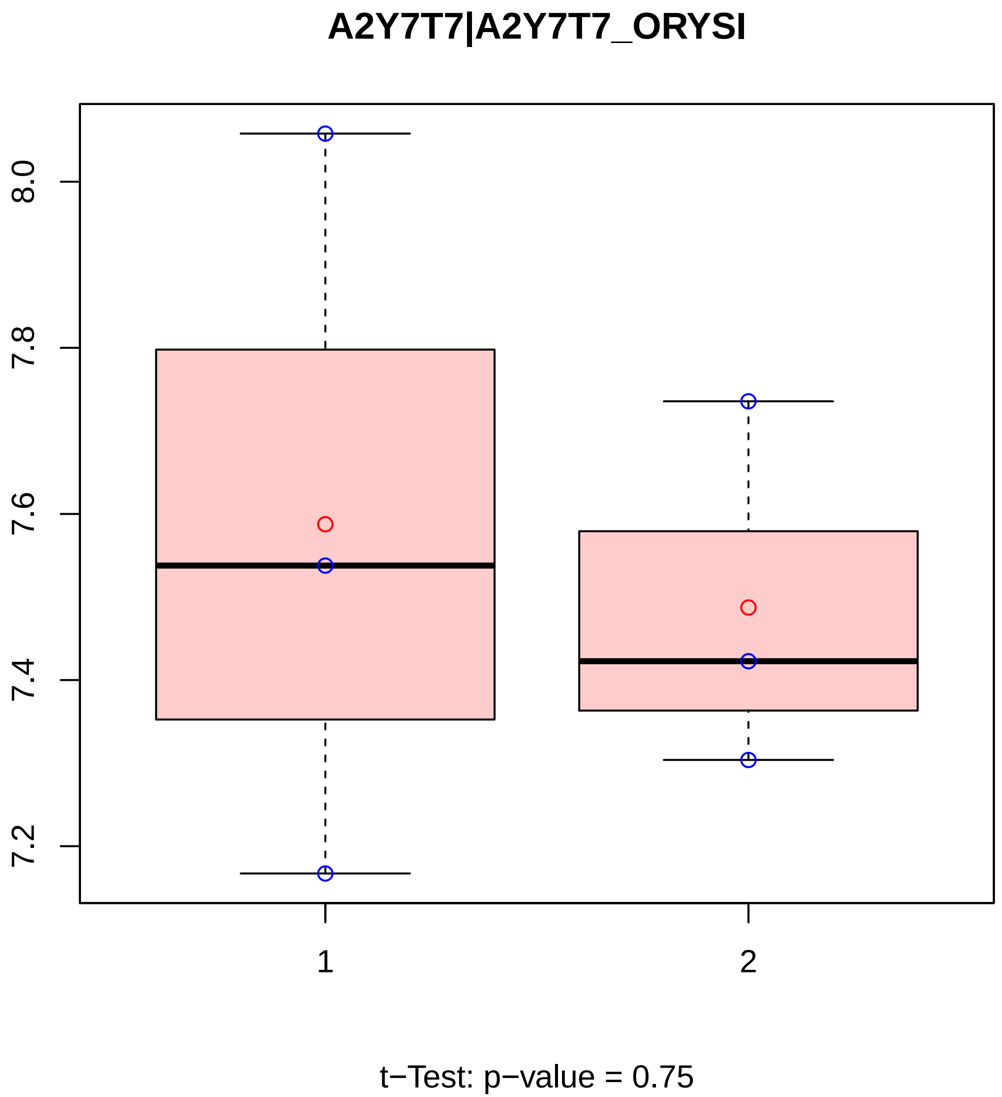
A2Y7T7|A2Y7T7_ORYSI
8.0
7.8
7.6
7.4
7.2
1
2
t−Test: p−value = 0.75

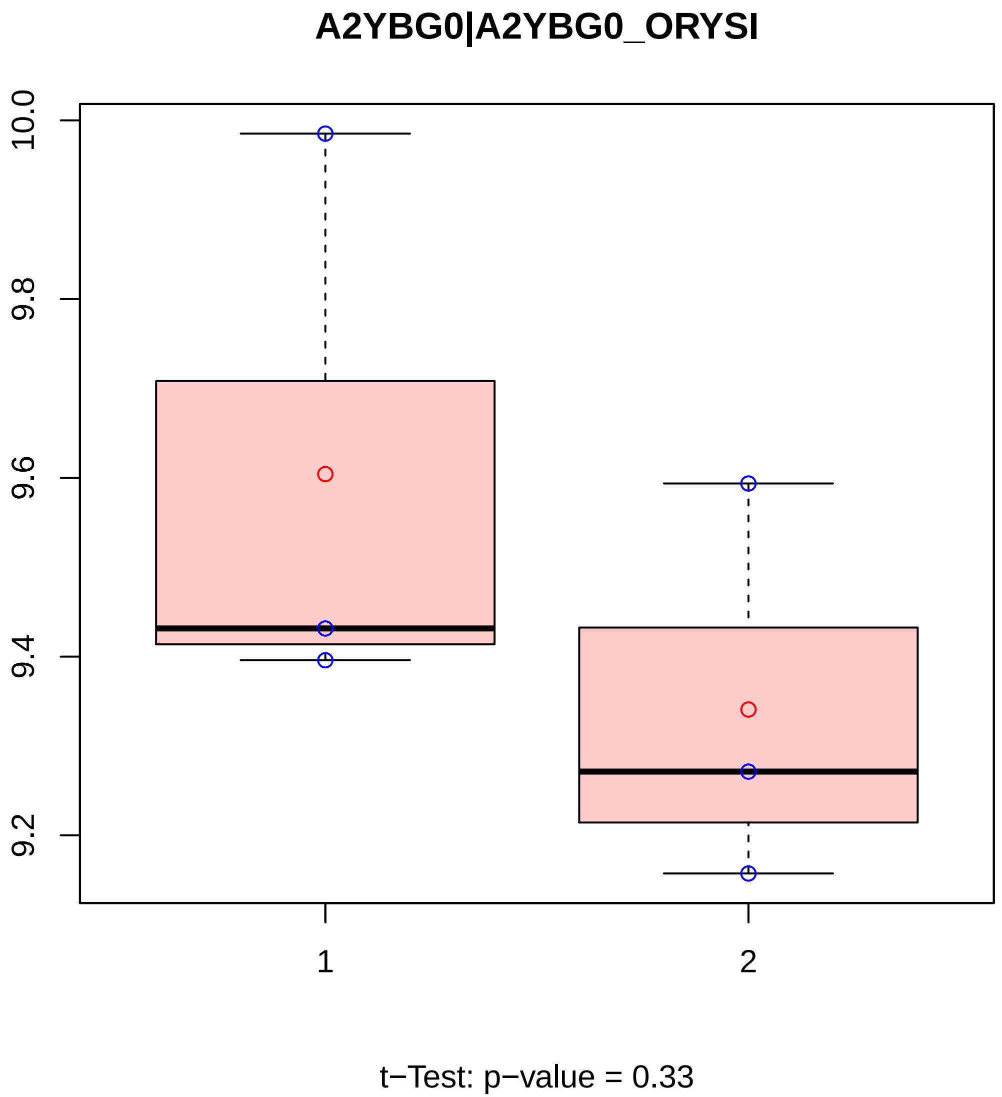
A2YBG0|A2YBG0_ORYSI
10.0
9.8
9.6
9.4
9.2
1
2
t−Test: p−value = 0.33

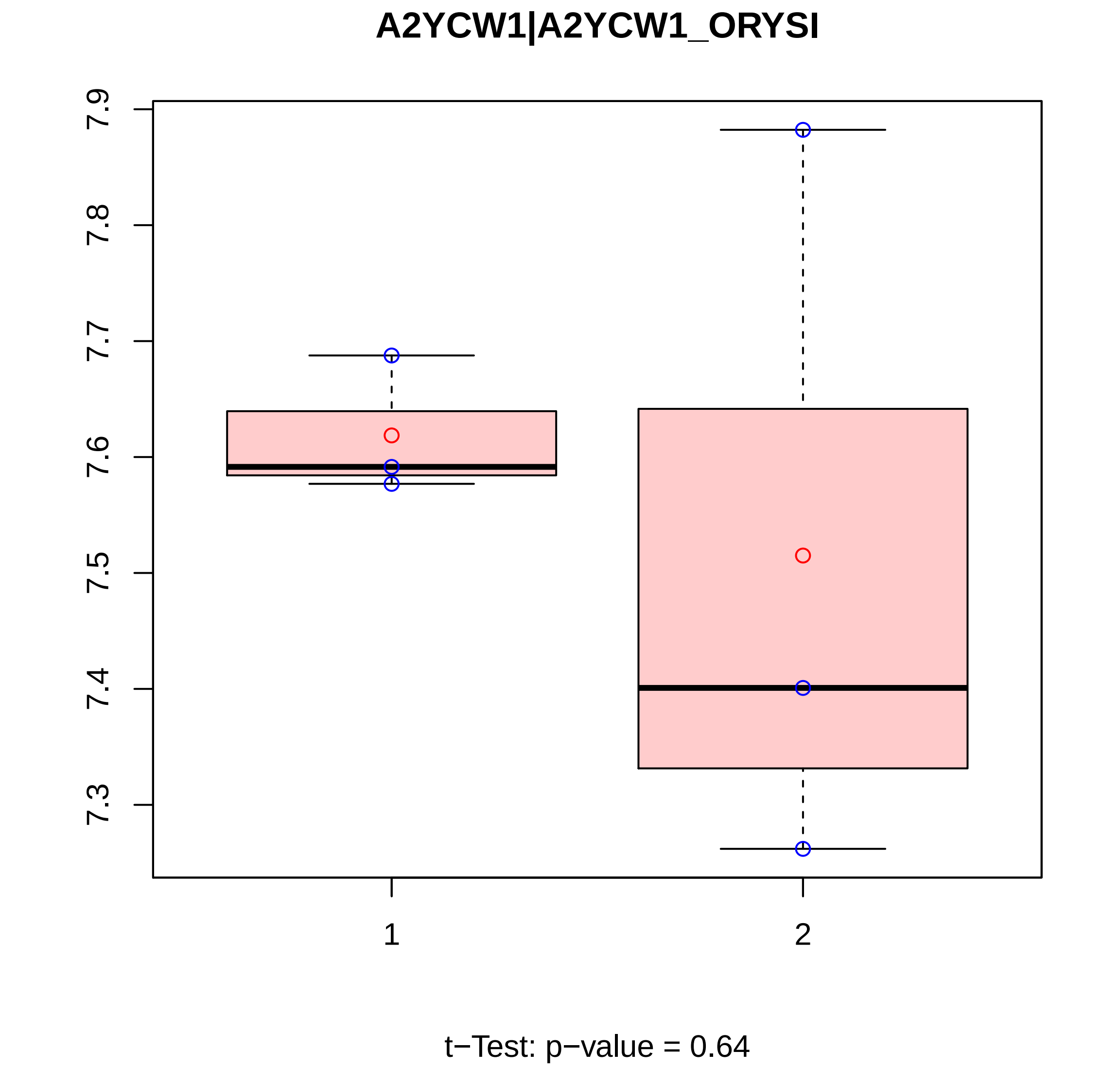

A2YCW1|A2YCW1_ORYSI
7.9
7.8
7.7
7.6
7.5
7.4
7.3
1
2
t−Test: p−value = 0.64

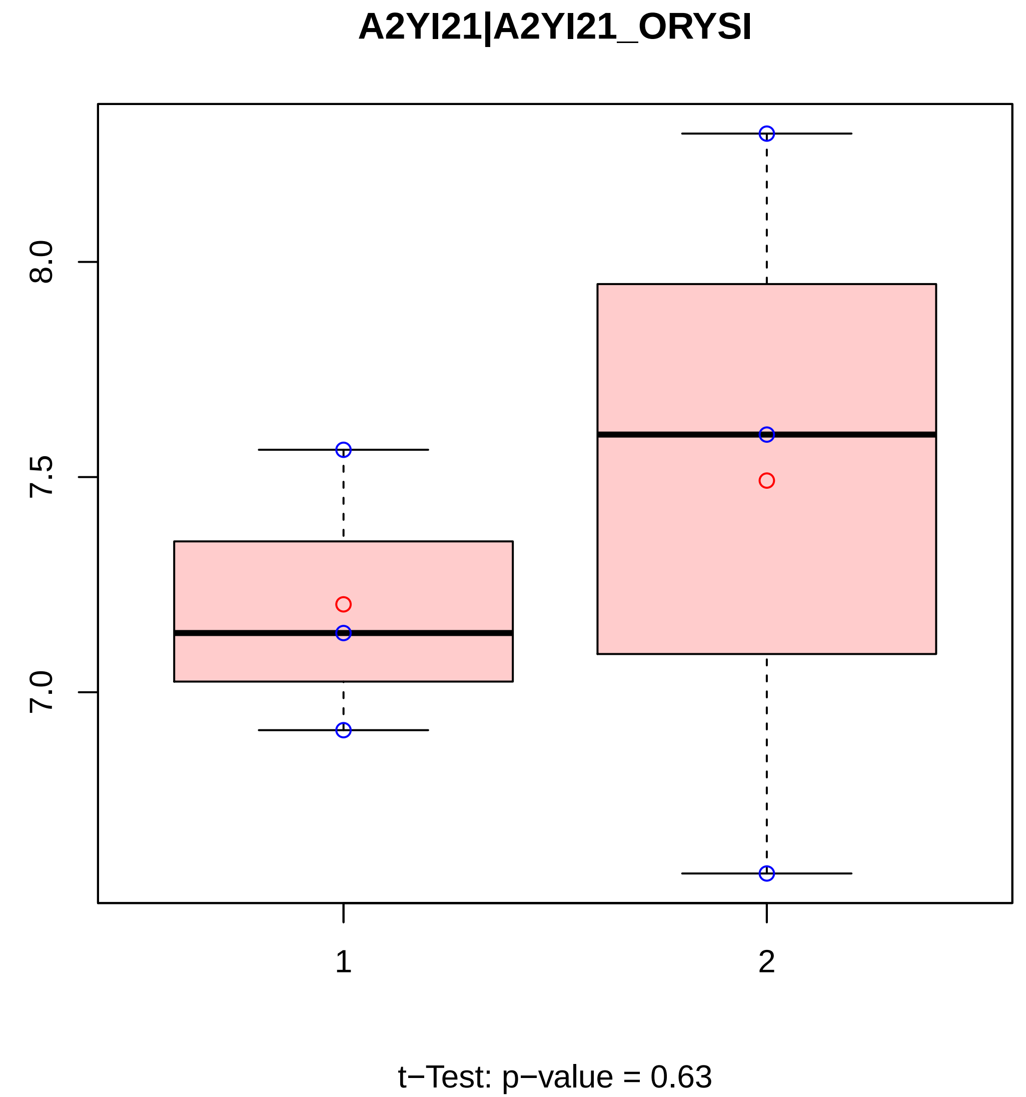
A2YI21|A2YI21_ORYSI
8.0
7.5
7.0
1
2
t−Test: p−value = 0.63

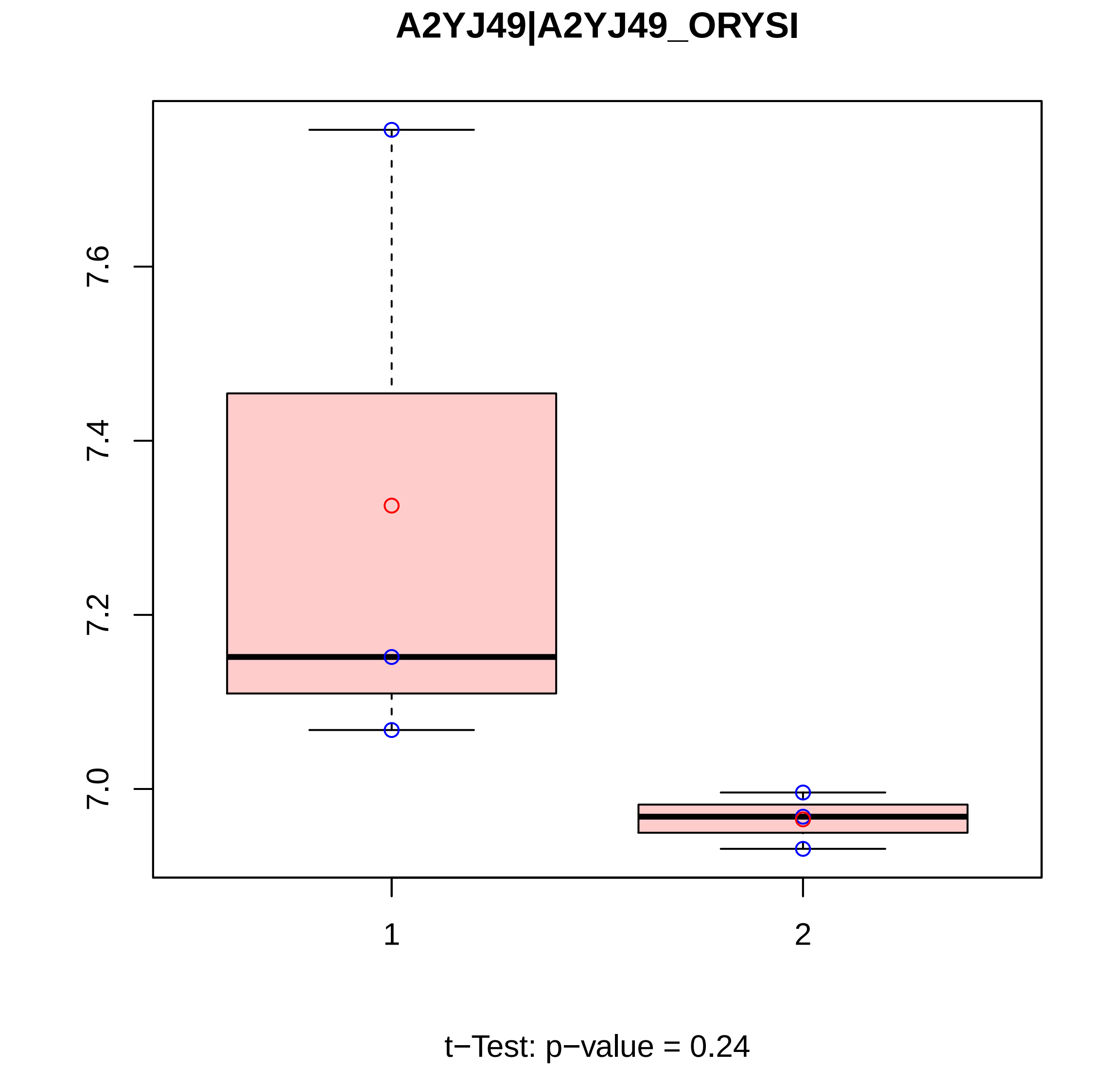
A2YJ49|A2YJ49_ORYSI
7.0
7.2
7.4
7.6
1
2
t−Test: p−value = 0.24

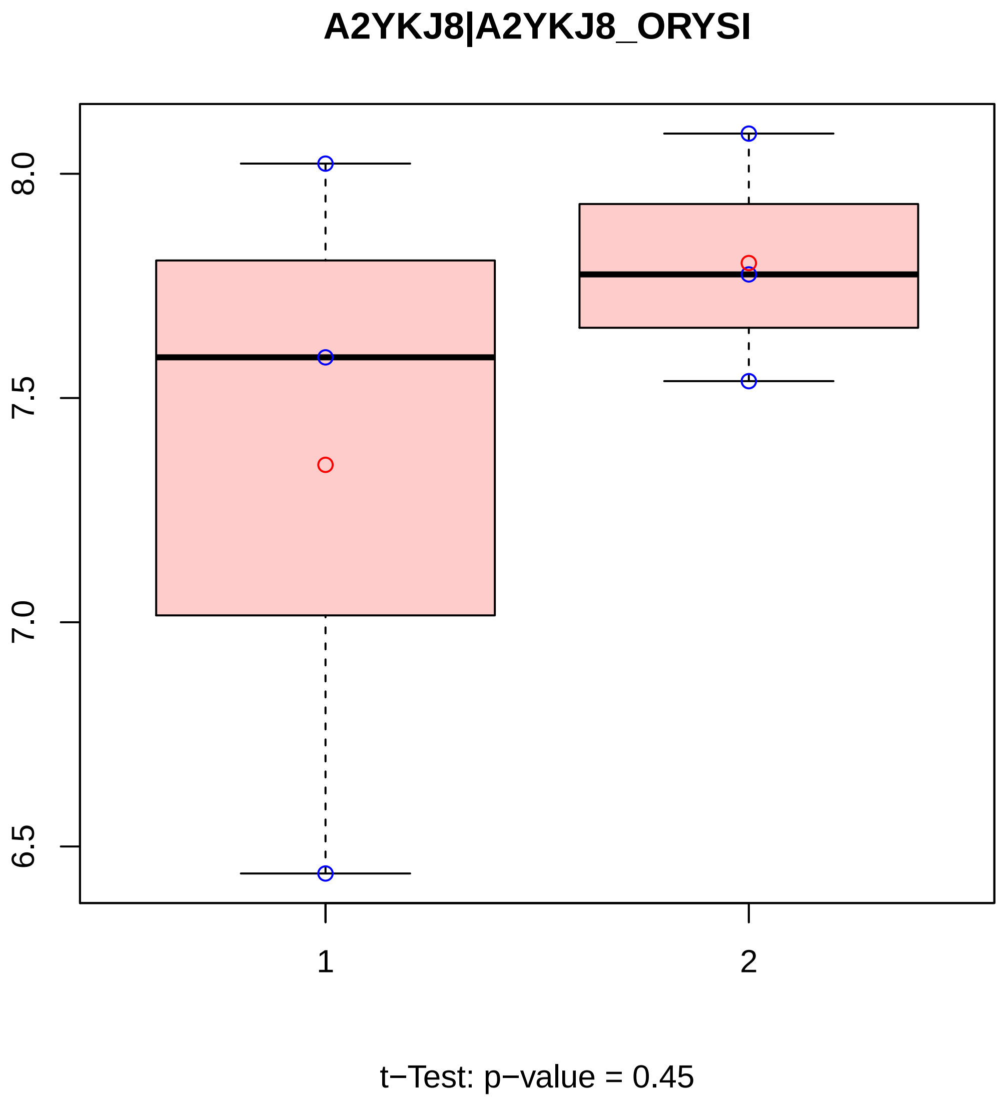

A2YKJ8|A2YKJ8_ORYSI
8.0
7.5
7.0
6.5
1
2
t−Test: p−value = 0.45

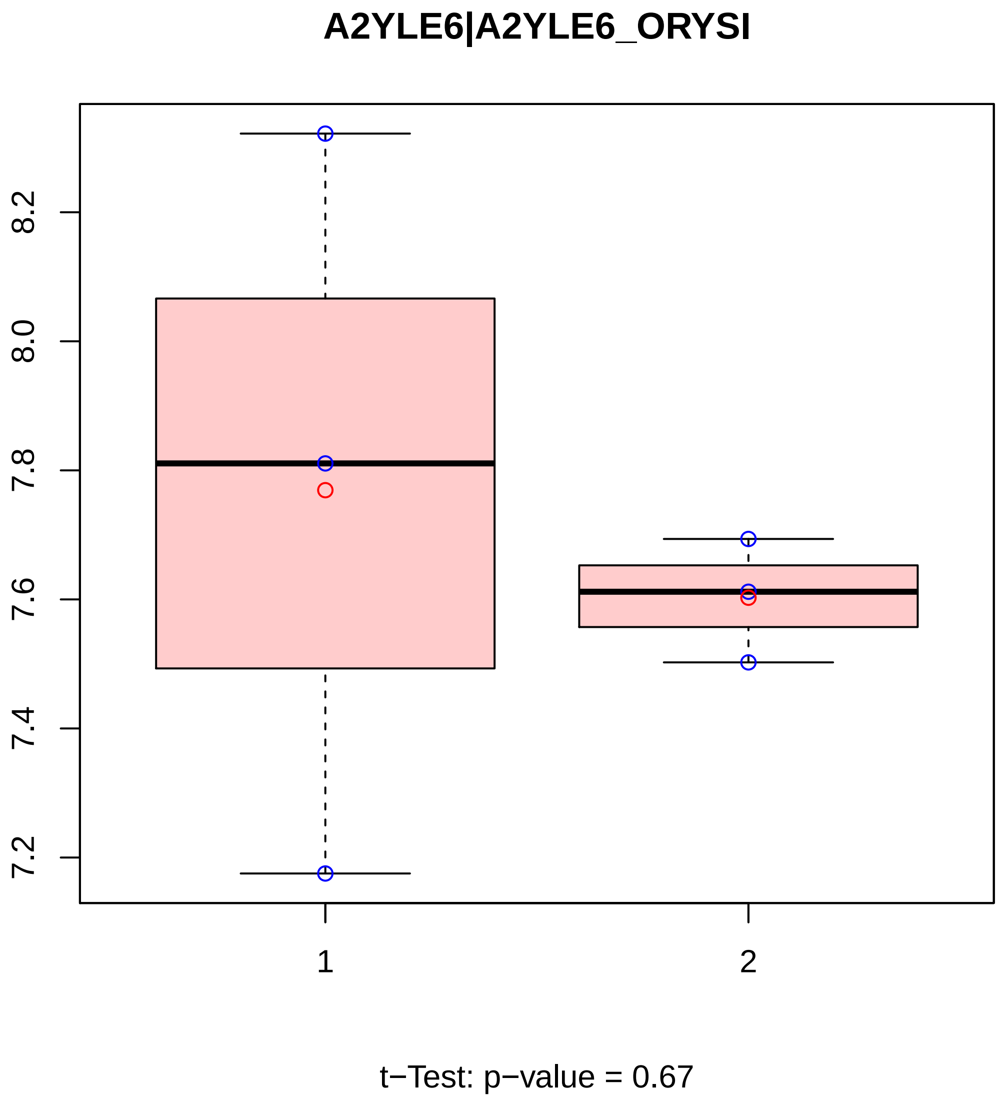

A2YLE6|A2YLE6_ORYSI
8.2
8.0
7.8
7.6
7.4
7.2
1
2
t−Test: p−value = 0.67

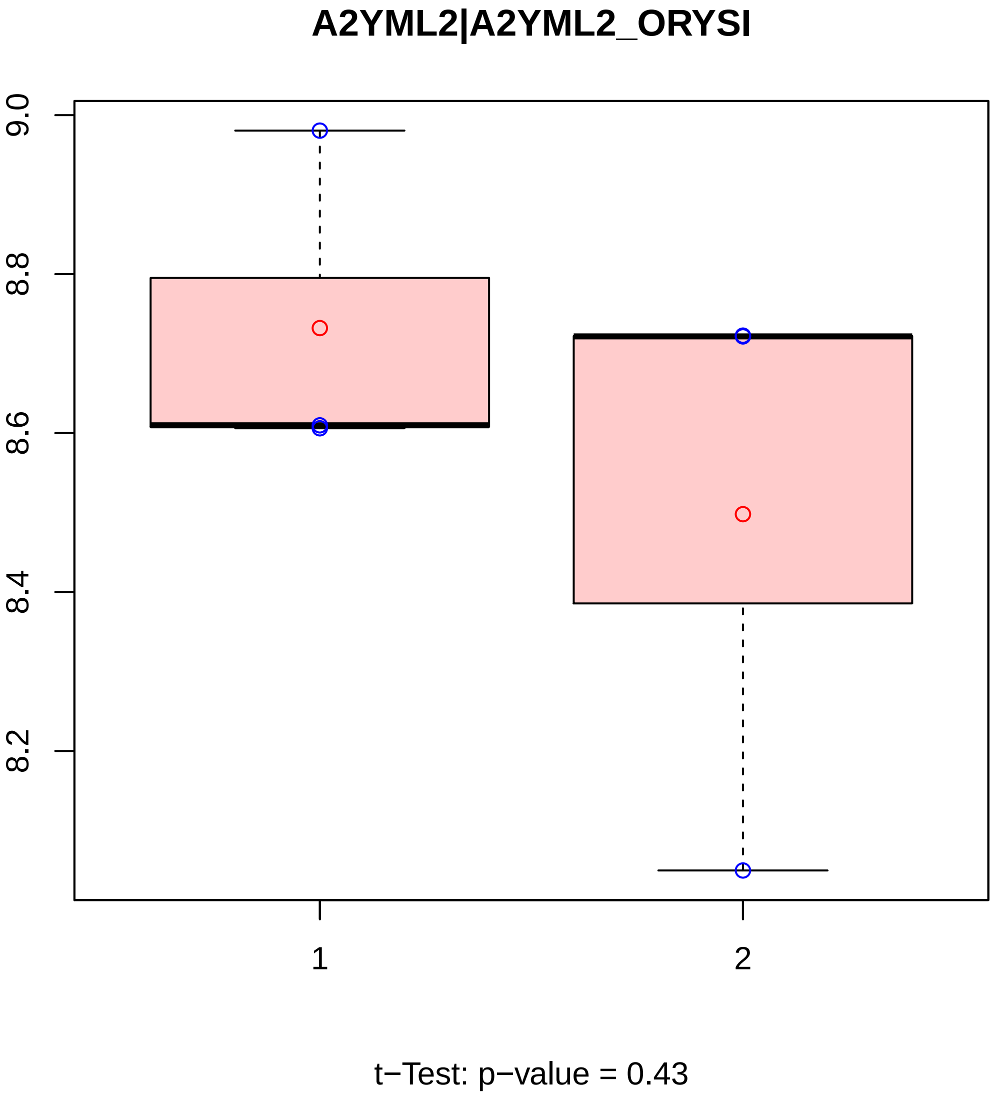

A2YML2|A2YML2_ORYSI
9.0
8.8
8.6
8.4
8.2
1
2
t−Test: p−value = 0.43

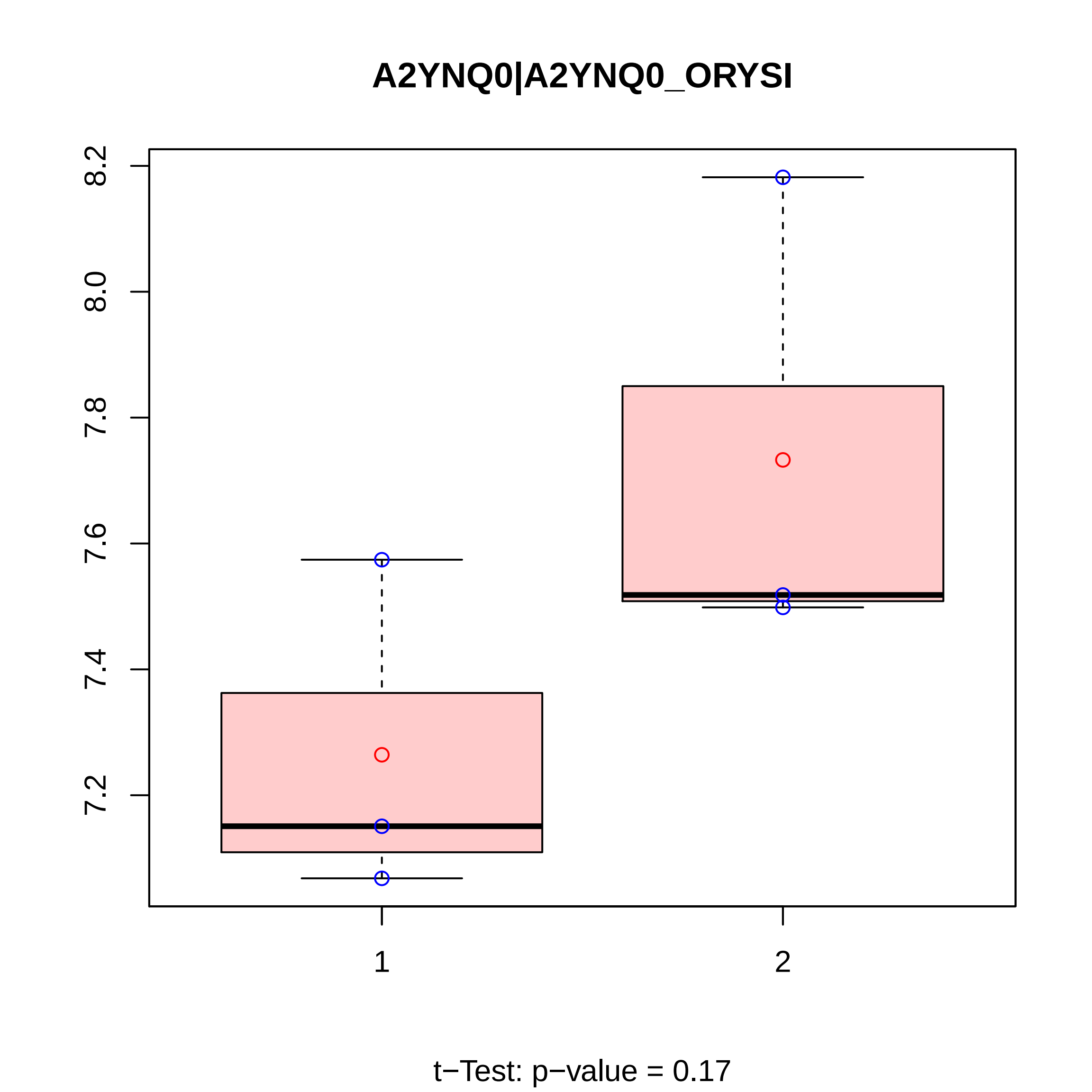
A2YNQ0|A2YNQ0_ORYSI
8.2
8.0
7.8
7.6
7.4
7.2
1
2
t−Test: p−value = 0.17

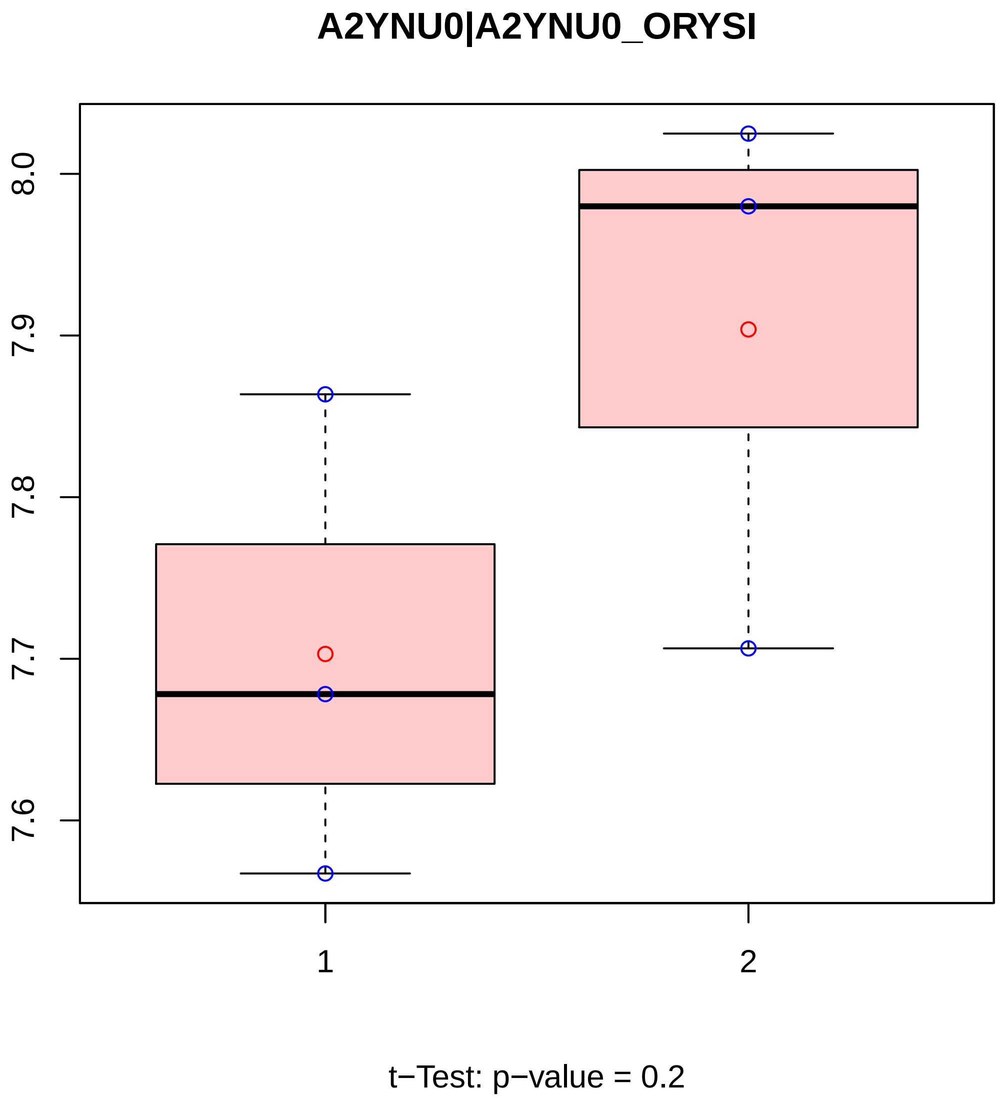

A2YNU0|A2YNU0_ORYSI
8.0
7.9
7.8
7.7
7.6
1
2
t−Test: p−value = 0.2

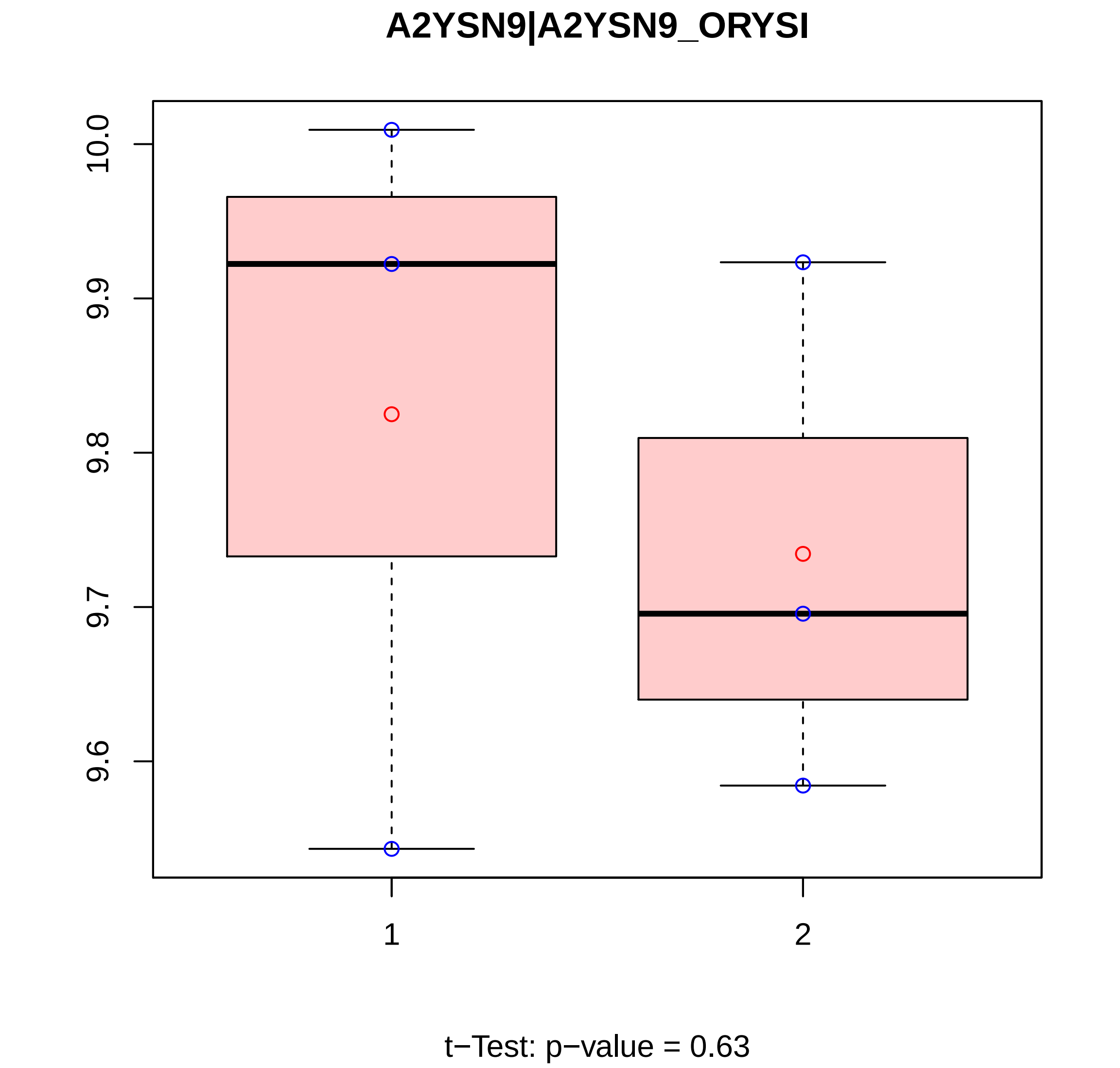
A2YSN9|A2YSN9_ORYSI
10.0
9.9
9.8
9.7
9.6
1
2
t−Test: p−value = 0.63

A2YSS8|A2YSS8_ORYSI

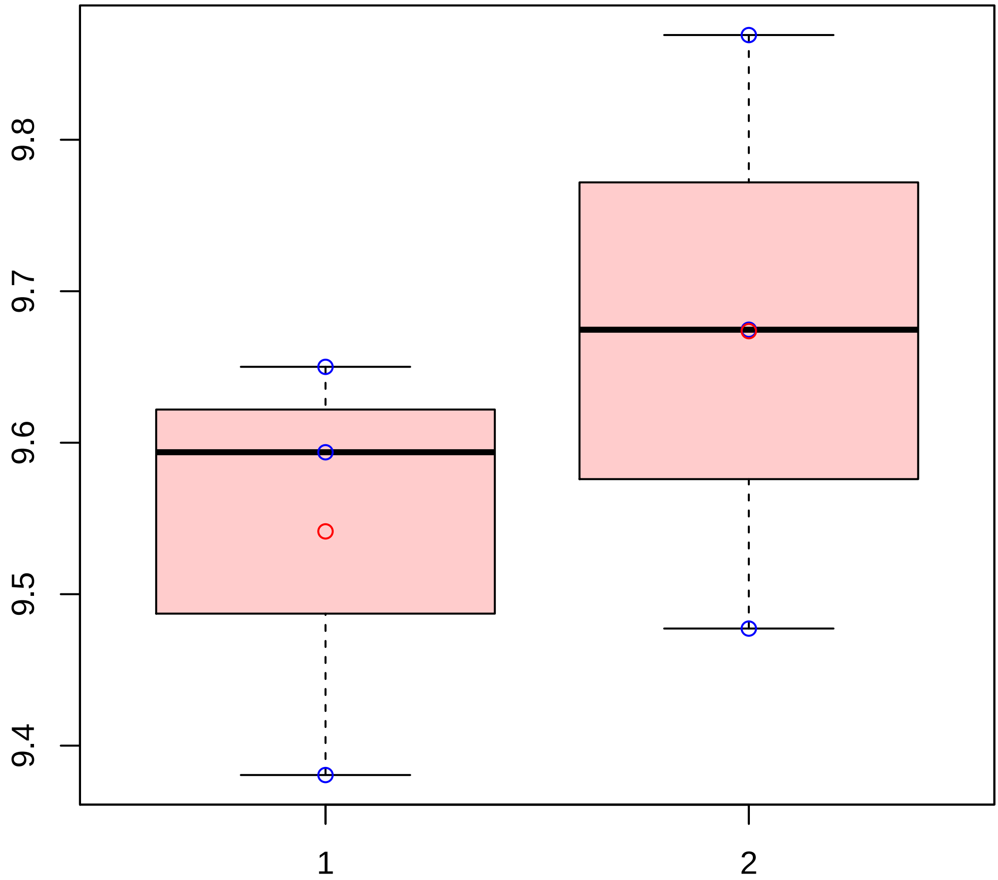

t−Test: p−value = 0.4

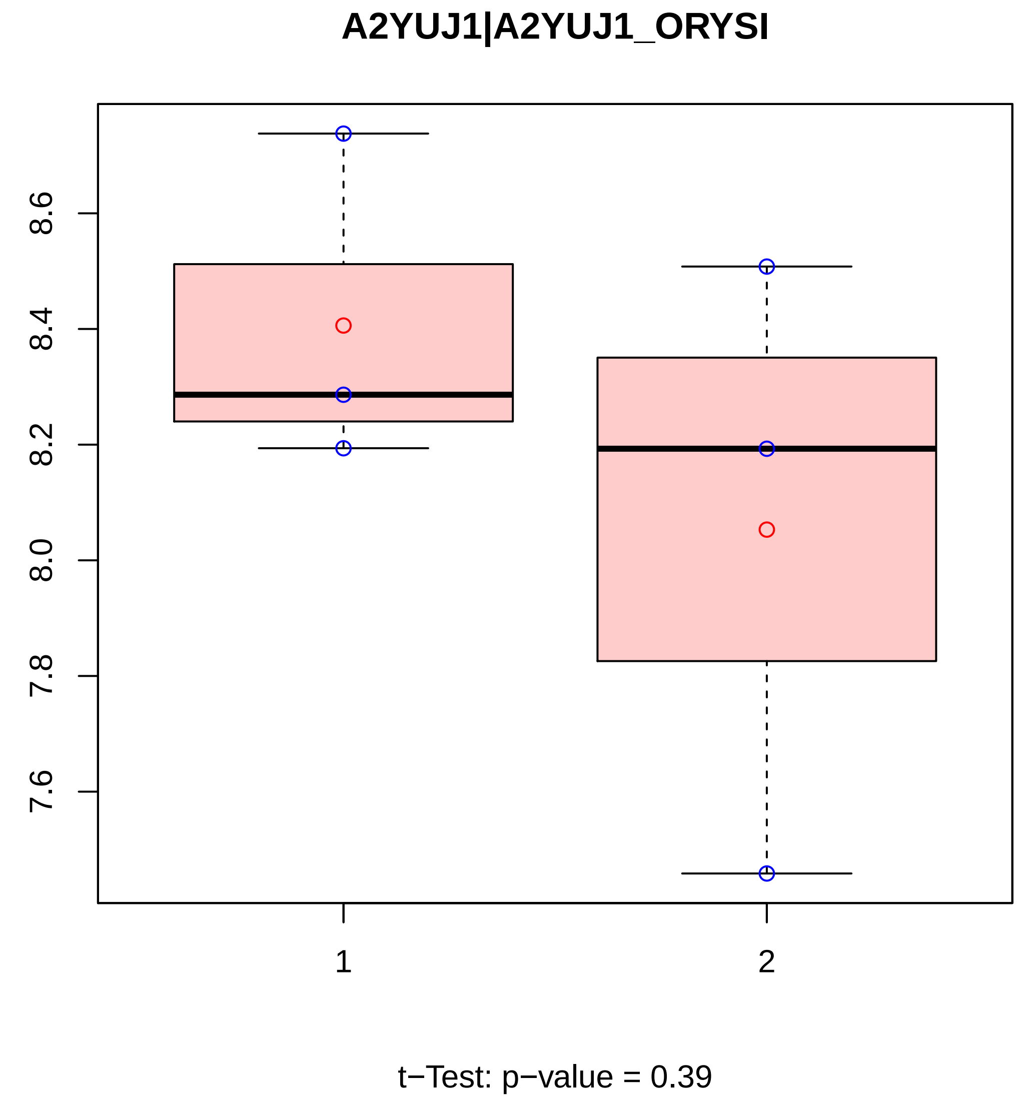

A2YUJ1|A2YUJ1_ORYSI
8.6
8.4
8.2
8.0
7.8
7.6
1
2
t−Test: p−value = 0.39

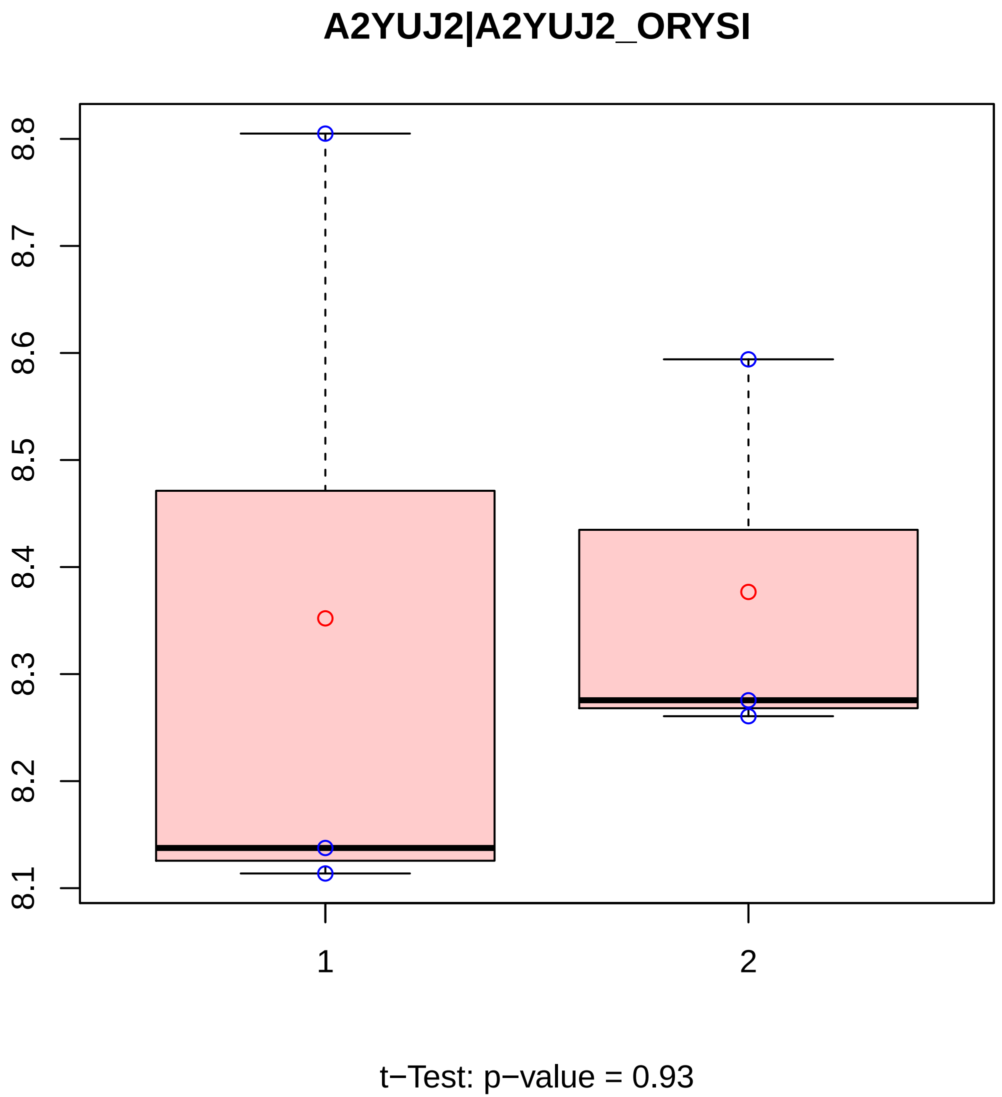
A2YUJ2|A2YUJ2_ORYSI
8.1
8.2
8.3
8.4
8.5
8.6
8.7
8.8
1
2
t−Test: p−value = 0.93

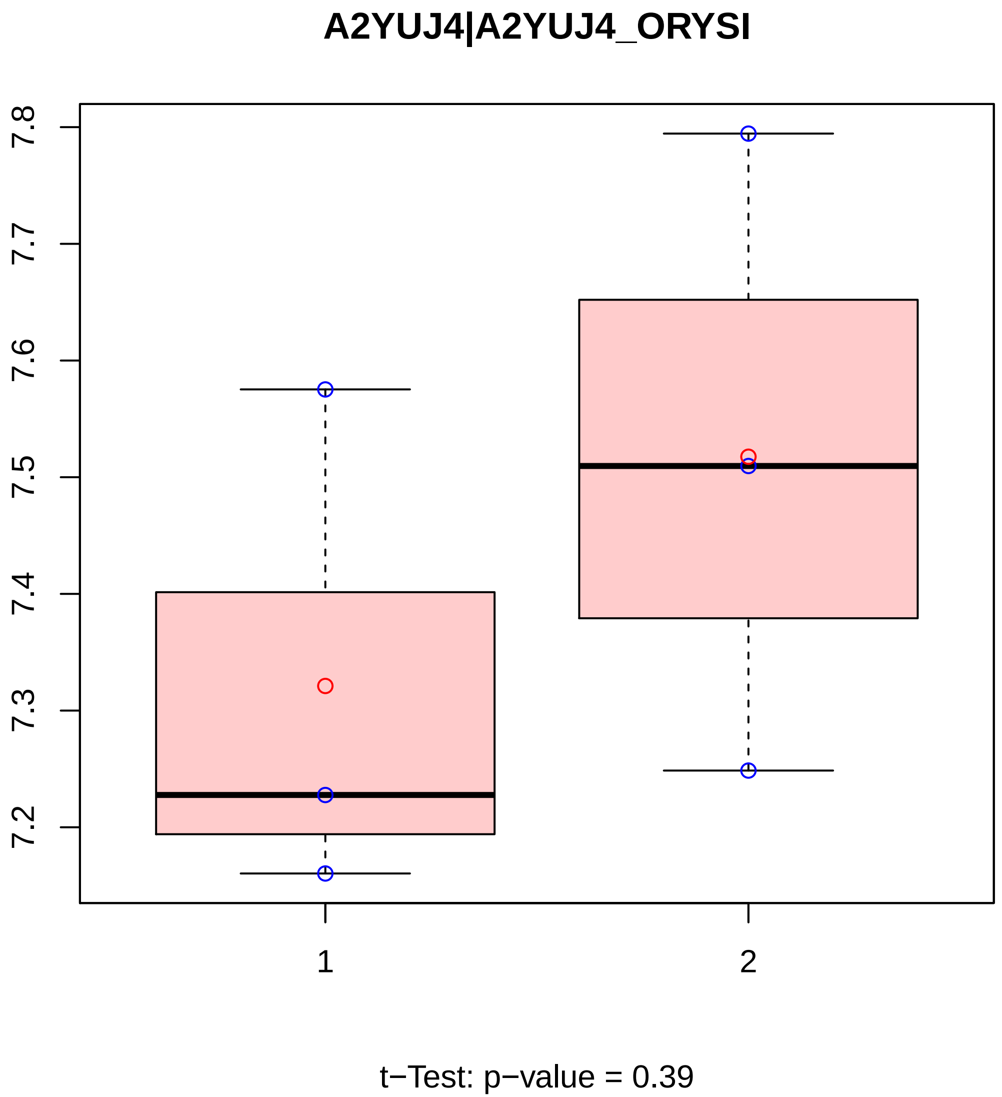

A2YUJ4|A2YUJ4_ORYSI
7.2
7.3
7.4
7.5
7.6
7.7
7.8
1
2
t−Test: p−value = 0.39

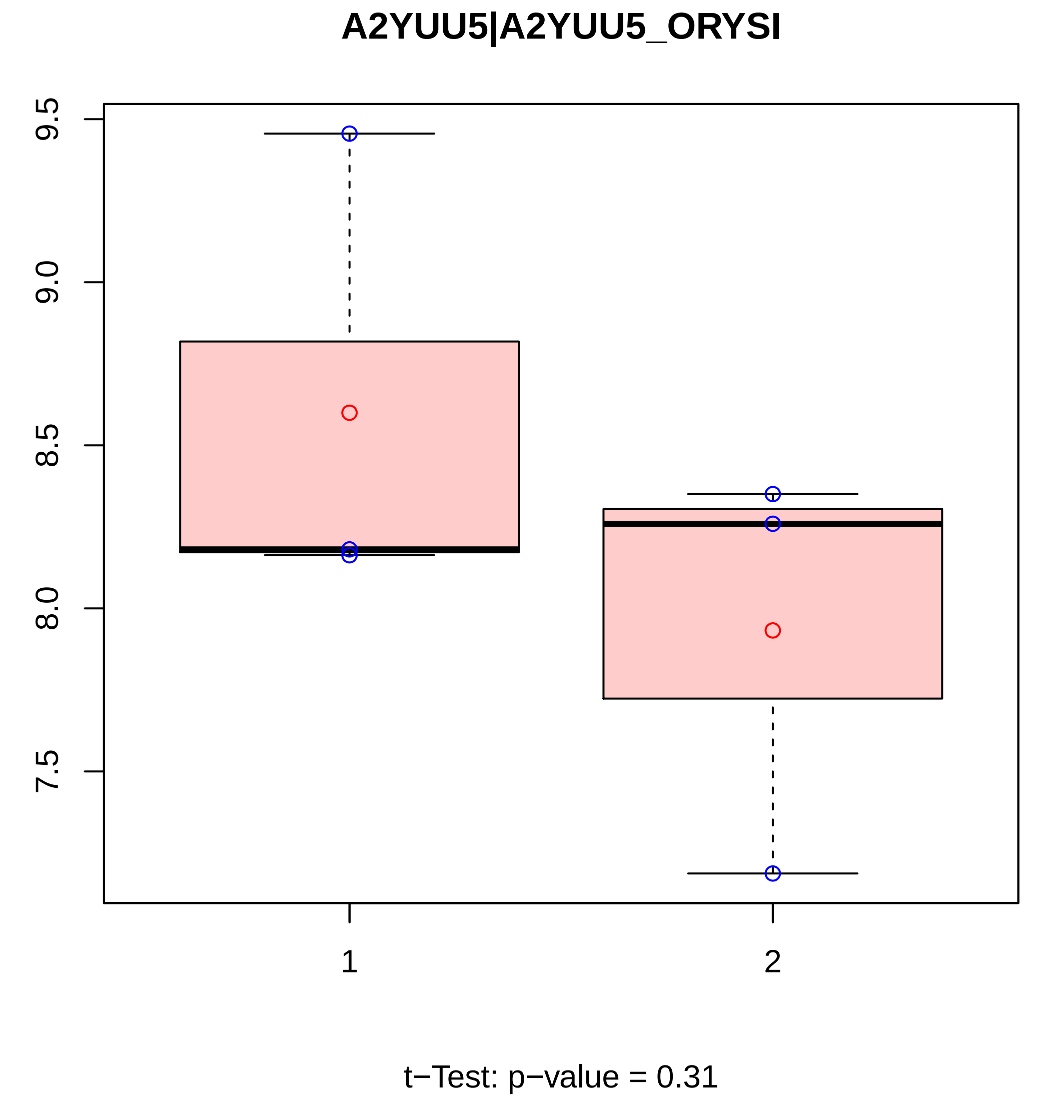

A2YUU5|A2YUU5_ORYSI
9.5
9.0
8.5
8.0
7.5
1
2
t−Test: p−value = 0.31

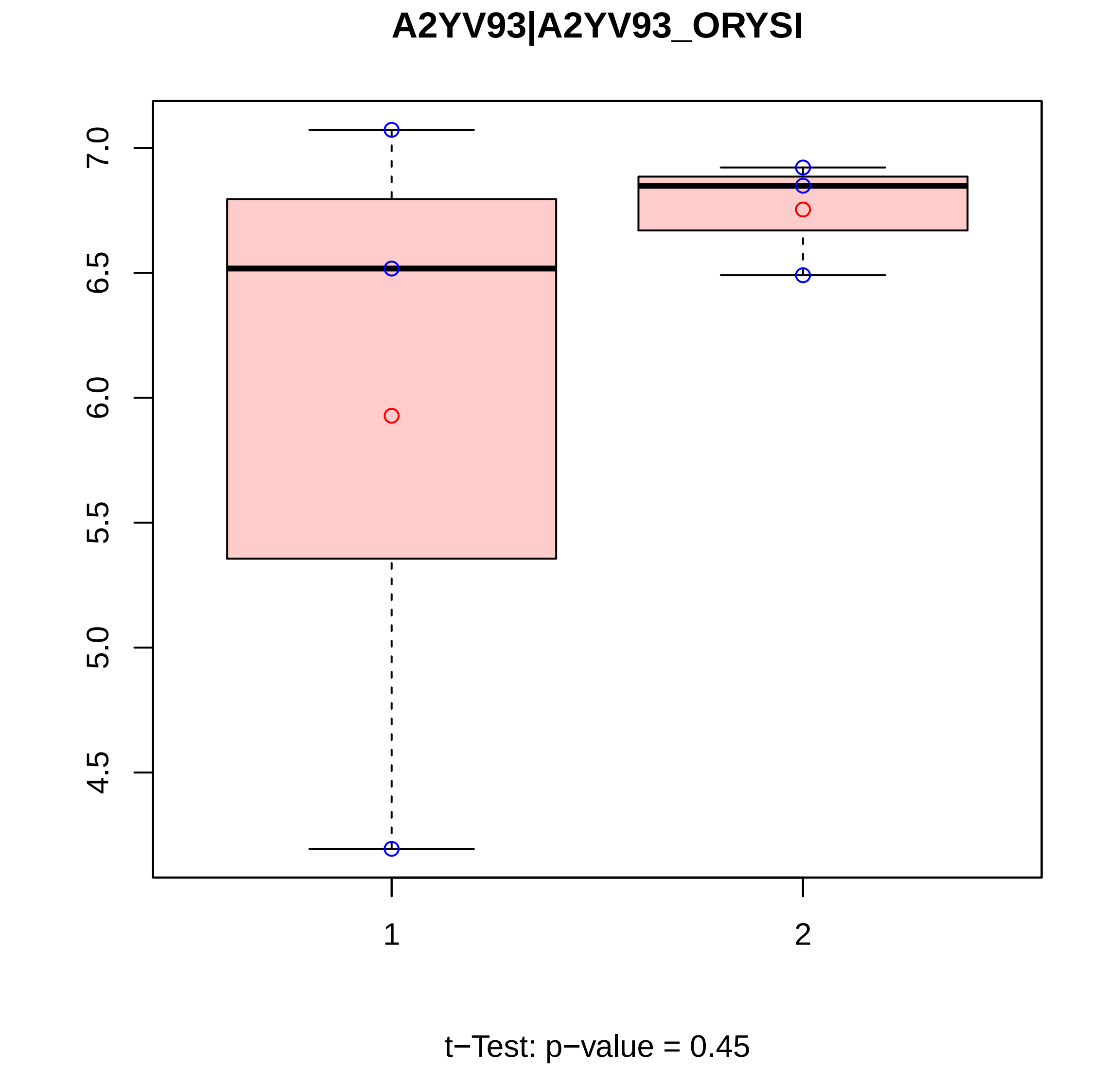

A2YV93|A2YV93_ORYSI
7.0
6.5
6.0
5.5
5.0
4.5
1
2
t−Test: p−value = 0.45

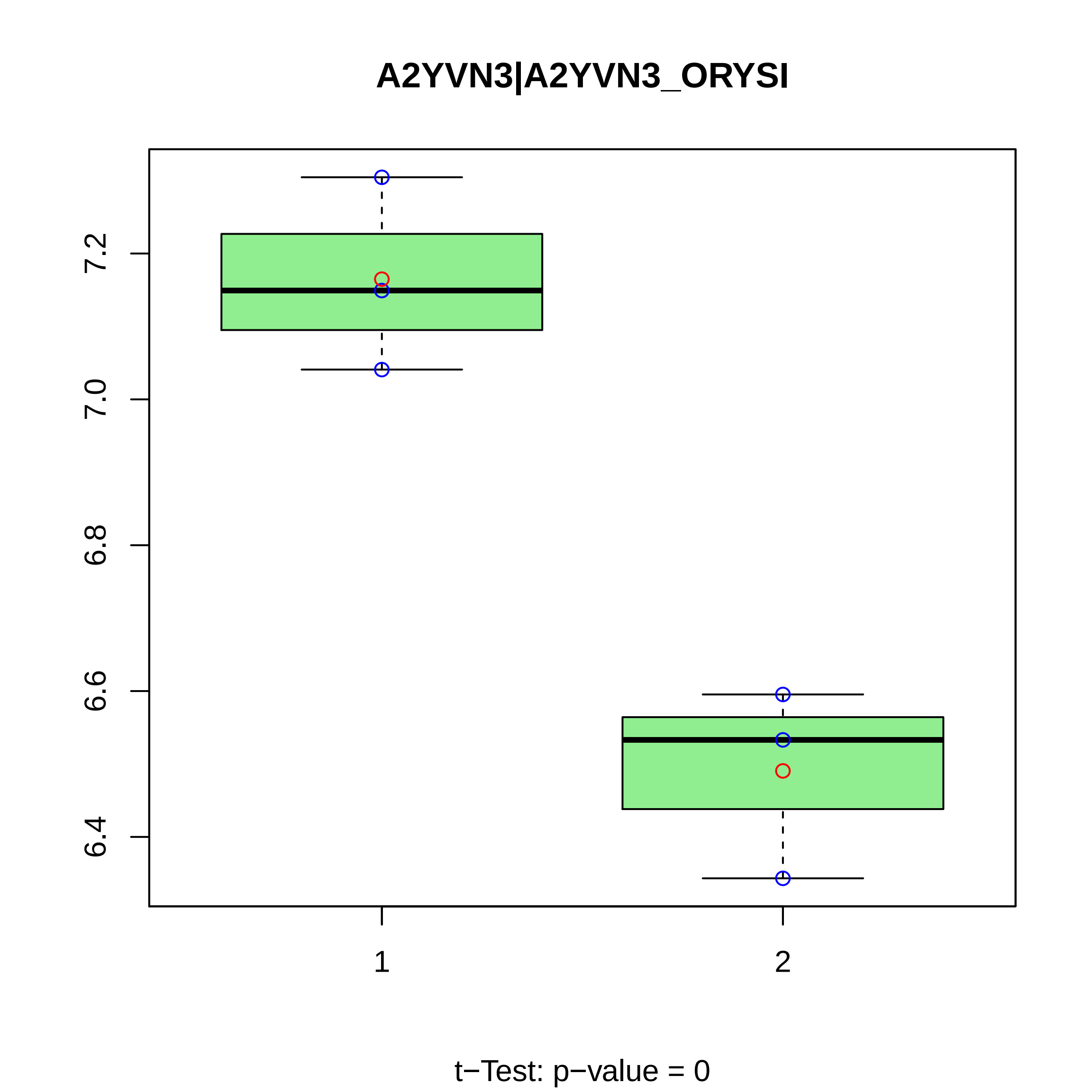

A2YVN3|A2YVN3_ORYSI
7.2
7.0
6.8
6.6
6.4
1
2
t−Test: p−value = 0

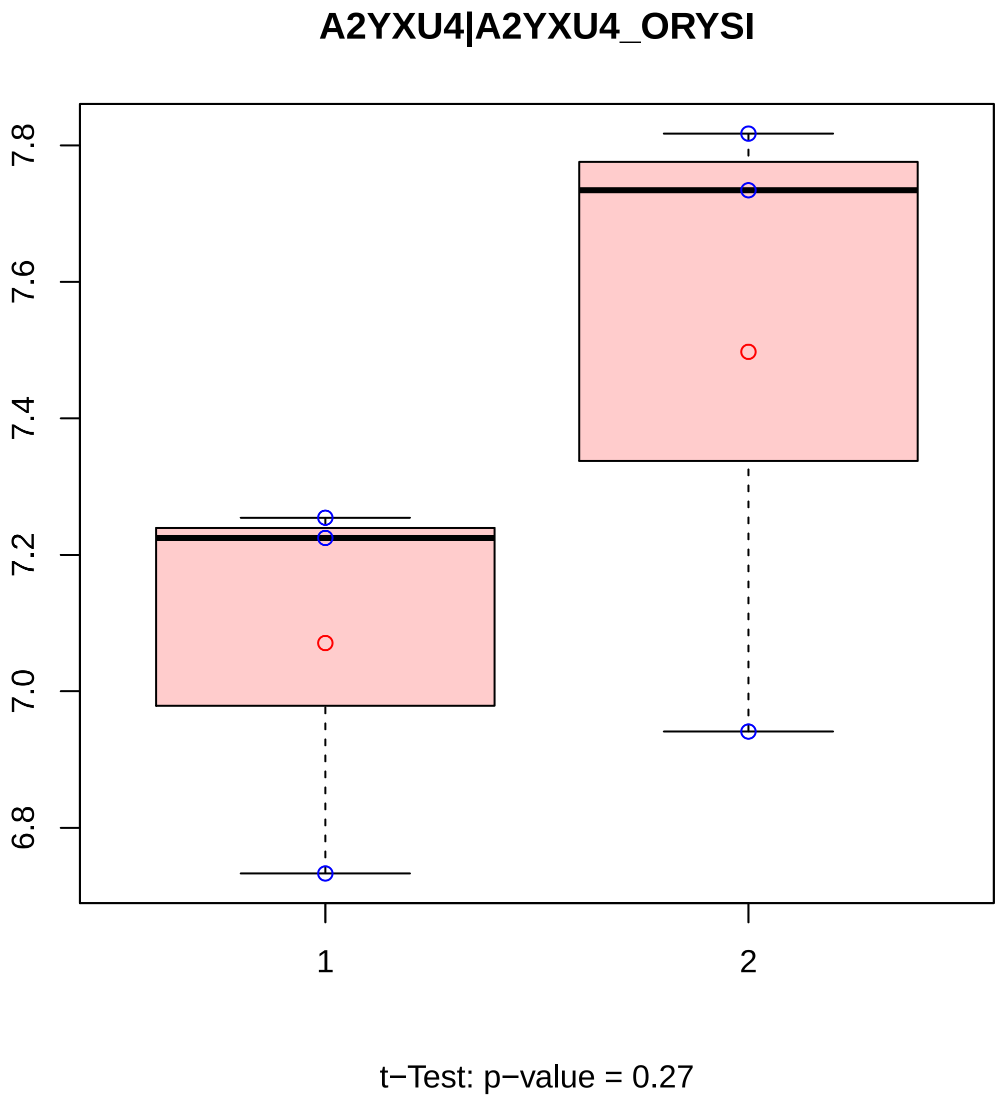

A2YXU4|A2YXU4_ORYSI
7.8
7.6
7.4
7.2
7.0
6.8
1
2
t−Test: p−value = 0.27

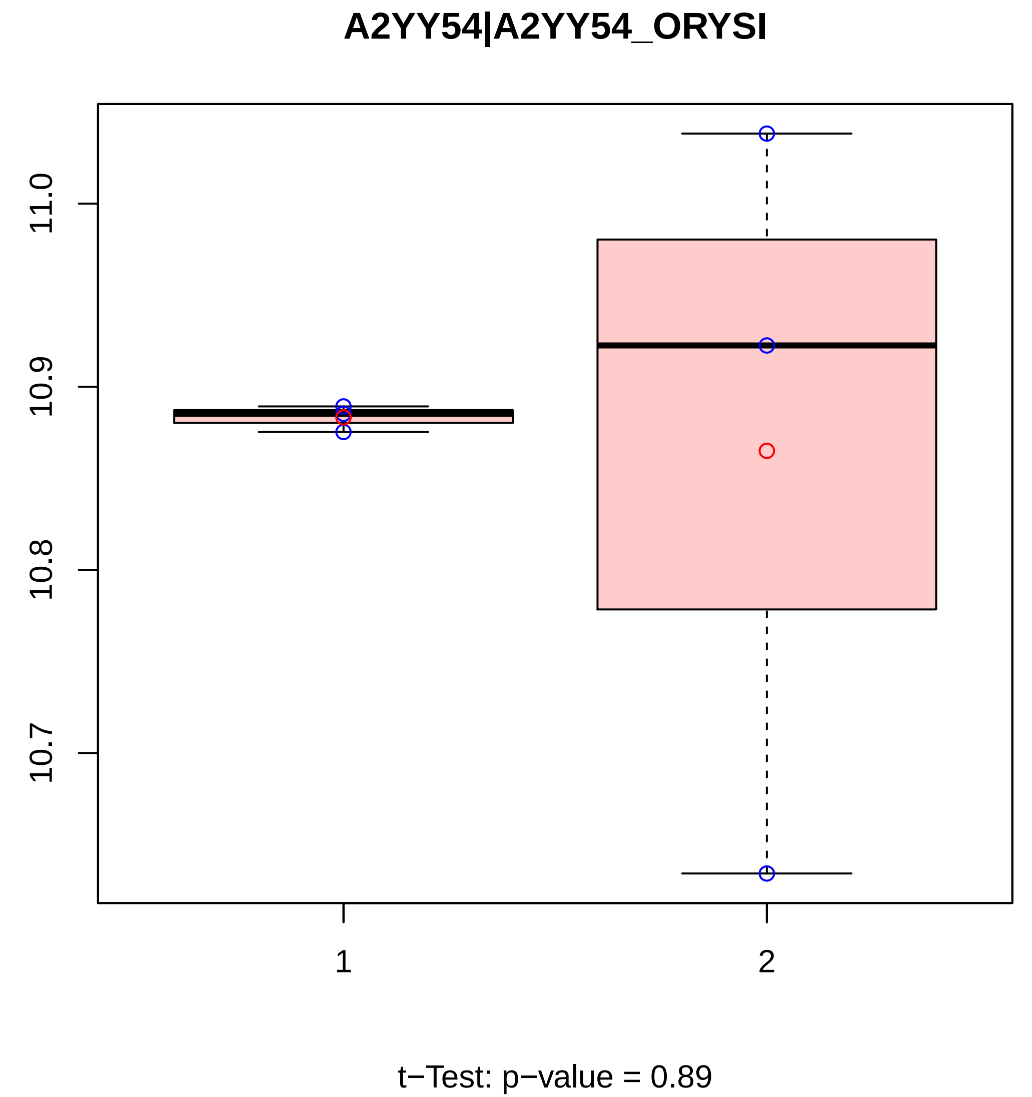
A2YY54|A2YY54_ORYSI
11.0
10.9
10.8
10.7
1
2
t−Test: p−value = 0.89

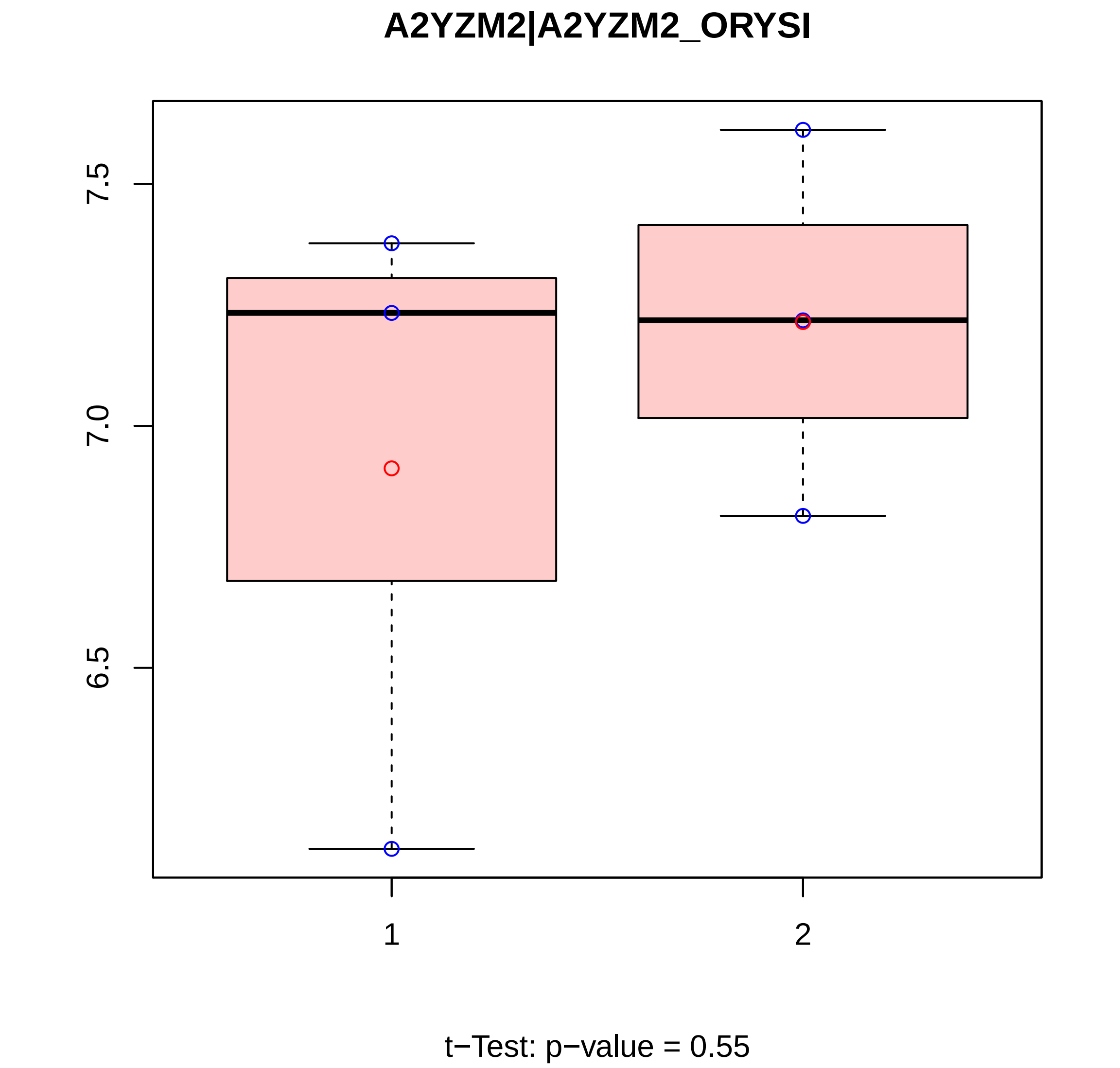

A2YZM2|A2YZM2_ORYSI
7.5
7.0
6.5
1
2
t−Test: p−value = 0.55

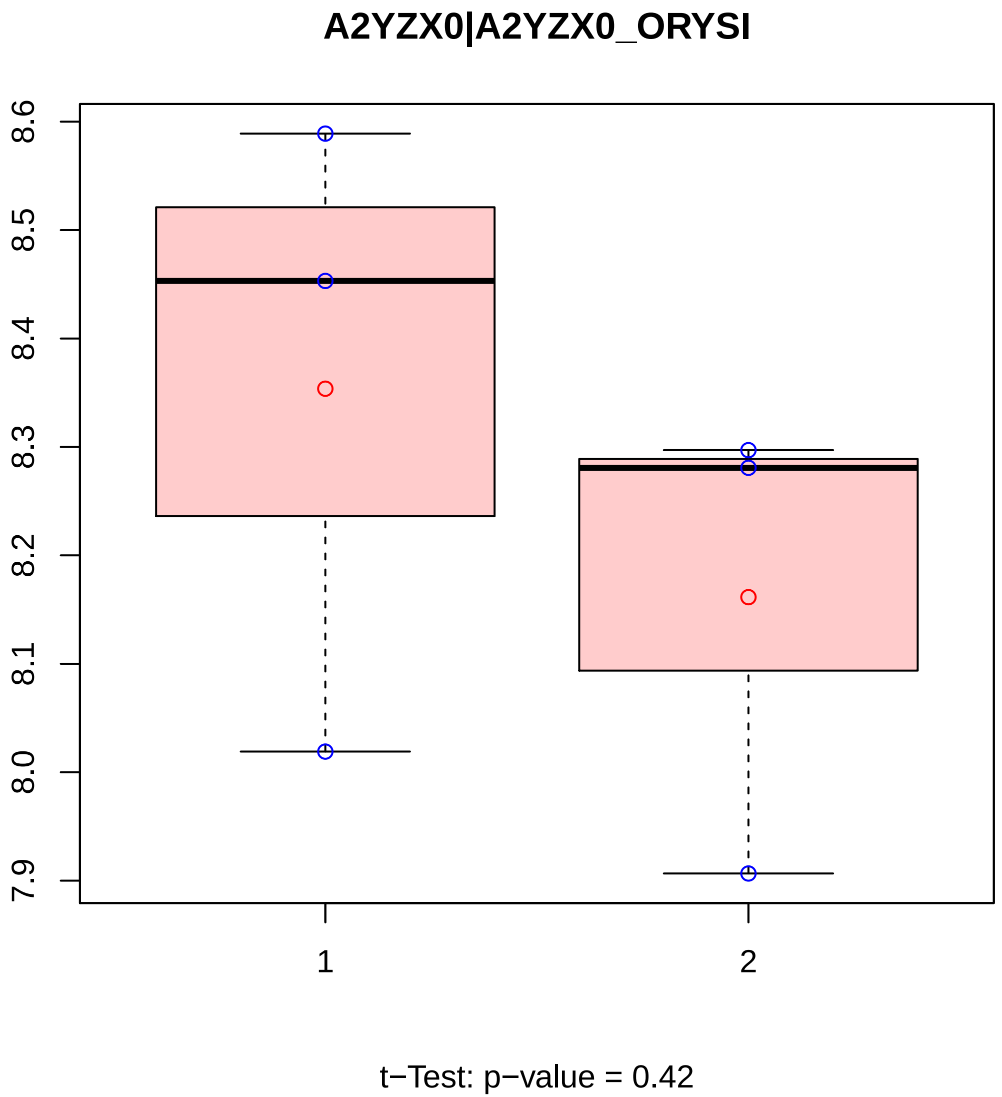

A2YZX0|A2YZX0_ORYSI
8.6
8.5
8.4
8.3
8.2
8.1
8.0
7.9
1
2
t−Test: p−value = 0.42

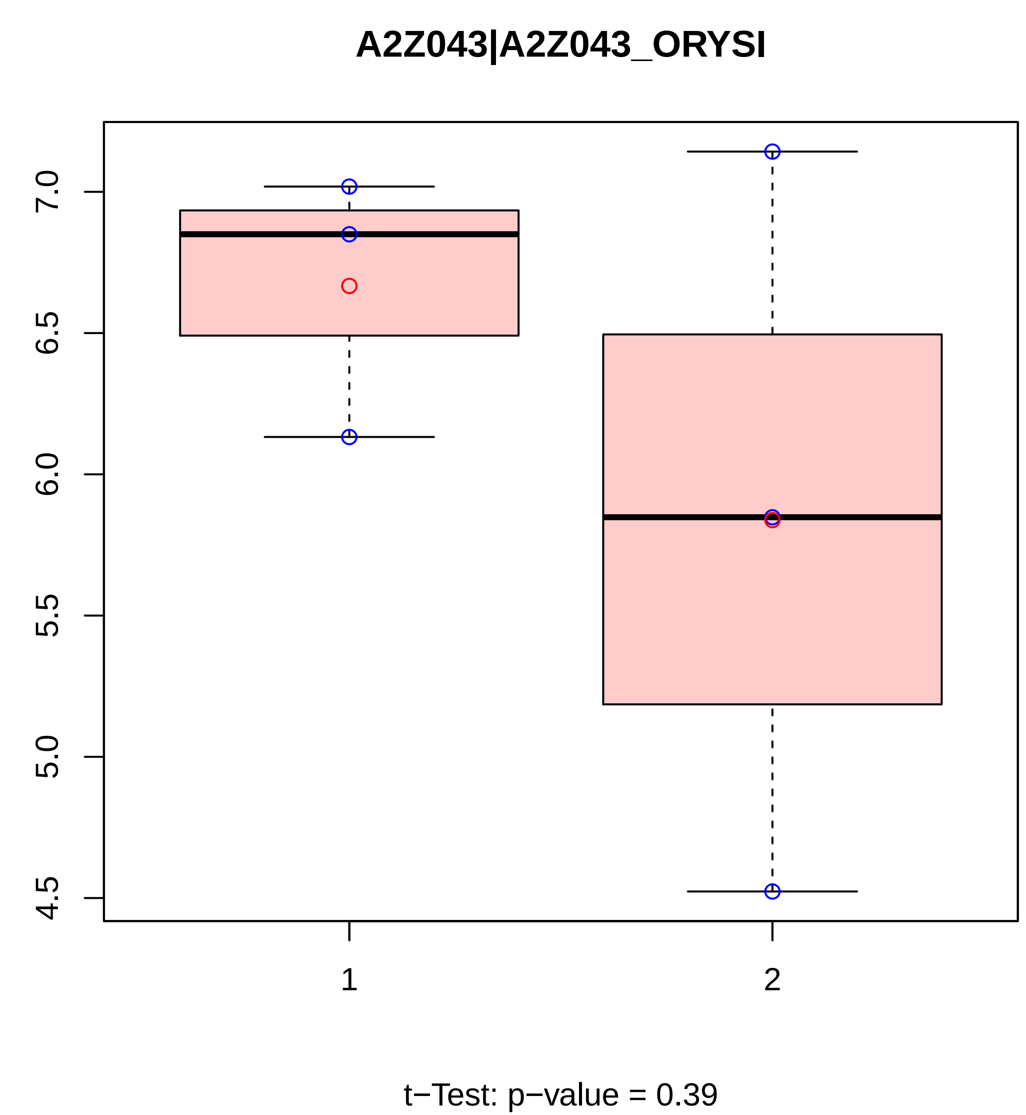
A2Z043|A2Z043_ORYSI
7.0
6.5
6.0
5.5
5.0
4.5
1
2
t−Test: p−value = 0.39

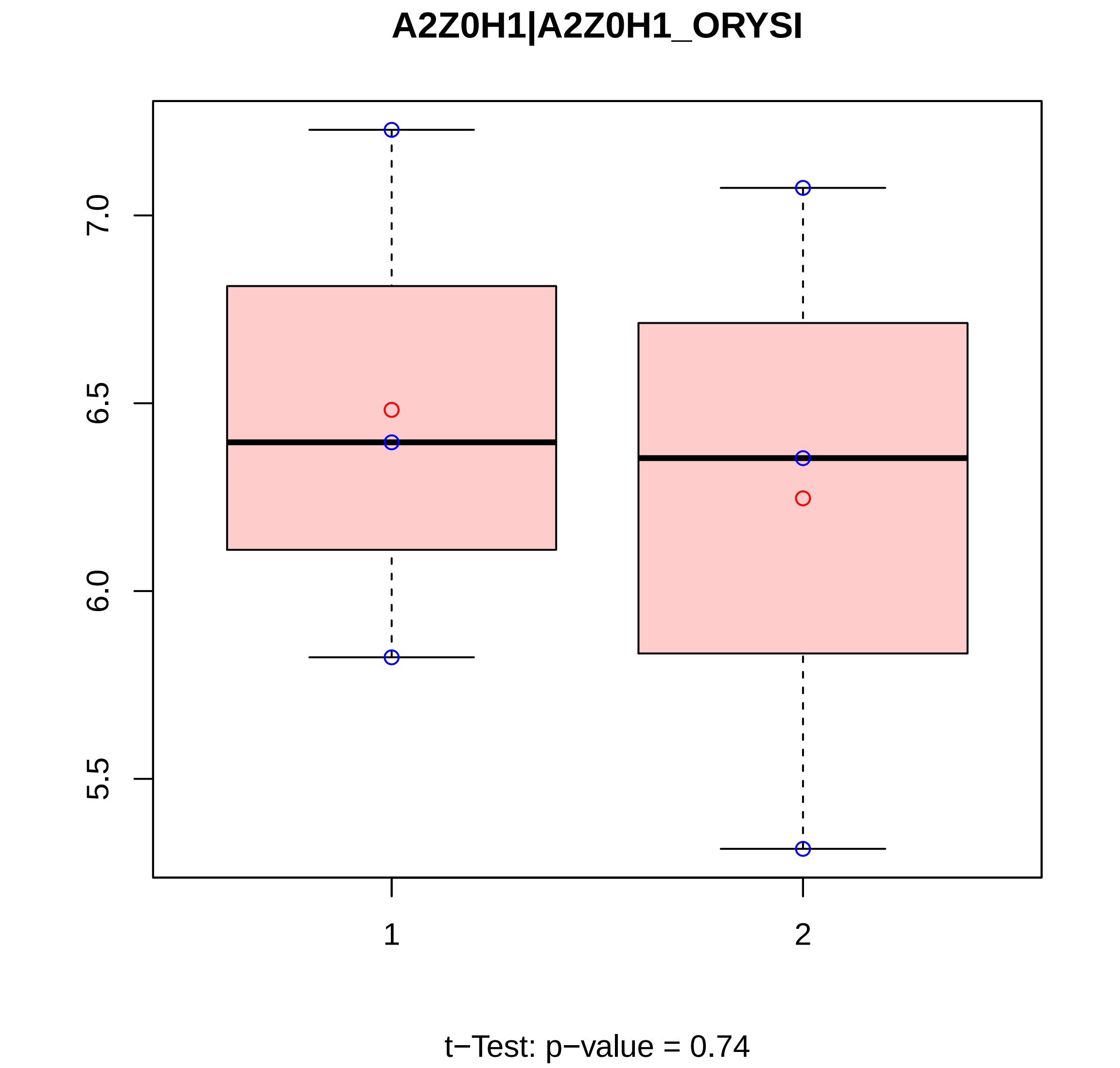
A2Z0H1|A2Z0H1_ORYSI
7.0
6.5
6.0
5.5
1
2
t−Test: p−value = 0.74

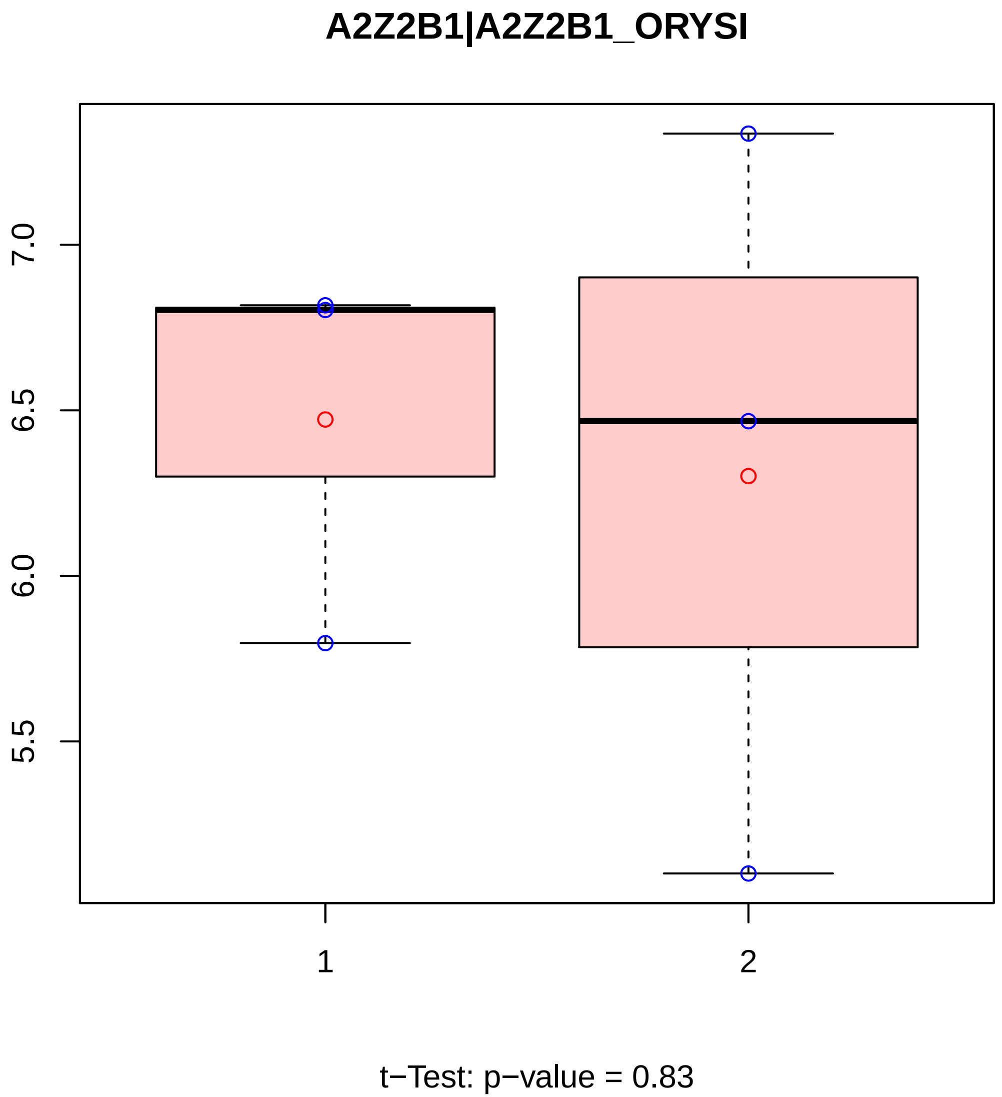

A2Z2B1|A2Z2B1_ORYSI
7.0
6.5
6.0
5.5
1
2
t−Test: p−value = 0.83

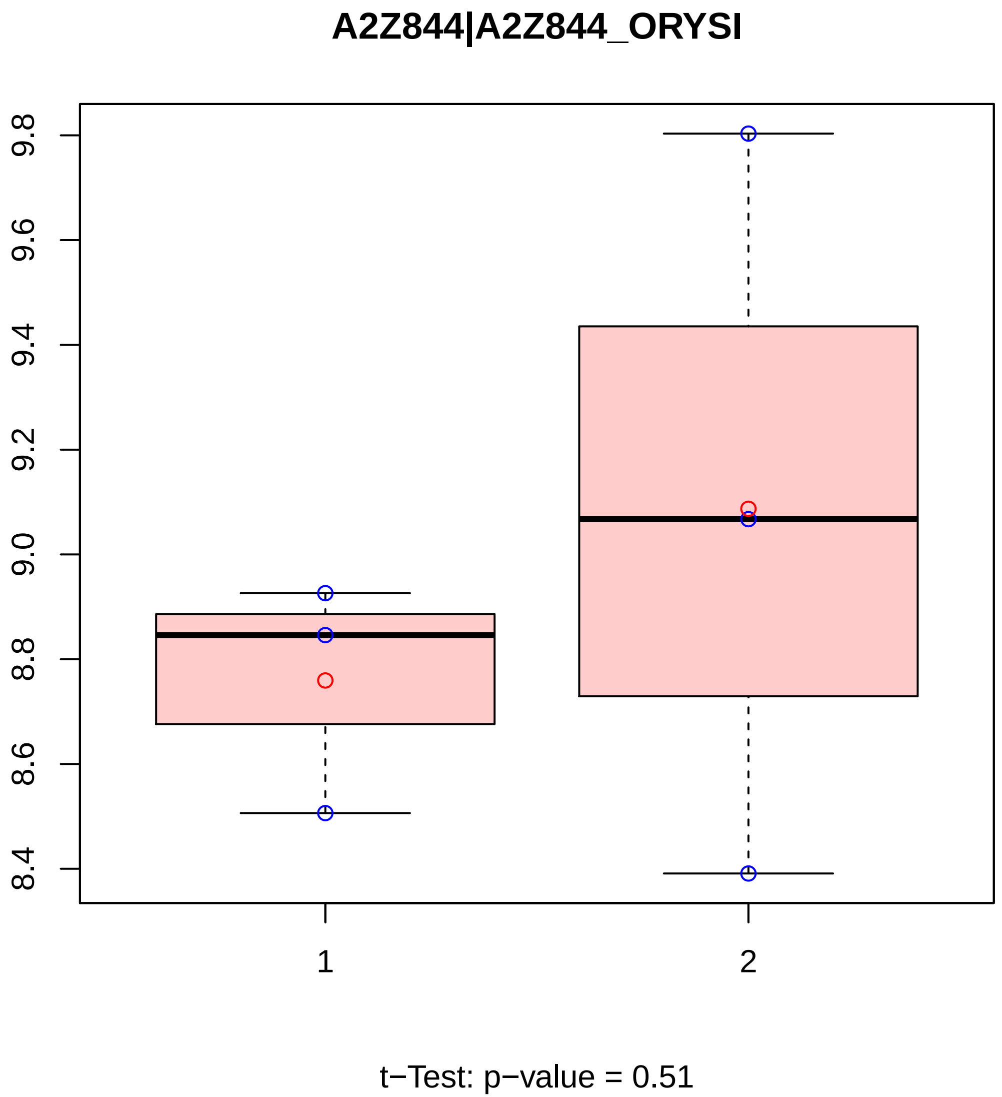

A2Z844|A2Z844_ORYSI
9.8
9.6
9.4
9.2
9.0
8.8
8.6
8.4
1
2
t−Test: p−value = 0.51

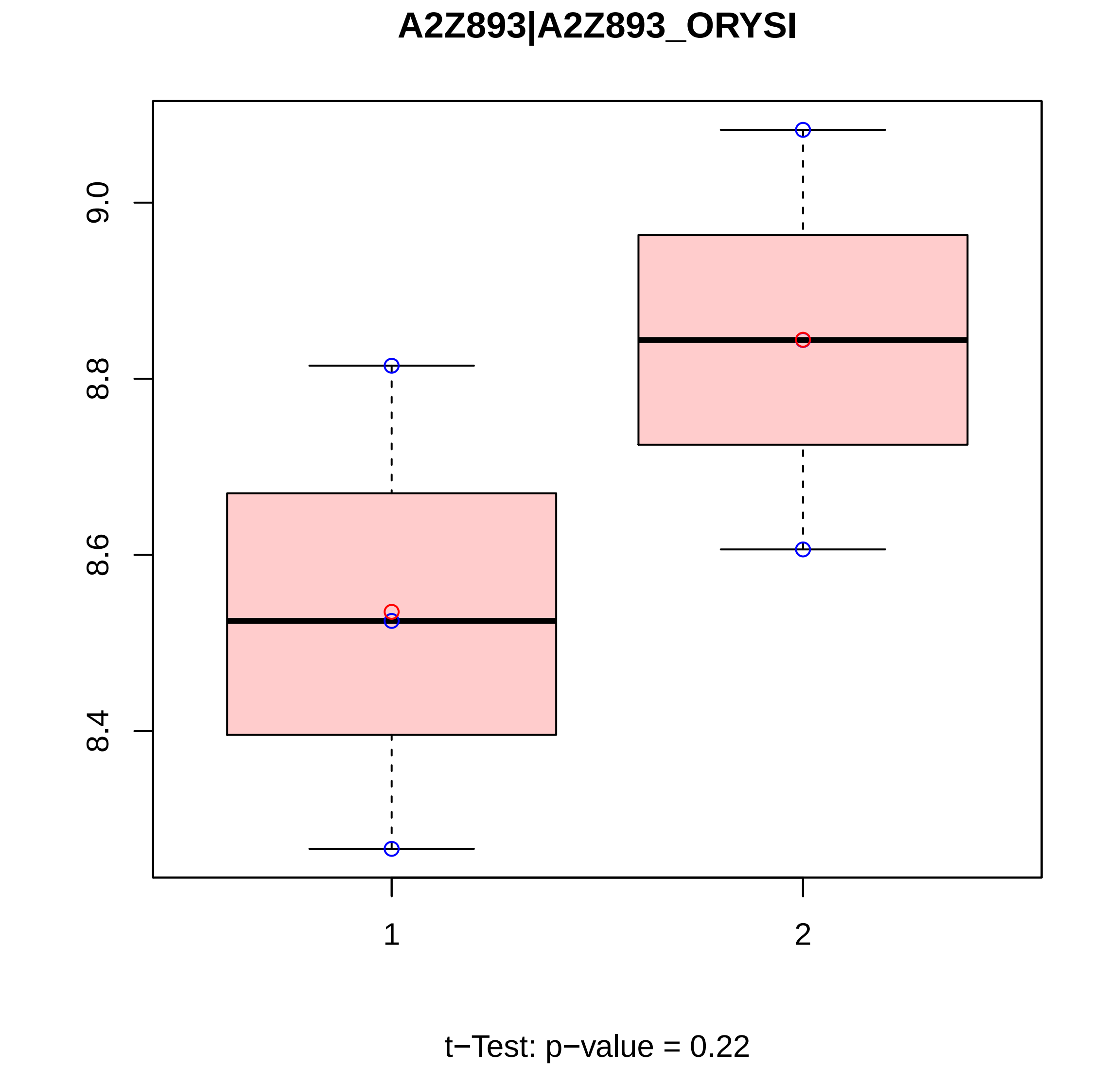

A2Z893|A2Z893_ORYSI
9.0
8.8
8.6
8.4
1
2
t−Test: p−value = 0.22

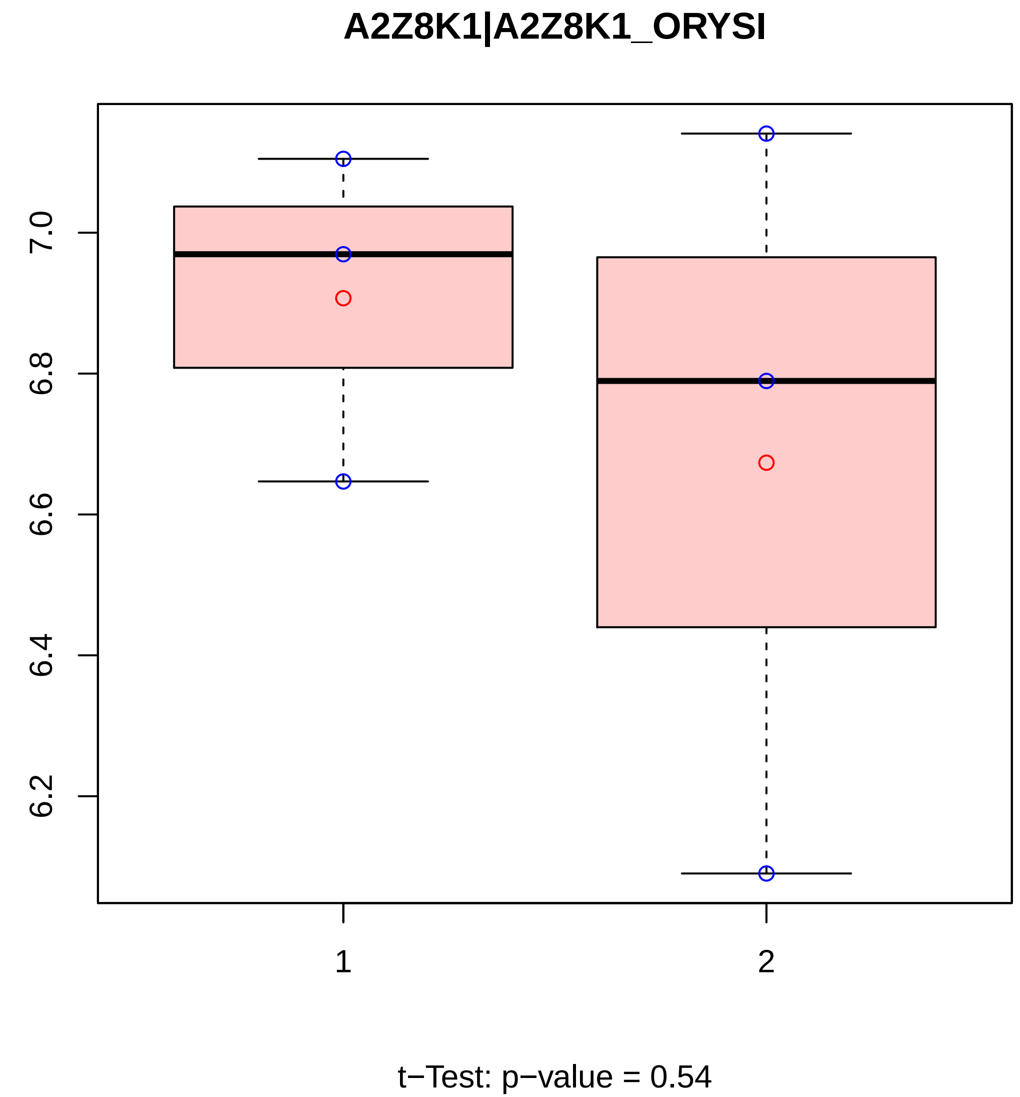
A2Z8K1|A2Z8K1_ORYSI
7.0
6.8
6.6
6.4
6.2
1
2
t−Test: p−value = 0.54

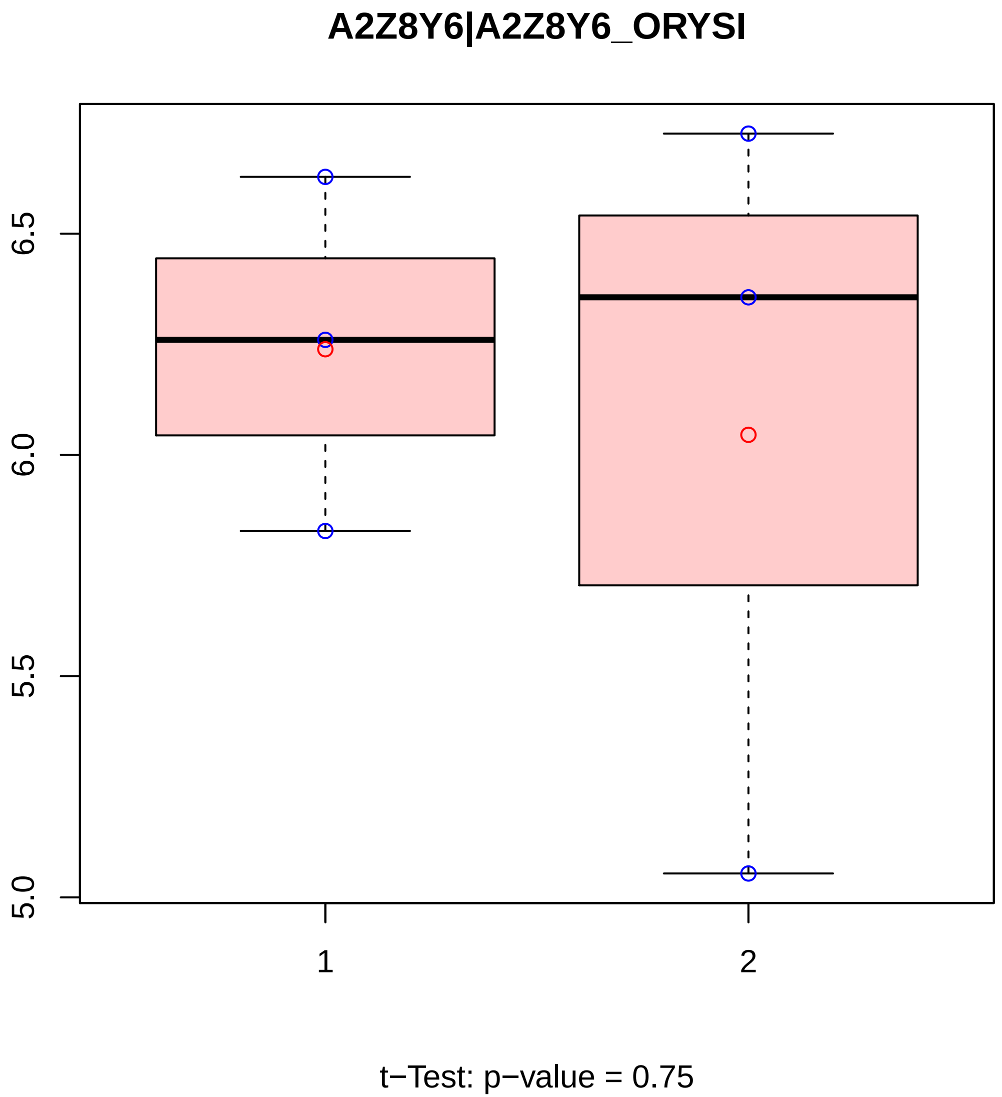
A2Z8Y6|A2Z8Y6_ORYSI
6.5
6.0
5.5
5.0
1
2
t−Test: p−value = 0.75

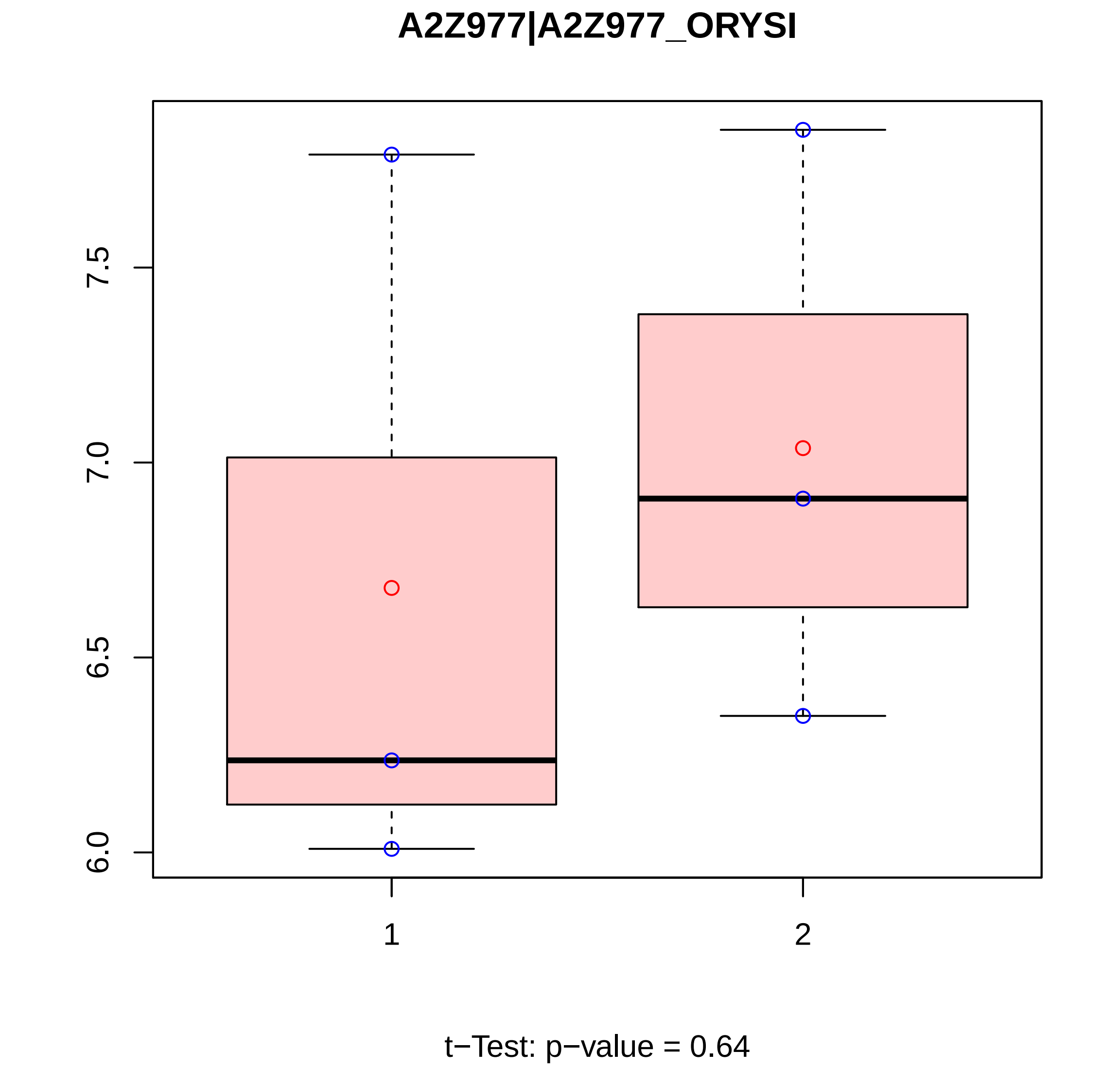
A2Z977|A2Z977_ORYSI
7.5
7.0
6.5
6.0
1
2
t−Test: p−value = 0.64

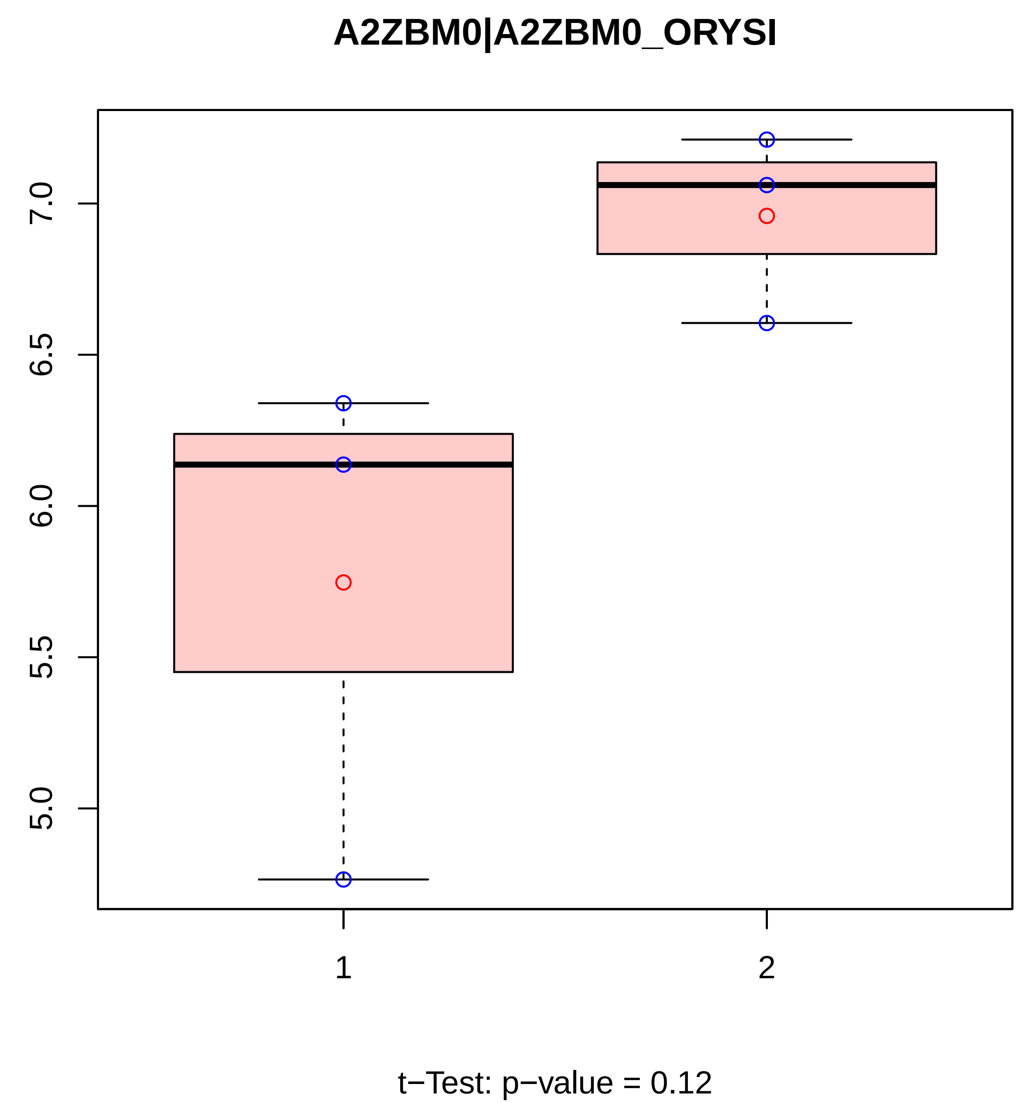
A2ZBM0|A2ZBM0_ORYSI
7.0
6.5
6.0
5.5
5.0
1
2
t−Test: p−value = 0.12

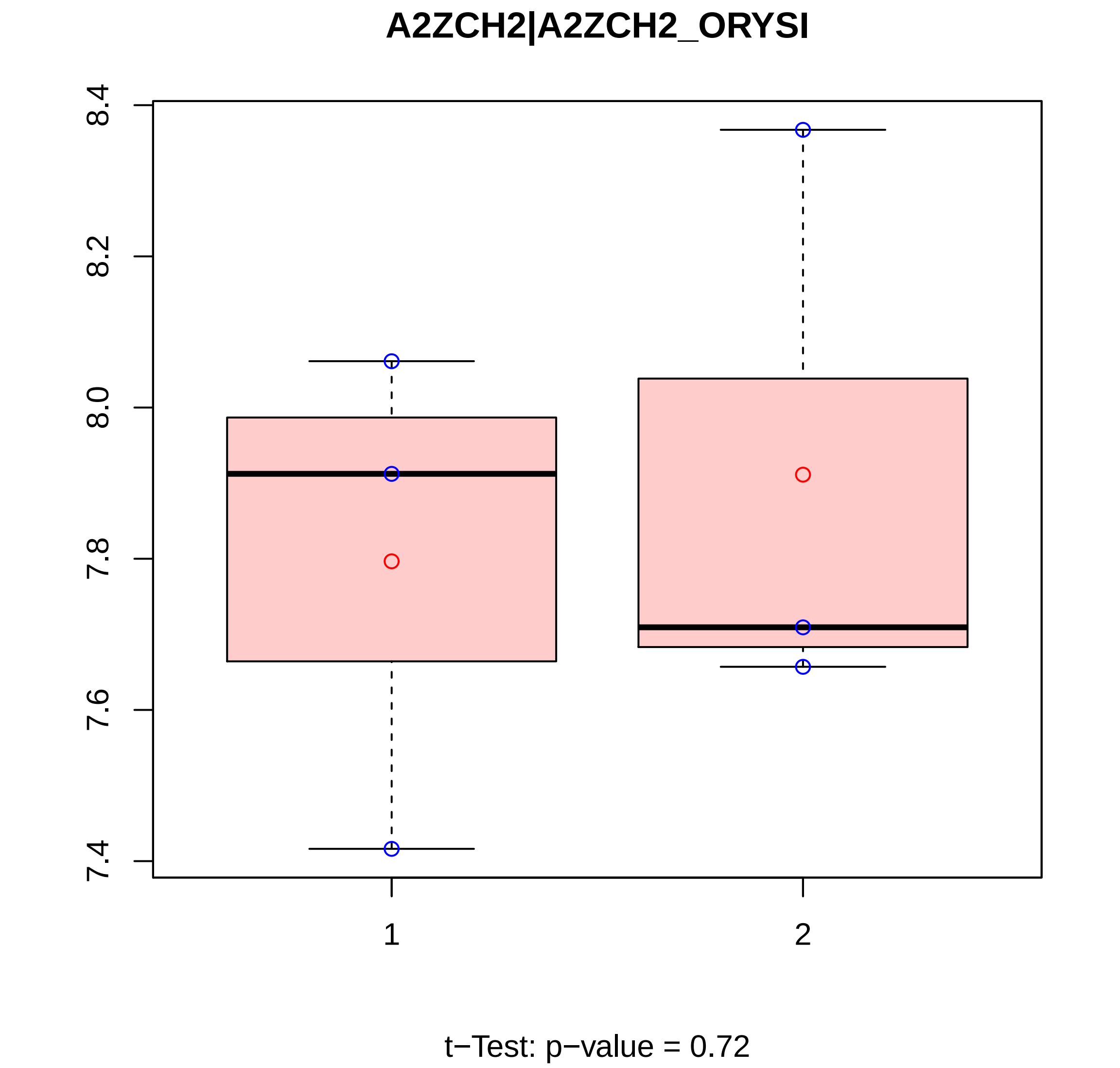
A2ZCH2|A2ZCH2_ORYSI
8.4
8.2
8.0
7.8
7.6
7.4
1
2
t−Test: p−value = 0.72

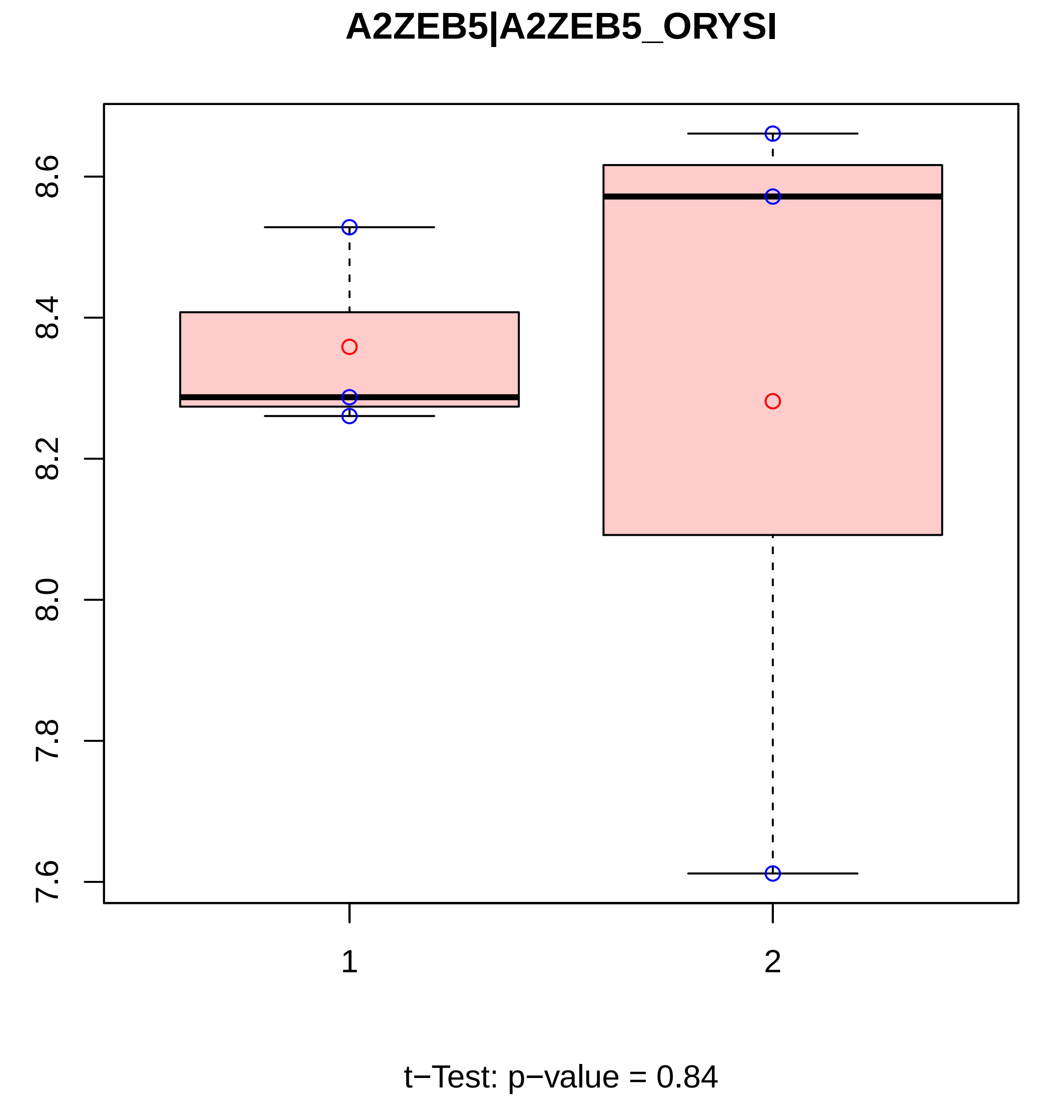
A2ZEB5|A2ZEB5_ORYSI
8.6
8.4
8.2
8.0
7.8
7.6
1
2
t−Test: p−value = 0.84

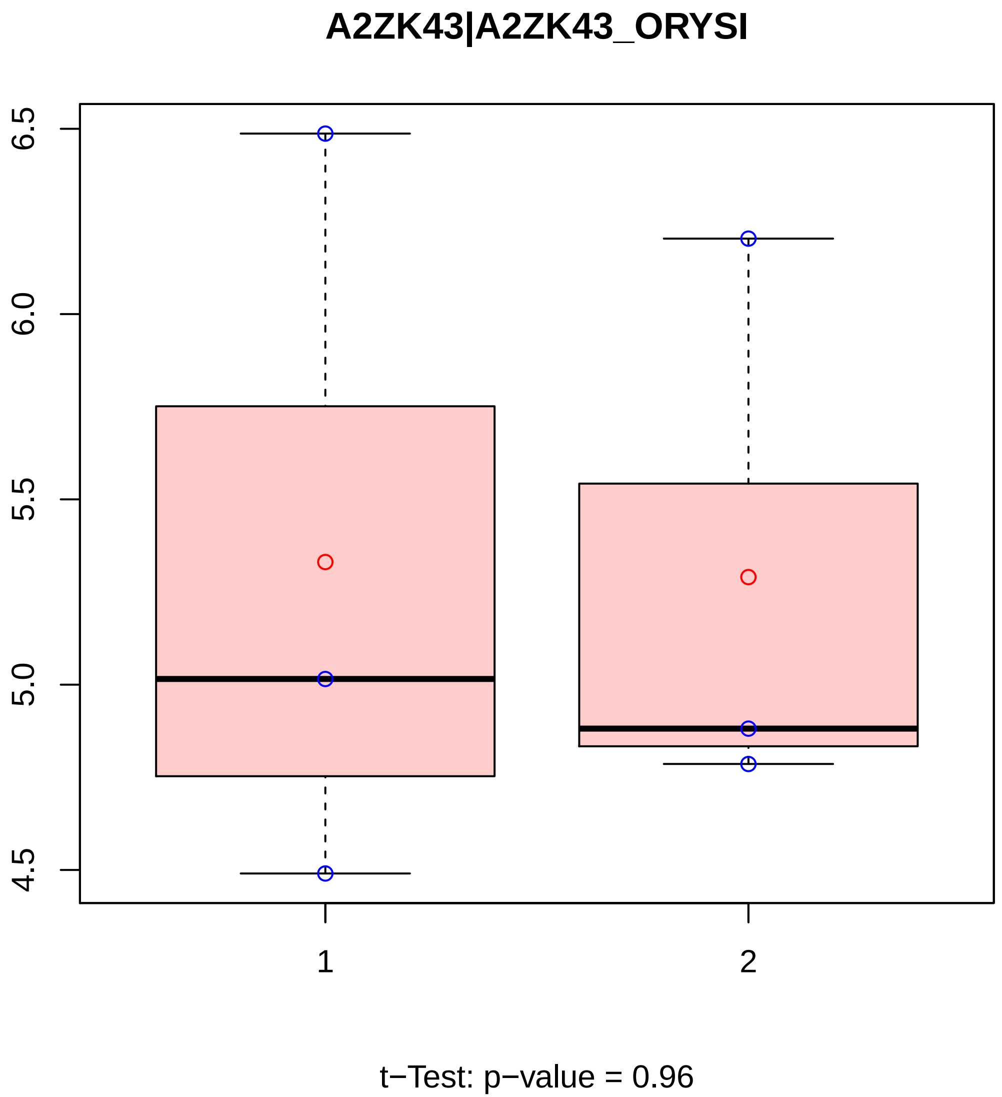
A2ZK43|A2ZK43_ORYSI
6.5
6.0
5.5
5.0
4.5
1
2
t−Test: p−value = 0.96

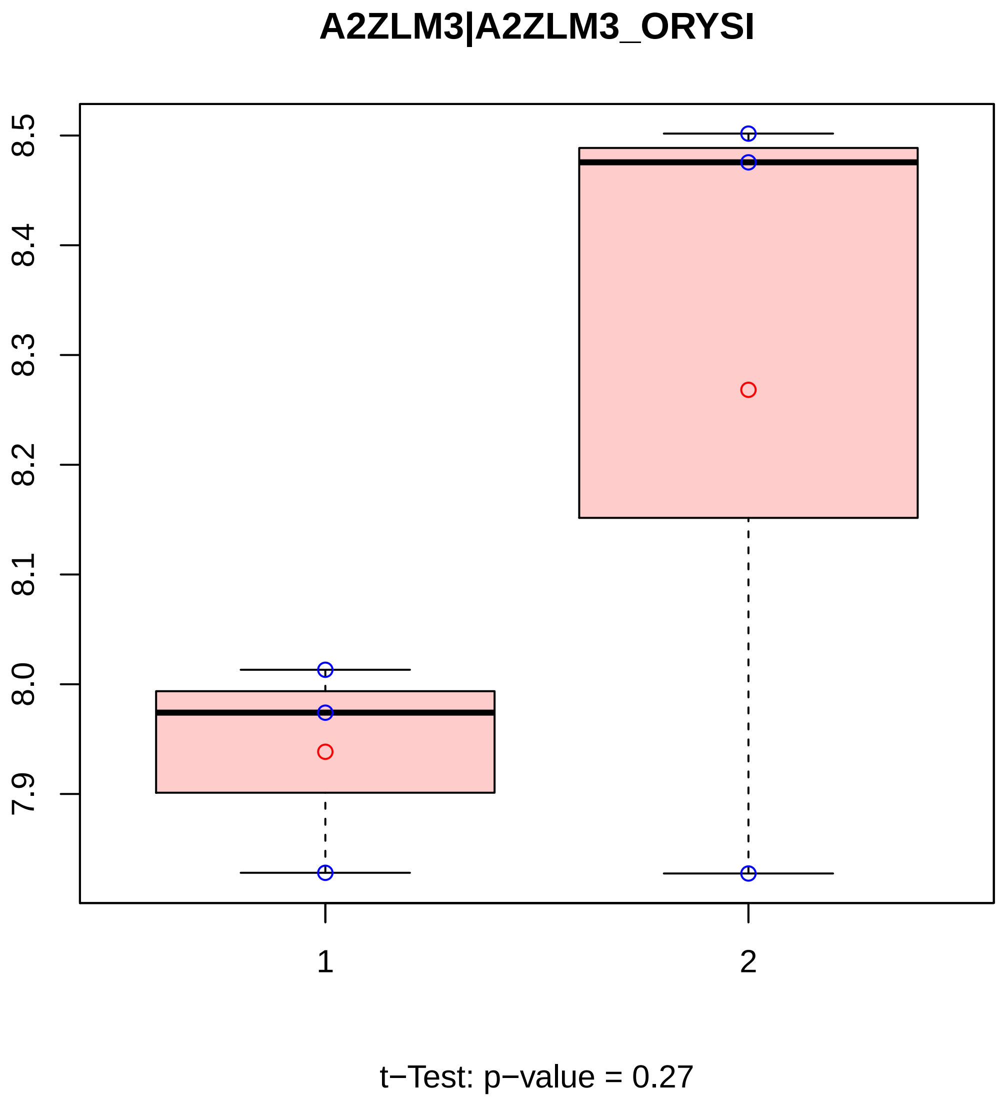

A2ZLM3|A2ZLM3_ORYSI
8.5
8.4
8.3
8.2
8.1
8.0
7.9
1
2
t−Test: p−value = 0.27

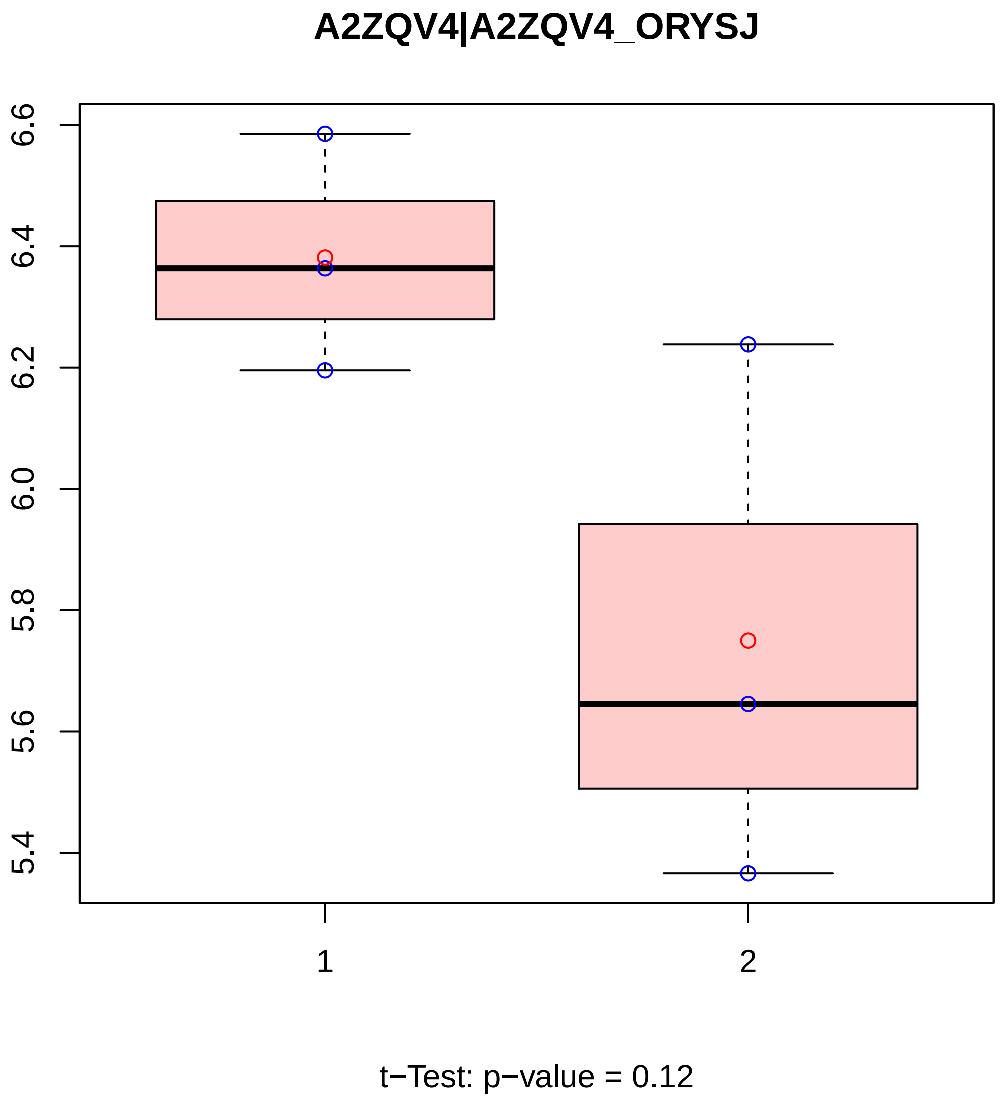
A2ZQV4|A2ZQV4_ORYSJ
6.6
6.4
6.2
6.0
5.8
5.6
5.4
1
2
t−Test: p−value = 0.12

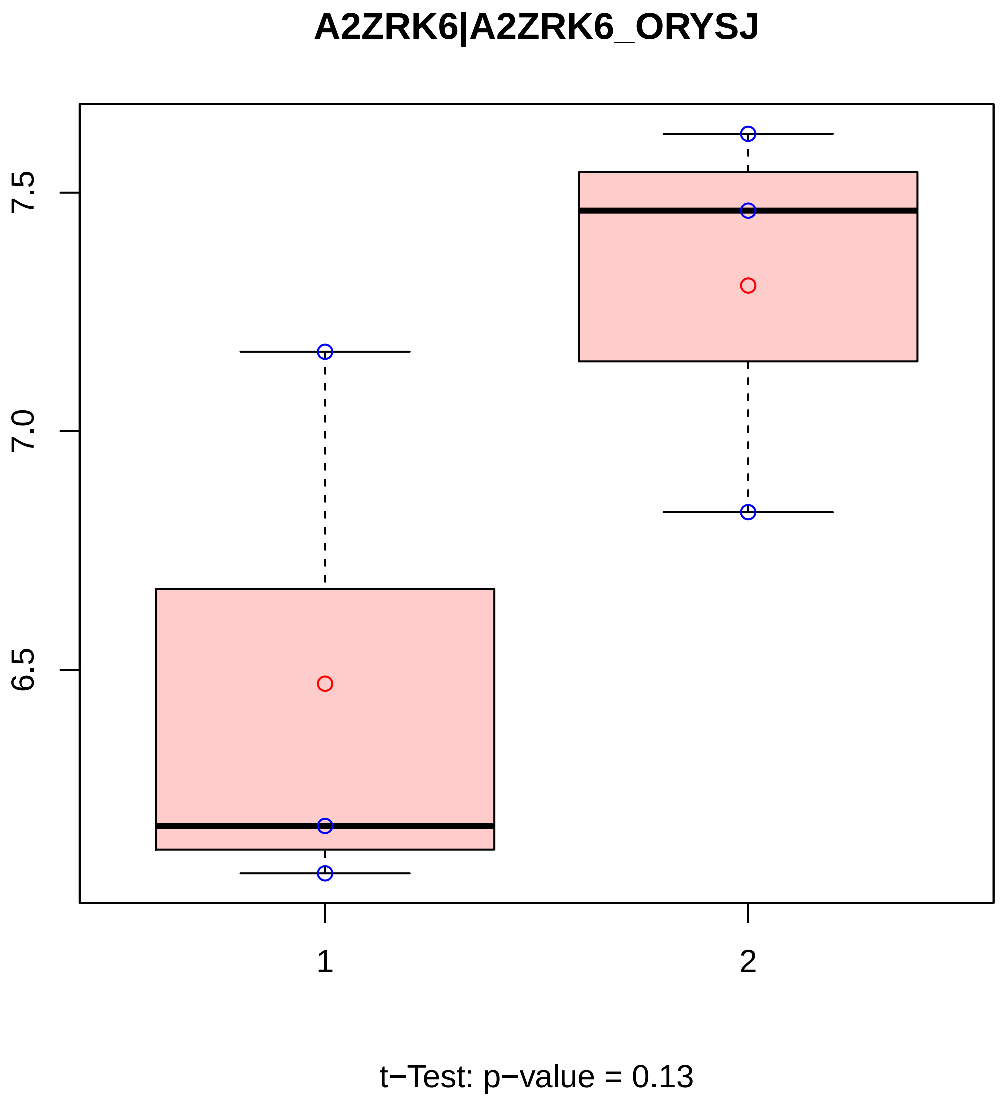
A2ZRK6|A2ZRK6_ORYSJ
7.5
7.0
6.5
1
2
t−Test: p−value = 0.13

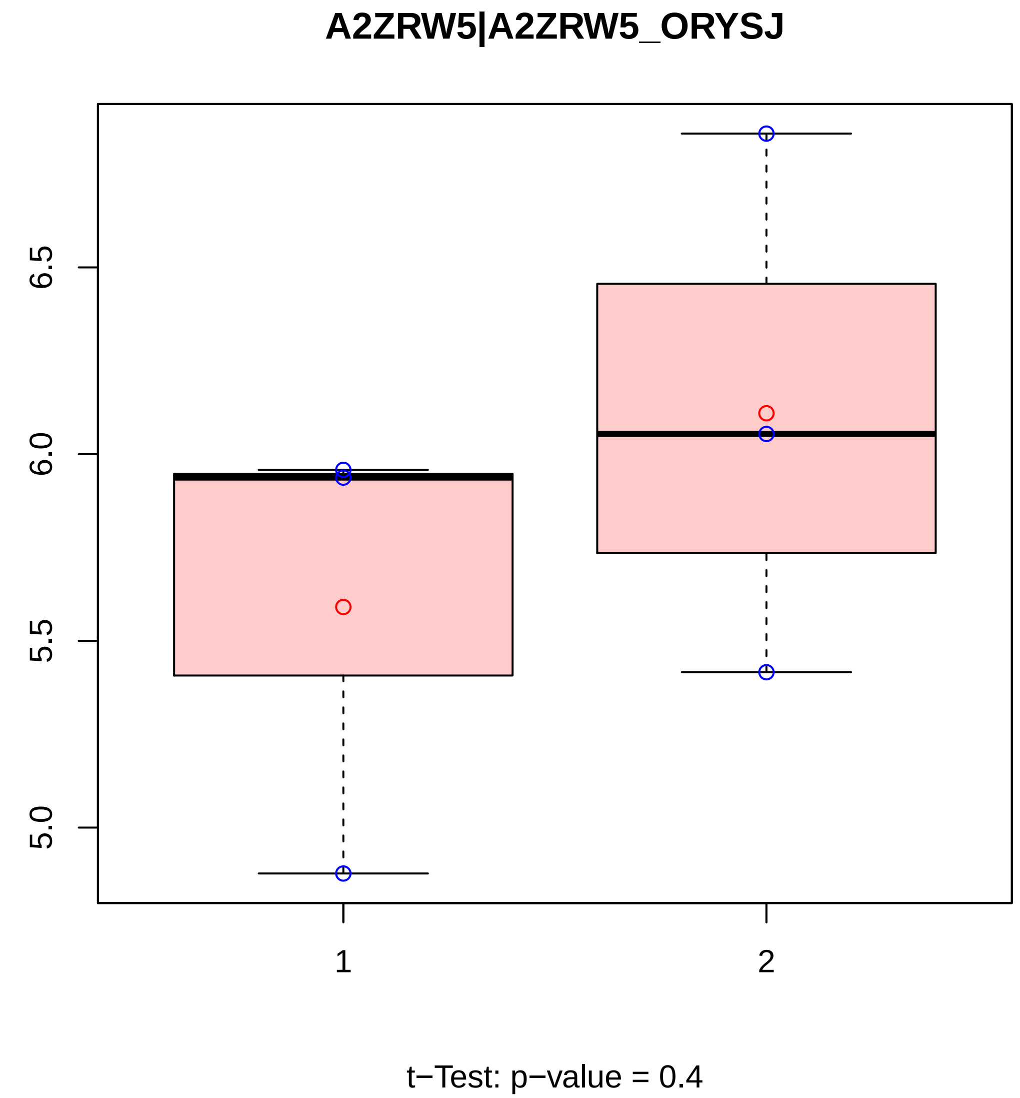

A2ZRW5|A2ZRW5_ORYSJ
6.5
6.0
5.5
5.0
1
2
t−Test: p−value = 0.4

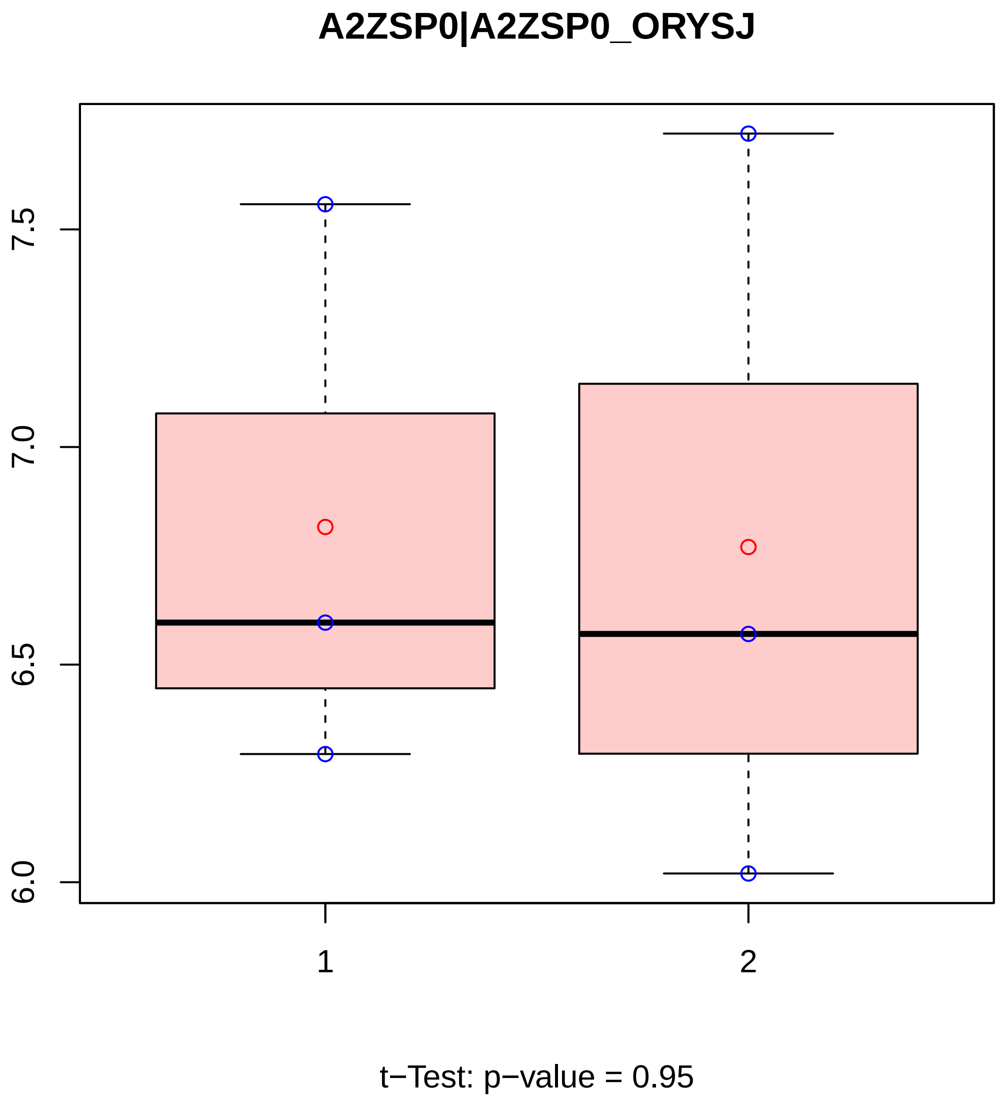

A2ZSP0|A2ZSP0_ORYSJ
7.5
7.0
6.5
6.0
1
2
t−Test: p−value = 0.95

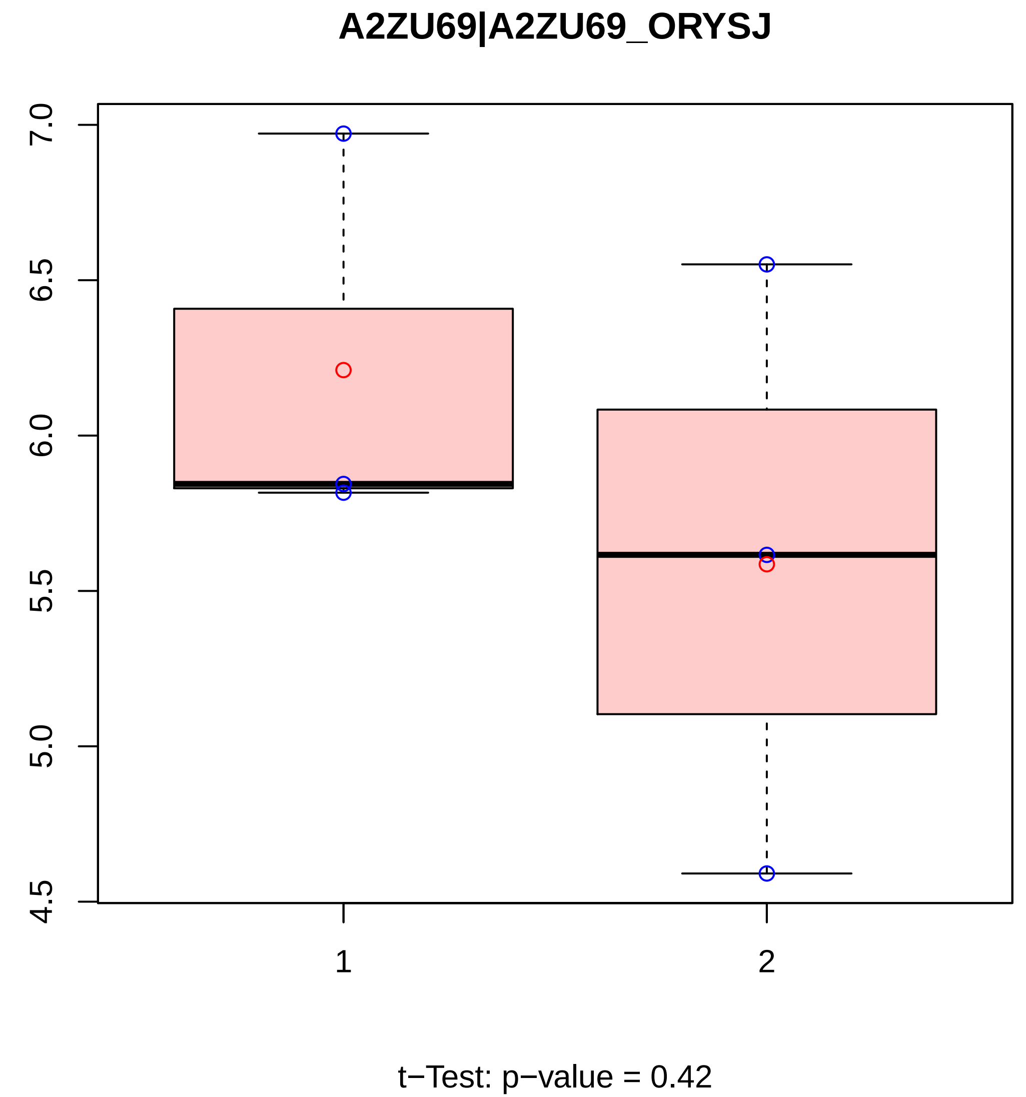

A2ZU69|A2ZU69_ORYSJ
7.0
6.5
6.0
5.5
5.0
4.5
1
2
t−Test: p−value = 0.42

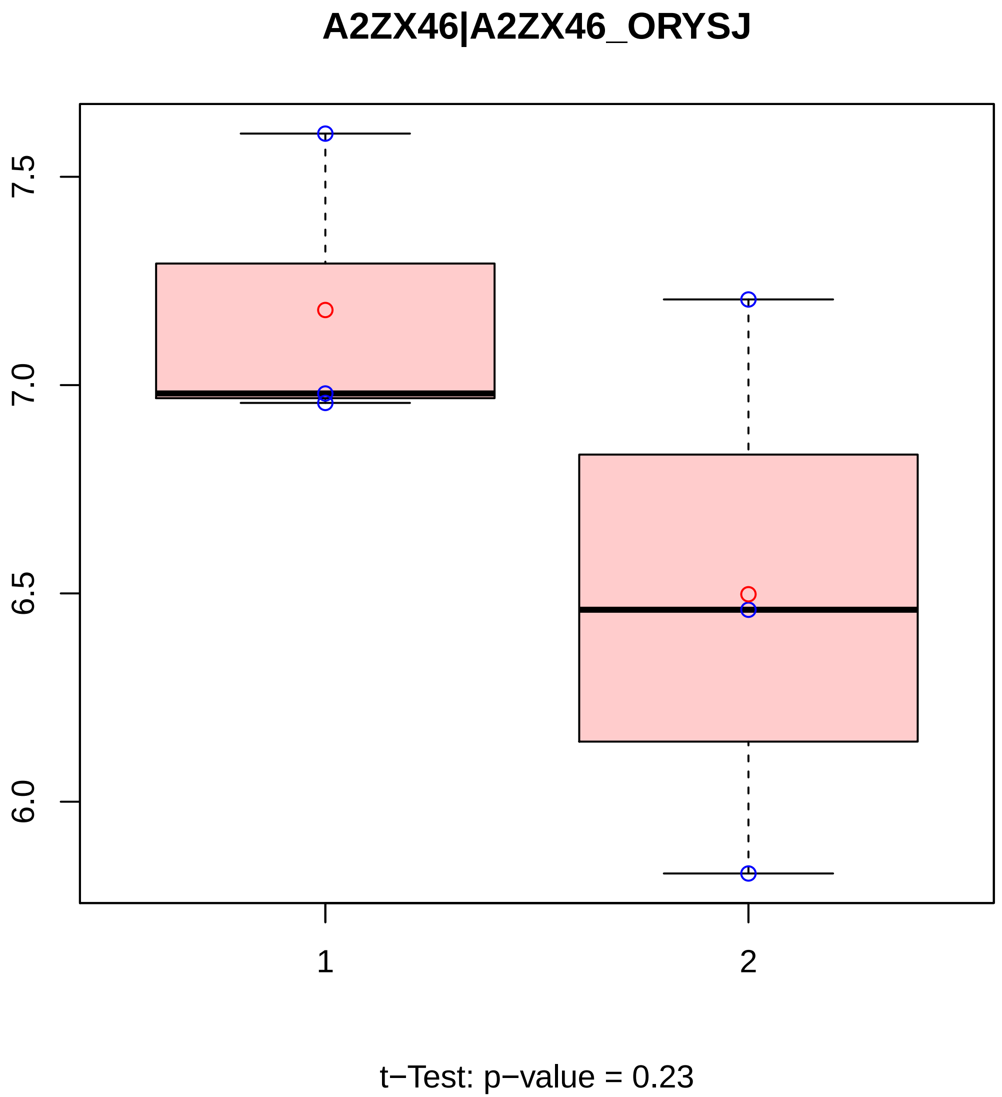
A2ZX46|A2ZX46_ORYSJ
7.5
7.0
6.5
6.0
1
2
t−Test: p−value = 0.23

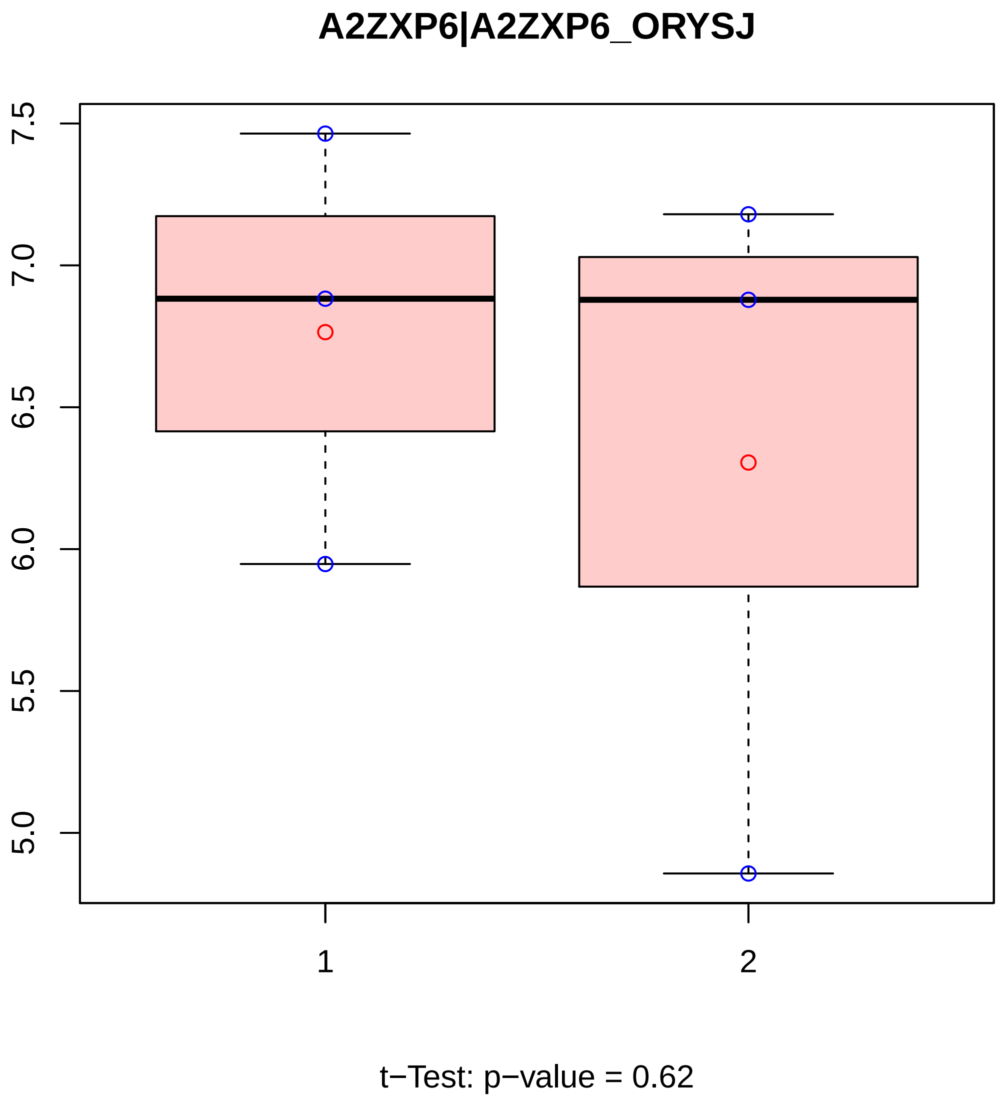
A2ZXP6|A2ZXP6_ORYSJ
7.5
7.0
6.5
6.0
5.5
5.0
1
2
t−Test: p−value = 0.62

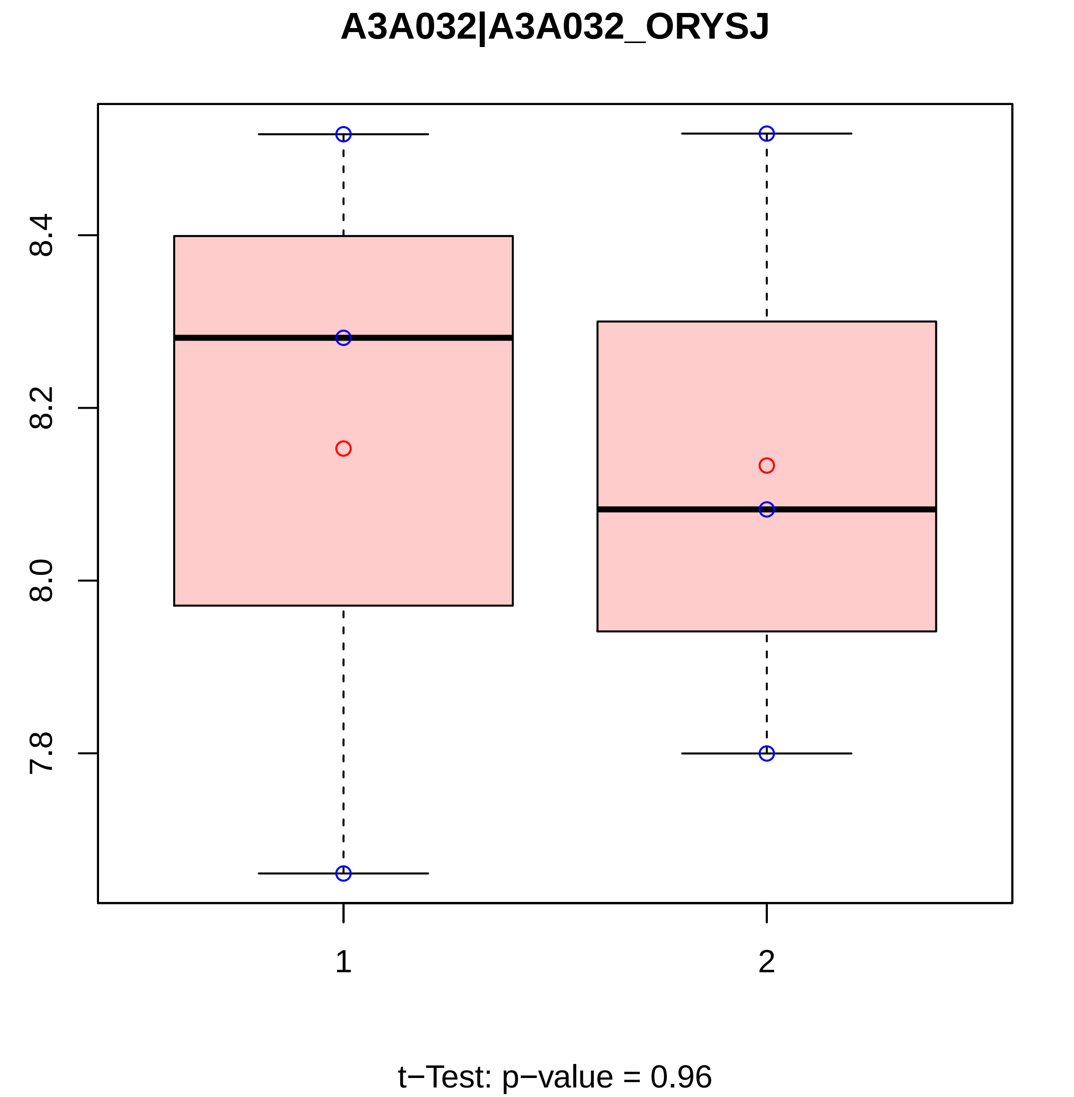

A3A032|A3A032_ORYSJ
8.4
8.2
8.0
7.8
1
2
t−Test: p−value = 0.96

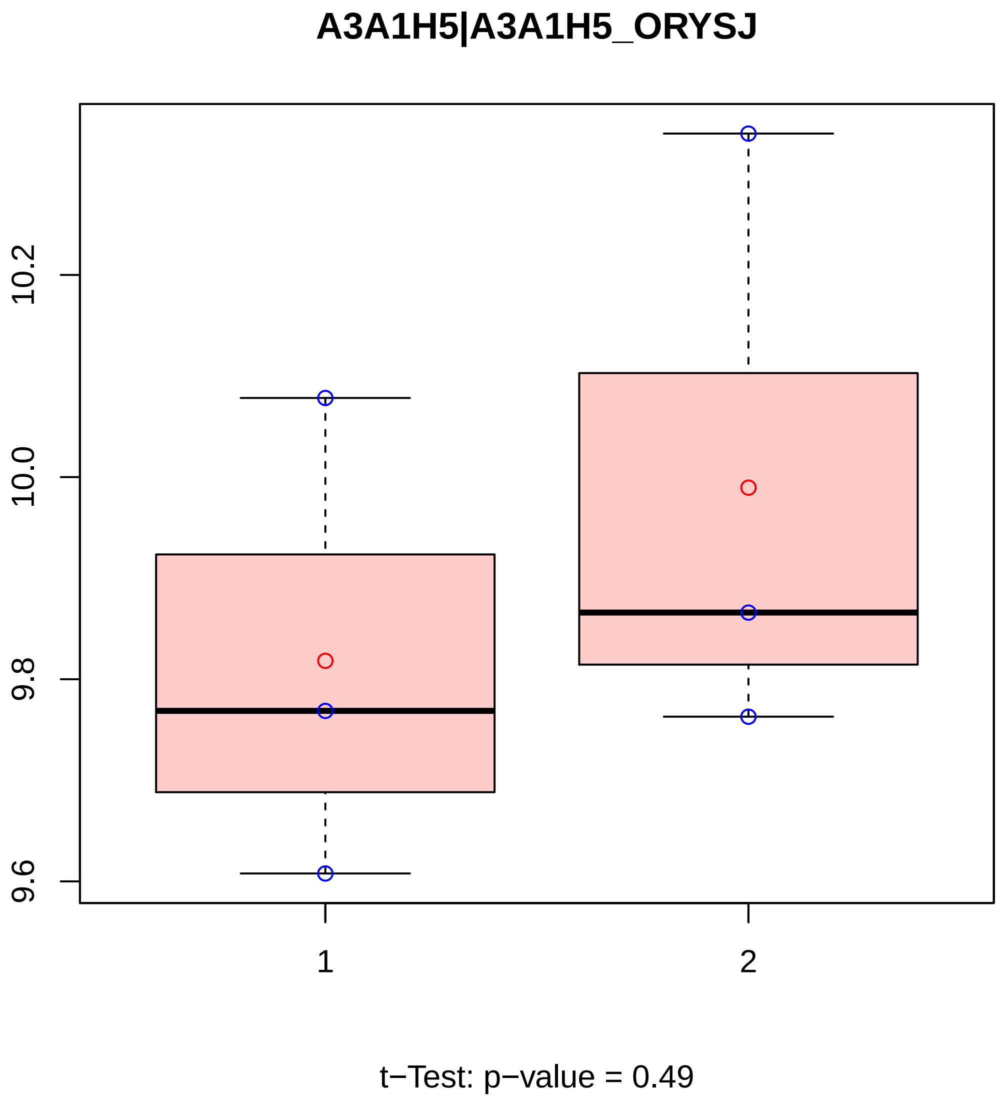
A3A1H5|A3A1H5_ORYSJ
10.2
10.0
9.8
9.6
1
2
t−Test: p−value = 0.49

A3A1V0|A3A1V0_ORYSJ

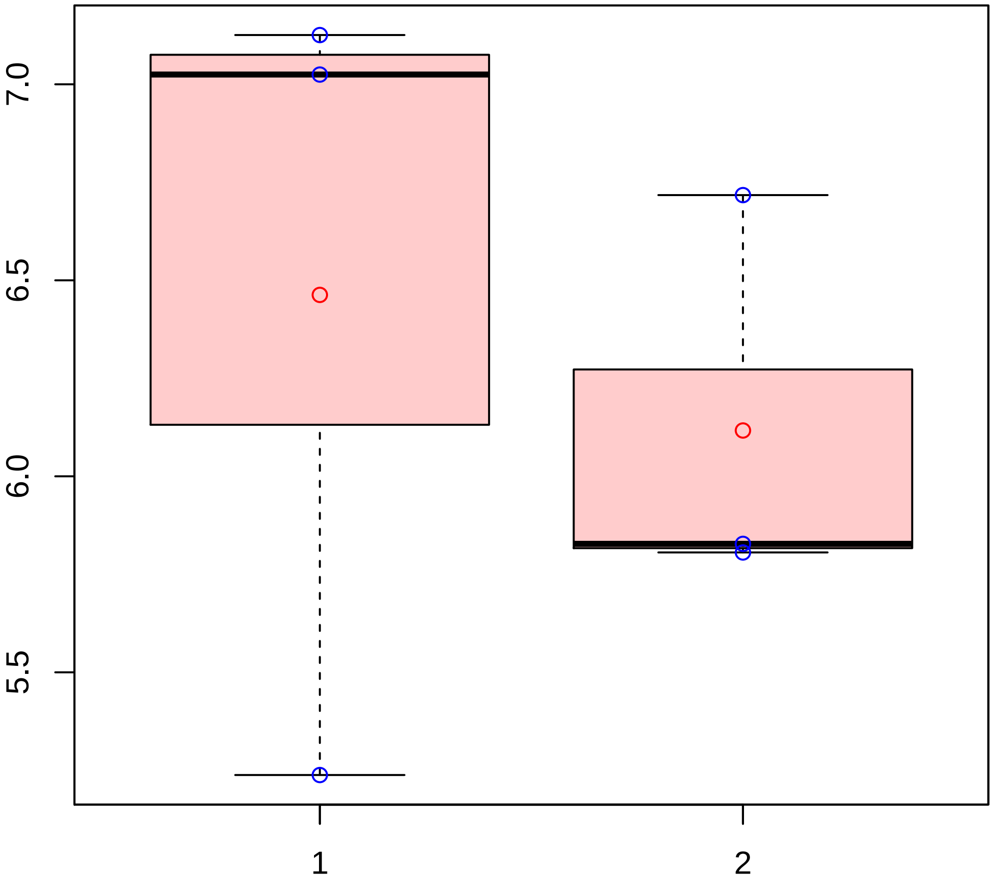

t−Test: p−value = 0.65

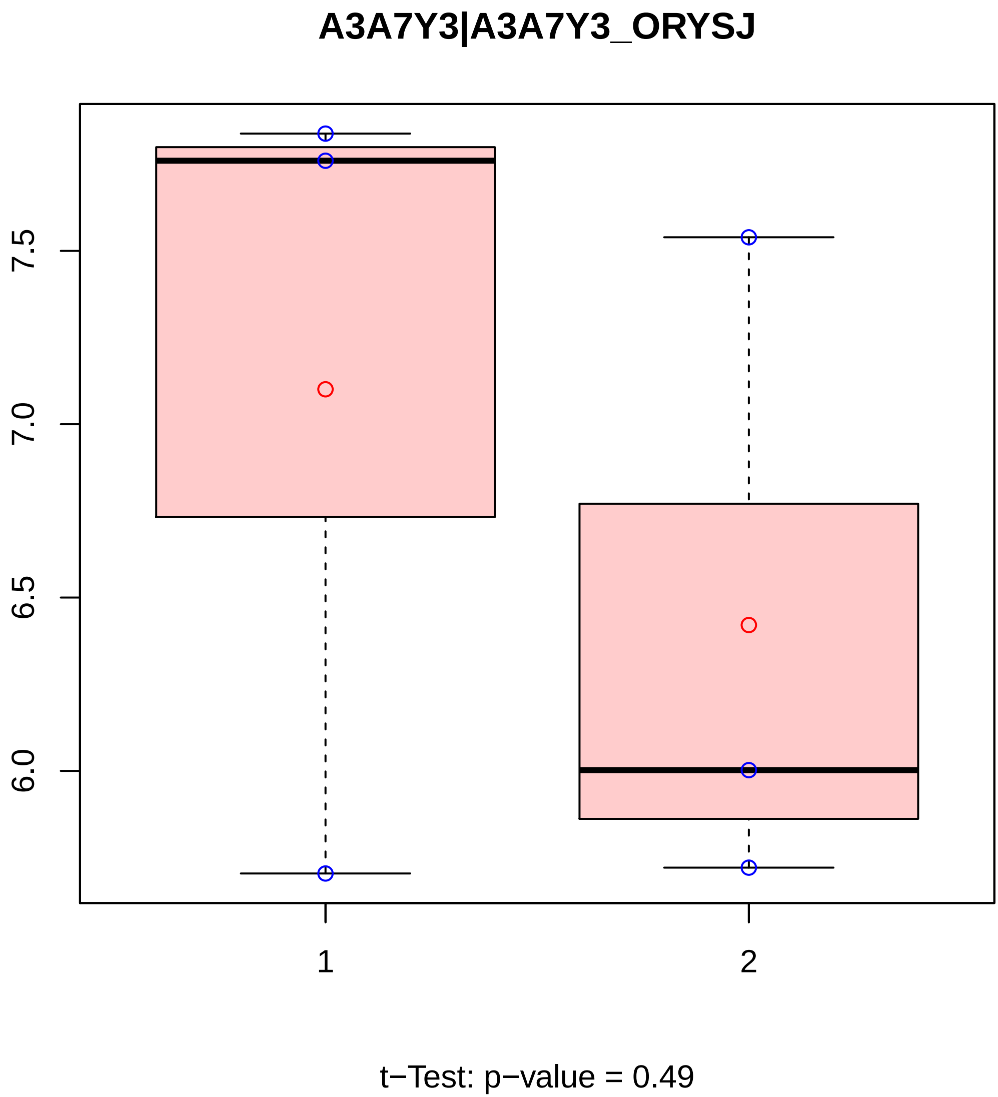

A3A7Y3|A3A7Y3_ORYSJ
7.5
7.0
6.5
6.0
1
2
t−Test: p−value = 0.49

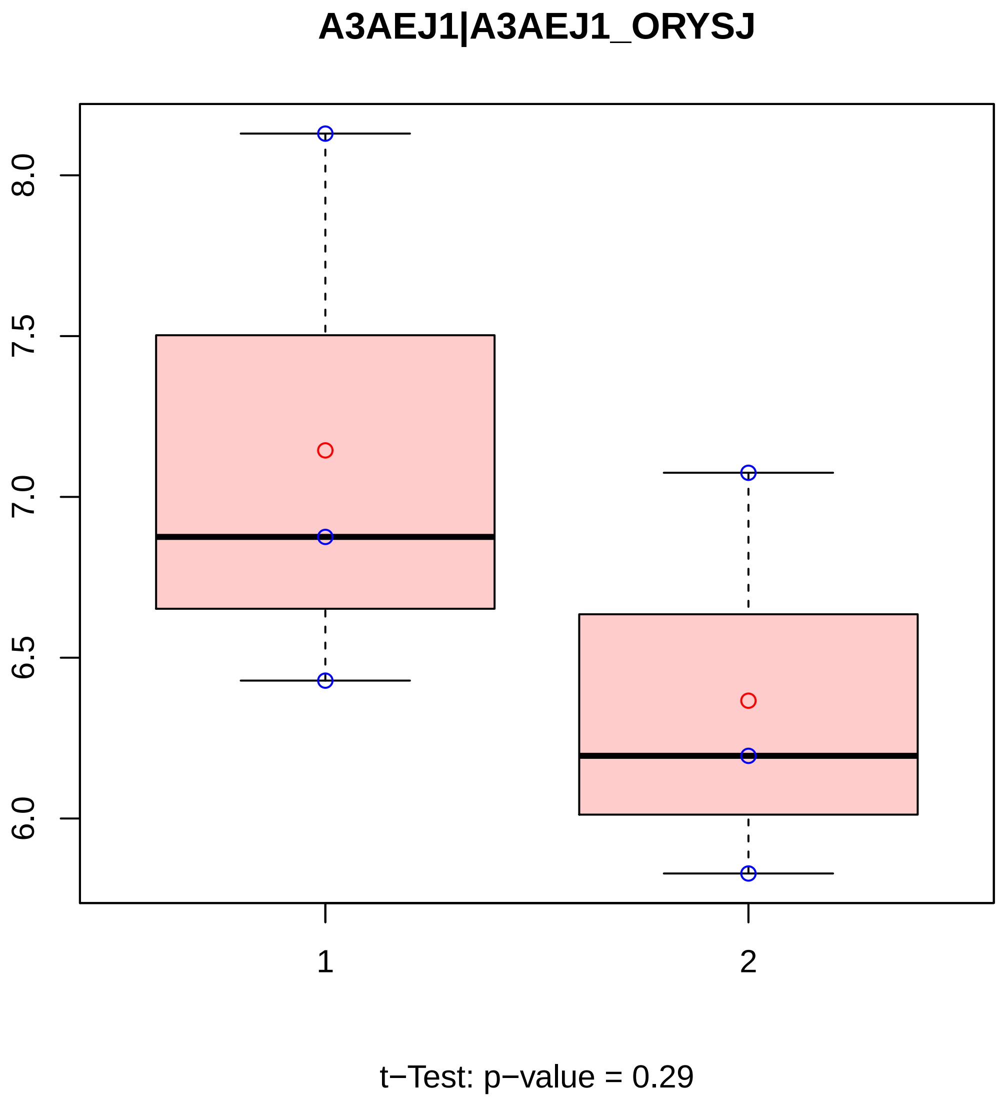

A3AEJ1|A3AEJ1_ORYSJ
8.0
7.5
7.0
6.5
6.0
1
2
t−Test: p−value = 0.29

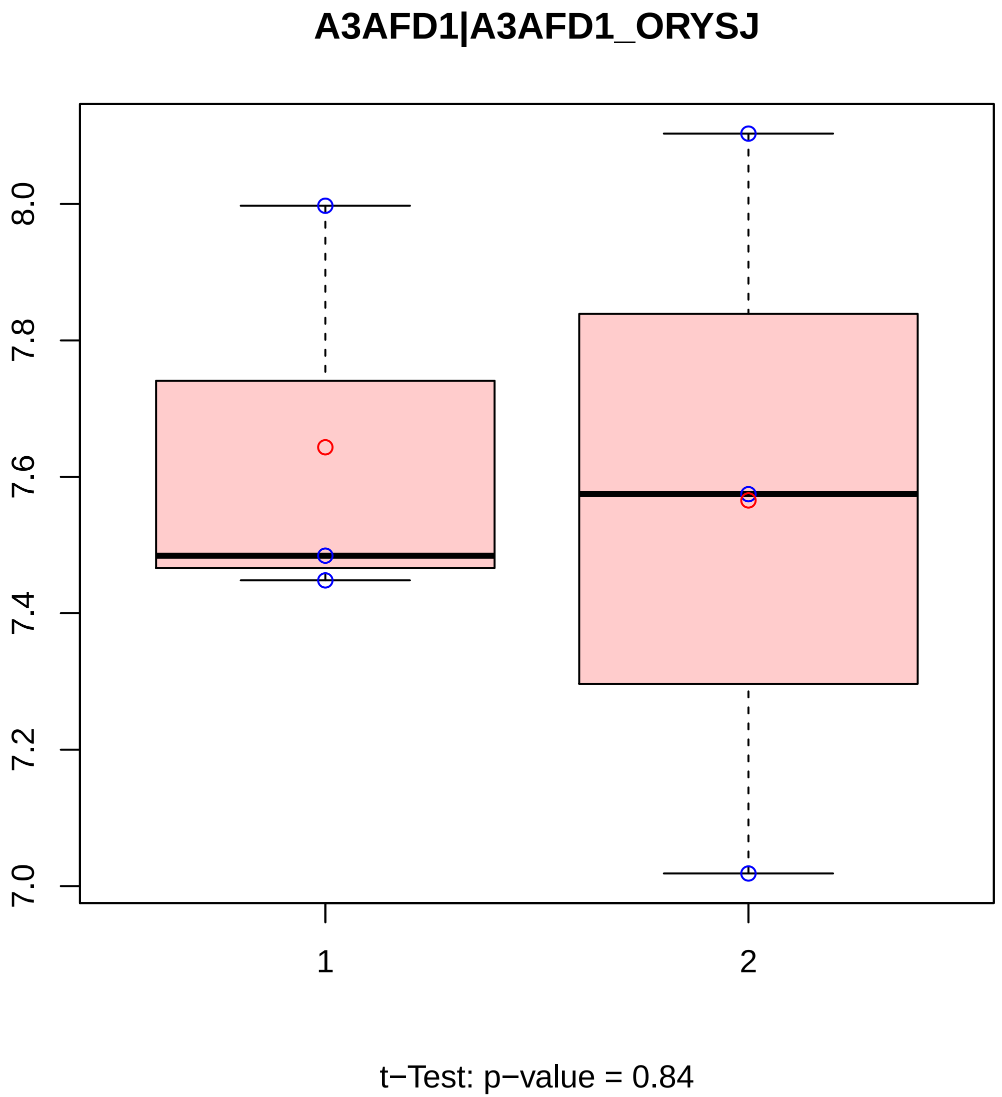
A3AFD1|A3AFD1_ORYSJ
8.0
7.8
7.6
7.4
7.2
7.0
1
2
t−Test: p−value = 0.84

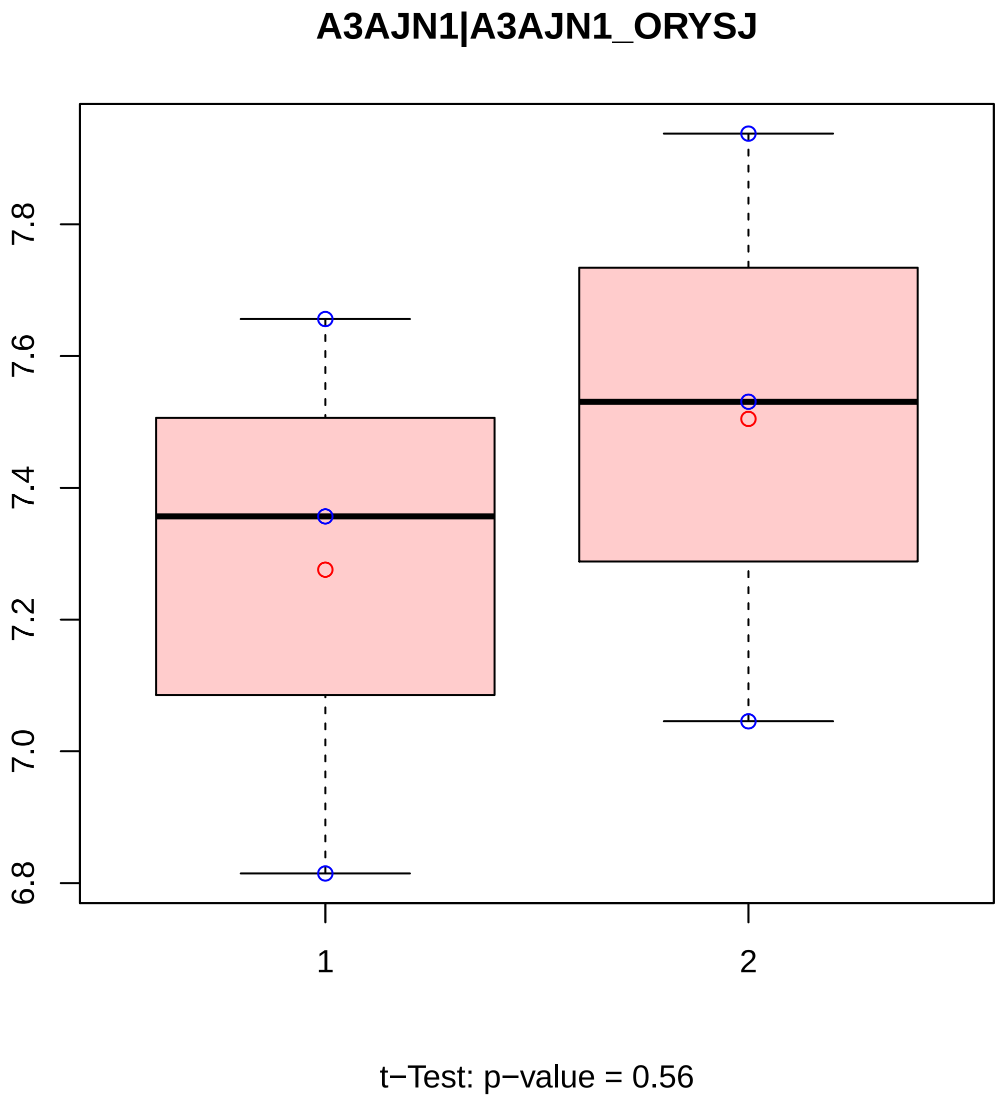
A3AJN1|A3AJN1_ORYSJ
6.8
7.0
7.2
7.4
7.6
7.8
1
2
t−Test: p−value = 0.56

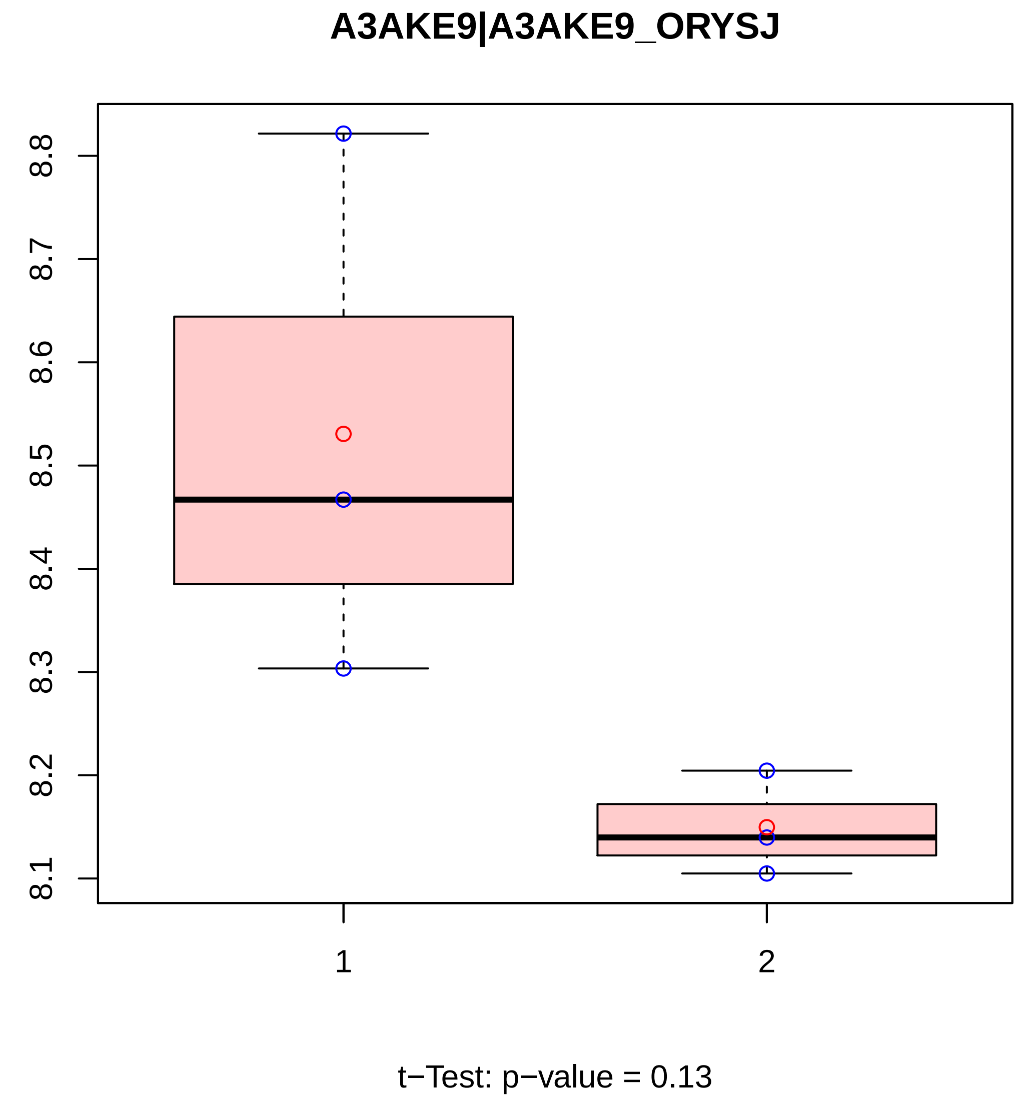

A3AKE9|A3AKE9_ORYSJ
8.1
8.2
8.3
8.4
8.5
8.6
8.7
8.8
1
2
t−Test: p−value = 0.13

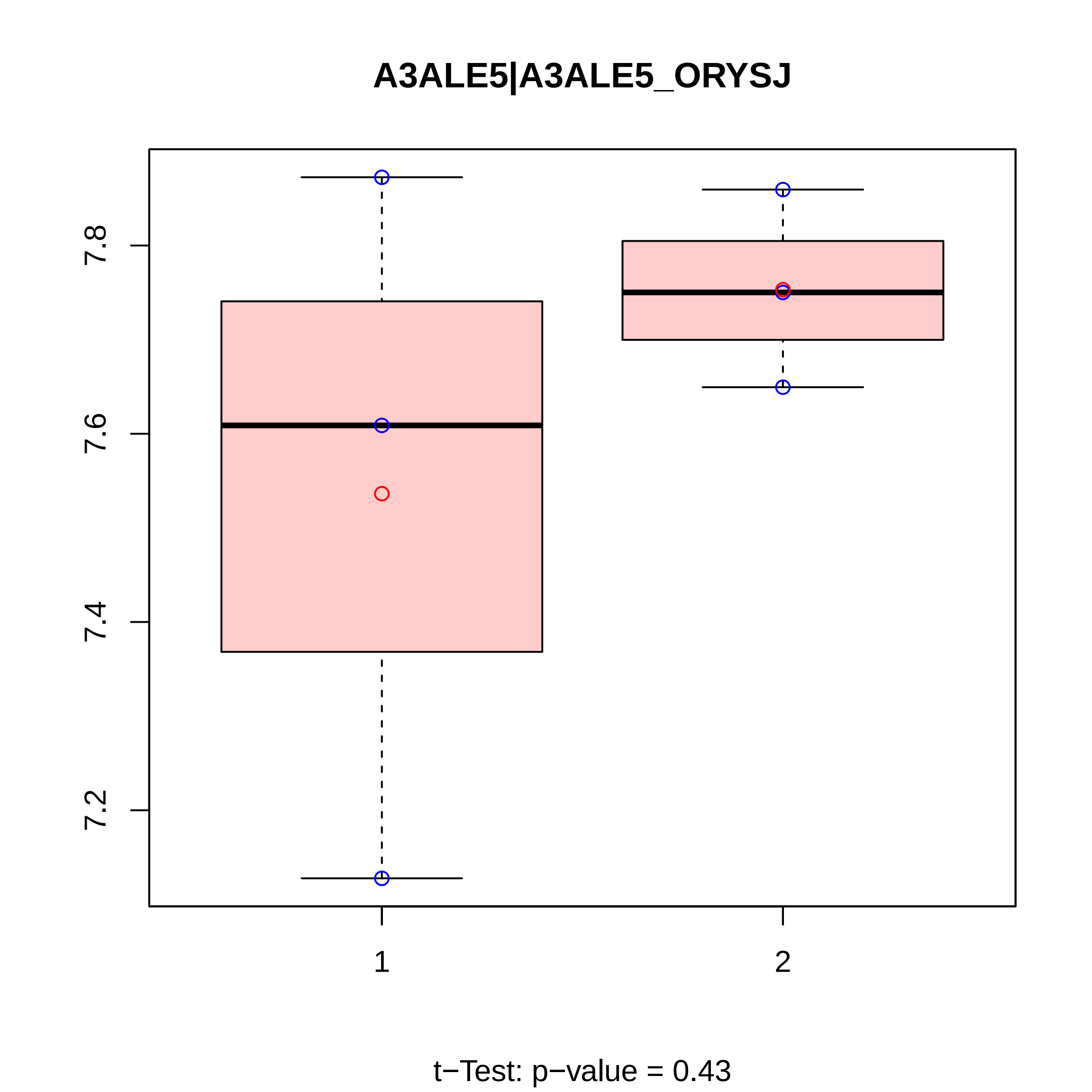
A3ALE5|A3ALE5_ORYSJ
7.2
7.4
7.6
7.8
1
2
t−Test: p−value = 0.43

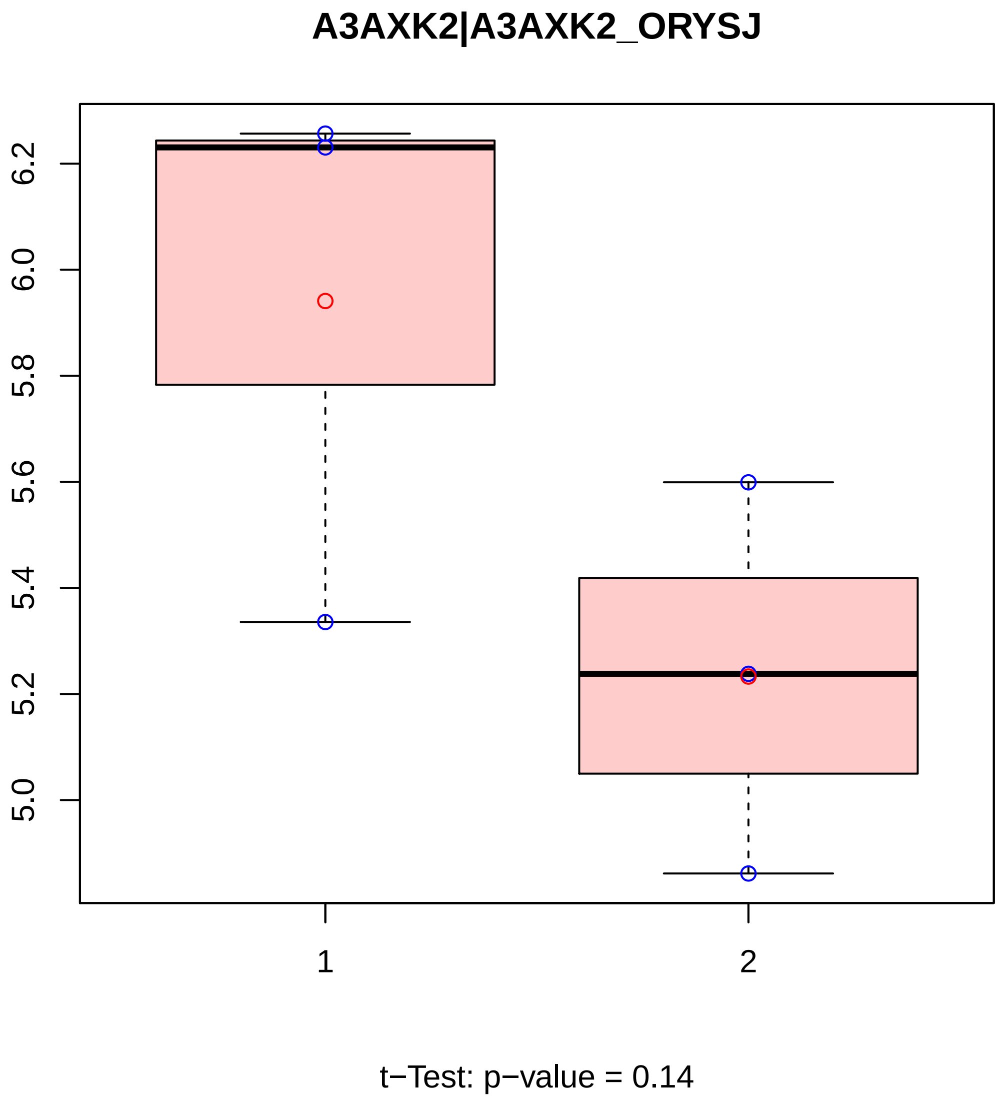

A3AXK2|A3AXK2_ORYSJ
6.2
6.0
5.8
5.6
5.4
5.2
5.0
1
2
t−Test: p−value = 0.14

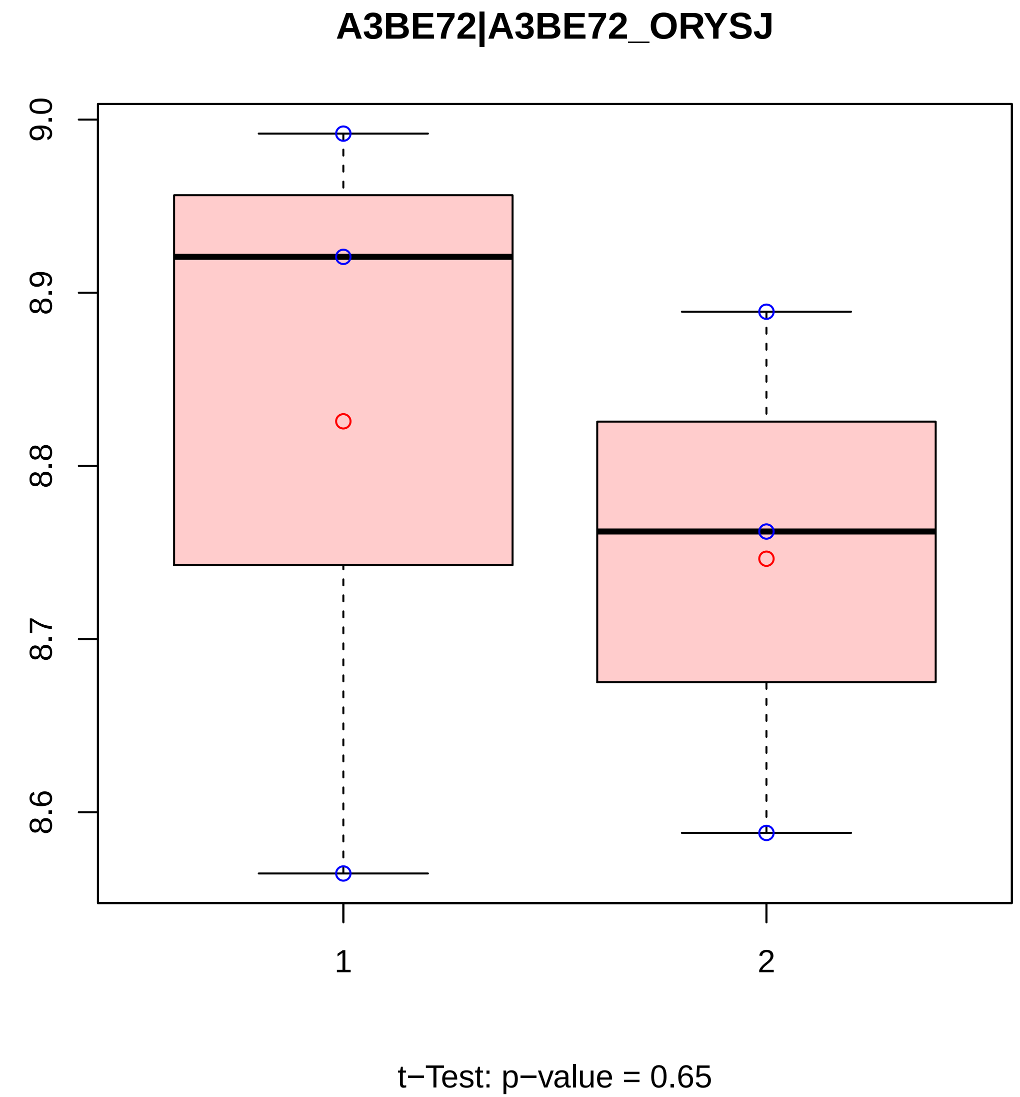

A3BE72|A3BE72_ORYSJ
9.0
8.9
8.8
8.7
8.6
1
2
t−Test: p−value = 0.65

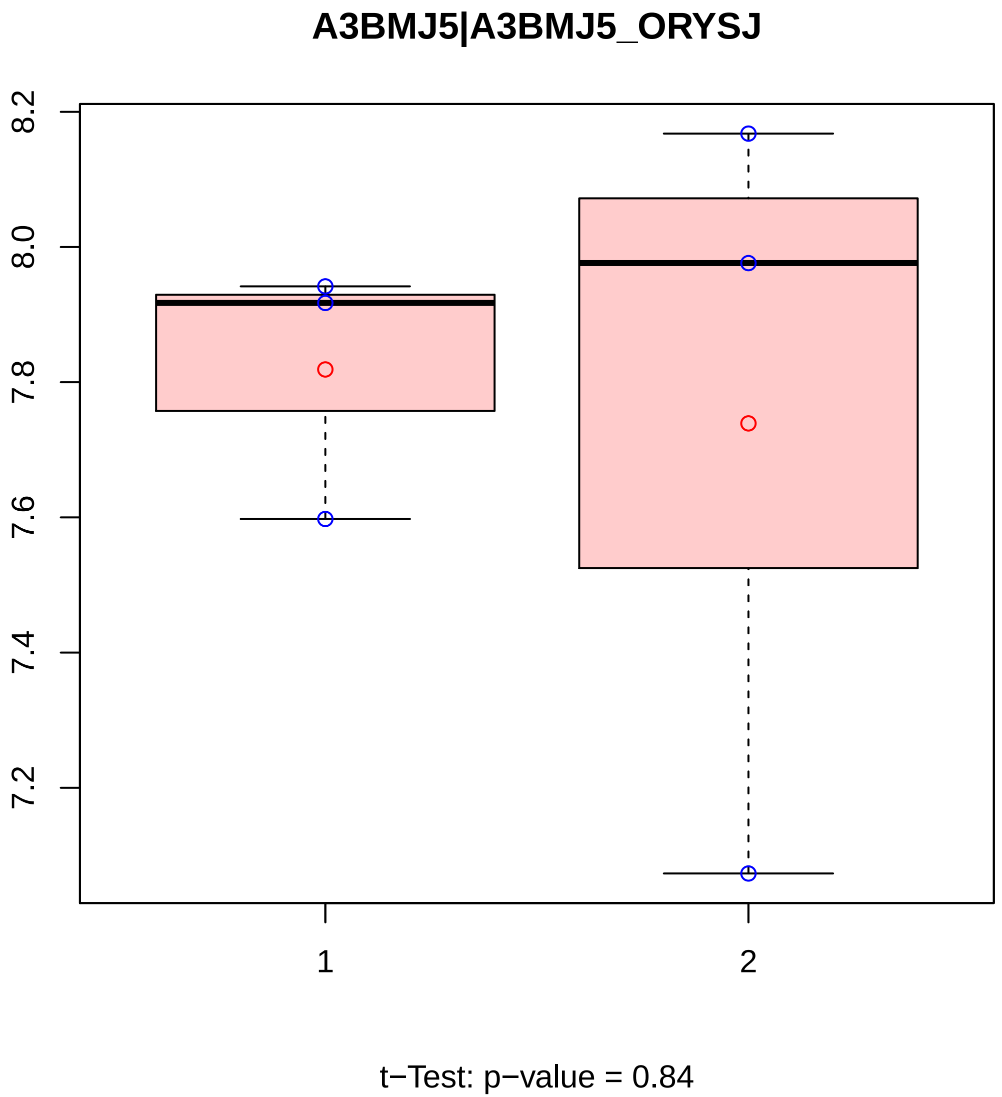

A3BMJ5|A3BMJ5_ORYSJ
8.2
8.0
7.8
7.6
7.4
7.2
1
2
t−Test: p−value = 0.84

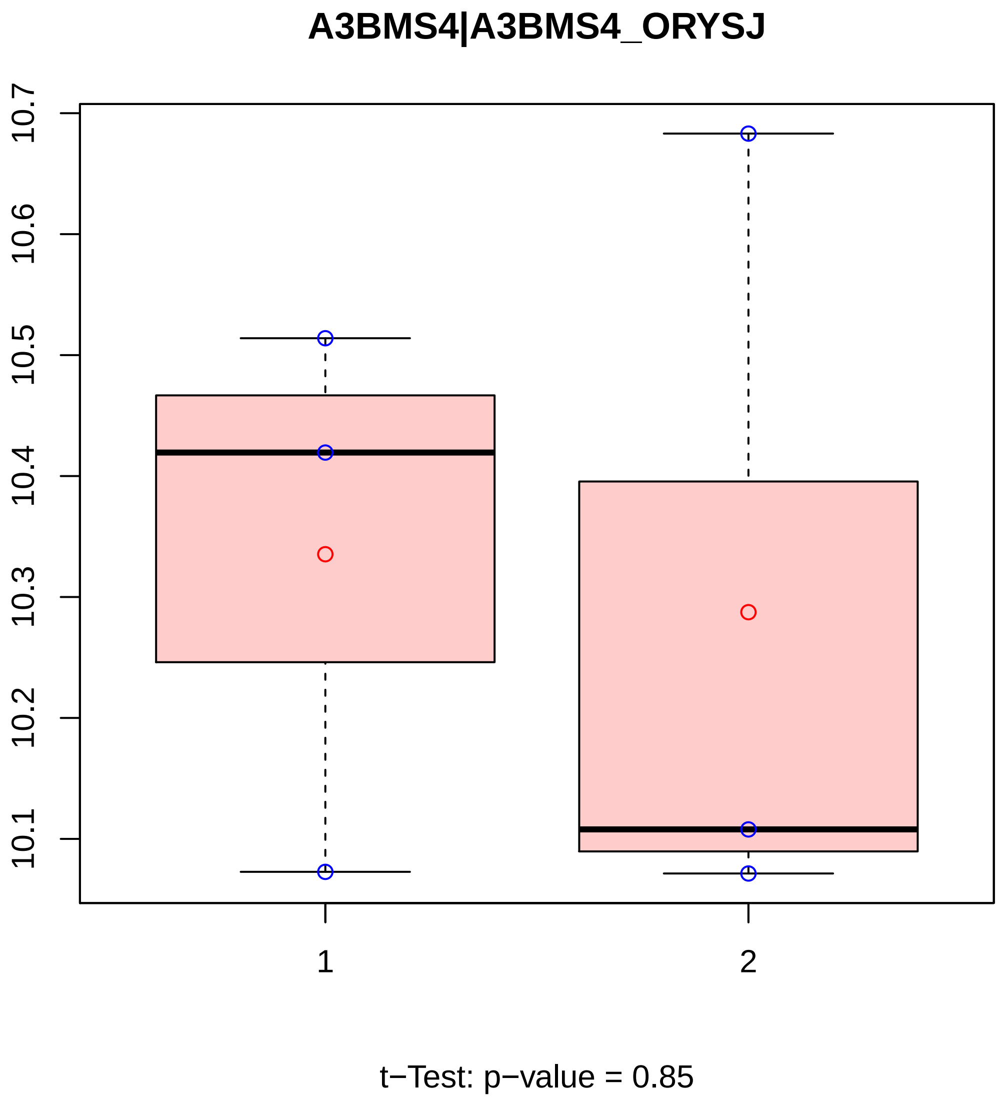
A3BMS4|A3BMS4_ORYSJ
10.7
10.6
10.5
10.4
10.3
10.2
10.1
1
2
t−Test: p−value = 0.85

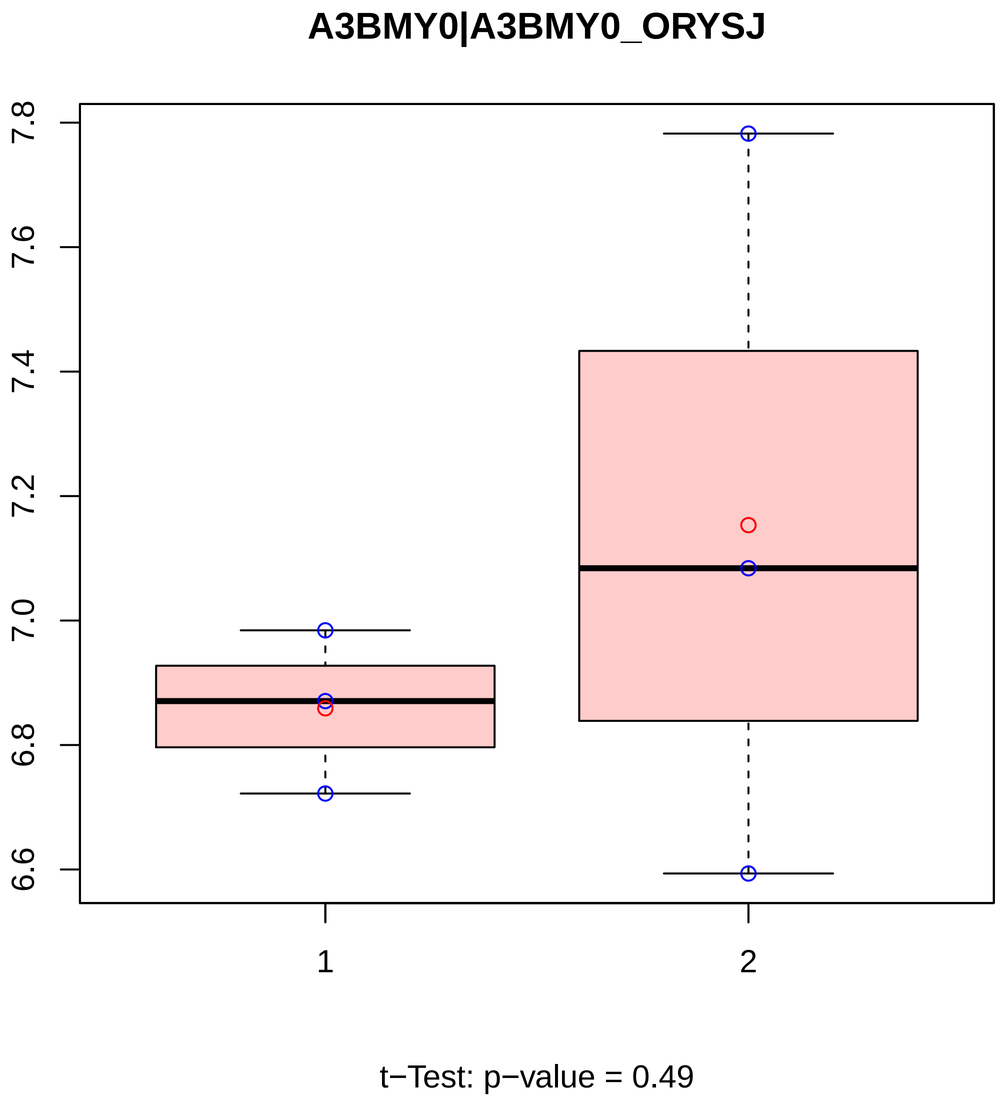
A3BMY0|A3BMY0_ORYSJ
7.8
7.6
7.4
7.2
7.0
6.8
6.6
1
2
t−Test: p−value = 0.49

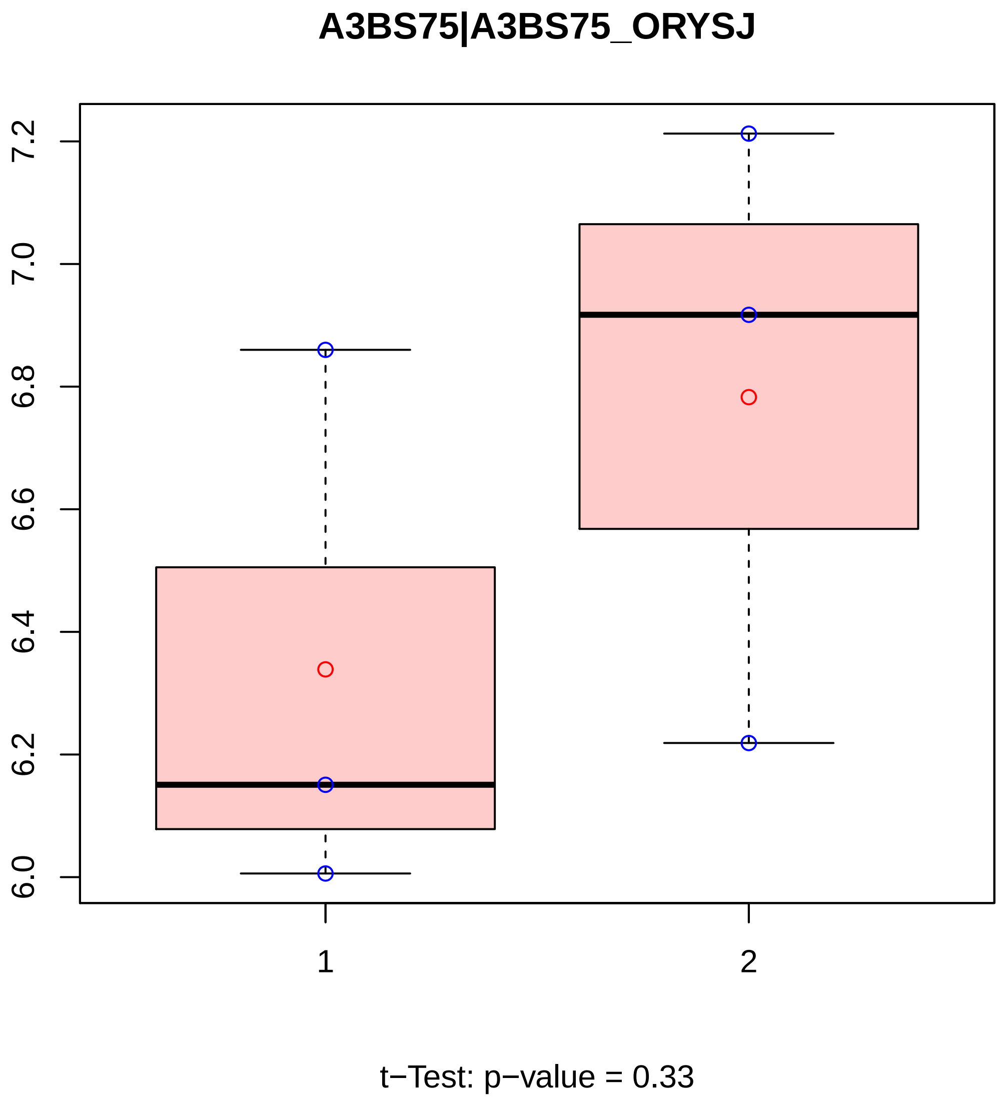

A3BS75|A3BS75_ORYSJ
7.2
7.0
6.8
6.6
6.4
6.2
6.0
1
2
t−Test: p−value = 0.33

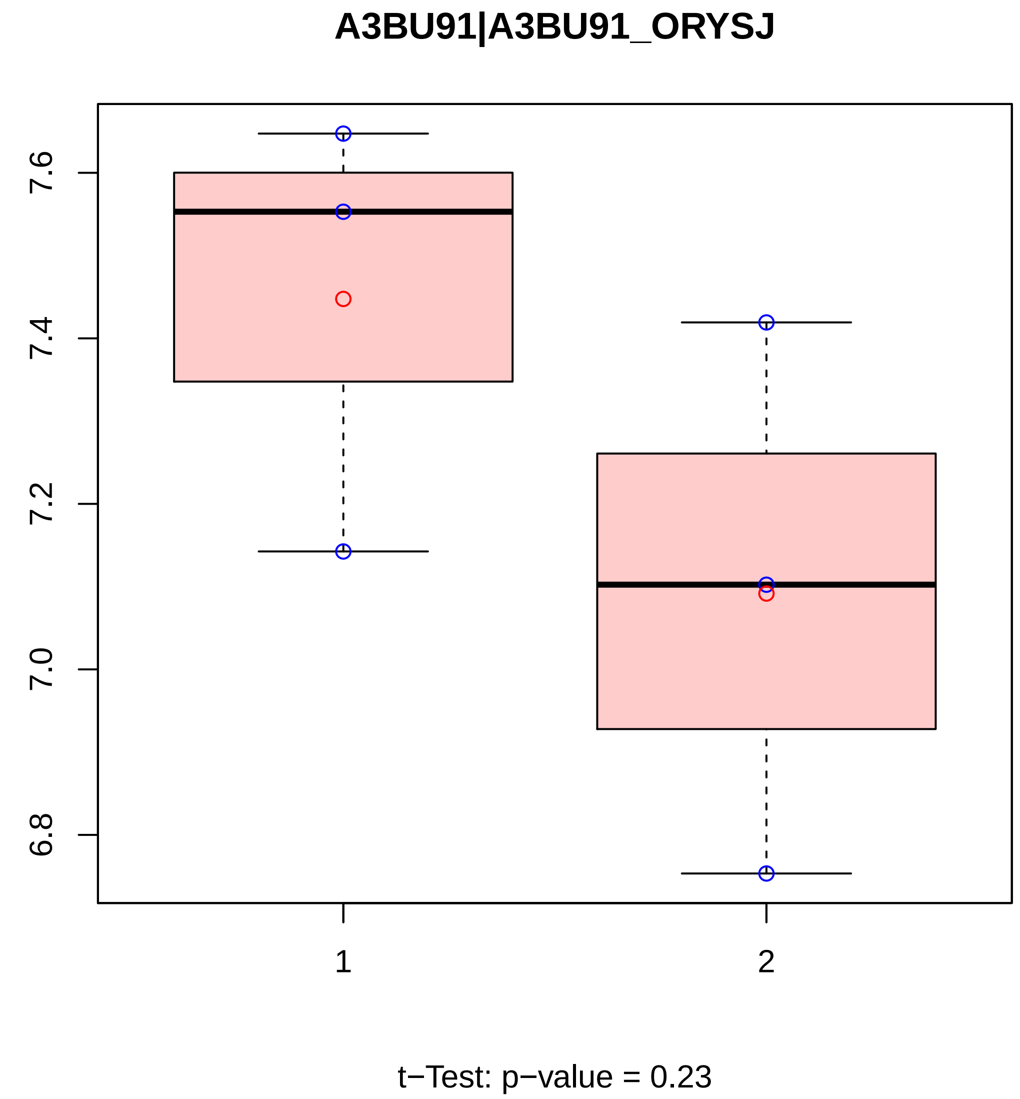
A3BU91|A3BU91_ORYSJ
7.6
7.4
7.2
7.0
6.8
1
2
t−Test: p−value = 0.23

A3BUT5|A3BUT5_ORYSJ

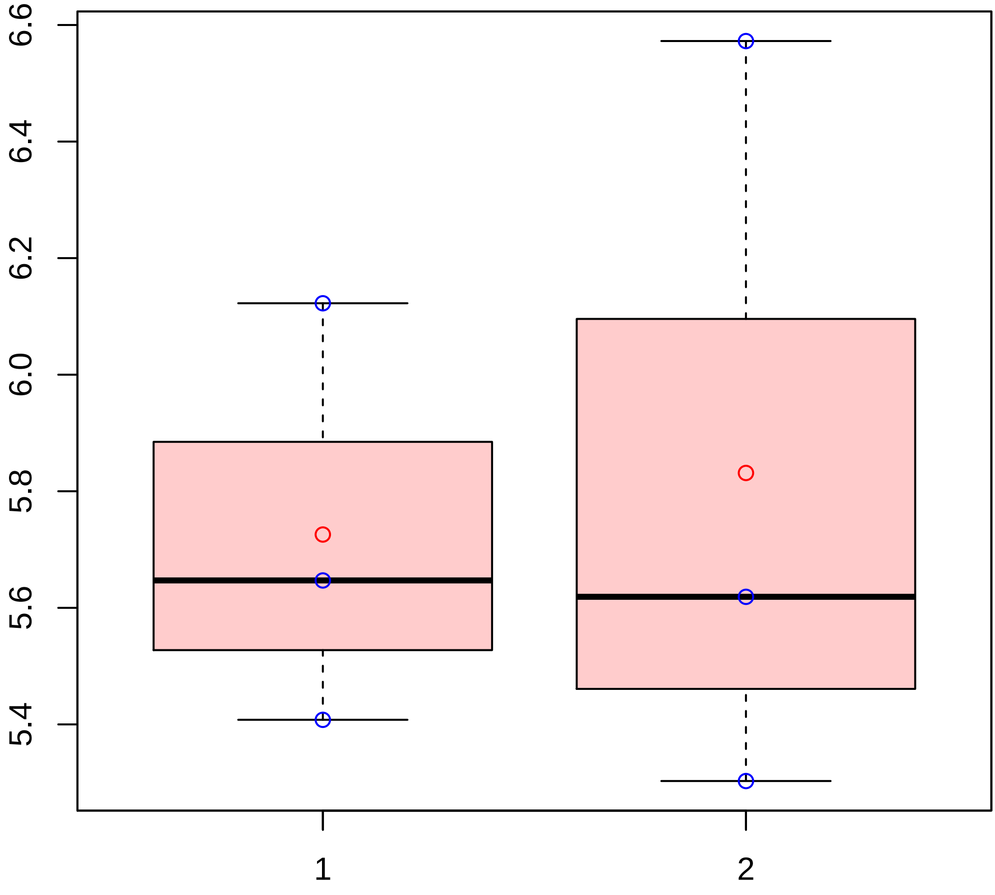

t−Test: p−value = 0.82

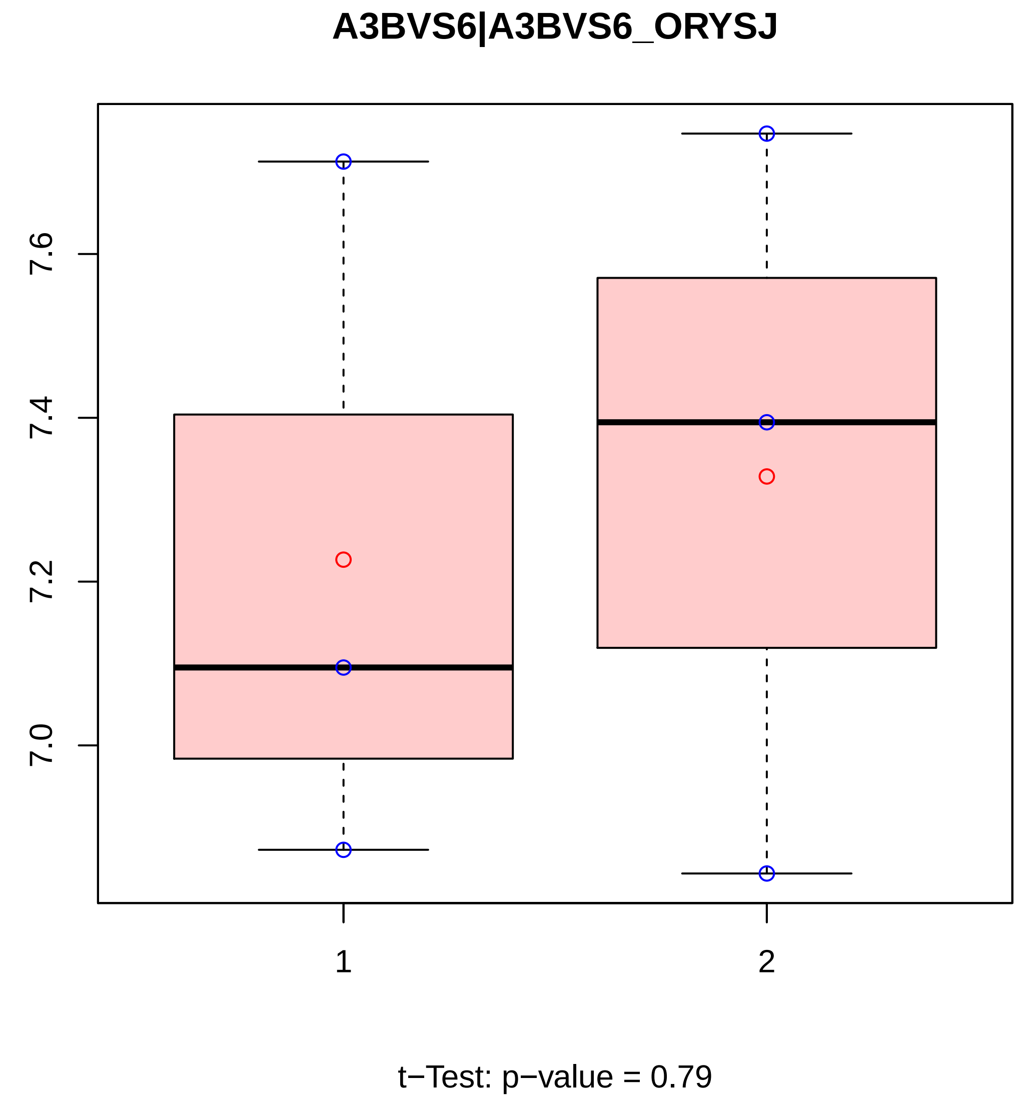
A3BVS6|A3BVS6_ORYSJ
7.0
7.2
7.4
7.6
1
2
t−Test: p−value = 0.79

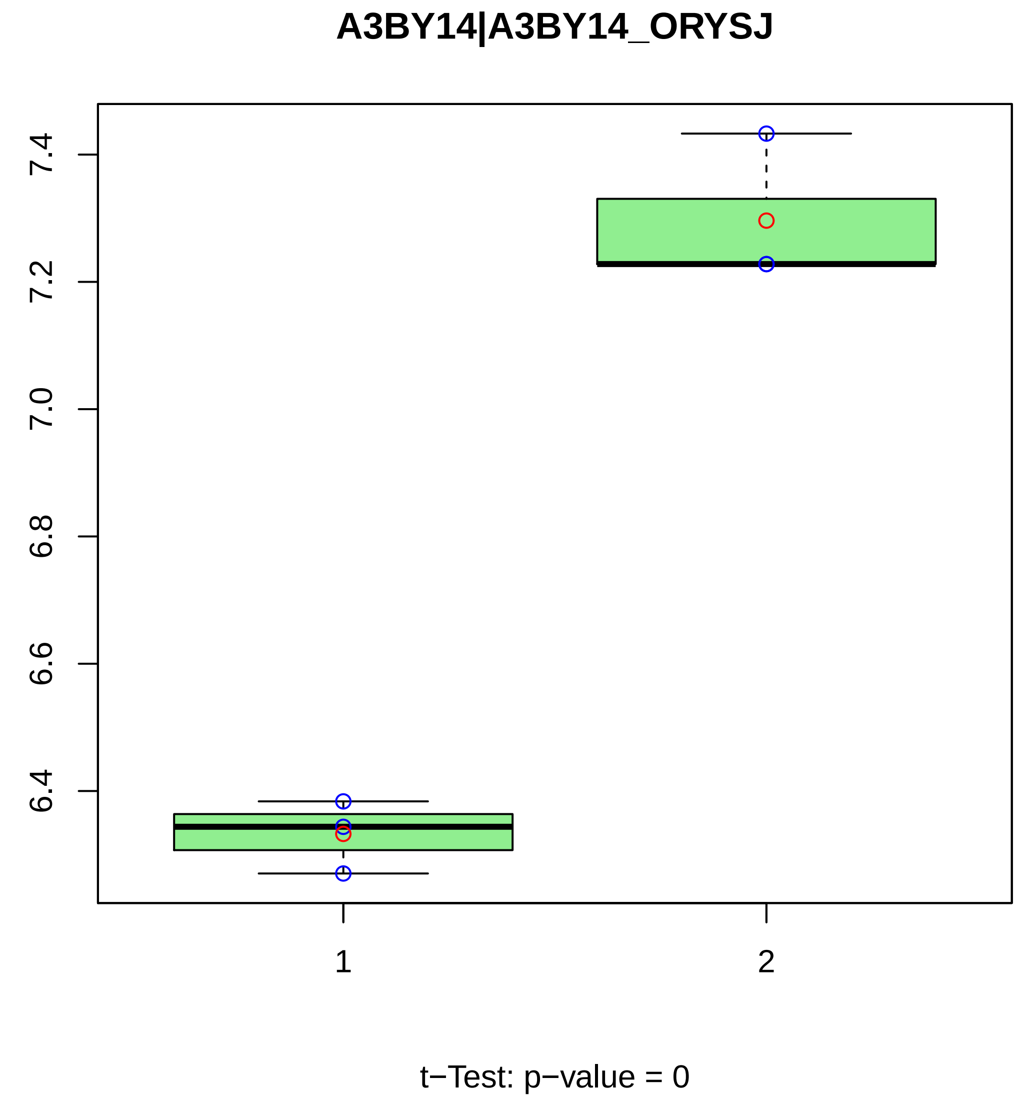
A3BY14|A3BY14_ORYSJ
7.4
7.2
7.0
6.8
6.6
6.4
1
2
t−Test: p−value = 0

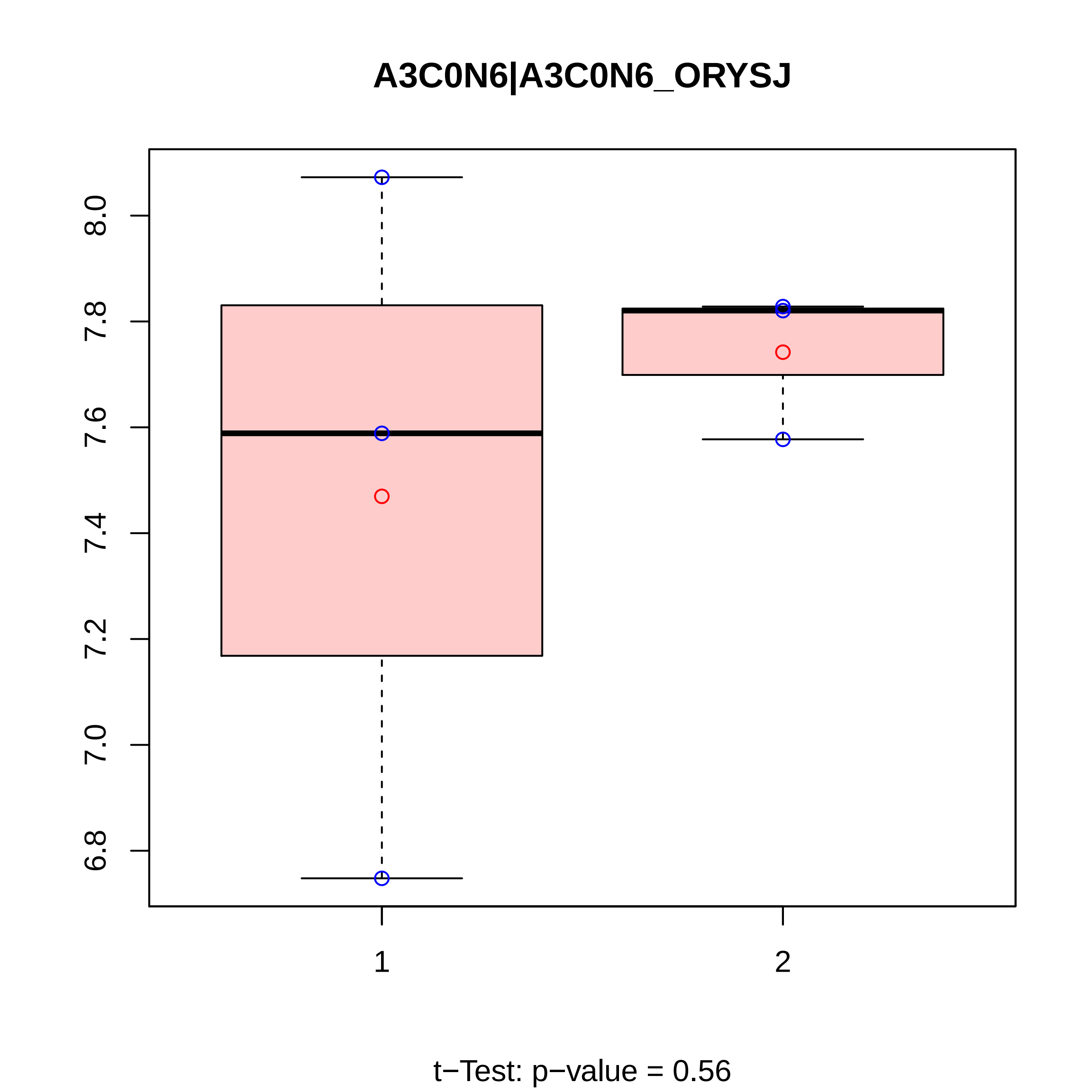
A3C0N6|A3C0N6_ORYSJ
8.0
7.8
7.6
7.4
7.2
7.0
6.8
1
2
t−Test: p−value = 0.56

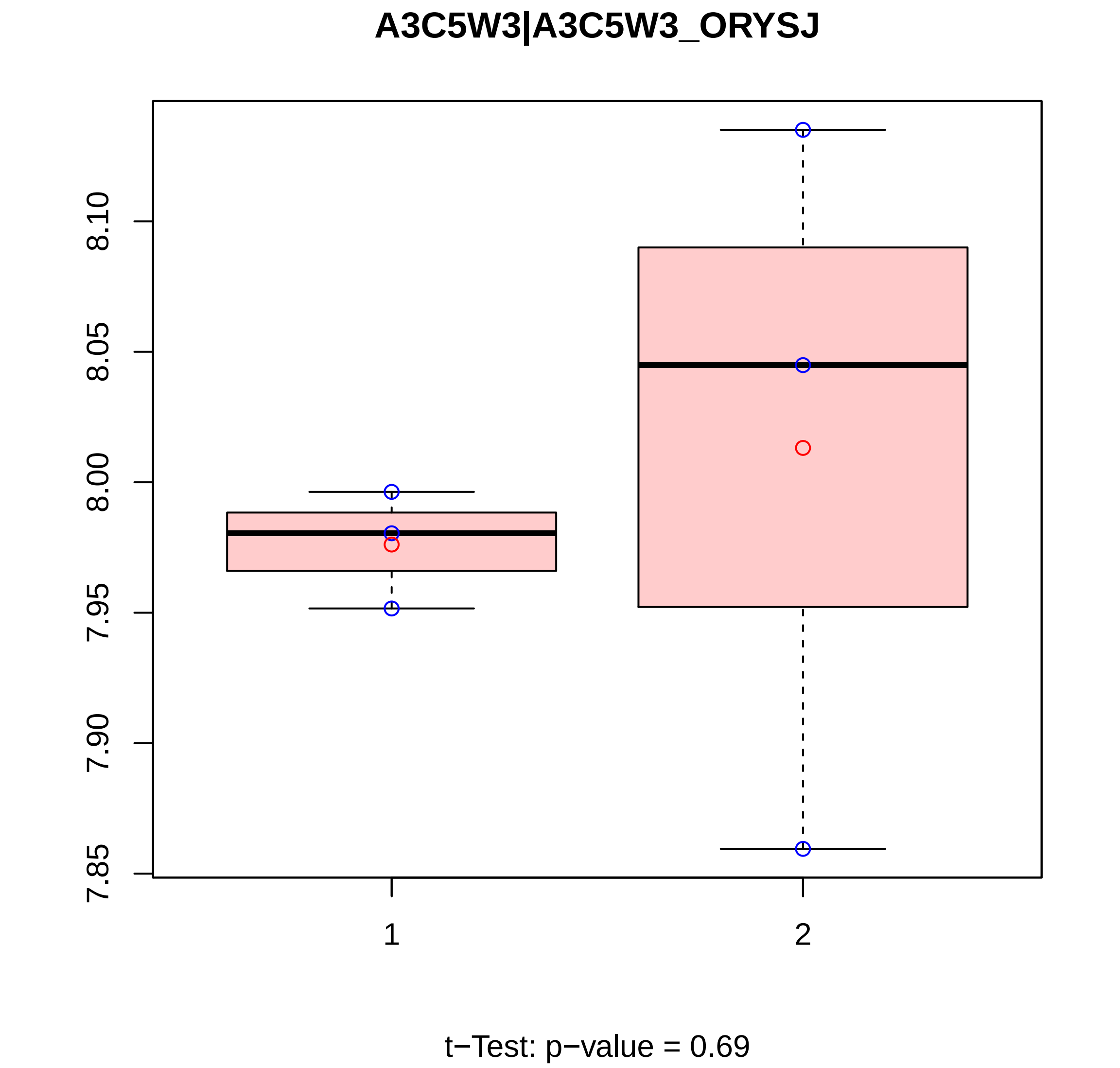

A3C5W3|A3C5W3_ORYSJ
8.10
8.05
8.00
7.95
7.90
7.85
1
2
t−Test: p−value = 0.69

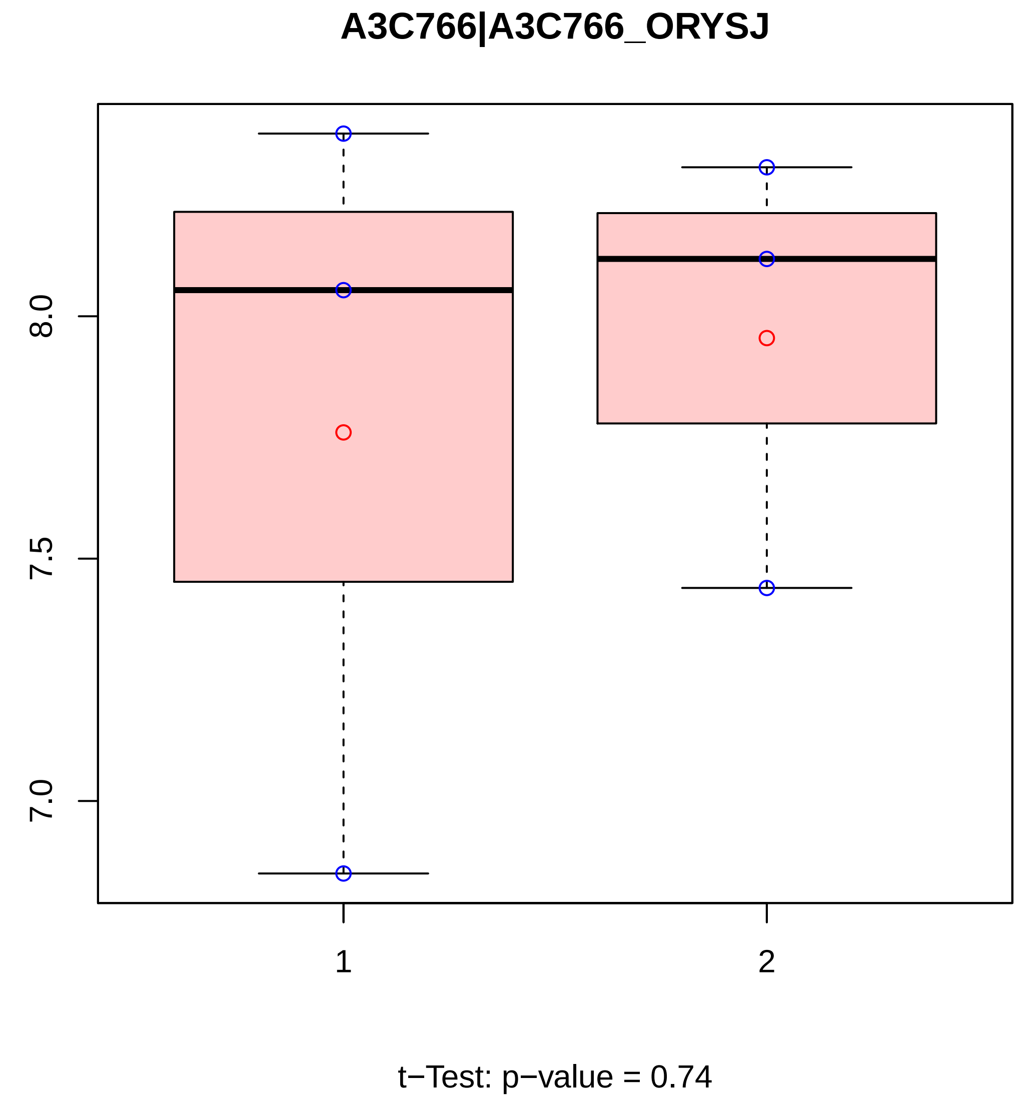
A3C766|A3C766_ORYSJ
8.0
7.5
7.0
1
2
t−Test: p−value = 0.74

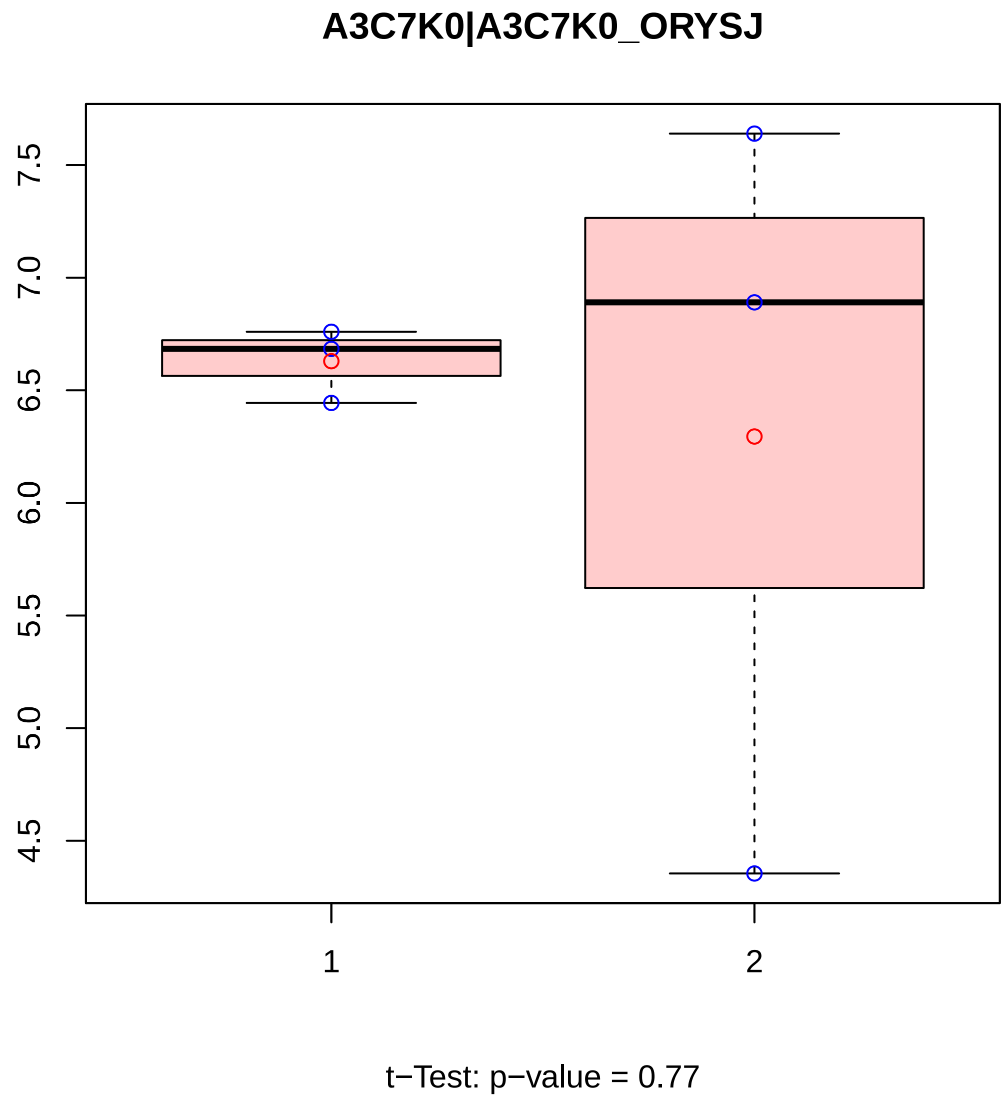
A3C7K0|A3C7K0_ORYSJ
7.5
7.0
6.5
6.0
5.5
5.0
4.5
1
2
t−Test: p−value = 0.77

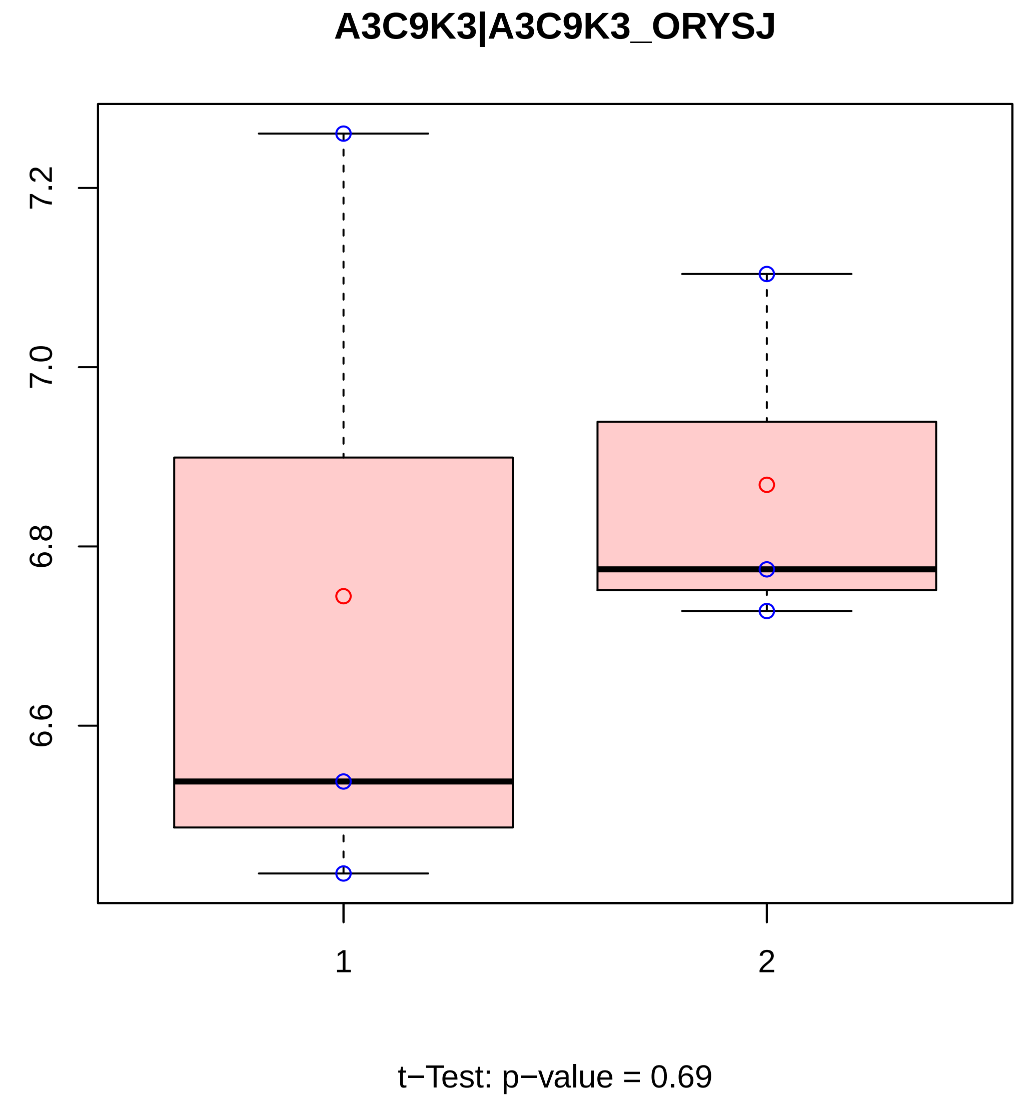
A3C9K3|A3C9K3_ORYSJ
7.2
7.0
6.8
6.6
1
2
t−Test: p−value = 0.69

A3CA11|A3CA11_ORYSJ

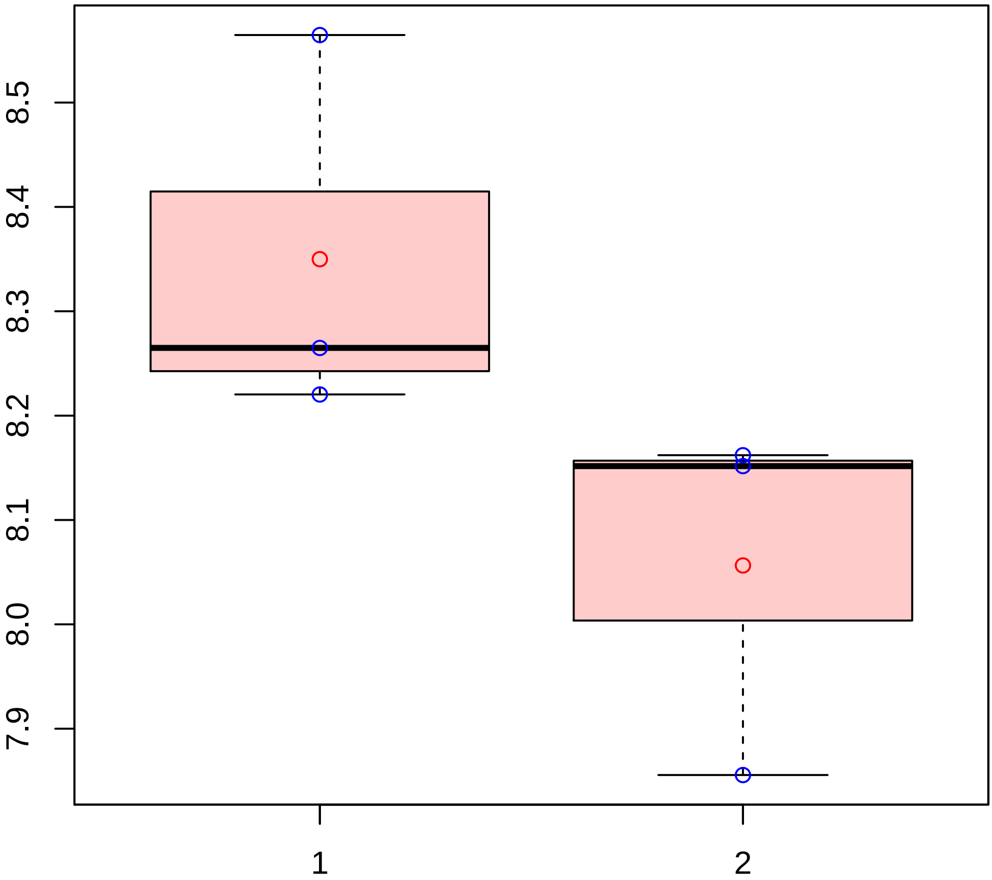

t−Test: p−value = 0.12

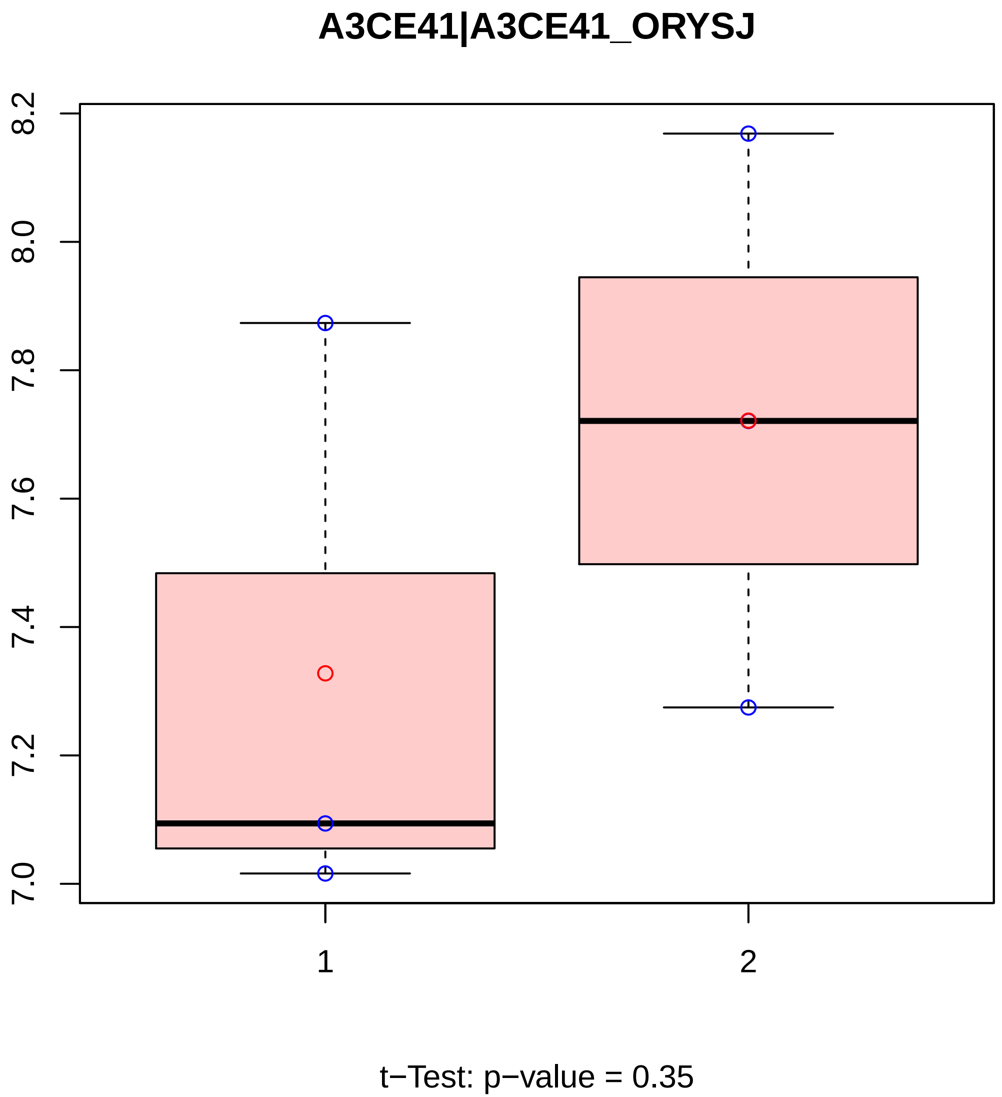
A3CE41|A3CE41_ORYSJ
8.2
8.0
7.8
7.6
7.4
7.2
7.0
1
2
t−Test: p−value = 0.35

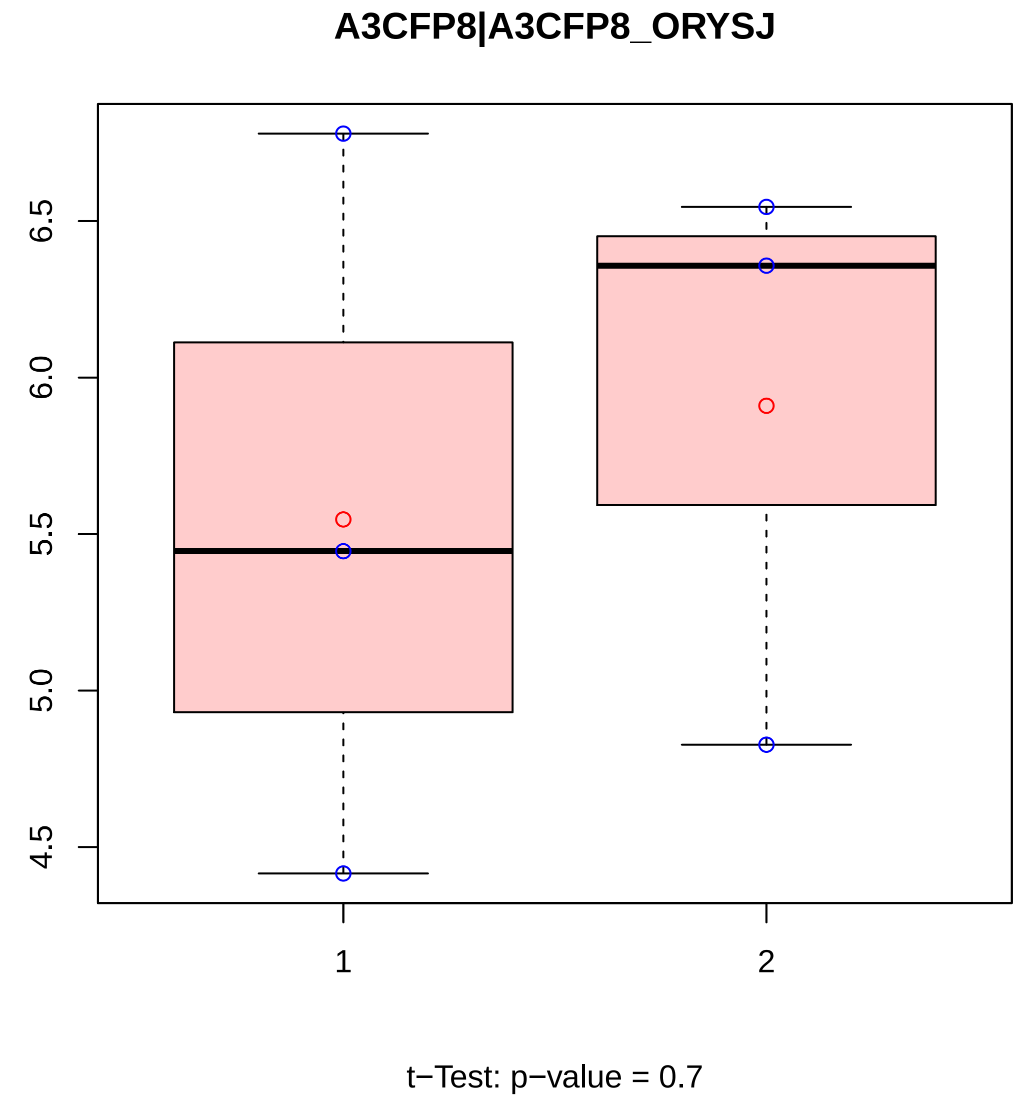
A3CFP8|A3CFP8_ORYSJ
6.5
6.0
5.5
5.0
4.5
1
2
t−Test: p−value = 0.7

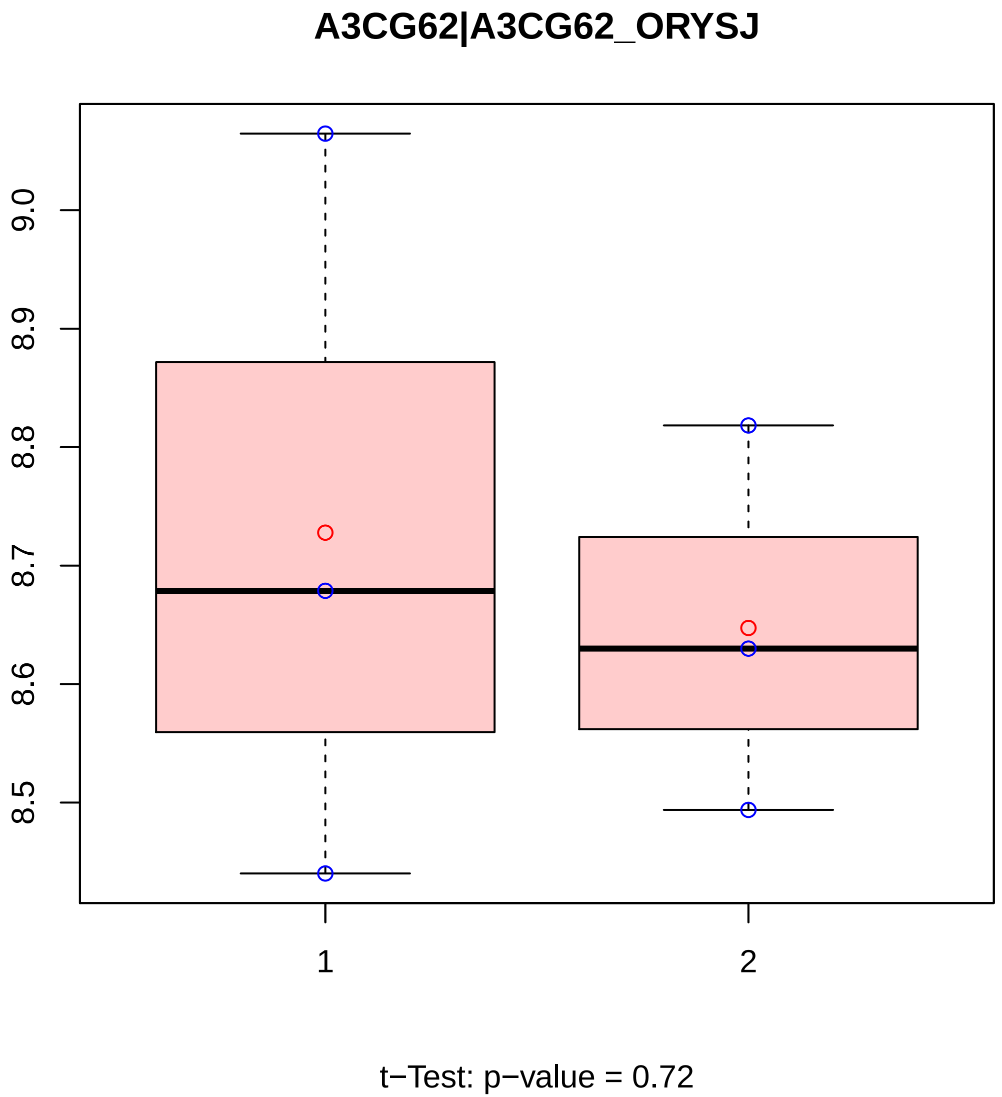

A3CG62|A3CG62_ORYSJ
9.0
8.9
8.8
8.7
8.6
8.5
1
2
t−Test: p−value = 0.72

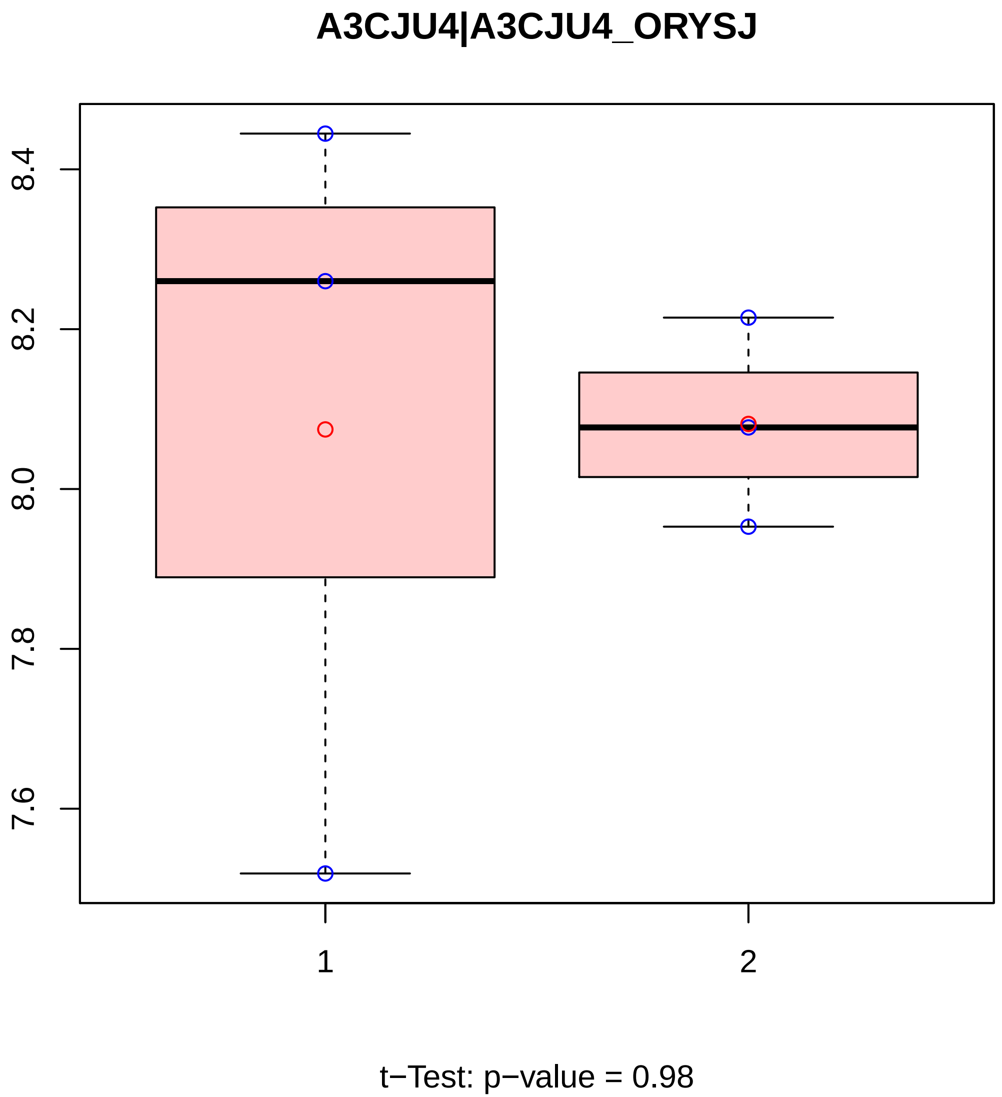

A3CJU4|A3CJU4_ORYSJ
8.4
8.2
8.0
7.8
7.6
1
2
t−Test: p−value = 0.98

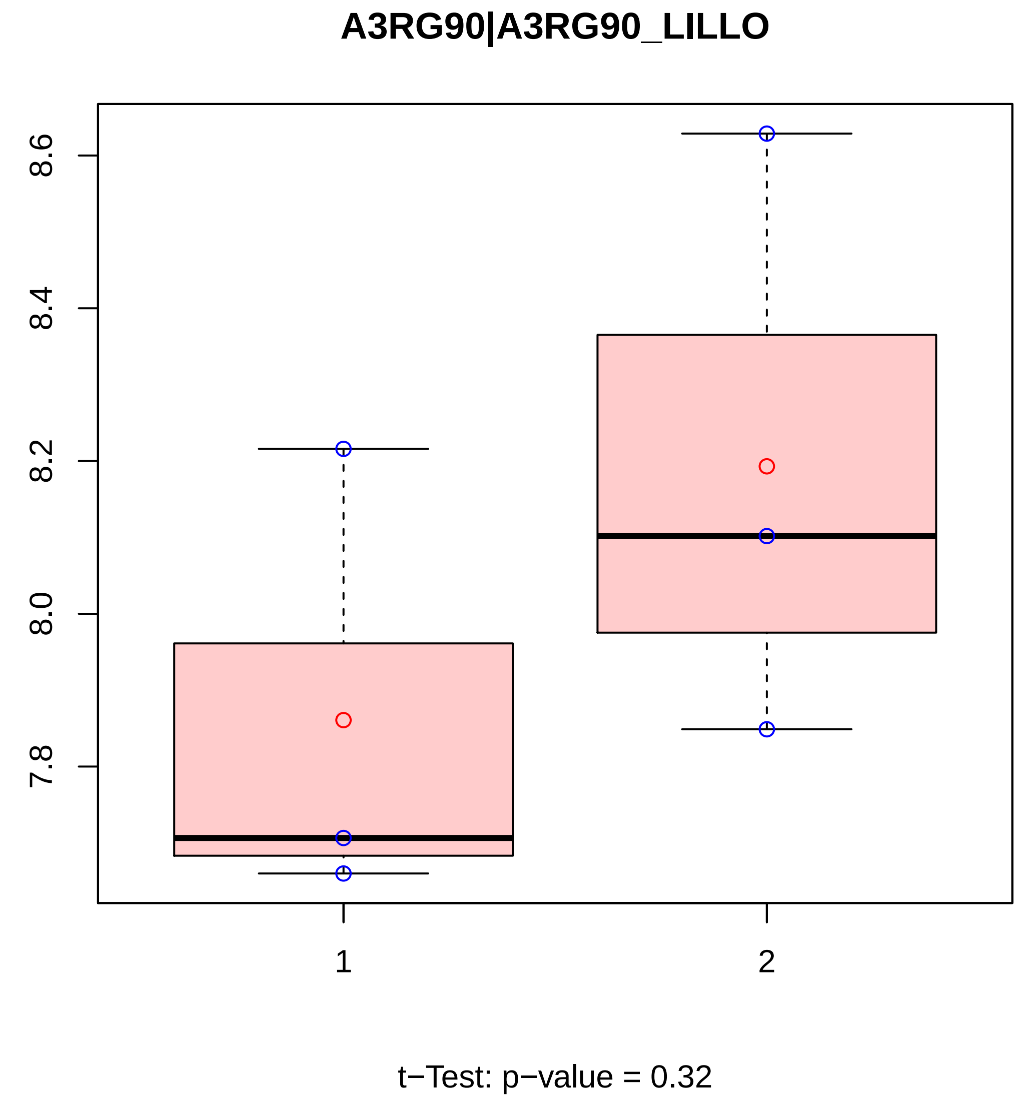

A3RG90|A3RG90_LILLO
8.6
8.4
8.2
8.0
7.8
1
2
t−Test: p−value = 0.32

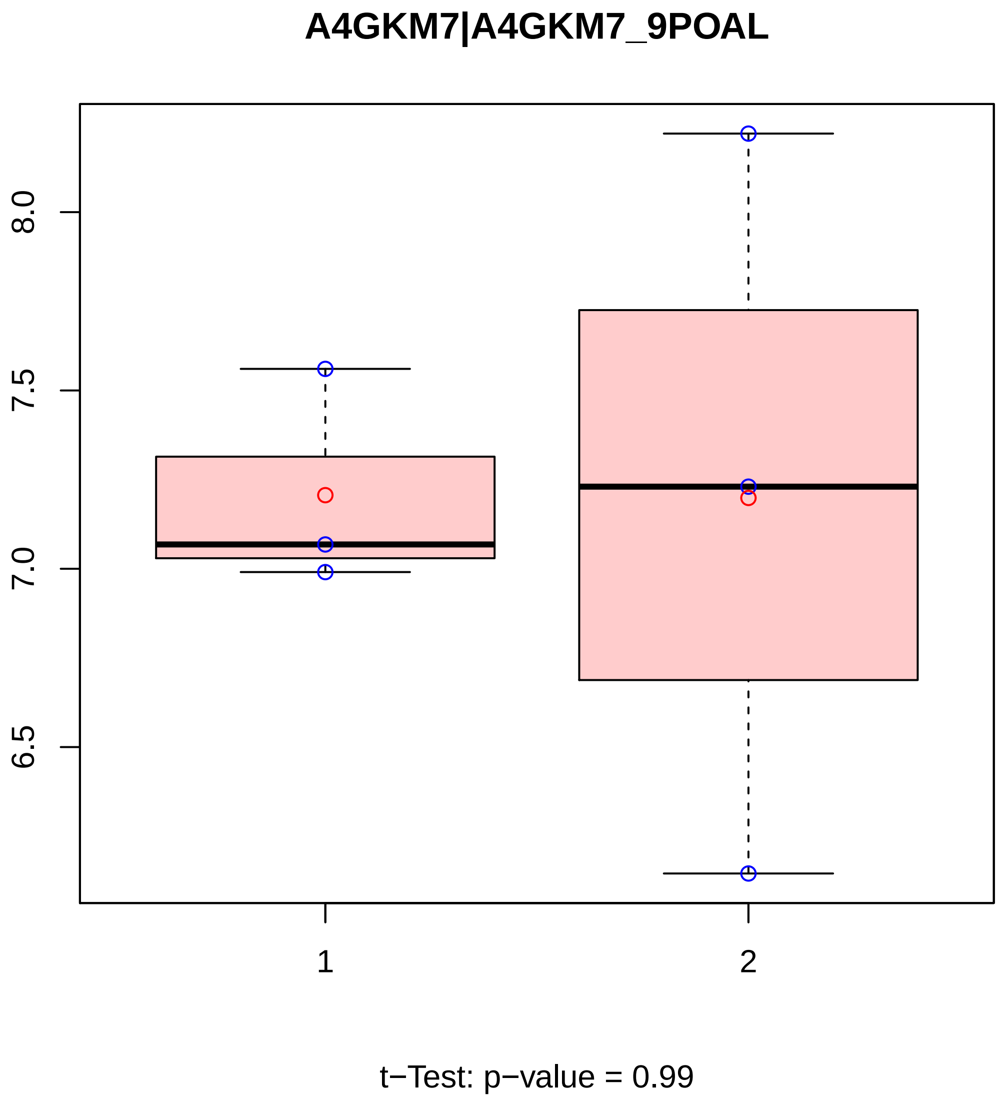
A4GKM7|A4GKM7_9POAL
8.0
7.5
7.0
6.5
1
2
t−Test: p−value = 0.99

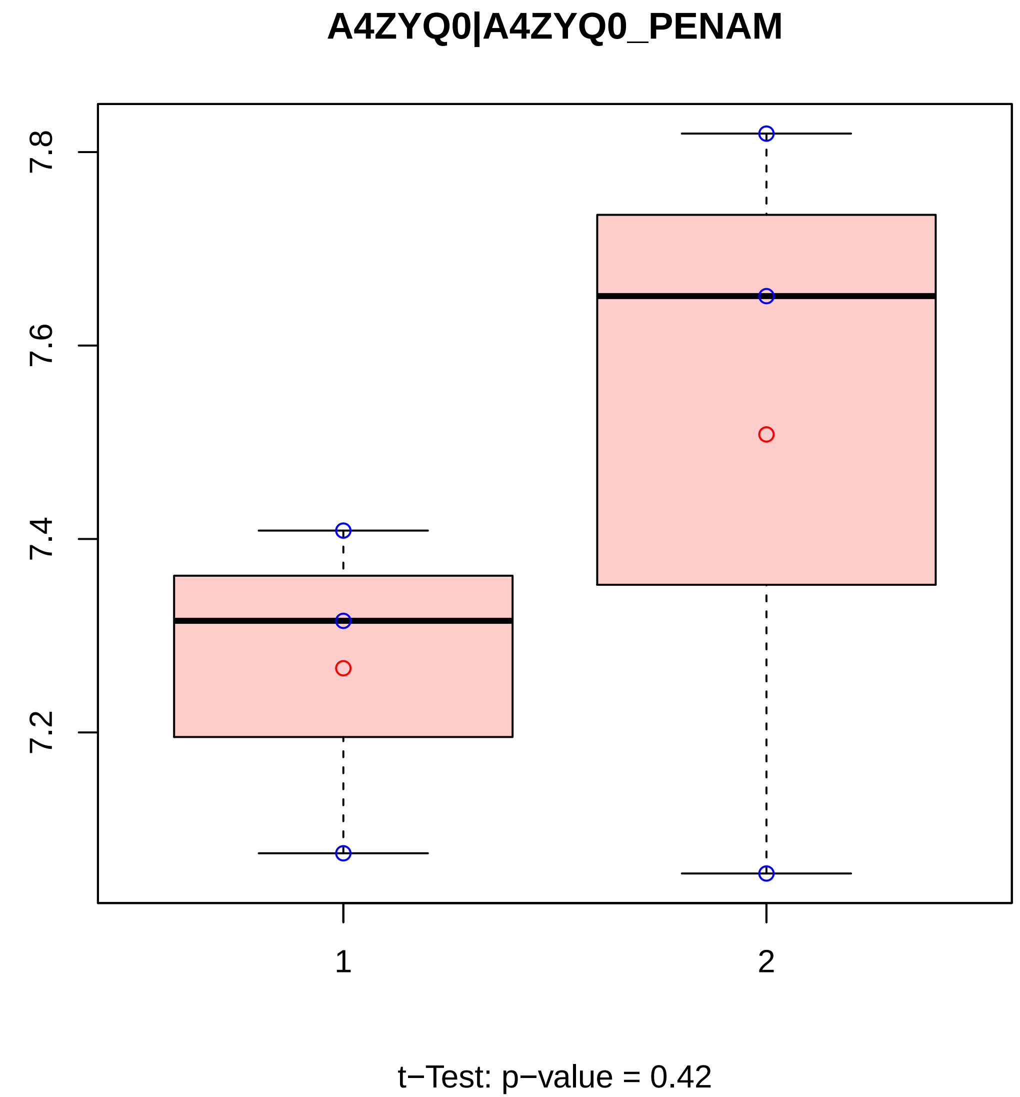
A4ZYQ0|A4ZYQ0_PENAM
7.2
7.4
7.6
7.8
1
2
t−Test: p−value = 0.42

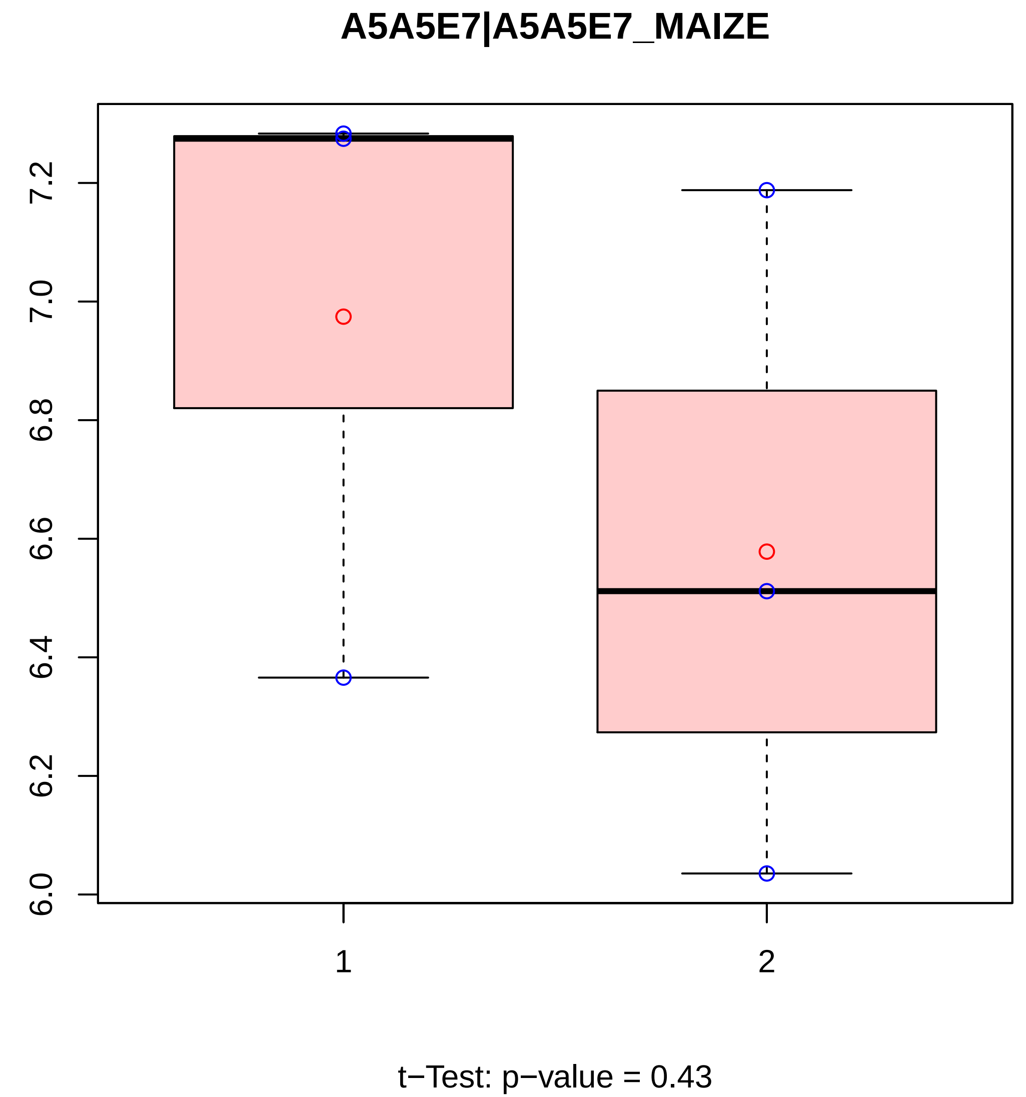
A5A5E7|A5A5E7_MAIZE
7.2
7.0
6.8
6.6
6.4
6.2
6.0
1
2
t−Test: p−value = 0.43

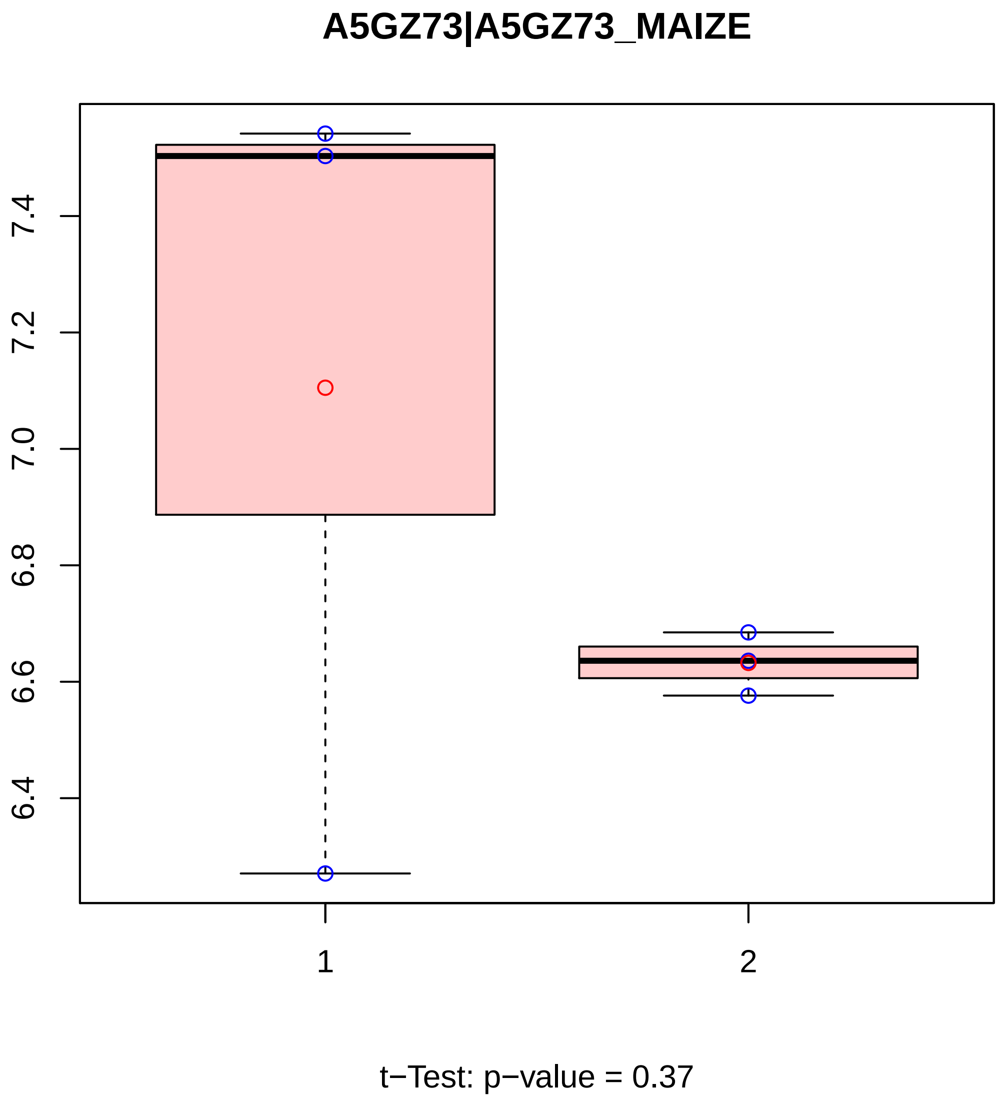

A5GZ73|A5GZ73_MAIZE
7.4
7.2
7.0
6.8
6.6
6.4
1
2
t−Test: p−value = 0.37

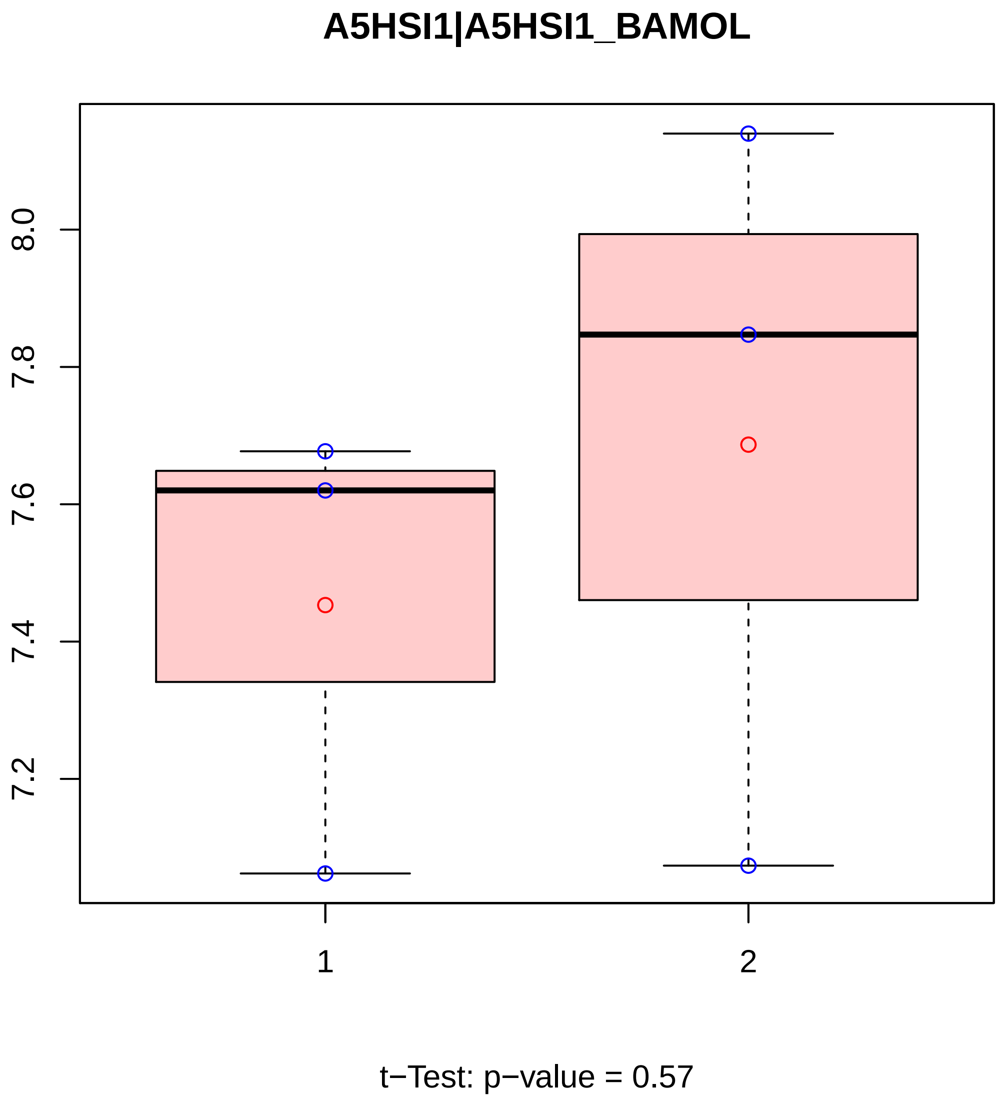

A5HSI1|A5HSI1_BAMOL
8.0
7.8
7.6
7.4
7.2
1
2
t−Test: p−value = 0.57

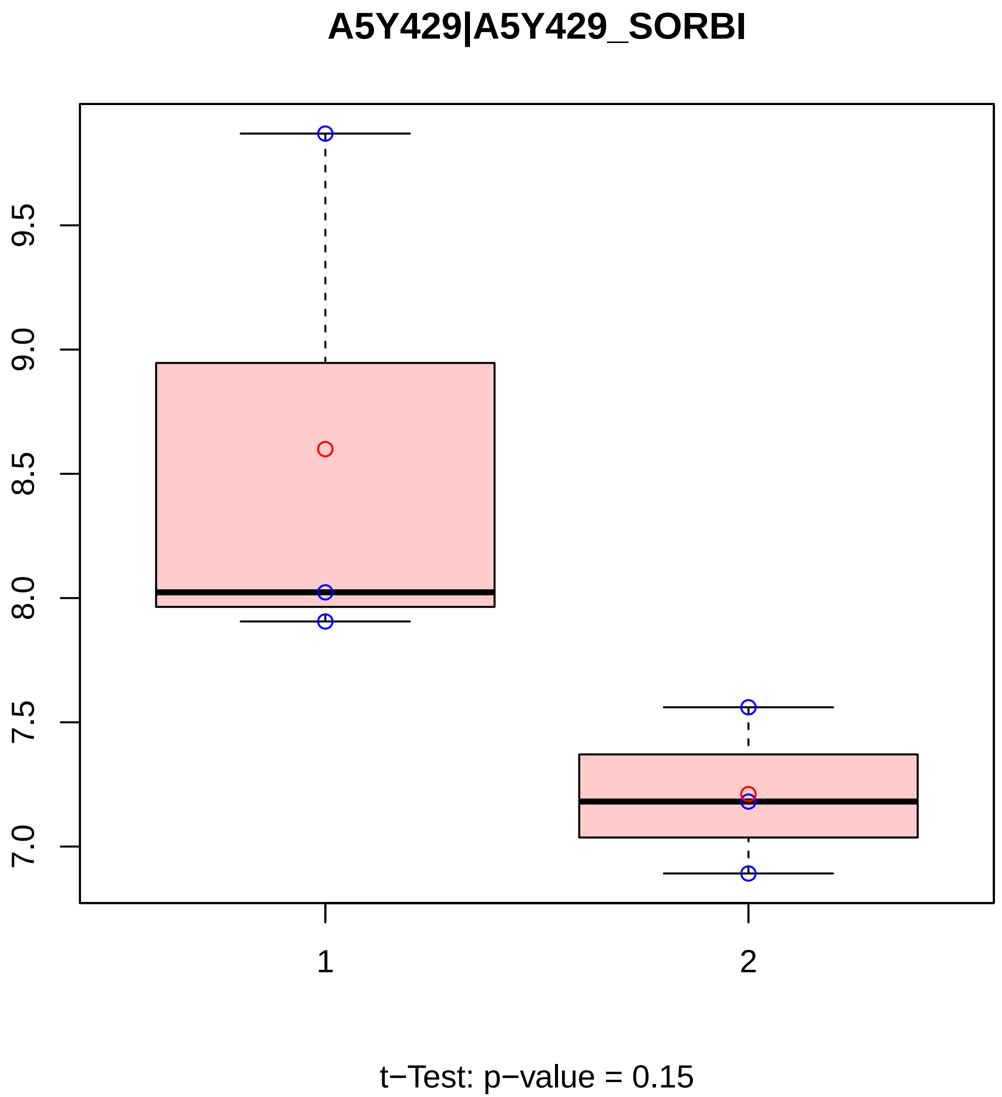
A5Y429|A5Y429_SORBI
9.5
9.0
8.5
8.0
7.5
7.0
1
2
t−Test: p−value = 0.15

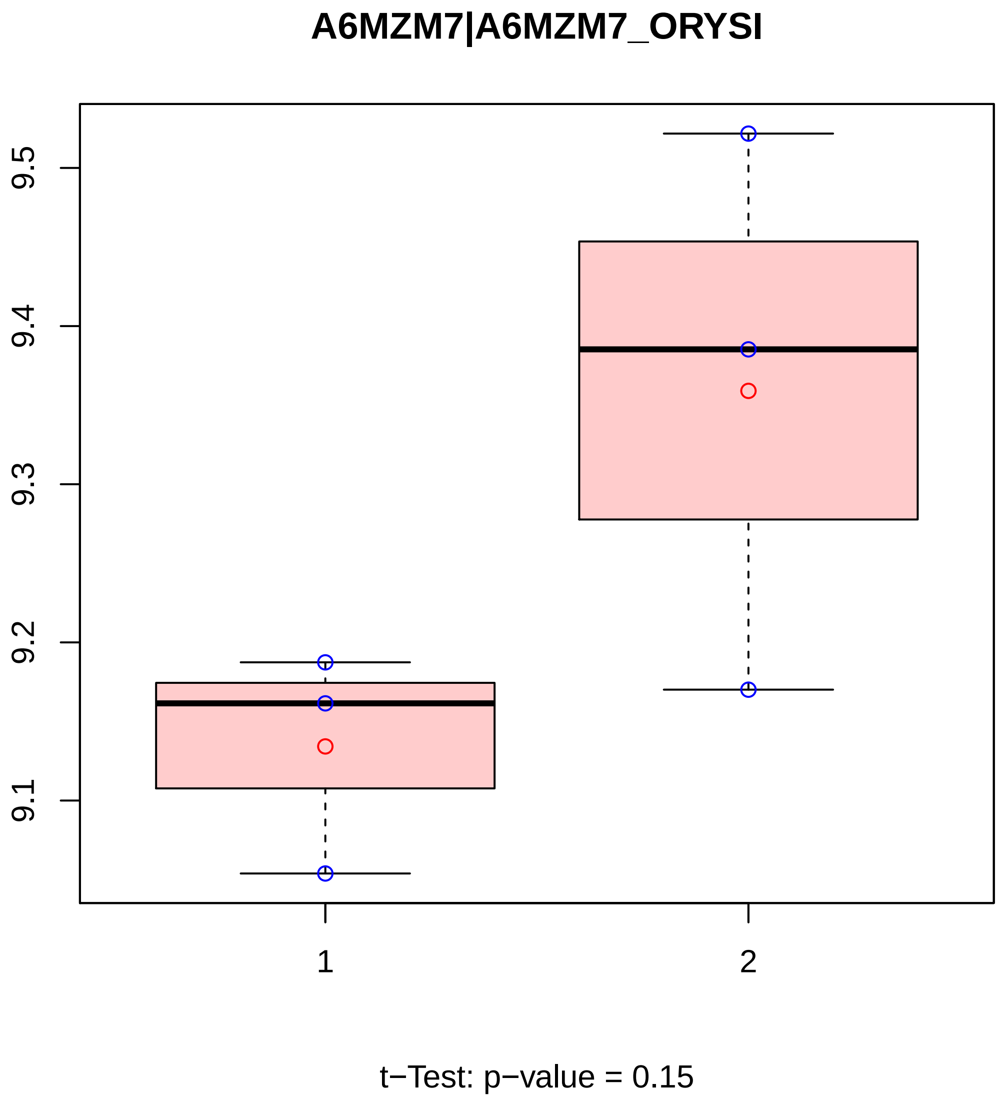
A6MZM7|A6MZM7_ORYSI
9.1
9.2
9.3
9.4
9.5
1
2
t−Test: p−value = 0.15

A6N038|A6N038_ORYSI

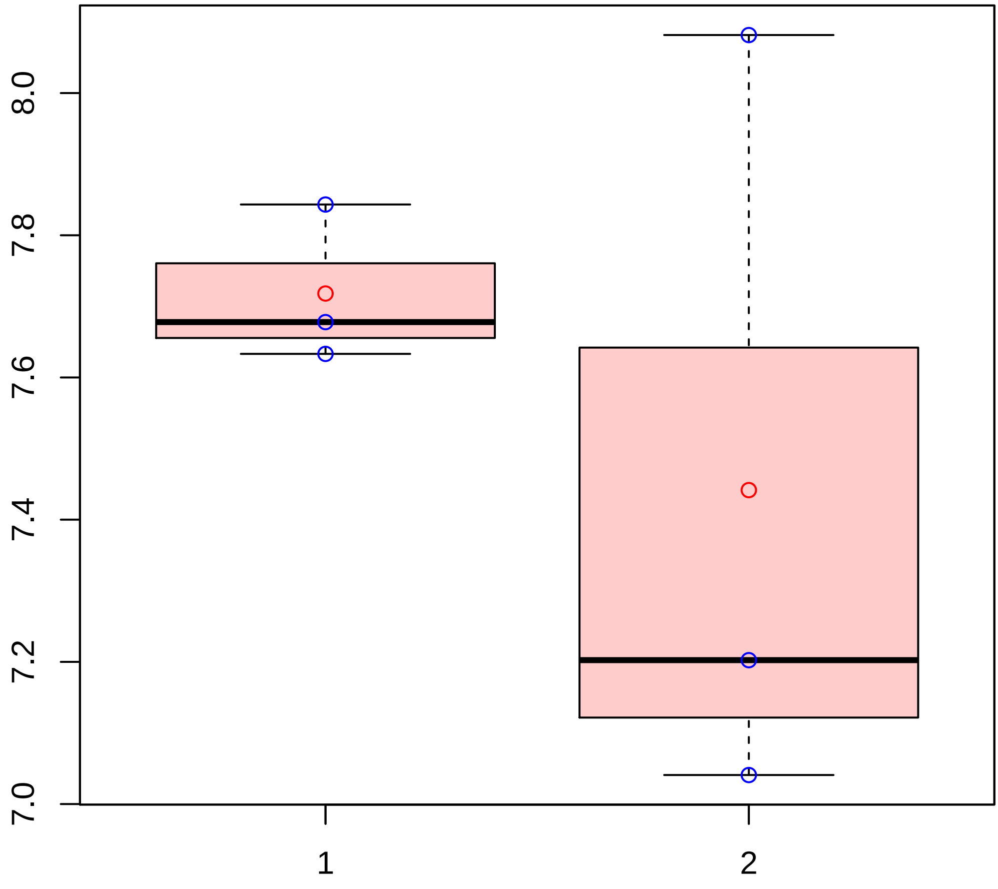

t−Test: p−value = 0.48

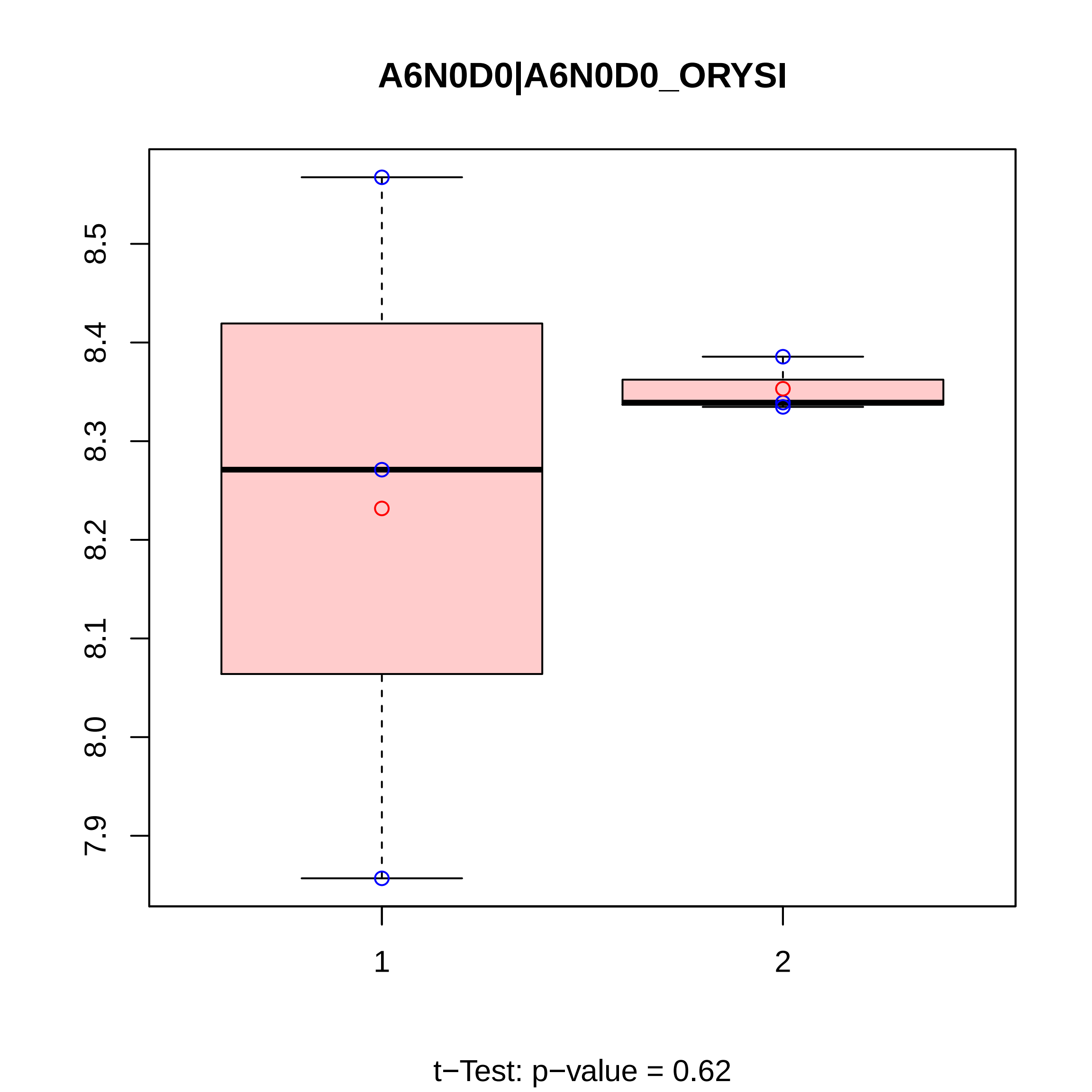
A6N0D0|A6N0D0_ORYSI
8.5
8.4
8.3
8.2
8.1
8.0
7.9
1
2
t−Test: p−value = 0.62

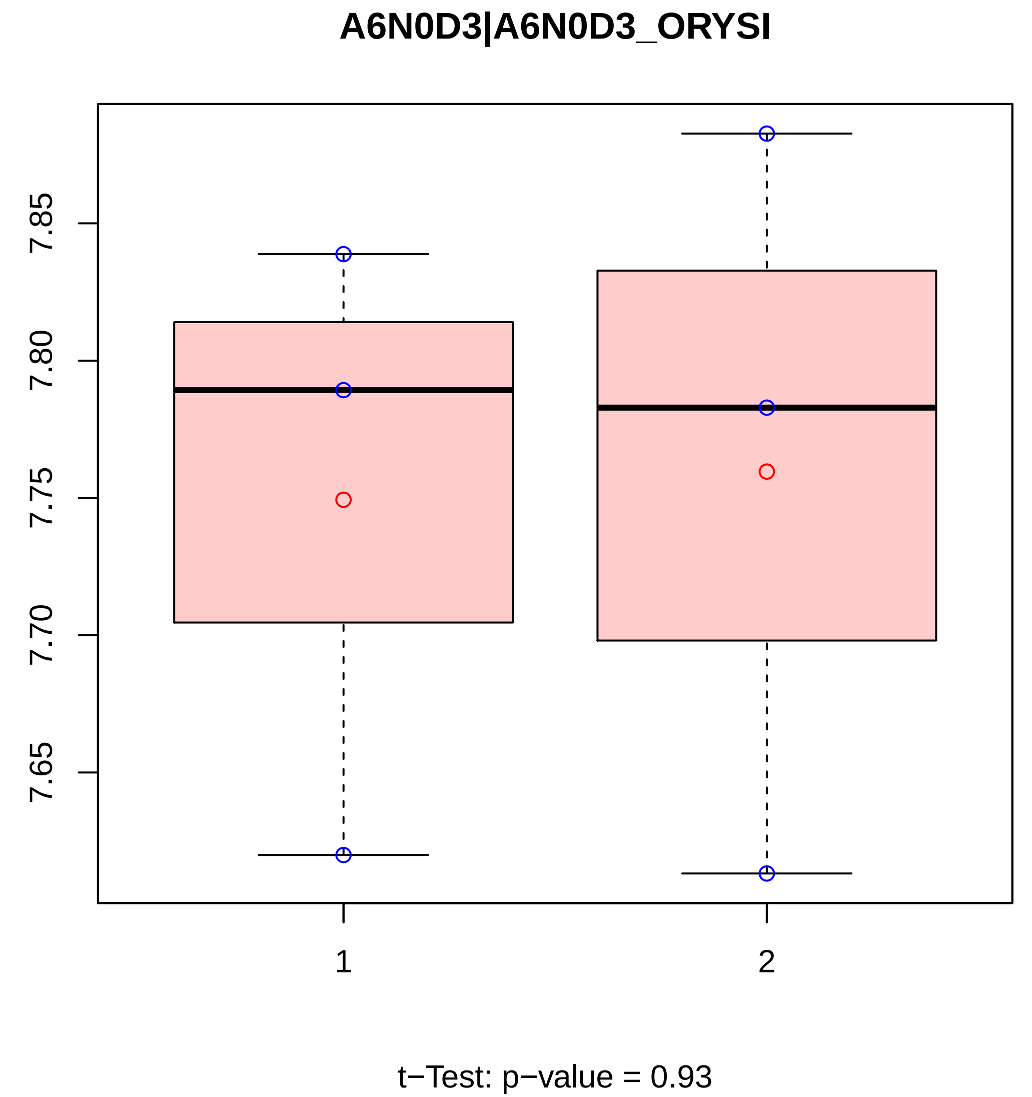
A6N0D3|A6N0D3_ORYSI
7.65
7.70
7.75
7.80
7.85
1
2
t−Test: p−value = 0.93

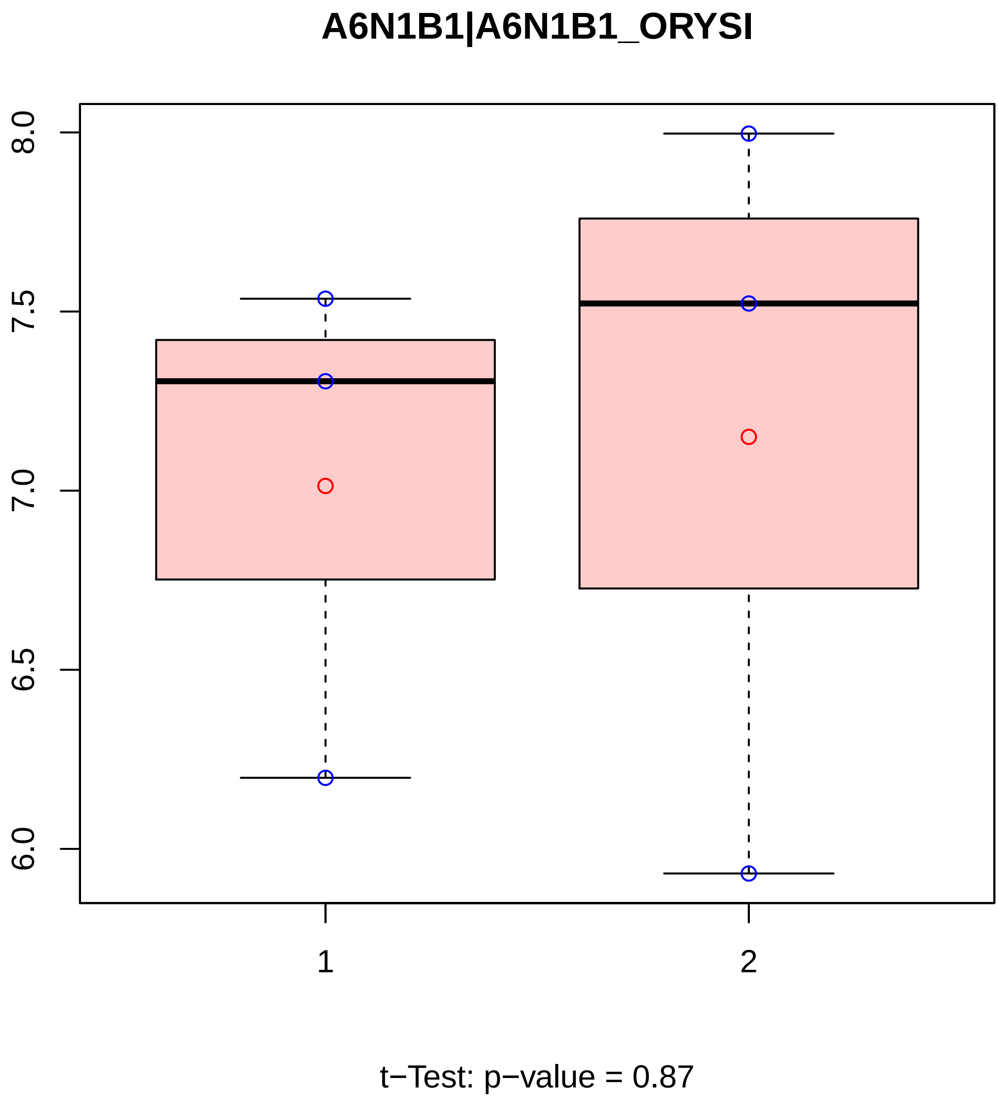
A6N1B1|A6N1B1_ORYSI
8.0
7.5
7.0
6.5
6.0
1
2
t−Test: p−value = 0.87

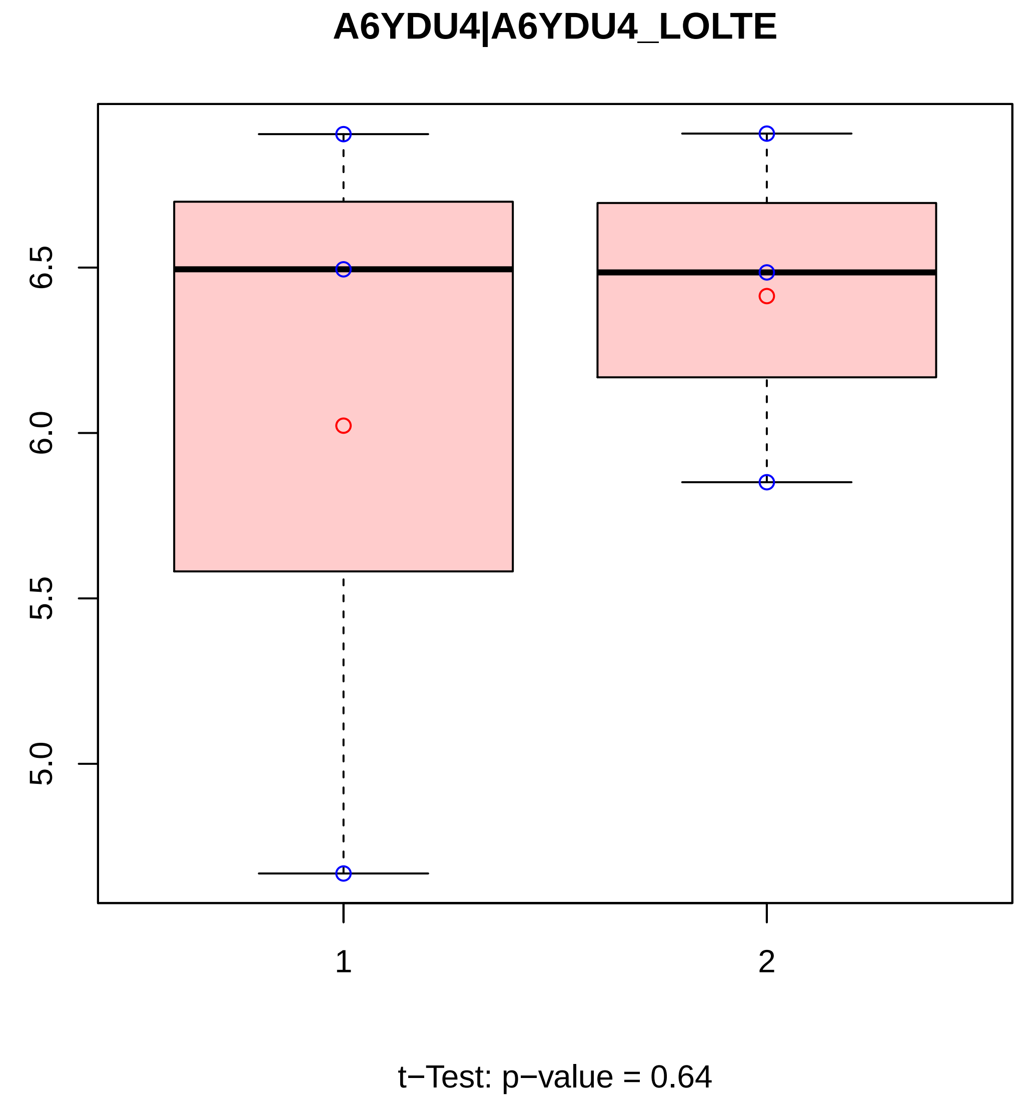

A6YDU4|A6YDU4_LOLTE
6.5
6.0
5.5
5.0
1
2
t−Test: p−value = 0.64

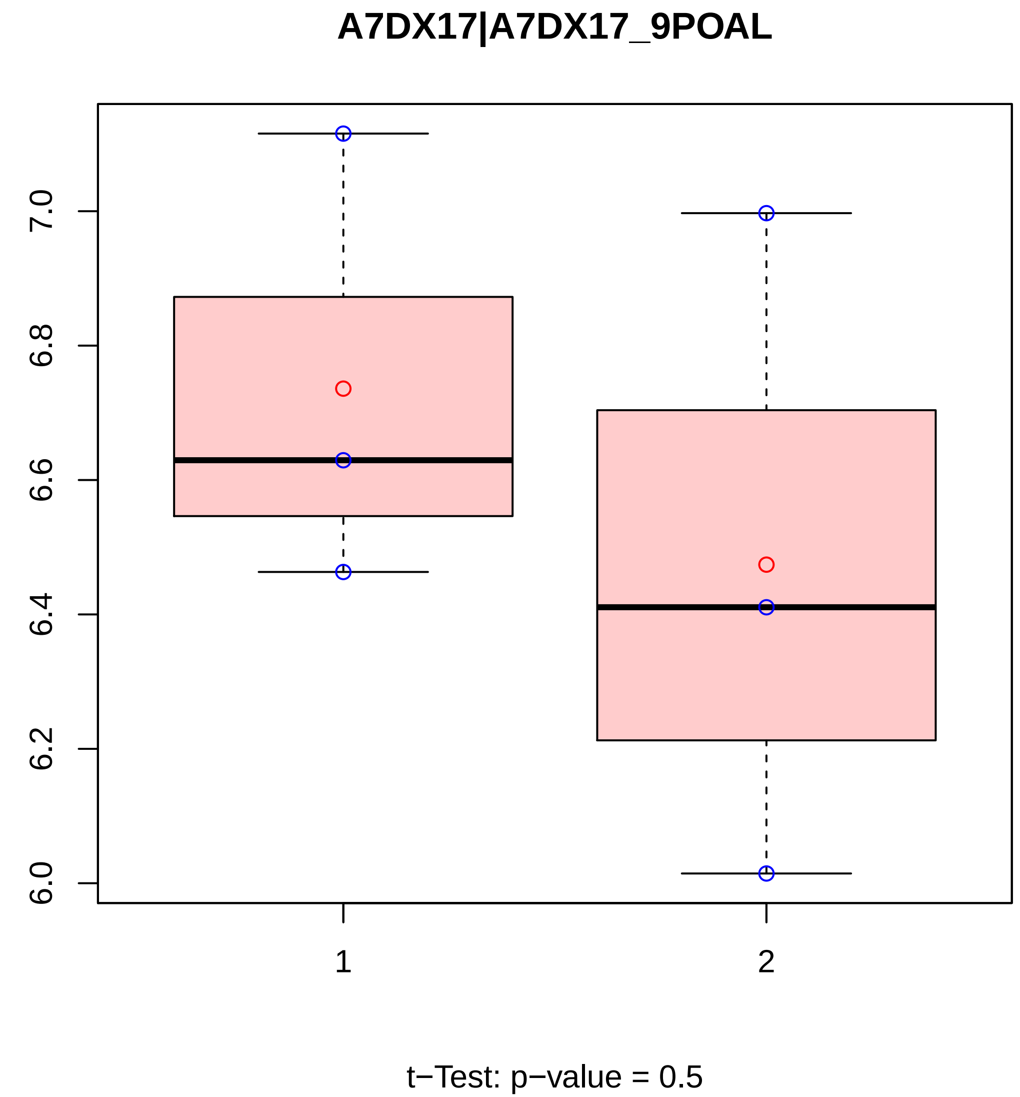

A7DX17|A7DX17_9POAL
7.0
6.8
6.6
6.4
6.2
6.0
1
2
t−Test: p−value = 0.5

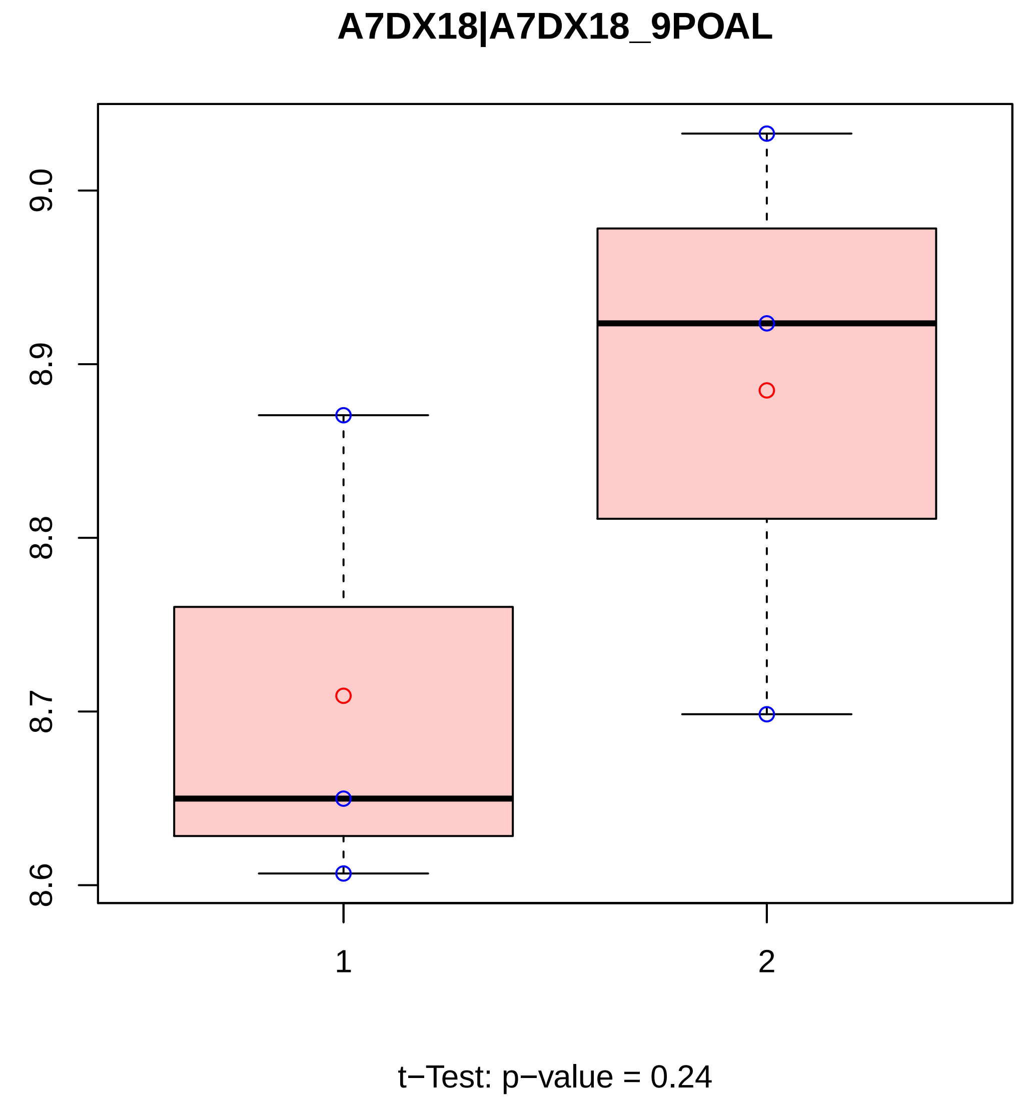
A7DX18|A7DX18_9POAL
9.0
8.9
8.8
8.7
8.6
1
2
t−Test: p−value = 0.24

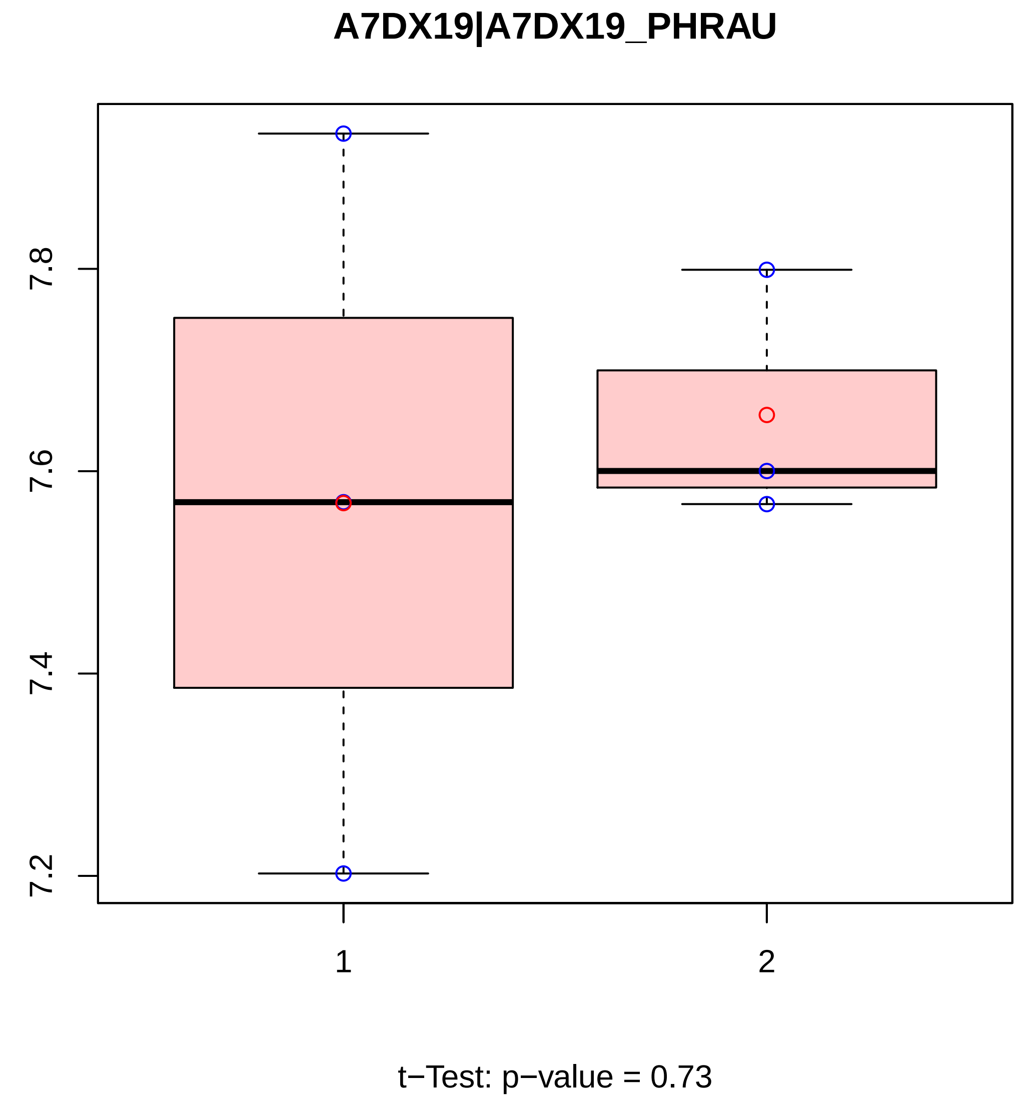
A7DX19|A7DX19_PHRAU
7.2
7.4
7.6
7.8
1
2
t−Test: p−value = 0.73

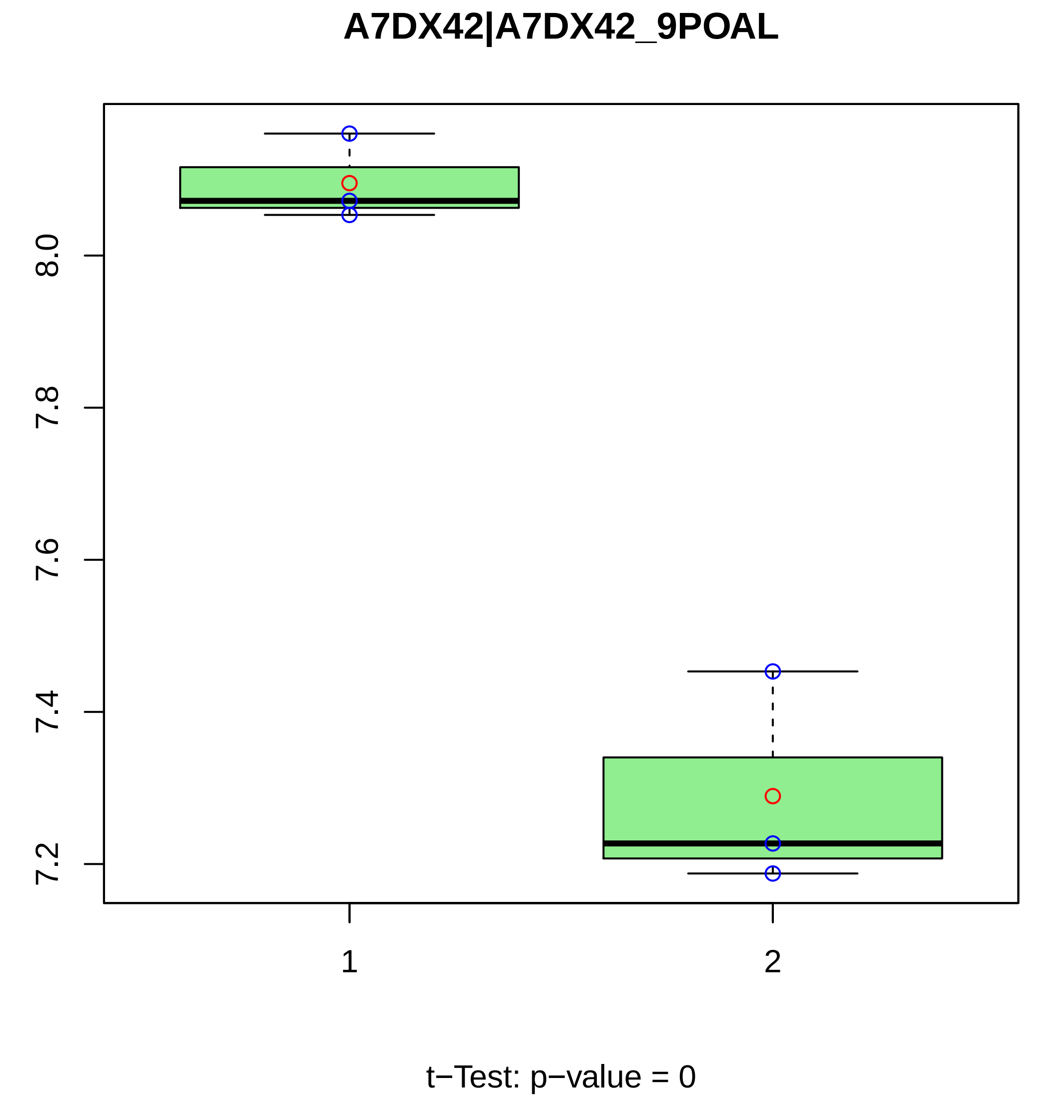

A7DX42|A7DX42_9POAL
8.0
7.8
7.6
7.4
7.2
1
2
t−Test: p−value = 0

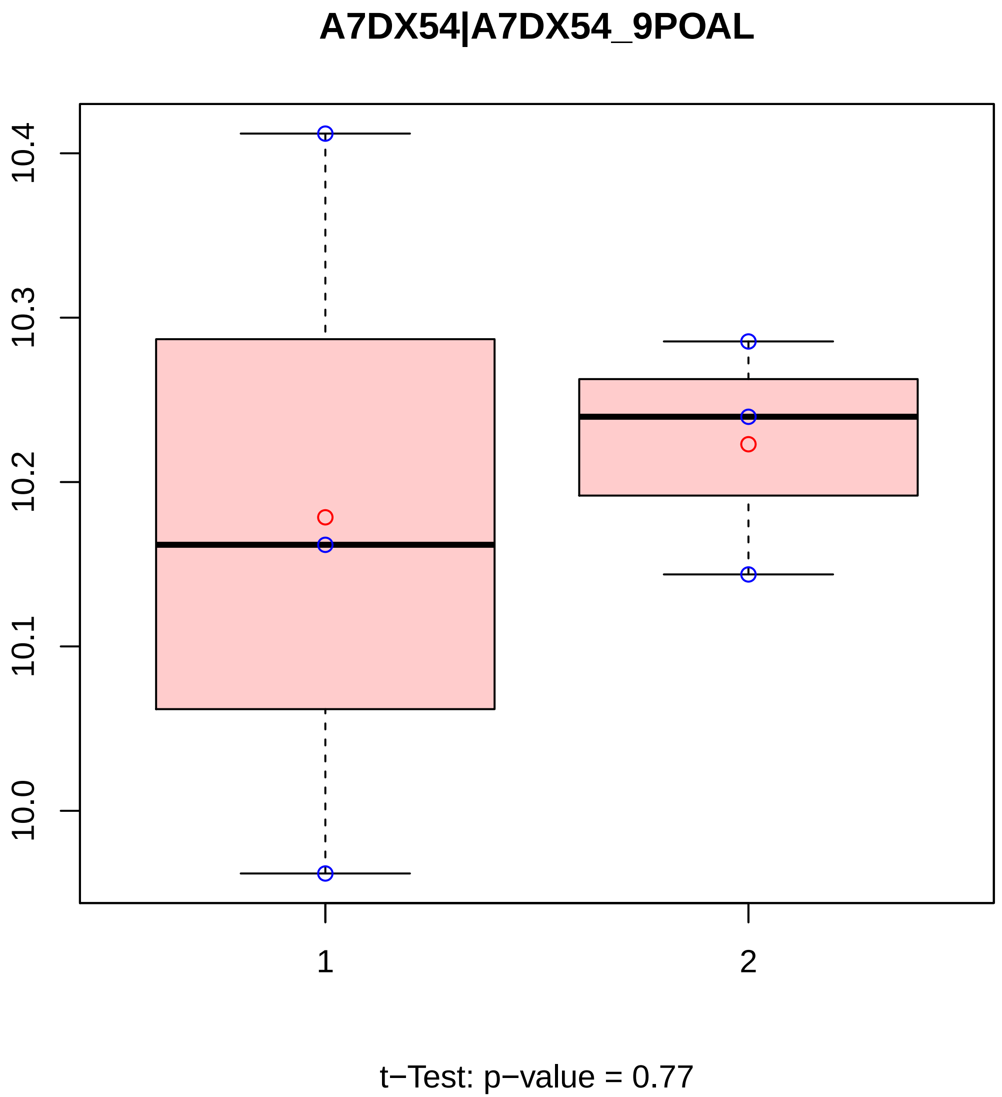

A7DX54|A7DX54_9POAL
10.4
10.3
10.2
10.1
10.0
1
2
t−Test: p−value = 0.77

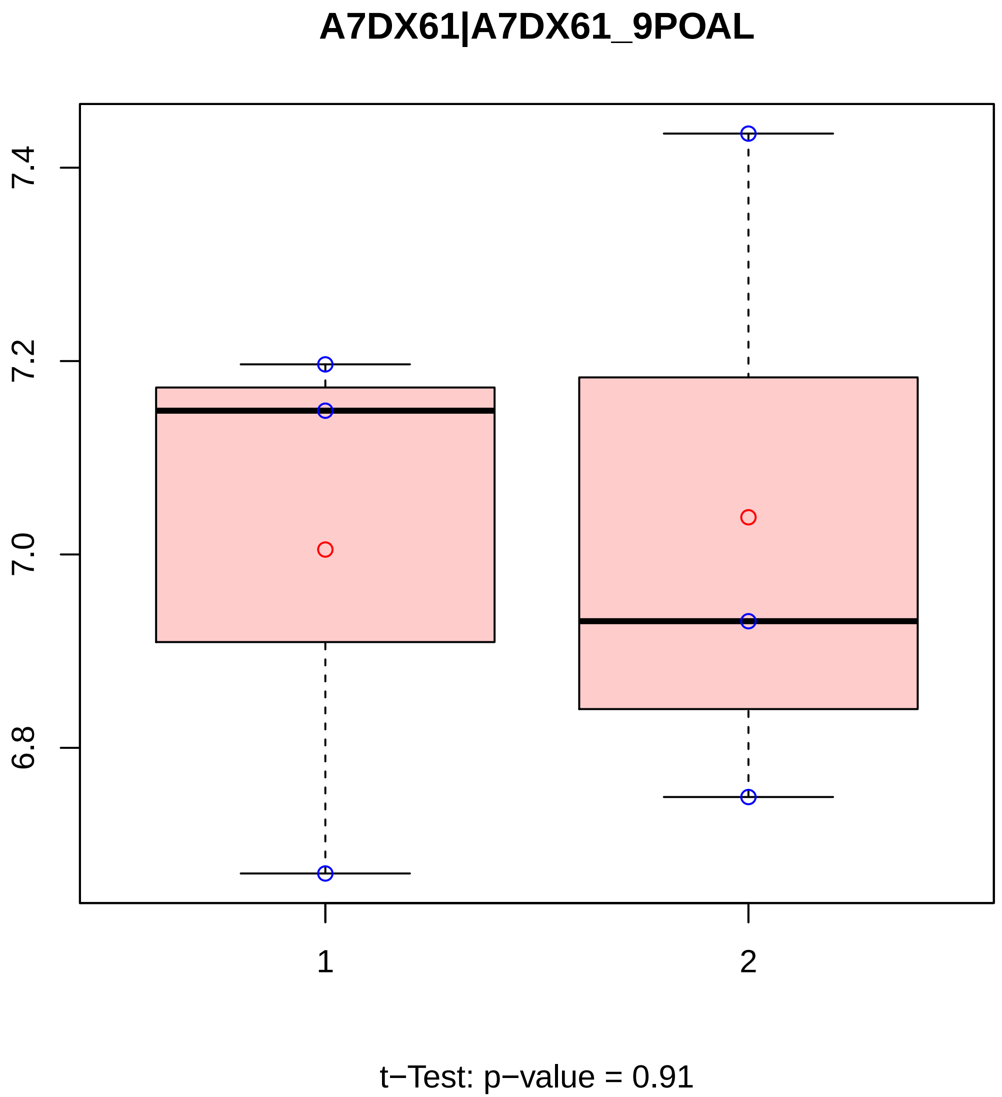
A7DX61|A7DX61_9POAL
7.4
7.2
7.0
6.8
1
2
t−Test: p−value = 0.91

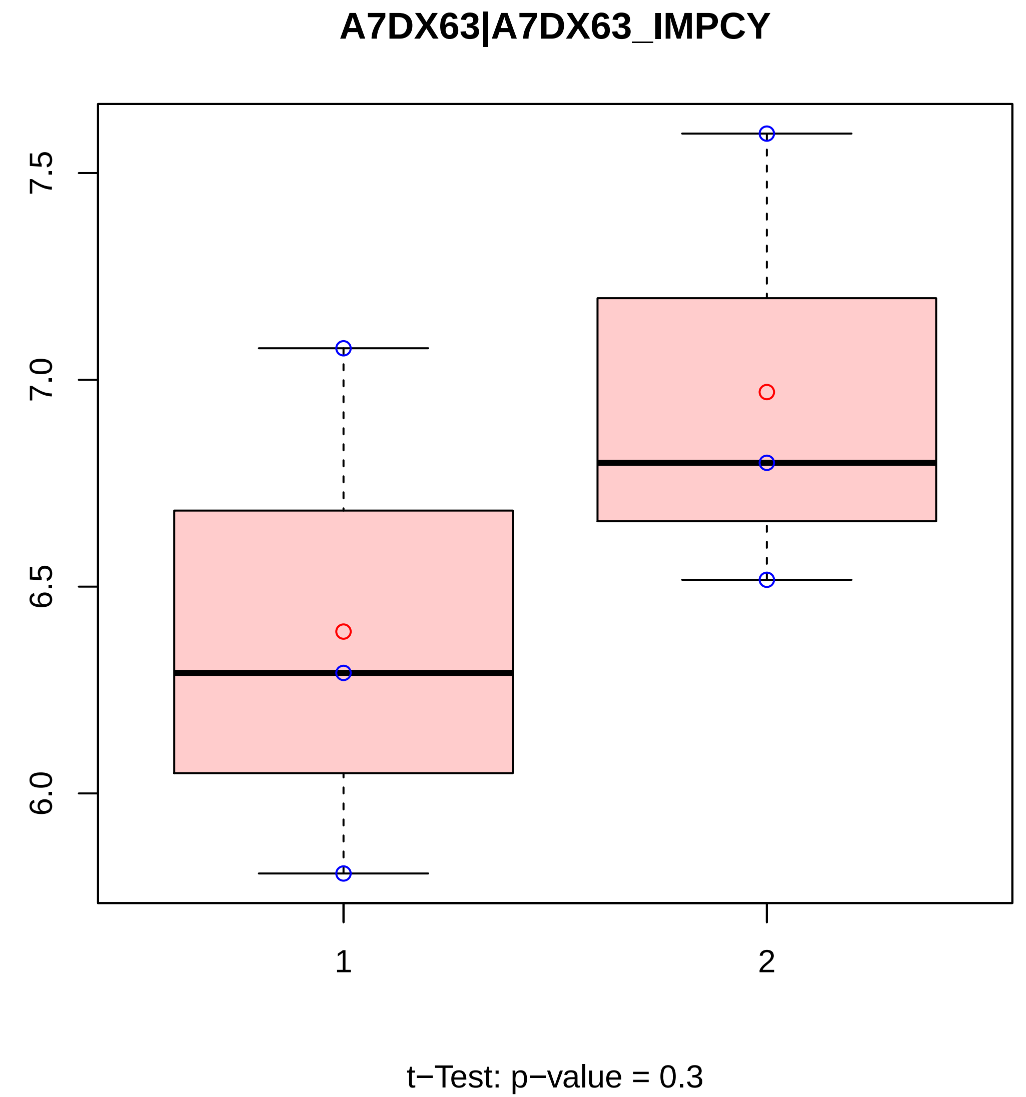
A7DX63|A7DX63_IMPCY
7.5
7.0
6.5
6.0
1
2
t−Test: p−value = 0.3

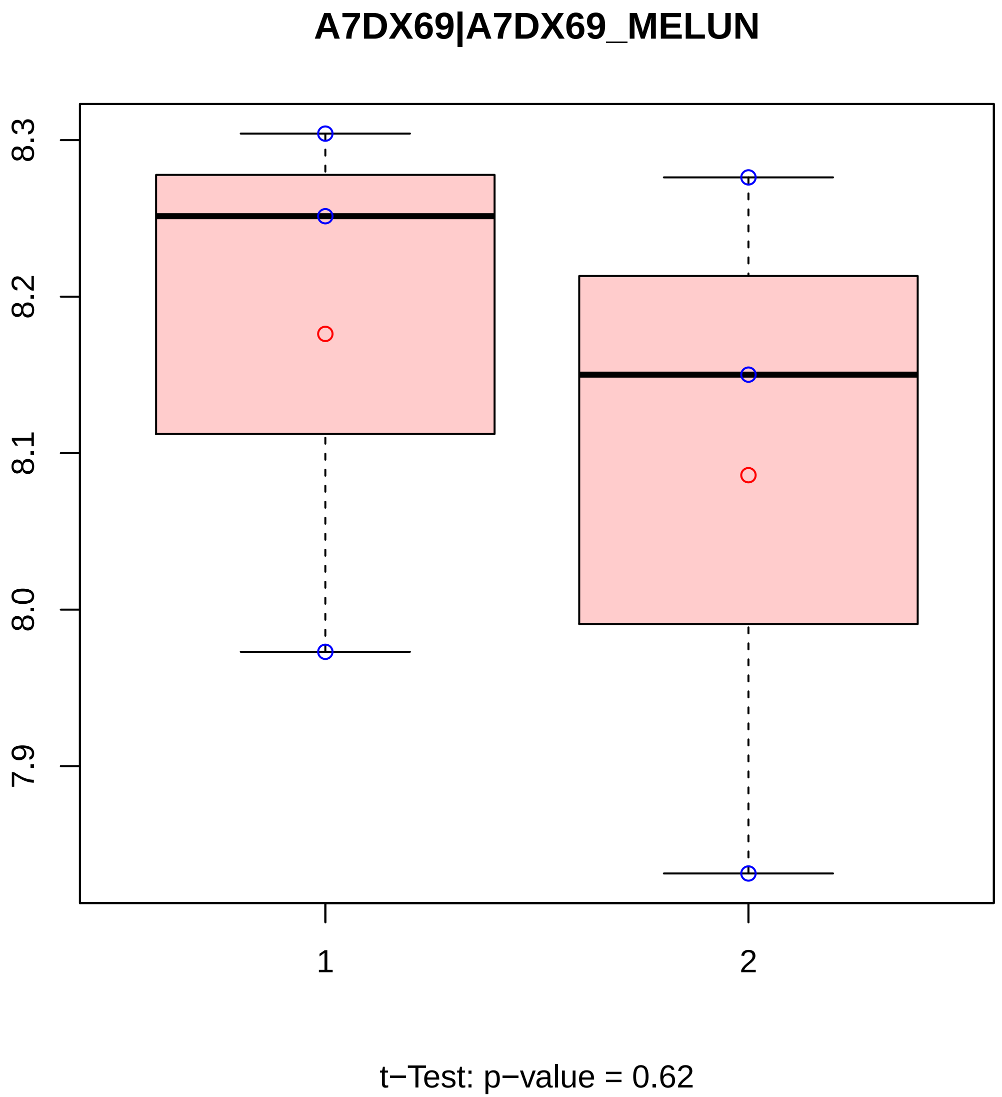
A7DX69|A7DX69_MELUN
8.3
8.2
8.1
8.0
7.9
1
2
t−Test: p−value = 0.62

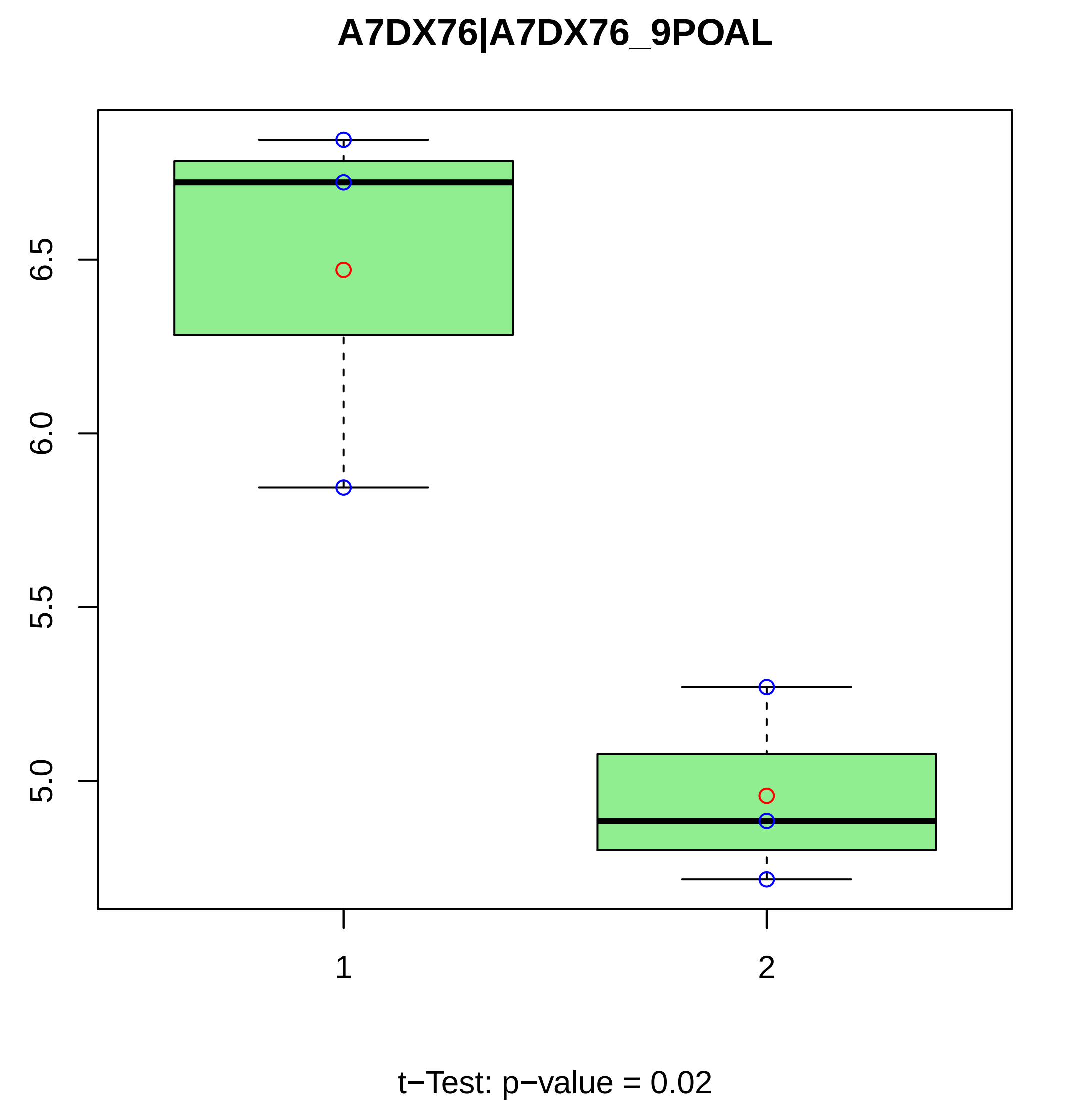
A7DX76|A7DX76_9POAL
6.5
6.0
5.5
5.0
1
2
t−Test: p−value = 0.02

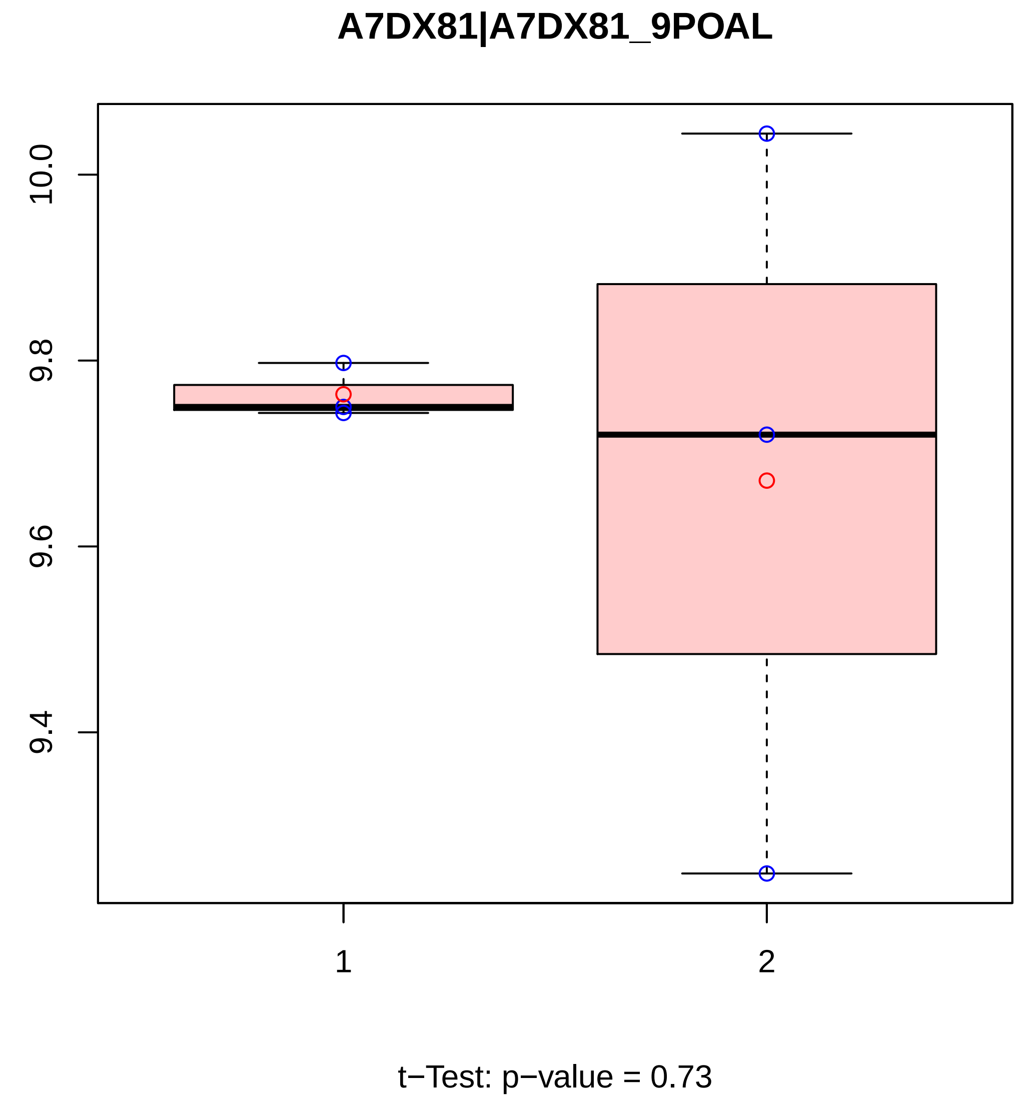
A7DX81|A7DX81_9POAL
10.0
9.8
9.6
9.4
1
2
t−Test: p−value = 0.73

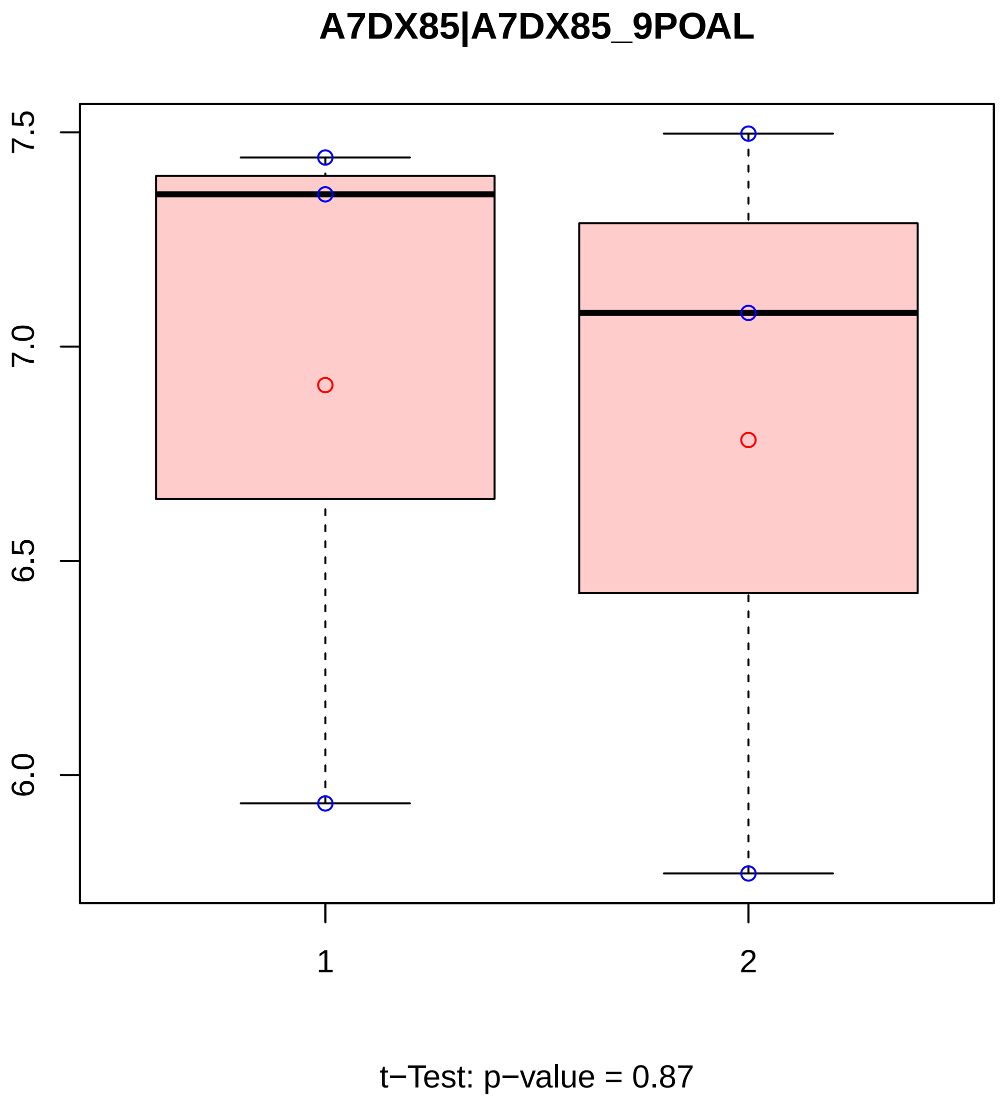

A7DX85|A7DX85_9POAL
7.5
7.0
6.5
6.0
1
2
t−Test: p−value = 0.87

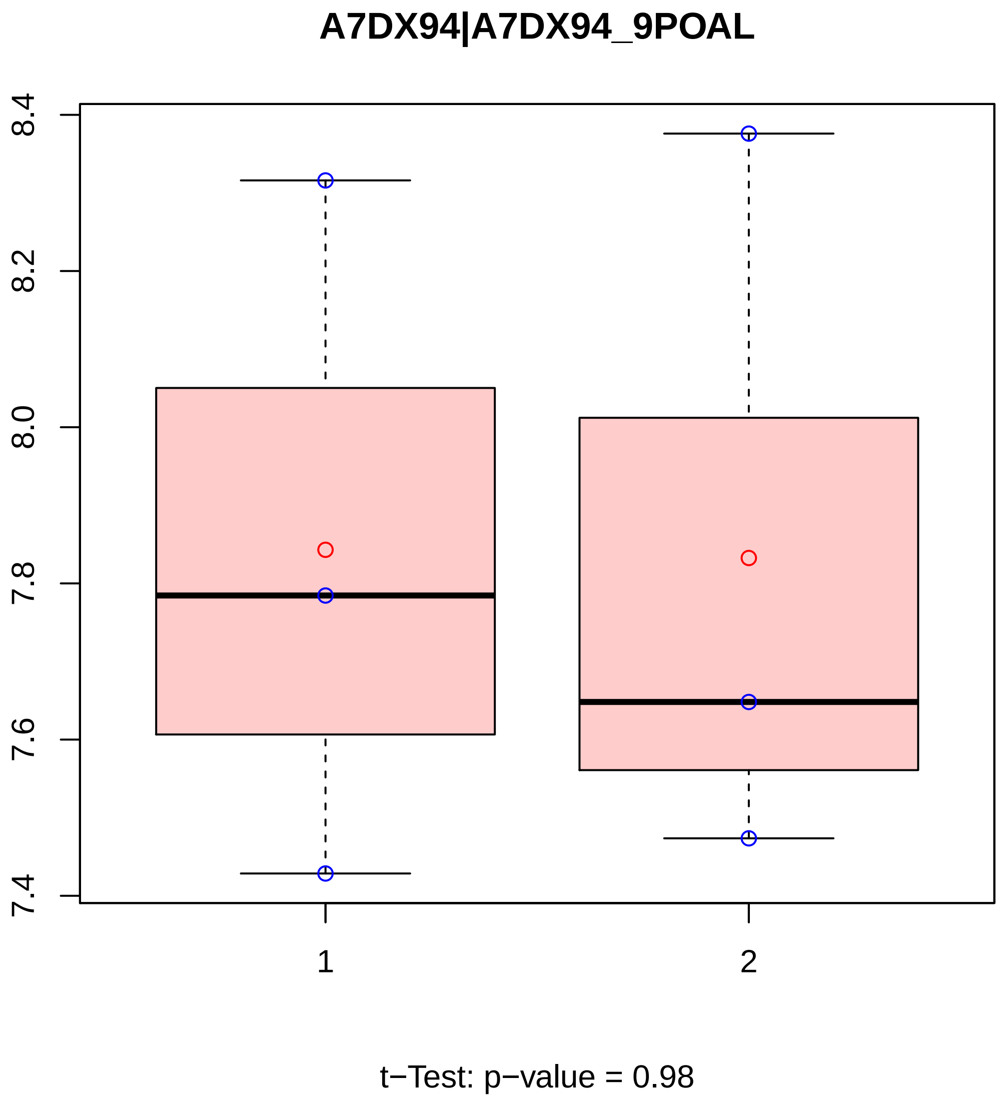
A7DX94|A7DX94_9POAL
8.4
8.2
8.0
7.8
7.6
7.4
1
2
t−Test: p−value = 0.98

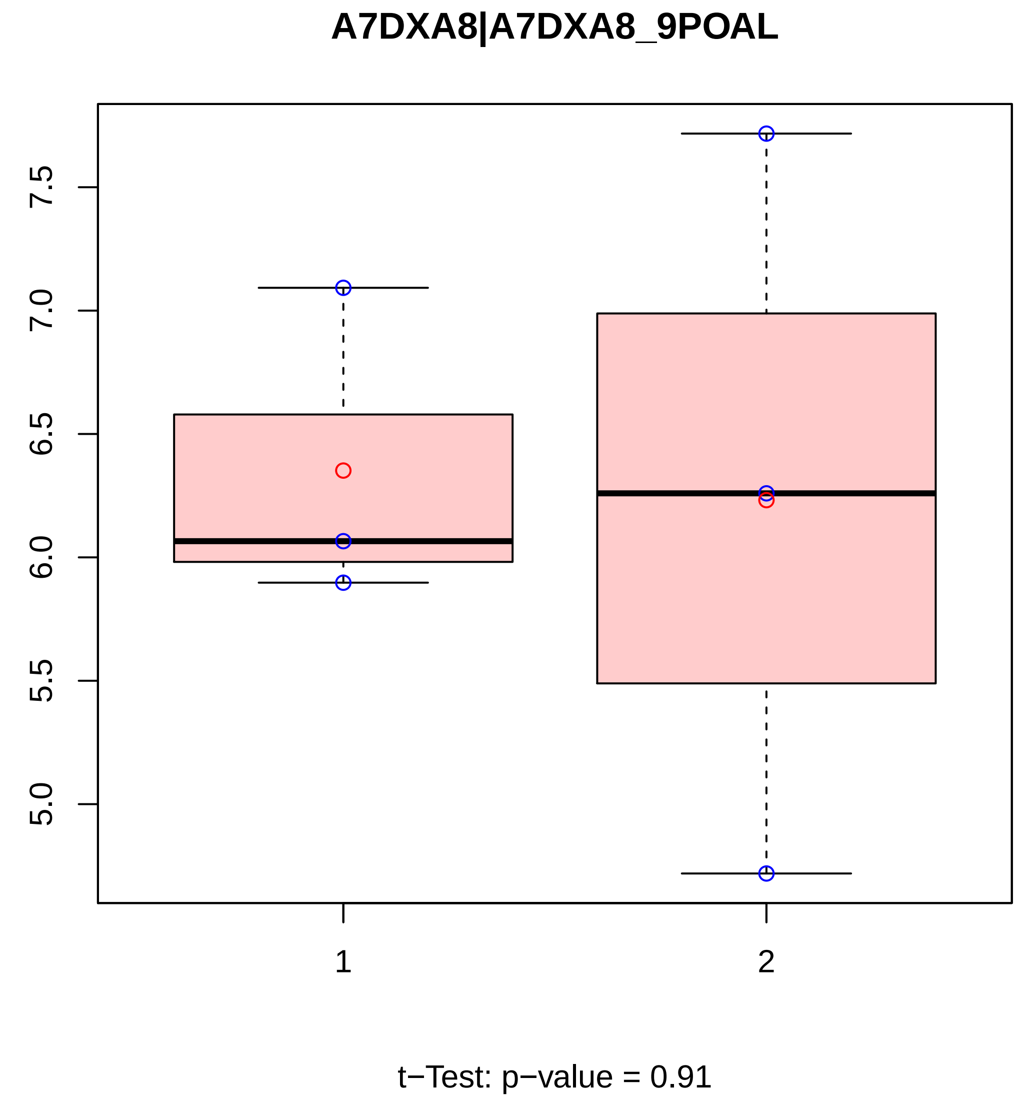
A7DXA8|A7DXA8_9POAL
7.5
7.0
6.5
6.0
5.5
5.0
1
2
t−Test: p−value = 0.91

A7DXB0|A7DXB0_9POAL

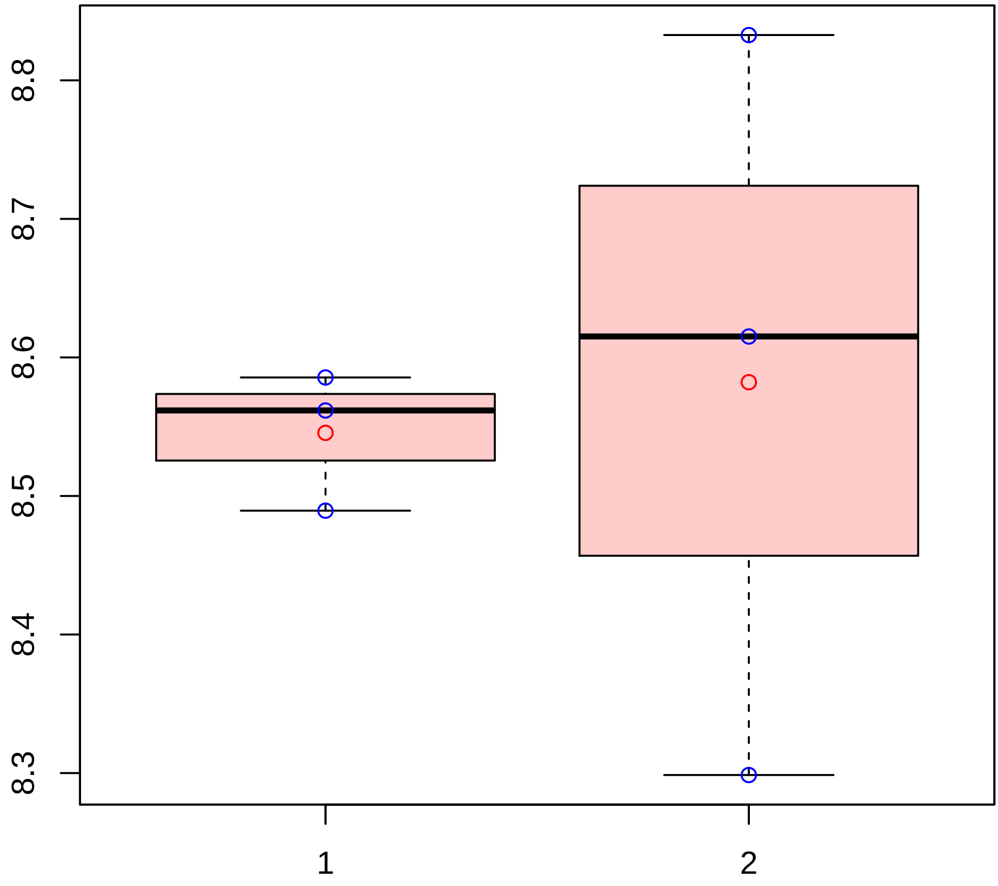

t−Test: p−value = 0.84

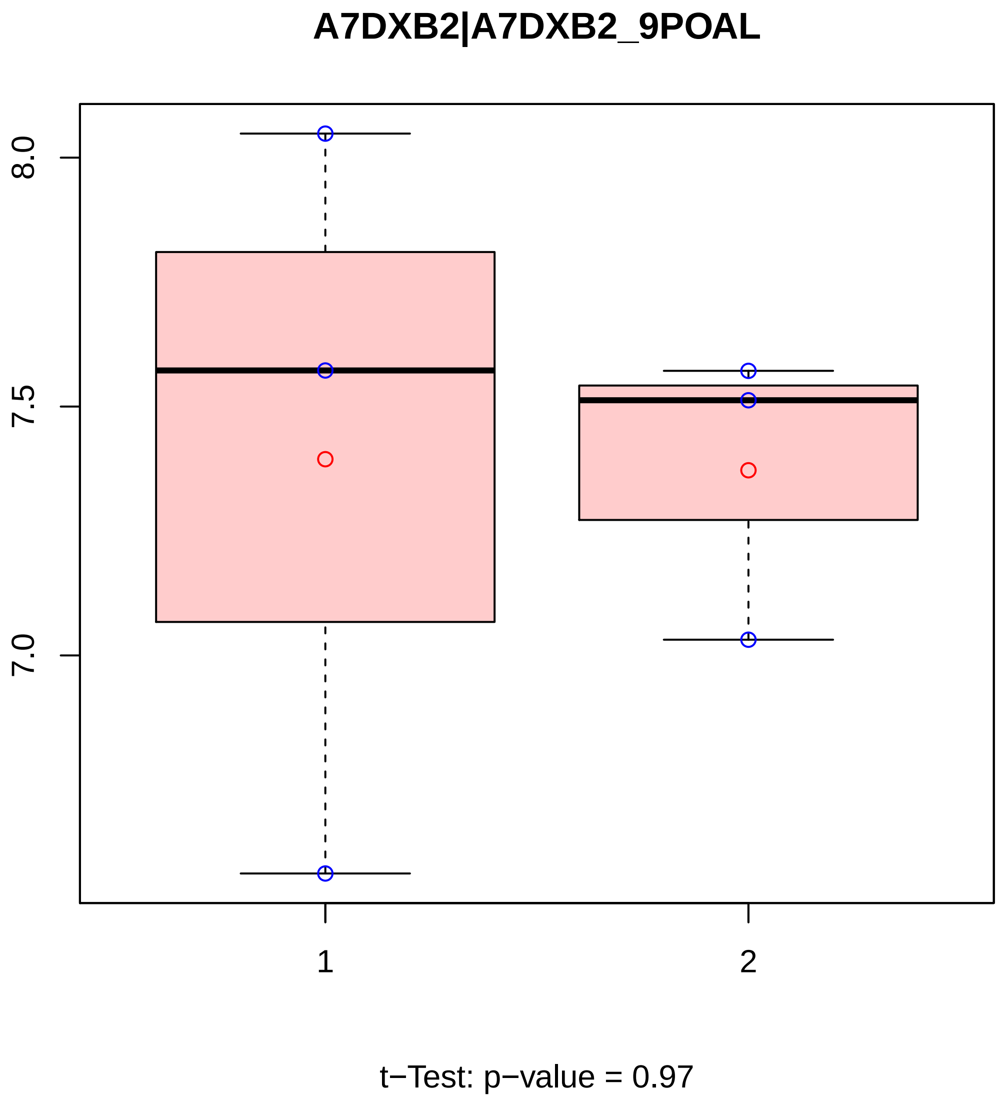
A7DXB2|A7DXB2_9POAL
8.0
7.5
7.0
1
2
t−Test: p−value = 0.97

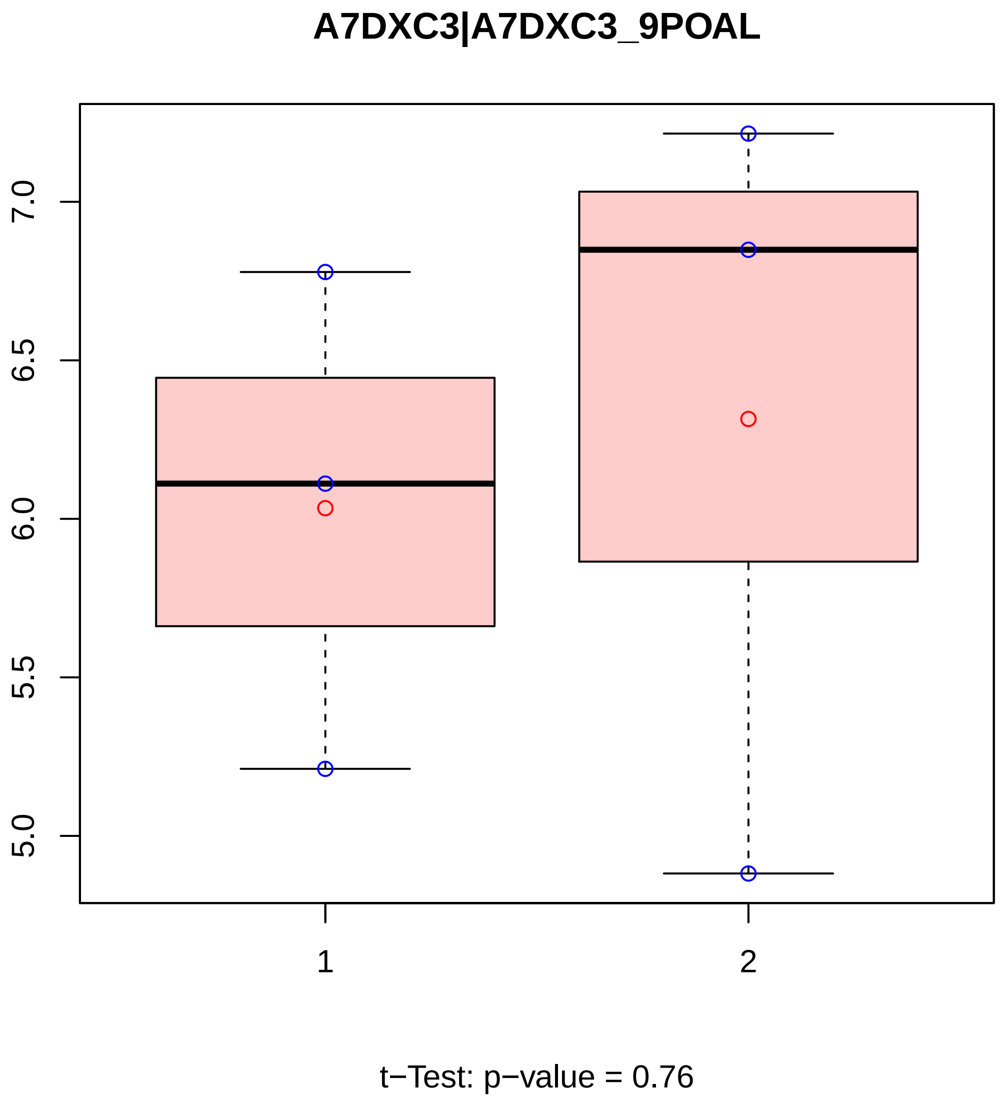
A7DXC3|A7DXC3_9POAL
7.0
6.5
6.0
5.5
5.0
1
2
t−Test: p−value = 0.76

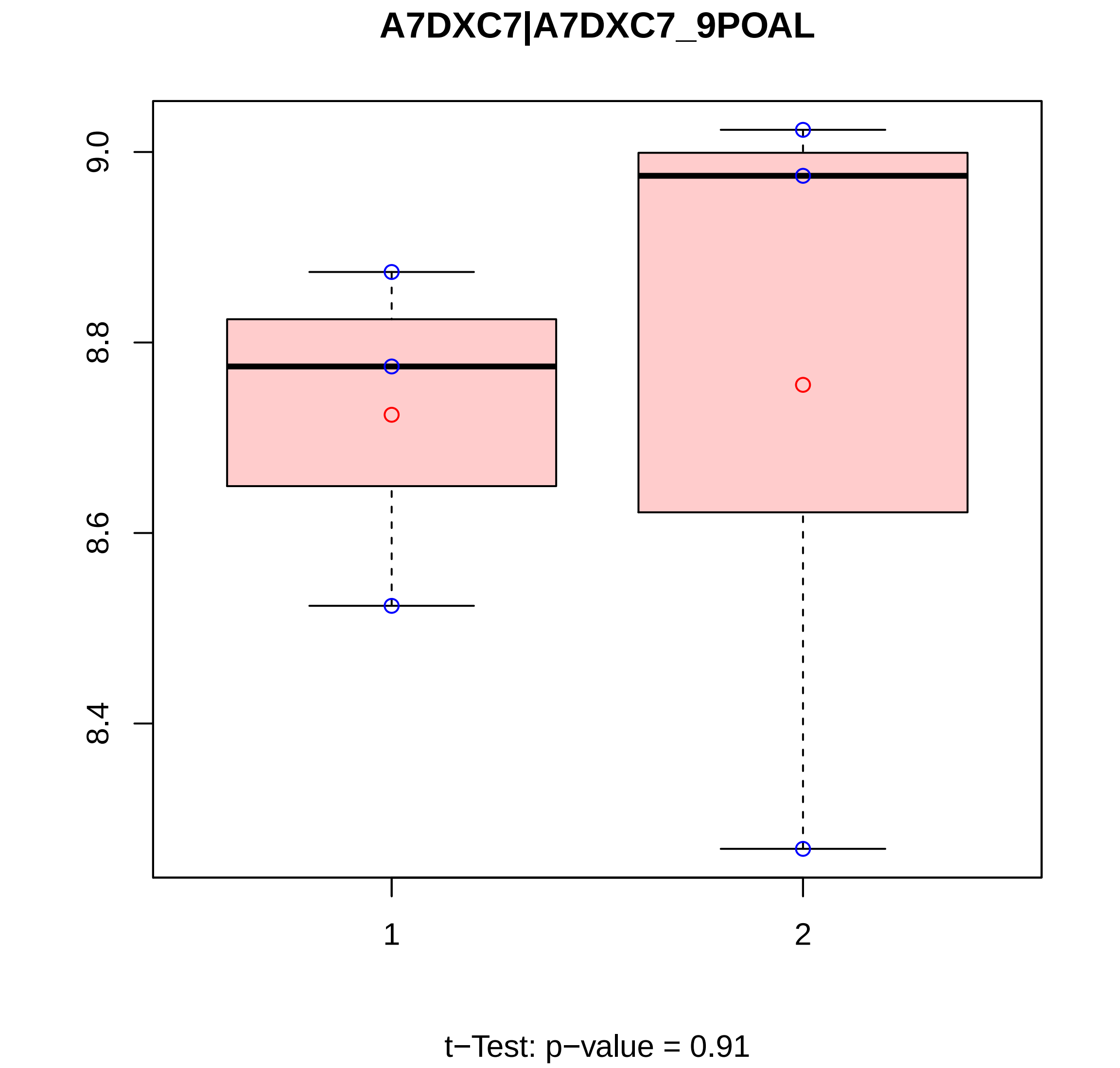

A7DXC7|A7DXC7_9POAL
9.0
8.8
8.6
8.4
1
2
t−Test: p−value = 0.91

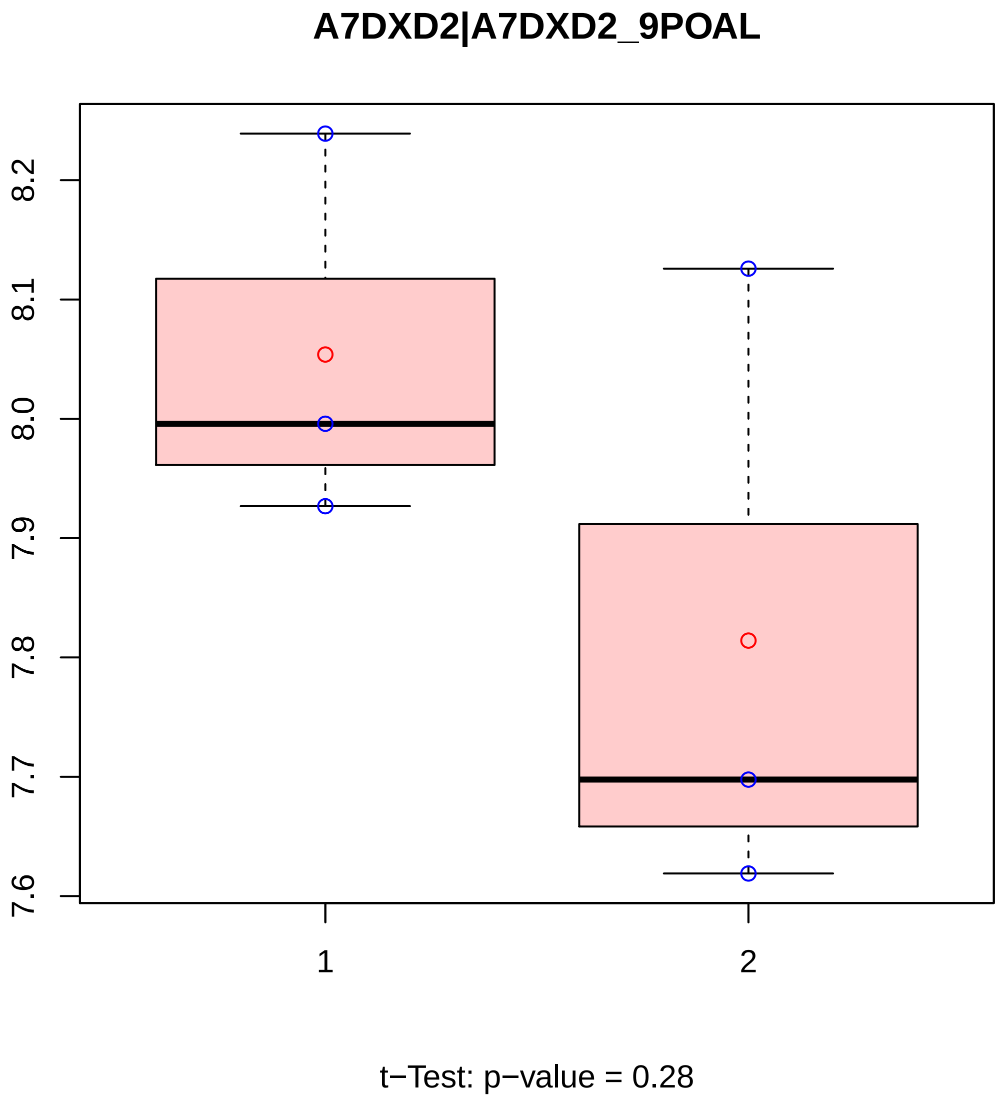

A7DXD2|A7DXD2_9POAL
8.2
8.1
8.0
7.9
7.8
7.7
7.6
1
2
t−Test: p−value = 0.28

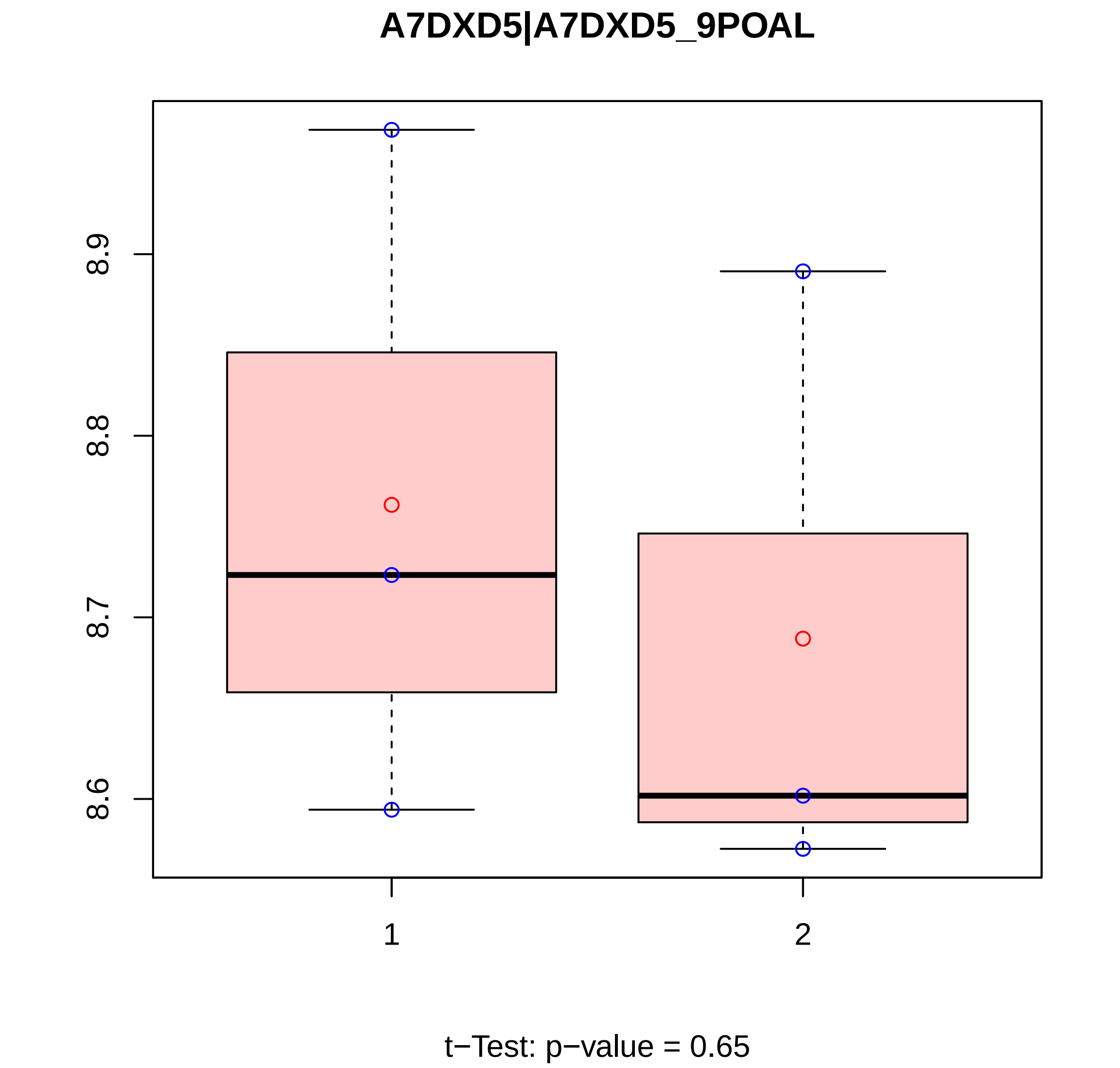
A7DXD5|A7DXD5_9POAL
8.6
8.7
8.8
8.9
1
2
t−Test: p−value = 0.65

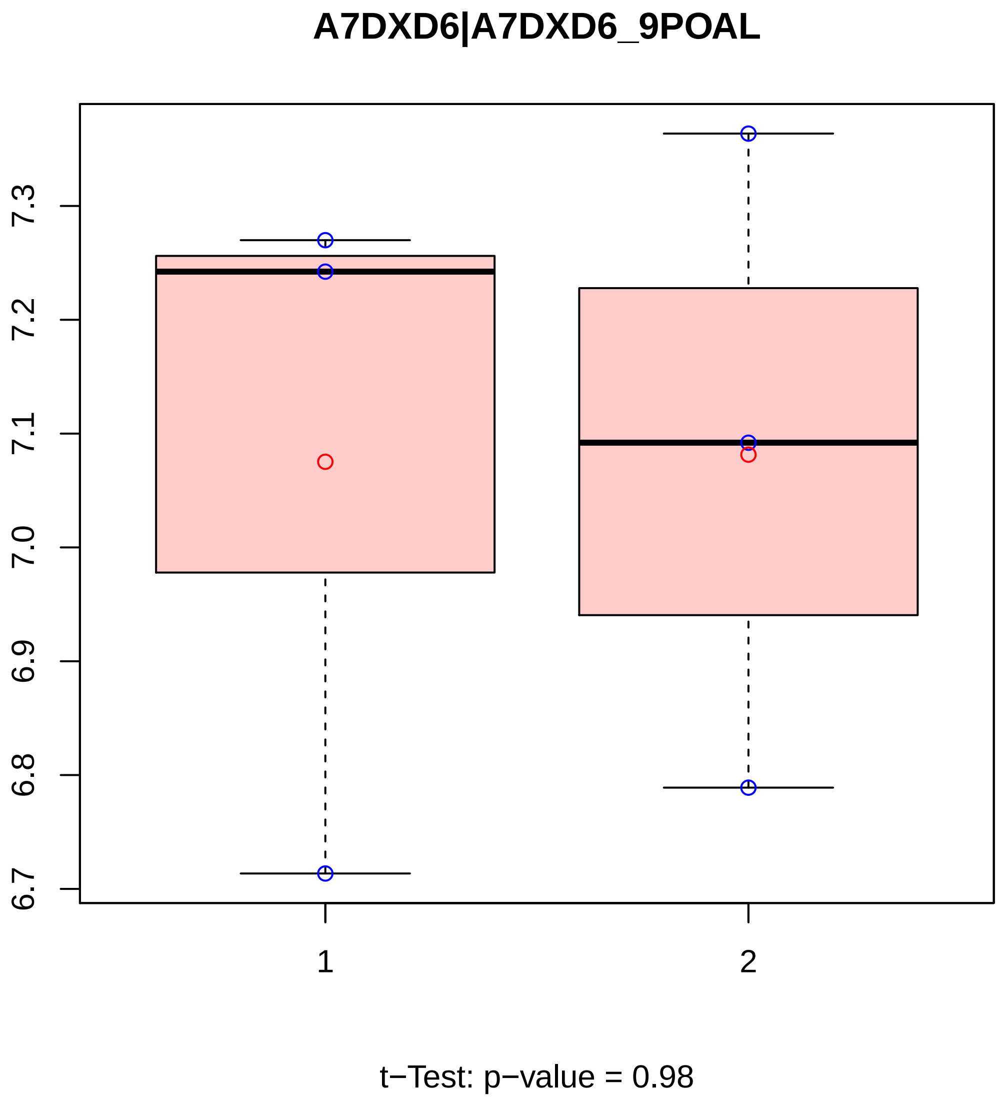
A7DXD6|A7DXD6_9POAL
7.3
7.2
7.1
7.0
6.9
6.8
6.7
1
2
t−Test: p−value = 0.98

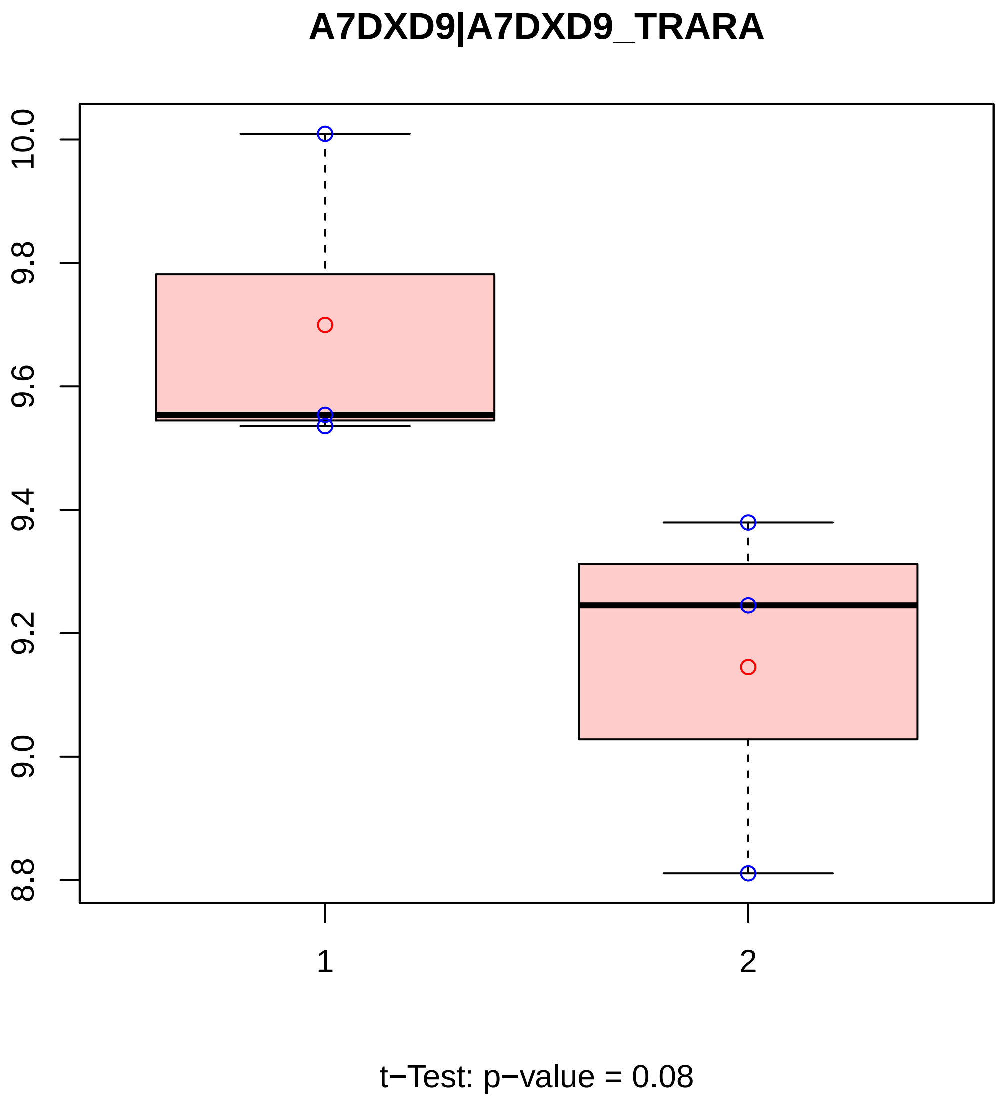
A7DXD9|A7DXD9_TRARA
10.0
9.8
9.6
9.4
9.2
9.0
8.8
1
2
t−Test: p−value = 0.08

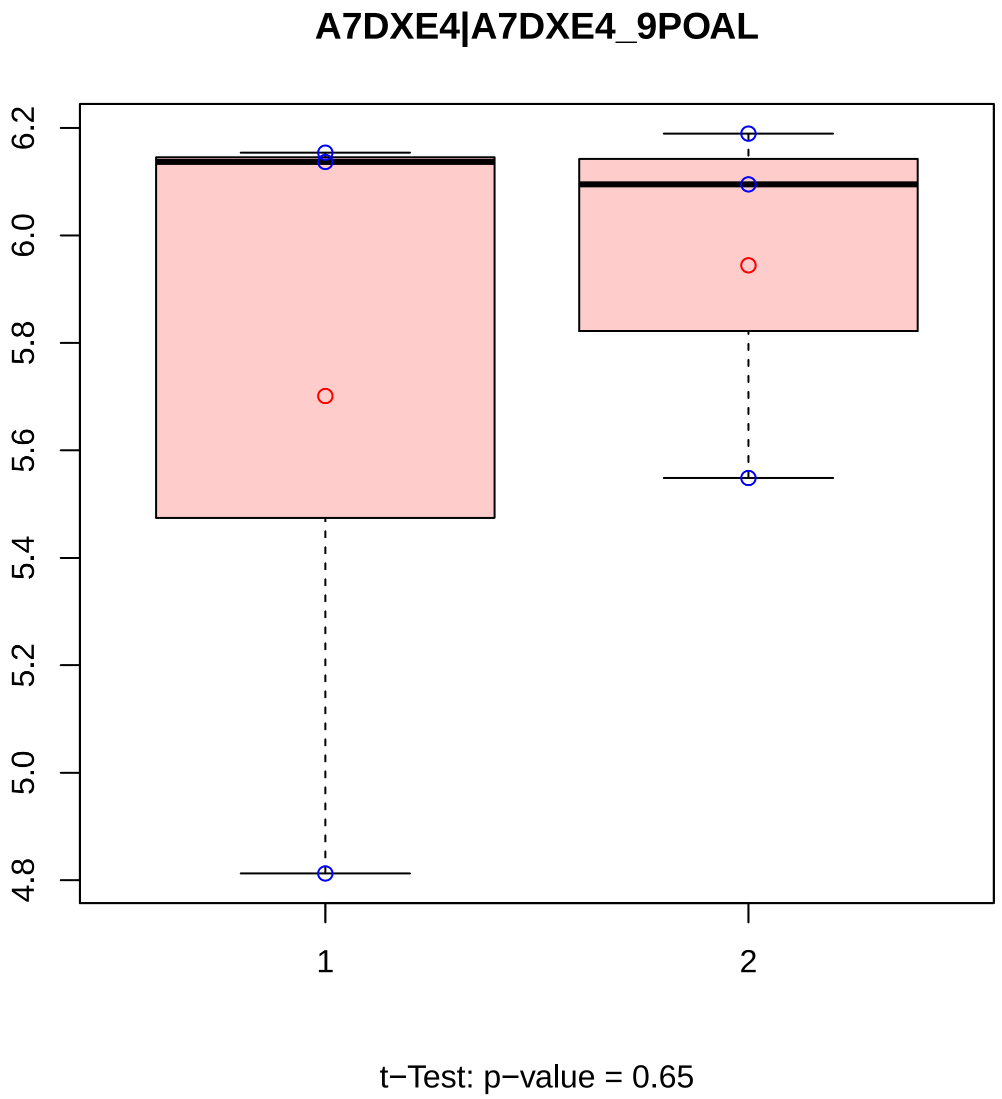
A7DXE4|A7DXE4_9POAL
6.2
6.0
5.8
5.6
5.4
5.2
5.0
4.8
1
2
t−Test: p−value = 0.65

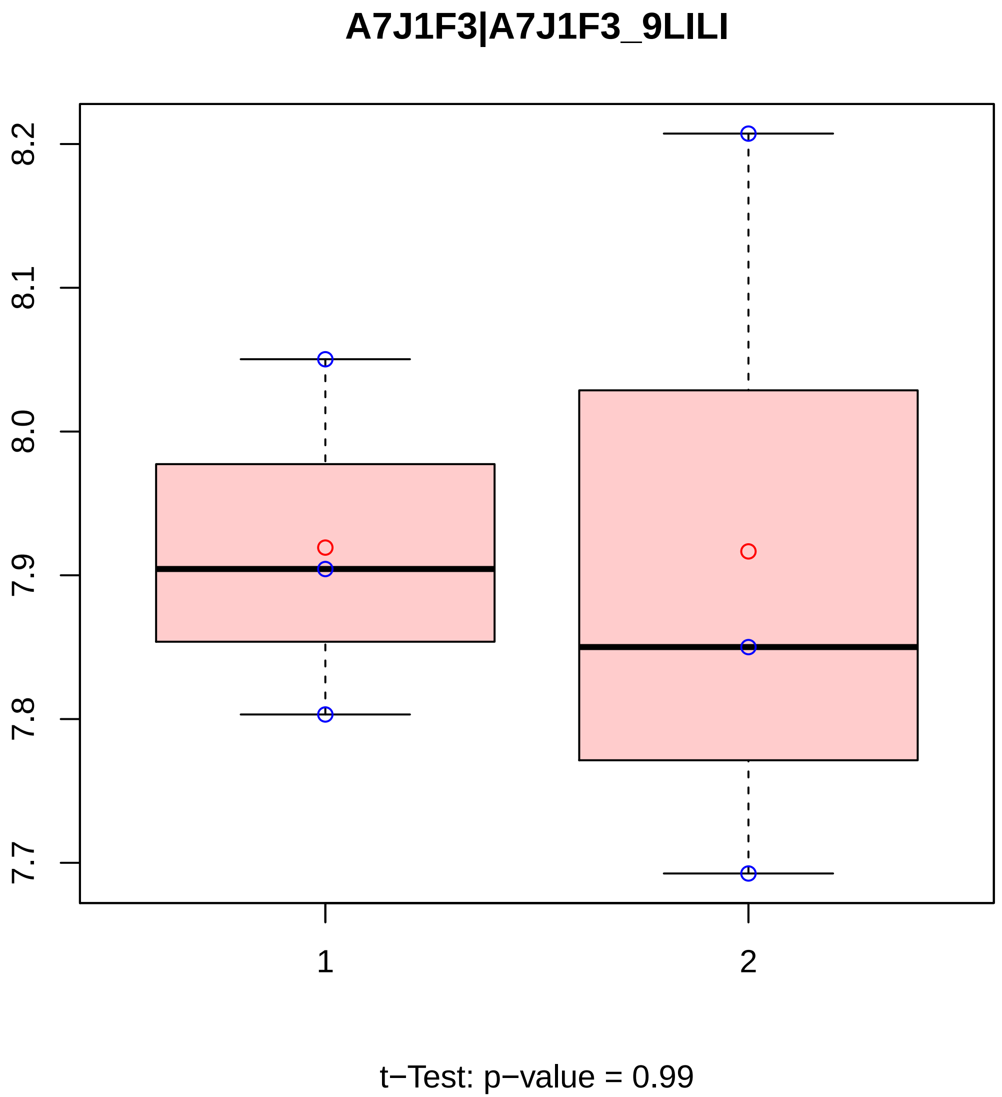
A7J1F3|A7J1F3_9LILI
8.2
8.1
8.0
7.9
7.8
7.7
1
2
t−Test: p−value = 0.99

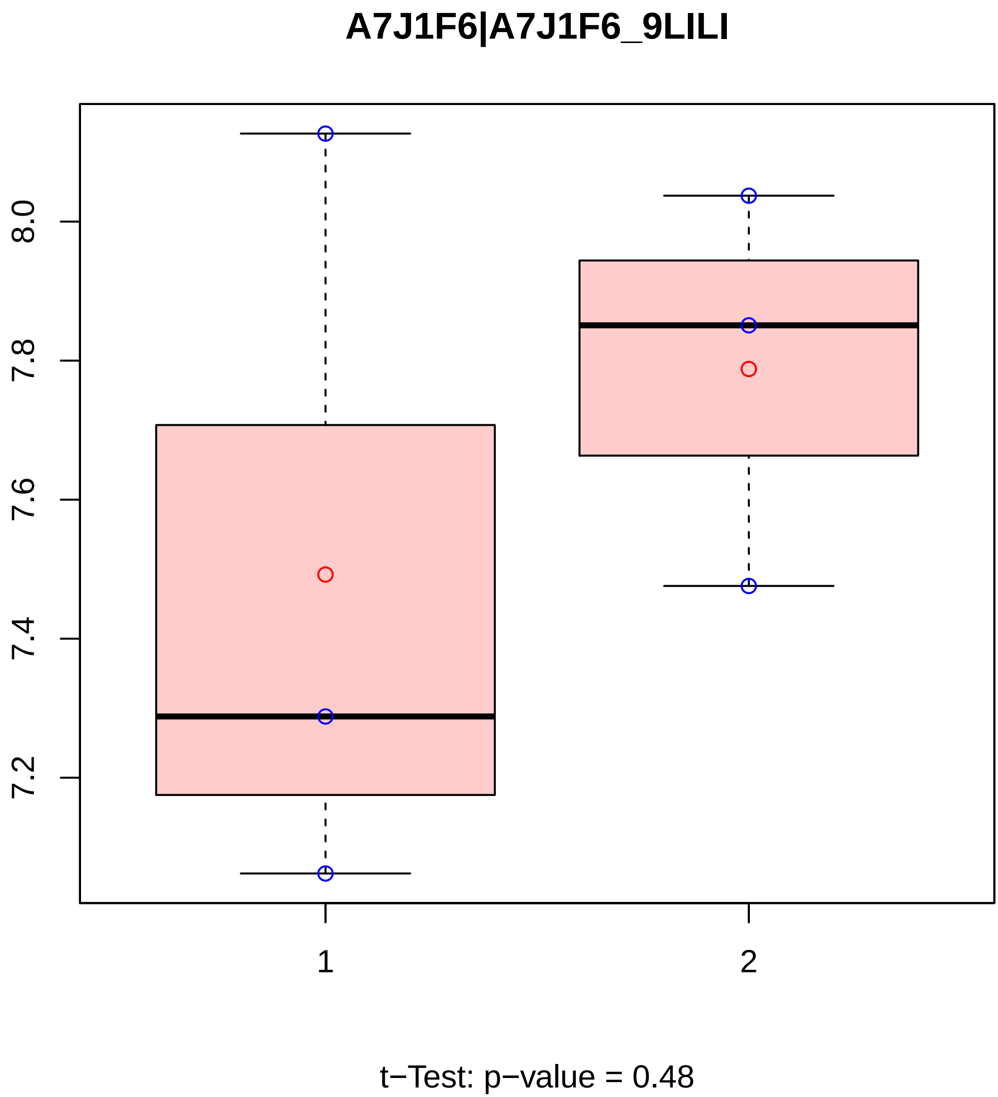

A7J1F6|A7J1F6_9LILI
8.0
7.8
7.6
7.4
7.2
1
2
t−Test: p−value = 0.48

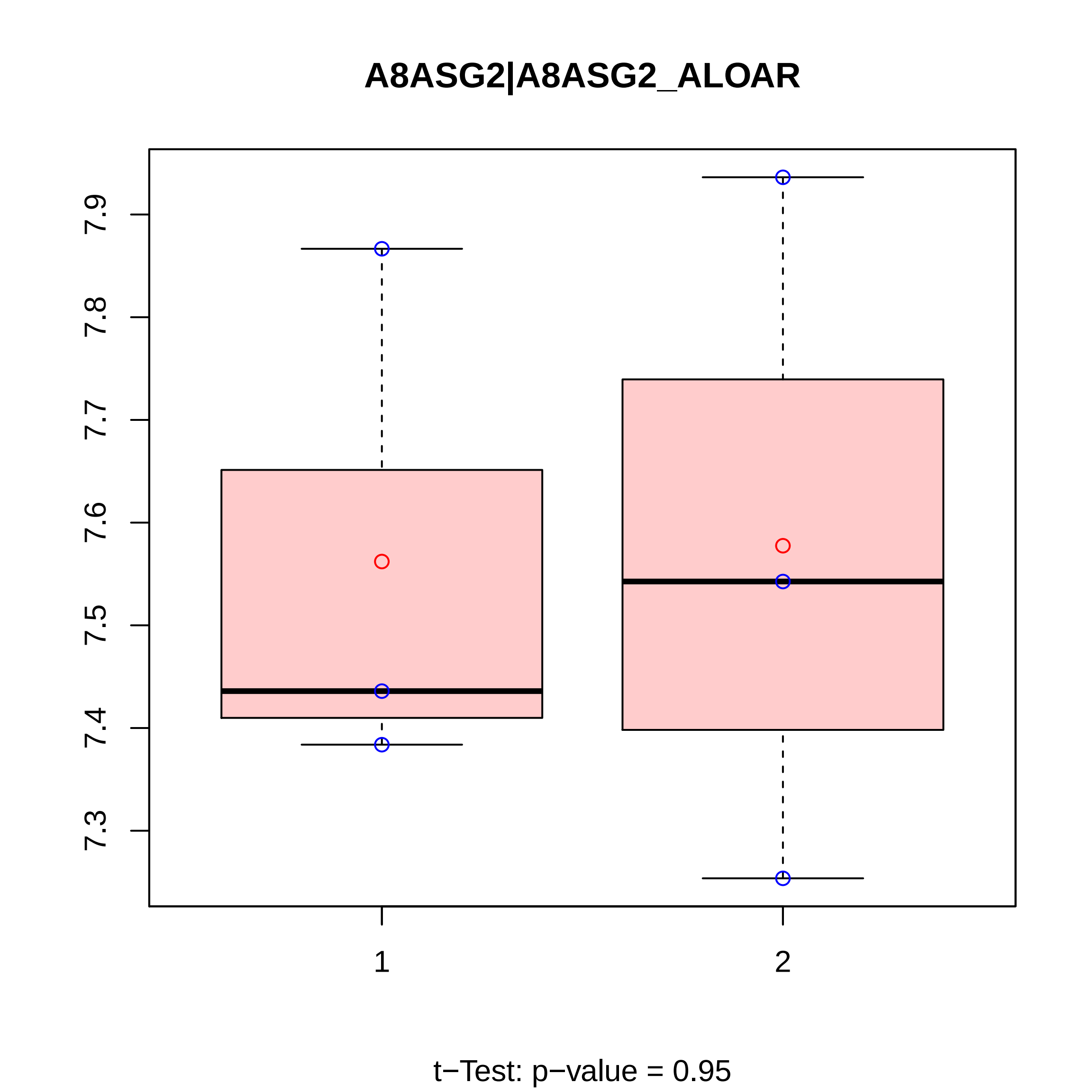
A8ASG2|A8ASG2_ALOAR
7.3
7.4
7.5
7.6
7.7
7.8
7.9
1
2
t−Test: p−value = 0.95

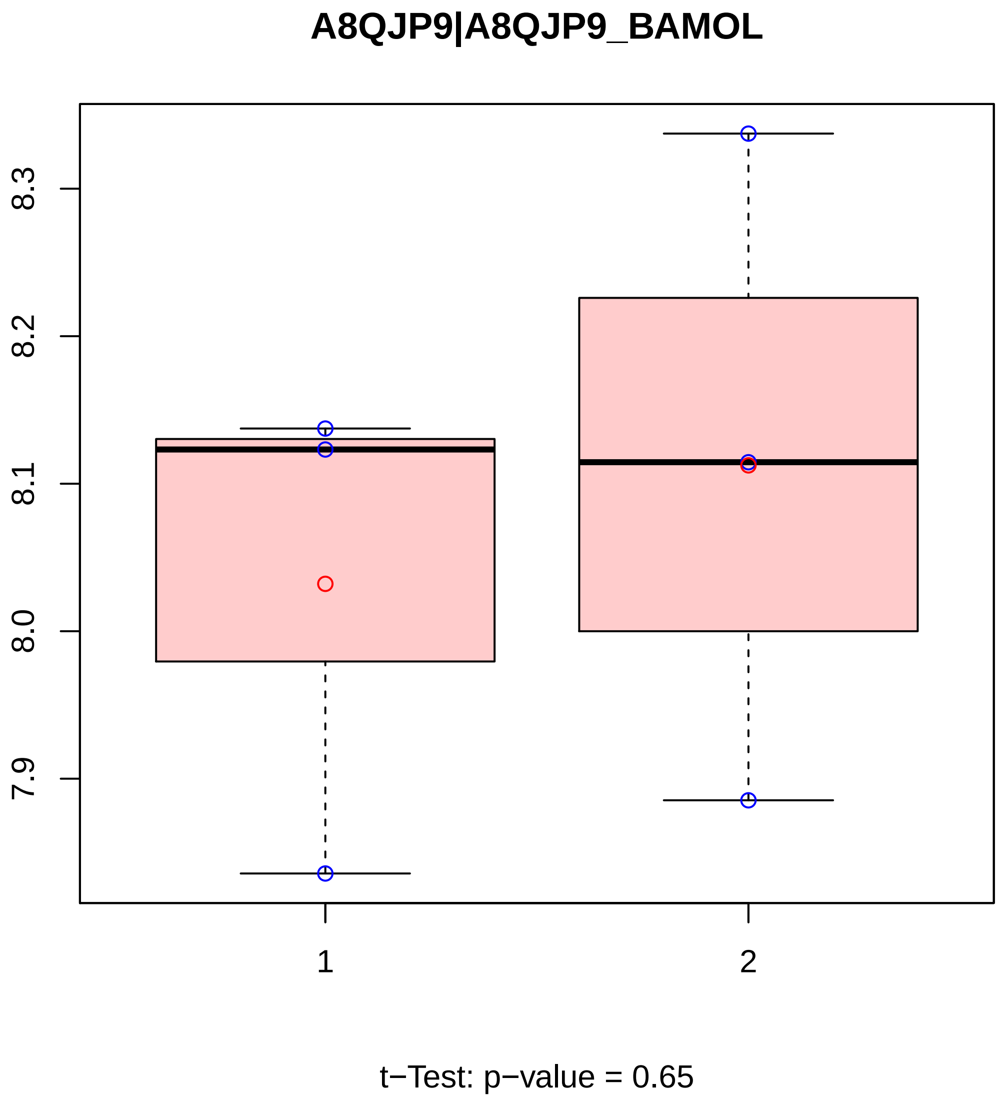
A8QJP9|A8QJP9_BAMOL
7.9
8.0
8.1
8.2
8.3
1
2
t−Test: p−value = 0.65

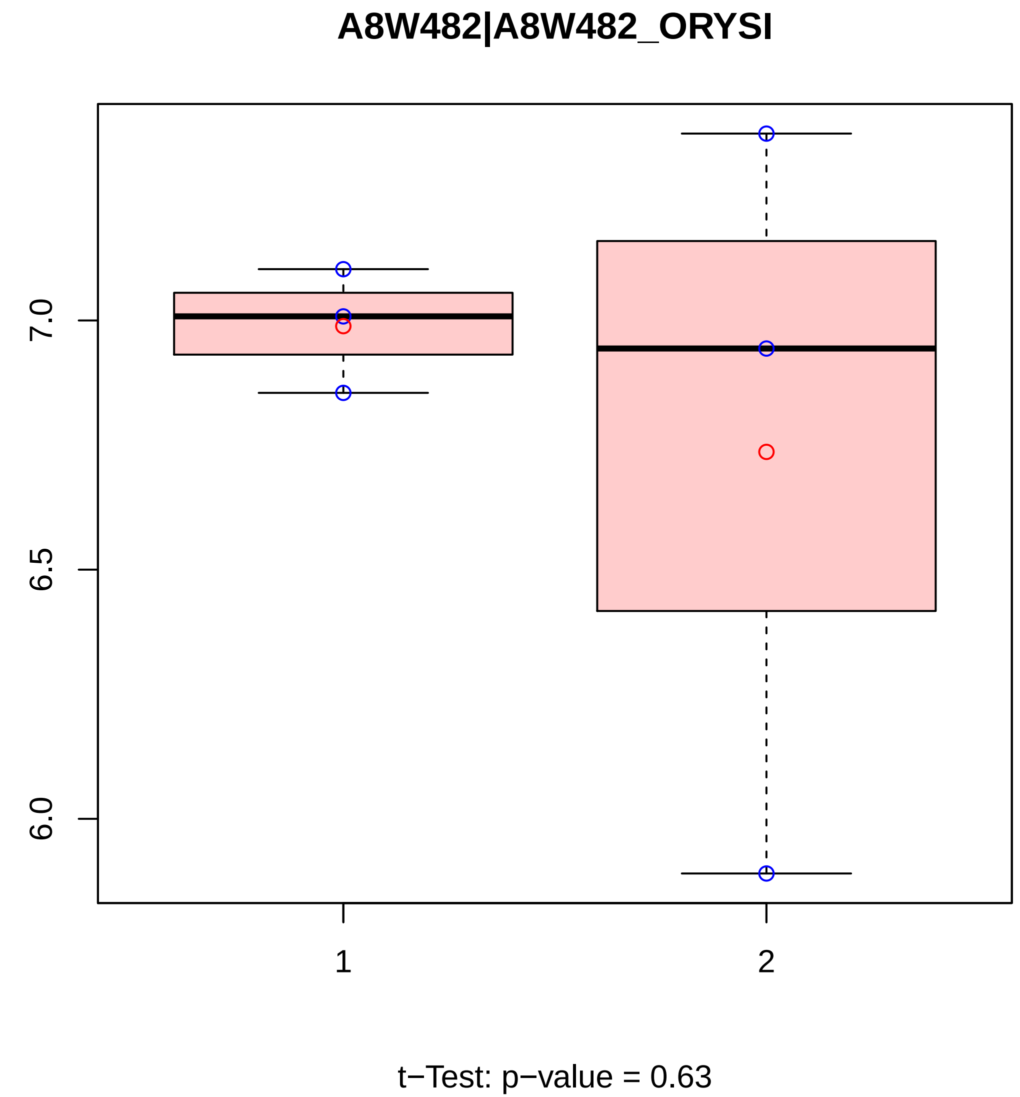

A8W482|A8W482_ORYSI
7.0
6.5
6.0
1
2
t−Test: p−value = 0.63

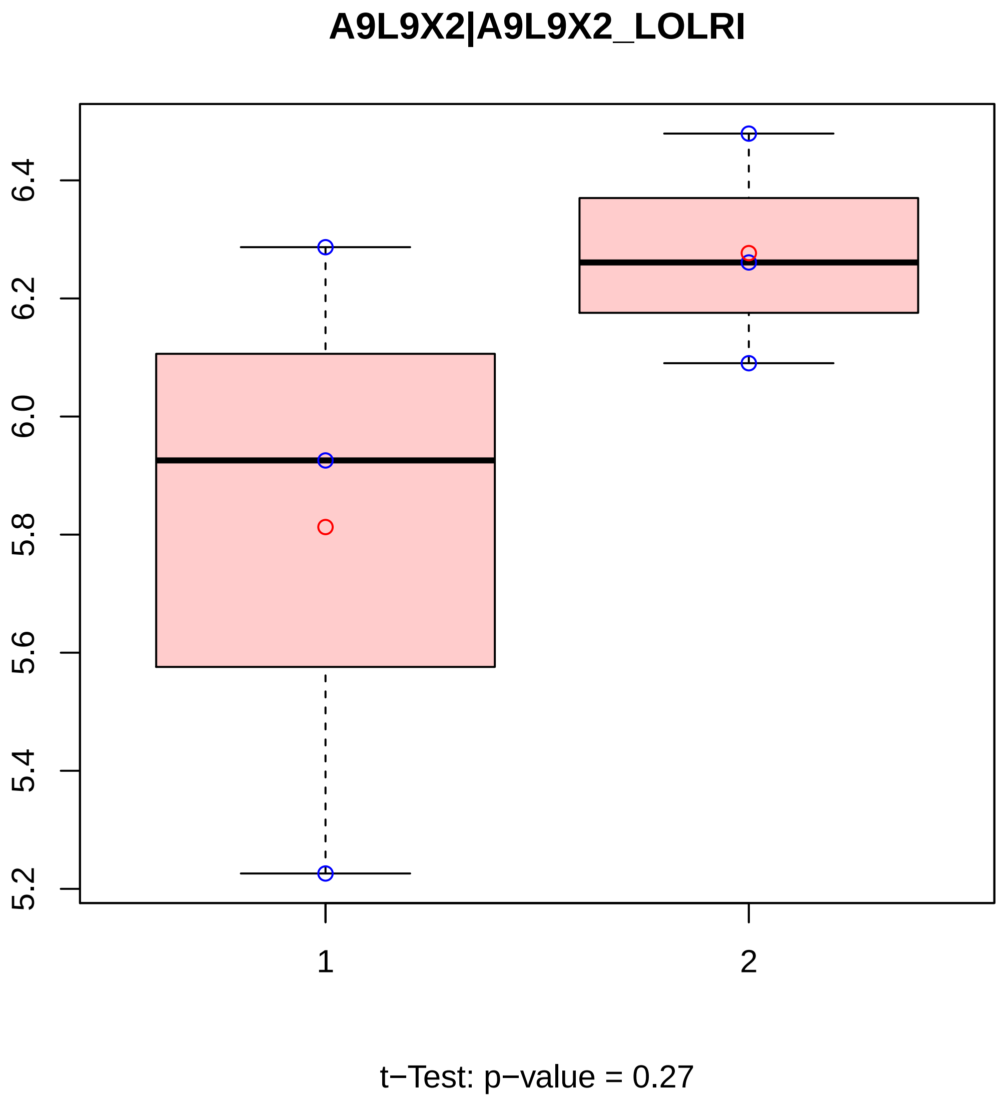

A9L9X2|A9L9X2_LOLRI
6.4
6.2
6.0
5.8
5.6
5.4
5.2
1
2
t−Test: p−value = 0.27

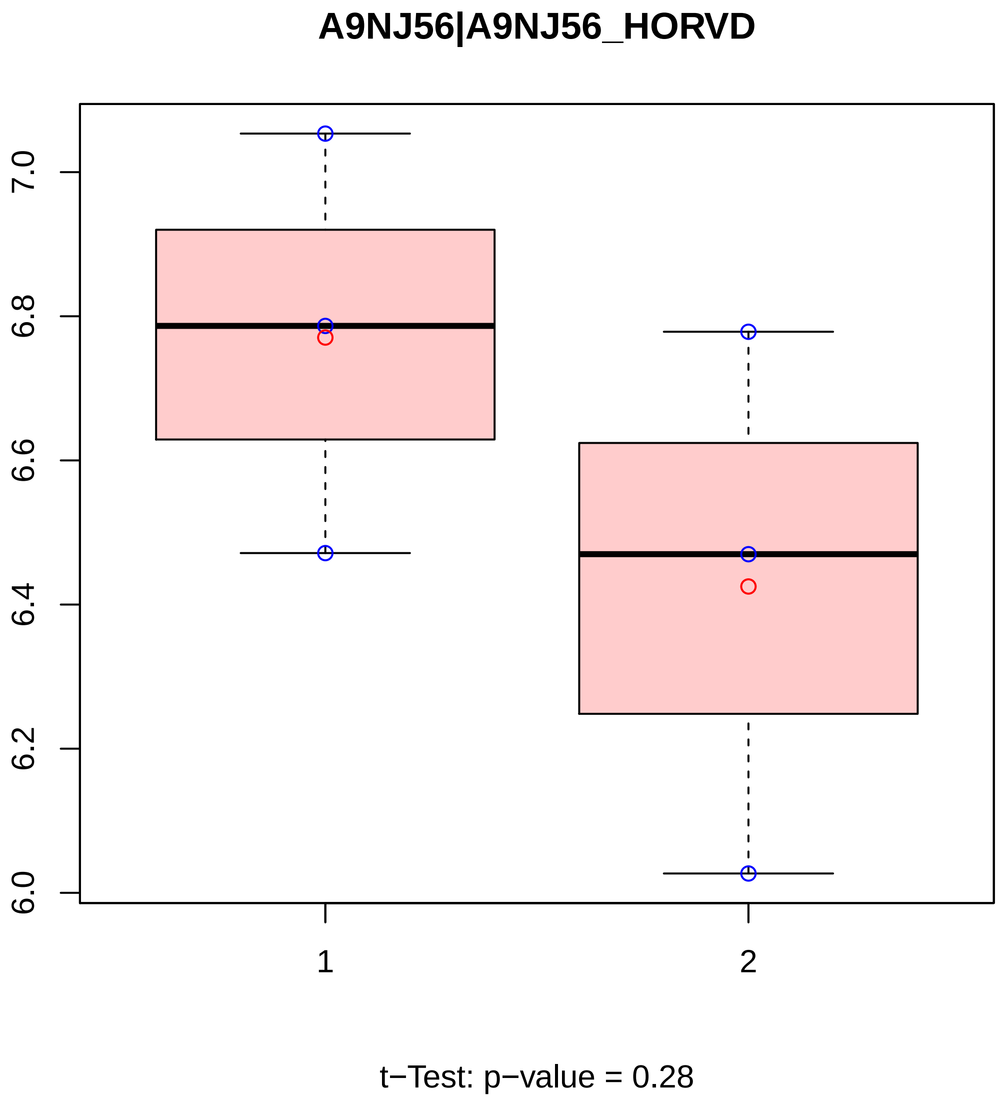

A9NJ56|A9NJ56_HORVD
7.0
6.8
6.6
6.4
6.2
6.0
1
2
t−Test: p−value = 0.28

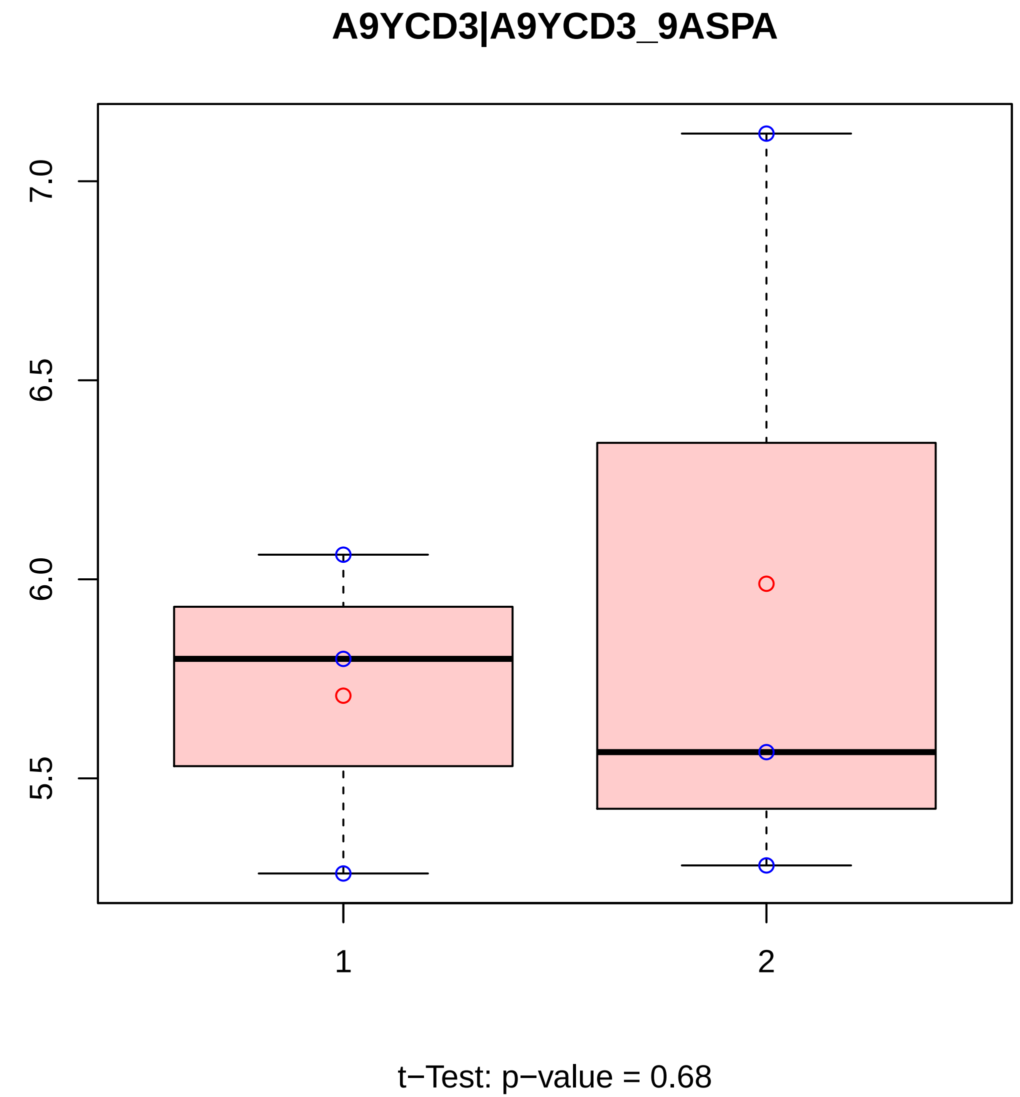
A9YCD3|A9YCD3_9ASPA
7.0
6.5
6.0
5.5
1
2
t−Test: p−value = 0.68

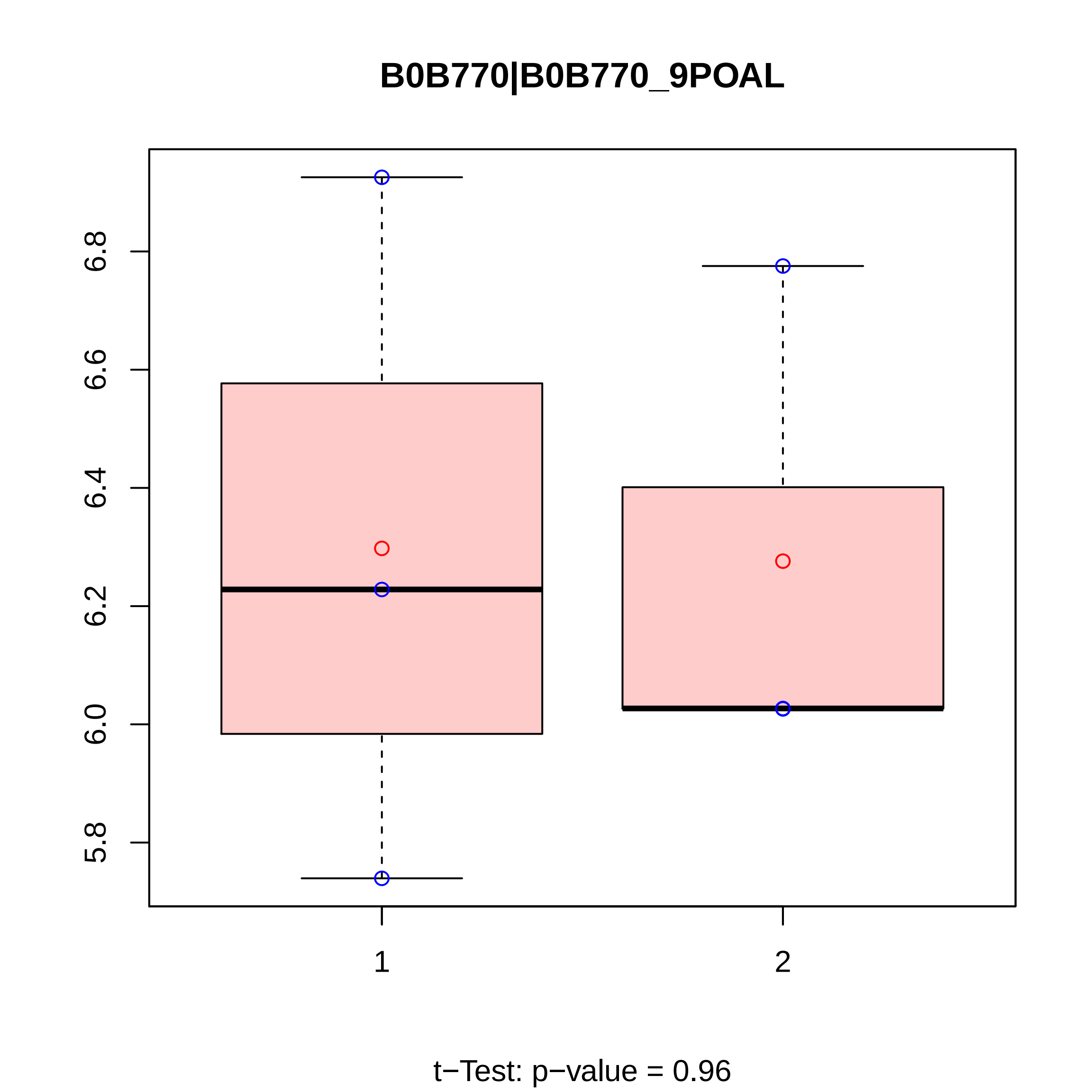
B0B770|B0B770_9POAL
6.8
6.6
6.4
6.2
6.0
5.8
1
2
t−Test: p−value = 0.96

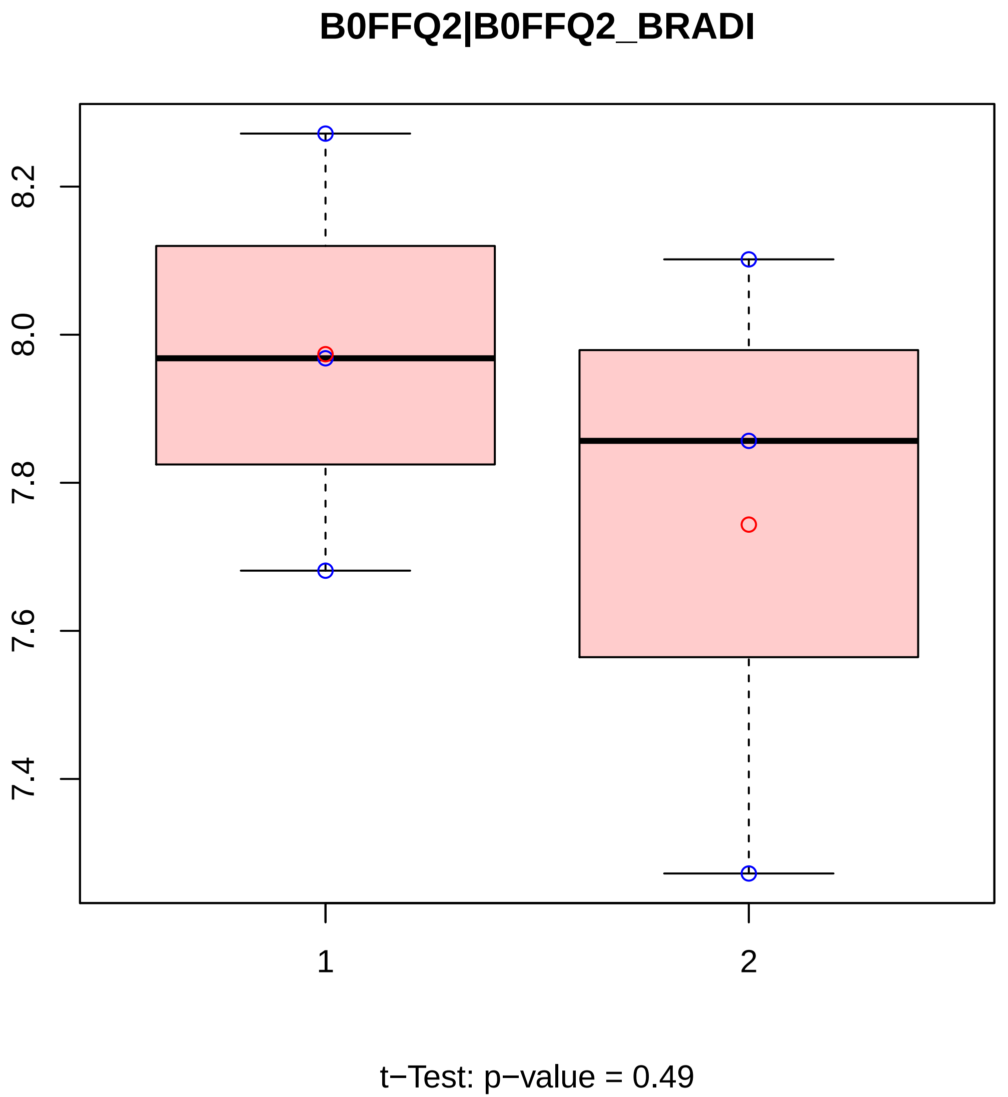

B0FFQ2|B0FFQ2_BRADI
8.2
8.0
7.8
7.6
7.4
1
2
t−Test: p−value = 0.49

B0YIB2|B0YIB2_9POAL

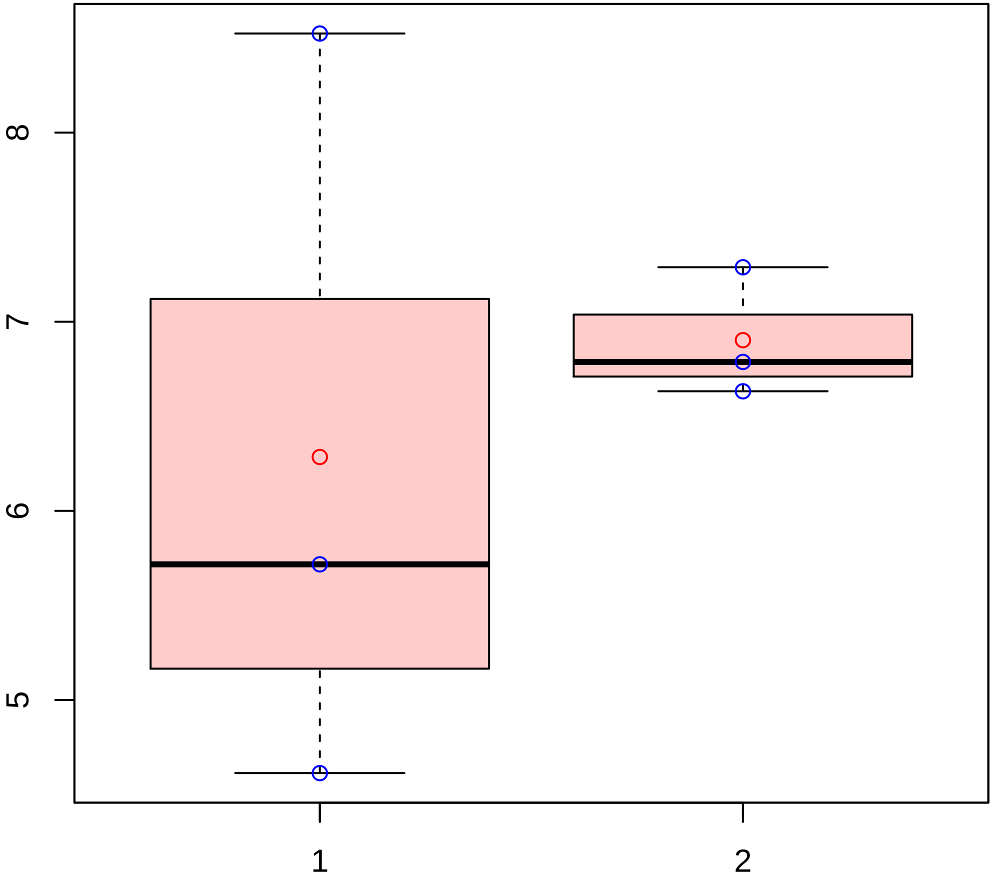

t−Test: p−value = 0.65

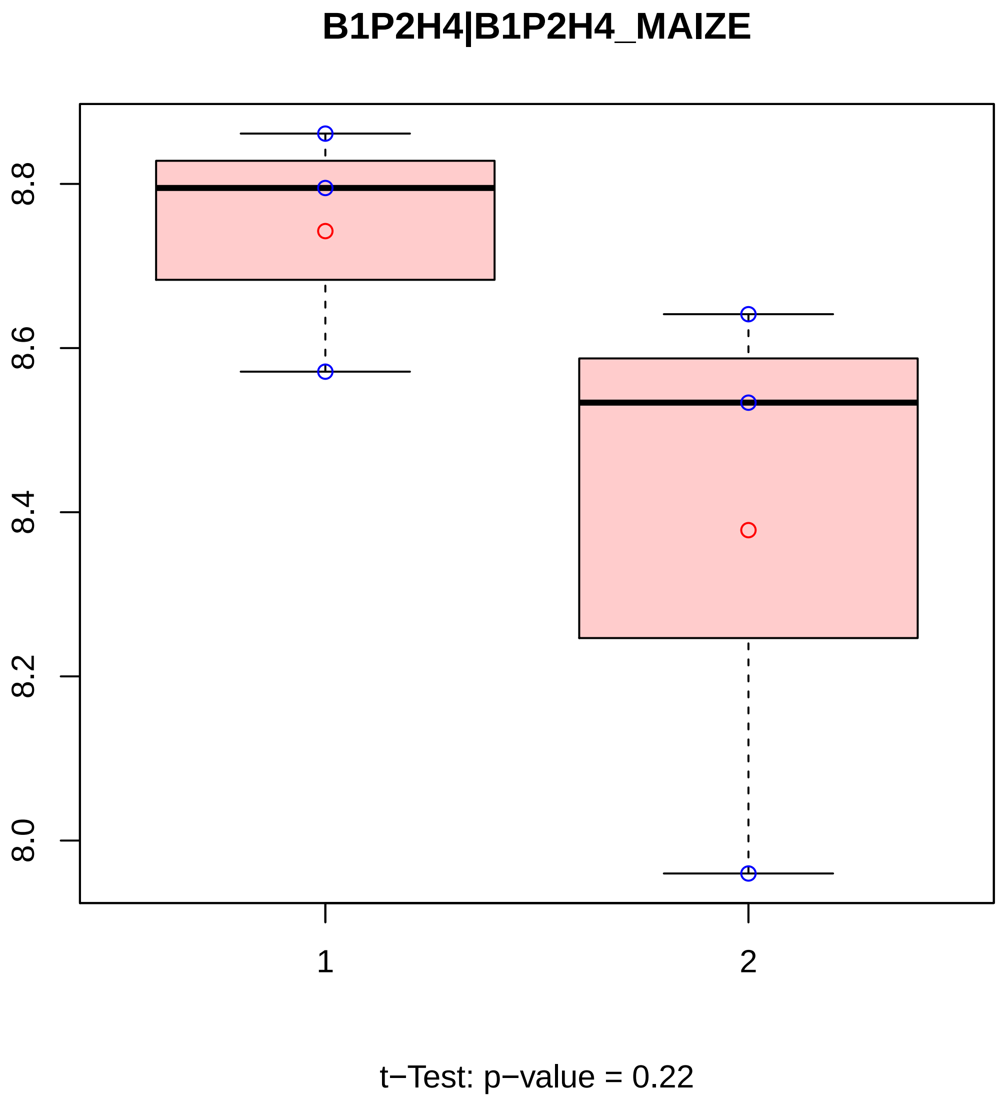
B1P2H4|B1P2H4_MAIZE
8.0
8.2
8.4
8.6
8.8
1
2
t−Test: p−value = 0.22

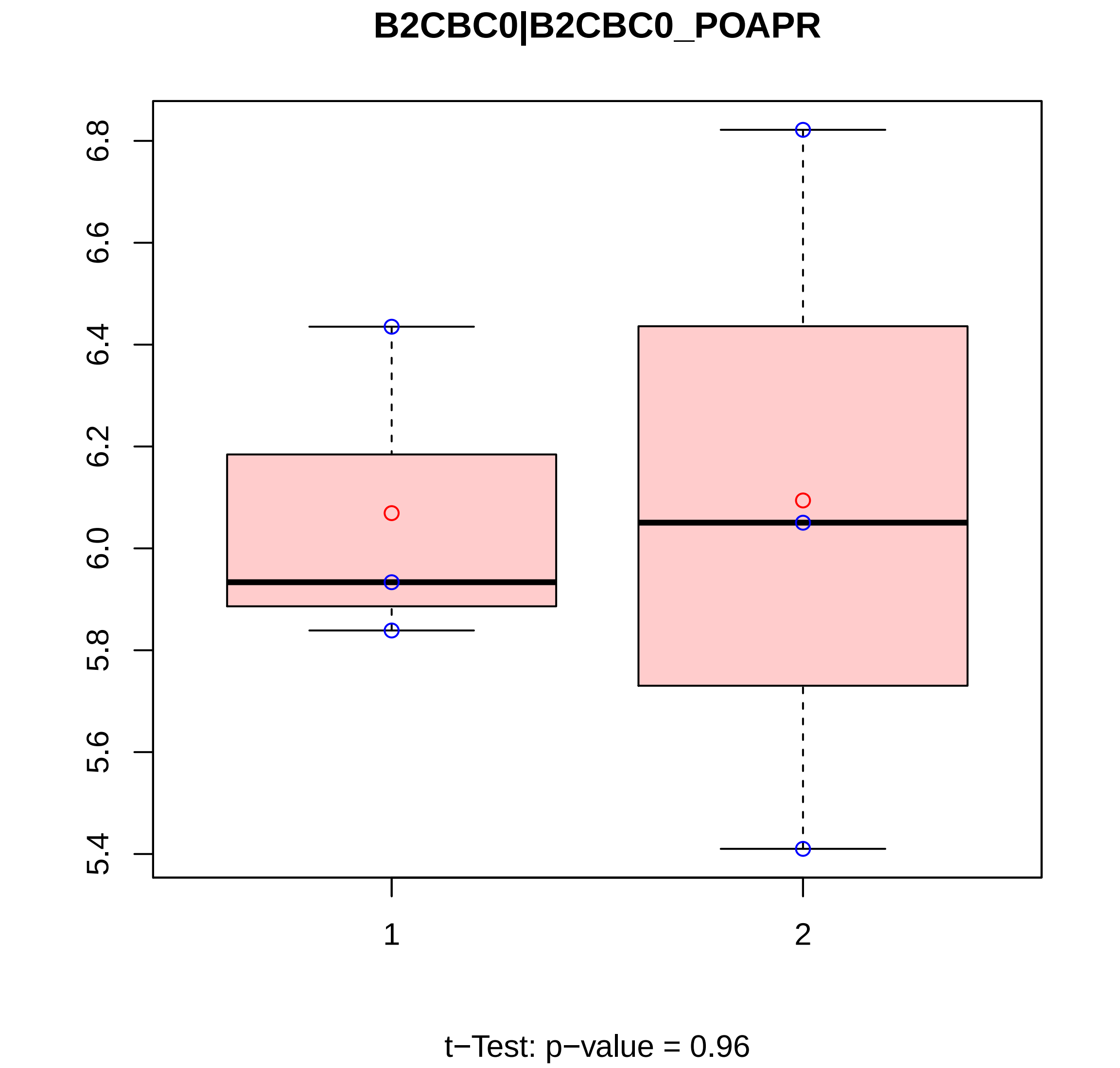
B2CBC0|B2CBC0_POAPR
6.8
6.6
6.4
6.2
6.0
5.8
5.6
5.4
1
2
t−Test: p−value = 0.96

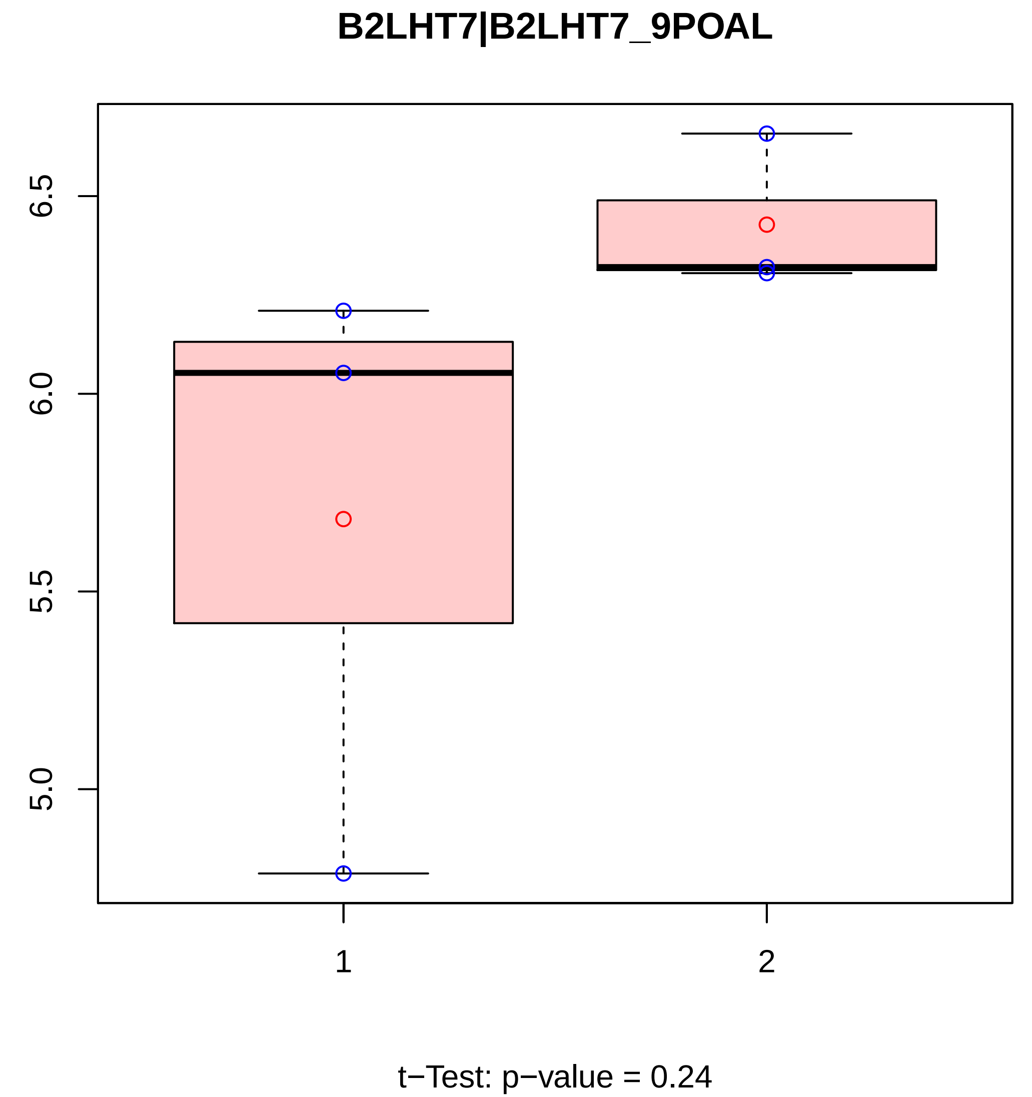
B2LHT7|B2LHT7_9POAL
6.5
6.0
5.5
5.0
1
2
t−Test: p−value = 0.24

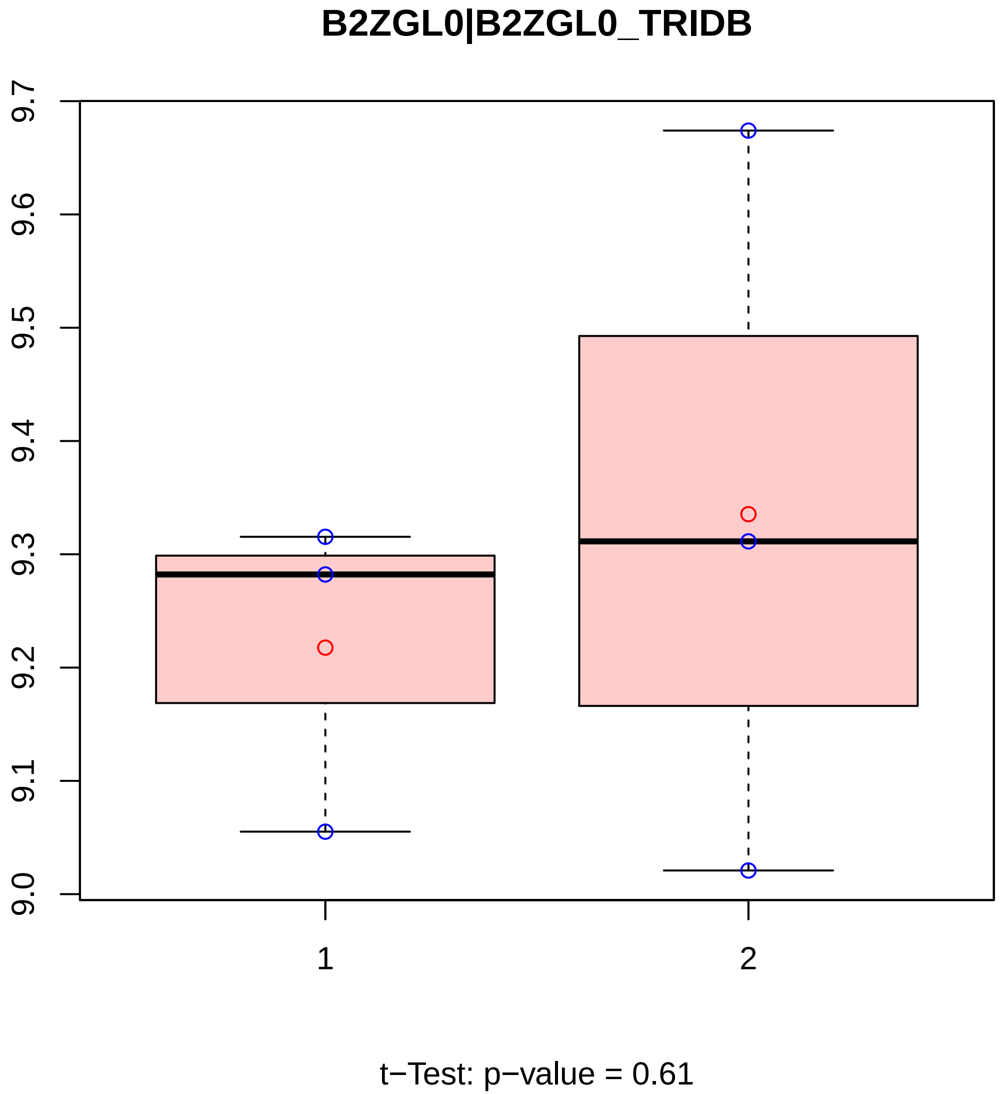

B2ZGL0|B2ZGL0_TRIDB
9.0
9.1
9.2
9.3
9.4
9.5
9.6
9.7
1
2
t−Test: p−value = 0.61

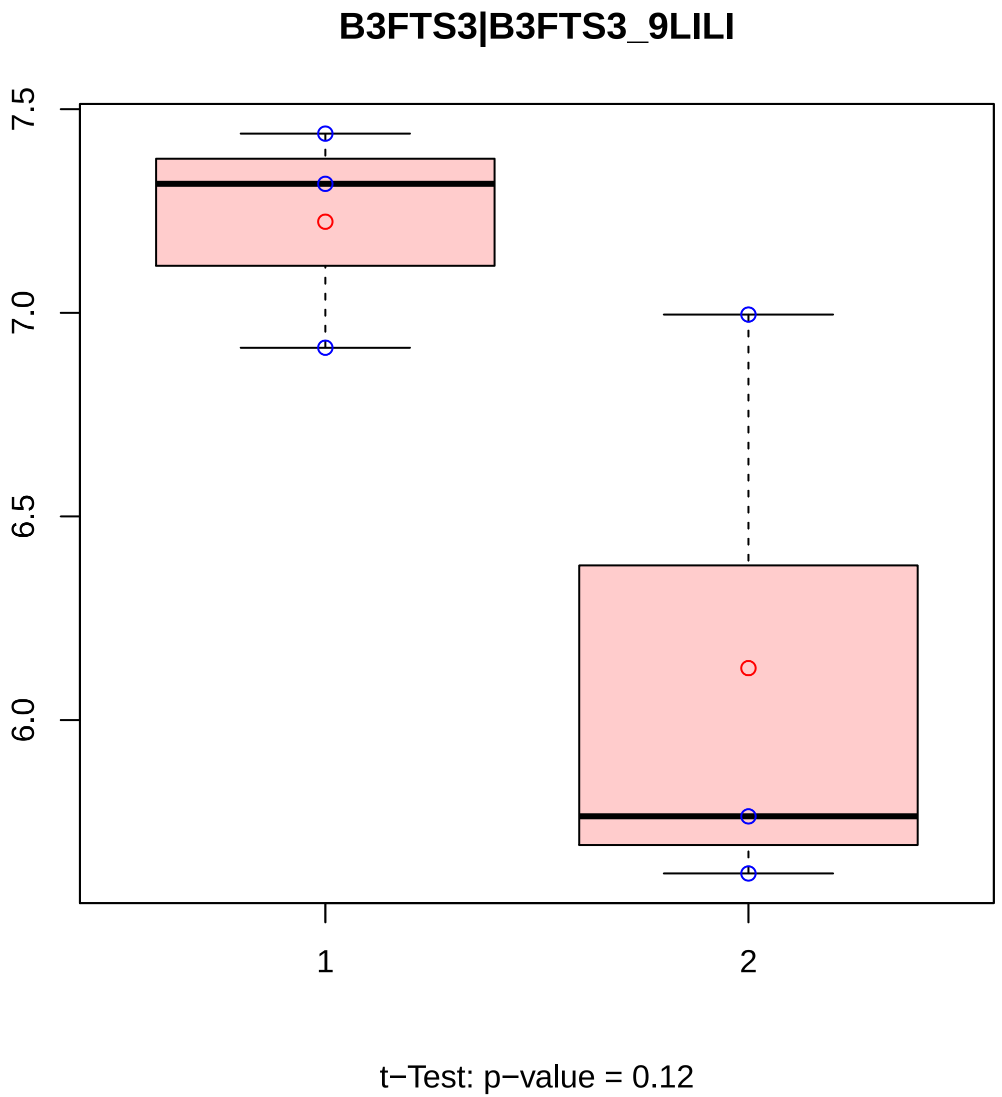

B3FTS3|B3FTS3_9LILI
7.5
7.0
6.5
6.0
1
2
t−Test: p−value = 0.12

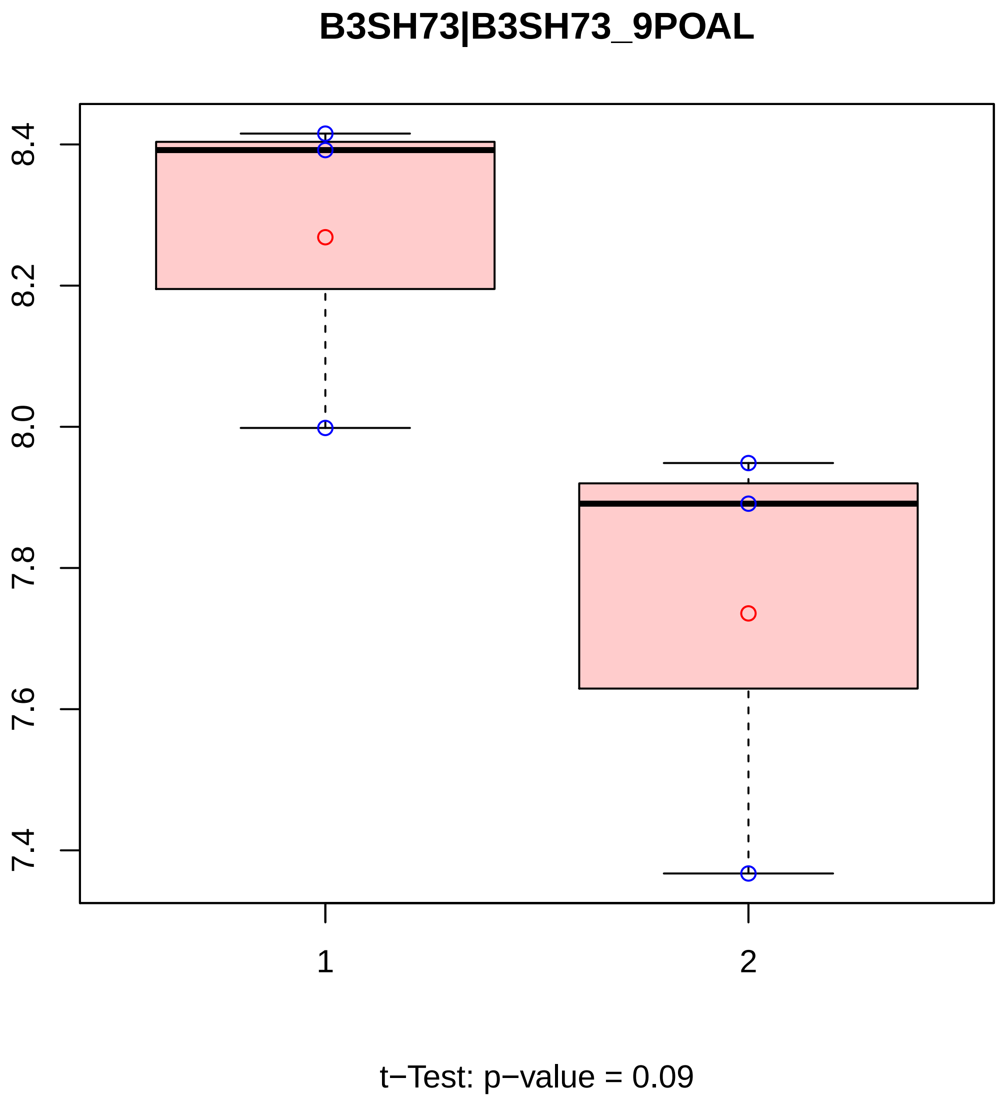
B3SH73|B3SH73_9POAL
8.4
8.2
8.0
7.8
7.6
7.4
1
2
t−Test: p−value = 0.09

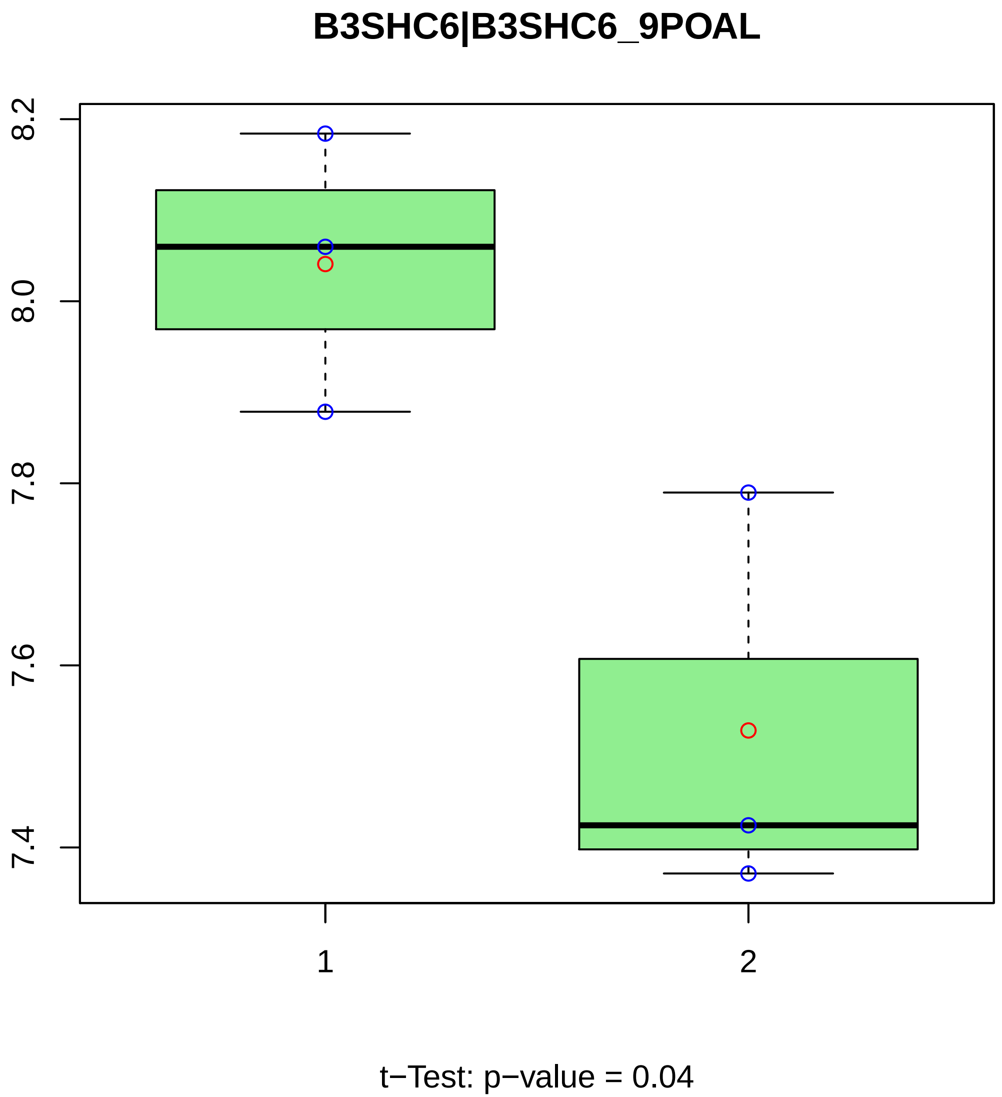
B3SHC6|B3SHC6_9POAL
8.2
8.0
7.8
7.6
7.4
1
2
t−Test: p−value = 0.04

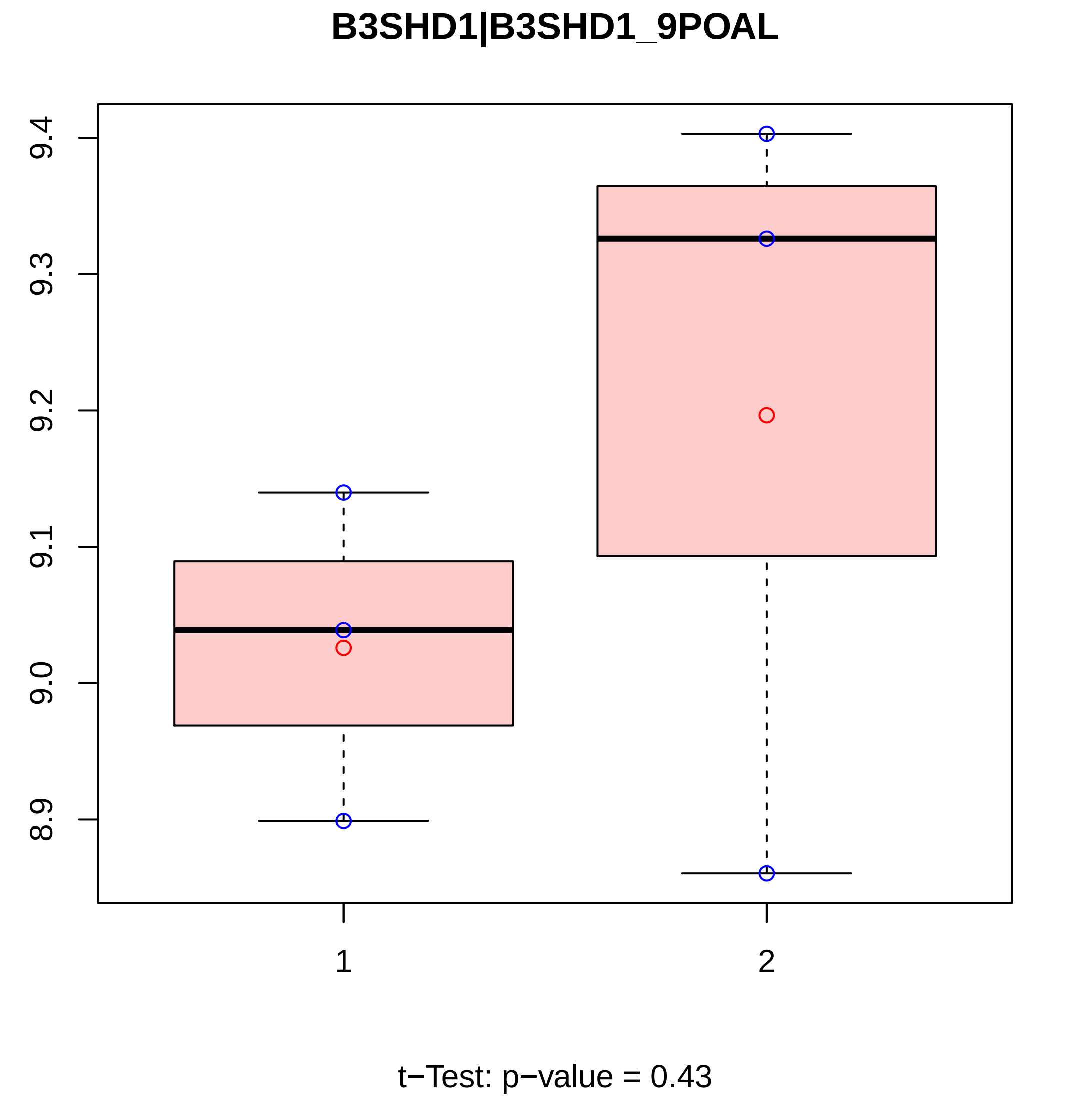
B3SHD1|B3SHD1_9POAL
9.4
9.3
9.2
9.1
9.0
8.9
1
2
t−Test: p−value = 0.43

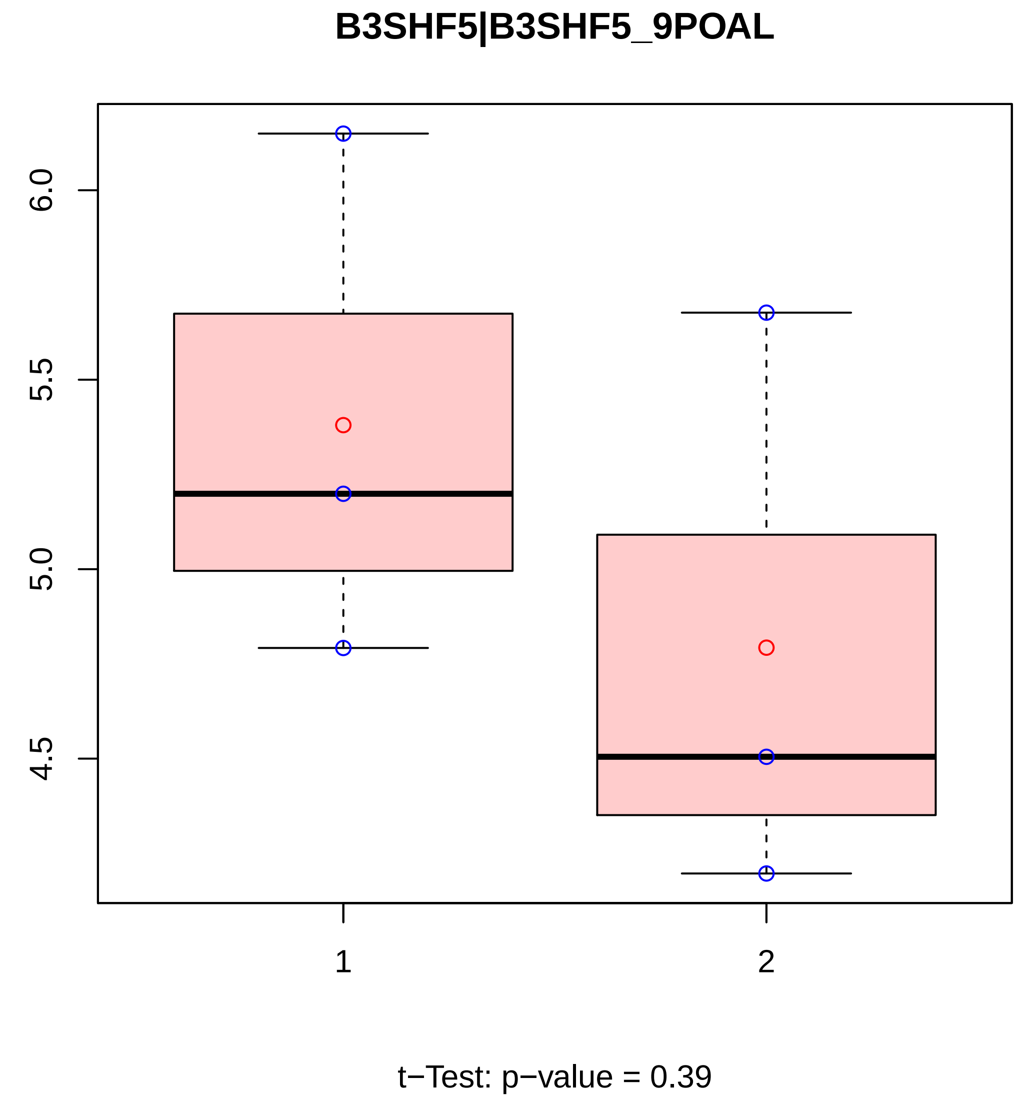
B3SHF5|B3SHF5_9POAL
6.0
5.5
5.0
4.5
1
2
t−Test: p−value = 0.39

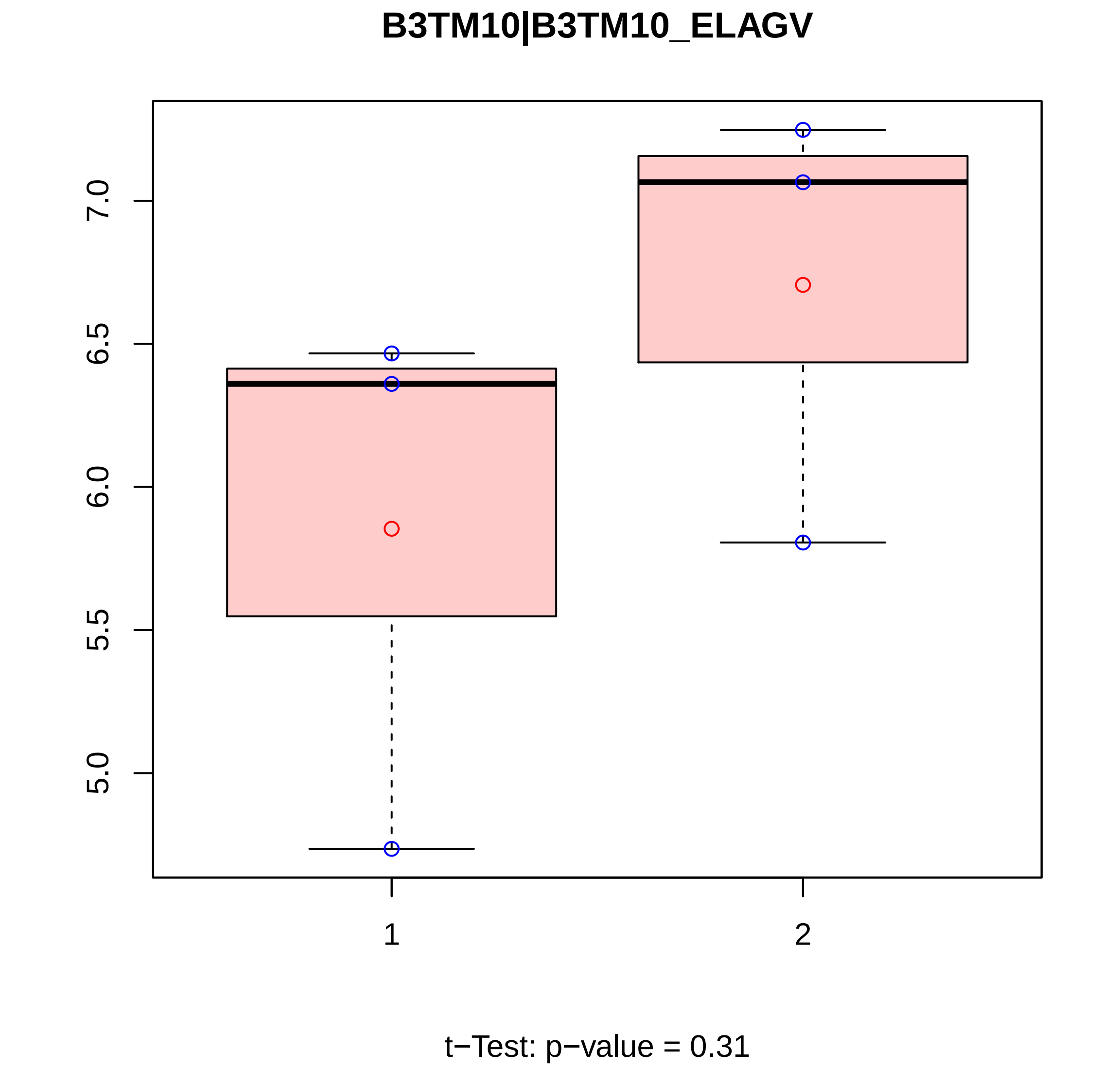

B3TM10|B3TM10_ELAGV
7.0
6.5
6.0
5.5
5.0
1
2
t−Test: p−value = 0.31

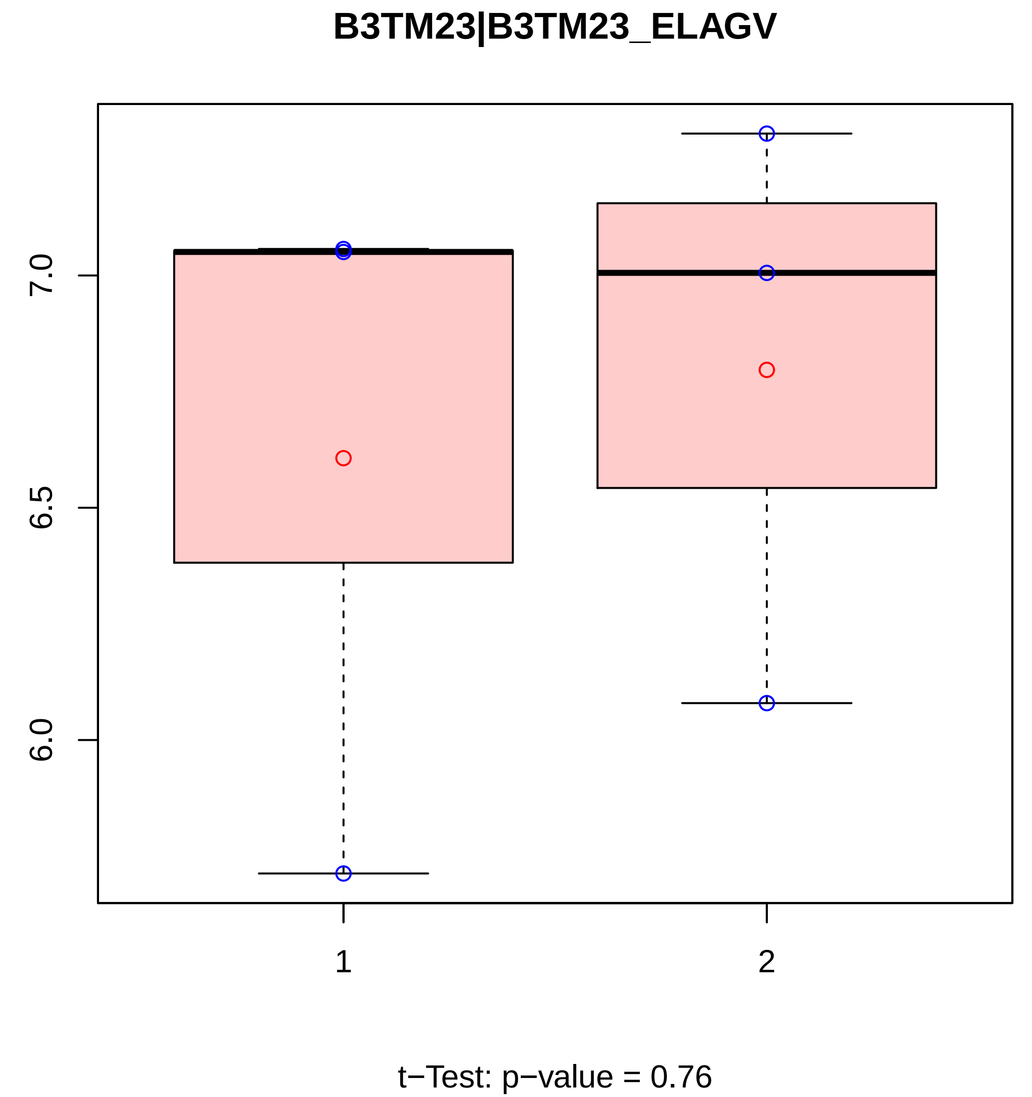

B3TM23|B3TM23_ELAGV
7.0
6.5
6.0
1
2
t−Test: p−value = 0.76

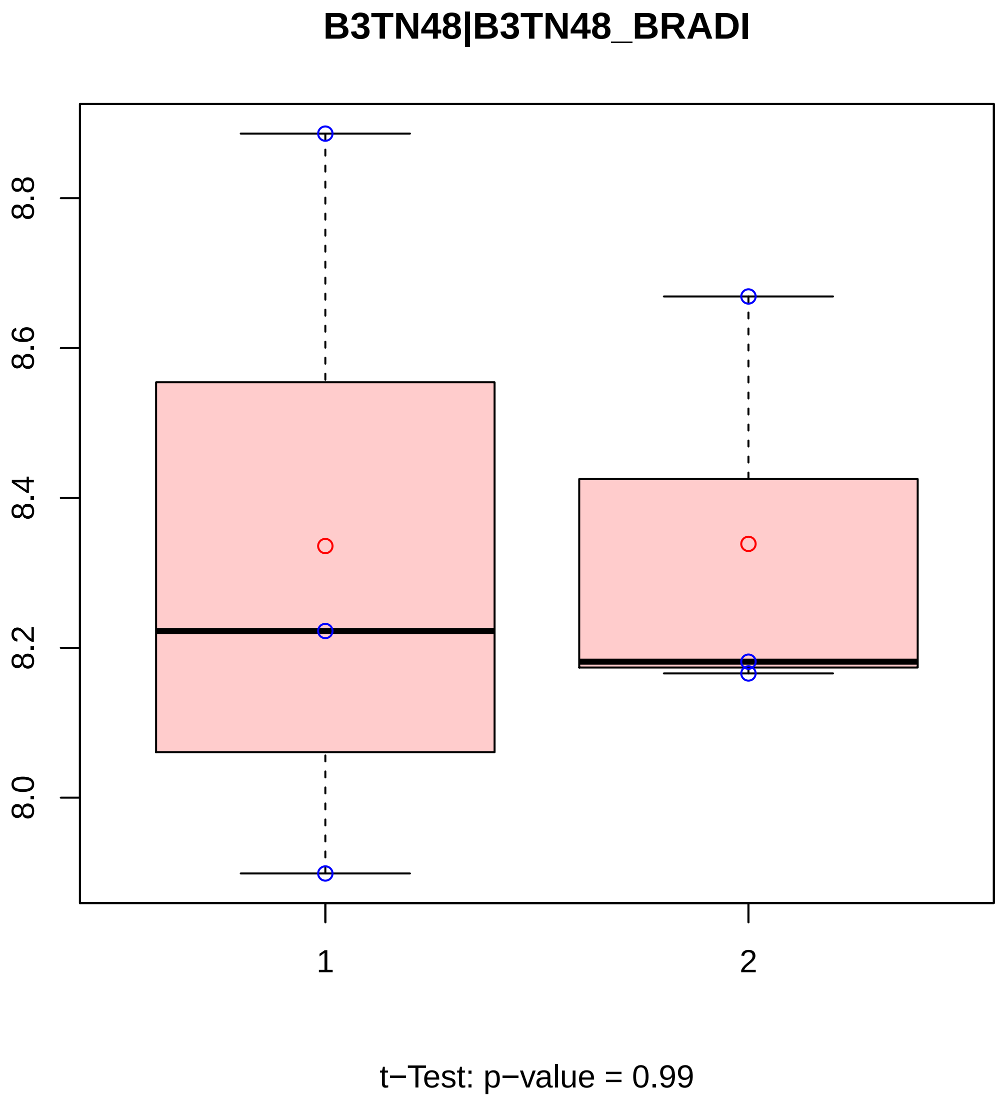

B3TN48|B3TN48_BRADI
8.0
8.2
8.4
8.6
8.8
1
2
t−Test: p−value = 0.99

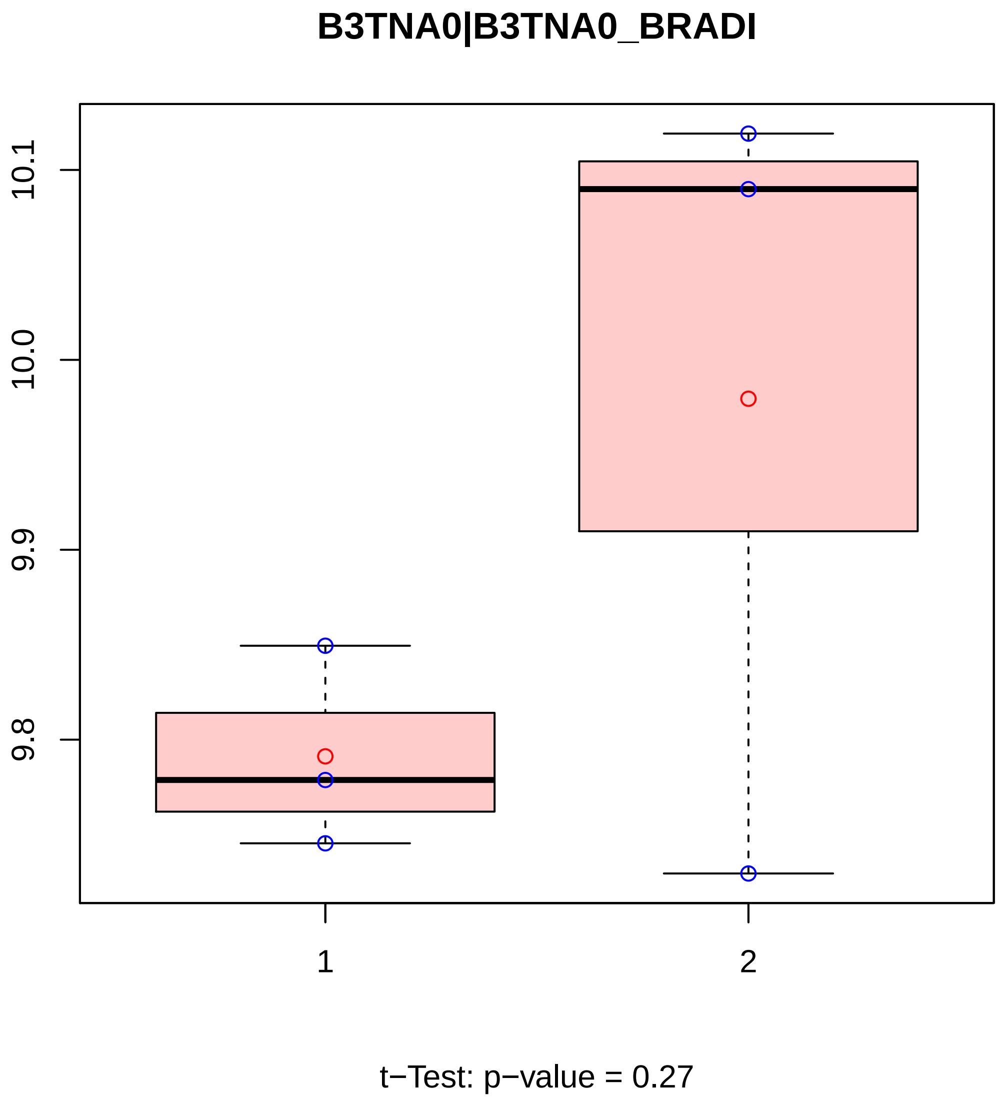

B3TNA0|B3TNA0_BRADI
10.1
10.0
9.9
9.8
1
2
t−Test: p−value = 0.27

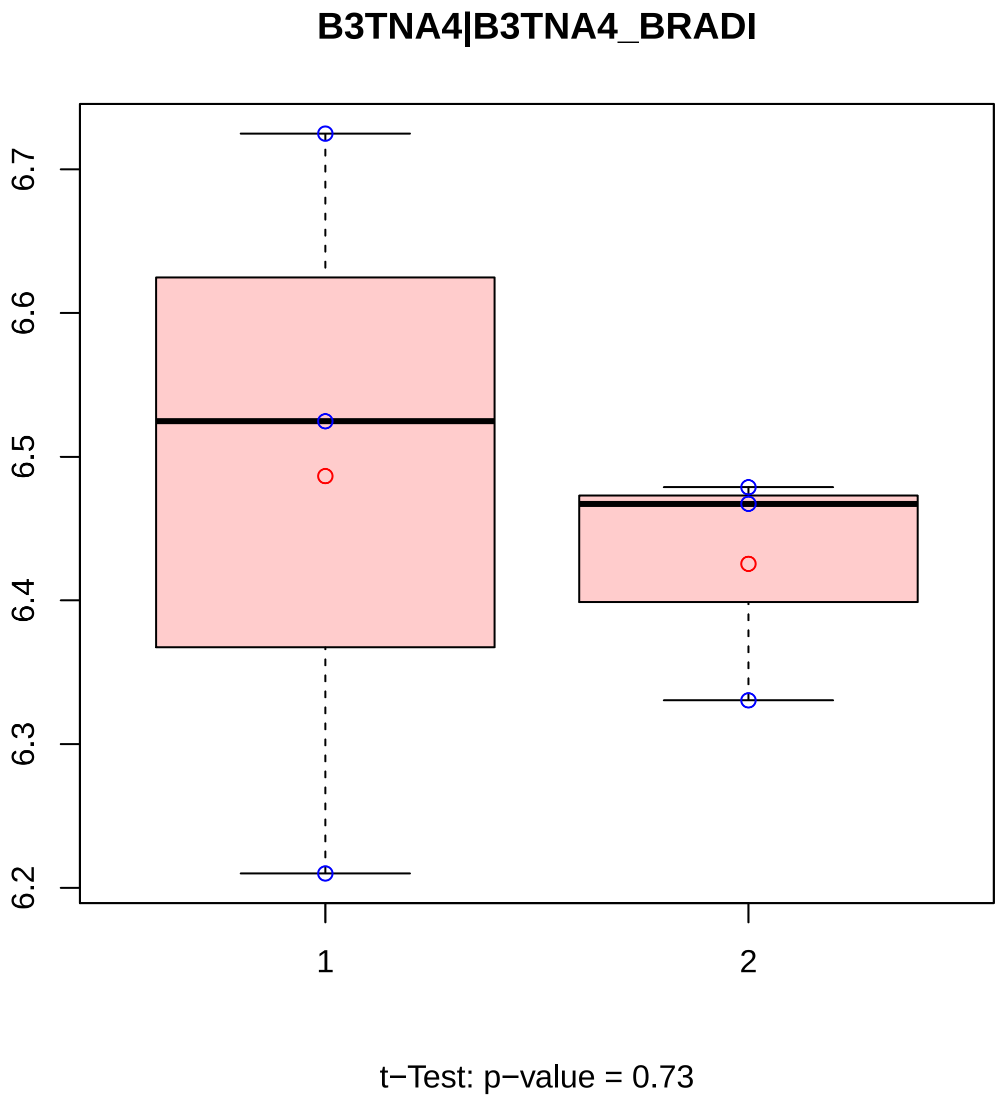

B3TNA4|B3TNA4_BRADI
6.2
6.3
6.4
6.5
6.6
6.7
1
2
t−Test: p−value = 0.73

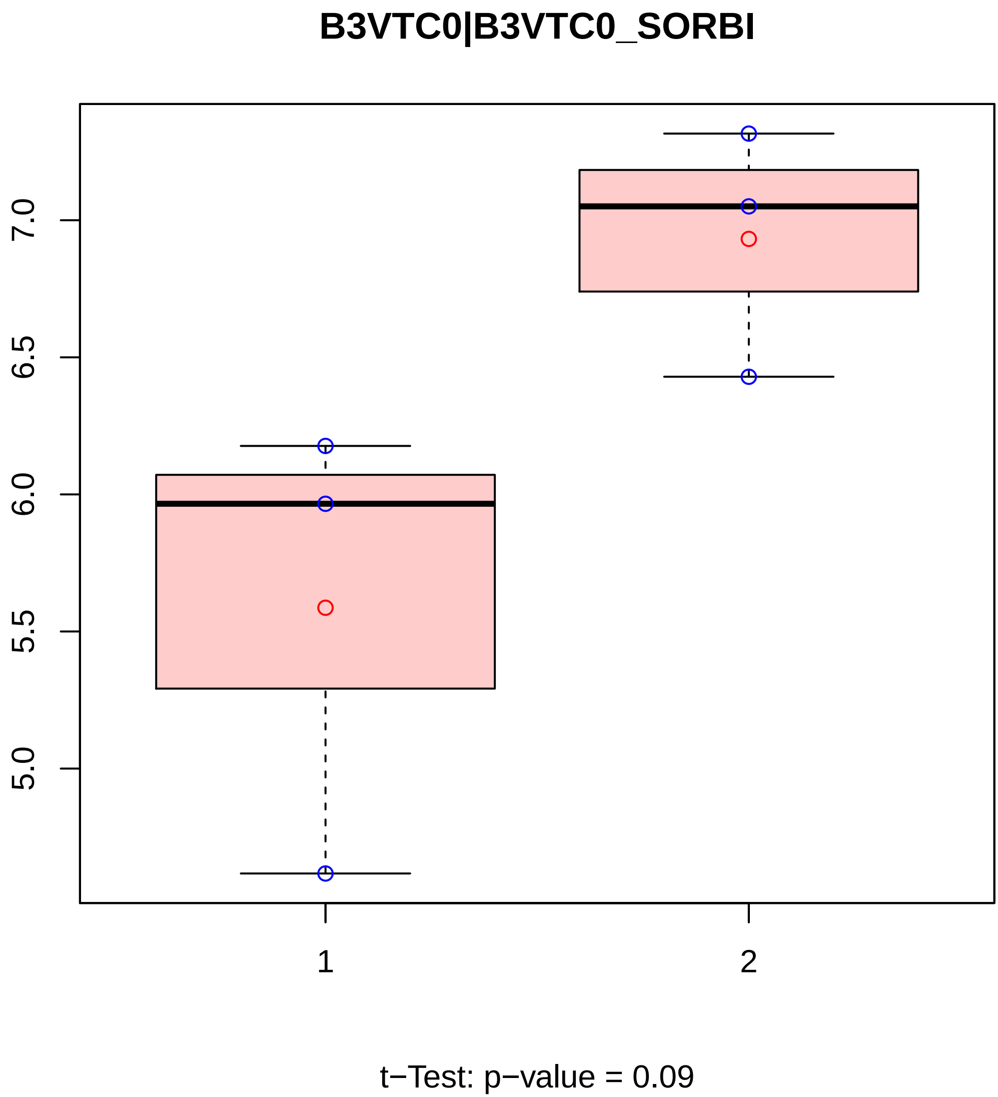

B3VTC0|B3VTC0_SORBI
7.0
6.5
6.0
5.5
5.0
1
2
t−Test: p−value = 0.09

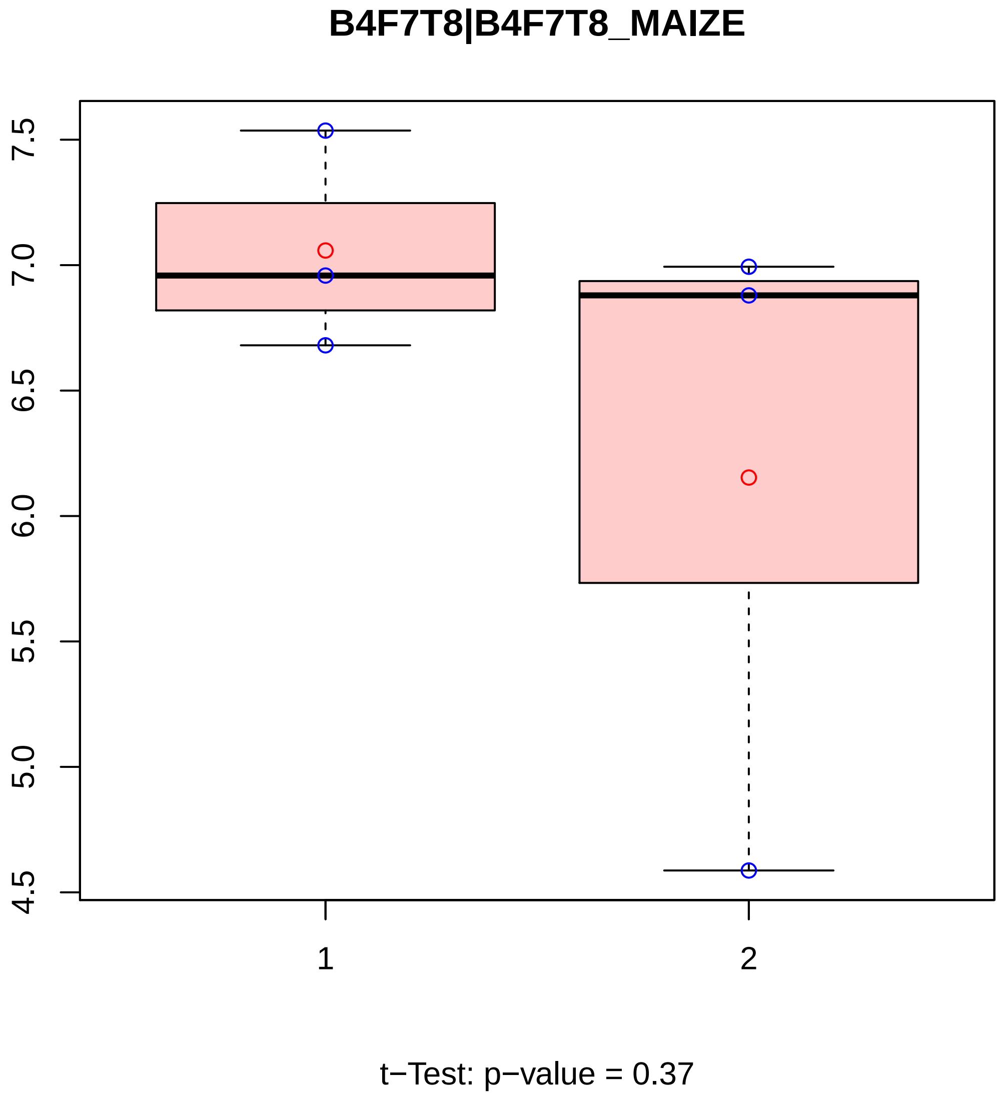

B4F7T8|B4F7T8_MAIZE
7.5
7.0
6.5
6.0
5.5
5.0
4.5
1
2
t−Test: p−value = 0.37

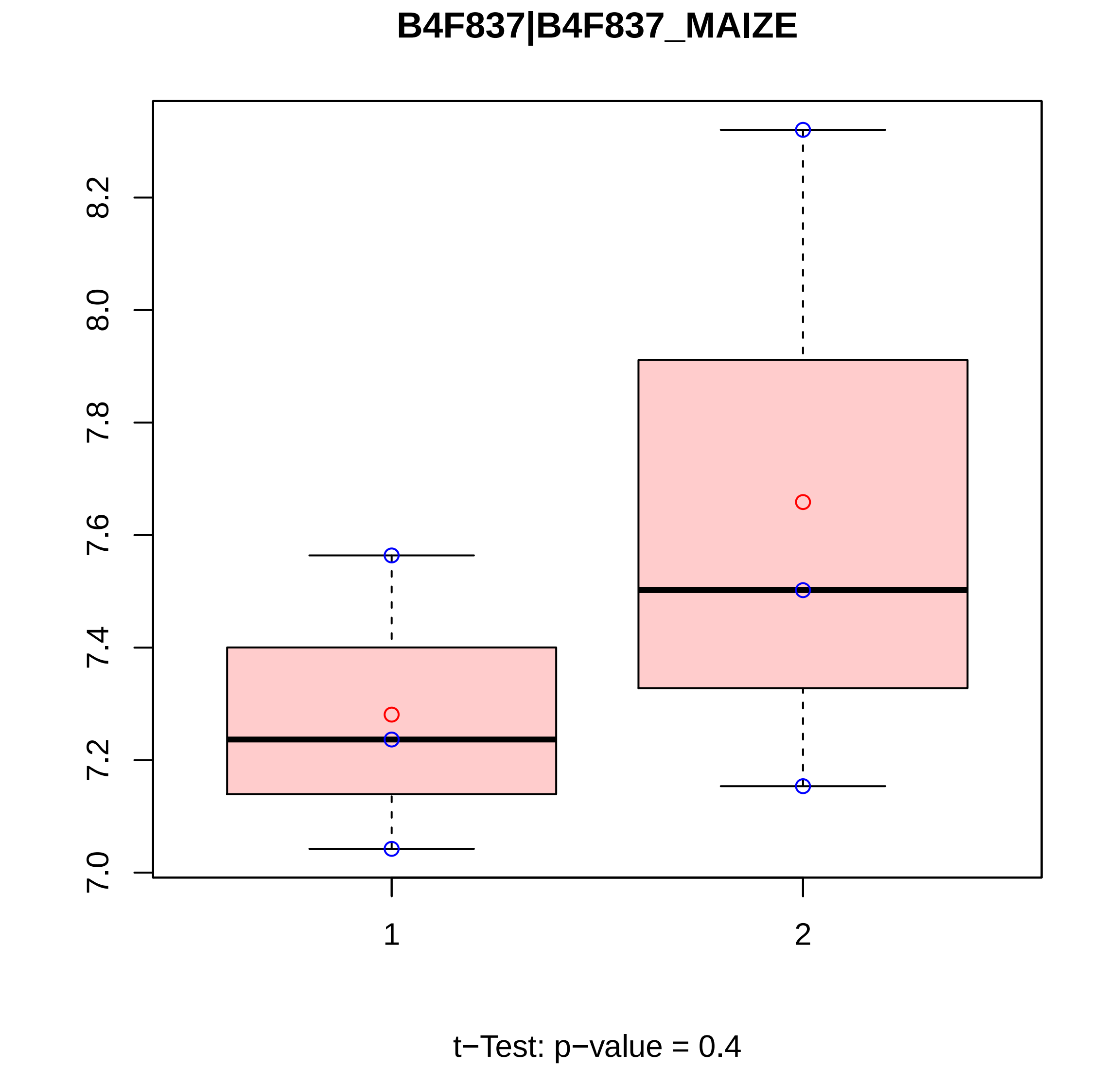
B4F837|B4F837_MAIZE
8.2
8.0
7.8
7.6
7.4
7.2
7.0
1
2
t−Test: p−value = 0.4

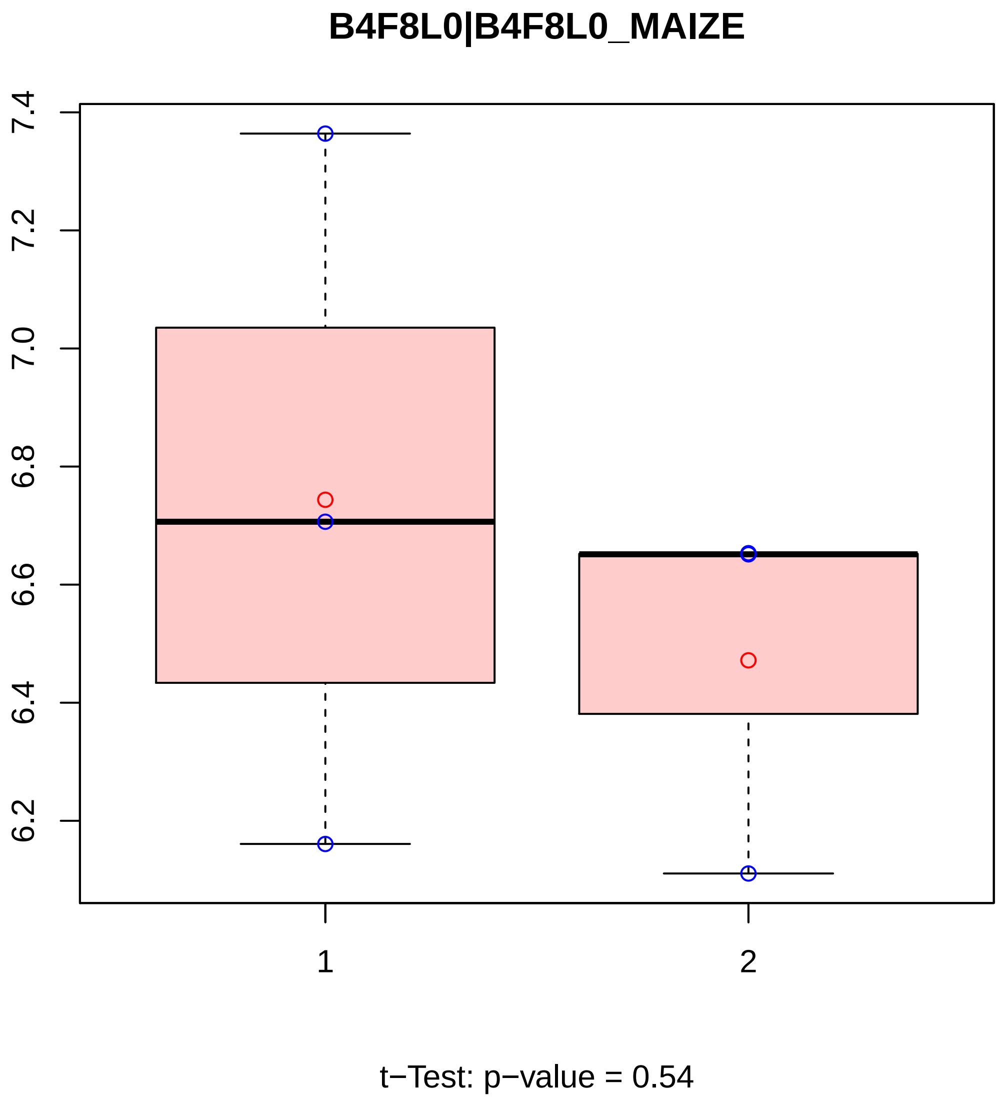
B4F8L0|B4F8L0_MAIZE
7.4
7.2
7.0
6.8
6.6
6.4
6.2
1
2
t−Test: p−value = 0.54

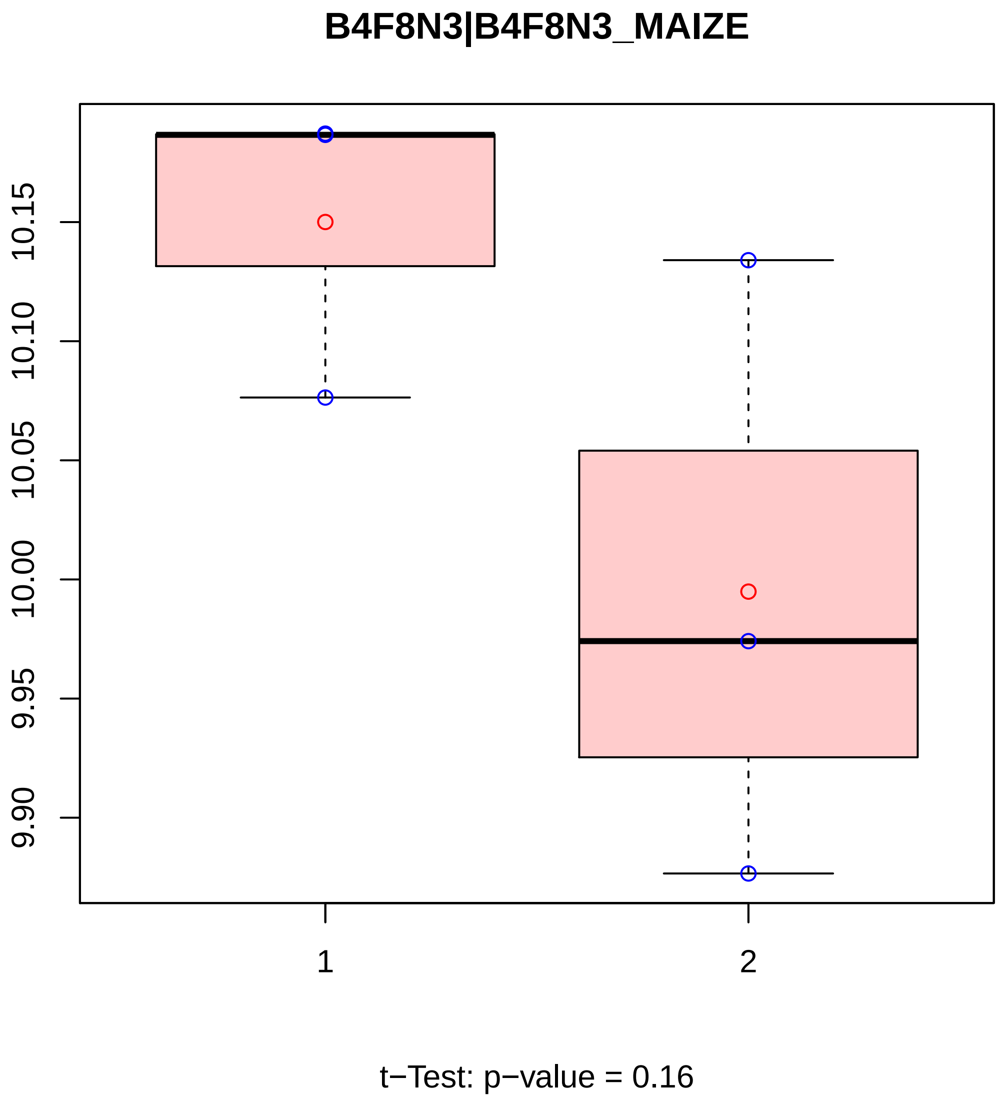

B4F8N3|B4F8N3_MAIZE
10.15
10.10
10.05
10.00
9.95
9.90
1
2
t−Test: p−value = 0.16

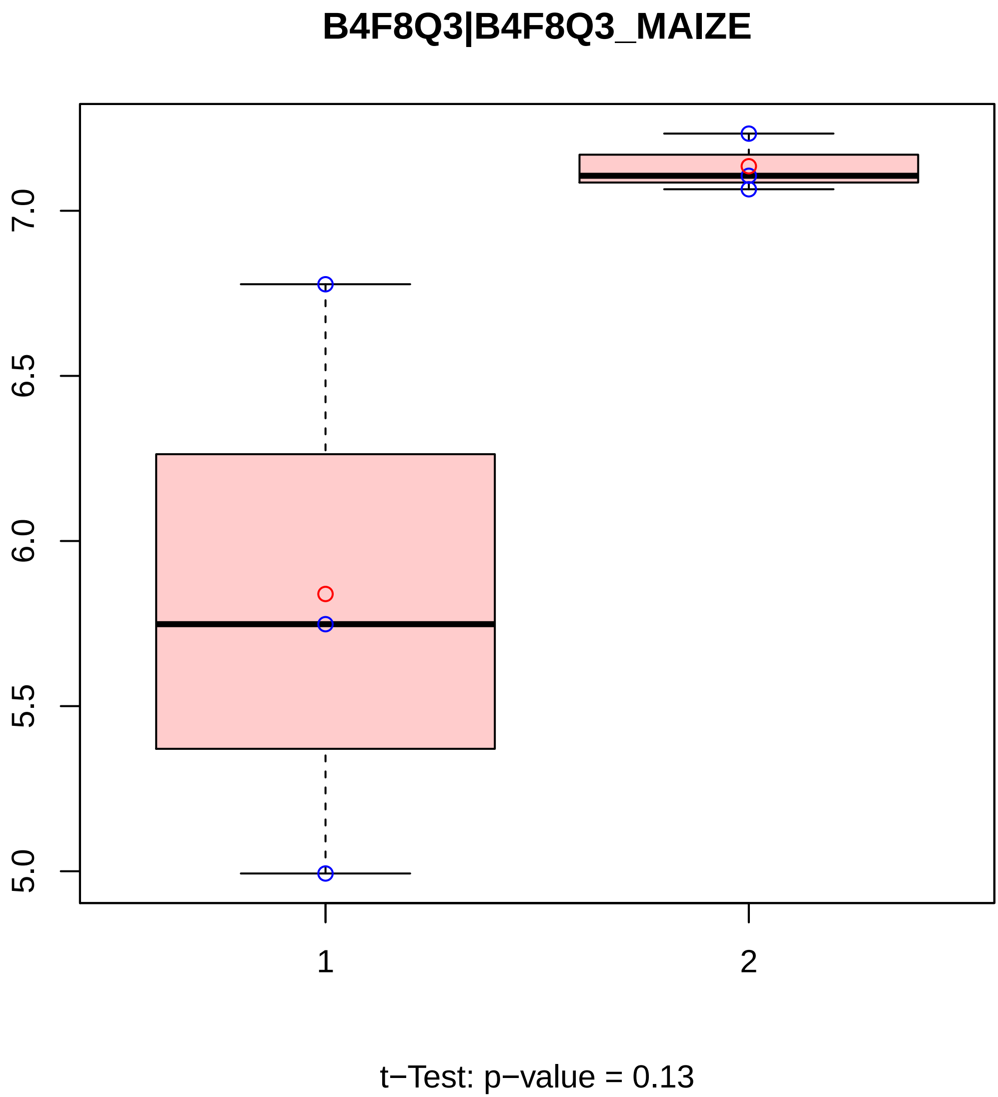
B4F8Q3|B4F8Q3_MAIZE
7.0
6.5
6.0
5.5
5.0
1
2
t−Test: p−value = 0.13

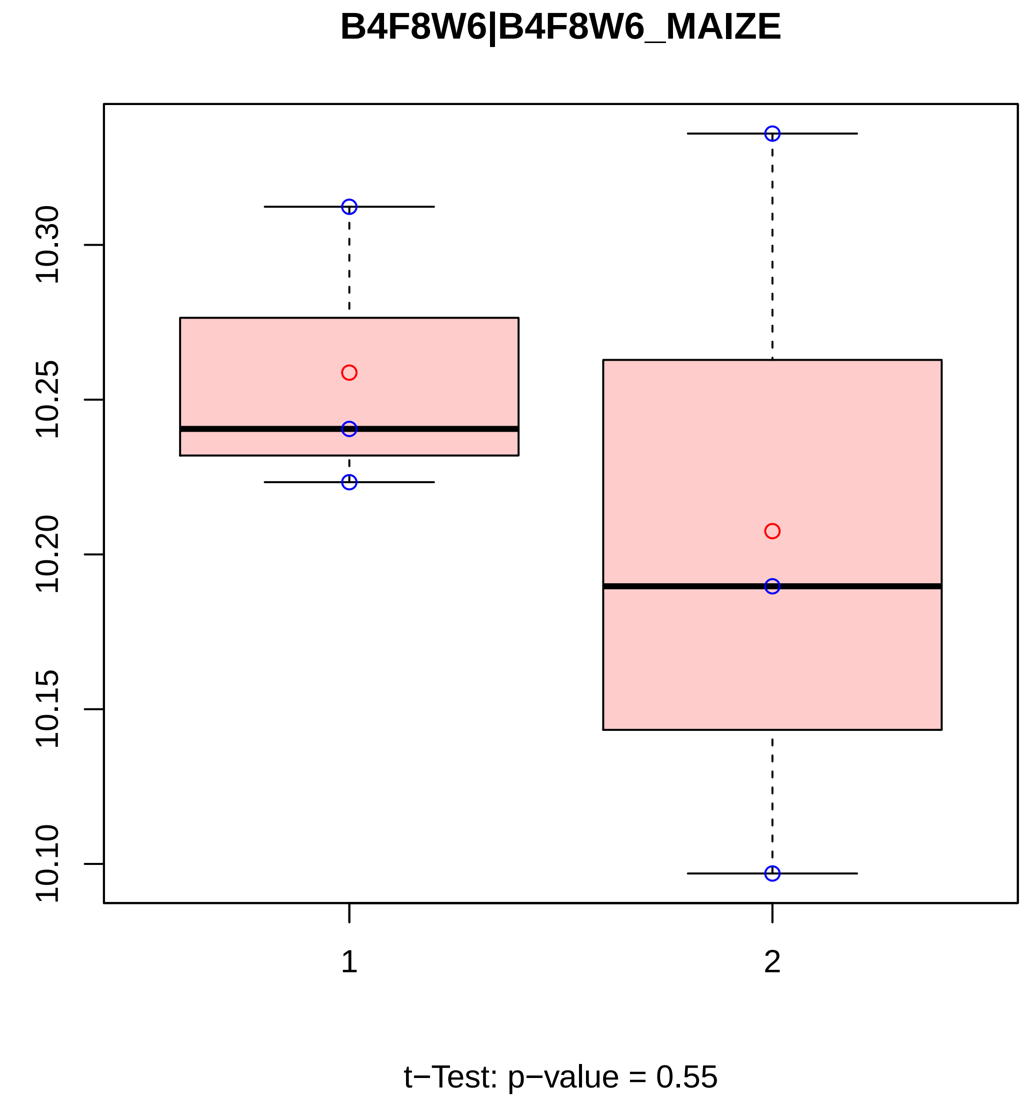

B4F8W6|B4F8W6_MAIZE
10.10
10.15
10.20
10.25
10.30
1
2
t−Test: p−value = 0.55

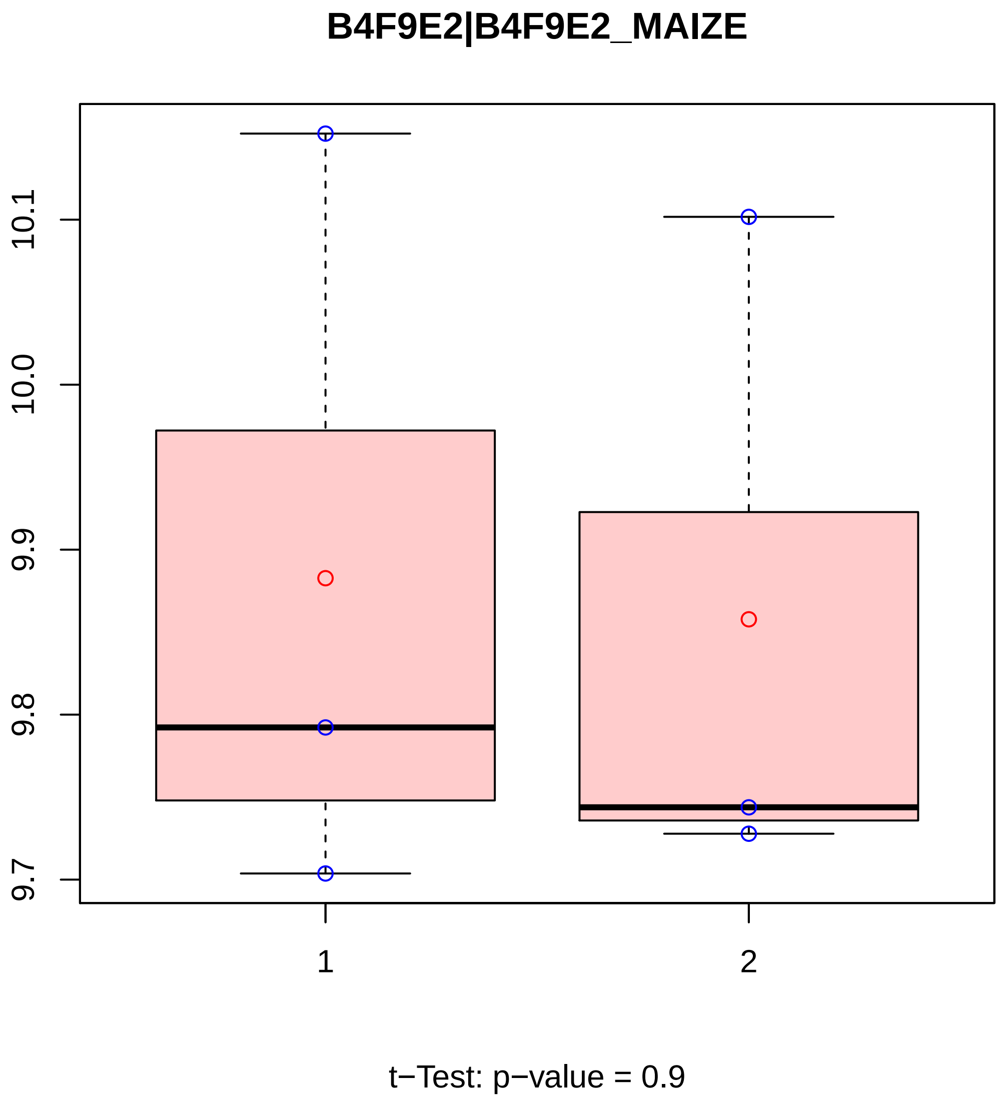
B4F9E2|B4F9E2_MAIZE
10.1
10.0
9.9
9.8
9.7
1
2
t−Test: p−value = 0.9

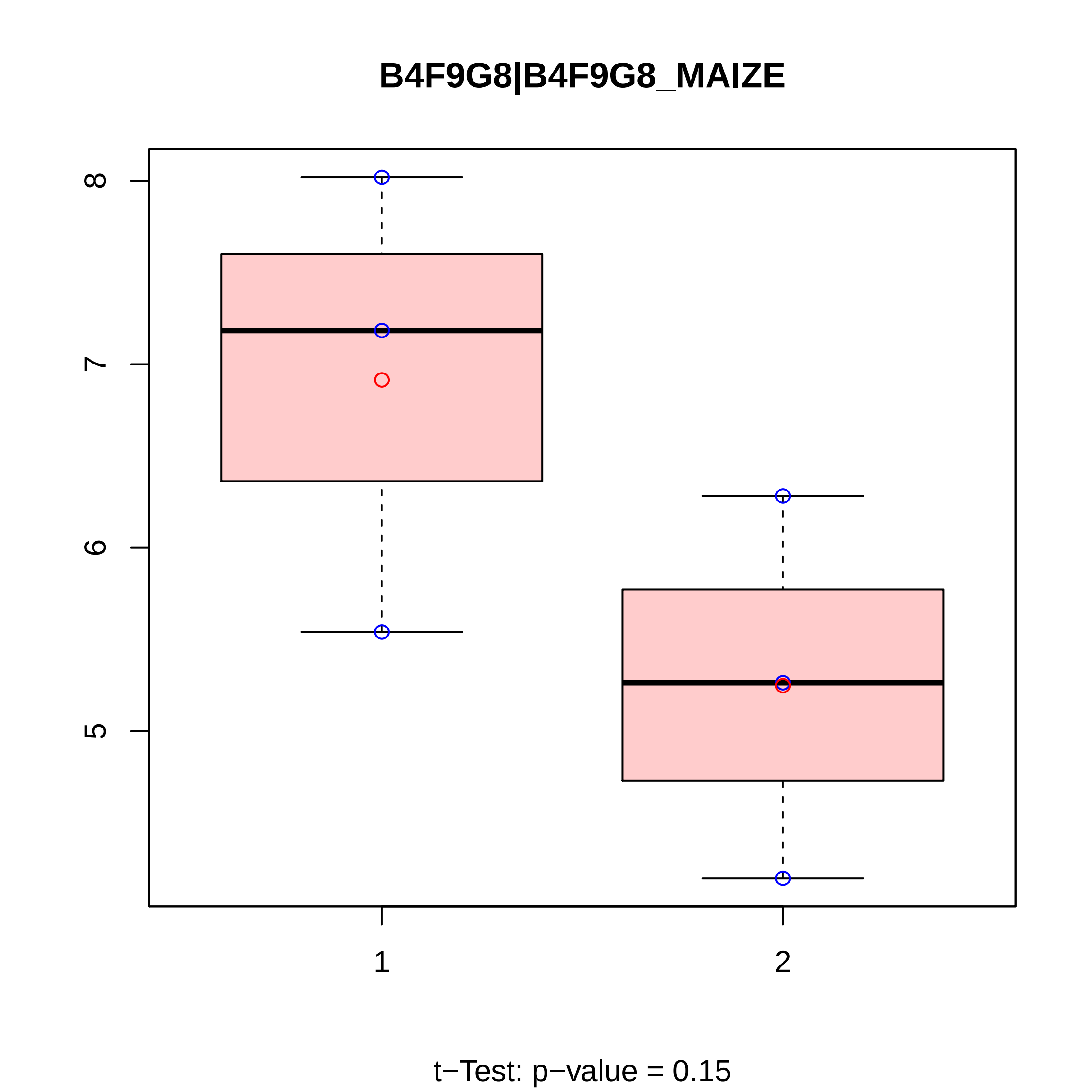

B4F9G8|B4F9G8_MAIZE
8
7
6
5
1
2
t−Test: p−value = 0.15

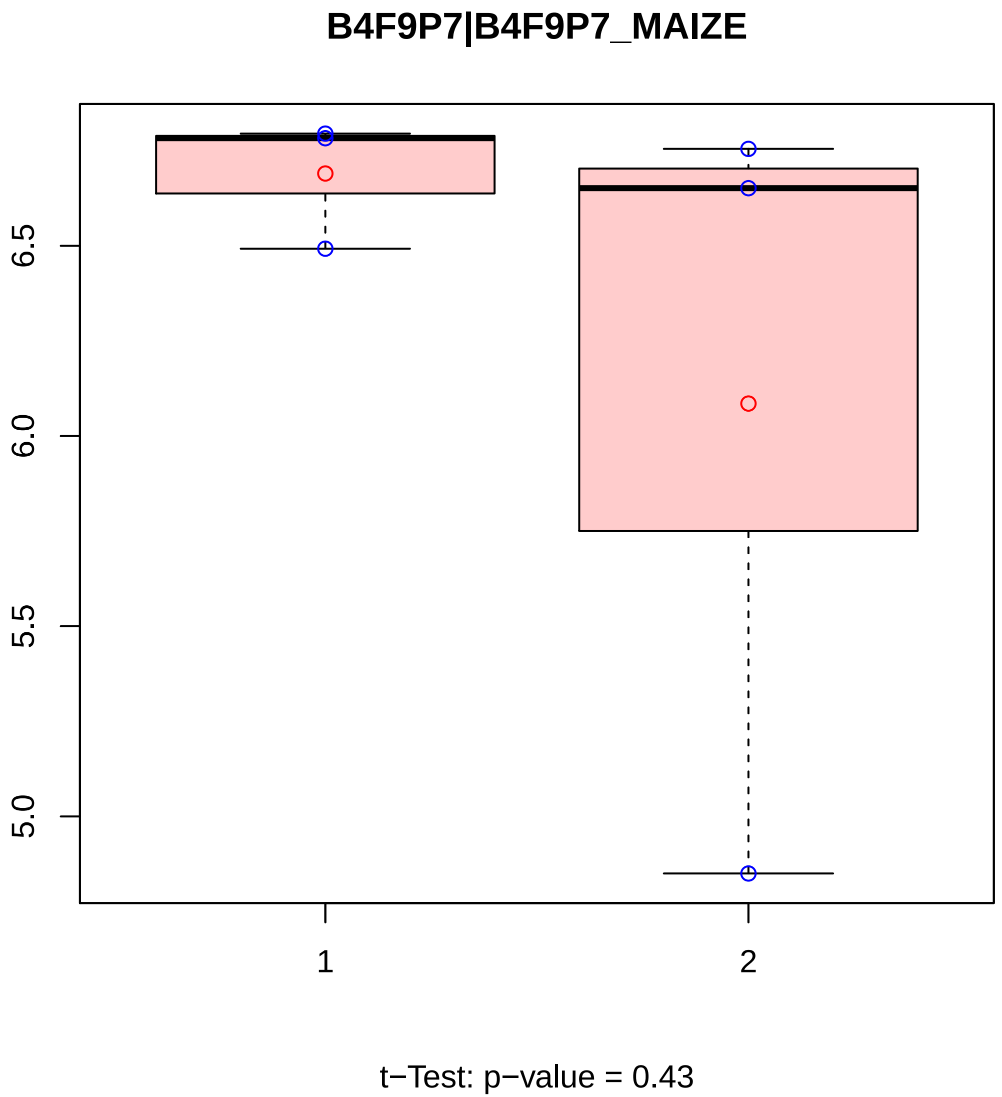

B4F9P7|B4F9P7_MAIZE
6.5
6.0
5.5
5.0
1
2
t−Test: p−value = 0.43

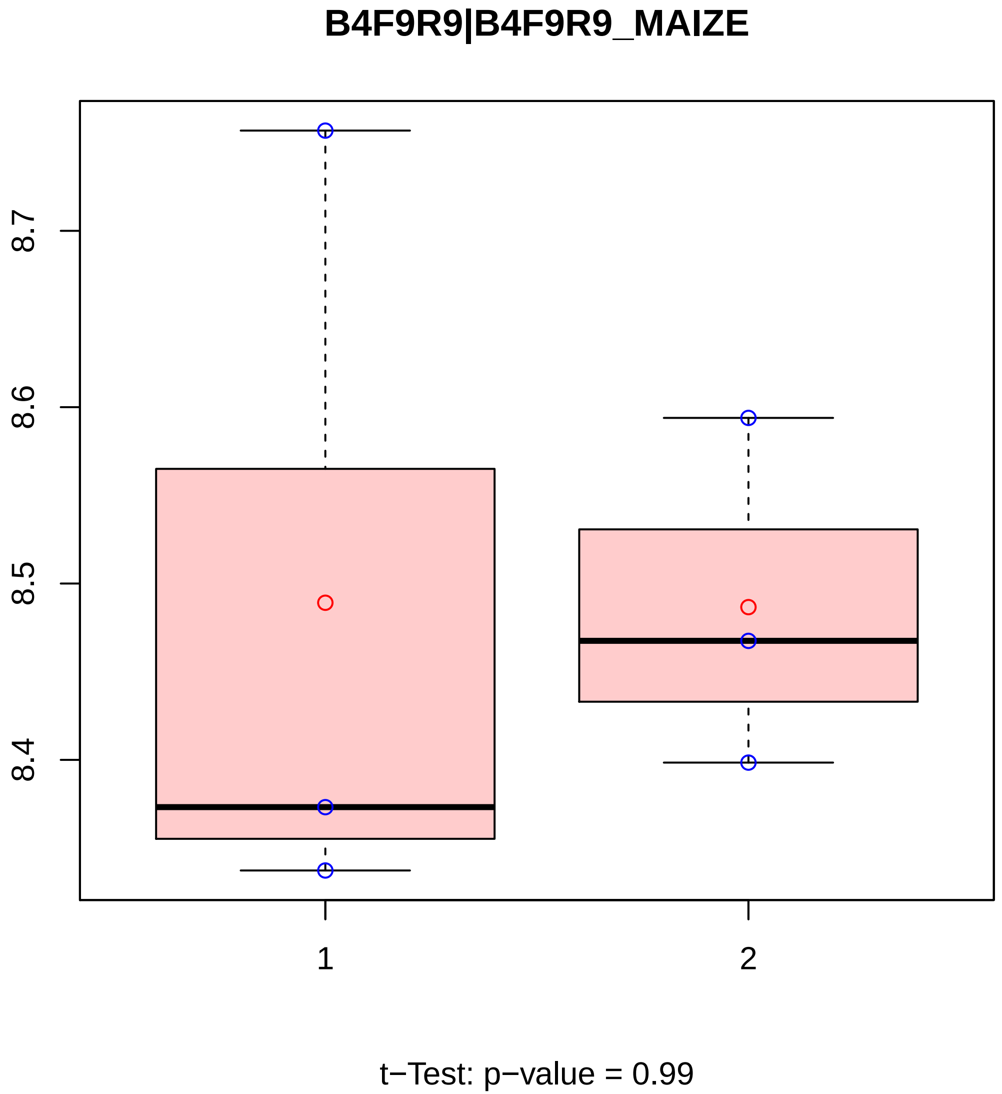
B4F9R9|B4F9R9_MAIZE
8.7
8.6
8.5
8.4
1
2
t−Test: p−value = 0.99

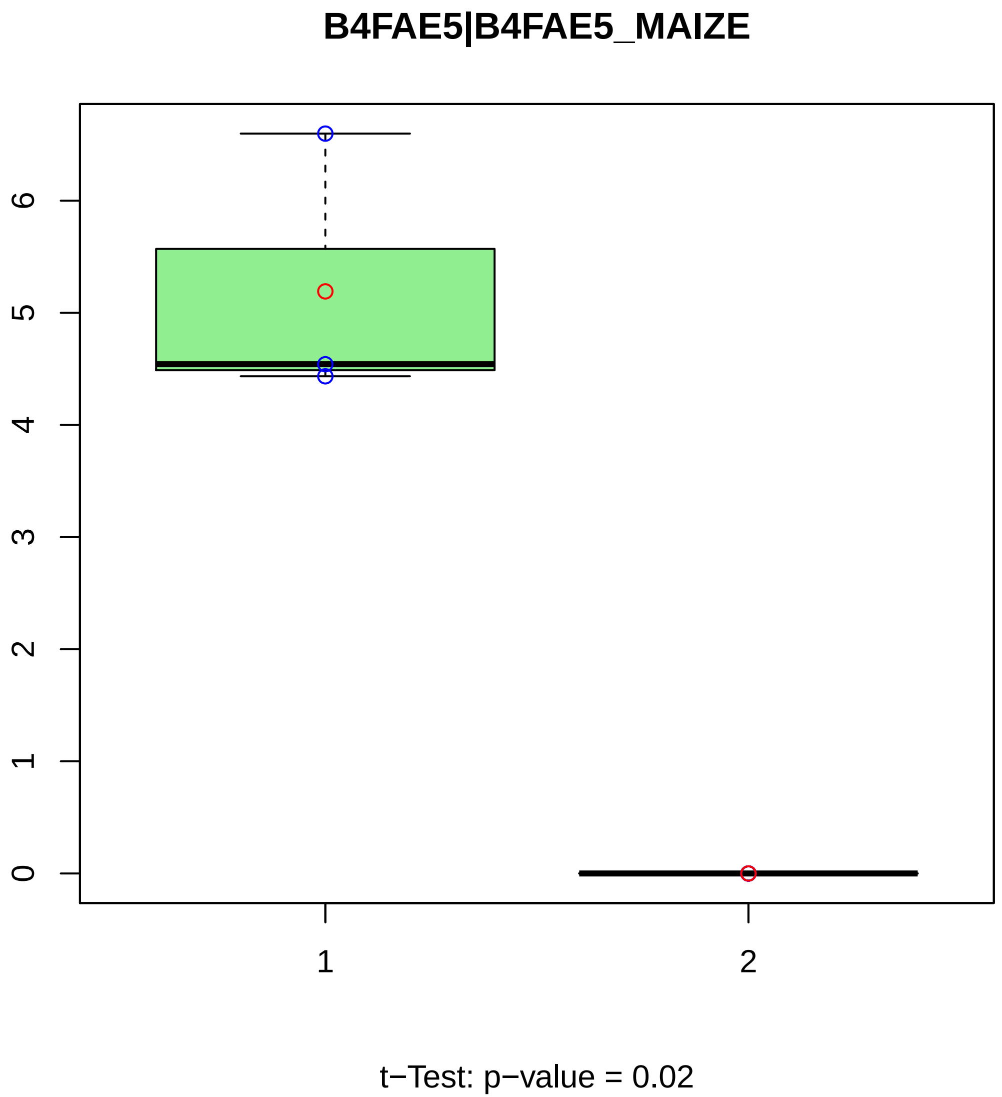
B4FAE5|B4FAE5_MAIZE
0
1
2
3
4
5
6
1
2
t−Test: p−value = 0.02

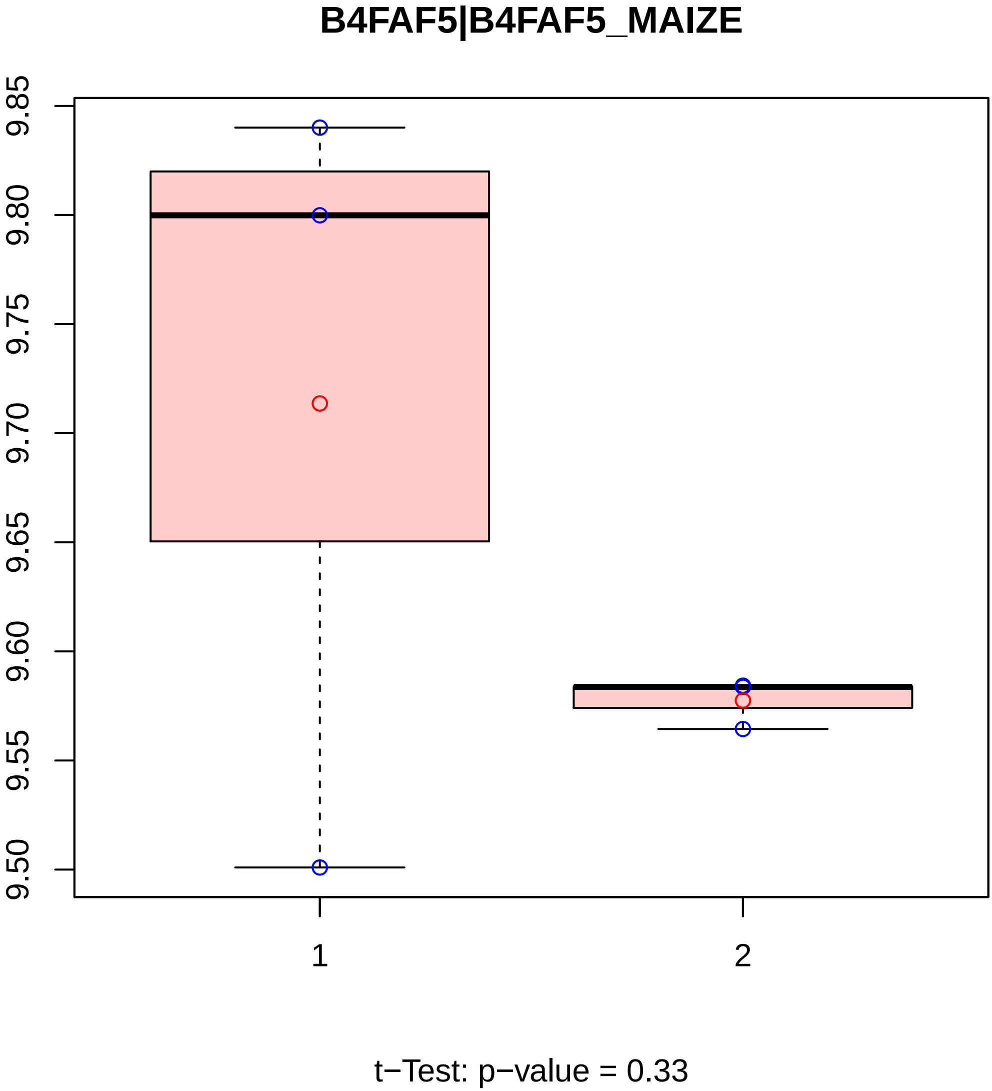

B4FAF5|B4FAF5_MAIZE
9.50
9.55
9.60
9.65
9.70
9.75
9.80
9.85
1
2
t−Test: p−value = 0.33

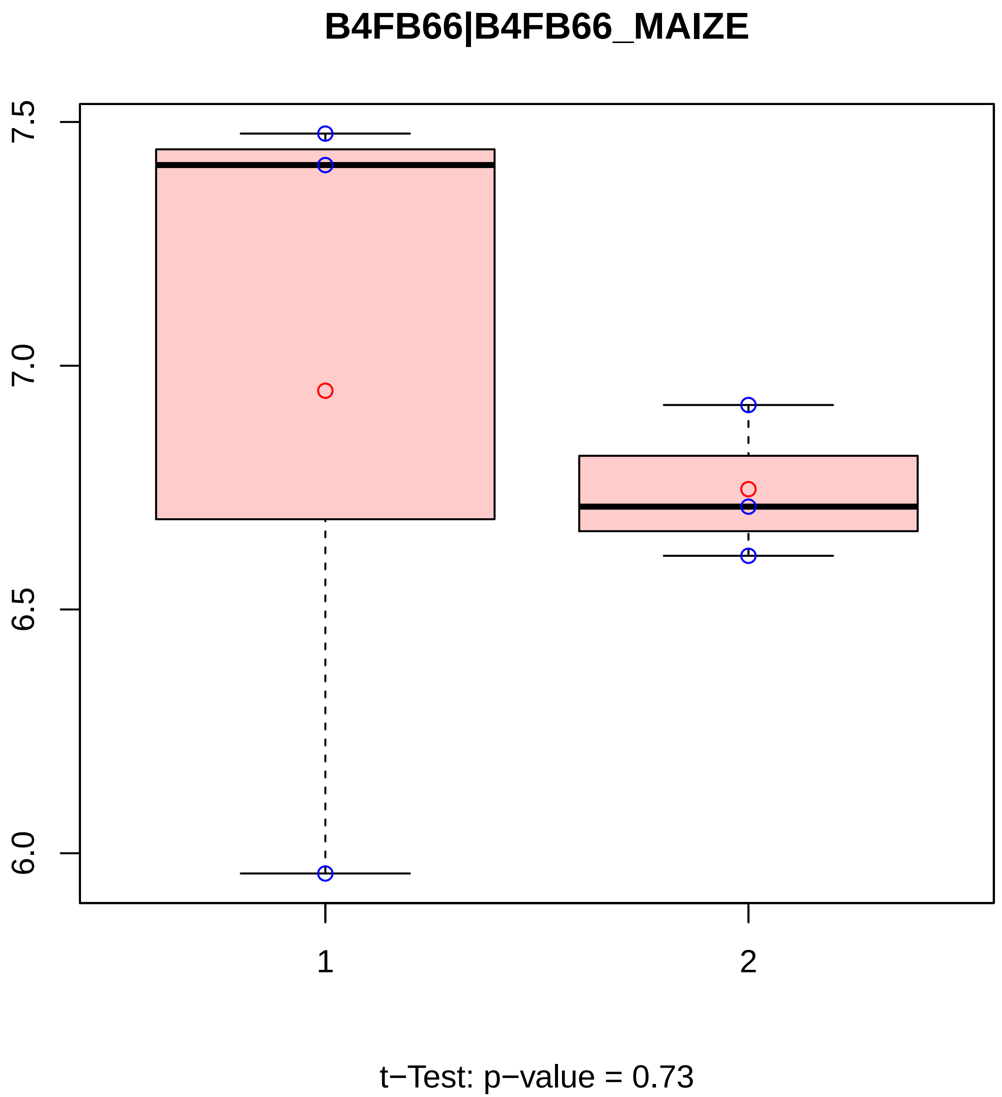
B4FB66|B4FB66_MAIZE
7.5
7.0
6.5
6.0
1
2
t−Test: p−value = 0.73

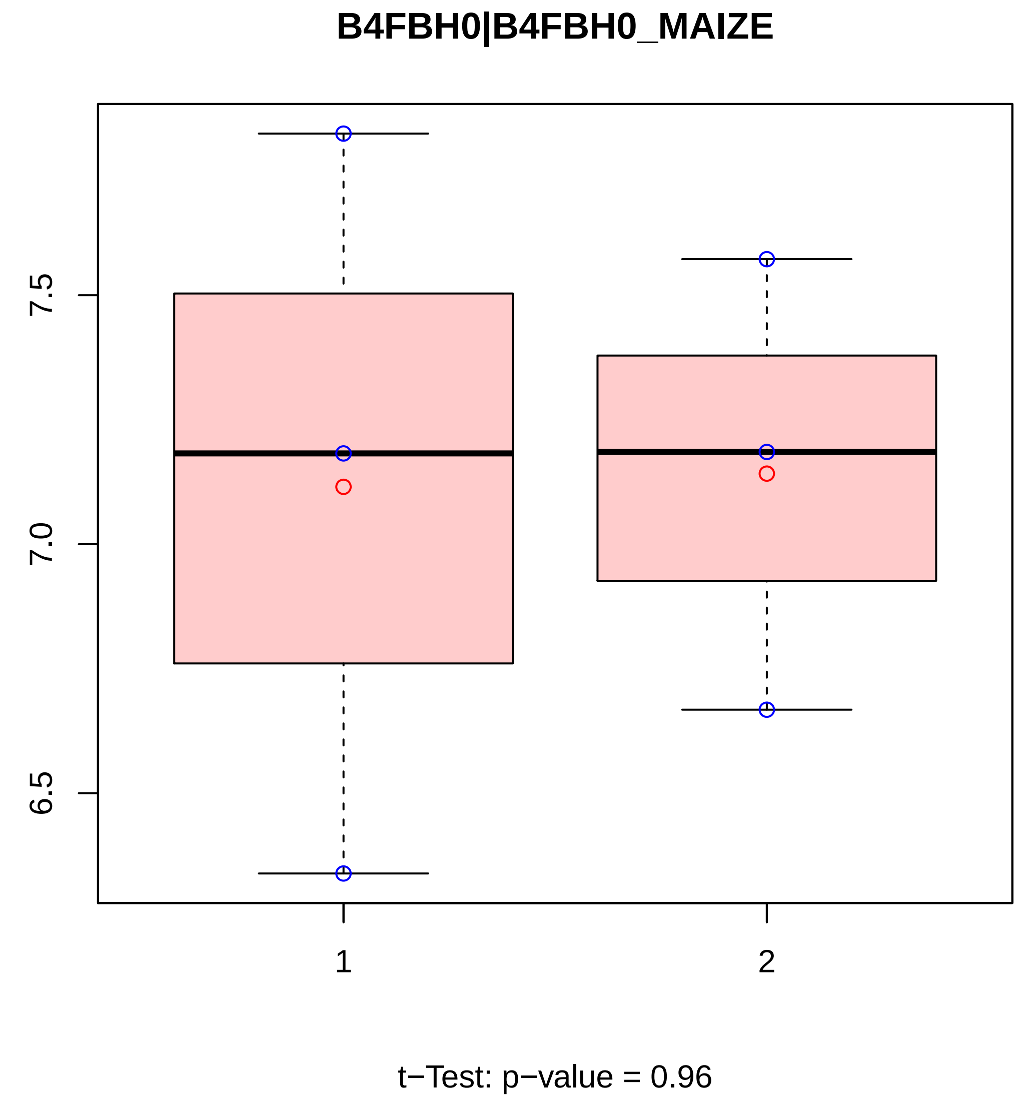

B4FBH0|B4FBH0_MAIZE
7.5
7.0
6.5
1
2
t−Test: p−value = 0.96

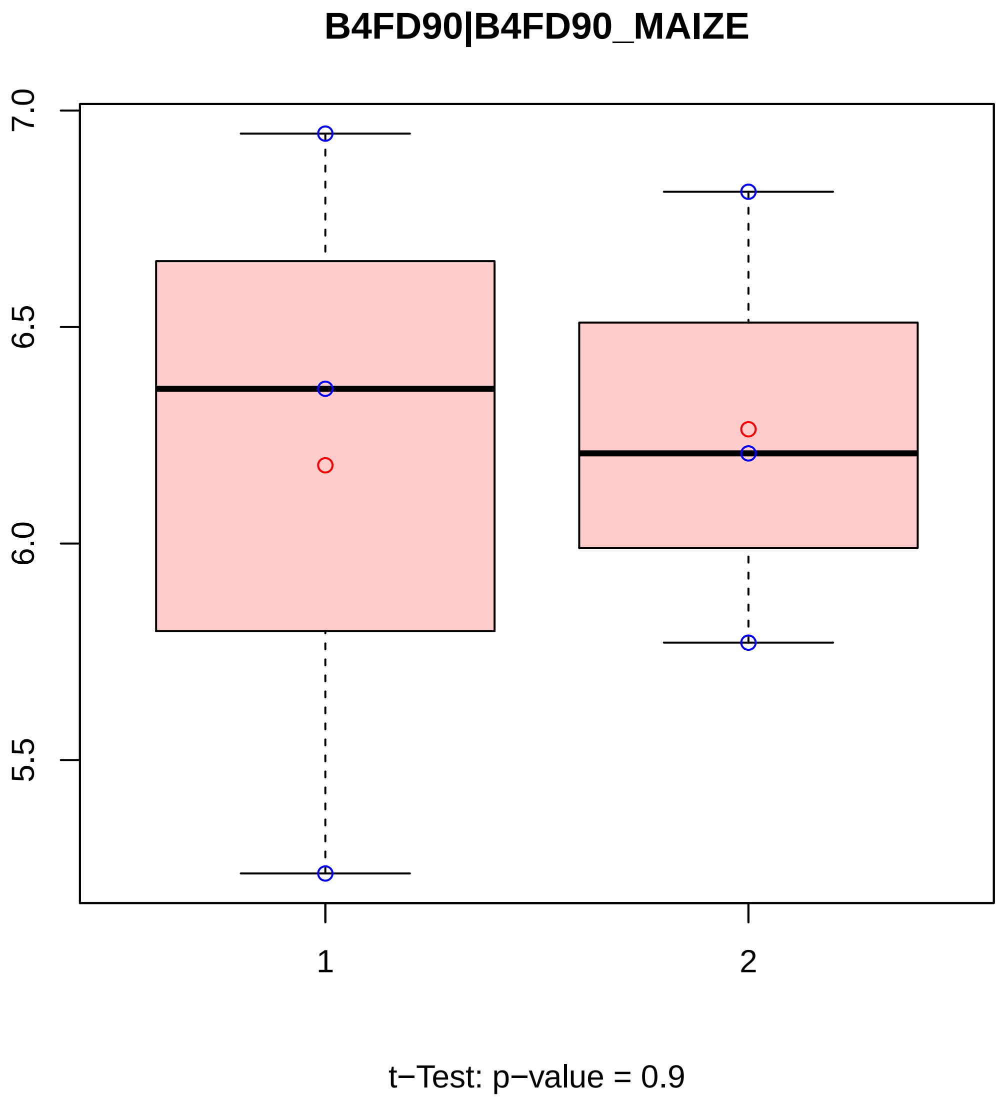

B4FD90|B4FD90_MAIZE
7.0
6.5
6.0
5.5
1
2
t−Test: p−value = 0.9

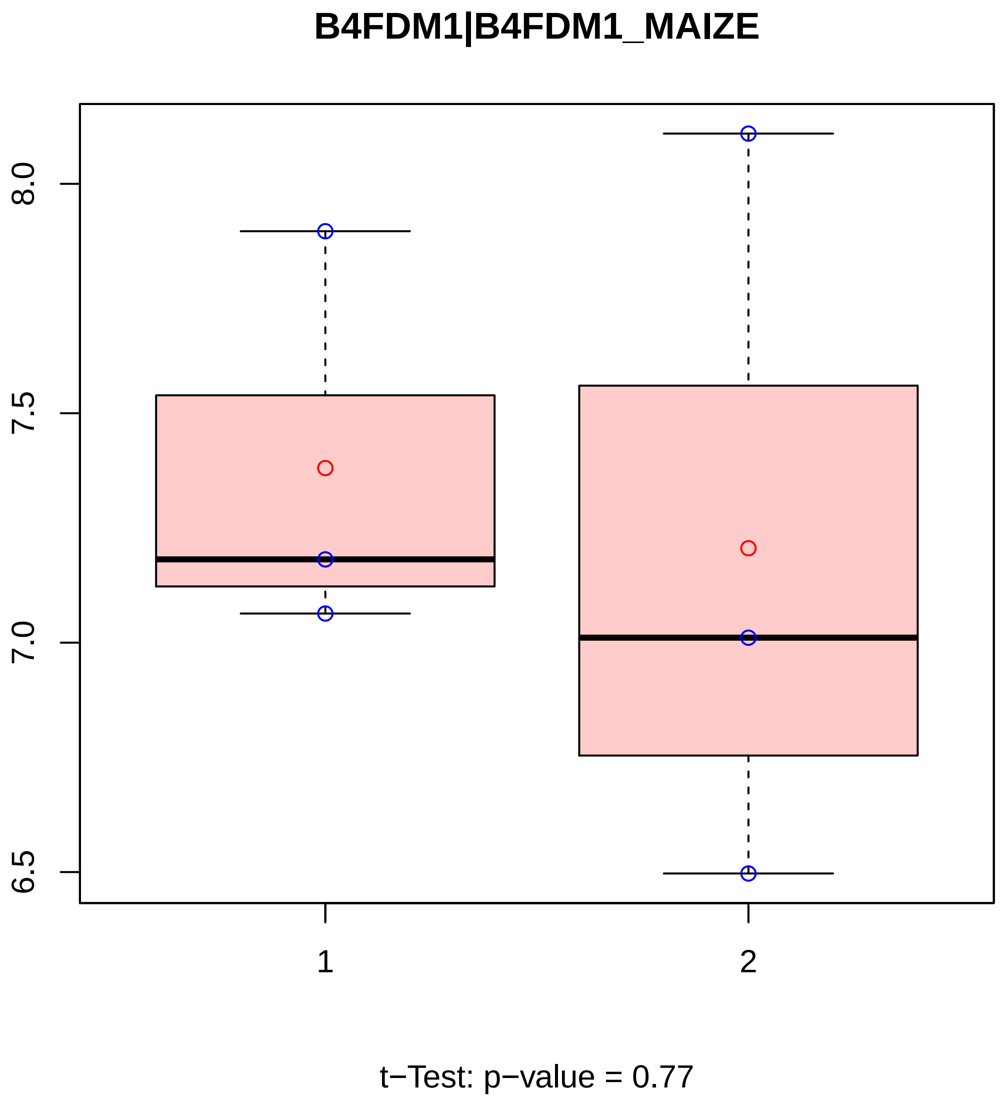
B4FDM1|B4FDM1_MAIZE
8.0
7.5
7.0
6.5
1
2
t−Test: p−value = 0.77

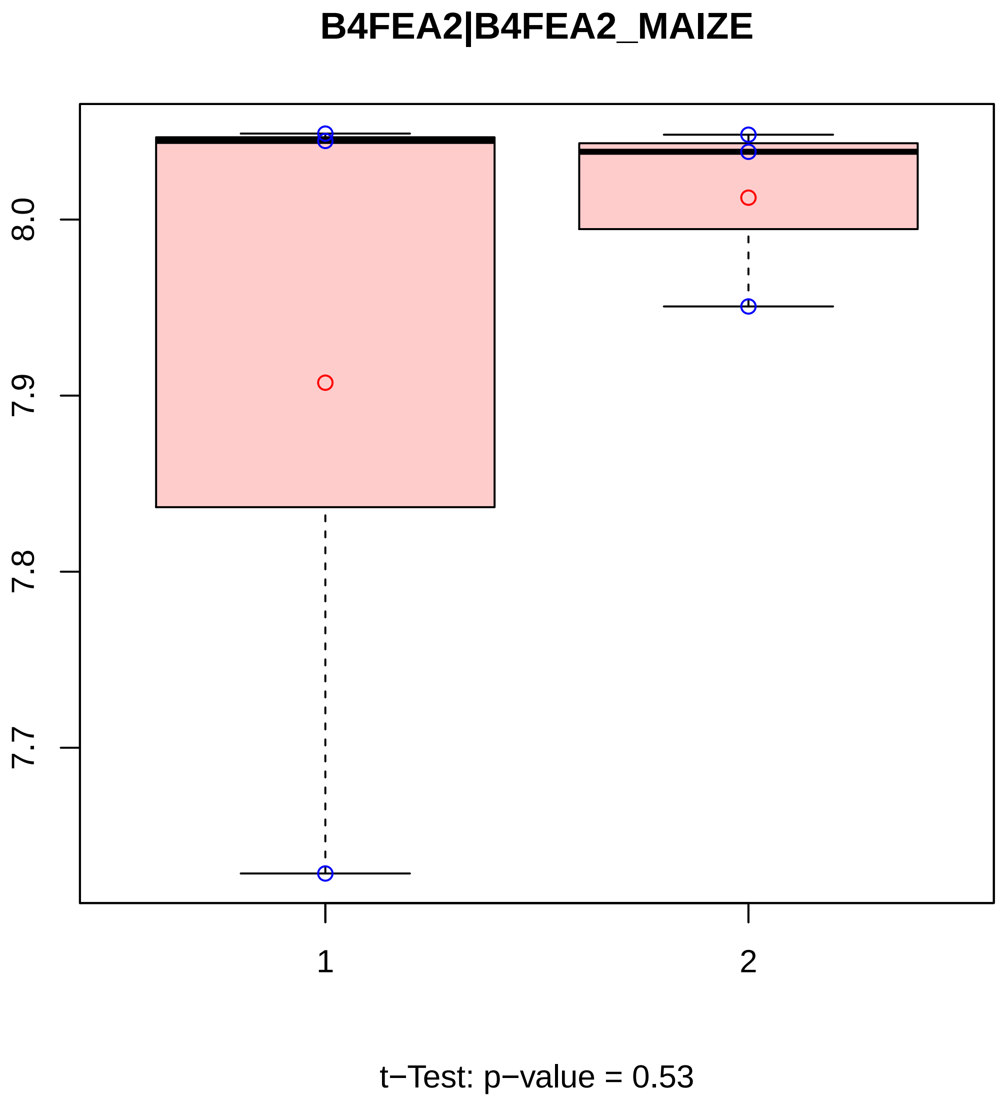

B4FEA2|B4FEA2_MAIZE
8.0
7.9
7.8
7.7
1
2
t−Test: p−value = 0.53

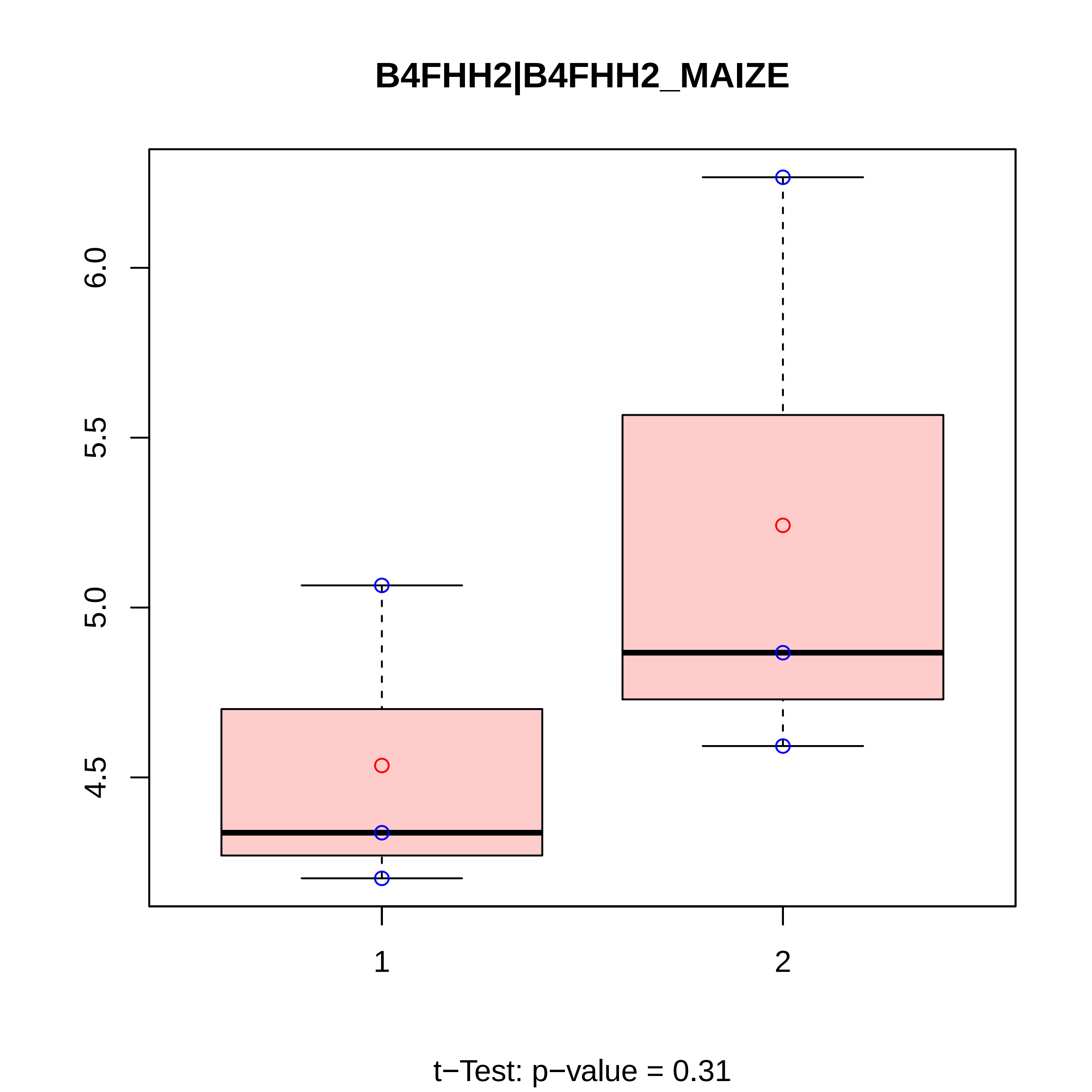

B4FHH2|B4FHH2_MAIZE
6.0
5.5
5.0
4.5
1
2
t−Test: p−value = 0.31

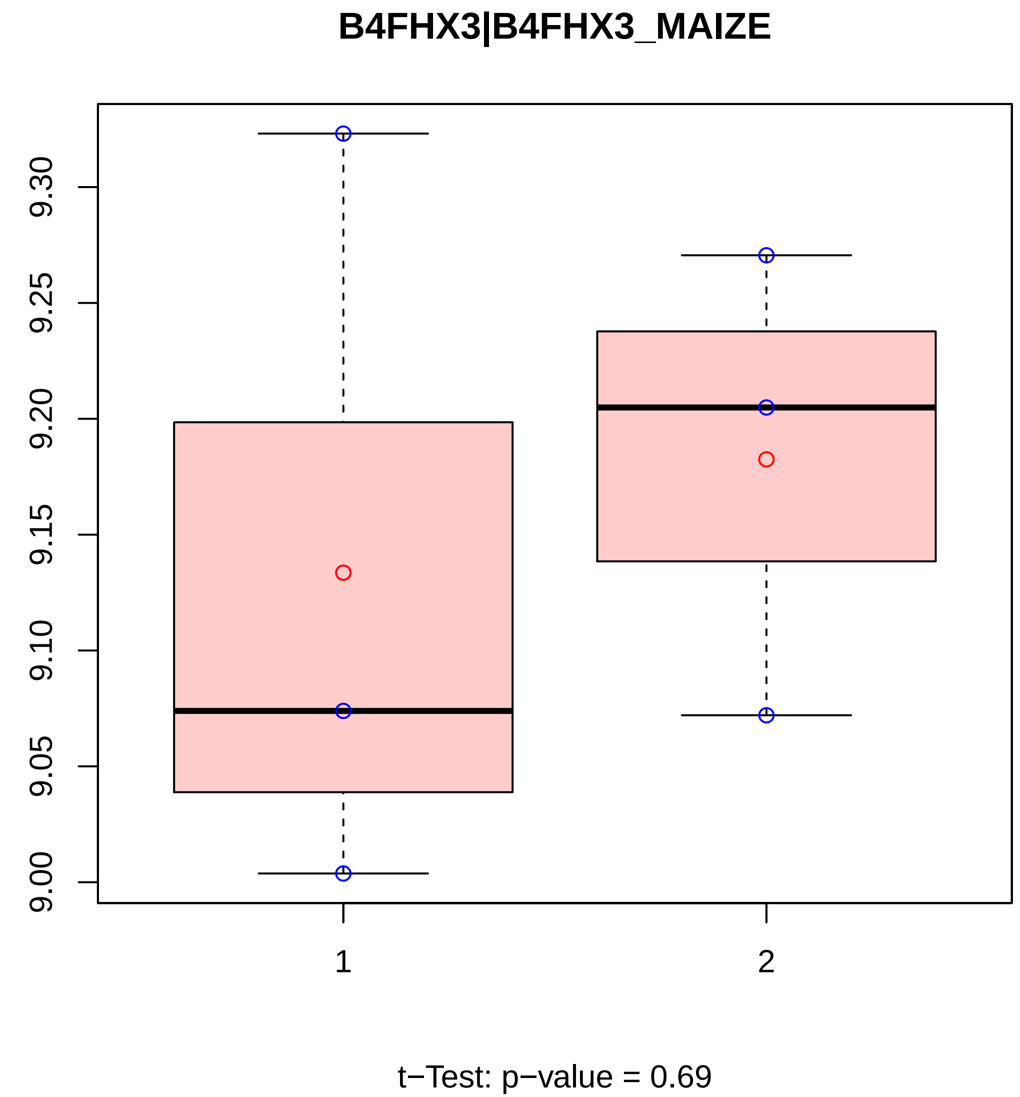
B4FHX3|B4FHX3_MAIZE
9.00
9.05
9.10
9.15
9.20
9.25
9.30
1
2
t−Test: p−value = 0.69

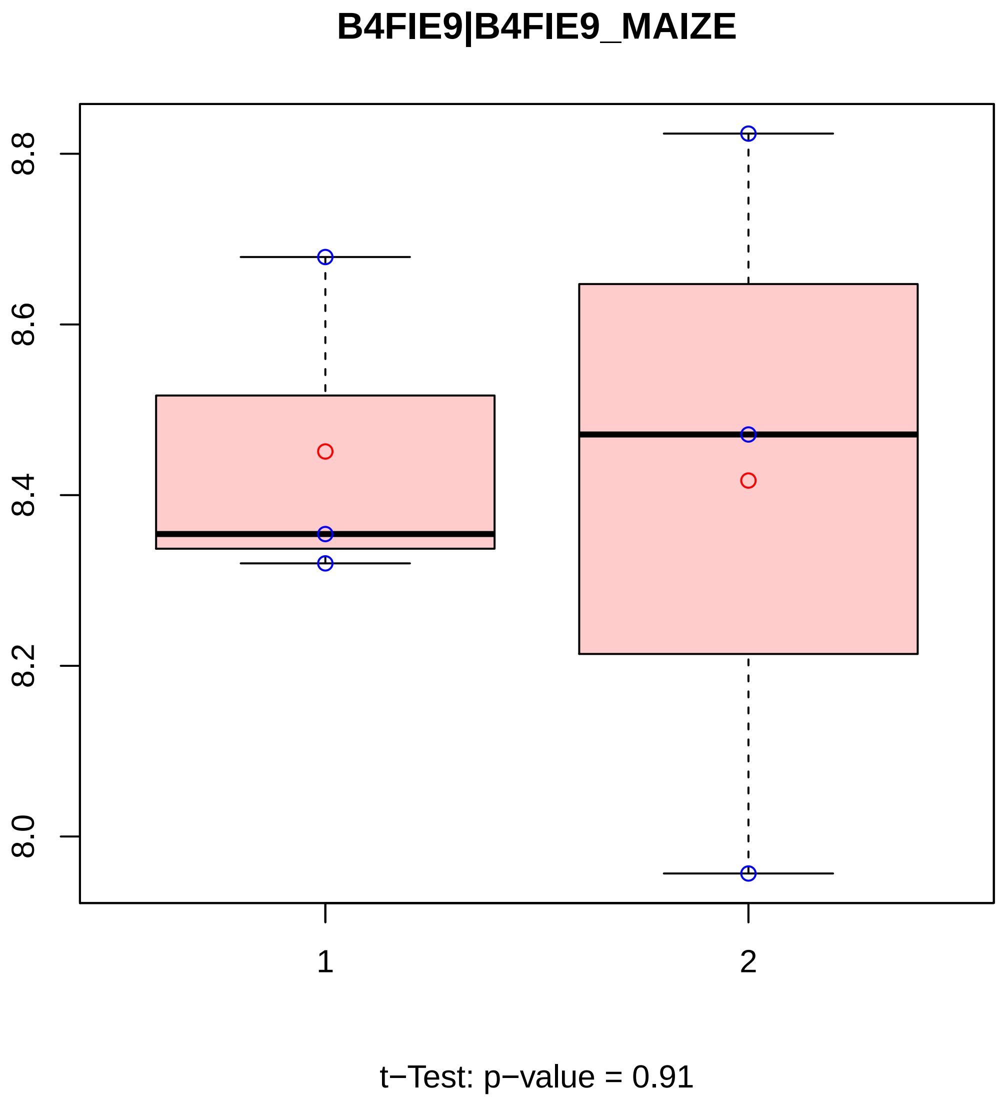
B4FIE9|B4FIE9_MAIZE
8.0
8.2
8.4
8.6
8.8
1
2
t−Test: p−value = 0.91

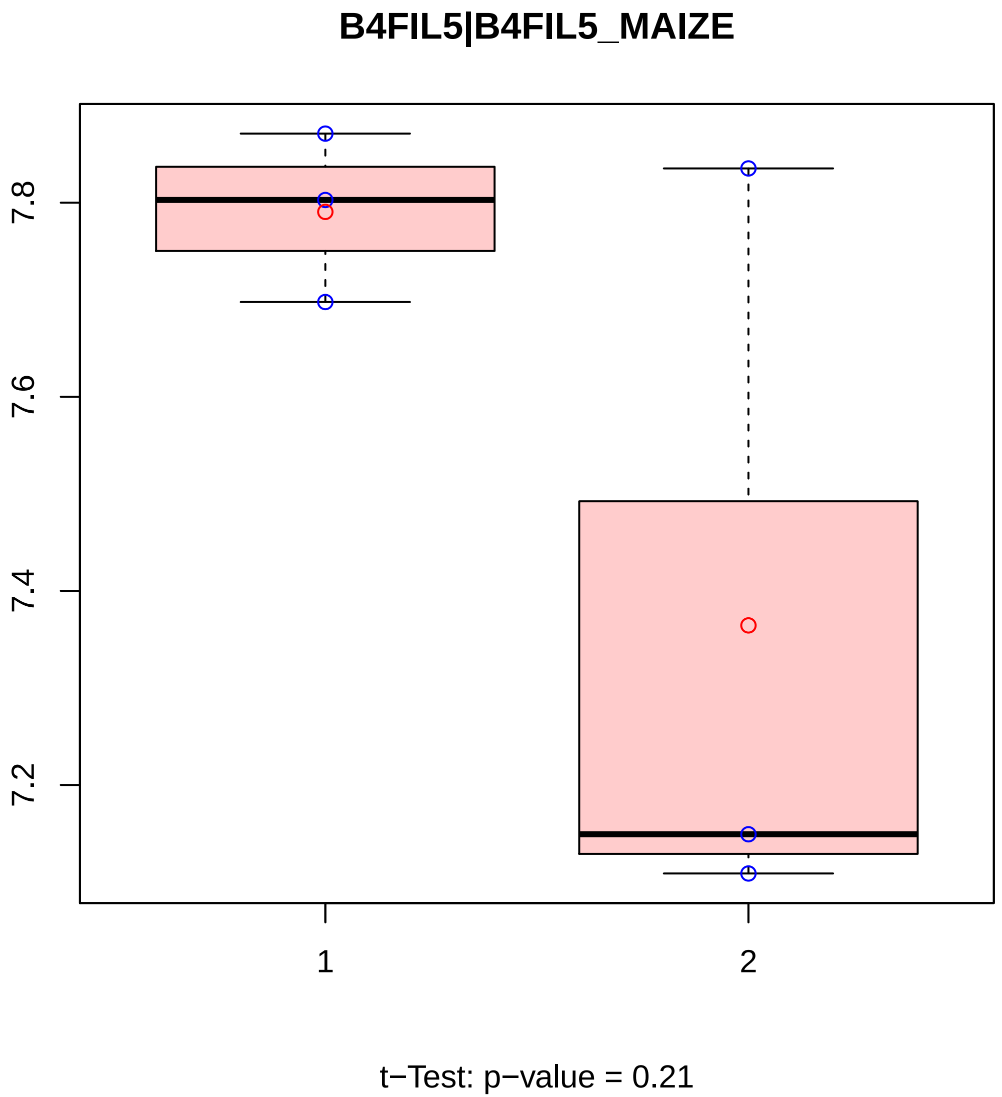

B4FIL5|B4FIL5_MAIZE
7.2
7.4
7.6
7.8
1
2
t−Test: p−value = 0.21

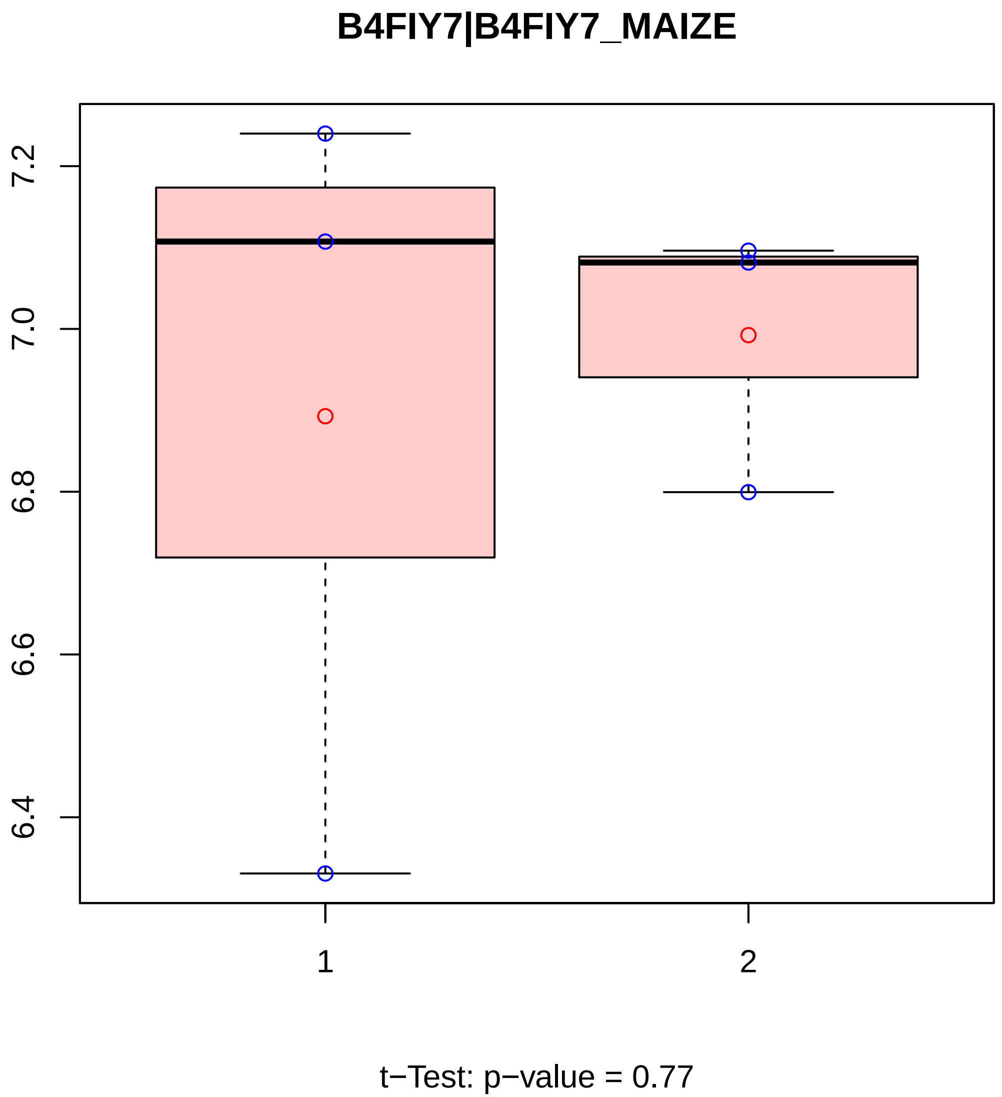
B4FIY7|B4FIY7_MAIZE
7.2
7.0
6.8
6.6
6.4
1
2
t−Test: p−value = 0.77

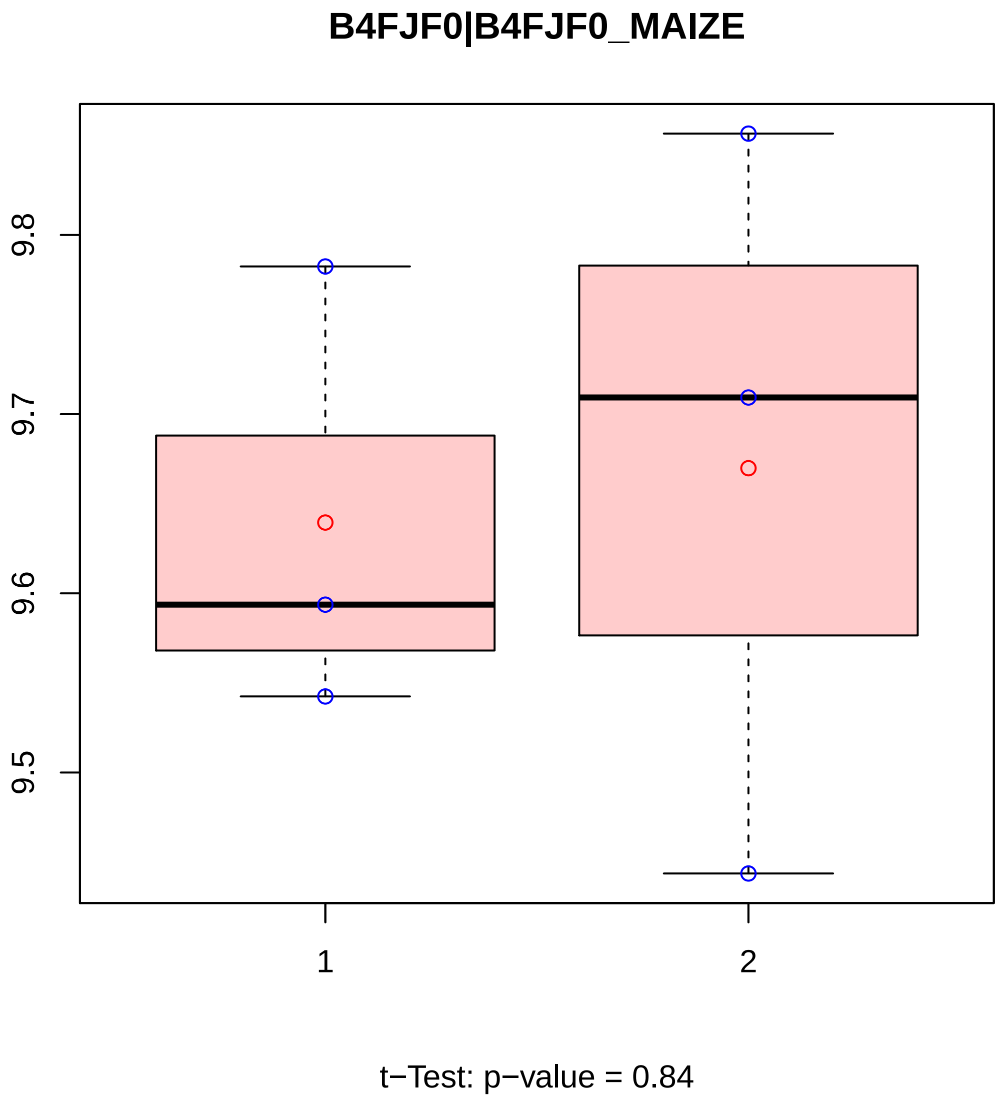
B4FJF0|B4FJF0_MAIZE
9.5
9.6
9.7
9.8
1
2
t−Test: p−value = 0.84

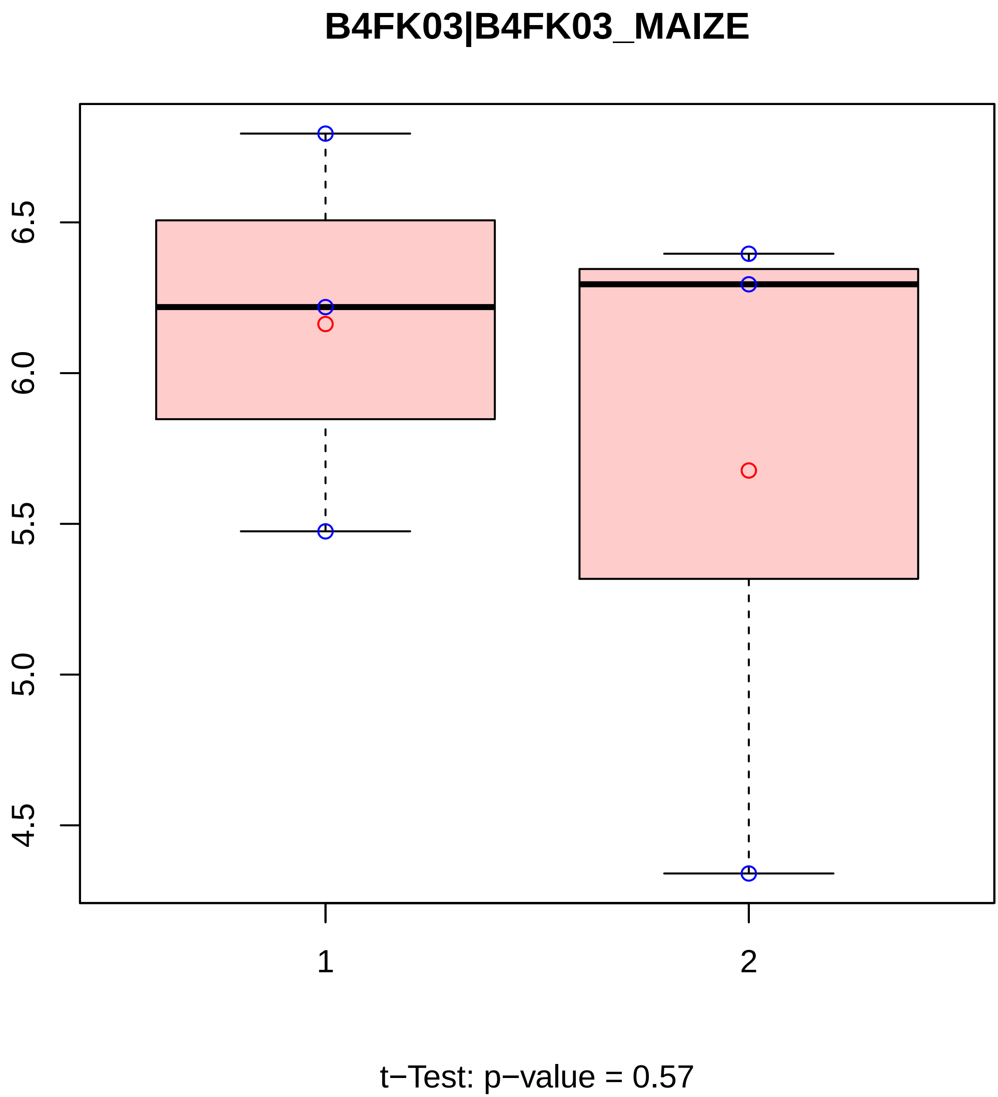
B4FK03|B4FK03_MAIZE
6.5
6.0
5.5
5.0
4.5
1
2
t−Test: p−value = 0.57

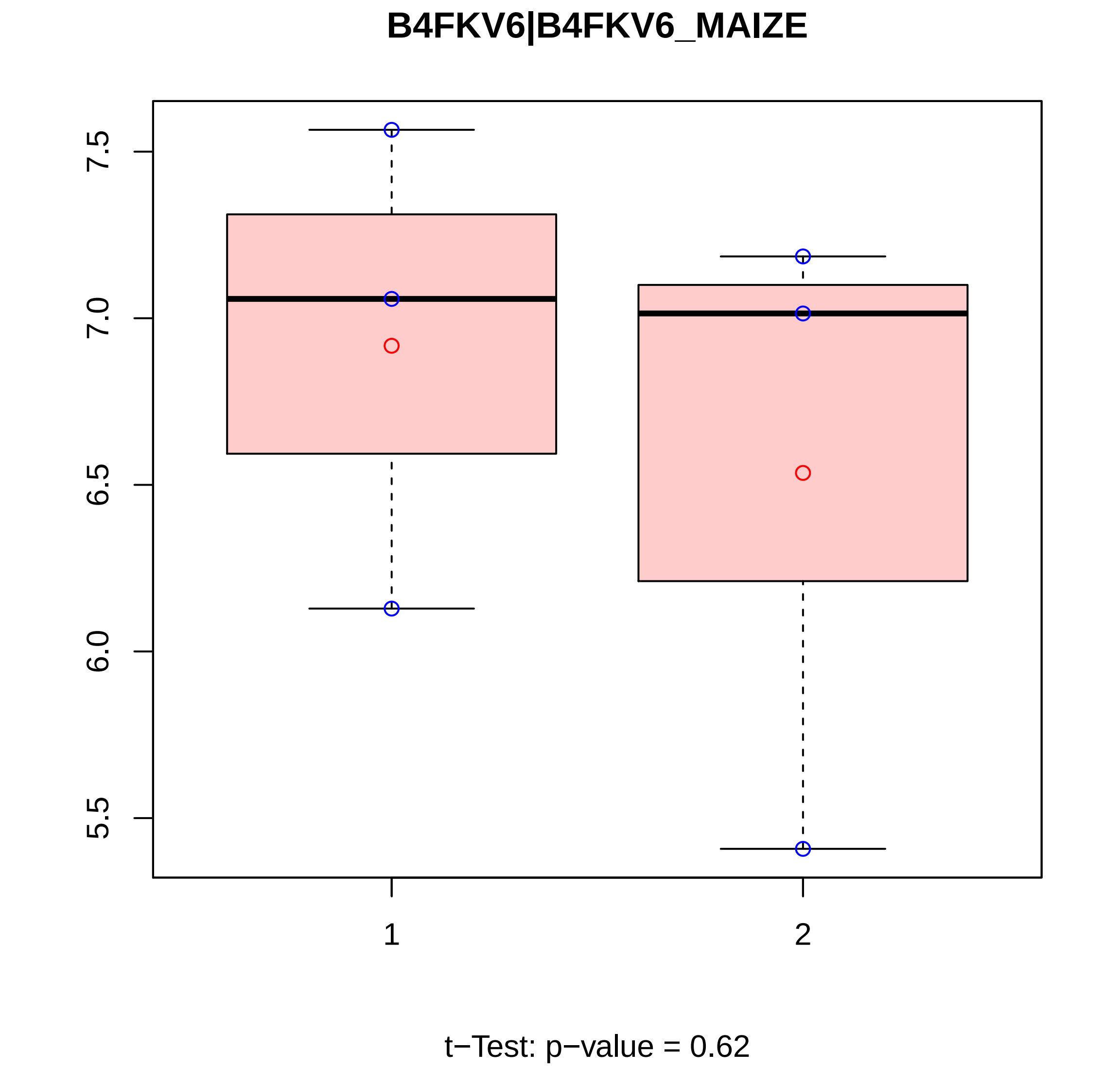
B4FKV6|B4FKV6_MAIZE
7.5
7.0
6.5
6.0
5.5
1
2
t−Test: p−value = 0.62

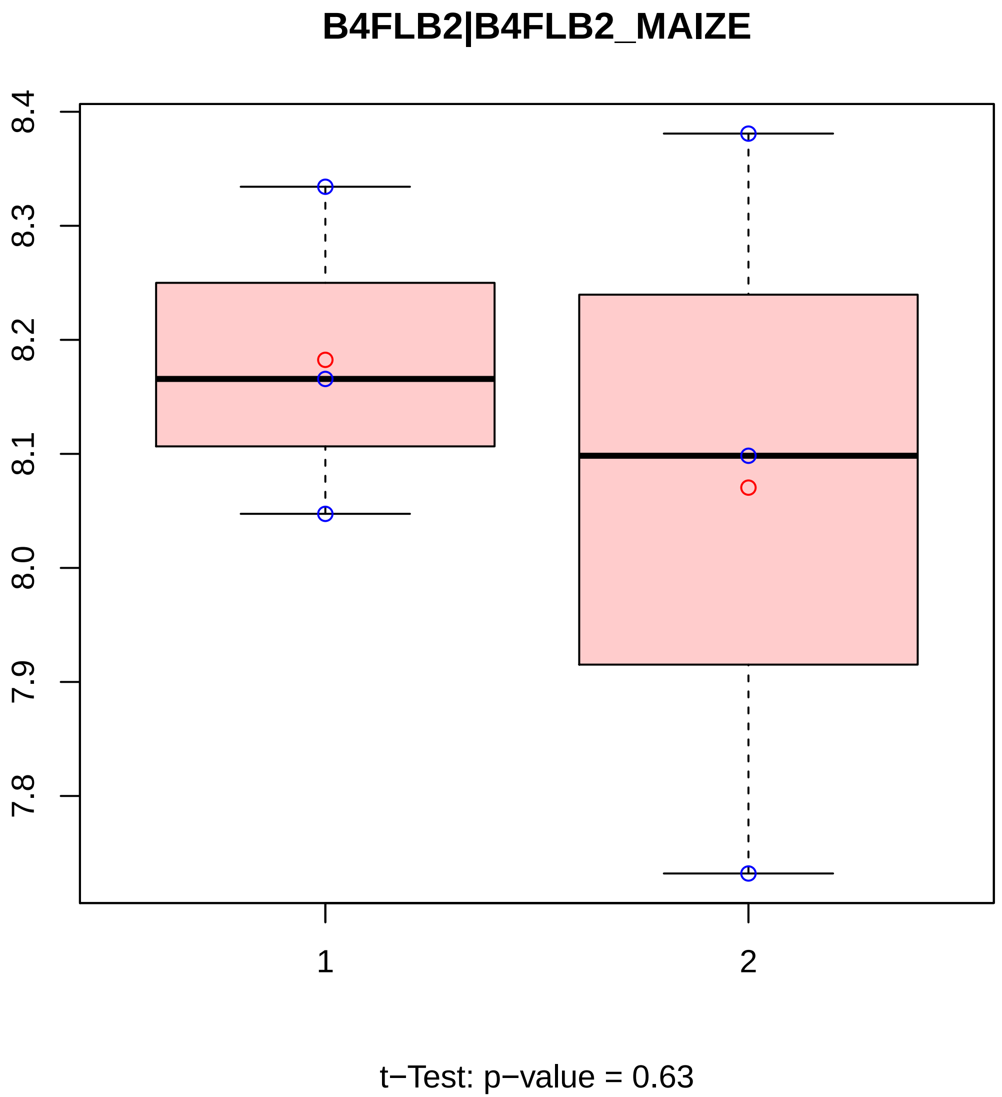
B4FLB2|B4FLB2_MAIZE
8.4
8.3
8.2
8.1
8.0
7.9
7.8
1
2
t−Test: p−value = 0.63

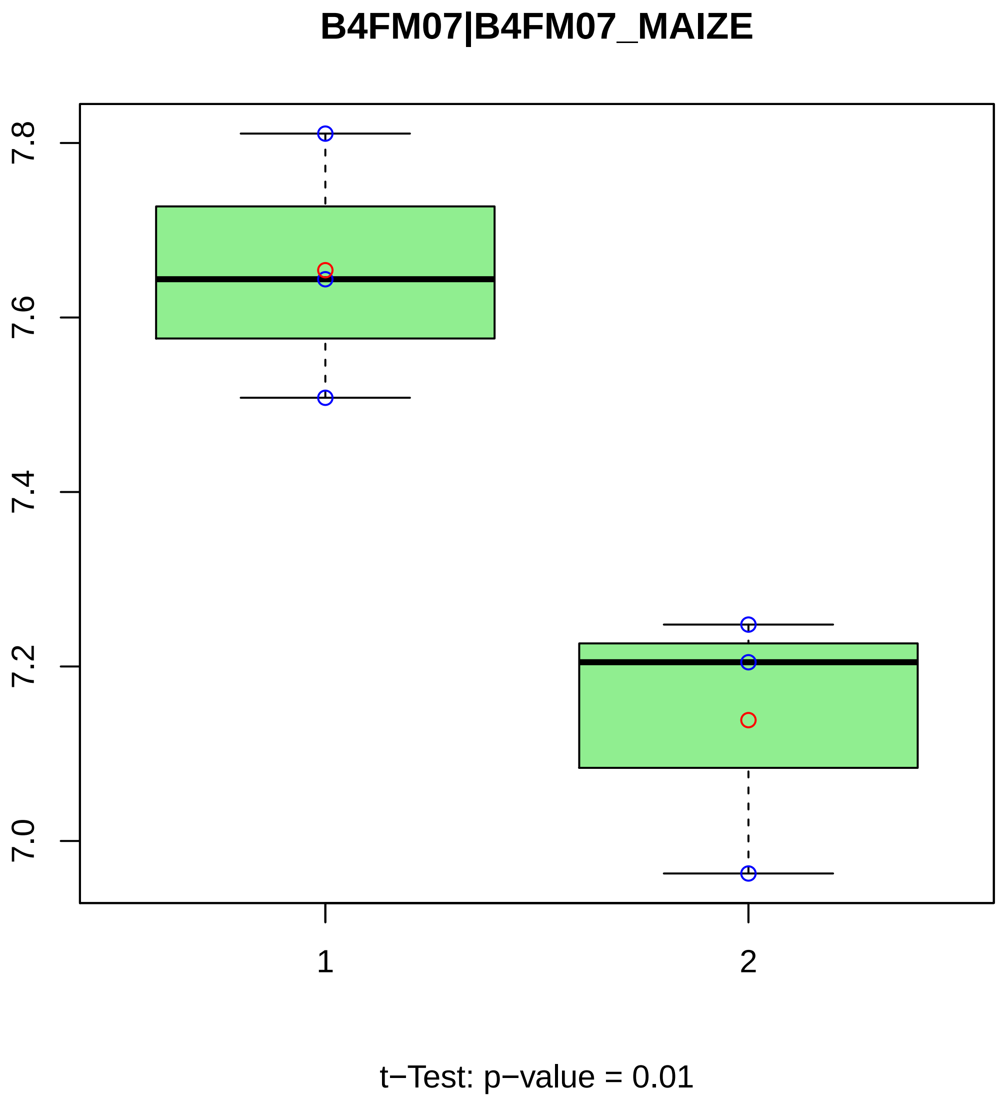
B4FM07|B4FM07_MAIZE
7.8
7.6
7.4
7.2
7.0
1
2
t−Test: p−value = 0.01

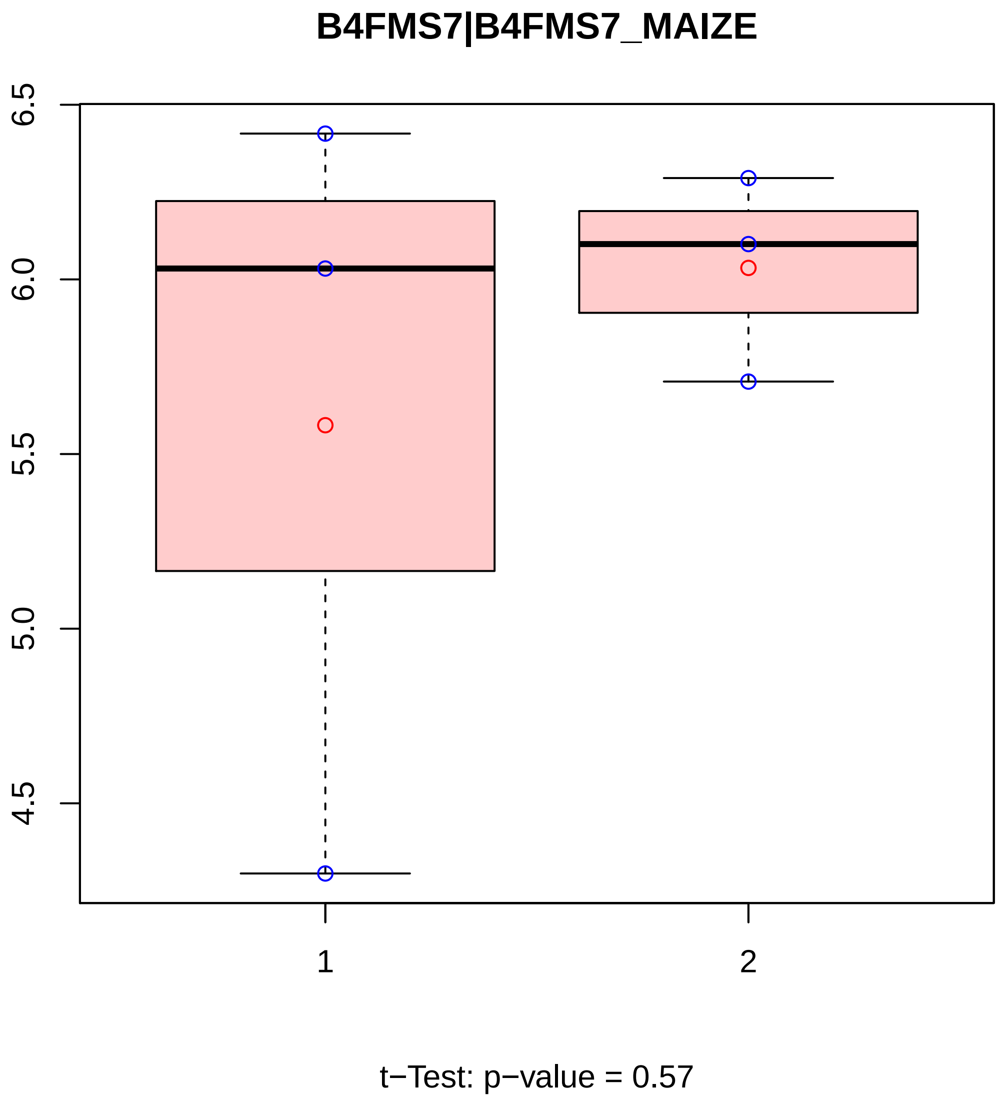

B4FMS7|B4FMS7_MAIZE
6.5
6.0
5.5
5.0
4.5
1
2
t−Test: p−value = 0.57

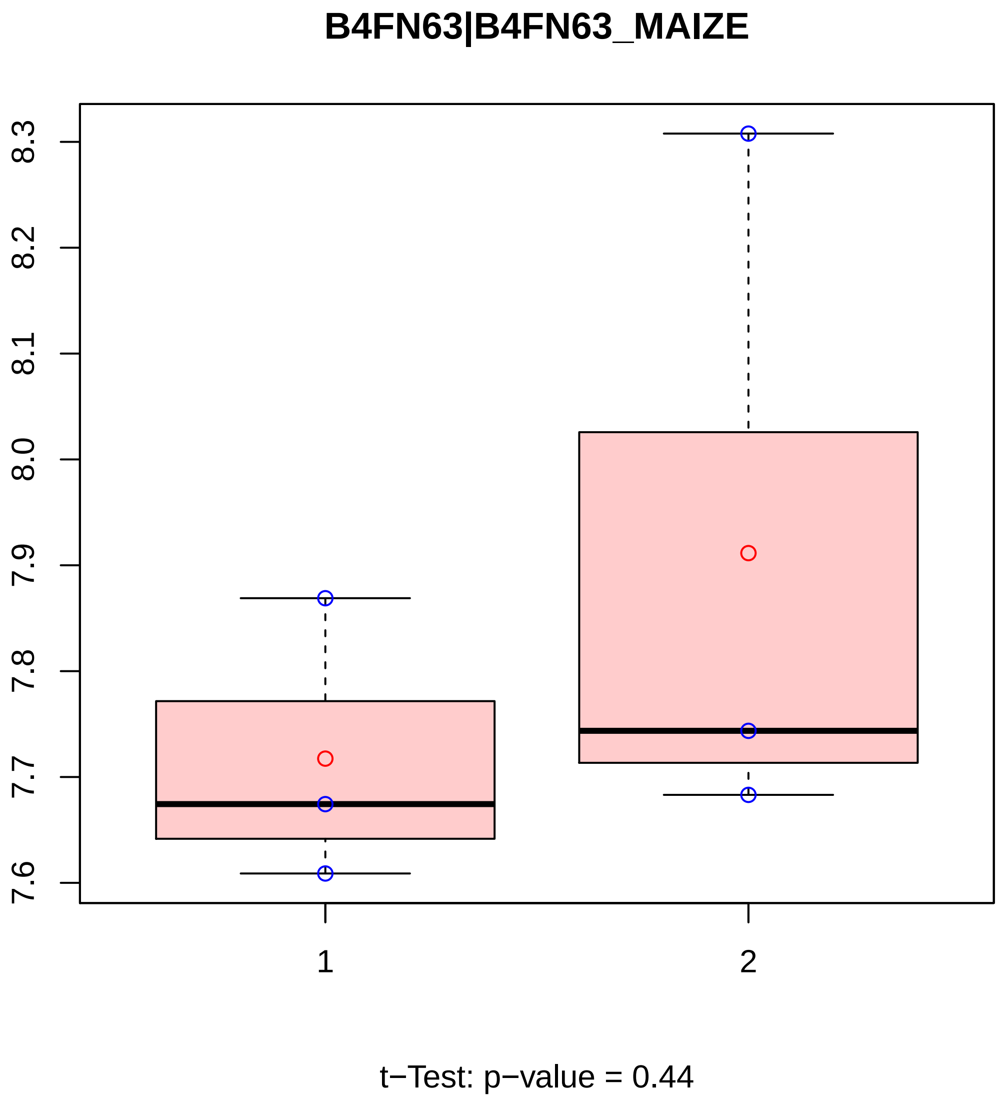

B4FN63|B4FN63_MAIZE
8.3
8.2
8.1
8.0
7.9
7.8
7.7
7.6
1
2
t−Test: p−value = 0.44

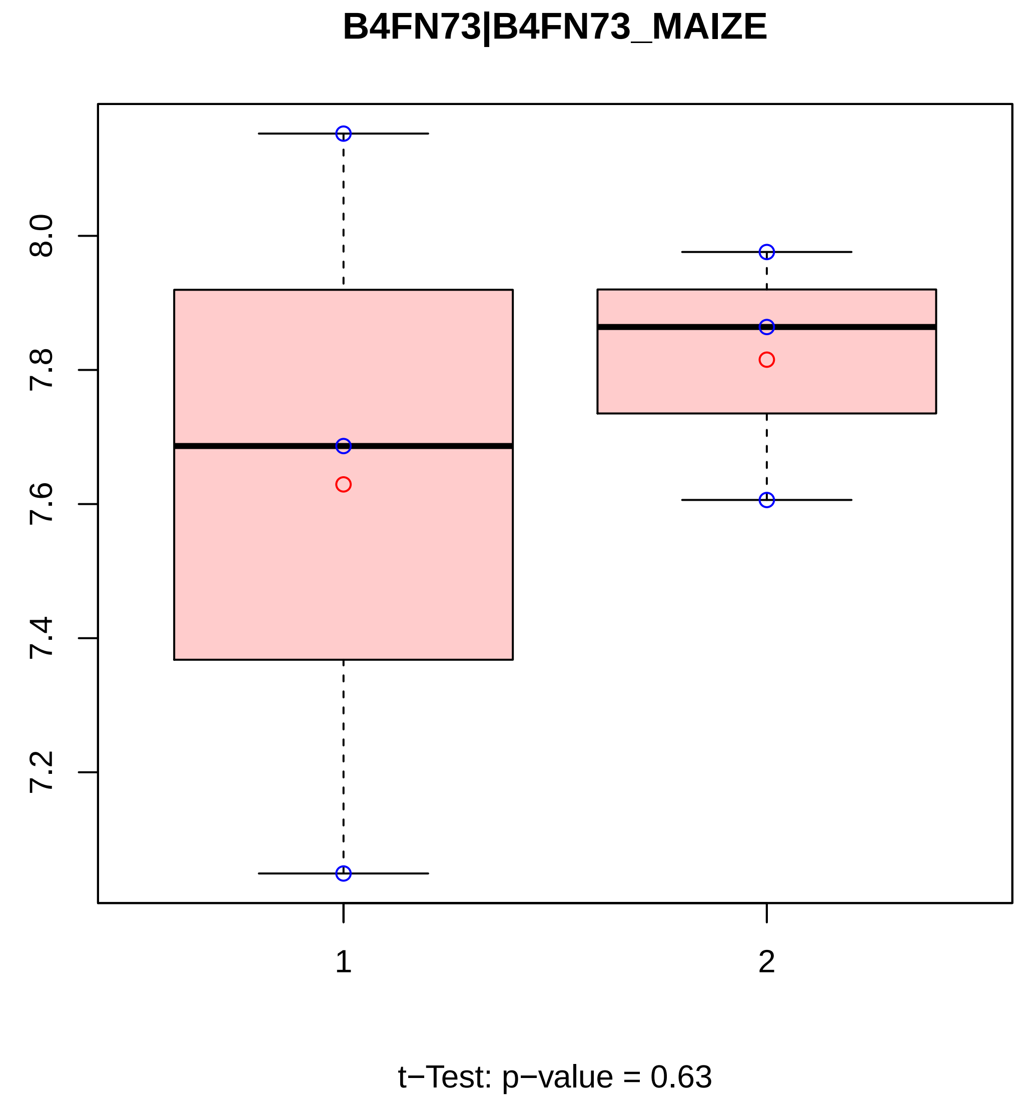

B4FN73|B4FN73_MAIZE
8.0
7.8
7.6
7.4
7.2
1
2
t−Test: p−value = 0.63

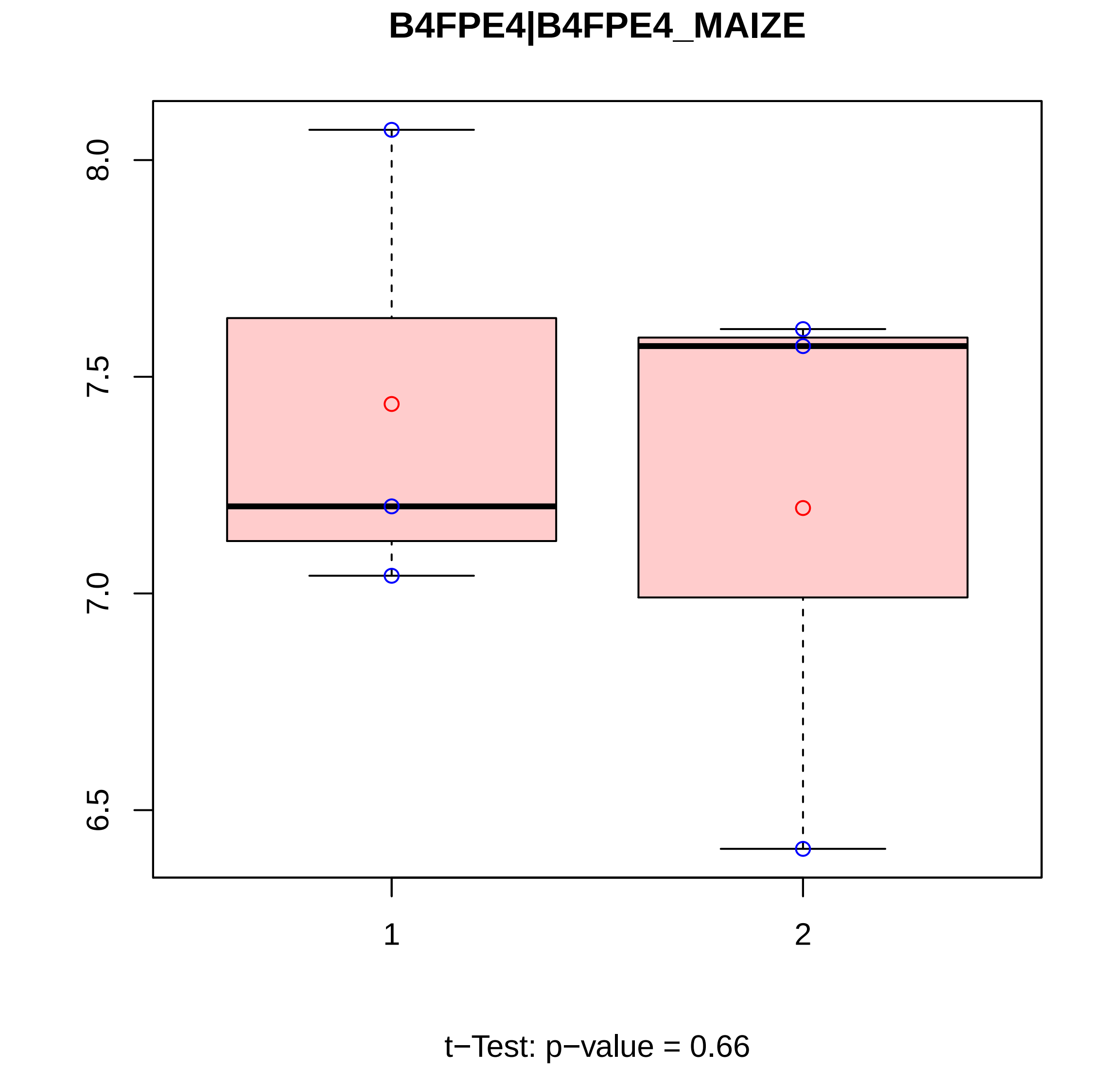

B4FPE4|B4FPE4_MAIZE
8.0
7.5
7.0
6.5
1
2
t−Test: p−value = 0.66

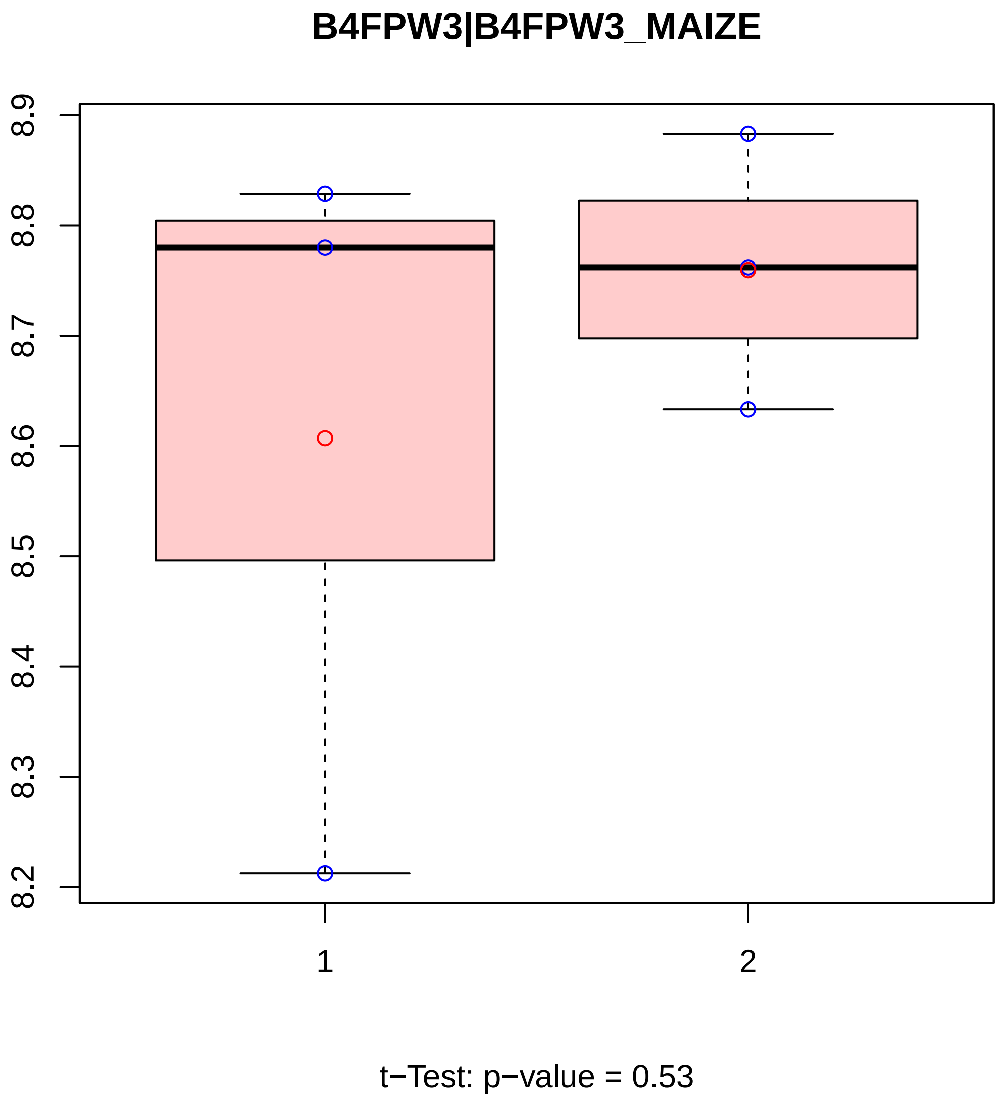
B4FPW3|B4FPW3_MAIZE
8.9
8.8
8.7
8.6
8.5
8.4
8.3
8.2
1
2
t−Test: p−value = 0.53

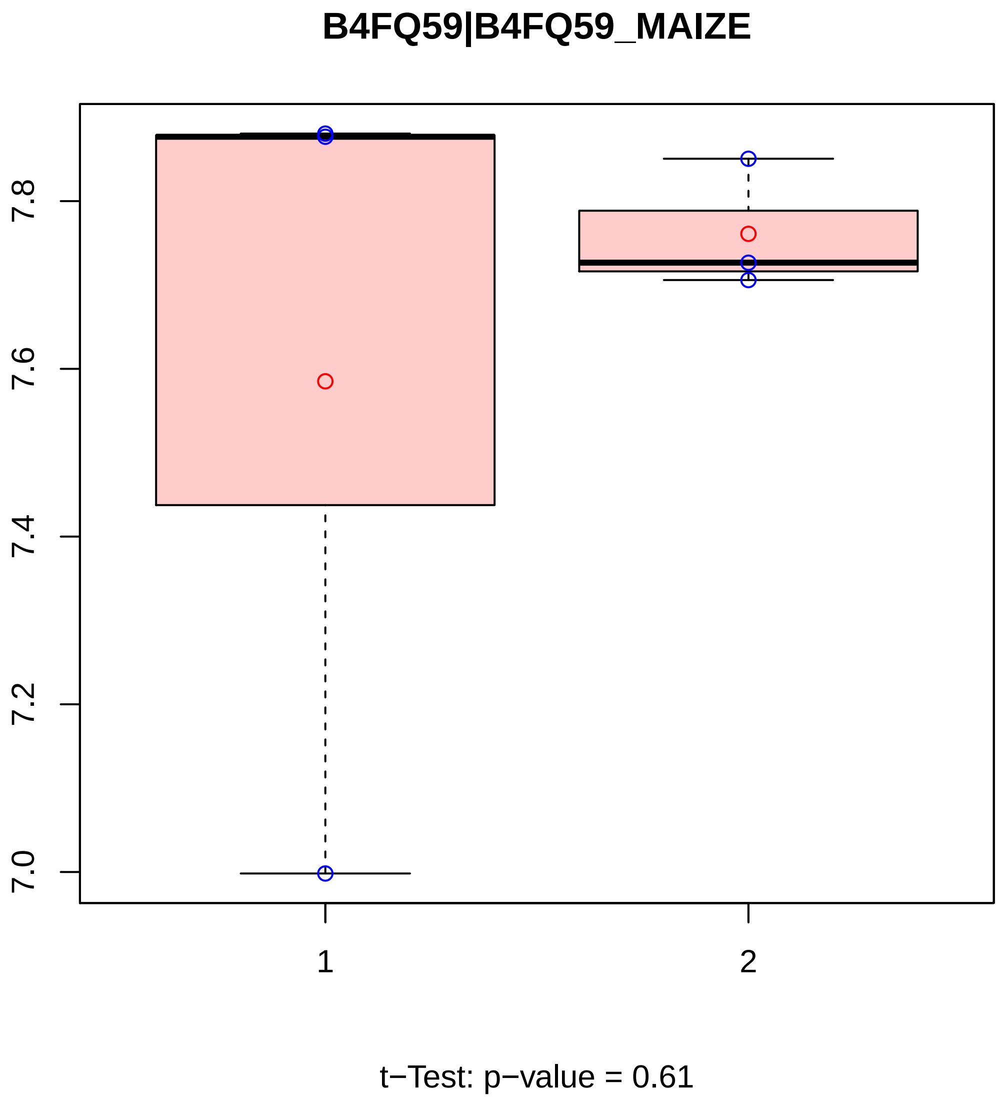
B4FQ59|B4FQ59_MAIZE
7.0
7.2
7.4
7.6
7.8
1
2
t−Test: p−value = 0.61

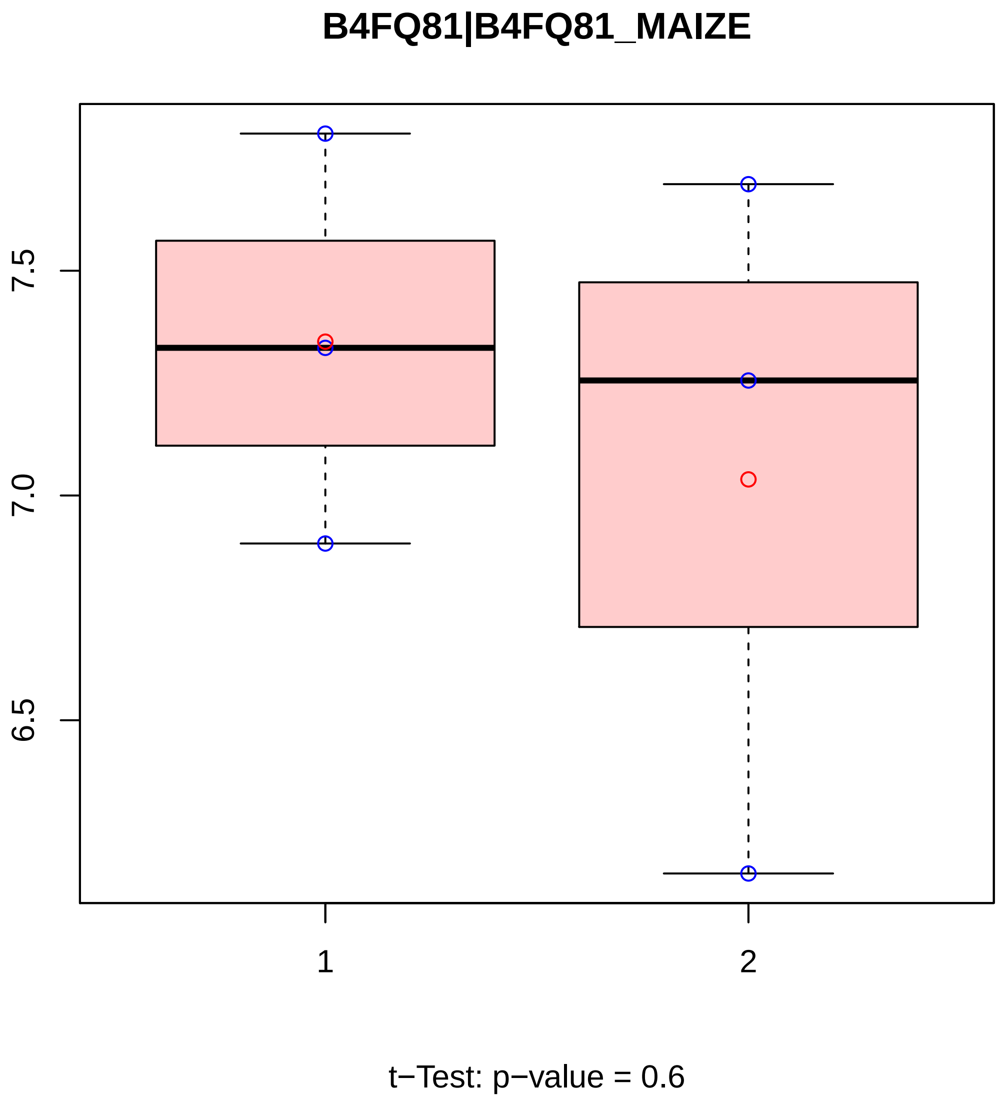

B4FQ81|B4FQ81_MAIZE
7.5
7.0
6.5
1
2
t−Test: p−value = 0.6

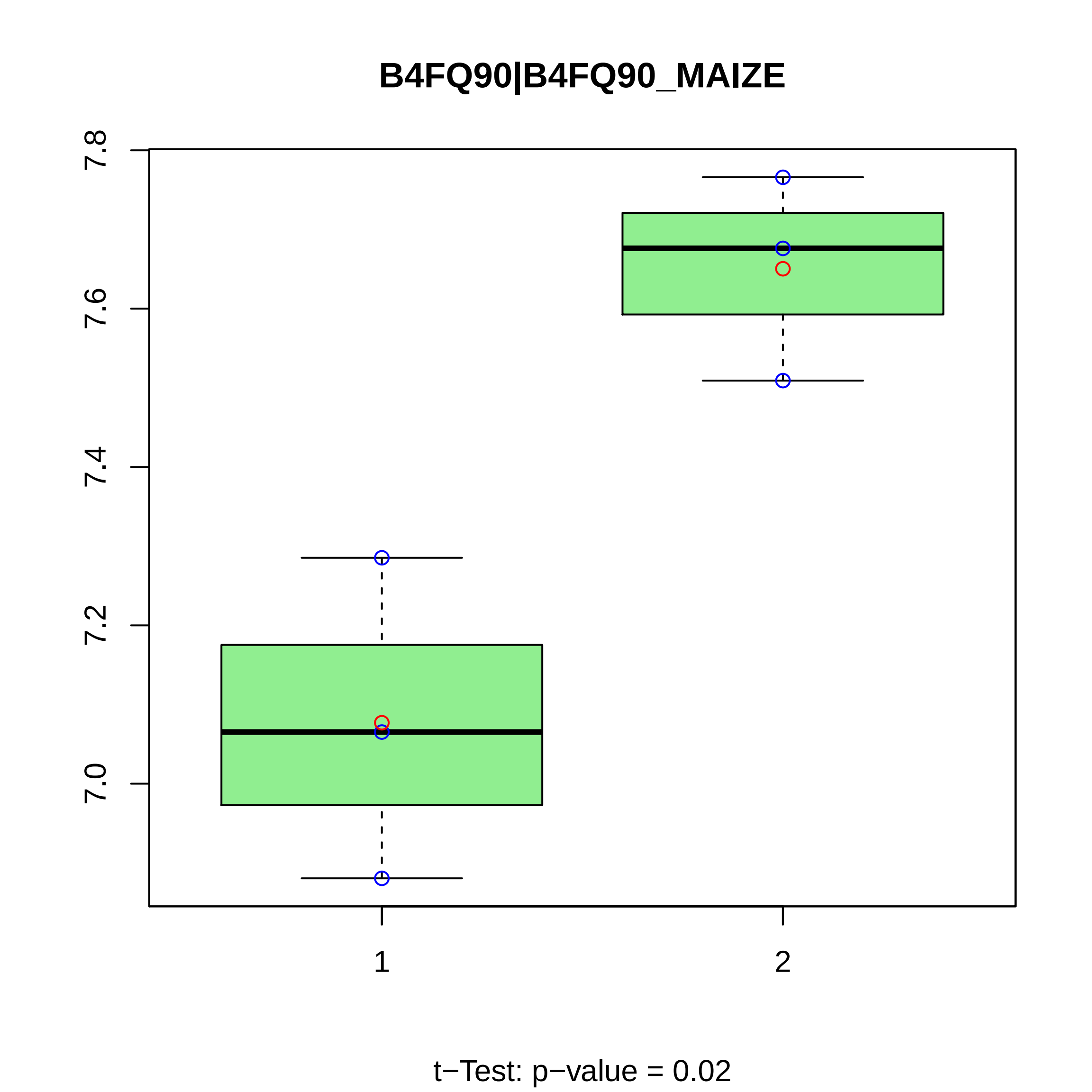

B4FQ90|B4FQ90_MAIZE
7.8
7.6
7.4
7.2
7.0
1
2
t−Test: p−value = 0.02

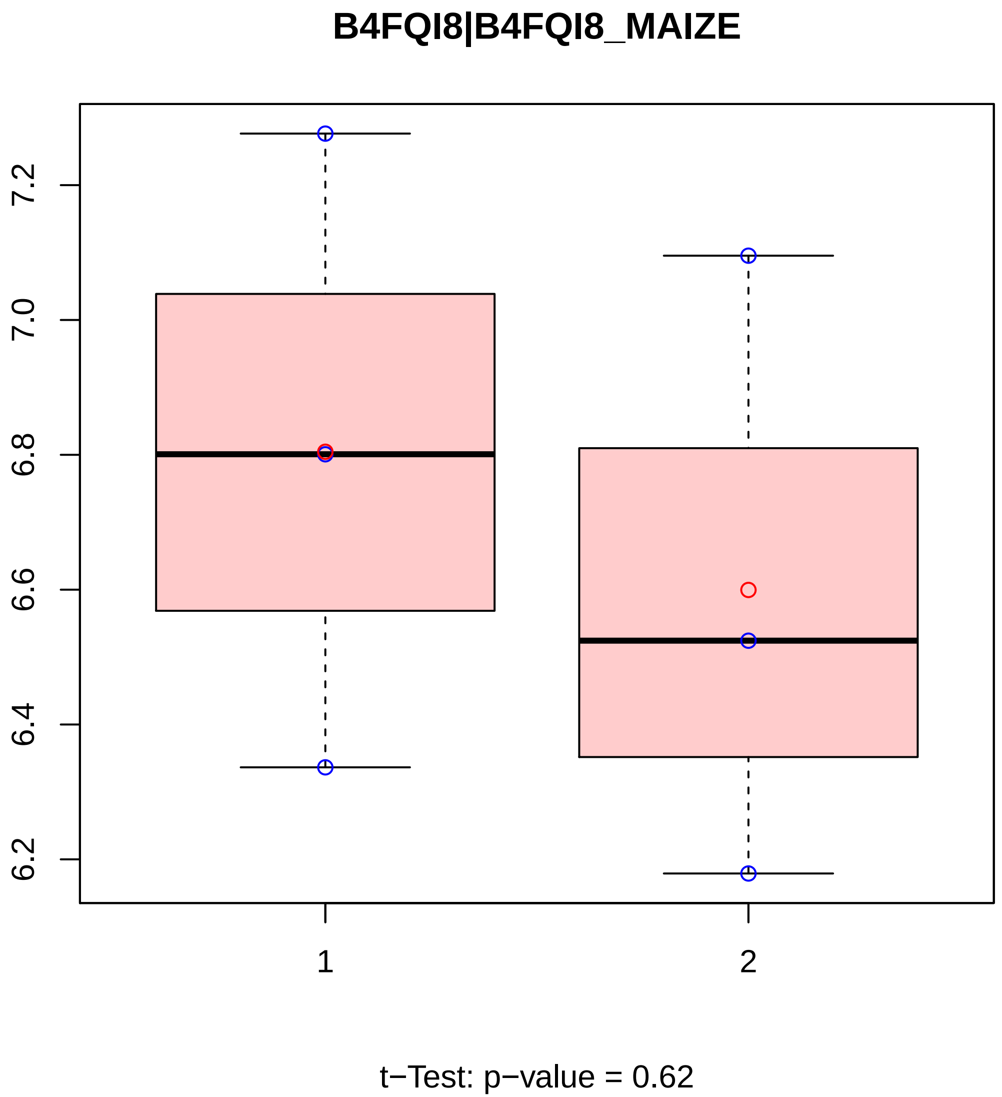

B4FQI8|B4FQI8_MAIZE
7.2
7.0
6.8
6.6
6.4
6.2
1
2
t−Test: p−value = 0.62

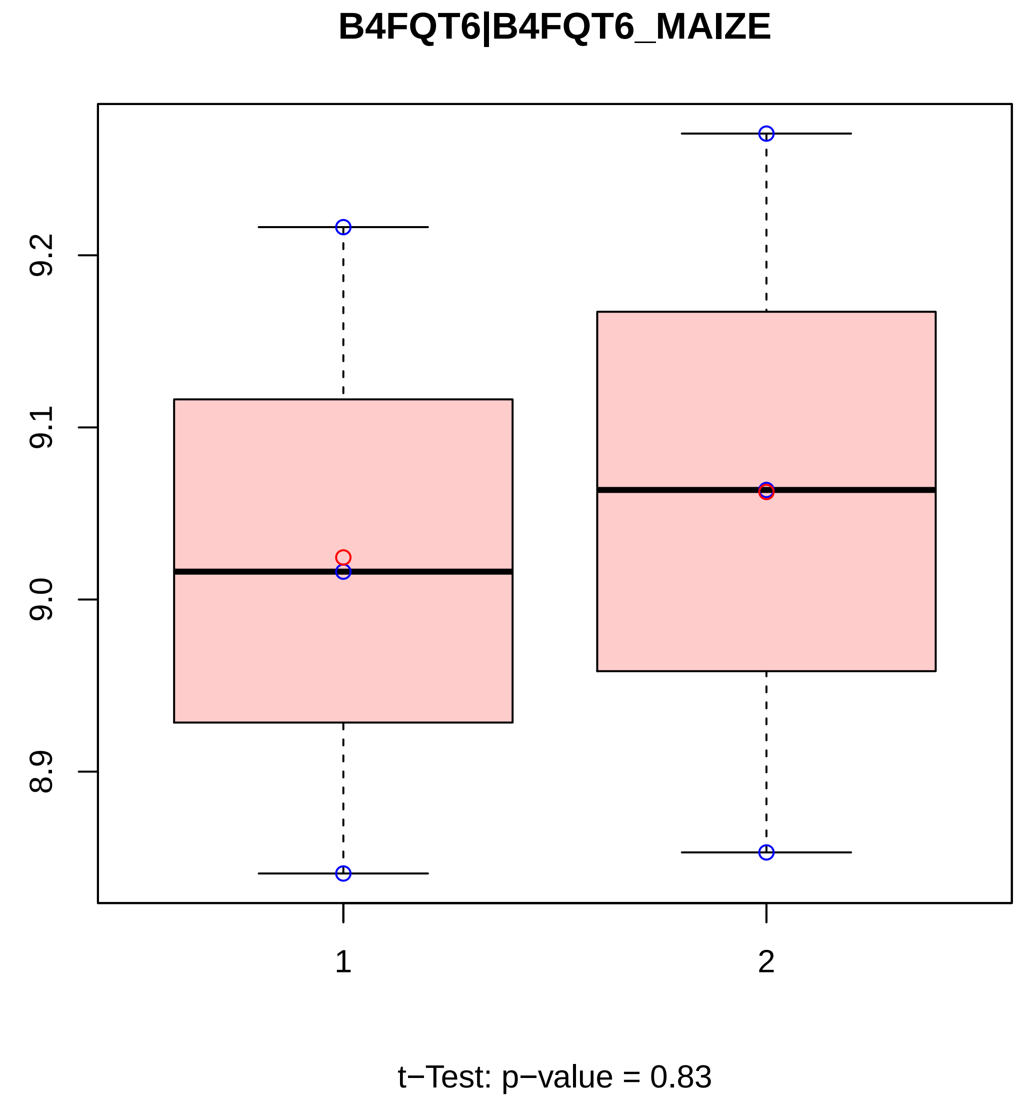

B4FQT6|B4FQT6_MAIZE
9.2
9.1
9.0
8.9
1
2
t−Test: p−value = 0.83

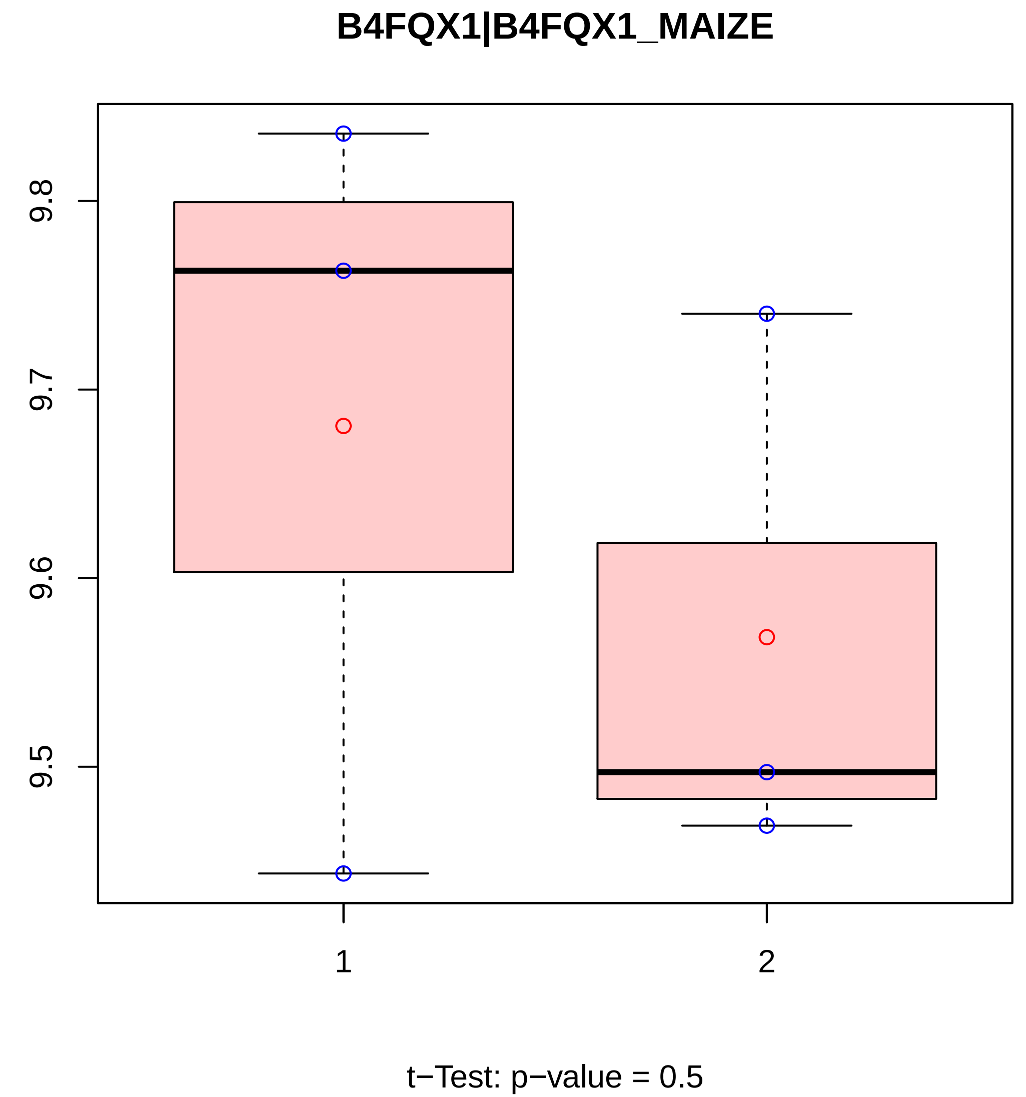
B4FQX1|B4FQX1_MAIZE
9.5
9.6
9.7
9.8
1
2
t−Test: p−value = 0.5

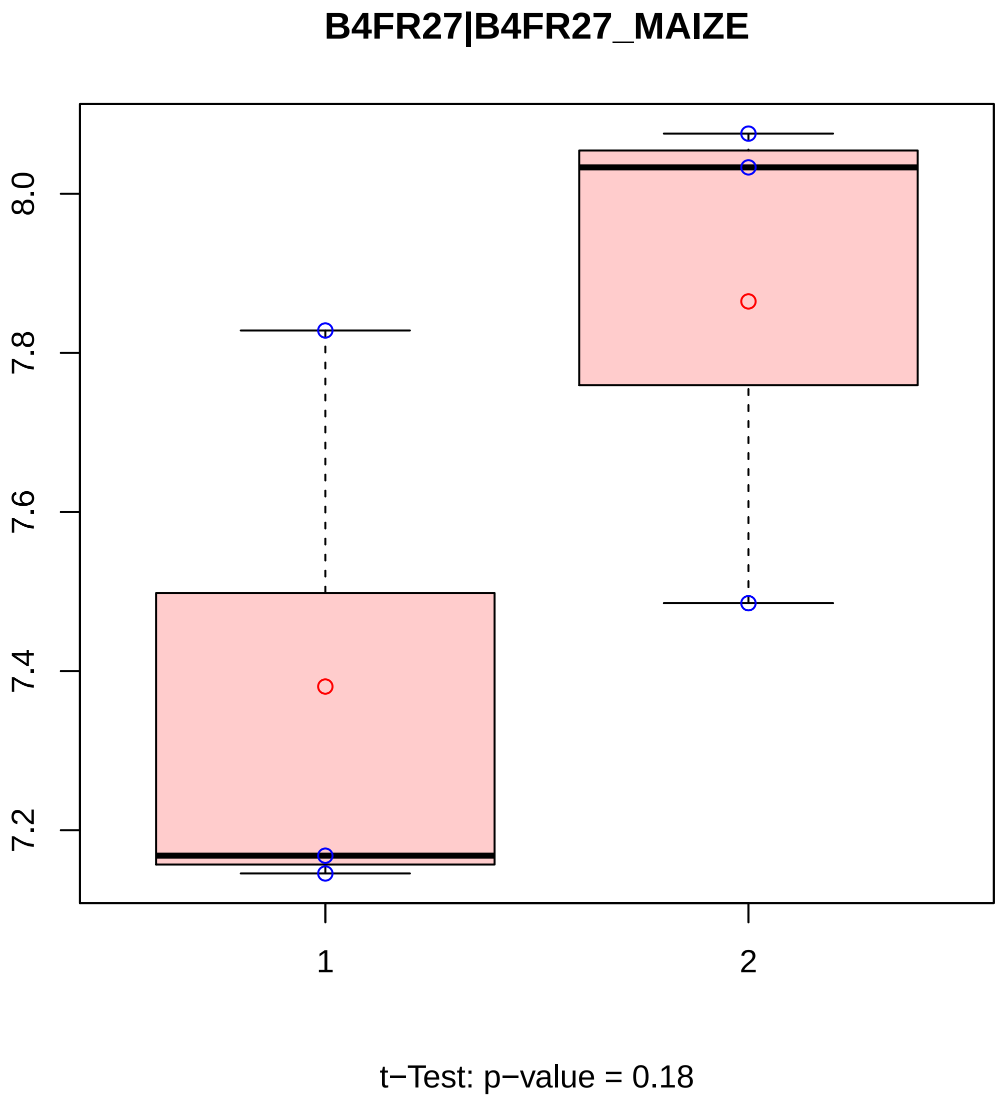
B4FR27|B4FR27_MAIZE
8.0
7.8
7.6
7.4
7.2
1
2
t−Test: p−value = 0.18

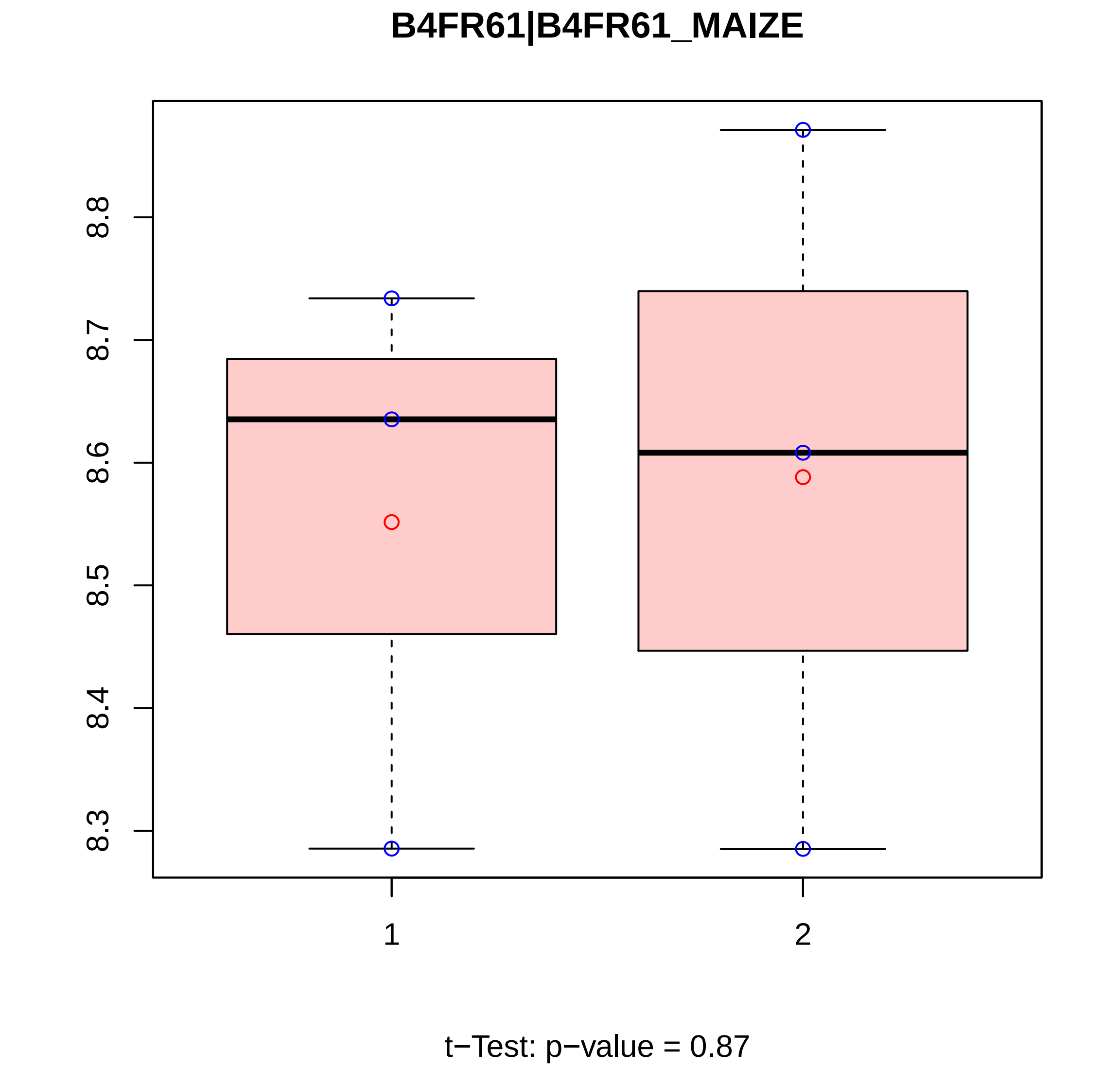

B4FR61|B4FR61_MAIZE
8.3
8.4
8.5
8.6
8.7
8.8
1
2
t−Test: p−value = 0.87

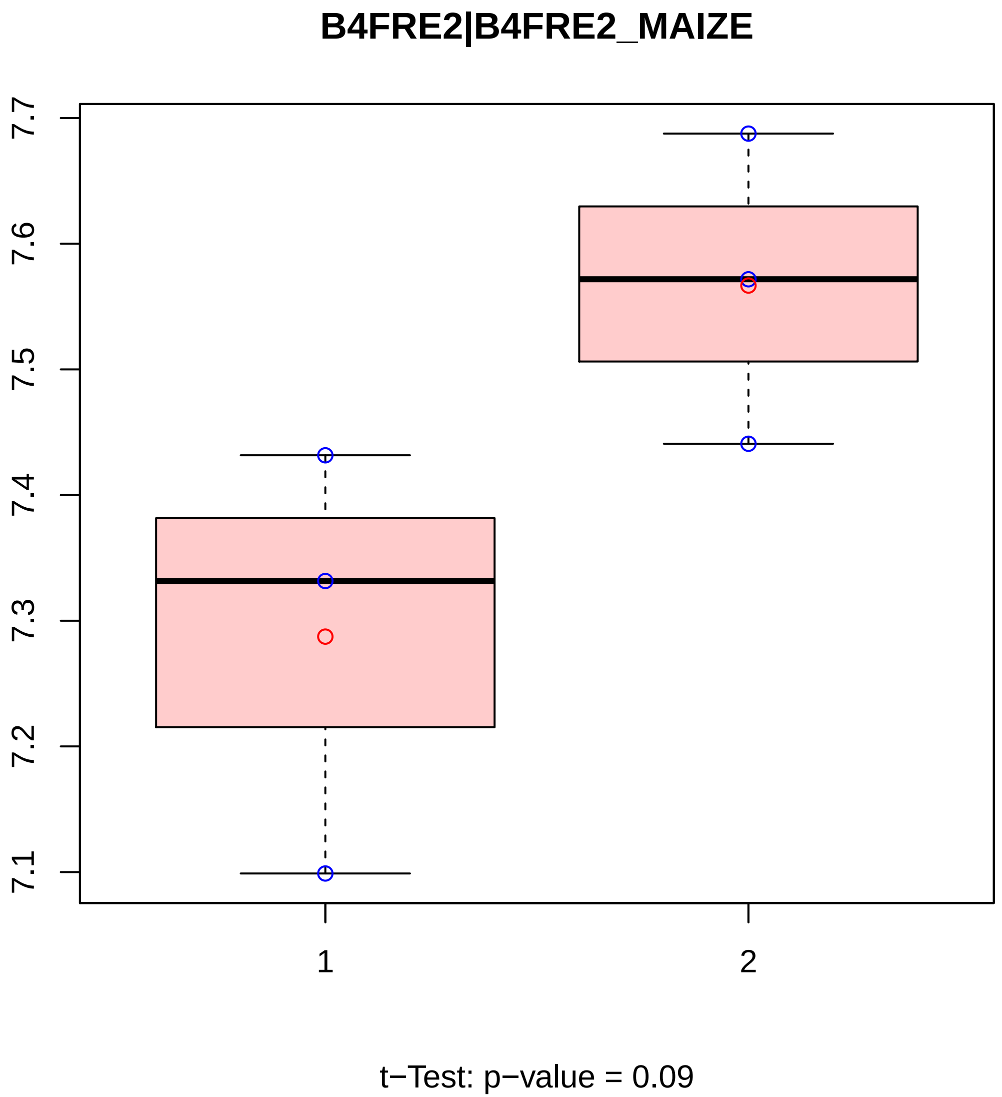
B4FRE2|B4FRE2_MAIZE
7.1
7.2
7.3
7.4
7.5
7.6
7.7
1
2
t−Test: p−value = 0.09

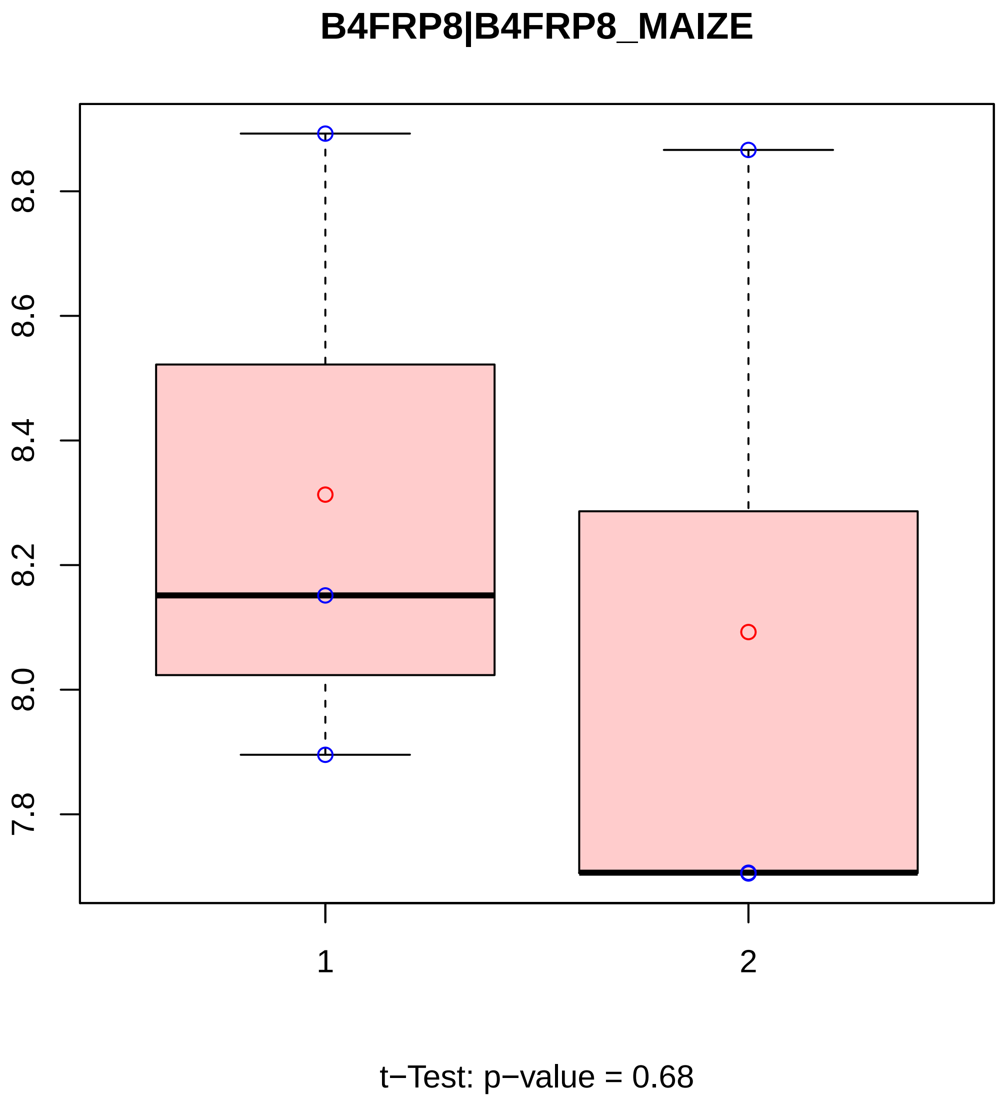
B4FRP8|B4FRP8_MAIZE
8.8
8.6
8.4
8.2
8.0
7.8
1
2
t−Test: p−value = 0.68

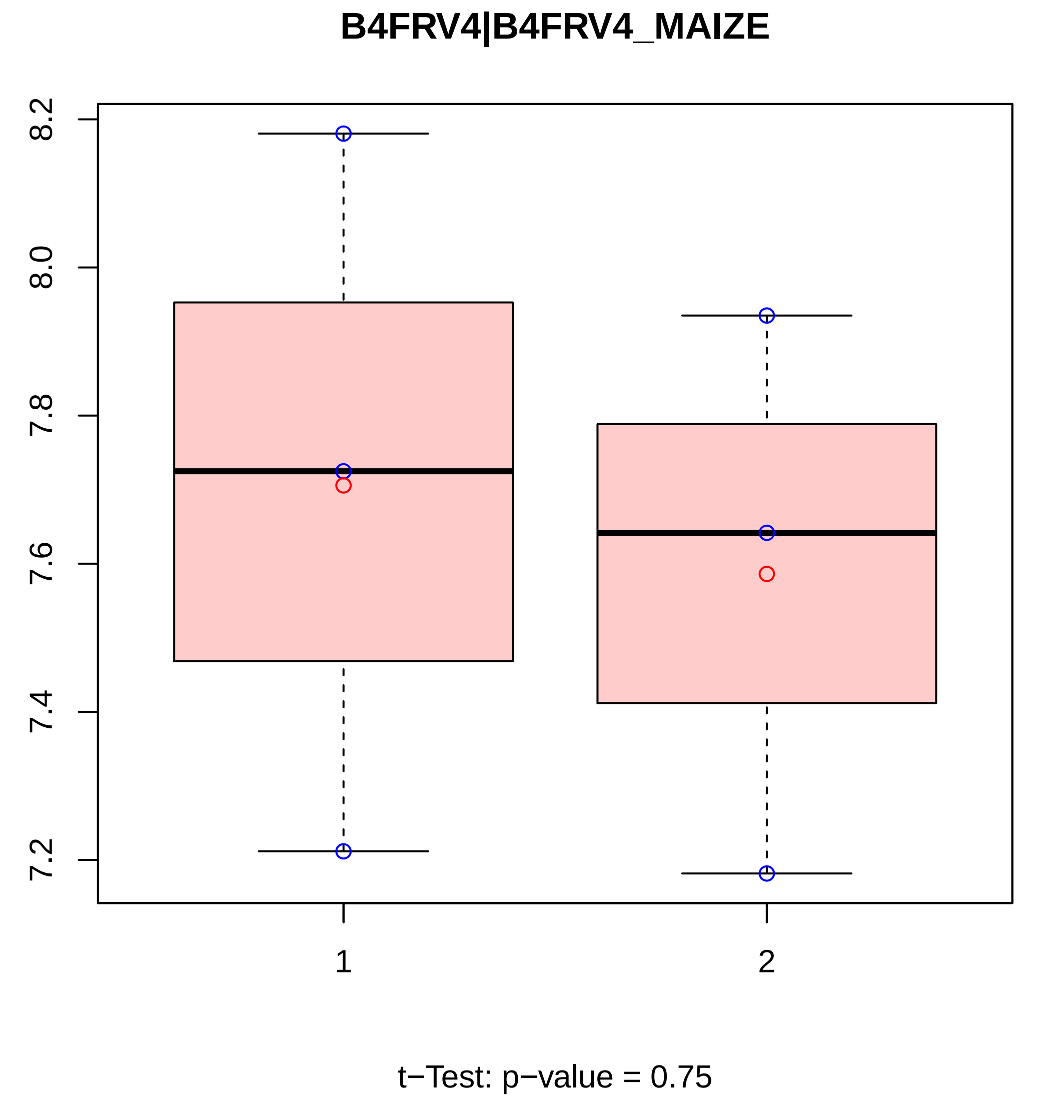
B4FRV4|B4FRV4_MAIZE
8.2
8.0
7.8
7.6
7.4
7.2
1
2
t−Test: p−value = 0.75

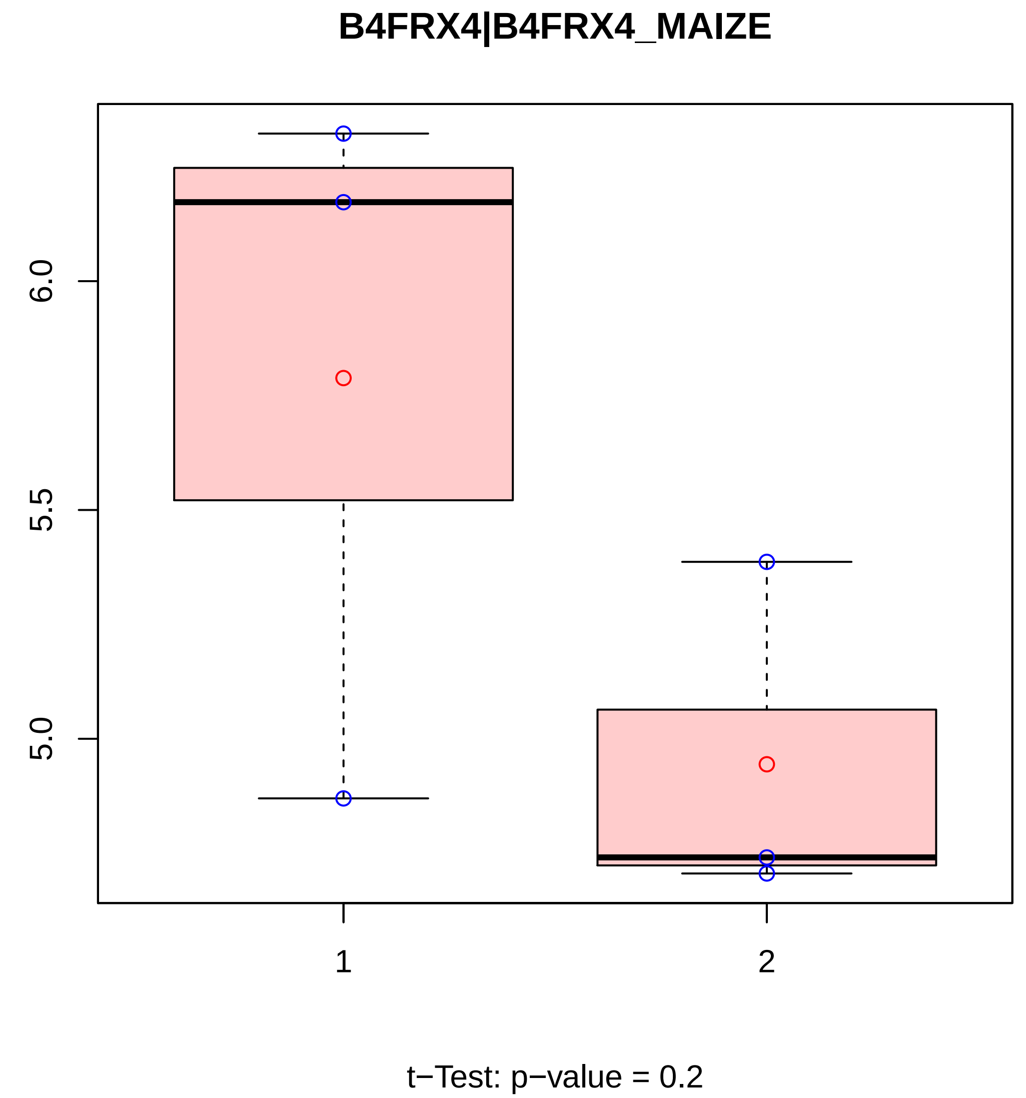

B4FRX4|B4FRX4_MAIZE
6.0
5.5
5.0
1
2
t−Test: p−value = 0.2

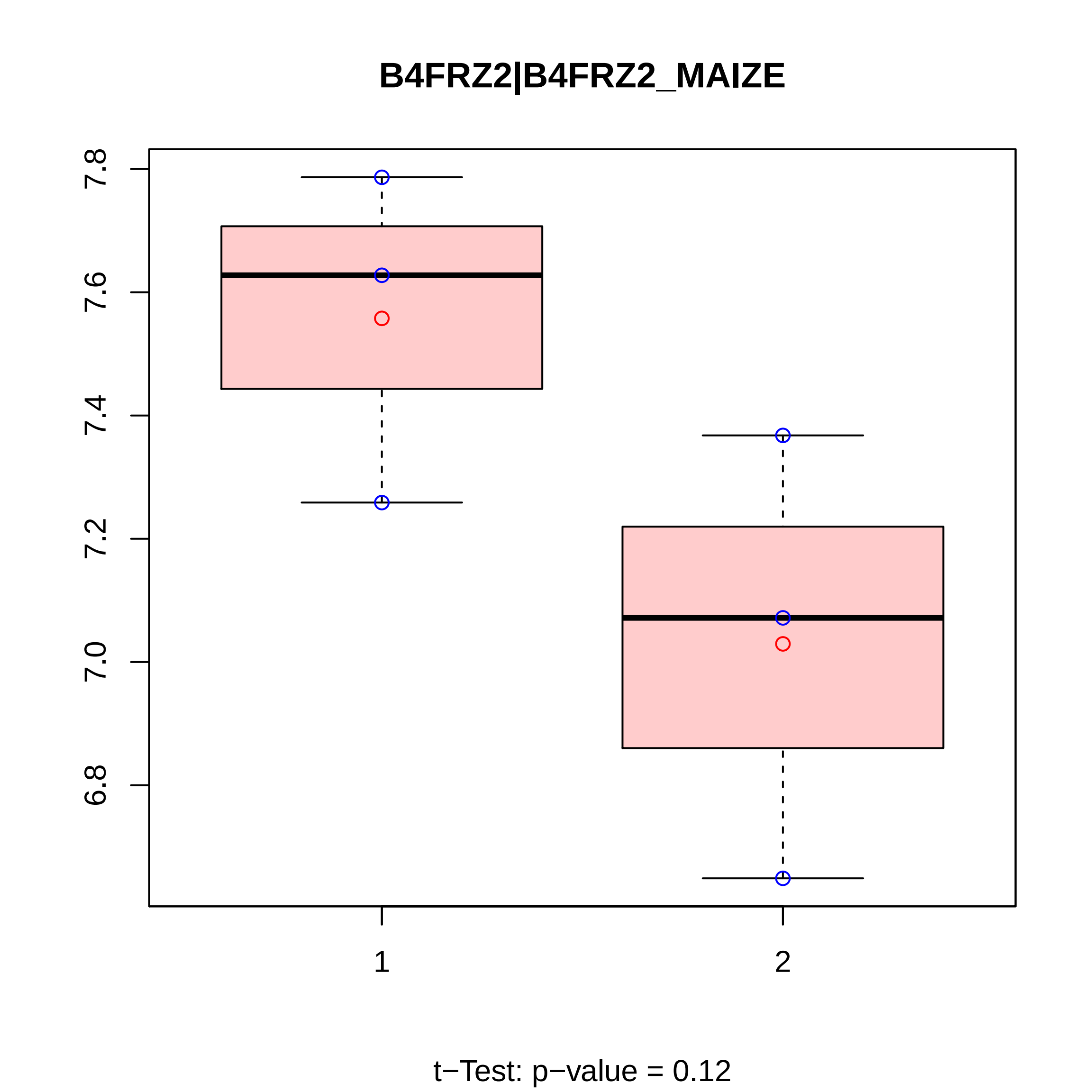
B4FRZ2|B4FRZ2_MAIZE
7.8
7.6
7.4
7.2
7.0
6.8
1
2
t−Test: p−value = 0.12

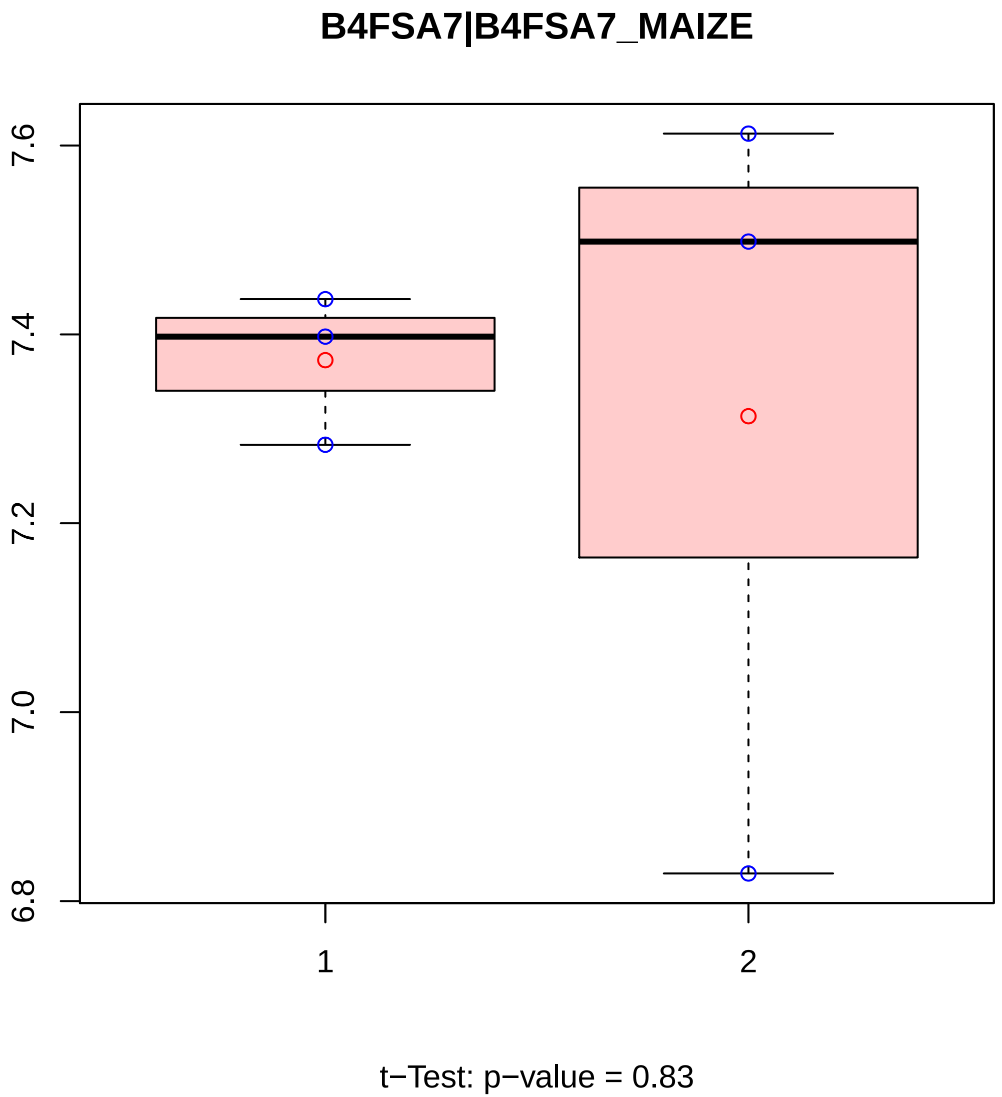

B4FSA7|B4FSA7_MAIZE
7.6
7.4
7.2
7.0
6.8
1
2
t−Test: p−value = 0.83

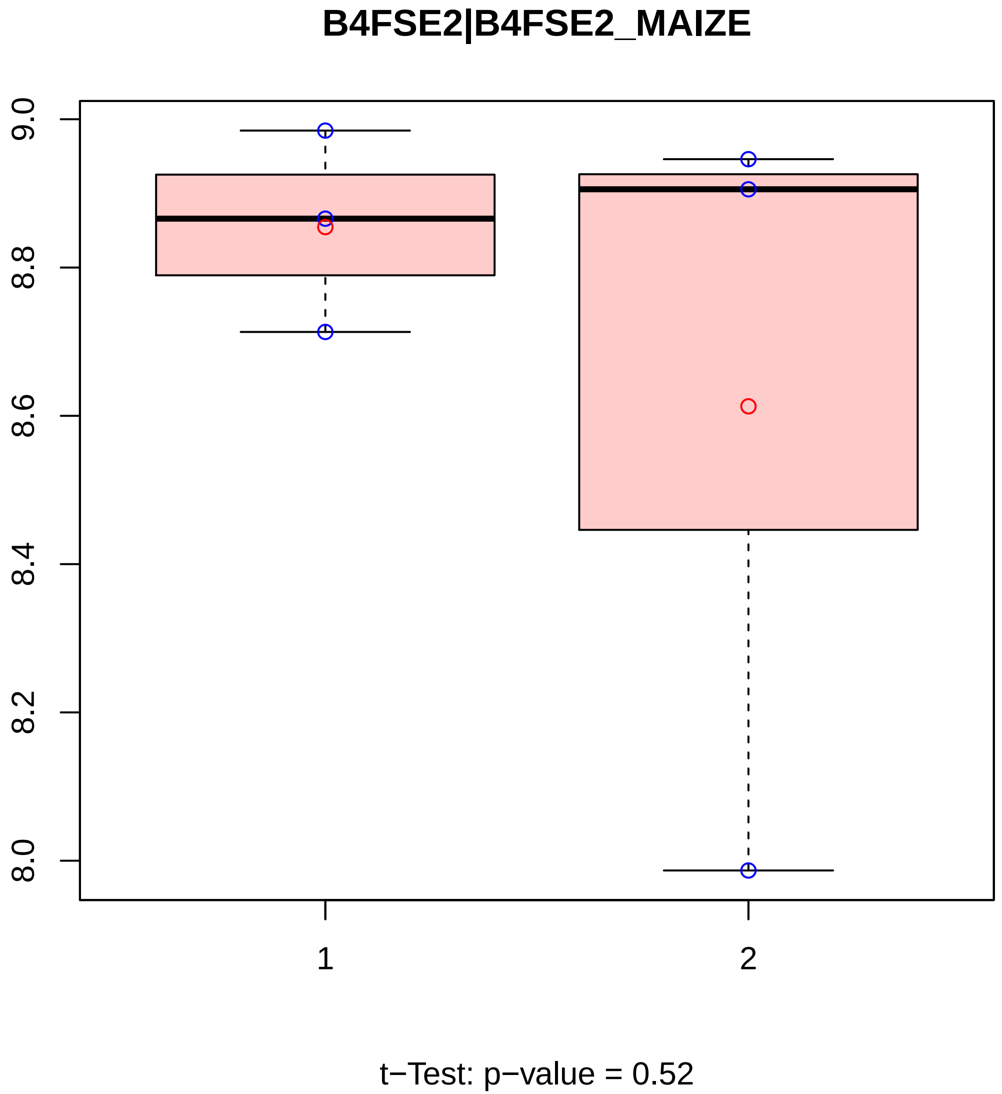
B4FSE2|B4FSE2_MAIZE
9.0
8.8
8.6
8.4
8.2
8.0
1
2
t−Test: p−value = 0.52

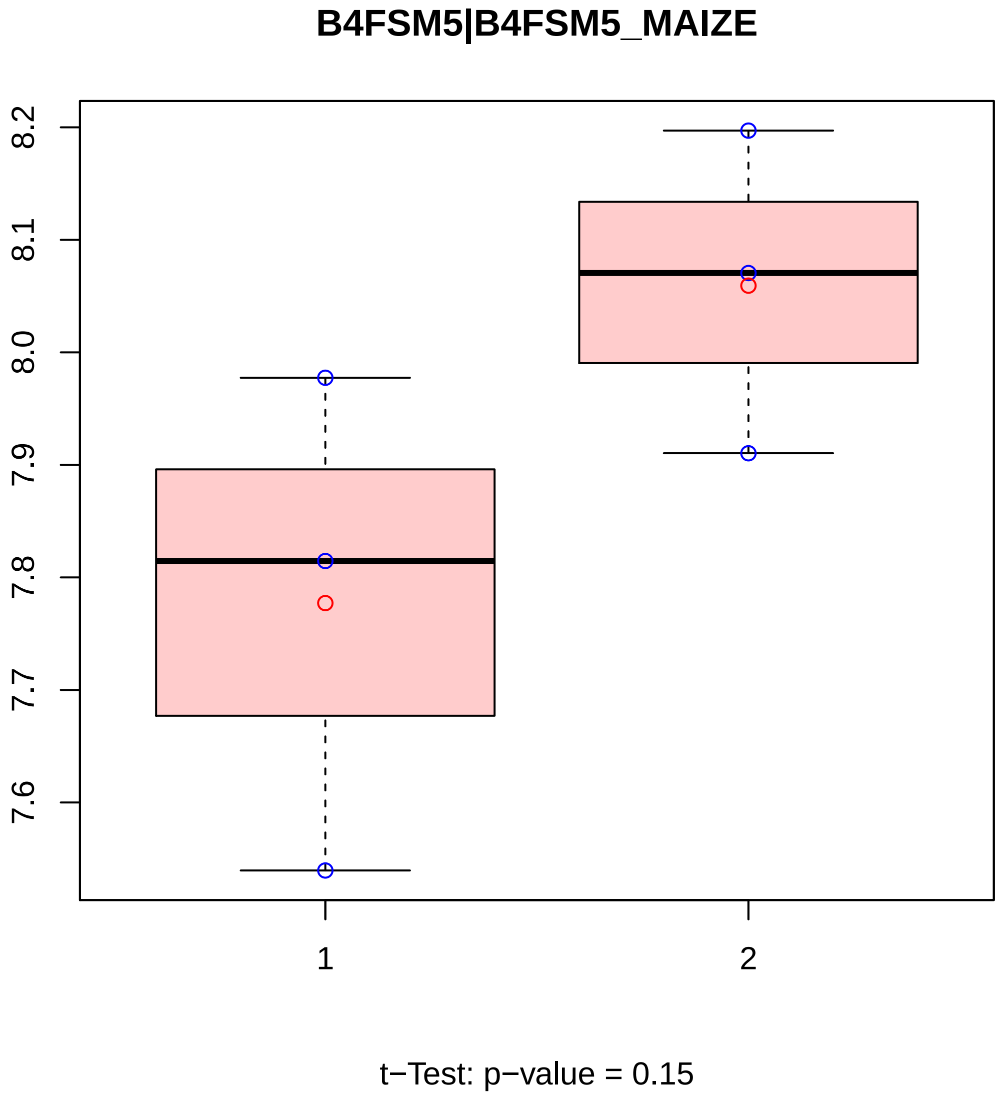

B4FSM5|B4FSM5_MAIZE
8.2
8.1
8.0
7.9
7.8
7.7
7.6
1
2
t−Test: p−value = 0.15

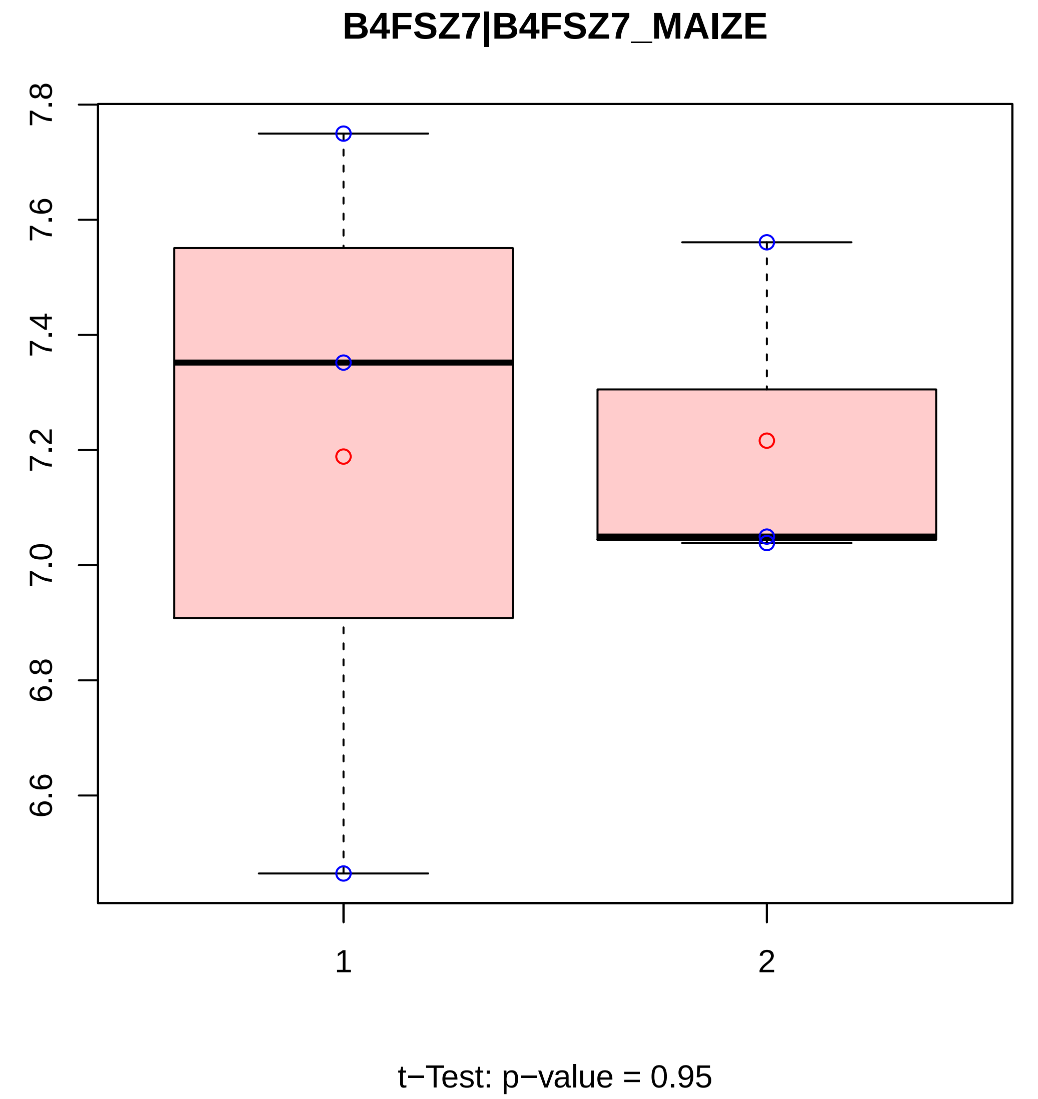
B4FSZ7|B4FSZ7_MAIZE
7.8
7.6
7.4
7.2
7.0
6.8
6.6
1
2
t−Test: p−value = 0.95

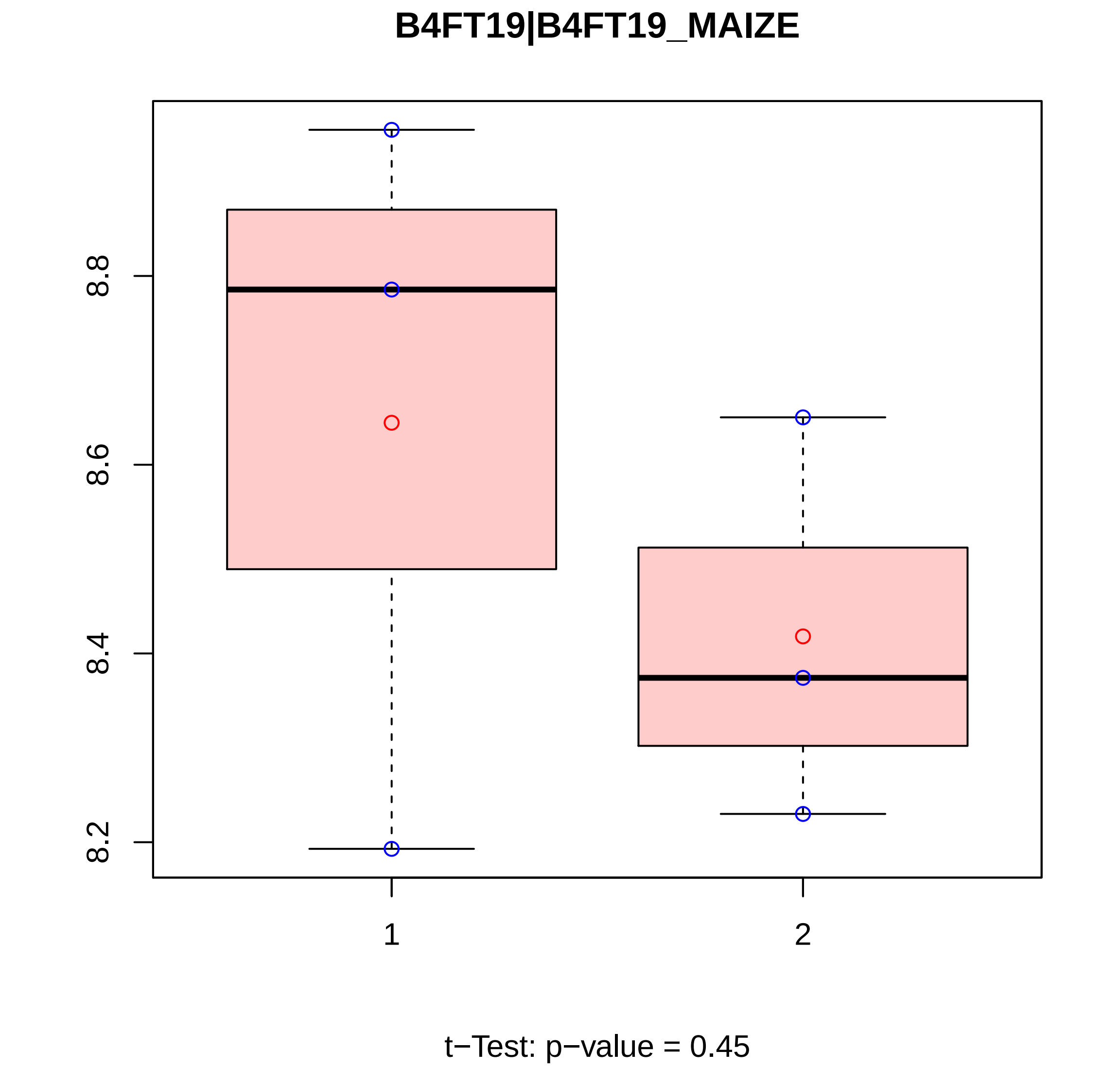
B4FT19|B4FT19_MAIZE
8.2
8.4
8.6
8.8
1
2
t−Test: p−value = 0.45

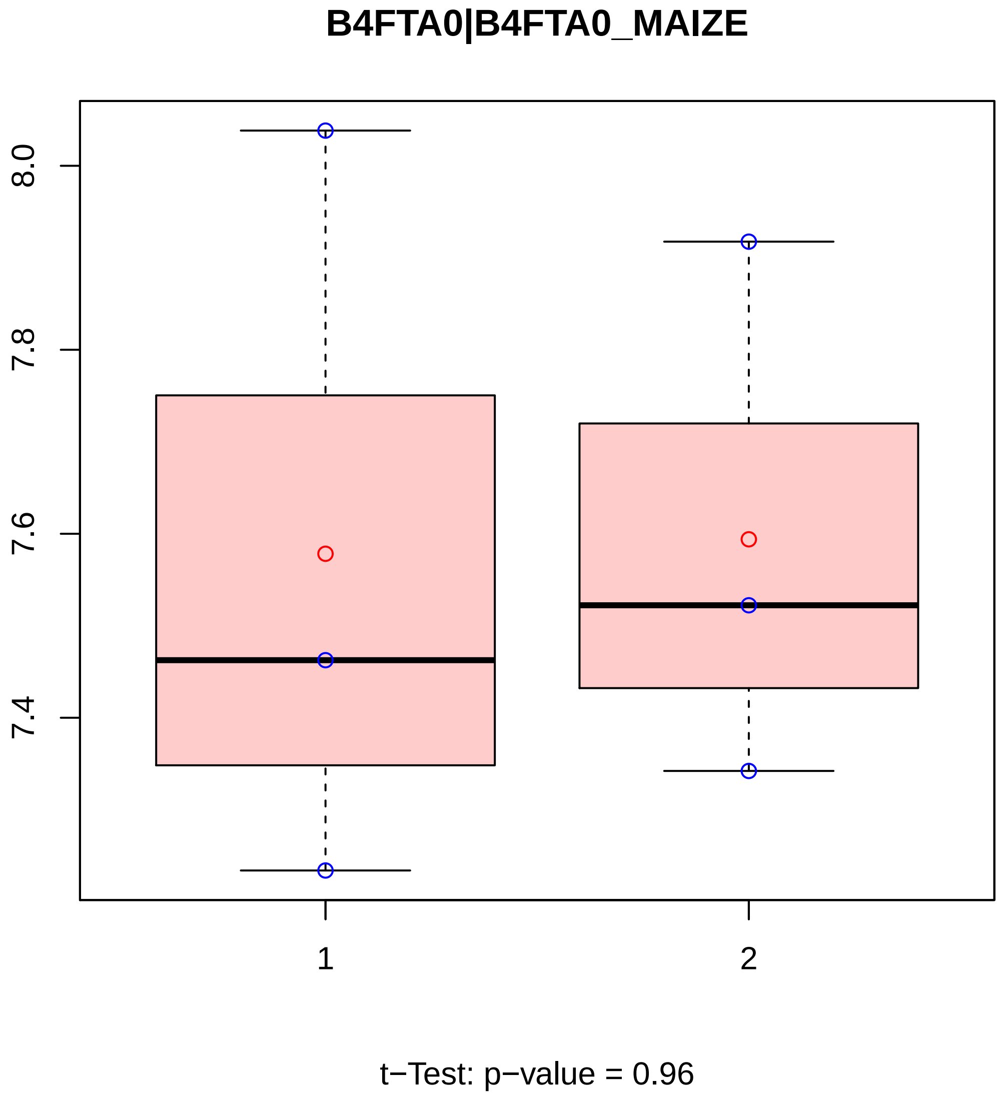
B4FTA0|B4FTA0_MAIZE
8.0
7.8
7.6
7.4
1
2
t−Test: p−value = 0.96

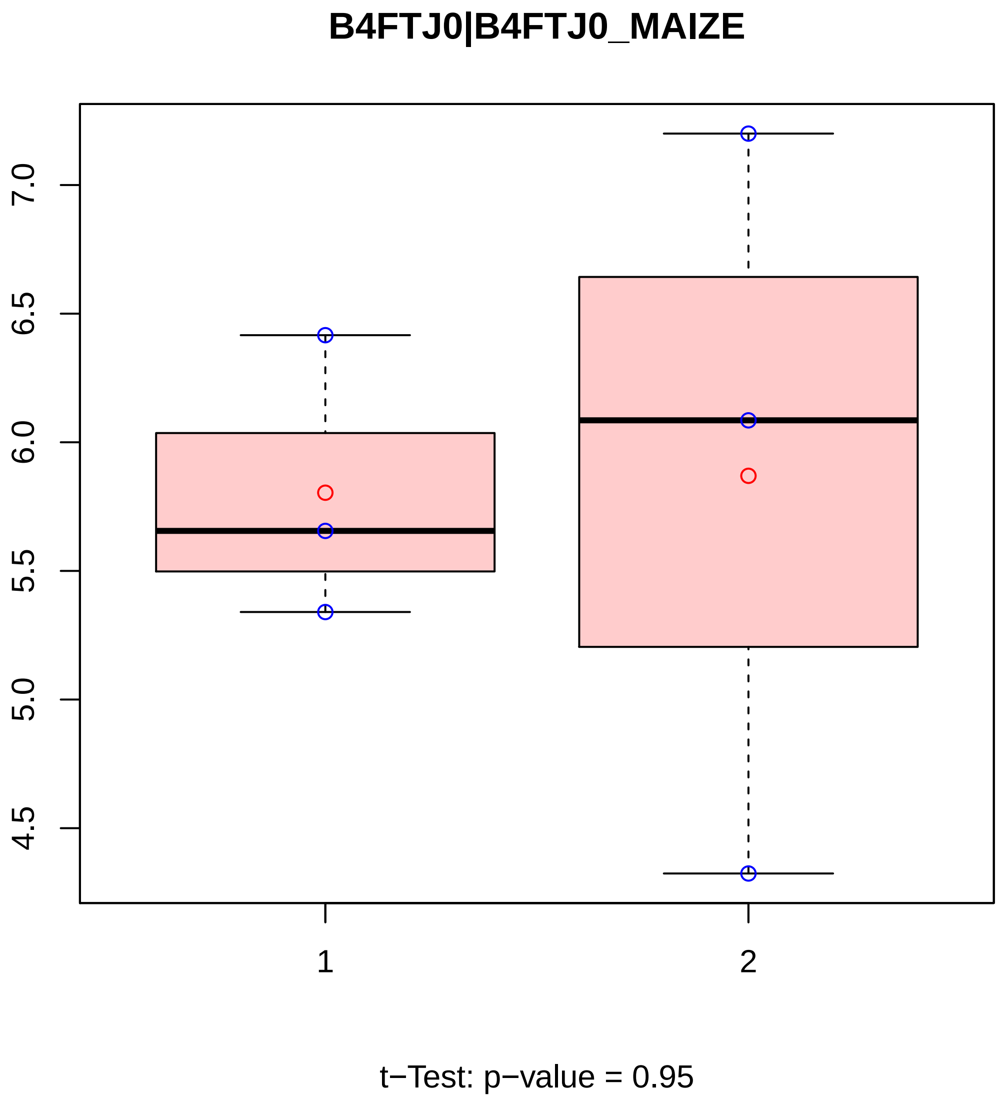

B4FTJ0|B4FTJ0_MAIZE
7.0
6.5
6.0
5.5
5.0
4.5
1
2
t−Test: p−value = 0.95

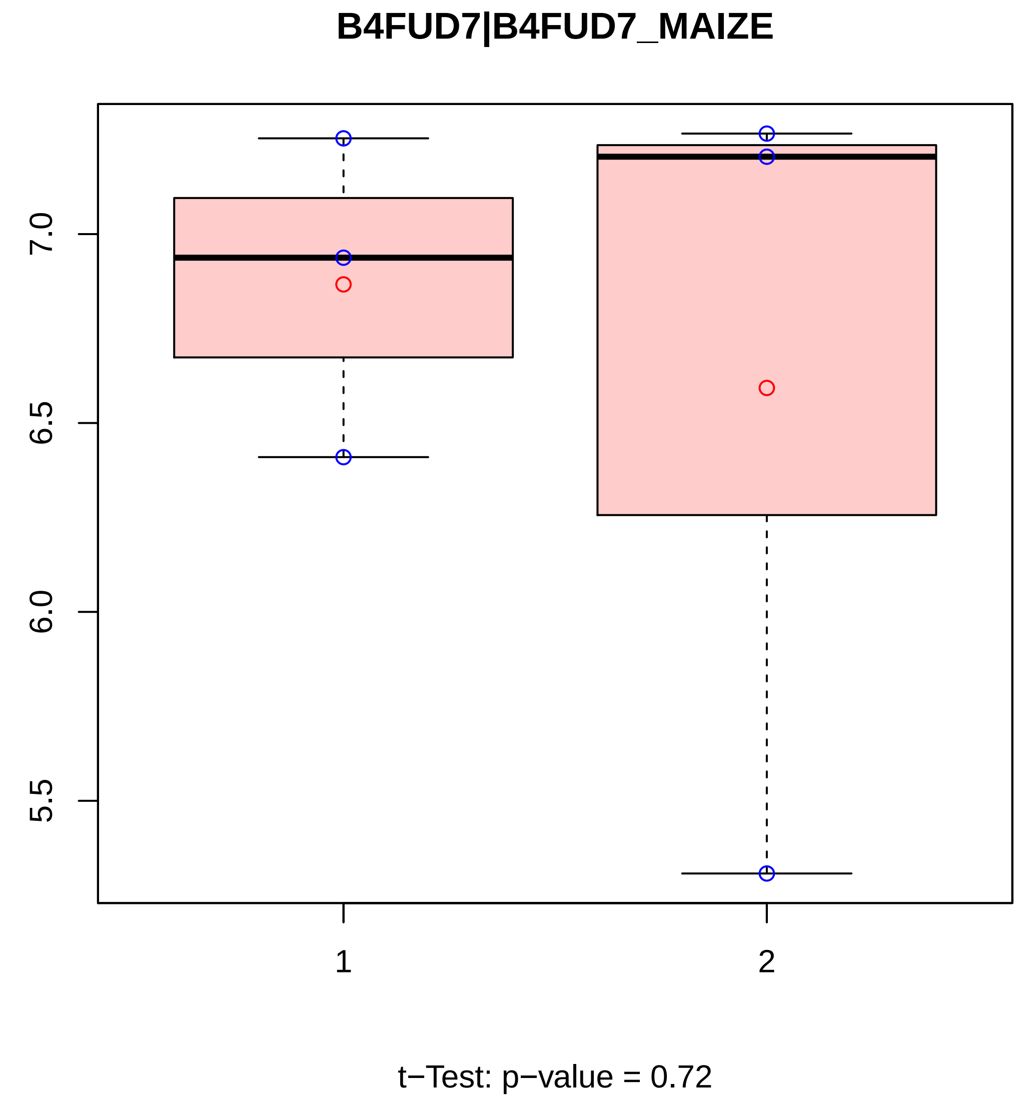
B4FUD7|B4FUD7_MAIZE
7.0
6.5
6.0
5.5
1
2
t−Test: p−value = 0.72

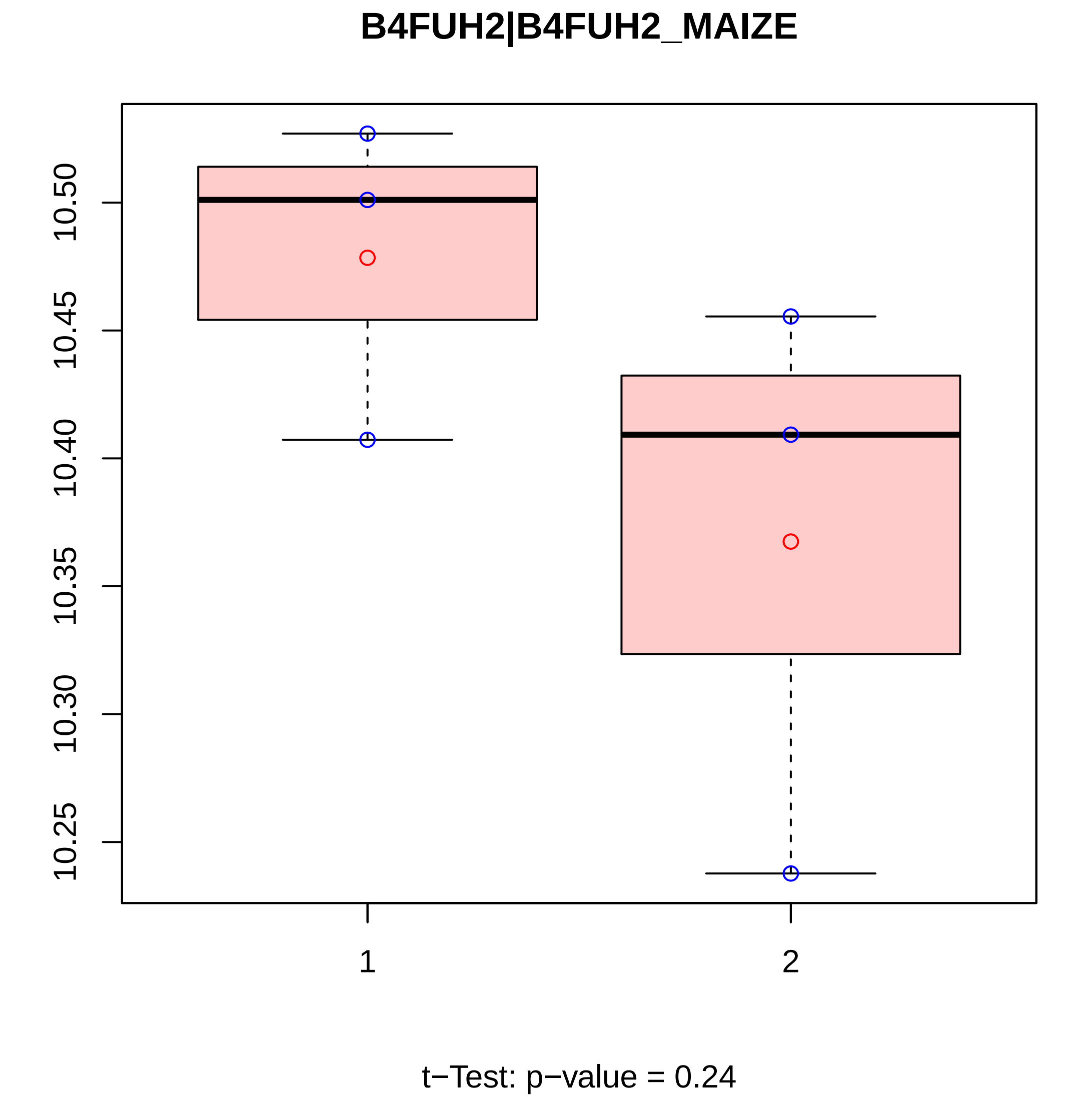

B4FUH2|B4FUH2_MAIZE
10.25
10.30
10.35
10.40
10.45
10.50
1
2
t−Test: p−value = 0.24

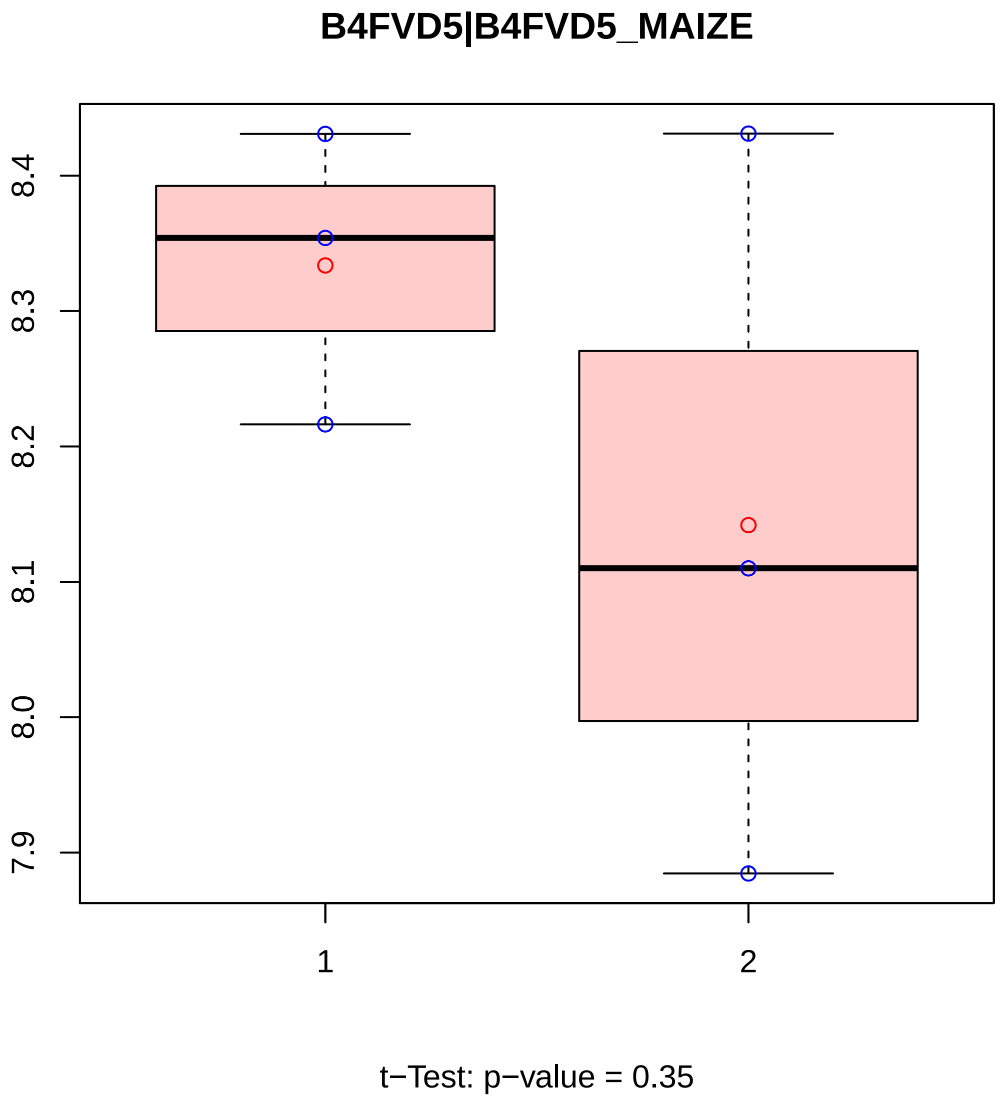
B4FVD5|B4FVD5_MAIZE
8.4
8.3
8.2
8.1
8.0
7.9
1
2
t−Test: p−value = 0.35

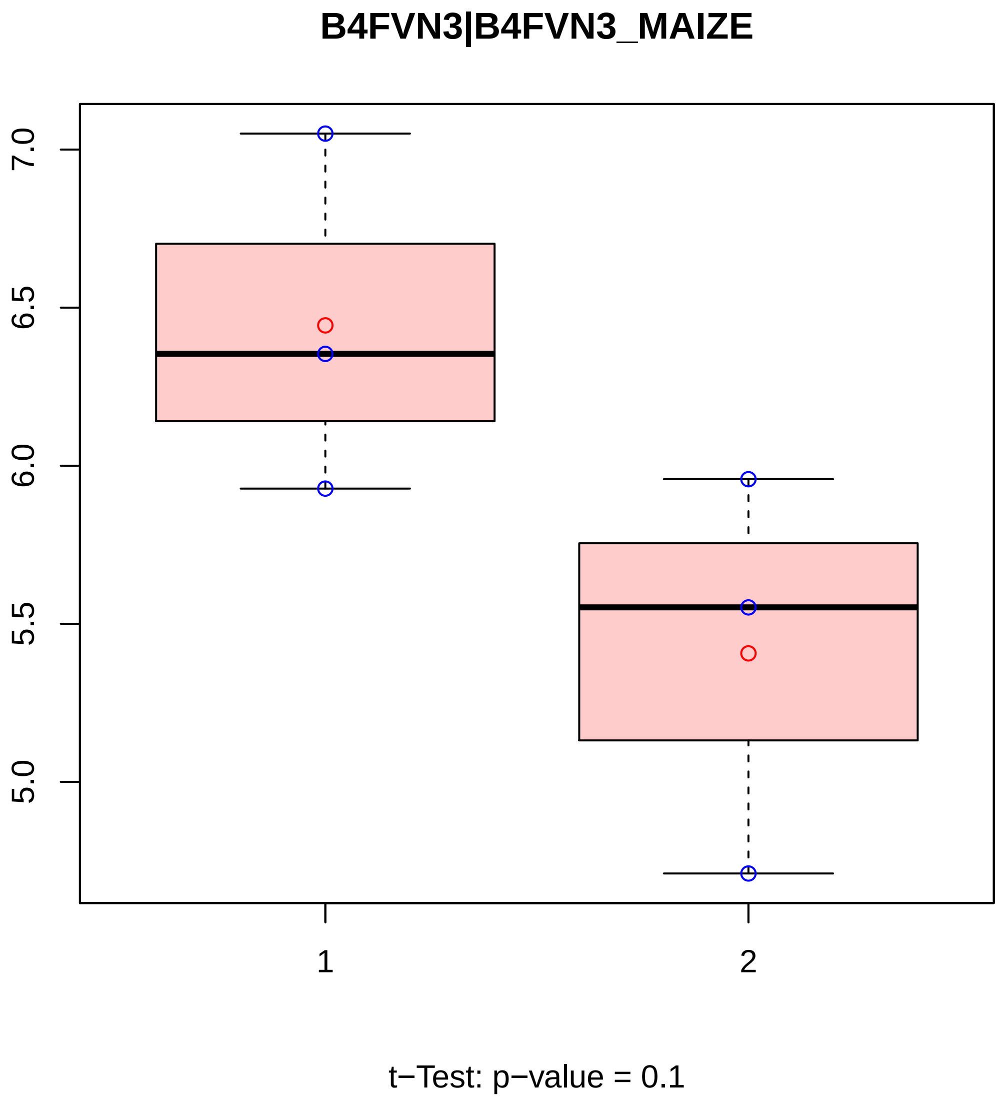
B4FVN3|B4FVN3_MAIZE
7.0
6.5
6.0
5.5
5.0
1
2
t−Test: p−value = 0.1

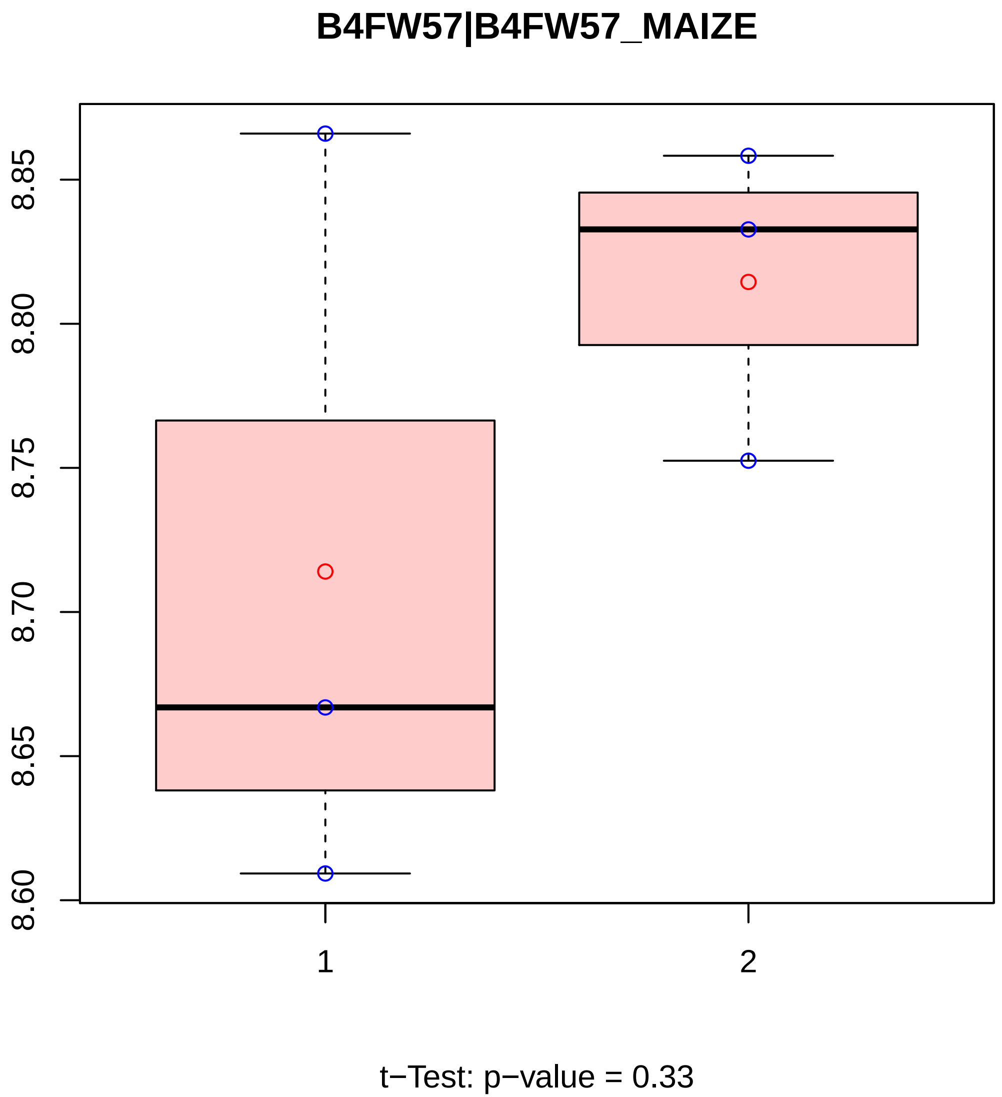
B4FW57|B4FW57_MAIZE
8.60
8.65
8.70
8.75
8.80
8.85
1
2
t−Test: p−value = 0.33

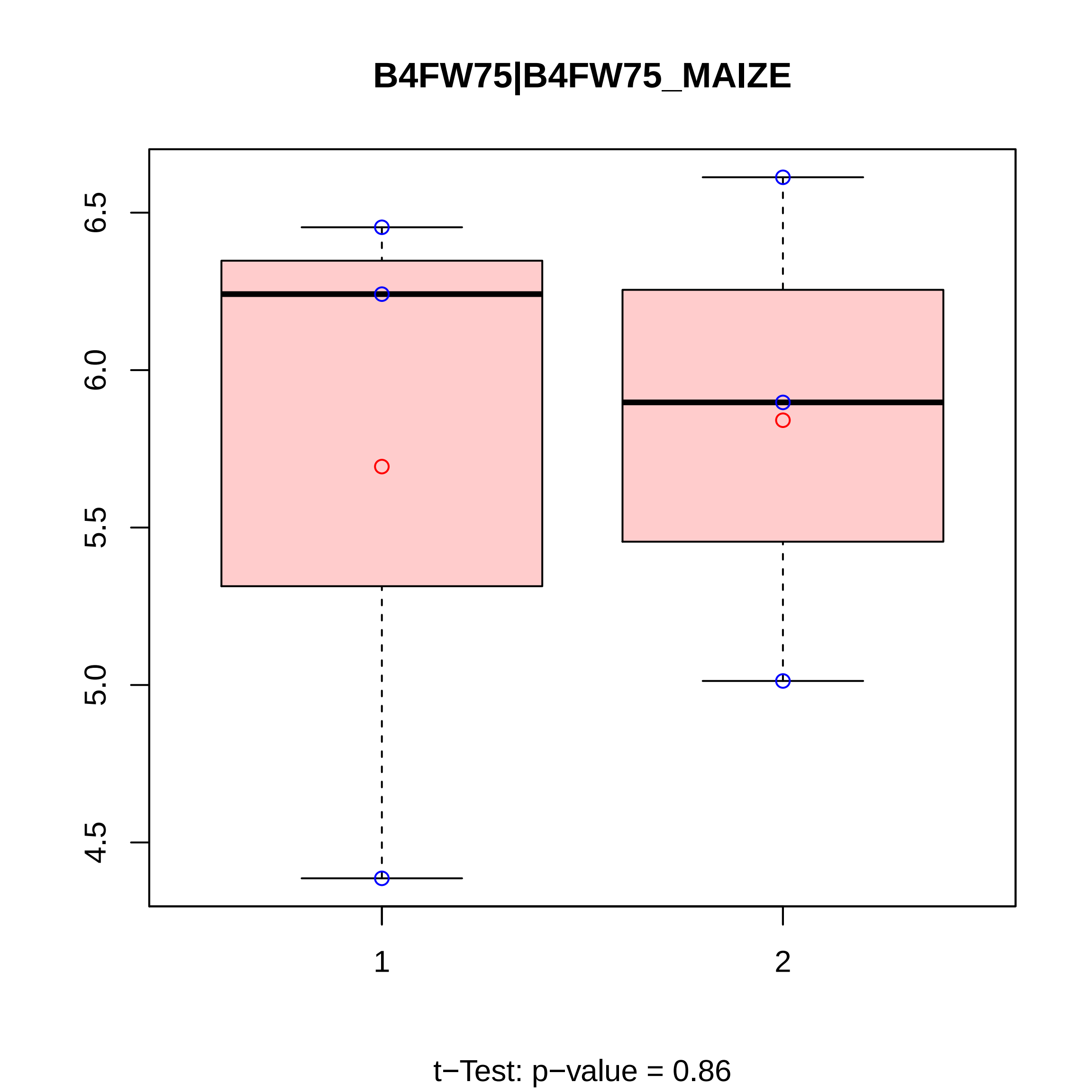
B4FW75|B4FW75_MAIZE
6.5
6.0
5.5
5.0
4.5
1
2
t−Test: p−value = 0.86

B4FWG2|B4FWG2_MAIZE

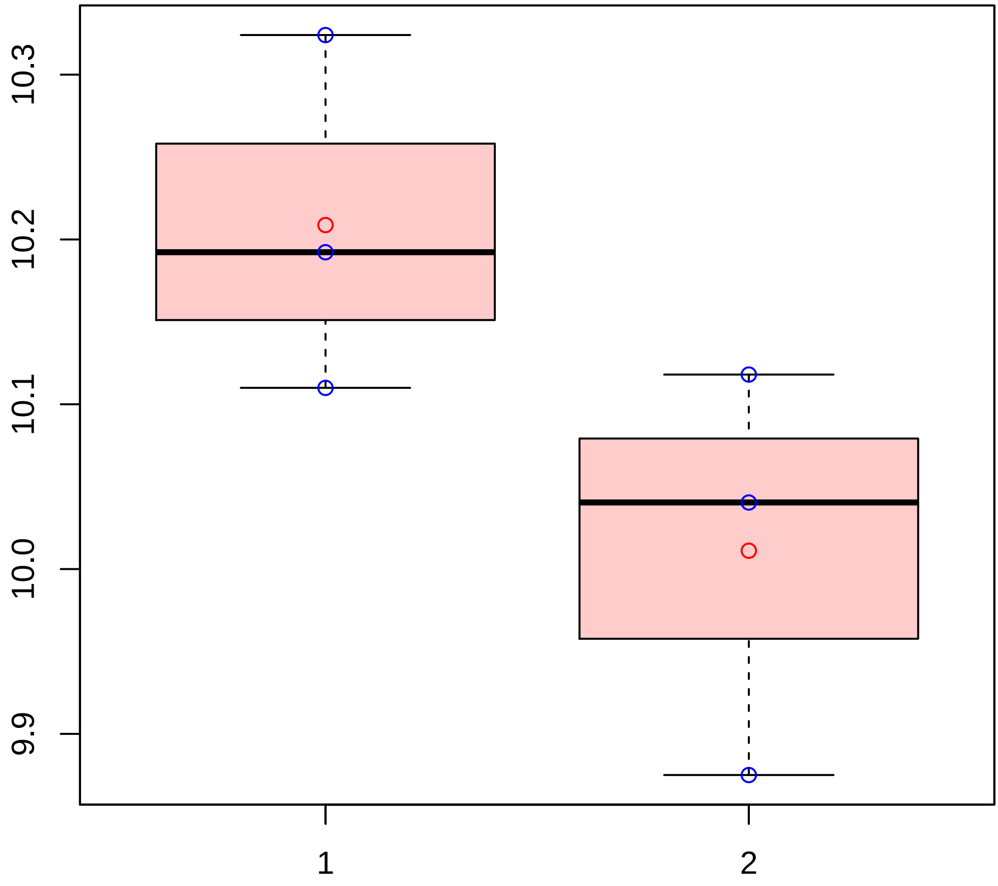

t−Test: p−value = 0.11

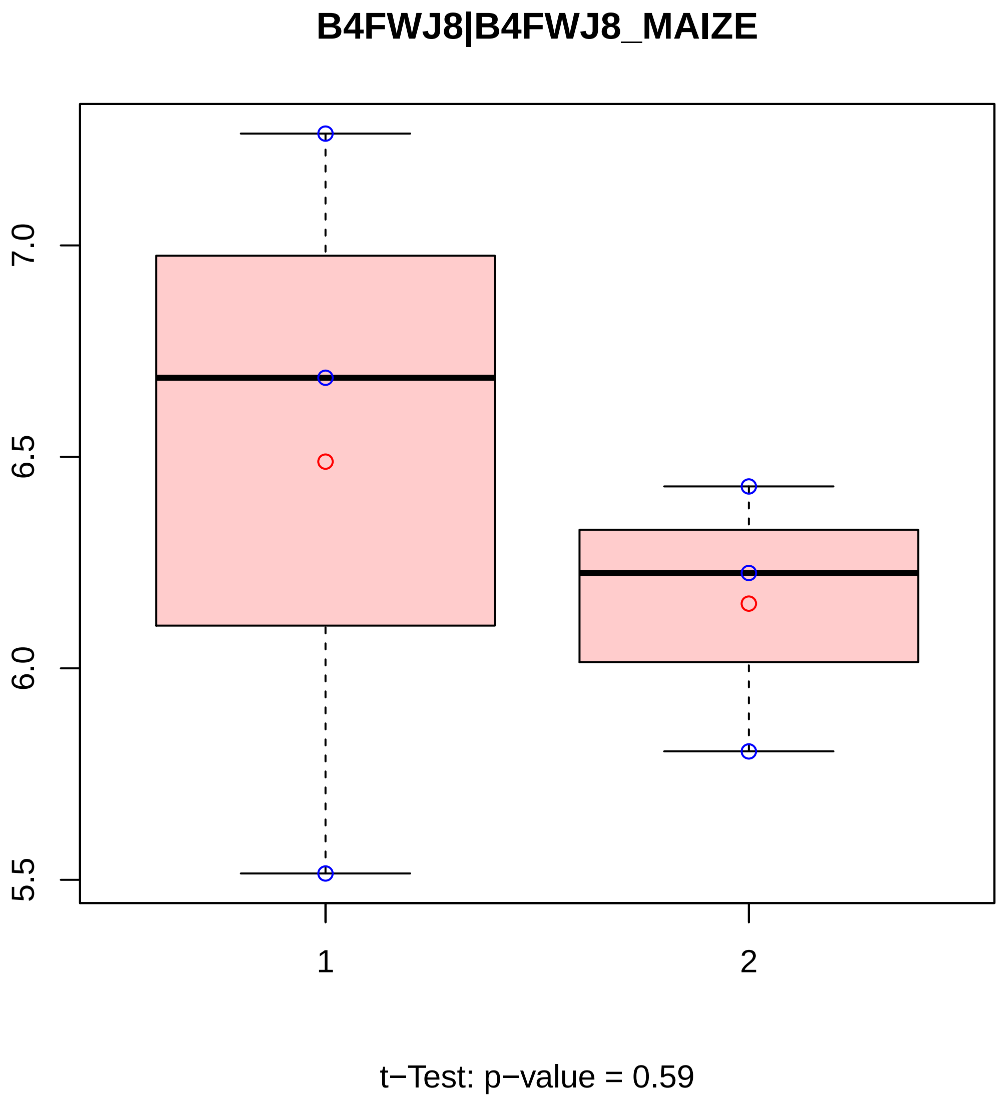

B4FWJ8|B4FWJ8_MAIZE
7.0
6.5
6.0
5.5
1
2
t−Test: p−value = 0.59

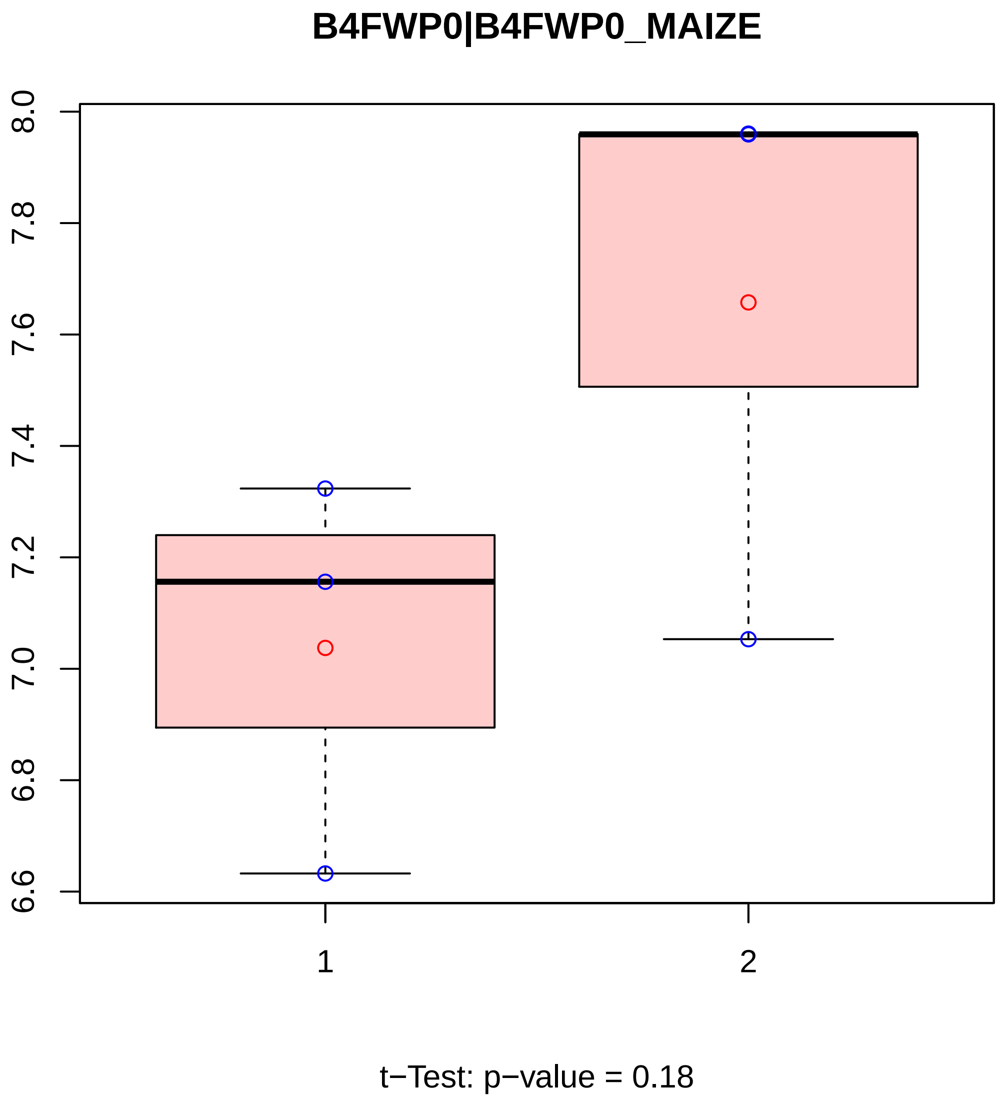

B4FWP0|B4FWP0_MAIZE
8.0
7.8
7.6
7.4
7.2
7.0
6.8
6.6
1
2
t−Test: p−value = 0.18

B4FXB0|B4FXB0_MAIZE

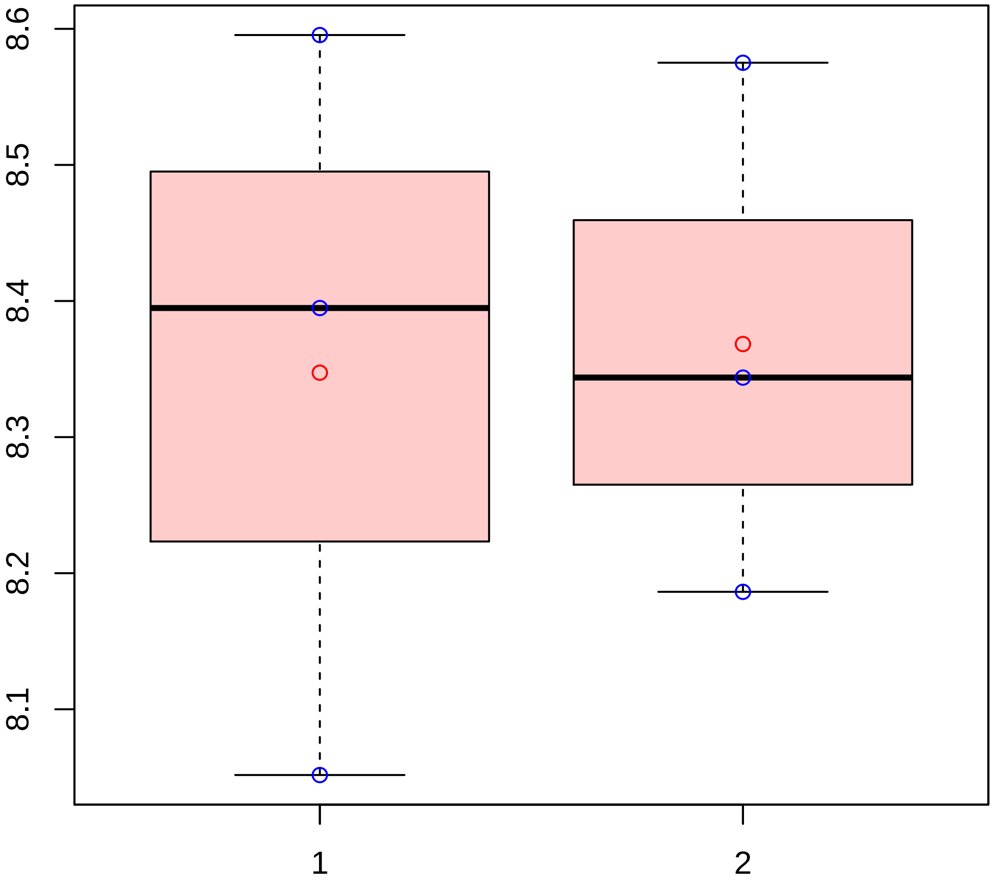

t−Test: p−value = 0.92

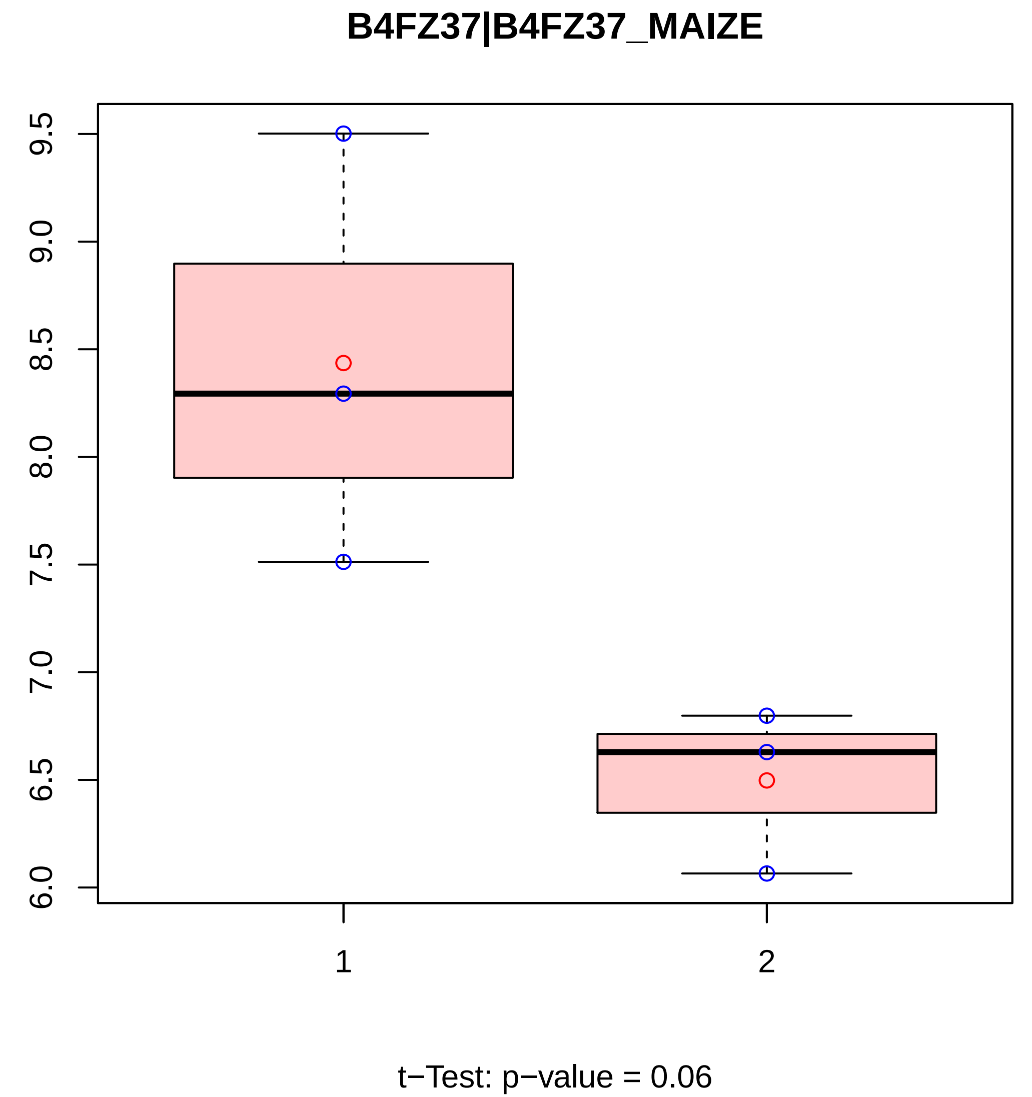
B4FZ37|B4FZ37_MAIZE
9.5
9.0
8.5
8.0
7.5
7.0
6.5
6.0
1
2
t−Test: p−value = 0.06

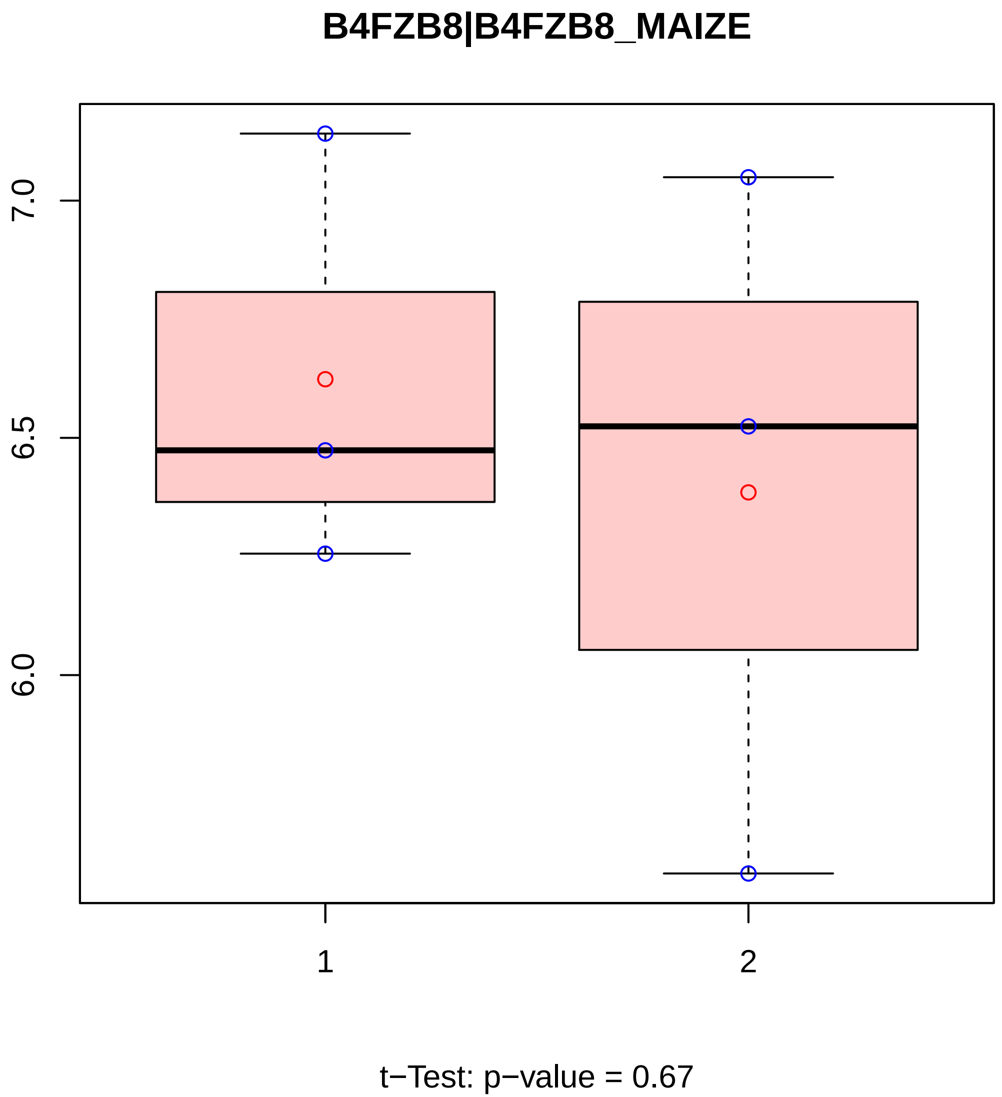

B4FZB8|B4FZB8_MAIZE
7.0
6.5
6.0
1
2
t−Test: p−value = 0.67

B4FZL4|B4FZL4_MAIZE

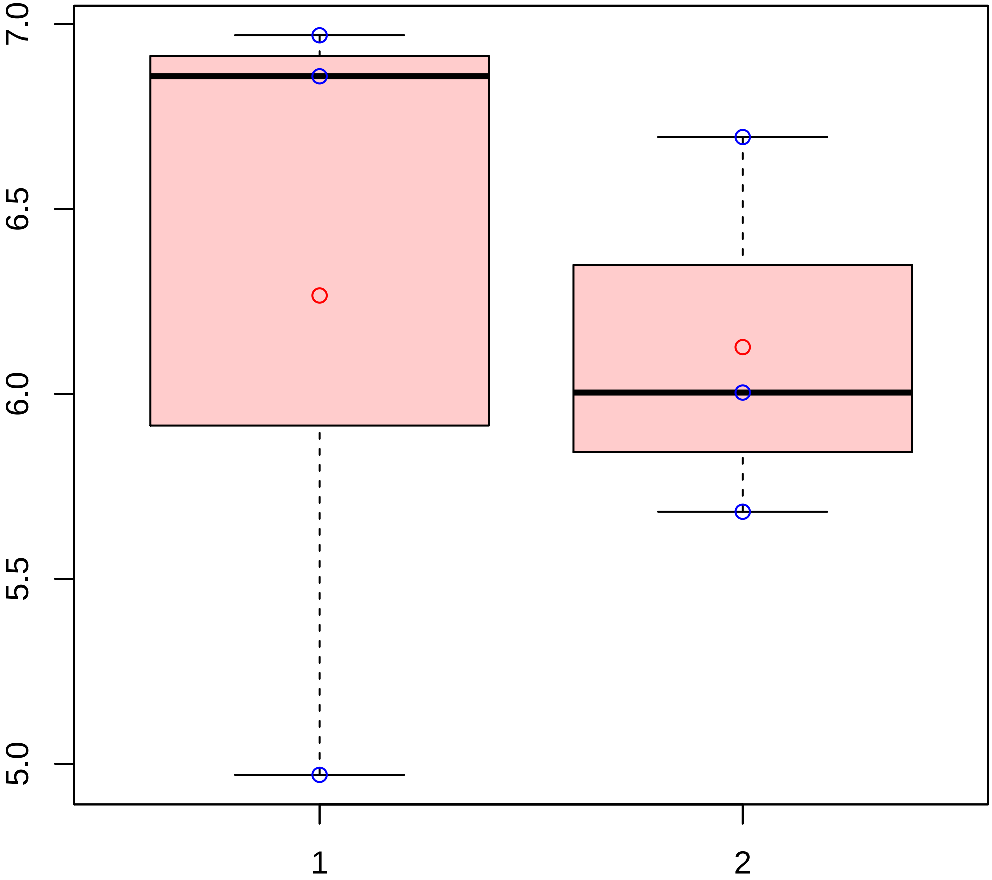

t−Test: p−value = 0.86

B4FZP0|B4FZP0_MAIZE

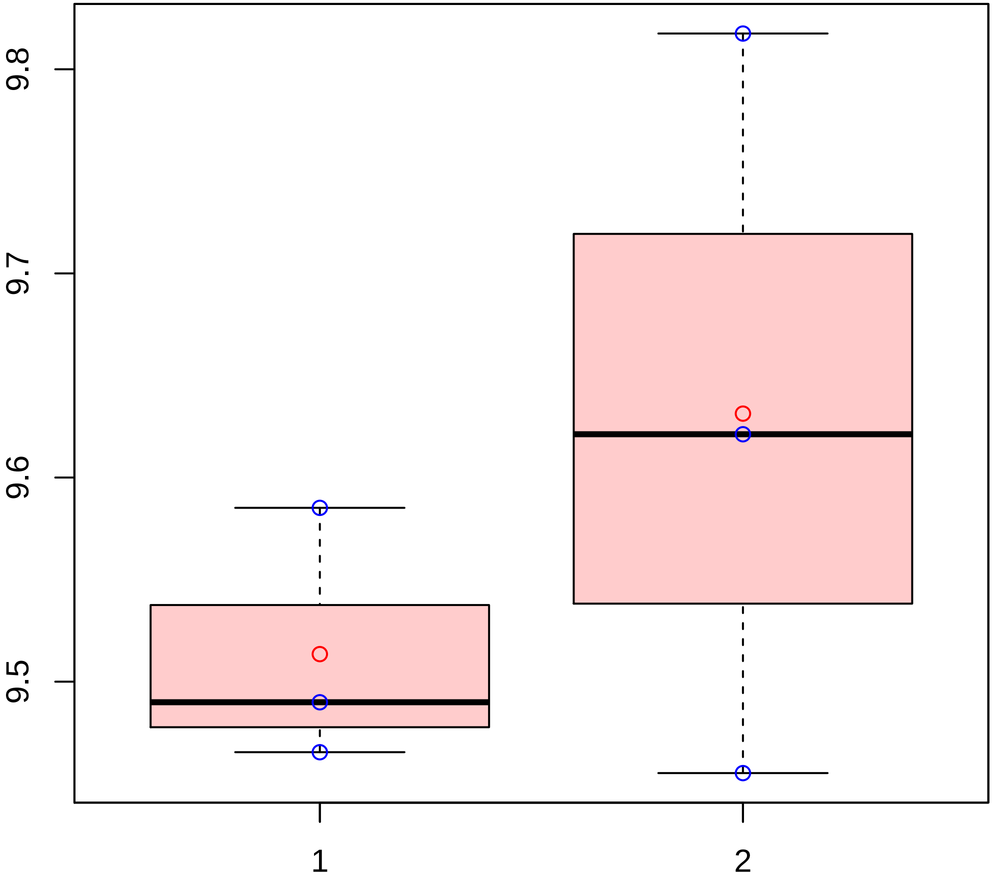

t−Test: p−value = 0.38

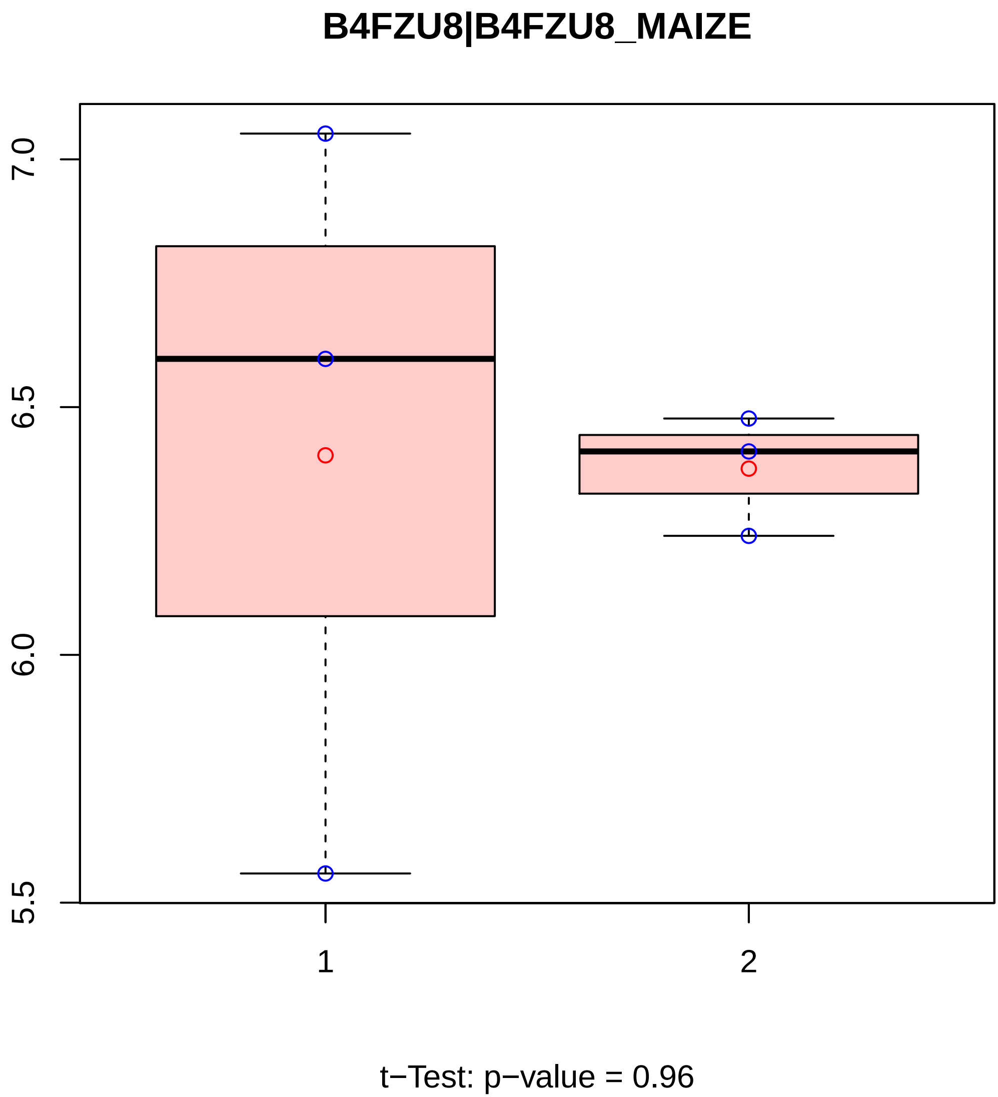
B4FZU8|B4FZU8_MAIZE
7.0
6.5
6.0
5.5
1
2
t−Test: p−value = 0.96

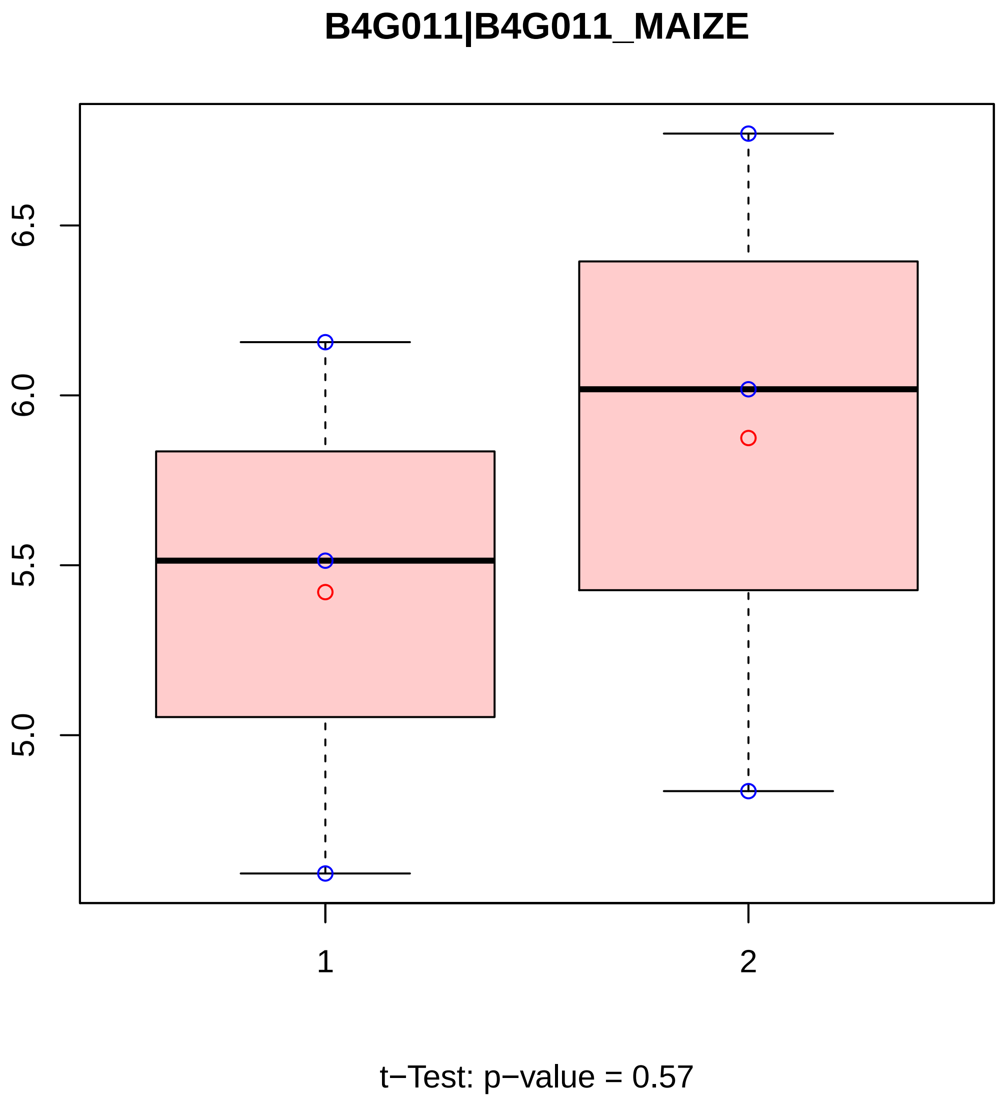
B4G011|B4G011_MAIZE
6.5
6.0
5.5
5.0
1
2
t−Test: p−value = 0.57

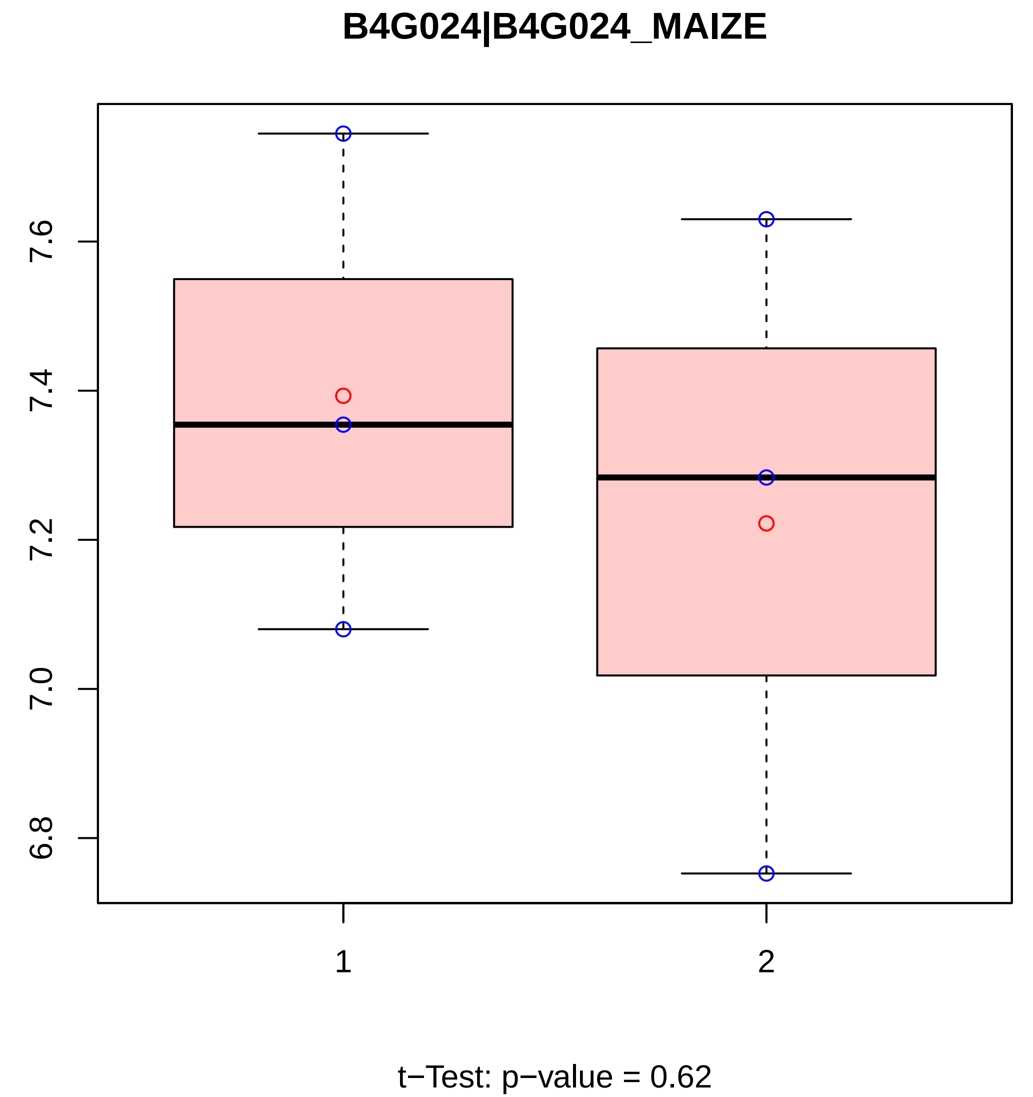
B4G024|B4G024_MAIZE
7.6
7.4
7.2
7.0
6.8
1
2
t−Test: p−value = 0.62

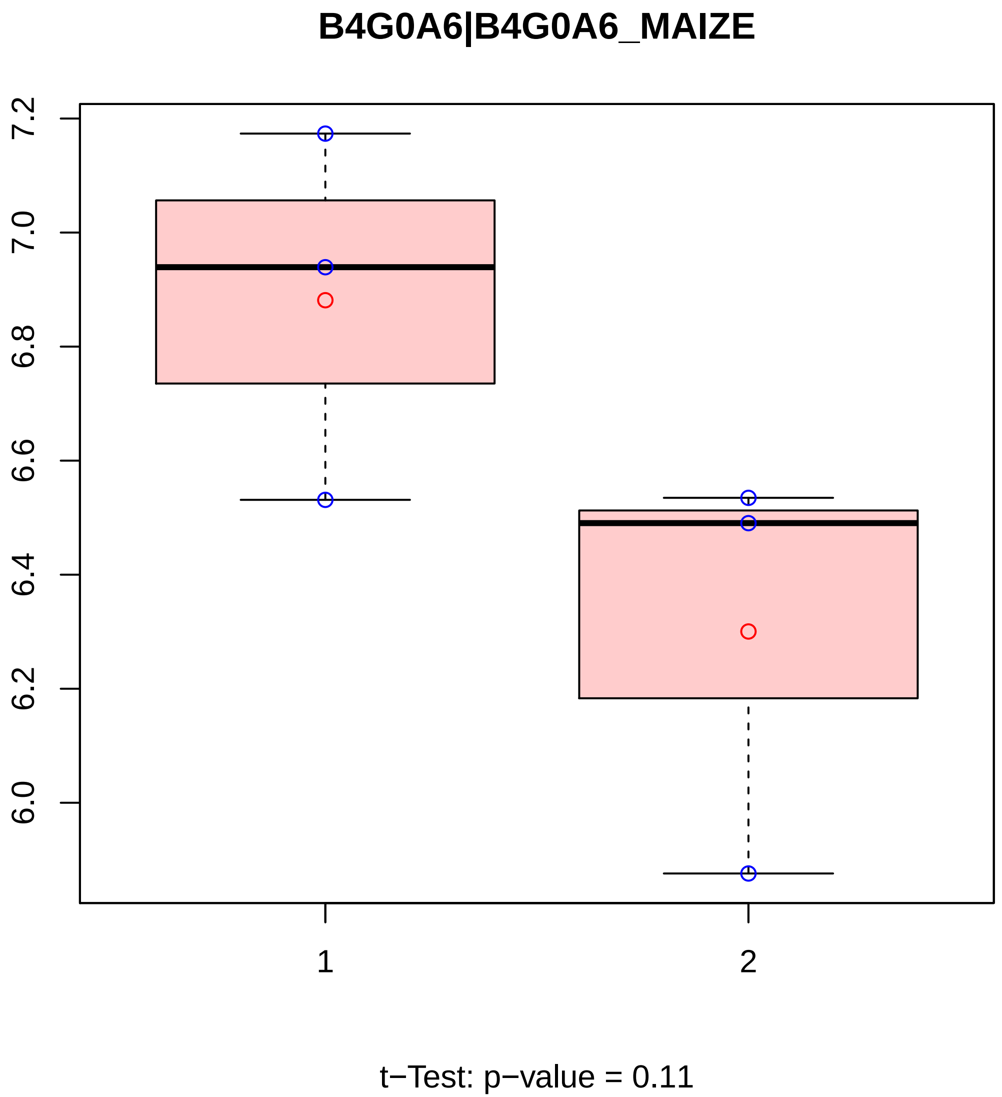
B4G0A6|B4G0A6_MAIZE
7.2
7.0
6.8
6.6
6.4
6.2
6.0
1
2
t−Test: p−value = 0.11

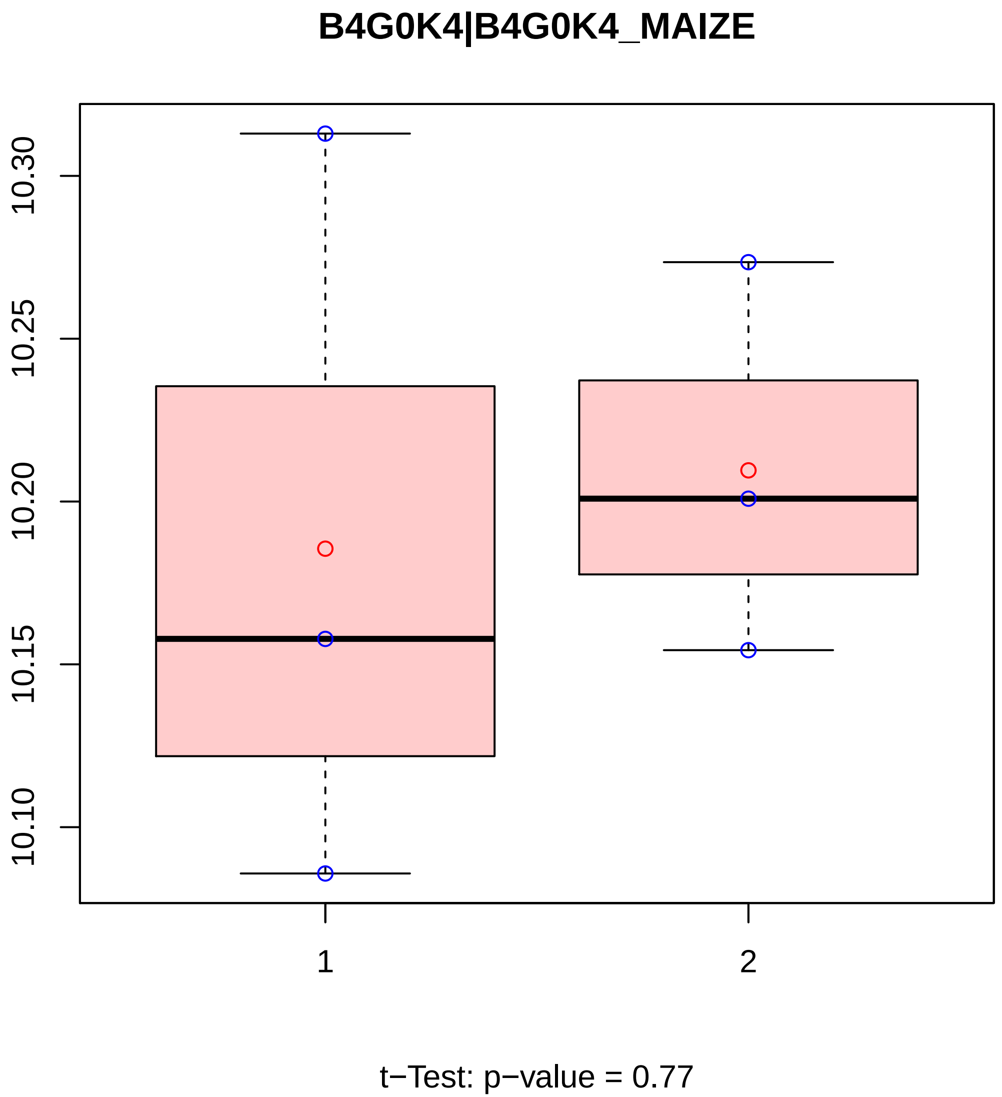
B4G0K4|B4G0K4_MAIZE
10.10
10.15
10.20
10.25
10.30
1
2
t−Test: p−value = 0.77

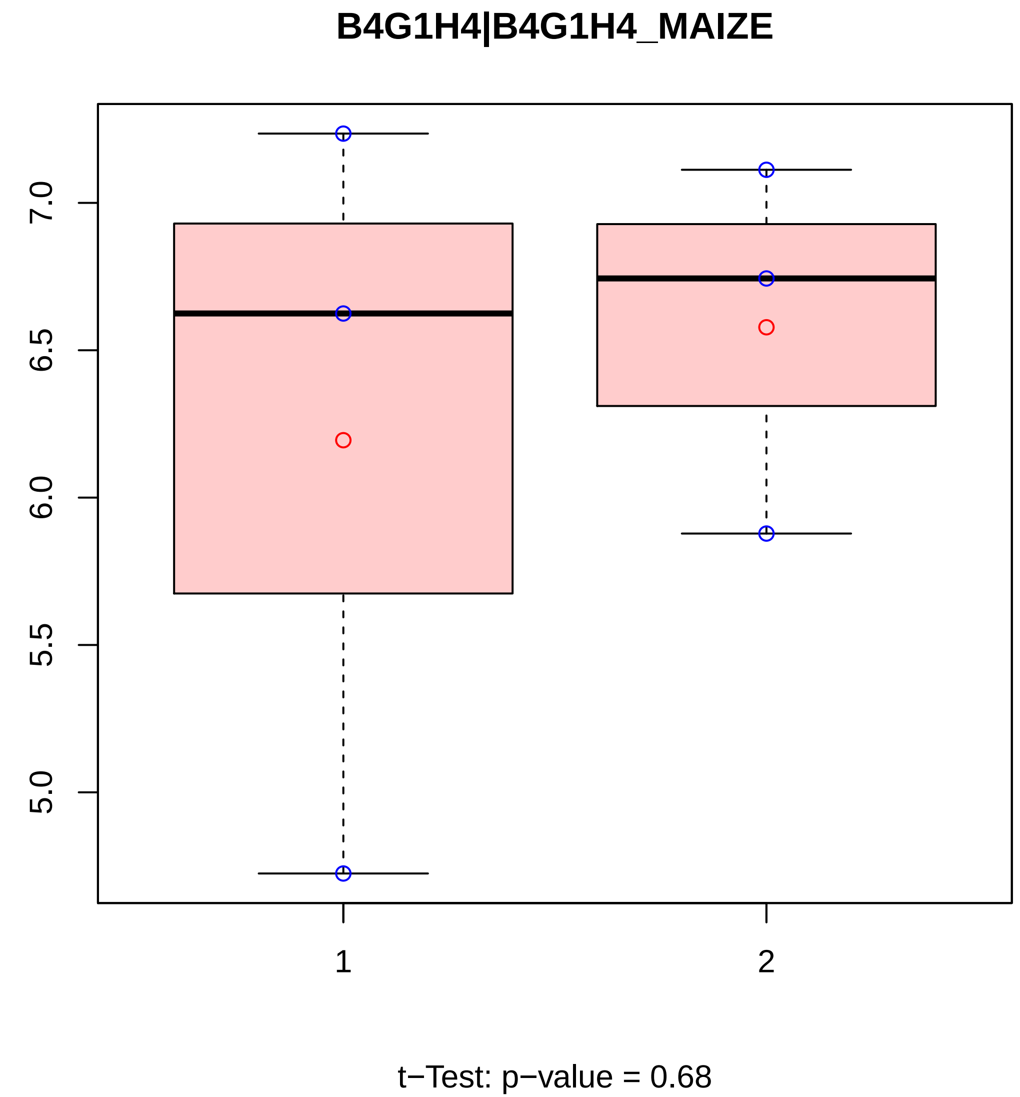
B4G1H4|B4G1H4_MAIZE
7.0
6.5
6.0
5.5
5.0
1
2
t−Test: p−value = 0.68

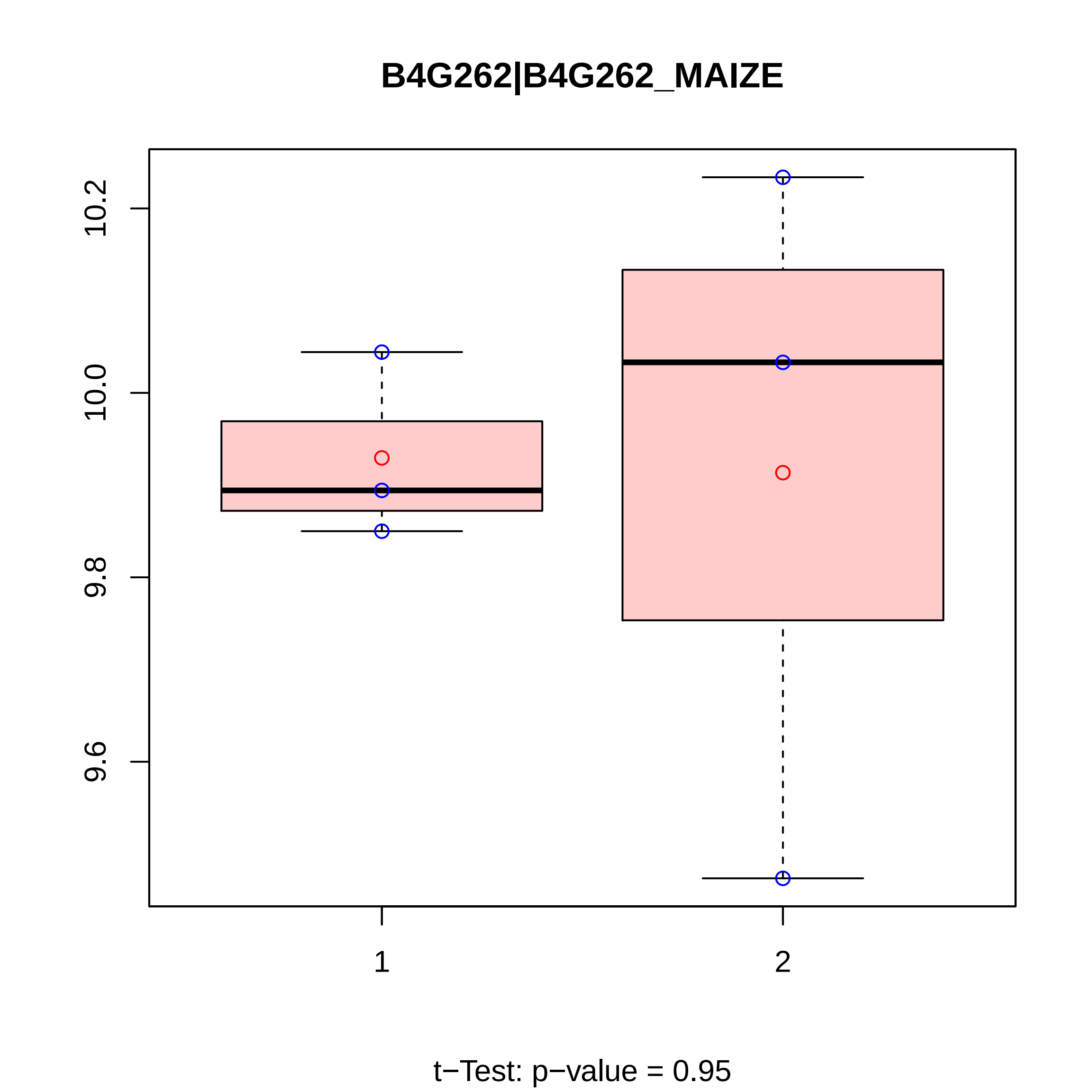

B4G262|B4G262_MAIZE
10.2
10.0
9.8
9.6
1
2
t−Test: p−value = 0.95

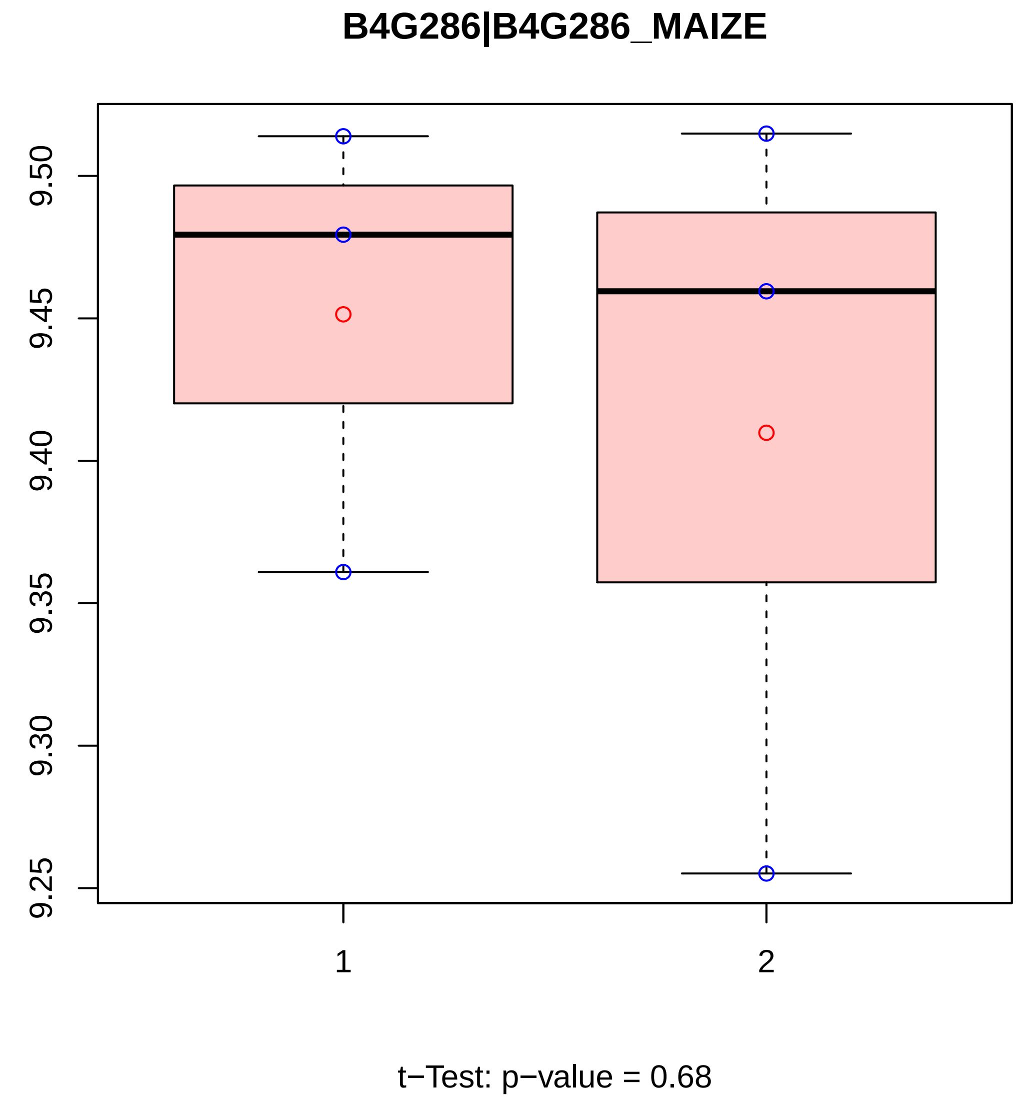
B4G286|B4G286_MAIZE
9.50
9.45
9.40
9.35
9.30
9.25
1
2
t−Test: p−value = 0.68

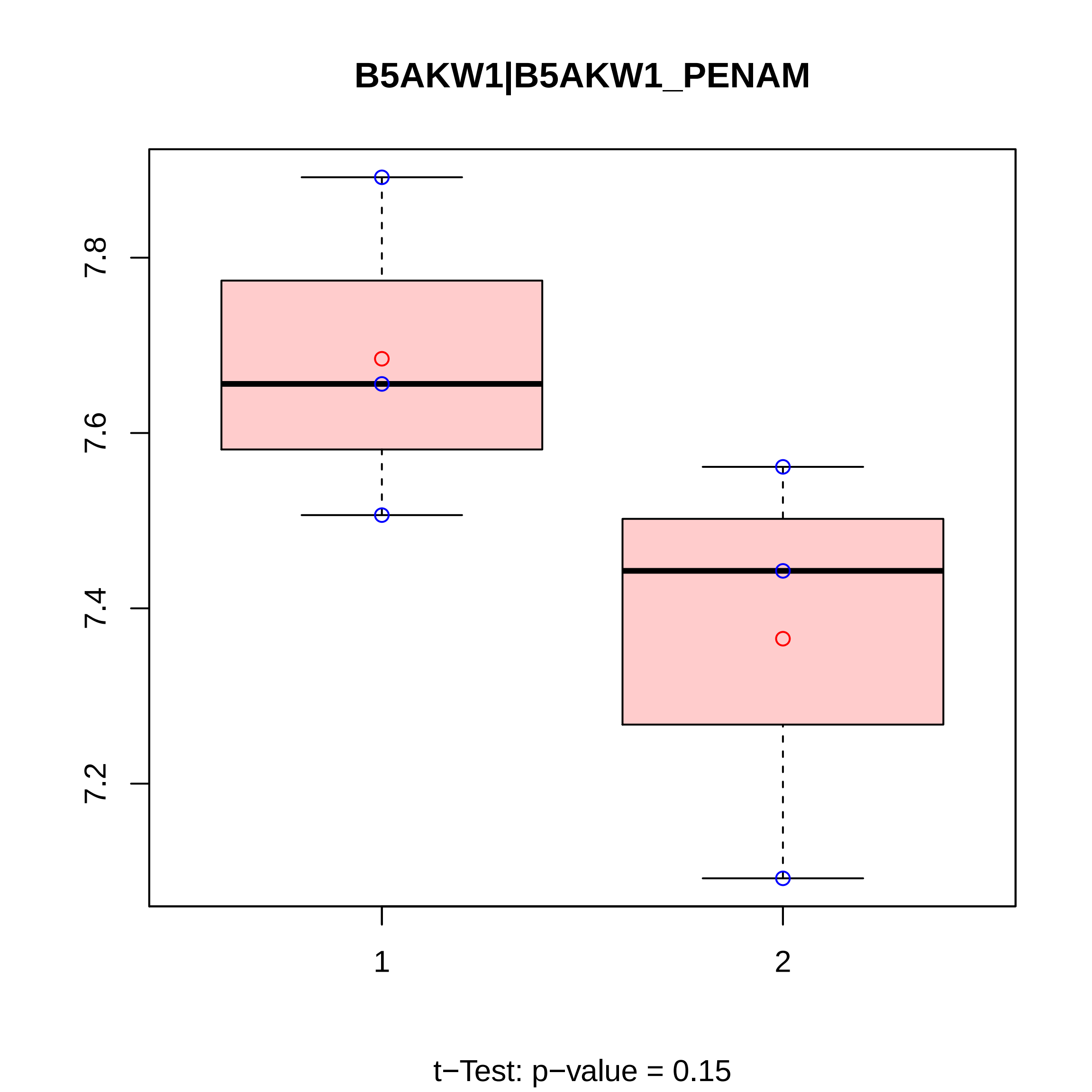

B5AKW1|B5AKW1_PENAM
7.2
7.4
7.6
7.8
1
2
t−Test: p−value = 0.15

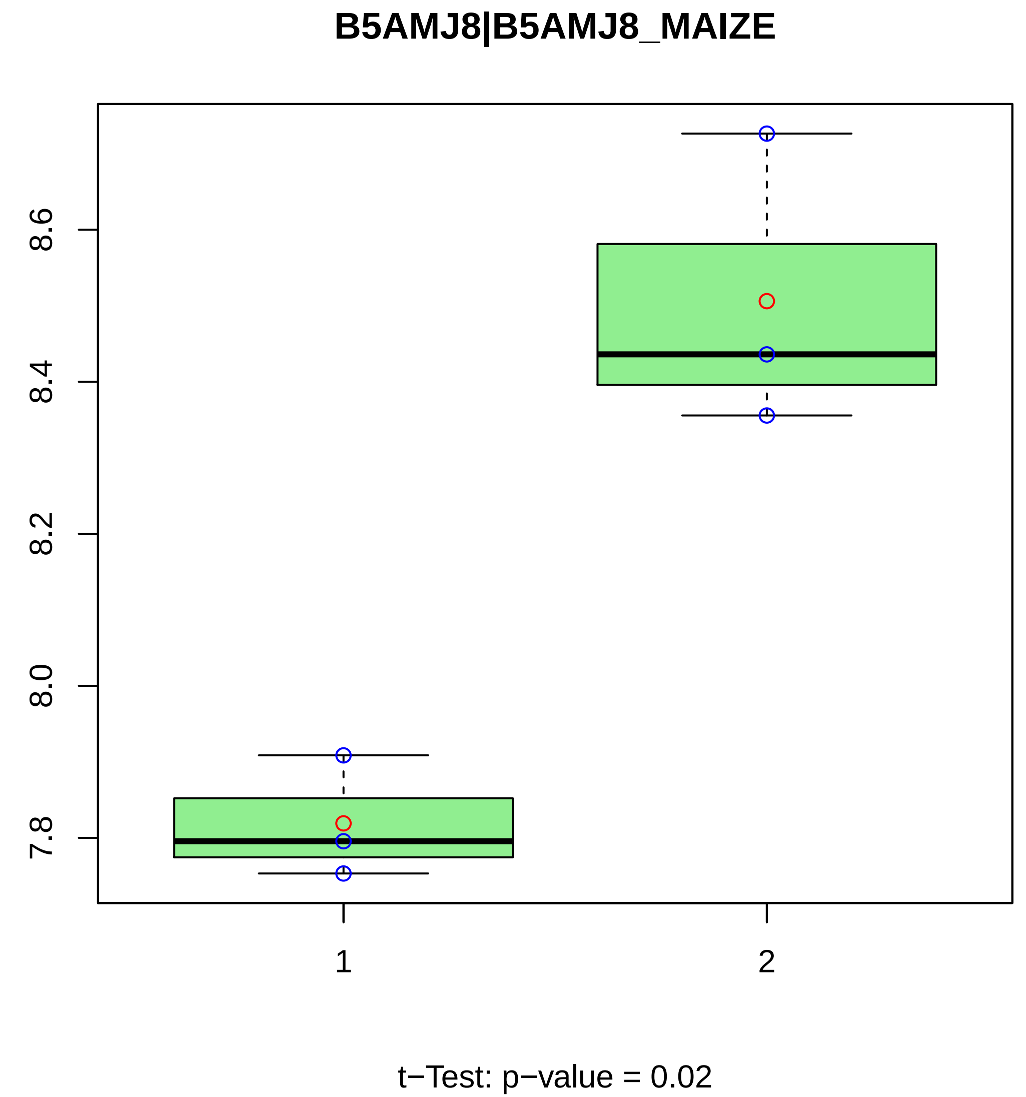
B5AMJ8|B5AMJ8_MAIZE
8.6
8.4
8.2
8.0
7.8
1
2
t−Test: p−value = 0.02

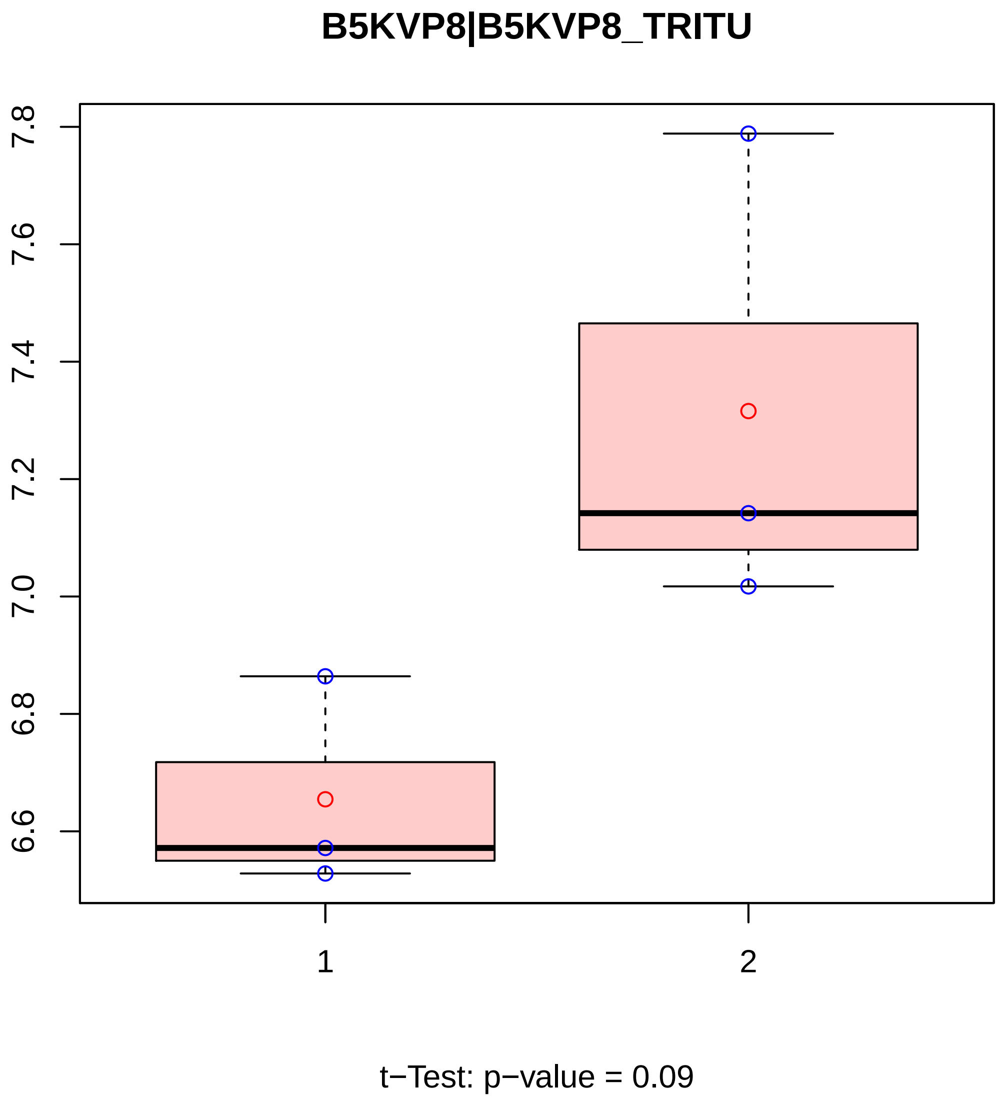
B5KVP8|B5KVP8_TRITU
7.8
7.6
7.4
7.2
7.0
6.8
6.6
1
2
t−Test: p−value = 0.09

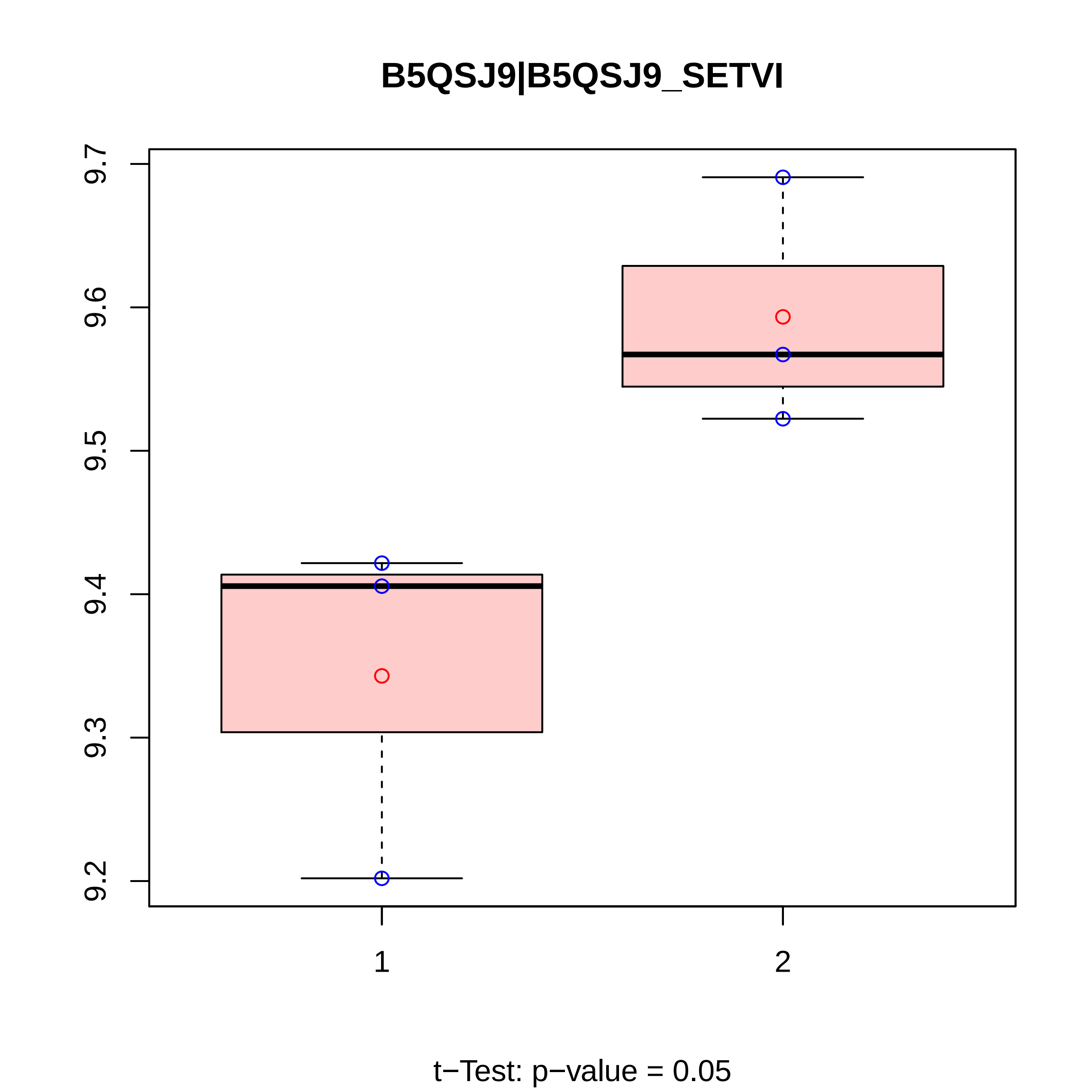
B5QSJ9|B5QSJ9_SETVI
9.7
9.6
9.5
9.4
9.3
9.2
1
2
t−Test: p−value = 0.05

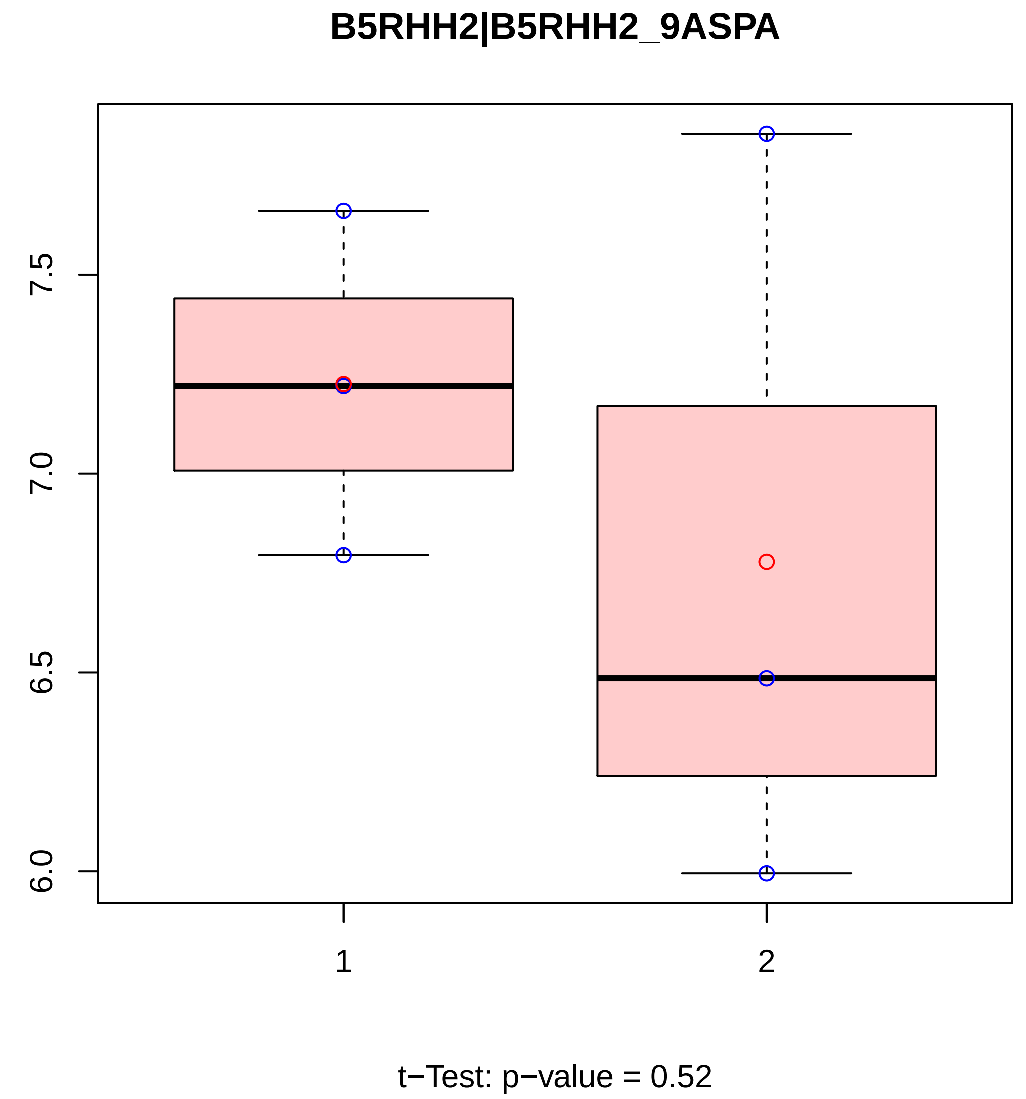
B5RHH2|B5RHH2_9ASPA
7.5
7.0
6.5
6.0
1
2
t−Test: p−value = 0.52

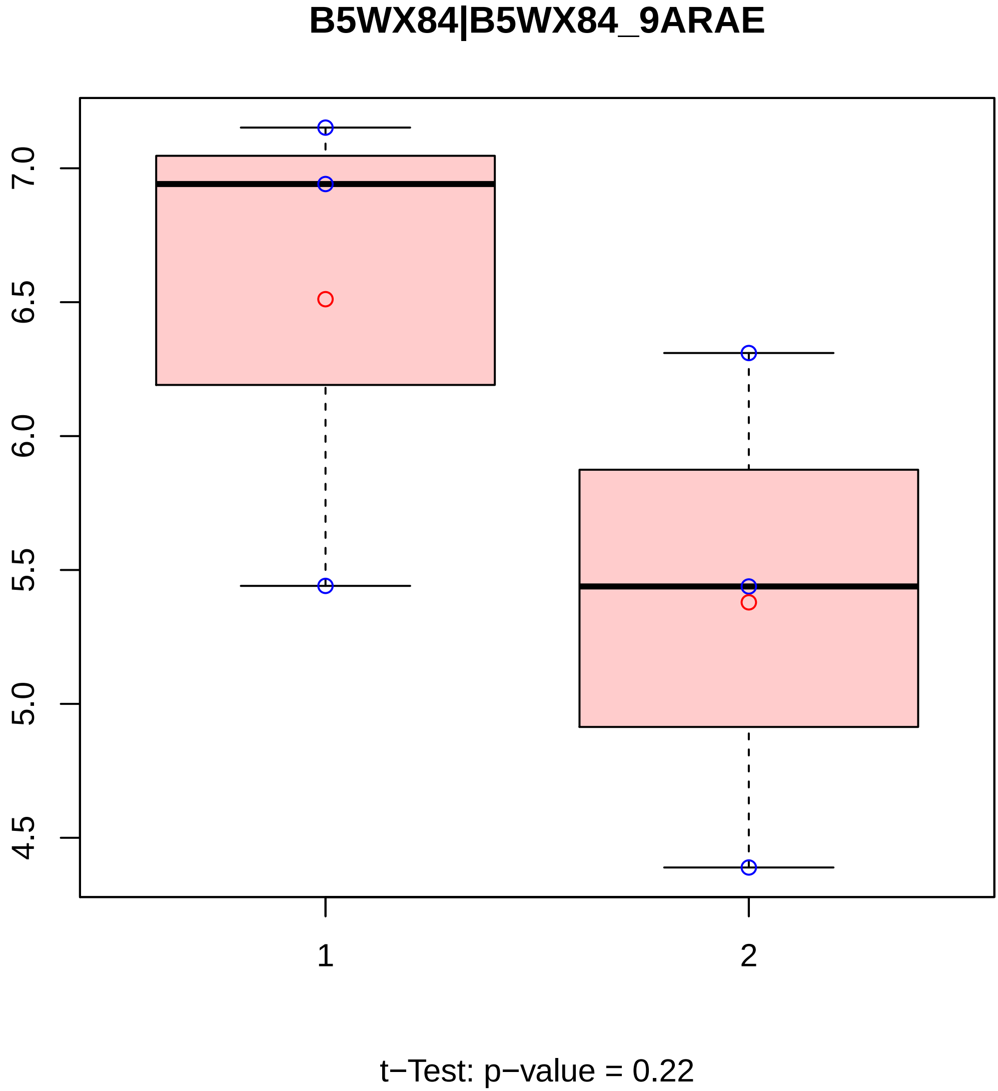
B5WX84|B5WX84_9ARAE
7.0
6.5
6.0
5.5
5.0
4.5
1
2
t−Test: p−value = 0.22

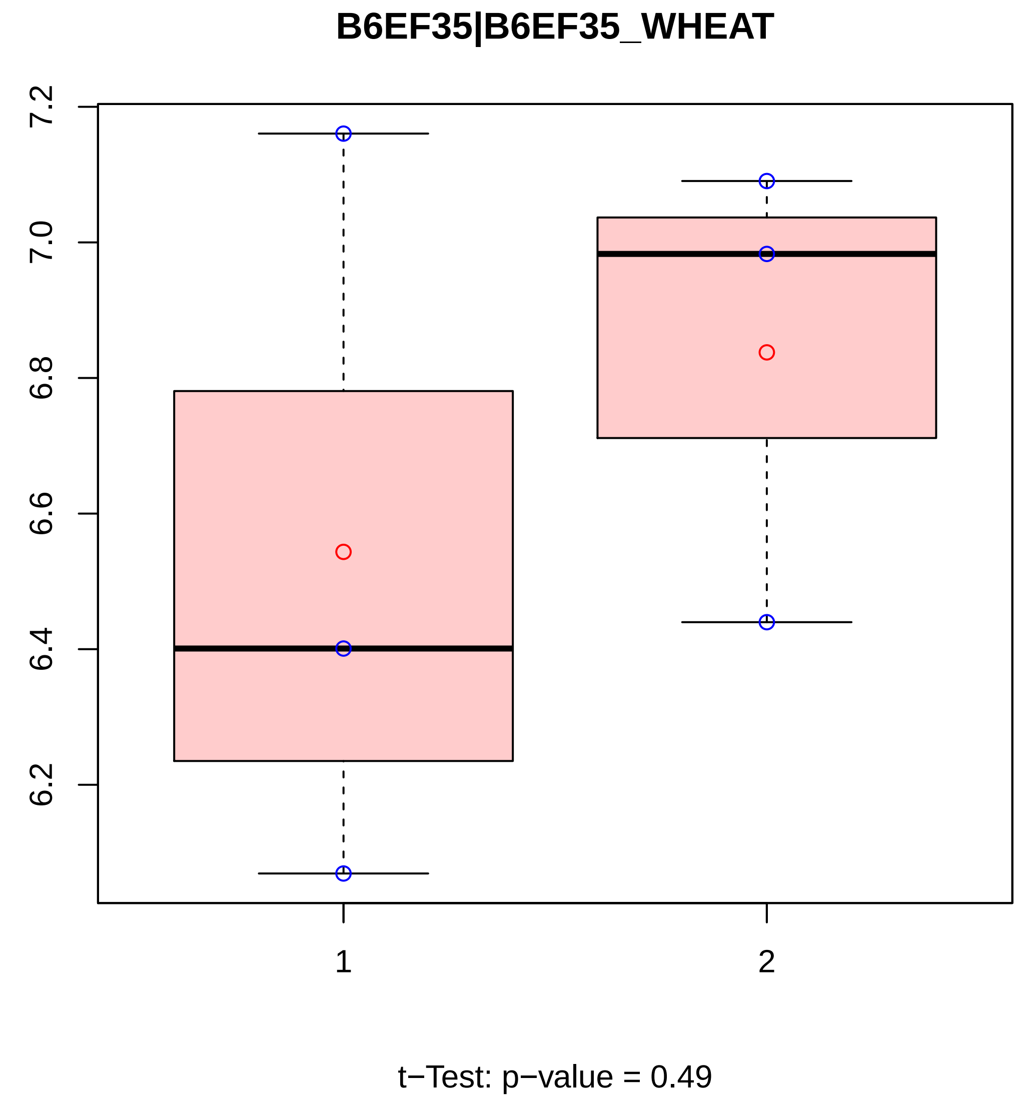
B6EF35|B6EF35_WHEAT
7.2
7.0
6.8
6.6
6.4
6.2
1
2
t−Test: p−value = 0.49

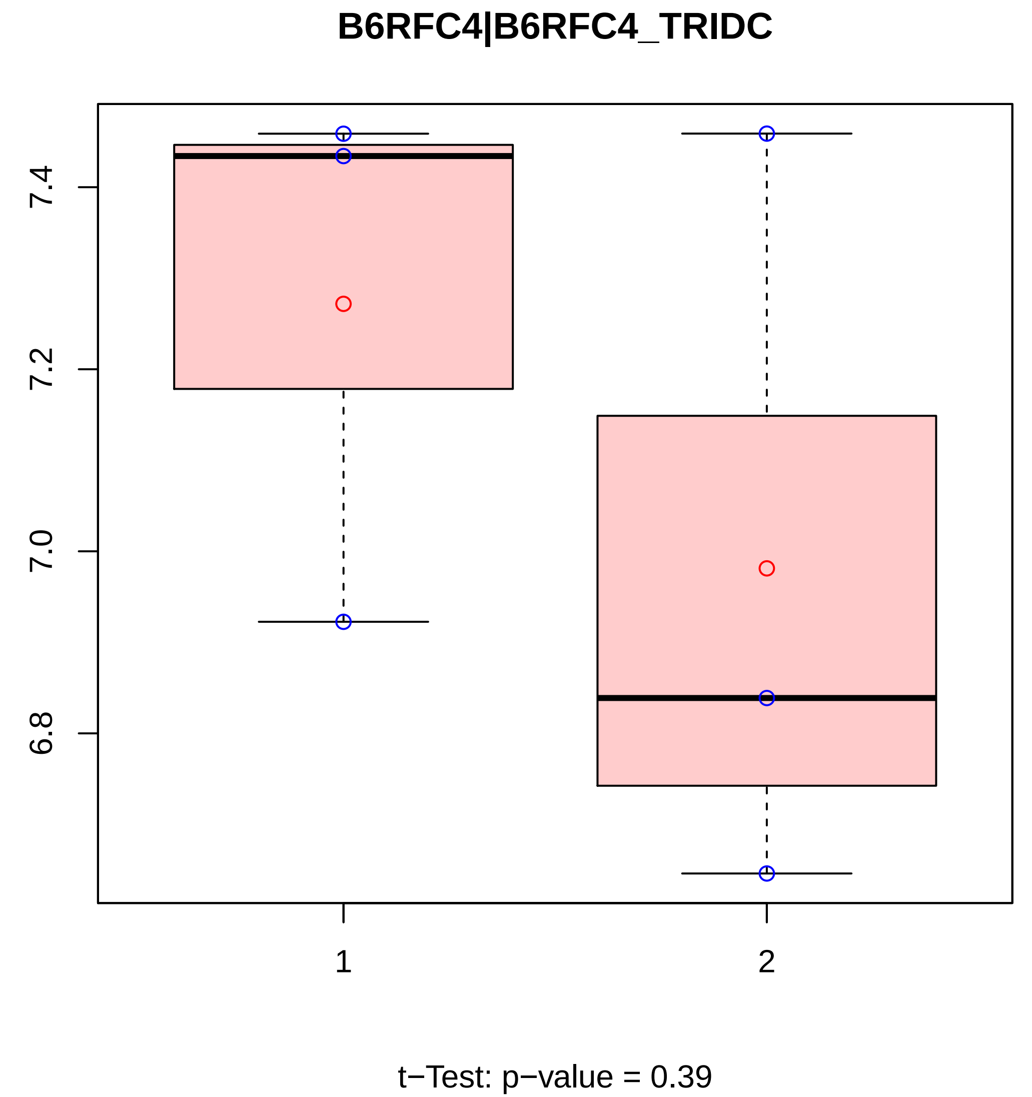

B6RFC4|B6RFC4_TRIDC
7.4
7.2
7.0
6.8
1
2
t−Test: p−value = 0.39

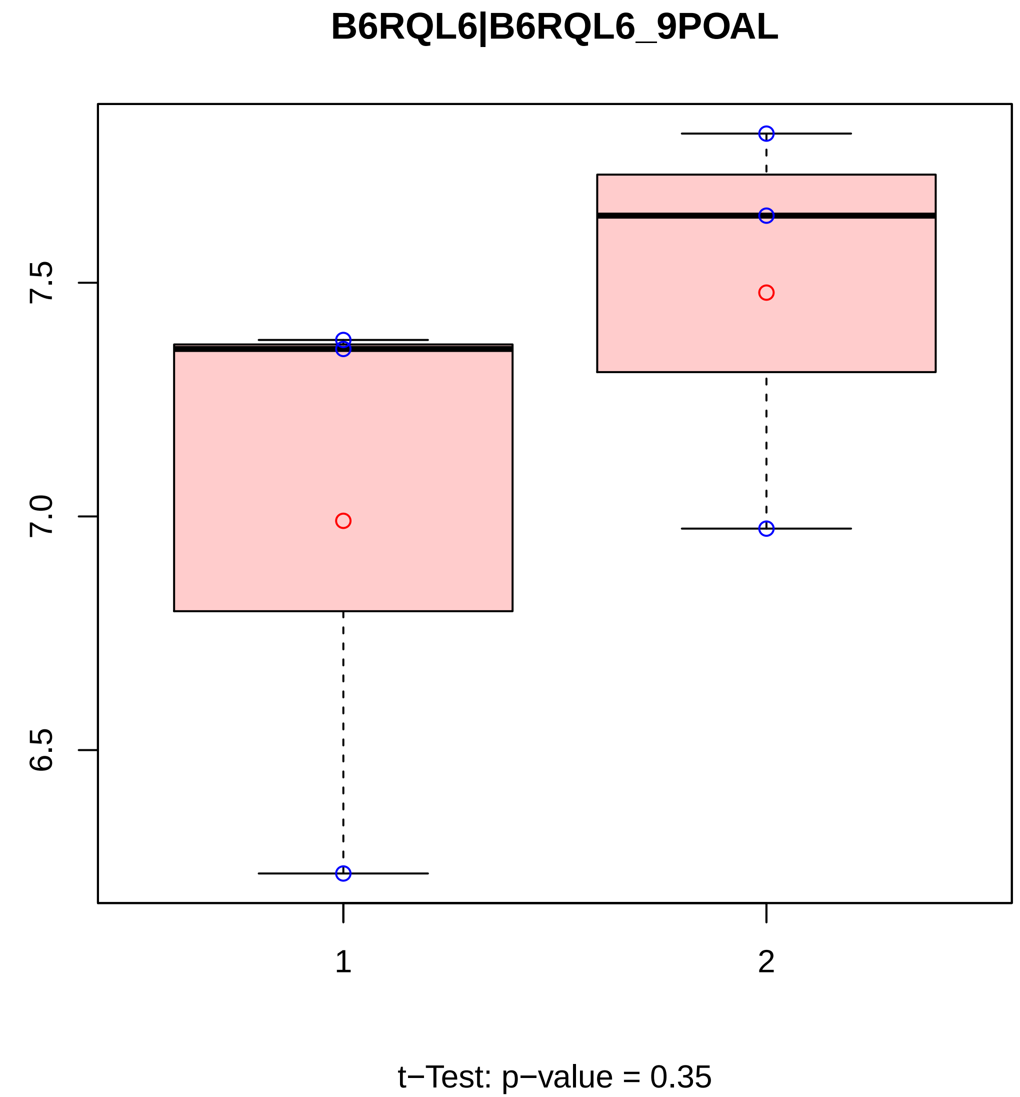

B6RQL6|B6RQL6_9POAL
7.5
7.0
6.5
1
2
t−Test: p−value = 0.35

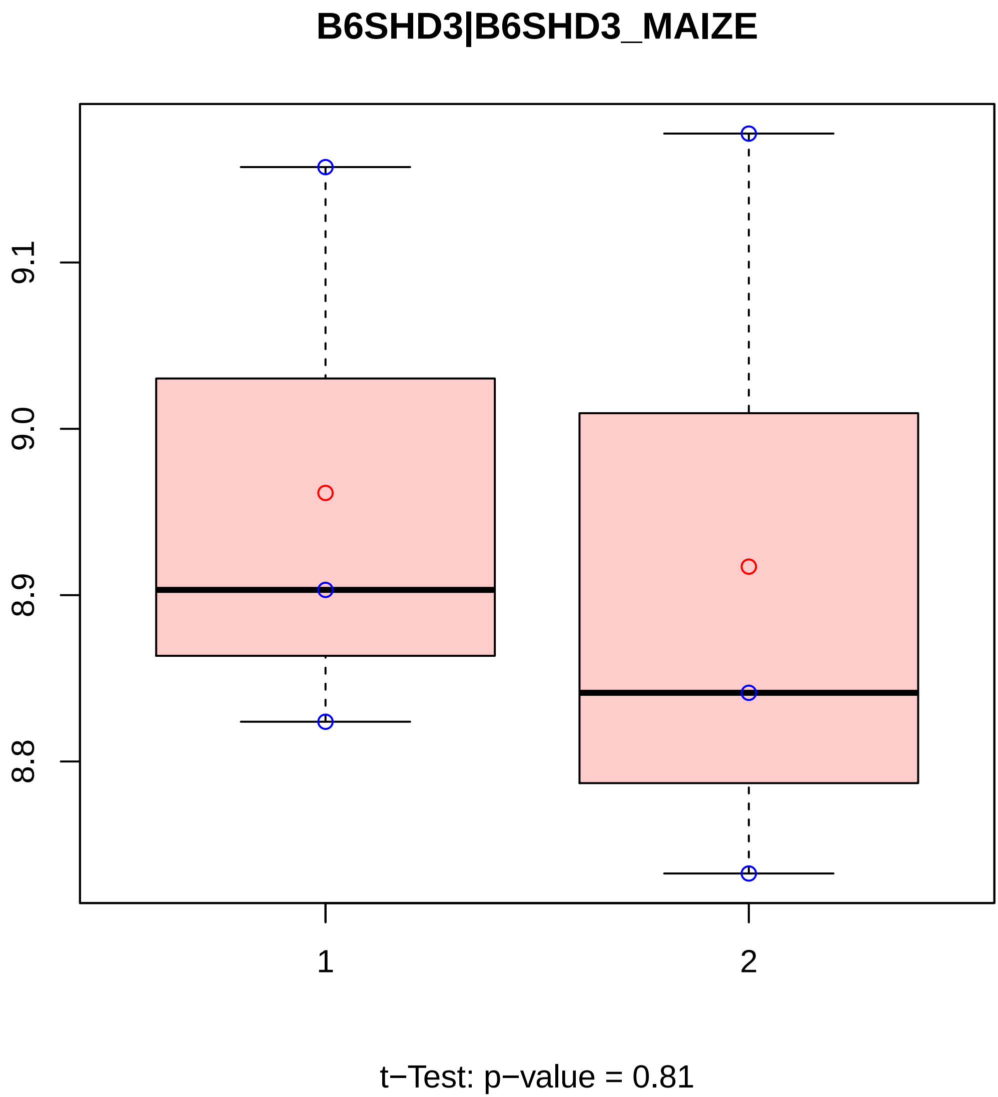

B6SHD3|B6SHD3_MAIZE
9.1
9.0
8.9
8.8
1
2
t−Test: p−value = 0.81

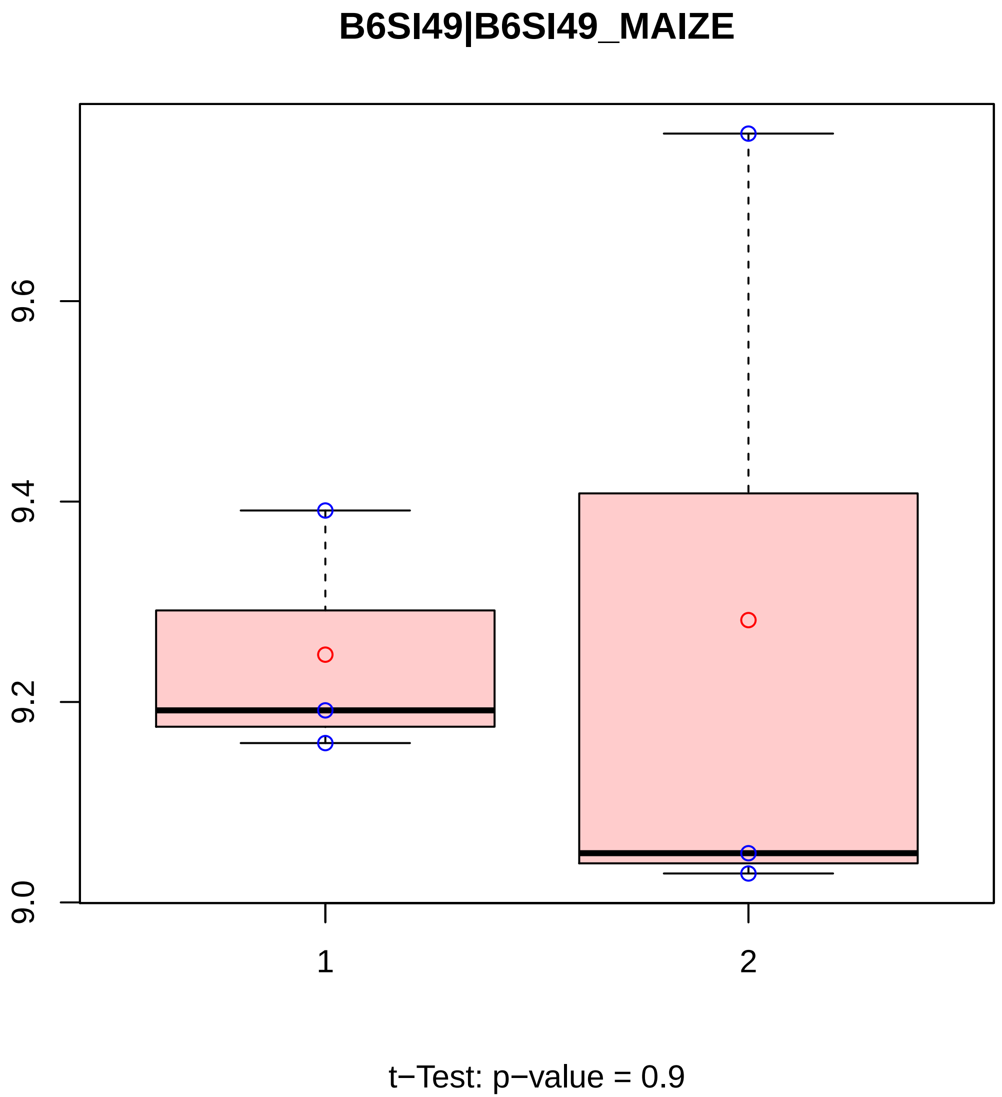
B6SI49|B6SI49_MAIZE
9.0
9.2
9.4
9.6
1
2
t−Test: p−value = 0.9

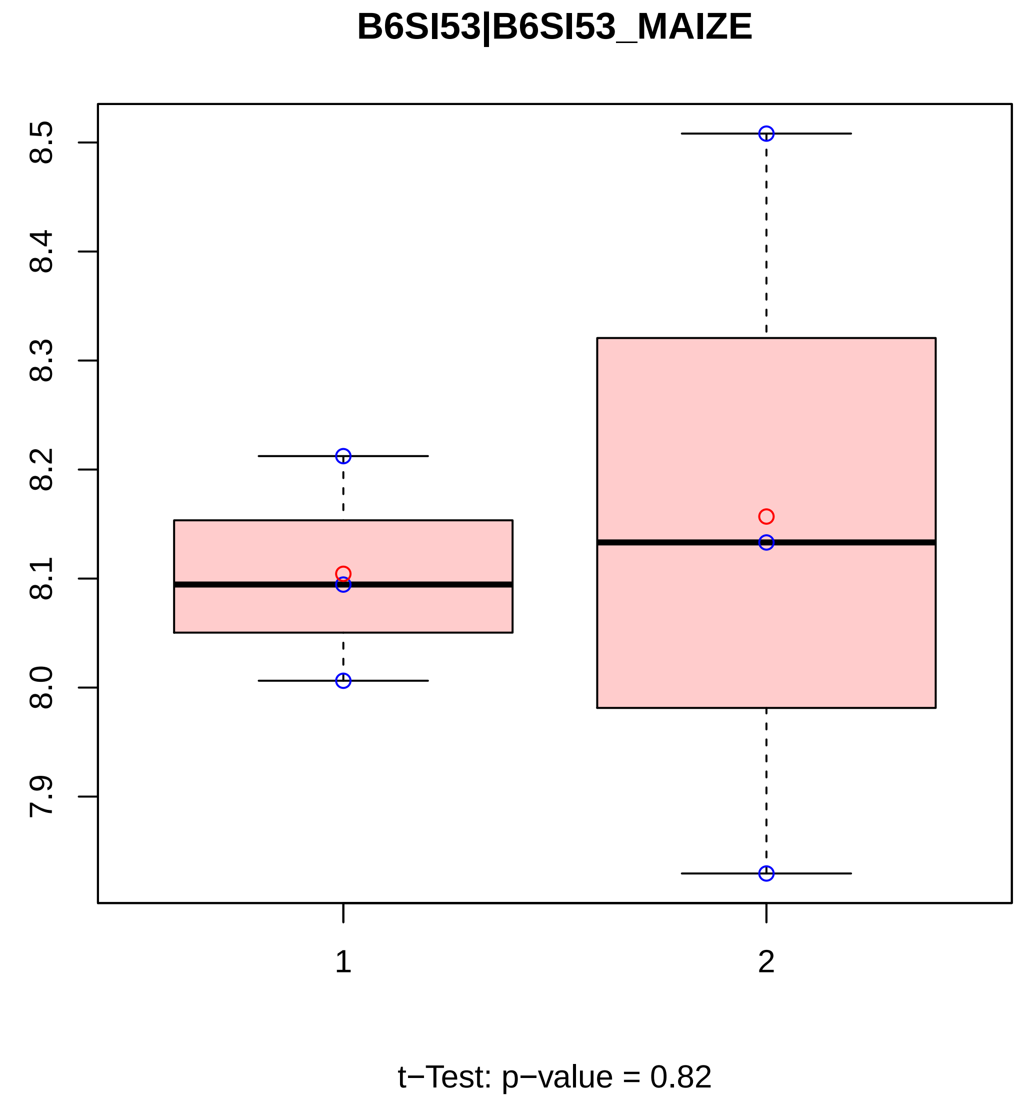
B6SI53|B6SI53_MAIZE
8.5
8.4
8.3
8.2
8.1
8.0
7.9
1
2
t−Test: p−value = 0.82

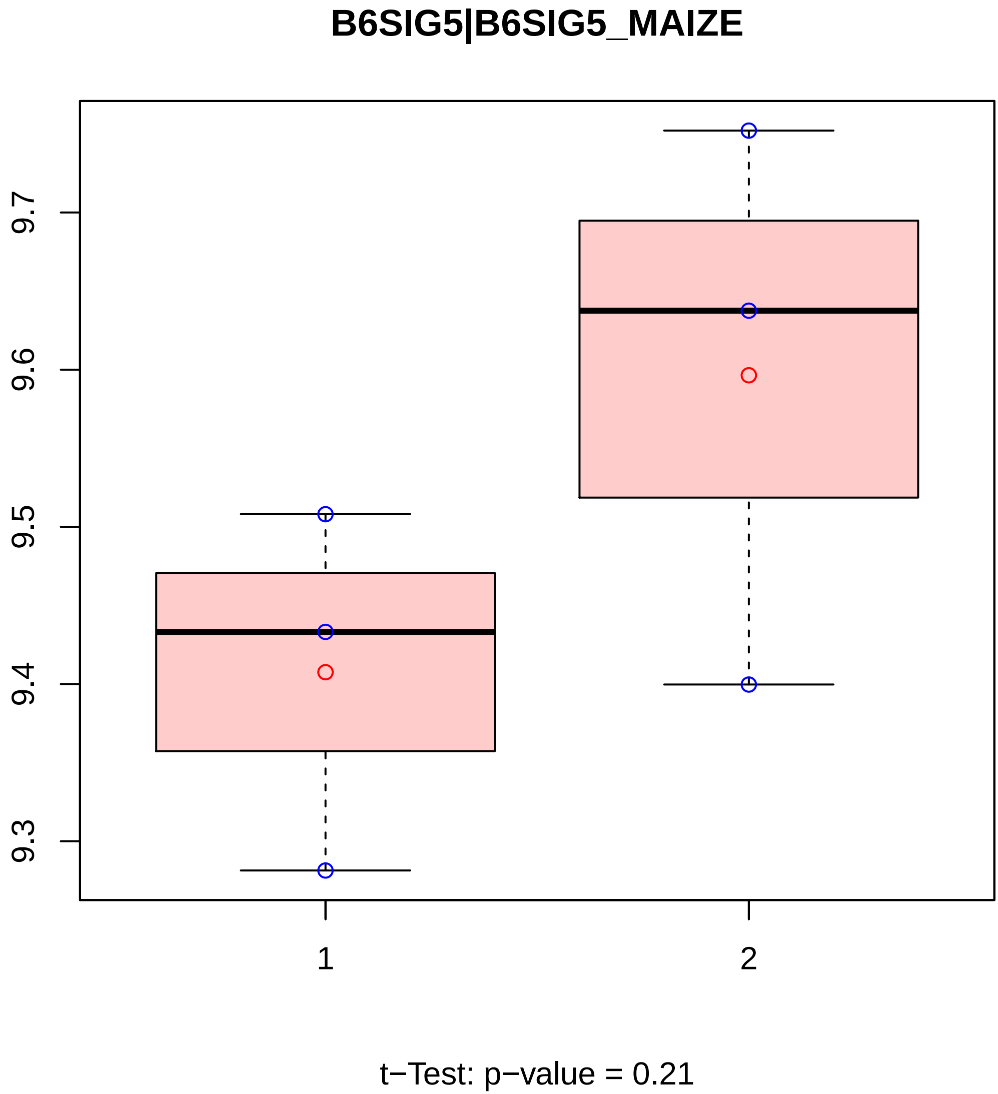
B6SIG5|B6SIG5_MAIZE
9.3
9.4
9.5
9.6
9.7
1
2
t−Test: p−value = 0.21

B6SIZ3|B6SIZ3_MAIZE

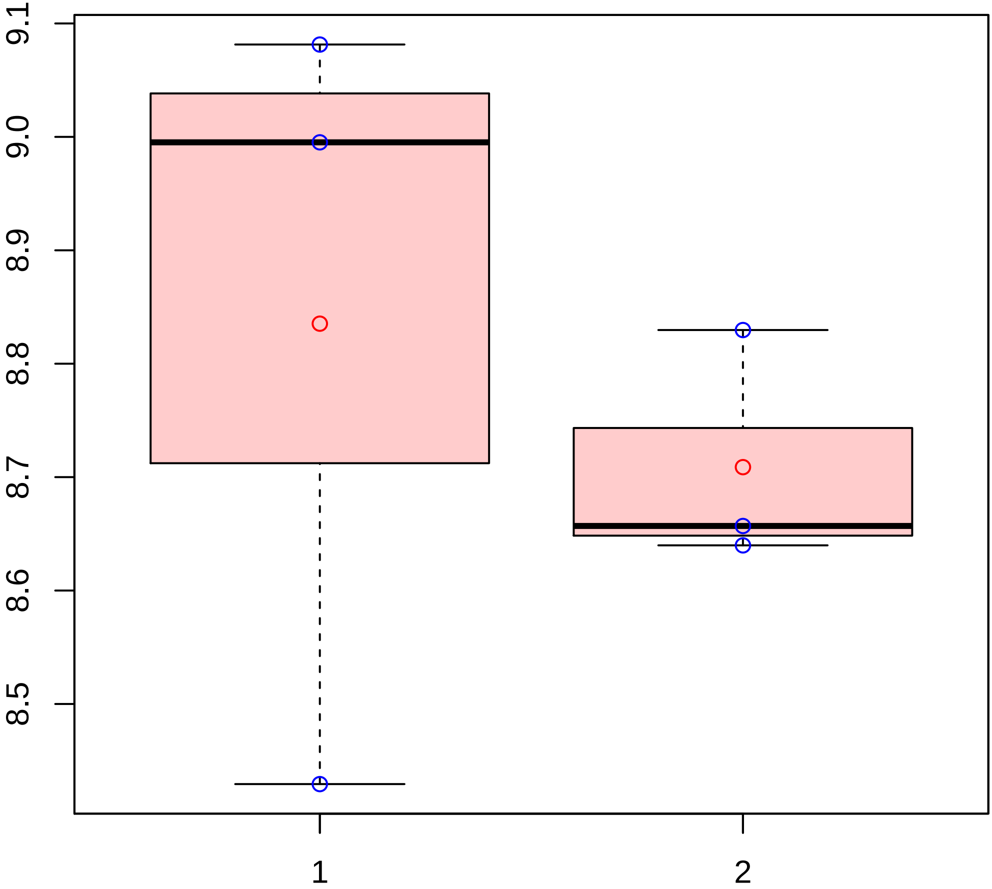

t−Test: p−value = 0.61

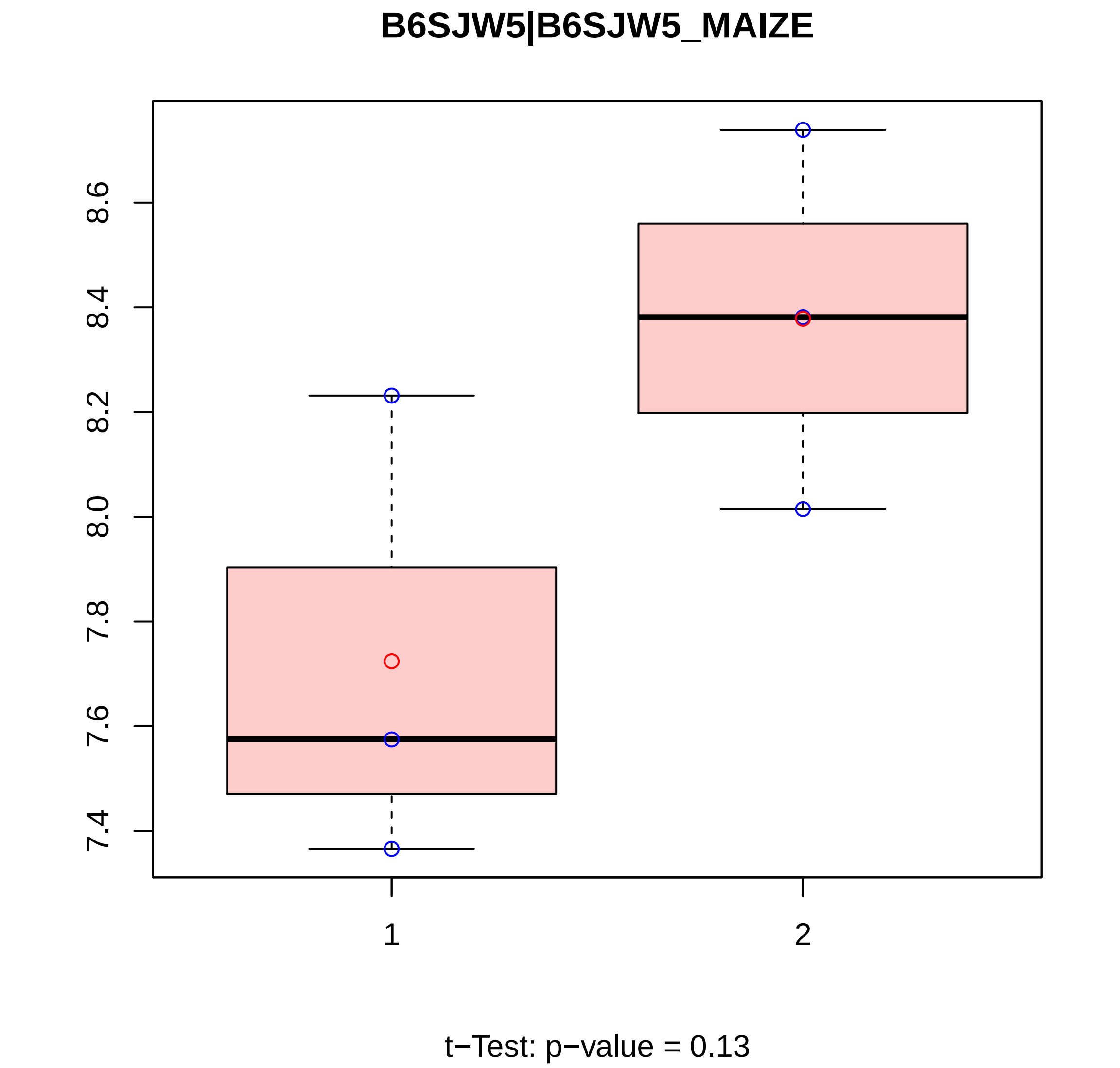

B6SJW5|B6SJW5_MAIZE
8.6
8.4
8.2
8.0
7.8
7.6
7.4
1
2
t−Test: p−value = 0.13

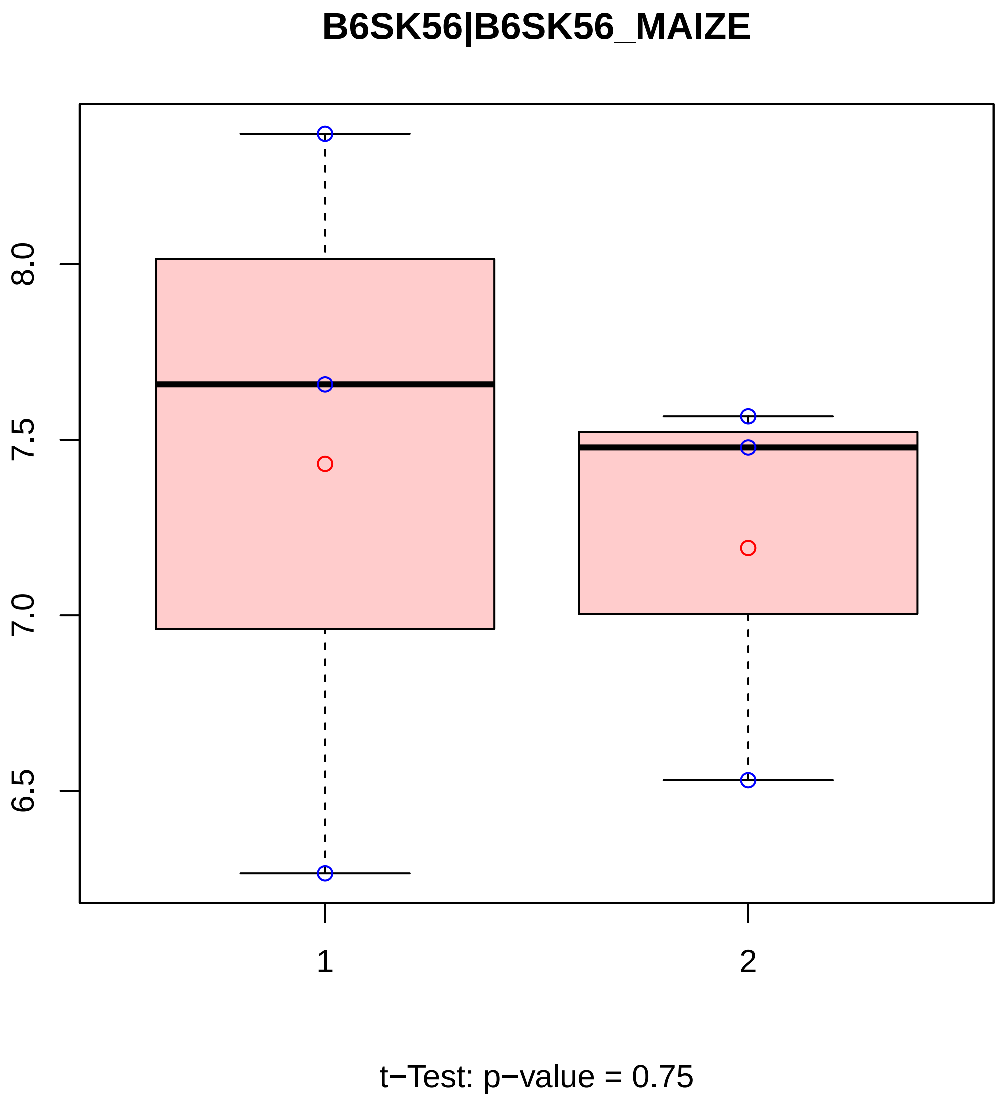
B6SK56|B6SK56_MAIZE
8.0
7.5
7.0
6.5
1
2
t−Test: p−value = 0.75

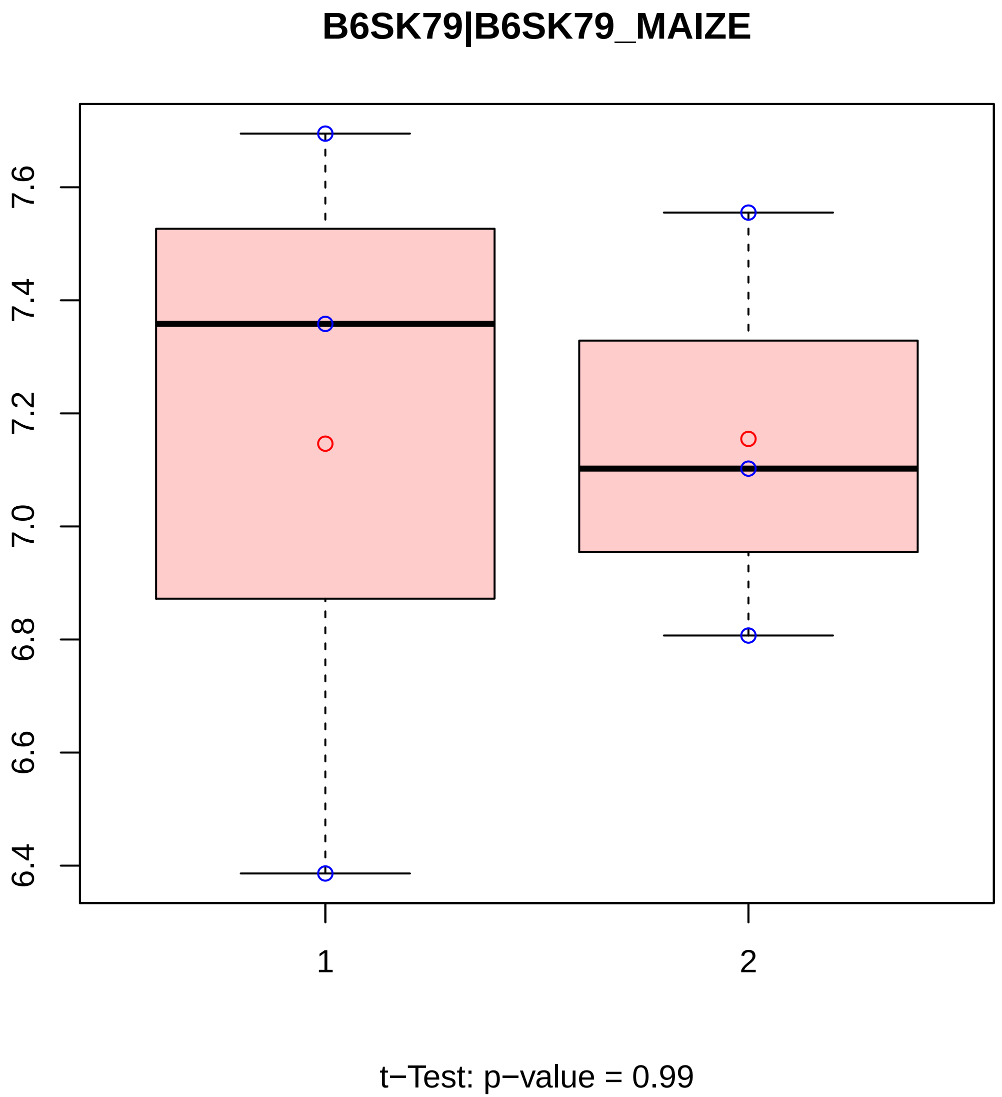

B6SK79|B6SK79_MAIZE
7.6
7.4
7.2
7.0
6.8
6.6
6.4
1
2
t−Test: p−value = 0.99

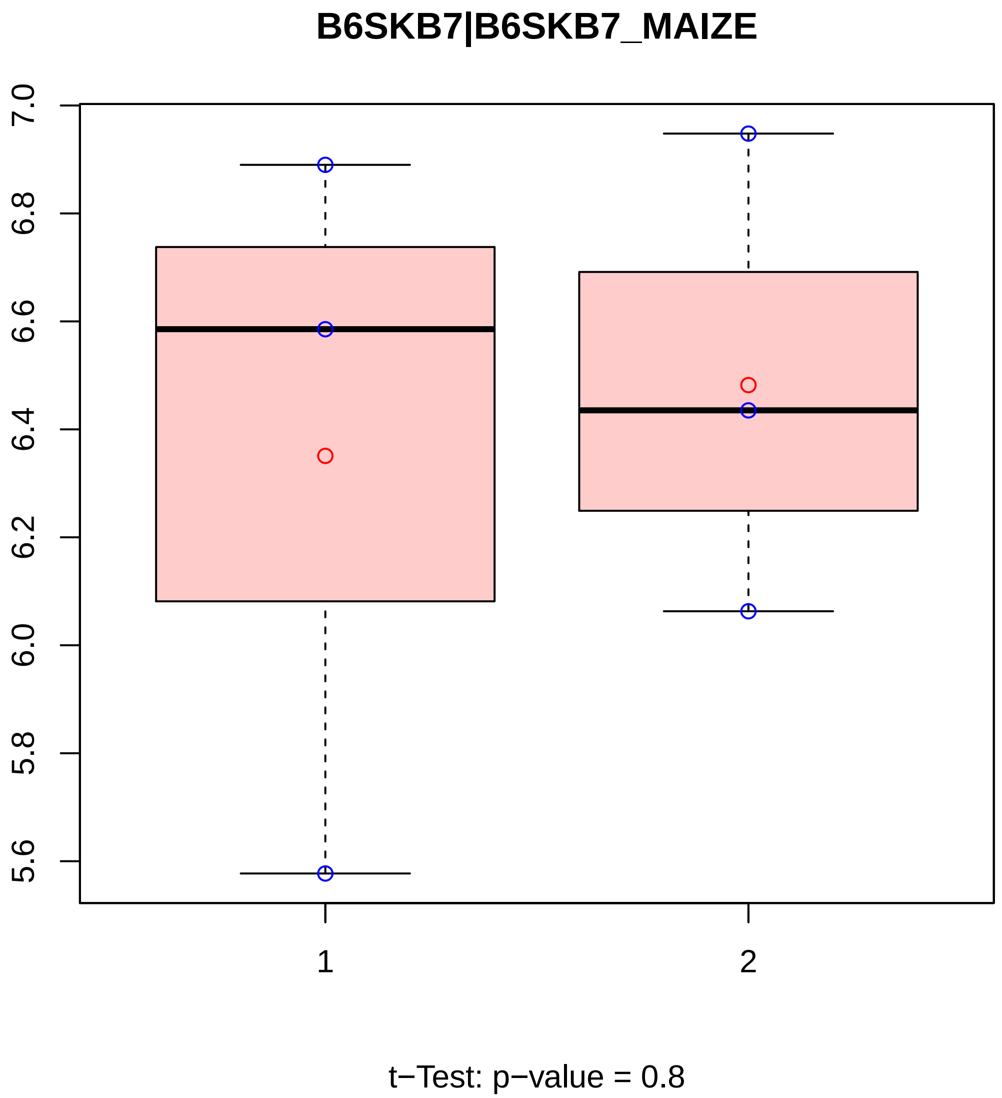
B6SKB7|B6SKB7_MAIZE
7.0
6.8
6.6
6.4
6.2
6.0
5.8
5.6
1
2
t−Test: p−value = 0.8

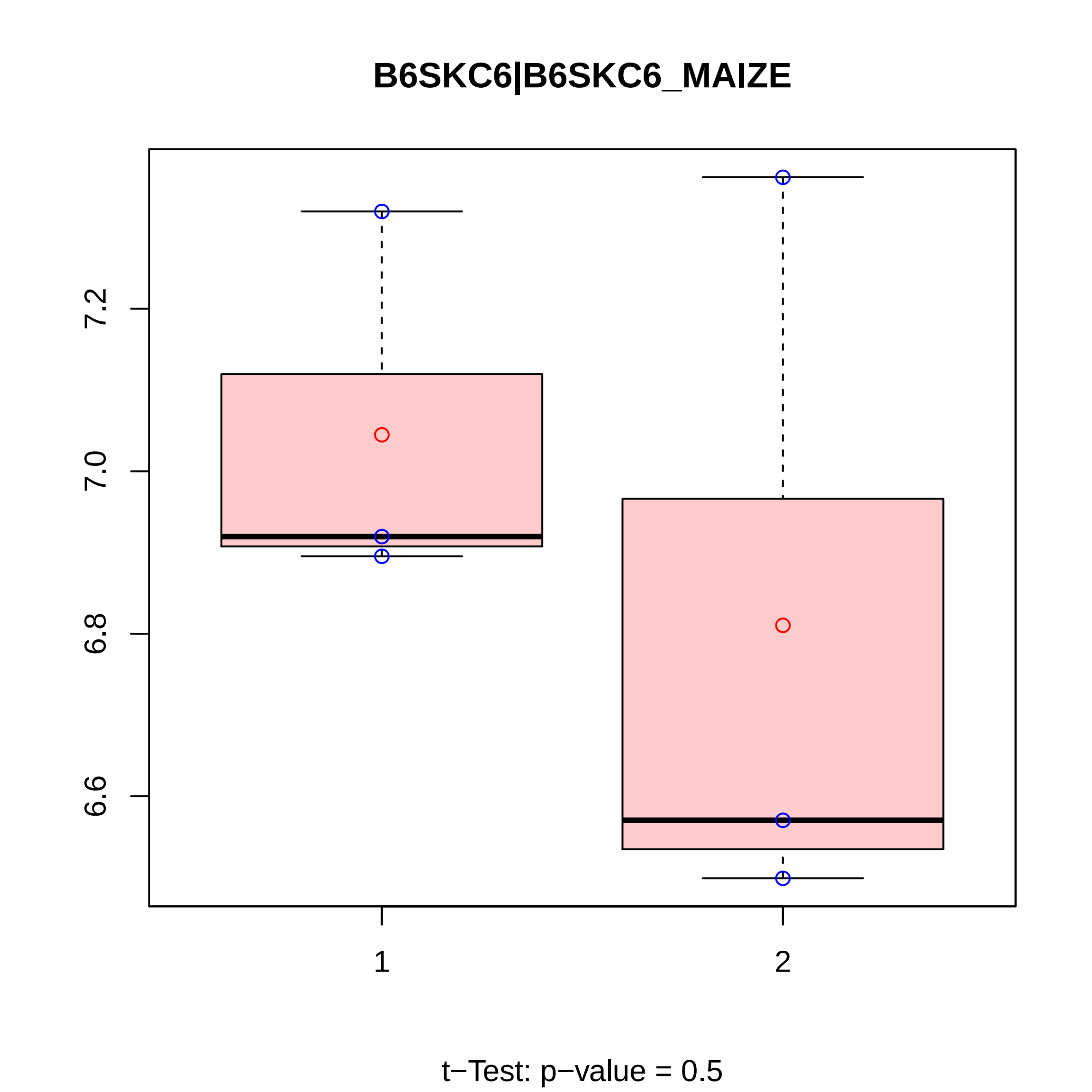

B6SKC6|B6SKC6_MAIZE
7.2
7.0
6.8
6.6
1
2
t−Test: p−value = 0.5

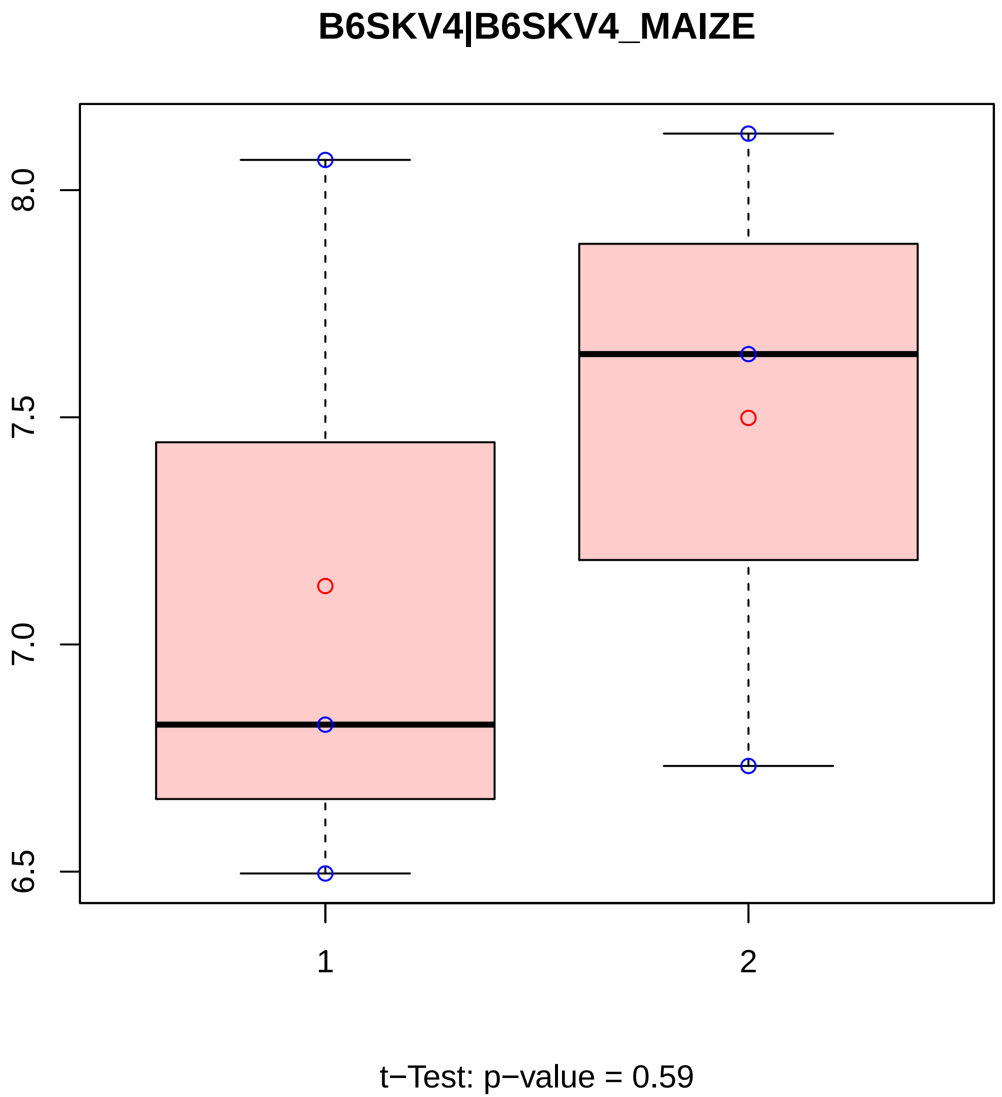
B6SKV4|B6SKV4_MAIZE
8.0
7.5
7.0
6.5
1
2
t−Test: p−value = 0.59

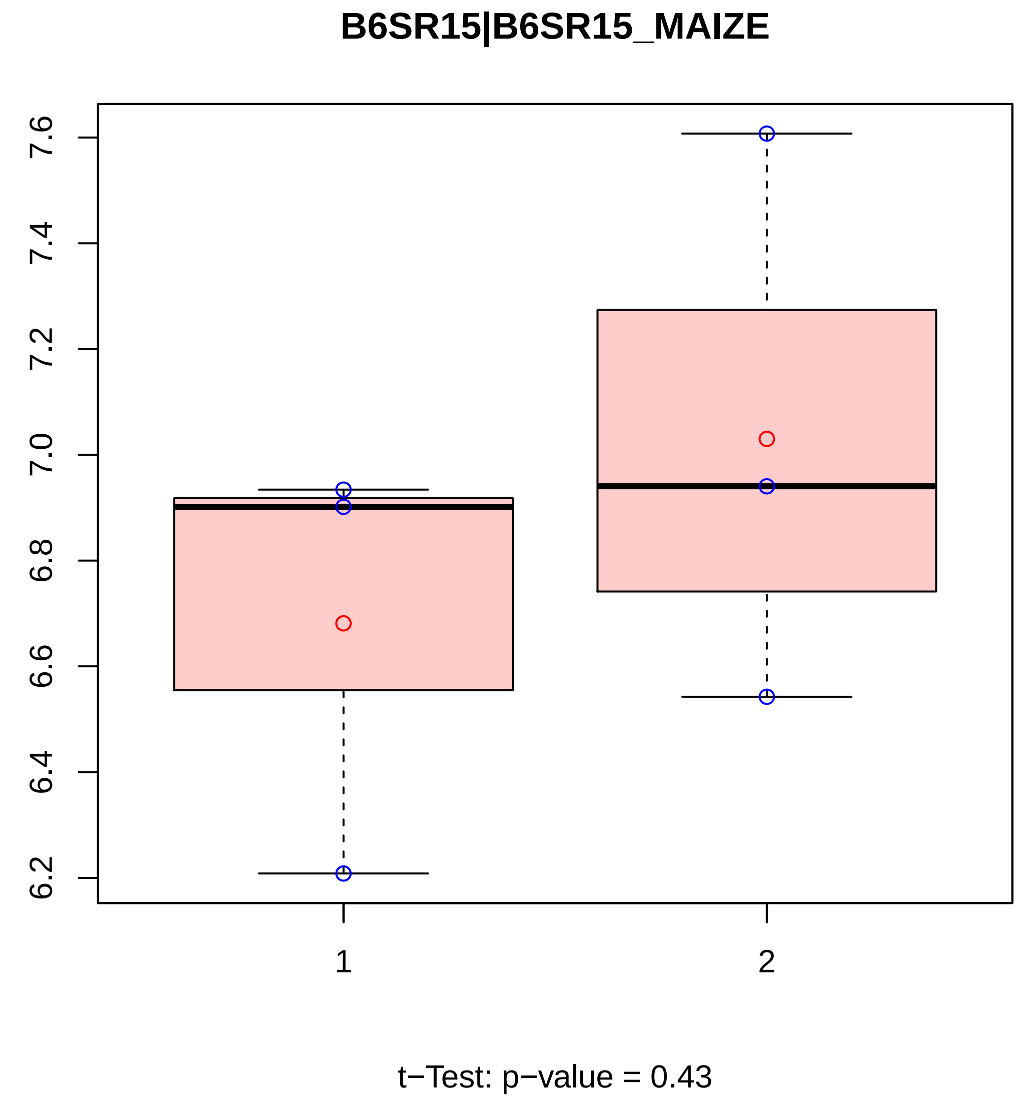

B6SR15|B6SR15_MAIZE
7.6
7.4
7.2
7.0
6.8
6.6
6.4
6.2
1
2
t−Test: p−value = 0.43

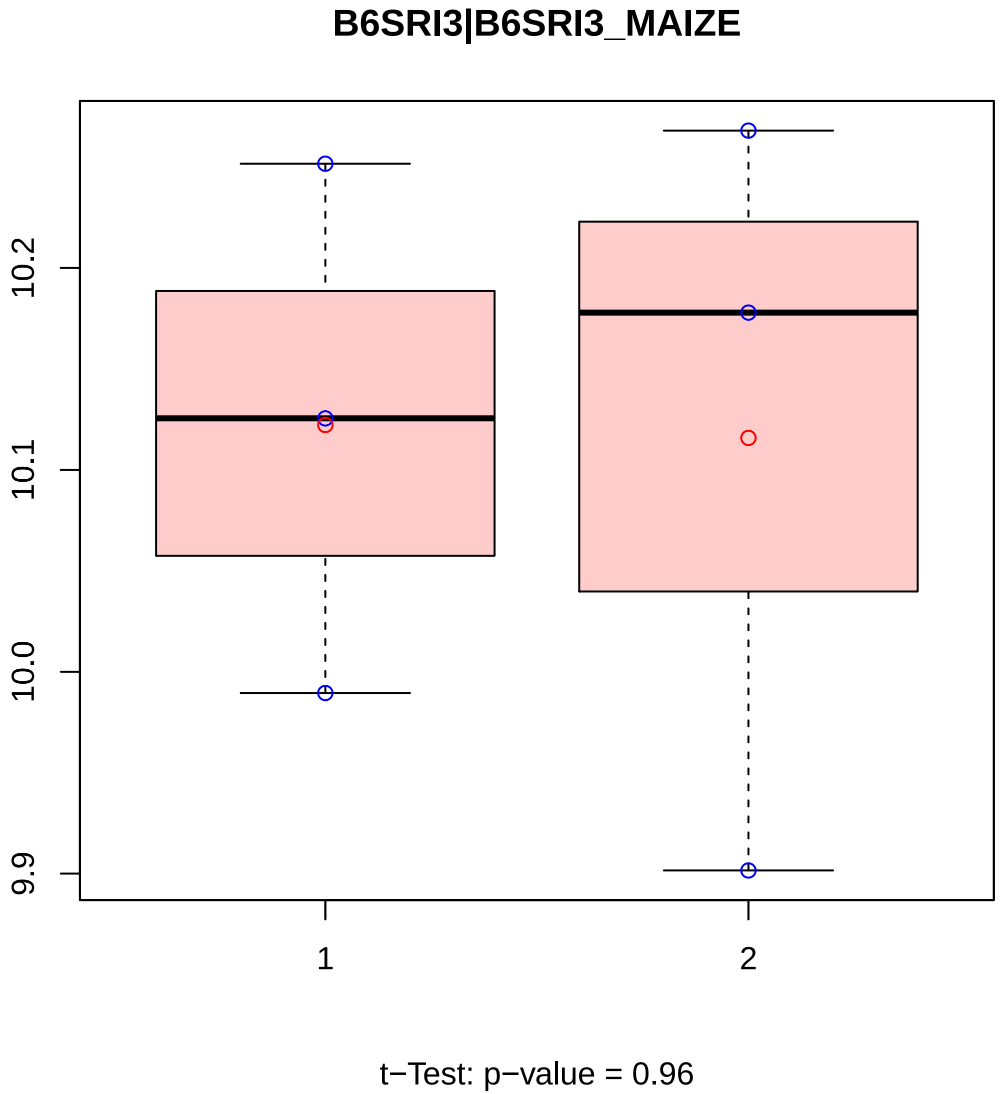
B6SRI3|B6SRI3_MAIZE
10.2
10.1
10.0
9.9
1
2
t−Test: p−value = 0.96

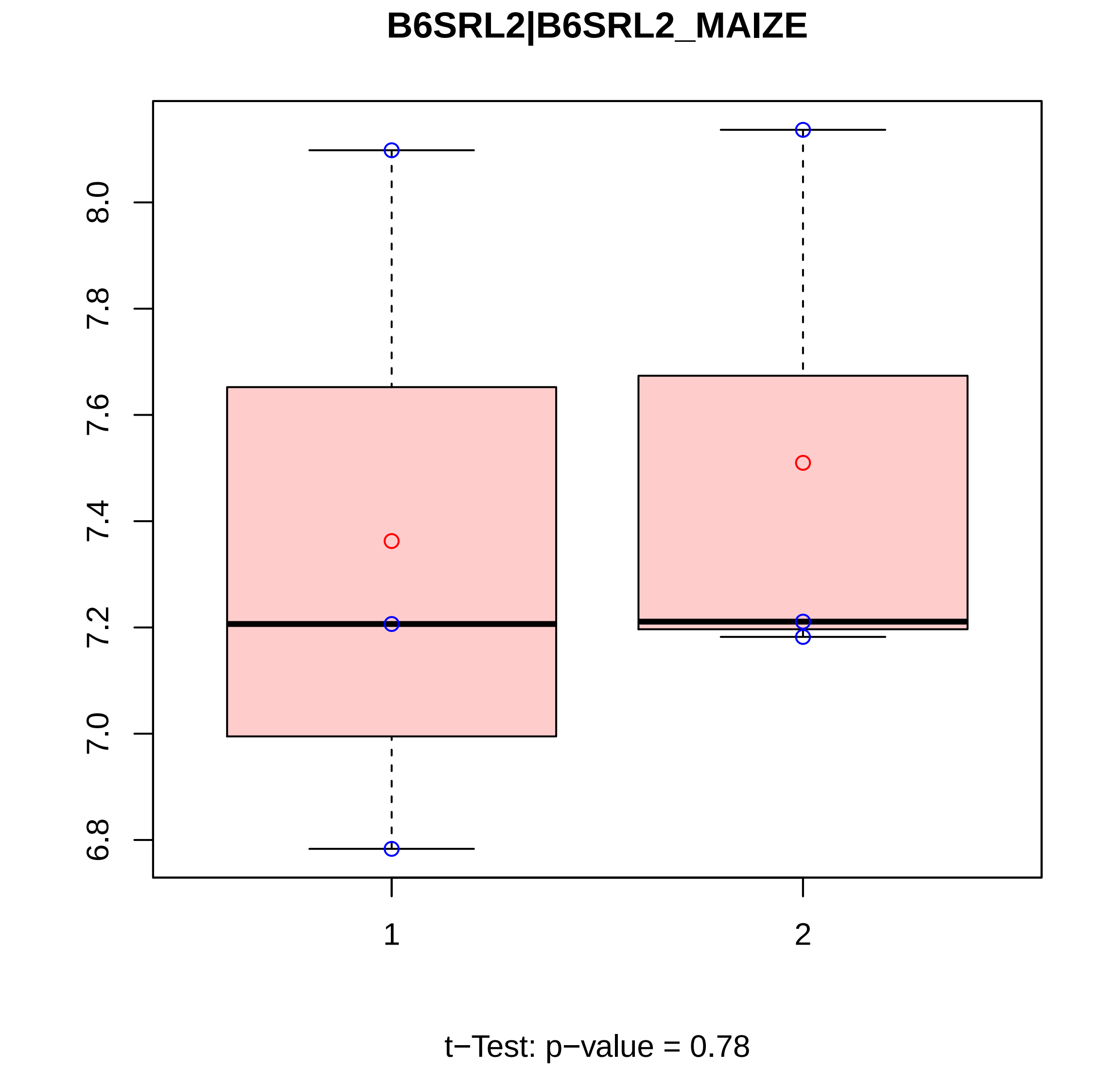
B6SRL2|B6SRL2_MAIZE
6.8
7.0
7.2
7.4
7.6
7.8
8.0
1
2
t−Test: p−value = 0.78

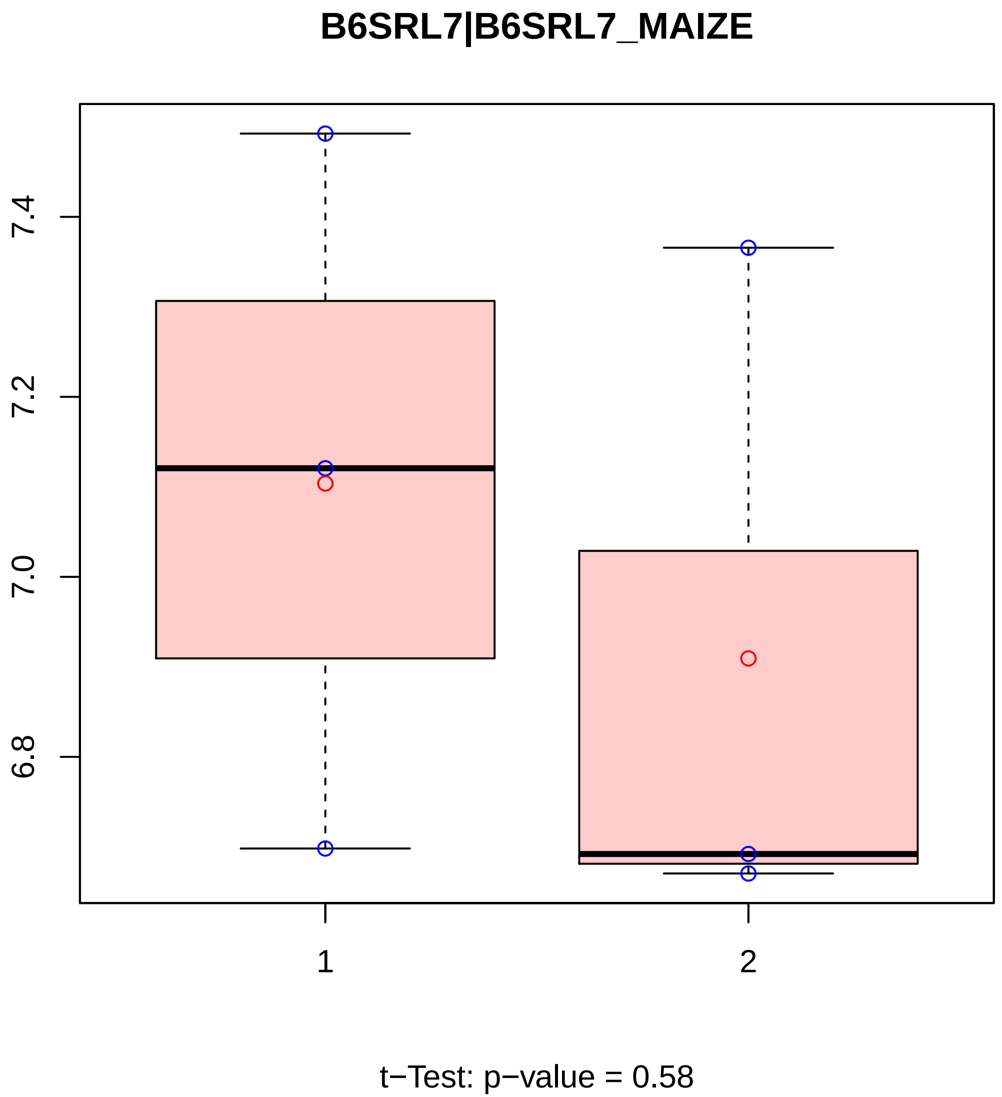
B6SRL7|B6SRL7_MAIZE
7.4
7.2
7.0
6.8
1
2
t−Test: p−value = 0.58

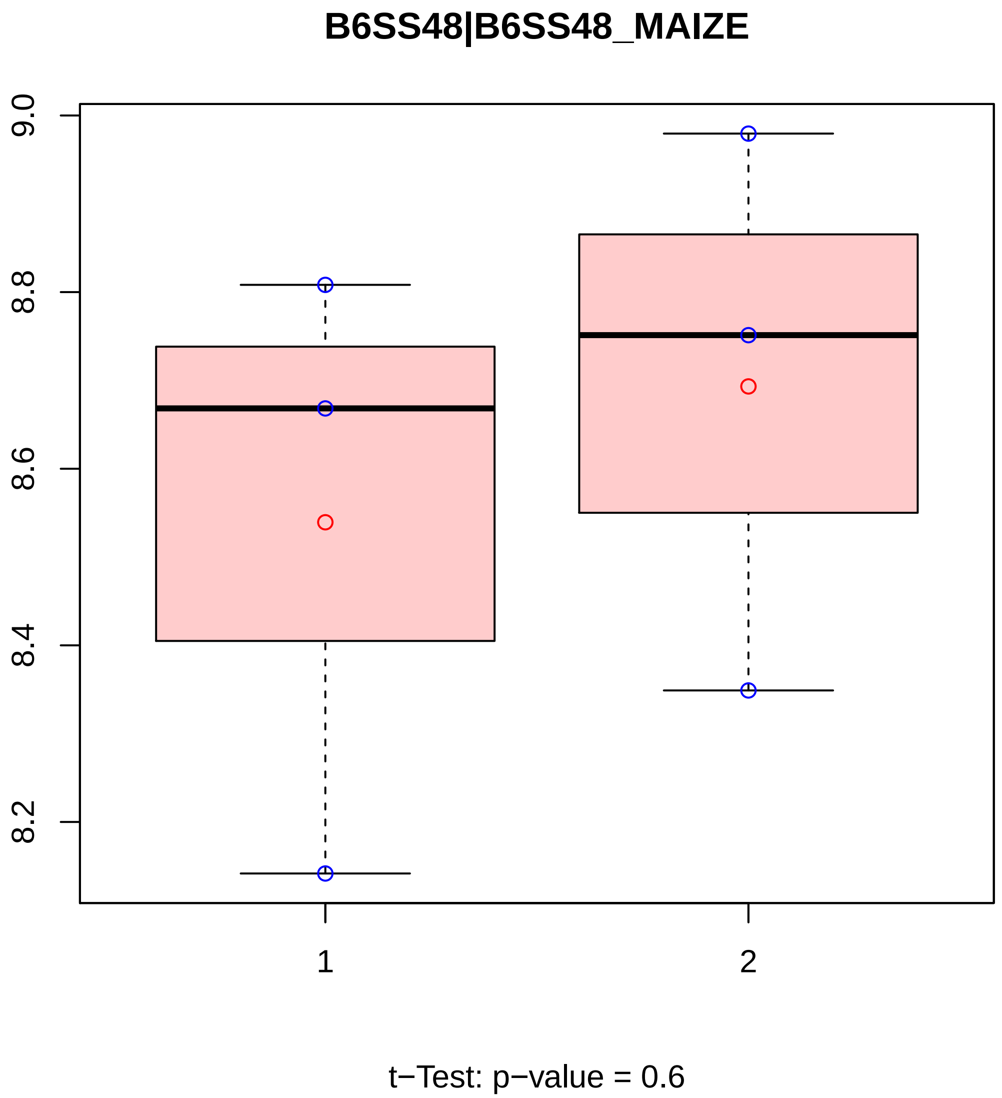

B6SS48|B6SS48_MAIZE
9.0
8.8
8.6
8.4
8.2
1
2
t−Test: p−value = 0.6

B6SSN3|B6SSN3_MAIZE

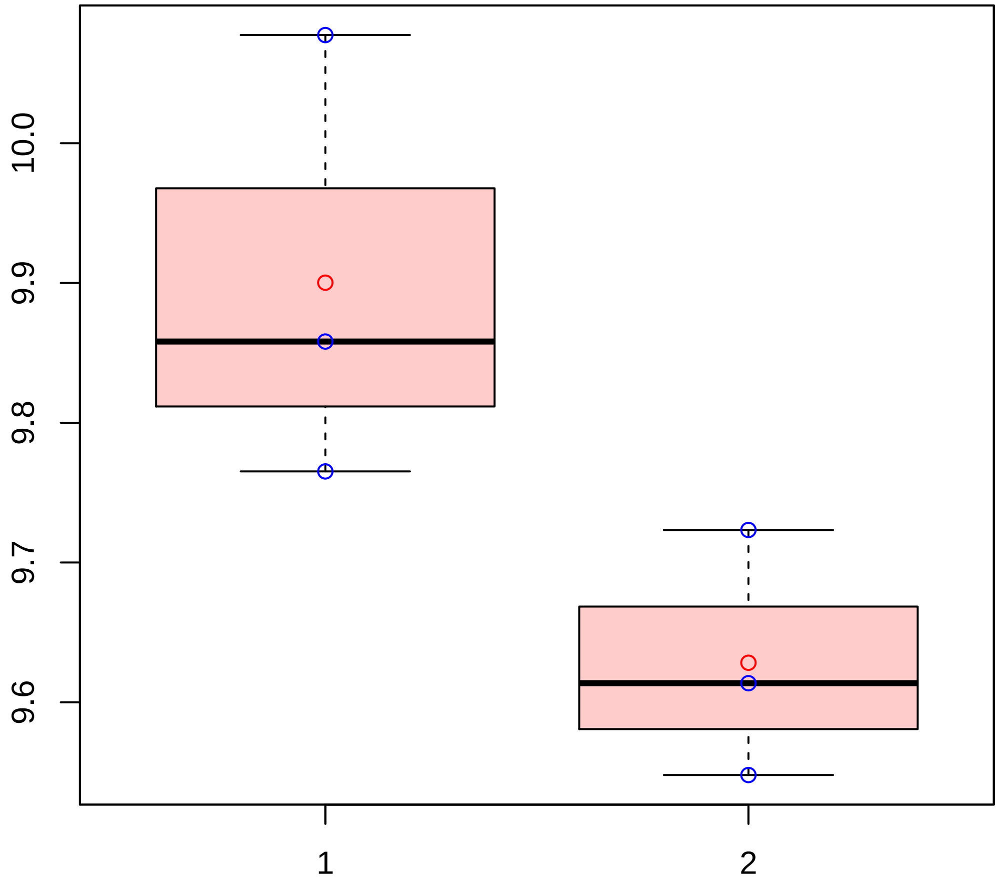

t−Test: p−value = 0.08

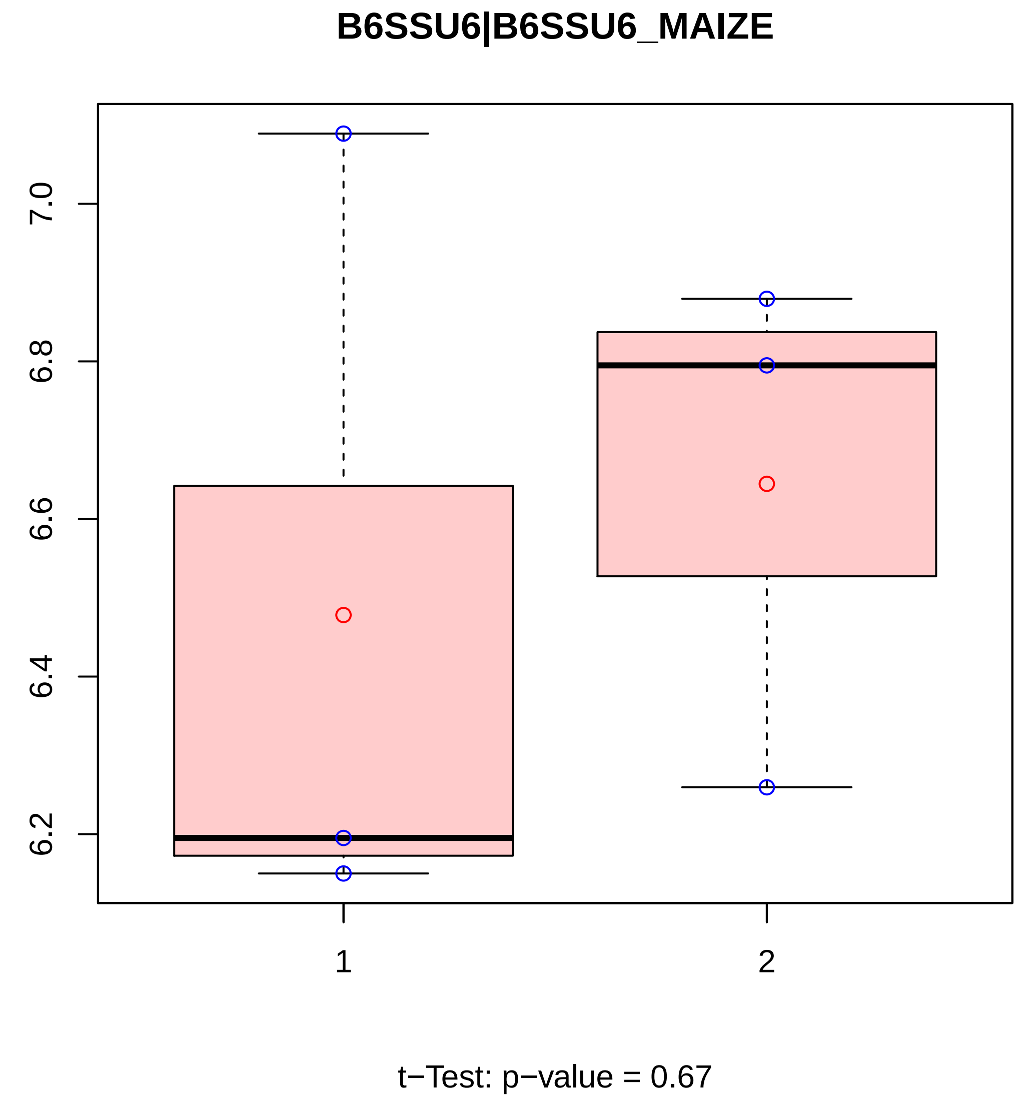
B6SSU6|B6SSU6_MAIZE
7.0
6.8
6.6
6.4
6.2
1
2
t−Test: p−value = 0.67

B6STN4|B6STN4_MAIZE

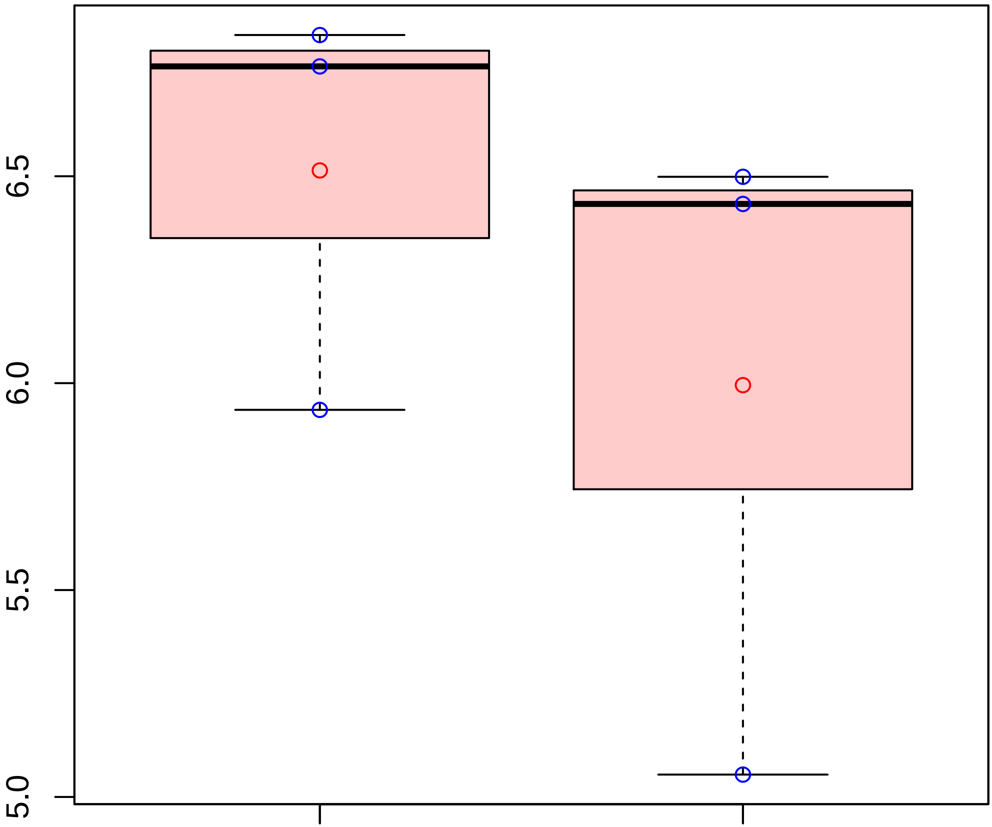

t−Test: p−value = 0.41

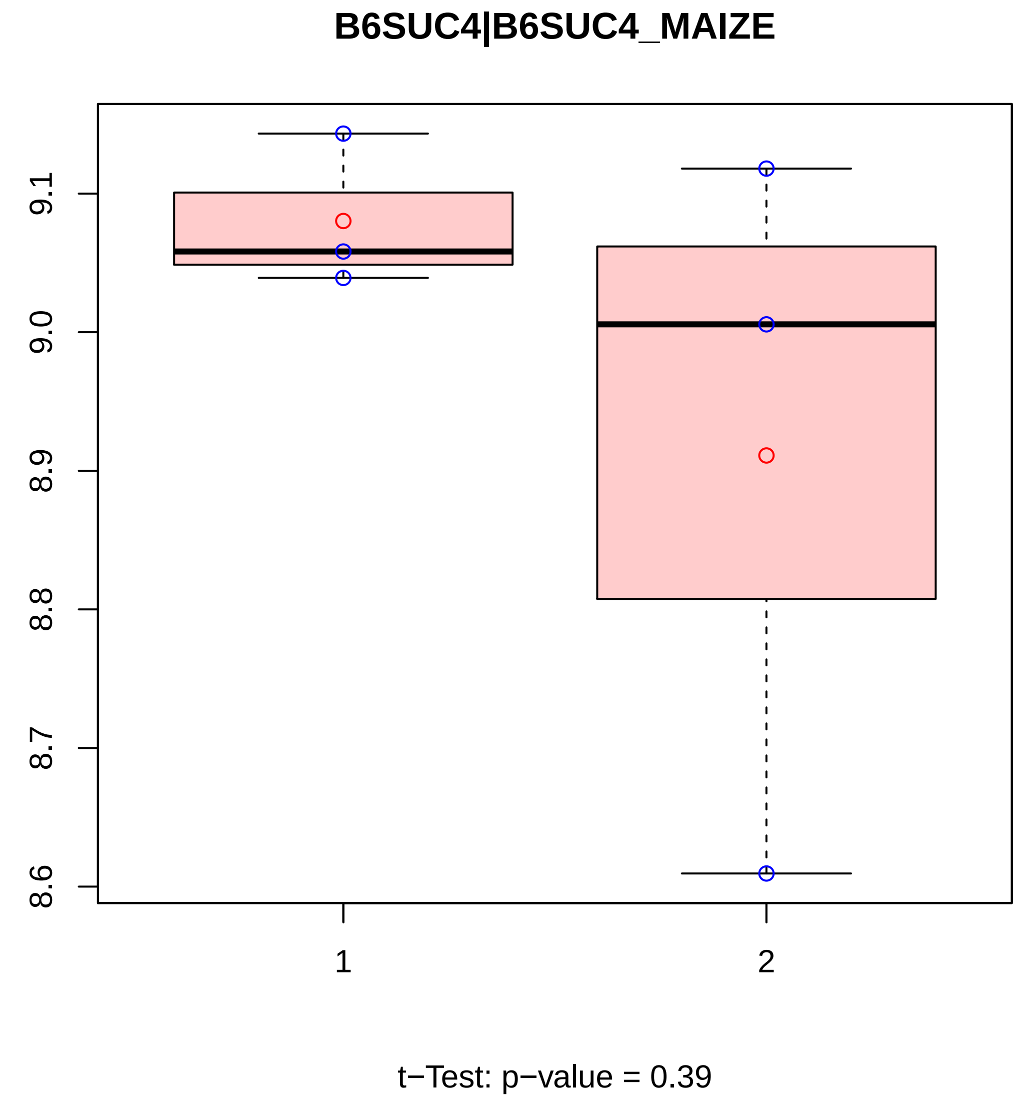
B6SUC4|B6SUC4_MAIZE
9.1
9.0
8.9
8.8
8.7
8.6
1
2
t−Test: p−value = 0.39

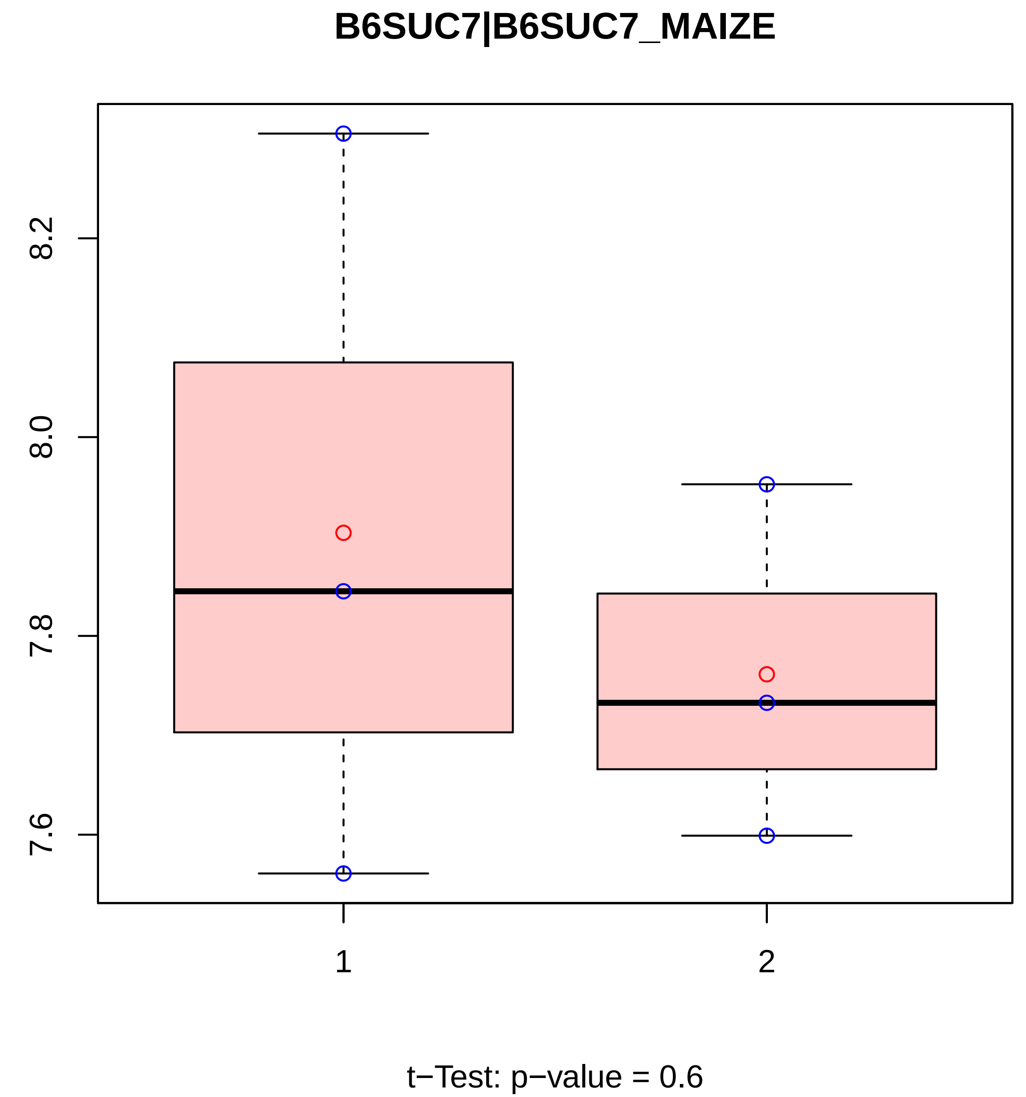
B6SUC7|B6SUC7_MAIZE
8.2
8.0
7.8
7.6
1
2
t−Test: p−value = 0.6

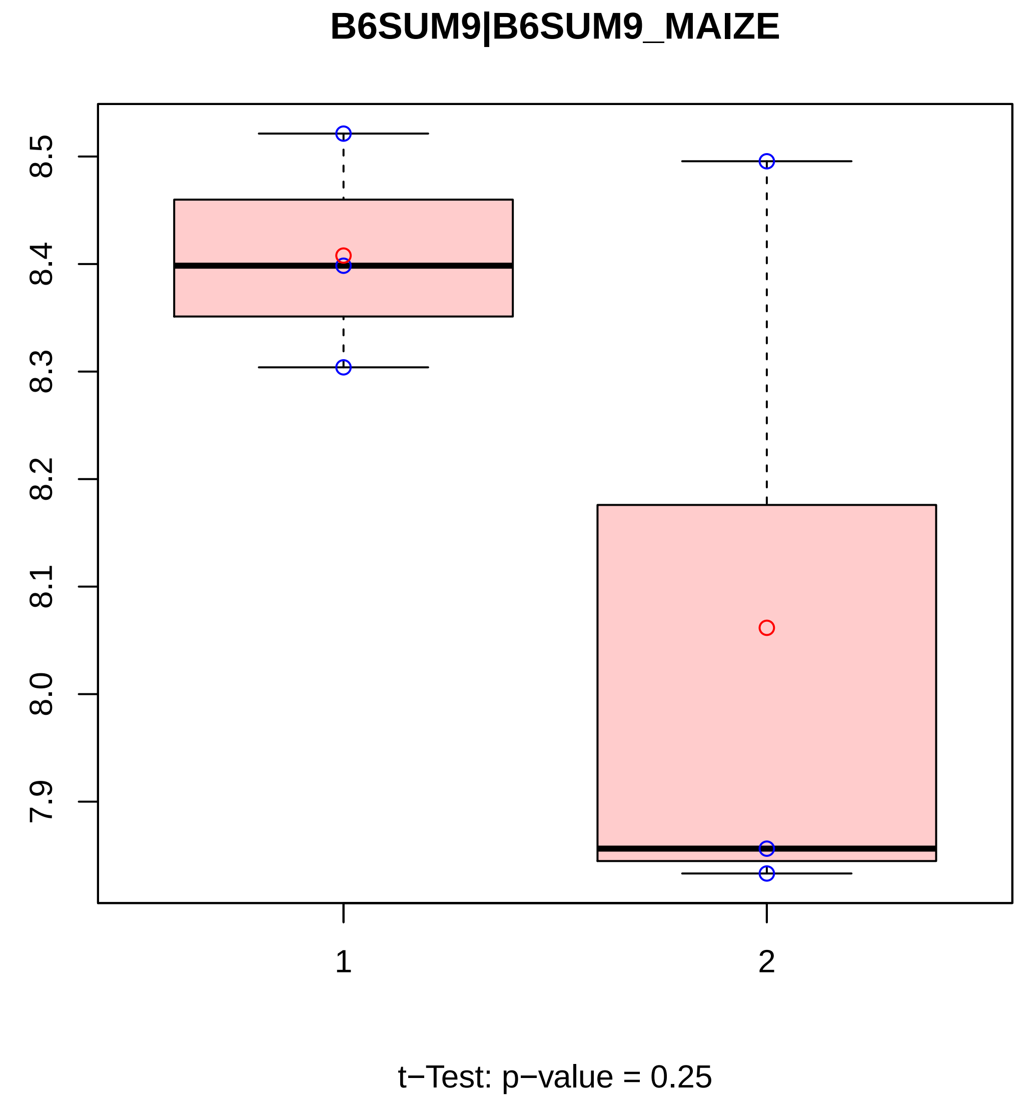
B6SUM9|B6SUM9_MAIZE
8.5
8.4
8.3
8.2
8.1
8.0
7.9
1
2
t−Test: p−value = 0.25

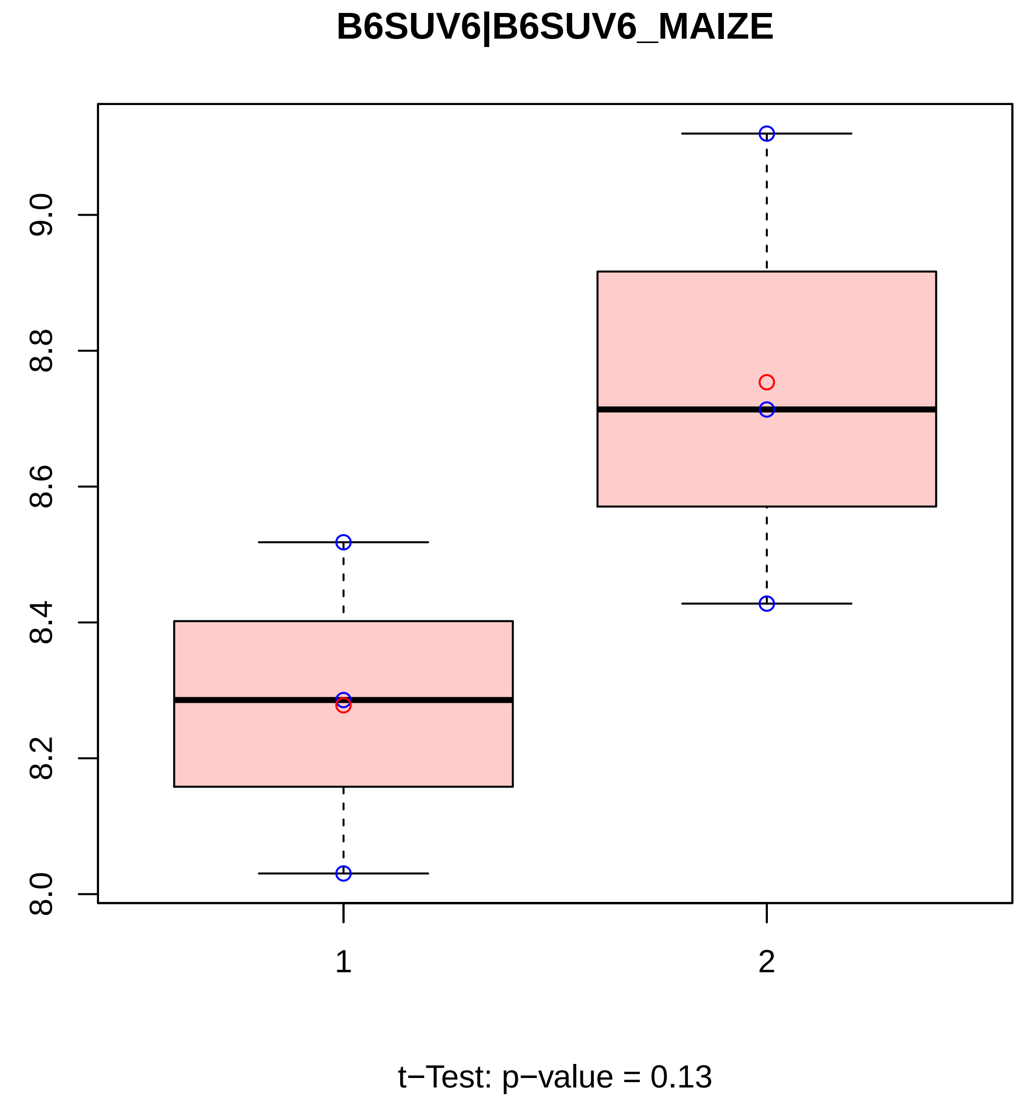

B6SUV6|B6SUV6_MAIZE
9.0
8.8
8.6
8.4
8.2
8.0
1
2
t−Test: p−value = 0.13

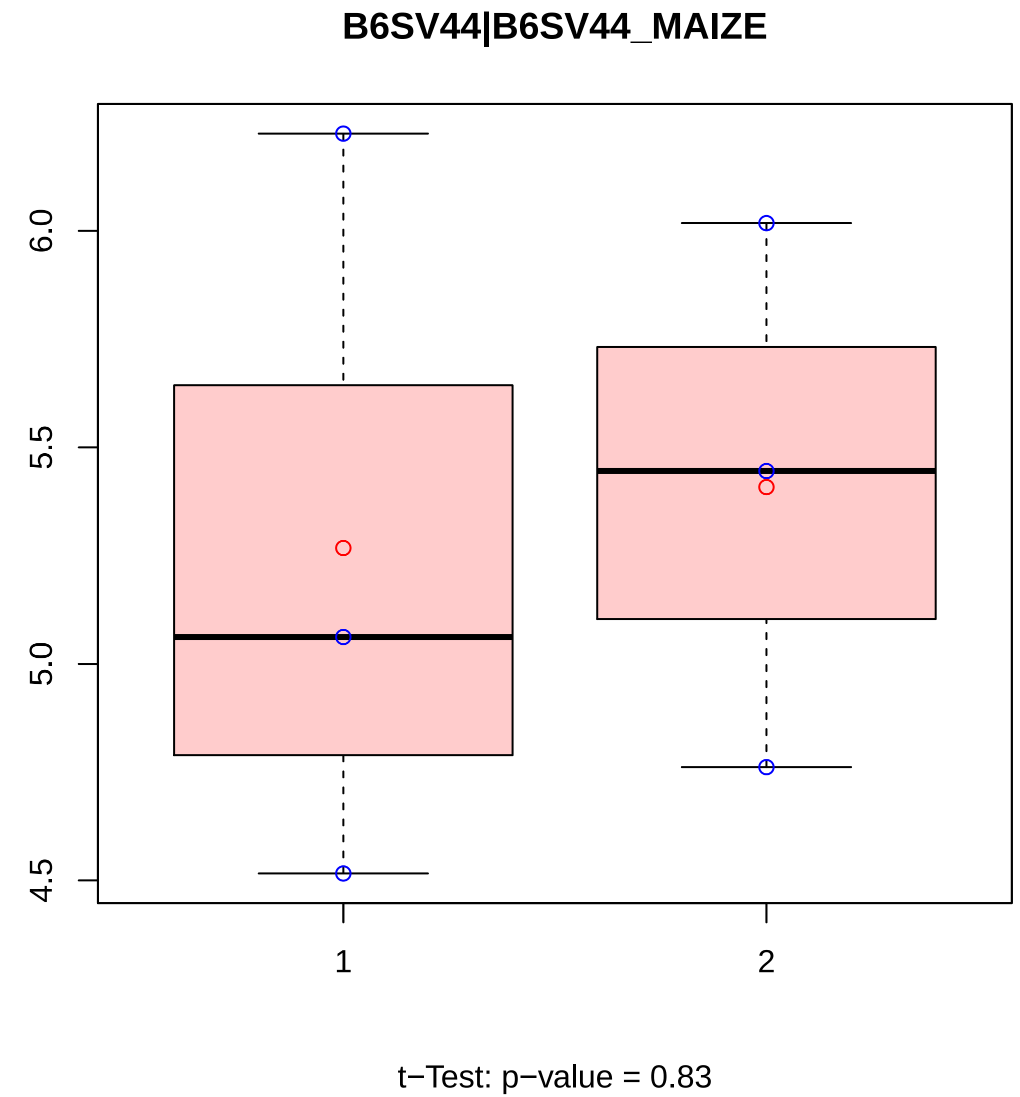

B6SV44|B6SV44_MAIZE
6.0
5.5
5.0
4.5
1
2
t−Test: p−value = 0.83

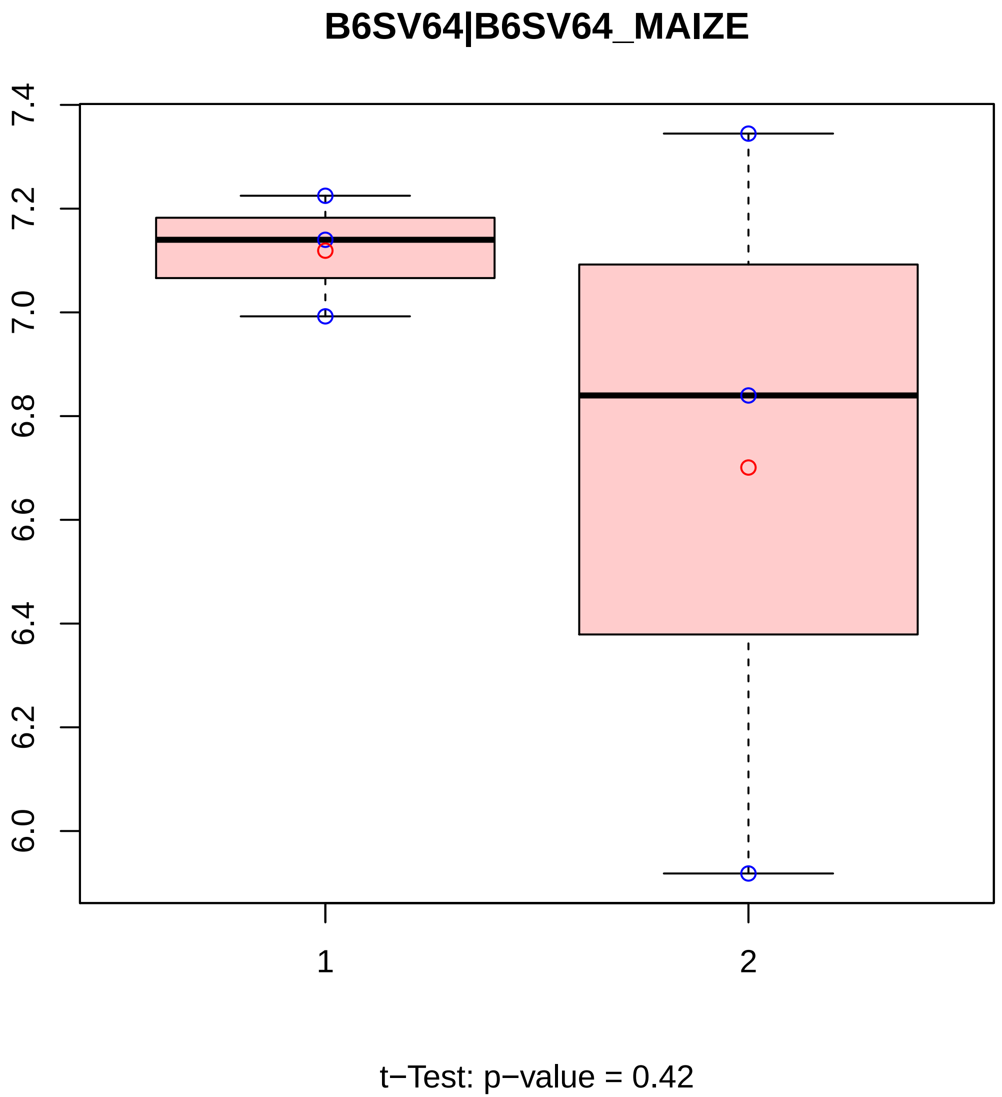
B6SV64|B6SV64_MAIZE
7.4
7.2
7.0
6.8
6.6
6.4
6.2
6.0
1
2
t−Test: p−value = 0.42

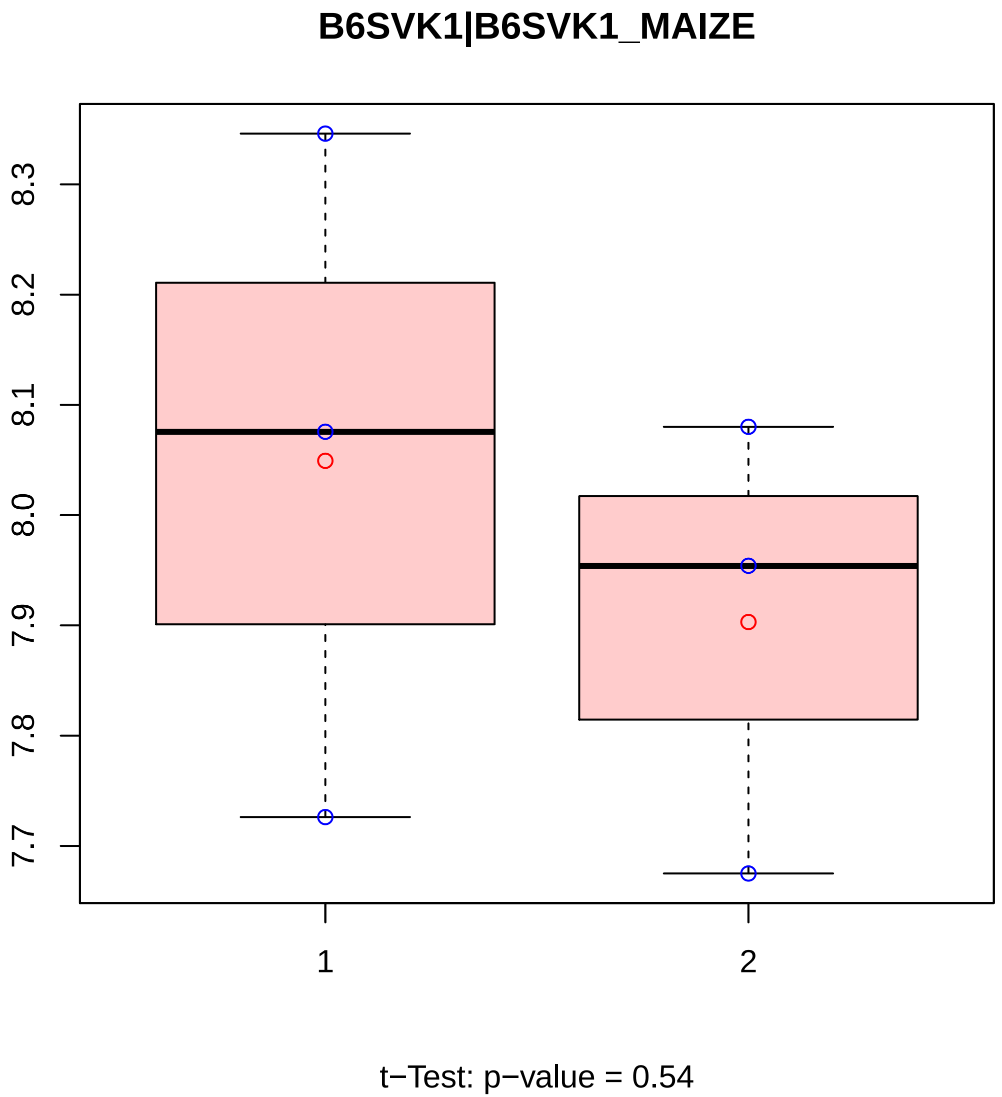
B6SVK1|B6SVK1_MAIZE
8.3
8.2
8.1
8.0
7.9
7.8
7.7
1
2
t−Test: p−value = 0.54

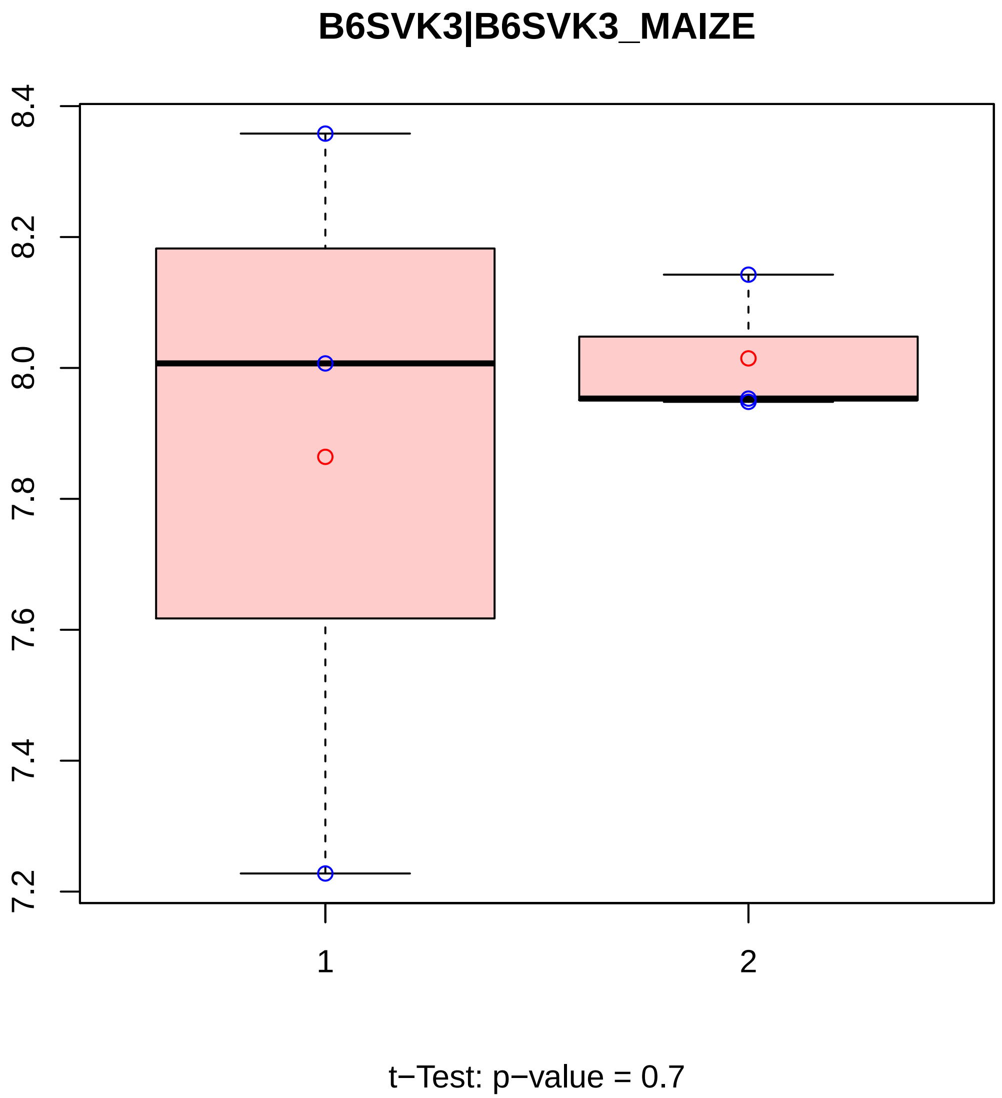

B6SVK3|B6SVK3_MAIZE
8.4
8.2
8.0
7.8
7.6
7.4
7.2
1
2
t−Test: p−value = 0.7

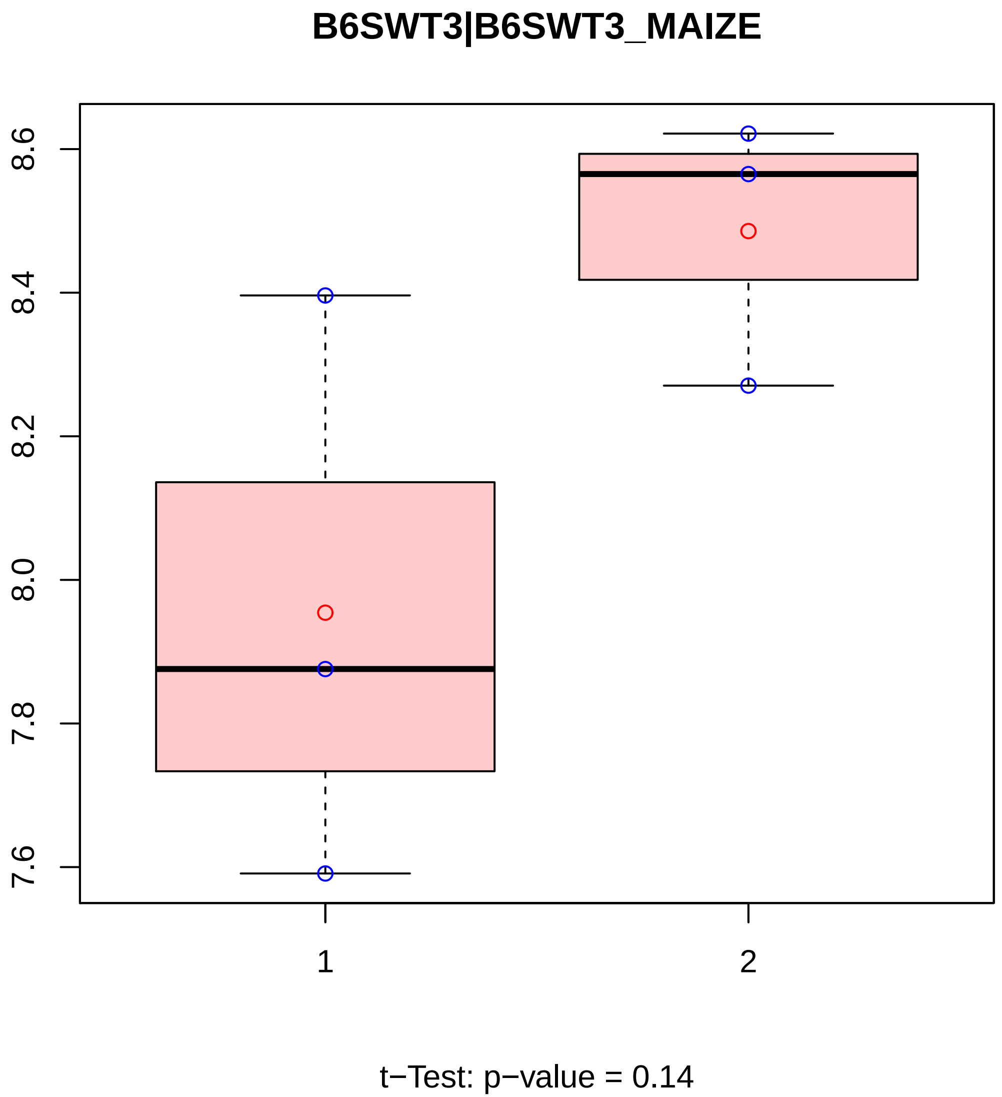

B6SWT3|B6SWT3_MAIZE
8.6
8.4
8.2
8.0
7.8
7.6
1
2
t−Test: p−value = 0.14

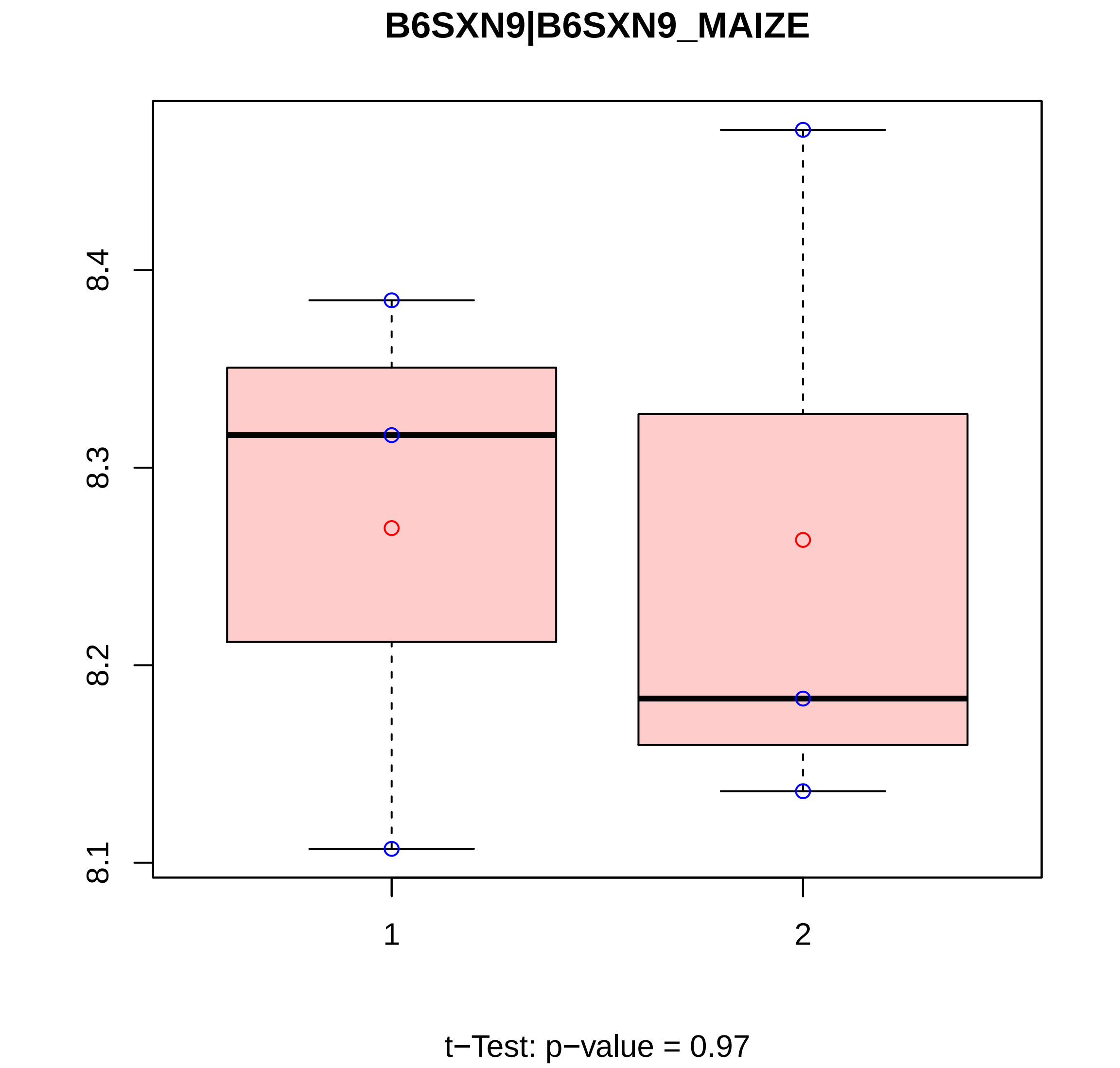
B6SXN9|B6SXN9_MAIZE
8.1
8.2
8.3
8.4
1
2
t−Test: p−value = 0.97

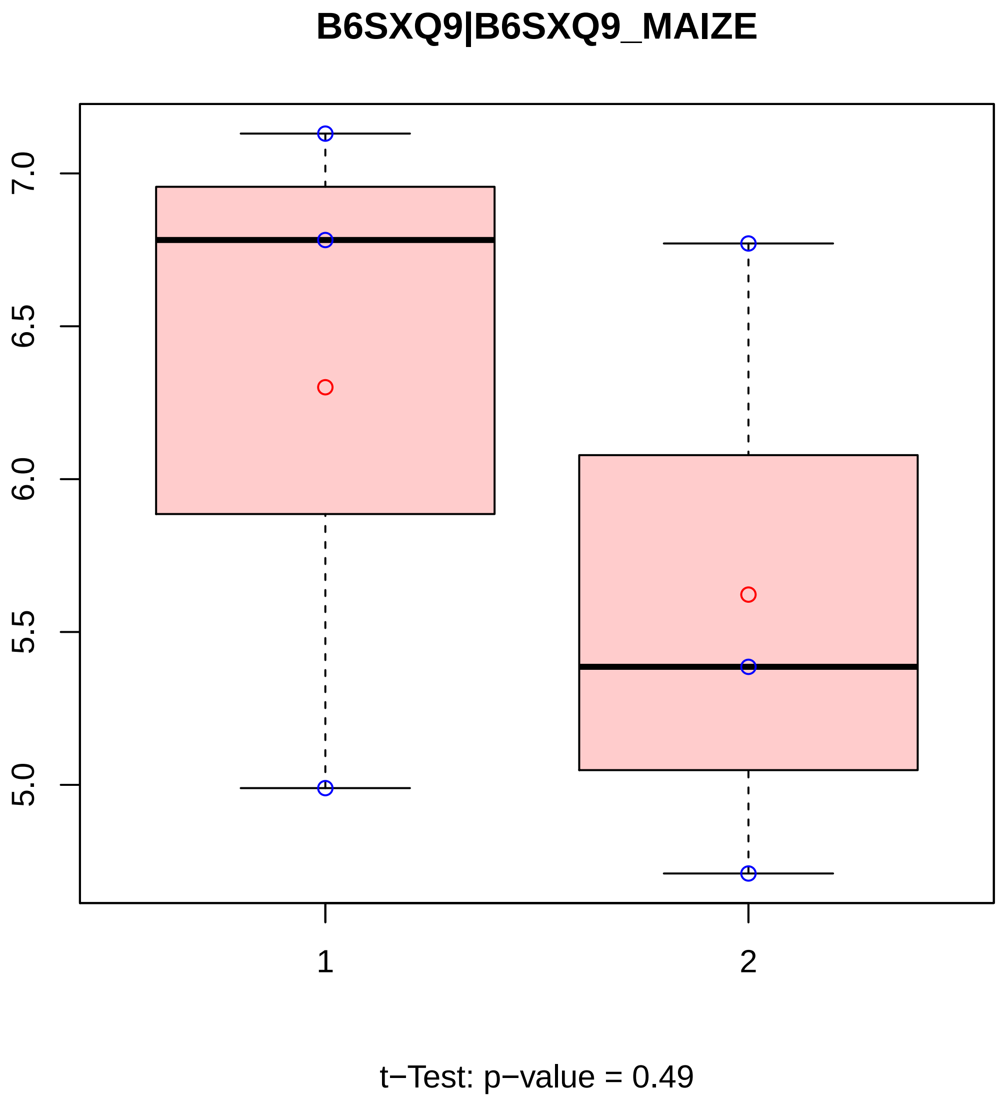
B6SXQ9|B6SXQ9_MAIZE
7.0
6.5
6.0
5.5
5.0
1
2
t−Test: p−value = 0.49

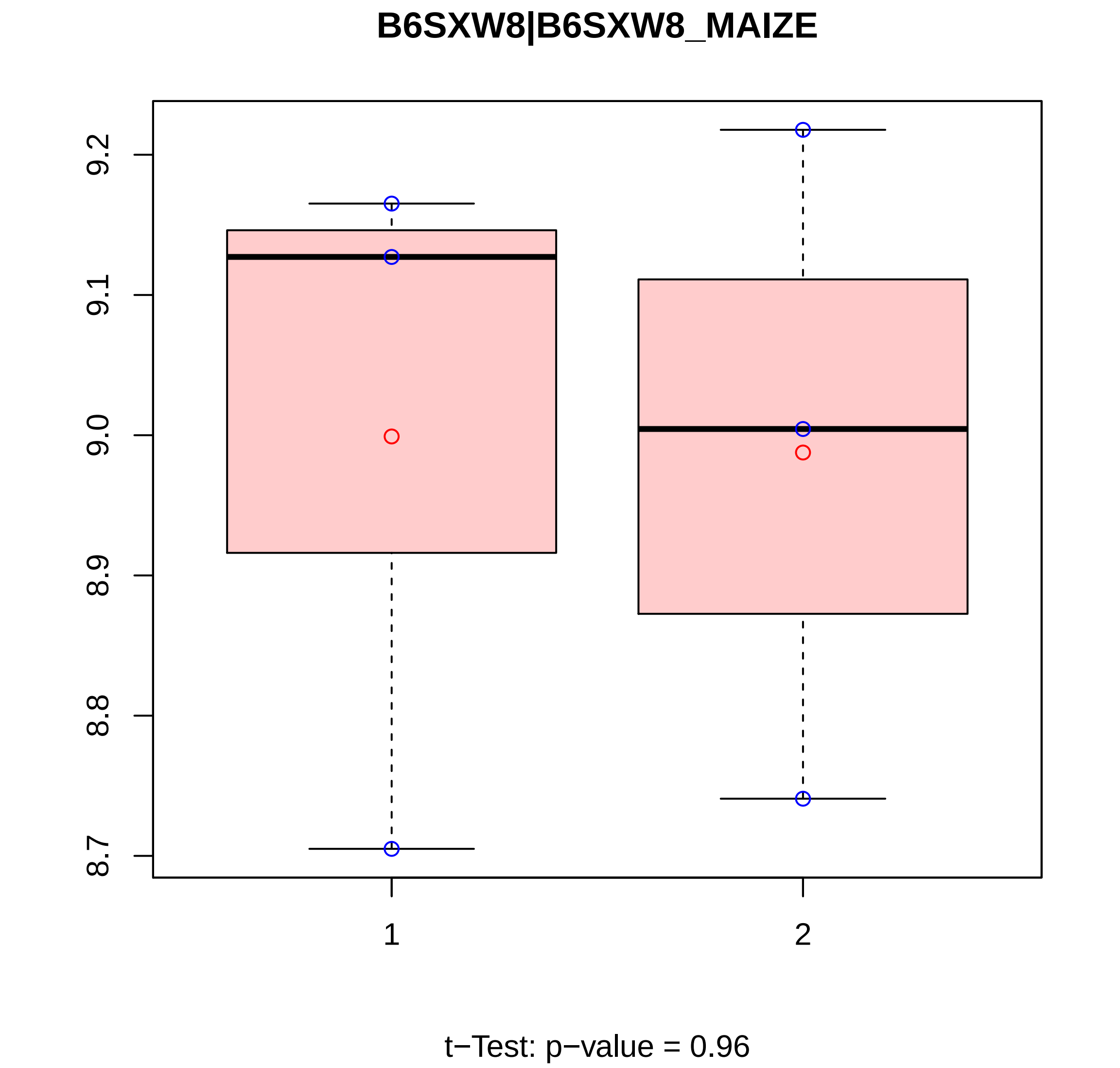

B6SXW8|B6SXW8_MAIZE
9.2
9.1
9.0
8.9
8.8
8.7
1
2
t−Test: p−value = 0.96

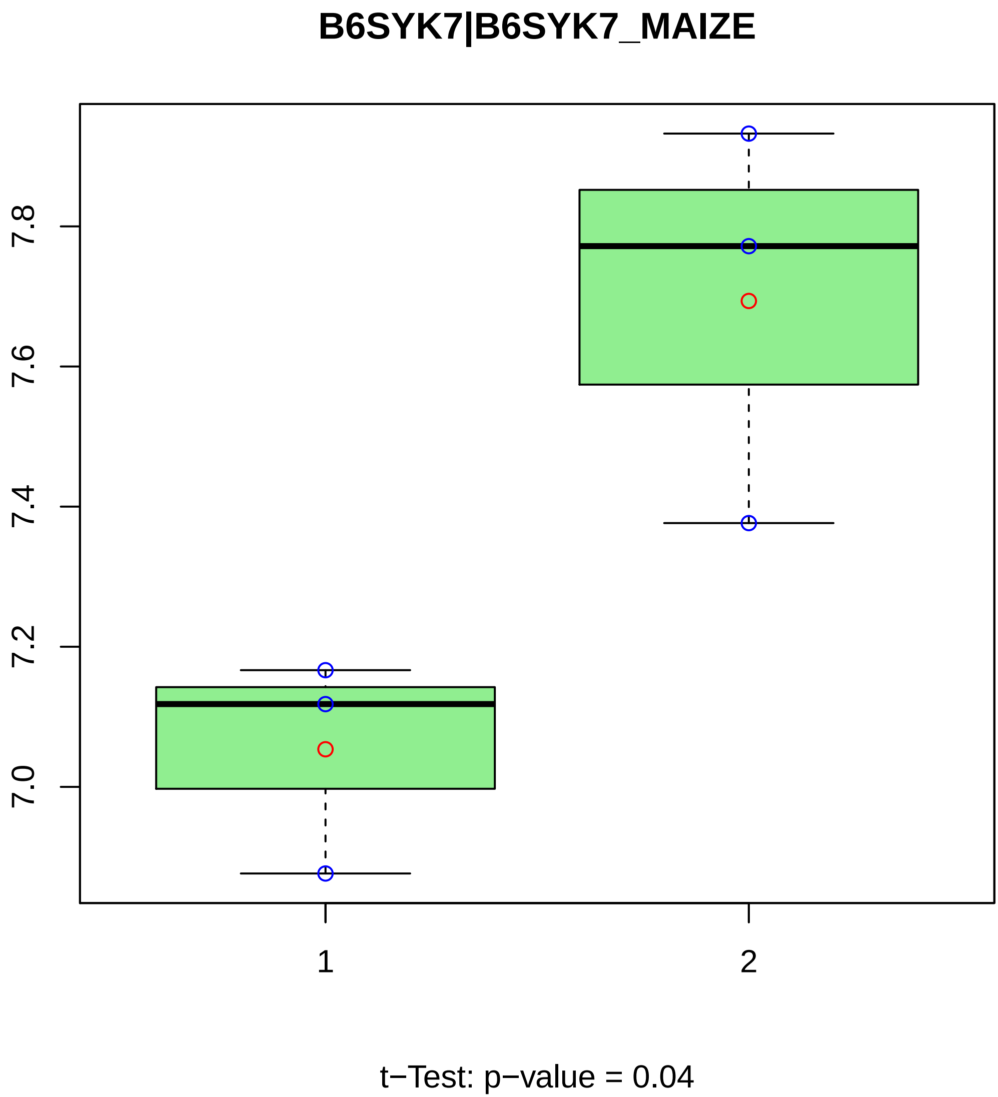
B6SYK7|B6SYK7_MAIZE
7.0
7.2
7.4
7.6
7.8
1
2
t−Test: p−value = 0.04

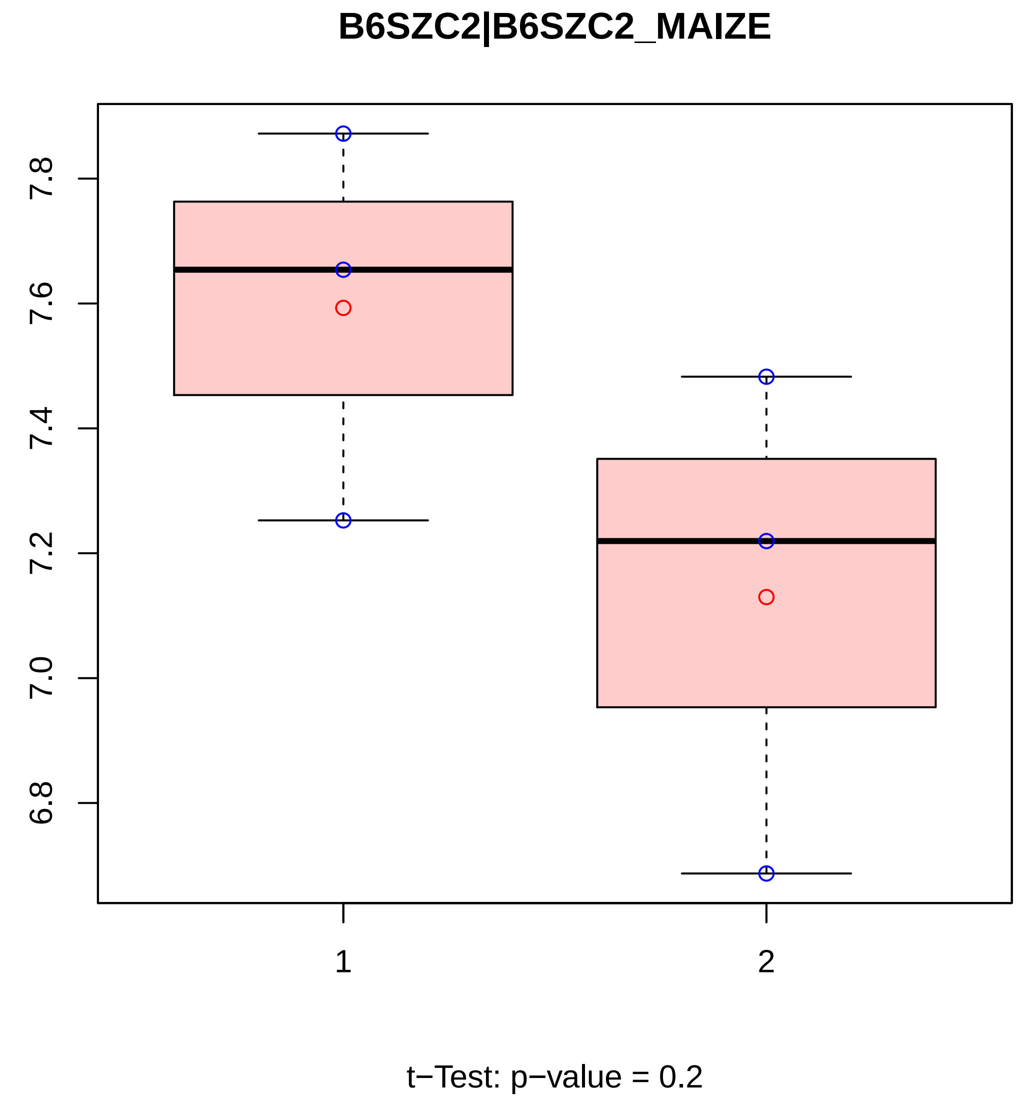
B6SZC2|B6SZC2_MAIZE
7.8
7.6
7.4
7.2
7.0
6.8
1
2
t−Test: p−value = 0.2

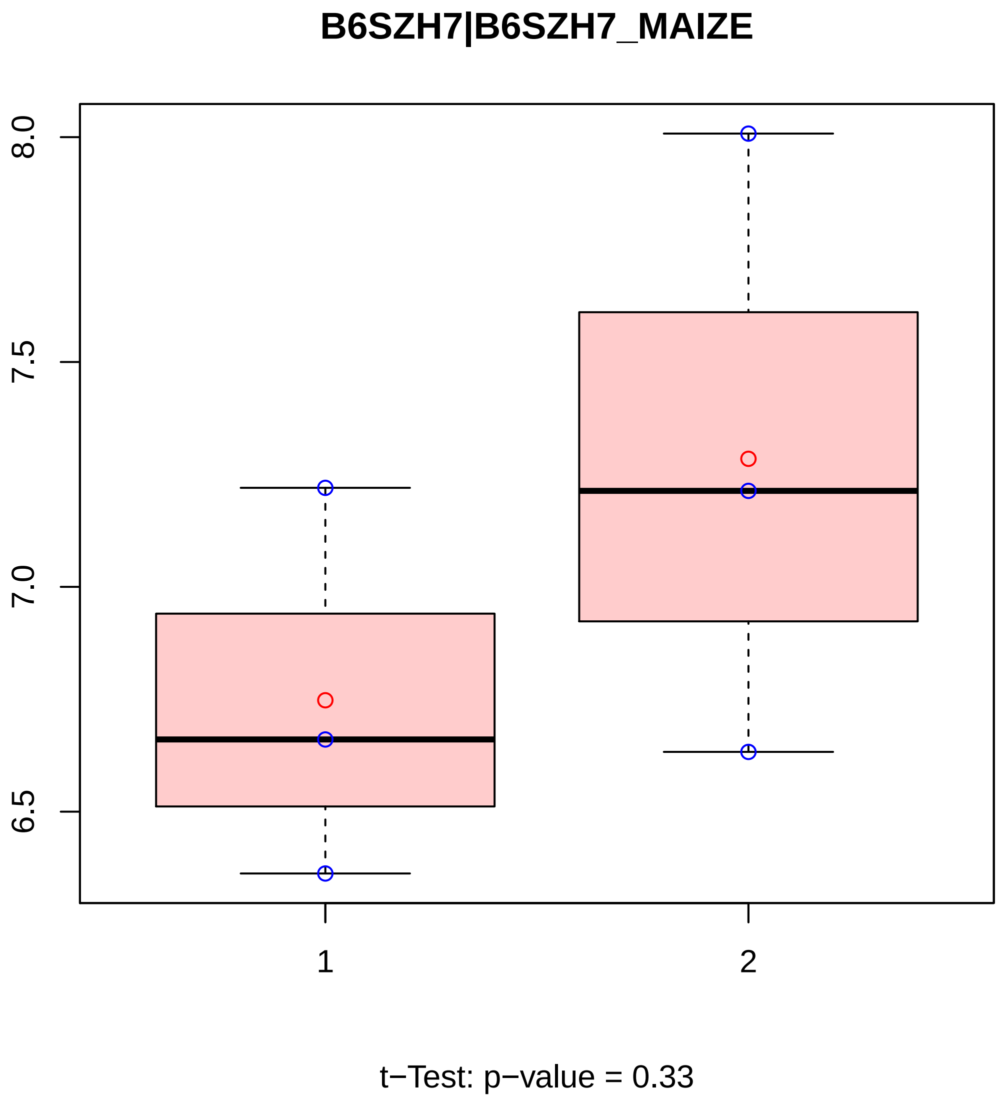

B6SZH7|B6SZH7_MAIZE
8.0
7.5
7.0
6.5
1
2
t−Test: p−value = 0.33

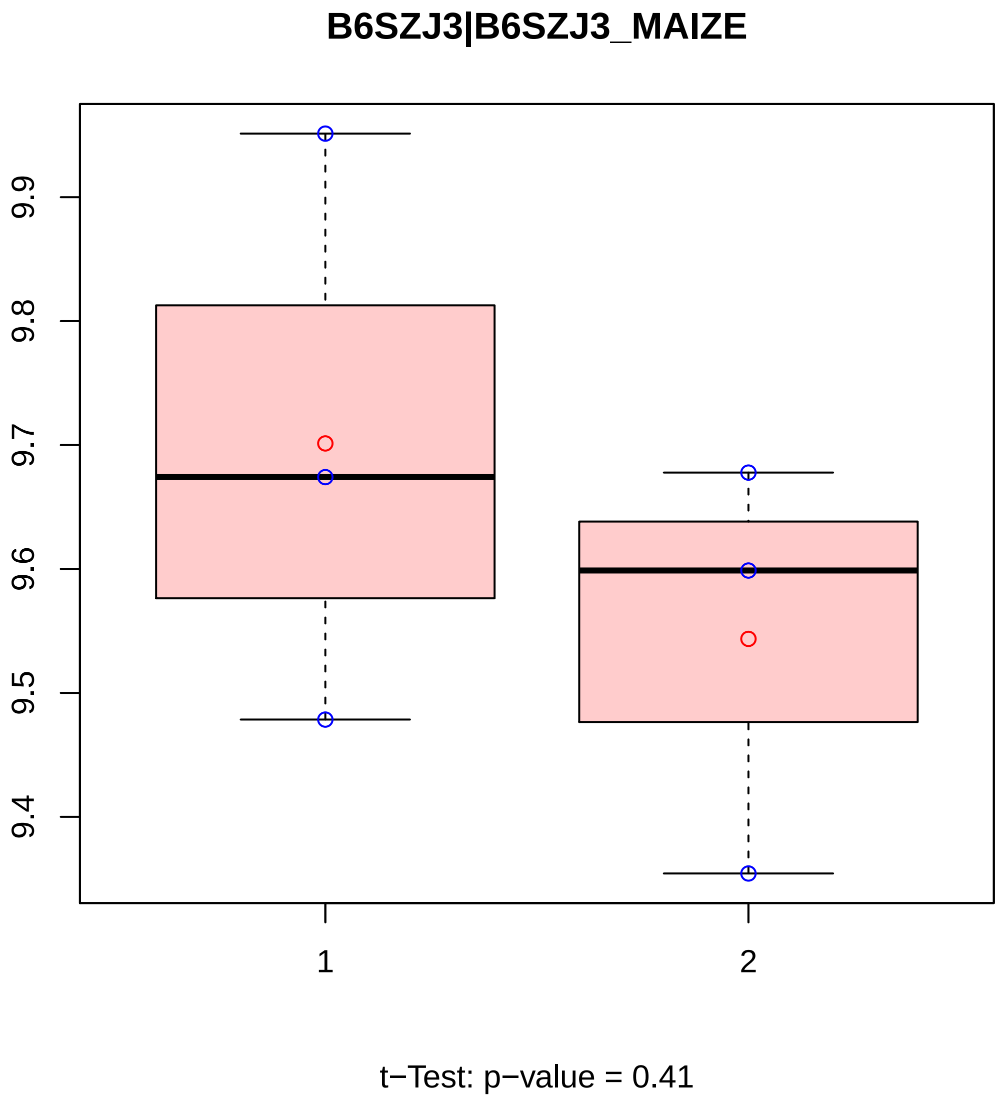
B6SZJ3|B6SZJ3_MAIZE
9.4
9.5
9.6
9.7
9.8
9.9
1
2
t−Test: p−value = 0.41

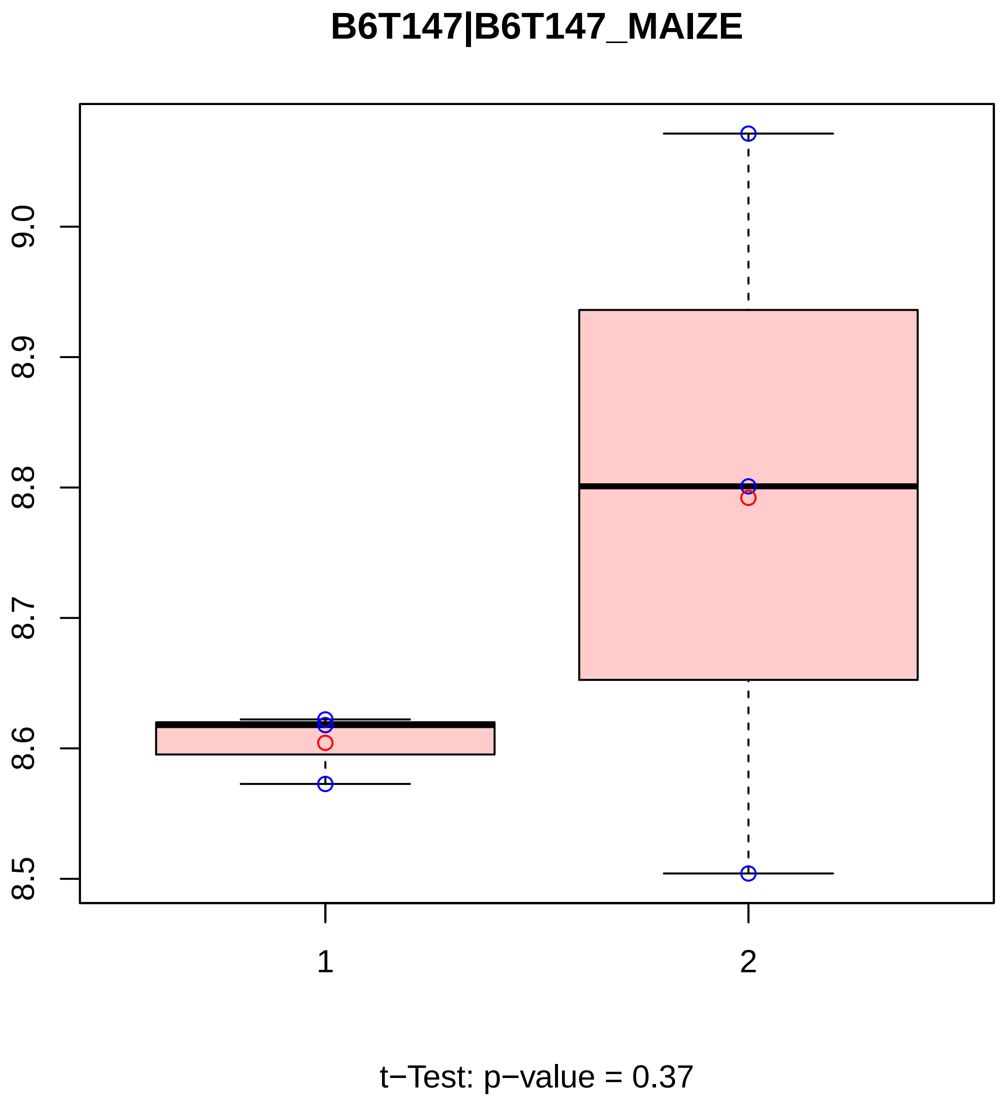

B6T147|B6T147_MAIZE
9.0
8.9
8.8
8.7
8.6
8.5
1
2
t−Test: p−value = 0.37

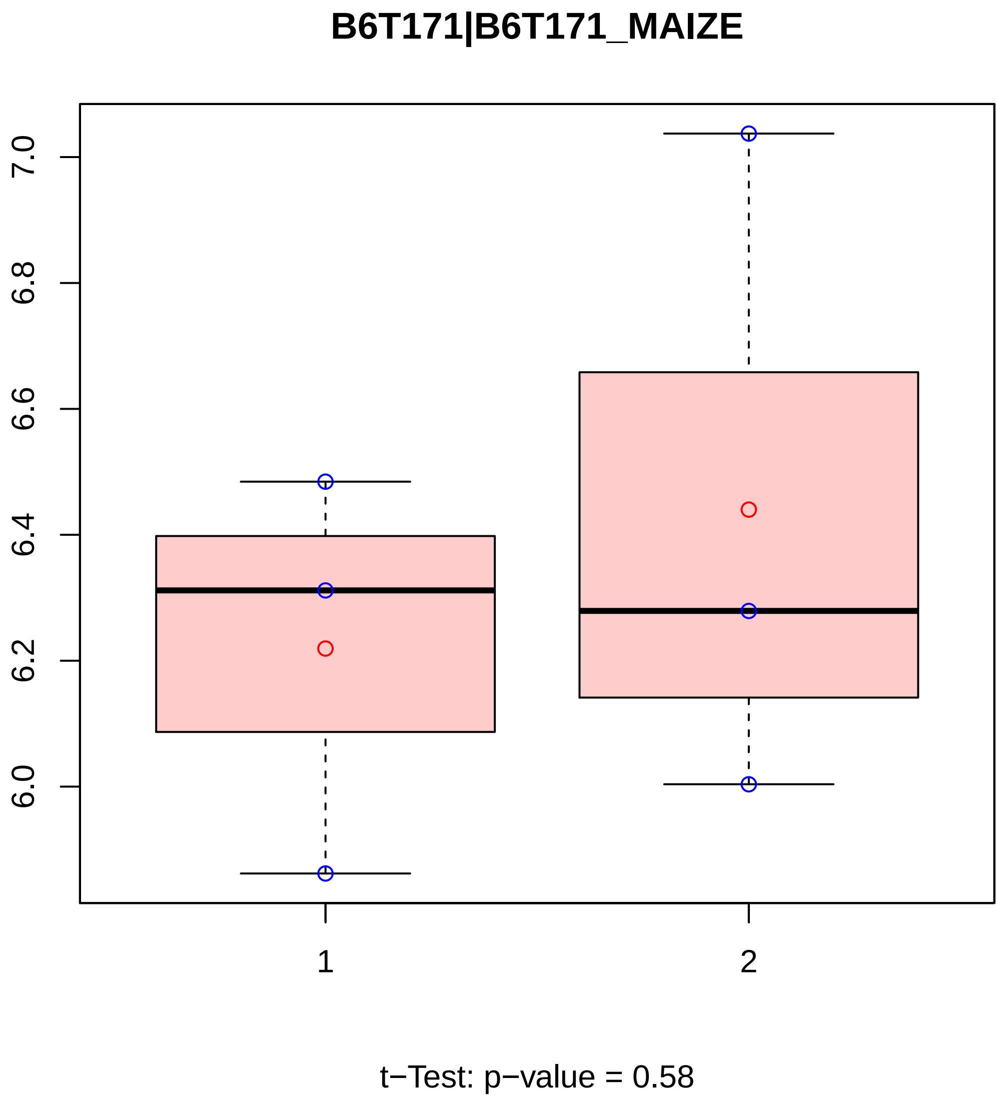
B6T171|B6T171_MAIZE
7.0
6.8
6.6
6.4
6.2
6.0
1
2
t−Test: p−value = 0.58

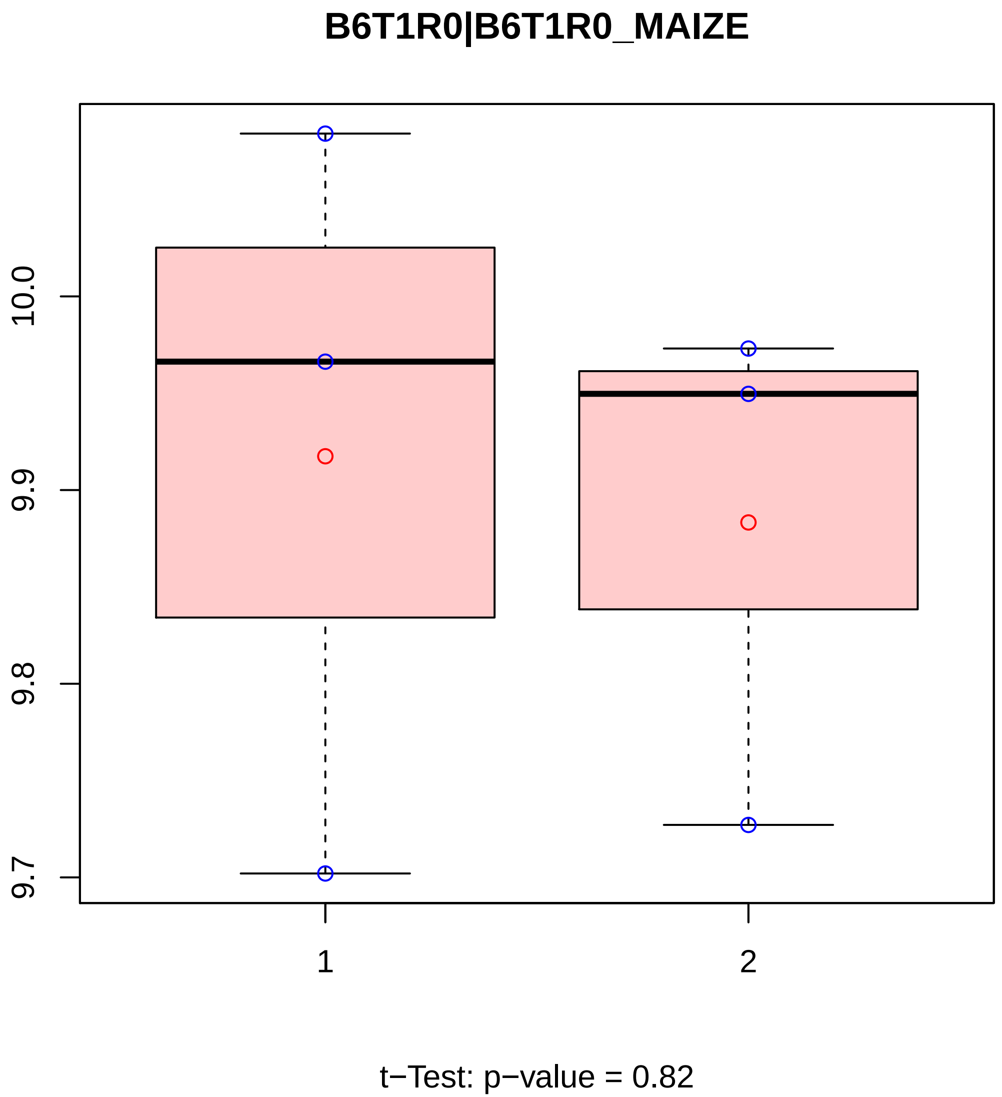

B6T1R0|B6T1R0_MAIZE
10.0
9.9
9.8
9.7
1
2
t−Test: p−value = 0.82

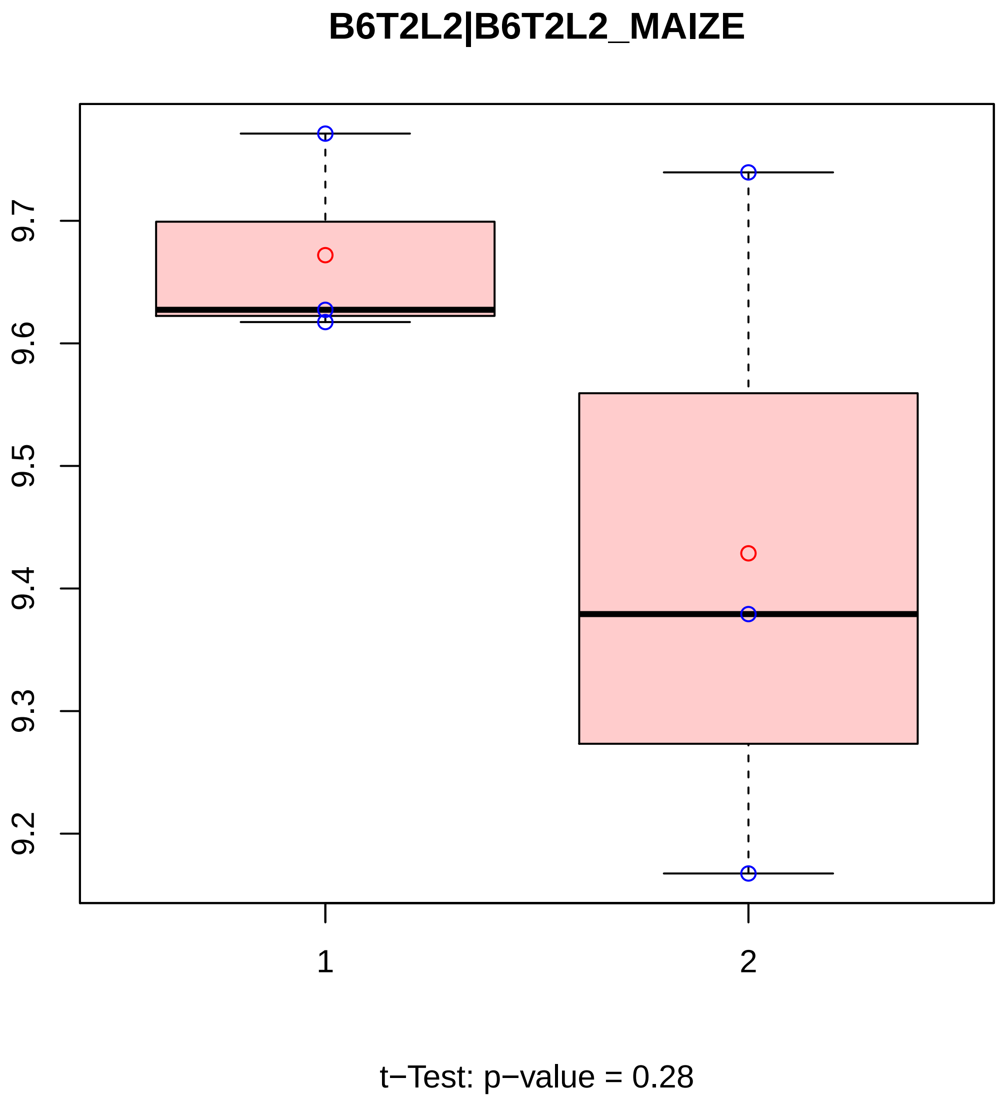
B6T2L2|B6T2L2_MAIZE
9.2
9.3
9.4
9.5
9.6
9.7
1
2
t−Test: p−value = 0.28

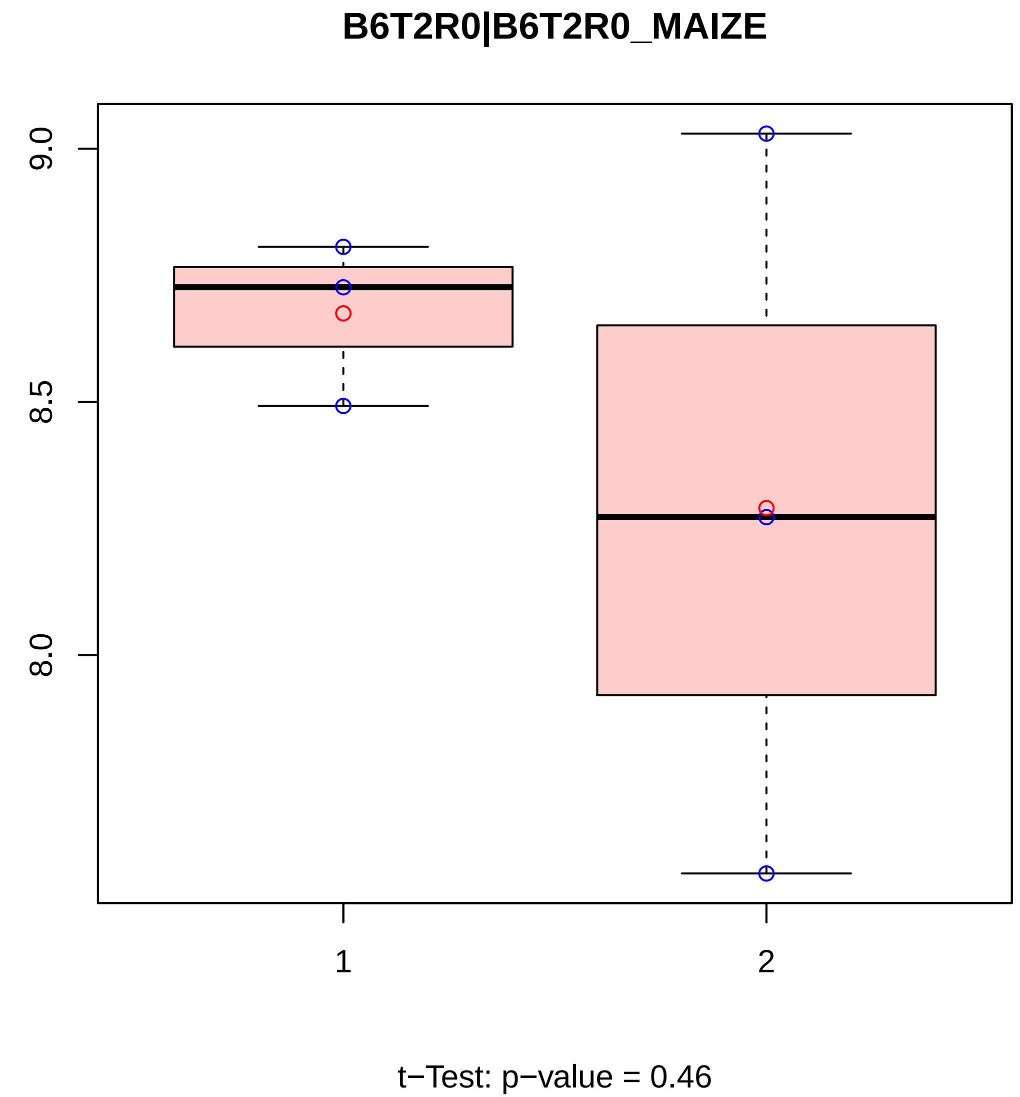
B6T2R0|B6T2R0_MAIZE
9.0
8.5
8.0
1
2
t−Test: p−value = 0.46

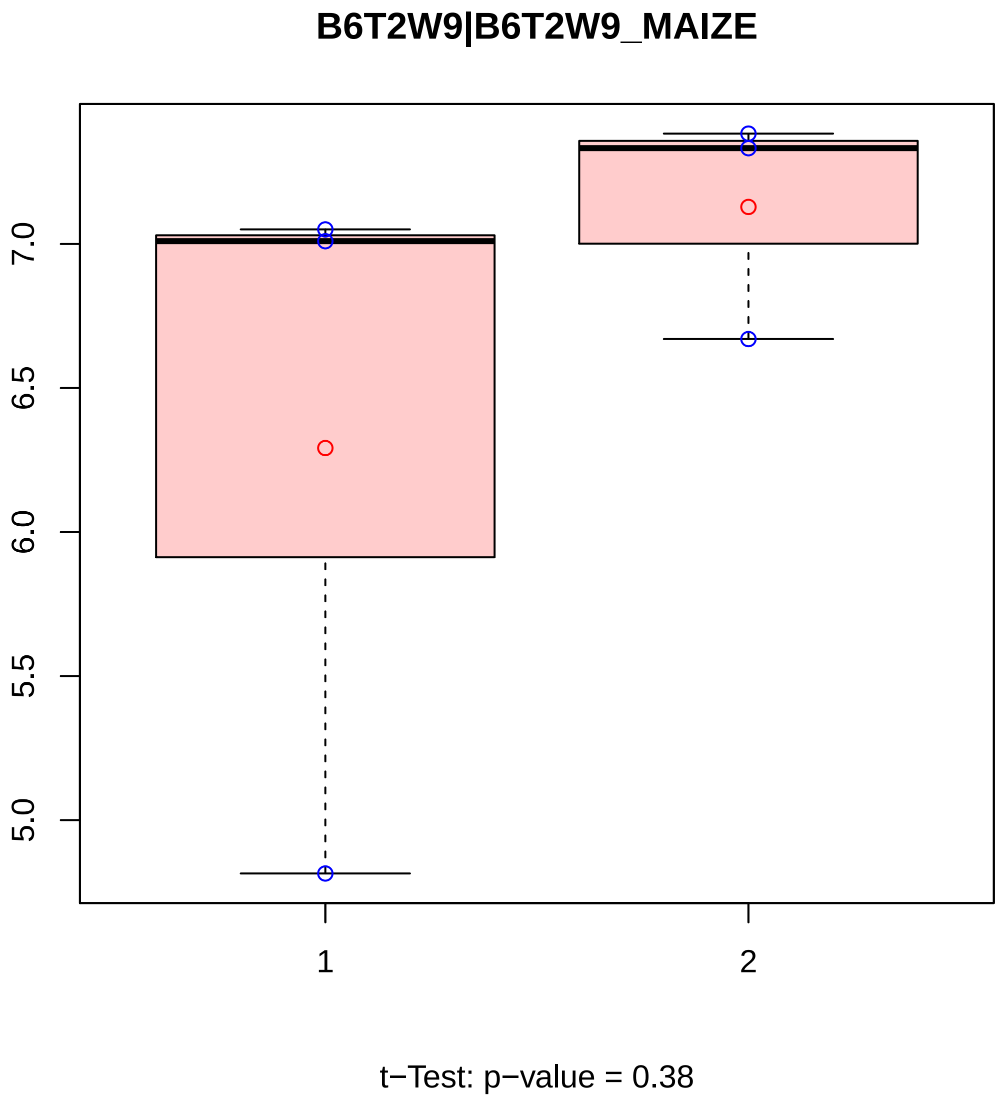
B6T2W9|B6T2W9_MAIZE
7.0
6.5
6.0
5.5
5.0
1
2
t−Test: p−value = 0.38

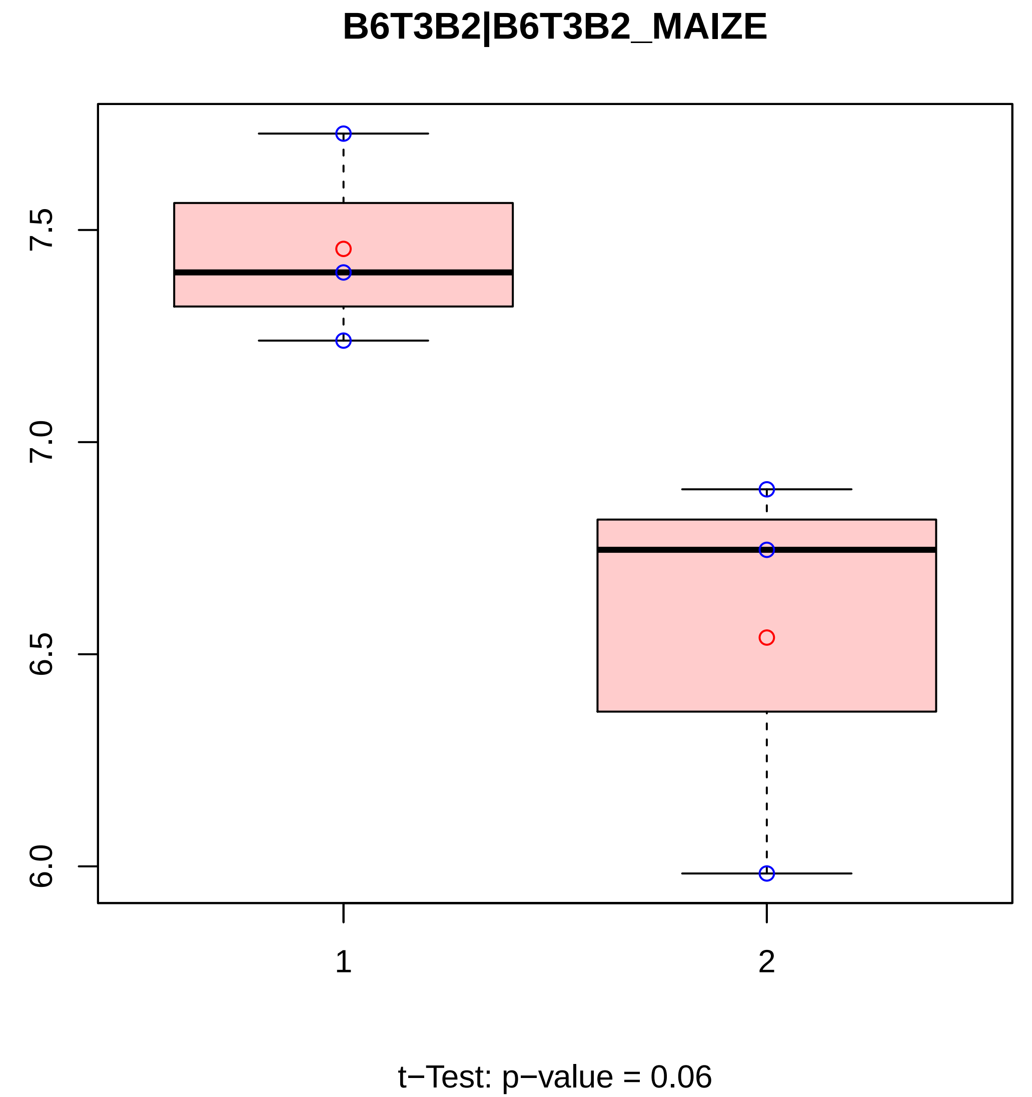

B6T3B2|B6T3B2_MAIZE
7.5
7.0
6.5
6.0
1
2
t−Test: p−value = 0.06

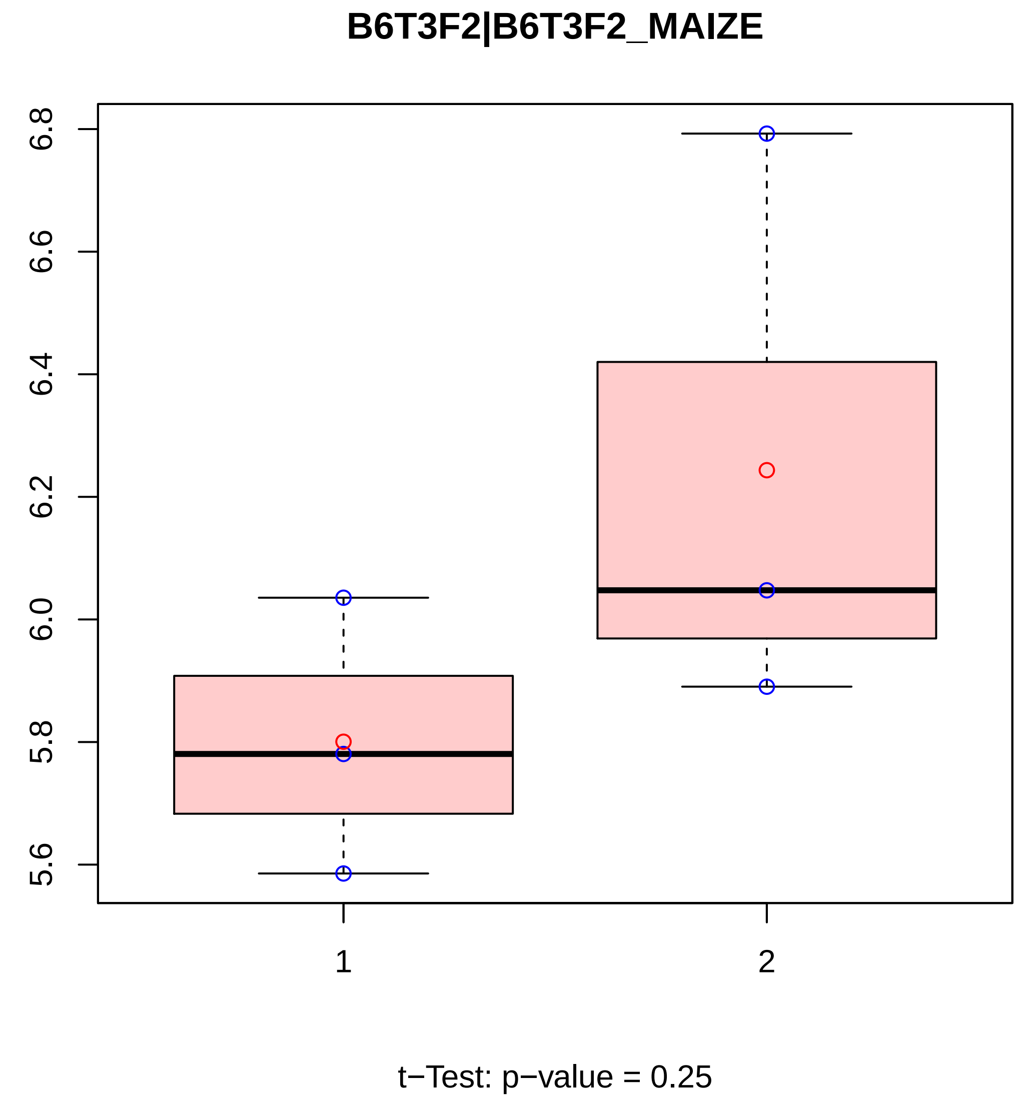
B6T3F2|B6T3F2_MAIZE
6.8
6.6
6.4
6.2
6.0
5.8
5.6
1
2
t−Test: p−value = 0.25

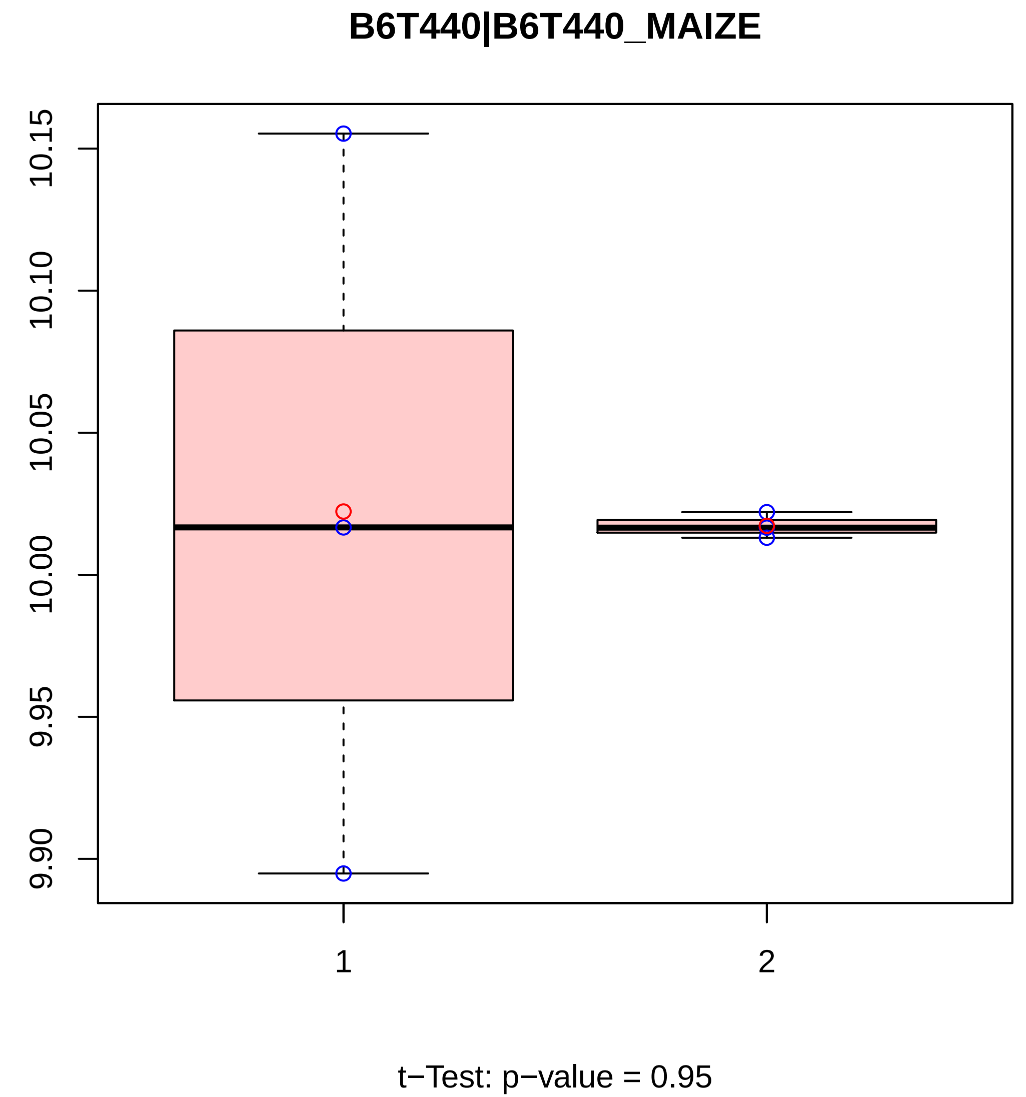
B6T440|B6T440_MAIZE
10.15
10.10
10.05
10.00
9.95
9.90
1
2
t−Test: p−value = 0.95

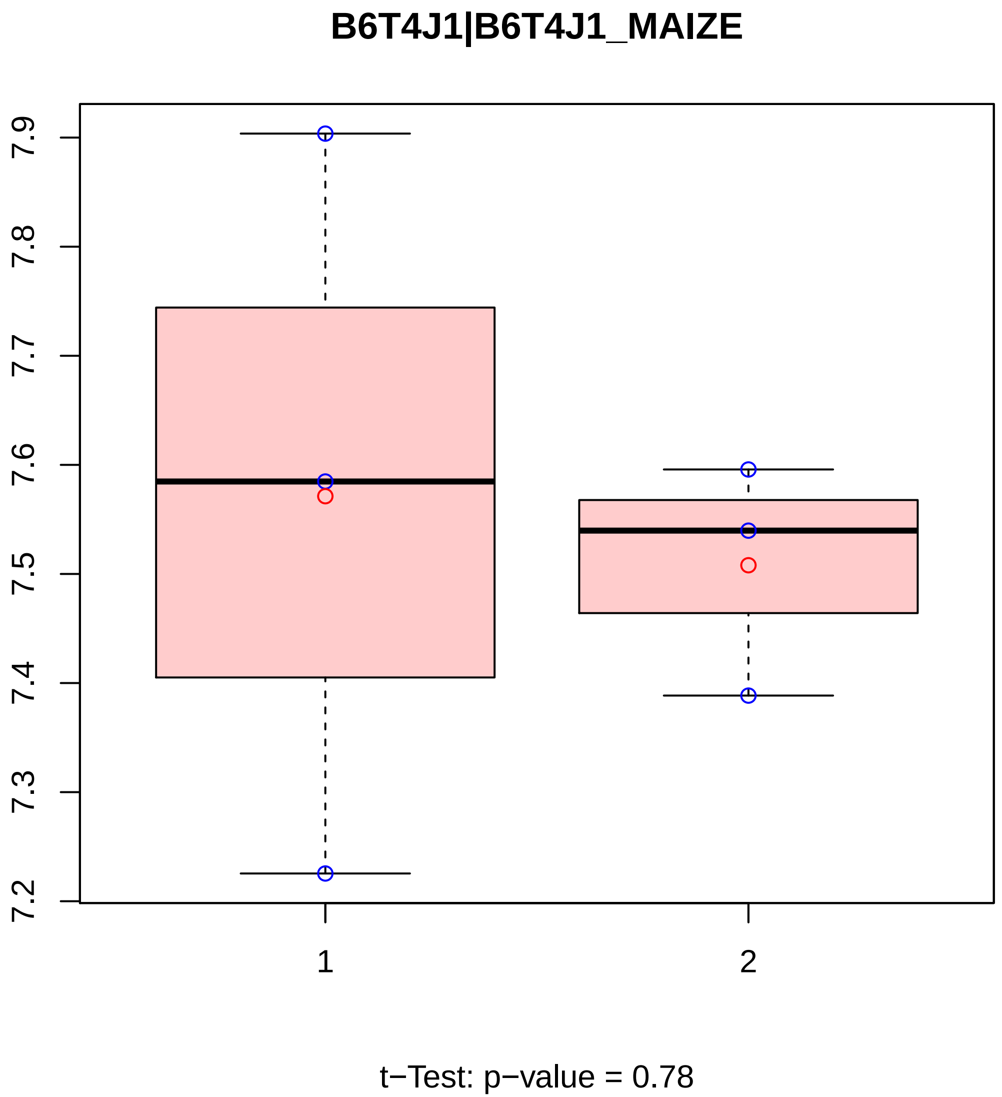
B6T4J1|B6T4J1_MAIZE
7.2
7.3
7.4
7.5
7.6
7.7
7.8
7.9
1
2
t−Test: p−value = 0.78

B6T4U8|B6T4U8_MAIZE

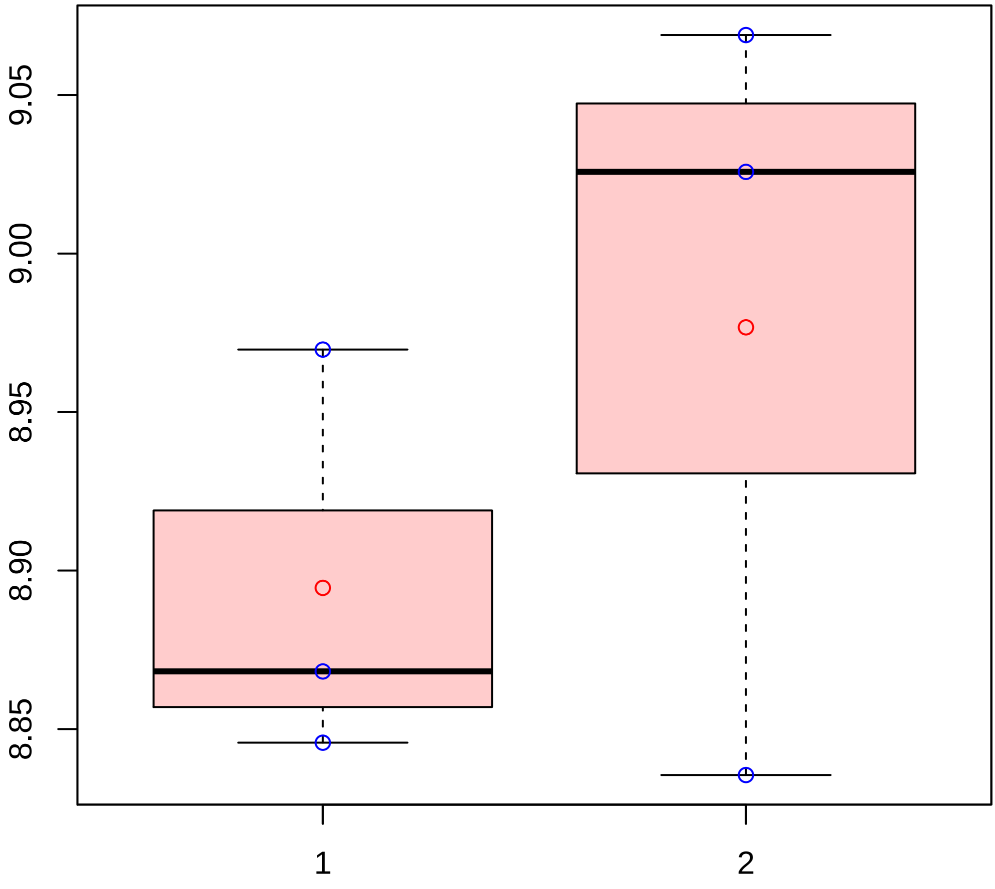

t−Test: p−value = 0.39

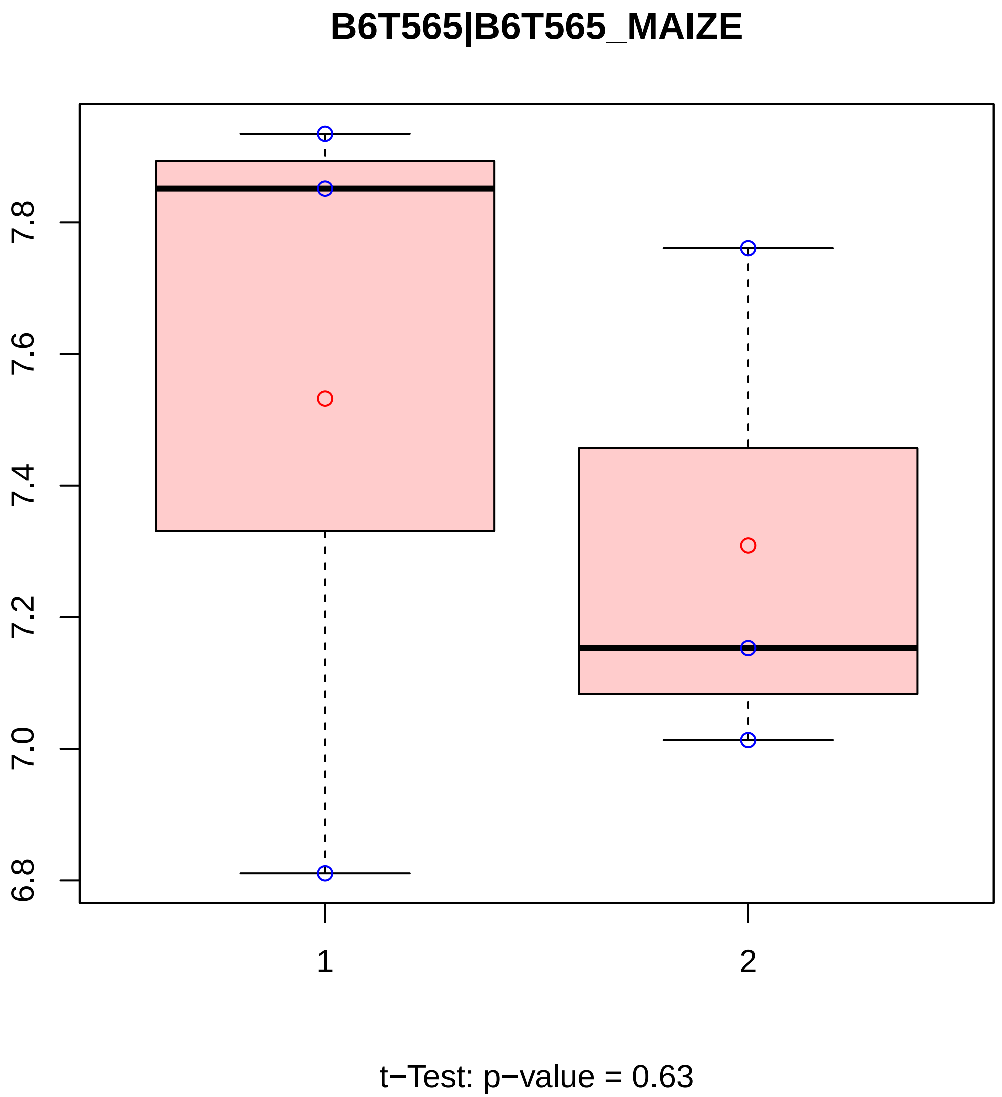
B6T565|B6T565_MAIZE
7.8
7.6
7.4
7.2
7.0
6.8
1
2
t−Test: p−value = 0.63

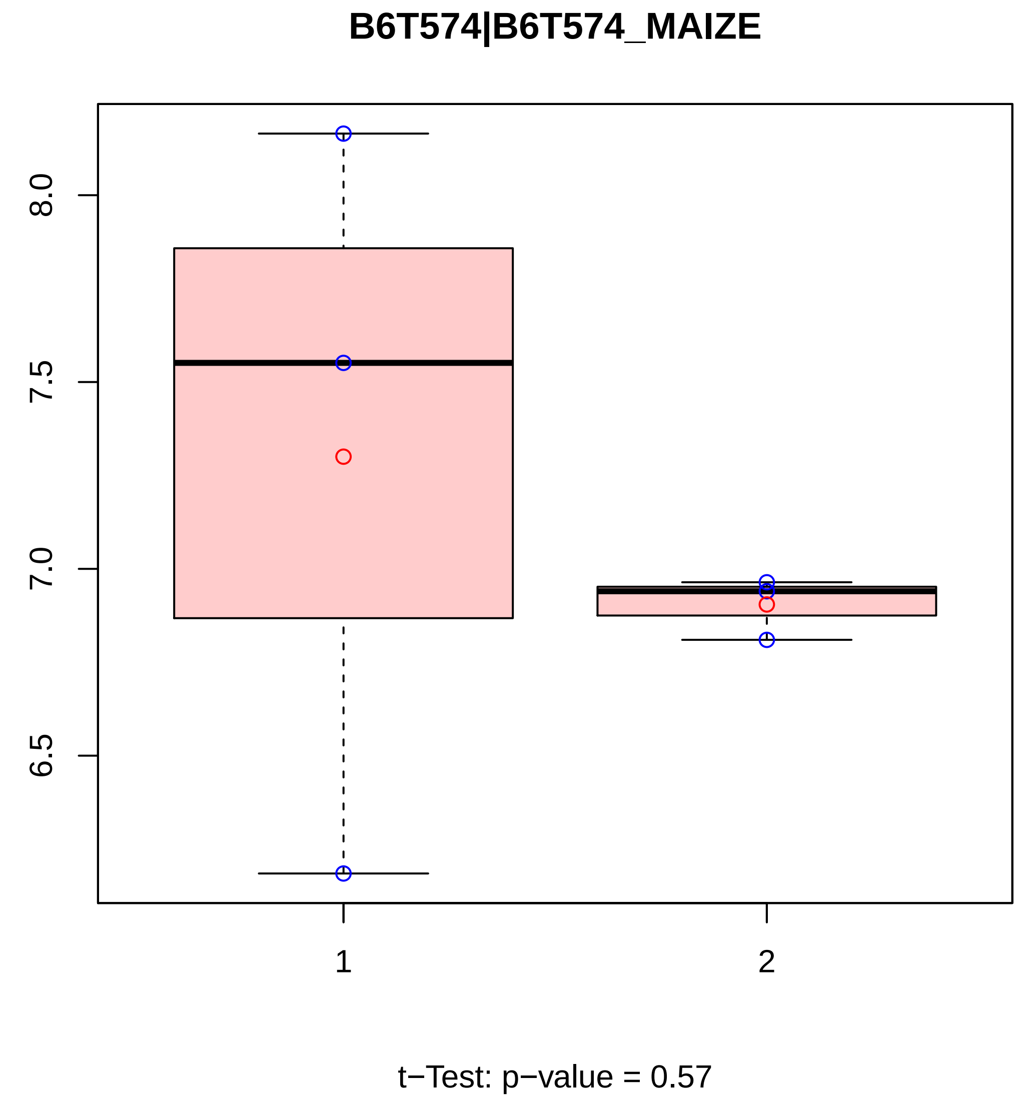
B6T574|B6T574_MAIZE
8.0
7.5
7.0
6.5
1
2
t−Test: p−value = 0.57

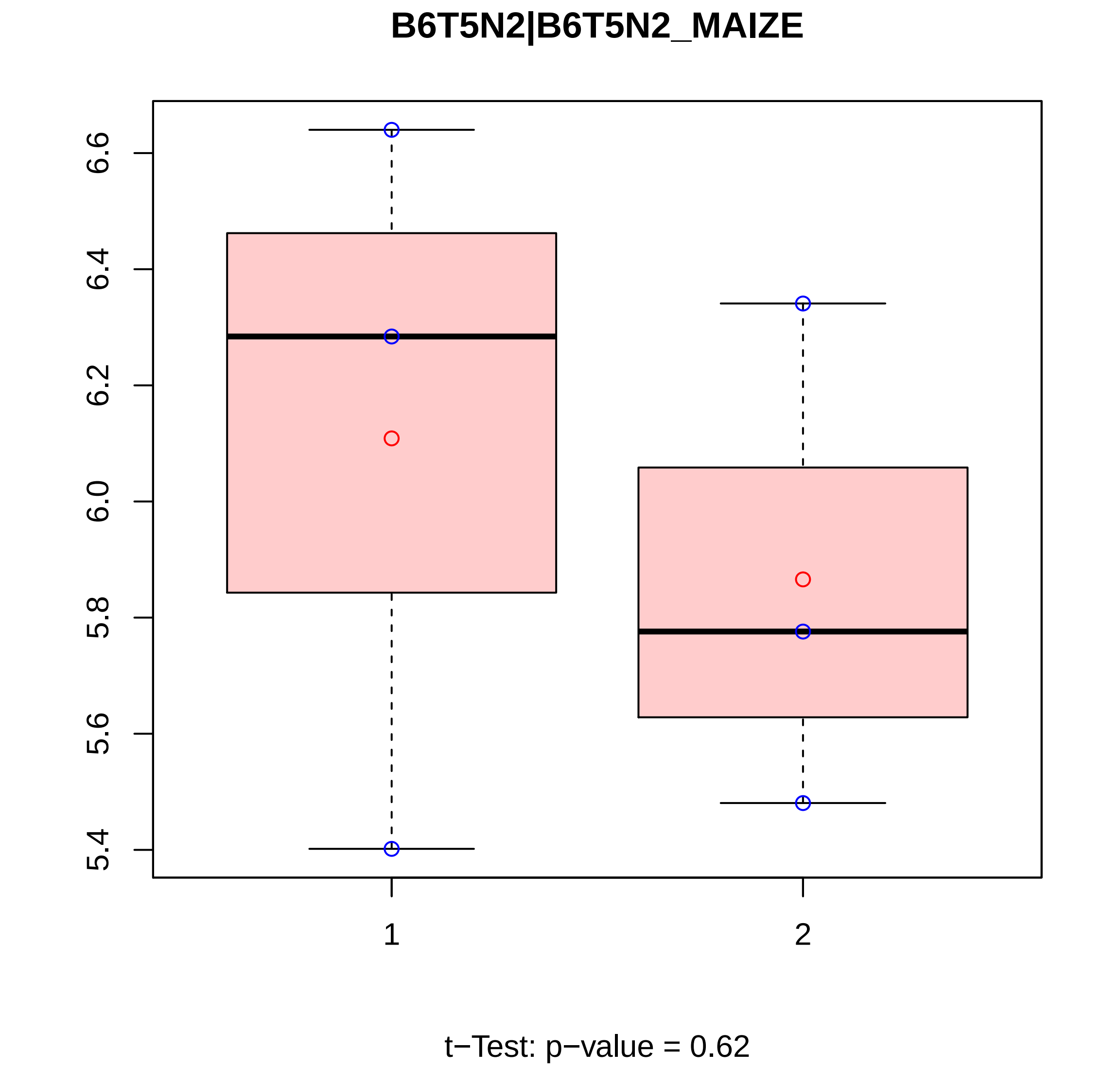
B6T5N2|B6T5N2_MAIZE
6.6
6.4
6.2
6.0
5.8
5.6
5.4
1
2
t−Test: p−value = 0.62

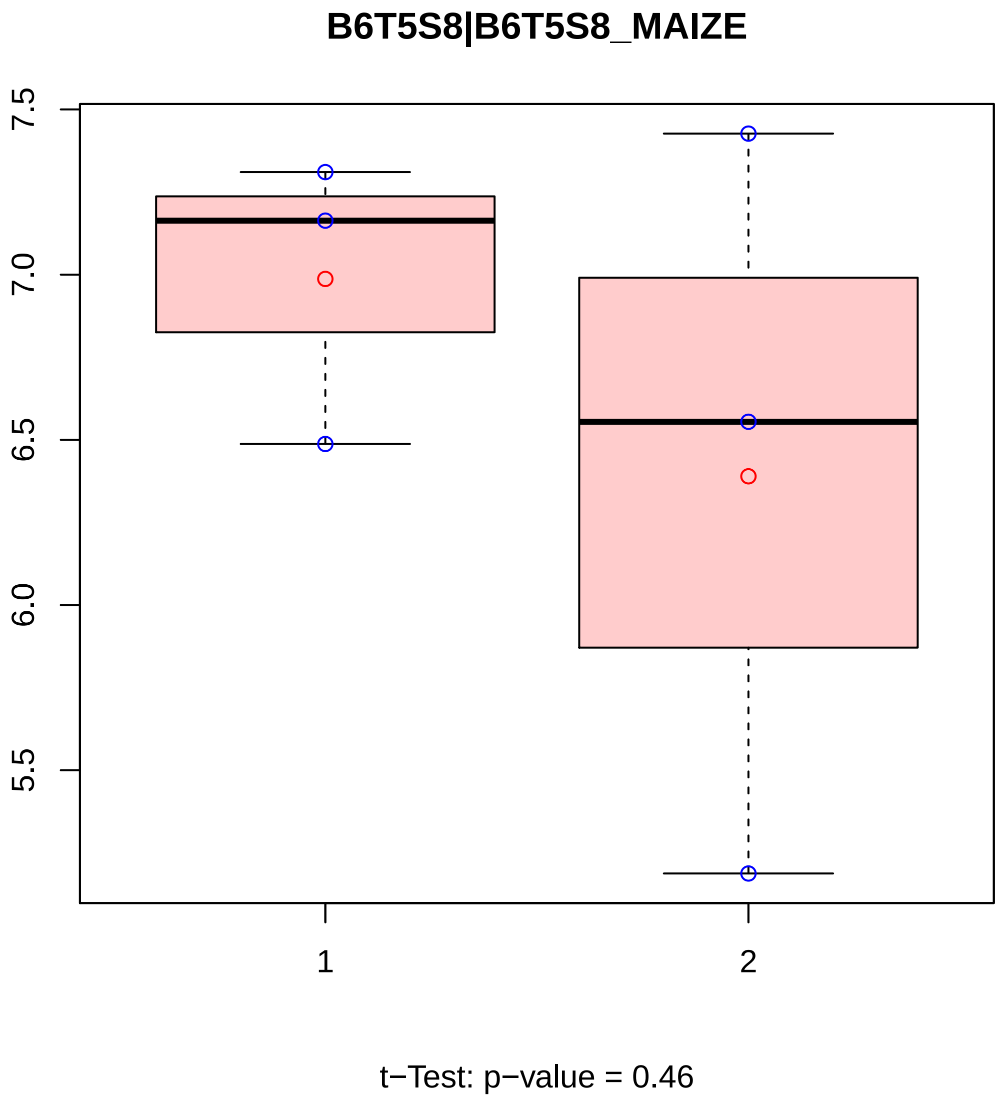

B6T5S8|B6T5S8_MAIZE
7.5
7.0
6.5
6.0
5.5
1
2
t−Test: p−value = 0.46

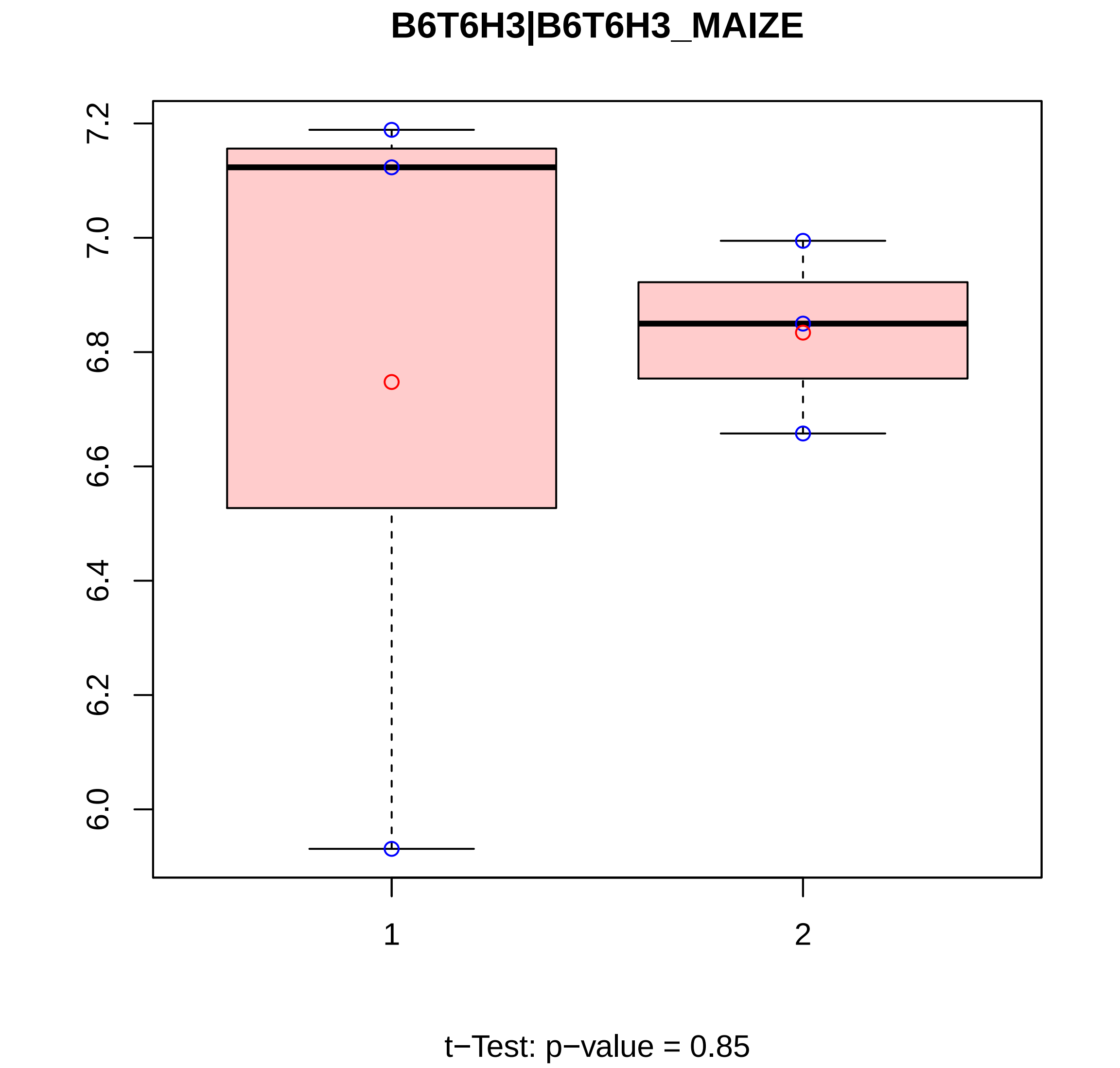

B6T6H3|B6T6H3_MAIZE
7.2
7.0
6.8
6.6
6.4
6.2
6.0
1
2
t−Test: p−value = 0.85

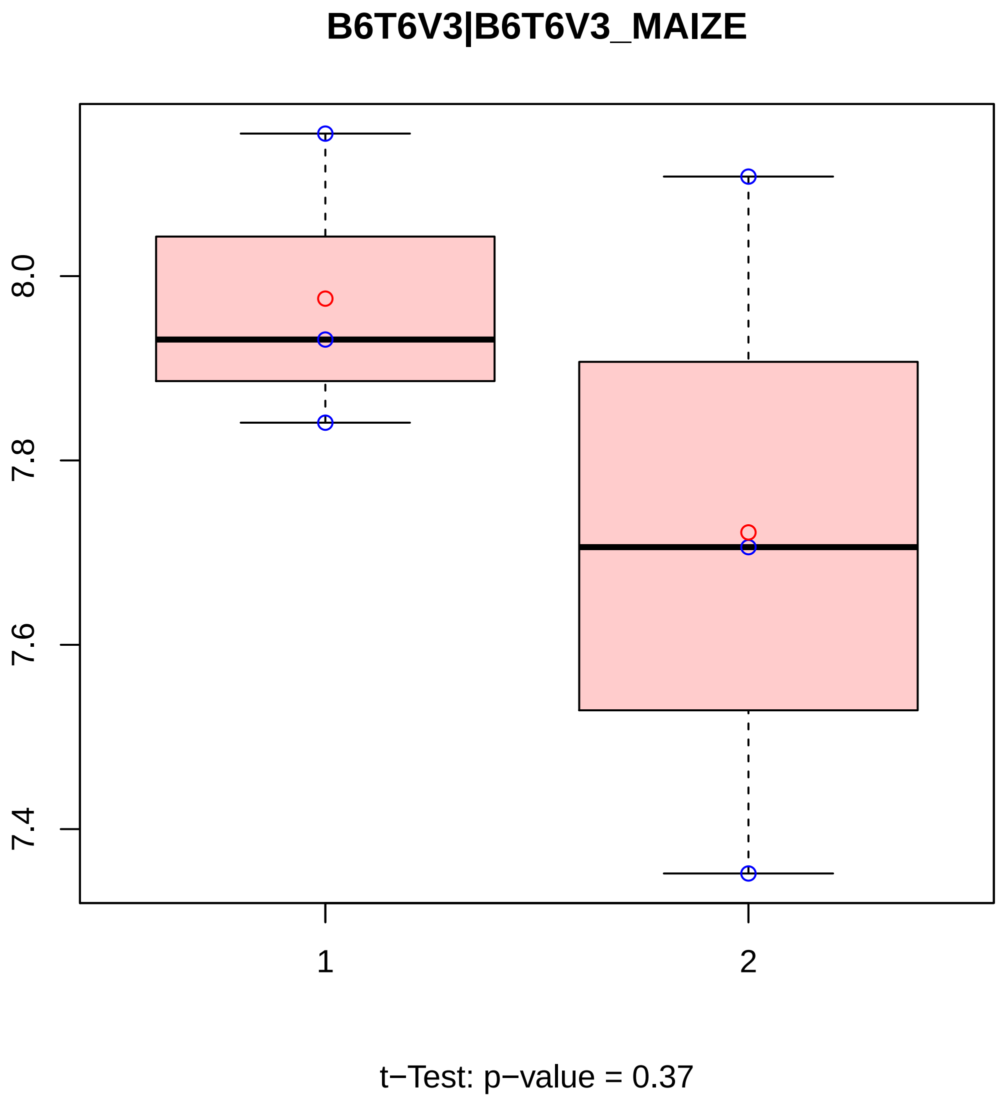
B6T6V3|B6T6V3_MAIZE
8.0
7.8
7.6
7.4
1
2
t−Test: p−value = 0.37

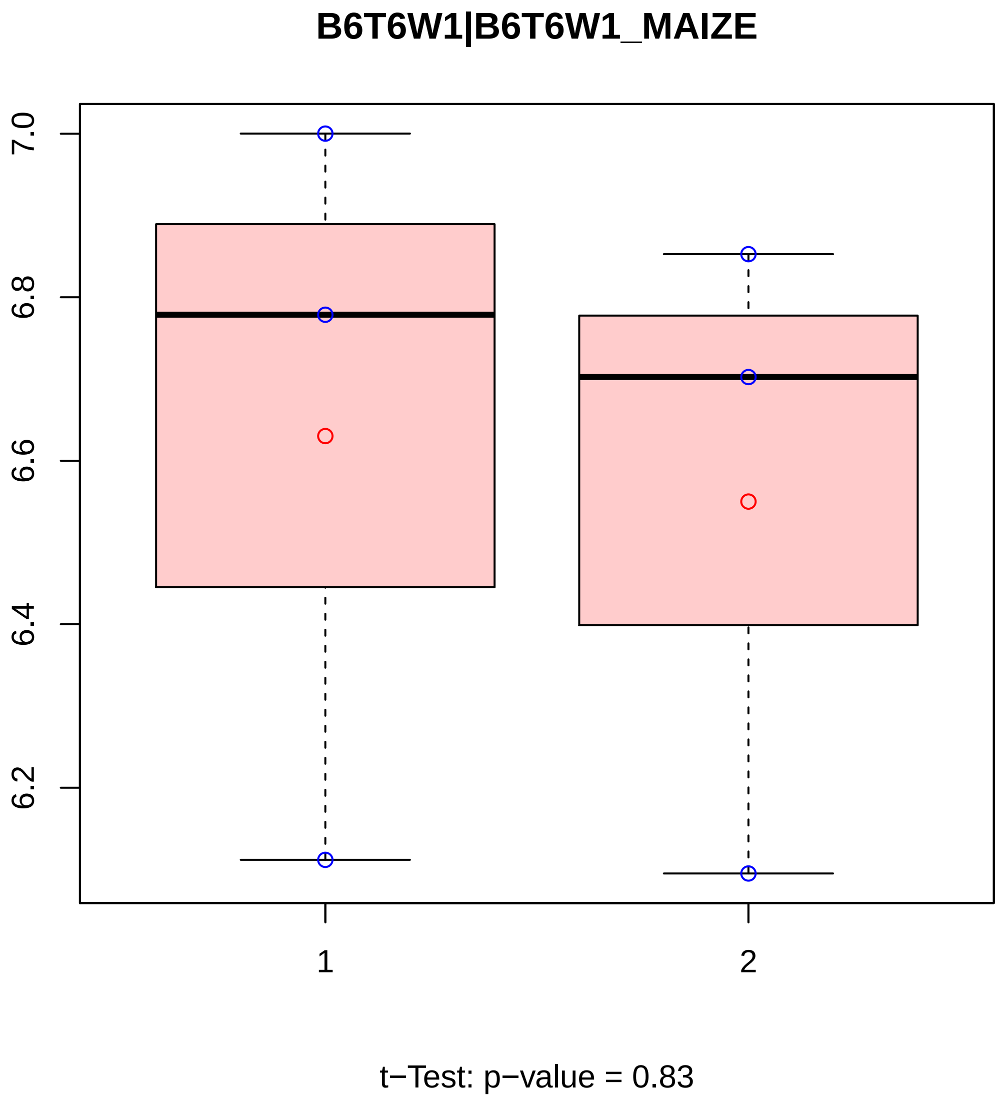

B6T6W1|B6T6W1_MAIZE
7.0
6.8
6.6
6.4
6.2
1
2
t−Test: p−value = 0.83

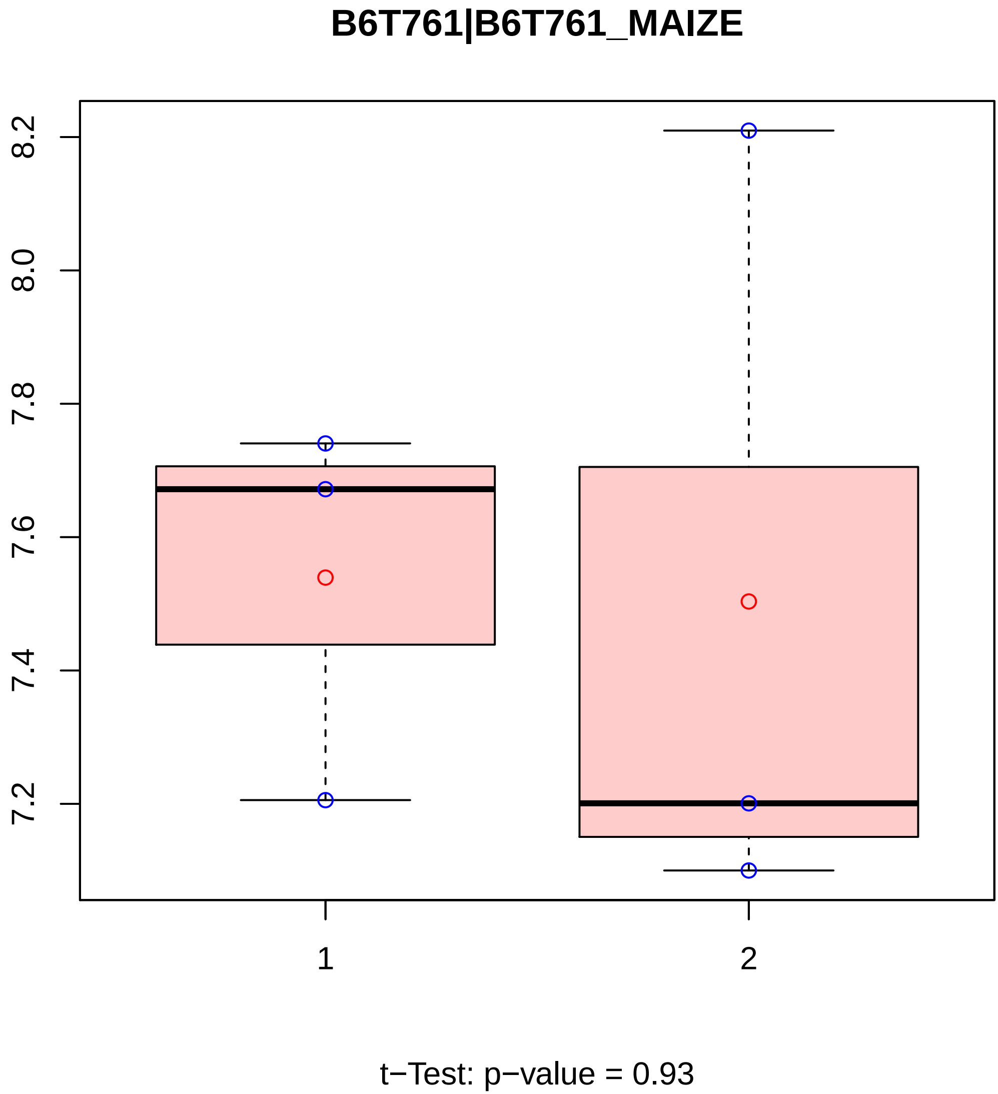
B6T761|B6T761_MAIZE
8.2
8.0
7.8
7.6
7.4
7.2
1
2
t−Test: p−value = 0.93

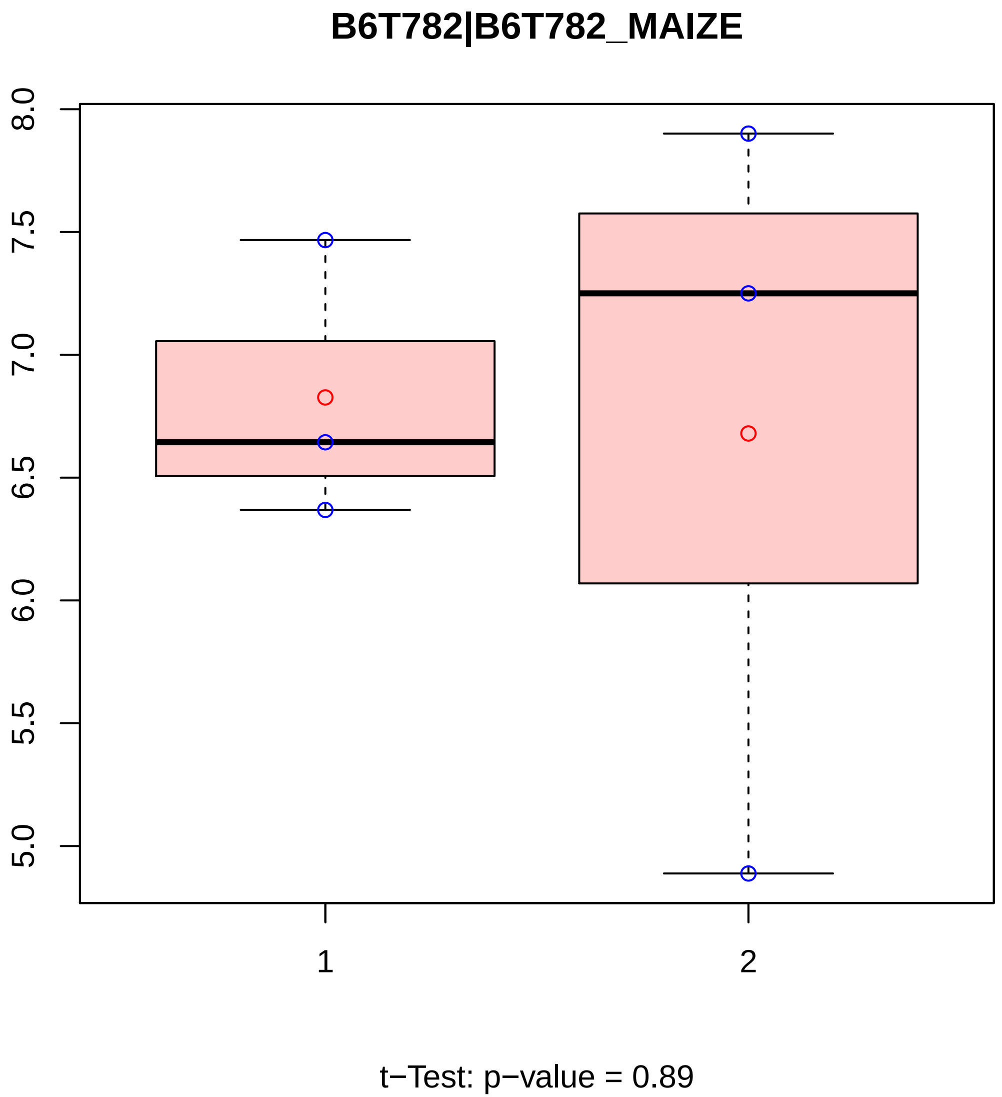
B6T782|B6T782_MAIZE
8.0
7.5
7.0
6.5
6.0
5.5
5.0
1
2
t−Test: p−value = 0.89

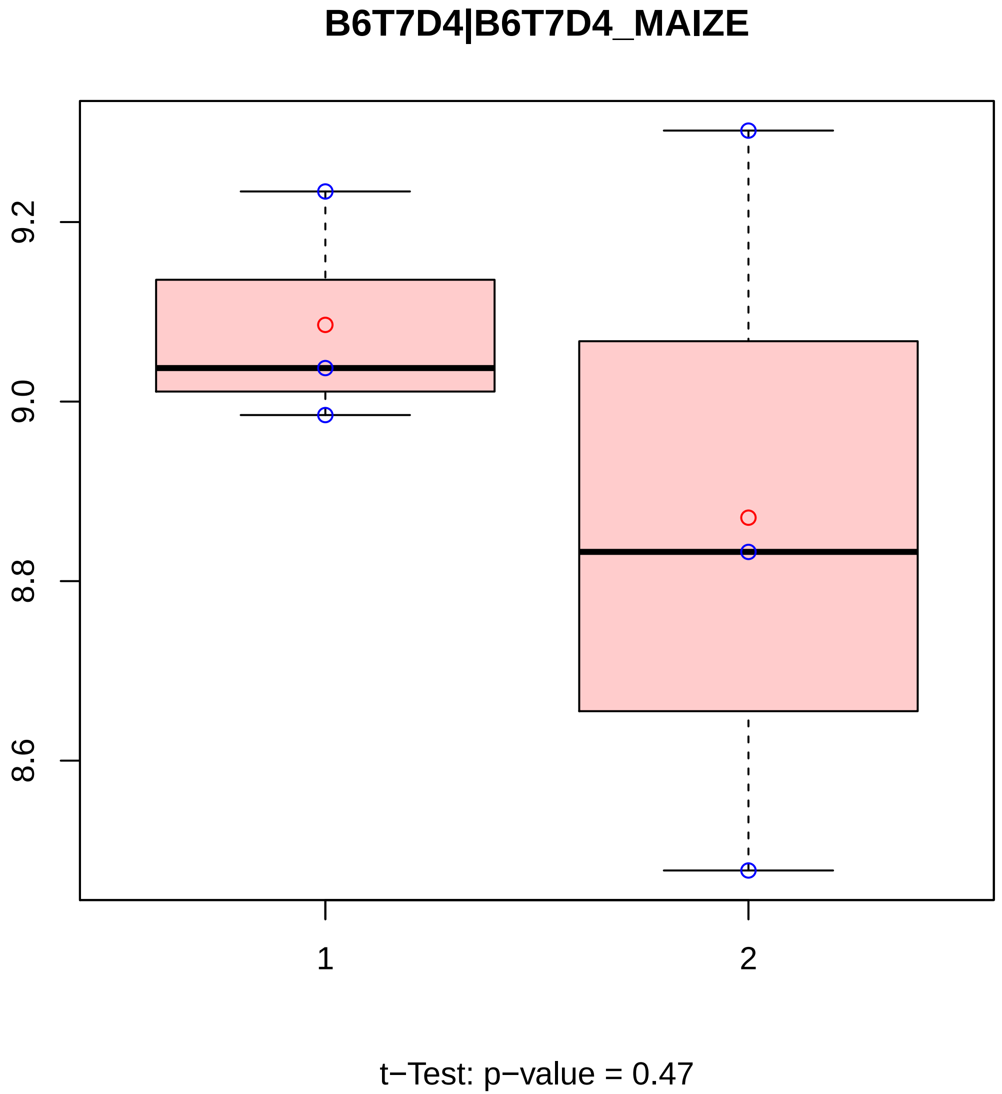
B6T7D4|B6T7D4_MAIZE
9.2
9.0
8.8
8.6
1
2
t−Test: p−value = 0.47

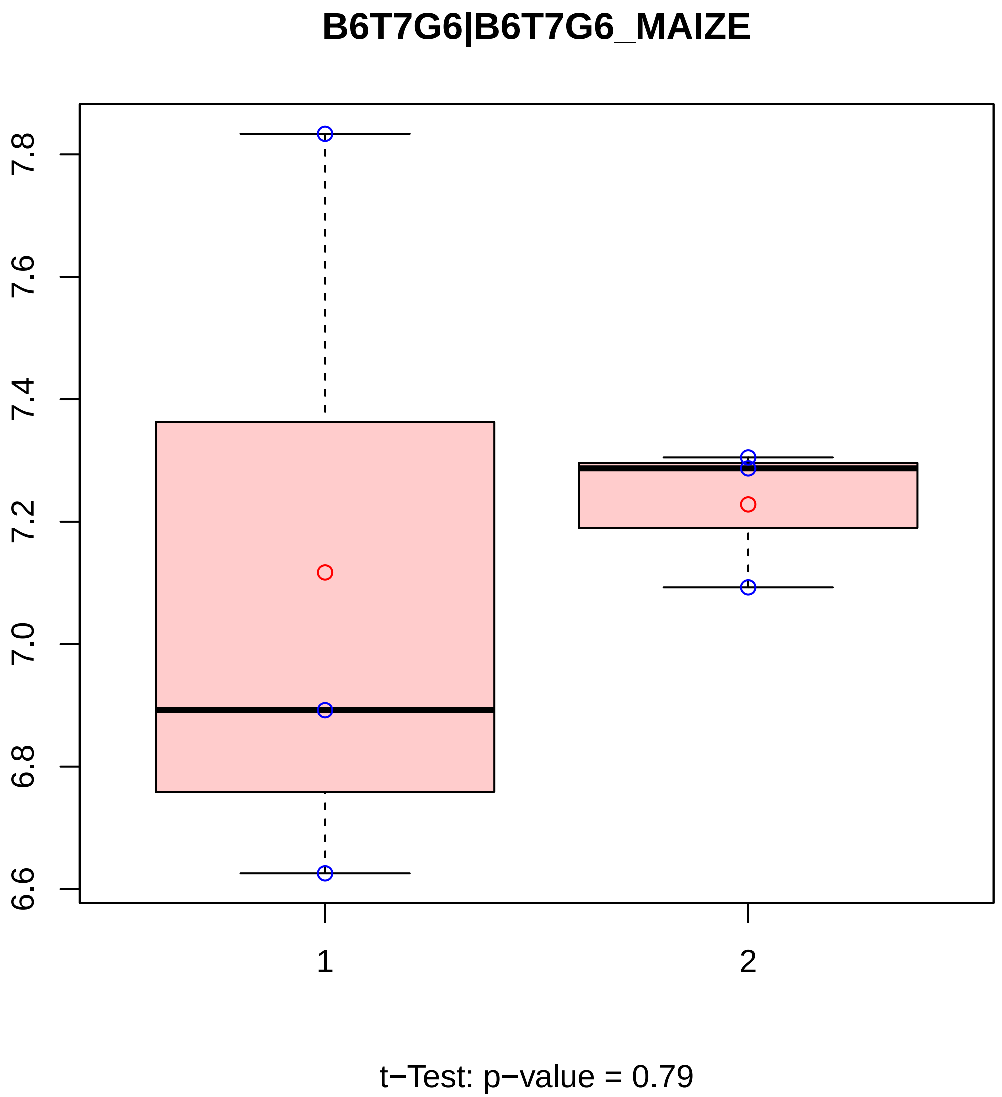
B6T7G6|B6T7G6_MAIZE
7.8
7.6
7.4
7.2
7.0
6.8
6.6
1
2
t−Test: p−value = 0.79

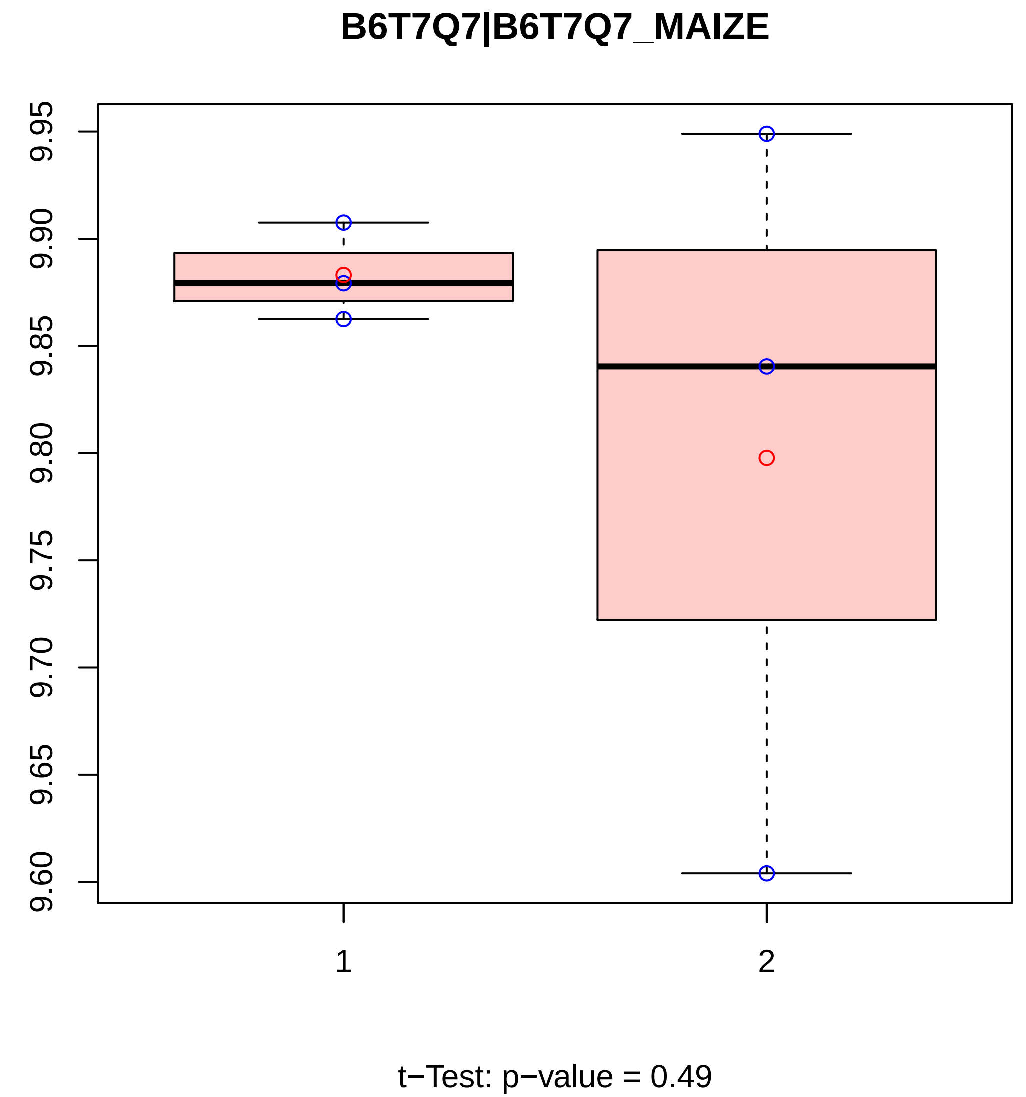

B6T7Q7|B6T7Q7_MAIZE
9.60
9.65
9.70
9.75
9.80
9.85
9.90
9.95
1
2
t−Test: p−value = 0.49

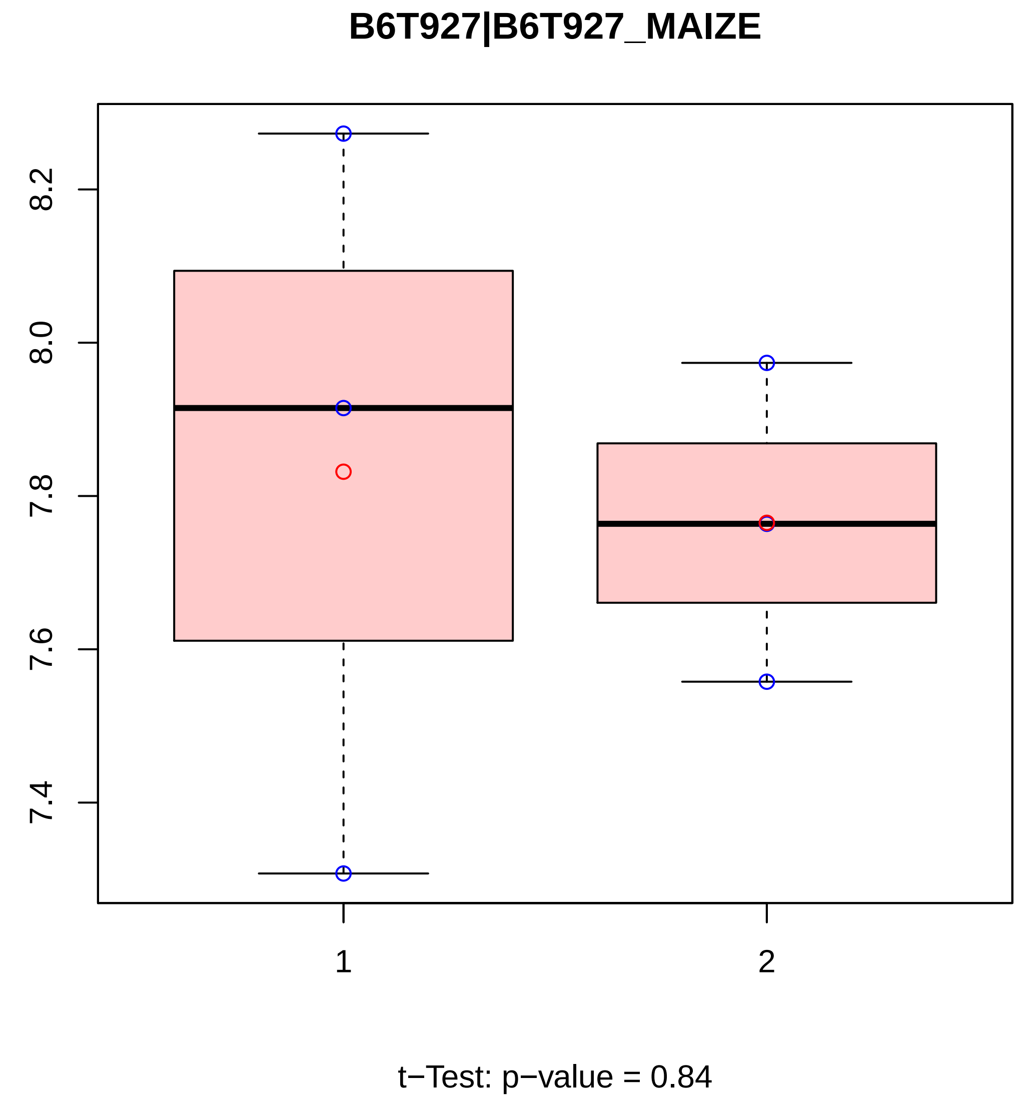
B6T927|B6T927_MAIZE
8.2
8.0
7.8
7.6
7.4
1
2
t−Test: p−value = 0.84

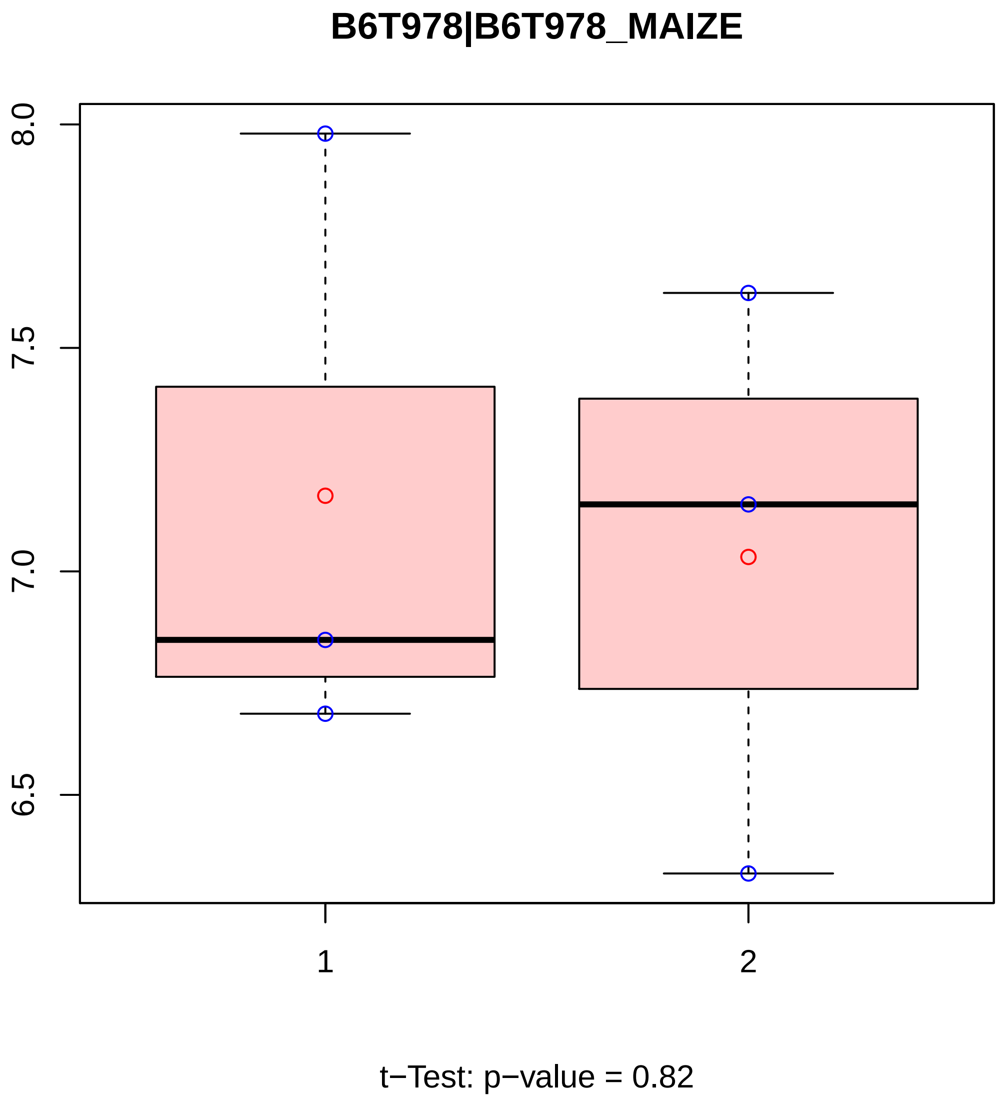

B6T978|B6T978_MAIZE
8.0
7.5
7.0
6.5
1
2
t−Test: p−value = 0.82

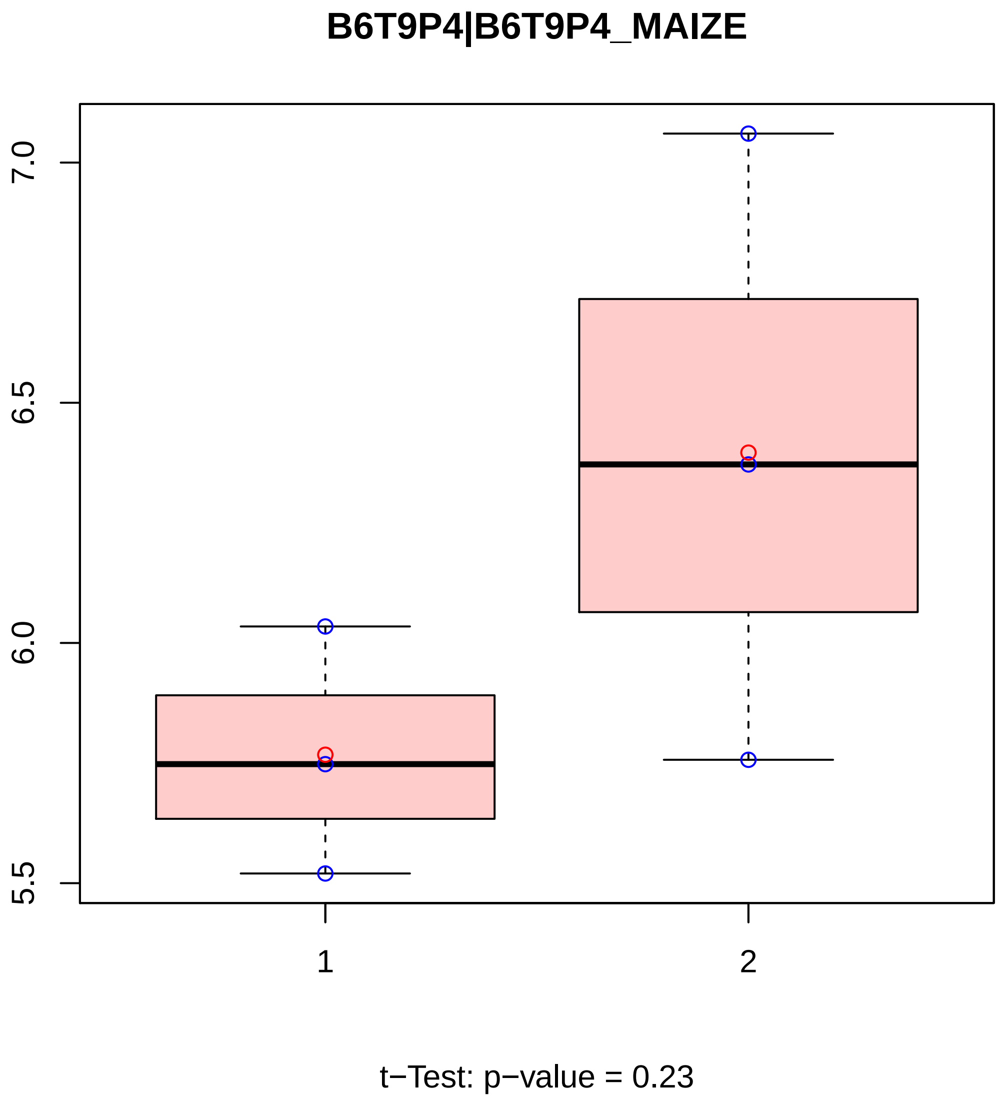
B6T9P4|B6T9P4_MAIZE
7.0
6.5
6.0
5.5
1
2
t−Test: p−value = 0.23

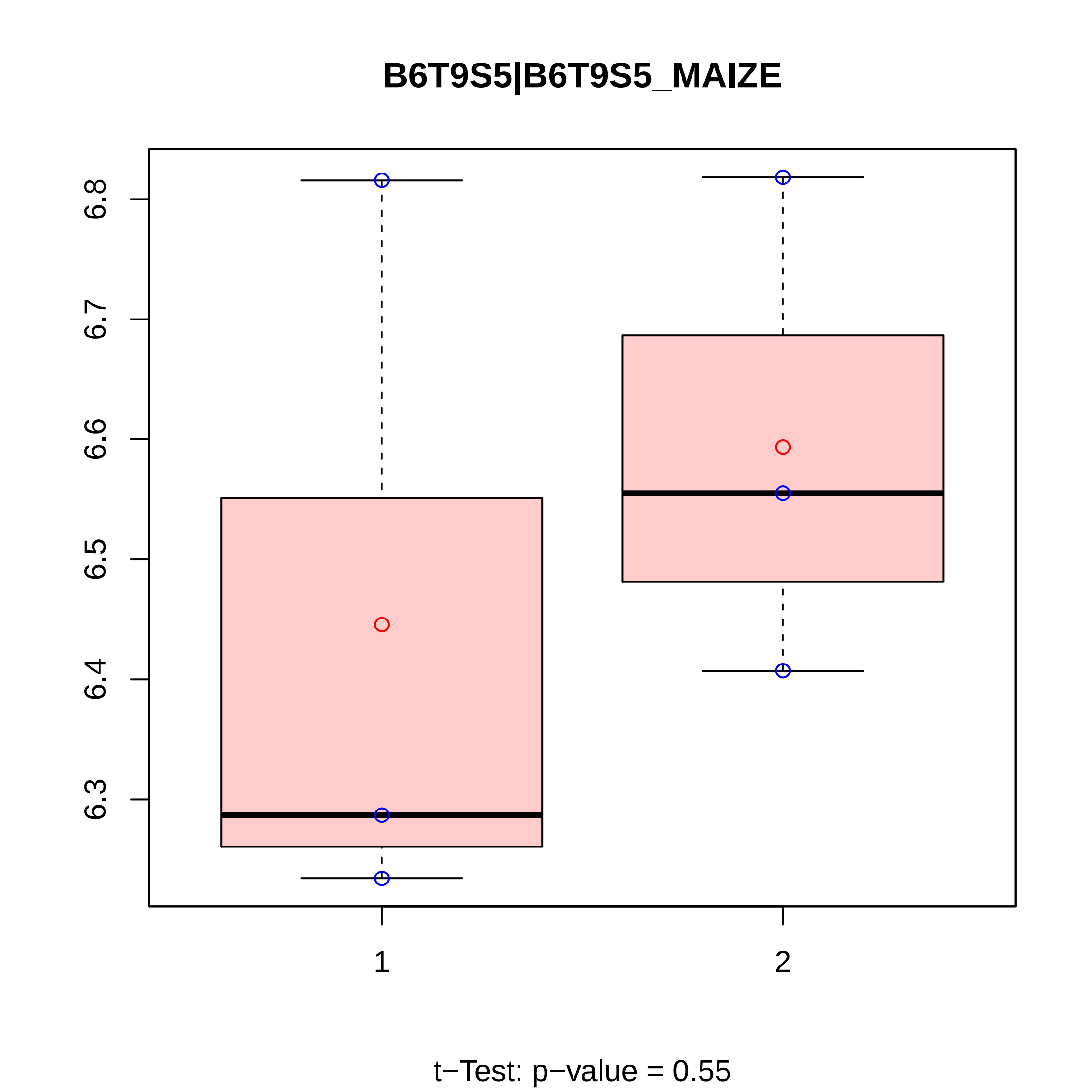

B6T9S5|B6T9S5_MAIZE
6.3
6.4
6.5
6.6
6.7
6.8
1
2
t−Test: p−value = 0.55

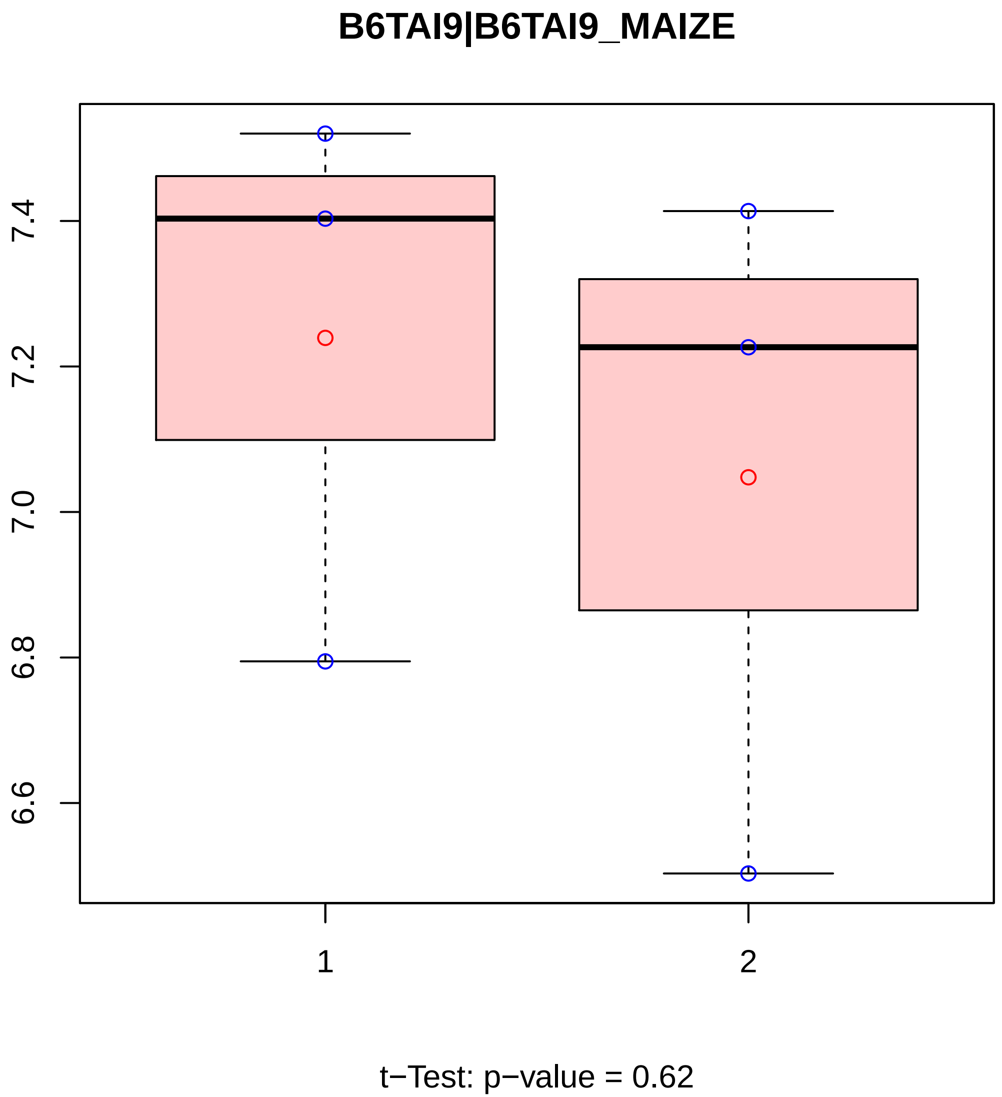
B6TAI9|B6TAI9_MAIZE
7.4
7.2
7.0
6.8
6.6
1
2
t−Test: p−value = 0.62

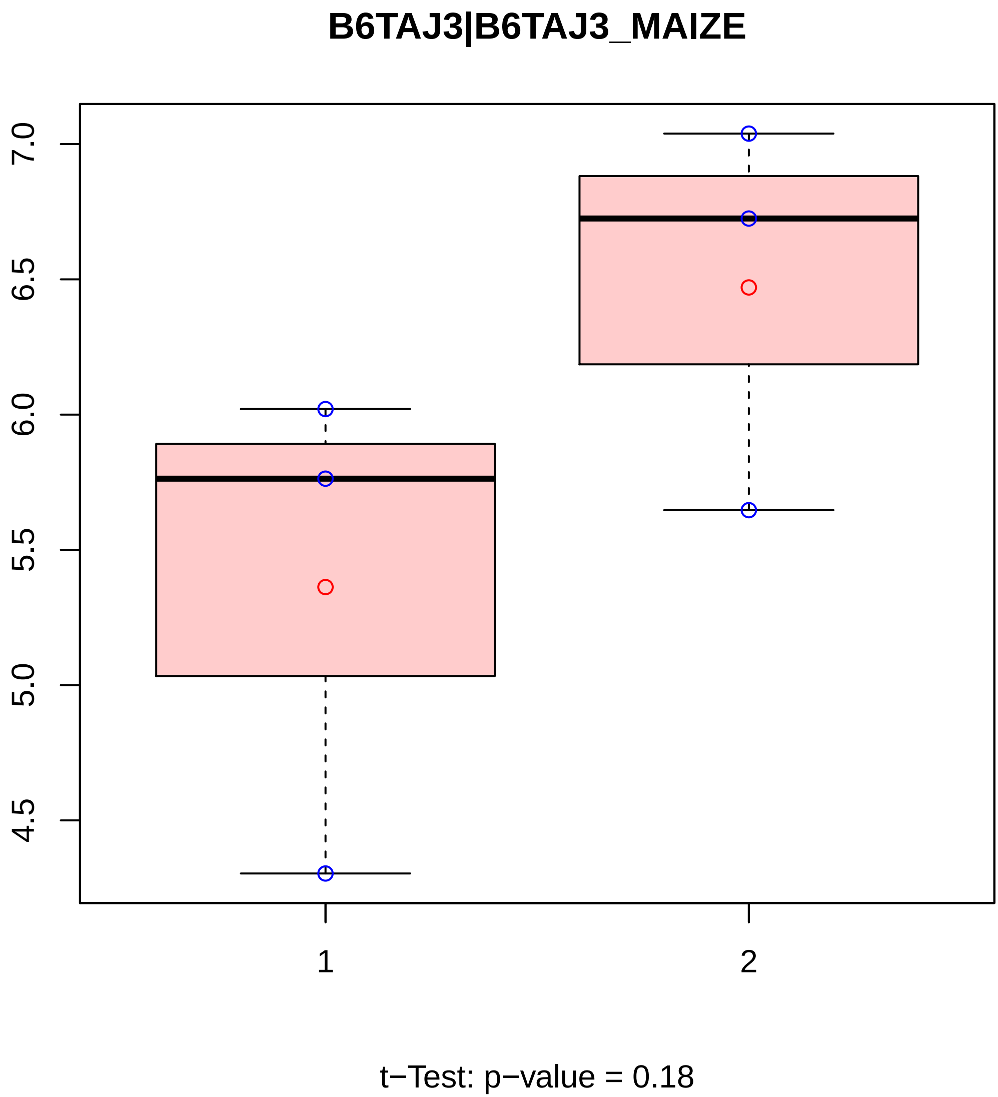
B6TAJ3|B6TAJ3_MAIZE
7.0
6.5
6.0
5.5
5.0
4.5
1
2
t−Test: p−value = 0.18

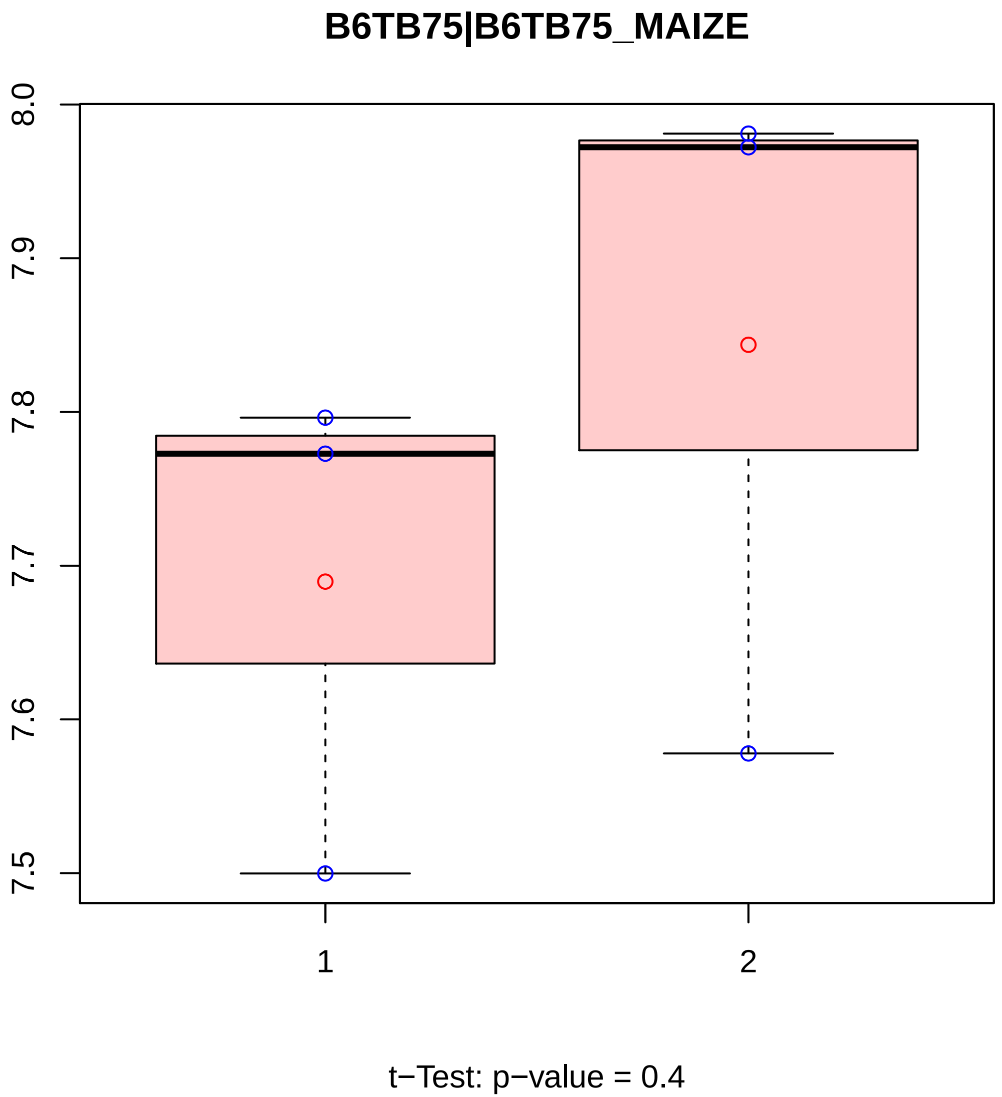
B6TB75|B6TB75_MAIZE
8.0
7.9
7.8
7.7
7.6
7.5
1
2
t−Test: p−value = 0.4

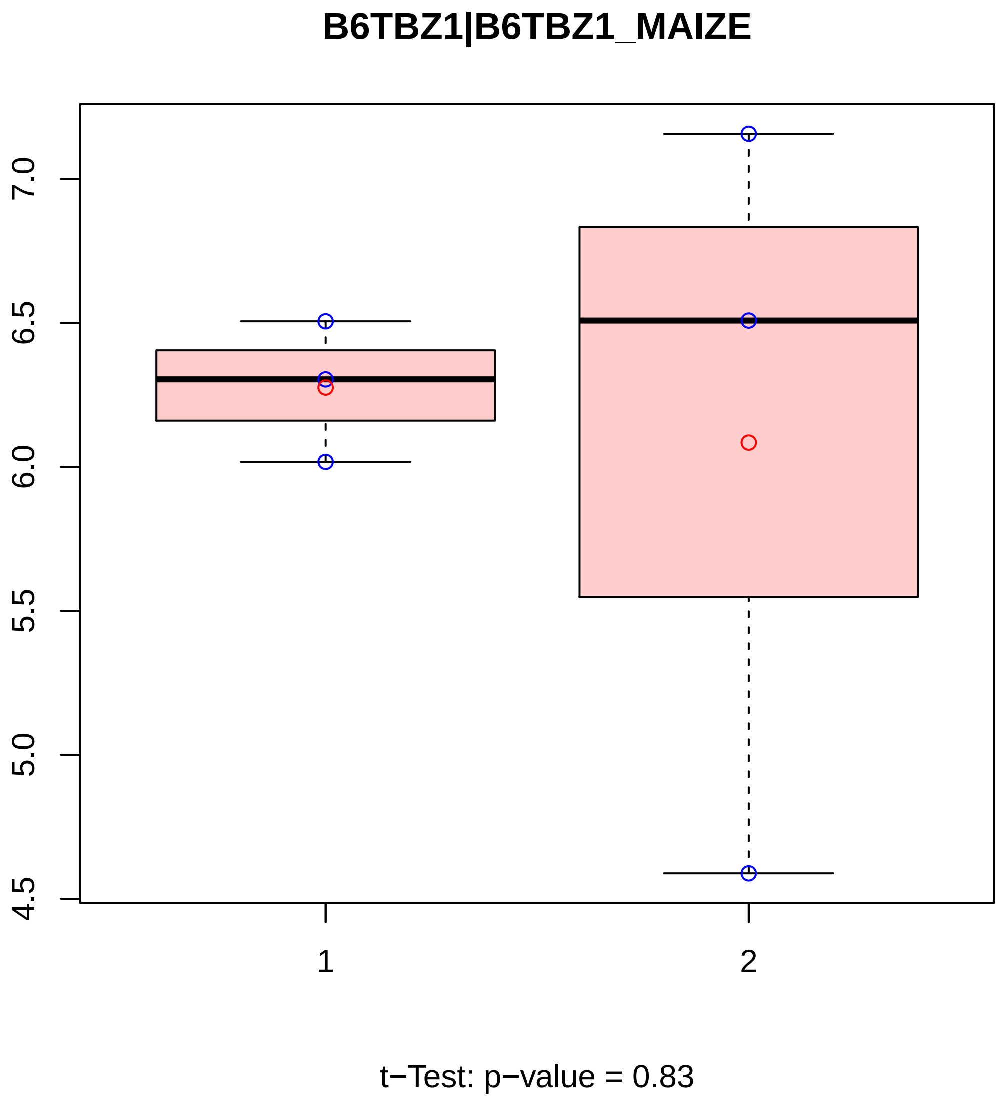
B6TBZ1|B6TBZ1_MAIZE
7.0
6.5
6.0
5.5
5.0
4.5
1
2
t−Test: p−value = 0.83

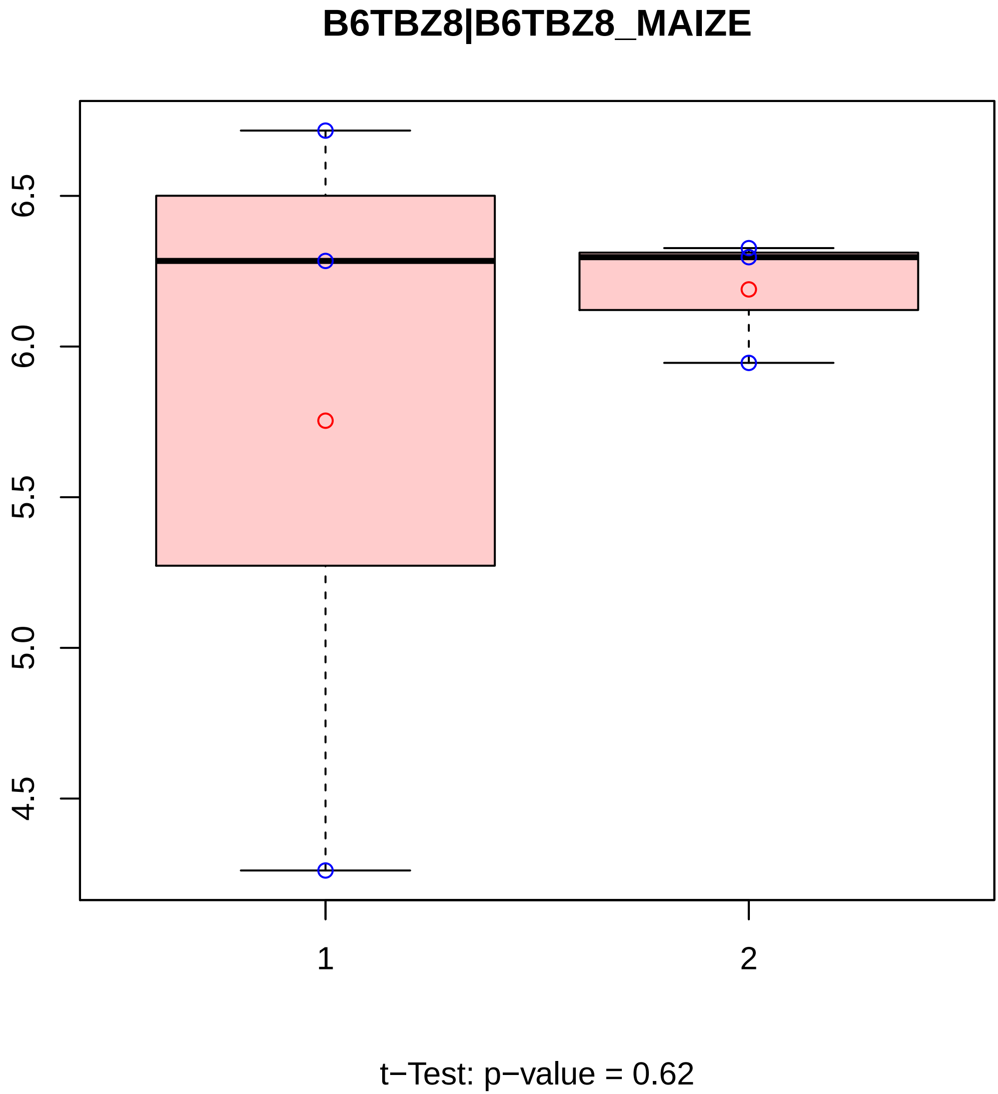

B6TBZ8|B6TBZ8_MAIZE
6.5
6.0
5.5
5.0
4.5
1
2
t−Test: p−value = 0.62

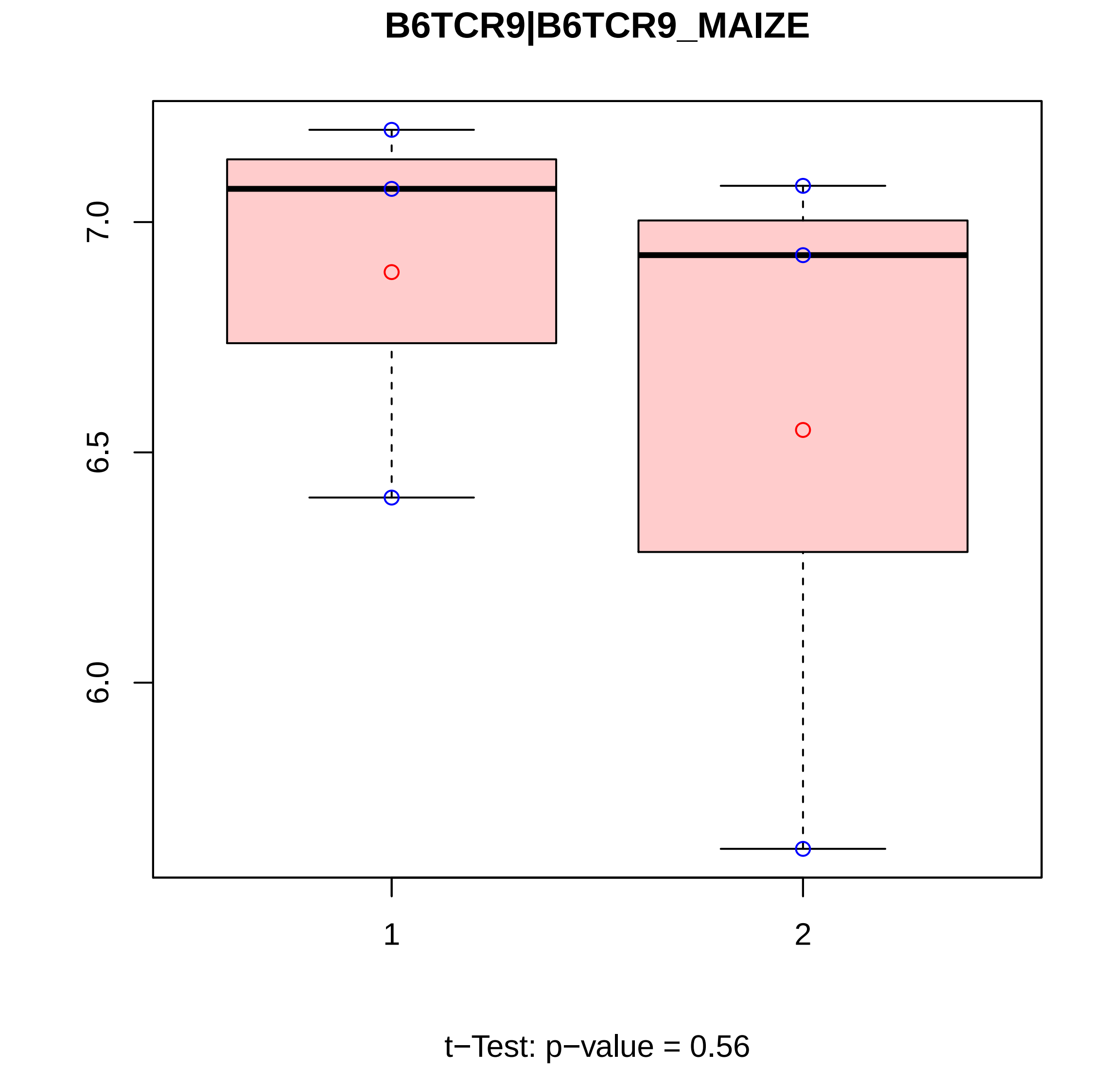
B6TCR9|B6TCR9_MAIZE
7.0
6.5
6.0
1
2
t−Test: p−value = 0.56

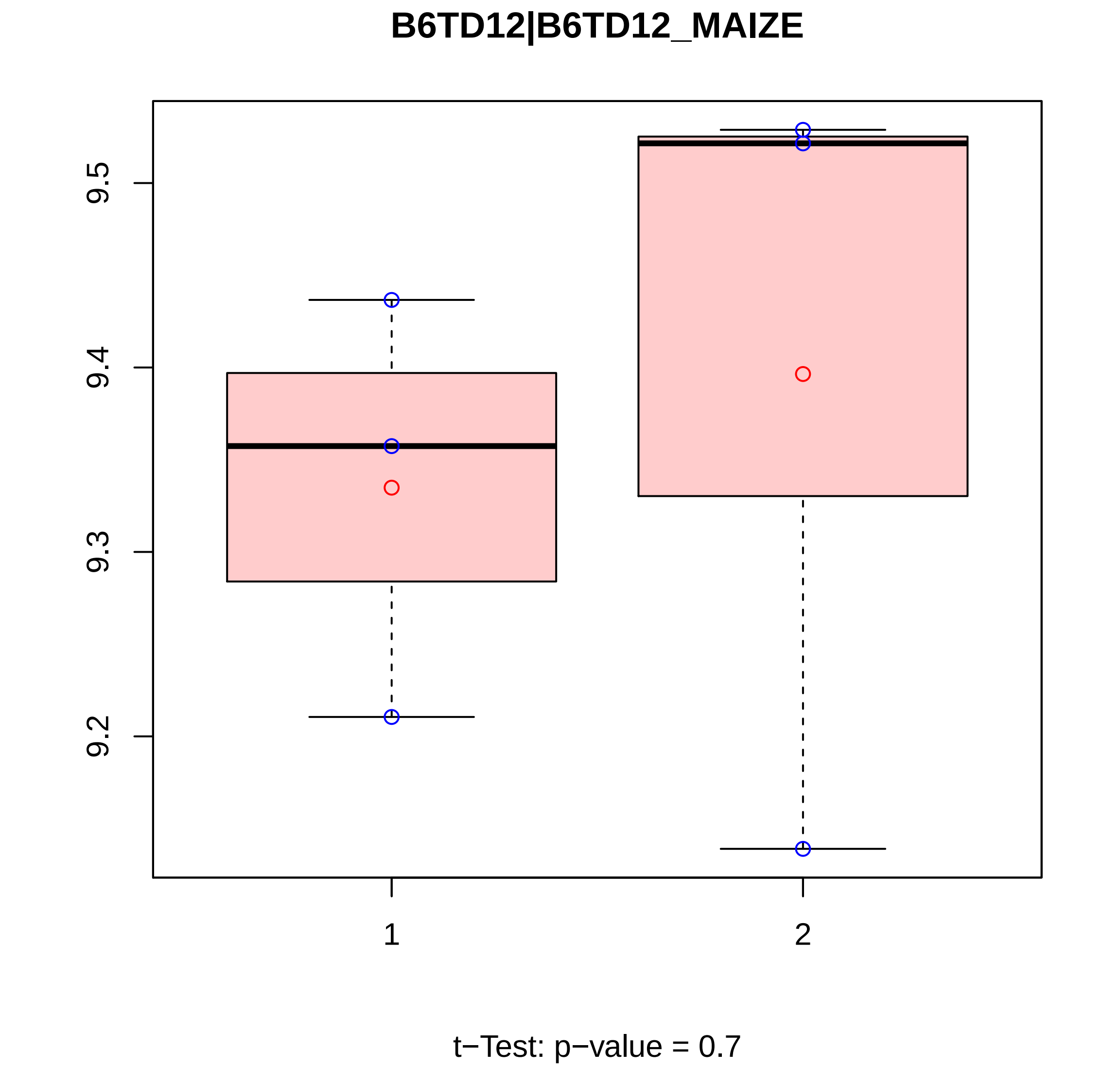

B6TD12|B6TD12_MAIZE
9.2
9.3
9.4
9.5
1
2
t−Test: p−value = 0.7

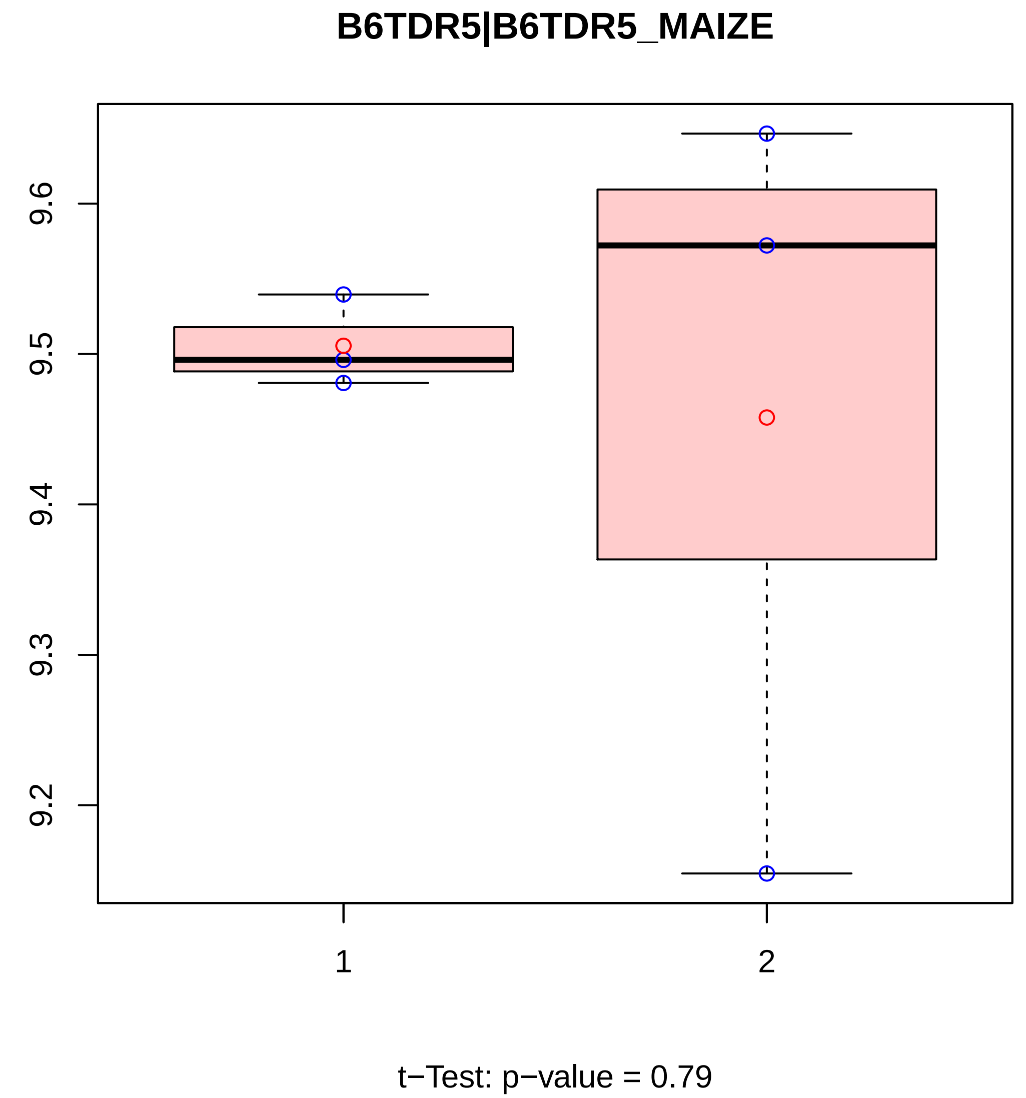
B6TDR5|B6TDR5_MAIZE
9.2
9.3
9.4
9.5
9.6
1
2
t−Test: p−value = 0.79

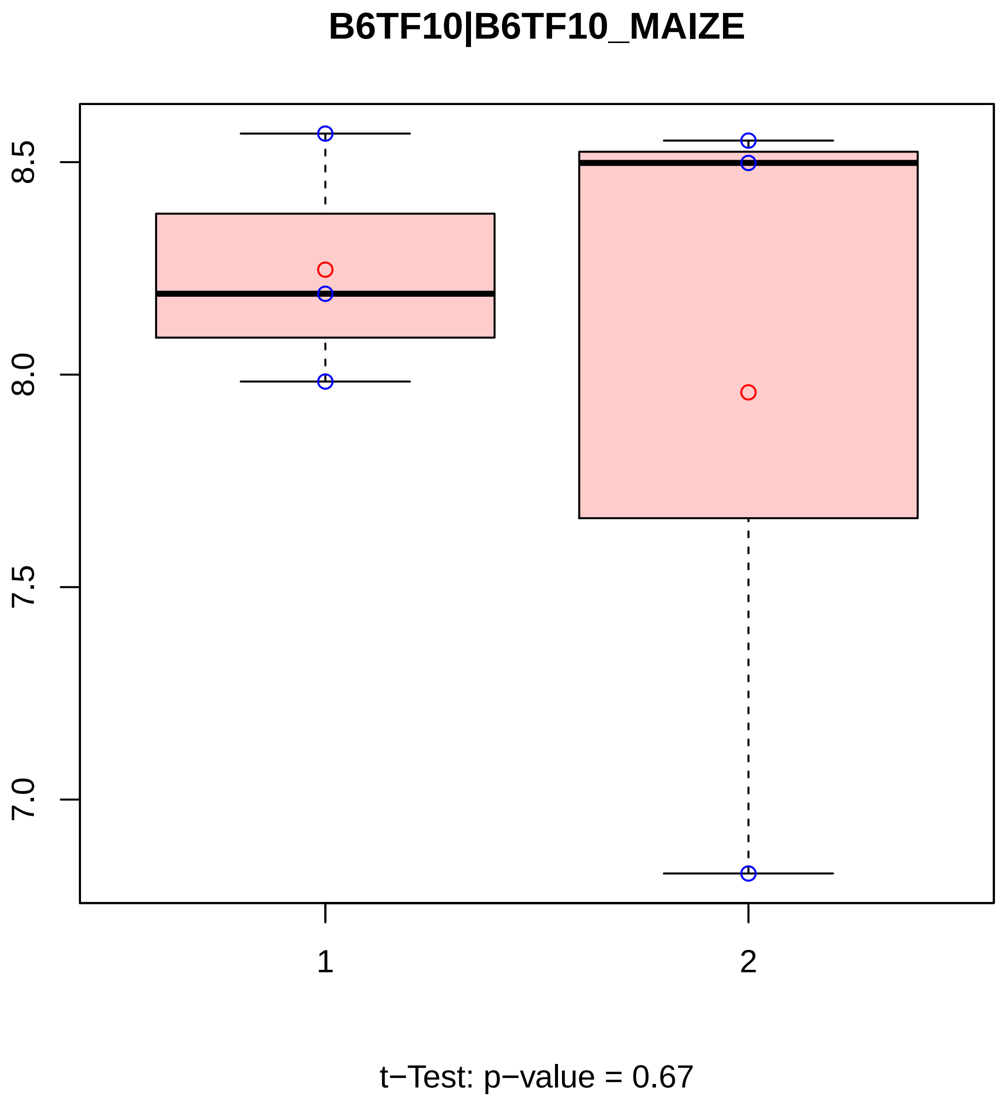
B6TF10|B6TF10_MAIZE
8.5
8.0
7.5
7.0
1
2
t−Test: p−value = 0.67

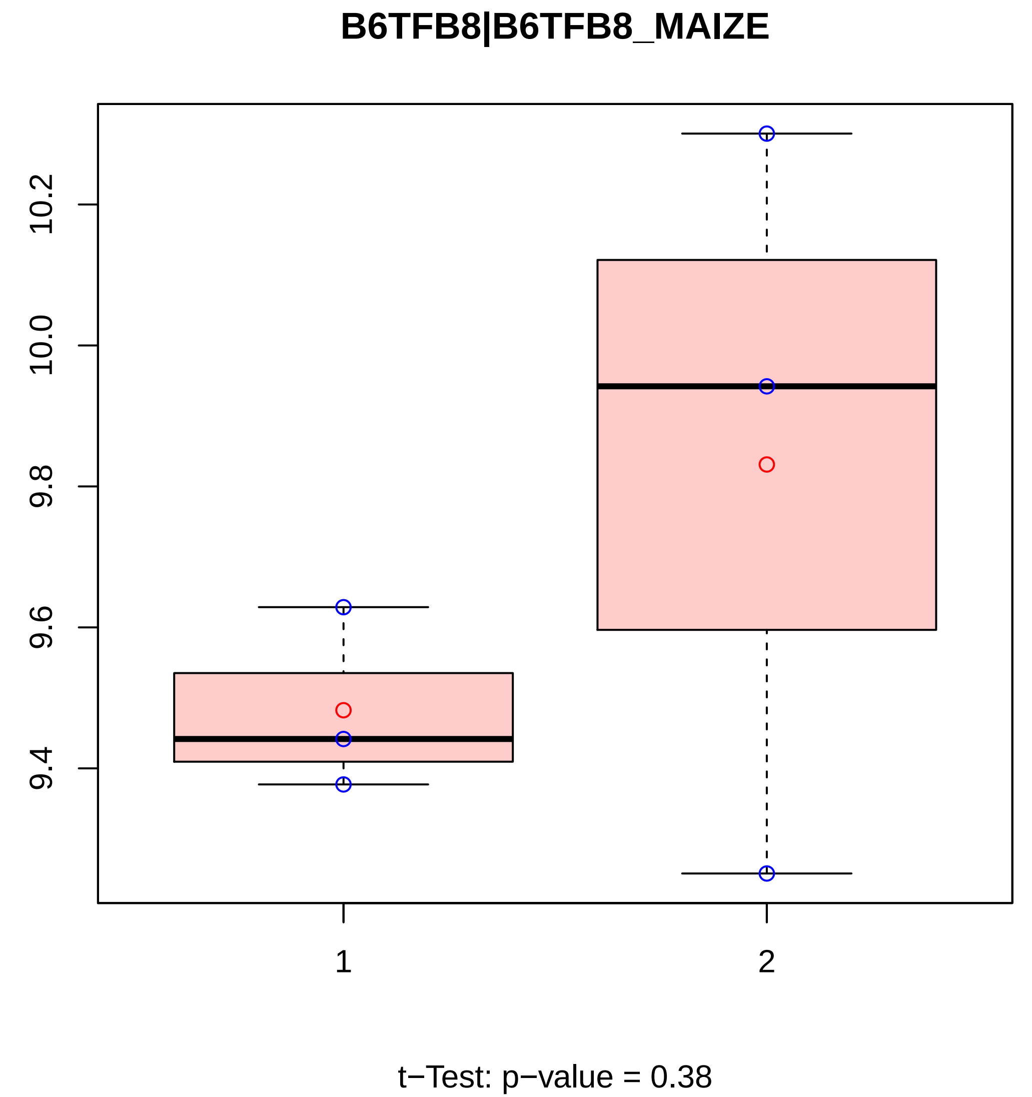

B6TFB8|B6TFB8_MAIZE
10.2
10.0
9.8
9.6
9.4
1
2
t−Test: p−value = 0.38

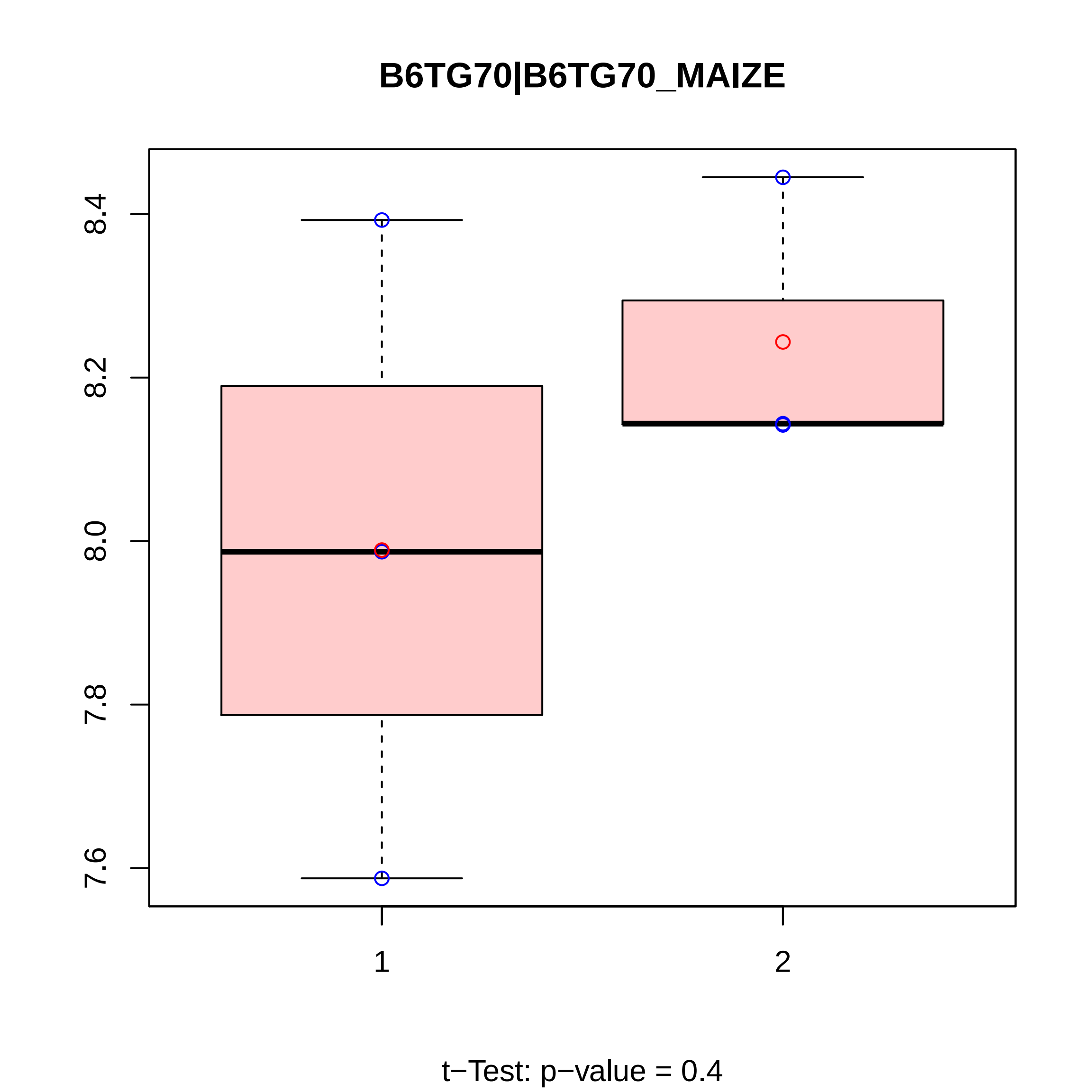

B6TG70|B6TG70_MAIZE
8.4
8.2
8.0
7.8
7.6
1
2
t−Test: p−value = 0.4

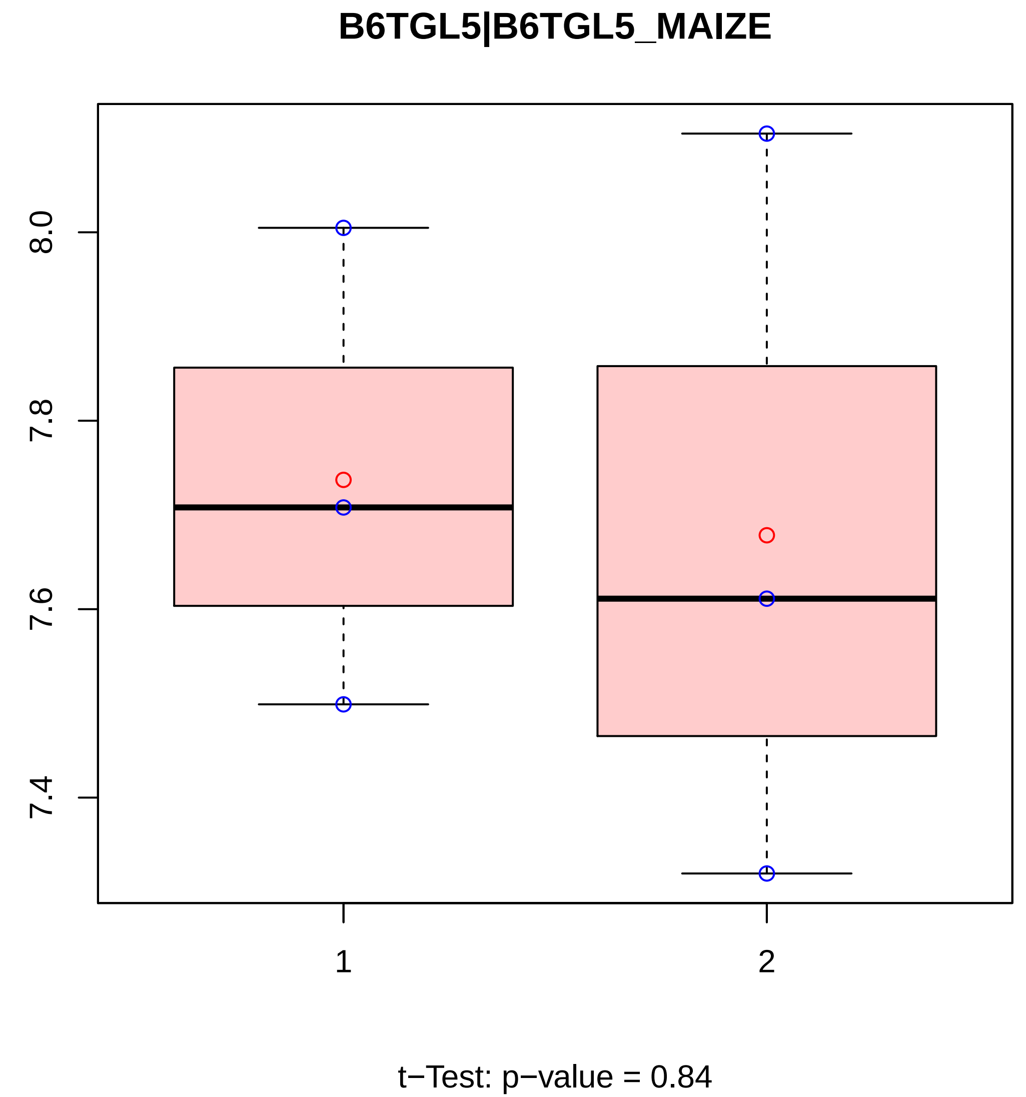

B6TGL5|B6TGL5_MAIZE
8.0
7.8
7.6
7.4
1
2
t−Test: p−value = 0.84

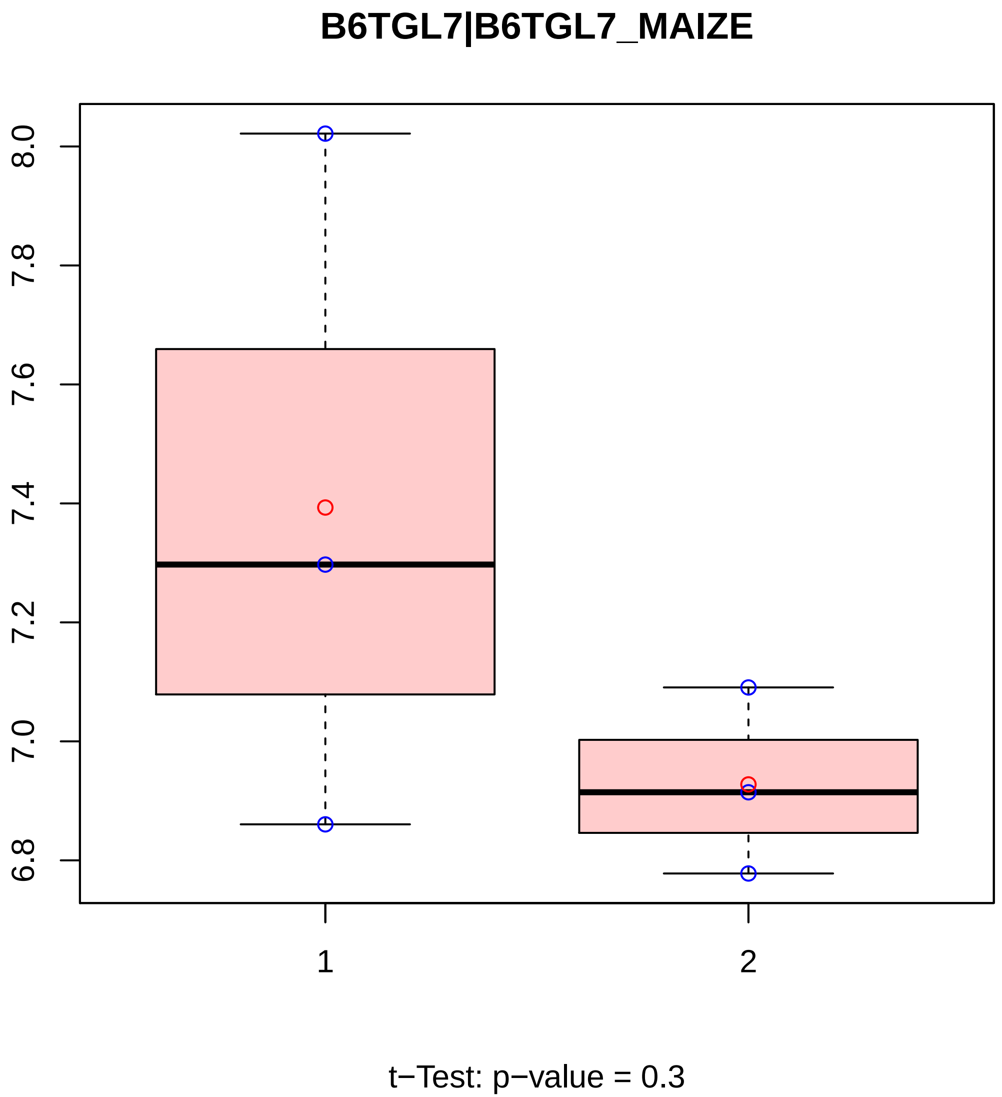

B6TGL7|B6TGL7_MAIZE
8.0
7.8
7.6
7.4
7.2
7.0
6.8
1
2
t−Test: p−value = 0.3

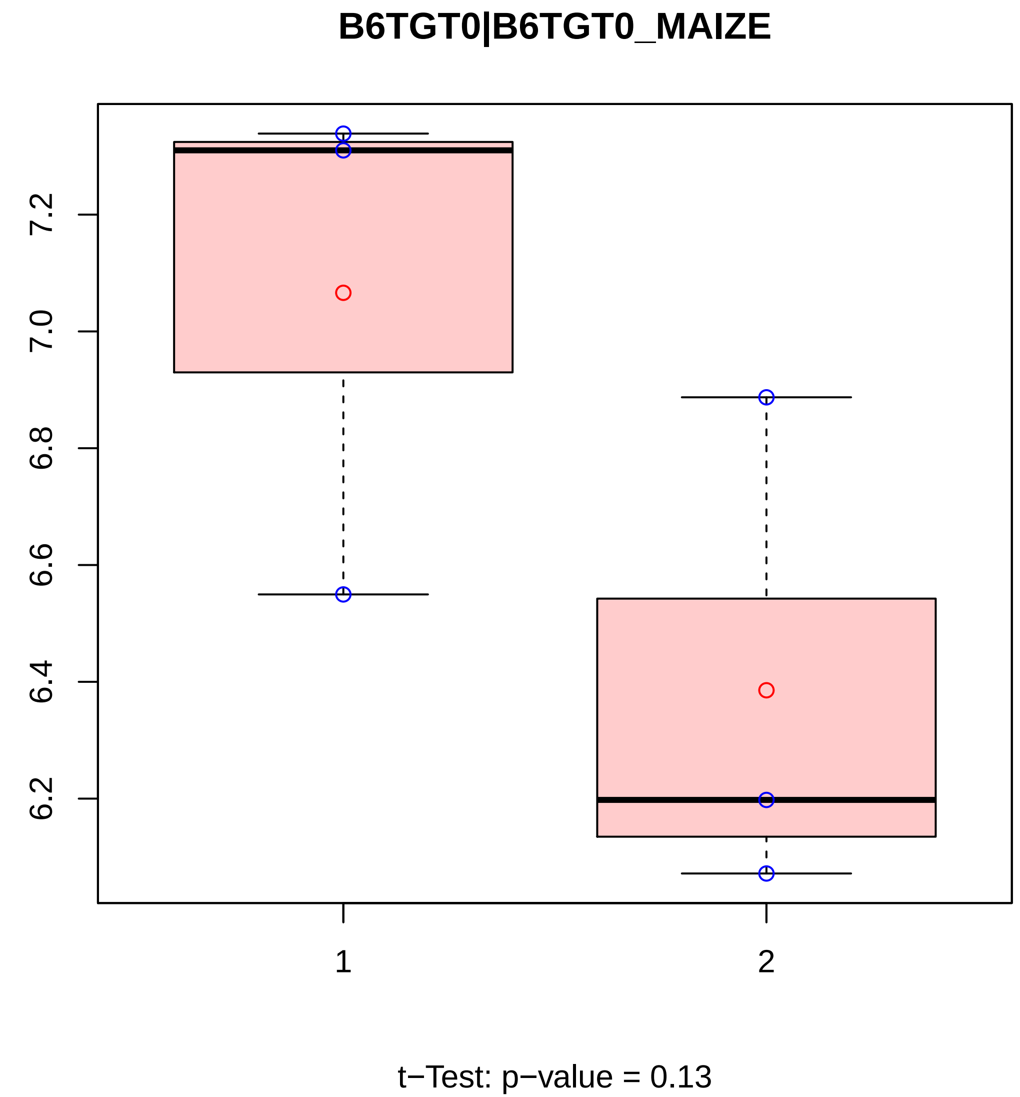
B6TGT0|B6TGT0_MAIZE
7.2
7.0
6.8
6.6
6.4
6.2
1
2
t−Test: p−value = 0.13

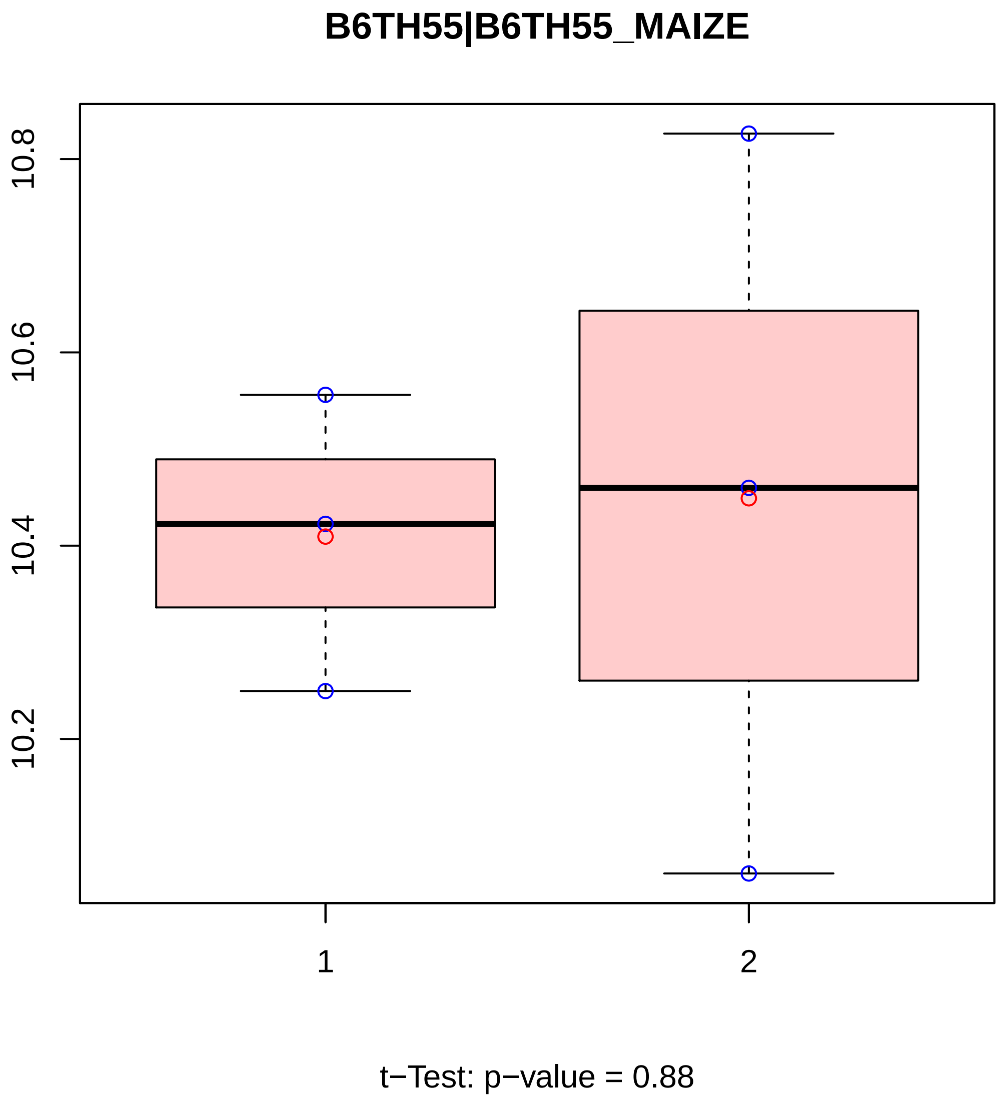

B6TH55|B6TH55_MAIZE
10.2
10.4
10.6
10.8
1
2
t−Test: p−value = 0.88

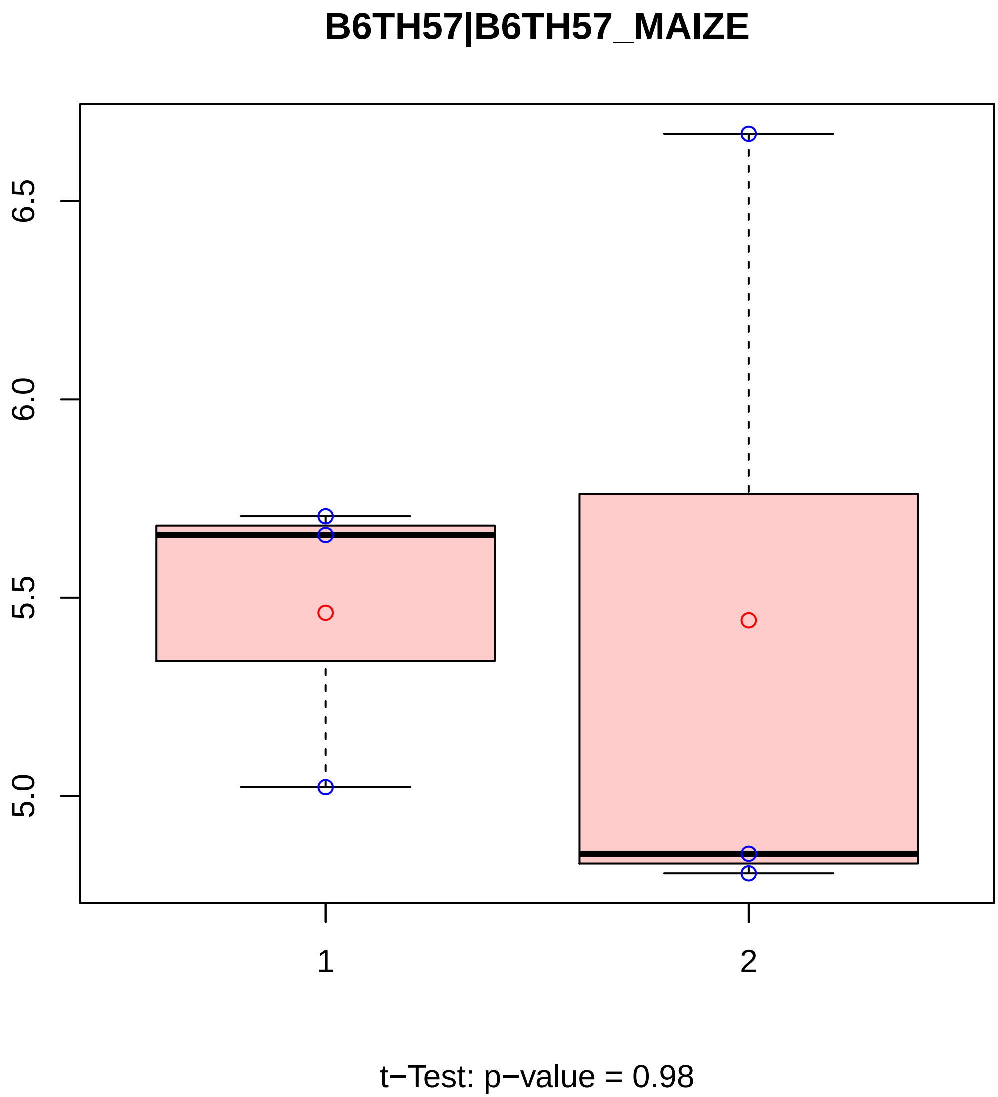
B6TH57|B6TH57_MAIZE
5.0
5.5
6.0
6.5
1
2
t−Test: p−value = 0.98

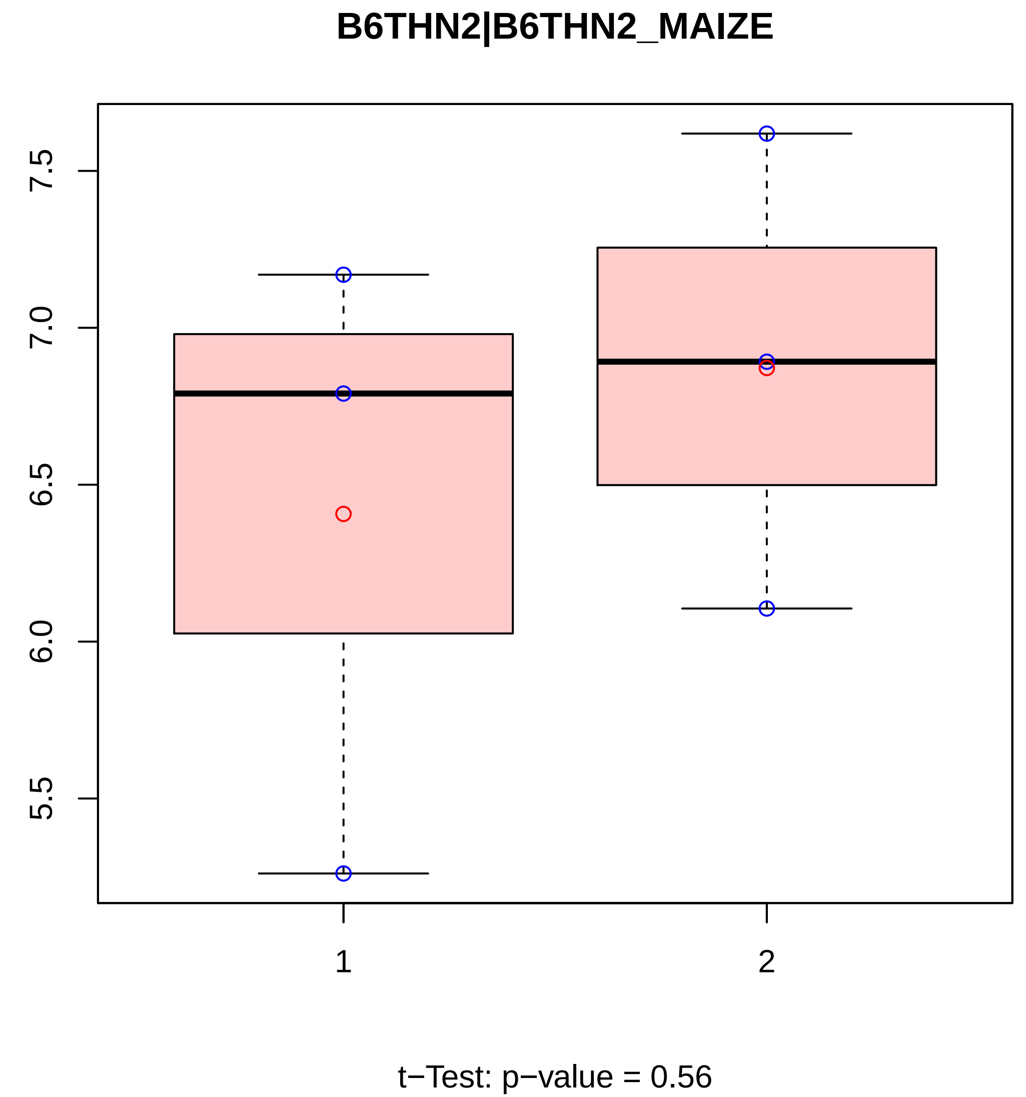
B6THN2|B6THN2_MAIZE
7.5
7.0
6.5
6.0
5.5
1
2
t−Test: p−value = 0.56

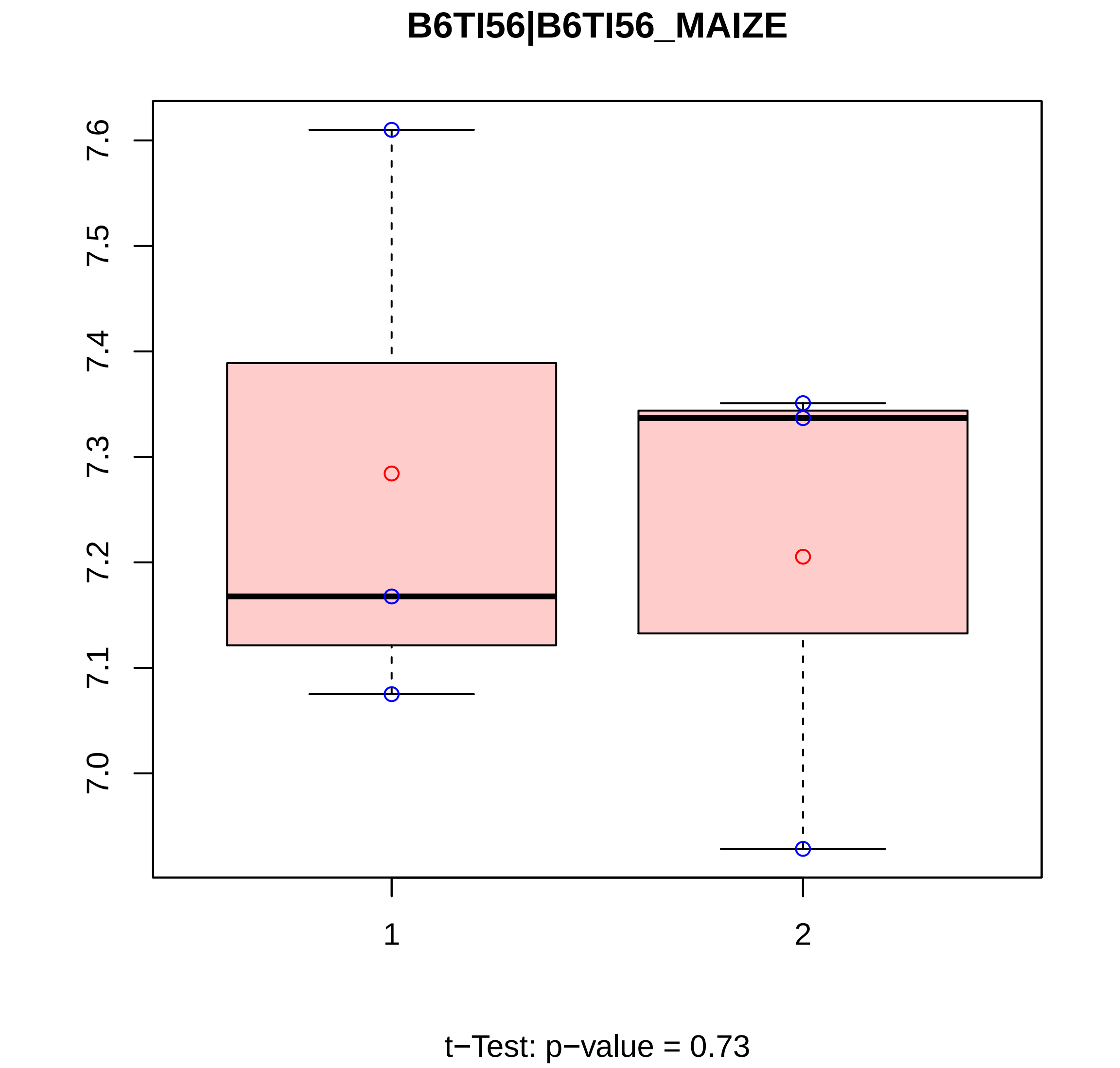
B6TI56|B6TI56_MAIZE
7.6
7.5
7.4
7.3
7.2
7.1
7.0
1
2
t−Test: p−value = 0.73

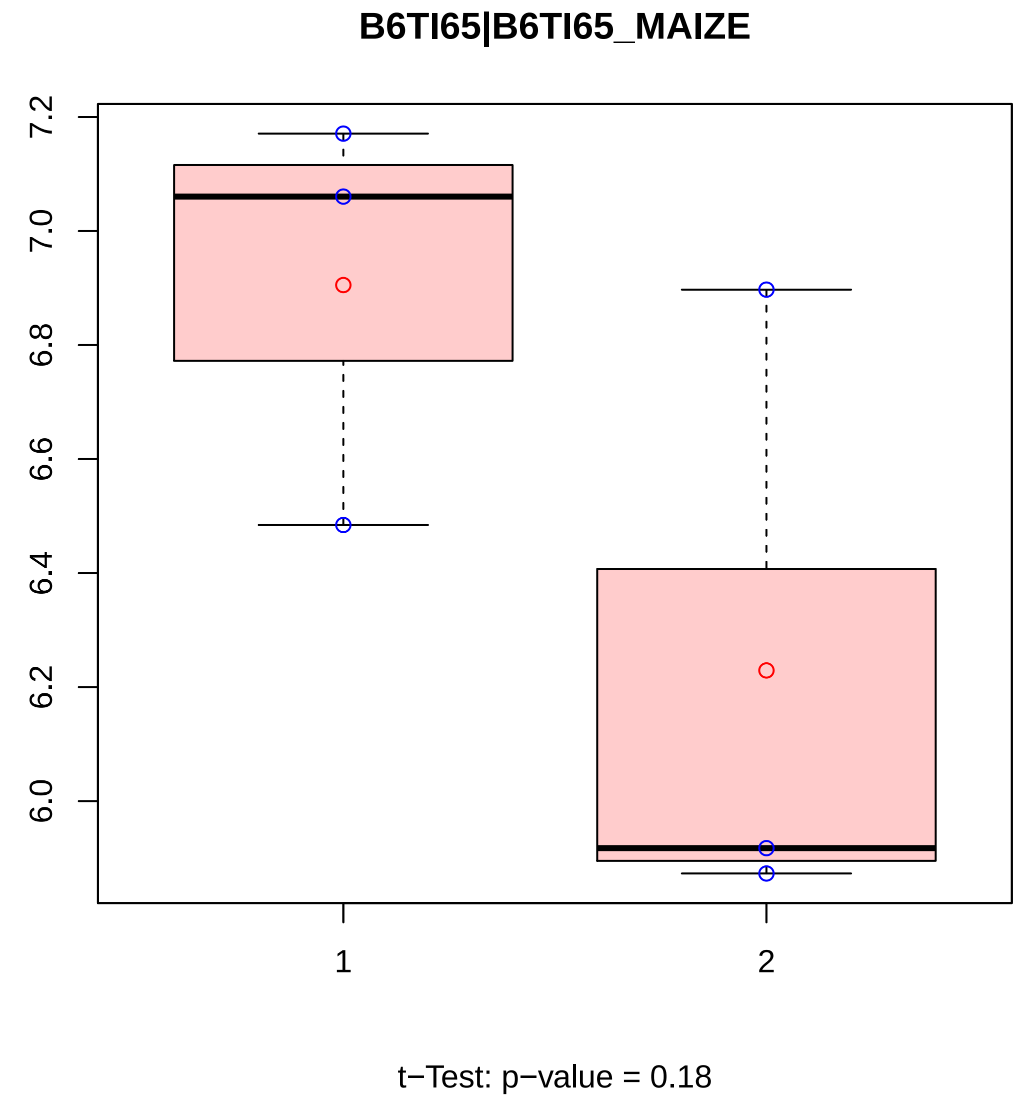

B6TI65|B6TI65_MAIZE
7.2
7.0
6.8
6.6
6.4
6.2
6.0
1
2
t−Test: p−value = 0.18

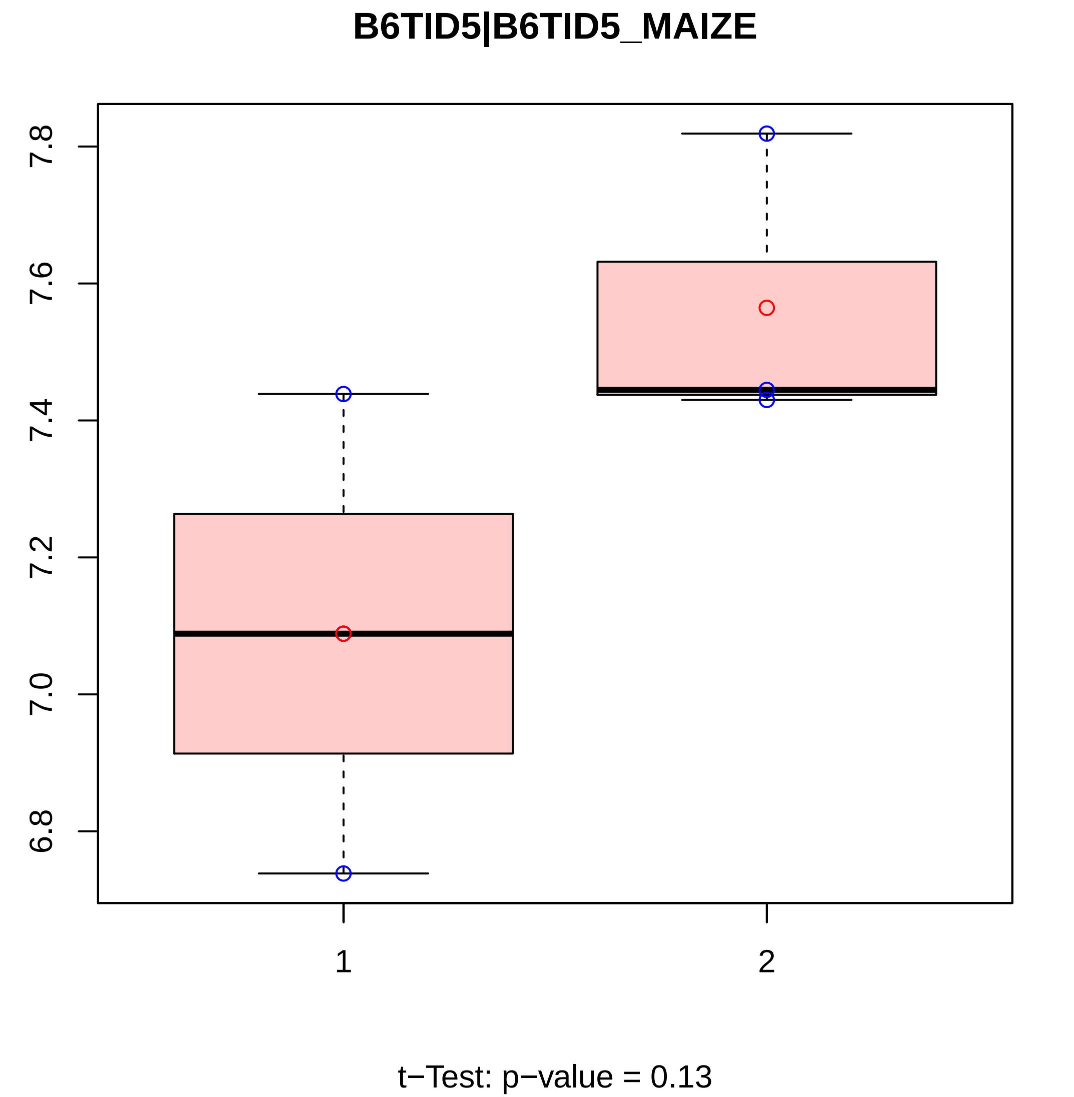
B6TID5|B6TID5_MAIZE
7.8
7.6
7.4
7.2
7.0
6.8
1
2
t−Test: p−value = 0.13

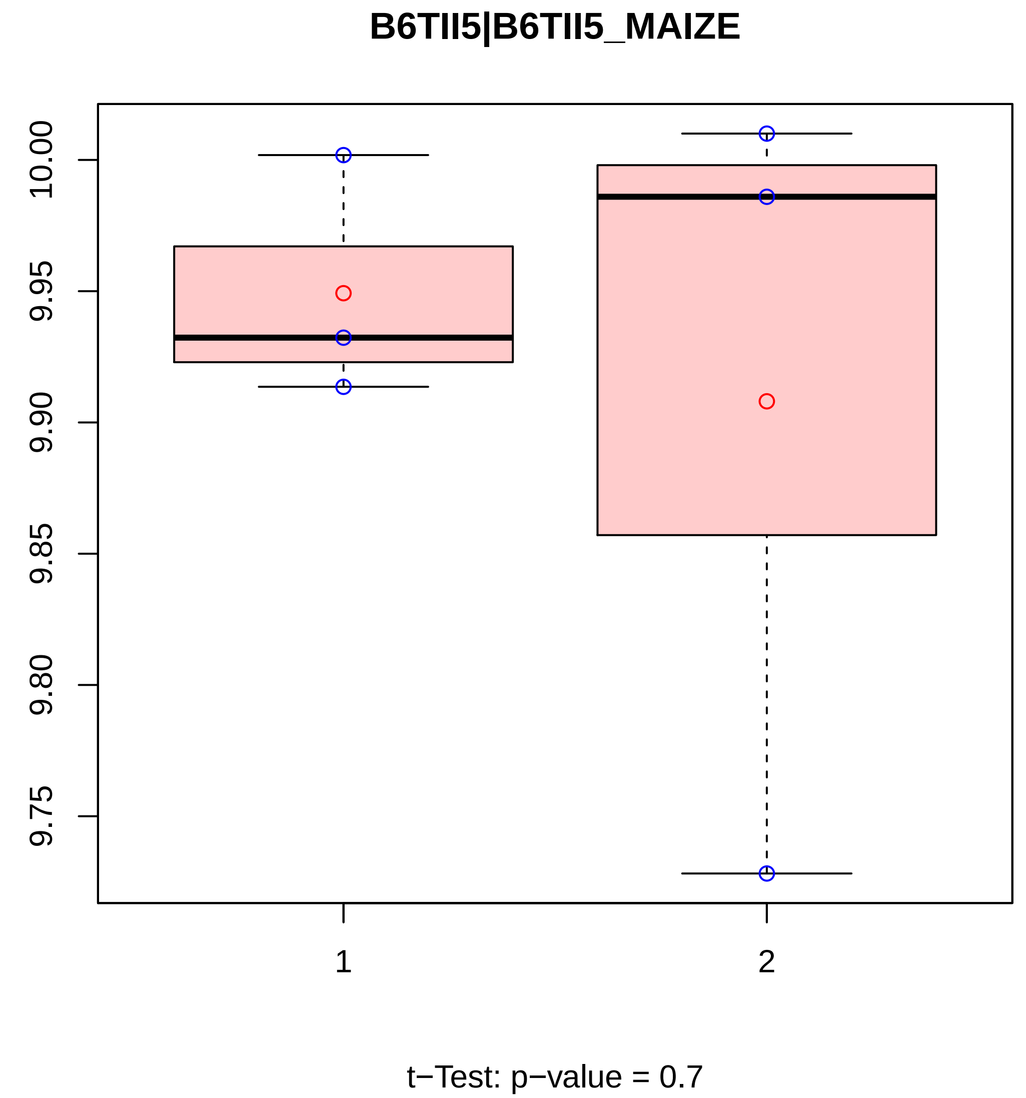
B6TII5|B6TII5_MAIZE
10.00
9.95
9.90
9.85
9.80
9.75
1
2
t−Test: p−value = 0.7

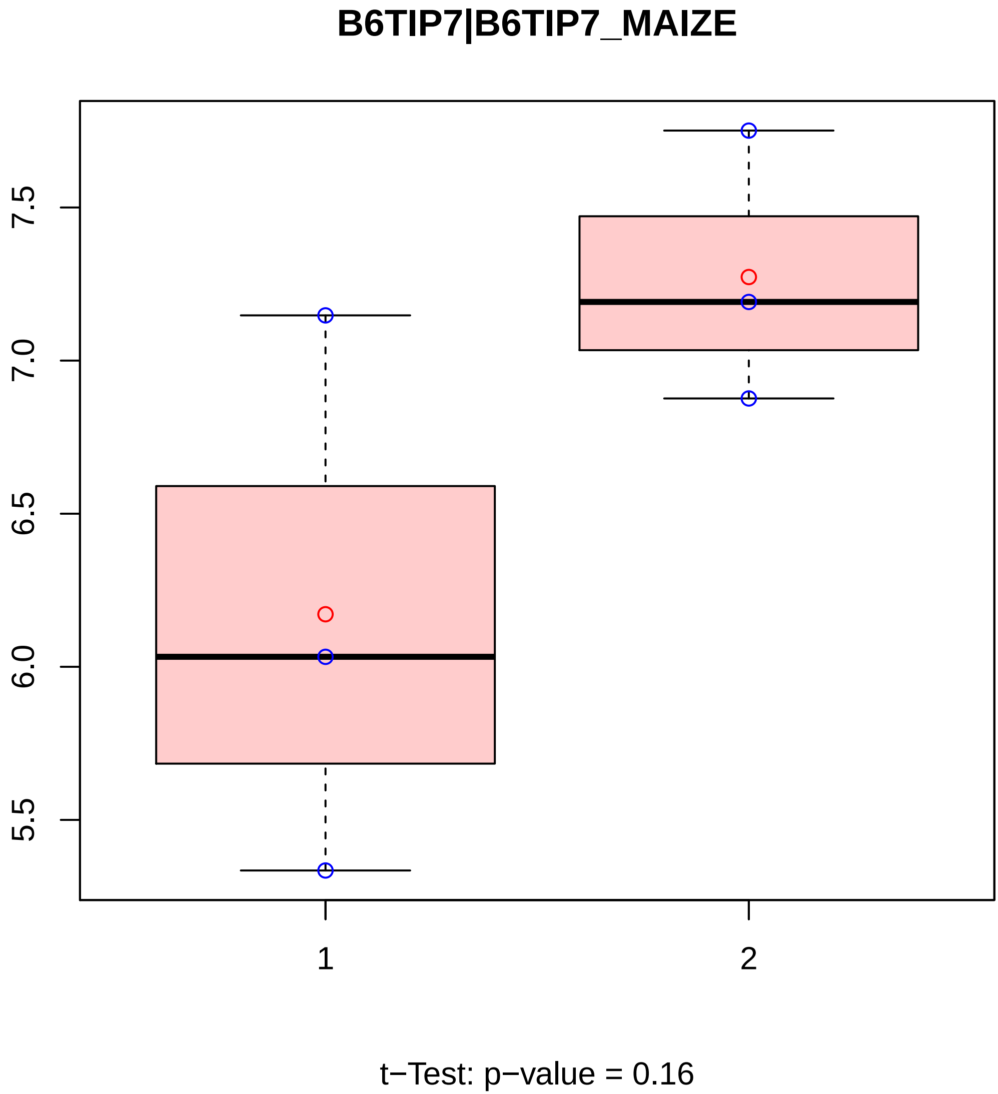
B6TIP7|B6TIP7_MAIZE
7.5
7.0
6.5
6.0
5.5
1
2
t−Test: p−value = 0.16

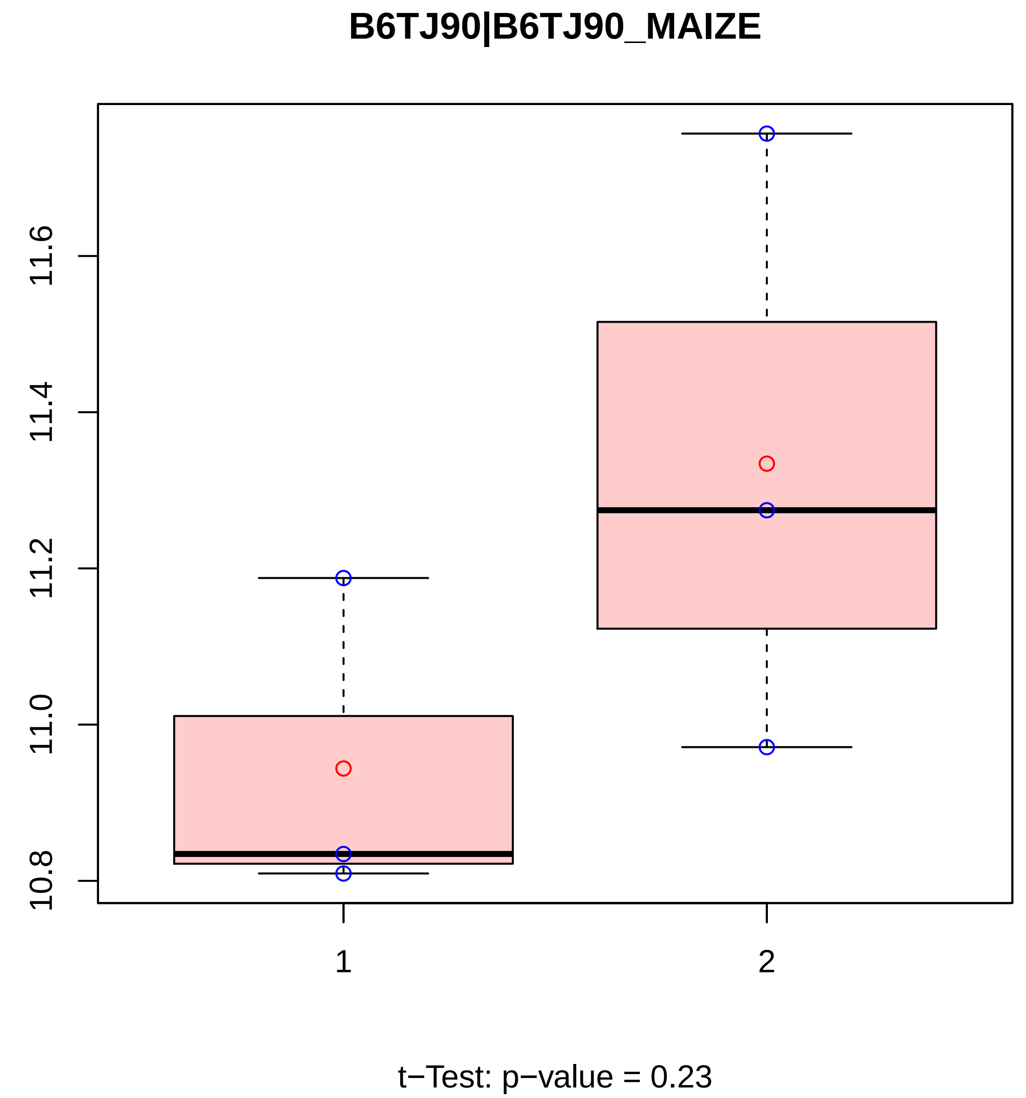

B6TJ90|B6TJ90_MAIZE
11.6
11.4
11.2
11.0
10.8
1
2
t−Test: p−value = 0.23

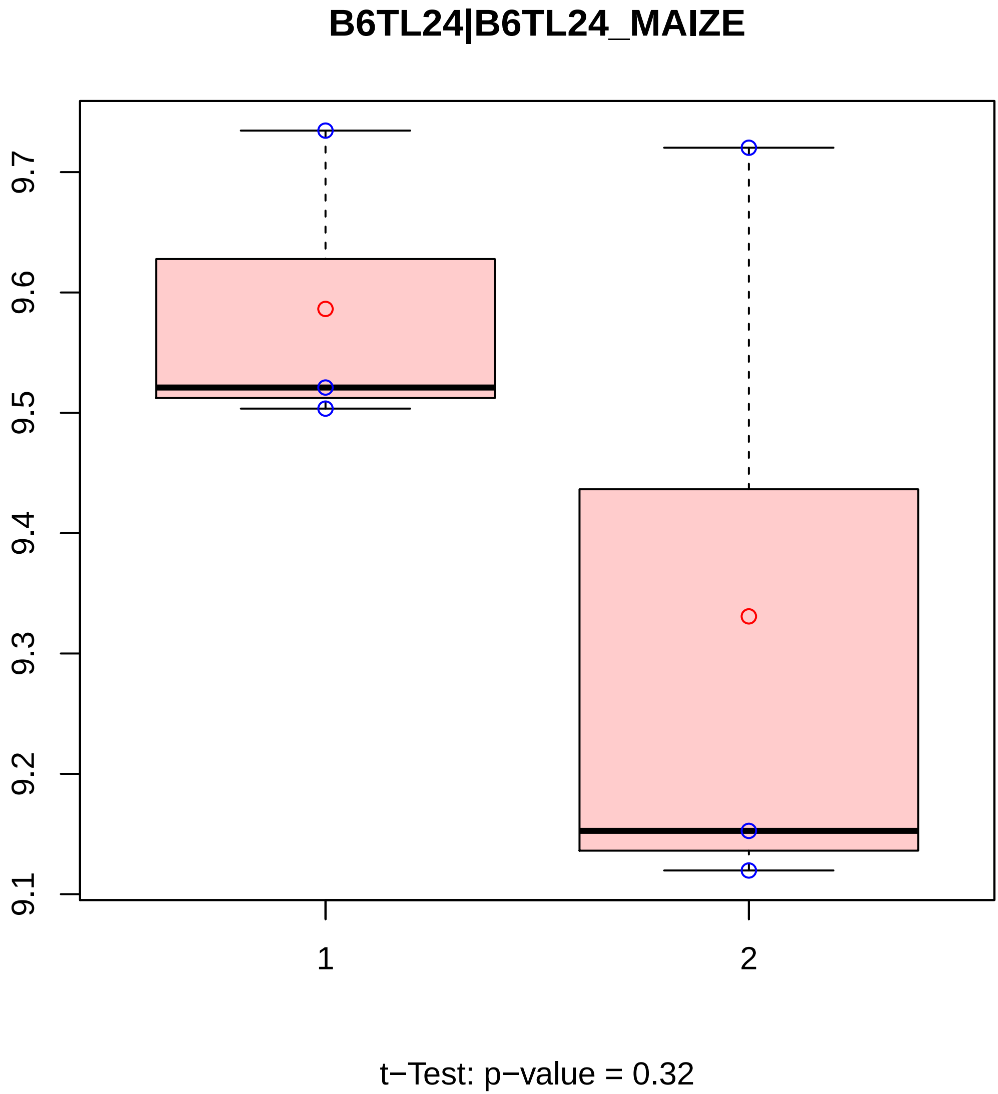

B6TL24|B6TL24_MAIZE
9.1
9.2
9.3
9.4
9.5
9.6
9.7
1
2
t−Test: p−value = 0.32

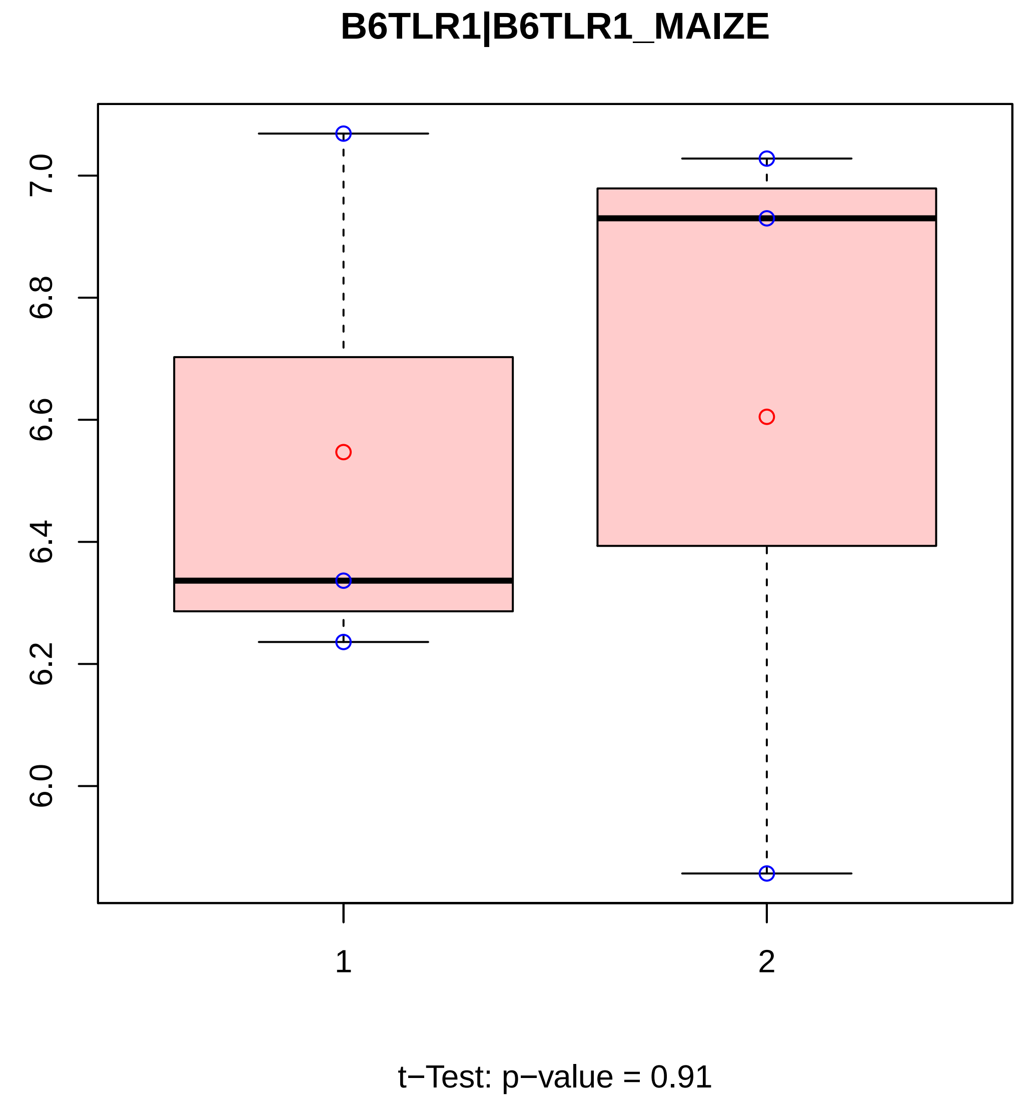

B6TLR1|B6TLR1_MAIZE
7.0
6.8
6.6
6.4
6.2
6.0
1
2
t−Test: p−value = 0.91

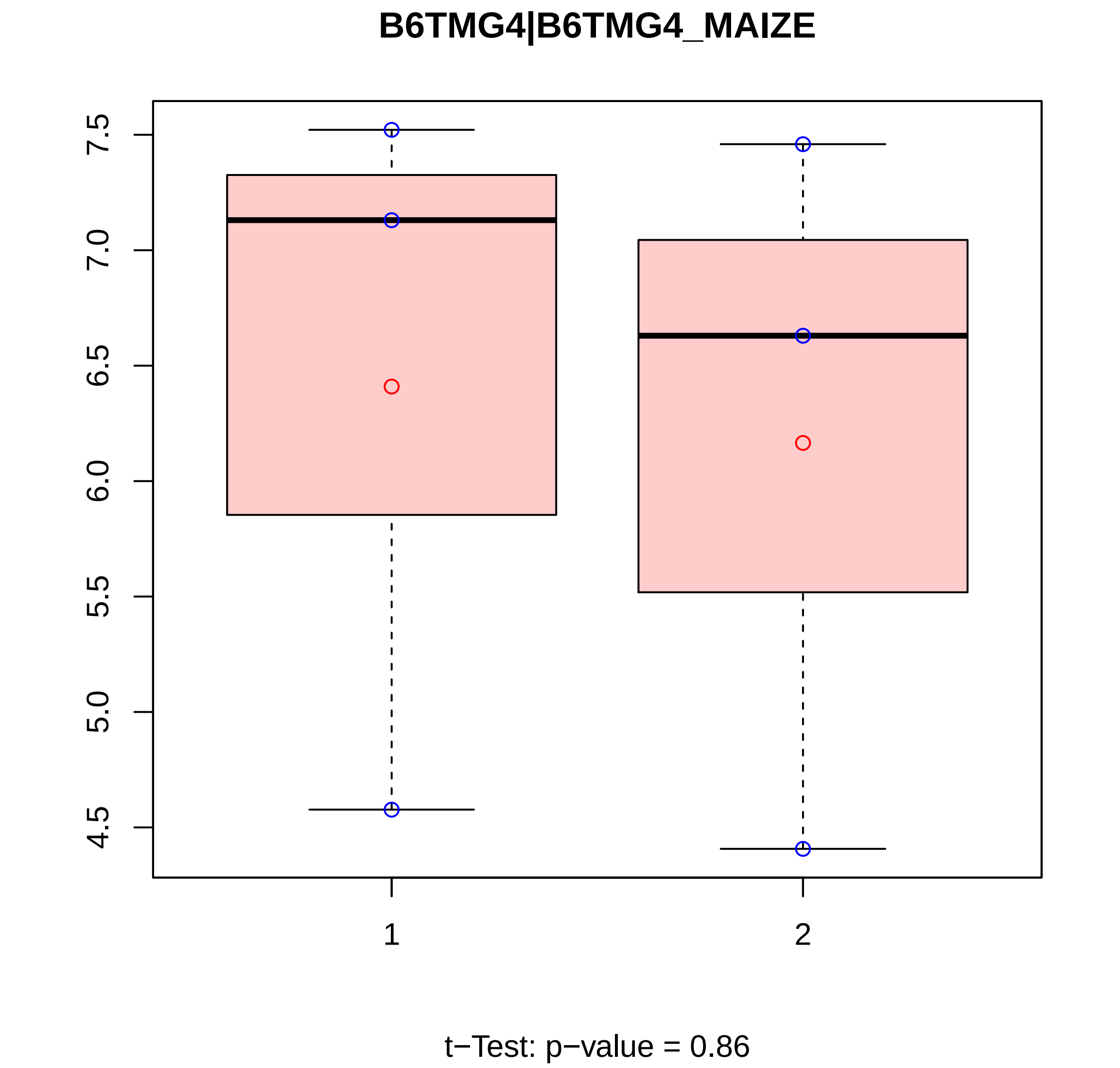

B6TMG4|B6TMG4_MAIZE
7.5
7.0
6.5
6.0
5.5
5.0
4.5
1
2
t−Test: p−value = 0.86

B6TMP9|B6TMP9_MAIZE

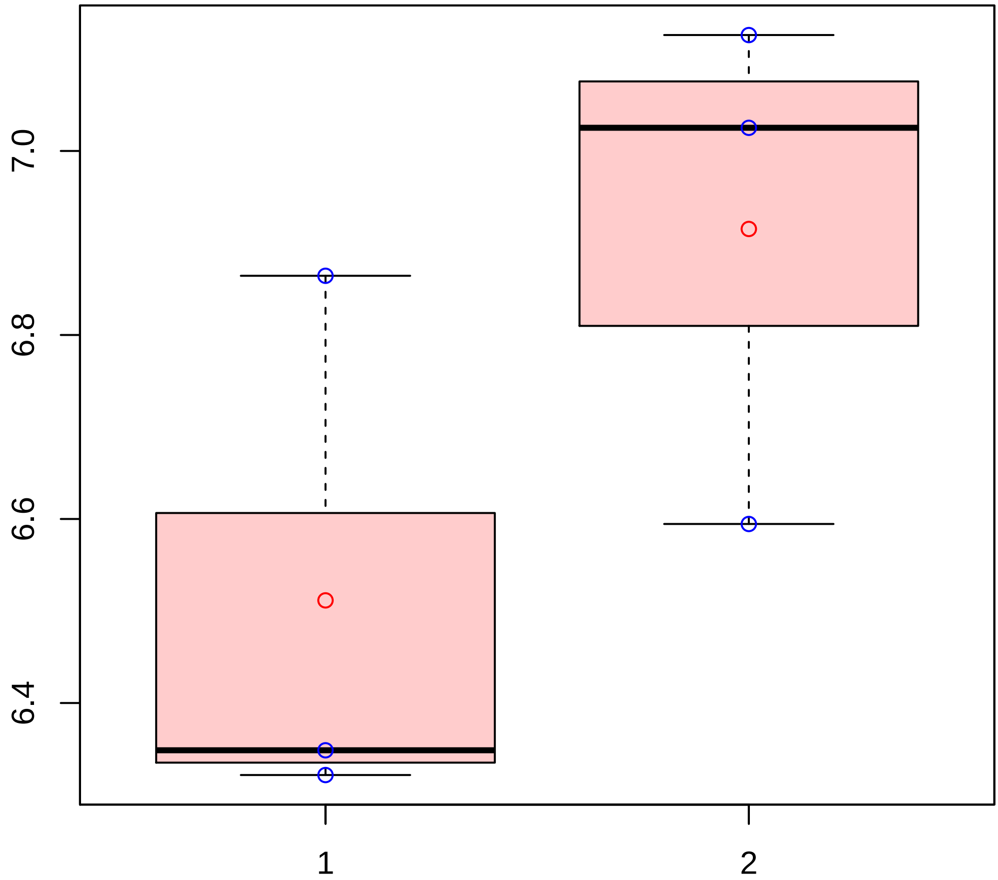

t−Test: p−value = 0.17

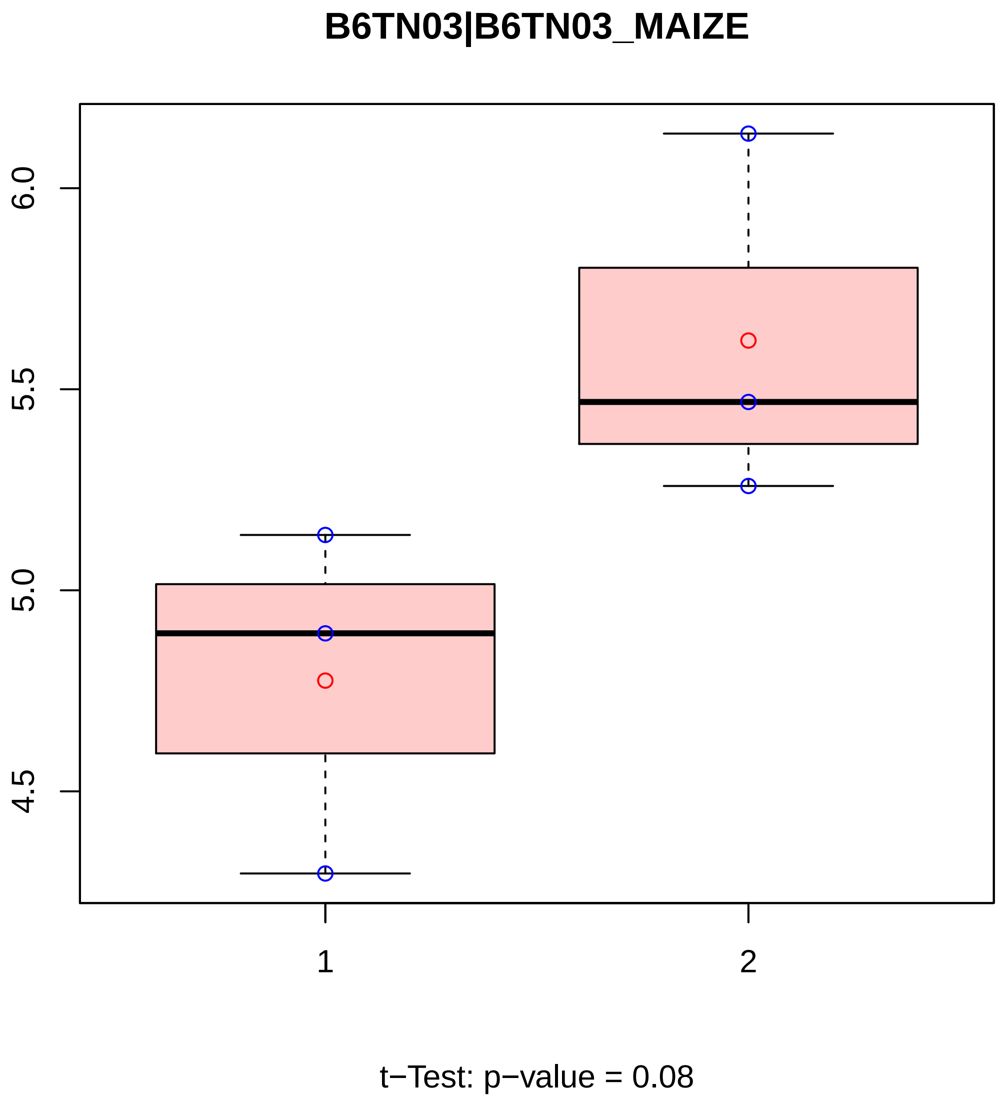

B6TN03|B6TN03_MAIZE
6.0
5.5
5.0
4.5
1
2
t−Test: p−value = 0.08

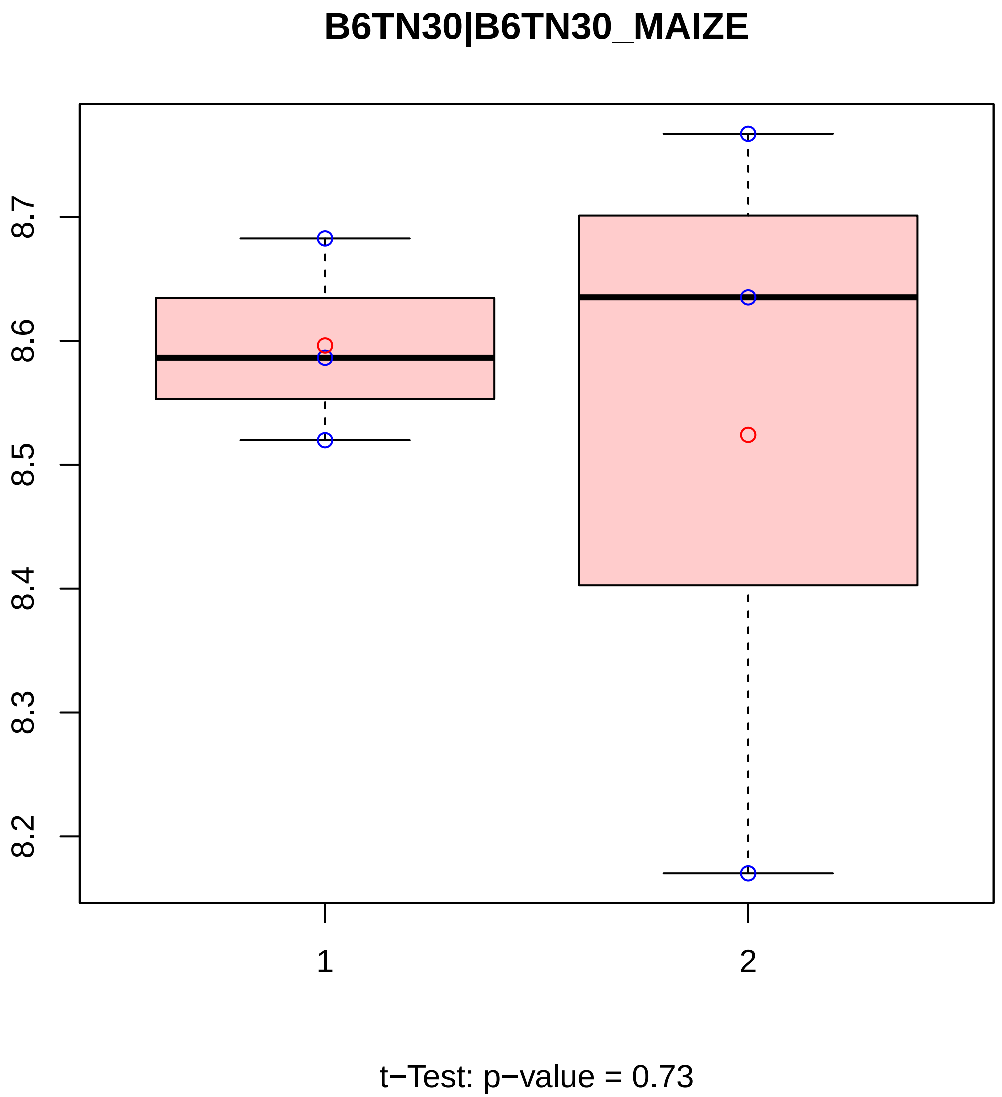
B6TN30|B6TN30_MAIZE
8.2
8.3
8.4
8.5
8.6
8.7
1
2
t−Test: p−value = 0.73

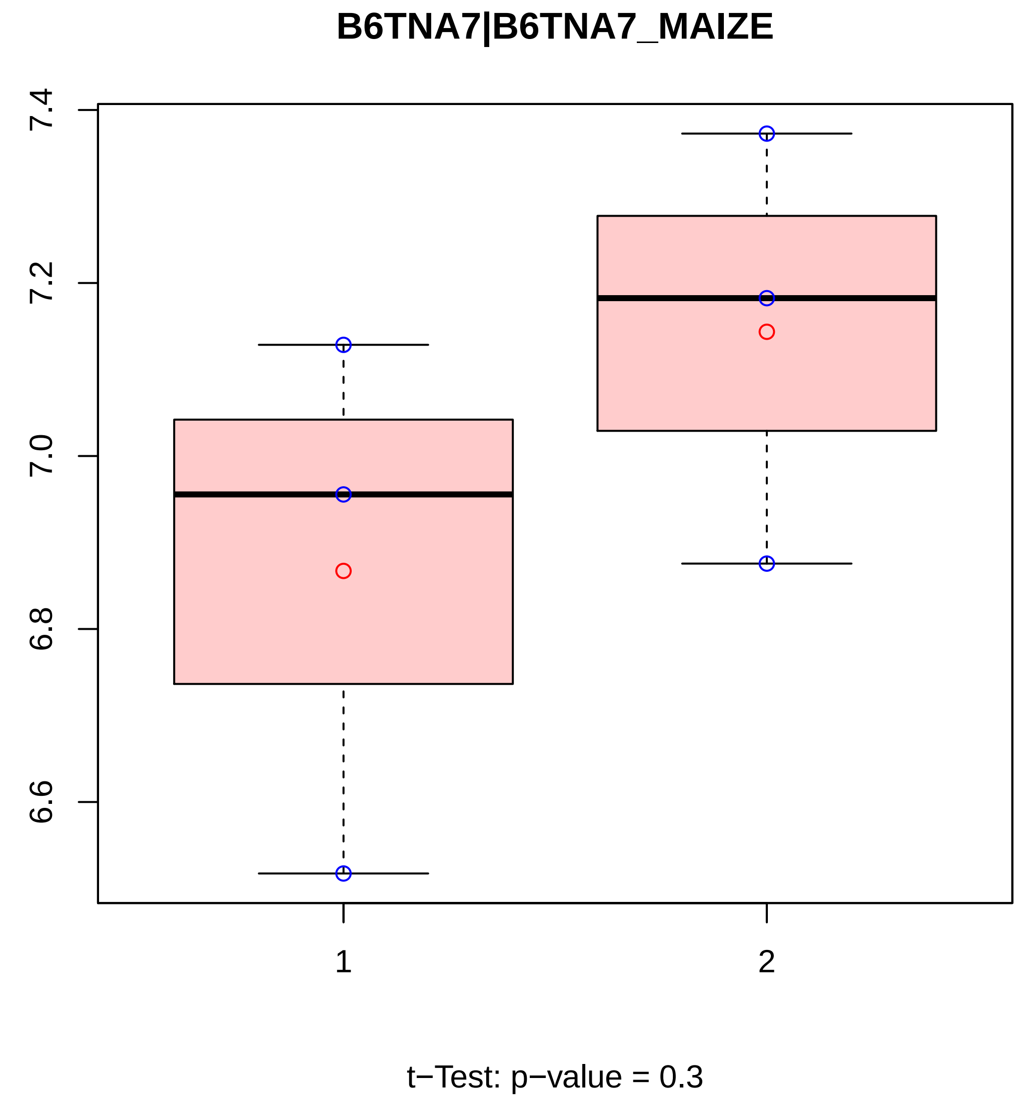

B6TNA7|B6TNA7_MAIZE
7.4
7.2
7.0
6.8
6.6
1
2
t−Test: p−value = 0.3

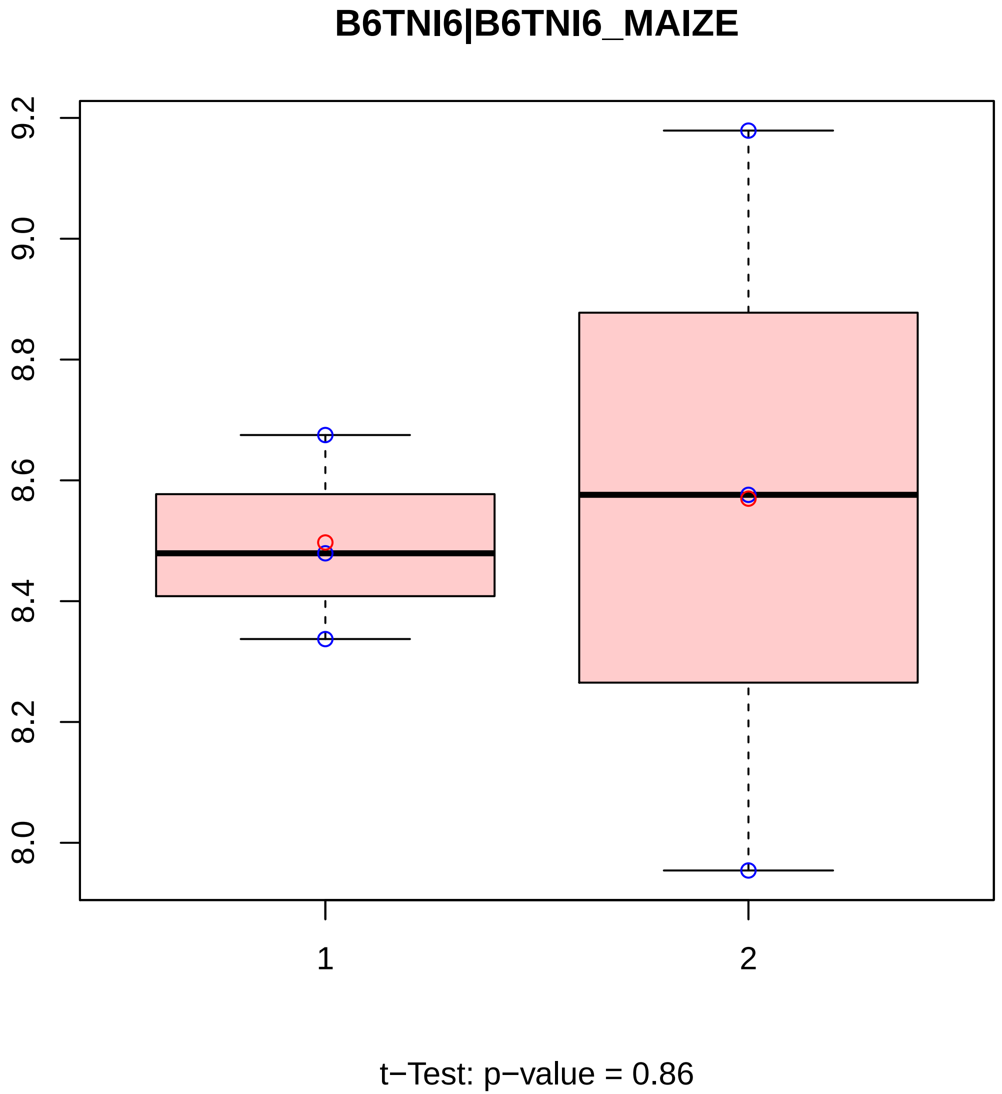

B6TNI6|B6TNI6_MAIZE
9.2
9.0
8.8
8.6
8.4
8.2
8.0
1
2
t−Test: p−value = 0.86

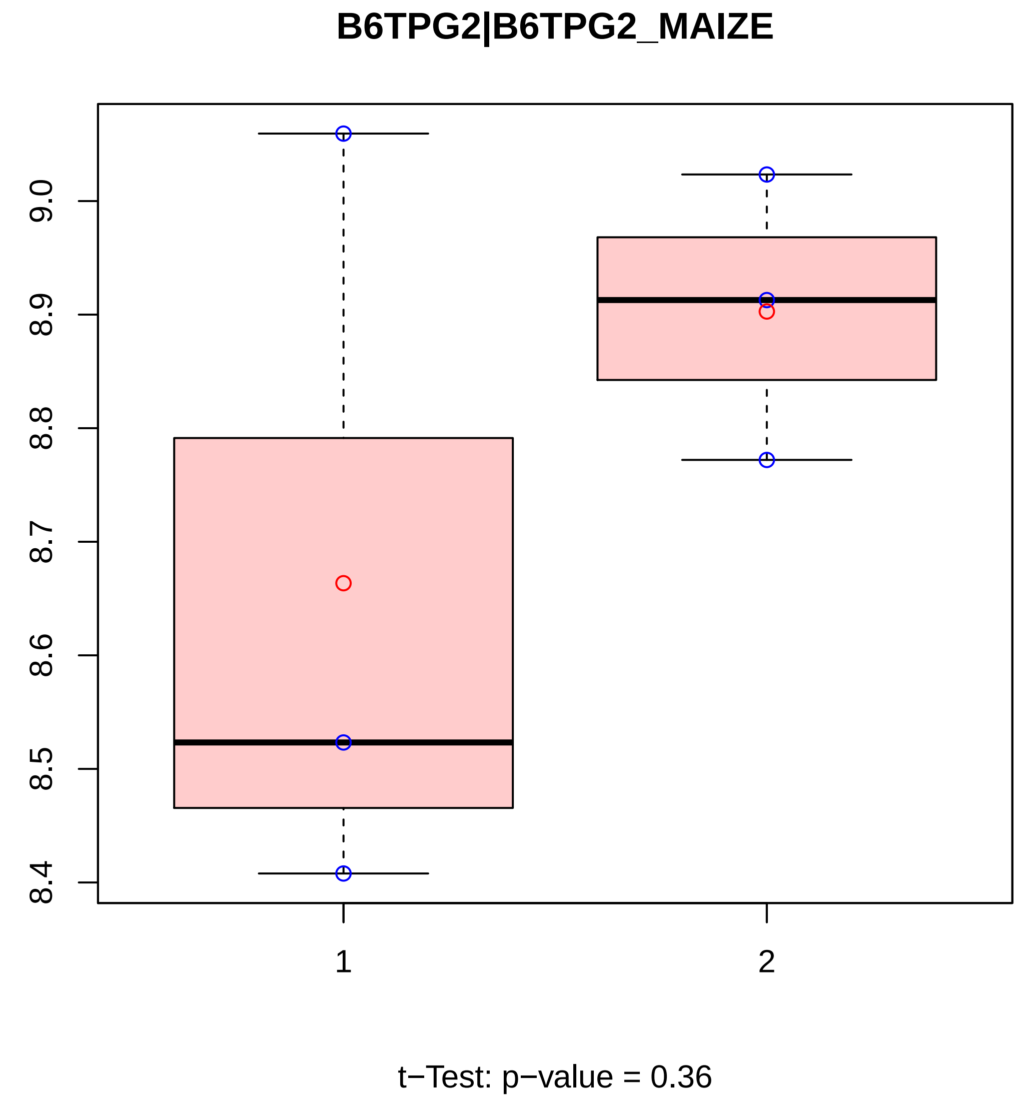
B6TPG2|B6TPG2_MAIZE
9.0
8.9
8.8
8.7
8.6
8.5
8.4
1
2
t−Test: p−value = 0.36

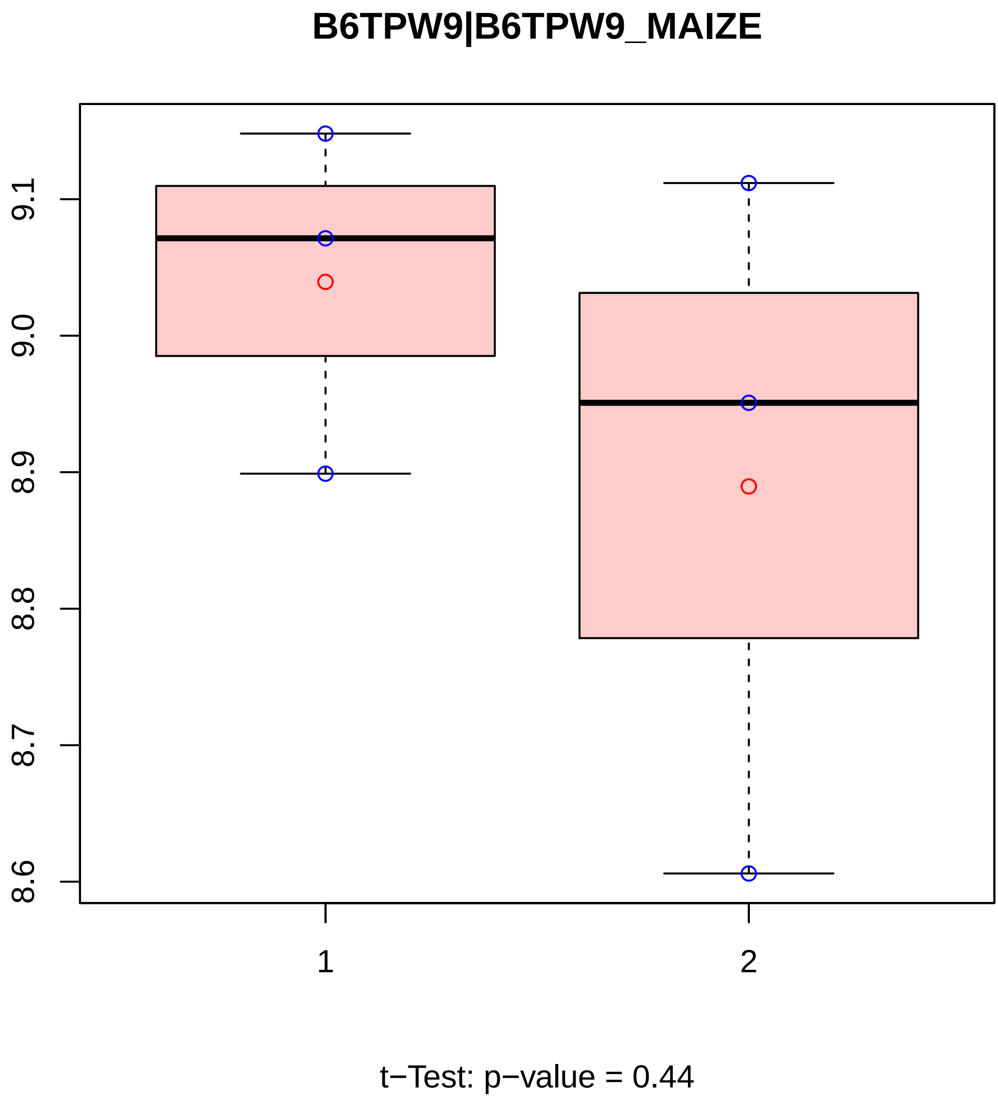

B6TPW9|B6TPW9_MAIZE
9.1
9.0
8.9
8.8
8.7
8.6
1
2
t−Test: p−value = 0.44

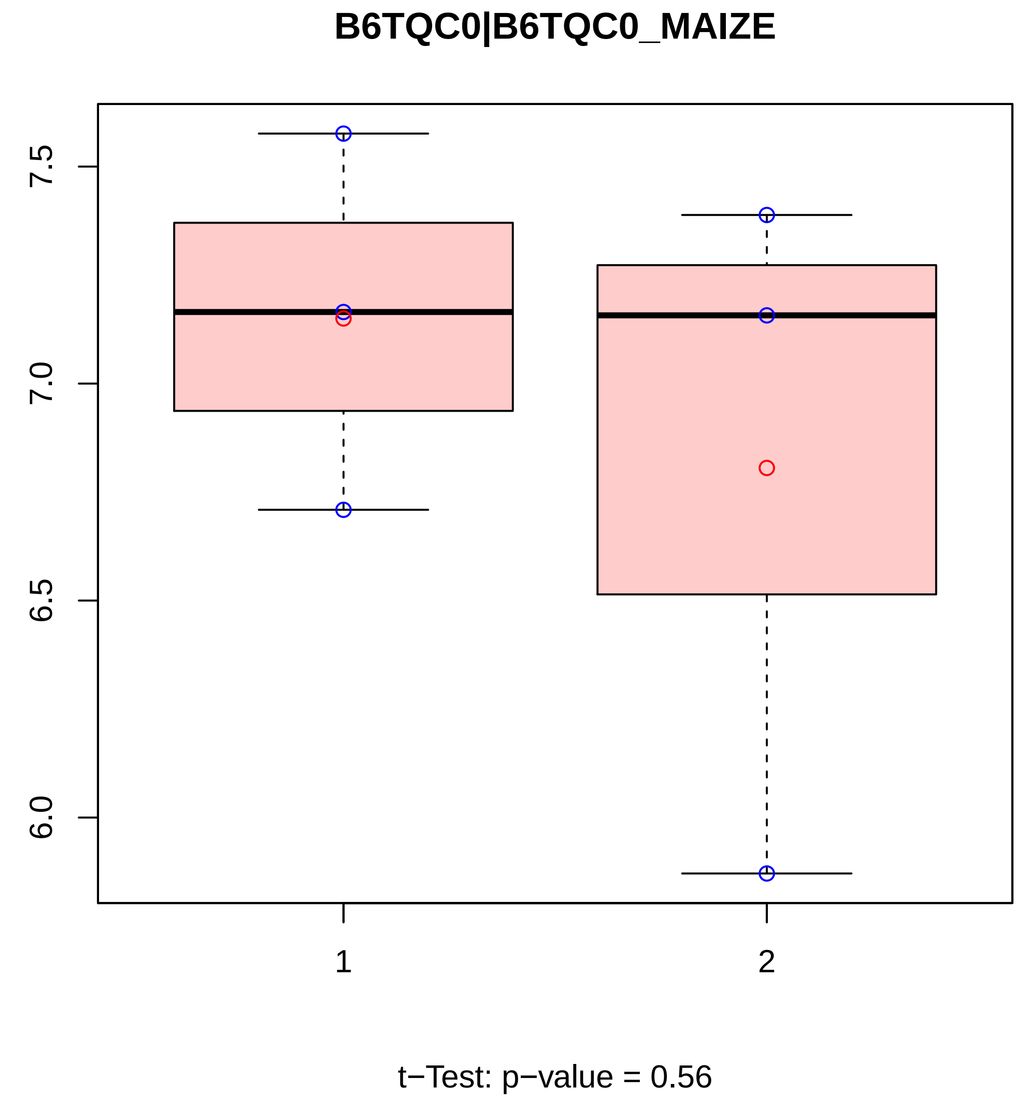
B6TQC0|B6TQC0_MAIZE
7.5
7.0
6.5
6.0
1
2
t−Test: p−value = 0.56

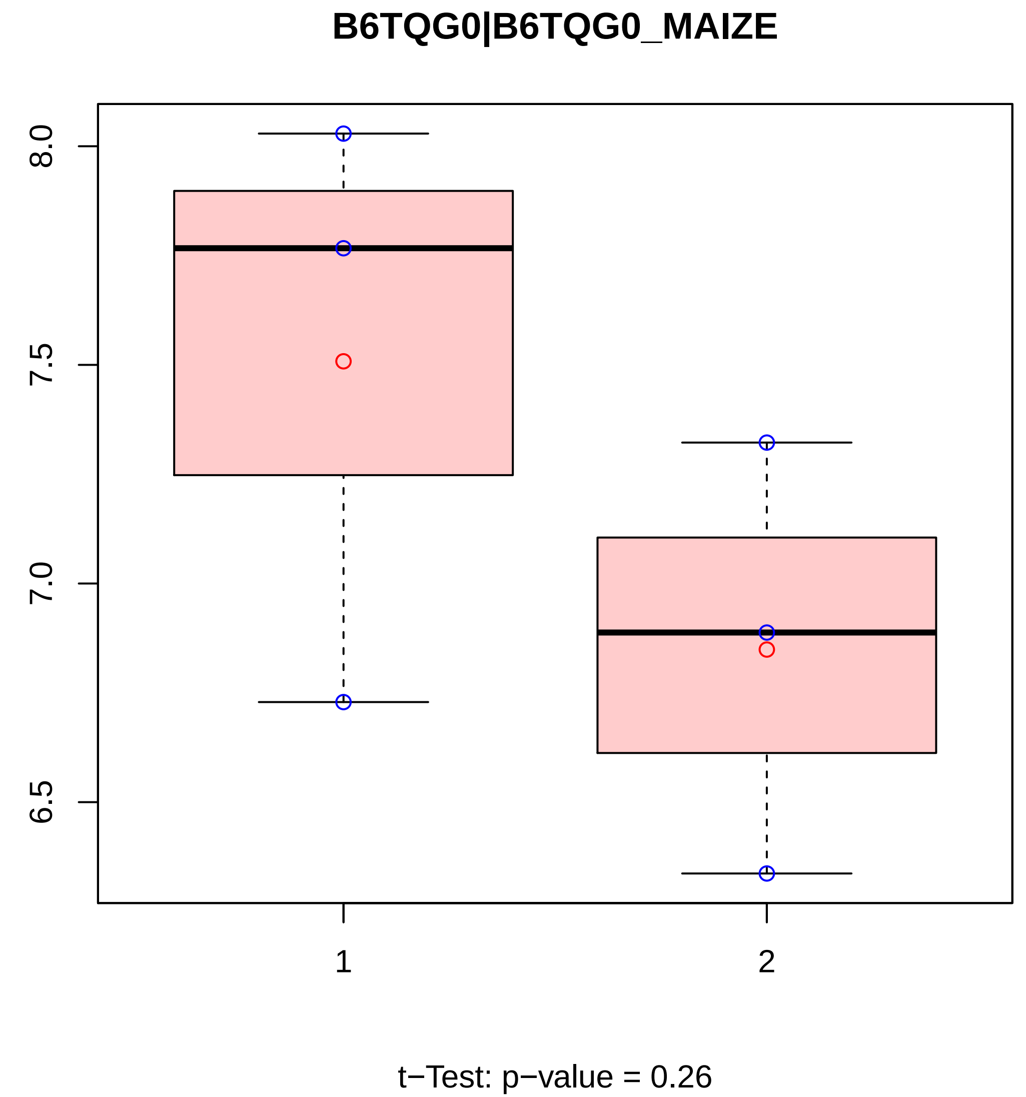
B6TQG0|B6TQG0_MAIZE
8.0
7.5
7.0
6.5
1
2
t−Test: p−value = 0.26

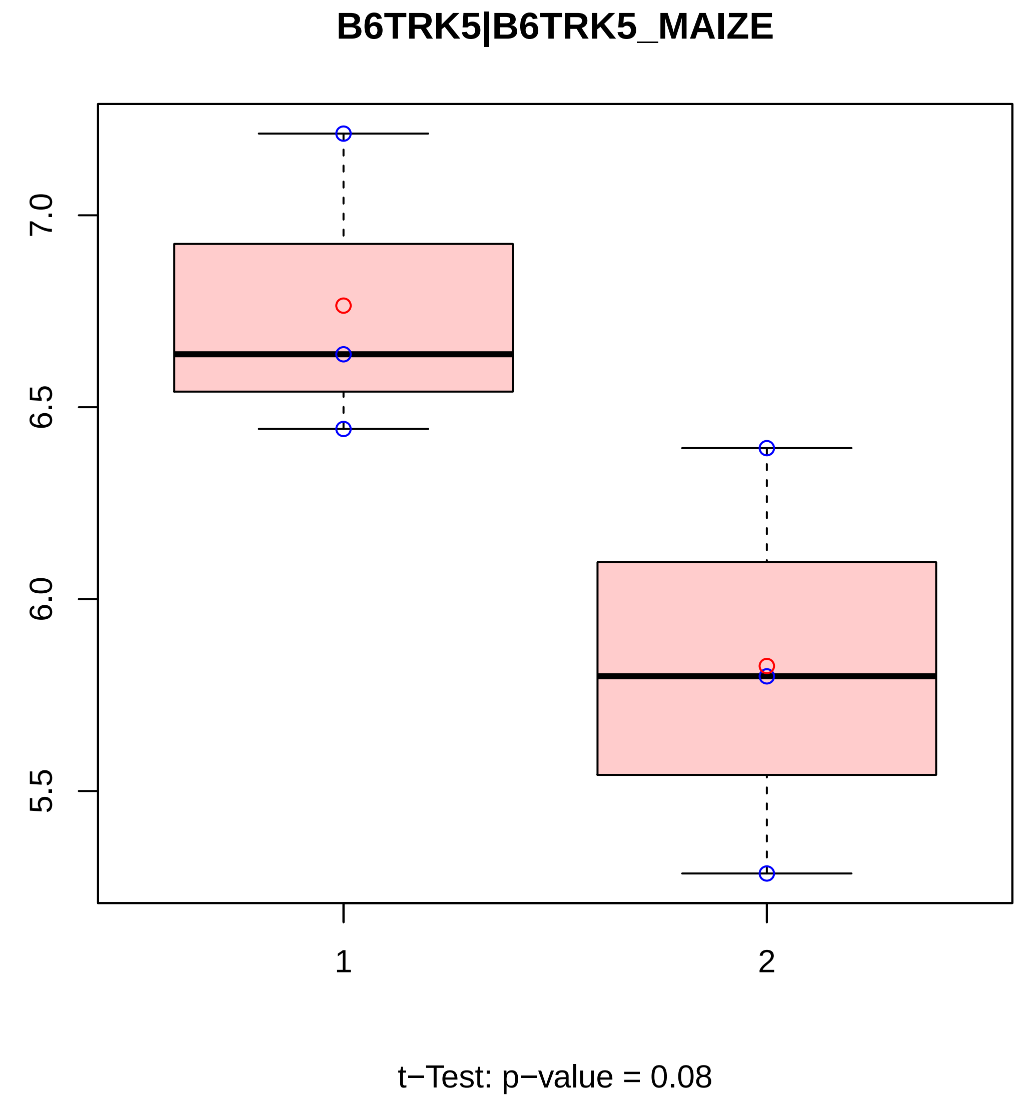
B6TRK5|B6TRK5_MAIZE
7.0
6.5
6.0
5.5
1
2
t−Test: p−value = 0.08

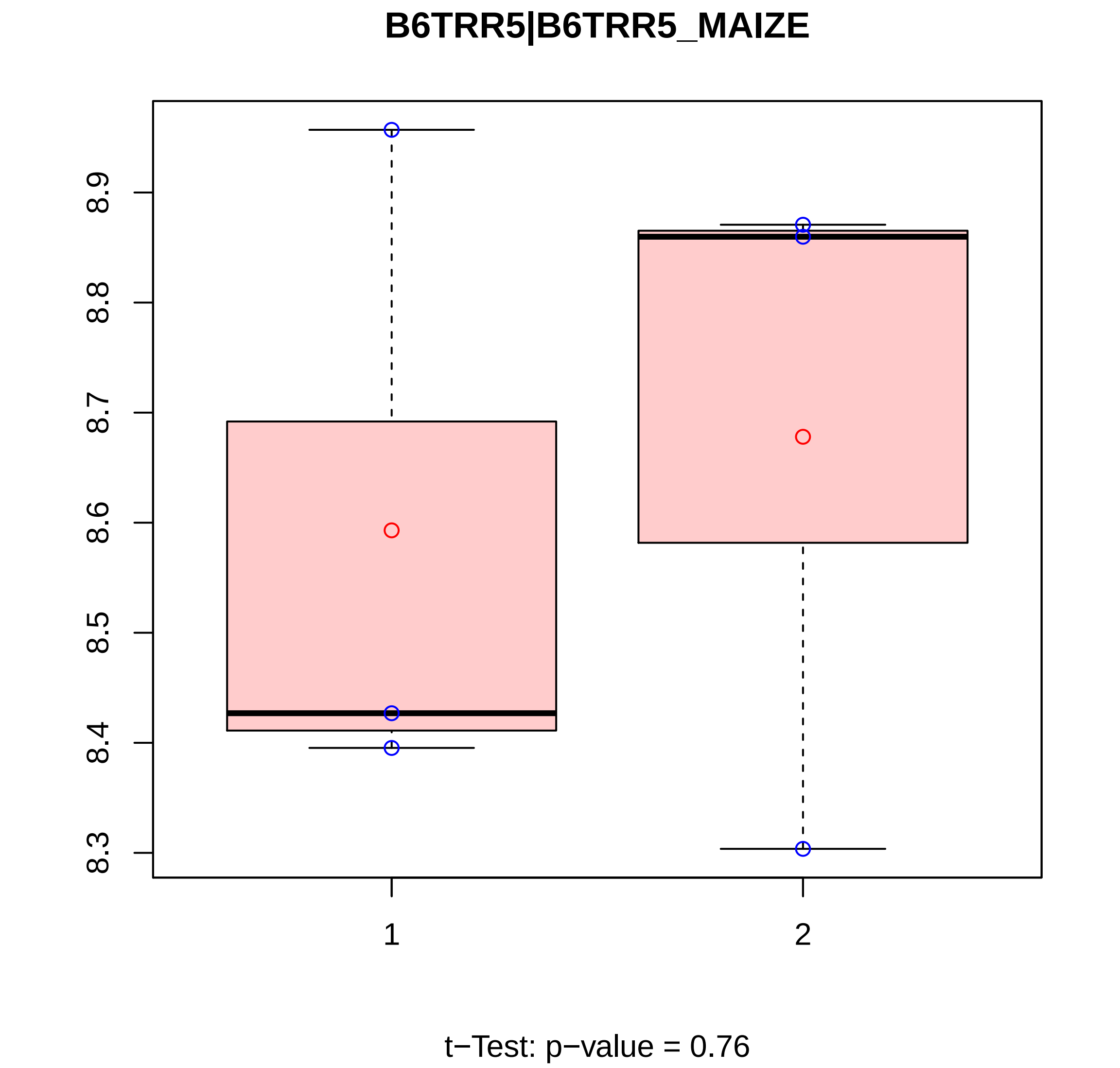

B6TRR5|B6TRR5_MAIZE
8.3
8.4
8.5
8.6
8.7
8.8
8.9
1
2
t−Test: p−value = 0.76

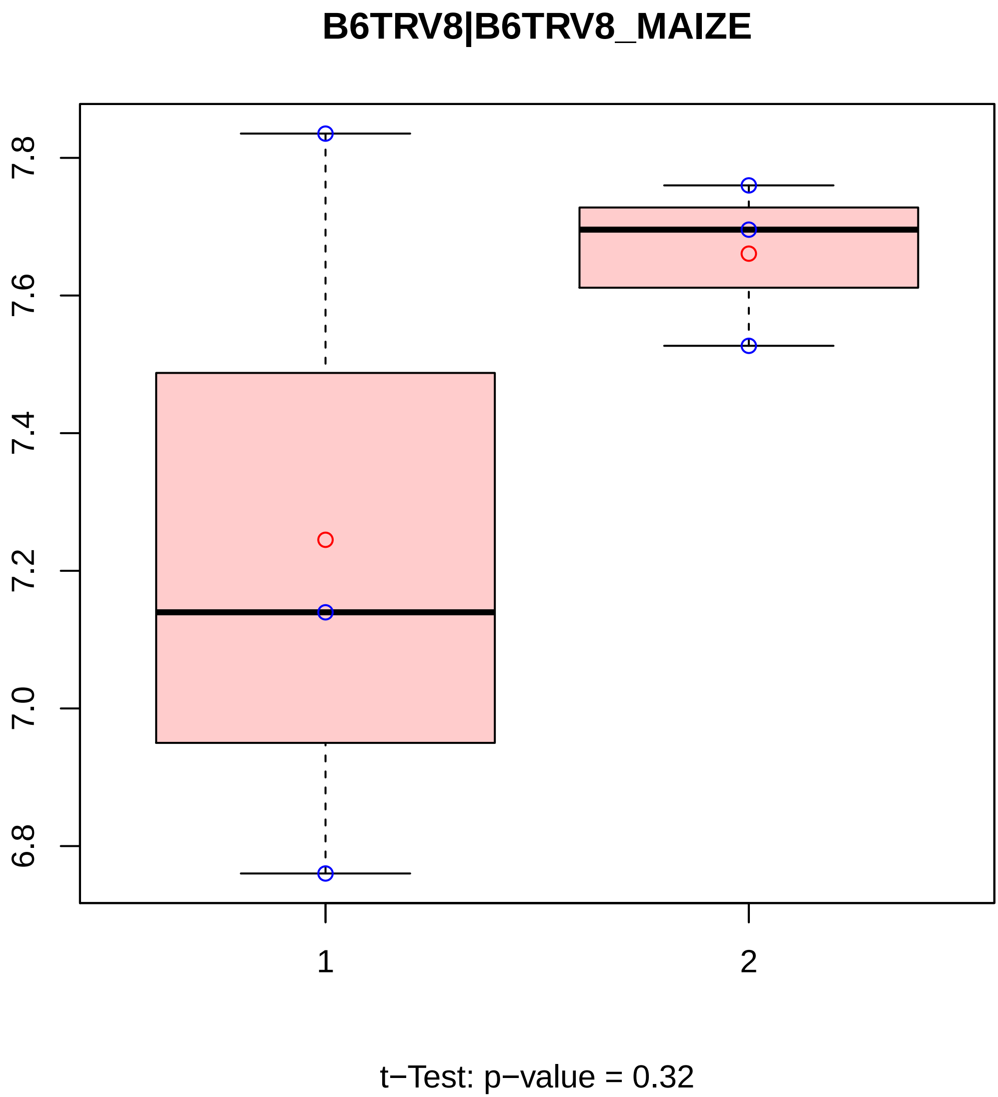

B6TRV8|B6TRV8_MAIZE
7.8
7.6
7.4
7.2
7.0
6.8
1
2
t−Test: p−value = 0.32

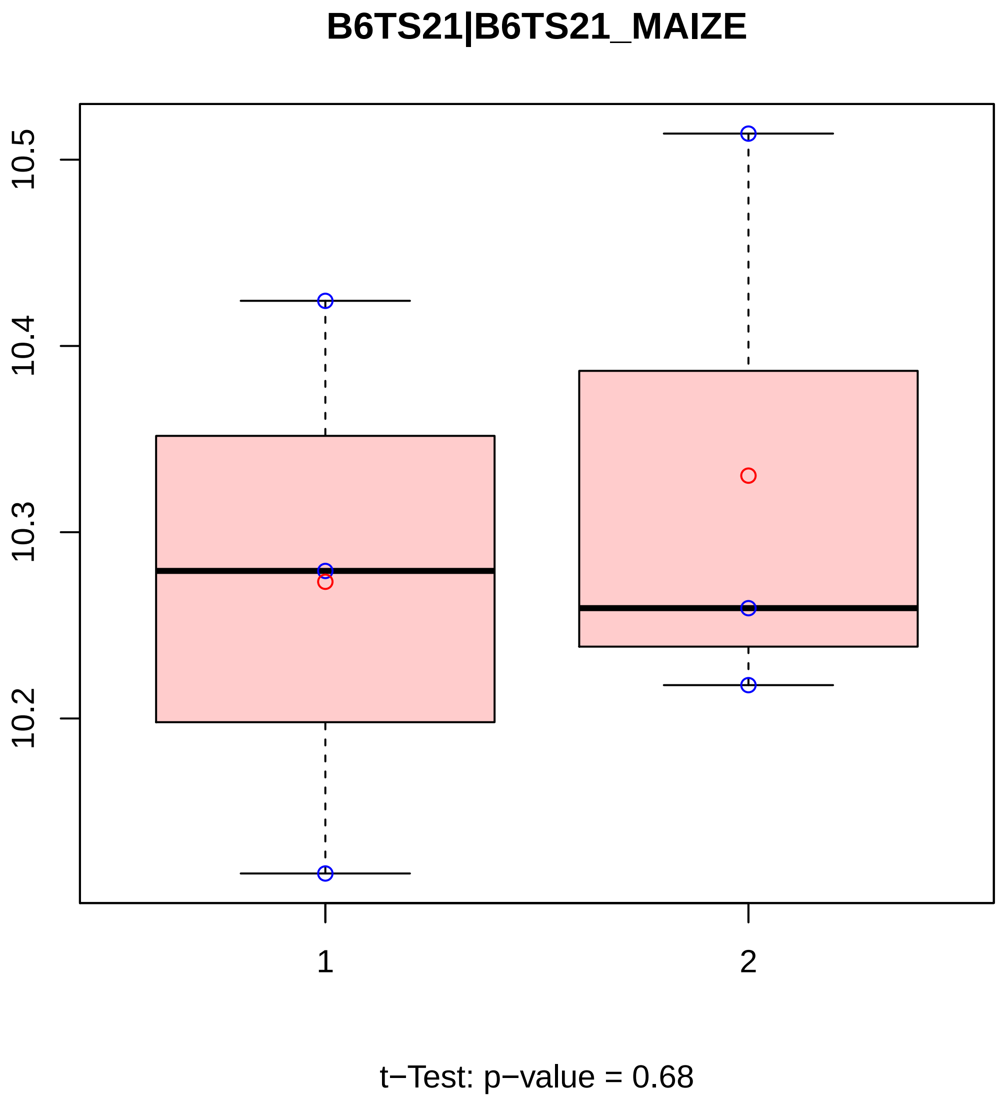
B6TS21|B6TS21_MAIZE
10.5
10.4
10.3
10.2
1
2
t−Test: p−value = 0.68

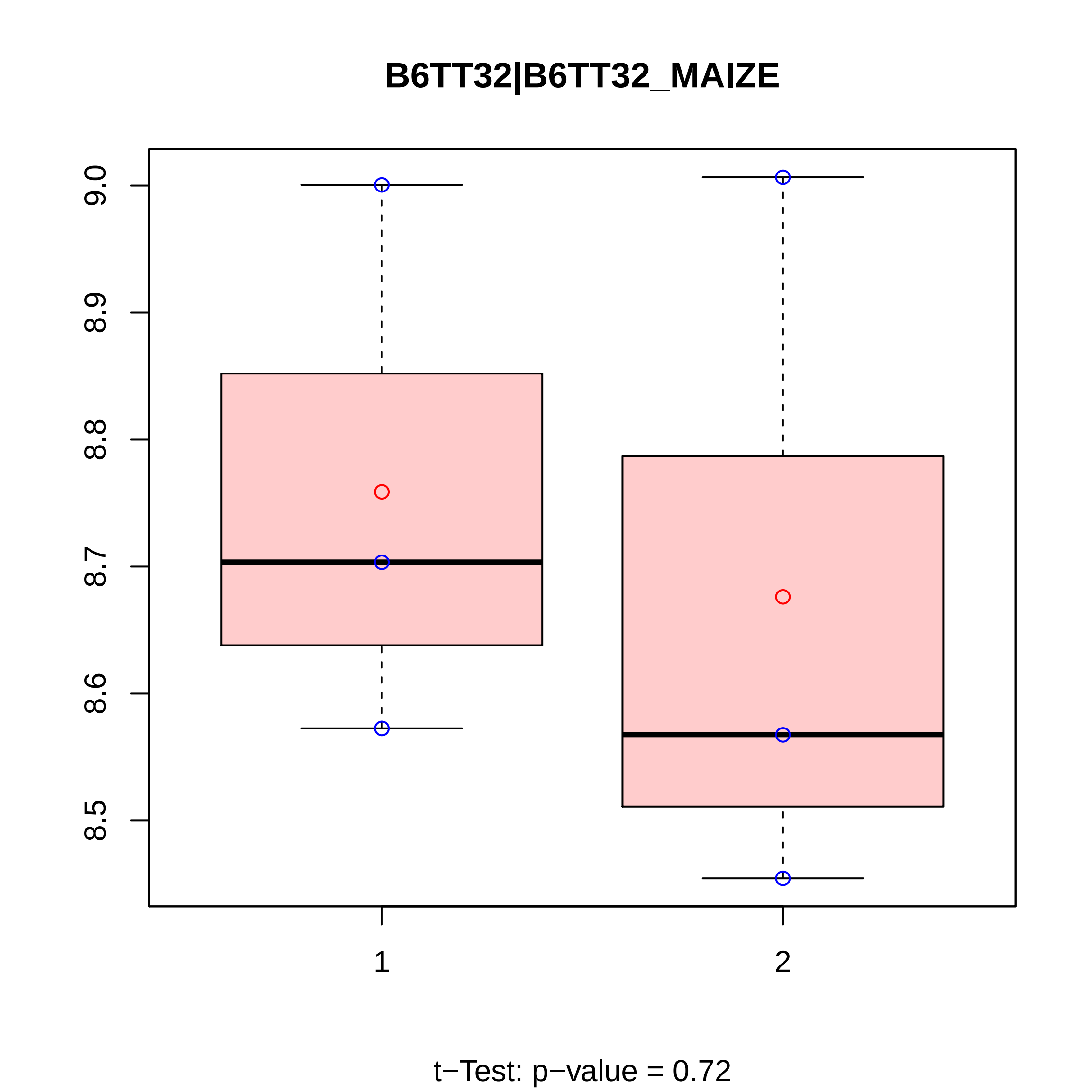
B6TT32|B6TT32_MAIZE
9.0
8.9
8.8
8.7
8.6
8.5
1
2
t−Test: p−value = 0.72

B6TUA2|B6TUA2_MAIZE

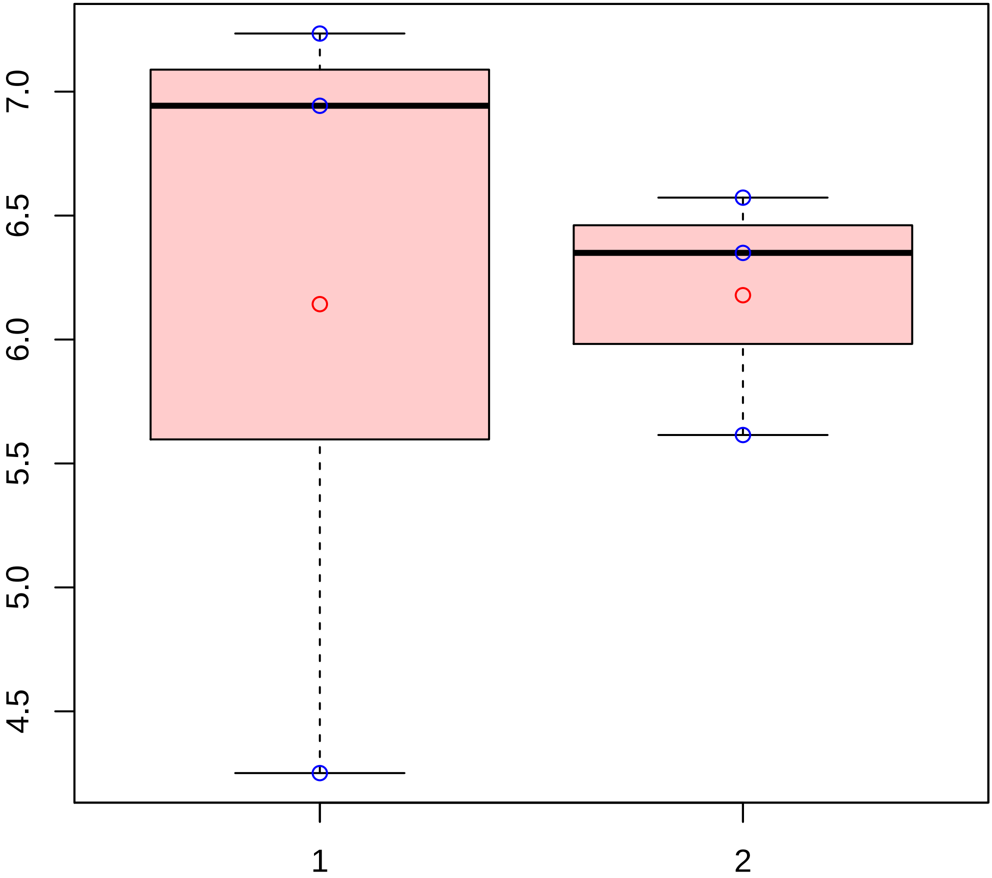

t−Test: p−value = 0.97

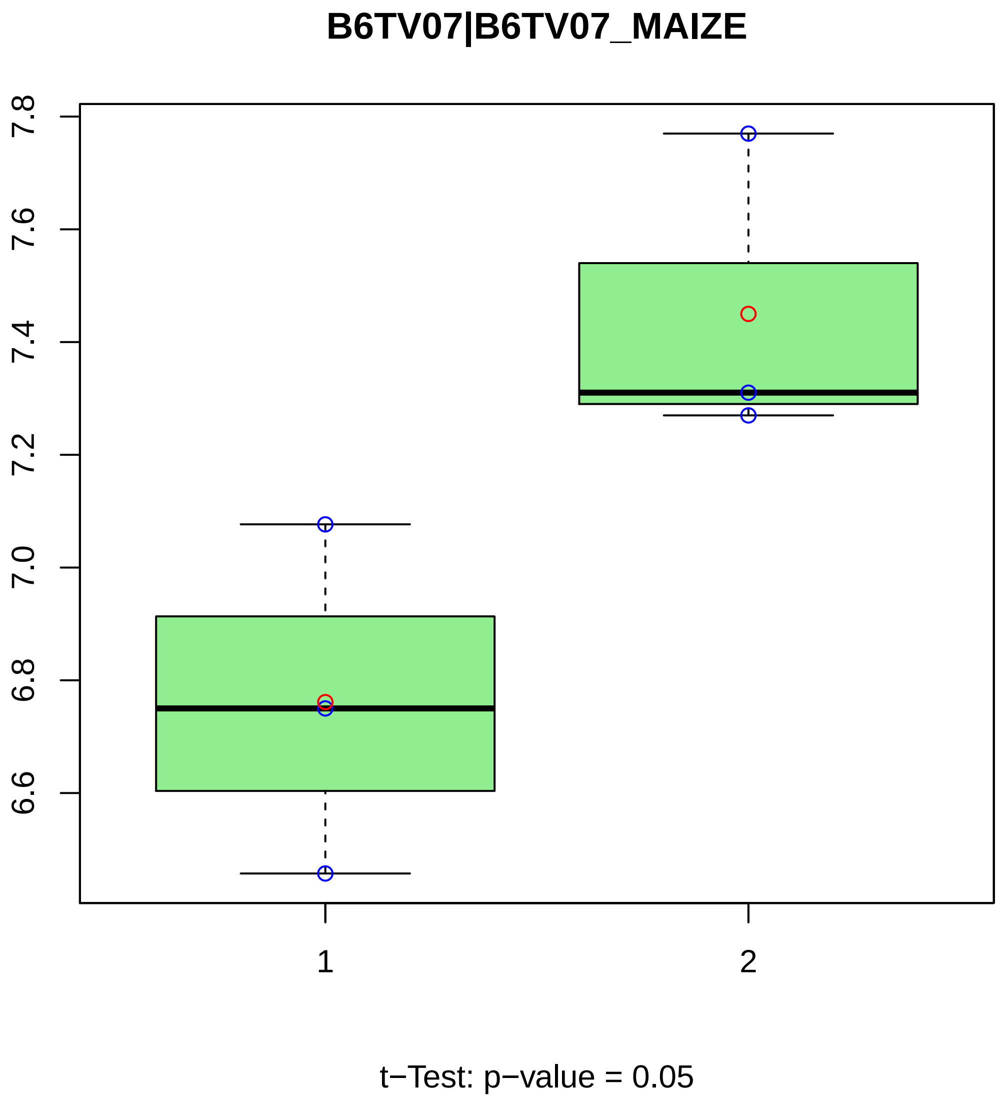
B6TV07|B6TV07_MAIZE
7.8
7.6
7.4
7.2
7.0
6.8
6.6
1
2
t−Test: p−value = 0.05

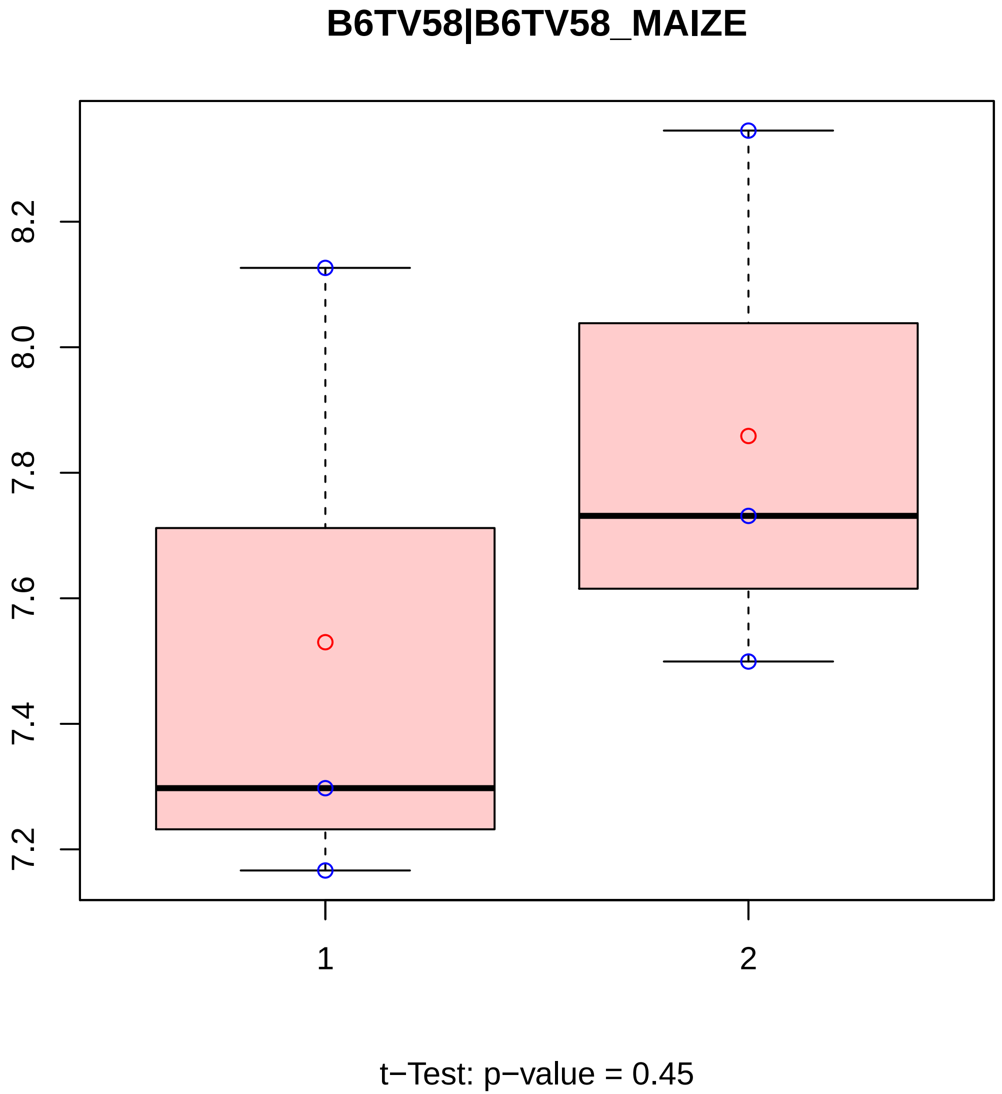
B6TV58|B6TV58_MAIZE
8.2
8.0
7.8
7.6
7.4
7.2
1
2
t−Test: p−value = 0.45

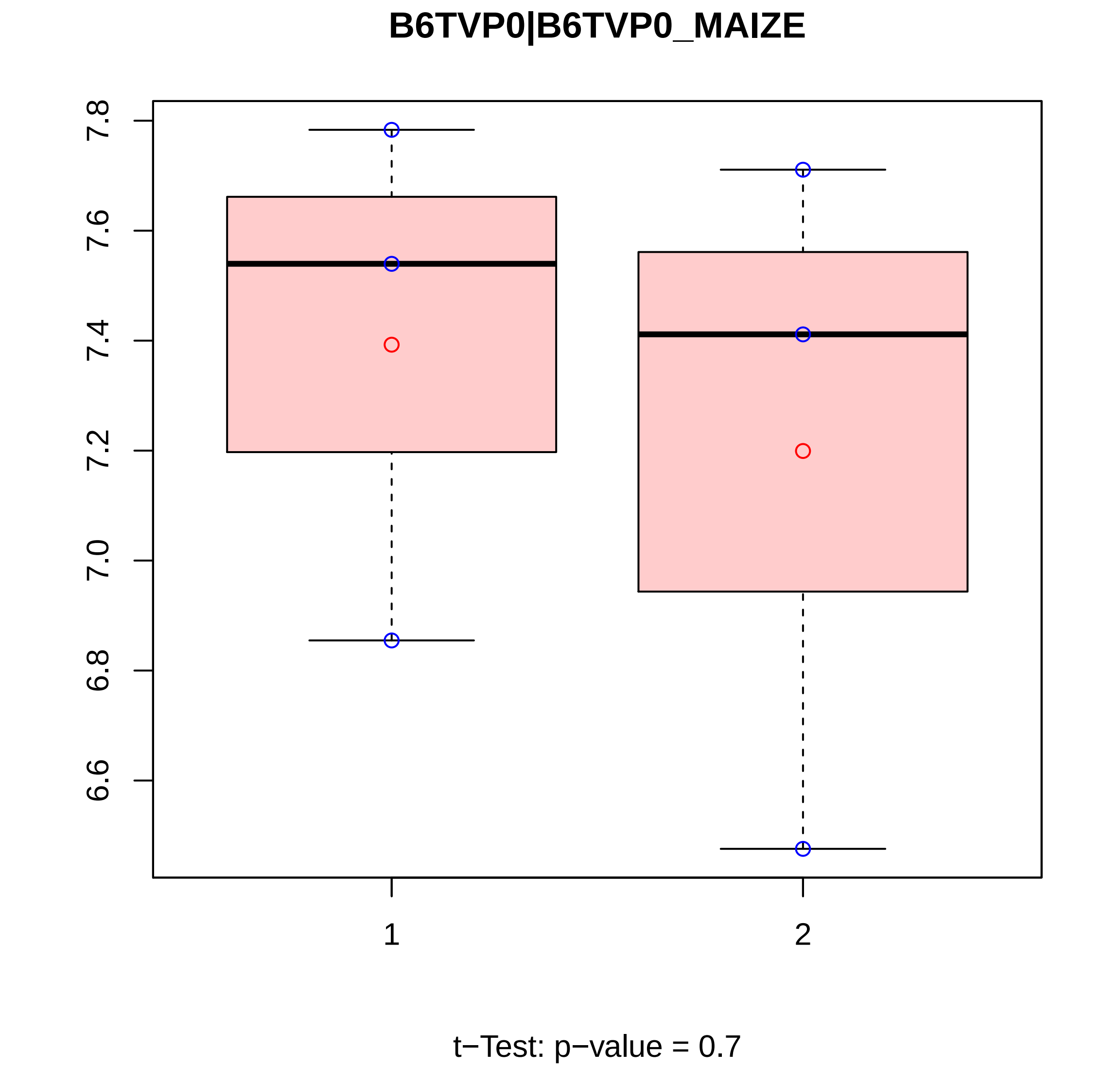

B6TVP0|B6TVP0_MAIZE
7.8
7.6
7.4
7.2
7.0
6.8
6.6
1
2
t−Test: p−value = 0.7

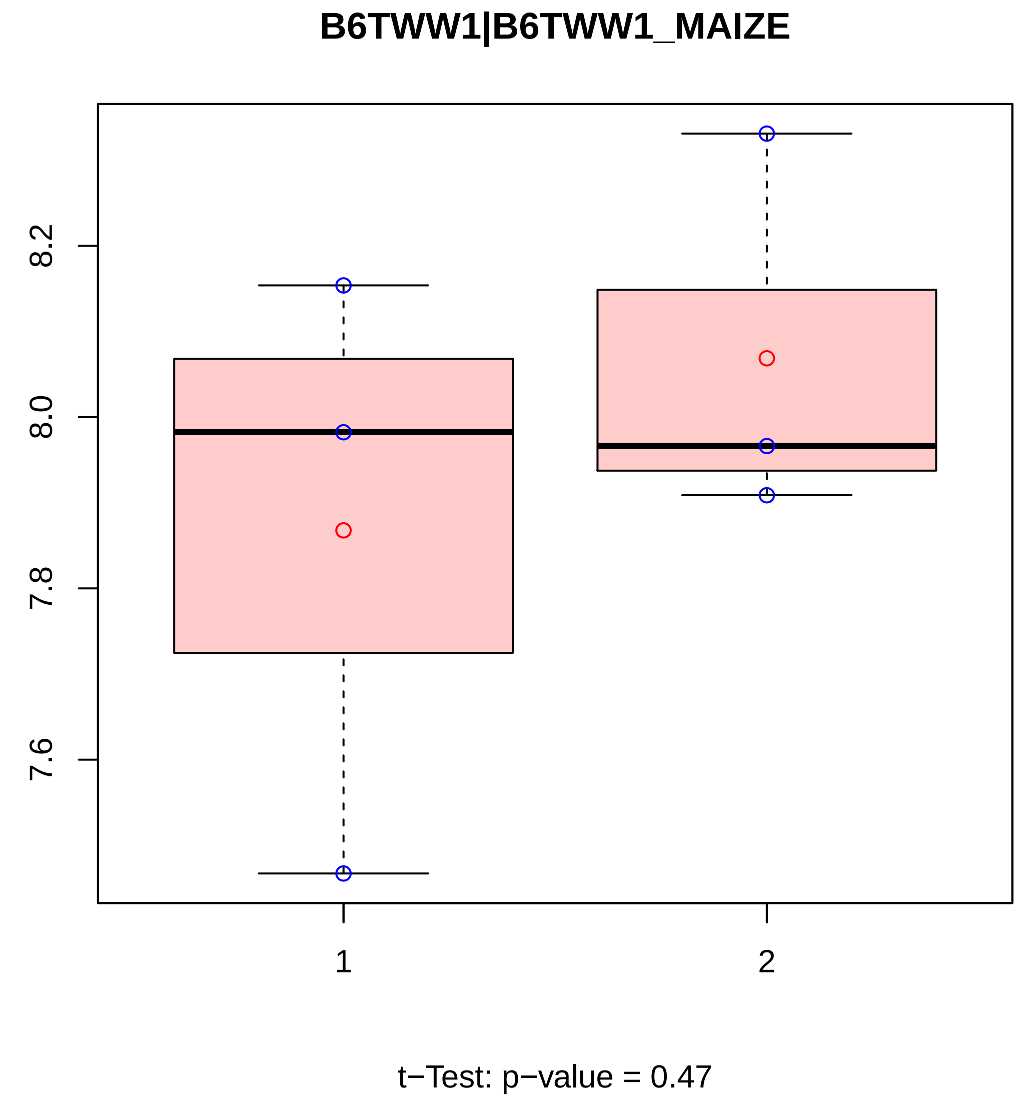

B6TWW1|B6TWW1_MAIZE
8.2
8.0
7.8
7.6
1
2
t−Test: p−value = 0.47

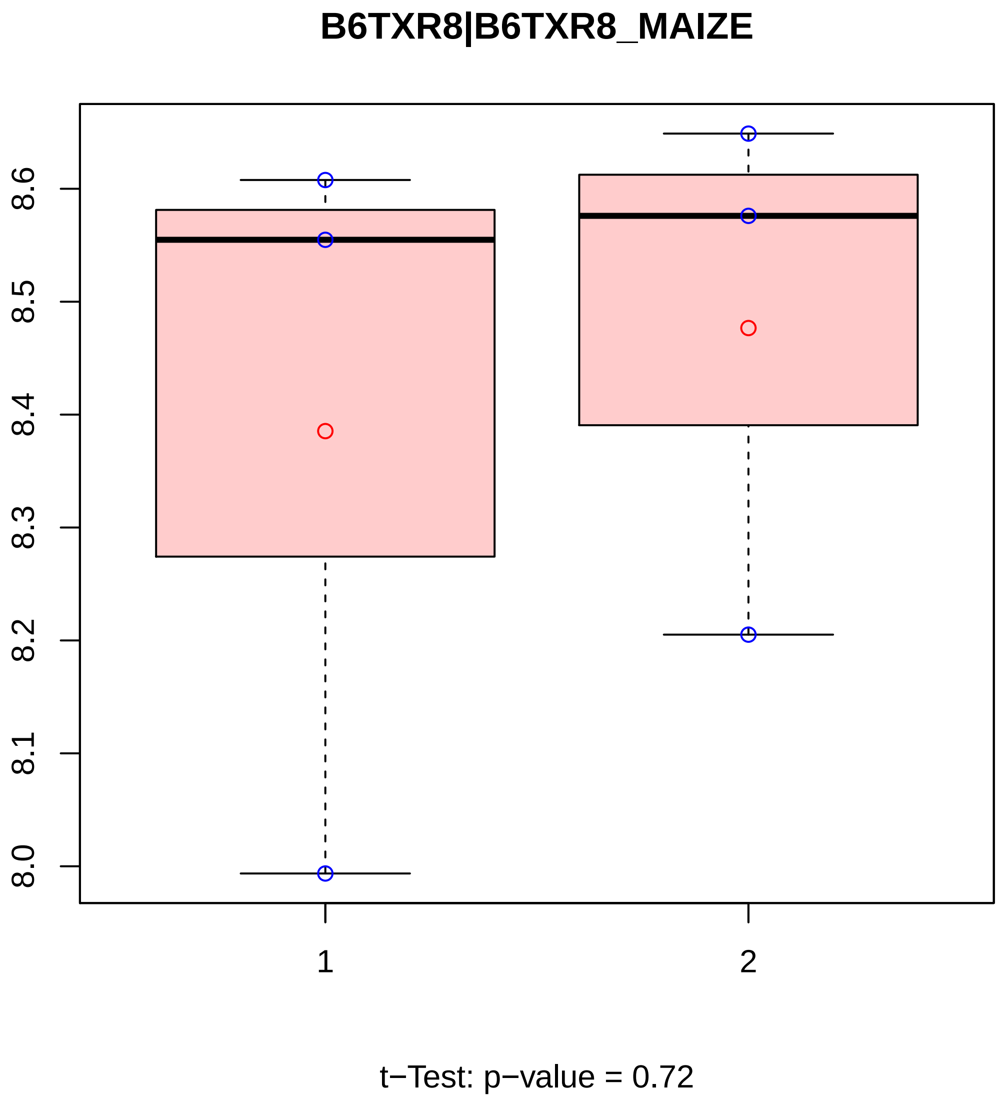
B6TXR8|B6TXR8_MAIZE
8.0
8.1
8.2
8.3
8.4
8.5
8.6
1
2
t−Test: p−value = 0.72

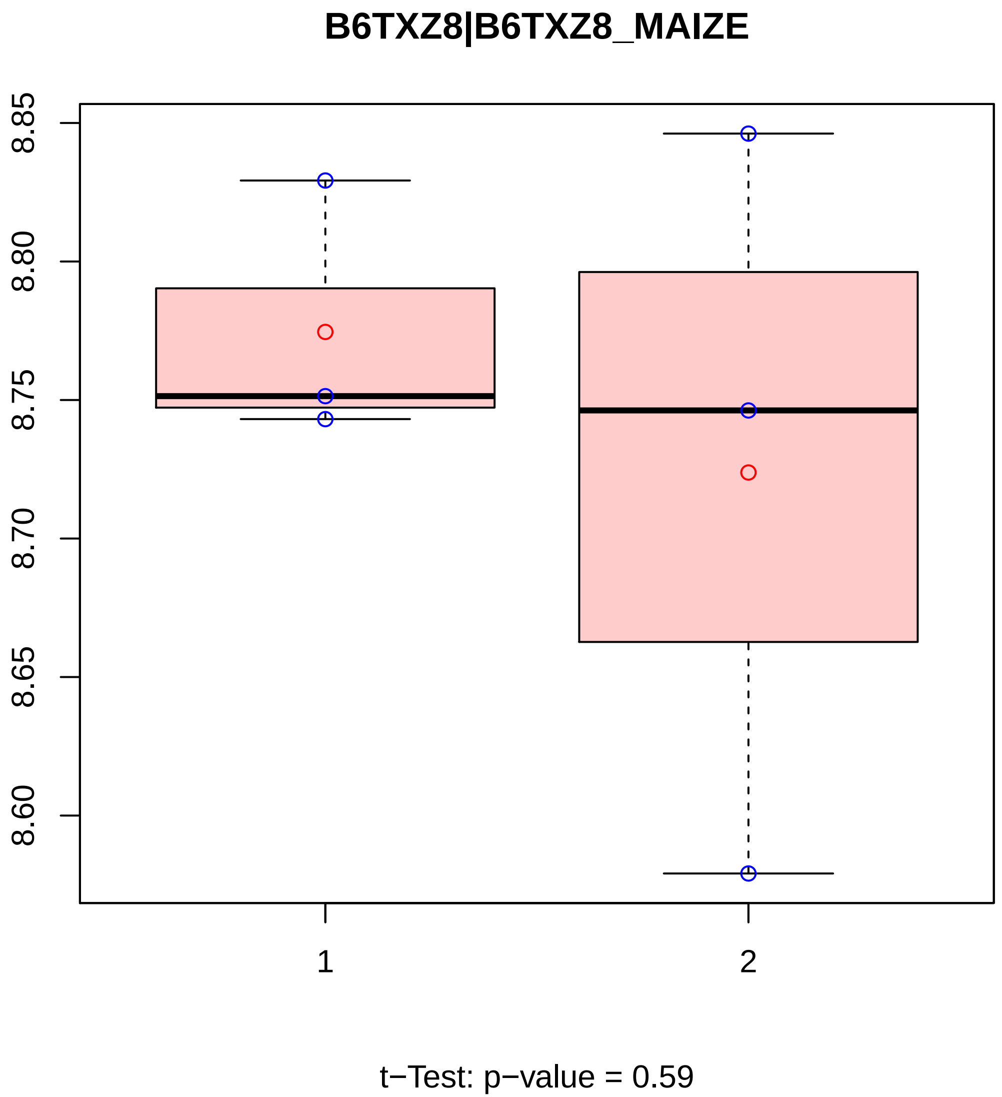
B6TXZ8|B6TXZ8_MAIZE
8.60
8.65
8.70
8.75
8.80
8.85
1
2
t−Test: p−value = 0.59

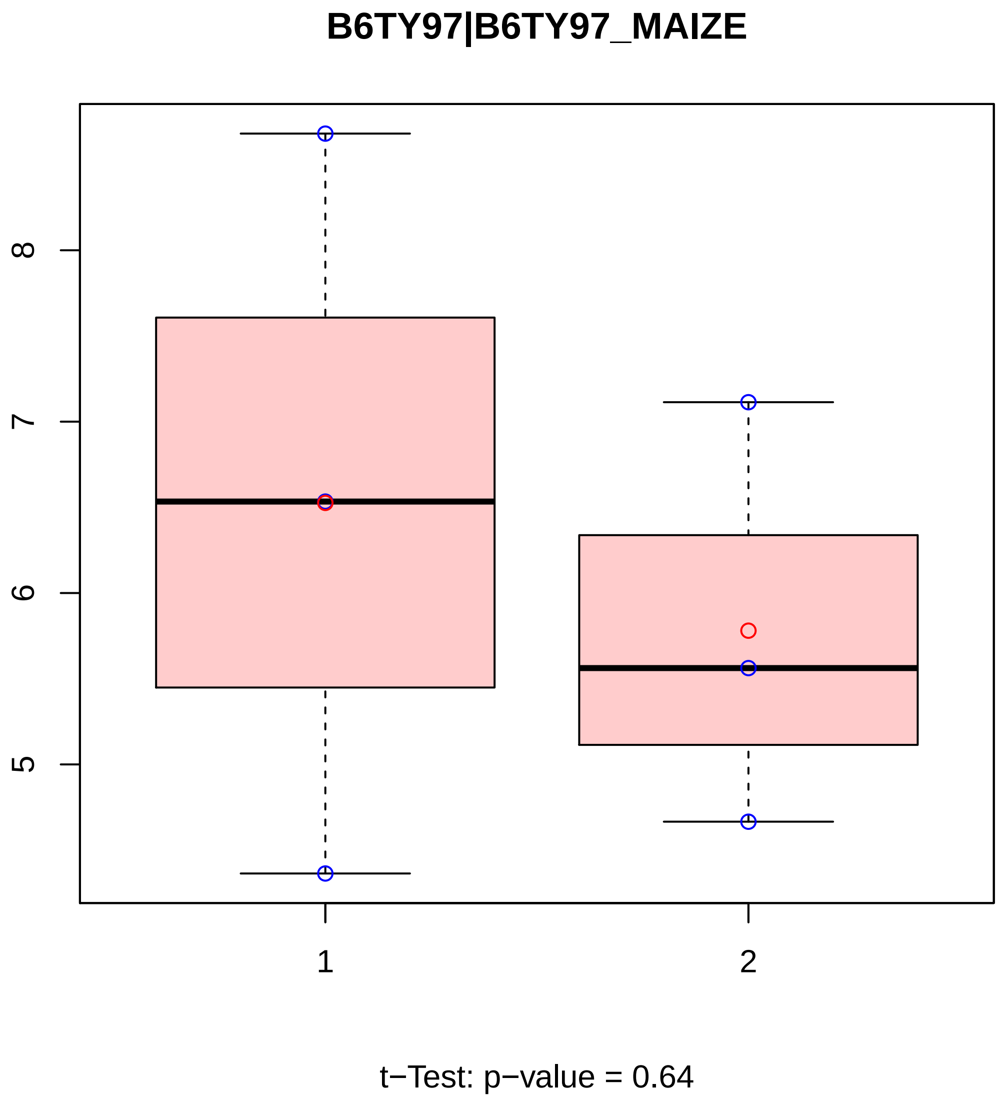

B6TY97|B6TY97_MAIZE
8
7
6
5
1
2
t−Test: p−value = 0.64

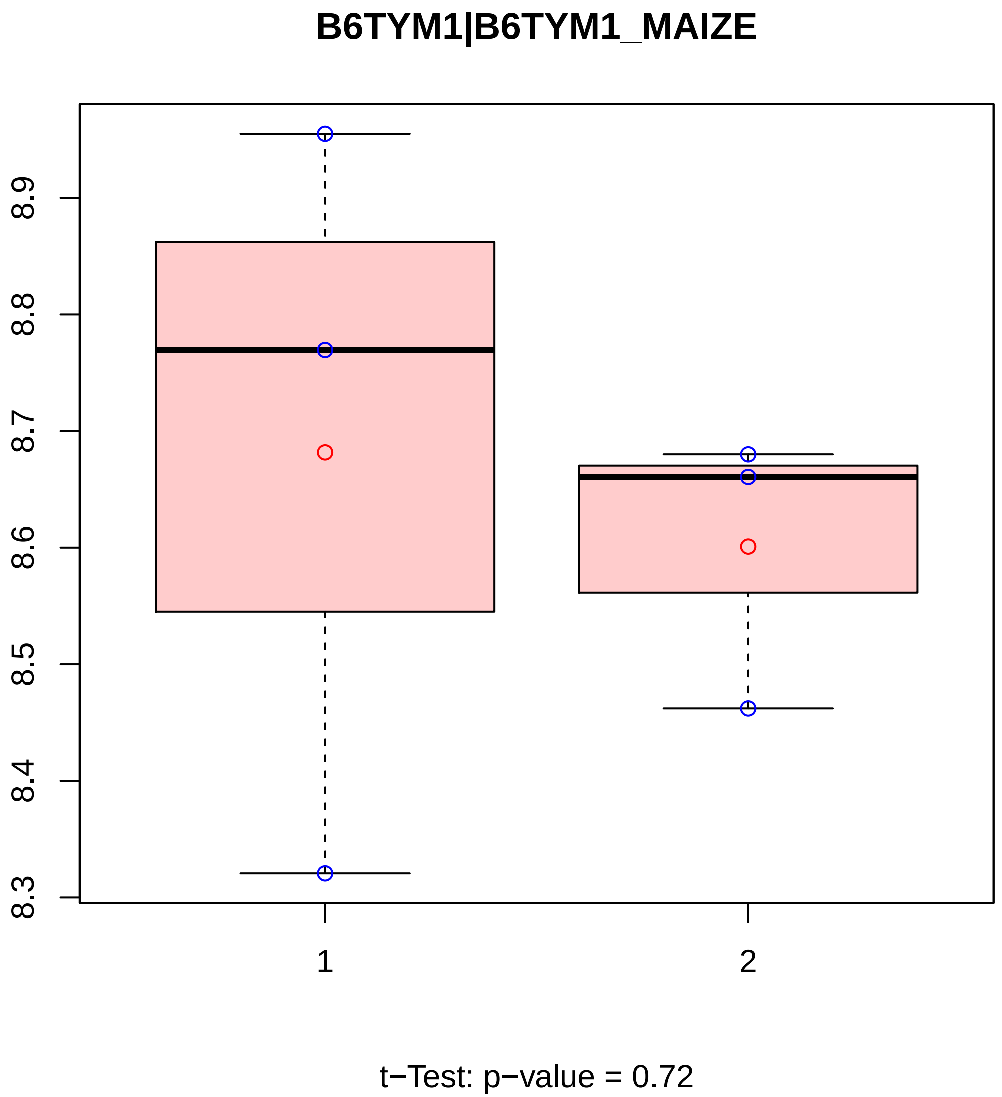

B6TYM1|B6TYM1_MAIZE
8.3
8.4
8.5
8.6
8.7
8.8
8.9
1
2
t−Test: p−value = 0.72

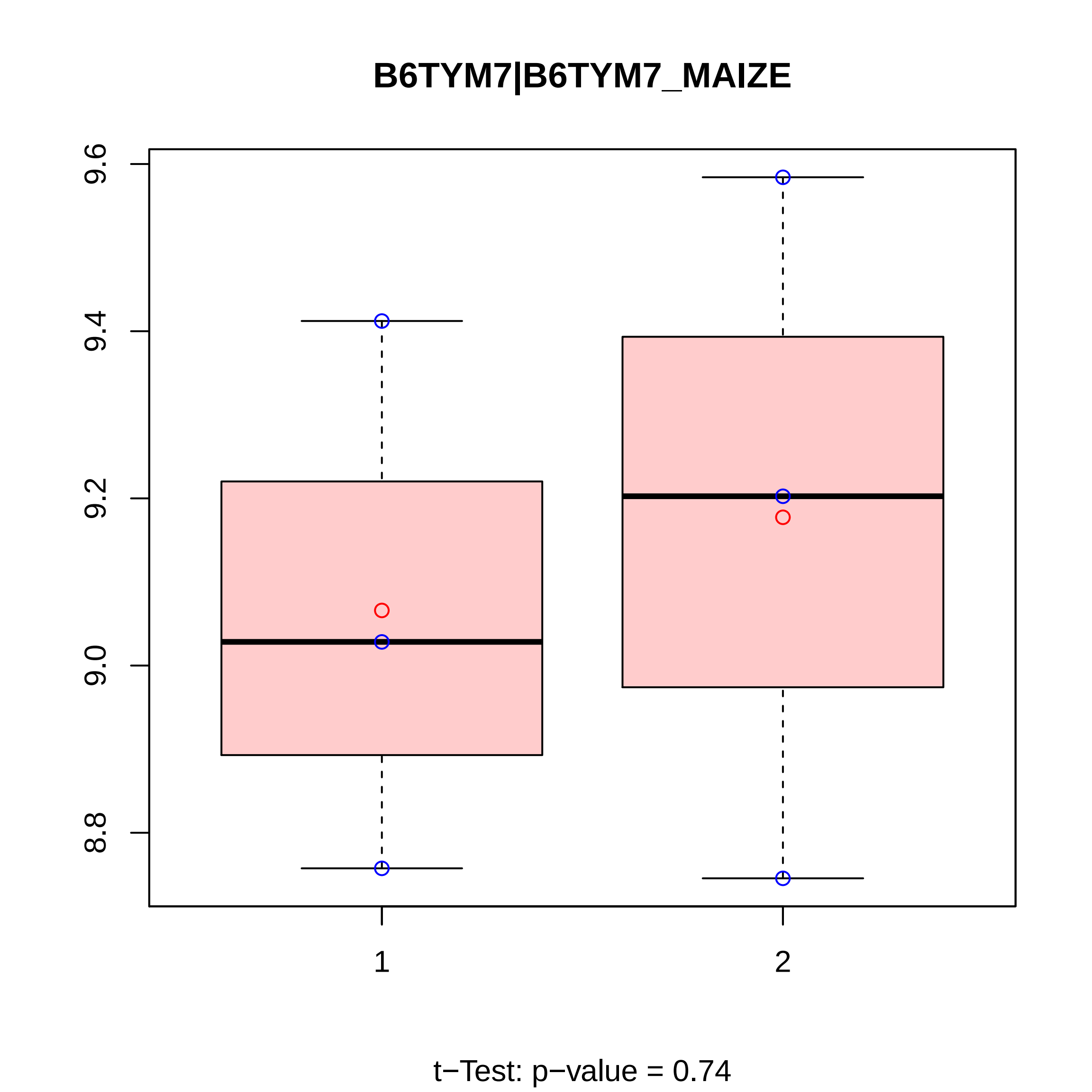

B6TYM7|B6TYM7_MAIZE
9.6
9.4
9.2
9.0
8.8
1
2
t−Test: p−value = 0.74

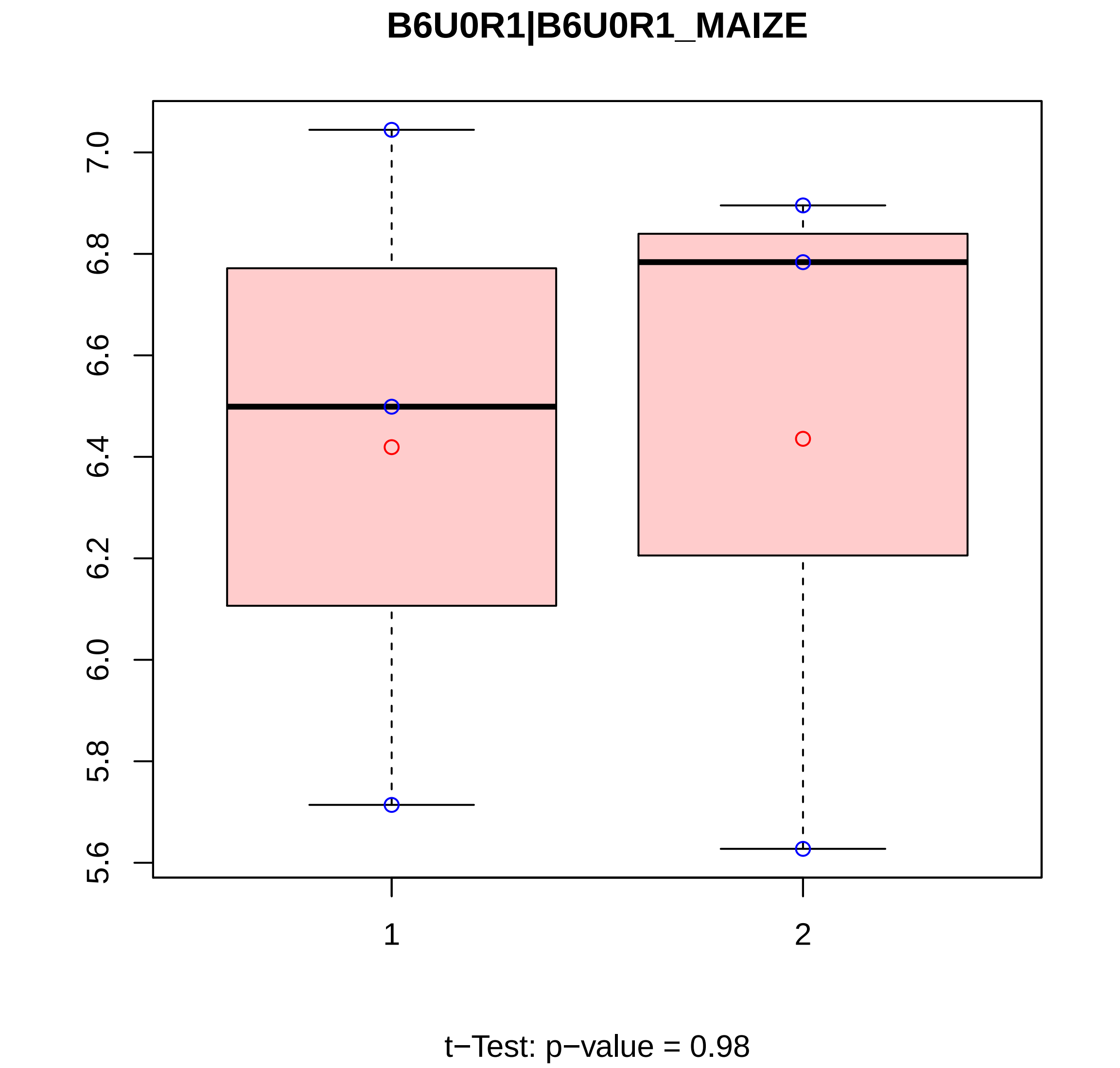
B6U0R1|B6U0R1_MAIZE
7.0
6.8
6.6
6.4
6.2
6.0
5.8
5.6
1
2
t−Test: p−value = 0.98

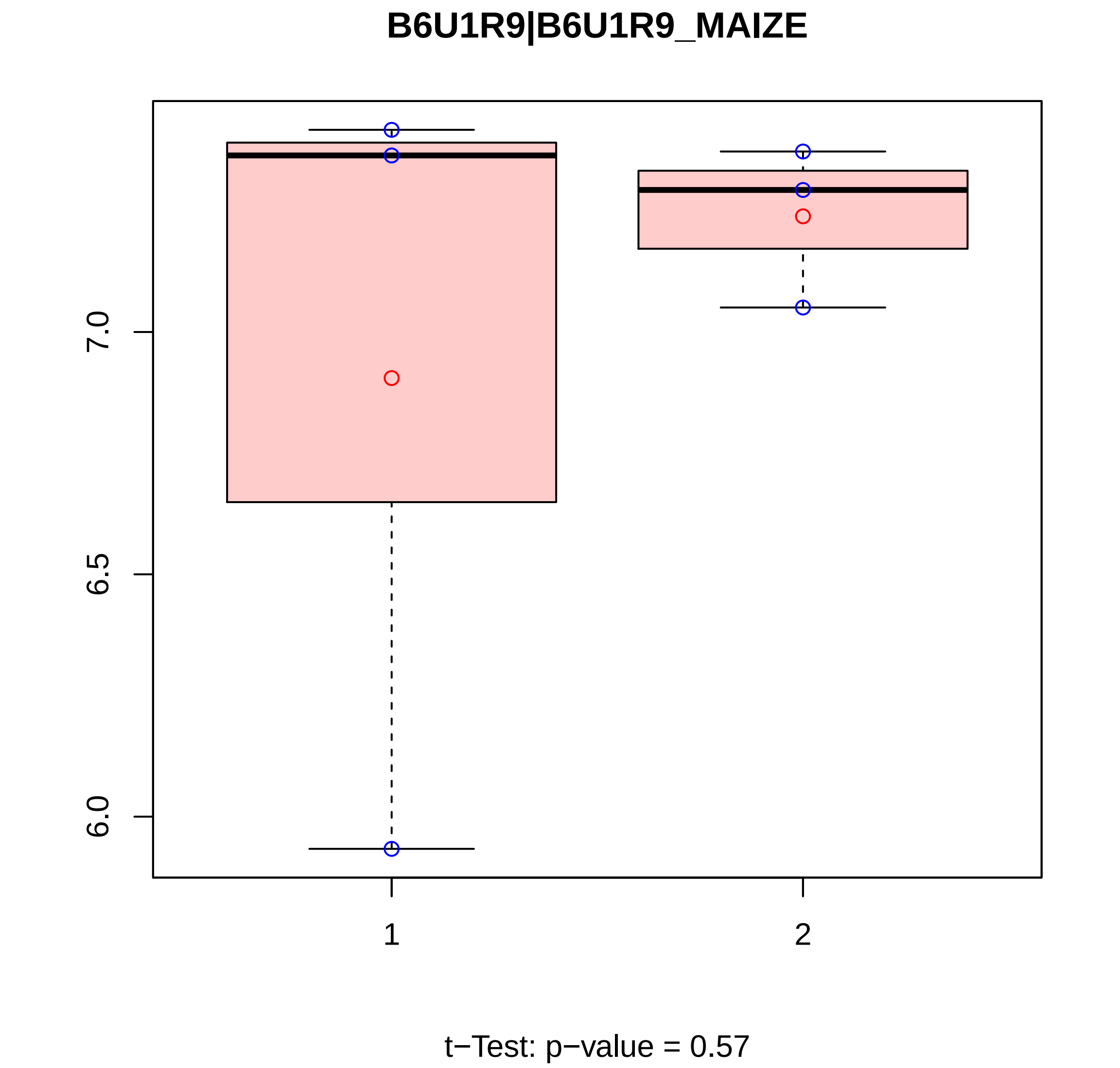

B6U1R9|B6U1R9_MAIZE
7.0
6.5
6.0
1
2
t−Test: p−value = 0.57

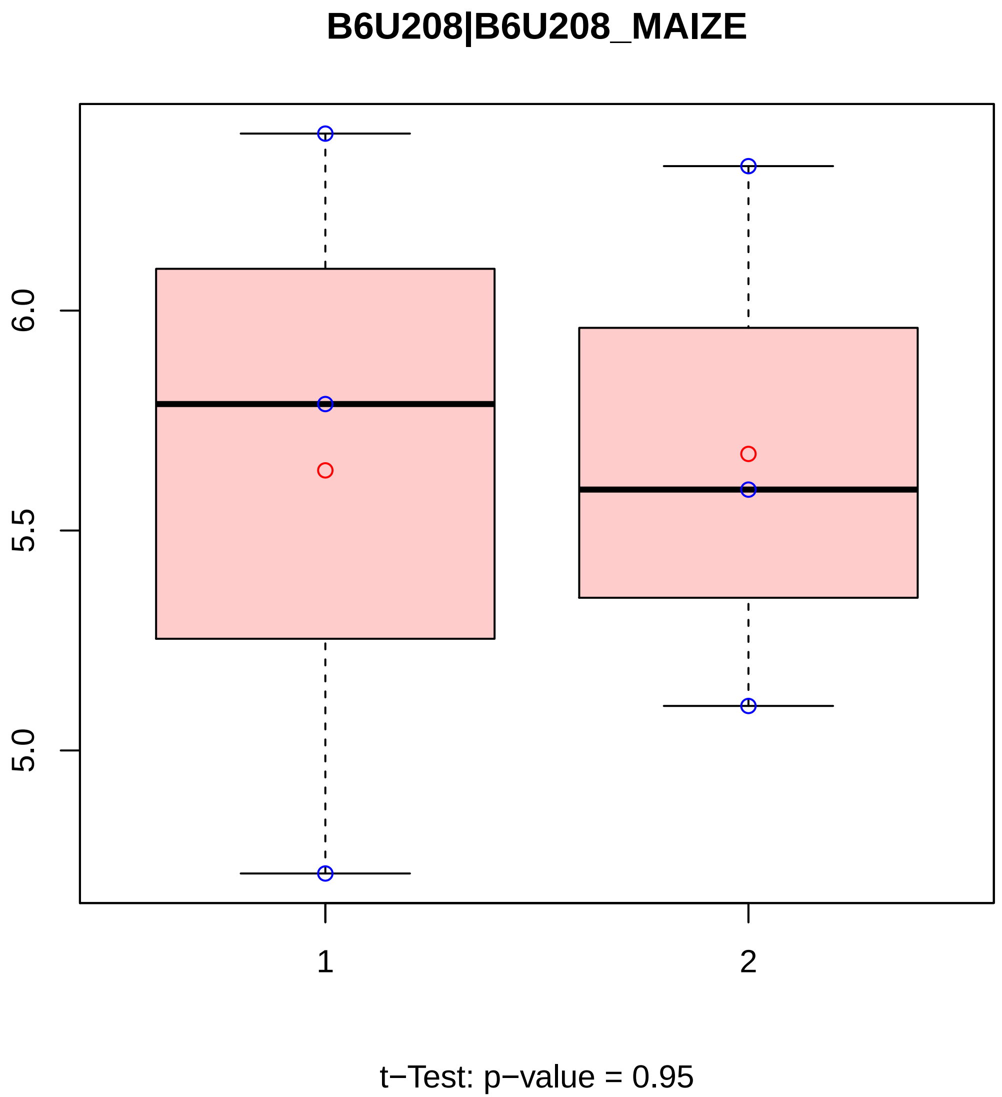
B6U208|B6U208_MAIZE
5.0
5.5
6.0
1
2
t−Test: p−value = 0.95

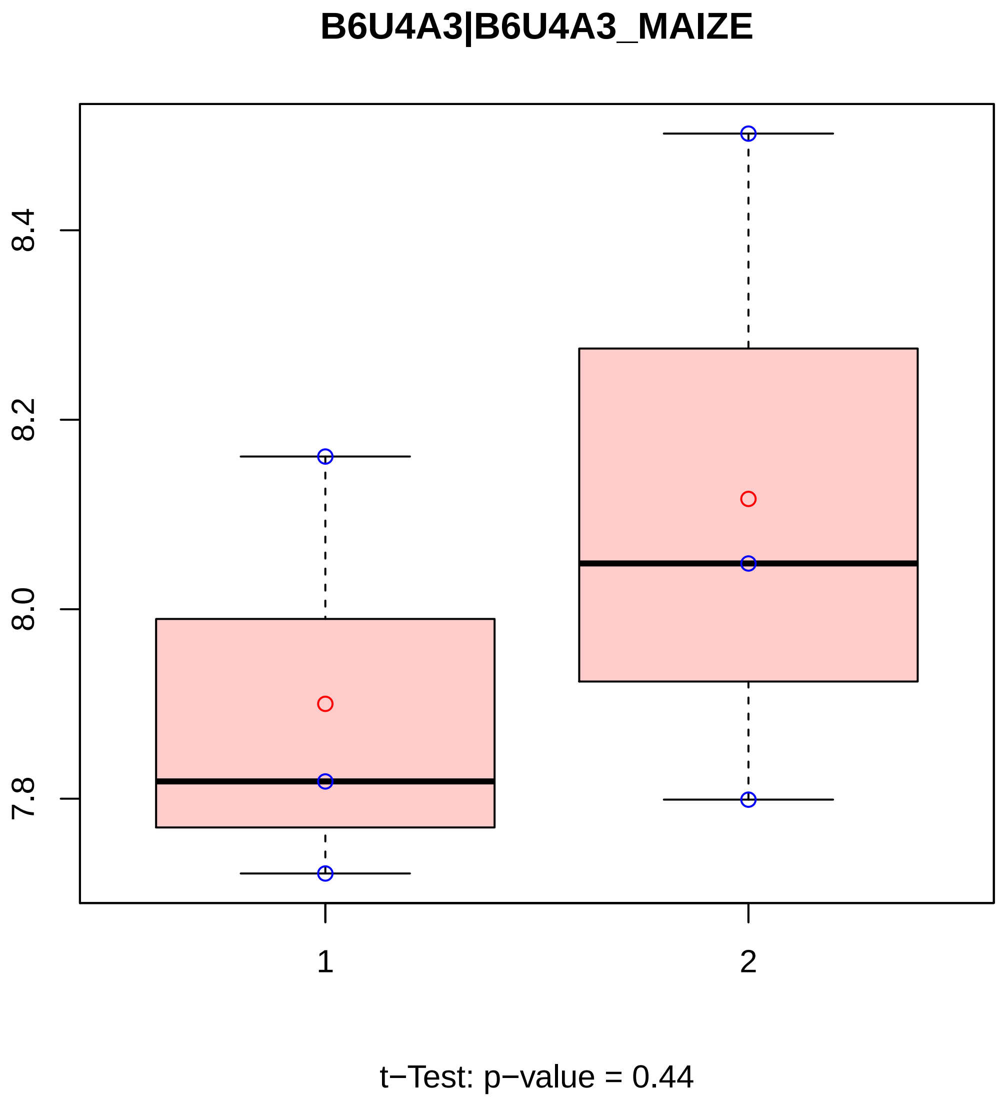

B6U4A3|B6U4A3_MAIZE
8.4
8.2
8.0
7.8
1
2
t−Test: p−value = 0.44

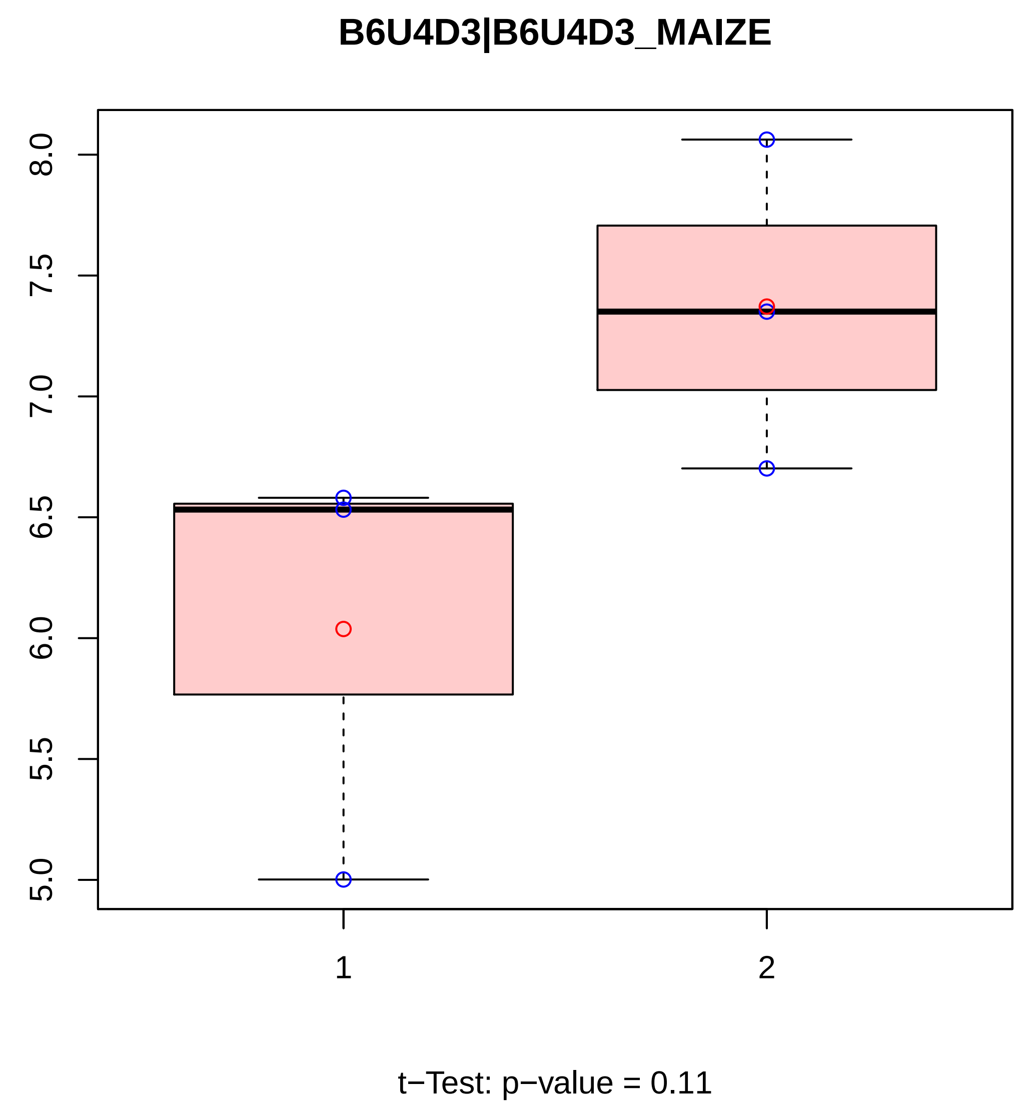

B6U4D3|B6U4D3_MAIZE
8.0
7.5
7.0
6.5
6.0
5.5
5.0
1
2
t−Test: p−value = 0.11

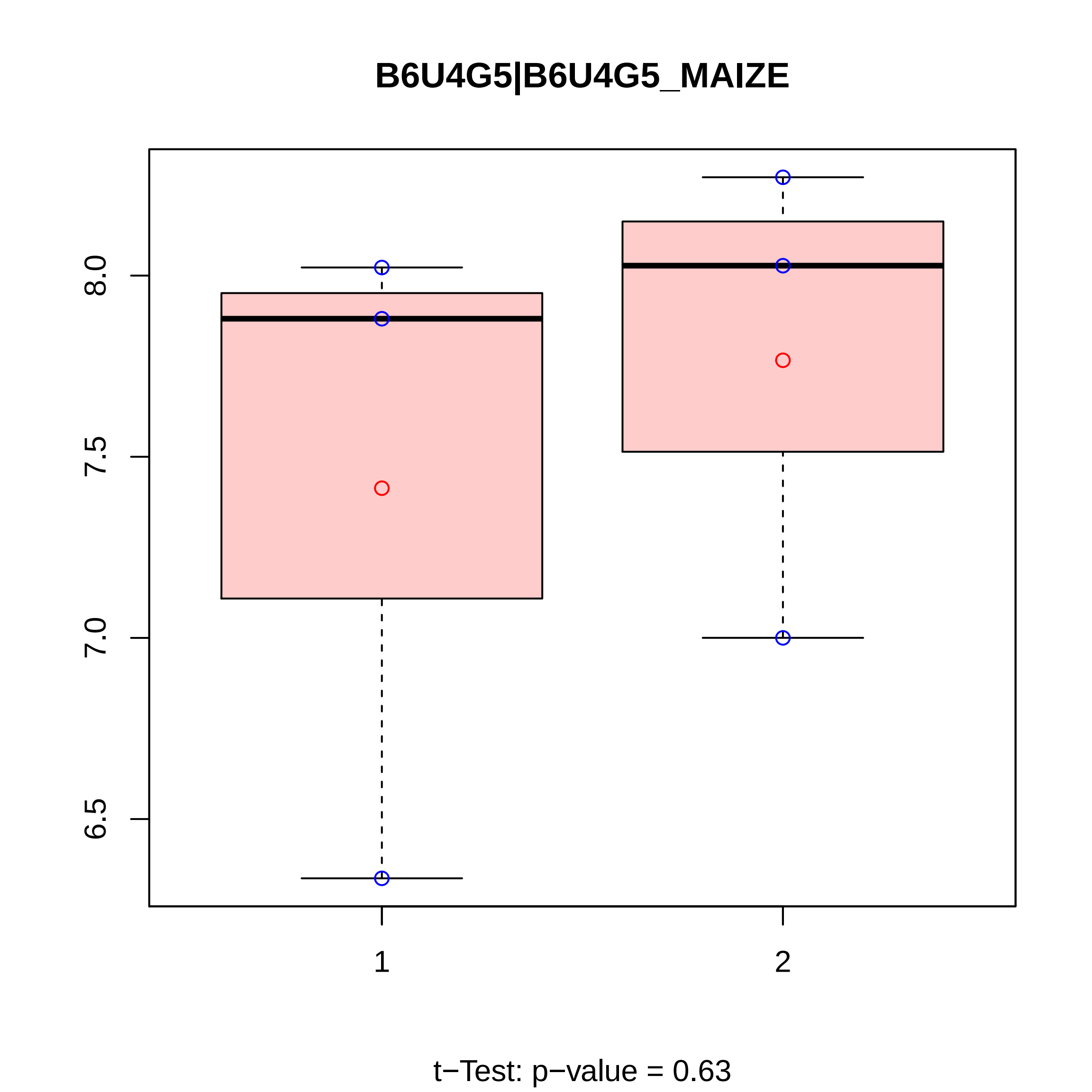
B6U4G5|B6U4G5_MAIZE
8.0
7.5
7.0
6.5
1
2
t−Test: p−value = 0.63

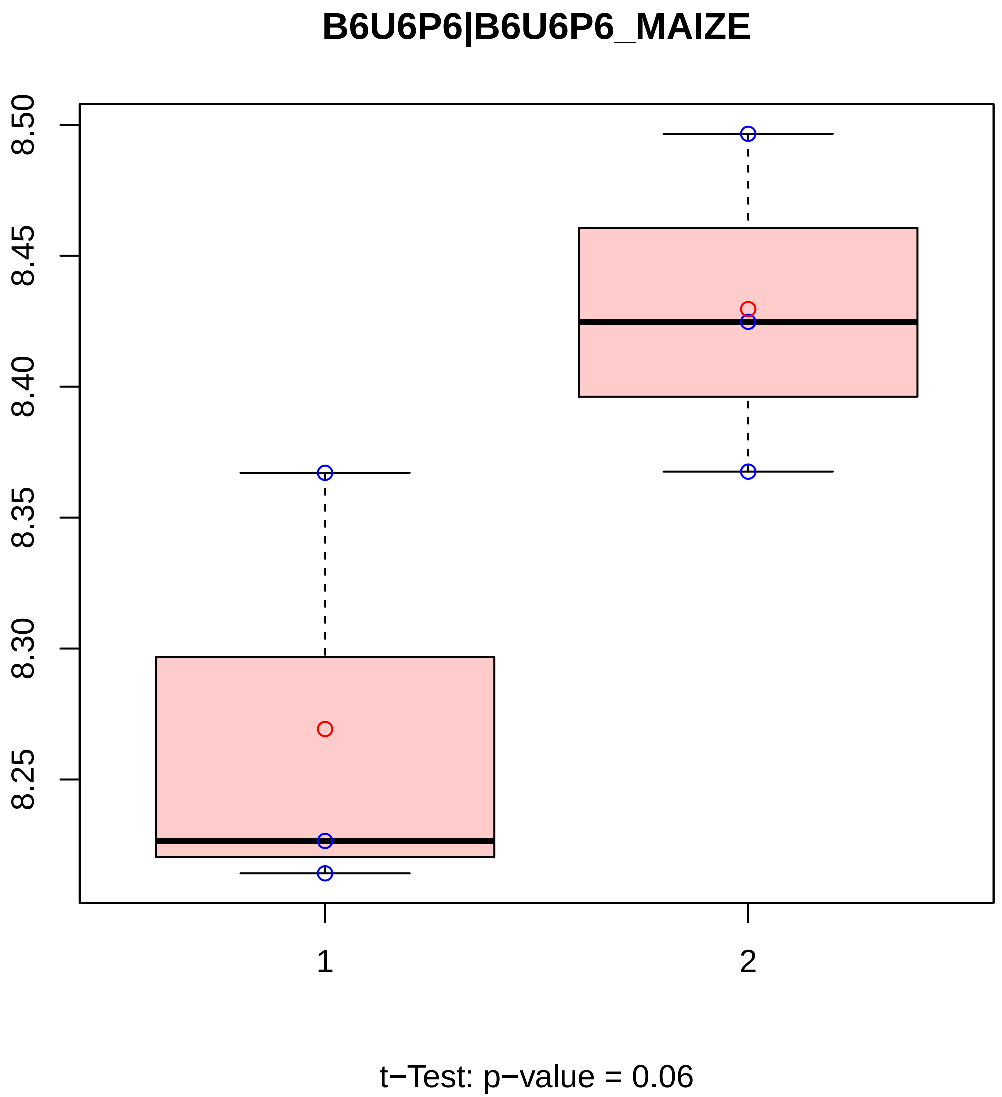
B6U6P6|B6U6P6_MAIZE
8.50
8.45
8.40
8.35
8.30
8.25
1
2
t−Test: p−value = 0.06

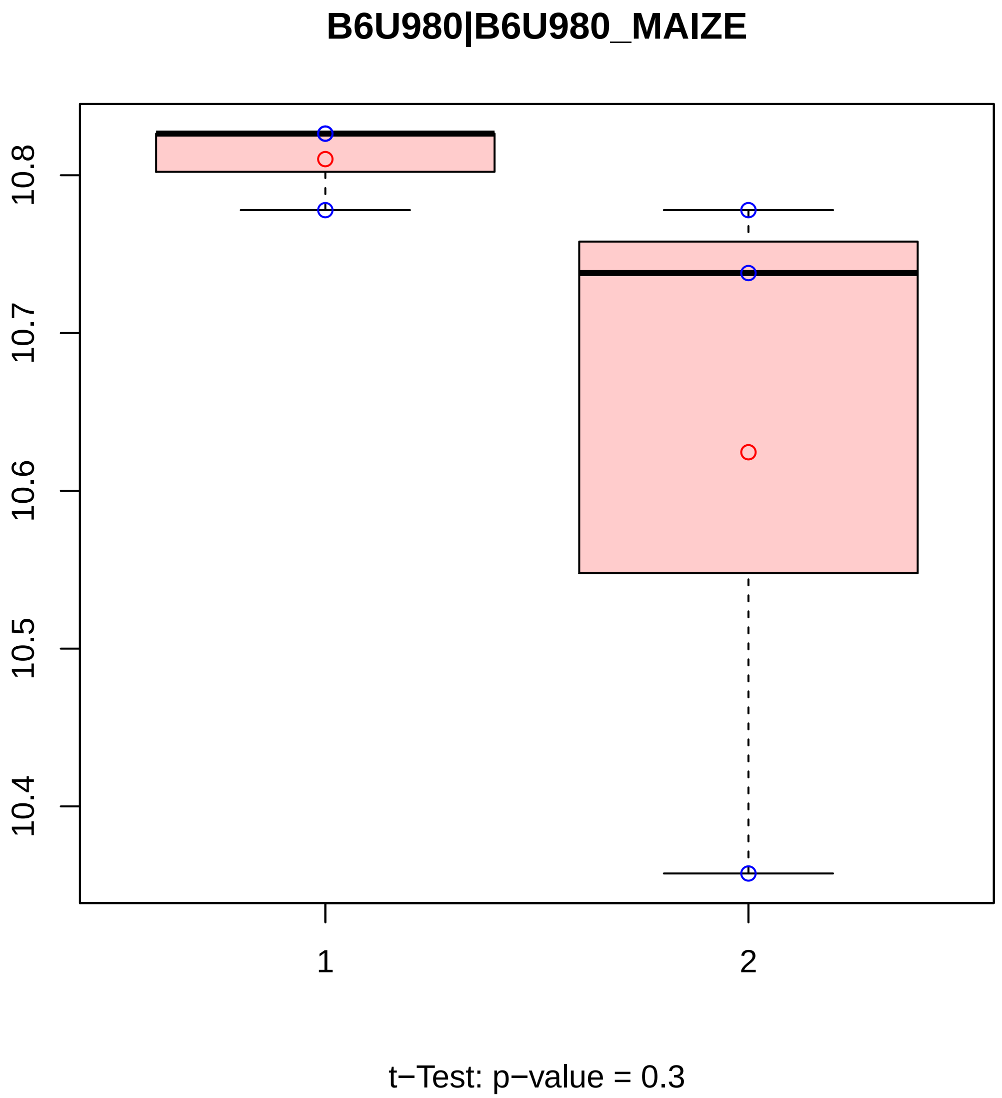
B6U980|B6U980_MAIZE
10.4
10.5
10.6
10.7
10.8
1
2
t−Test: p−value = 0.3

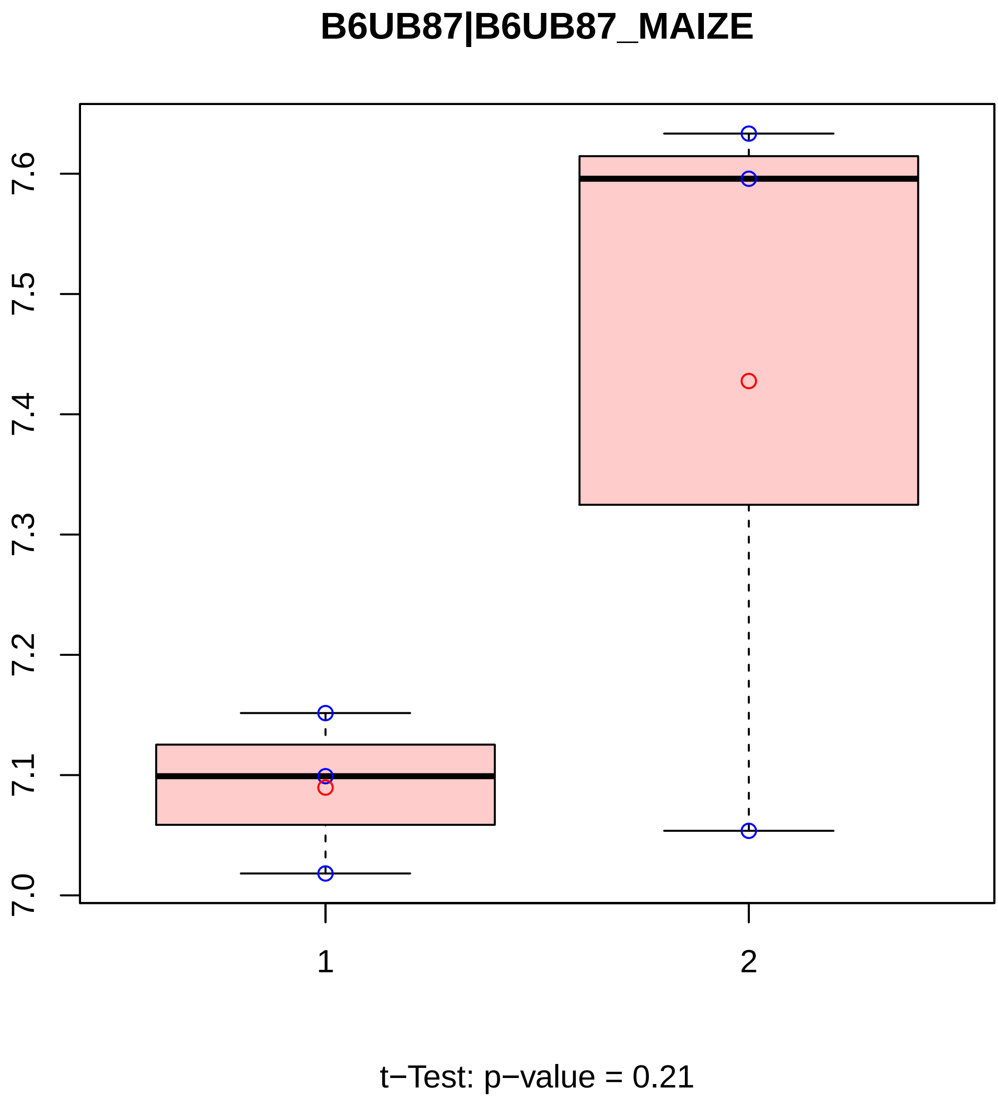

B6UB87|B6UB87_MAIZE
7.0
7.1
7.2
7.3
7.4
7.5
7.6
1
2
t−Test: p−value = 0.21

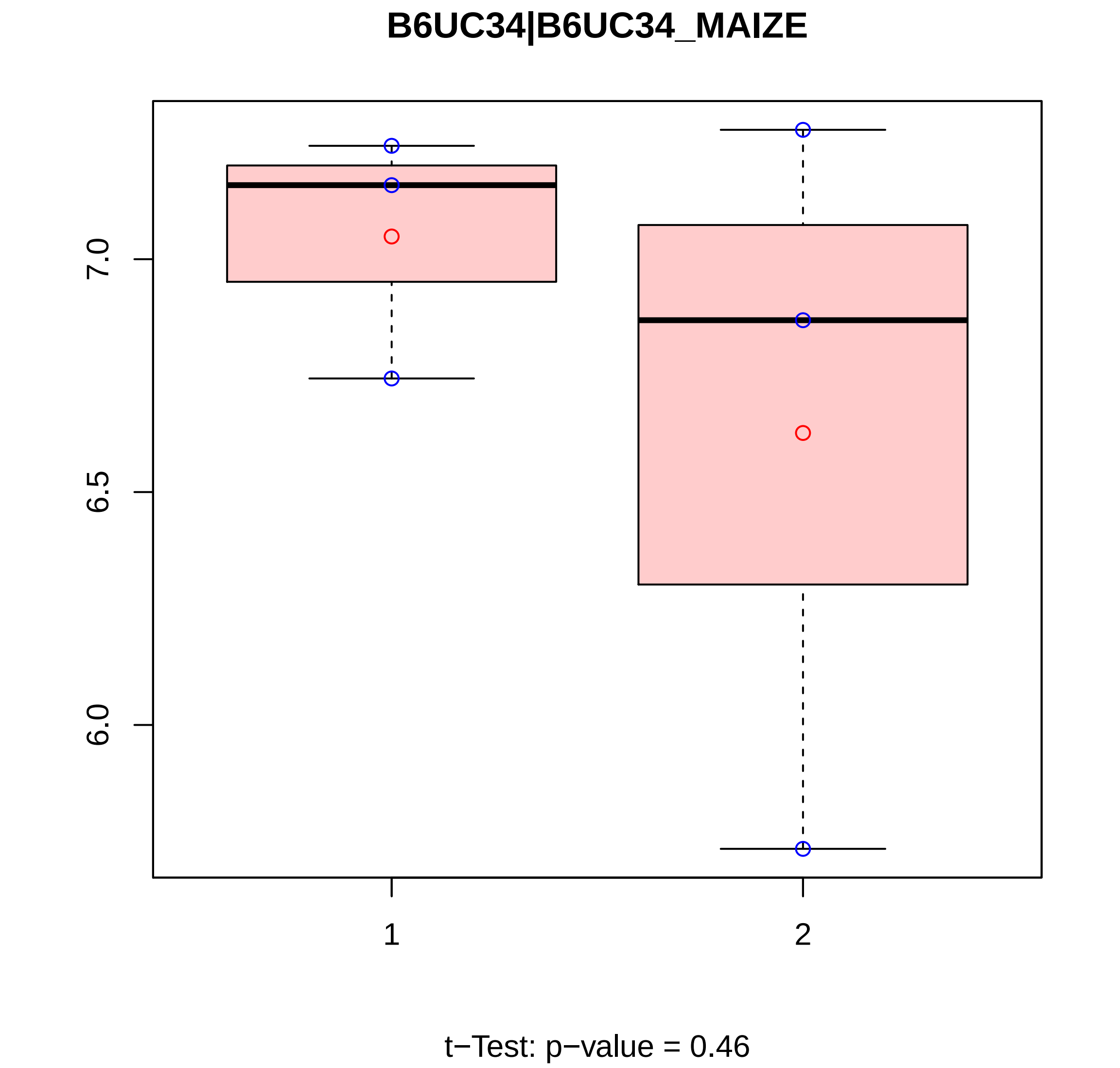
B6UC34|B6UC34_MAIZE
7.0
6.5
6.0
1
2
t−Test: p−value = 0.46

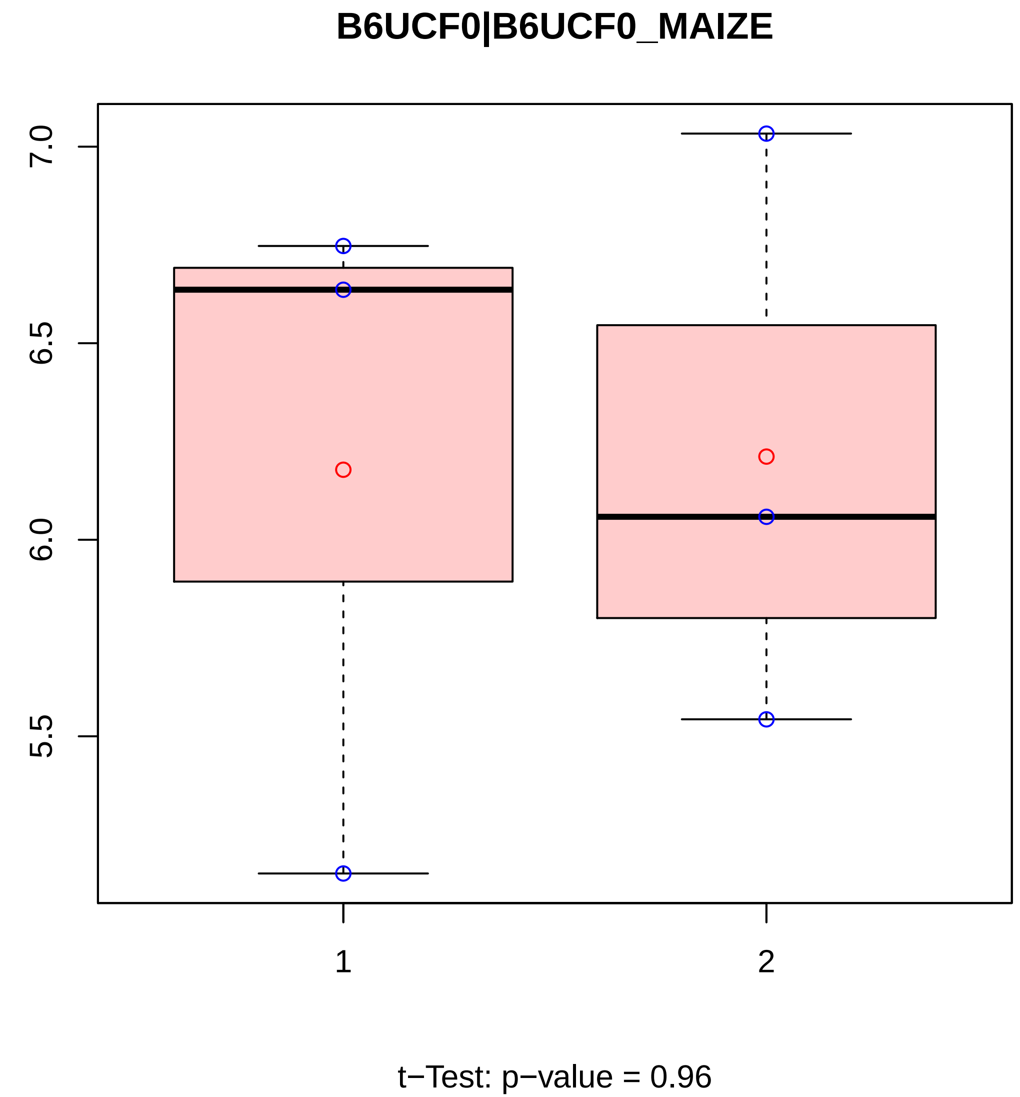
B6UCF0|B6UCF0_MAIZE
7.0
6.5
6.0
5.5
1
2
t−Test: p−value = 0.96

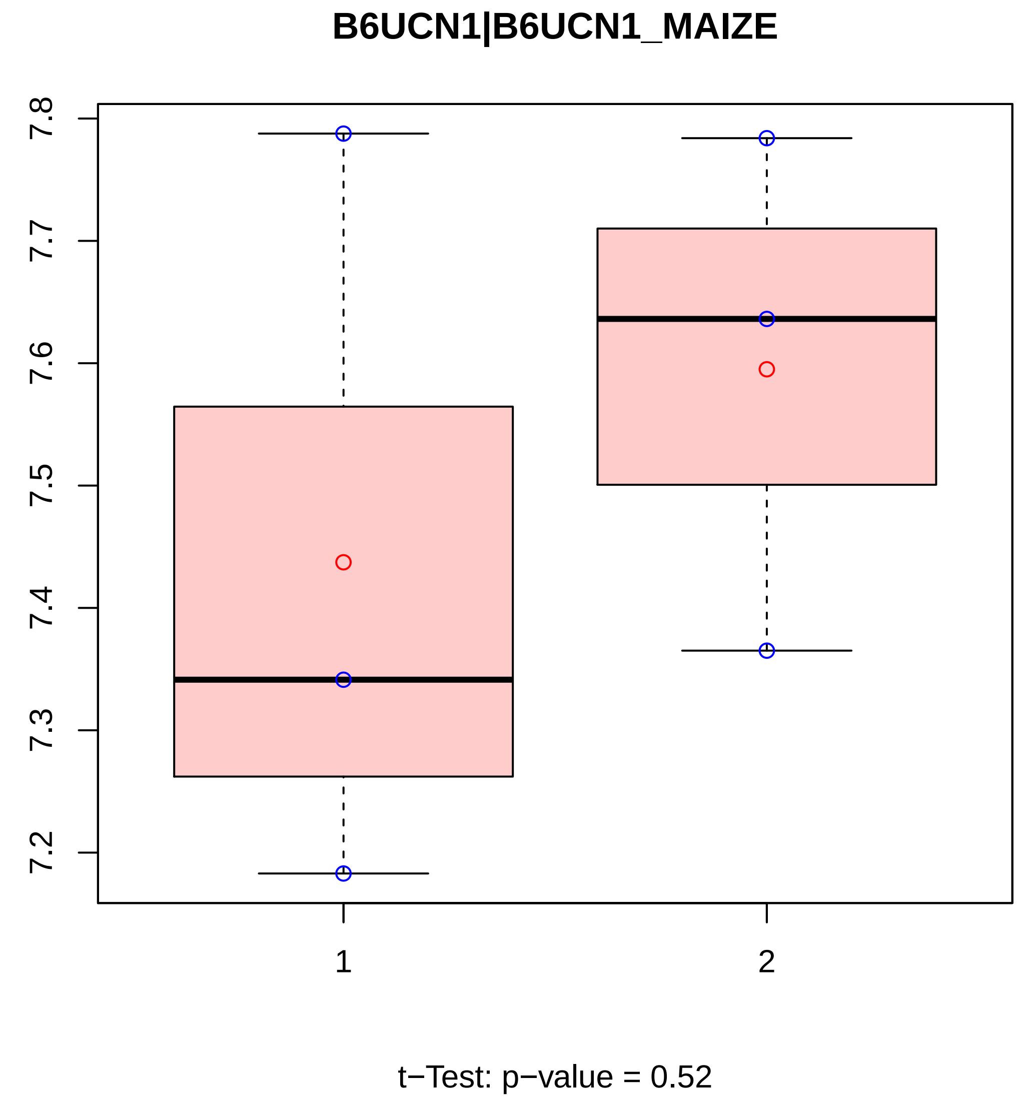
B6UCN1|B6UCN1_MAIZE
7.2
7.3
7.4
7.5
7.6
7.7
7.8
1
2
t−Test: p−value = 0.52

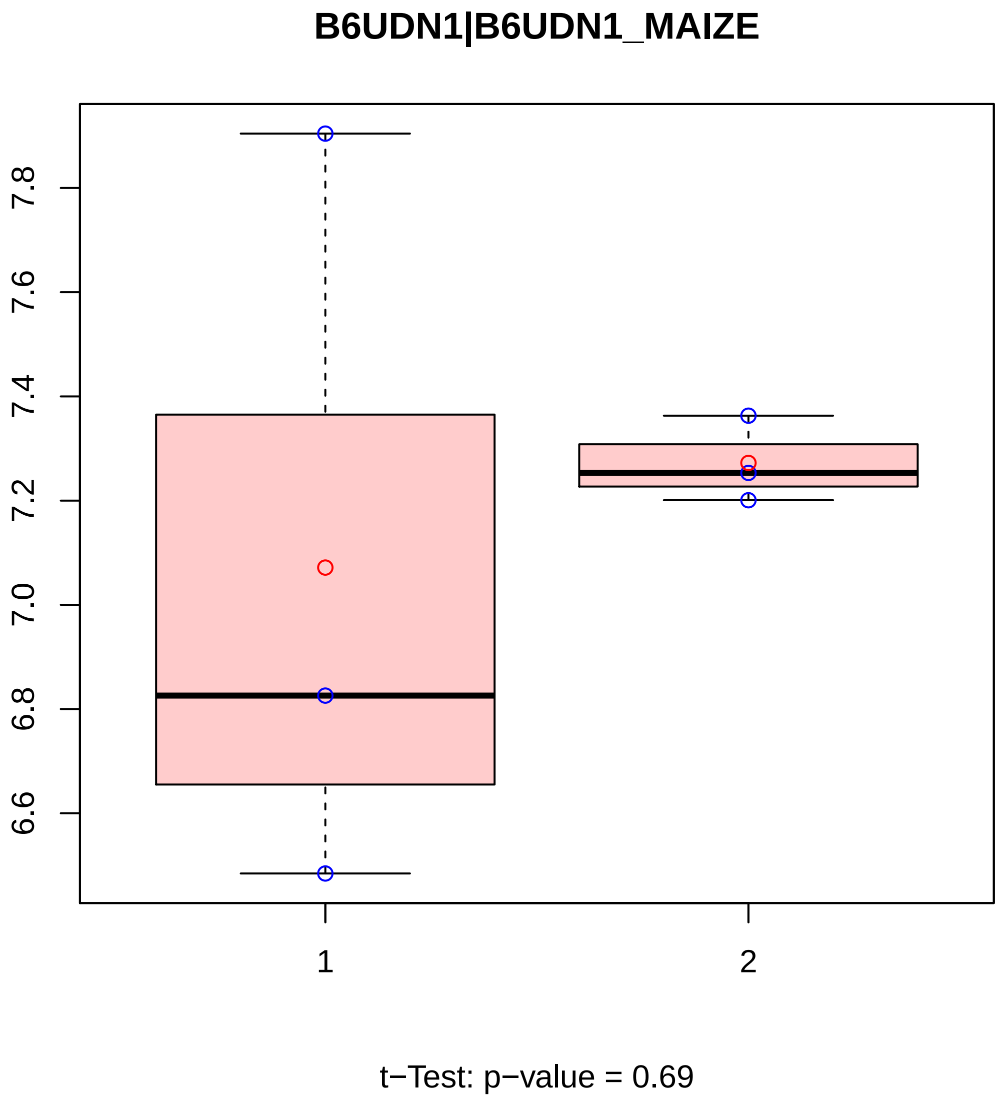
B6UDN1|B6UDN1_MAIZE
6.6
6.8
7.0
7.2
7.4
7.6
7.8
1
2
t−Test: p−value = 0.69

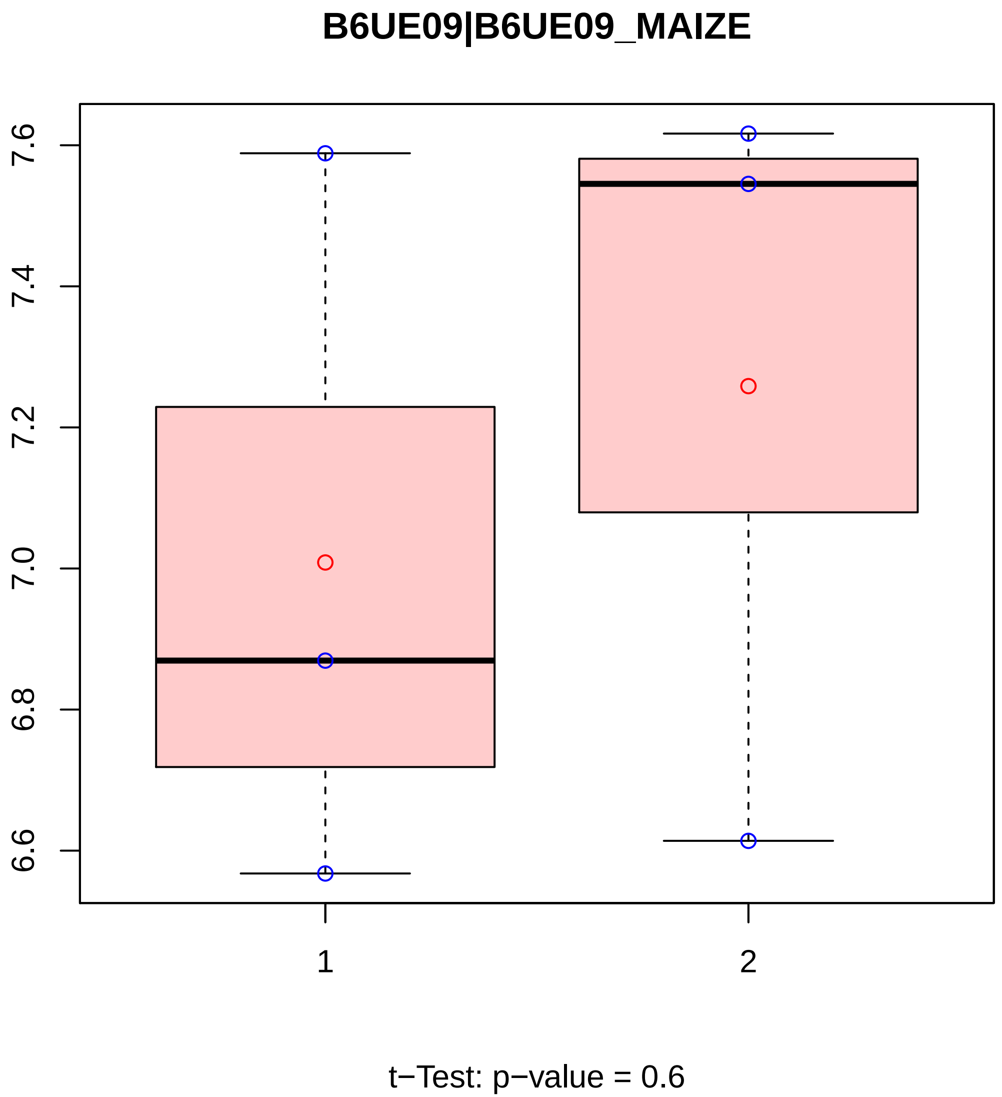

B6UE09|B6UE09_MAIZE
7.6
7.4
7.2
7.0
6.8
6.6
1
2
t−Test: p−value = 0.6

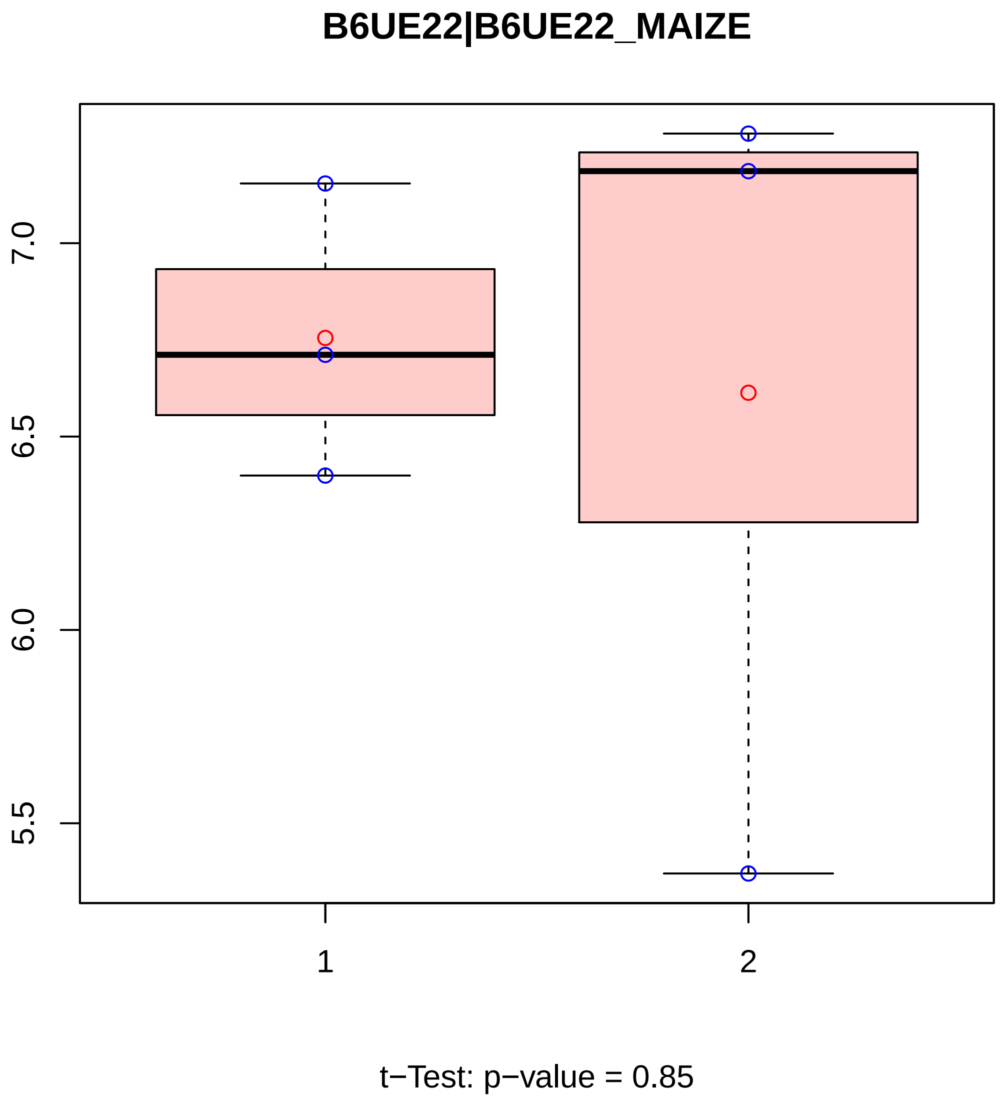

B6UE22|B6UE22_MAIZE
7.0
6.5
6.0
5.5
1
2
t−Test: p−value = 0.85

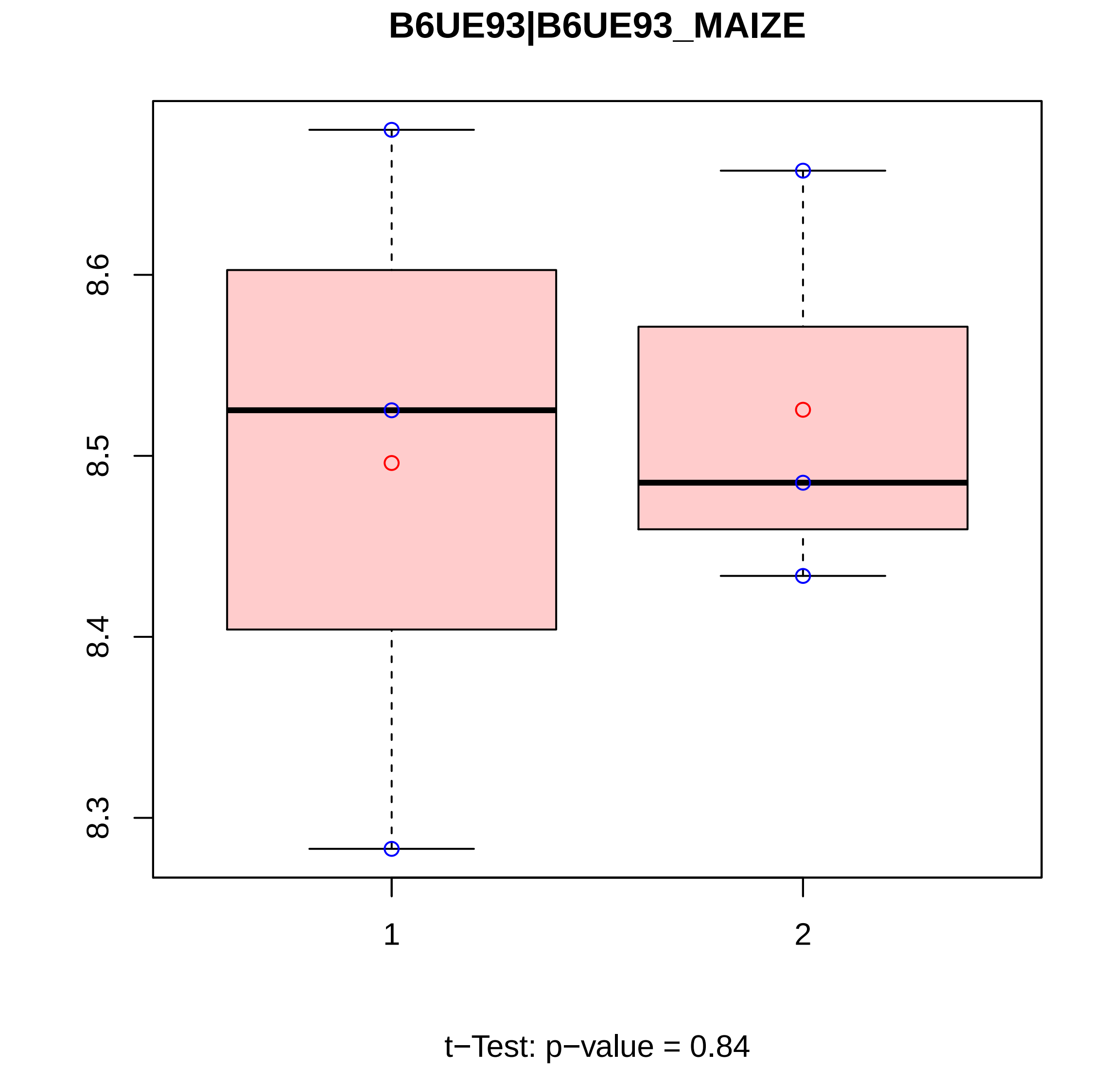
B6UE93|B6UE93_MAIZE
8.3
8.4
8.5
8.6
1
2
t−Test: p−value = 0.84

B6UF55|B6UF55_MAIZE

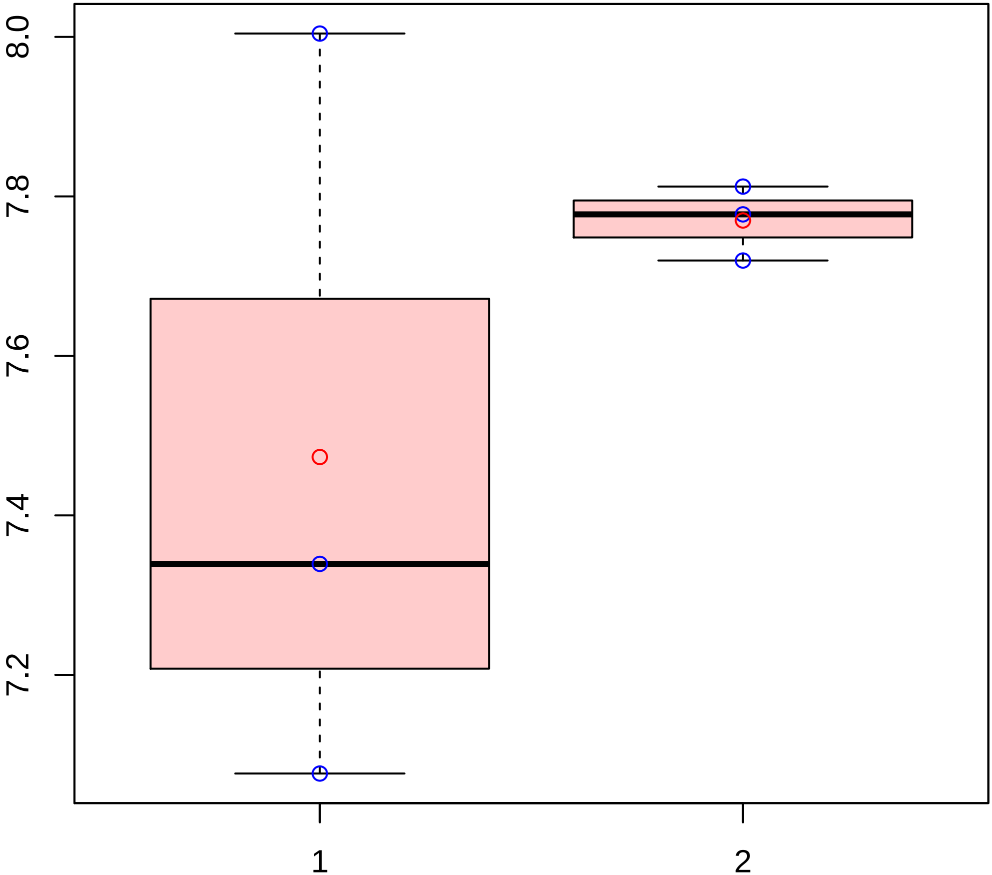

t−Test: p−value = 0.4

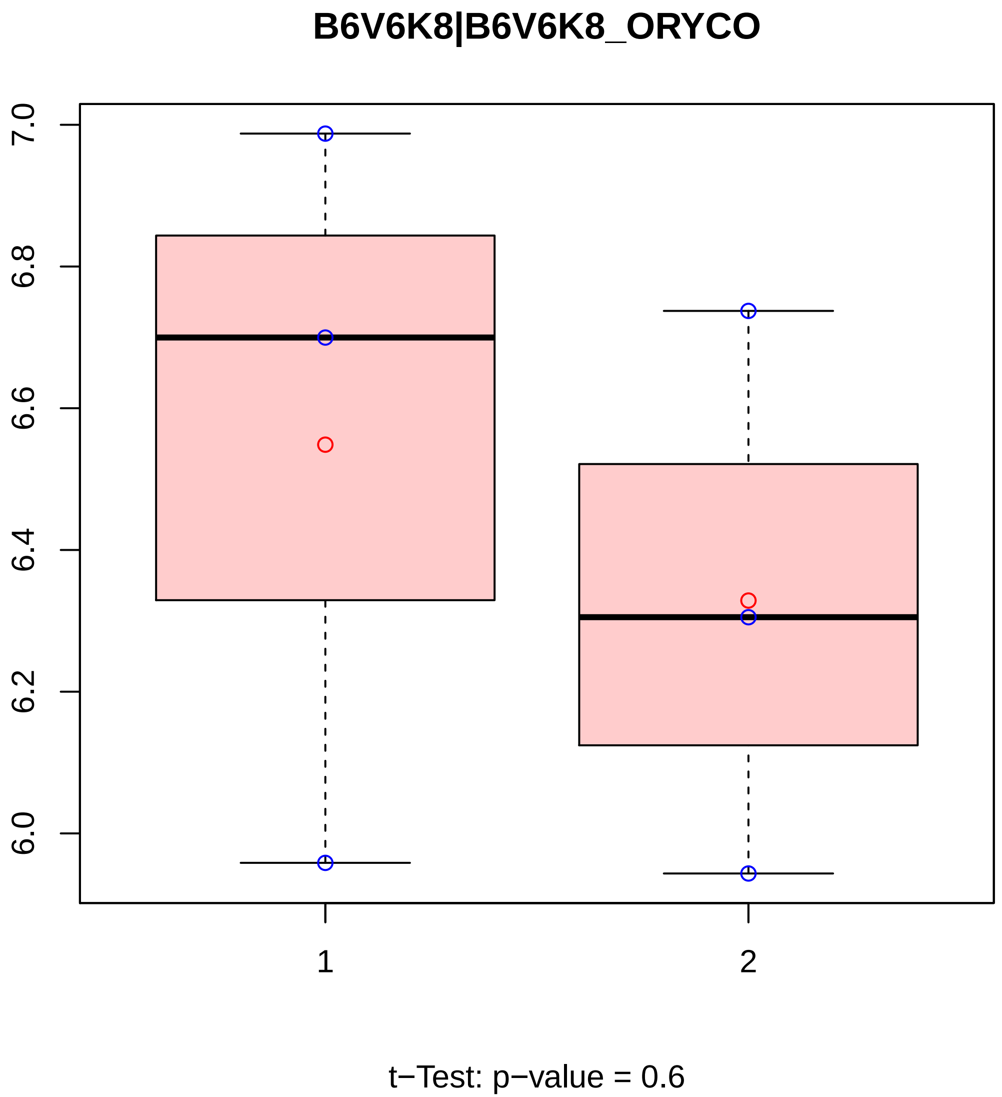
B6V6K8|B6V6K8_ORYCO
7.0
6.8
6.6
6.4
6.2
6.0
1
2
t−Test: p−value = 0.6

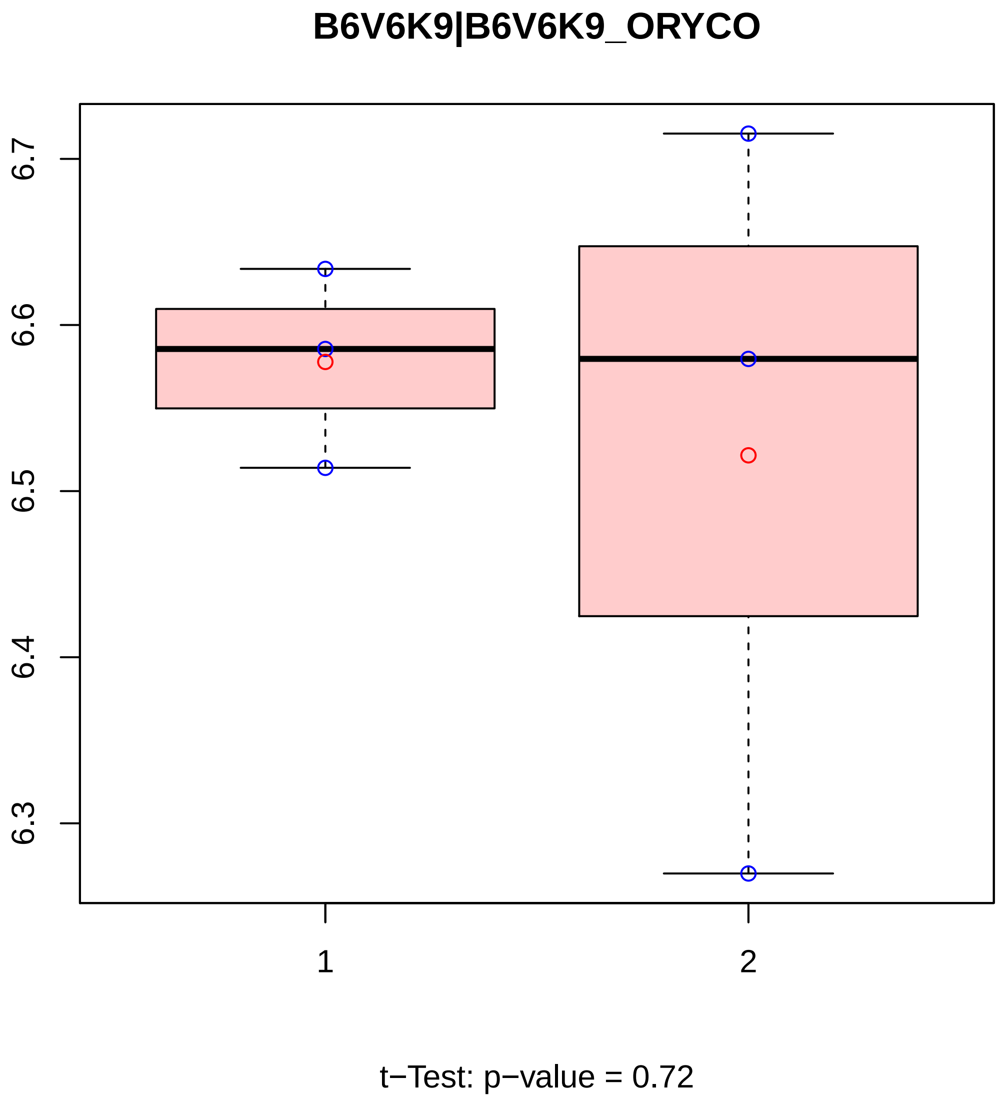

B6V6K9|B6V6K9_ORYCO
6.3
6.4
6.5
6.6
6.7
1
2
t−Test: p−value = 0.72

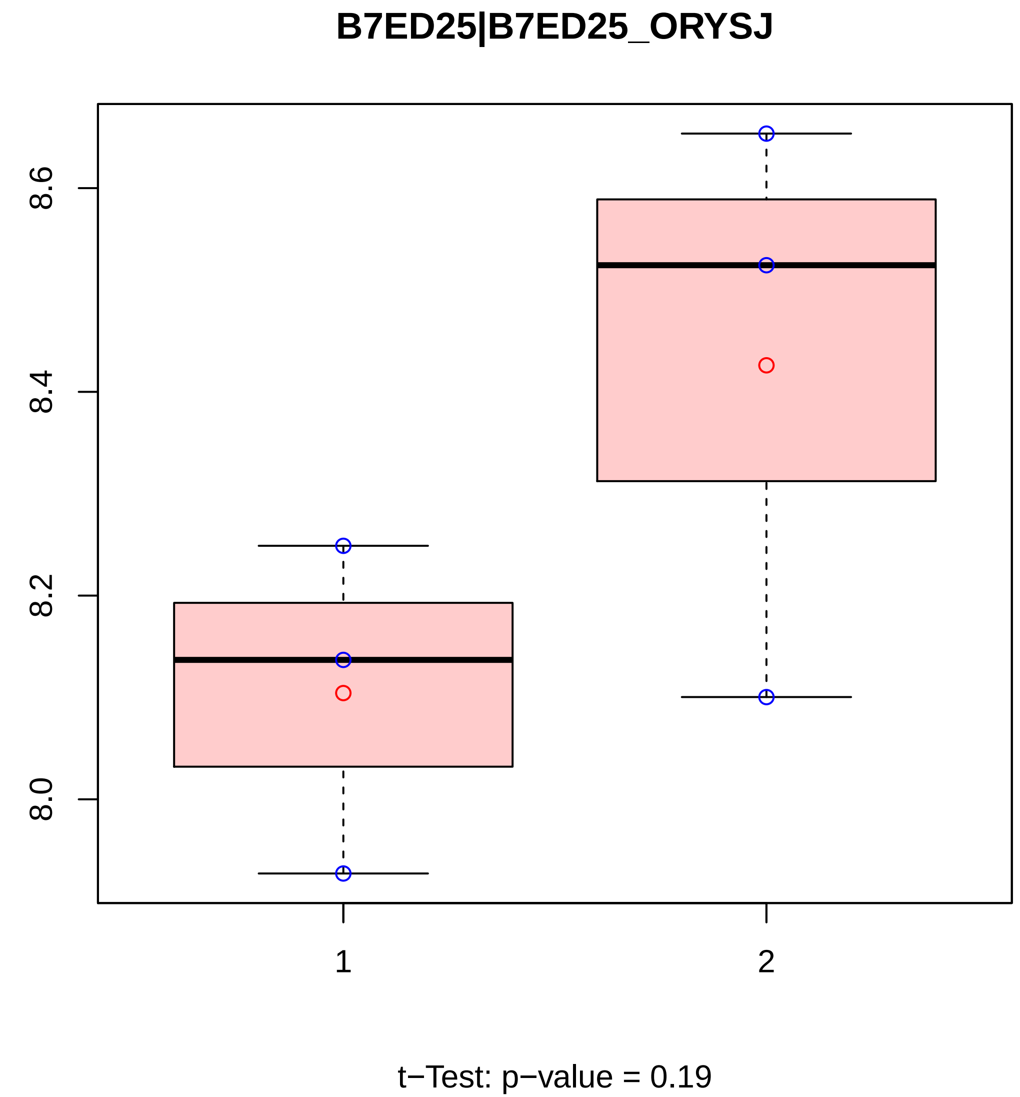
B7ED25|B7ED25_ORYSJ
8.0
8.2
8.4
8.6
1
2
t−Test: p−value = 0.19

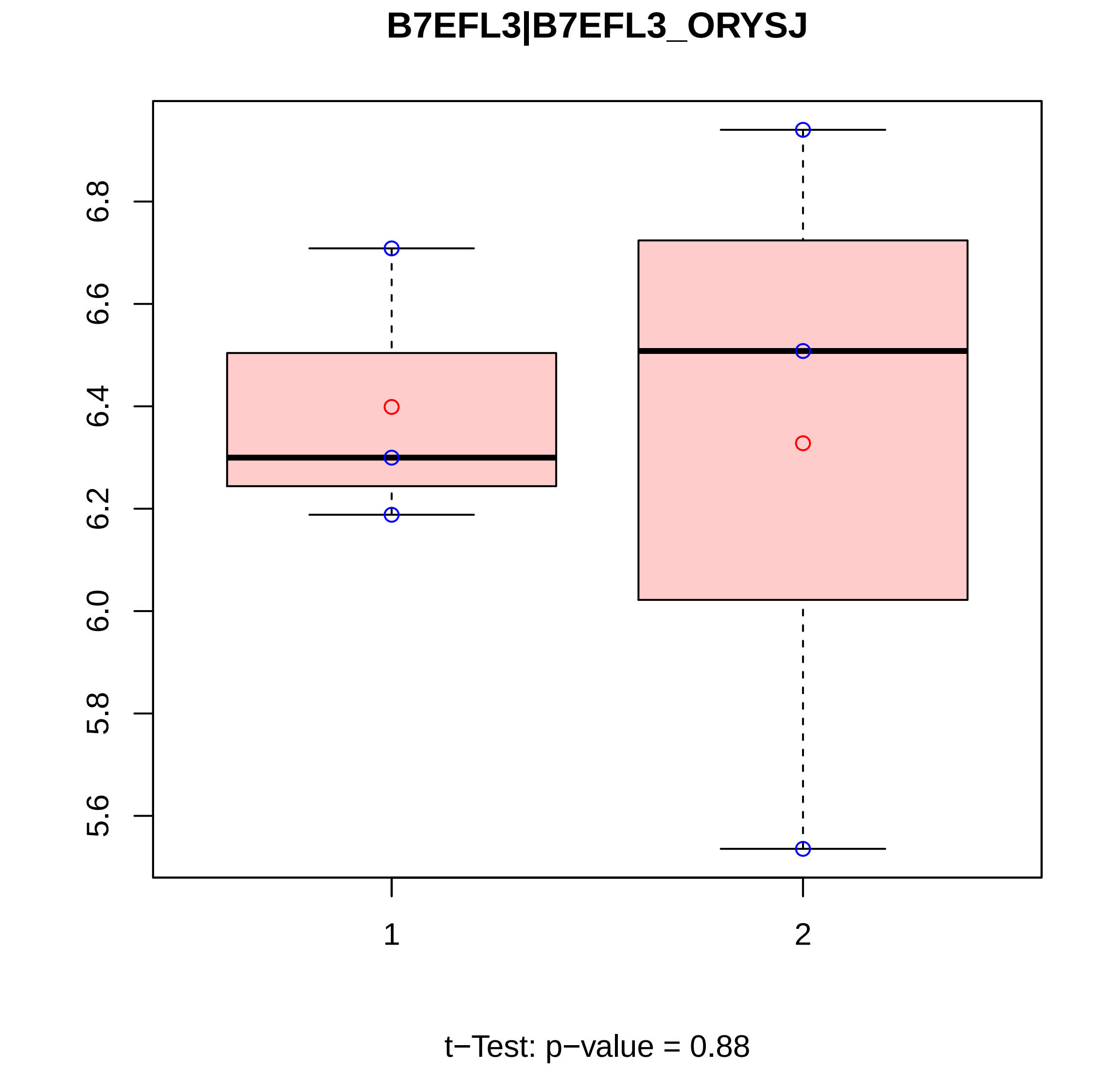

B7EFL3|B7EFL3_ORYSJ
6.8
6.6
6.4
6.2
6.0
5.8
5.6
1
2
t−Test: p−value = 0.88

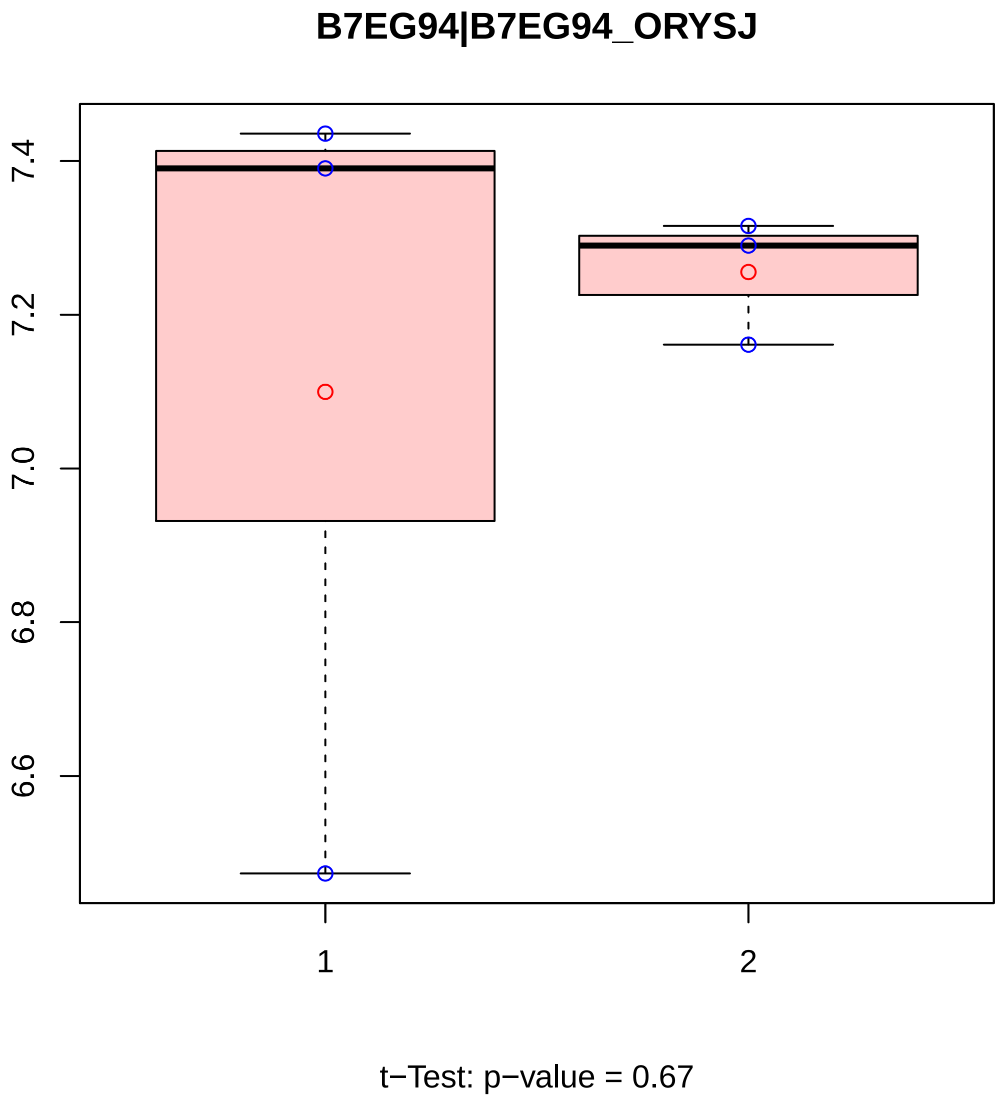

B7EG94|B7EG94_ORYSJ
7.4
7.2
7.0
6.8
6.6
1
2
t−Test: p−value = 0.67

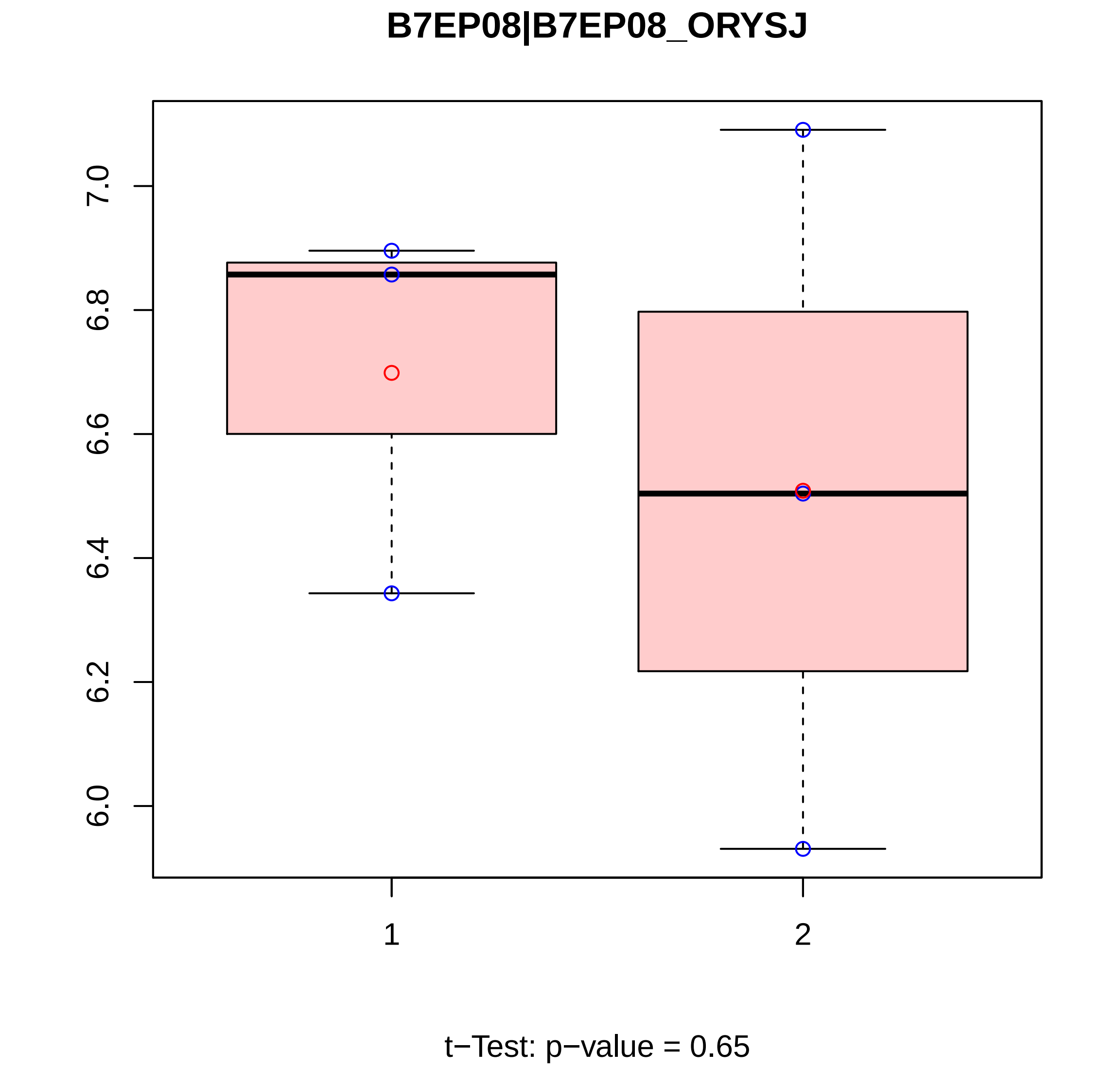

B7EP08|B7EP08_ORYSJ
7.0
6.8
6.6
6.4
6.2
6.0
1
2
t−Test: p−value = 0.65

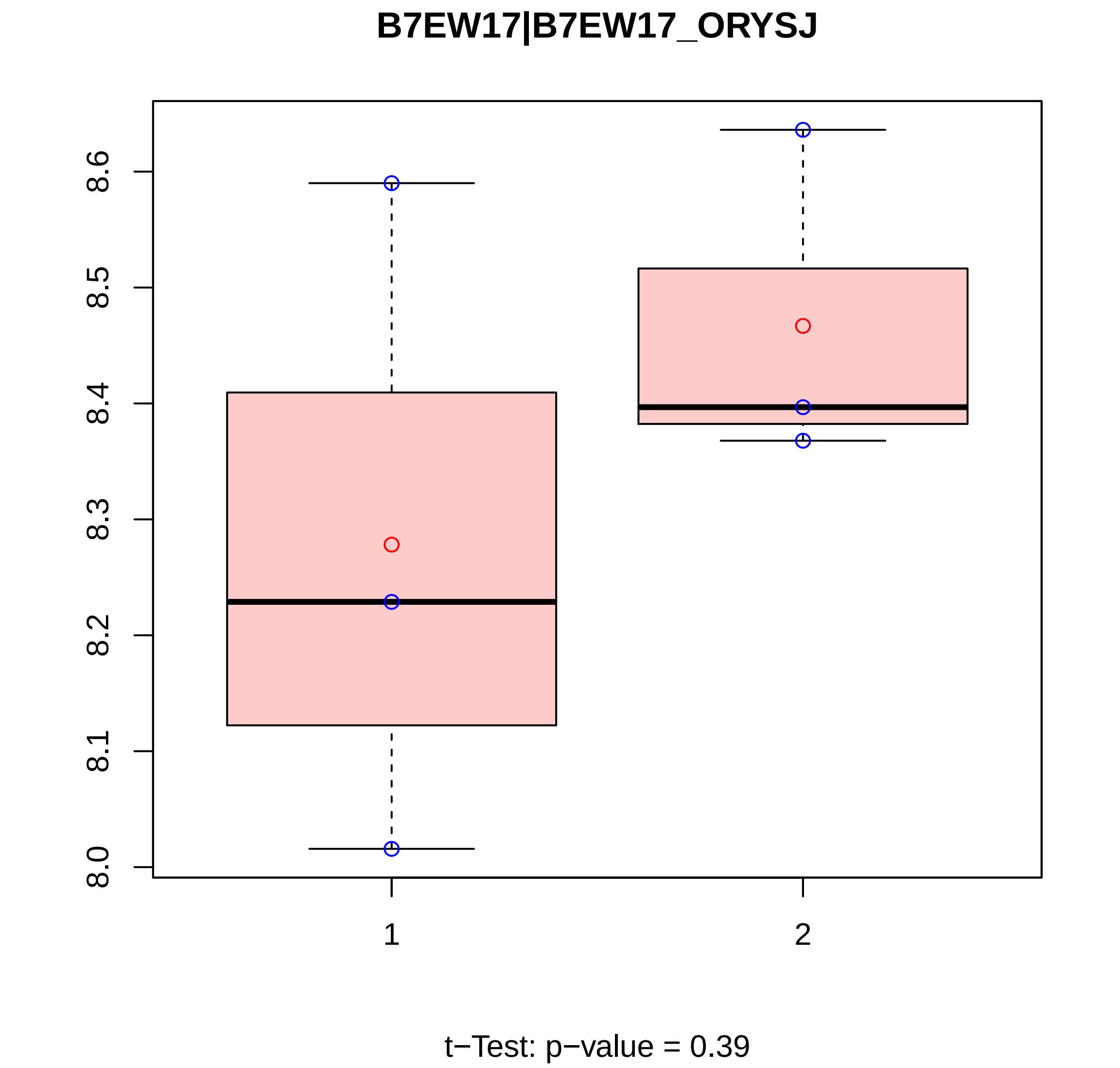

B7EW17|B7EW17_ORYSJ
8.0
8.1
8.2
8.3
8.4
8.5
8.6
1
2
t−Test: p−value = 0.39

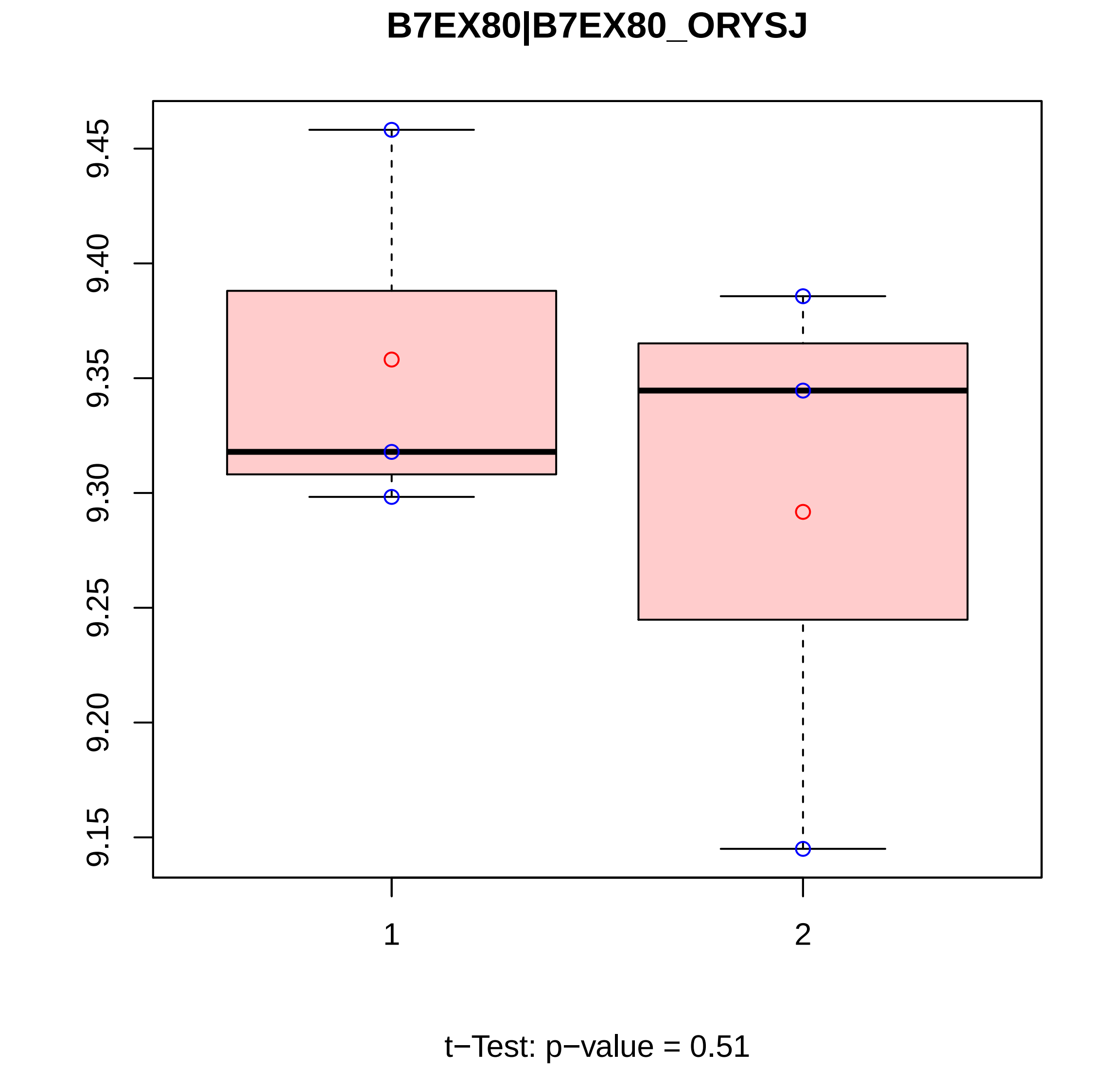

B7EX80|B7EX80_ORYSJ
9.45
9.40
9.35
9.30
9.25
9.20
9.15
1
2
t−Test: p−value = 0.51

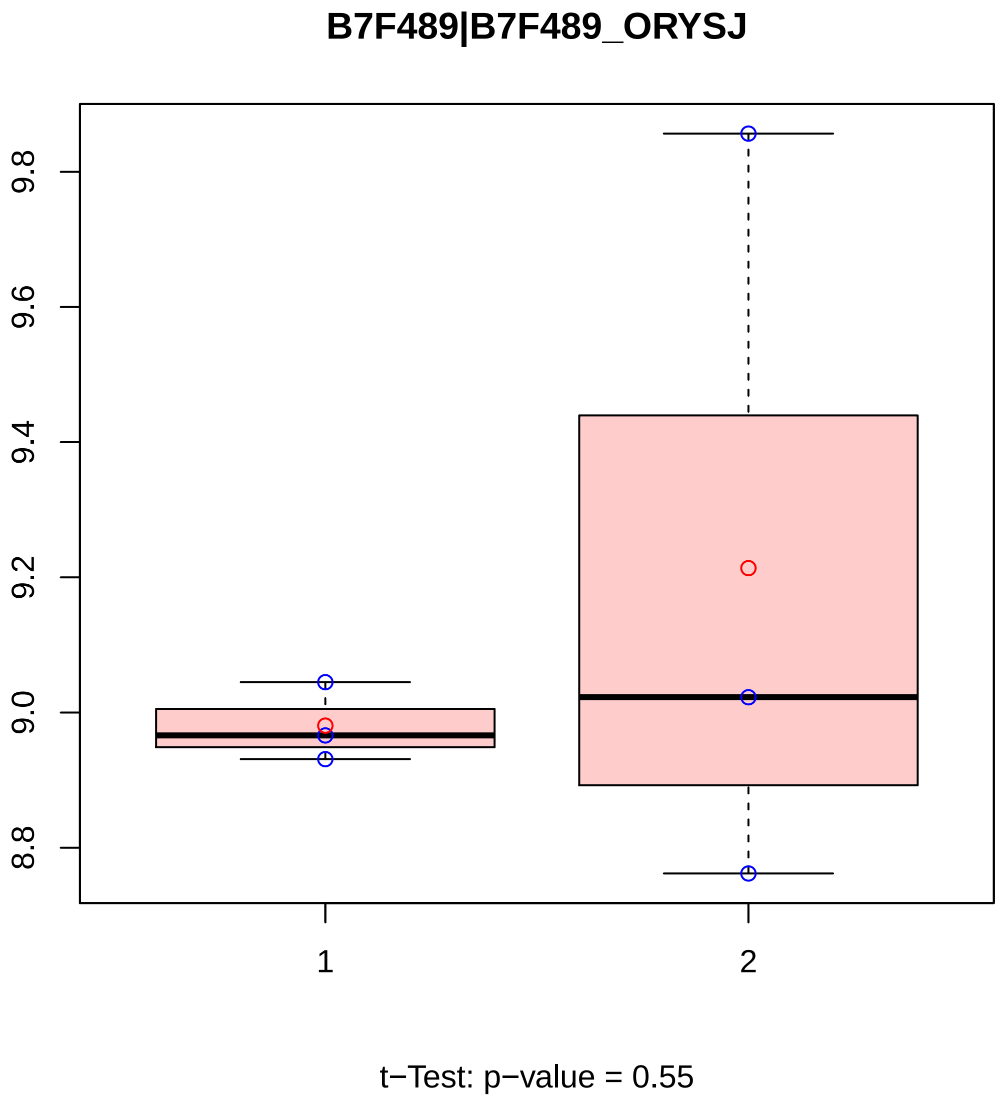

B7F489|B7F489_ORYSJ
9.8
9.6
9.4
9.2
9.0
8.8
1
2
t−Test: p−value = 0.55

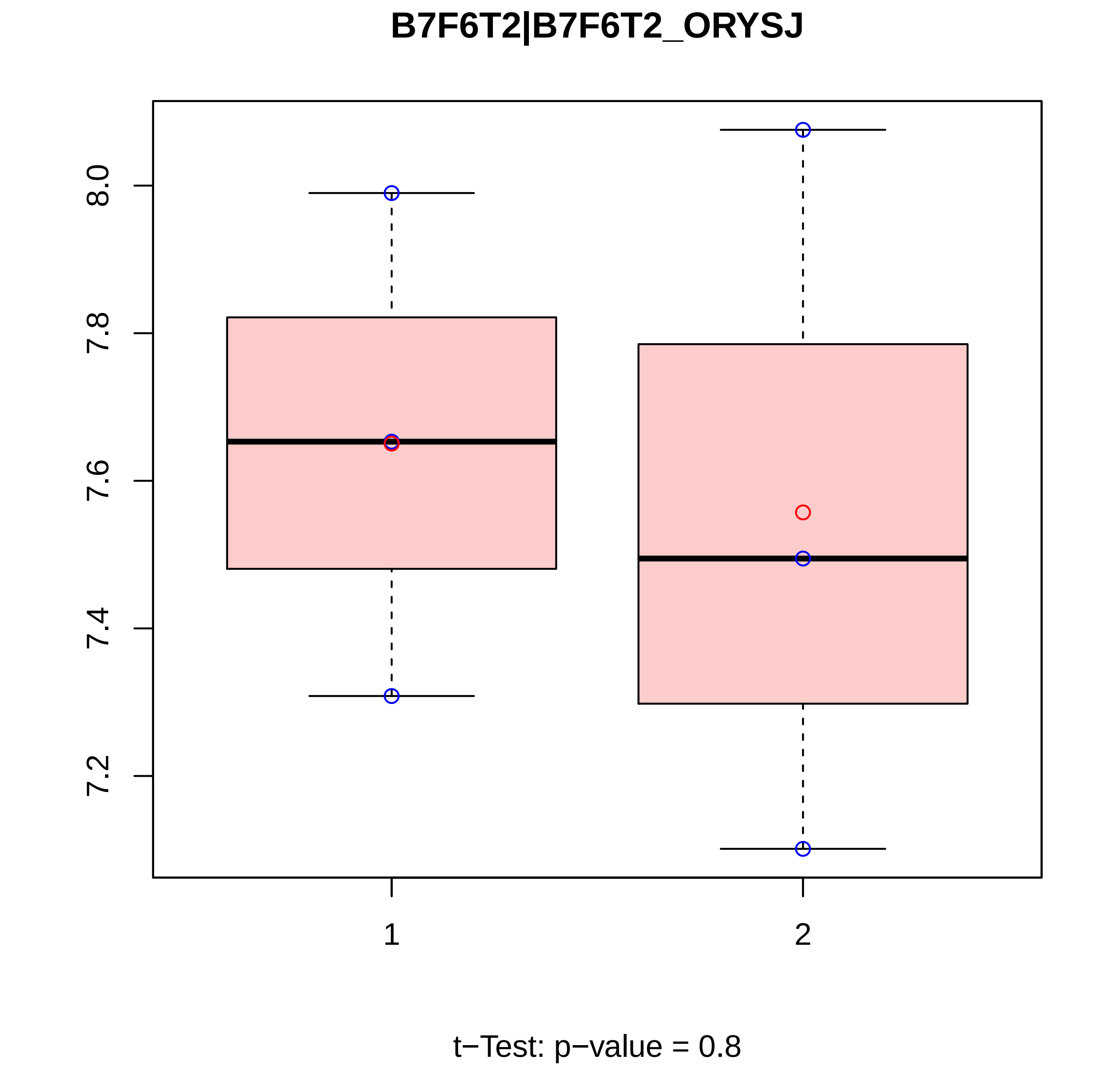
B7F6T2|B7F6T2_ORYSJ
8.0
7.8
7.6
7.4
7.2
1
2
t−Test: p−value = 0.8

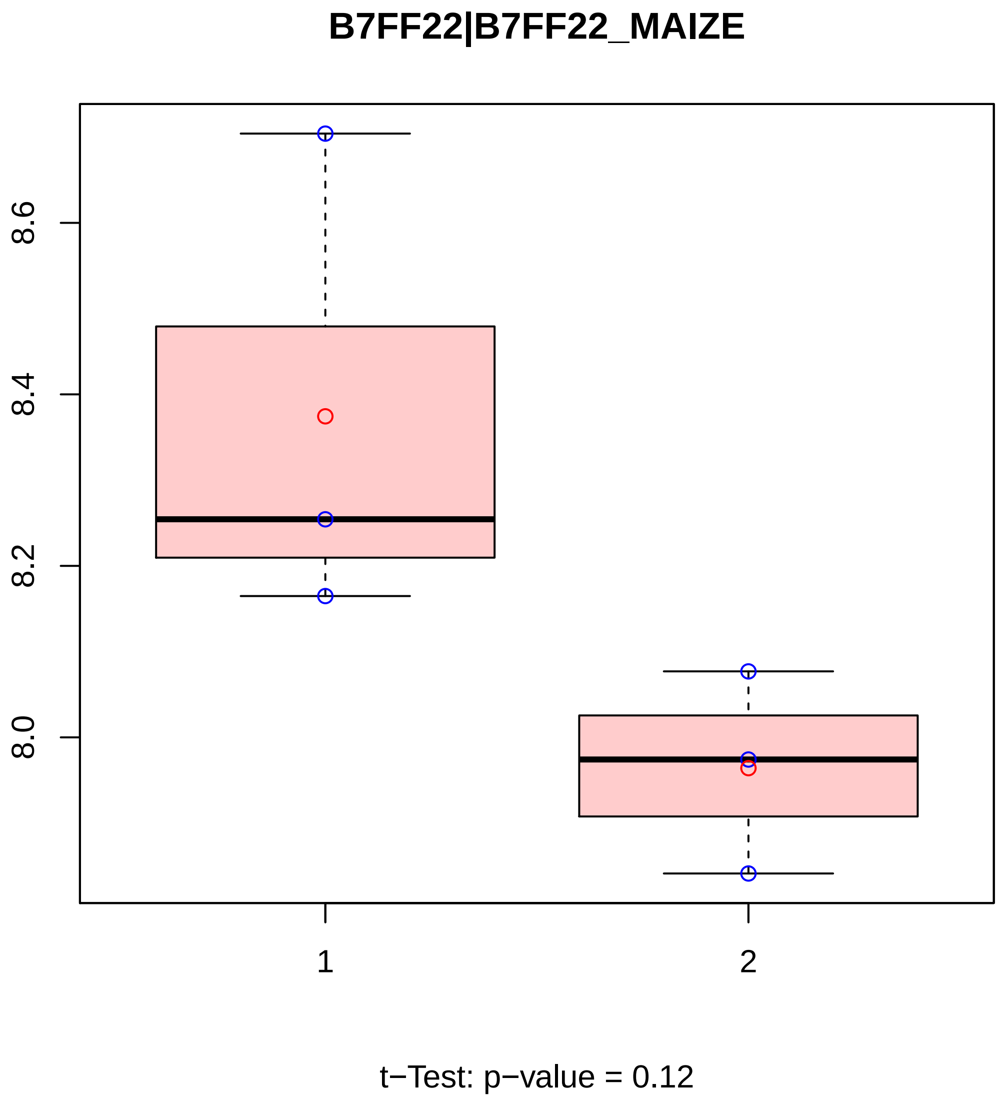

B7FF22|B7FF22_MAIZE
8.6
8.4
8.2
8.0
1
2
t−Test: p−value = 0.12

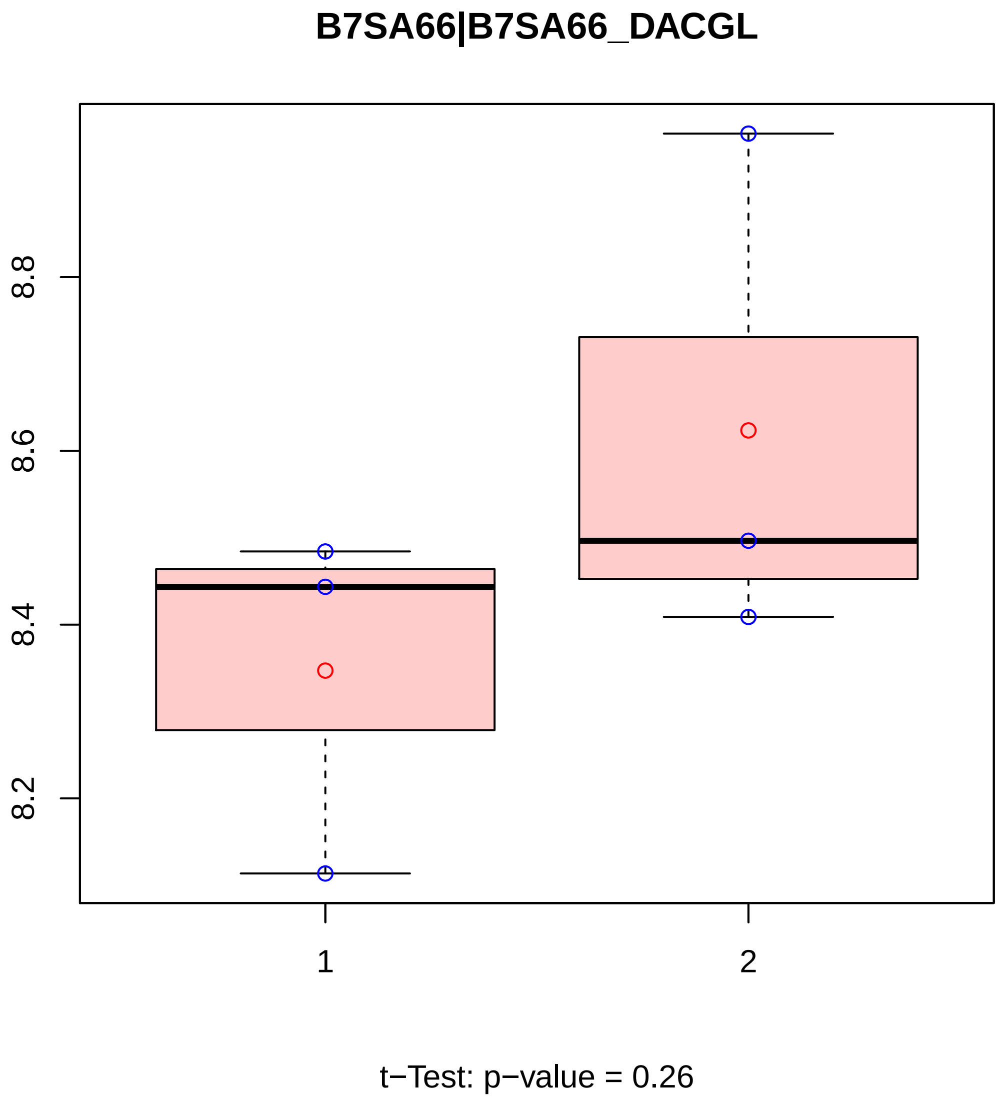
B7SA66|B7SA66_DACGL
8.2
8.4
8.6
8.8
1
2
t−Test: p−value = 0.26

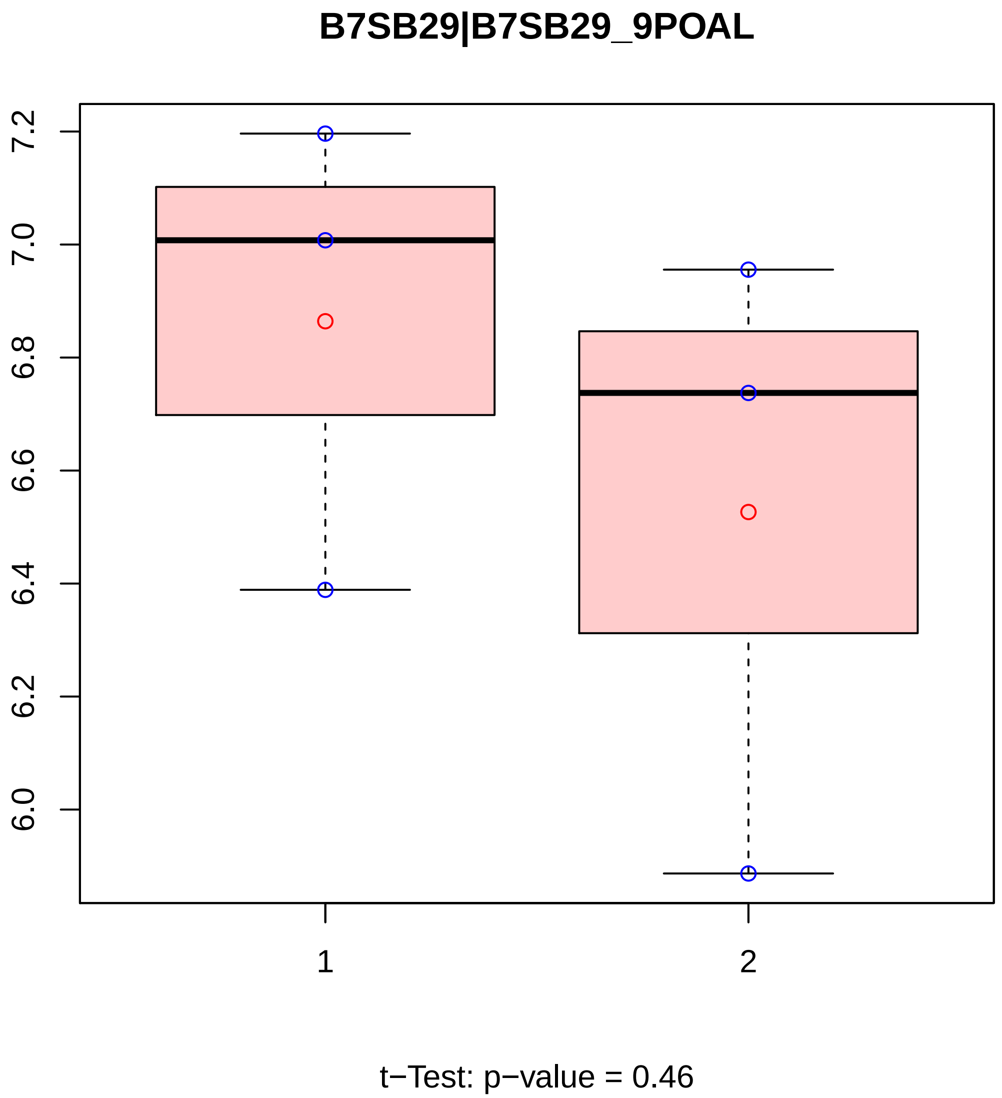
B7SB29|B7SB29_9POAL
7.2
7.0
6.8
6.6
6.4
6.2
6.0
1
2
t−Test: p−value = 0.46

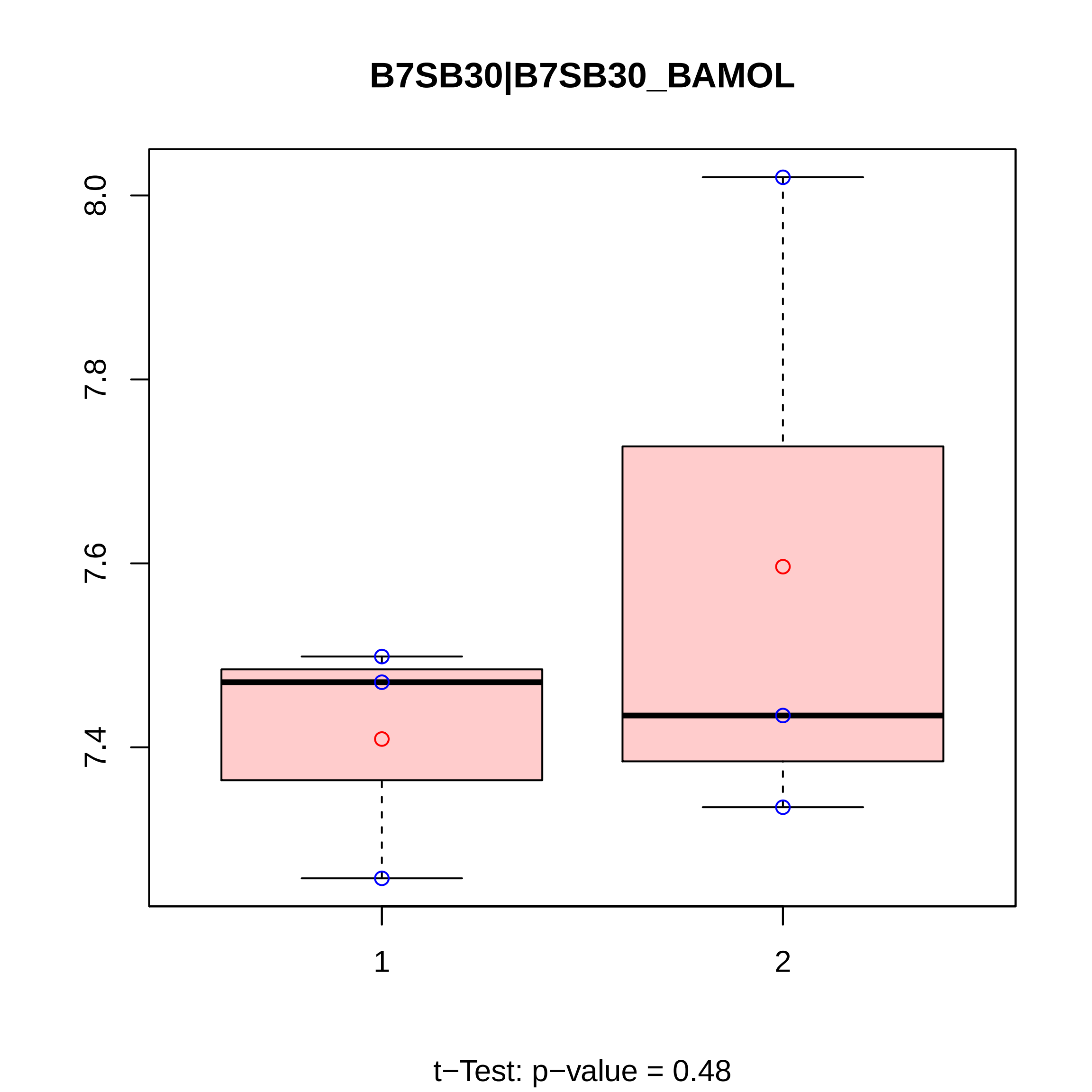

B7SB30|B7SB30_BAMOL
8.0
7.8
7.6
7.4
1
2
t−Test: p−value = 0.48

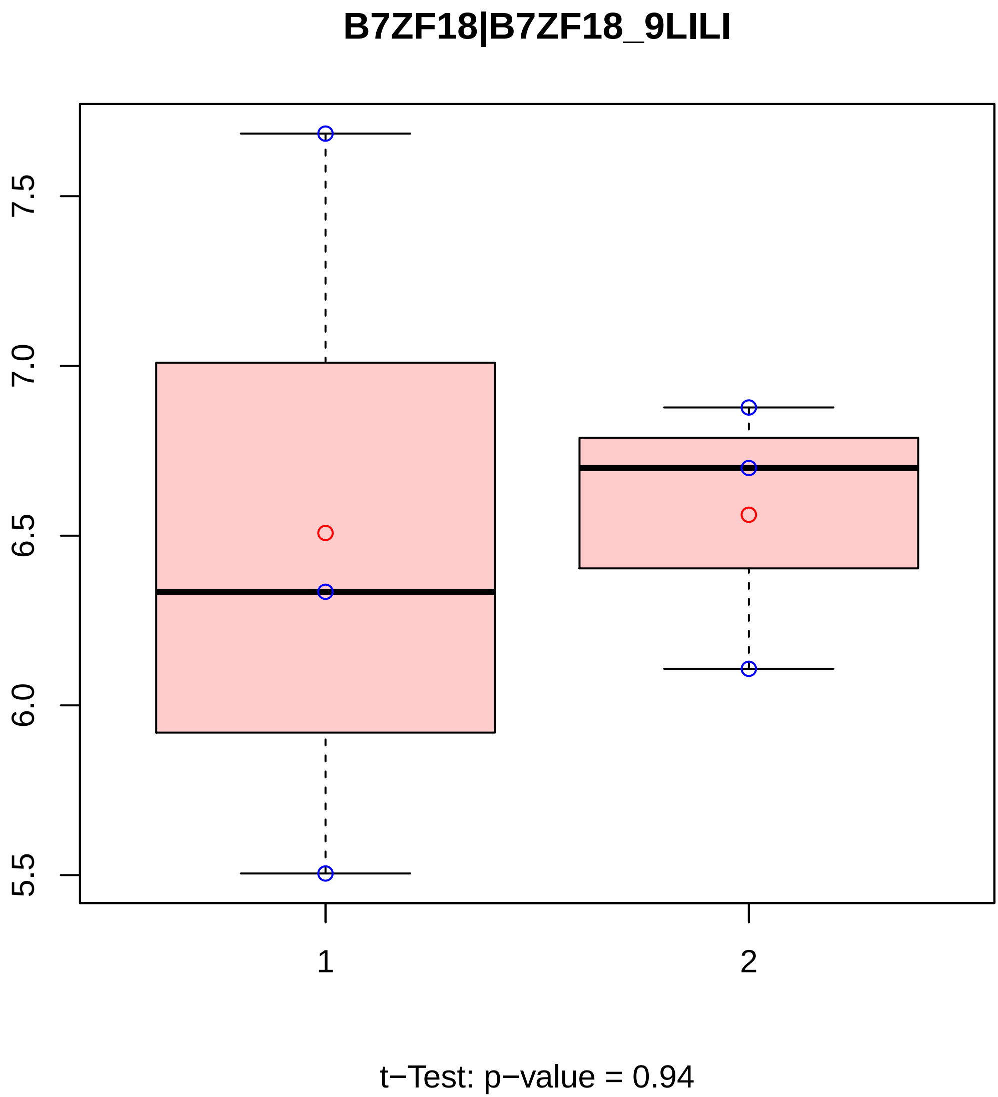
B7ZF18|B7ZF18_9LILI
7.5
7.0
6.5
6.0
5.5
1
2
t−Test: p−value = 0.94

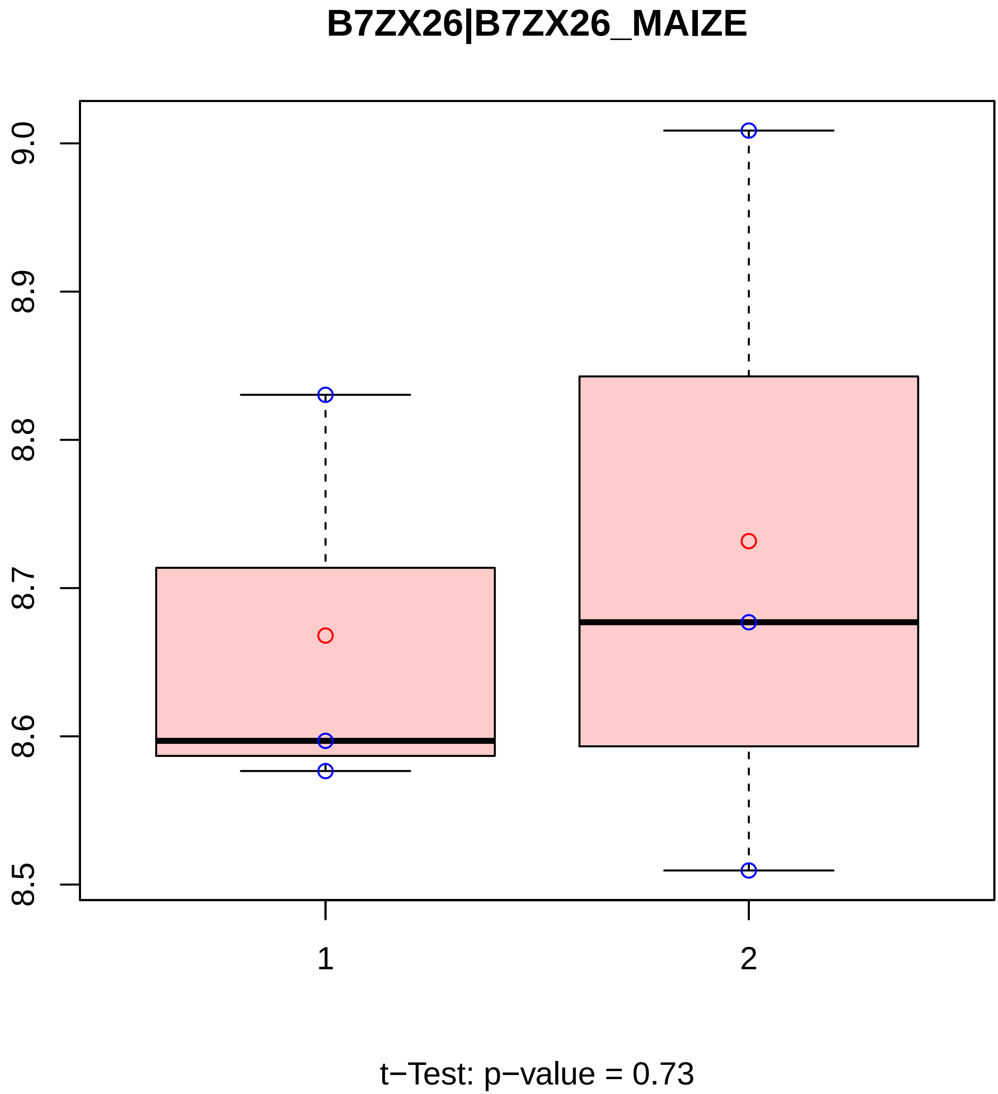
B7ZX26|B7ZX26_MAIZE
9.0
8.9
8.8
8.7
8.6
8.5
1
2
t−Test: p−value = 0.73

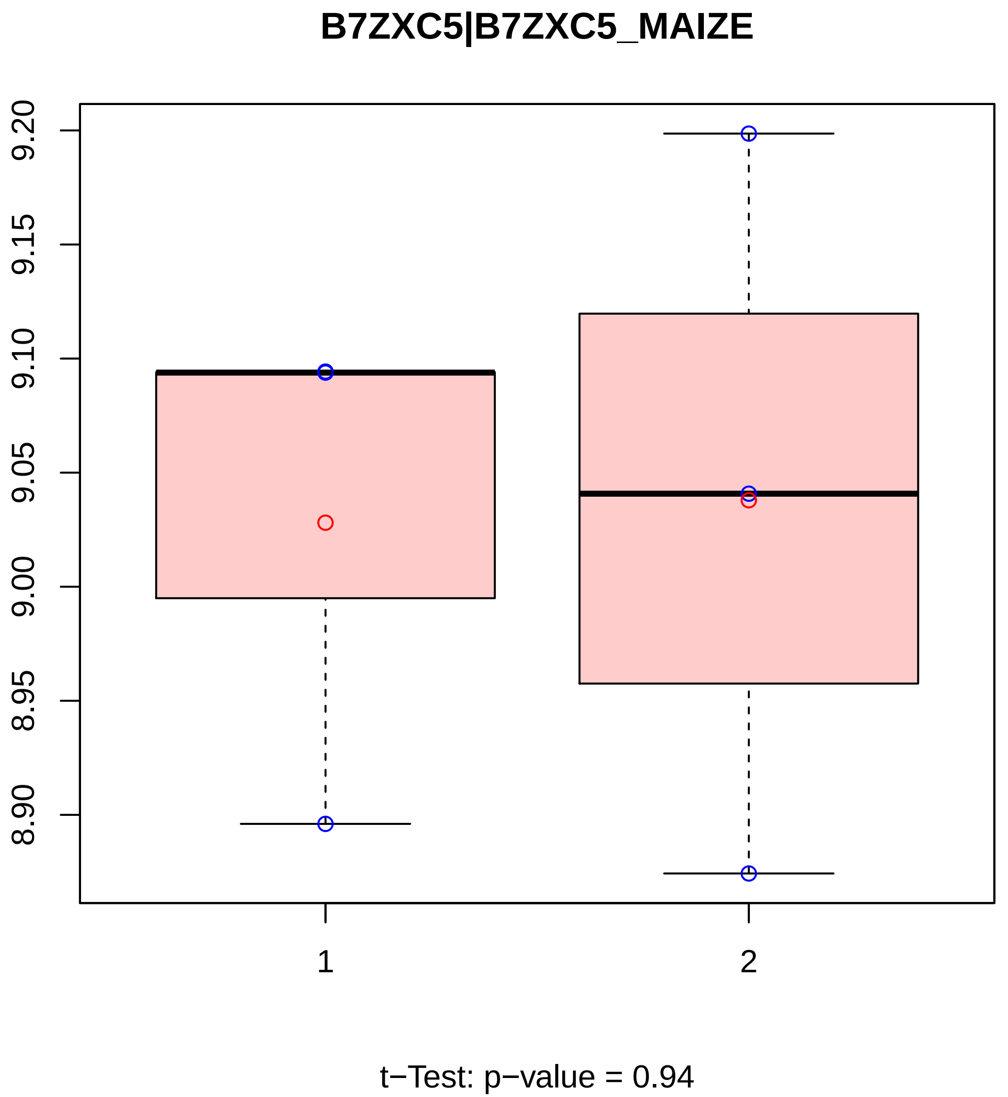

B7ZXC5|B7ZXC5_MAIZE
9.20
9.15
9.10
9.05
9.00
8.95
8.90
1
2
t−Test: p−value = 0.94

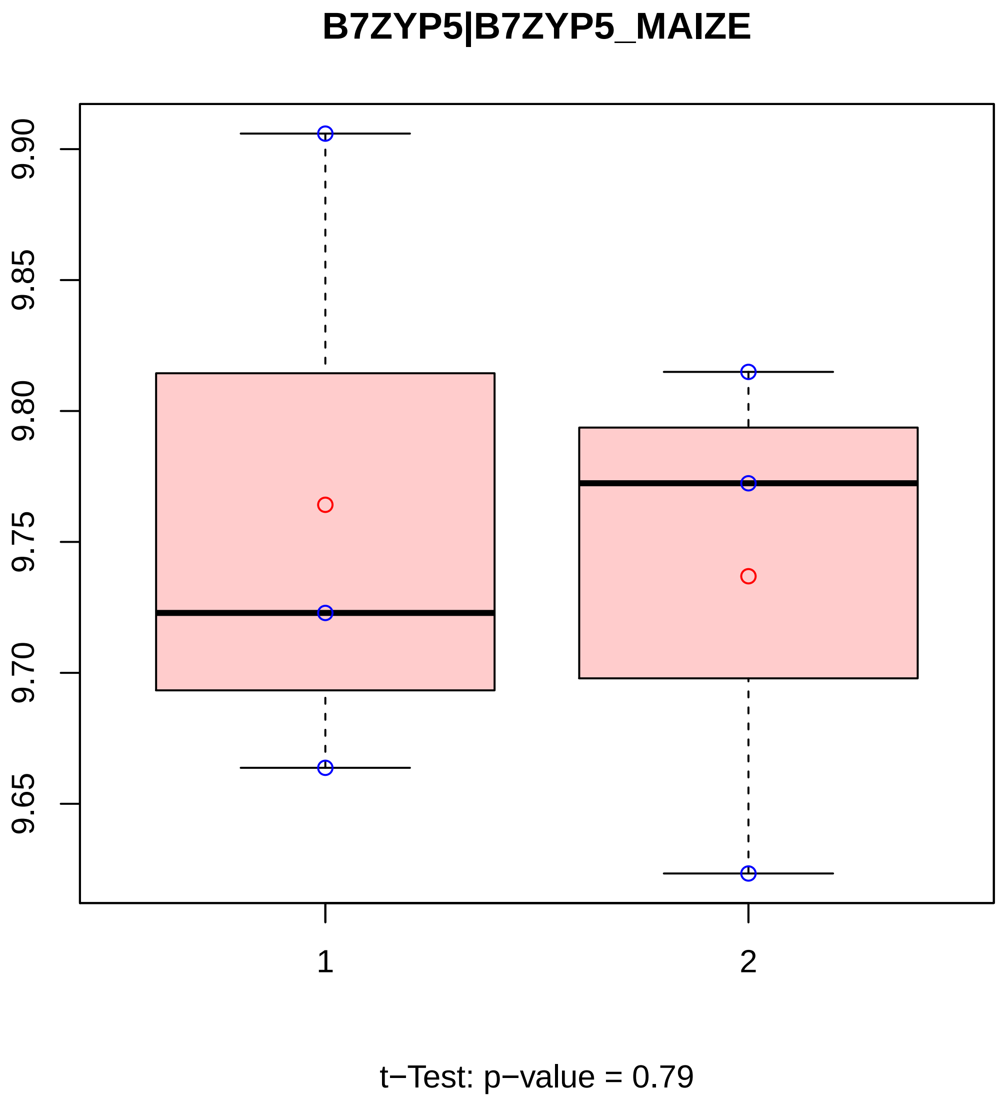
B7ZYP5|B7ZYP5_MAIZE
9.90
9.85
9.80
9.75
9.70
9.65
1
2
t−Test: p−value = 0.79

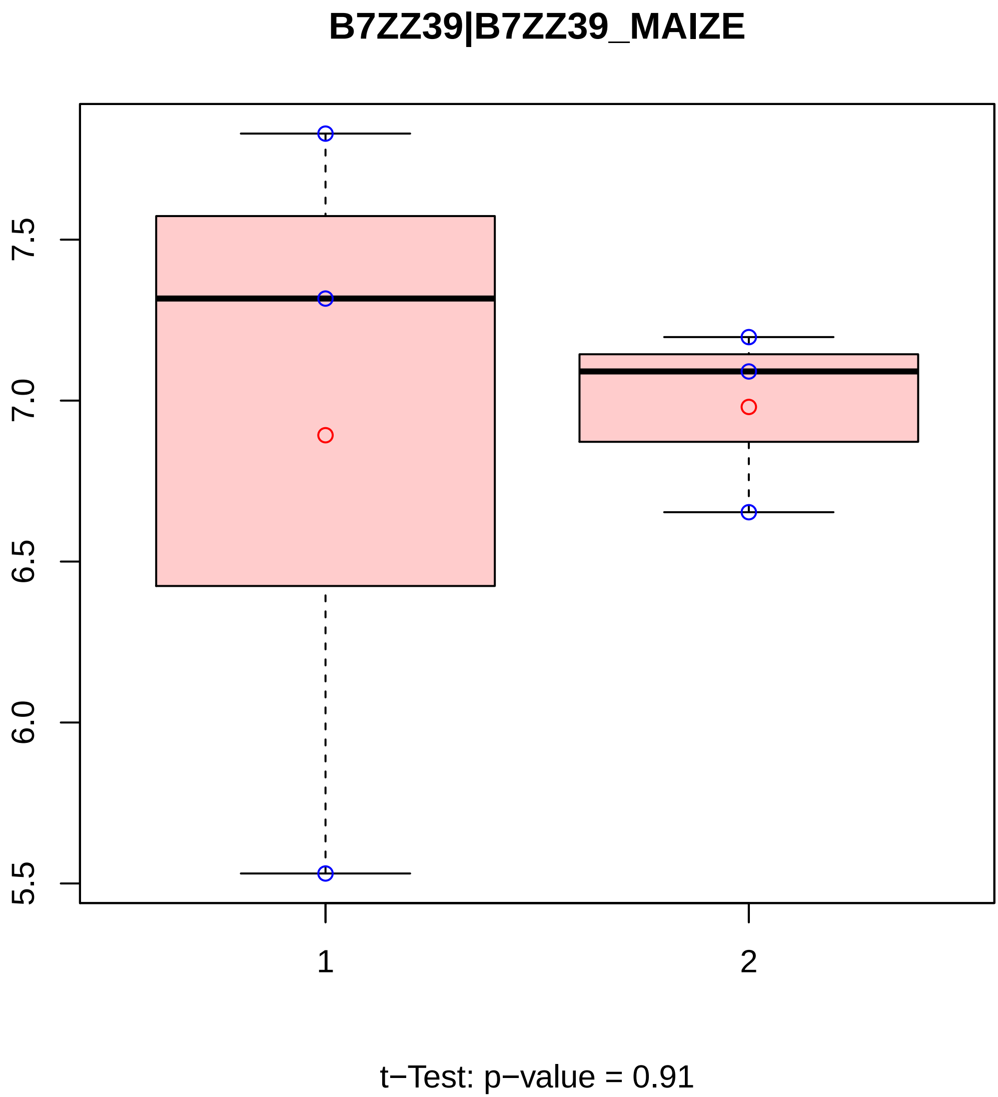
B7ZZ39|B7ZZ39_MAIZE
7.5
7.0
6.5
6.0
5.5
1
2
t−Test: p−value = 0.91

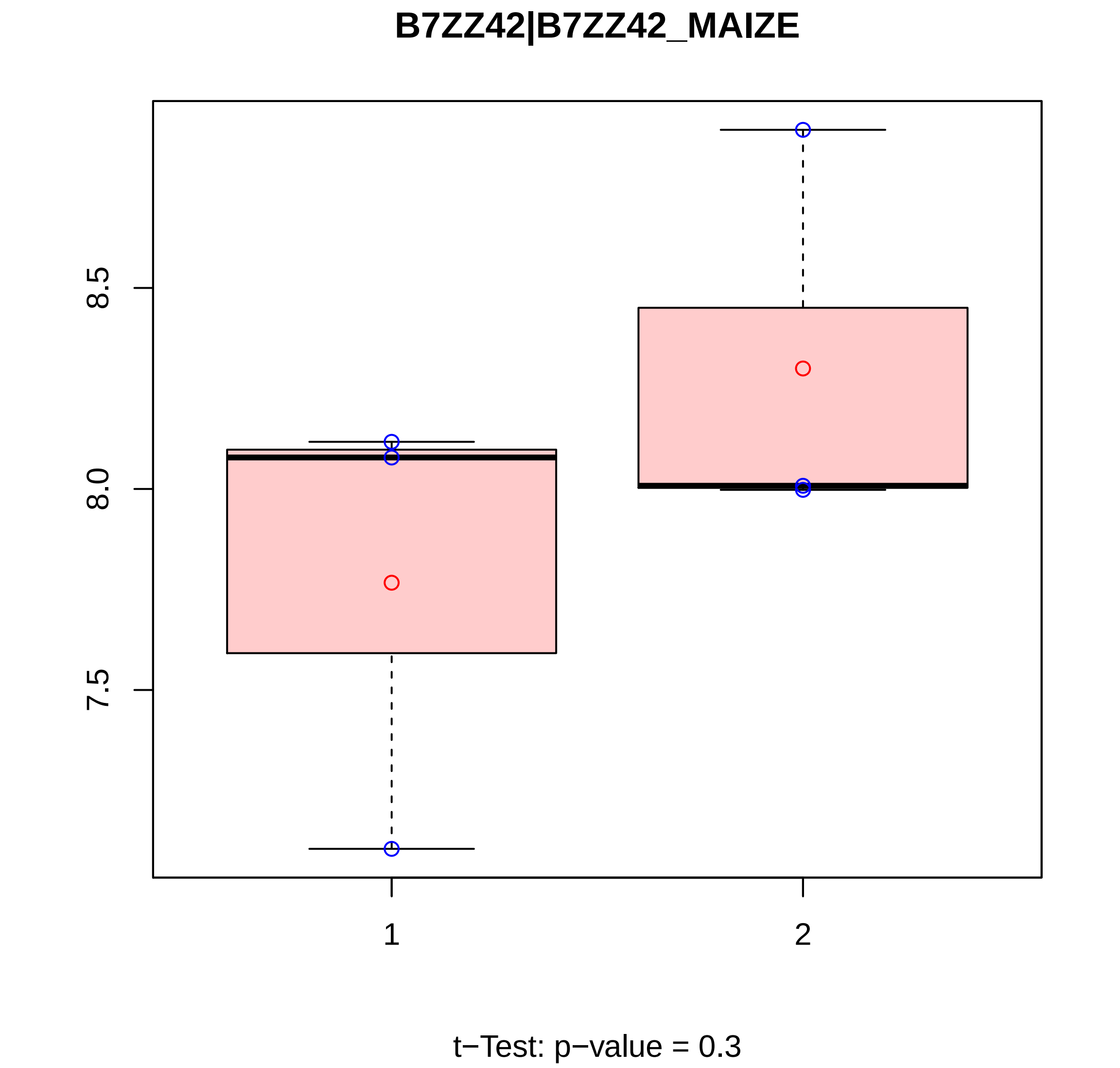
B7ZZ42|B7ZZ42_MAIZE
8.5
8.0
7.5
1
2
t−Test: p−value = 0.3

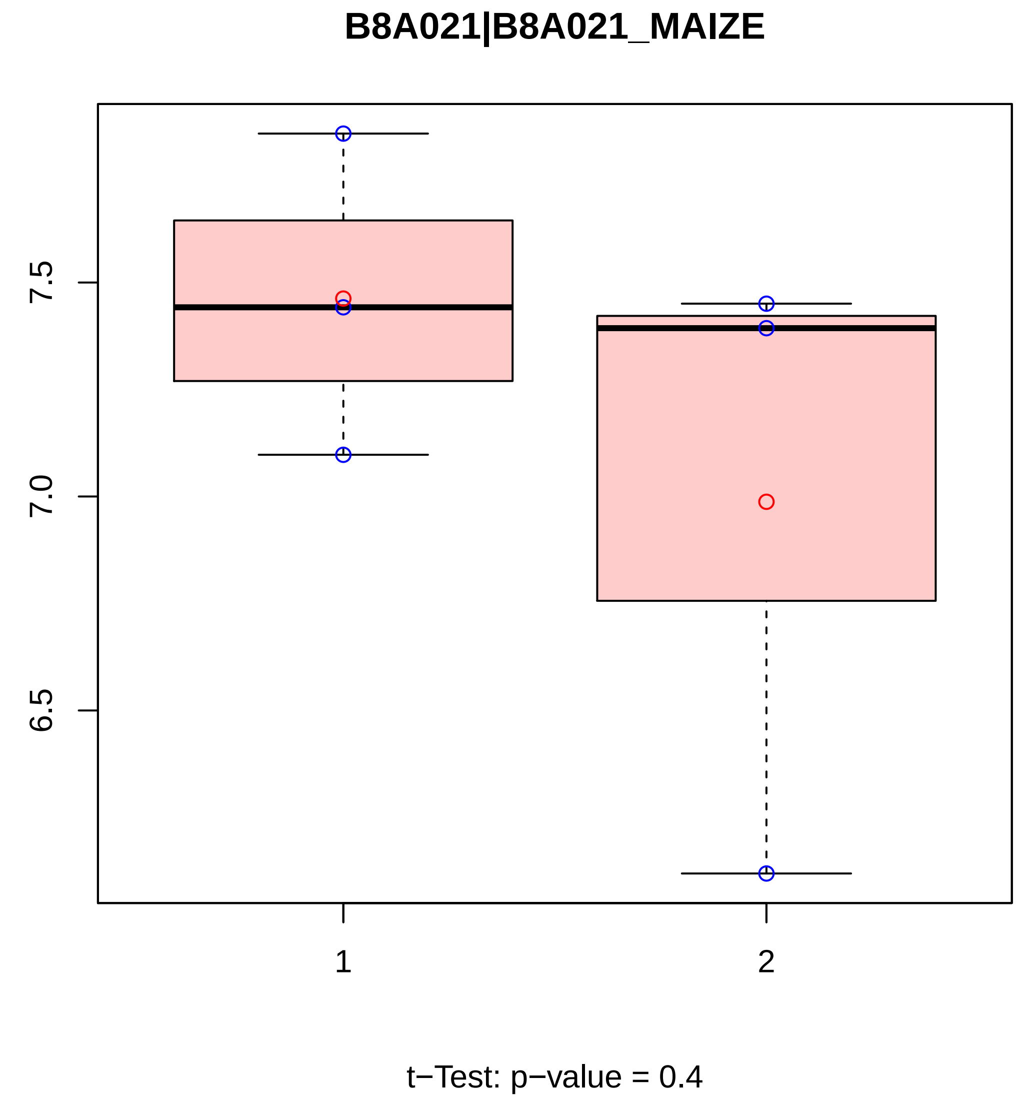

B8A021|B8A021_MAIZE
7.5
7.0
6.5
1
2
t−Test: p−value = 0.4

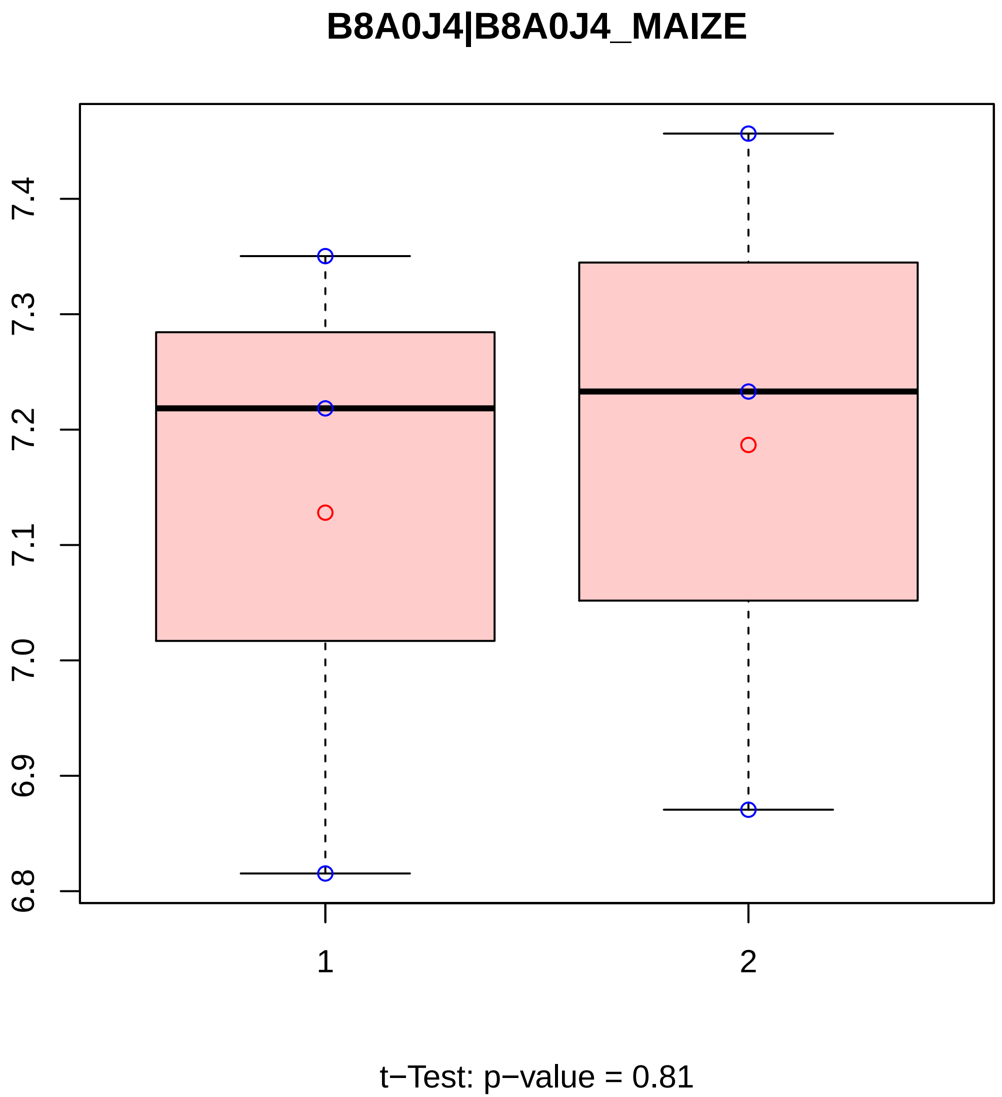
B8A0J4|B8A0J4_MAIZE
7.4
7.3
7.2
7.1
7.0
6.9
6.8
1
2
t−Test: p−value = 0.81

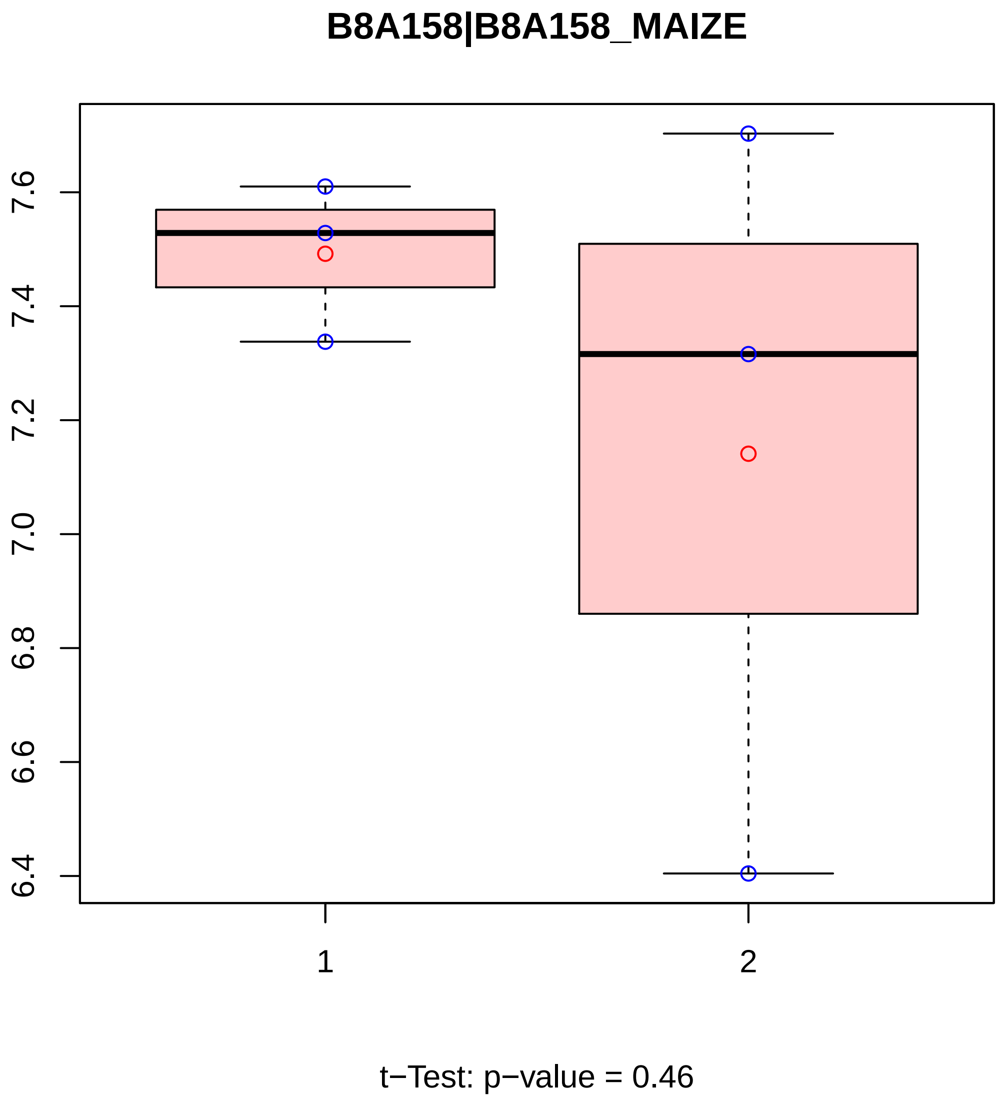

B8A158|B8A158_MAIZE
7.6
7.4
7.2
7.0
6.8
6.6
6.4
1
2
t−Test: p−value = 0.46

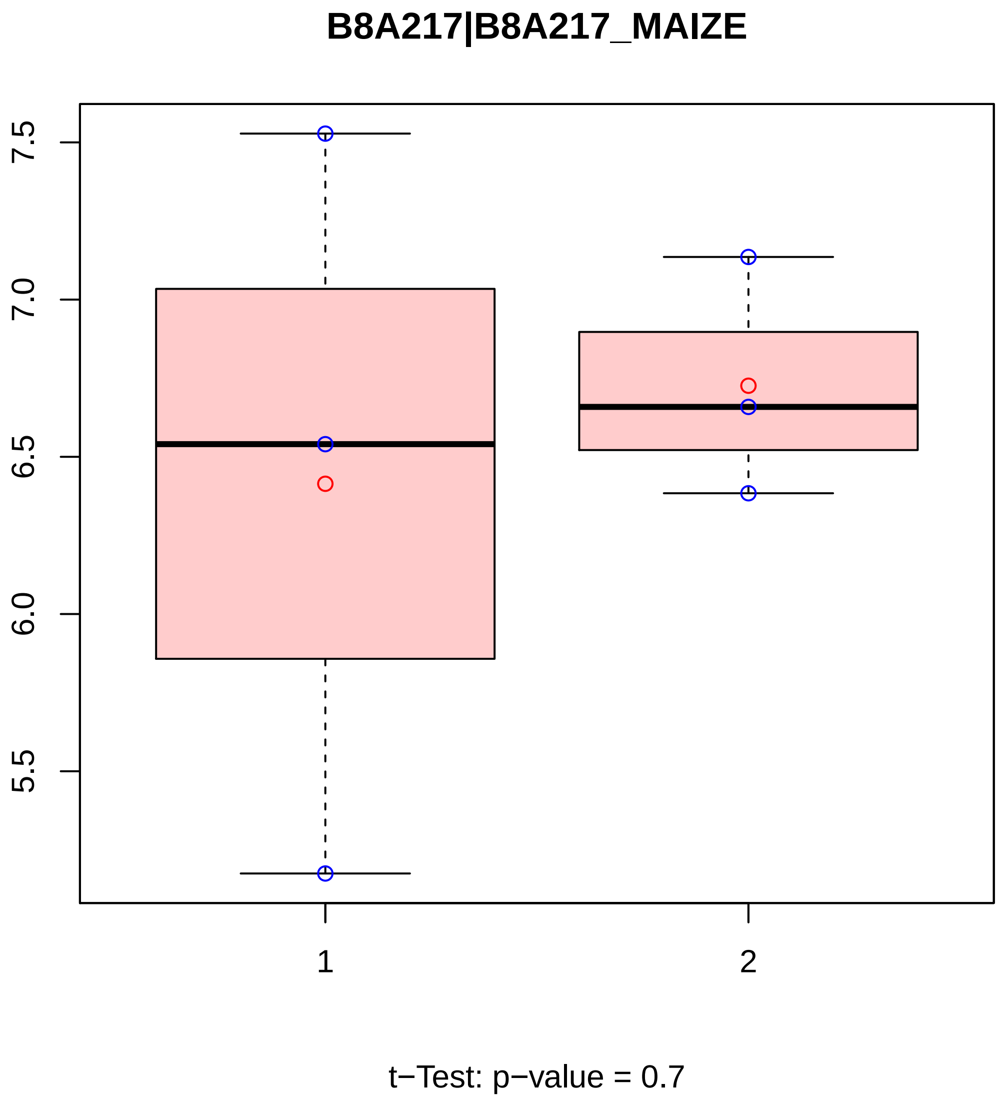
B8A217|B8A217_MAIZE
7.5
7.0
6.5
6.0
5.5
1
2
t−Test: p−value = 0.7

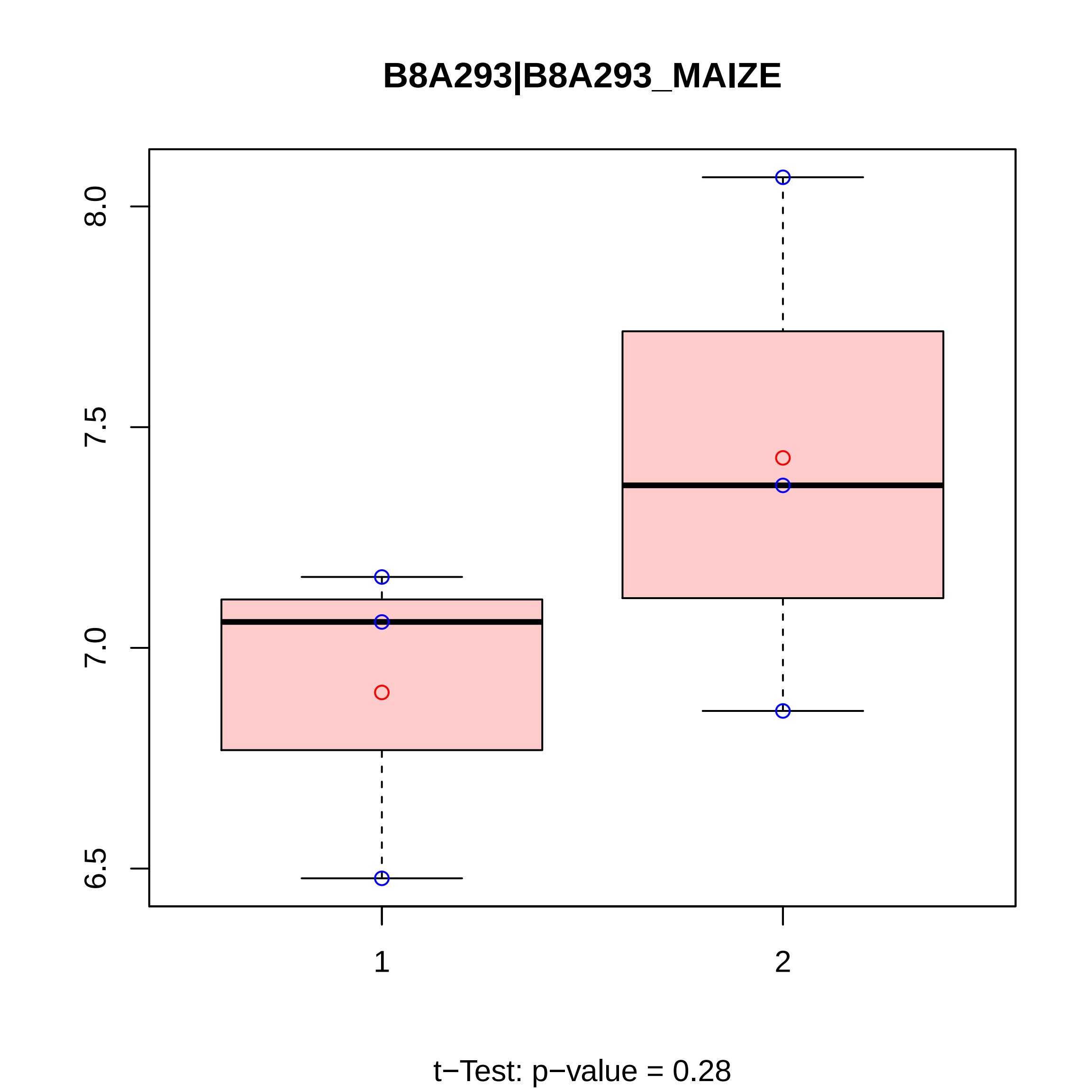

B8A293|B8A293_MAIZE
8.0
7.5
7.0
6.5
1
2
t−Test: p−value = 0.28

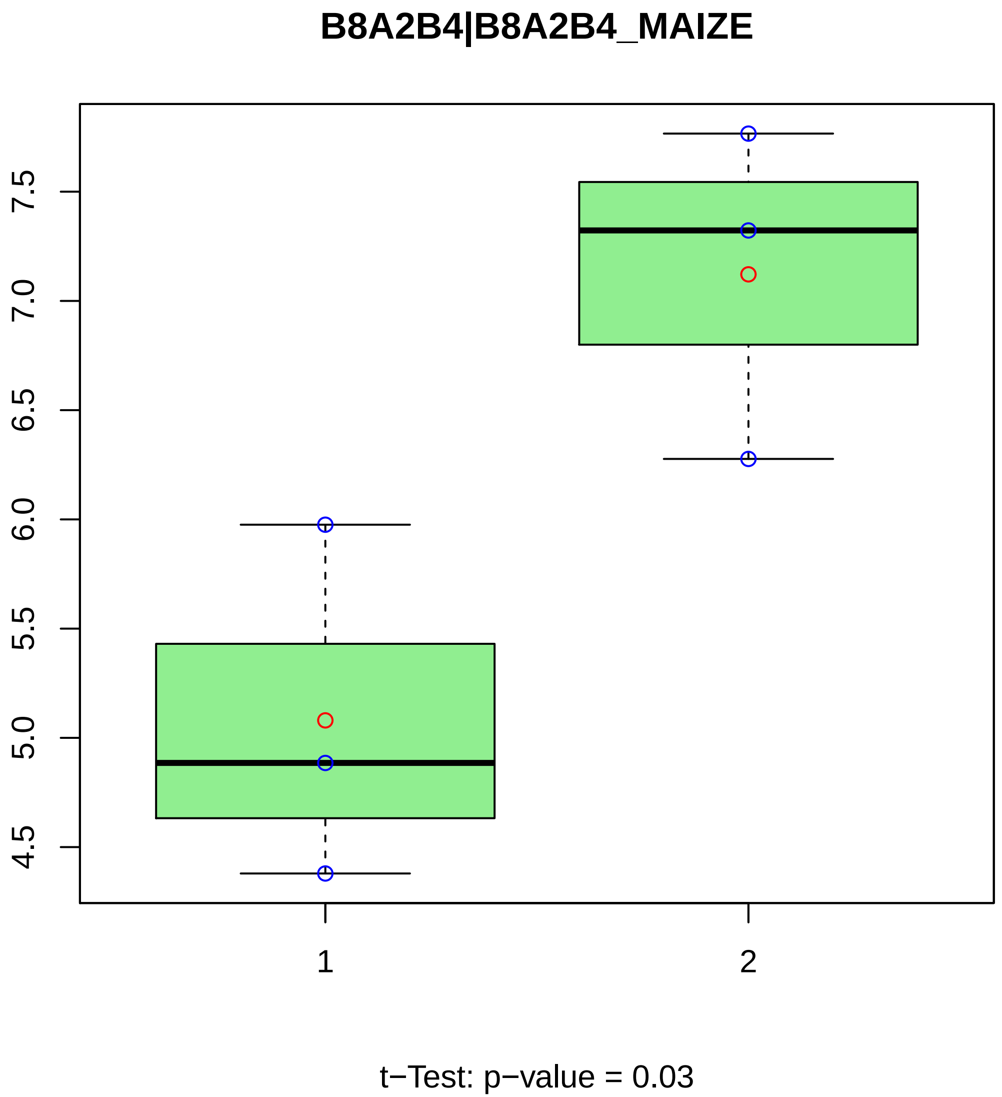
B8A2B4|B8A2B4_MAIZE
7.5
7.0
6.5
6.0
5.5
5.0
4.5
1
2
t−Test: p−value = 0.03

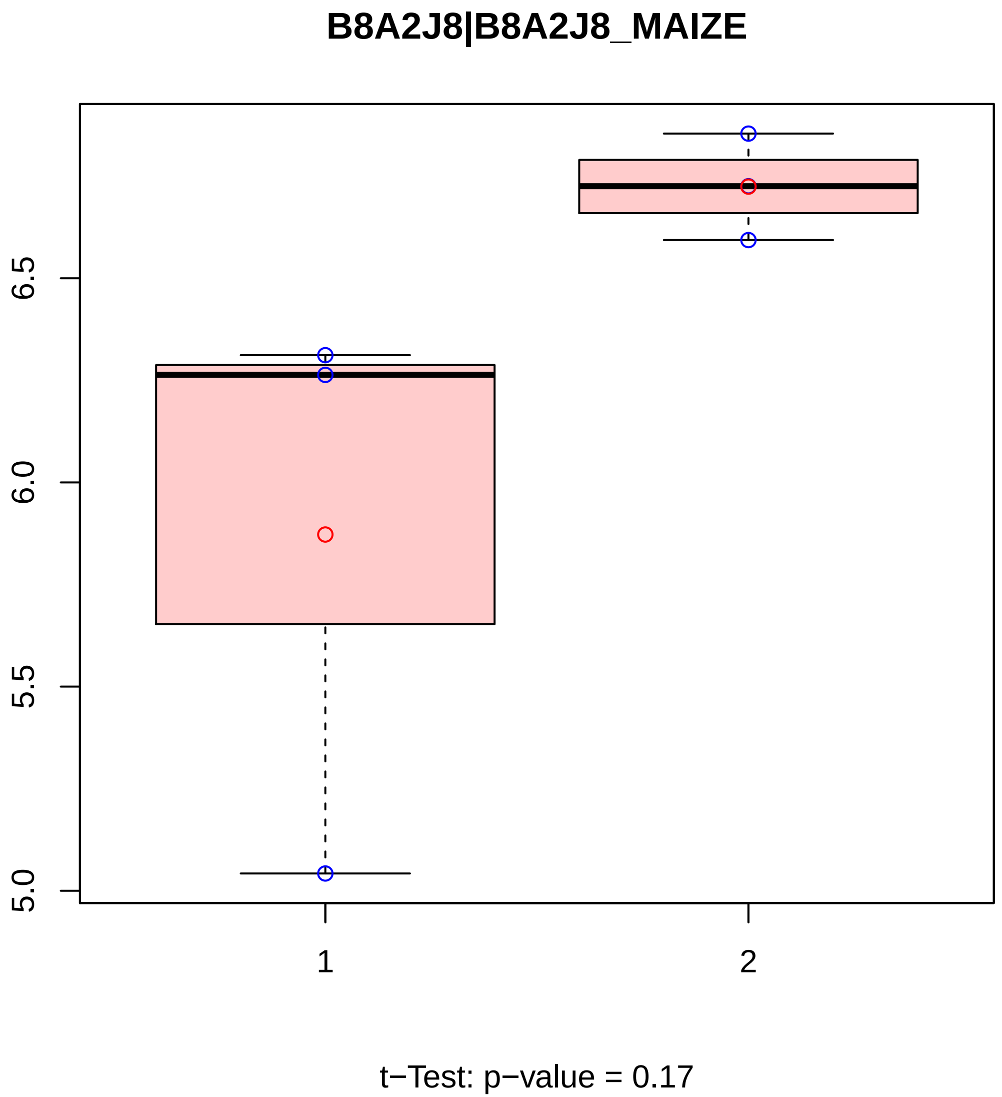

B8A2J8|B8A2J8_MAIZE
5.0
5.5
6.0
6.5
1
2
t−Test: p−value = 0.17

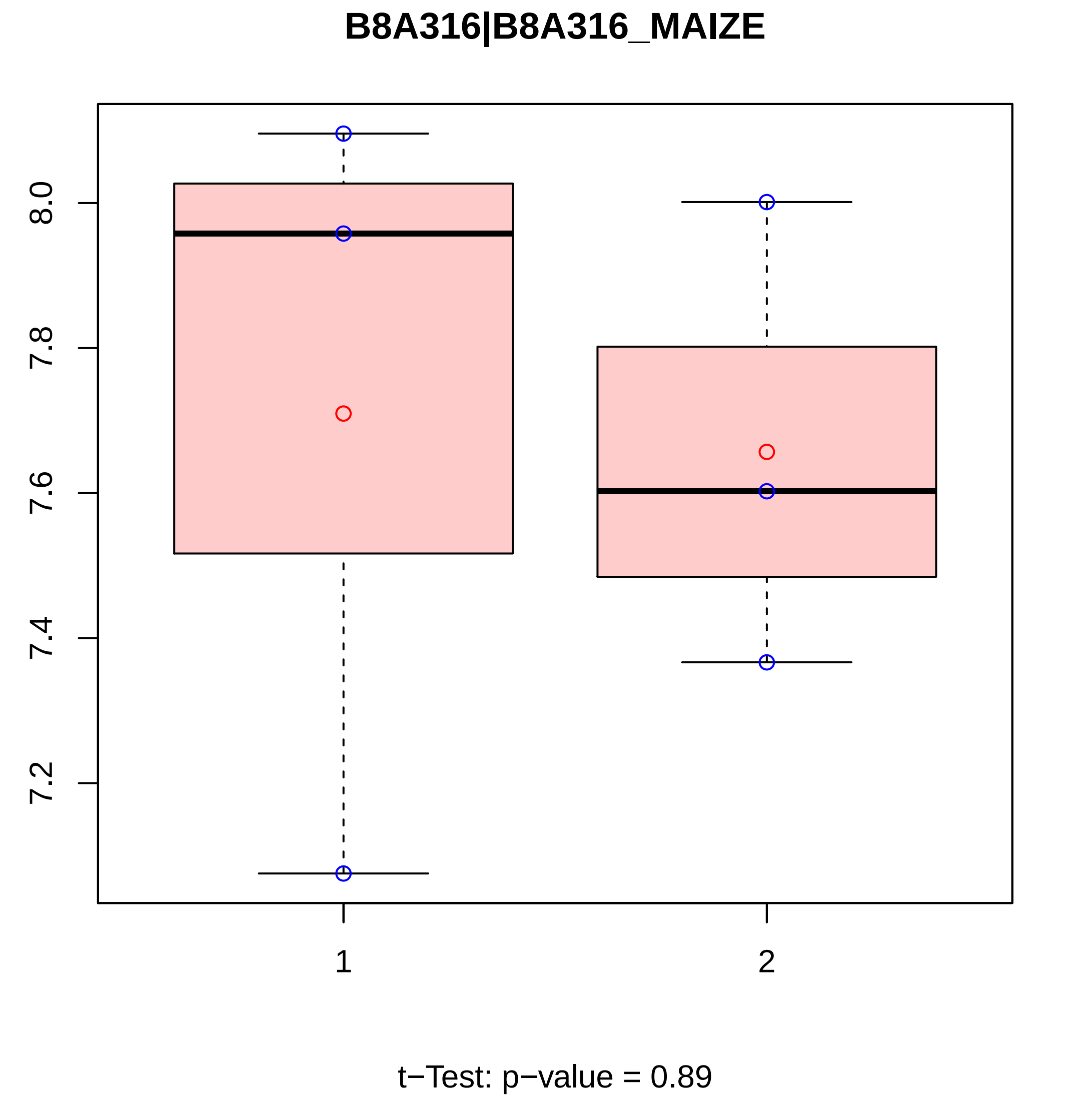
B8A316|B8A316_MAIZE
8.0
7.8
7.6
7.4
7.2
1
2
t−Test: p−value = 0.89

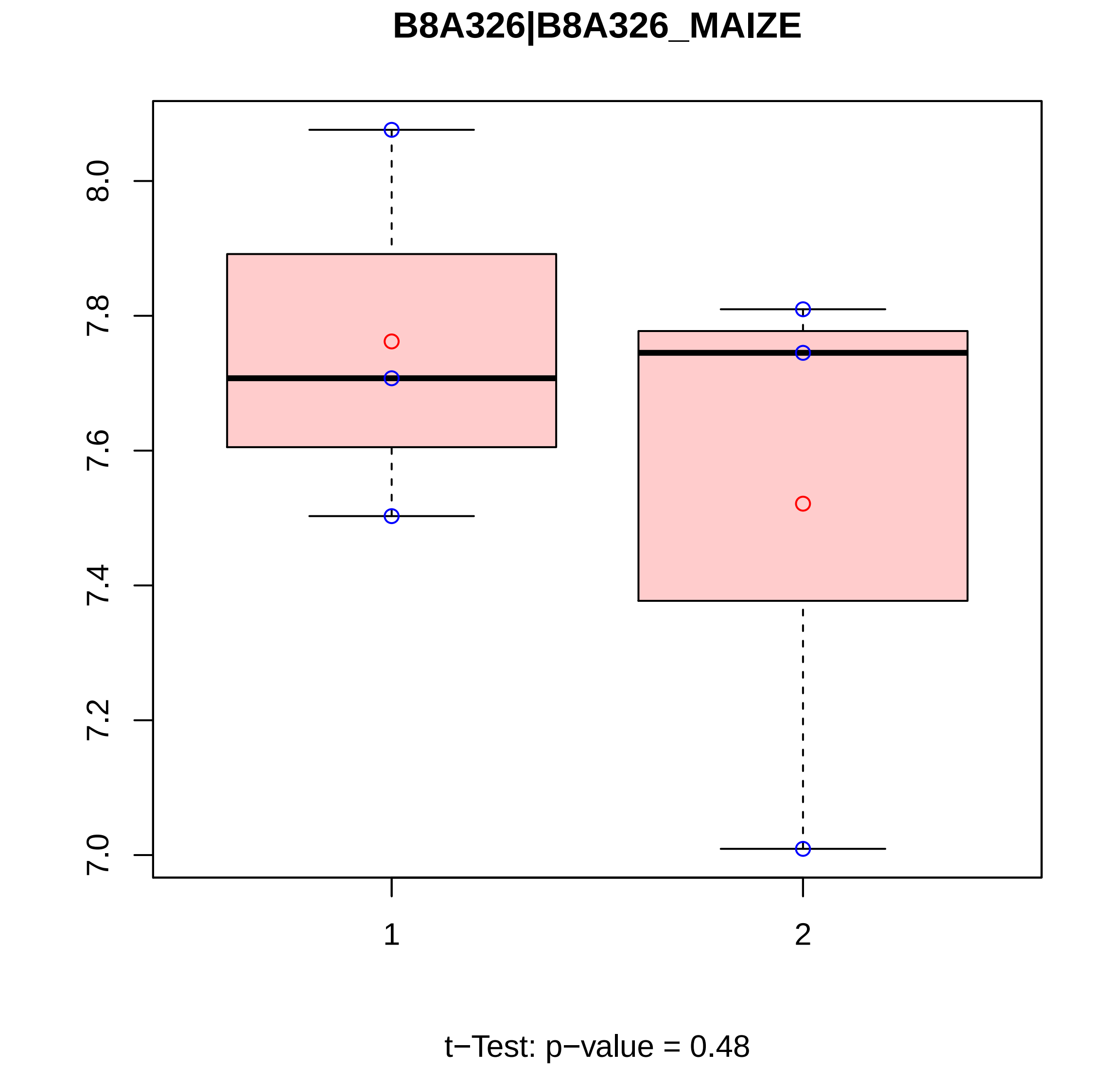

B8A326|B8A326_MAIZE
8.0
7.8
7.6
7.4
7.2
7.0
1
2
t−Test: p−value = 0.48

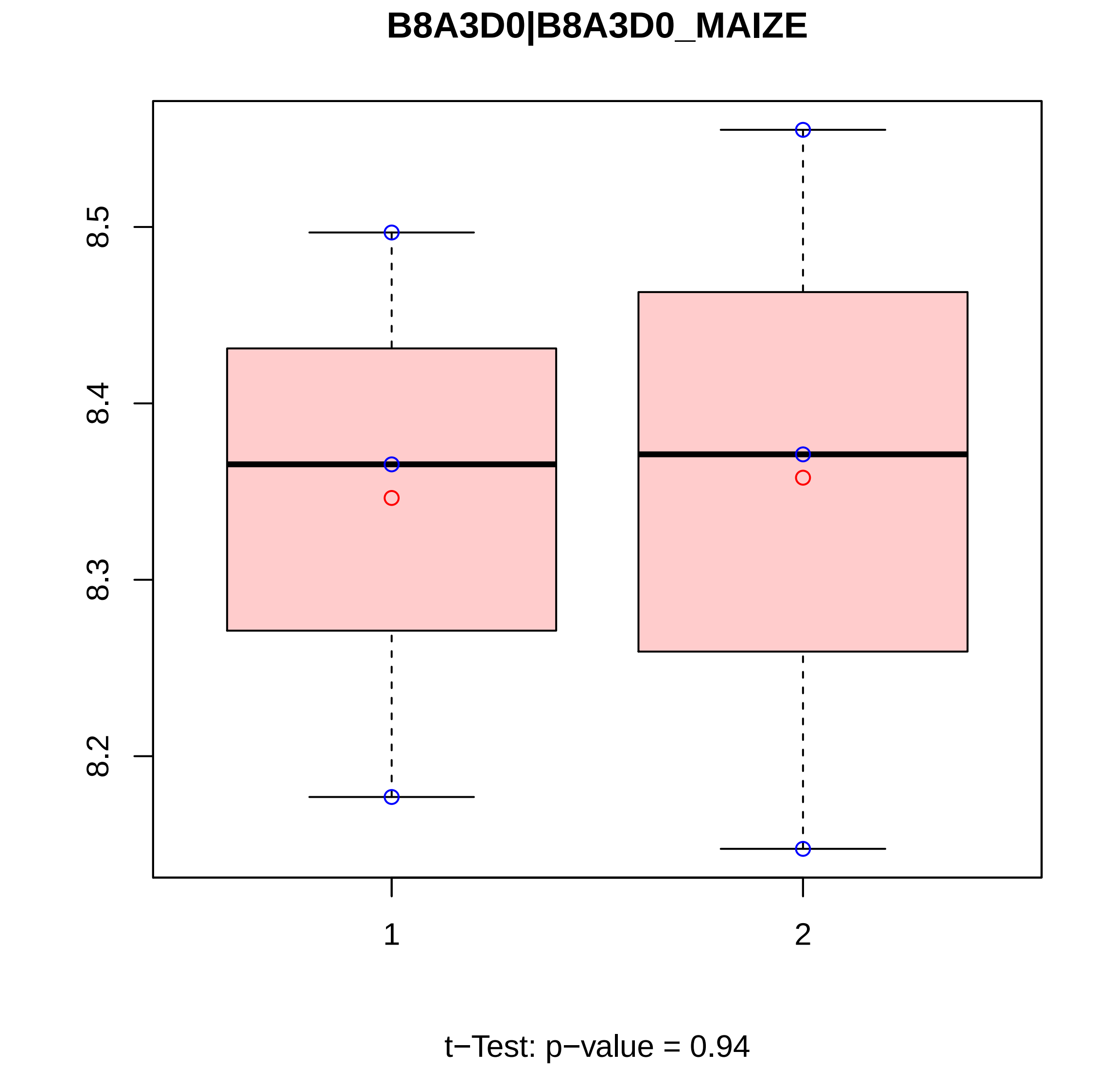
B8A3D0|B8A3D0_MAIZE
8.2
8.3
8.4
8.5
1
2
t−Test: p−value = 0.94

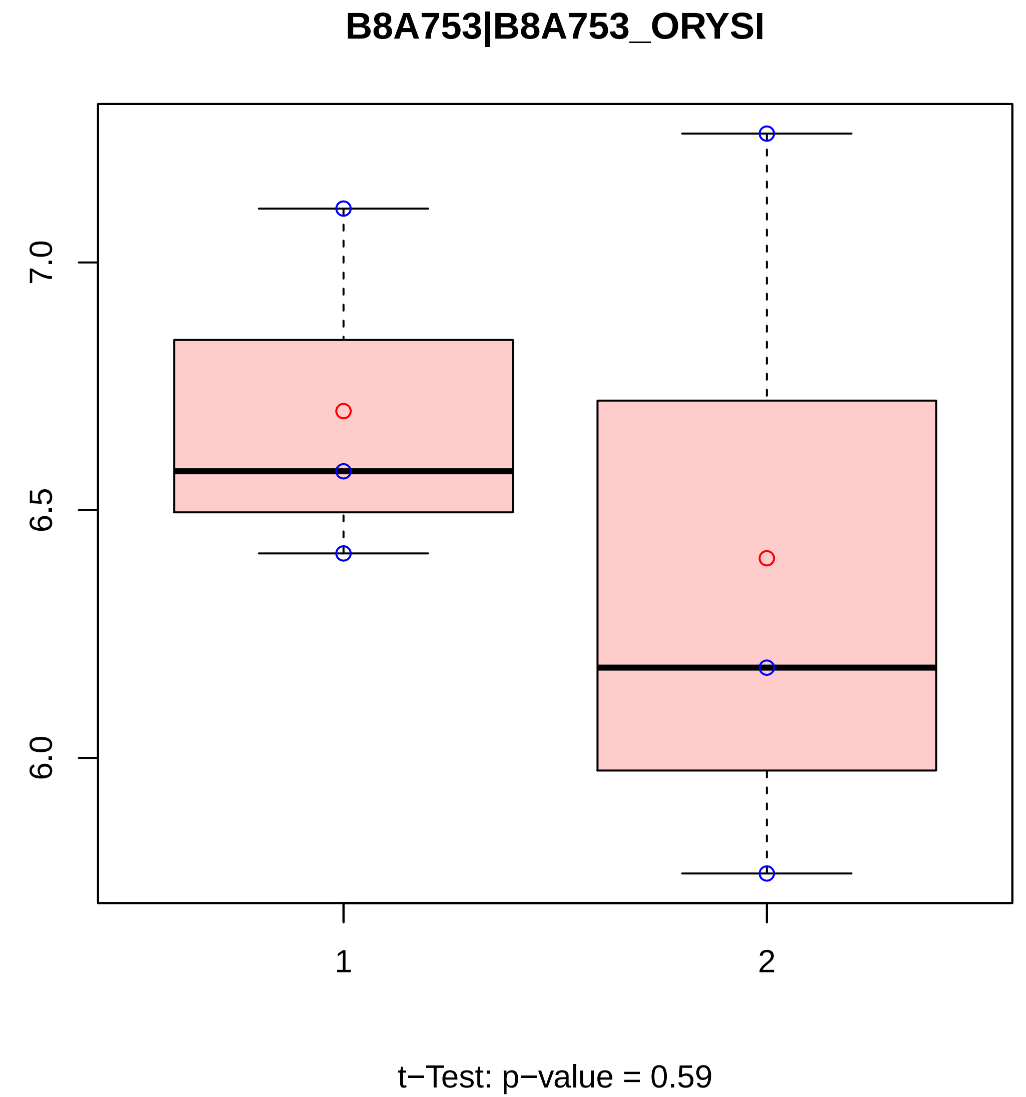
B8A753|B8A753_ORYSI
7.0
6.5
6.0
1
2
t−Test: p−value = 0.59

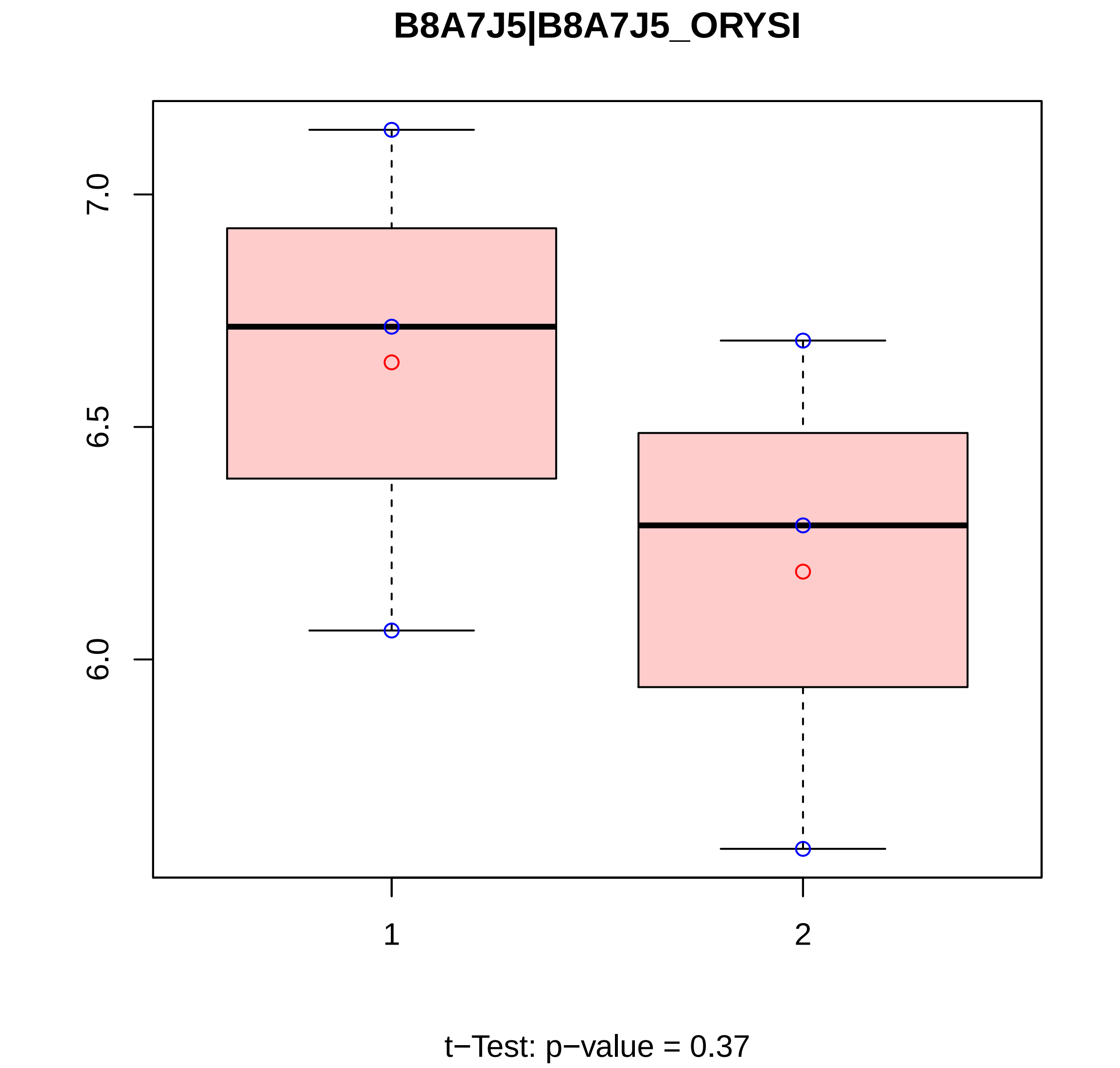
B8A7J5|B8A7J5_ORYSI
7.0
6.5
6.0
1
2
t−Test: p−value = 0.37

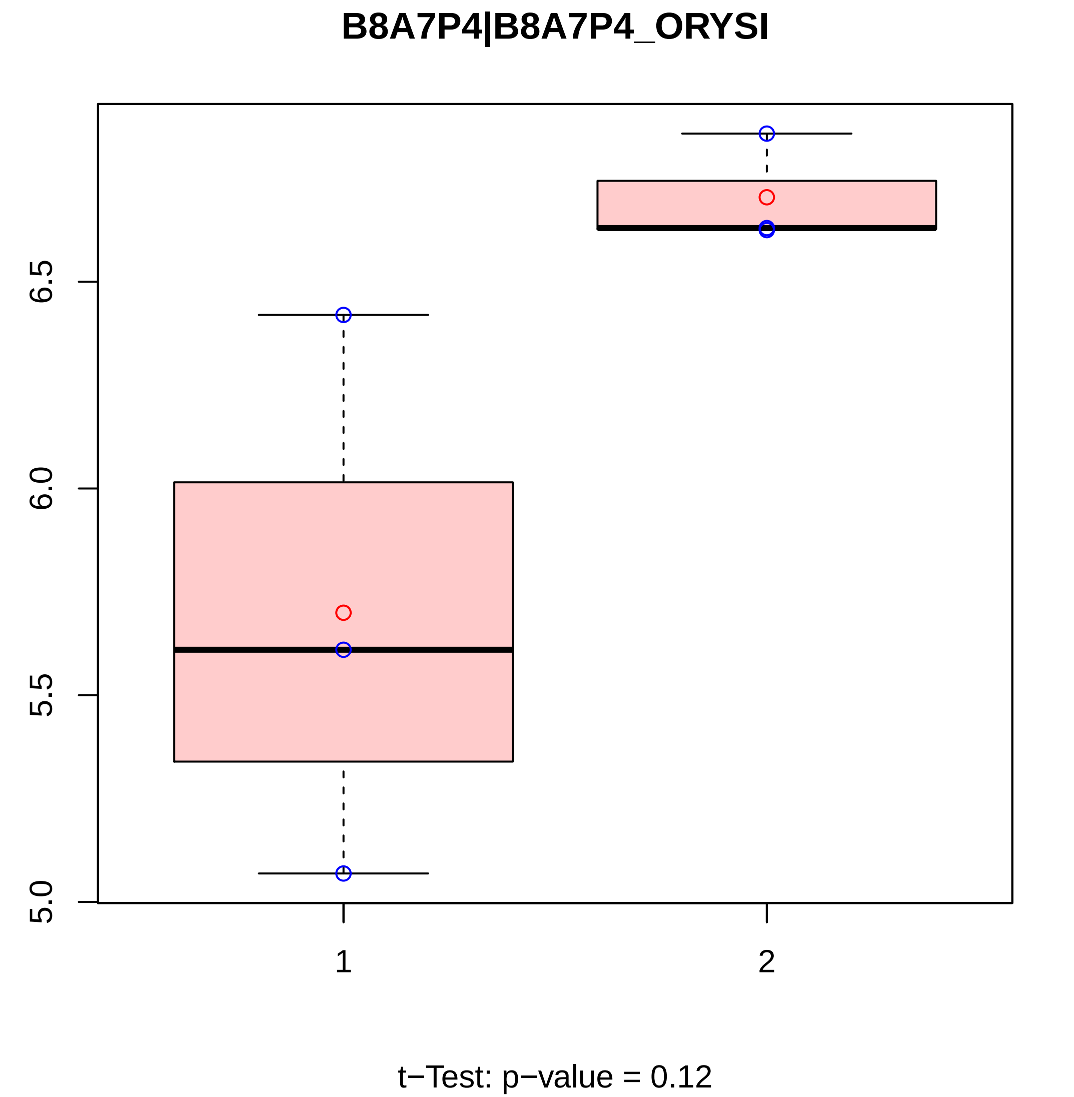
B8A7P4|B8A7P4_ORYSI
5.0
5.5
6.0
6.5
1
2
t−Test: p−value = 0.12

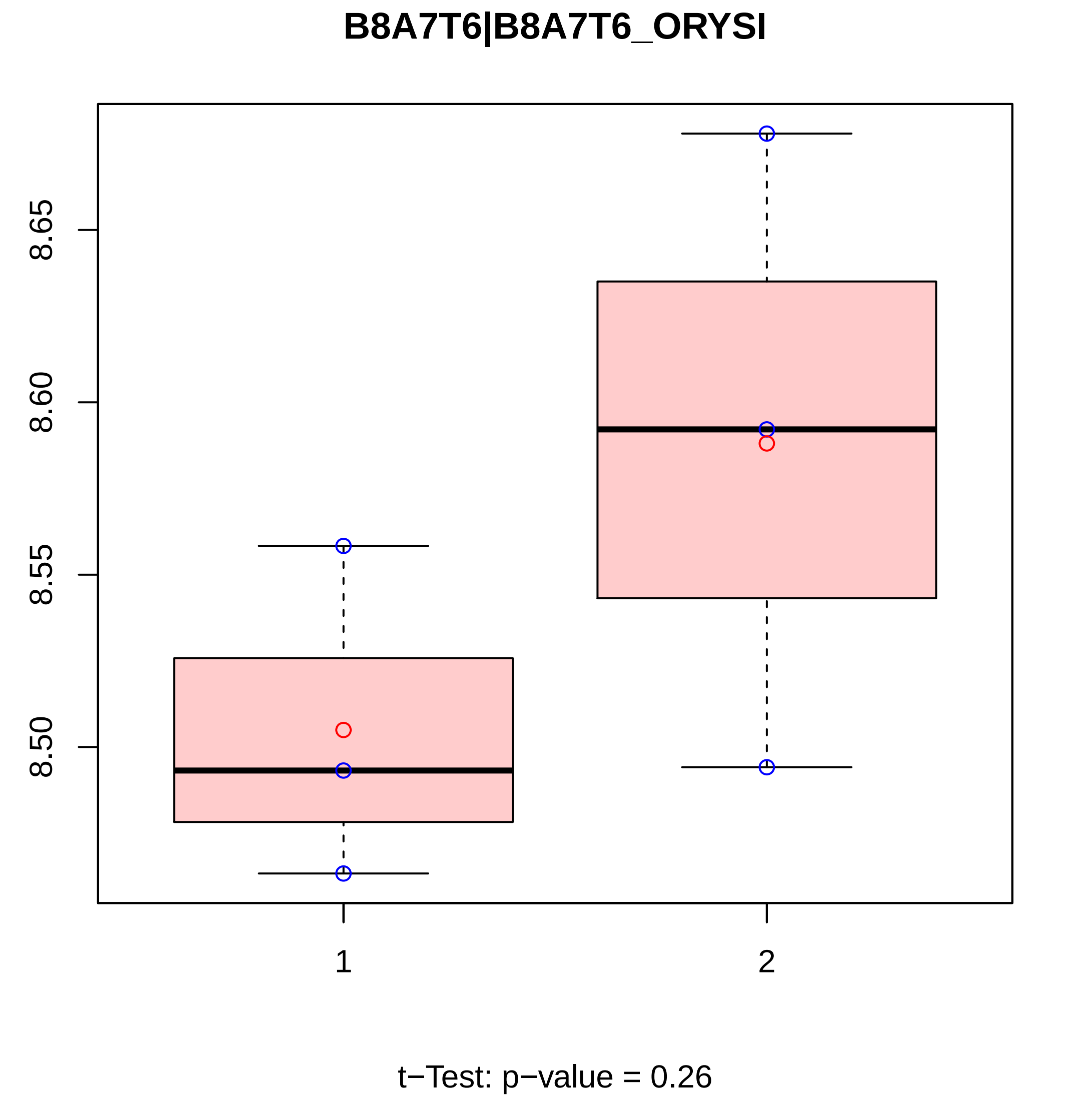
B8A7T6|B8A7T6_ORYSI
8.50
8.55
8.60
8.65
1
2
t−Test: p−value = 0.26

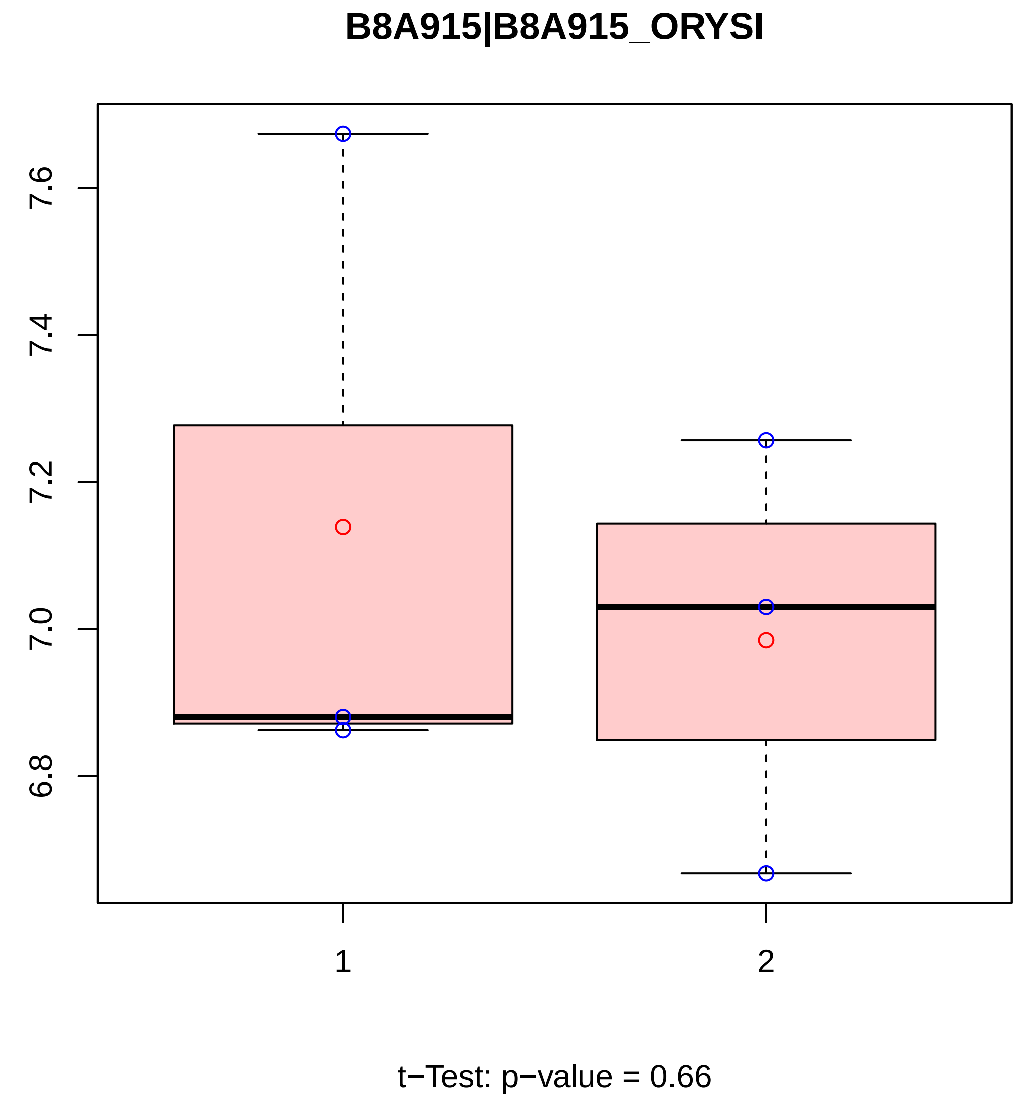

B8A915|B8A915_ORYSI
7.6
7.4
7.2
7.0
6.8
1
2
t−Test: p−value = 0.66

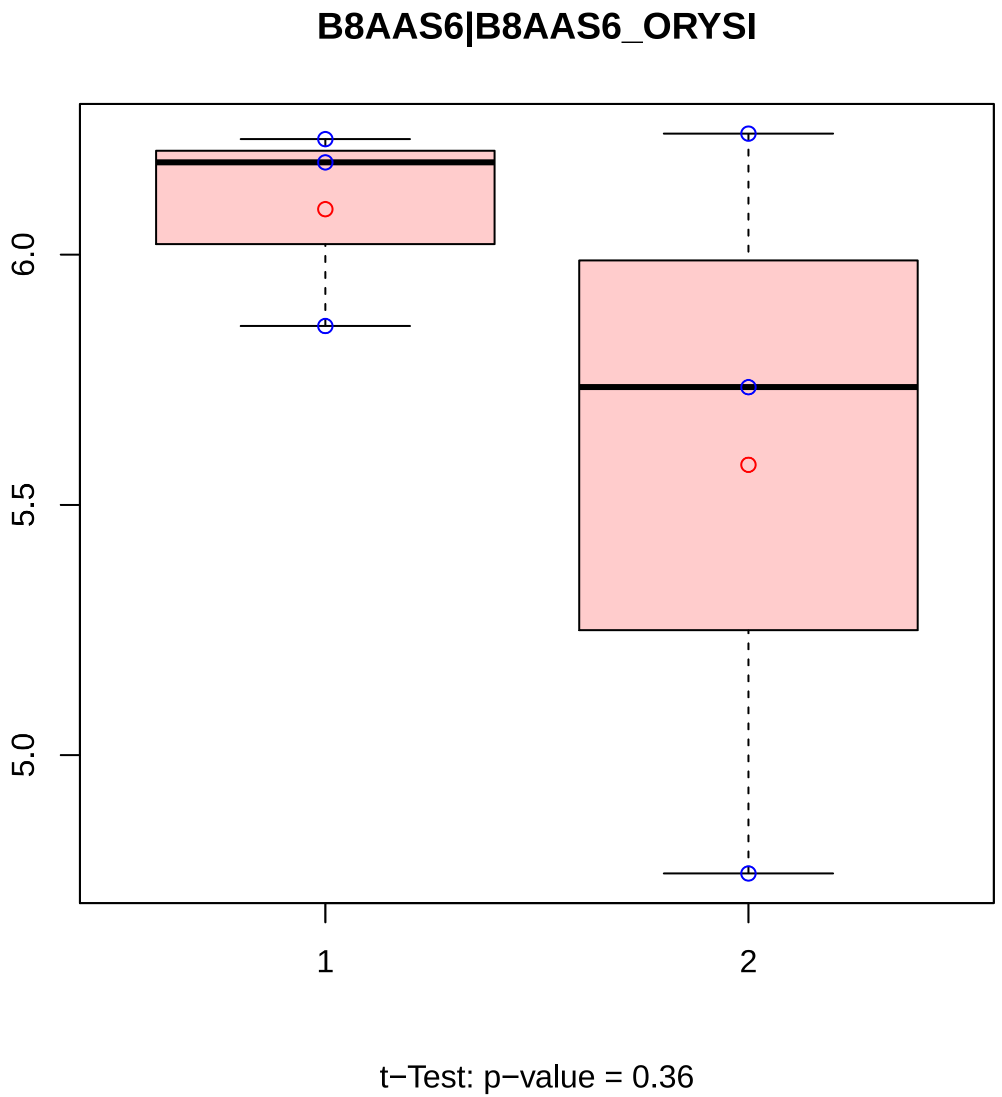
B8AAS6|B8AAS6_ORYSI
6.0
5.5
5.0
1
2
t−Test: p−value = 0.36

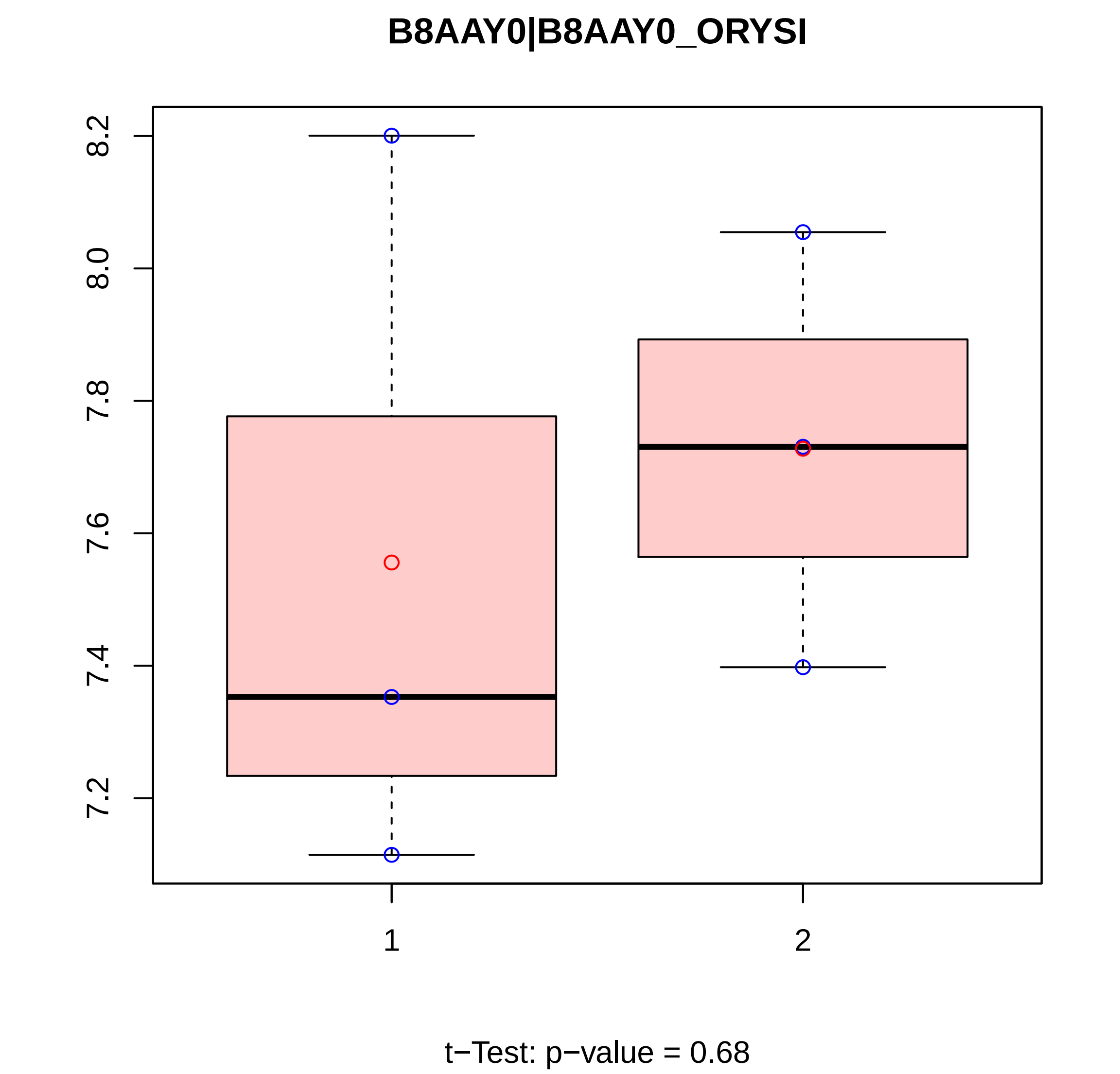

B8AAY0|B8AAY0_ORYSI
8.2
8.0
7.8
7.6
7.4
7.2
1
2
t−Test: p−value = 0.68

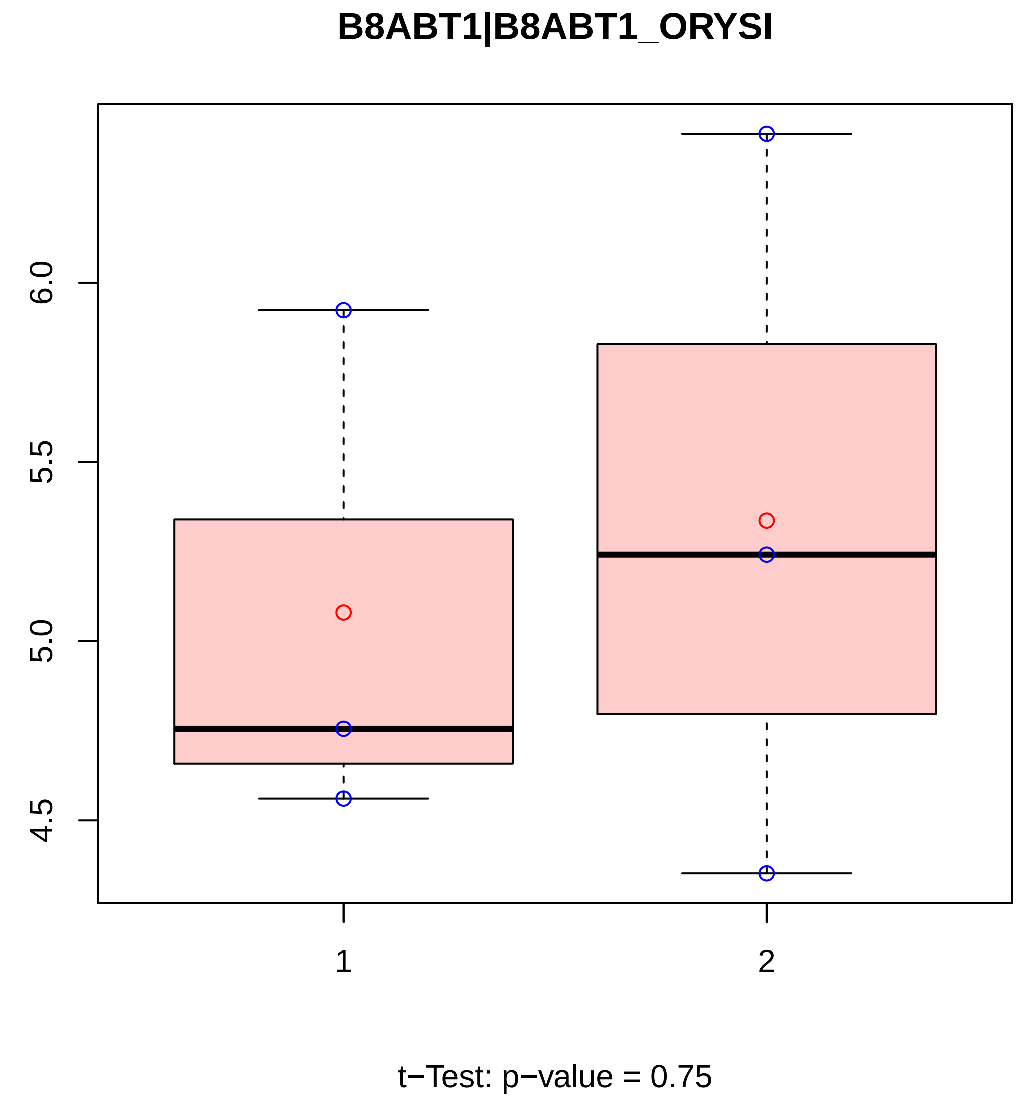
B8ABT1|B8ABT1_ORYSI
6.0
5.5
5.0
4.5
1
2
t−Test: p−value = 0.75

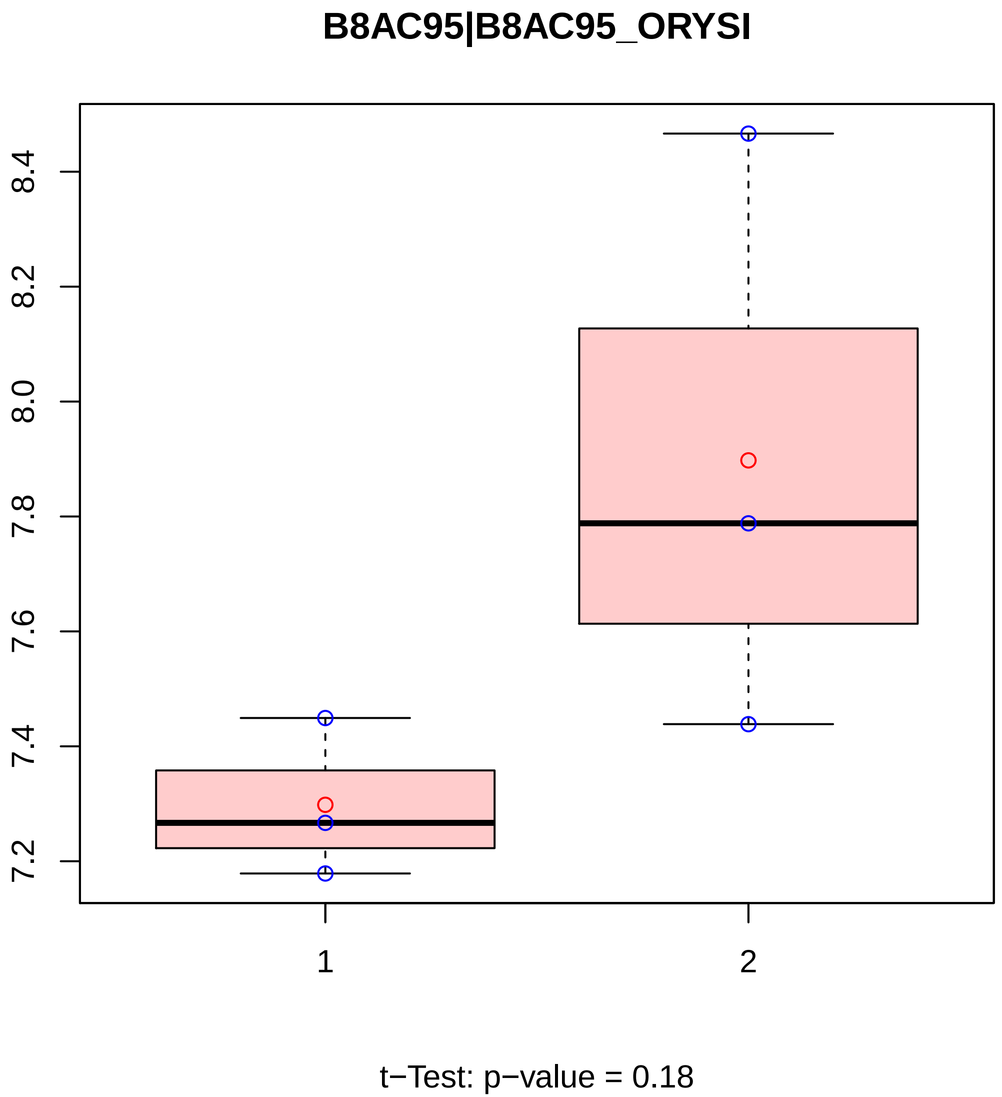
B8AC95|B8AC95_ORYSI
8.4
8.2
8.0
7.8
7.6
7.4
7.2
1
2
t−Test: p−value = 0.18

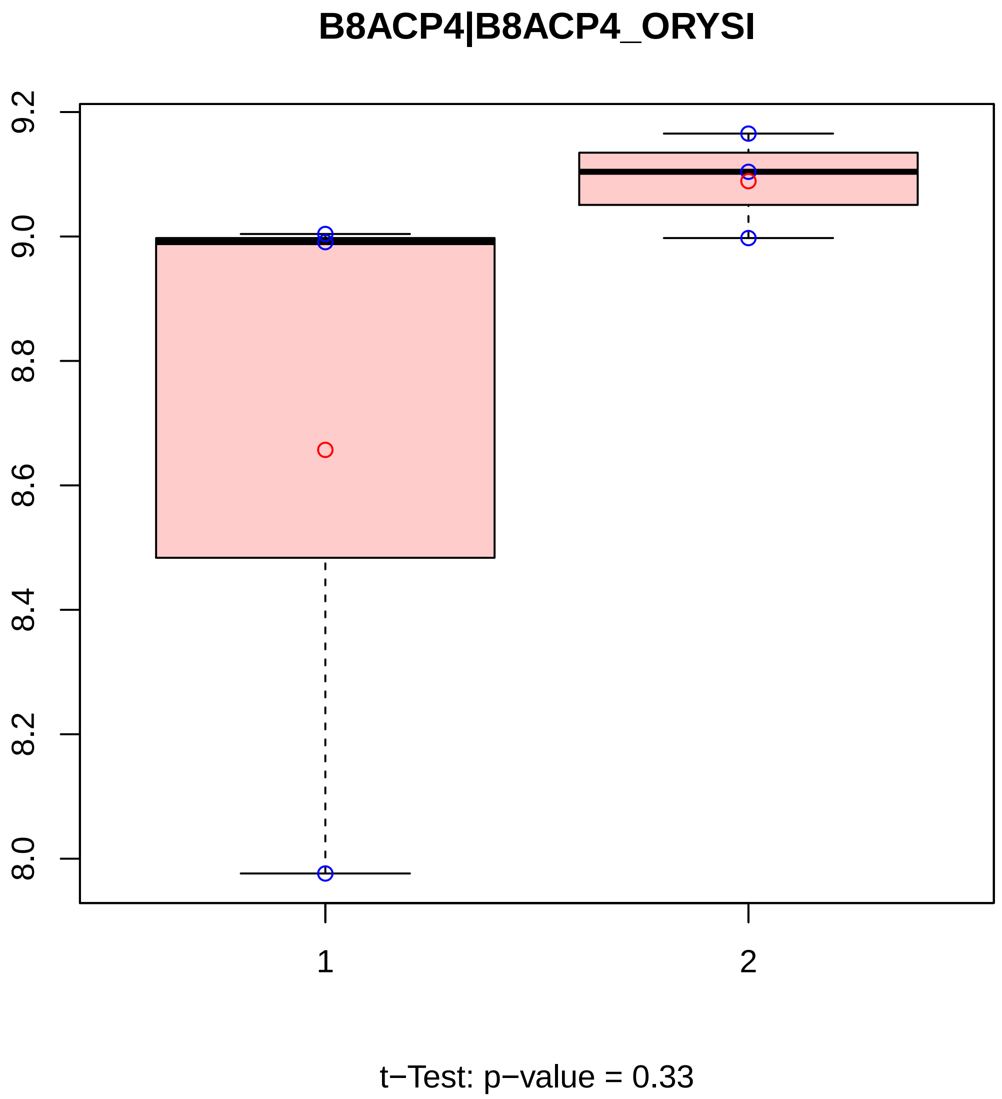
B8ACP4|B8ACP4_ORYSI
9.2
9.0
8.8
8.6
8.4
8.2
8.0
1
2
t−Test: p−value = 0.33

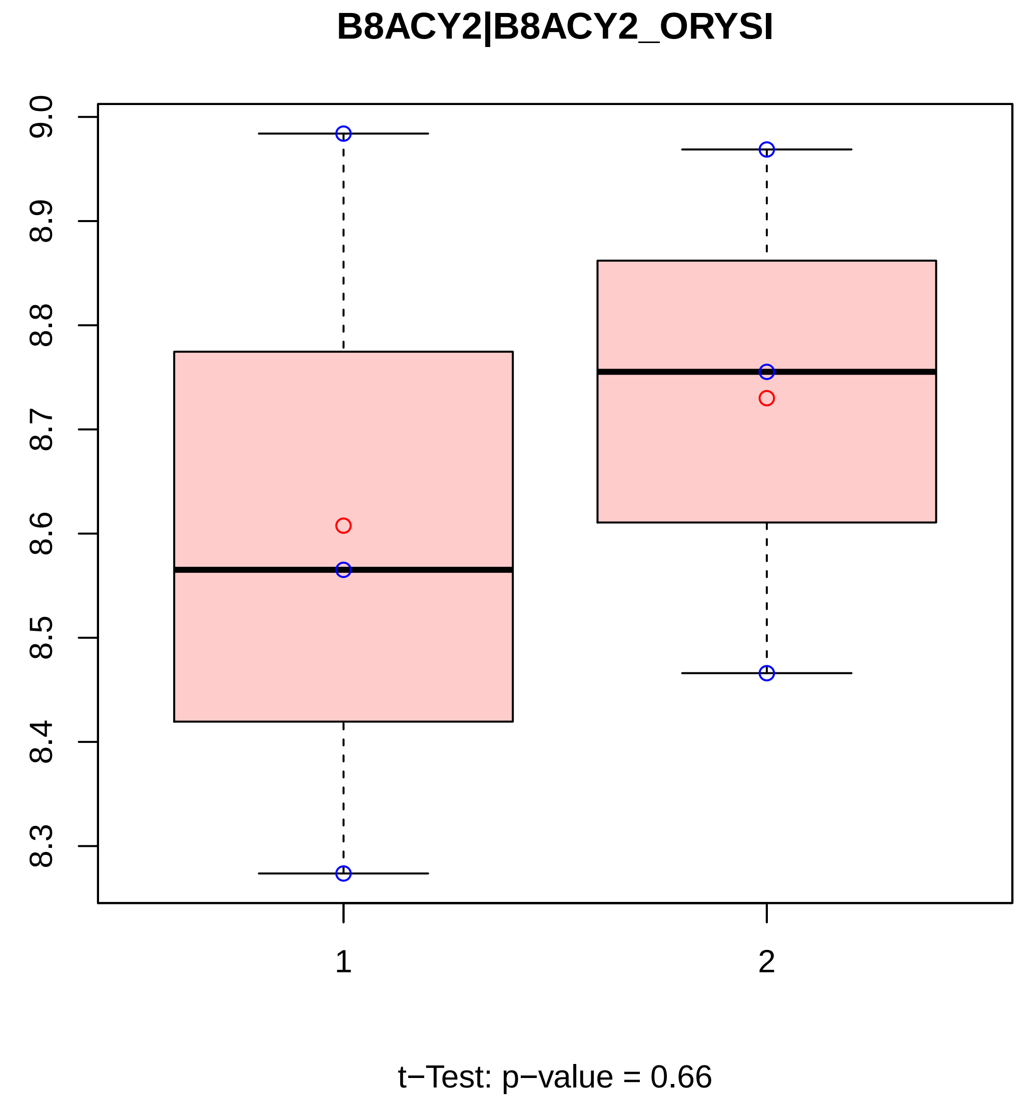
B8ACY2|B8ACY2_ORYSI
9.0
8.9
8.8
8.7
8.6
8.5
8.4
8.3
1
2
t−Test: p−value = 0.66

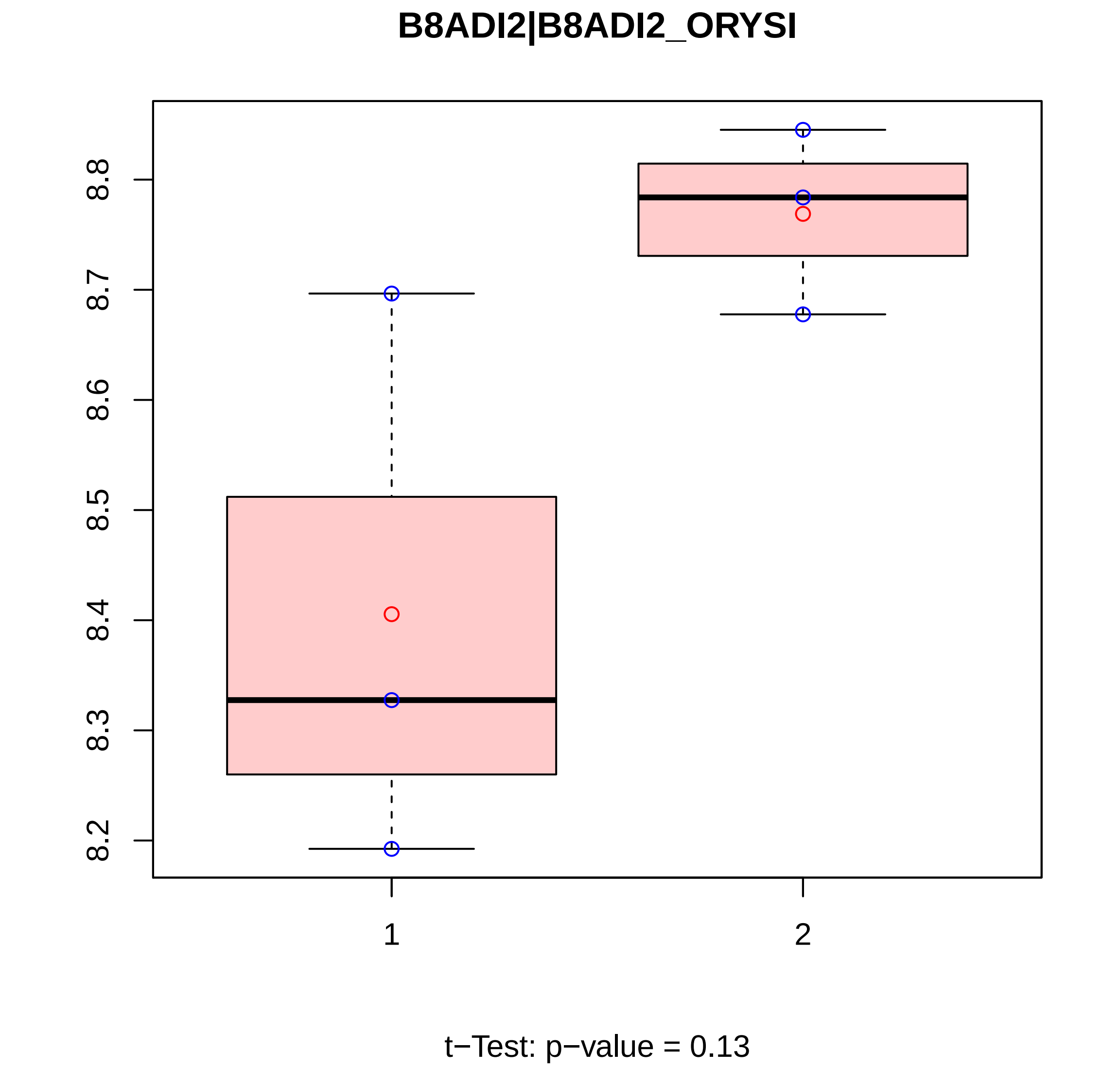
B8ADI2|B8ADI2_ORYSI
8.2
8.3
8.4
8.5
8.6
8.7
8.8
1
2
t−Test: p−value = 0.13

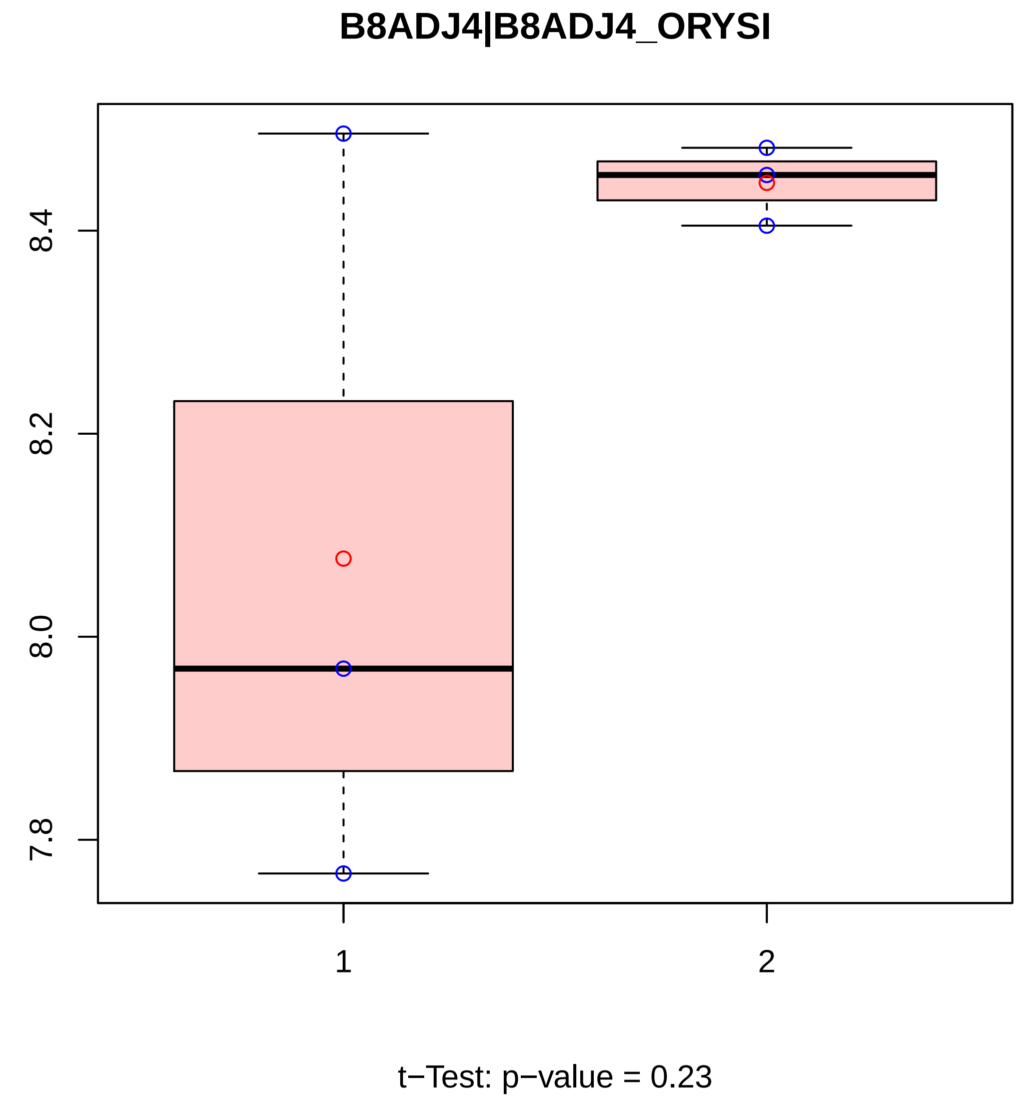

B8ADJ4|B8ADJ4_ORYSI
8.4
8.2
8.0
7.8
1
2
t−Test: p−value = 0.23

B8AEK7|B8AEK7_ORYSI

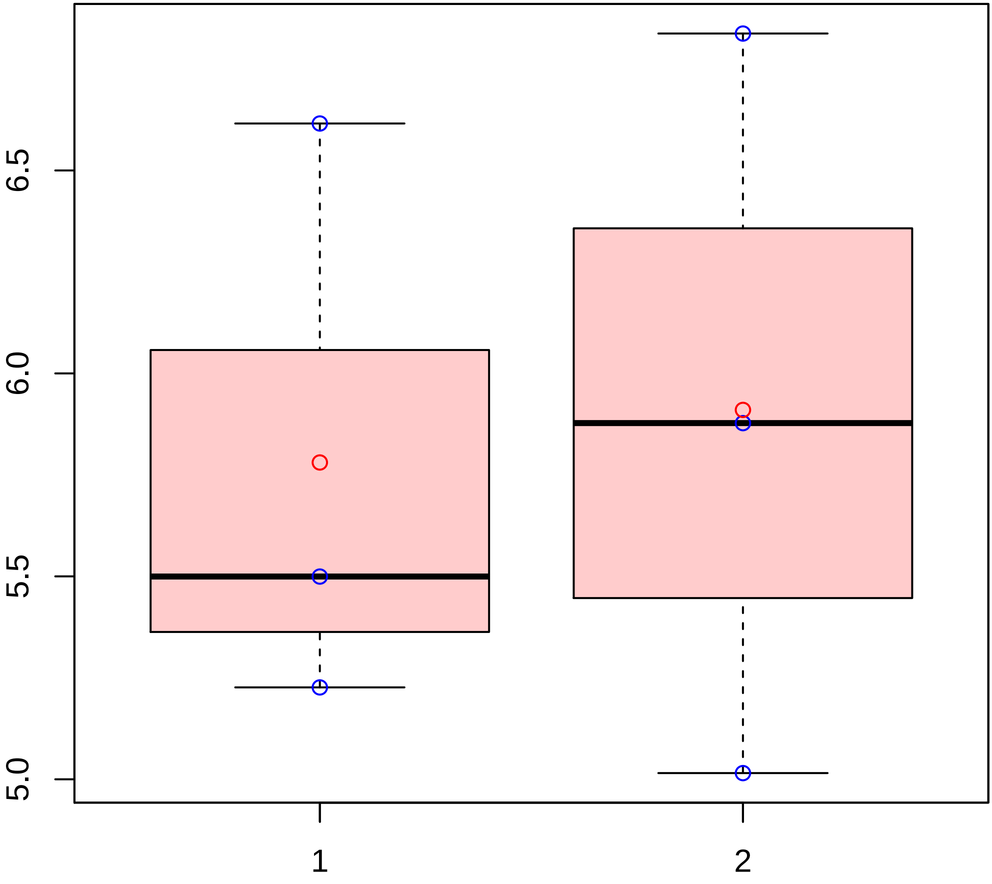

t−Test: p−value = 0.86

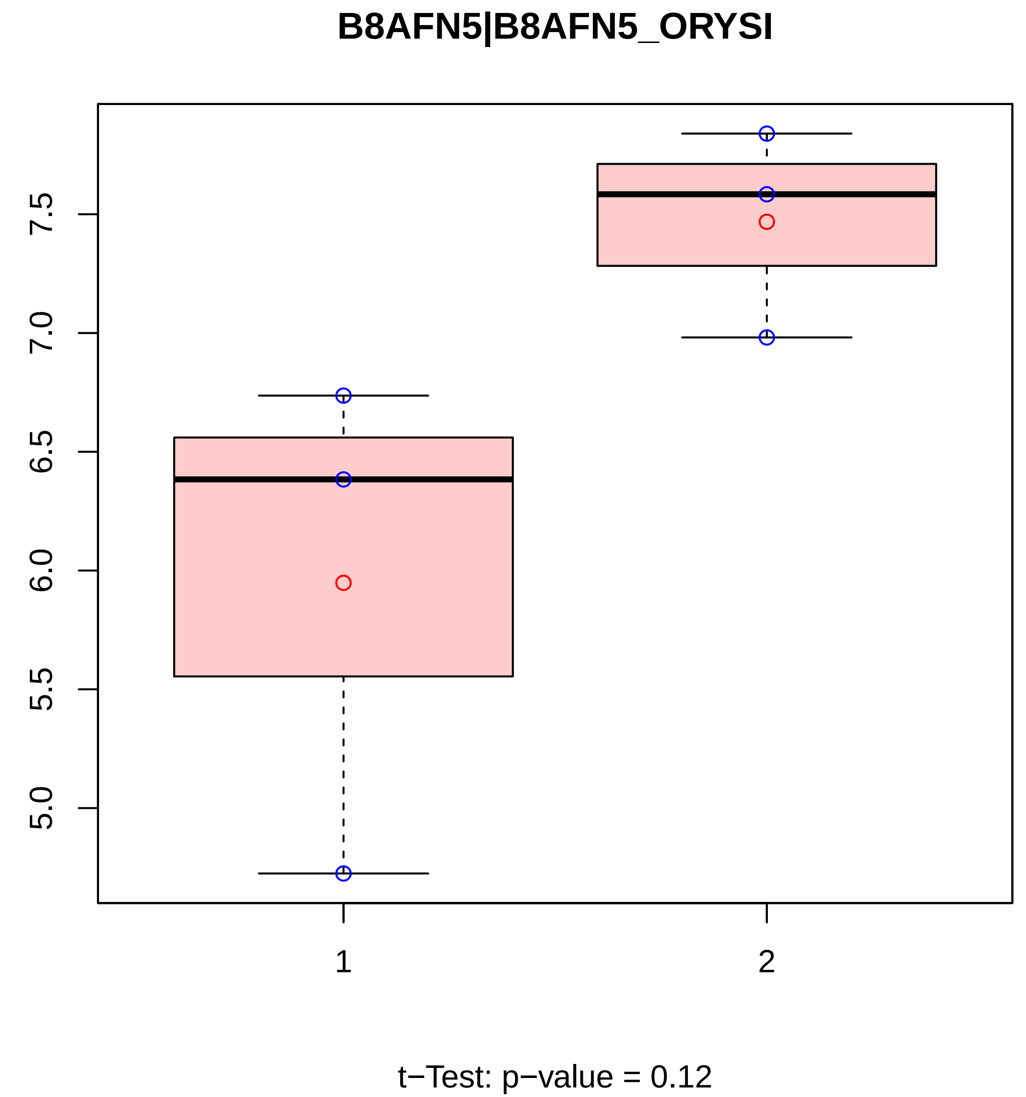
B8AFN5|B8AFN5_ORYSI
5.0
5.5
6.0
6.5
7.0
7.5
1
2
t−Test: p−value = 0.12

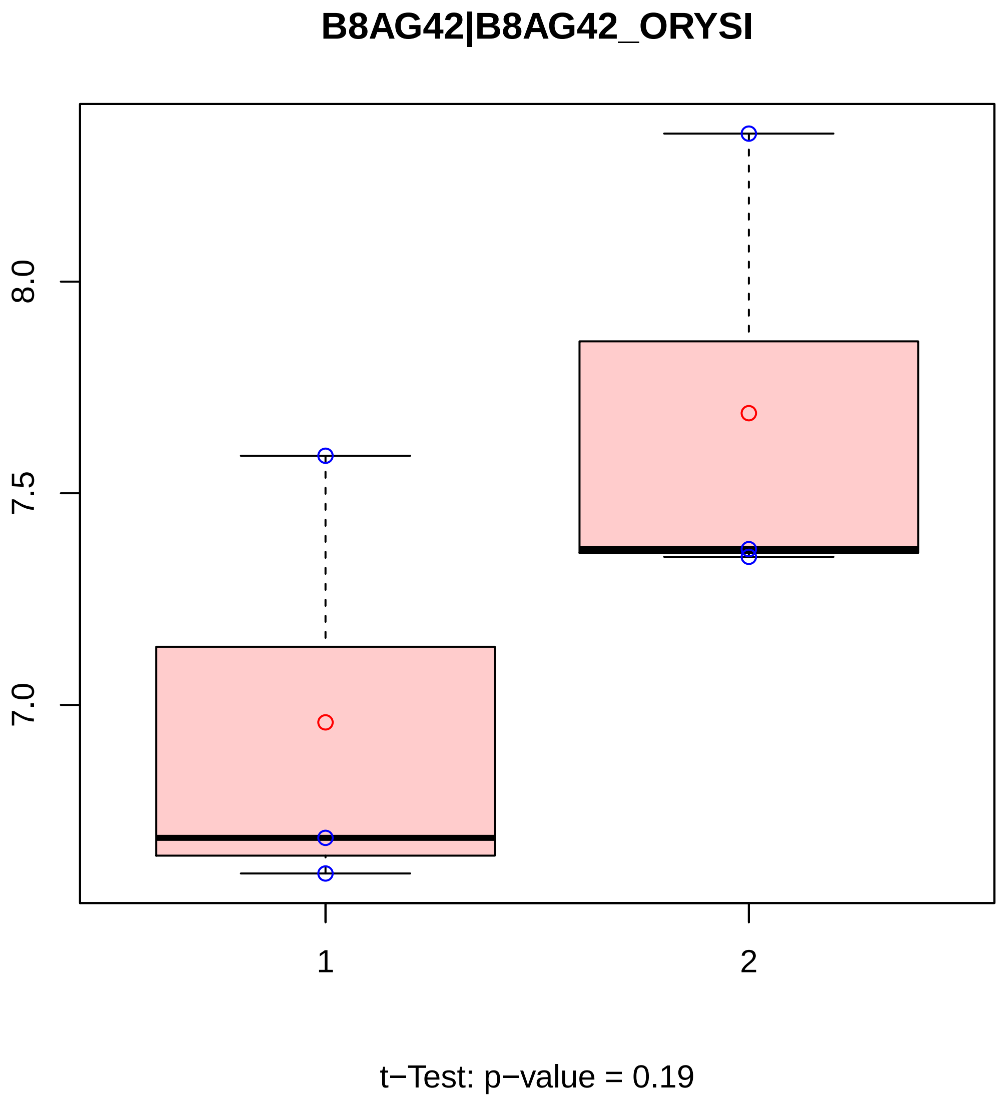

B8AG42|B8AG42_ORYSI
8.0
7.5
7.0
1
2
t−Test: p−value = 0.19

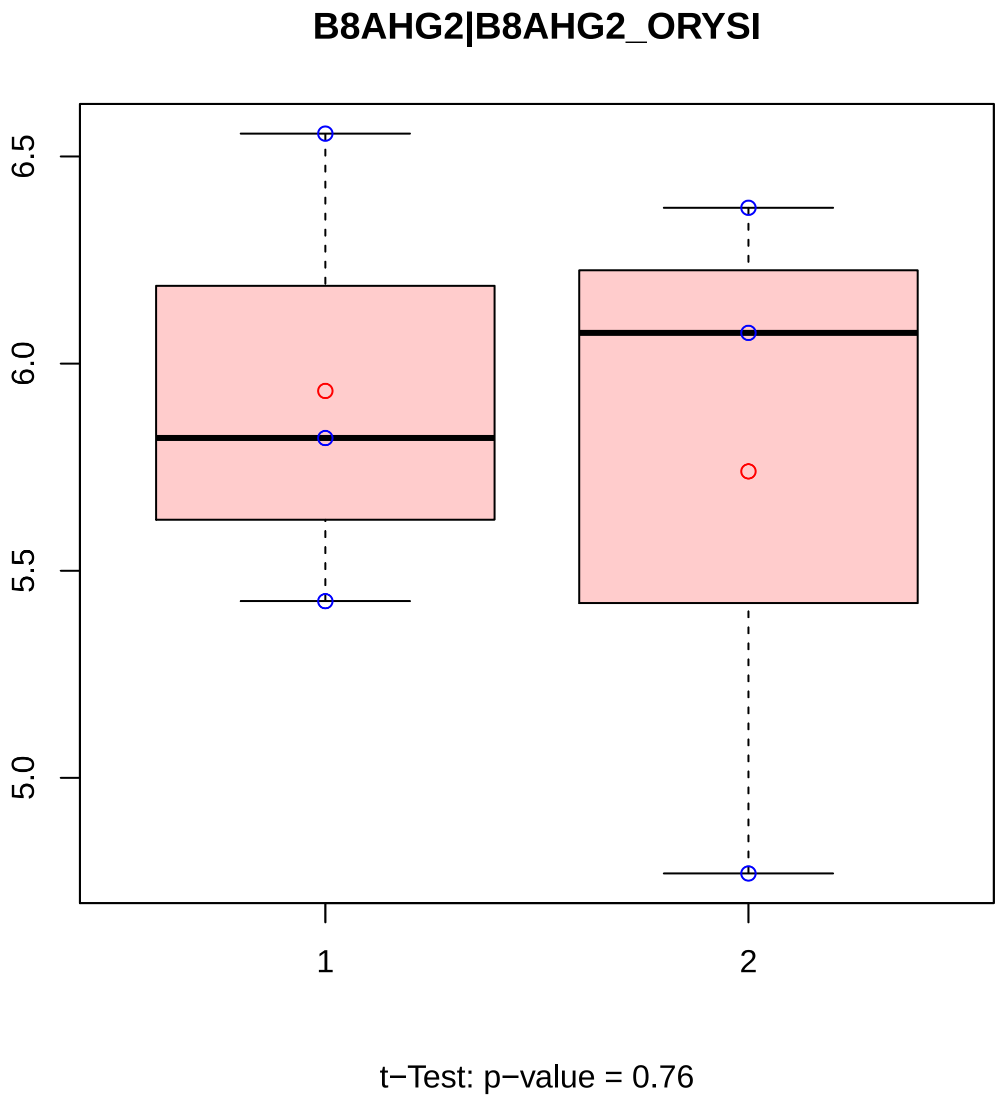
B8AHG2|B8AHG2_ORYSI
6.5
6.0
5.5
5.0
1
2
t−Test: p−value = 0.76

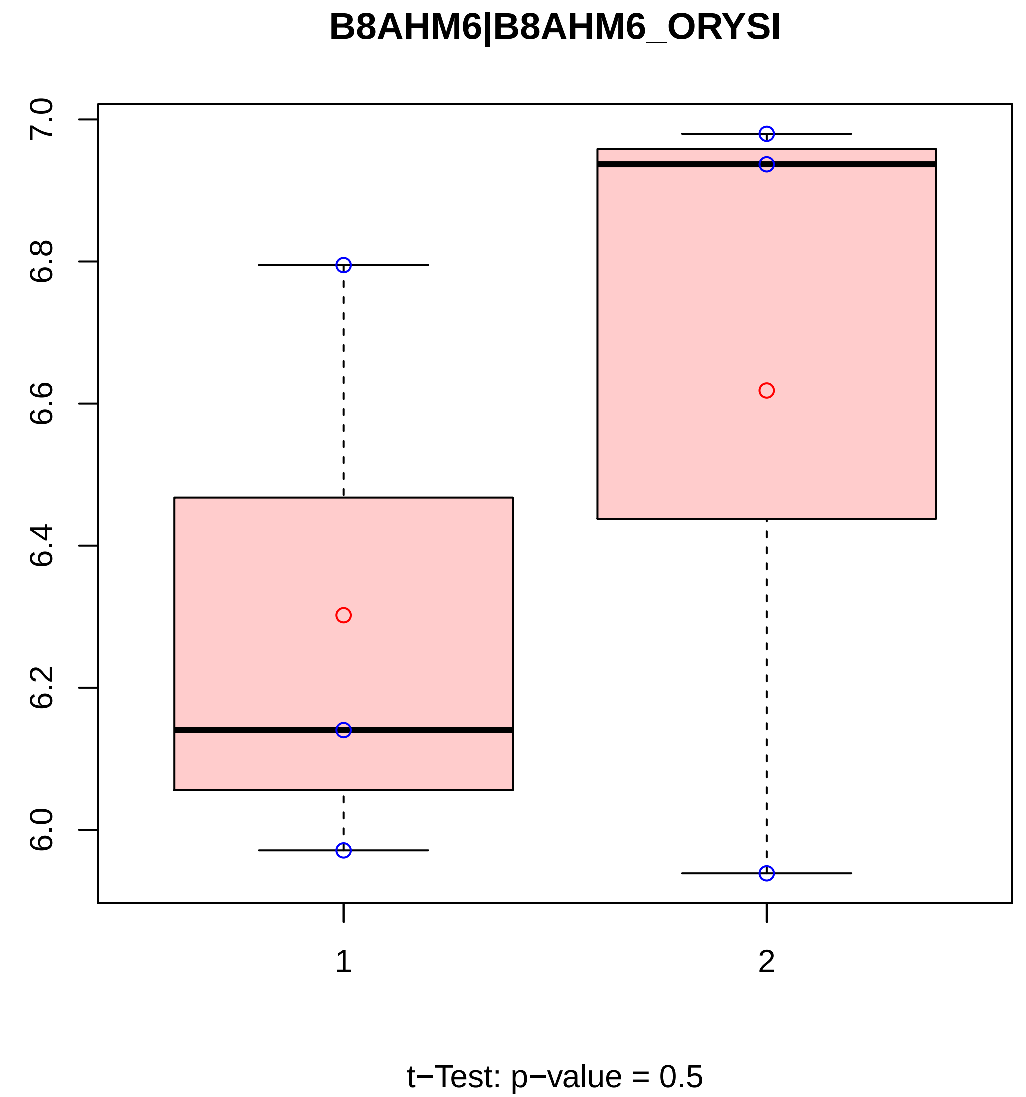
B8AHM6|B8AHM6_ORYSI
7.0
6.8
6.6
6.4
6.2
6.0
1
2
t−Test: p−value = 0.5

B8AII9|B8AII9_ORYSI

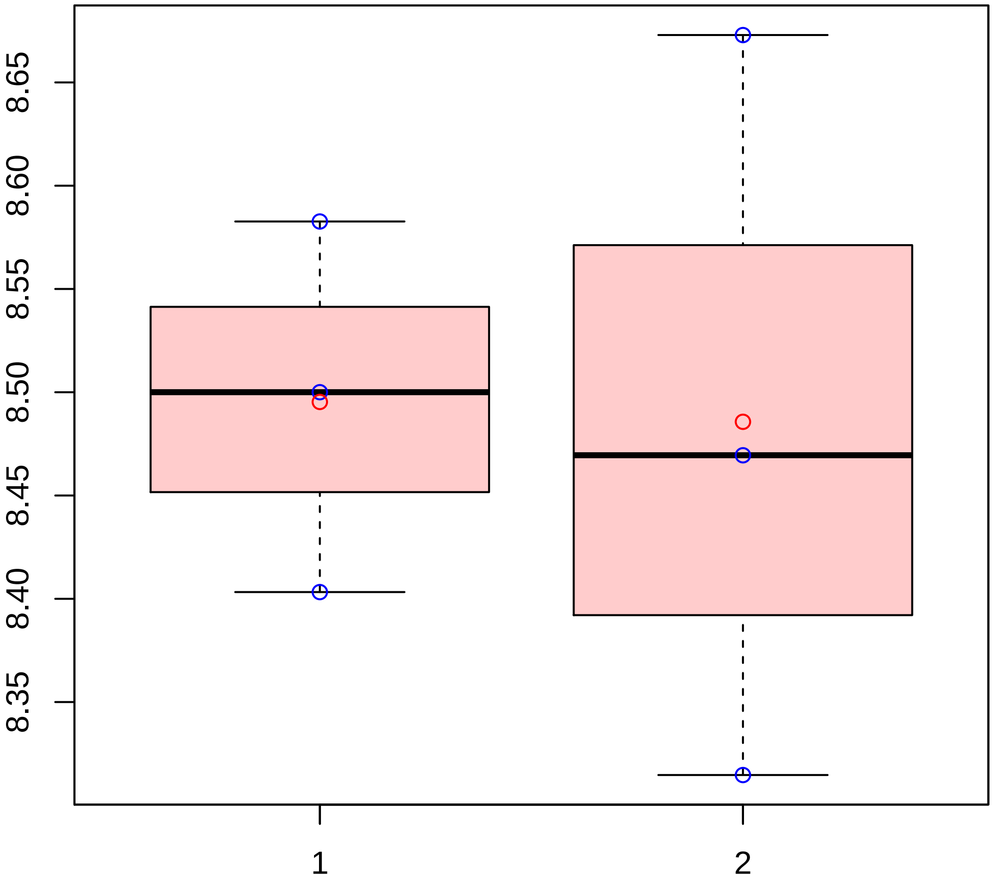

t−Test: p−value = 0.94

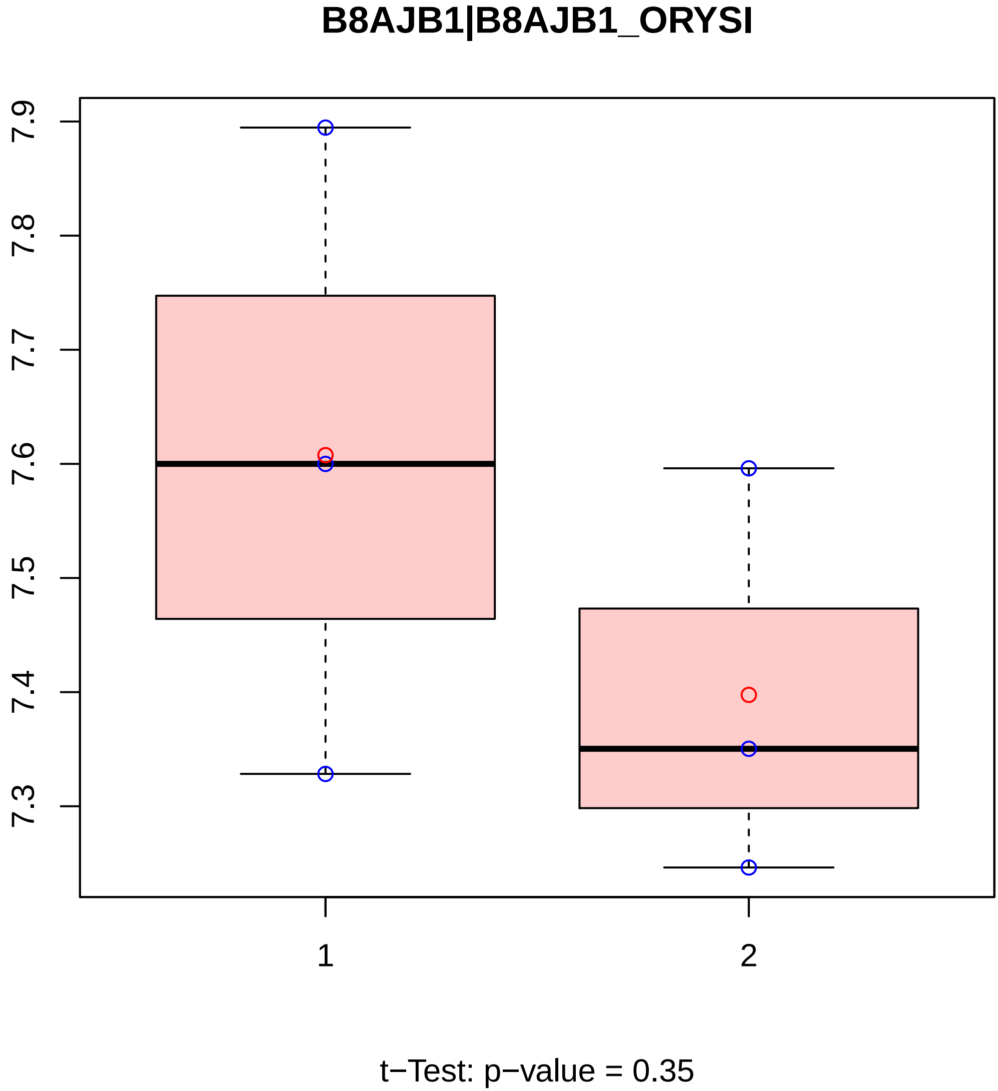
B8AJB1|B8AJB1_ORYSI
7.3
7.4
7.5
7.6
7.7
7.8
7.9
1
2
t−Test: p−value = 0.35

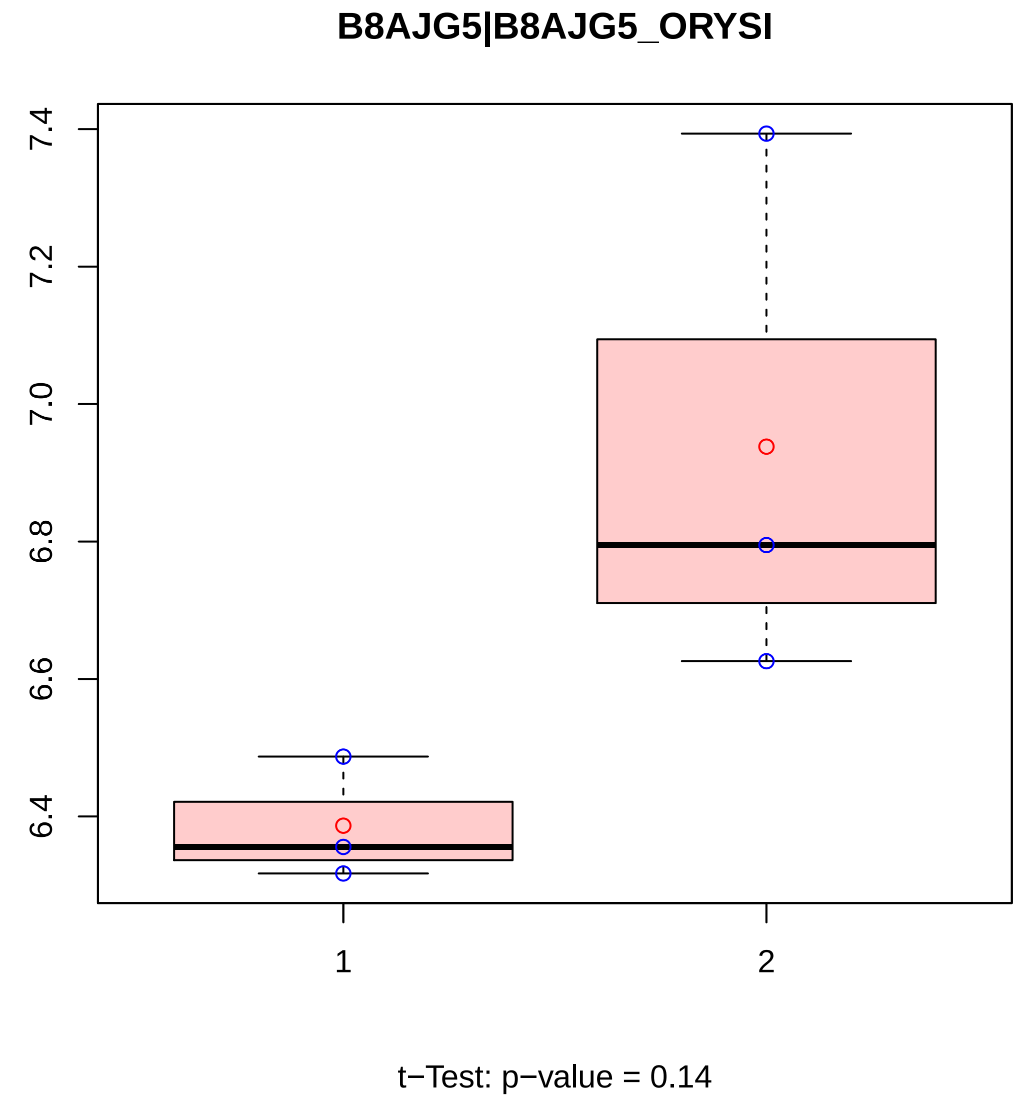
B8AJG5|B8AJG5_ORYSI
7.4
7.2
7.0
6.8
6.6
6.4
1
2
t−Test: p−value = 0.14

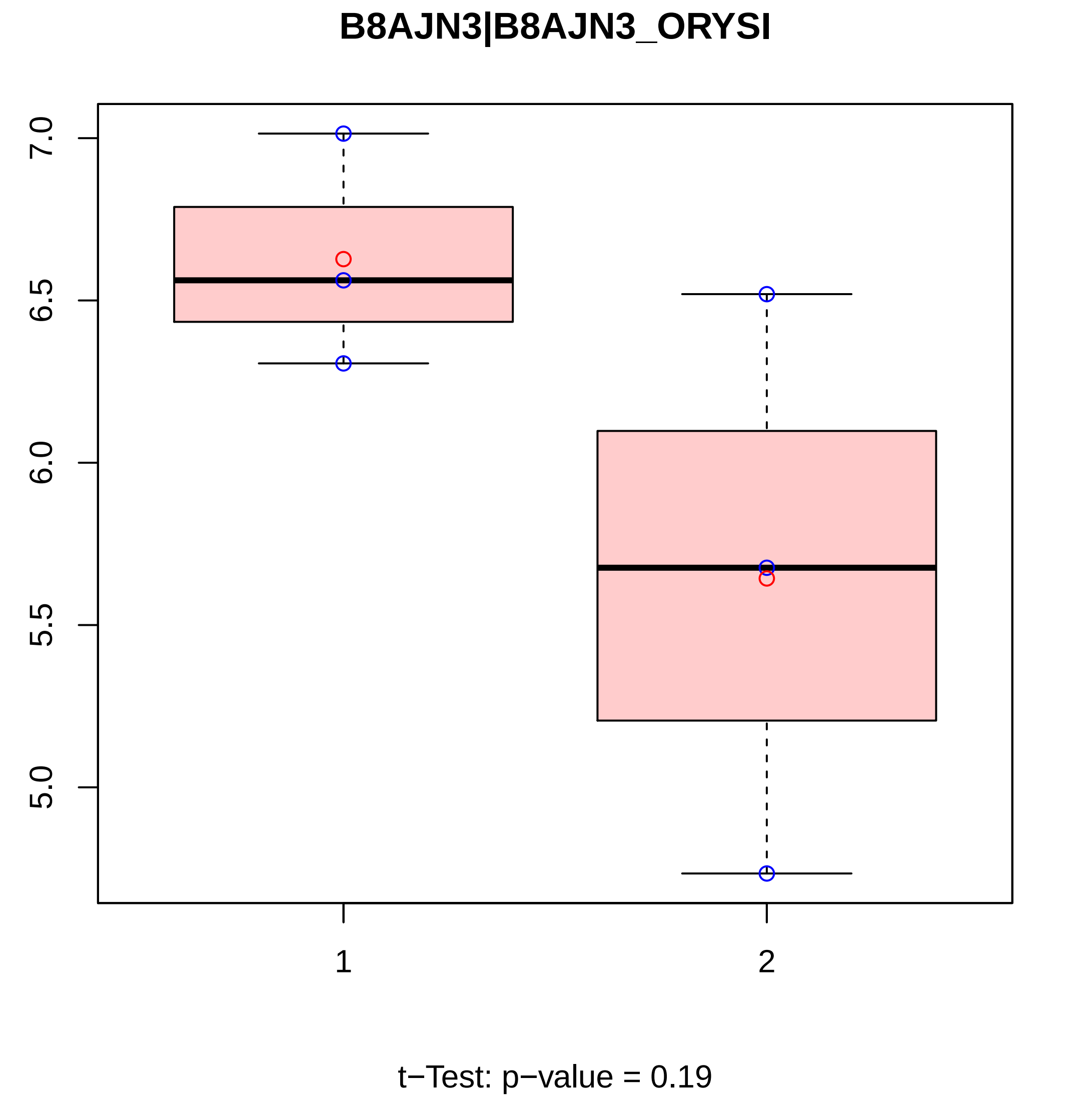
B8AJN3|B8AJN3_ORYSI
7.0
6.5
6.0
5.5
5.0
1
2
t−Test: p−value = 0.19

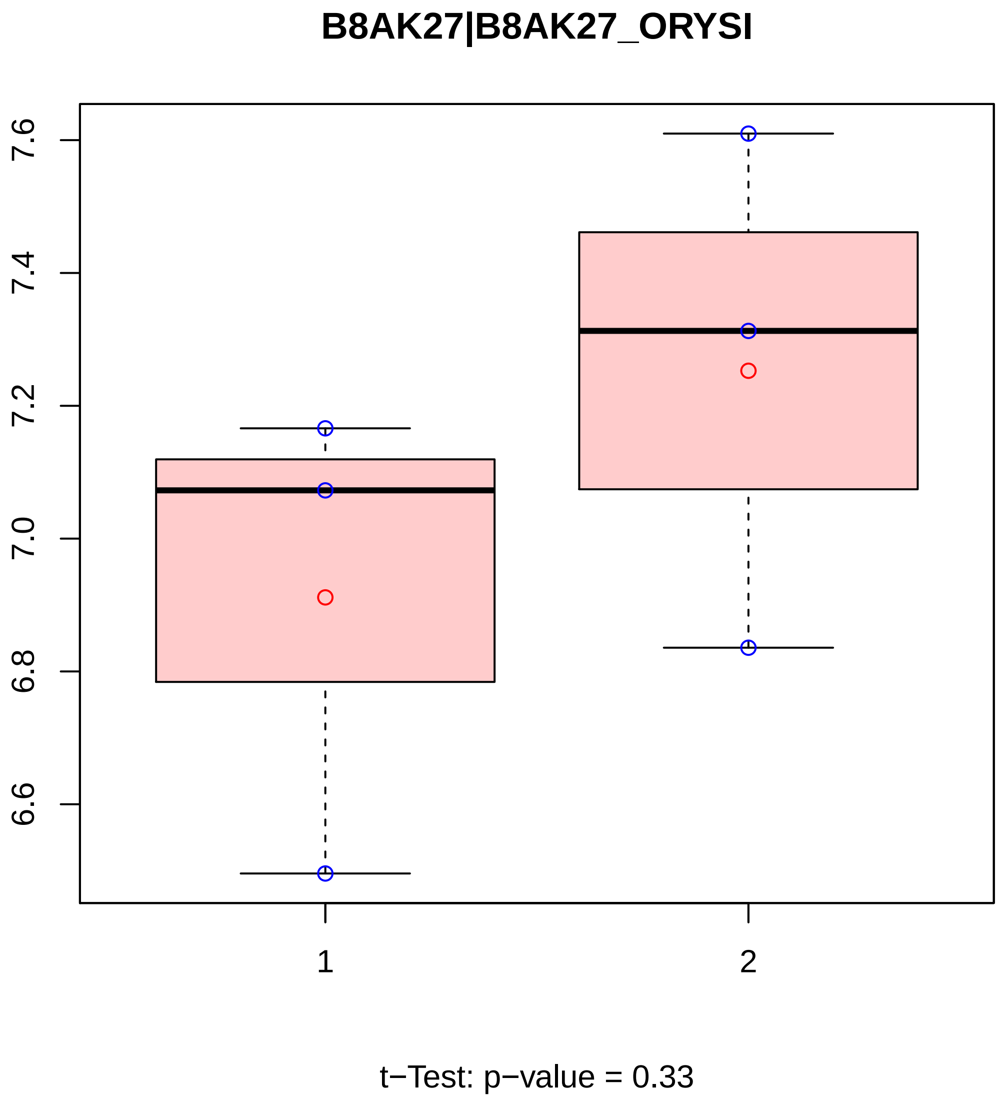
B8AK27|B8AK27_ORYSI
7.6
7.4
7.2
7.0
6.8
6.6
1
2
t−Test: p−value = 0.33

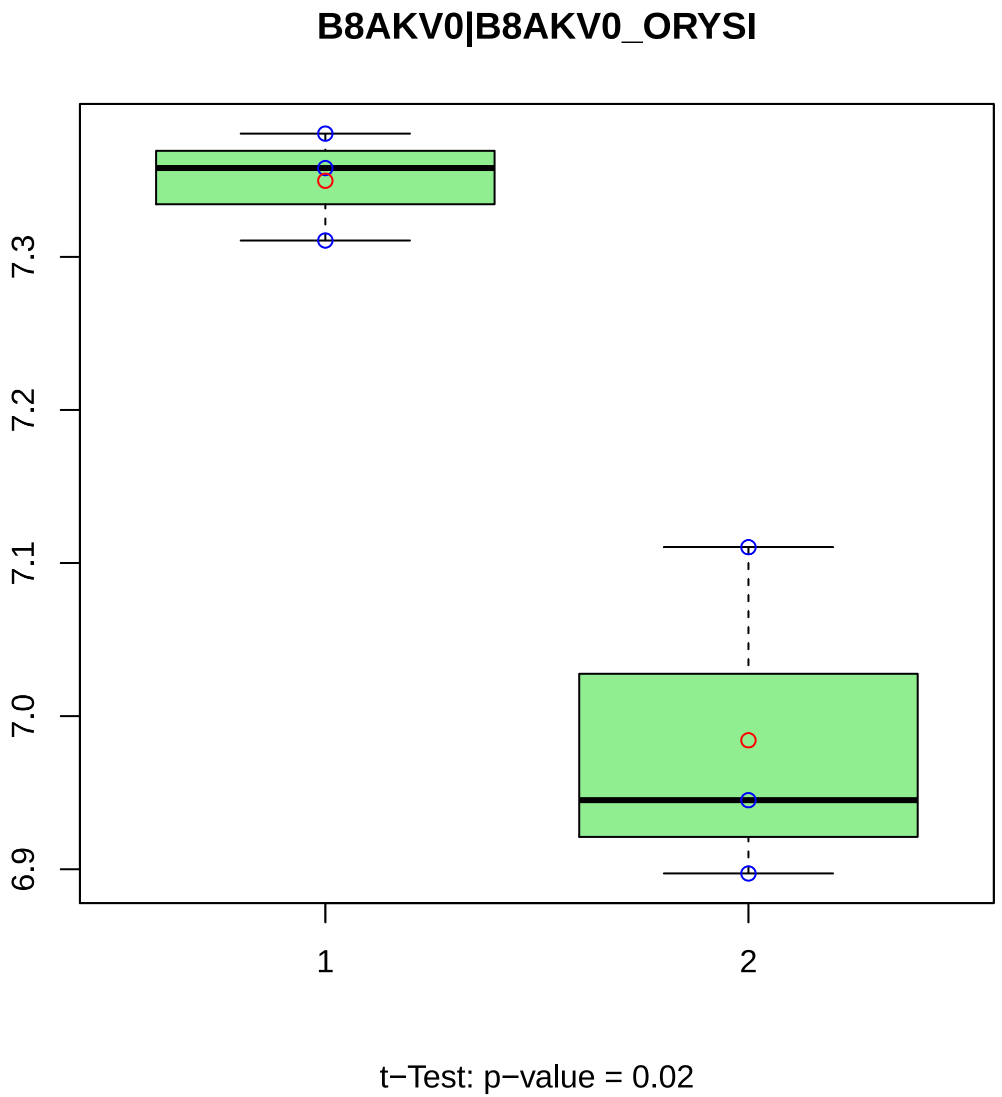

B8AKV0|B8AKV0_ORYSI
7.3
7.2
7.1
7.0
6.9
1
2
t−Test: p−value = 0.02

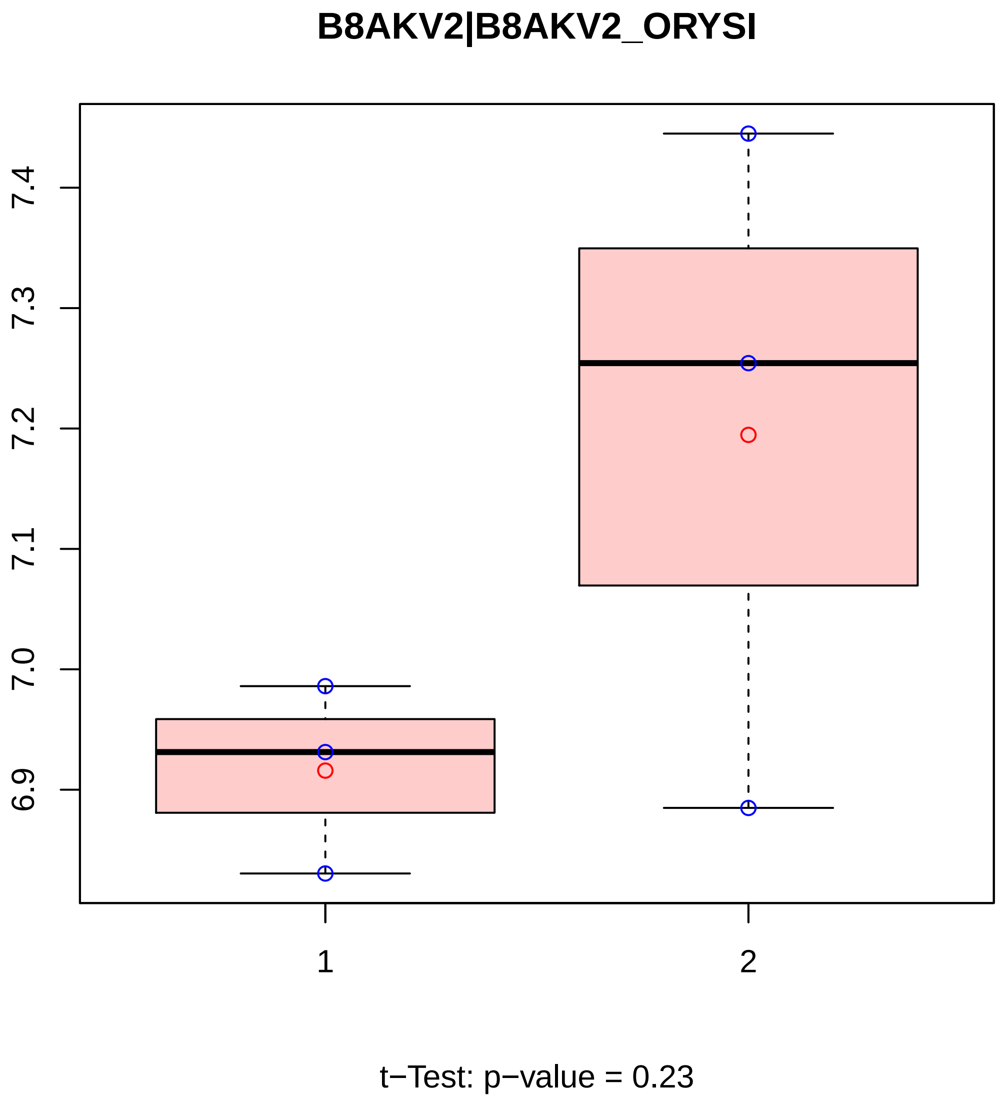
B8AKV2|B8AKV2_ORYSI
7.4
7.3
7.2
7.1
7.0
6.9
1
2
t−Test: p−value = 0.23

B8AKX5|B8AKX5_ORYSI

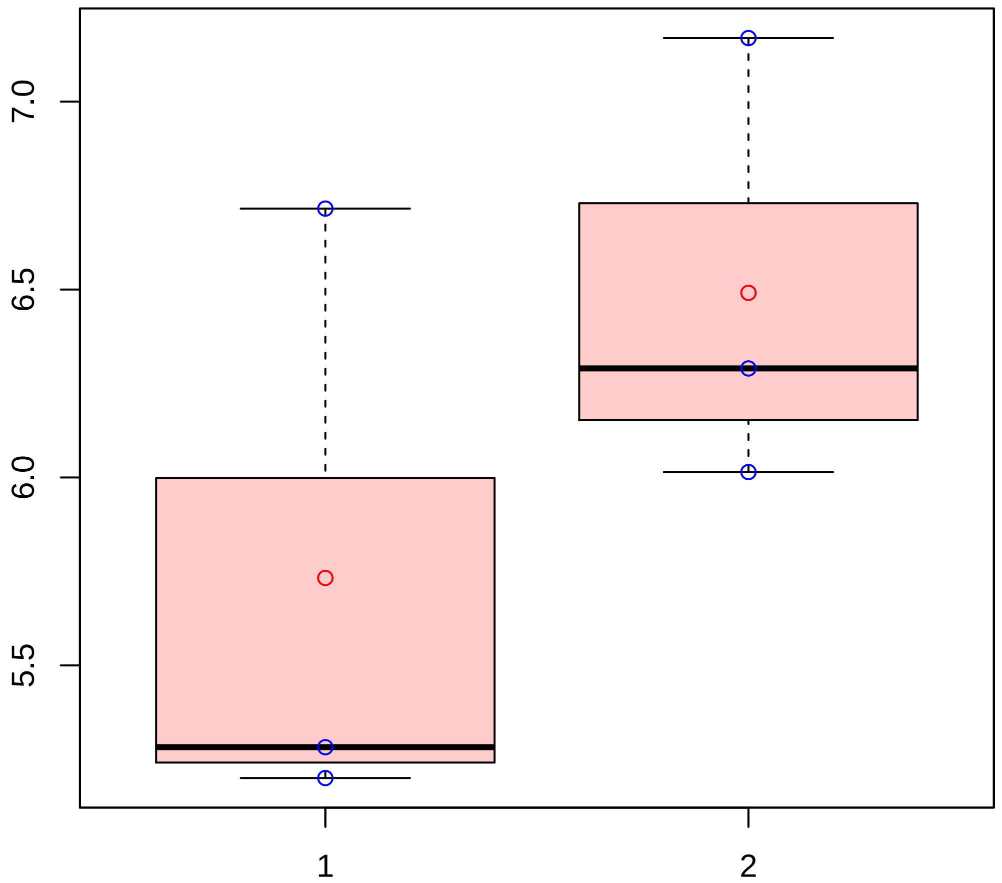

t−Test: p−value = 0.28

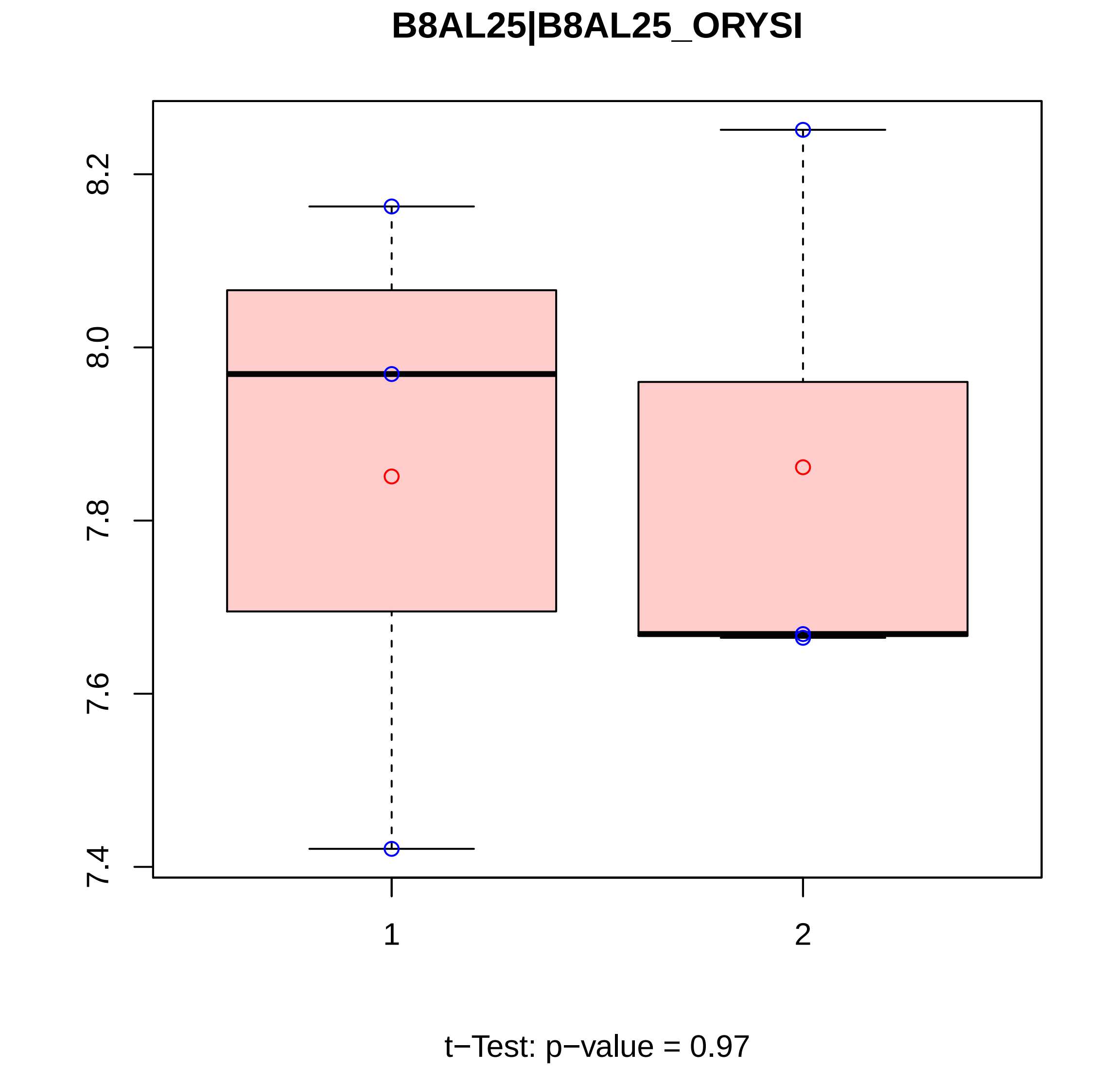
B8AL25|B8AL25_ORYSI
8.2
8.0
7.8
7.6
7.4
1
2
t−Test: p−value = 0.97

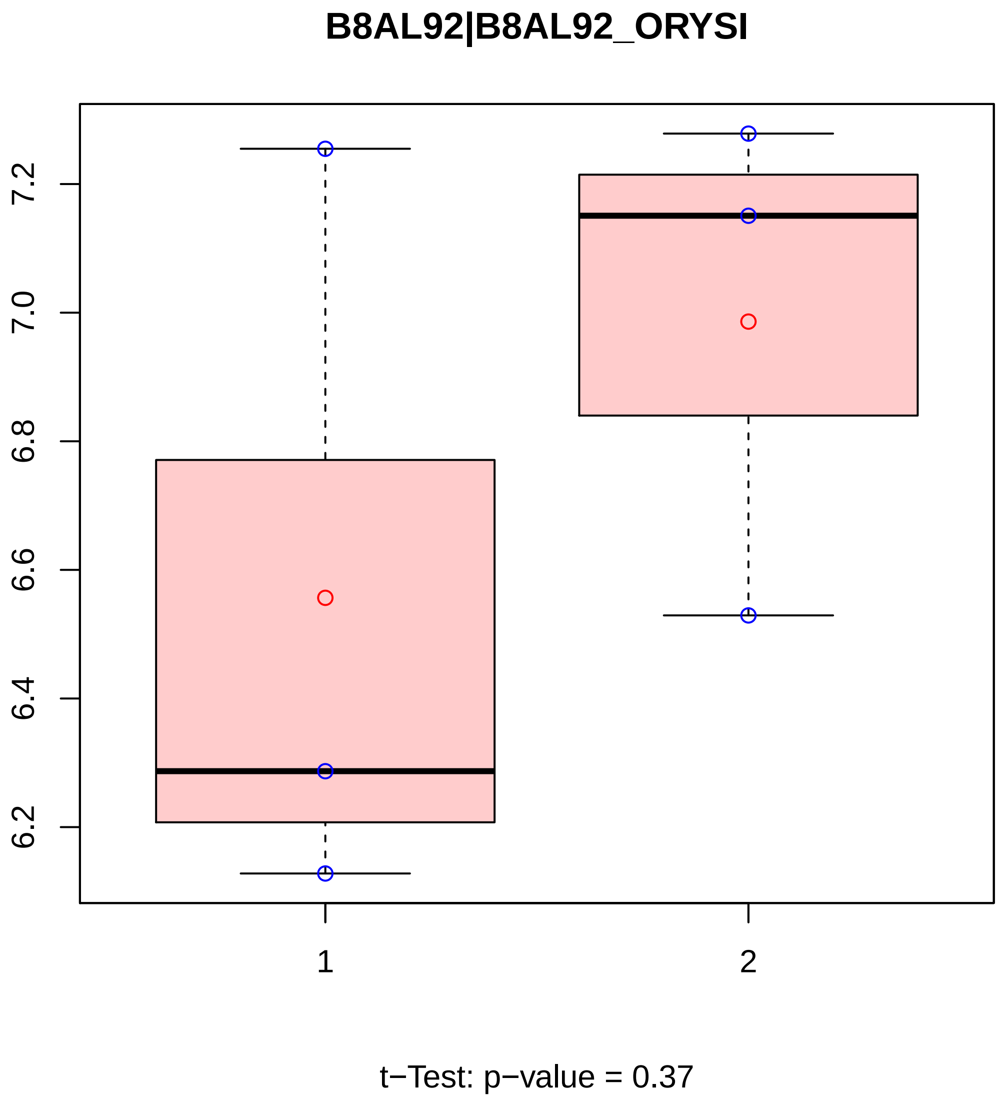

B8AL92|B8AL92_ORYSI
7.2
7.0
6.8
6.6
6.4
6.2
1
2
t−Test: p−value = 0.37

B8ALH3|B8ALH3_ORYSI

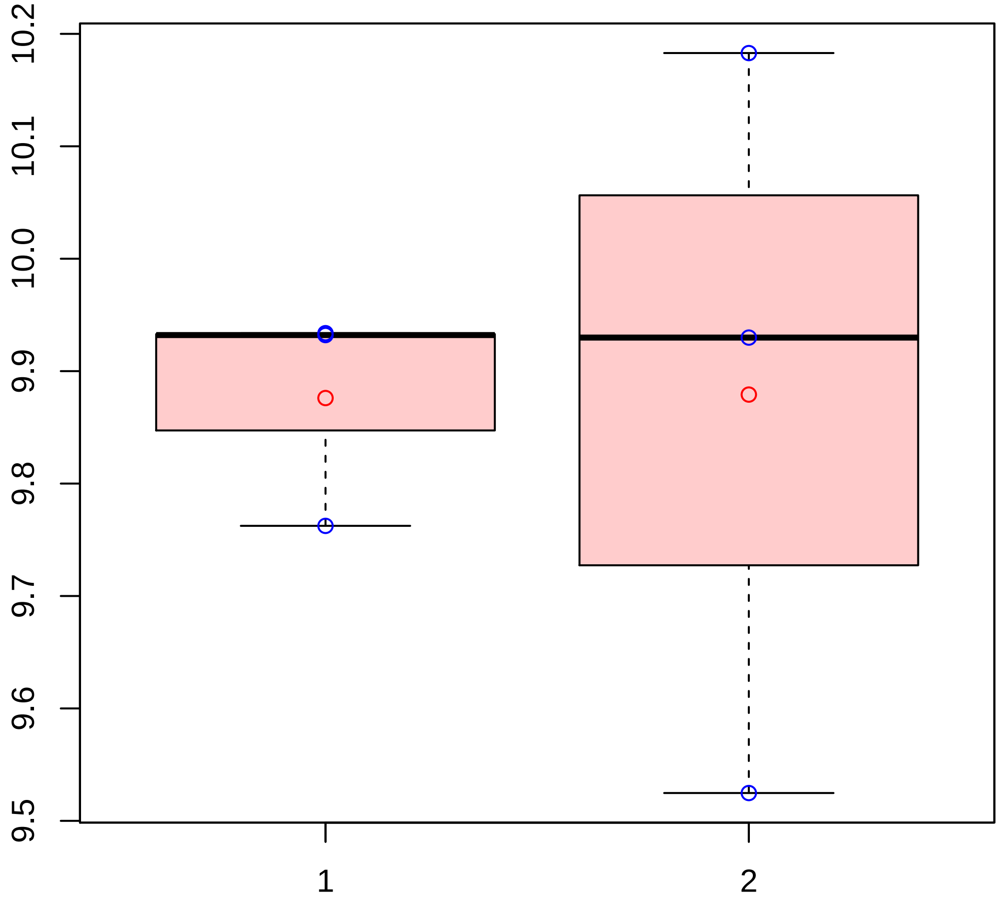

t−Test: p−value = 0.99

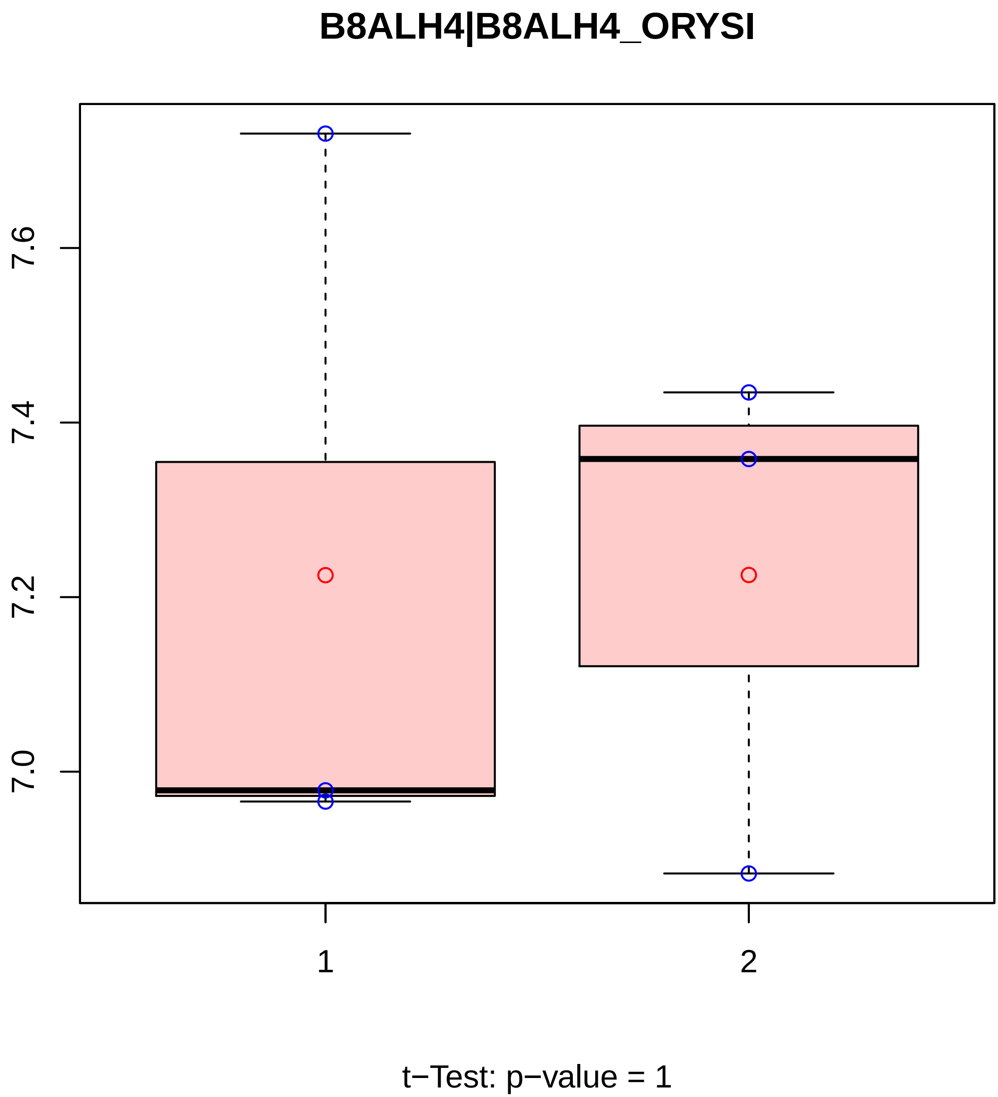

B8ALH4|B8ALH4_ORYSI
7.0
7.2
7.4
7.6
1
2
t−Test: p−value = 1

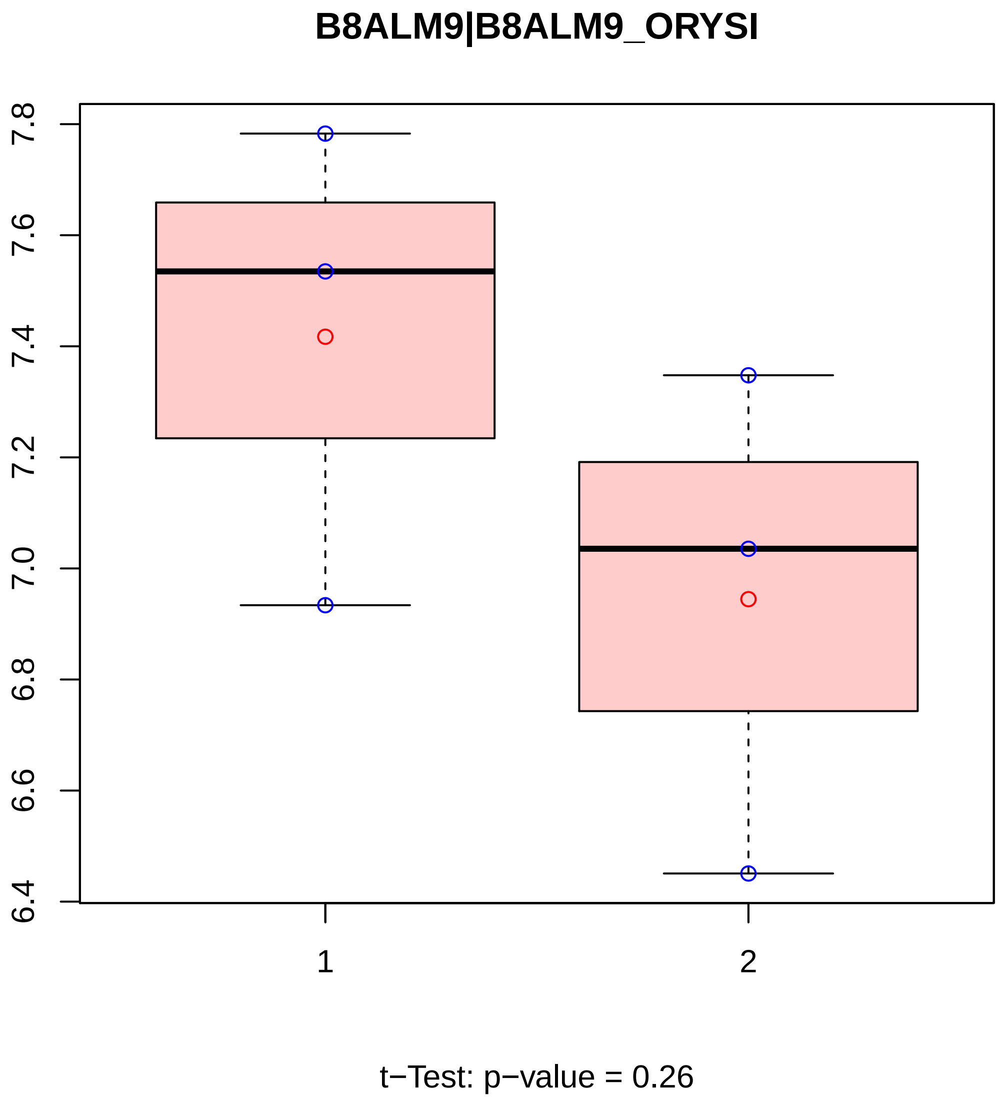

B8ALM9|B8ALM9_ORYSI
7.8
7.6
7.4
7.2
7.0
6.8
6.6
6.4
1
2
t−Test: p−value = 0.26

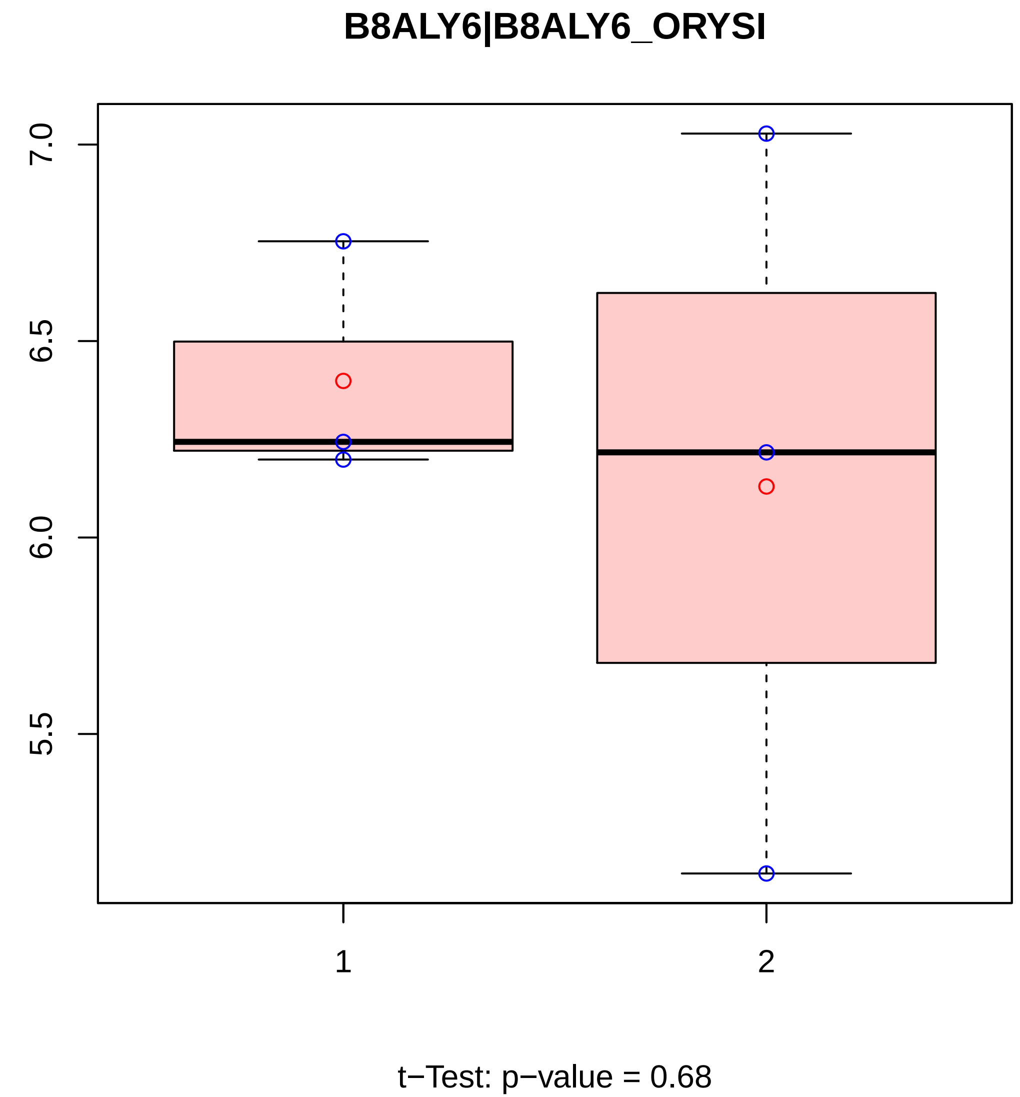
B8ALY6|B8ALY6_ORYSI
7.0
6.5
6.0
5.5
1
2
t−Test: p−value = 0.68

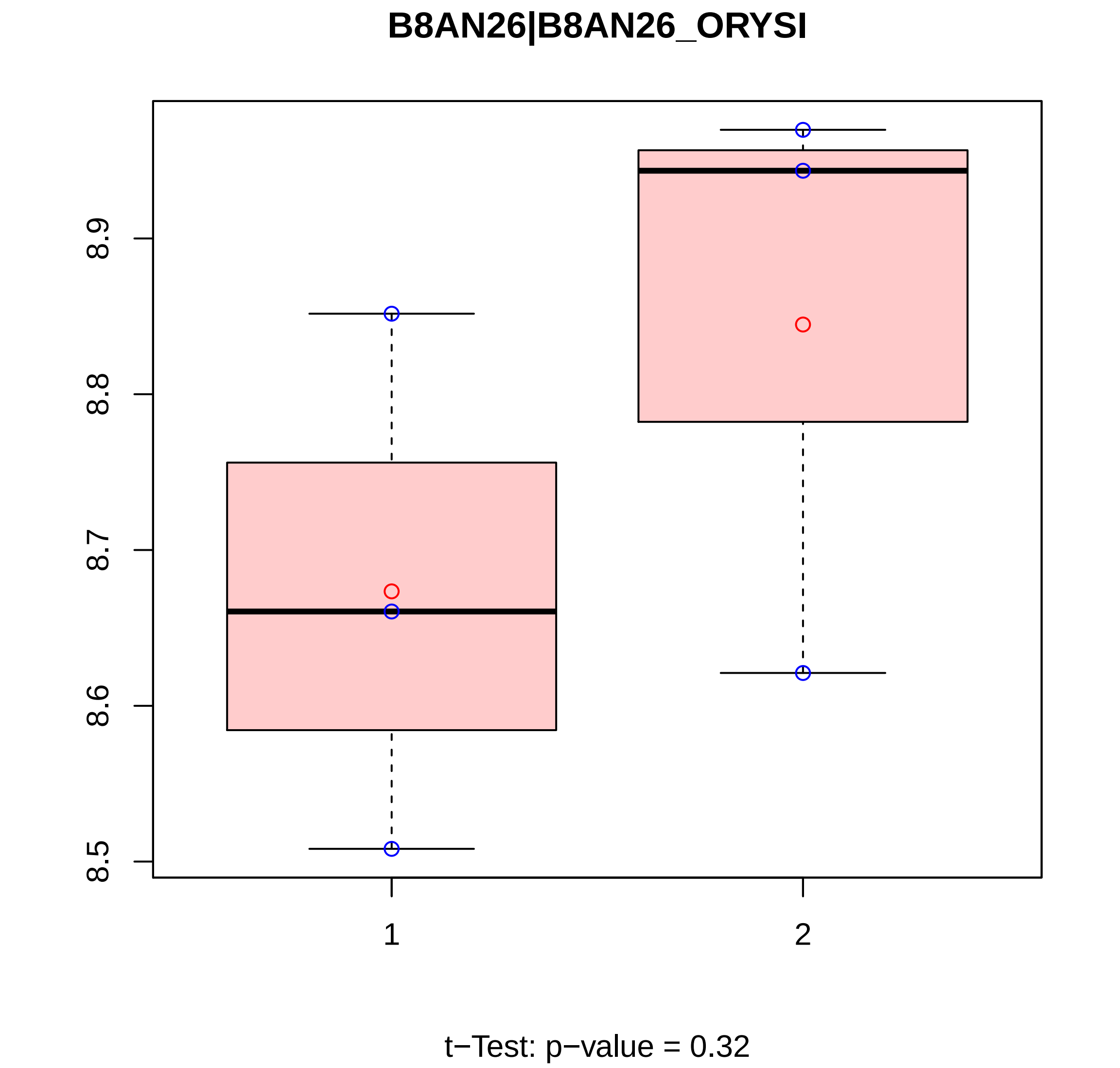

B8AN26|B8AN26_ORYSI
8.5
8.6
8.7
8.8
8.9
1
2
t−Test: p−value = 0.32

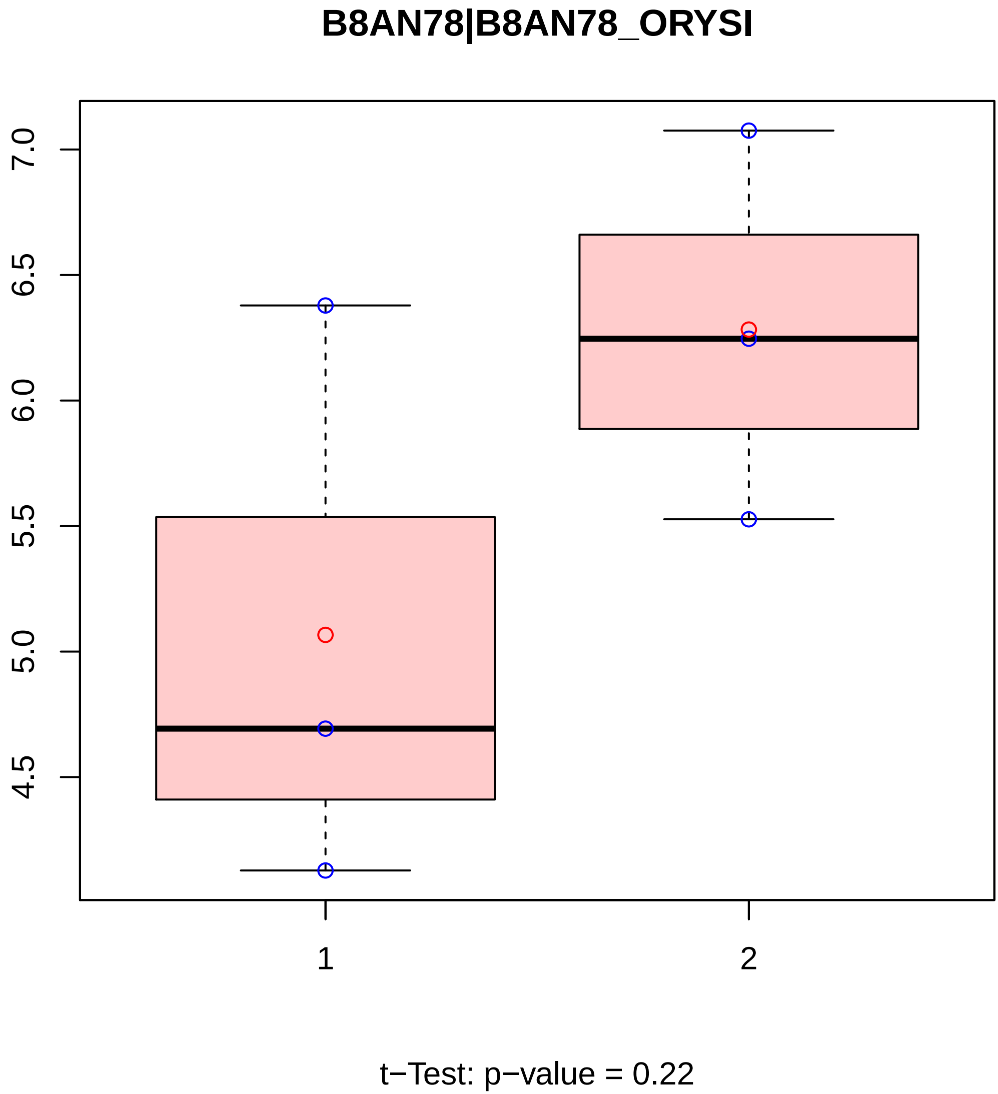
B8AN78|B8AN78_ORYSI
7.0
6.5
6.0
5.5
5.0
4.5
1
2
t−Test: p−value = 0.22

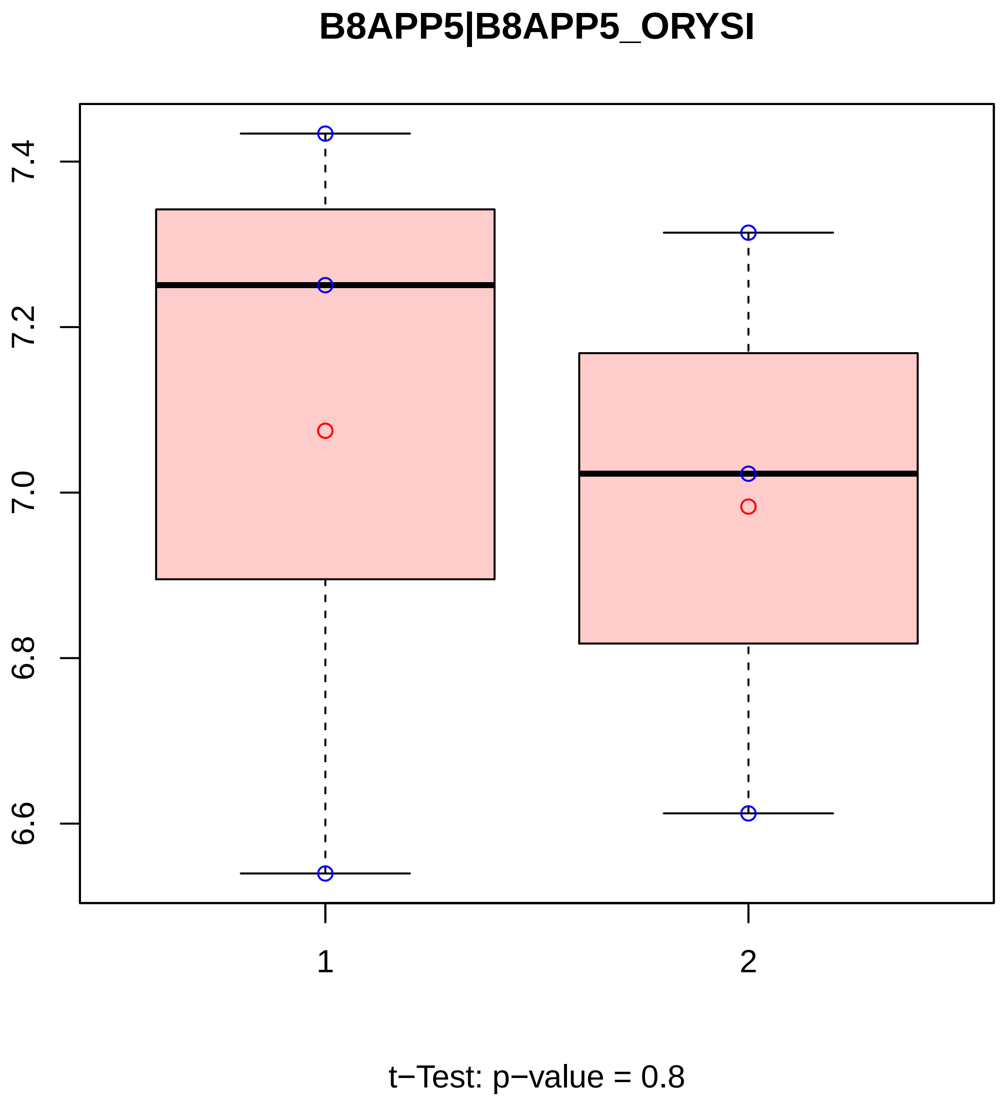

B8APP5|B8APP5_ORYSI
7.4
7.2
7.0
6.8
6.6
1
2
t−Test: p−value = 0.8

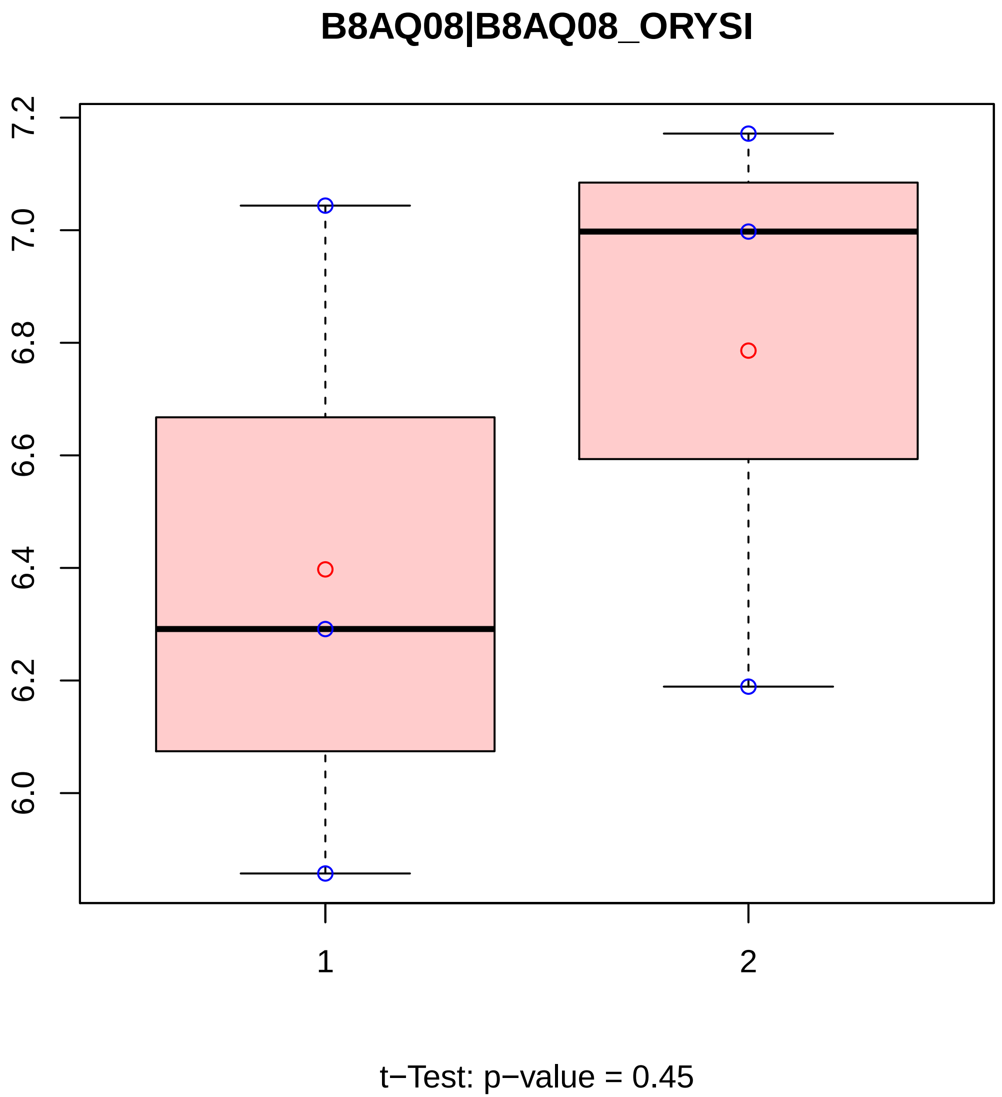

B8AQ08|B8AQ08_ORYSI
6.0
6.2
6.4
6.6
6.8
7.0
7.2
1
2
t−Test: p−value = 0.45

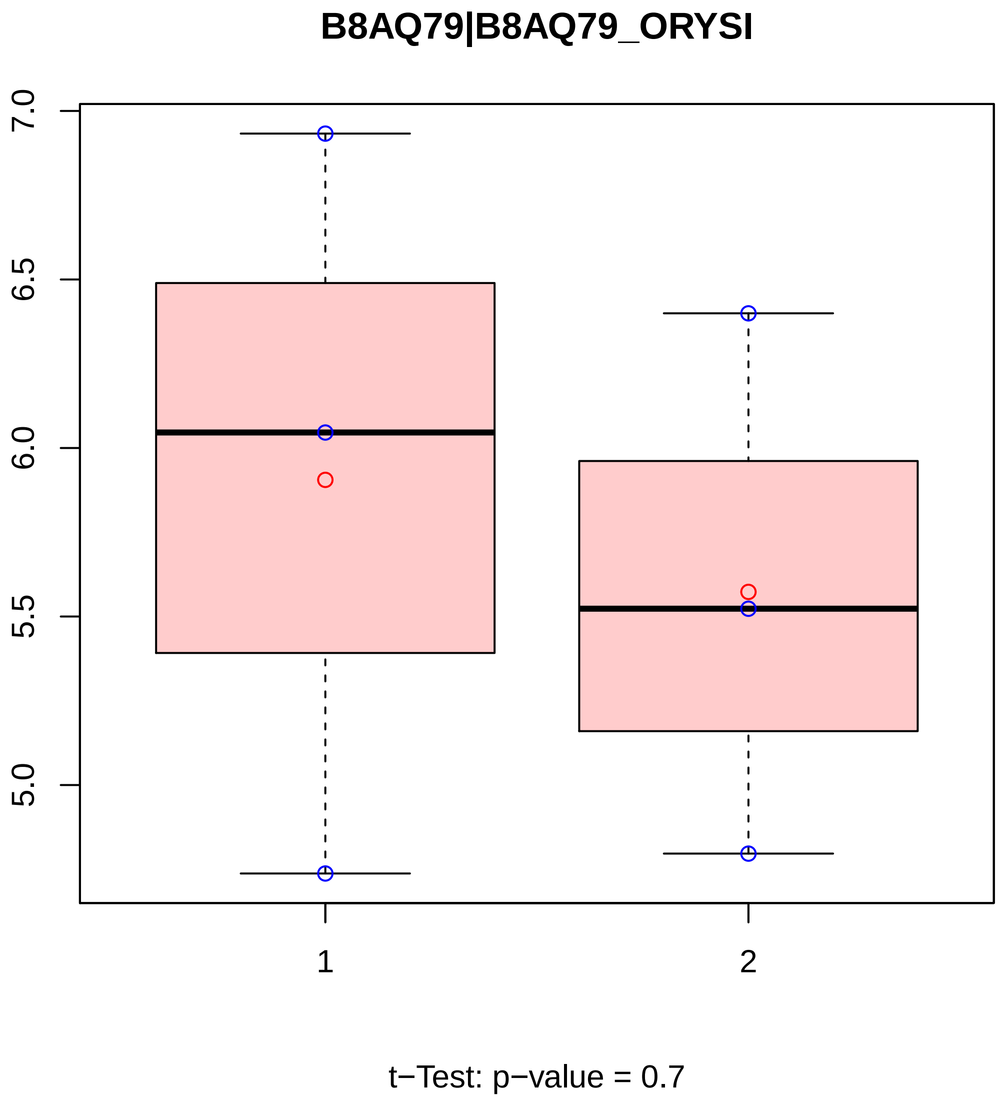
B8AQ79|B8AQ79_ORYSI
7.0
6.5
6.0
5.5
5.0
1
2
t−Test: p−value = 0.7

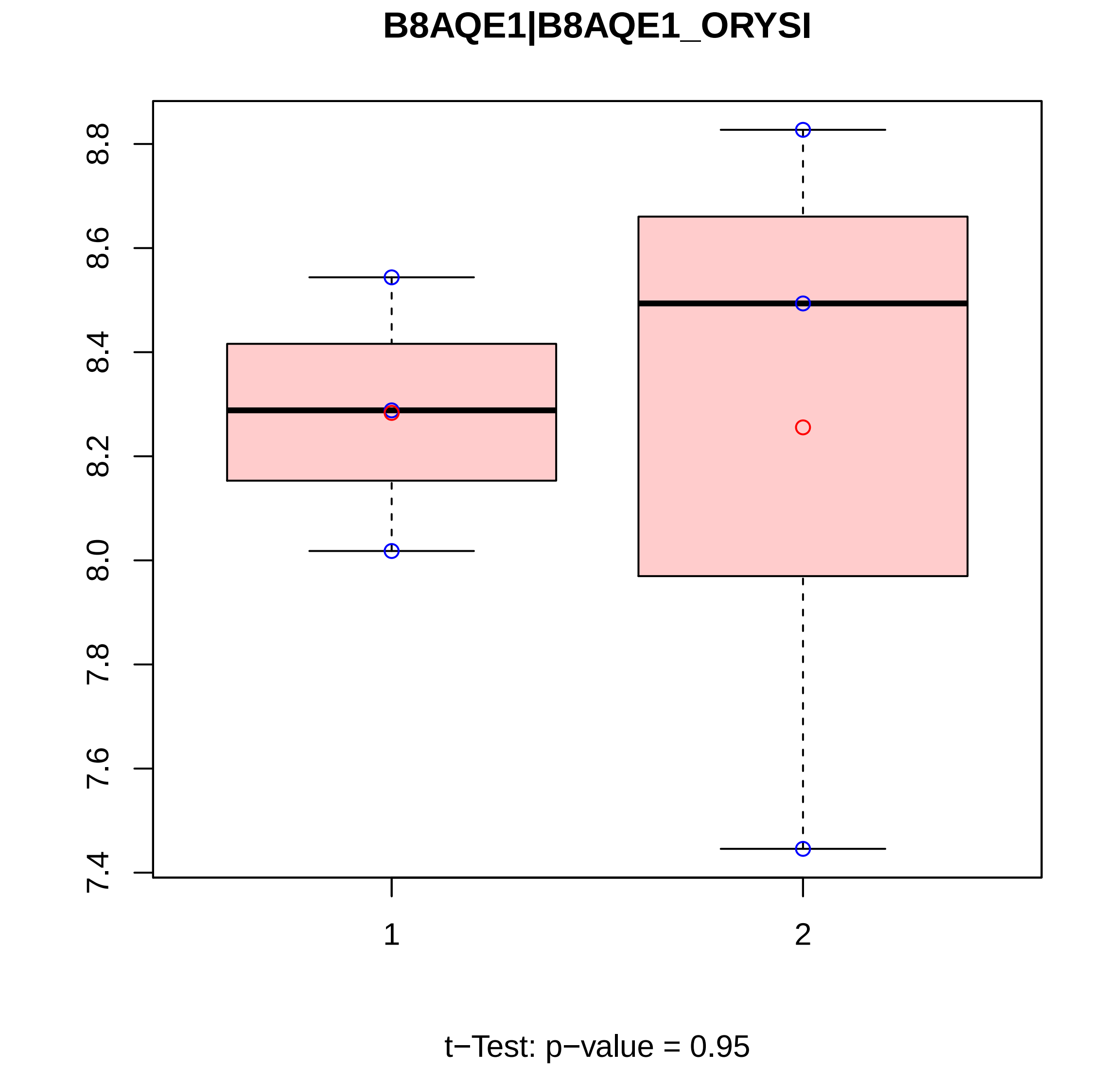

B8AQE1|B8AQE1_ORYSI
8.8
8.6
8.4
8.2
8.0
7.8
7.6
7.4
1
2
t−Test: p−value = 0.95

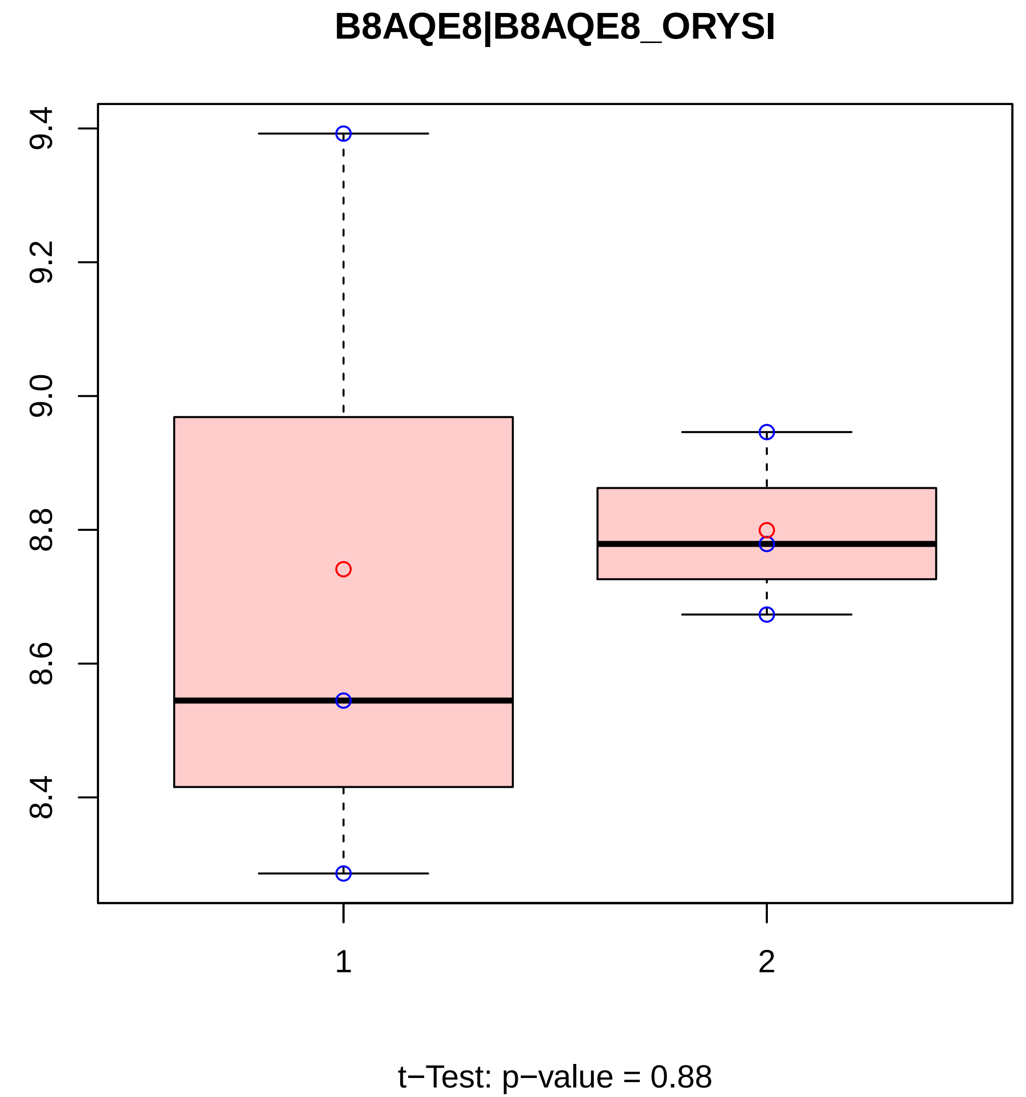
B8AQE8|B8AQE8_ORYSI
9.4
9.2
9.0
8.8
8.6
8.4
1
2
t−Test: p−value = 0.88

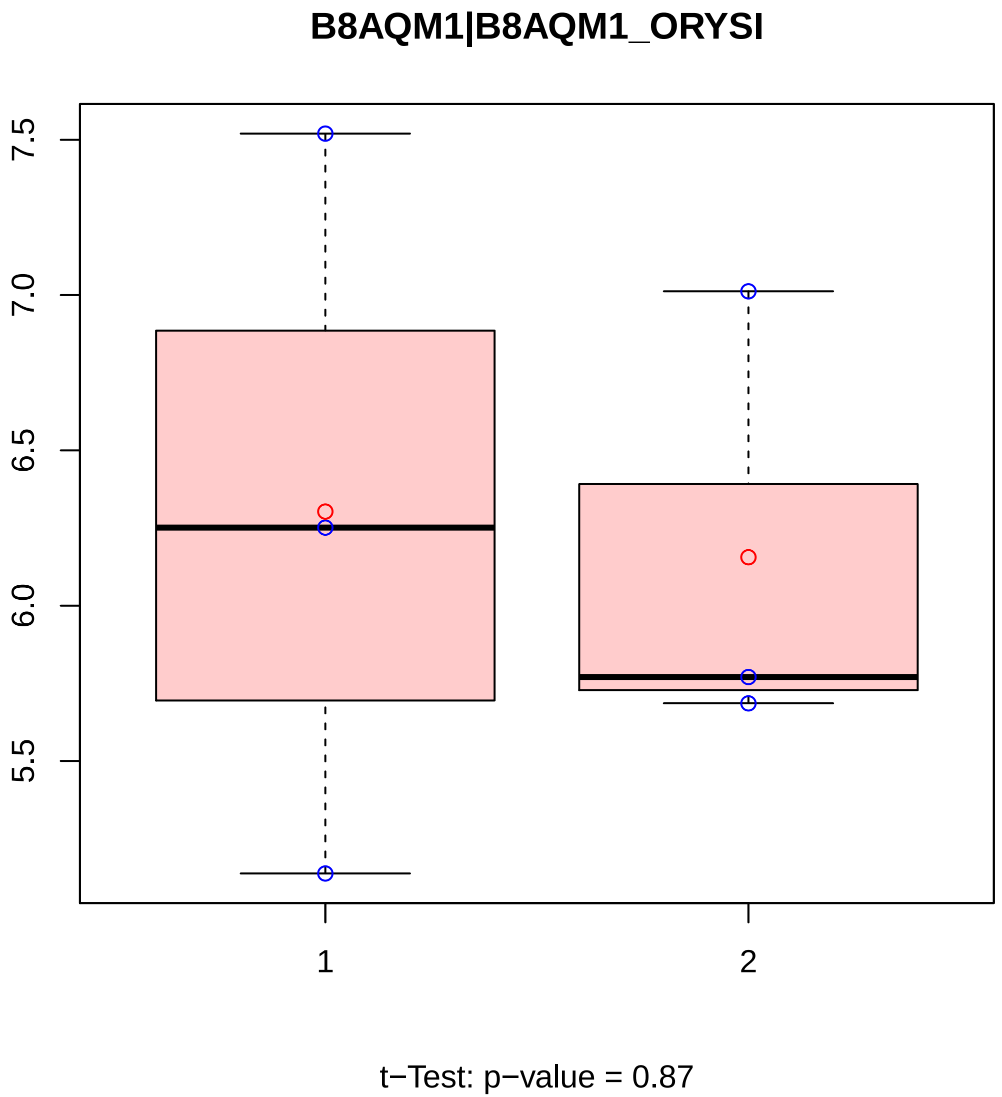

B8AQM1|B8AQM1_ORYSI
7.5
7.0
6.5
6.0
5.5
1
2
t−Test: p−value = 0.87

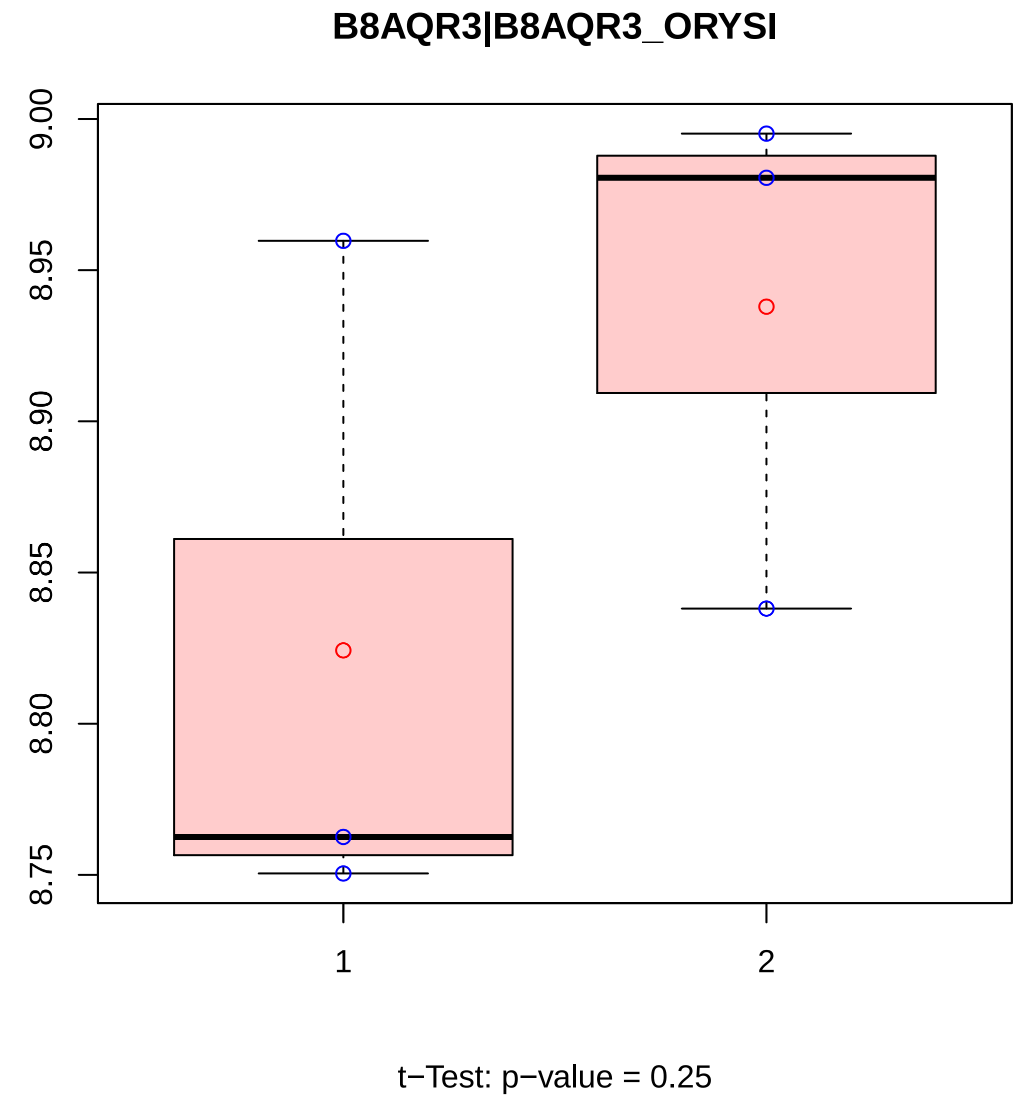
B8AQR3|B8AQR3_ORYSI
9.00
8.95
8.90
8.85
8.80
8.75
1
2
t−Test: p−value = 0.25

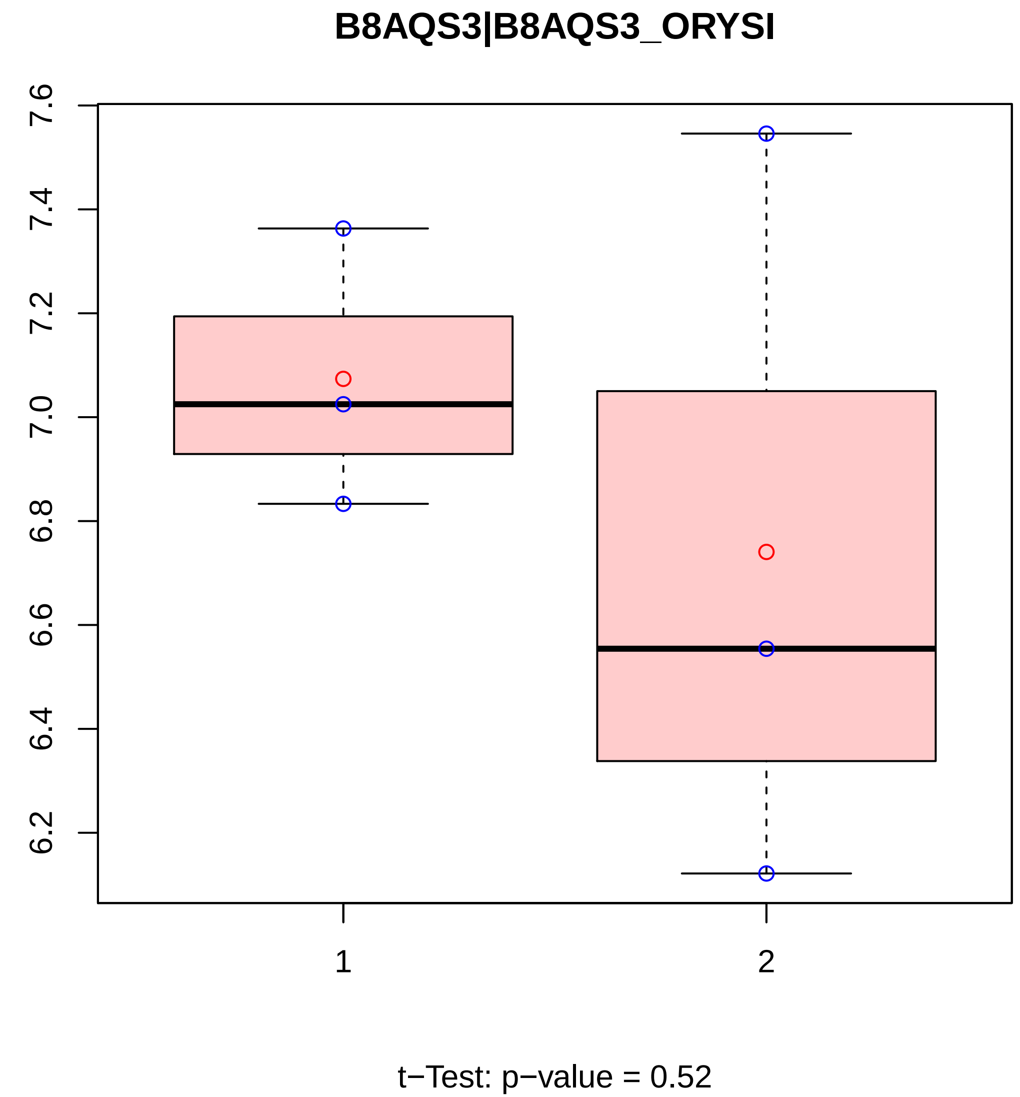
B8AQS3|B8AQS3_ORYSI
7.6
7.4
7.2
7.0
6.8
6.6
6.4
6.2
1
2
t−Test: p−value = 0.52

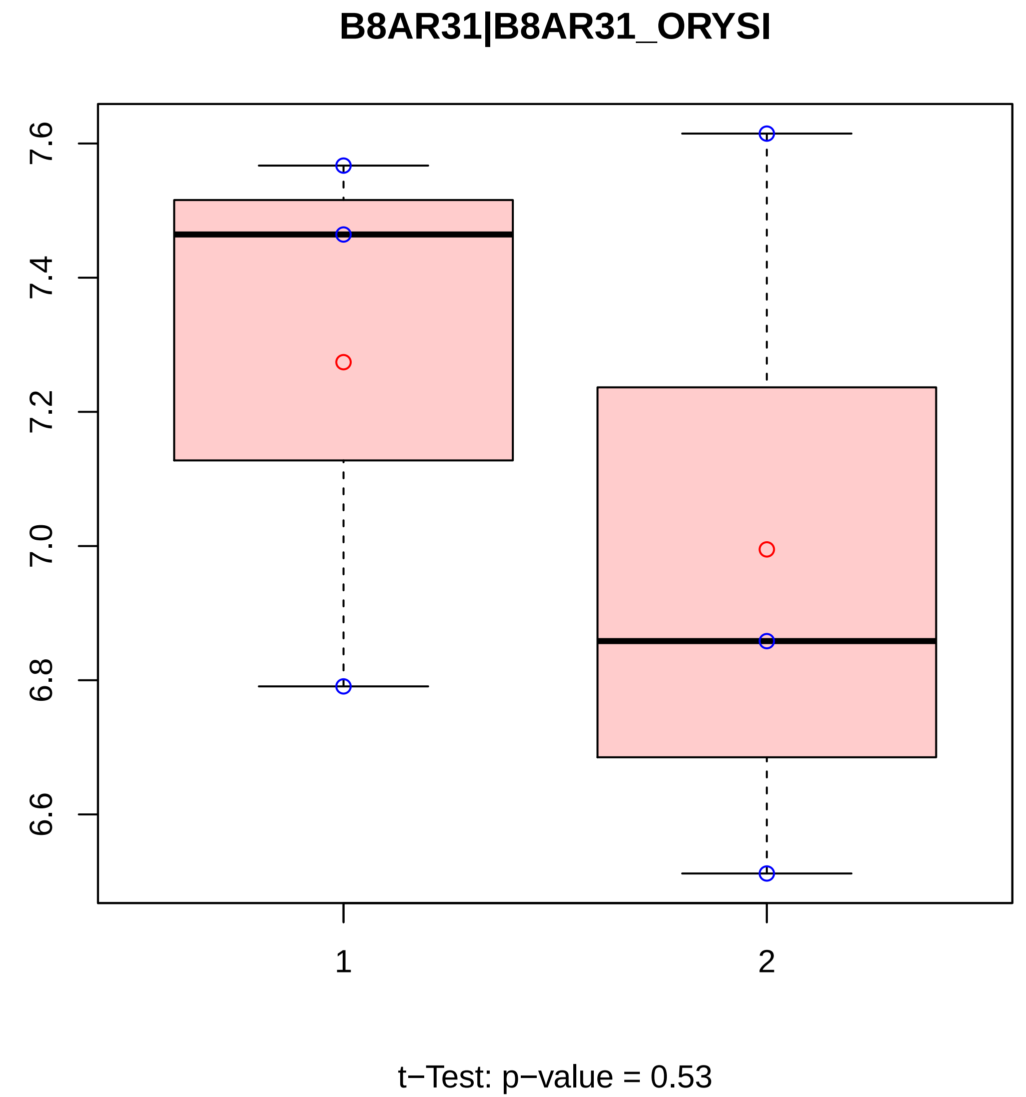
B8AR31|B8AR31_ORYSI
7.6
7.4
7.2
7.0
6.8
6.6
1
2
t−Test: p−value = 0.53

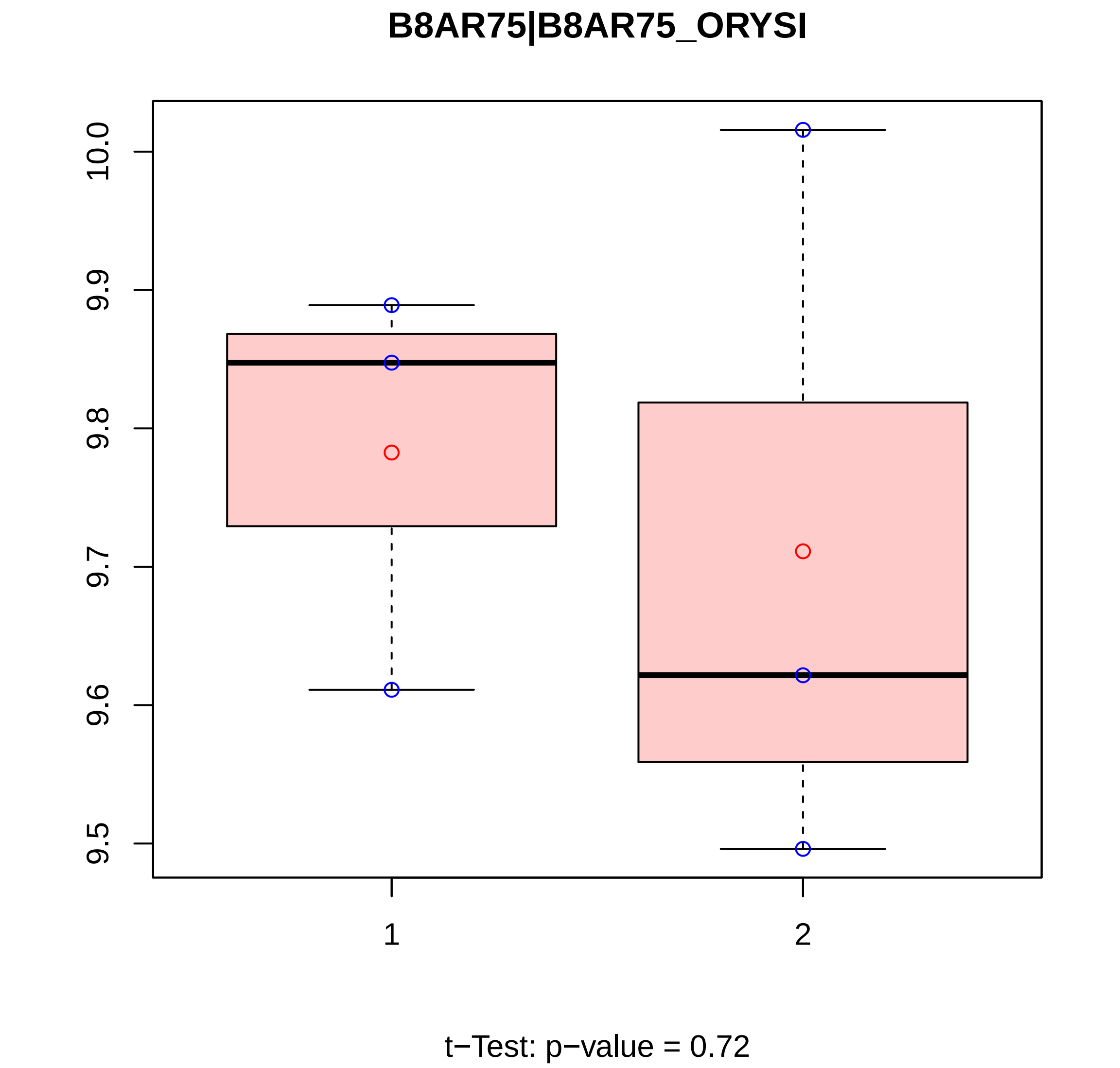

B8AR75|B8AR75_ORYSI
10.0
9.9
9.8
9.7
9.6
9.5
1
2
t−Test: p−value = 0.72

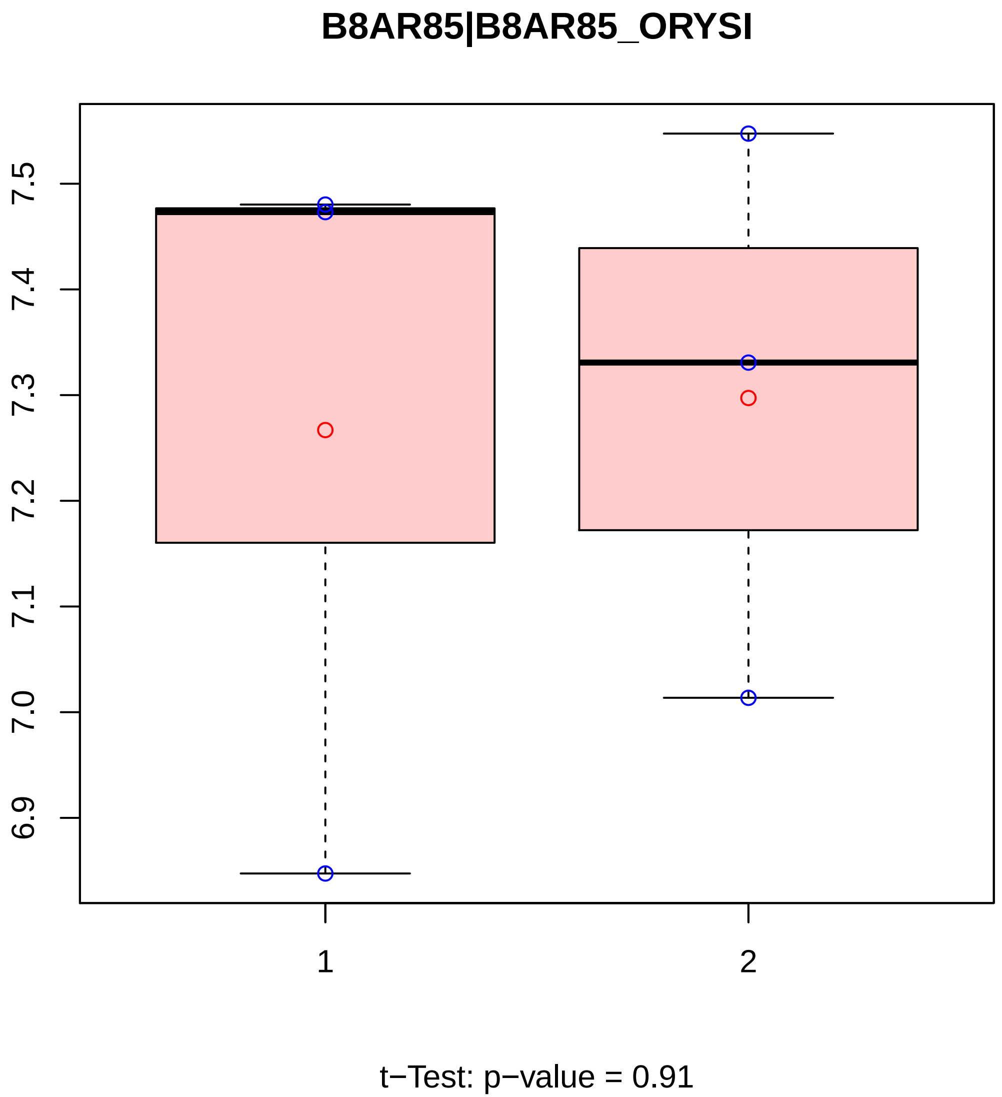
B8AR85|B8AR85_ORYSI
7.5
7.4
7.3
7.2
7.1
7.0
6.9
1
2
t−Test: p−value = 0.91

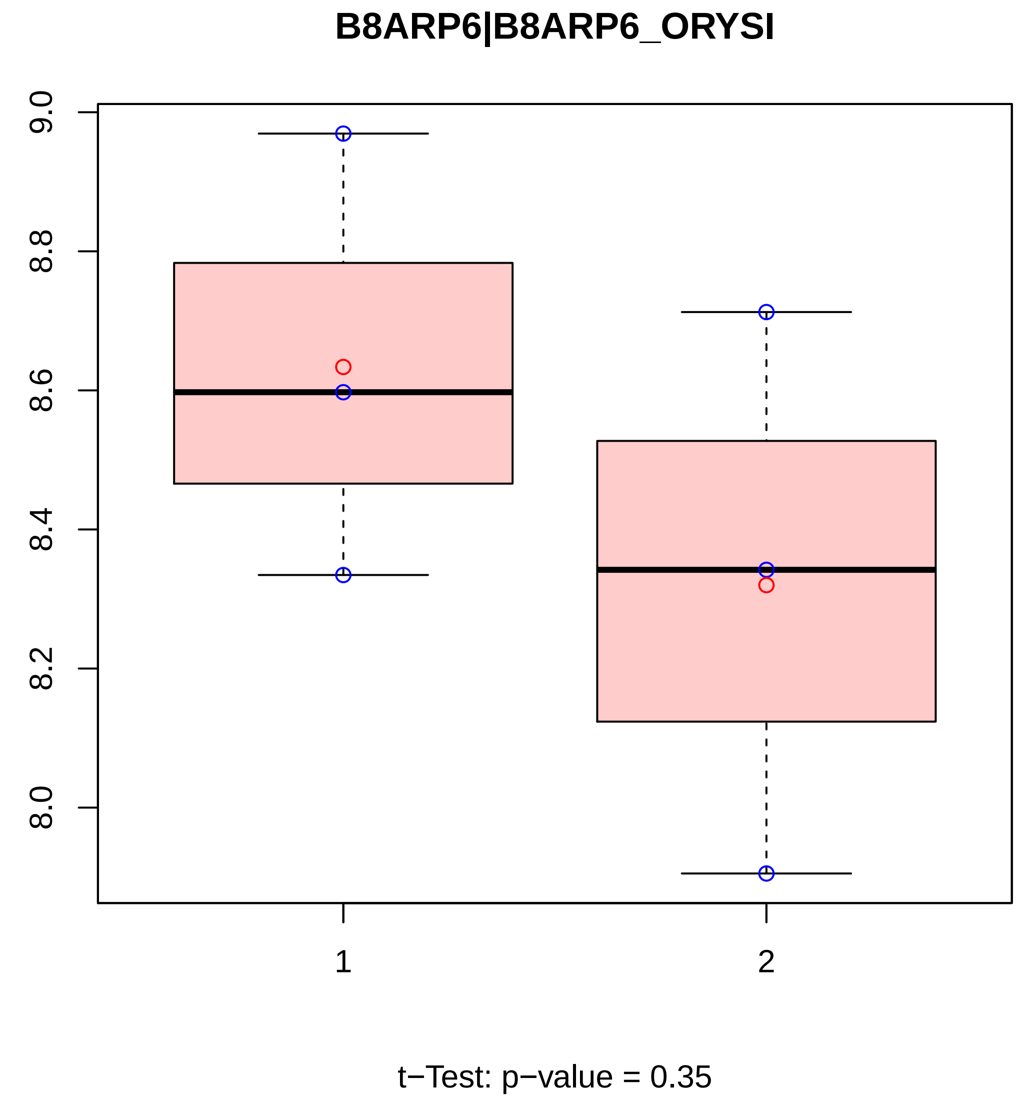

B8ARP6|B8ARP6_ORYSI
9.0
8.8
8.6
8.4
8.2
8.0
1
2
t−Test: p−value = 0.35

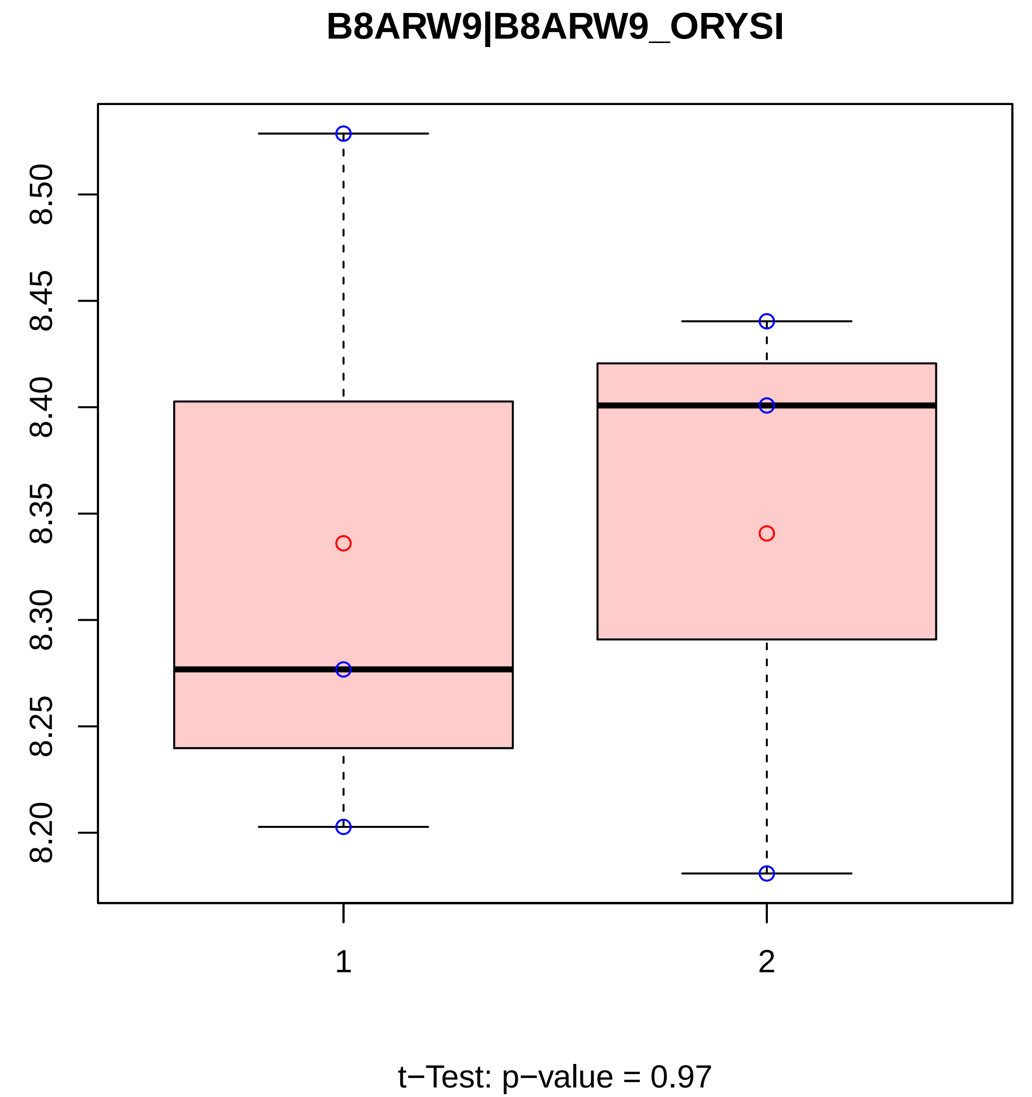

B8ARW9|B8ARW9_ORYSI
8.20
8.25
8.30
8.35
8.40
8.45
8.50
1
2
t−Test: p−value = 0.97

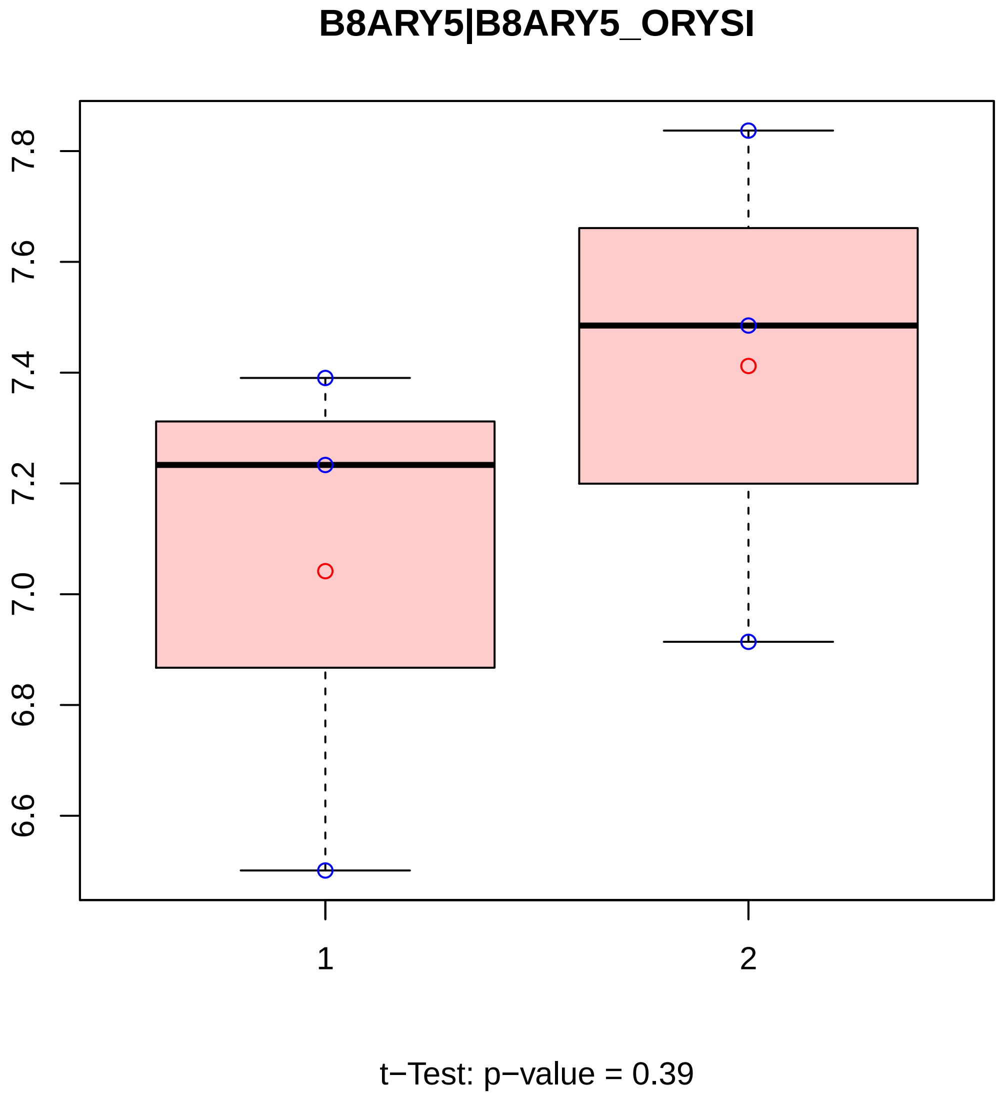

B8ARY5|B8ARY5_ORYSI
7.8
7.6
7.4
7.2
7.0
6.8
6.6
1
2
t−Test: p−value = 0.39

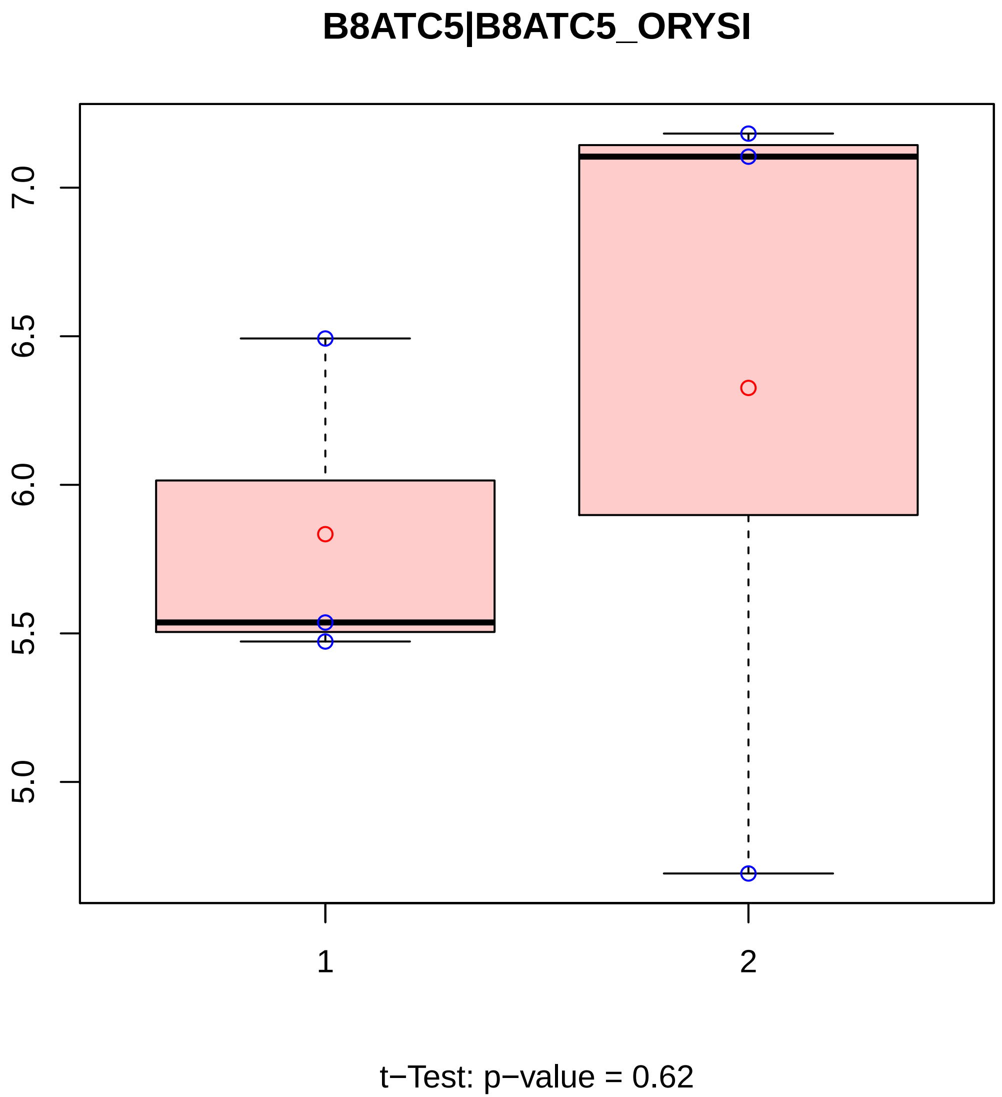

B8ATC5|B8ATC5_ORYSI
7.0
6.5
6.0
5.5
5.0
1
2
t−Test: p−value = 0.62

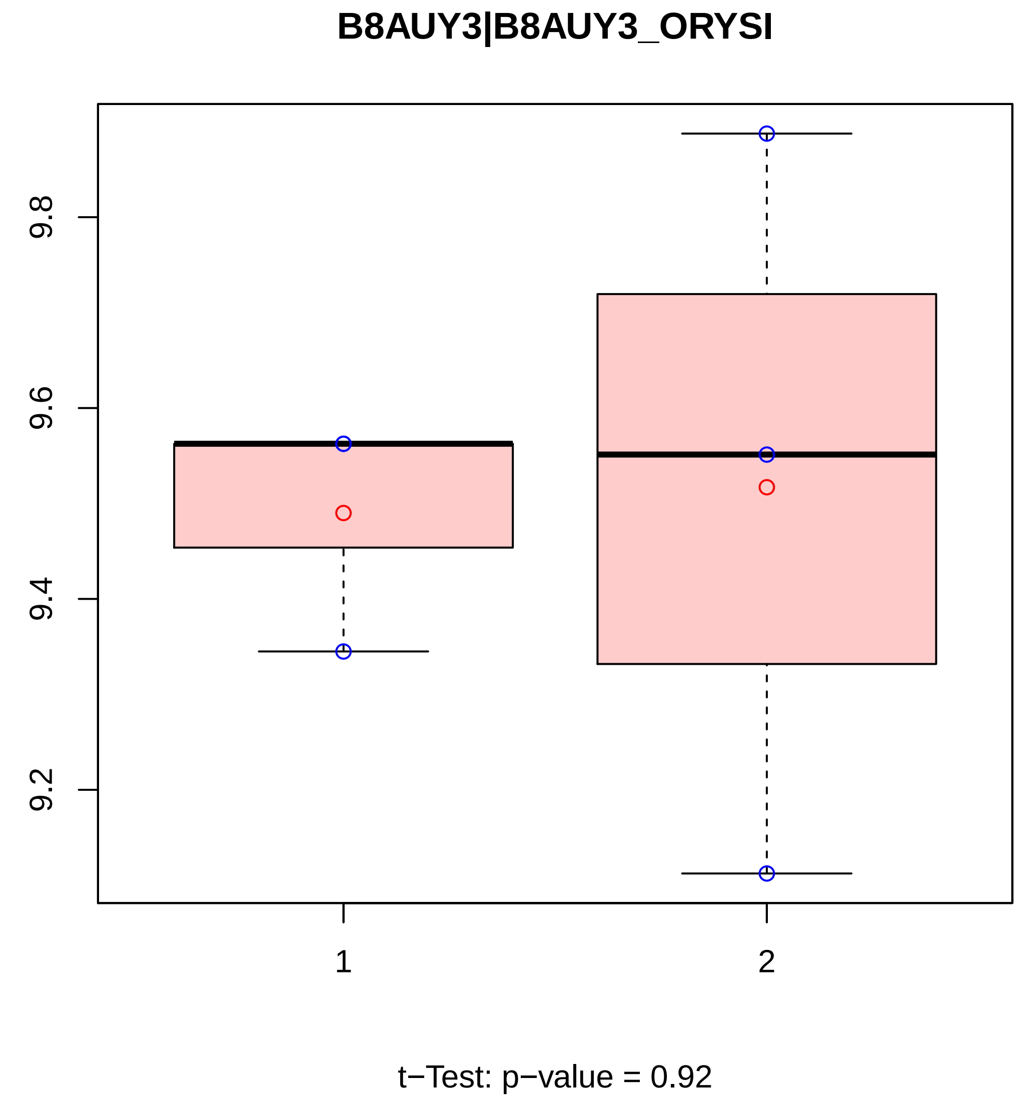
B8AUY3|B8AUY3_ORYSI
9.8
9.6
9.4
9.2
1
2
t−Test: p−value = 0.92

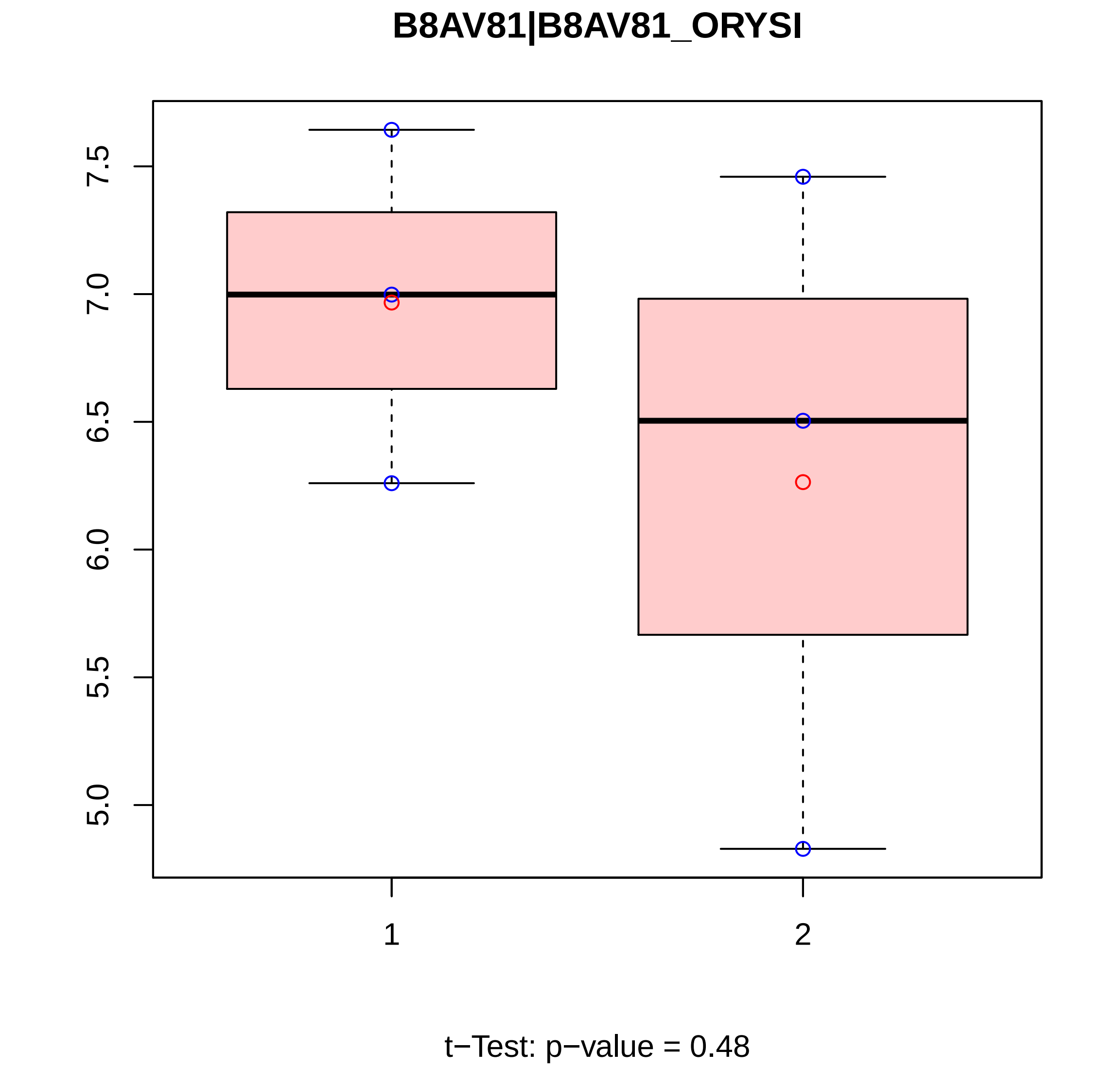

B8AV81|B8AV81_ORYSI
7.5
7.0
6.5
6.0
5.5
5.0
1
2
t−Test: p−value = 0.48

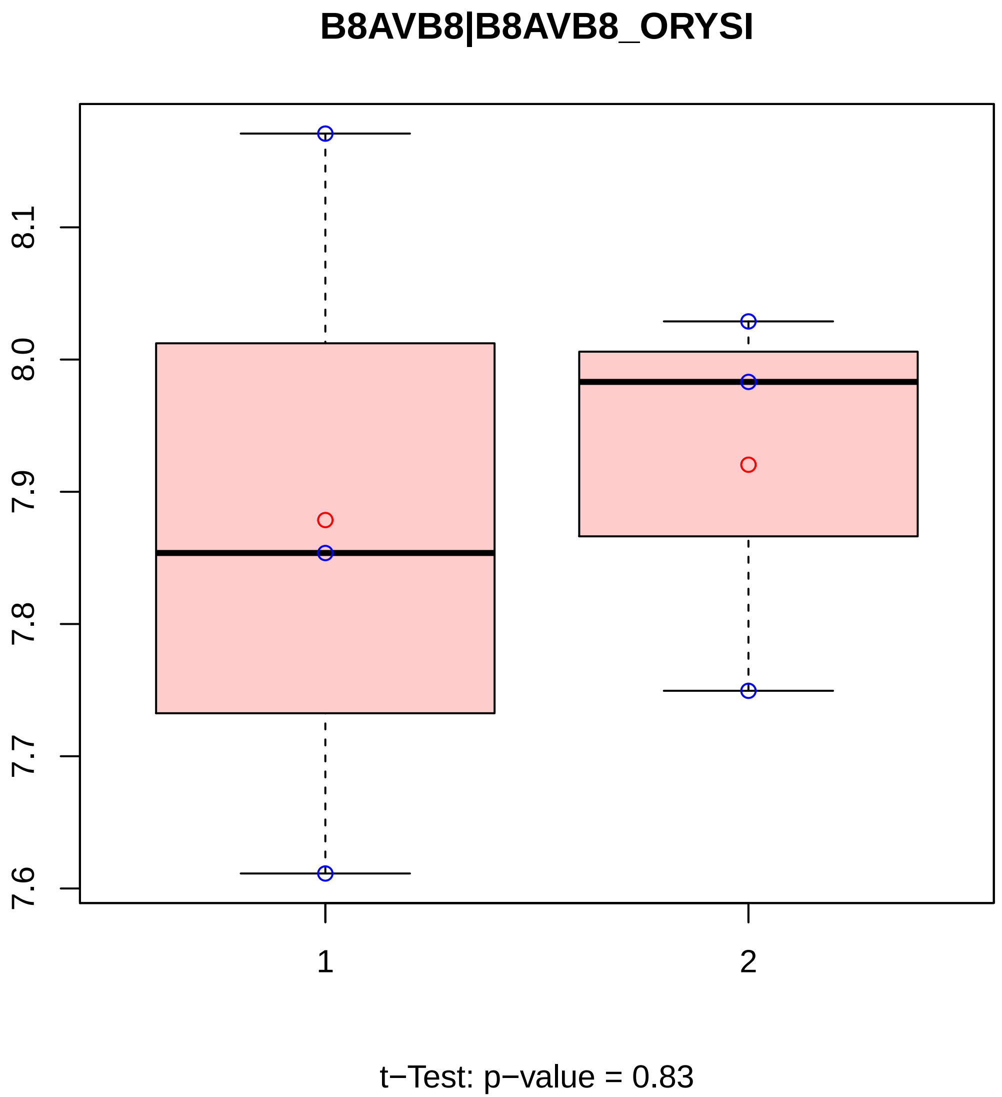
B8AVB8|B8AVB8_ORYSI
8.1
8.0
7.9
7.8
7.7
7.6
1
2
t−Test: p−value = 0.83

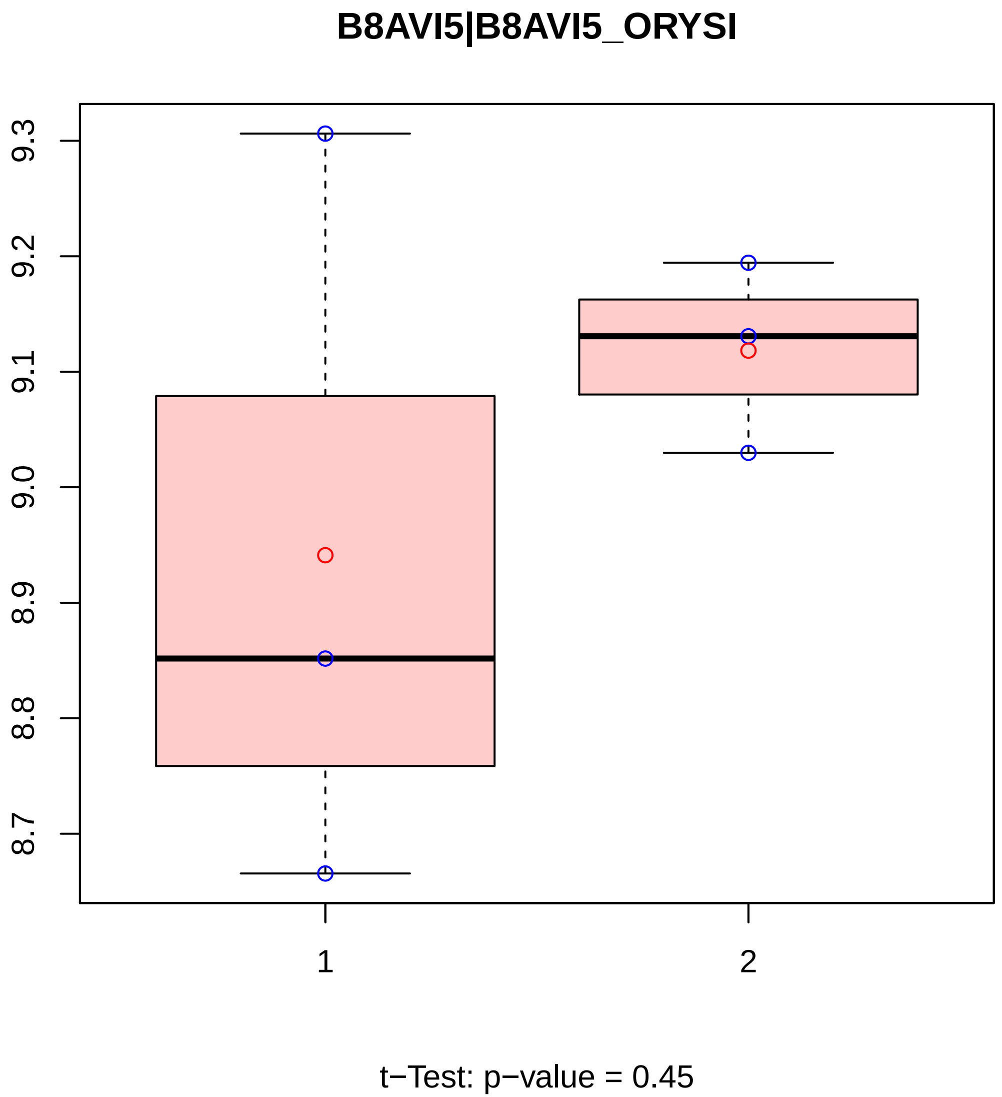
B8AVI5|B8AVI5_ORYSI
9.3
9.2
9.1
9.0
8.9
8.8
8.7
1
2
t−Test: p−value = 0.45

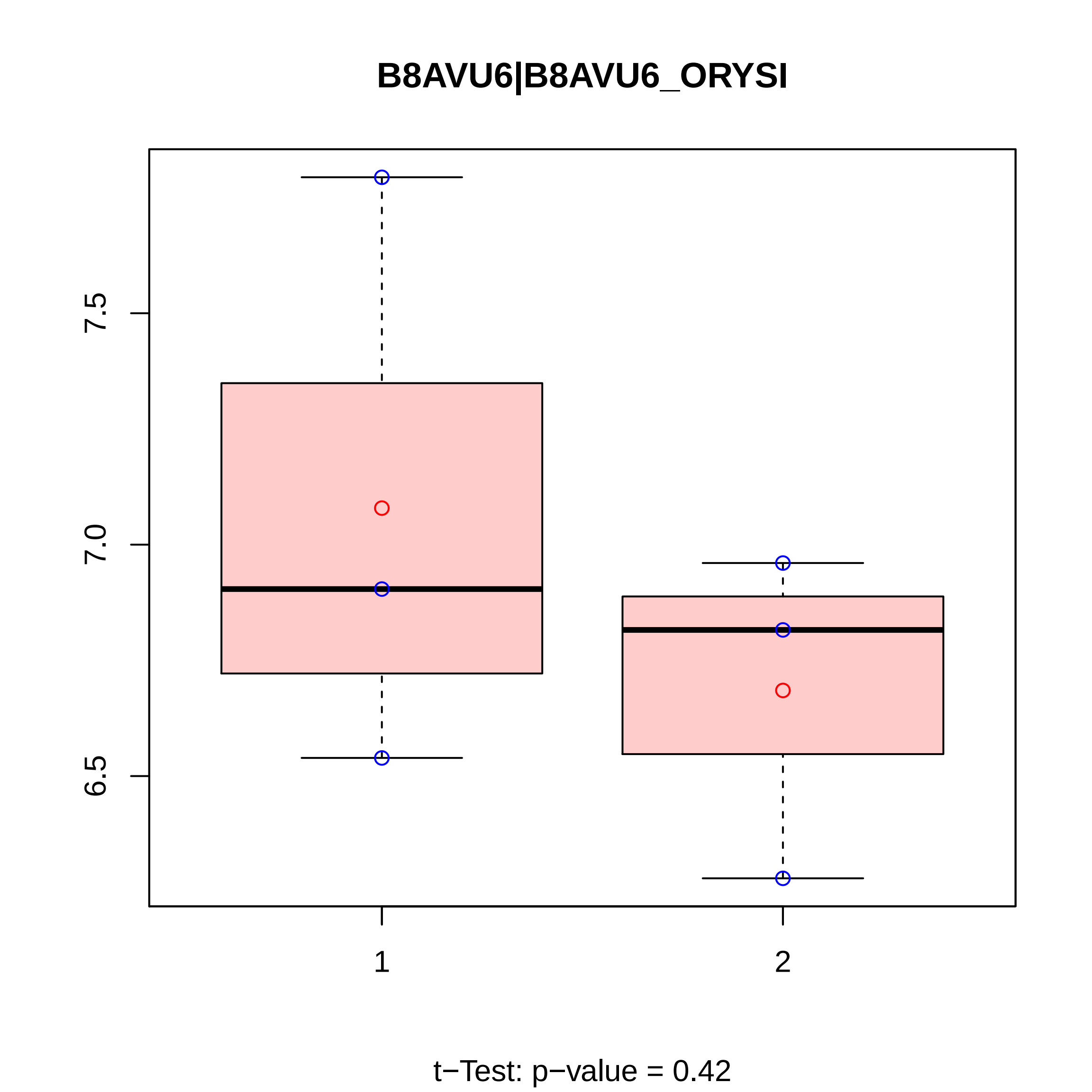

B8AVU6|B8AVU6_ORYSI
7.5
7.0
6.5
1
2
t−Test: p−value = 0.42

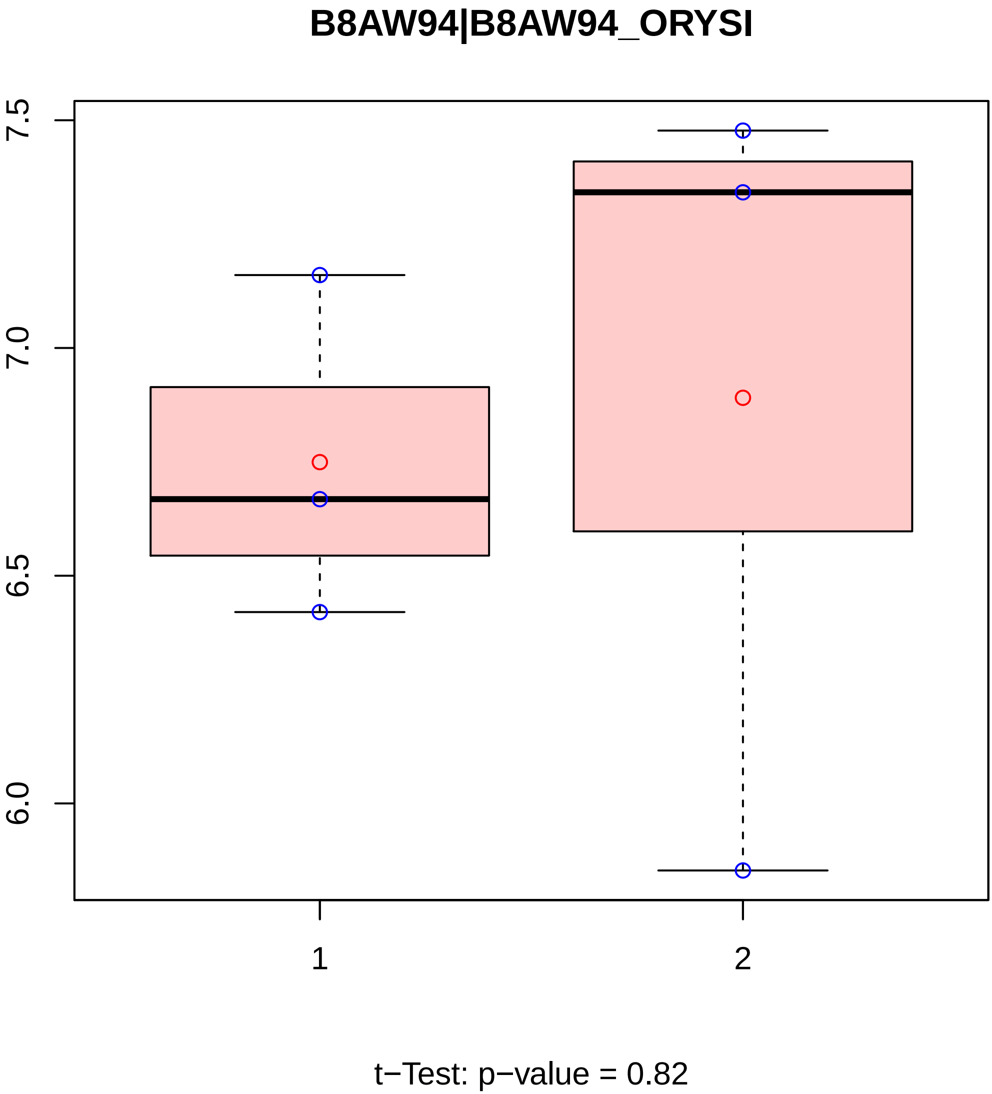

B8AW94|B8AW94_ORYSI
7.5
7.0
6.5
6.0
1
2
t−Test: p−value = 0.82

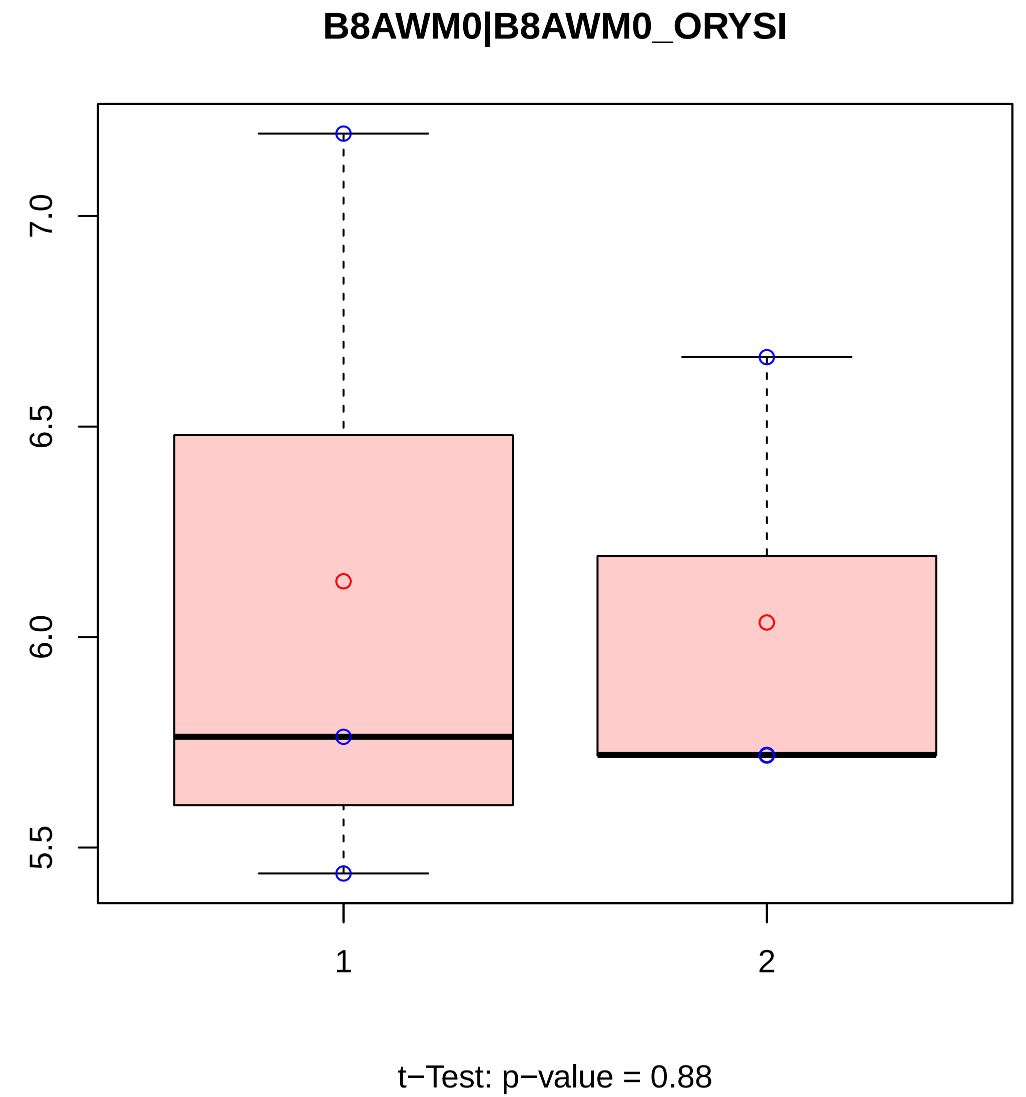
B8AWM0|B8AWM0_ORYSI
7.0
6.5
6.0
5.5
1
2
t−Test: p−value = 0.88

B8AXS1|B8AXS1_ORYSI

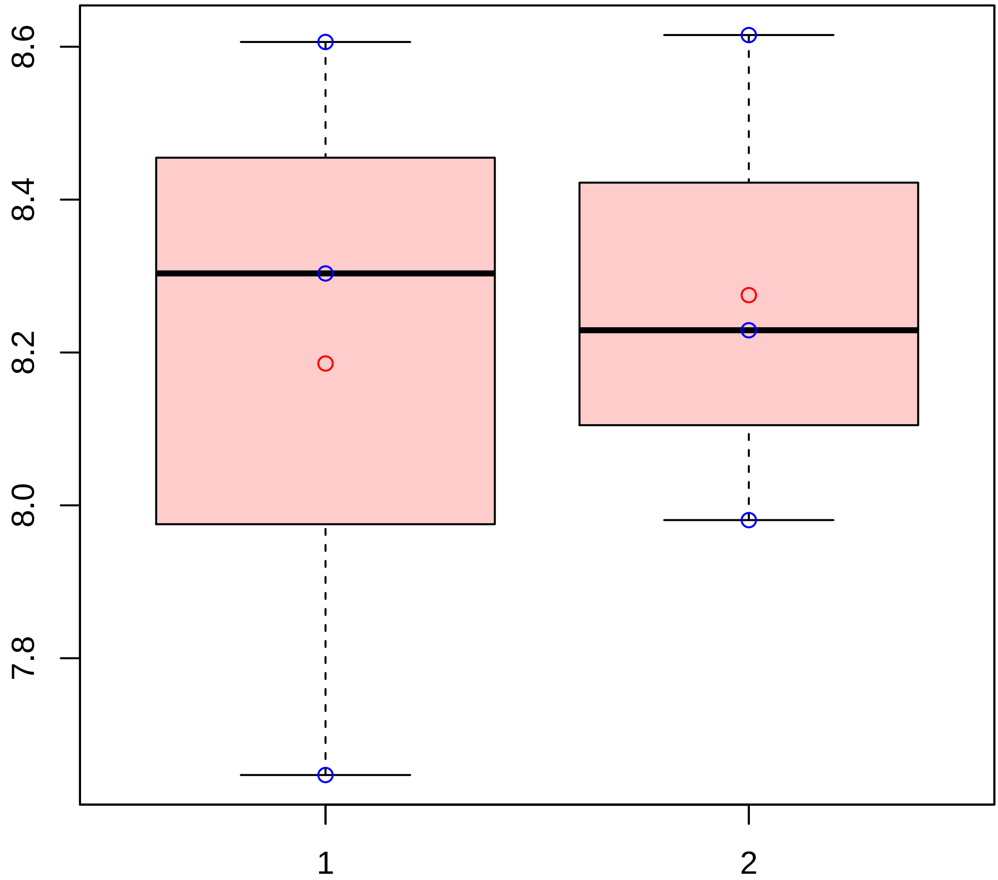

t−Test: p−value = 0.81

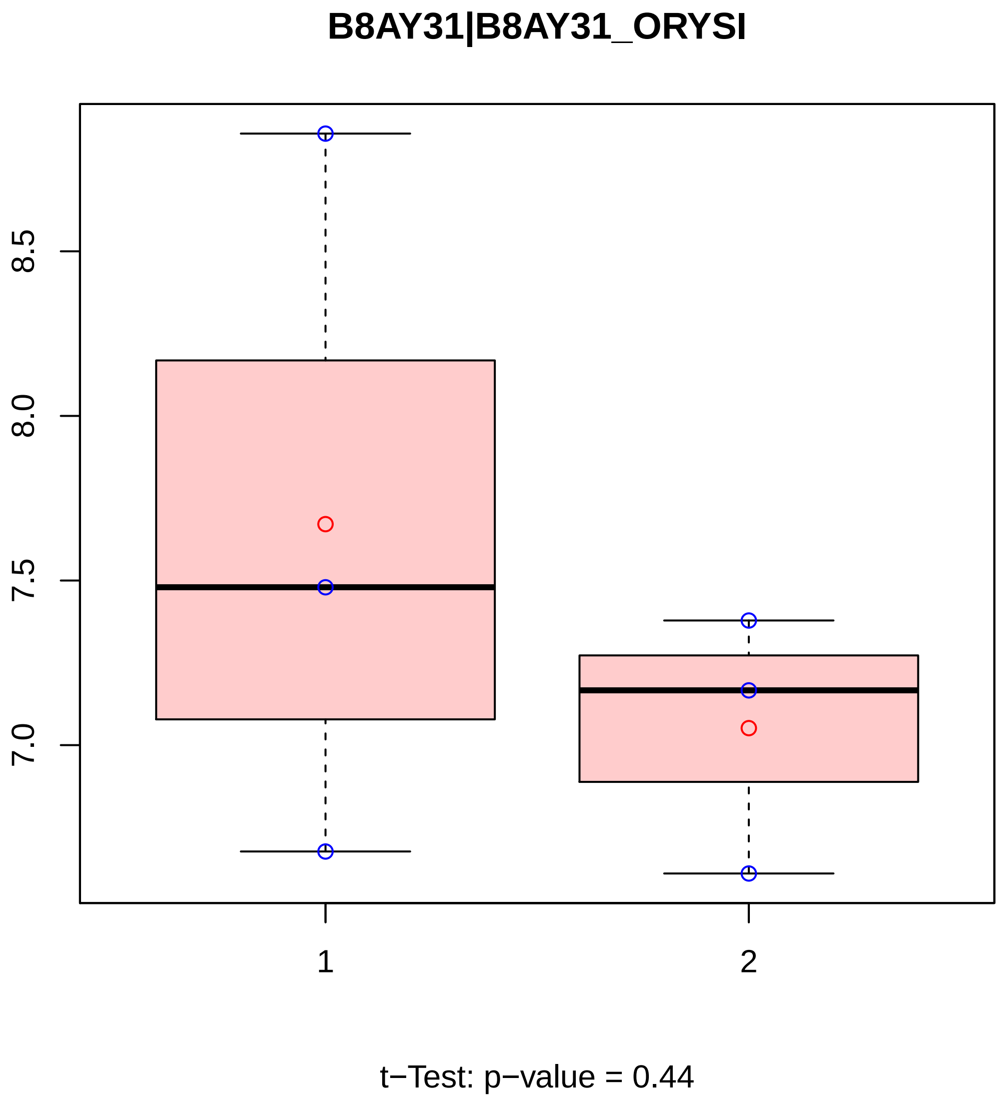
B8AY31|B8AY31_ORYSI
8.5
8.0
7.5
7.0
1
2
t−Test: p−value = 0.44

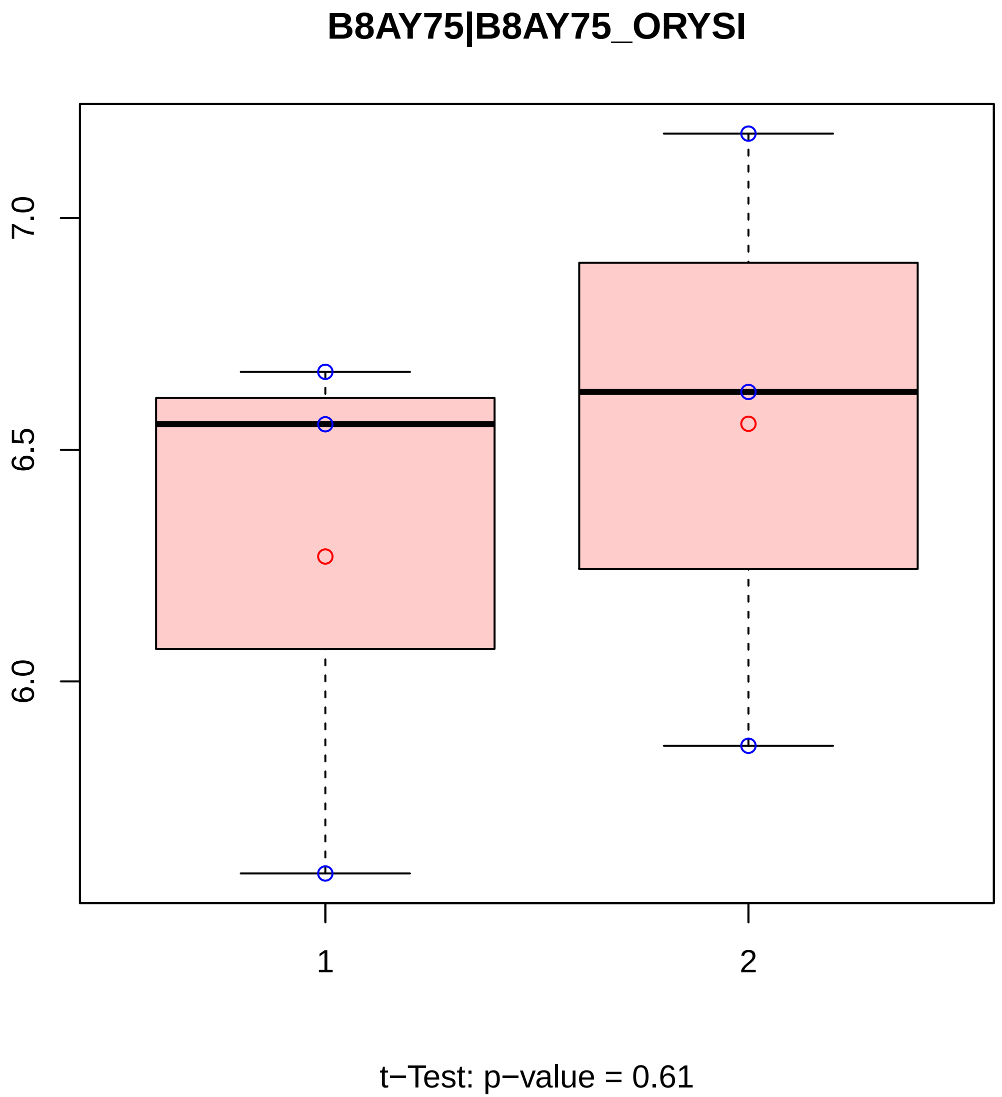
B8AY75|B8AY75_ORYSI
7.0
6.5
6.0
1
2
t−Test: p−value = 0.61

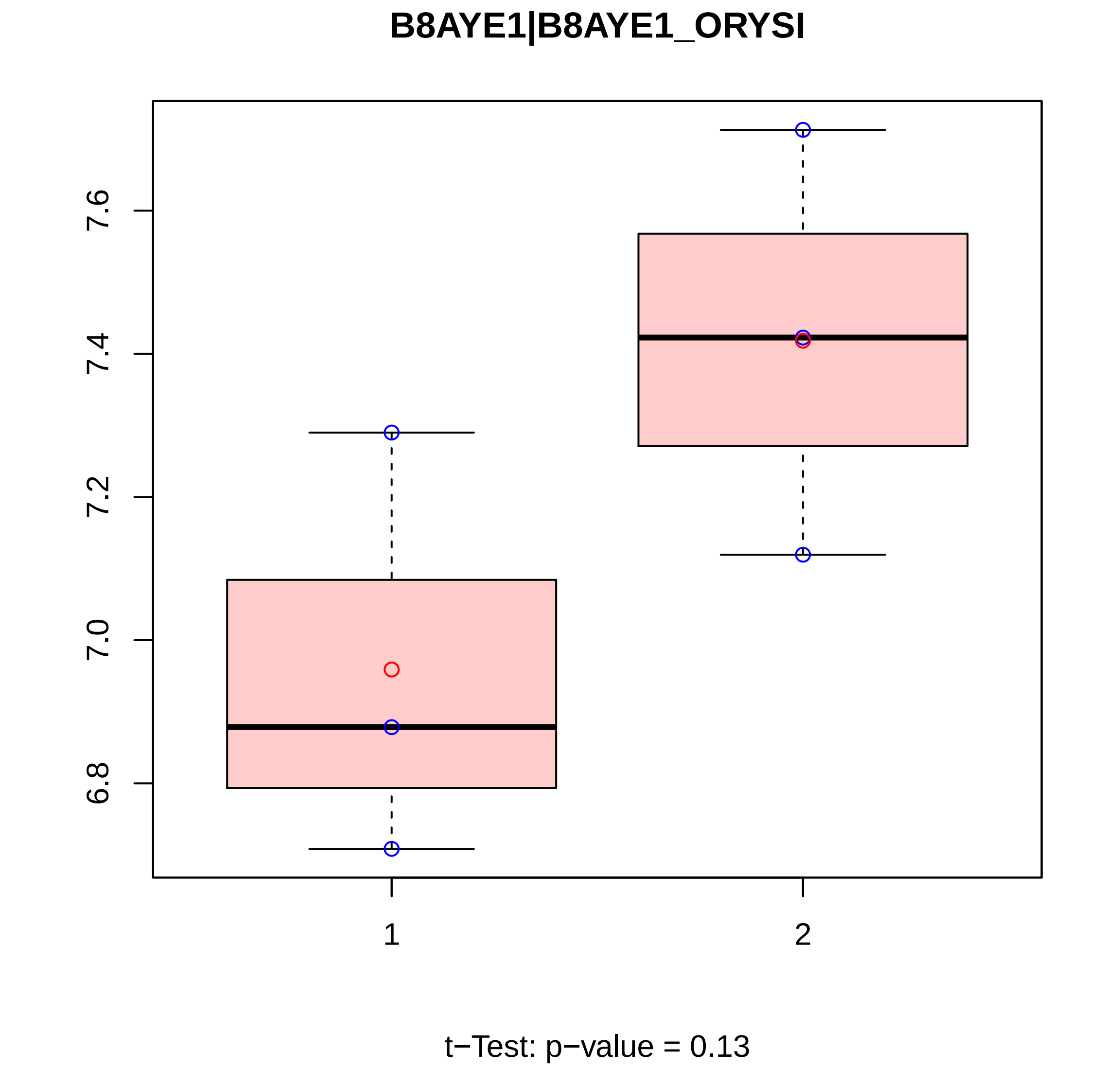
B8AYE1|B8AYE1_ORYSI
7.6
7.4
7.2
7.0
6.8
1
2
t−Test: p−value = 0.13

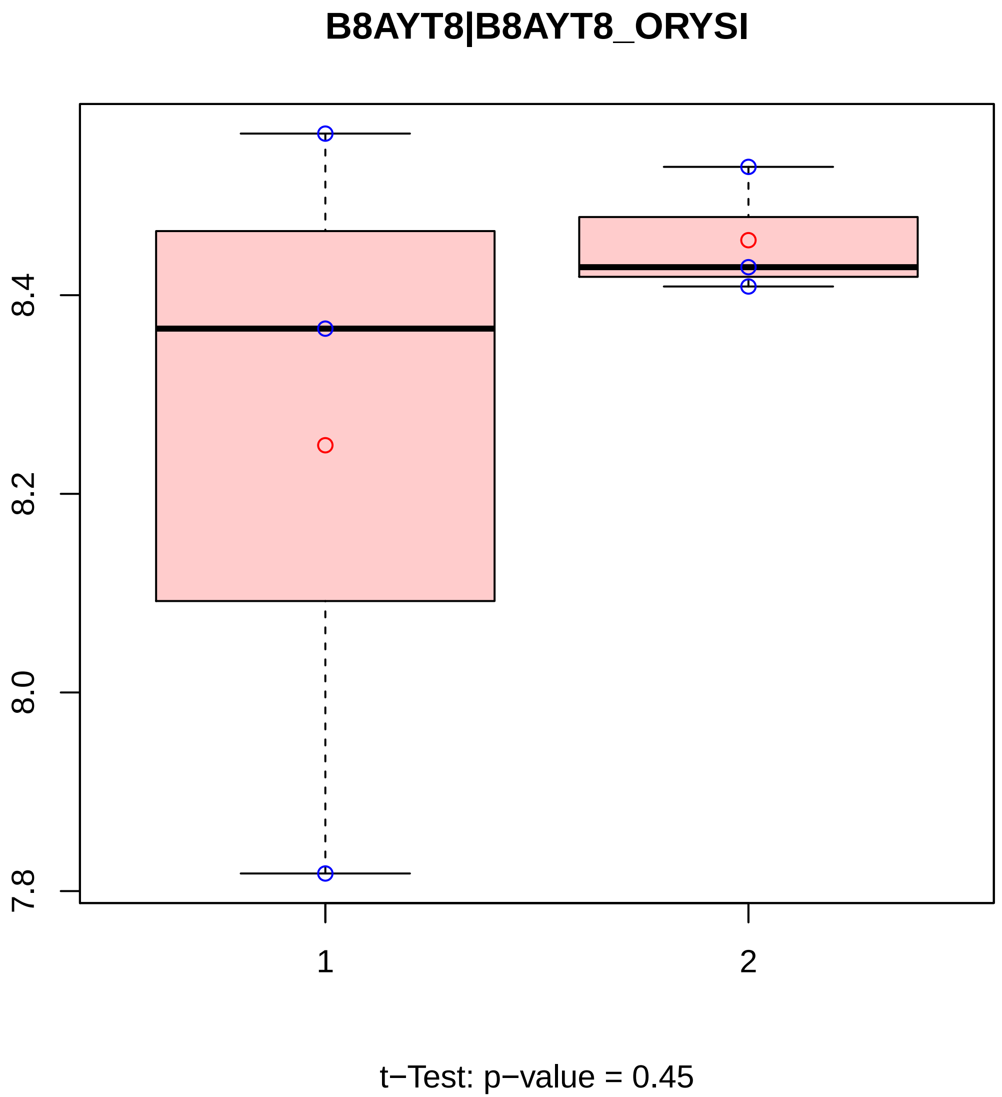
B8AYT8|B8AYT8_ORYSI
8.4
8.2
8.0
7.8
1
2
t−Test: p−value = 0.45

B8AZ97|B8AZ97_ORYSI

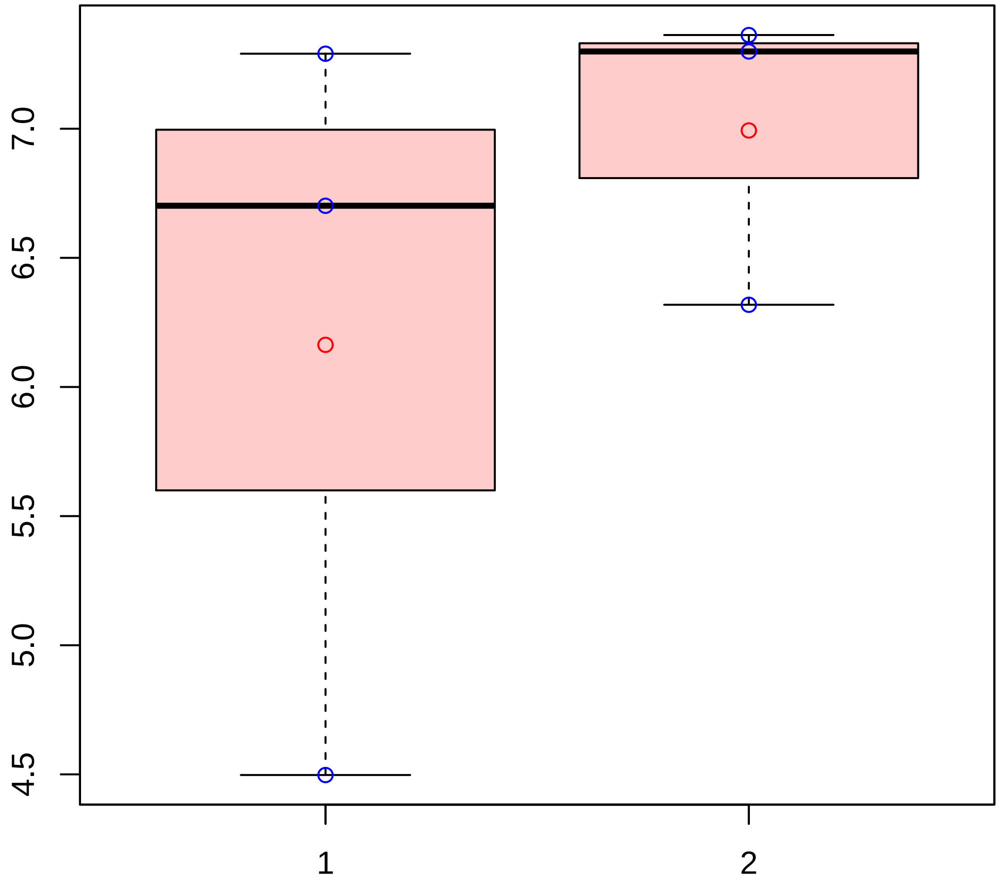

t−Test: p−value = 0.44

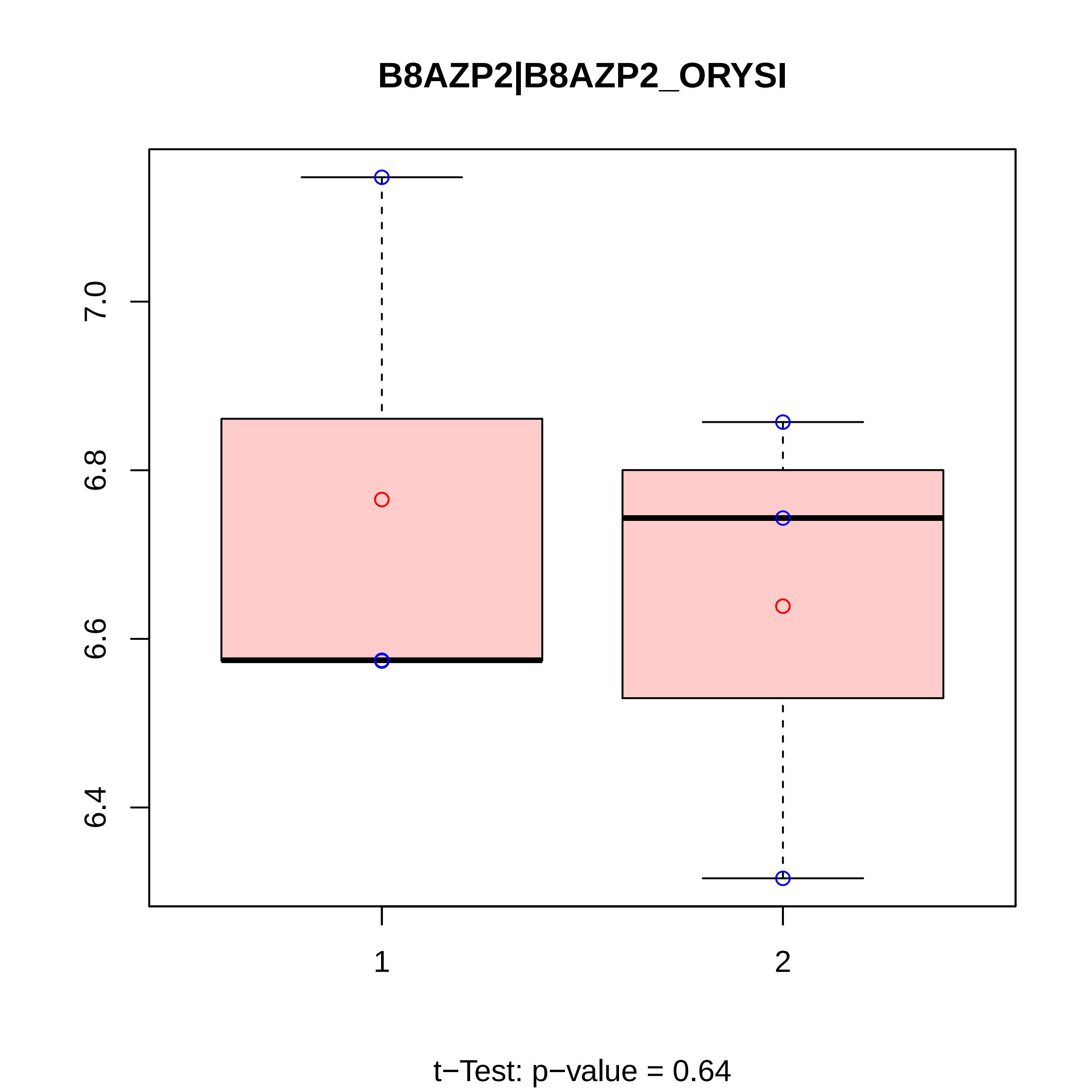
B8AZP2|B8AZP2_ORYSI
7.0
6.8
6.6
6.4
1
2
t−Test: p−value = 0.64

B8B025|B8B025_ORYSI

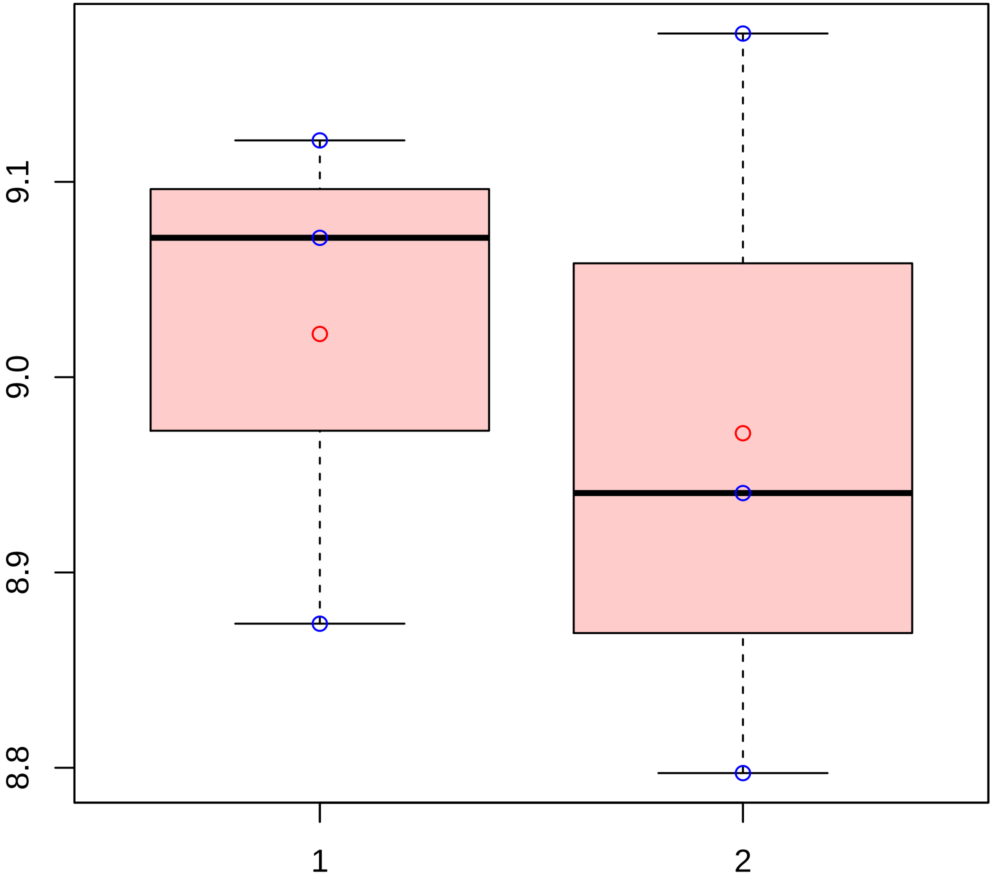

t−Test: p−value = 0.73

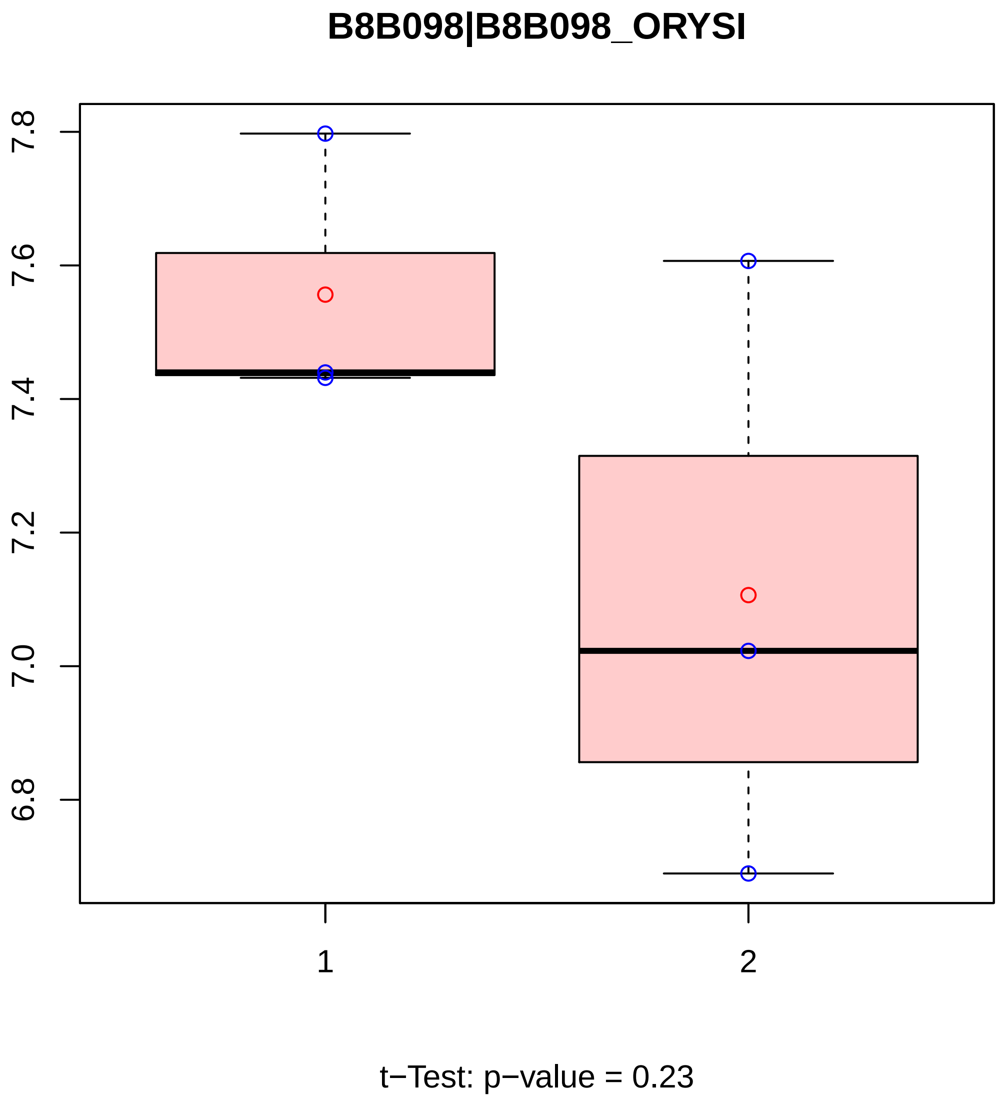
B8B098|B8B098_ORYSI
7.8
7.6
7.4
7.2
7.0
6.8
1
2
t−Test: p−value = 0.23

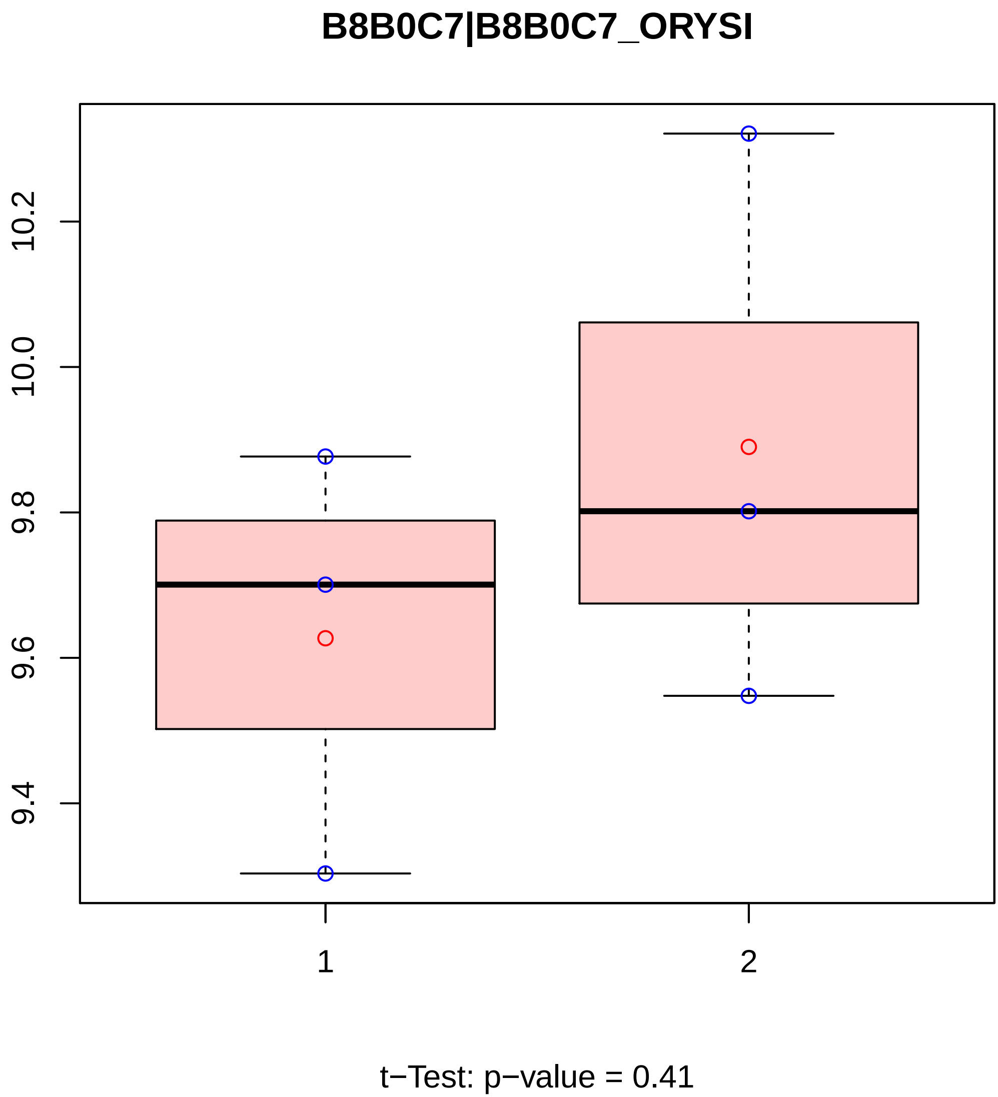
B8B0C7|B8B0C7_ORYSI
10.2
10.0
9.8
9.6
9.4
1
2
t−Test: p−value = 0.41

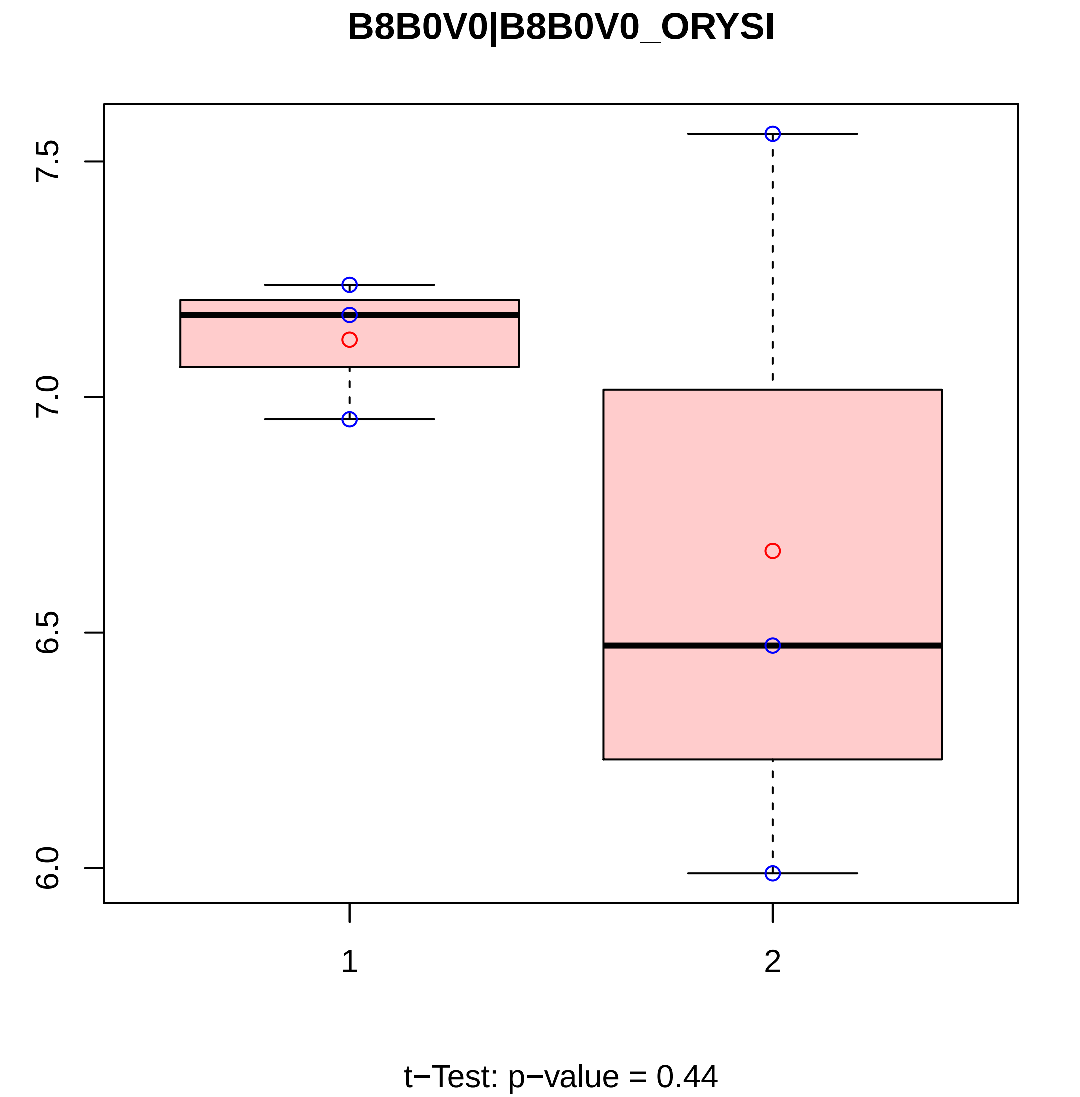

B8B0V0|B8B0V0_ORYSI
7.5
7.0
6.5
6.0
1
2
t−Test: p−value = 0.44

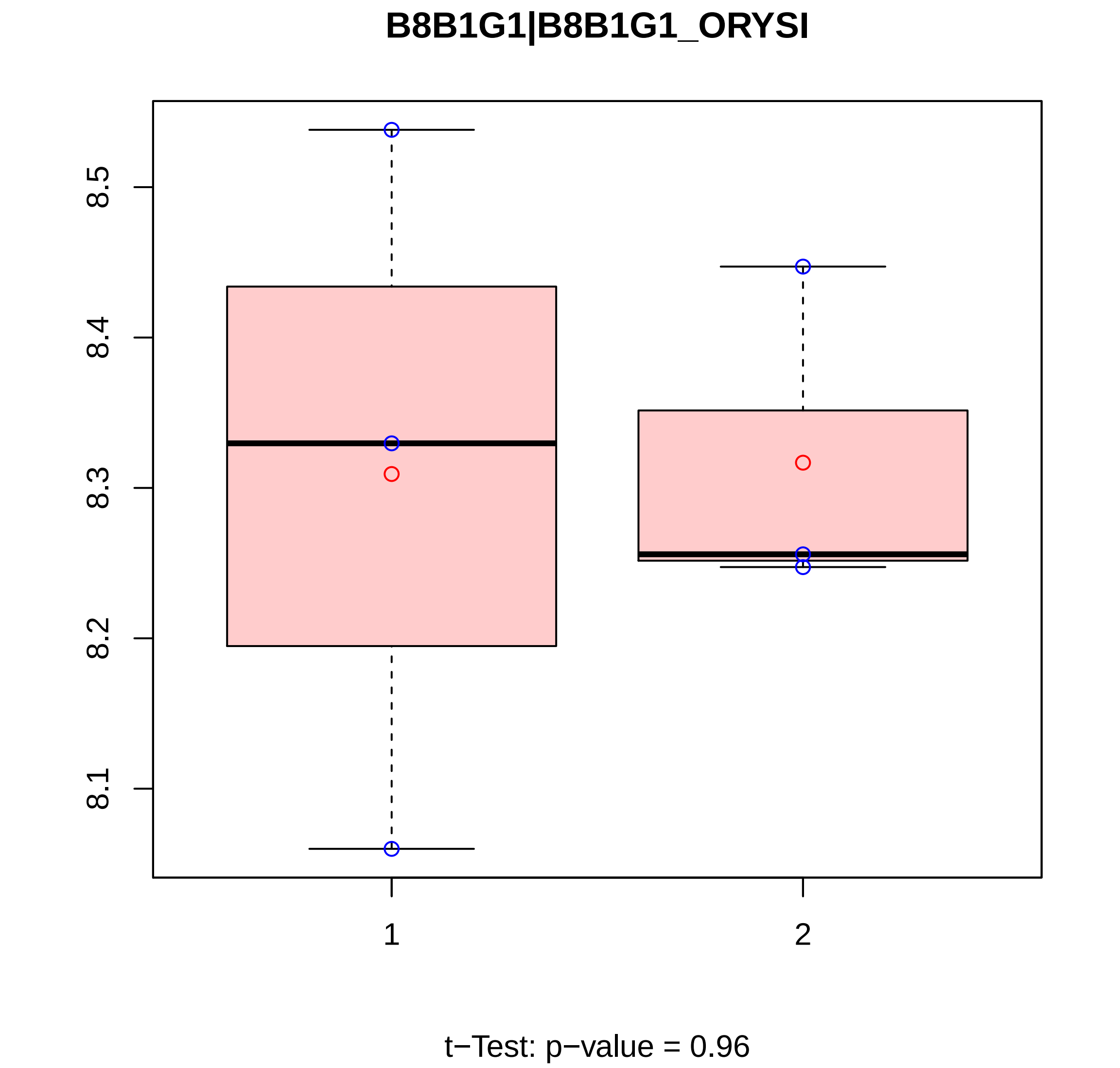
B8B1G1|B8B1G1_ORYSI
8.1
8.2
8.3
8.4
8.5
1
2
t−Test: p−value = 0.96

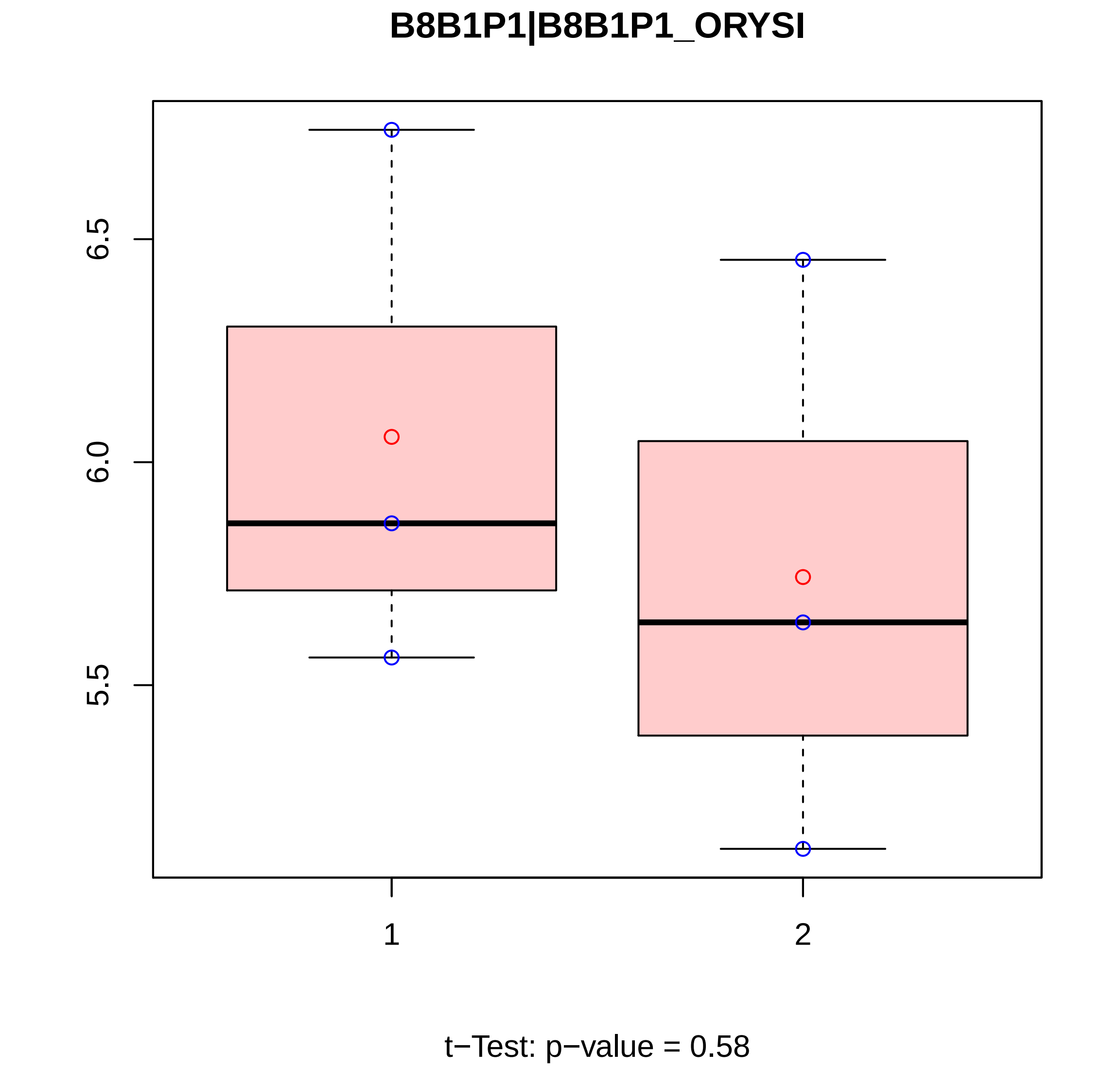

B8B1P1|B8B1P1_ORYSI
6.5
6.0
5.5
1
2
t−Test: p−value = 0.58

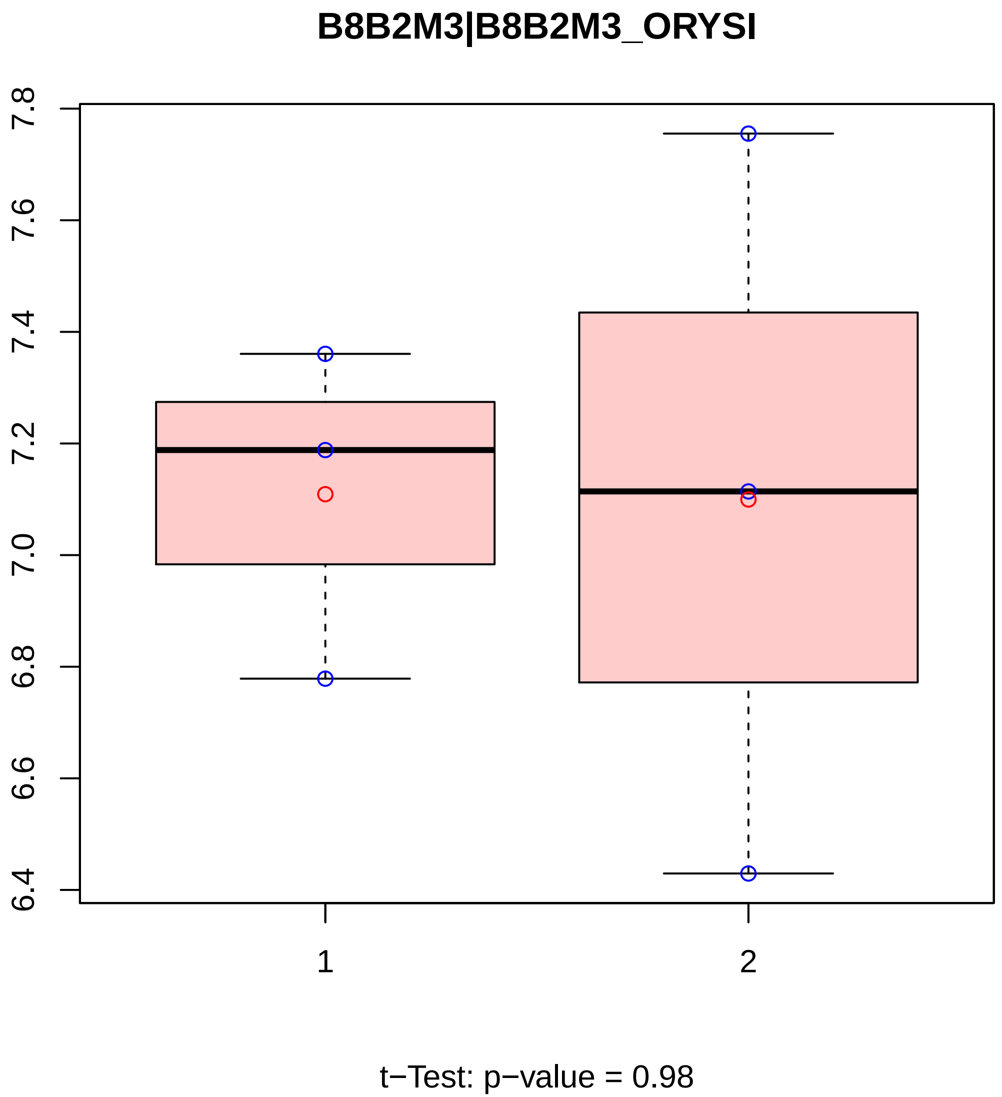
B8B2M3|B8B2M3_ORYSI
7.8
7.6
7.4
7.2
7.0
6.8
6.6
6.4
1
2
t−Test: p−value = 0.98

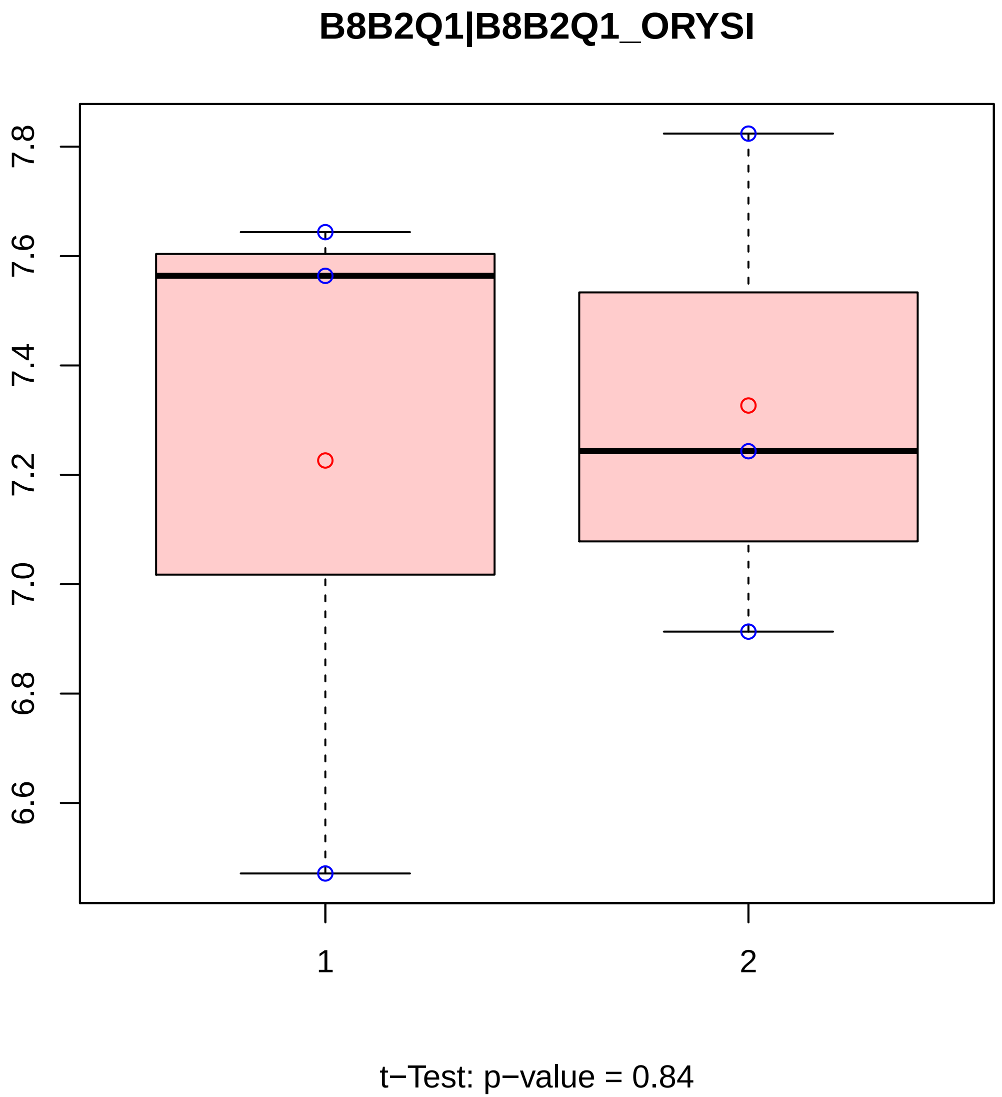
B8B2Q1|B8B2Q1_ORYSI
7.8
7.6
7.4
7.2
7.0
6.8
6.6
1
2
t−Test: p−value = 0.84

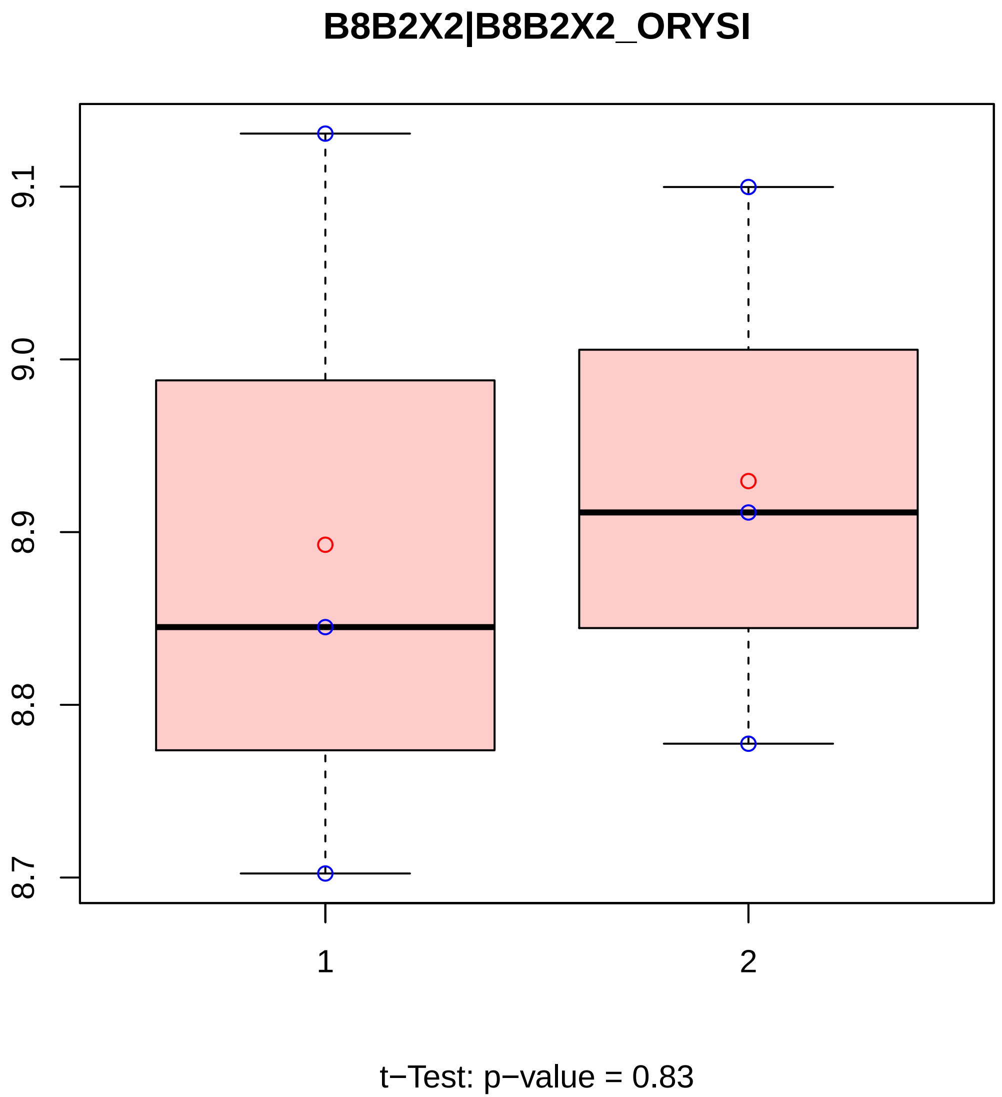
B8B2X2|B8B2X2_ORYSI
9.1
9.0
8.9
8.8
8.7
1
2
t−Test: p−value = 0.83

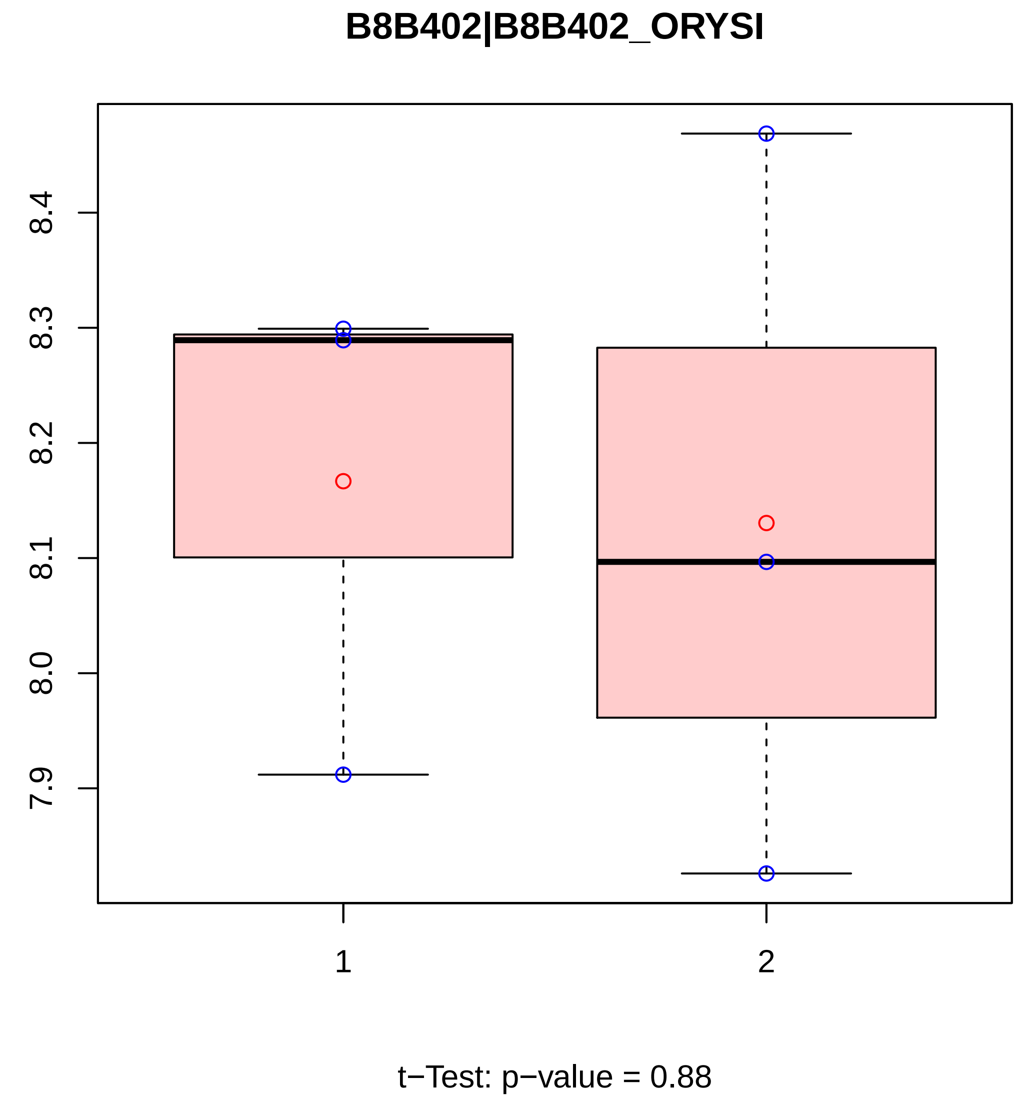
B8B402|B8B402_ORYSI
8.4
8.3
8.2
8.1
8.0
7.9
1
2
t−Test: p−value = 0.88

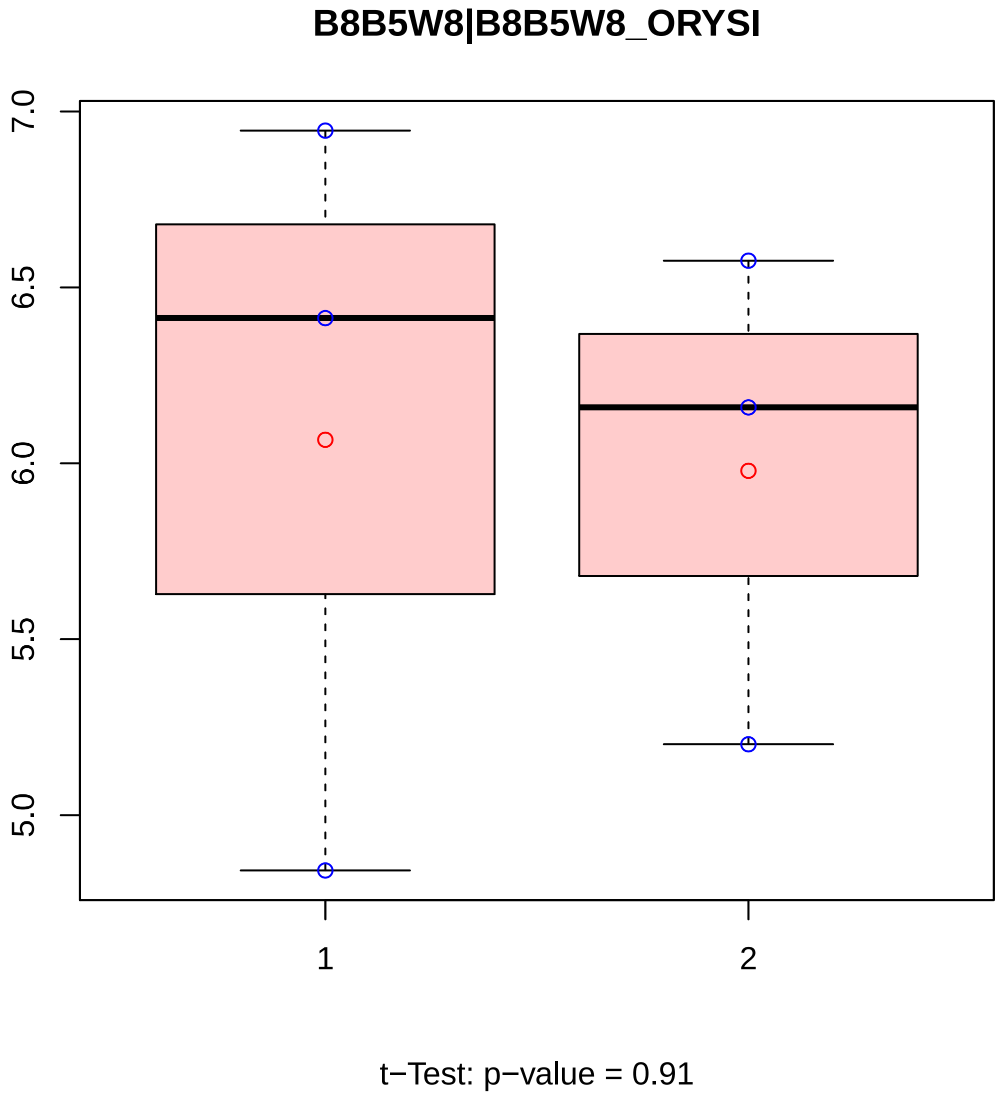

B8B5W8|B8B5W8_ORYSI
7.0
6.5
6.0
5.5
5.0
1
2
t−Test: p−value = 0.91

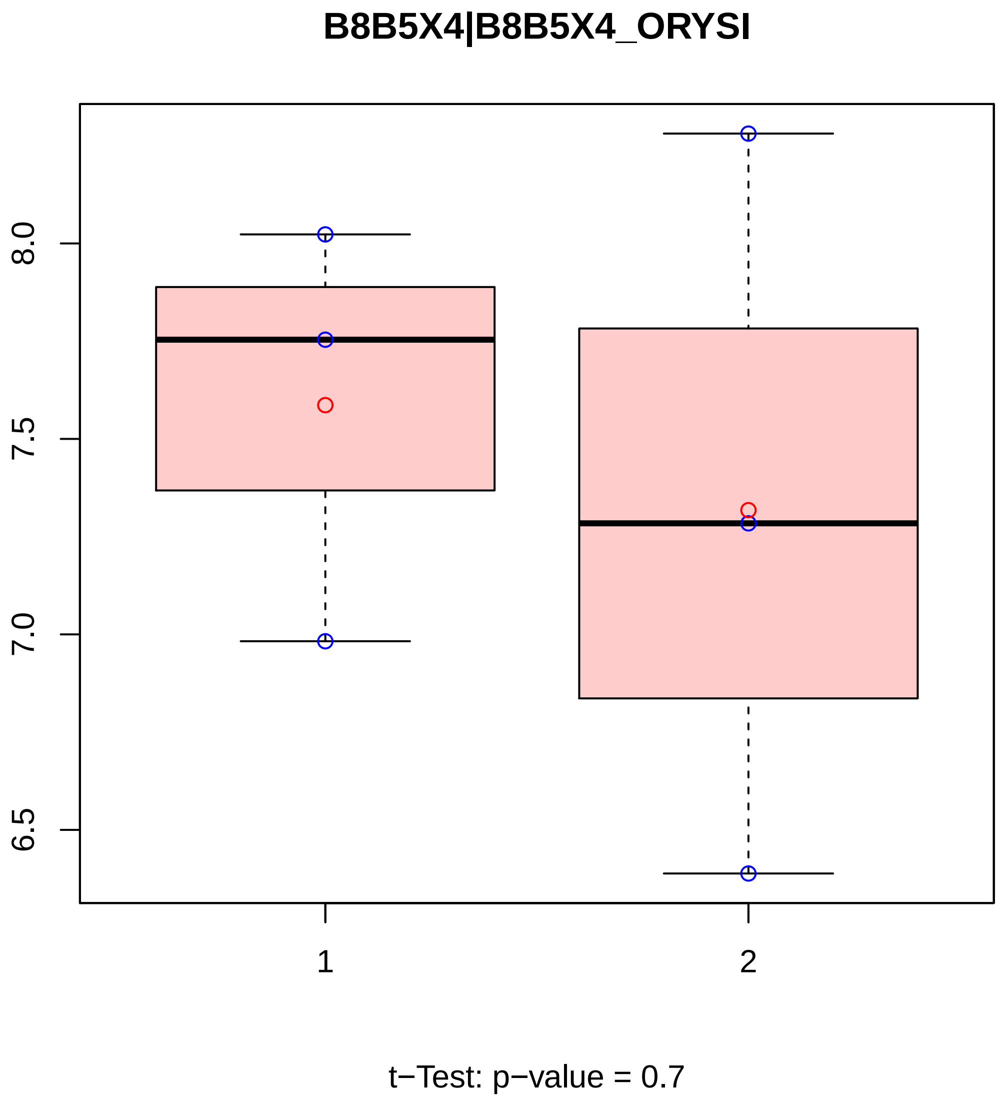
B8B5X4|B8B5X4_ORYSI
8.0
7.5
7.0
6.5
1
2
t−Test: p−value = 0.7

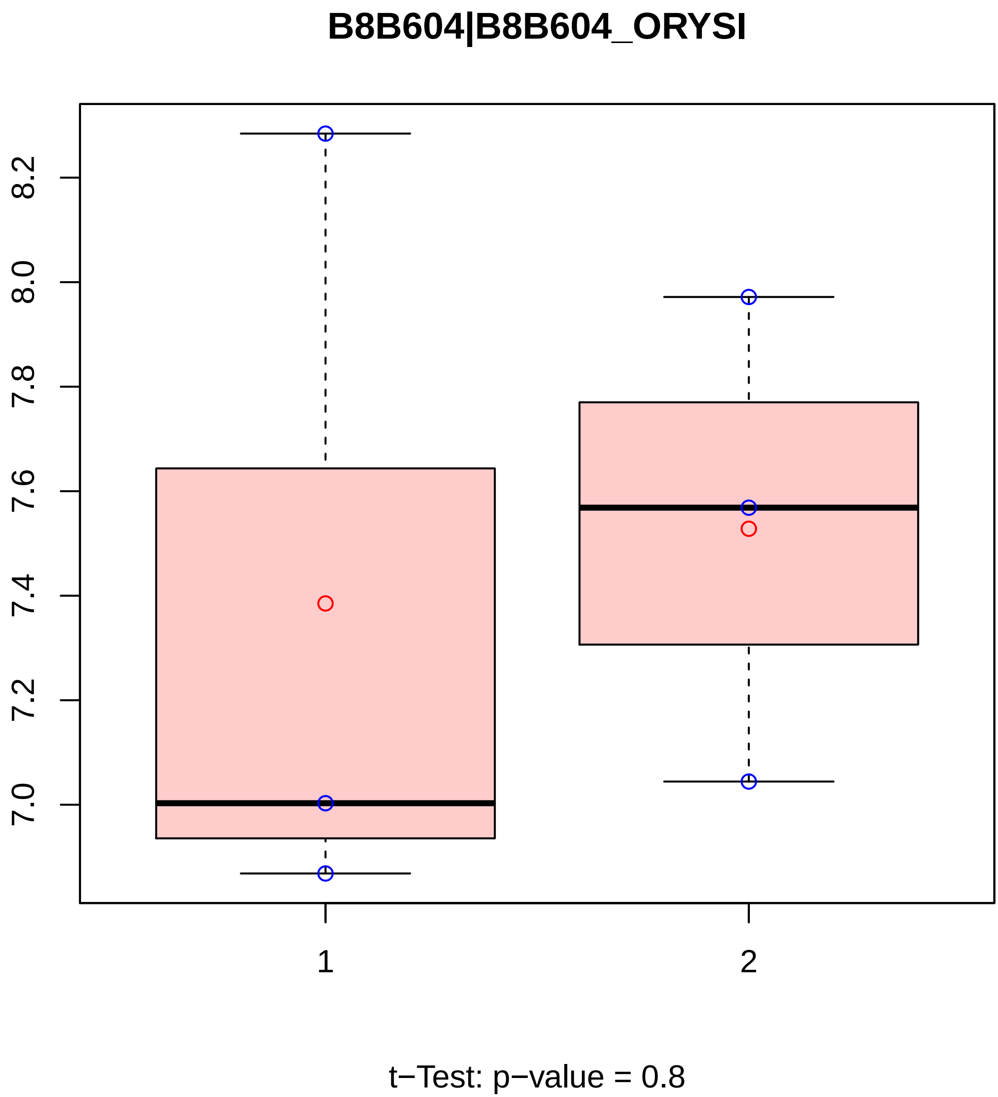

B8B604|B8B604_ORYSI
8.2
8.0
7.8
7.6
7.4
7.2
7.0
1
2
t−Test: p−value = 0.8

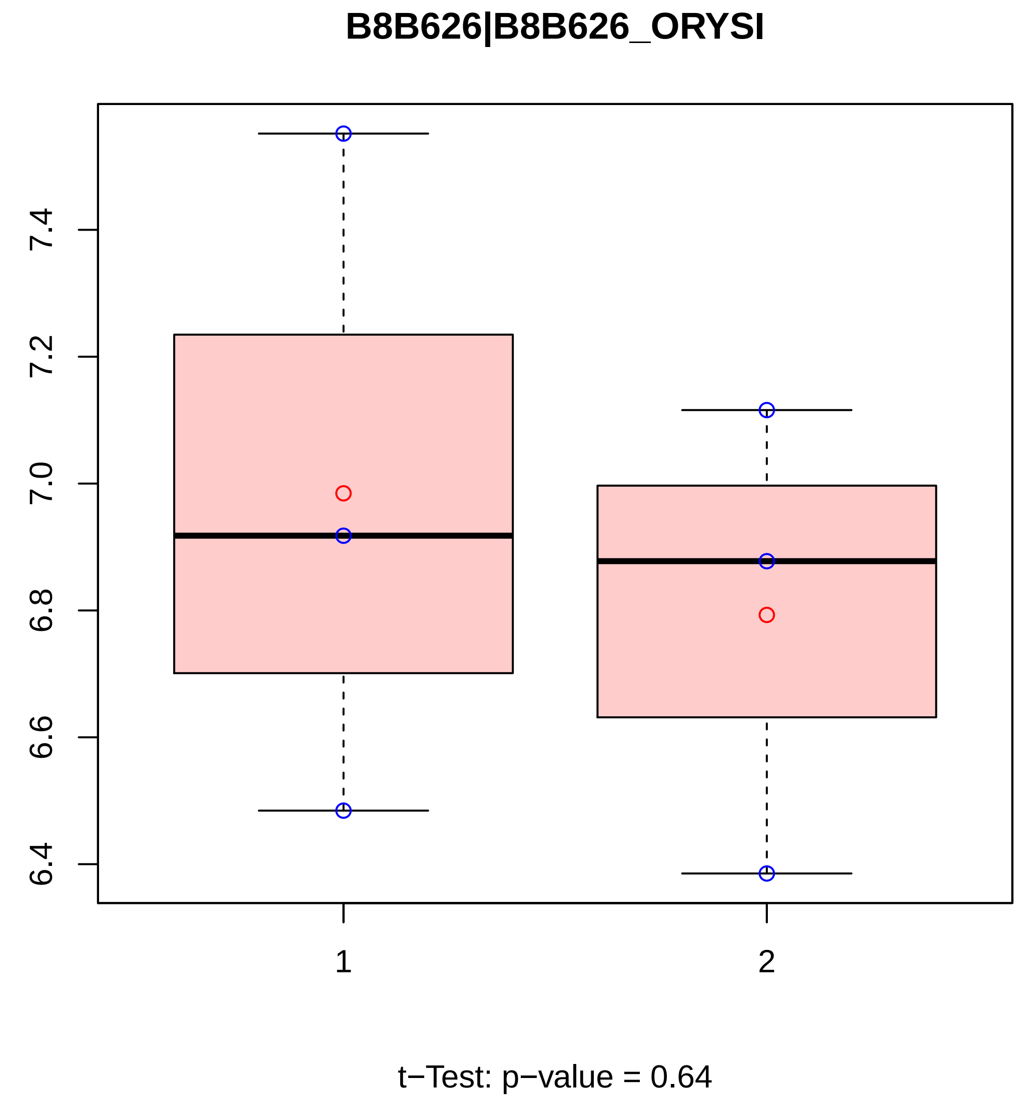

B8B626|B8B626_ORYSI
7.4
7.2
7.0
6.8
6.6
6.4
1
2
t−Test: p−value = 0.64

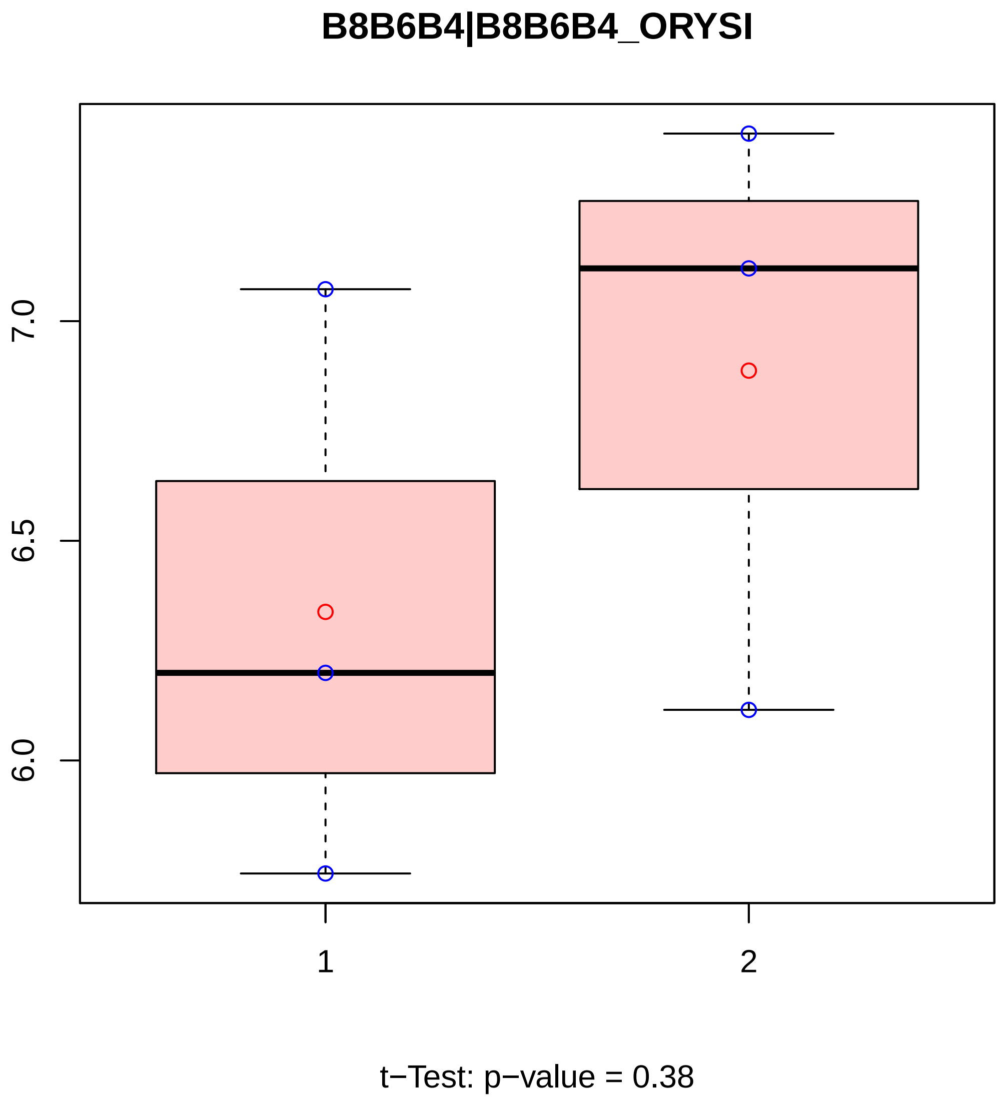

B8B6B4|B8B6B4_ORYSI
7.0
6.5
6.0
1
2
t−Test: p−value = 0.38

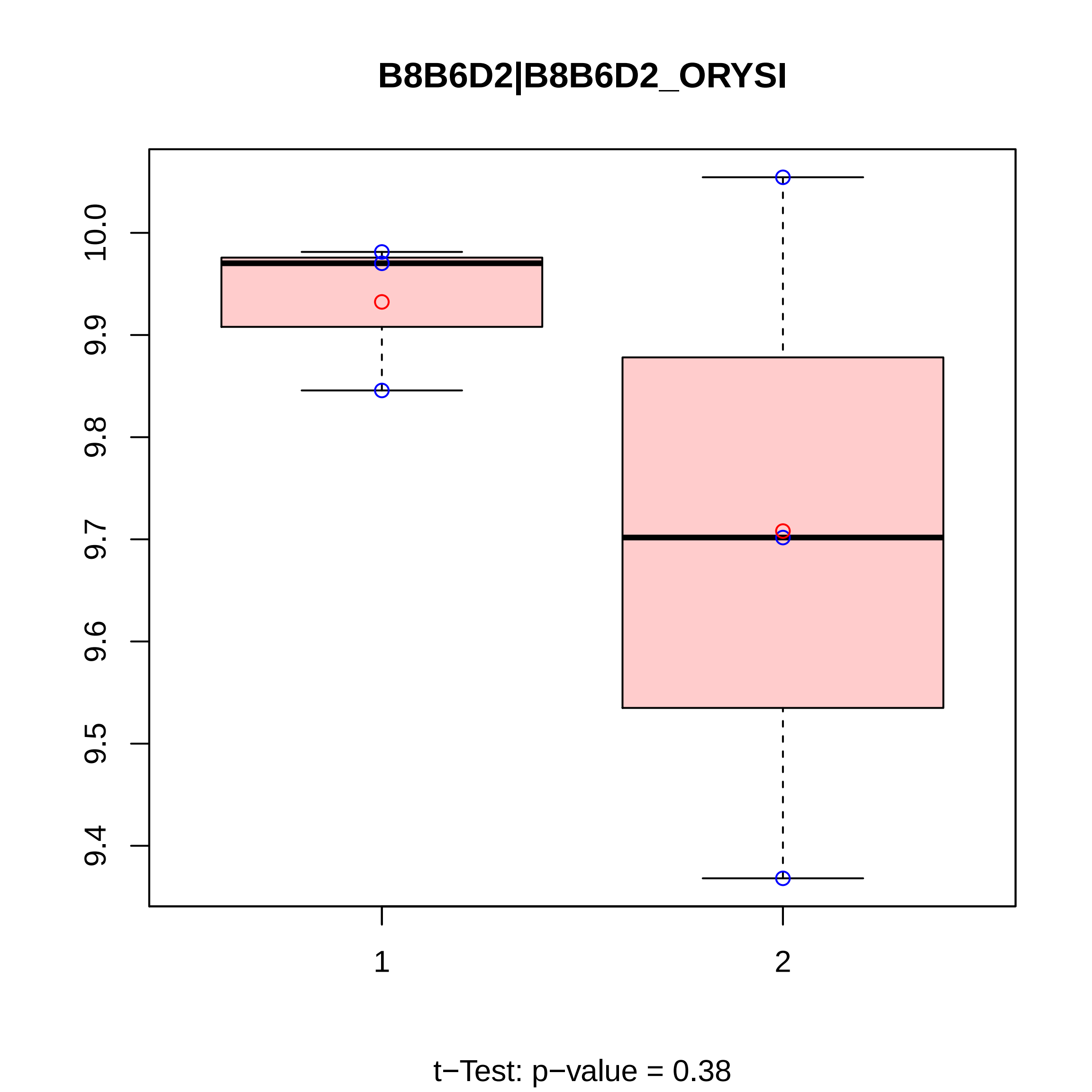

B8B6D2|B8B6D2_ORYSI
10.0
9.9
9.8
9.7
9.6
9.5
9.4
1
2
t−Test: p−value = 0.38

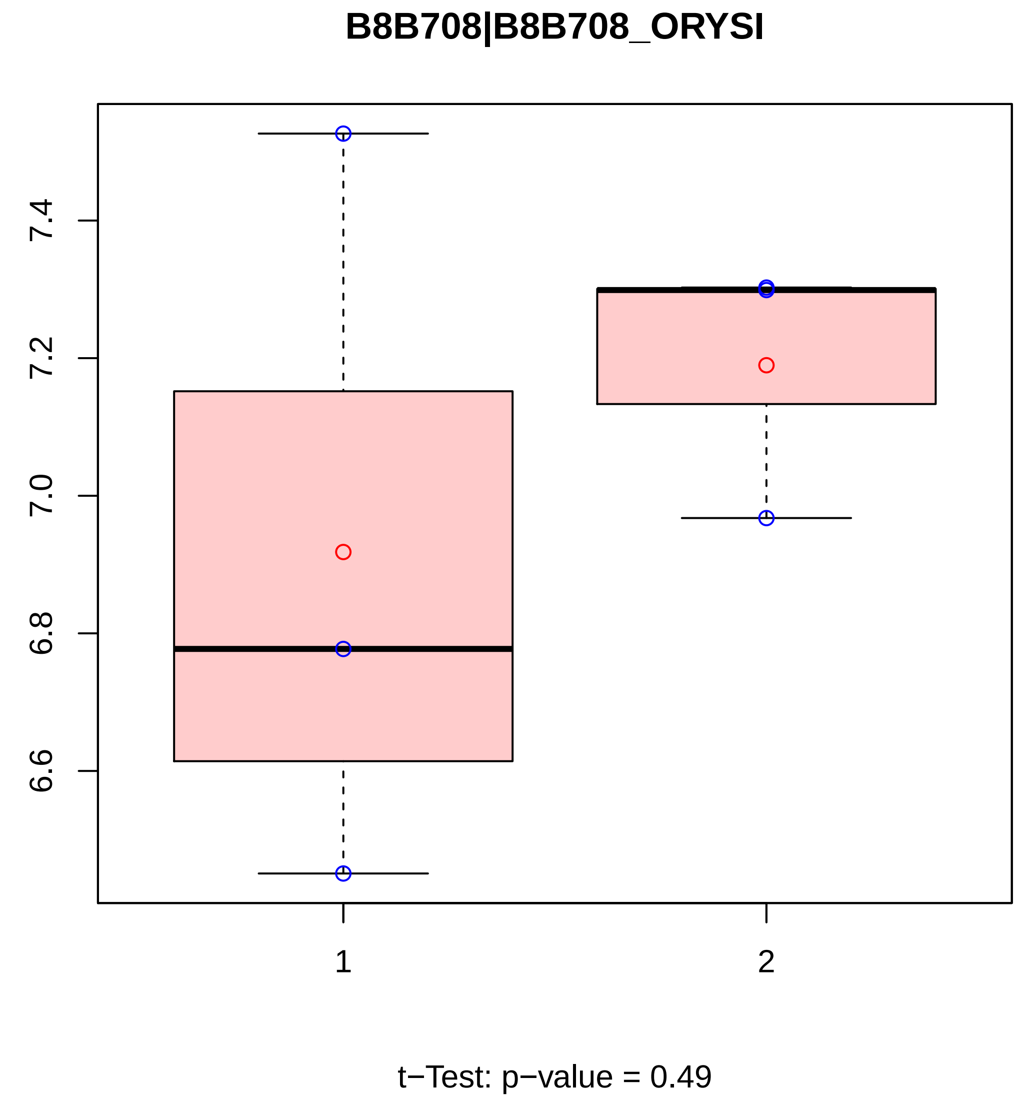
B8B708|B8B708_ORYSI
7.4
7.2
7.0
6.8
6.6
1
2
t−Test: p−value = 0.49

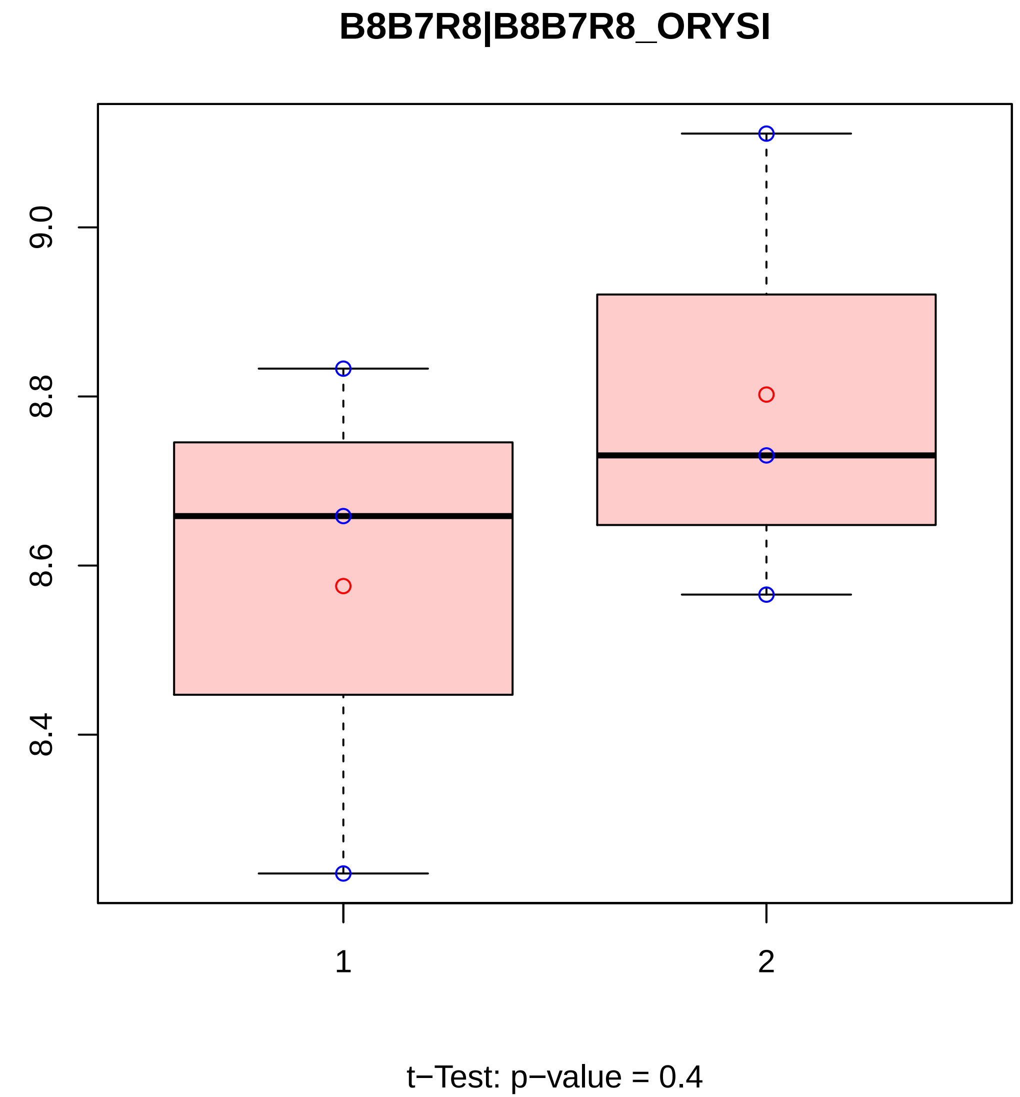
B8B7R8|B8B7R8_ORYSI
9.0
8.8
8.6
8.4
1
2
t−Test: p−value = 0.4

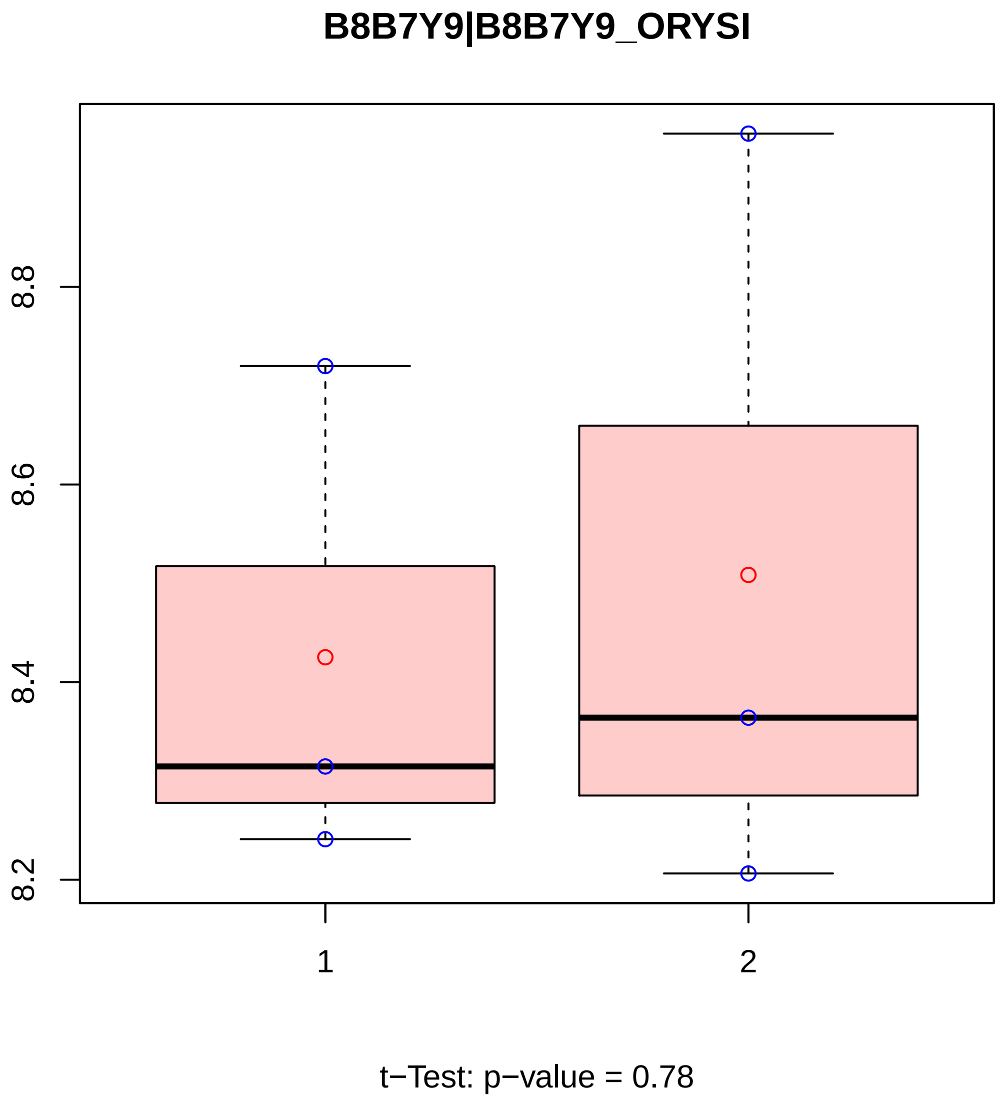
B8B7Y9|B8B7Y9_ORYSI
8.2
8.4
8.6
8.8
1
2
t−Test: p−value = 0.78

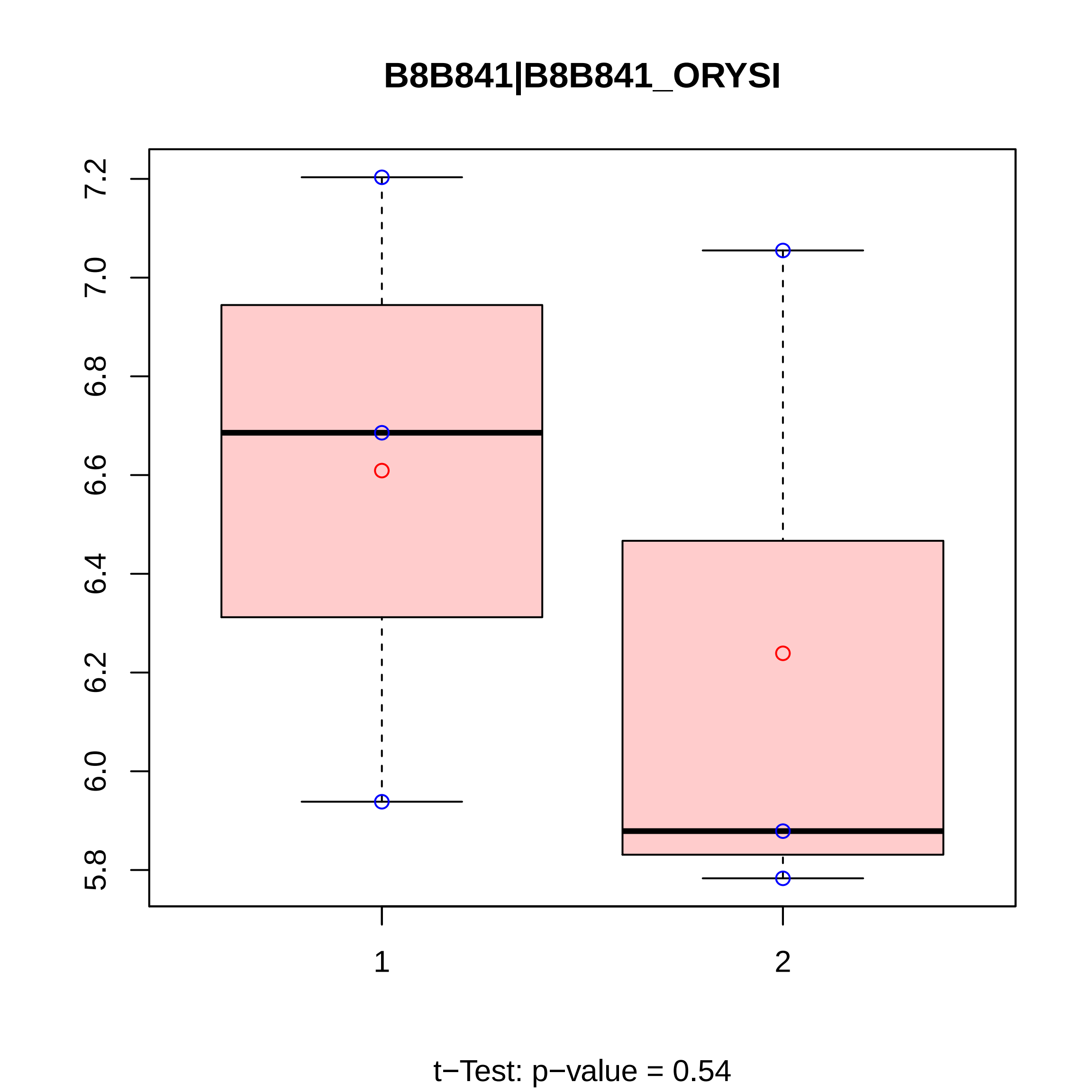

B8B841|B8B841_ORYSI
7.2
7.0
6.8
6.6
6.4
6.2
6.0
5.8
1
2
t−Test: p−value = 0.54

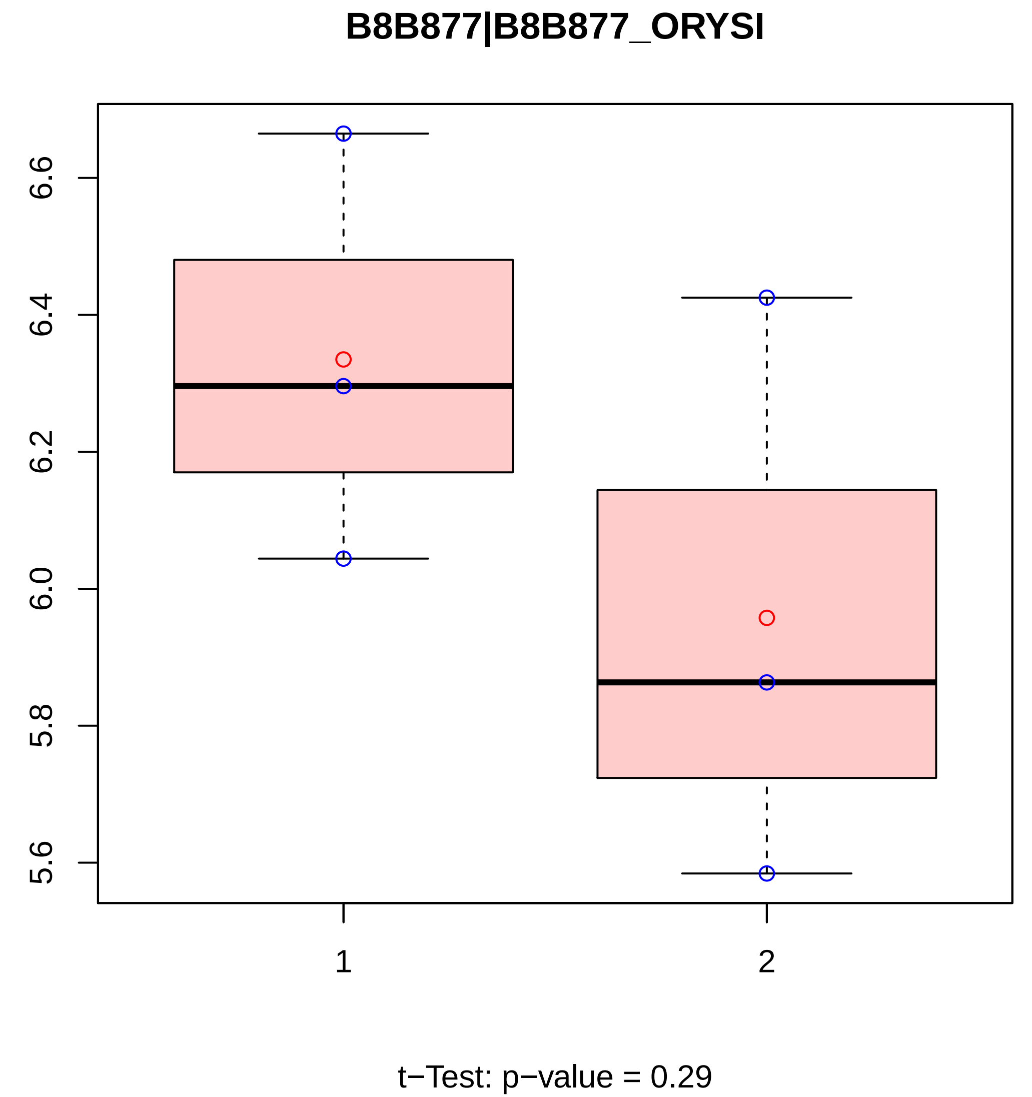

B8B877|B8B877_ORYSI
6.6
6.4
6.2
6.0
5.8
5.6
1
2
t−Test: p−value = 0.29

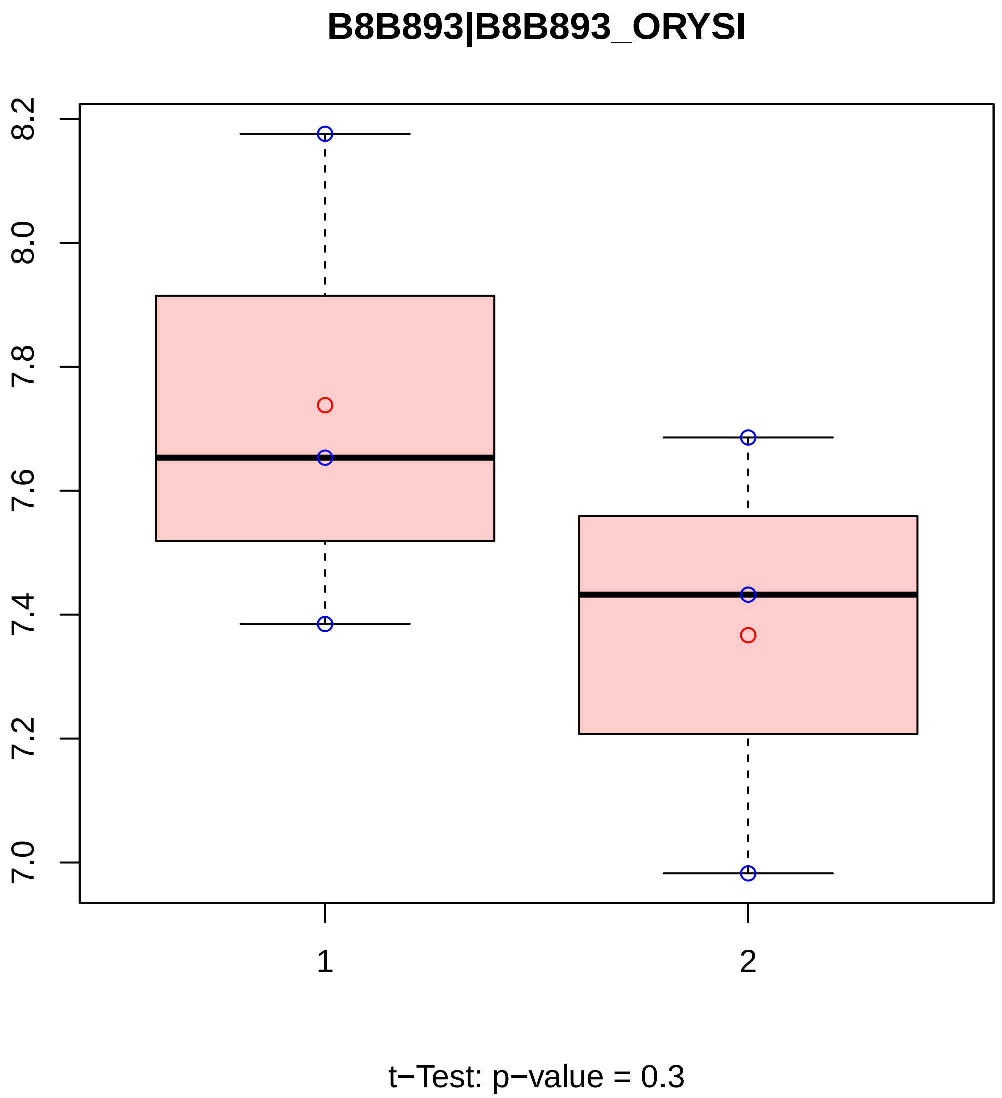
B8B893|B8B893_ORYSI
8.2
8.0
7.8
7.6
7.4
7.2
7.0
1
2
t−Test: p−value = 0.3

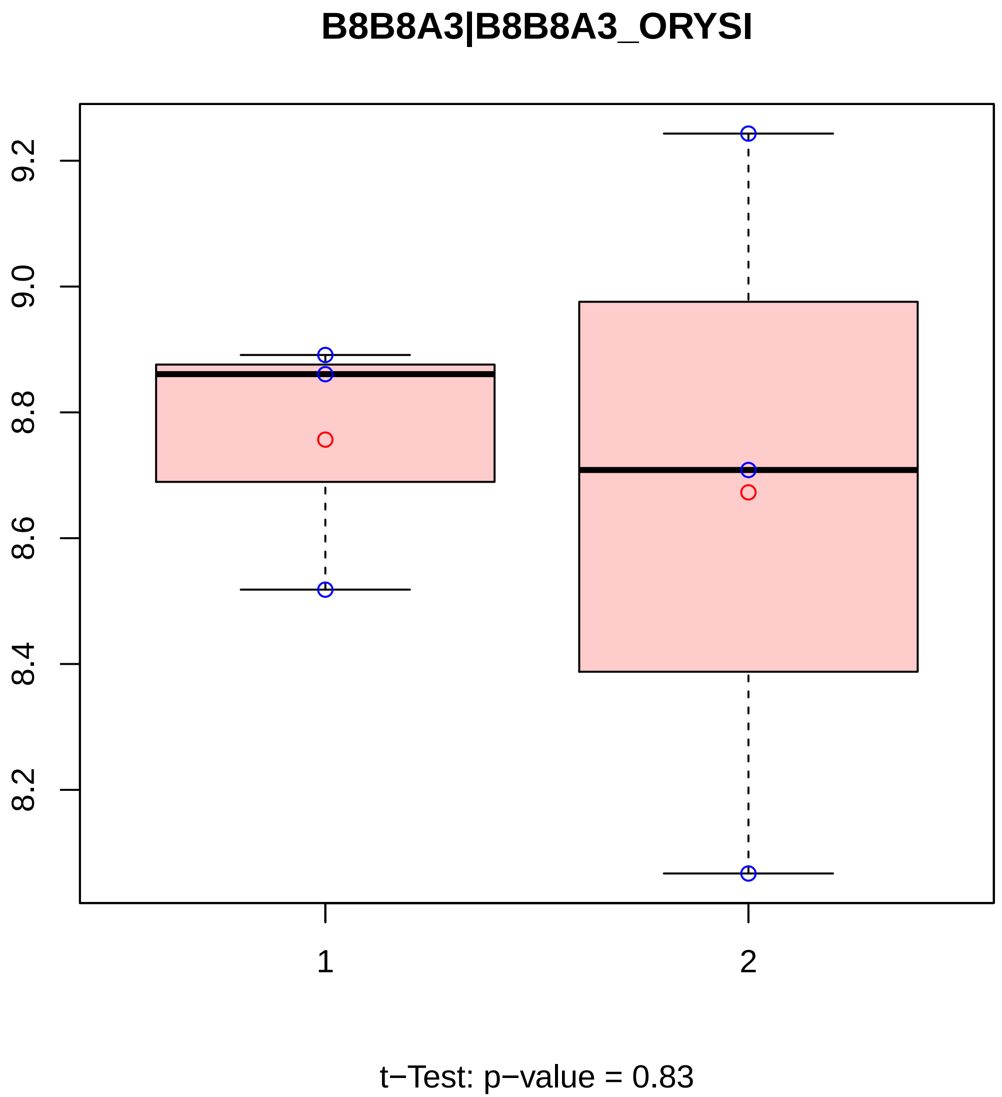

B8B8A3|B8B8A3_ORYSI
9.2
9.0
8.8
8.6
8.4
8.2
1
2
t−Test: p−value = 0.83

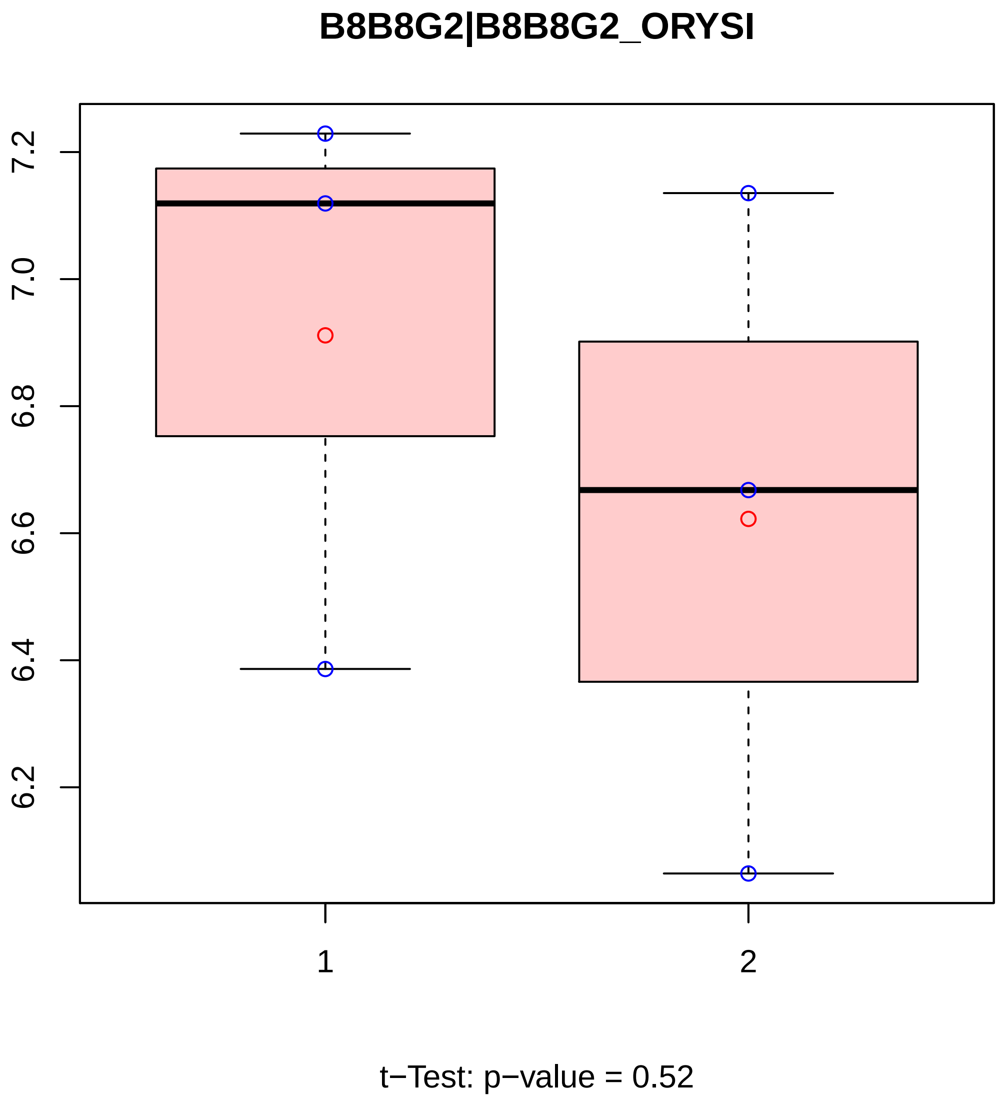

B8B8G2|B8B8G2_ORYSI
7.2
7.0
6.8
6.6
6.4
6.2
1
2
t−Test: p−value = 0.52

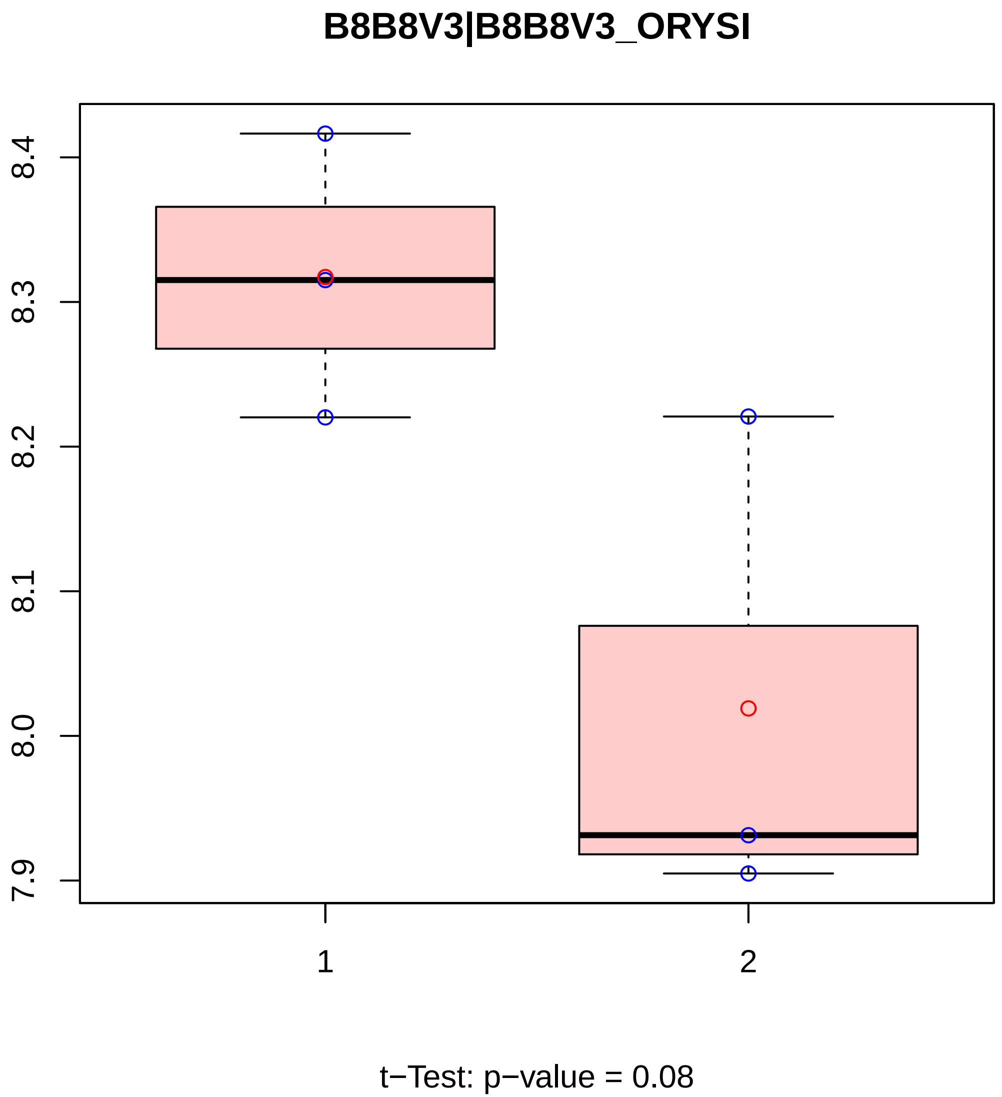
B8B8V3|B8B8V3_ORYSI
8.4
8.3
8.2
8.1
8.0
7.9
1
2
t−Test: p−value = 0.08

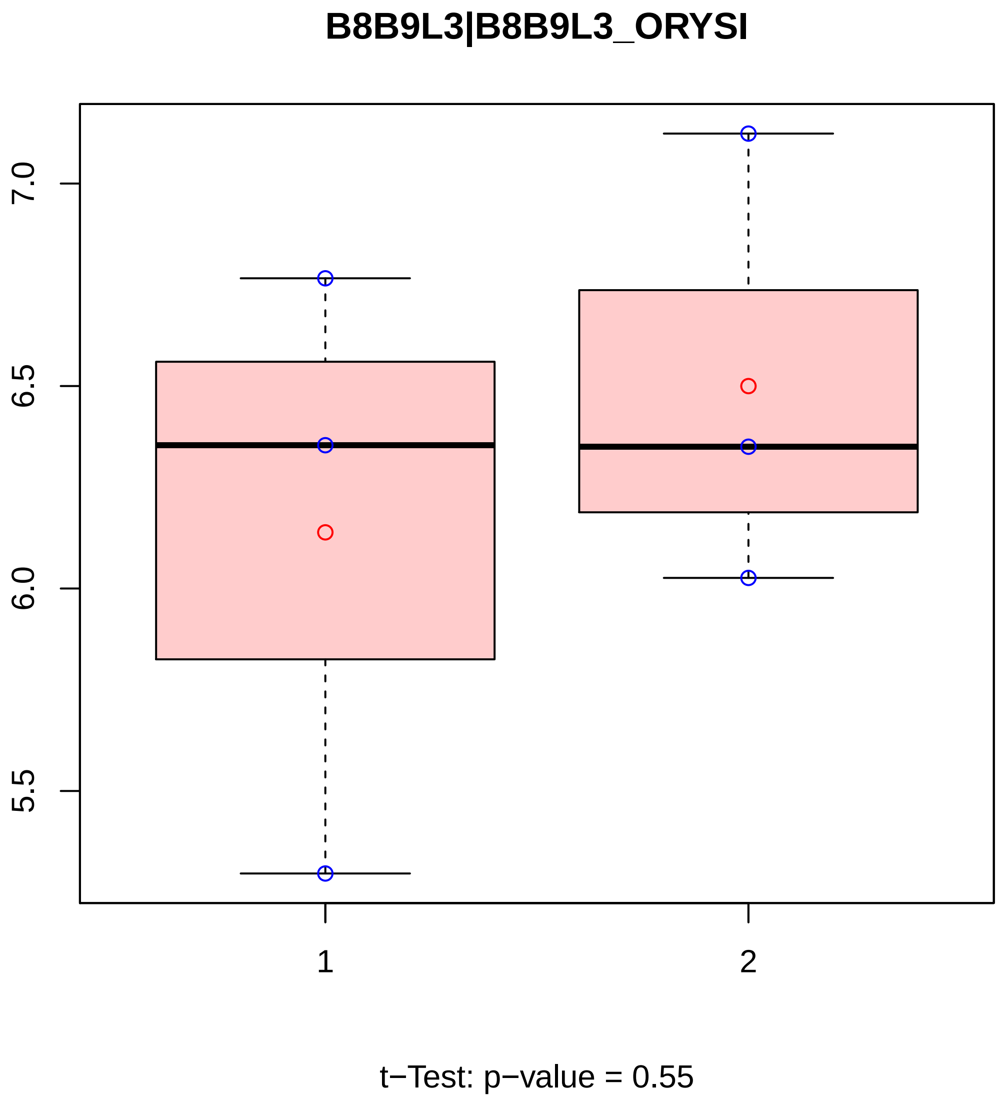
B8B9L3|B8B9L3_ORYSI
7.0
6.5
6.0
5.5
1
2
t−Test: p−value = 0.55

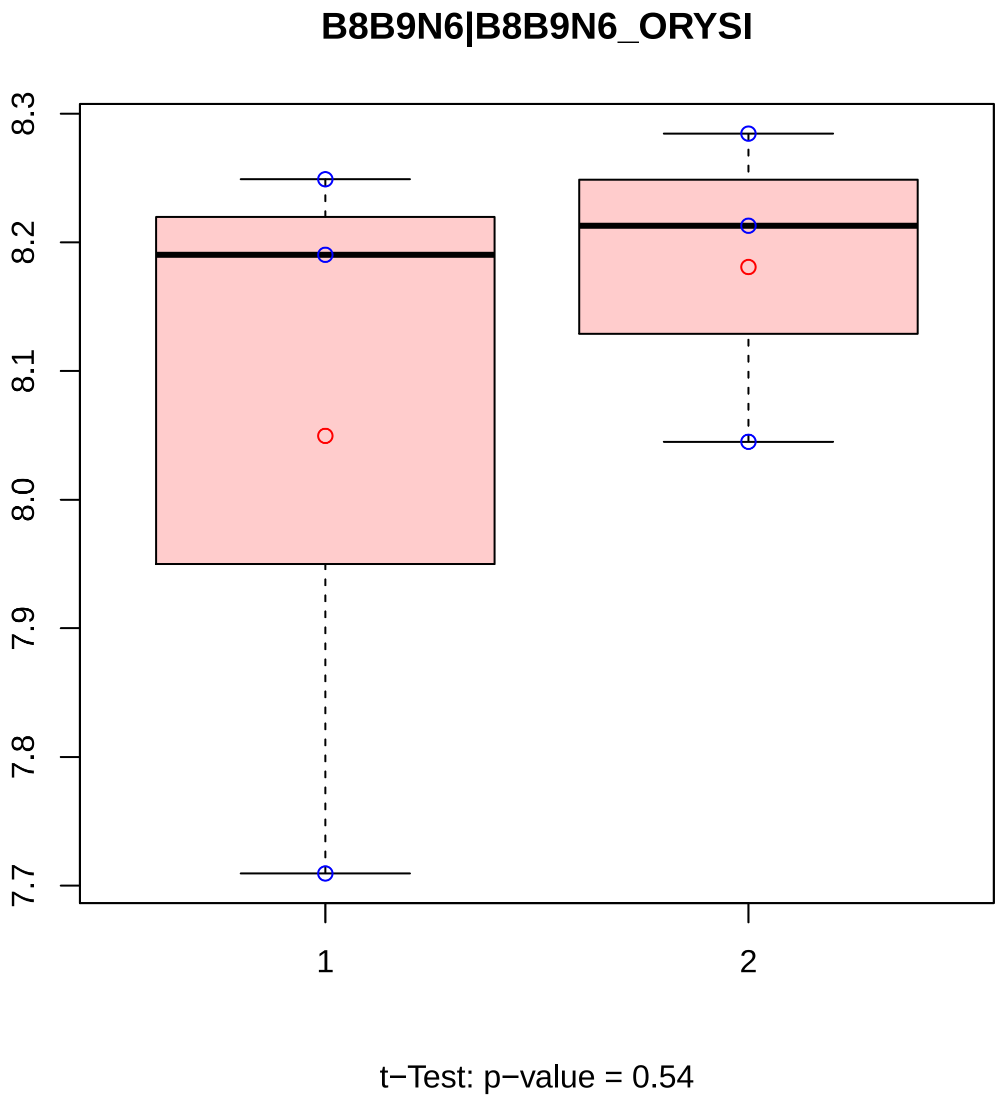

B8B9N6|B8B9N6_ORYSI
8.3
8.2
8.1
8.0
7.9
7.8
7.7
1
2
t−Test: p−value = 0.54

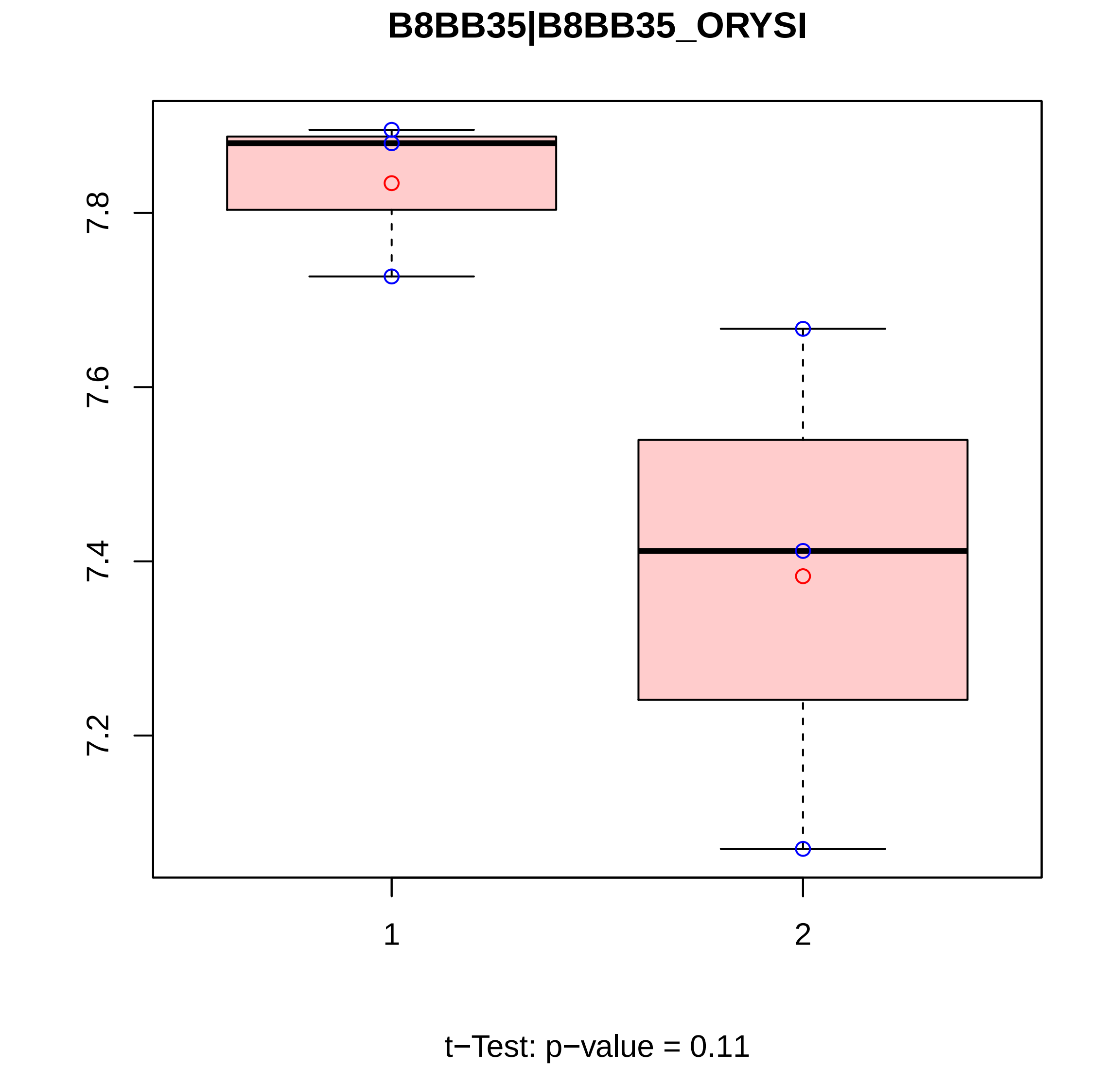
B8BB35|B8BB35_ORYSI
7.2
7.4
7.6
7.8
1
2
t−Test: p−value = 0.11

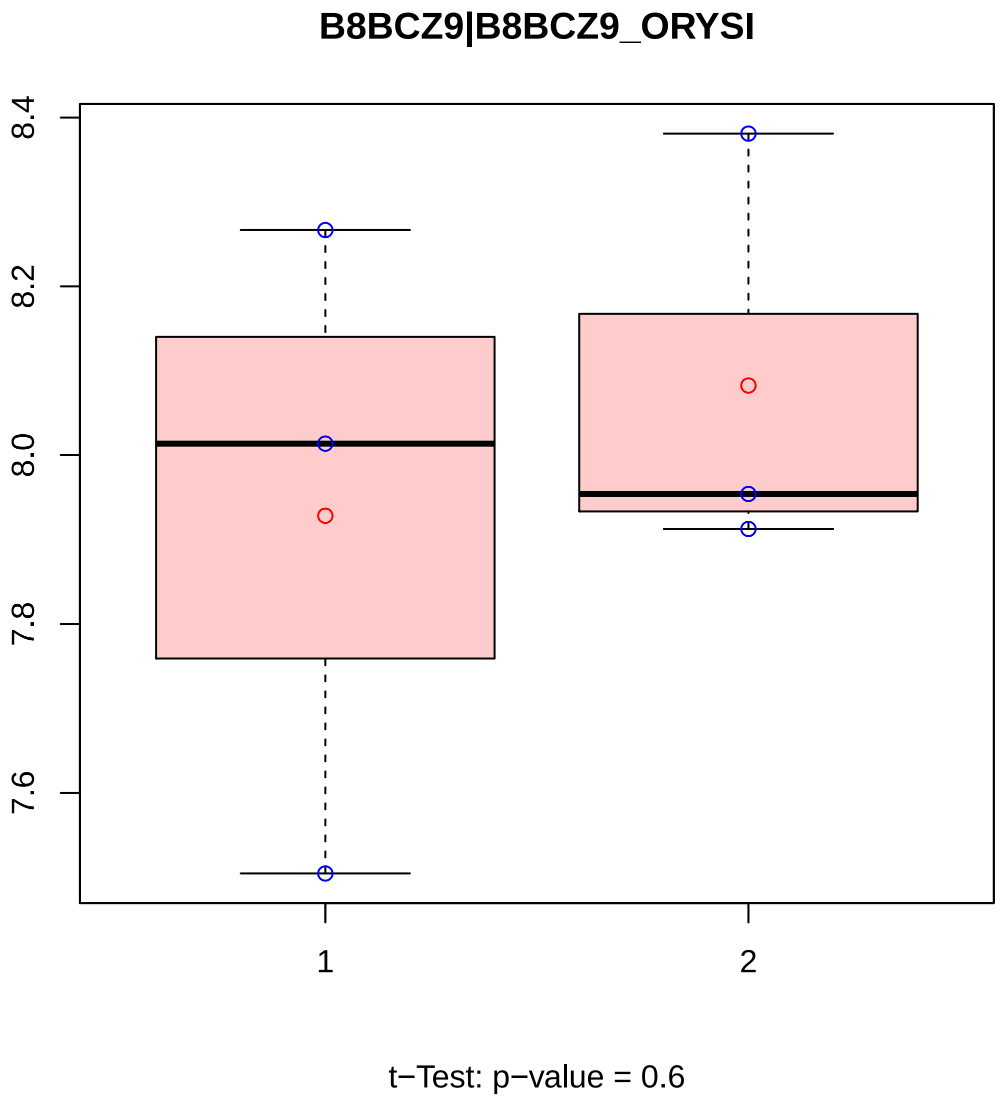
B8BCZ9|B8BCZ9_ORYSI
8.4
8.2
8.0
7.8
7.6
1
2
t−Test: p−value = 0.6

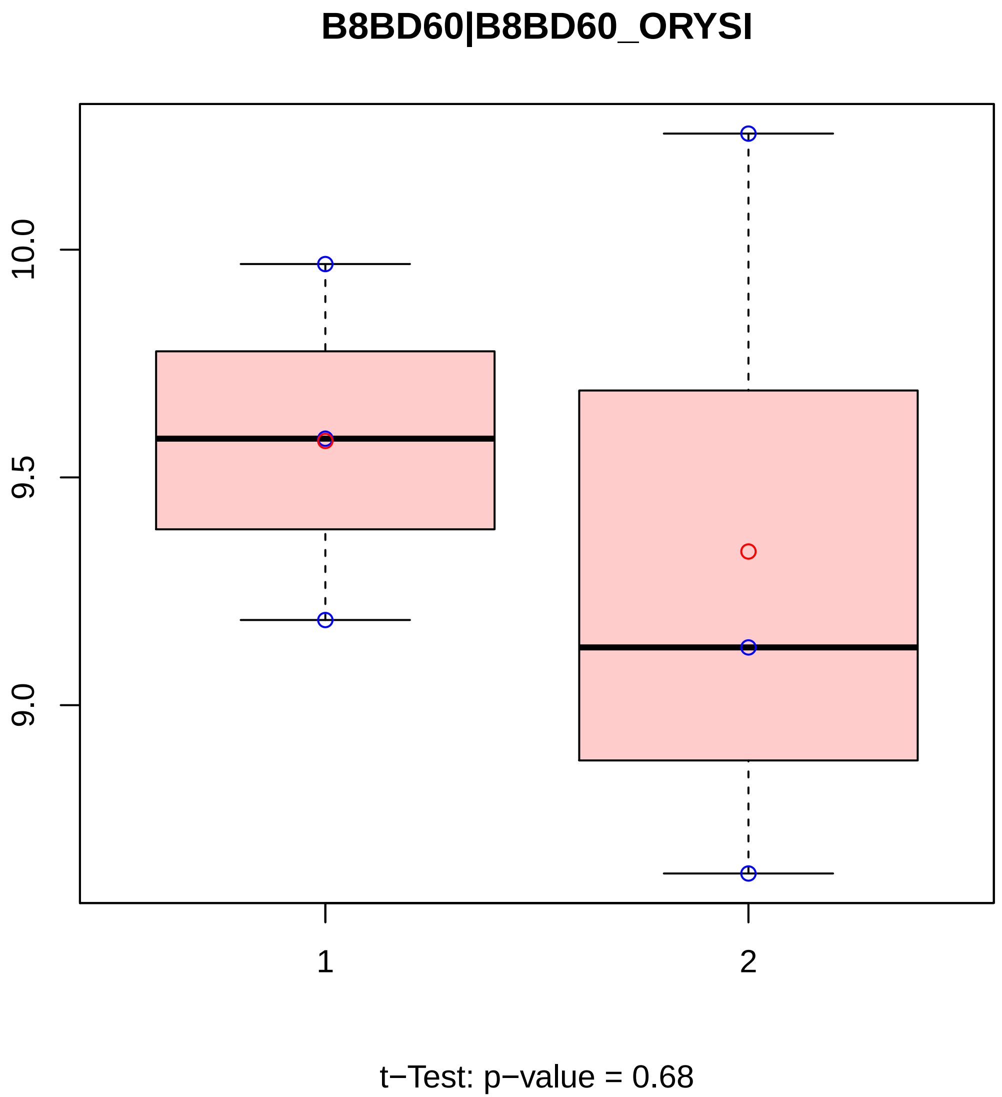

B8BD60|B8BD60_ORYSI
10.0
9.5
9.0
1
2
t−Test: p−value = 0.68

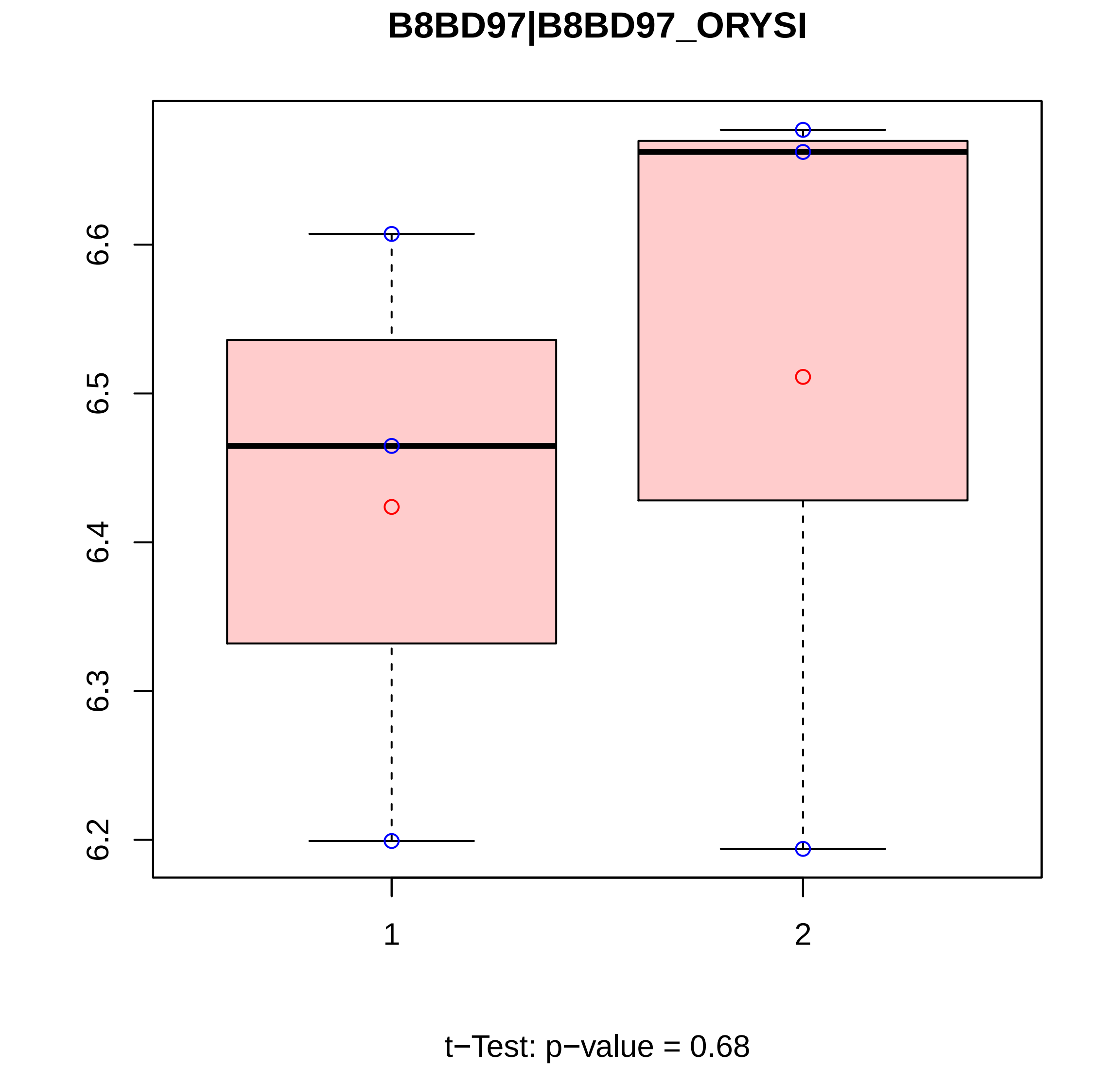
B8BD97|B8BD97_ORYSI
6.2
6.3
6.4
6.5
6.6
1
2
t−Test: p−value = 0.68

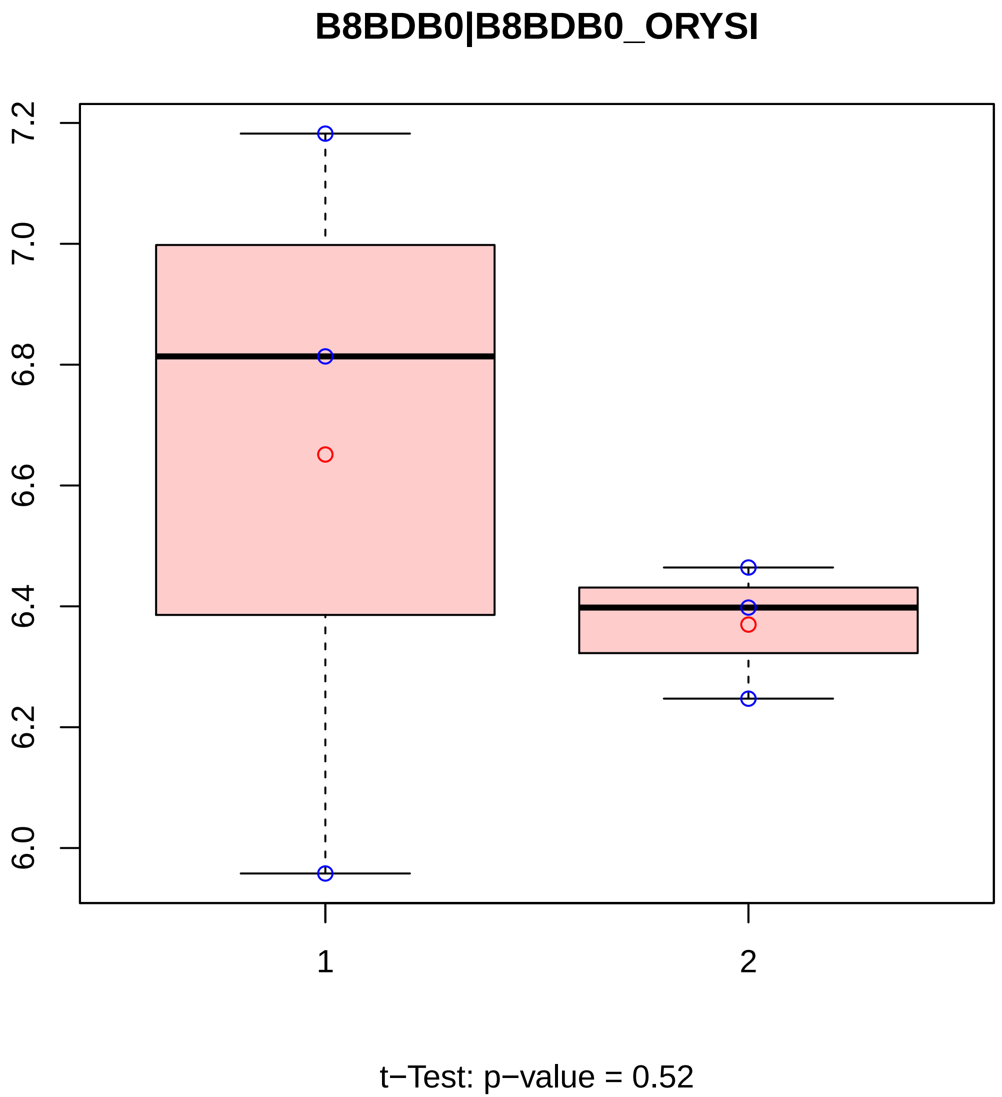

B8BDB0|B8BDB0_ORYSI
7.2
7.0
6.8
6.6
6.4
6.2
6.0
1
2
t−Test: p−value = 0.52

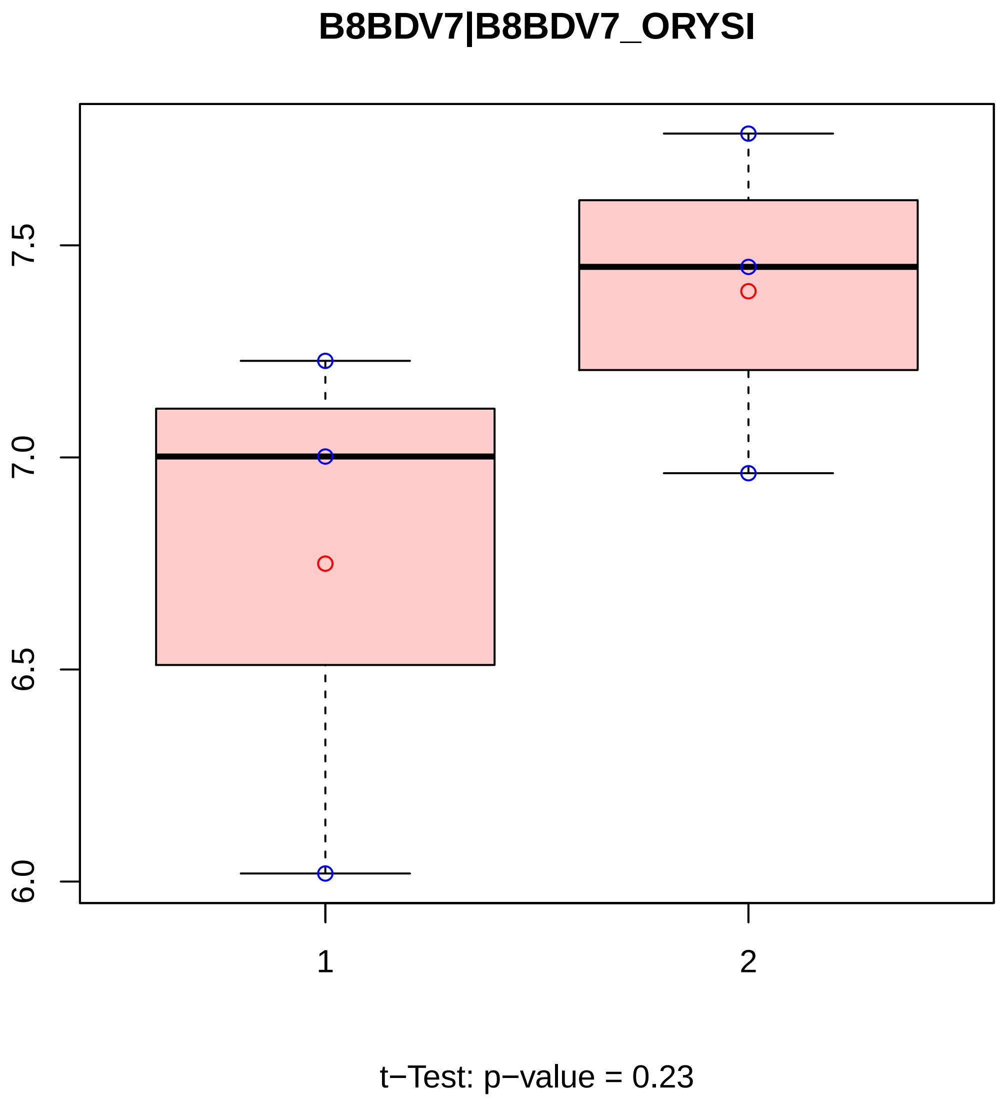

B8BDV7|B8BDV7_ORYSI
7.5
7.0
6.5
6.0
1
2
t−Test: p−value = 0.23

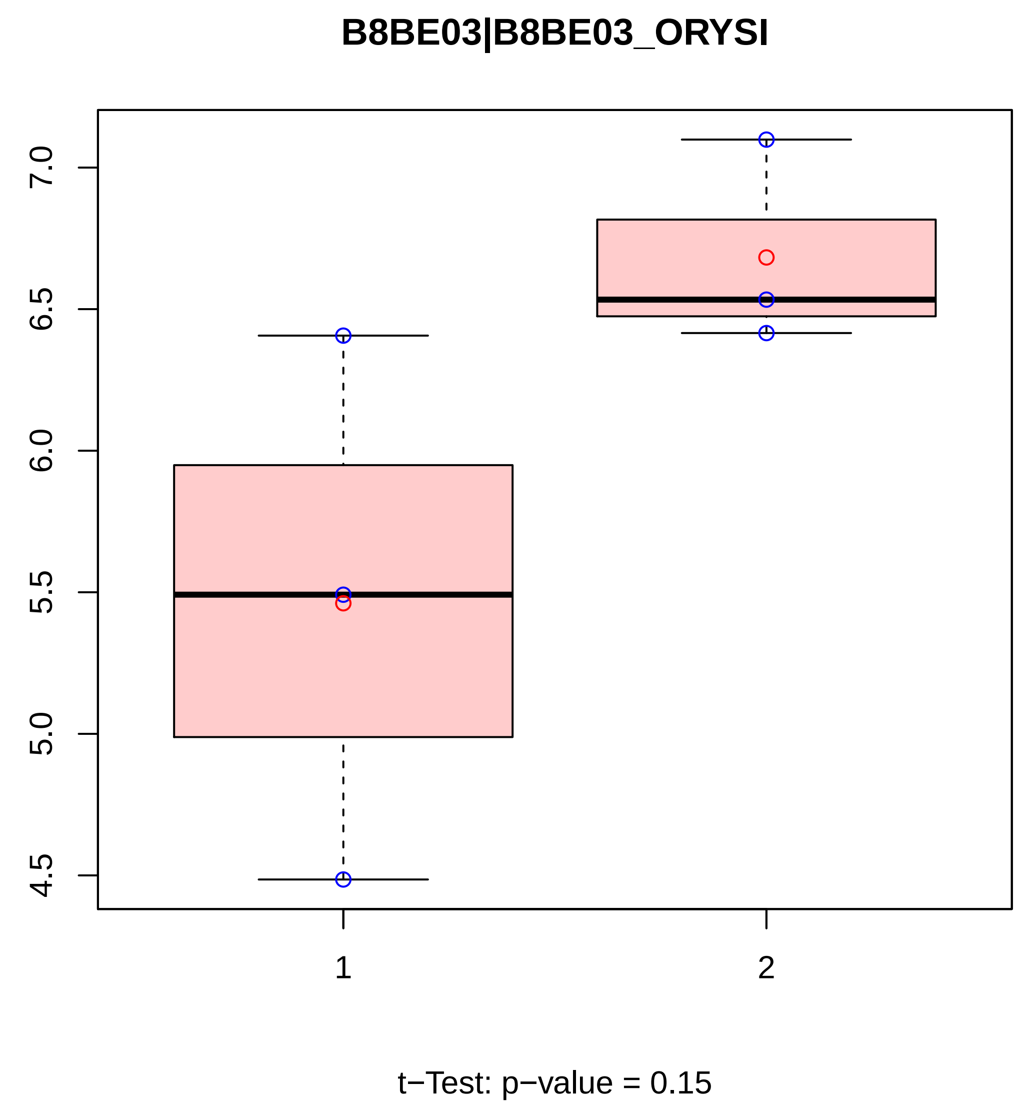
B8BE03|B8BE03_ORYSI
4.5
5.0
5.5
6.0
6.5
7.0
1
2
t−Test: p−value = 0.15

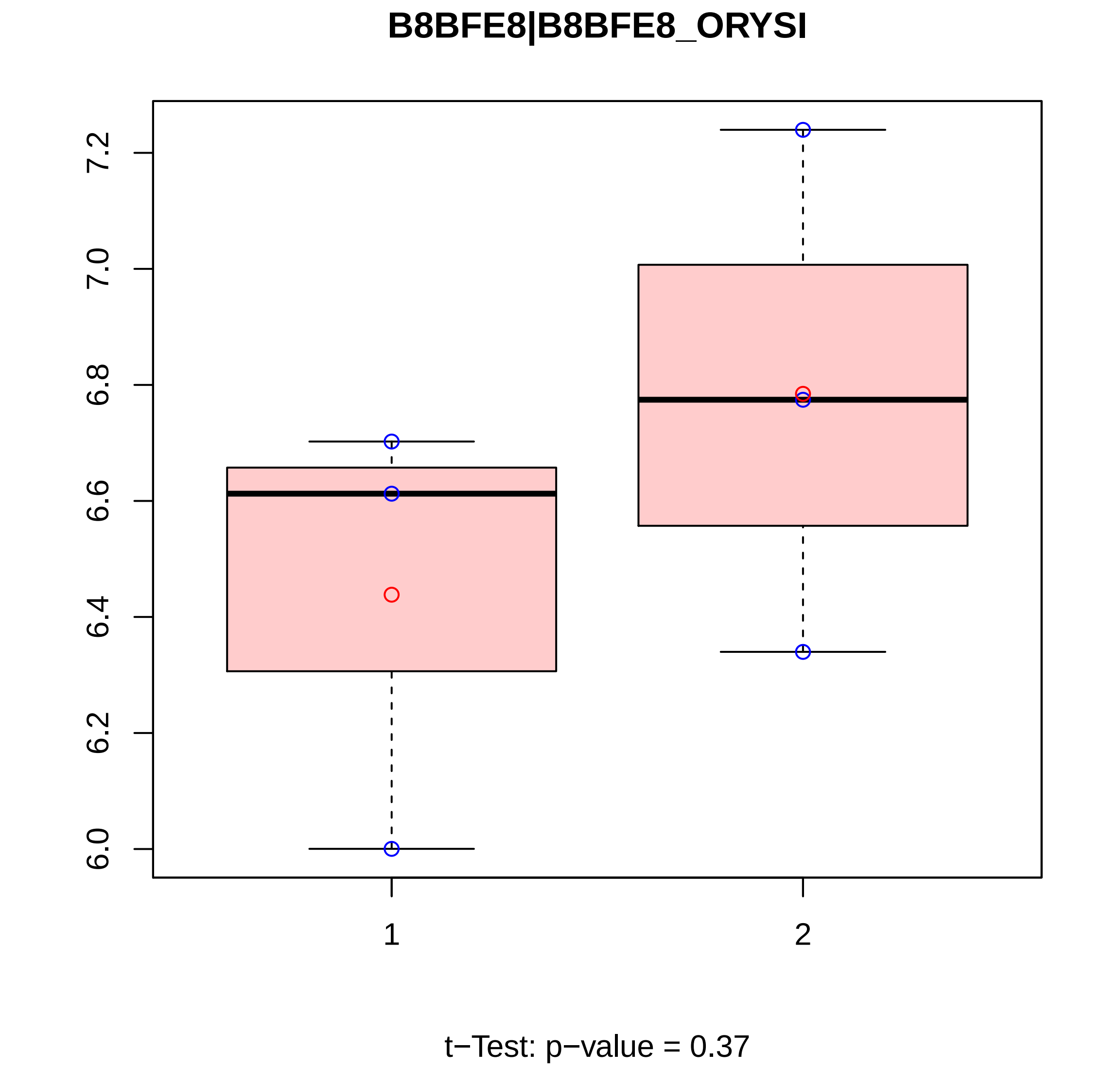
B8BFE8|B8BFE8_ORYSI
6.0
6.2
6.4
6.6
6.8
7.0
7.2
1
2
t−Test: p−value = 0.37

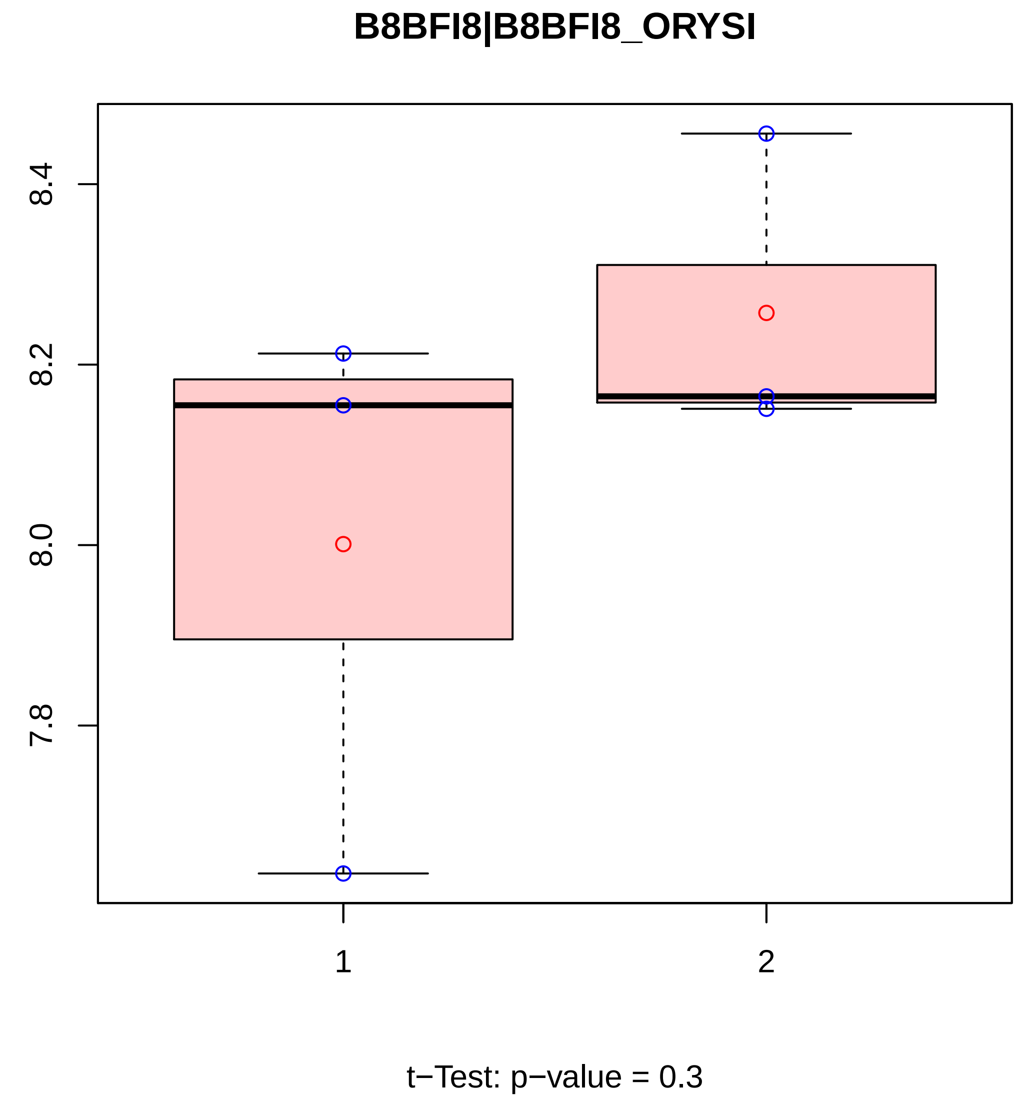
B8BFI8|B8BFI8_ORYSI
8.4
8.2
8.0
7.8
1
2
t−Test: p−value = 0.3

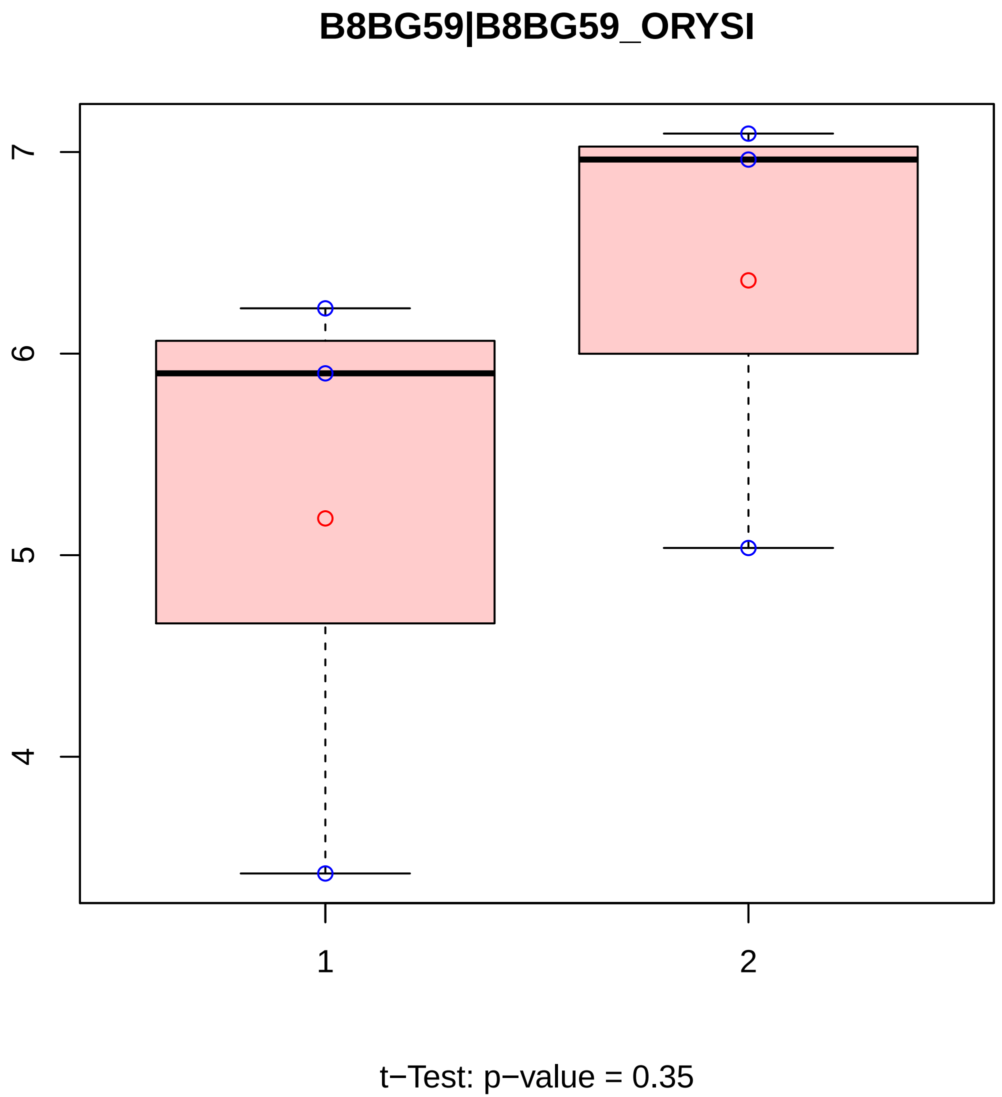

B8BG59|B8BG59_ORYSI
7
6
5
4
1
2
t−Test: p−value = 0.35

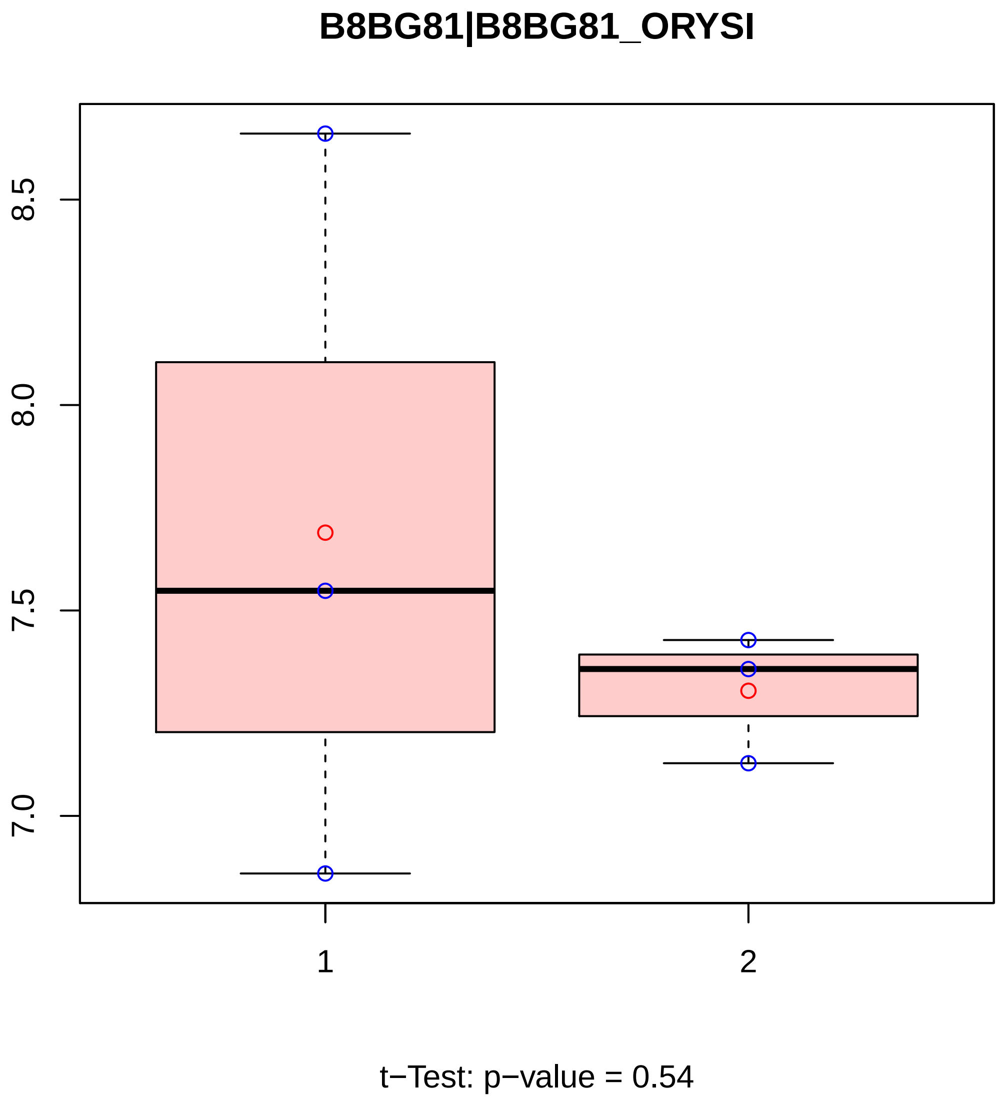

B8BG81|B8BG81_ORYSI
8.5
8.0
7.5
7.0
1
2
t−Test: p−value = 0.54

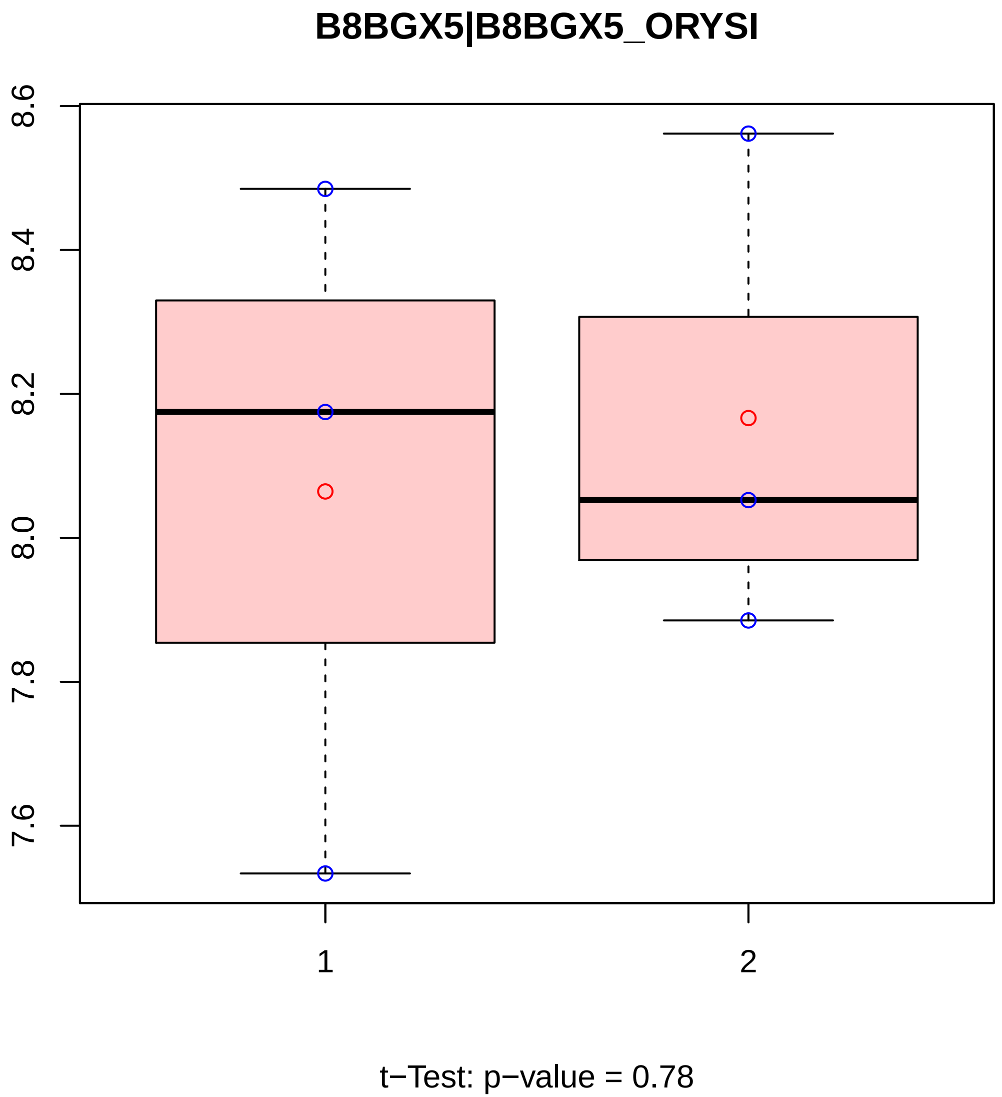
B8BGX5|B8BGX5_ORYSI
8.6
8.4
8.2
8.0
7.8
7.6
1
2
t−Test: p−value = 0.78

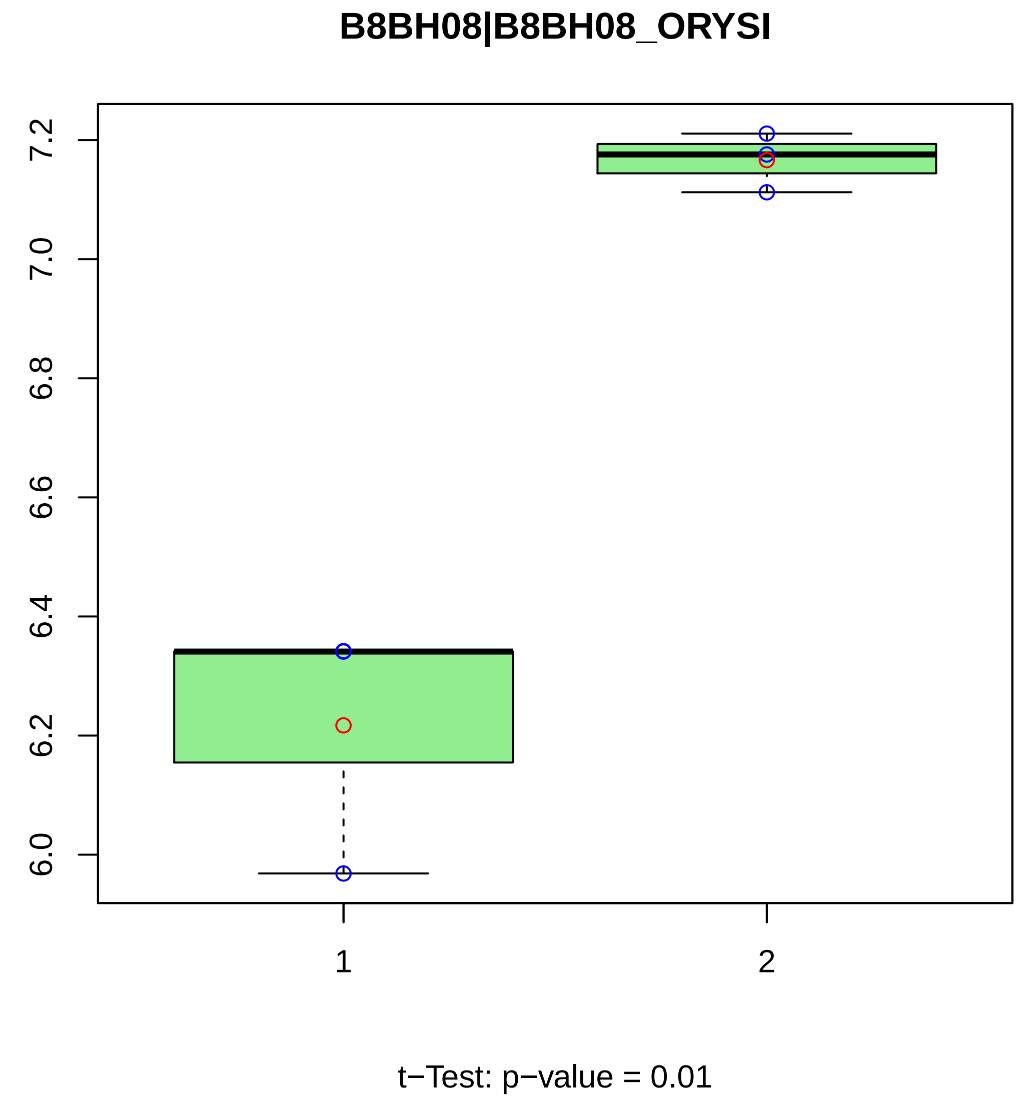

B8BH08|B8BH08_ORYSI
7.2
7.0
6.8
6.6
6.4
6.2
6.0
1
2
t−Test: p−value = 0.01

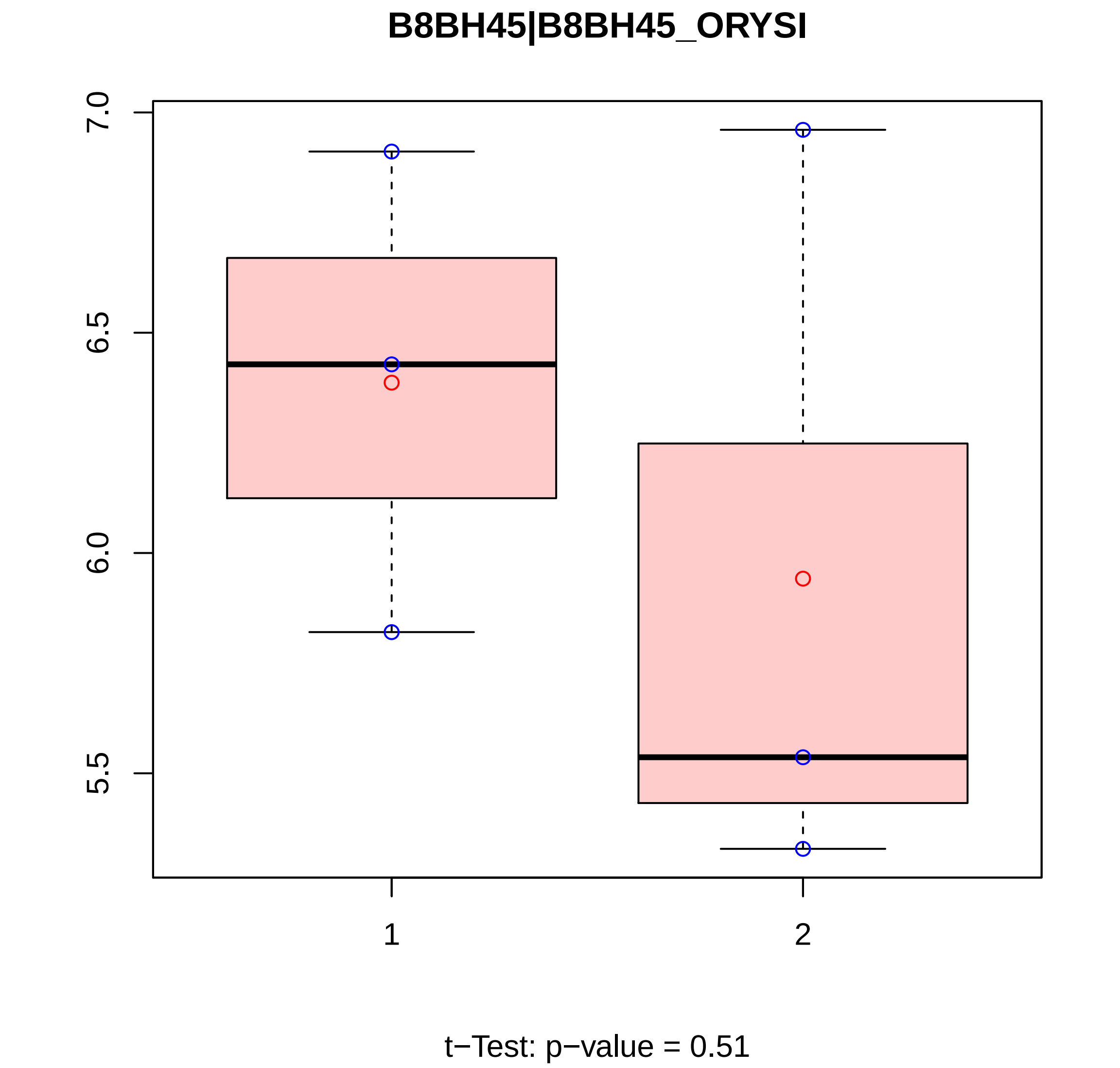
B8BH45|B8BH45_ORYSI
7.0
6.5
6.0
5.5
1
2
t−Test: p−value = 0.51

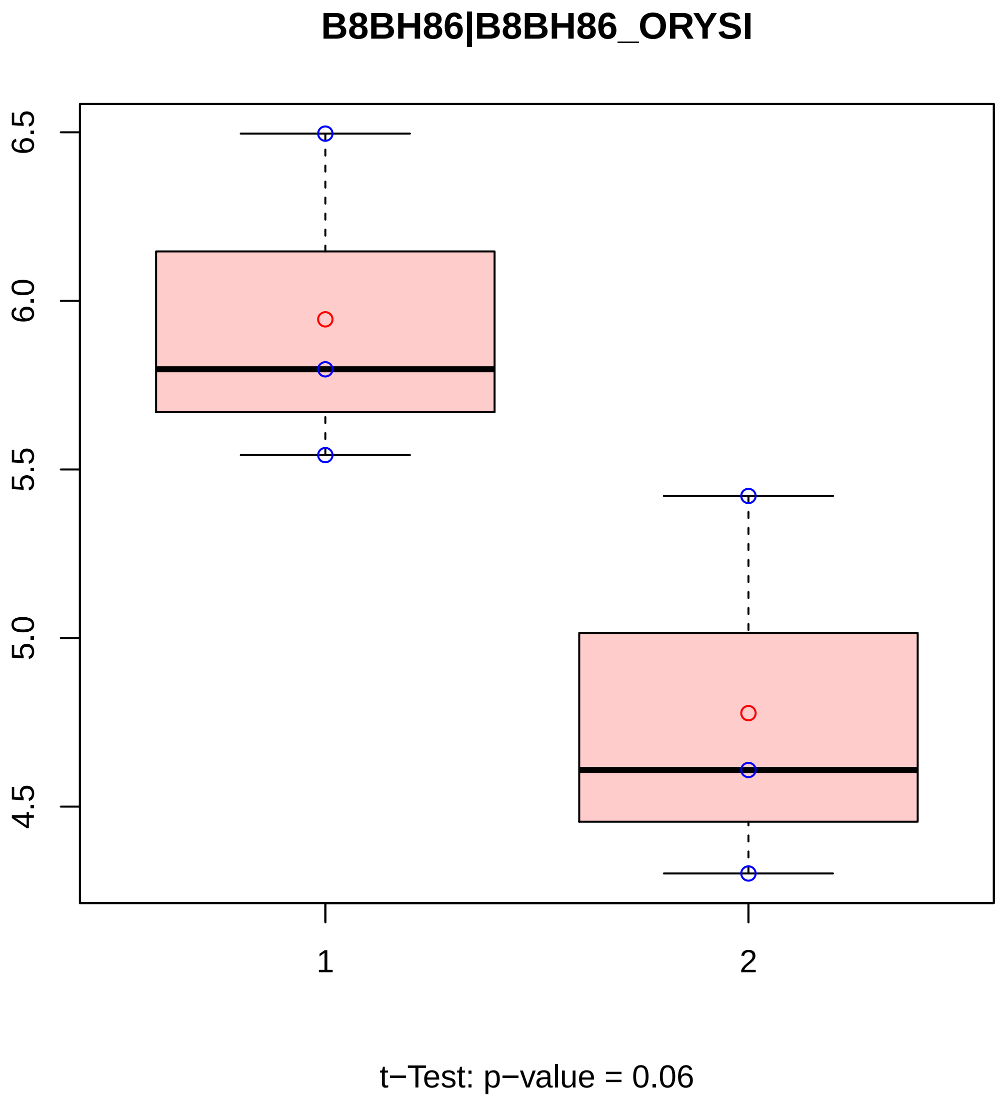

B8BH86|B8BH86_ORYSI
6.5
6.0
5.5
5.0
4.5
1
2
t−Test: p−value = 0.06

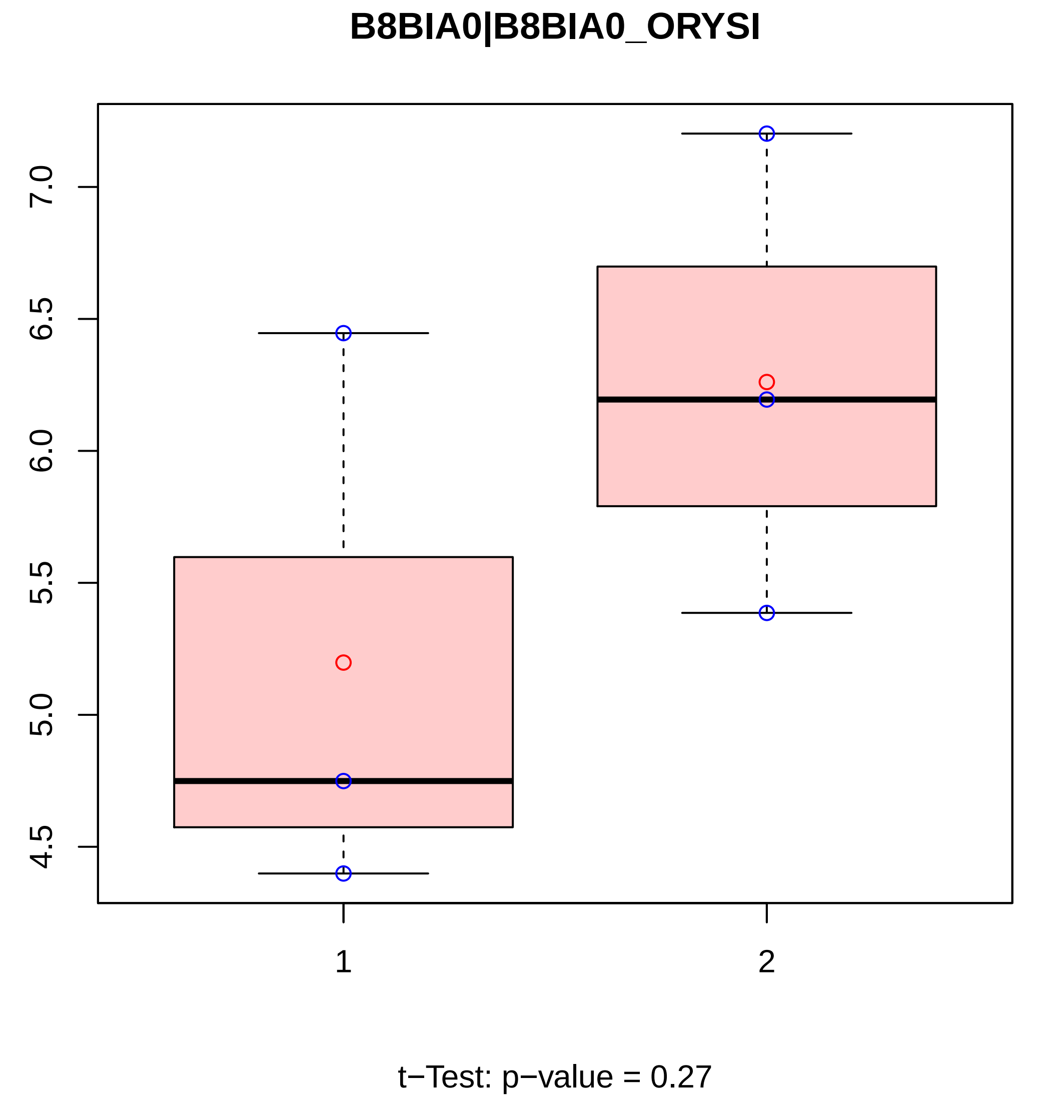
B8BIA0|B8BIA0_ORYSI
4.5
5.0
5.5
6.0
6.5
7.0
1
2
t−Test: p−value = 0.27

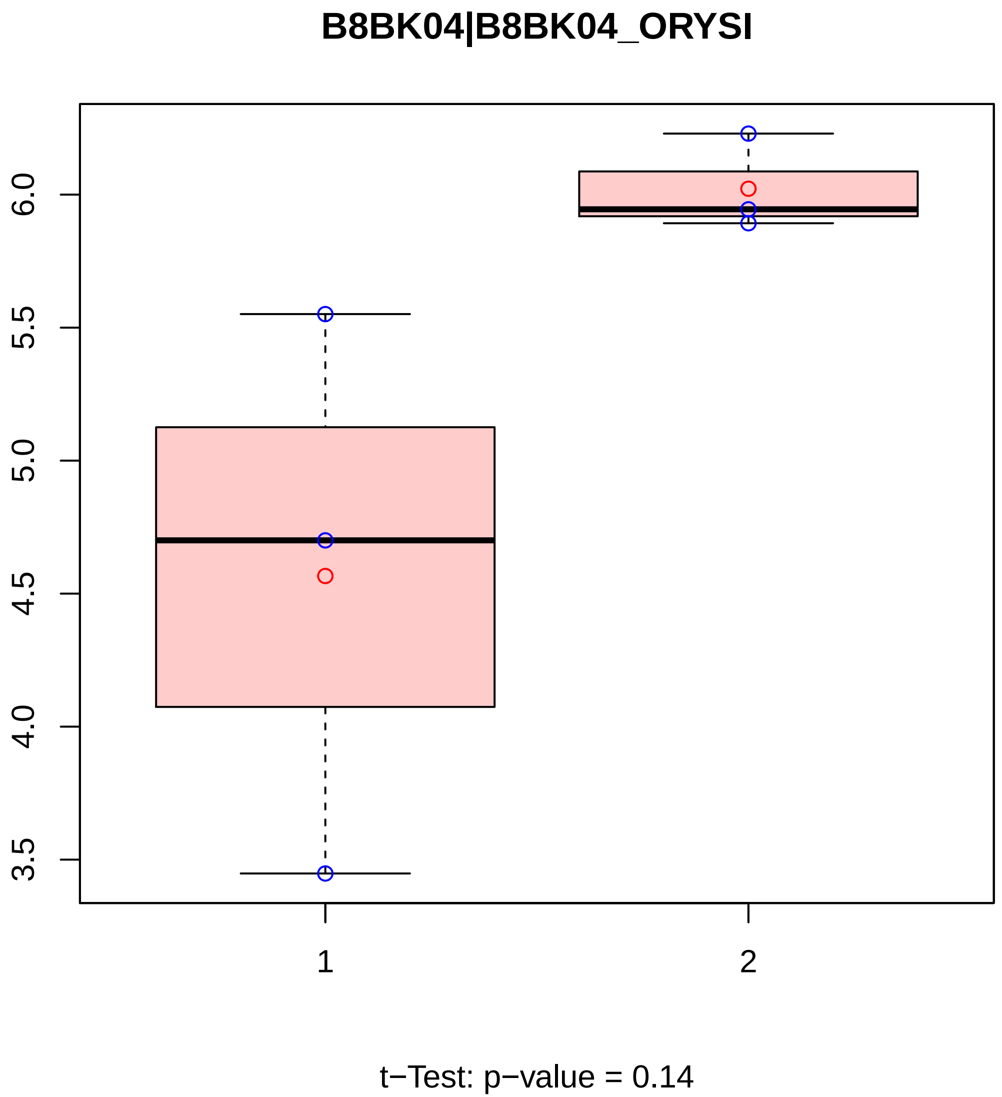

B8BK04|B8BK04_ORYSI
6.0
5.5
5.0
4.5
4.0
3.5
1
2
t−Test: p−value = 0.14

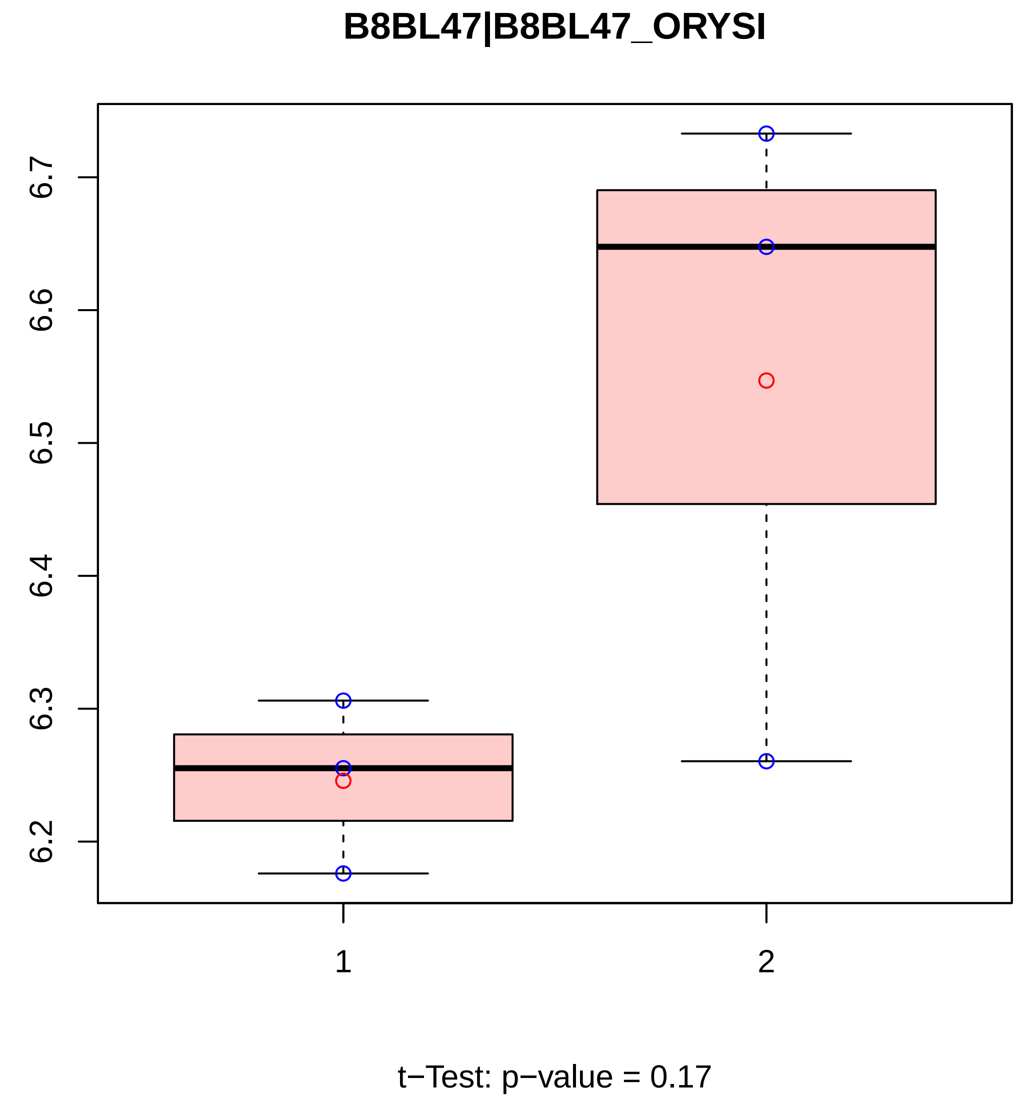

B8BL47|B8BL47_ORYSI
6.2
6.3
6.4
6.5
6.6
6.7
1
2
t−Test: p−value = 0.17

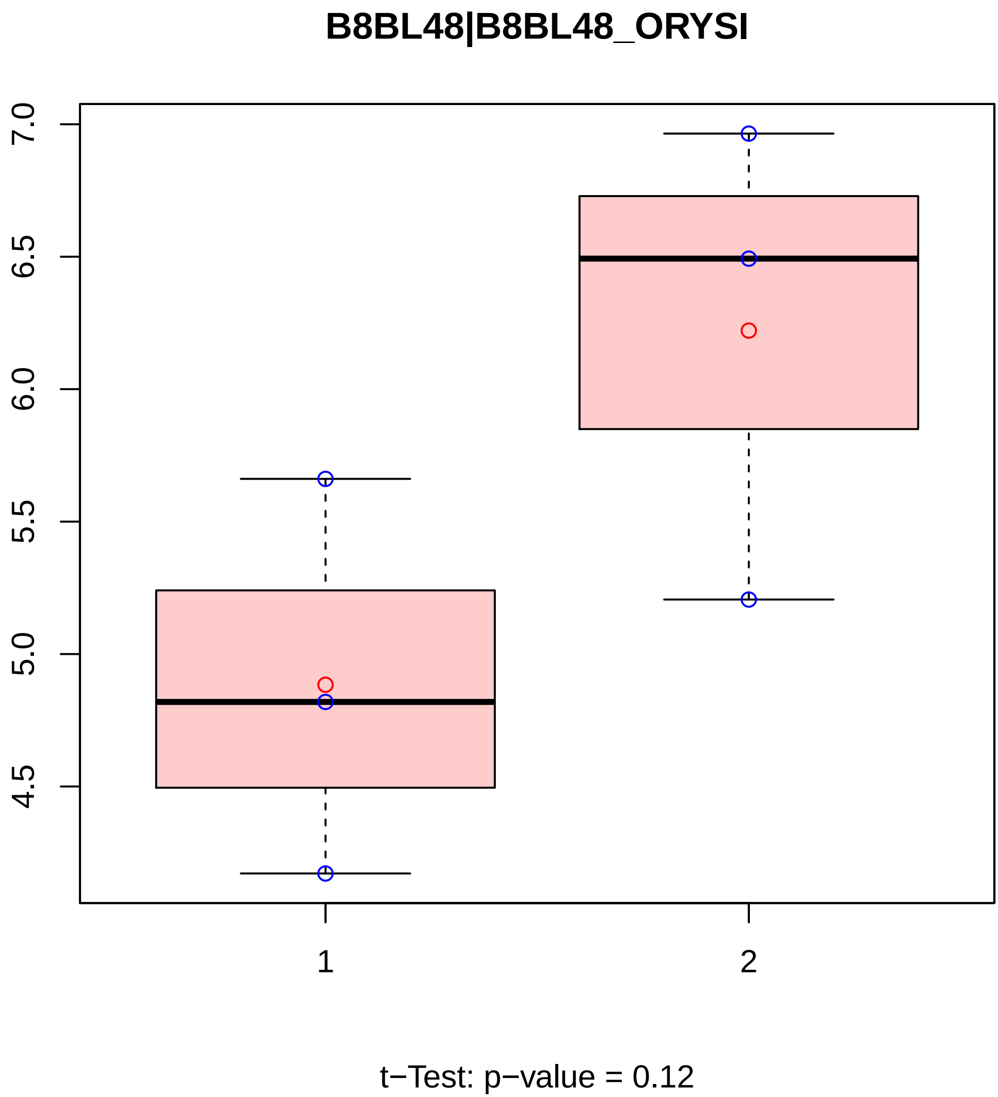
B8BL48|B8BL48_ORYSI
7.0
6.5
6.0
5.5
5.0
4.5
1
2
t−Test: p−value = 0.12

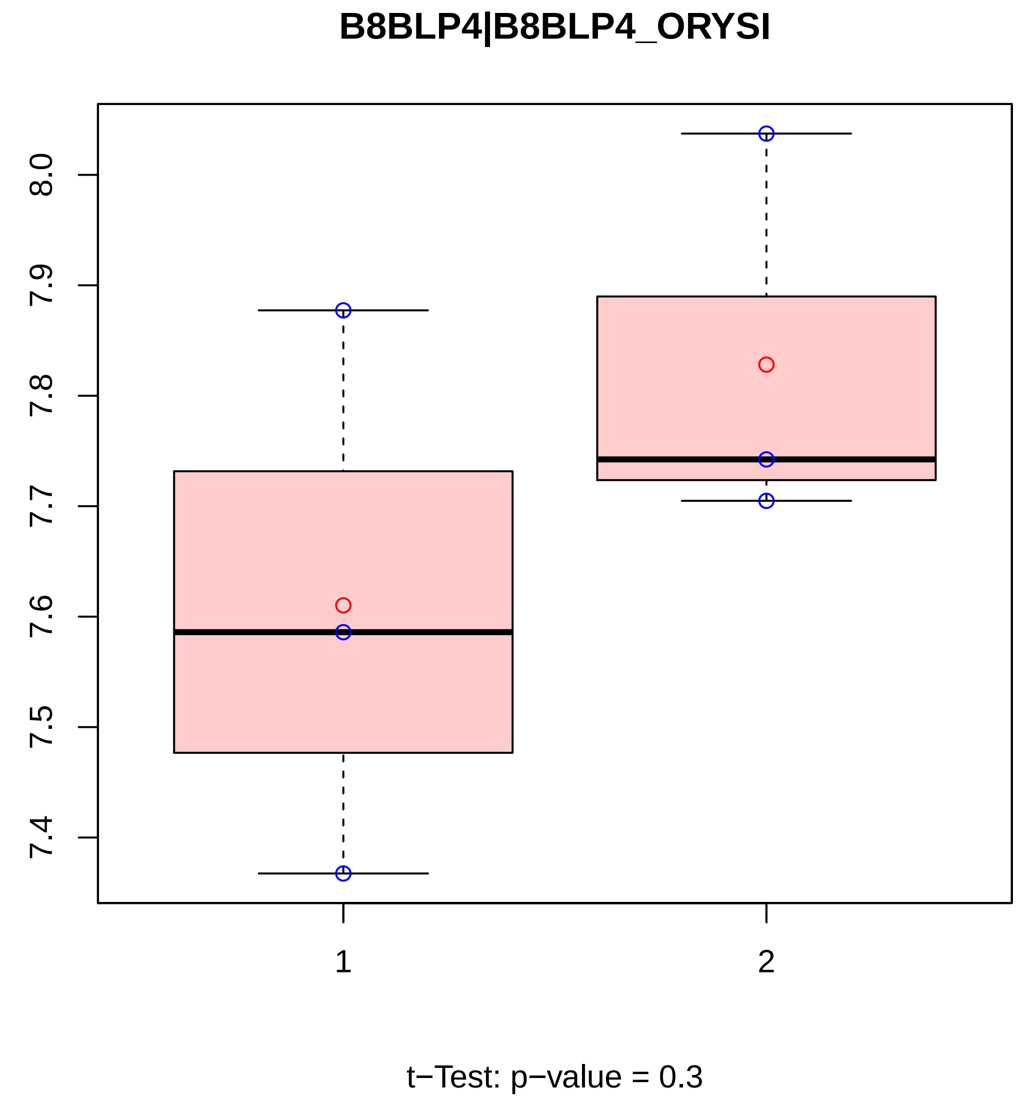
B8BLP4|B8BLP4_ORYSI
8.0
7.9
7.8
7.7
7.6
7.5
7.4
1
2
t−Test: p−value = 0.3

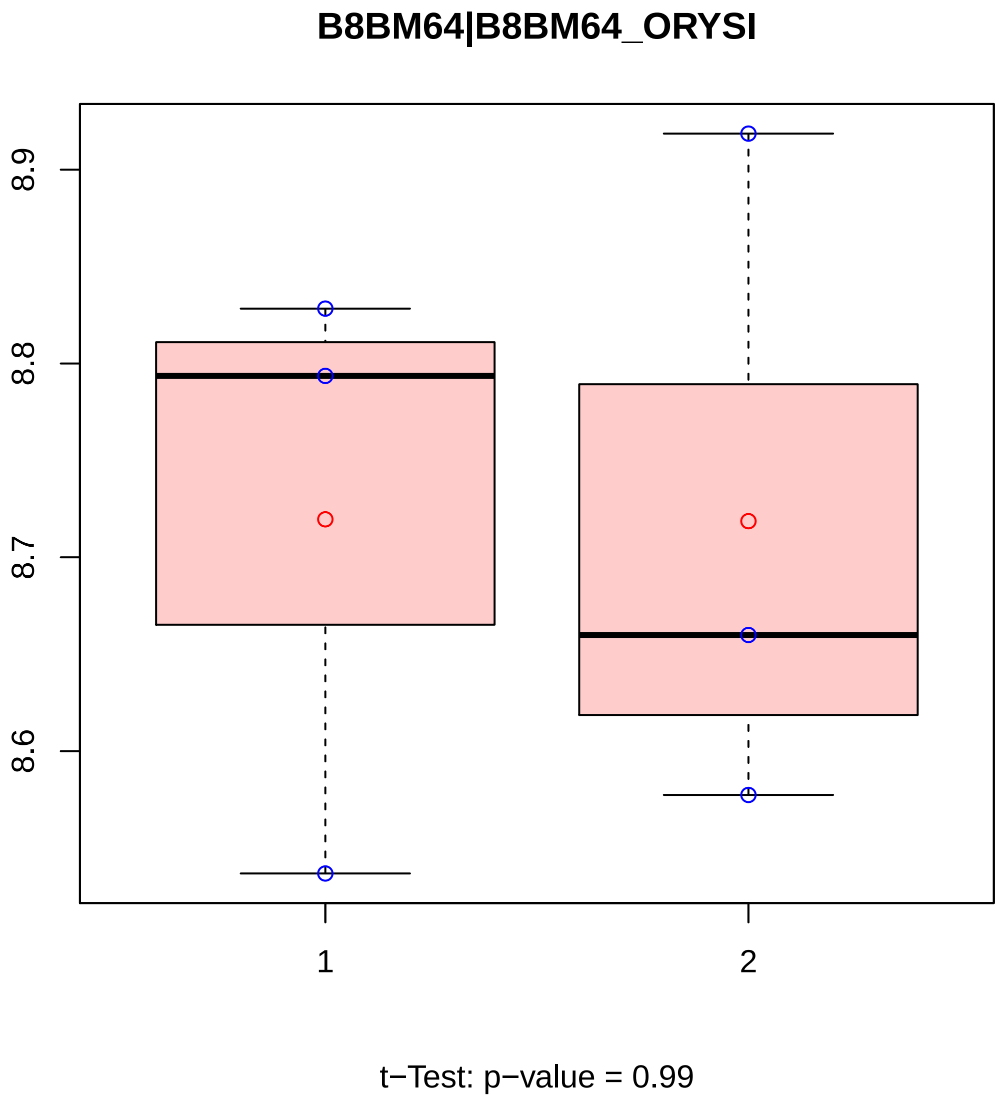
B8BM64|B8BM64_ORYSI
8.9
8.8
8.7
8.6
1
2
t−Test: p−value = 0.99

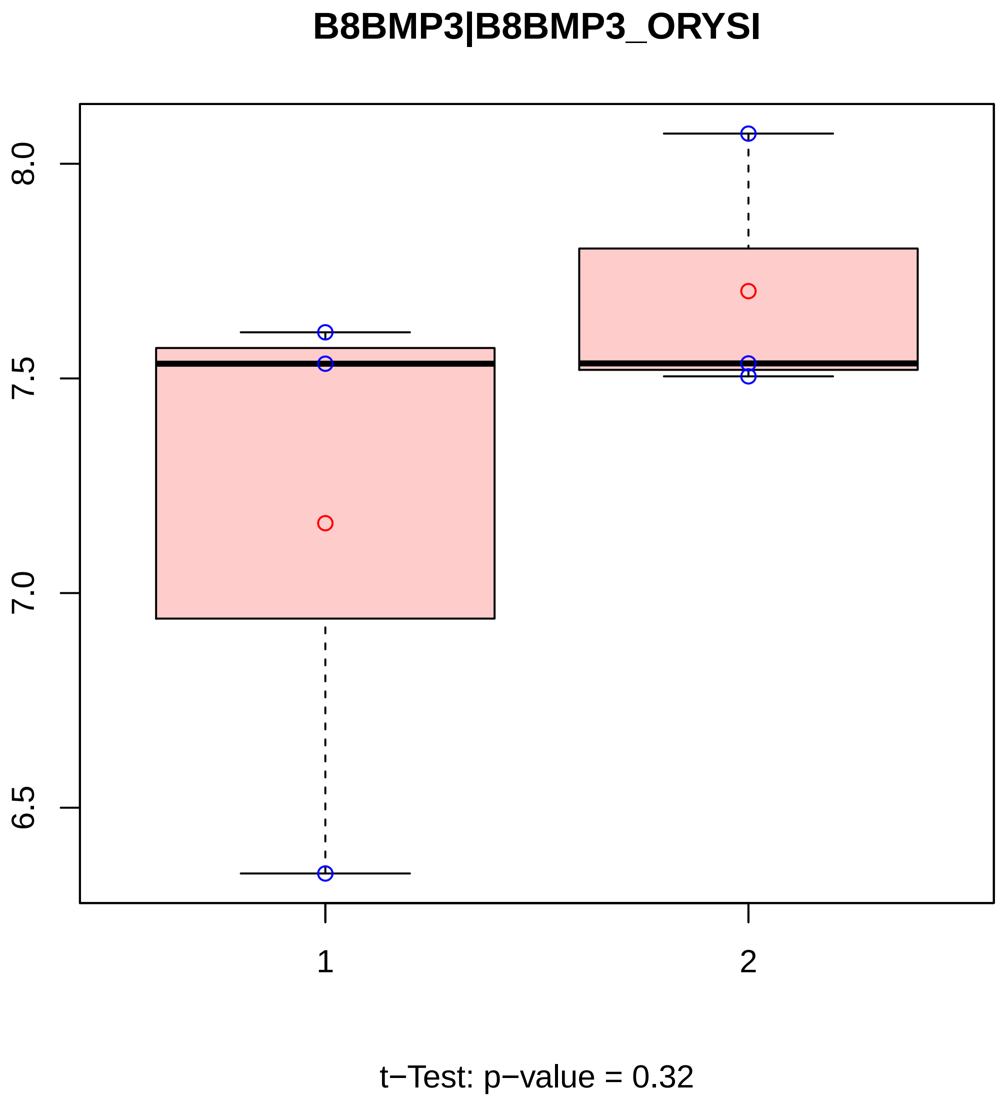
B8BMP3|B8BMP3_ORYSI
8.0
7.5
7.0
6.5
1
2
t−Test: p−value = 0.32

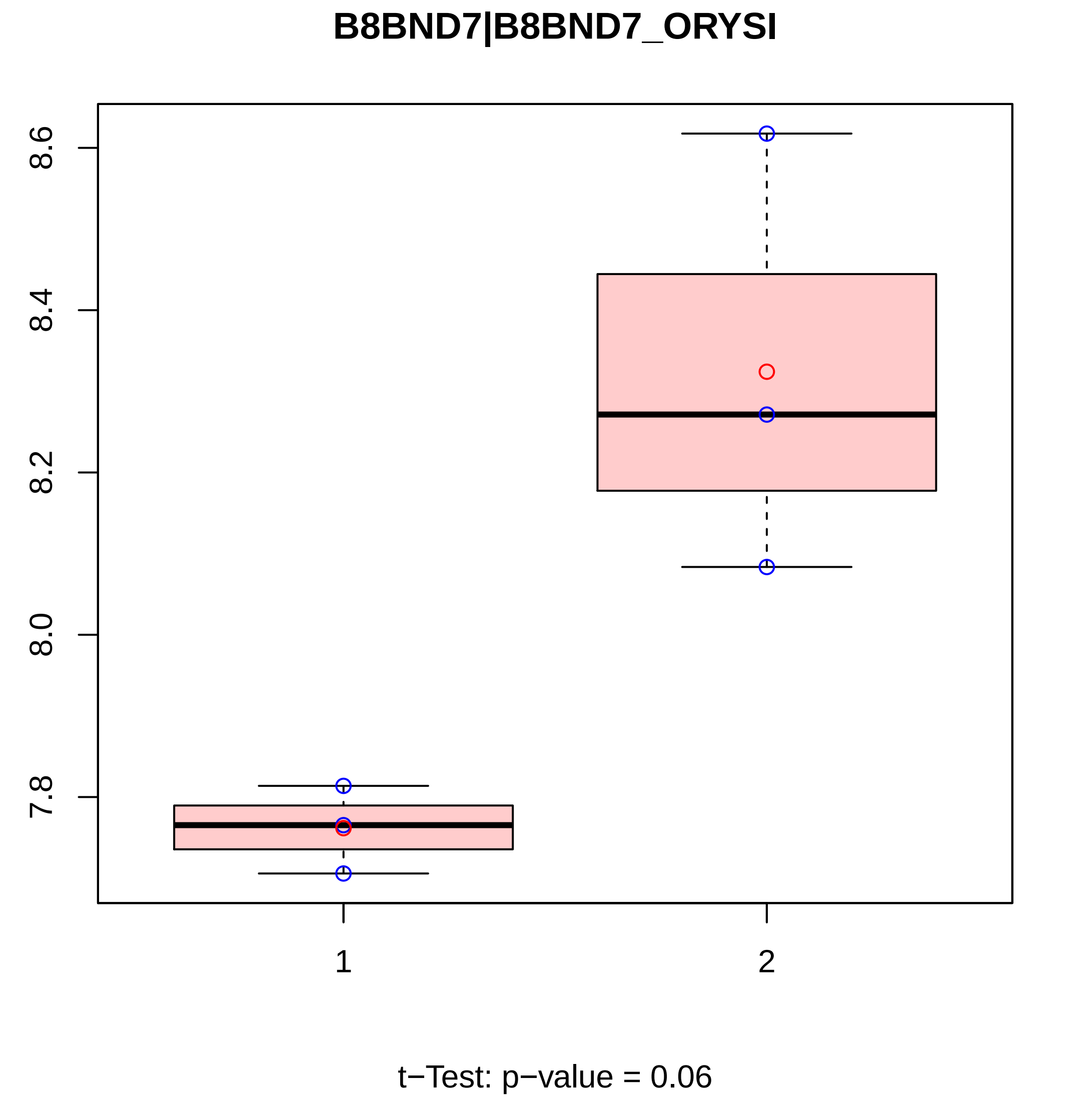
B8BND7|B8BND7_ORYSI
8.6
8.4
8.2
8.0
7.8
1
2
t−Test: p−value = 0.06

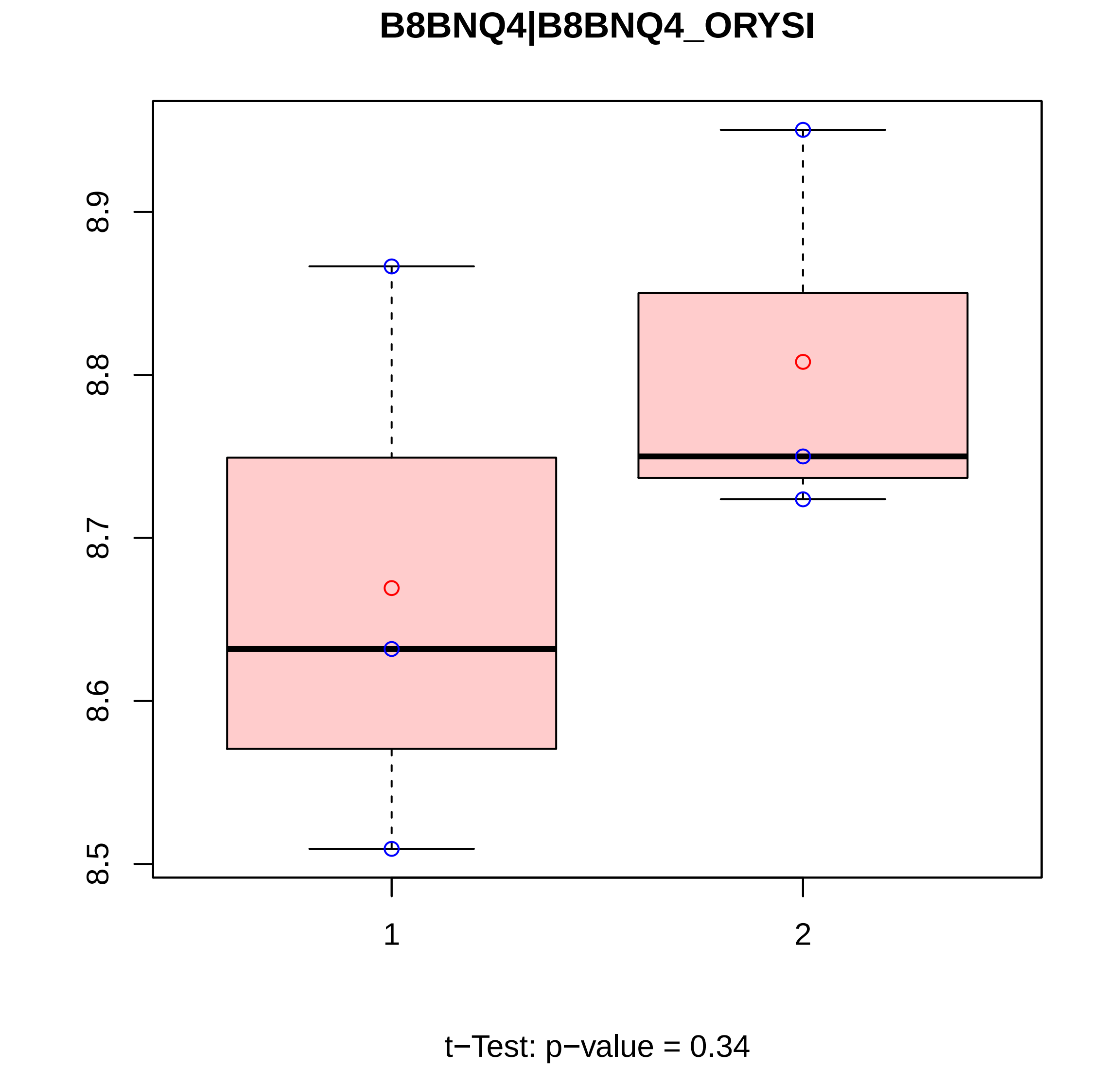
B8BNQ4|B8BNQ4_ORYSI
8.5
8.6
8.7
8.8
8.9
1
2
t−Test: p−value = 0.34

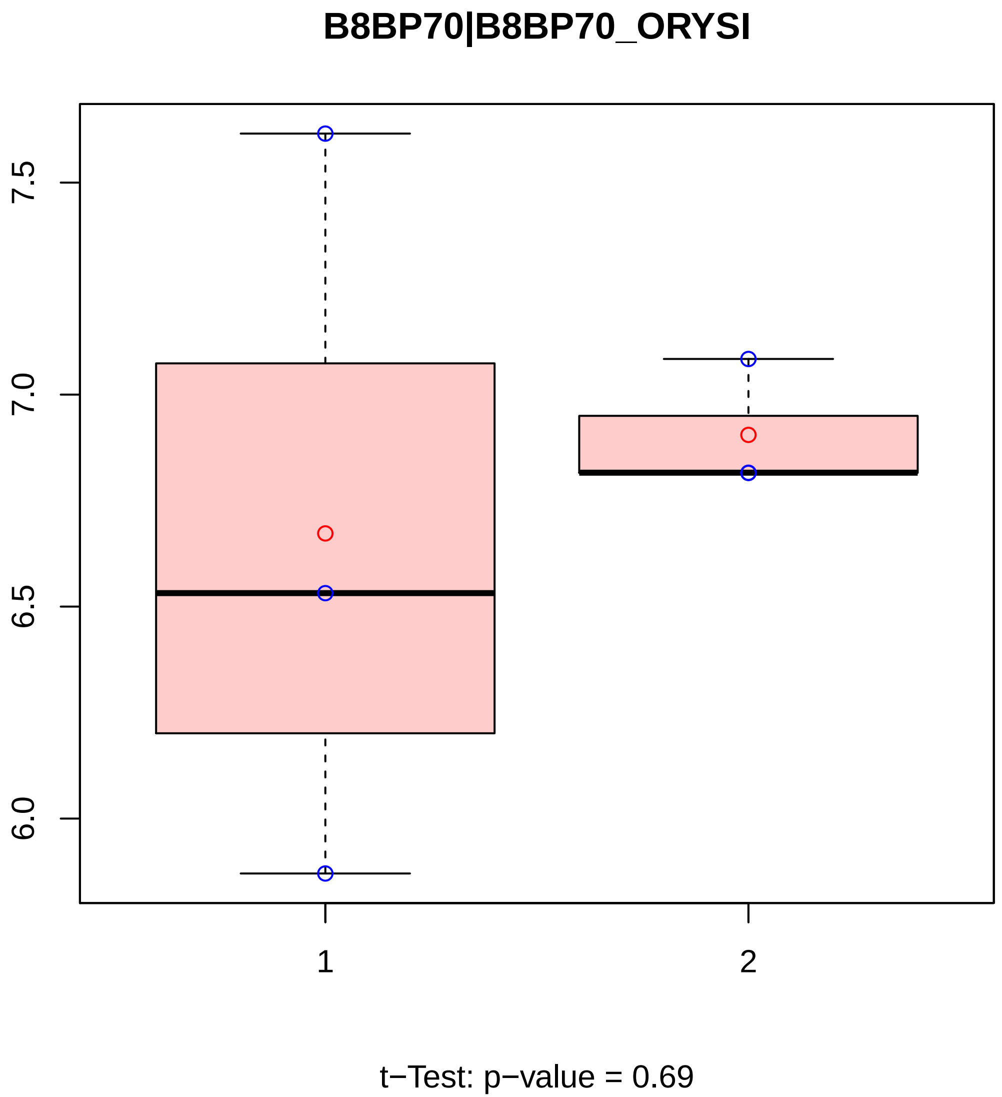
B8BP70|B8BP70_ORYSI
7.5
7.0
6.5
6.0
1
2
t−Test: p−value = 0.69

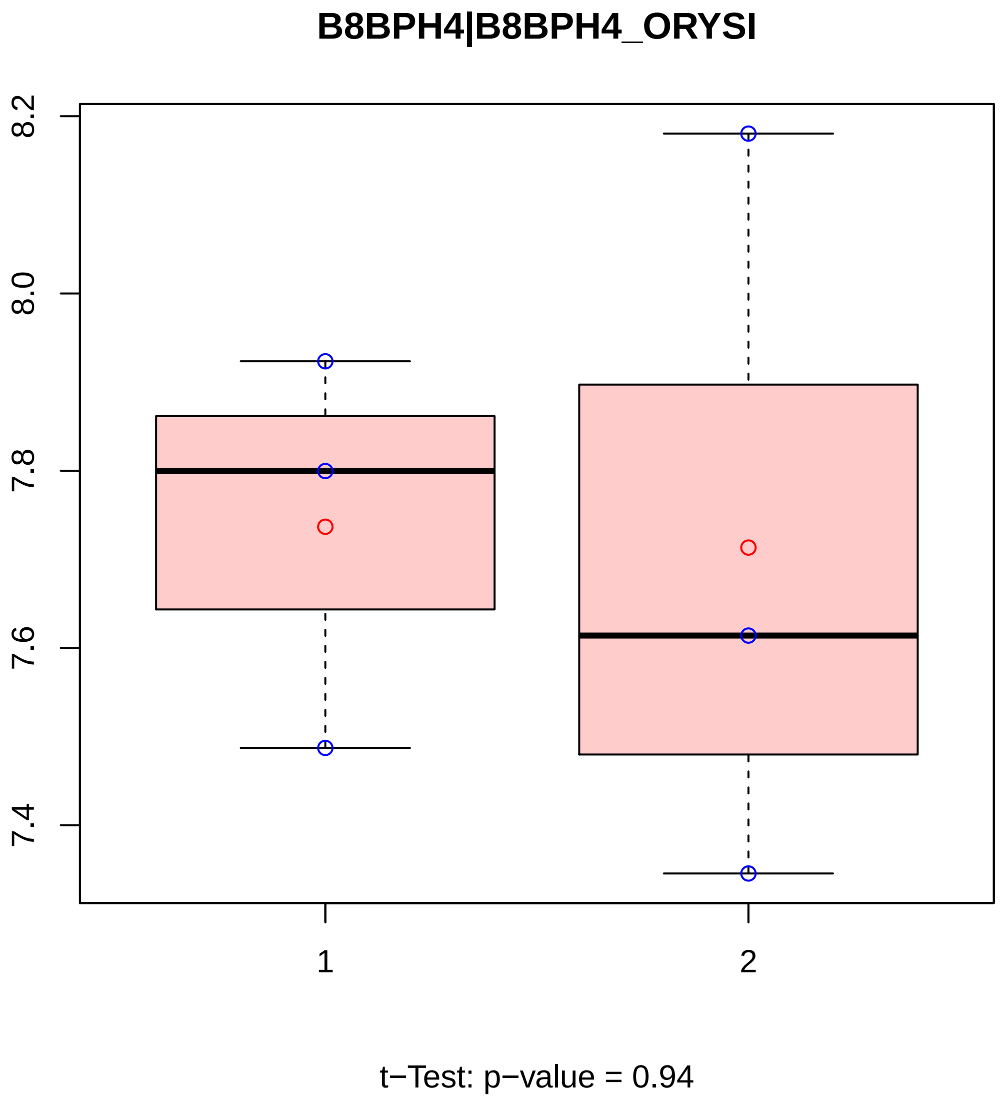

B8BPH4|B8BPH4_ORYSI
8.2
8.0
7.8
7.6
7.4
1
2
t−Test: p−value = 0.94

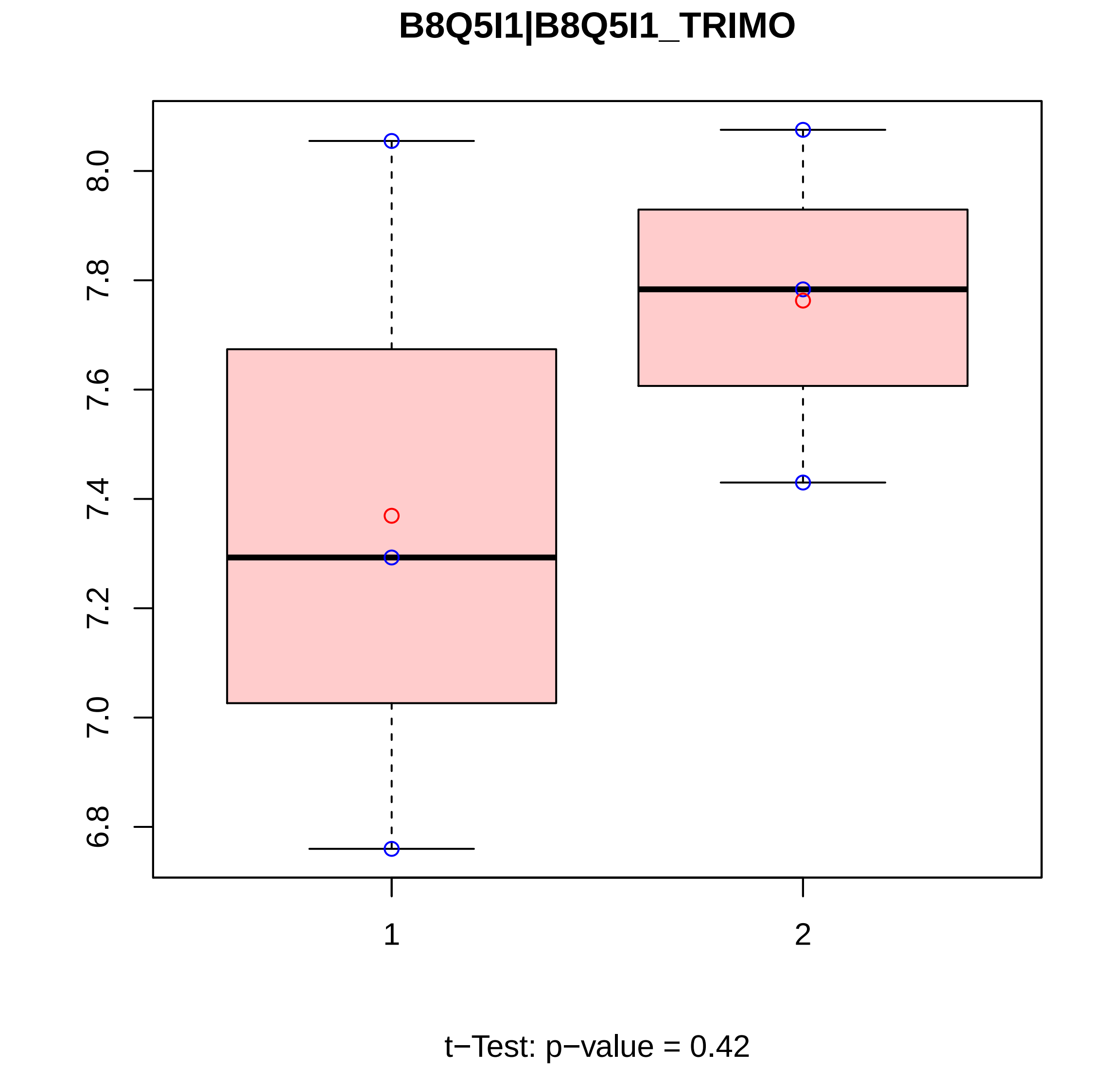

B8Q5I1|B8Q5I1_TRIMO
8.0
7.8
7.6
7.4
7.2
7.0
6.8
1
2
t−Test: p−value = 0.42

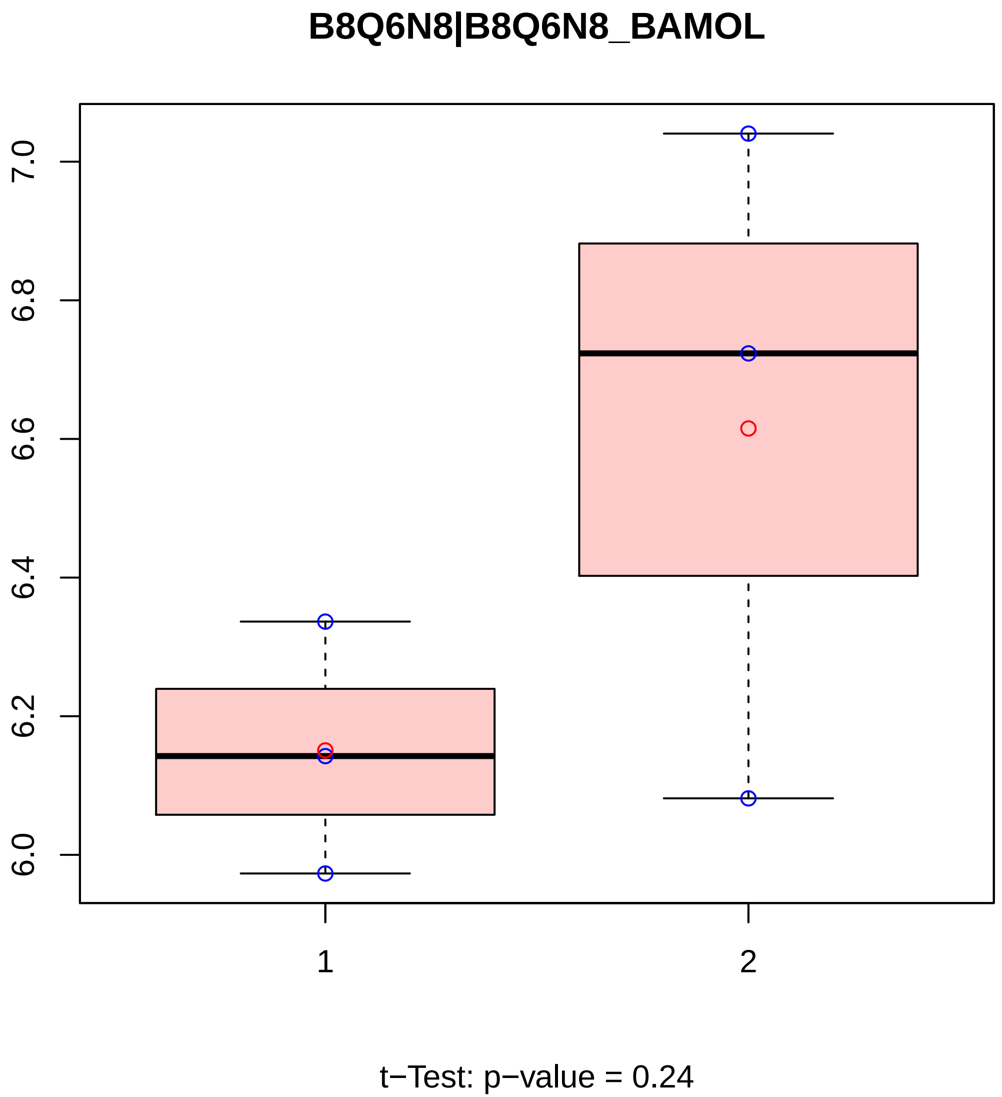

B8Q6N8|B8Q6N8_BAMOL
6.0
6.2
6.4
6.6
6.8
7.0
1
2
t−Test: p−value = 0.24

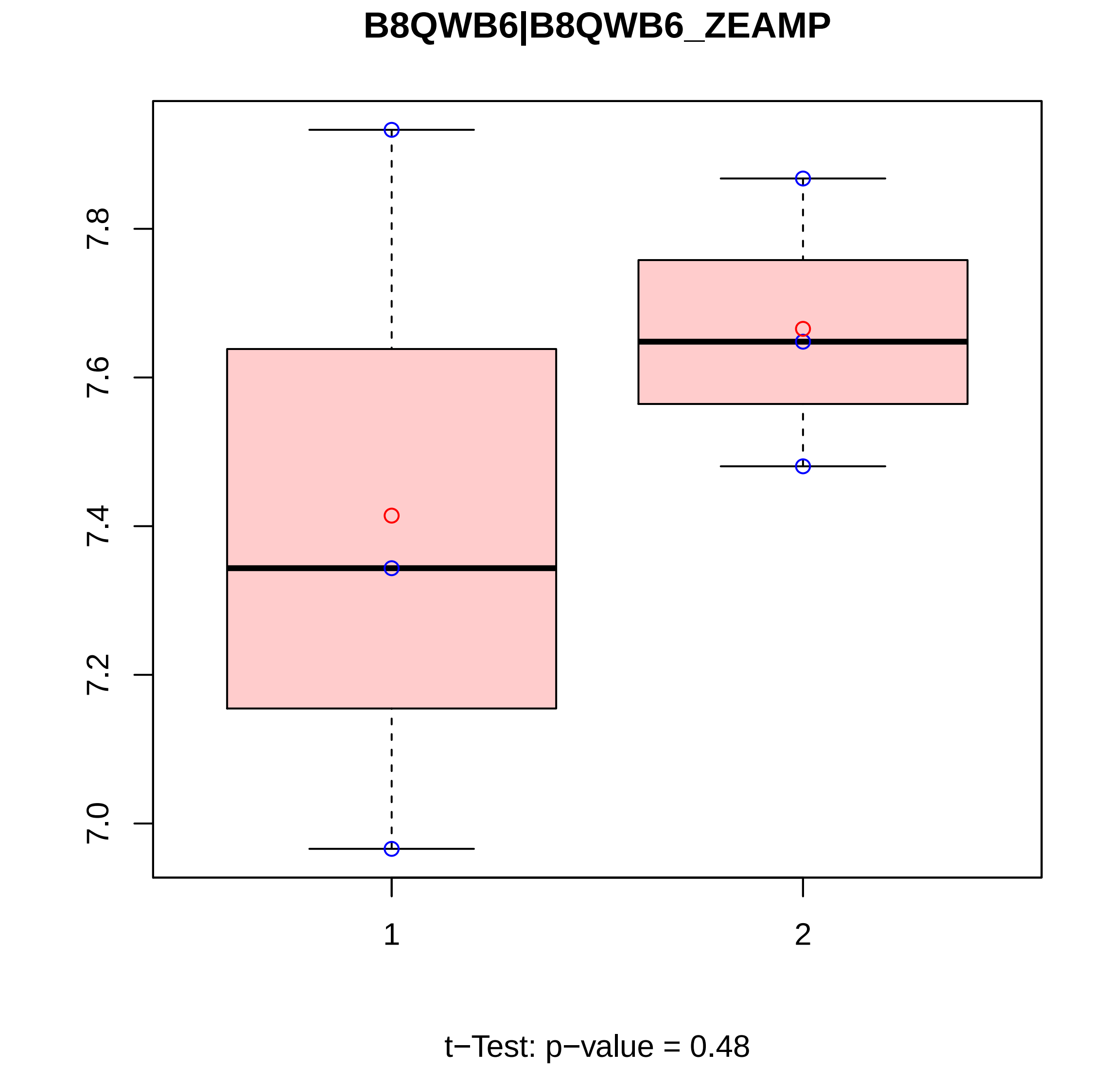
B8QWB6|B8QWB6_ZEAMP
7.0
7.2
7.4
7.6
7.8
1
2
t−Test: p−value = 0.48

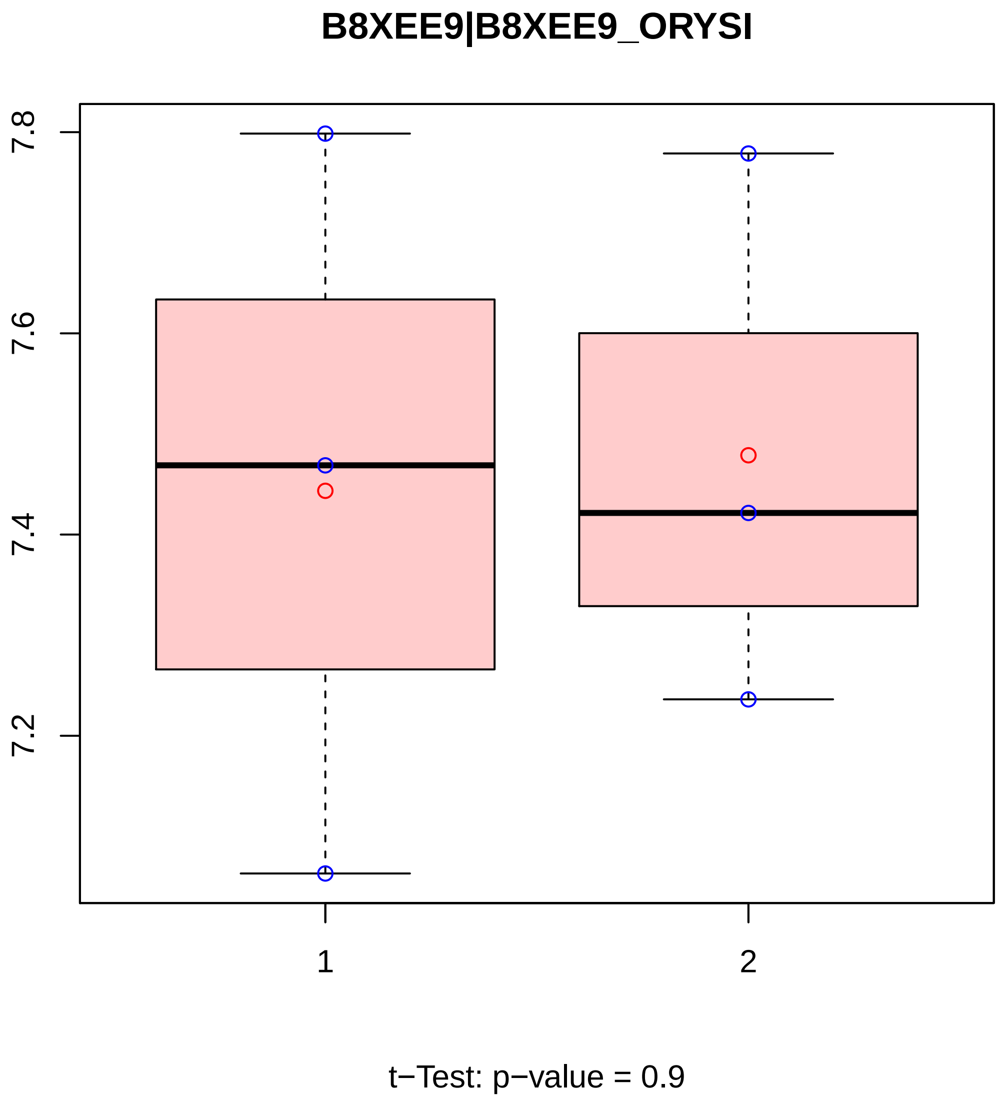
B8XEE9|B8XEE9_ORYSI
7.8
7.6
7.4
7.2
1
2
t−Test: p−value = 0.9

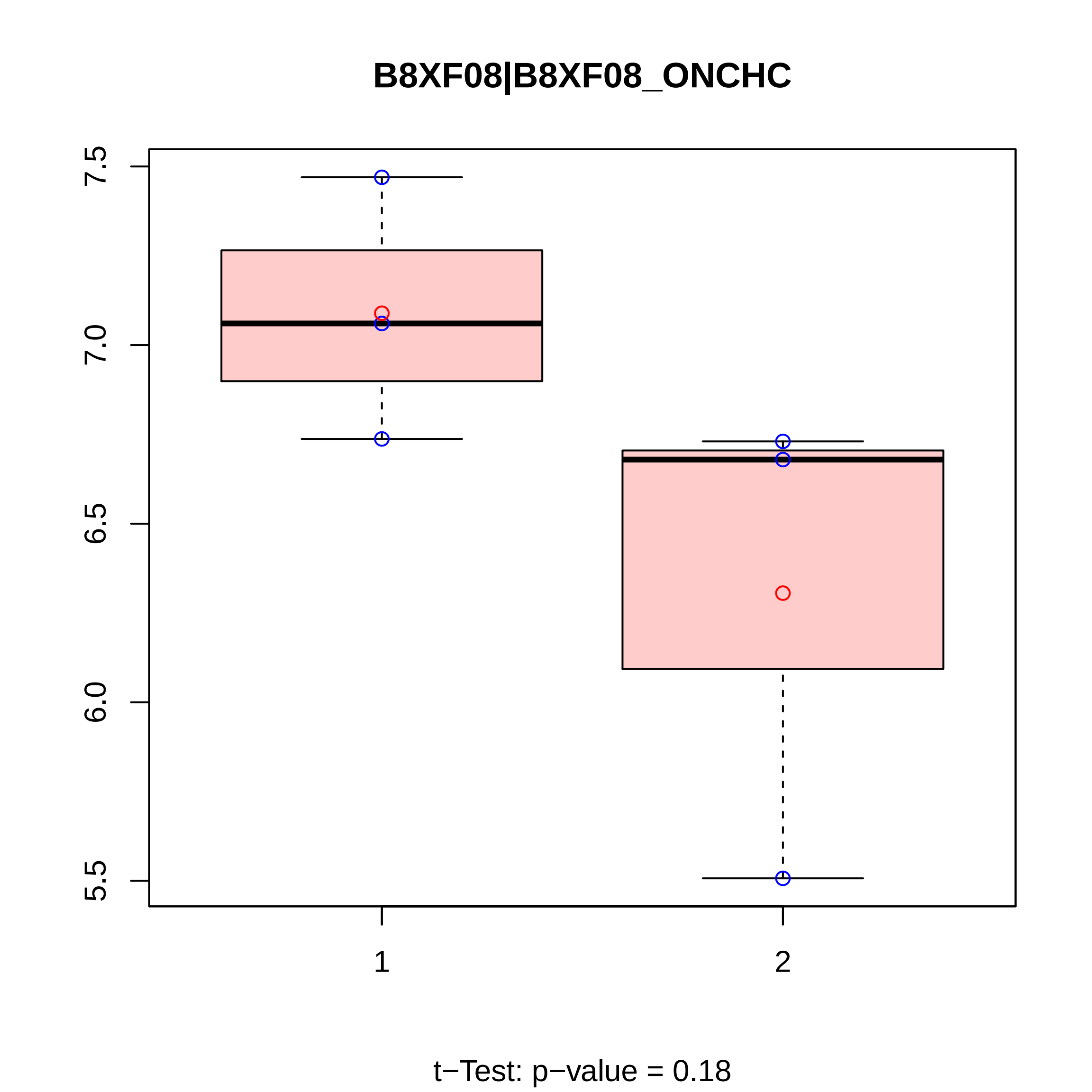
B8XF08|B8XF08_ONCHC
7.5
7.0
6.5
6.0
5.5
1
2
t−Test: p−value = 0.18

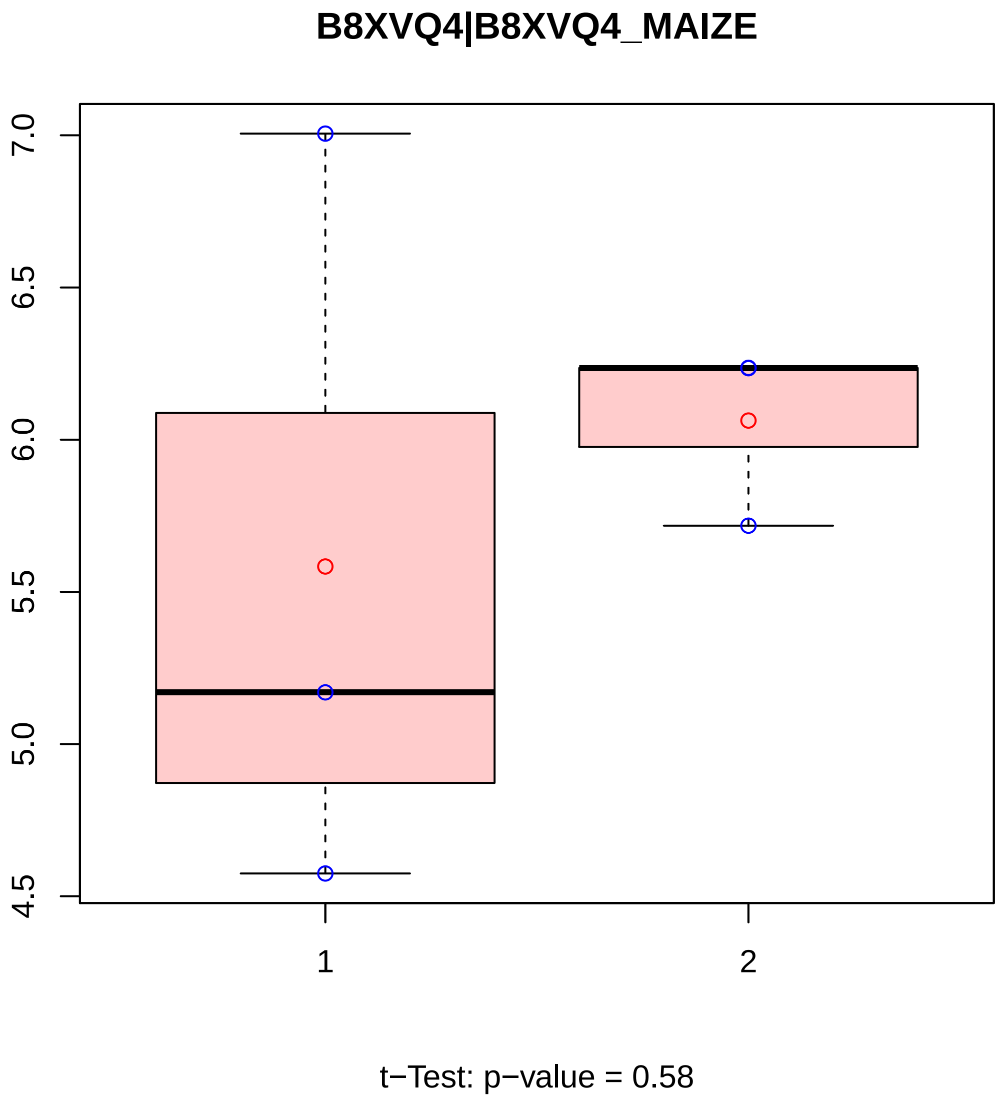
B8XVQ4|B8XVQ4_MAIZE
7.0
6.5
6.0
5.5
5.0
4.5
1
2
t−Test: p−value = 0.58

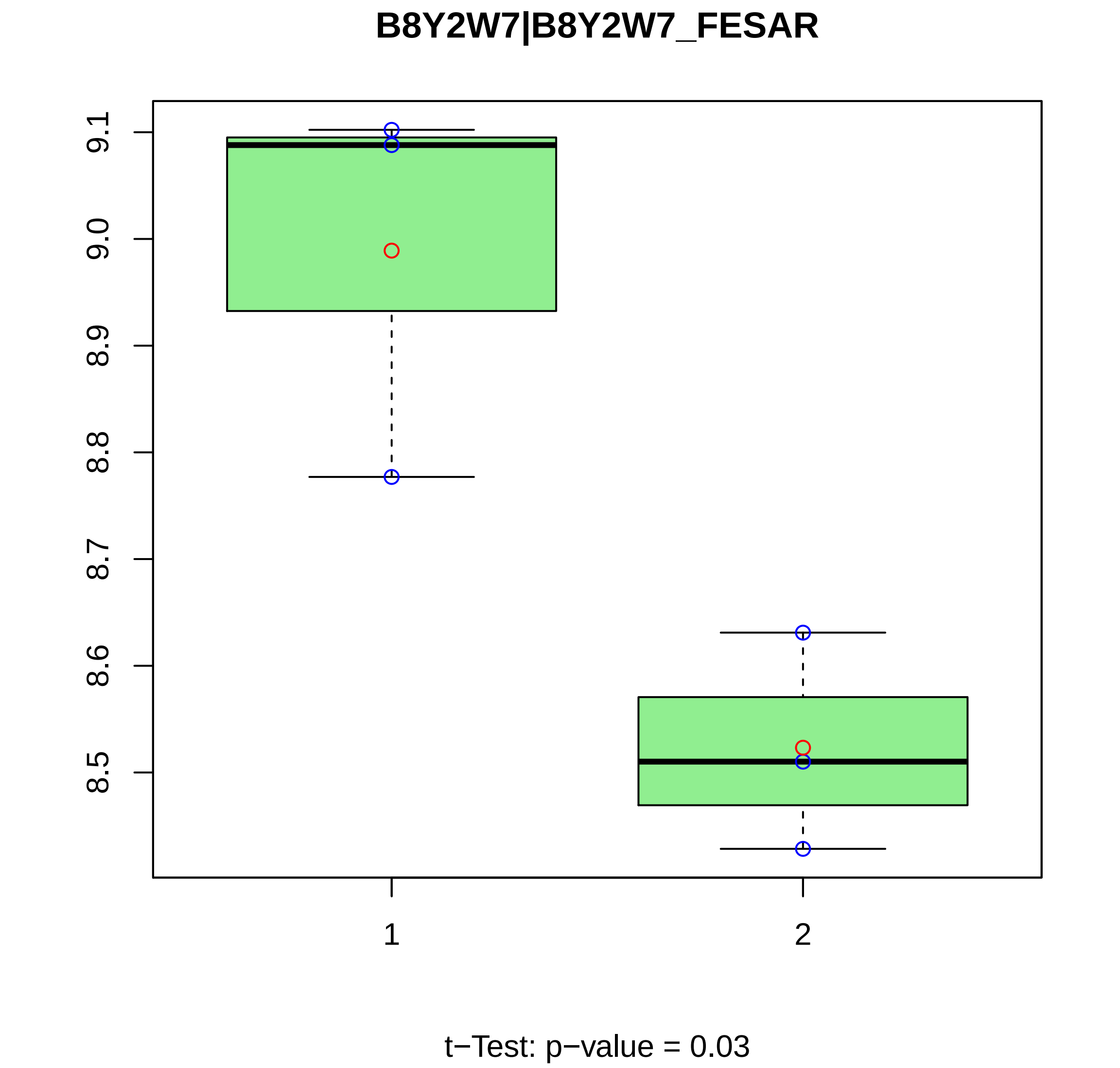
B8Y2W7|B8Y2W7_FESAR
9.1
9.0
8.9
8.8
8.7
8.6
8.5
1
2
t−Test: p−value = 0.03

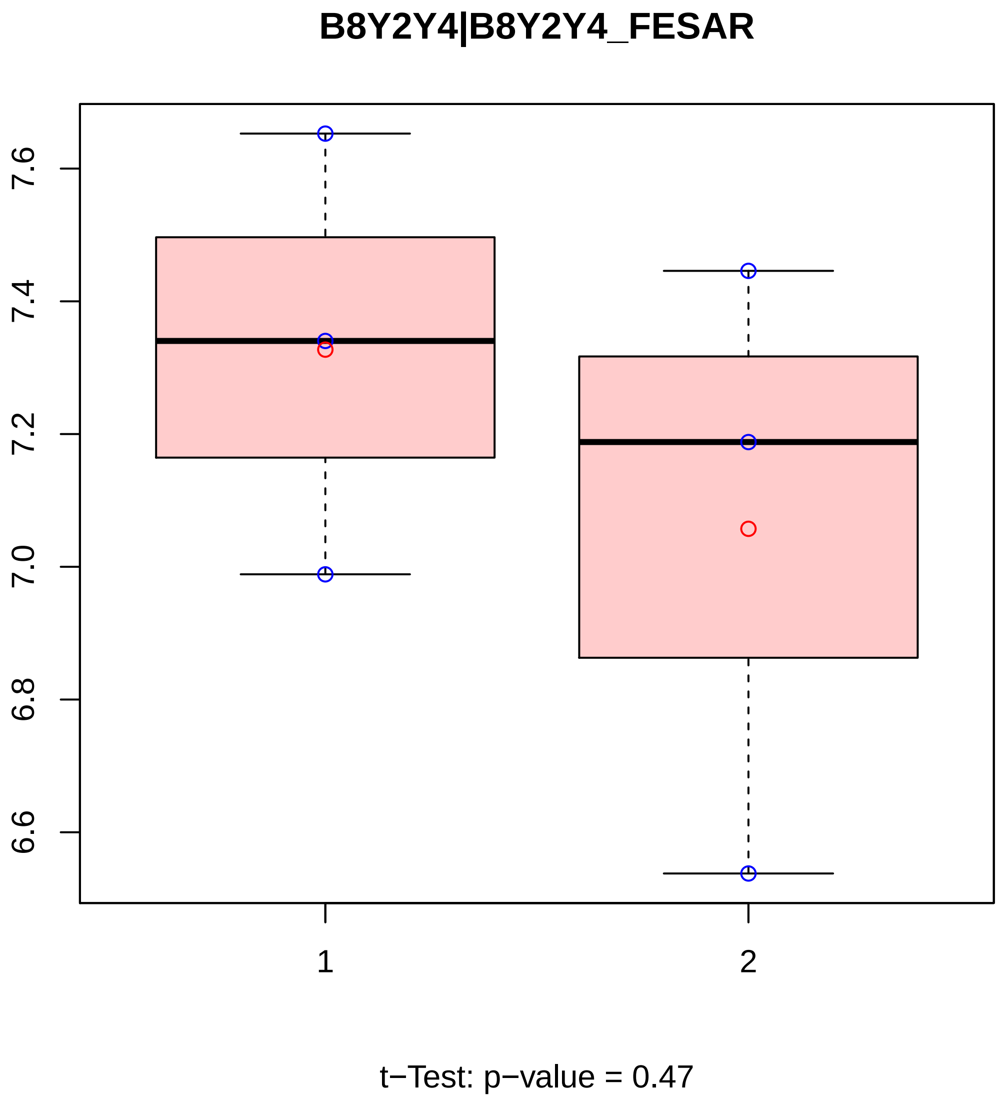

B8Y2Y4|B8Y2Y4_FESAR
7.6
7.4
7.2
7.0
6.8
6.6
1
2
t−Test: p−value = 0.47

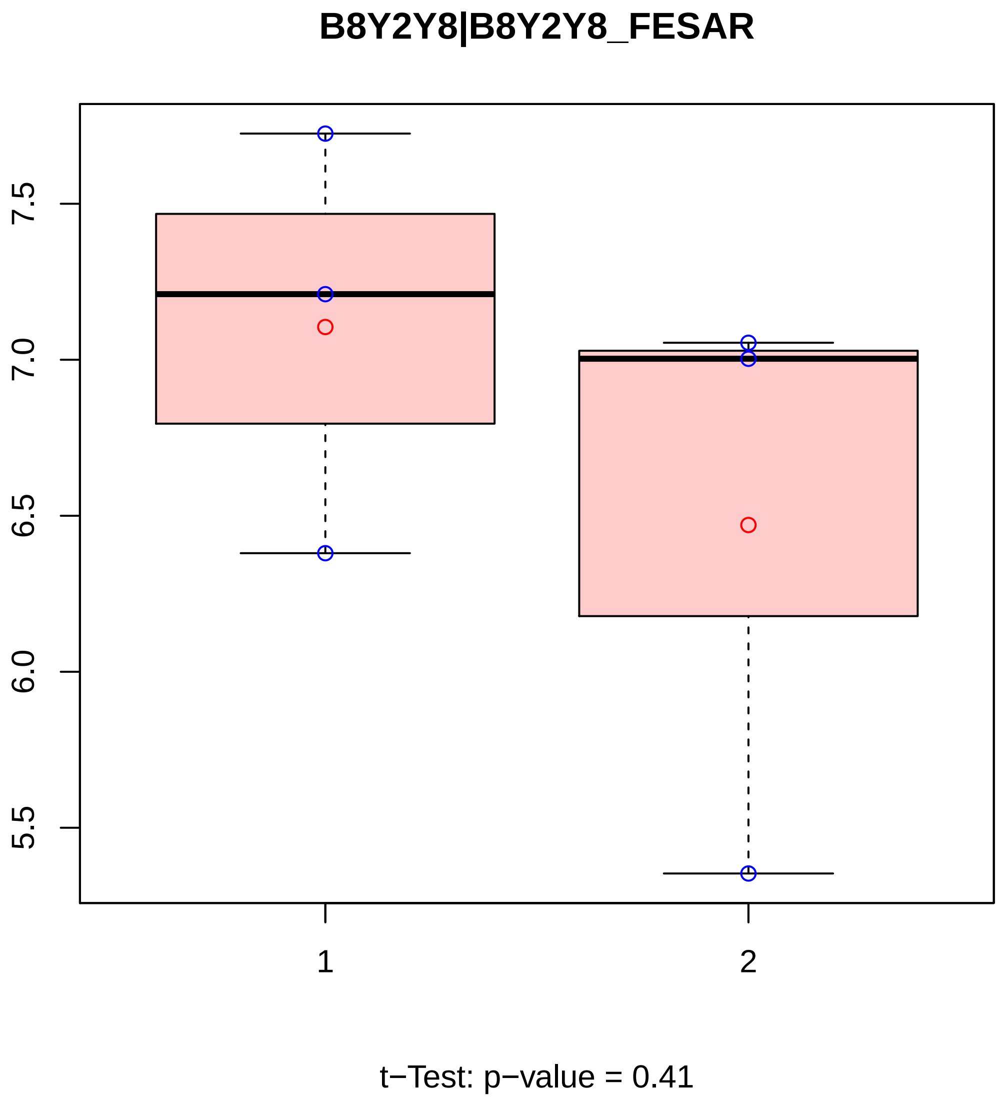
B8Y2Y8|B8Y2Y8_FESAR
7.5
7.0
6.5
6.0
5.5
1
2
t−Test: p−value = 0.41

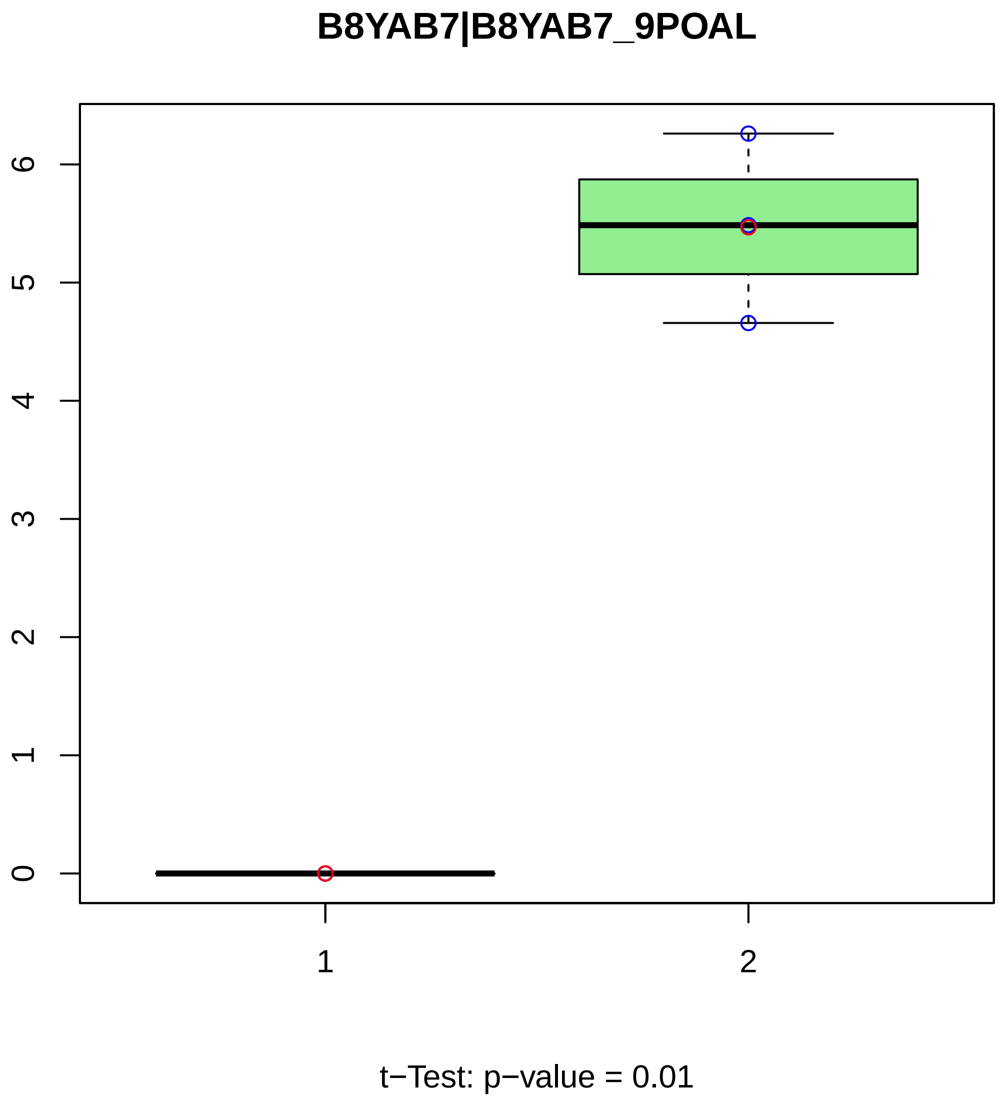

B8YAB7|B8YAB7_9POAL
0
1
2
3
4
5
6
1
2
t−Test: p−value = 0.01

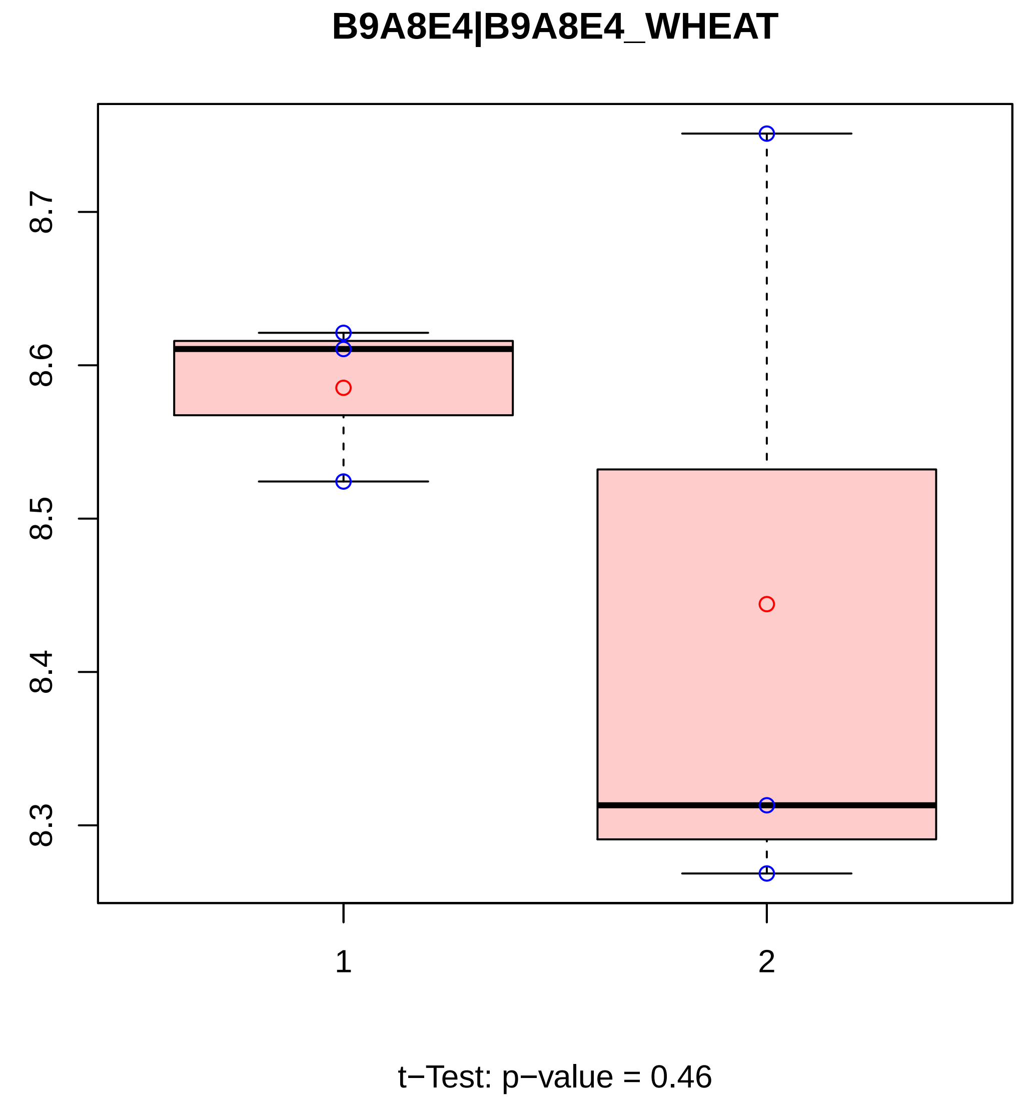
B9A8E4|B9A8E4_WHEAT
8.7
8.6
8.5
8.4
8.3
1
2
t−Test: p−value = 0.46

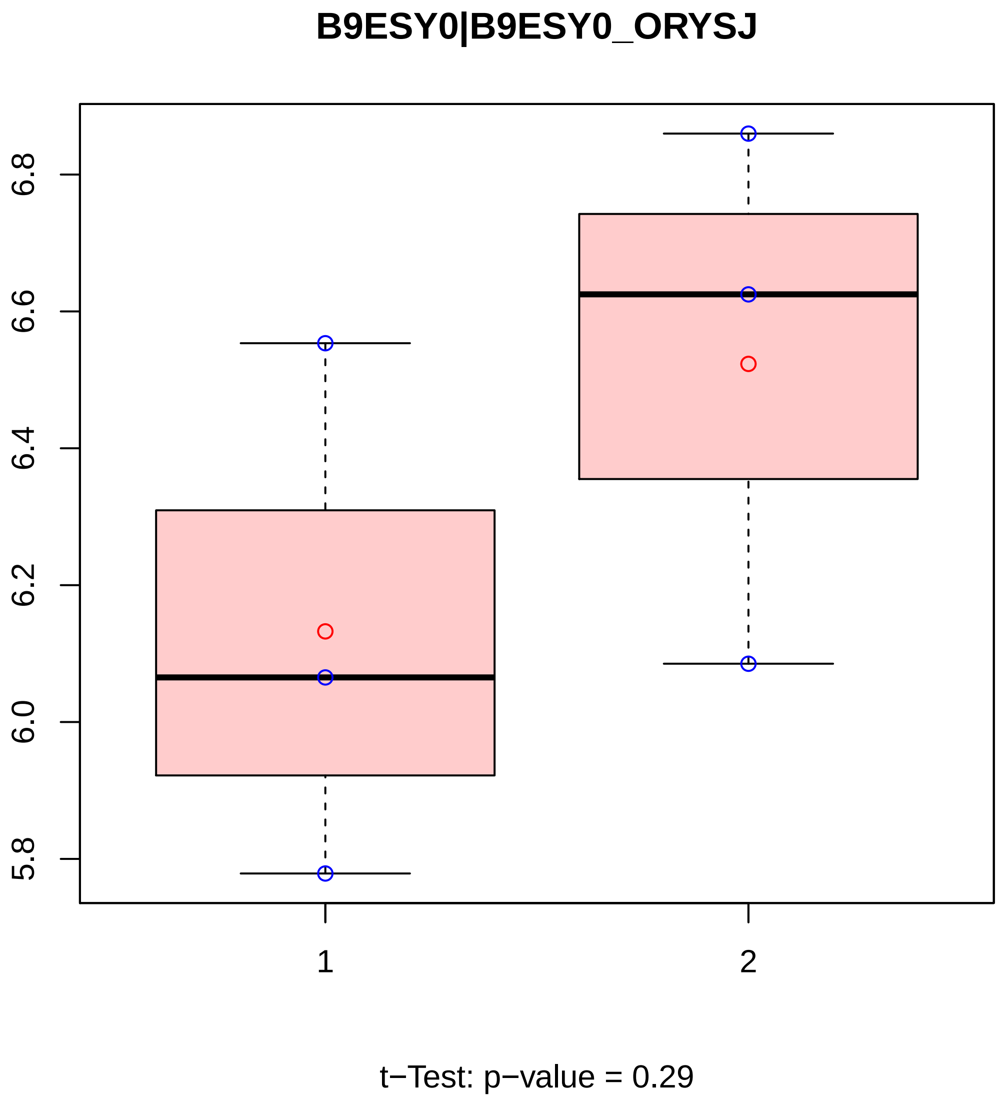
B9ESY0|B9ESY0_ORYSJ
6.8
6.6
6.4
6.2
6.0
5.8
1
2
t−Test: p−value = 0.29

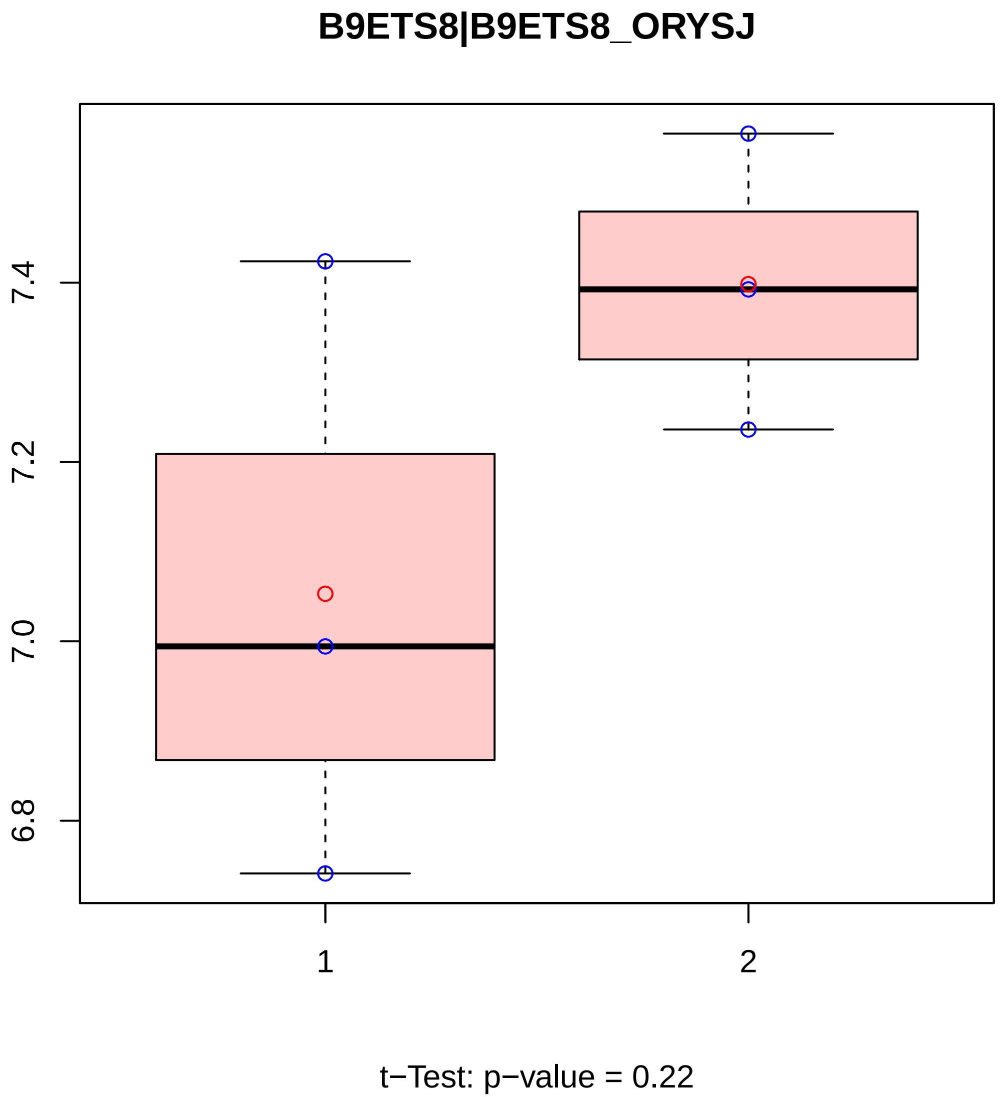

B9ETS8|B9ETS8_ORYSJ
7.4
7.2
7.0
6.8
1
2
t−Test: p−value = 0.22

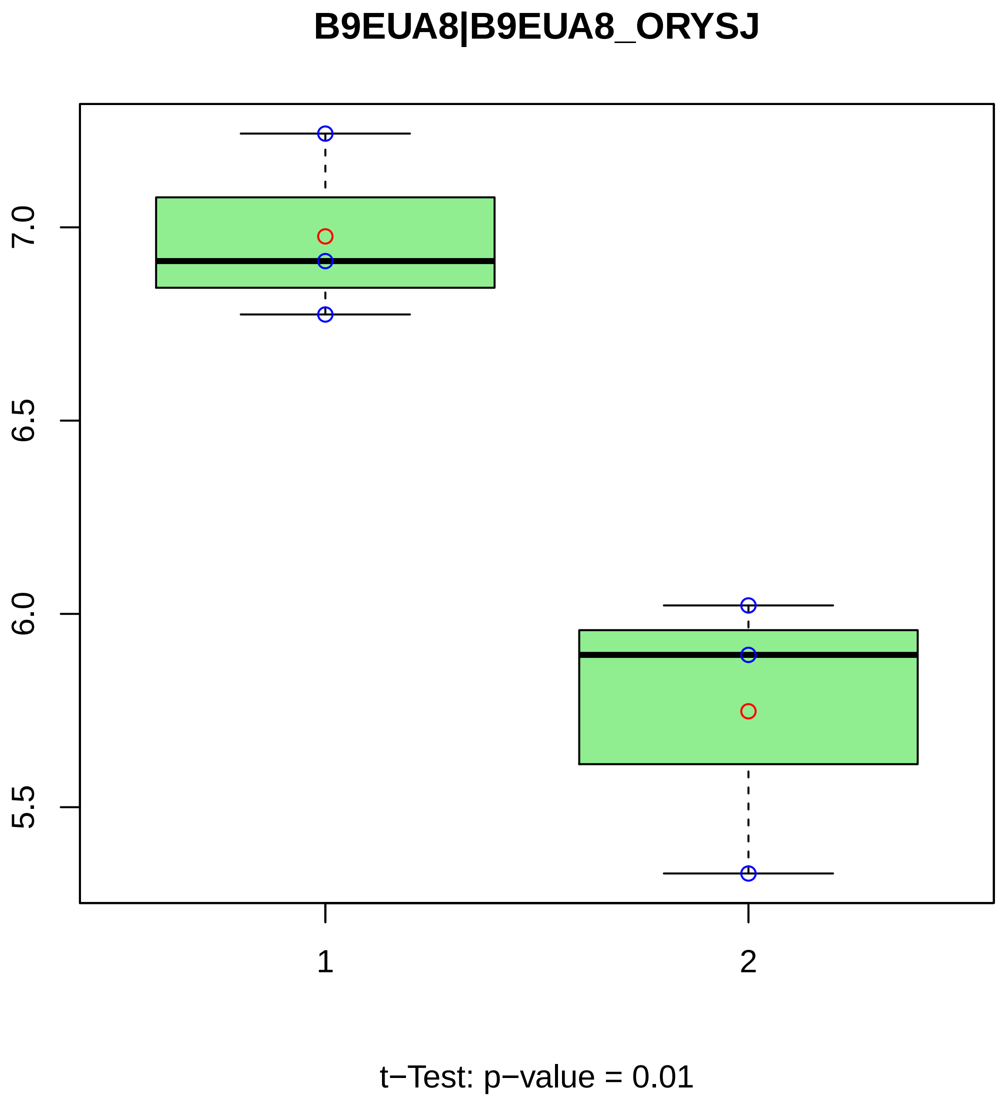
B9EUA8|B9EUA8_ORYSJ
7.0
6.5
6.0
5.5
1
2
t−Test: p−value = 0.01

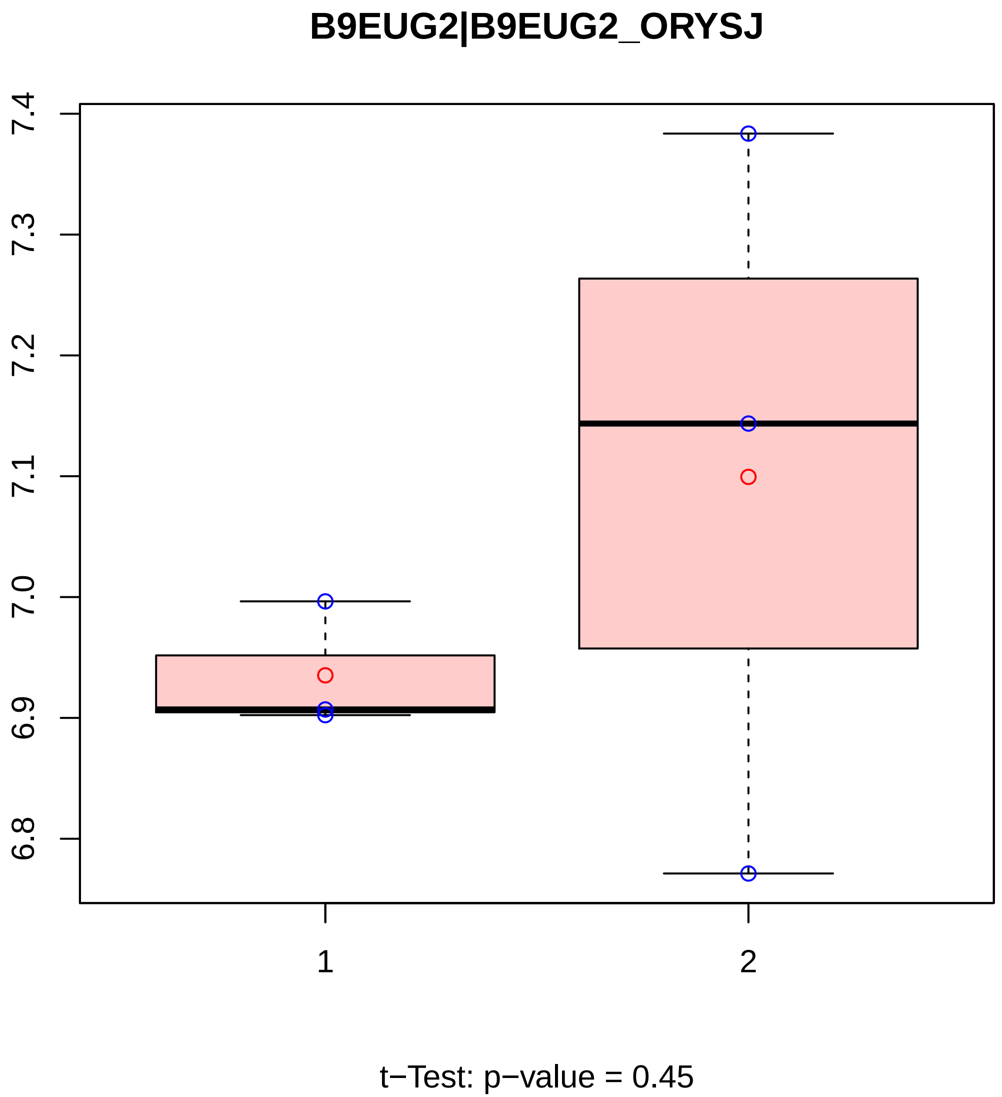
B9EUG2|B9EUG2_ORYSJ
7.4
7.3
7.2
7.1
7.0
6.9
6.8
1
2
t−Test: p−value = 0.45

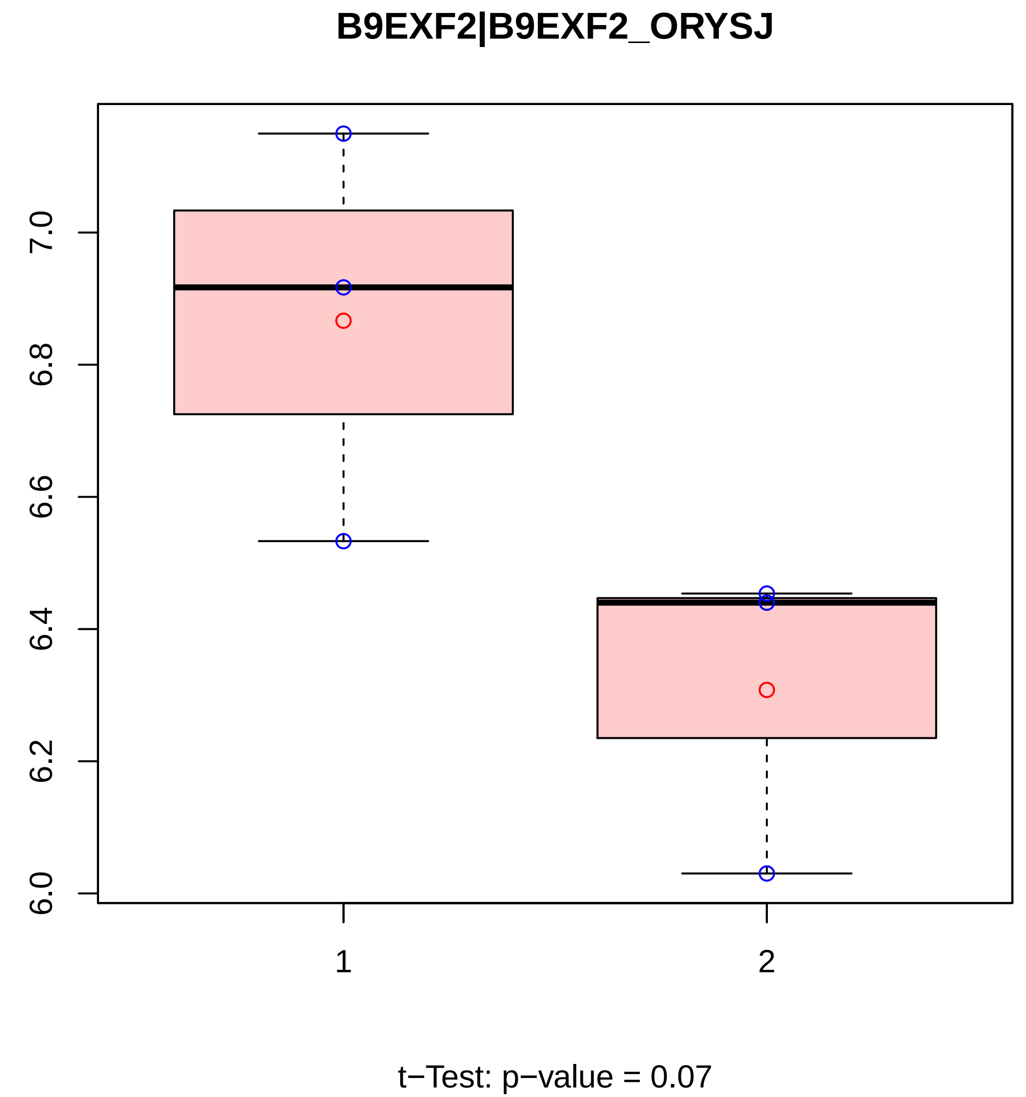
B9EXF2|B9EXF2_ORYSJ
7.0
6.8
6.6
6.4
6.2
6.0
1
2
t−Test: p−value = 0.07

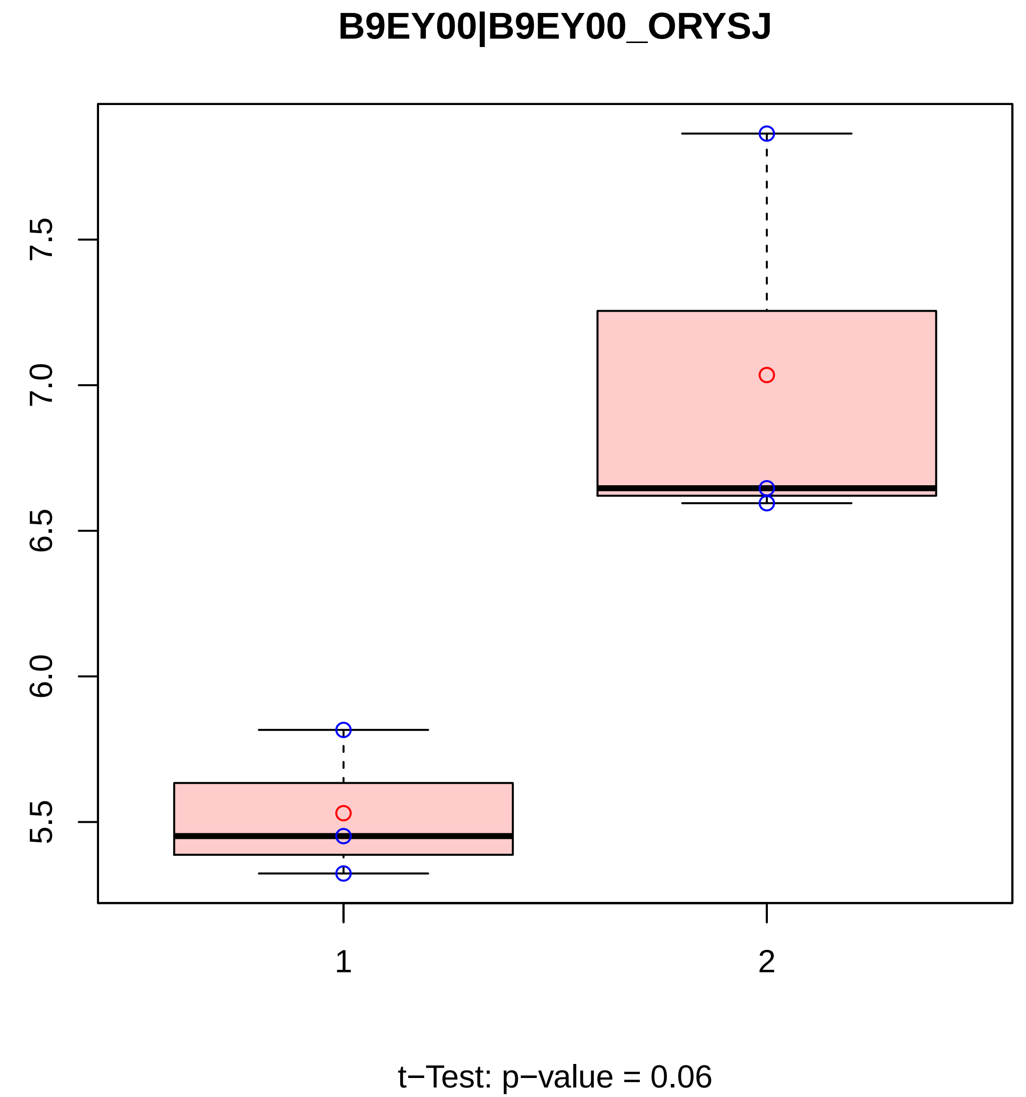
B9EY00|B9EY00_ORYSJ
7.5
7.0
6.5
6.0
5.5
1
2
t−Test: p−value = 0.06

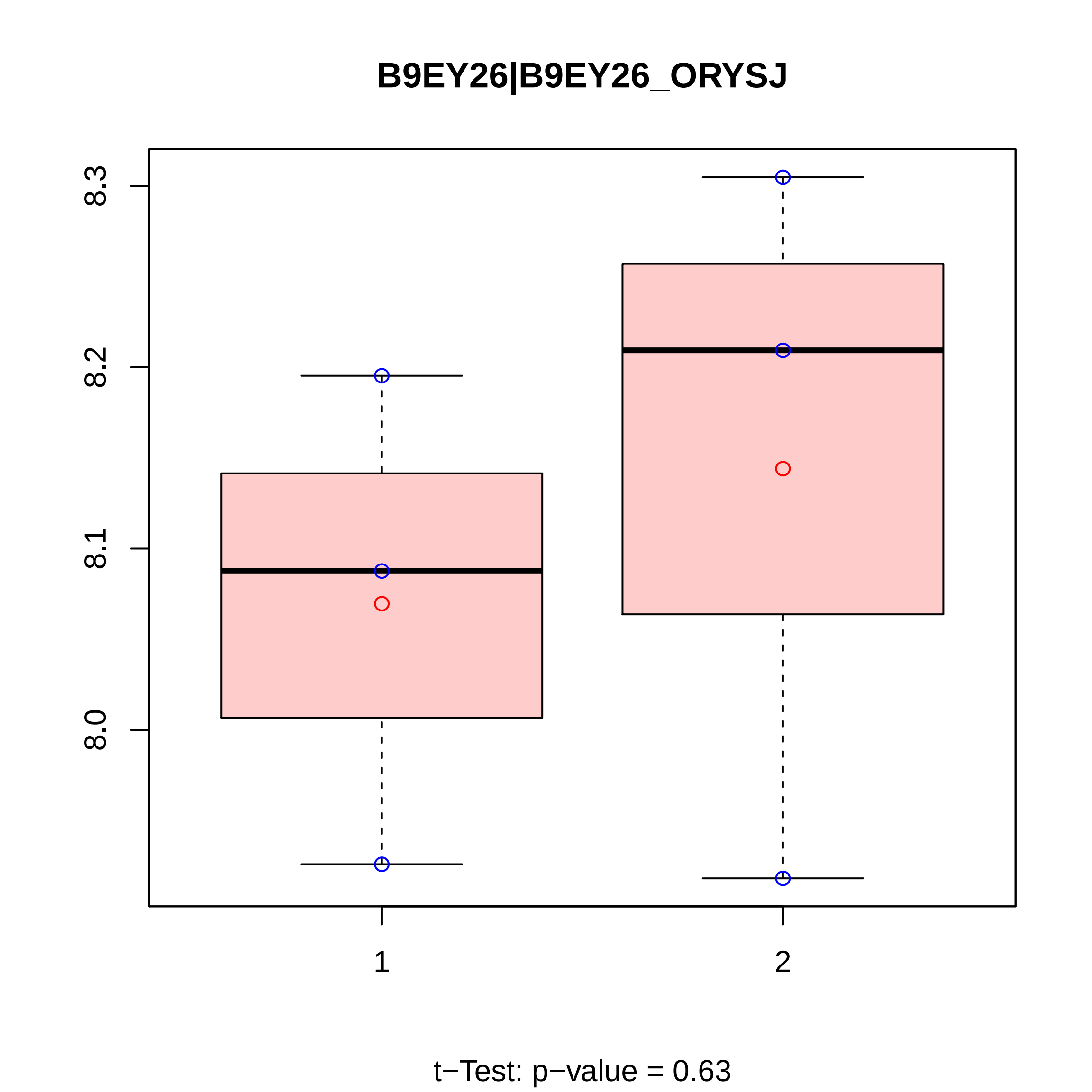

B9EY26|B9EY26_ORYSJ
8.0
8.1
8.2
8.3
1
2
t−Test: p−value = 0.63

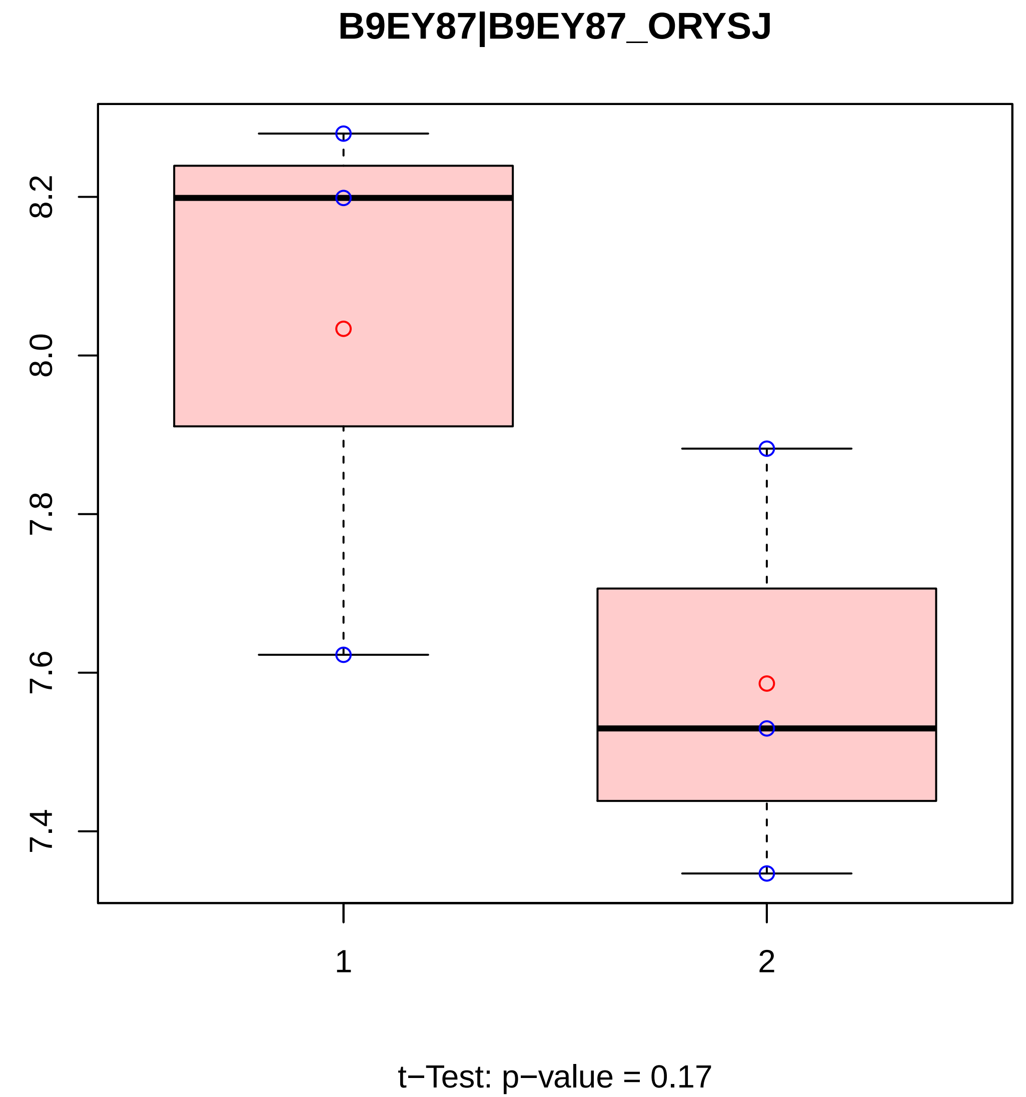

B9EY87|B9EY87_ORYSJ
8.2
8.0
7.8
7.6
7.4
1
2
t−Test: p−value = 0.17

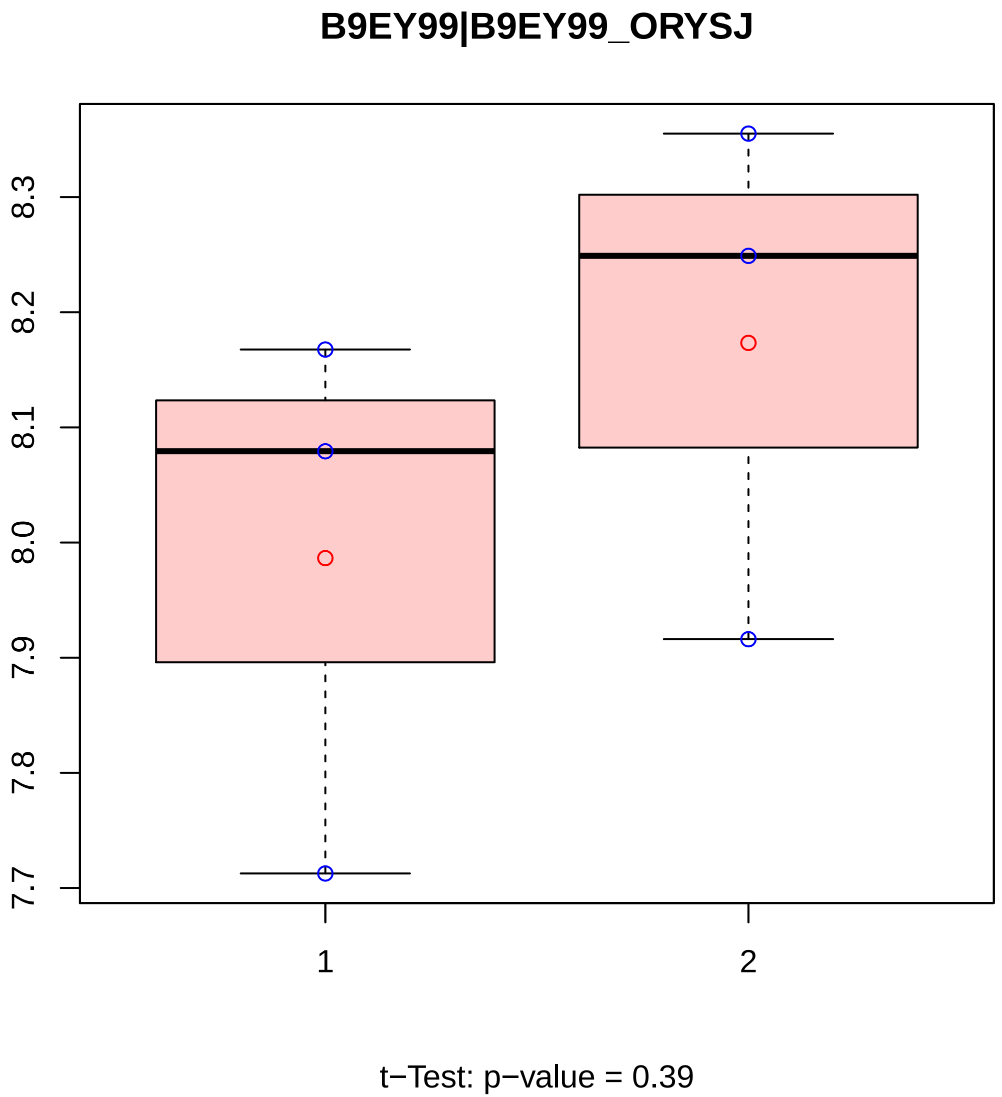
B9EY99|B9EY99_ORYSJ
8.3
8.2
8.1
8.0
7.9
7.8
7.7
1
2
t−Test: p−value = 0.39

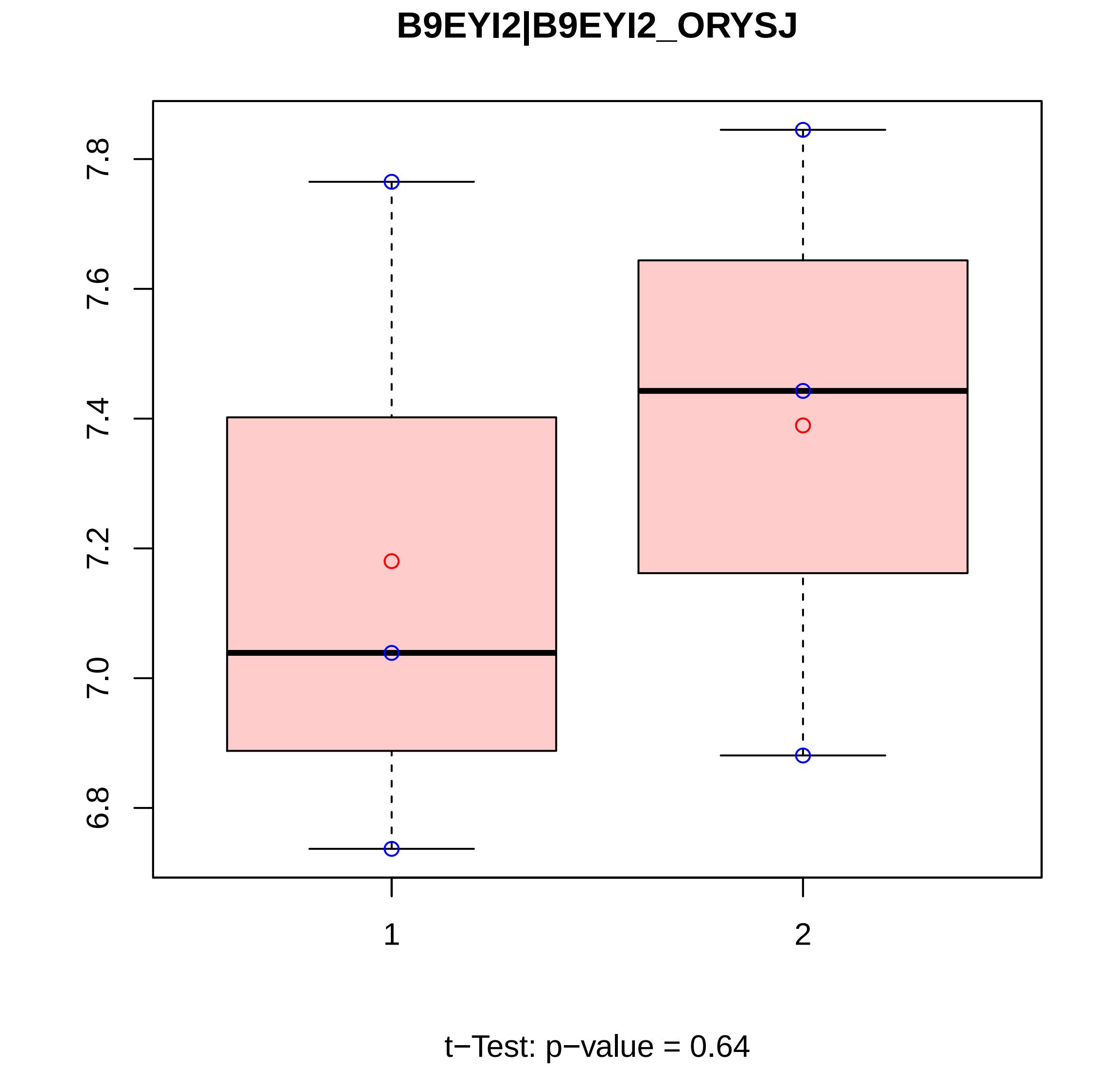
B9EYI2|B9EYI2_ORYSJ
7.8
7.6
7.4
7.2
7.0
6.8
1
2
t−Test: p−value = 0.64

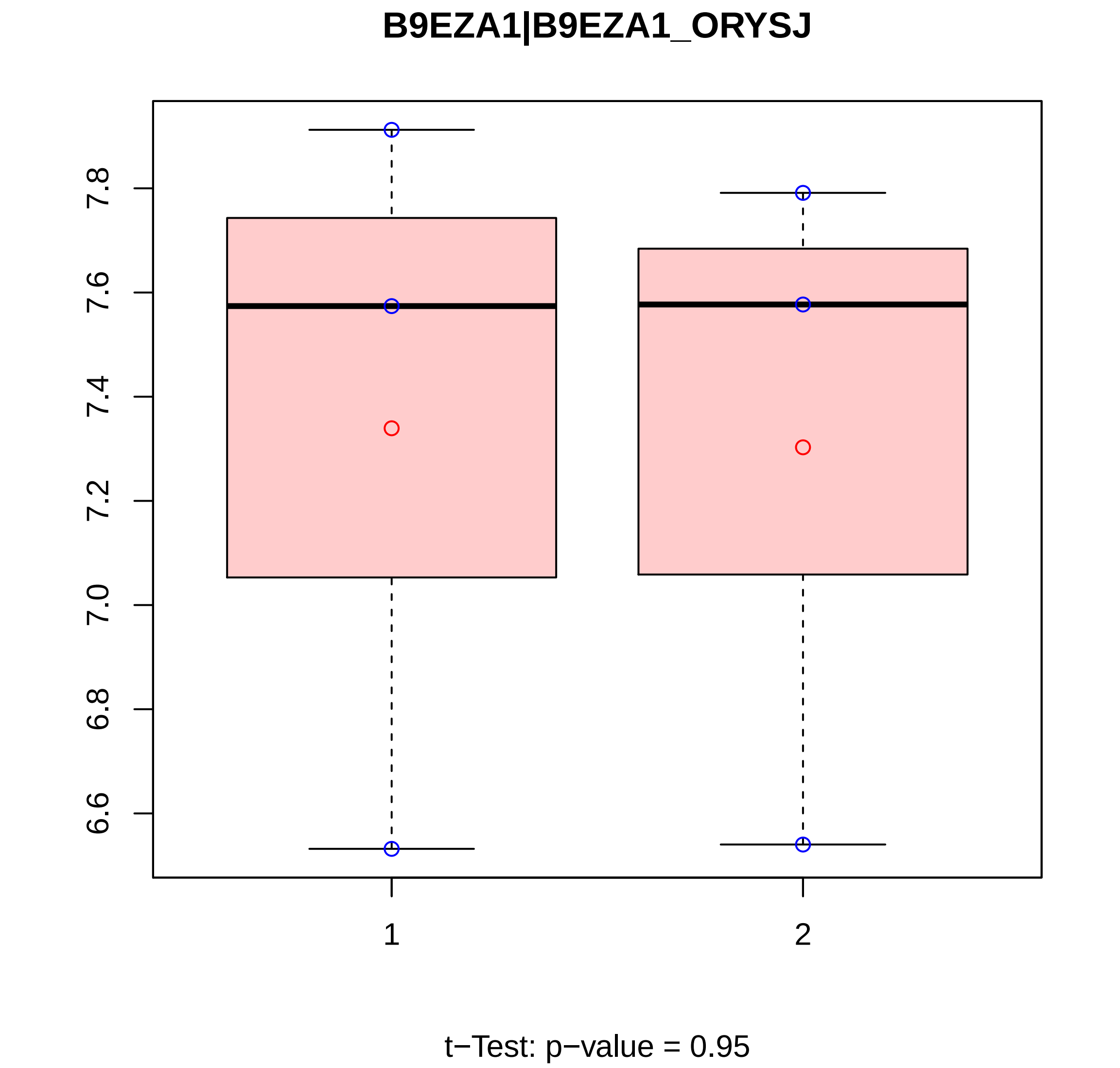
B9EZA1|B9EZA1_ORYSJ
7.8
7.6
7.4
7.2
7.0
6.8
6.6
1
2
t−Test: p−value = 0.95

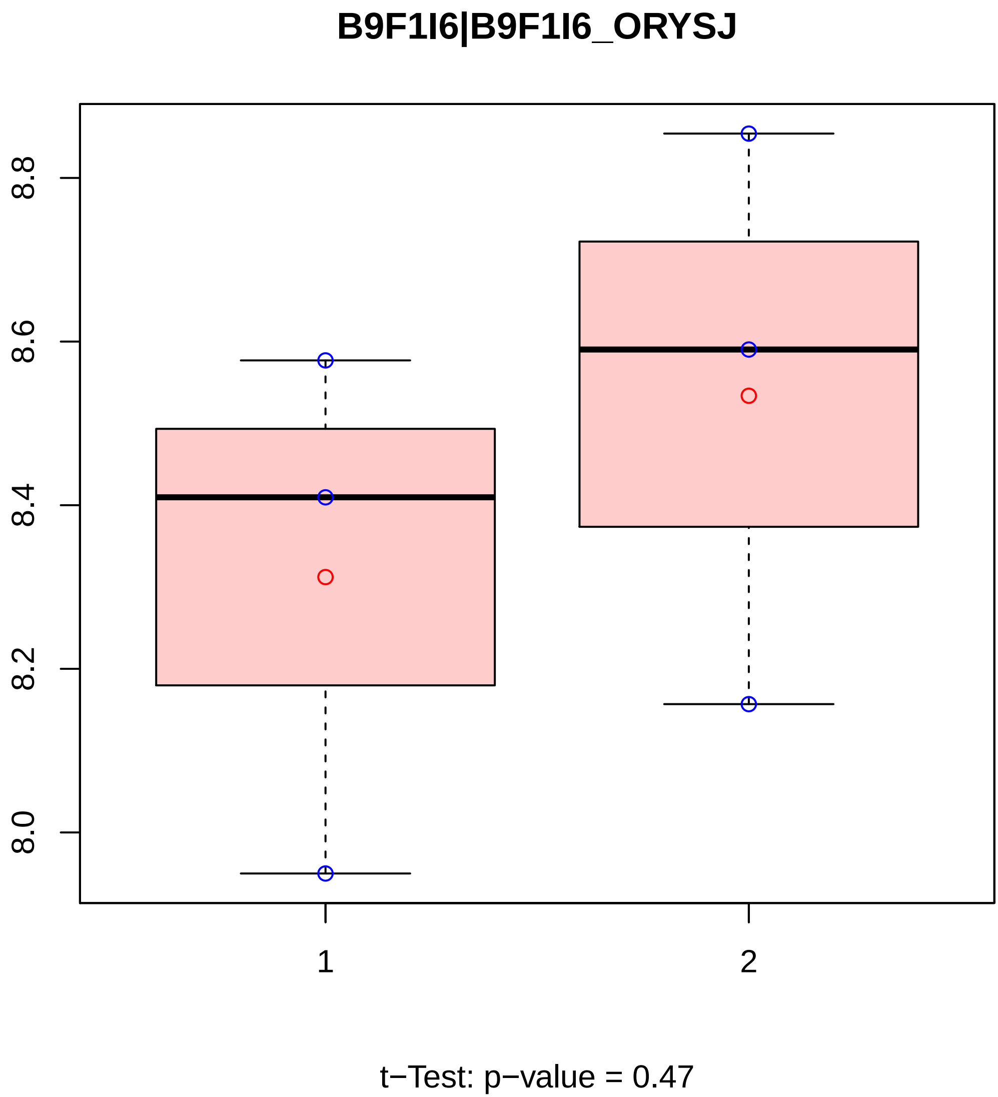
B9F1I6|B9F1I6_ORYSJ
8.0
8.2
8.4
8.6
8.8
1
2
t−Test: p−value = 0.47

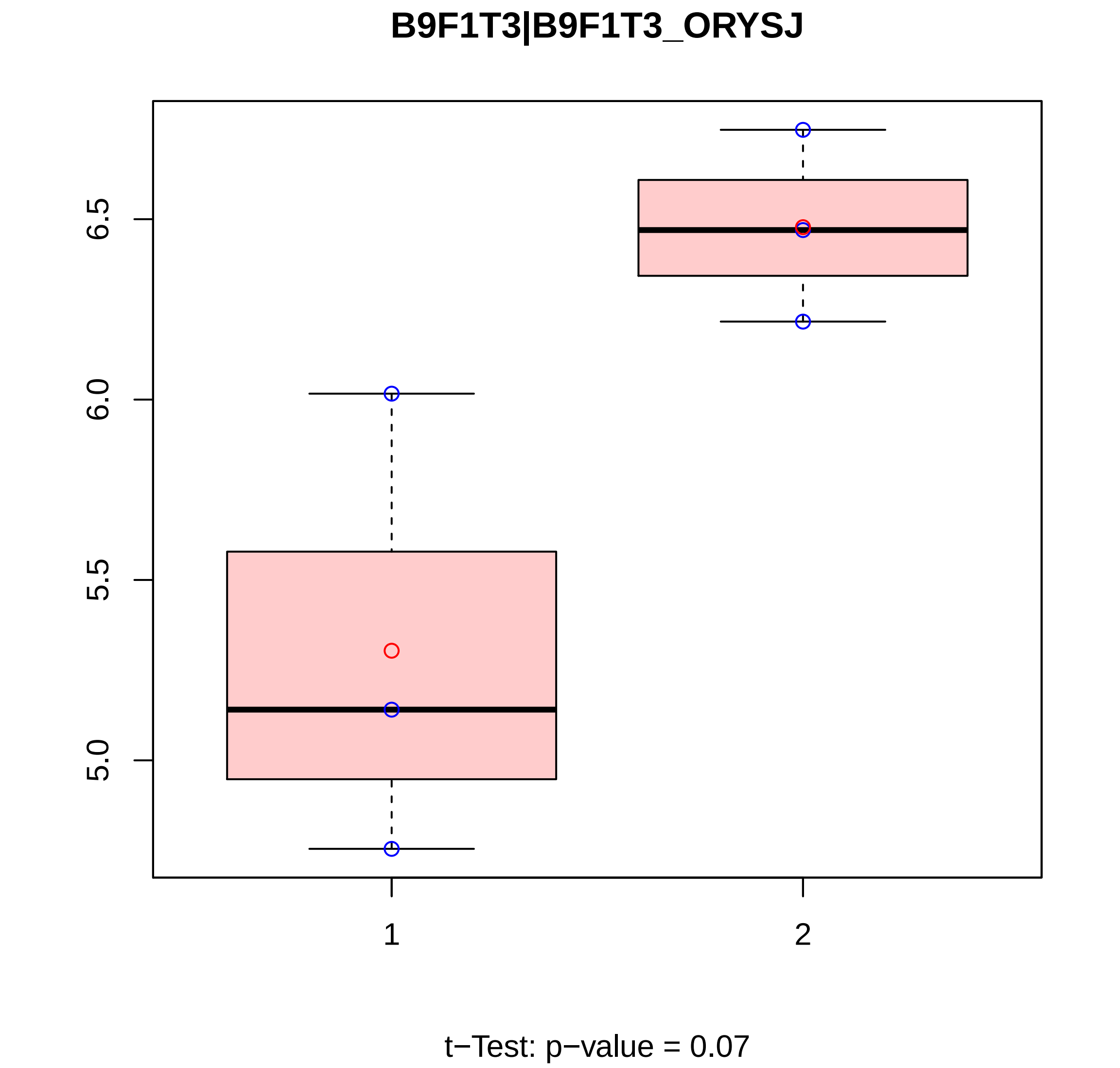

B9F1T3|B9F1T3_ORYSJ
6.5
6.0
5.5
5.0
1
2
t−Test: p−value = 0.07

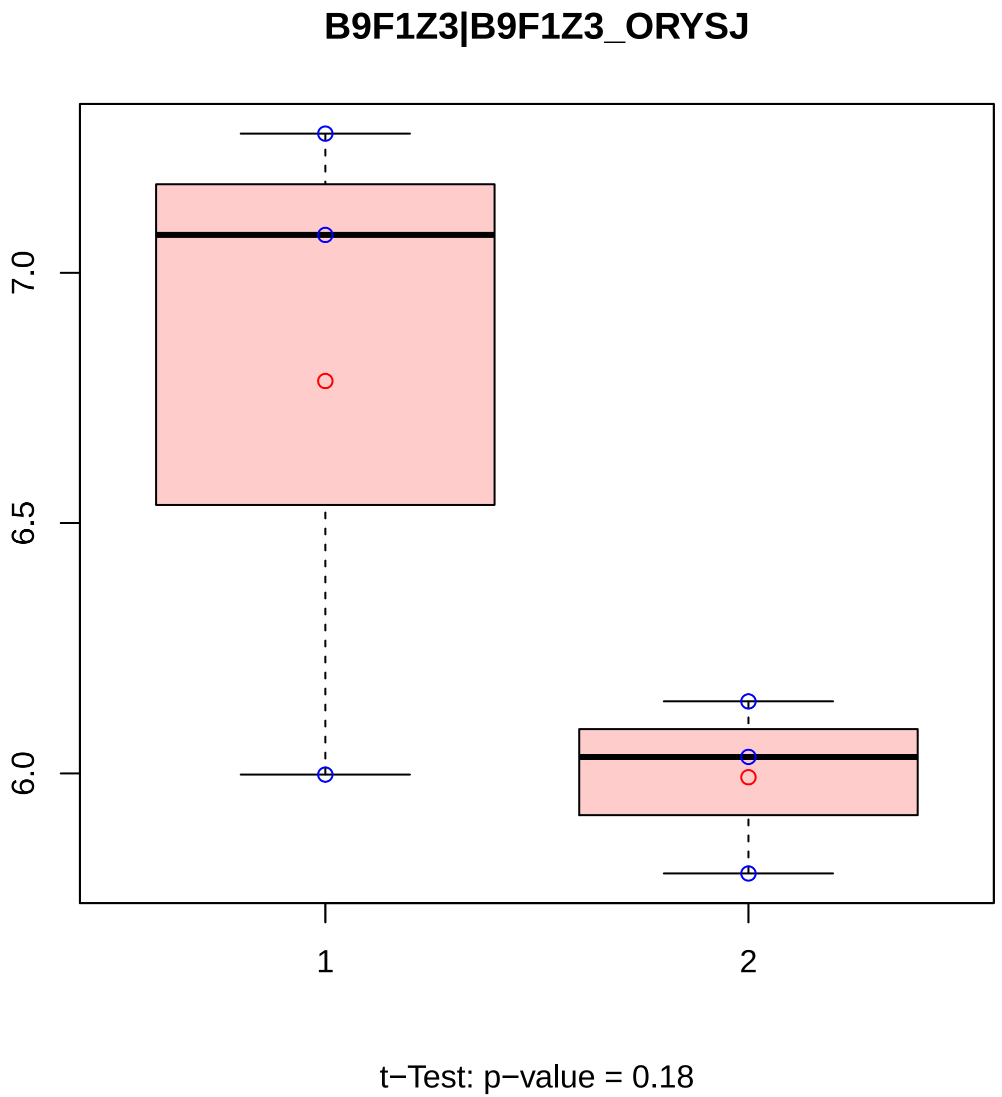

B9F1Z3|B9F1Z3_ORYSJ
7.0
6.5
6.0
1
2
t−Test: p−value = 0.18

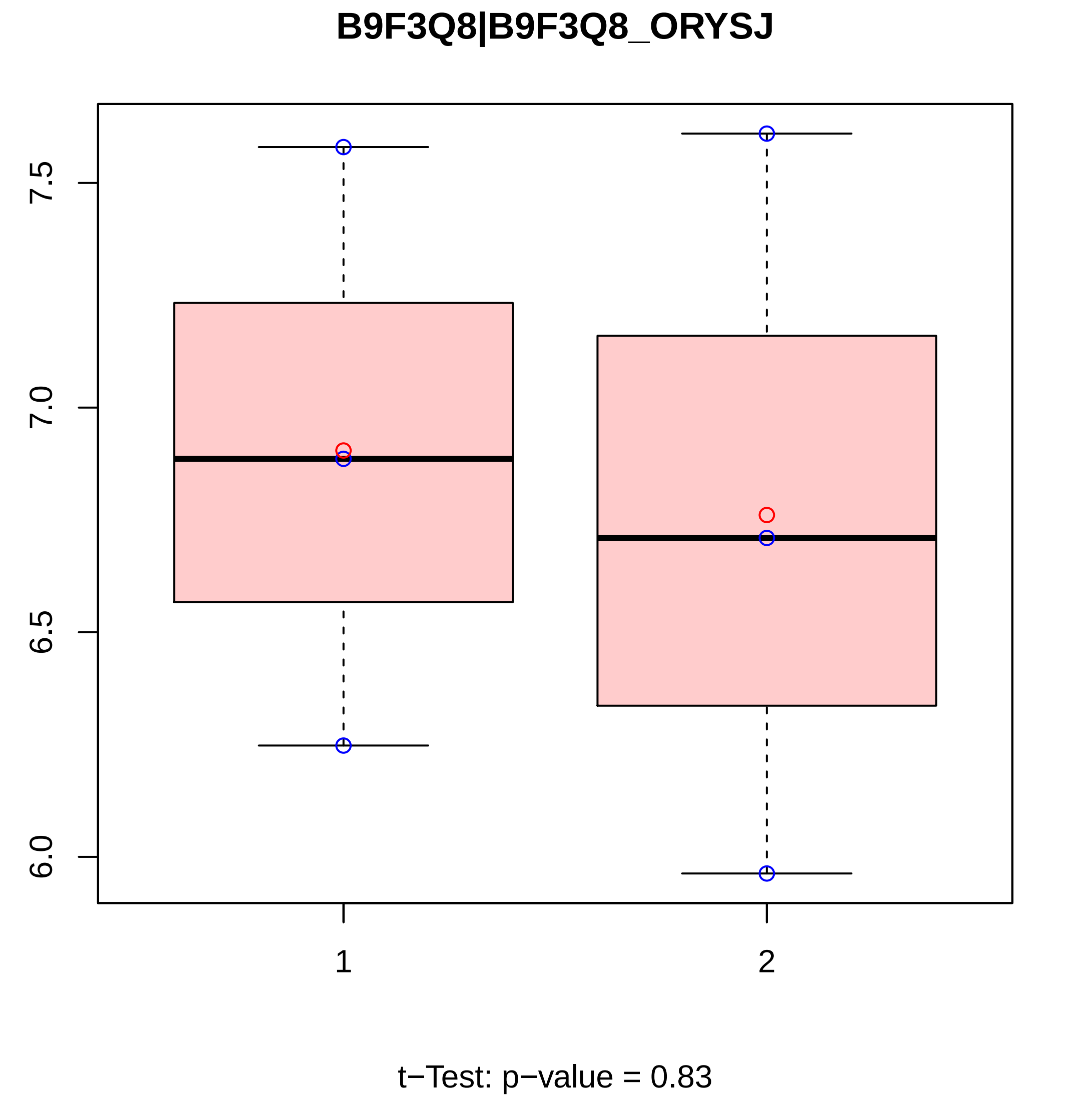
B9F3Q8|B9F3Q8_ORYSJ
7.5
7.0
6.5
6.0
1
2
t−Test: p−value = 0.83

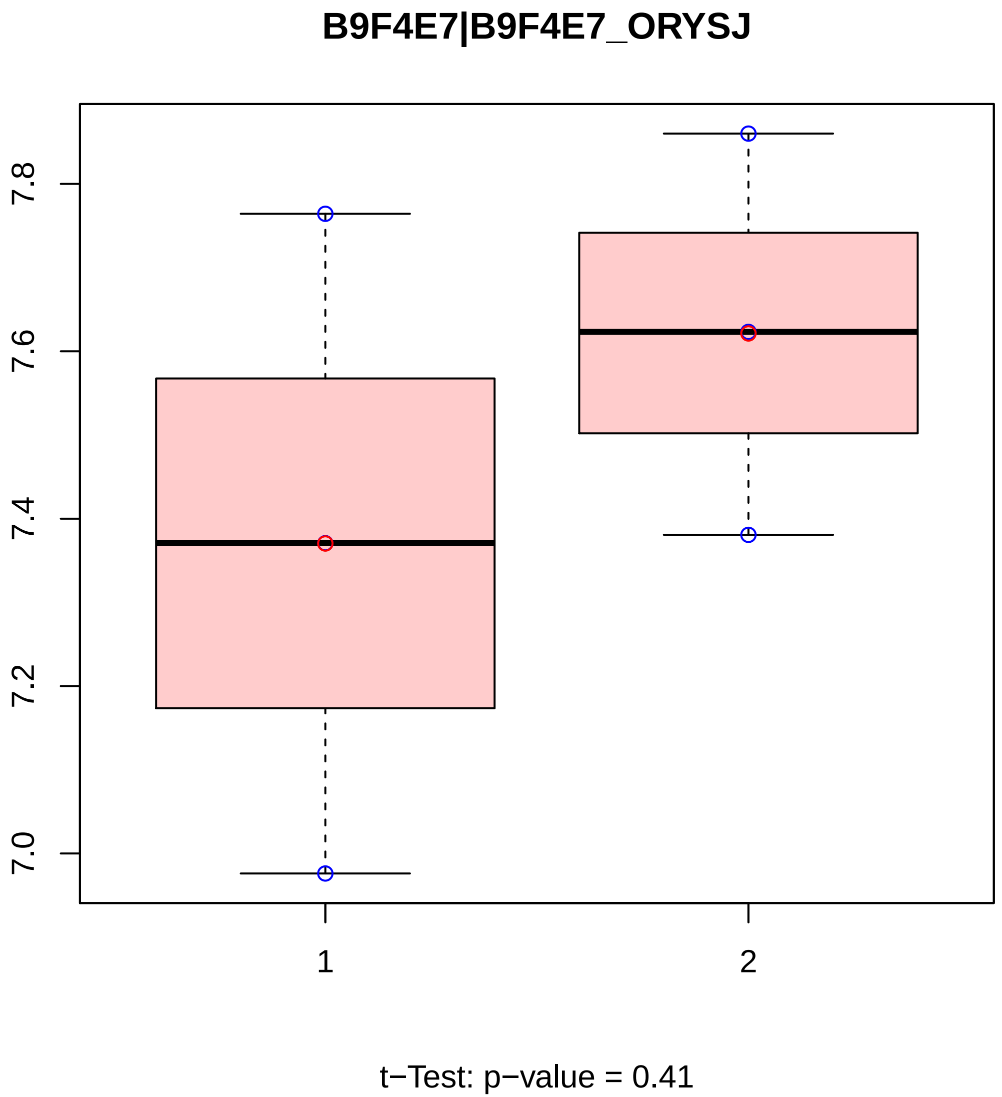

B9F4E7|B9F4E7_ORYSJ
7.0
7.2
7.4
7.6
7.8
1
2
t−Test: p−value = 0.41

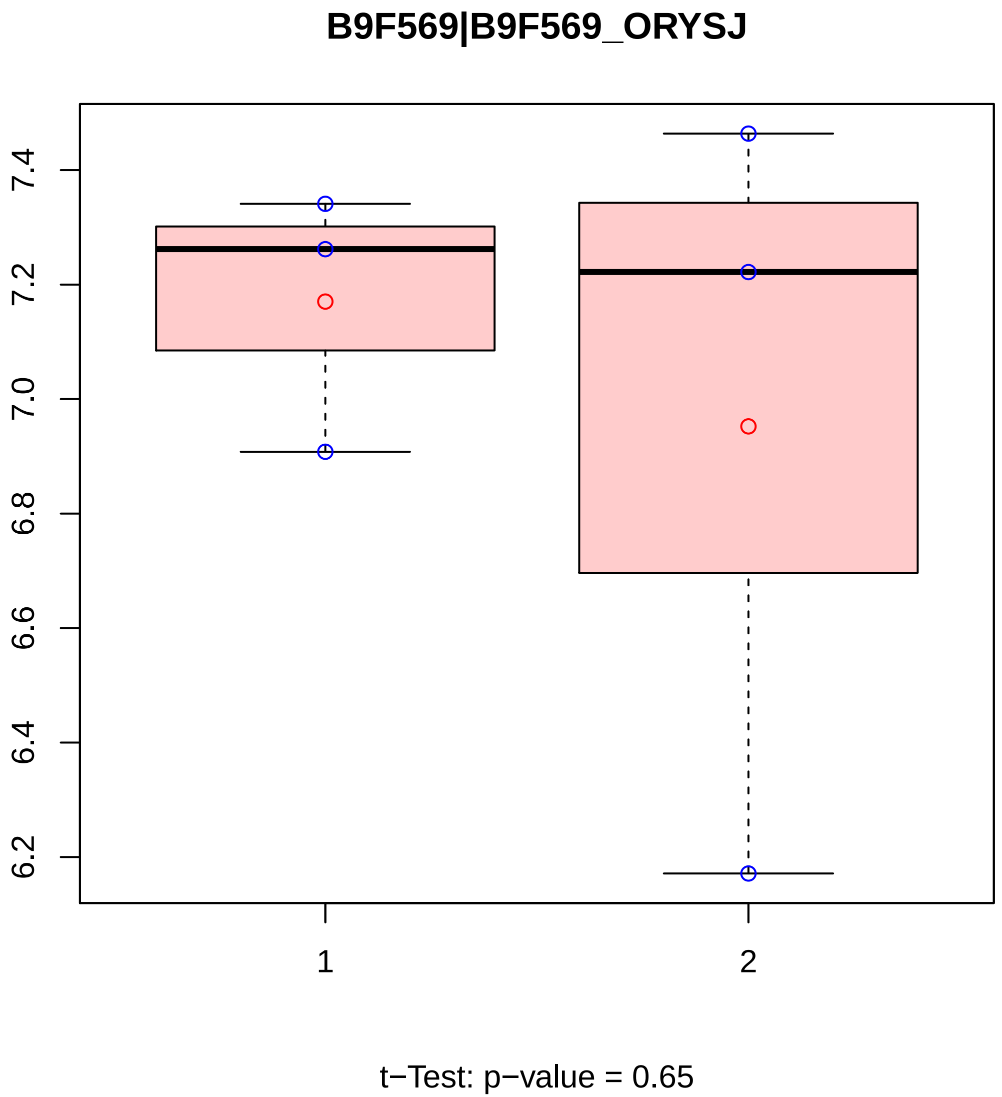
B9F569|B9F569_ORYSJ
7.4
7.2
7.0
6.8
6.6
6.4
6.2
1
2
t−Test: p−value = 0.65

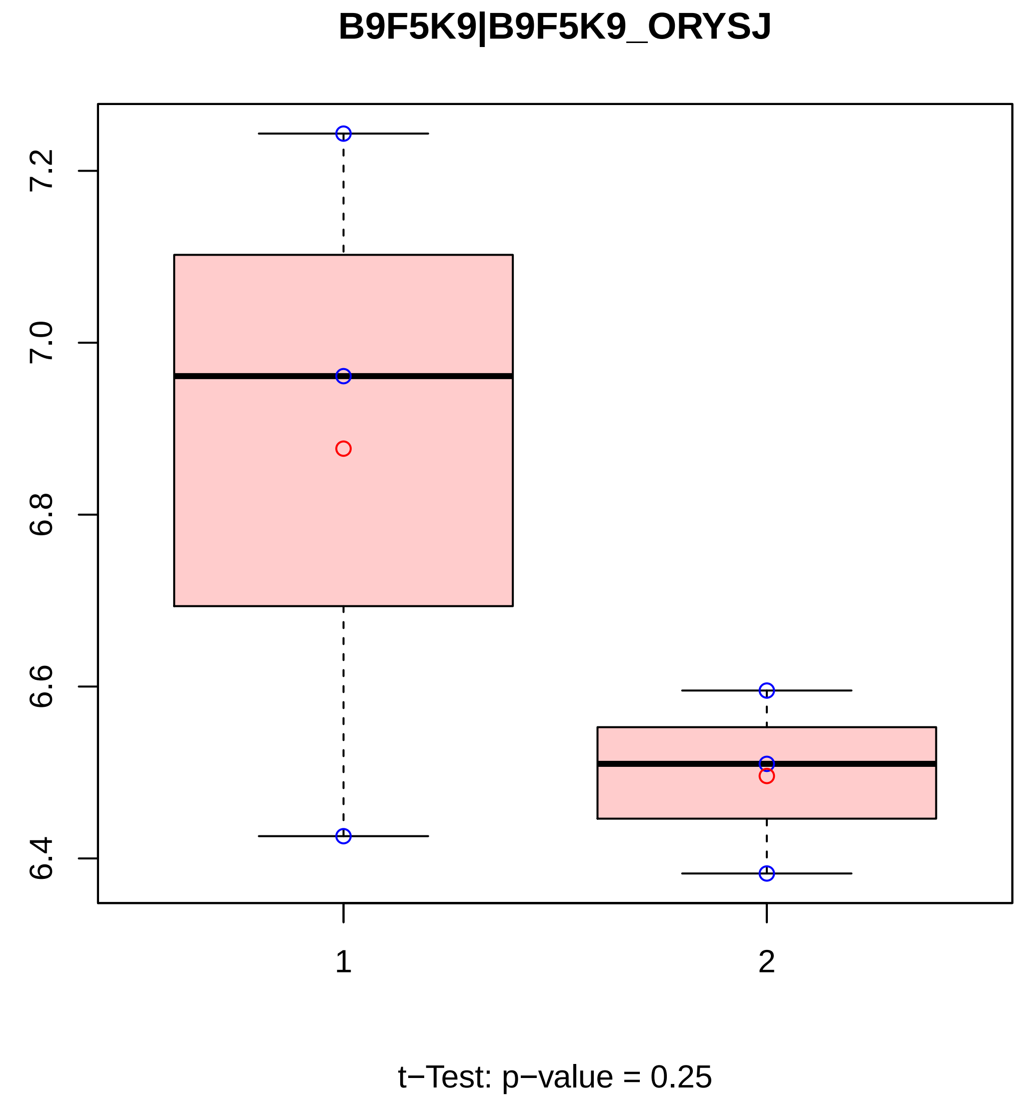
B9F5K9|B9F5K9_ORYSJ
7.2
7.0
6.8
6.6
6.4
1
2
t−Test: p−value = 0.25

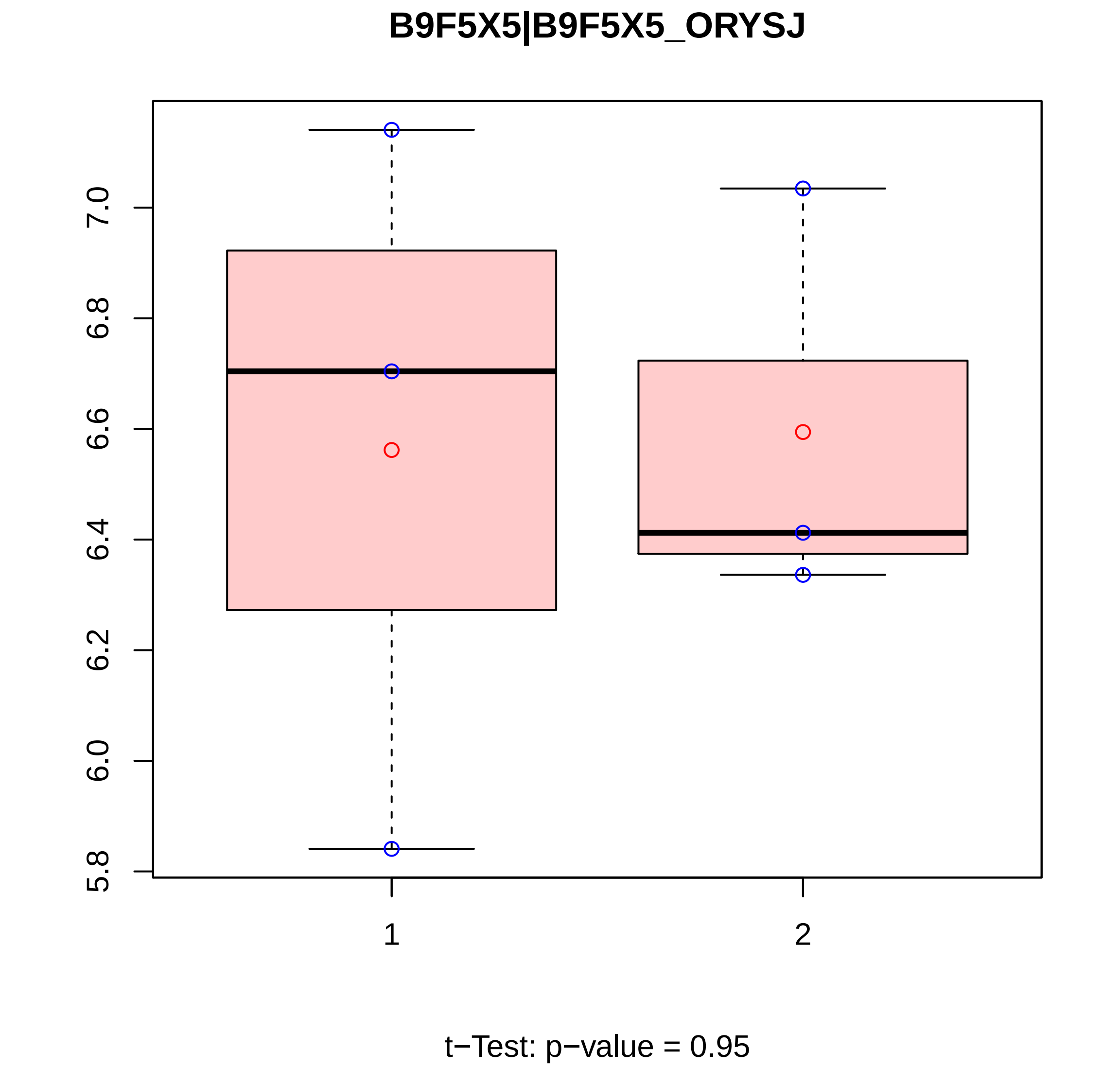

B9F5X5|B9F5X5_ORYSJ
7.0
6.8
6.6
6.4
6.2
6.0
5.8
1
2
t−Test: p−value = 0.95

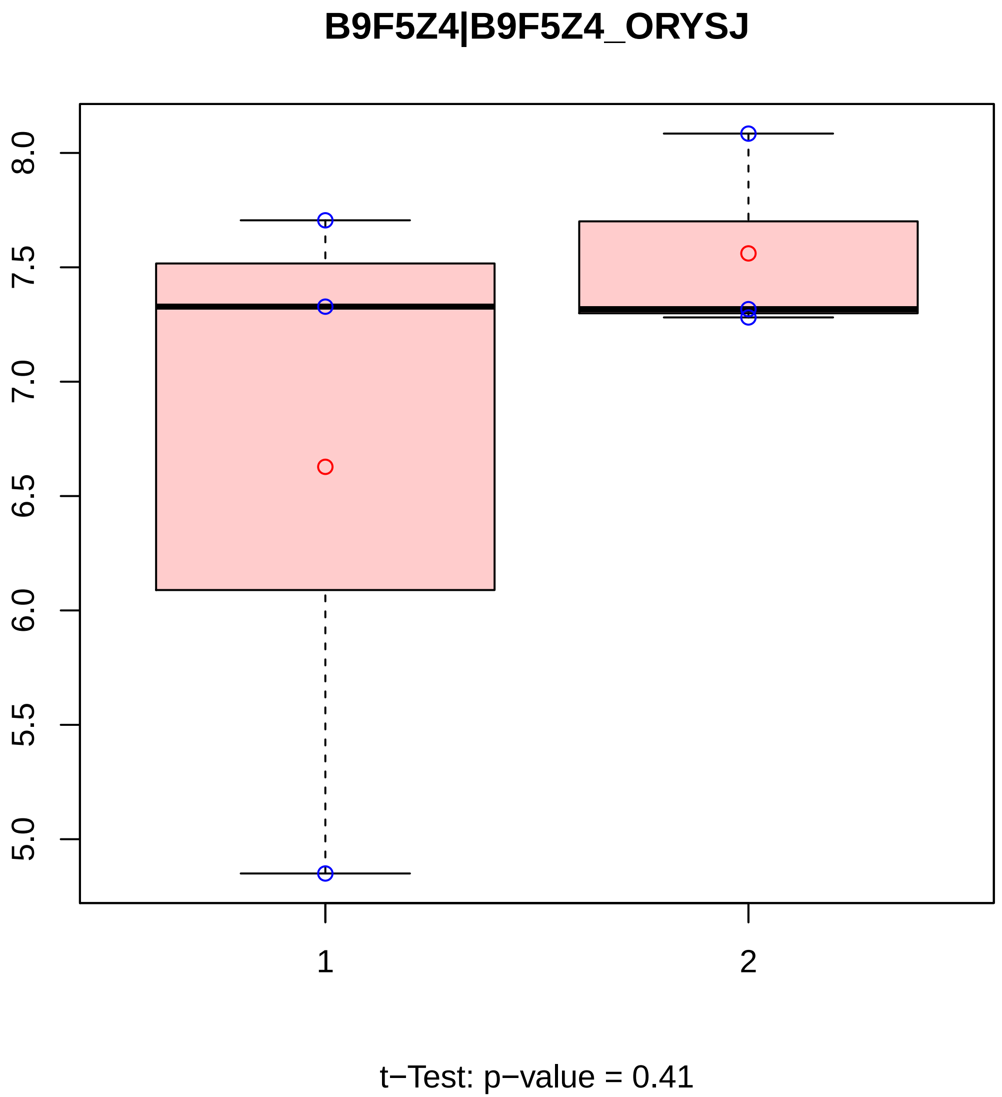

B9F5Z4|B9F5Z4_ORYSJ
8.0
7.5
7.0
6.5
6.0
5.5
5.0
1
2
t−Test: p−value = 0.41

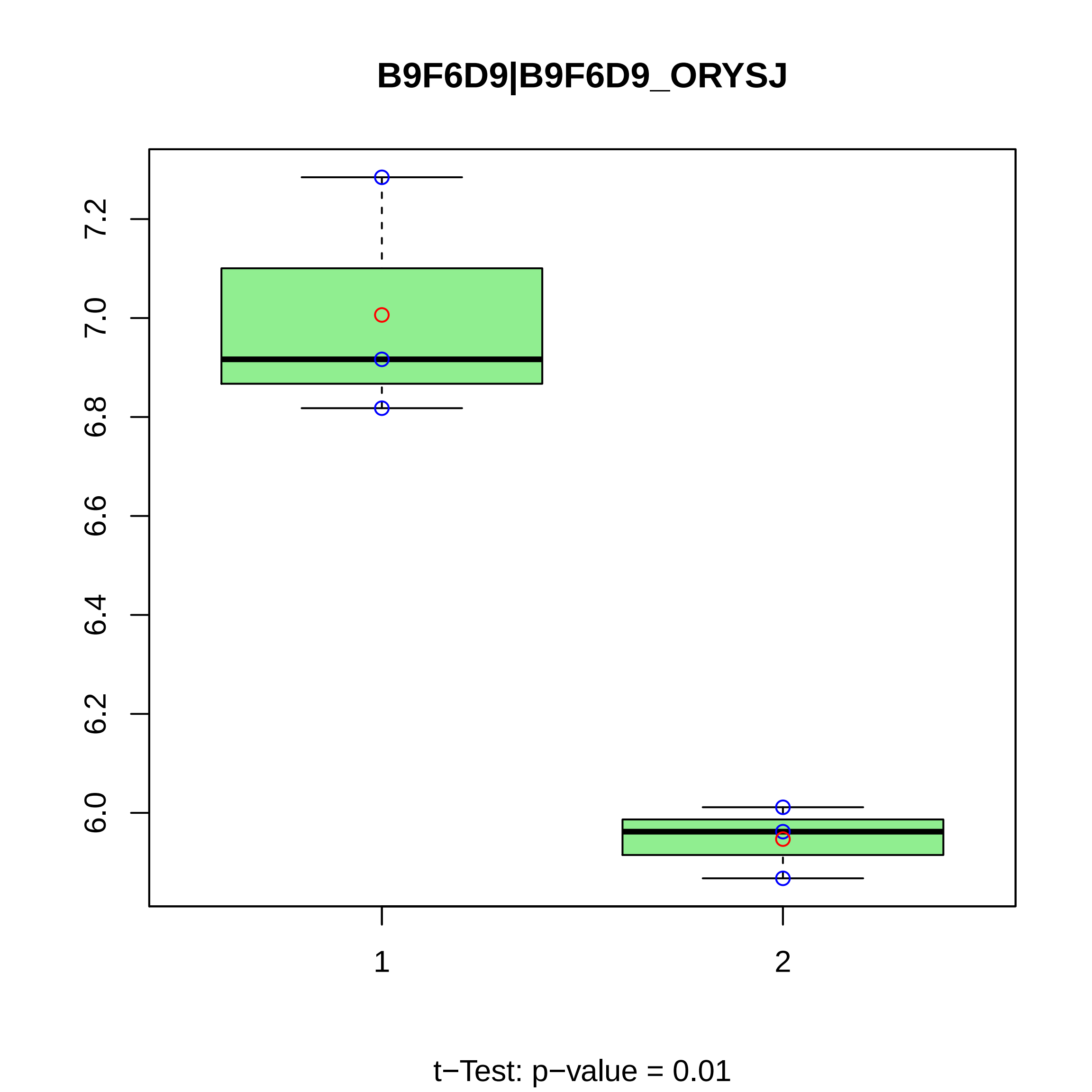

B9F6D9|B9F6D9_ORYSJ
7.2
7.0
6.8
6.6
6.4
6.2
6.0
1
2
t−Test: p−value = 0.01

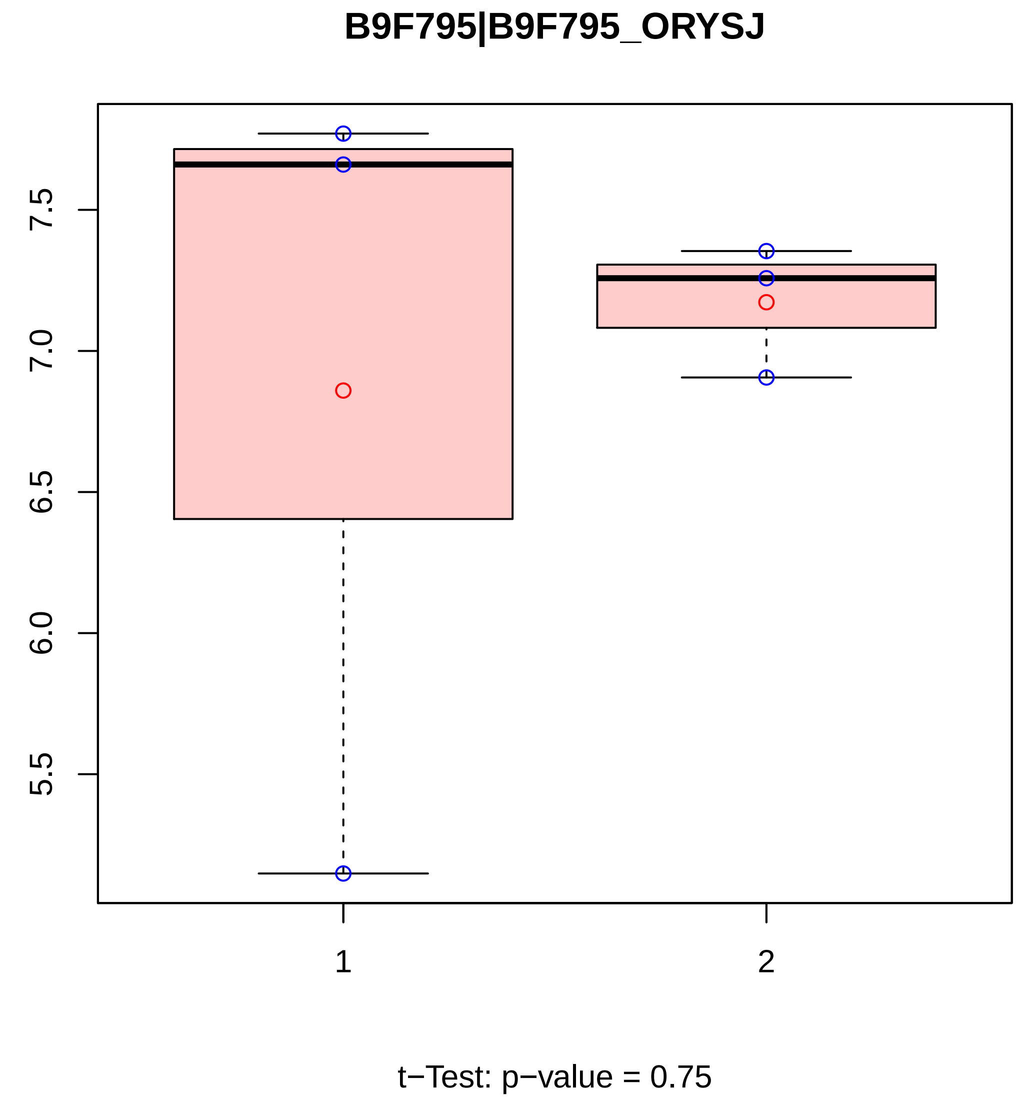

B9F795|B9F795_ORYSJ
7.5
7.0
6.5
6.0
5.5
1
2
t−Test: p−value = 0.75

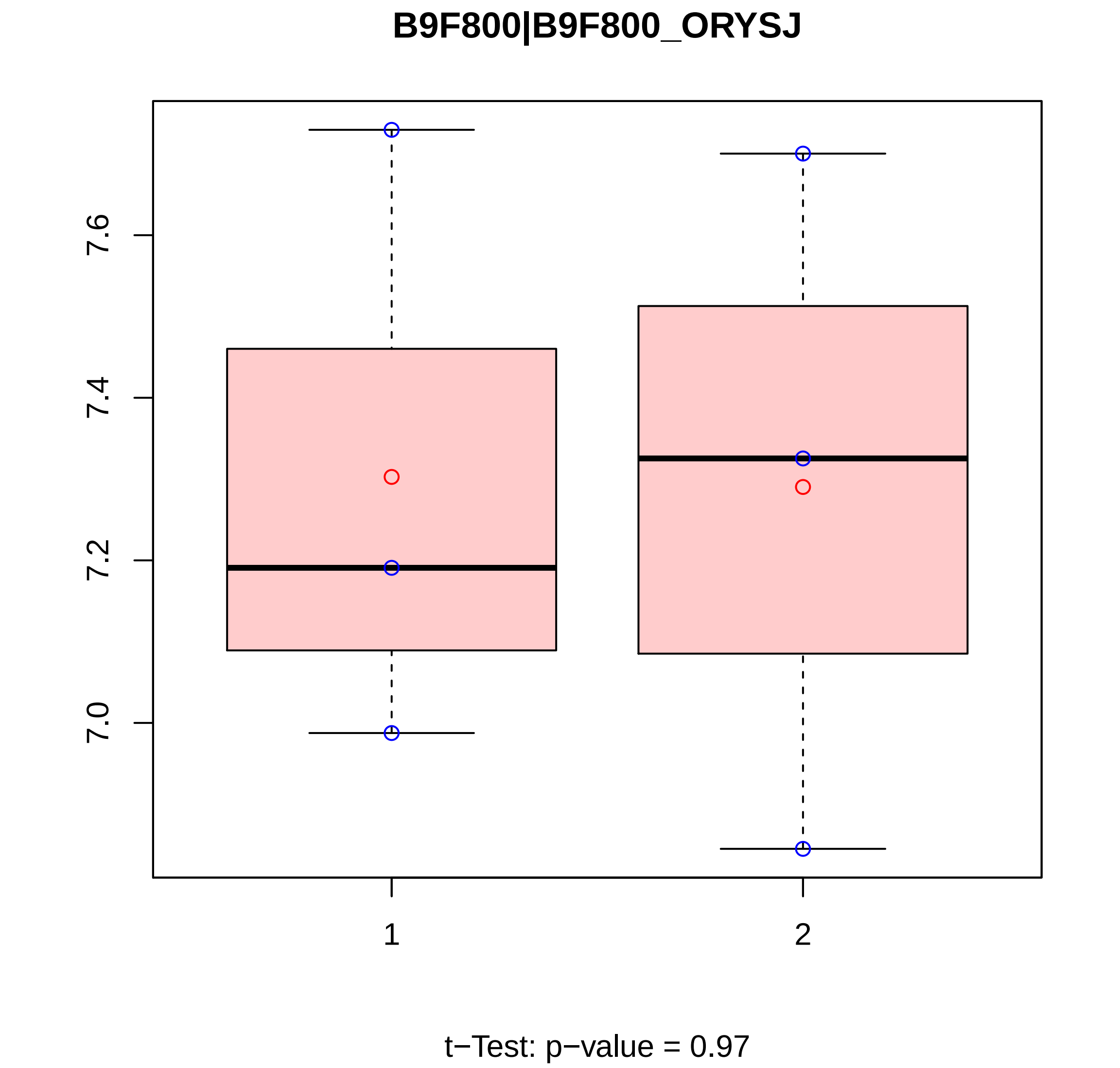
B9F800|B9F800_ORYSJ
7.0
7.2
7.4
7.6
1
2
t−Test: p−value = 0.97

B9F8C3|B9F8C3_ORYSJ

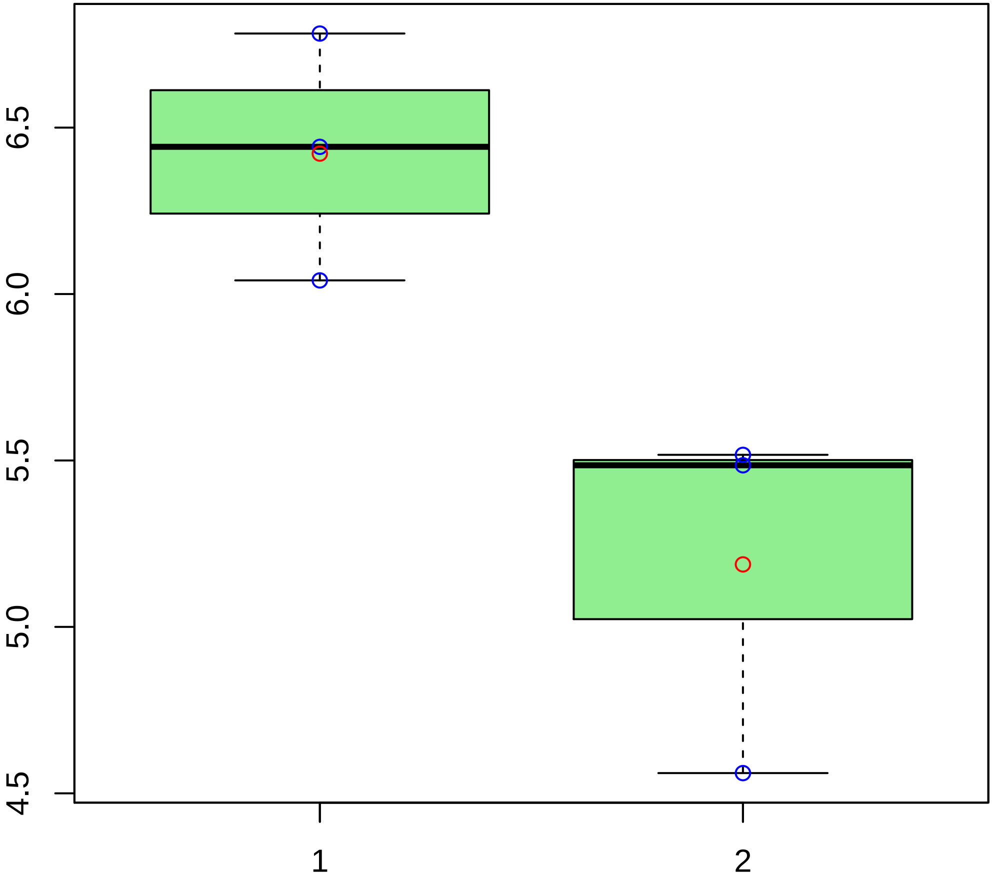

t−Test: p−value = 0.04

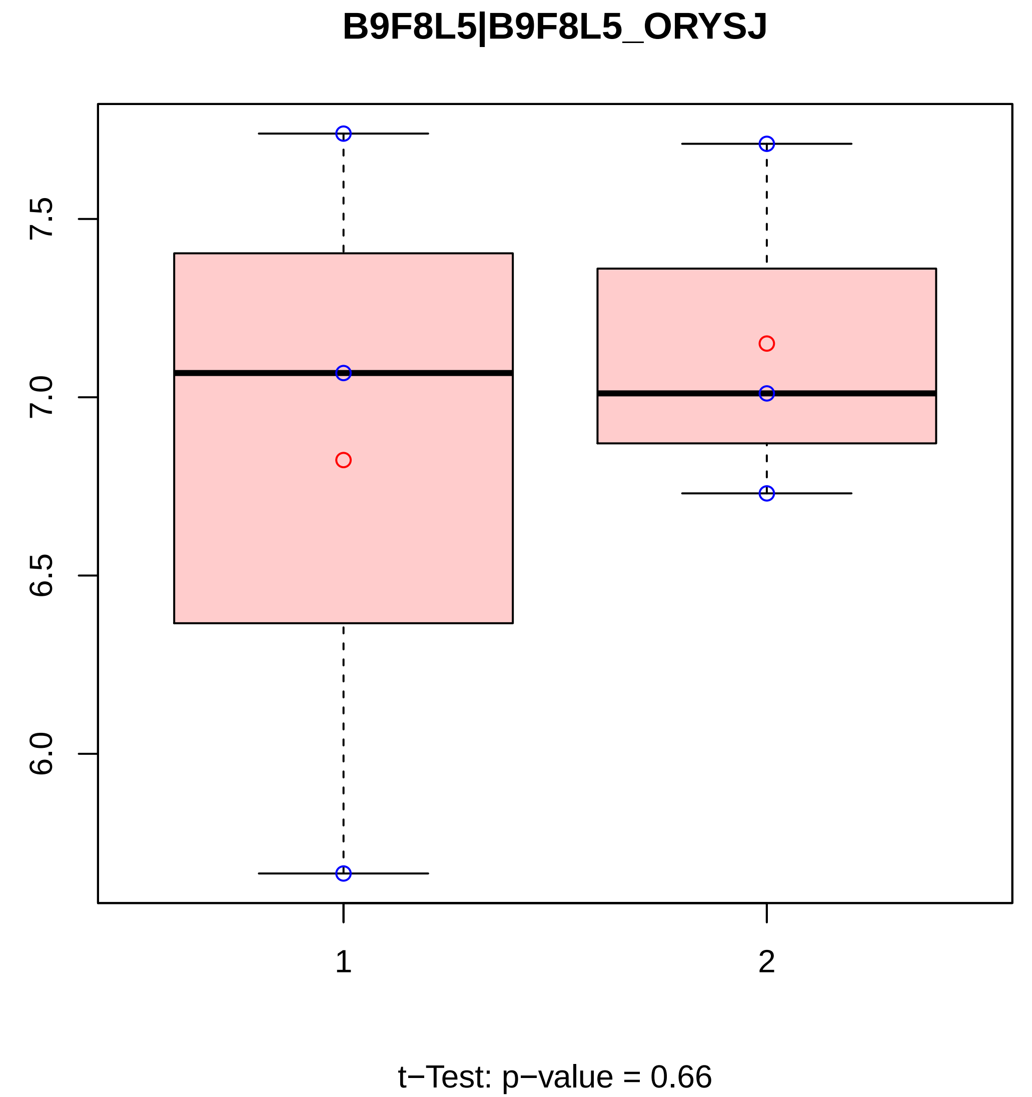
B9F8L5|B9F8L5_ORYSJ
7.5
7.0
6.5
6.0
1
2
t−Test: p−value = 0.66

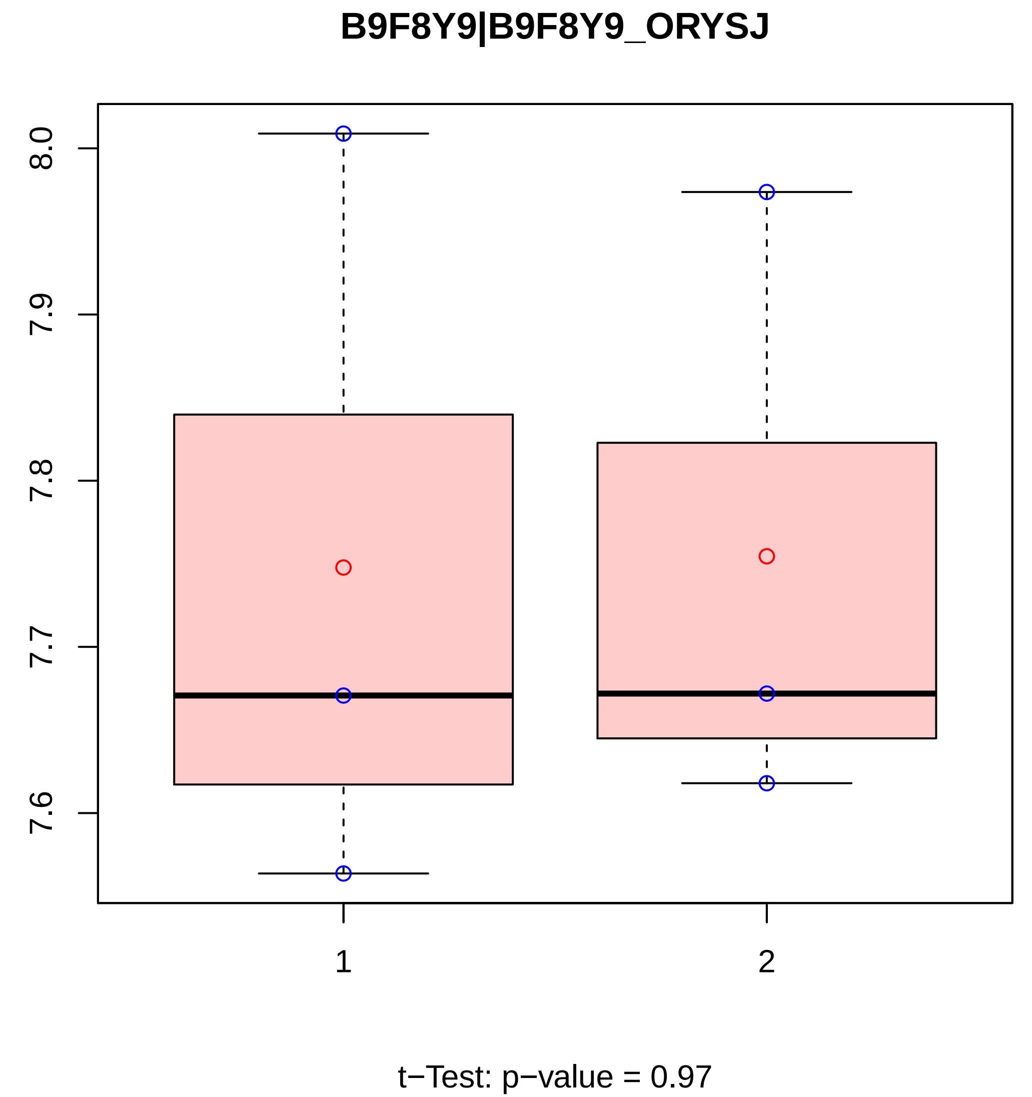
B9F8Y9|B9F8Y9_ORYSJ
8.0
7.9
7.8
7.7
7.6
1
2
t−Test: p−value = 0.97

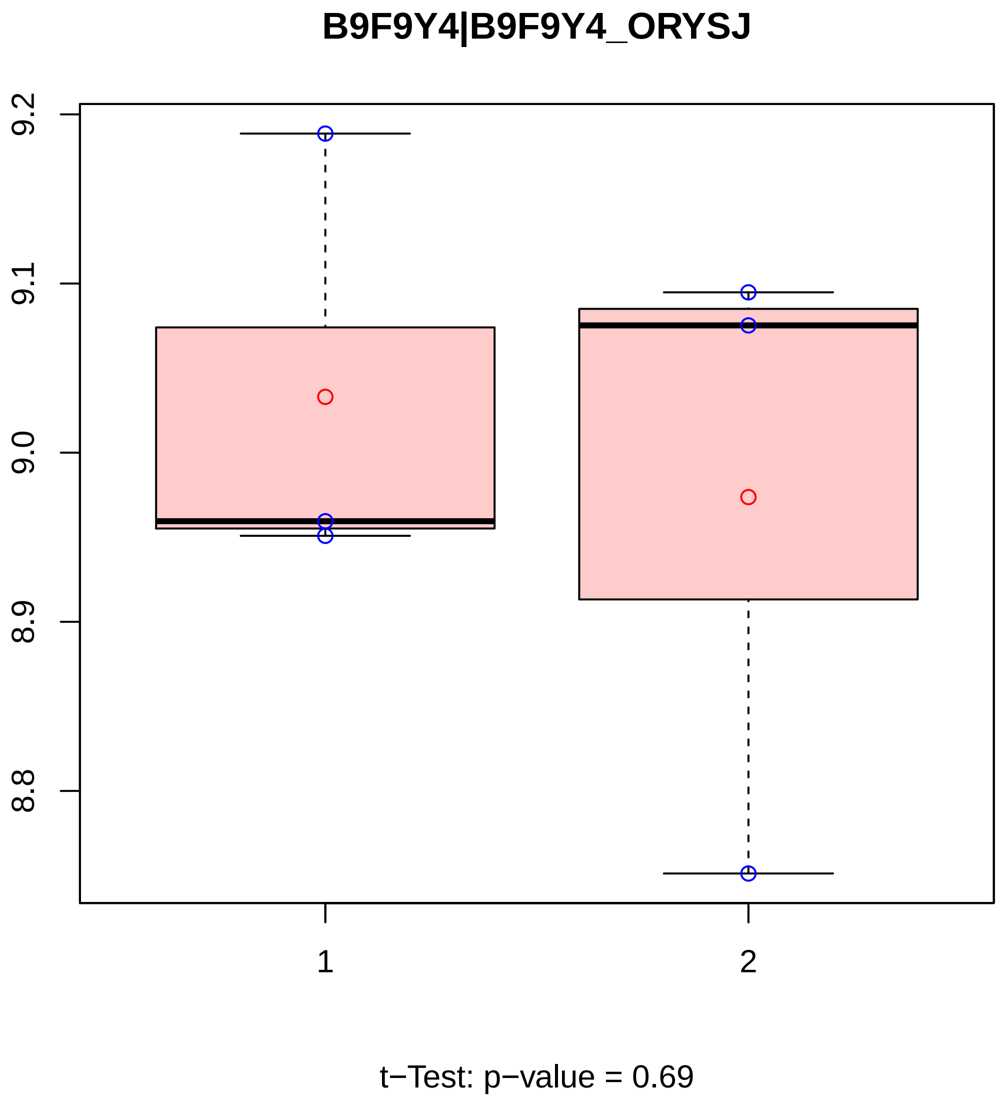

B9F9Y4|B9F9Y4_ORYSJ
9.2
9.1
9.0
8.9
8.8
1
2
t−Test: p−value = 0.69

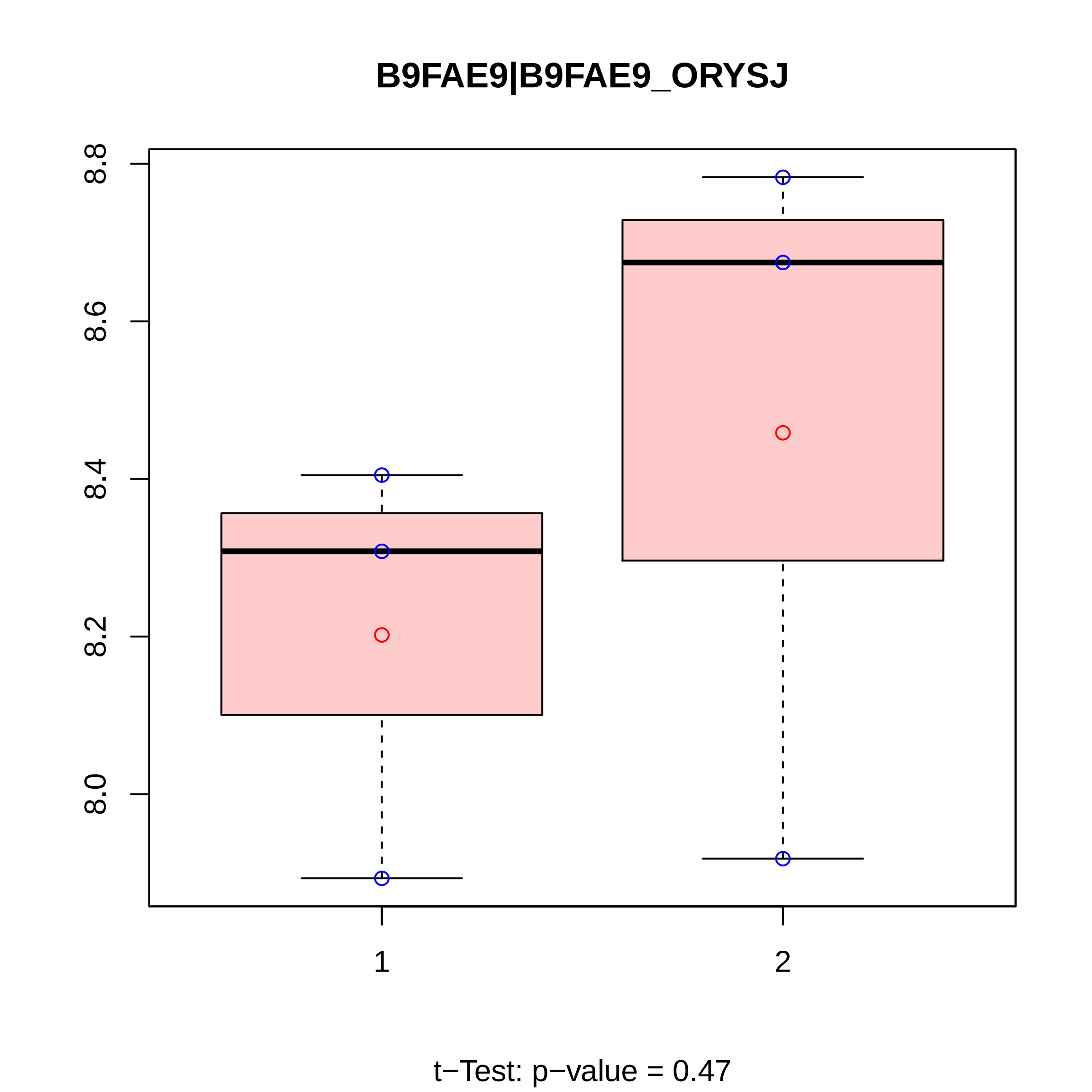

B9FAE9|B9FAE9_ORYSJ
8.0
8.2
8.4
8.6
8.8
1
2
t−Test: p−value = 0.47

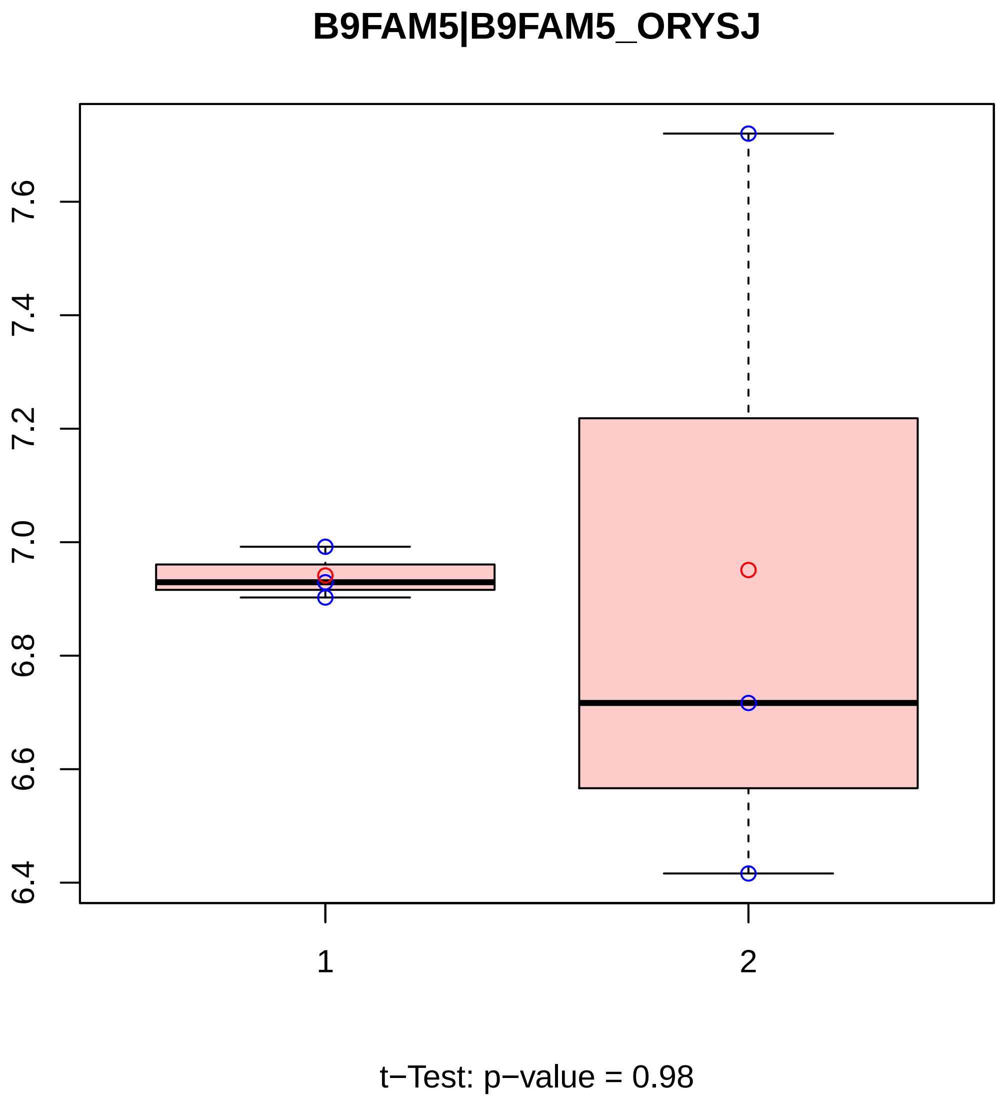
B9FAM5|B9FAM5_ORYSJ
7.6
7.4
7.2
7.0
6.8
6.6
6.4
1
2
t−Test: p−value = 0.98

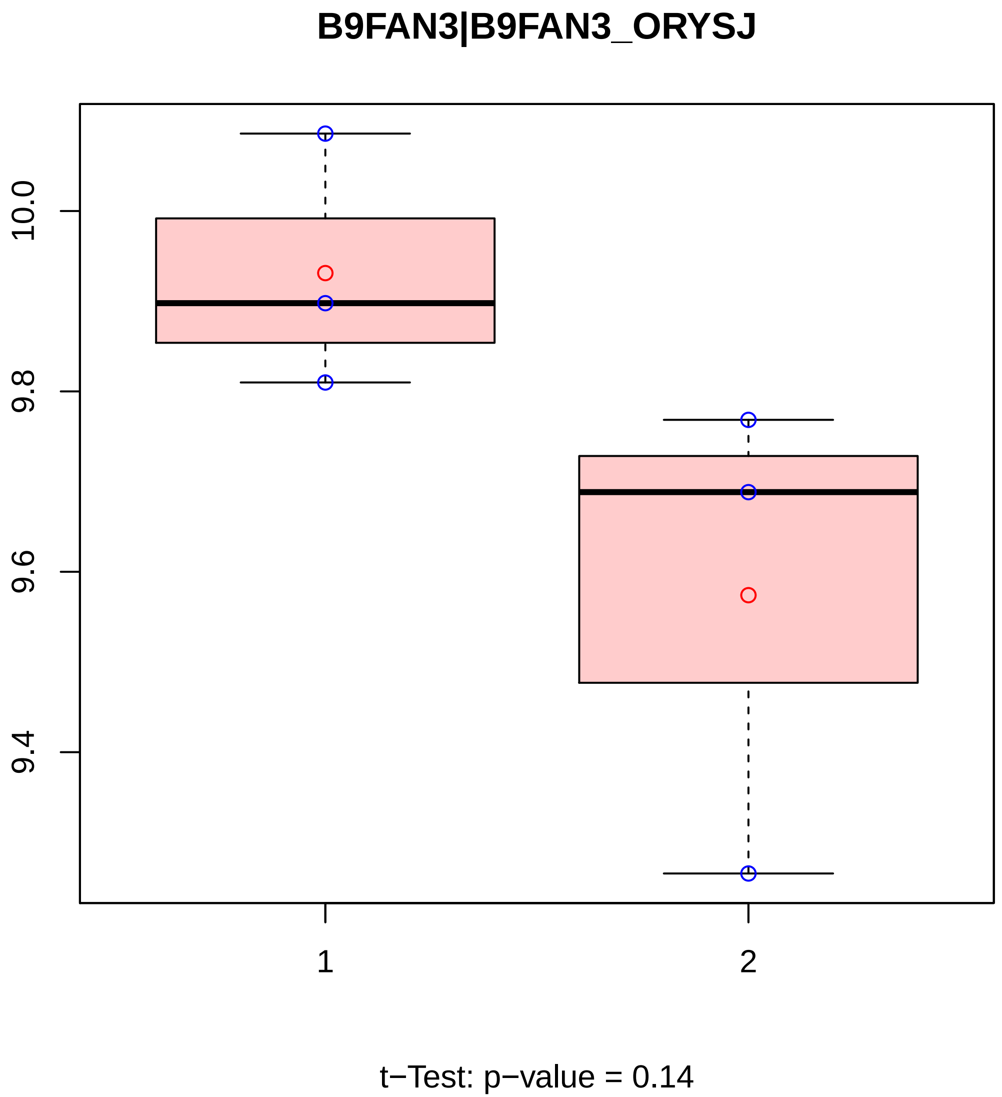

B9FAN3|B9FAN3_ORYSJ
10.0
9.8
9.6
9.4
1
2
t−Test: p−value = 0.14

B9FAZ4|B9FAZ4_ORYSJ

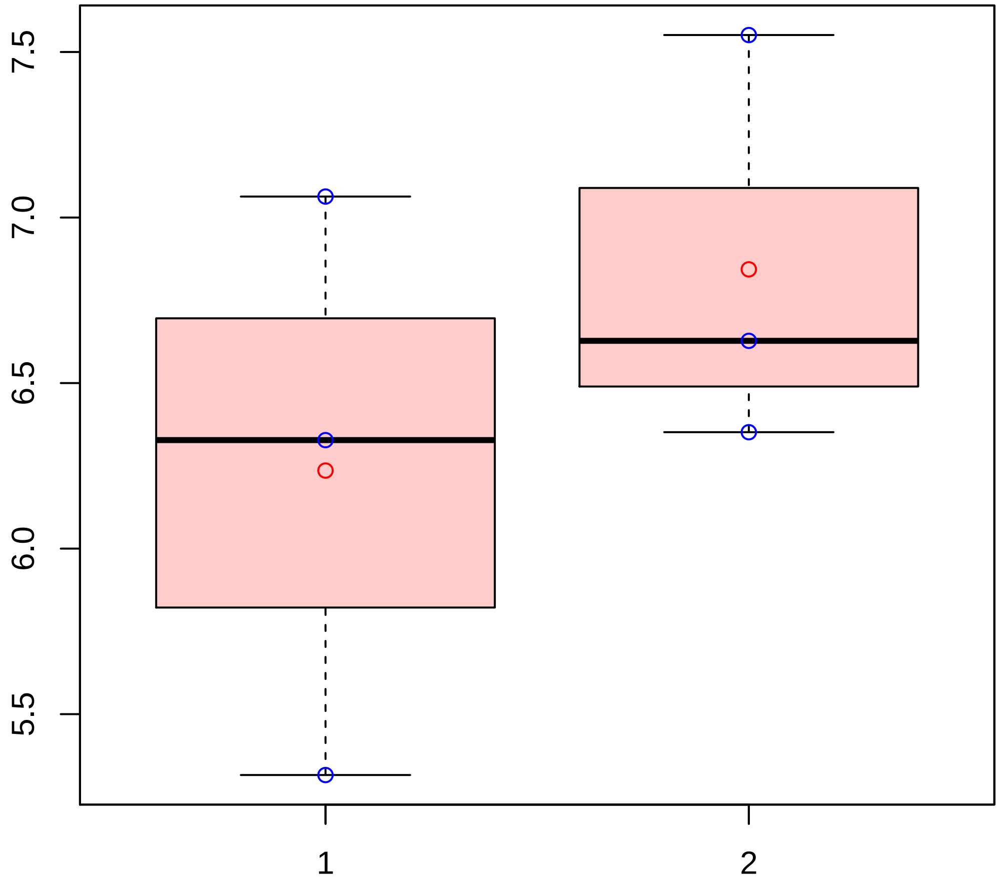

t−Test: p−value = 0.39

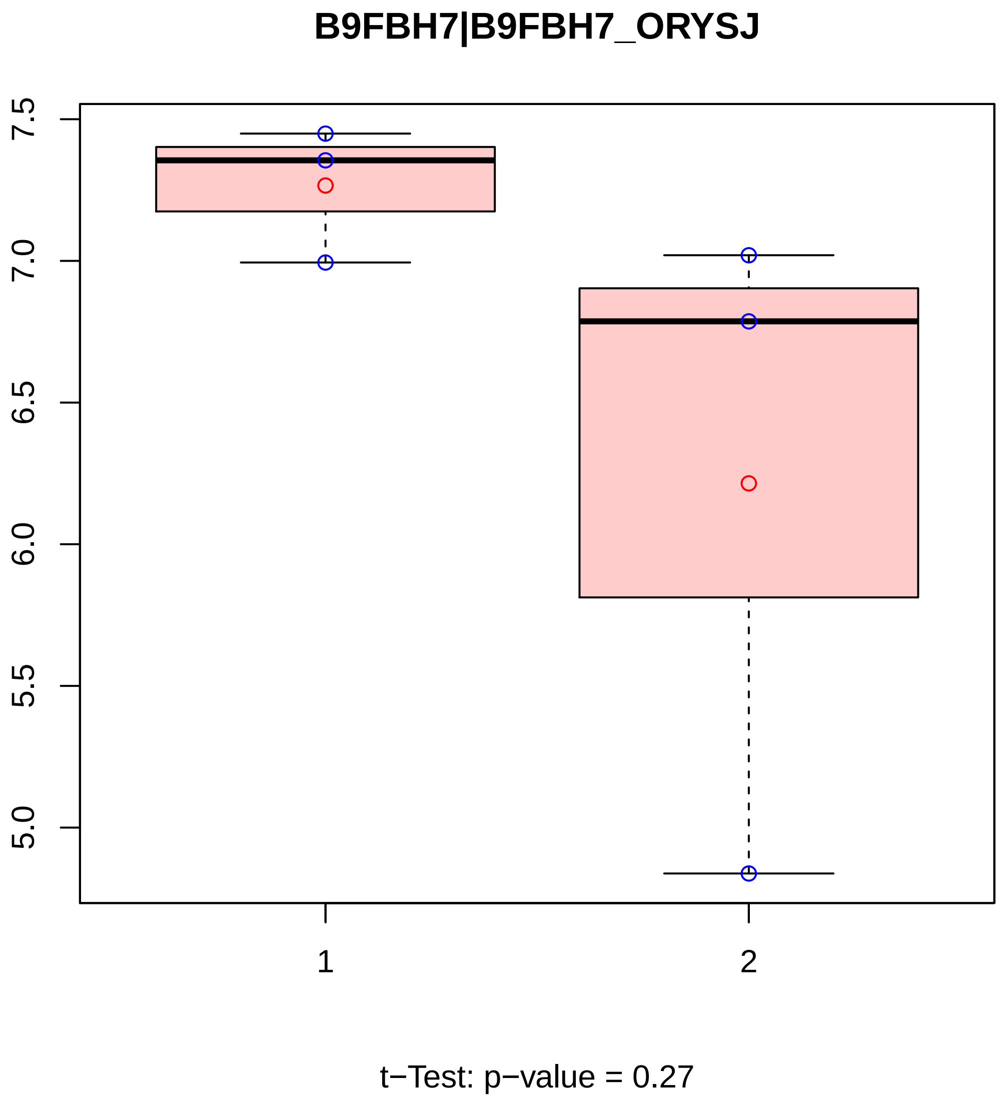
B9FBH7|B9FBH7_ORYSJ
7.5
7.0
6.5
6.0
5.5
5.0
1
2
t−Test: p−value = 0.27

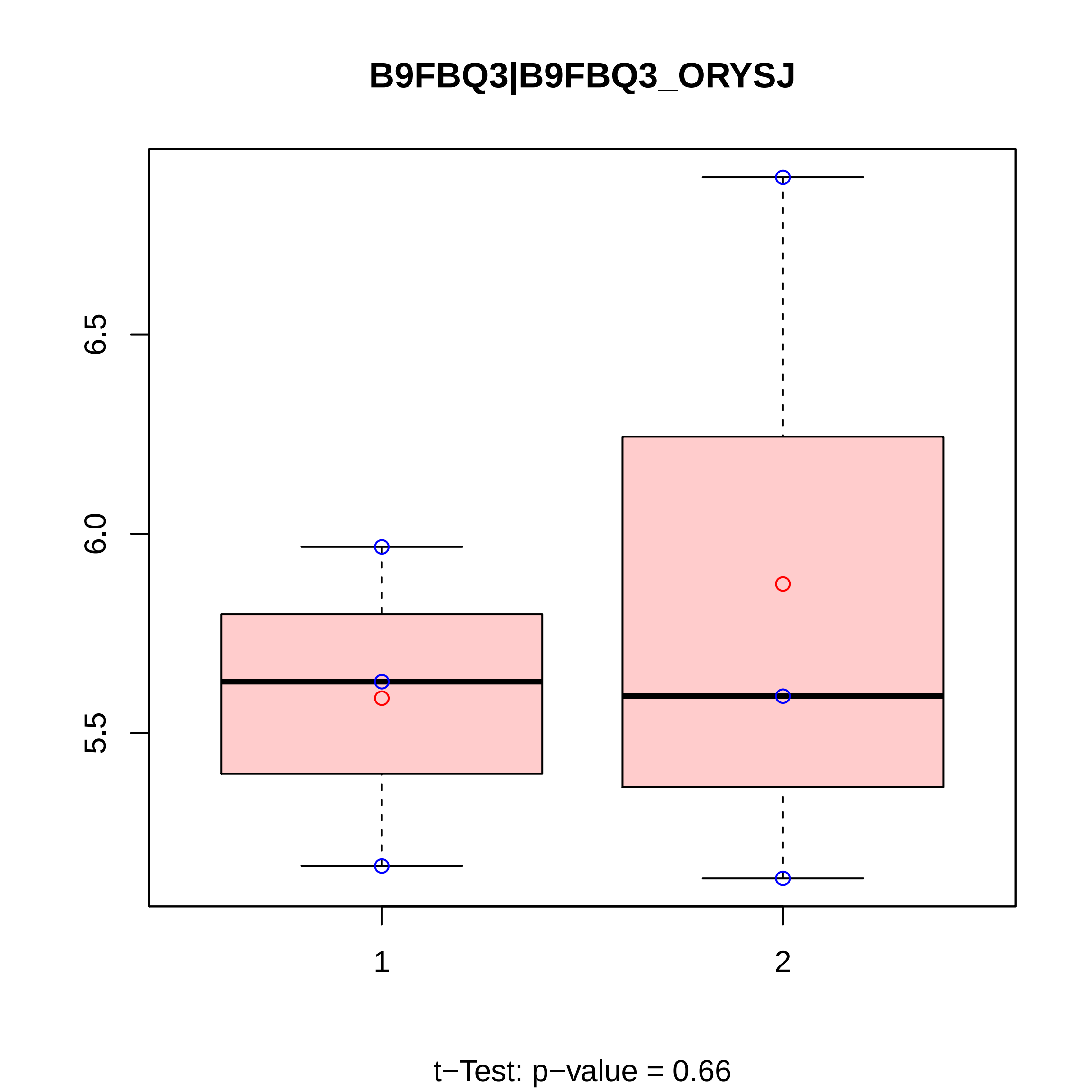
B9FBQ3|B9FBQ3_ORYSJ
6.5
6.0
5.5
1
2
t−Test: p−value = 0.66

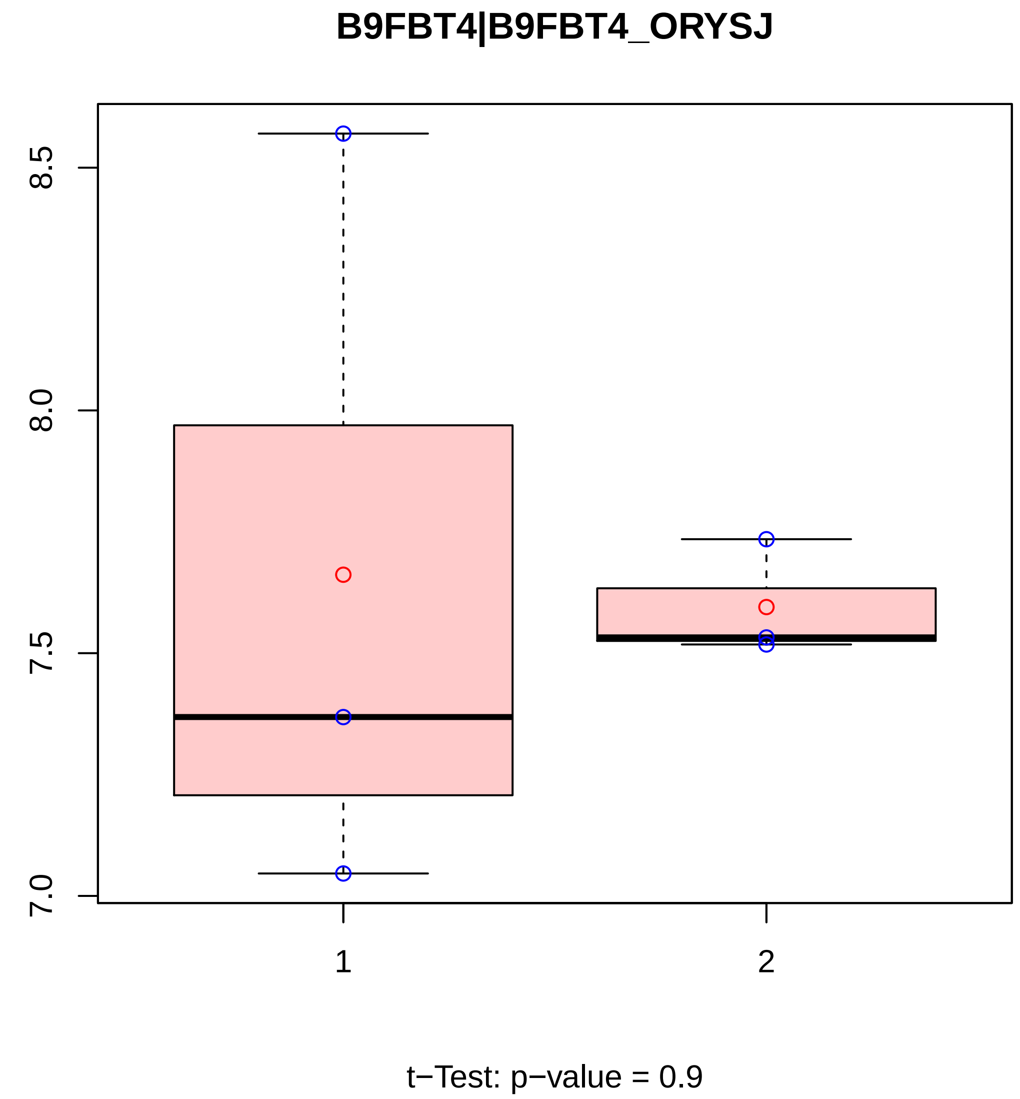

B9FBT4|B9FBT4_ORYSJ
8.5
8.0
7.5
7.0
1
2
t−Test: p−value = 0.9

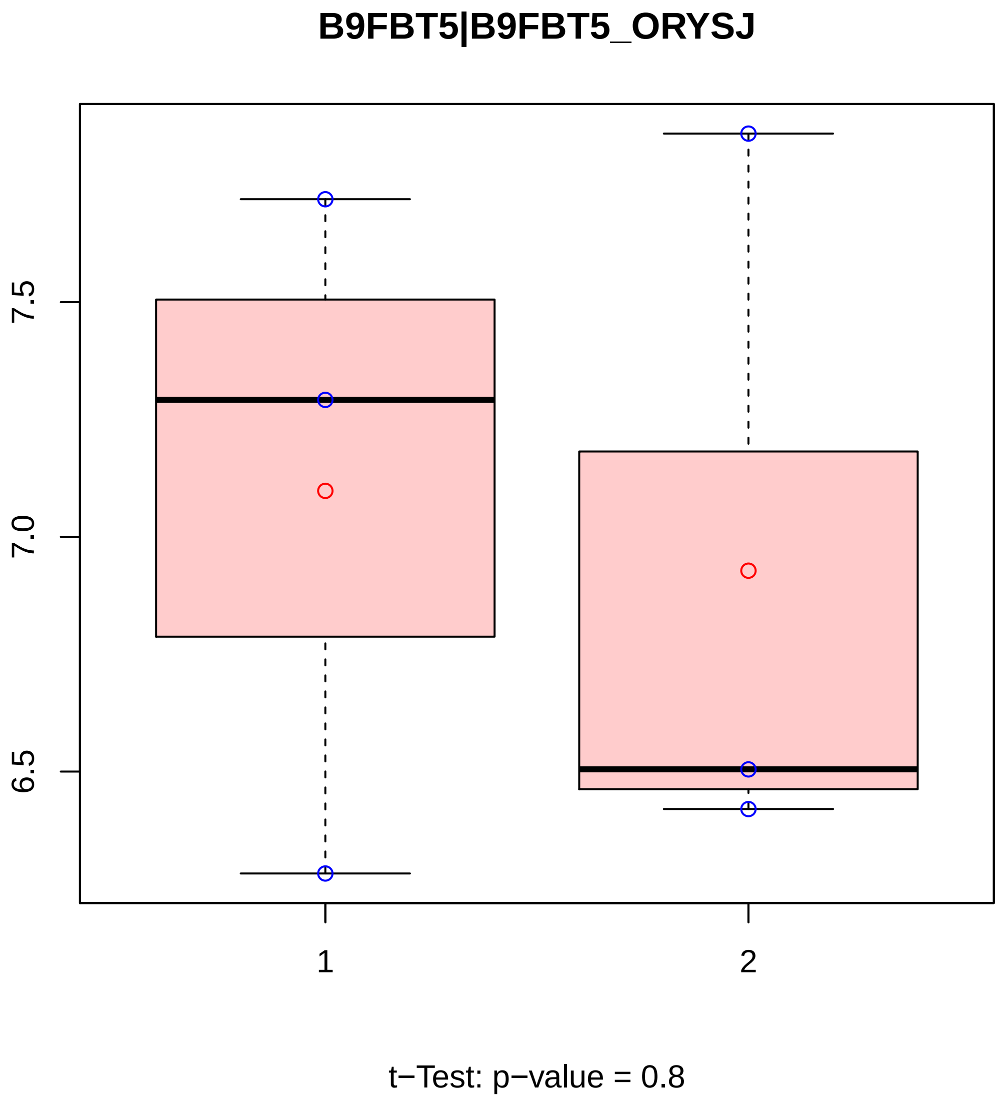

B9FBT5|B9FBT5_ORYSJ
7.5
7.0
6.5
1
2
t−Test: p−value = 0.8

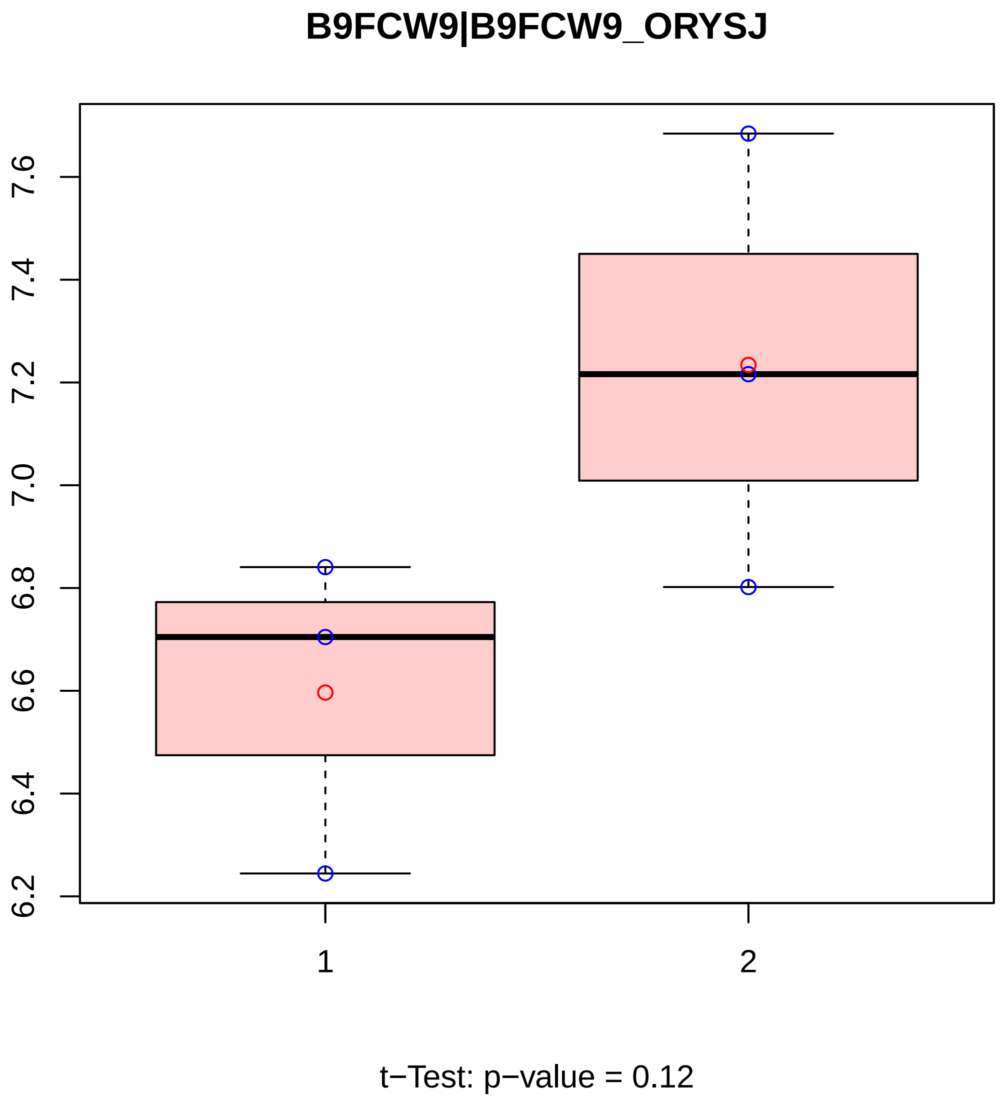

B9FCW9|B9FCW9_ORYSJ
7.6
7.4
7.2
7.0
6.8
6.6
6.4
6.2
1
2
t−Test: p−value = 0.12

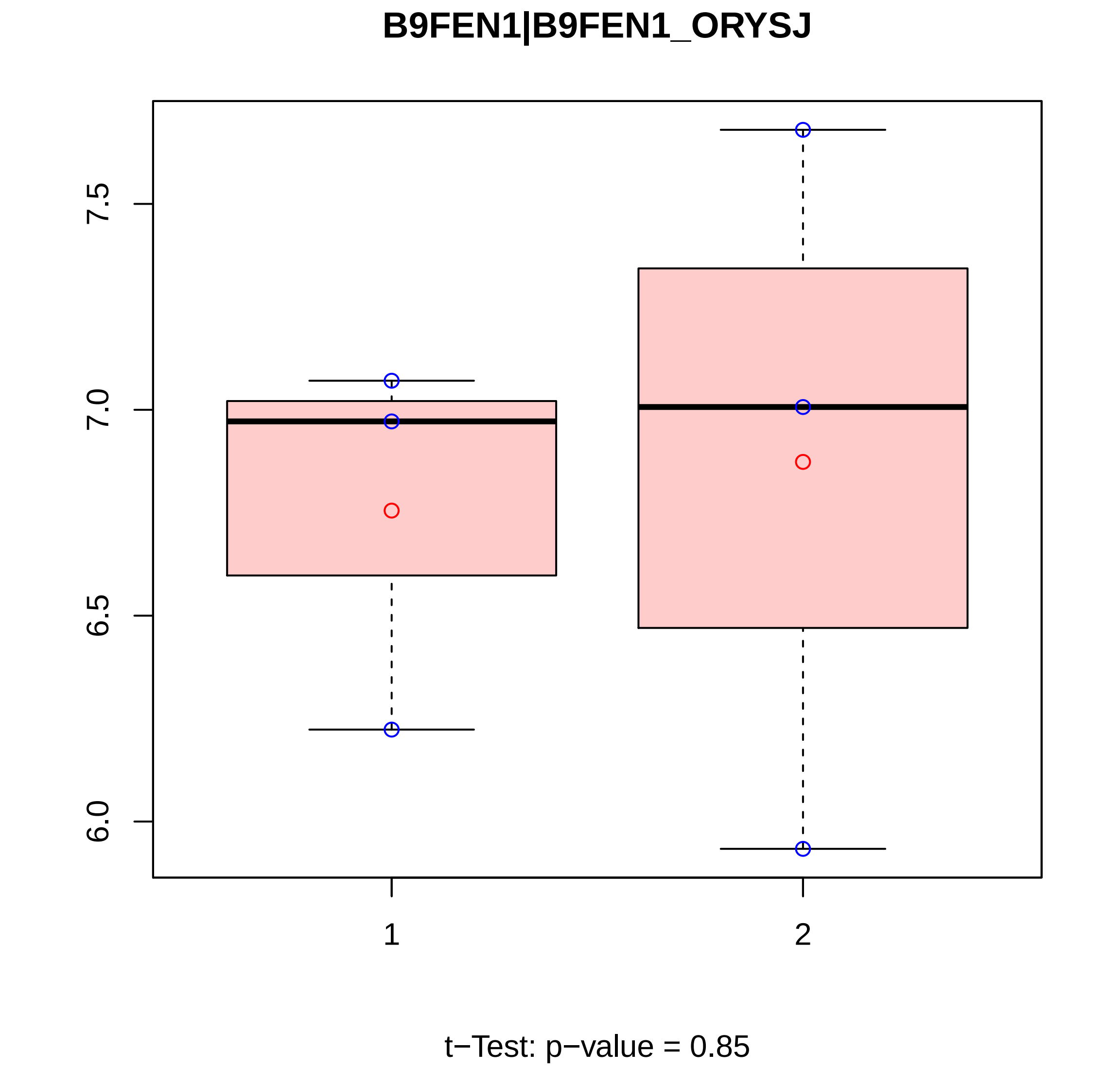
B9FEN1|B9FEN1_ORYSJ
7.5
7.0
6.5
6.0
1
2
t−Test: p−value = 0.85

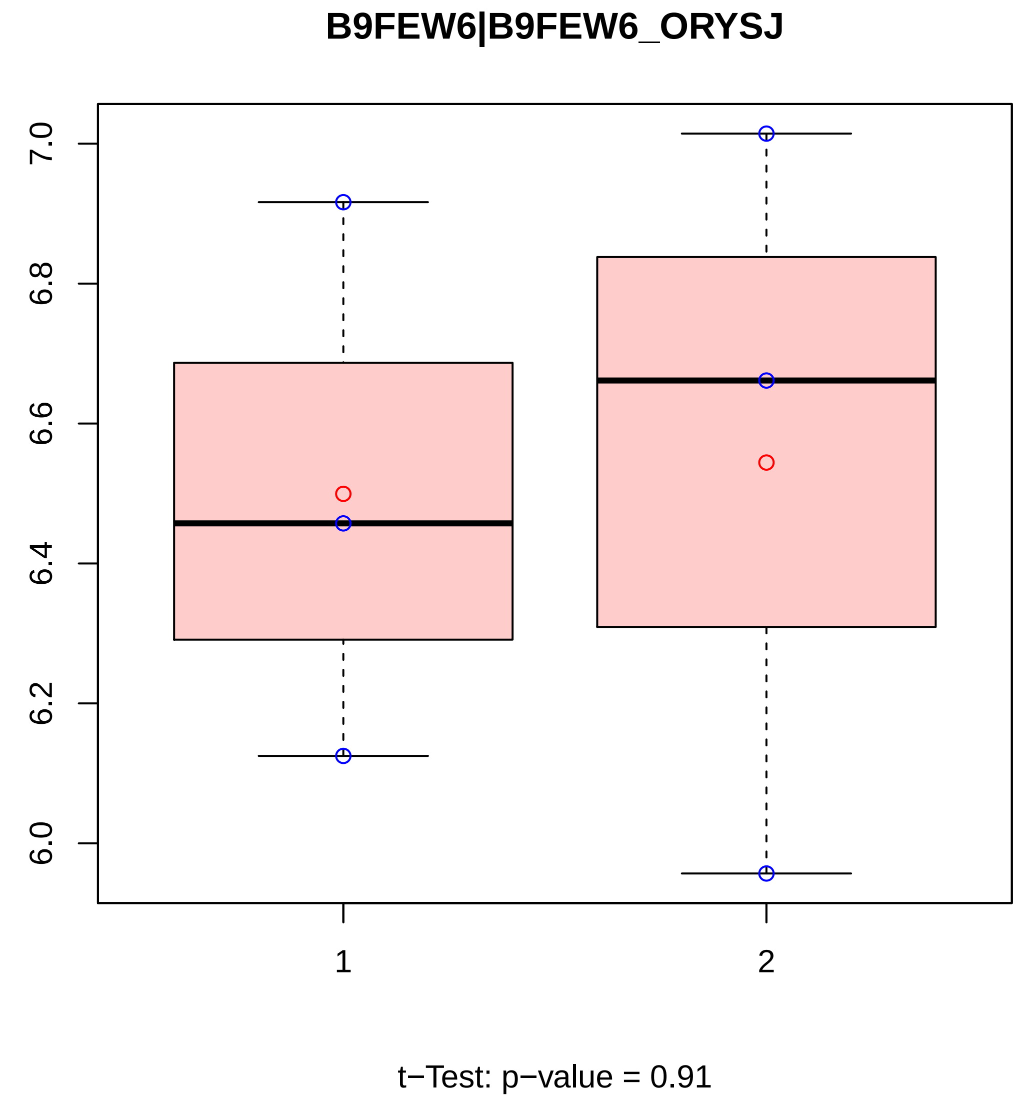
B9FEW6|B9FEW6_ORYSJ
7.0
6.8
6.6
6.4
6.2
6.0
1
2
t−Test: p−value = 0.91

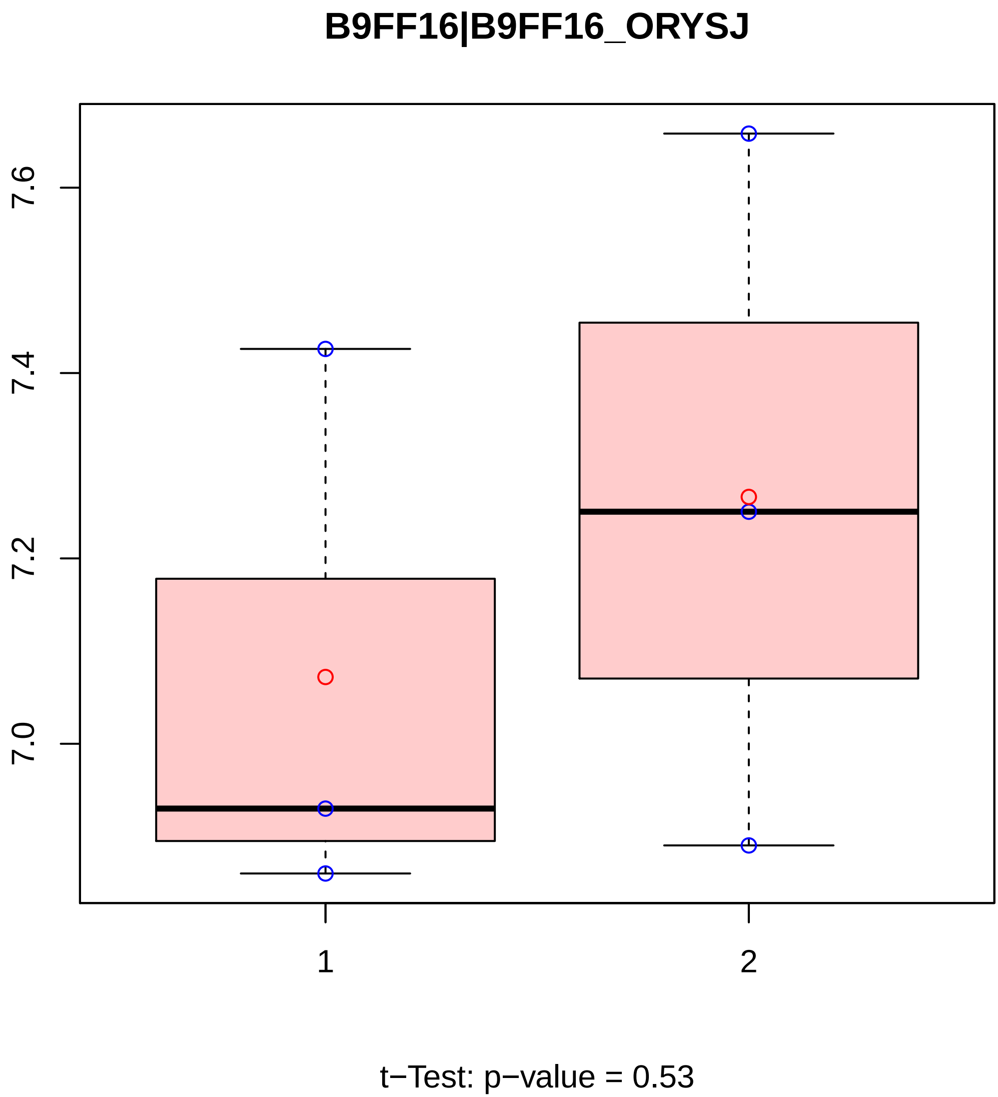
B9FF16|B9FF16_ORYSJ
7.0
7.2
7.4
7.6
1
2
t−Test: p−value = 0.53

B9FGE6|B9FGE6_ORYSJ

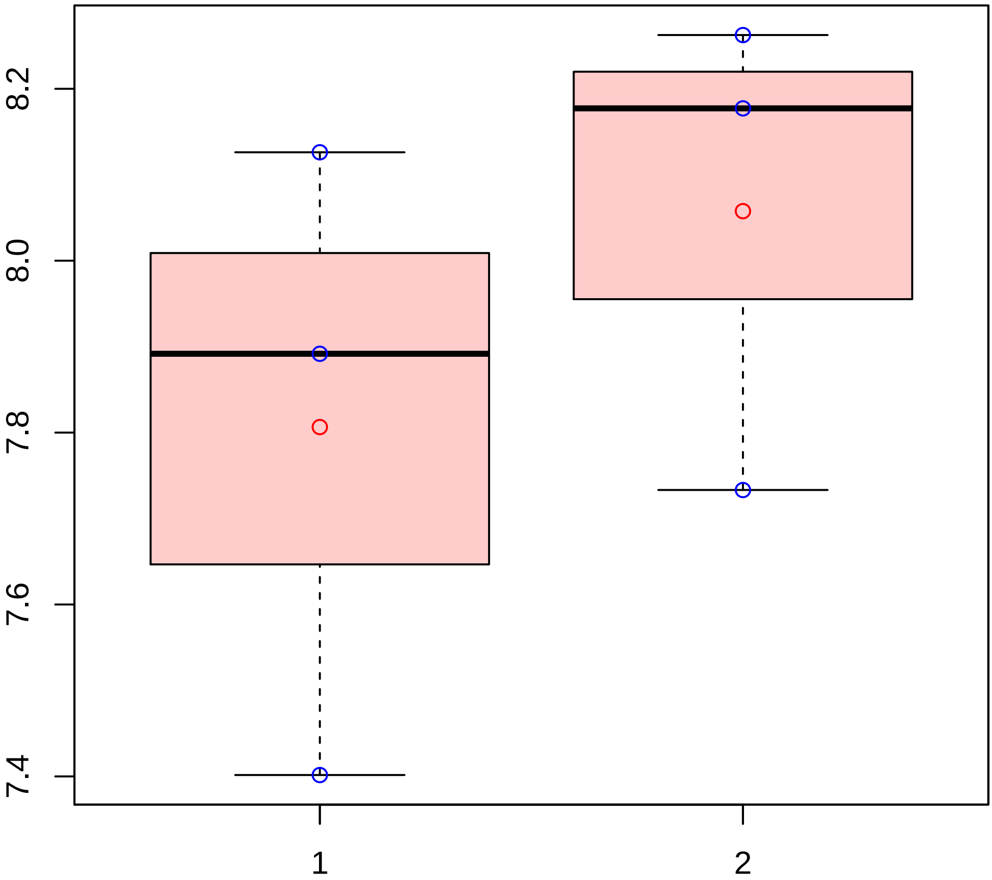

t−Test: p−value = 0.41

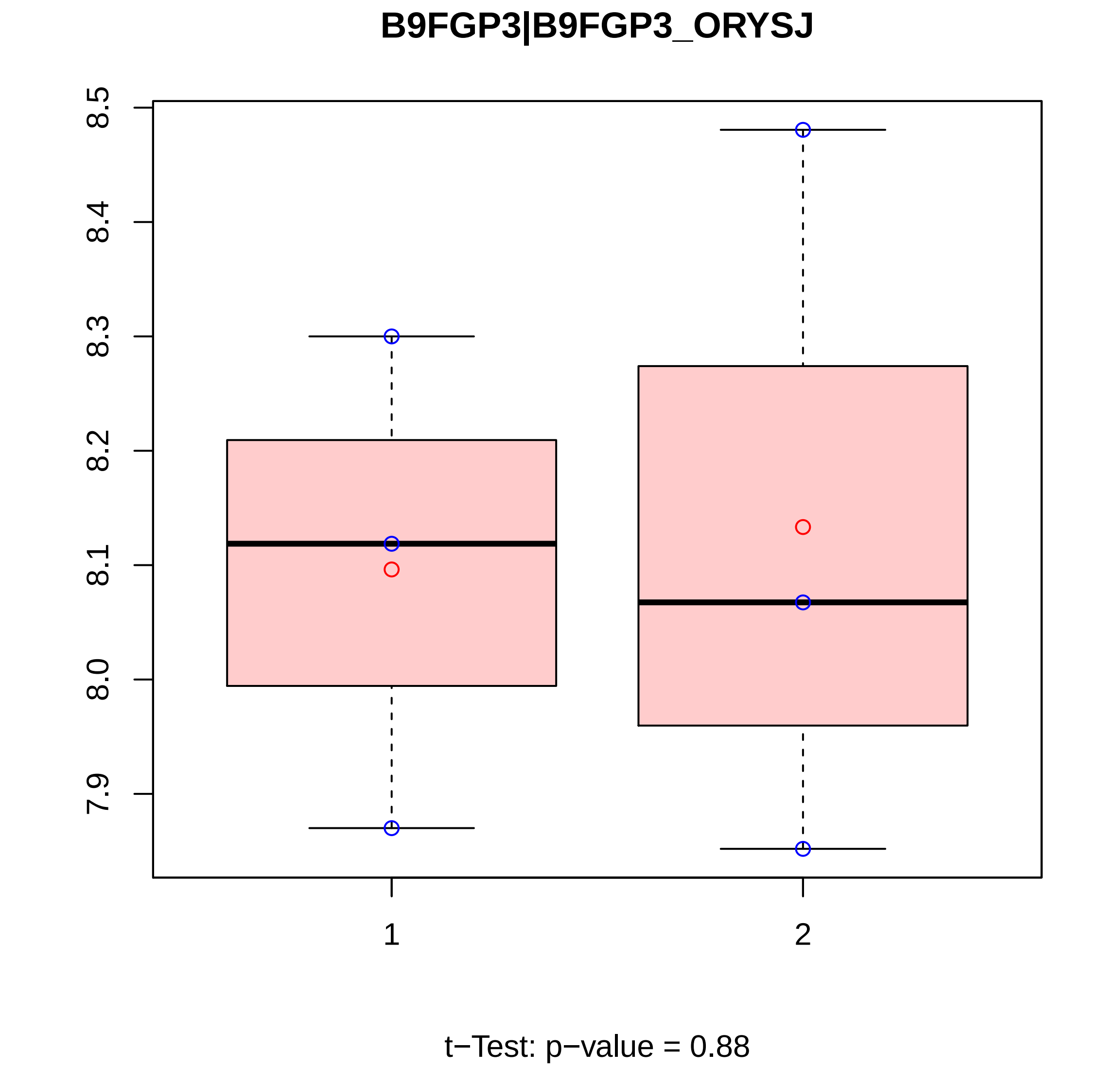
B9FGP3|B9FGP3_ORYSJ
8.5
8.4
8.3
8.2
8.1
8.0
7.9
1
2
t−Test: p−value = 0.88

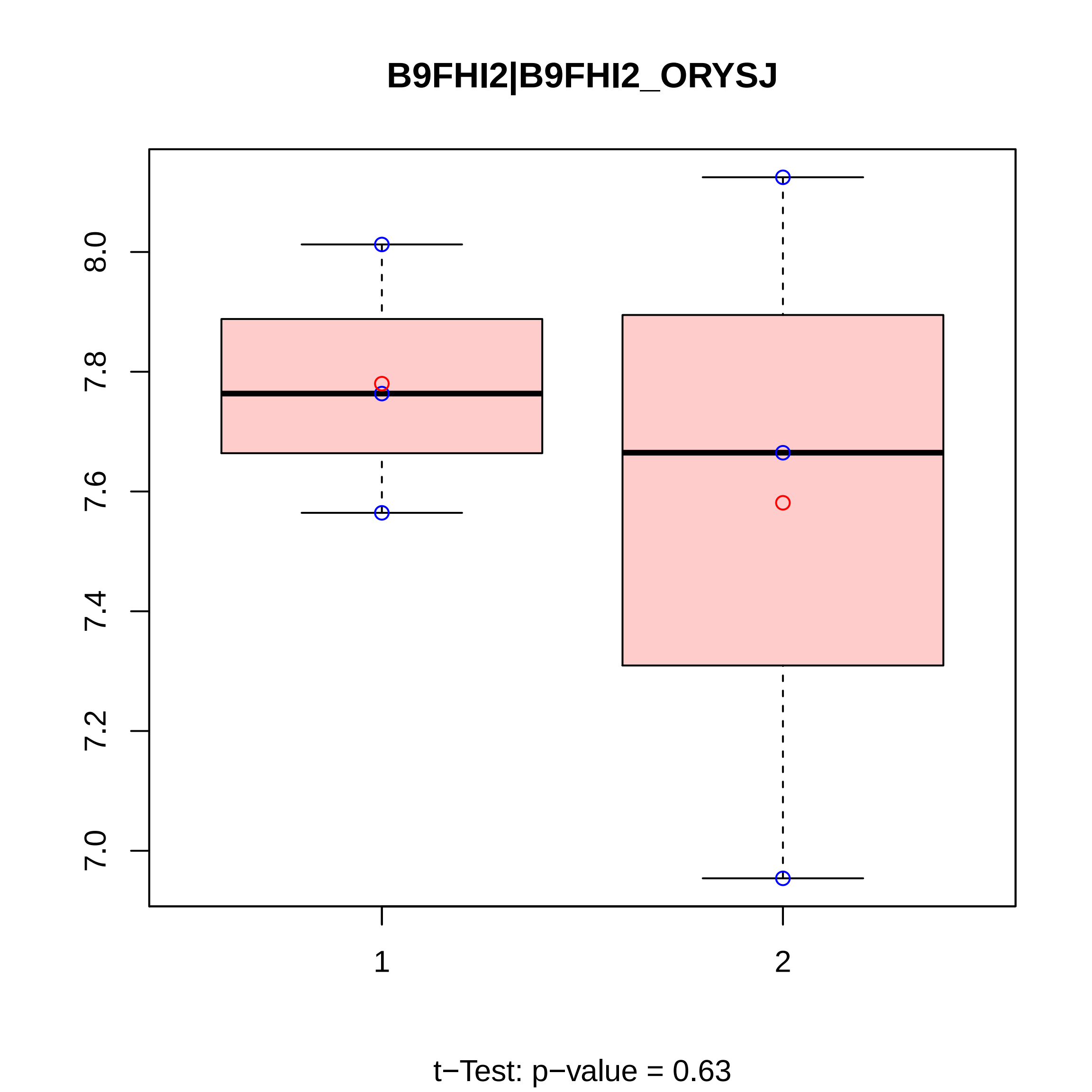
B9FHI2|B9FHI2_ORYSJ
8.0
7.8
7.6
7.4
7.2
7.0
1
2
t−Test: p−value = 0.63

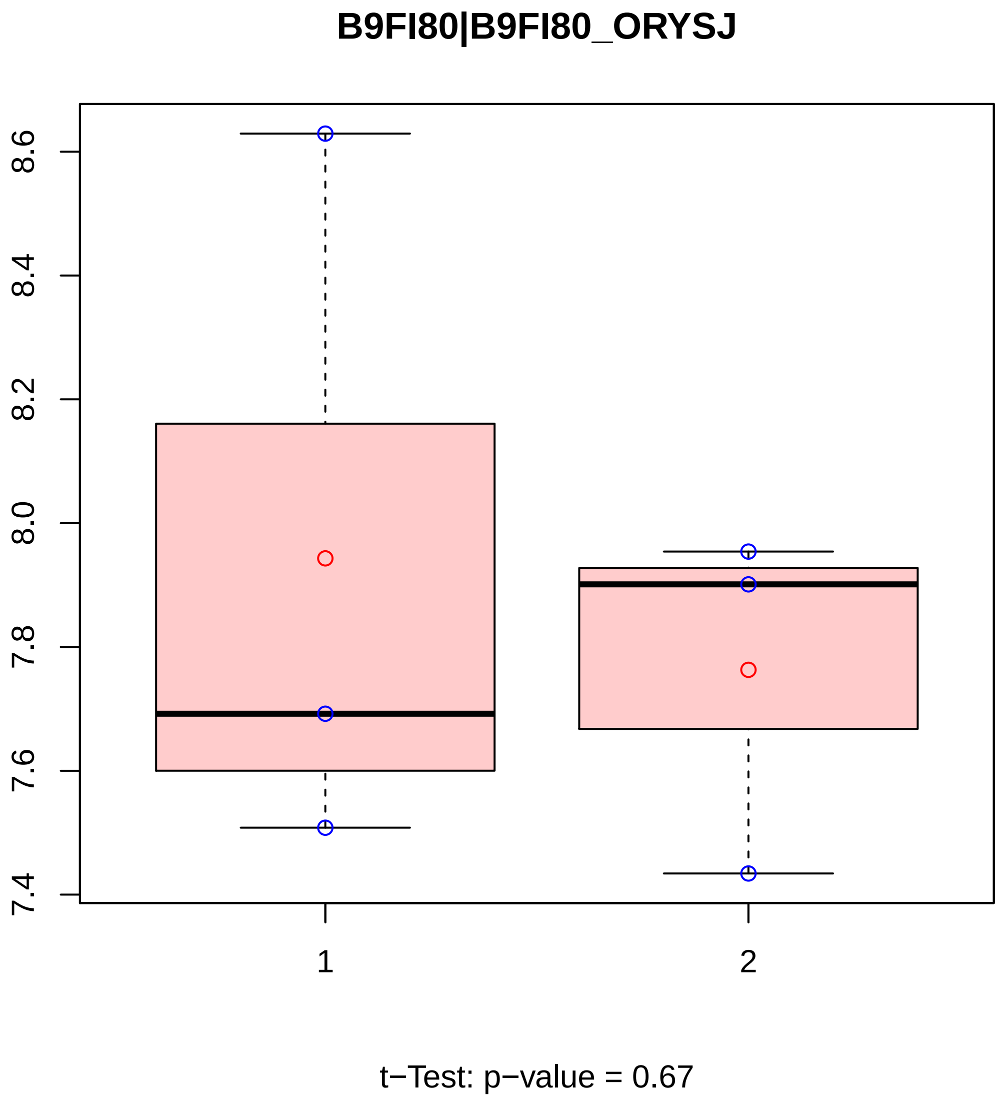

B9FI80|B9FI80_ORYSJ
8.6
8.4
8.2
8.0
7.8
7.6
7.4
1
2
t−Test: p−value = 0.67

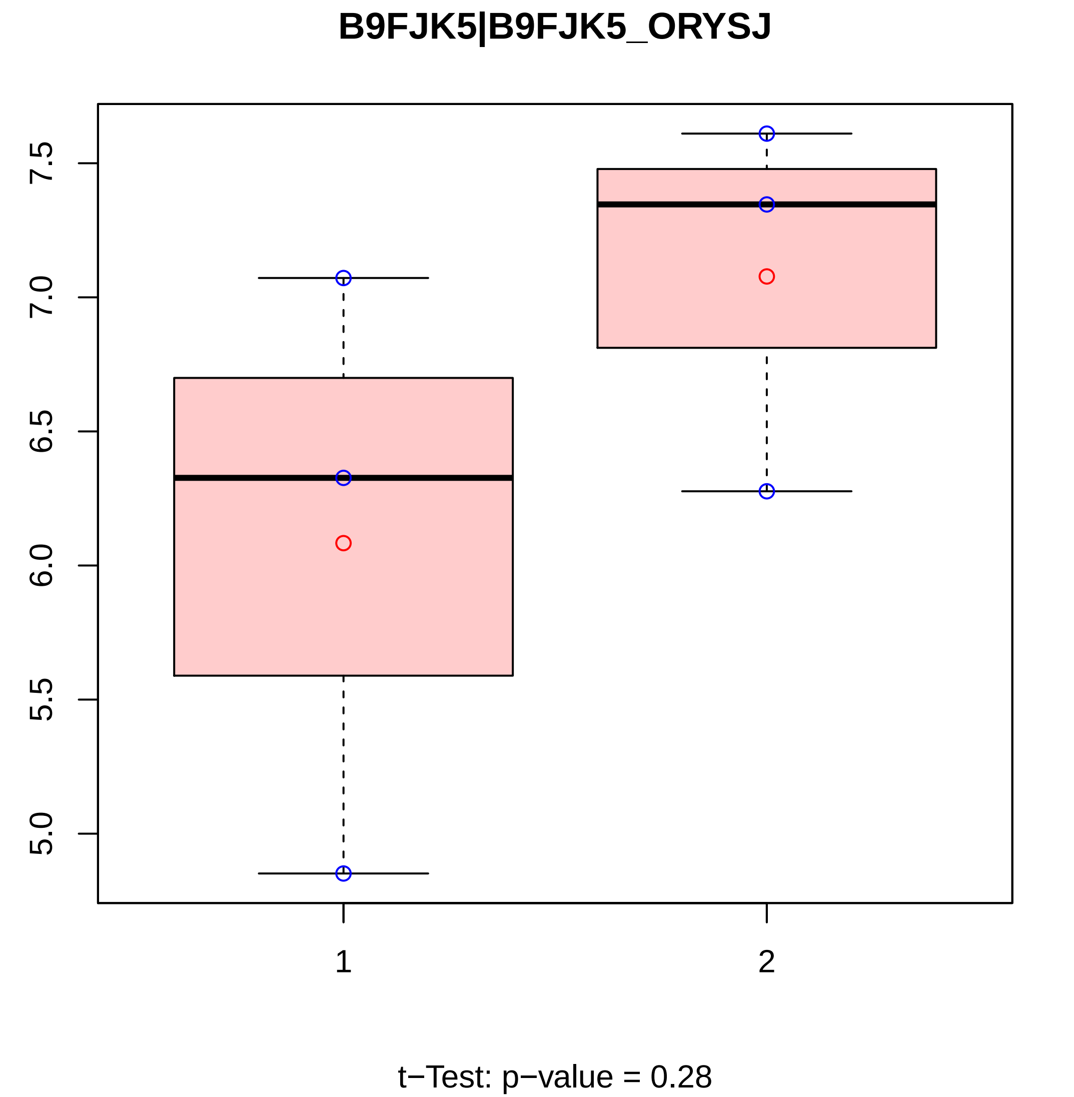
B9FJK5|B9FJK5_ORYSJ
7.5
7.0
6.5
6.0
5.5
5.0
1
2
t−Test: p−value = 0.28

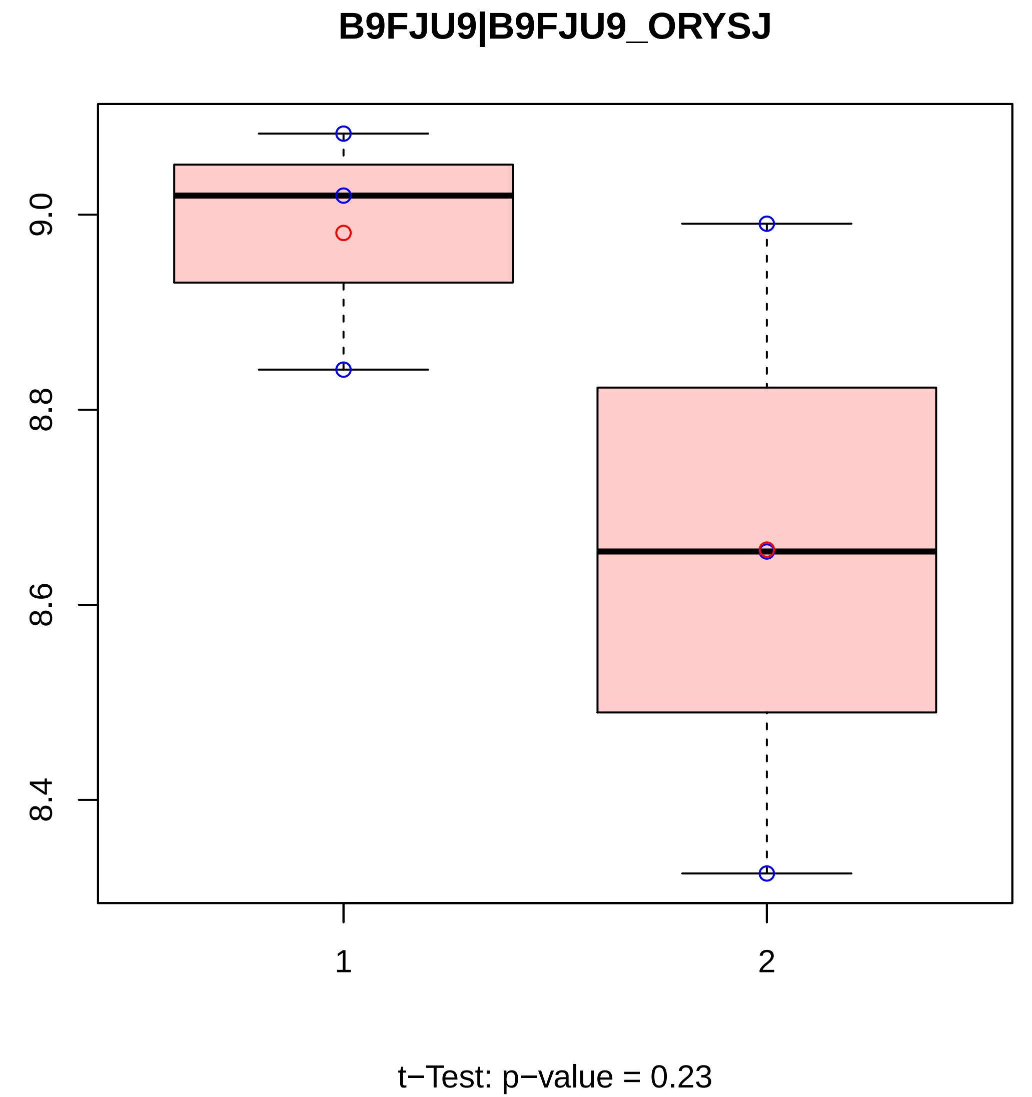
B9FJU9|B9FJU9_ORYSJ
9.0
8.8
8.6
8.4
1
2
t−Test: p−value = 0.23

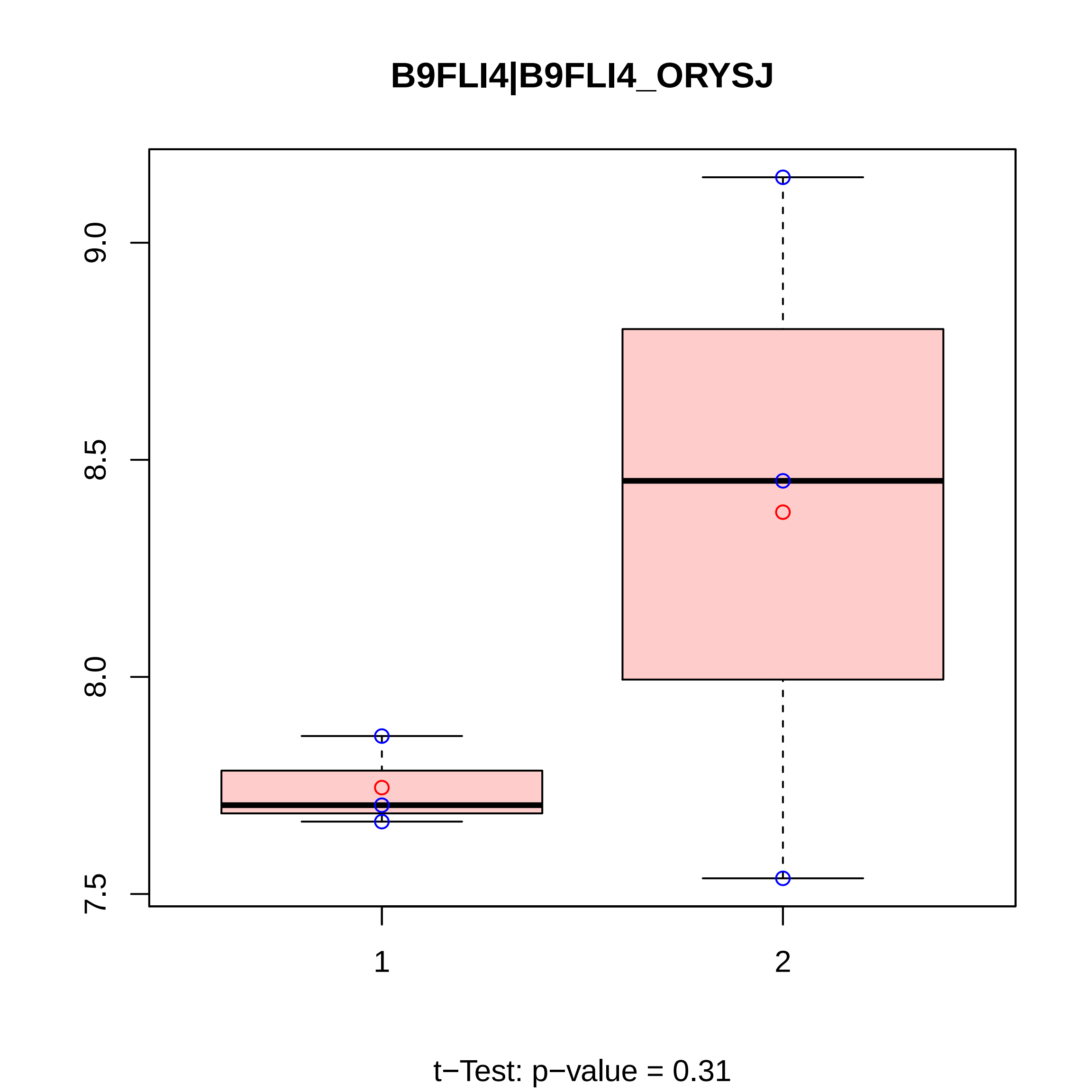
B9FLI4|B9FLI4_ORYSJ
9.0
8.5
8.0
7.5
1
2
t−Test: p−value = 0.31

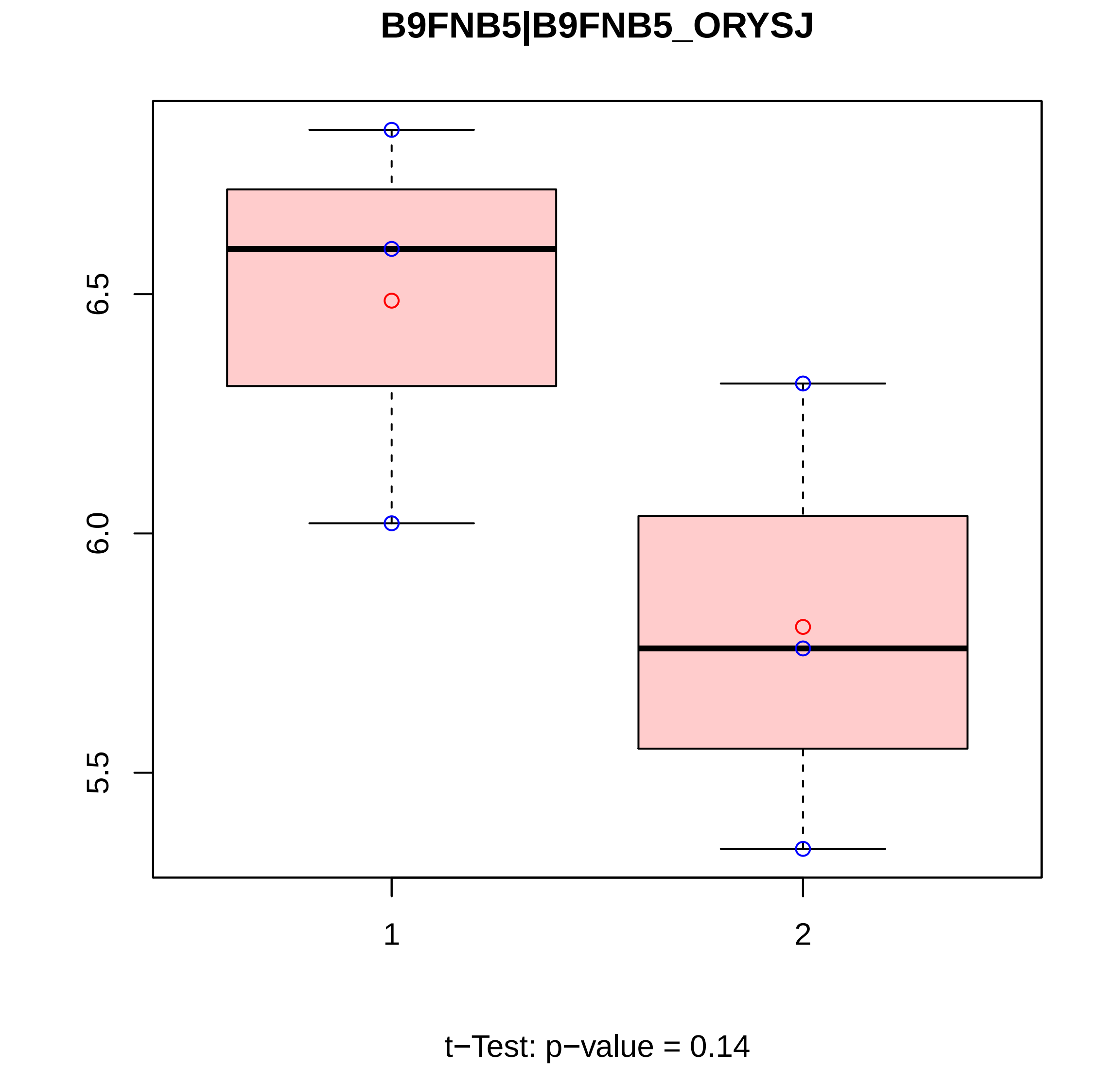

B9FNB5|B9FNB5_ORYSJ
6.5
6.0
5.5
1
2
t−Test: p−value = 0.14

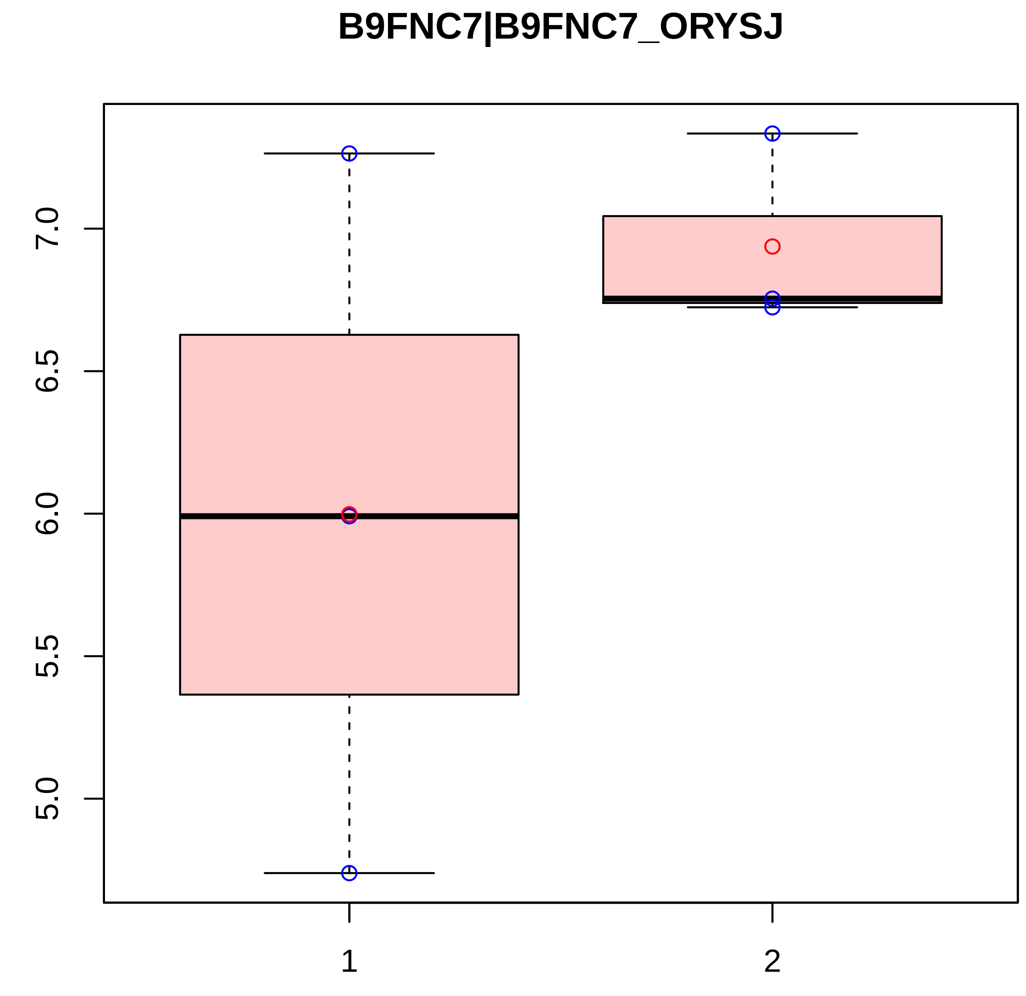

t−Test: p−value = 0.33

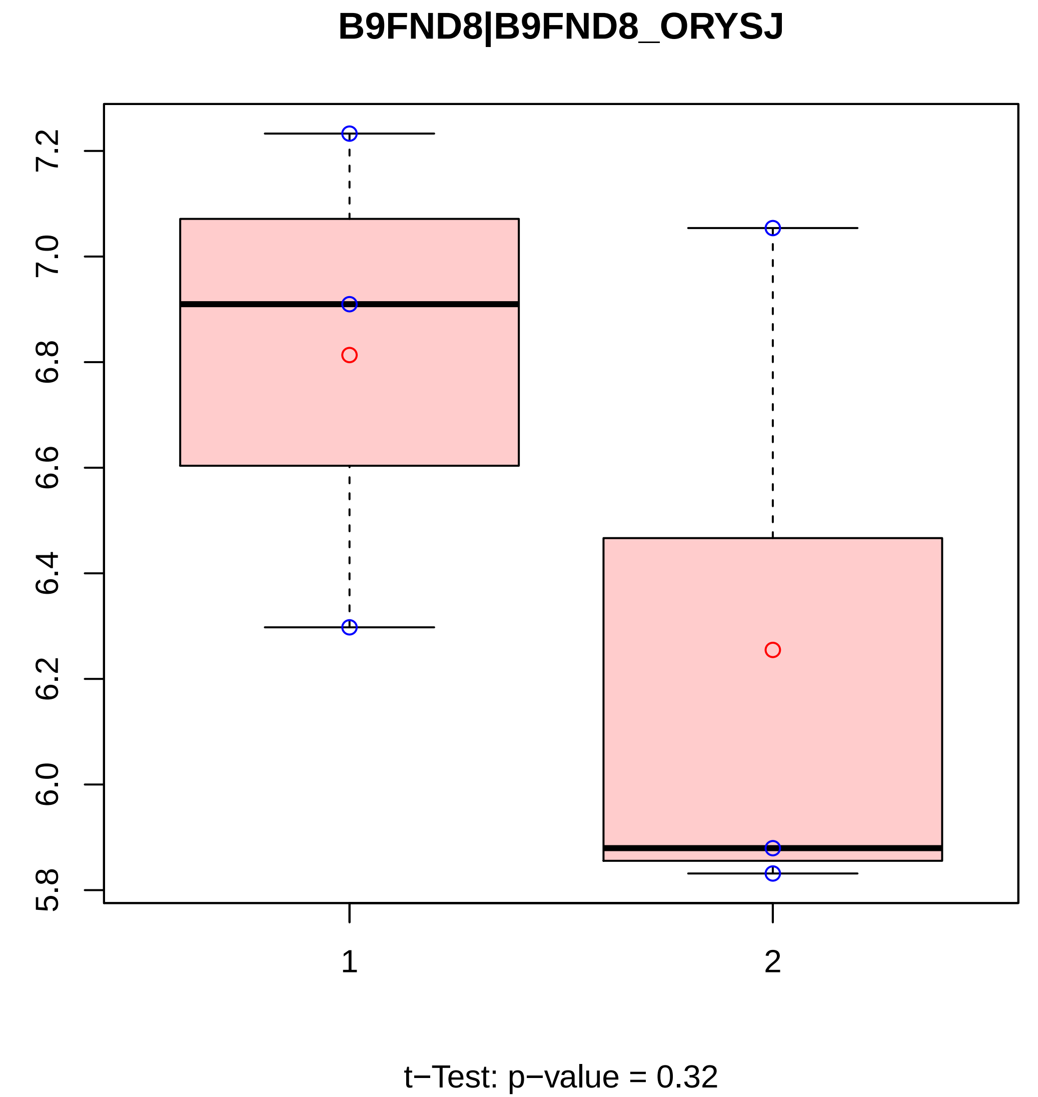
B9FND8|B9FND8_ORYSJ
7.2
7.0
6.8
6.6
6.4
6.2
6.0
5.8
1
2
t−Test: p−value = 0.32

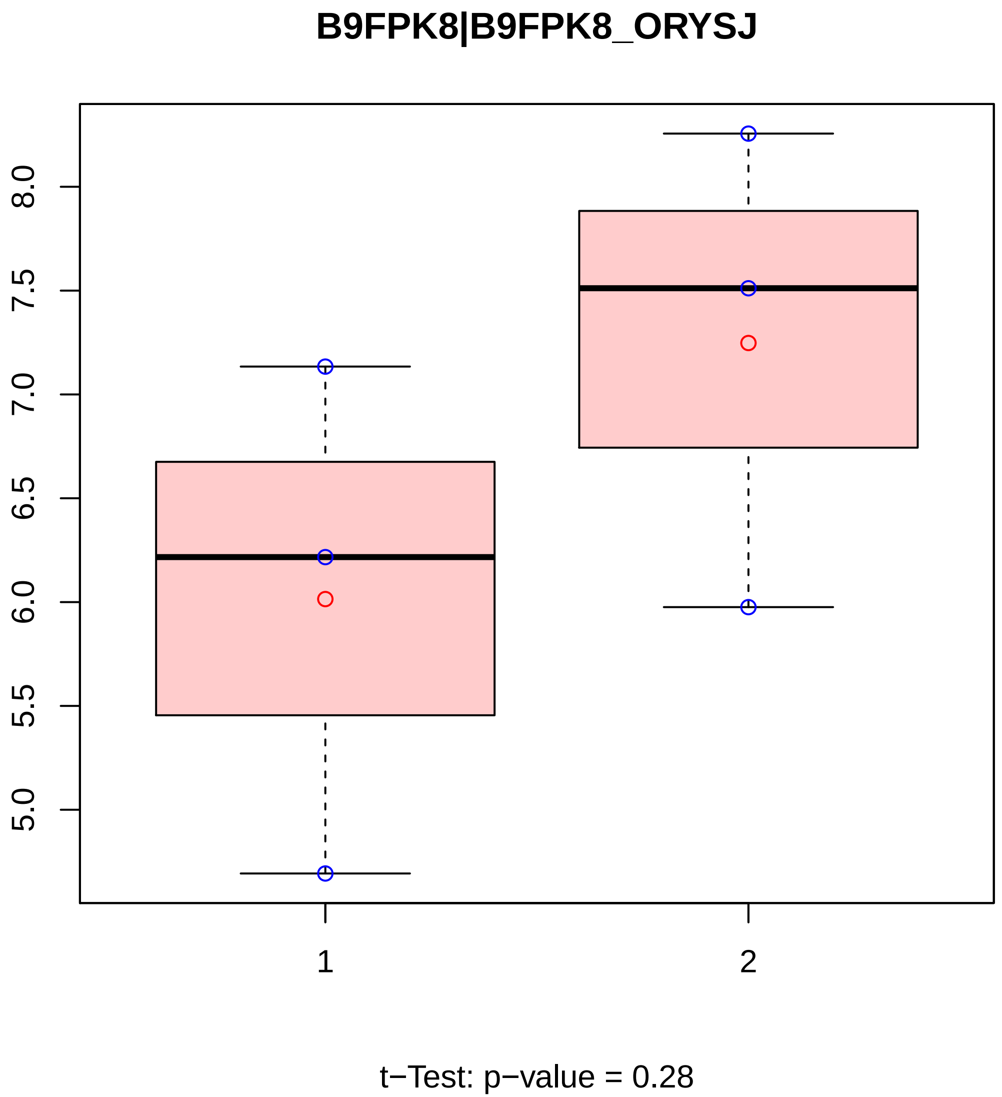

B9FPK8|B9FPK8_ORYSJ
8.0
7.5
7.0
6.5
6.0
5.5
5.0
1
2
t−Test: p−value = 0.28

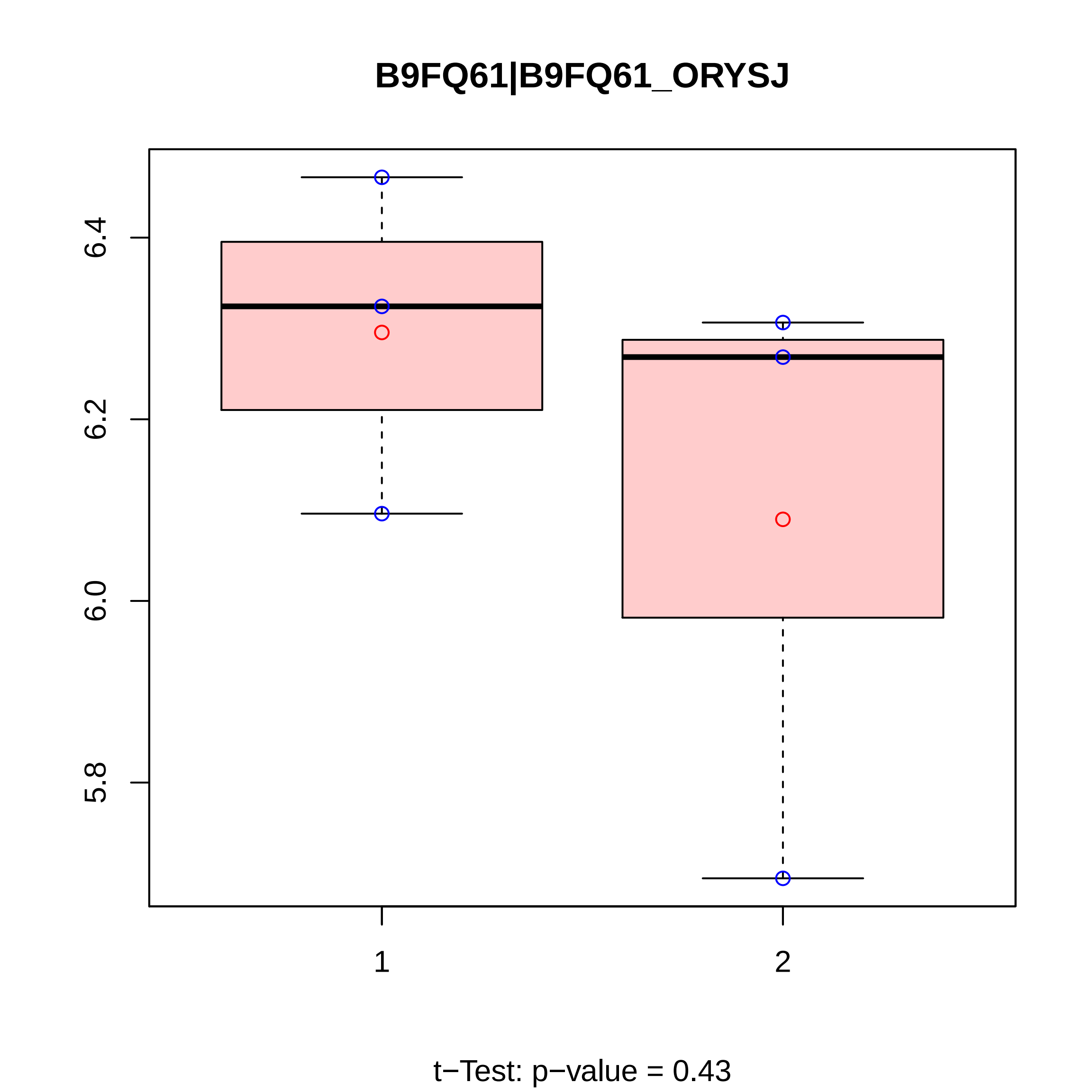

B9FQ61|B9FQ61_ORYSJ
6.4
6.2
6.0
5.8
1
2
t−Test: p−value = 0.43

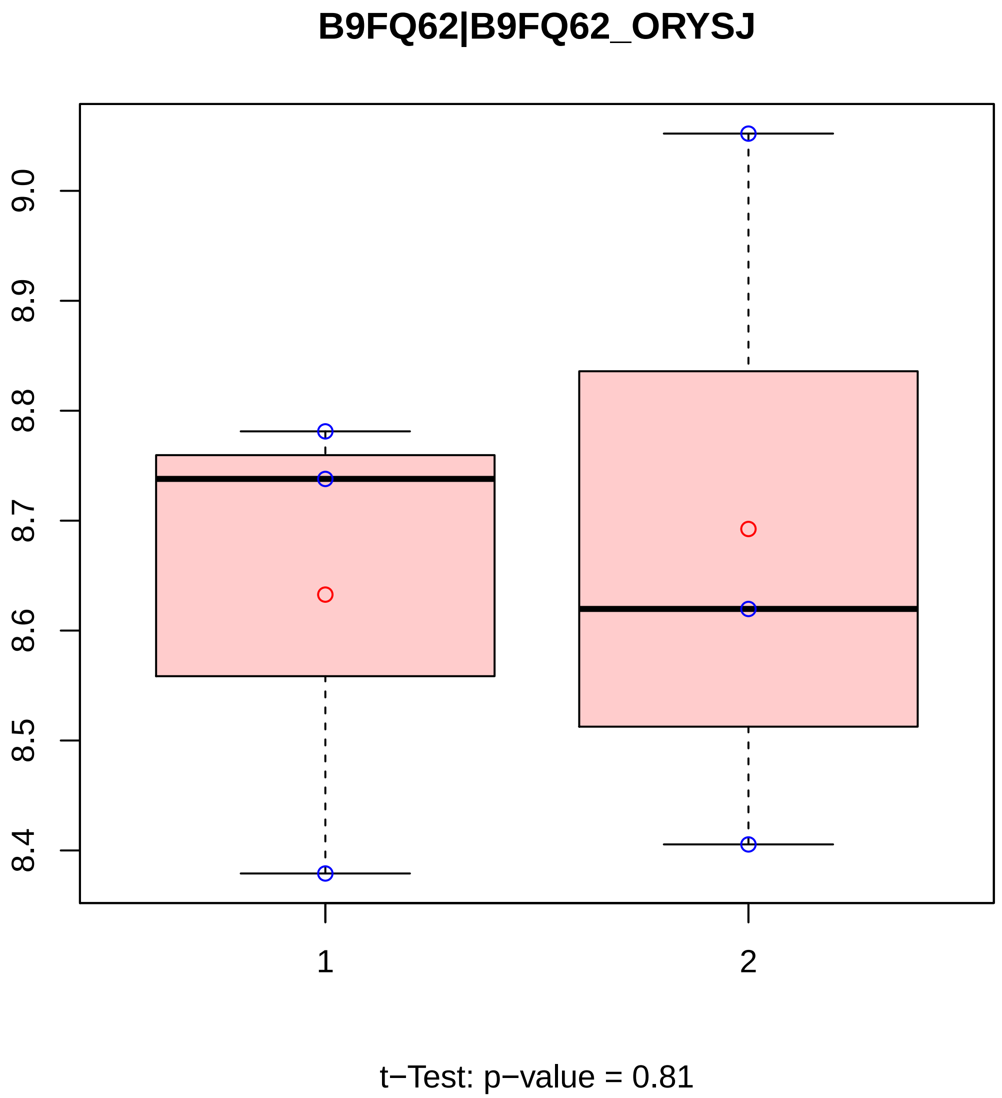

B9FQ62|B9FQ62_ORYSJ
9.0
8.9
8.8
8.7
8.6
8.5
8.4
1
2
t−Test: p−value = 0.81

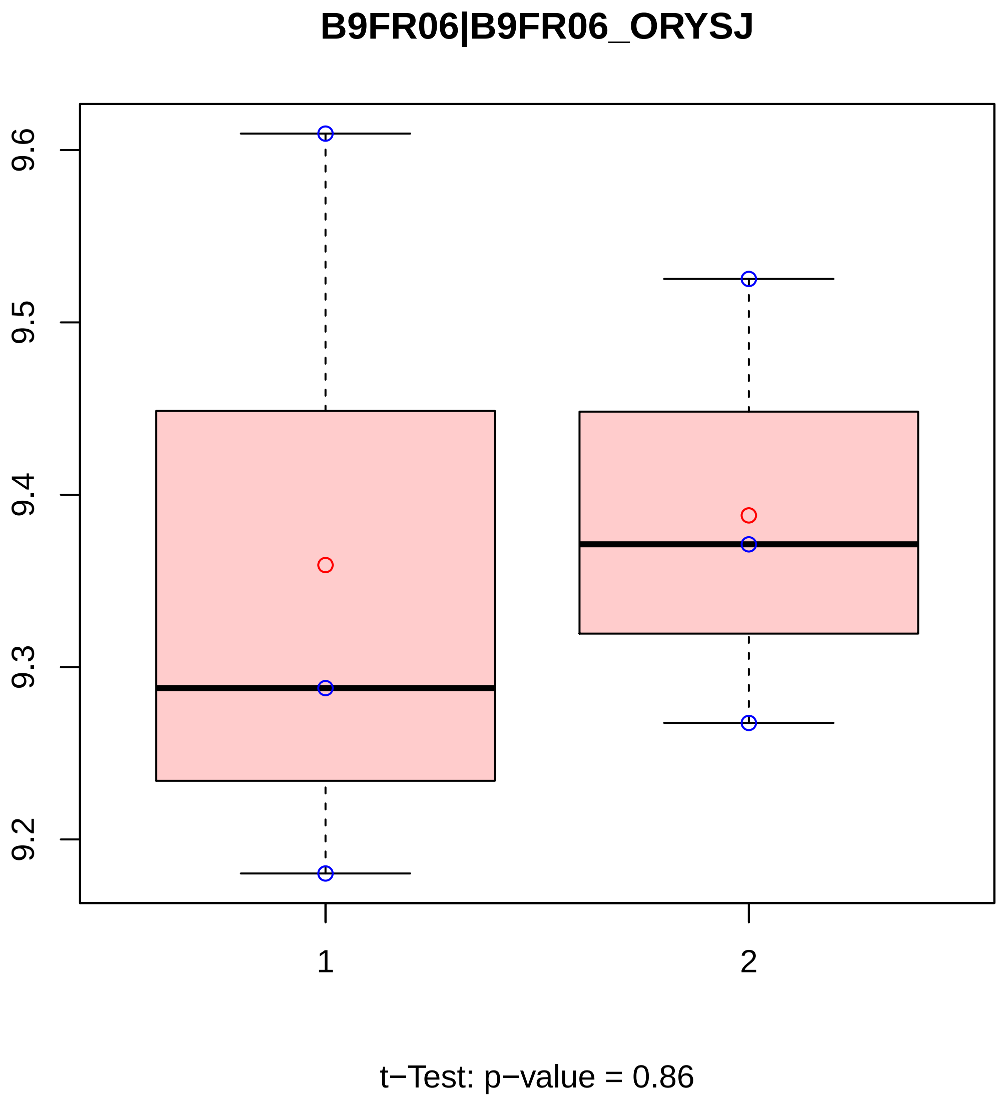
B9FR06|B9FR06_ORYSJ
9.6
9.5
9.4
9.3
9.2
1
2
t−Test: p−value = 0.86

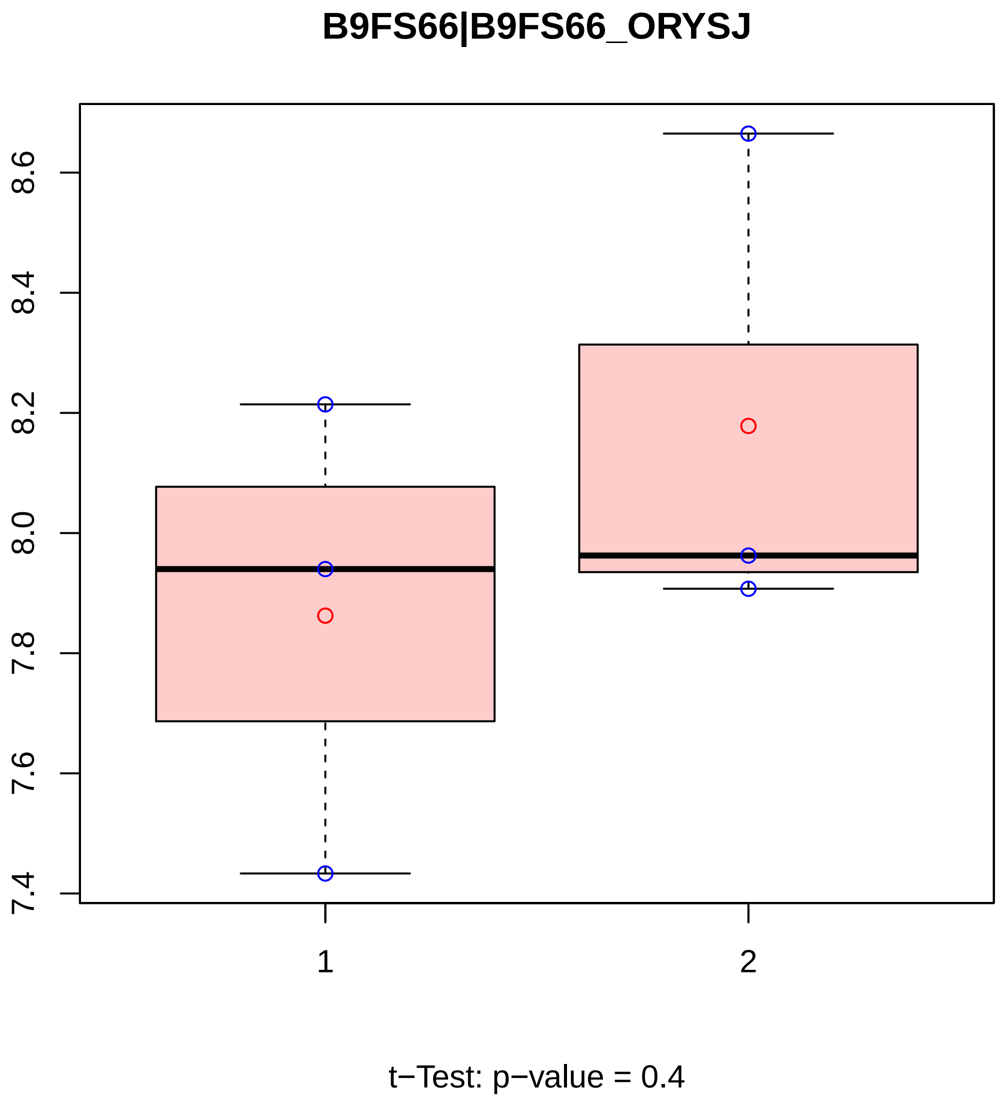

B9FS66|B9FS66_ORYSJ
8.6
8.4
8.2
8.0
7.8
7.6
7.4
1
2
t−Test: p−value = 0.4

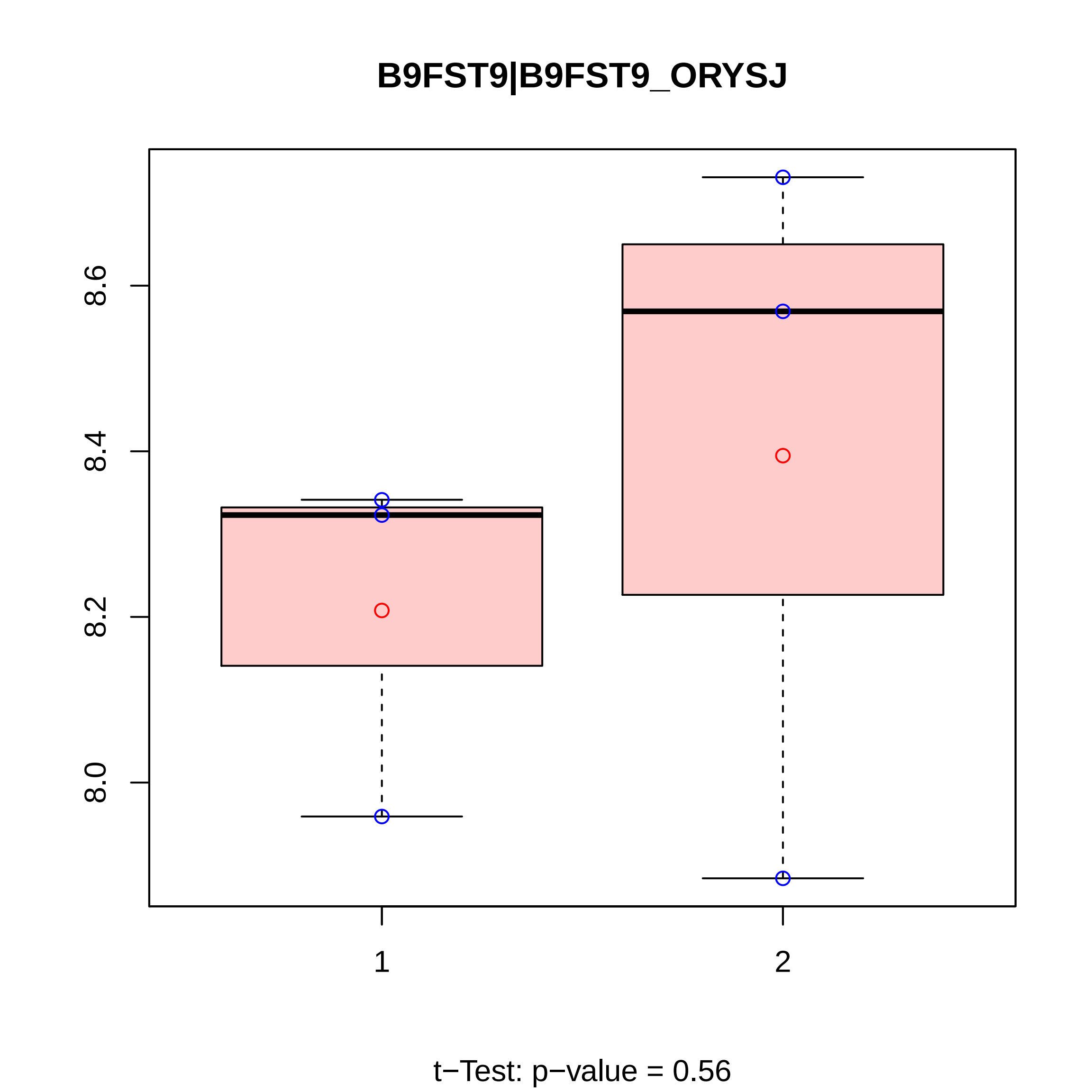
B9FST9|B9FST9_ORYSJ
8.0
8.2
8.4
8.6
1
2
t−Test: p−value = 0.56

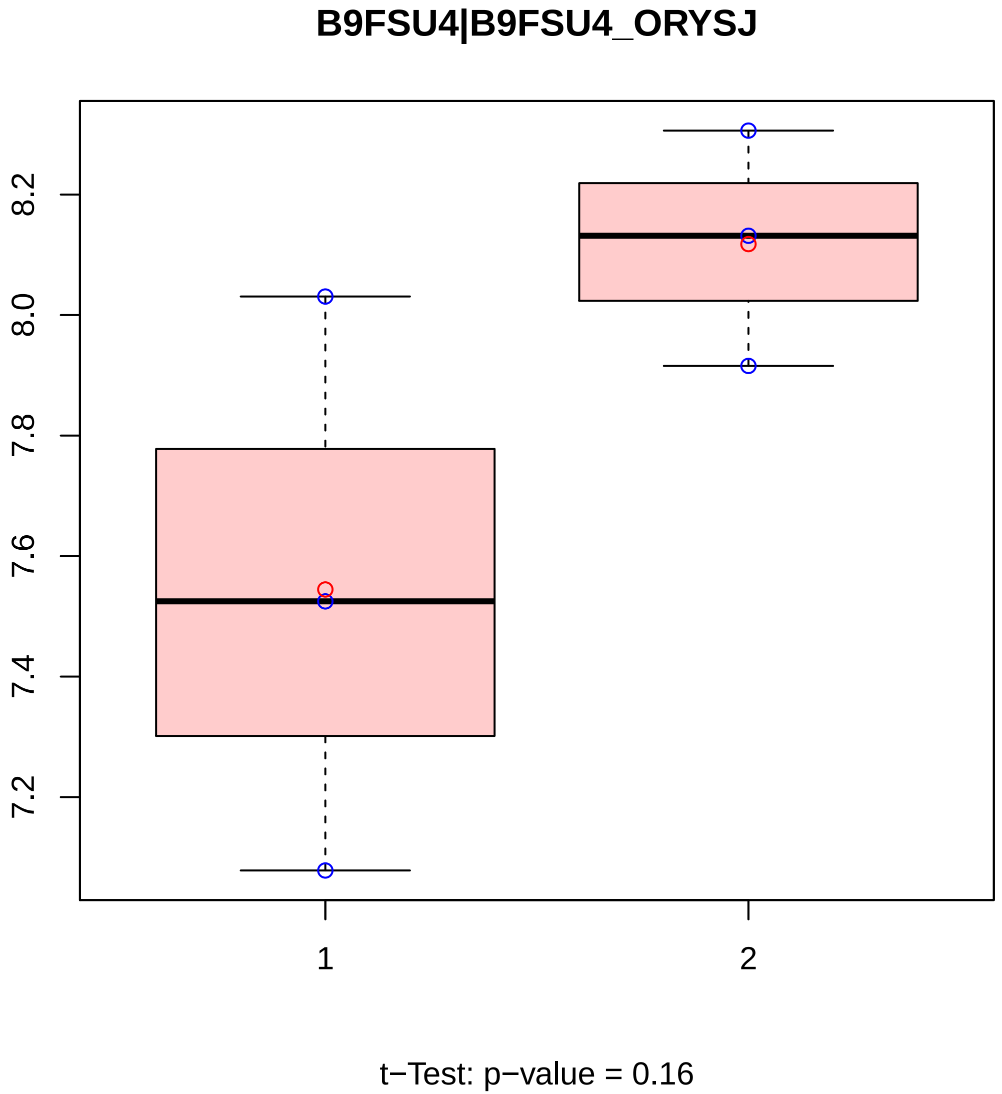
B9FSU4|B9FSU4_ORYSJ
8.2
8.0
7.8
7.6
7.4
7.2
1
2
t−Test: p−value = 0.16

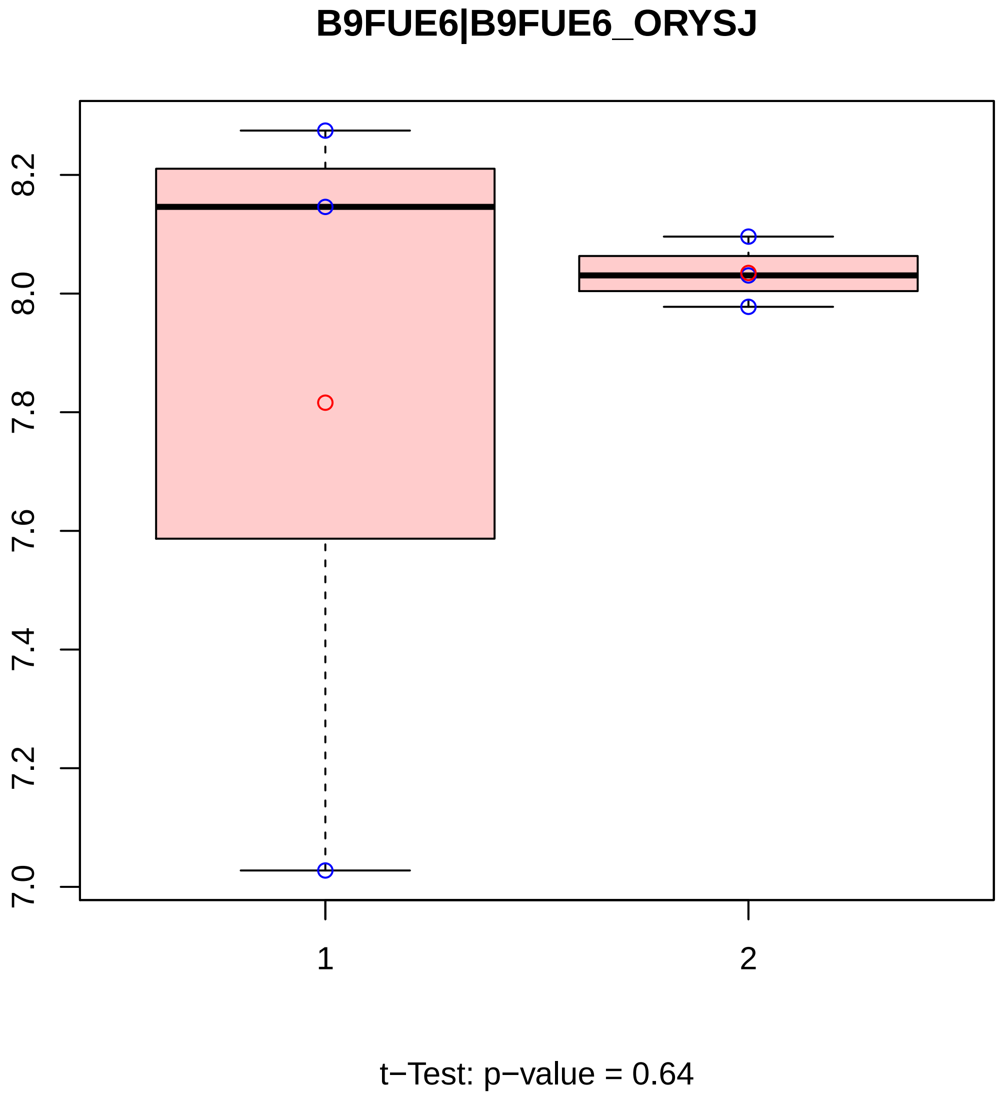

B9FUE6|B9FUE6_ORYSJ
8.2
8.0
7.8
7.6
7.4
7.2
7.0
1
2
t−Test: p−value = 0.64

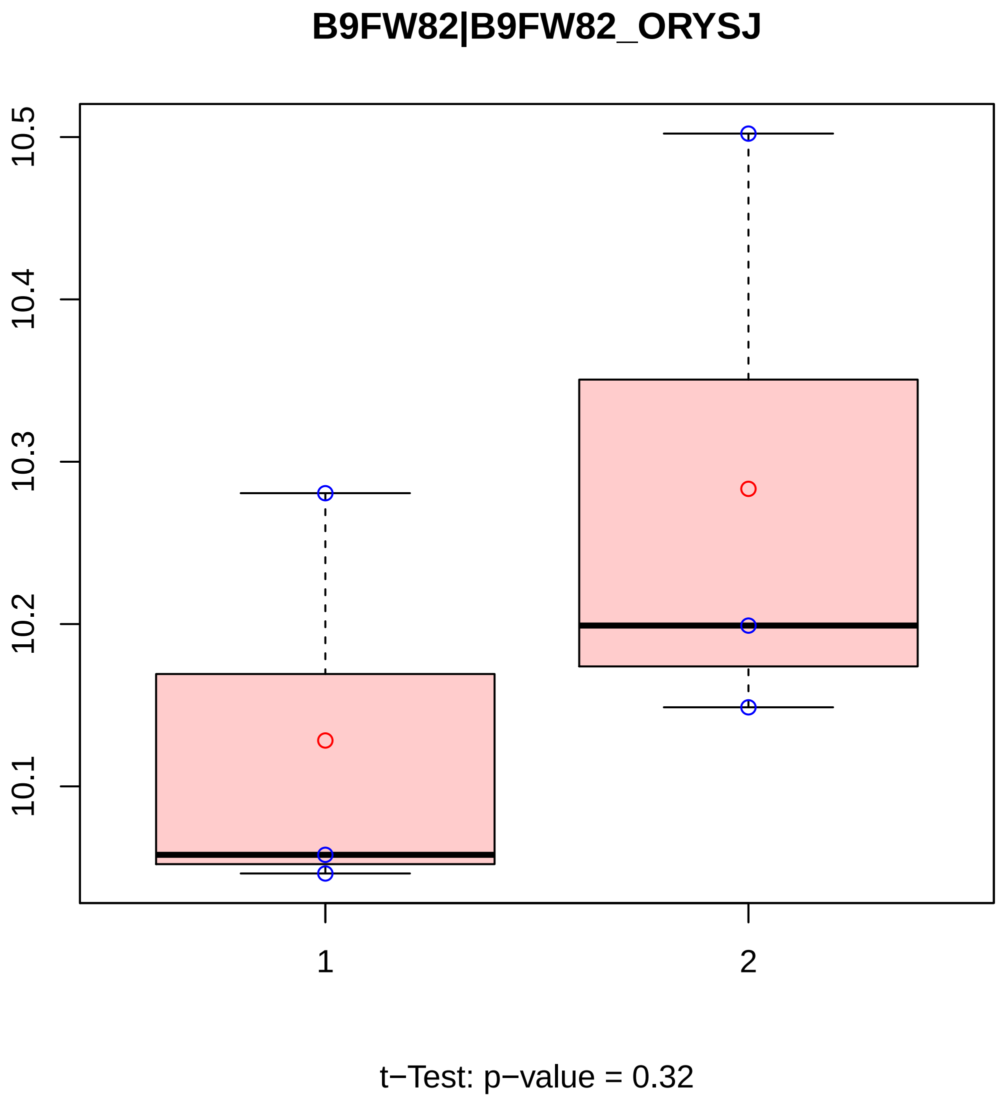
B9FW82|B9FW82_ORYSJ
10.1
10.2
10.3
10.4
10.5
1
2
t−Test: p−value = 0.32

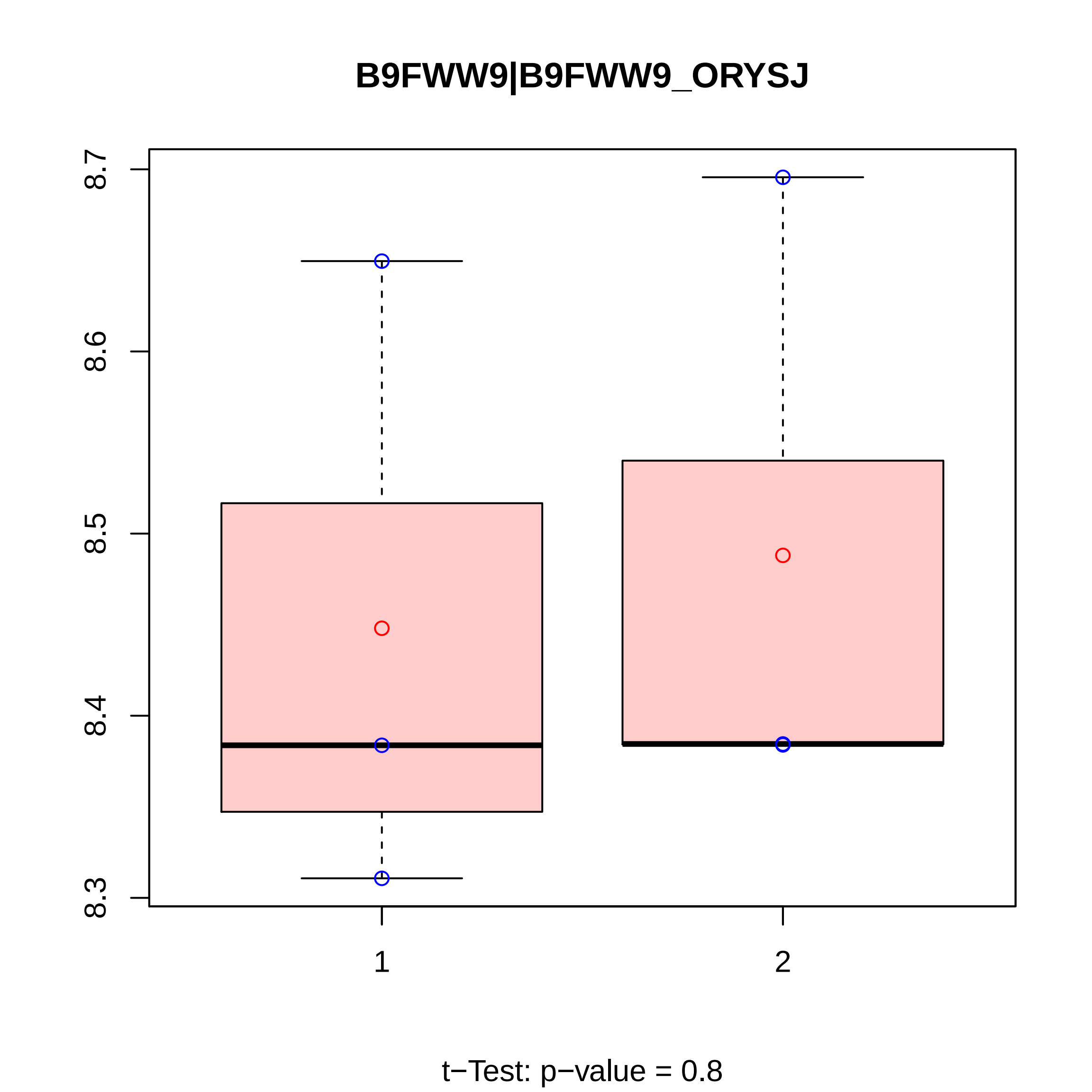

B9FWW9|B9FWW9_ORYSJ
8.3
8.4
8.5
8.6
8.7
1
2
t−Test: p−value = 0.8

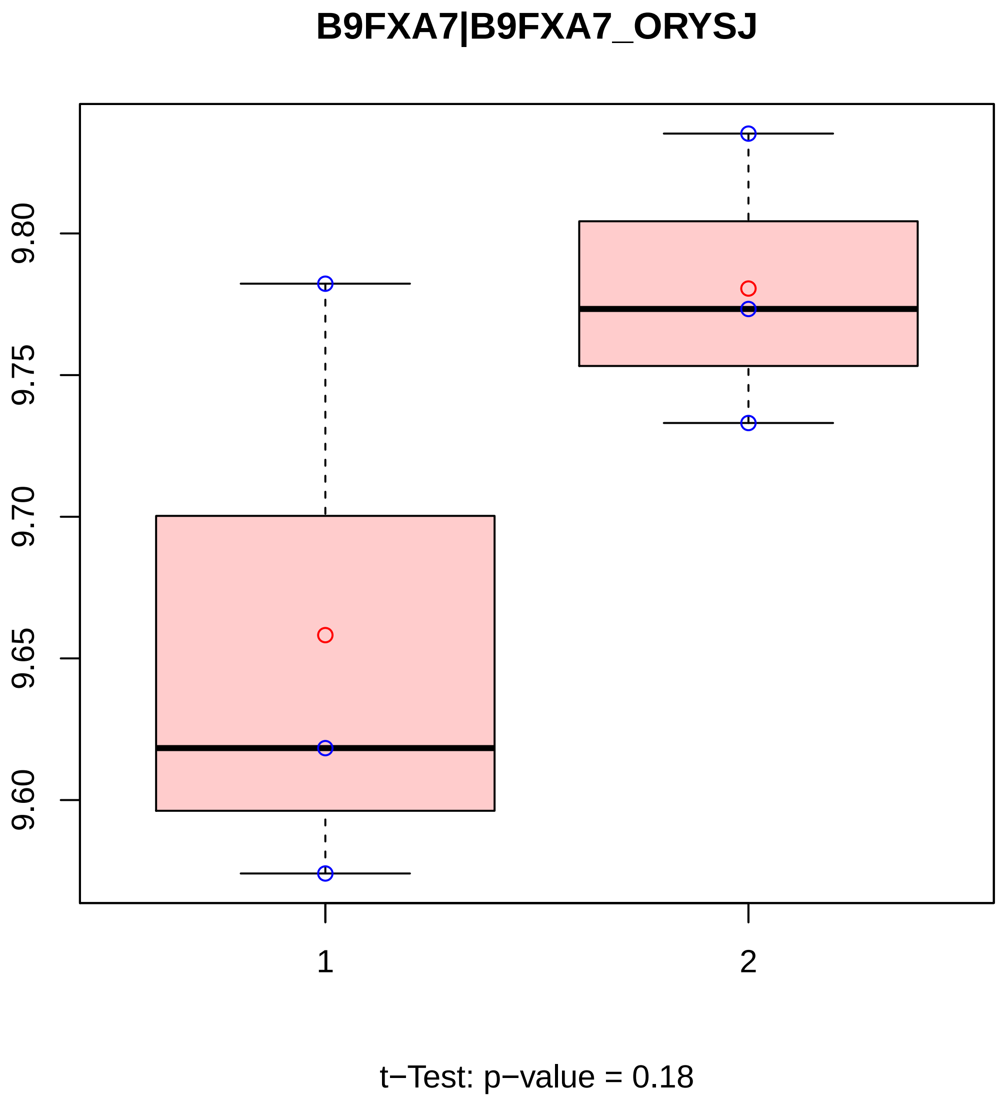

B9FXA7|B9FXA7_ORYSJ
9.60
9.65
9.70
9.75
9.80
1
2
t−Test: p−value = 0.18

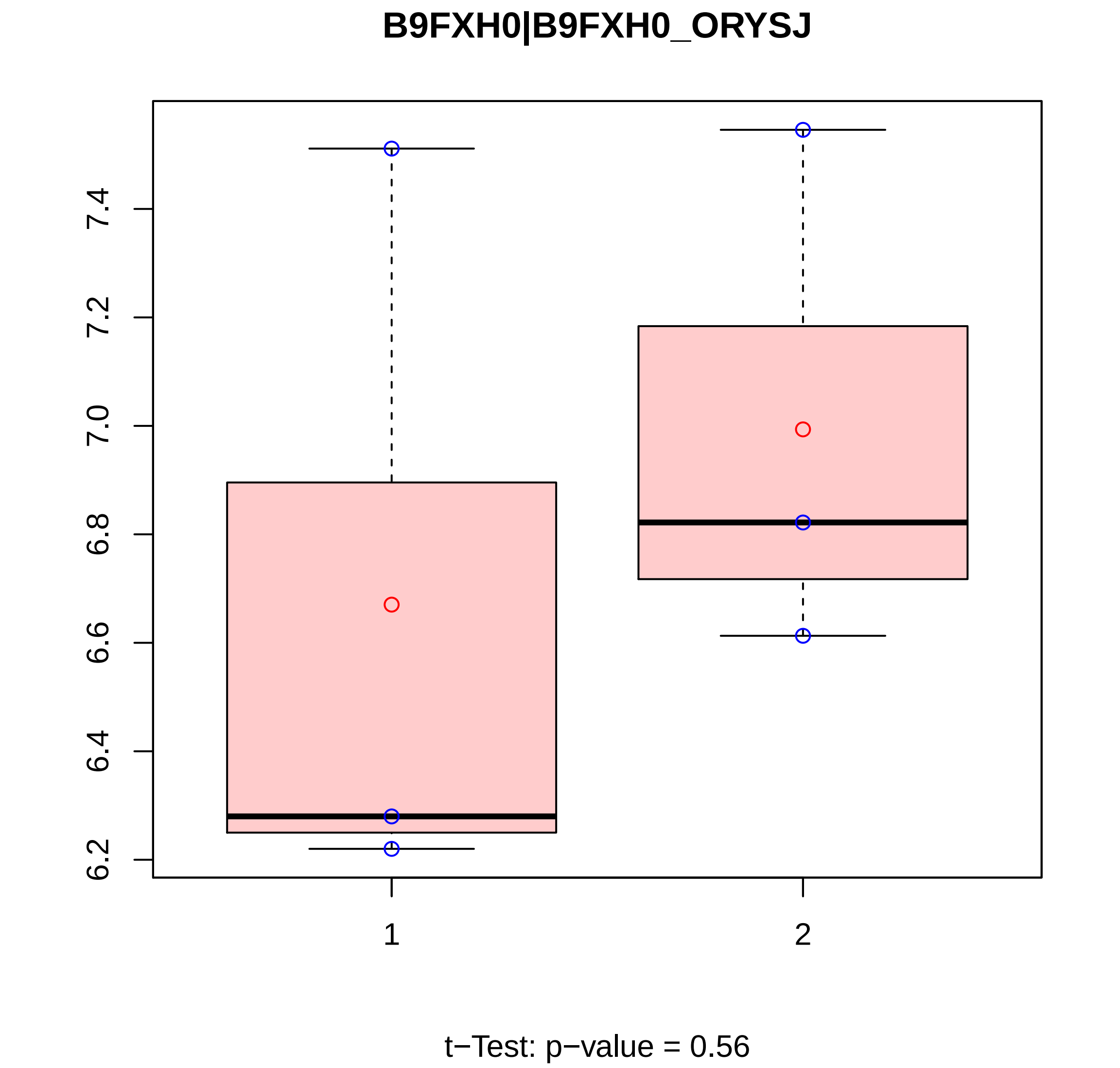

B9FXH0|B9FXH0_ORYSJ
7.4
7.2
7.0
6.8
6.6
6.4
6.2
1
2
t−Test: p−value = 0.56

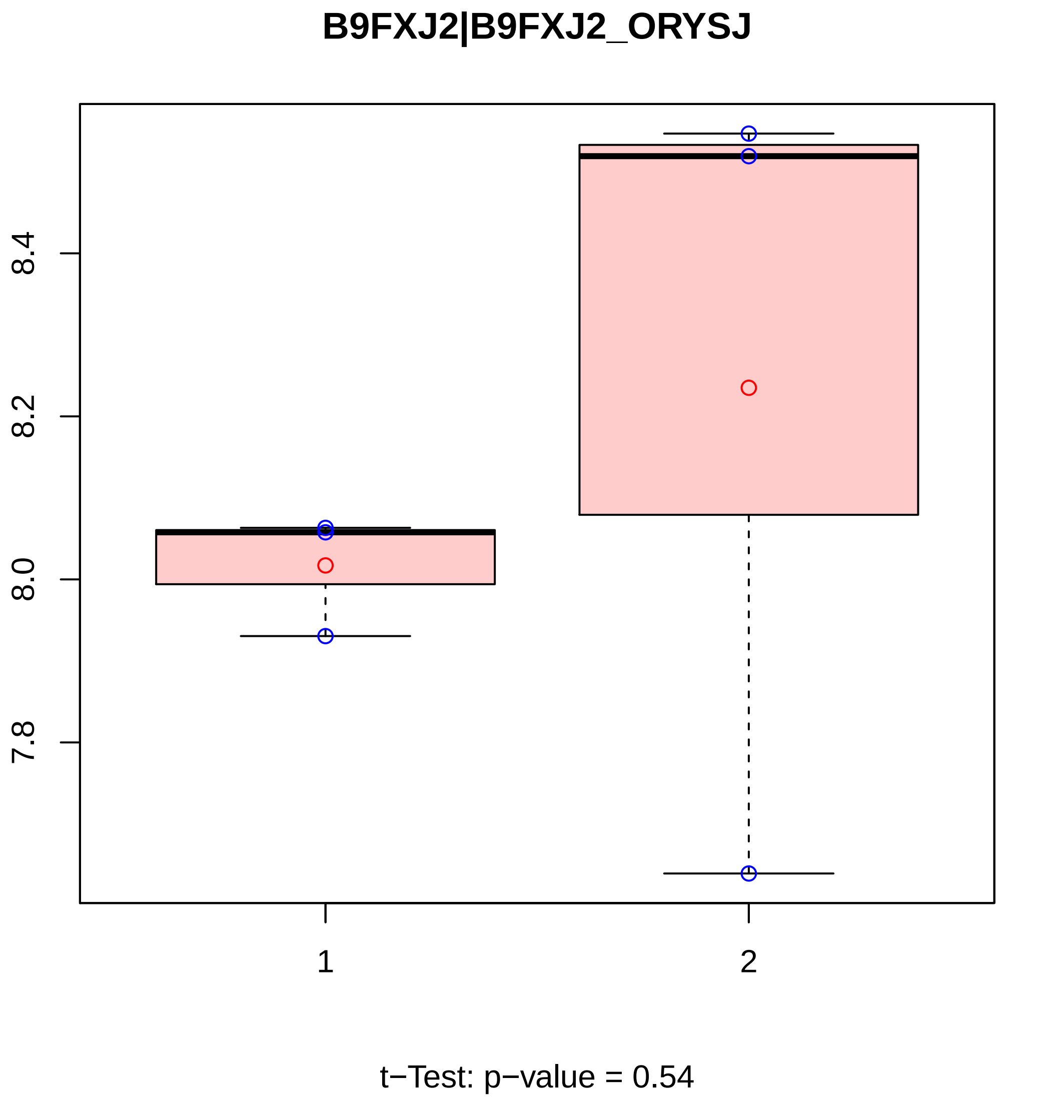
B9FXJ2|B9FXJ2_ORYSJ
8.4
8.2
8.0
7.8
1
2
t−Test: p−value = 0.54

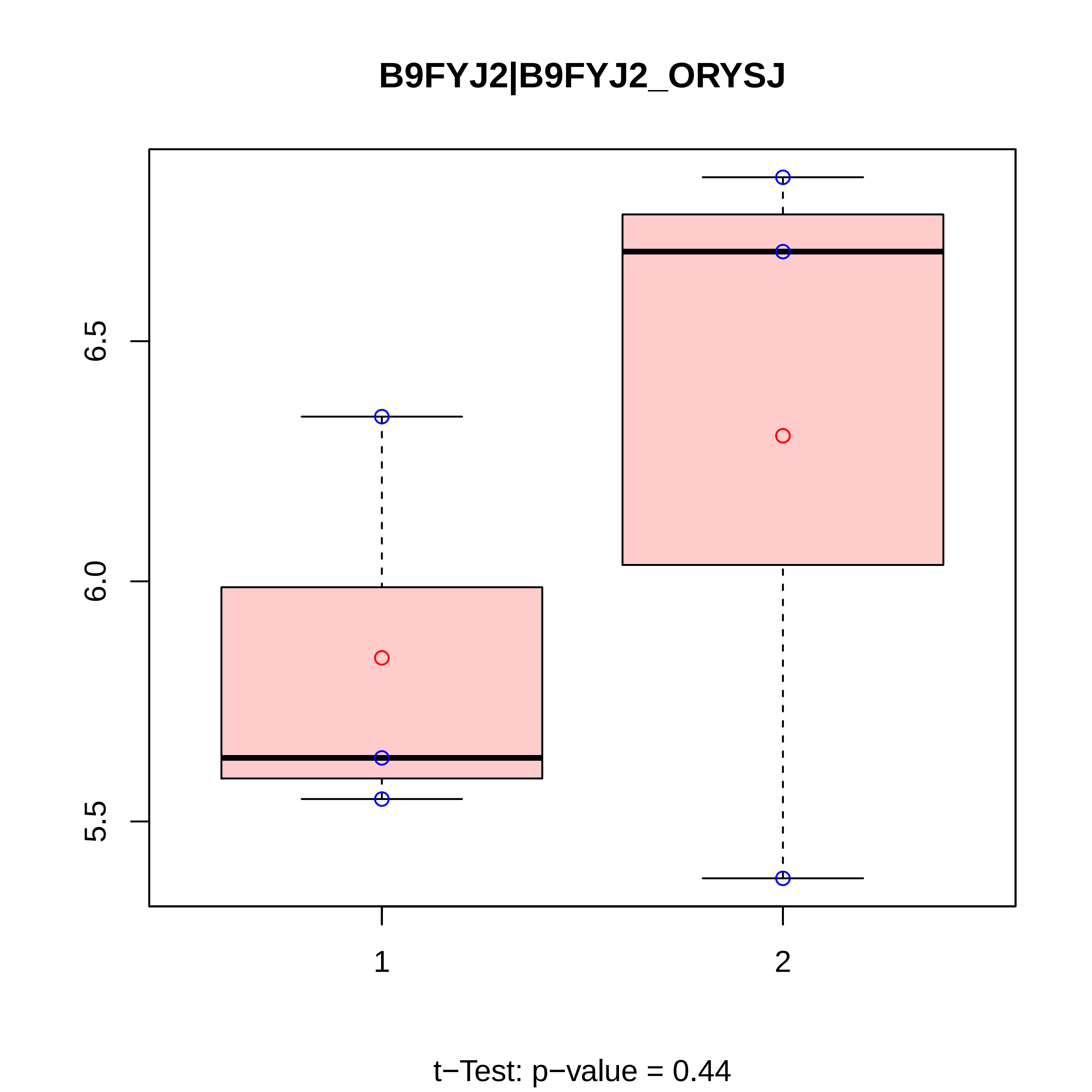

B9FYJ2|B9FYJ2_ORYSJ
6.5
6.0
5.5
1
2
t−Test: p−value = 0.44

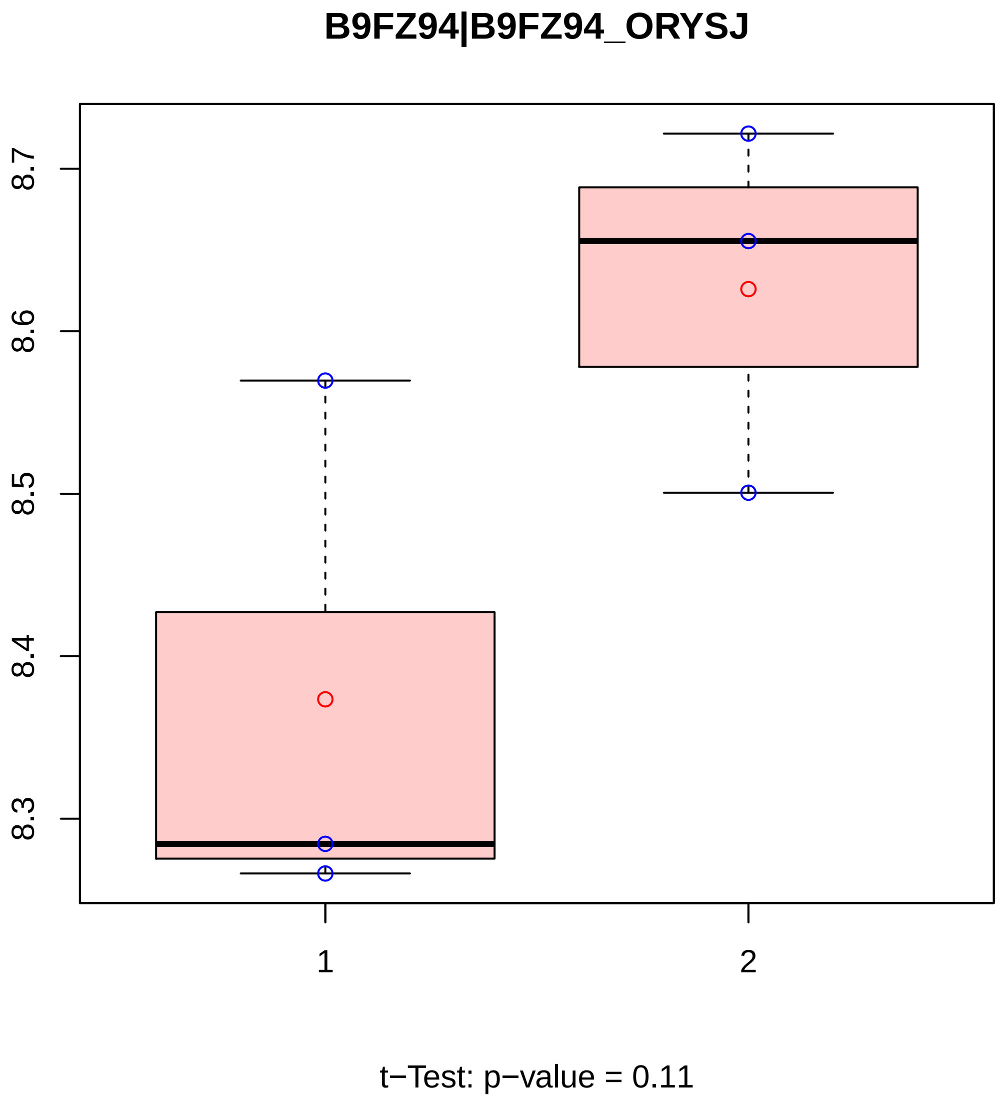
B9FZ94|B9FZ94_ORYSJ
8.3
8.4
8.5
8.6
8.7
1
2
t−Test: p−value = 0.11

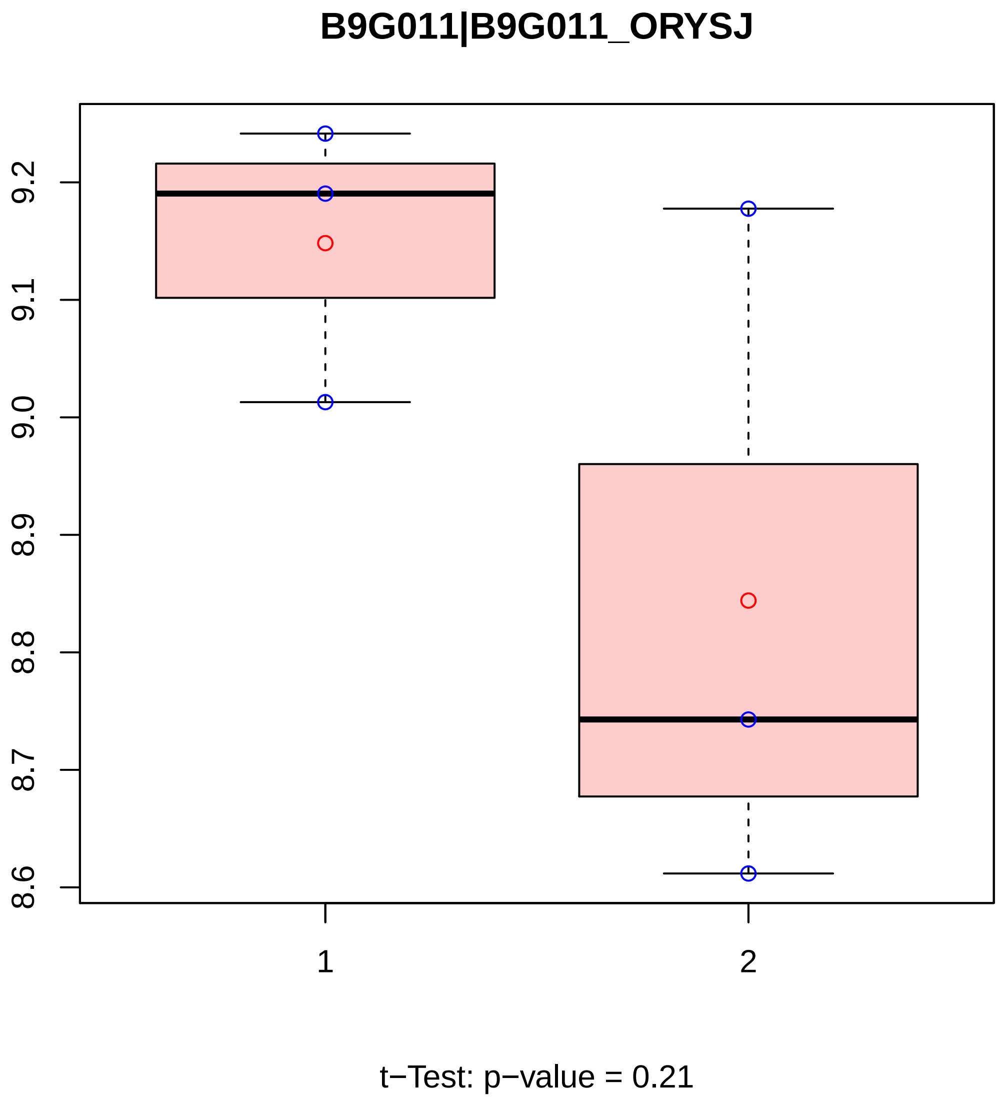

B9G011|B9G011_ORYSJ
9.2
9.1
9.0
8.9
8.8
8.7
8.6
1
2
t−Test: p−value = 0.21

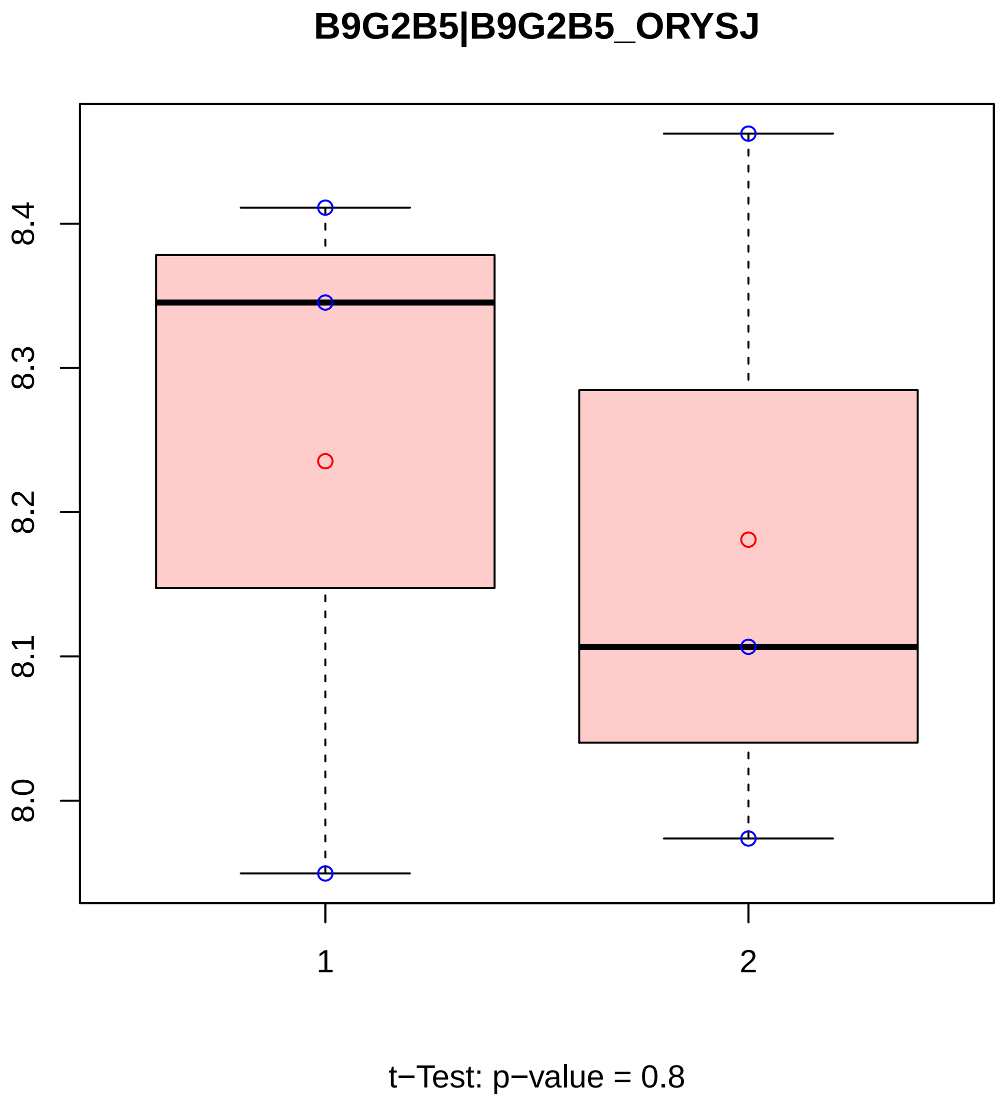

B9G2B5|B9G2B5_ORYSJ
8.0
8.1
8.2
8.3
8.4
1
2
t−Test: p−value = 0.8

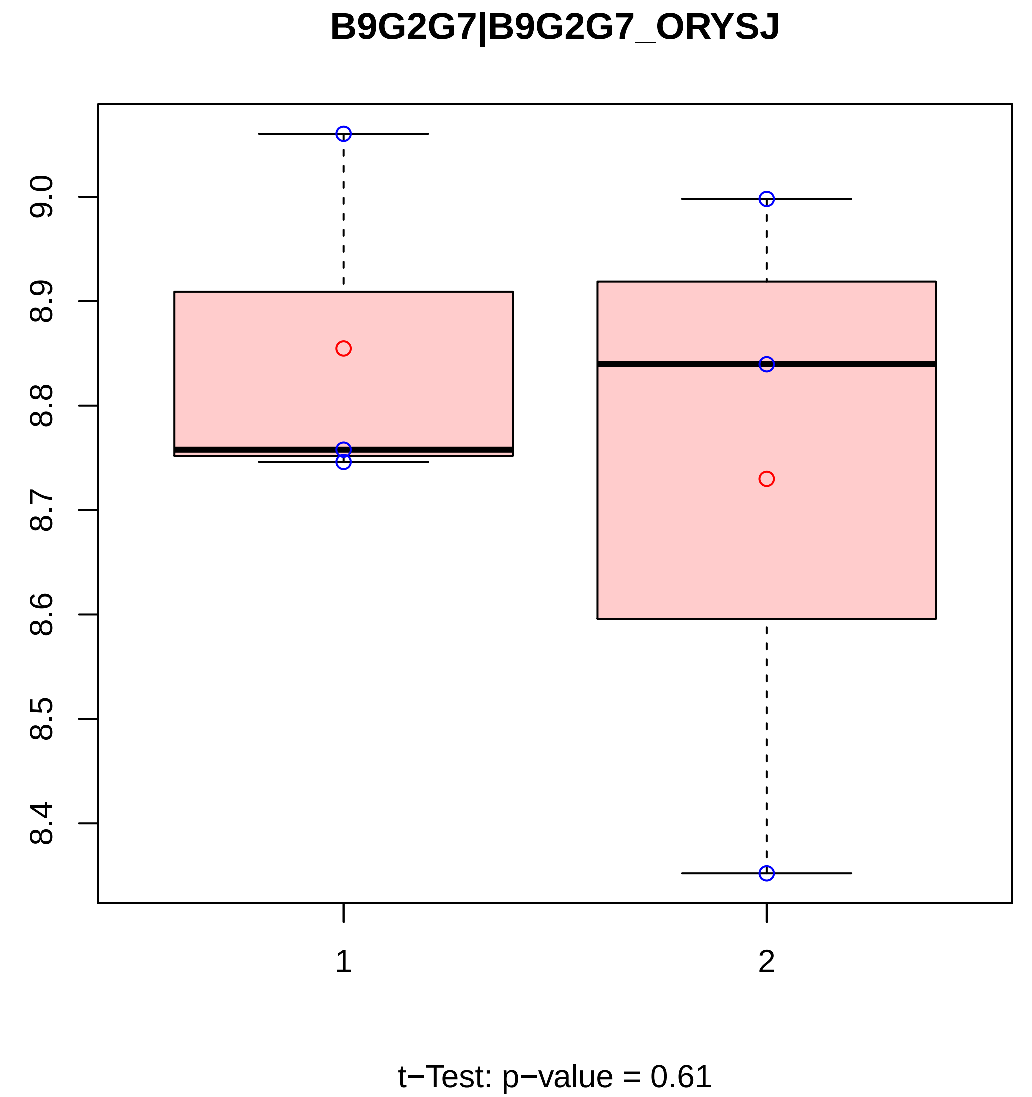
B9G2G7|B9G2G7_ORYSJ
9.0
8.9
8.8
8.7
8.6
8.5
8.4
1
2
t−Test: p−value = 0.61

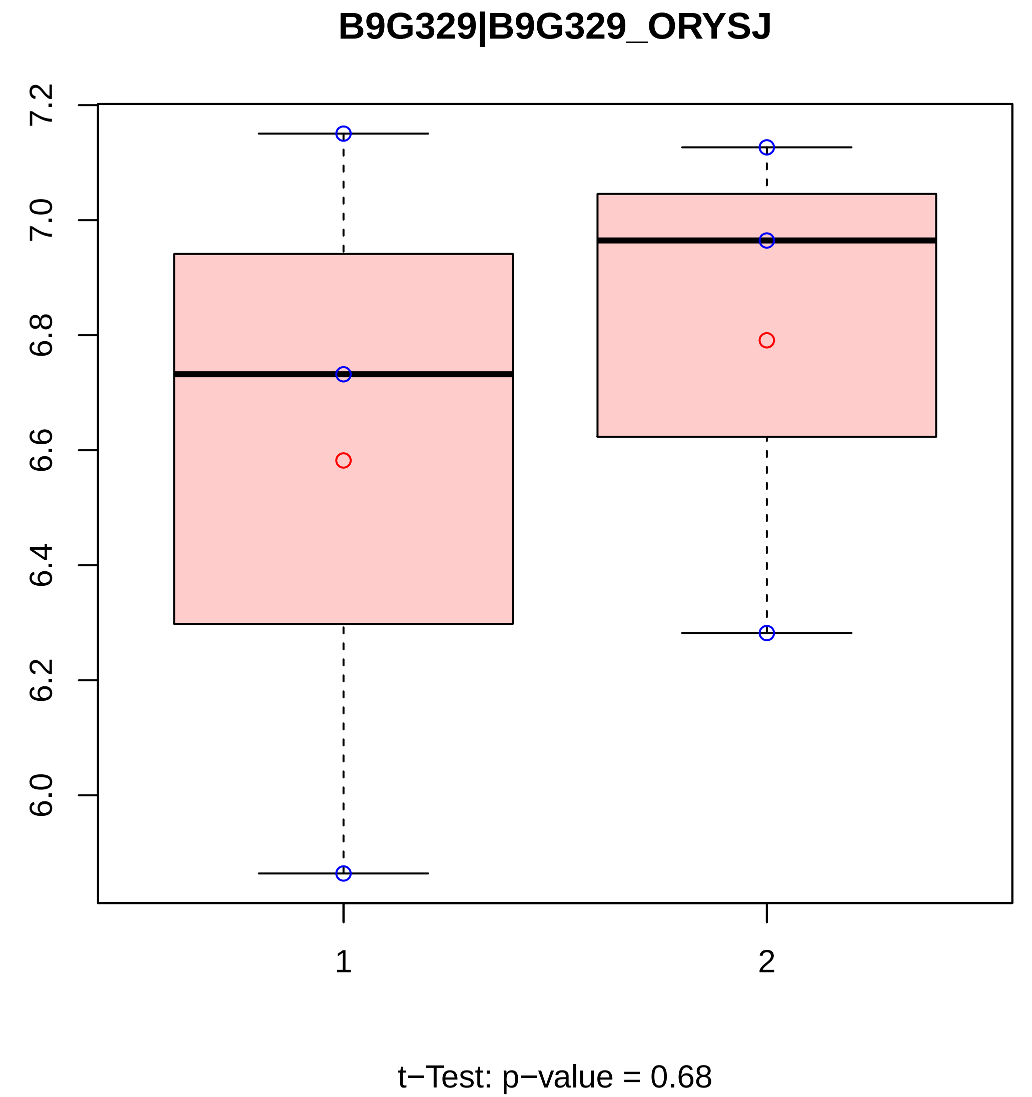

B9G329|B9G329_ORYSJ
7.2
7.0
6.8
6.6
6.4
6.2
6.0
1
2
t−Test: p−value = 0.68

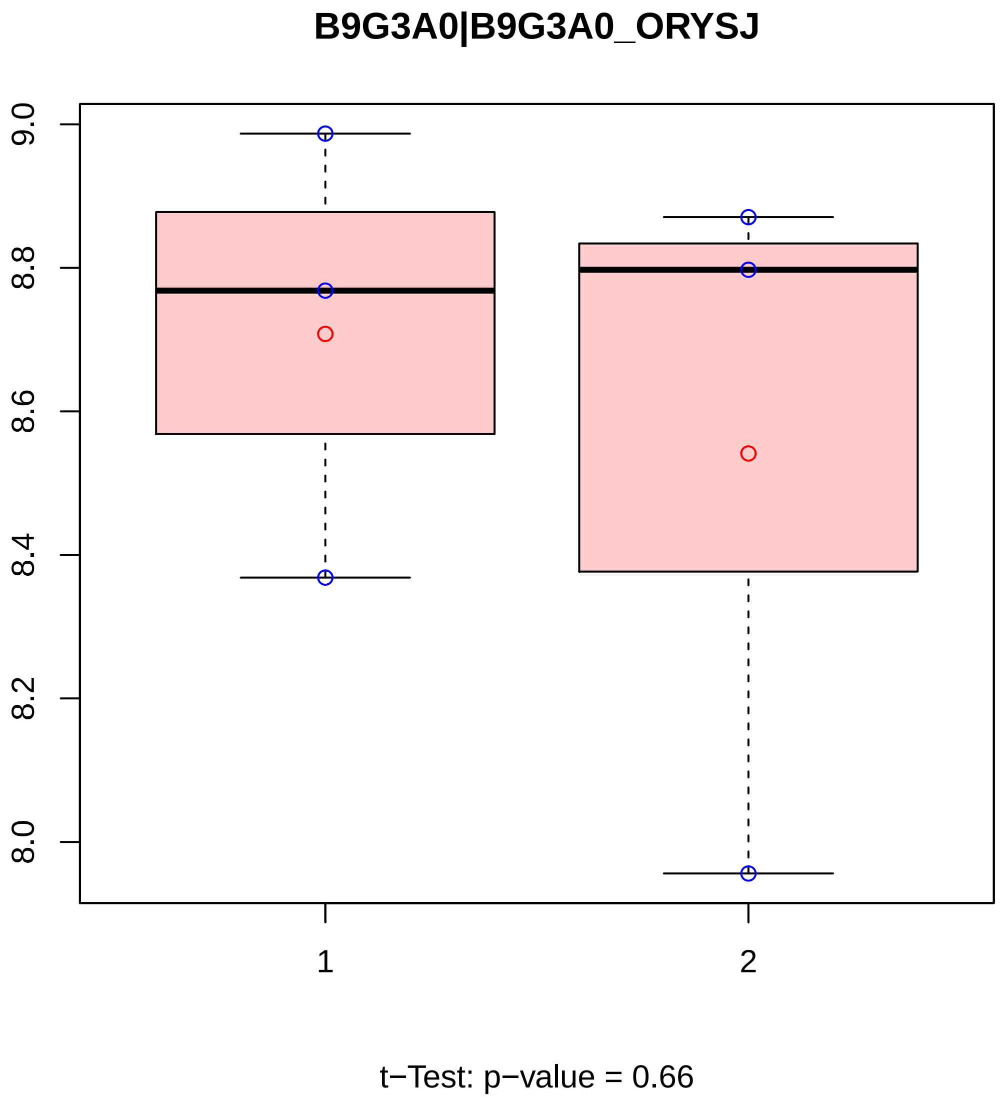
B9G3A0|B9G3A0_ORYSJ
9.0
8.8
8.6
8.4
8.2
8.0
1
2
t−Test: p−value = 0.66

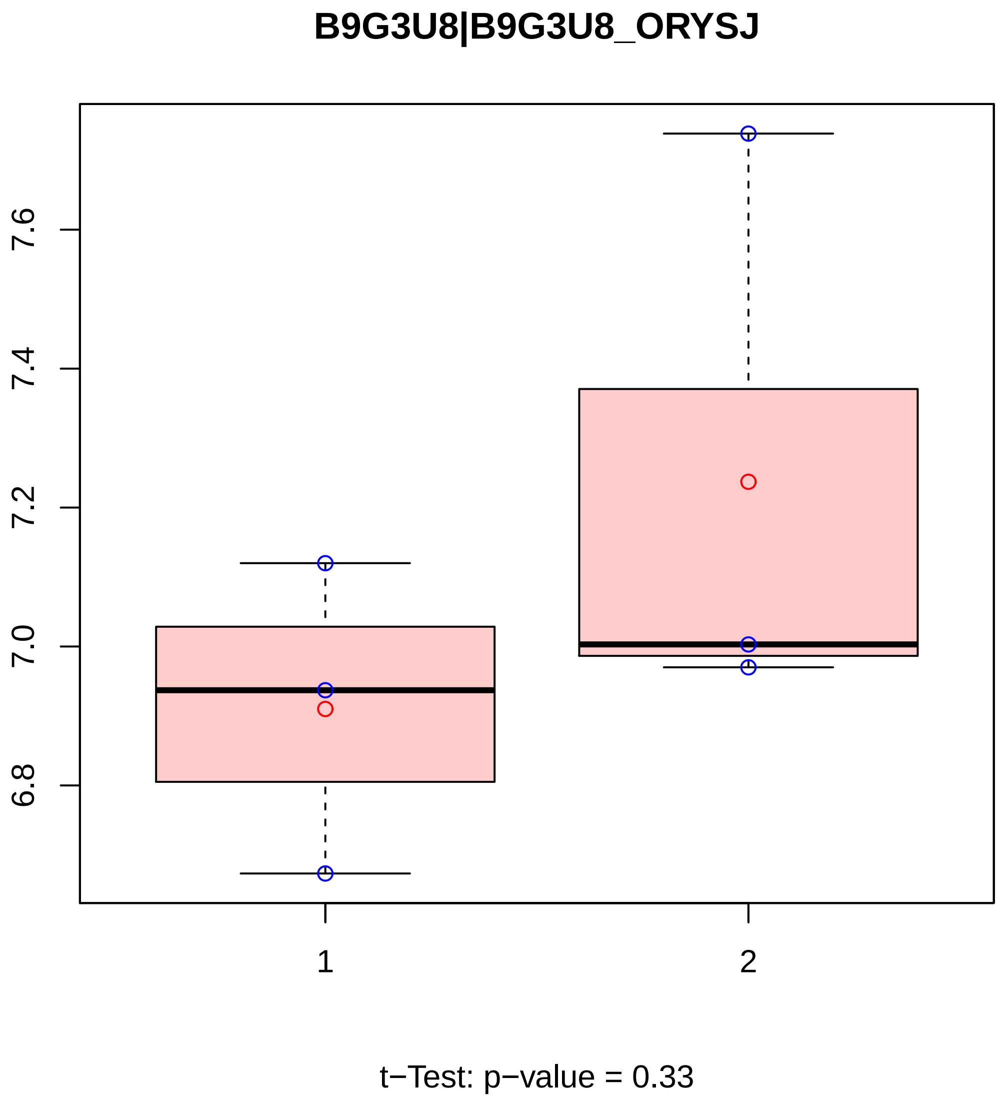

B9G3U8|B9G3U8_ORYSJ
7.6
7.4
7.2
7.0
6.8
1
2
t−Test: p−value = 0.33

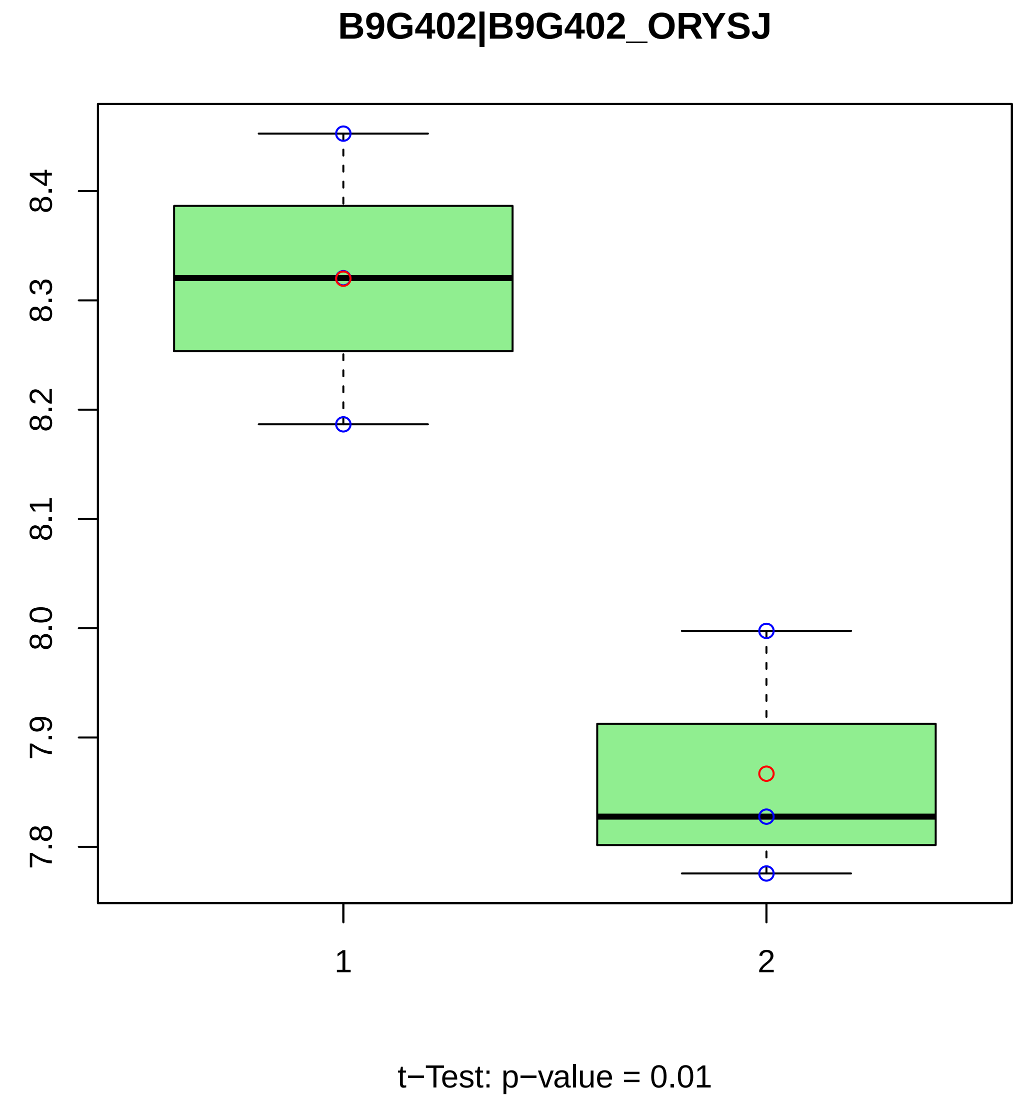
B9G402|B9G402_ORYSJ
8.4
8.3
8.2
8.1
8.0
7.9
7.8
1
2
t−Test: p−value = 0.01

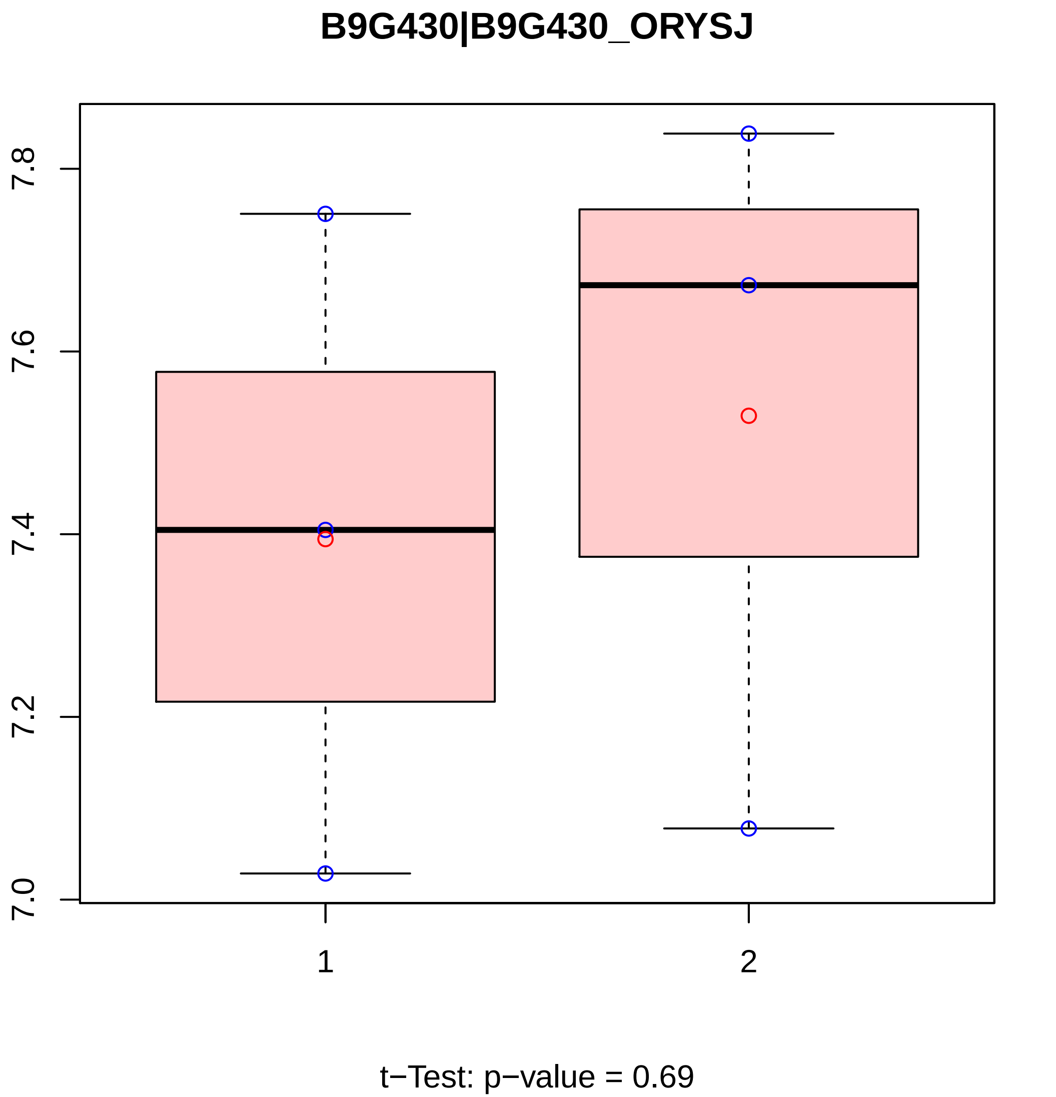
B9G430|B9G430_ORYSJ
7.0
7.2
7.4
7.6
7.8
1
2
t−Test: p−value = 0.69

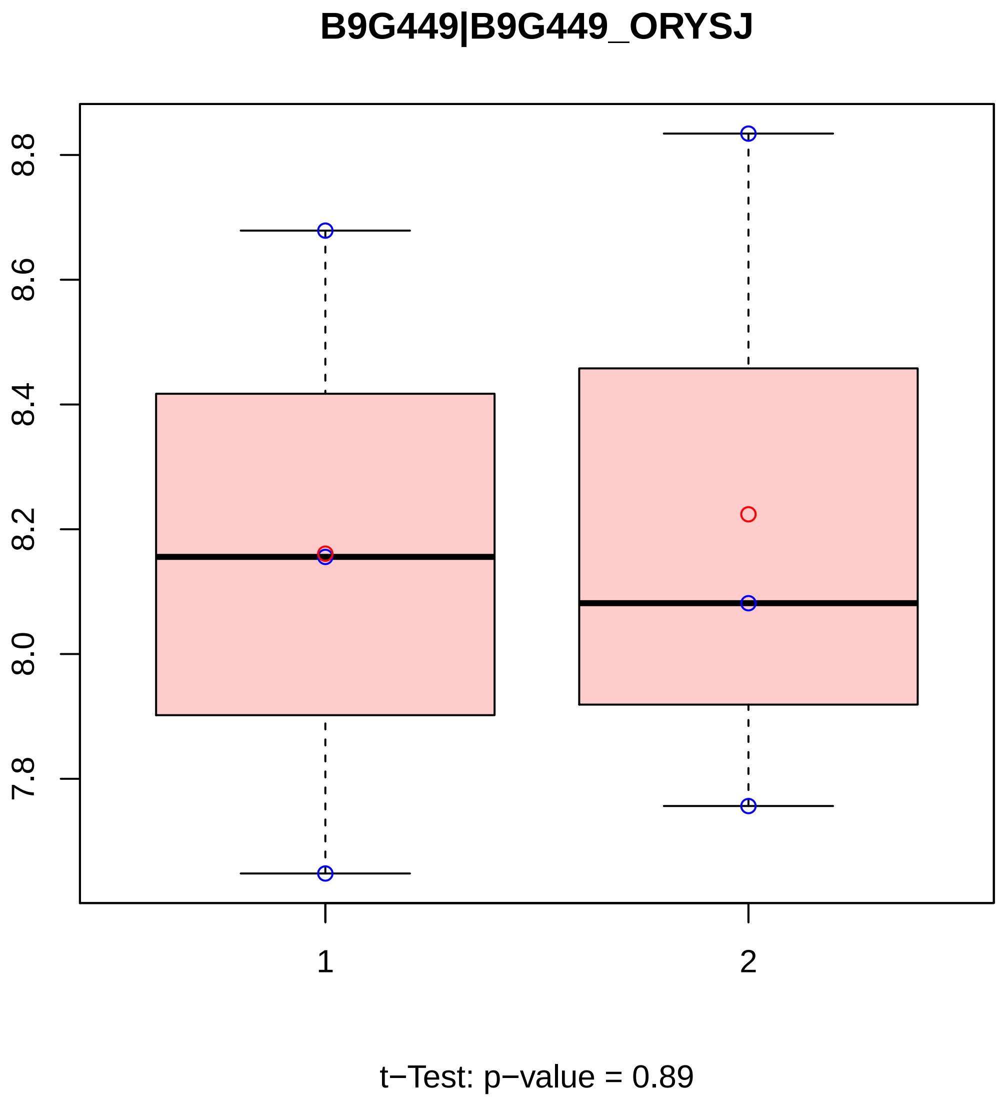
B9G449|B9G449_ORYSJ
8.8
8.6
8.4
8.2
8.0
7.8
1
2
t−Test: p−value = 0.89

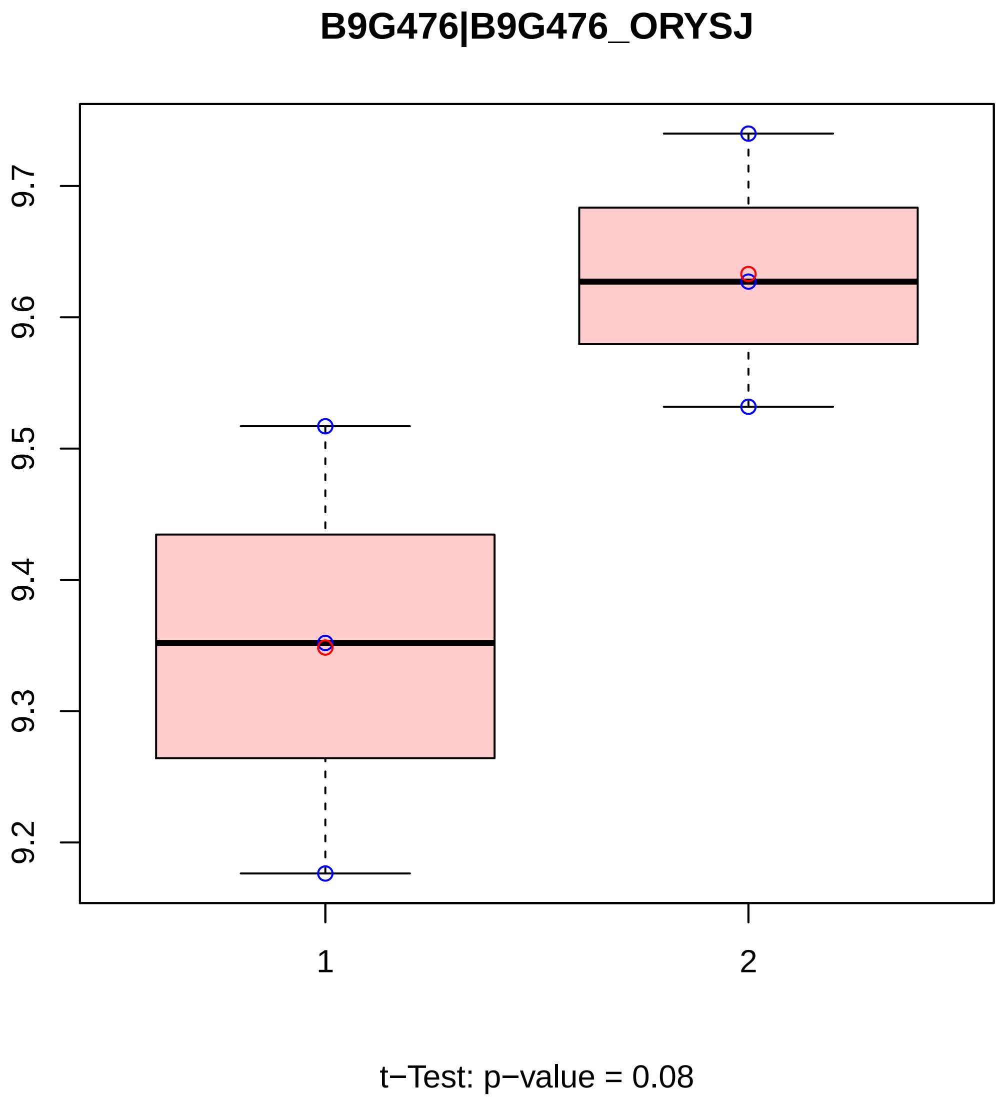
B9G476|B9G476_ORYSJ
9.2
9.3
9.4
9.5
9.6
9.7
1
2
t−Test: p−value = 0.08

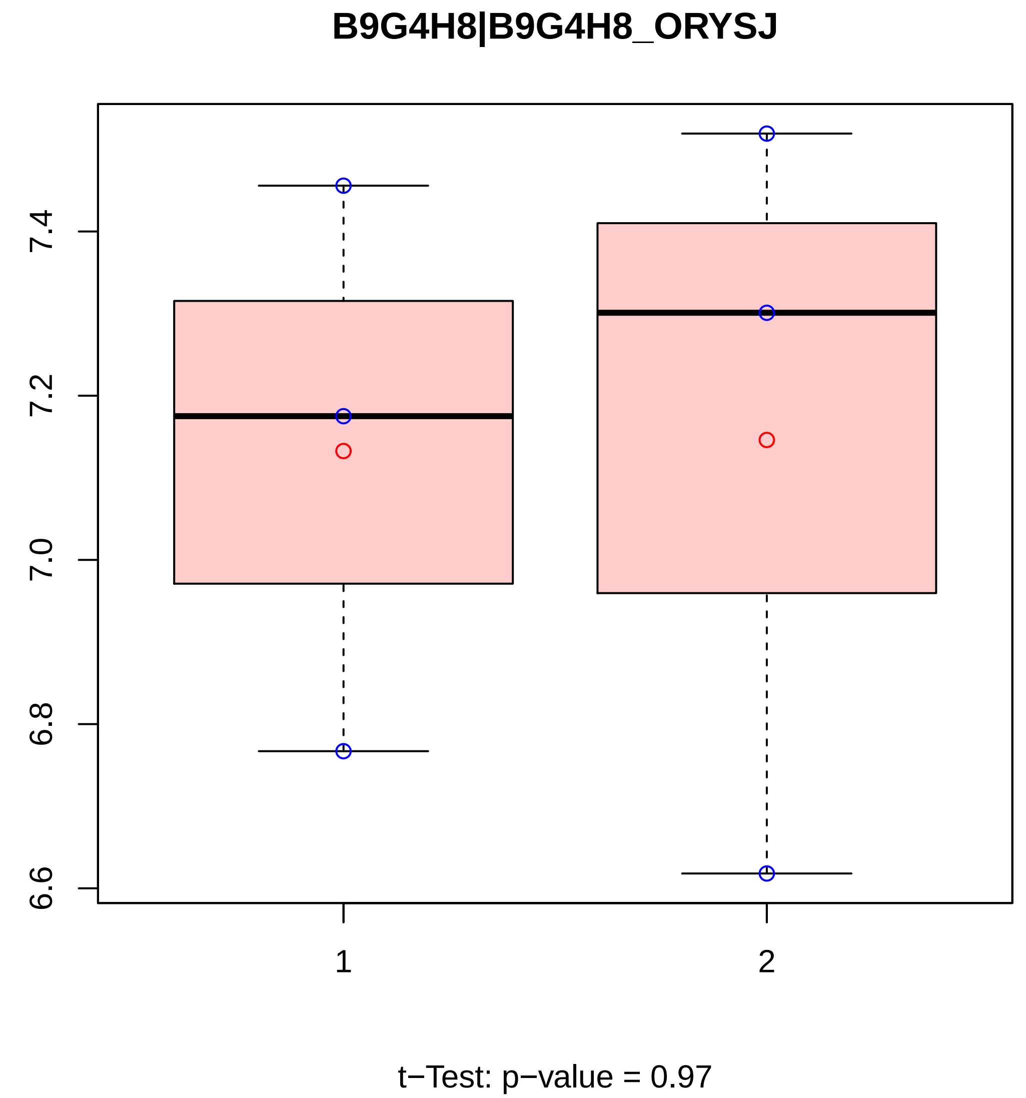
B9G4H8|B9G4H8_ORYSJ
7.4
7.2
7.0
6.8
6.6
1
2
t−Test: p−value = 0.97

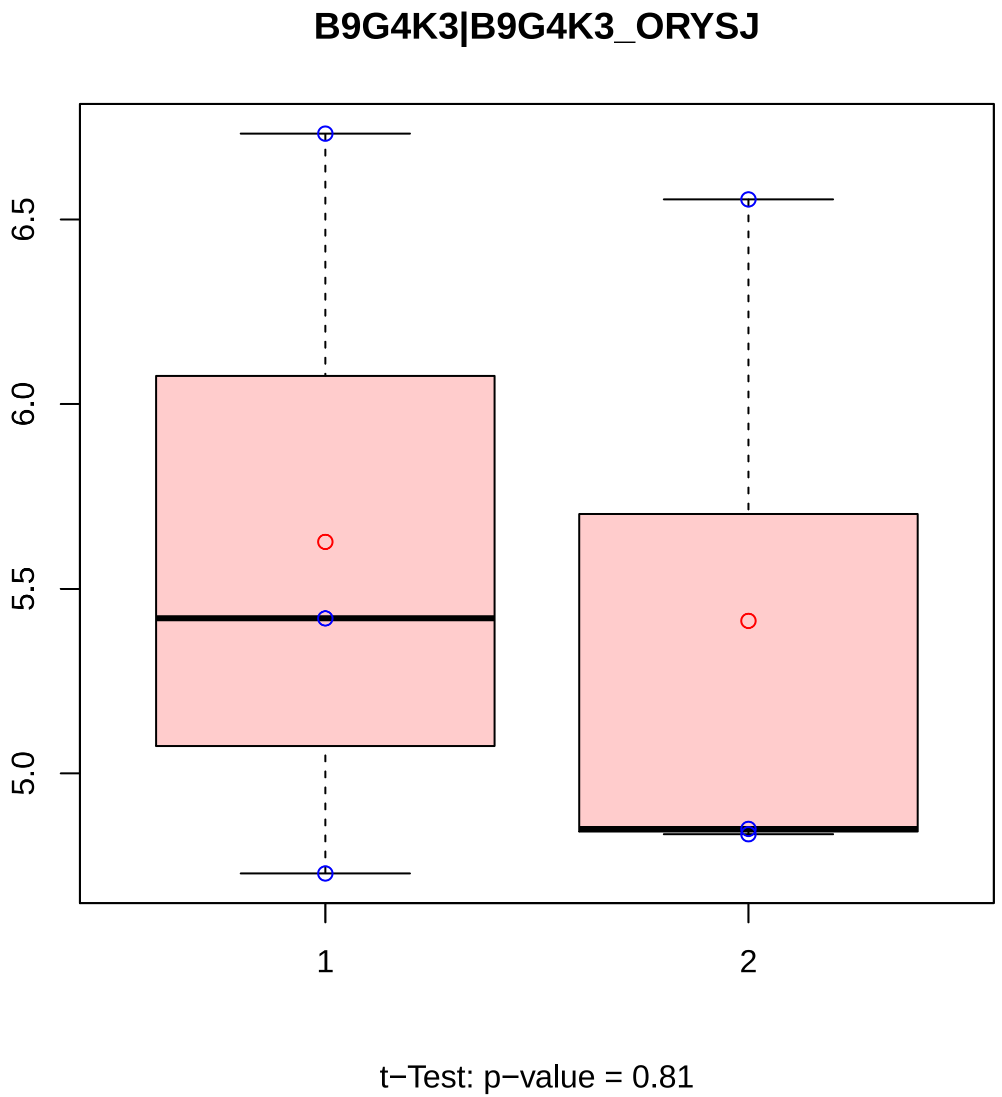

B9G4K3|B9G4K3_ORYSJ
6.5
6.0
5.5
5.0
1
2
t−Test: p−value = 0.81

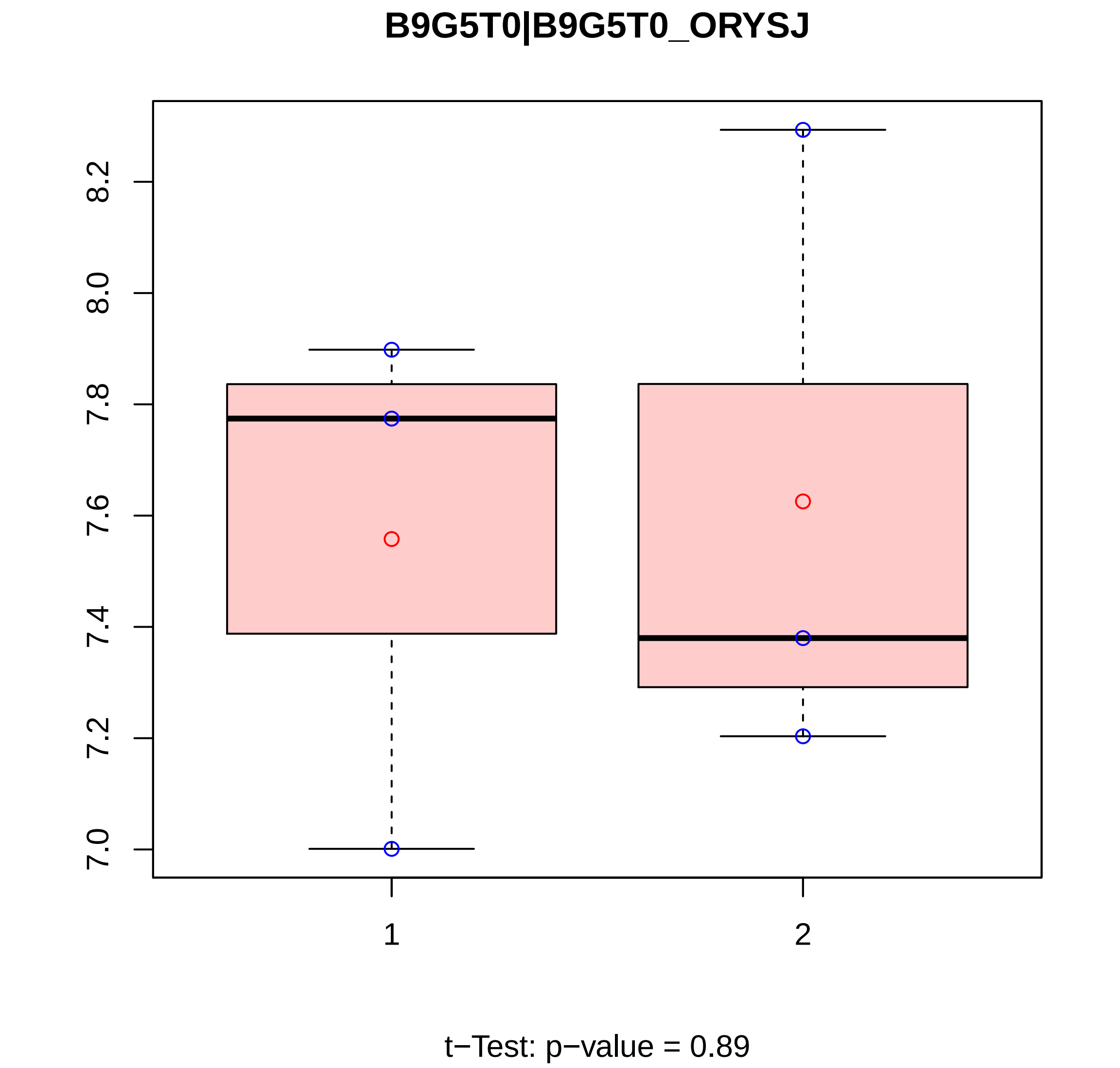

B9G5T0|B9G5T0_ORYSJ
7.0
7.2
7.4
7.6
7.8
8.0
8.2
1
2
t−Test: p−value = 0.89

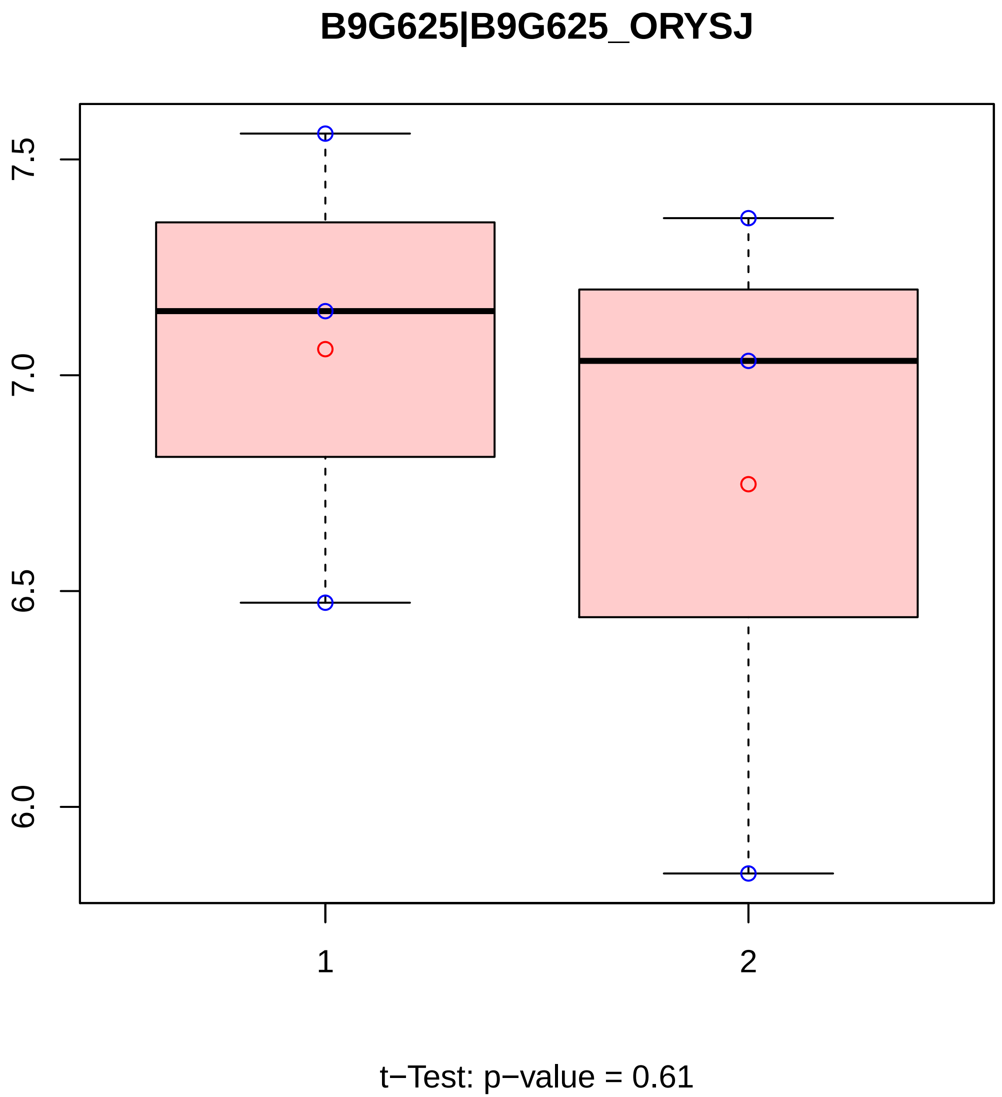
B9G625|B9G625_ORYSJ
7.5
7.0
6.5
6.0
1
2
t−Test: p−value = 0.61

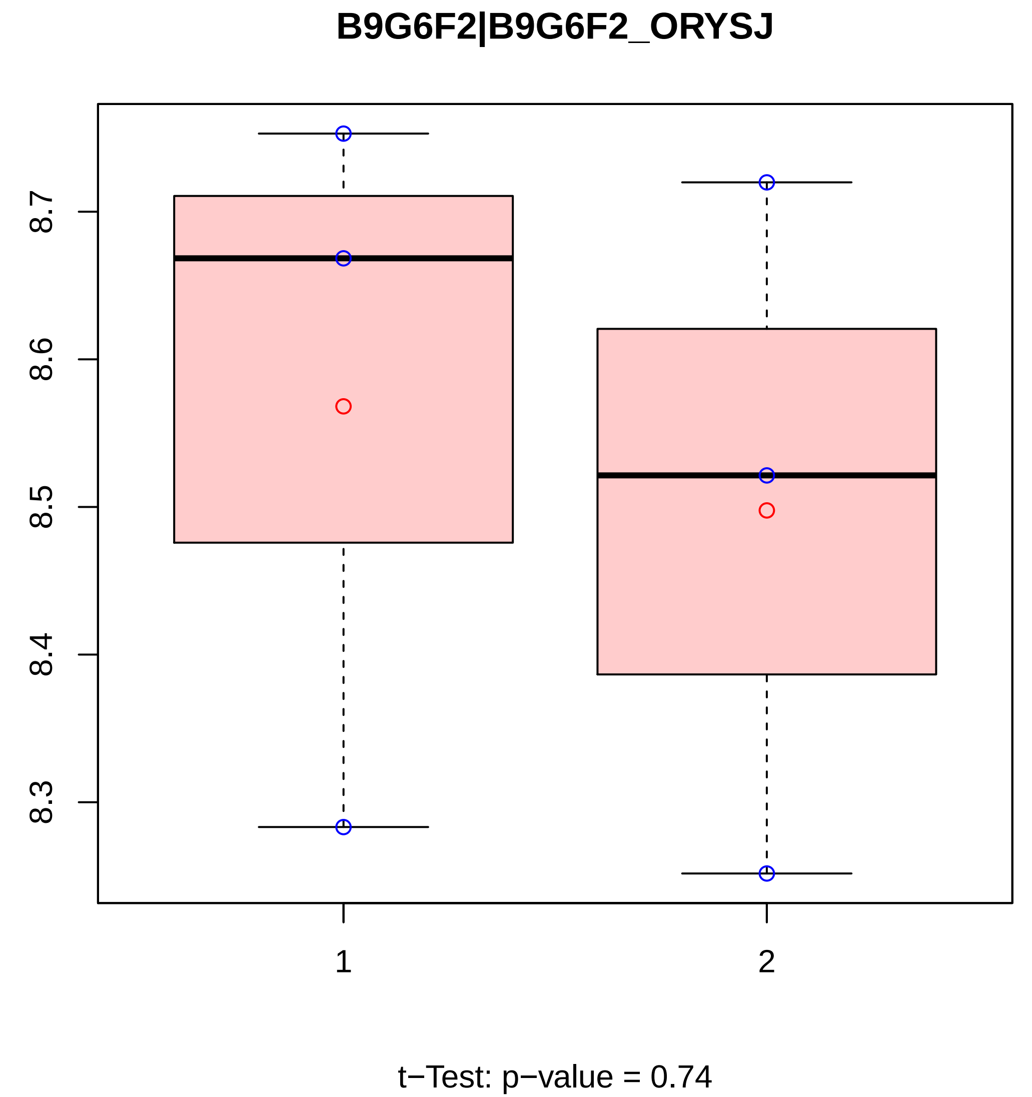
B9G6F2|B9G6F2_ORYSJ
8.3
8.4
8.5
8.6
8.7
1
2
t−Test: p−value = 0.74

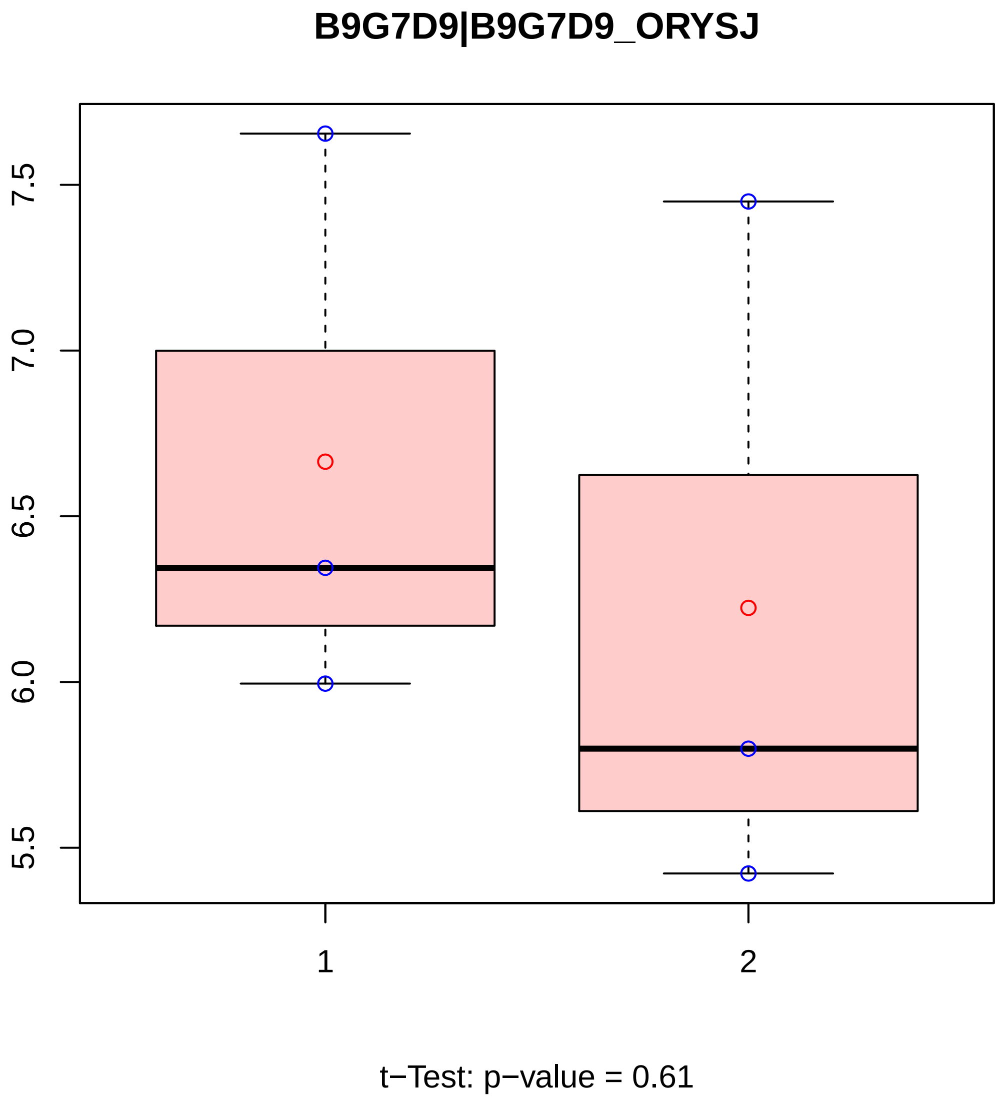

B9G7D9|B9G7D9_ORYSJ
7.5
7.0
6.5
6.0
5.5
1
2
t−Test: p−value = 0.61

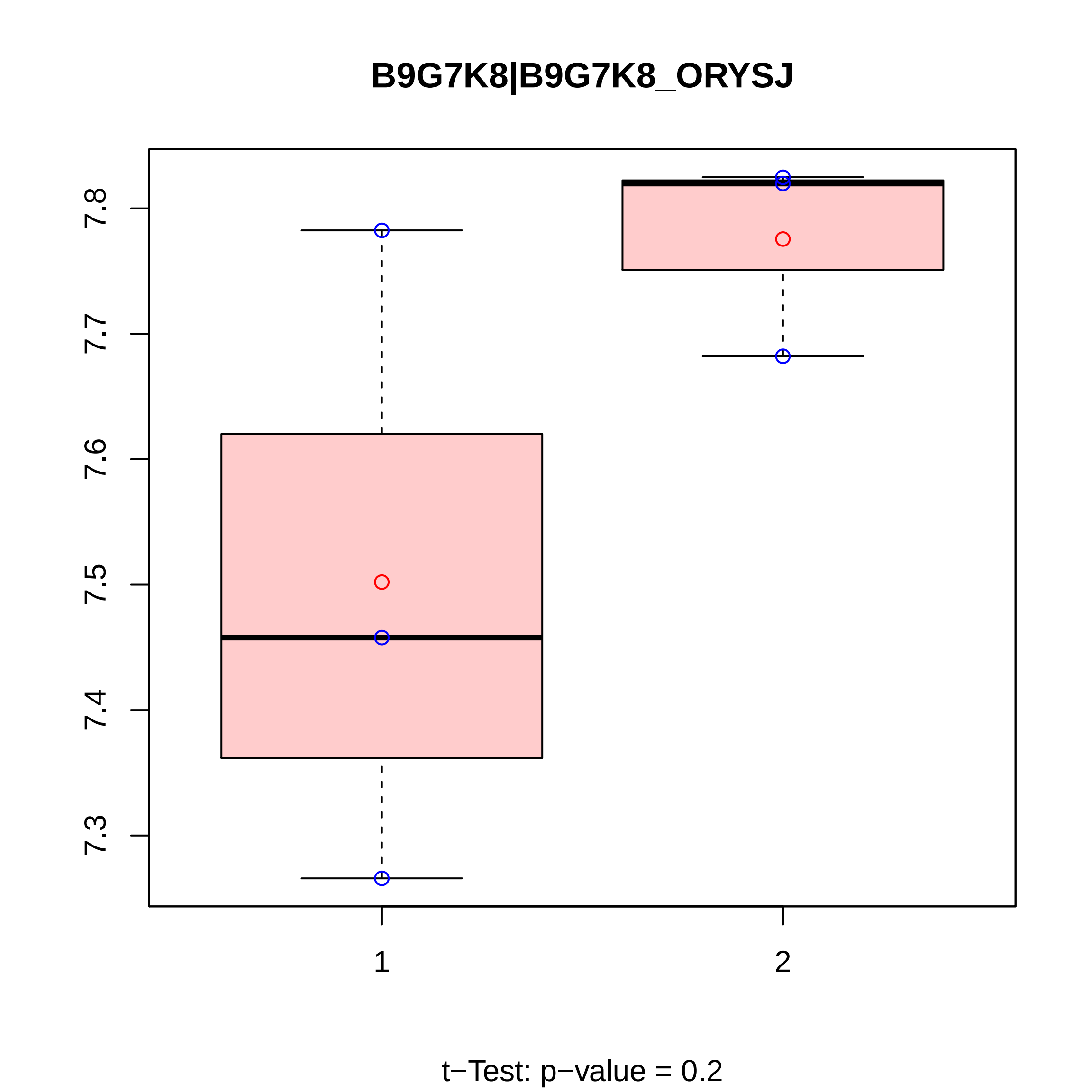
B9G7K8|B9G7K8_ORYSJ
7.3
7.4
7.5
7.6
7.7
7.8
1
2
t−Test: p−value = 0.2

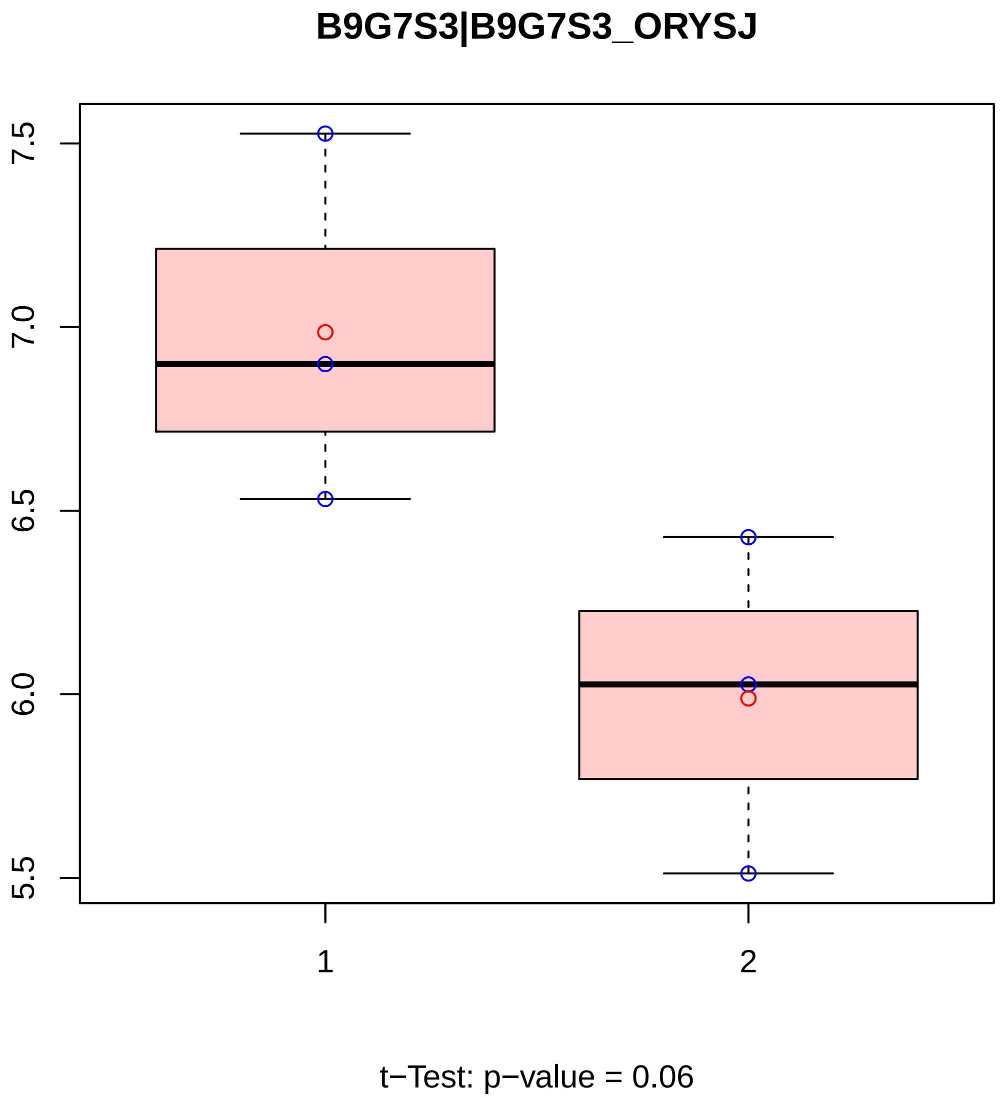

B9G7S3|B9G7S3_ORYSJ
7.5
7.0
6.5
6.0
5.5
1
2
t−Test: p−value = 0.06

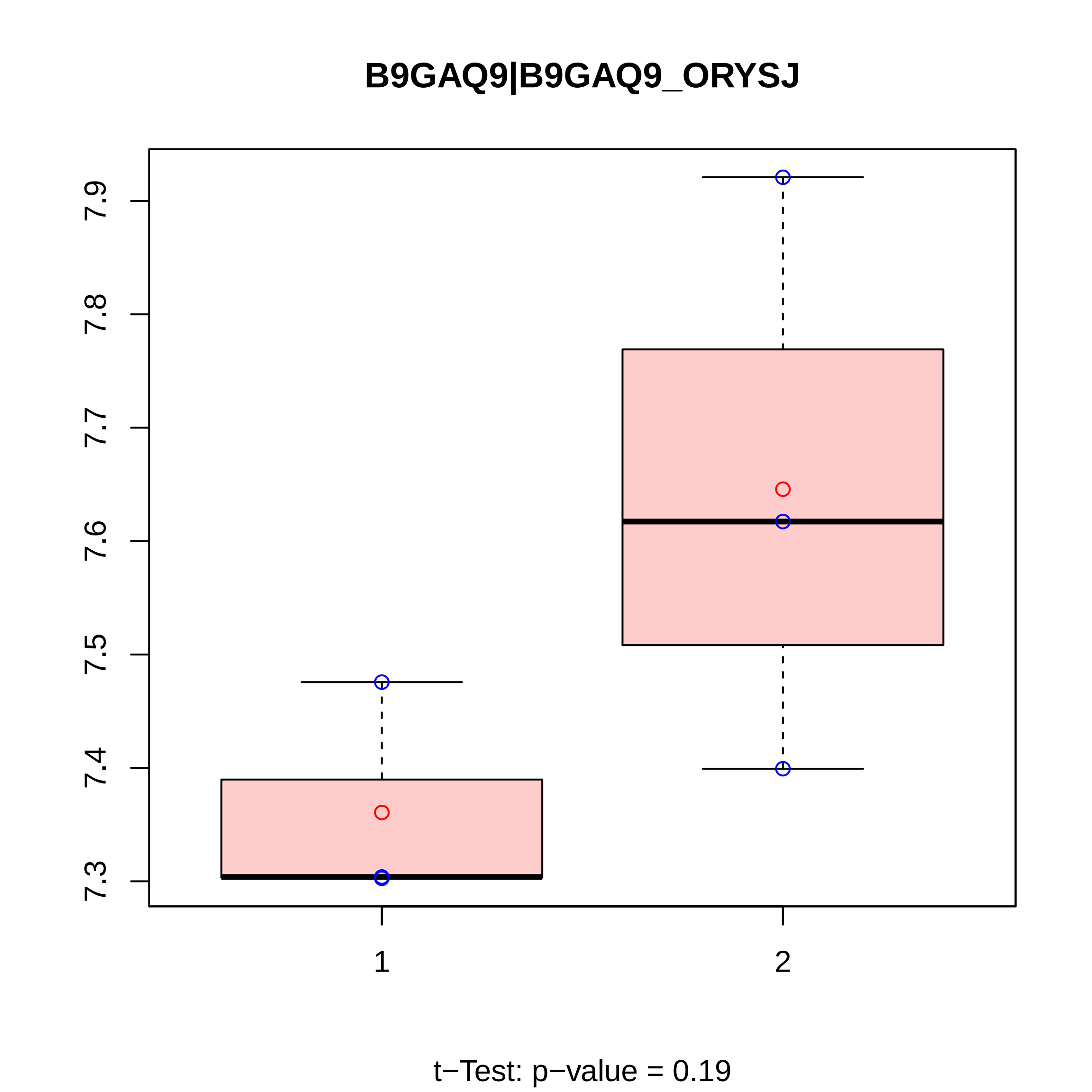

B9GAQ9|B9GAQ9_ORYSJ
7.3
7.4
7.5
7.6
7.7
7.8
7.9
1
2
t−Test: p−value = 0.19

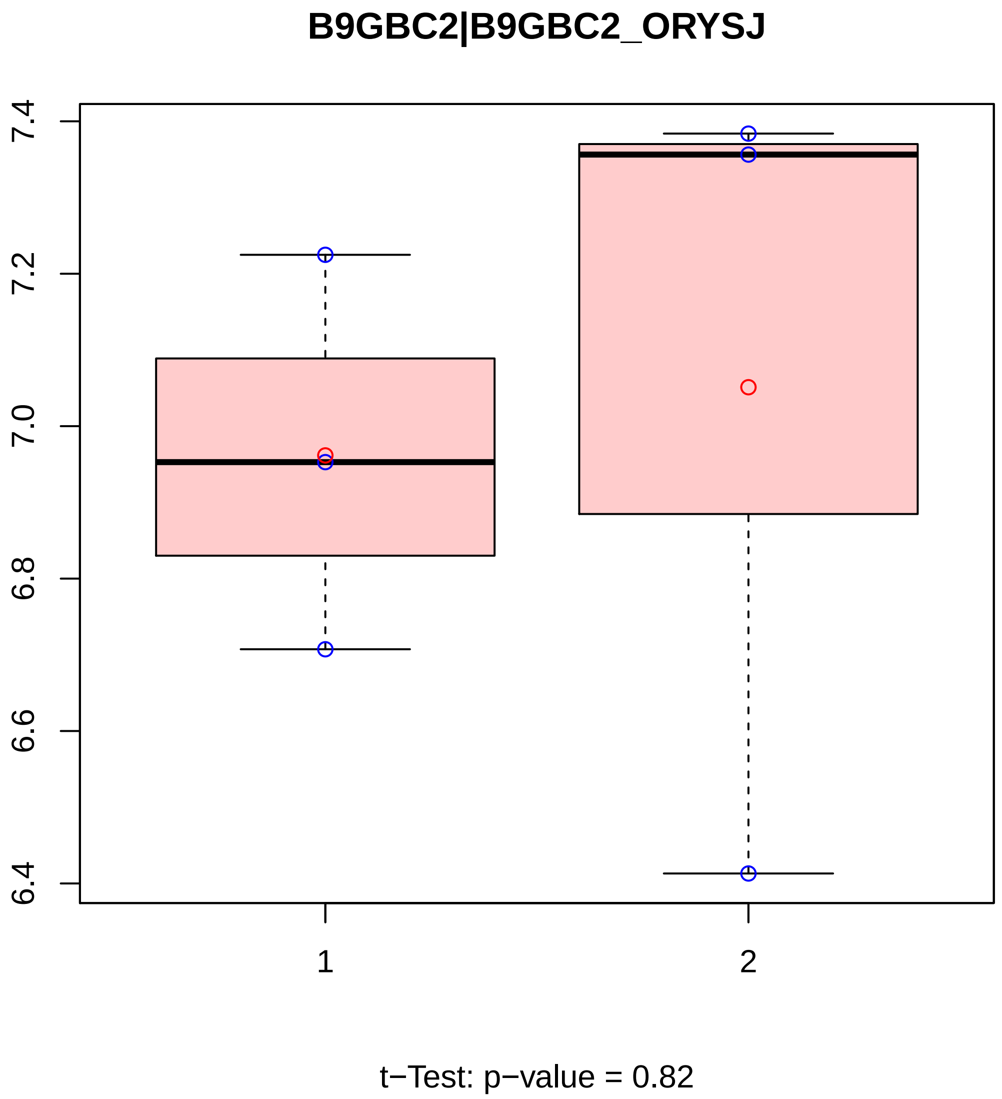

B9GBC2|B9GBC2_ORYSJ
7.4
7.2
7.0
6.8
6.6
6.4
1
2
t−Test: p−value = 0.82

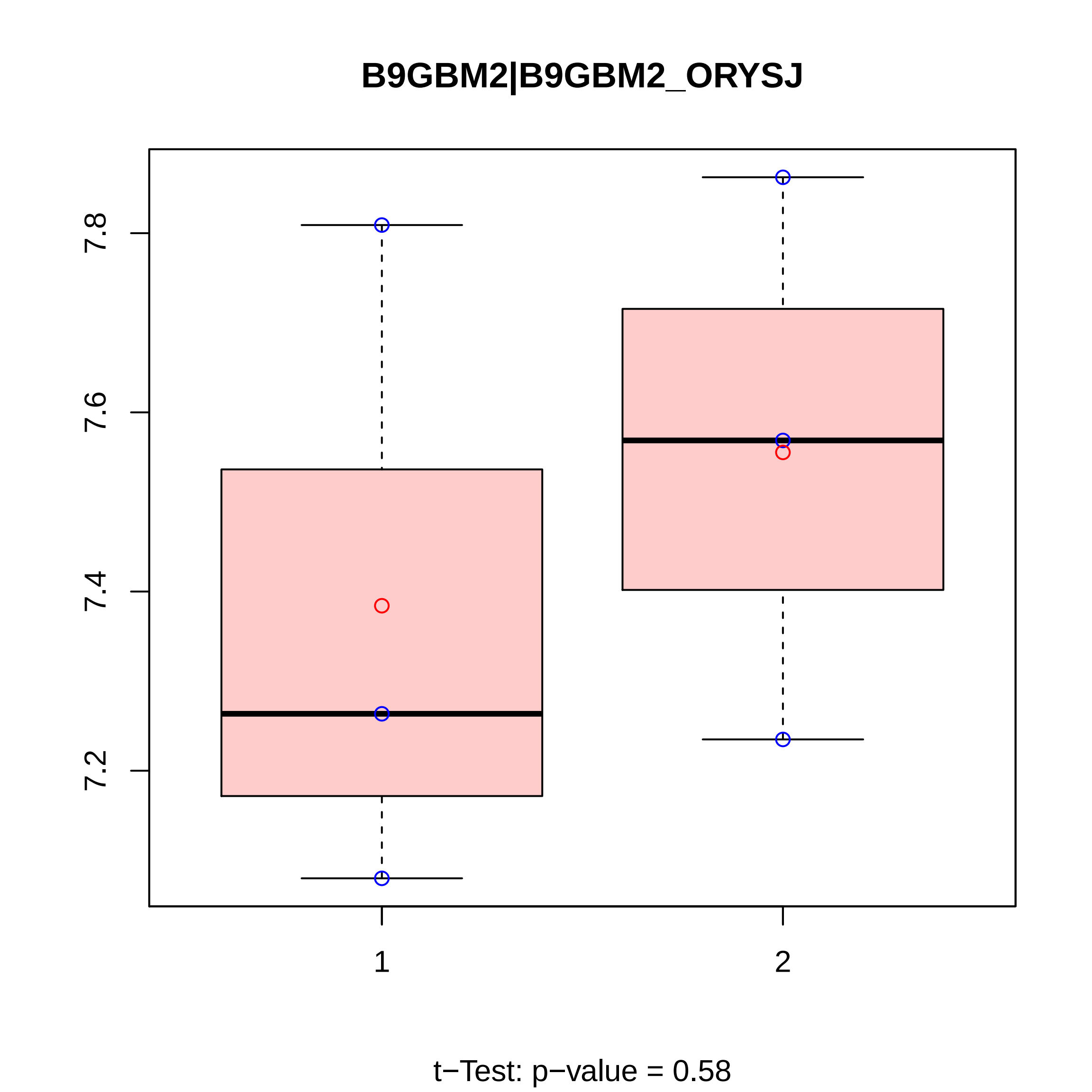
B9GBM2|B9GBM2_ORYSJ
7.2
7.4
7.6
7.8
1
2
t−Test: p−value = 0.58

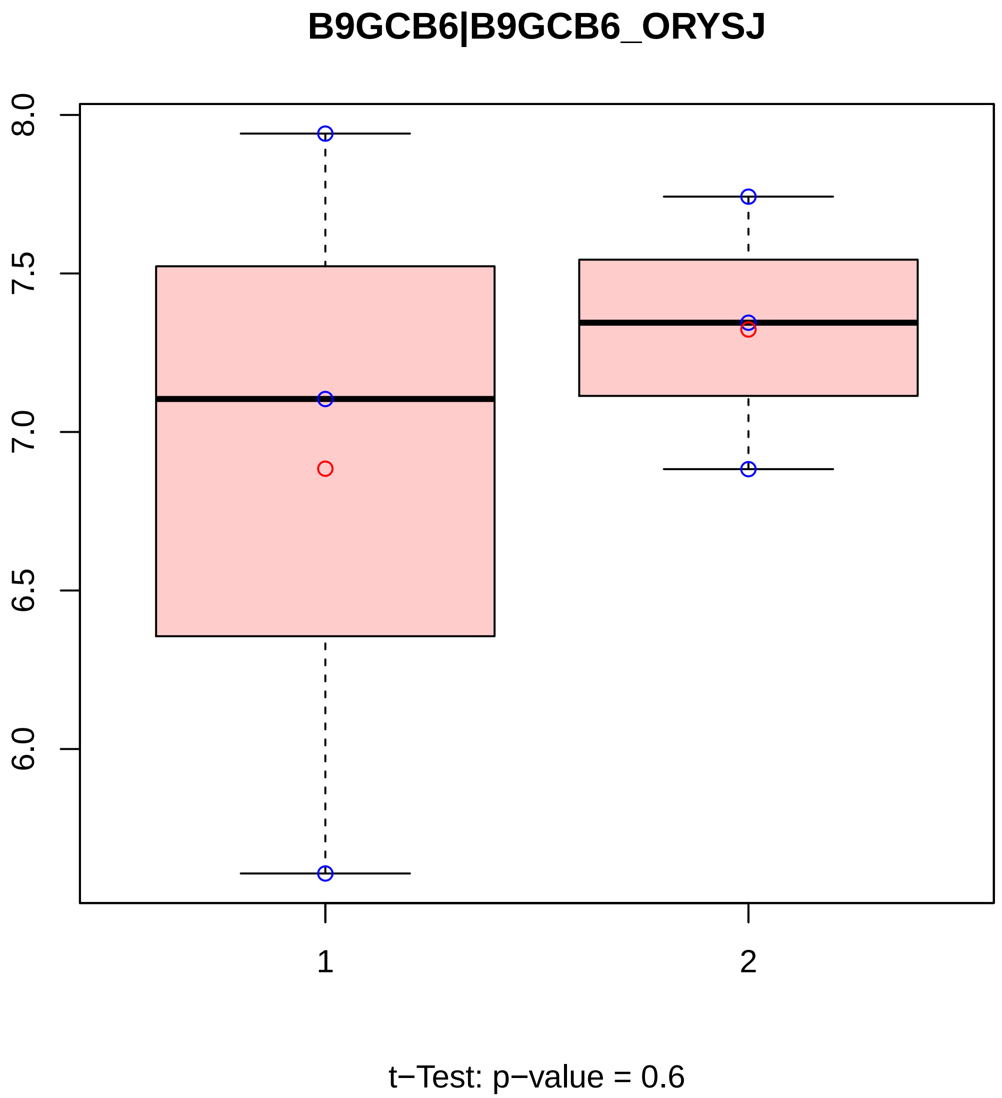

B9GCB6|B9GCB6_ORYSJ
8.0
7.5
7.0
6.5
6.0
1
2
t−Test: p−value = 0.6

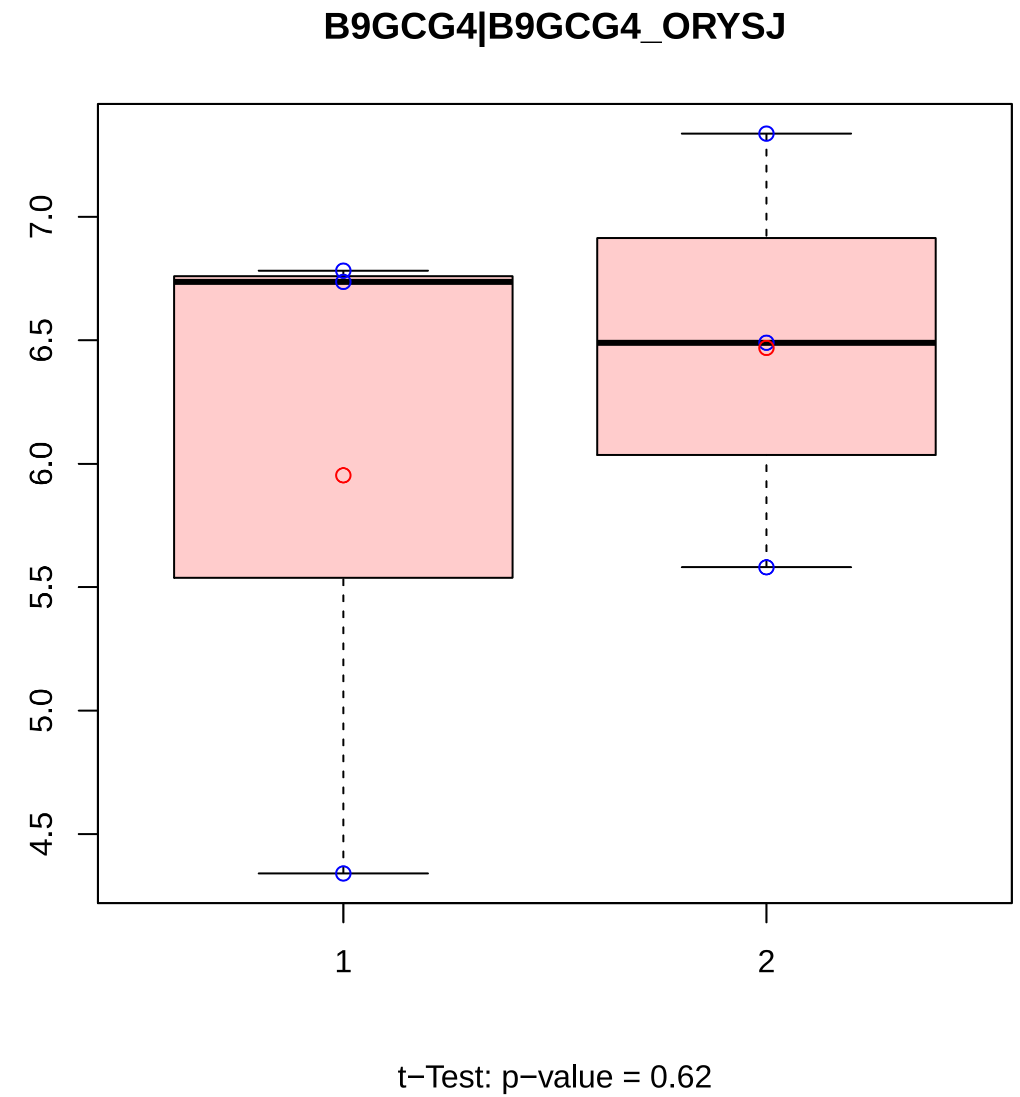
B9GCG4|B9GCG4_ORYSJ
7.0
6.5
6.0
5.5
5.0
4.5
1
2
t−Test: p−value = 0.62

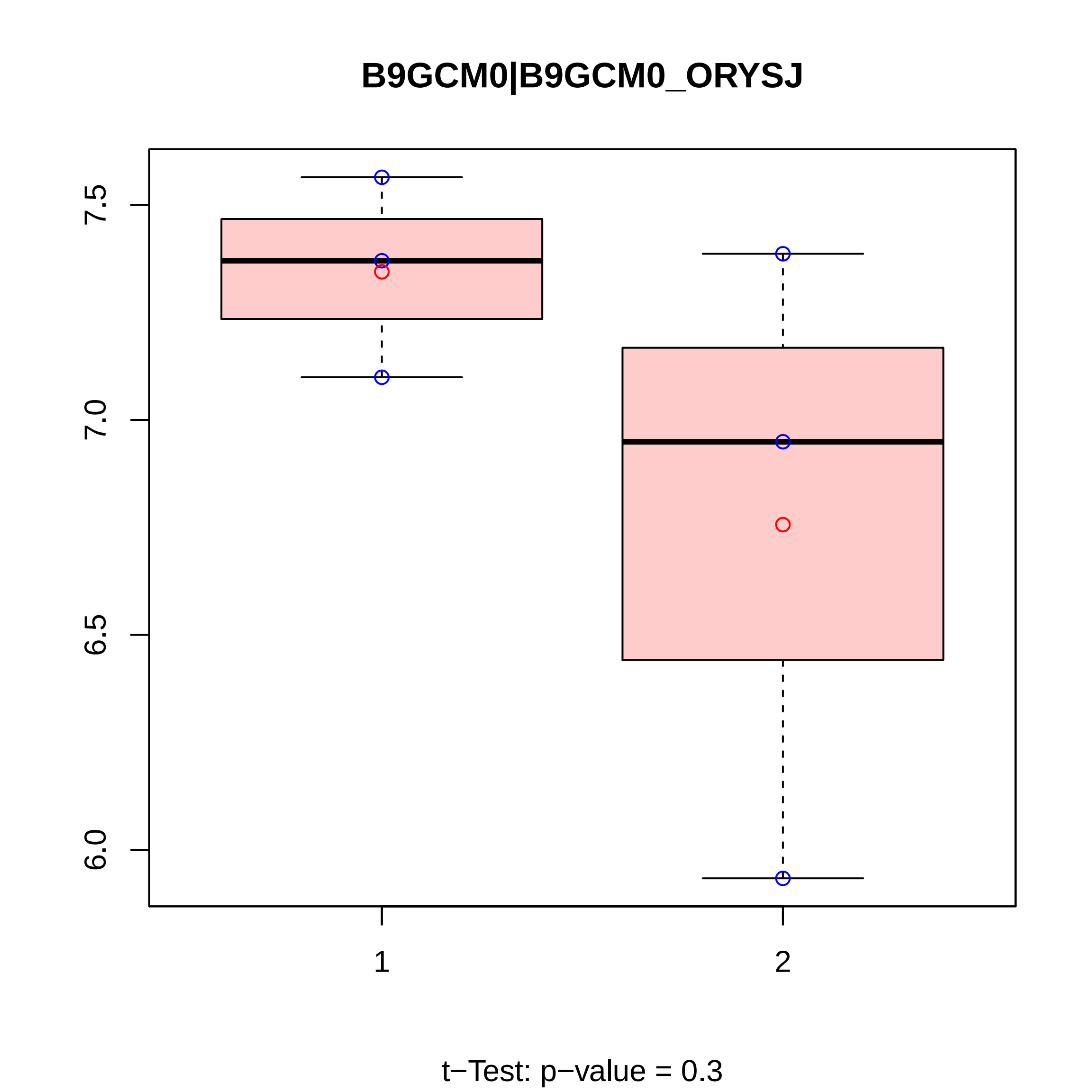

B9GCM0|B9GCM0_ORYSJ
7.5
7.0
6.5
6.0
1
2
t−Test: p−value = 0.3

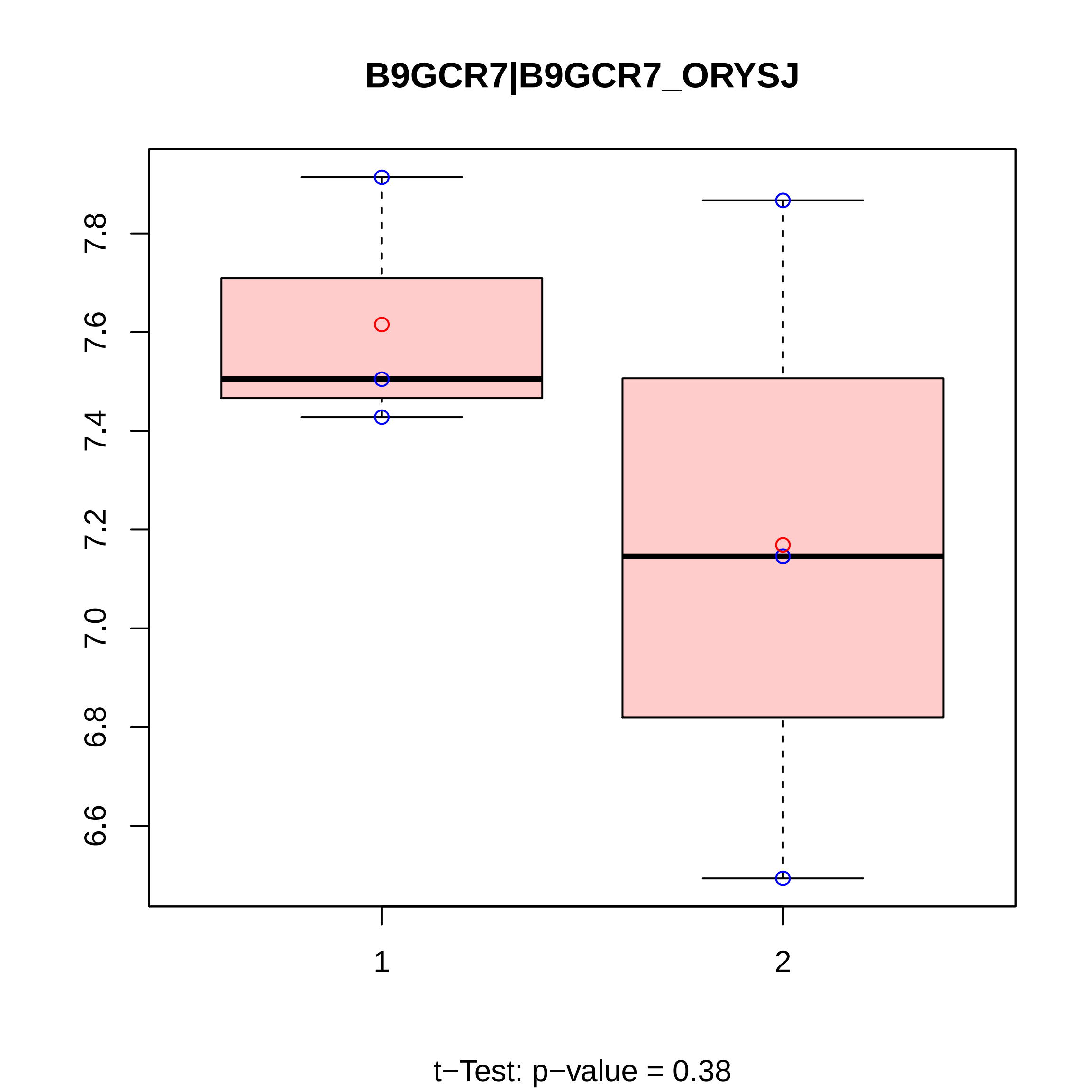
B9GCR7|B9GCR7_ORYSJ
7.8
7.6
7.4
7.2
7.0
6.8
6.6
1
2
t−Test: p−value = 0.38

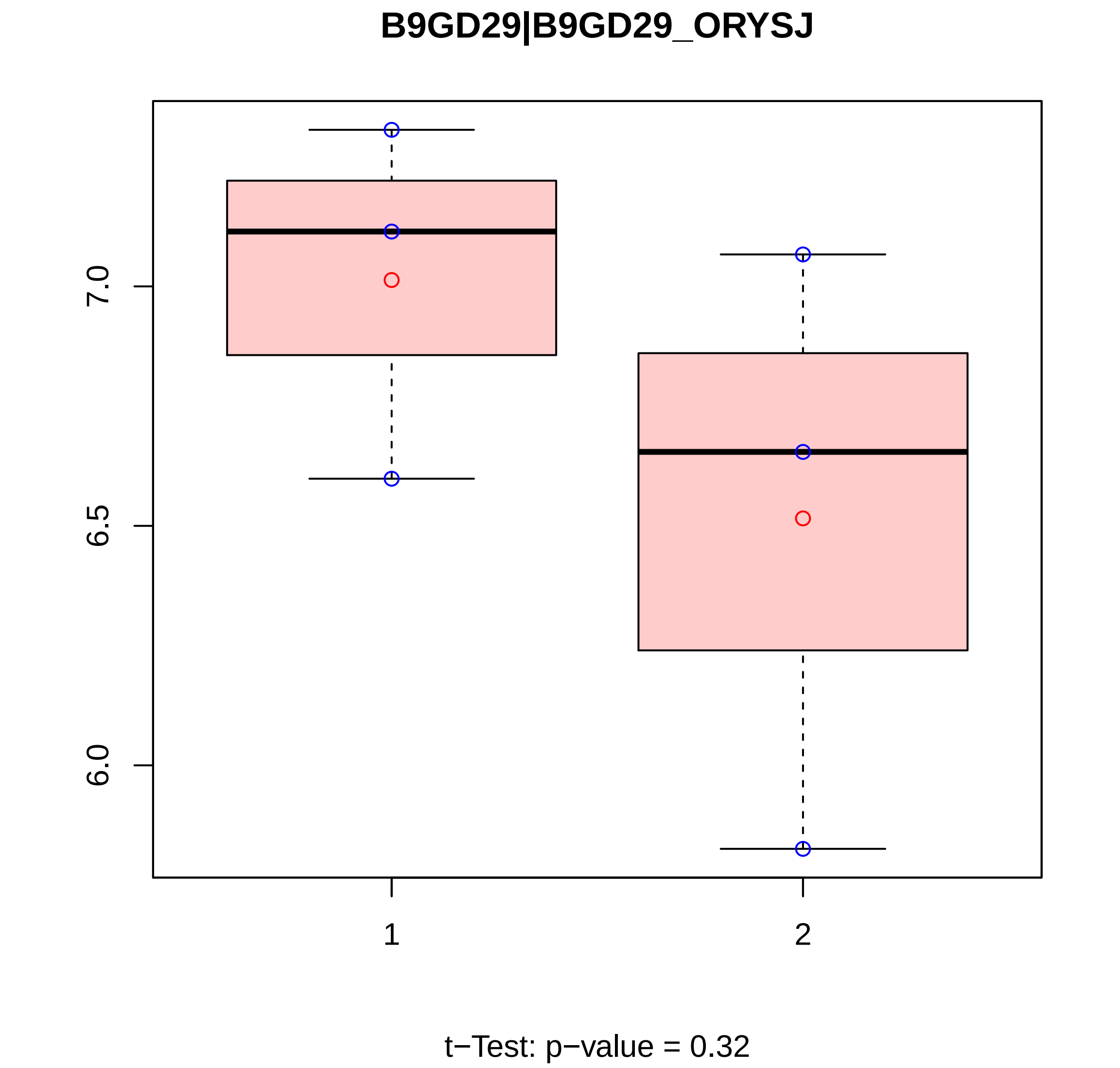
B9GD29|B9GD29_ORYSJ
7.0
6.5
6.0
1
2
t−Test: p−value = 0.32

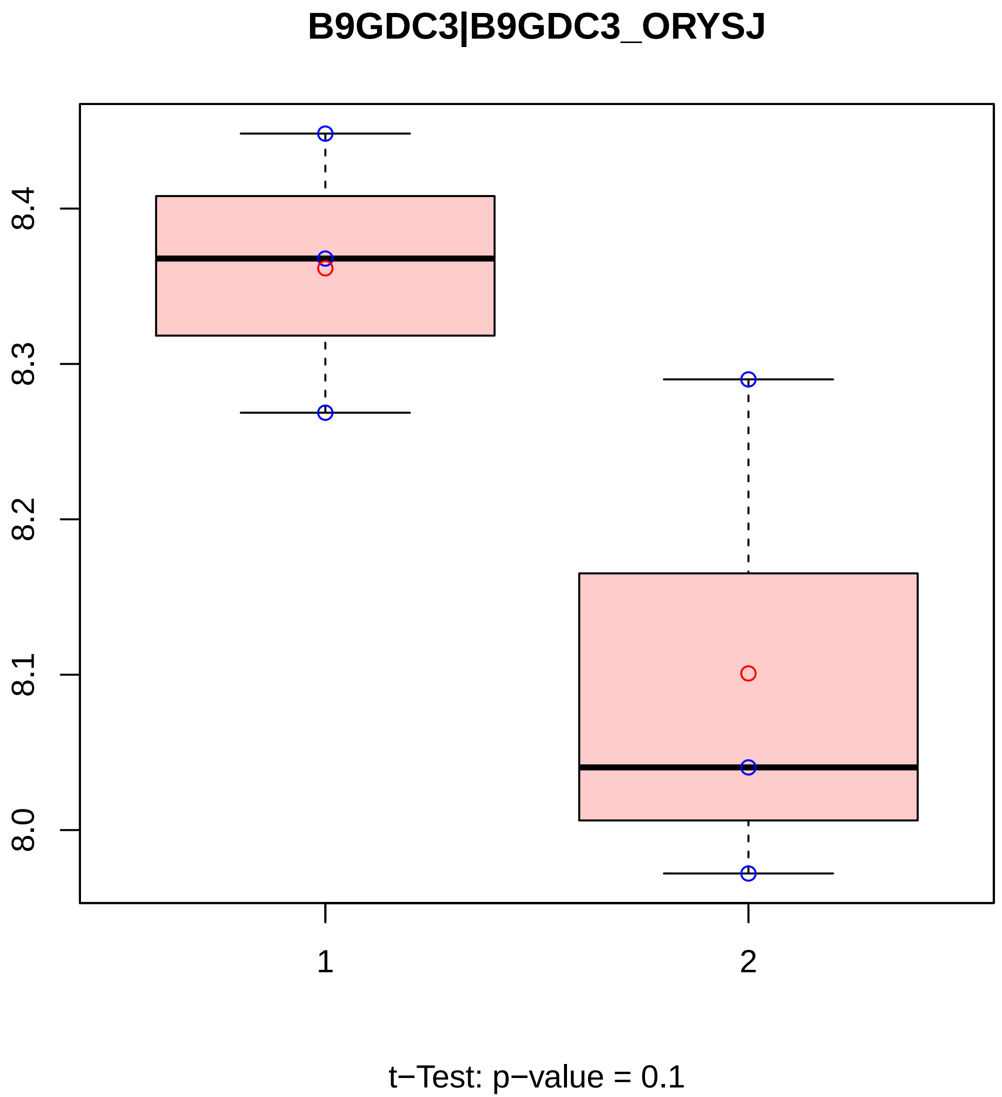
B9GDC3|B9GDC3_ORYSJ
8.4
8.3
8.2
8.1
8.0
1
2
t−Test: p−value = 0.1

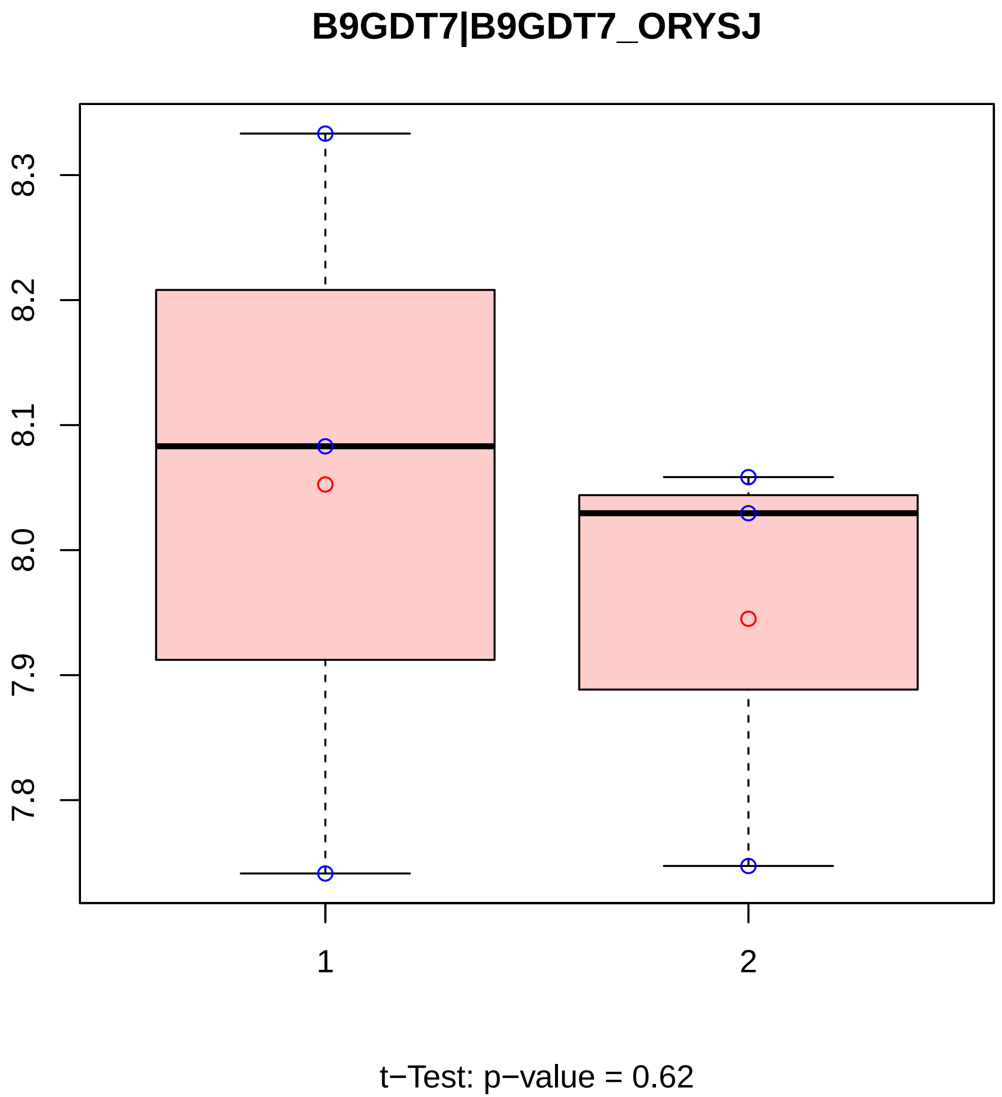
B9GDT7|B9GDT7_ORYSJ
8.3
8.2
8.1
8.0
7.9
7.8
1
2
t−Test: p−value = 0.62

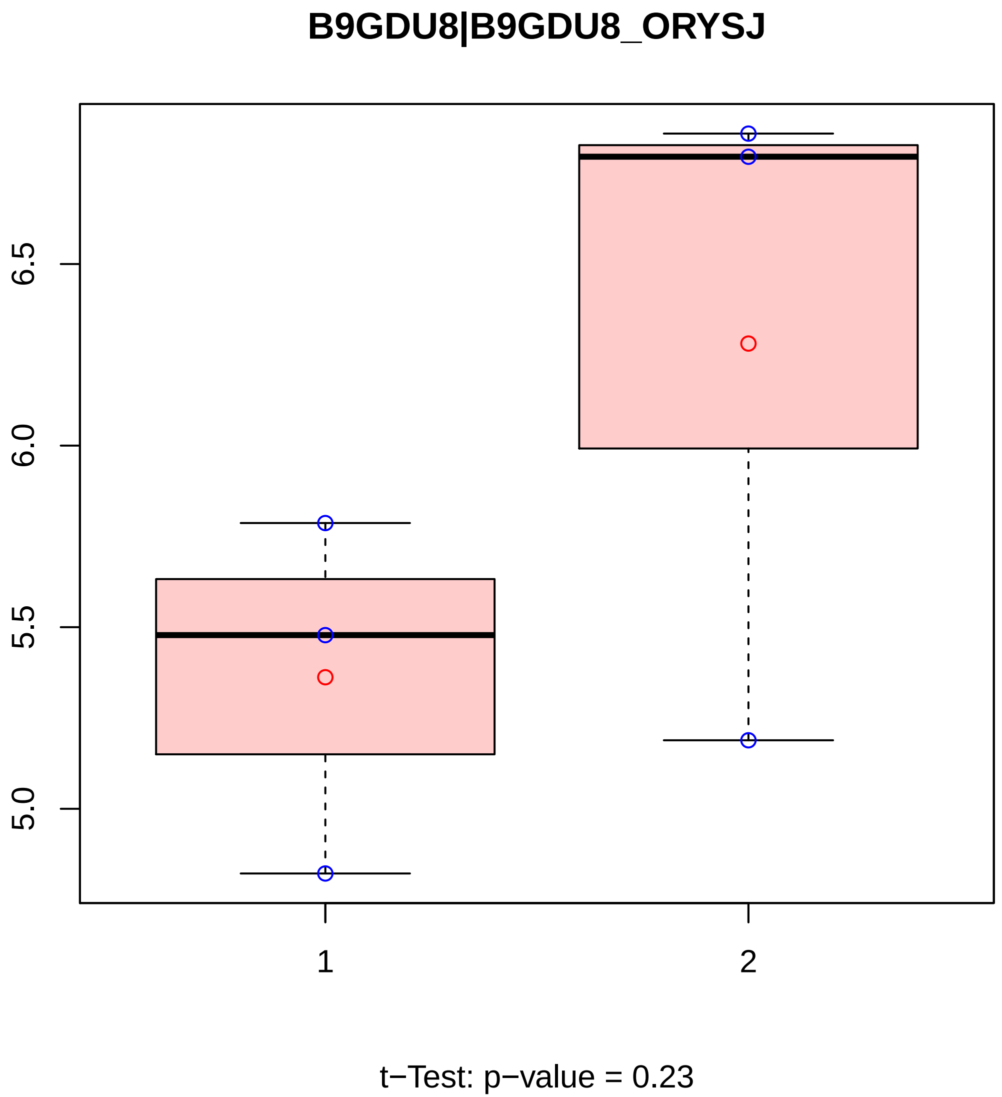
B9GDU8|B9GDU8_ORYSJ
5.0
5.5
6.0
6.5
1
2
t−Test: p−value = 0.23

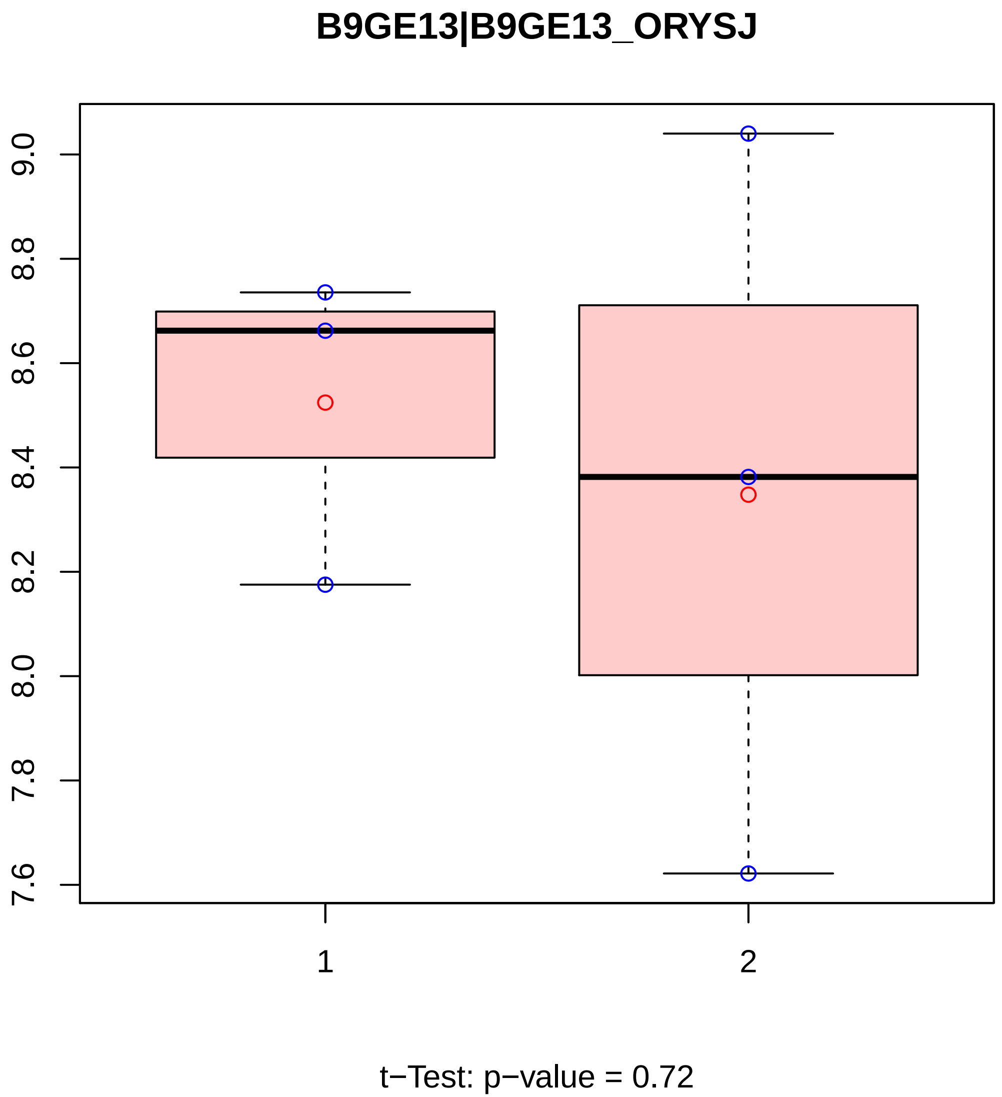
B9GE13|B9GE13_ORYSJ
9.0
8.8
8.6
8.4
8.2
8.0
7.8
7.6
1
2
t−Test: p−value = 0.72

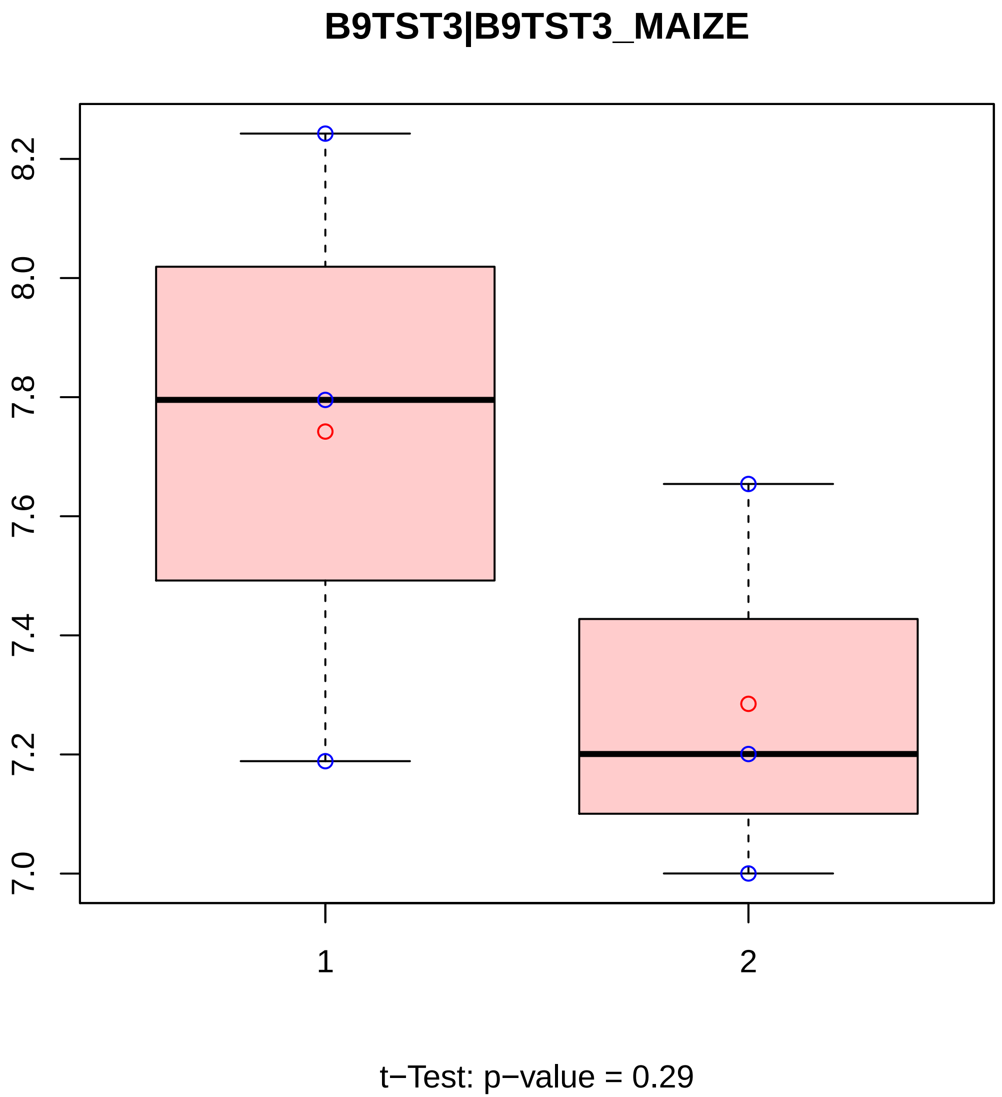
B9TST3|B9TST3_MAIZE
8.2
8.0
7.8
7.6
7.4
7.2
7.0
1
2
t−Test: p−value = 0.29

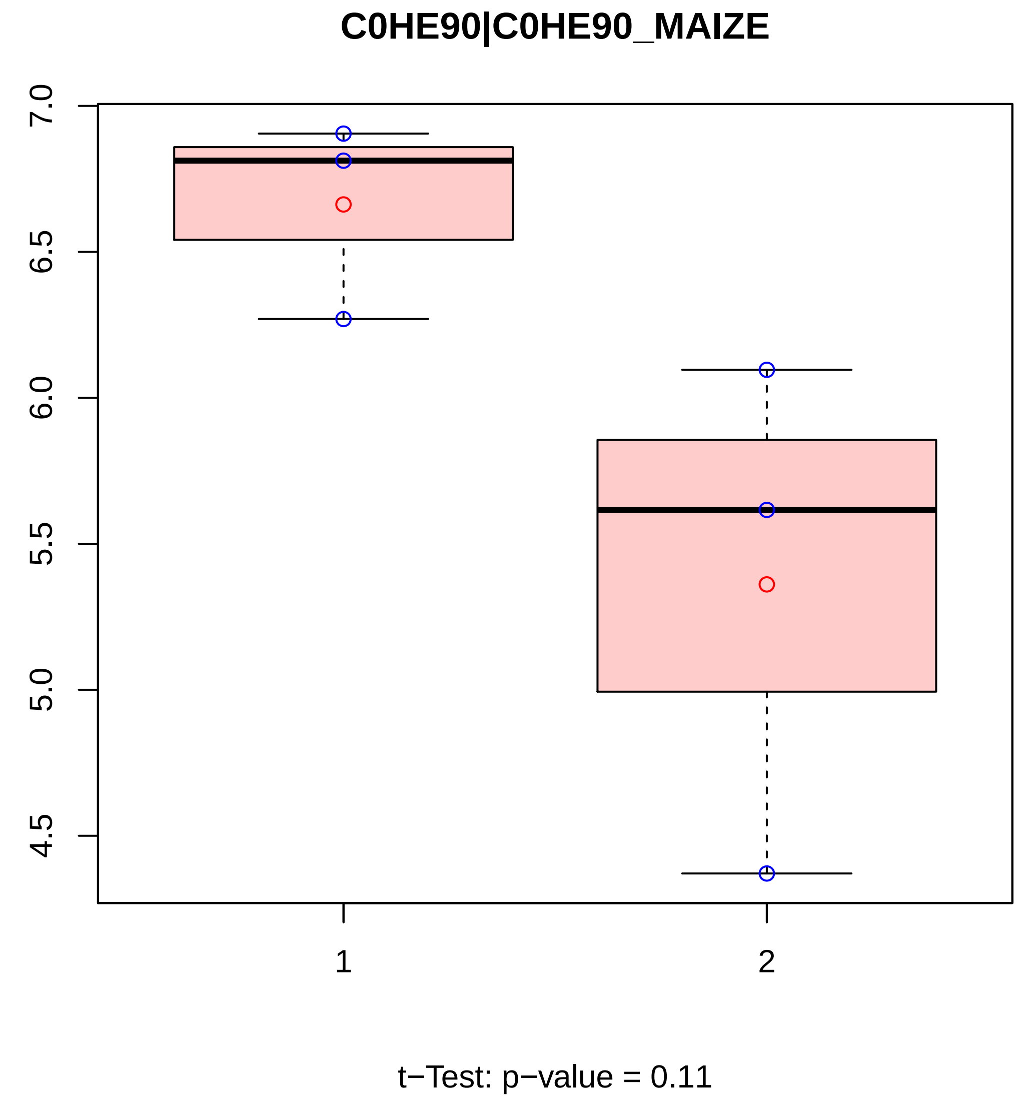
C0HE90|C0HE90_MAIZE
7.0
6.5
6.0
5.5
5.0
4.5
1
2
t−Test: p−value = 0.11

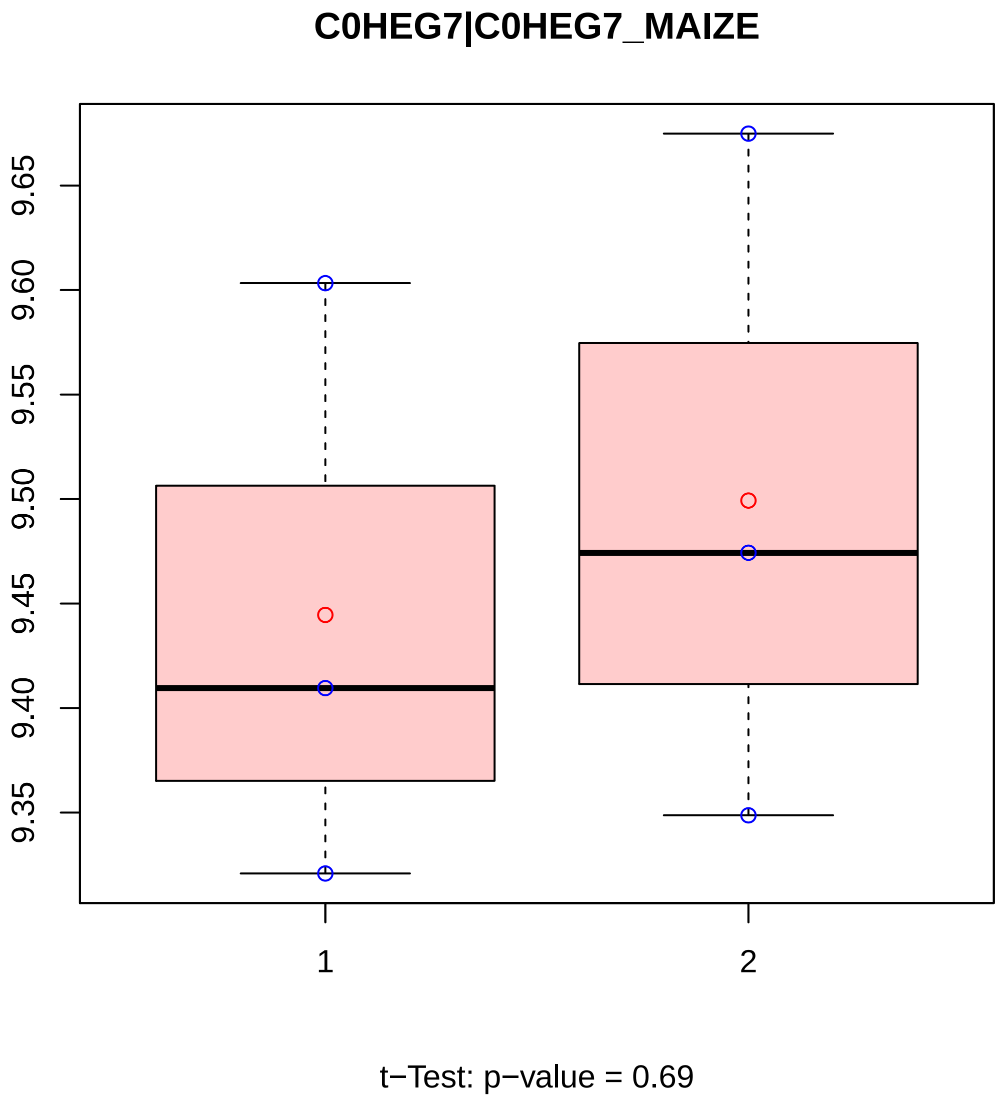
C0HEG7|C0HEG7_MAIZE
9.35
9.40
9.45
9.50
9.55
9.60
9.65
1
2
t−Test: p−value = 0.69

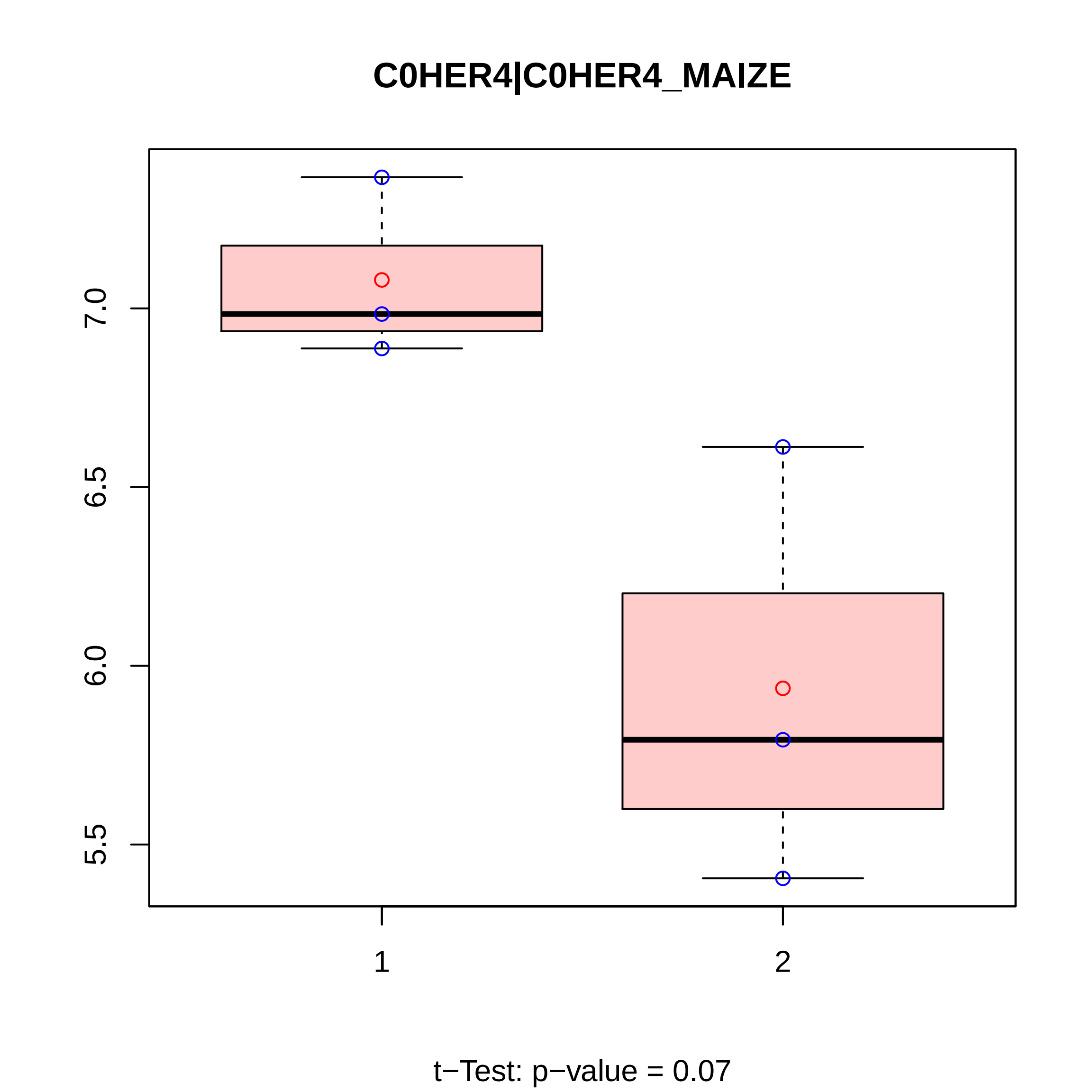

C0HER4|C0HER4_MAIZE
7.0
6.5
6.0
5.5
1
2
t−Test: p−value = 0.07

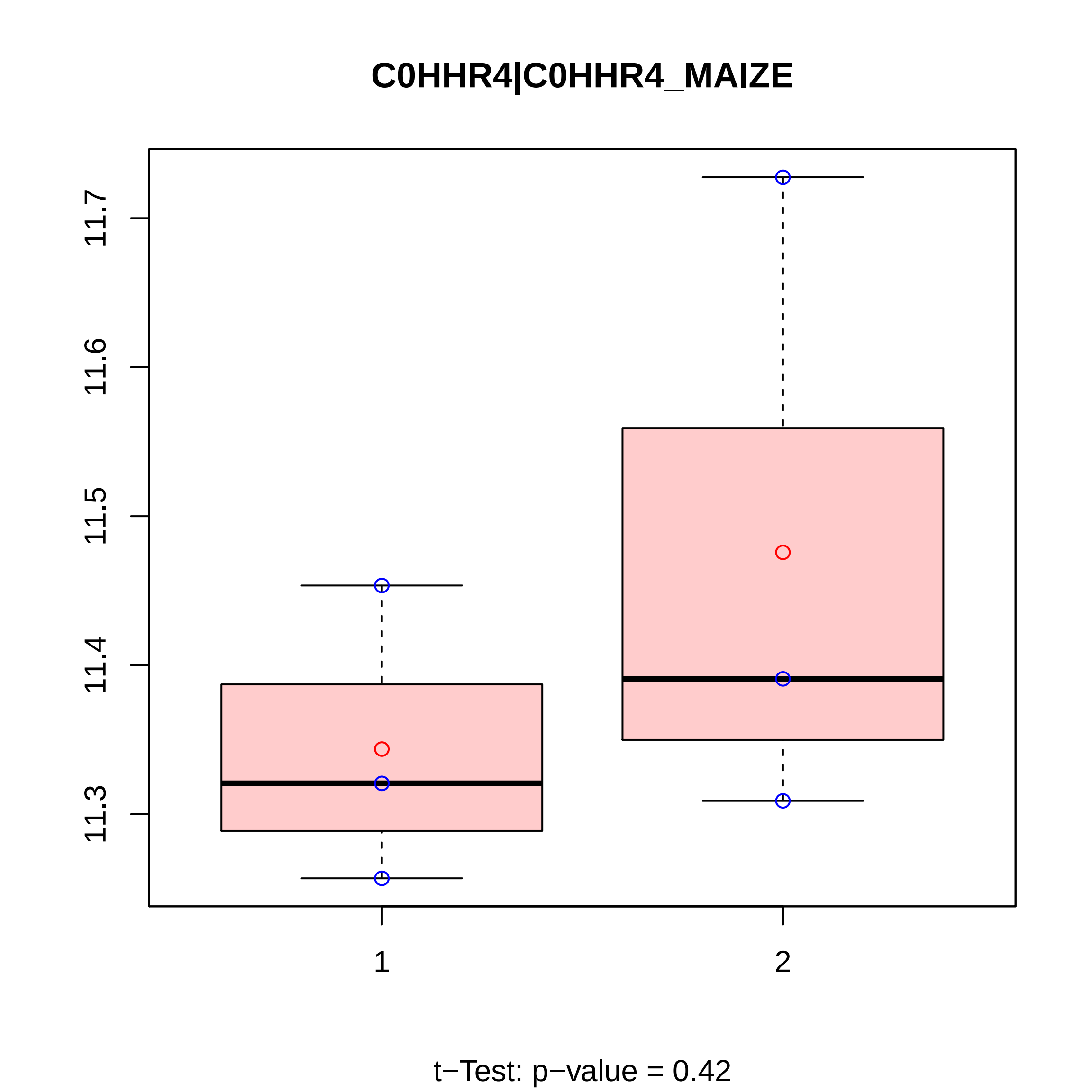

C0HHR4|C0HHR4_MAIZE
11.7
11.6
11.5
11.4
11.3
1
2
t−Test: p−value = 0.42

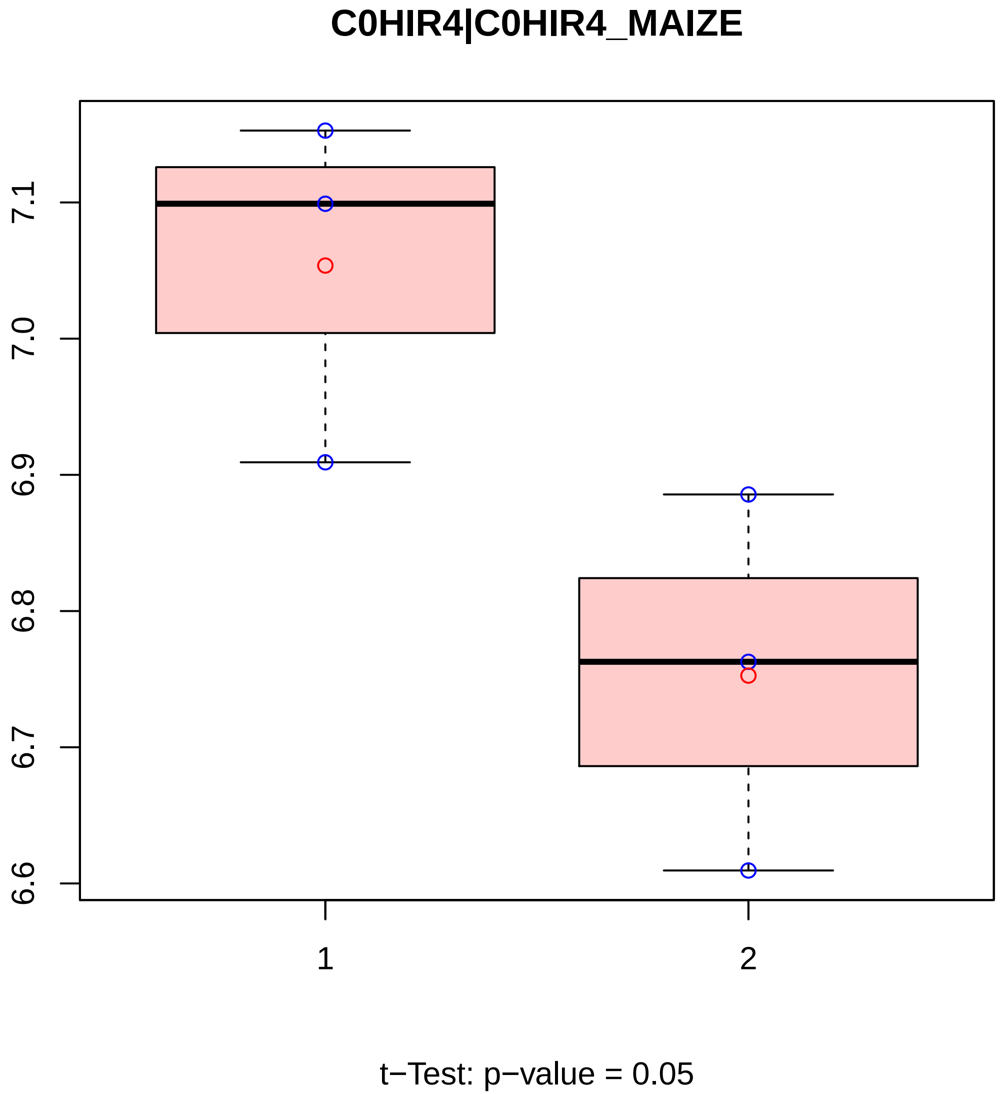
C0HIR4|C0HIR4_MAIZE
7.1
7.0
6.9
6.8
6.7
6.6
1
2
t−Test: p−value = 0.05

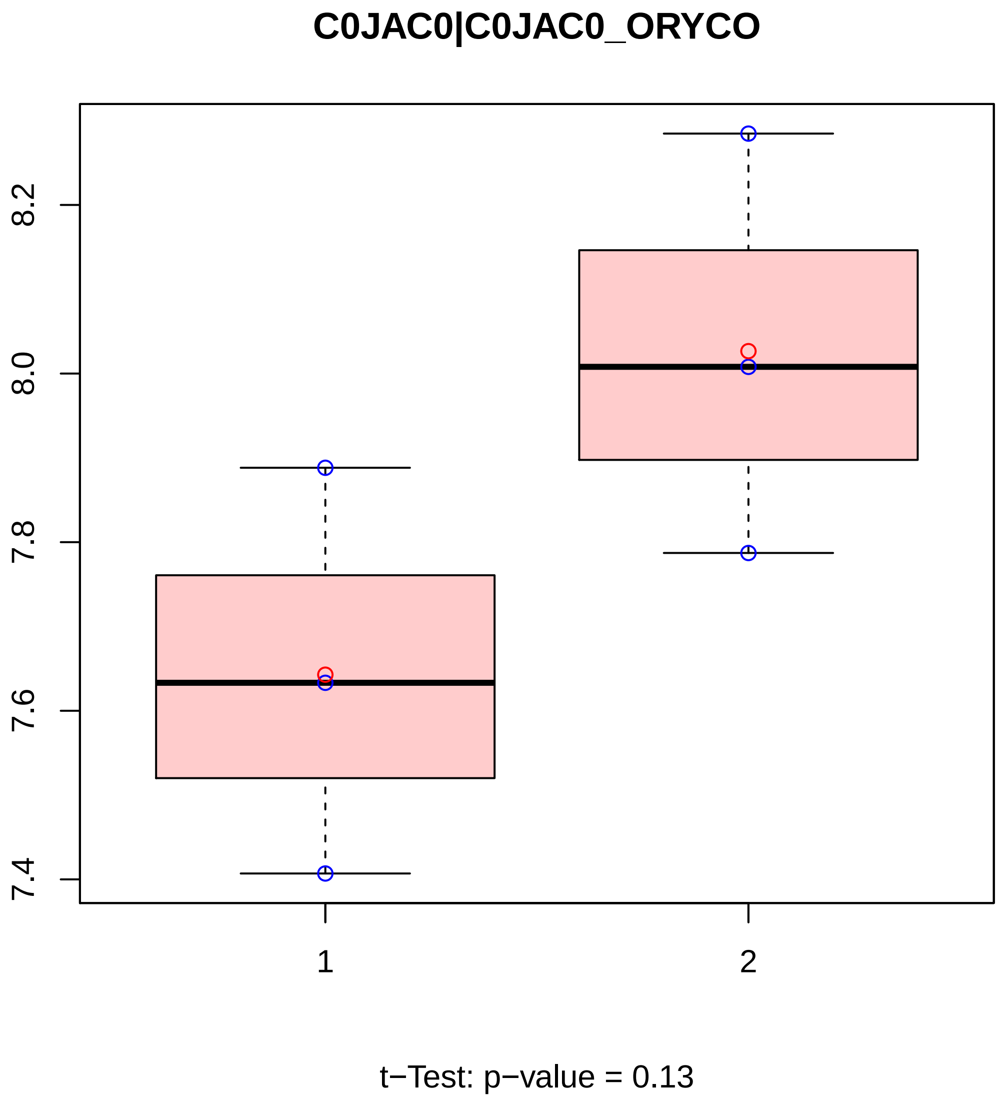
C0JAC0|C0JAC0_ORYCO
8.2
8.0
7.8
7.6
7.4
1
2
t−Test: p−value = 0.13

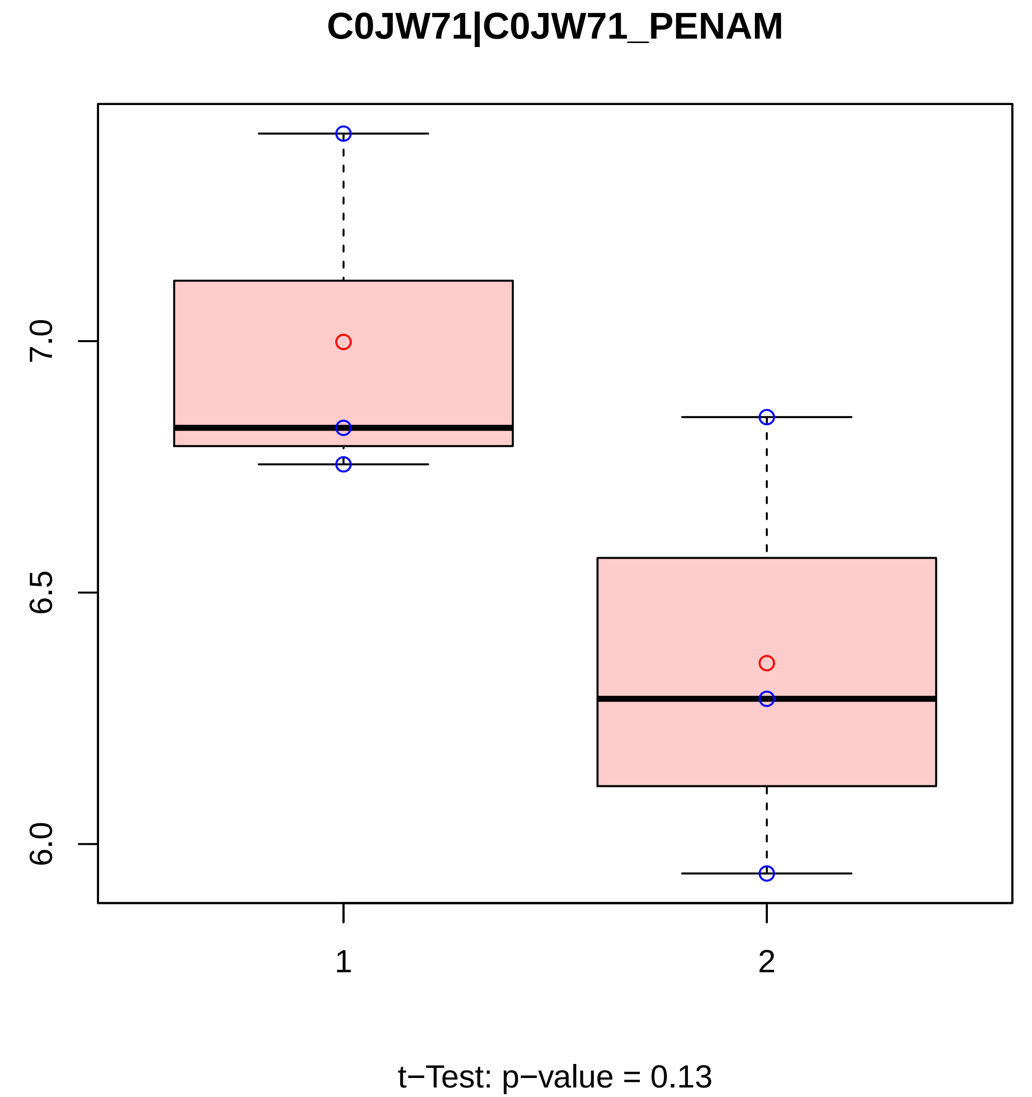

C0JW71|C0JW71_PENAM
7.0
6.5
6.0
1
2
t−Test: p−value = 0.13

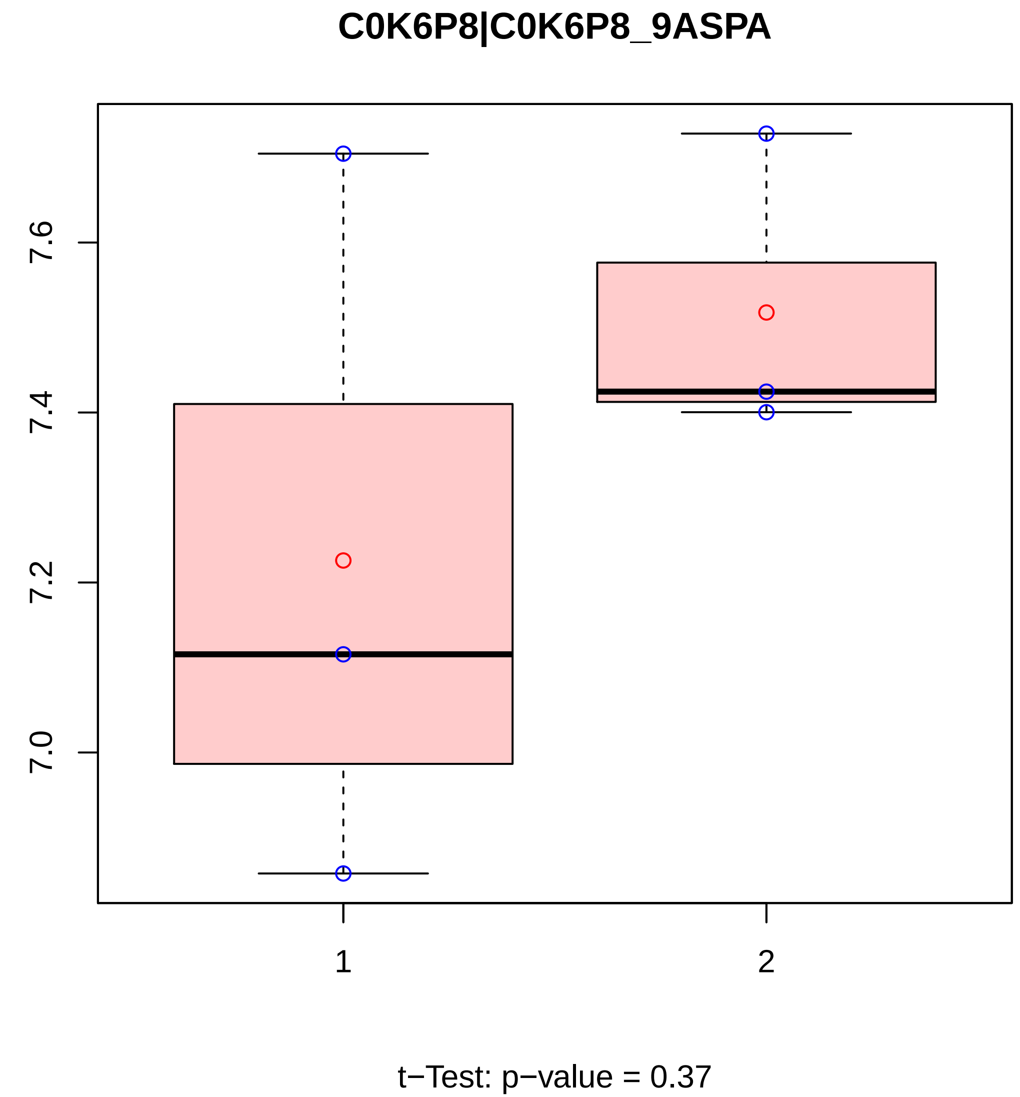

C0K6P8|C0K6P8_9ASPA
7.6
7.4
7.2
7.0
1
2
t−Test: p−value = 0.37

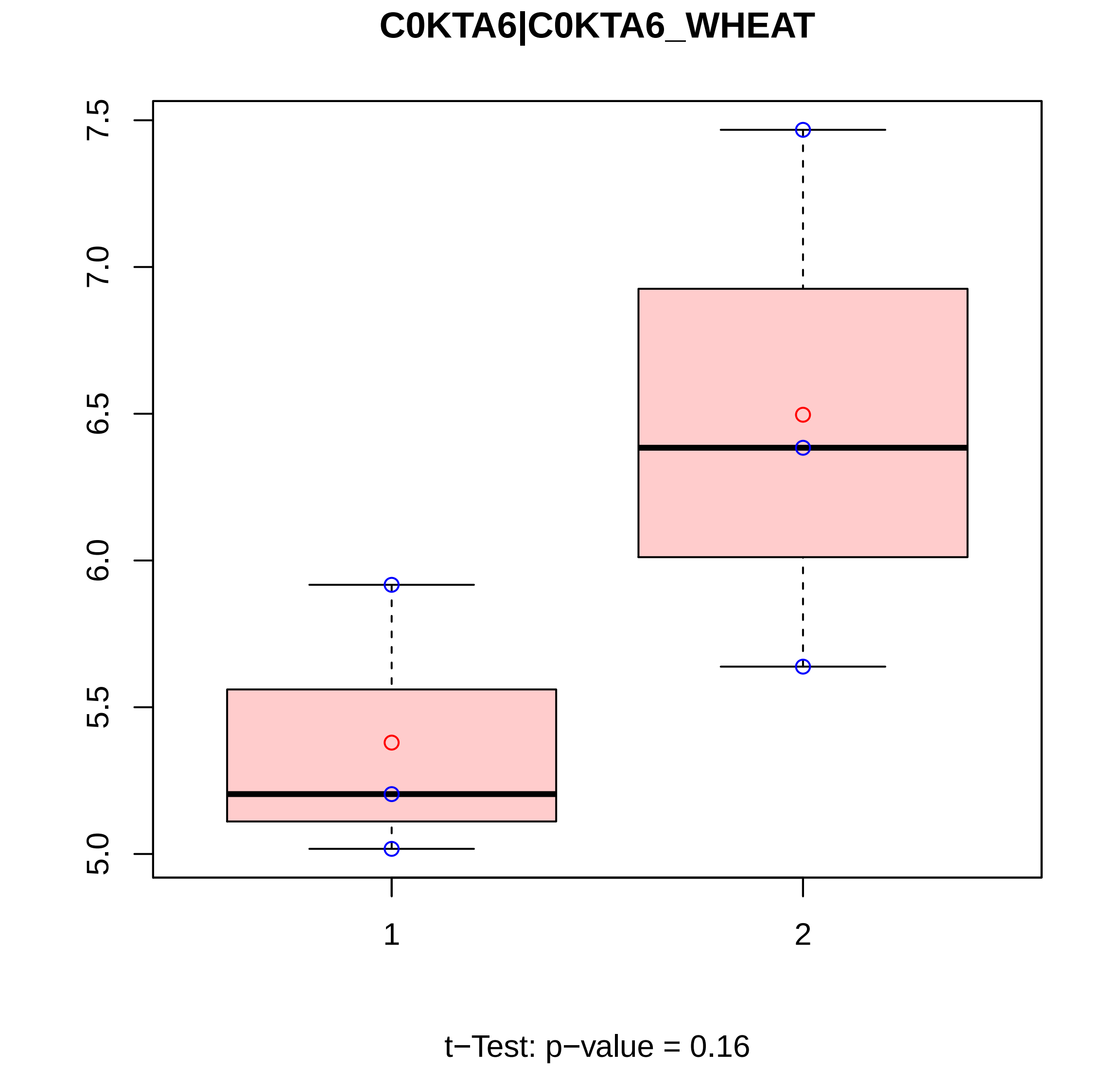
C0KTA6|C0KTA6_WHEAT
7.5
7.0
6.5
6.0
5.5
5.0
1
2
t−Test: p−value = 0.16

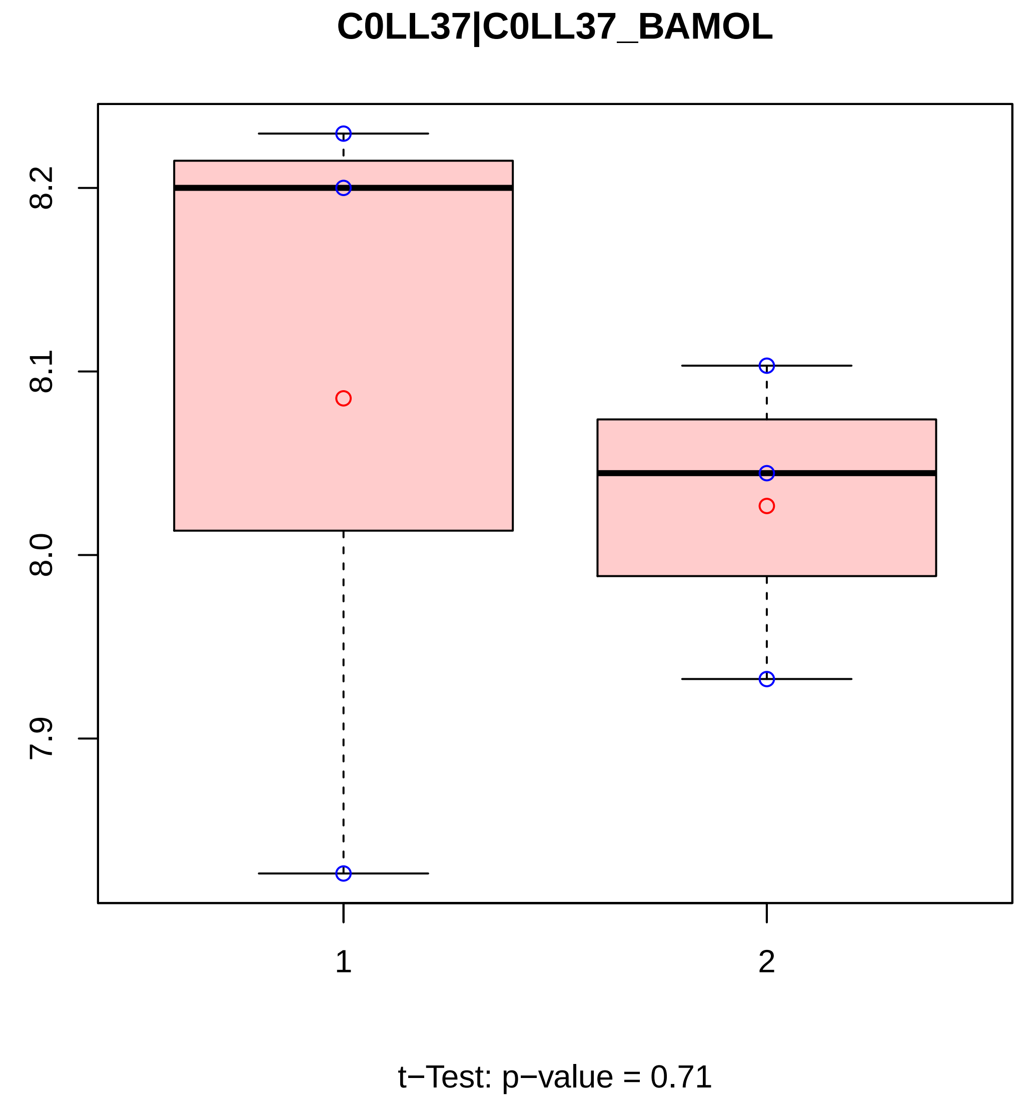
C0LL37|C0LL37_BAMOL
8.2
8.1
8.0
7.9
1
2
t−Test: p−value = 0.71

C0P2Y3|C0P2Y3_MAIZE

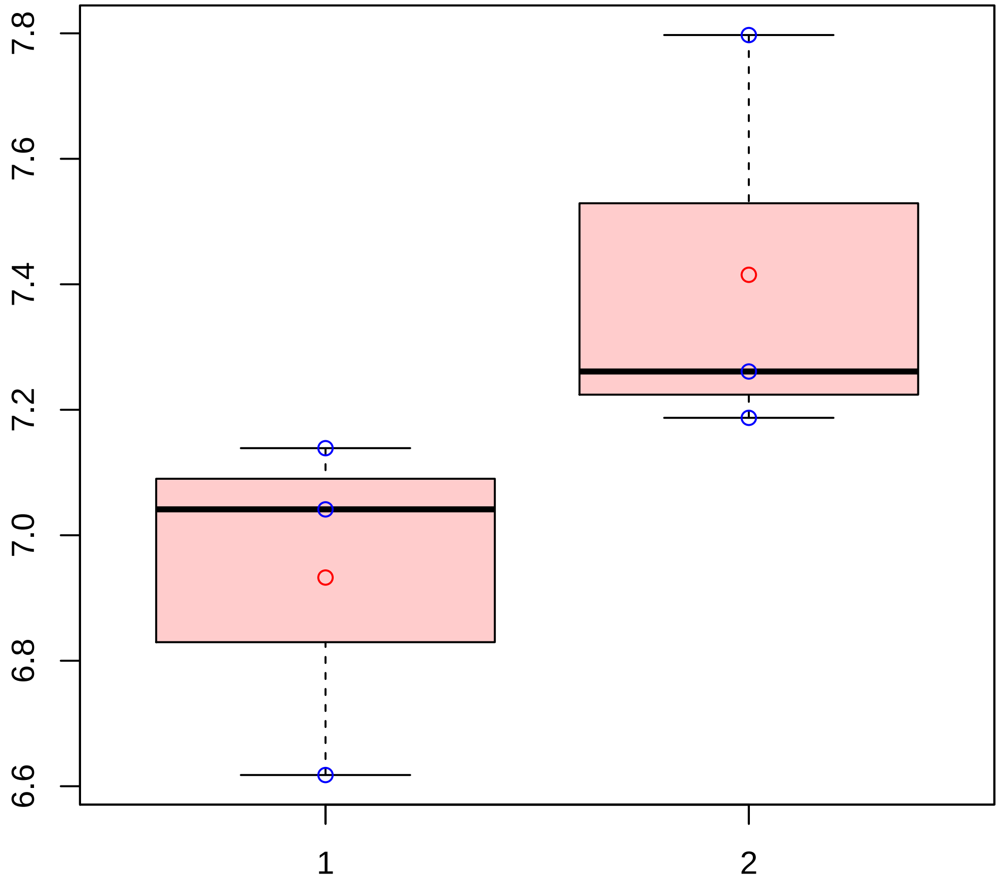

t−Test: p−value = 0.13

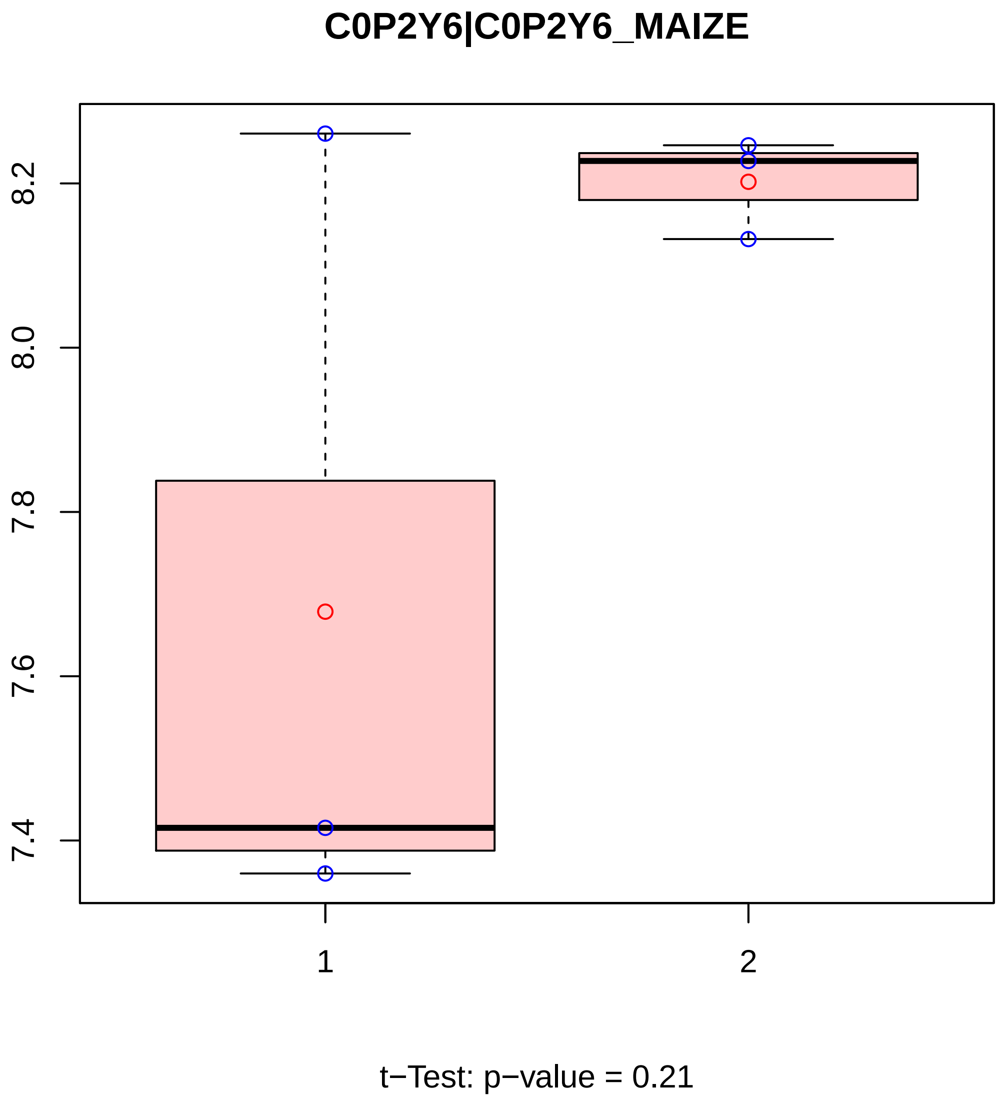
C0P2Y6|C0P2Y6_MAIZE
8.2
8.0
7.8
7.6
7.4
1
2
t−Test: p−value = 0.21

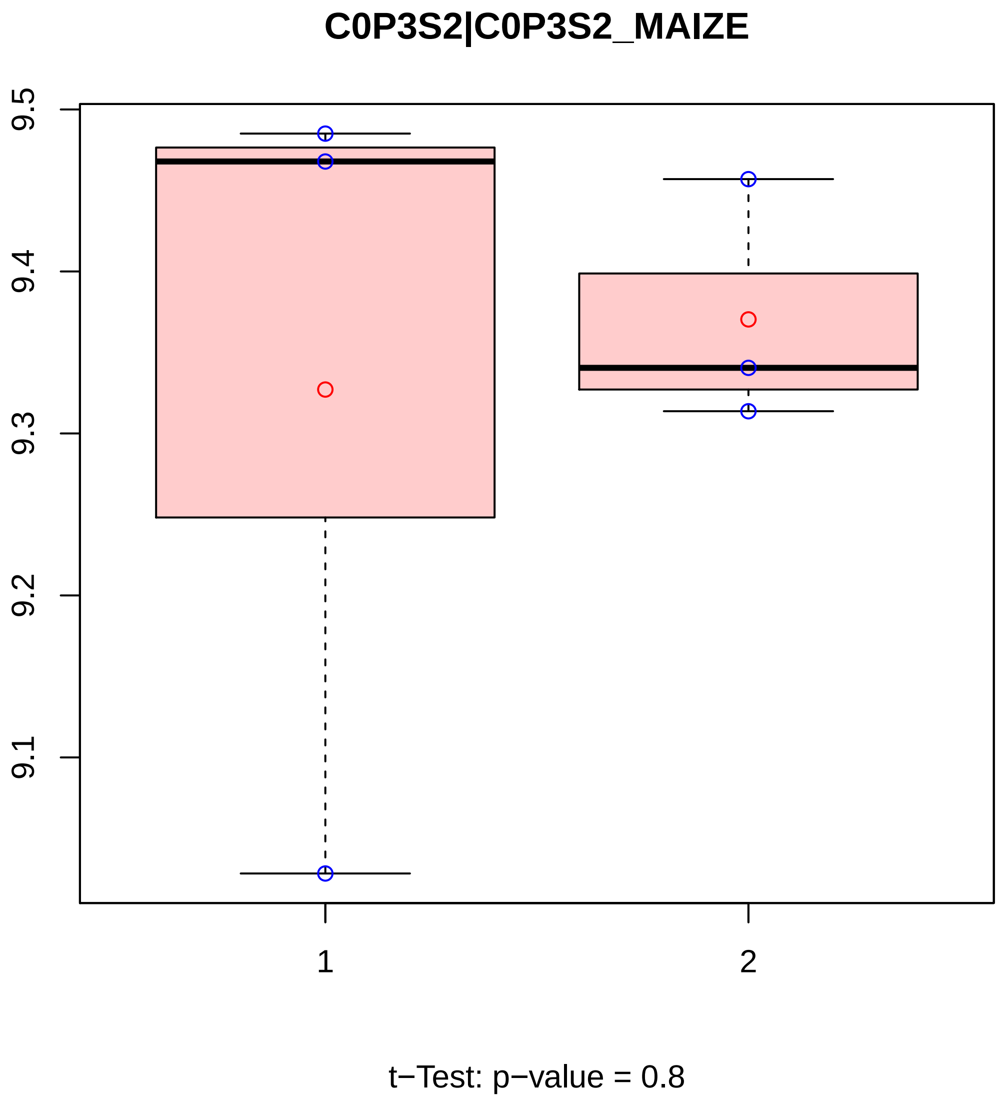

C0P3S2|C0P3S2_MAIZE
9.5
9.4
9.3
9.2
9.1
1
2
t−Test: p−value = 0.8

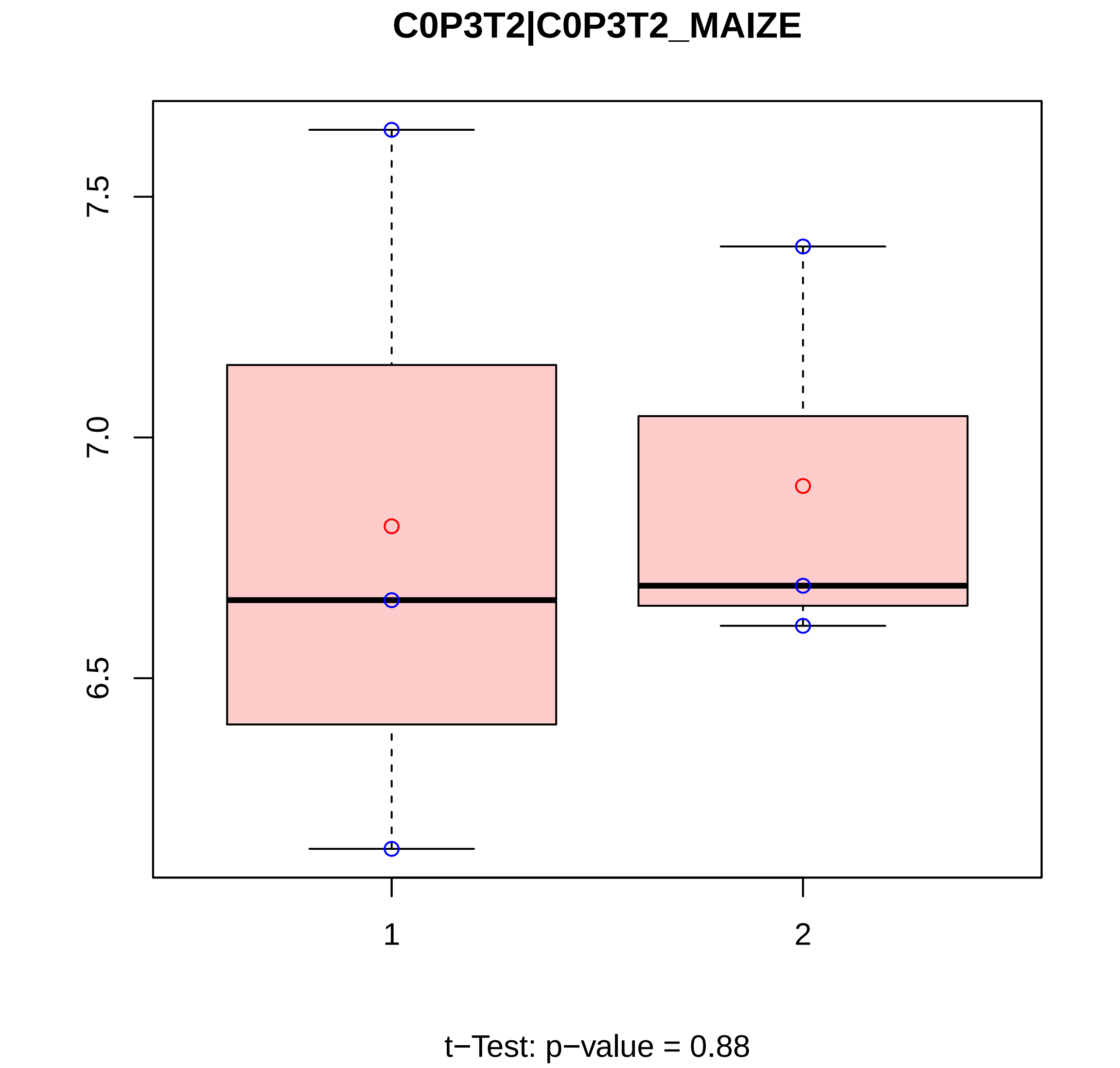

C0P3T2|C0P3T2_MAIZE
7.5
7.0
6.5
1
2
t−Test: p−value = 0.88

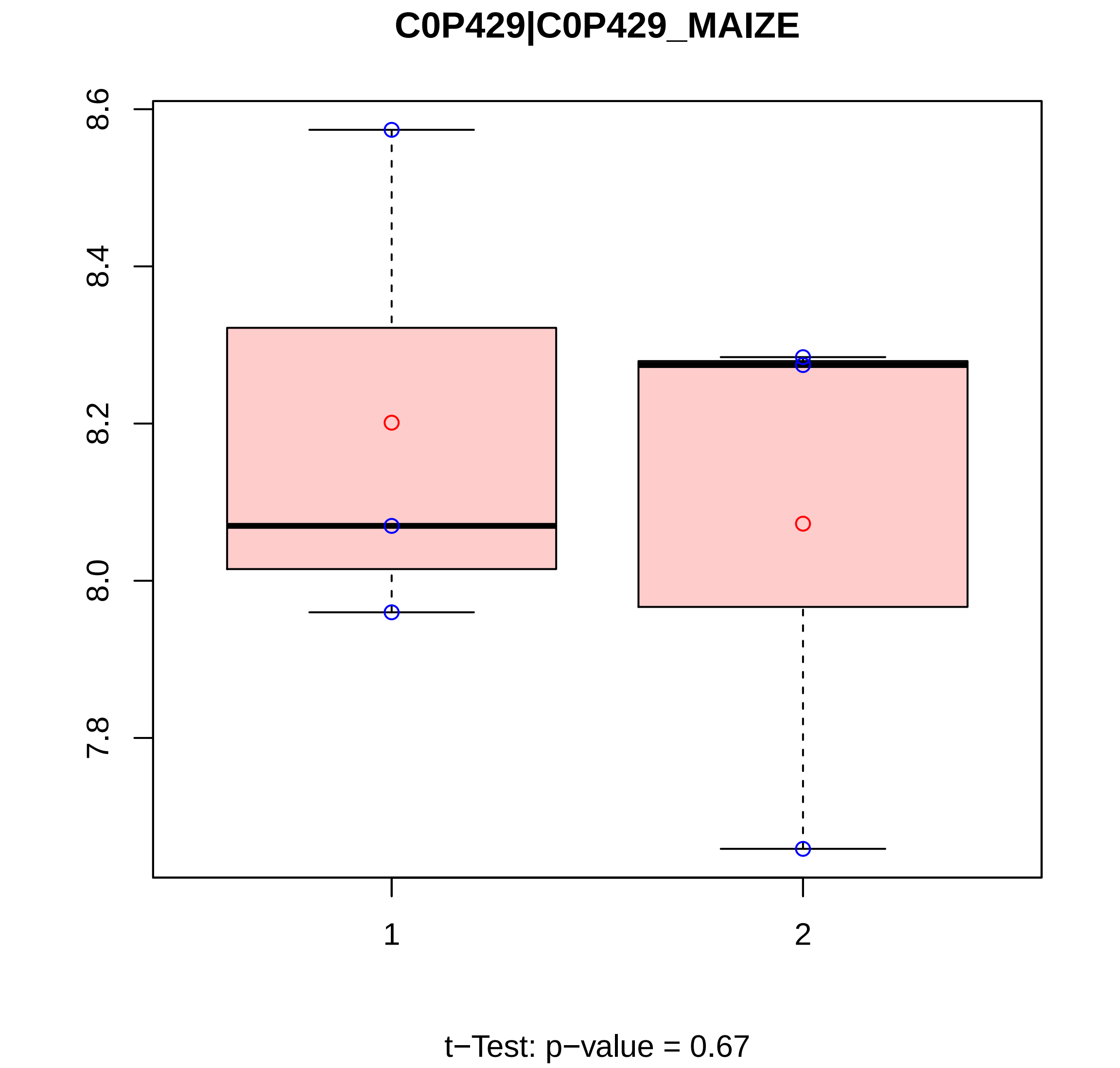

C0P429|C0P429_MAIZE
8.6
8.4
8.2
8.0
7.8
1
2
t−Test: p−value = 0.67

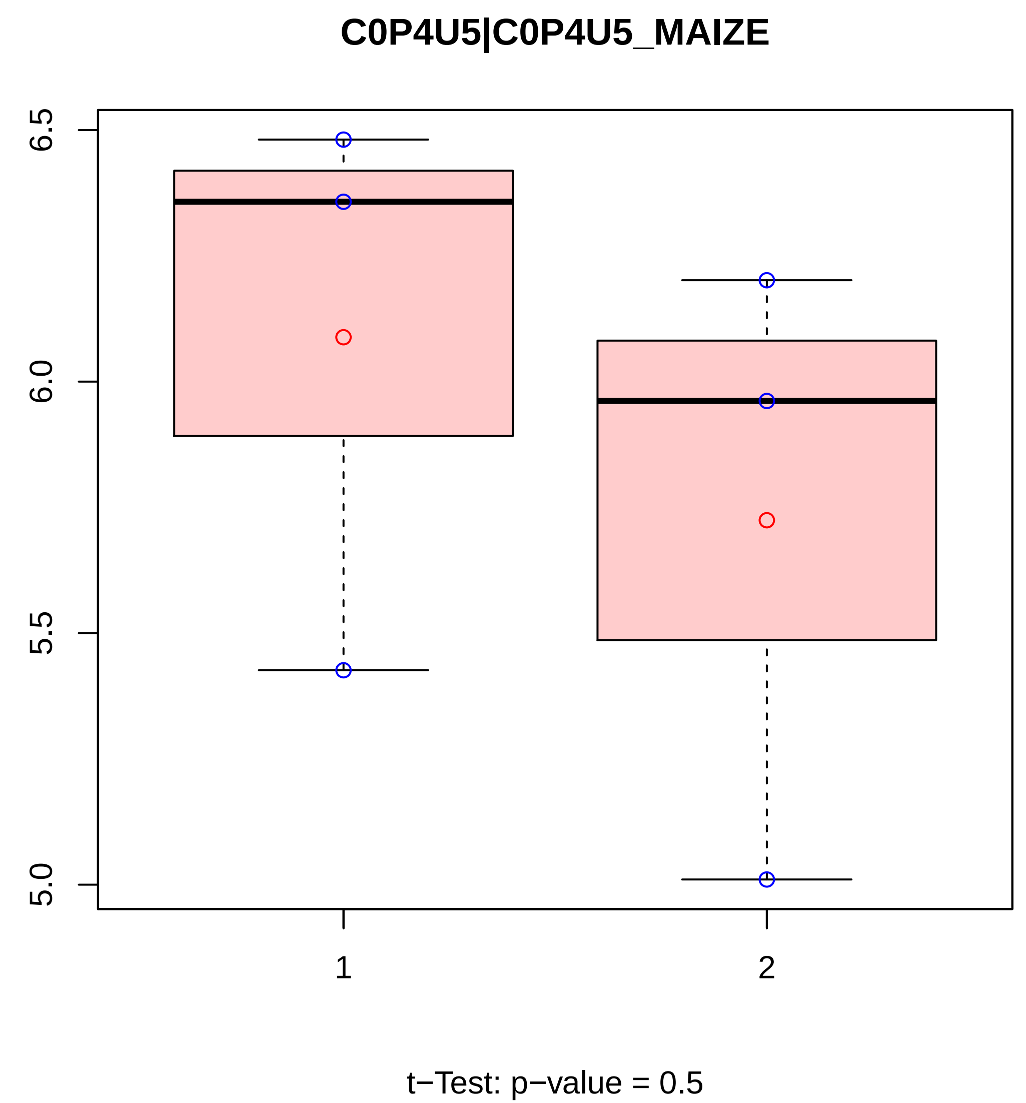
C0P4U5|C0P4U5_MAIZE
6.5
6.0
5.5
5.0
1
2
t−Test: p−value = 0.5

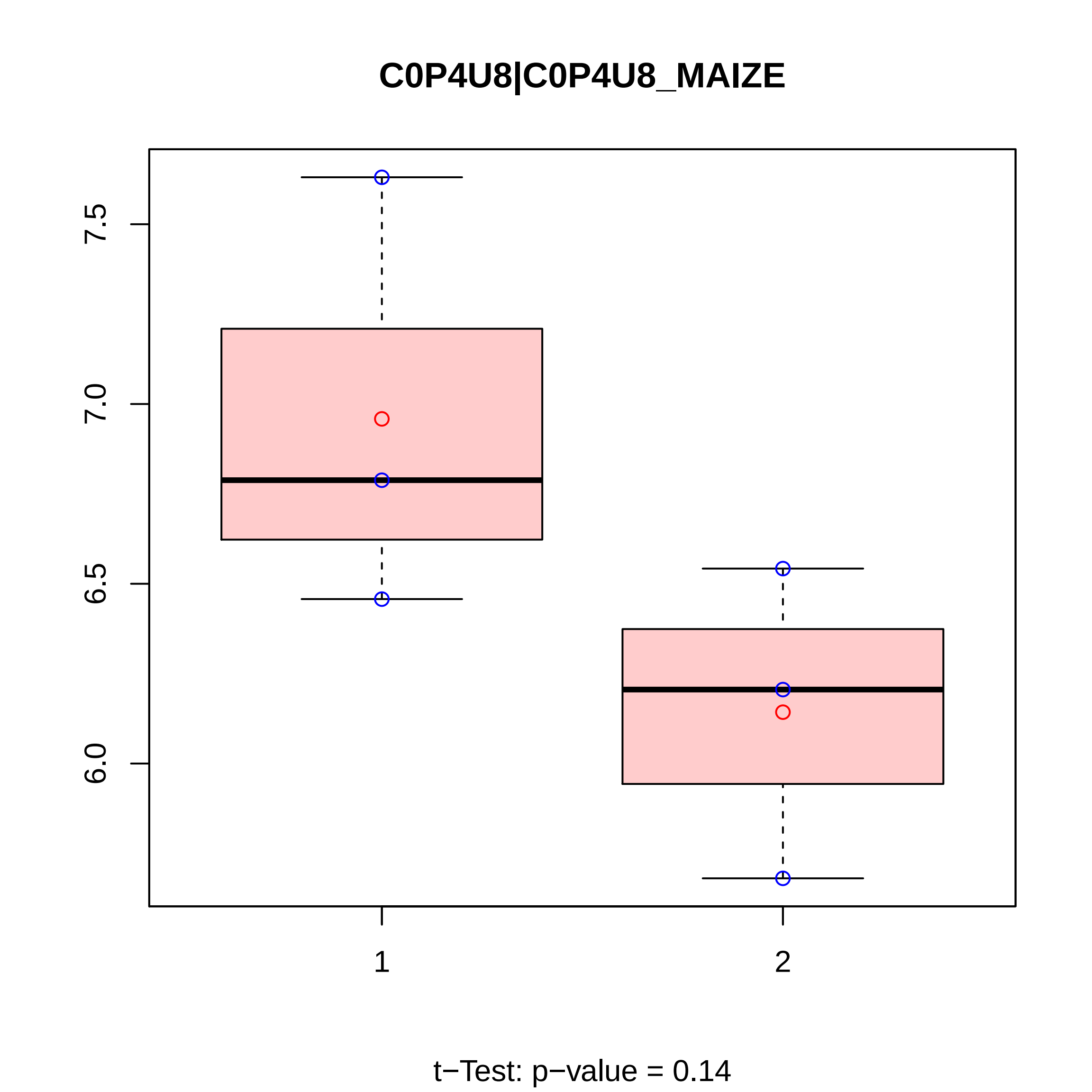
C0P4U8|C0P4U8_MAIZE
7.5
7.0
6.5
6.0
1
2
t−Test: p−value = 0.14

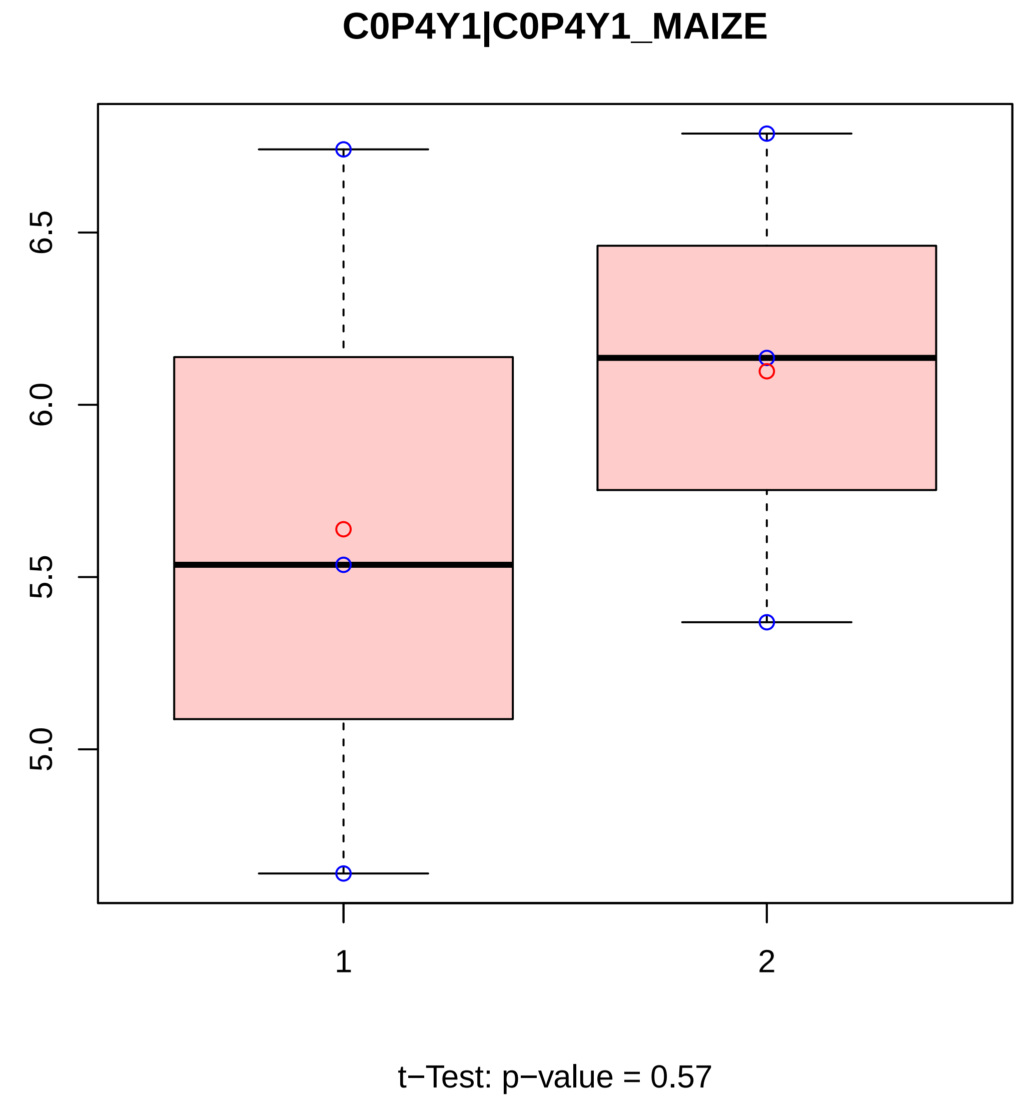
C0P4Y1|C0P4Y1_MAIZE
6.5
6.0
5.5
5.0
1
2
t−Test: p−value = 0.57

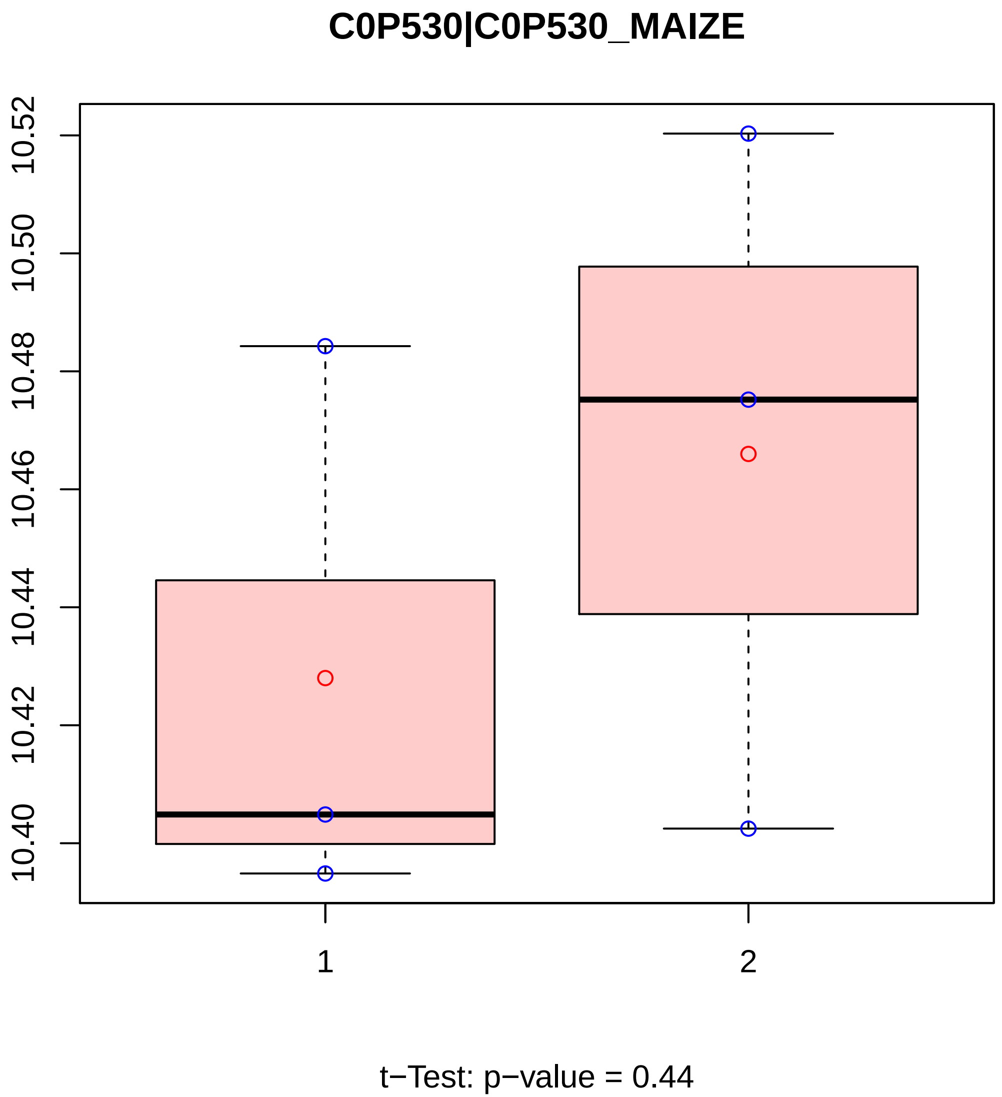
C0P530|C0P530_MAIZE
10.40
10.42
10.44
10.46
10.48
10.50
10.52
1
2
t−Test: p−value = 0.44

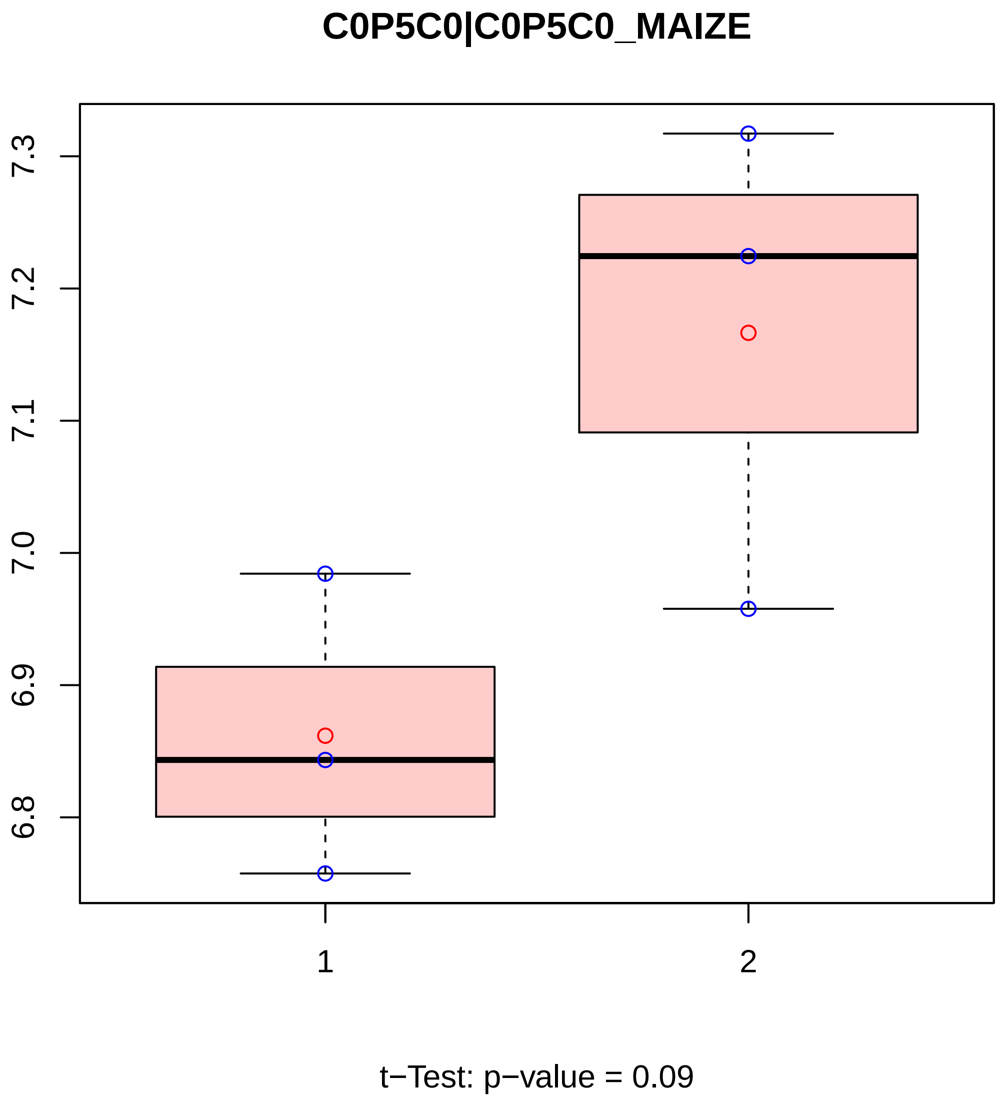

C0P5C0|C0P5C0_MAIZE
7.3
7.2
7.1
7.0
6.9
6.8
1
2
t−Test: p−value = 0.09

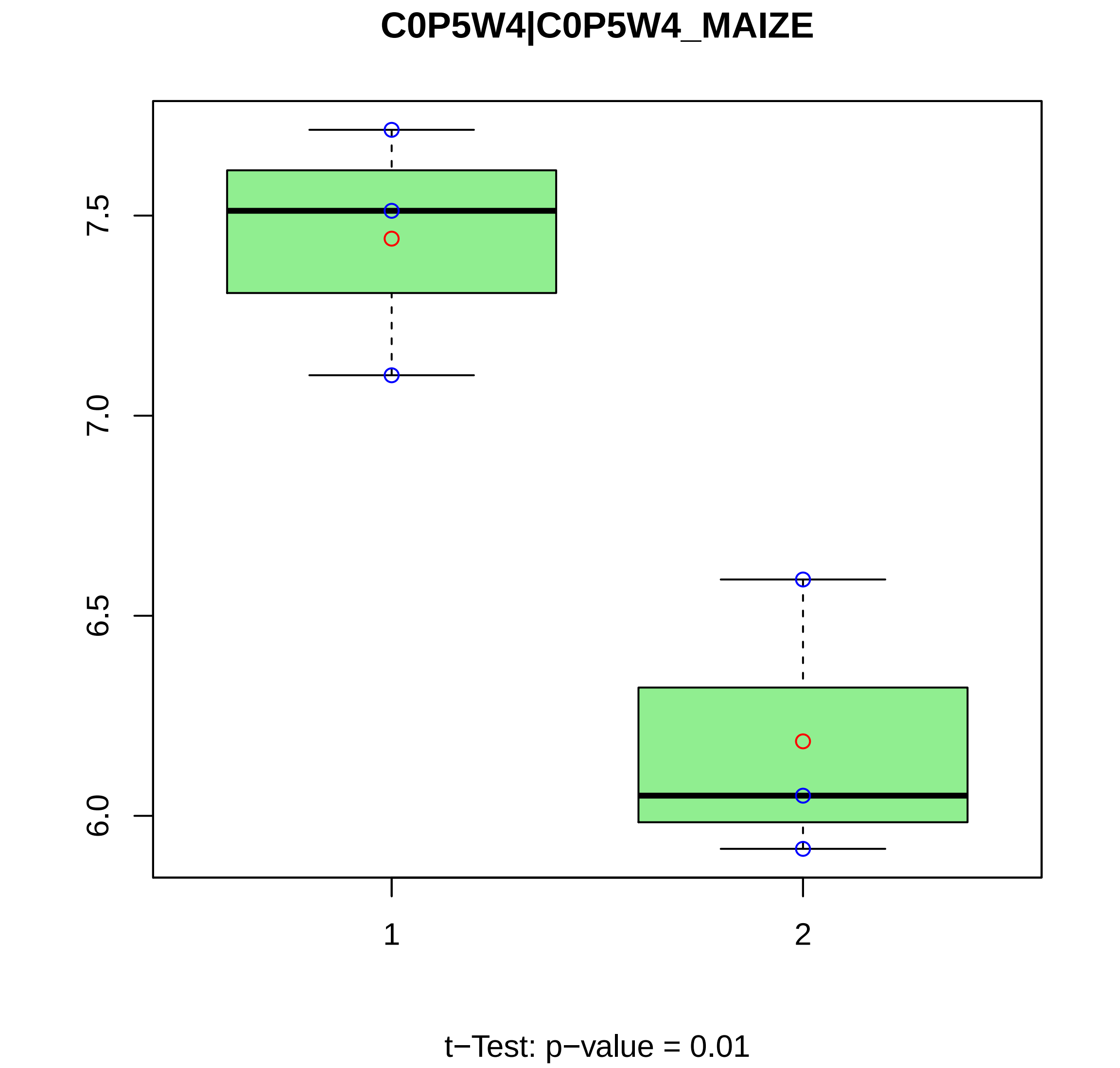
C0P5W4|C0P5W4_MAIZE
7.5
7.0
6.5
6.0
1
2
t−Test: p−value = 0.01

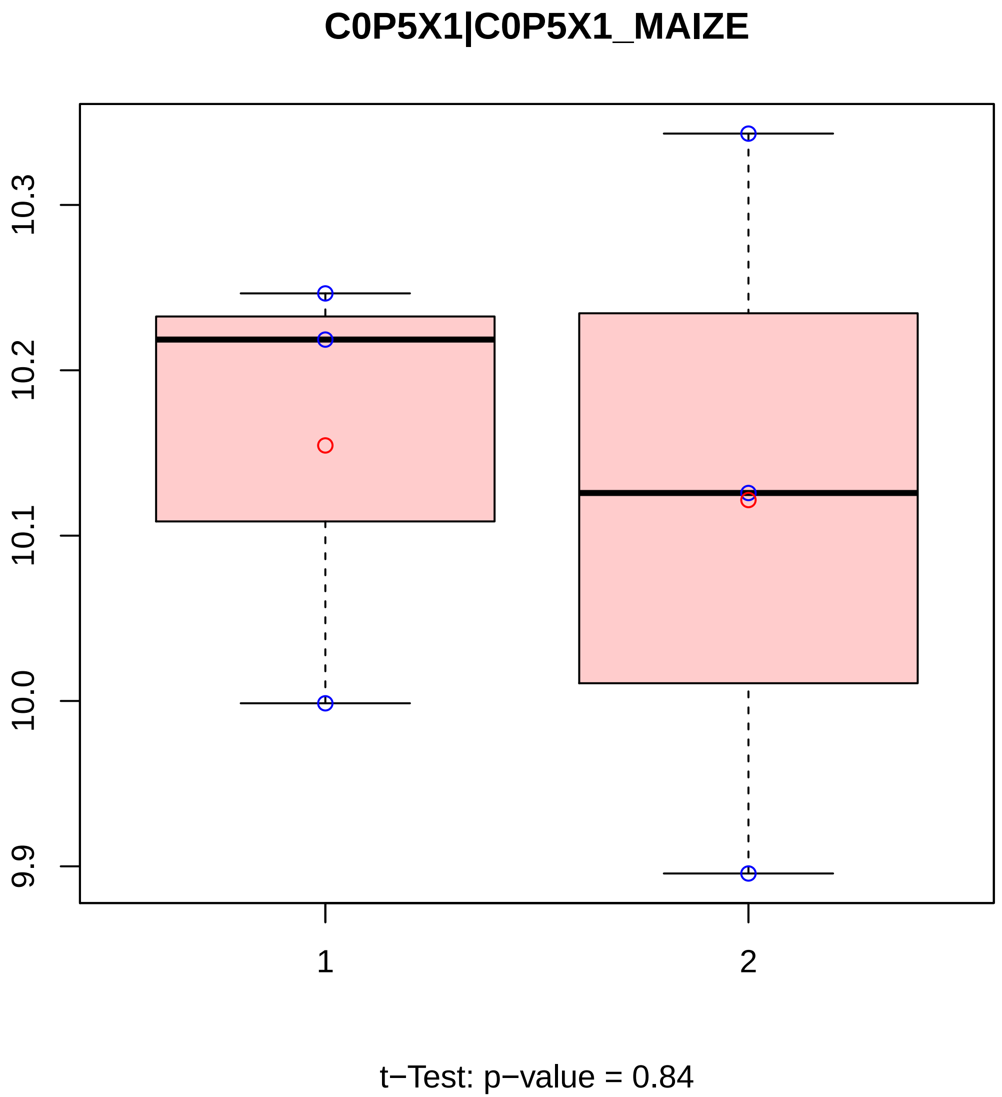
C0P5X1|C0P5X1_MAIZE
10.3
10.2
10.1
10.0
9.9
1
2
t−Test: p−value = 0.84

C0P5Y3|C0P5Y3_MAIZE

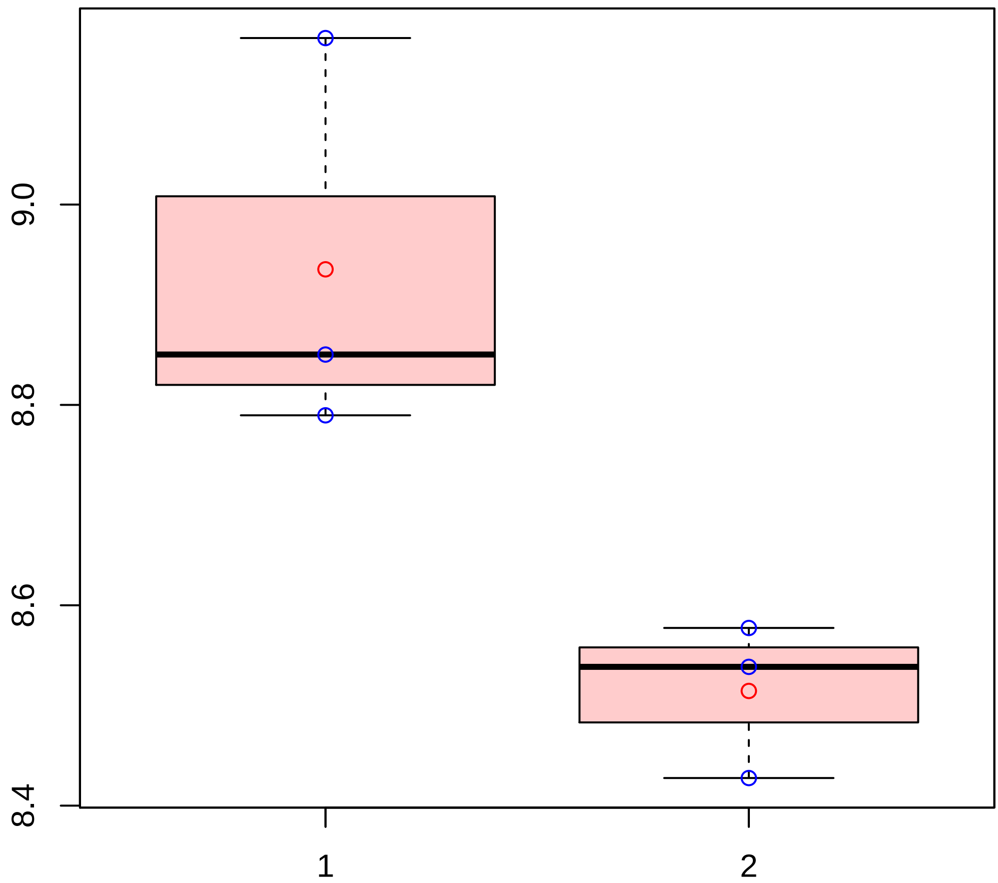

t−Test: p−value = 0.05

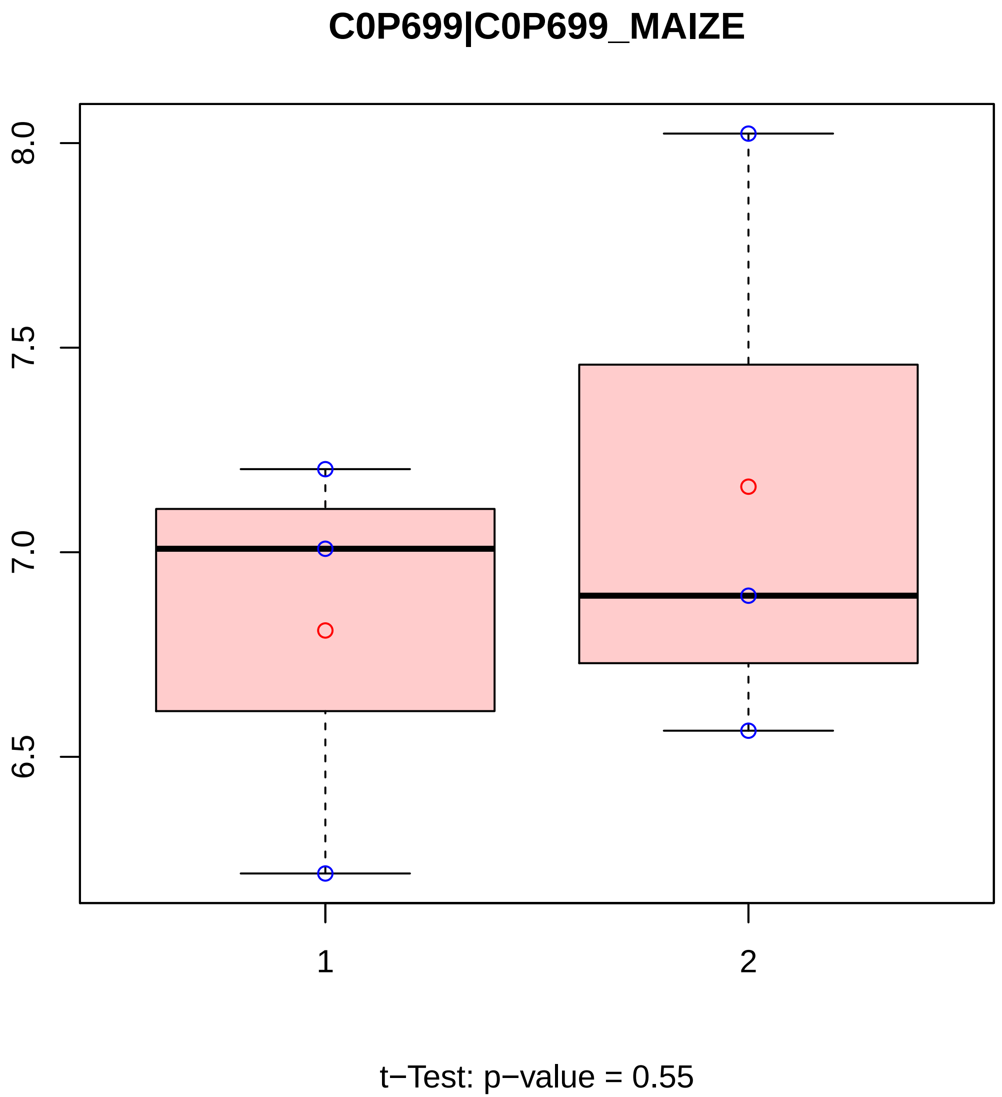
C0P699|C0P699_MAIZE
8.0
7.5
7.0
6.5
1
2
t−Test: p−value = 0.55

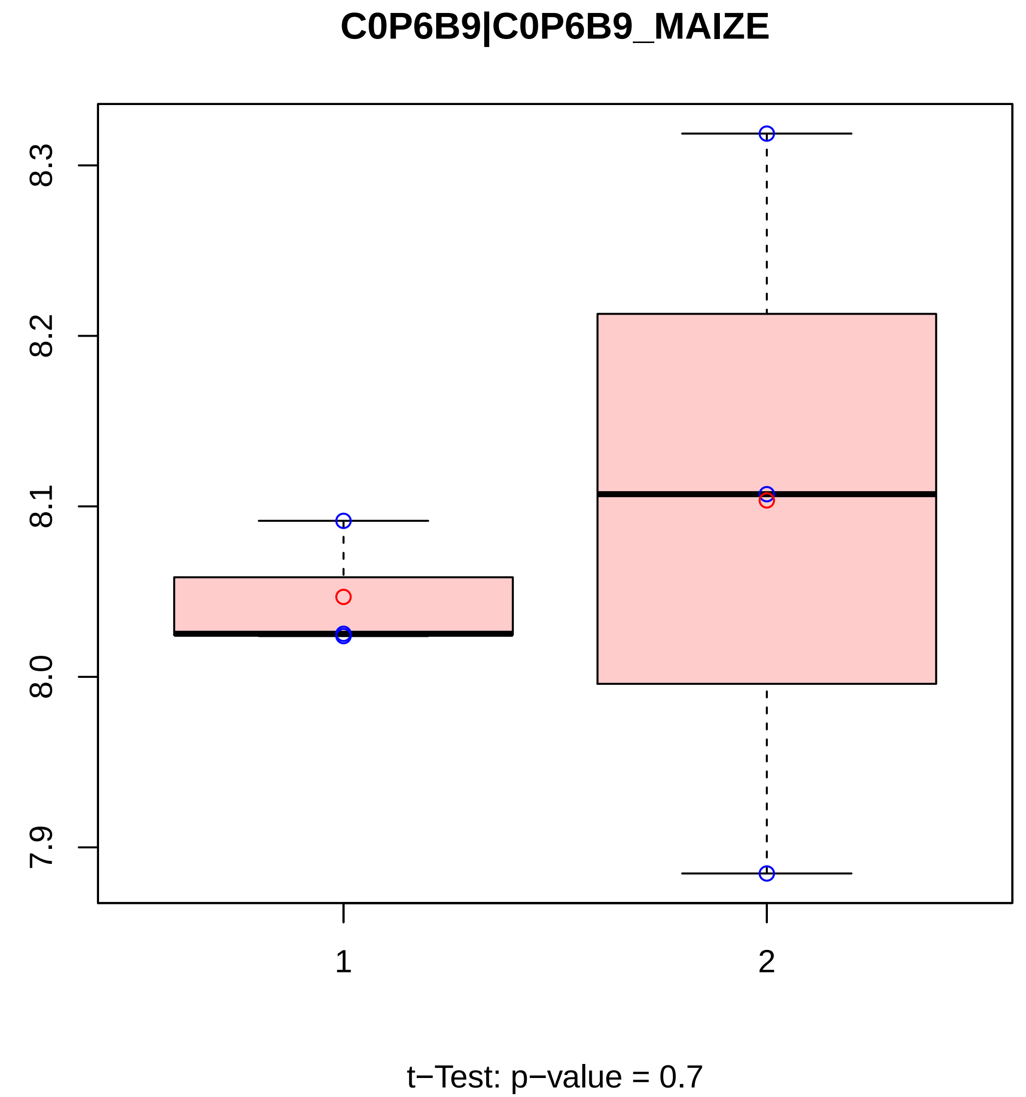

C0P6B9|C0P6B9_MAIZE
8.3
8.2
8.1
8.0
7.9
1
2
t−Test: p−value = 0.7

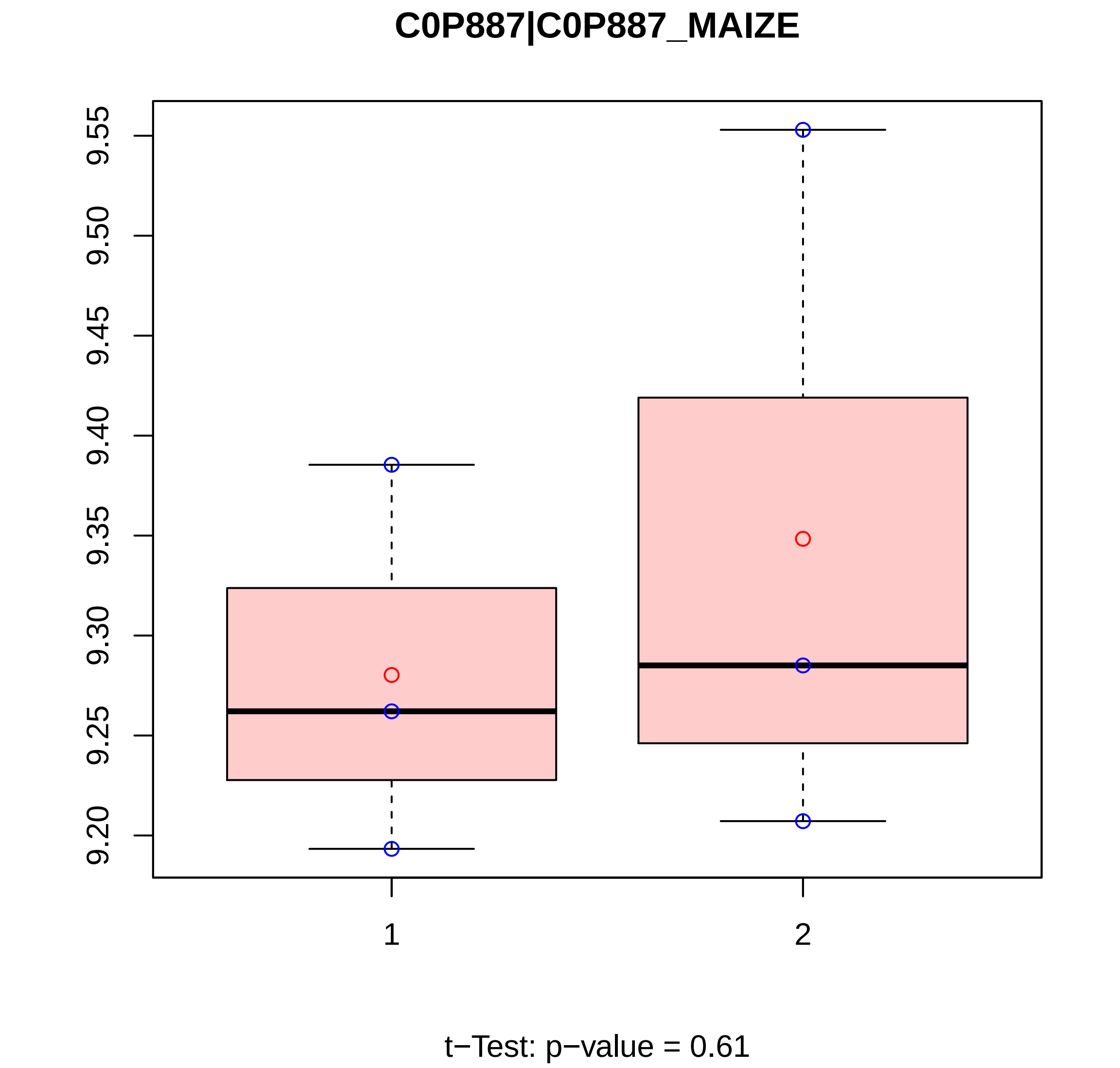
C0P887|C0P887_MAIZE
9.20
9.25
9.30
9.35
9.40
9.45
9.50
9.55
1
2
t−Test: p−value = 0.61

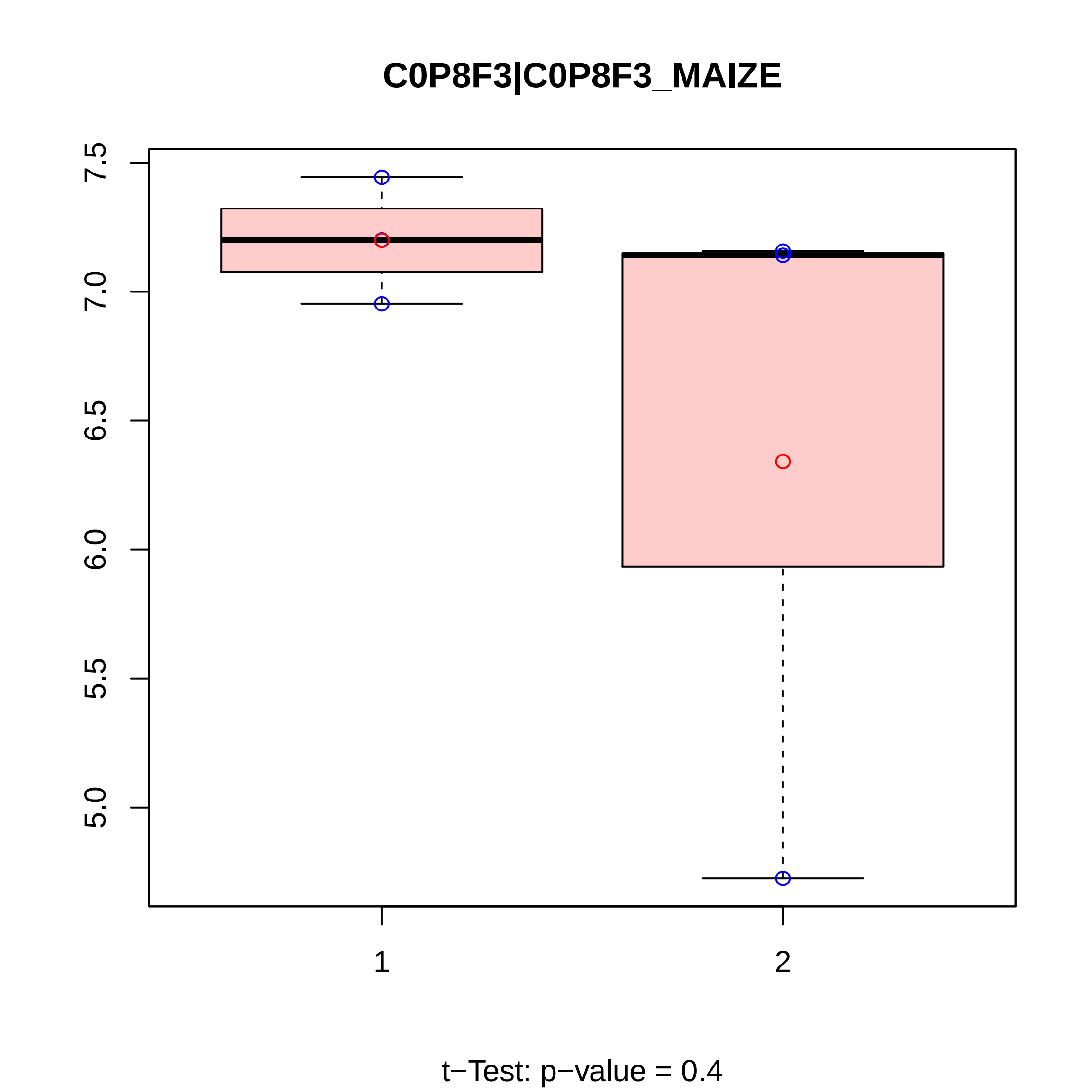

C0P8F3|C0P8F3_MAIZE
7.5
7.0
6.5
6.0
5.5
5.0
1
2
t−Test: p−value = 0.4

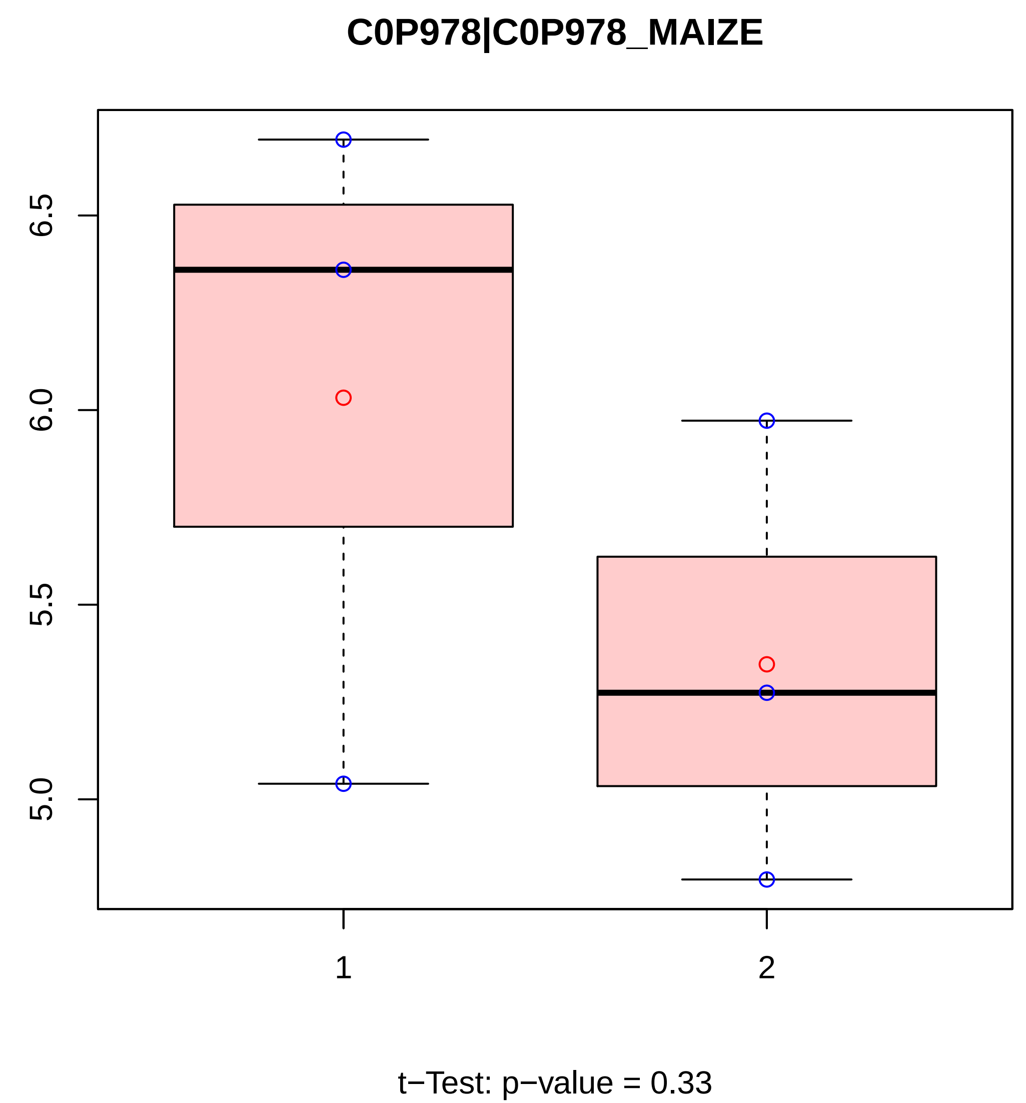
C0P978|C0P978_MAIZE
6.5
6.0
5.5
5.0
1
2
t−Test: p−value = 0.33

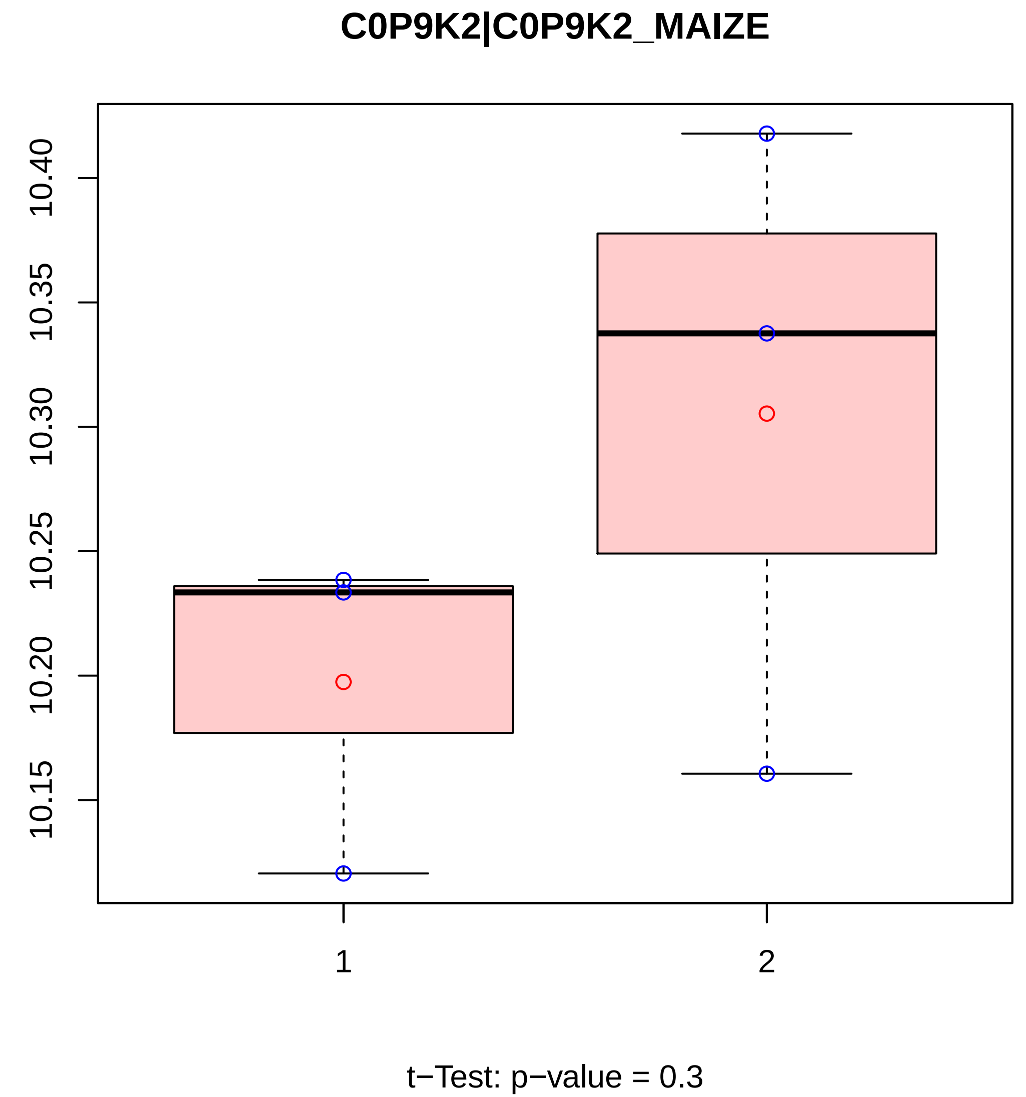
C0P9K2|C0P9K2_MAIZE
10.15
10.20
10.25
10.30
10.35
10.40
1
2
t−Test: p−value = 0.3

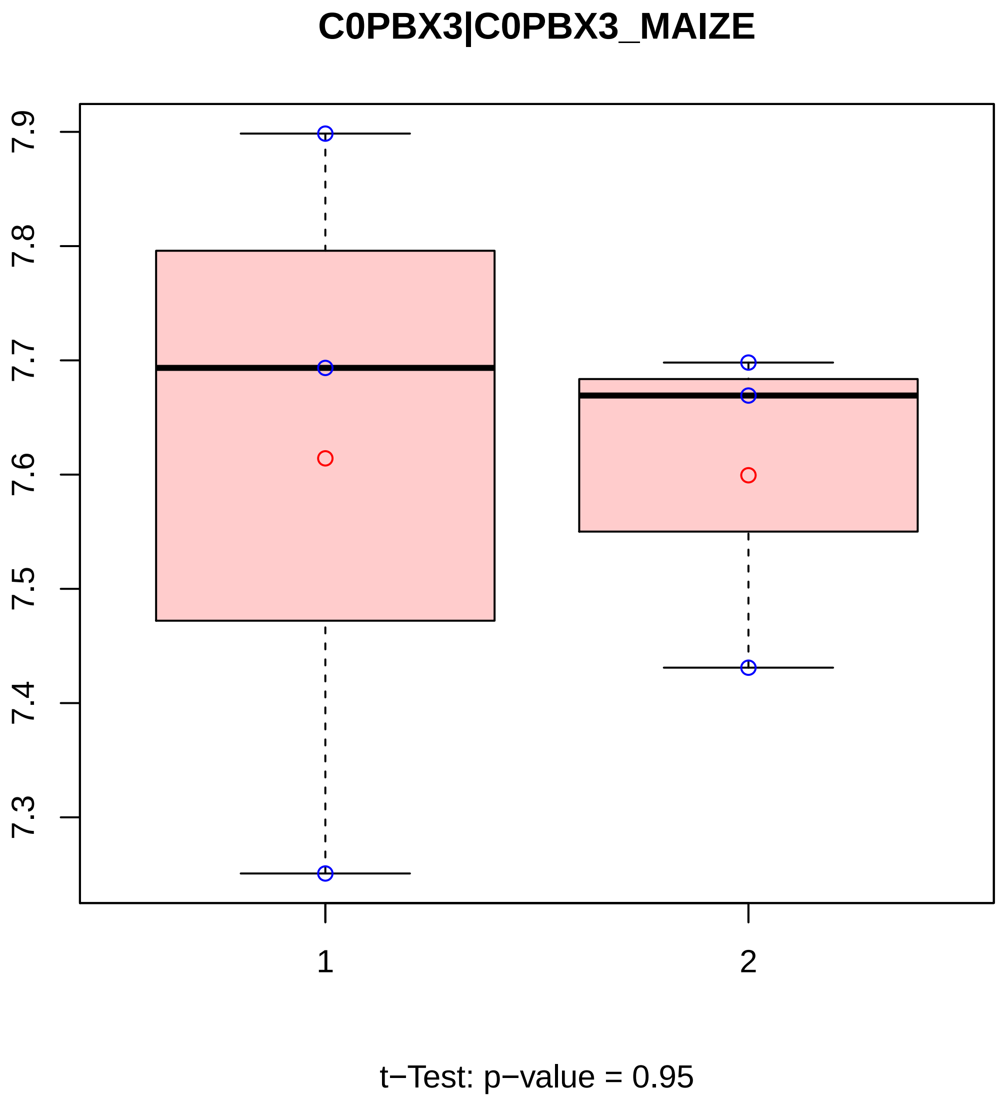
C0PBX3|C0PBX3_MAIZE
7.3
7.4
7.5
7.6
7.7
7.8
7.9
1
2
t−Test: p−value = 0.95

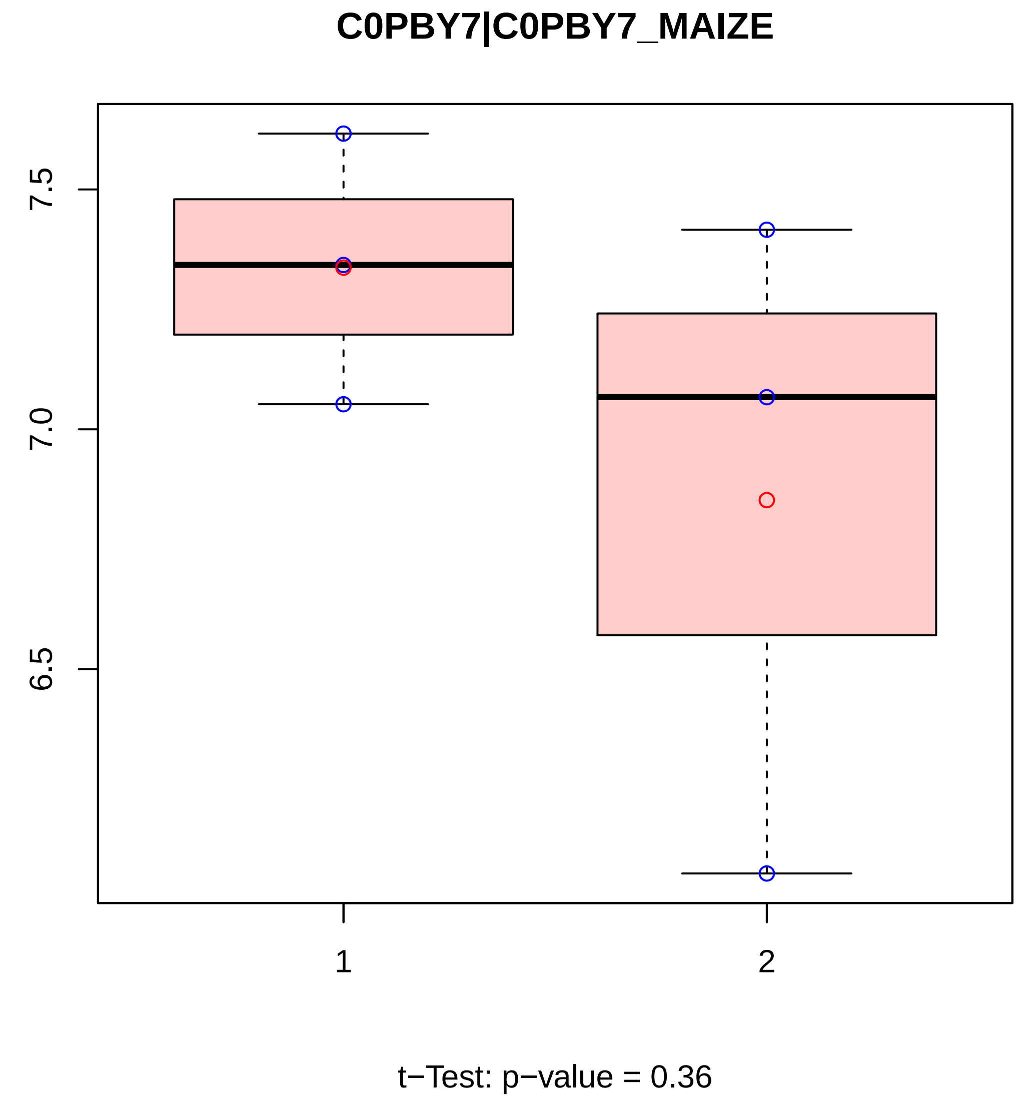

C0PBY7|C0PBY7_MAIZE
7.5
7.0
6.5
1
2
t−Test: p−value = 0.36

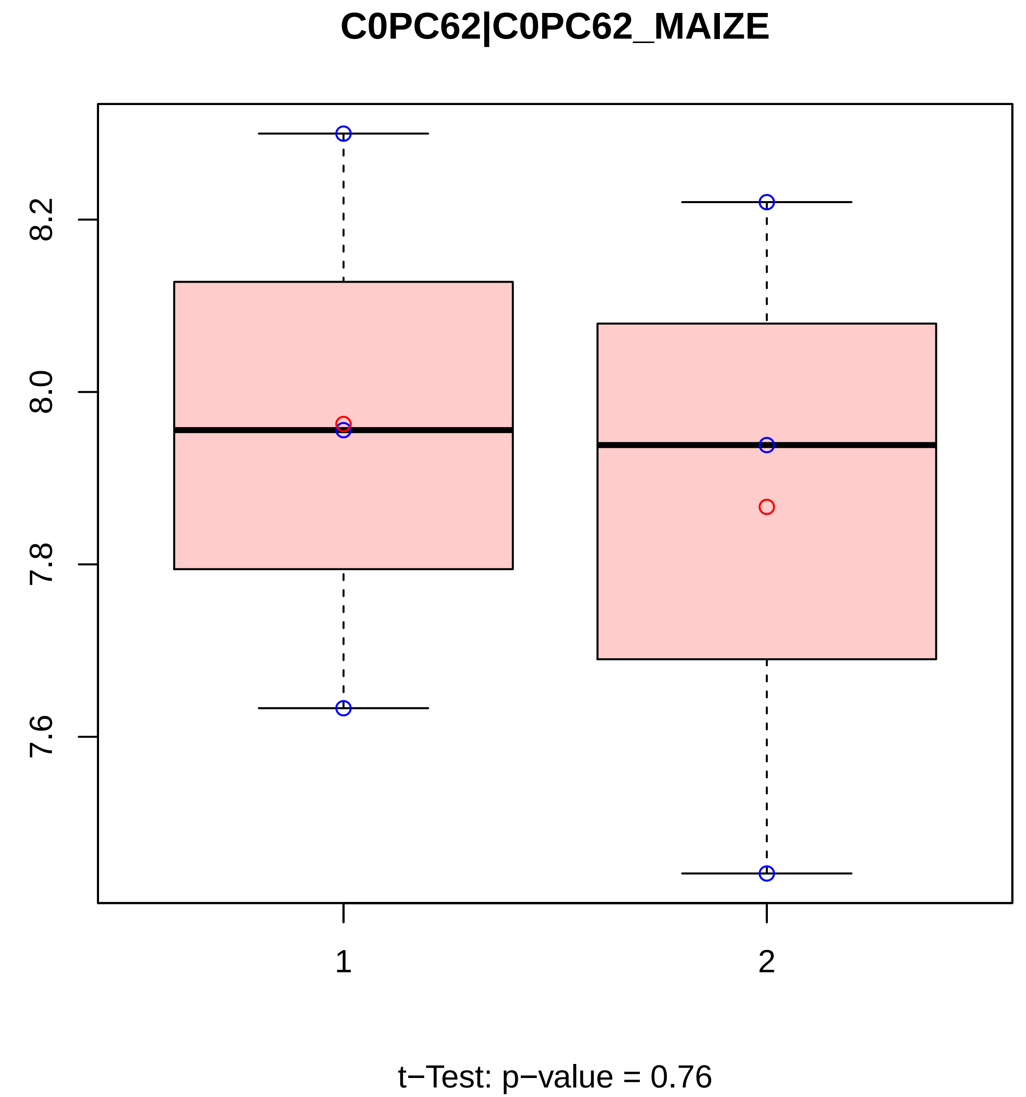
C0PC62|C0PC62_MAIZE
8.2
8.0
7.8
7.6
1
2
t−Test: p−value = 0.76

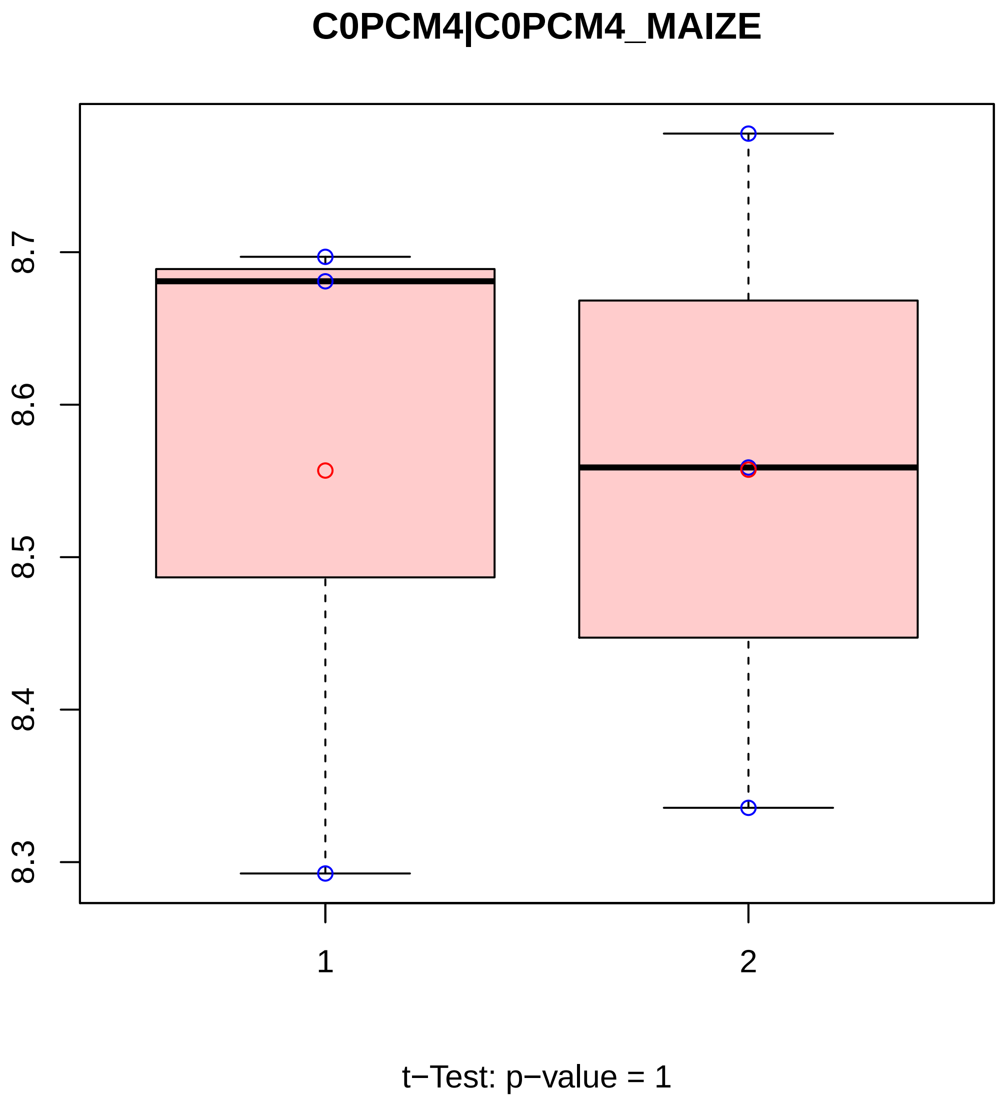
C0PCM4|C0PCM4_MAIZE
8.3
8.4
8.5
8.6
8.7
1
2
t−Test: p−value = 1

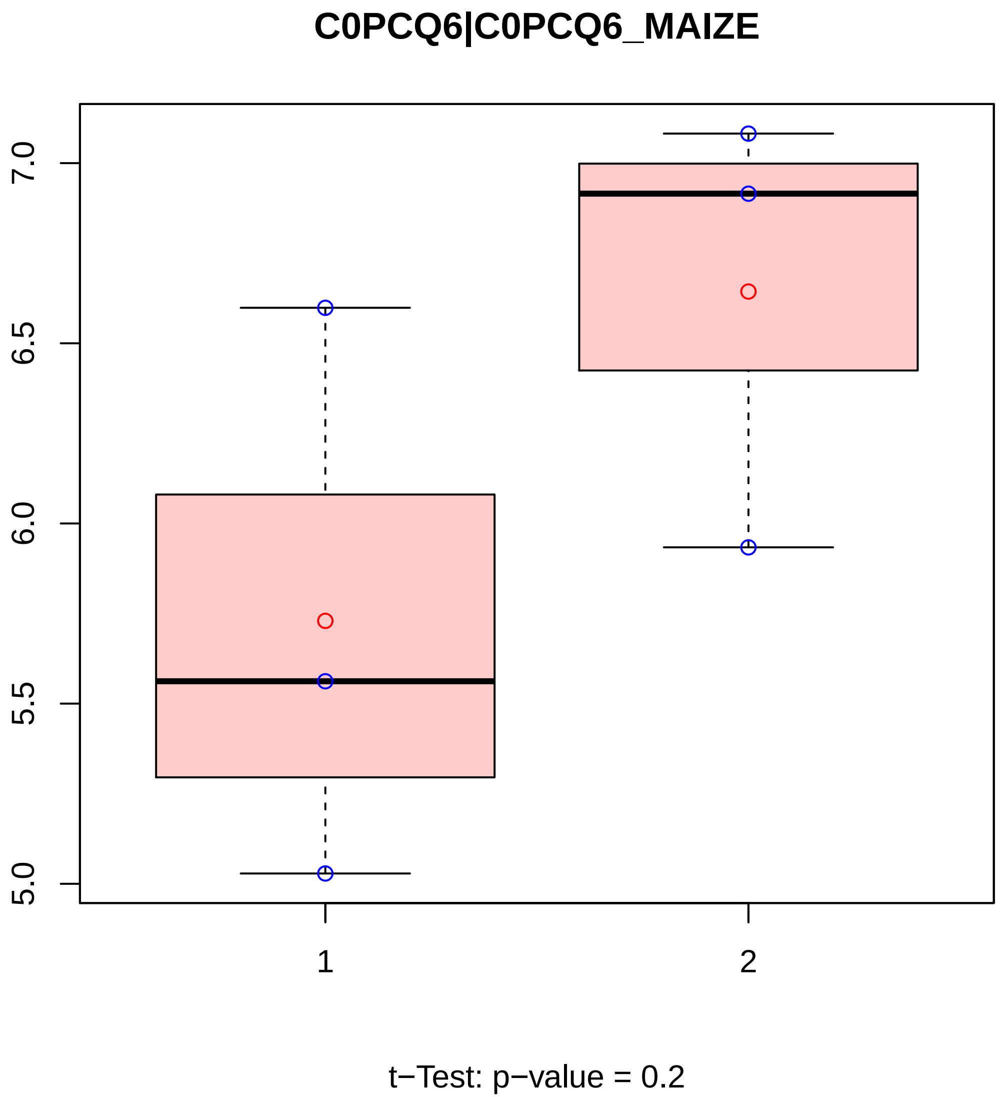
C0PCQ6|C0PCQ6_MAIZE
7.0
6.5
6.0
5.5
5.0
1
2
t−Test: p−value = 0.2

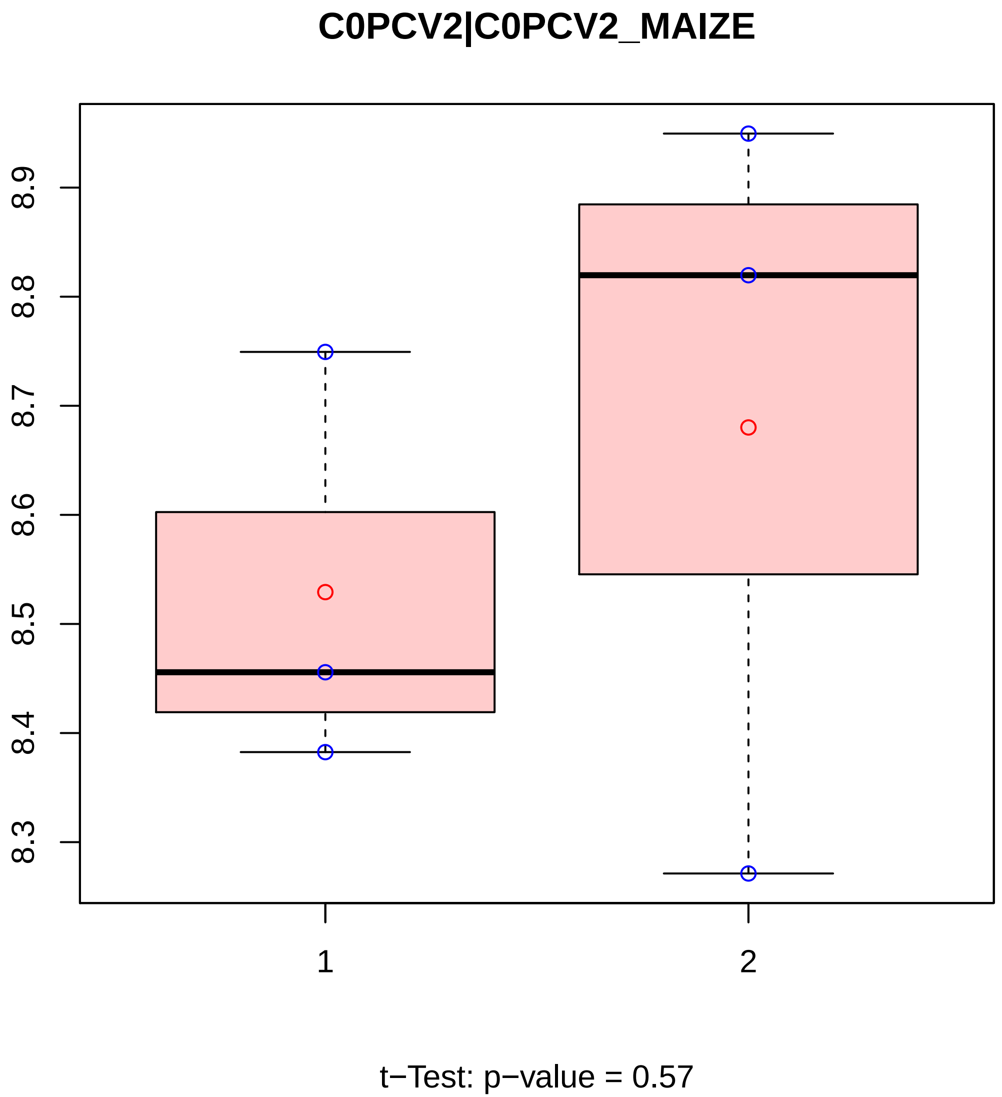

C0PCV2|C0PCV2_MAIZE
8.3
8.4
8.5
8.6
8.7
8.8
8.9
1
2
t−Test: p−value = 0.57

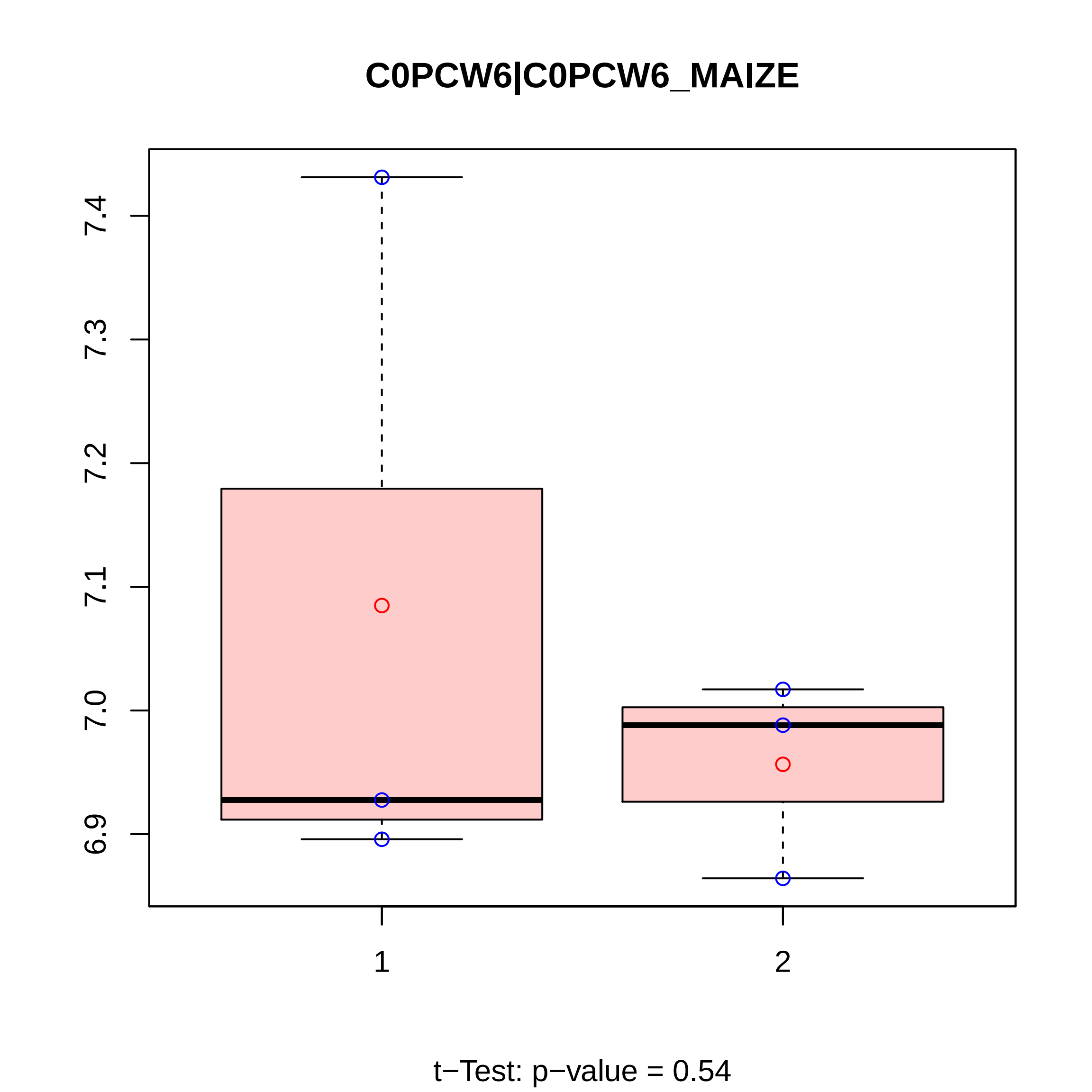
C0PCW6|C0PCW6_MAIZE
7.4
7.3
7.2
7.1
7.0
6.9
1
2
t−Test: p−value = 0.54

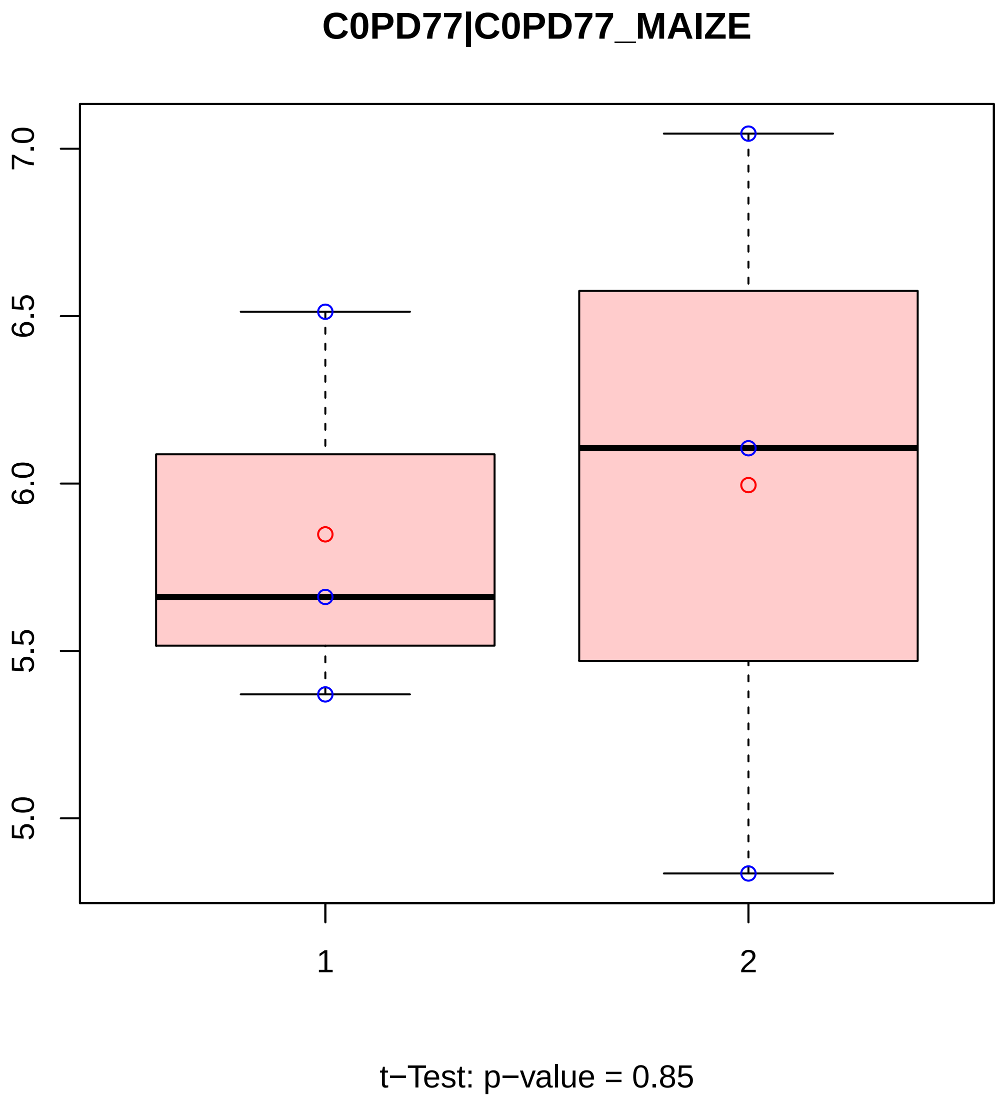
C0PD77|C0PD77_MAIZE
7.0
6.5
6.0
5.5
5.0
1
2
t−Test: p−value = 0.85

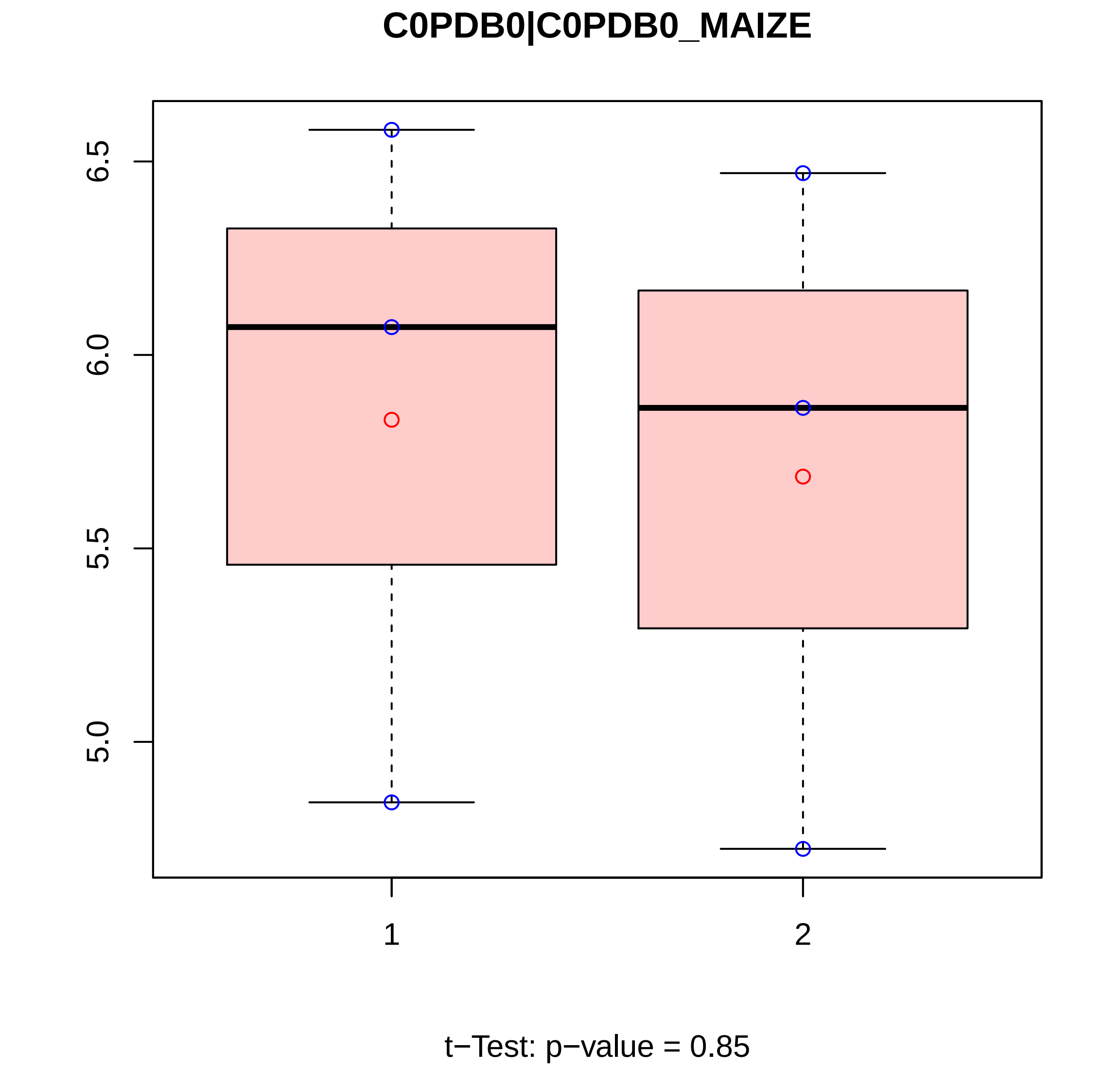
C0PDB0|C0PDB0_MAIZE
6.5
6.0
5.5
5.0
1
2
t−Test: p−value = 0.85

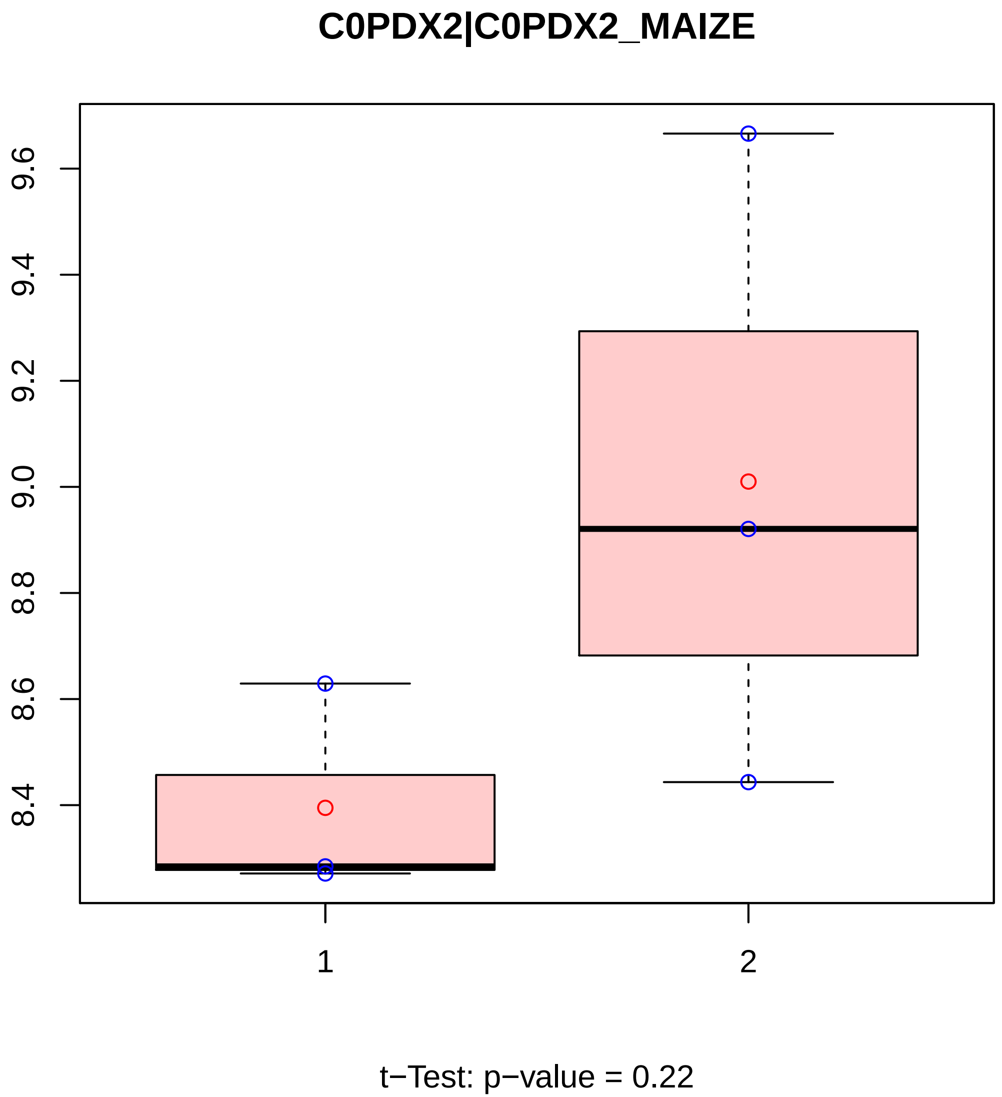
C0PDX2|C0PDX2_MAIZE
9.6
9.4
9.2
9.0
8.8
8.6
8.4
1
2
t−Test: p−value = 0.22

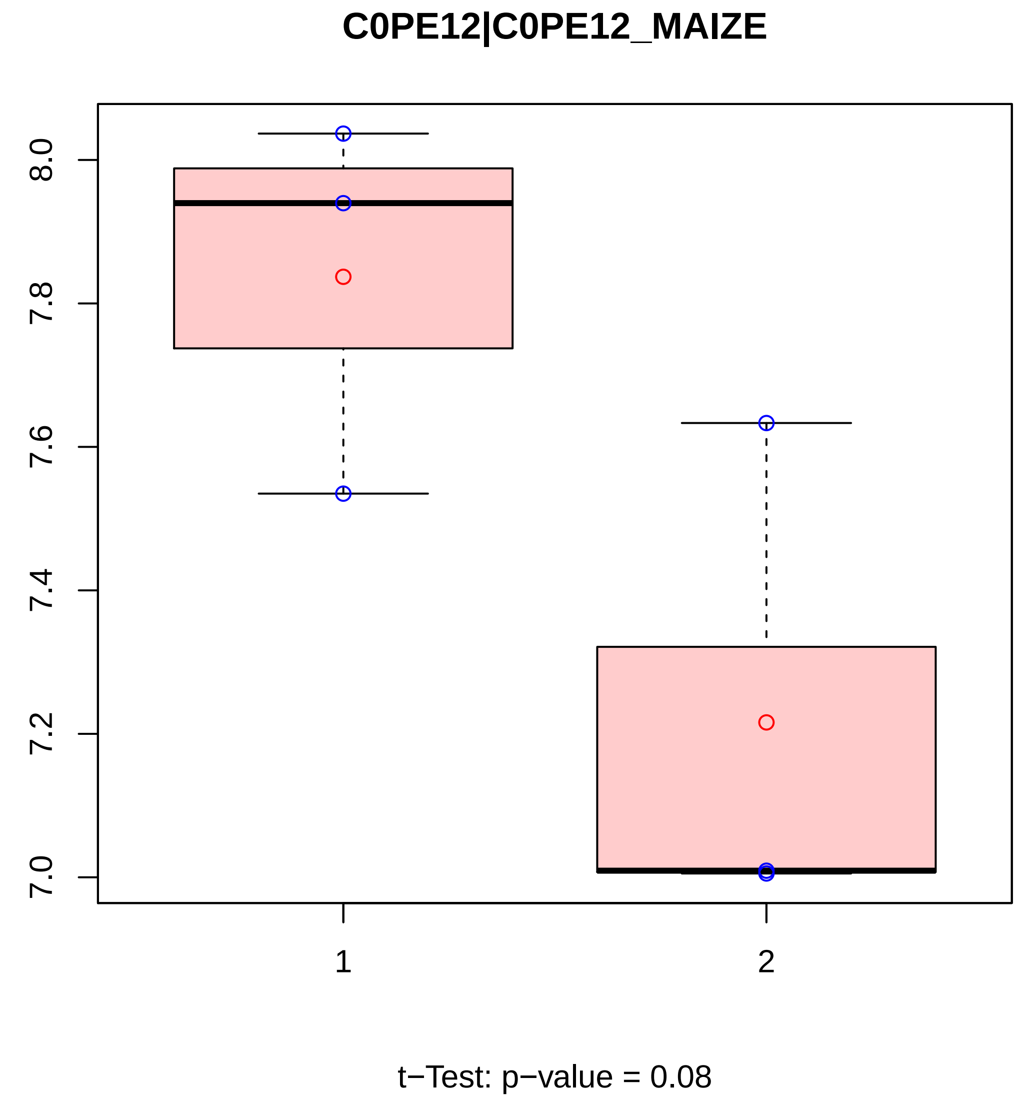
C0PE12|C0PE12_MAIZE
8.0
7.8
7.6
7.4
7.2
7.0
1
2
t−Test: p−value = 0.08

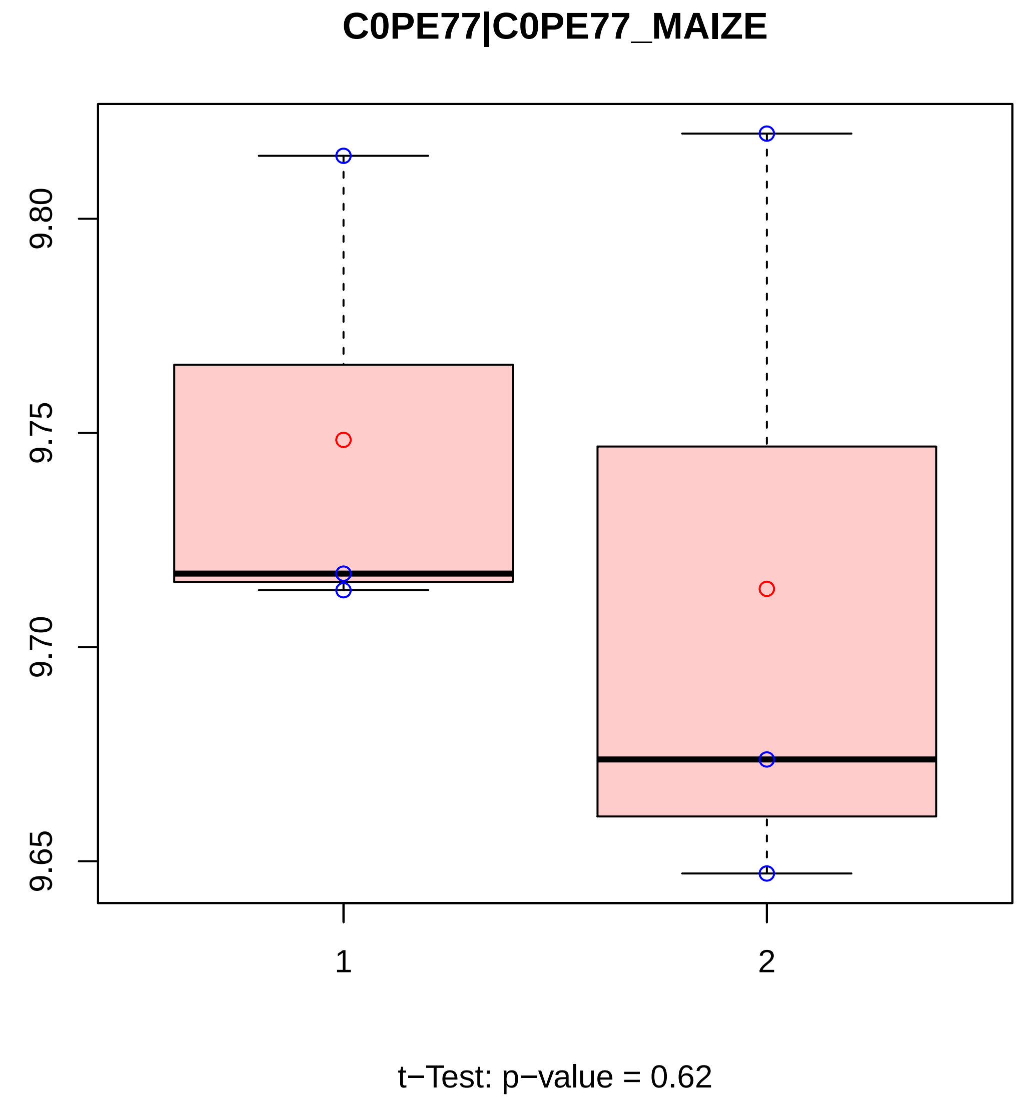
C0PE77|C0PE77_MAIZE
9.80
9.75
9.70
9.65
1
2
t−Test: p−value = 0.62

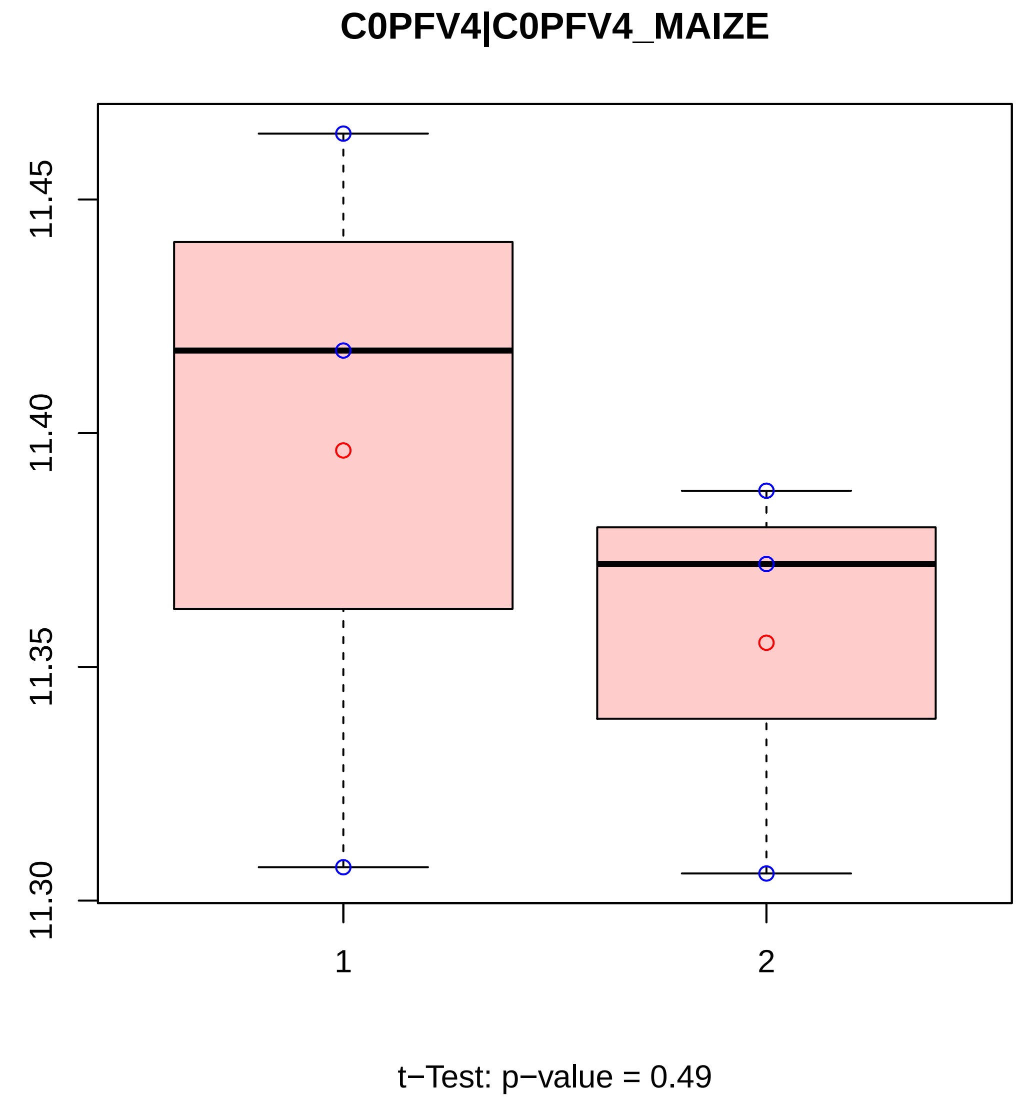
C0PFV4|C0PFV4_MAIZE
11.45
11.40
11.35
11.30
1
2
t−Test: p−value = 0.49

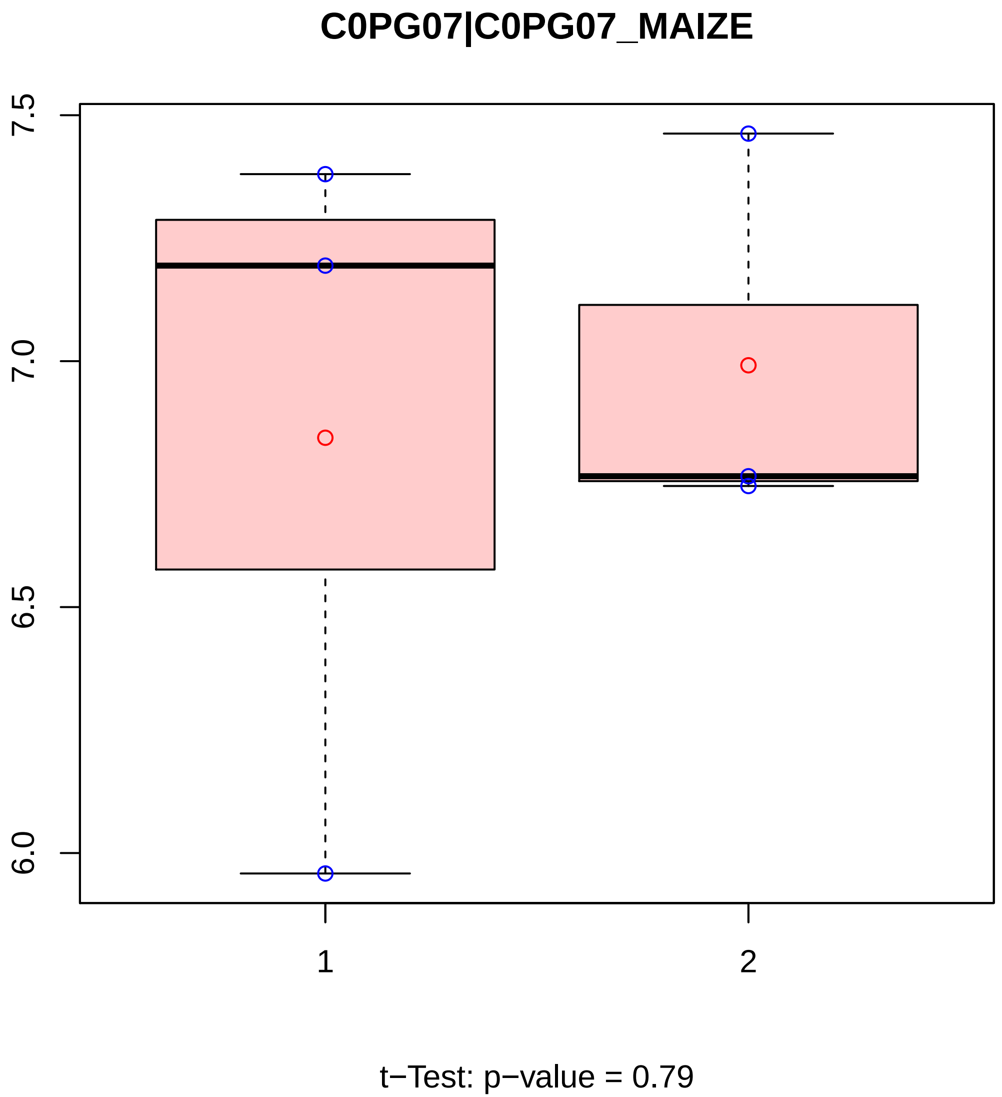

C0PG07|C0PG07_MAIZE
7.5
7.0
6.5
6.0
1
2
t−Test: p−value = 0.79

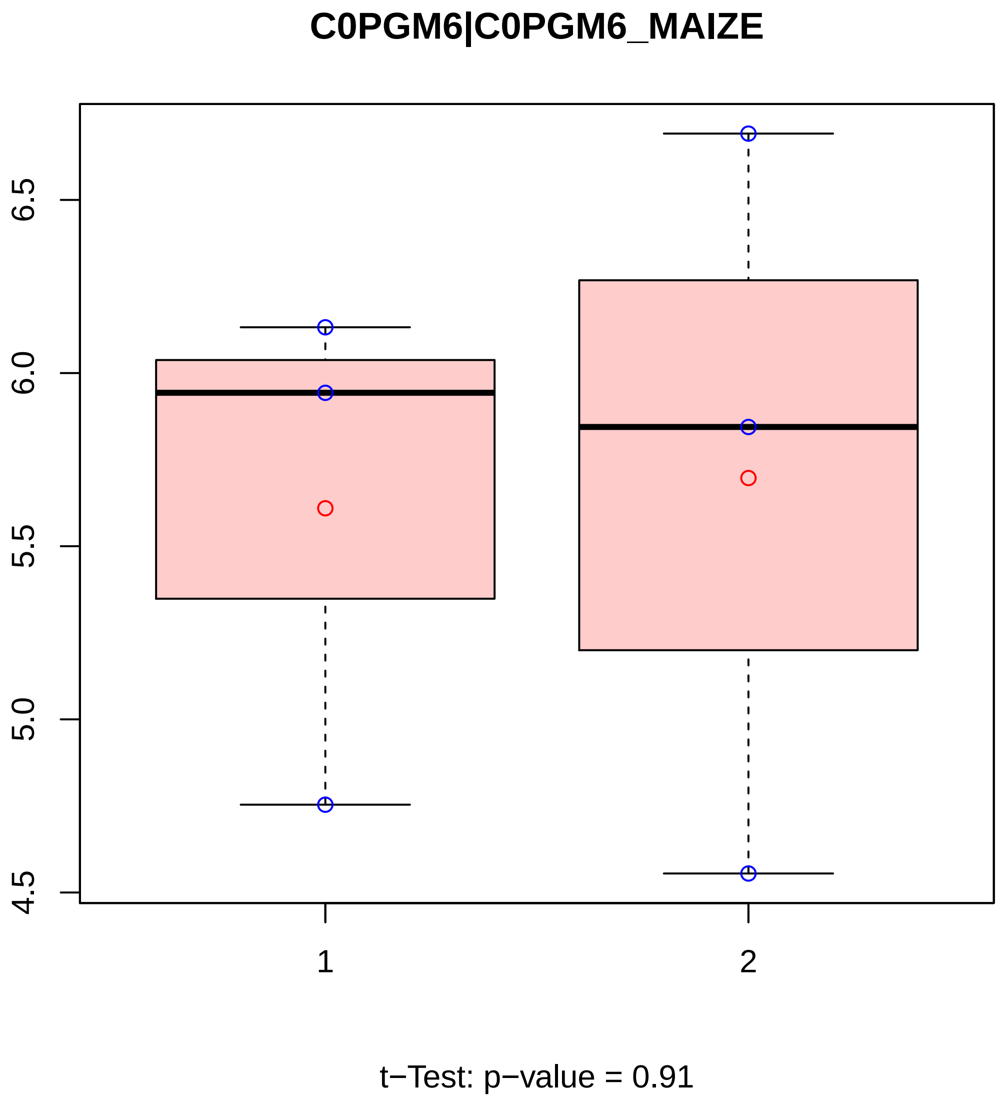
C0PGM6|C0PGM6_MAIZE
6.5
6.0
5.5
5.0
4.5
1
2
t−Test: p−value = 0.91

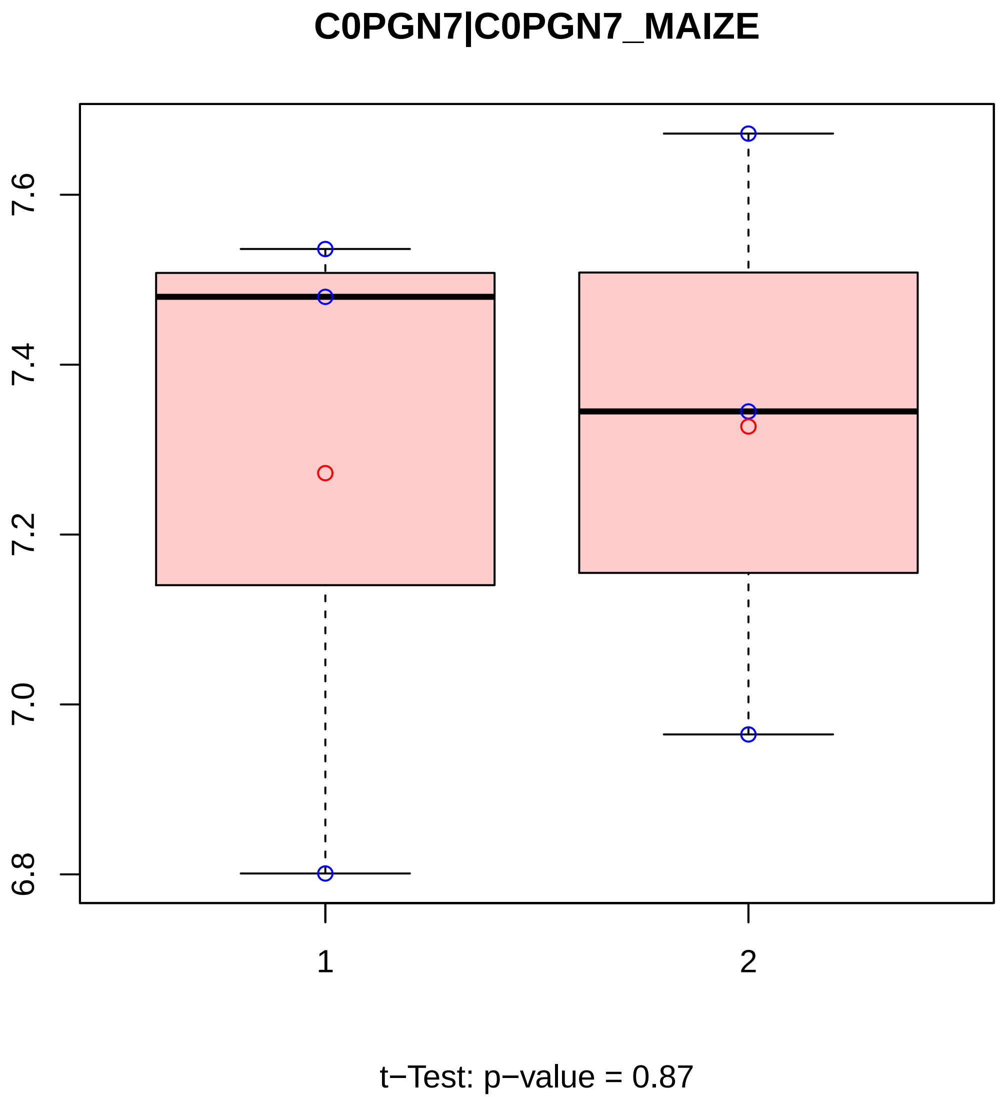
C0PGN7|C0PGN7_MAIZE
7.6
7.4
7.2
7.0
6.8
1
2
t−Test: p−value = 0.87

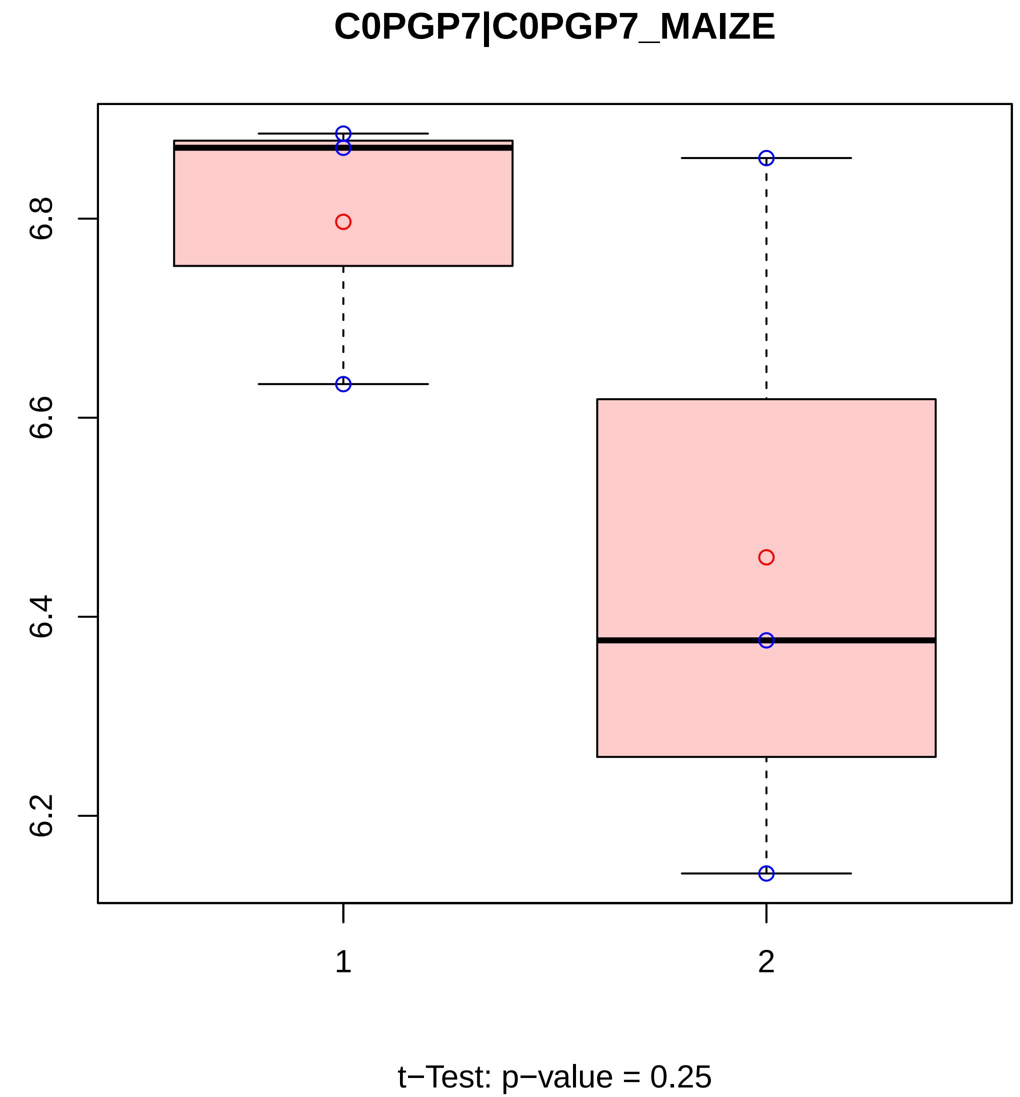
C0PGP7|C0PGP7_MAIZE
6.2
6.4
6.6
6.8
1
2
t−Test: p−value = 0.25

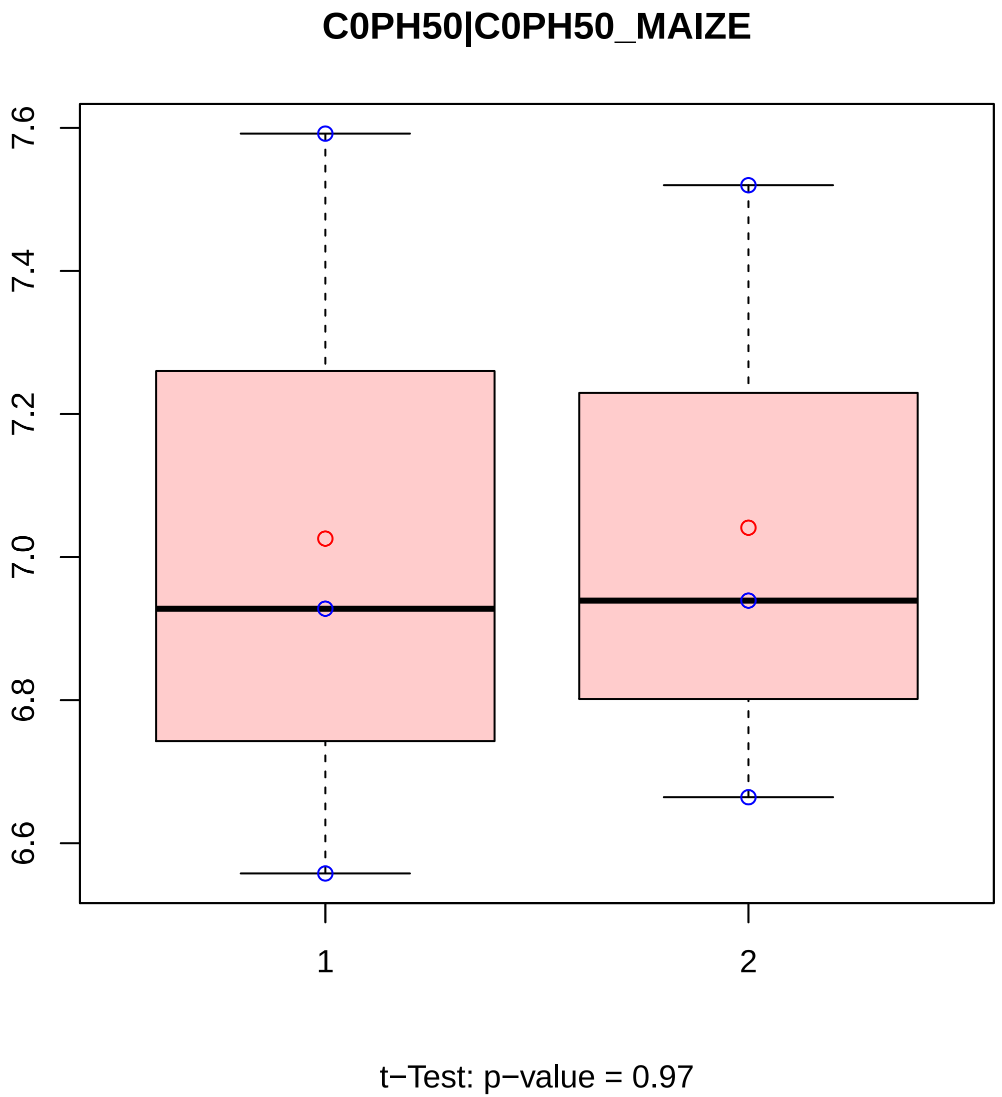
C0PH50|C0PH50_MAIZE
7.6
7.4
7.2
7.0
6.8
6.6
1
2
t−Test: p−value = 0.97

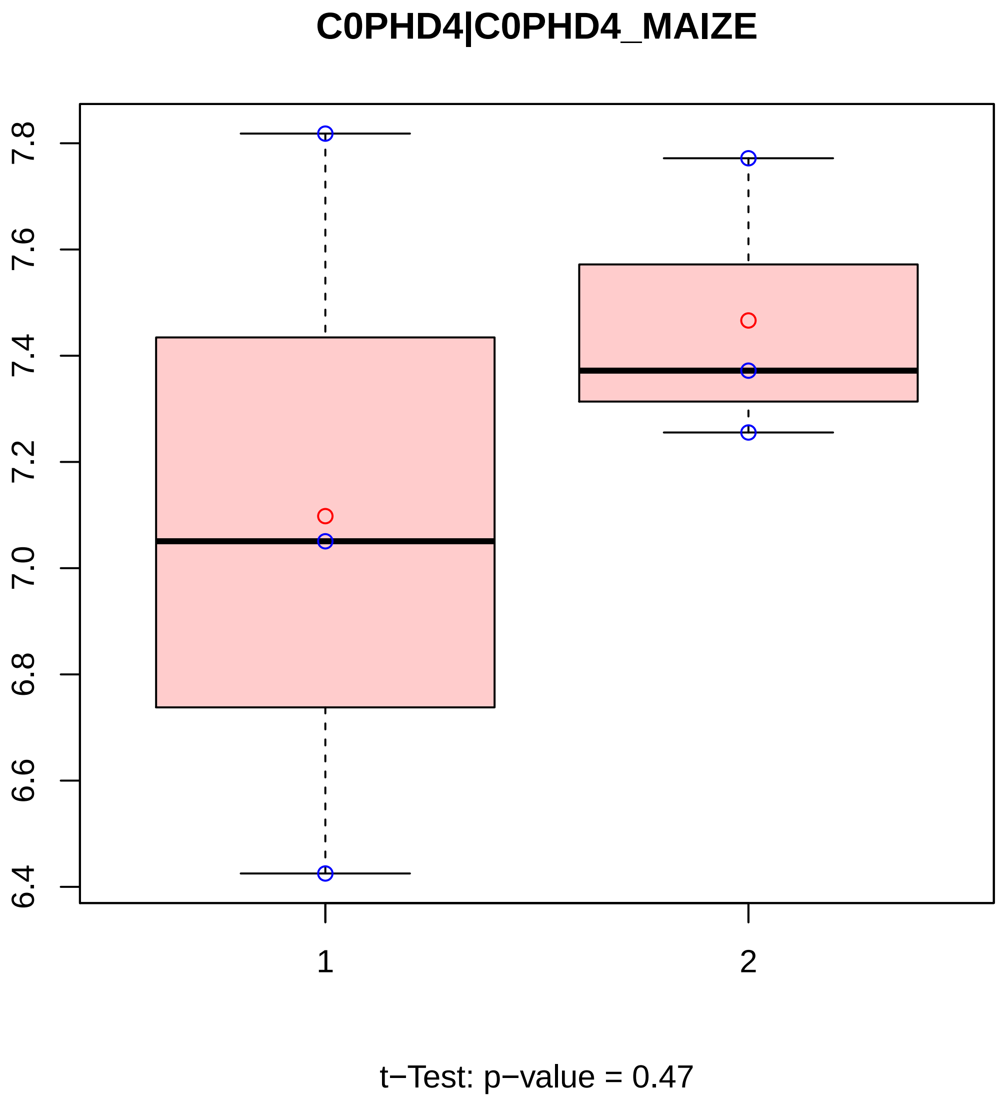
C0PHD4|C0PHD4_MAIZE
7.8
7.6
7.4
7.2
7.0
6.8
6.6
6.4
1
2
t−Test: p−value = 0.47

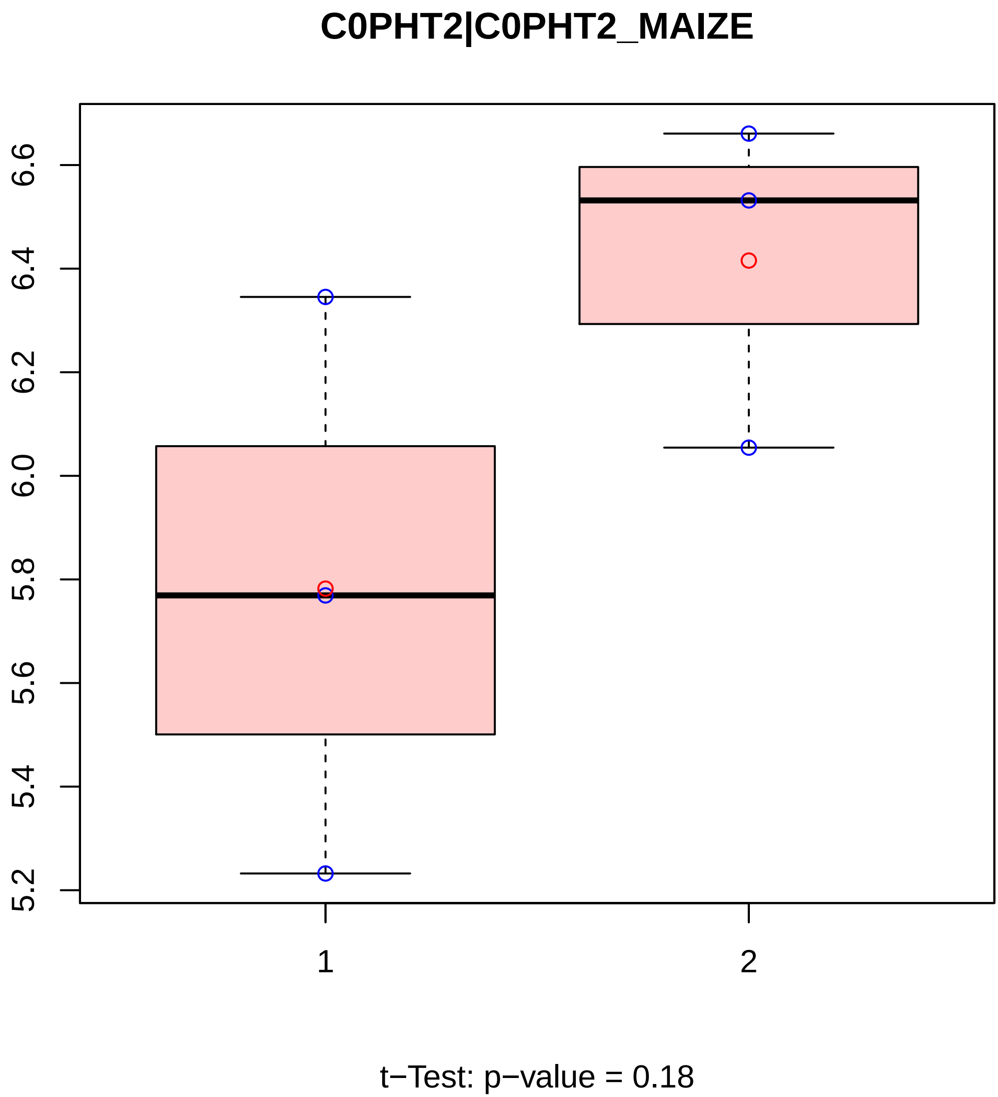
C0PHT2|C0PHT2_MAIZE
6.6
6.4
6.2
6.0
5.8
5.6
5.4
5.2
1
2
t−Test: p−value = 0.18

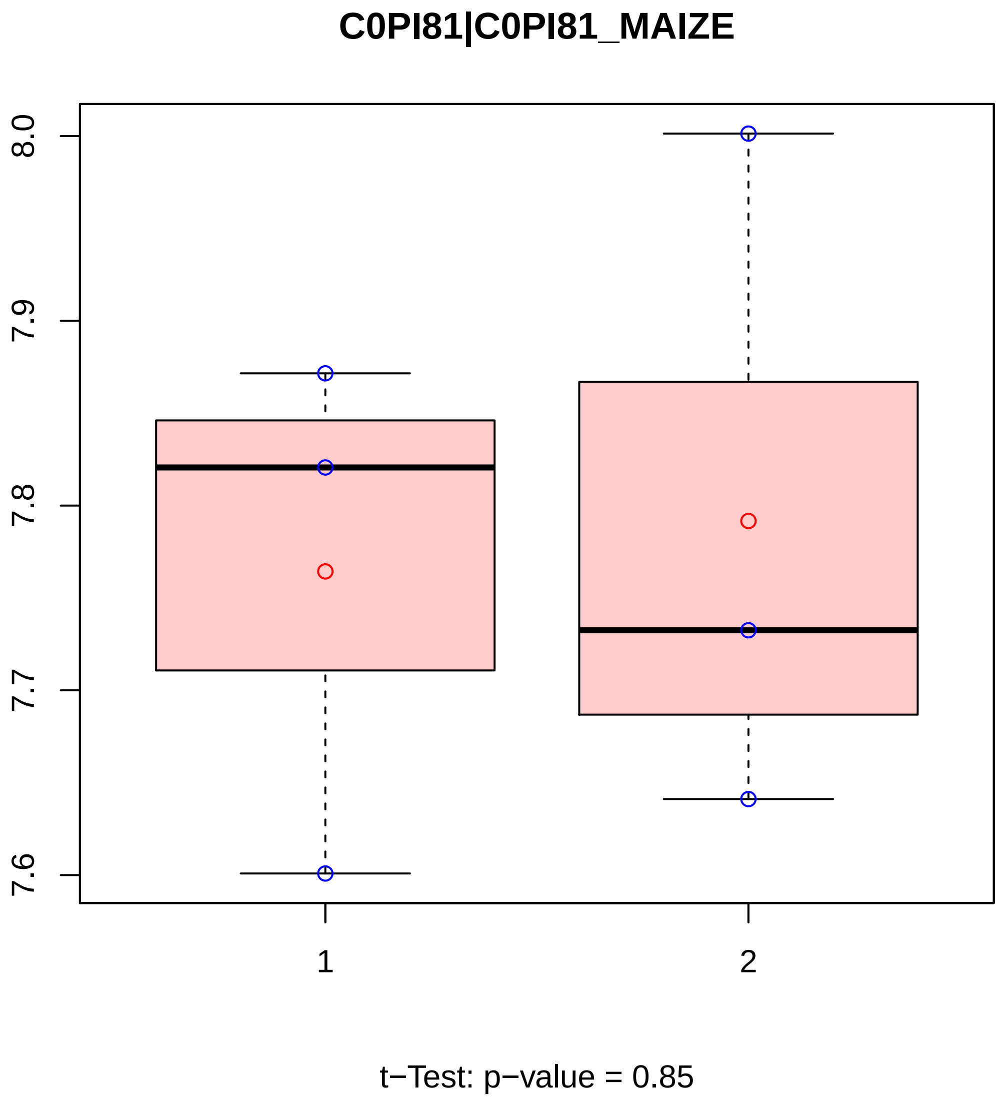
C0PI81|C0PI81_MAIZE
8.0
7.9
7.8
7.7
7.6
1
2
t−Test: p−value = 0.85

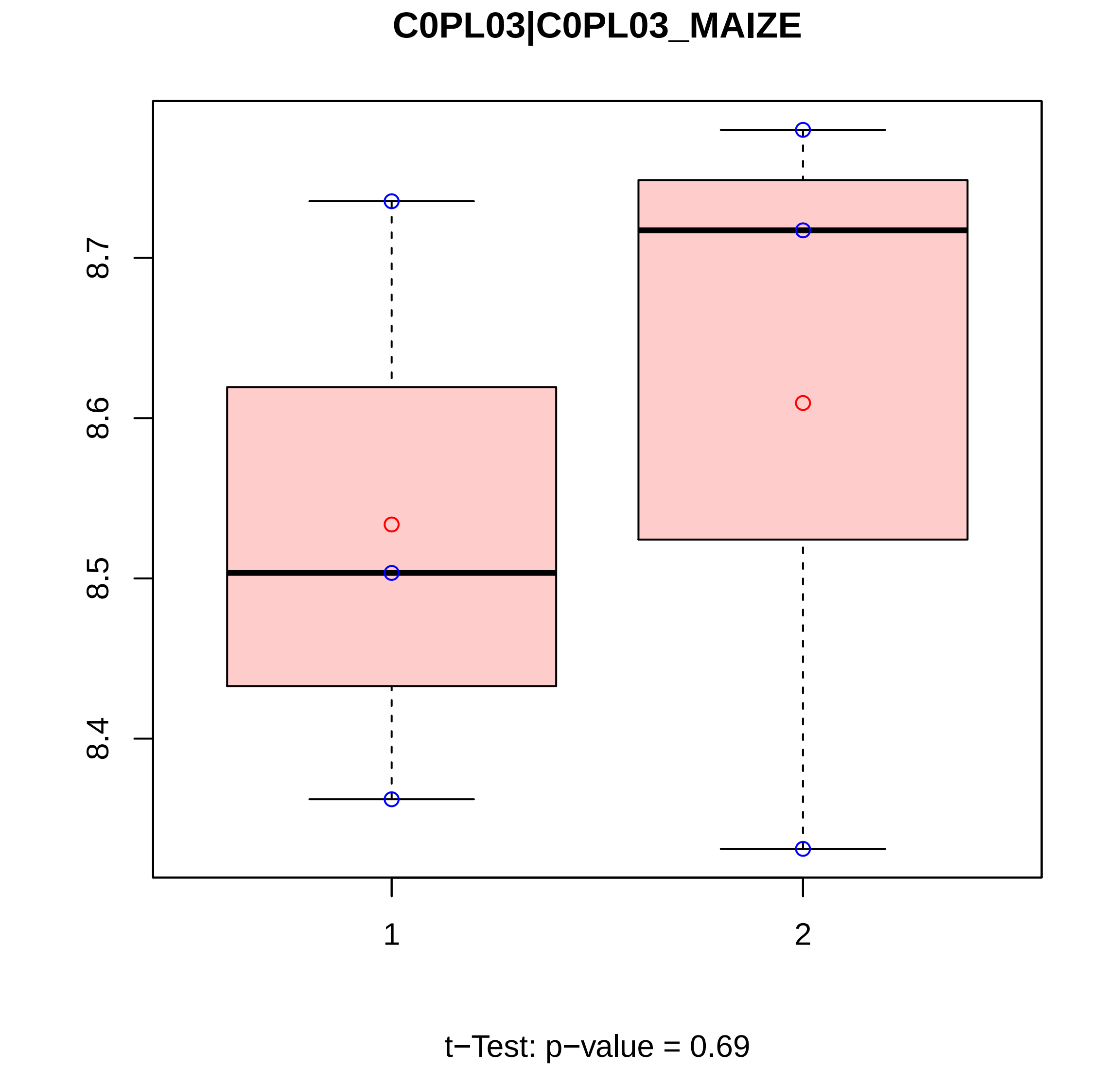

C0PL03|C0PL03_MAIZE
8.4
8.5
8.6
8.7
1
2
t−Test: p−value = 0.69

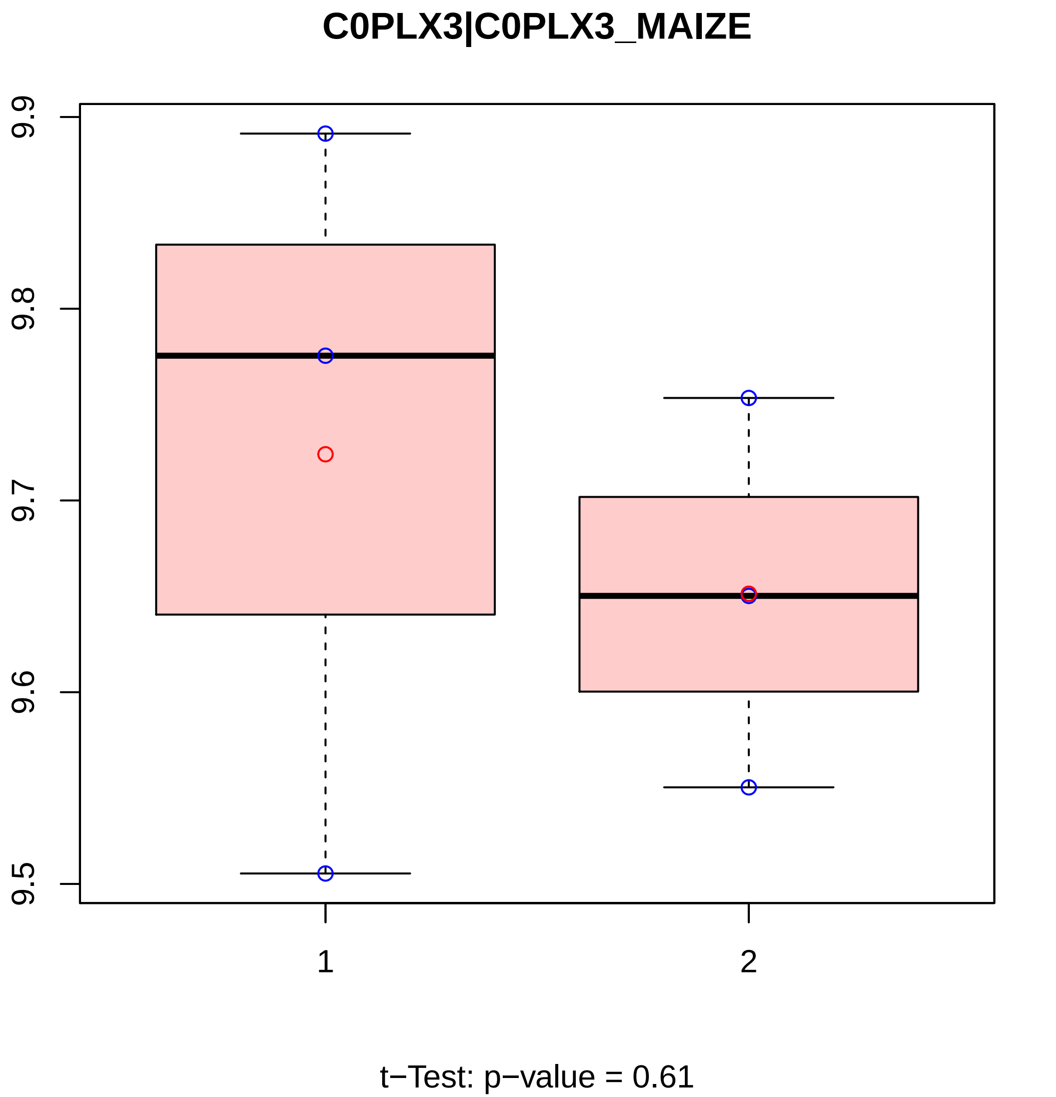
C0PLX3|C0PLX3_MAIZE
9.5
9.6
9.7
9.8
9.9
1
2
t−Test: p−value = 0.61

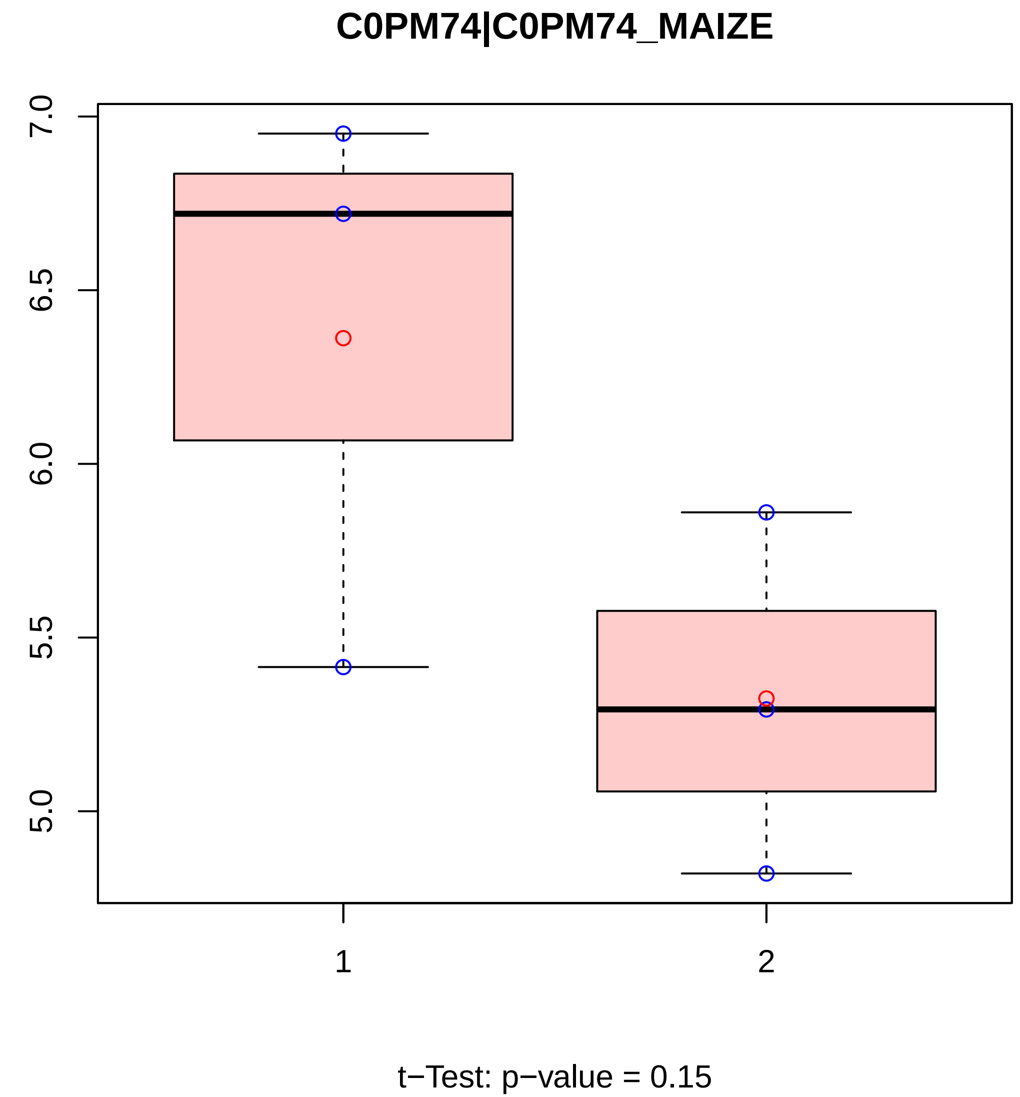
C0PM74|C0PM74_MAIZE
7.0
6.5
6.0
5.5
5.0
1
2
t−Test: p−value = 0.15

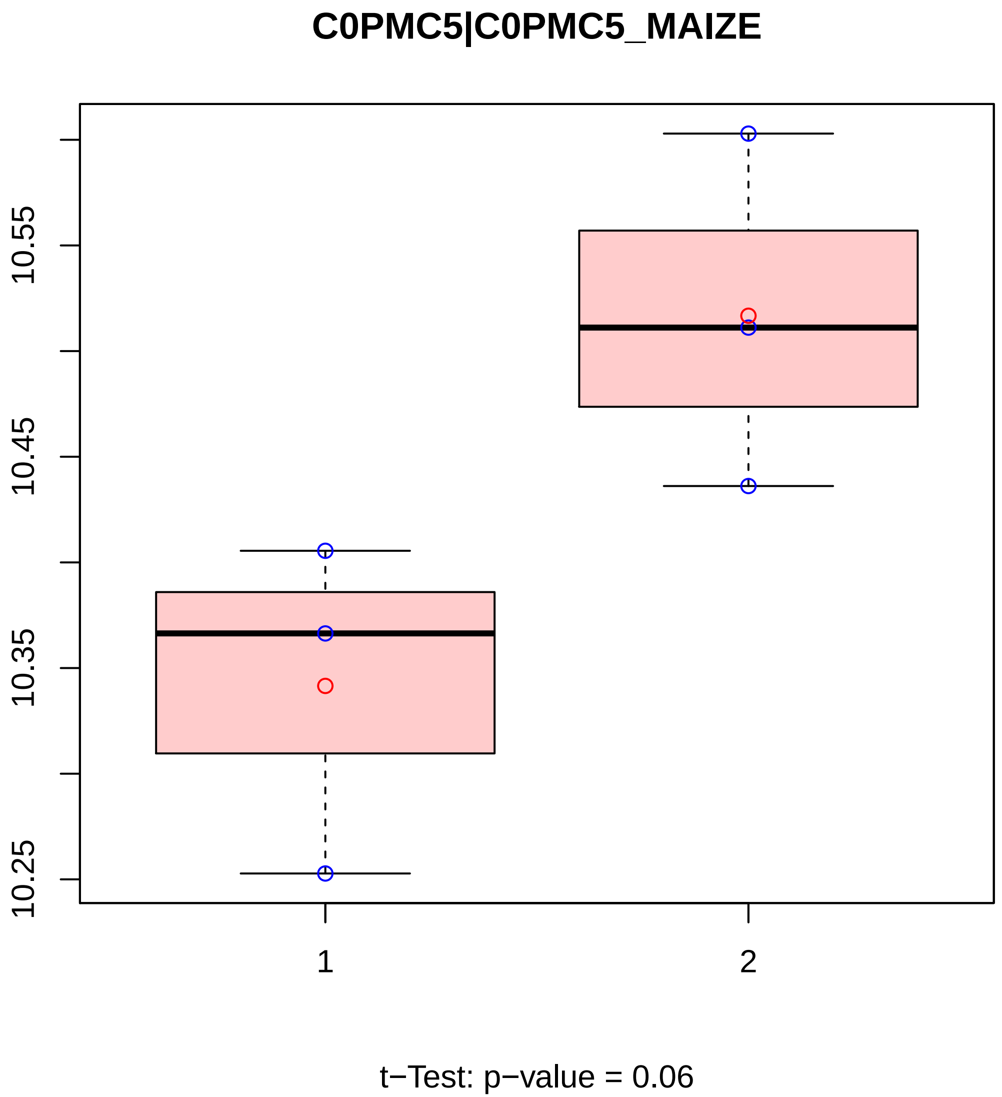

C0PMC5|C0PMC5_MAIZE
10.25
10.35
10.45
10.55
1
2
t−Test: p−value = 0.06

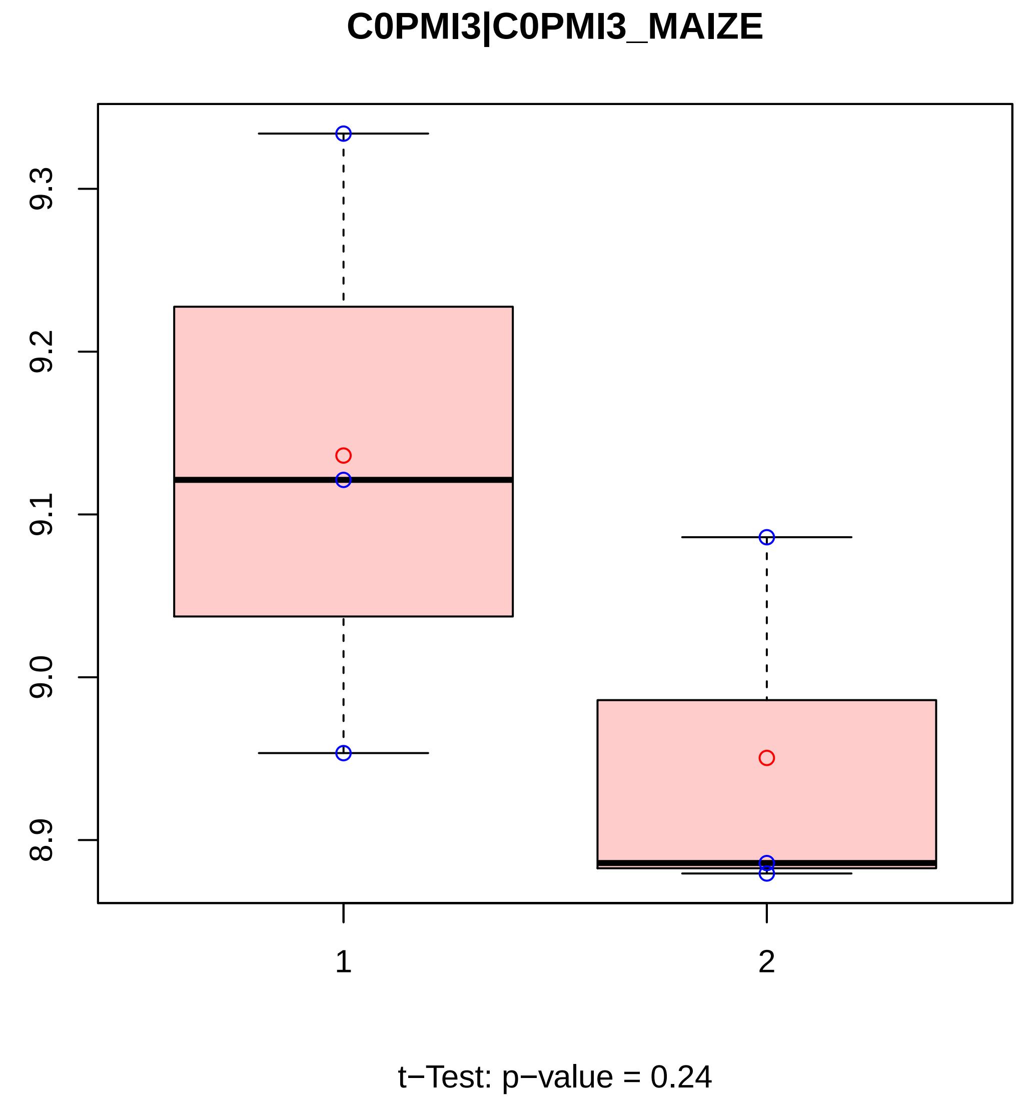

C0PMI3|C0PMI3_MAIZE
9.3
9.2
9.1
9.0
8.9
1
2
t−Test: p−value = 0.24

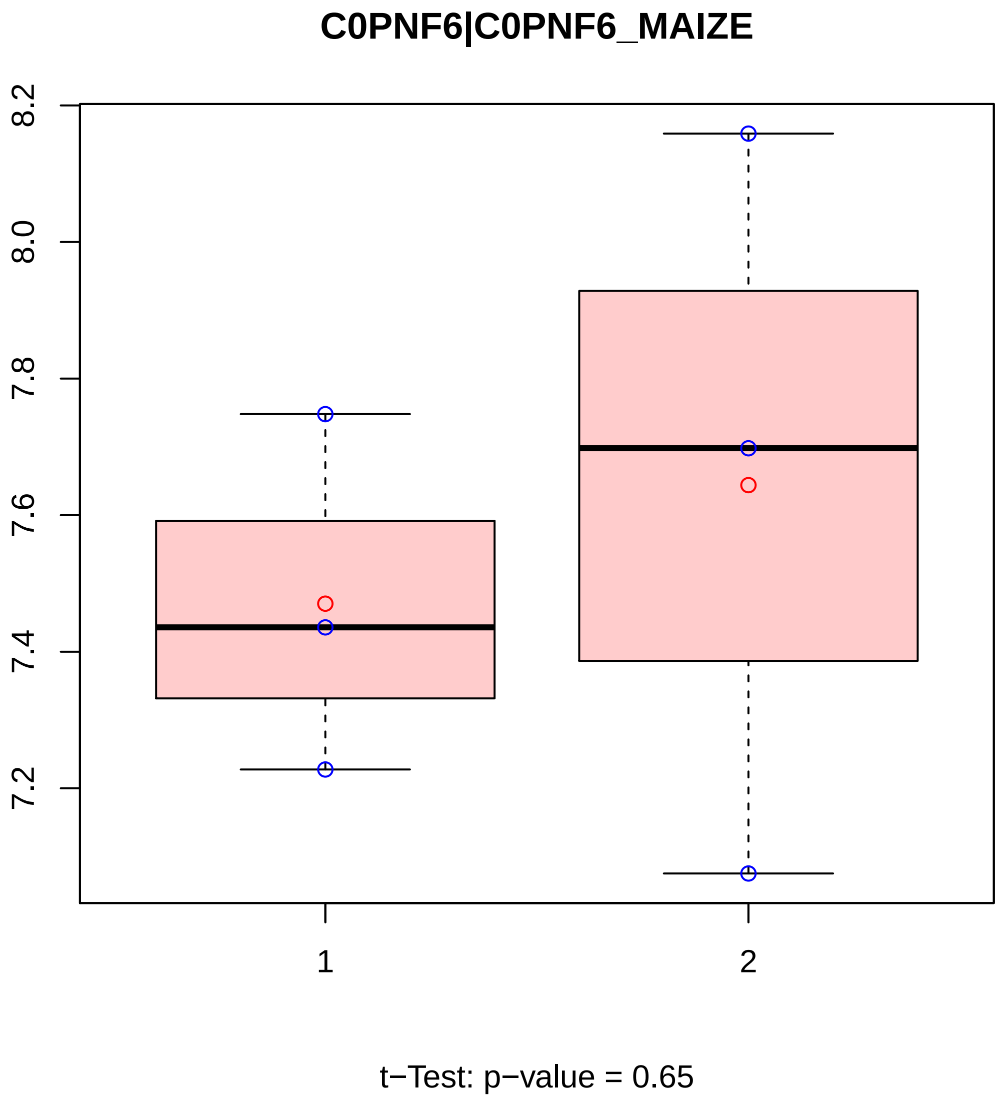
C0PNF6|C0PNF6_MAIZE
8.2
8.0
7.8
7.6
7.4
7.2
1
2
t−Test: p−value = 0.65

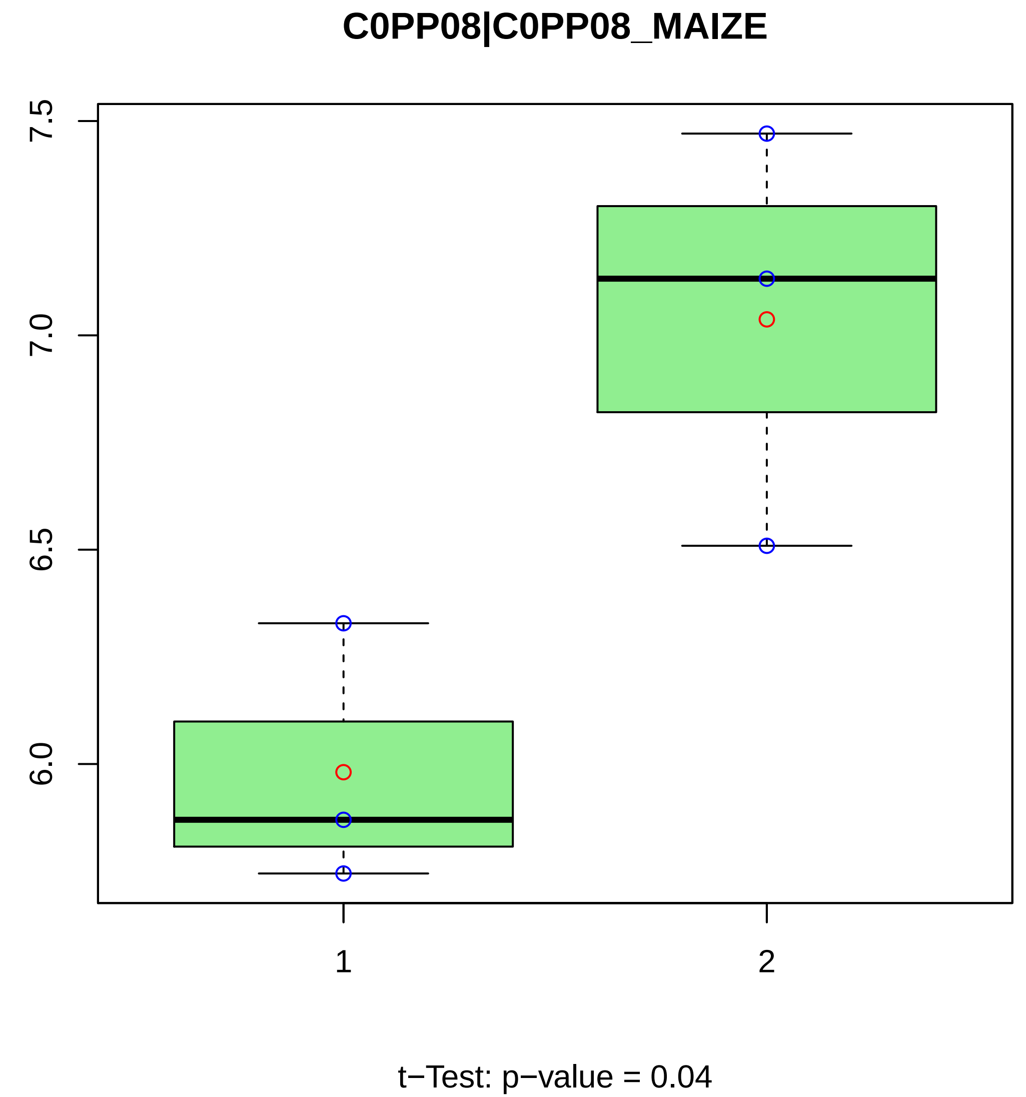
C0PP08|C0PP08_MAIZE
7.5
7.0
6.5
6.0
1
2
t−Test: p−value = 0.04

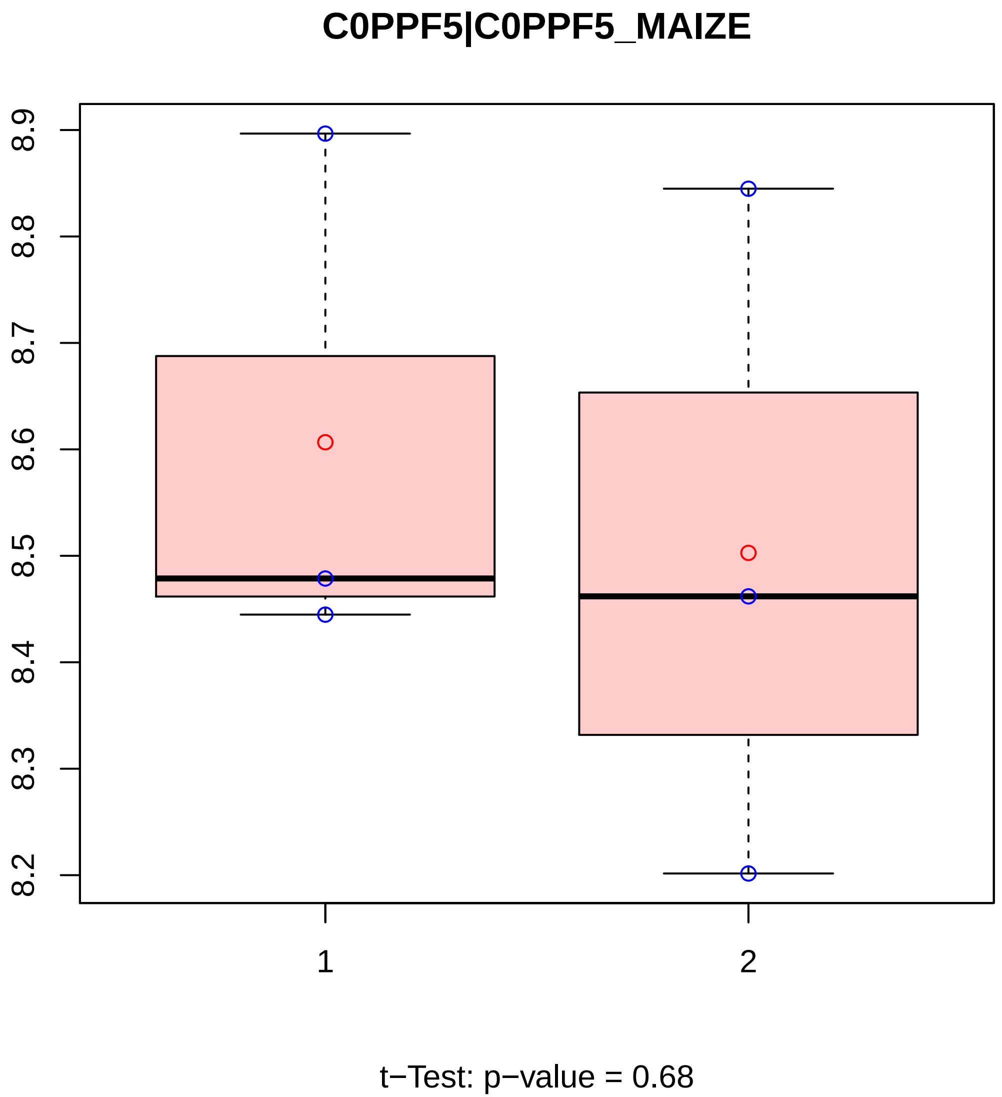
C0PPF5|C0PPF5_MAIZE
8.2
8.3
8.4
8.5
8.6
8.7
8.8
8.9
1
2
t−Test: p−value = 0.68

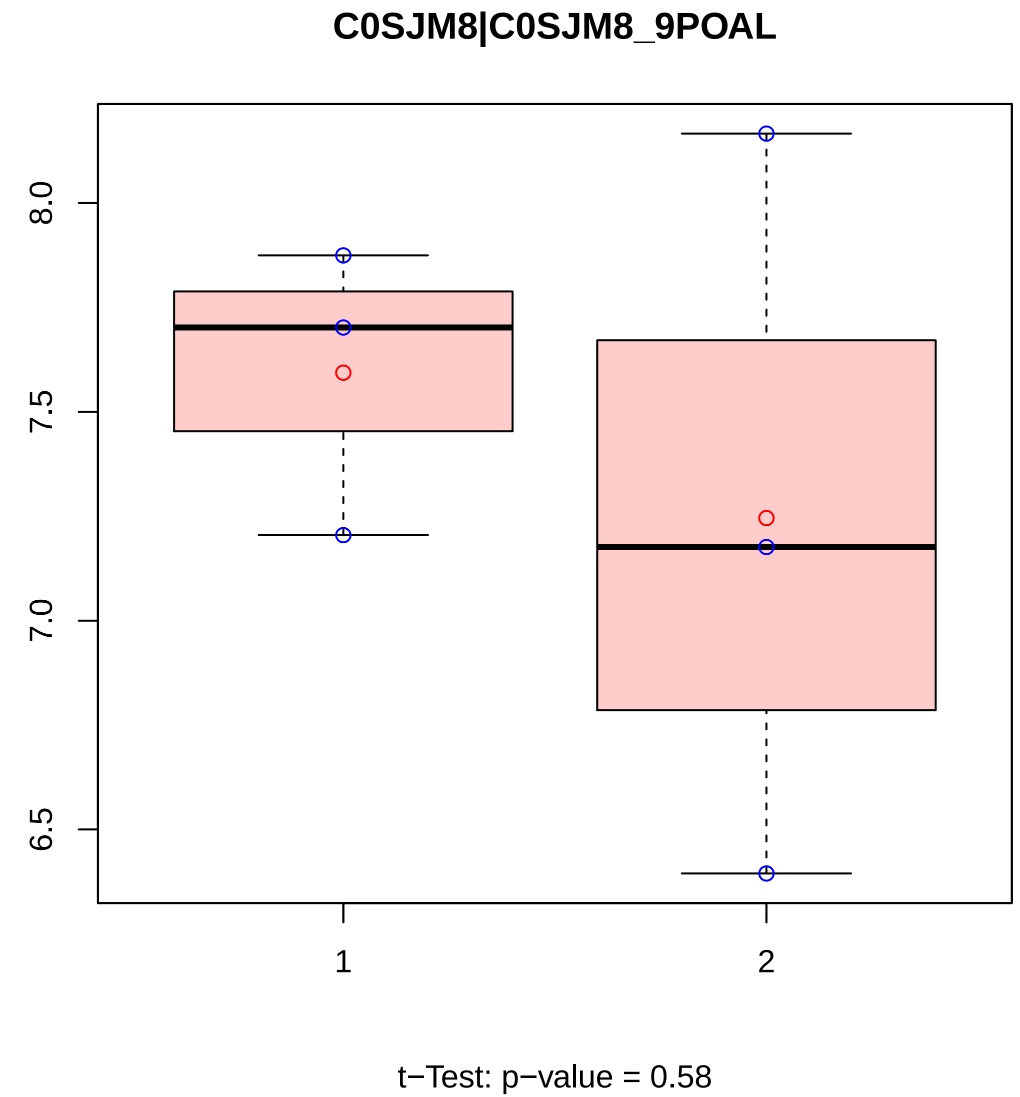
C0SJM8|C0SJM8_9POAL
8.0
7.5
7.0
6.5
1
2
t−Test: p−value = 0.58

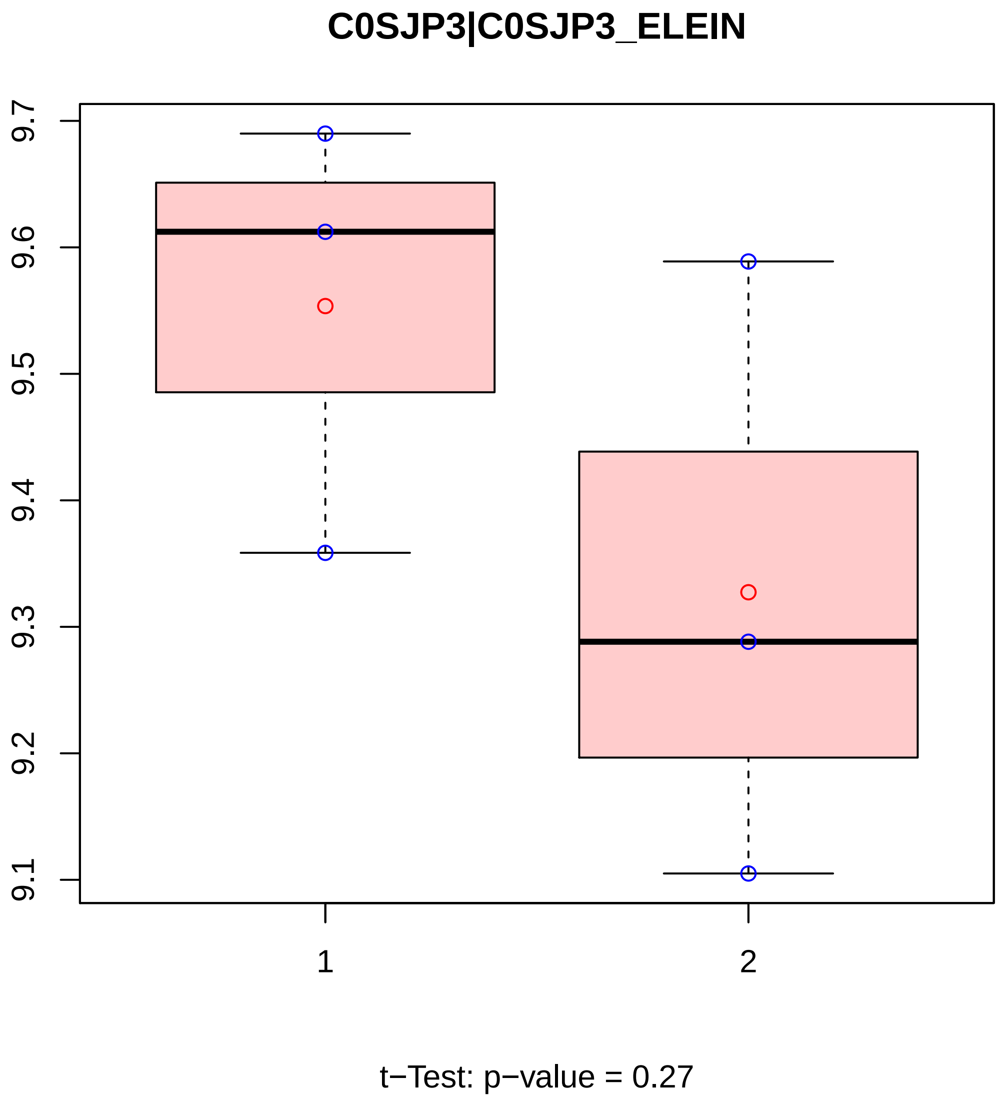
C0SJP3|C0SJP3_ELEIN
9.7
9.6
9.5
9.4
9.3
9.2
9.1
1
2
t−Test: p−value = 0.27

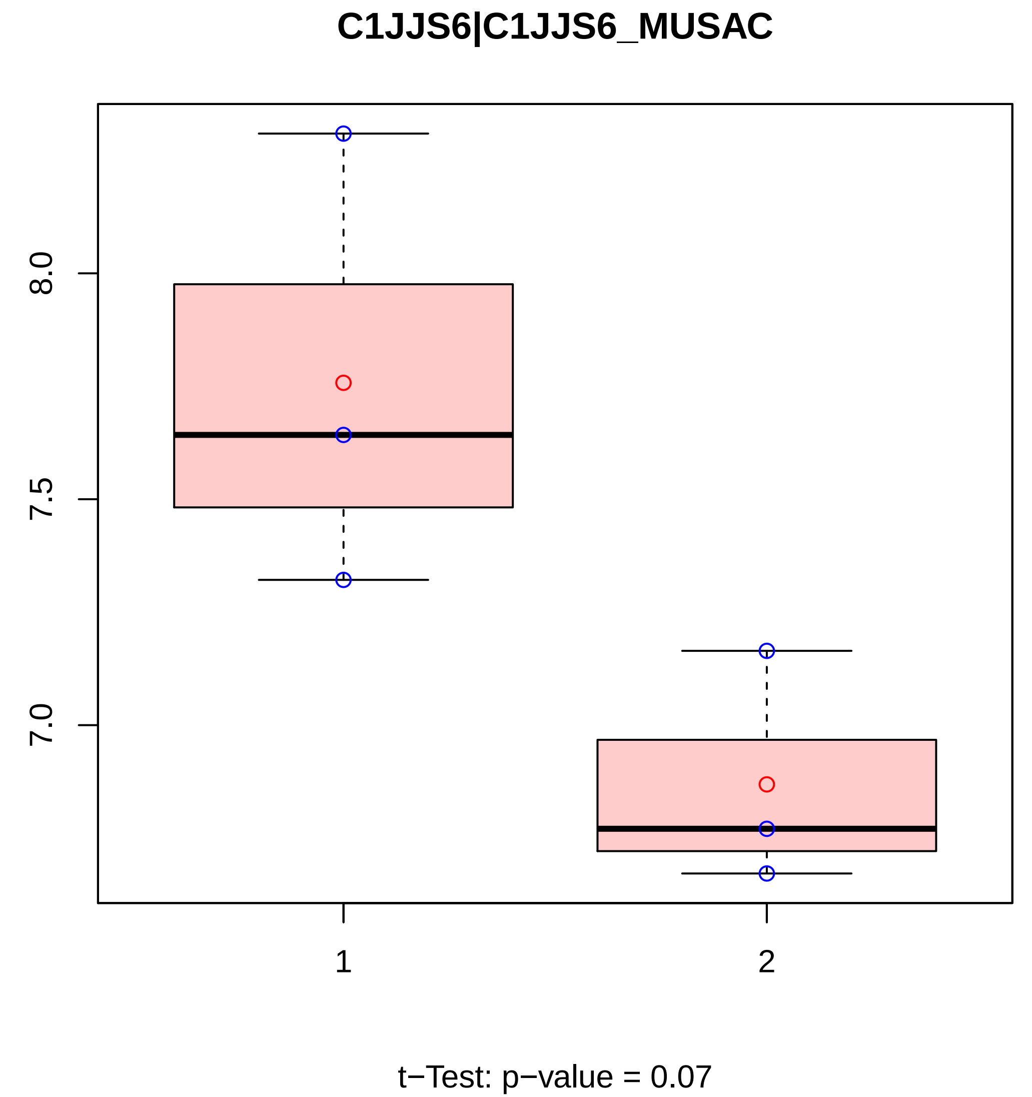
C1JJS6|C1JJS6_MUSAC
8.0
7.5
7.0
1
2
t−Test: p−value = 0.07

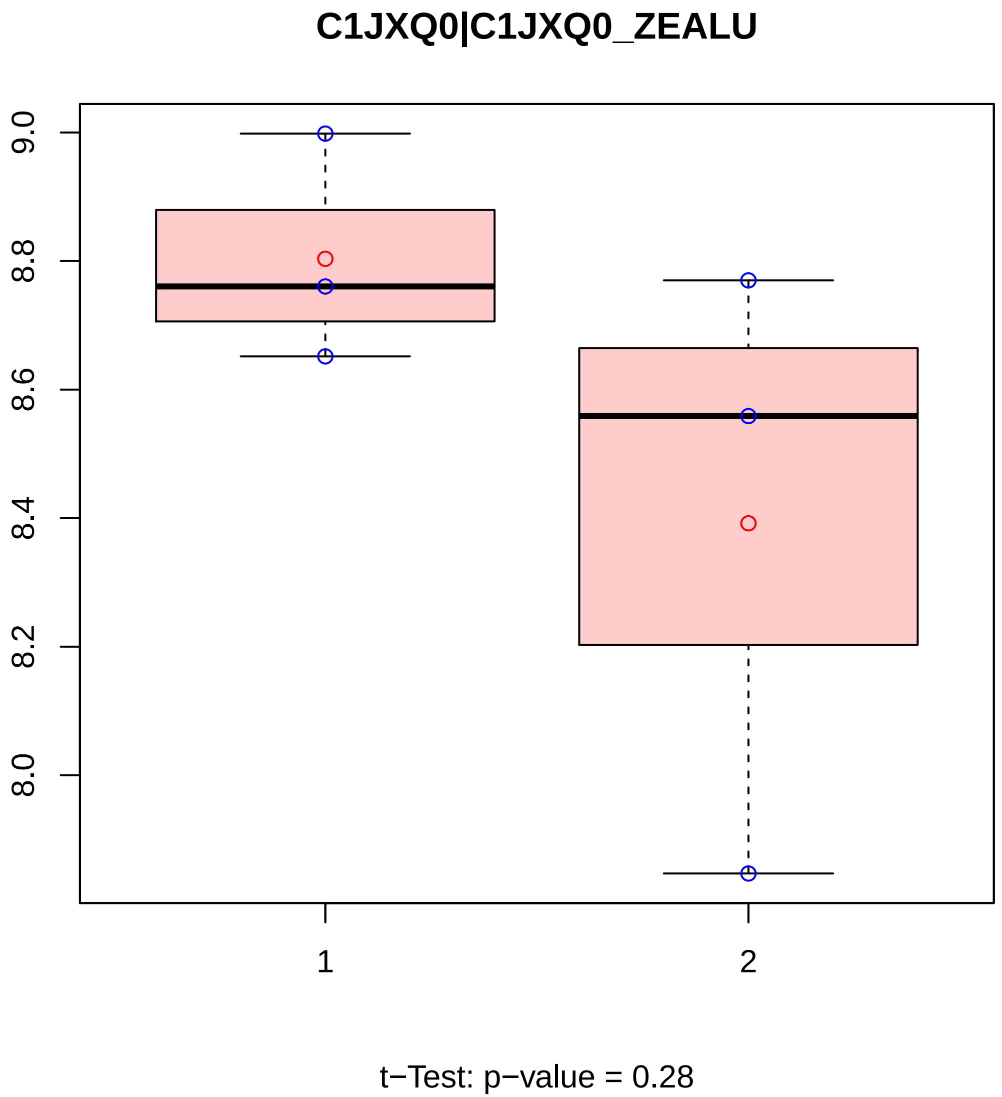

C1JXQ0|C1JXQ0_ZEALU
9.0
8.8
8.6
8.4
8.2
8.0
1
2
t−Test: p−value = 0.28

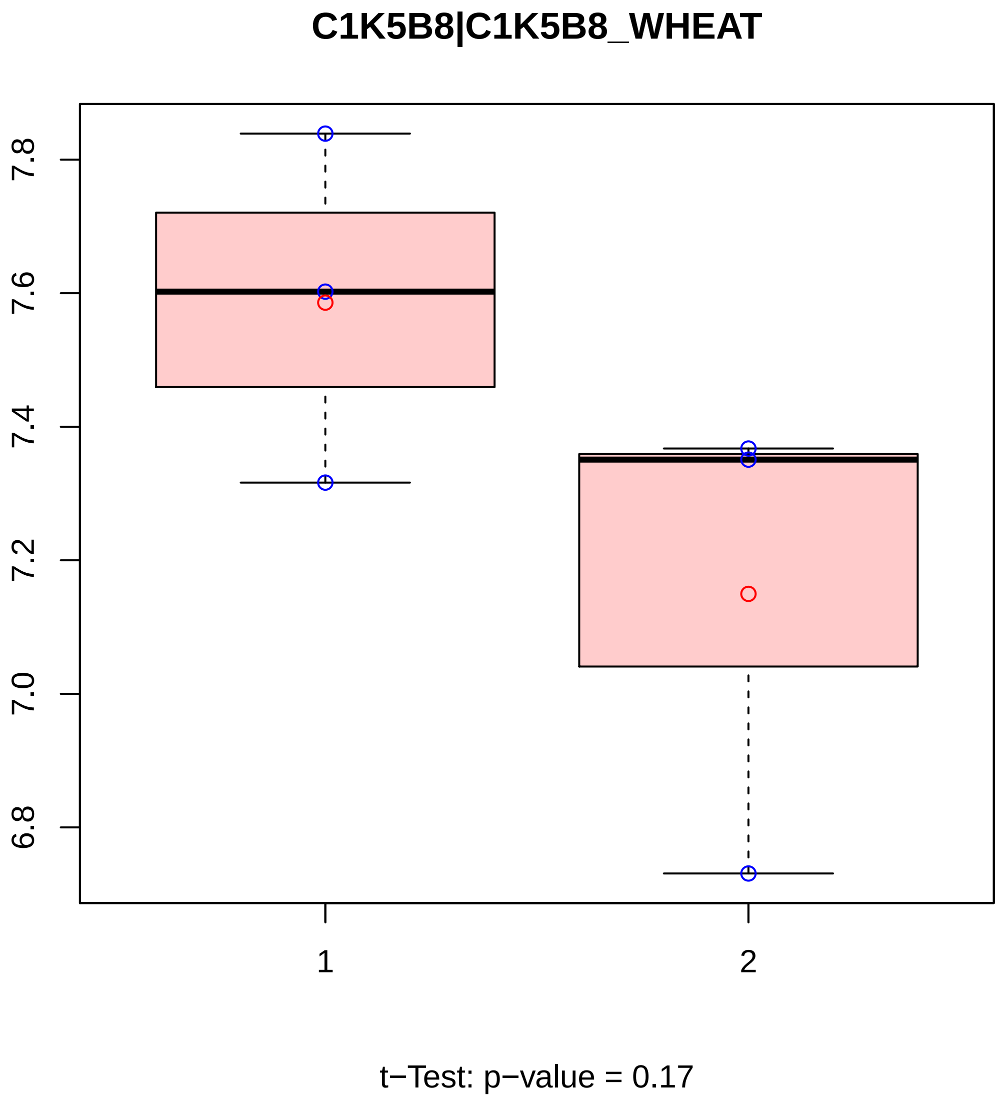
C1K5B8|C1K5B8_WHEAT
7.8
7.6
7.4
7.2
7.0
6.8
1
2
t−Test: p−value = 0.17

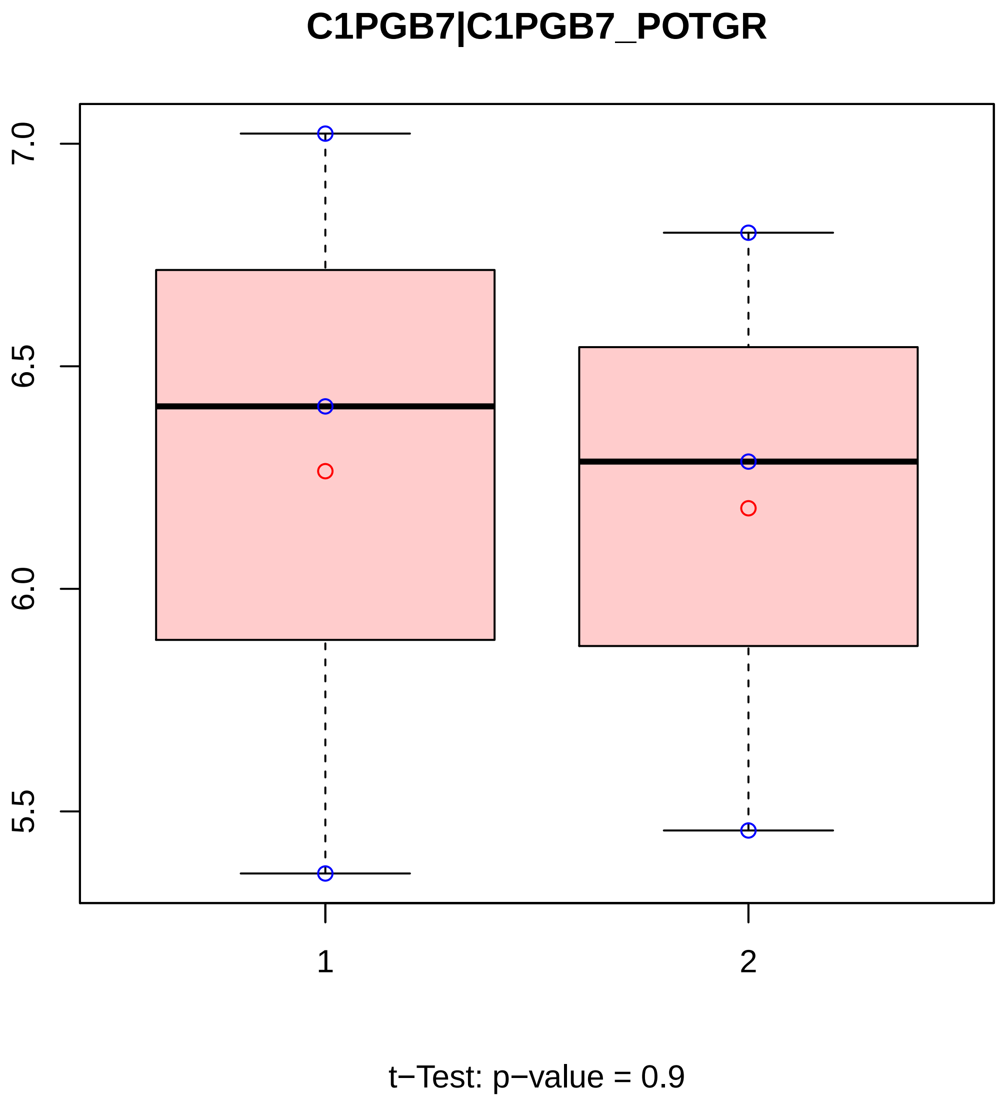

C1PGB7|C1PGB7_POTGR
7.0
6.5
6.0
5.5
1
2
t−Test: p−value = 0.9

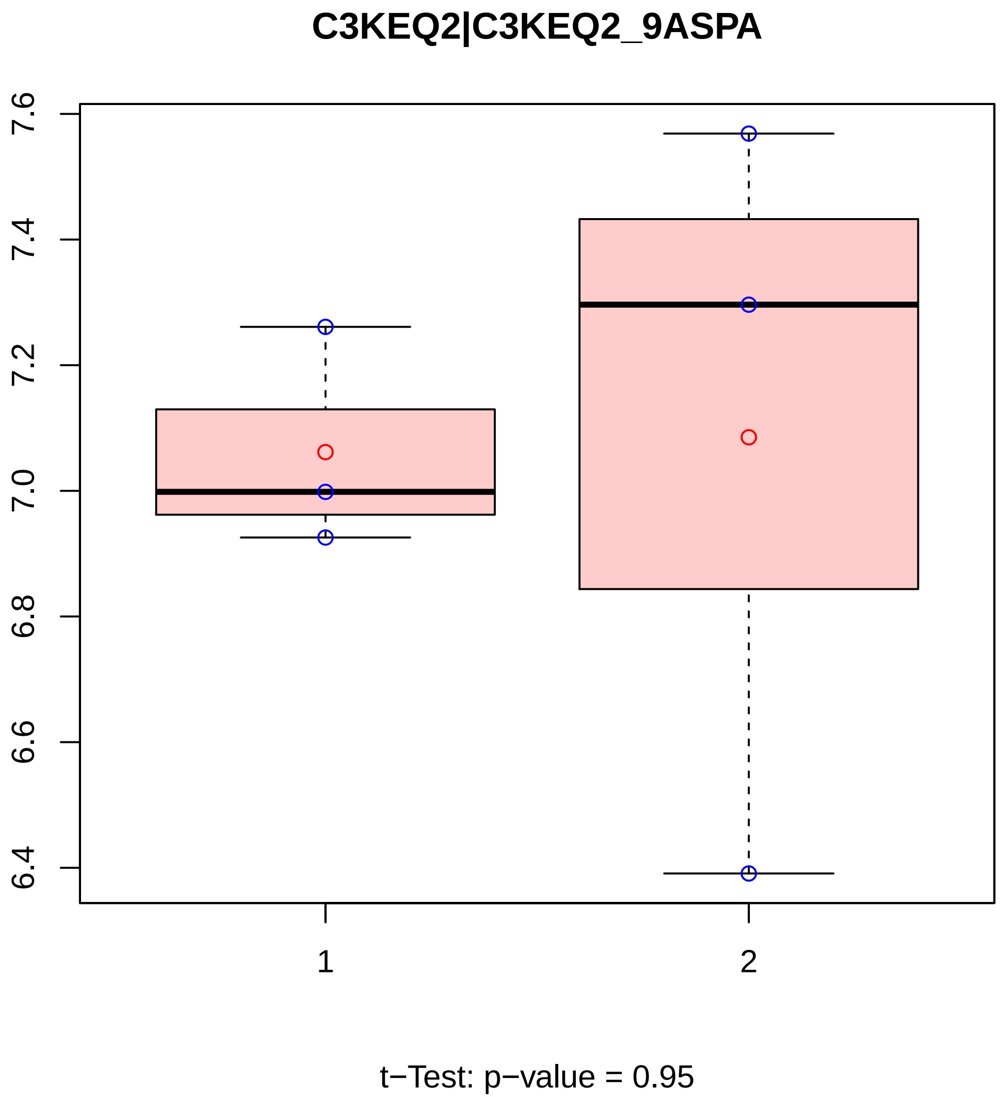
C3KEQ2|C3KEQ2_9ASPA
7.6
7.4
7.2
7.0
6.8
6.6
6.4
1
2
t−Test: p−value = 0.95

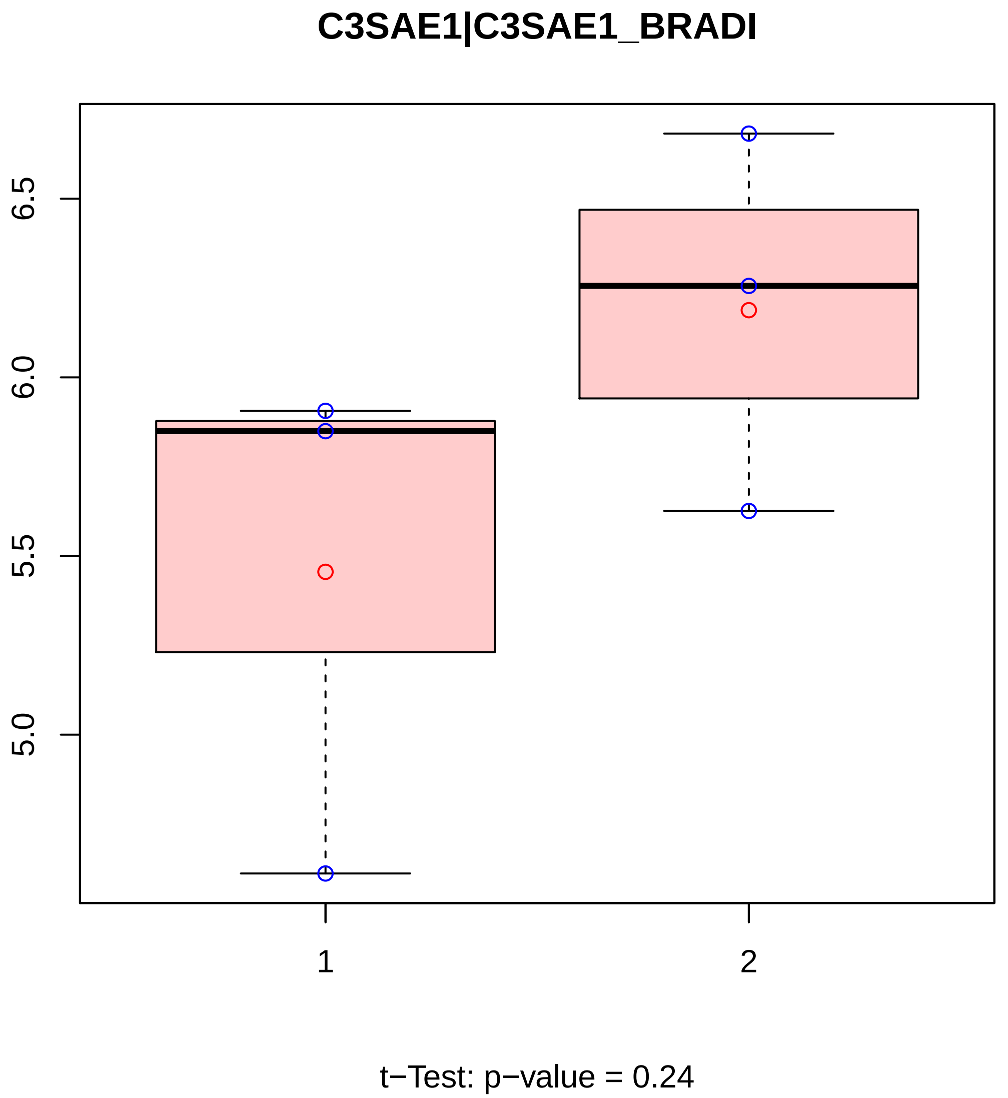
C3SAE1|C3SAE1_BRADI
6.5
6.0
5.5
5.0
1
2
t−Test: p−value = 0.24

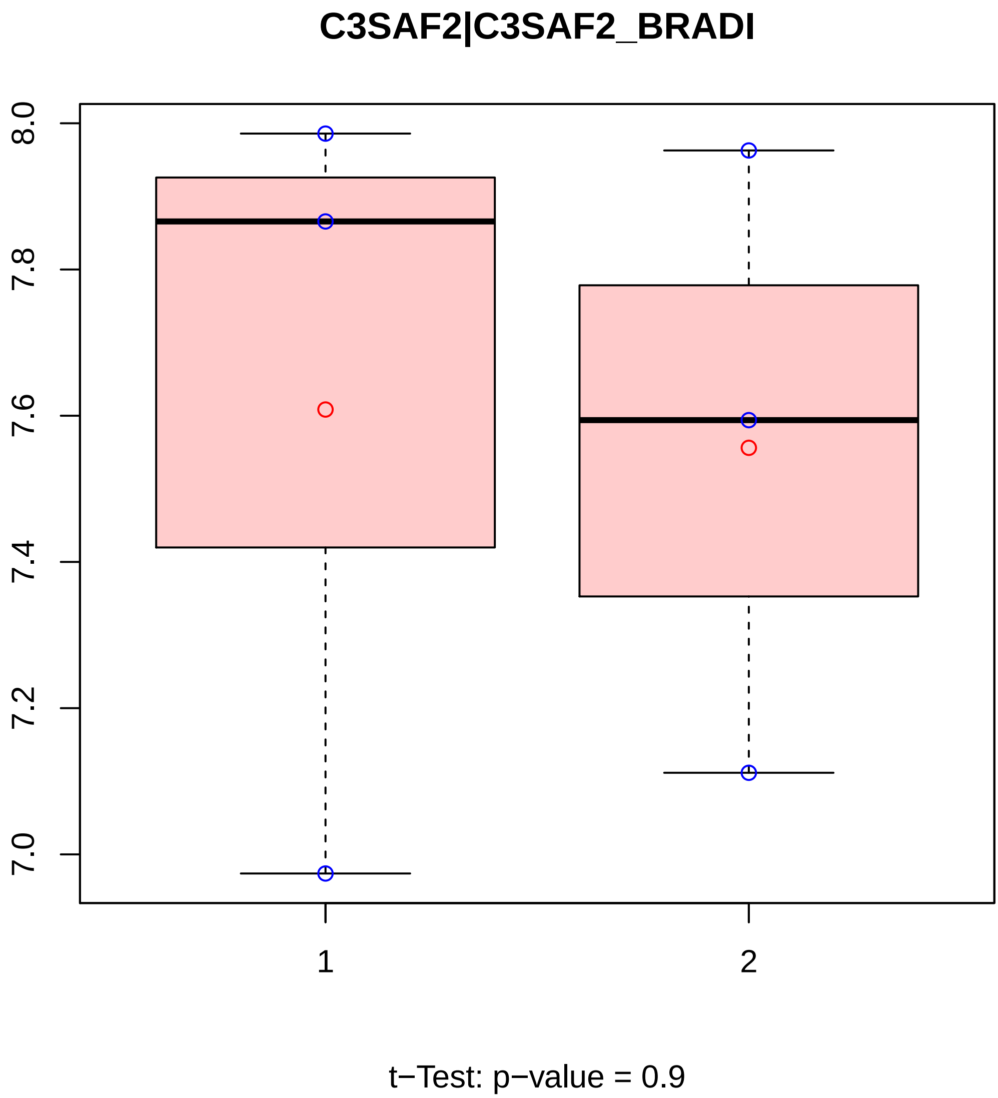

C3SAF2|C3SAF2_BRADI
8.0
7.8
7.6
7.4
7.2
7.0
1
2
t−Test: p−value = 0.9

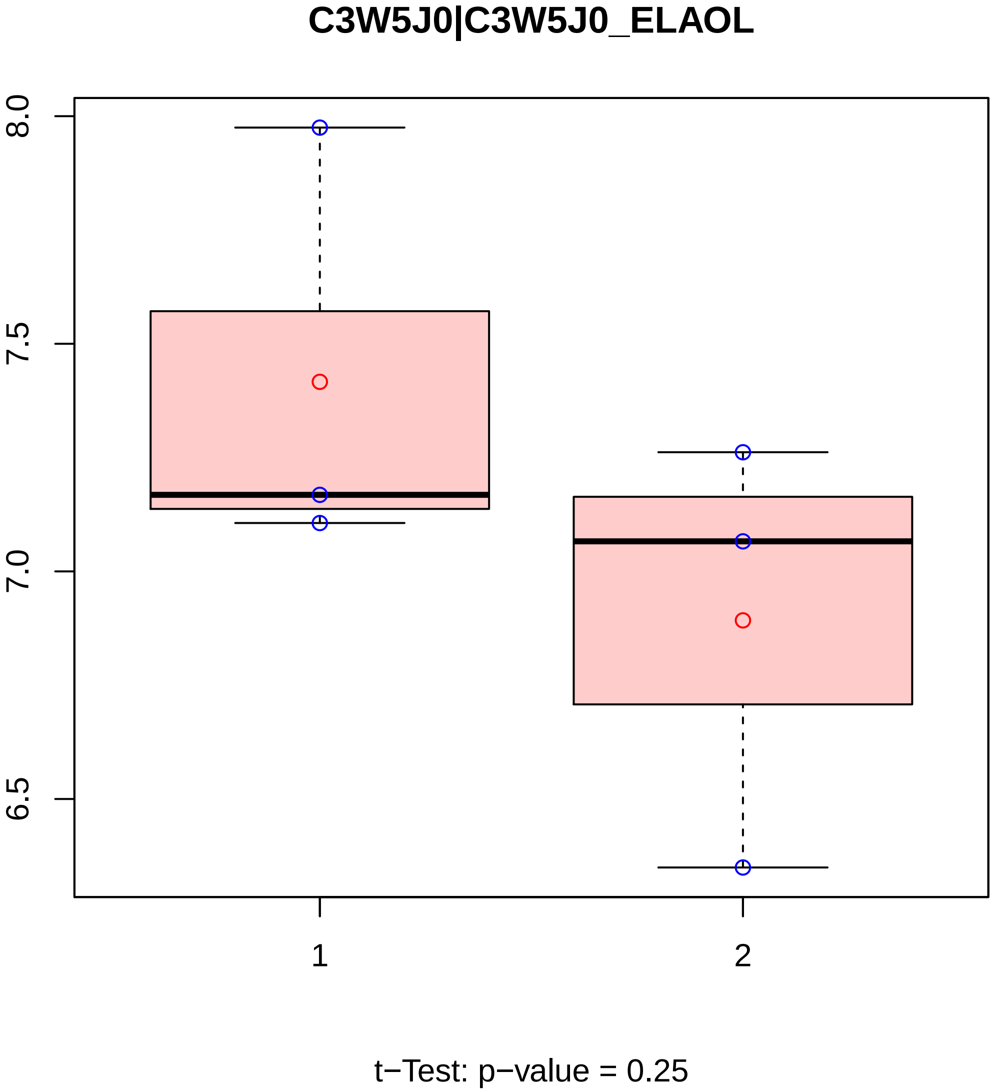

C3W5J0|C3W5J0_ELAOL
8.0
7.5
7.0
6.5
1
2
t−Test: p−value = 0.25

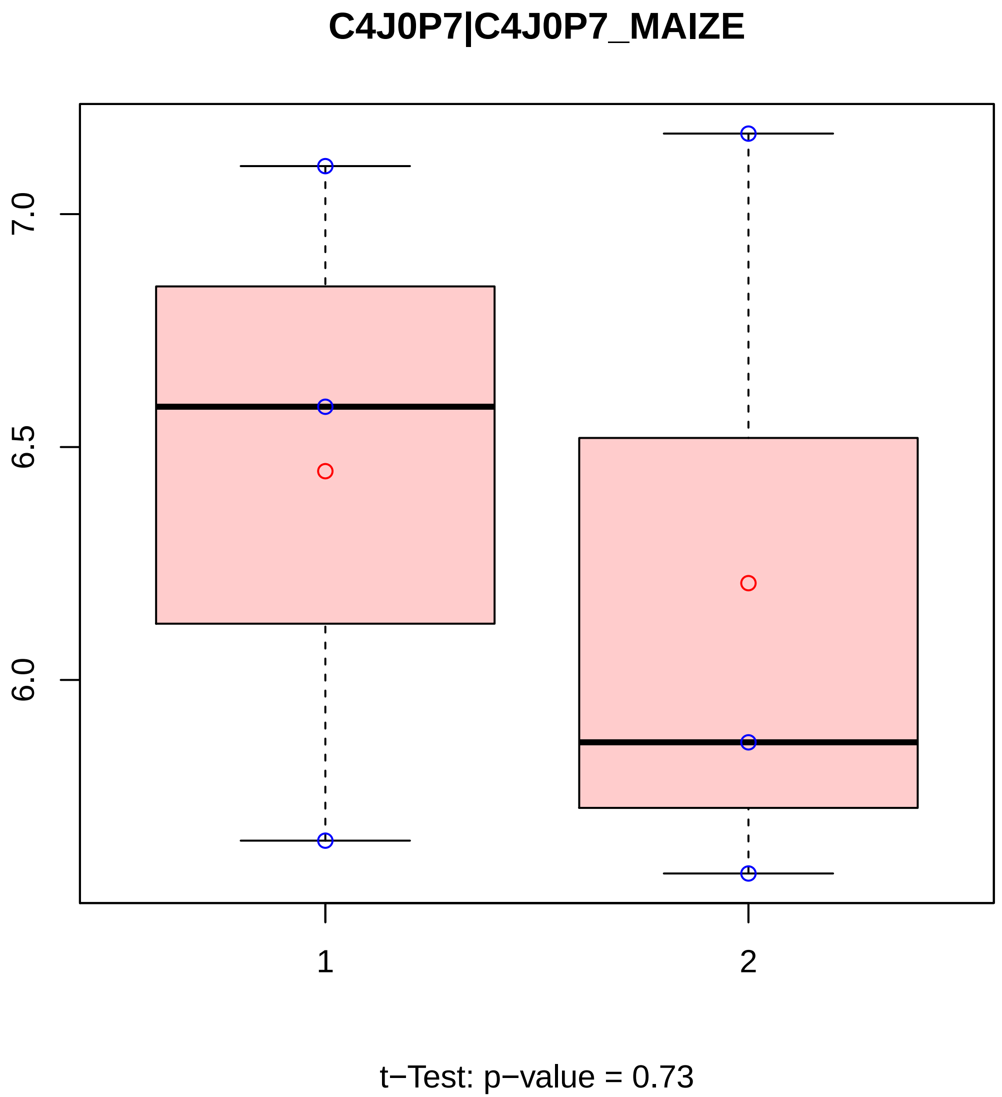
C4J0P7|C4J0P7_MAIZE
7.0
6.5
6.0
1
2
t−Test: p−value = 0.73

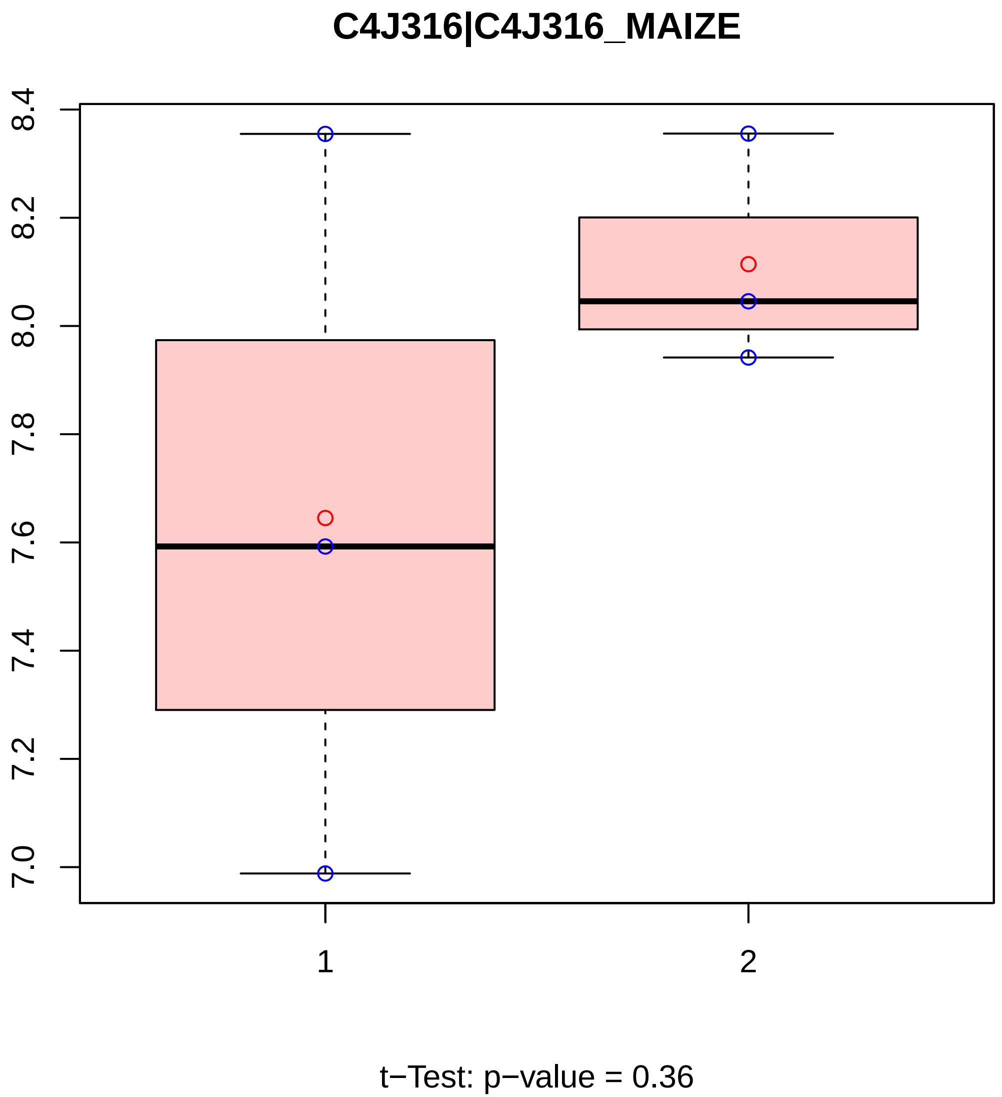
C4J316|C4J316_MAIZE
8.4
8.2
8.0
7.8
7.6
7.4
7.2
7.0
1
2
t−Test: p−value = 0.36

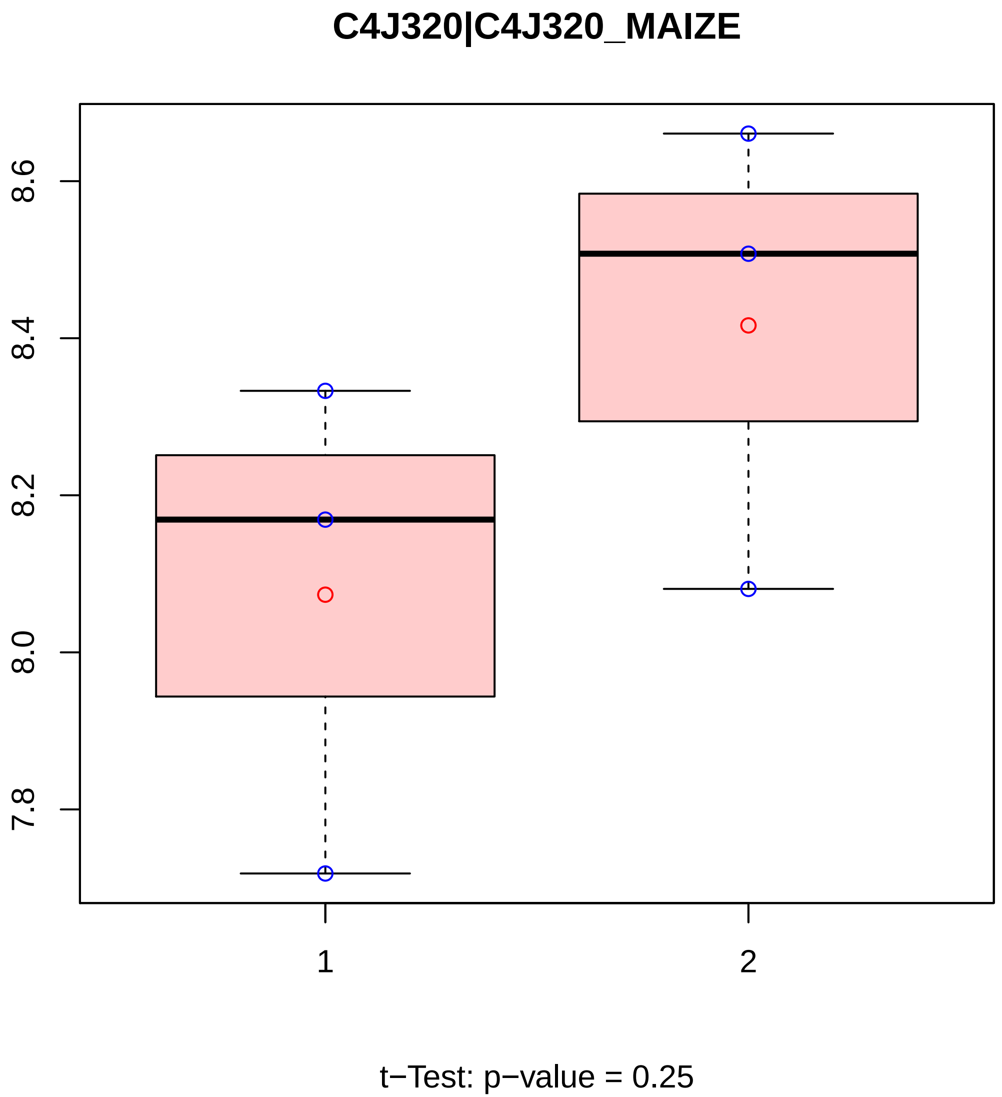
C4J320|C4J320_MAIZE
7.8
8.0
8.2
8.4
8.6
1
2
t−Test: p−value = 0.25

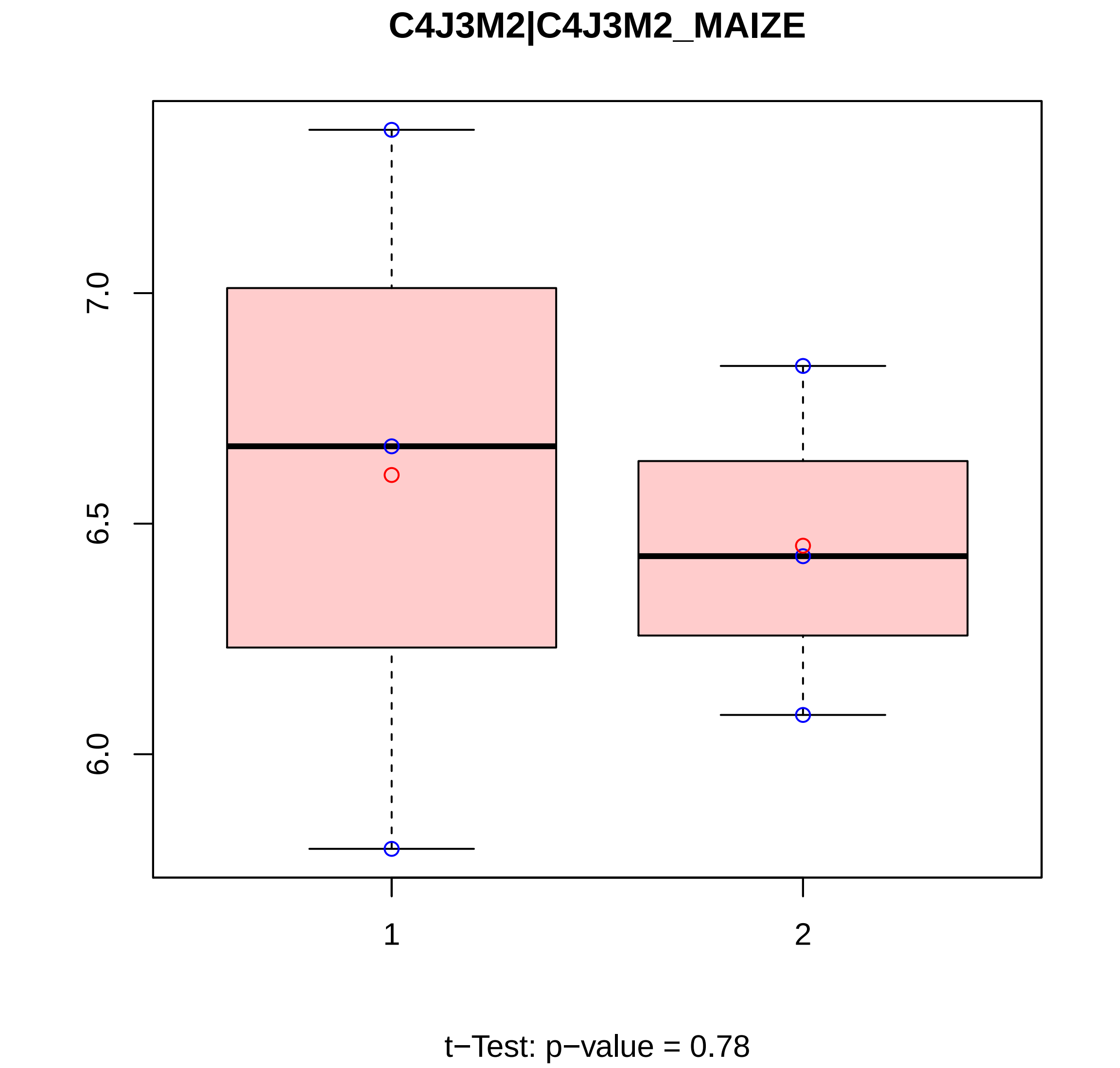

C4J3M2|C4J3M2_MAIZE
7.0
6.5
6.0
1
2
t−Test: p−value = 0.78

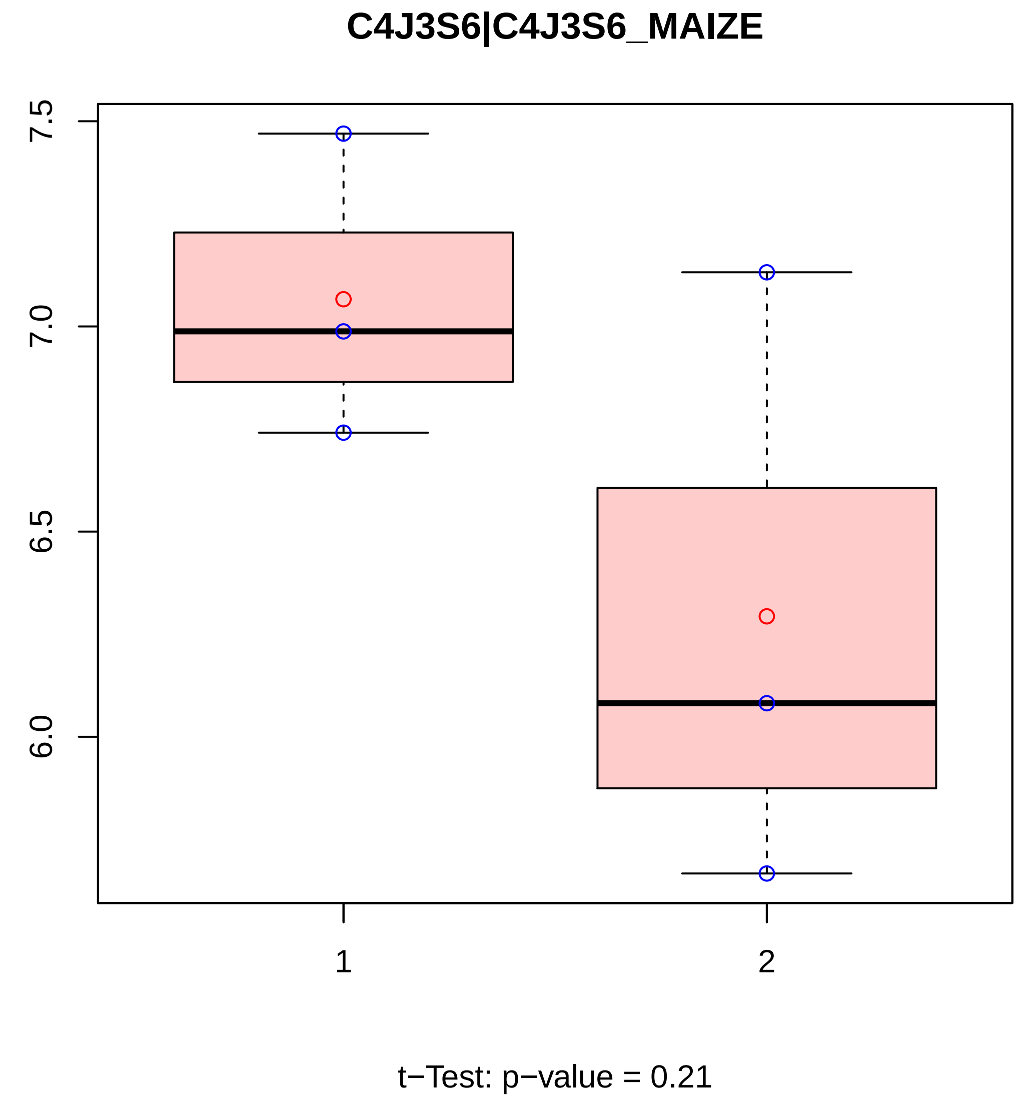

C4J3S6|C4J3S6_MAIZE
7.5
7.0
6.5
6.0
1
2
t−Test: p−value = 0.21

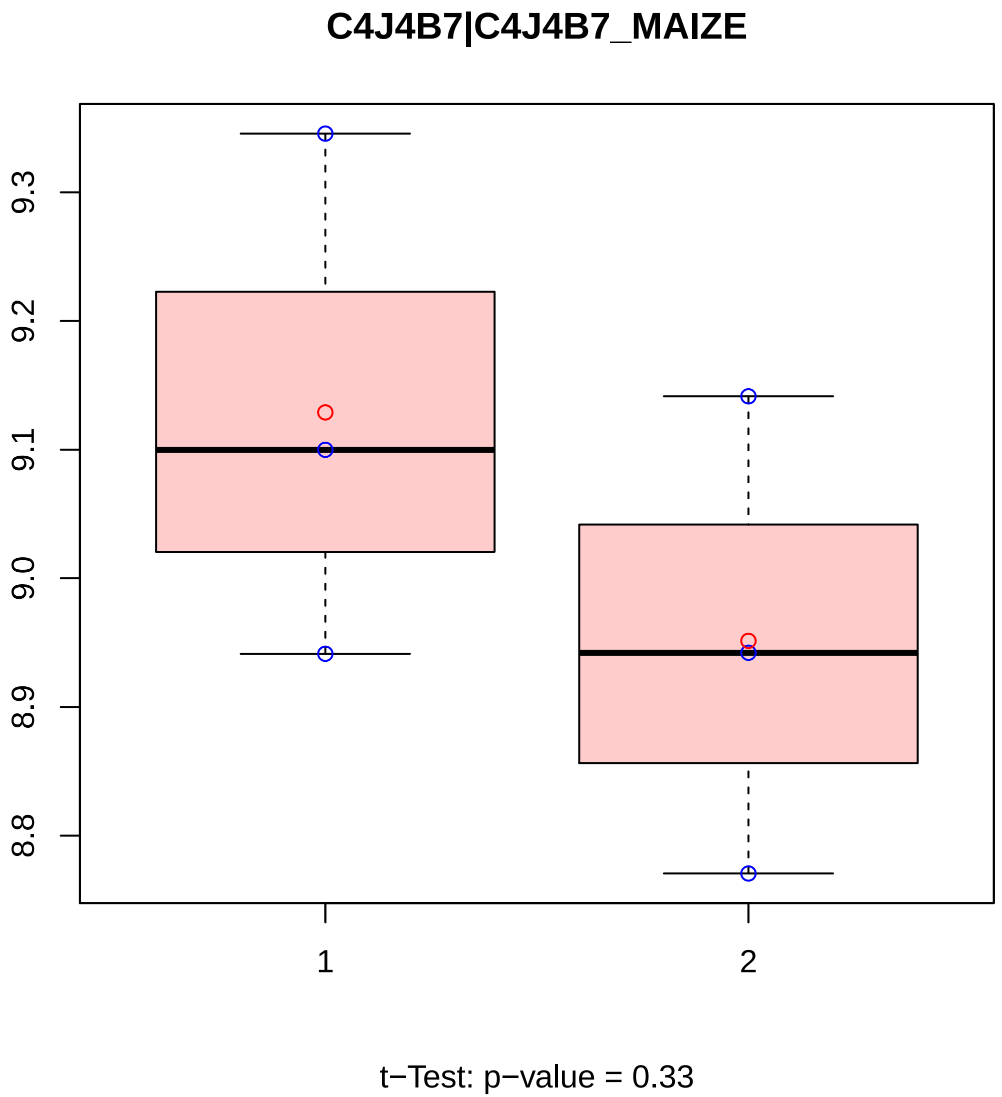
C4J4B7|C4J4B7_MAIZE
9.3
9.2
9.1
9.0
8.9
8.8
1
2
t−Test: p−value = 0.33

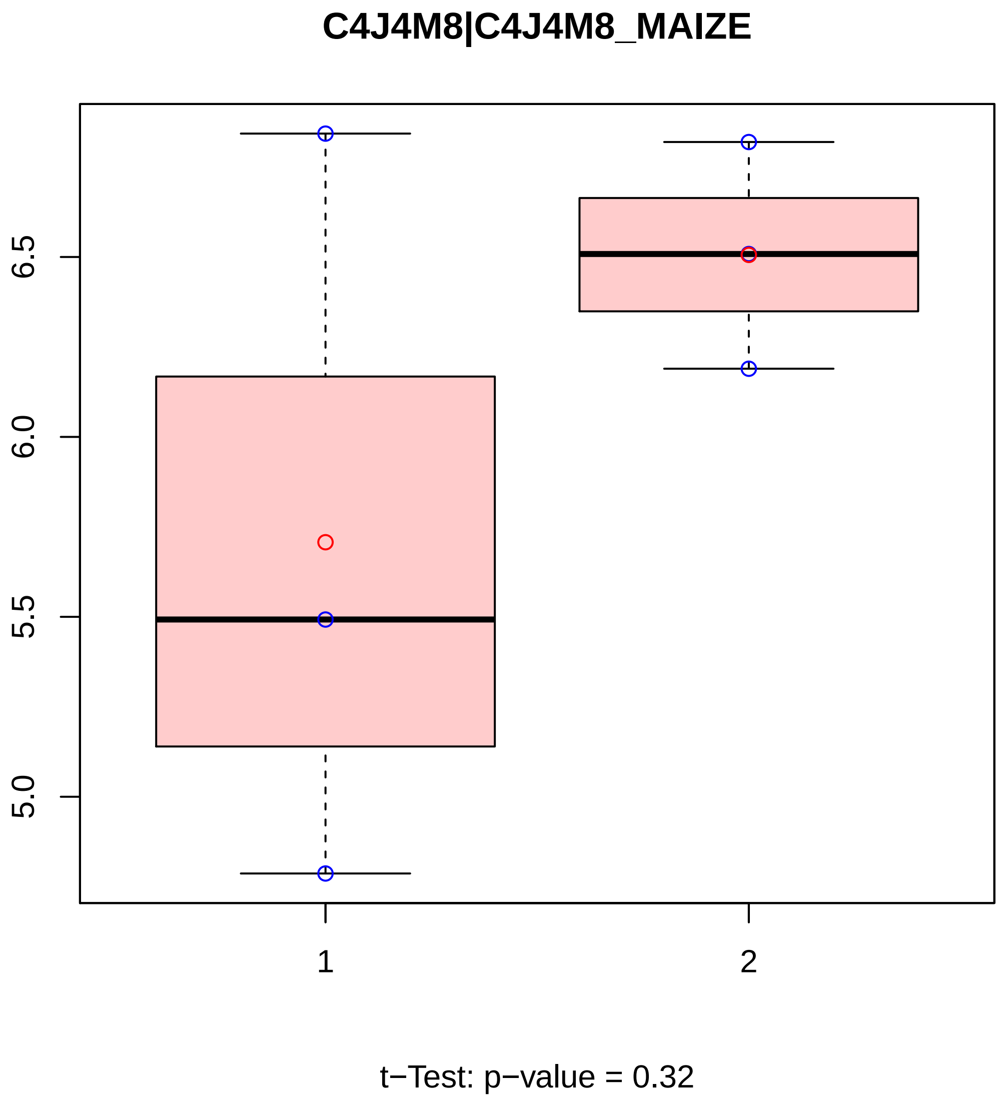
C4J4M8|C4J4M8_MAIZE
5.0
5.5
6.0
6.5
1
2
t−Test: p−value = 0.32

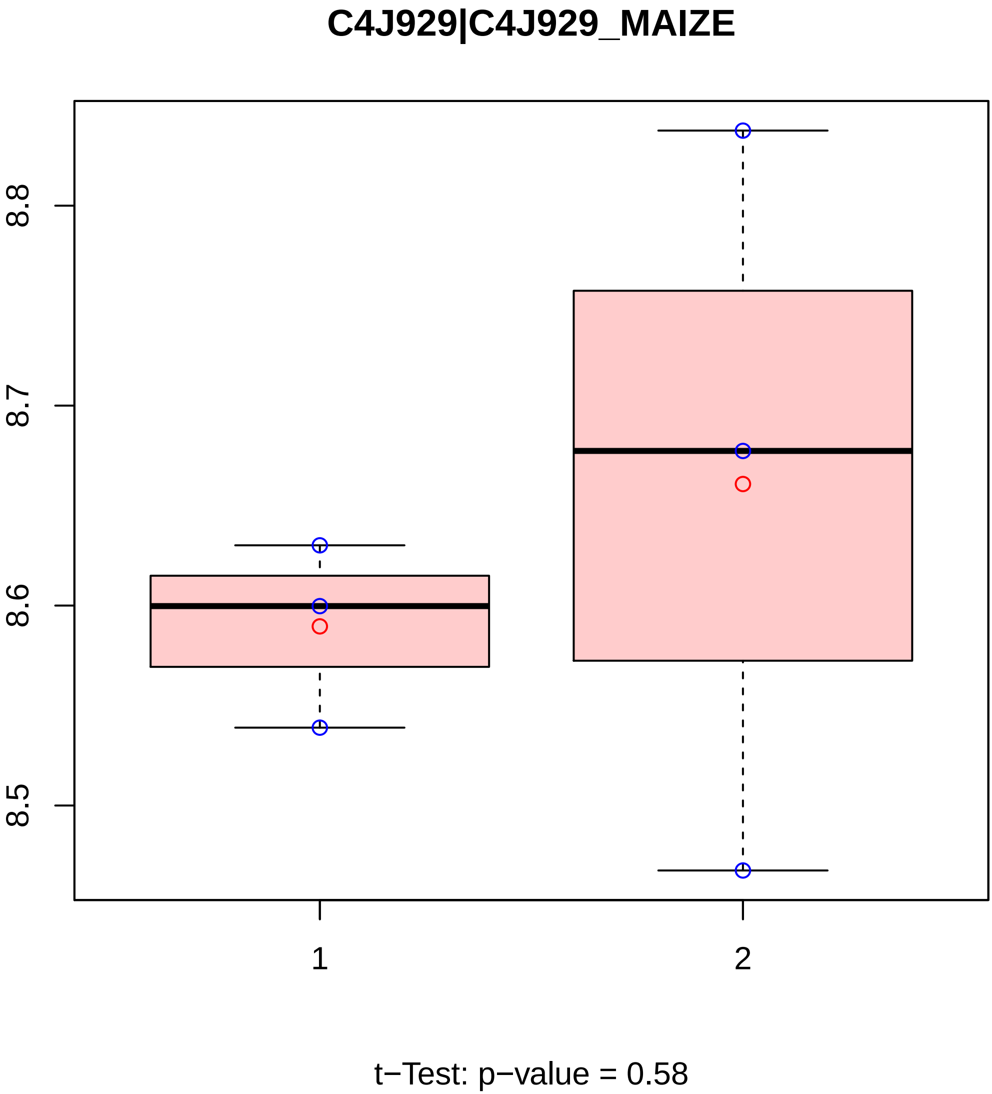

C4J929|C4J929_MAIZE
8.5
8.6
8.7
8.8
1
2
t−Test: p−value = 0.58

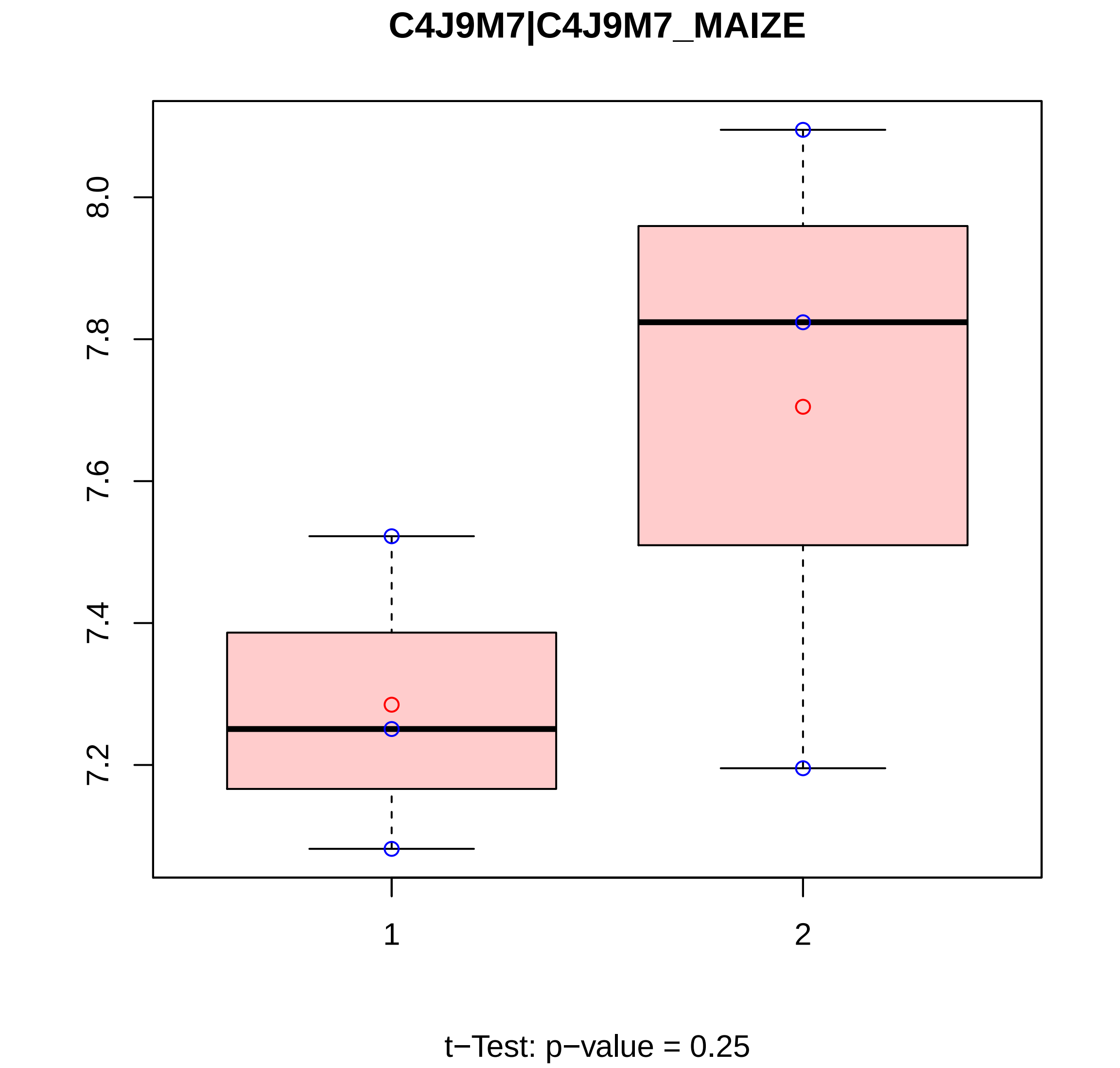

C4J9M7|C4J9M7_MAIZE
8.0
7.8
7.6
7.4
7.2
1
2
t−Test: p−value = 0.25

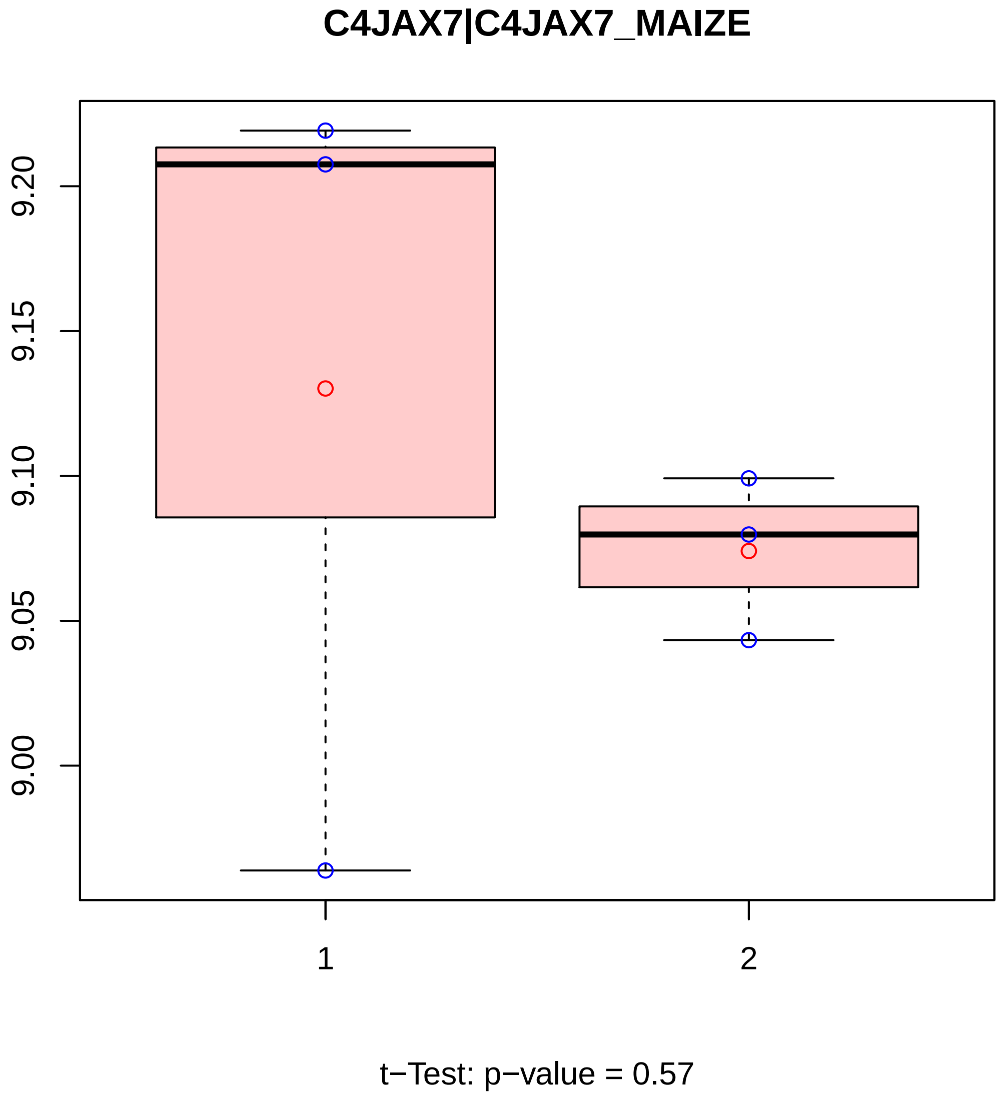
C4JAX7|C4JAX7_MAIZE
9.00
9.05
9.10
9.15
9.20
1
2
t−Test: p−value = 0.57

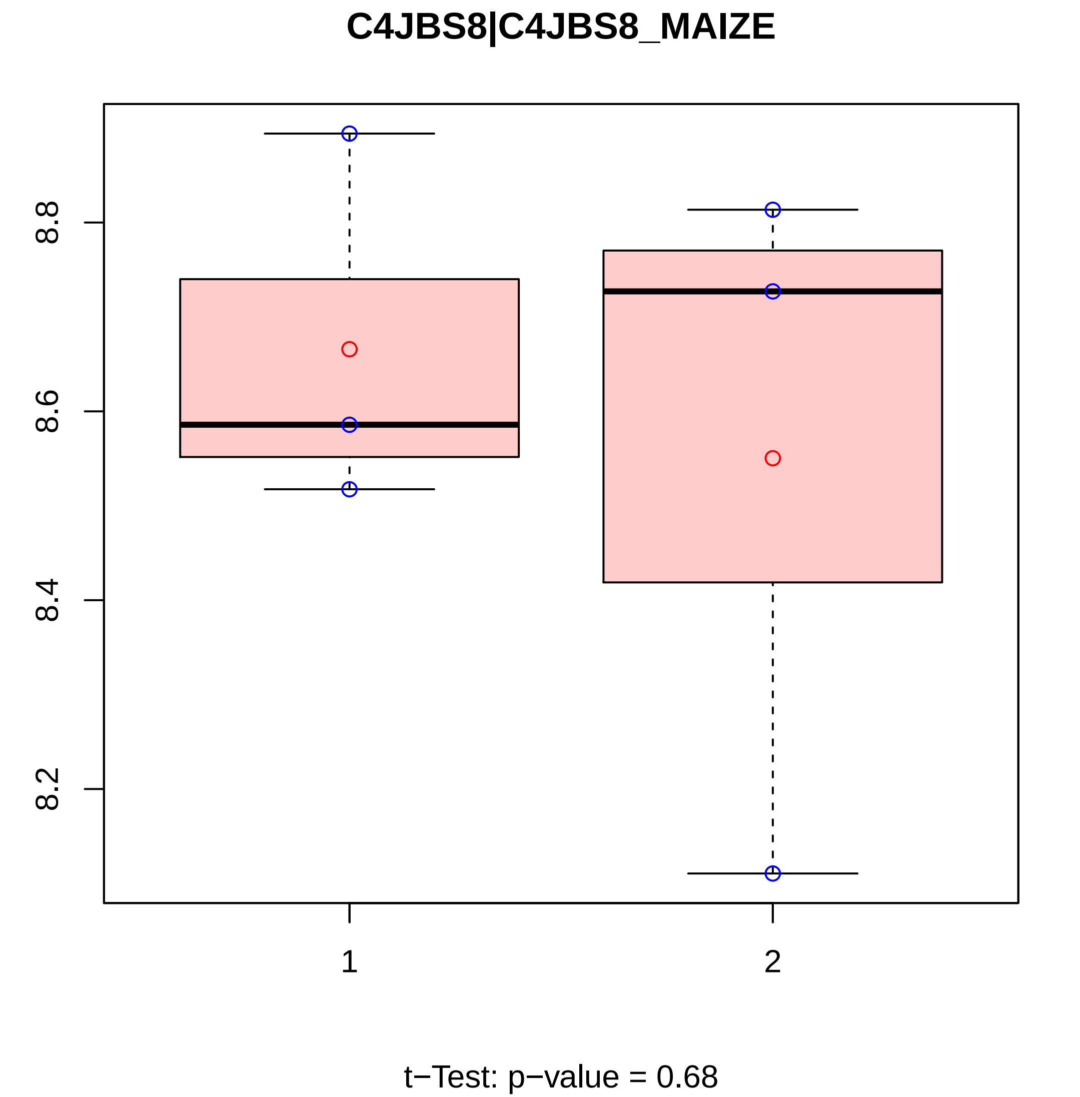
C4JBS8|C4JBS8_MAIZE
8.8
8.6
8.4
8.2
1
2
t−Test: p−value = 0.68

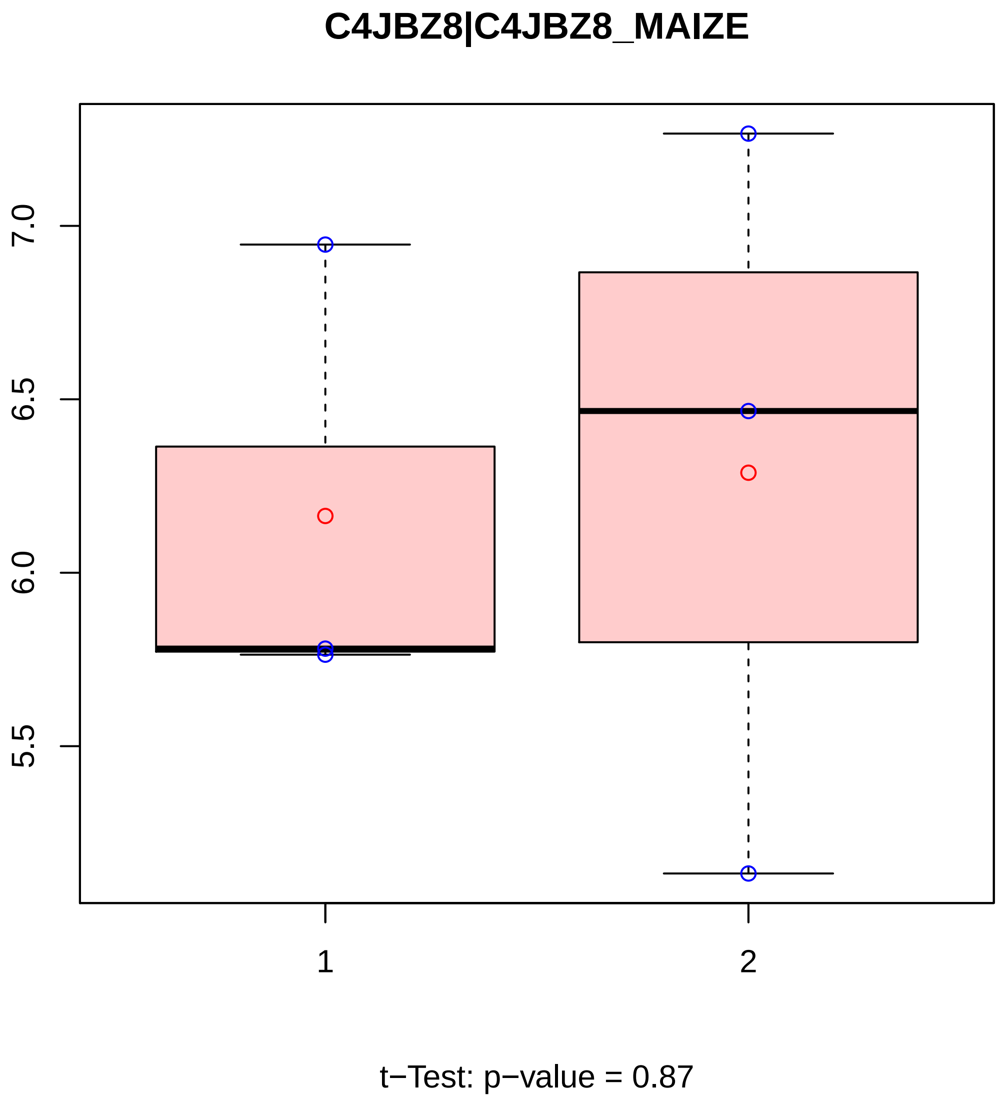

C4JBZ8|C4JBZ8_MAIZE
7.0
6.5
6.0
5.5
1
2
t−Test: p−value = 0.87

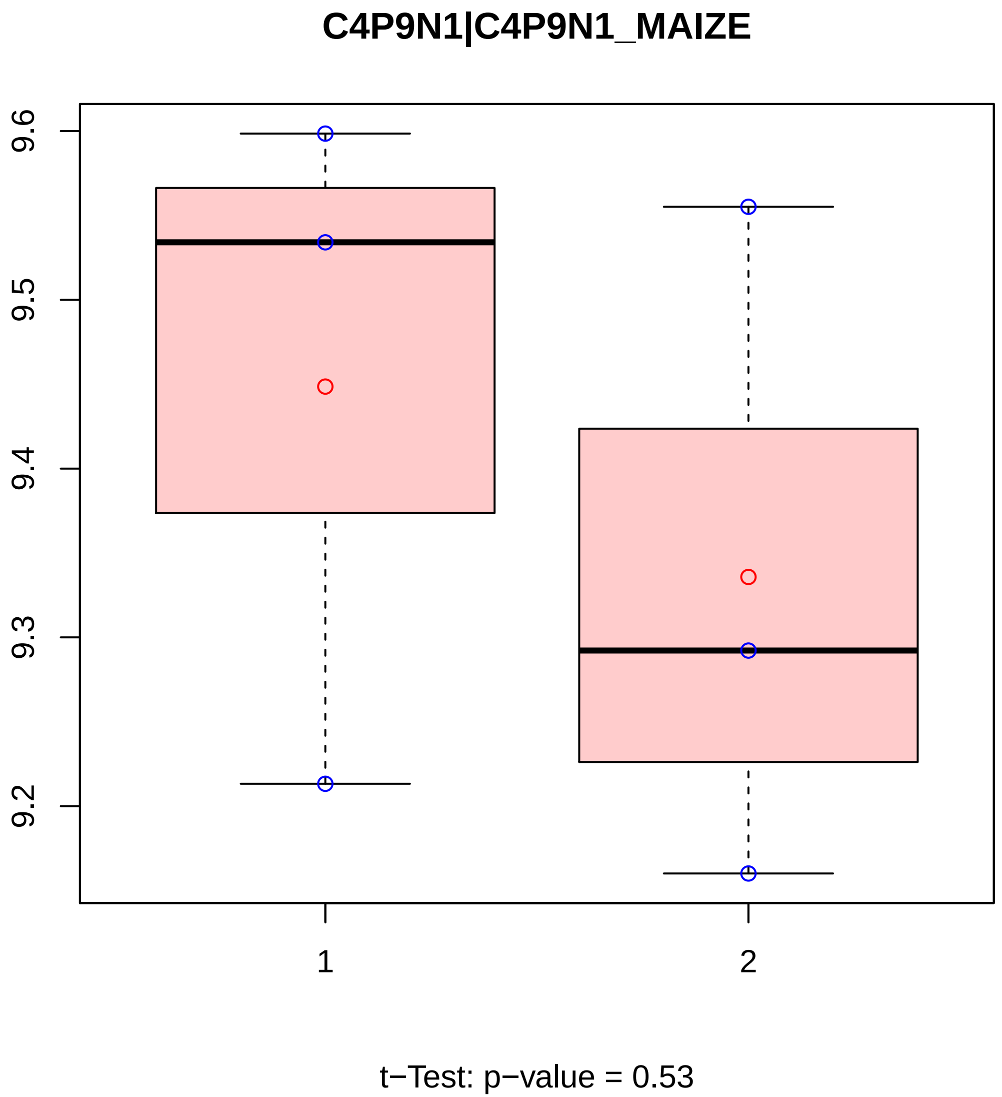
C4P9N1|C4P9N1_MAIZE
9.6
9.5
9.4
9.3
9.2
1
2
t−Test: p−value = 0.53

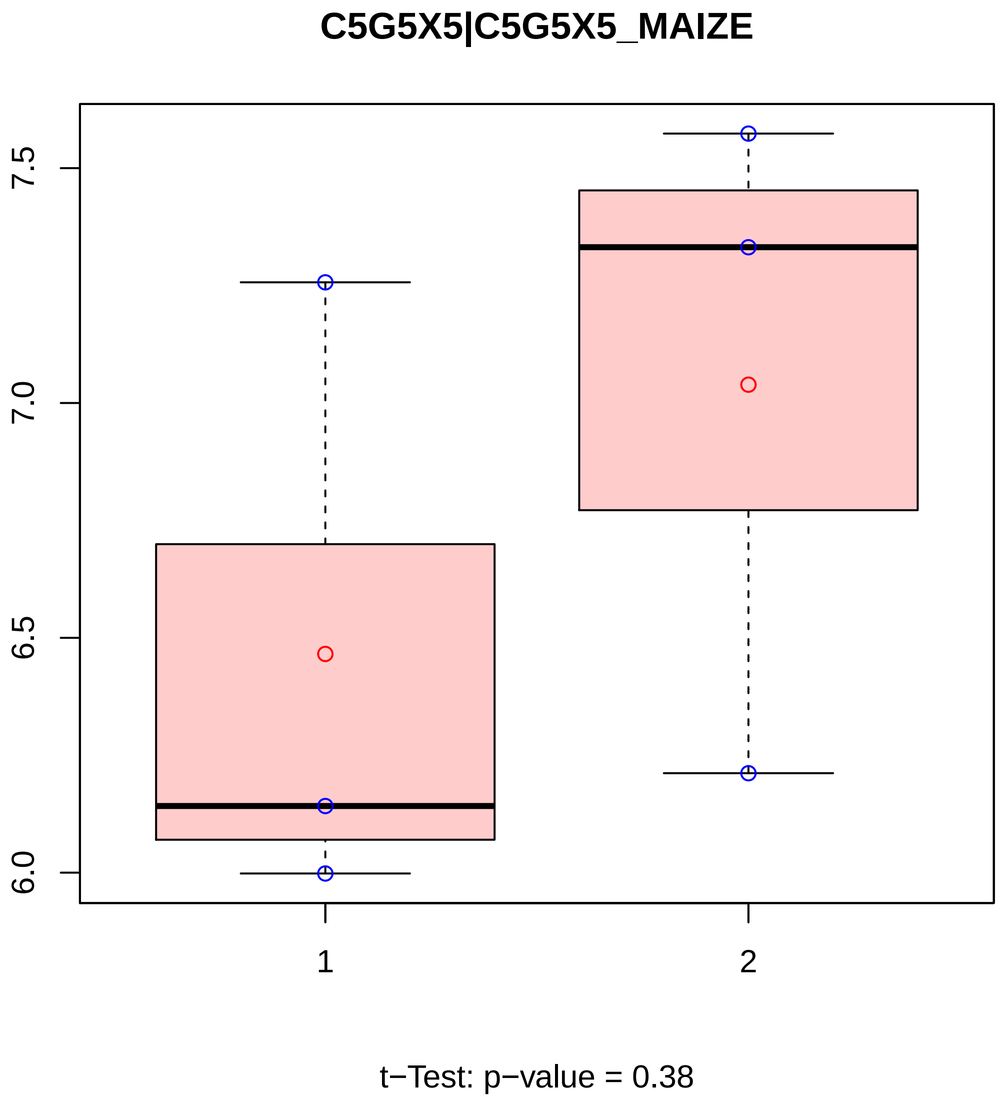

C5G5X5|C5G5X5_MAIZE
7.5
7.0
6.5
6.0
1
2
t−Test: p−value = 0.38

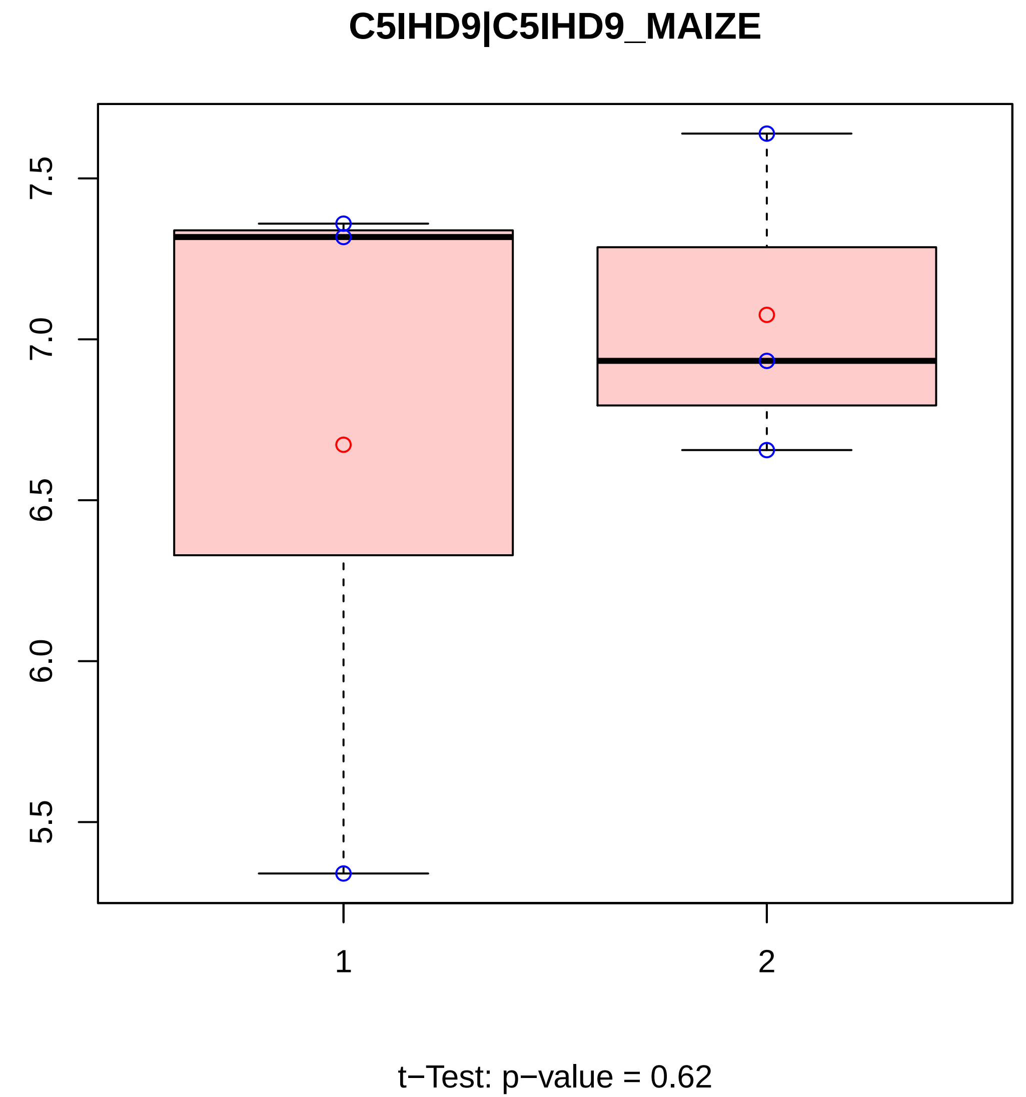

C5IHD9|C5IHD9_MAIZE
7.5
7.0
6.5
6.0
5.5
1
2
t−Test: p−value = 0.62

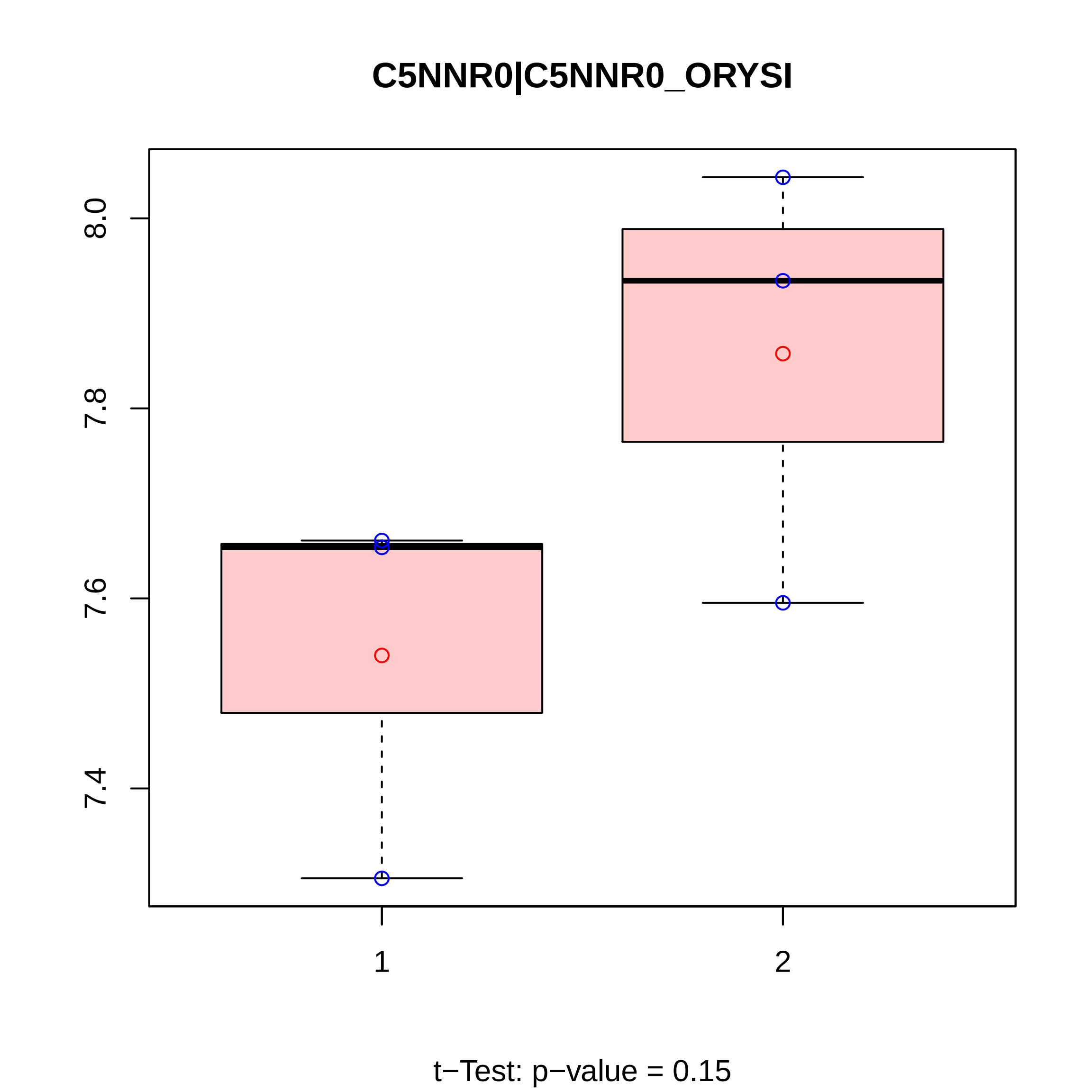
C5NNR0|C5NNR0_ORYSI
8.0
7.8
7.6
7.4
1
2
t−Test: p−value = 0.15

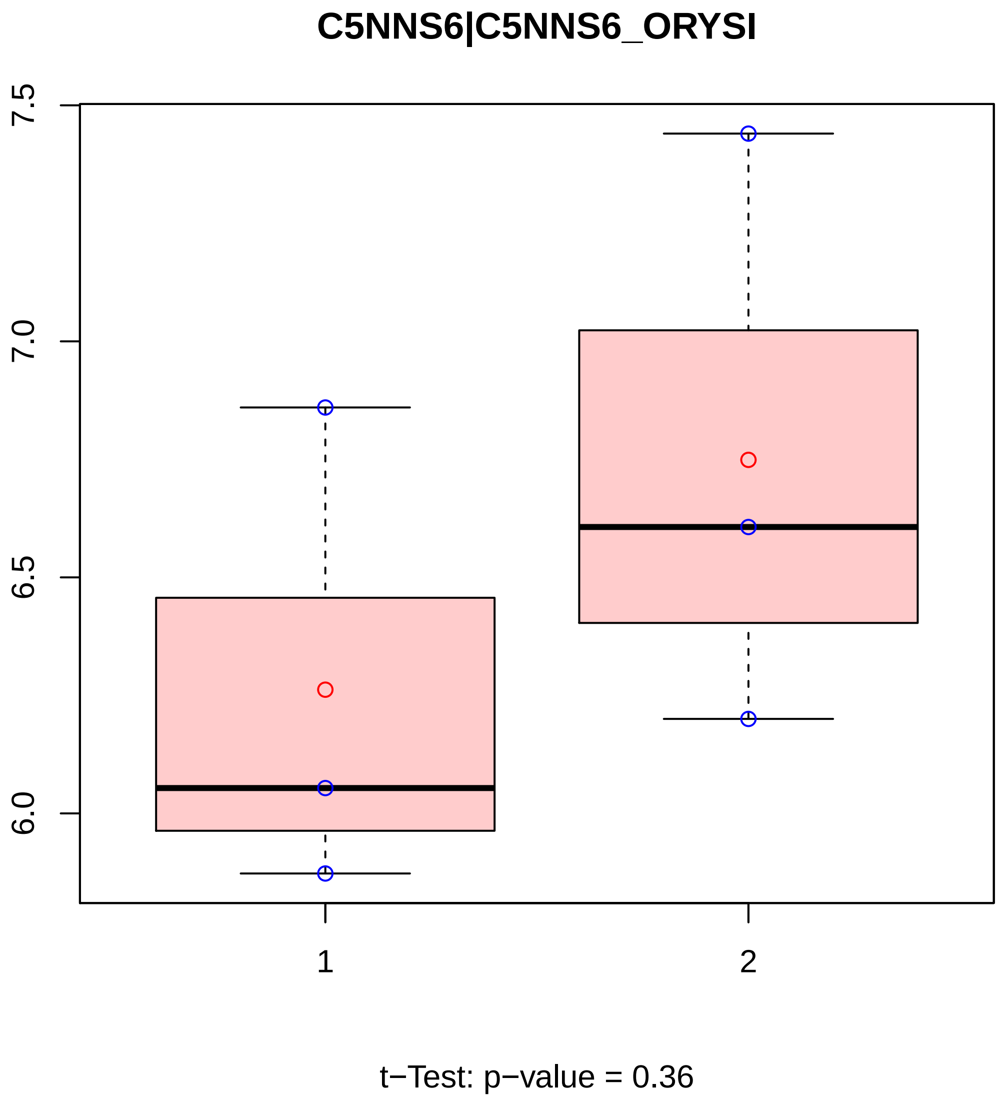
C5NNS6|C5NNS6_ORYSI
7.5
7.0
6.5
6.0
1
2
t−Test: p−value = 0.36

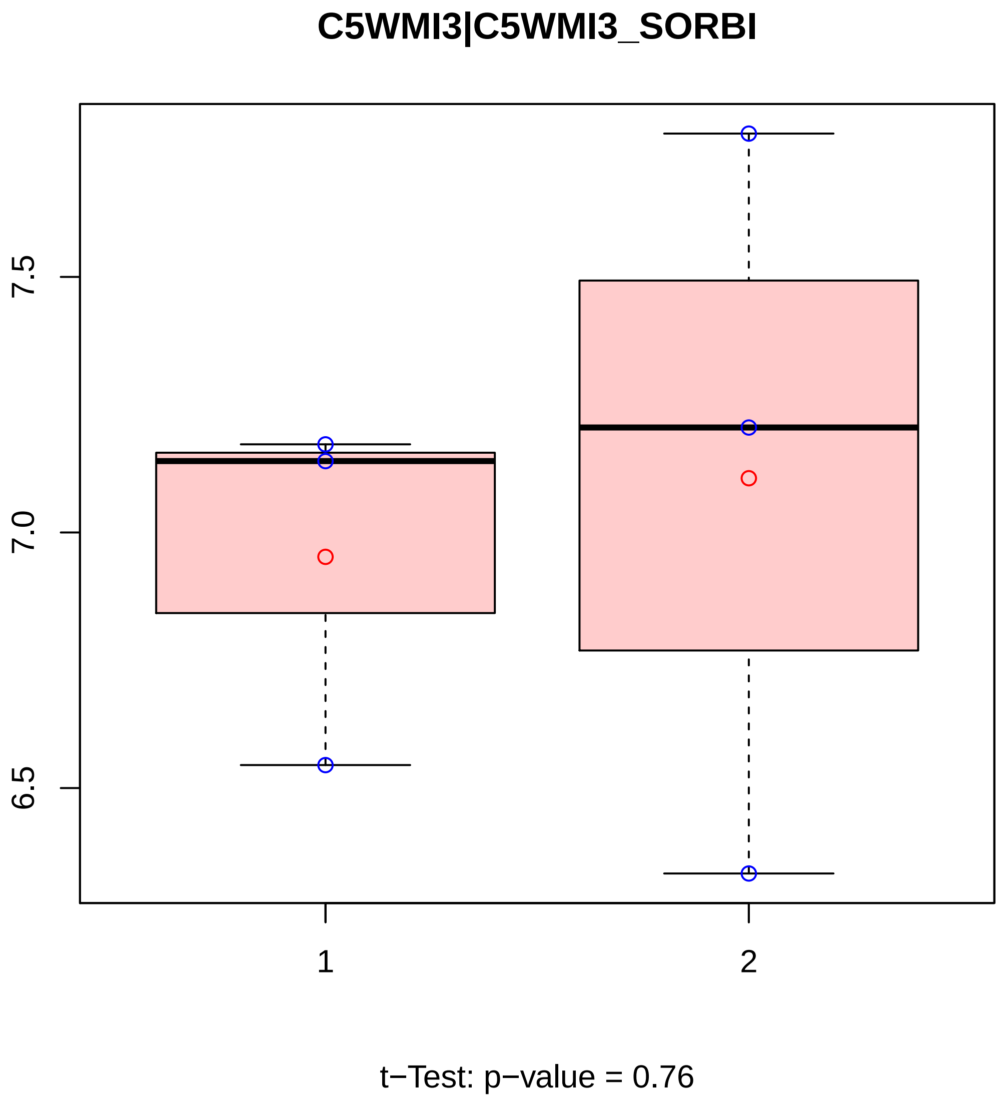
C5WMI3|C5WMI3_SORBI
7.5
7.0
6.5
1
2
t−Test: p−value = 0.76

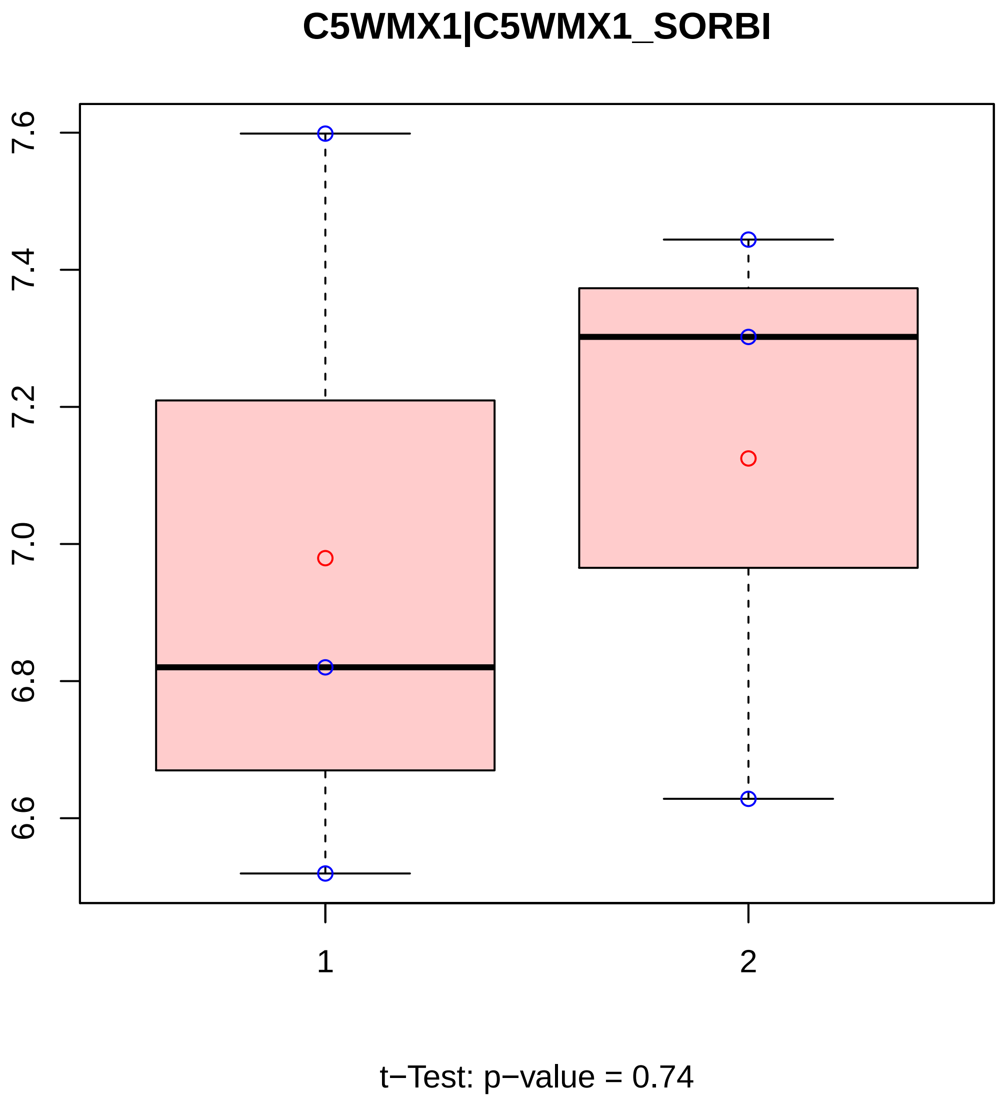

C5WMX1|C5WMX1_SORBI
7.6
7.4
7.2
7.0
6.8
6.6
1
2
t−Test: p−value = 0.74

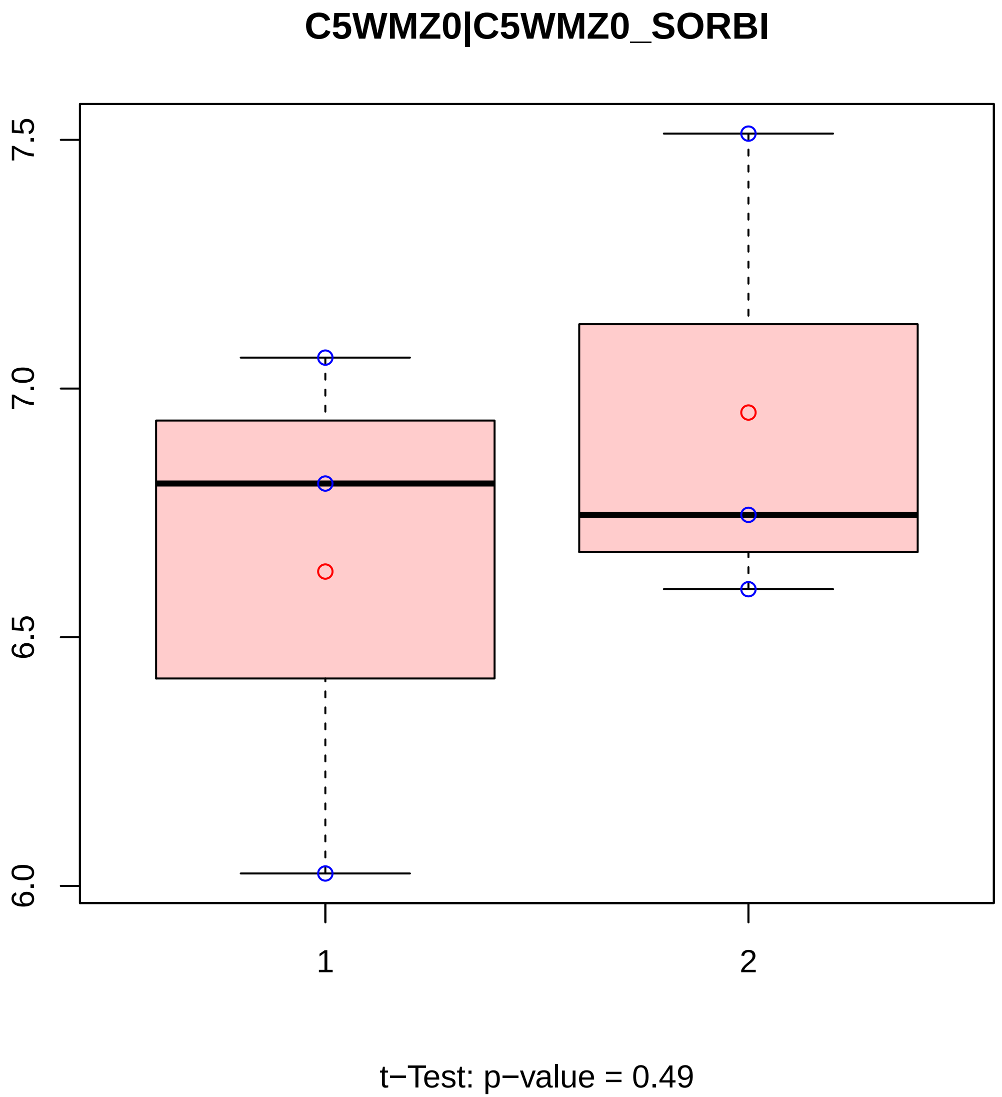

C5WMZ0|C5WMZ0_SORBI
7.5
7.0
6.5
6.0
1
2
t−Test: p−value = 0.49

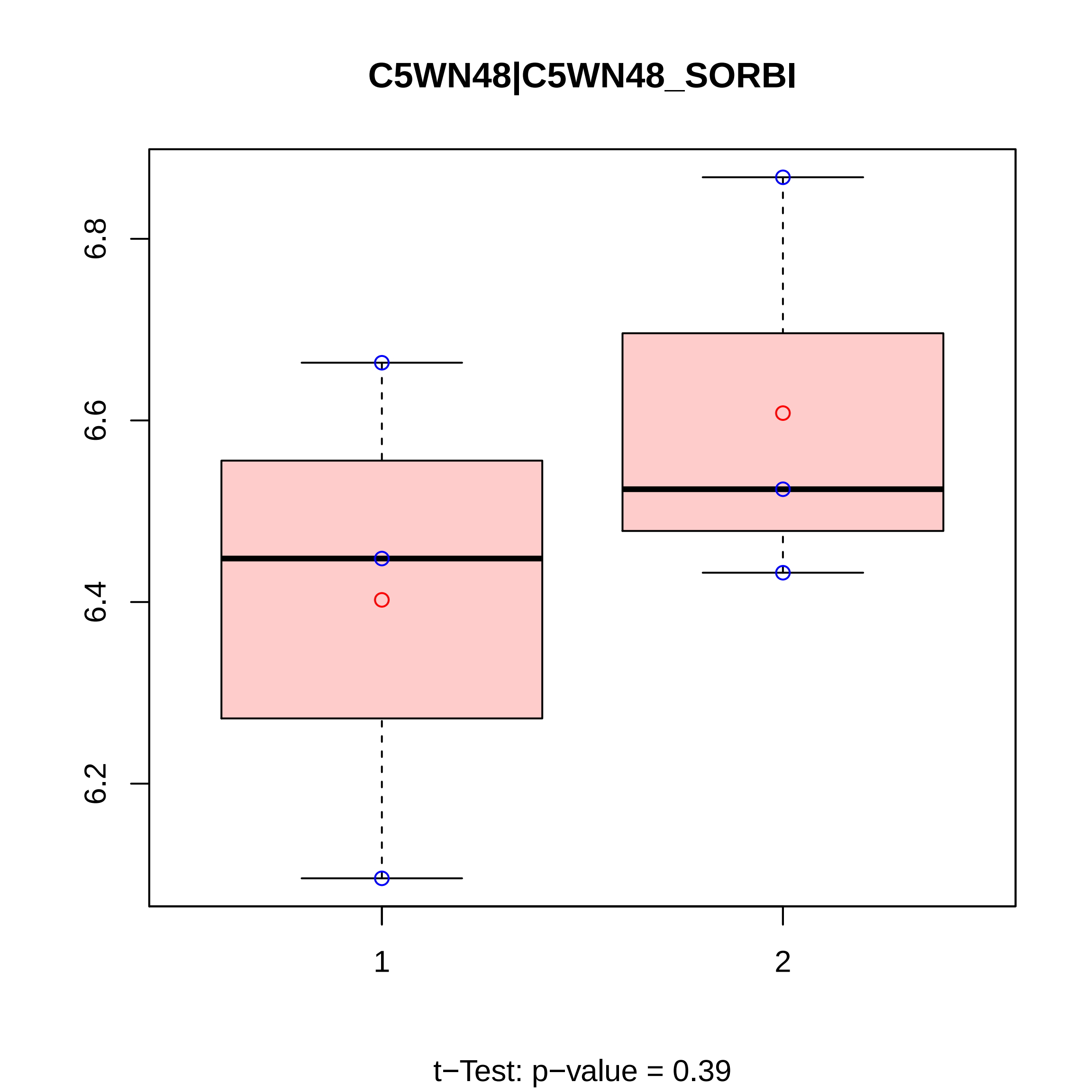

C5WN48|C5WN48_SORBI
6.2
6.4
6.6
6.8
1
2
t−Test: p−value = 0.39

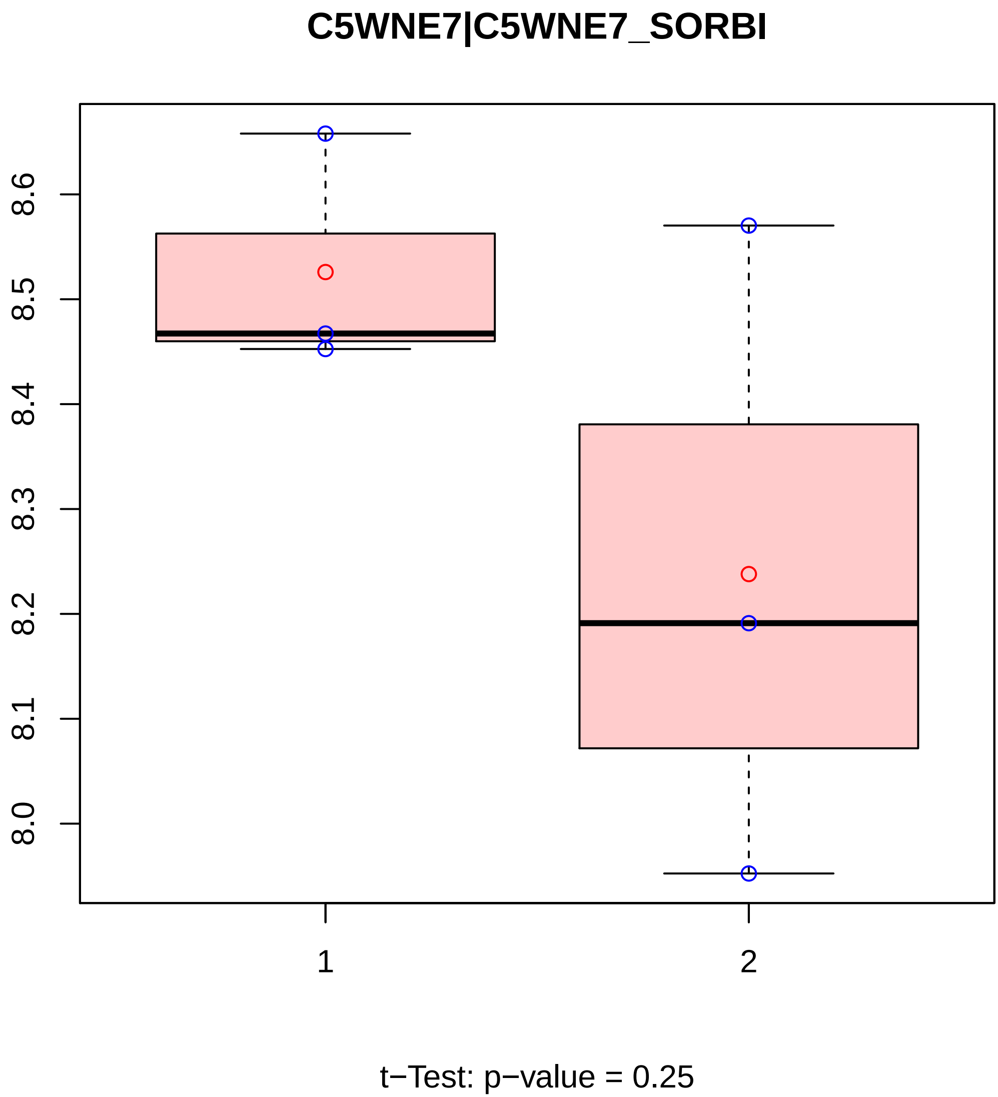
C5WNE7|C5WNE7_SORBI
8.0
8.1
8.2
8.3
8.4
8.5
8.6
1
2
t−Test: p−value = 0.25

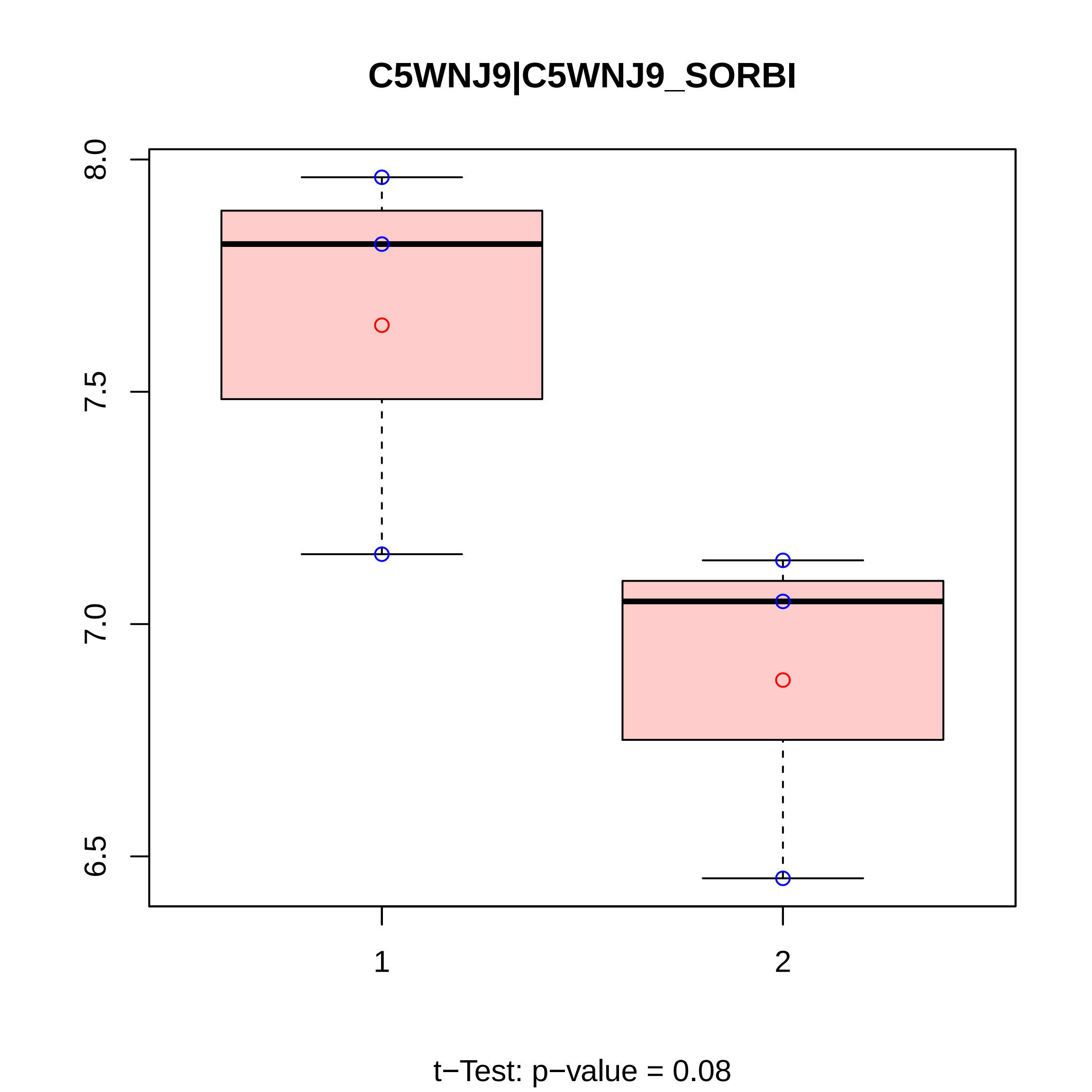
C5WNJ9|C5WNJ9_SORBI
8.0
7.5
7.0
6.5
1
2
t−Test: p−value = 0.08

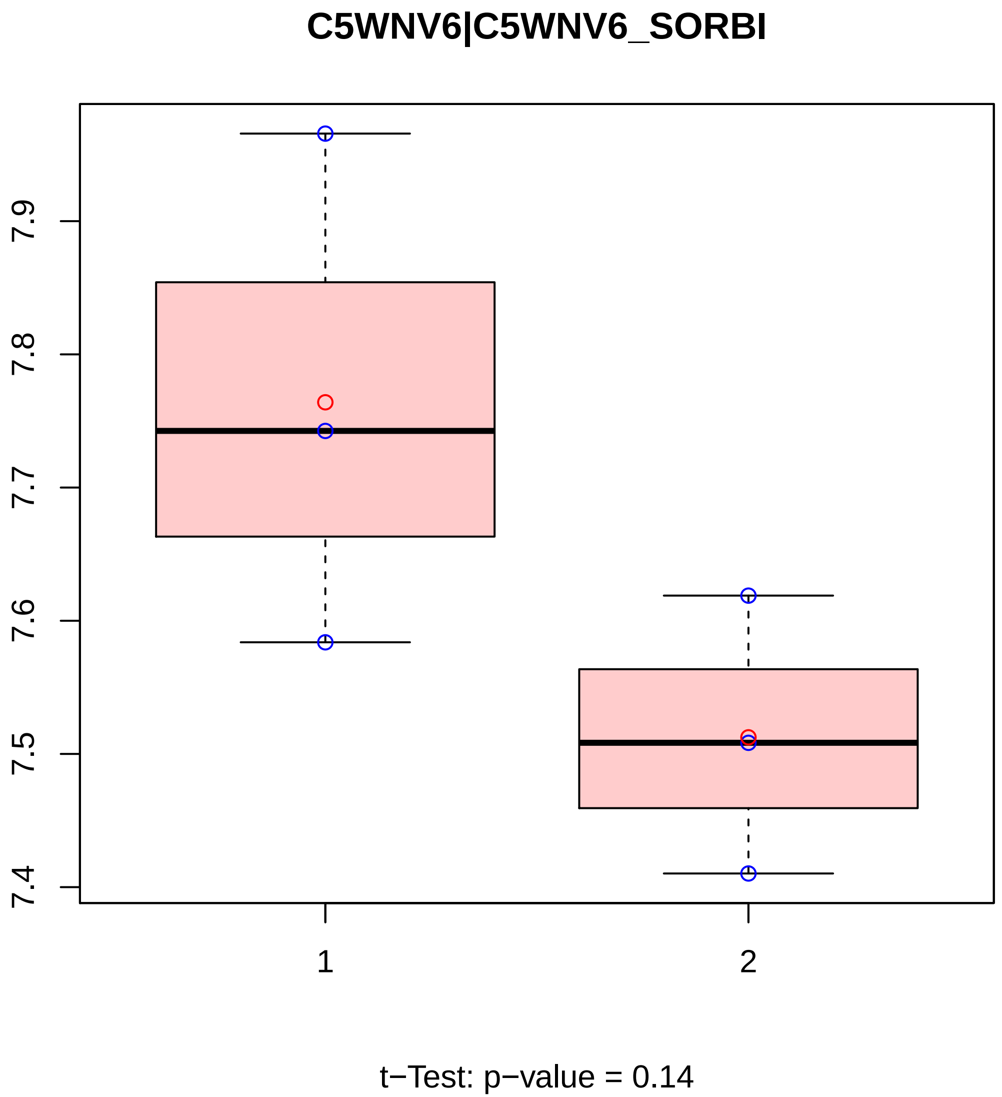
C5WNV6|C5WNV6_SORBI
7.4
7.5
7.6
7.7
7.8
7.9
1
2
t−Test: p−value = 0.14

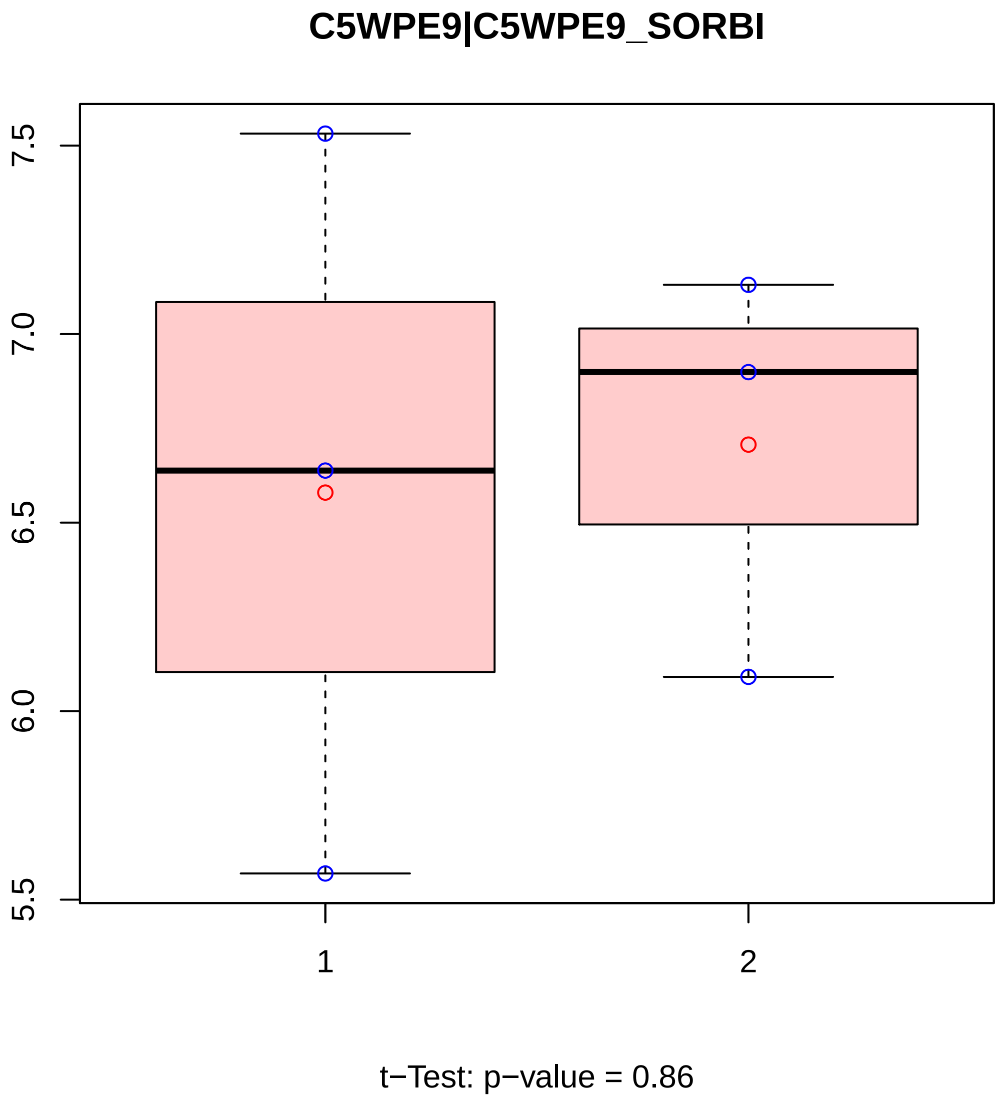
C5WPE9|C5WPE9_SORBI
7.5
7.0
6.5
6.0
5.5
1
2
t−Test: p−value = 0.86

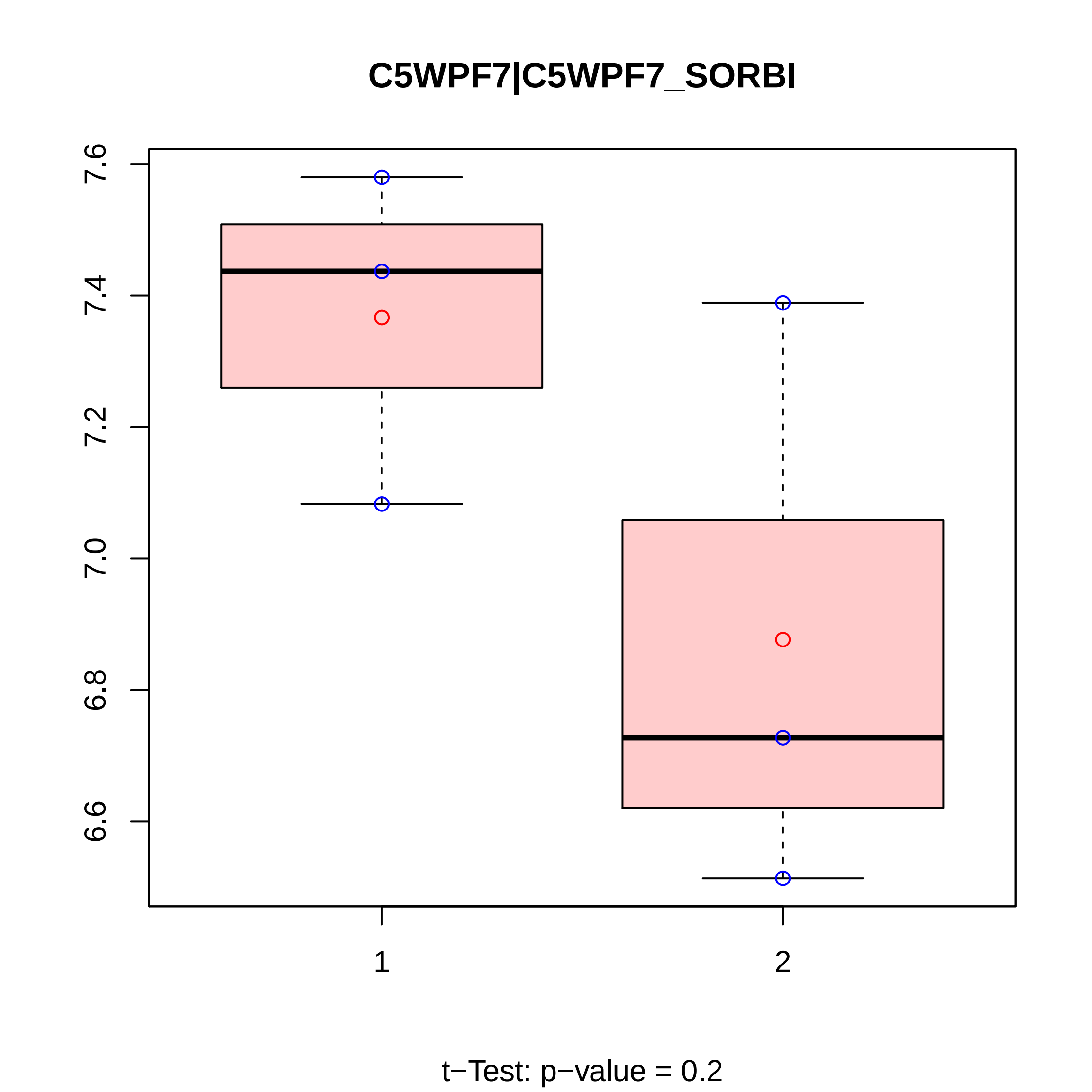
C5WPF7|C5WPF7_SORBI
7.6
7.4
7.2
7.0
6.8
6.6
1
2
t−Test: p−value = 0.2

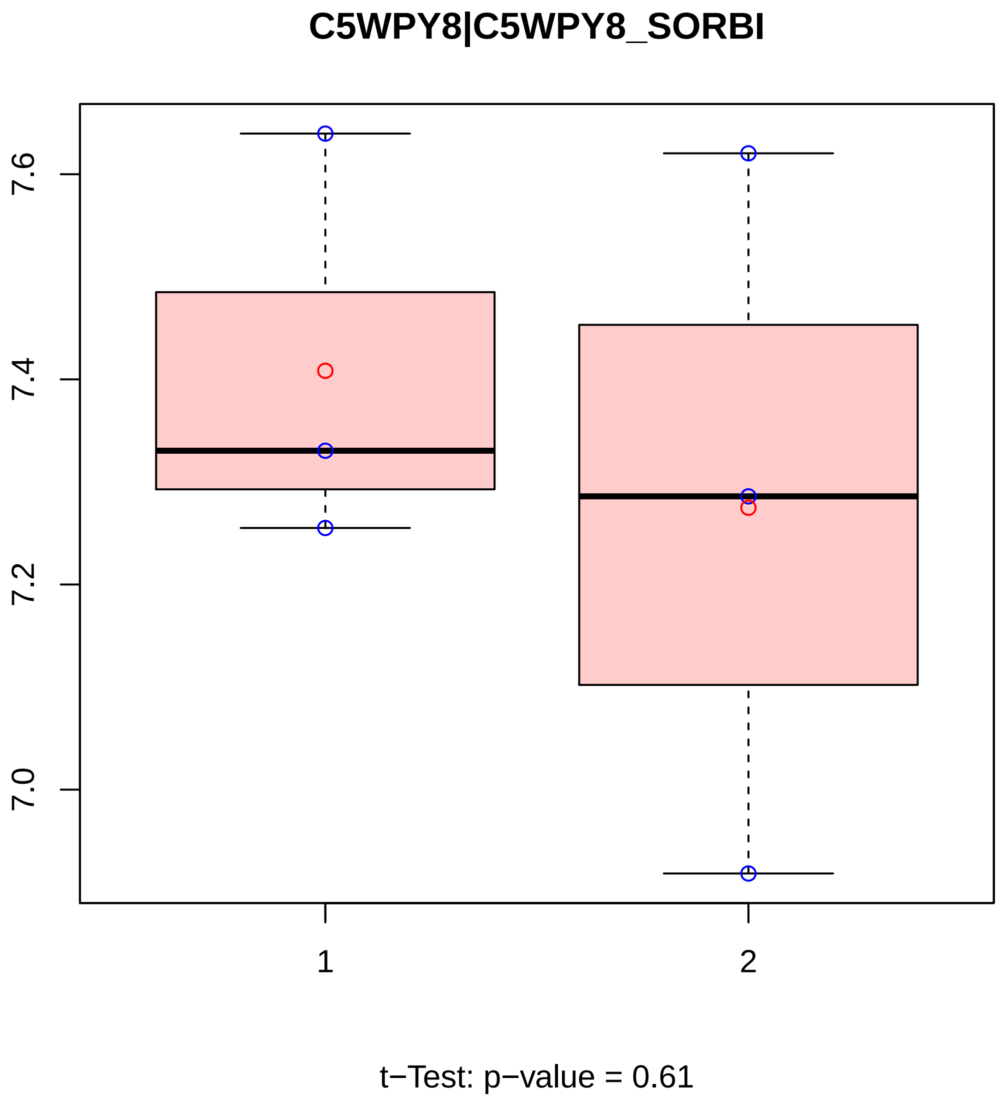

C5WPY8|C5WPY8_SORBI
7.6
7.4
7.2
7.0
1
2
t−Test: p−value = 0.61

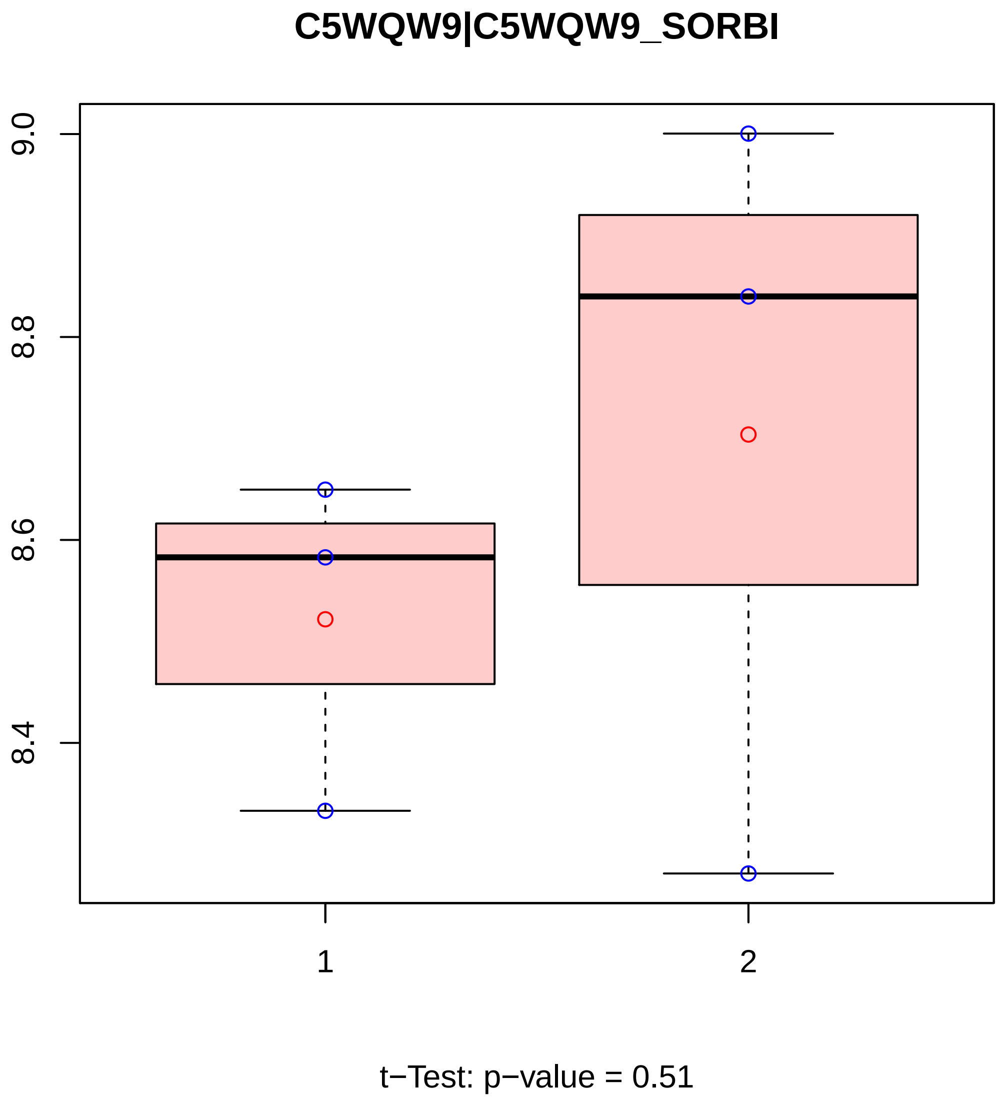
C5WQW9|C5WQW9_SORBI
9.0
8.8
8.6
8.4
1
2
t−Test: p−value = 0.51

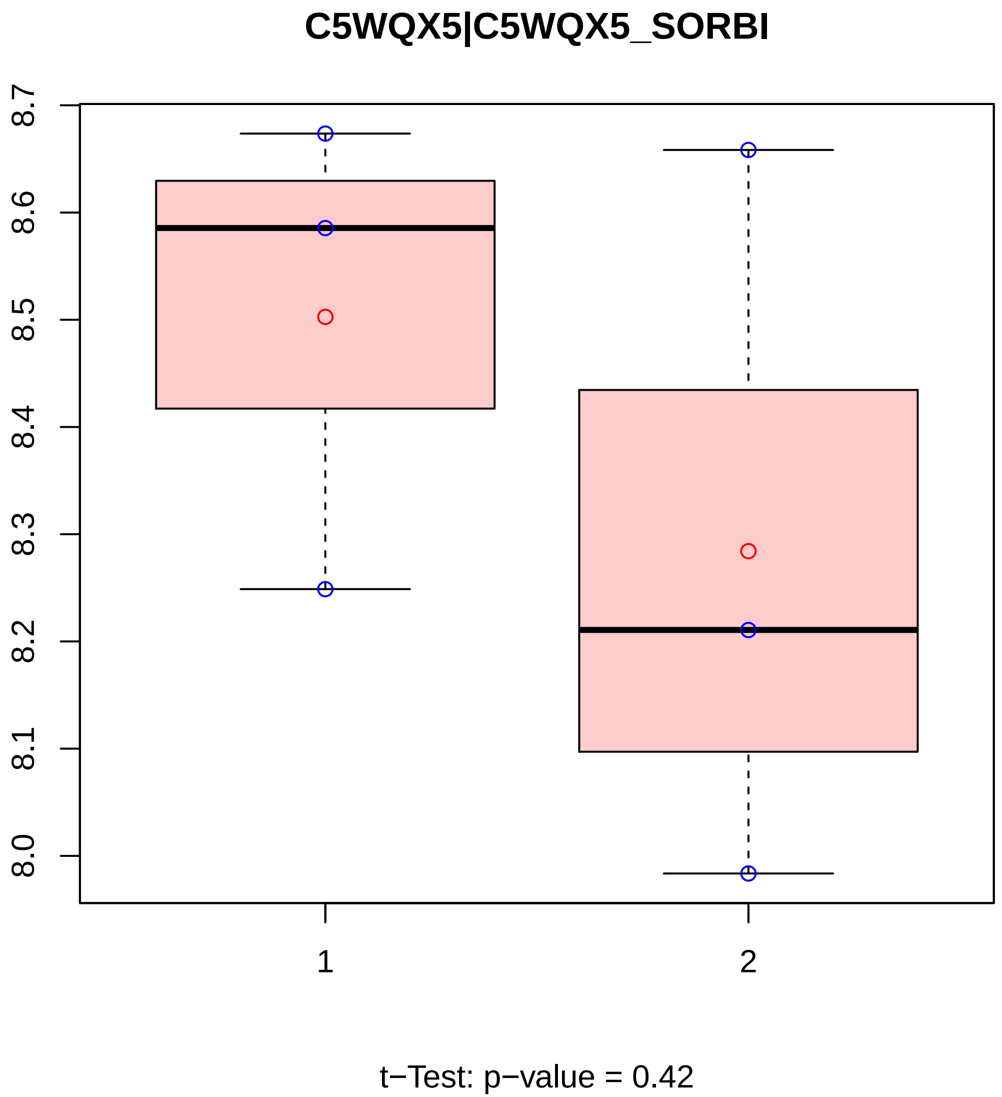
C5WQX5|C5WQX5_SORBI
8.0
8.1
8.2
8.3
8.4
8.5
8.6
8.7
1
2
t−Test: p−value = 0.42

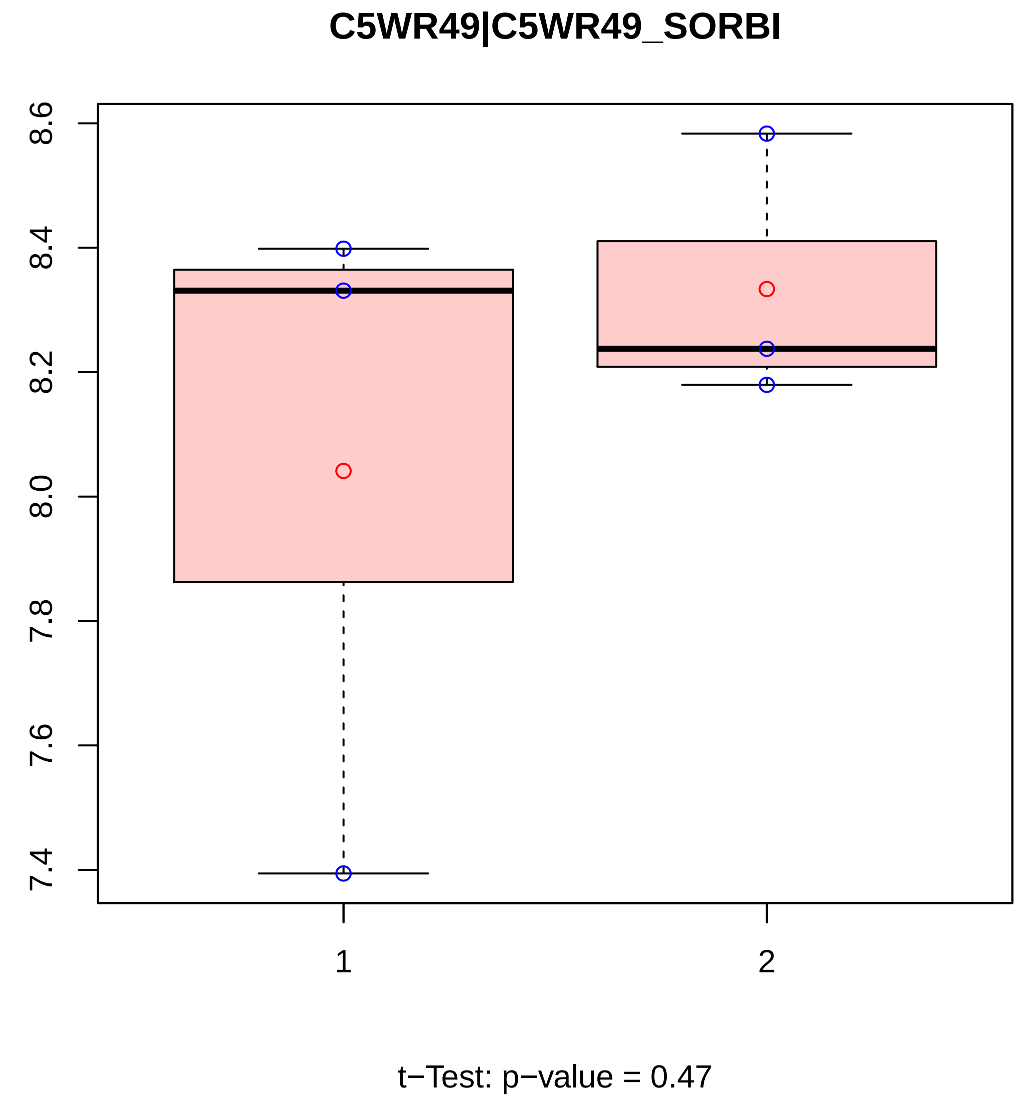
C5WR49|C5WR49_SORBI
8.6
8.4
8.2
8.0
7.8
7.6
7.4
1
2
t−Test: p−value = 0.47

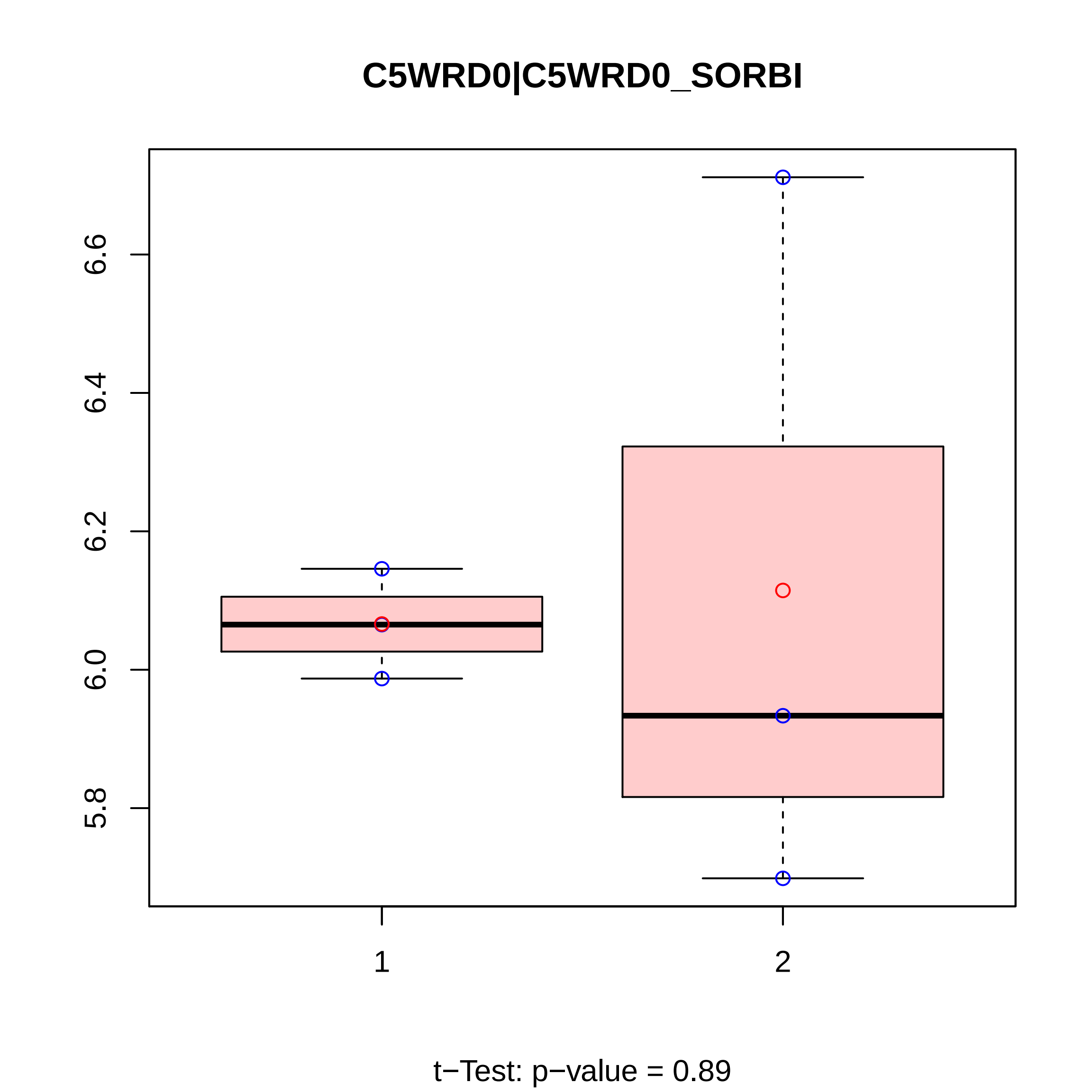
C5WRD0|C5WRD0_SORBI
6.6
6.4
6.2
6.0
5.8
1
2
t−Test: p−value = 0.89

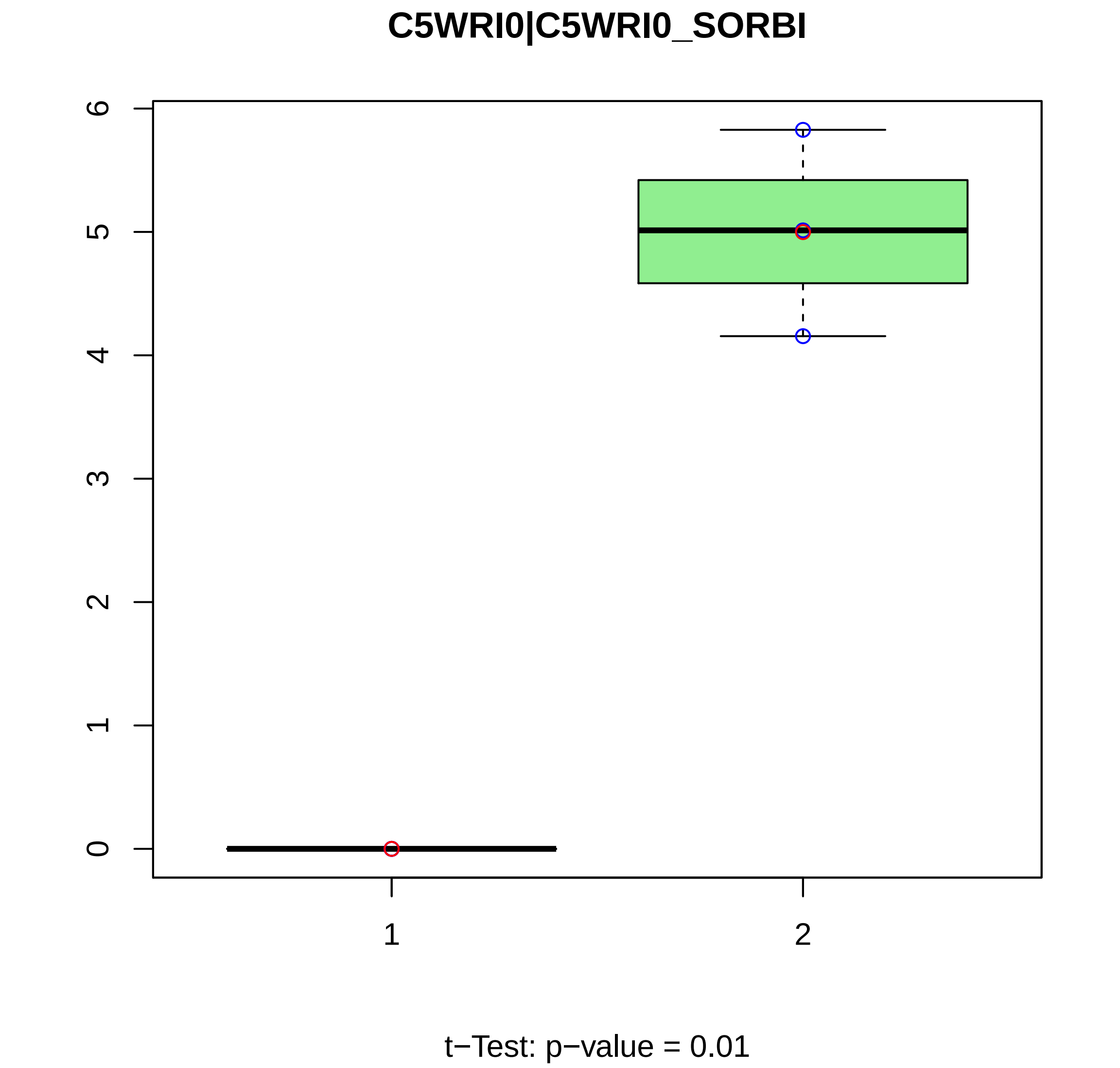

C5WRI0|C5WRI0_SORBI
0
1
2
3
4
5
6
1
2
t−Test: p−value = 0.01

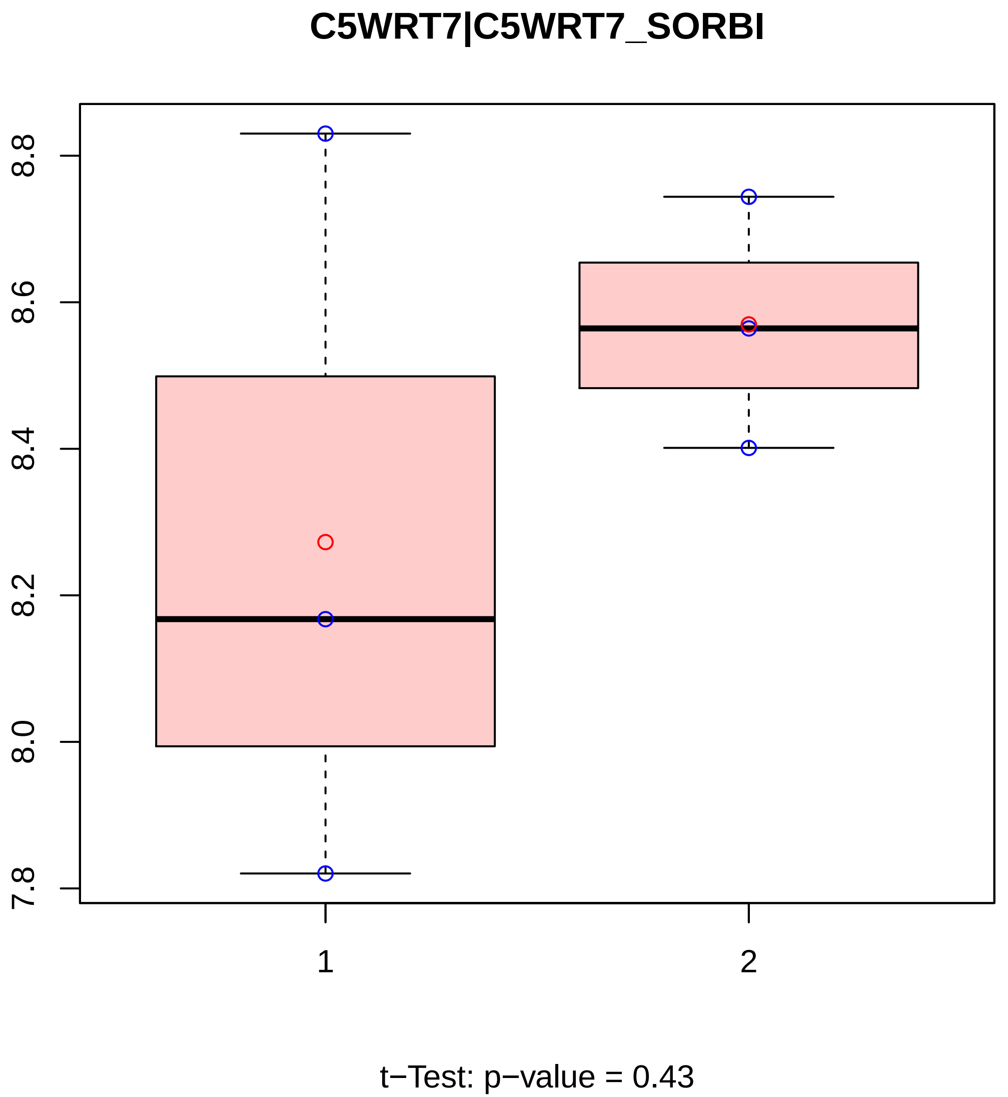

C5WRT7|C5WRT7_SORBI
8.8
8.6
8.4
8.2
8.0
7.8
1
2
t−Test: p−value = 0.43

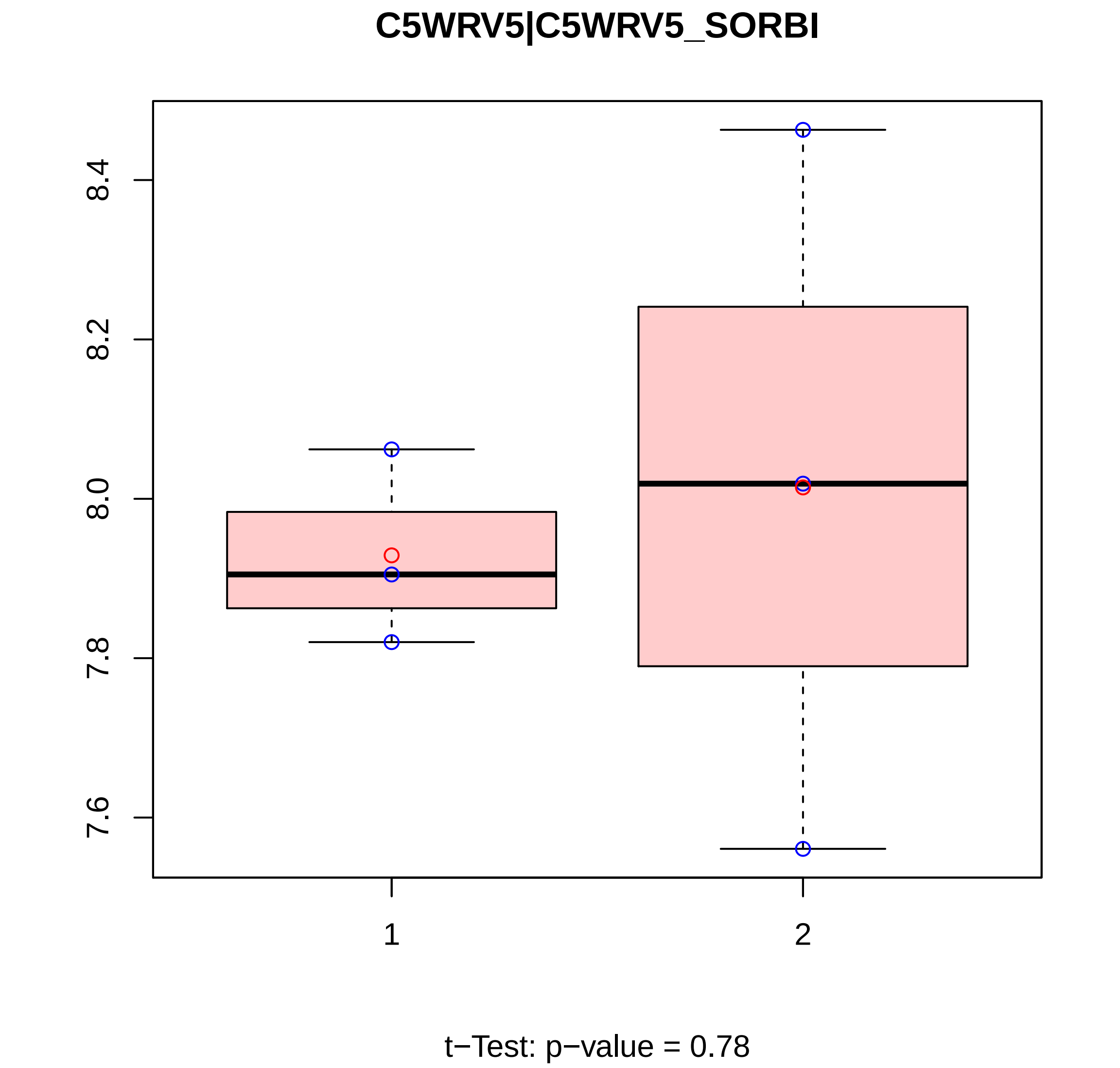

C5WRV5|C5WRV5_SORBI
8.4
8.2
8.0
7.8
7.6
1
2
t−Test: p−value = 0.78

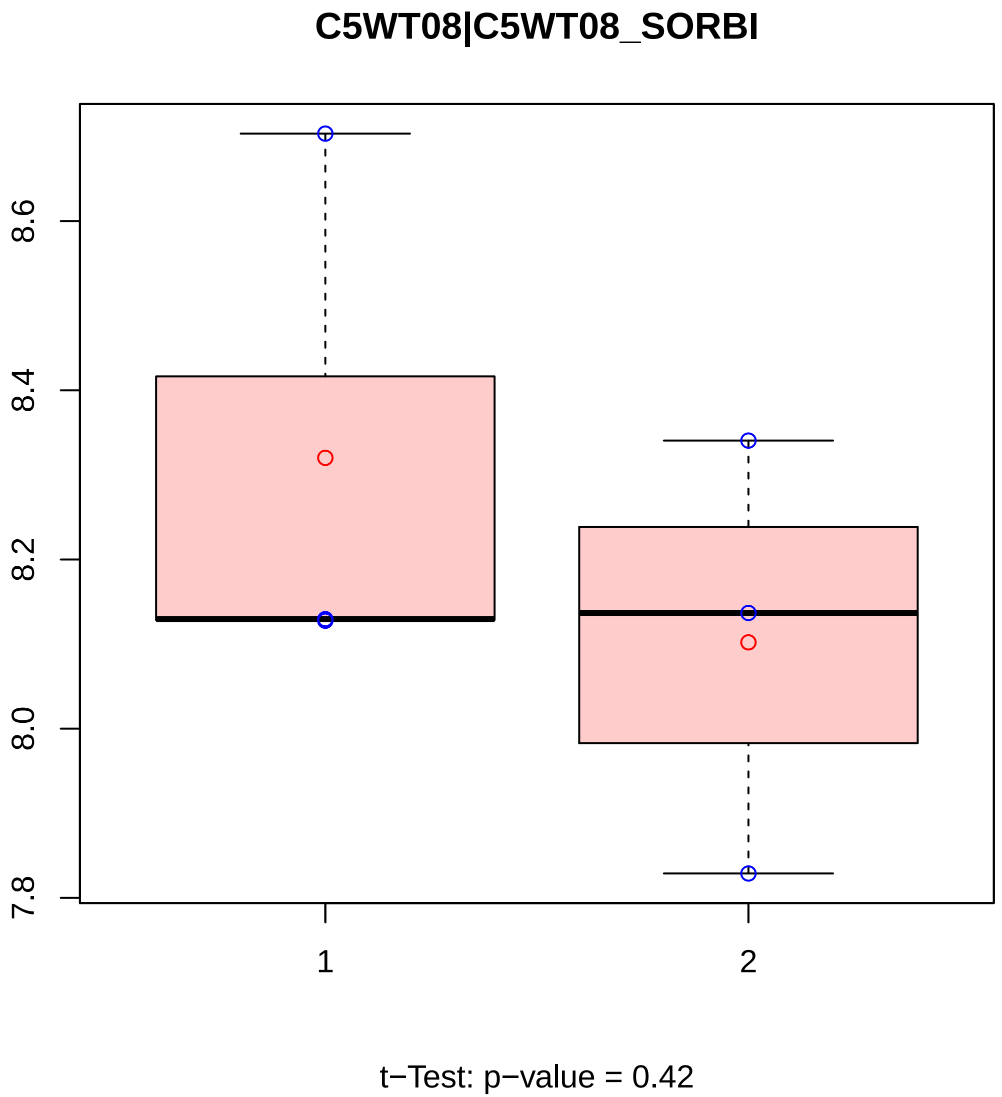
C5WT08|C5WT08_SORBI
8.6
8.4
8.2
8.0
7.8
1
2
t−Test: p−value = 0.42

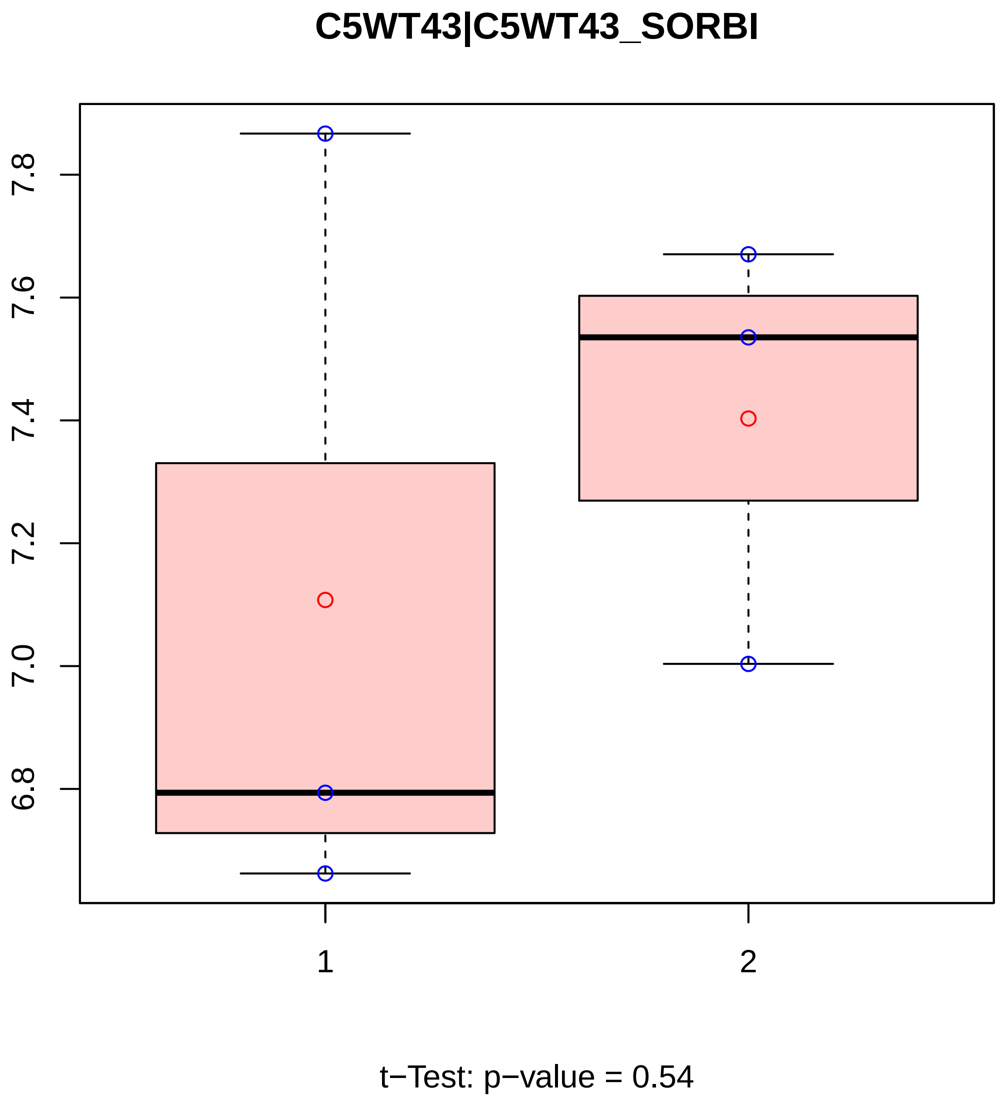

C5WT43|C5WT43_SORBI
7.8
7.6
7.4
7.2
7.0
6.8
1
2
t−Test: p−value = 0.54

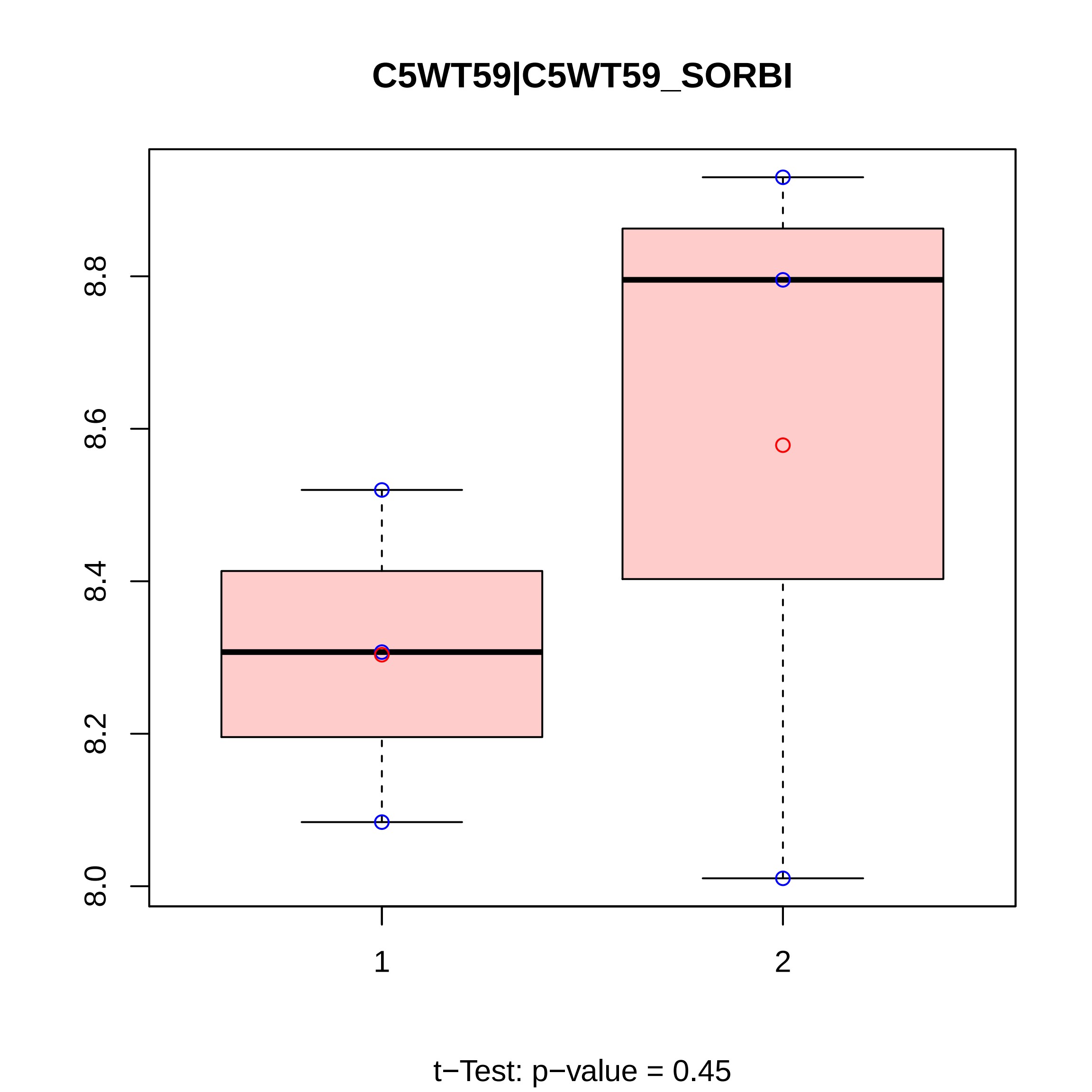

C5WT59|C5WT59_SORBI
8.0
8.2
8.4
8.6
8.8
1
2
t−Test: p−value = 0.45

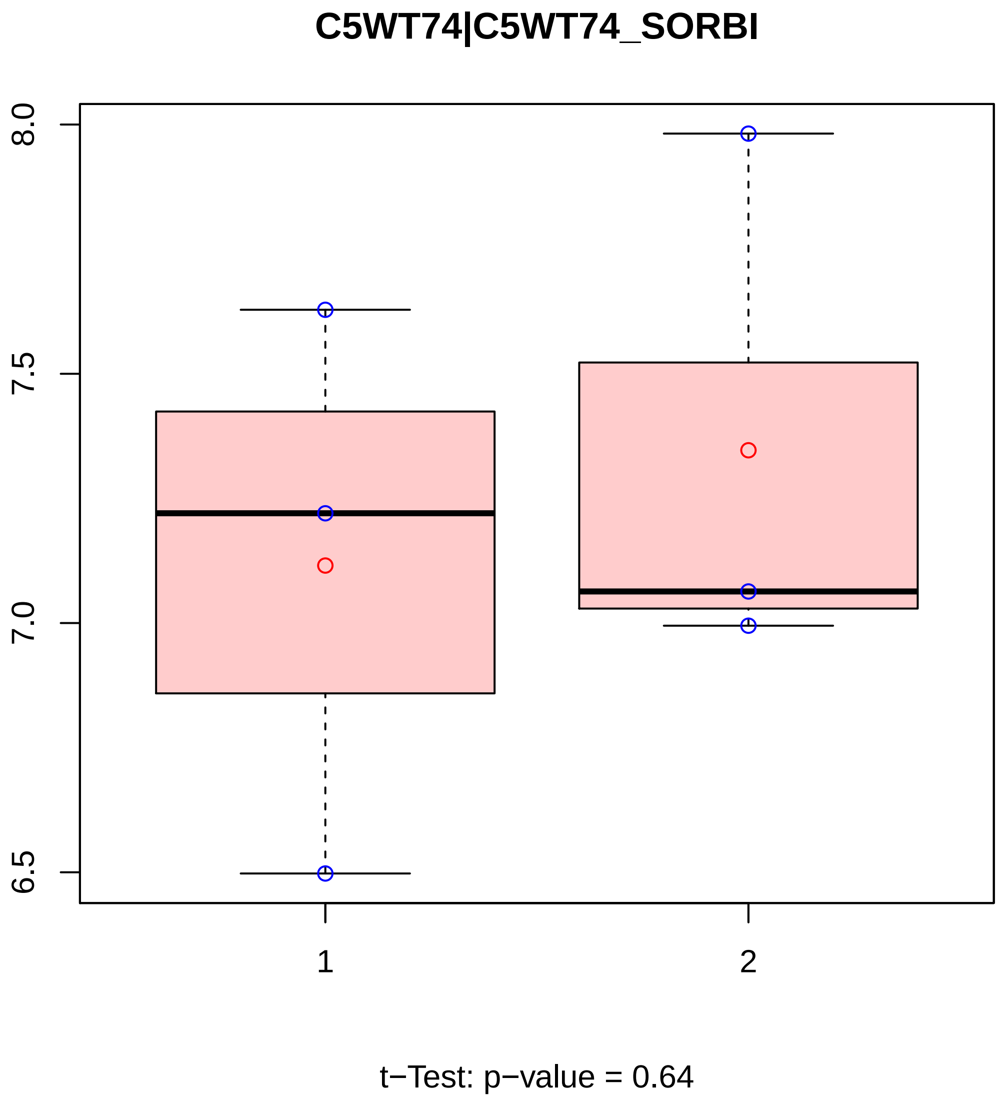
C5WT74|C5WT74_SORBI
8.0
7.5
7.0
6.5
1
2
t−Test: p−value = 0.64

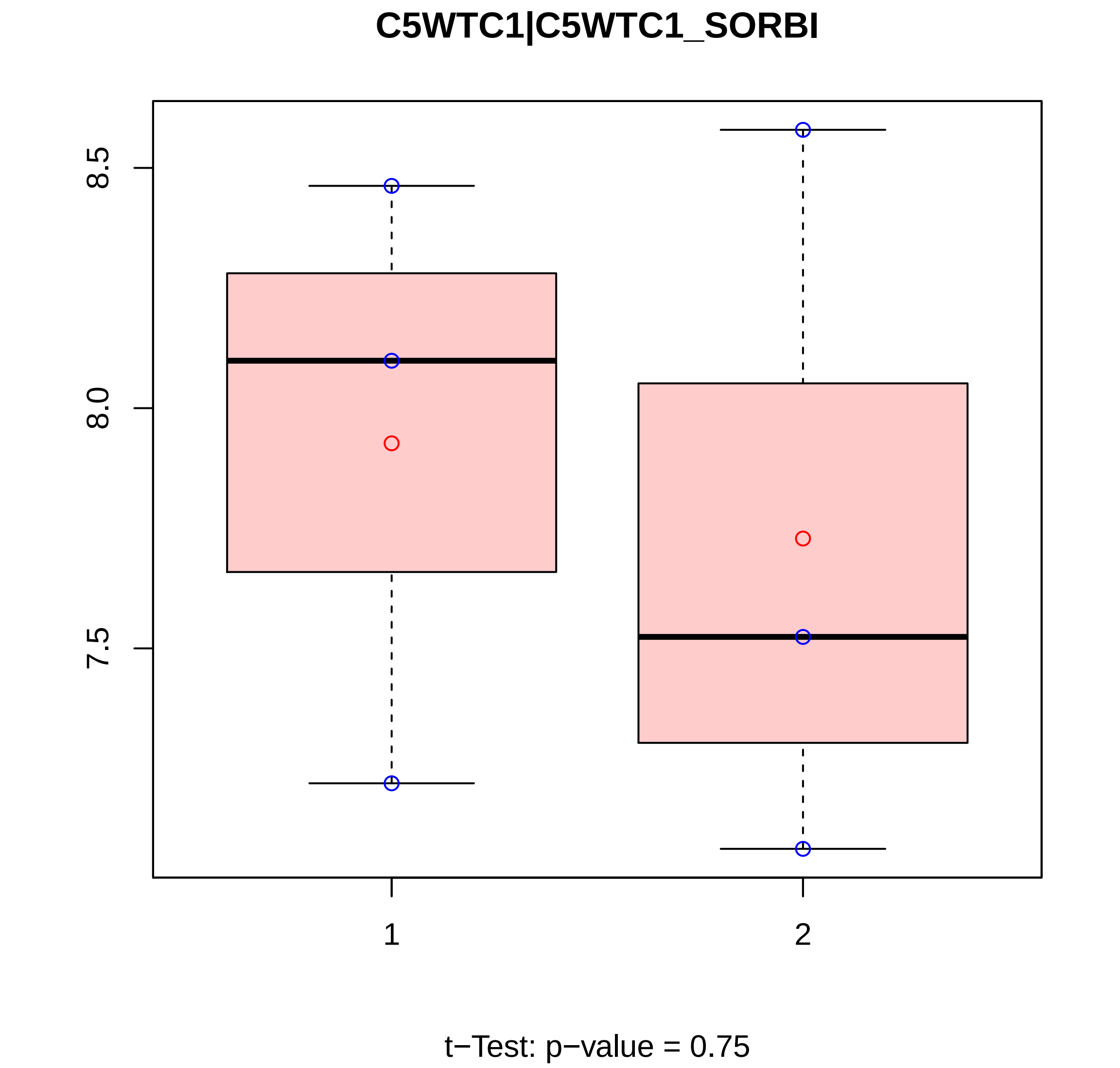

C5WTC1|C5WTC1_SORBI
8.5
8.0
7.5
1
2
t−Test: p−value = 0.75

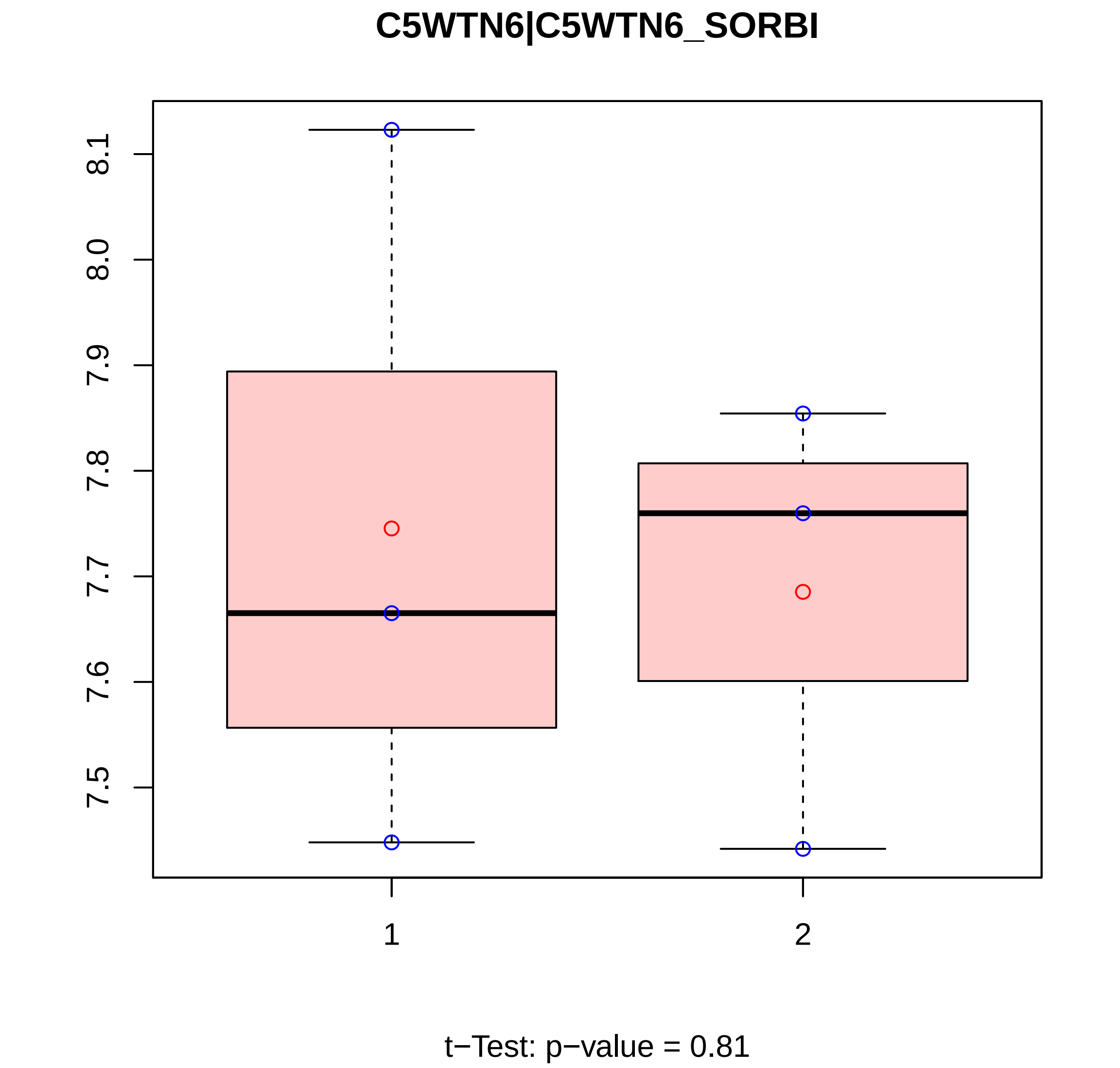
C5WTN6|C5WTN6_SORBI
8.1
8.0
7.9
7.8
7.7
7.6
7.5
1
2
t−Test: p−value = 0.81

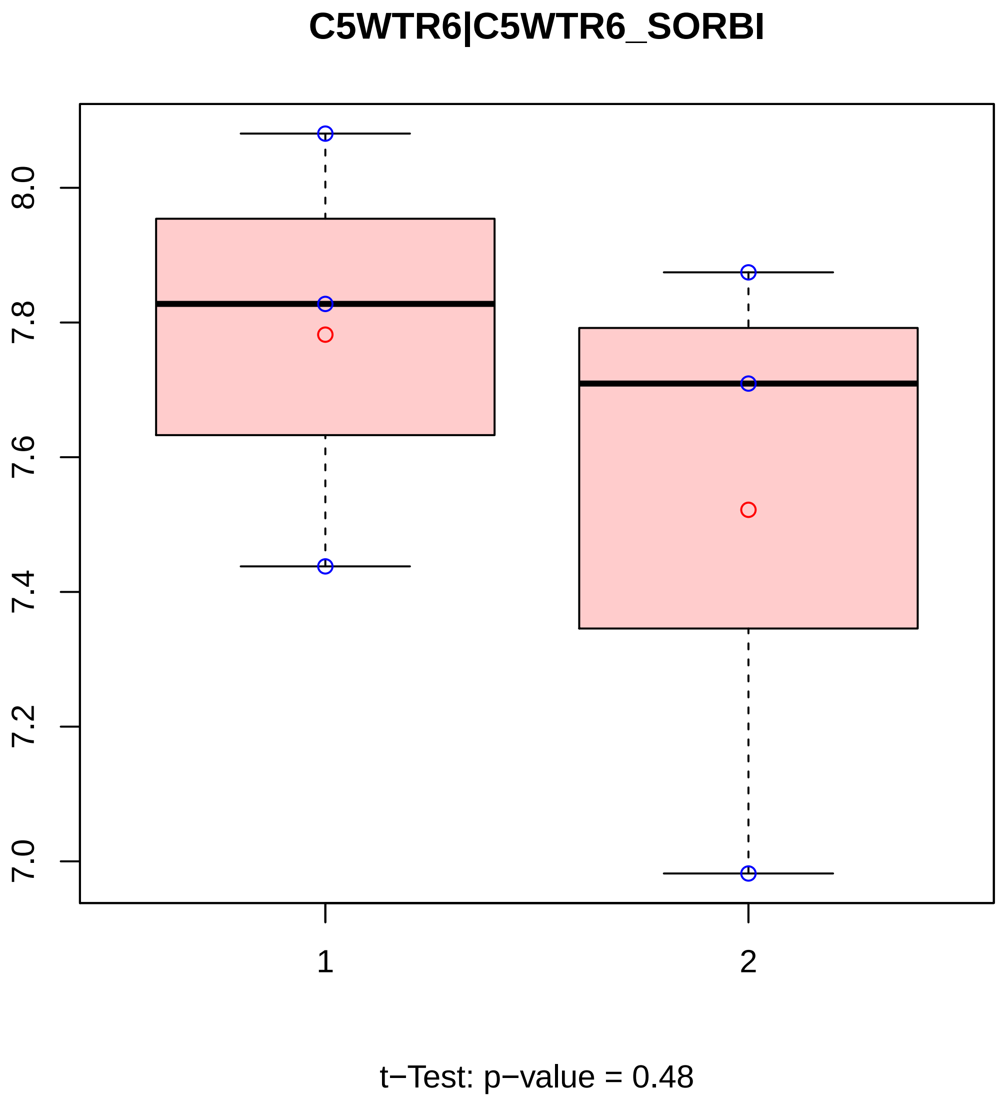
C5WTR6|C5WTR6_SORBI
8.0
7.8
7.6
7.4
7.2
7.0
1
2
t−Test: p−value = 0.48

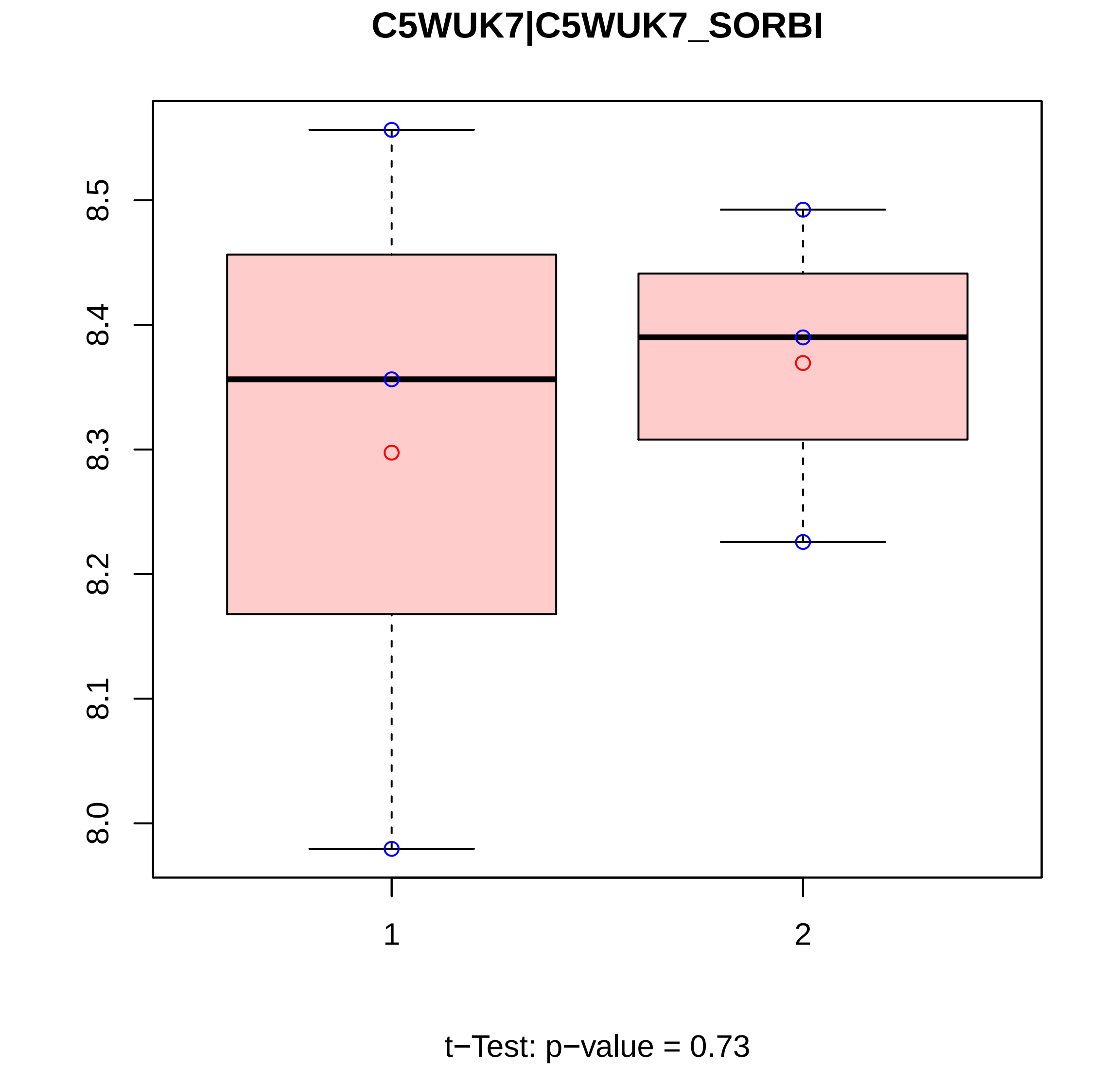

C5WUK7|C5WUK7_SORBI
8.0
8.1
8.2
8.3
8.4
8.5
1
2
t−Test: p−value = 0.73

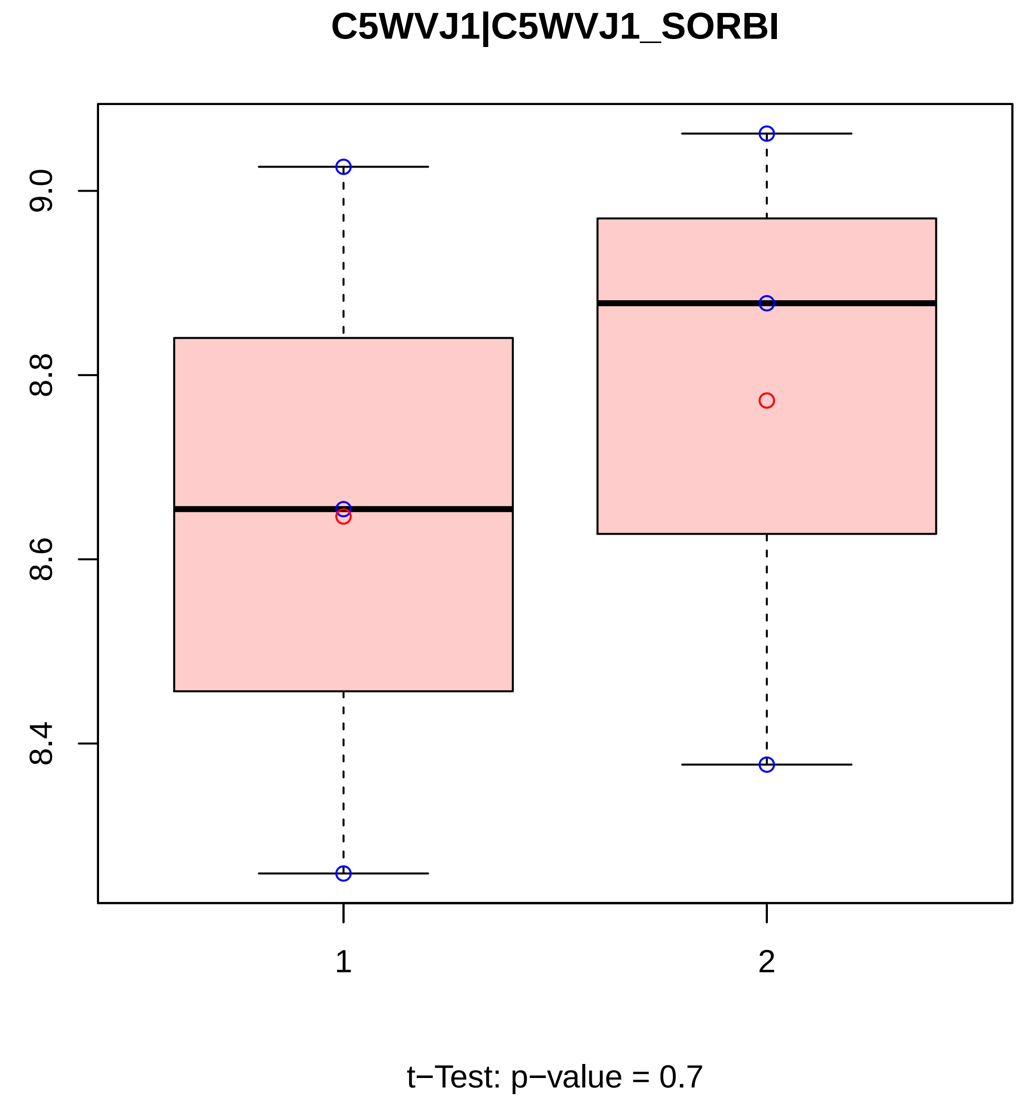
C5WVJ1|C5WVJ1_SORBI
9.0
8.8
8.6
8.4
1
2
t−Test: p−value = 0.7

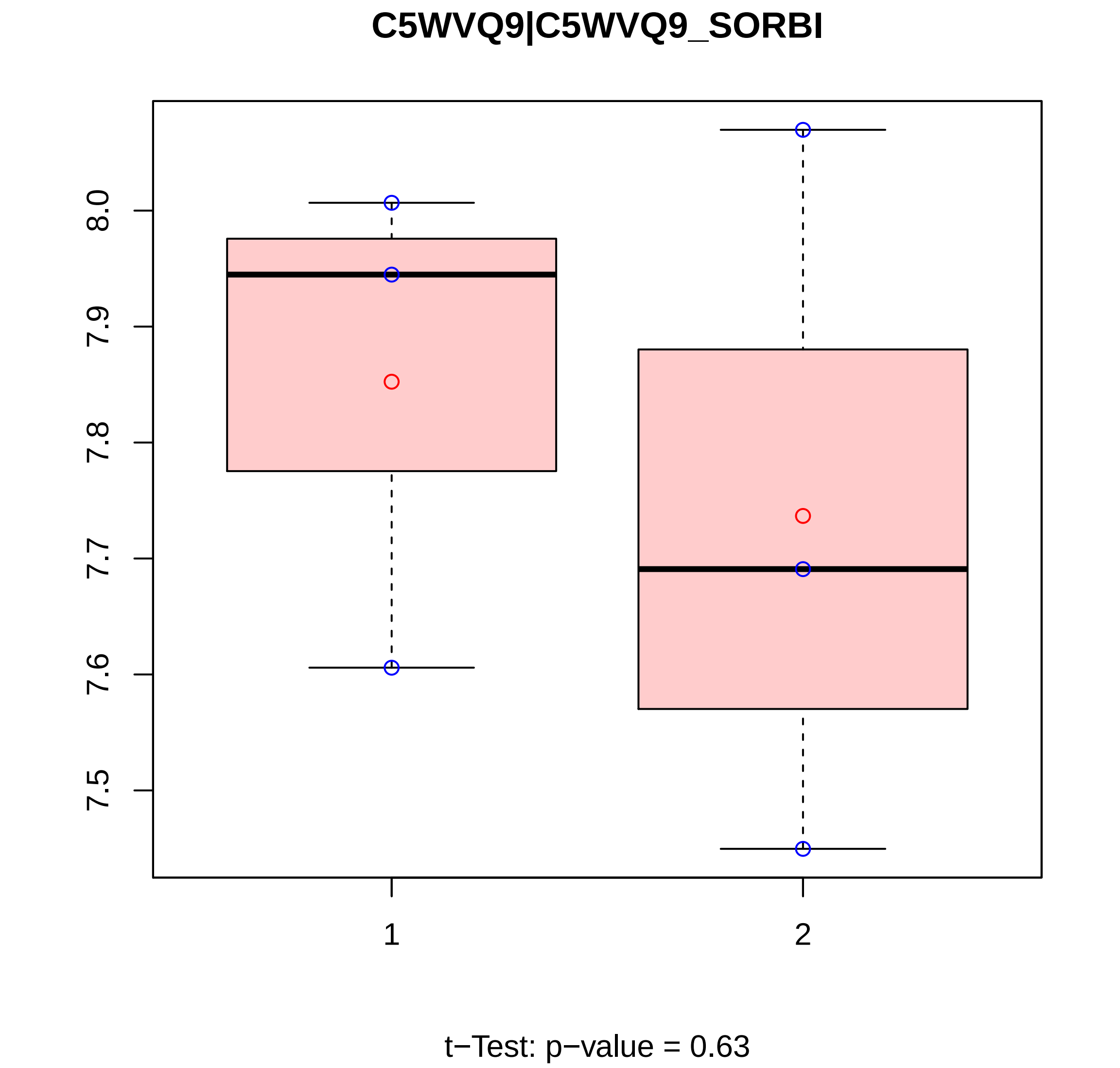
C5WVQ9|C5WVQ9_SORBI
8.0
7.9
7.8
7.7
7.6
7.5
1
2
t−Test: p−value = 0.63

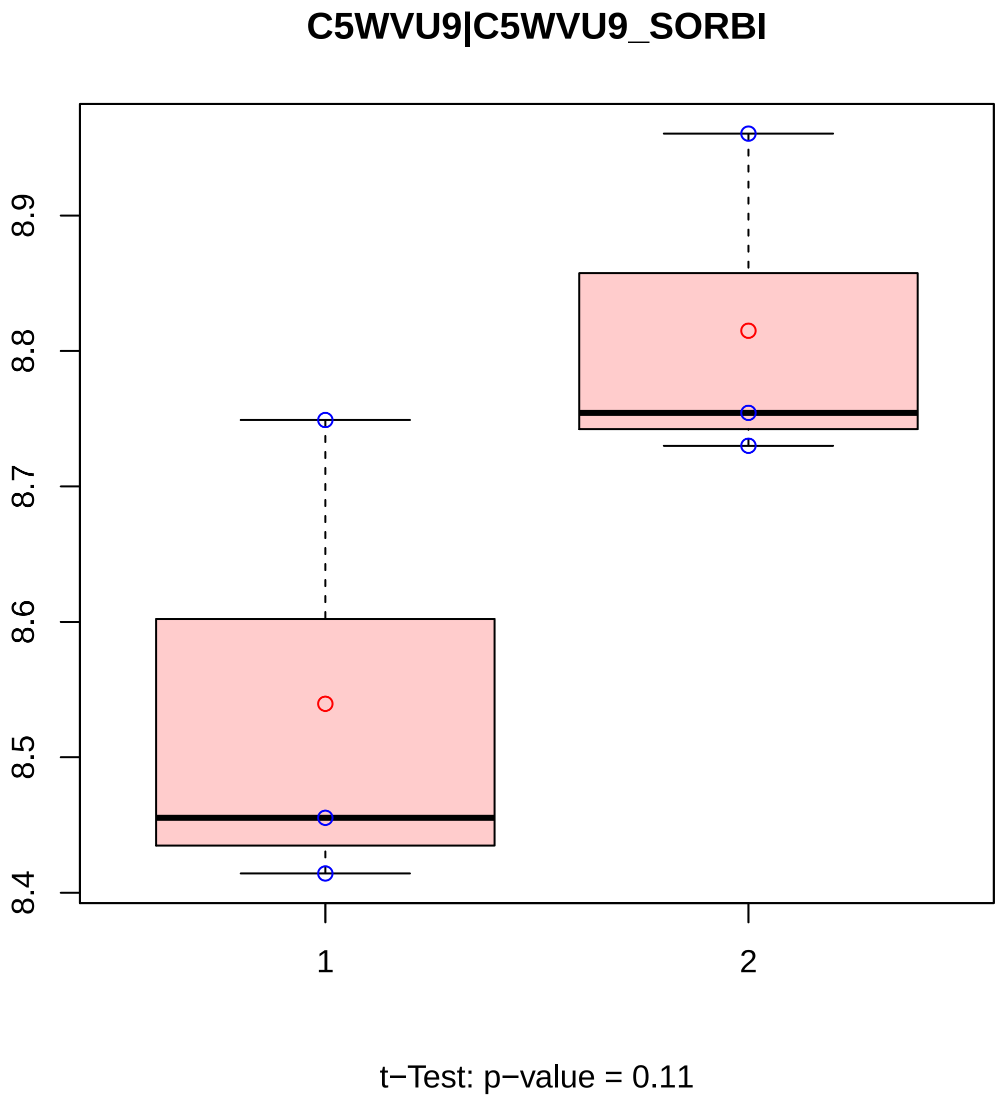
C5WVU9|C5WVU9_SORBI
8.4
8.5
8.6
8.7
8.8
8.9
1
2
t−Test: p−value = 0.11

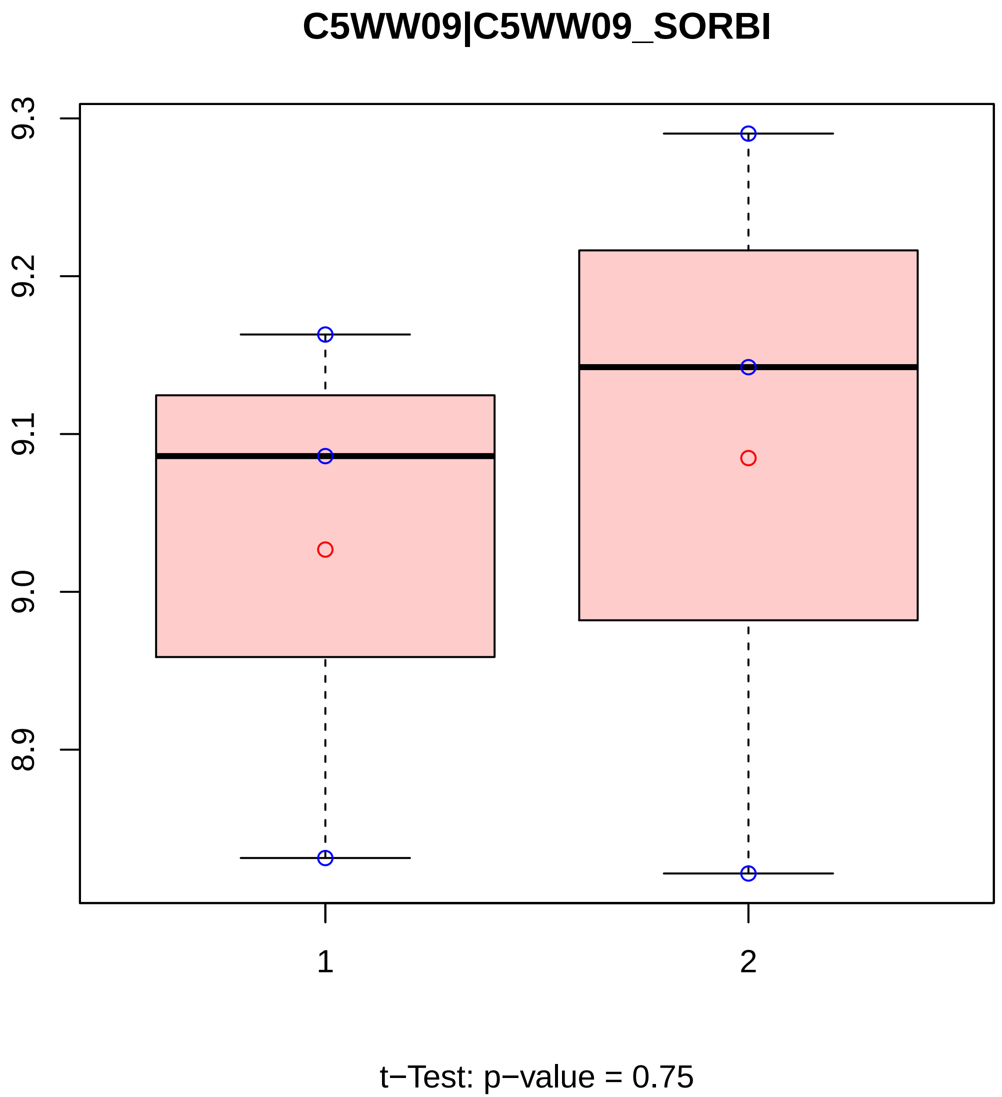
C5WW09|C5WW09_SORBI
9.3
9.2
9.1
9.0
8.9
1
2
t−Test: p−value = 0.75

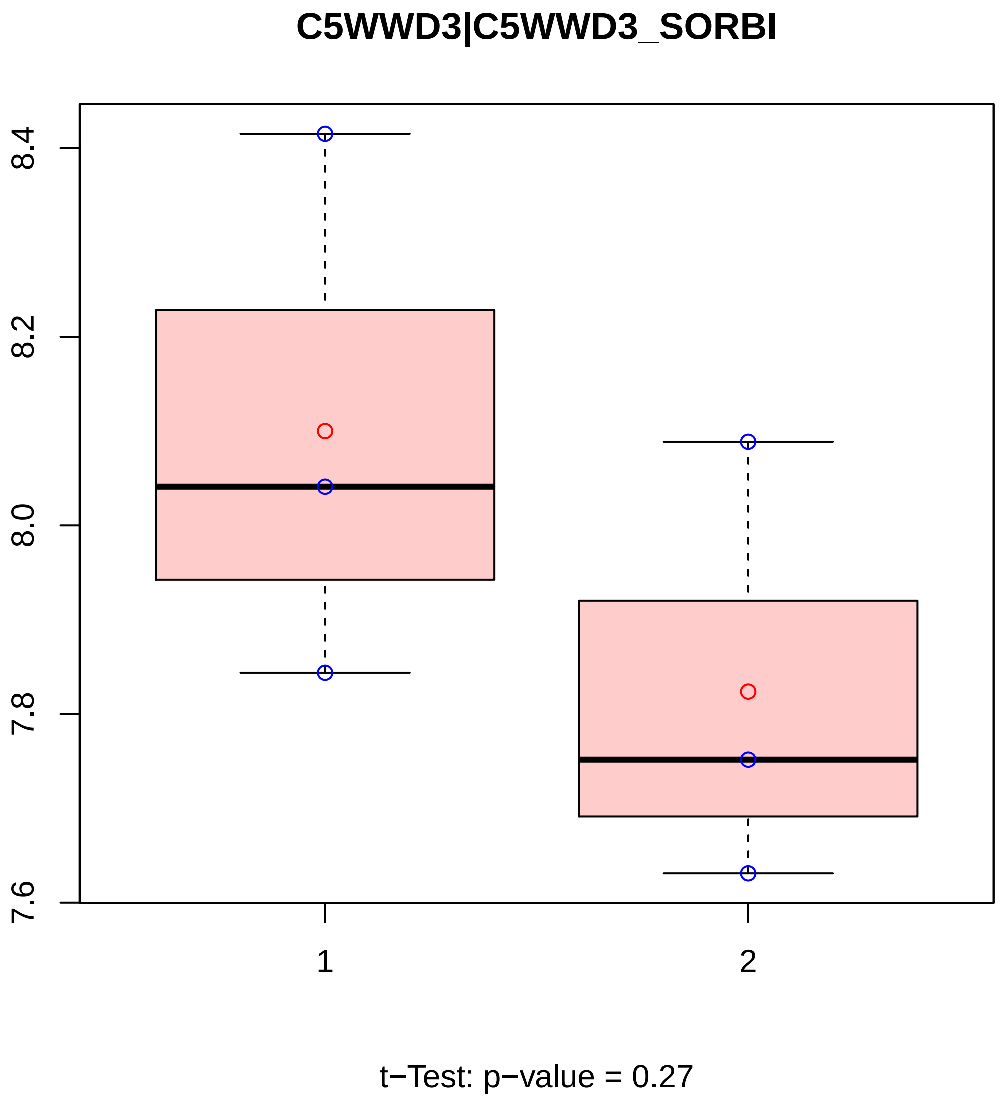
C5WWD3|C5WWD3_SORBI
8.4
8.2
8.0
7.8
7.6
1
2
t−Test: p−value = 0.27

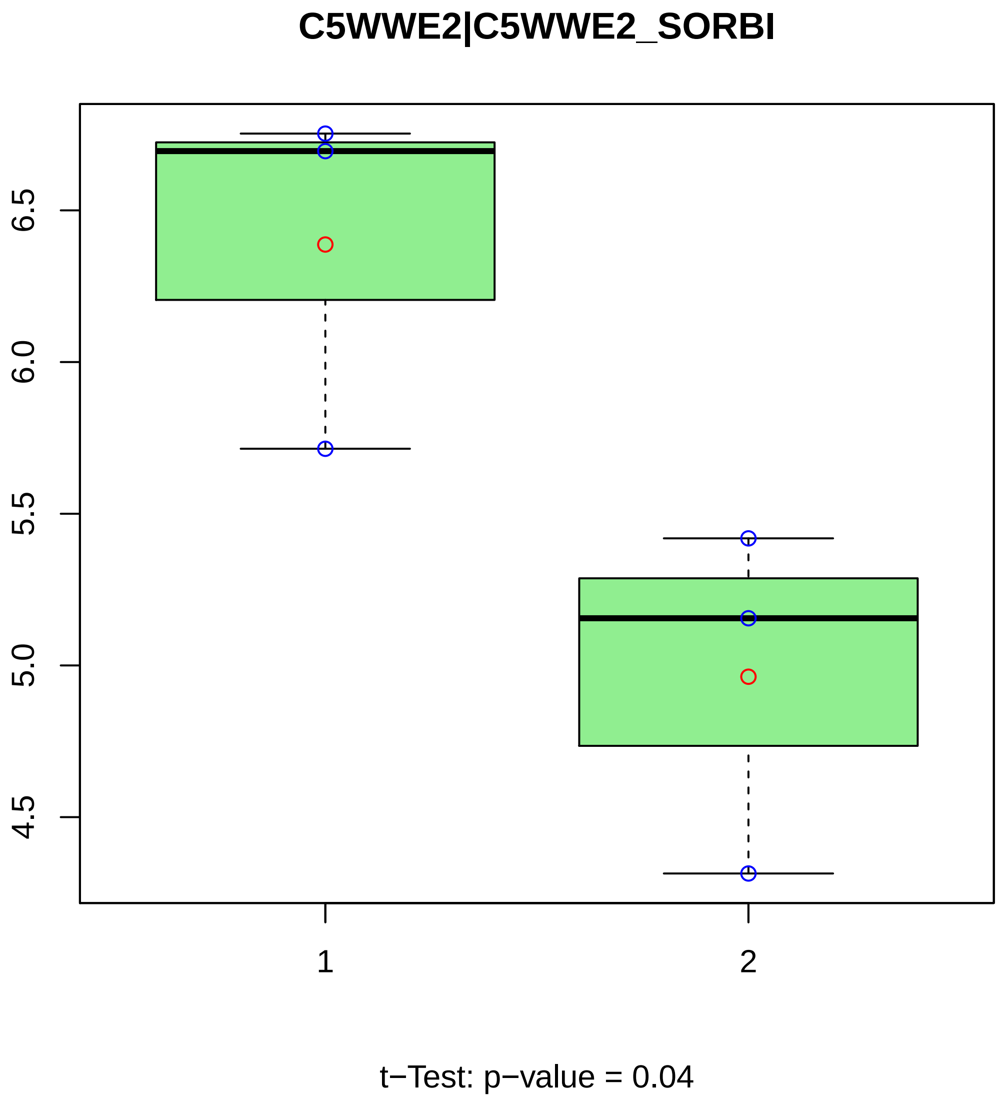

C5WWE2|C5WWE2_SORBI
6.5
6.0
5.5
5.0
4.5
1
2
t−Test: p−value = 0.04

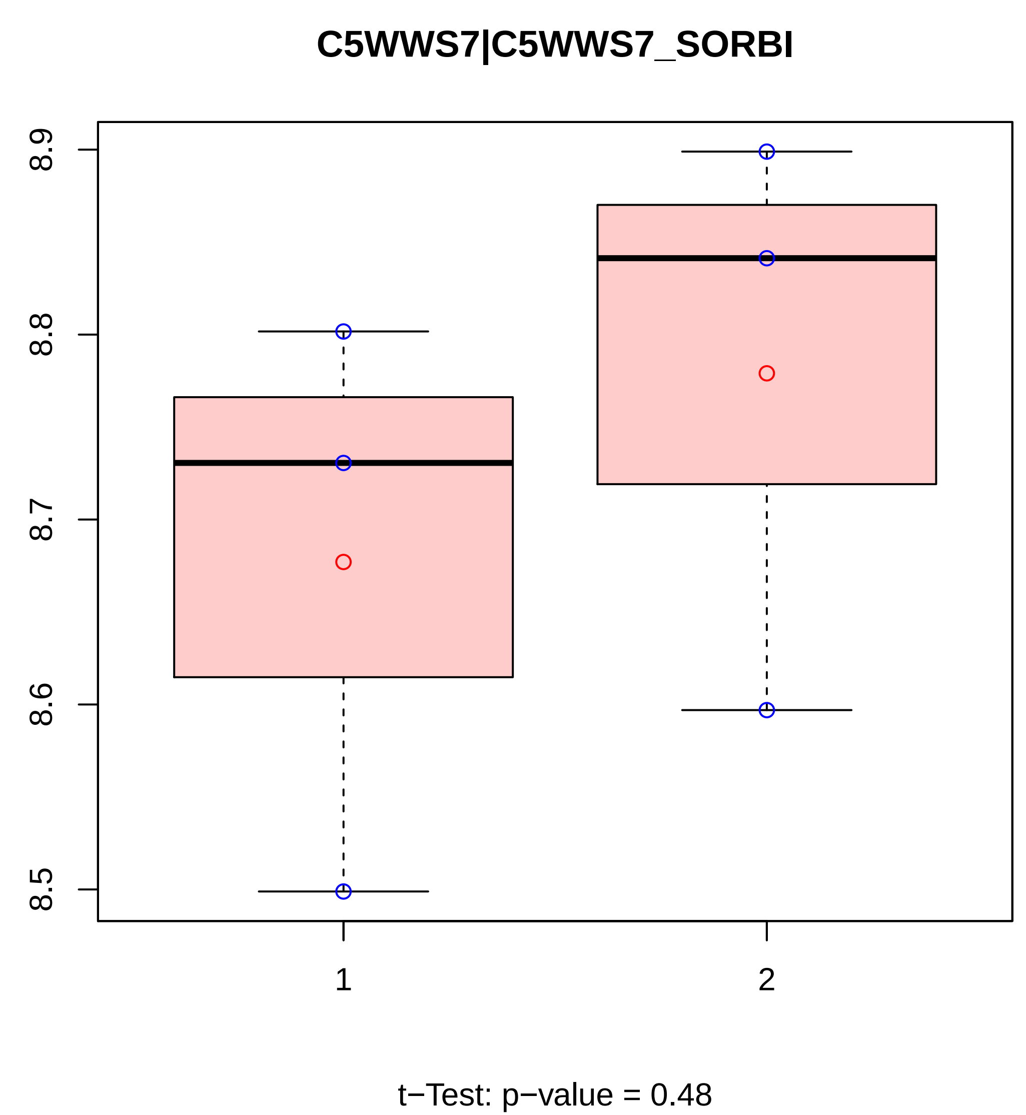
C5WWS7|C5WWS7_SORBI
8.5
8.6
8.7
8.8
8.9
1
2
t−Test: p−value = 0.48

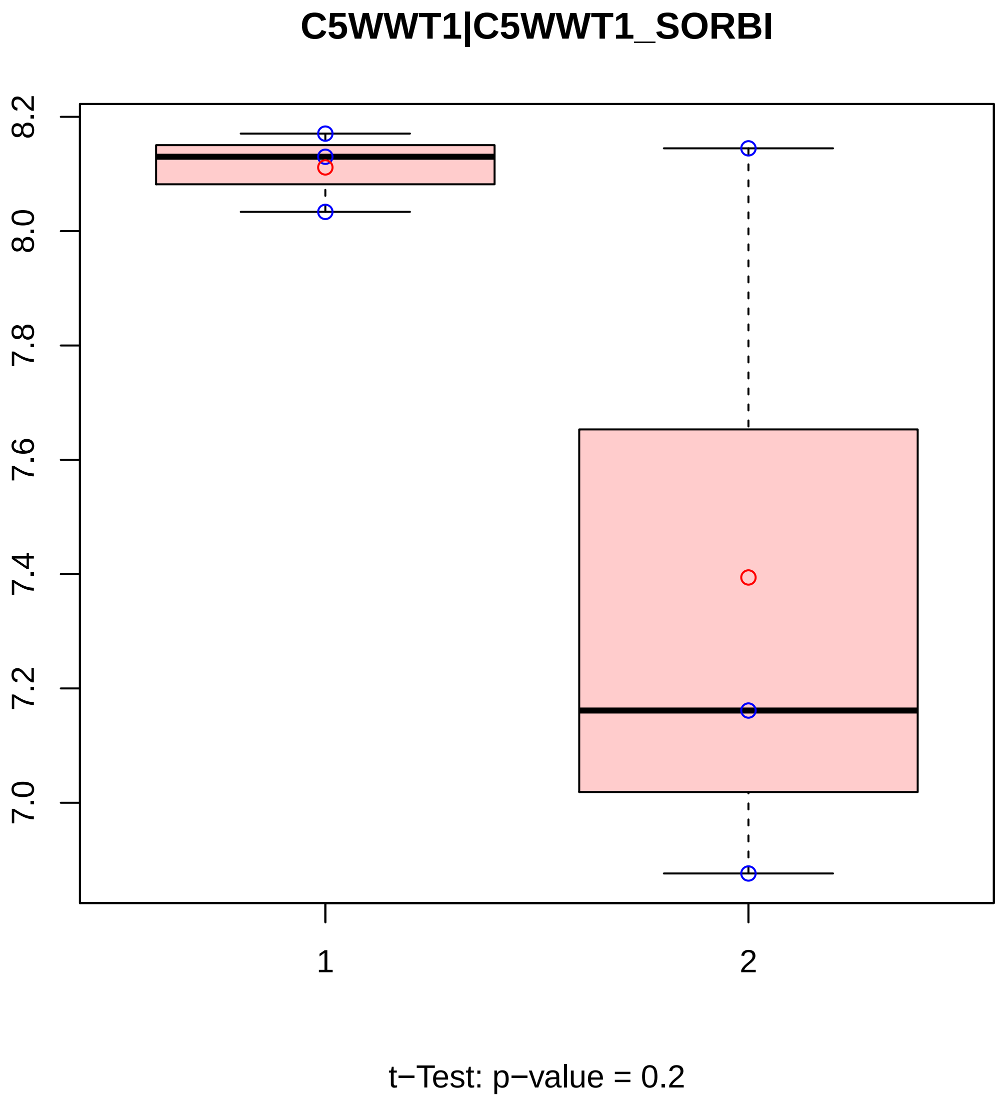
C5WWT1|C5WWT1_SORBI
8.2
8.0
7.8
7.6
7.4
7.2
7.0
1
2
t−Test: p−value = 0.2

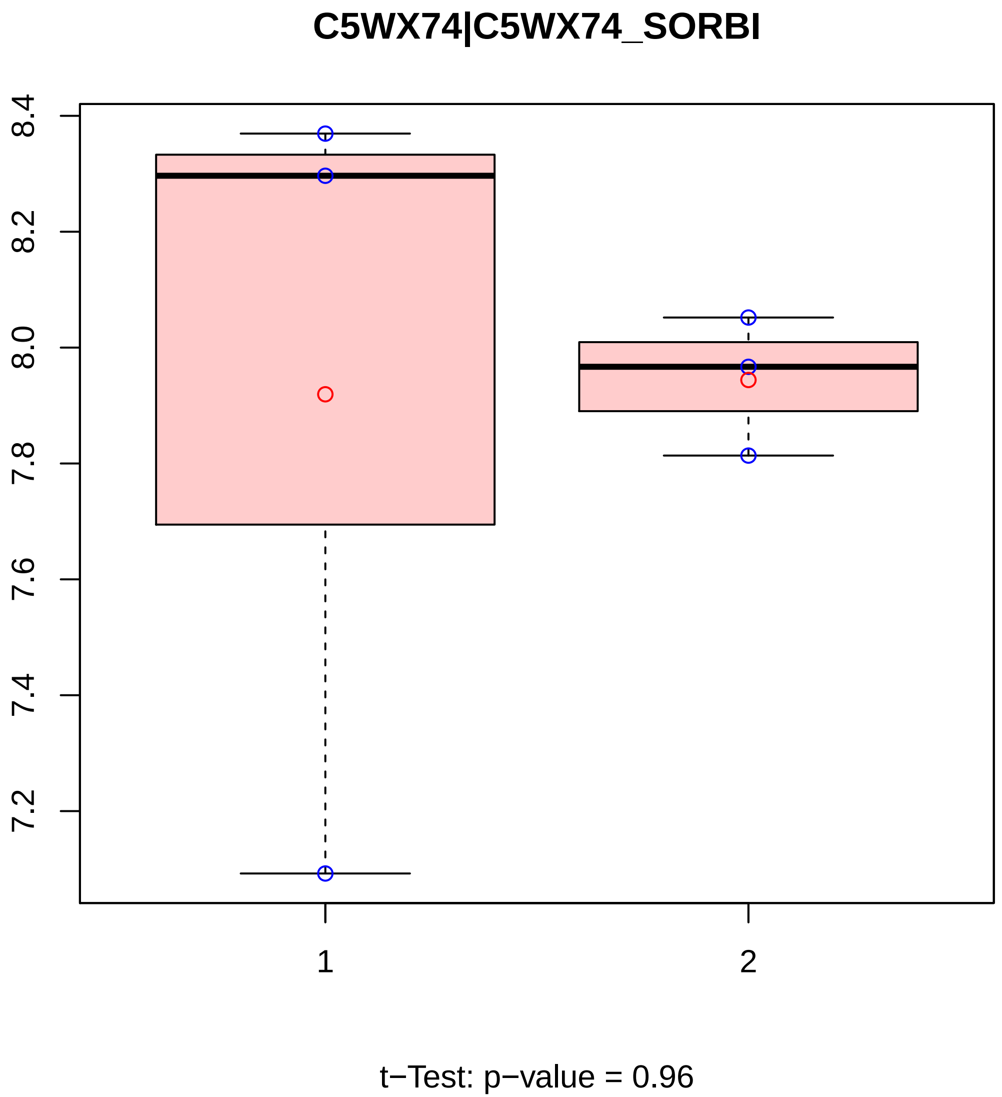
C5WX74|C5WX74_SORBI
8.4
8.2
8.0
7.8
7.6
7.4
7.2
1
2
t−Test: p−value = 0.96

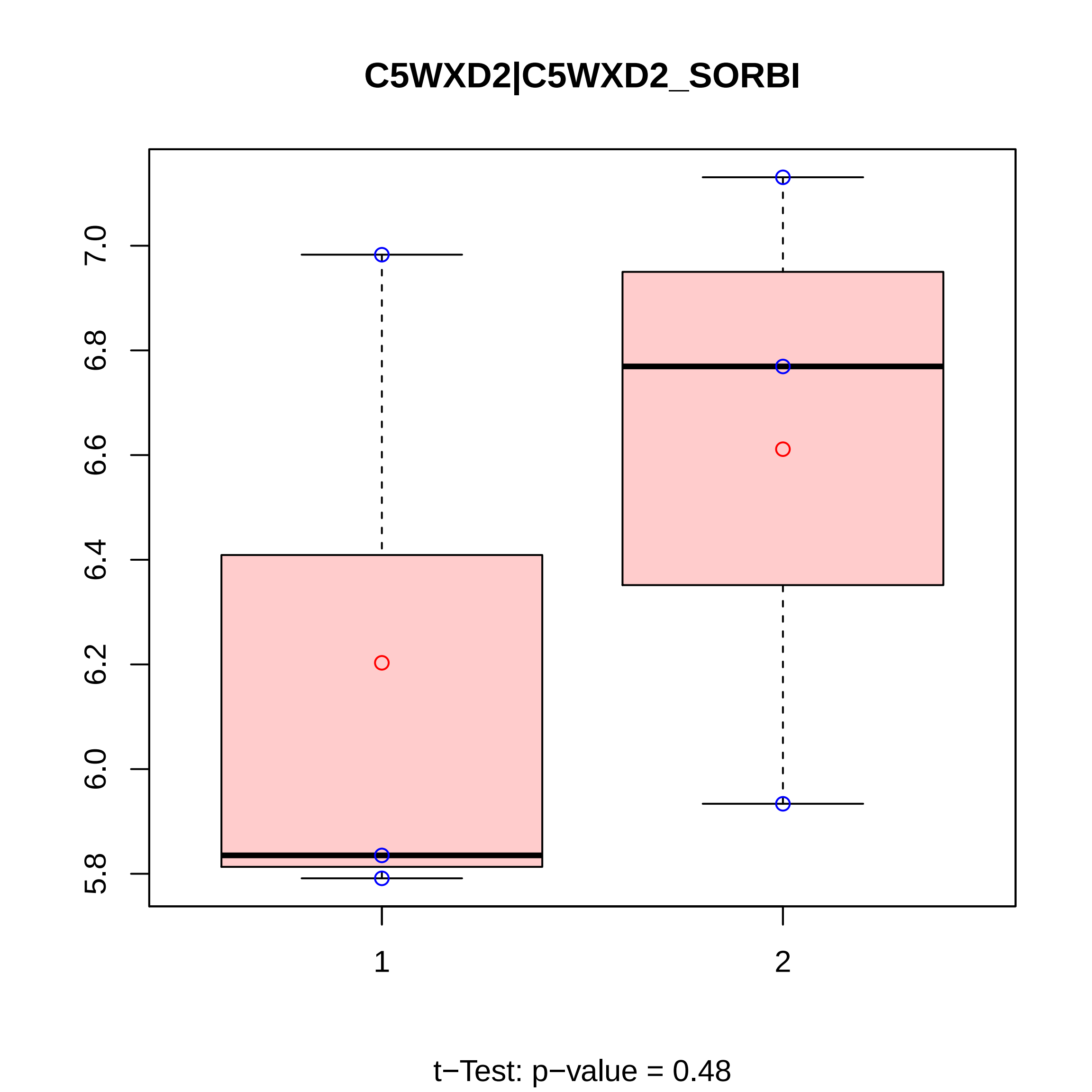
C5WXD2|C5WXD2_SORBI
7.0
6.8
6.6
6.4
6.2
6.0
5.8
1
2
t−Test: p−value = 0.48

C5WY19|C5WY19_SORBI

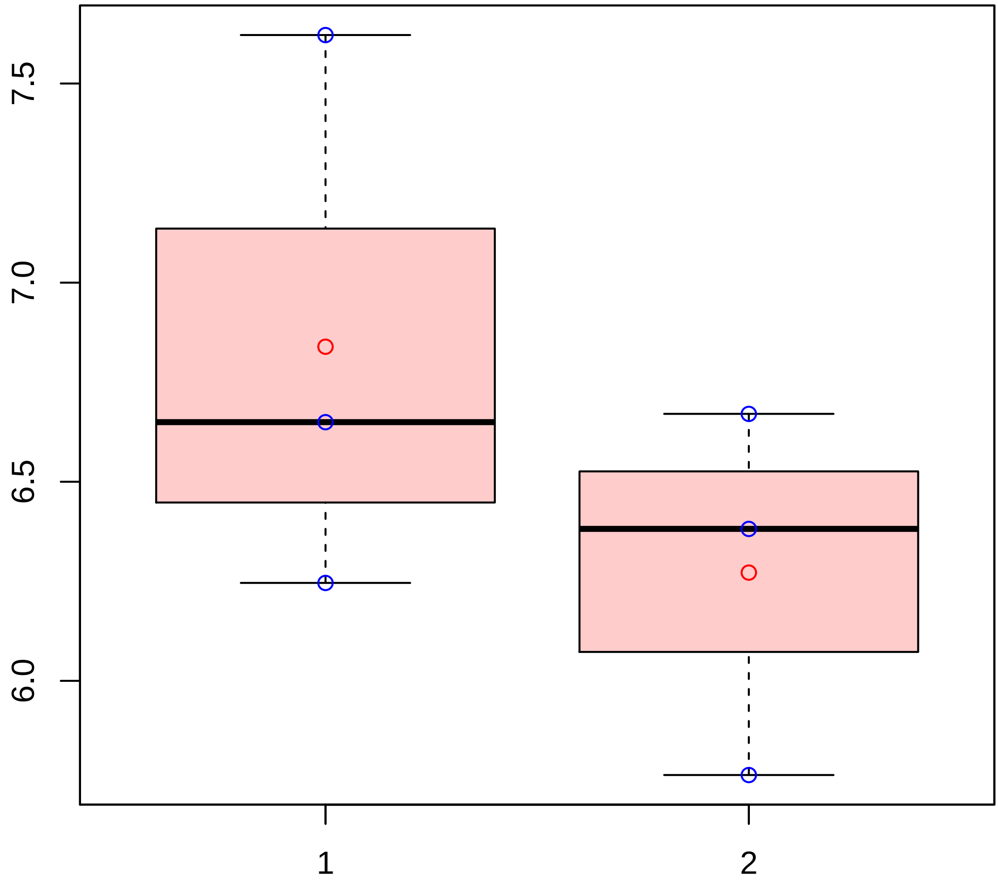

t−Test: p−value = 0.32

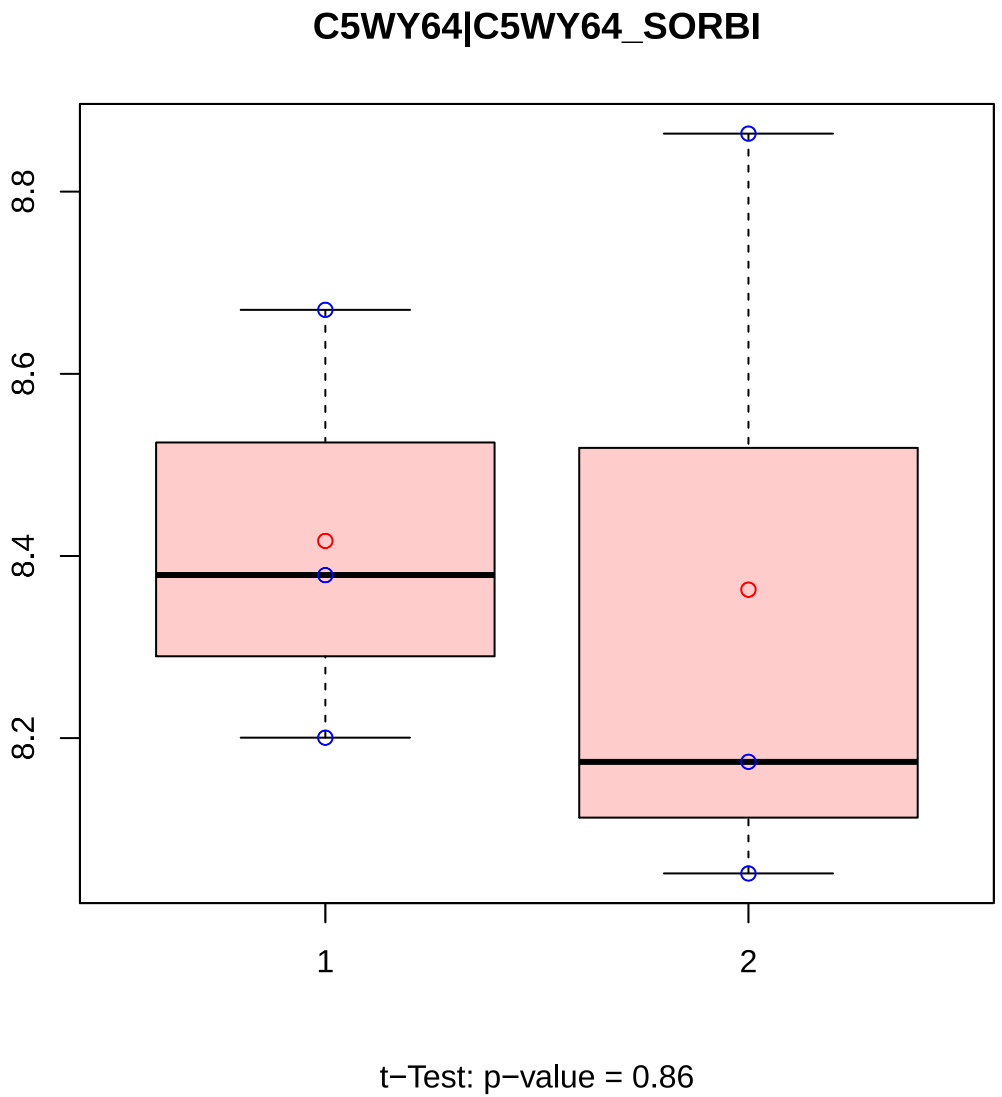

C5WY64|C5WY64_SORBI
8.8
8.6
8.4
8.2
1
2
t−Test: p−value = 0.86

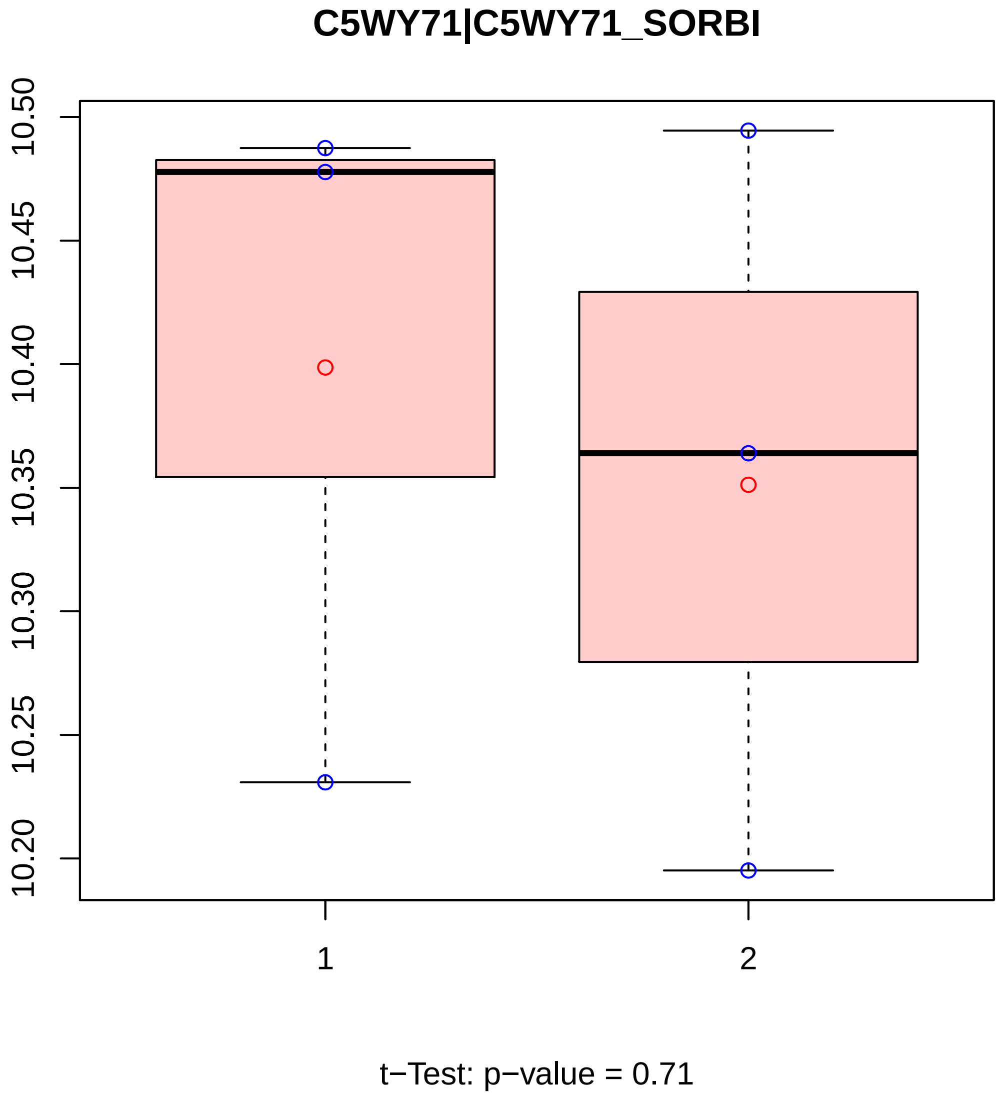
C5WY71|C5WY71_SORBI
10.50
10.45
10.40
10.35
10.30
10.25
10.20
1
2
t−Test: p−value = 0.71

C5WYF2|C5WYF2_SORBI

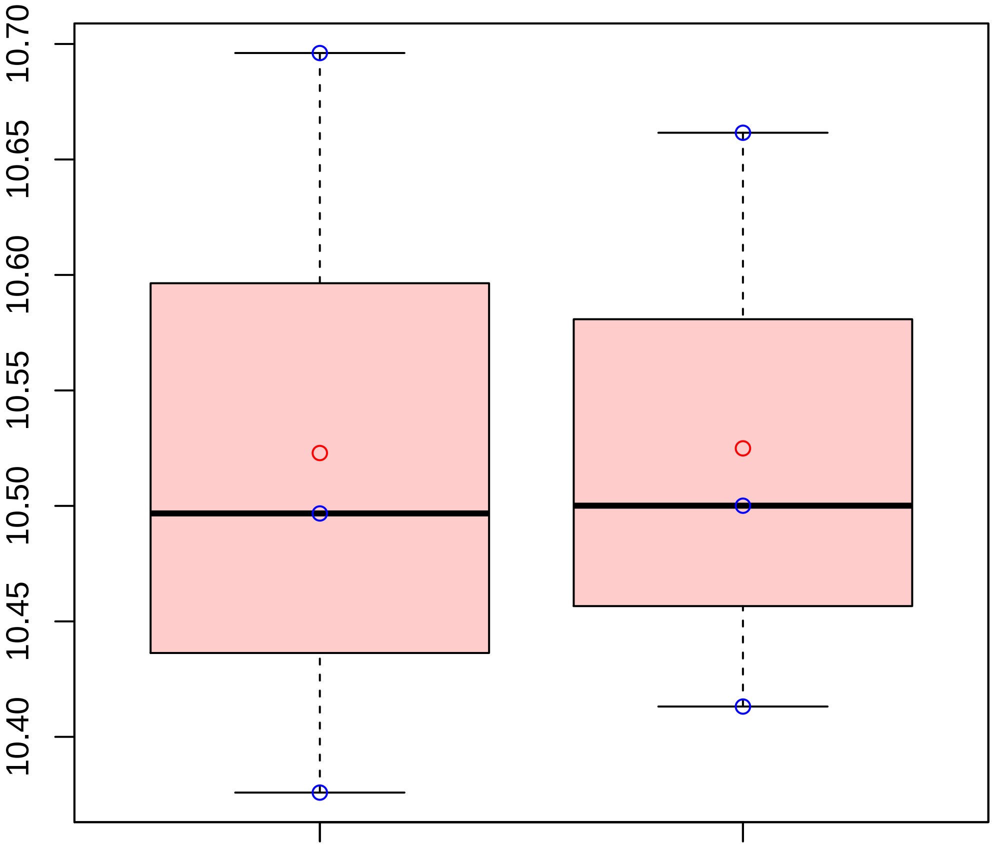

t−Test: p−value = 0.99

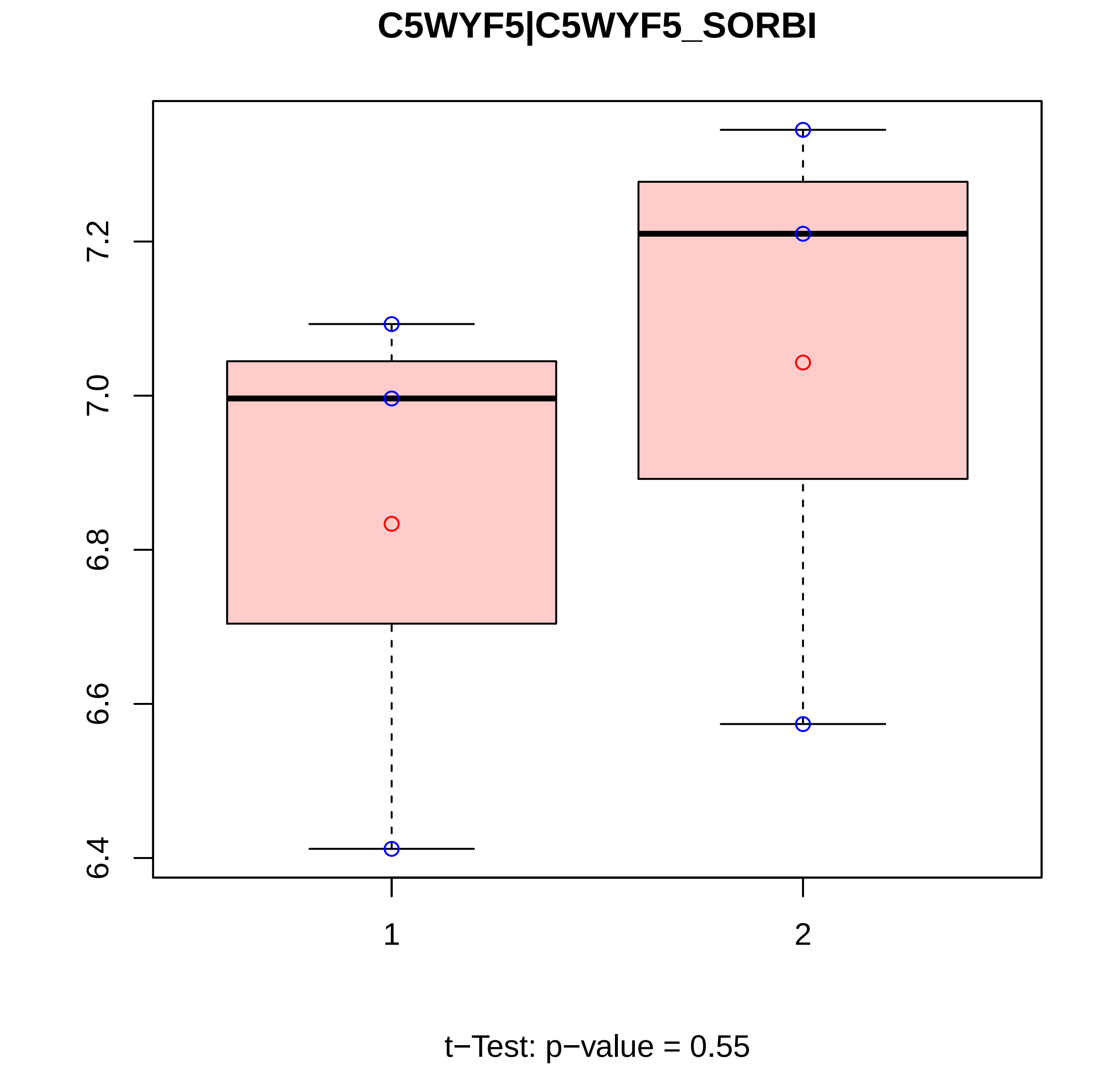
C5WYF5|C5WYF5_SORBI
7.2
7.0
6.8
6.6
6.4
1
2
t−Test: p−value = 0.55

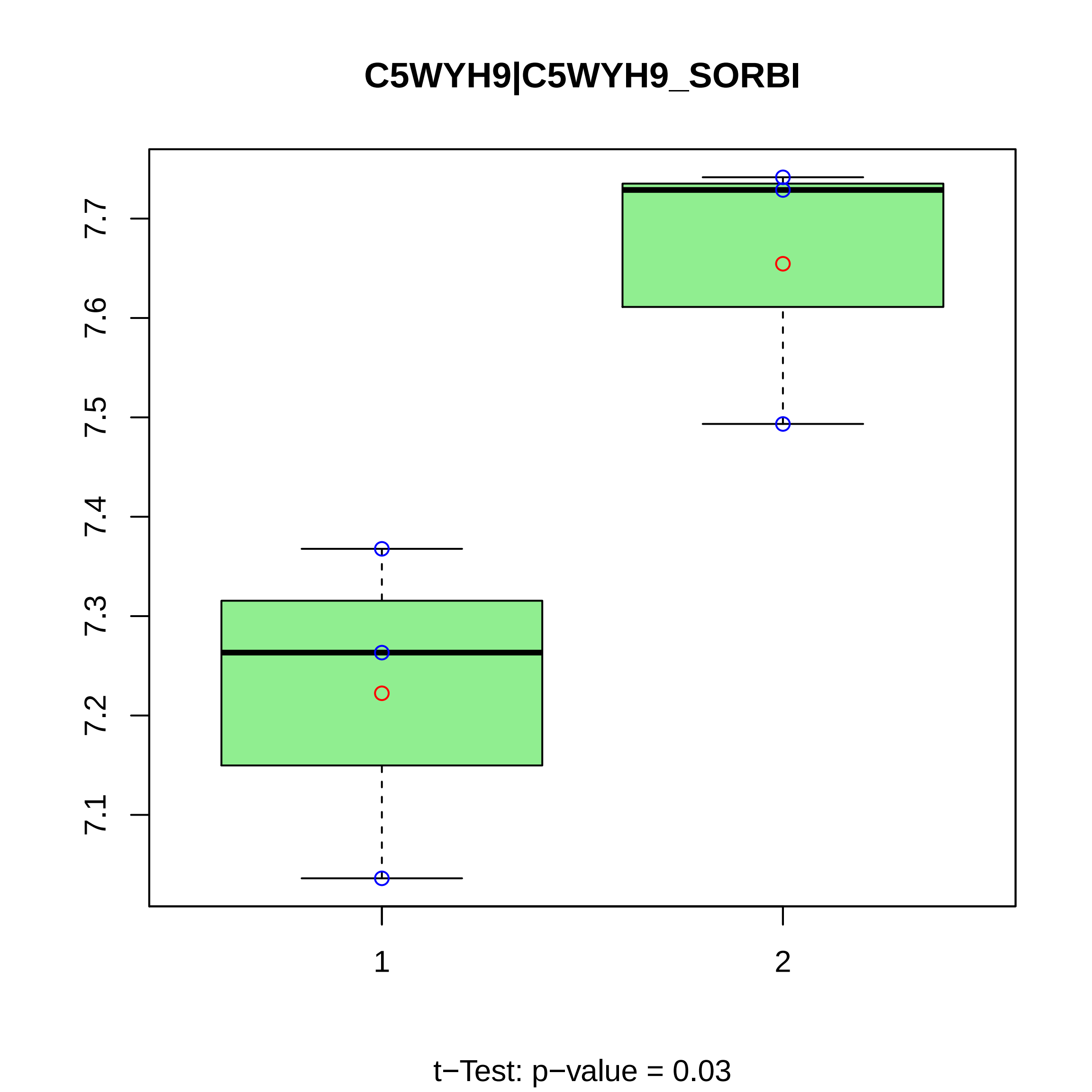
C5WYH9|C5WYH9_SORBI
7.1
7.2
7.3
7.4
7.5
7.6
7.7
1
2
t−Test: p−value = 0.03

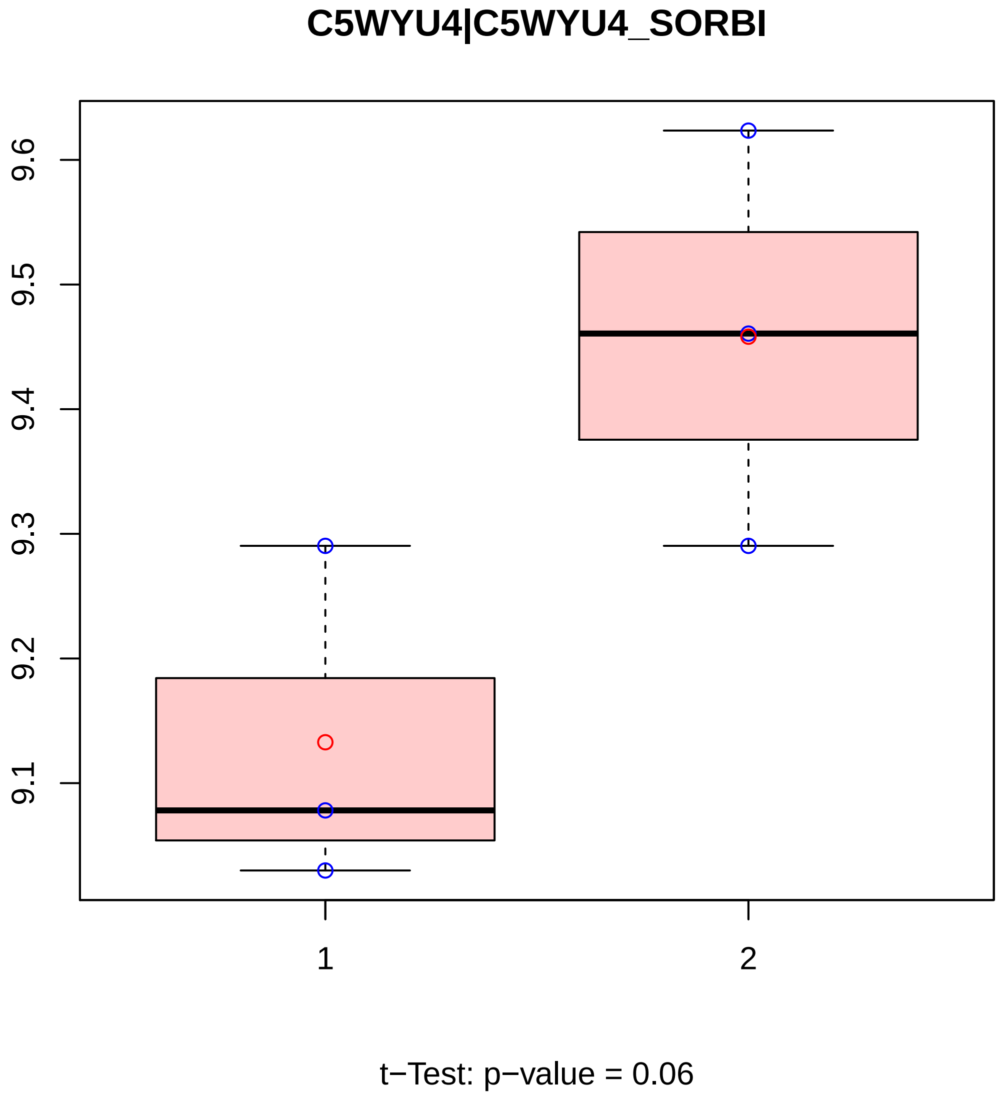
C5WYU4|C5WYU4_SORBI
9.1
9.2
9.3
9.4
9.5
9.6
1
2
t−Test: p−value = 0.06

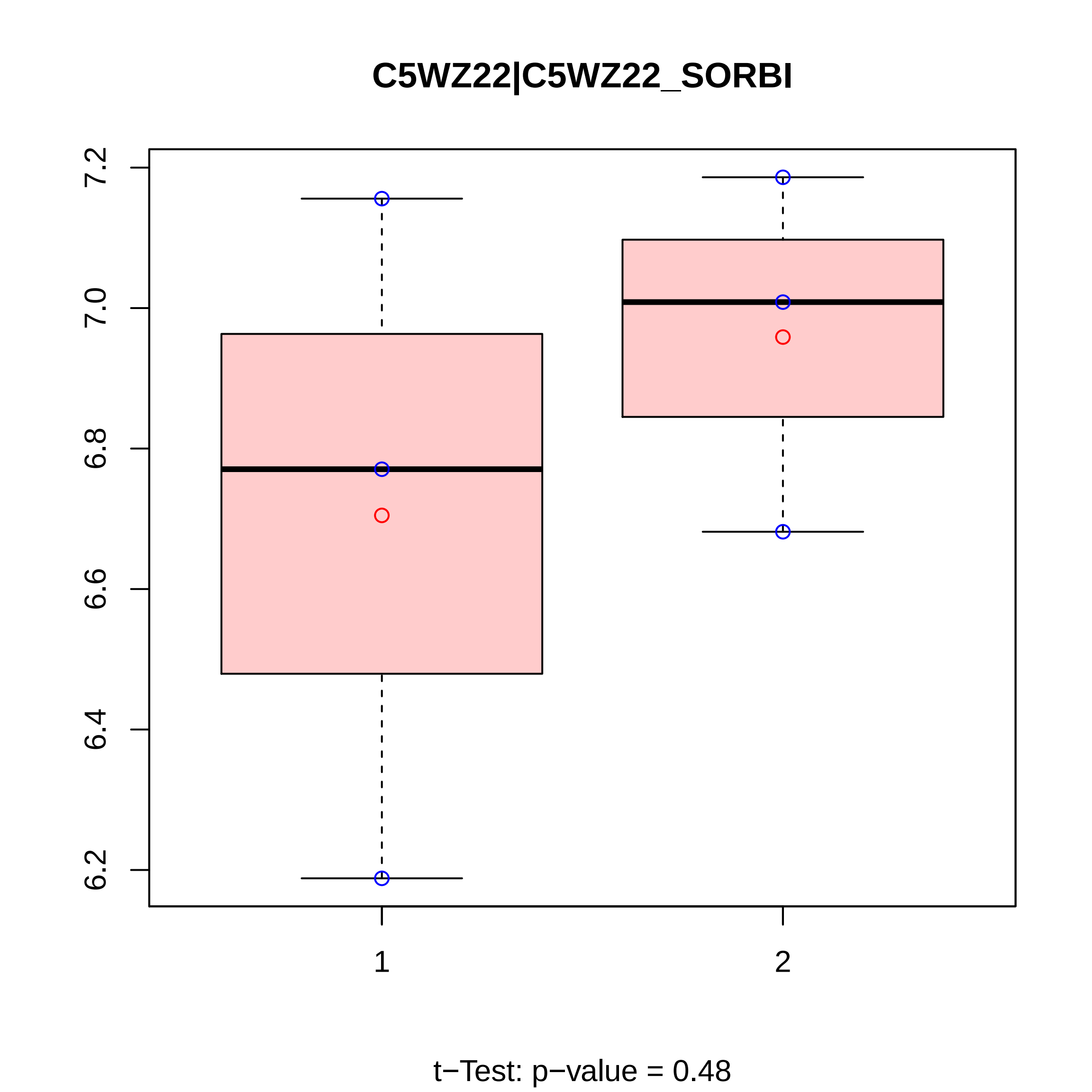

C5WZ22|C5WZ22_SORBI
7.2
7.0
6.8
6.6
6.4
6.2
1
2
t−Test: p−value = 0.48

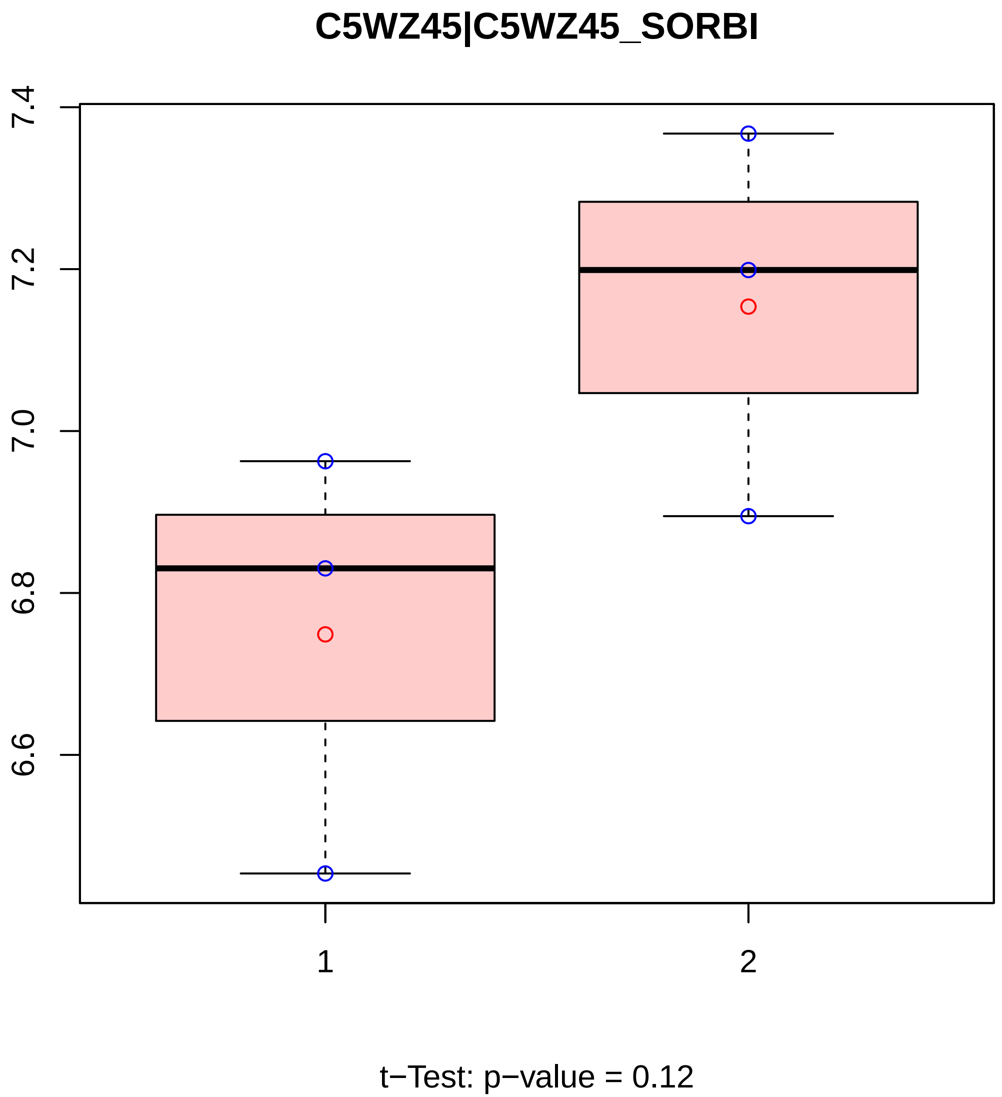
C5WZ45|C5WZ45_SORBI
7.4
7.2
7.0
6.8
6.6
1
2
t−Test: p−value = 0.12

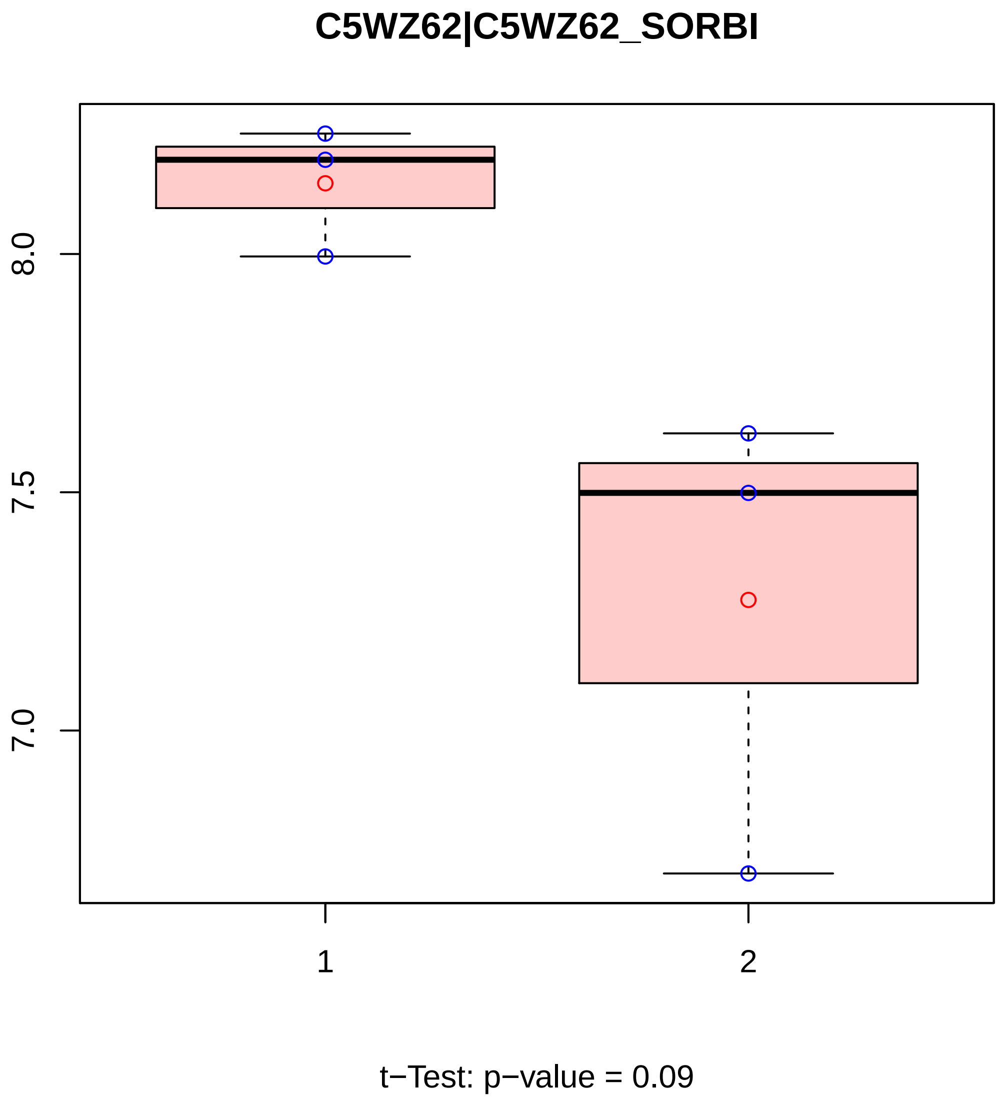
C5WZ62|C5WZ62_SORBI
8.0
7.5
7.0
1
2
t−Test: p−value = 0.09

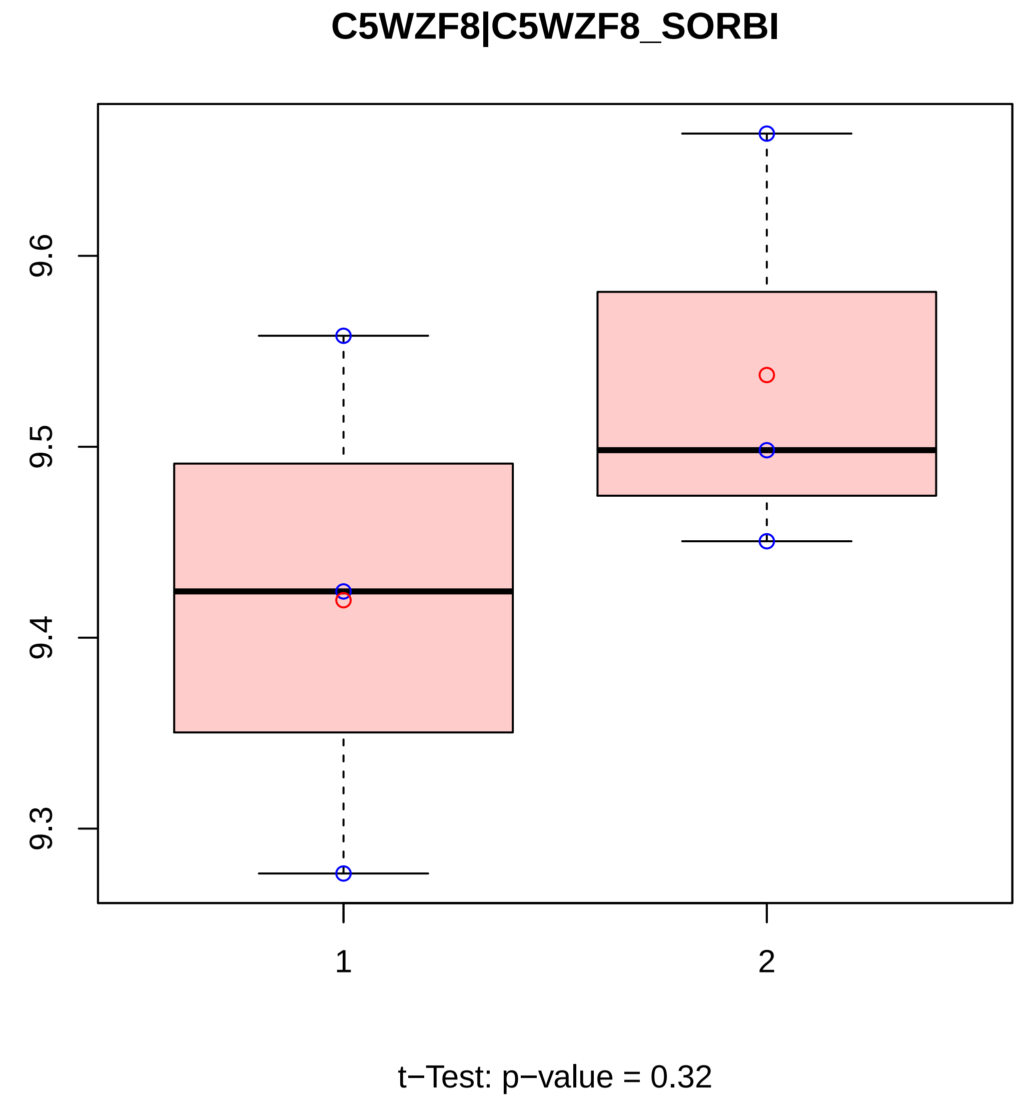
C5WZF8|C5WZF8_SORBI
9.3
9.4
9.5
9.6
1
2
t−Test: p−value = 0.32

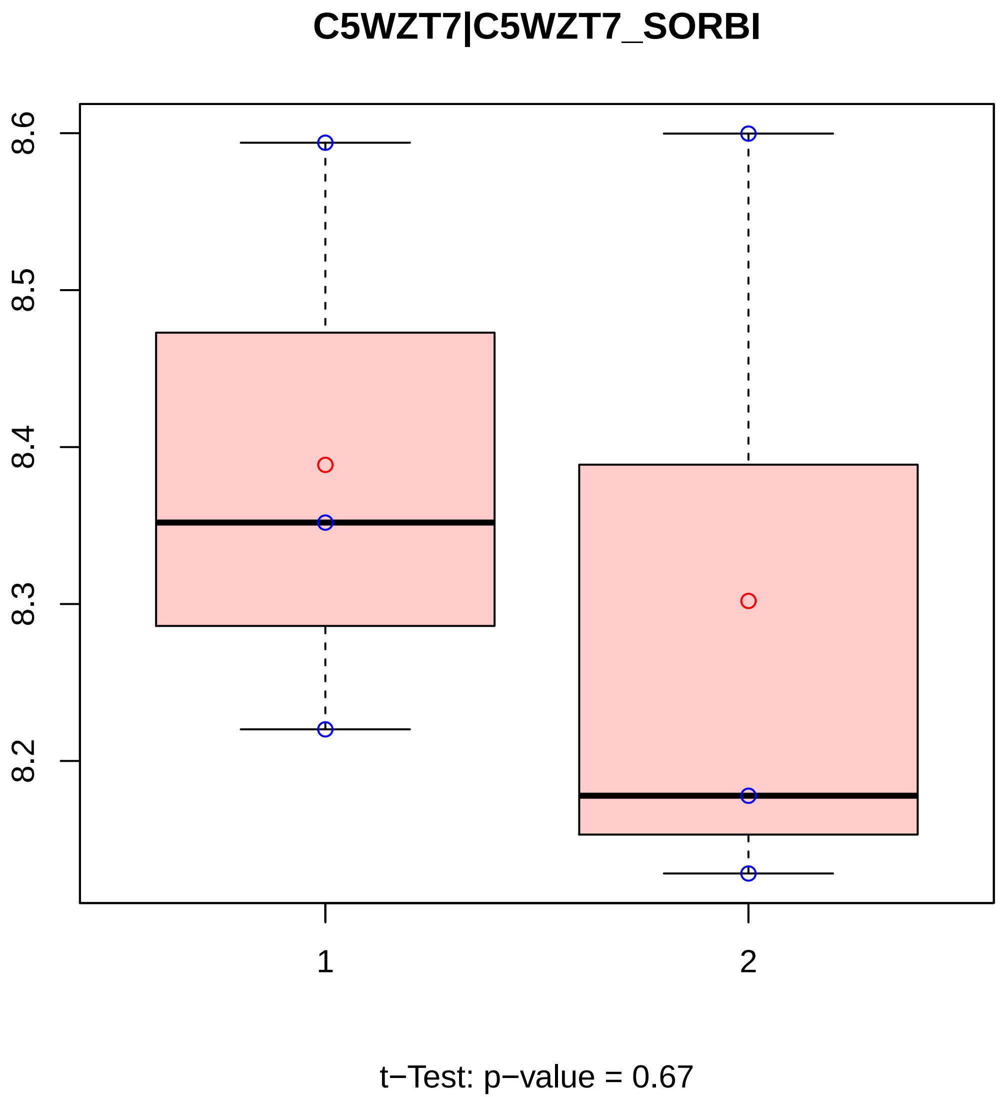
C5WZT7|C5WZT7_SORBI
8.2
8.3
8.4
8.5
8.6
1
2
t−Test: p−value = 0.67

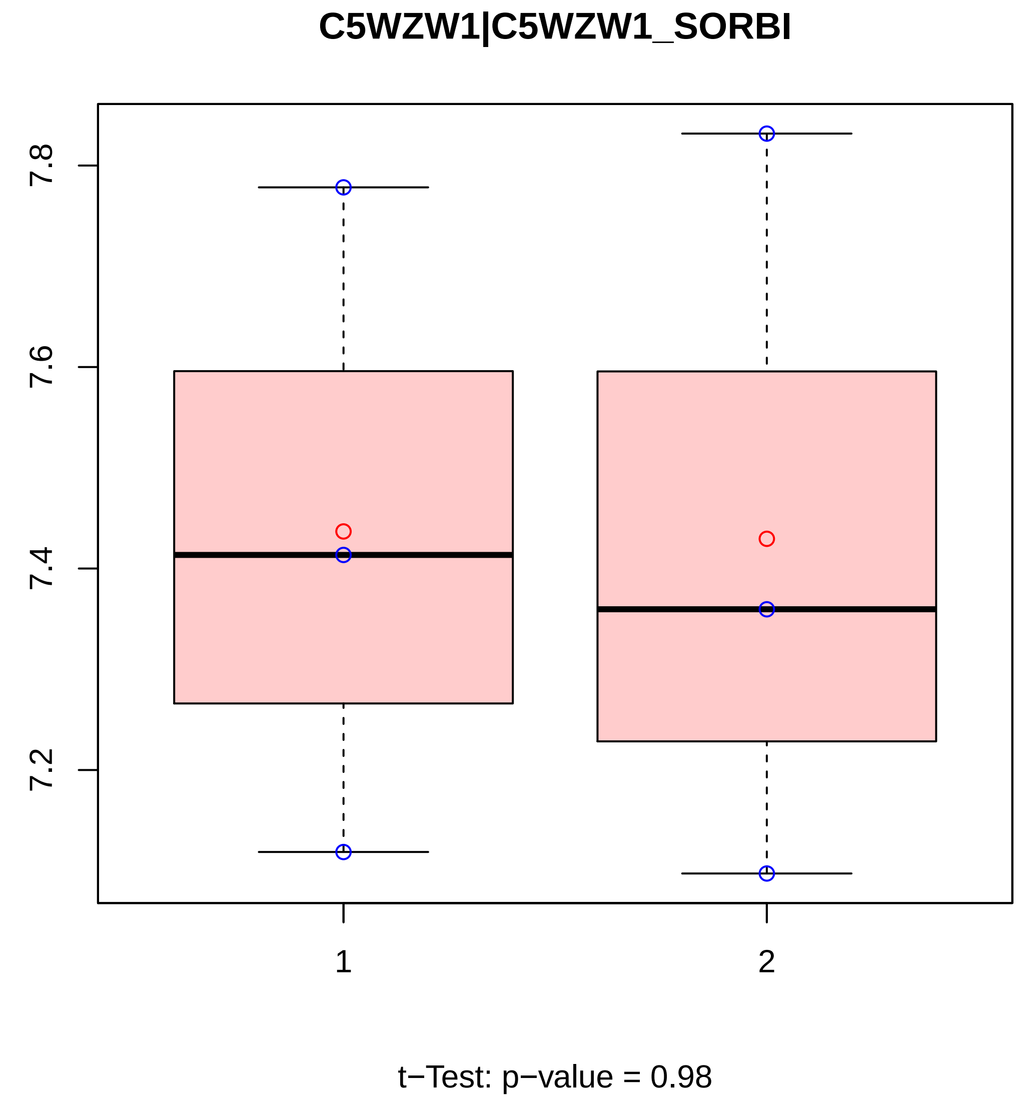

C5WZW1|C5WZW1_SORBI
7.2
7.4
7.6
7.8
1
2
t−Test: p−value = 0.98

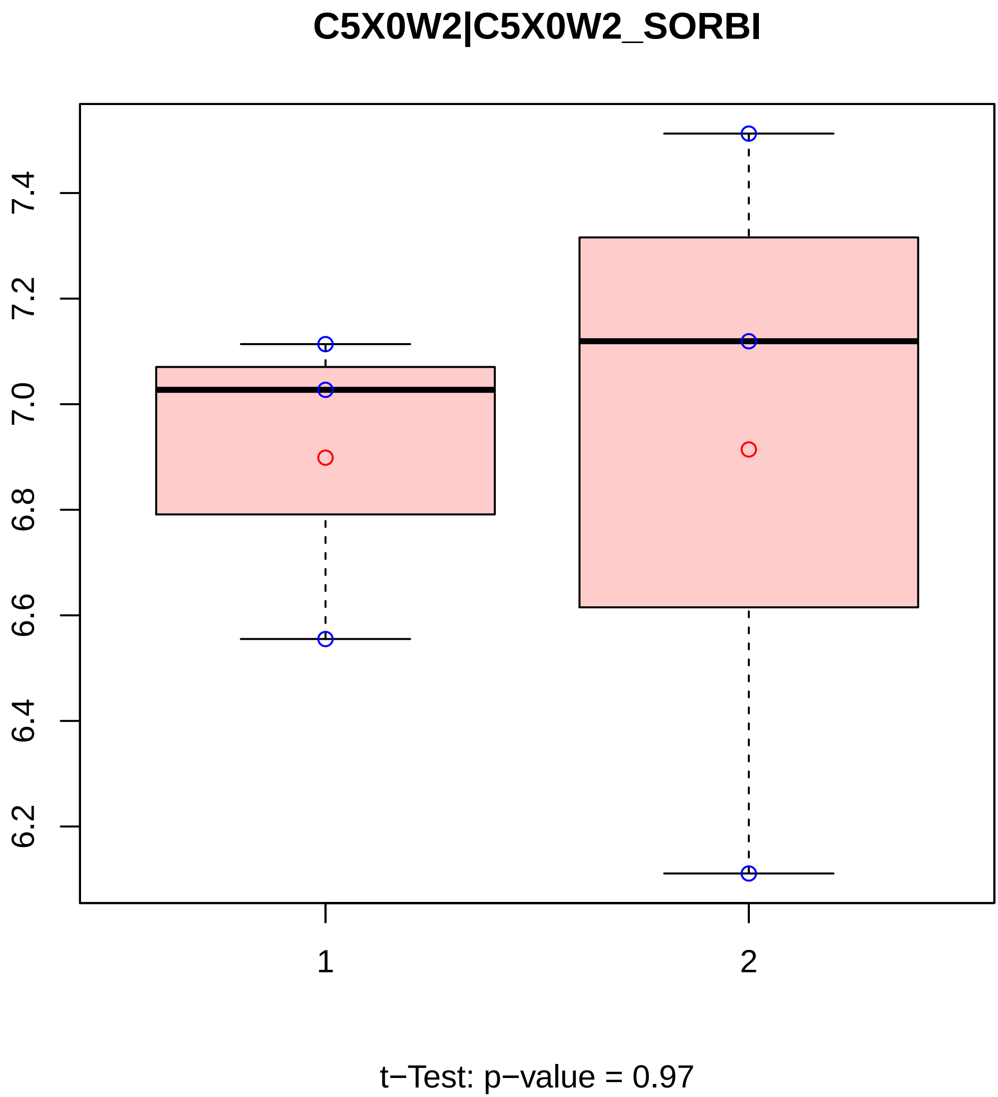

C5X0W2|C5X0W2_SORBI
7.4
7.2
7.0
6.8
6.6
6.4
6.2
1
2
t−Test: p−value = 0.97

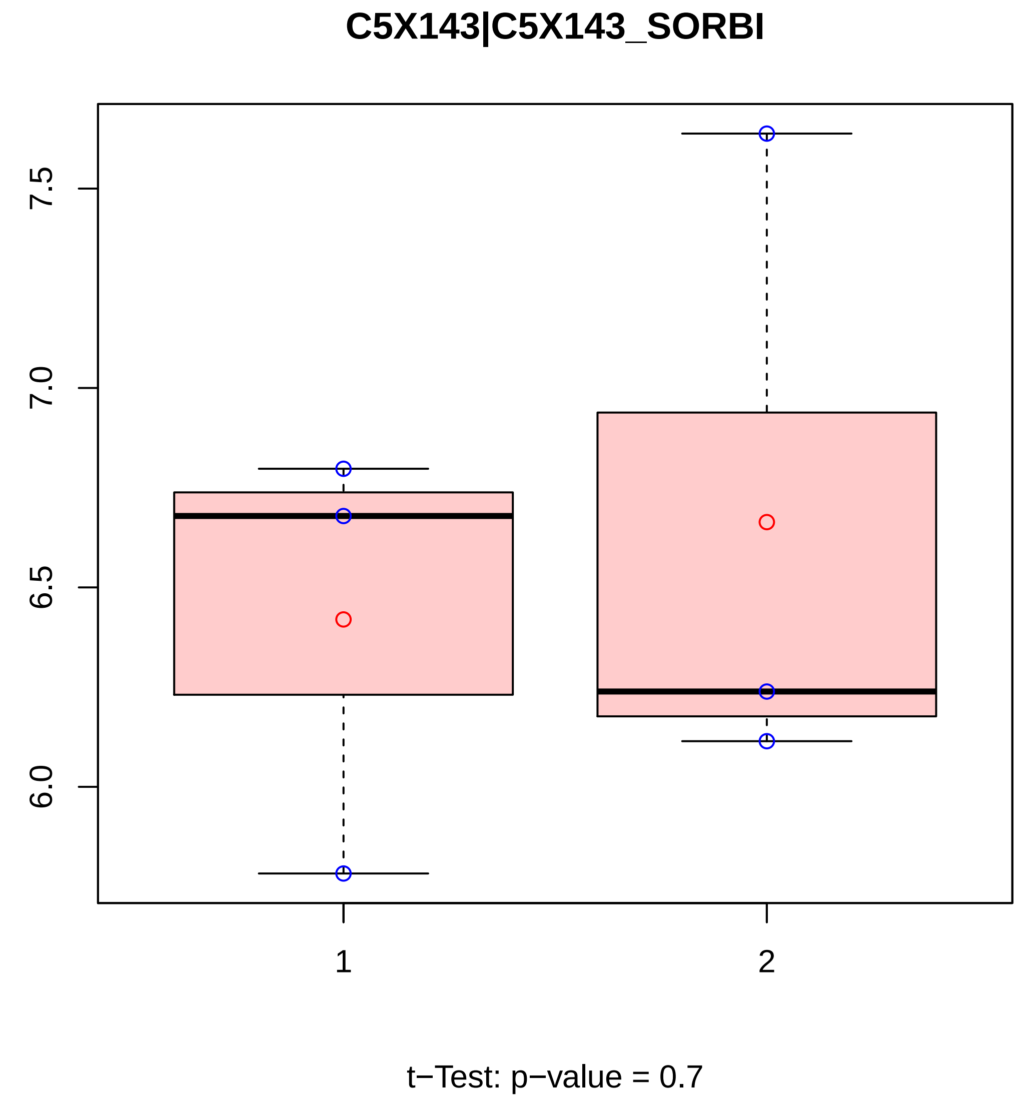
C5X143|C5X143_SORBI
7.5
7.0
6.5
6.0
1
2
t−Test: p−value = 0.7

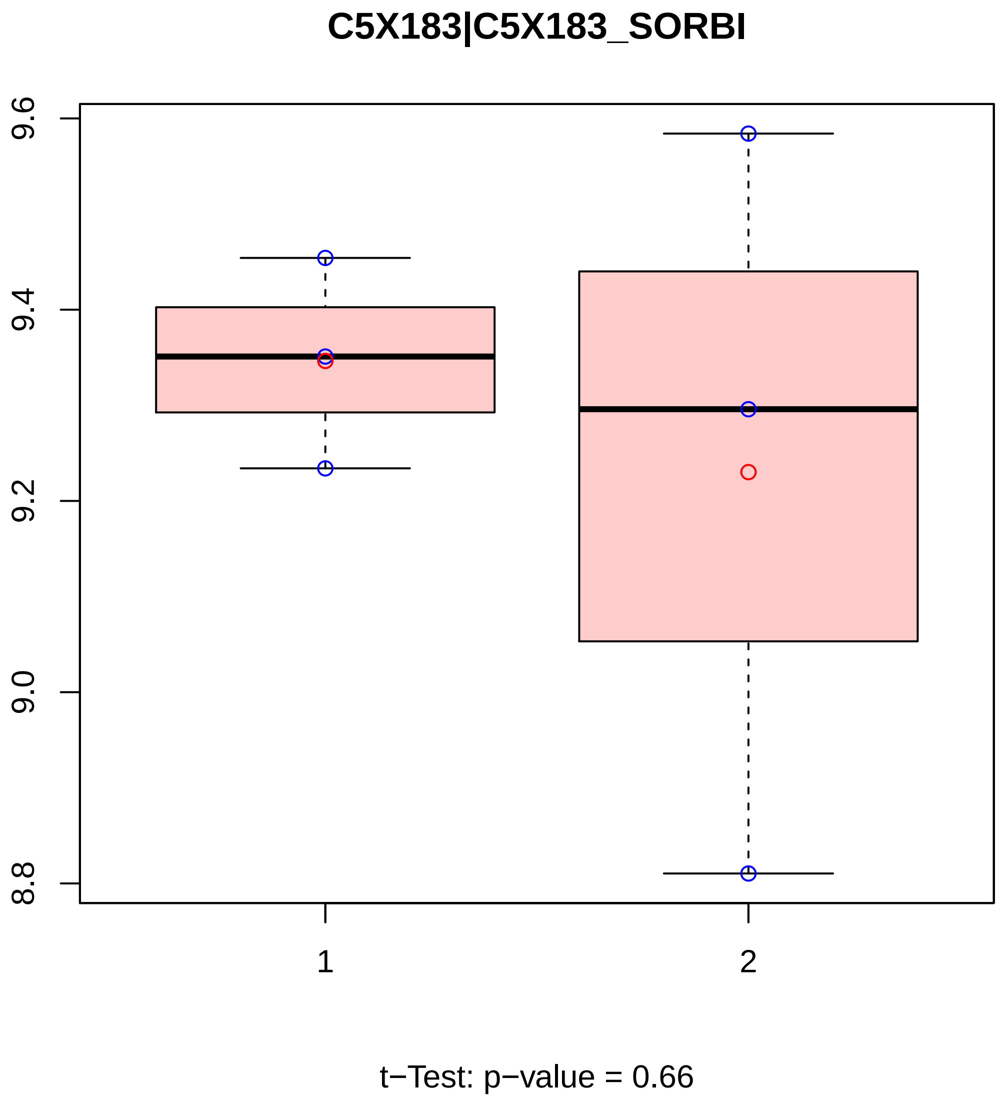
C5X183|C5X183_SORBI
9.6
9.4
9.2
9.0
8.8
1
2
t−Test: p−value = 0.66

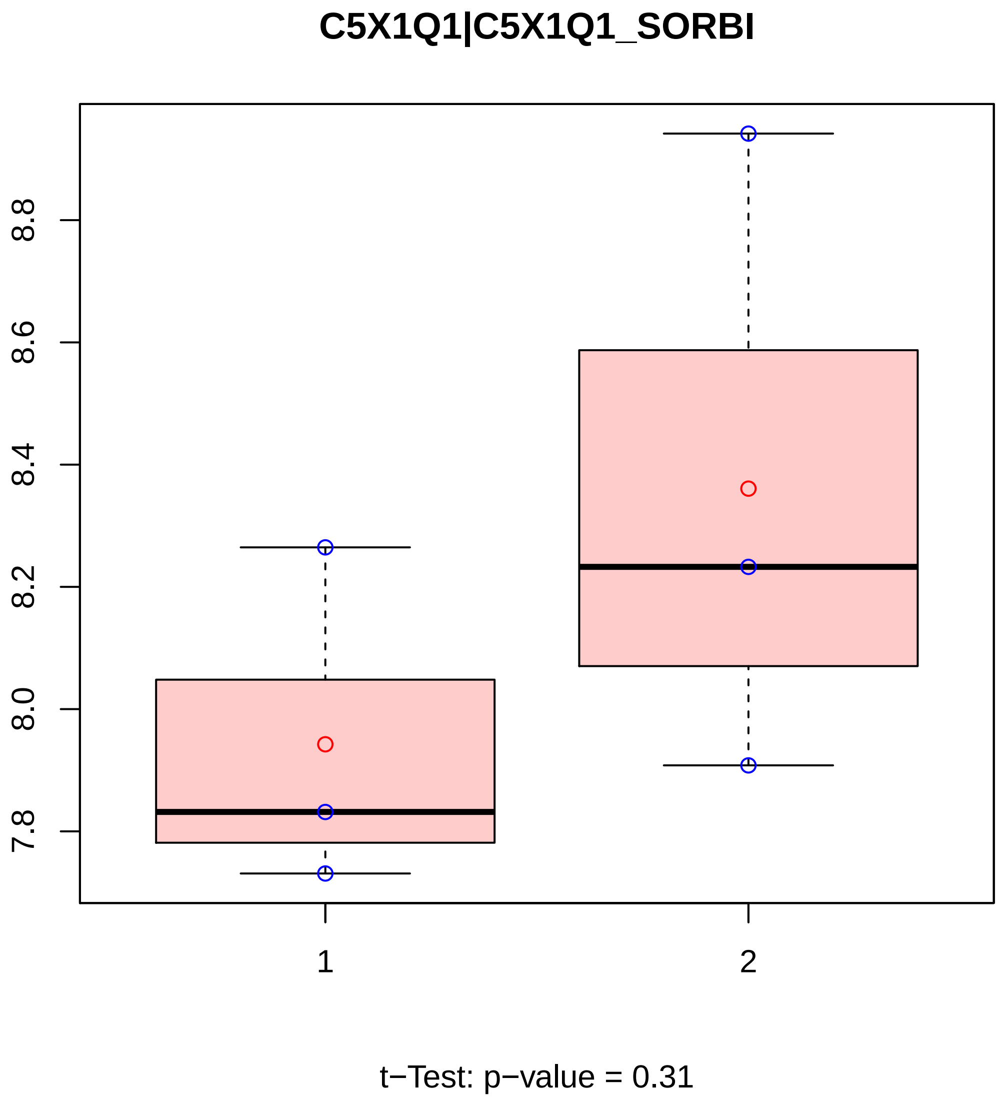

C5X1Q1|C5X1Q1_SORBI
8.8
8.6
8.4
8.2
8.0
7.8
1
2
t−Test: p−value = 0.31

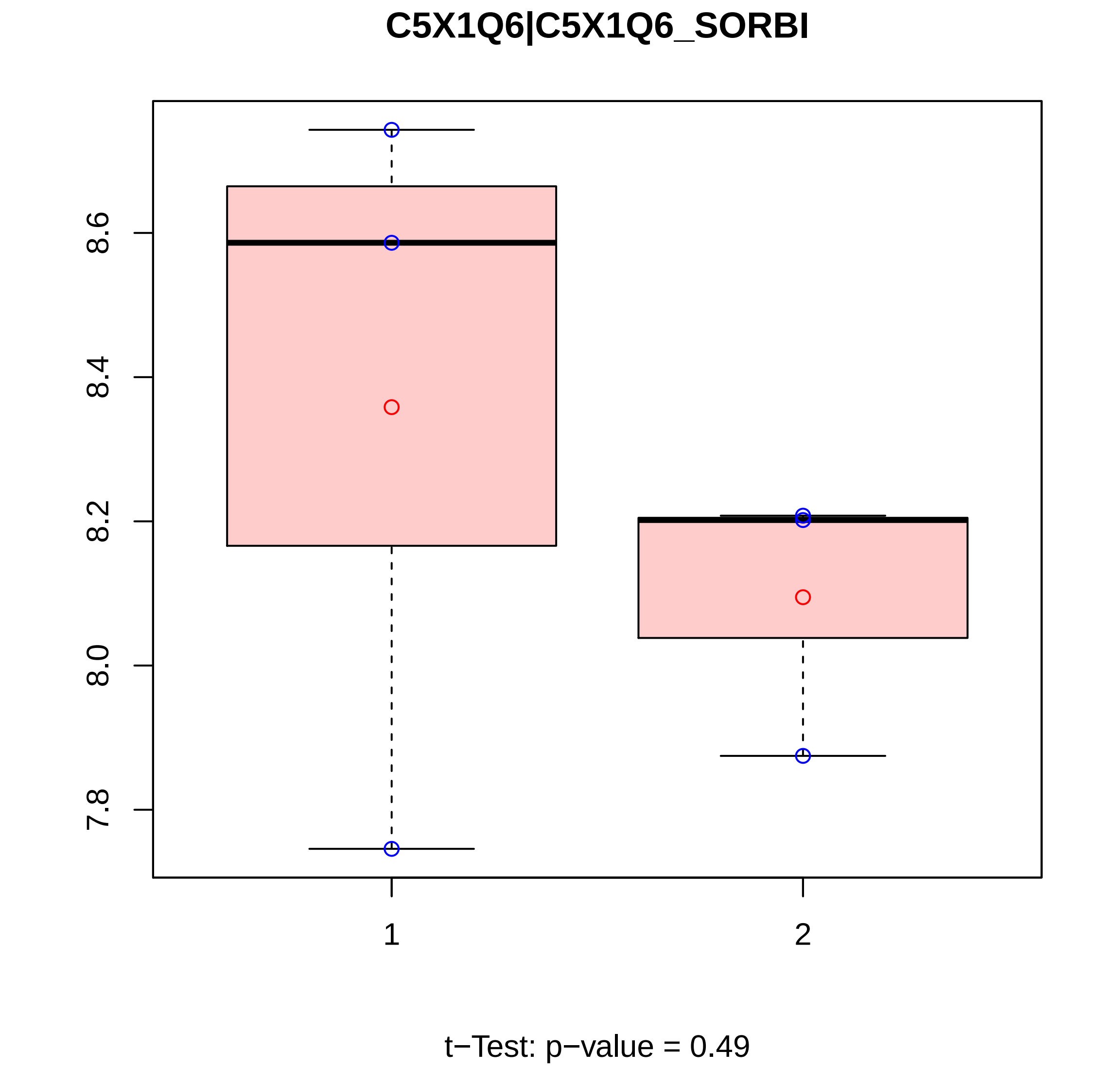

C5X1Q6|C5X1Q6_SORBI
8.6
8.4
8.2
8.0
7.8
1
2
t−Test: p−value = 0.49

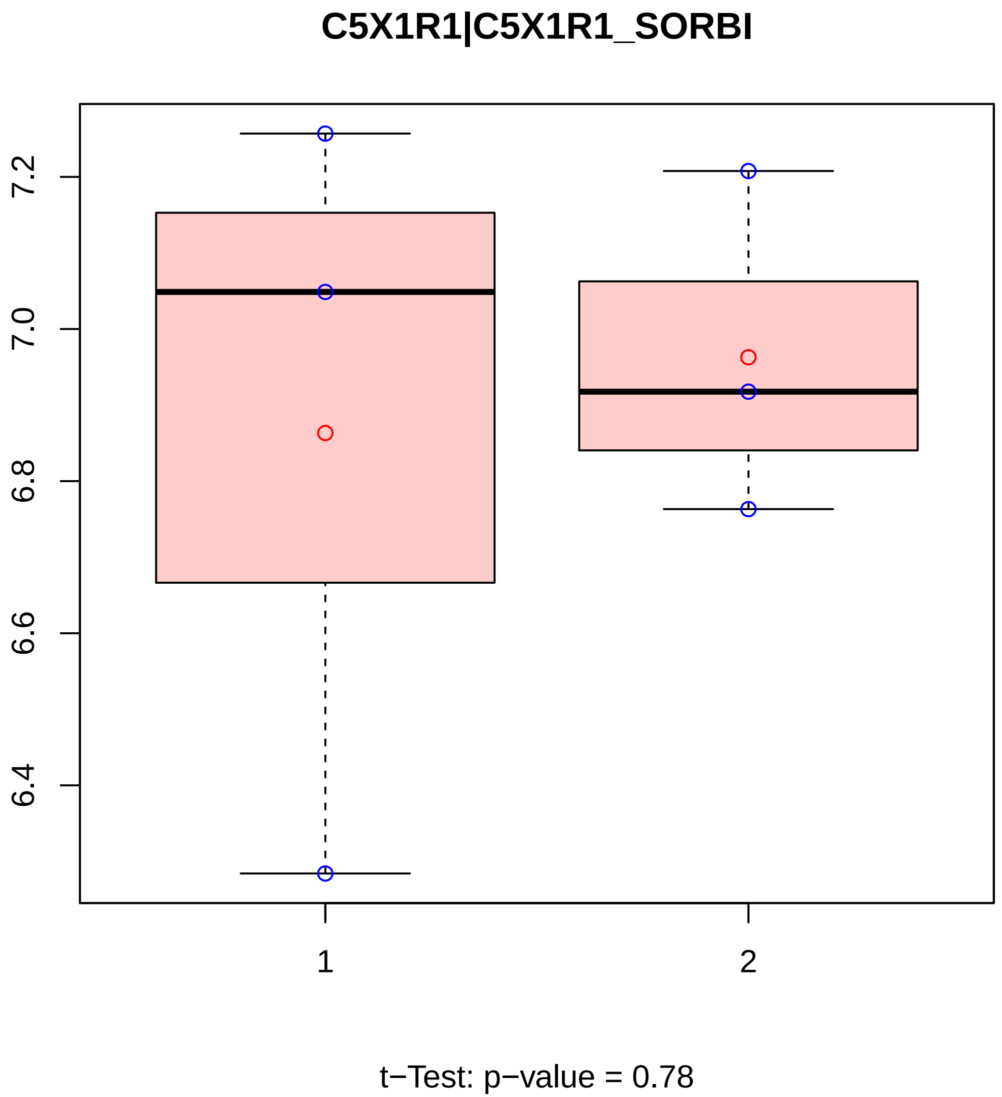
C5X1R1|C5X1R1_SORBI
7.2
7.0
6.8
6.6
6.4
1
2
t−Test: p−value = 0.78

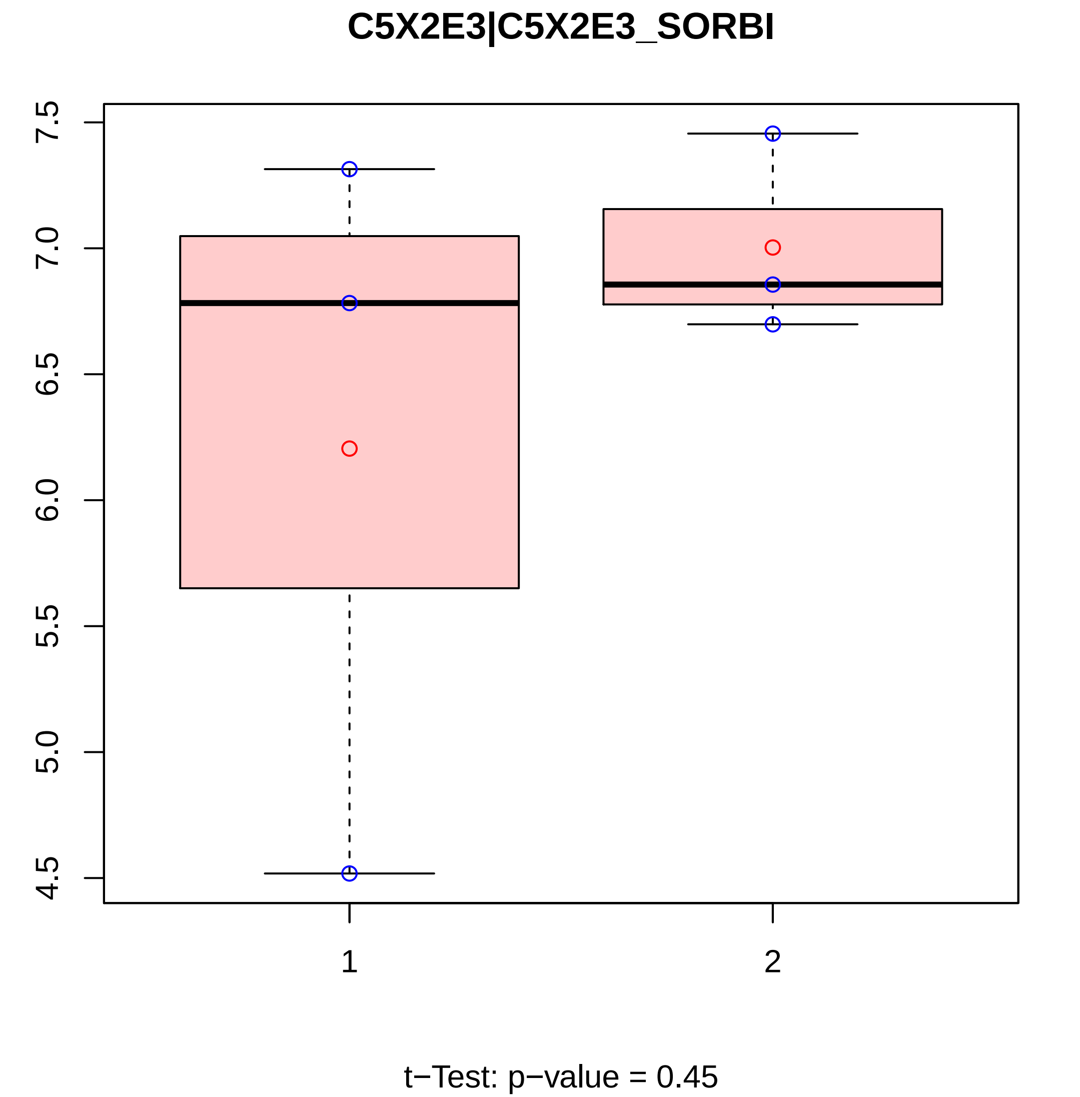

C5X2E3|C5X2E3_SORBI
7.5
7.0
6.5
6.0
5.5
5.0
4.5
1
2
t−Test: p−value = 0.45

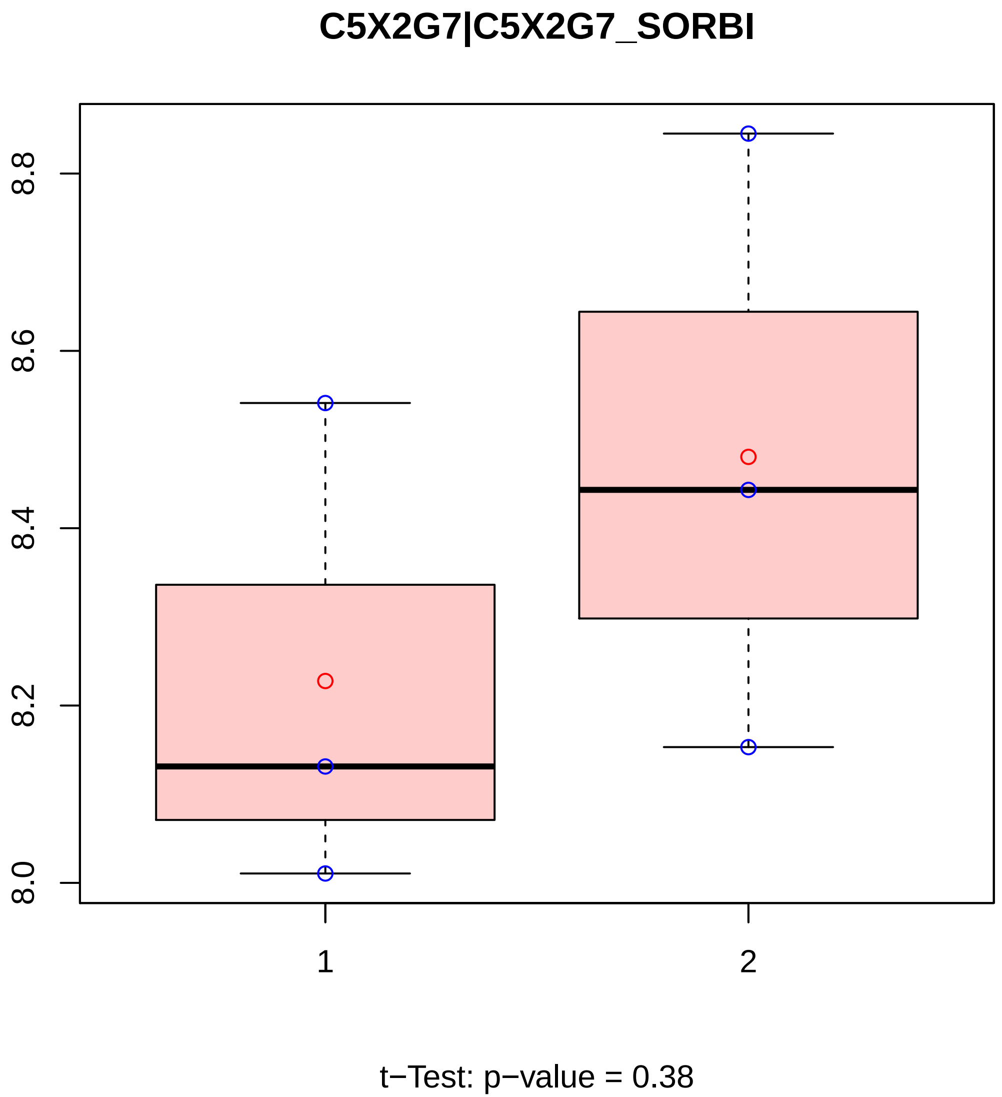
C5X2G7|C5X2G7_SORBI
8.0
8.2
8.4
8.6
8.8
1
2
t−Test: p−value = 0.38

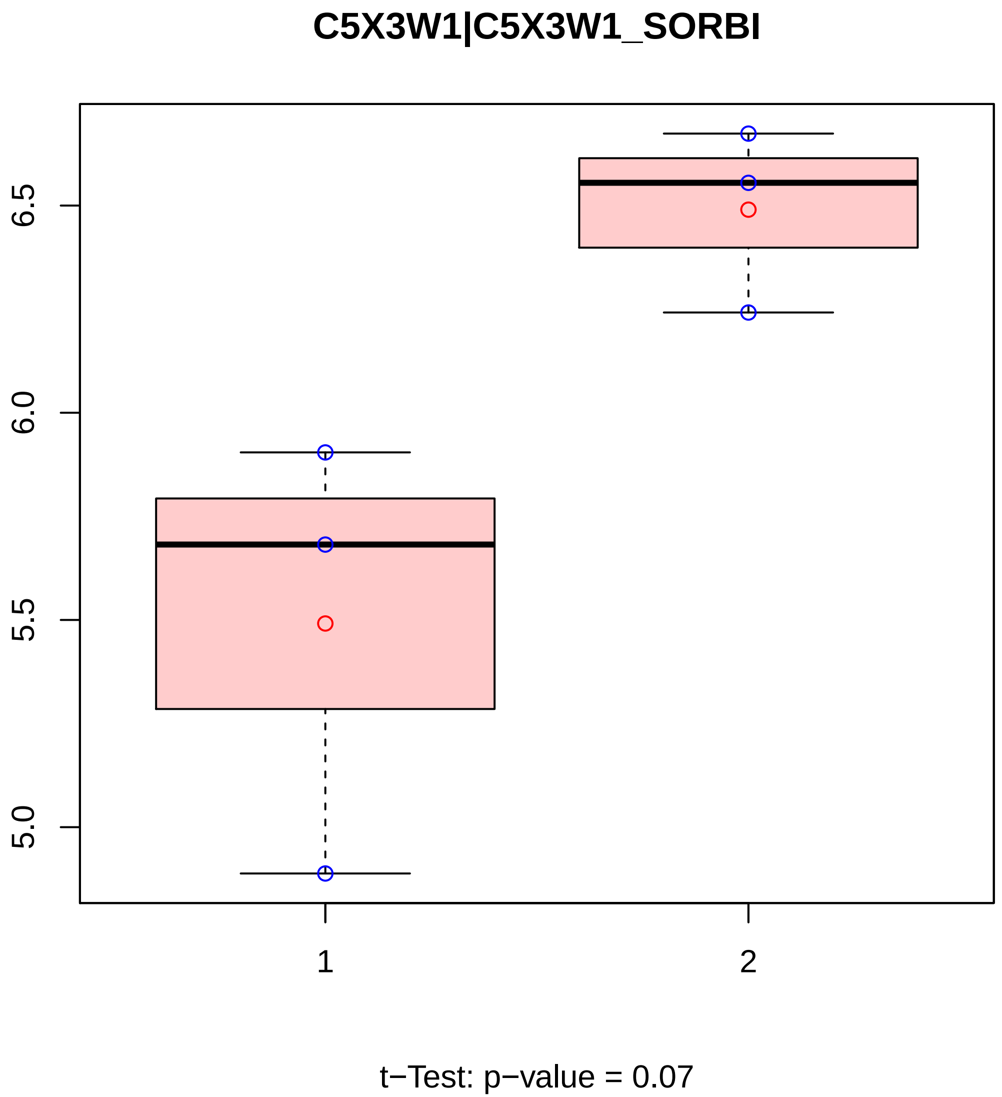

C5X3W1|C5X3W1_SORBI
6.5
6.0
5.5
5.0
1
2
t−Test: p−value = 0.07

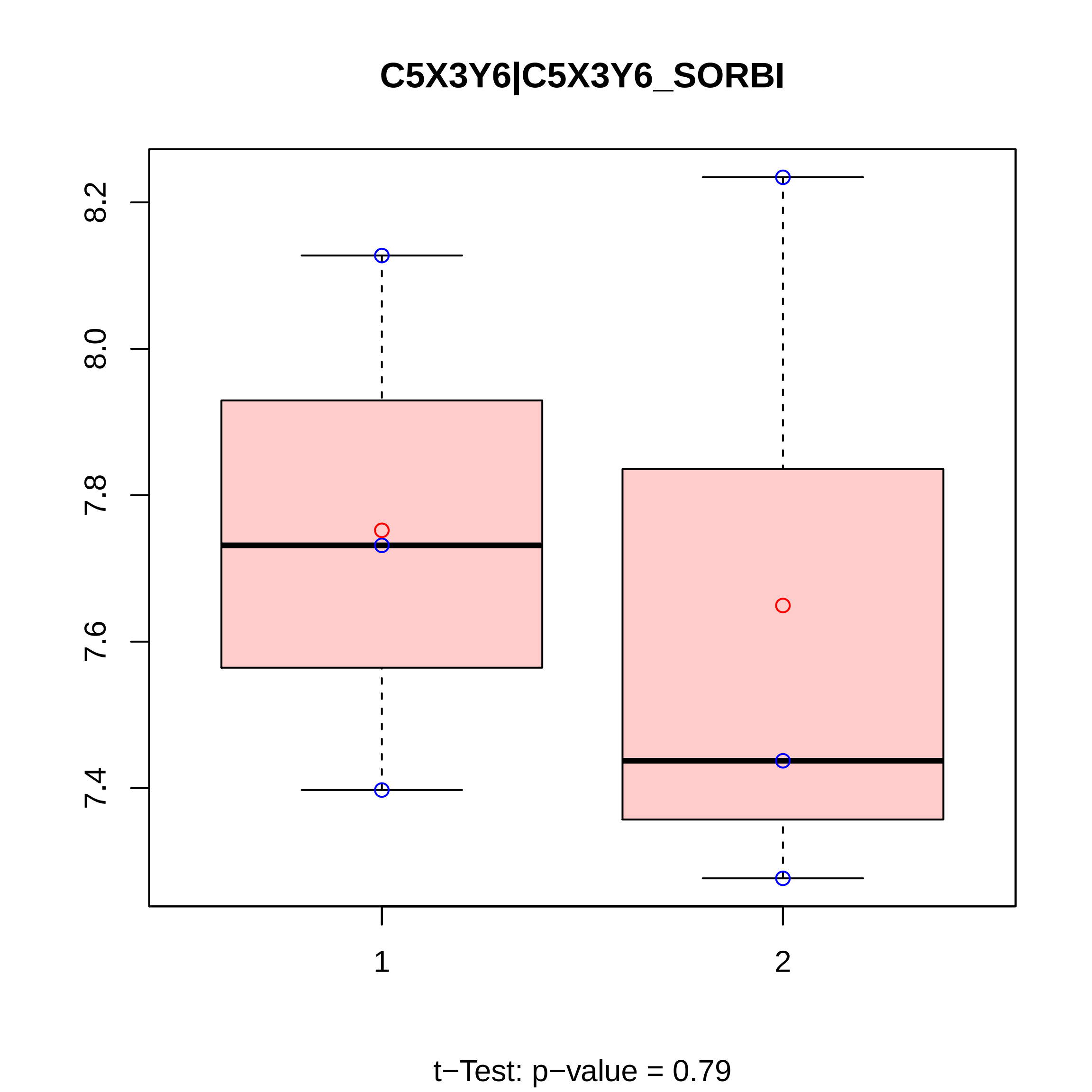

C5X3Y6|C5X3Y6_SORBI
8.2
8.0
7.8
7.6
7.4
1
2
t−Test: p−value = 0.79

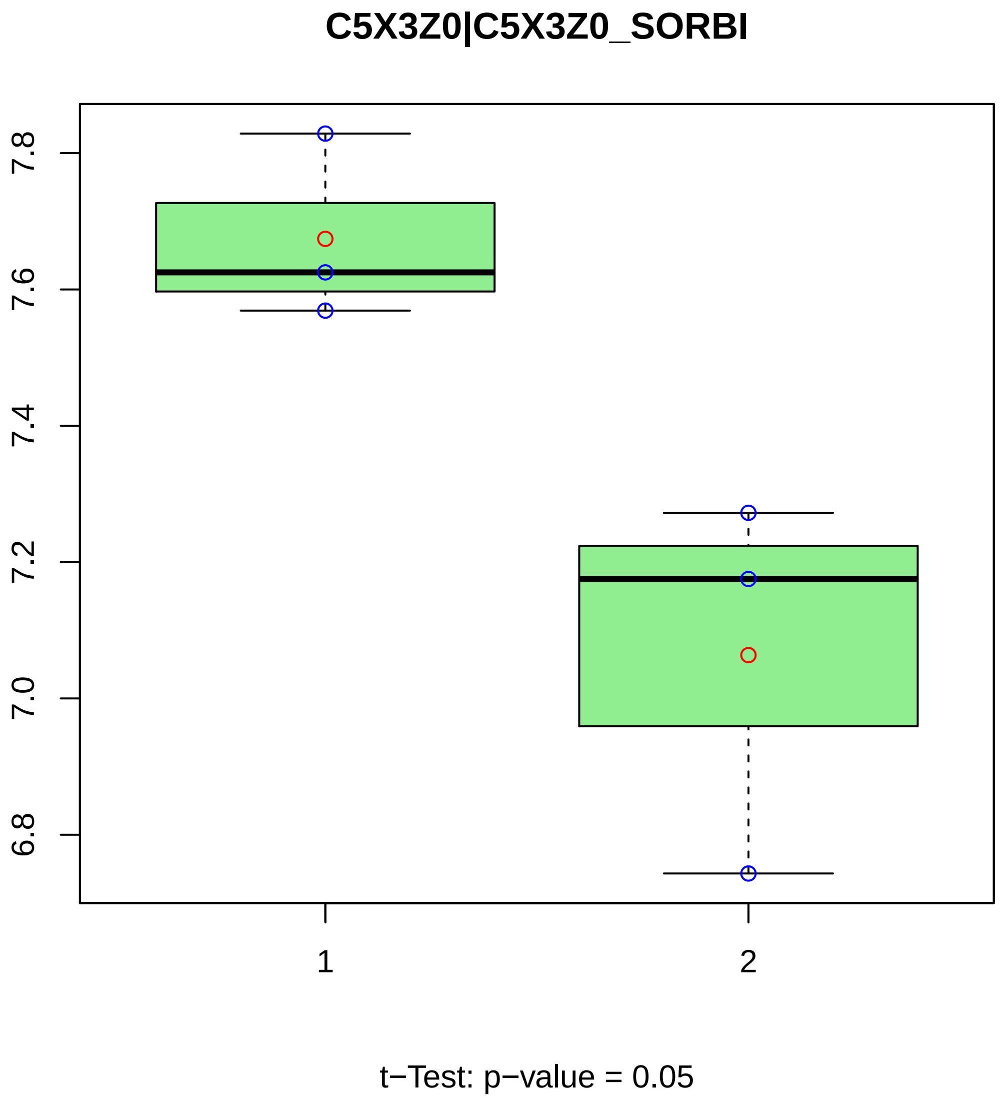

C5X3Z0|C5X3Z0_SORBI
7.8
7.6
7.4
7.2
7.0
6.8
1
2
t−Test: p−value = 0.05

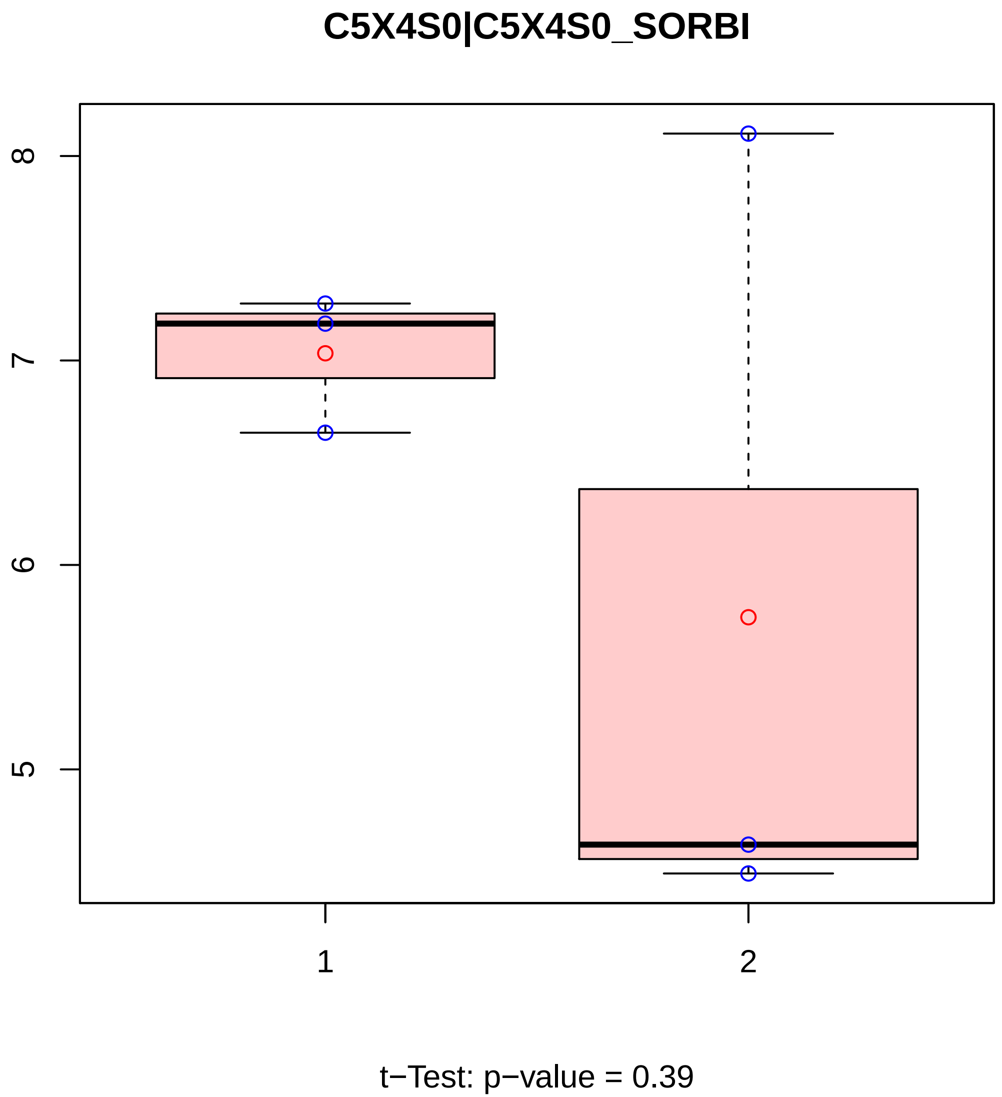
C5X4S0|C5X4S0_SORBI
8
7
6
5
1
2
t−Test: p−value = 0.39

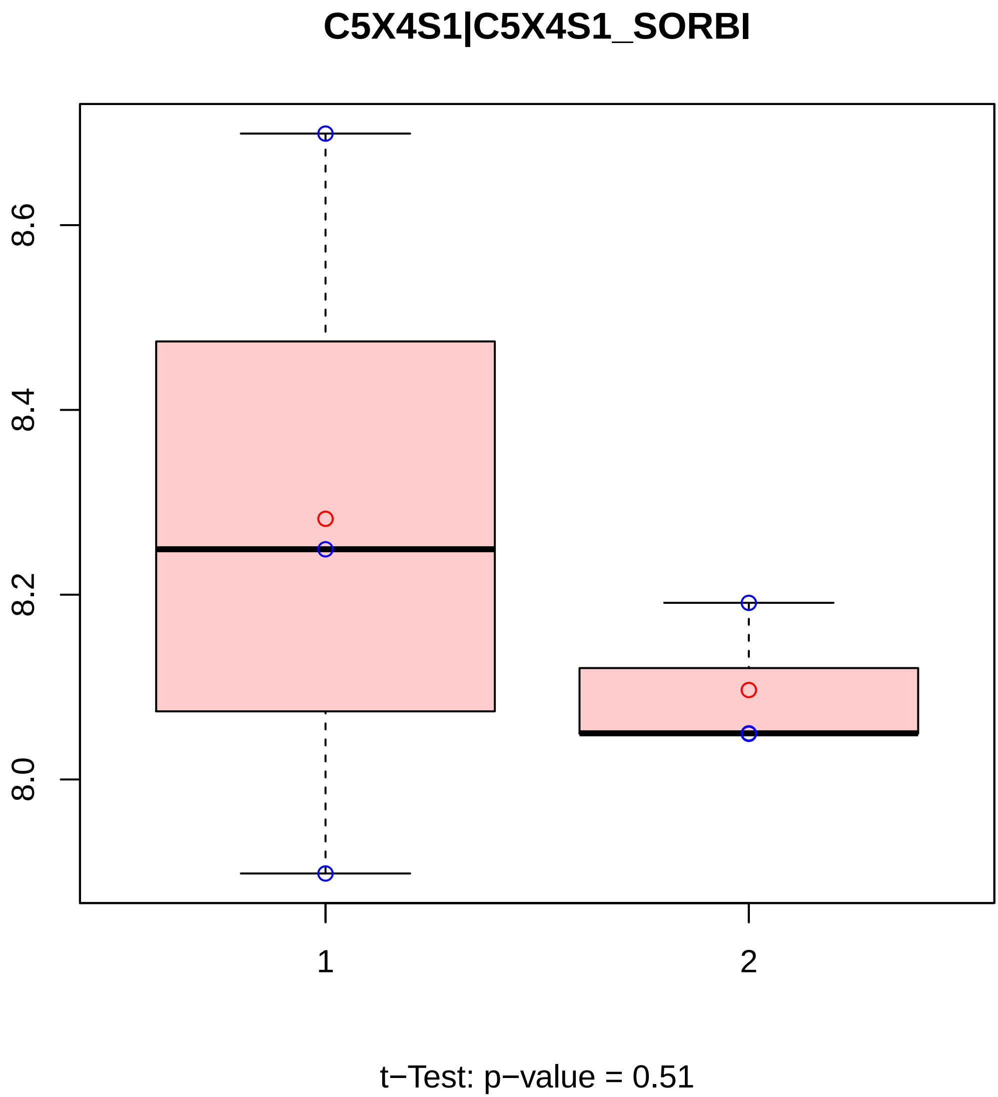

C5X4S1|C5X4S1_SORBI
8.0
8.2
8.4
8.6
1
2
t−Test: p−value = 0.51

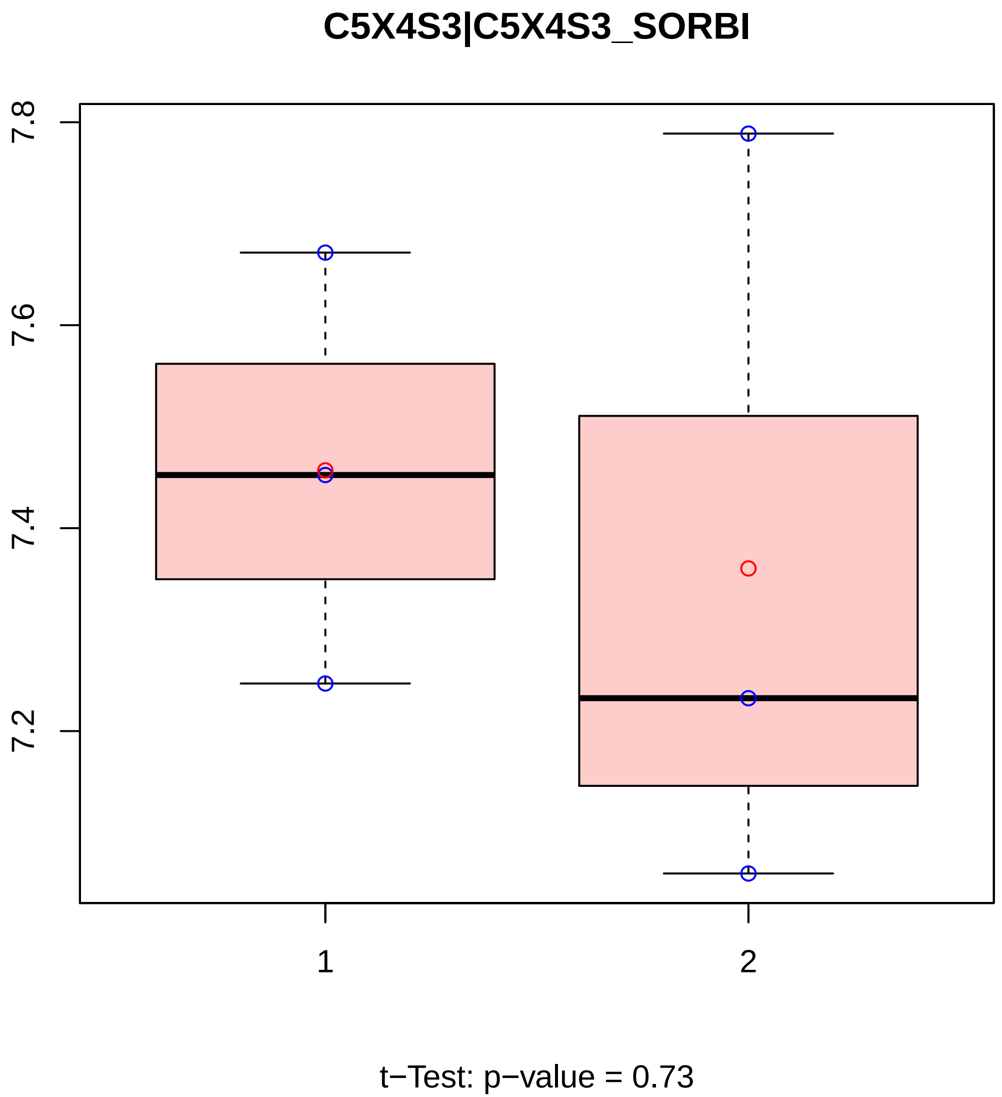
C5X4S3|C5X4S3_SORBI
7.8
7.6
7.4
7.2
1
2
t−Test: p−value = 0.73

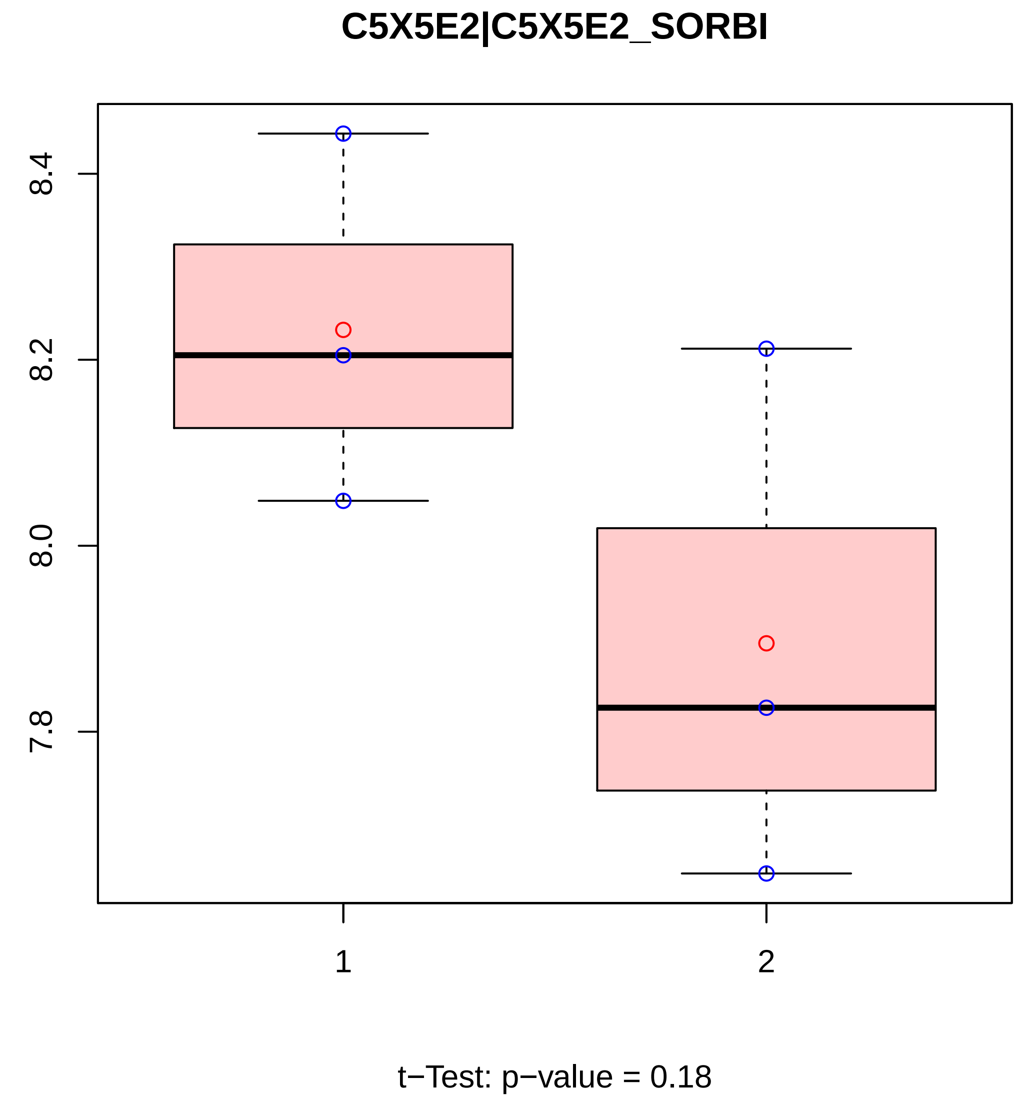
C5X5E2|C5X5E2_SORBI
8.4
8.2
8.0
7.8
1
2
t−Test: p−value = 0.18

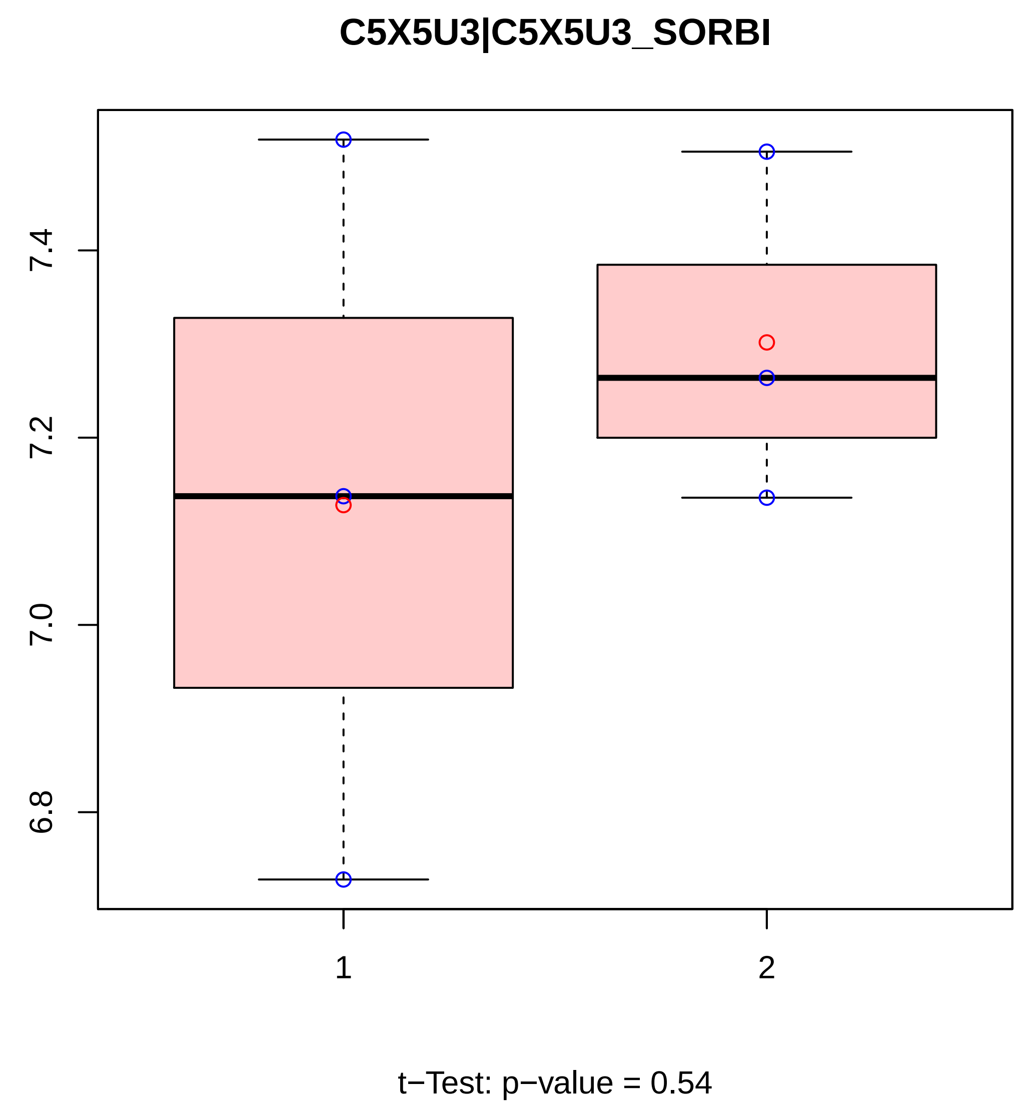

C5X5U3|C5X5U3_SORBI
7.4
7.2
7.0
6.8
1
2
t−Test: p−value = 0.54

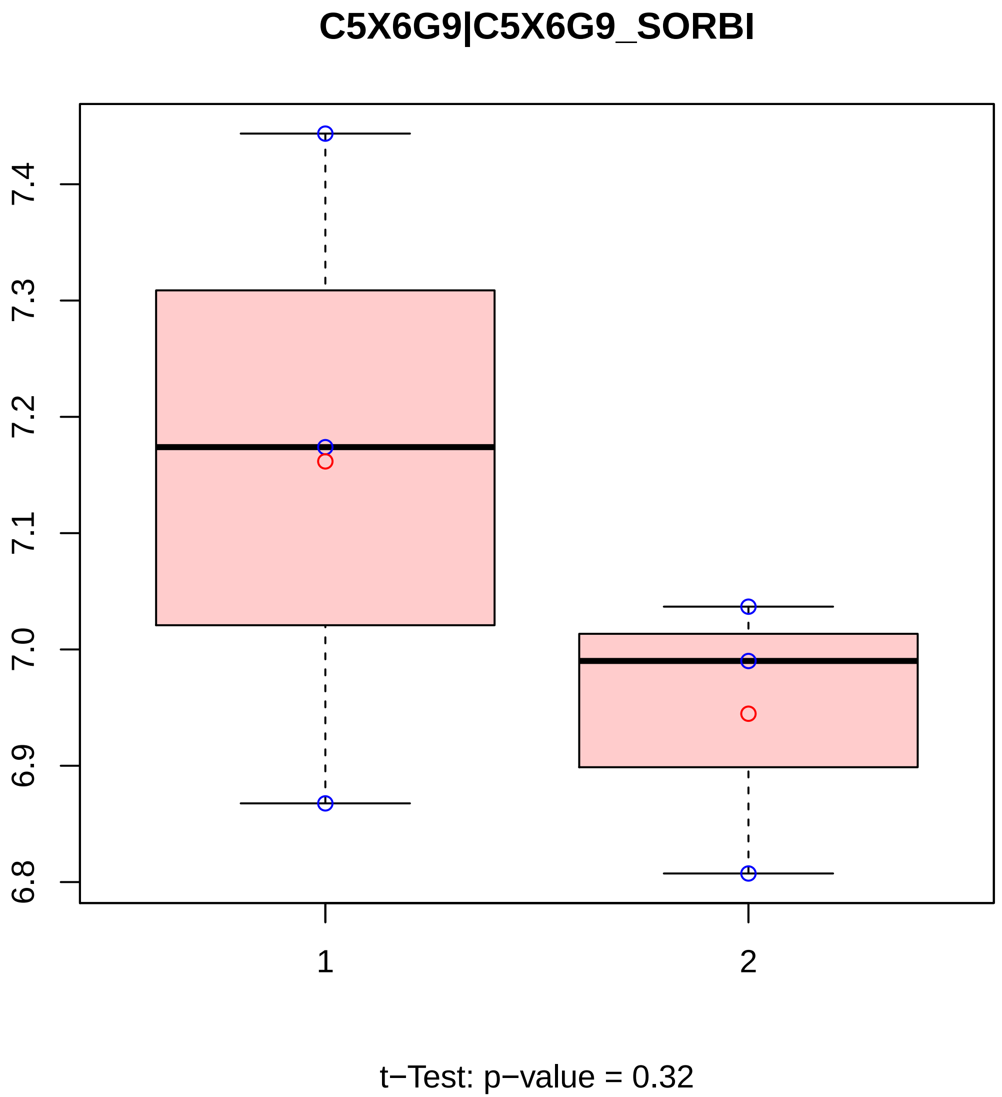
C5X6G9|C5X6G9_SORBI
7.4
7.3
7.2
7.1
7.0
6.9
6.8
1
2
t−Test: p−value = 0.32

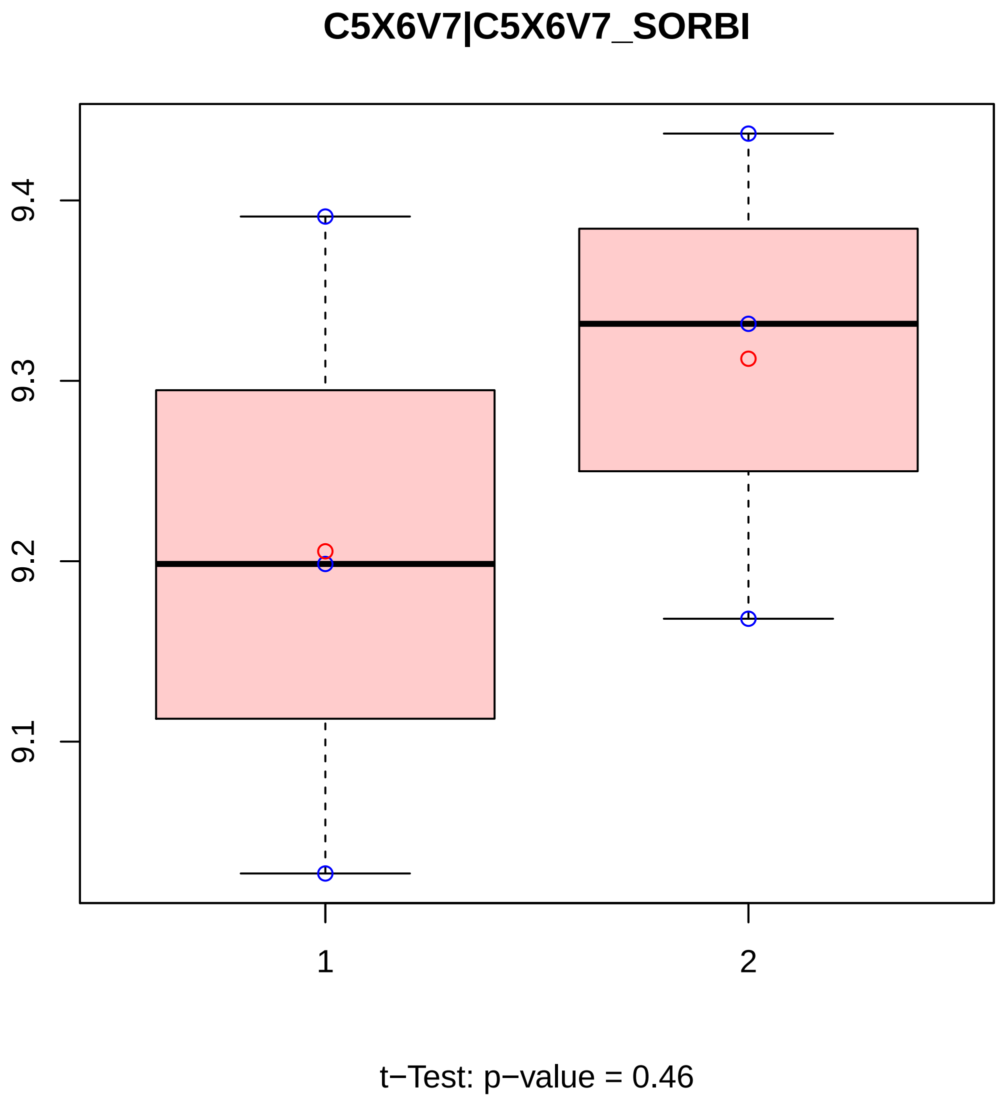
C5X6V7|C5X6V7_SORBI
9.1
9.2
9.3
9.4
1
2
t−Test: p−value = 0.46

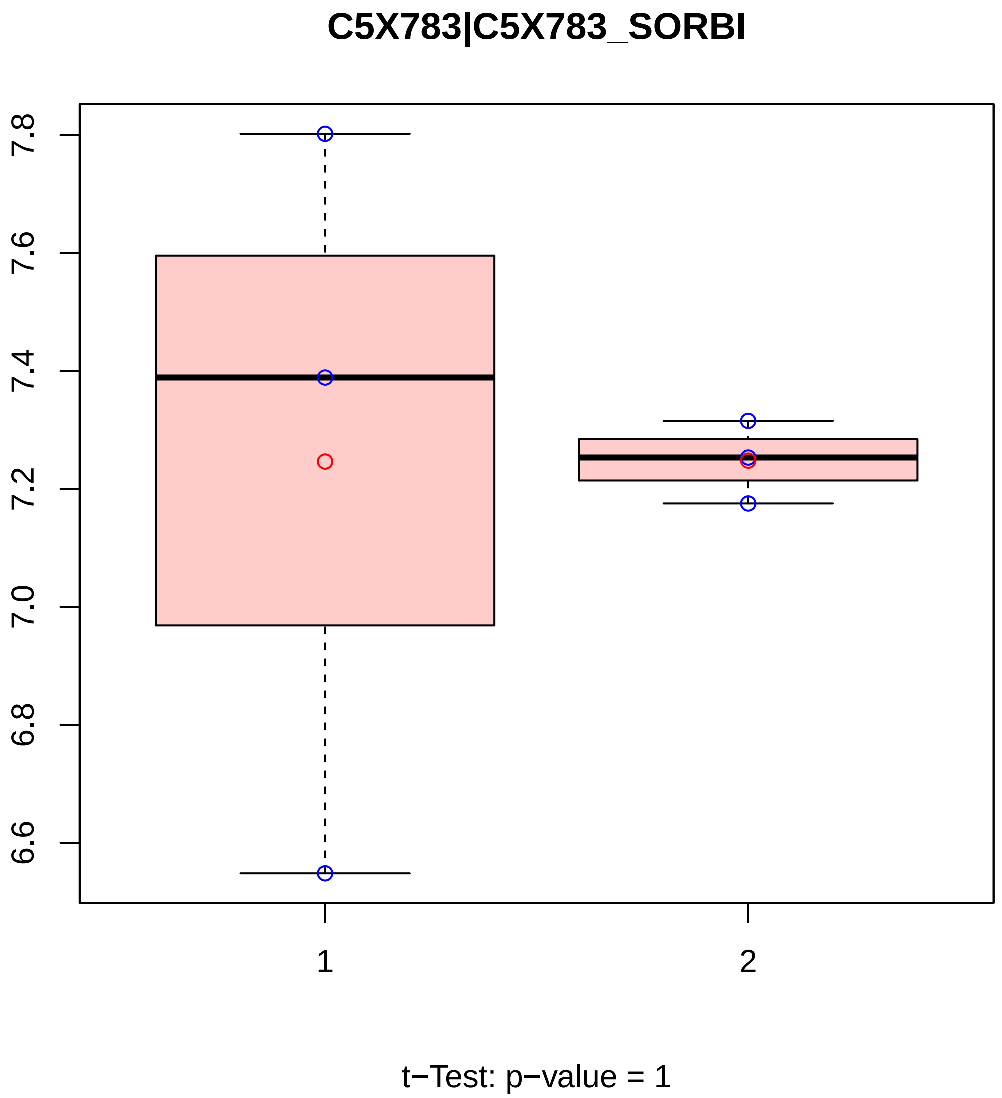
C5X783|C5X783_SORBI
7.8
7.6
7.4
7.2
7.0
6.8
6.6
1
2
t−Test: p−value = 1

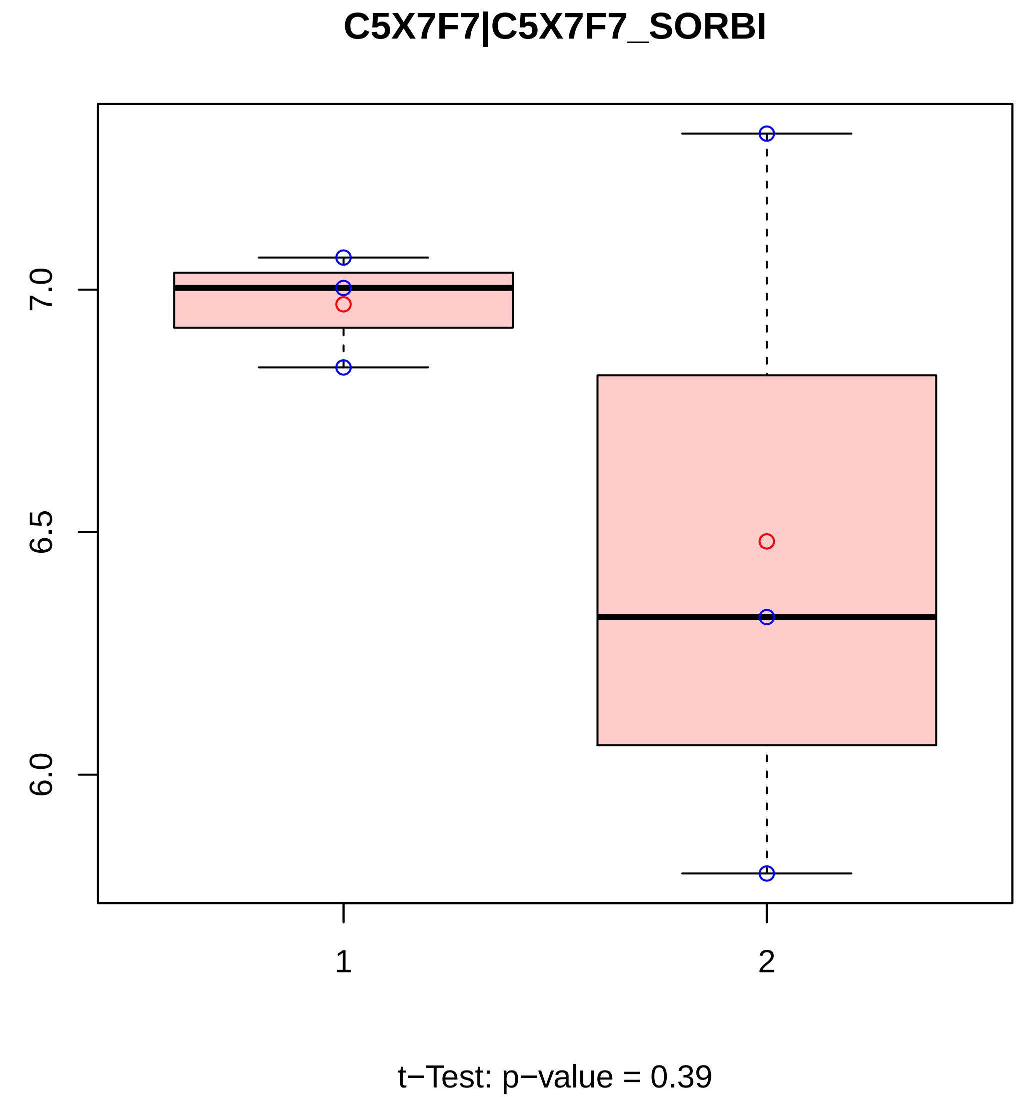
C5X7F7|C5X7F7_SORBI
7.0
6.5
6.0
1
2
t−Test: p−value = 0.39

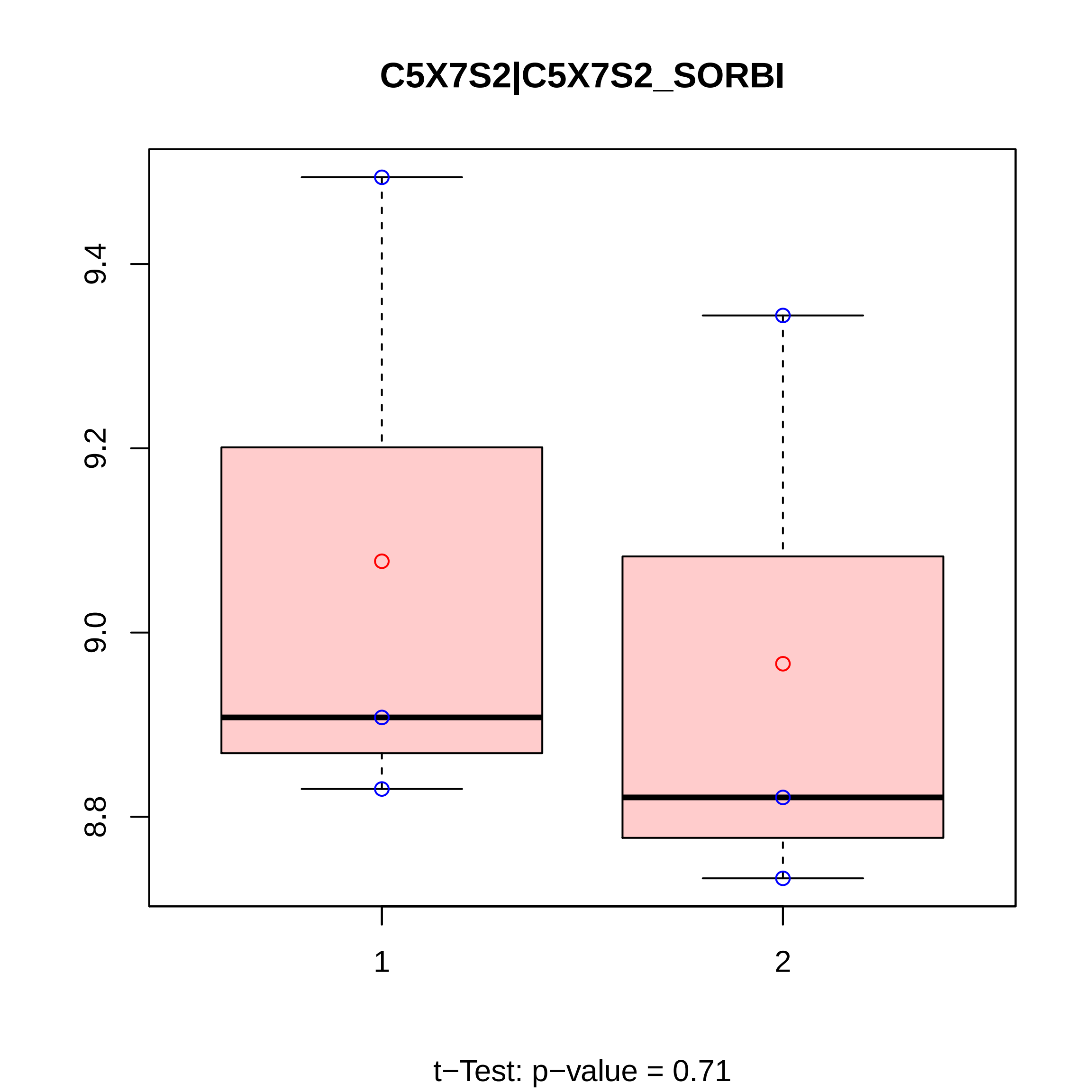

C5X7S2|C5X7S2_SORBI
9.4
9.2
9.0
8.8
1
2
t−Test: p−value = 0.71

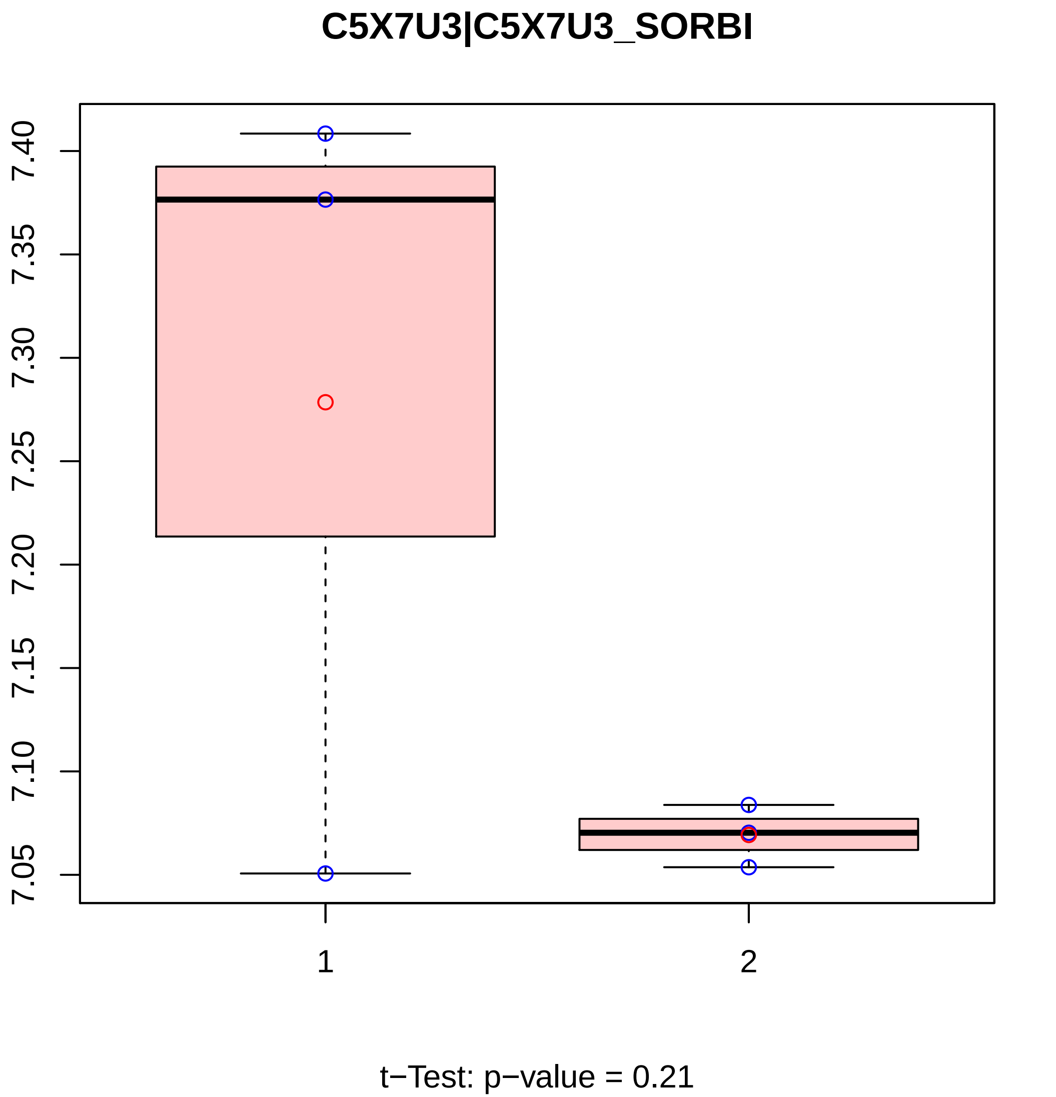
C5X7U3|C5X7U3_SORBI
7.40
7.35
7.30
7.25
7.20
7.15
7.10
7.05
1
2
t−Test: p−value = 0.21

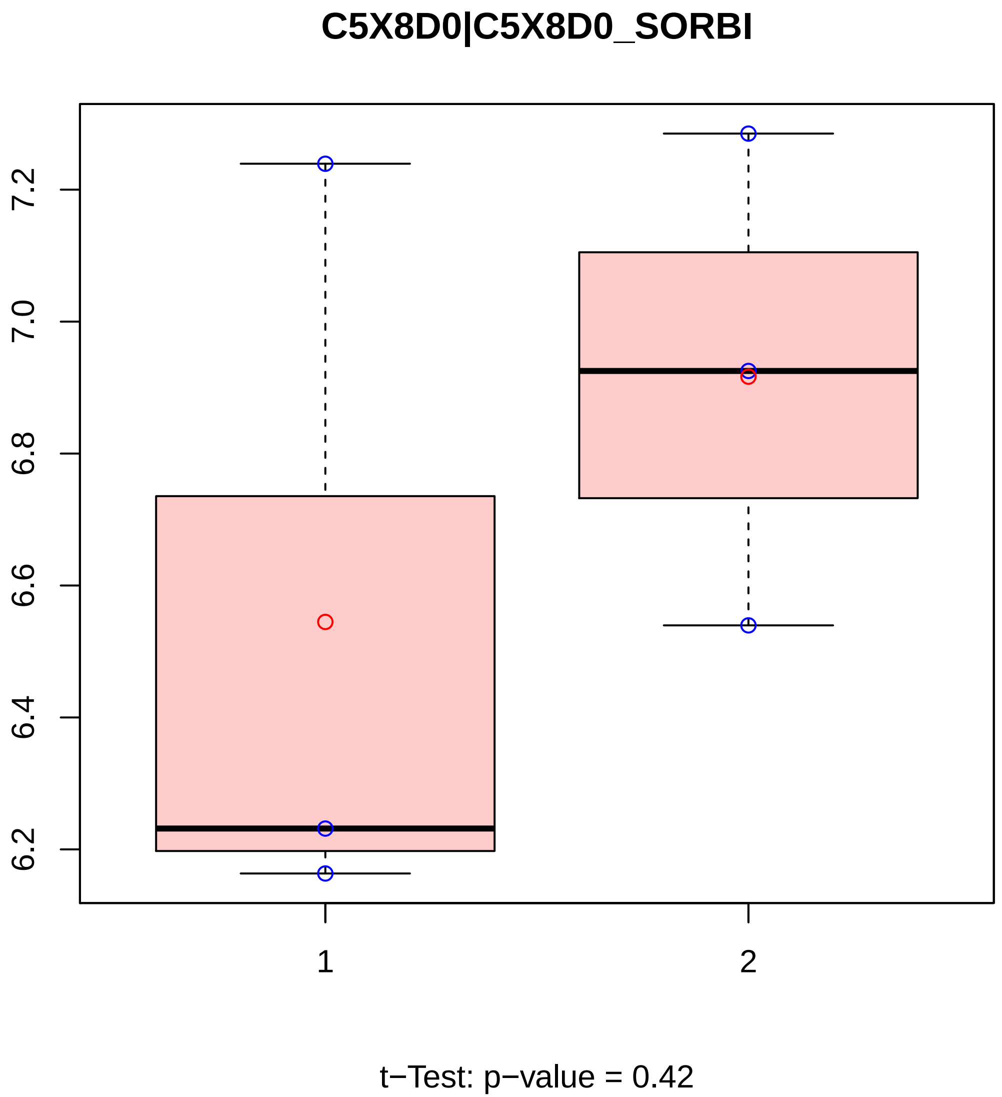
C5X8D0|C5X8D0_SORBI
7.2
7.0
6.8
6.6
6.4
6.2
1
2
t−Test: p−value = 0.42

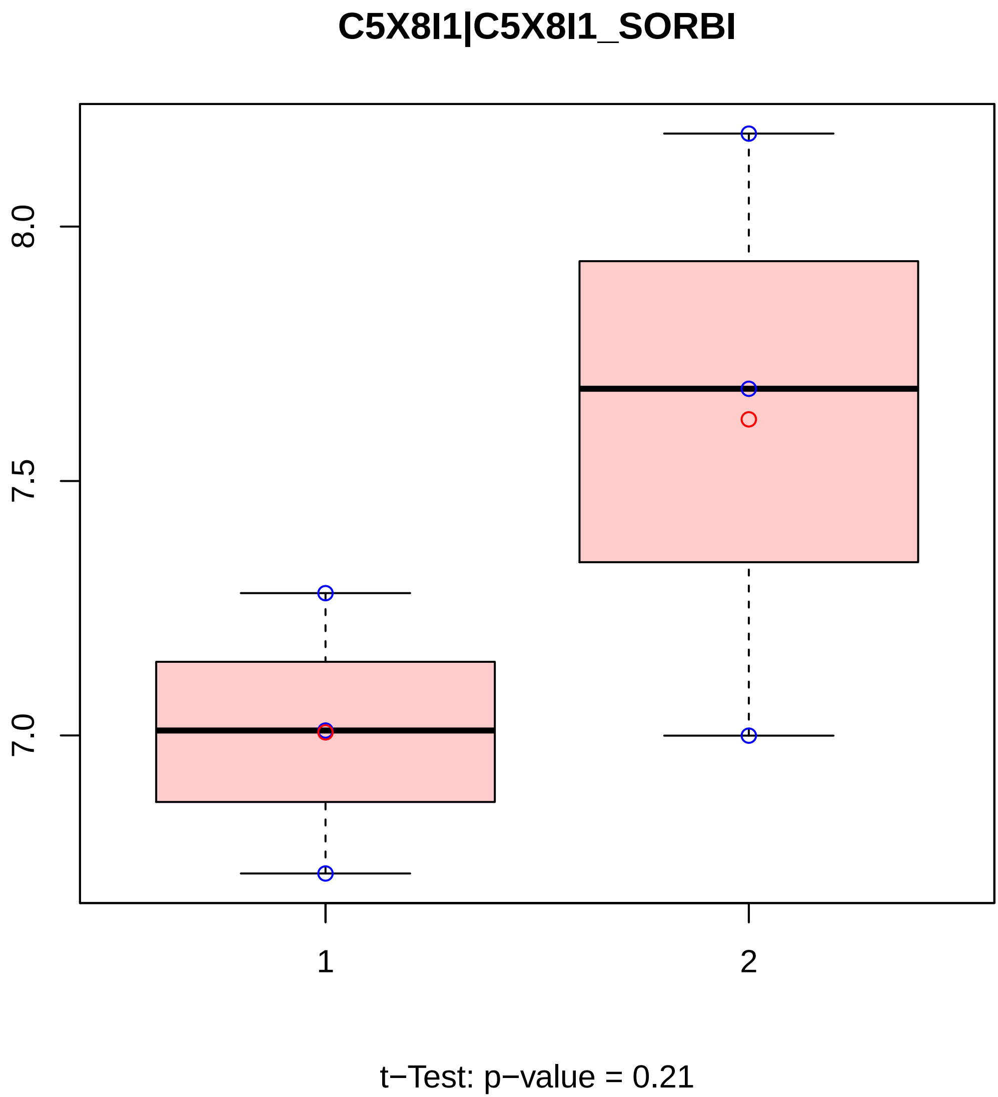
C5X8I1|C5X8I1_SORBI
8.0
7.5
7.0
1
2
t−Test: p−value = 0.21

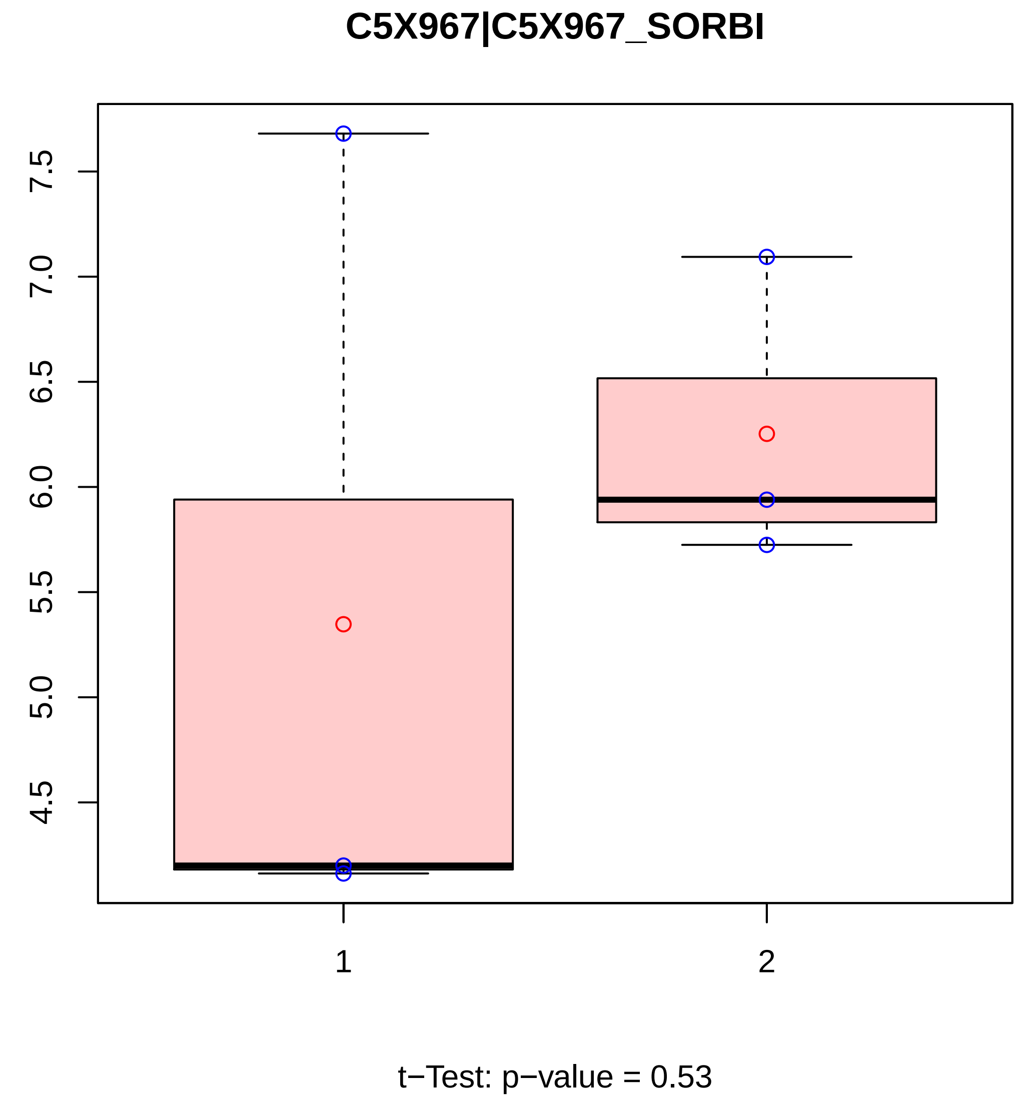

C5X967|C5X967_SORBI
7.5
7.0
6.5
6.0
5.5
5.0
4.5
1
2
t−Test: p−value = 0.53

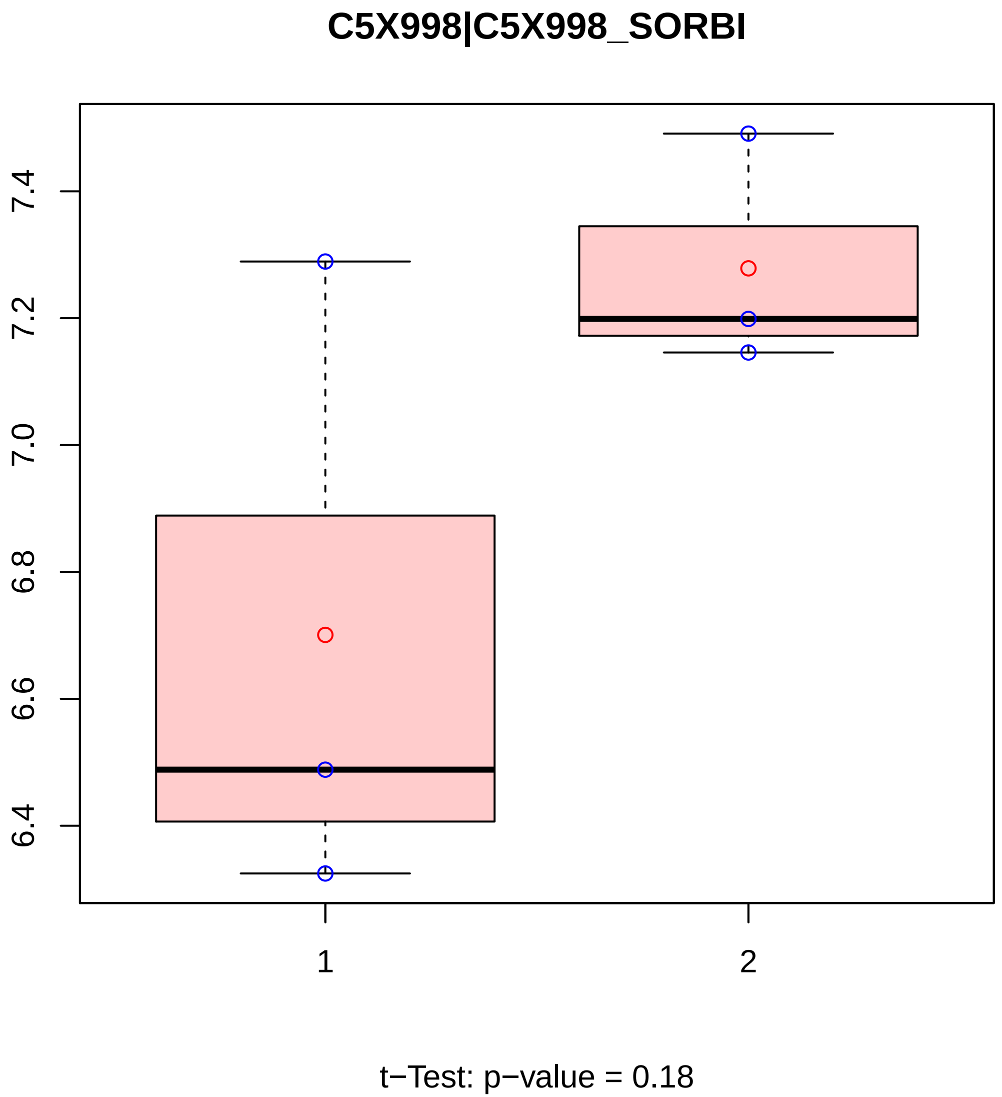
C5X998|C5X998_SORBI
7.4
7.2
7.0
6.8
6.6
6.4
1
2
t−Test: p−value = 0.18

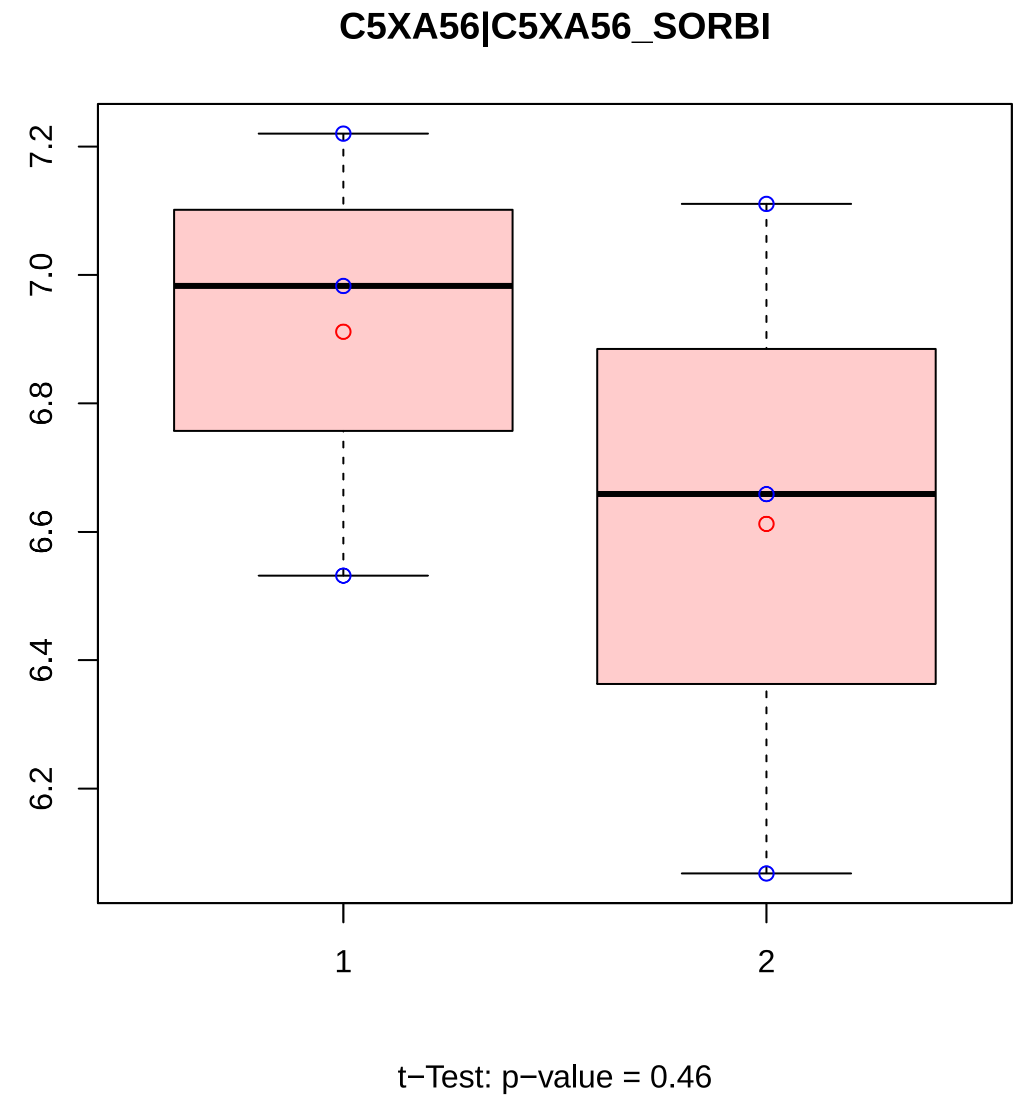
C5XA56|C5XA56_SORBI
7.2
7.0
6.8
6.6
6.4
6.2
1
2
t−Test: p−value = 0.46

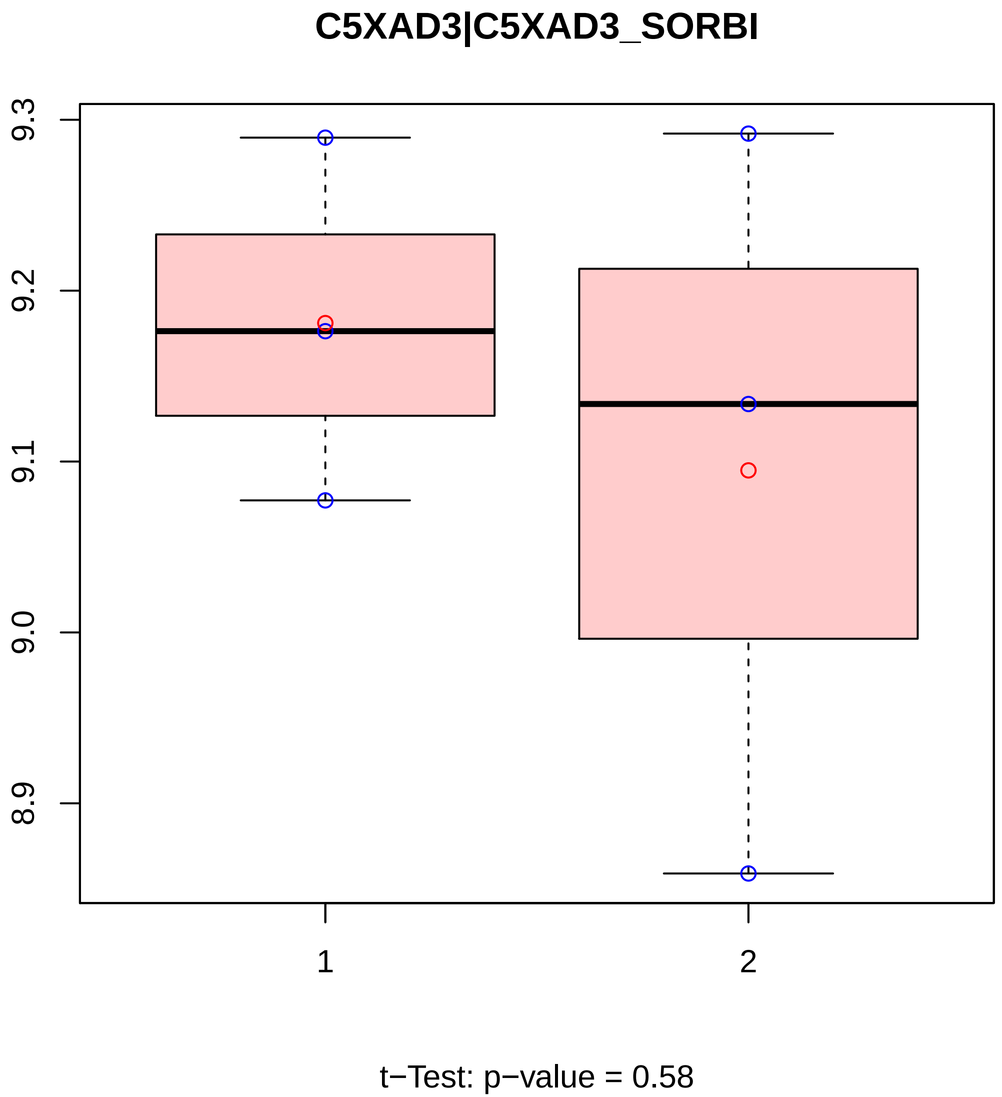

C5XAD3|C5XAD3_SORBI
9.3
9.2
9.1
9.0
8.9
1
2
t−Test: p−value = 0.58

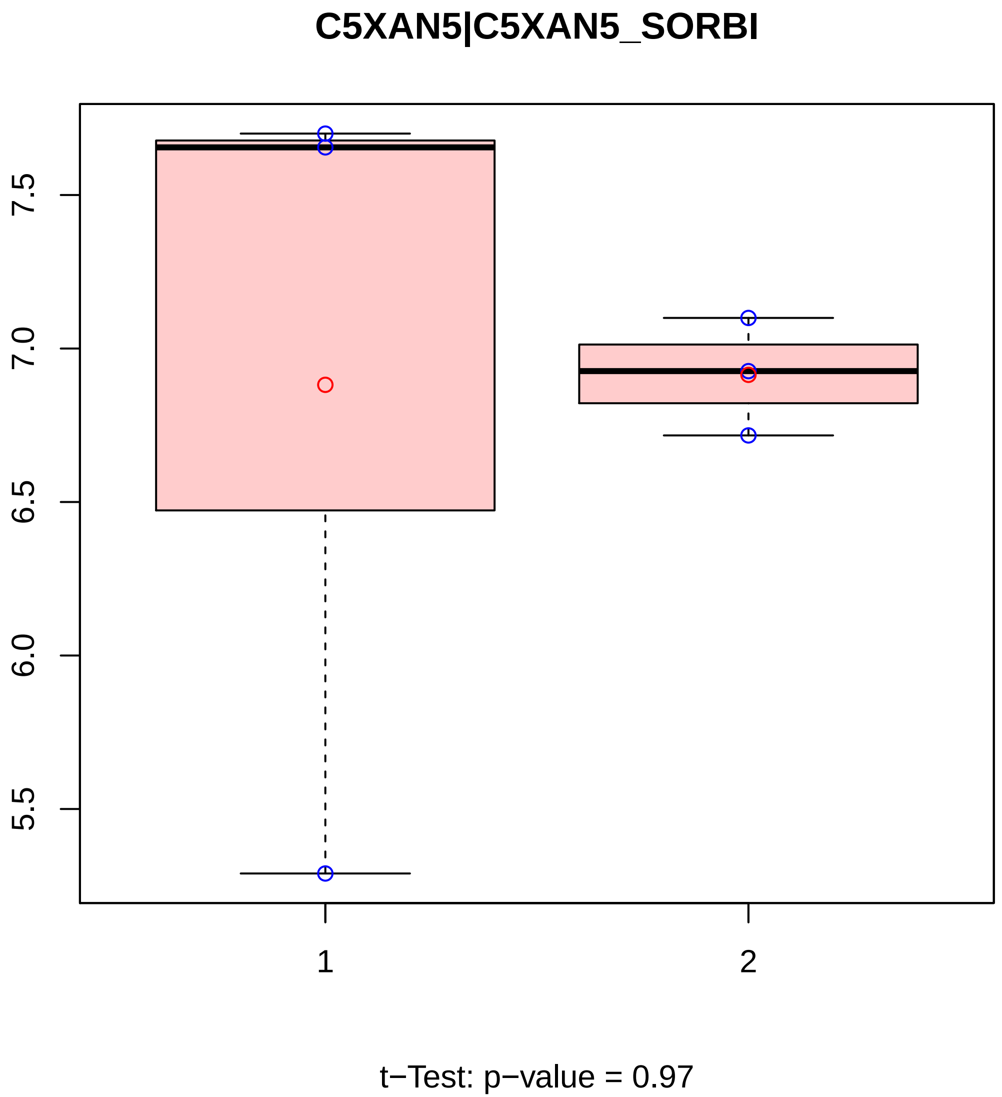
C5XAN5|C5XAN5_SORBI
7.5
7.0
6.5
6.0
5.5
1
2
t−Test: p−value = 0.97

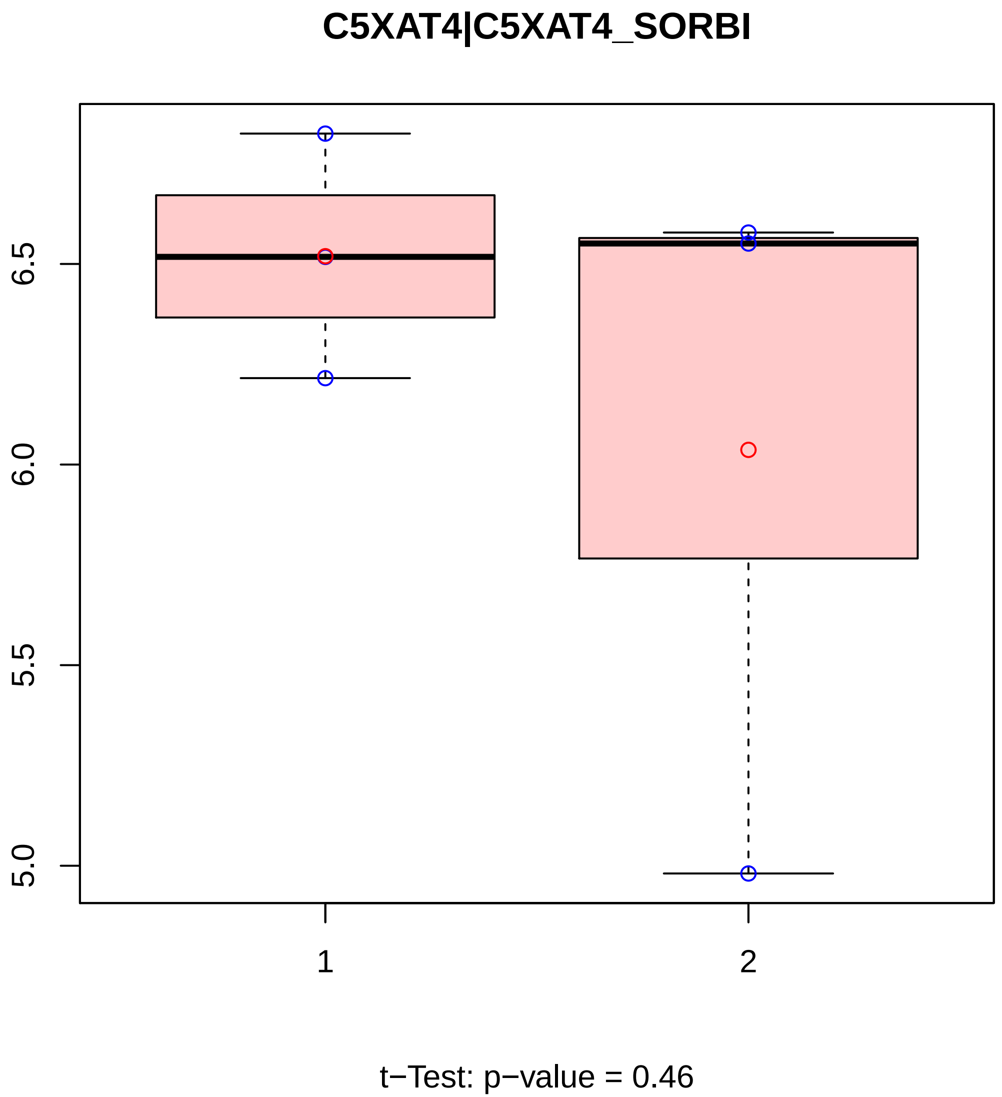

C5XAT4|C5XAT4_SORBI
5.0
5.5
6.0
6.5
1
2
t−Test: p−value = 0.46

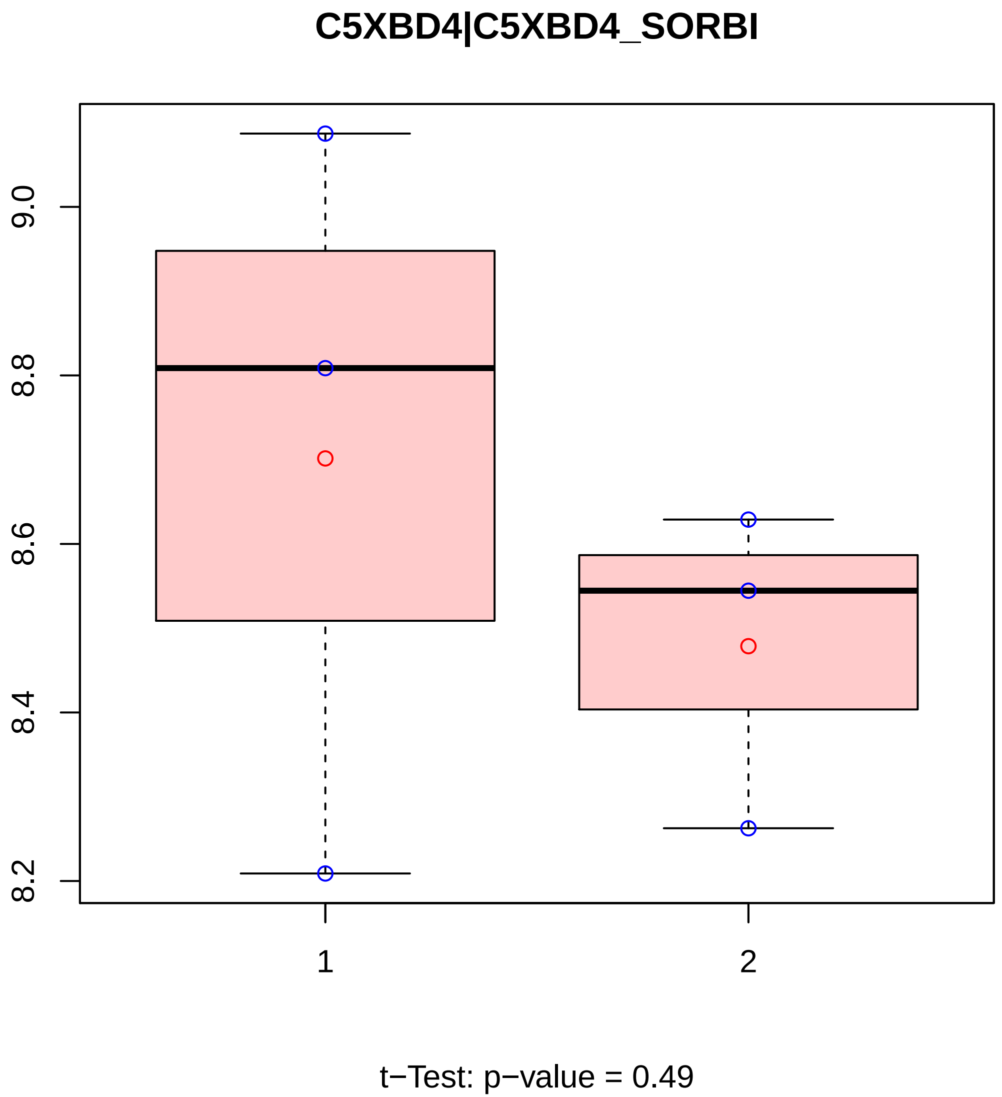
C5XBD4|C5XBD4_SORBI
9.0
8.8
8.6
8.4
8.2
1
2
t−Test: p−value = 0.49

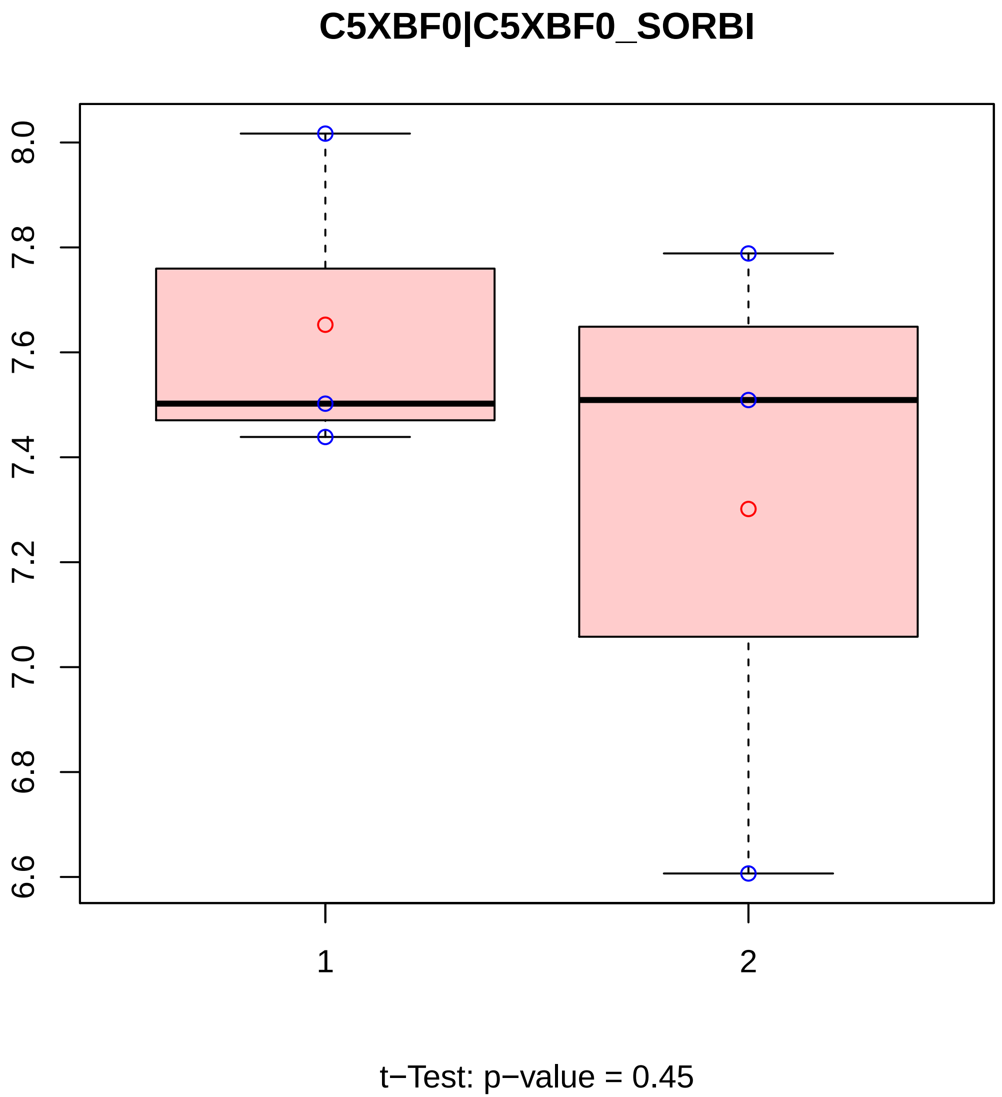
C5XBF0|C5XBF0_SORBI
8.0
7.8
7.6
7.4
7.2
7.0
6.8
6.6
1
2
t−Test: p−value = 0.45

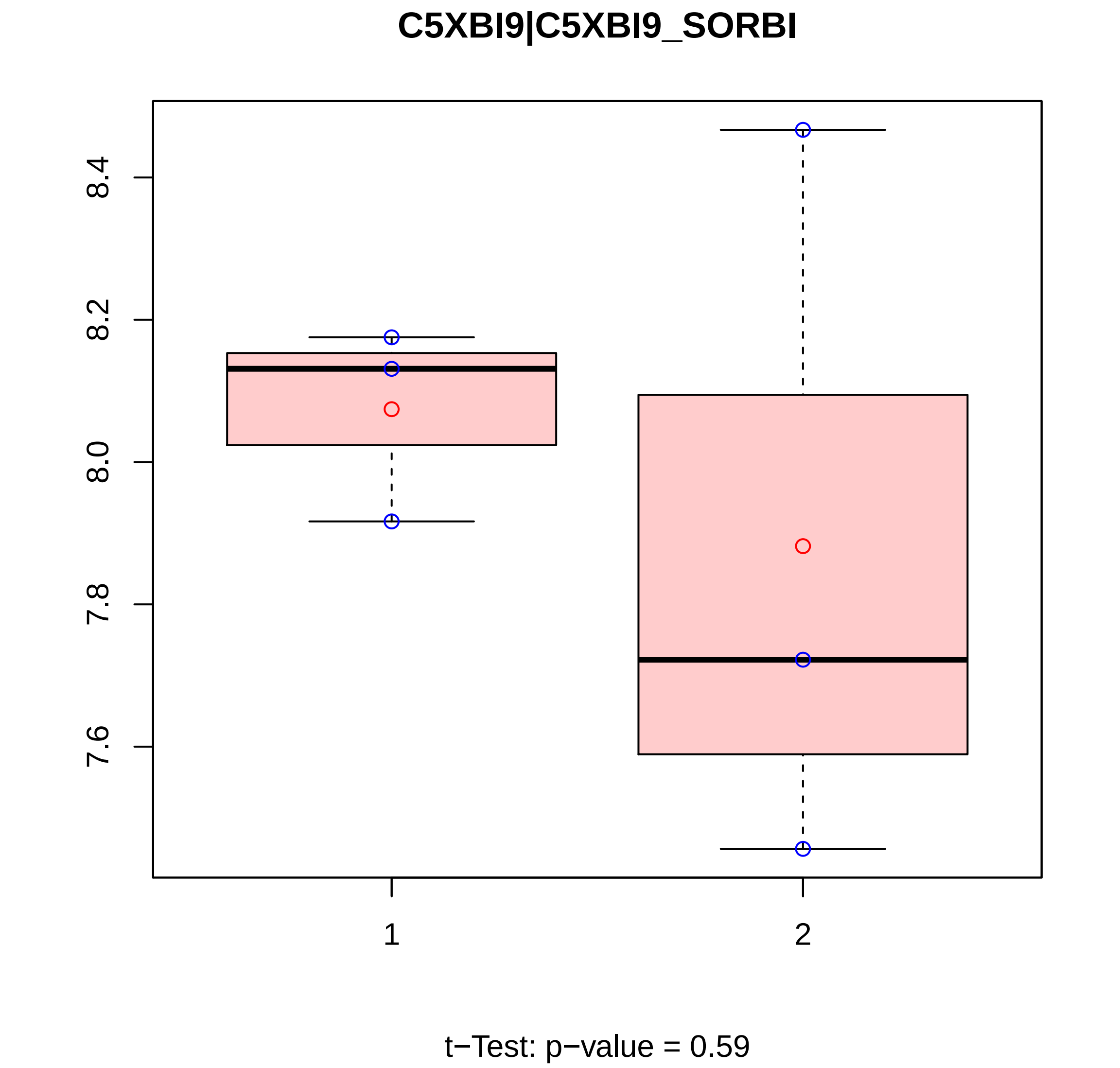

C5XBI9|C5XBI9_SORBI
7.6
7.8
8.0
8.2
8.4
1
2
t−Test: p−value = 0.59

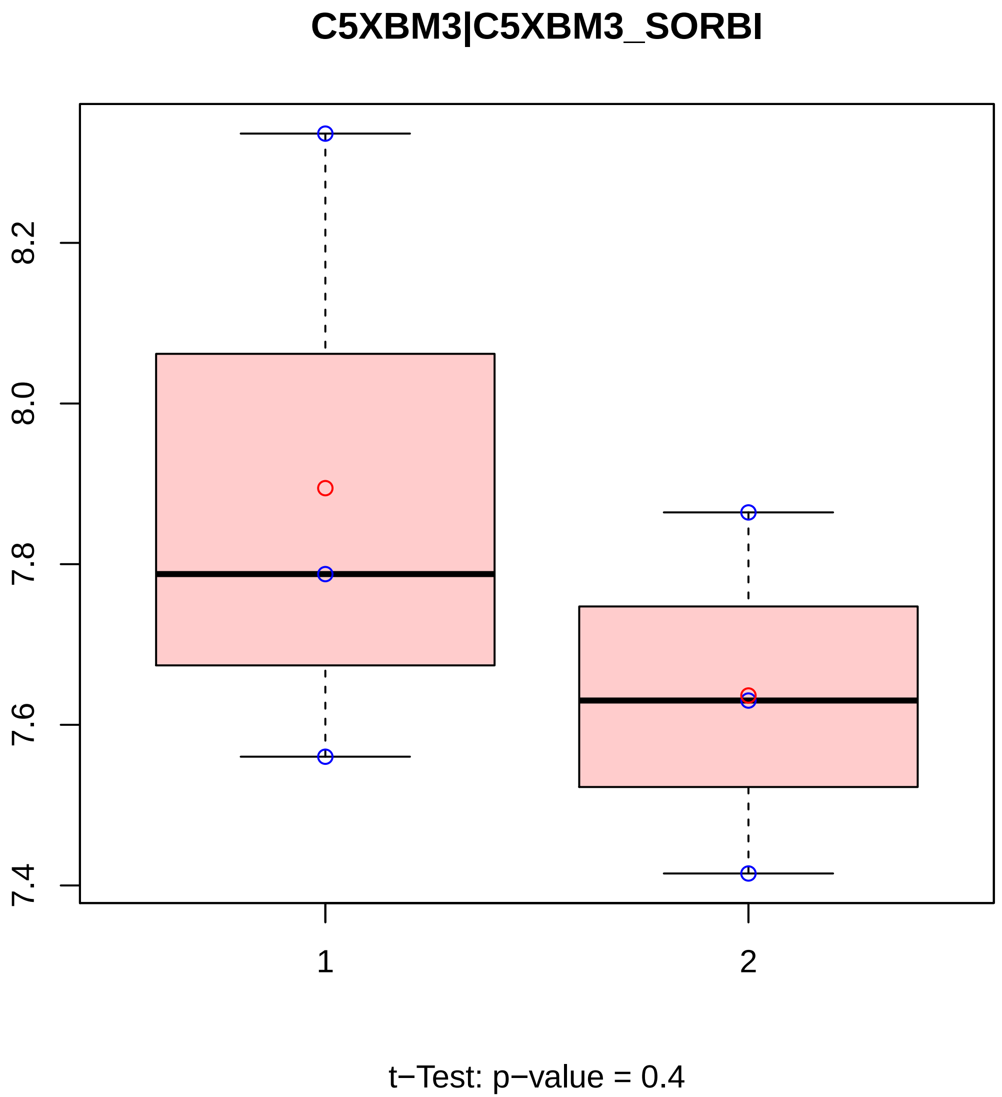
C5XBM3|C5XBM3_SORBI
8.2
8.0
7.8
7.6
7.4
1
2
t−Test: p−value = 0.4

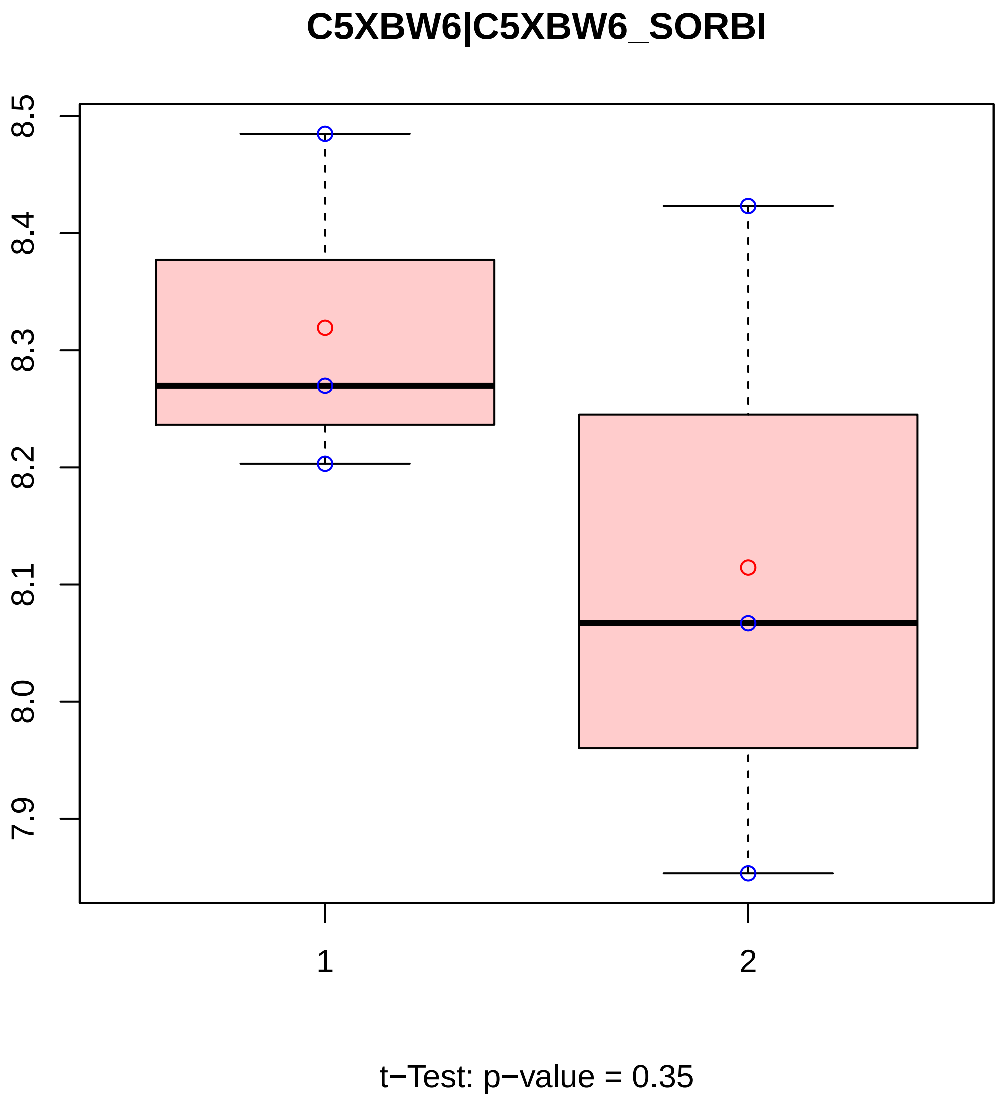
C5XBW6|C5XBW6_SORBI
8.5
8.4
8.3
8.2
8.1
8.0
7.9
1
2
t−Test: p−value = 0.35

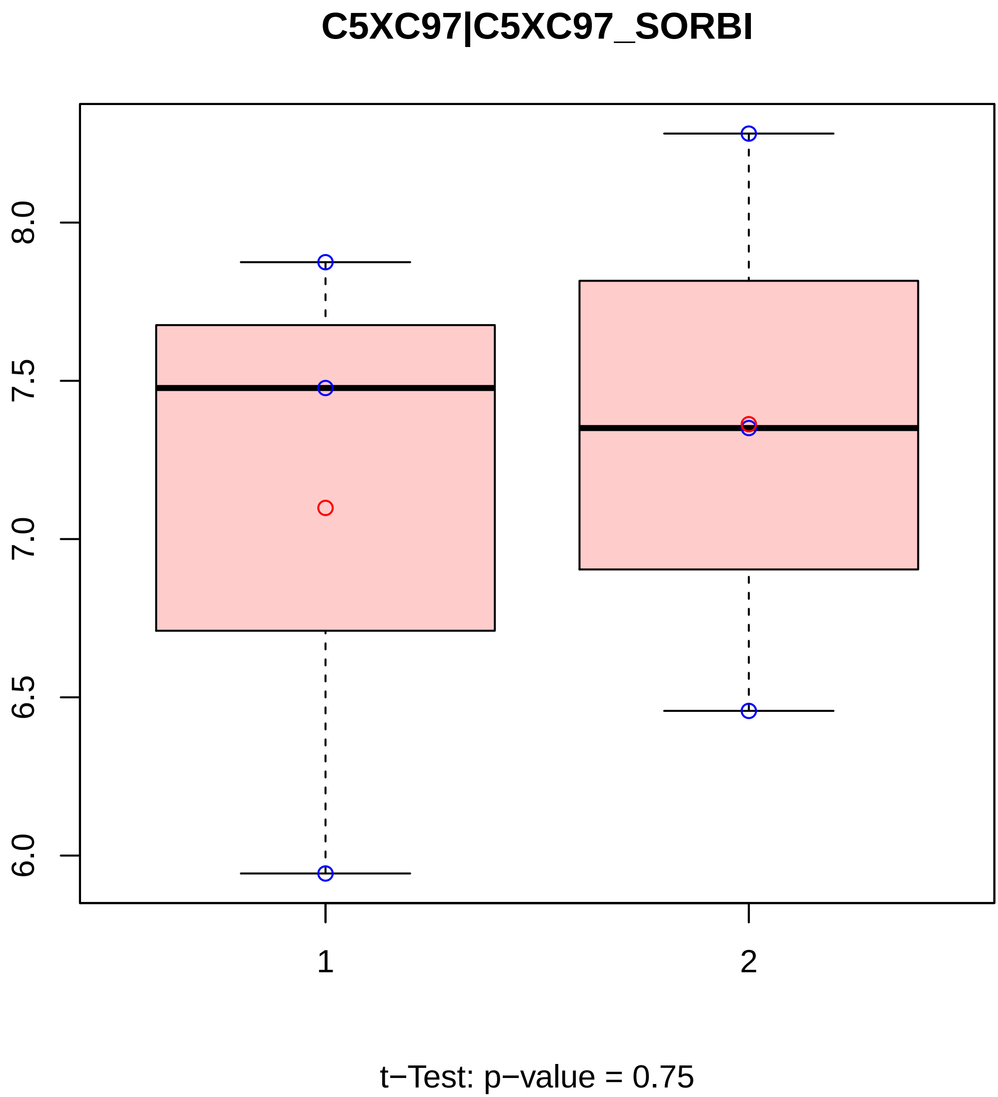
C5XC97|C5XC97_SORBI
6.0
6.5
7.0
7.5
8.0
1
2
t−Test: p−value = 0.75

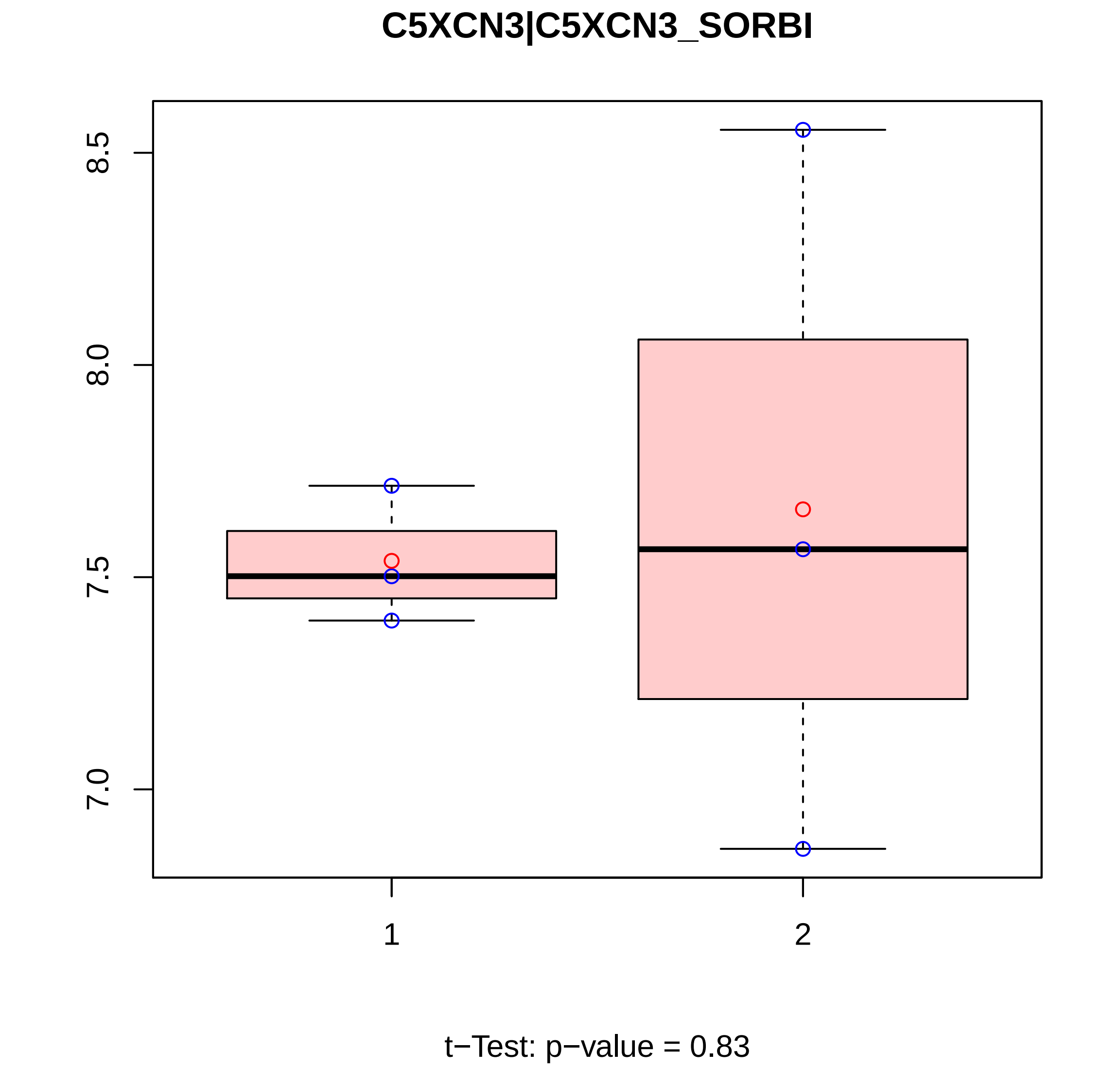
C5XCN3|C5XCN3_SORBI
8.5
8.0
7.5
7.0
1
2
t−Test: p−value = 0.83

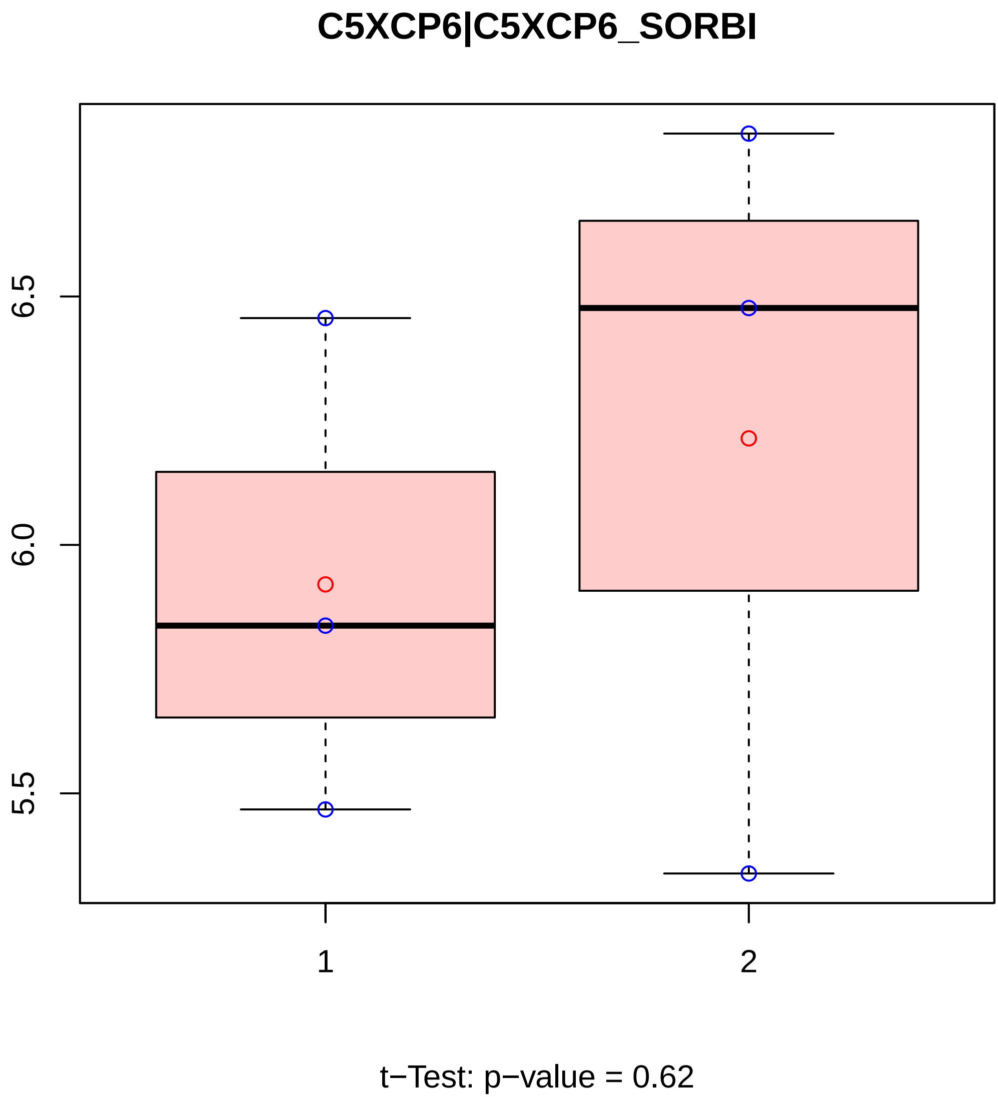
C5XCP6|C5XCP6_SORBI
6.5
6.0
5.5
1
2
t−Test: p−value = 0.62

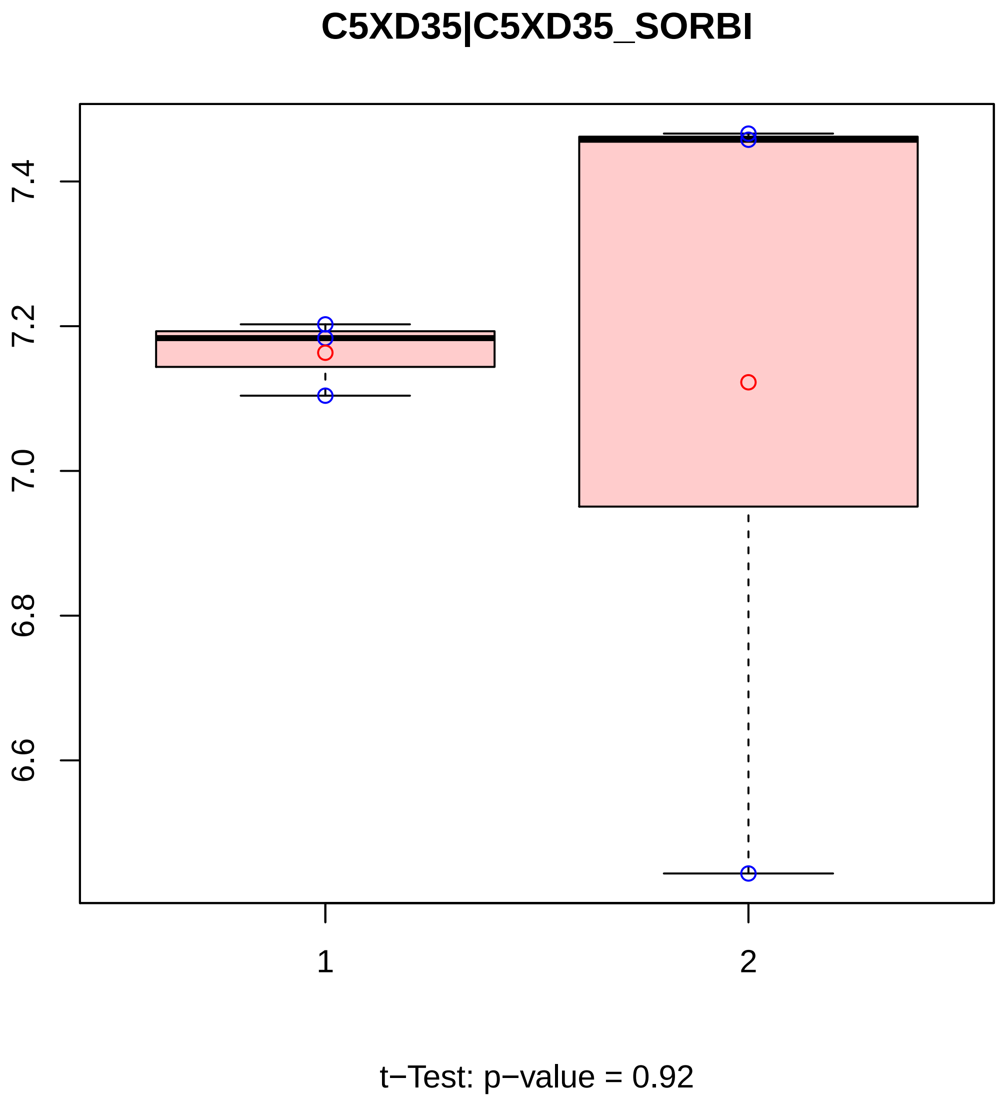

C5XD35|C5XD35_SORBI
7.4
7.2
7.0
6.8
6.6
1
2
t−Test: p−value = 0.92

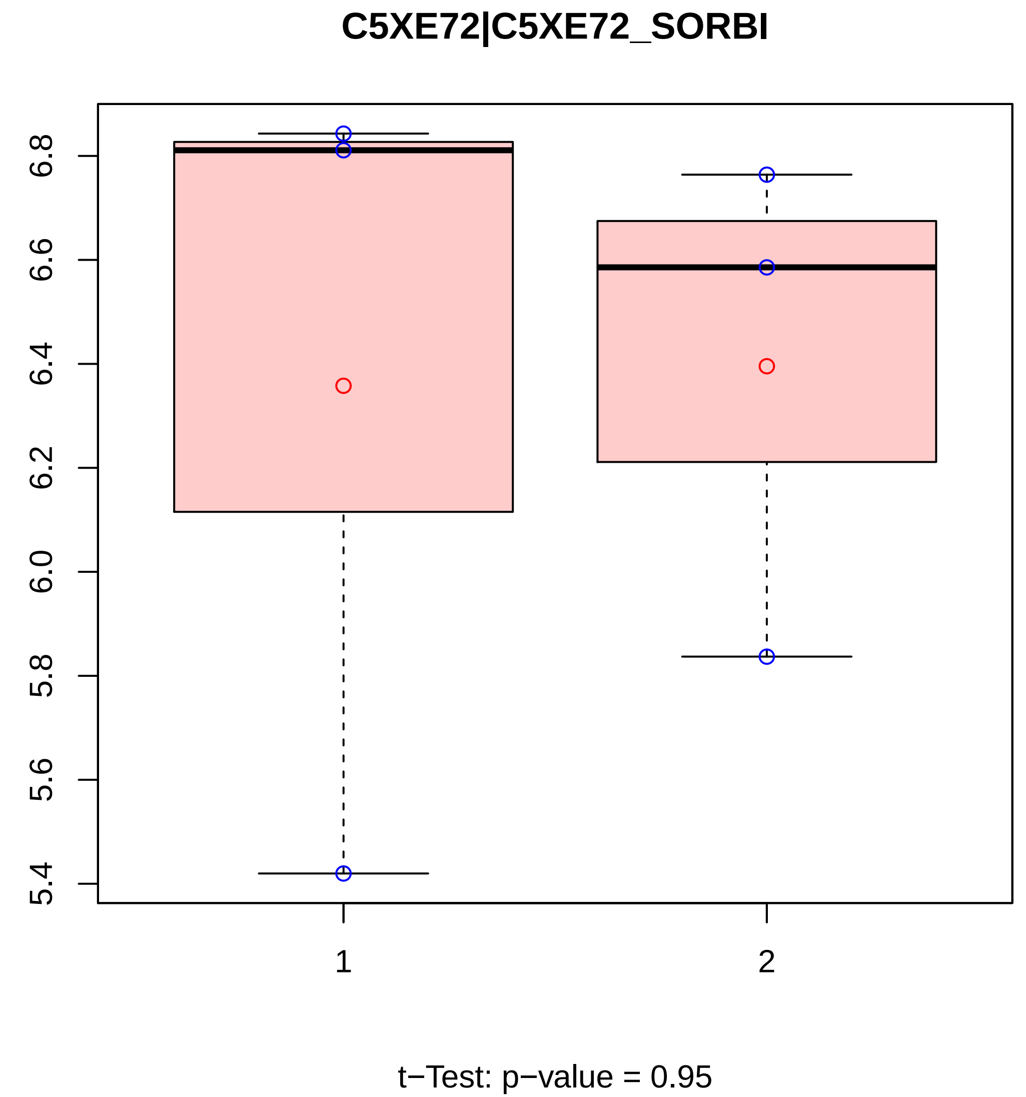
C5XE72|C5XE72_SORBI
6.8
6.6
6.4
6.2
6.0
5.8
5.6
5.4
1
2
t−Test: p−value = 0.95

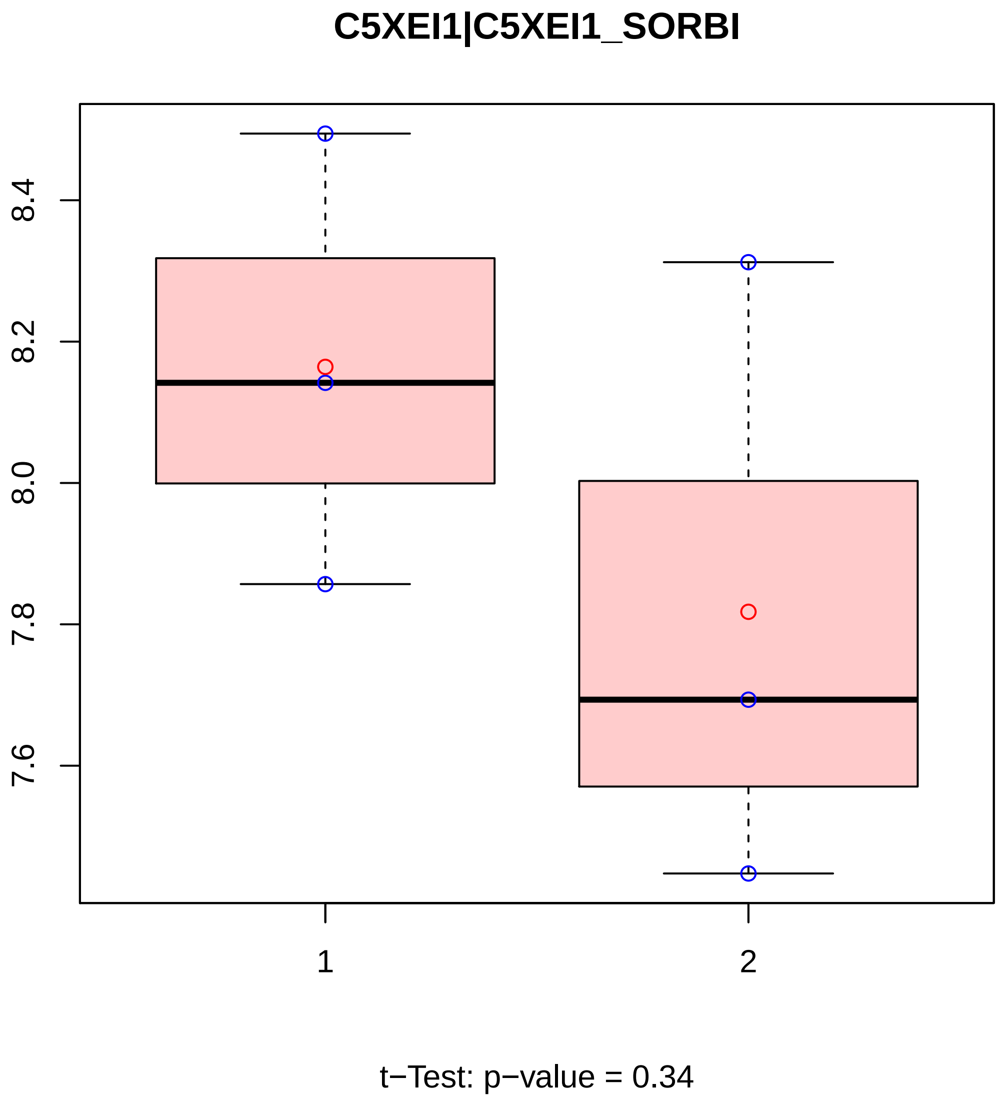
C5XEI1|C5XEI1_SORBI
8.4
8.2
8.0
7.8
7.6
1
2
t−Test: p−value = 0.34

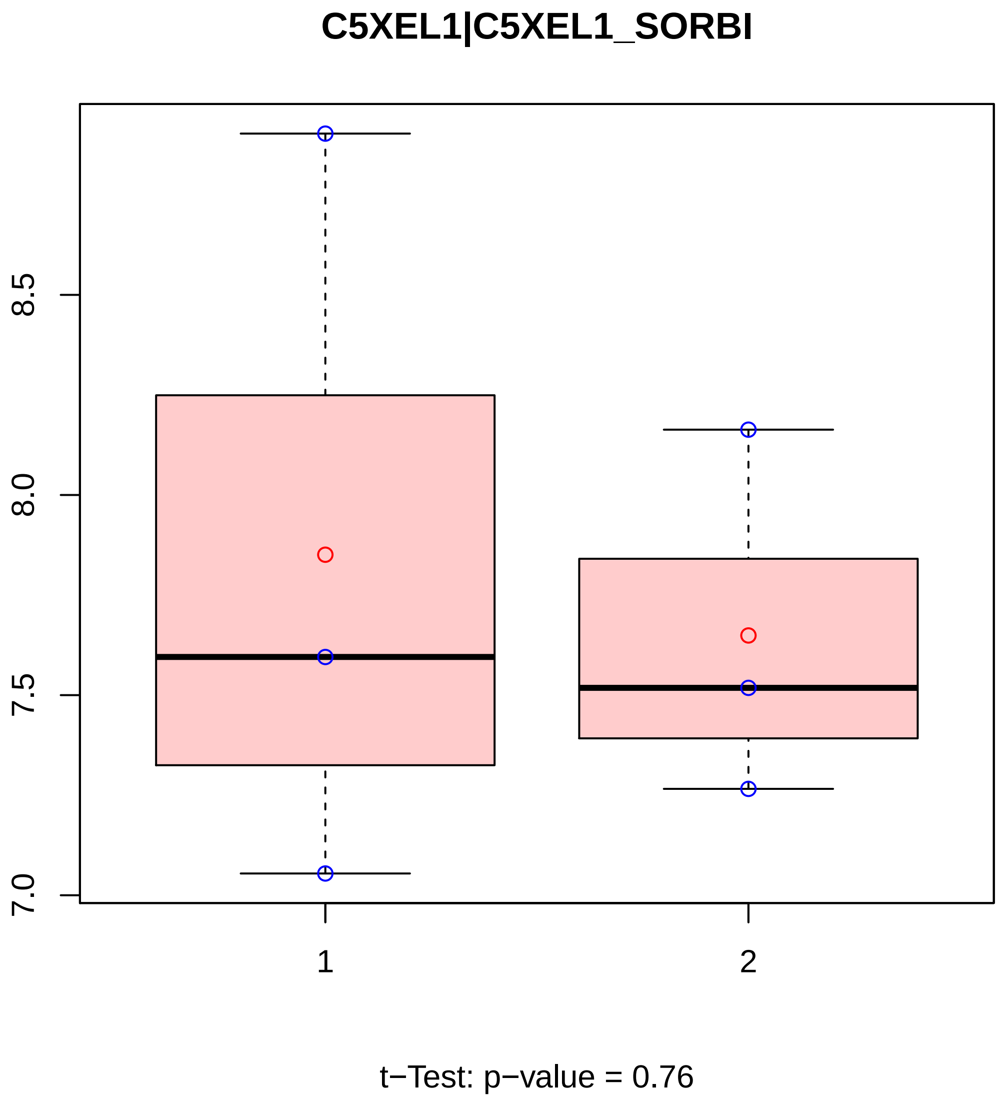
C5XEL1|C5XEL1_SORBI
8.5
8.0
7.5
7.0
1
2
t−Test: p−value = 0.76

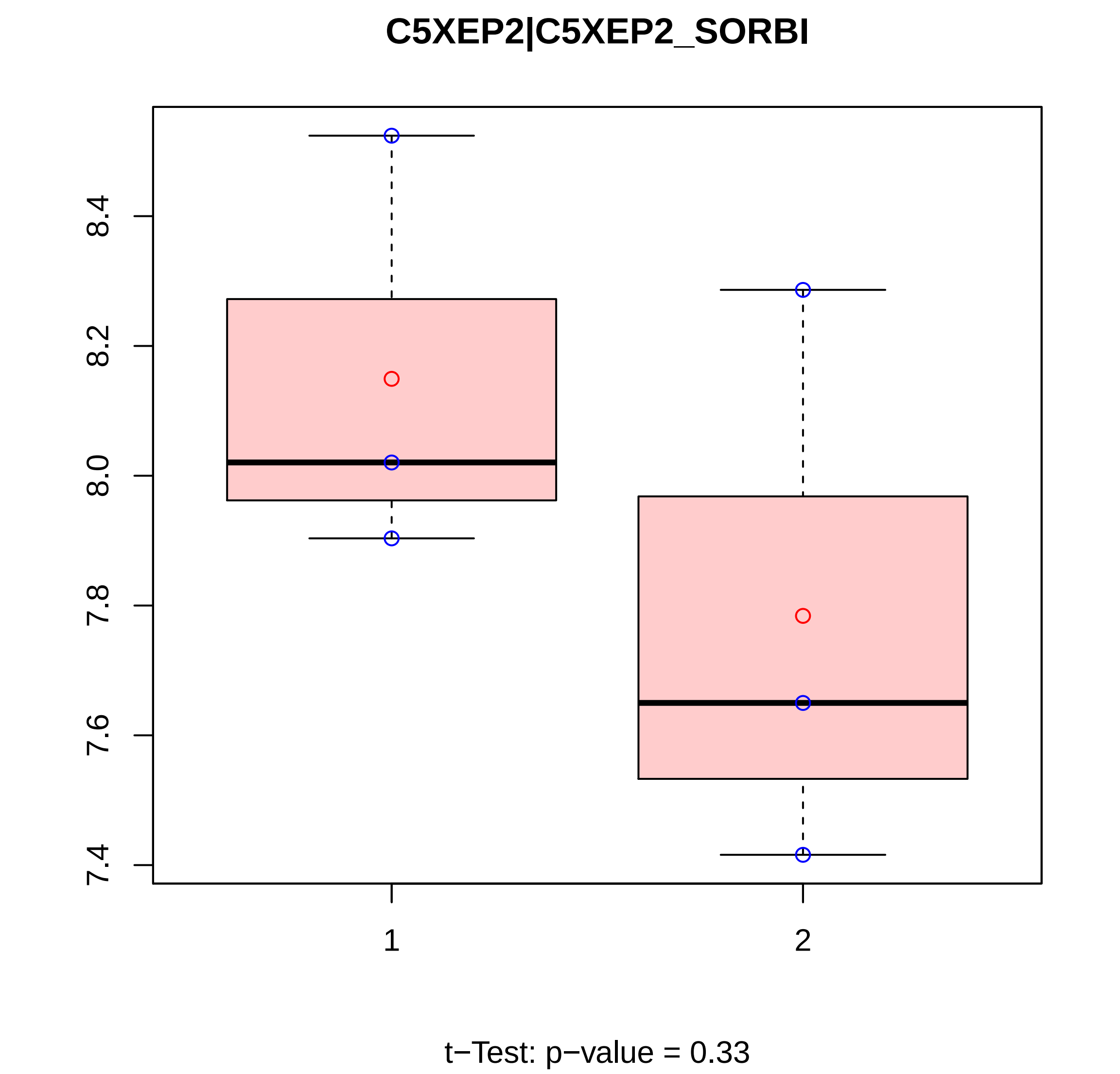
C5XEP2|C5XEP2_SORBI
8.4
8.2
8.0
7.8
7.6
7.4
1
2
t−Test: p−value = 0.33

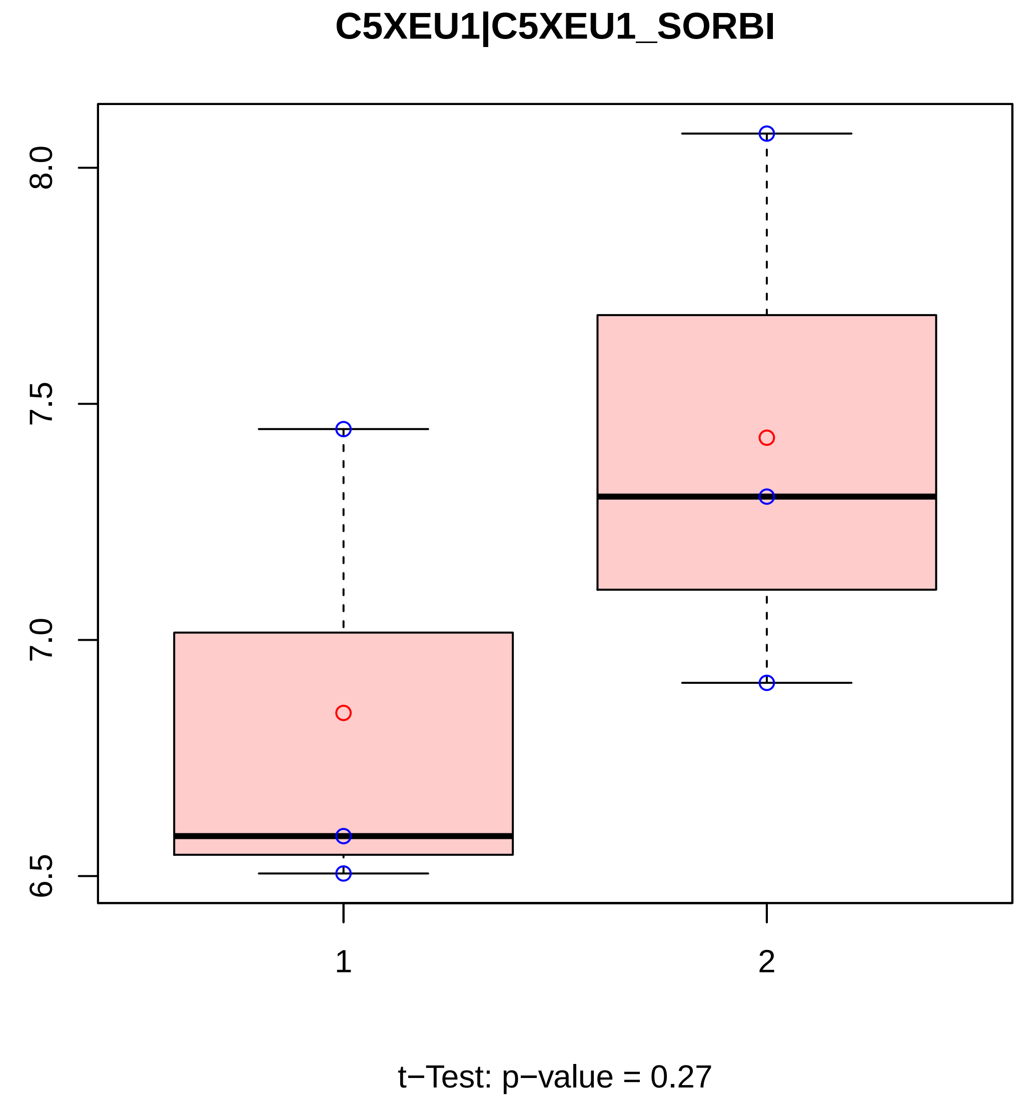
C5XEU1|C5XEU1_SORBI
8.0
7.5
7.0
6.5
1
2
t−Test: p−value = 0.27

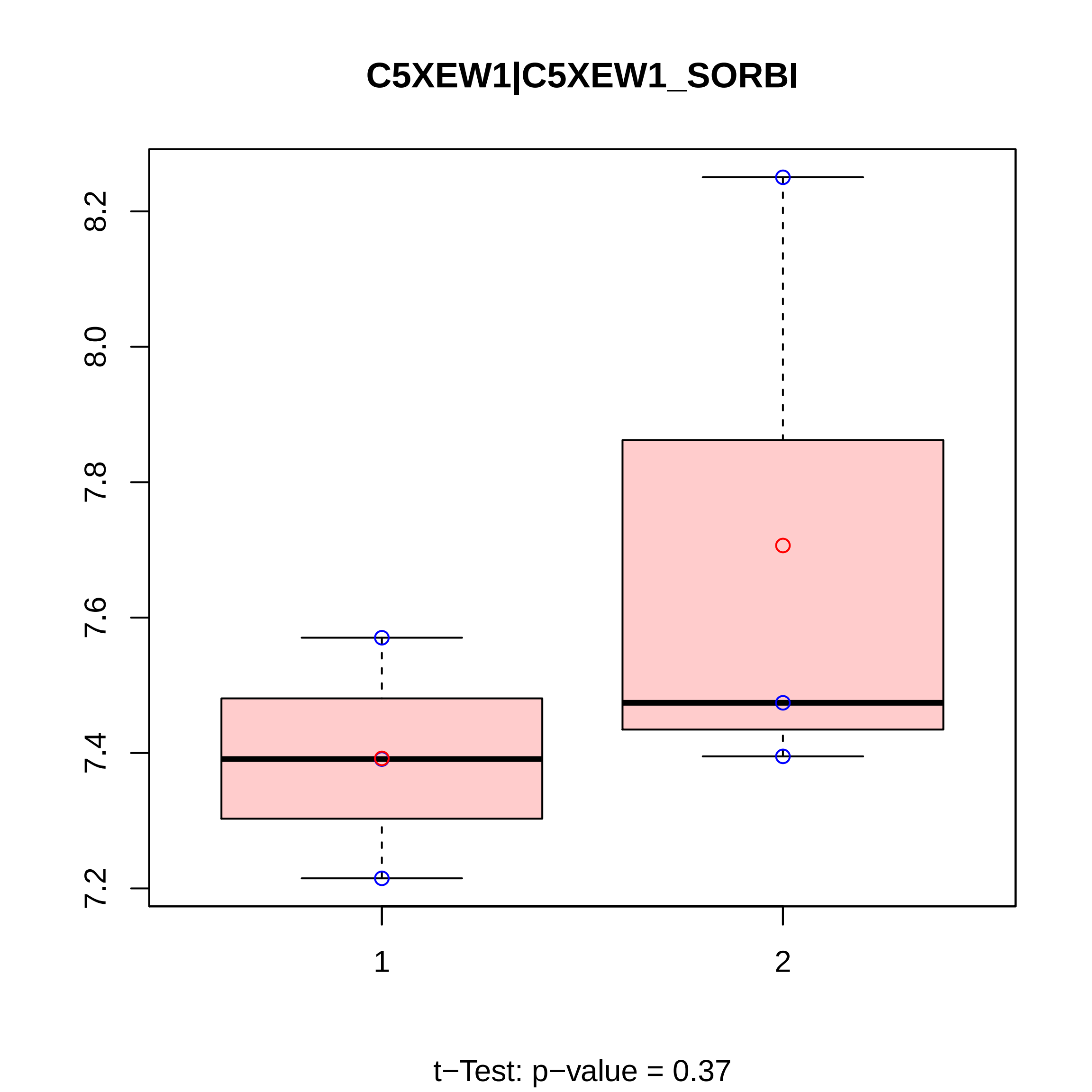
C5XEW1|C5XEW1_SORBI
7.2
7.4
7.6
7.8
8.0
8.2
1
2
t−Test: p−value = 0.37

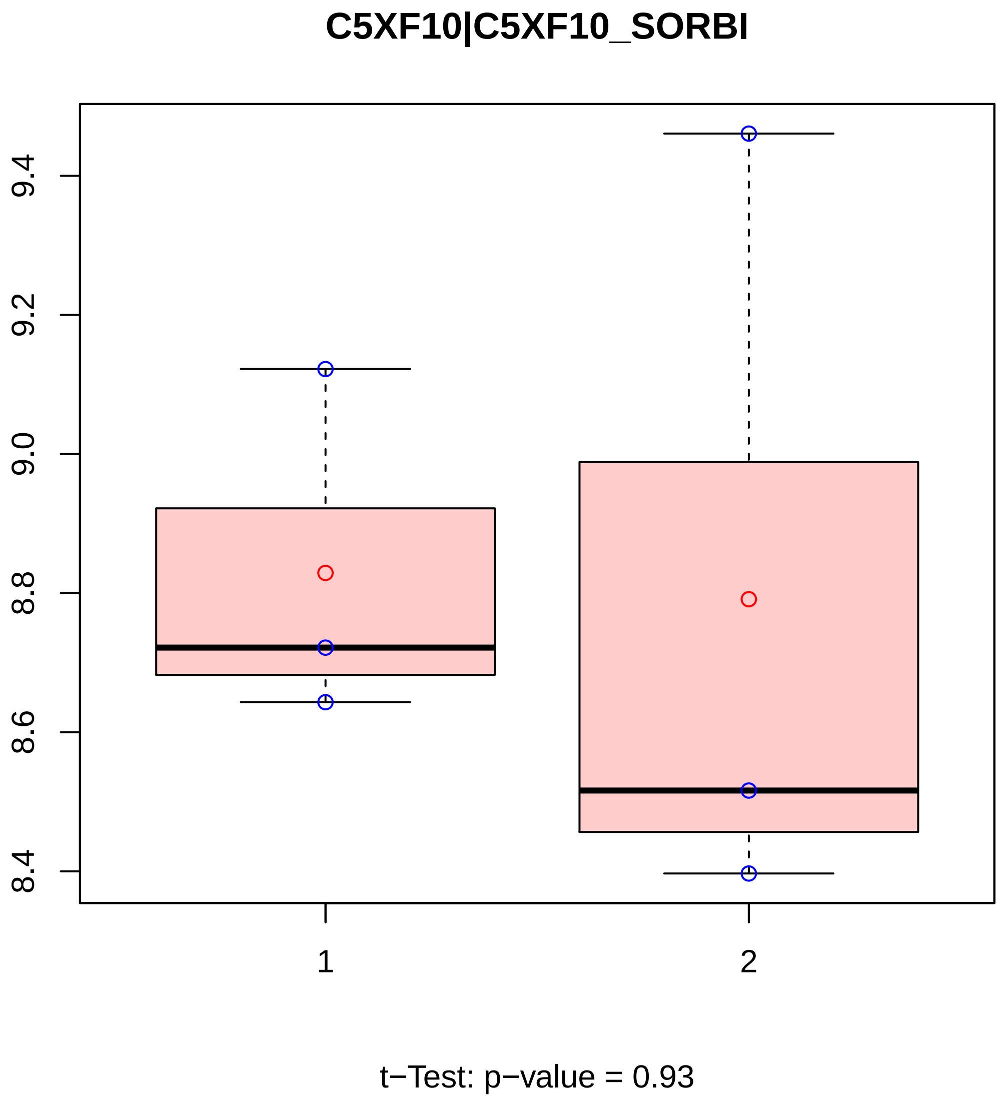

C5XF10|C5XF10_SORBI
9.4
9.2
9.0
8.8
8.6
8.4
1
2
t−Test: p−value = 0.93

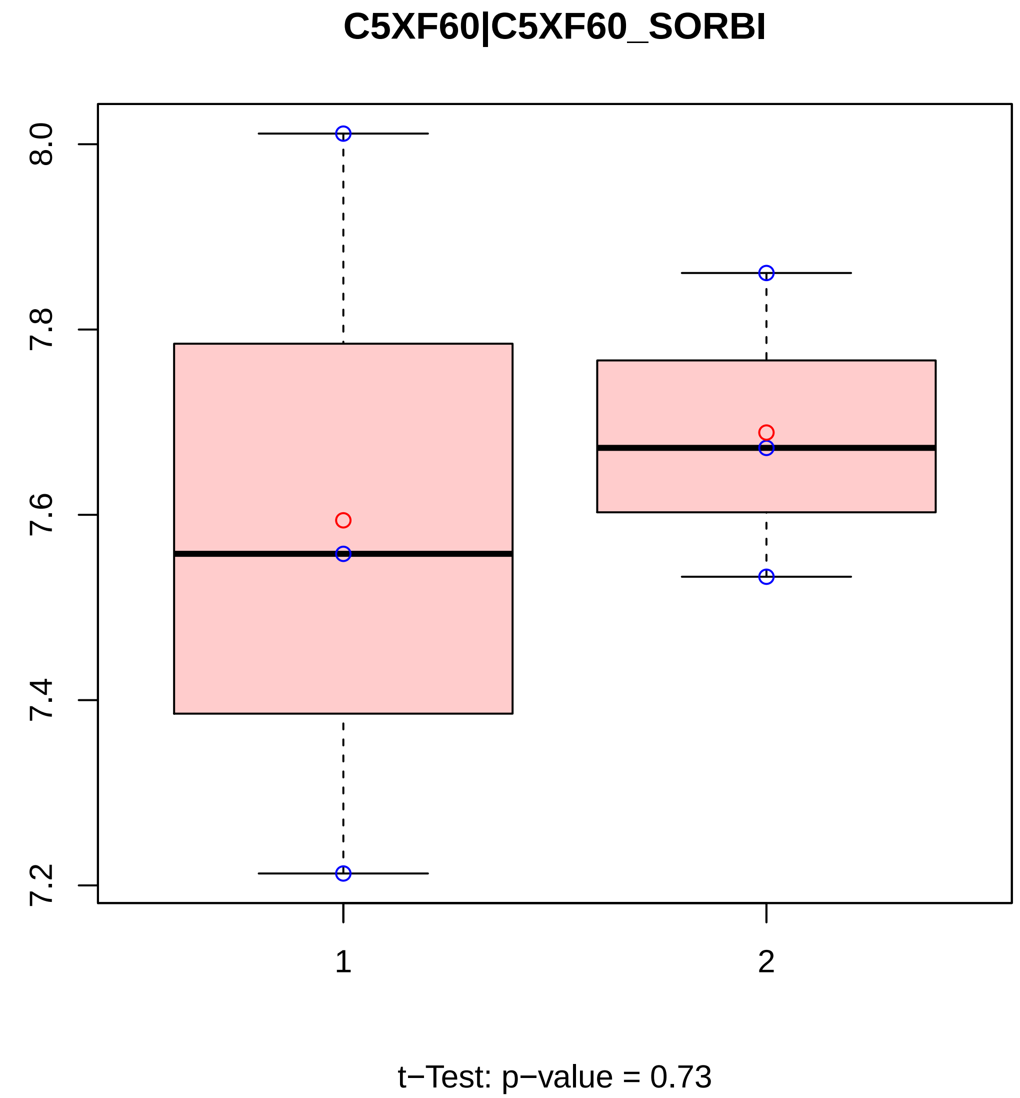
C5XF60|C5XF60_SORBI
8.0
7.8
7.6
7.4
7.2
1
2
t−Test: p−value = 0.73

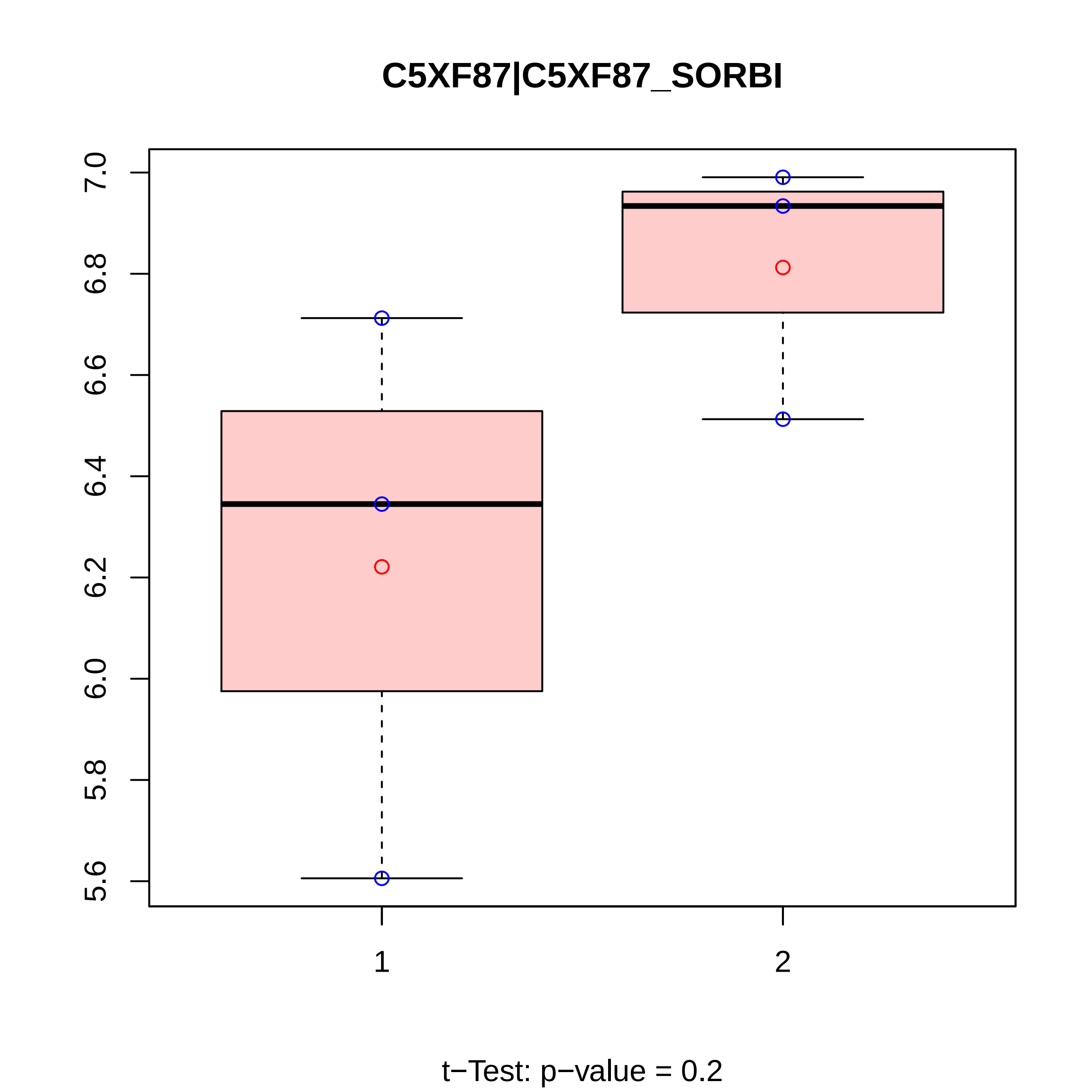
C5XF87|C5XF87_SORBI
7.0
6.8
6.6
6.4
6.2
6.0
5.8
5.6
1
2
t−Test: p−value = 0.2

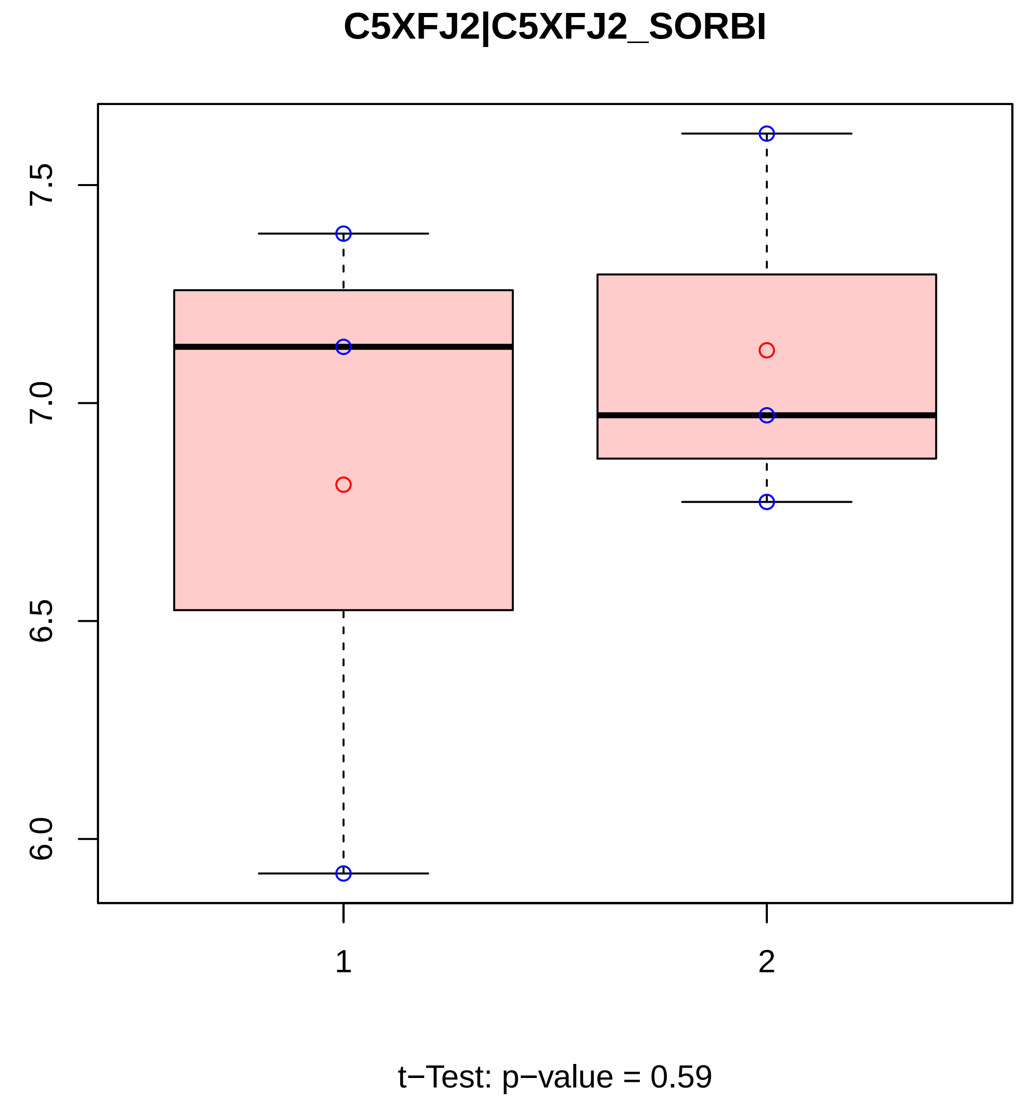
C5XFJ2|C5XFJ2_SORBI
6.0
6.5
7.0
7.5
1
2
t−Test: p−value = 0.59

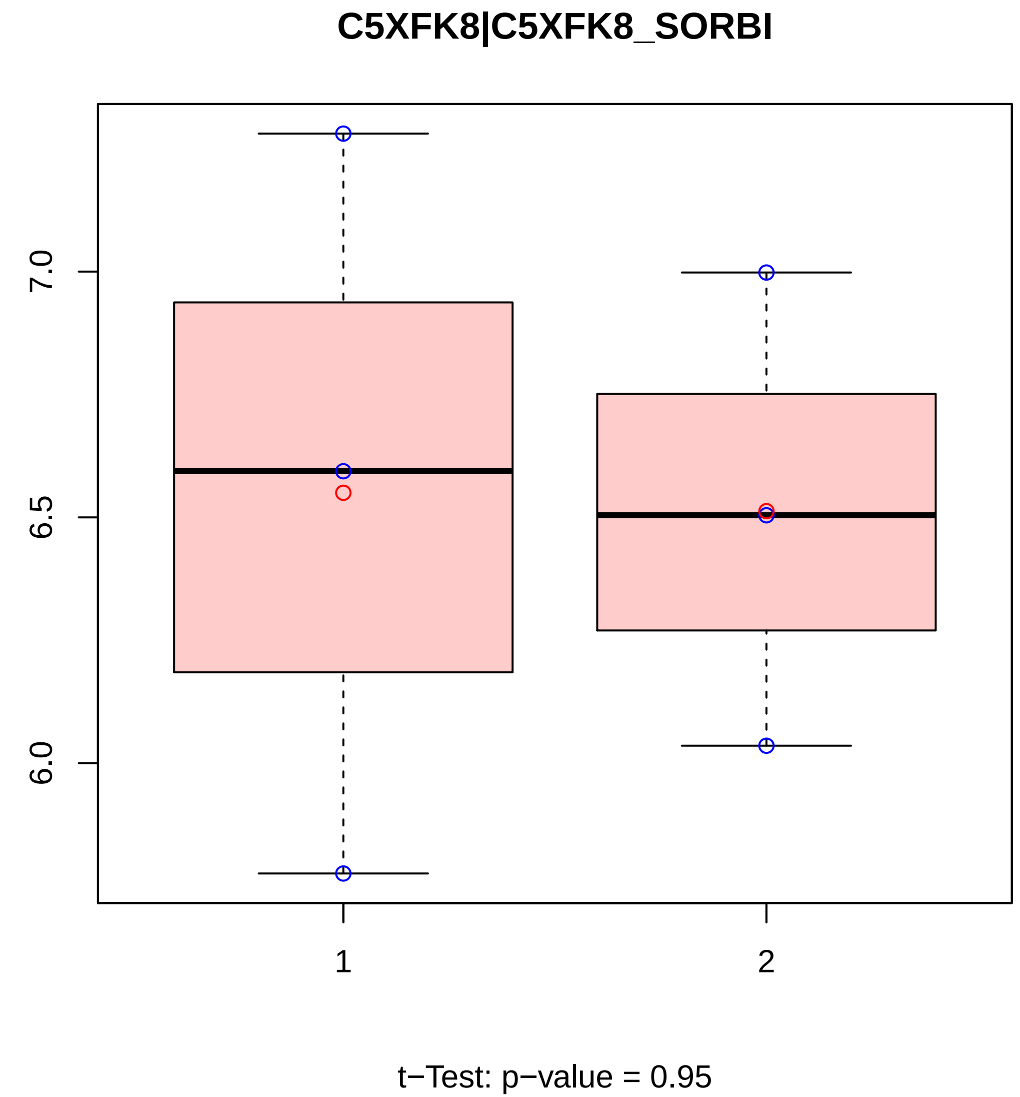
C5XFK8|C5XFK8_SORBI
7.0
6.5
6.0
1
2
t−Test: p−value = 0.95

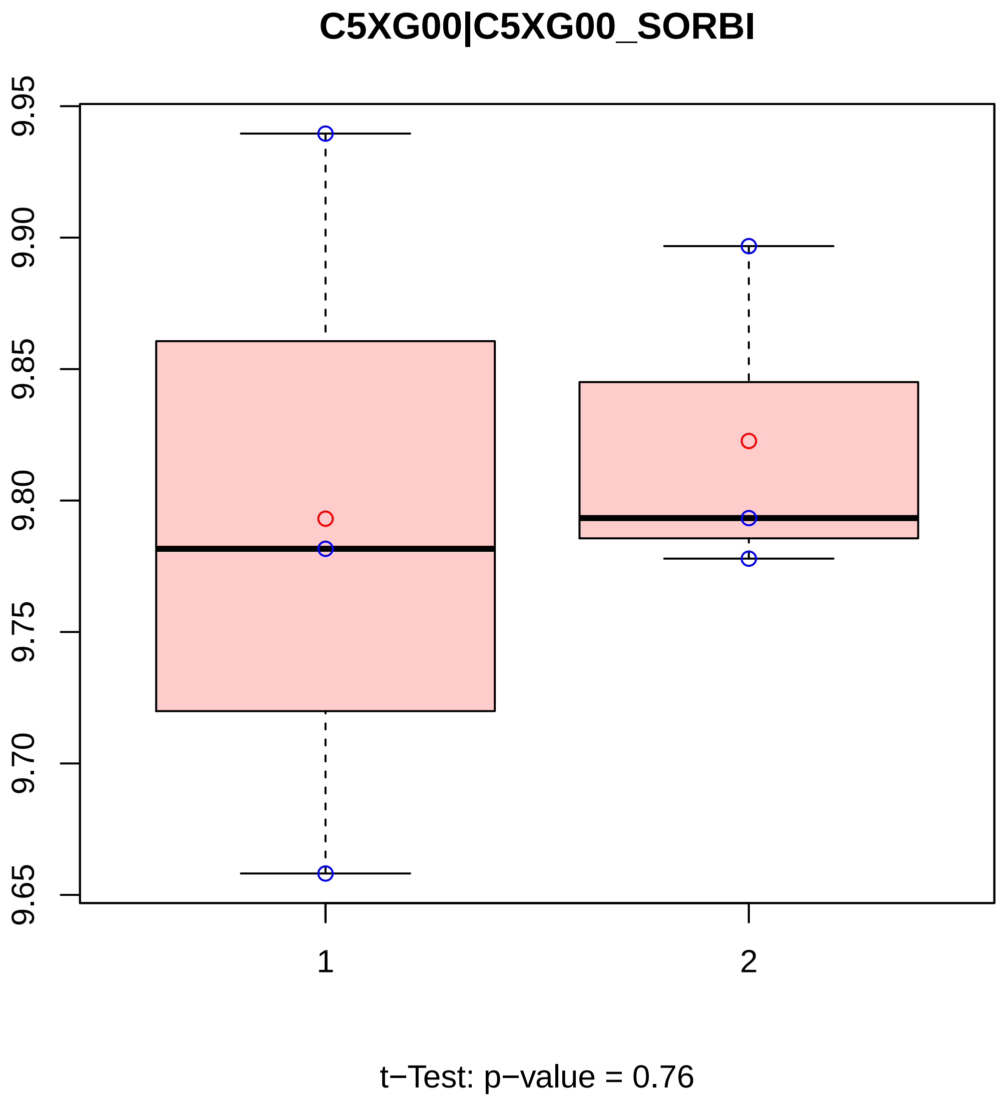

C5XG00|C5XG00_SORBI
9.95
9.90
9.85
9.80
9.75
9.70
9.65
1
2
t−Test: p−value = 0.76

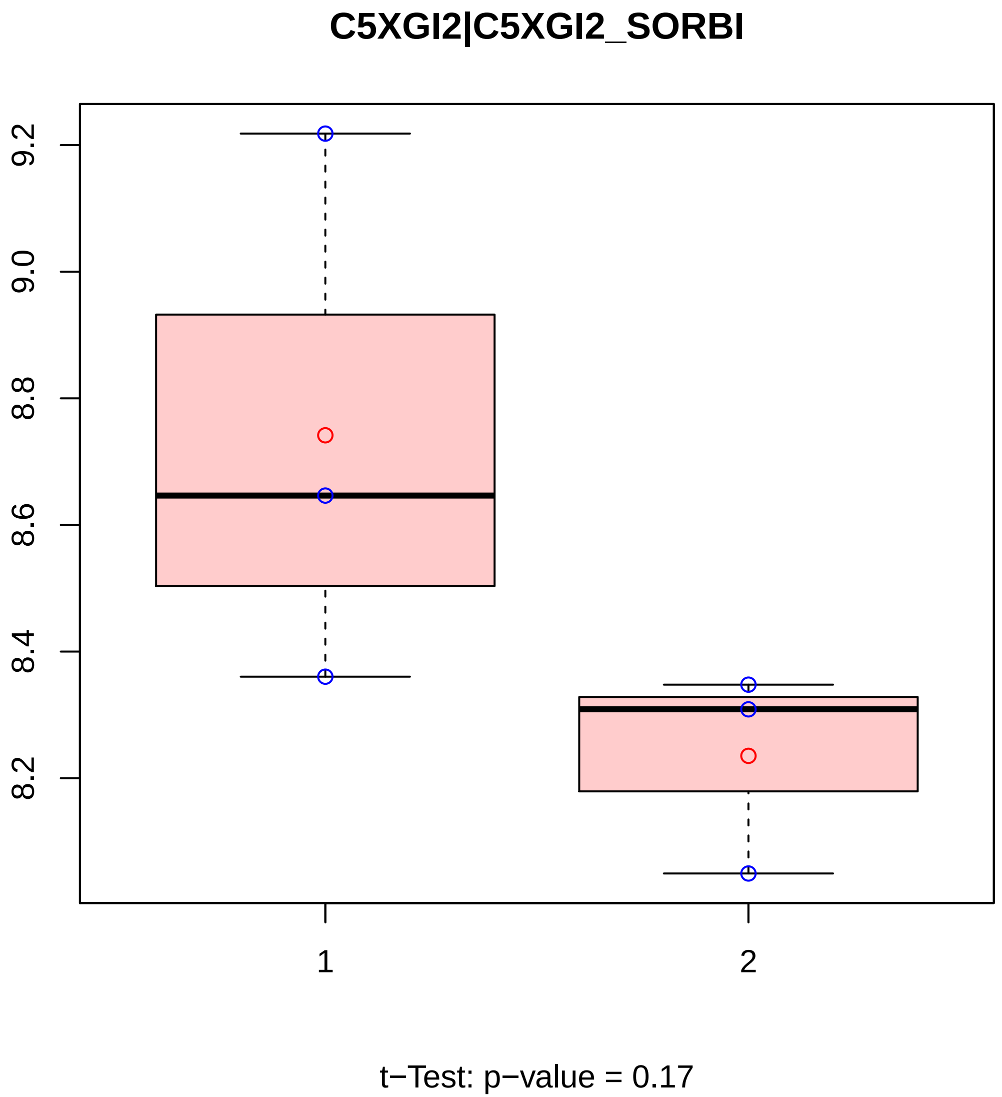
C5XGI2|C5XGI2_SORBI
9.2
9.0
8.8
8.6
8.4
8.2
1
2
t−Test: p−value = 0.17

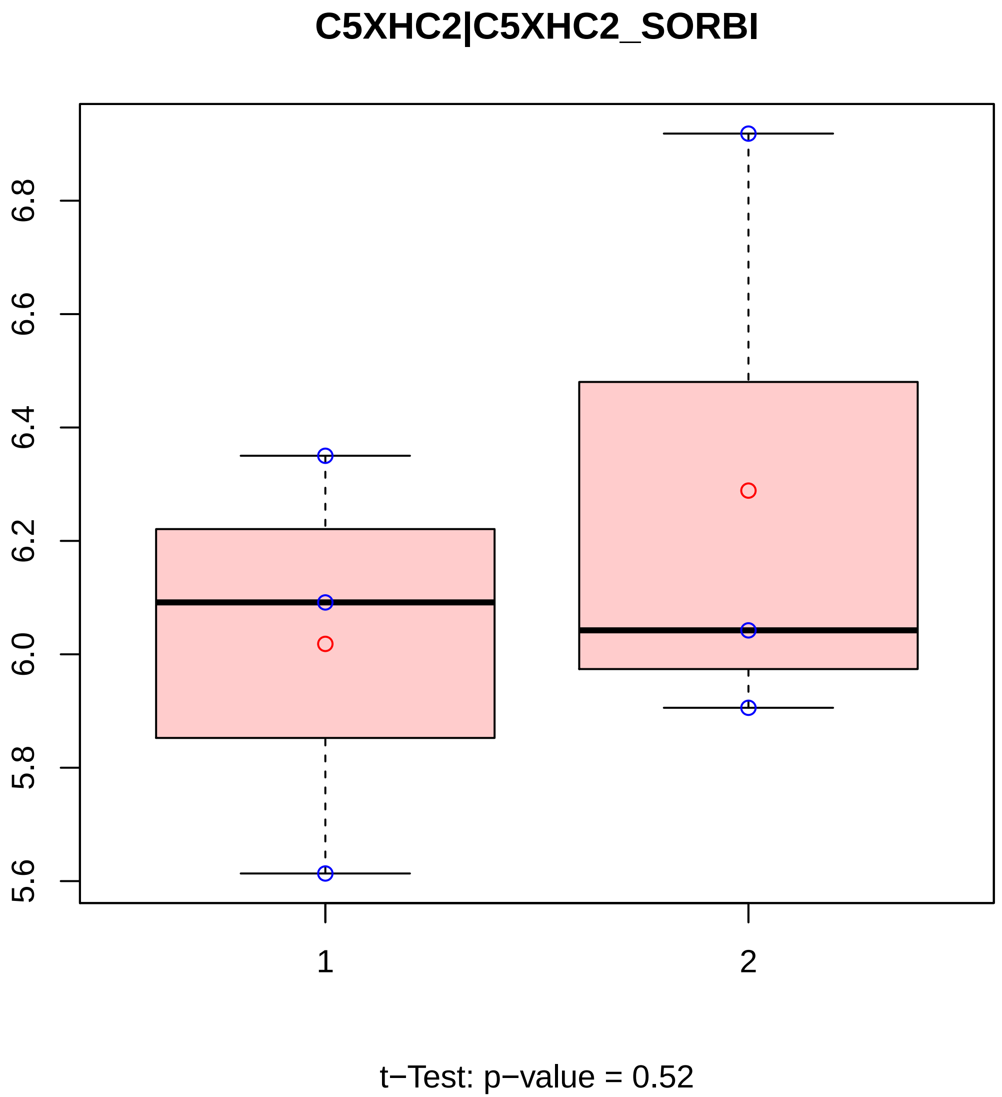
C5XHC2|C5XHC2_SORBI
5.6
5.8
6.0
6.2
6.4
6.6
6.8
1
2
t−Test: p−value = 0.52

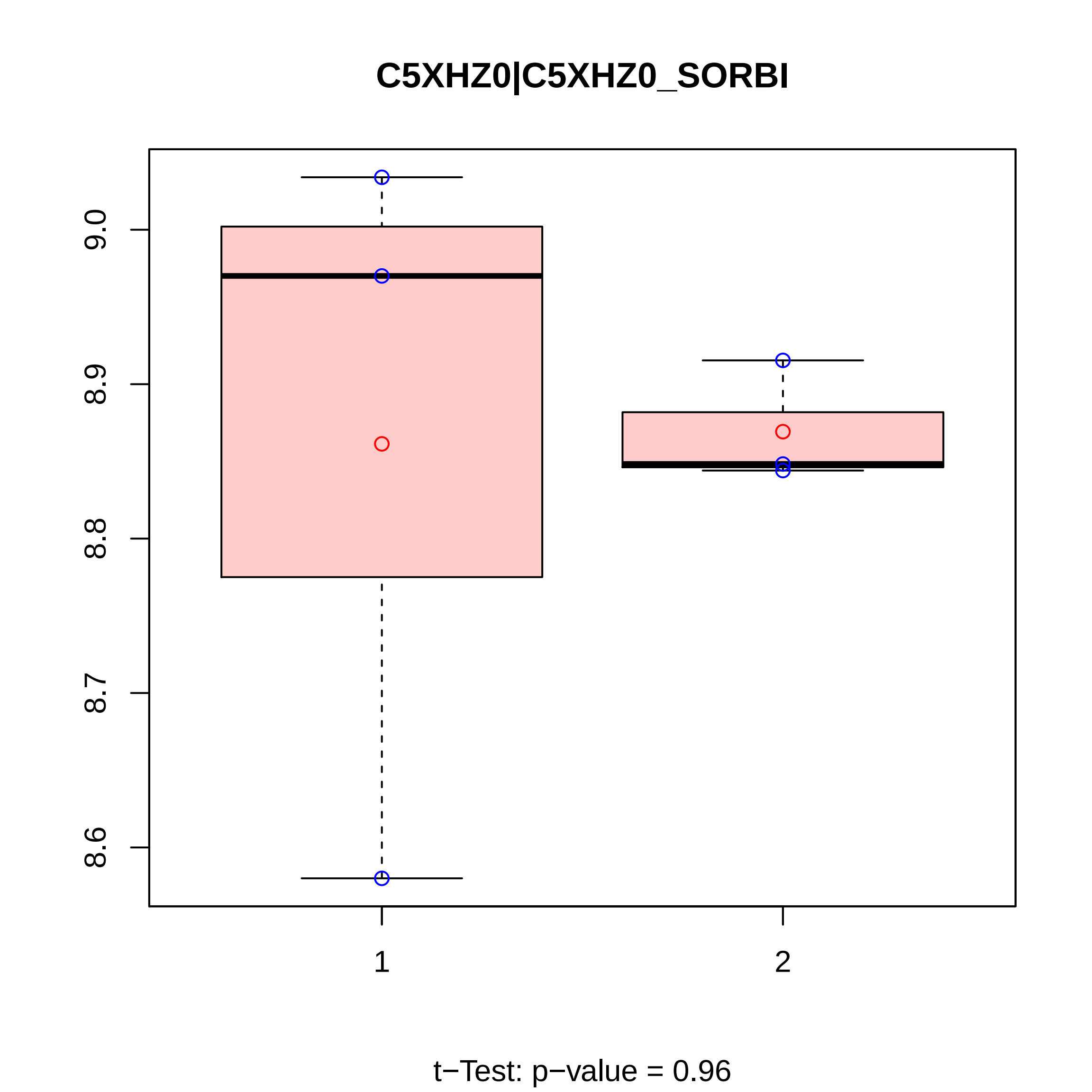
C5XHZ0|C5XHZ0_SORBI
9.0
8.9
8.8
8.7
8.6
1
2
t−Test: p−value = 0.96

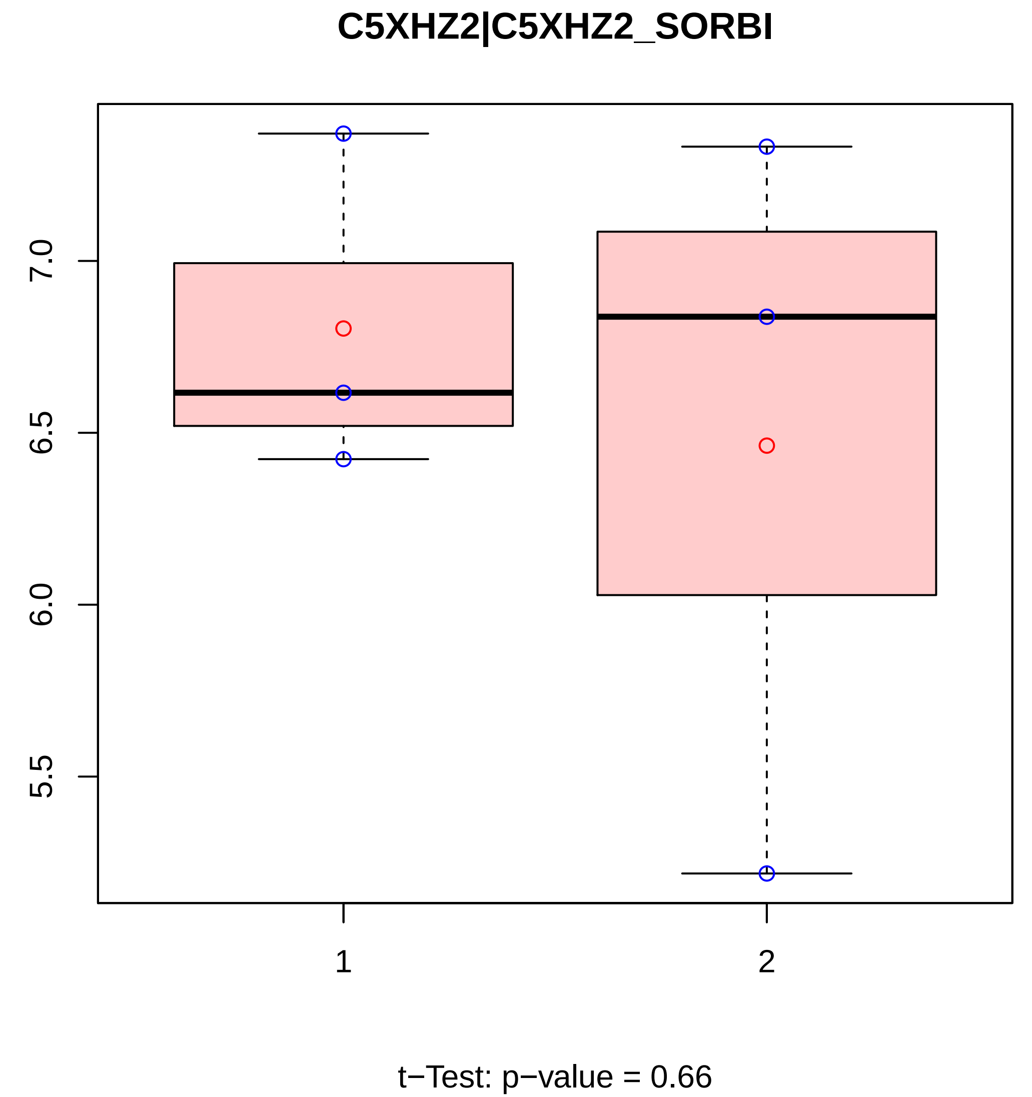
C5XHZ2|C5XHZ2_SORBI
7.0
6.5
6.0
5.5
1
2
t−Test: p−value = 0.66

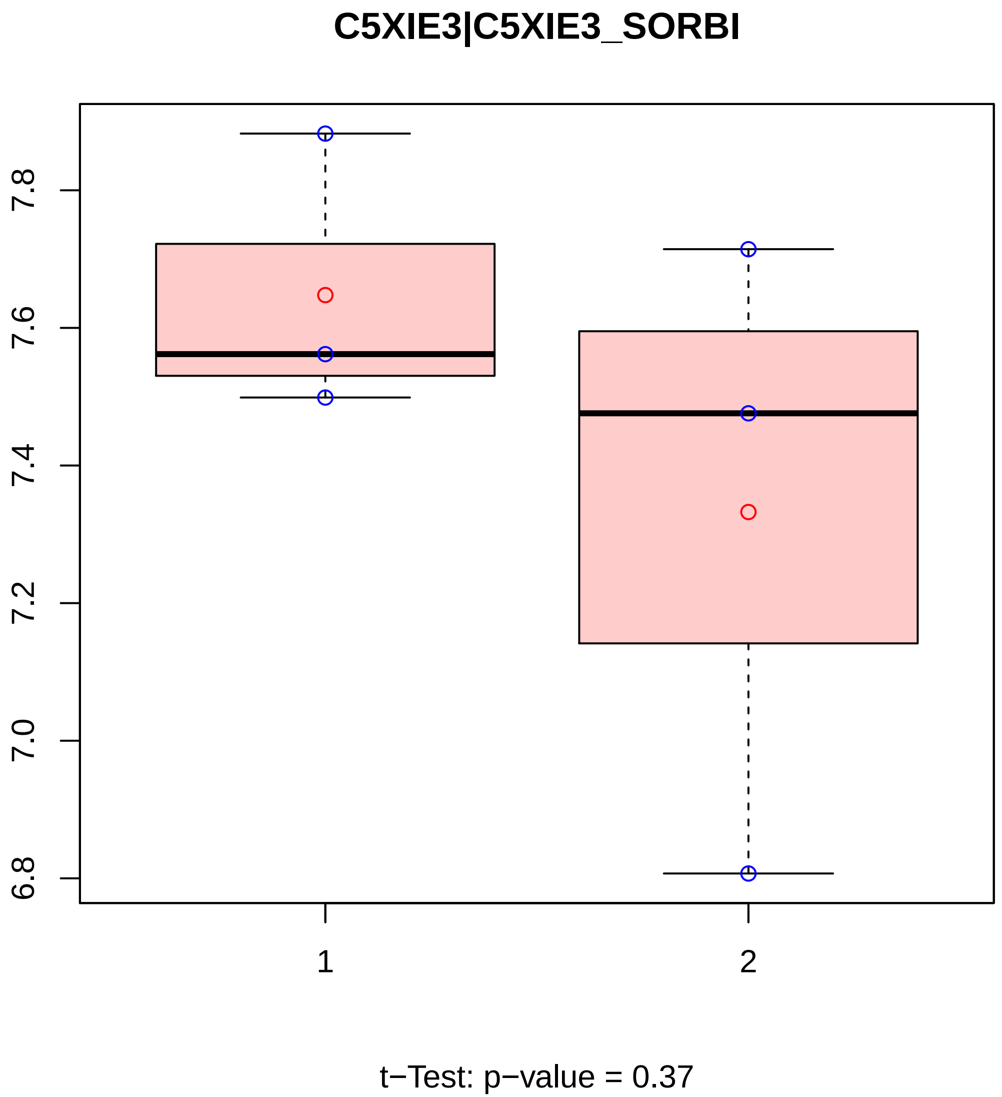

C5XIE3|C5XIE3_SORBI
7.8
7.6
7.4
7.2
7.0
6.8
1
2
t−Test: p−value = 0.37

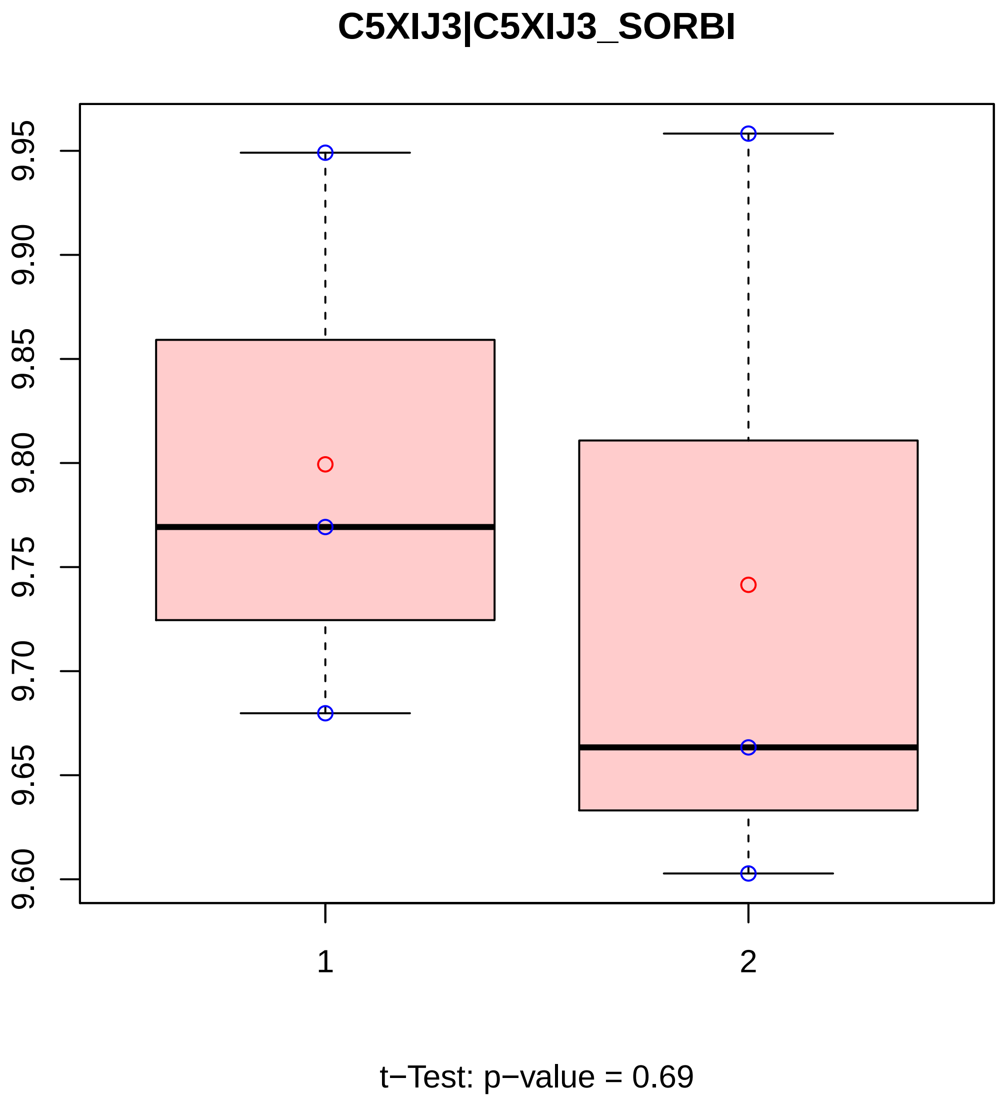

C5XIJ3|C5XIJ3_SORBI
9.60
9.65
9.70
9.75
9.80
9.85
9.90
9.95
1
2
t−Test: p−value = 0.69

C5XIL2|C5XIL2_SORBI

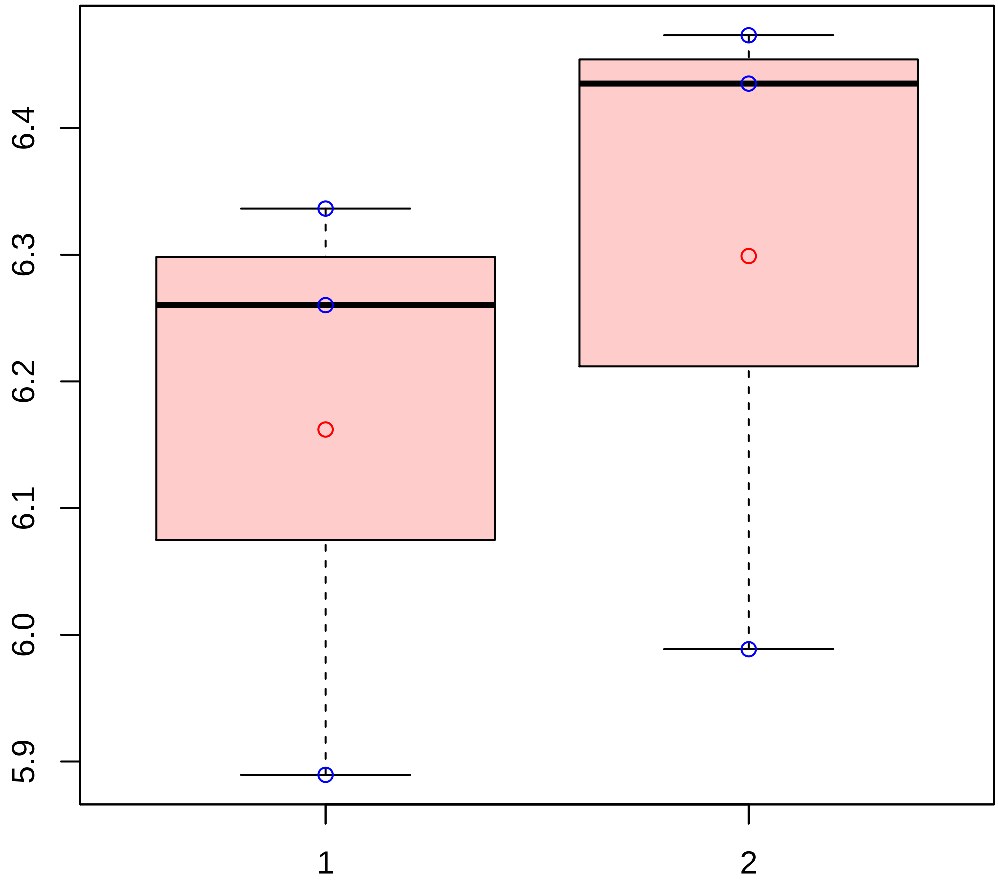

t−Test: p−value = 0.55

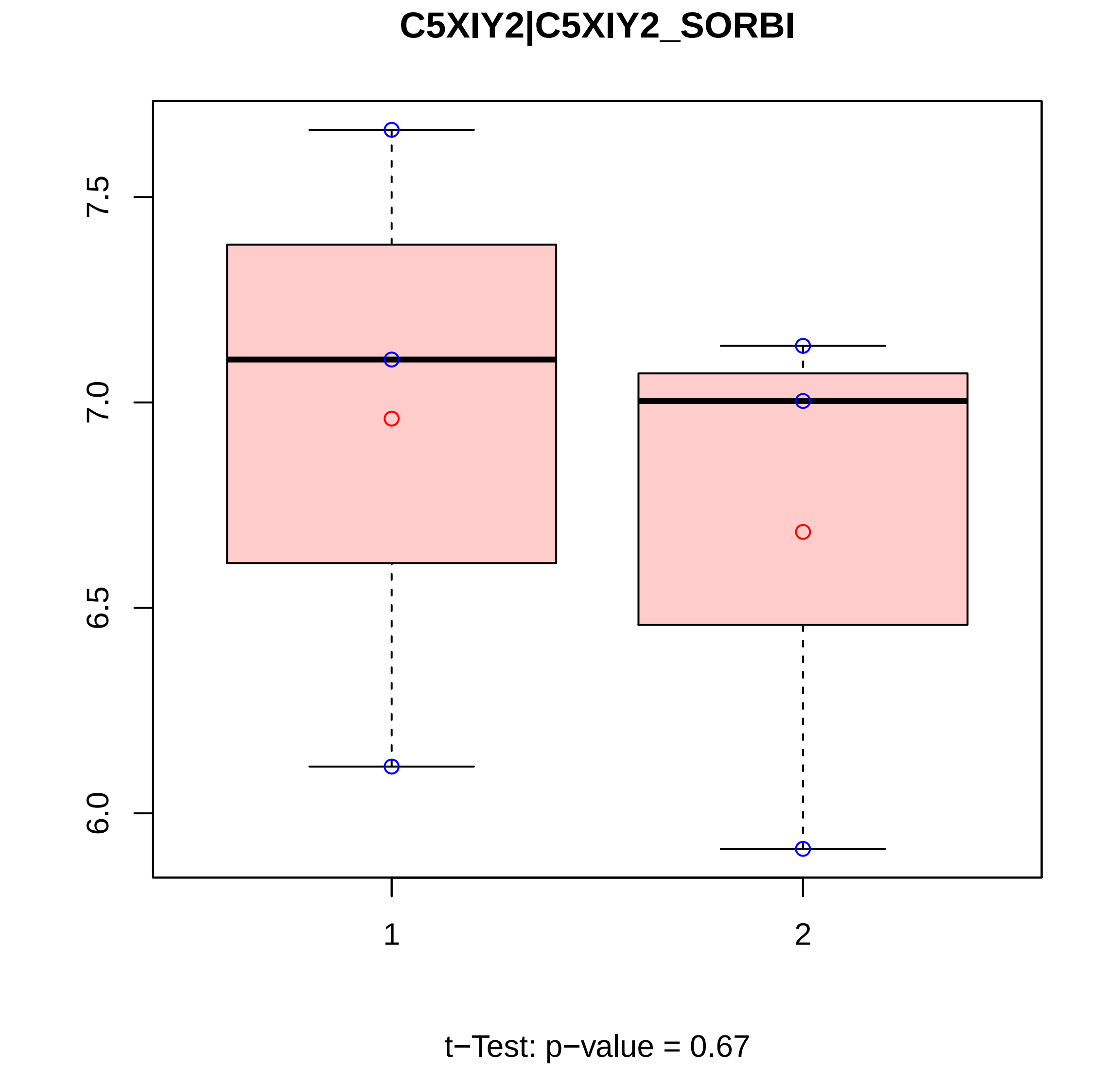
C5XIY2|C5XIY2_SORBI
7.5
7.0
6.5
6.0
1
2
t−Test: p−value = 0.67

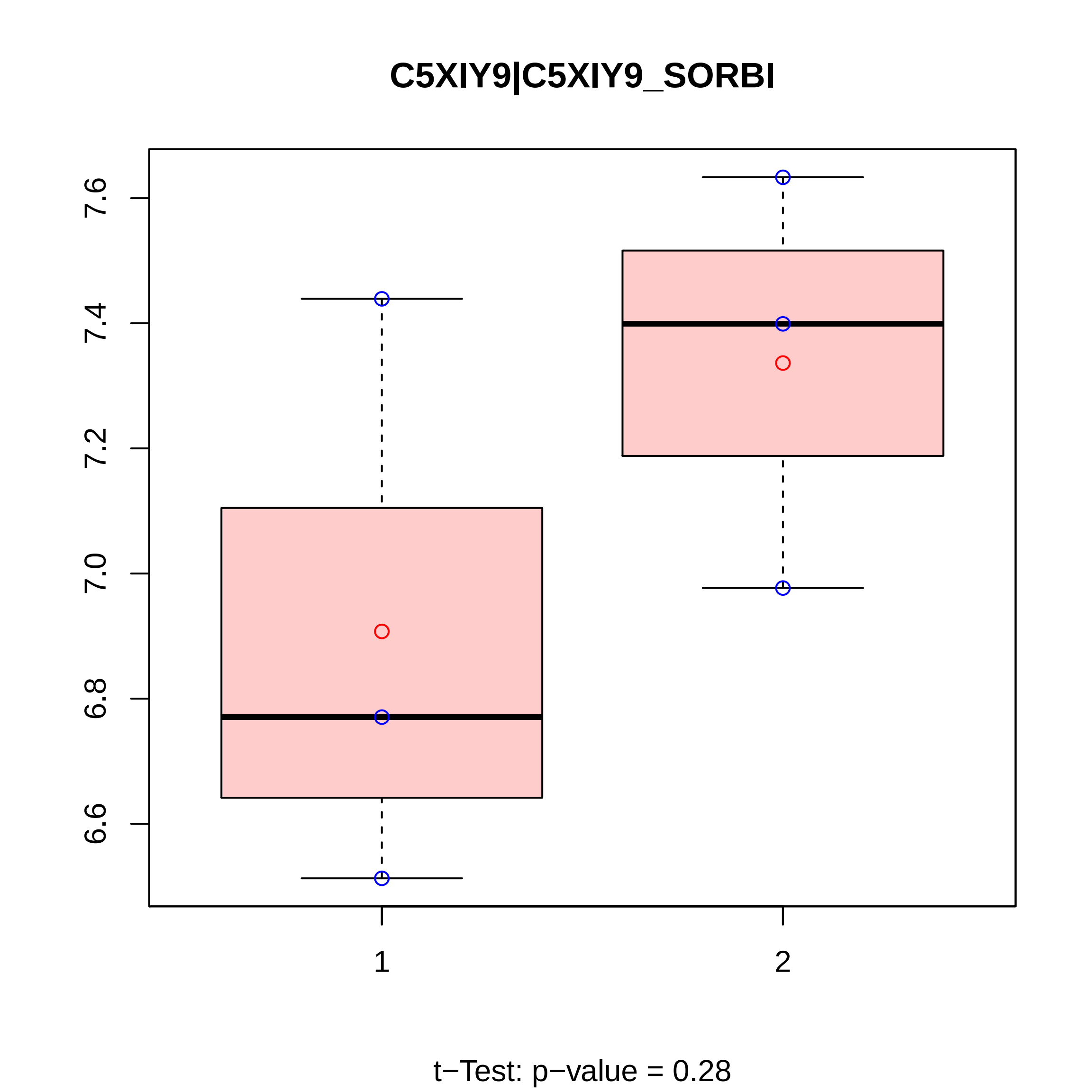

C5XIY9|C5XIY9_SORBI
7.6
7.4
7.2
7.0
6.8
6.6
1
2
t−Test: p−value = 0.28

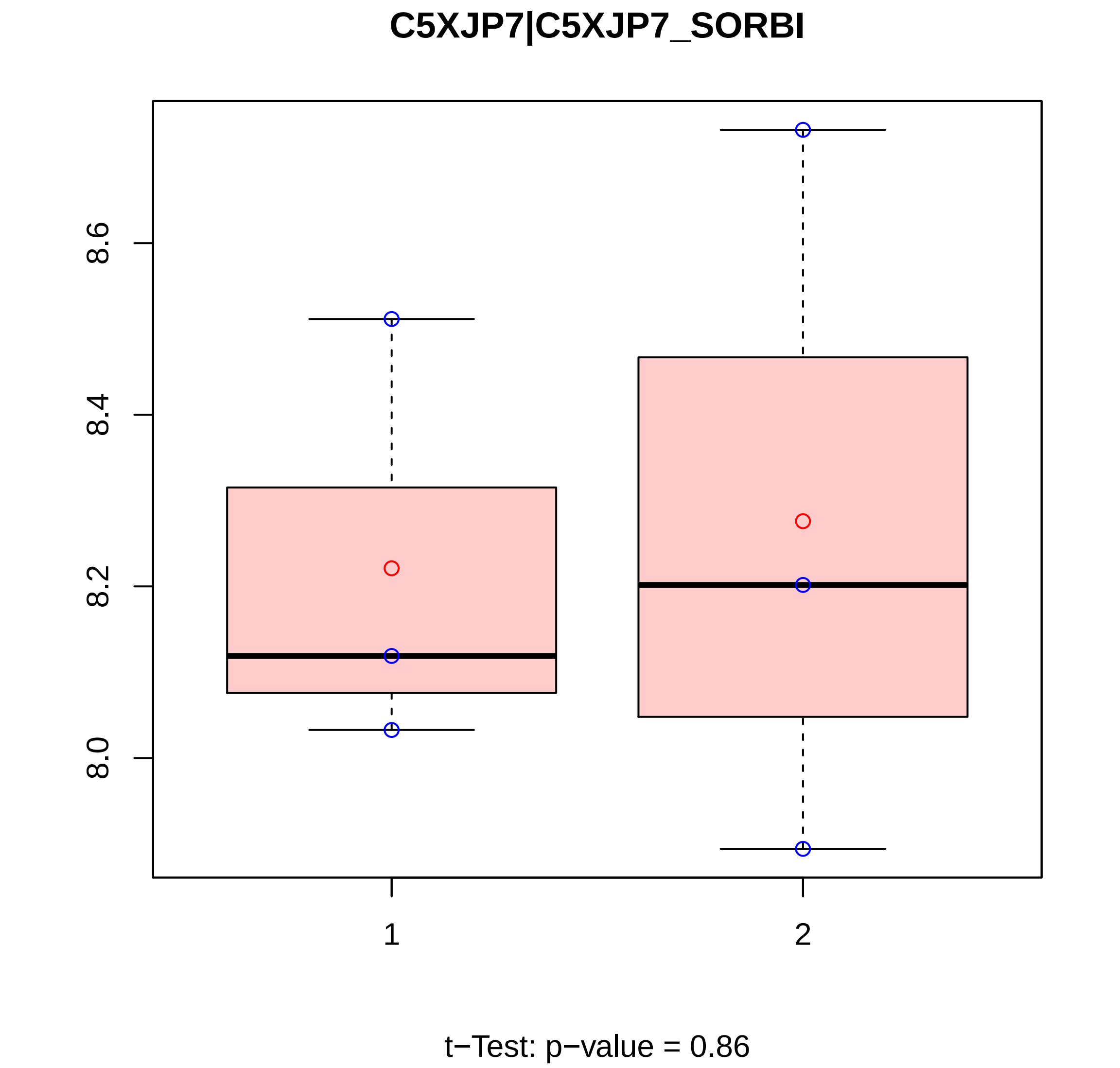
C5XJP7|C5XJP7_SORBI
8.0
8.2
8.4
8.6
1
2
t−Test: p−value = 0.86

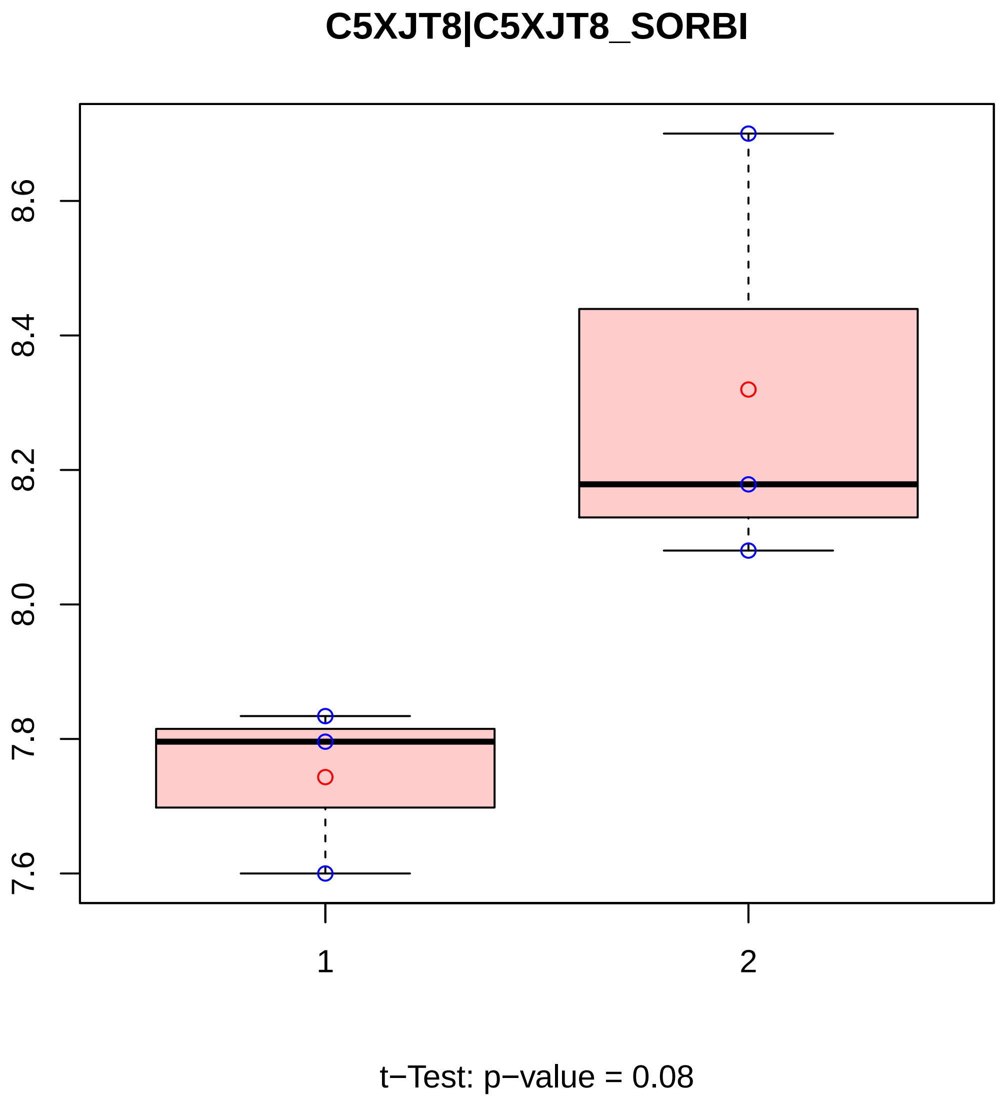
C5XJT8|C5XJT8_SORBI
8.6
8.4
8.2
8.0
7.8
7.6
1
2
t−Test: p−value = 0.08

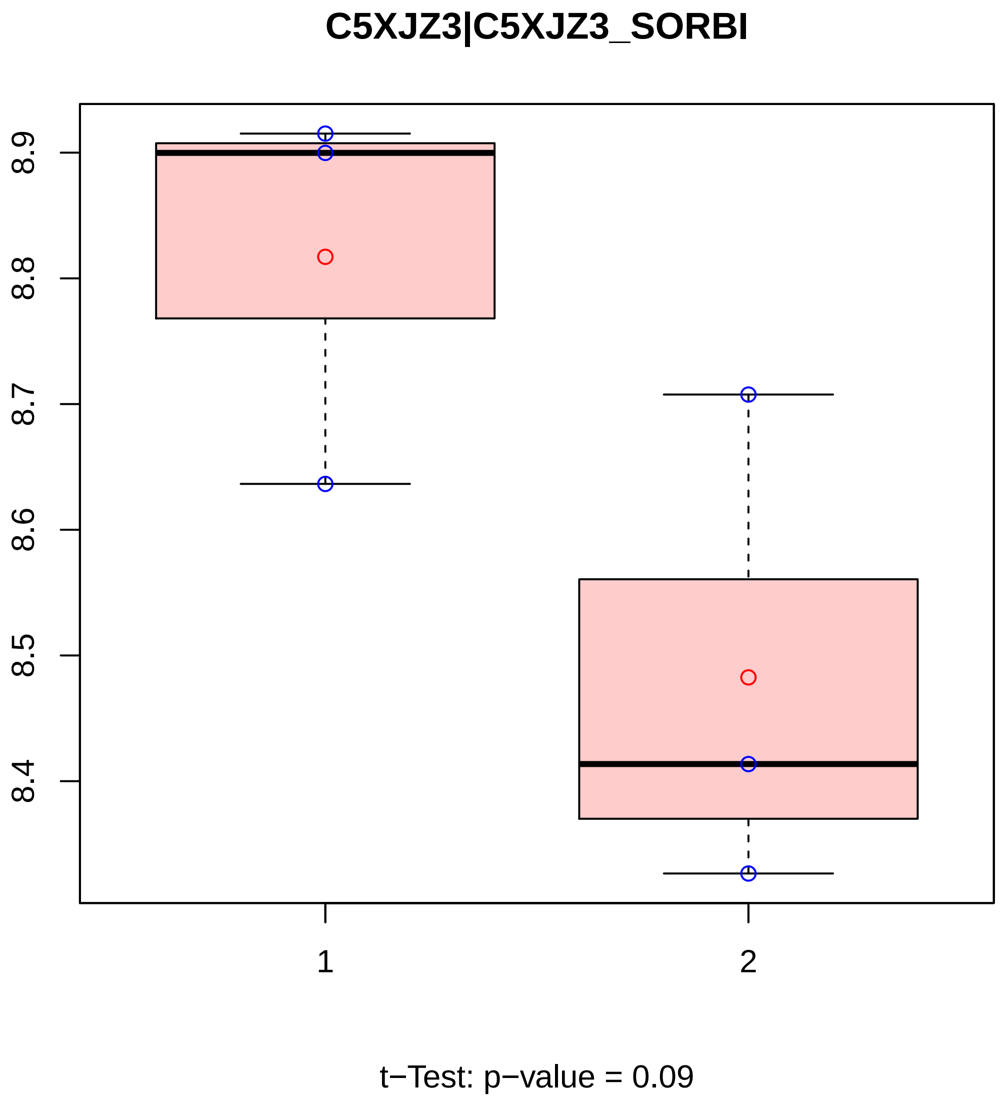
C5XJZ3|C5XJZ3_SORBI
8.4
8.5
8.6
8.7
8.8
8.9
1
2
t−Test: p−value = 0.09

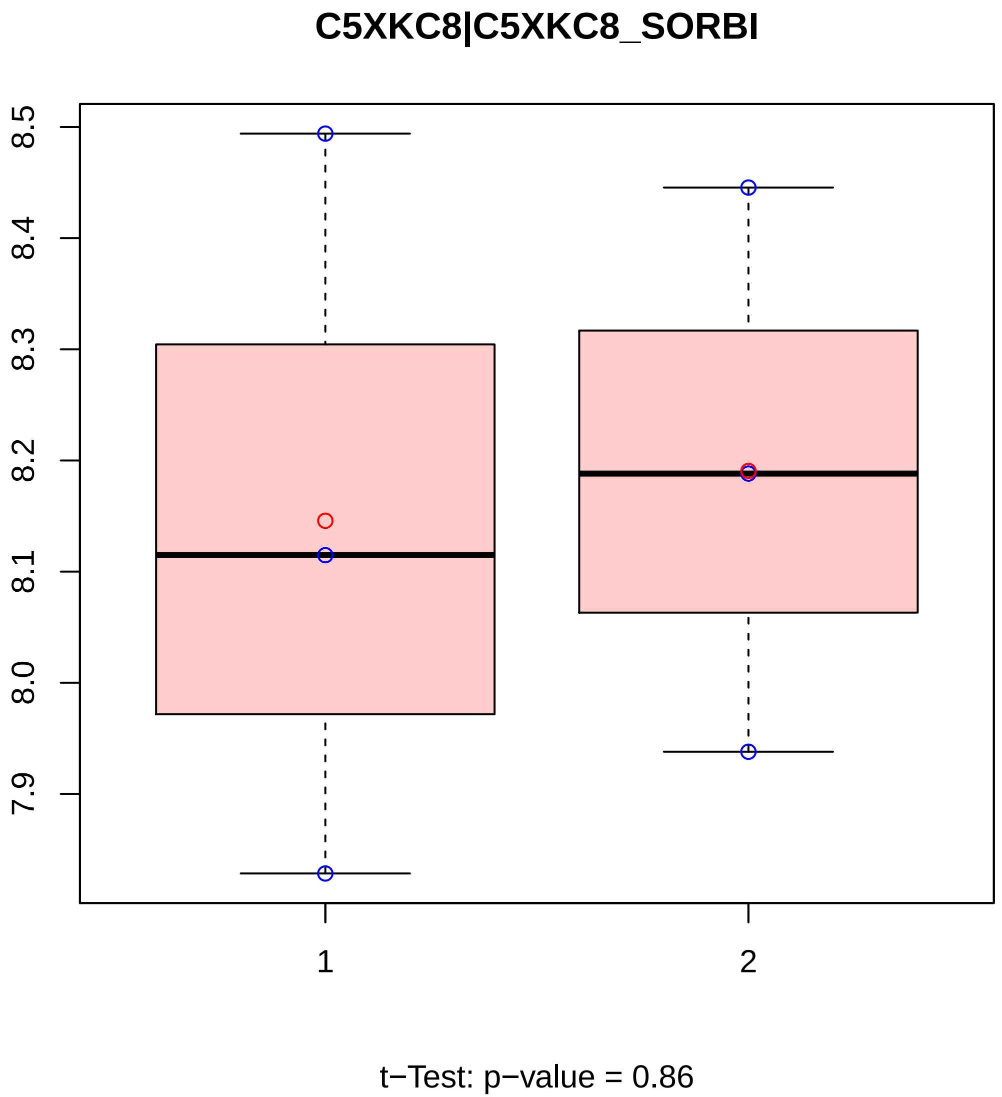

C5XKC8|C5XKC8_SORBI
8.5
8.4
8.3
8.2
8.1
8.0
7.9
1
2
t−Test: p−value = 0.86

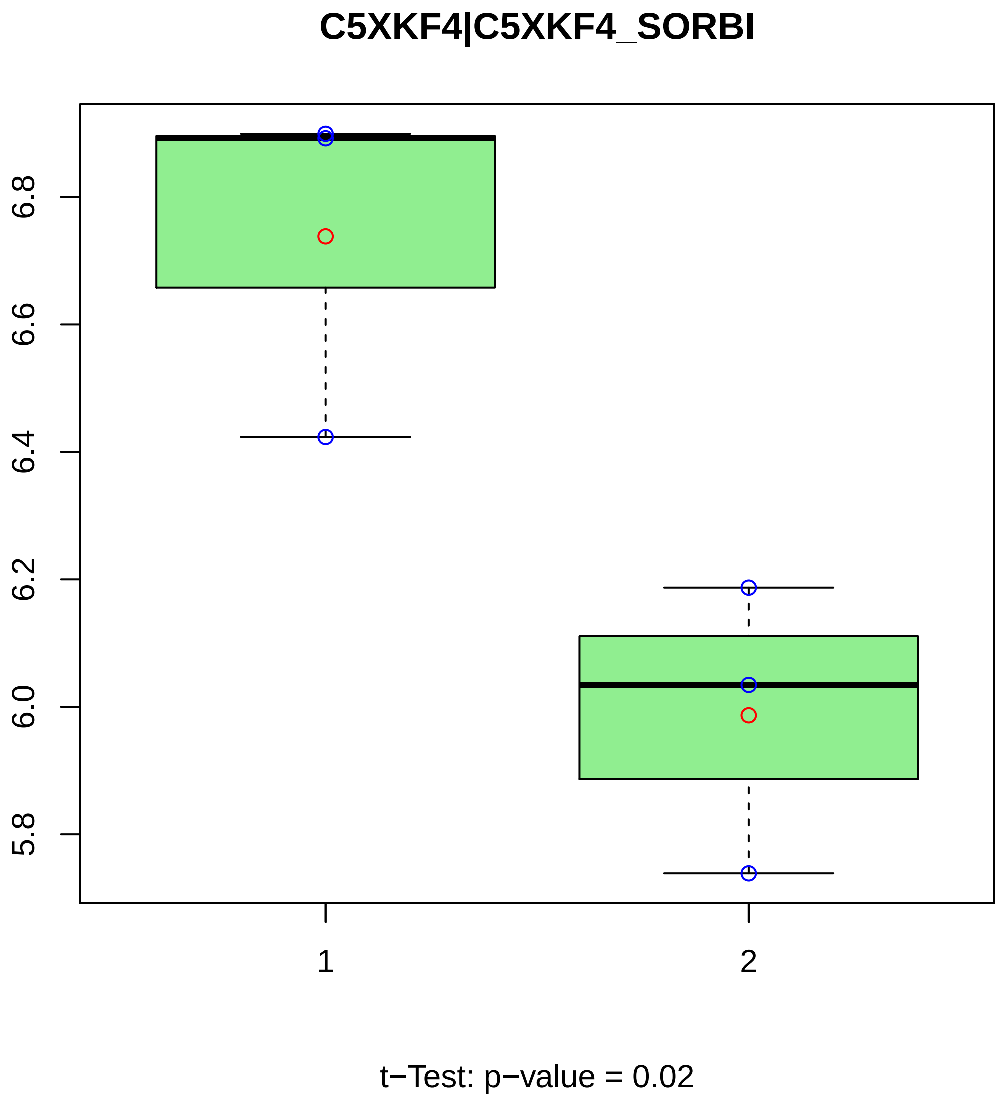

C5XKF4|C5XKF4_SORBI
6.8
6.6
6.4
6.2
6.0
5.8
1
2
t−Test: p−value = 0.02

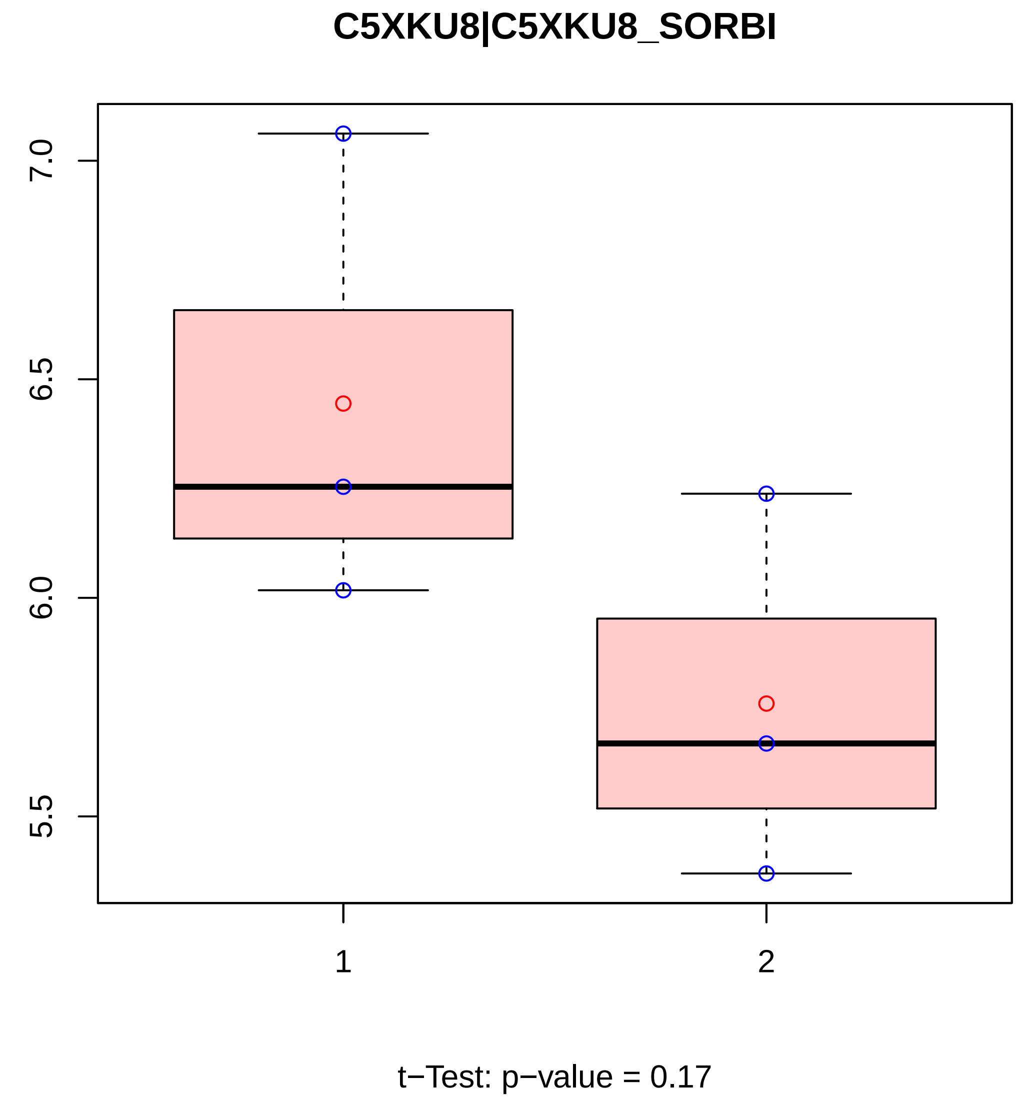
C5XKU8|C5XKU8_SORBI
7.0
6.5
6.0
5.5
1
2
t−Test: p−value = 0.17

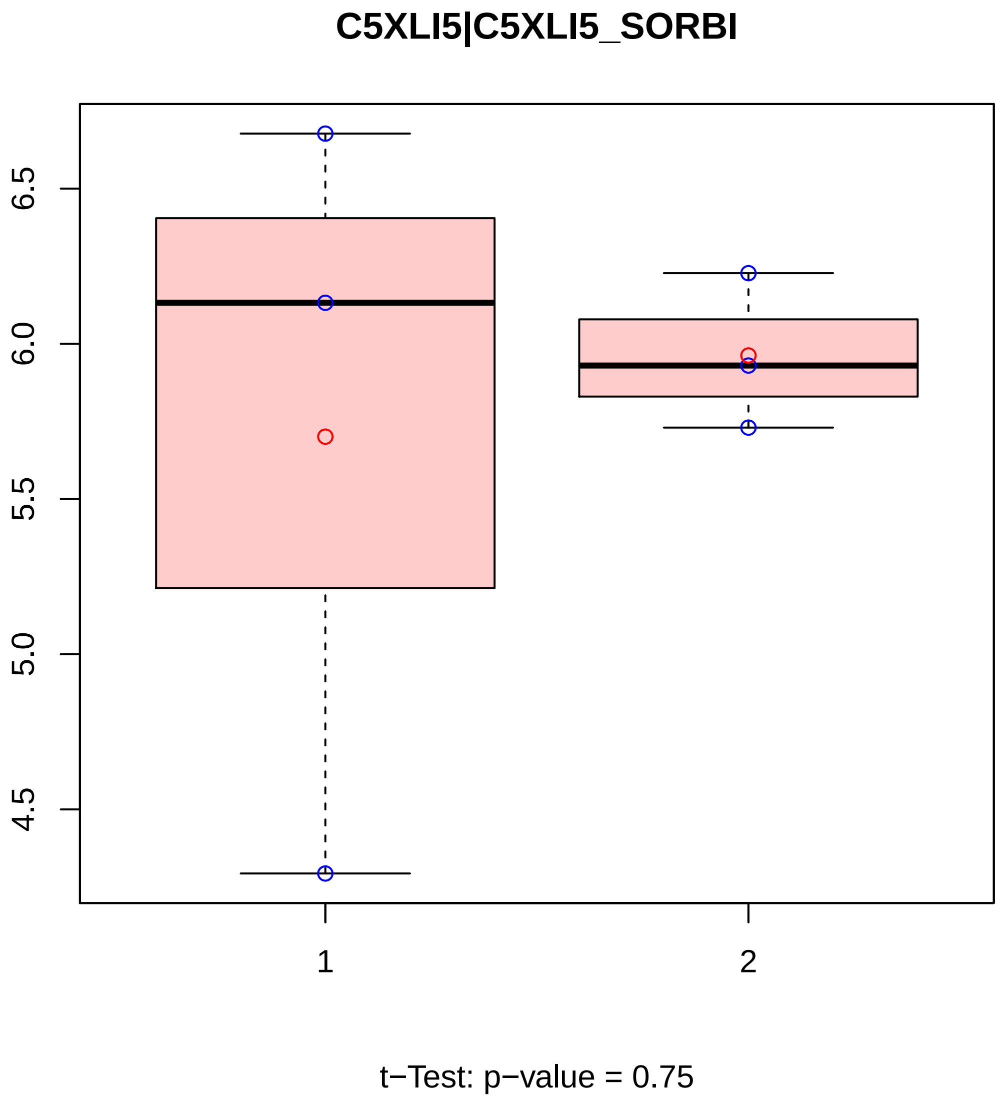
C5XLI5|C5XLI5_SORBI
6.5
6.0
5.5
5.0
4.5
1
2
t−Test: p−value = 0.75

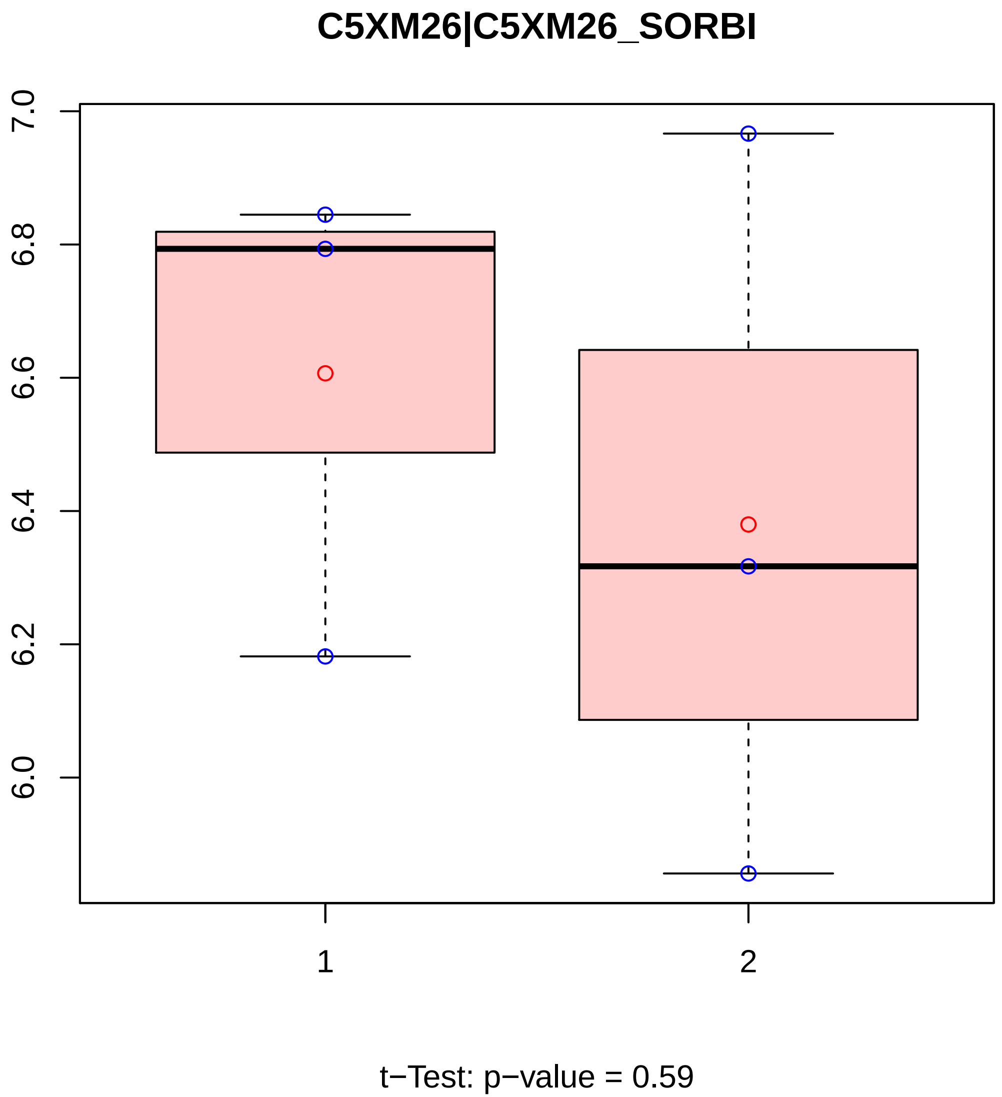

C5XM26|C5XM26_SORBI
7.0
6.8
6.6
6.4
6.2
6.0
1
2
t−Test: p−value = 0.59

C5XN42|C5XN42_SORBI

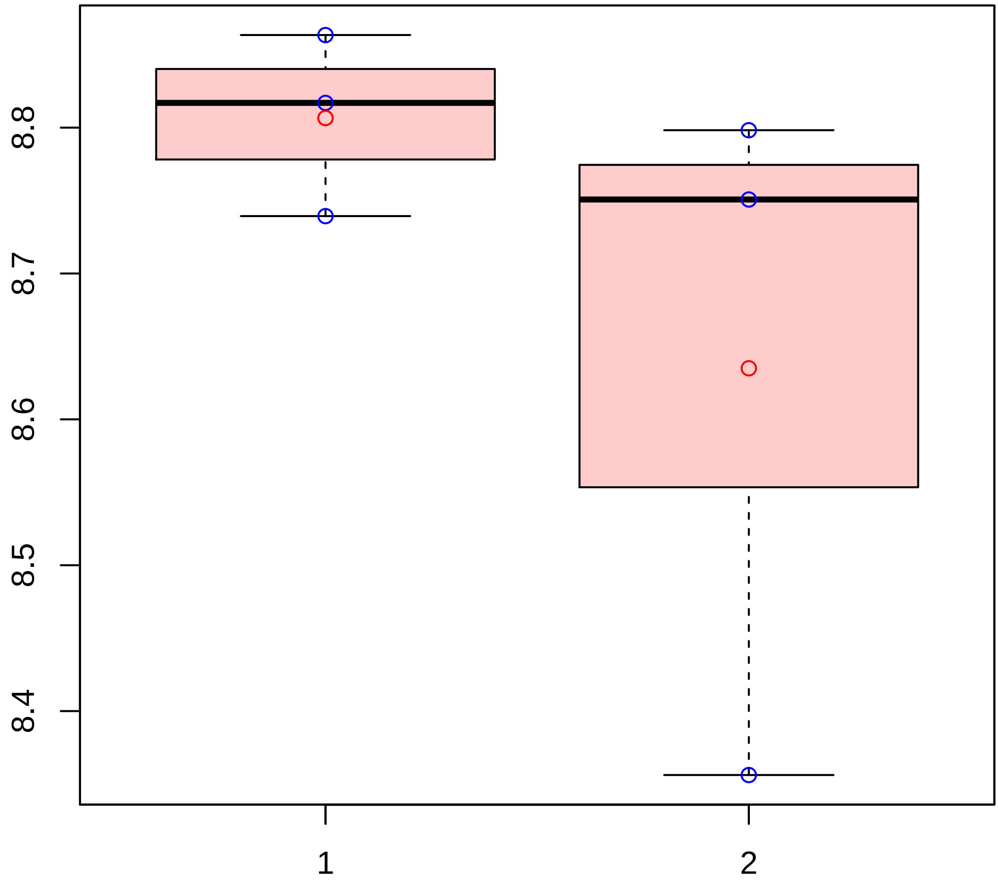

t−Test: p−value = 0.35

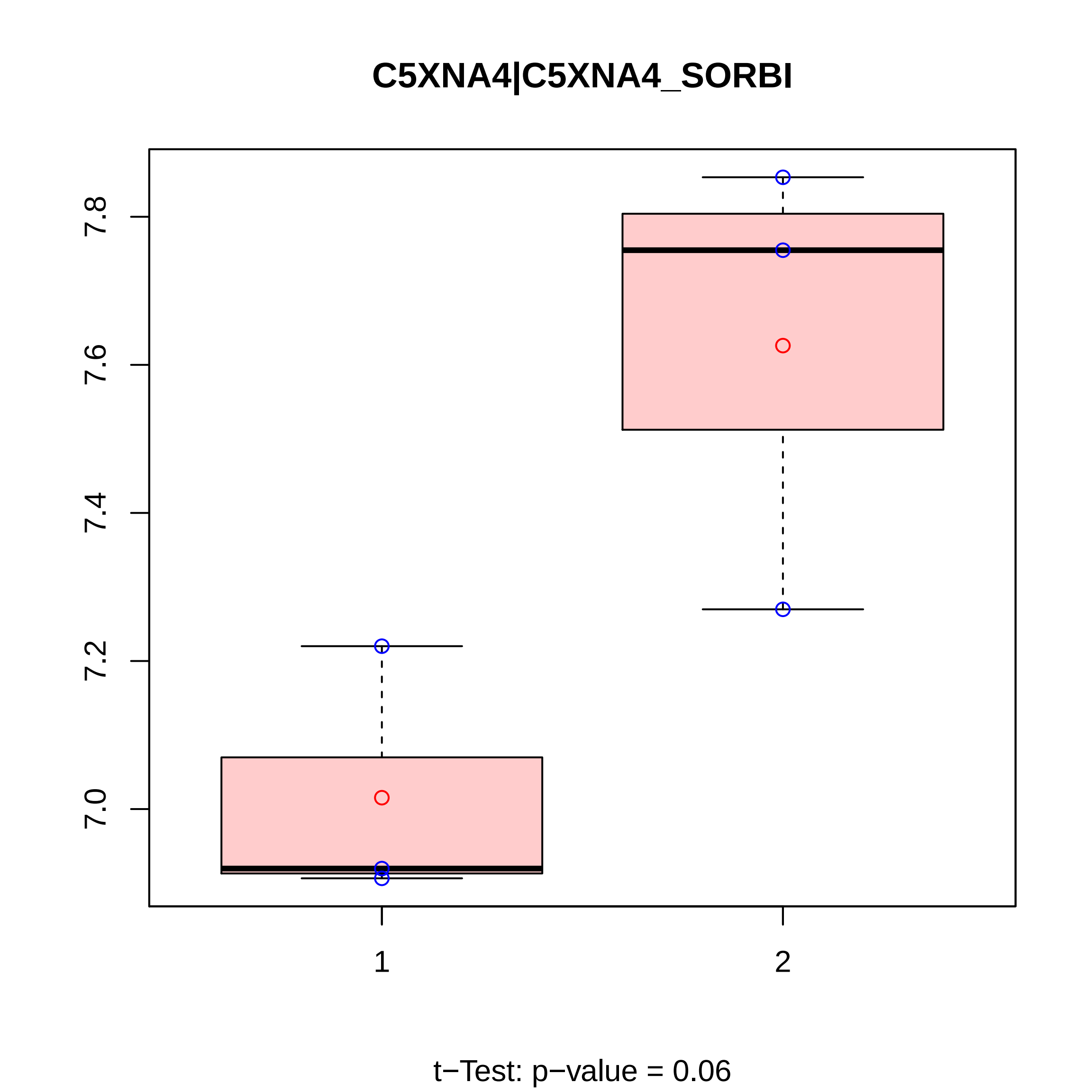
C5XNA4|C5XNA4_SORBI
7.0
7.2
7.4
7.6
7.8
1
2
t−Test: p−value = 0.06

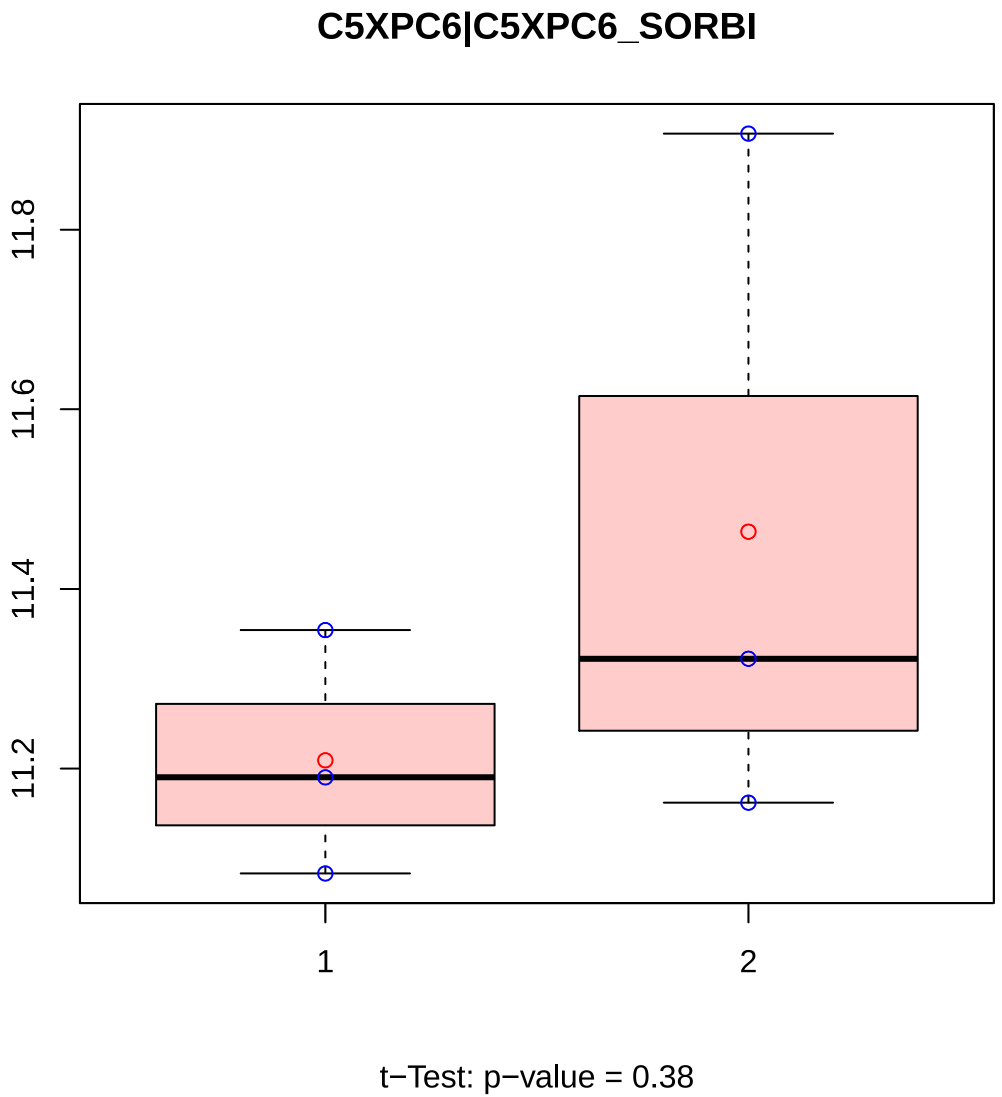
C5XPC6|C5XPC6_SORBI
11.2
11.4
11.6
11.8
1
2
t−Test: p−value = 0.38

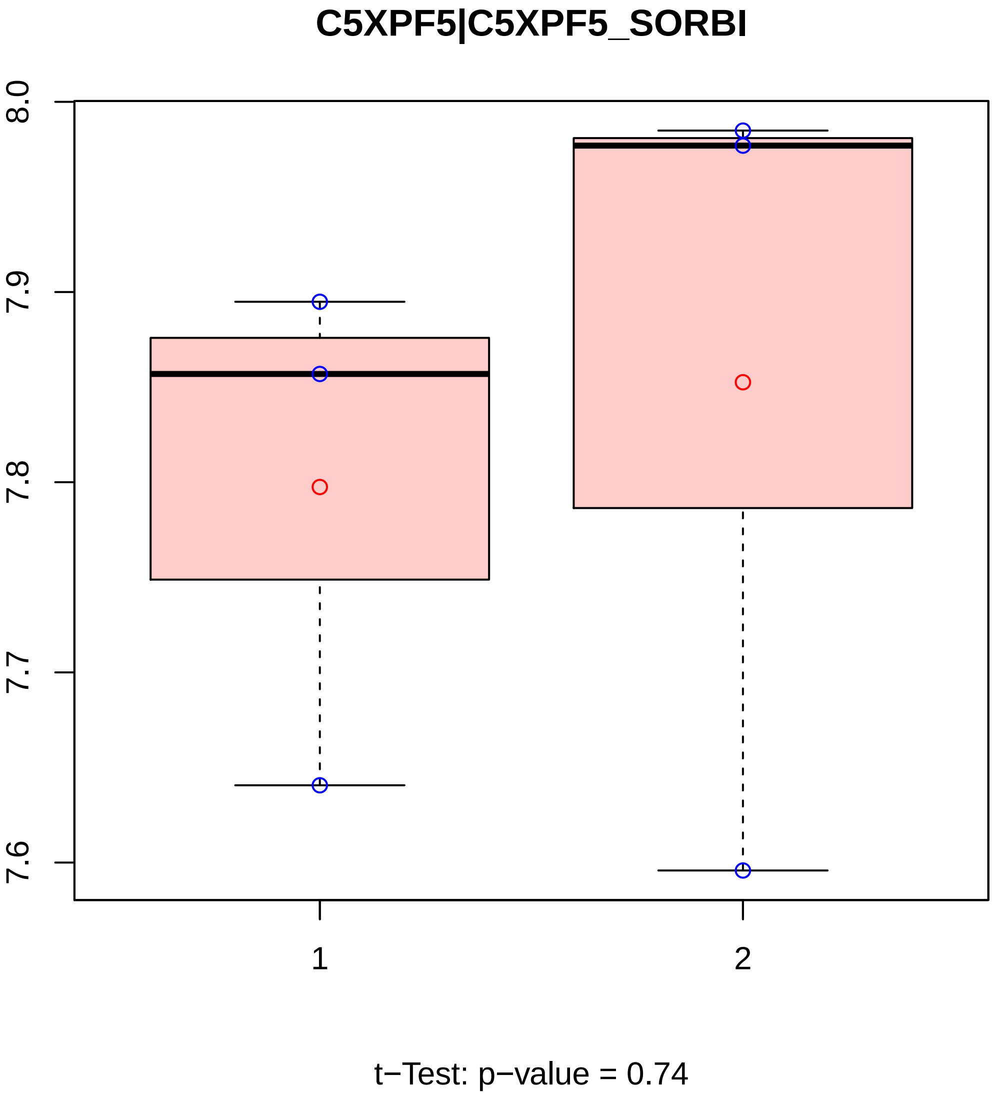

C5XPF5|C5XPF5_SORBI
8.0
7.9
7.8
7.7
7.6
1
2
t−Test: p−value = 0.74

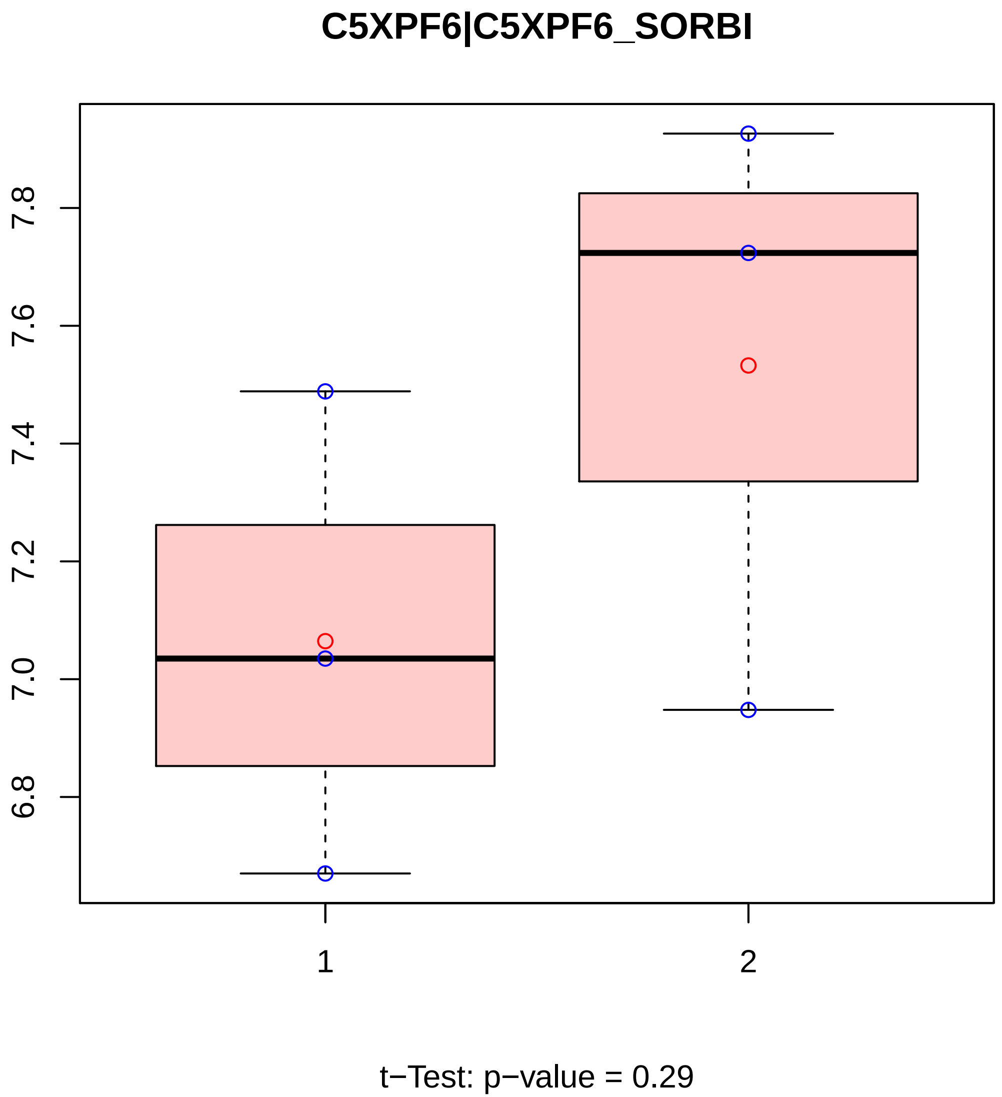
C5XPF6|C5XPF6_SORBI
6.8
7.0
7.2
7.4
7.6
7.8
1
2
t−Test: p−value = 0.29

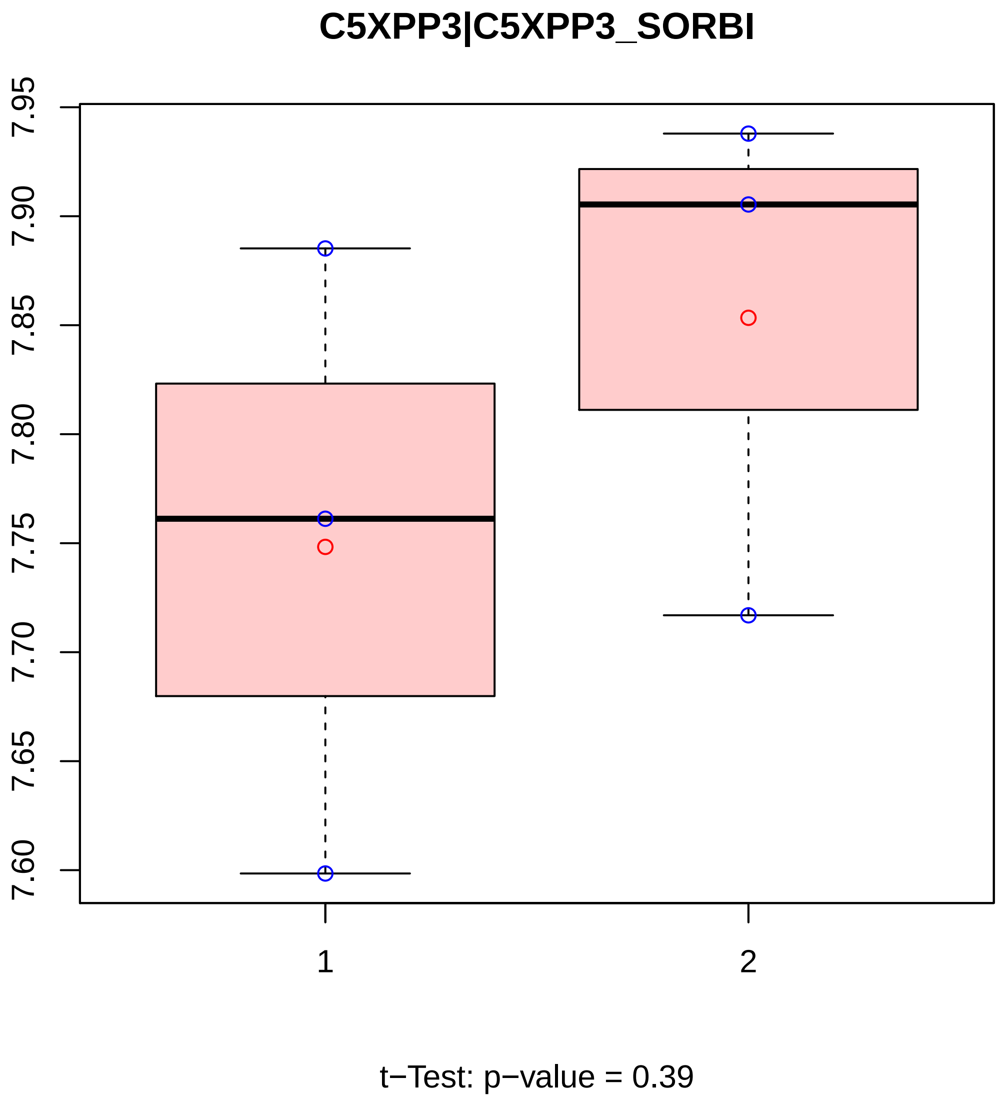
C5XPP3|C5XPP3_SORBI
7.95
7.90
7.85
7.80
7.75
7.70
7.65
7.60
1
2
t−Test: p−value = 0.39

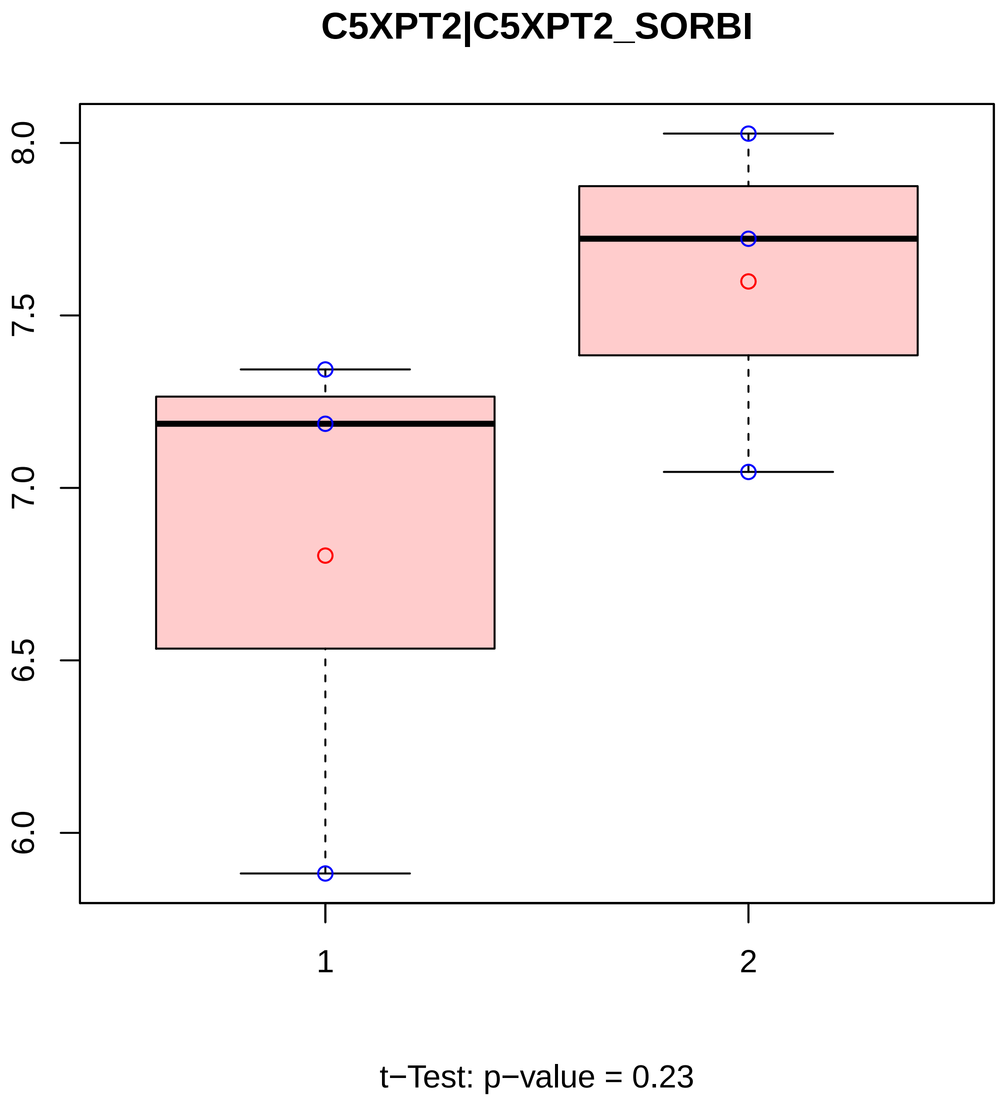
C5XPT2|C5XPT2_SORBI
8.0
7.5
7.0
6.5
6.0
1
2
t−Test: p−value = 0.23

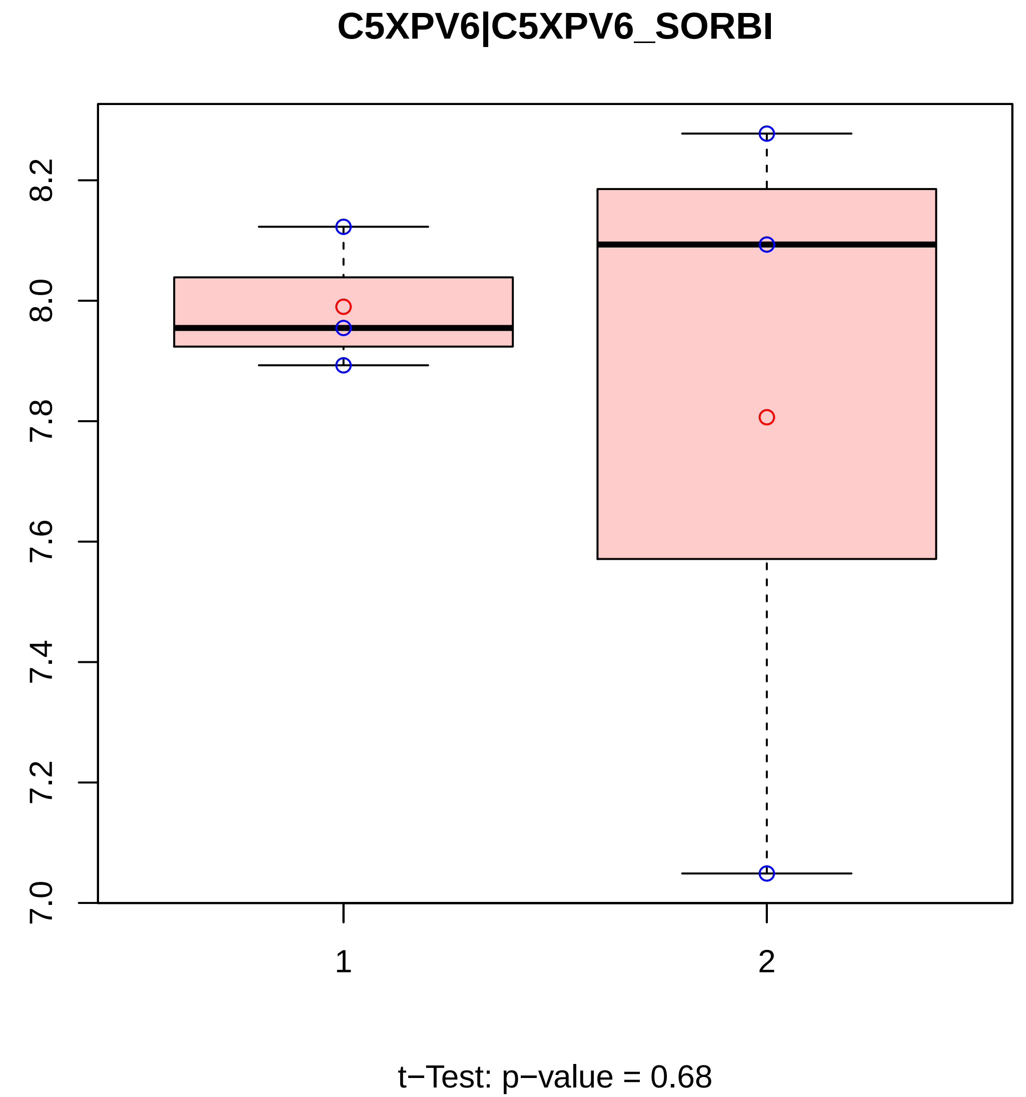

C5XPV6|C5XPV6_SORBI
7.0
7.2
7.4
7.6
7.8
8.0
8.2
1
2
t−Test: p−value = 0.68

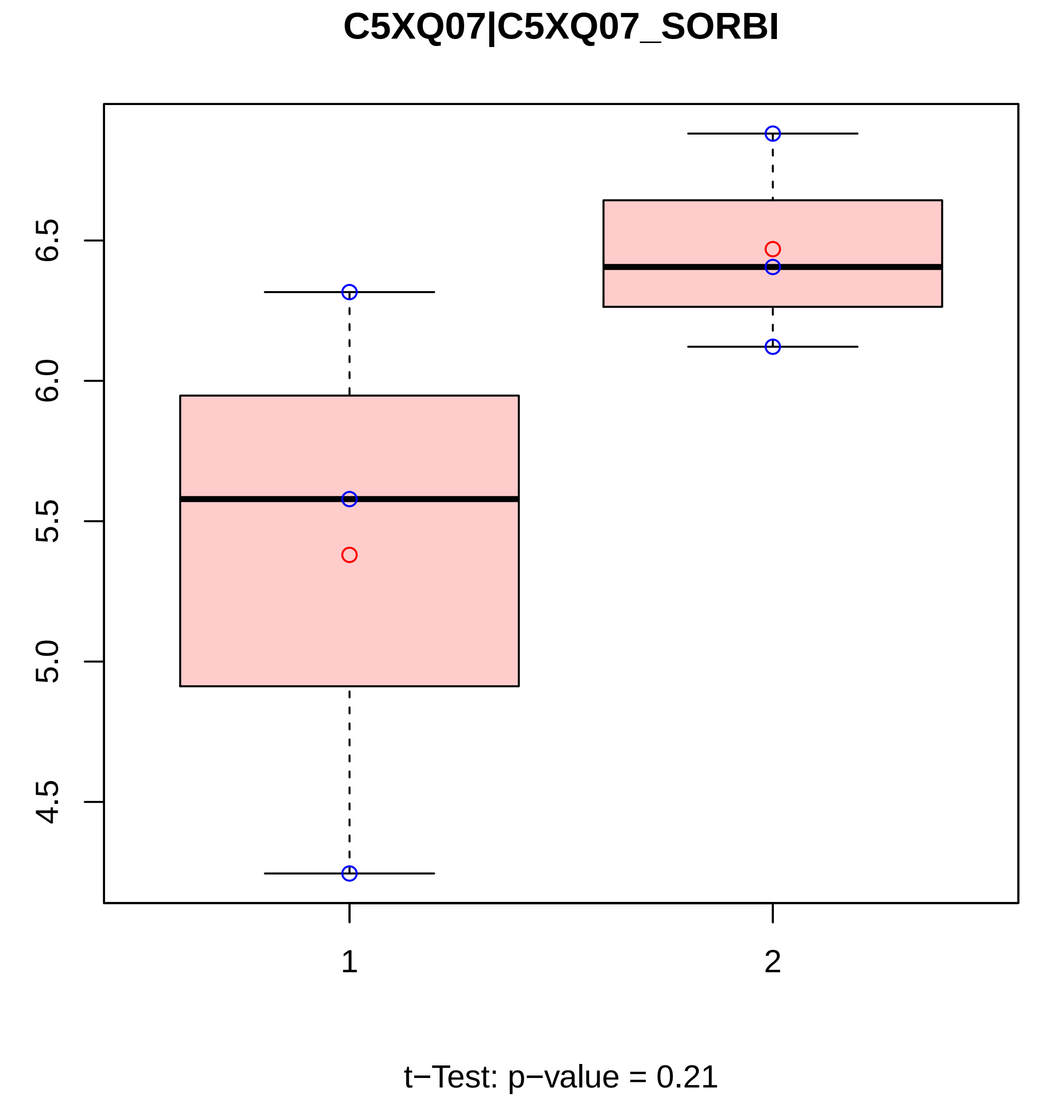
C5XQ07|C5XQ07_SORBI
6.5
6.0
5.5
5.0
4.5
1
2
t−Test: p−value = 0.21

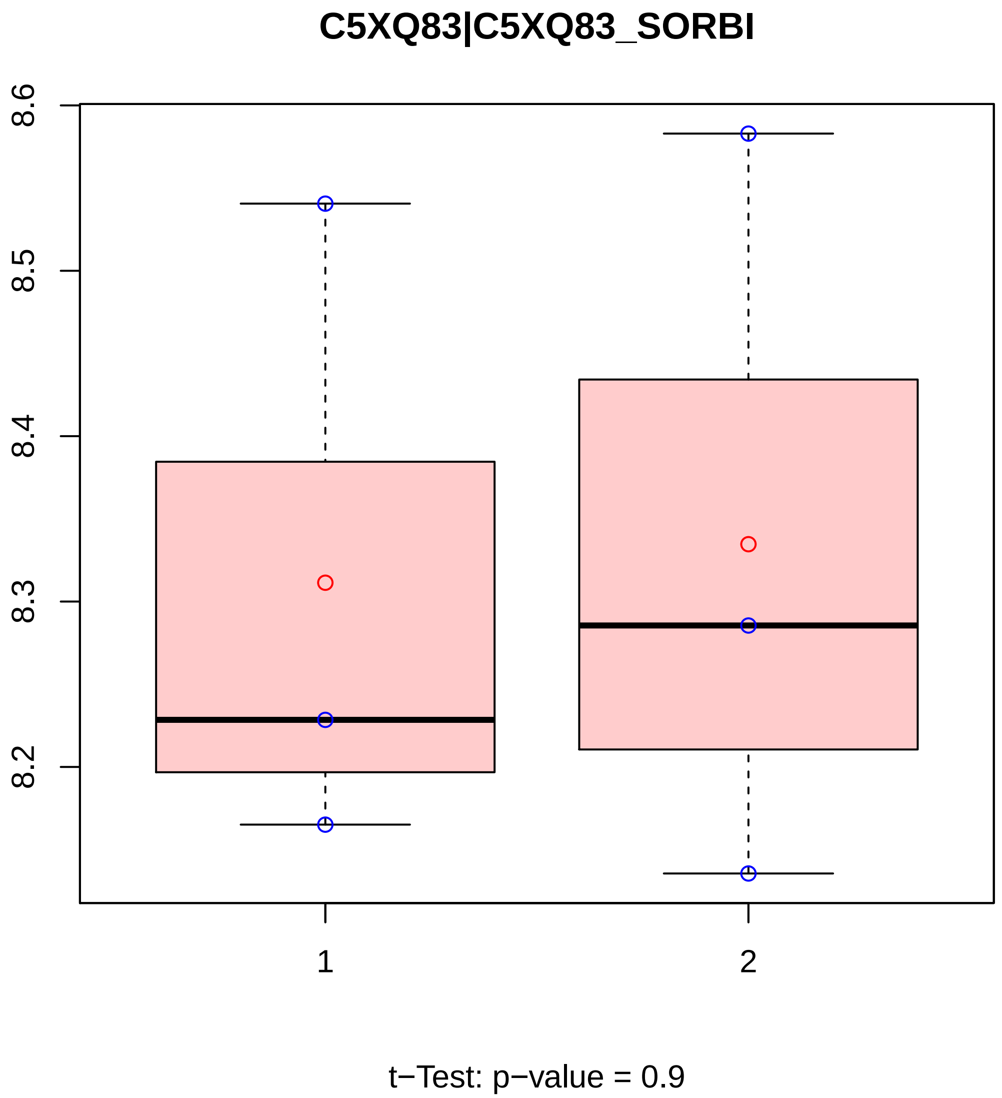
C5XQ83|C5XQ83_SORBI
8.6
8.5
8.4
8.3
8.2
1
2
t−Test: p−value = 0.9

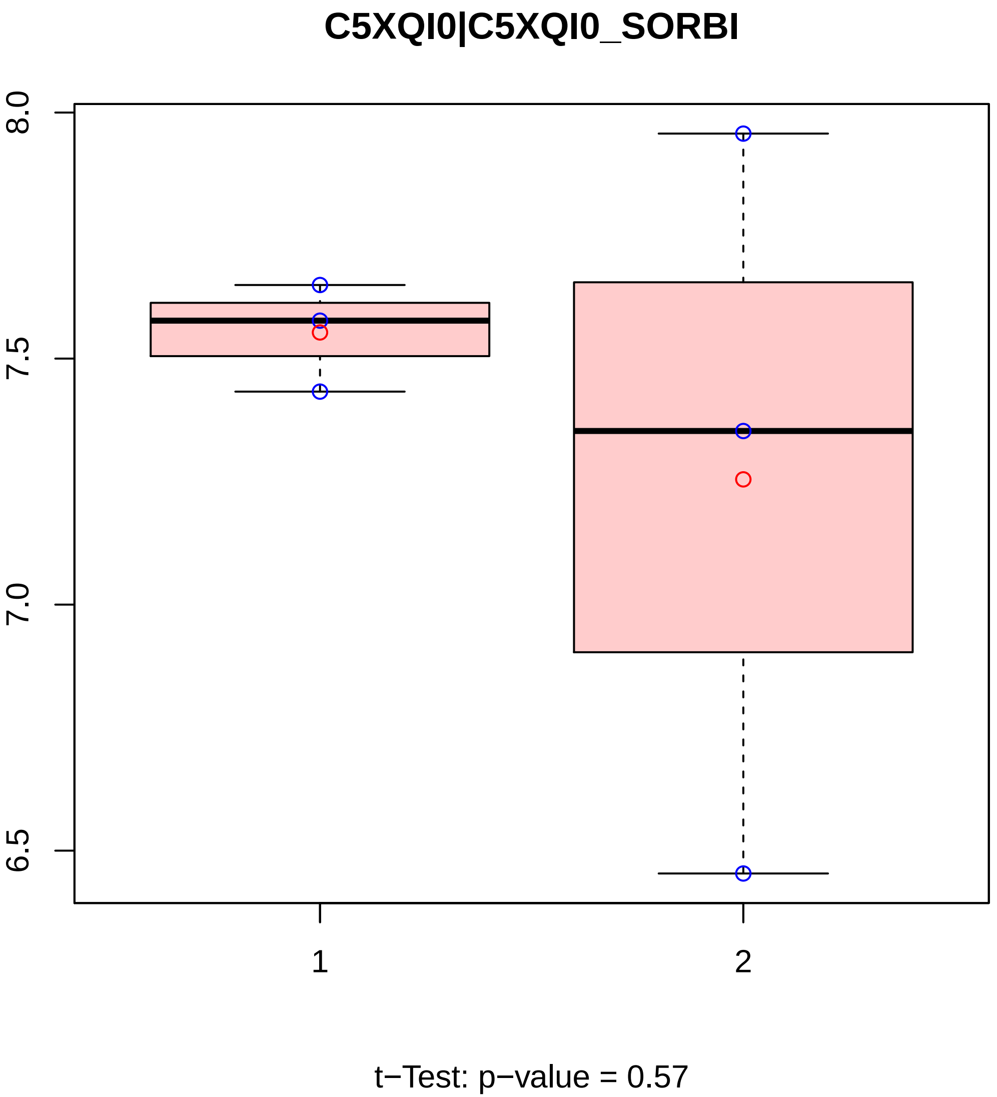
C5XQI0|C5XQI0_SORBI
8.0
7.5
7.0
6.5
1
2
t−Test: p−value = 0.57

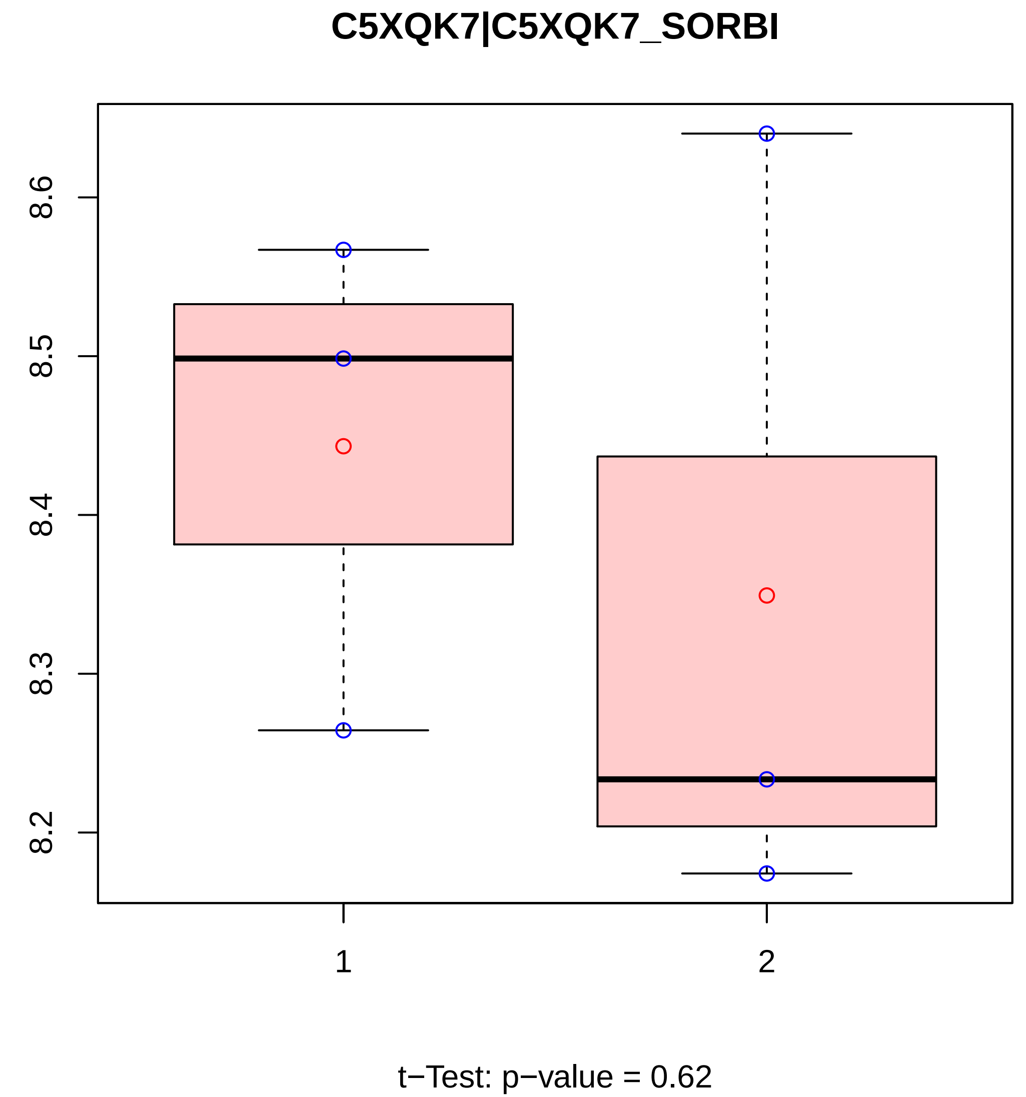
C5XQK7|C5XQK7_SORBI
8.2
8.3
8.4
8.5
8.6
1
2
t−Test: p−value = 0.62

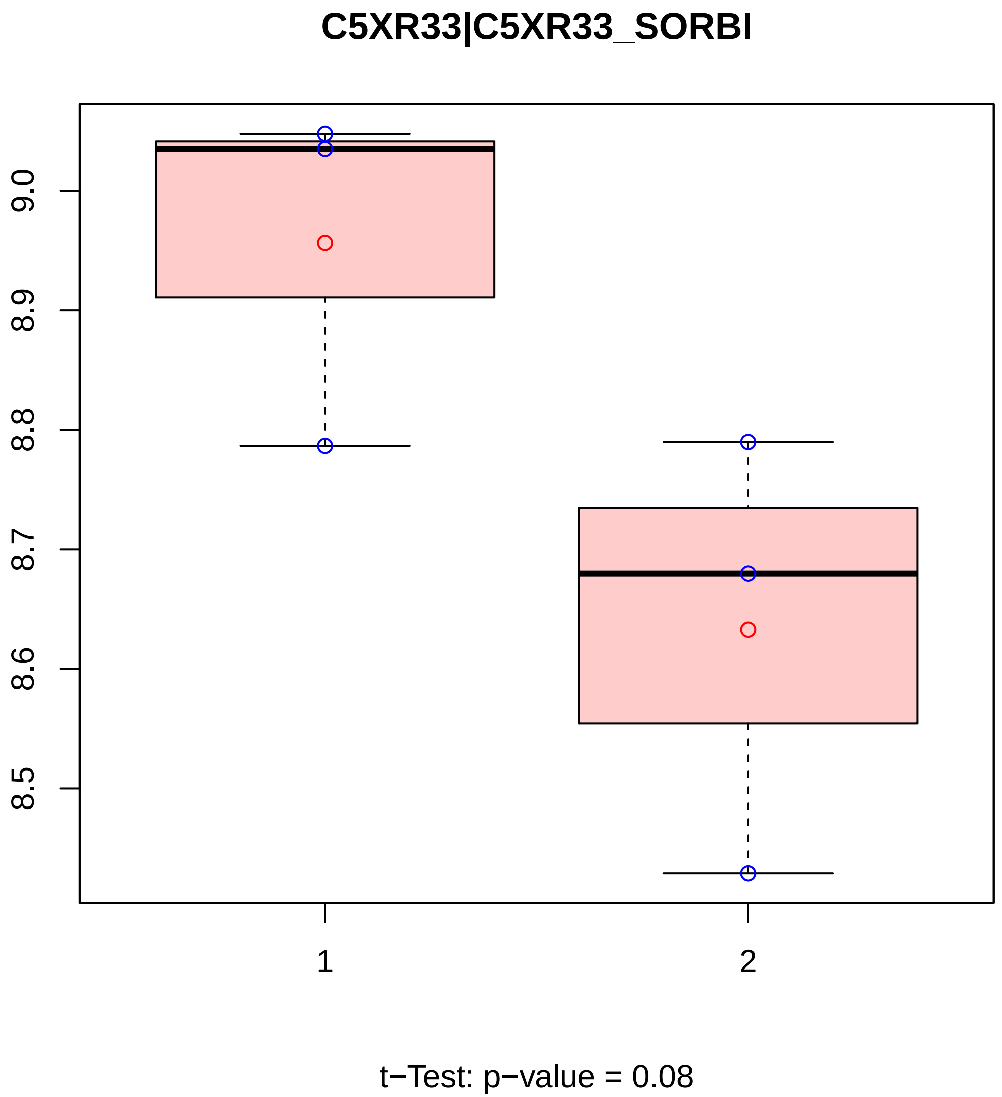
C5XR33|C5XR33_SORBI
9.0
8.9
8.8
8.7
8.6
8.5
1
2
t−Test: p−value = 0.08

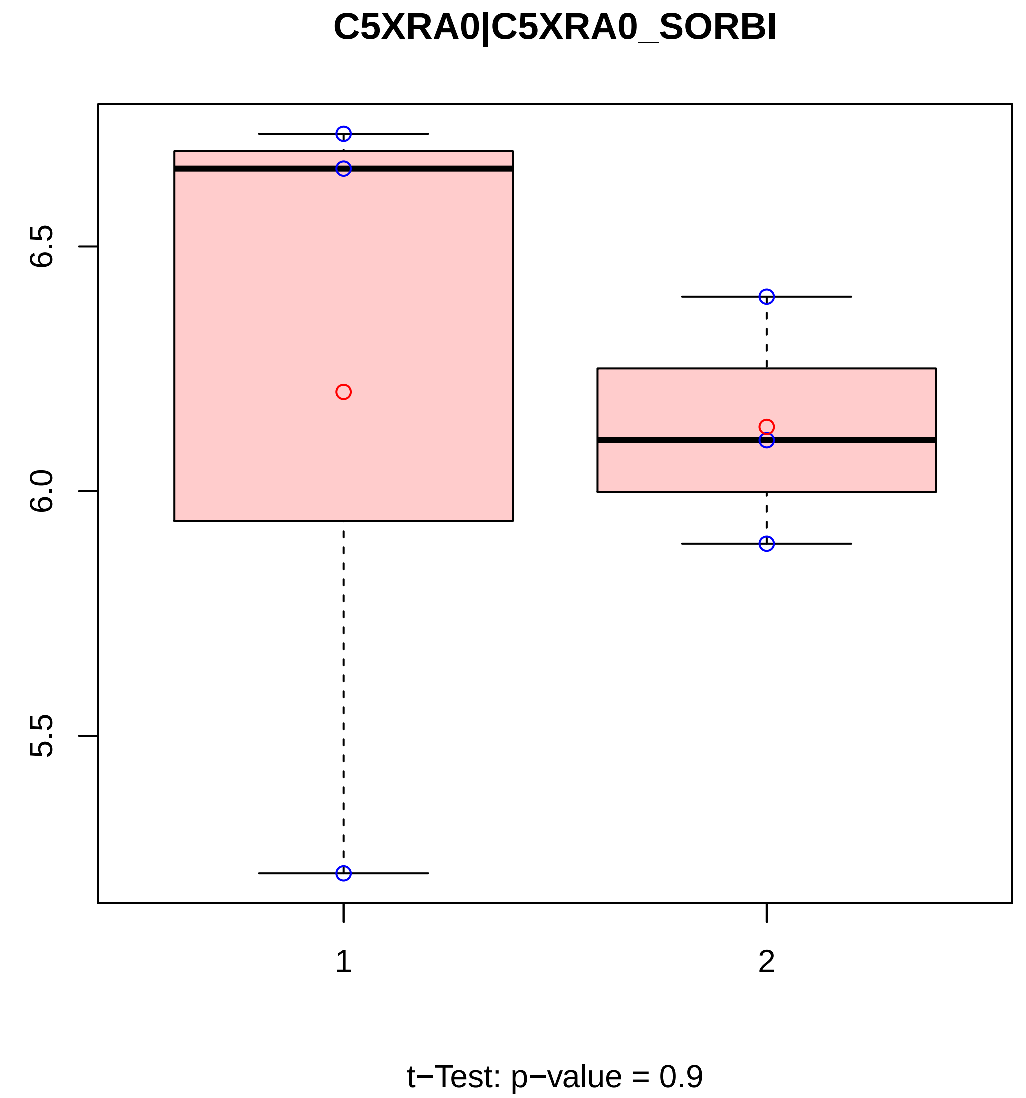

C5XRA0|C5XRA0_SORBI
6.5
6.0
5.5
1
2
t−Test: p−value = 0.9

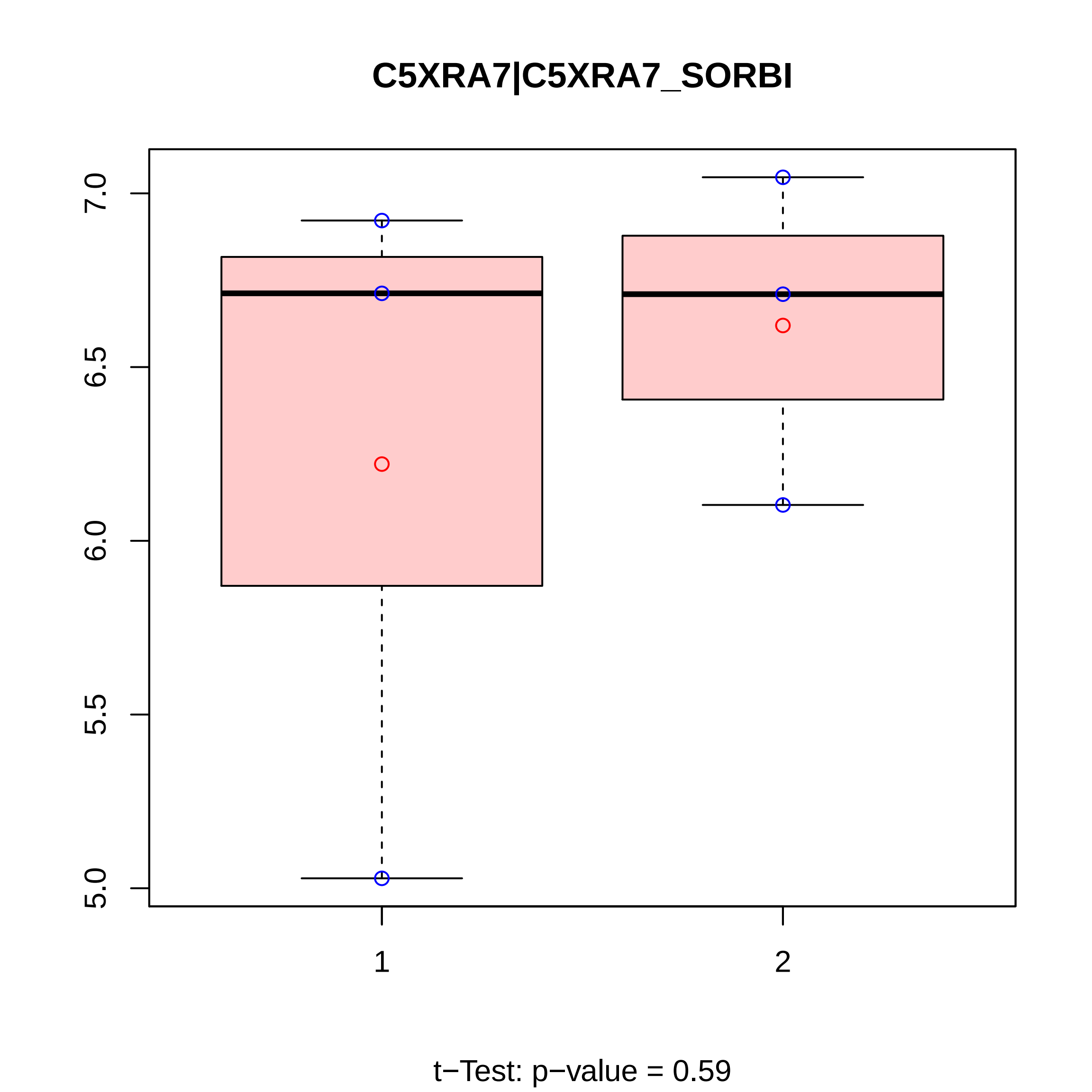

C5XRA7|C5XRA7_SORBI
7.0
6.5
6.0
5.5
5.0
1
2
t−Test: p−value = 0.59

C5XSE2|C5XSE2_SORBI

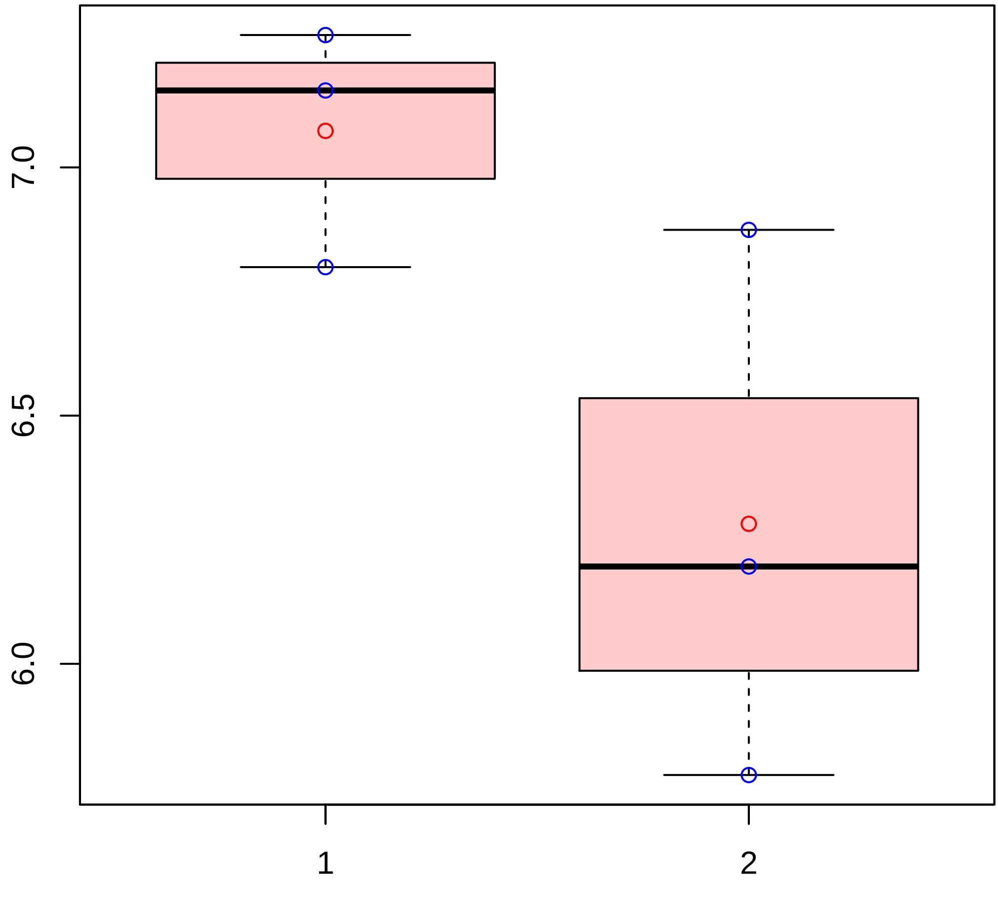

t−Test: p−value = 0.12

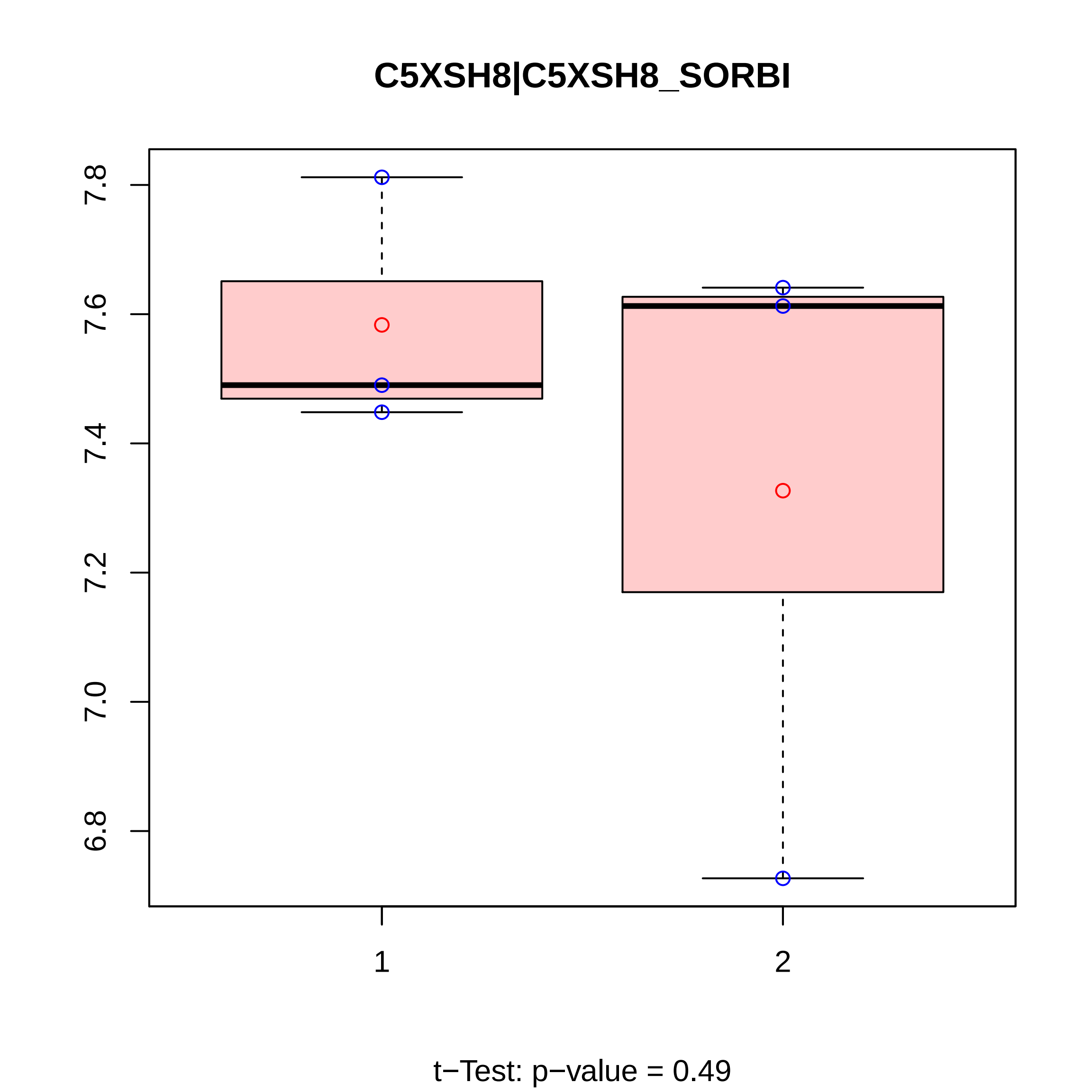

C5XSH8|C5XSH8_SORBI
7.8
7.6
7.4
7.2
7.0
6.8
1
2
t−Test: p−value = 0.49

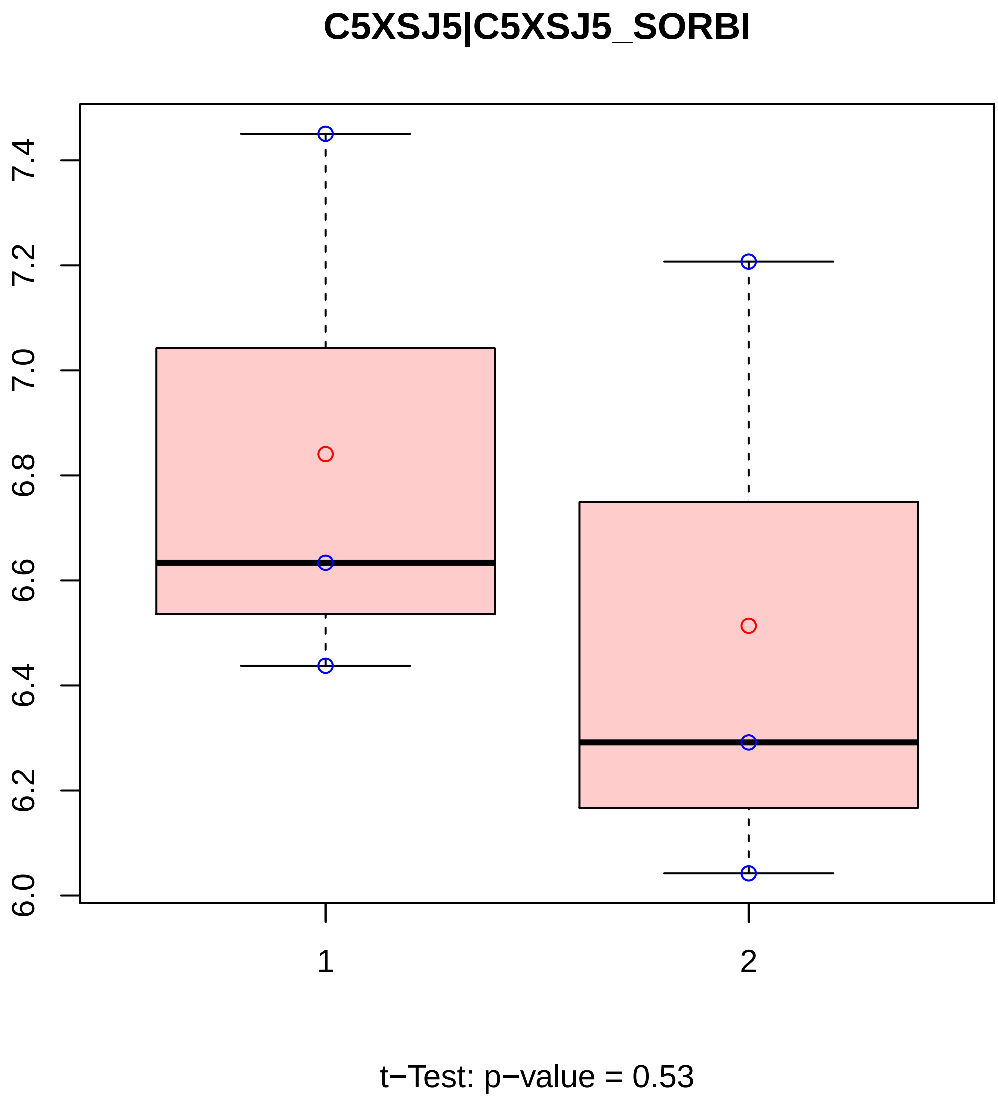

C5XSJ5|C5XSJ5_SORBI
6.0
6.2
6.4
6.6
6.8
7.0
7.2
7.4
1
2
t−Test: p−value = 0.53

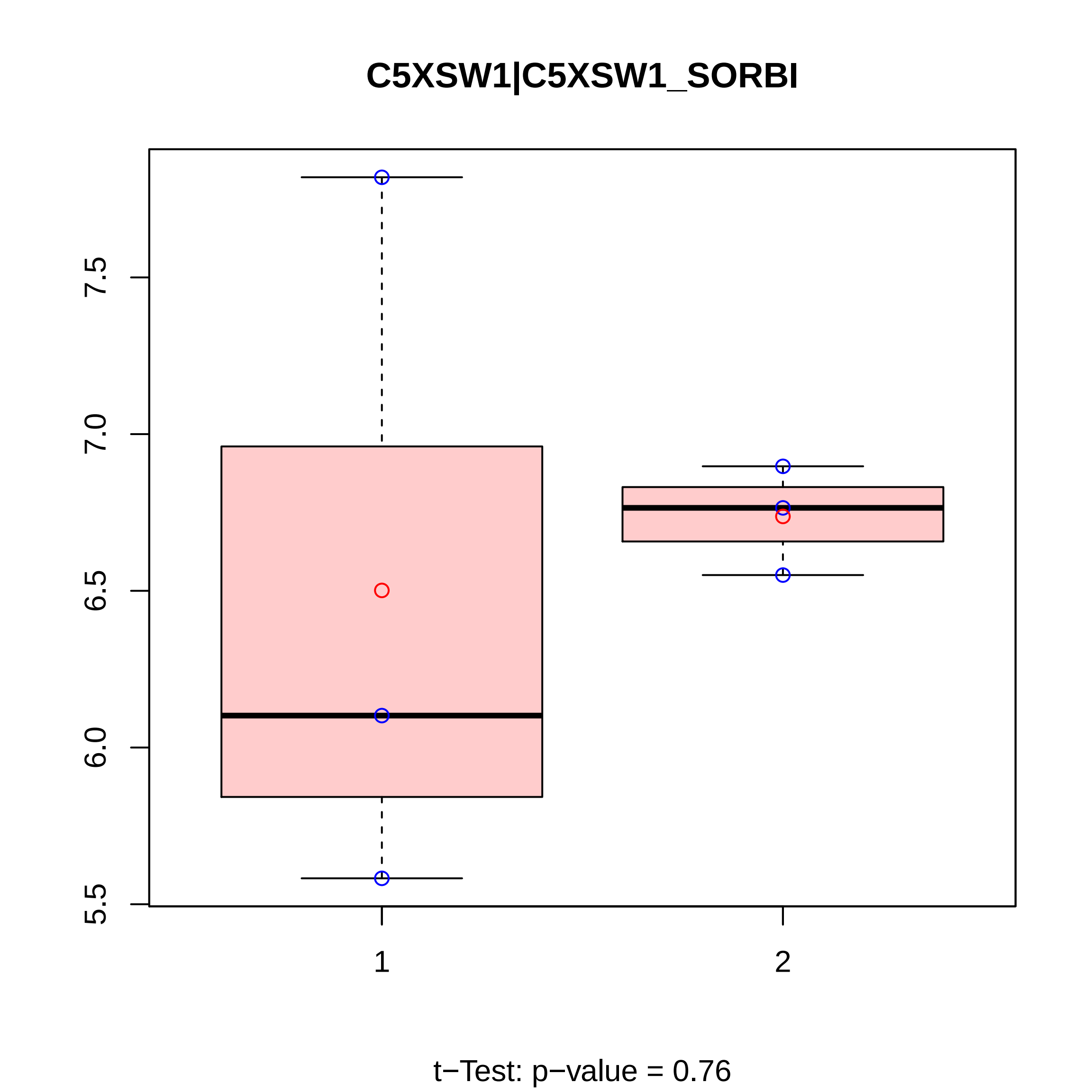
C5XSW1|C5XSW1_SORBI
7.5
7.0
6.5
6.0
5.5
1
2
t−Test: p−value = 0.76

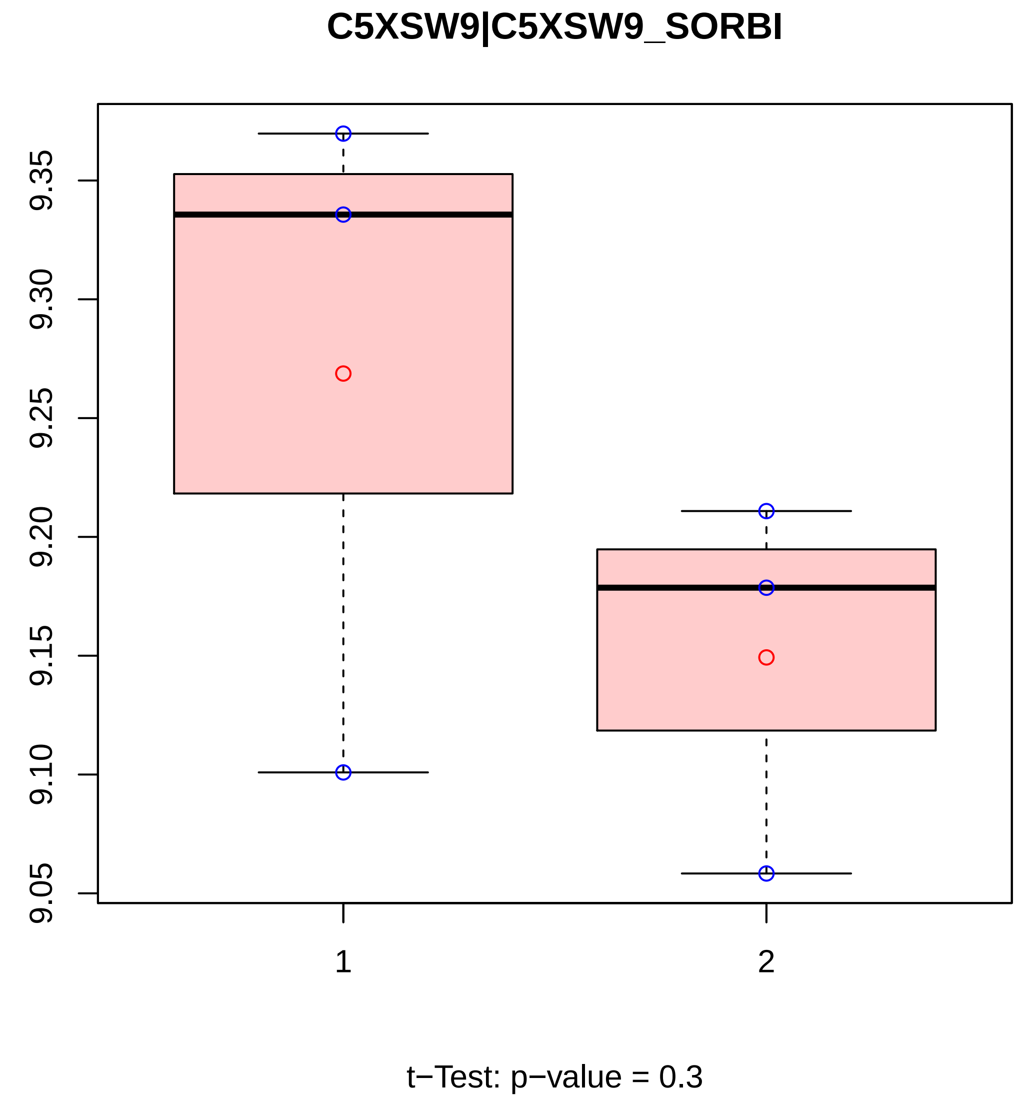
C5XSW9|C5XSW9_SORBI
9.05
9.10
9.15
9.20
9.25
9.30
9.35
1
2
t−Test: p−value = 0.3

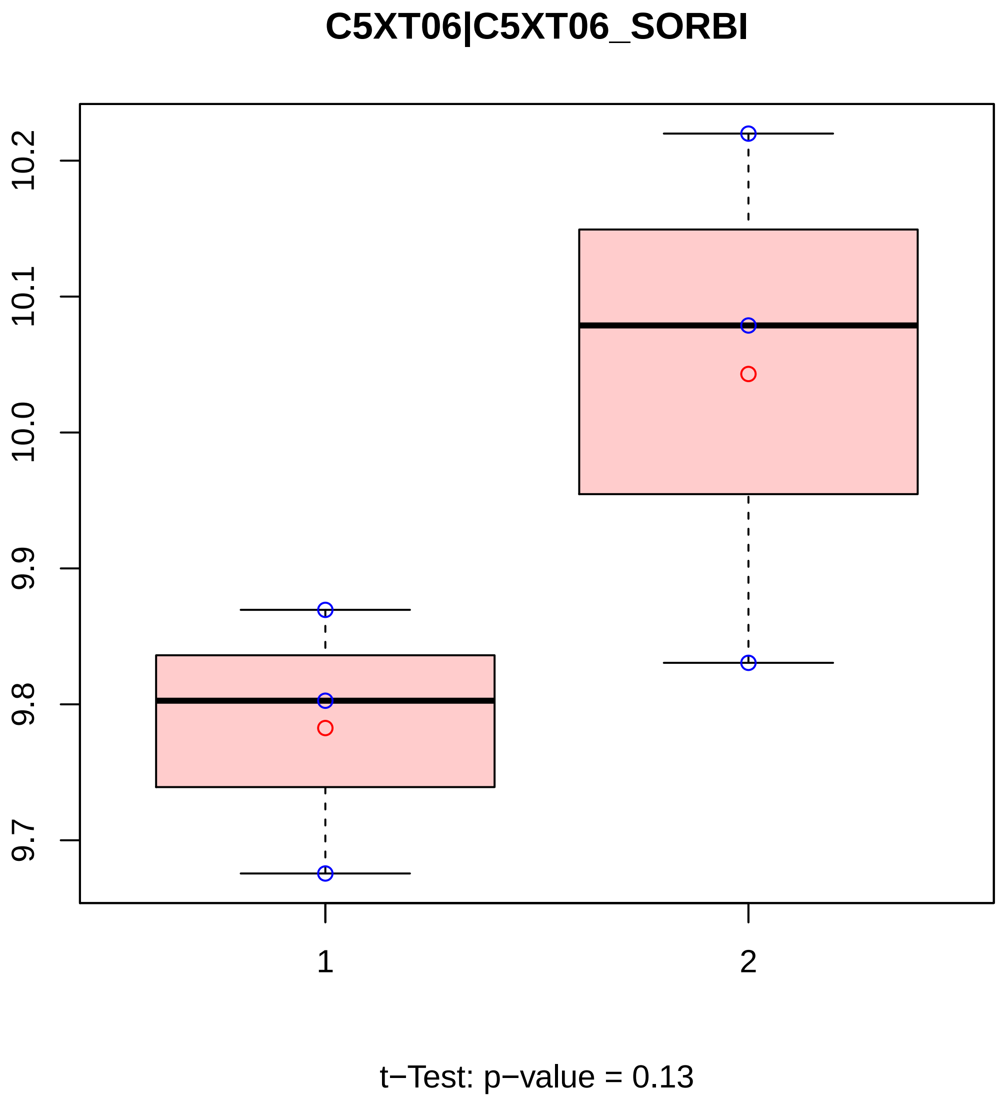
C5XT06|C5XT06_SORBI
10.2
10.1
10.0
9.9
9.8
9.7
1
2
t−Test: p−value = 0.13

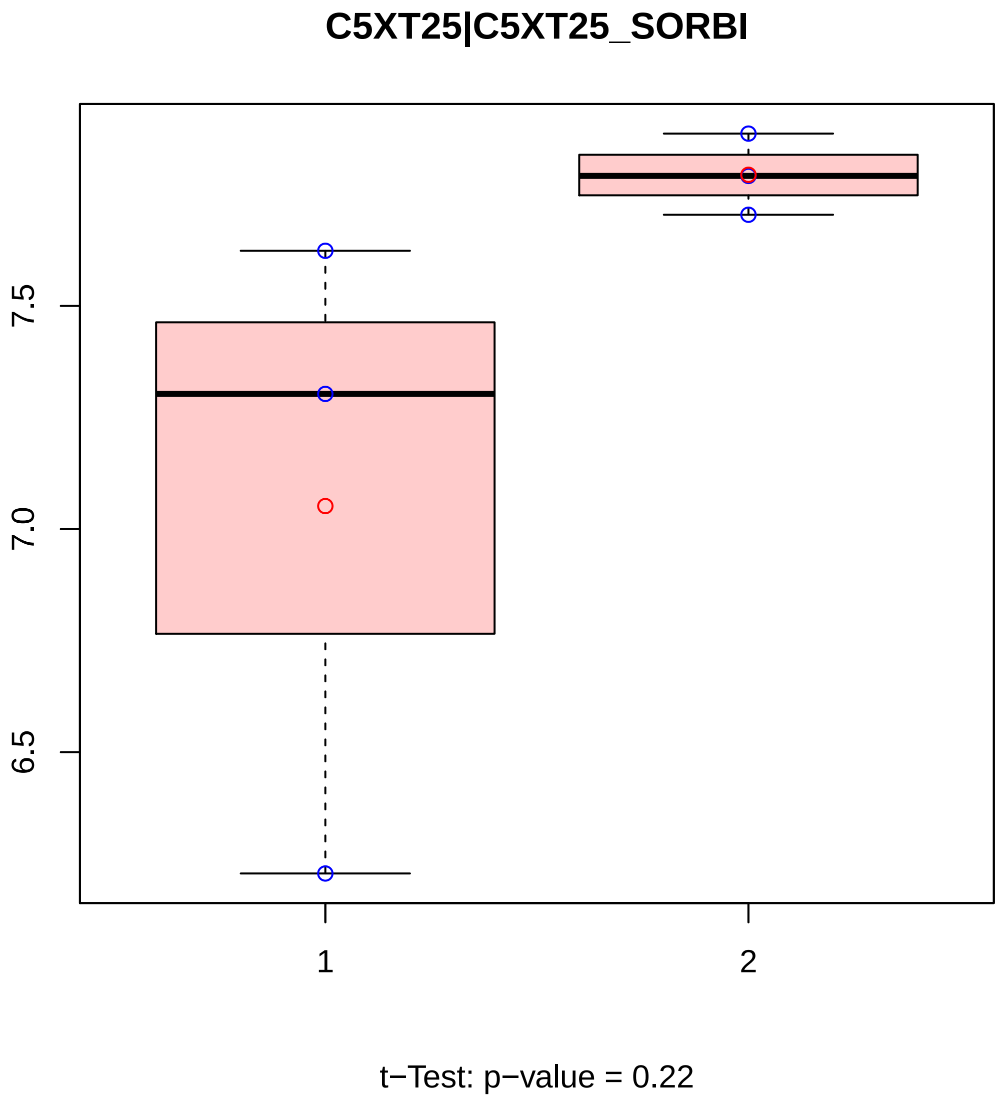
C5XT25|C5XT25_SORBI
7.5
7.0
6.5
1
2
t−Test: p−value = 0.22

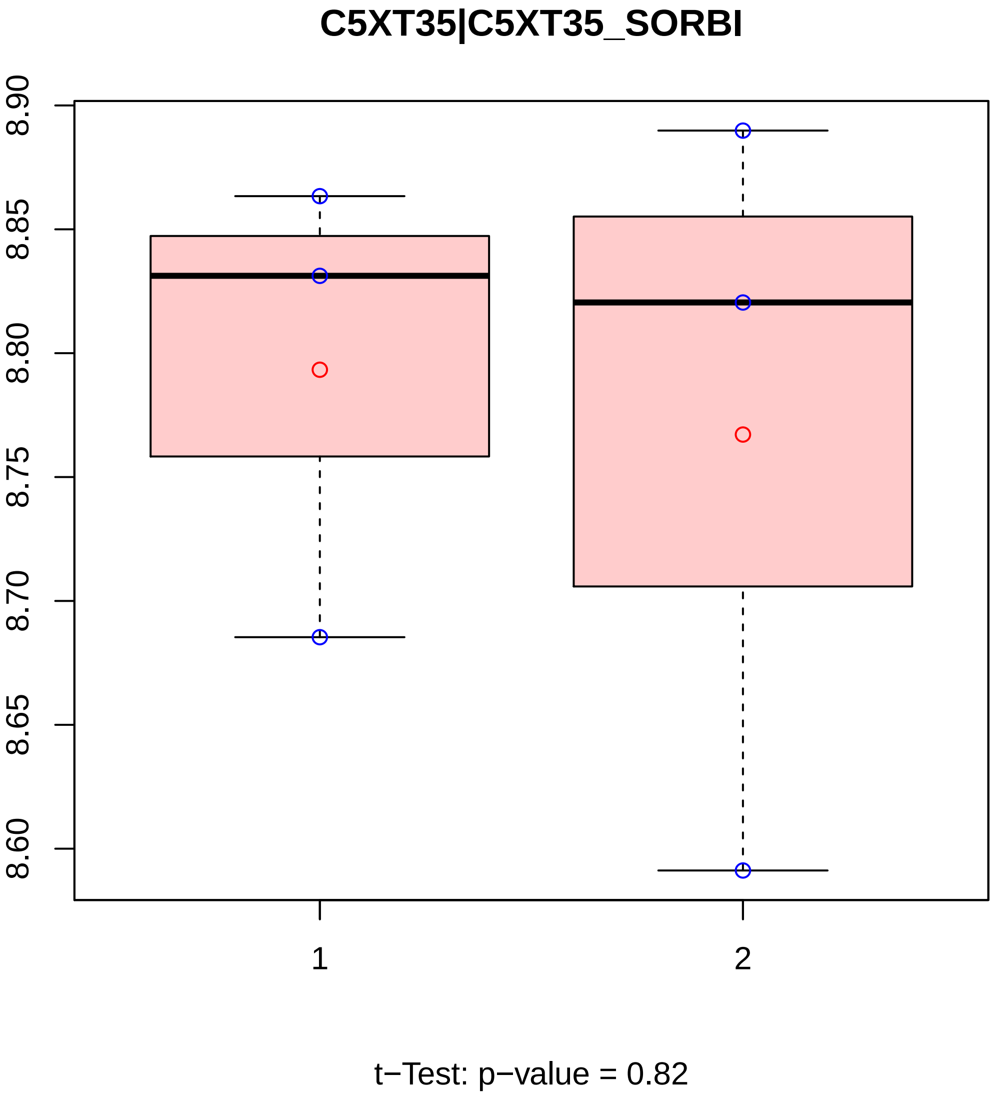

C5XT35|C5XT35_SORBI
8.60
8.65
8.70
8.75
8.80
8.85
8.90
1
2
t−Test: p−value = 0.82

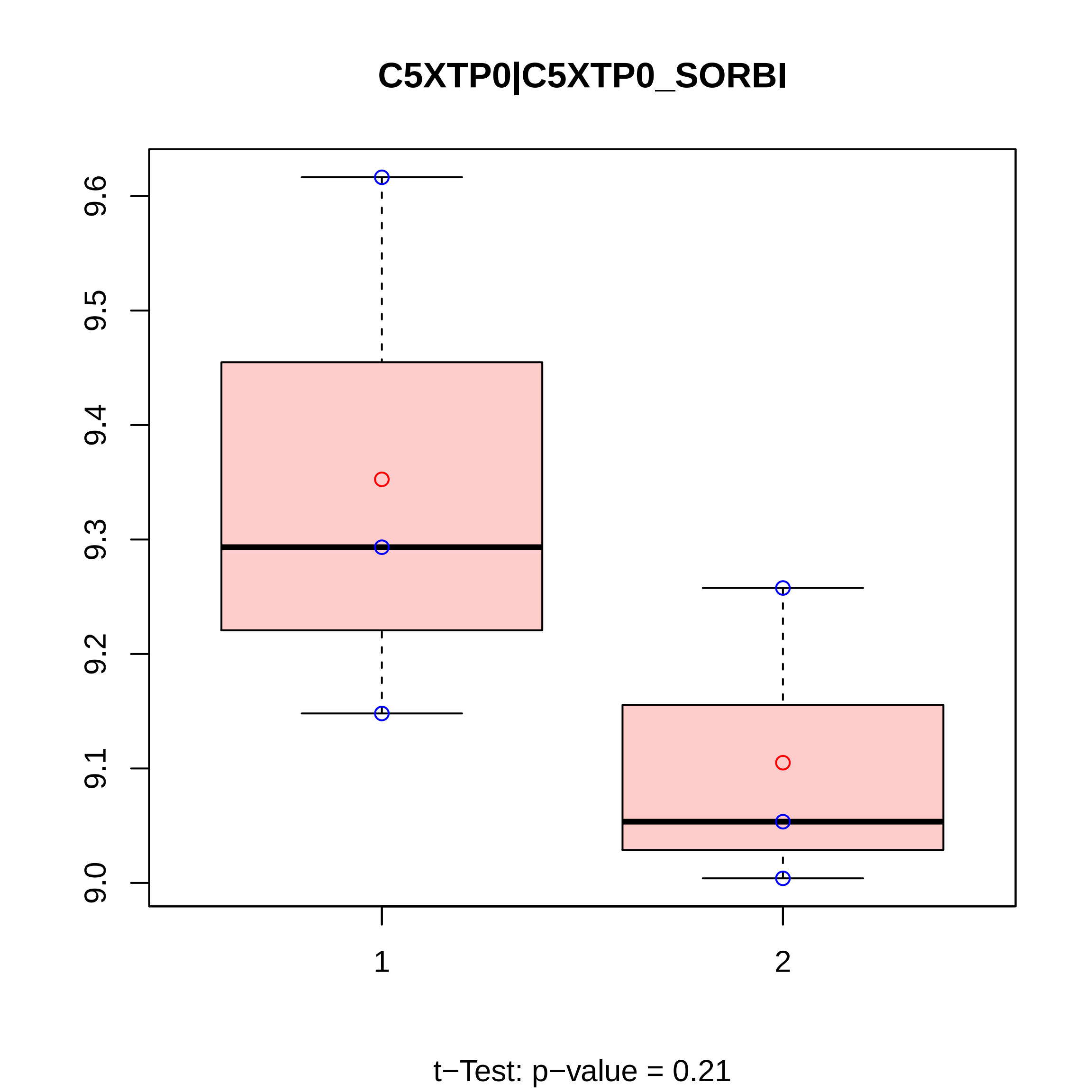

C5XTP0|C5XTP0_SORBI
9.0
9.1
9.2
9.3
9.4
9.5
9.6
1
2
t−Test: p−value = 0.21

C5XTY9|C5XTY9_SORBI

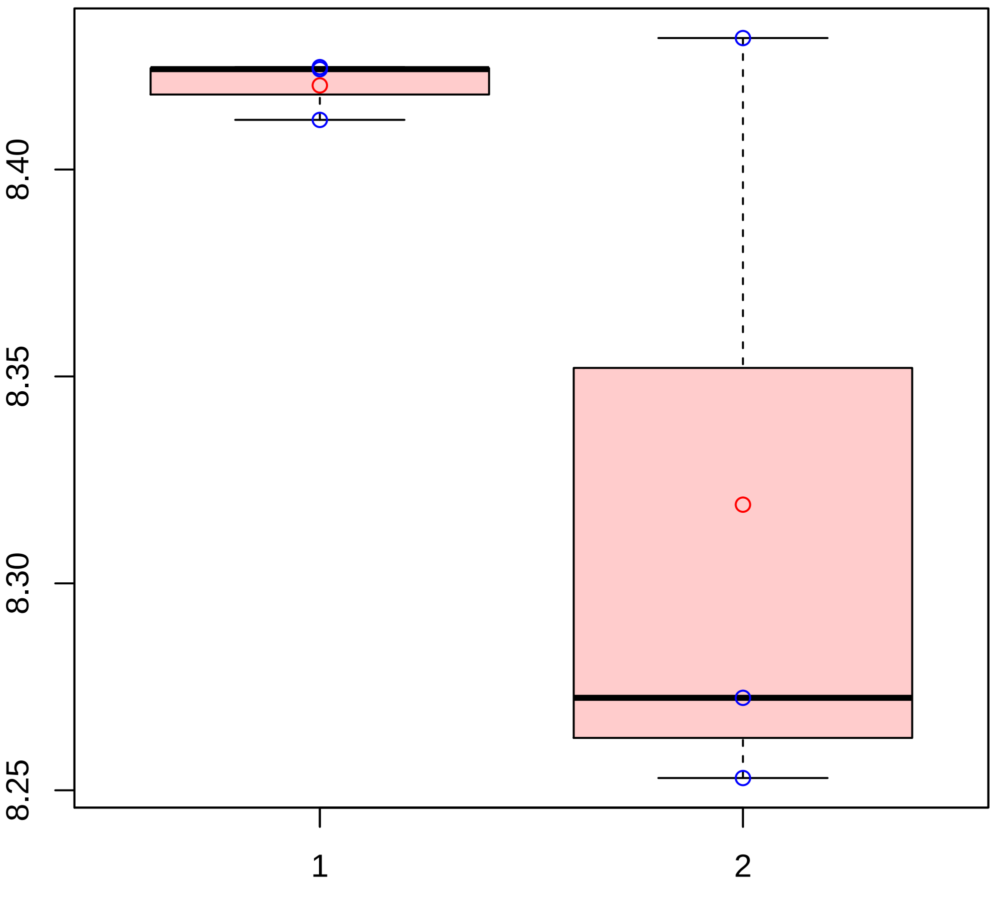

t−Test: p−value = 0.22

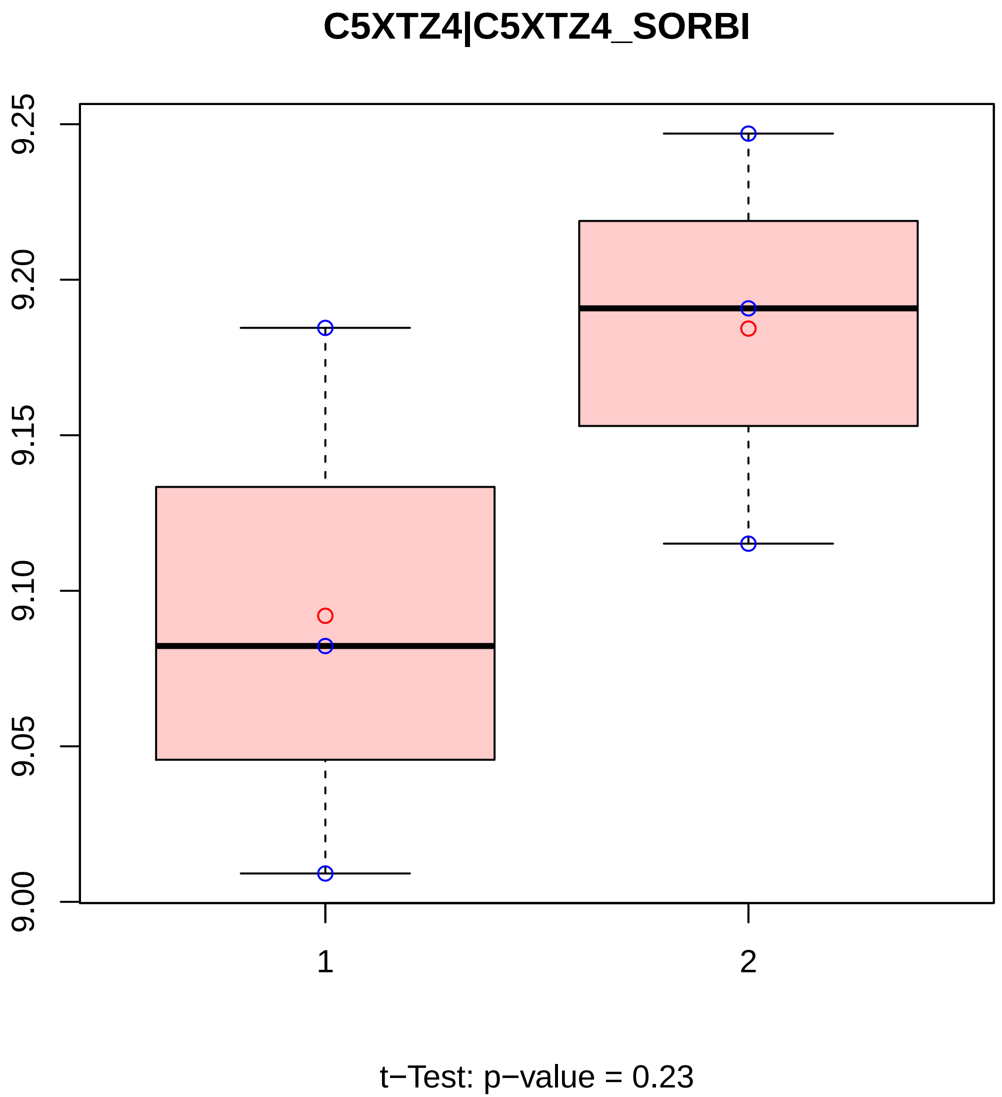
C5XTZ4|C5XTZ4_SORBI
9.00
9.05
9.10
9.15
9.20
9.25
1
2
t−Test: p−value = 0.23

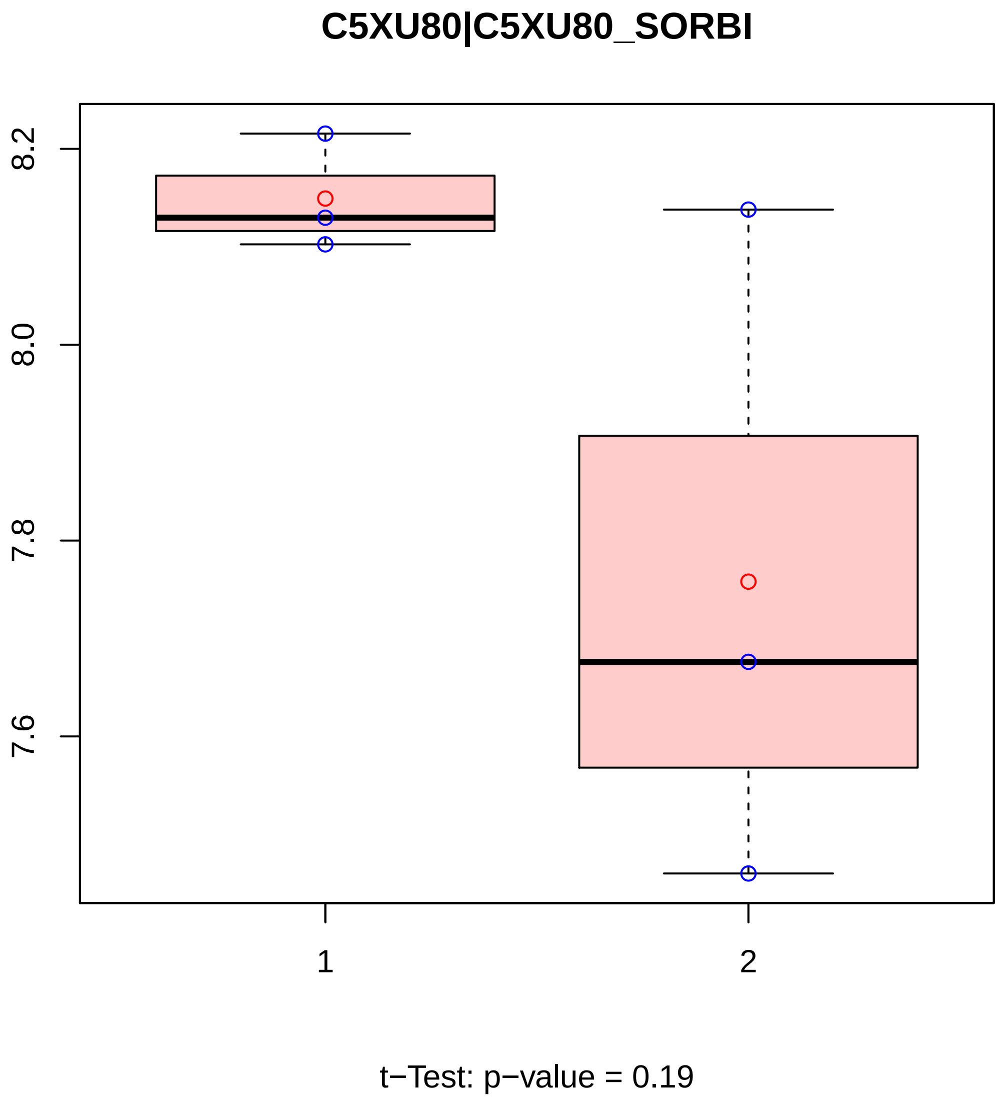

C5XU80|C5XU80_SORBI
8.2
8.0
7.8
7.6
1
2
t−Test: p−value = 0.19

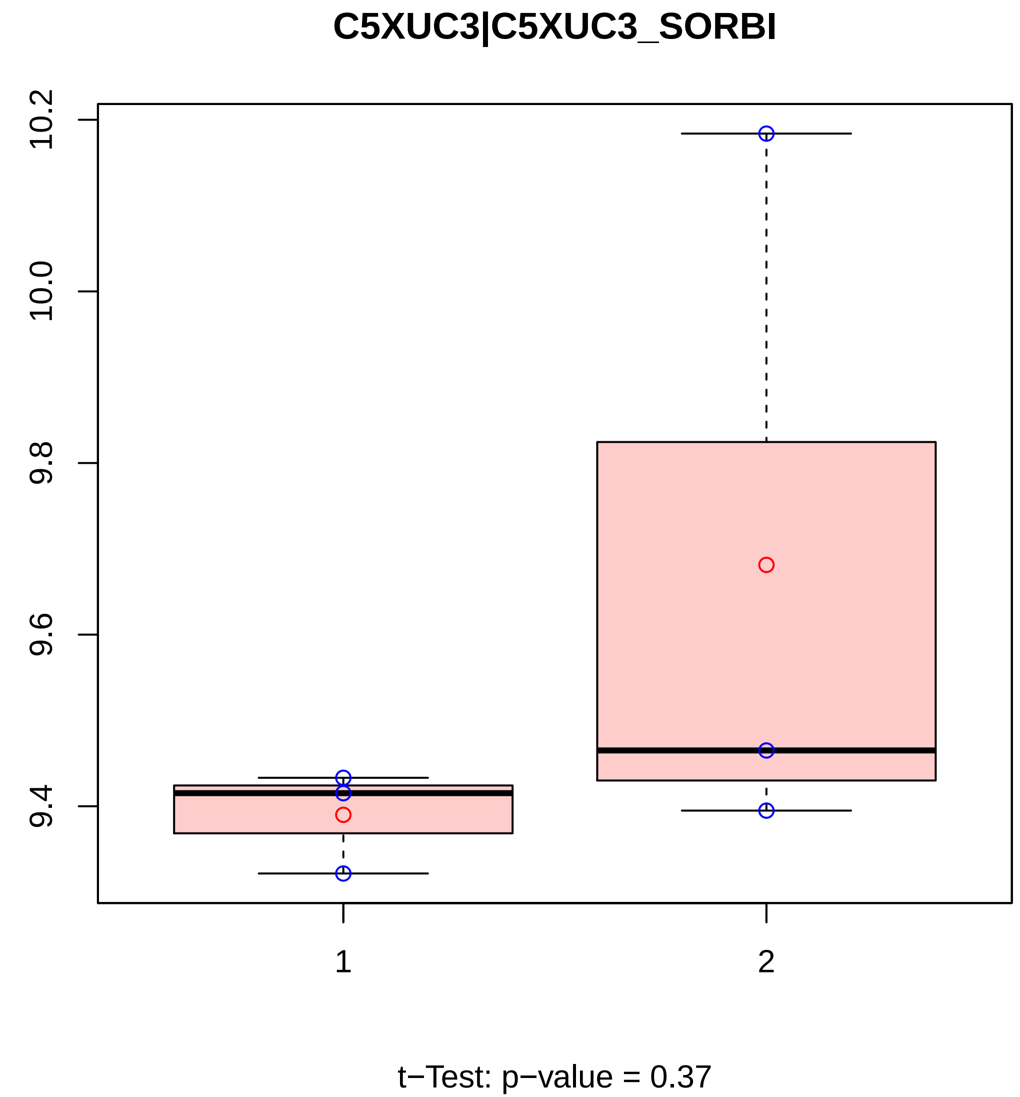

C5XUC3|C5XUC3_SORBI
10.2
10.0
9.8
9.6
9.4
1
2
t−Test: p−value = 0.37

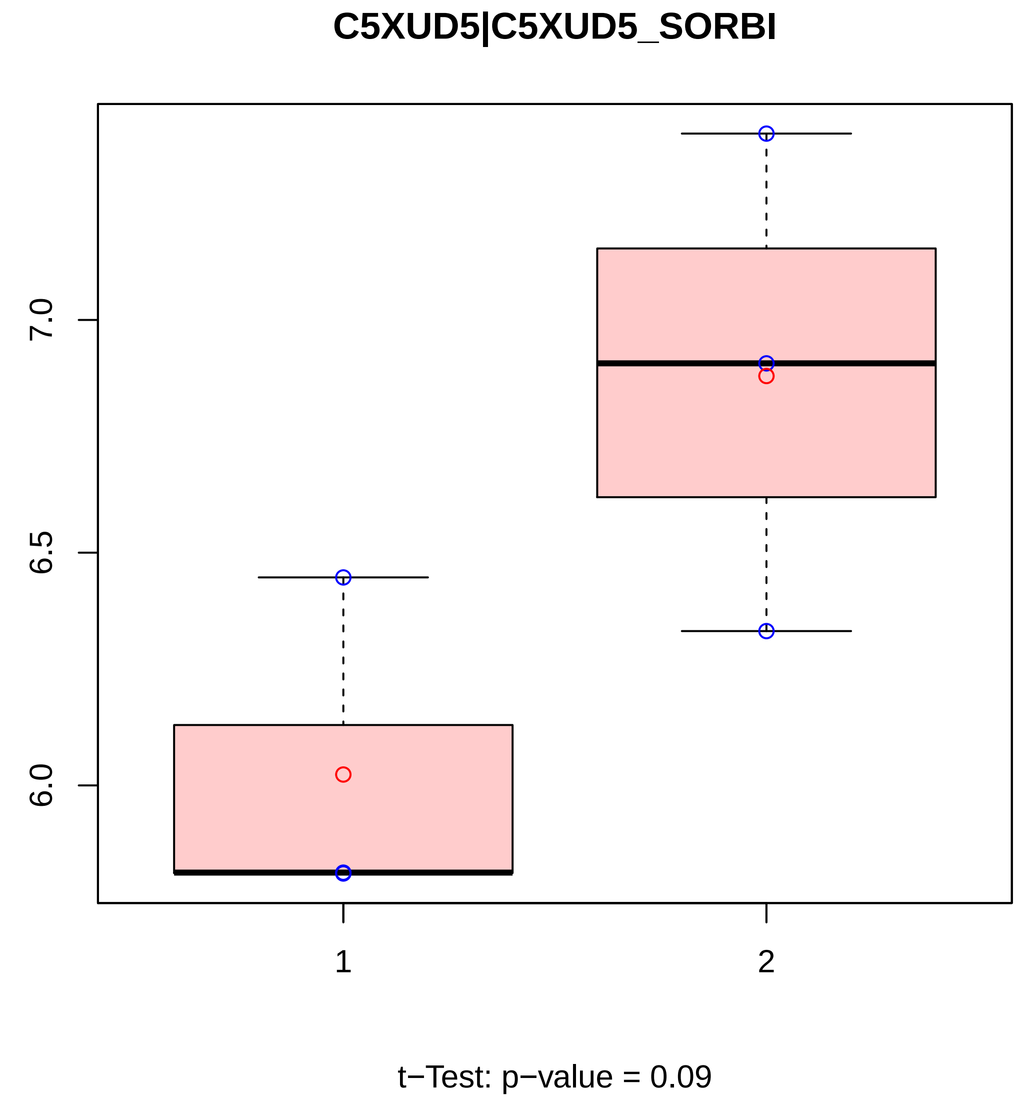

C5XUD5|C5XUD5_SORBI
7.0
6.5
6.0
1
2
t−Test: p−value = 0.09

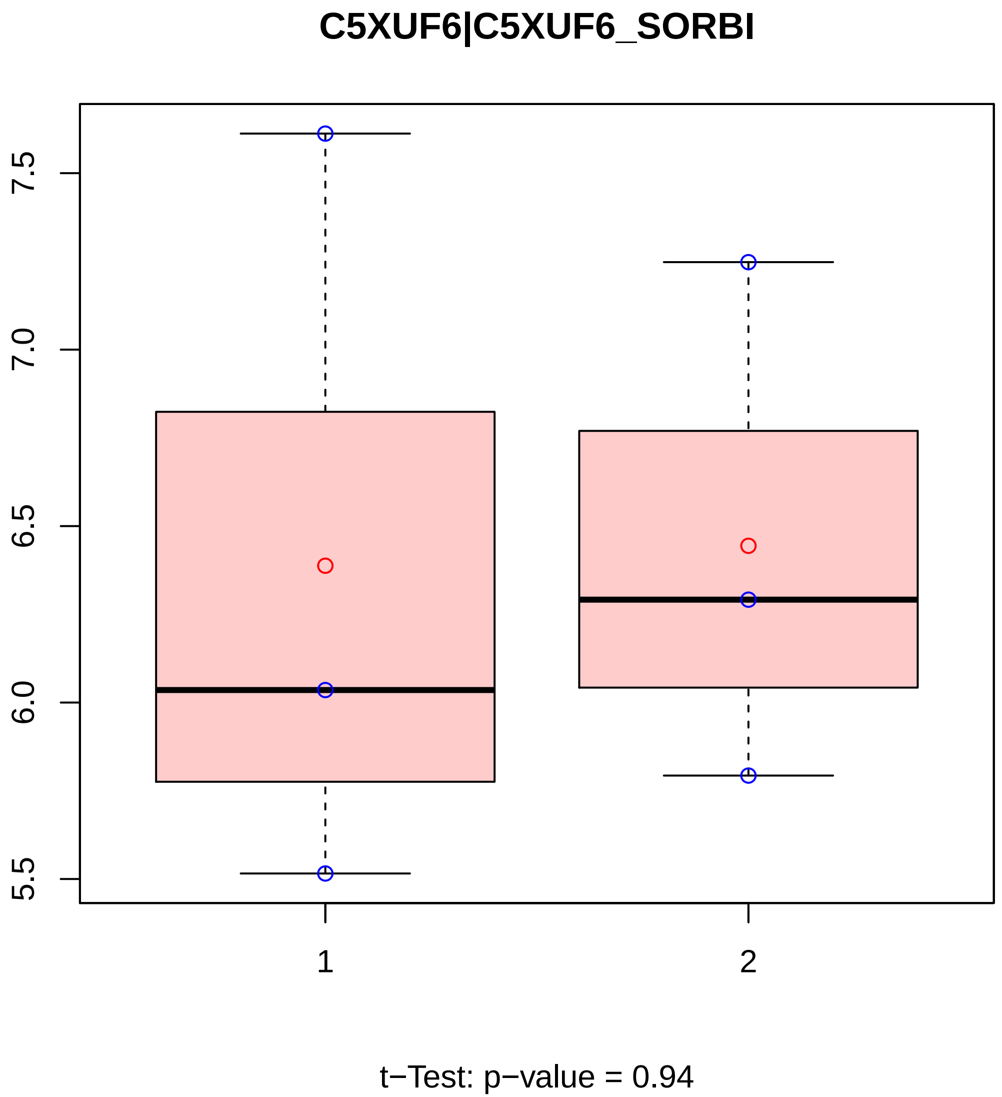

C5XUF6|C5XUF6_SORBI
7.5
7.0
6.5
6.0
5.5
1
2
t−Test: p−value = 0.94

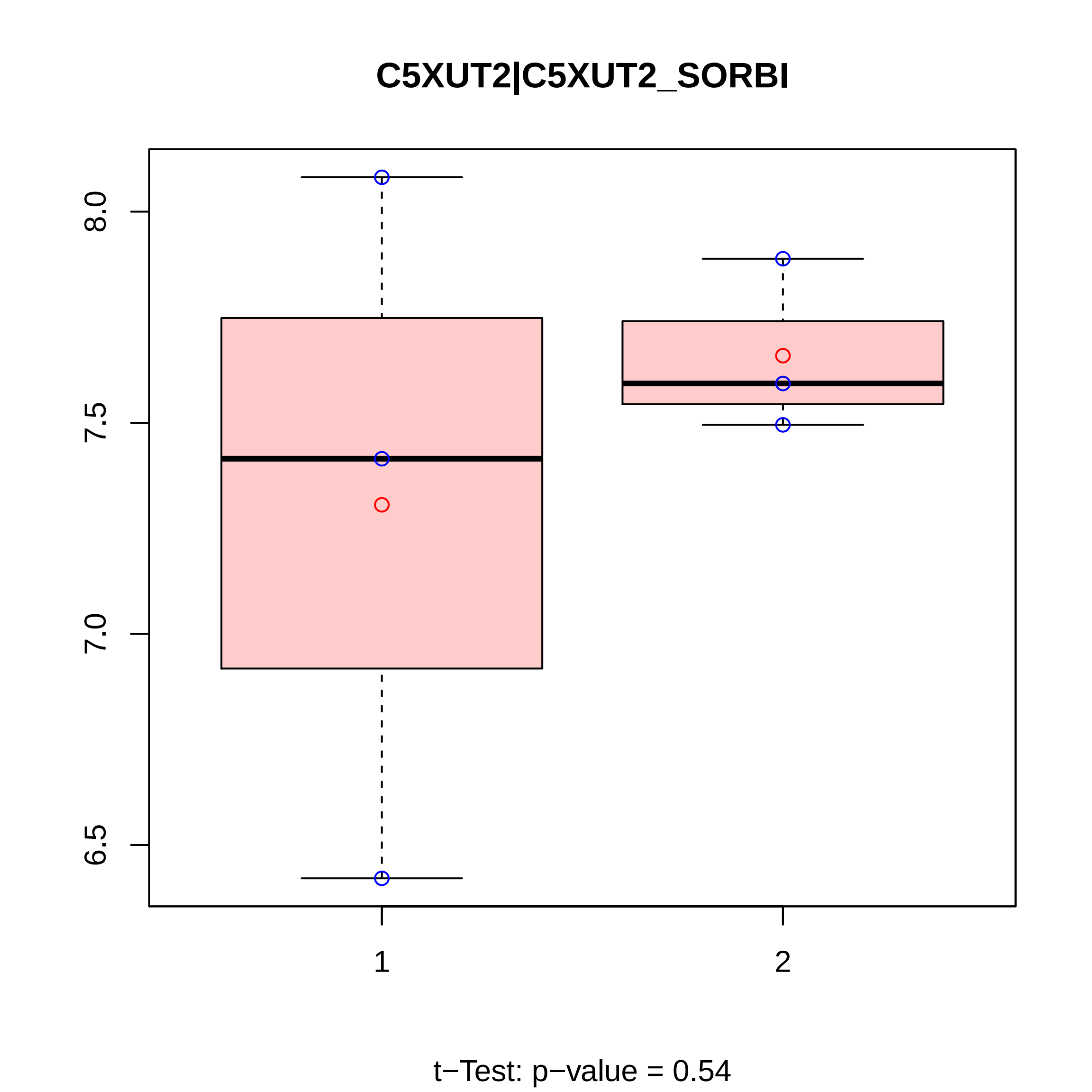
C5XUT2|C5XUT2_SORBI
8.0
7.5
7.0
6.5
1
2
t−Test: p−value = 0.54

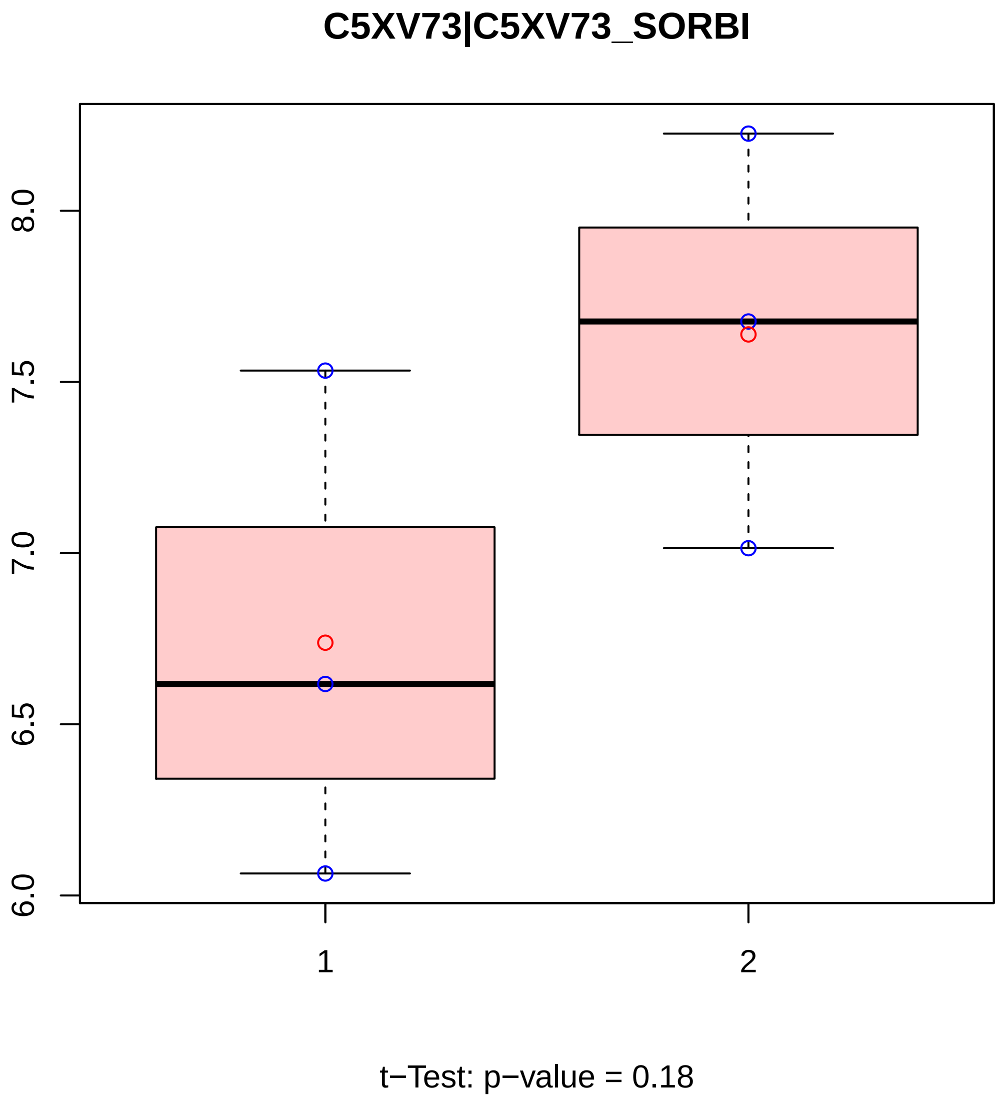
C5XV73|C5XV73_SORBI
6.0
6.5
7.0
7.5
8.0
1
2
t−Test: p−value = 0.18

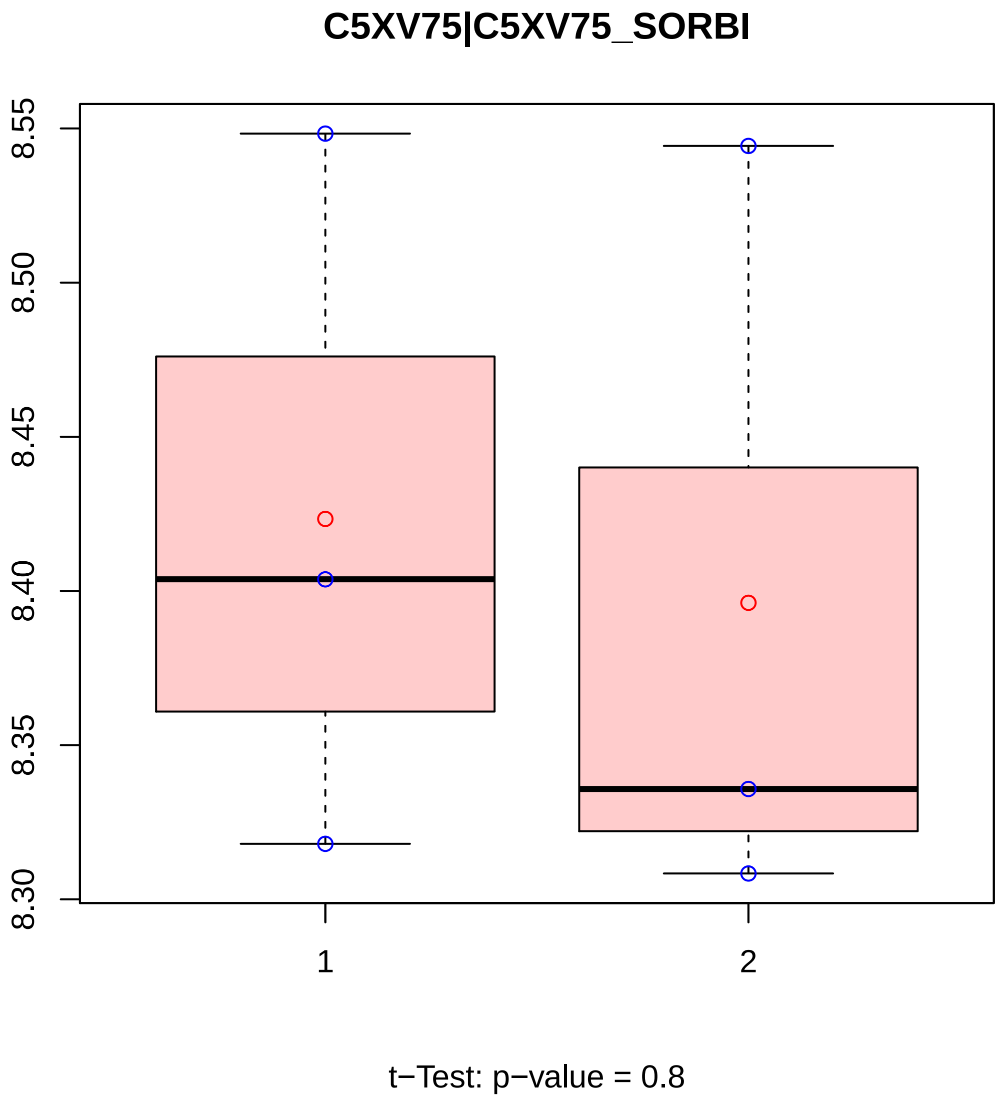

C5XV75|C5XV75_SORBI
8.55
8.50
8.45
8.40
8.35
8.30
1
2
t−Test: p−value = 0.8

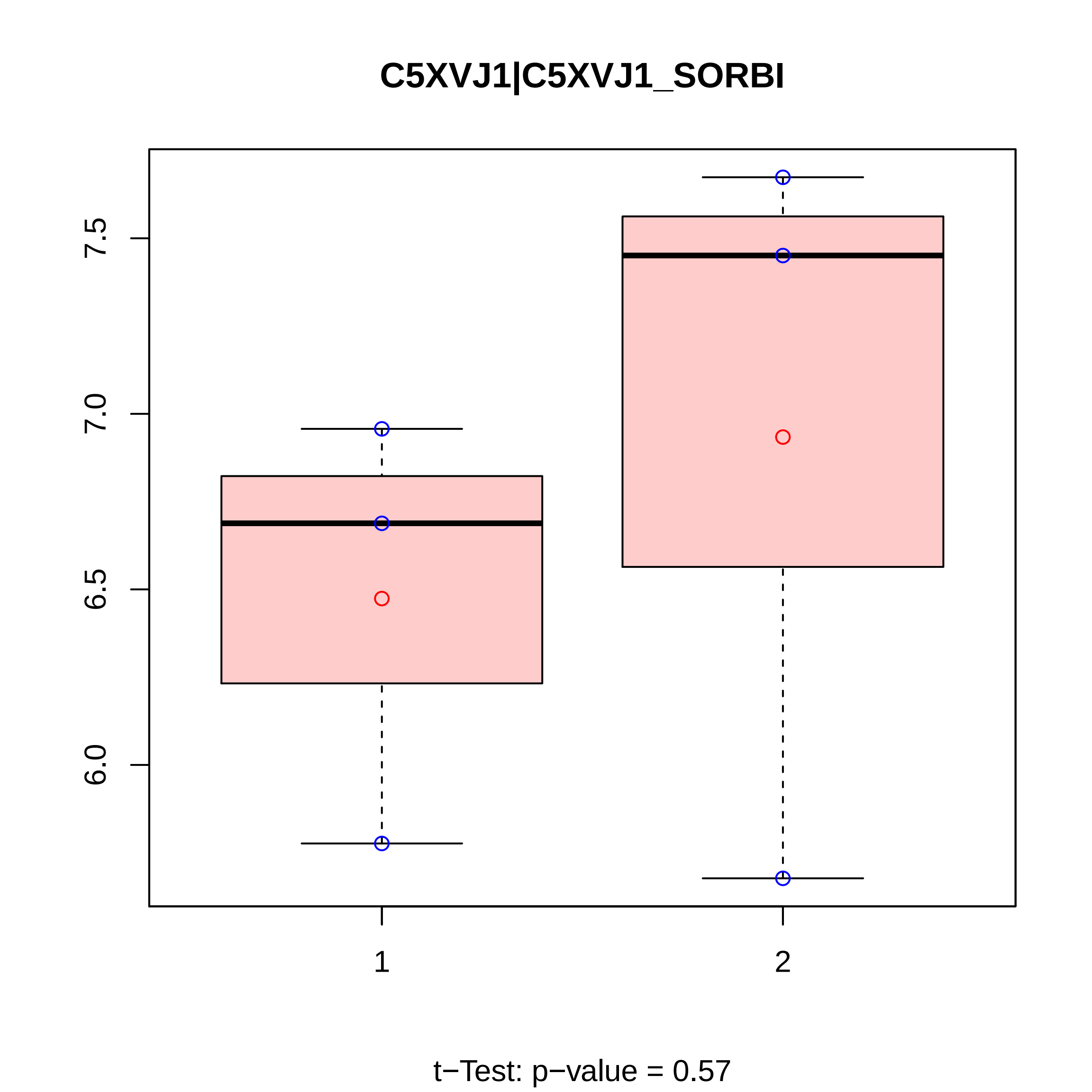

C5XVJ1|C5XVJ1_SORBI
7.5
7.0
6.5
6.0
1
2
t−Test: p−value = 0.57

C5XVY5|C5XVY5_SORBI

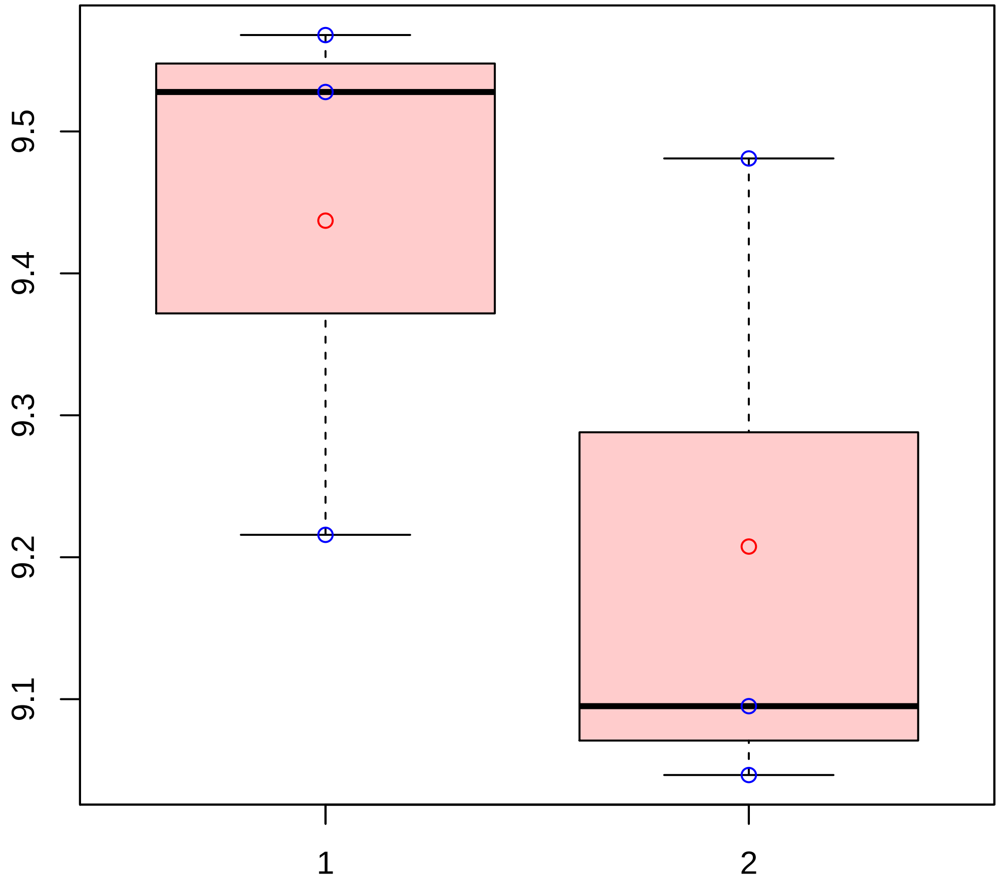

t−Test: p−value = 0.27

C5XW31|C5XW31_SORBI

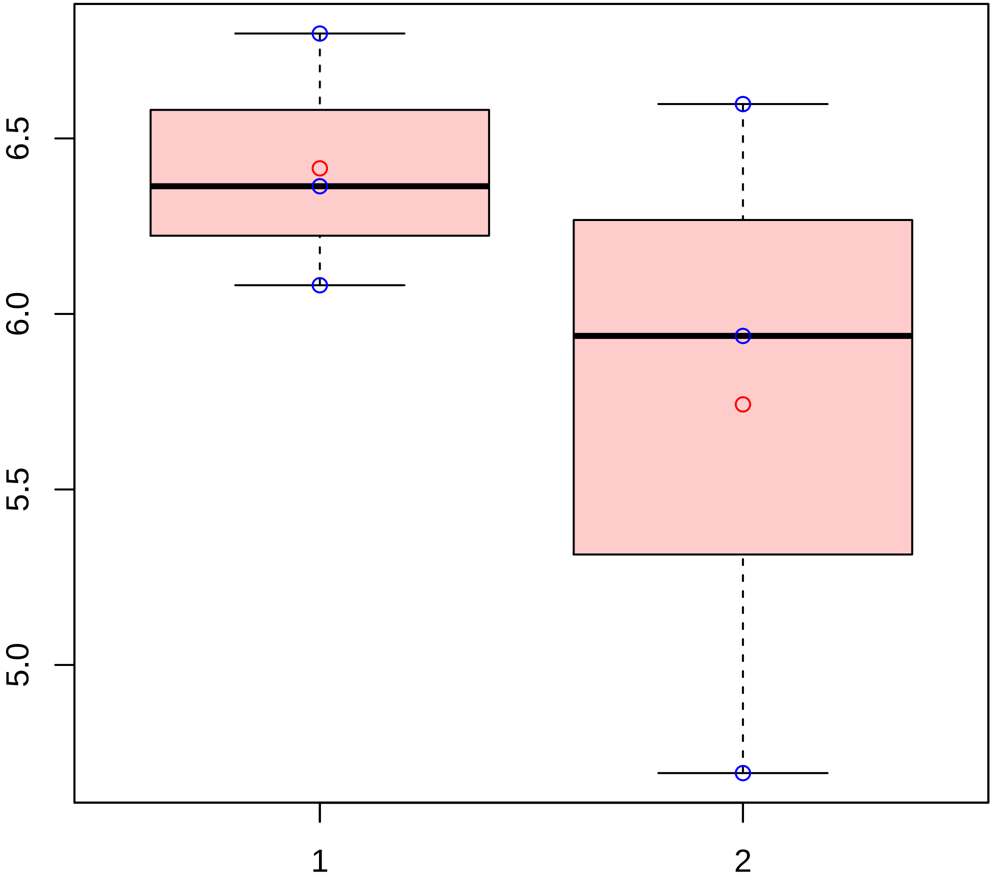

t−Test: p−value = 0.35

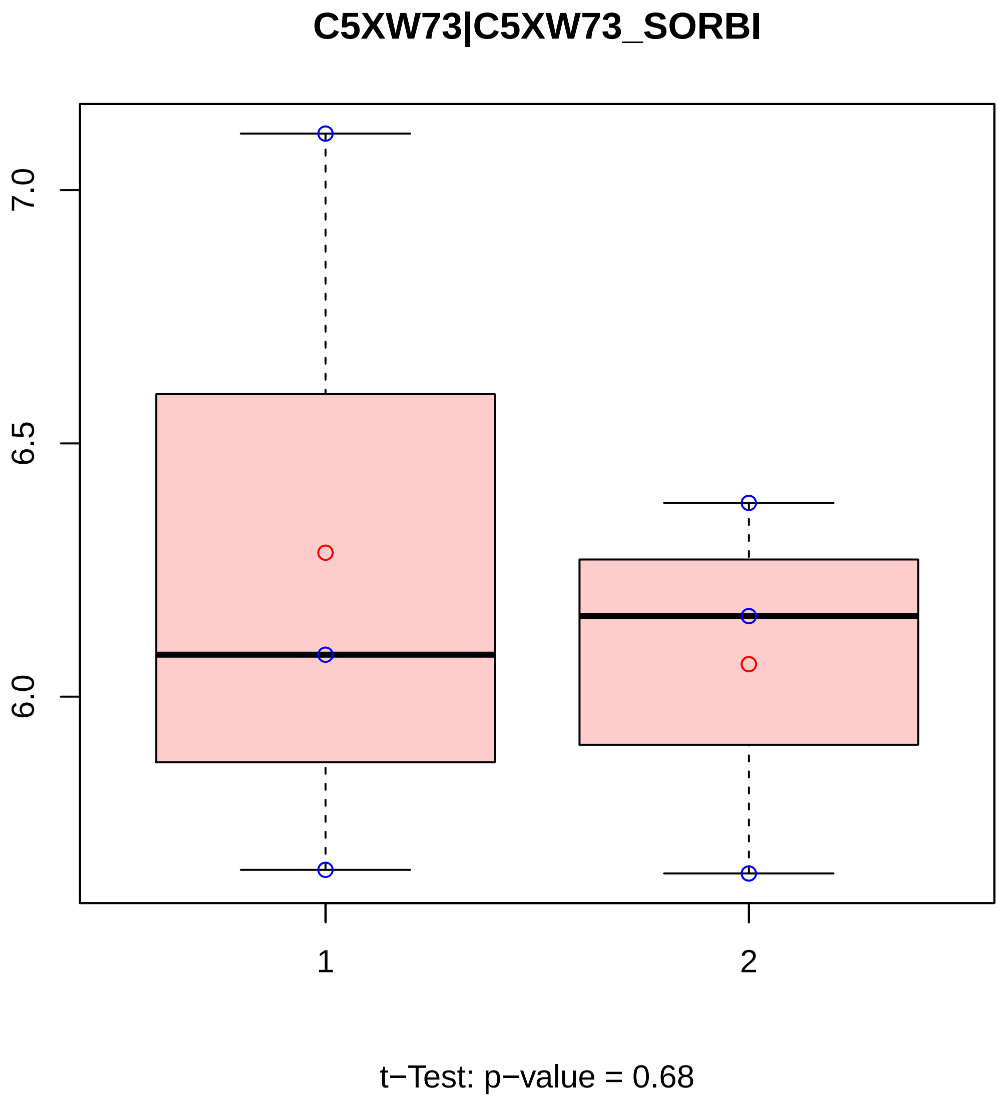
C5XW73|C5XW73_SORBI
7.0
6.5
6.0
1
2
t−Test: p−value = 0.68

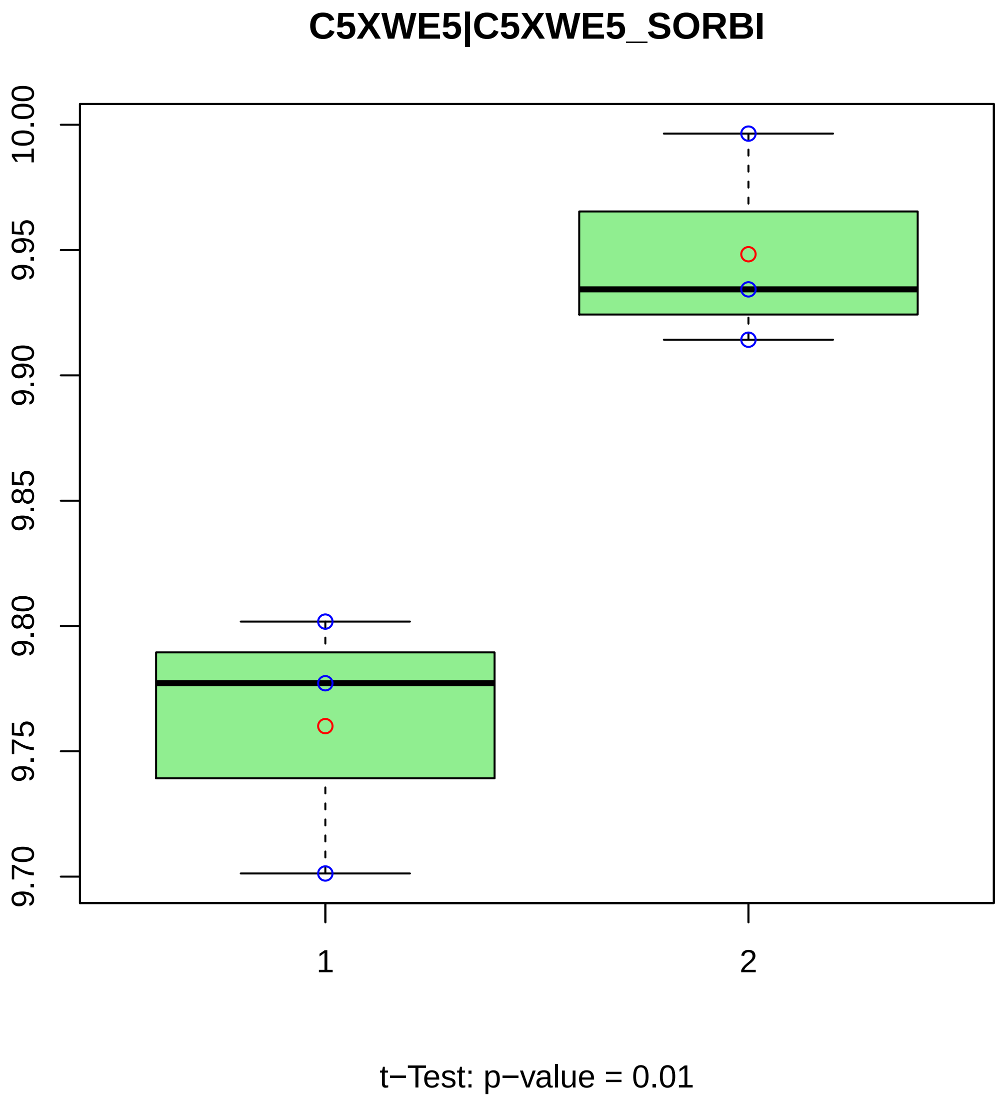

C5XWE5|C5XWE5_SORBI
10.00
9.95
9.90
9.85
9.80
9.75
9.70
1
2
t−Test: p−value = 0.01

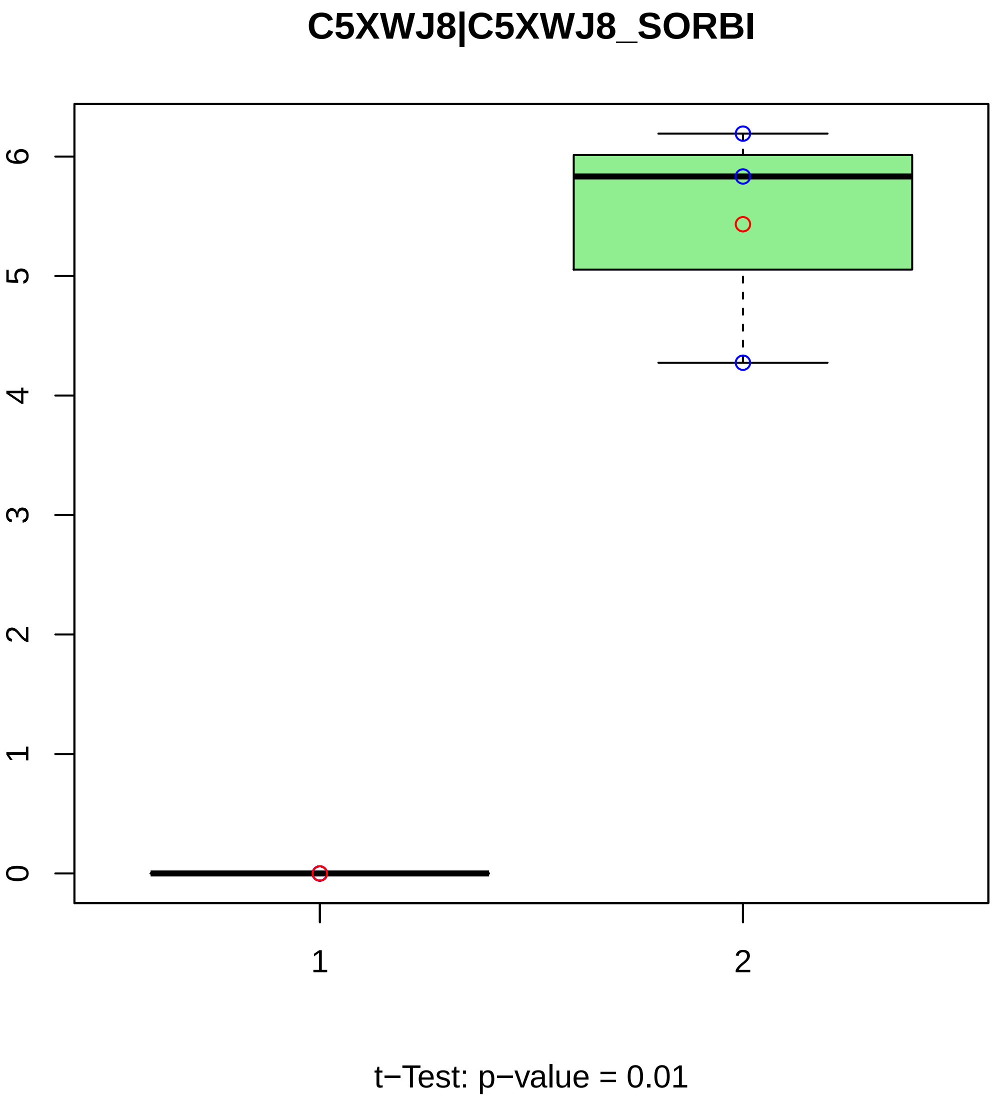
C5XWJ8|C5XWJ8_SORBI
0
1
2
3
4
5
6
1
2
t−Test: p−value = 0.01

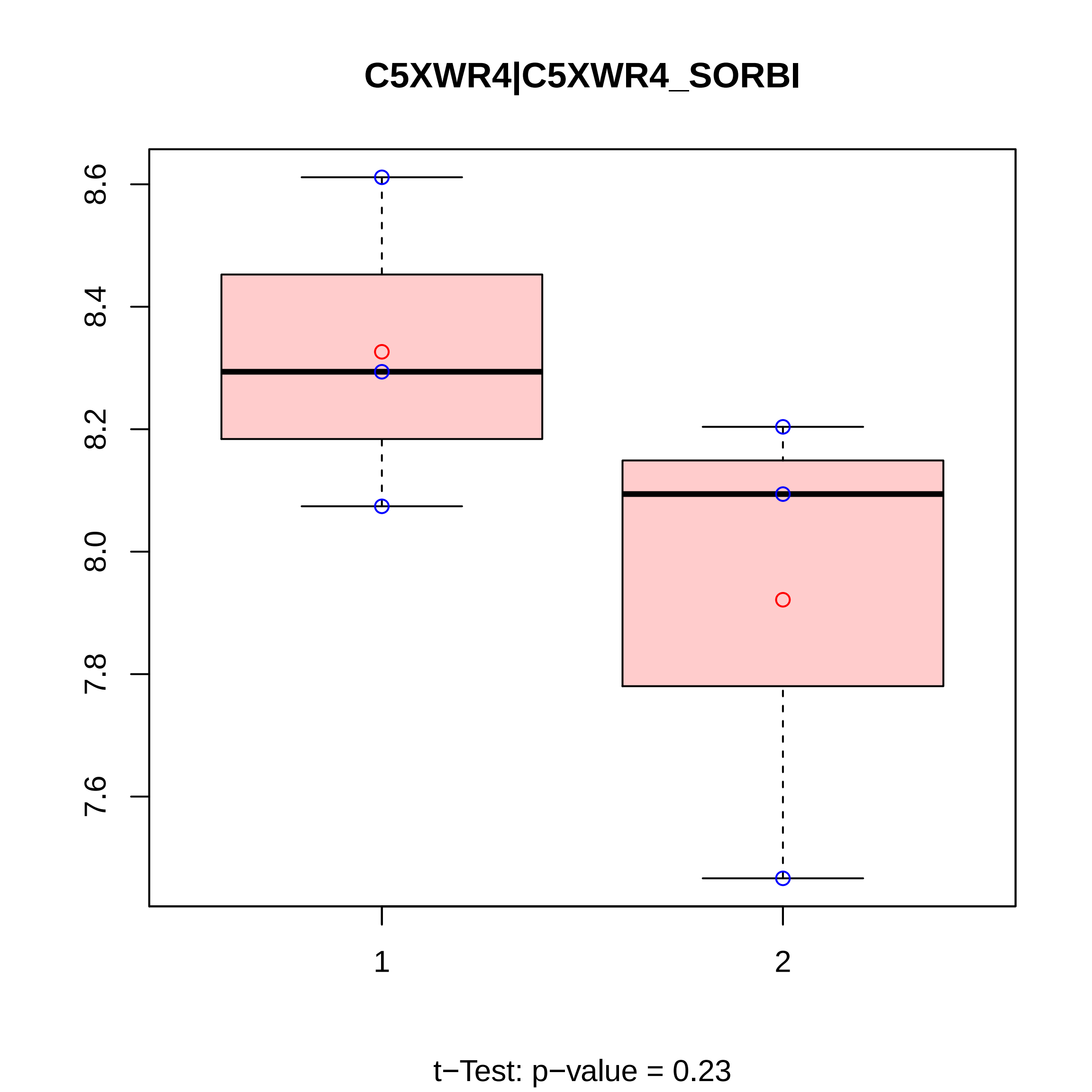

C5XWR4|C5XWR4_SORBI
8.6
8.4
8.2
8.0
7.8
7.6
1
2
t−Test: p−value = 0.23

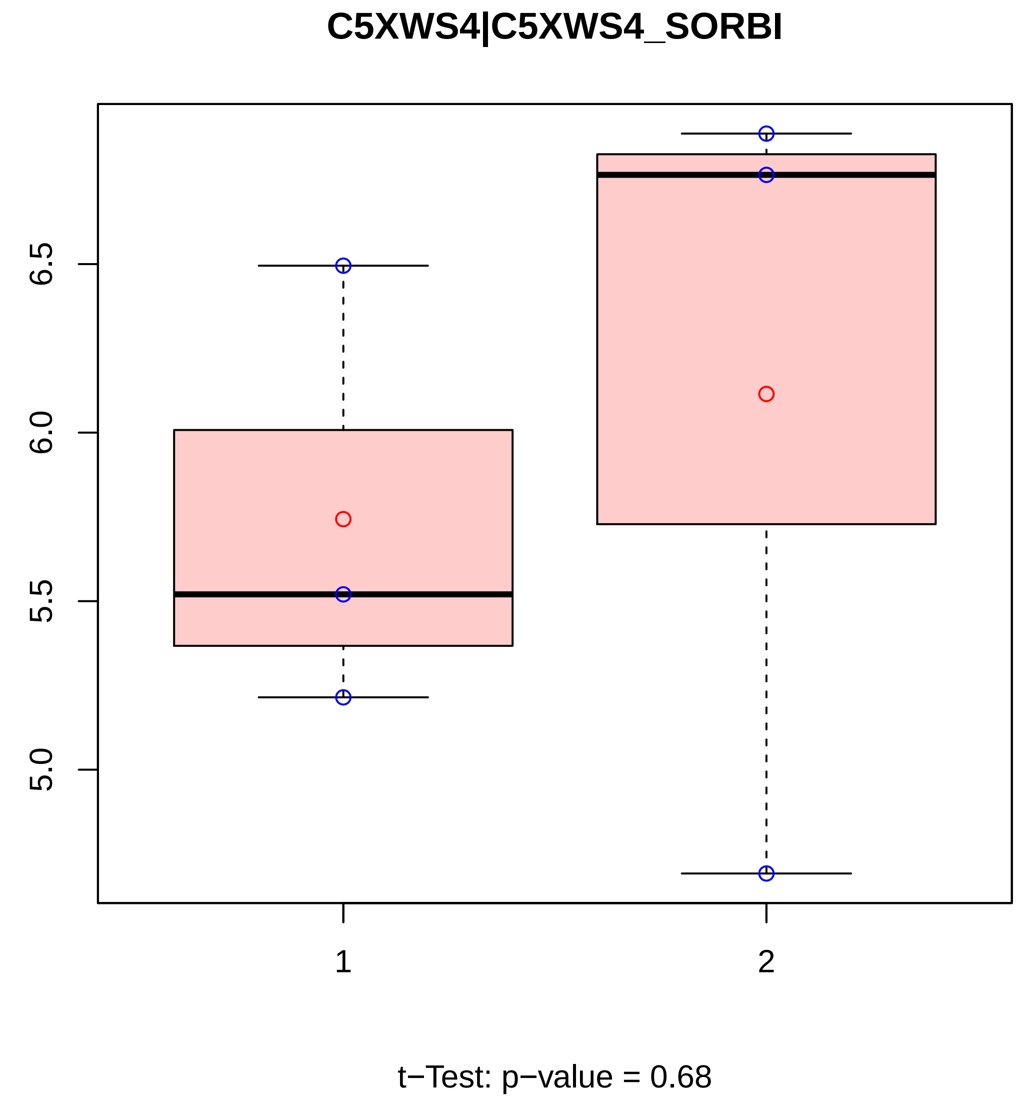

C5XWS4|C5XWS4_SORBI
6.5
6.0
5.5
5.0
1
2
t−Test: p−value = 0.68

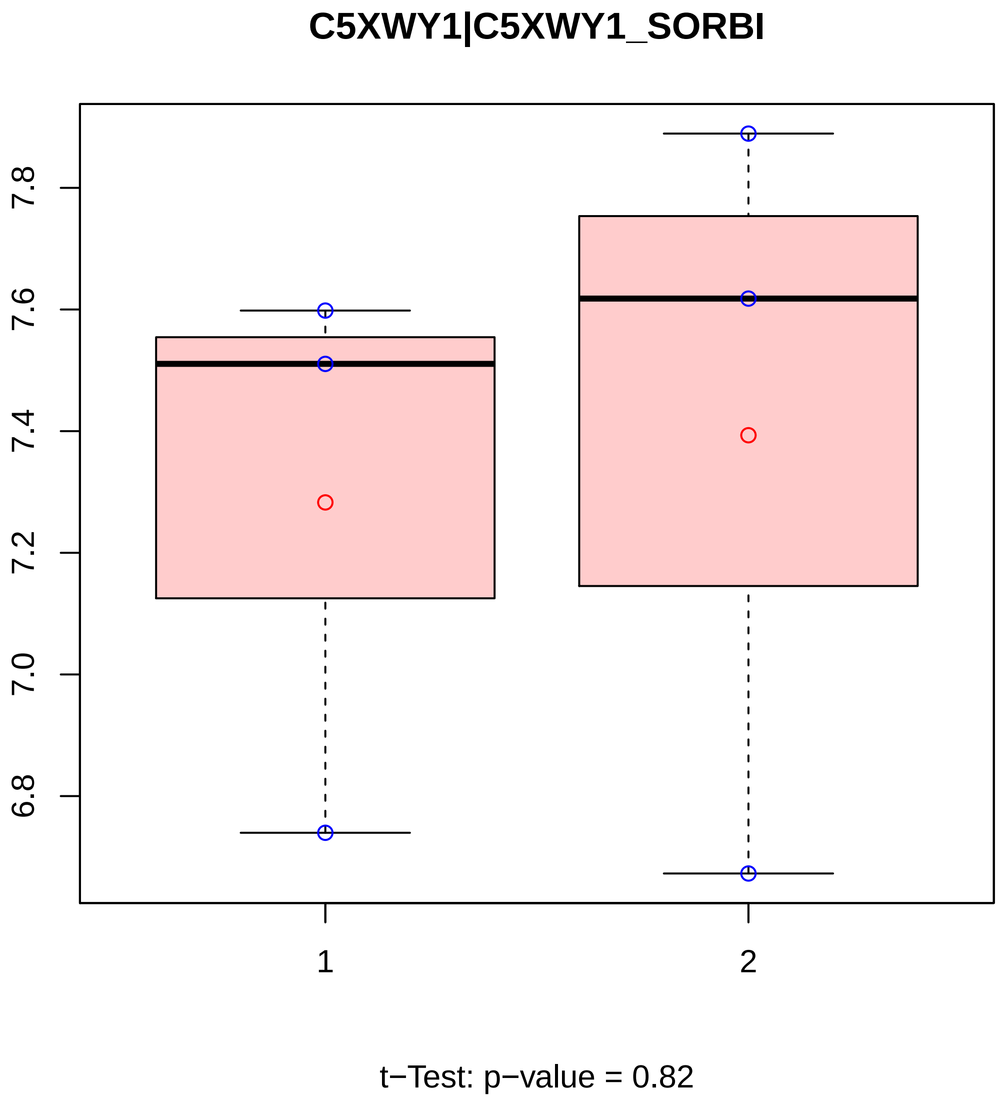

C5XWY1|C5XWY1_SORBI
7.8
7.6
7.4
7.2
7.0
6.8
1
2
t−Test: p−value = 0.82

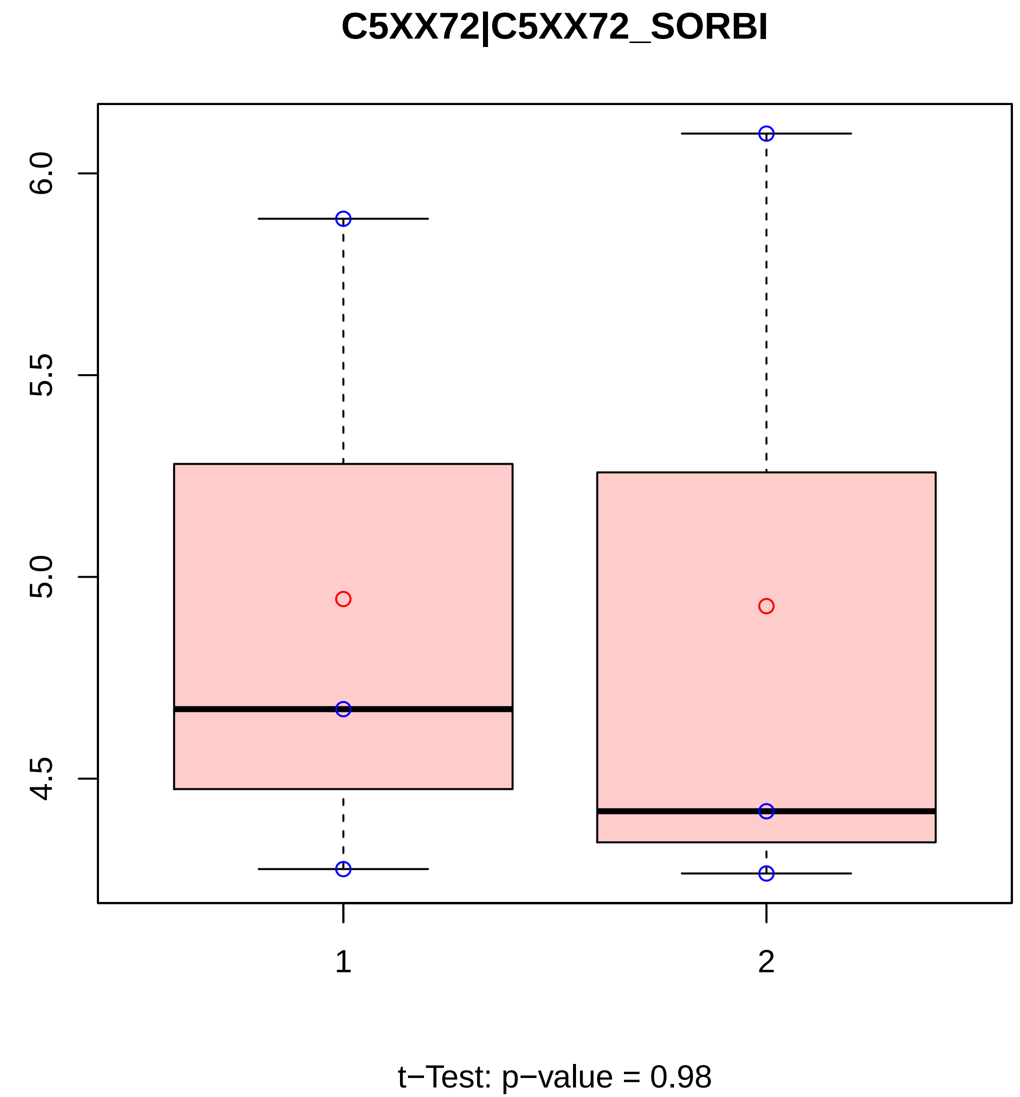
C5XX72|C5XX72_SORBI
6.0
5.5
5.0
4.5
1
2
t−Test: p−value = 0.98

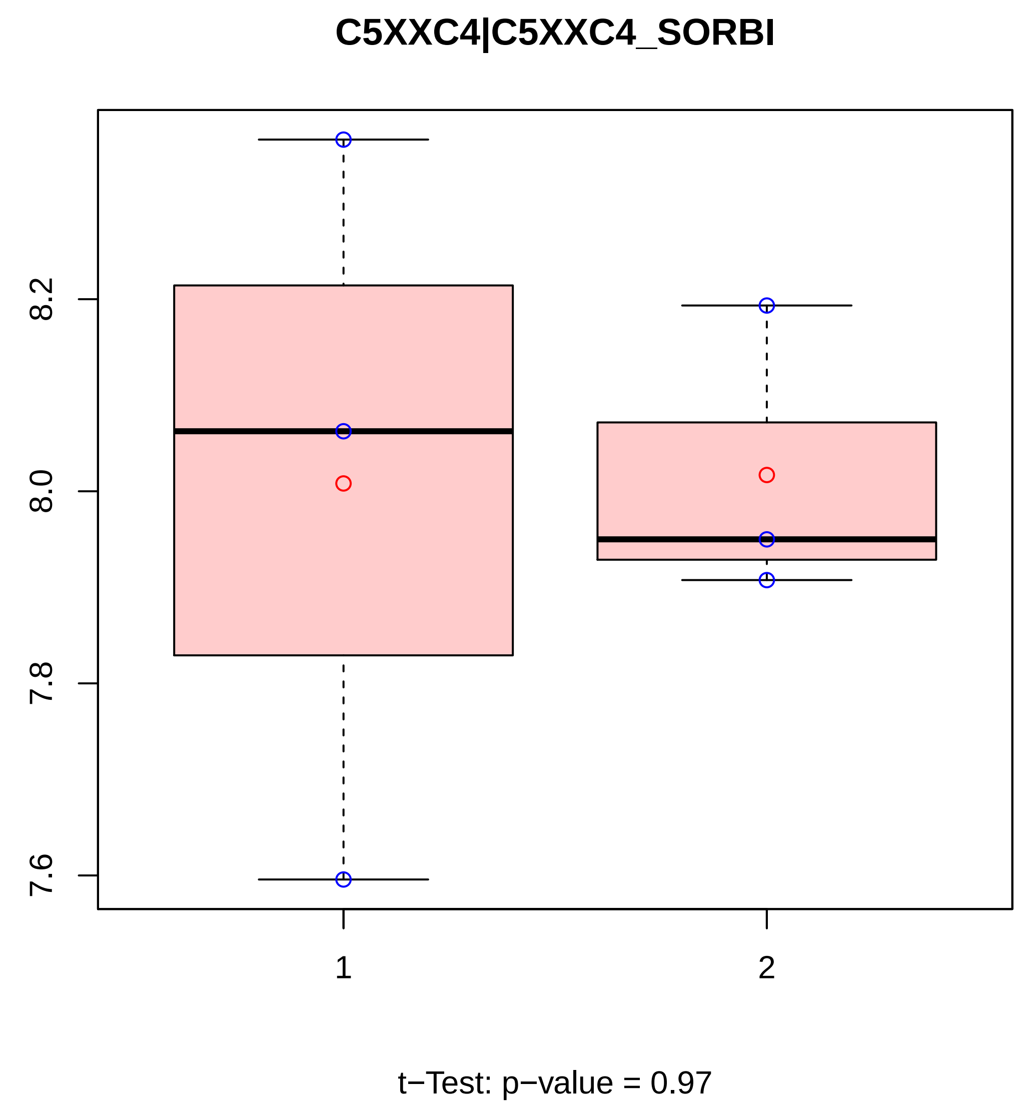
C5XXC4|C5XXC4_SORBI
8.2
8.0
7.8
7.6
1
2
t−Test: p−value = 0.97

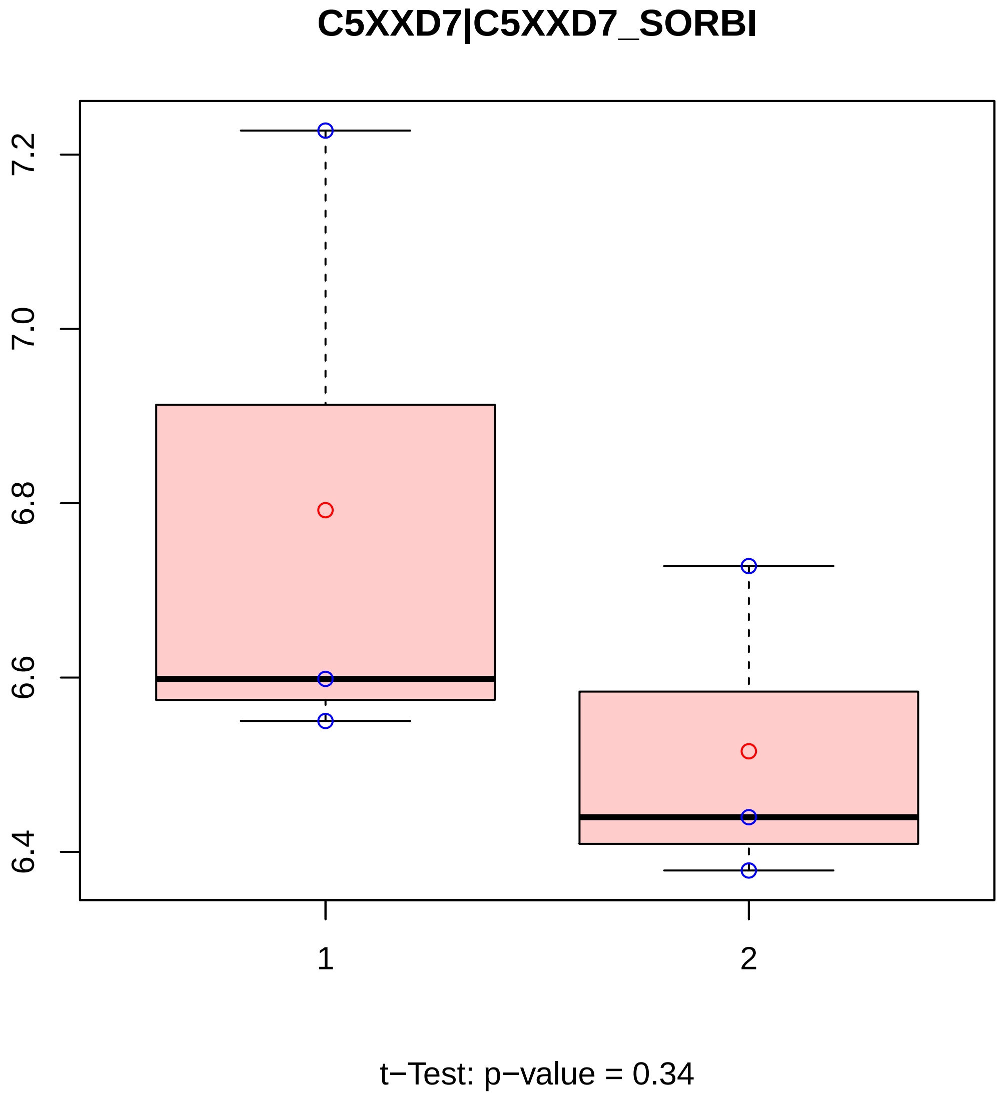
C5XXD7|C5XXD7_SORBI
7.2
7.0
6.8
6.6
6.4
1
2
t−Test: p−value = 0.34

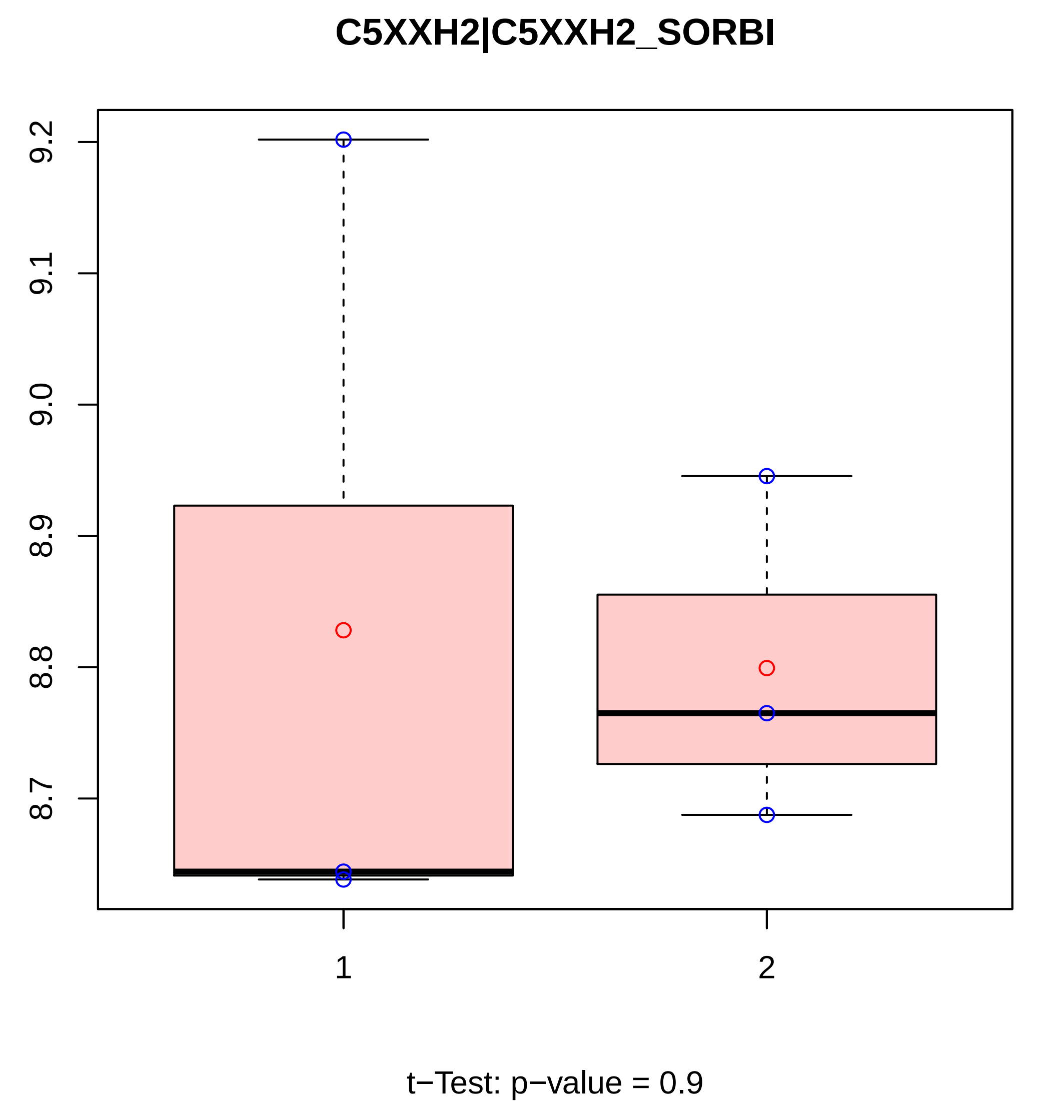

C5XXH2|C5XXH2_SORBI
9.2
9.1
9.0
8.9
8.8
8.7
1
2
t−Test: p−value = 0.9

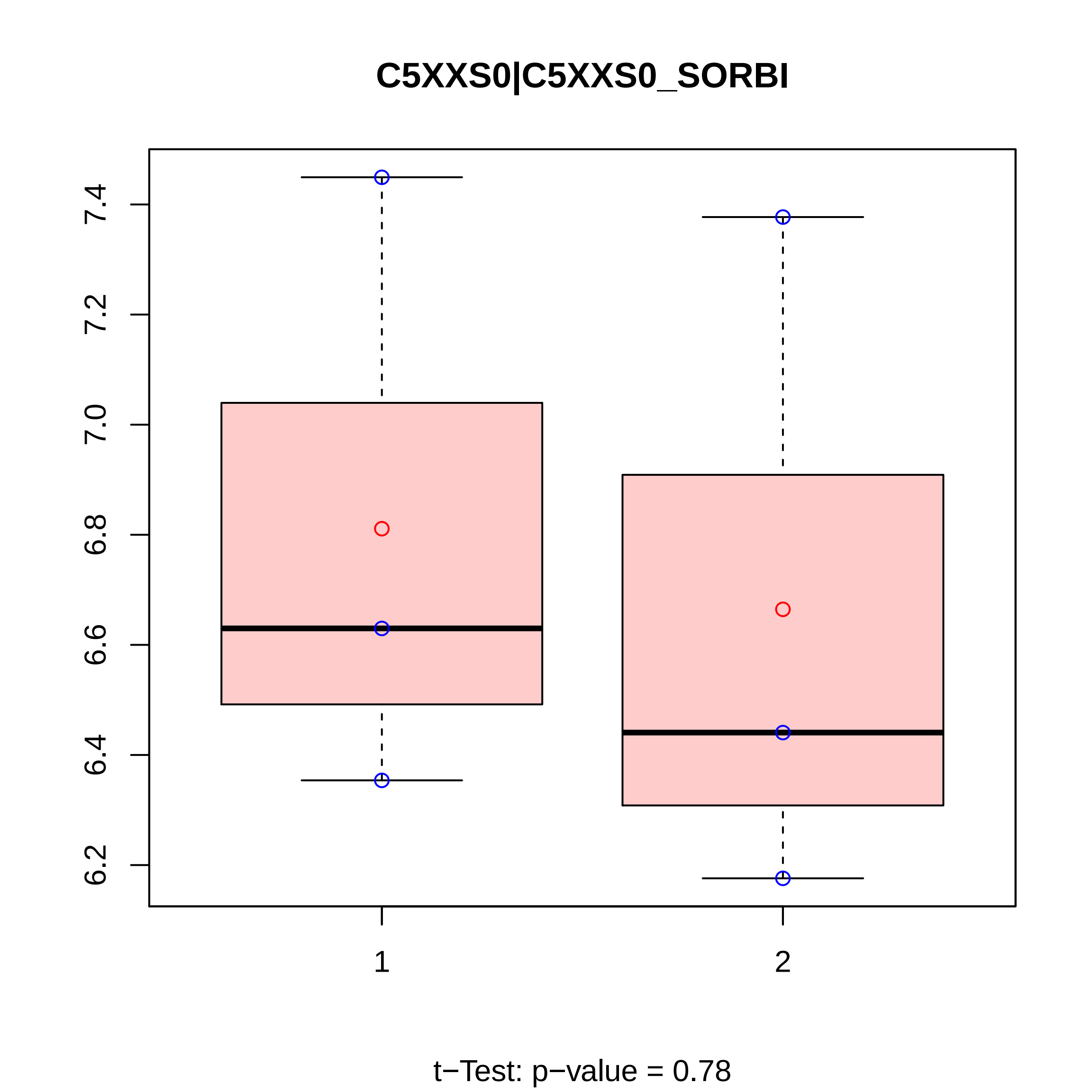

C5XXS0|C5XXS0_SORBI
7.4
7.2
7.0
6.8
6.6
6.4
6.2
1
2
t−Test: p−value = 0.78

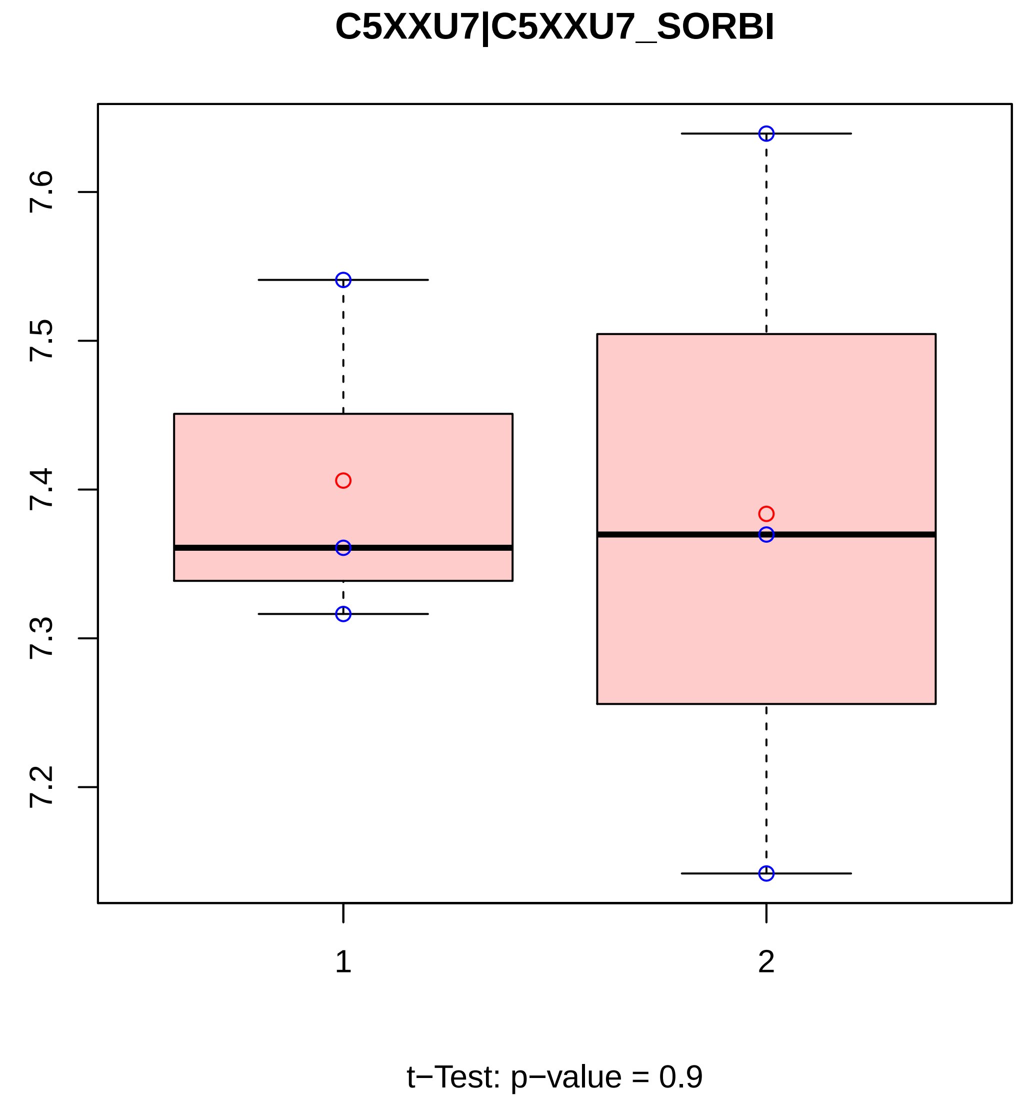
C5XXU7|C5XXU7_SORBI
7.2
7.3
7.4
7.5
7.6
1
2
t−Test: p−value = 0.9

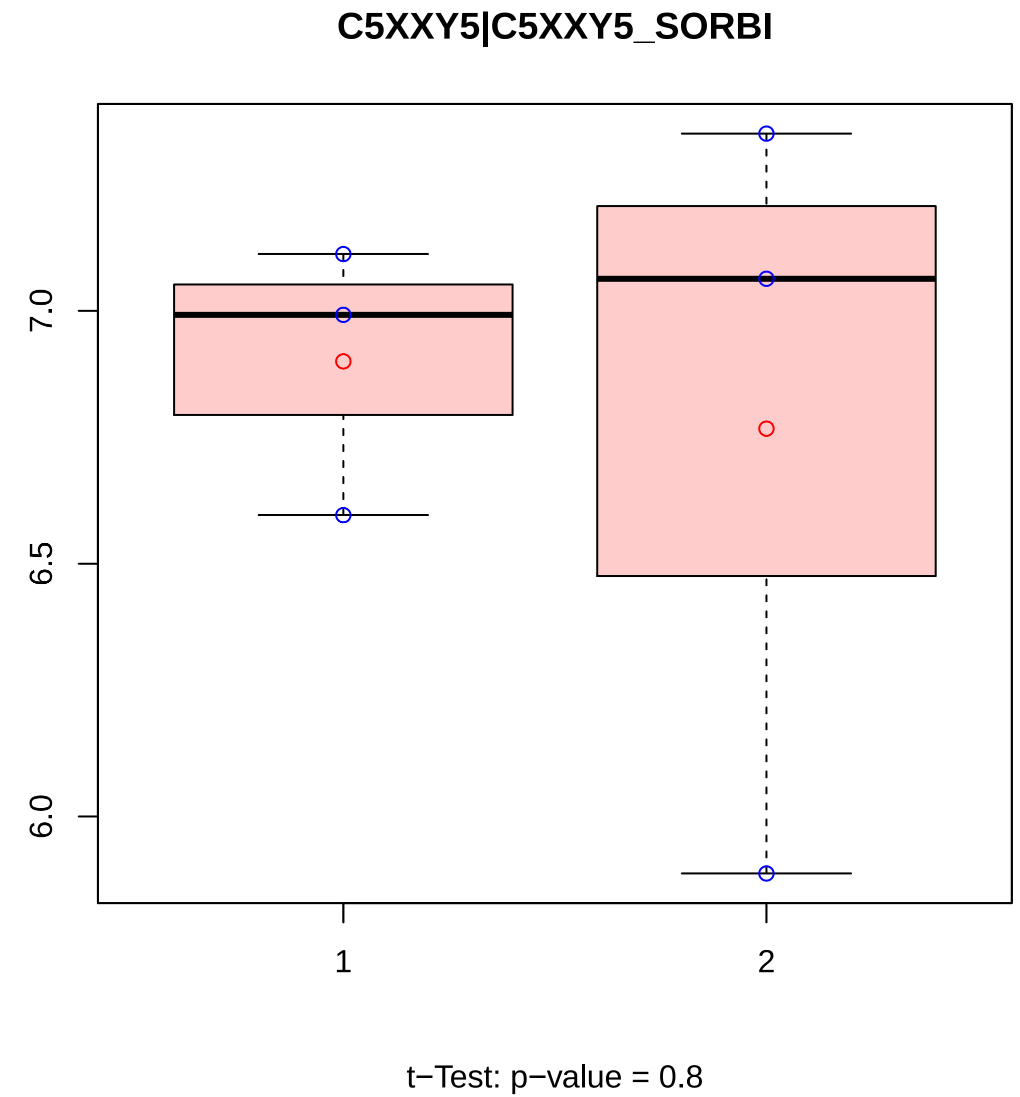

C5XXY5|C5XXY5_SORBI
7.0
6.5
6.0
1
2
t−Test: p−value = 0.8

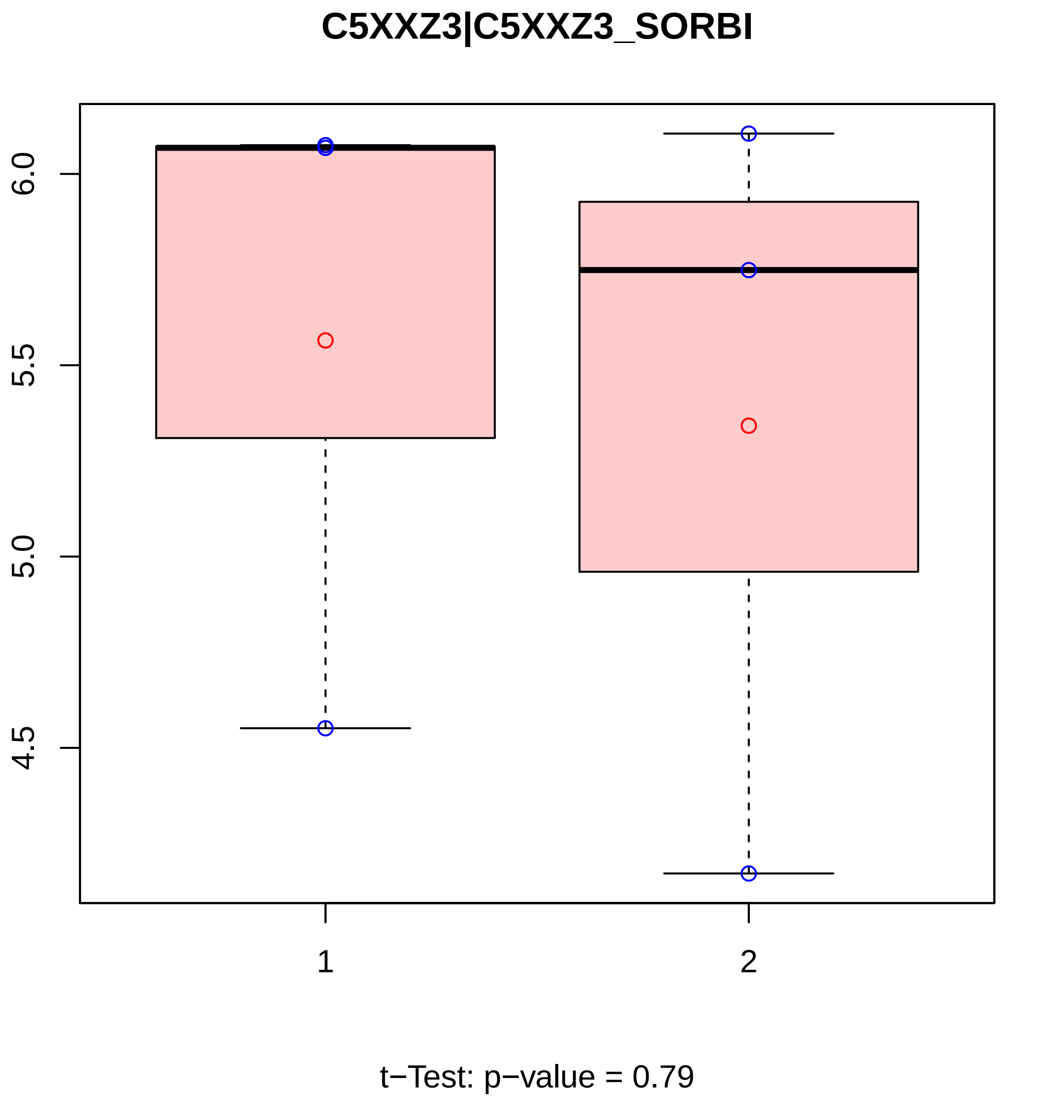

C5XXZ3|C5XXZ3_SORBI
6.0
5.5
5.0
4.5
1
2
t−Test: p−value = 0.79

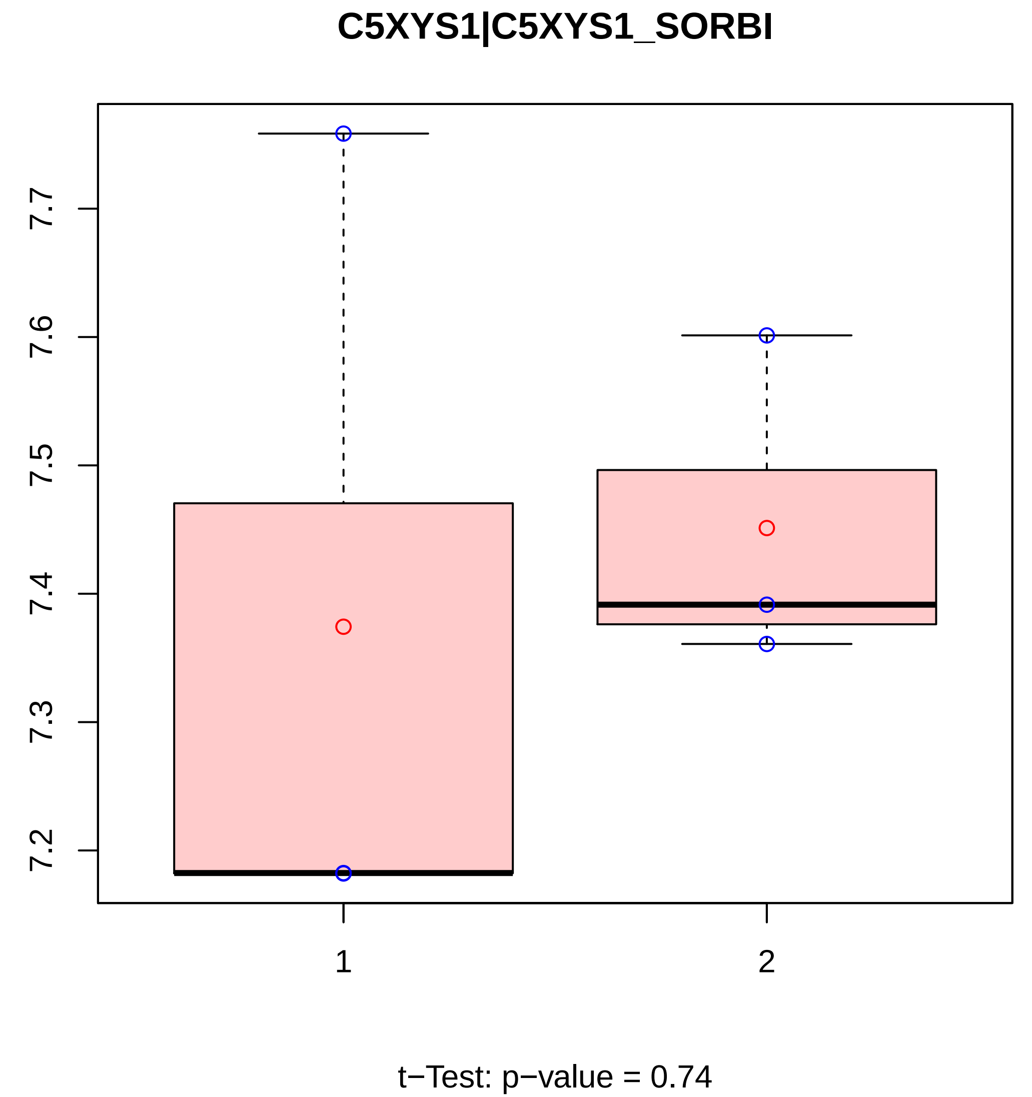

C5XYS1|C5XYS1_SORBI
7.2
7.3
7.4
7.5
7.6
7.7
1
2
t−Test: p−value = 0.74

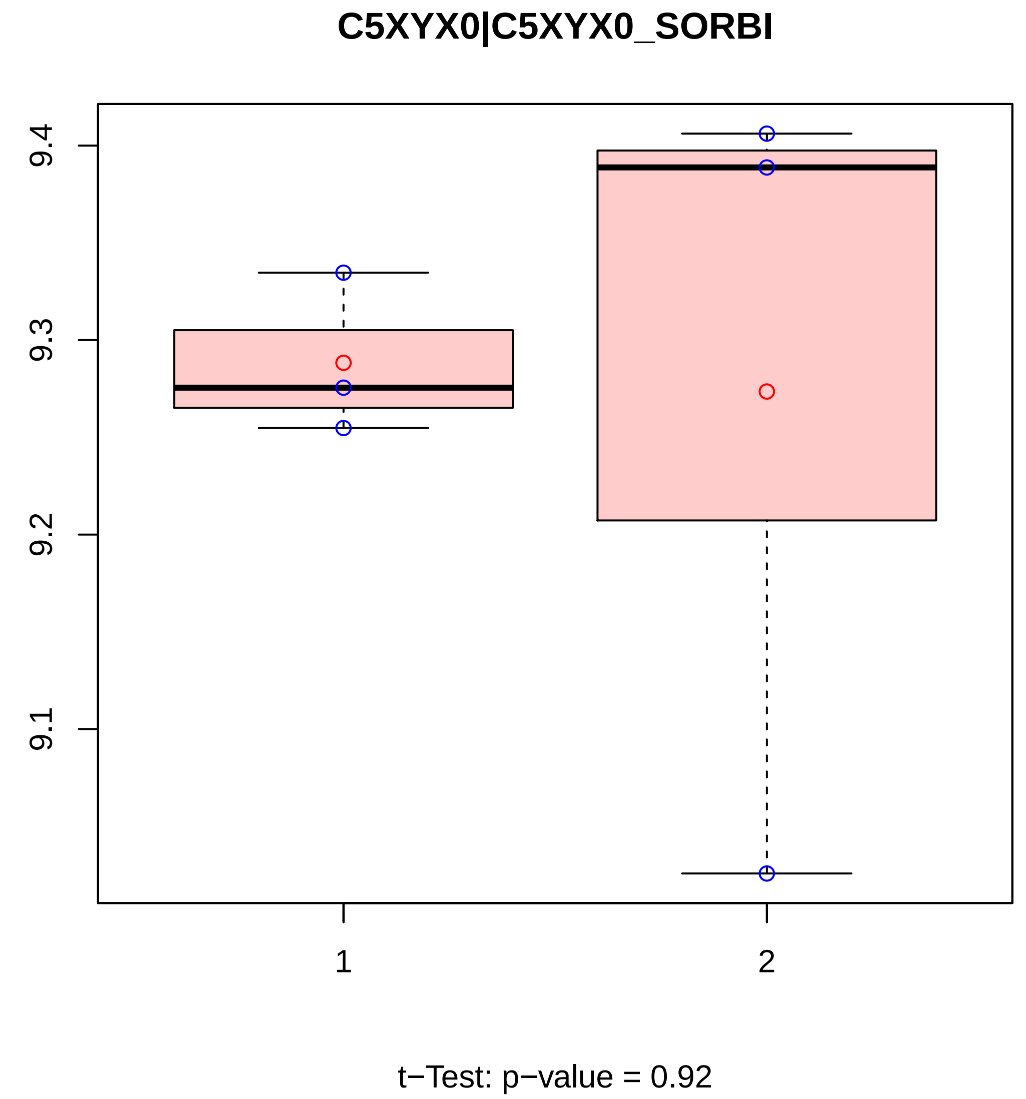
C5XYX0|C5XYX0_SORBI
9.1
9.2
9.3
9.4
1
2
t−Test: p−value = 0.92

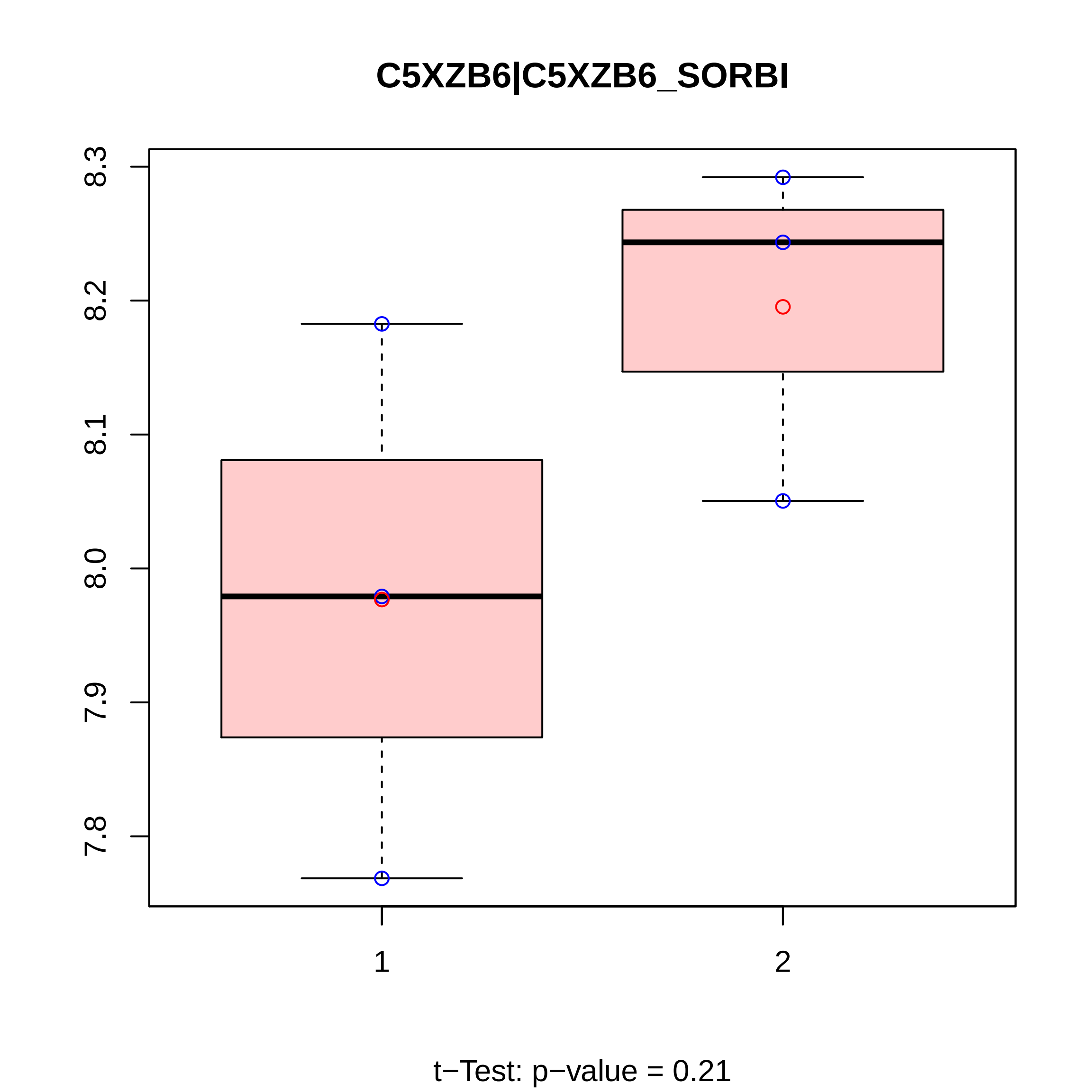
C5XZB6|C5XZB6_SORBI
8.3
8.2
8.1
8.0
7.9
7.8
1
2
t−Test: p−value = 0.21

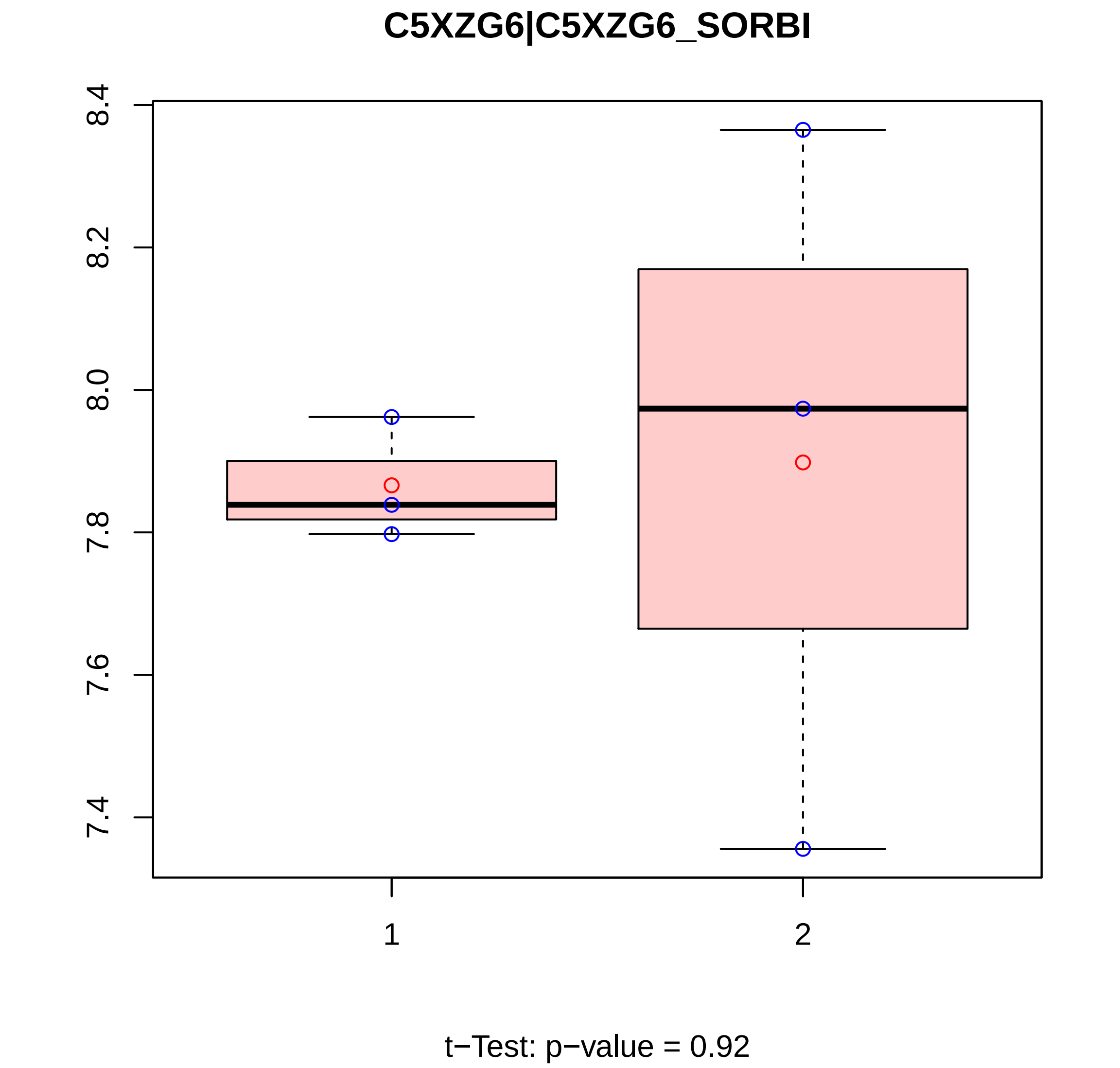
C5XZG6|C5XZG6_SORBI
8.4
8.2
8.0
7.8
7.6
7.4
1
2
t−Test: p−value = 0.92

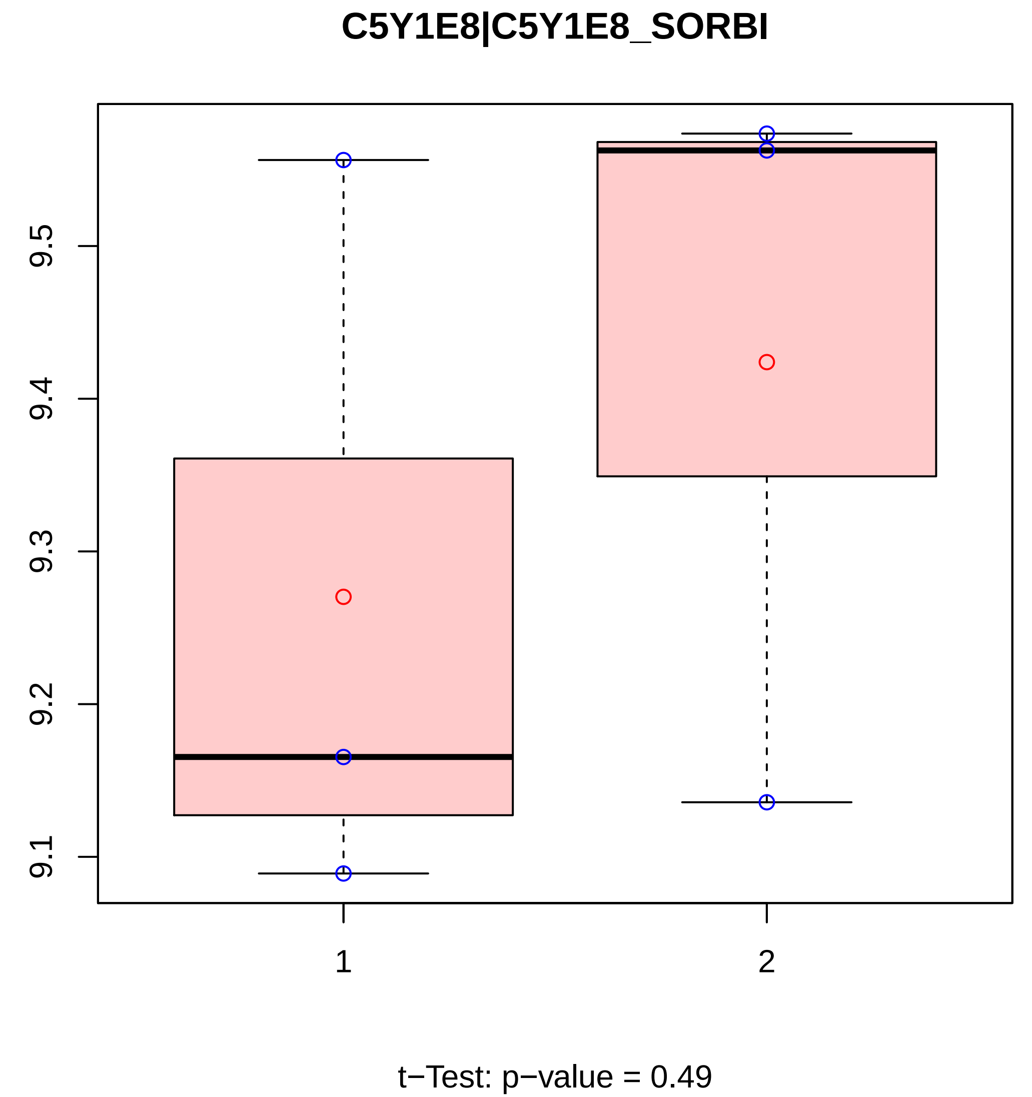

C5Y1E8|C5Y1E8_SORBI
9.1
9.2
9.3
9.4
9.5
1
2
t−Test: p−value = 0.49

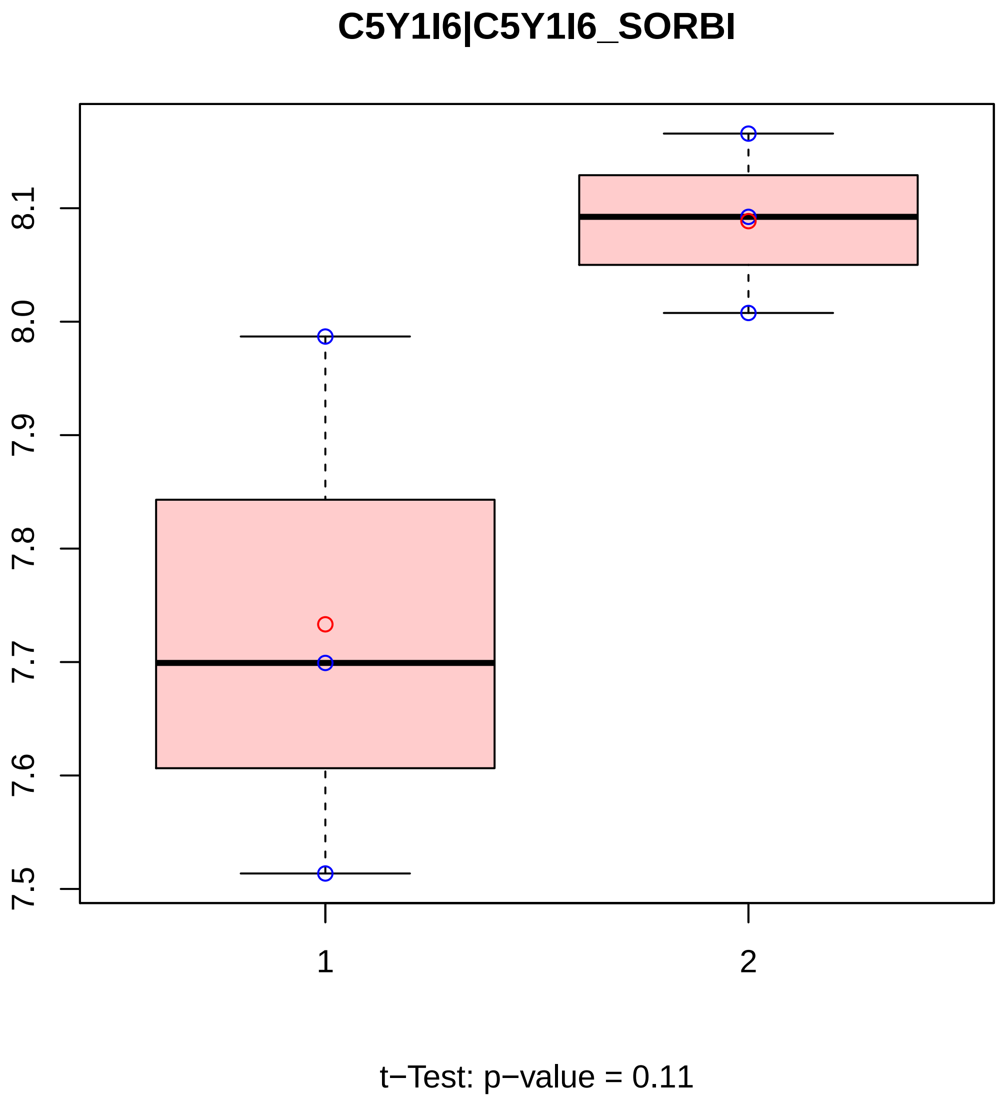
C5Y1I6|C5Y1I6_SORBI
8.1
8.0
7.9
7.8
7.7
7.6
7.5
1
2
t−Test: p−value = 0.11

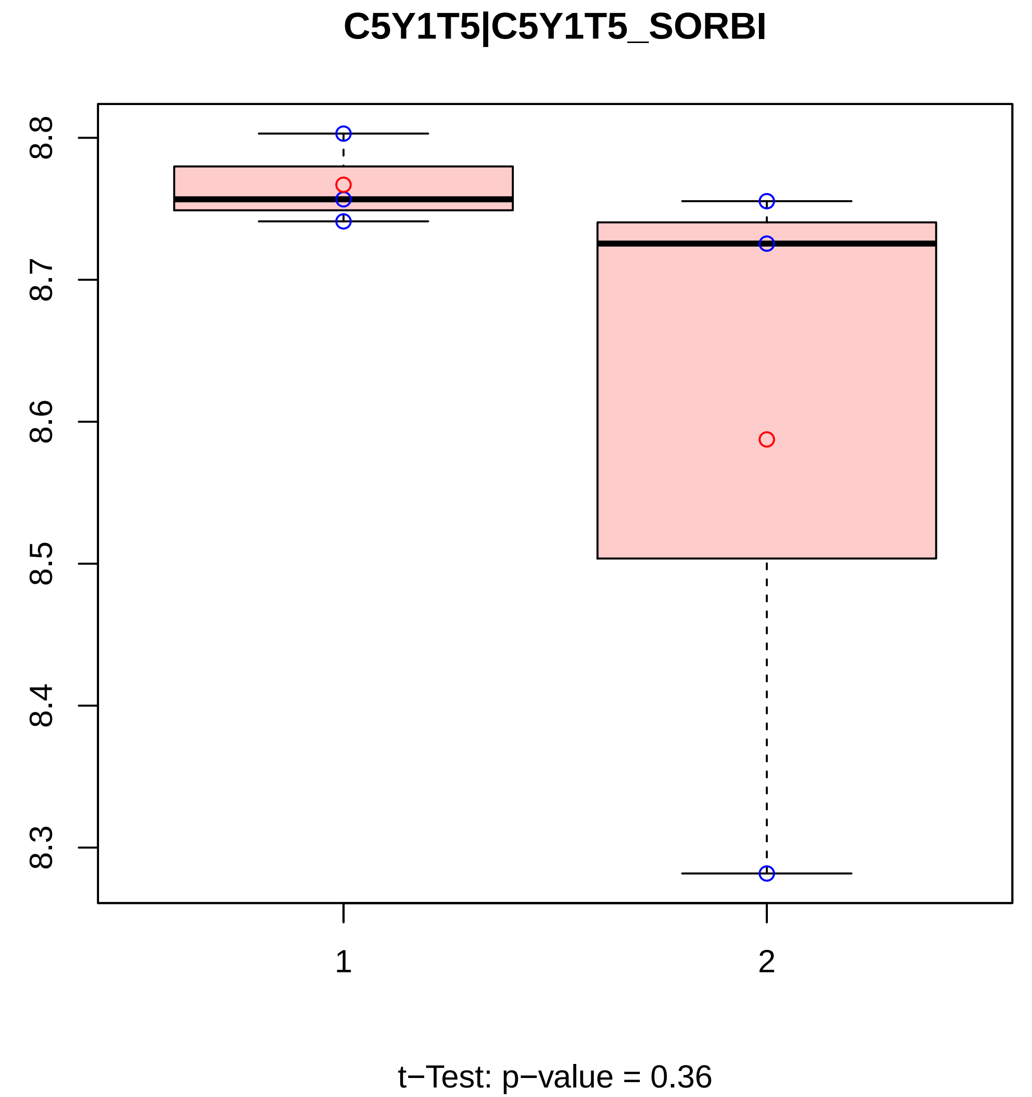

C5Y1T5|C5Y1T5_SORBI
8.8
8.7
8.6
8.5
8.4
8.3
1
2
t−Test: p−value = 0.36

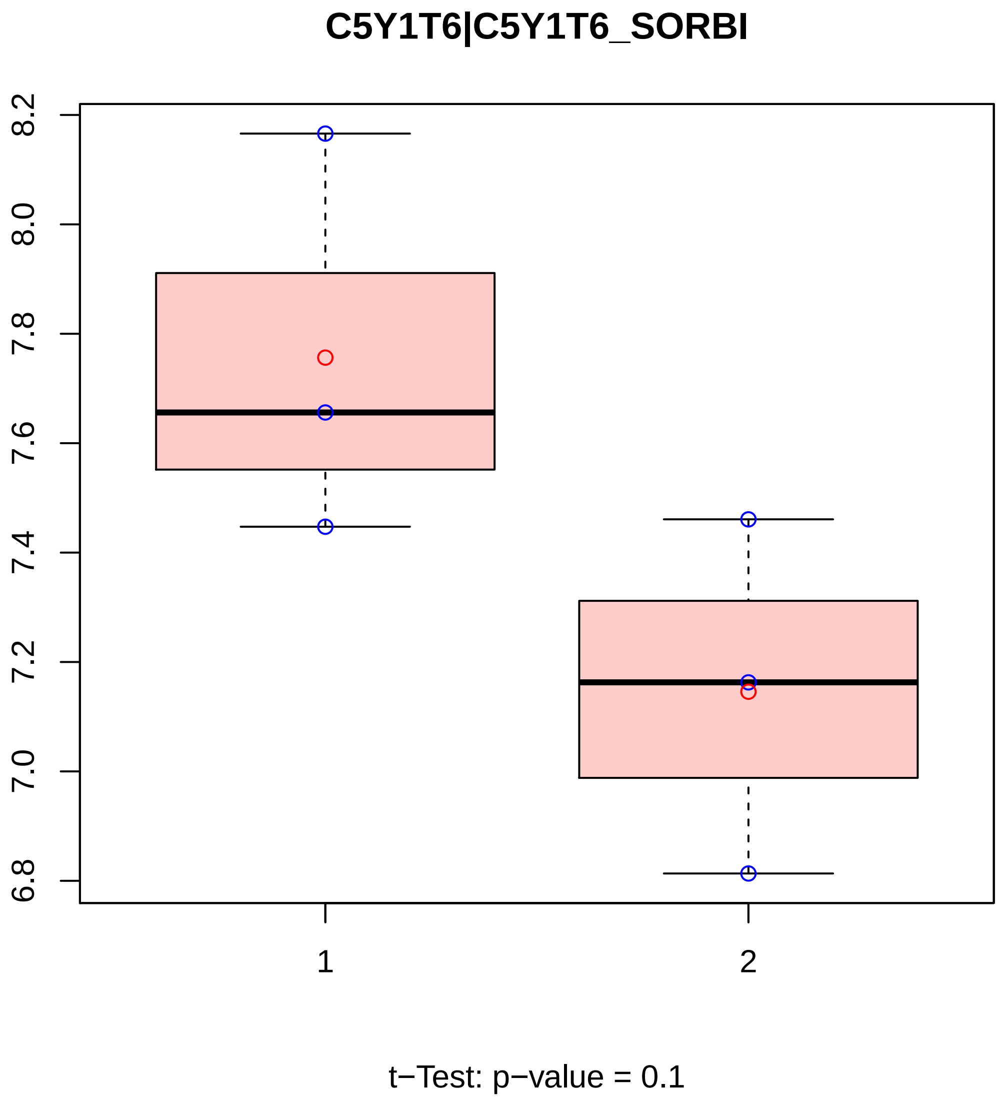

C5Y1T6|C5Y1T6_SORBI
8.2
8.0
7.8
7.6
7.4
7.2
7.0
6.8
1
2
t−Test: p−value = 0.1

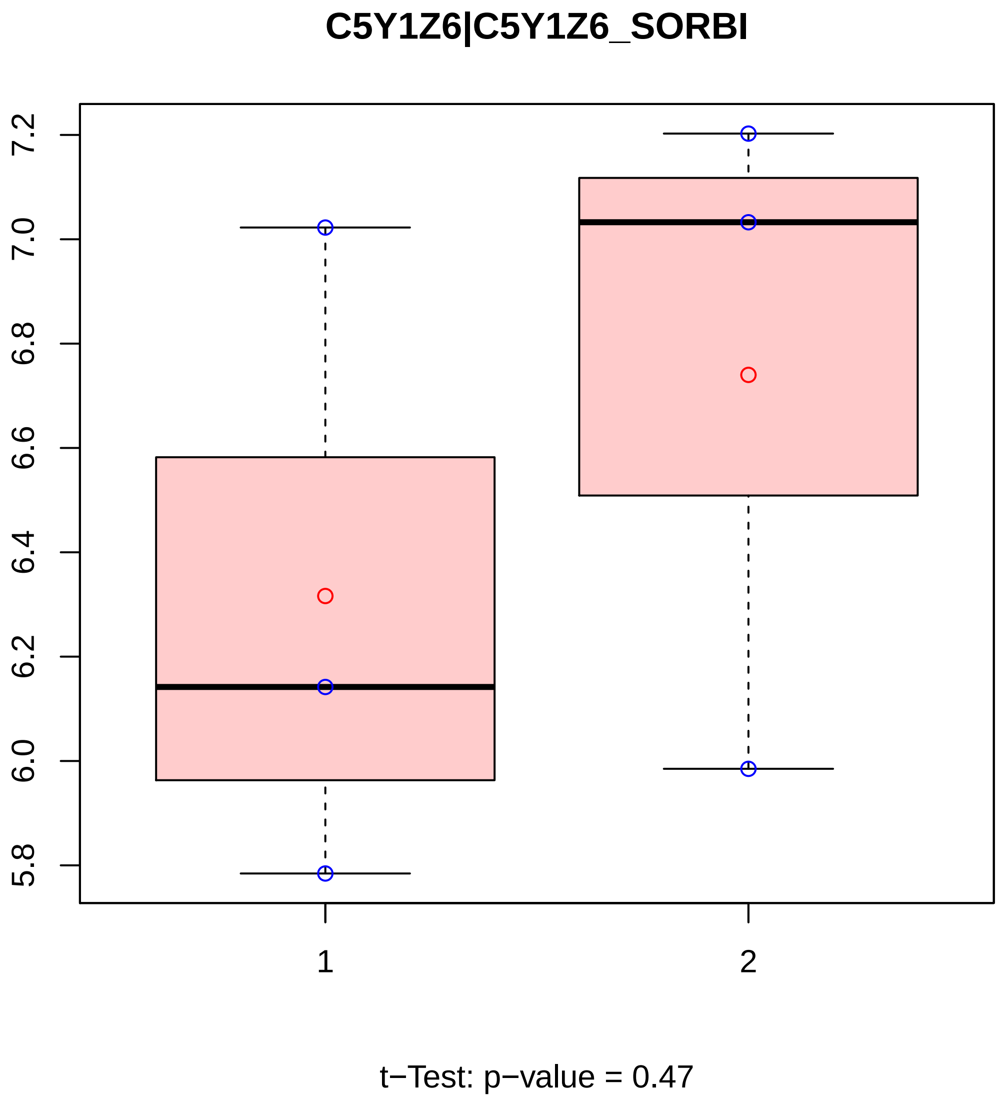
C5Y1Z6|C5Y1Z6_SORBI
7.2
7.0
6.8
6.6
6.4
6.2
6.0
5.8
1
2
t−Test: p−value = 0.47

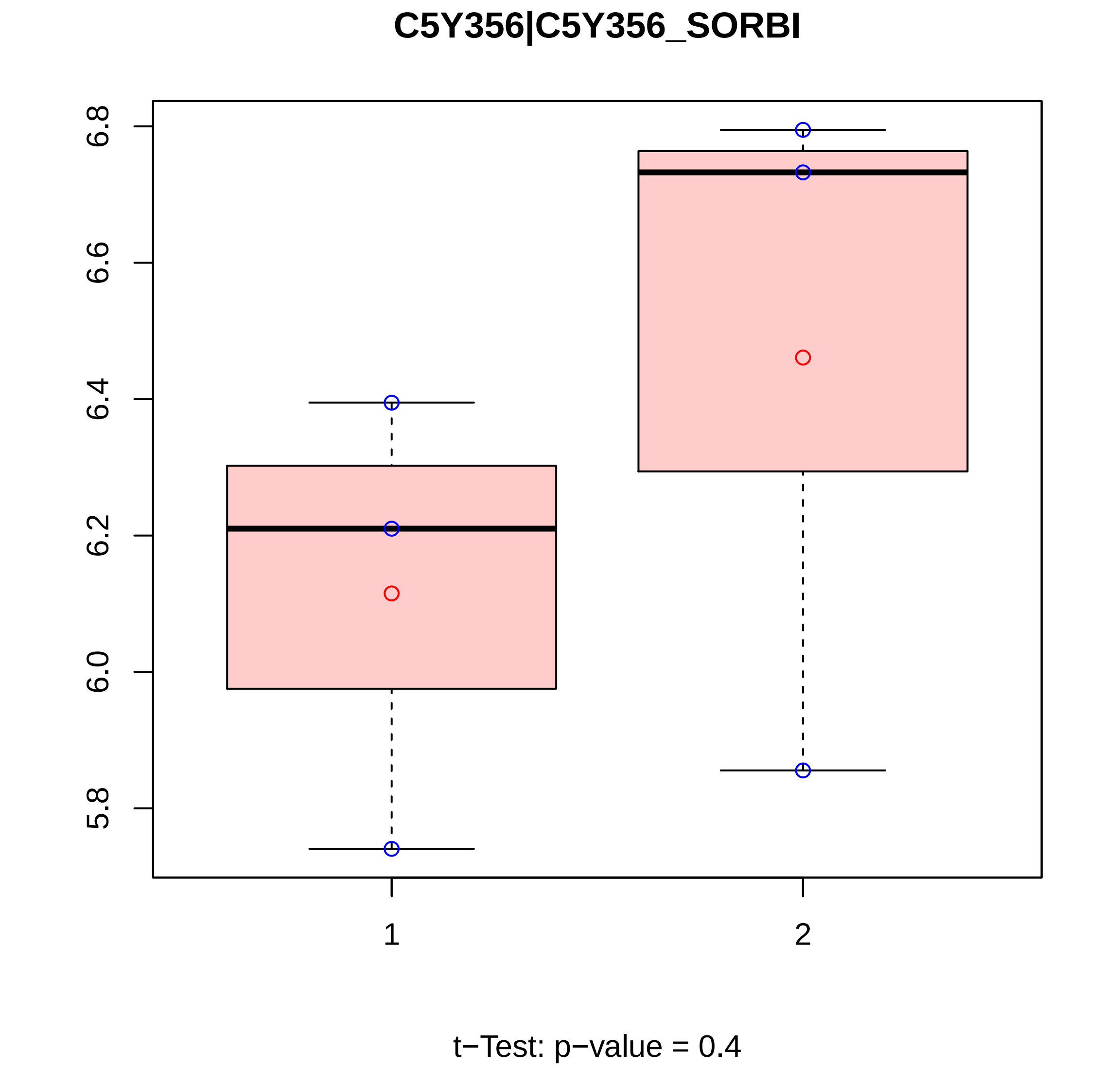

C5Y356|C5Y356_SORBI
6.8
6.6
6.4
6.2
6.0
5.8
1
2
t−Test: p−value = 0.4

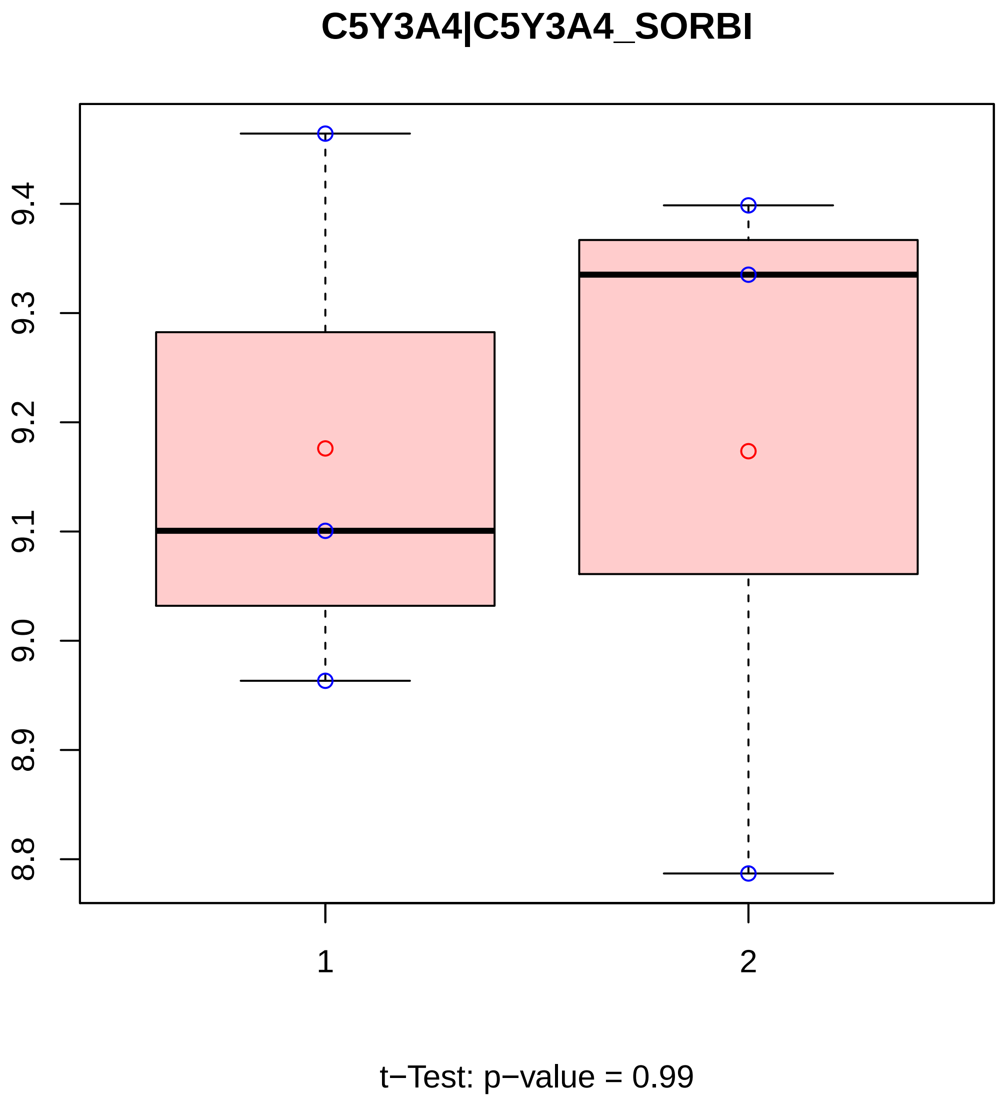

C5Y3A4|C5Y3A4_SORBI
9.4
9.3
9.2
9.1
9.0
8.9
8.8
1
2
t−Test: p−value = 0.99

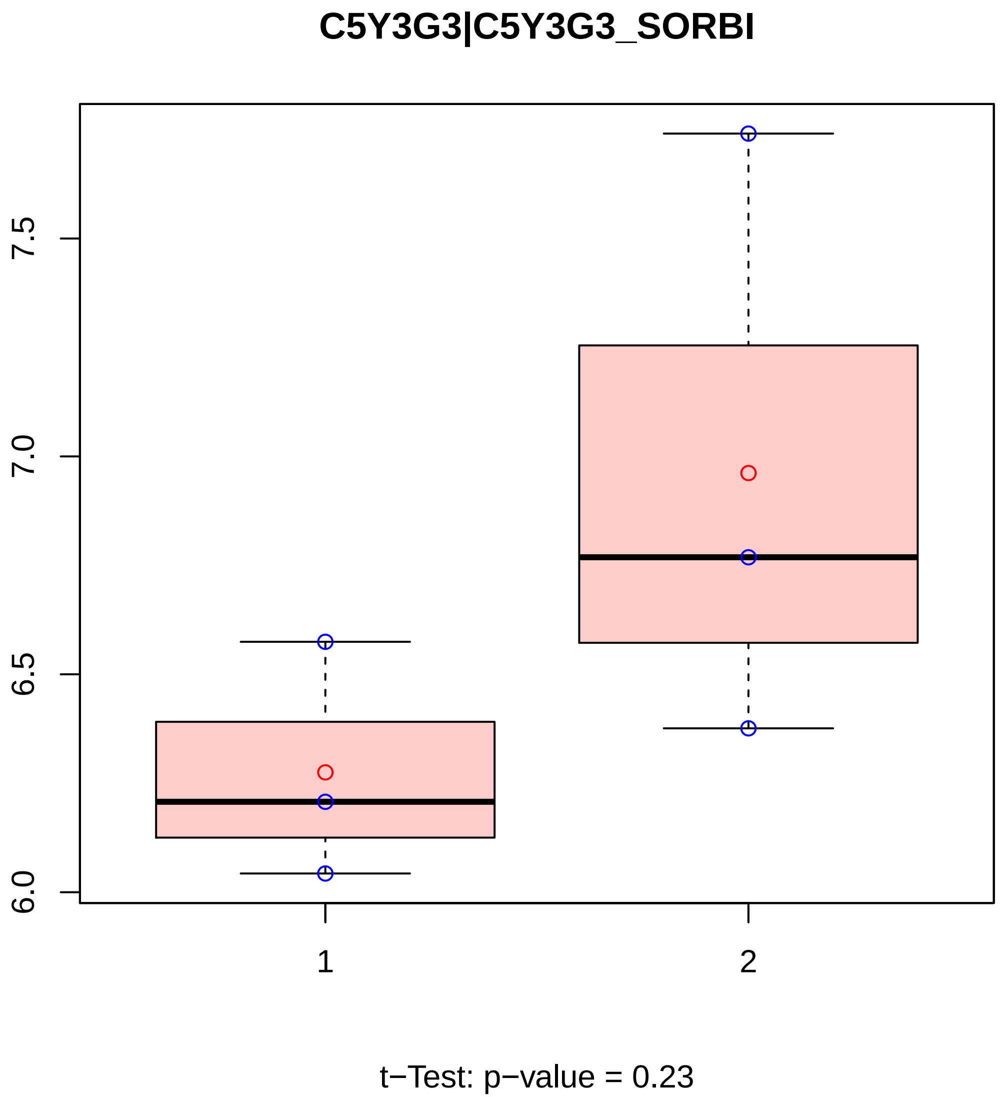
C5Y3G3|C5Y3G3_SORBI
6.0
6.5
7.0
7.5
1
2
t−Test: p−value = 0.23

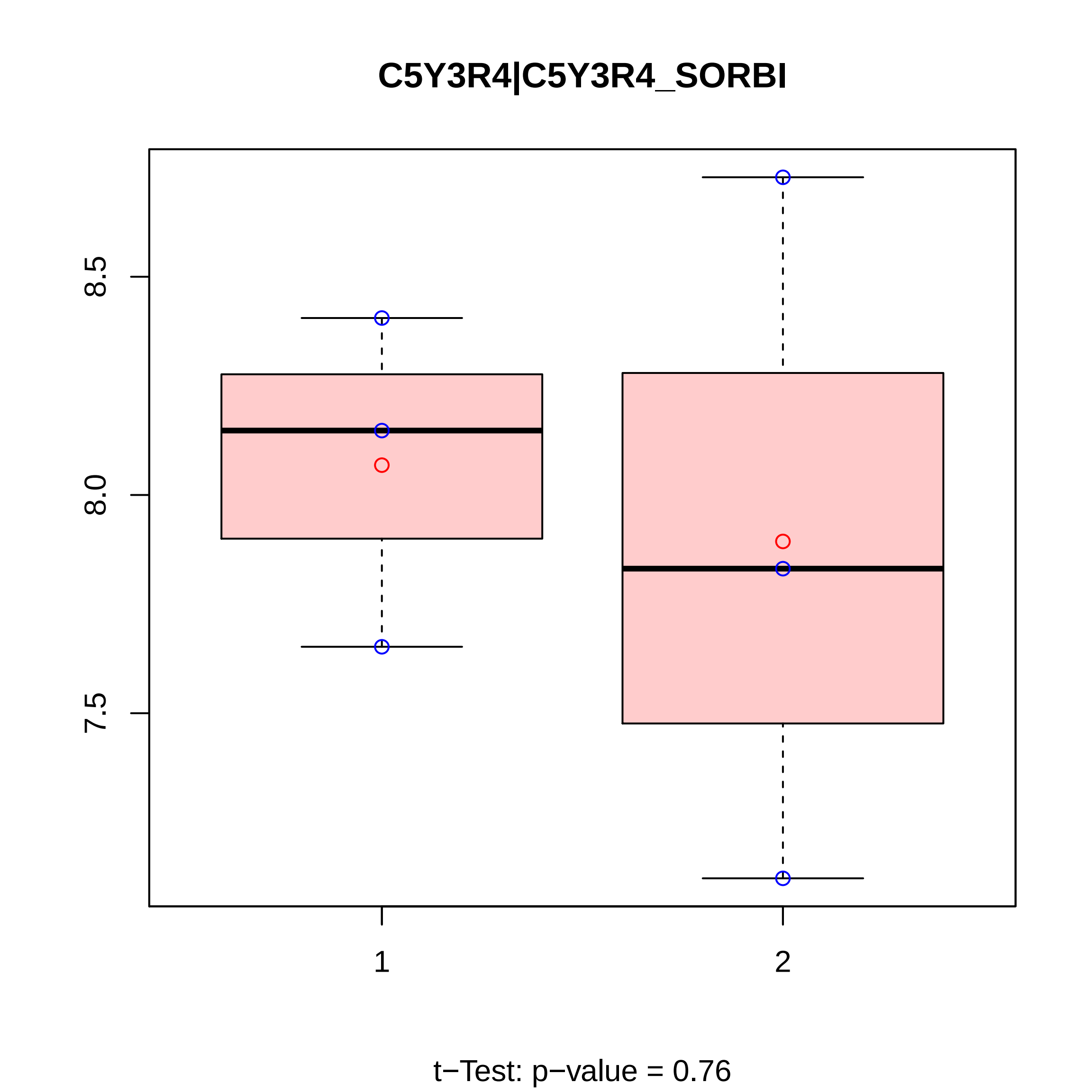

C5Y3R4|C5Y3R4_SORBI
8.5
8.0
7.5
1
2
t−Test: p−value = 0.76

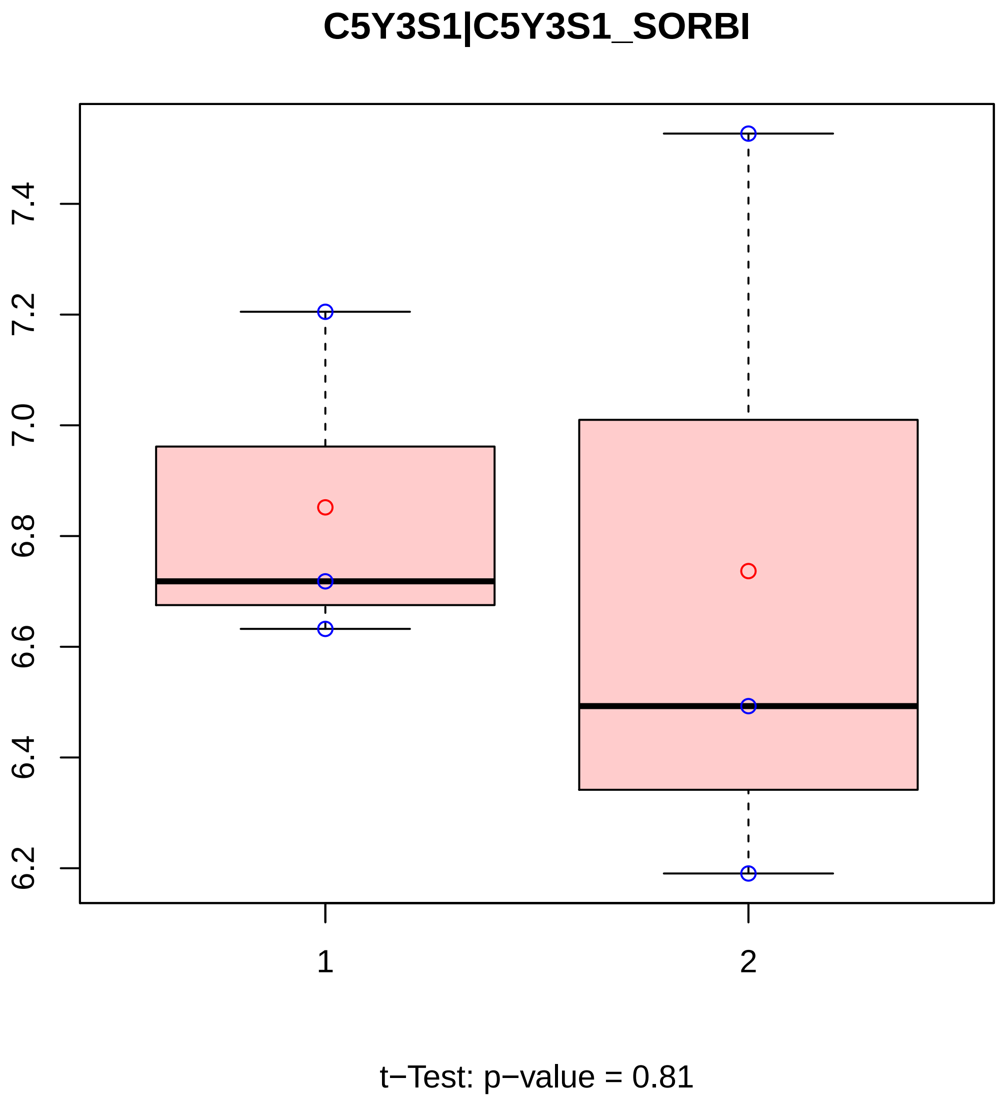

C5Y3S1|C5Y3S1_SORBI
7.4
7.2
7.0
6.8
6.6
6.4
6.2
1
2
t−Test: p−value = 0.81

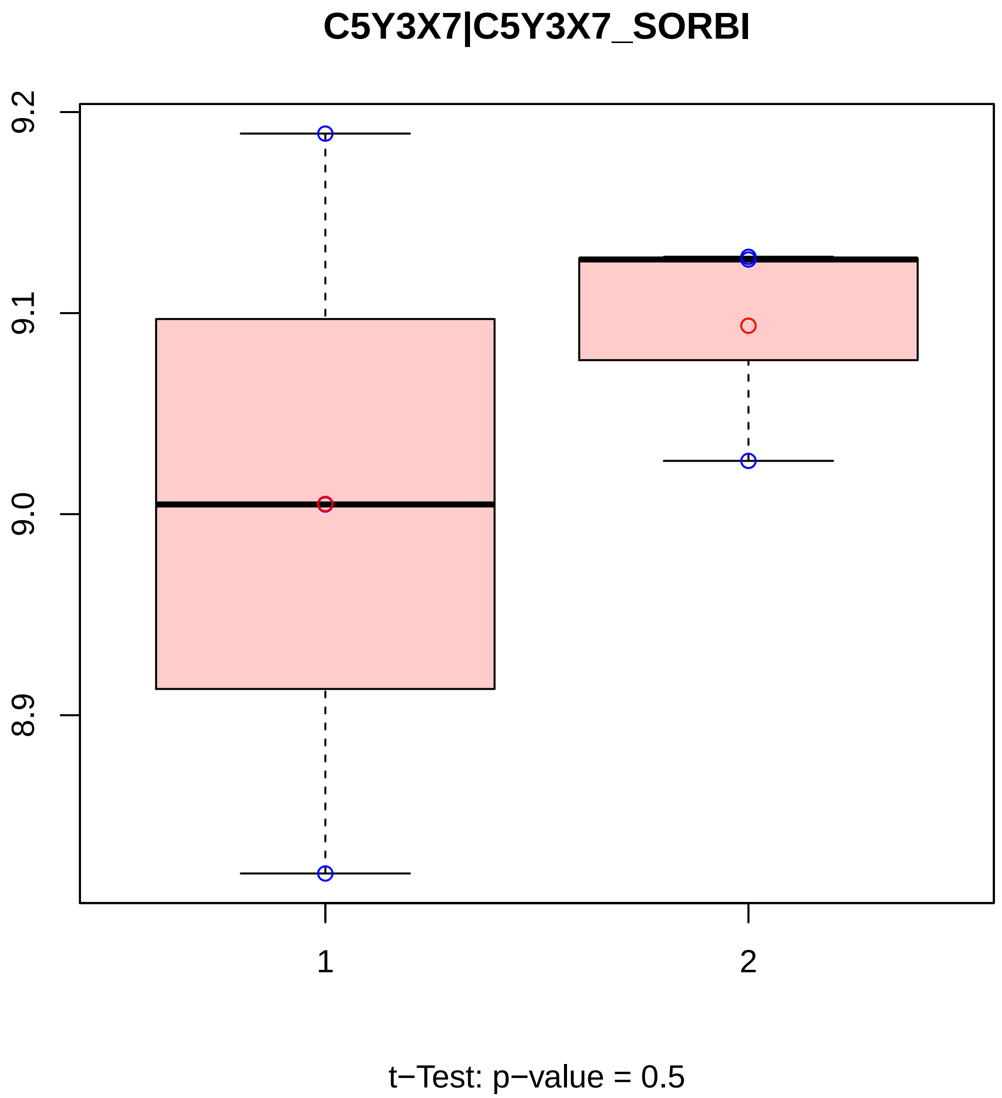
C5Y3X7|C5Y3X7_SORBI
9.2
9.1
9.0
8.9
1
2
t−Test: p−value = 0.5

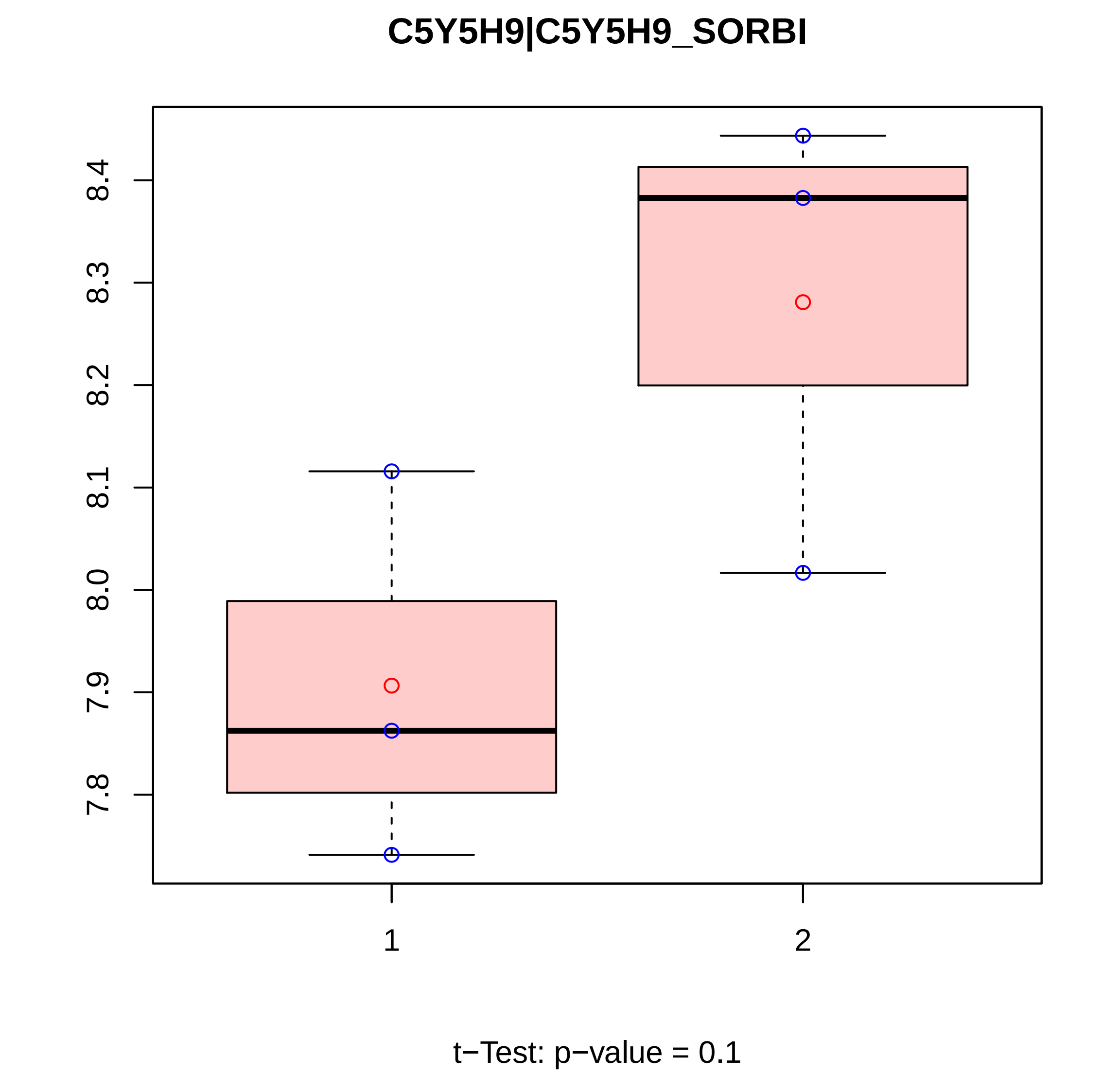
C5Y5H9|C5Y5H9_SORBI
8.4
8.3
8.2
8.1
8.0
7.9
7.8
1
2
t−Test: p−value = 0.1

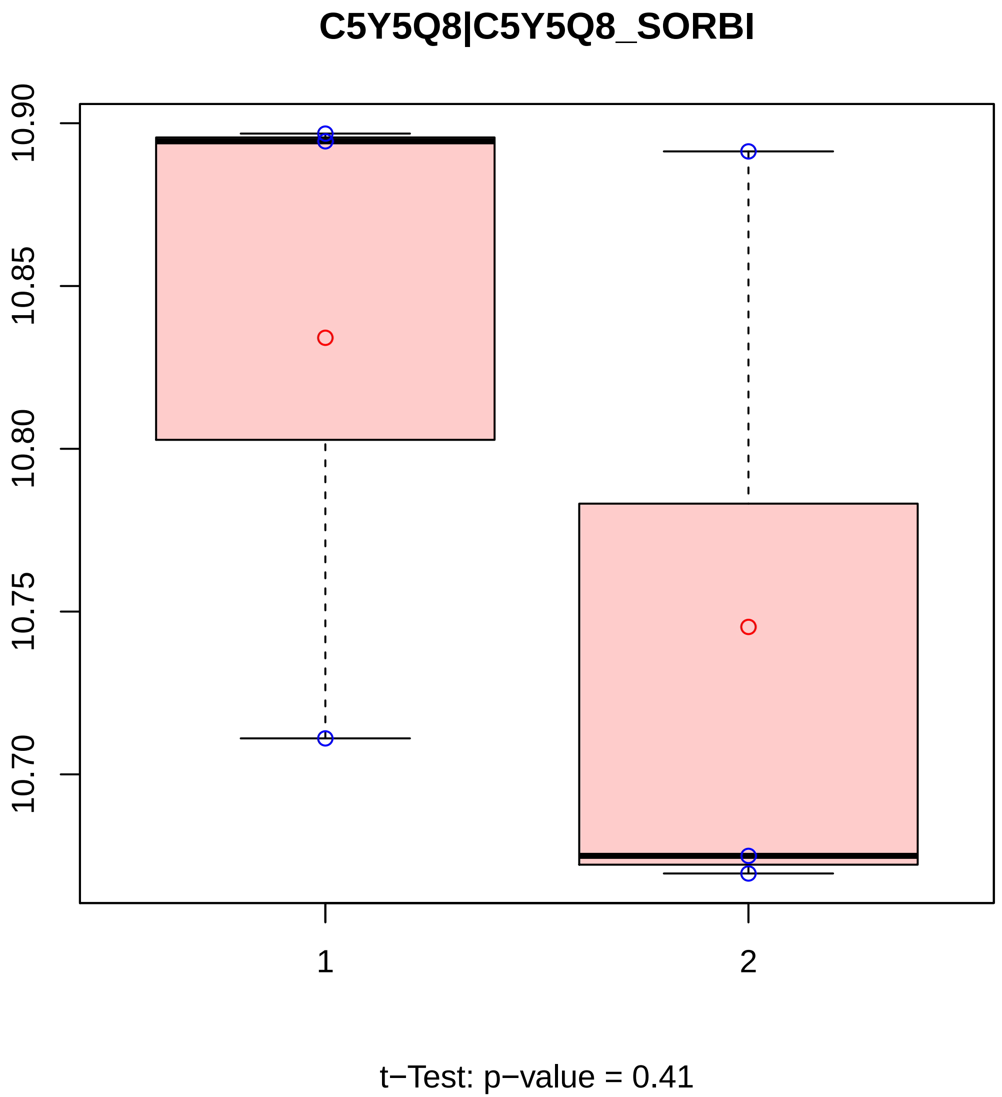
C5Y5Q8|C5Y5Q8_SORBI
10.90
10.85
10.80
10.75
10.70
1
2
t−Test: p−value = 0.41

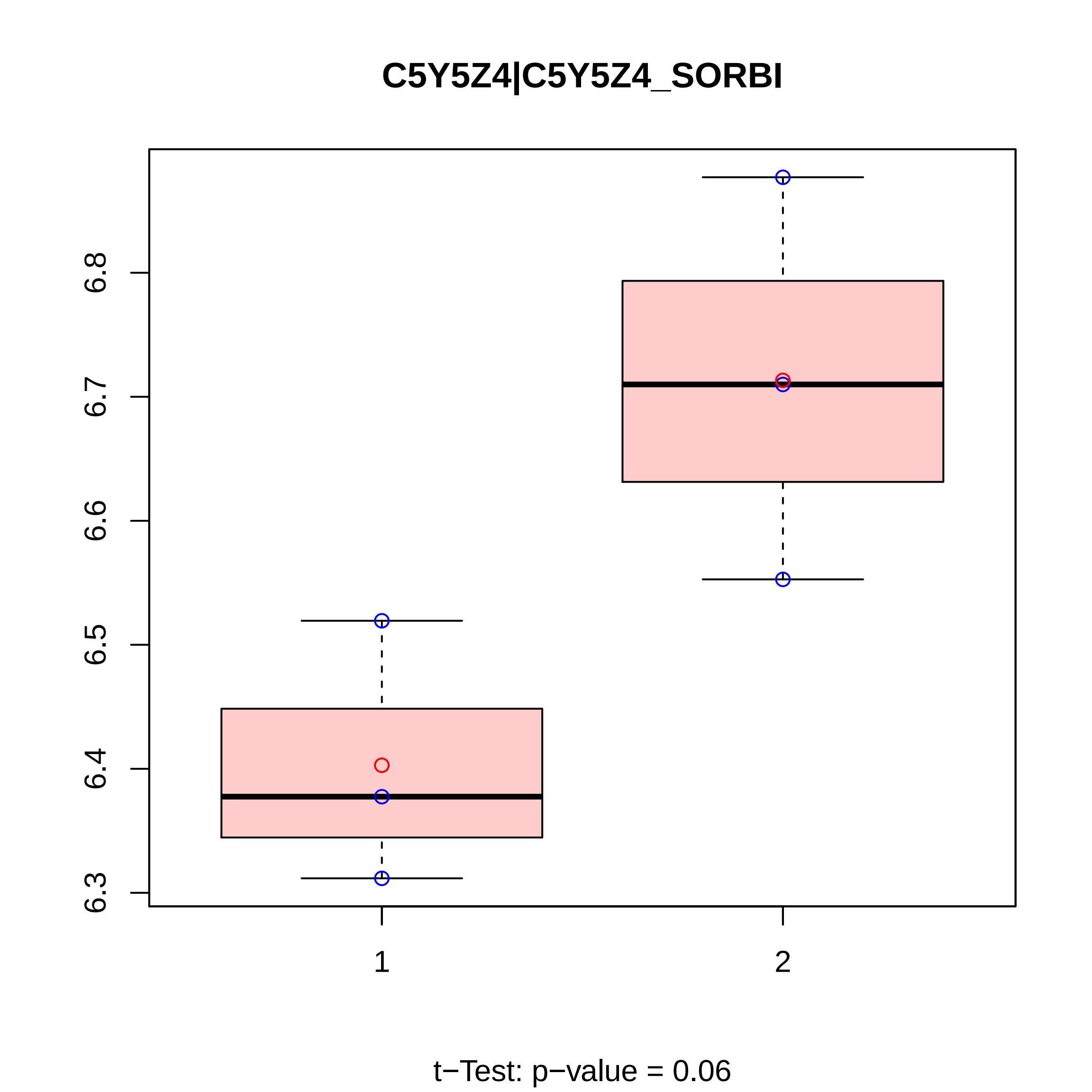
C5Y5Z4|C5Y5Z4_SORBI
6.3
6.4
6.5
6.6
6.7
6.8
1
2
t−Test: p−value = 0.06

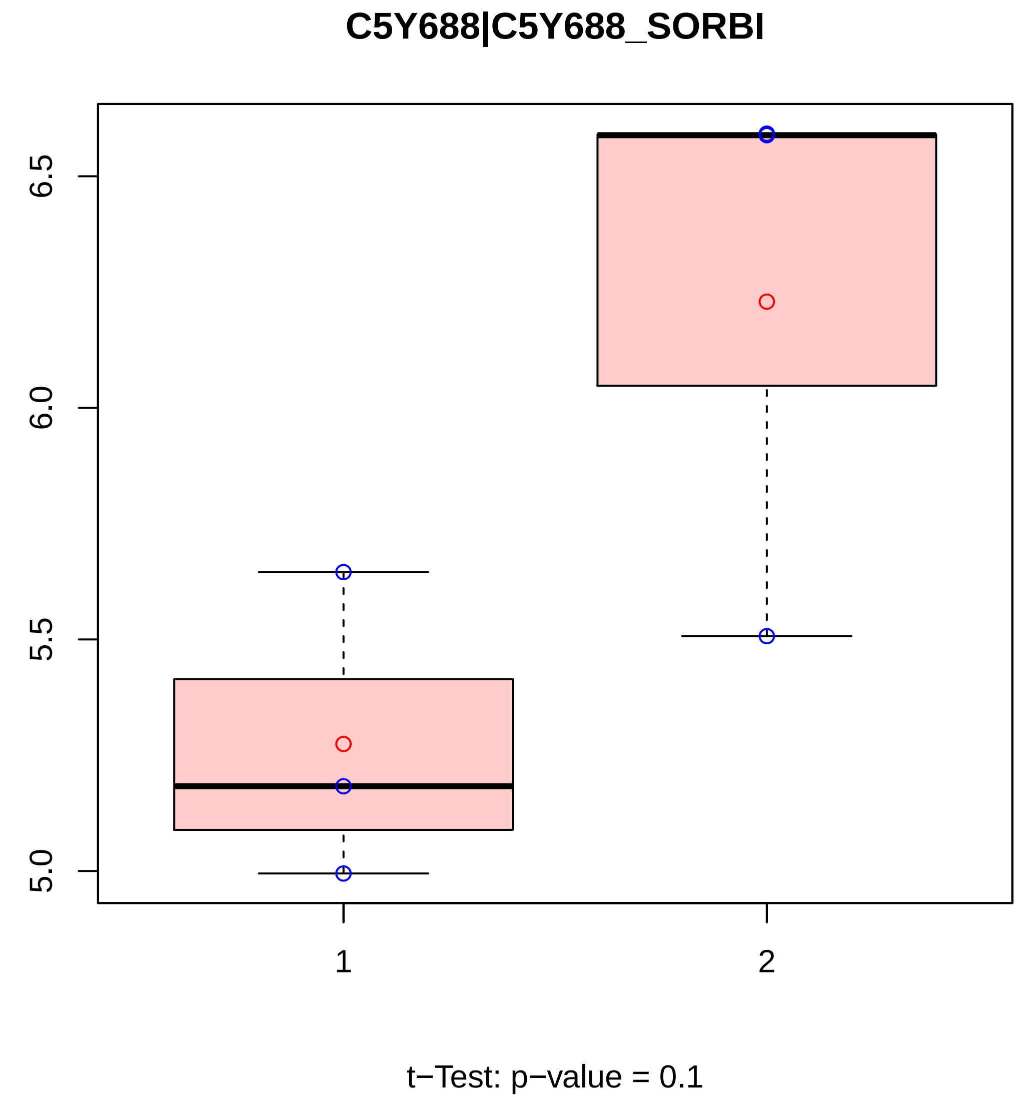
C5Y688|C5Y688_SORBI
6.5
6.0
5.5
5.0
1
2
t−Test: p−value = 0.1

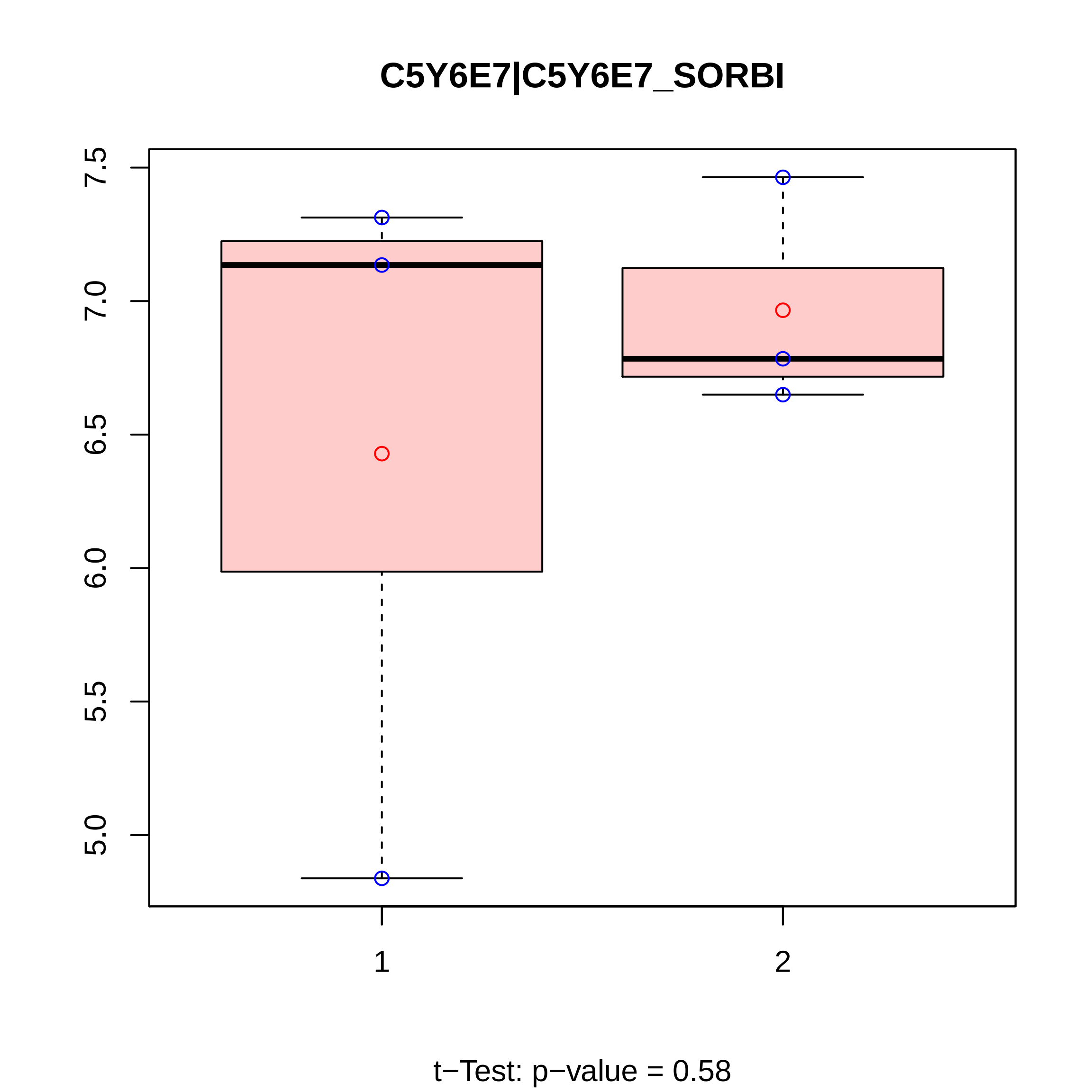

C5Y6E7|C5Y6E7_SORBI
7.5
7.0
6.5
6.0
5.5
5.0
1
2
t−Test: p−value = 0.58

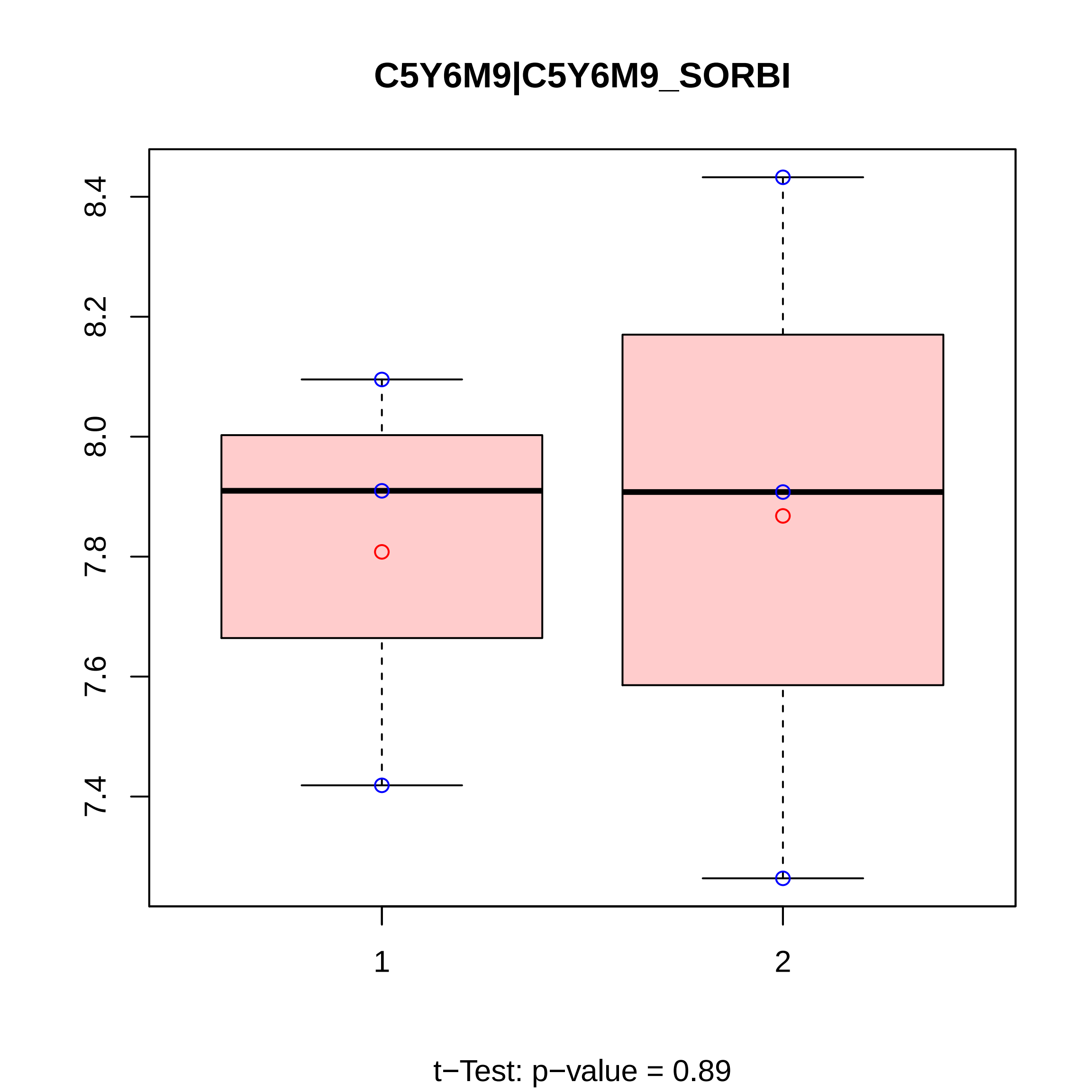
C5Y6M9|C5Y6M9_SORBI
8.4
8.2
8.0
7.8
7.6
7.4
1
2
t−Test: p−value = 0.89

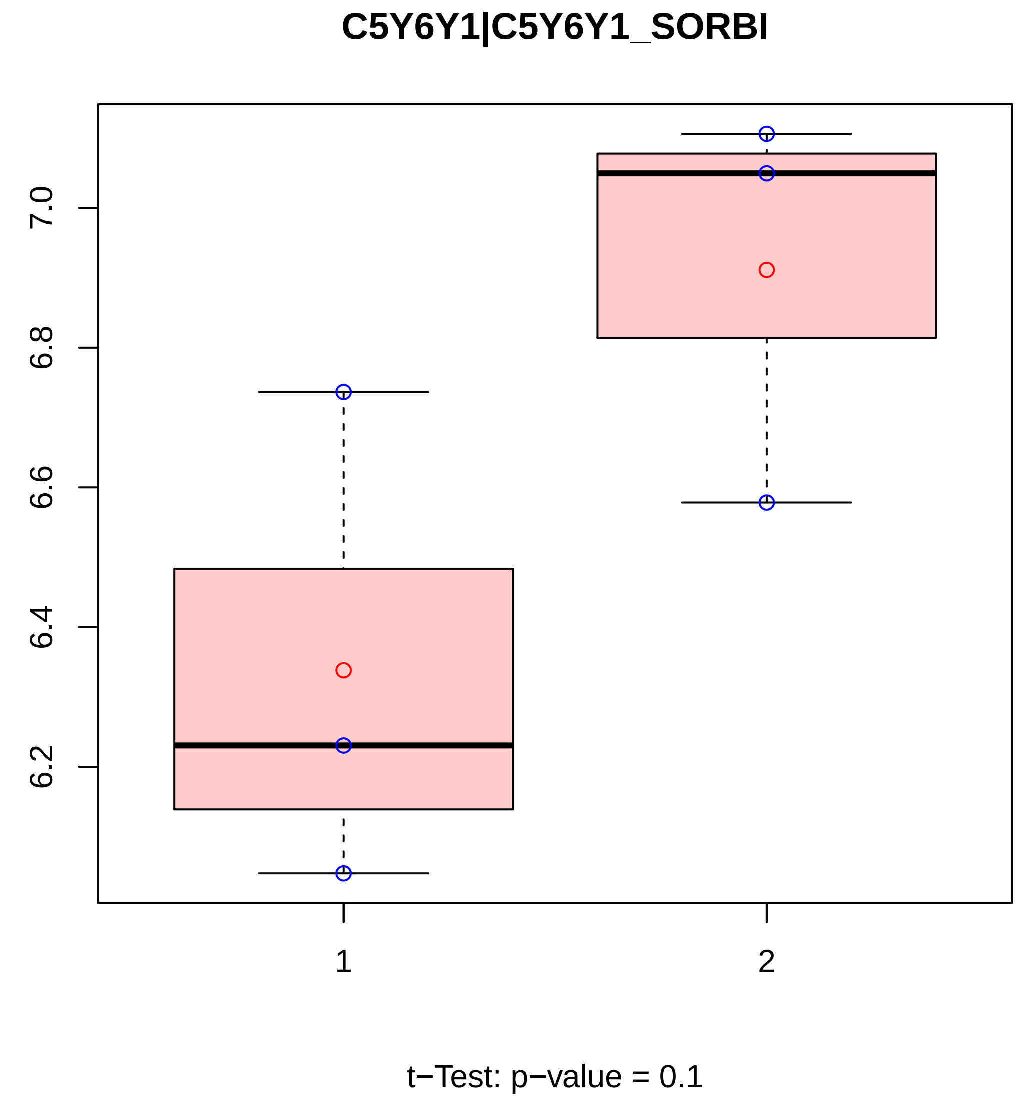
C5Y6Y1|C5Y6Y1_SORBI
7.0
6.8
6.6
6.4
6.2
1
2
t−Test: p−value = 0.1

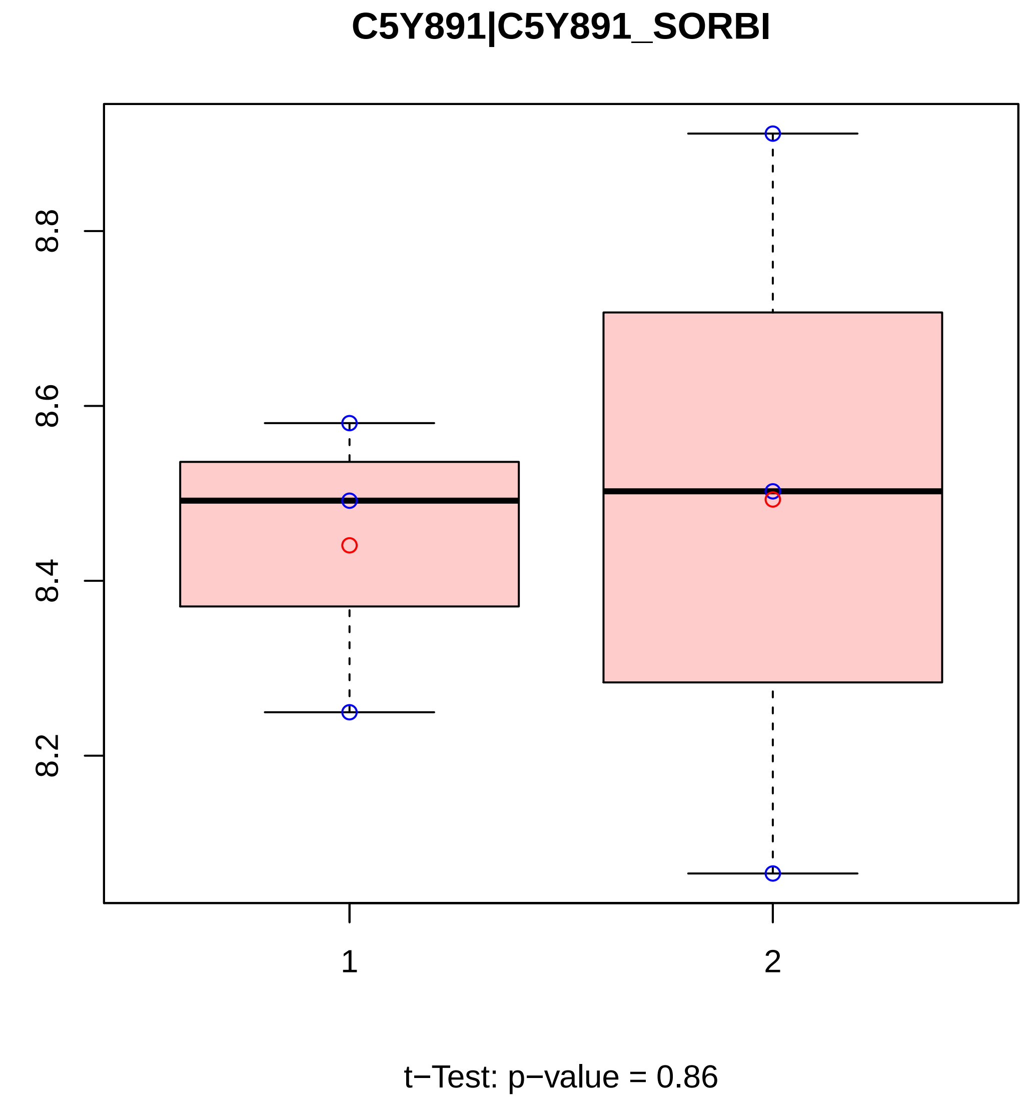
C5Y891|C5Y891_SORBI
8.2
8.4
8.6
8.8
1
2
t−Test: p−value = 0.86

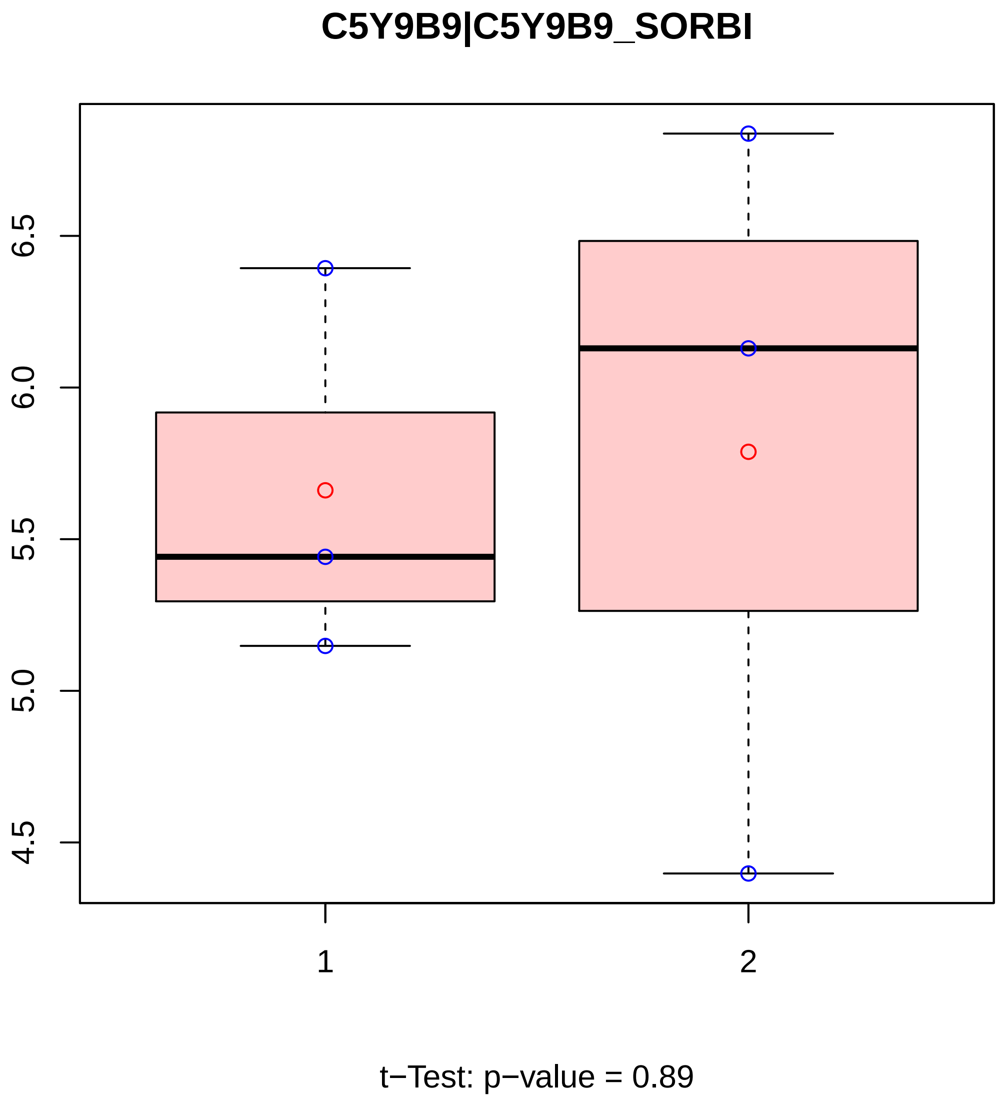
C5Y9B9|C5Y9B9_SORBI
6.5
6.0
5.5
5.0
4.5
1
2
t−Test: p−value = 0.89

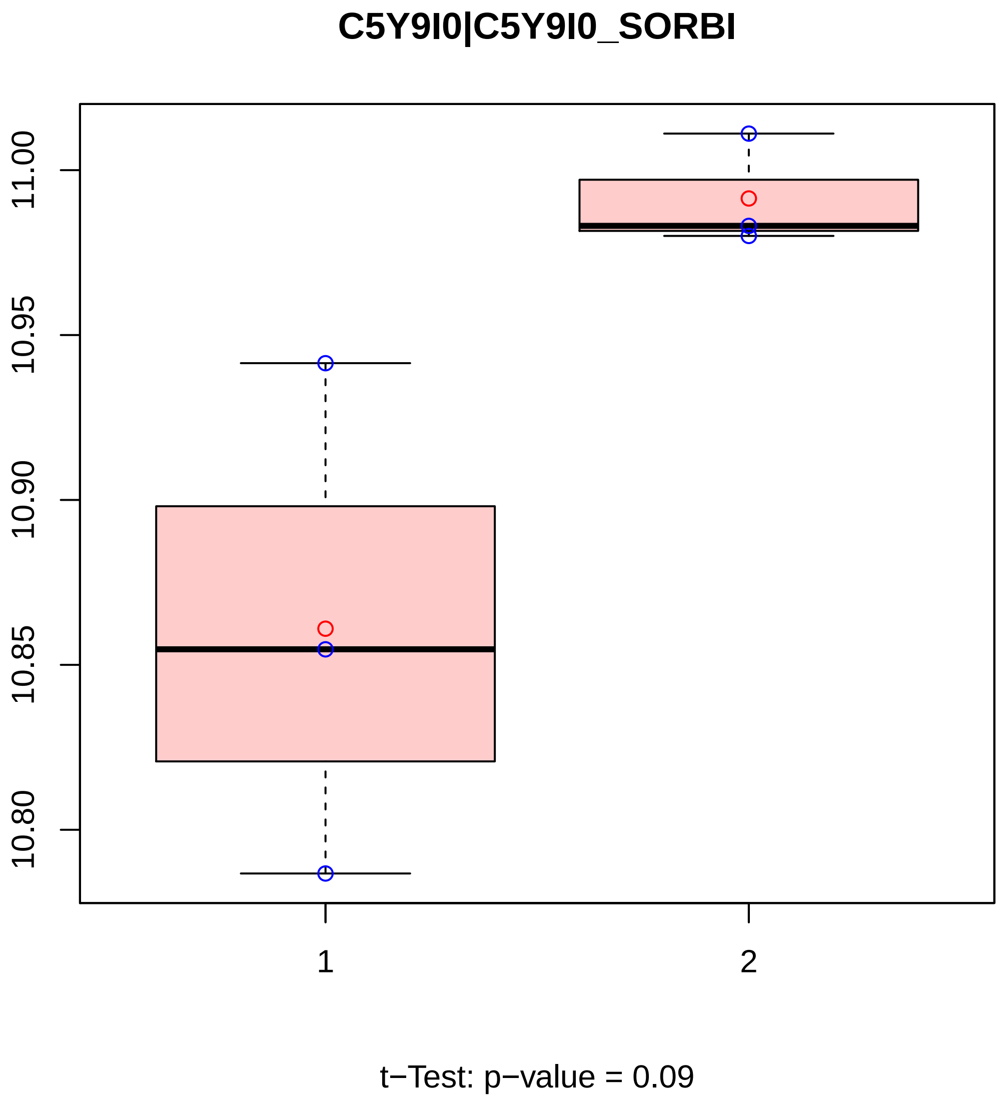
C5Y9I0|C5Y9I0_SORBI
11.00
10.95
10.90
10.85
10.80
1
2
t−Test: p−value = 0.09

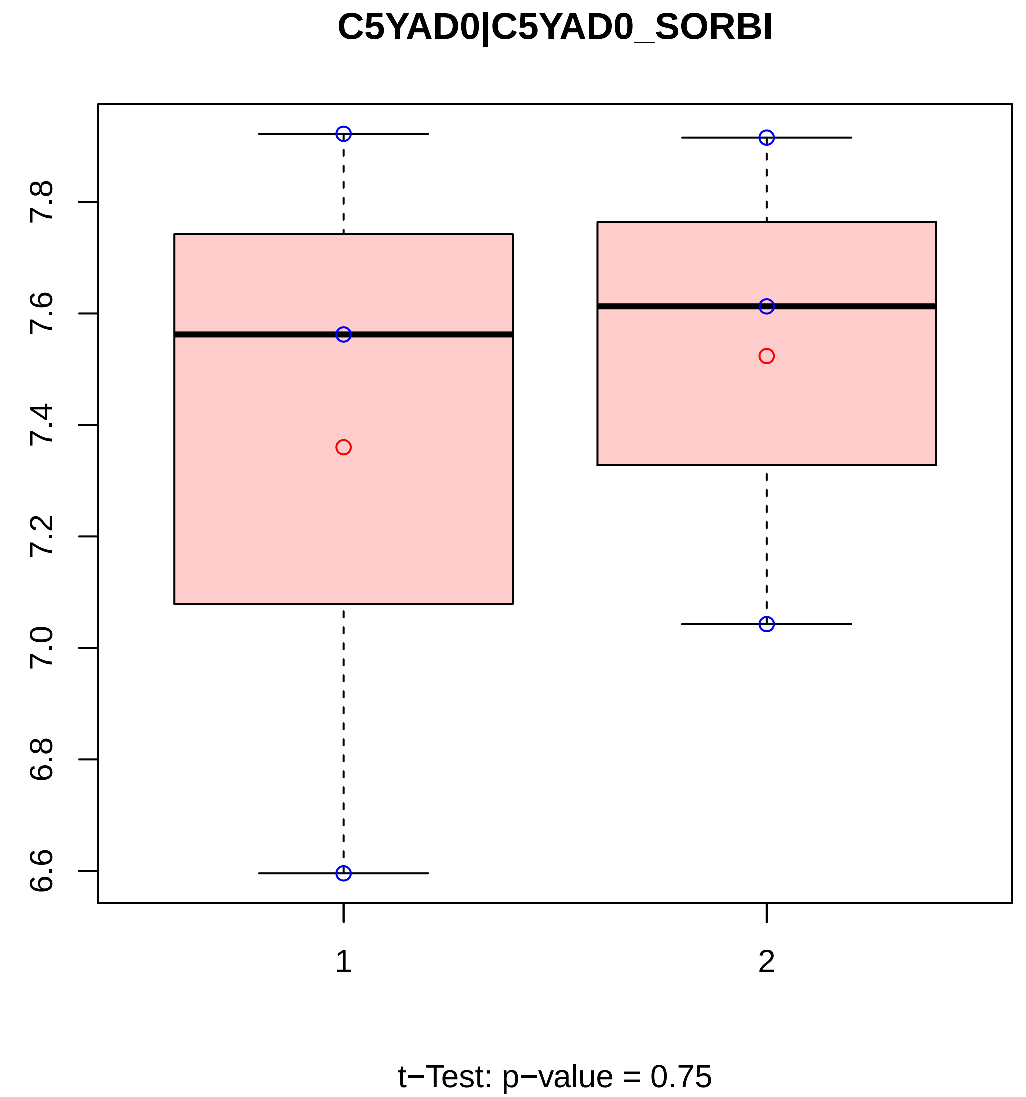
C5YAD0|C5YAD0_SORBI
7.8
7.6
7.4
7.2
7.0
6.8
6.6
1
2
t−Test: p−value = 0.75

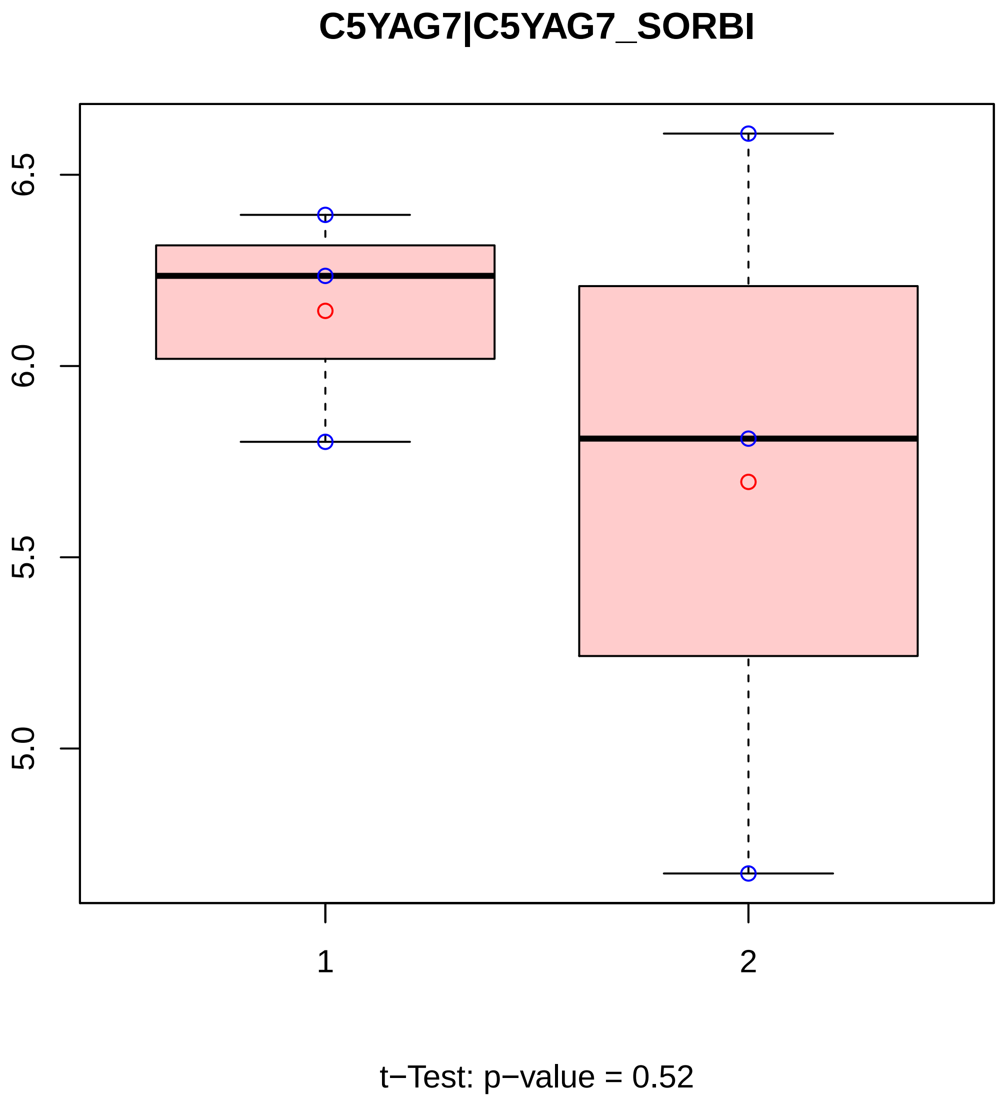
C5YAG7|C5YAG7_SORBI
6.5
6.0
5.5
5.0
1
2
t−Test: p−value = 0.52

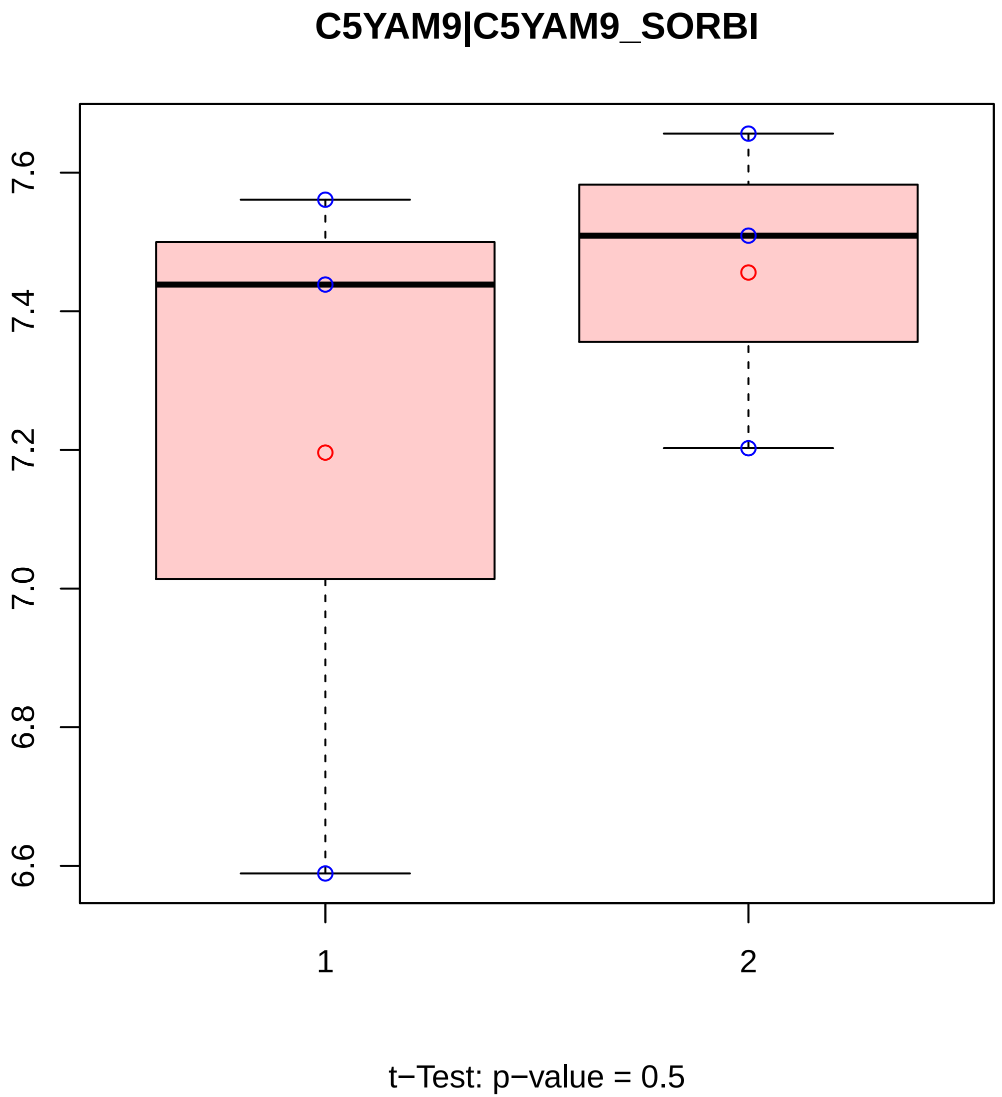
C5YAM9|C5YAM9_SORBI
7.6
7.4
7.2
7.0
6.8
6.6
1
2
t−Test: p−value = 0.5

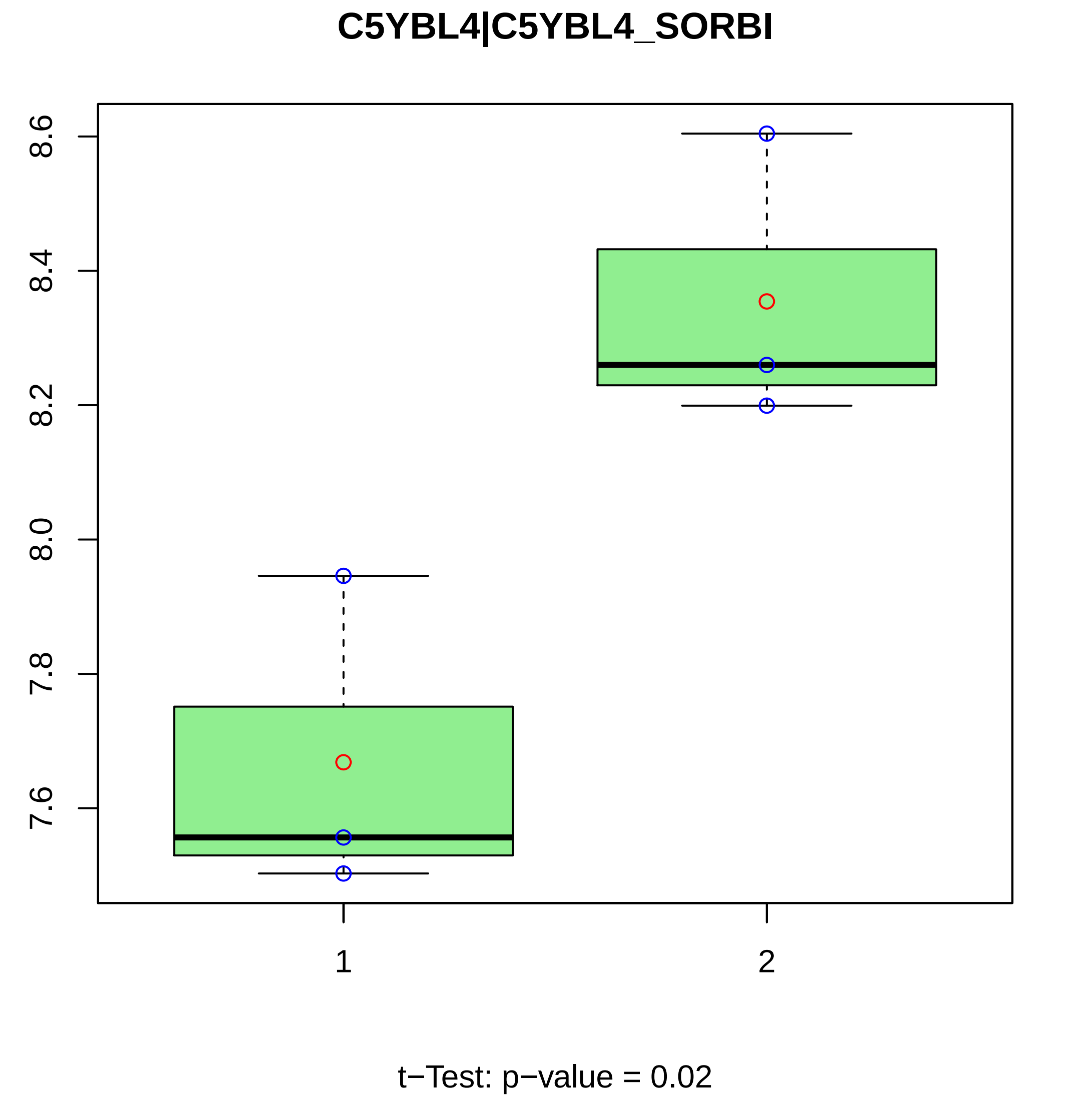

C5YBL4|C5YBL4_SORBI
8.6
8.4
8.2
8.0
7.8
7.6
1
2
t−Test: p−value = 0.02

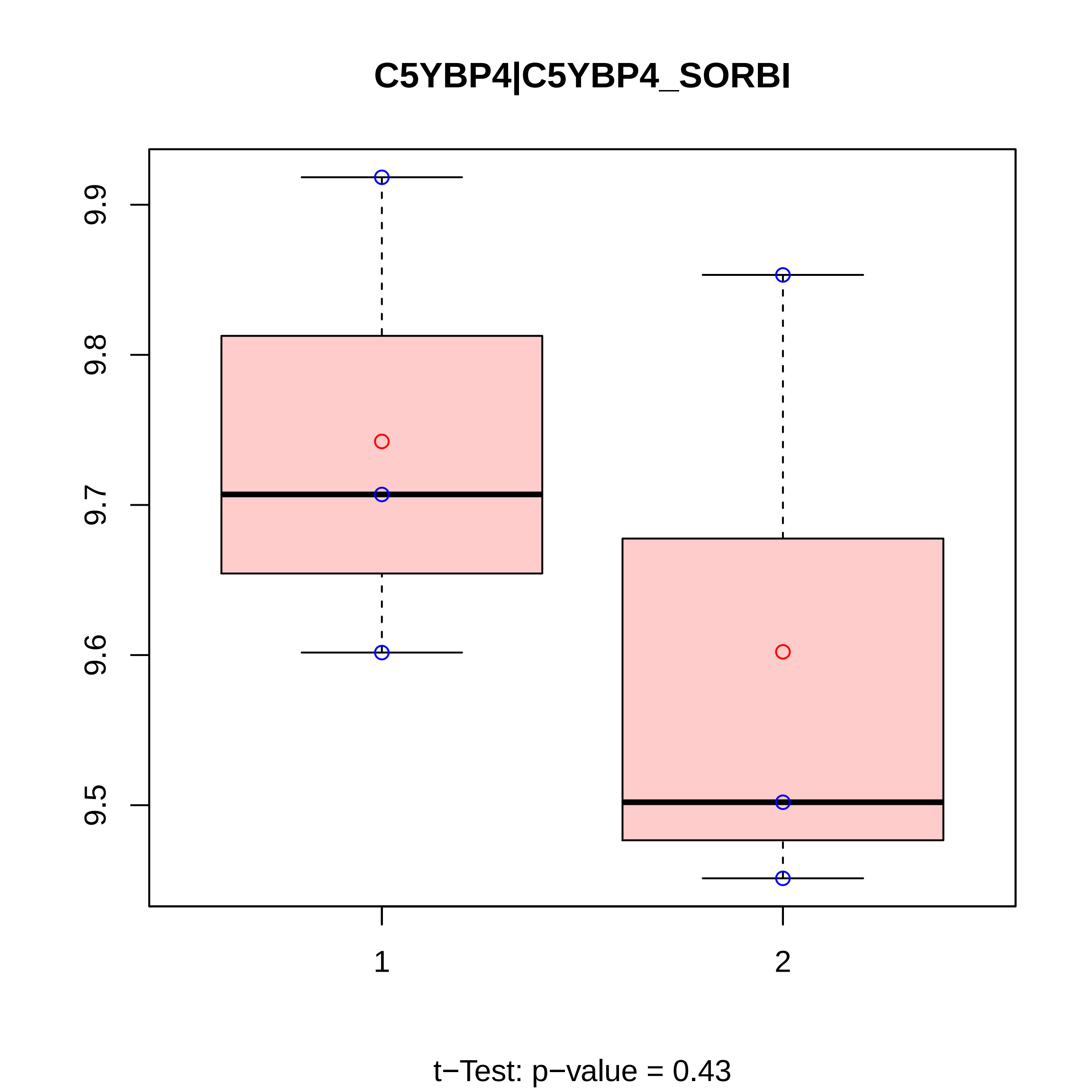

C5YBP4|C5YBP4_SORBI
9.9
9.8
9.7
9.6
9.5
1
2
t−Test: p−value = 0.43

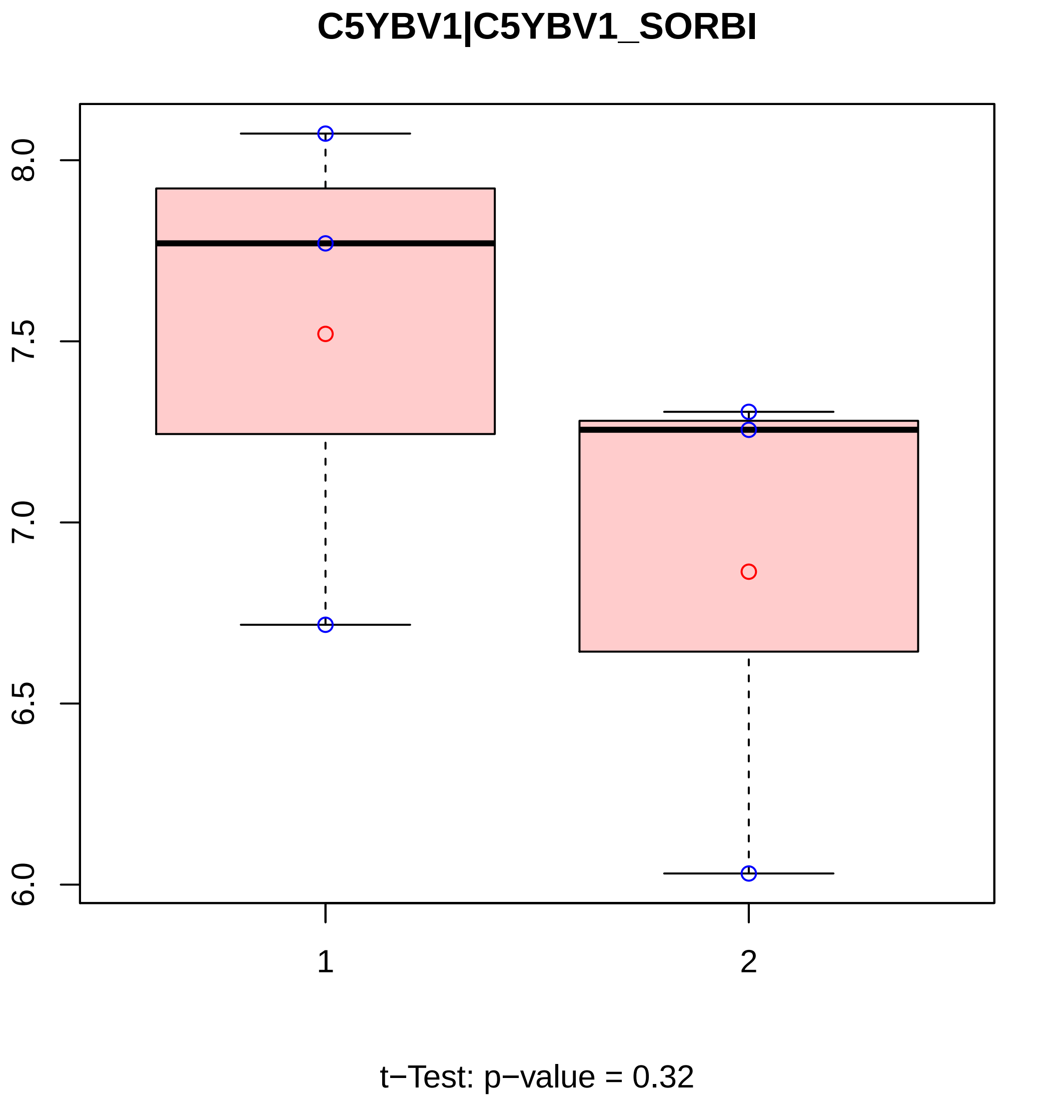

C5YBV1|C5YBV1_SORBI
8.0
7.5
7.0
6.5
6.0
1
2
t−Test: p−value = 0.32

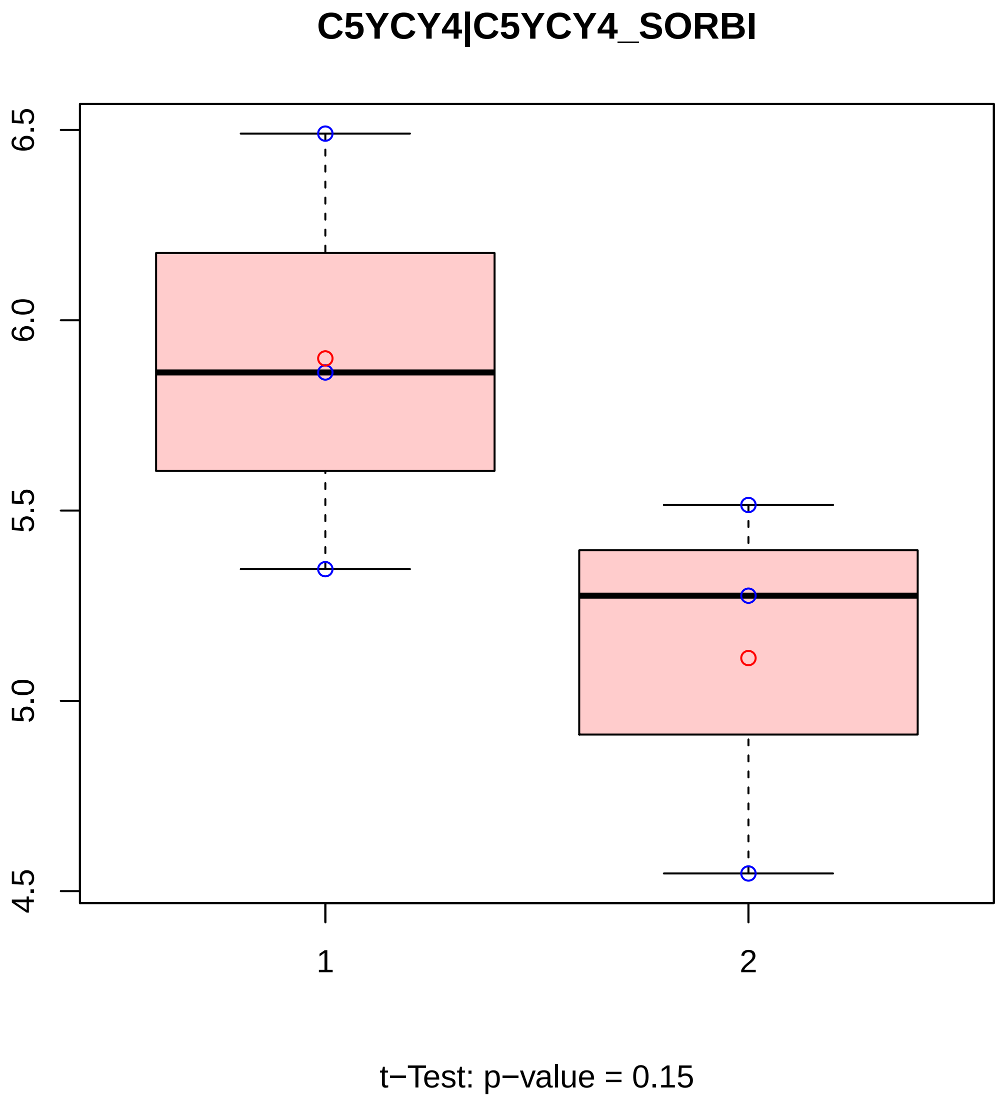
C5YCY4|C5YCY4_SORBI
6.5
6.0
5.5
5.0
4.5
1
2
t−Test: p−value = 0.15

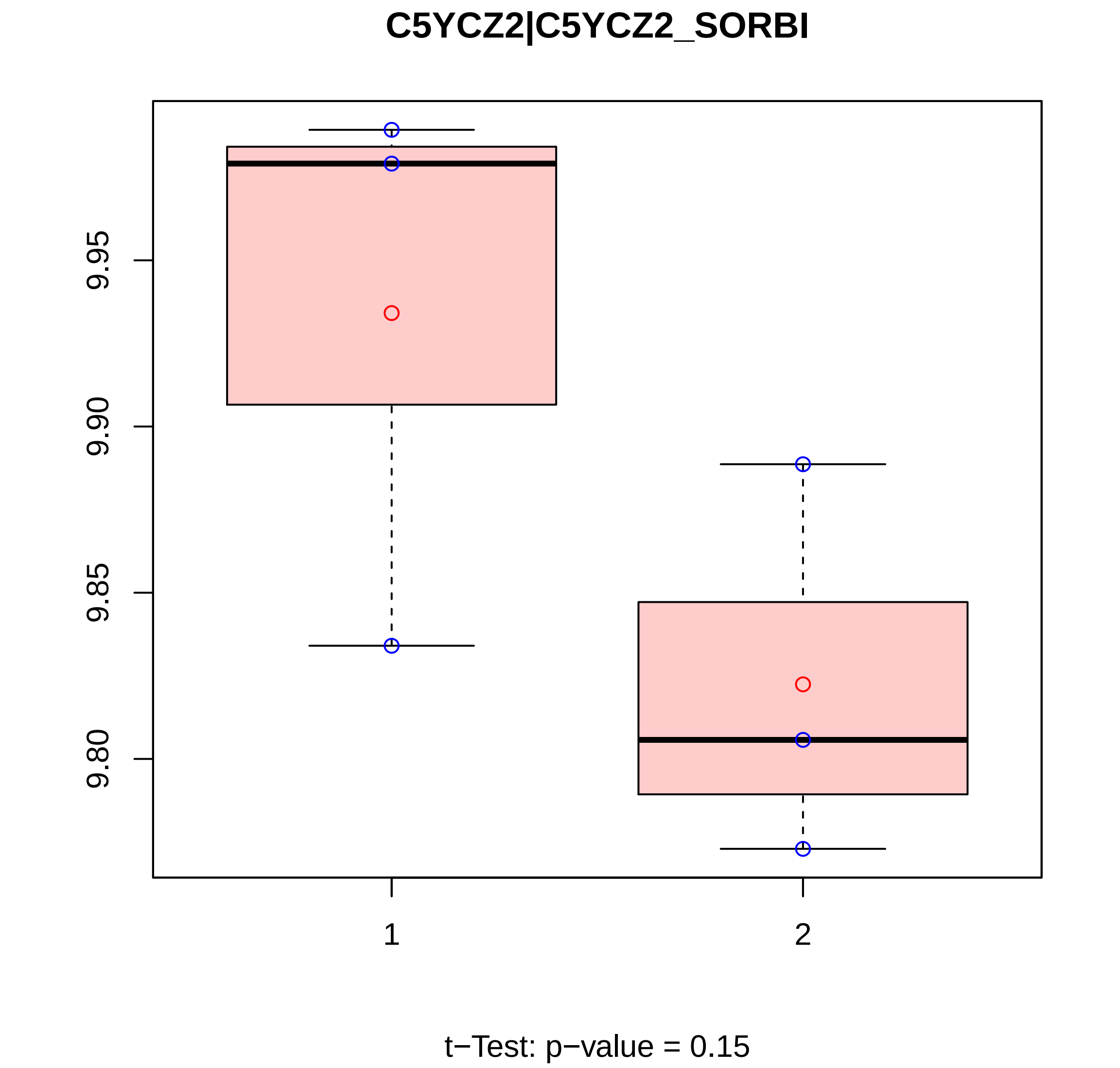

C5YCZ2|C5YCZ2_SORBI
9.80
9.85
9.90
9.95
1
2
t−Test: p−value = 0.15

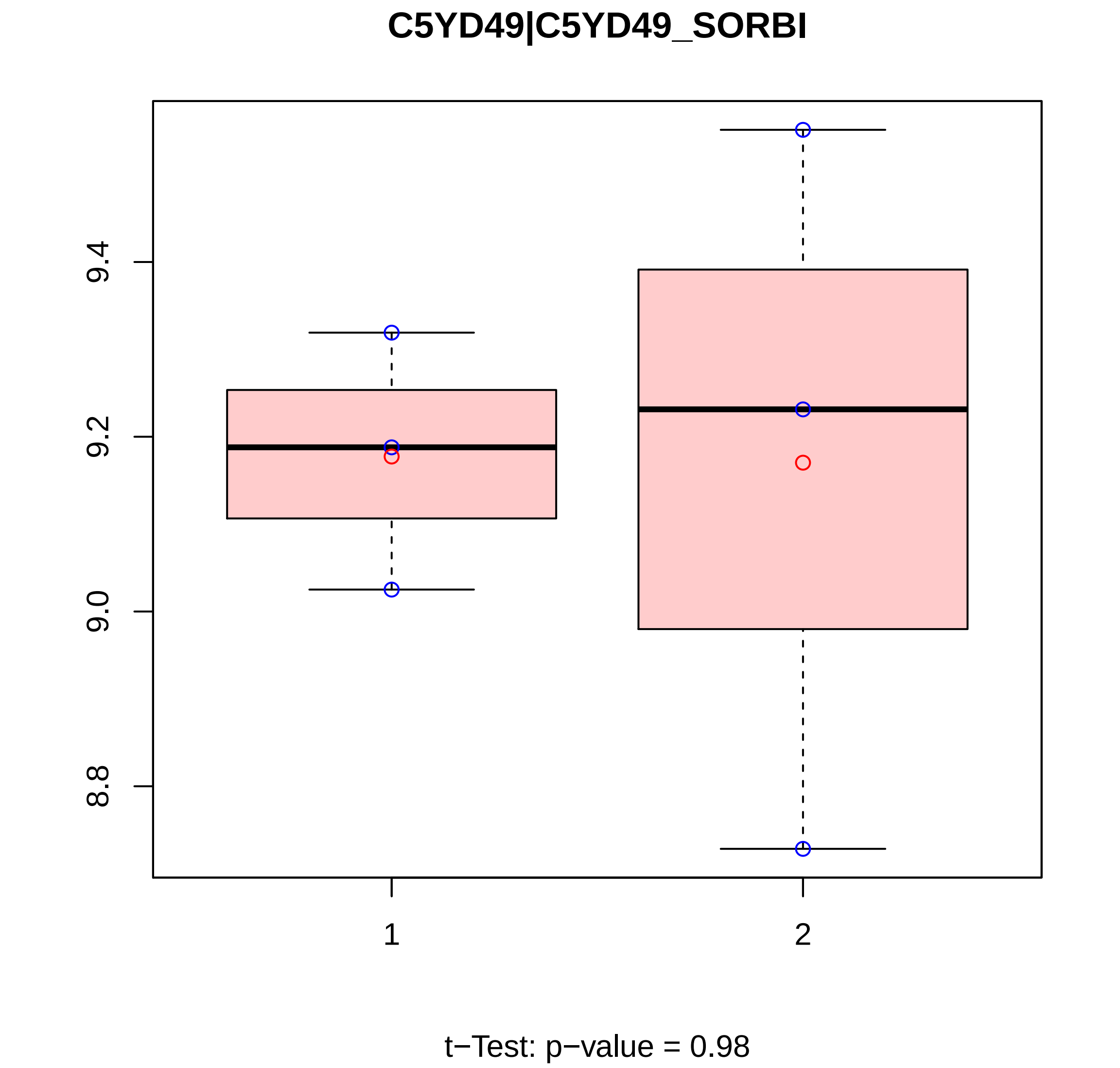
C5YD49|C5YD49_SORBI
9.4
9.2
9.0
8.8
1
2
t−Test: p−value = 0.98

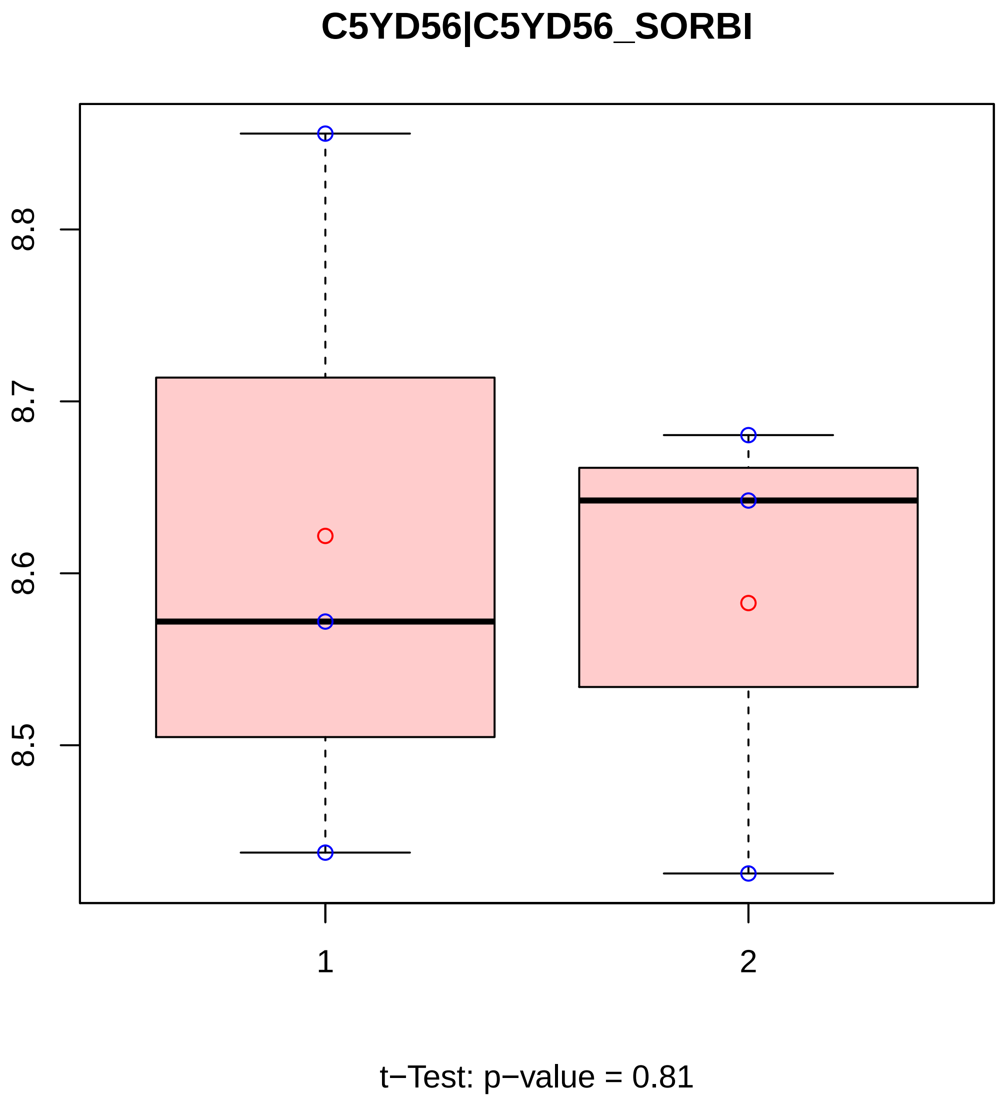
C5YD56|C5YD56_SORBI
8.5
8.6
8.7
8.8
1
2
t−Test: p−value = 0.81

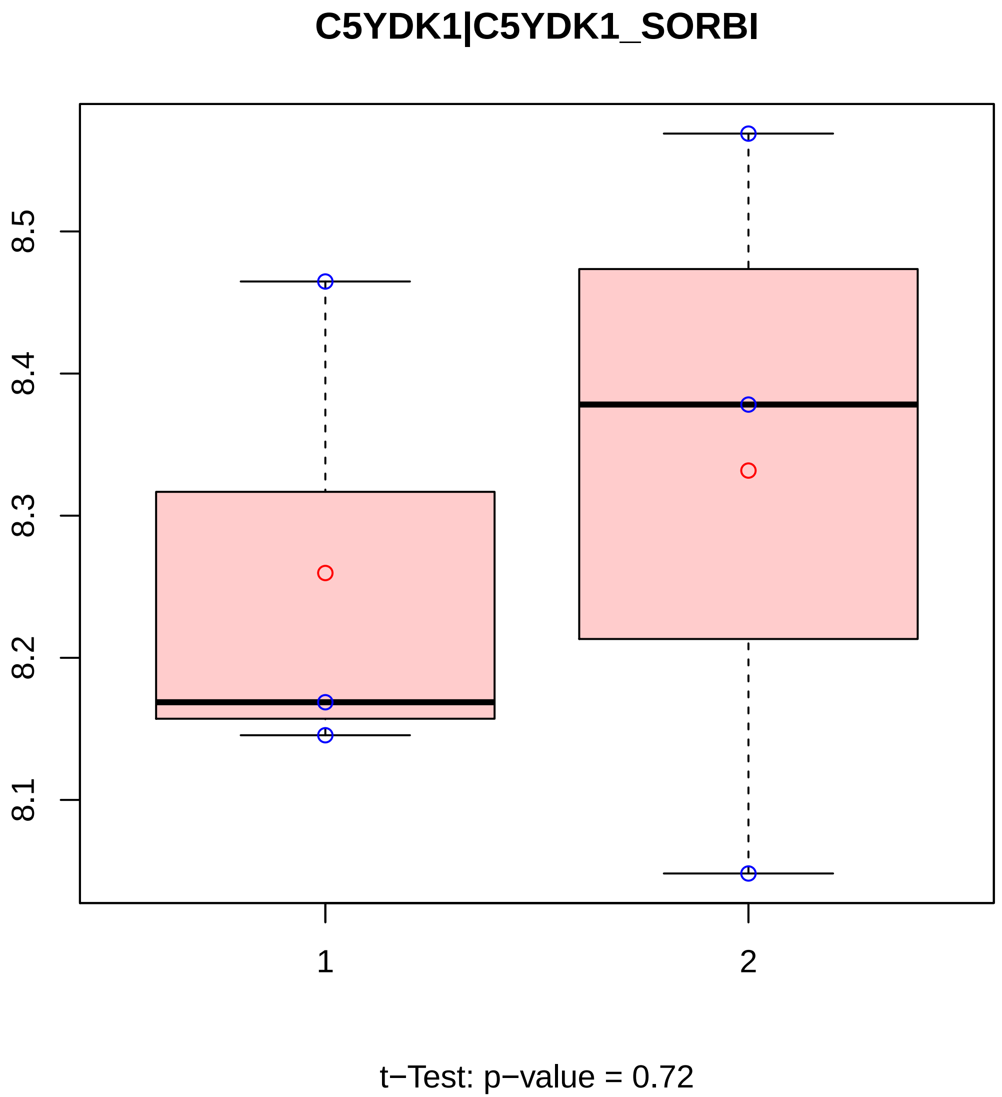

C5YDK1|C5YDK1_SORBI
8.1
8.2
8.3
8.4
8.5
1
2
t−Test: p−value = 0.72

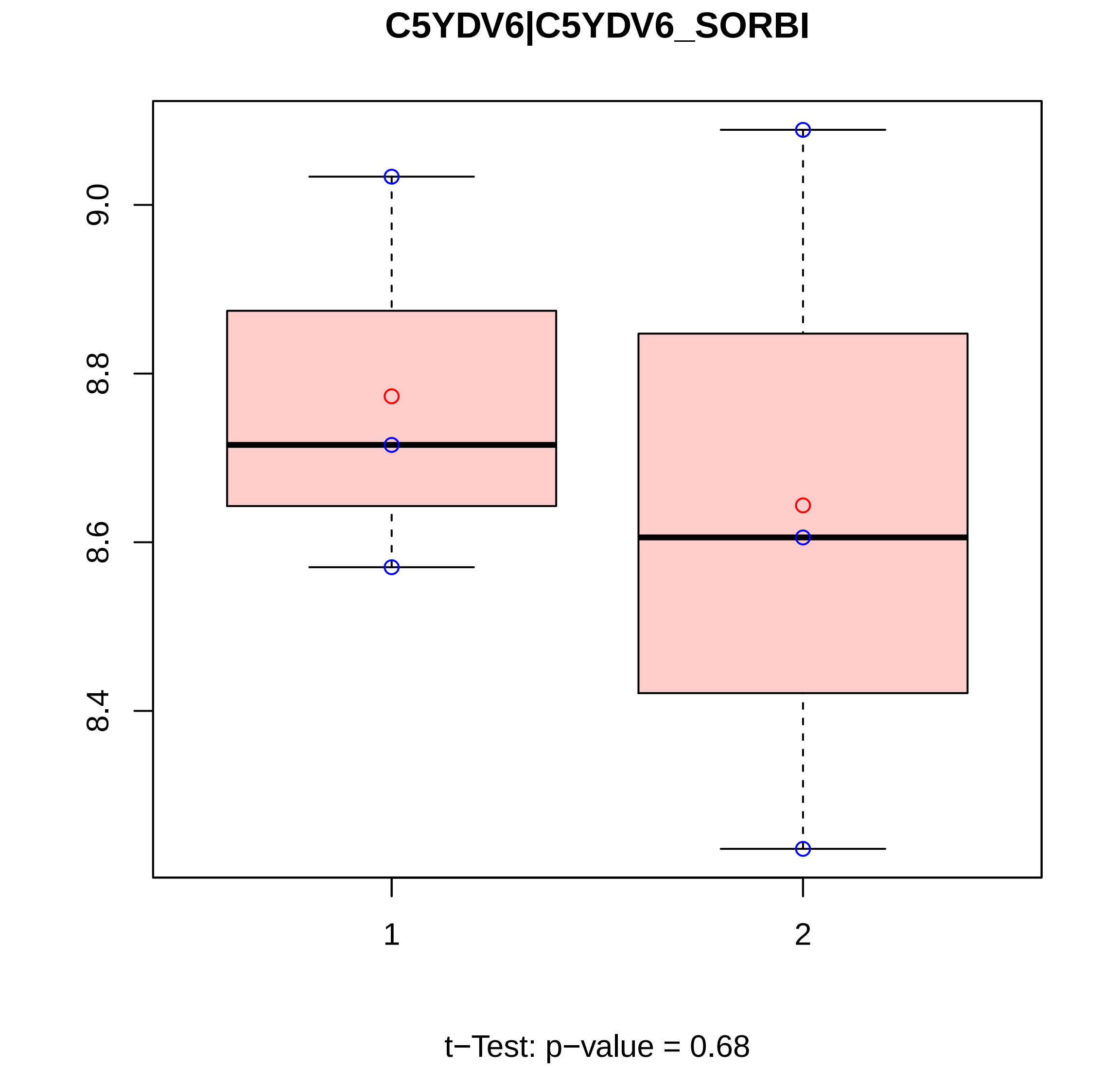

C5YDV6|C5YDV6_SORBI
9.0
8.8
8.6
8.4
1
2
t−Test: p−value = 0.68

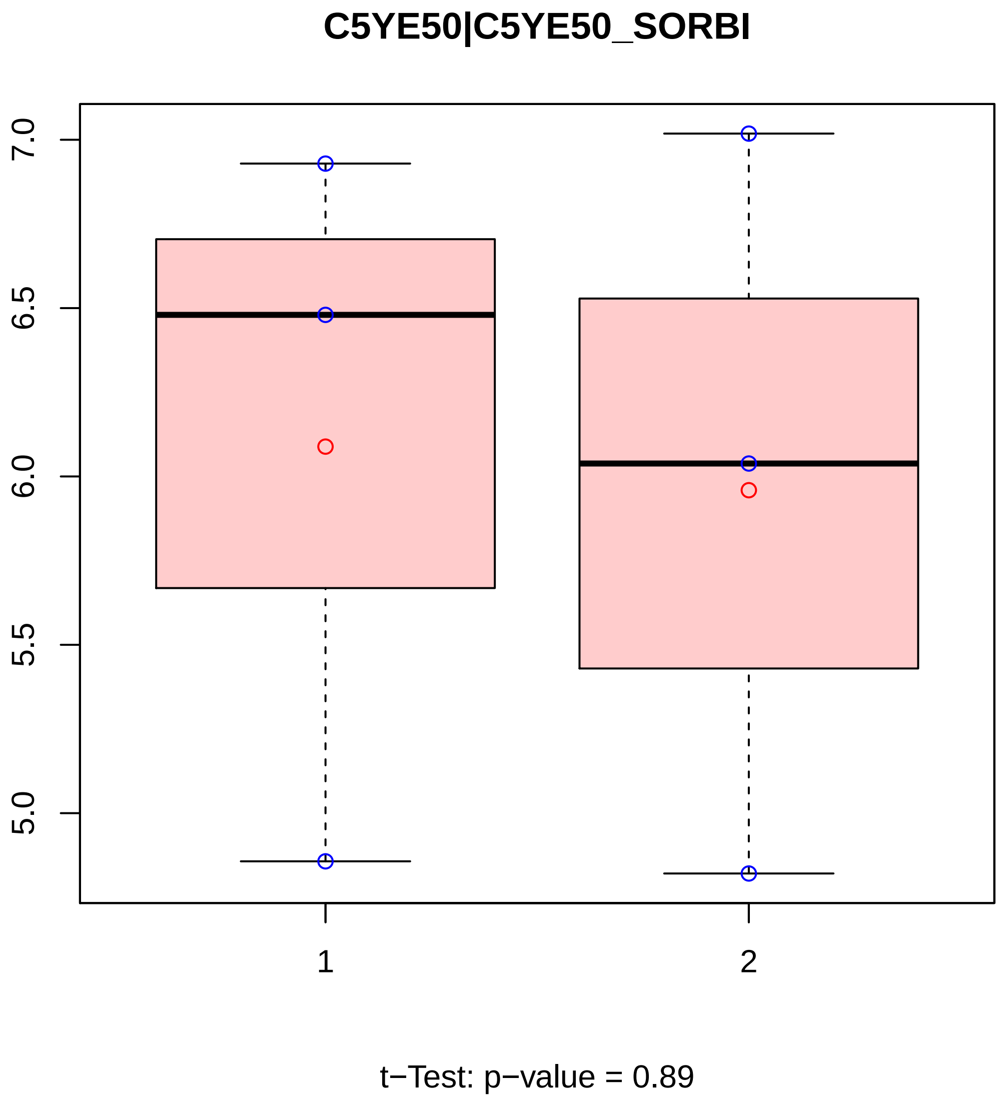
C5YE50|C5YE50_SORBI
7.0
6.5
6.0
5.5
5.0
1
2
t−Test: p−value = 0.89

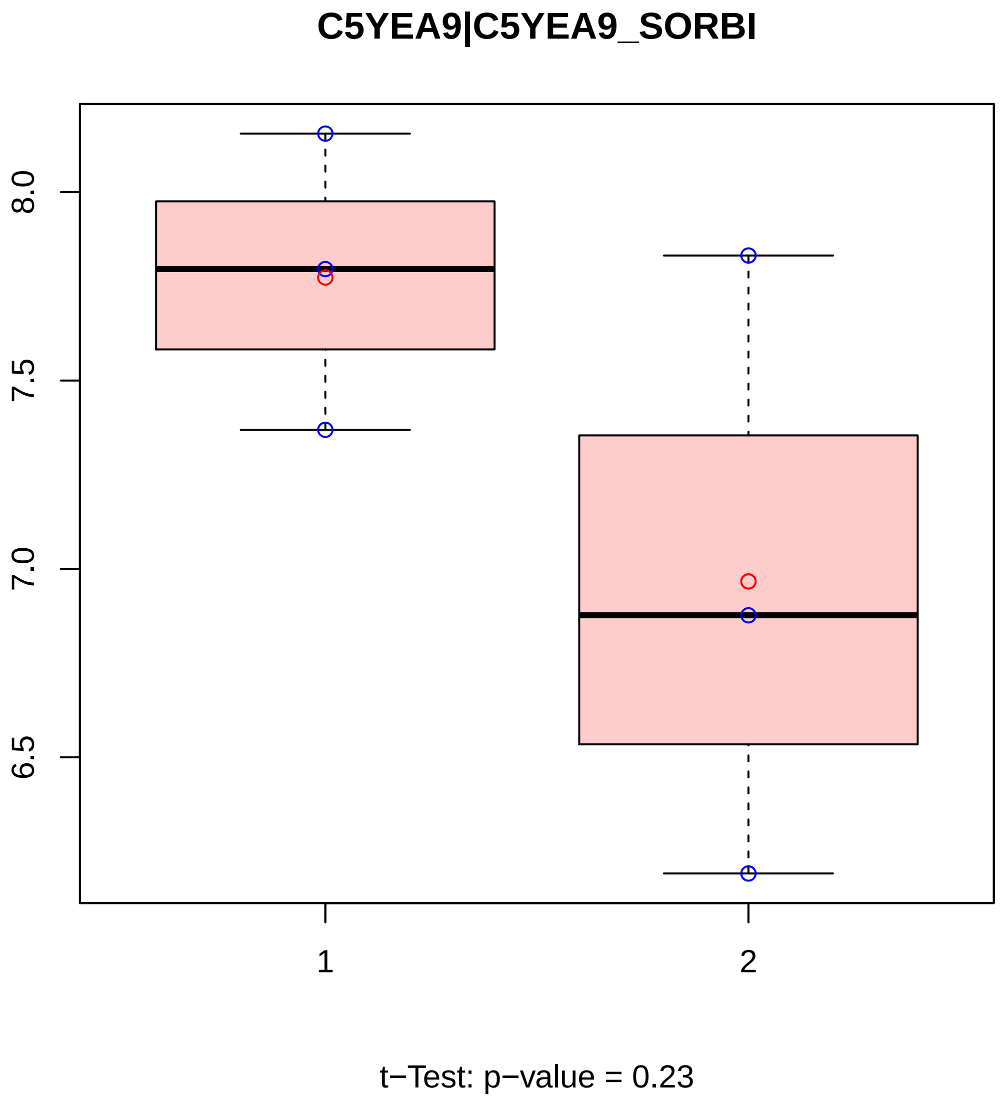

C5YEA9|C5YEA9_SORBI
8.0
7.5
7.0
6.5
1
2
t−Test: p−value = 0.23

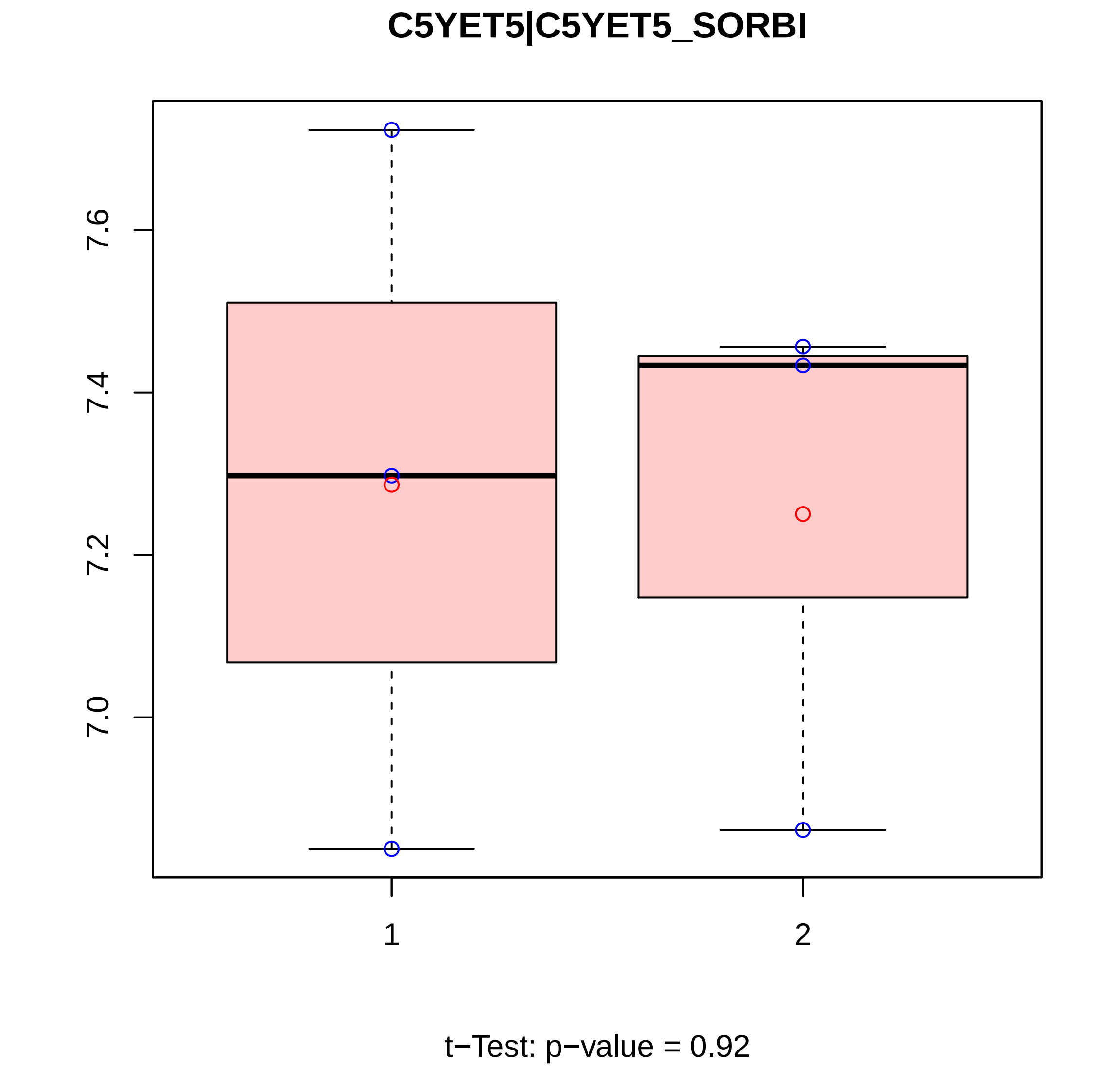
C5YET5|C5YET5_SORBI
7.0
7.2
7.4
7.6
1
2
t−Test: p−value = 0.92

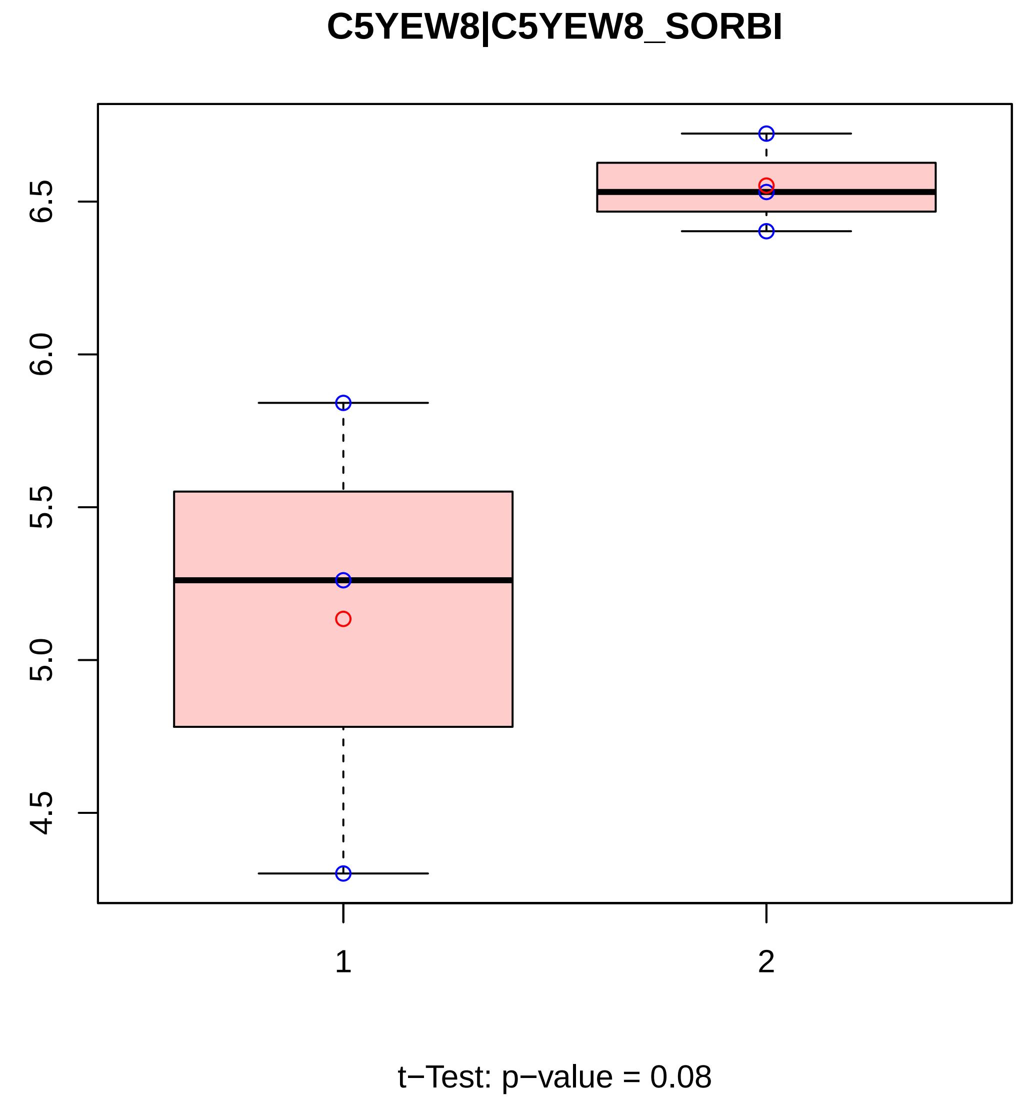

C5YEW8|C5YEW8_SORBI
6.5
6.0
5.5
5.0
4.5
1
2
t−Test: p−value = 0.08

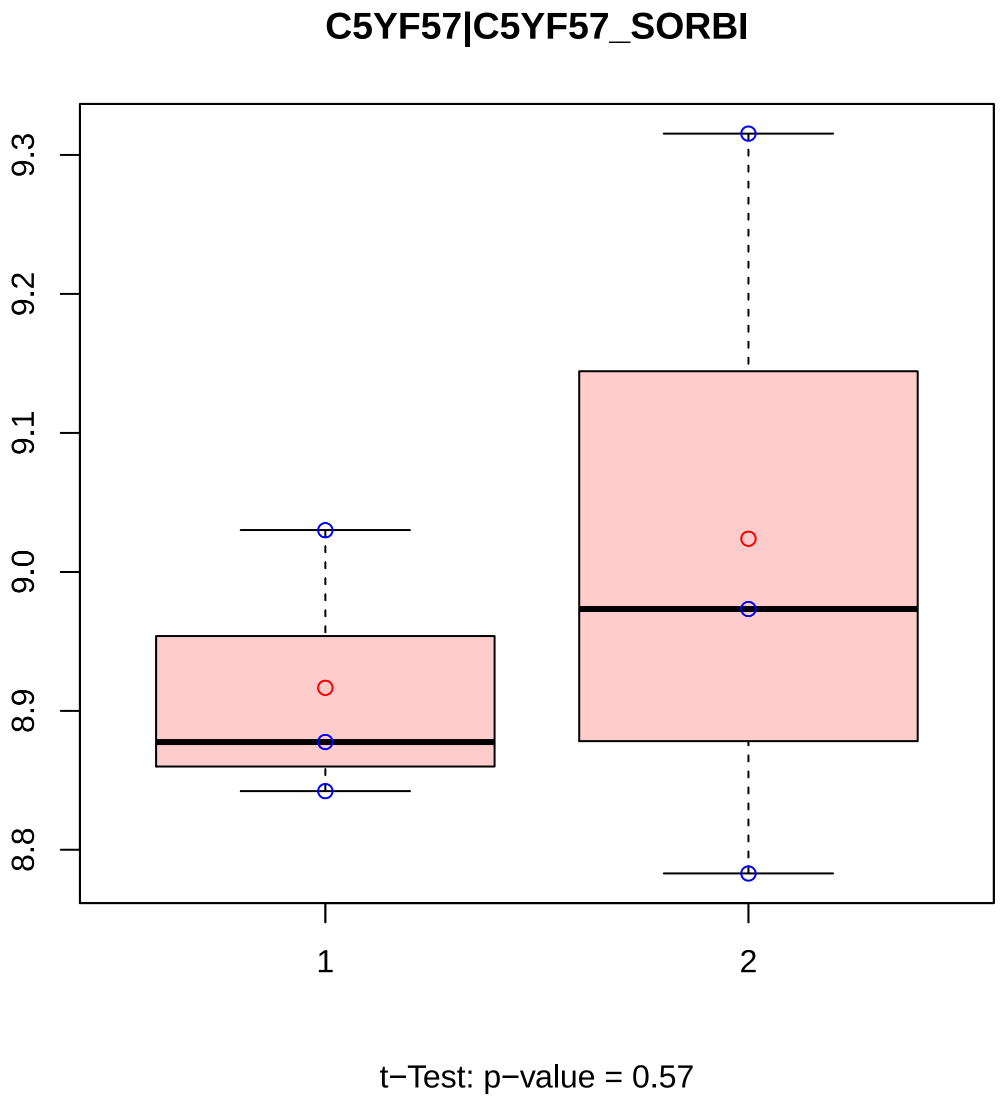
C5YF57|C5YF57_SORBI
9.3
9.2
9.1
9.0
8.9
8.8
1
2
t−Test: p−value = 0.57

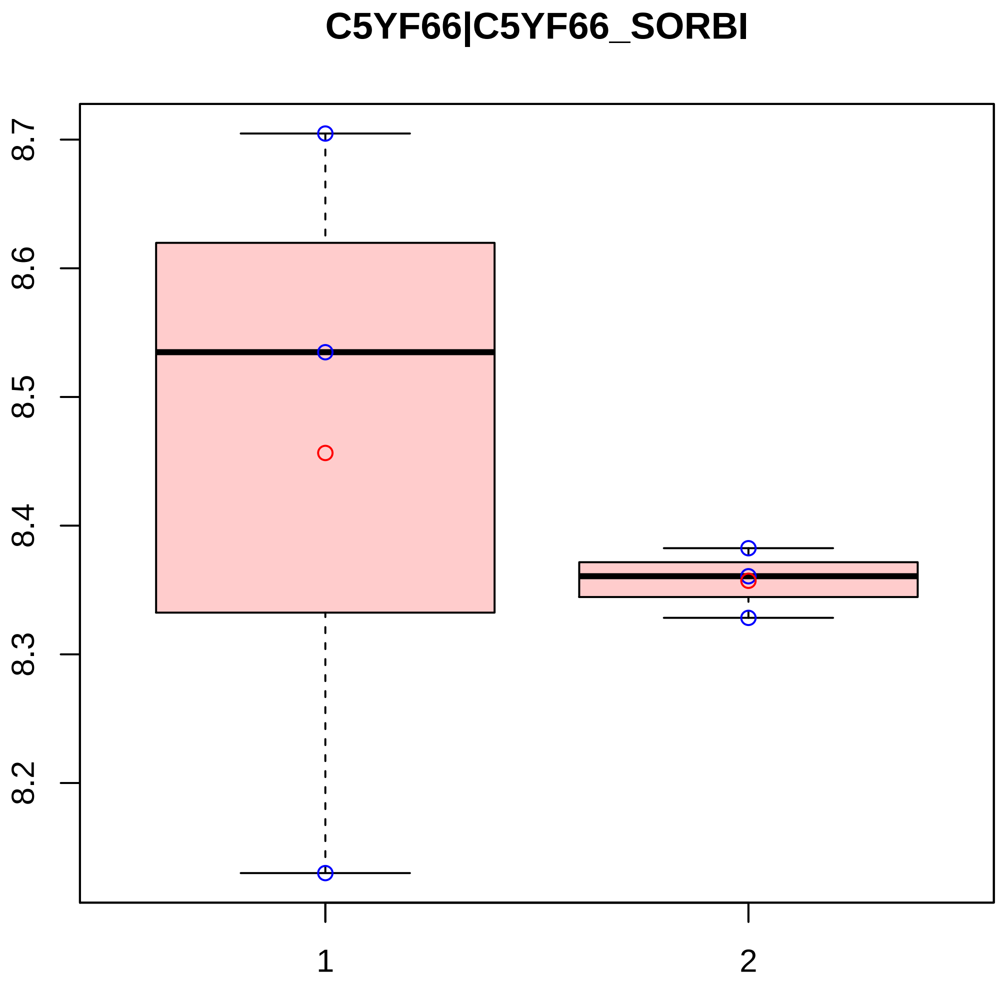

t−Test: p−value = 0.62

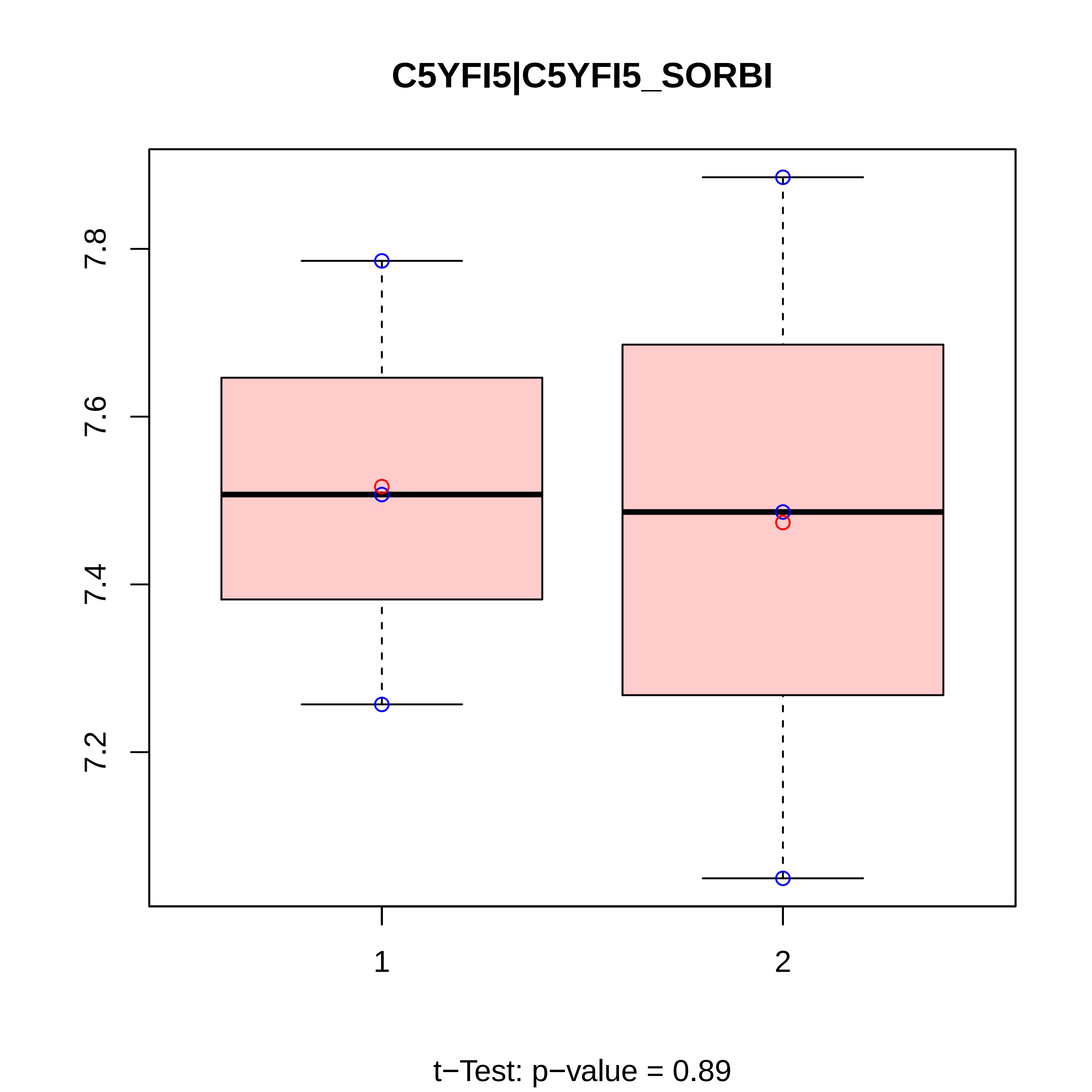
C5YFI5|C5YFI5_SORBI
7.2
7.4
7.6
7.8
1
2
t−Test: p−value = 0.89

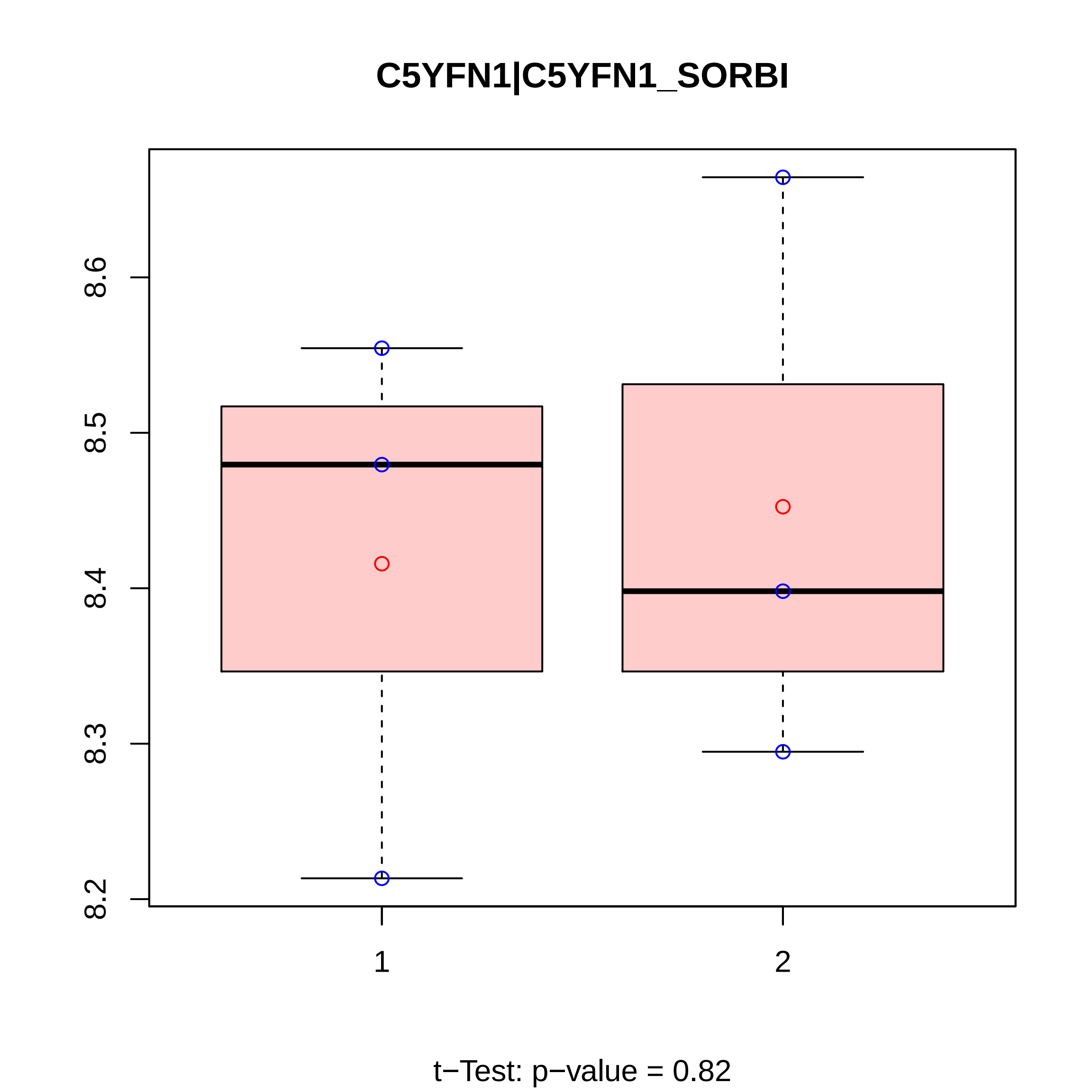
C5YFN1|C5YFN1_SORBI
8.2
8.3
8.4
8.5
8.6
1
2
t−Test: p−value = 0.82

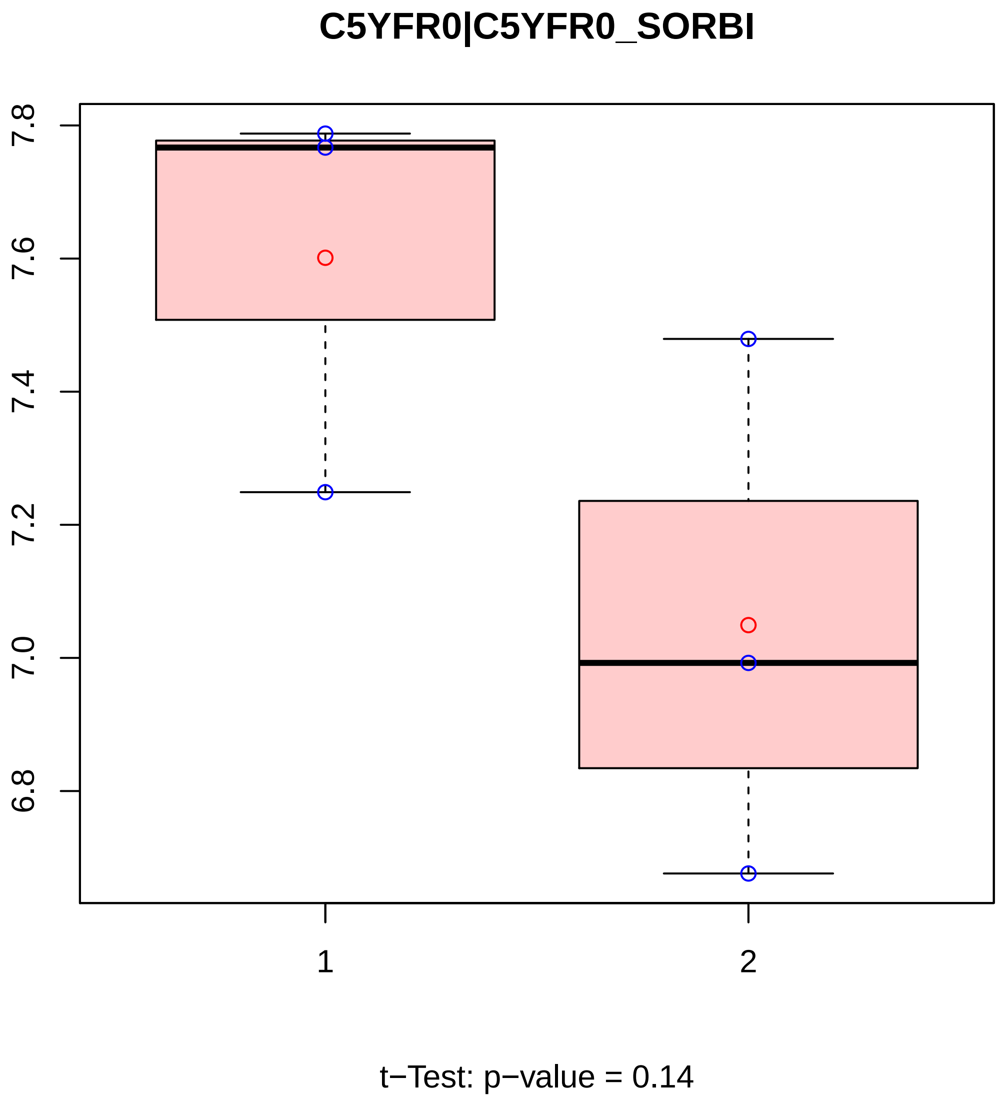

C5YFR0|C5YFR0_SORBI
7.8
7.6
7.4
7.2
7.0
6.8
1
2
t−Test: p−value = 0.14

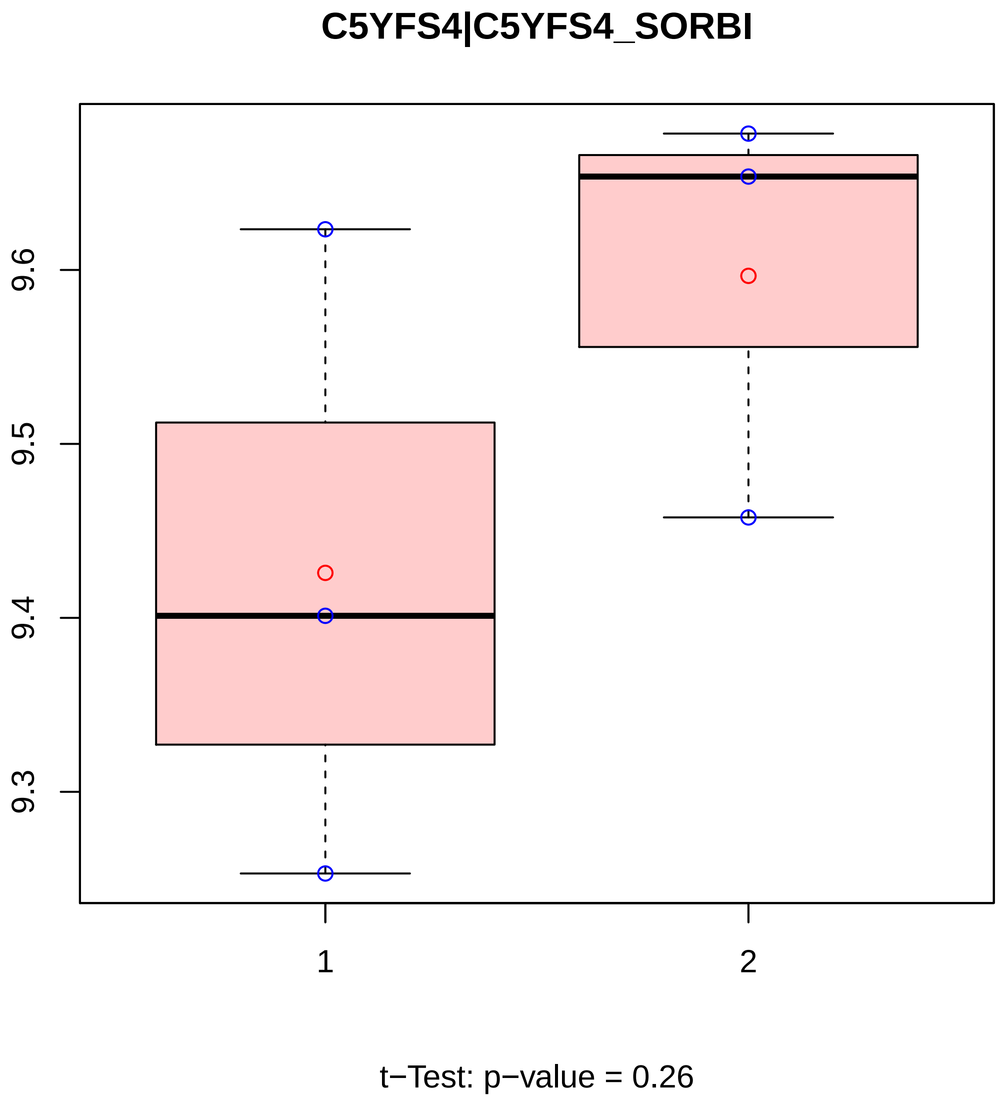

C5YFS4|C5YFS4_SORBI
9.3
9.4
9.5
9.6
1
2
t−Test: p−value = 0.26

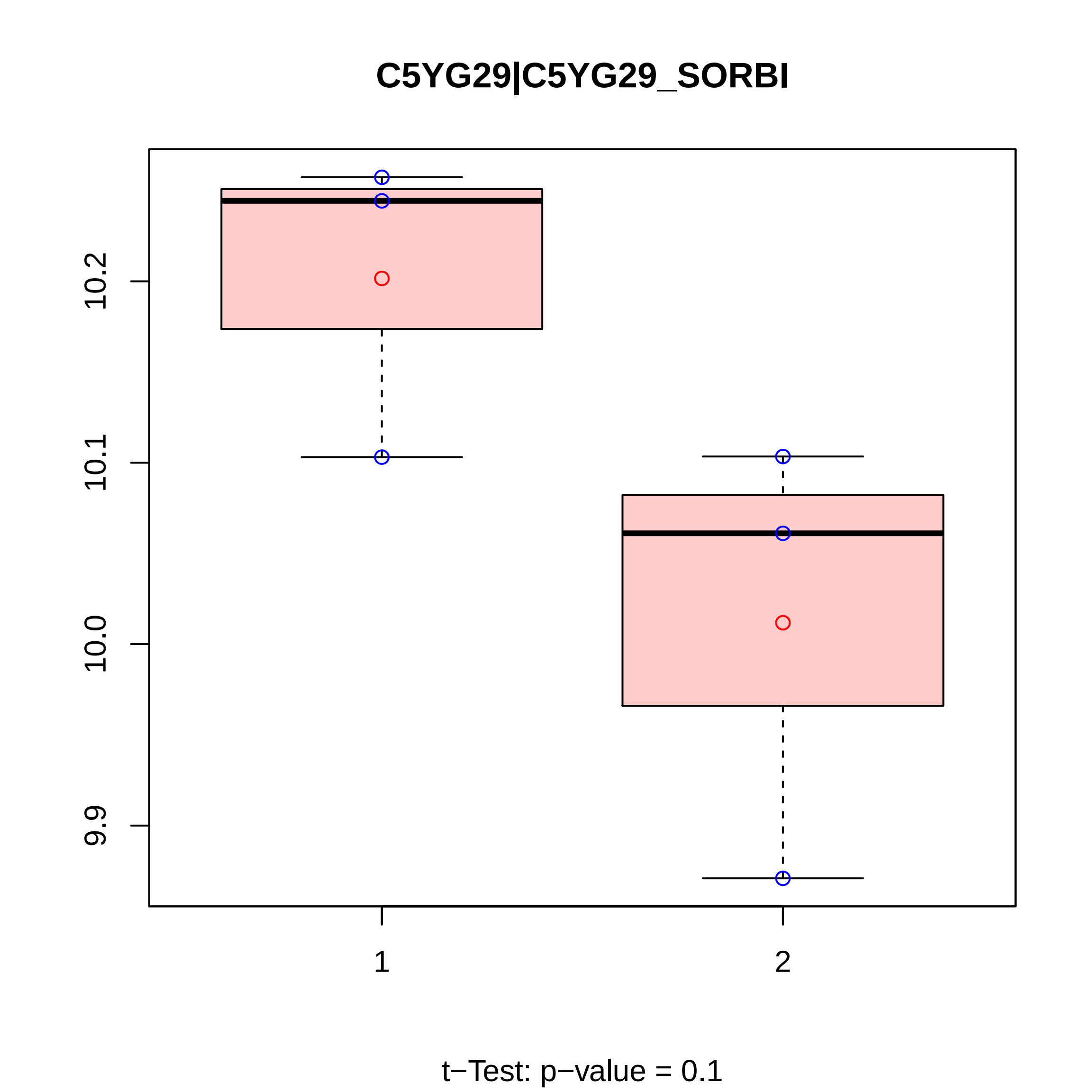

C5YG29|C5YG29_SORBI
10.2
10.1
10.0
9.9
1
2
t−Test: p−value = 0.1

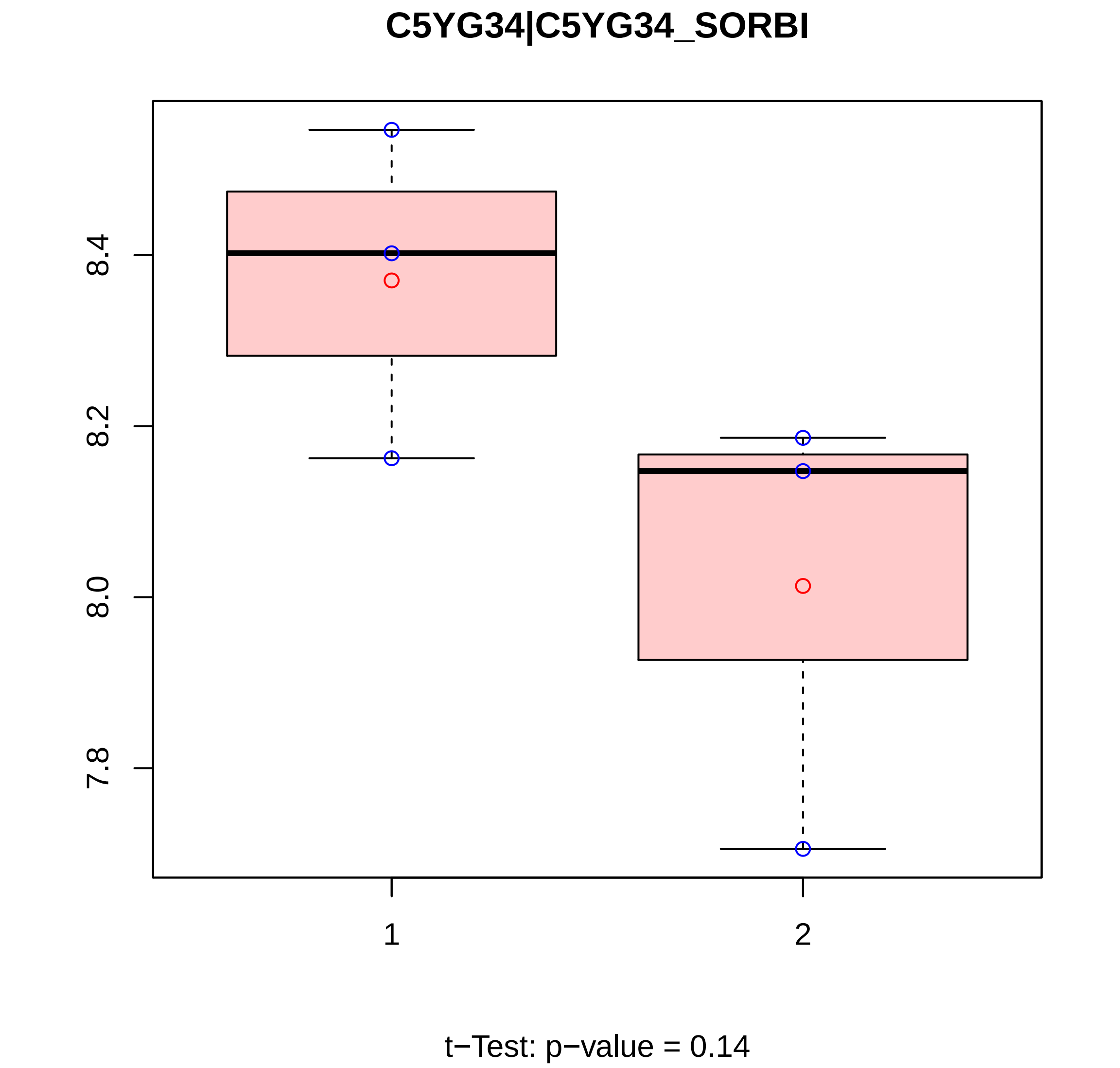
C5YG34|C5YG34_SORBI
8.4
8.2
8.0
7.8
1
2
t−Test: p−value = 0.14

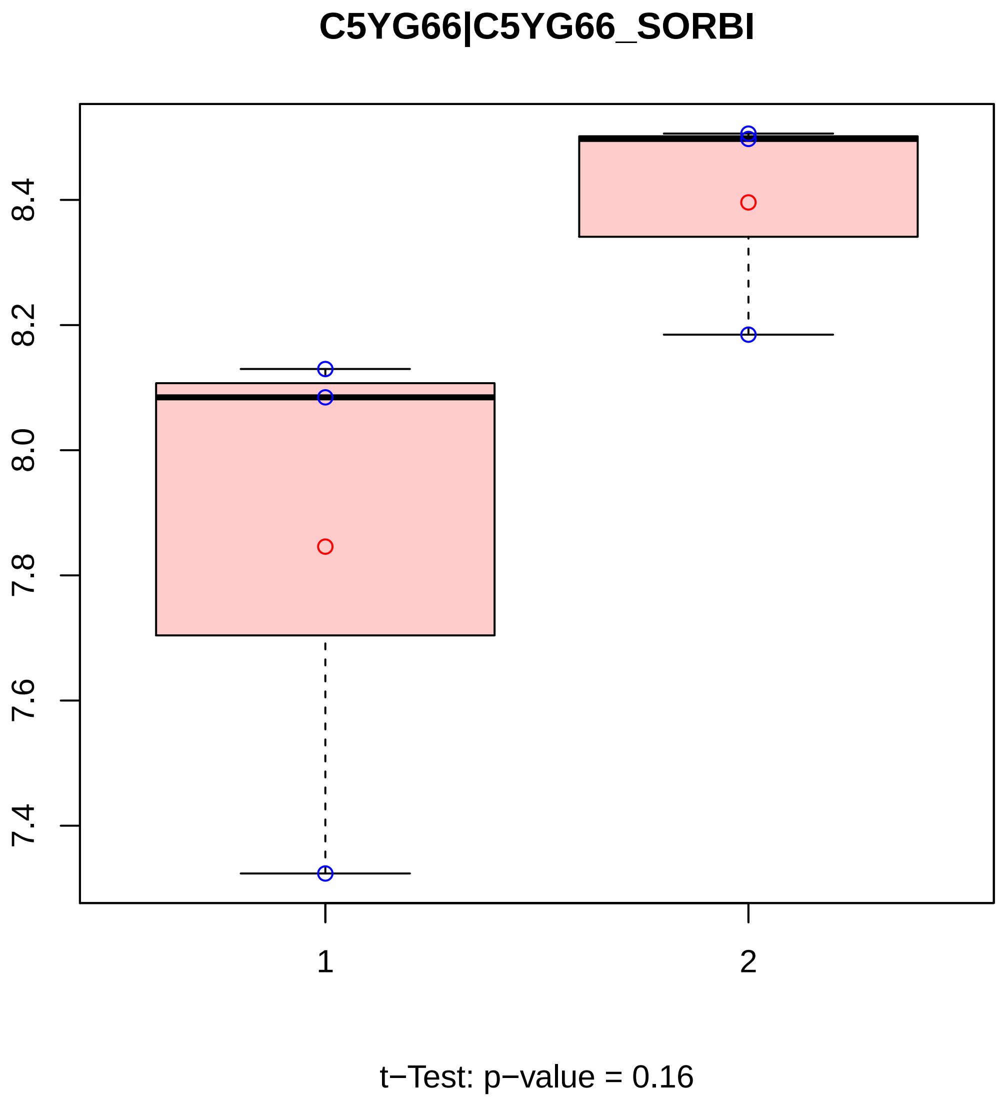

C5YG66|C5YG66_SORBI
8.4
8.2
8.0
7.8
7.6
7.4
1
2
t−Test: p−value = 0.16

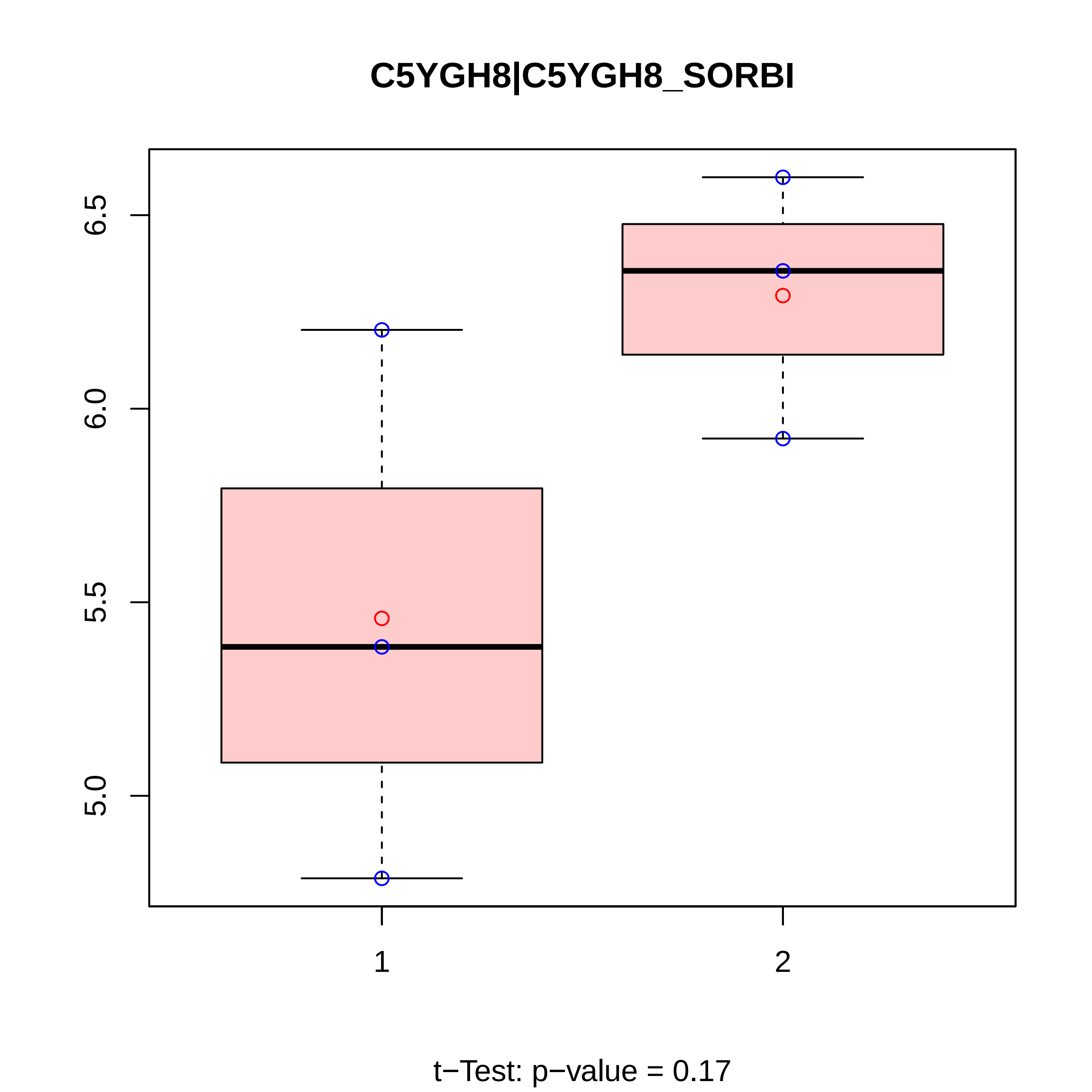
C5YGH8|C5YGH8_SORBI
6.5
6.0
5.5
5.0
1
2
t−Test: p−value = 0.17

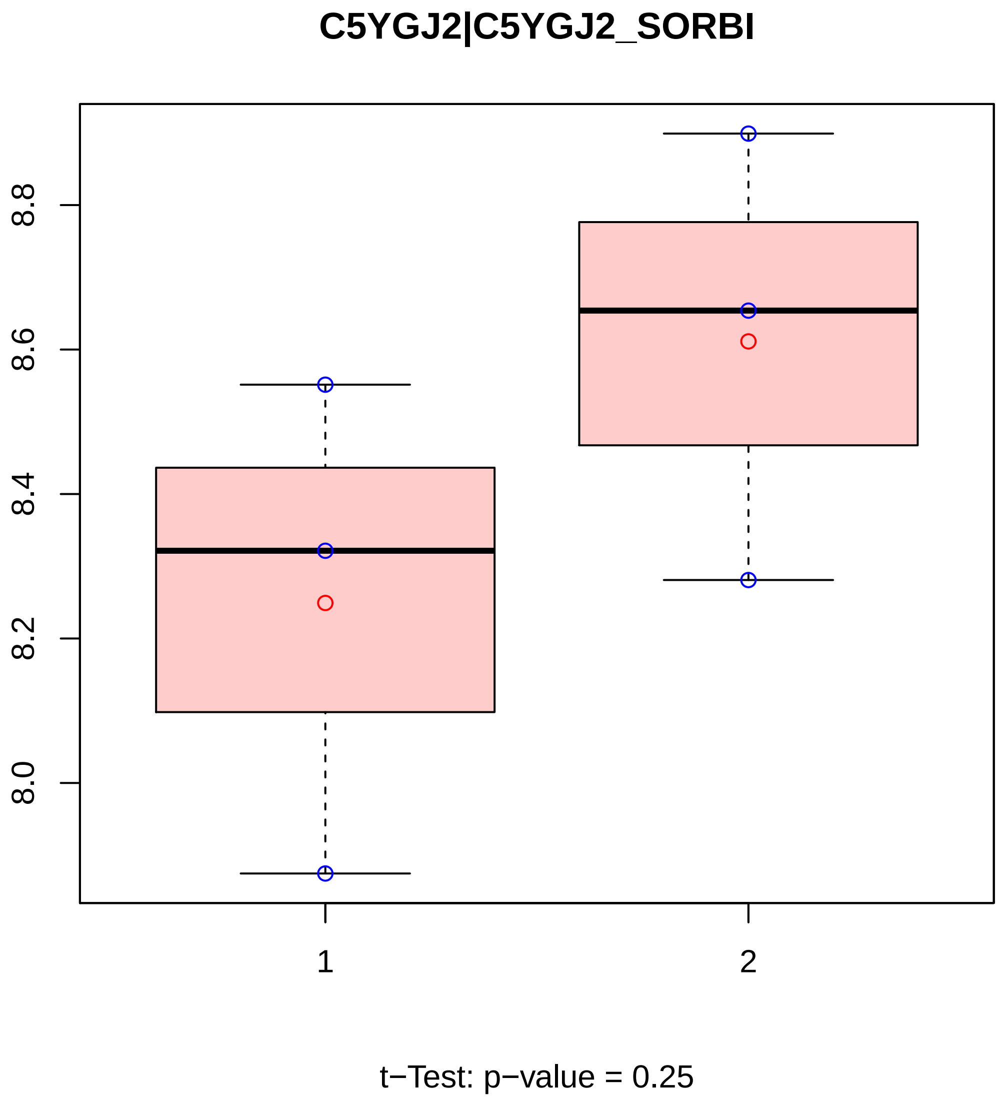

C5YGJ2|C5YGJ2_SORBI
8.0
8.2
8.4
8.6
8.8
1
2
t−Test: p−value = 0.25

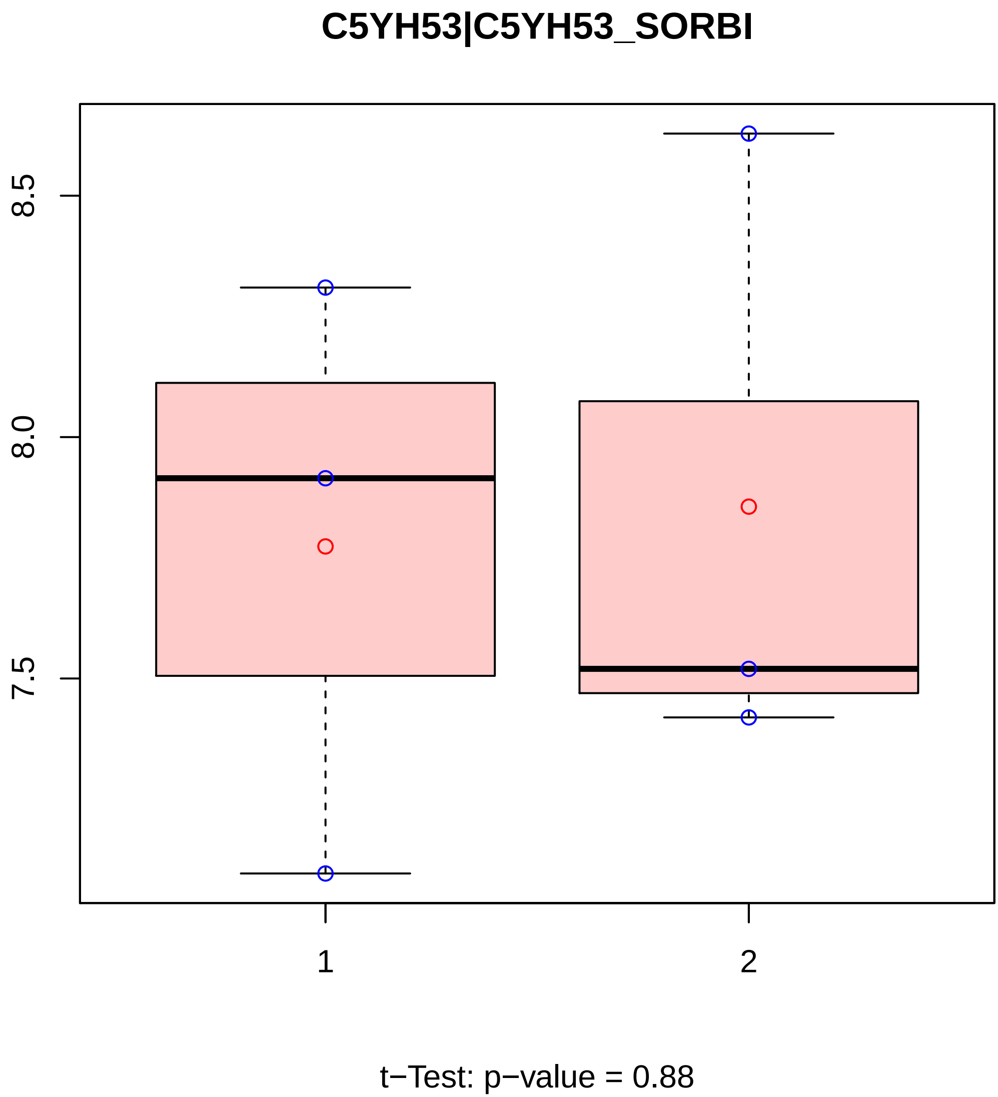

C5YH53|C5YH53_SORBI
8.5
8.0
7.5
1
2
t−Test: p−value = 0.88

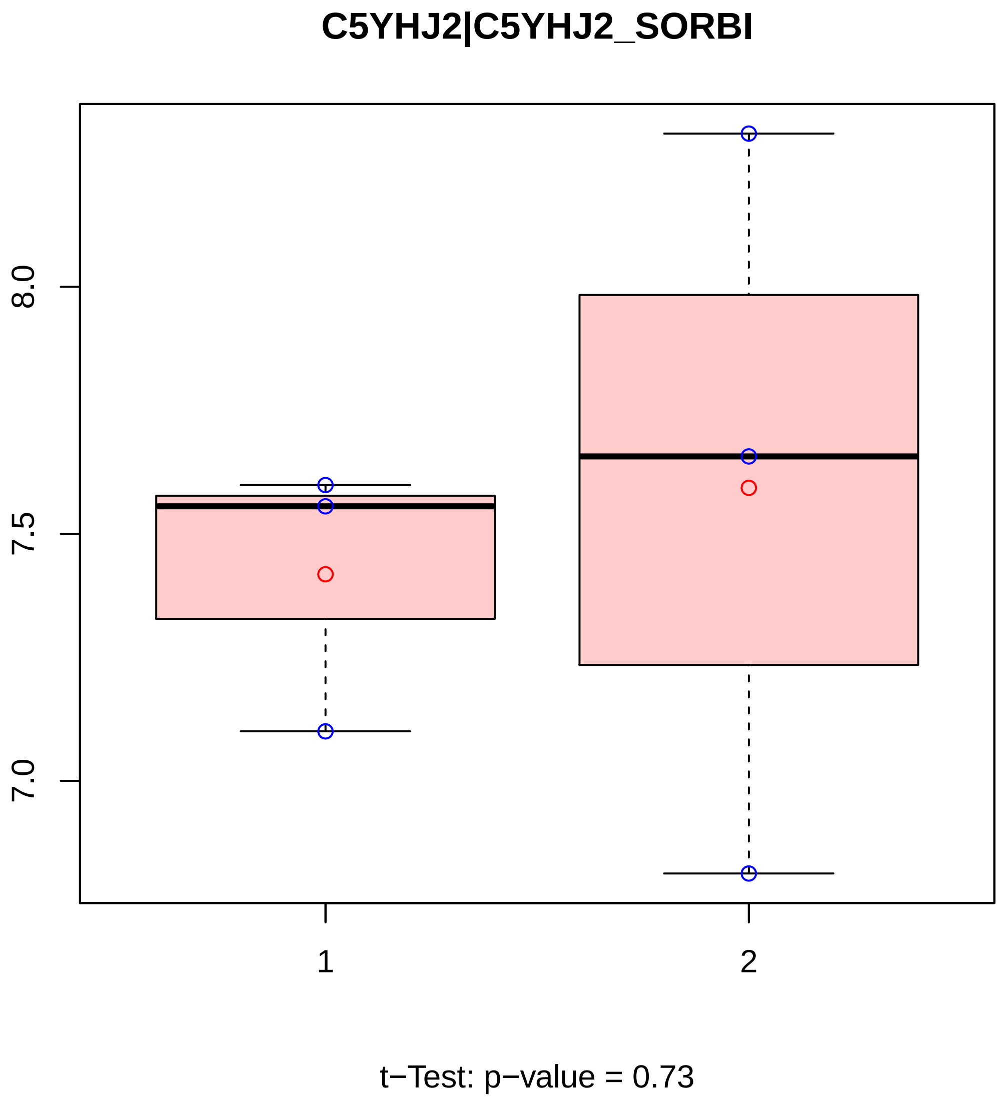
C5YHJ2|C5YHJ2_SORBI
7.0
7.5
8.0
1
2
t−Test: p−value = 0.73

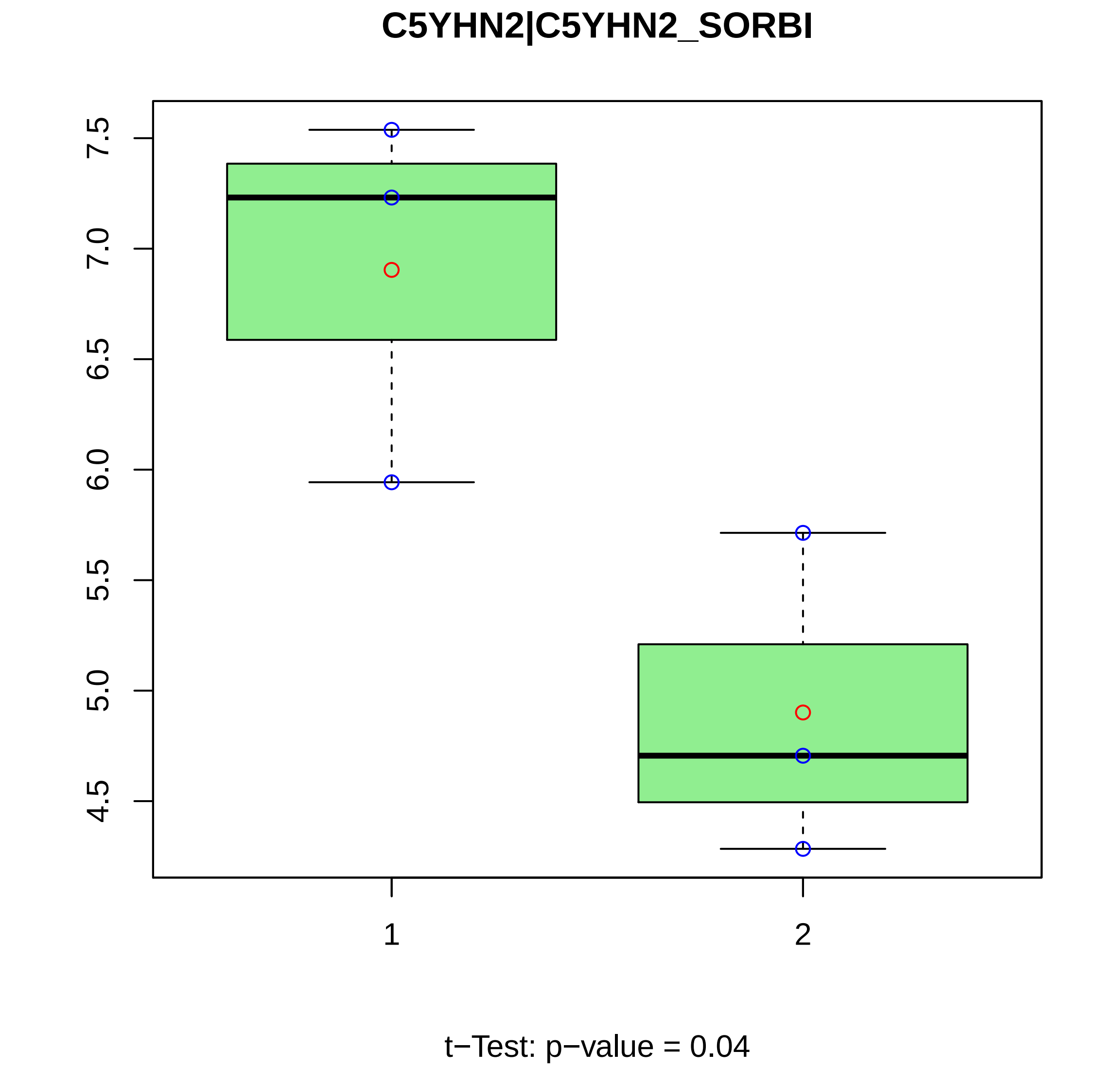

C5YHN2|C5YHN2_SORBI
7.5
7.0
6.5
6.0
5.5
5.0
4.5
1
2
t−Test: p−value = 0.04

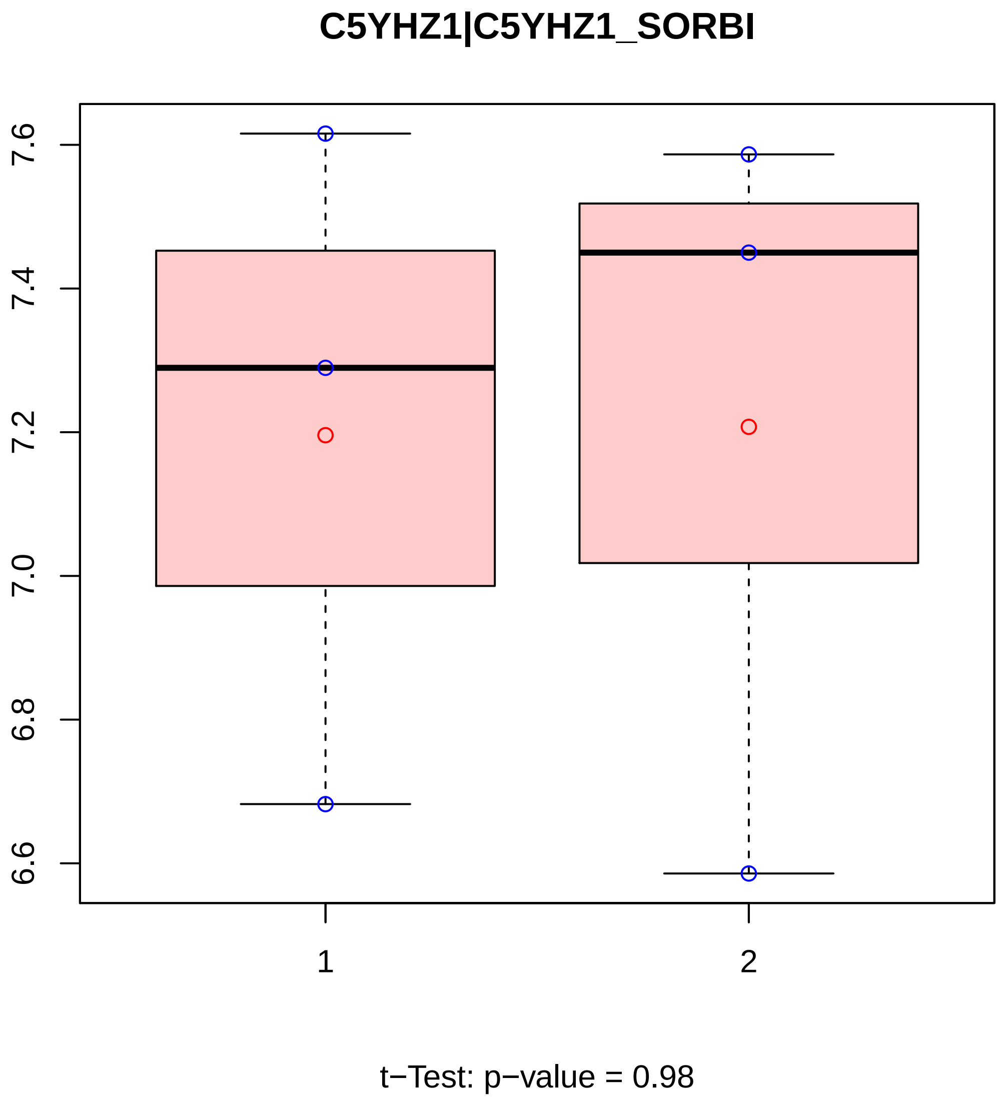
C5YHZ1|C5YHZ1_SORBI
7.6
7.4
7.2
7.0
6.8
6.6
1
2
t−Test: p−value = 0.98

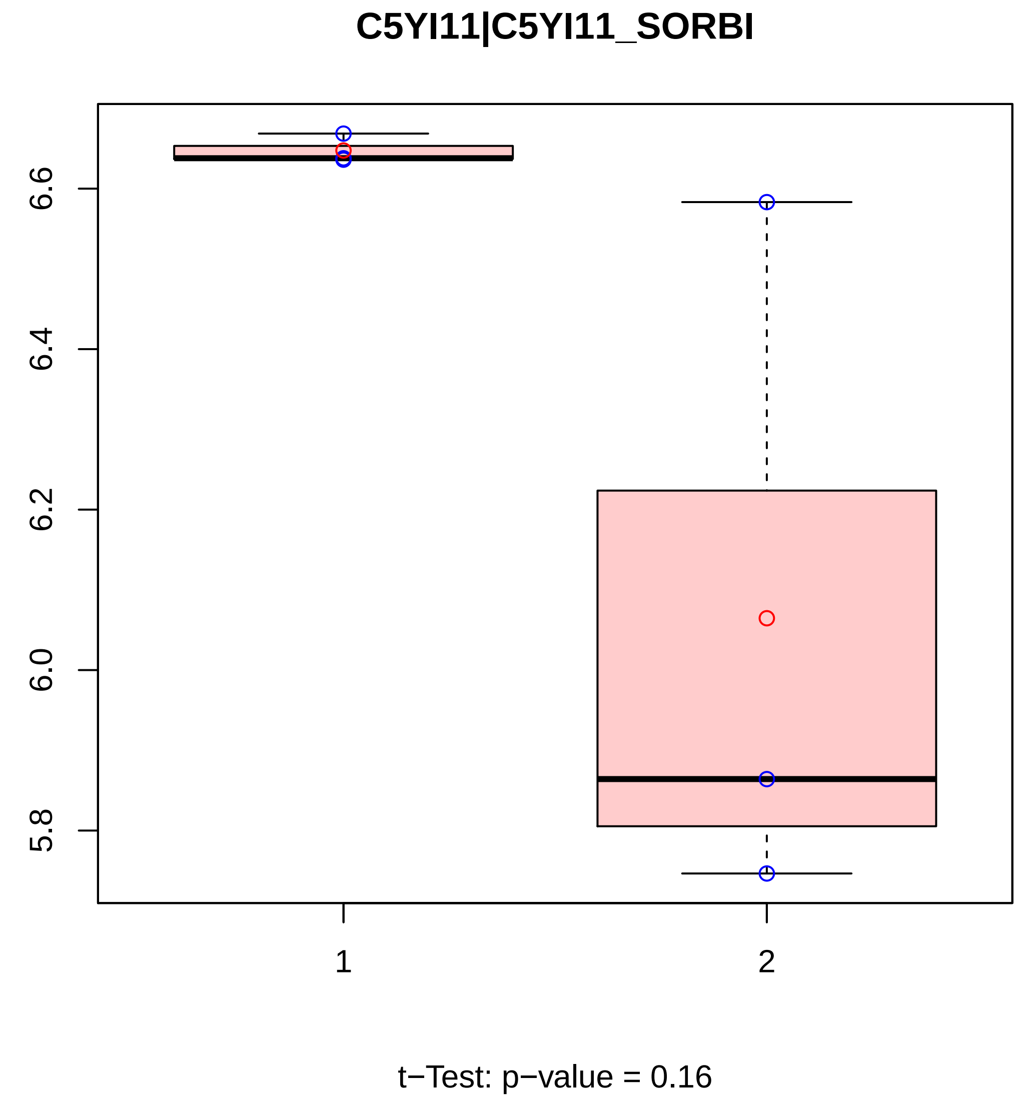
C5YI11|C5YI11_SORBI
6.6
6.4
6.2
6.0
5.8
1
2
t−Test: p−value = 0.16

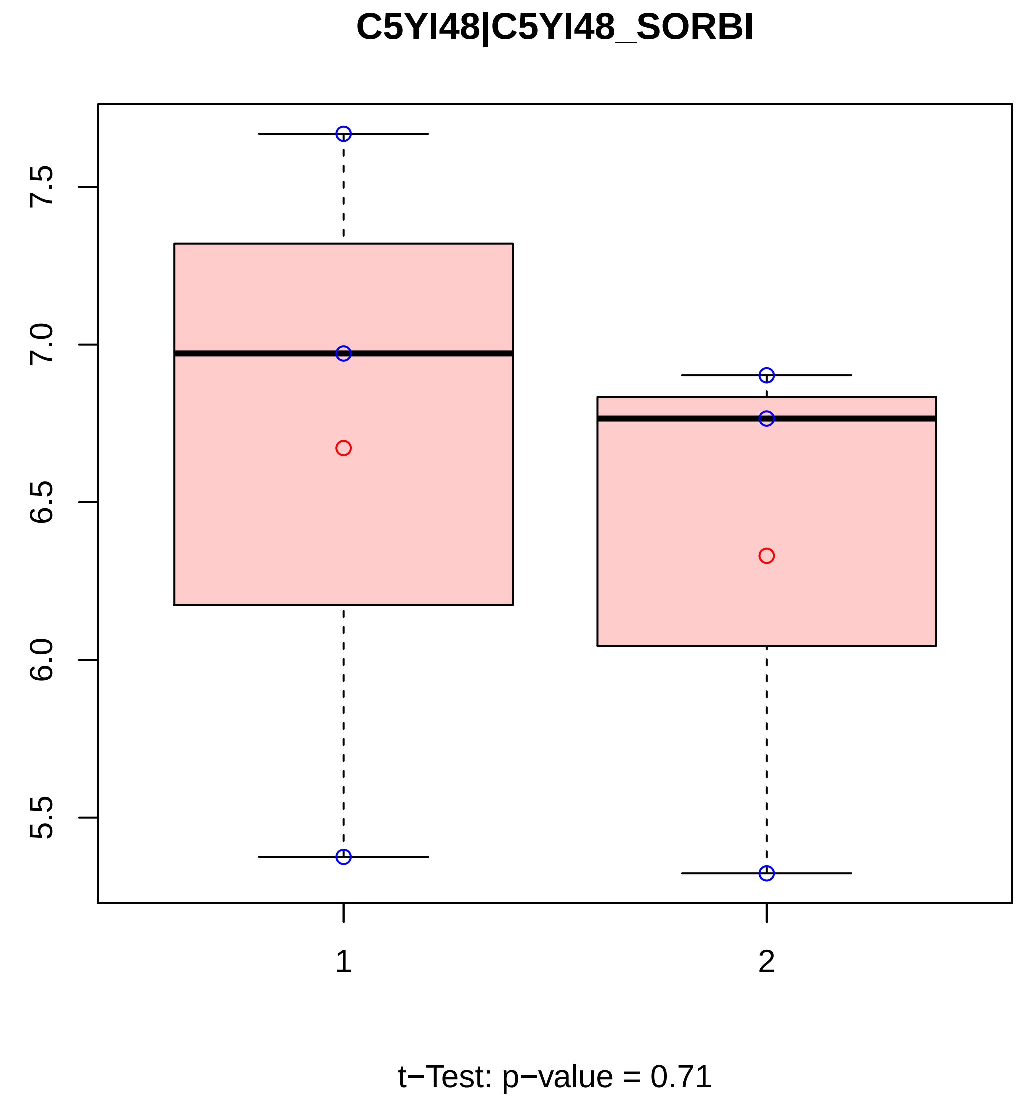
C5YI48|C5YI48_SORBI
7.5
7.0
6.5
6.0
5.5
1
2
t−Test: p−value = 0.71

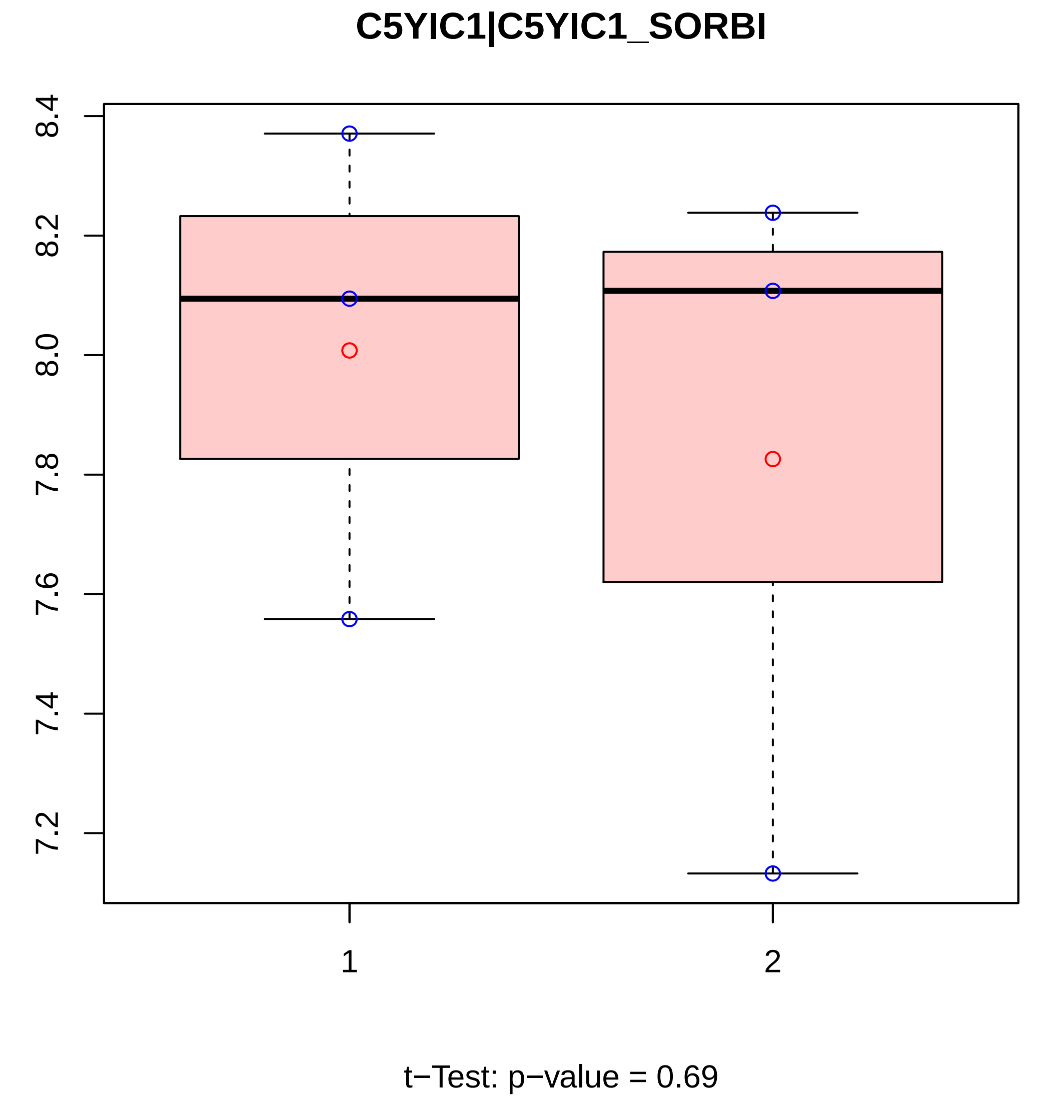
C5YIC1|C5YIC1_SORBI
8.4
8.2
8.0
7.8
7.6
7.4
7.2
1
2
t−Test: p−value = 0.69

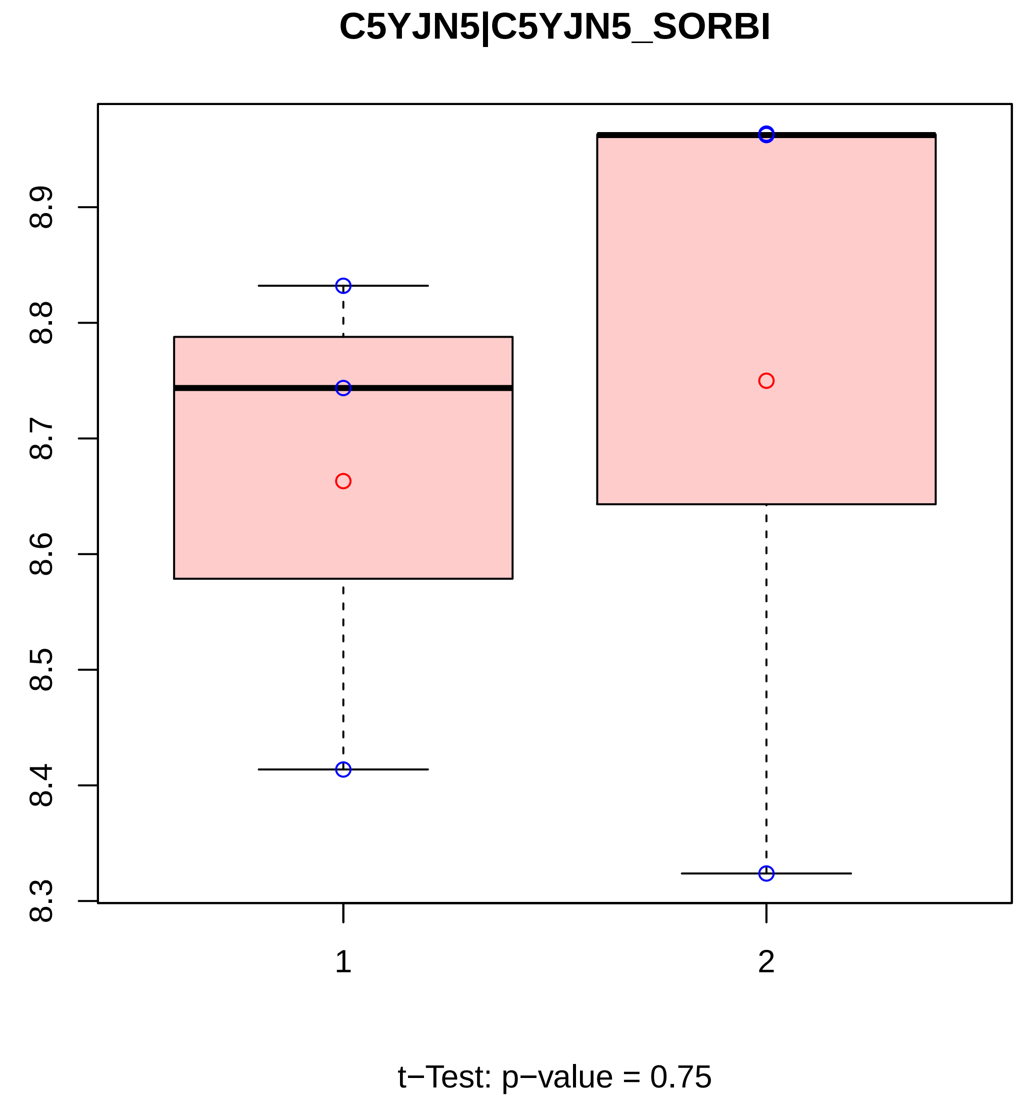
C5YJN5|C5YJN5_SORBI
8.3
8.4
8.5
8.6
8.7
8.8
8.9
1
2
t−Test: p−value = 0.75

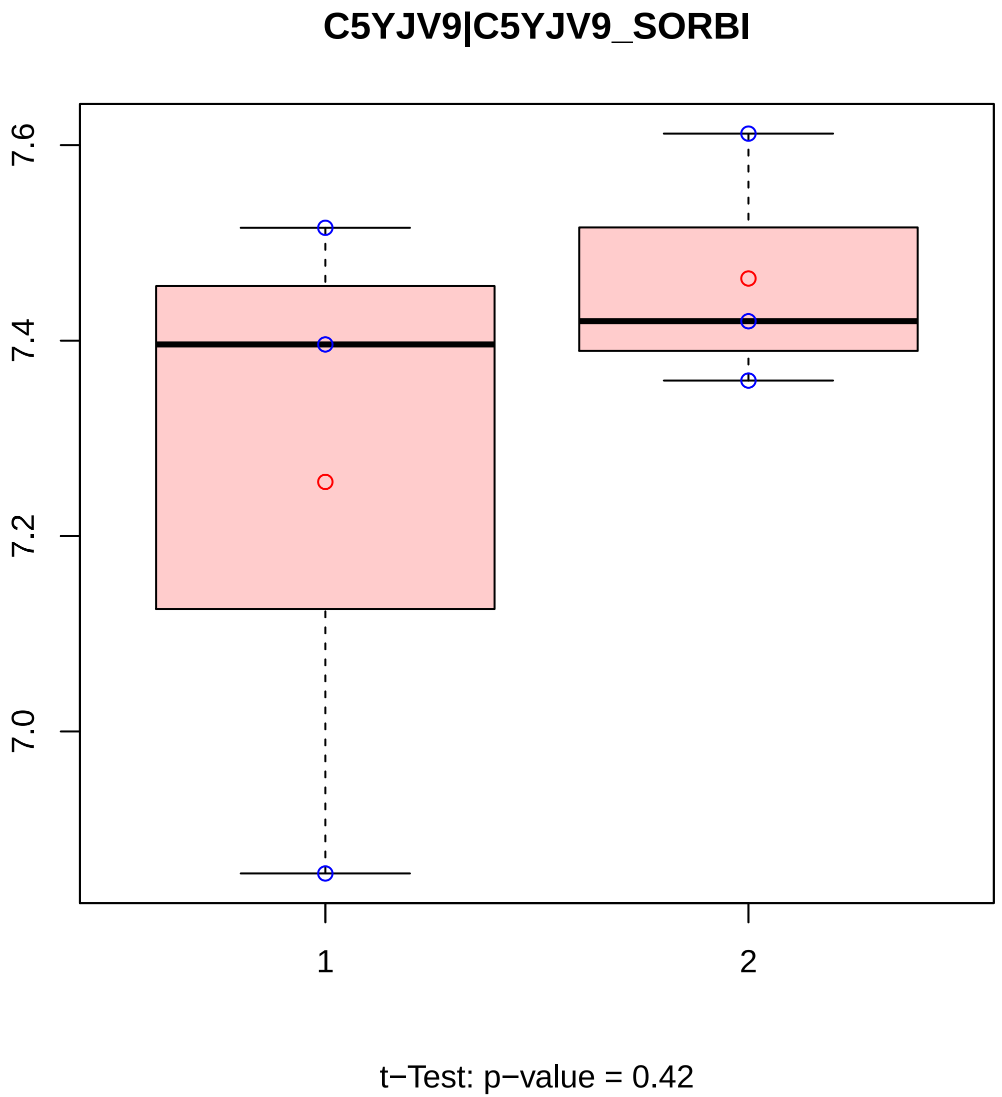

C5YJV9|C5YJV9_SORBI
7.6
7.4
7.2
7.0
1
2
t−Test: p−value = 0.42

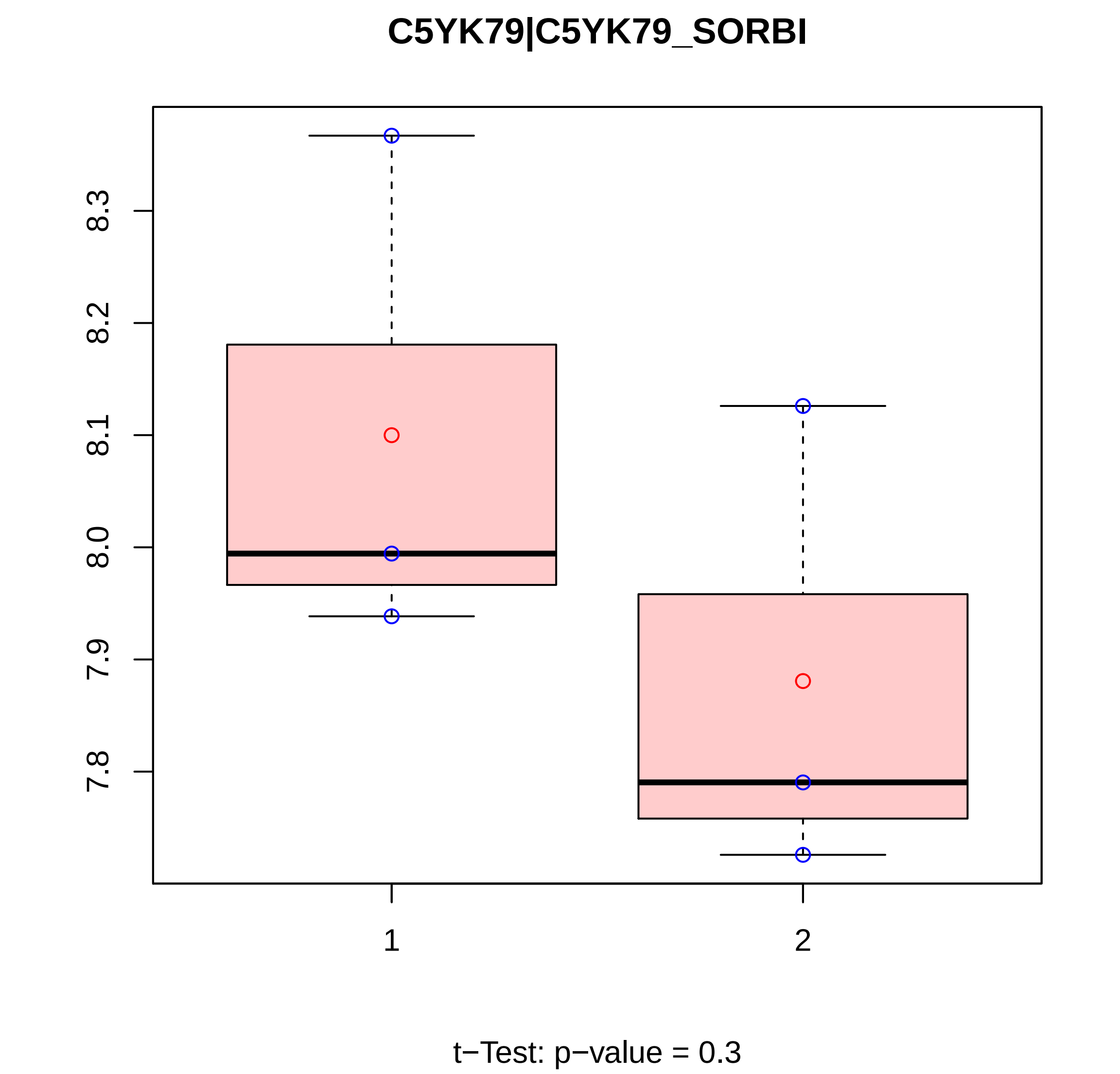
C5YK79|C5YK79_SORBI
8.3
8.2
8.1
8.0
7.9
7.8
1
2
t−Test: p−value = 0.3

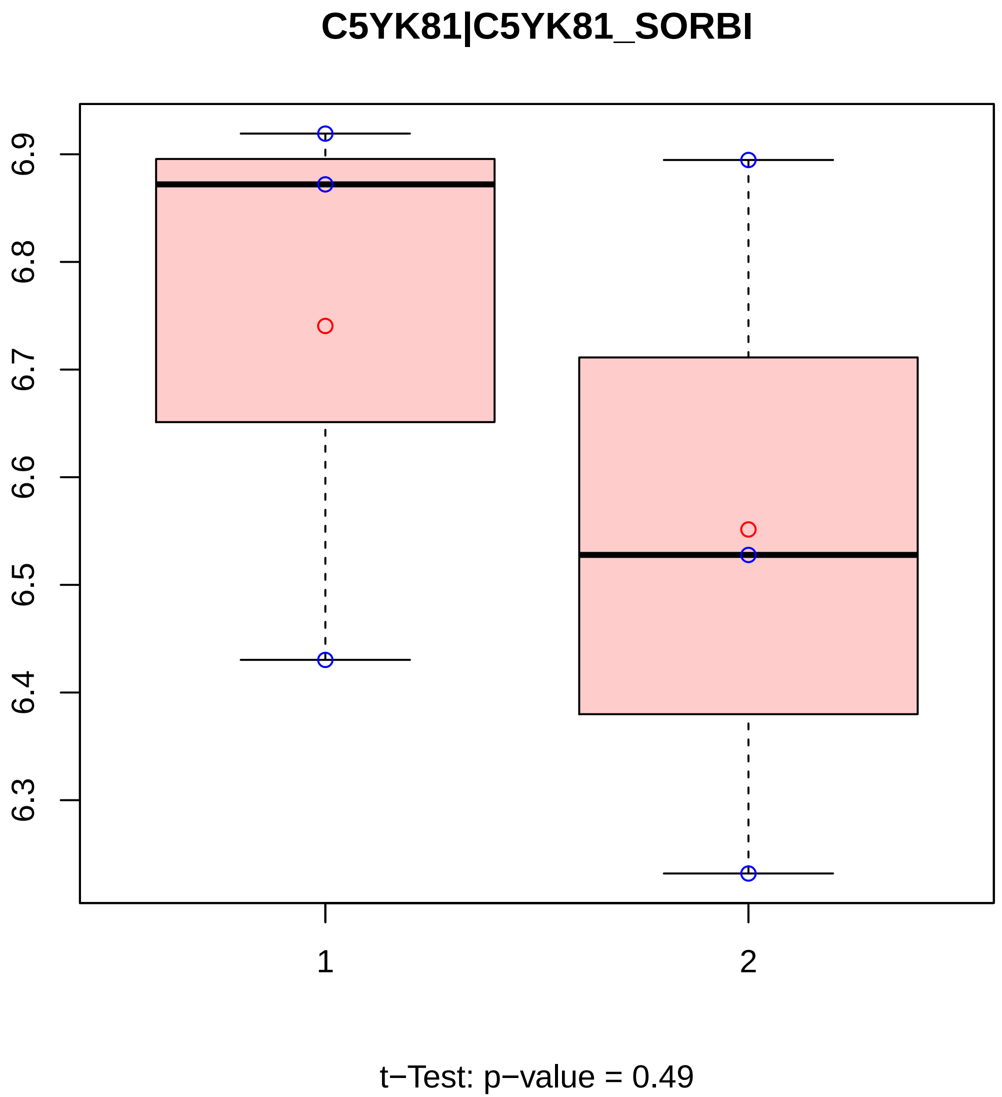

C5YK81|C5YK81_SORBI
6.3
6.4
6.5
6.6
6.7
6.8
6.9
1
2
t−Test: p−value = 0.49

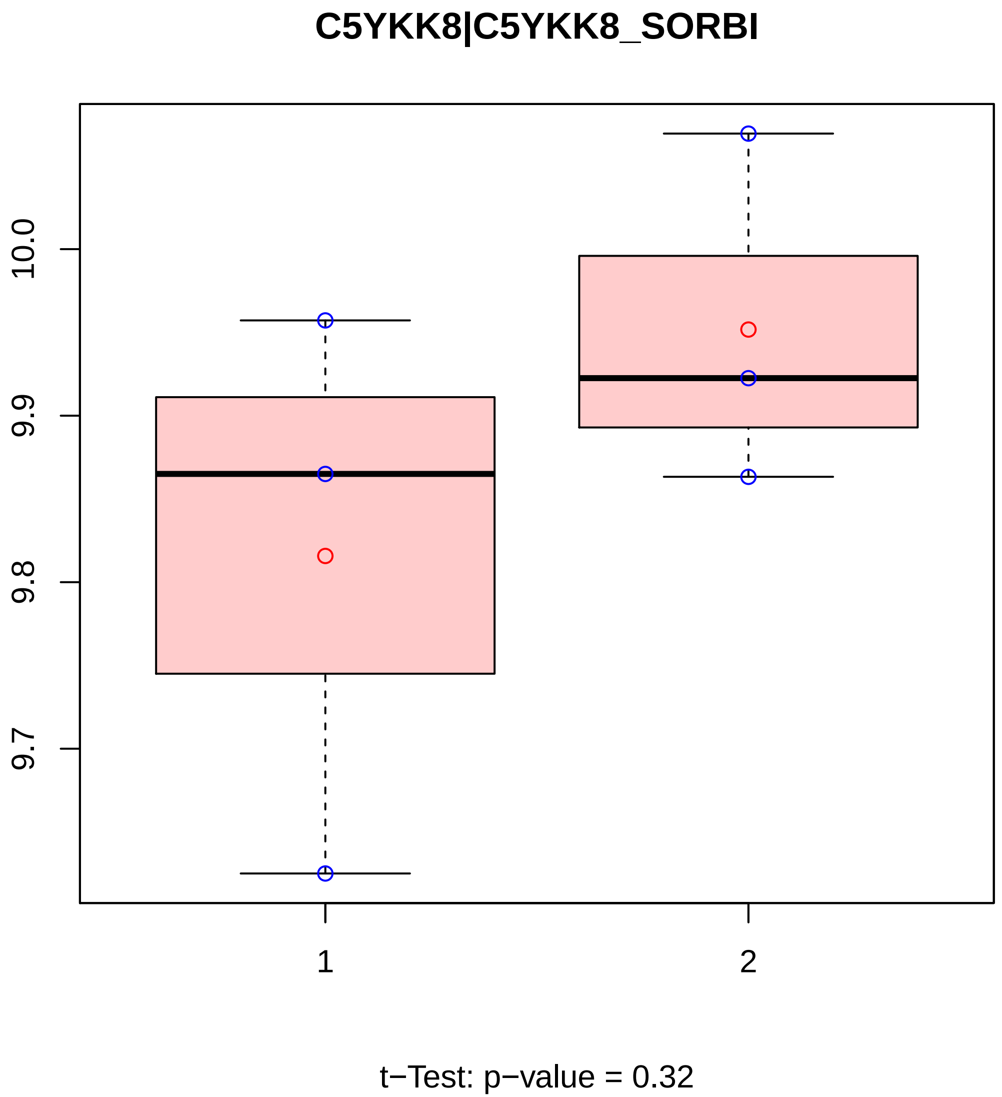
C5YKK8|C5YKK8_SORBI
10.0
9.9
9.8
9.7
1
2
t−Test: p−value = 0.32

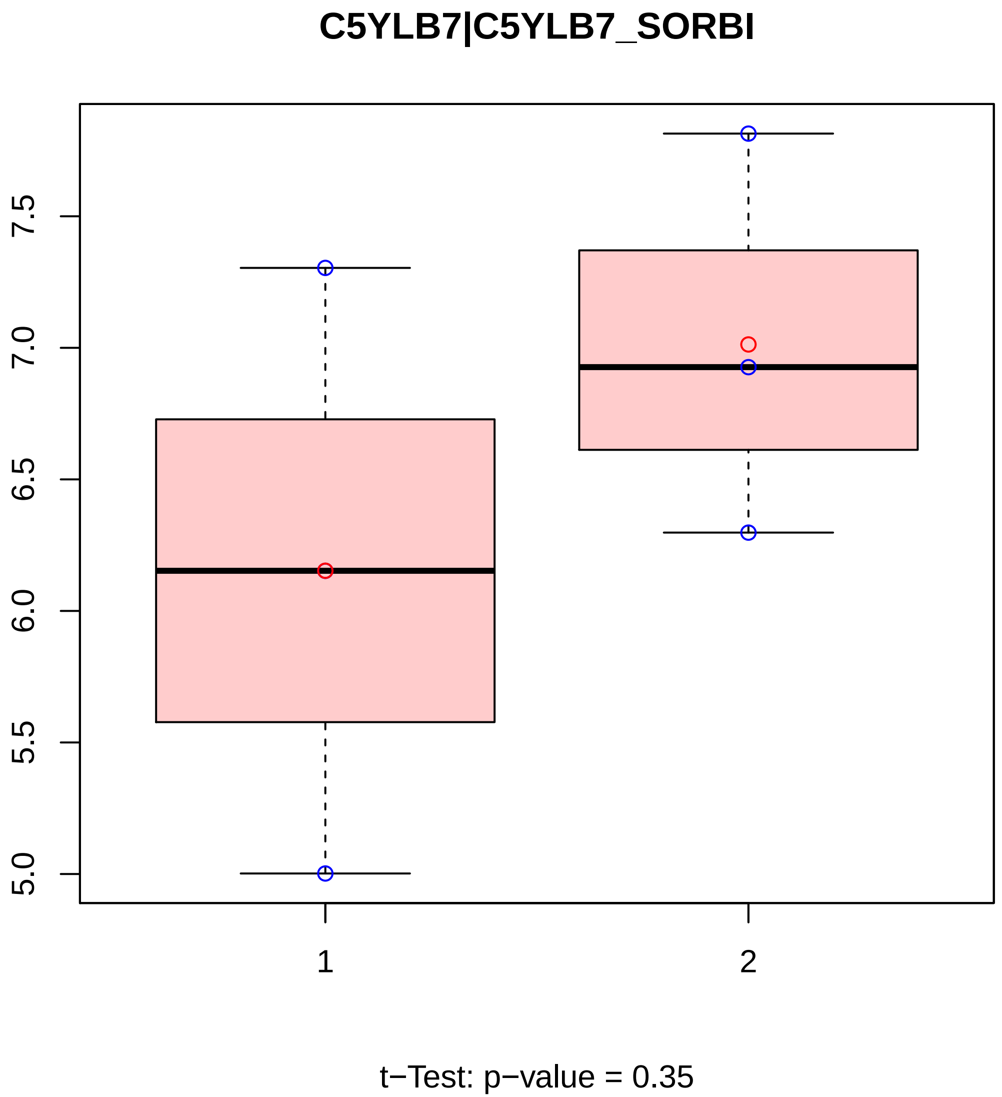

C5YLB7|C5YLB7_SORBI
7.5
7.0
6.5
6.0
5.5
5.0
1
2
t−Test: p−value = 0.35

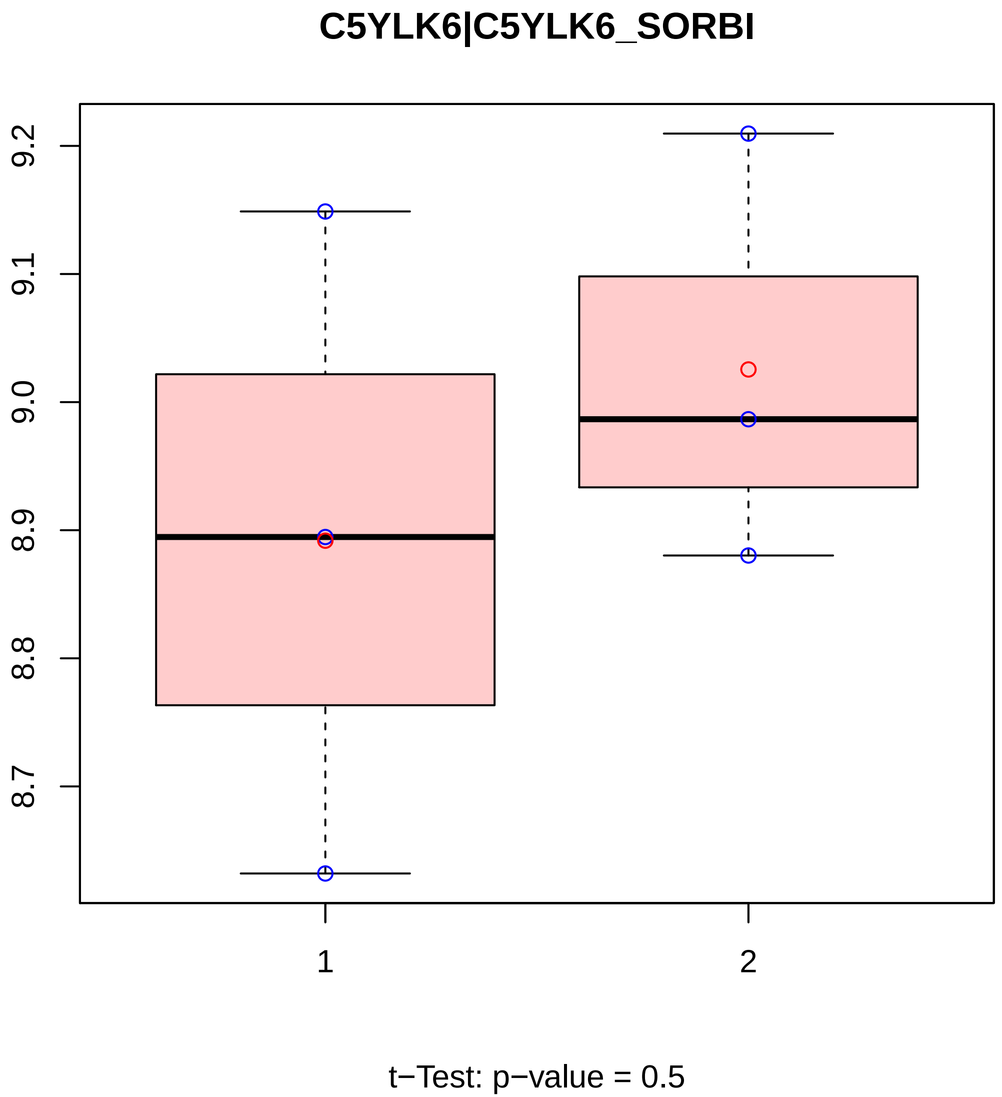
C5YLK6|C5YLK6_SORBI
9.2
9.1
9.0
8.9
8.8
8.7
1
2
t−Test: p−value = 0.5

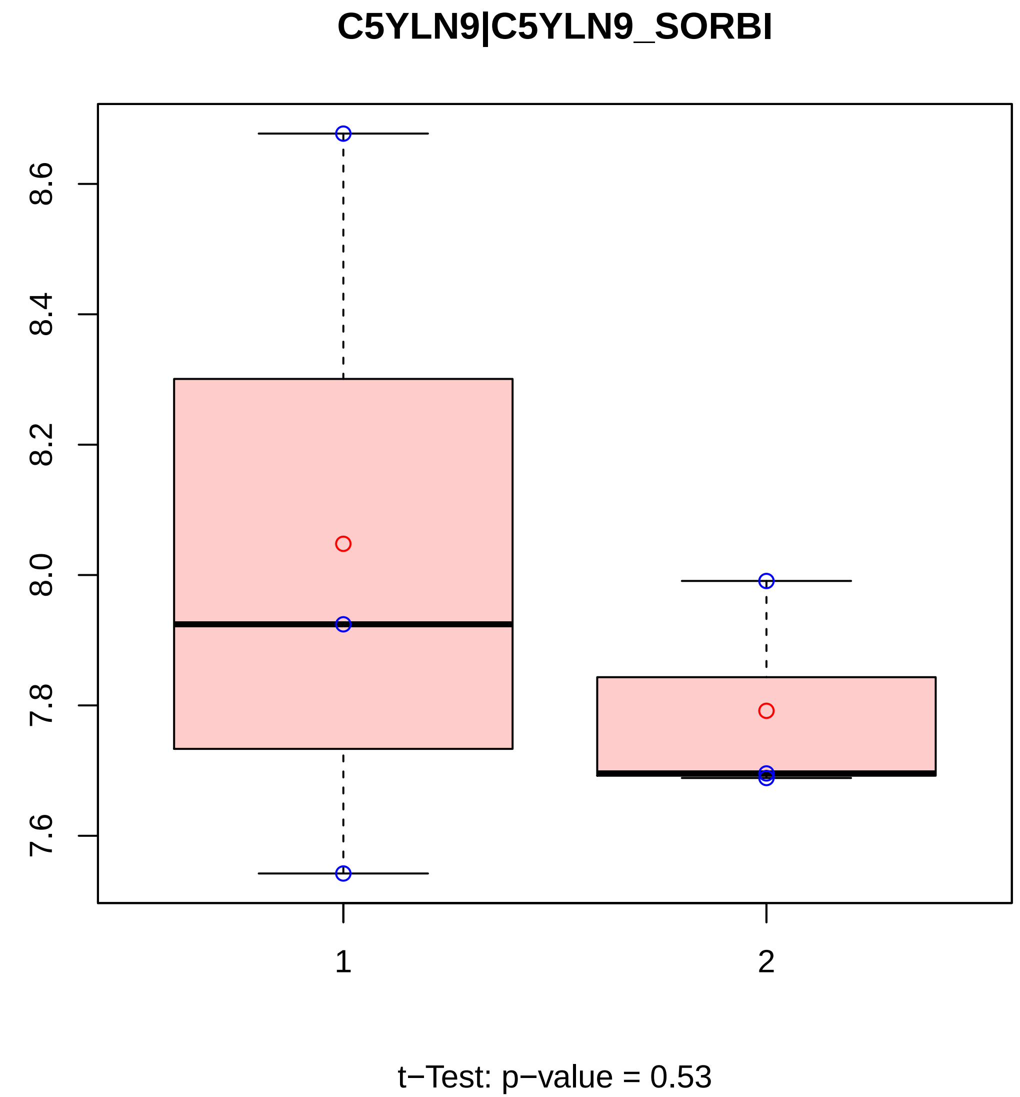
C5YLN9|C5YLN9_SORBI
8.6
8.4
8.2
8.0
7.8
7.6
1
2
t−Test: p−value = 0.53

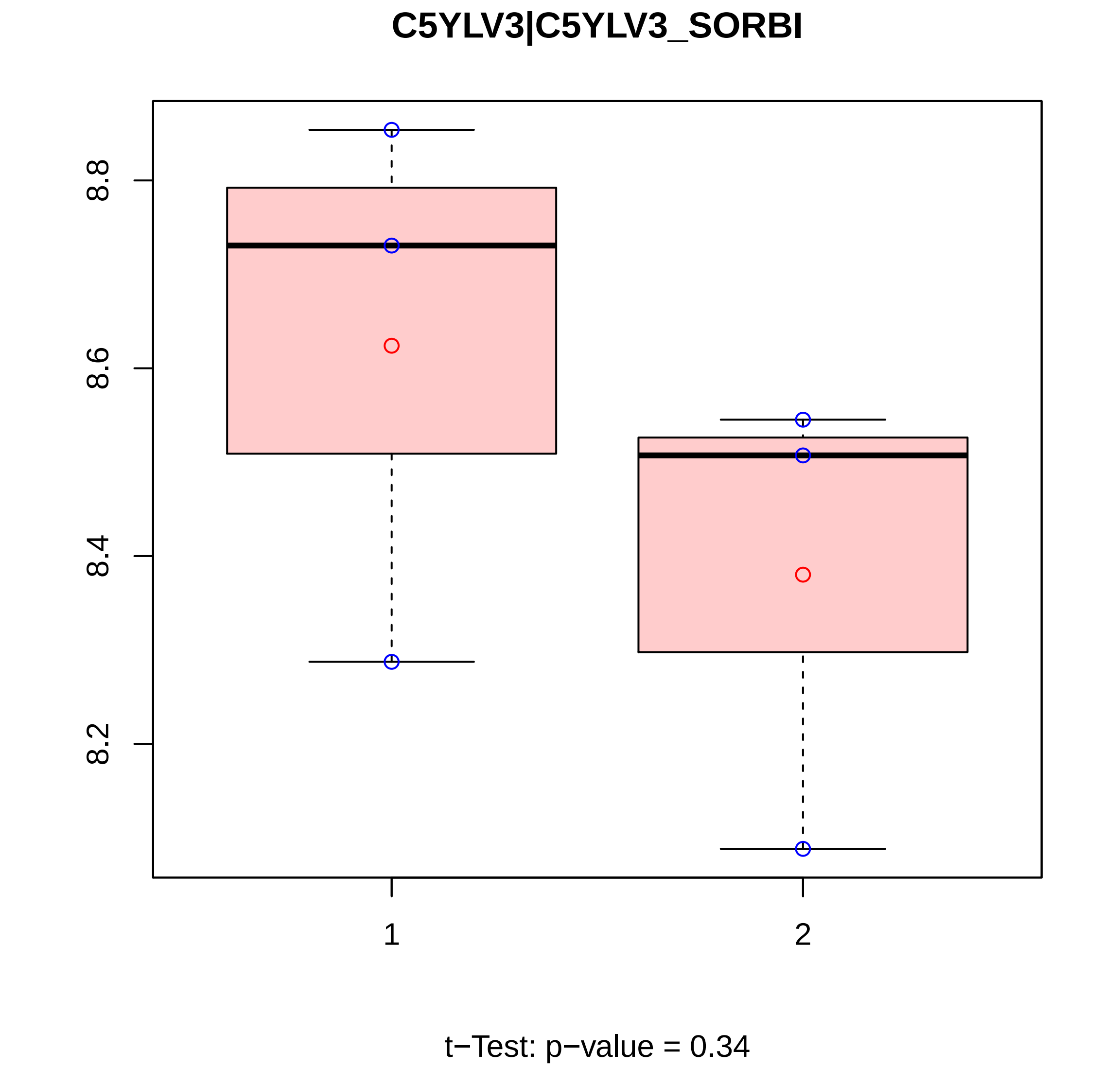
C5YLV3|C5YLV3_SORBI
8.2
8.4
8.6
8.8
1
2
t−Test: p−value = 0.34

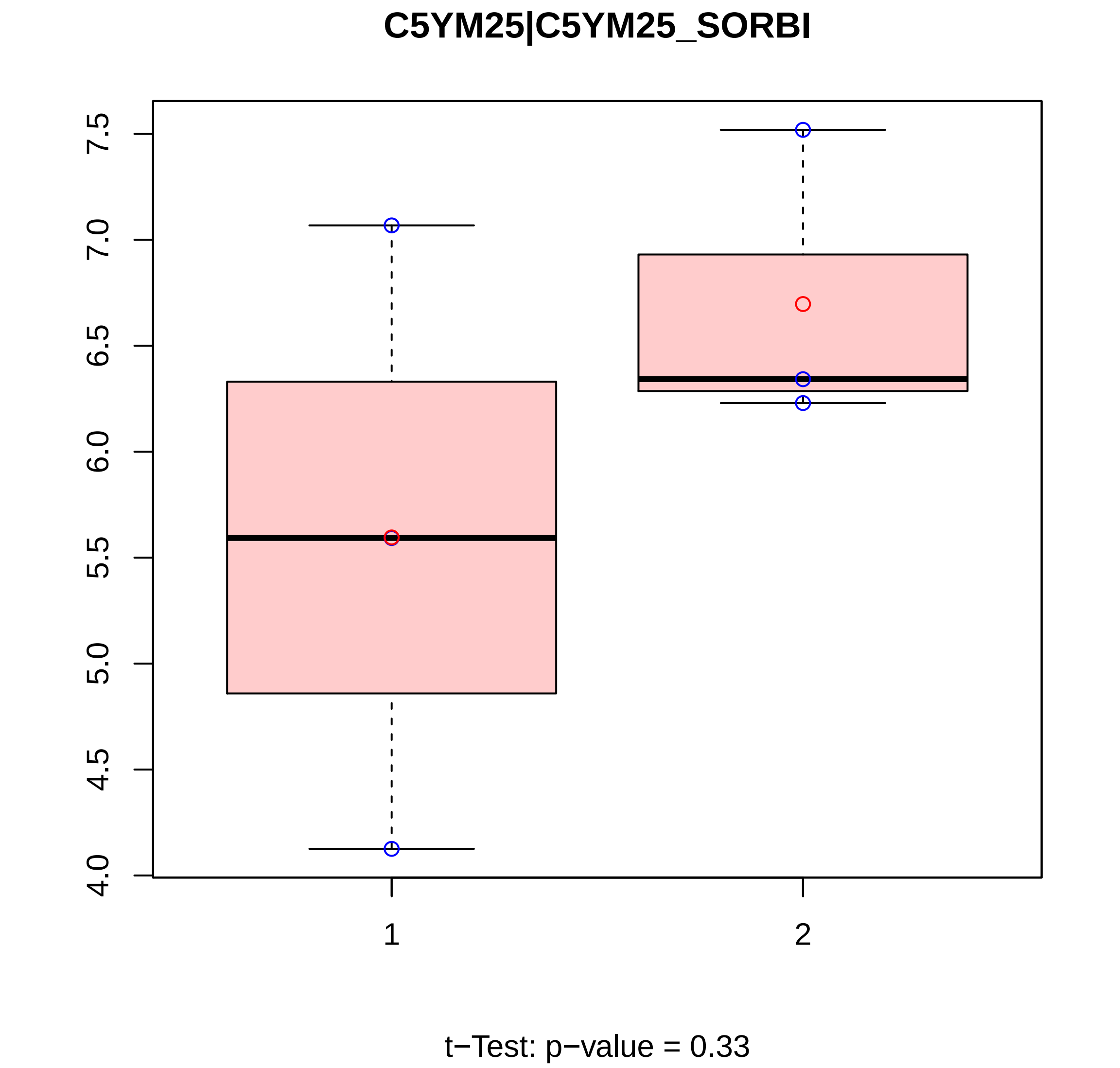
C5YM25|C5YM25_SORBI
7.5
7.0
6.5
6.0
5.5
5.0
4.5
4.0
1
2
t−Test: p−value = 0.33

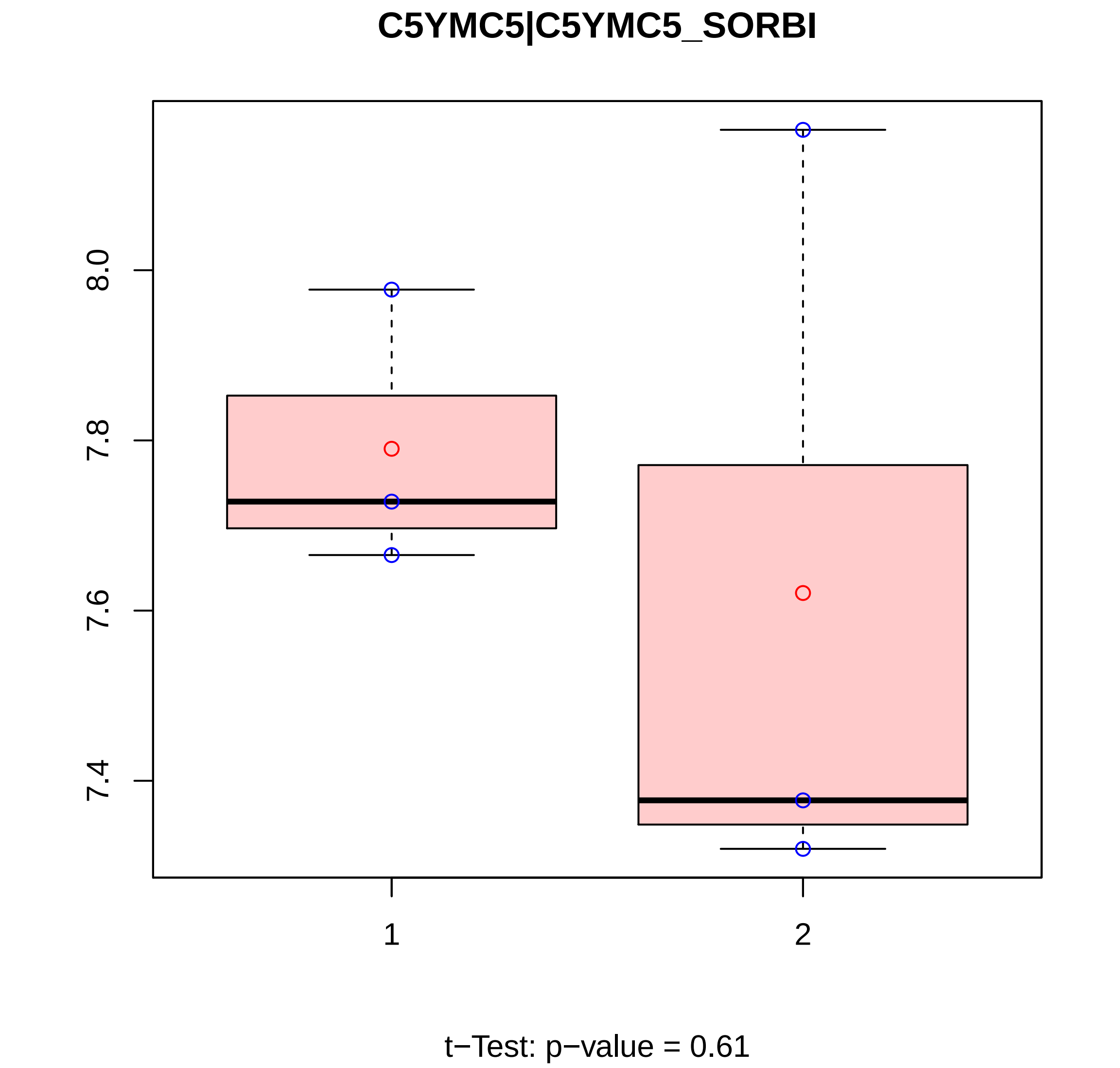

C5YMC5|C5YMC5_SORBI
8.0
7.8
7.6
7.4
1
2
t−Test: p−value = 0.61

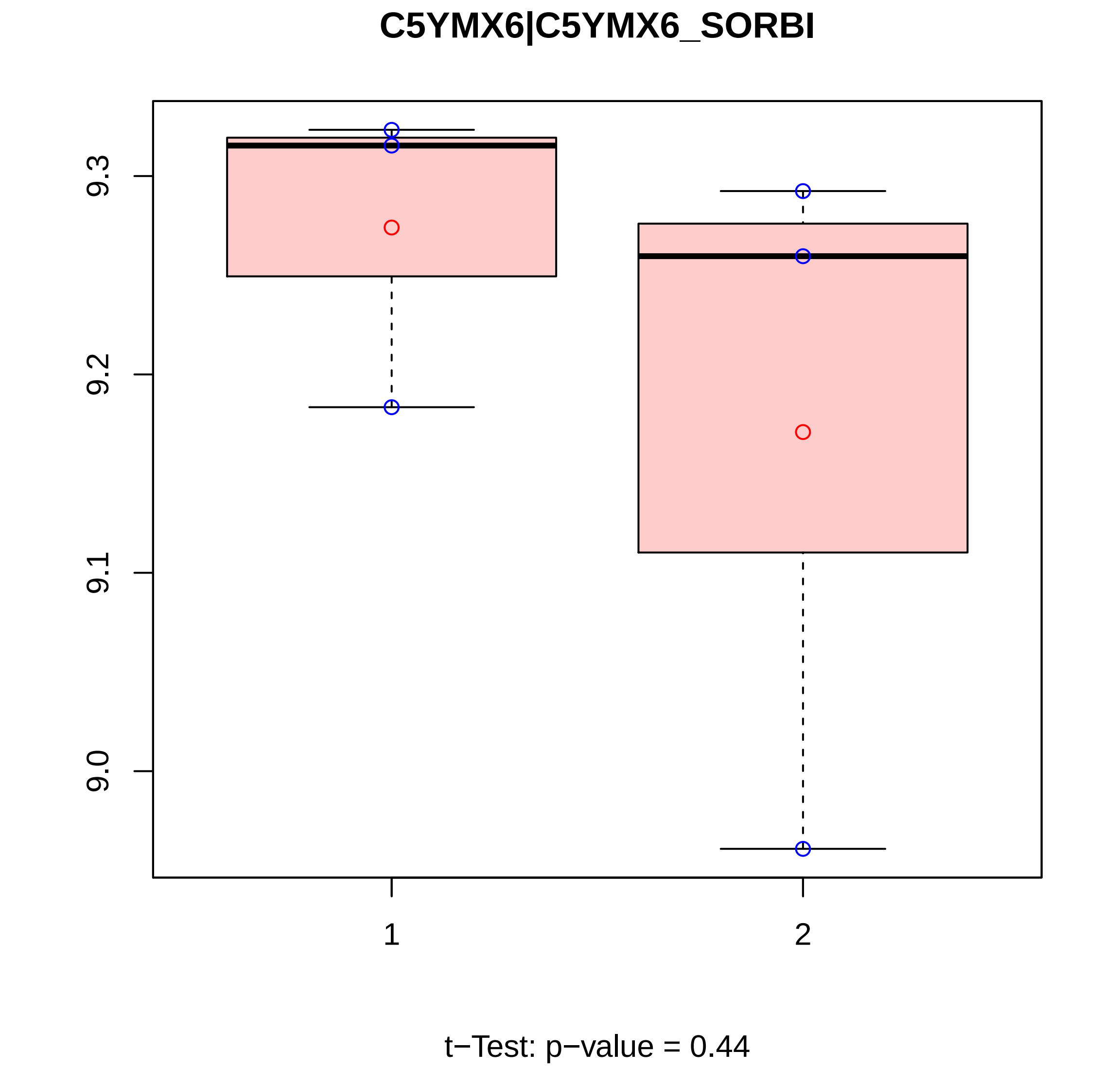

C5YMX6|C5YMX6_SORBI
9.0
9.1
9.2
9.3
1
2
t−Test: p−value = 0.44

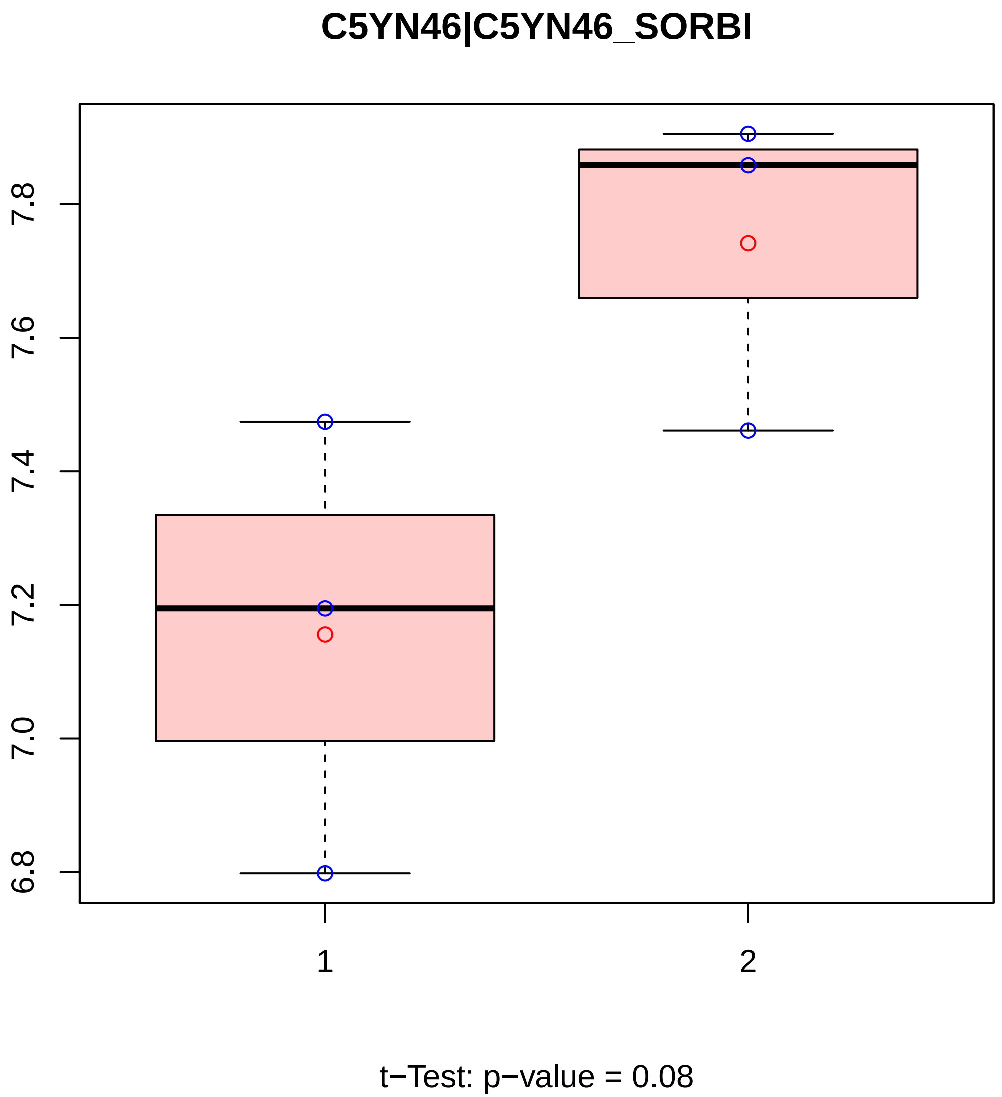
C5YN46|C5YN46_SORBI
7.8
7.6
7.4
7.2
7.0
6.8
1
2
t−Test: p−value = 0.08

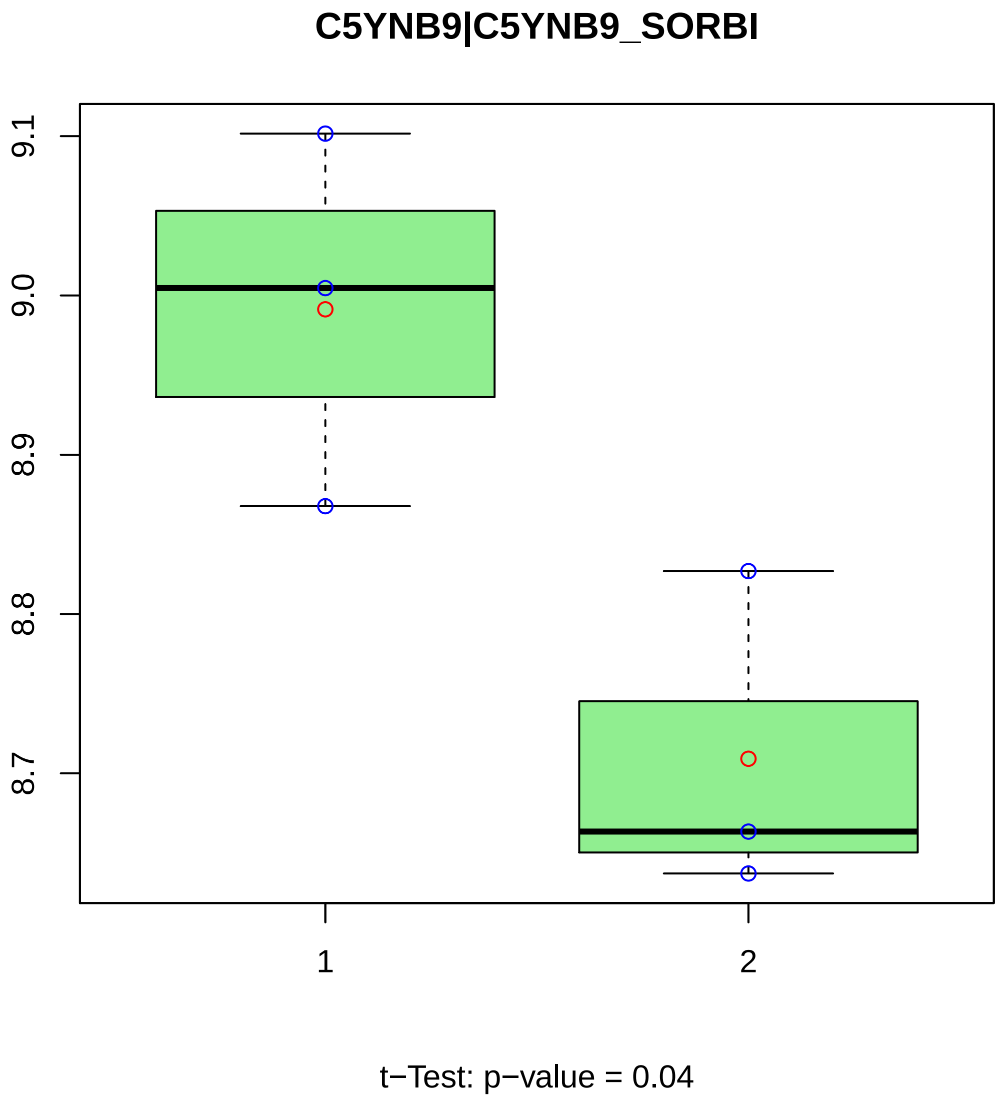

C5YNB9|C5YNB9_SORBI
9.1
9.0
8.9
8.8
8.7
1
2
t−Test: p−value = 0.04

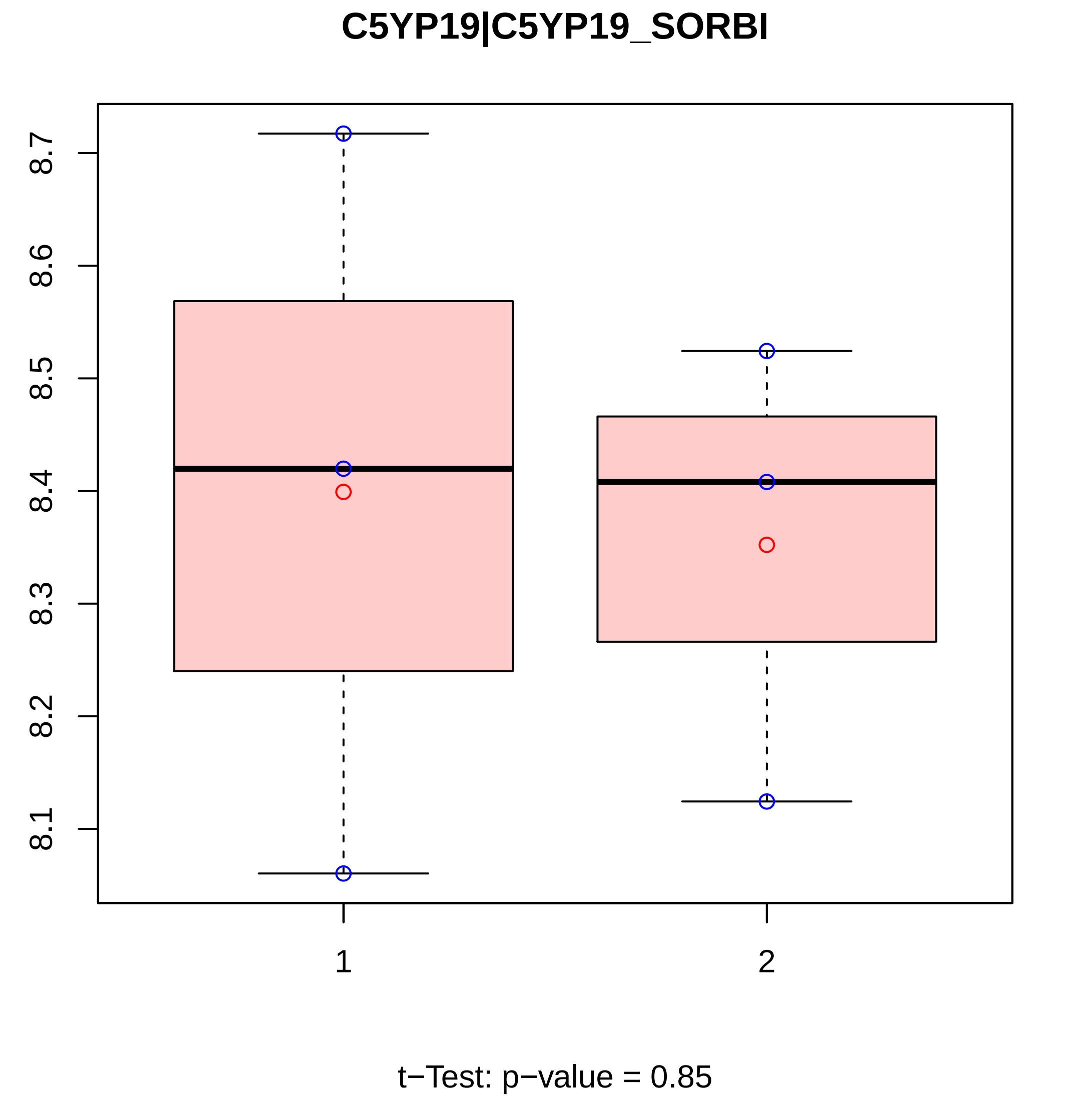
C5YP19|C5YP19_SORBI
8.7
8.6
8.5
8.4
8.3
8.2
8.1
1
2
t−Test: p−value = 0.85

C5YPL6|C5YPL6_SORBI

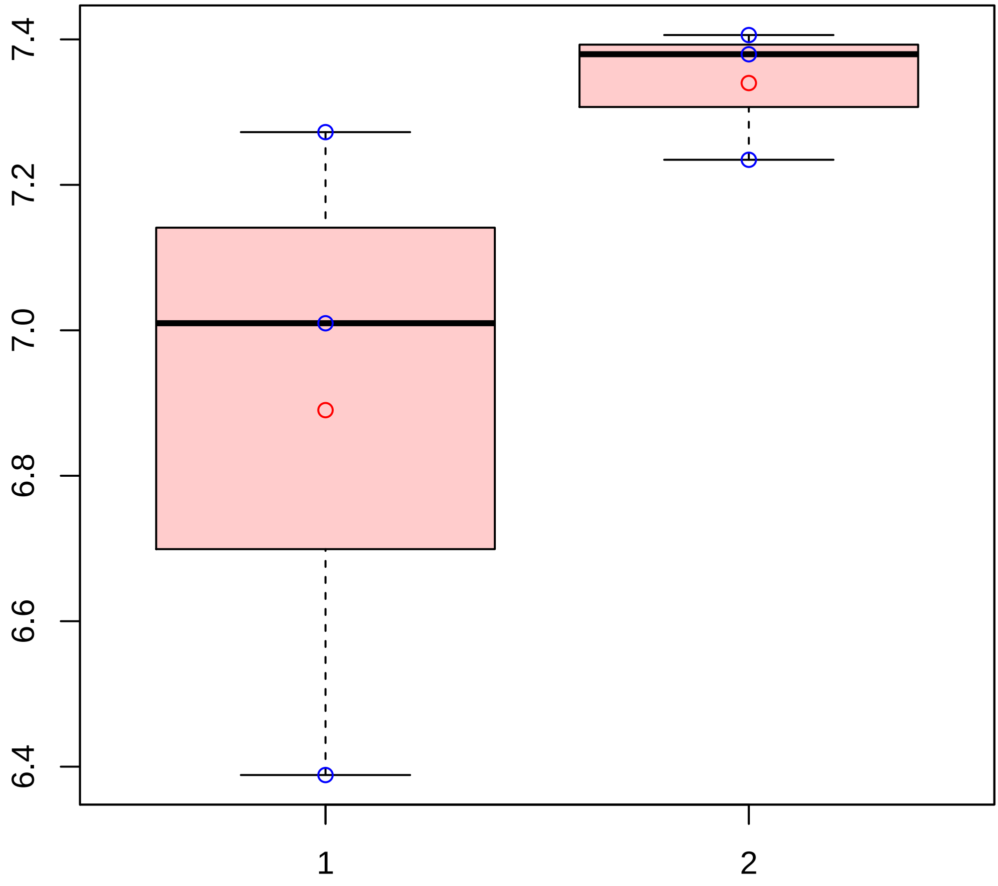

t−Test: p−value = 0.23

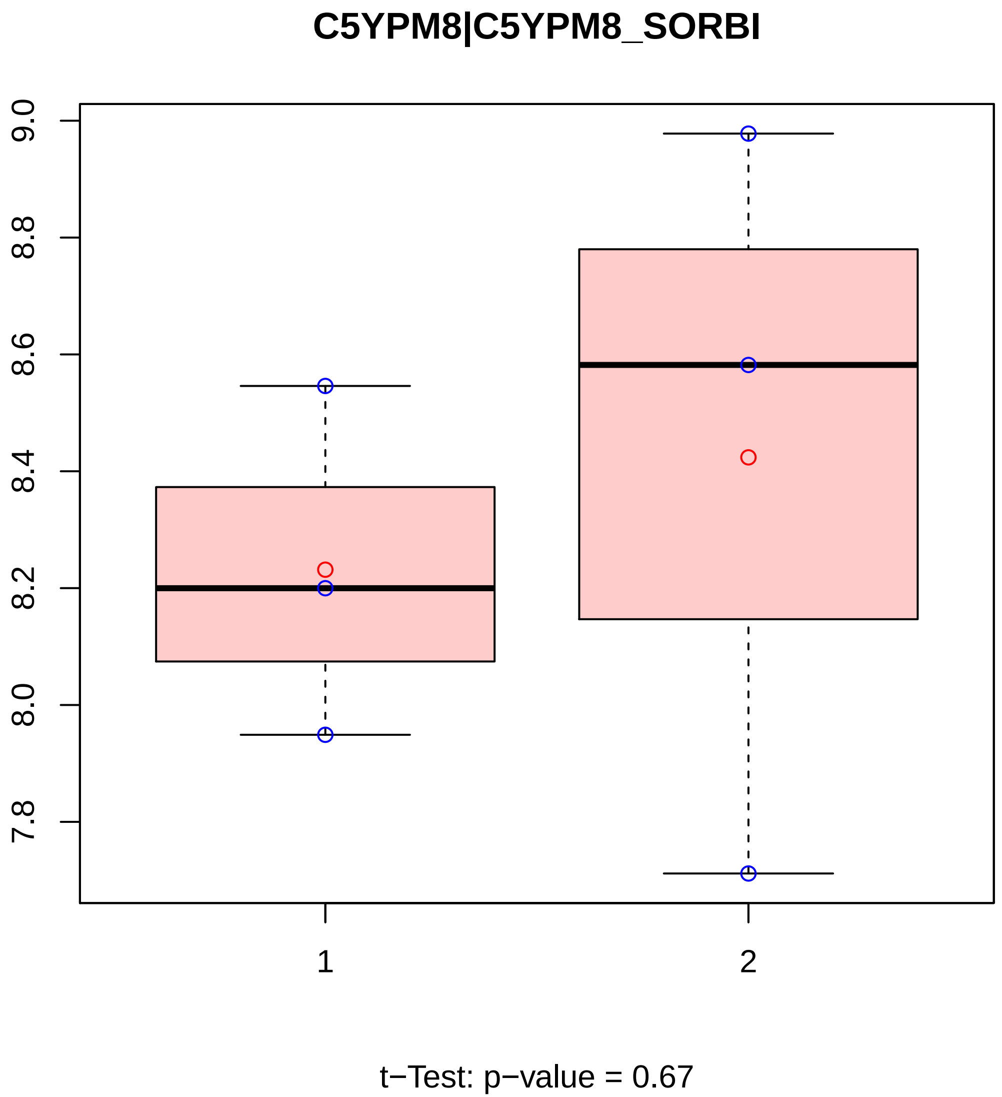
C5YPM8|C5YPM8_SORBI
9.0
8.8
8.6
8.4
8.2
8.0
7.8
1
2
t−Test: p−value = 0.67

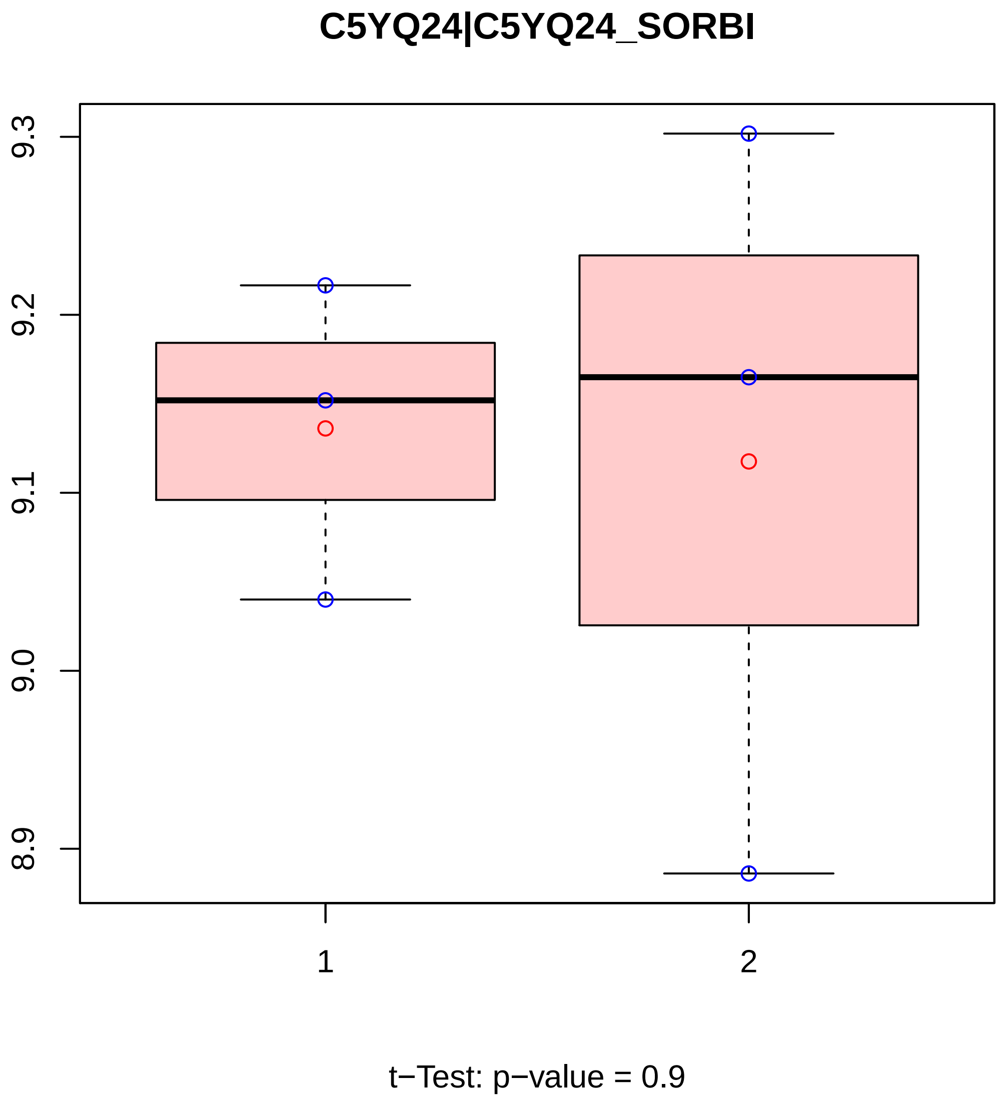
C5YQ24|C5YQ24_SORBI
9.3
9.2
9.1
9.0
8.9
1
2
t−Test: p−value = 0.9

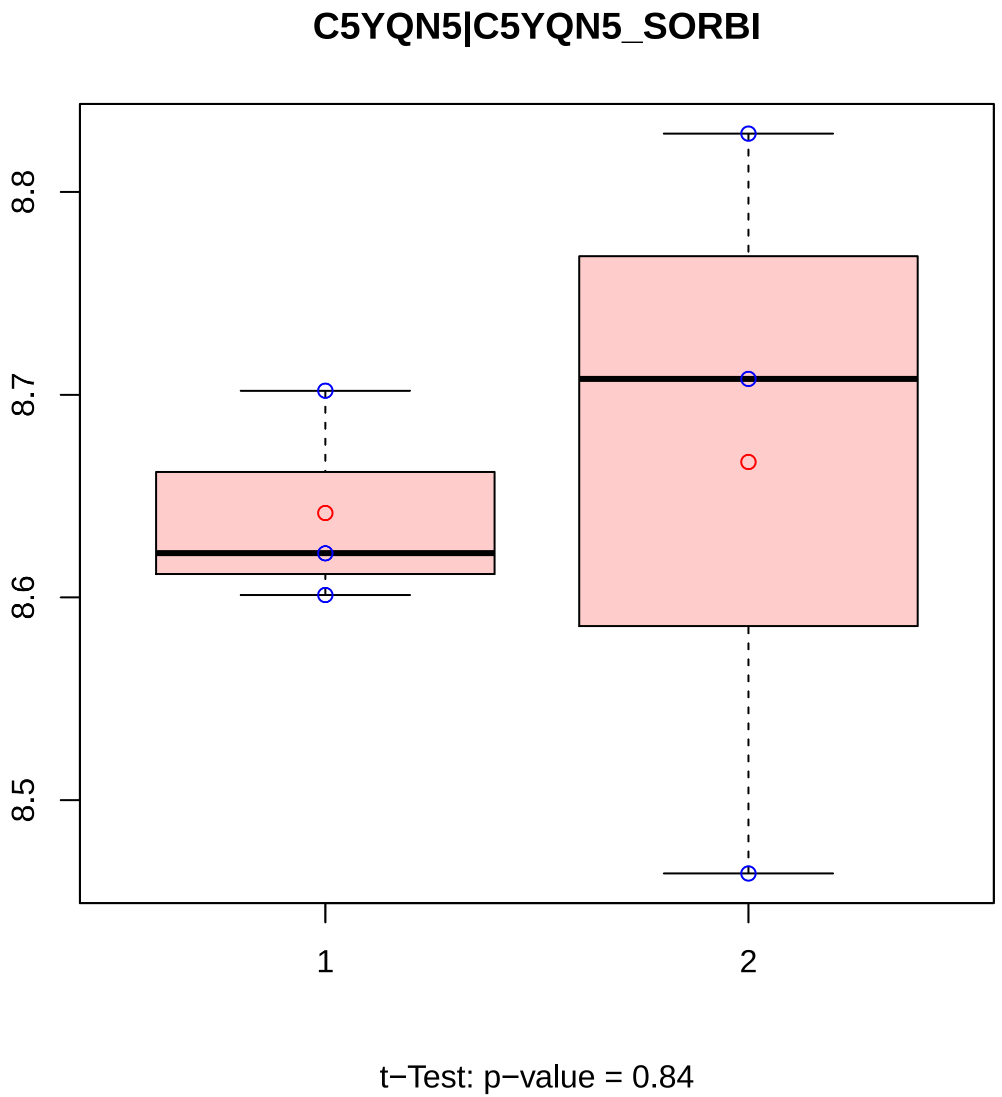
C5YQN5|C5YQN5_SORBI
8.5
8.6
8.7
8.8
1
2
t−Test: p−value = 0.84

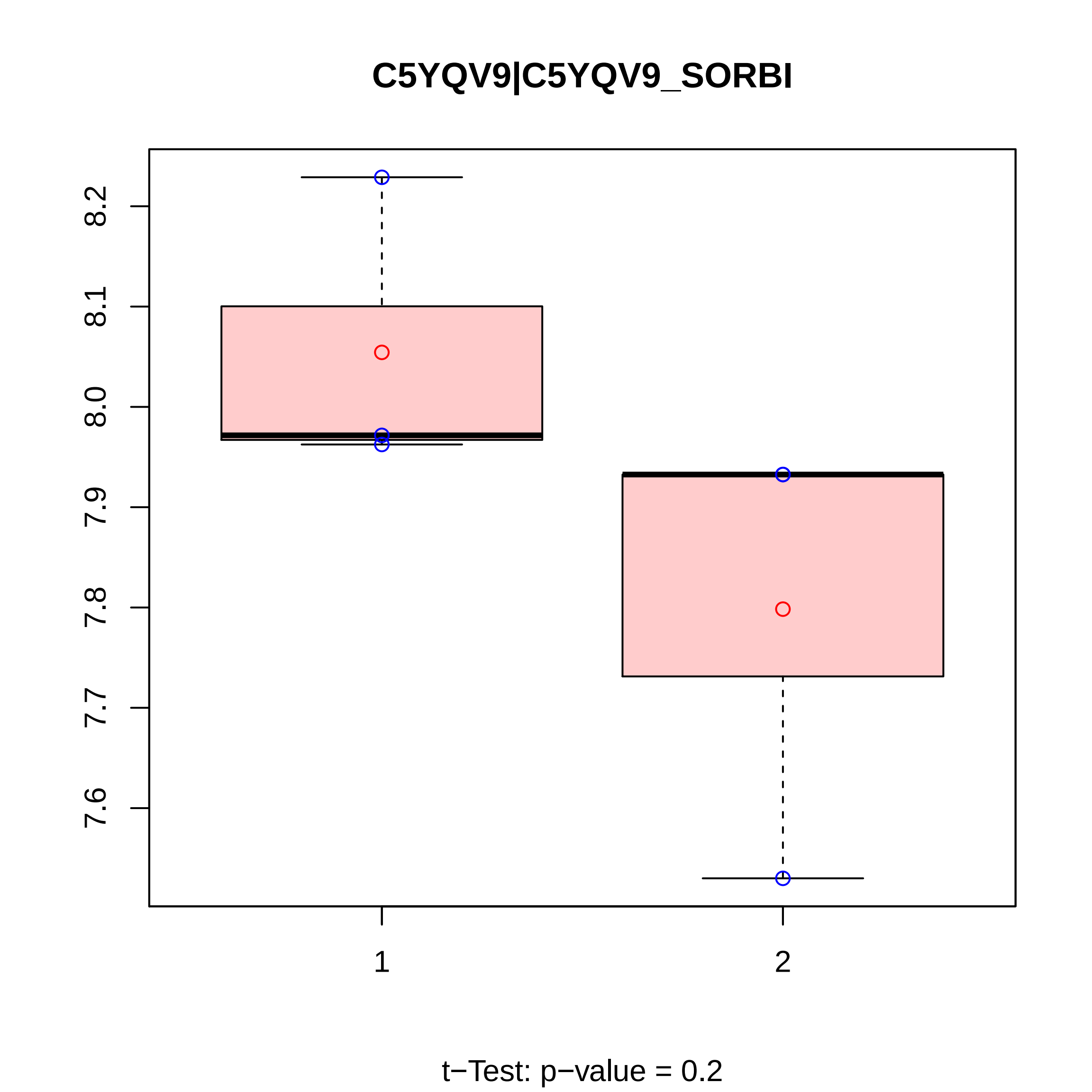

C5YQV9|C5YQV9_SORBI
8.2
8.1
8.0
7.9
7.8
7.7
7.6
1
2
t−Test: p−value = 0.2

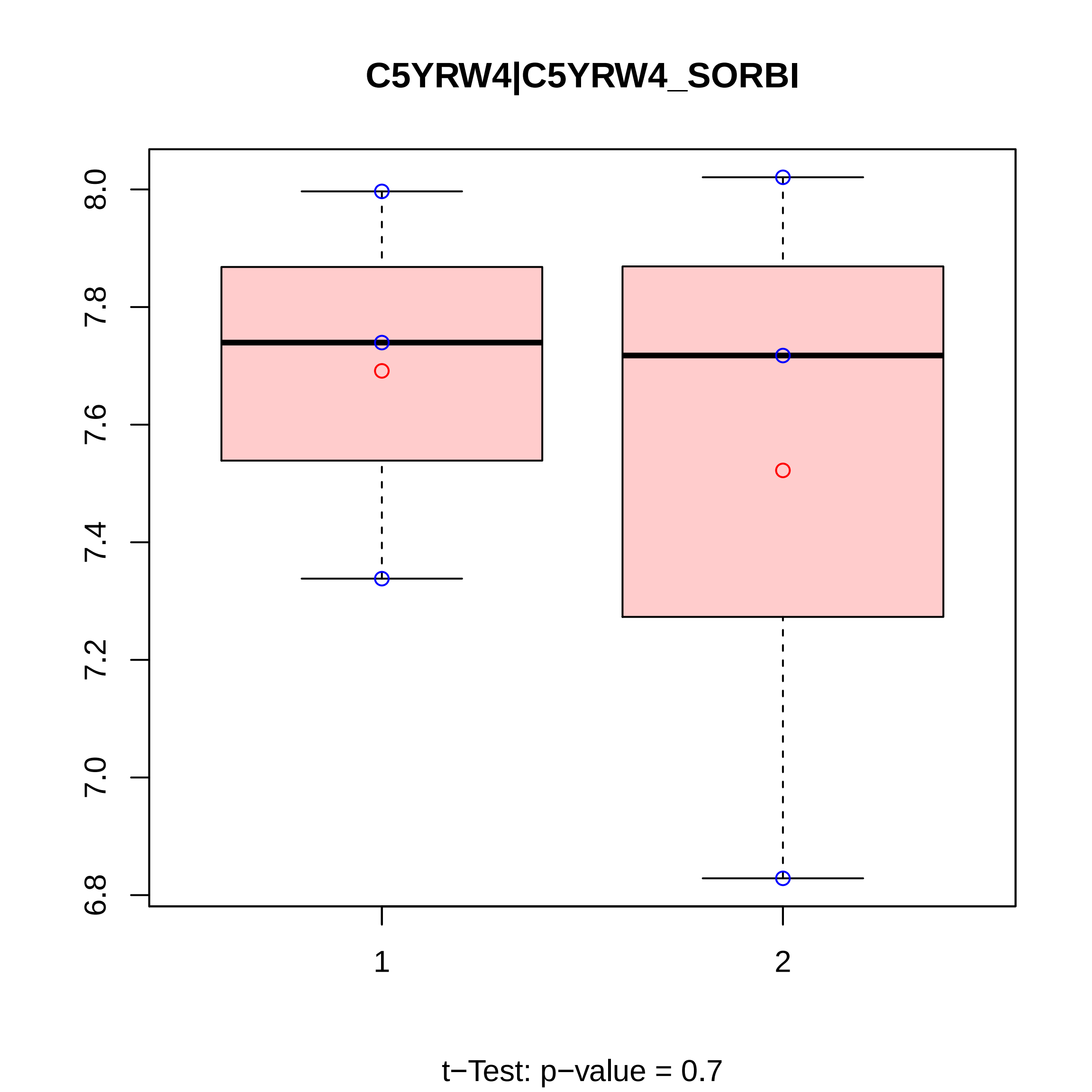
C5YRW4|C5YRW4_SORBI
8.0
7.8
7.6
7.4
7.2
7.0
6.8
1
2
t−Test: p−value = 0.7

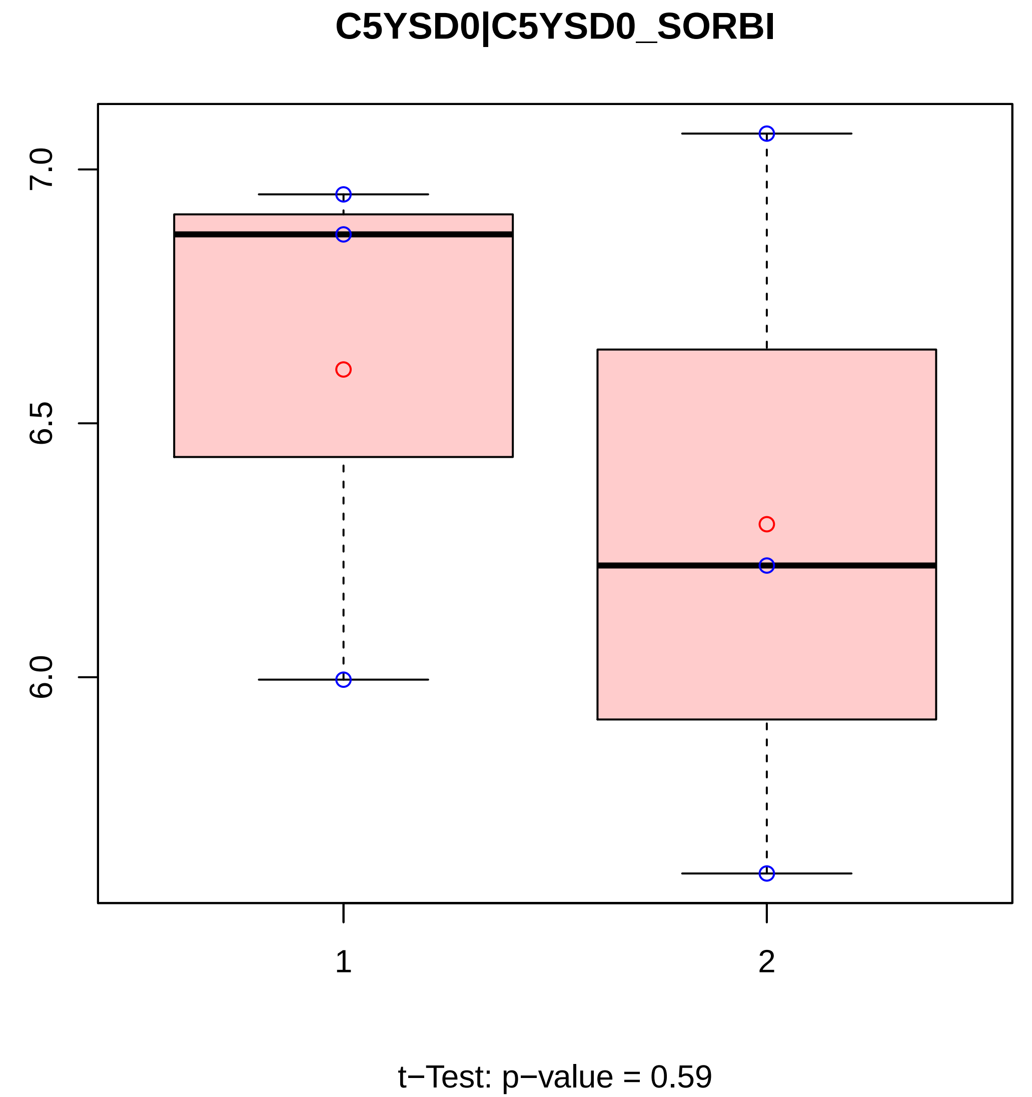
C5YSD0|C5YSD0_SORBI
7.0
6.5
6.0
1
2
t−Test: p−value = 0.59

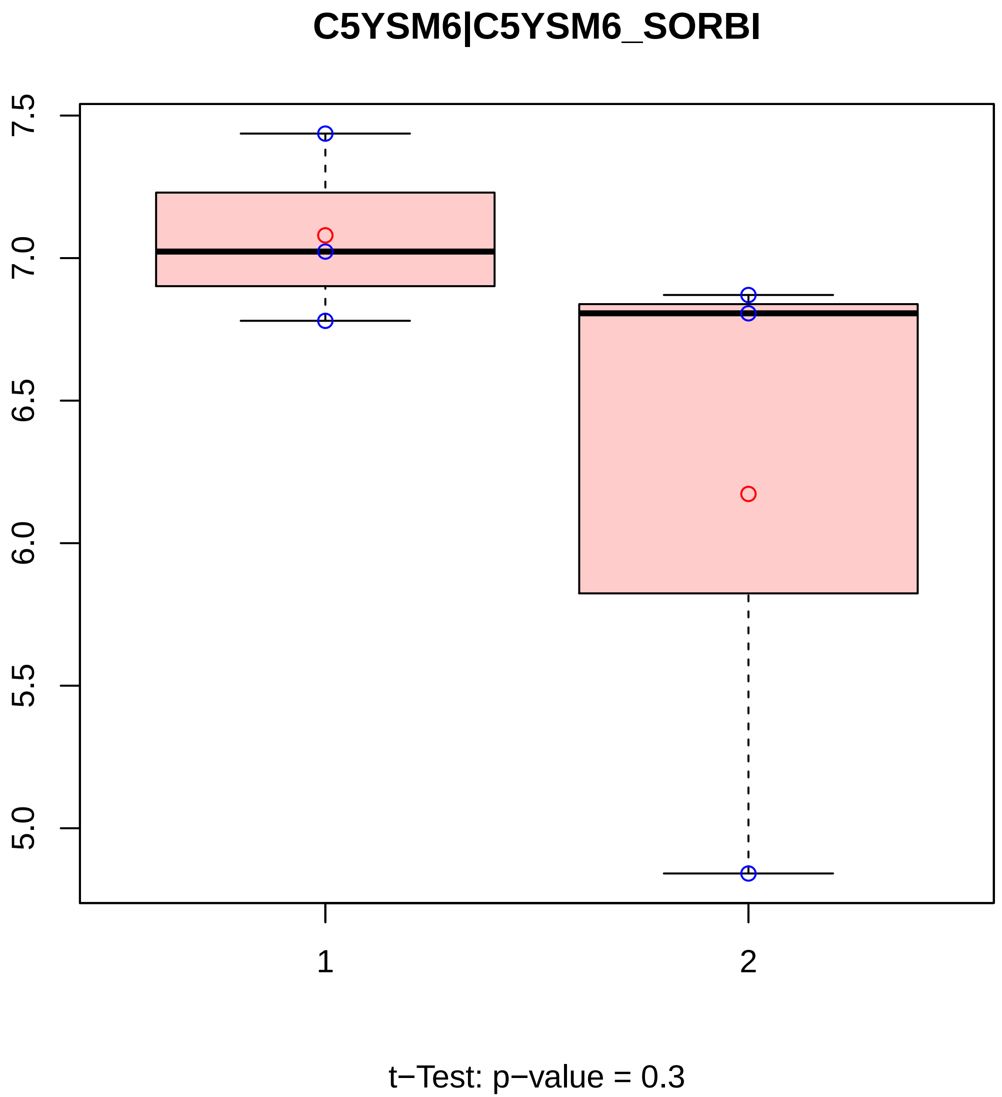

C5YSM6|C5YSM6_SORBI
7.5
7.0
6.5
6.0
5.5
5.0
1
2
t−Test: p−value = 0.3

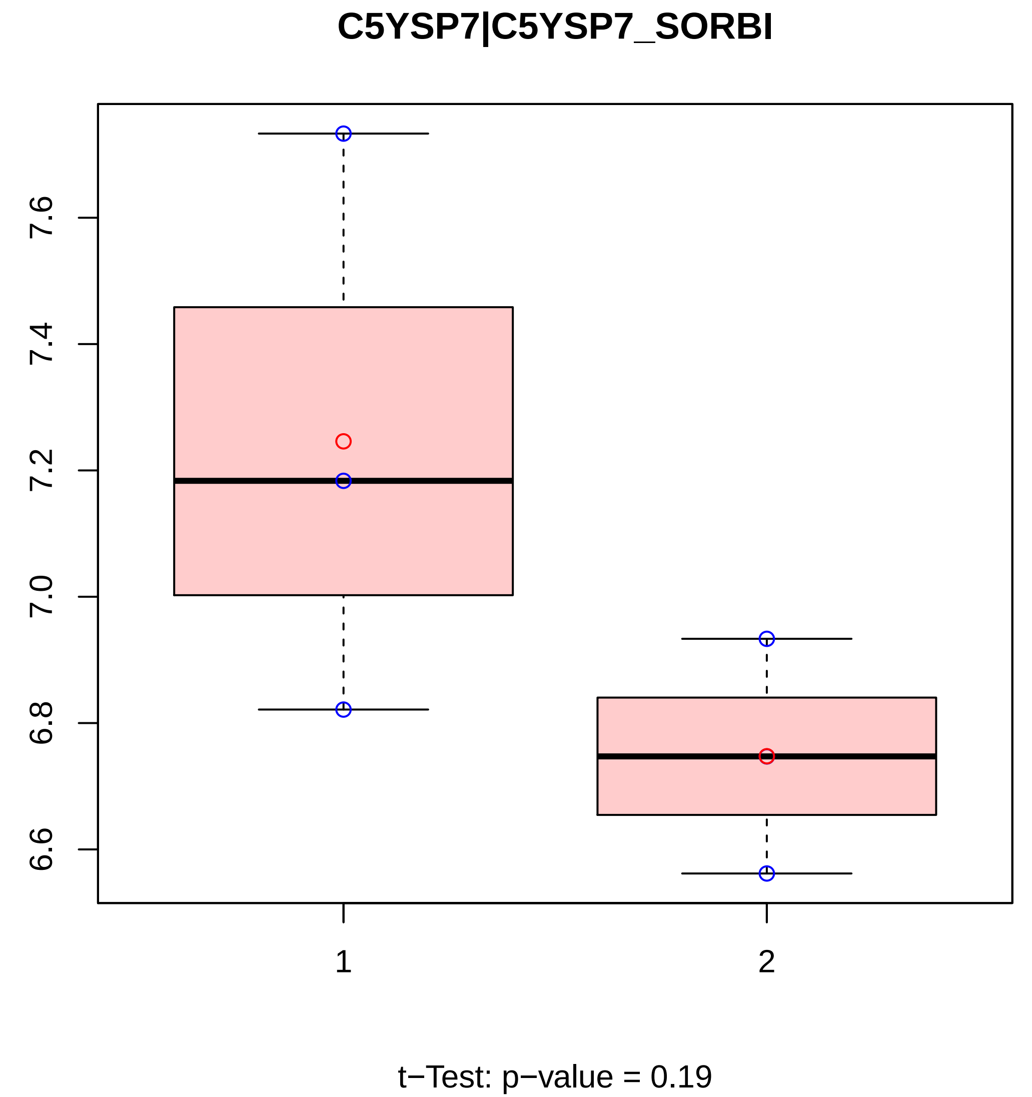

C5YSP7|C5YSP7_SORBI
7.6
7.4
7.2
7.0
6.8
6.6
1
2
t−Test: p−value = 0.19

C5YSU1|C5YSU1_SORBI

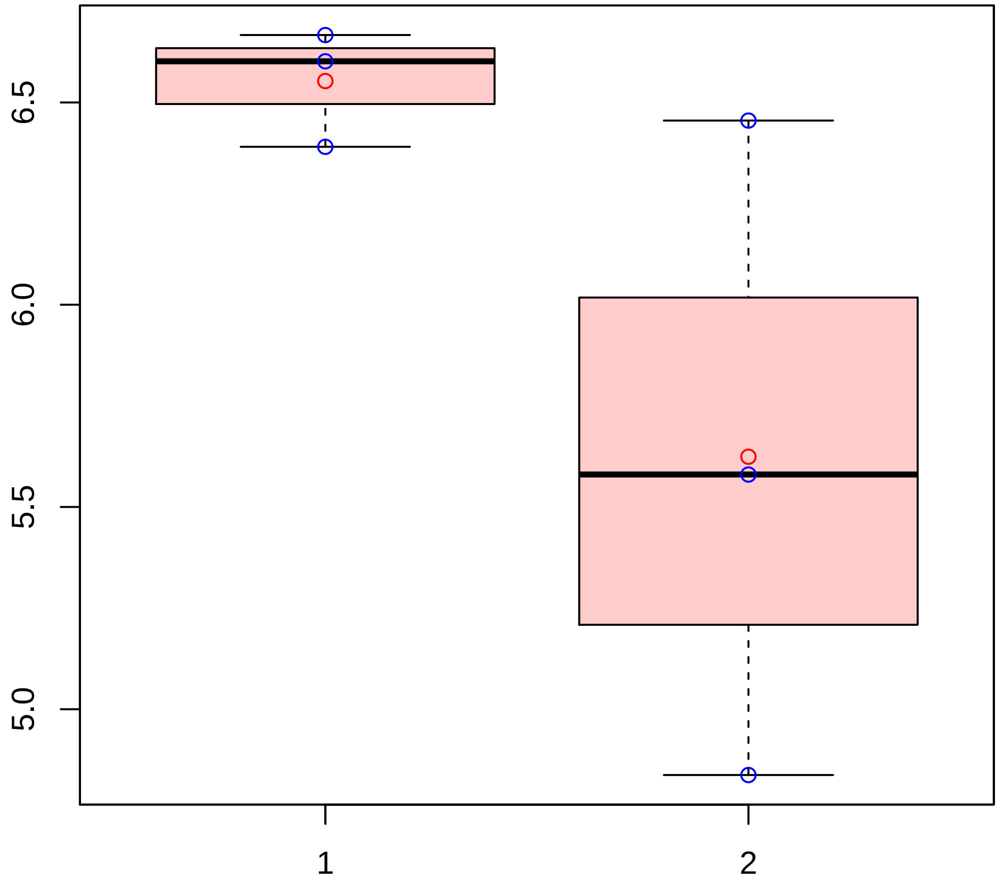

t−Test: p−value = 0.18

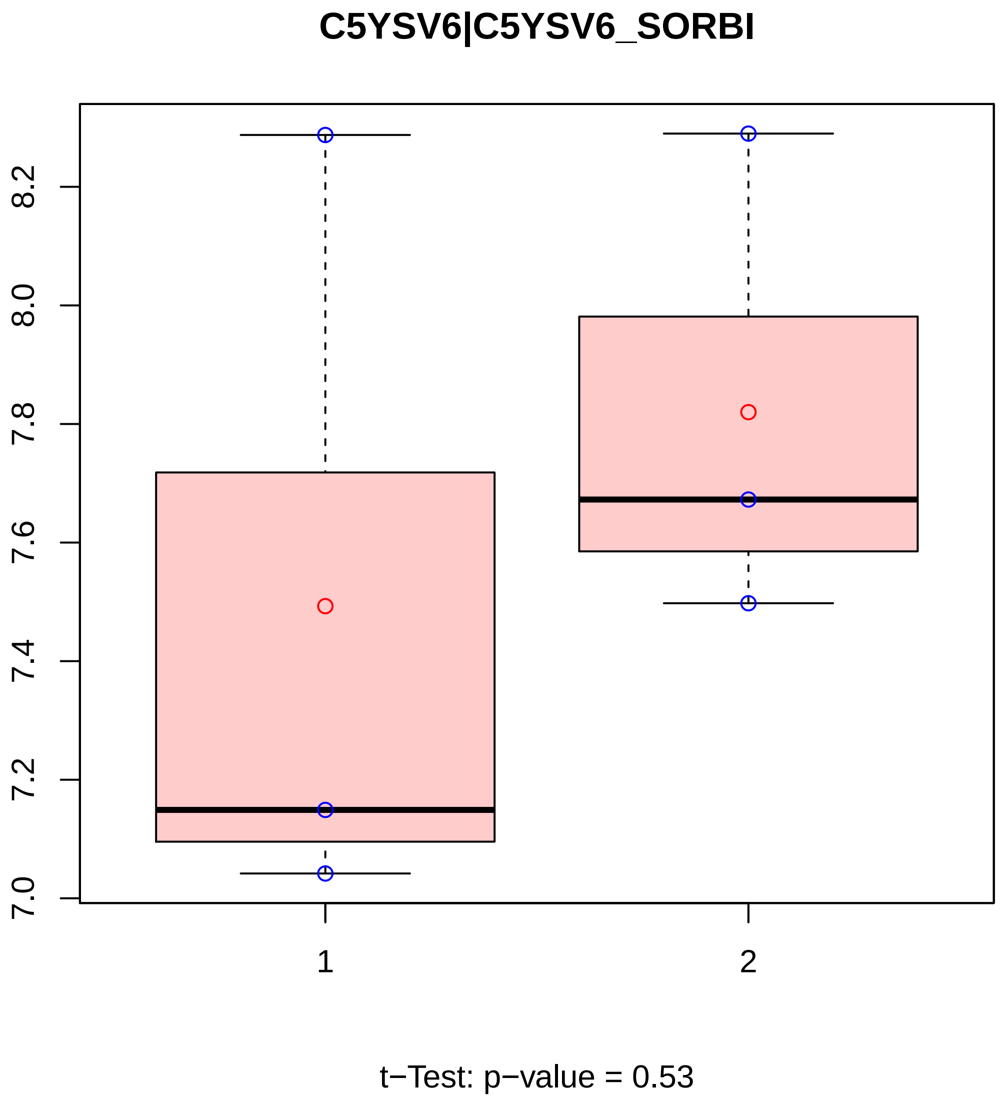
C5YSV6|C5YSV6_SORBI
7.0
7.2
7.4
7.6
7.8
8.0
8.2
1
2
t−Test: p−value = 0.53

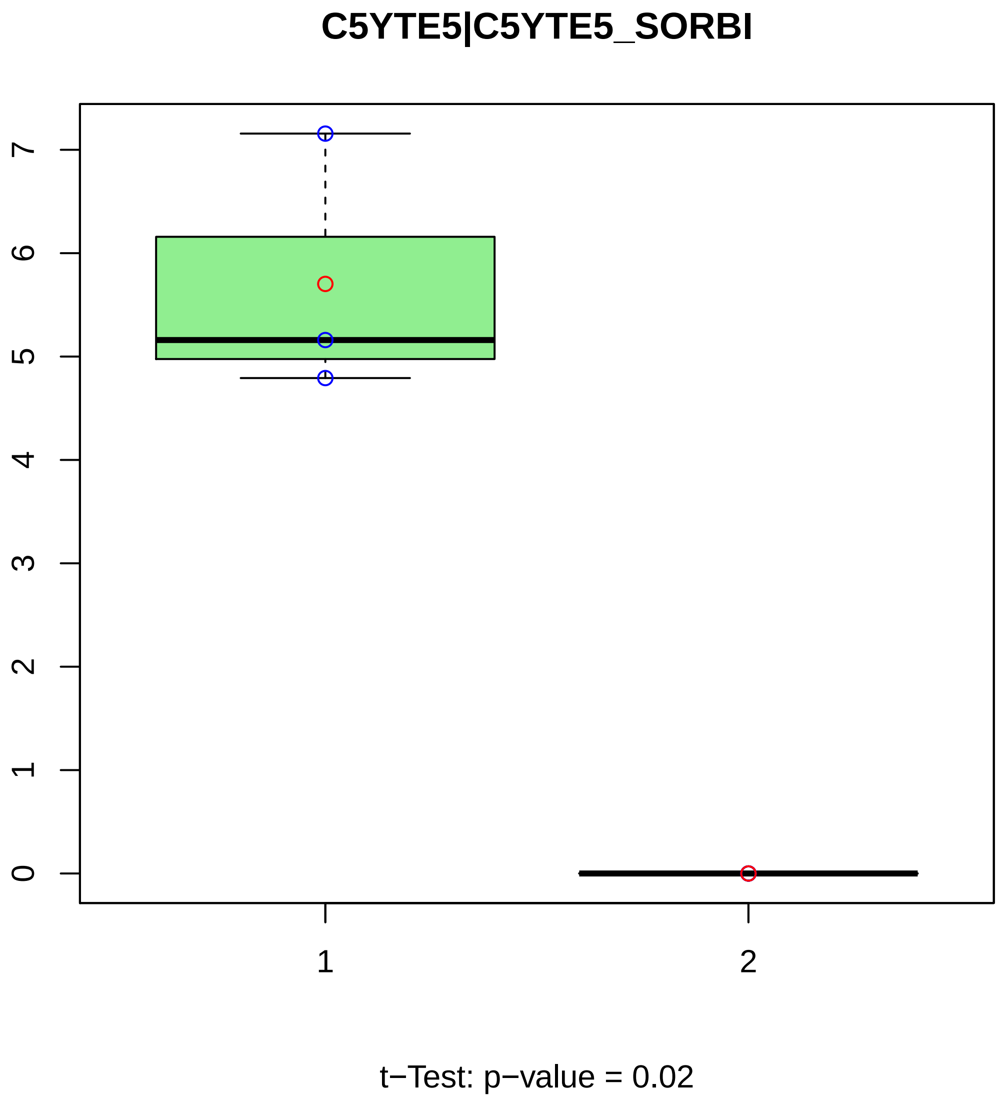

C5YTE5|C5YTE5_SORBI
0
1
2
3
4
5
6
7
1
2
t−Test: p−value = 0.02

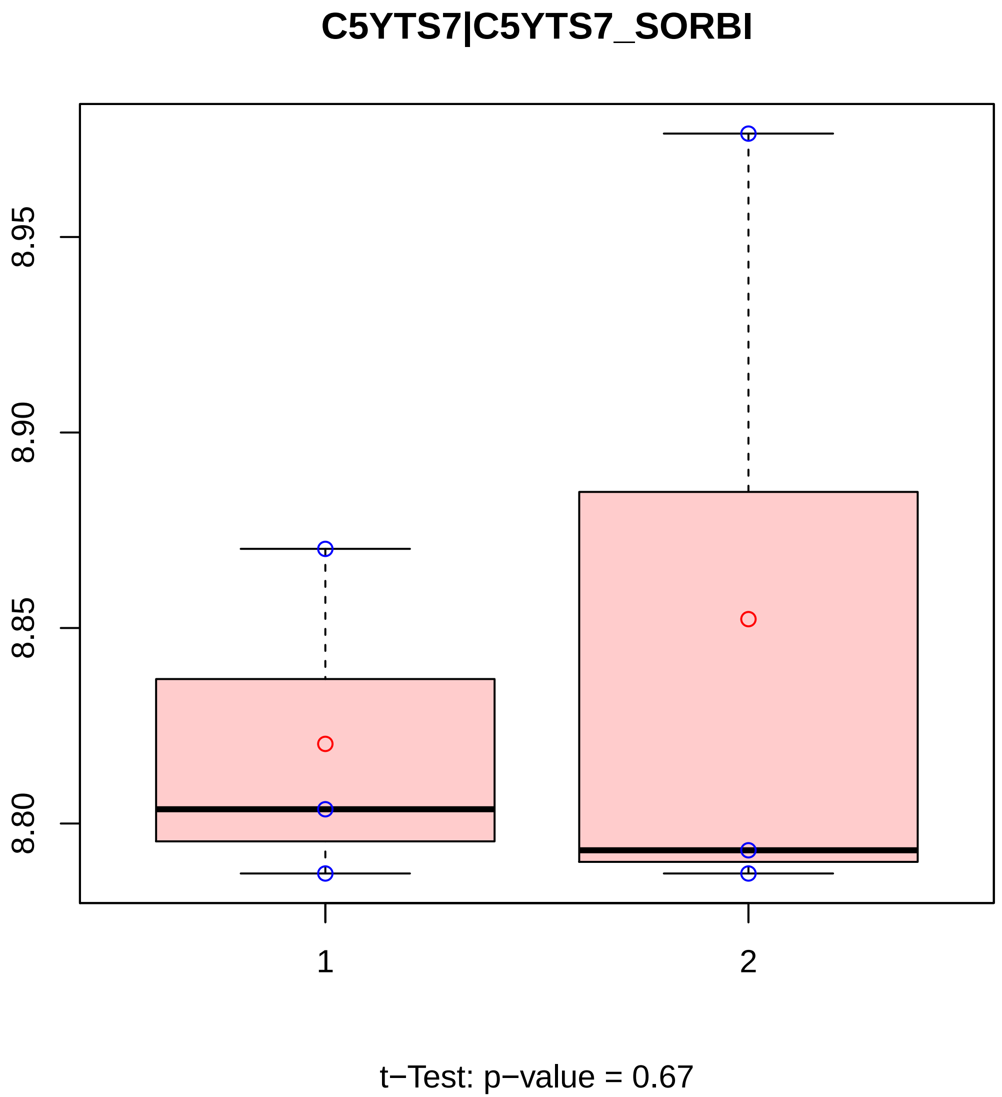

C5YTS7|C5YTS7_SORBI
8.80
8.85
8.90
8.95
1
2
t−Test: p−value = 0.67

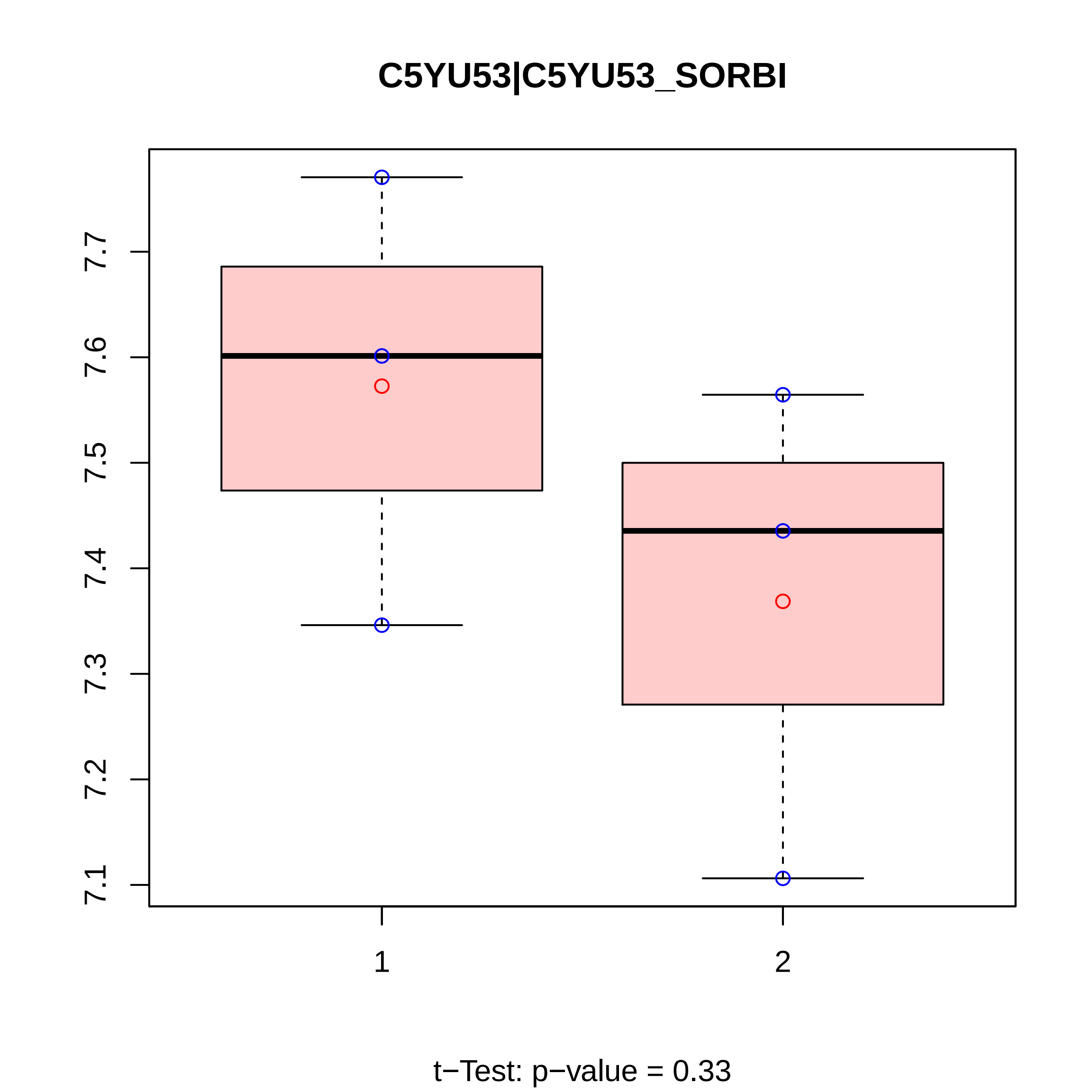
C5YU53|C5YU53_SORBI
7.1
7.2
7.3
7.4
7.5
7.6
7.7
1
2
t−Test: p−value = 0.33

C5YU58|C5YU58_SORBI

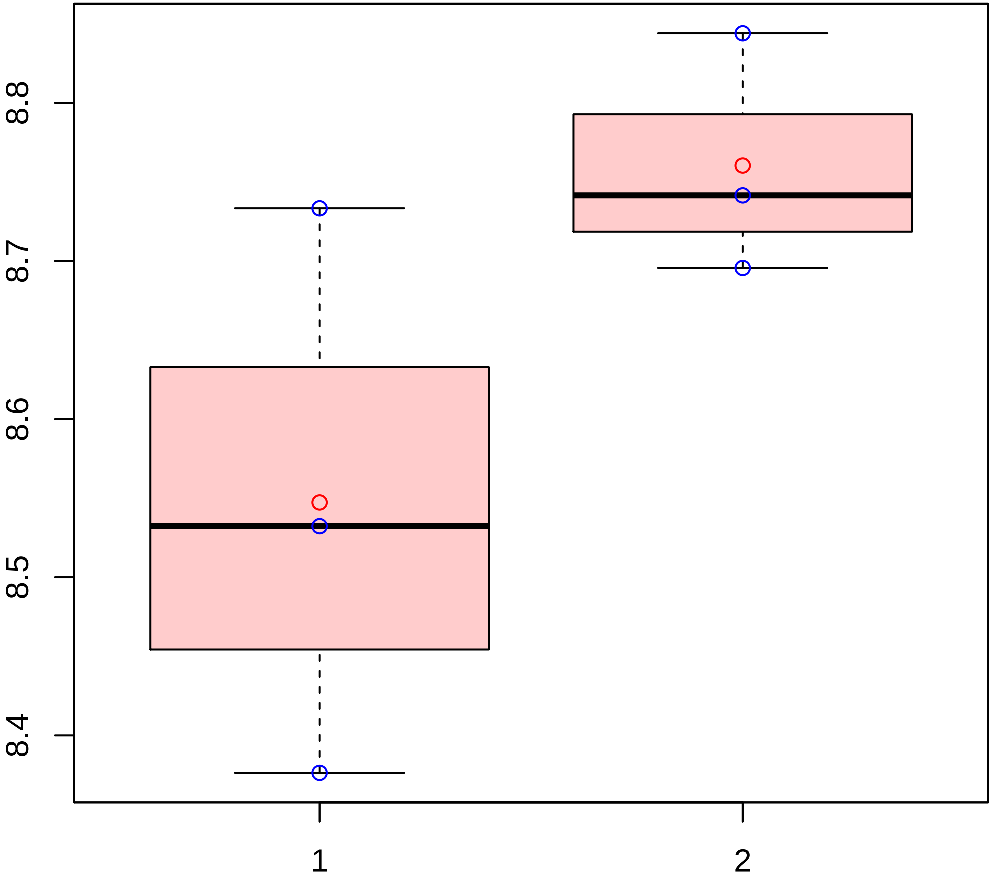

t−Test: p−value = 0.16

C5YU66|C5YU66_SORBI

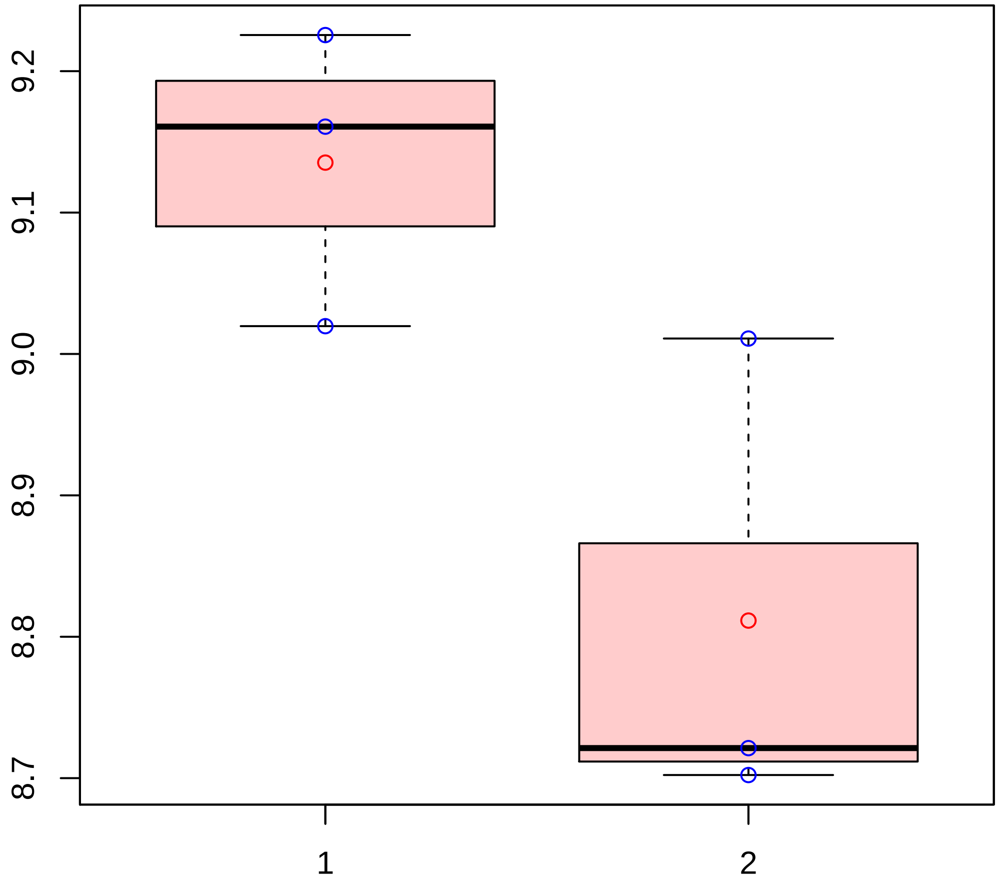

t−Test: p−value = 0.06

C5YUI6|C5YUI6_SORBI

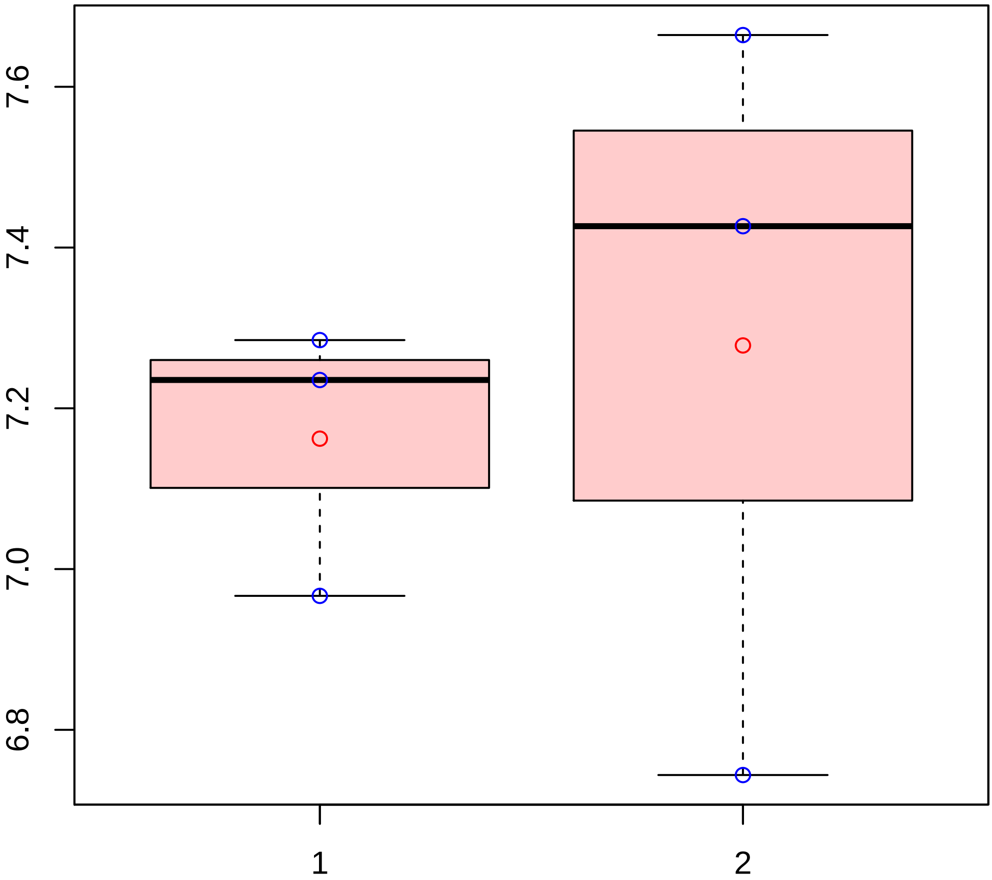

t−Test: p−value = 0.72

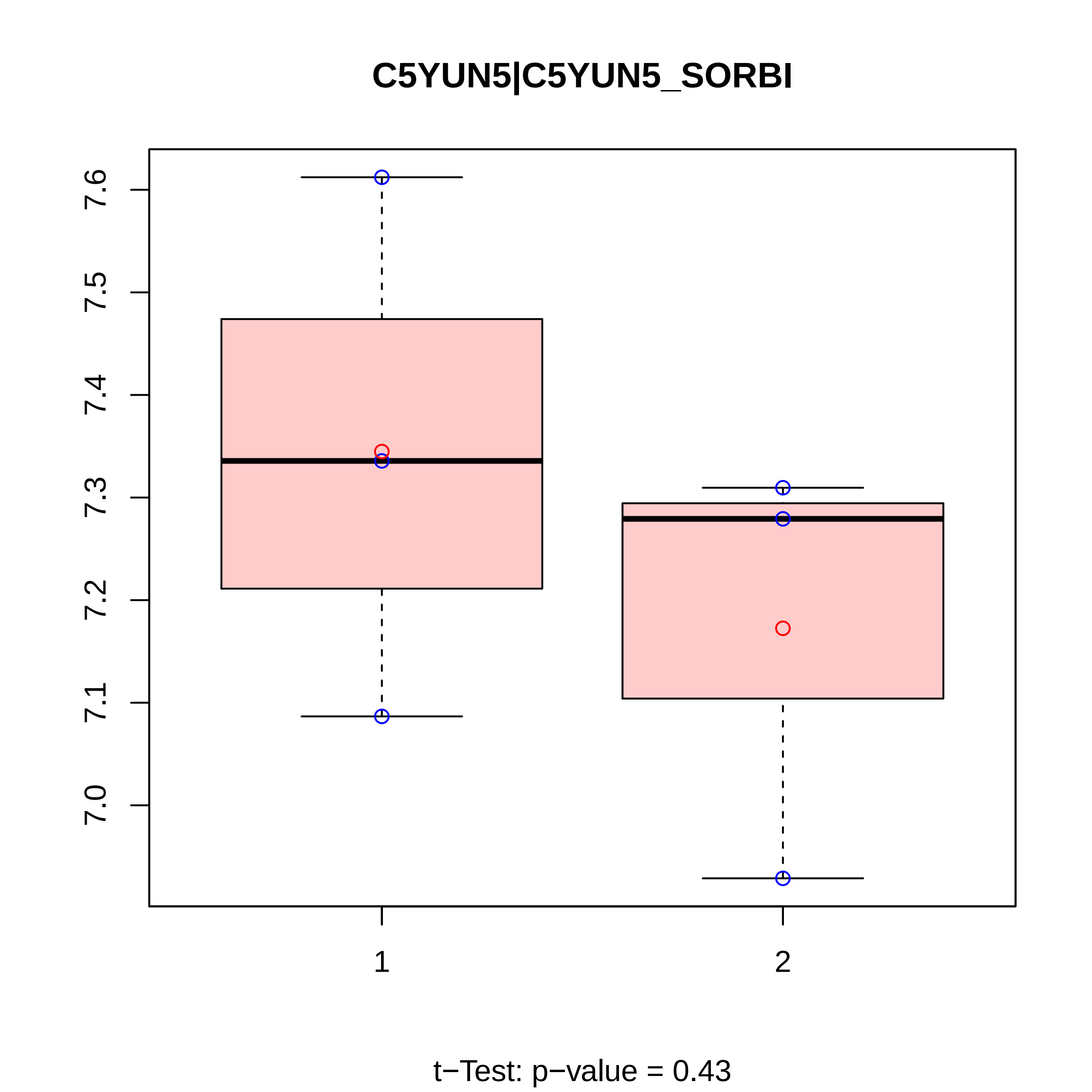
C5YUN5|C5YUN5_SORBI
7.6
7.5
7.4
7.3
7.2
7.1
7.0
1
2
t−Test: p−value = 0.43

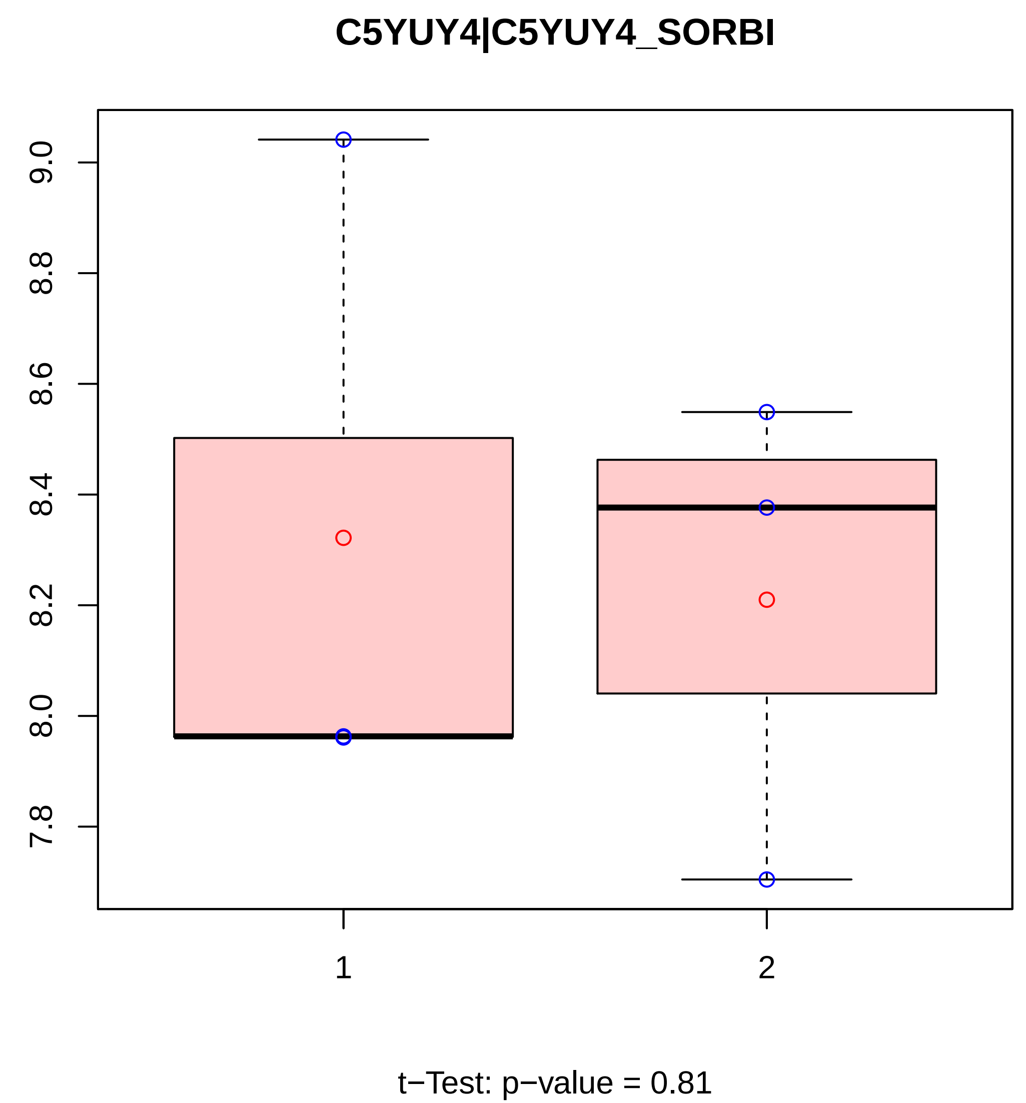
C5YUY4|C5YUY4_SORBI
9.0
8.8
8.6
8.4
8.2
8.0
7.8
1
2
t−Test: p−value = 0.81

C5YV18|C5YV18_SORBI

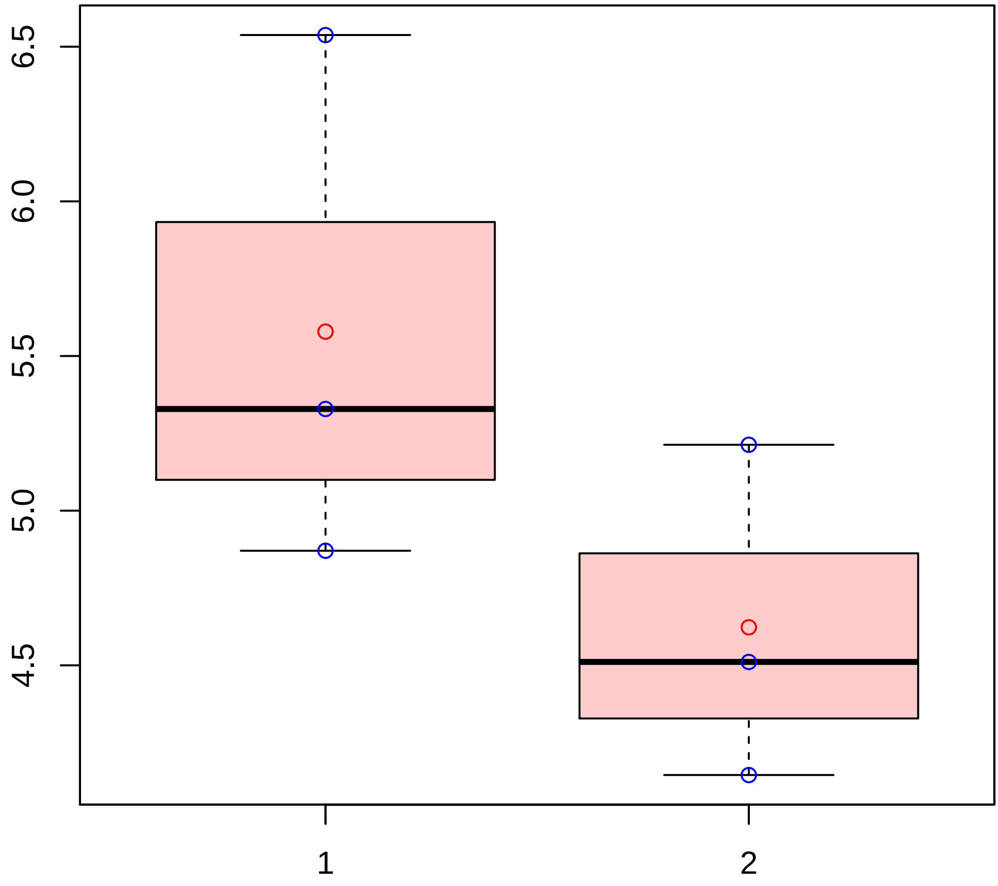

t−Test: p−value = 0.19

C5YV65|C5YV65_SORBI

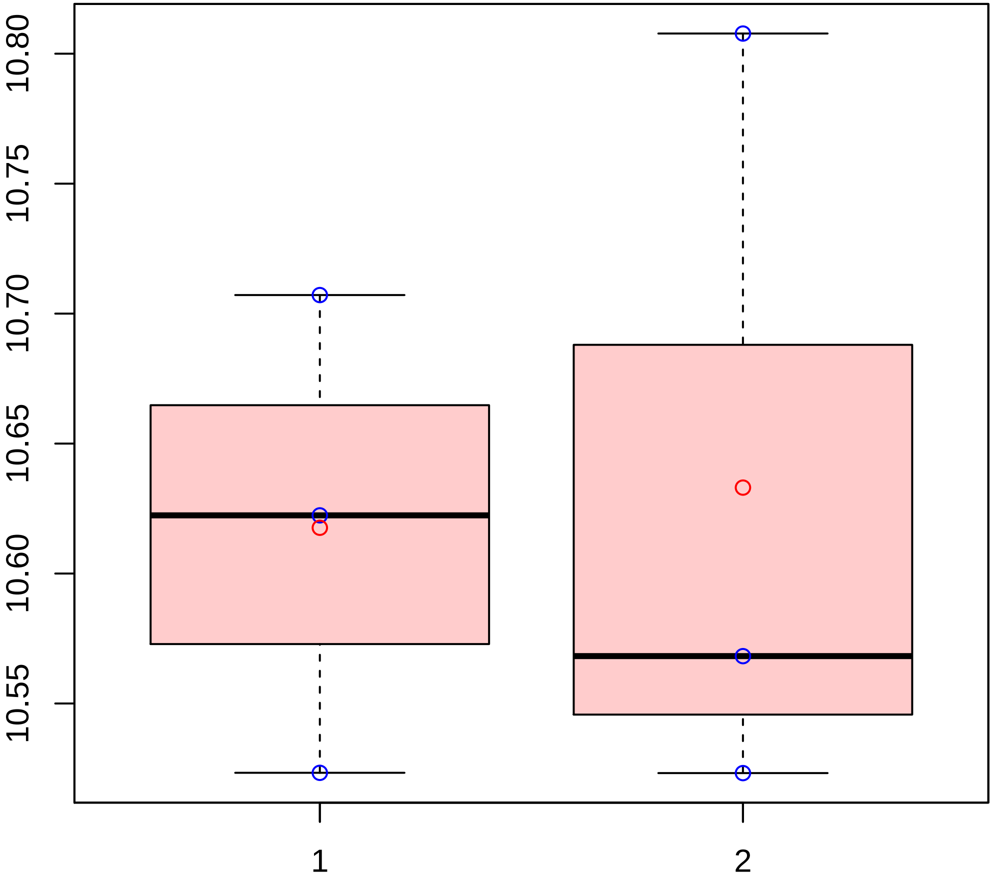

t−Test: p−value = 0.89

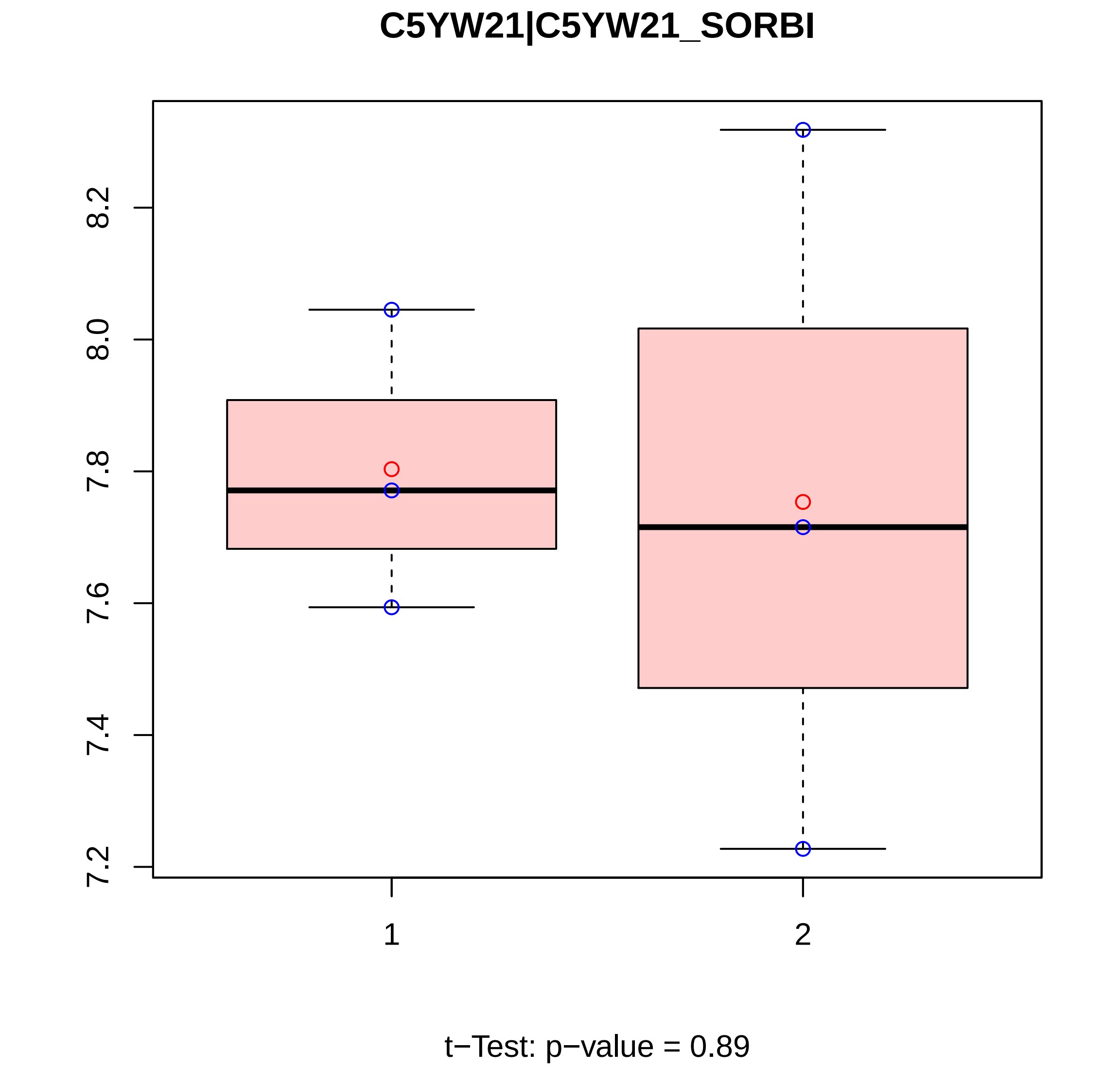
C5YW21|C5YW21_SORBI
8.2
8.0
7.8
7.6
7.4
7.2
1
2
t−Test: p−value = 0.89

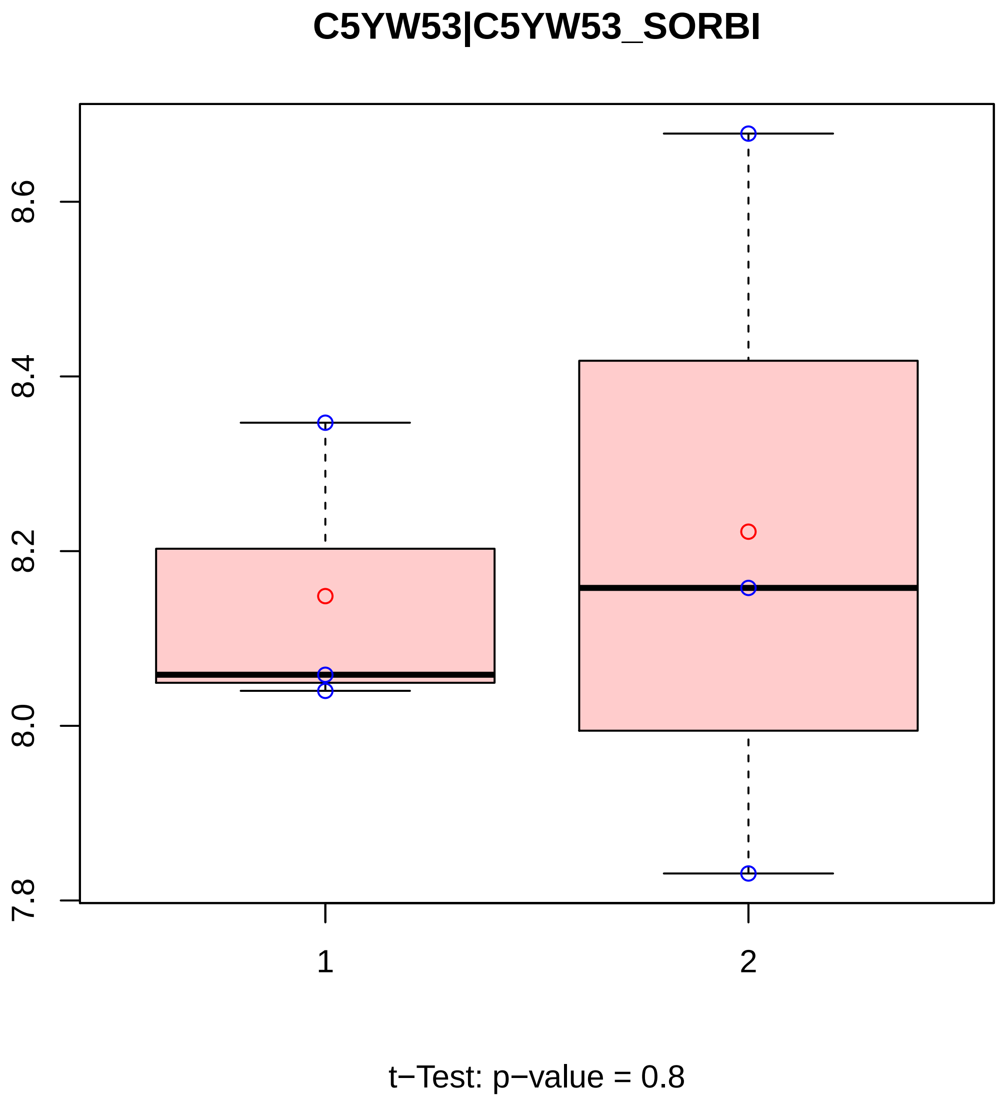
C5YW53|C5YW53_SORBI
8.6
8.4
8.2
8.0
7.8
1
2
t−Test: p−value = 0.8

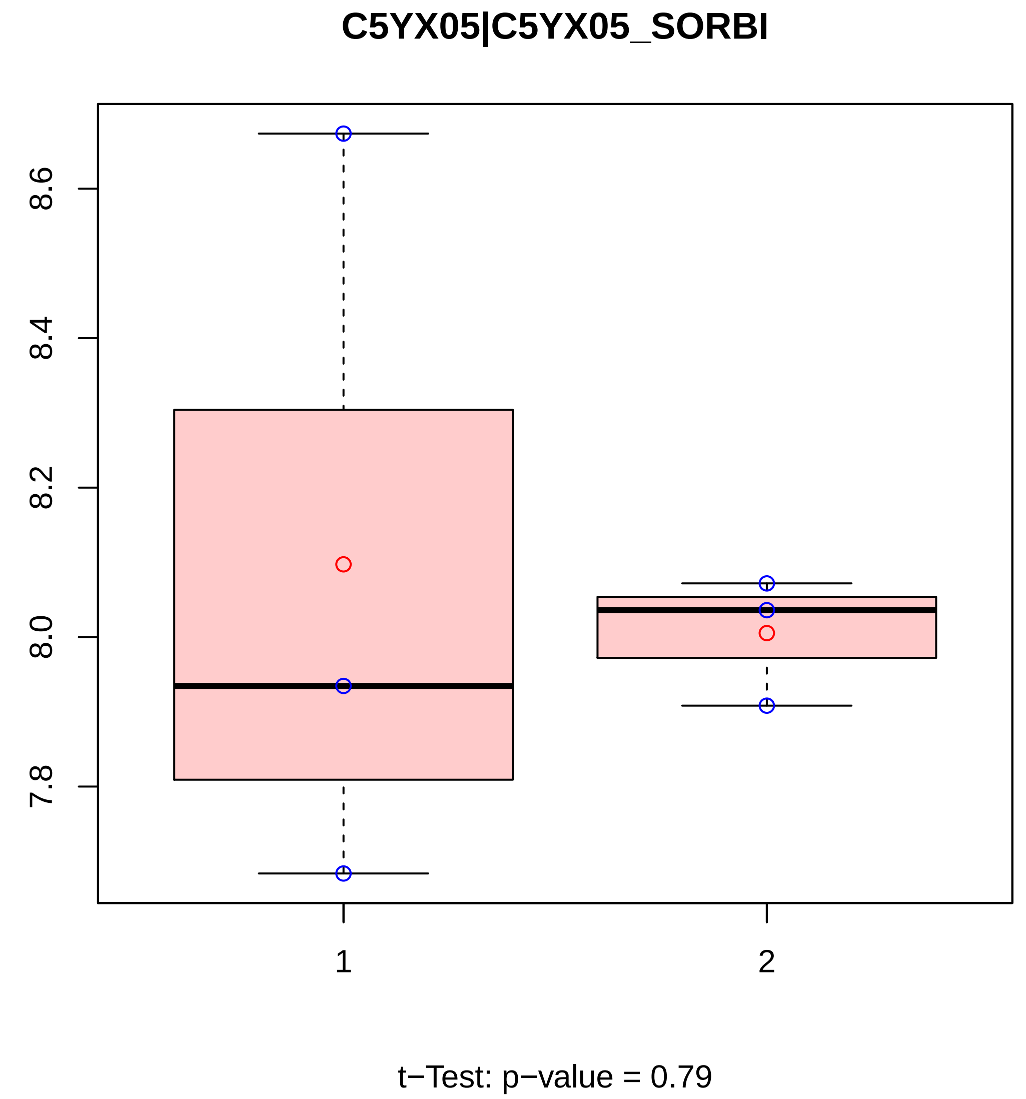
C5YX05|C5YX05_SORBI
8.6
8.4
8.2
8.0
7.8
1
2
t−Test: p−value = 0.79

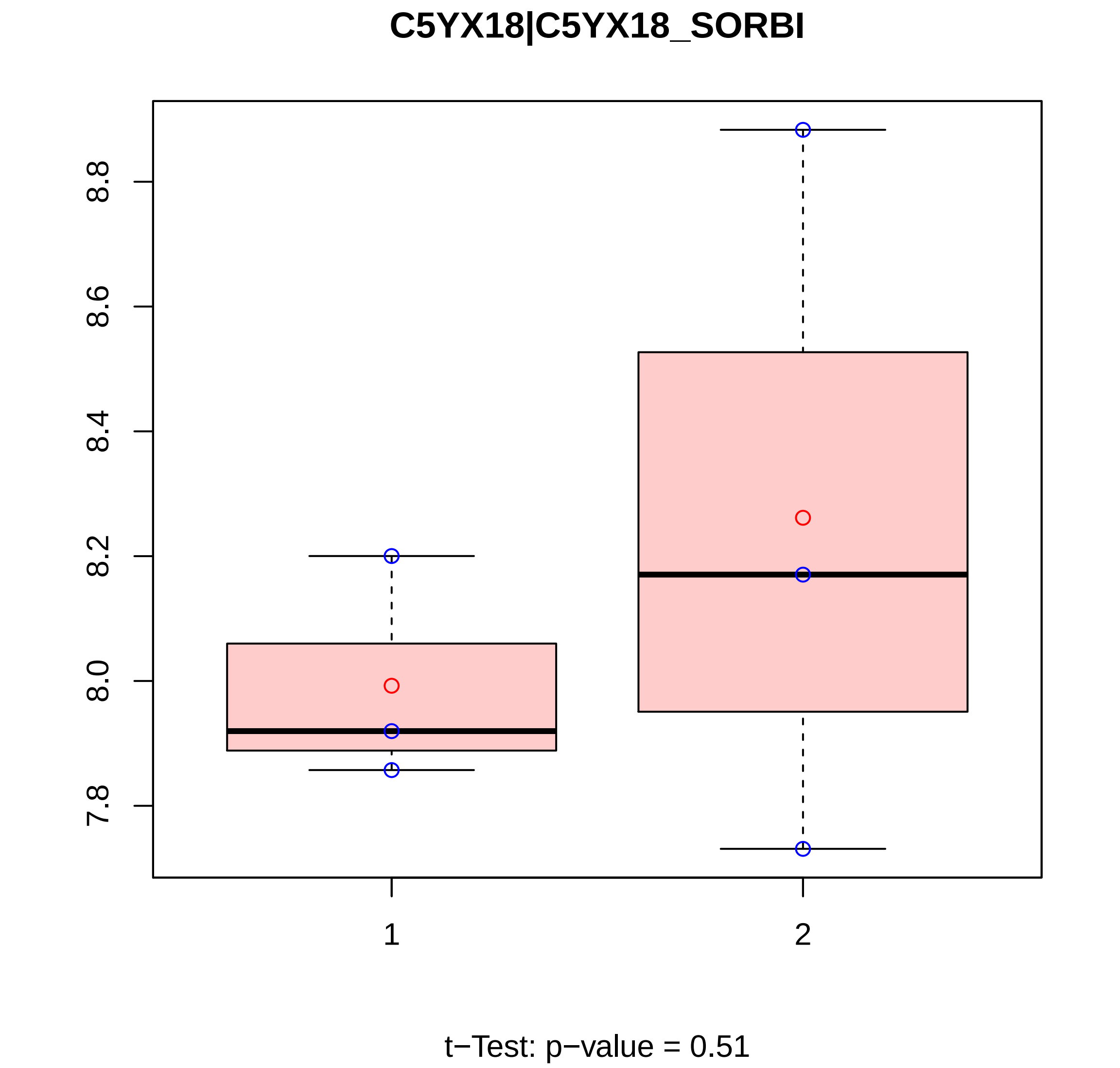
C5YX18|C5YX18_SORBI
8.8
8.6
8.4
8.2
8.0
7.8
1
2
t−Test: p−value = 0.51

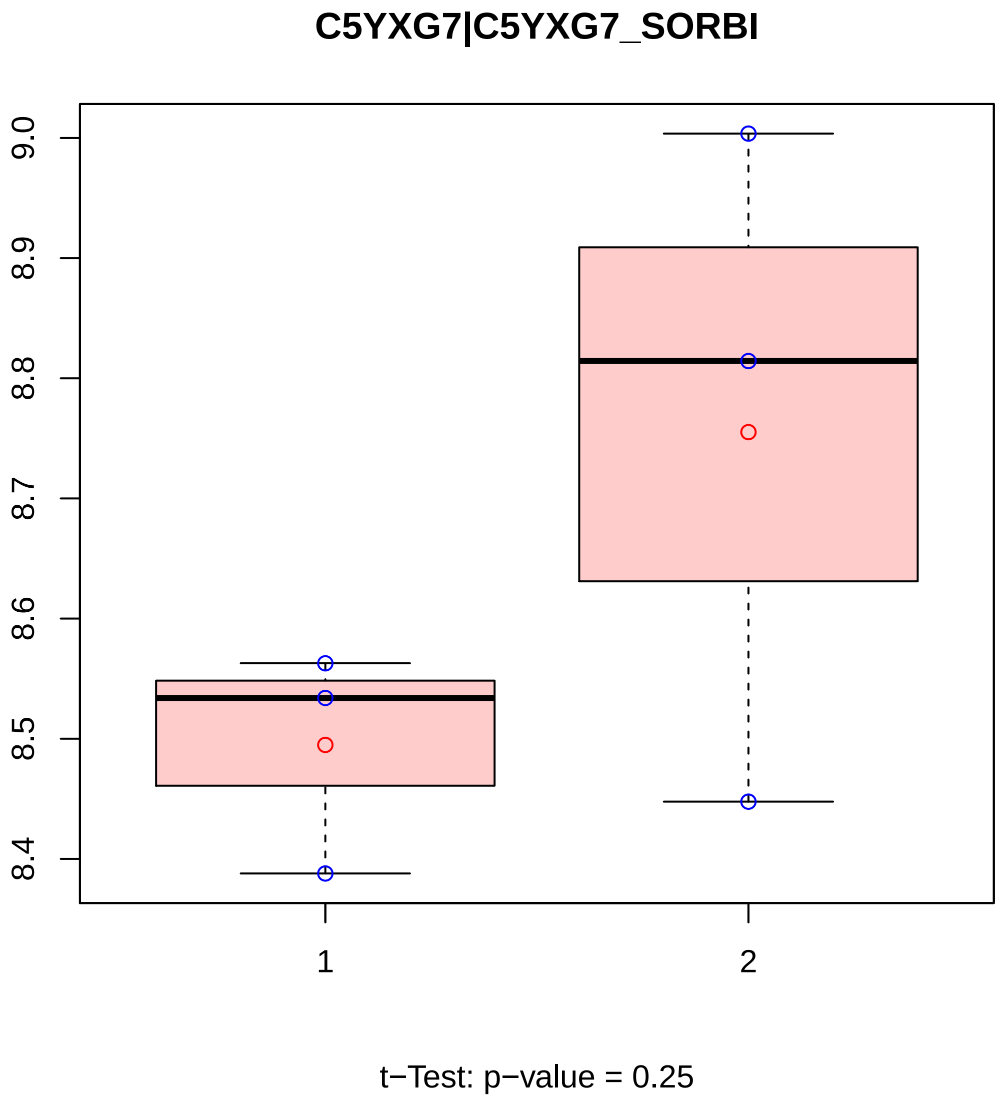
C5YXG7|C5YXG7_SORBI
9.0
8.9
8.8
8.7
8.6
8.5
8.4
1
2
t−Test: p−value = 0.25

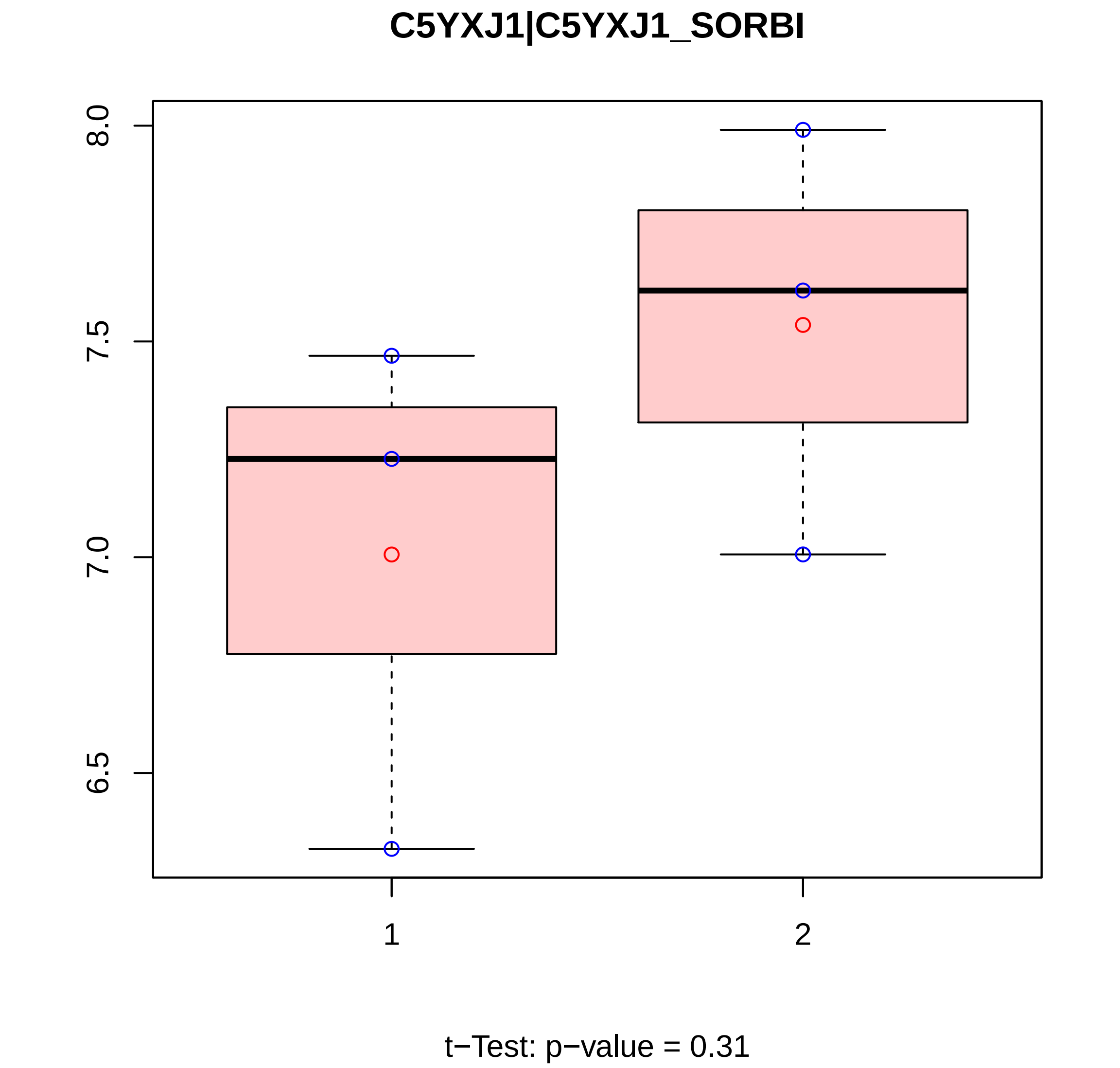
C5YXJ1|C5YXJ1_SORBI
8.0
7.5
7.0
6.5
1
2
t−Test: p−value = 0.31

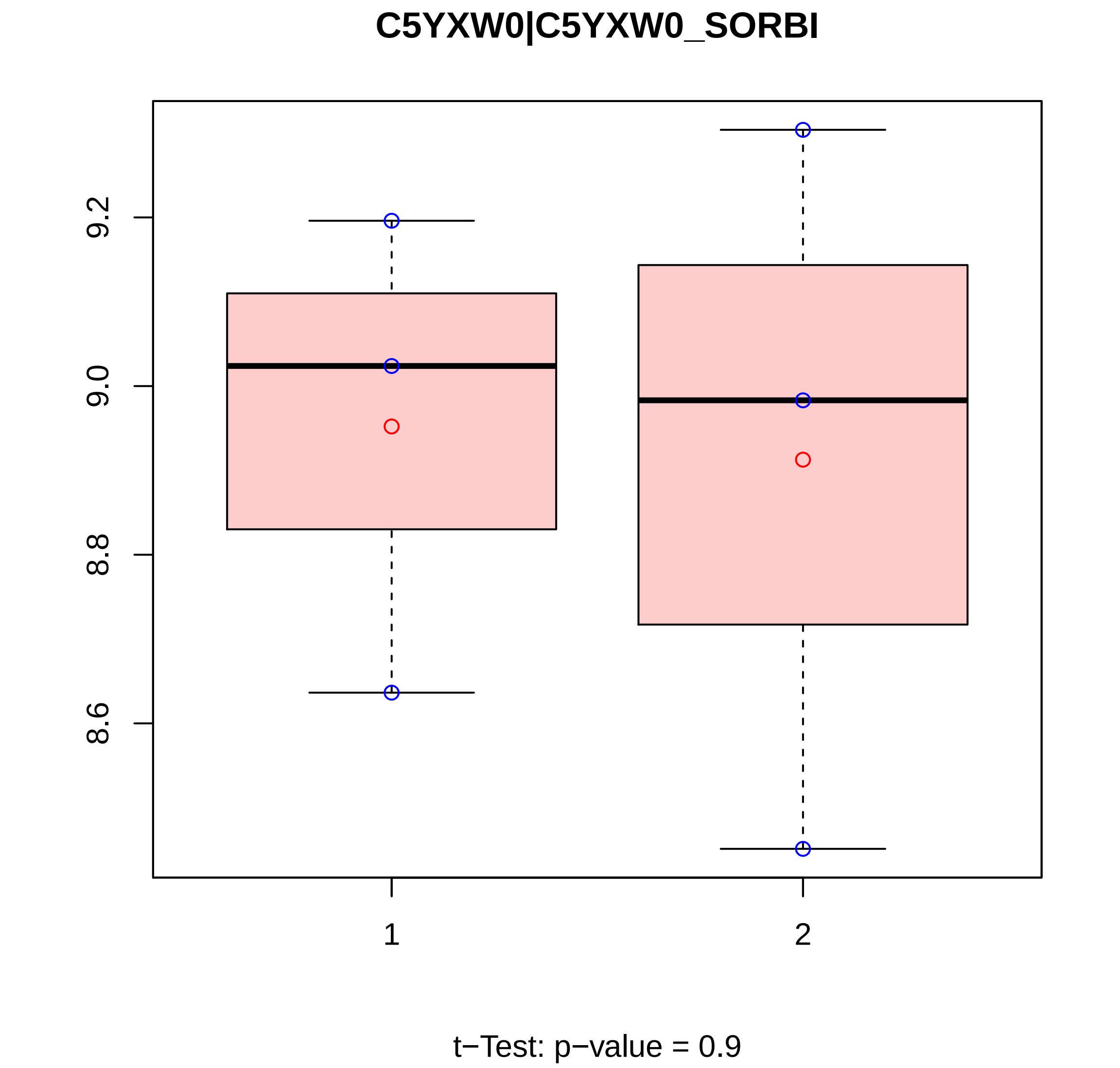
C5YXW0|C5YXW0_SORBI
9.2
9.0
8.8
8.6
1
2
t−Test: p−value = 0.9

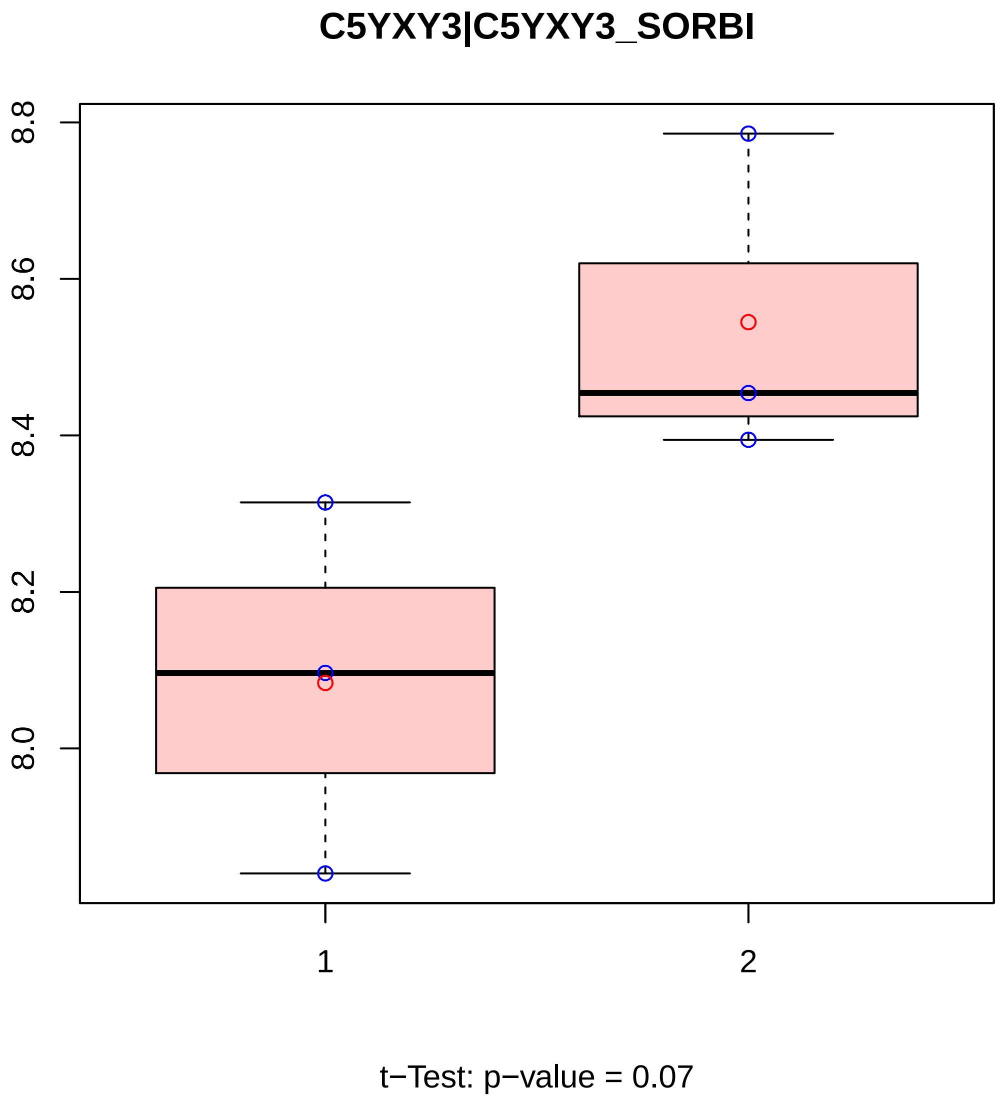
C5YXY3|C5YXY3_SORBI
8.0
8.2
8.4
8.6
8.8
1
2
t−Test: p−value = 0.07

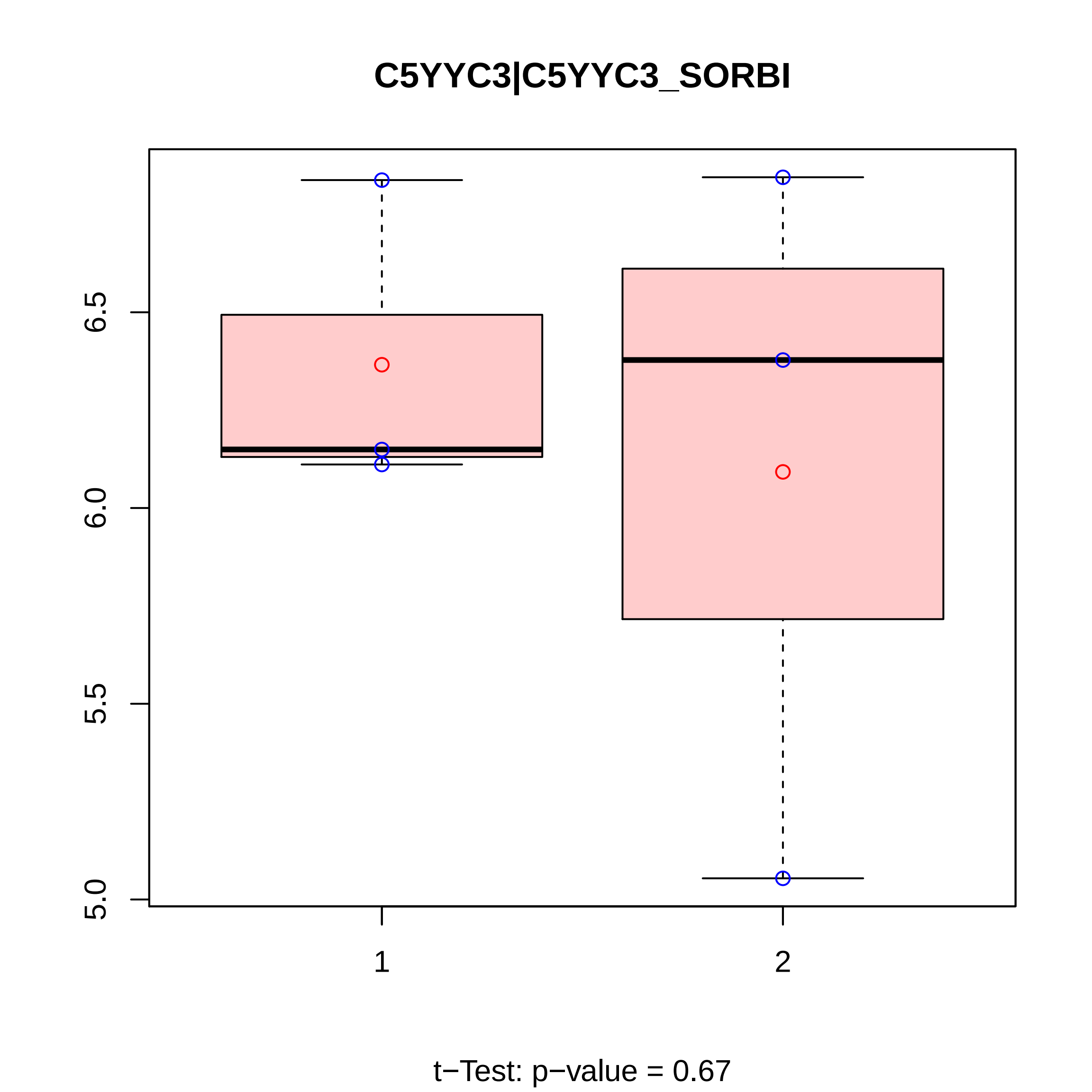
C5YYC3|C5YYC3_SORBI
6.5
6.0
5.5
5.0
1
2
t−Test: p−value = 0.67

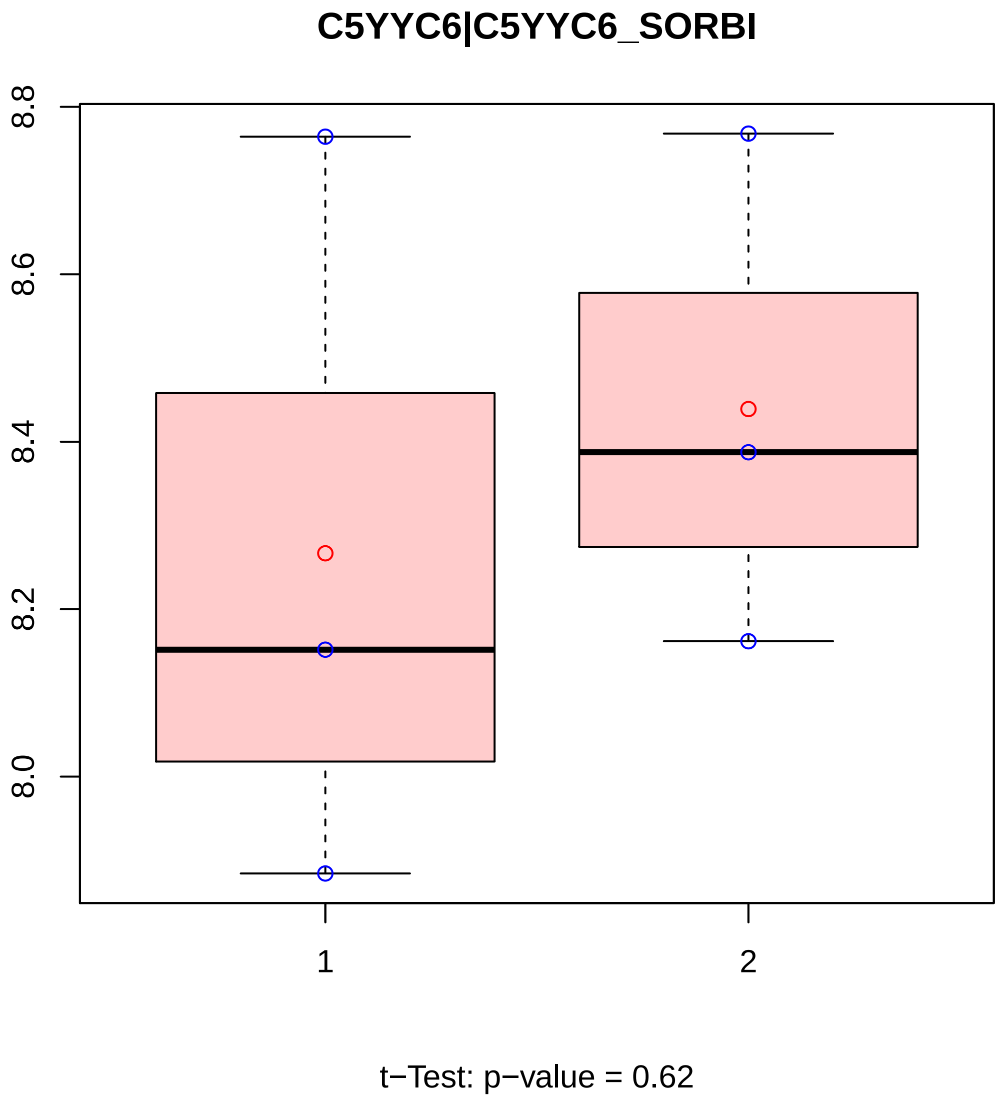

C5YYC6|C5YYC6_SORBI
8.0
8.2
8.4
8.6
8.8
1
2
t−Test: p−value = 0.62

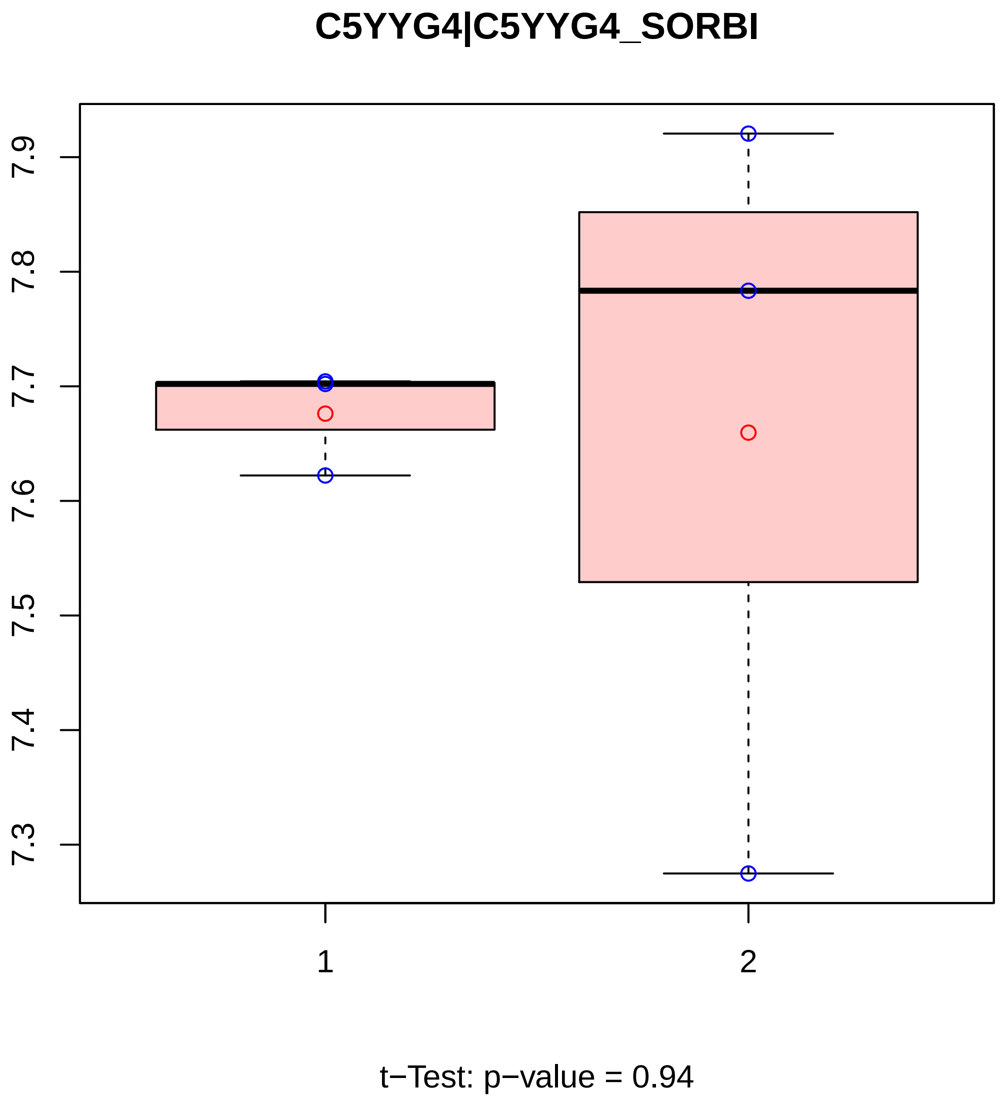
C5YYG4|C5YYG4_SORBI
7.3
7.4
7.5
7.6
7.7
7.8
7.9
1
2
t−Test: p−value = 0.94

C5YYK9|C5YYK9_SORBI

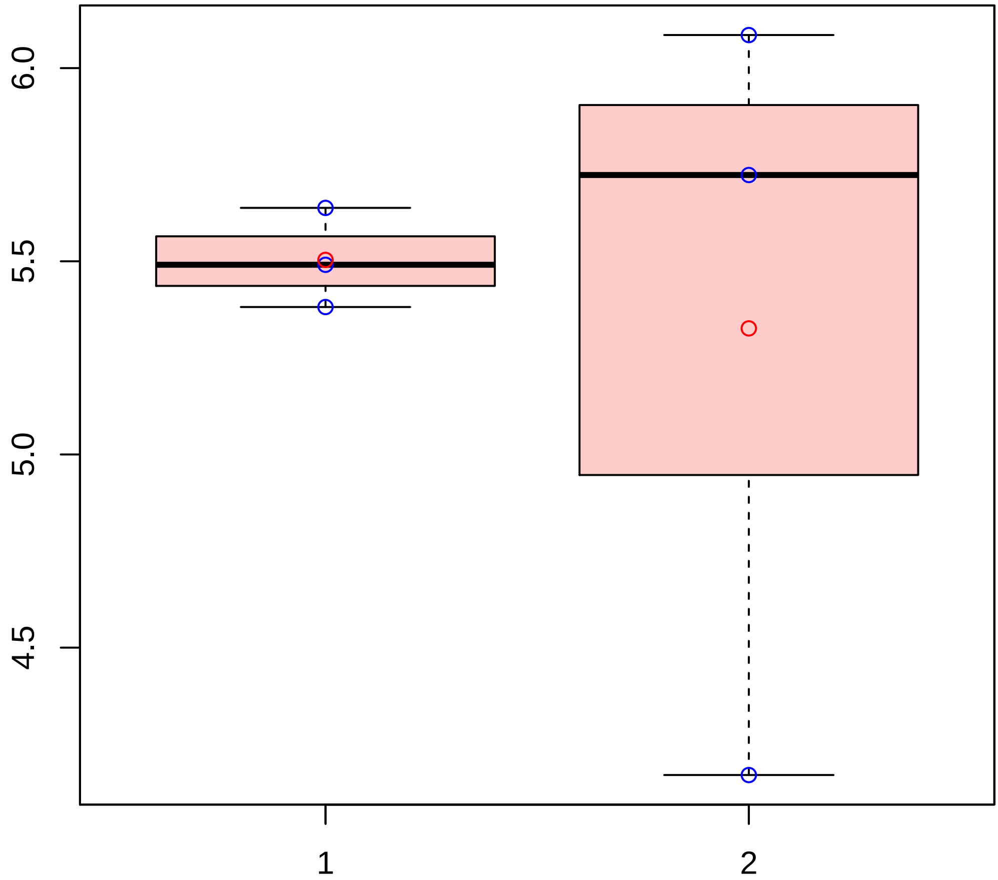

t−Test: p−value = 0.79

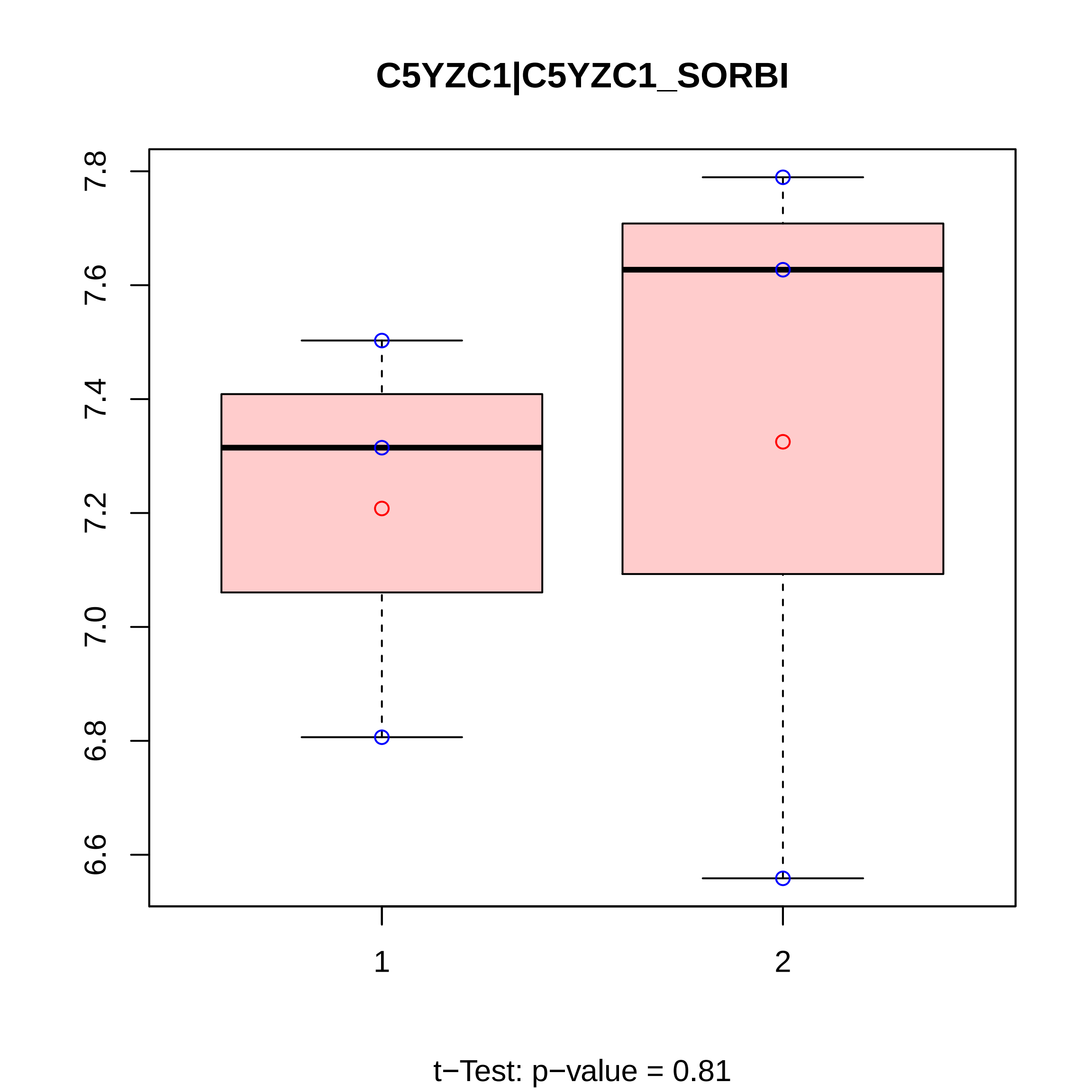

C5YZC1|C5YZC1_SORBI
7.8
7.6
7.4
7.2
7.0
6.8
6.6
1
2
t−Test: p−value = 0.81

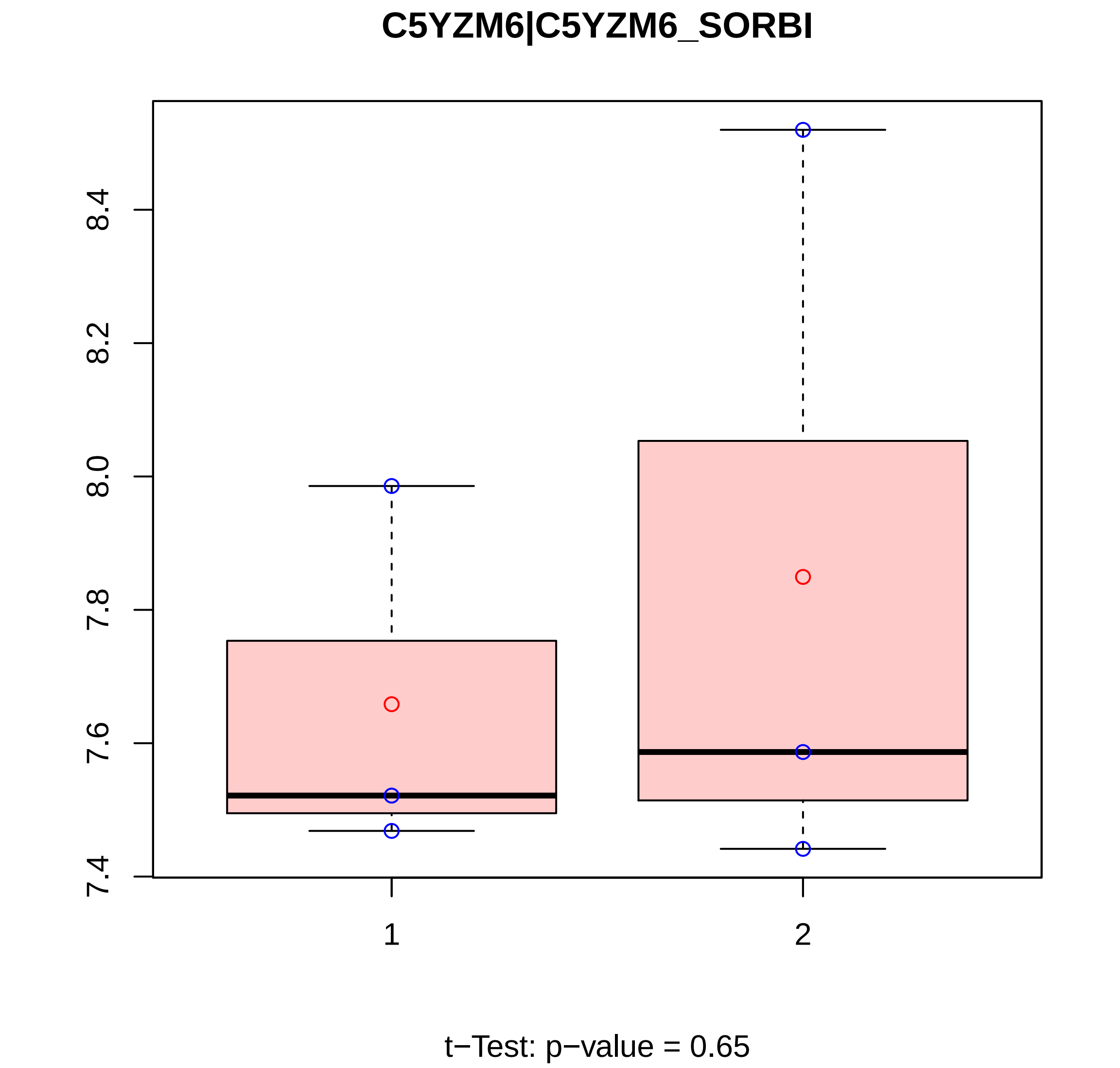
C5YZM6|C5YZM6_SORBI
8.4
8.2
8.0
7.8
7.6
7.4
1
2
t−Test: p−value = 0.65

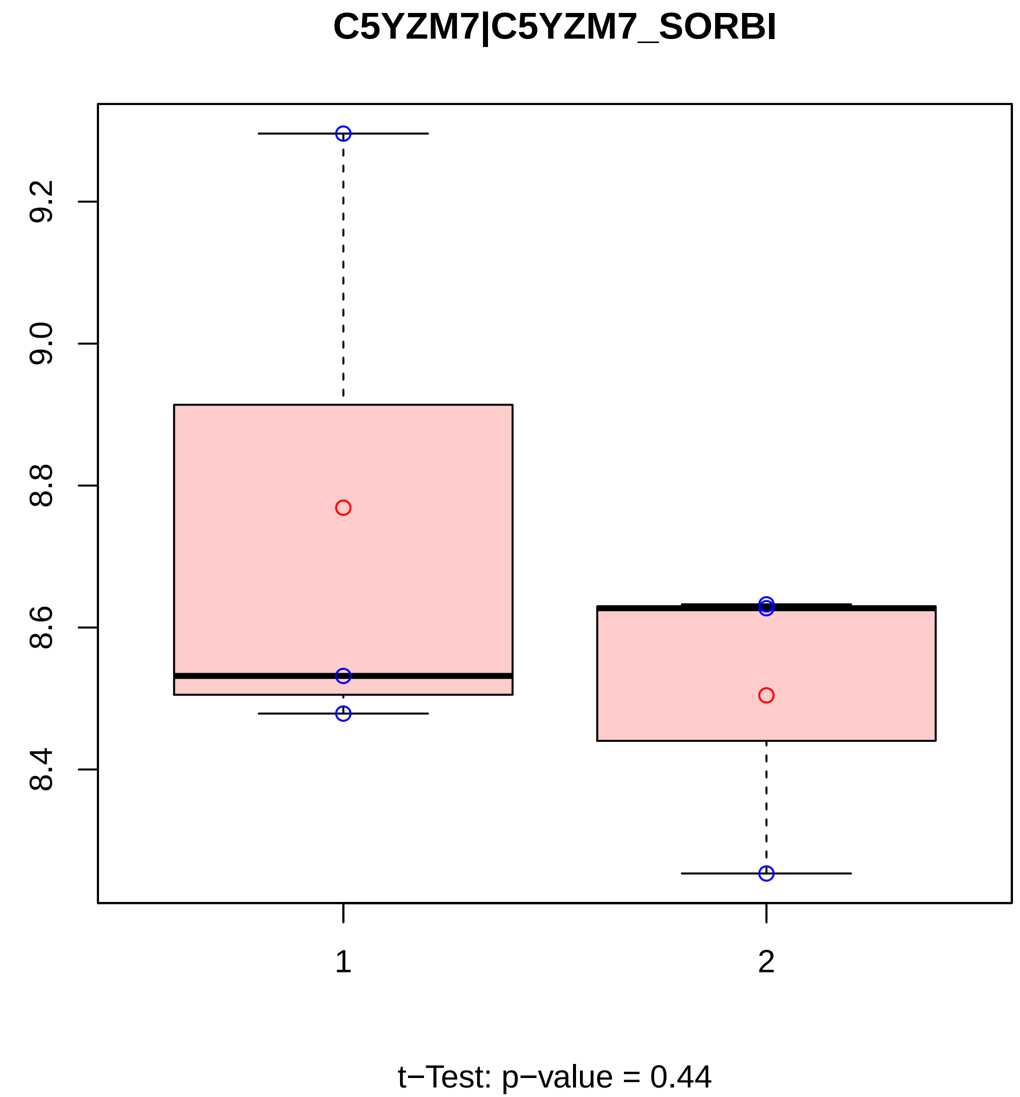
C5YZM7|C5YZM7_SORBI
9.2
9.0
8.8
8.6
8.4
1
2
t−Test: p−value = 0.44

C5Z147|C5Z147_SORBI

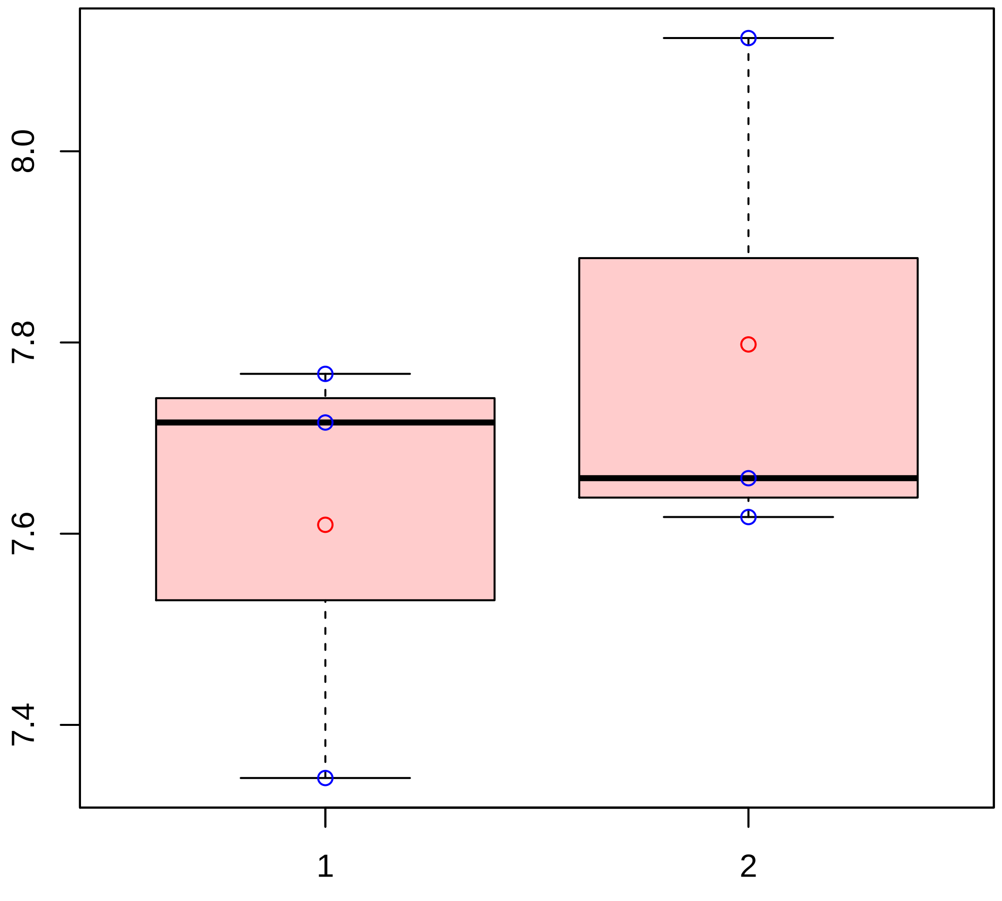

t−Test: p−value = 0.42

C5Z1D3|C5Z1D3_SORBI

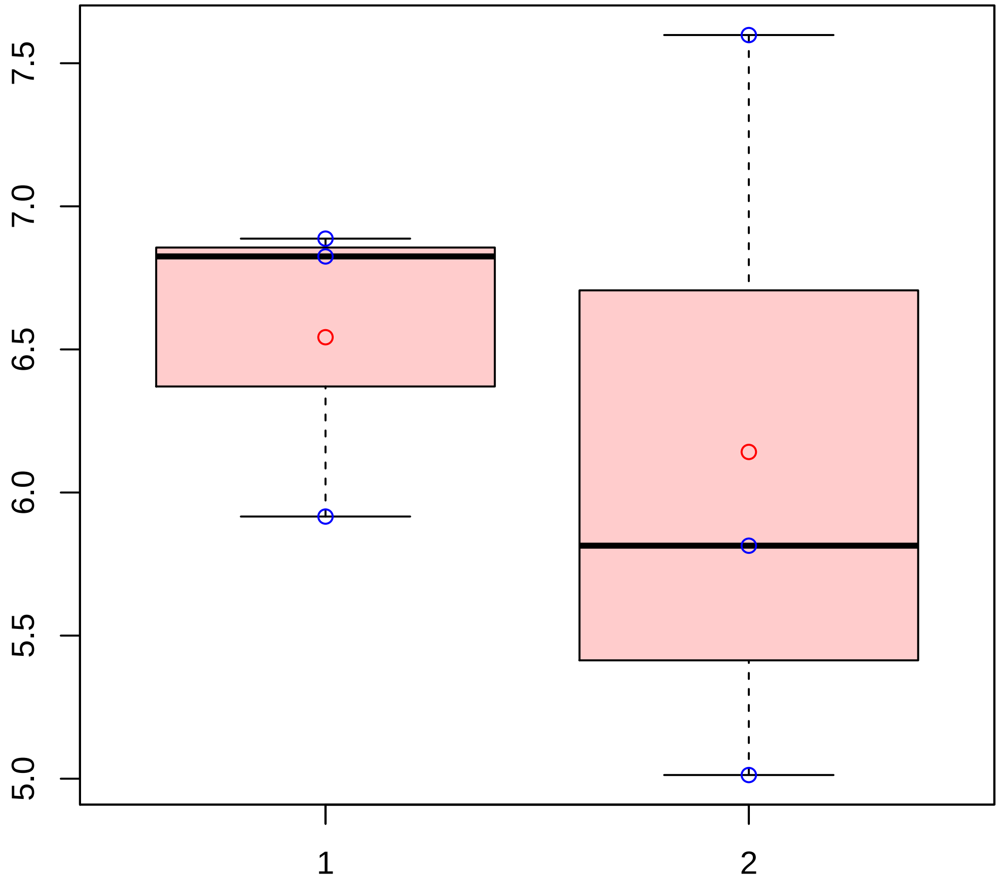

t−Test: p−value = 0.66

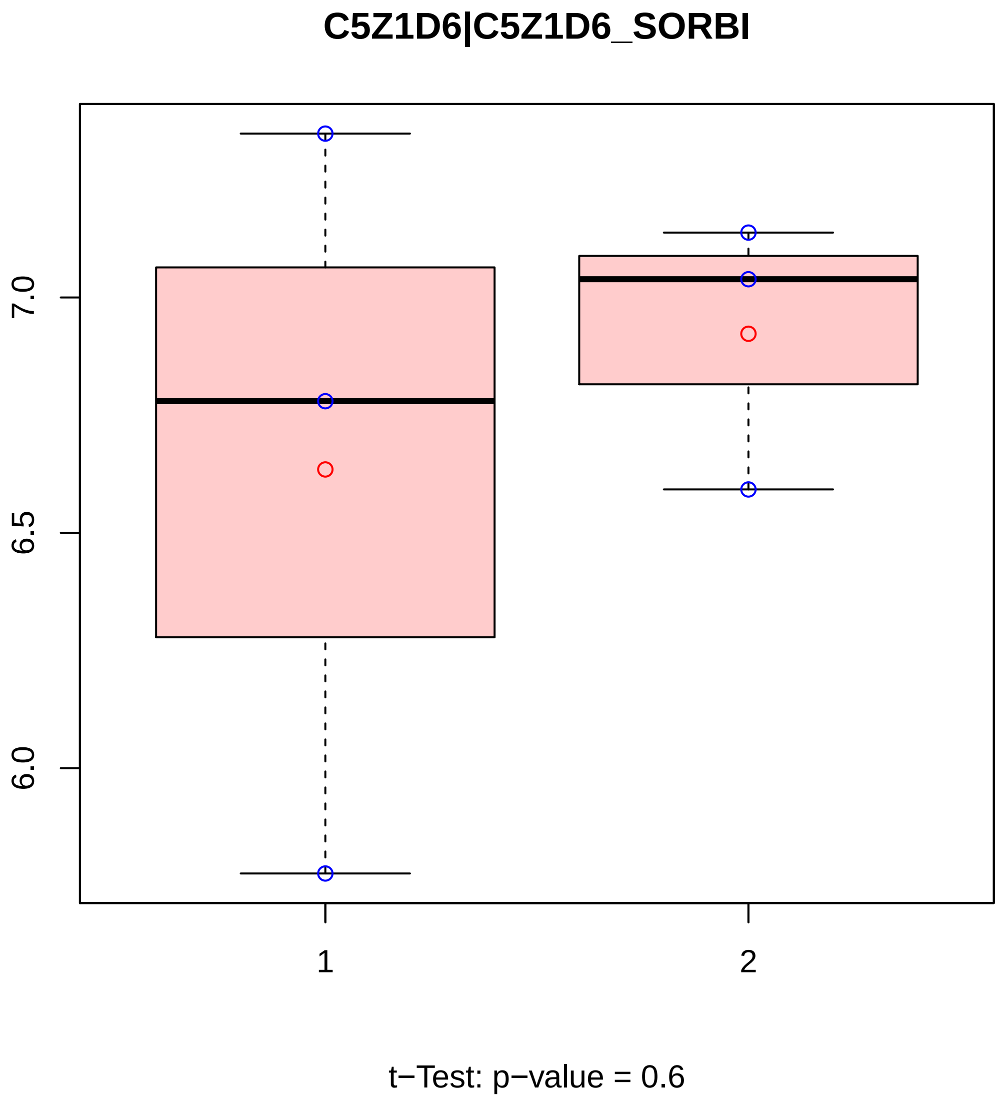
C5Z1D6|C5Z1D6_SORBI
7.0
6.5
6.0
1
2
t−Test: p−value = 0.6

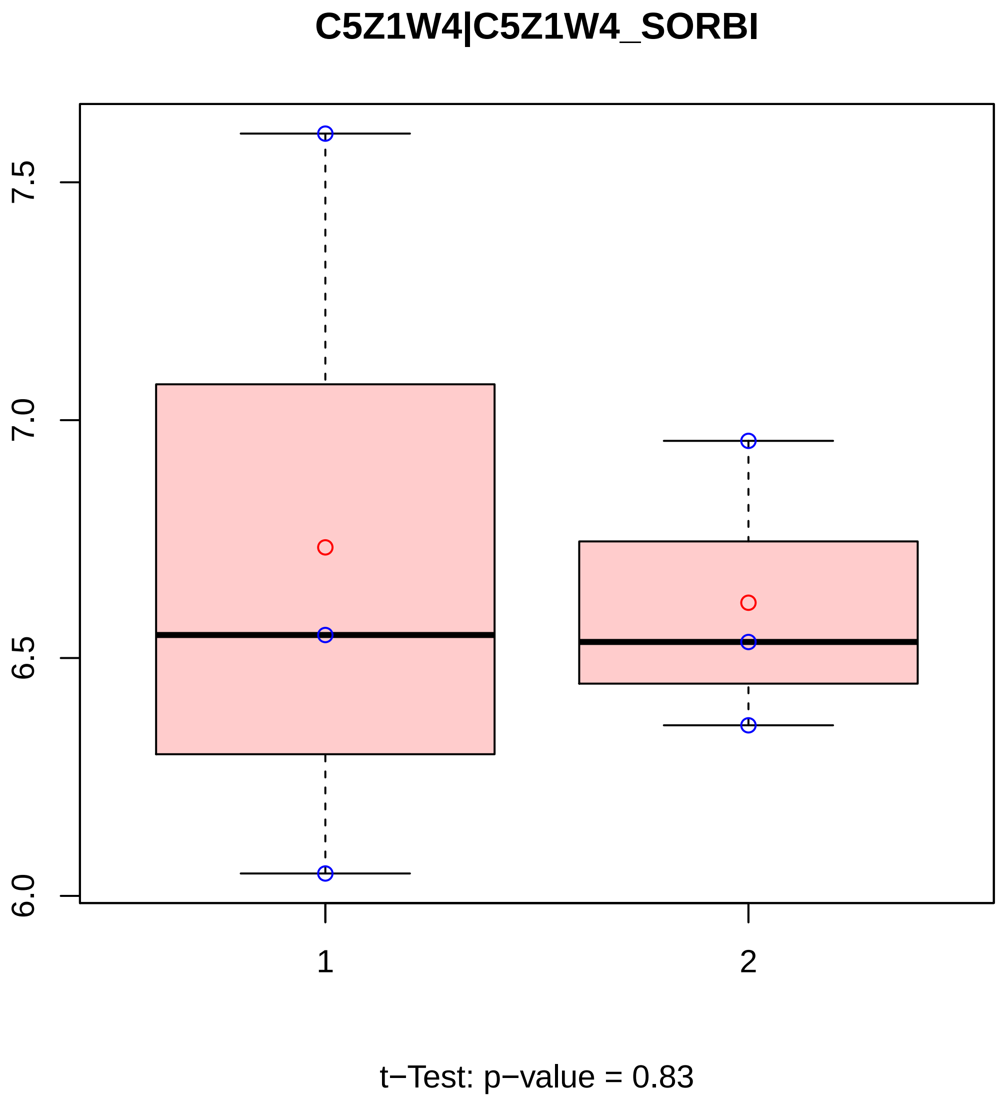

C5Z1W4|C5Z1W4_SORBI
7.5
7.0
6.5
6.0
1
2
t−Test: p−value = 0.83

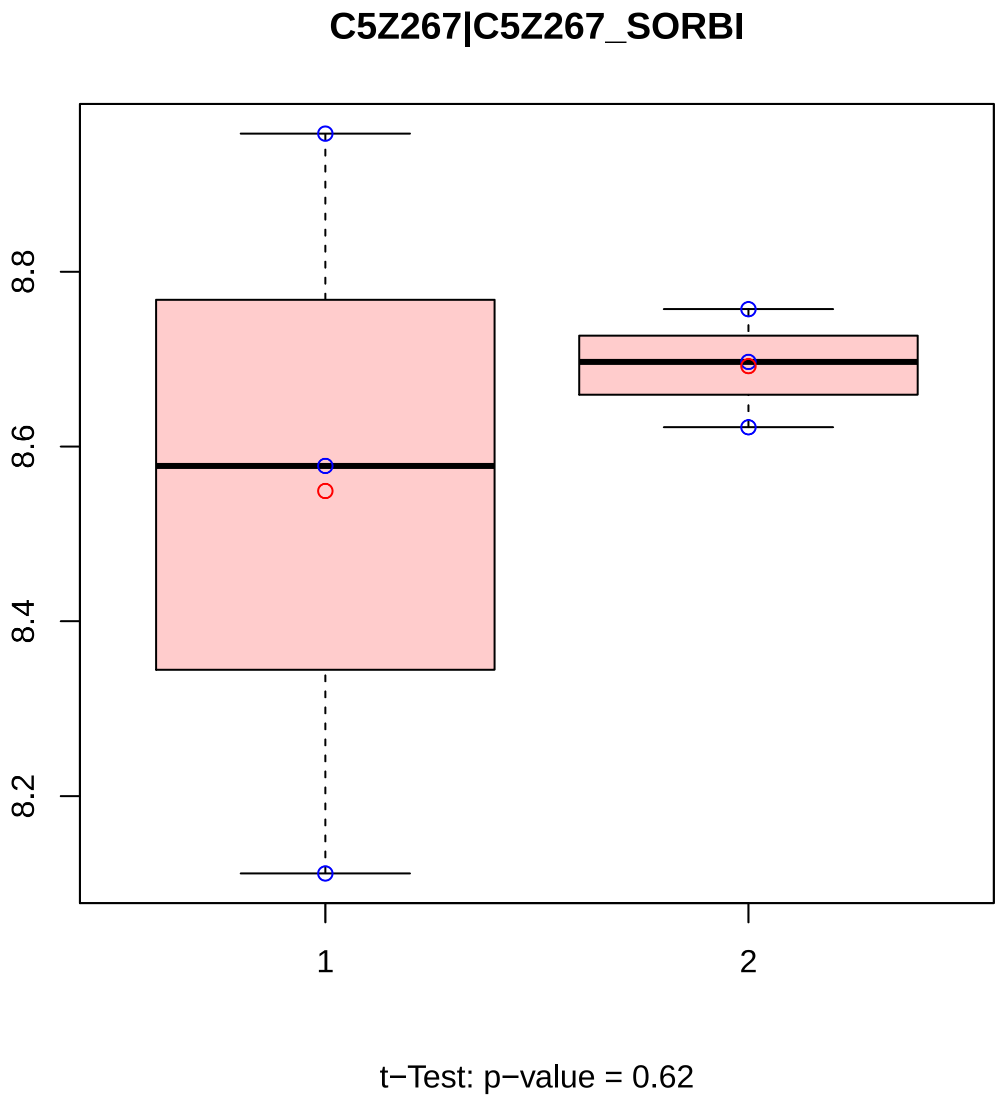
C5Z267|C5Z267_SORBI
8.2
8.4
8.6
8.8
1
2
t−Test: p−value = 0.62

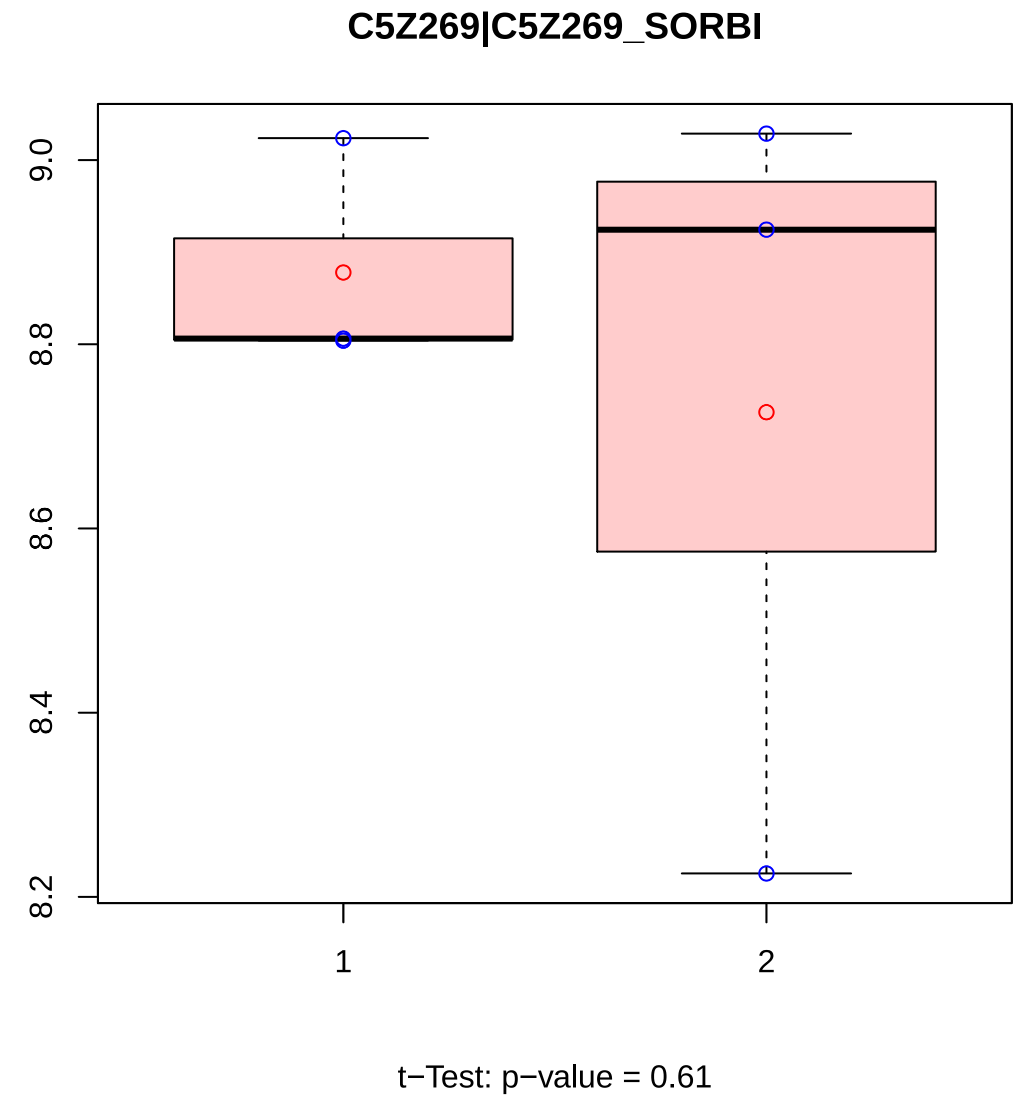
C5Z269|C5Z269_SORBI
9.0
8.8
8.6
8.4
8.2
1
2
t−Test: p−value = 0.61

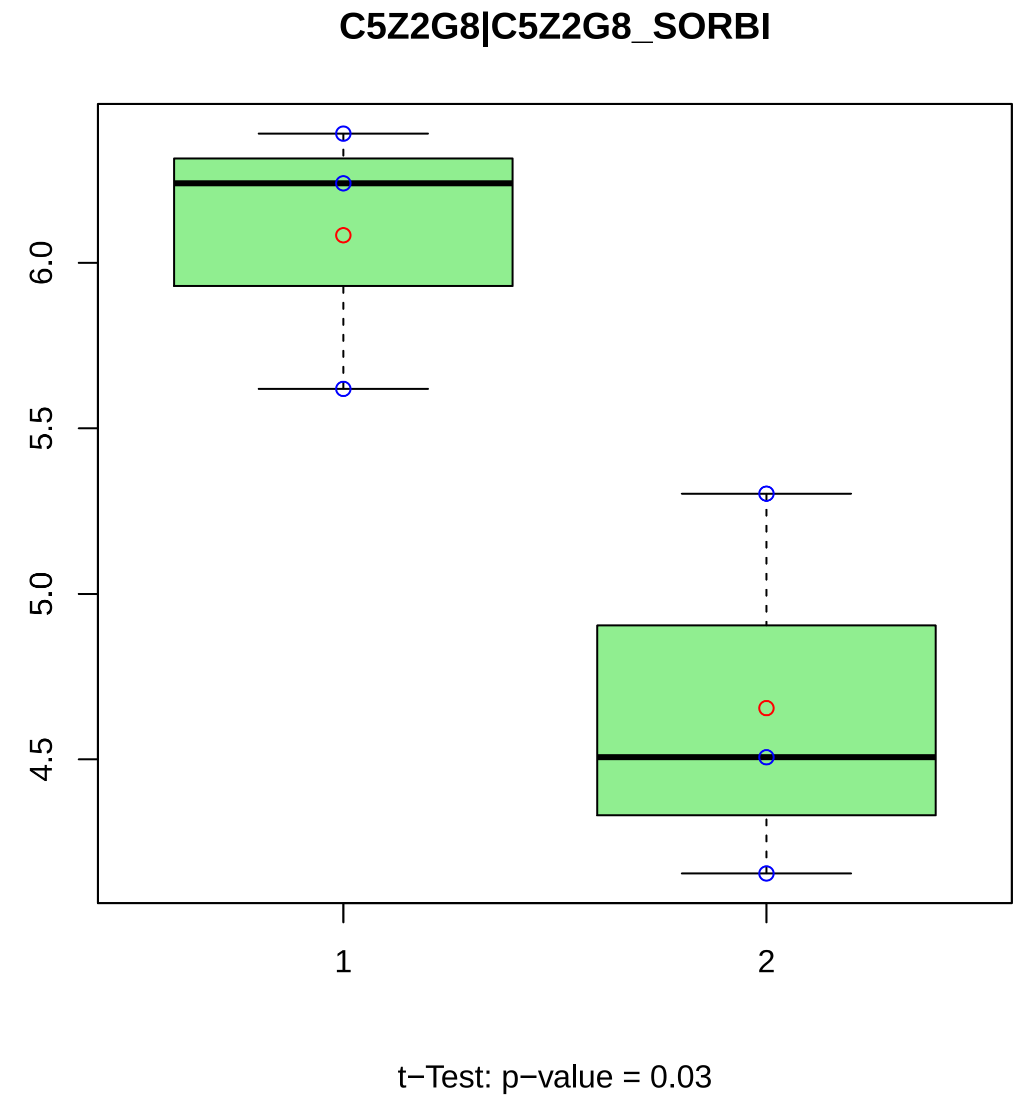

C5Z2G8|C5Z2G8_SORBI
6.0
5.5
5.0
4.5
1
2
t−Test: p−value = 0.03

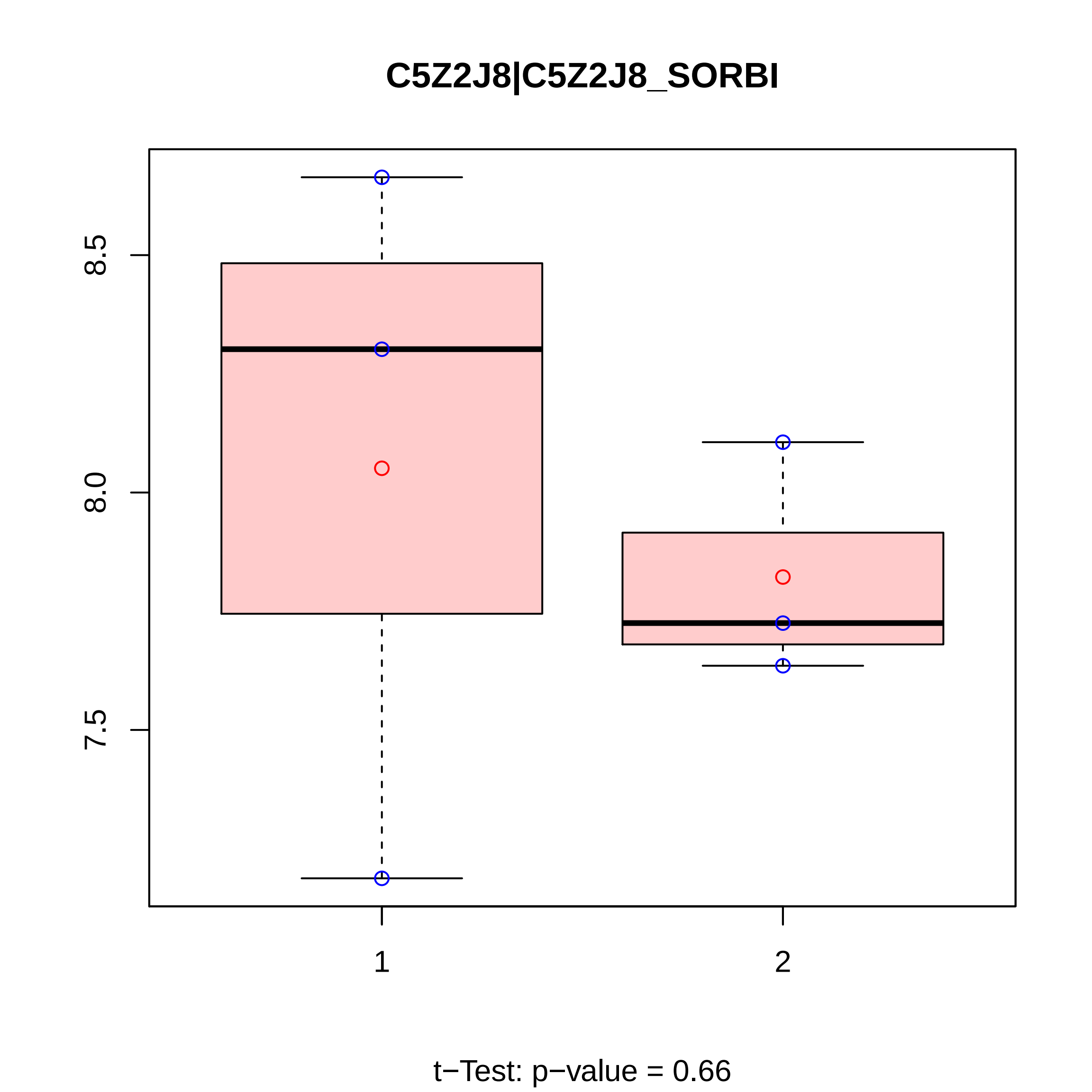
C5Z2J8|C5Z2J8_SORBI
8.5
8.0
7.5
1
2
t−Test: p−value = 0.66

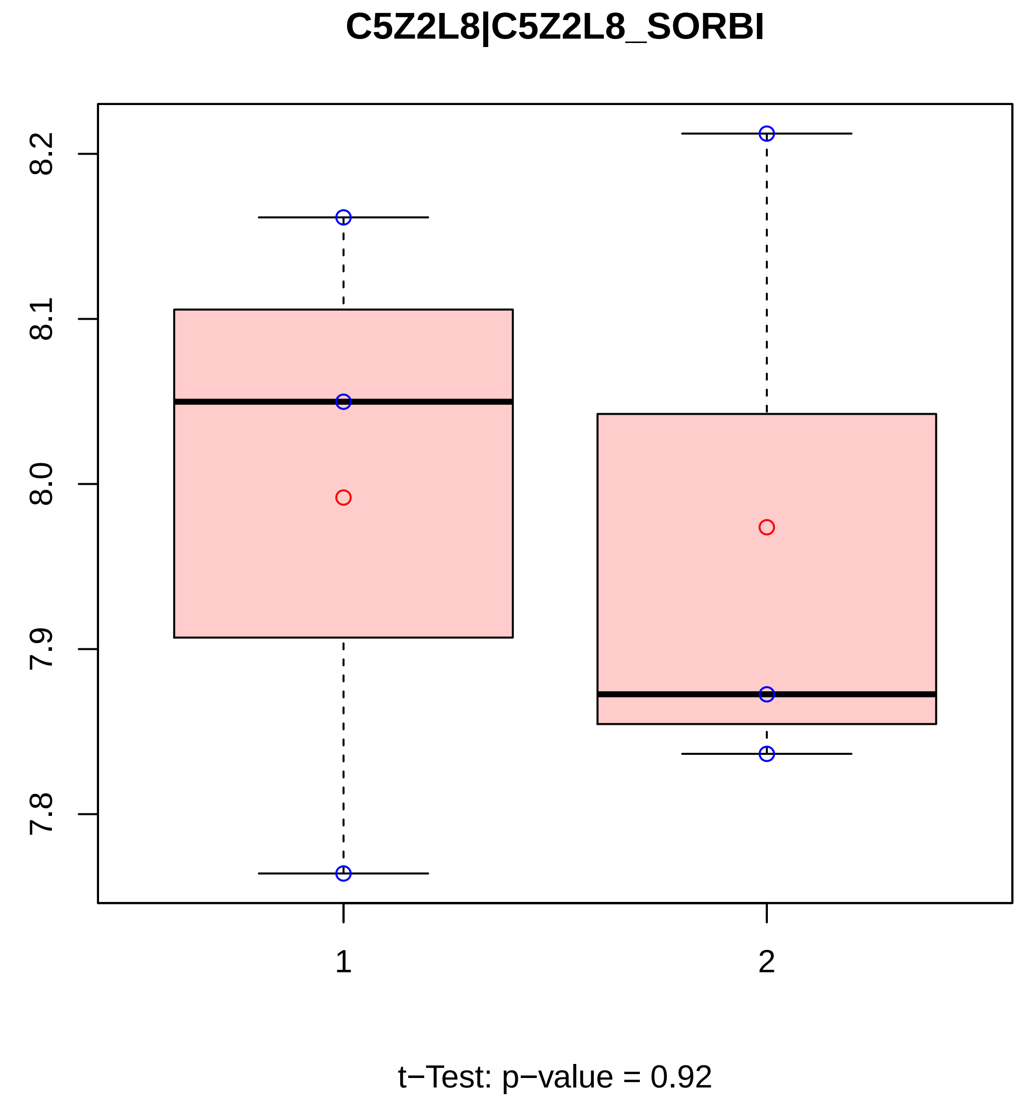

C5Z2L8|C5Z2L8_SORBI
8.2
8.1
8.0
7.9
7.8
1
2
t−Test: p−value = 0.92

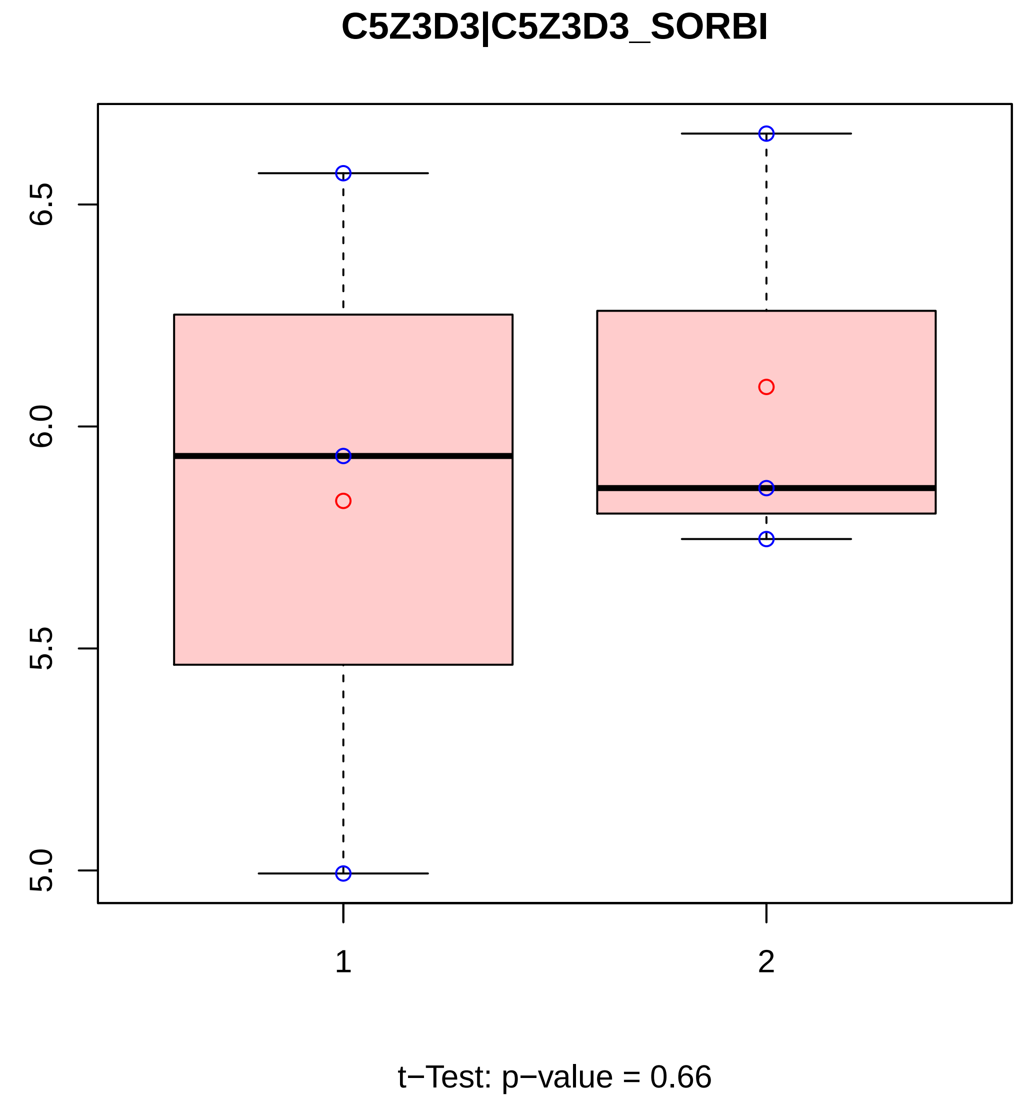
C5Z3D3|C5Z3D3_SORBI
5.0
5.5
6.0
6.5
1
2
t−Test: p−value = 0.66

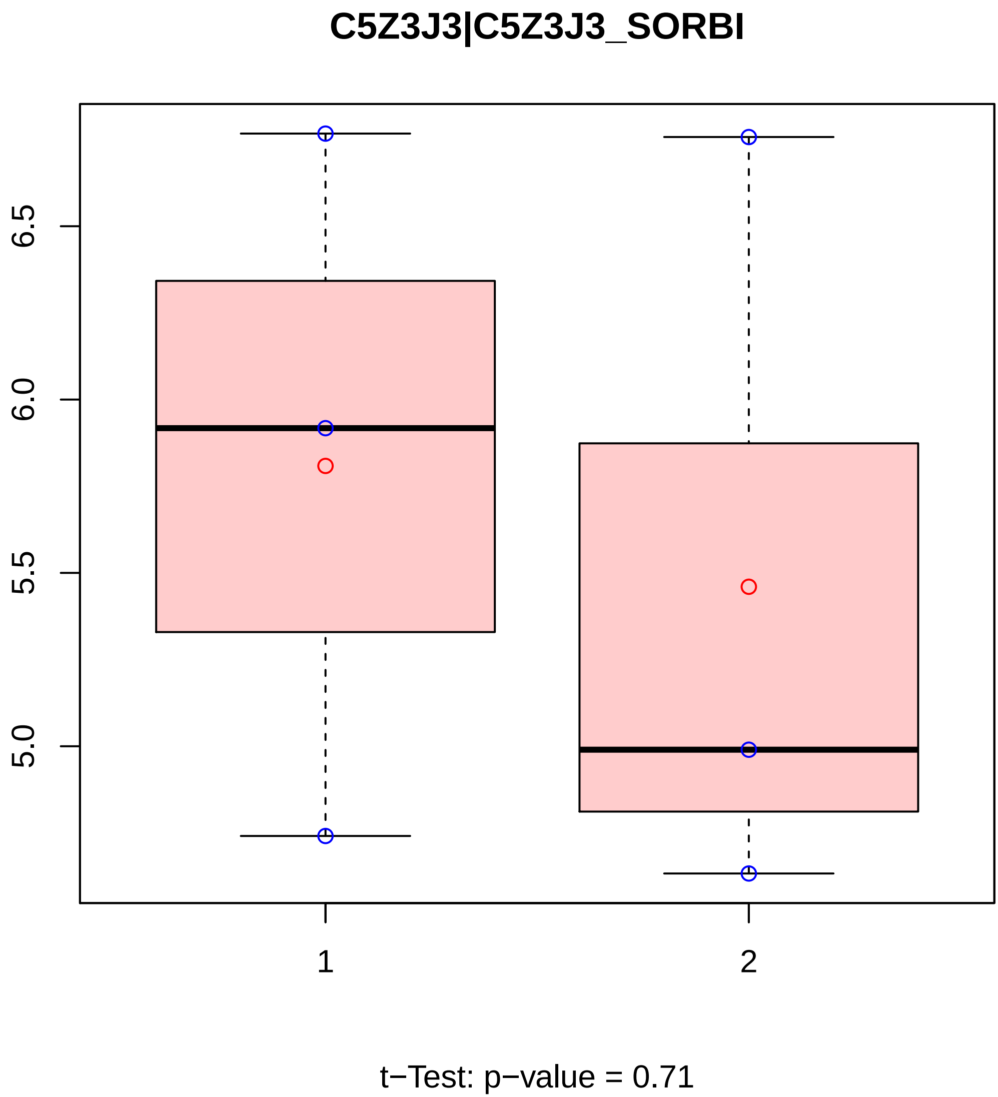
C5Z3J3|C5Z3J3_SORBI
6.5
6.0
5.5
5.0
1
2
t−Test: p−value = 0.71

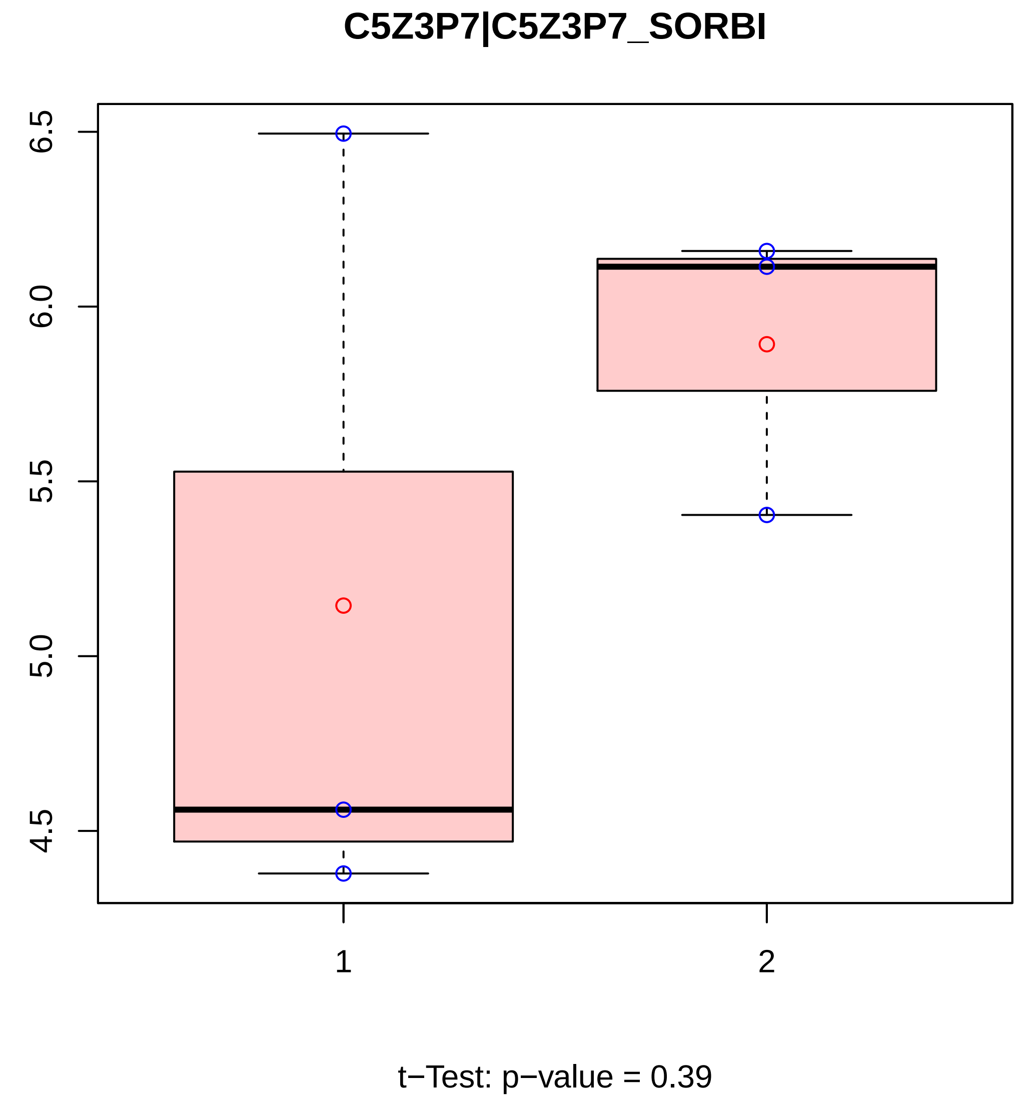
C5Z3P7|C5Z3P7_SORBI
6.5
6.0
5.5
5.0
4.5
1
2
t−Test: p−value = 0.39

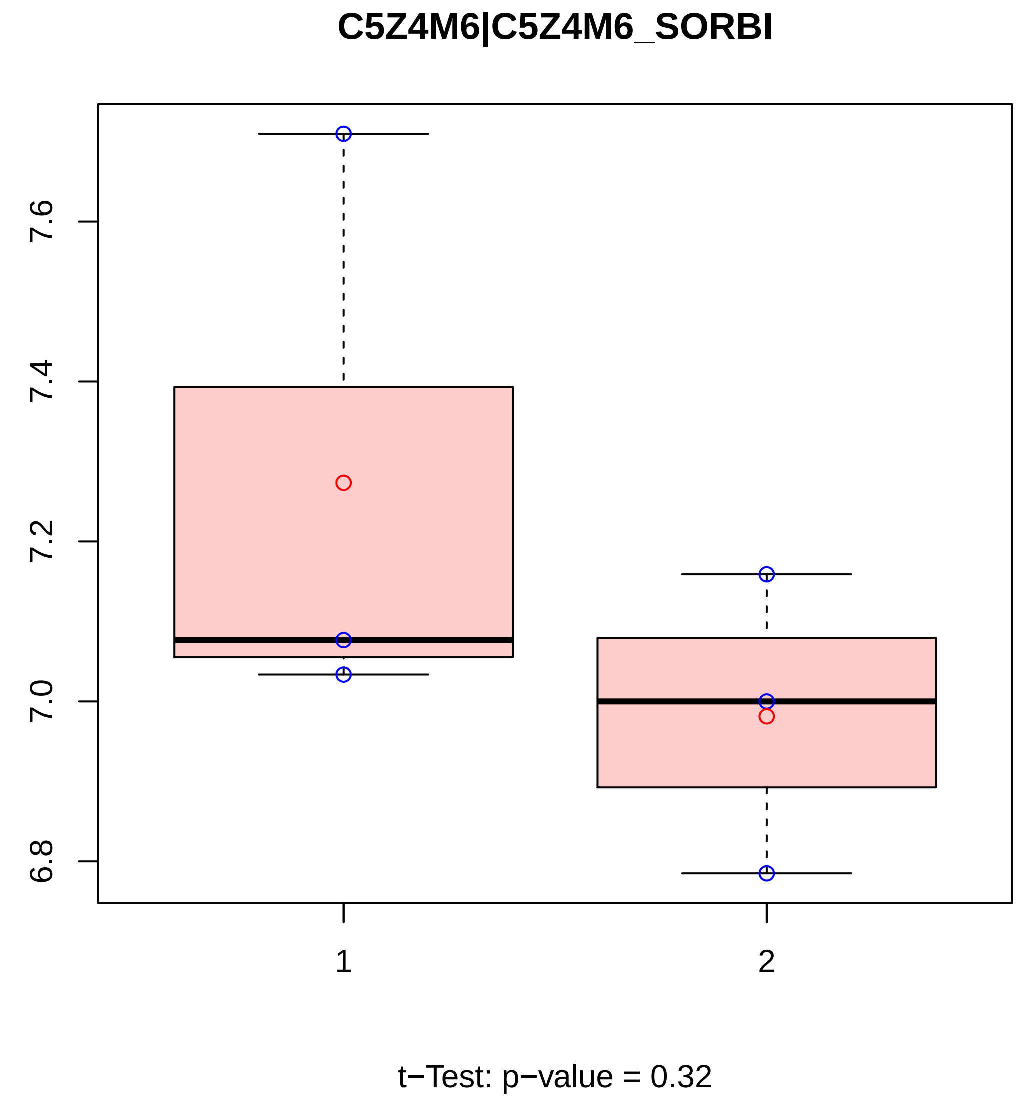
C5Z4M6|C5Z4M6_SORBI
7.6
7.4
7.2
7.0
6.8
1
2
t−Test: p−value = 0.32

C5Z4Y2|C5Z4Y2_SORBI

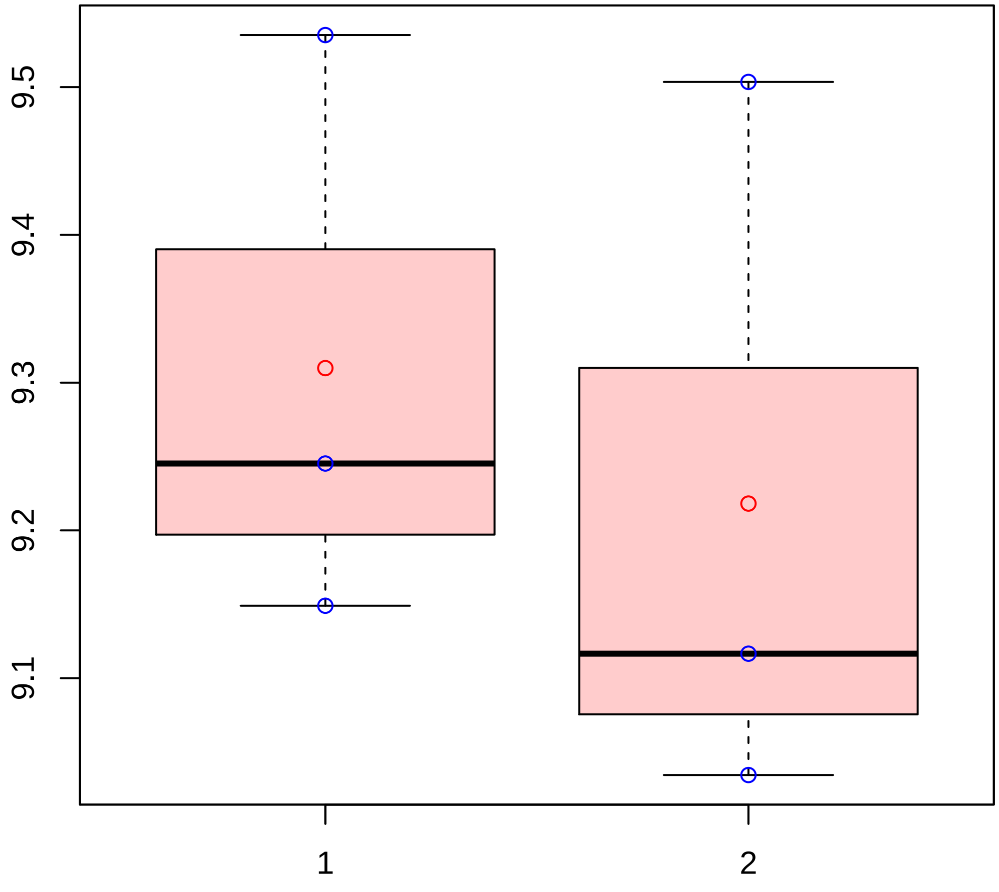

t−Test: p−value = 0.65

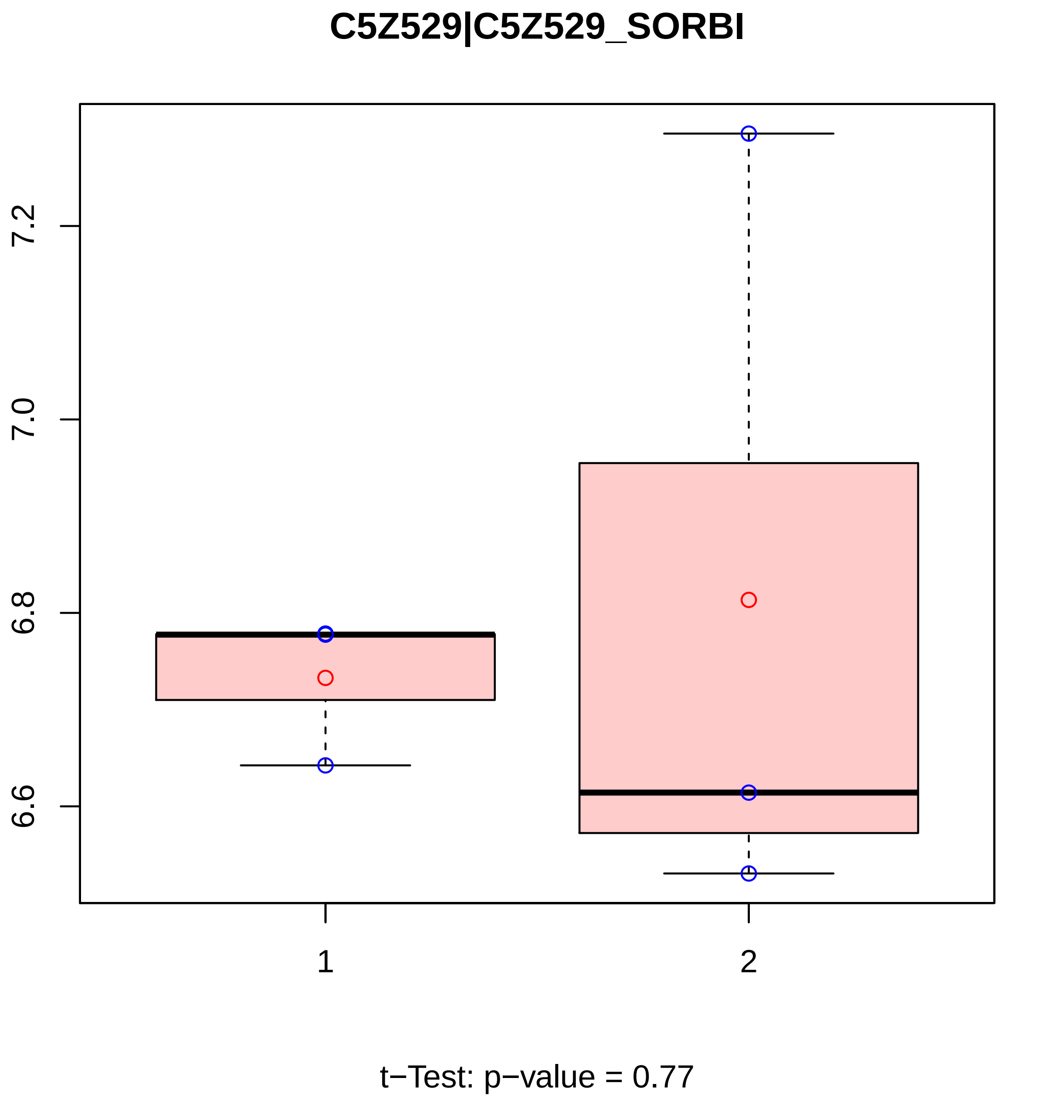
C5Z529|C5Z529_SORBI
7.2
7.0
6.8
6.6
1
2
t−Test: p−value = 0.77

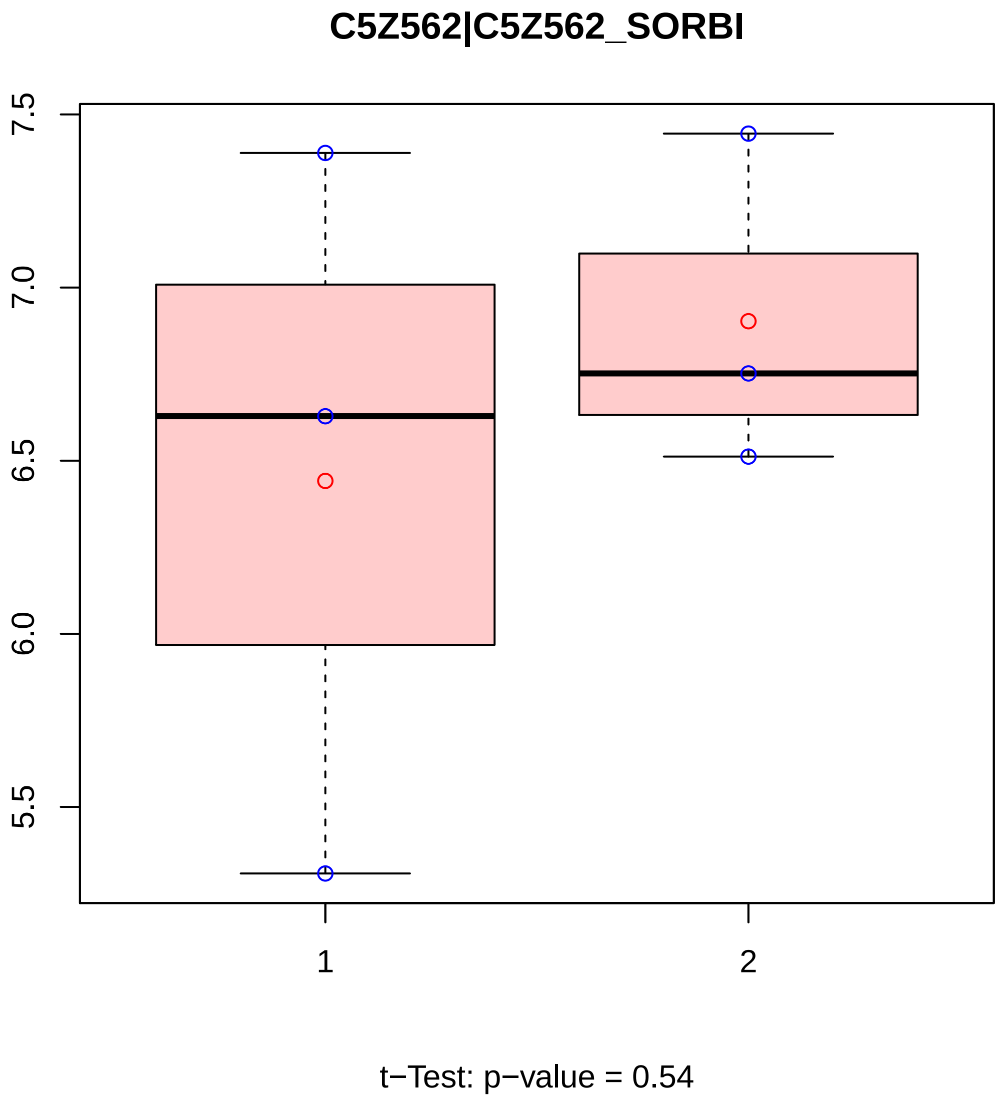
C5Z562|C5Z562_SORBI
7.5
7.0
6.5
6.0
5.5
1
2
t−Test: p−value = 0.54

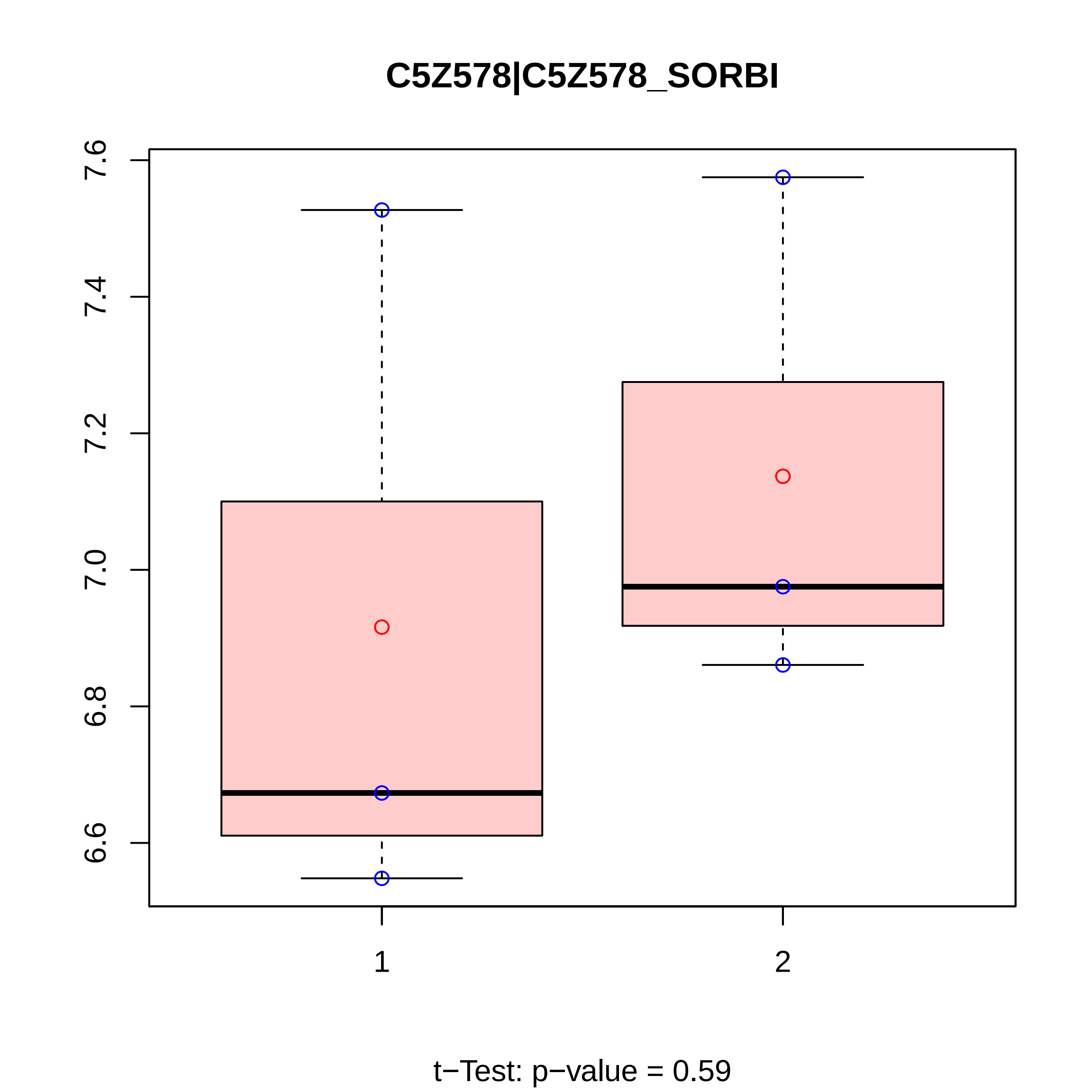
C5Z578|C5Z578_SORBI
7.6
7.4
7.2
7.0
6.8
6.6
1
2
t−Test: p−value = 0.59

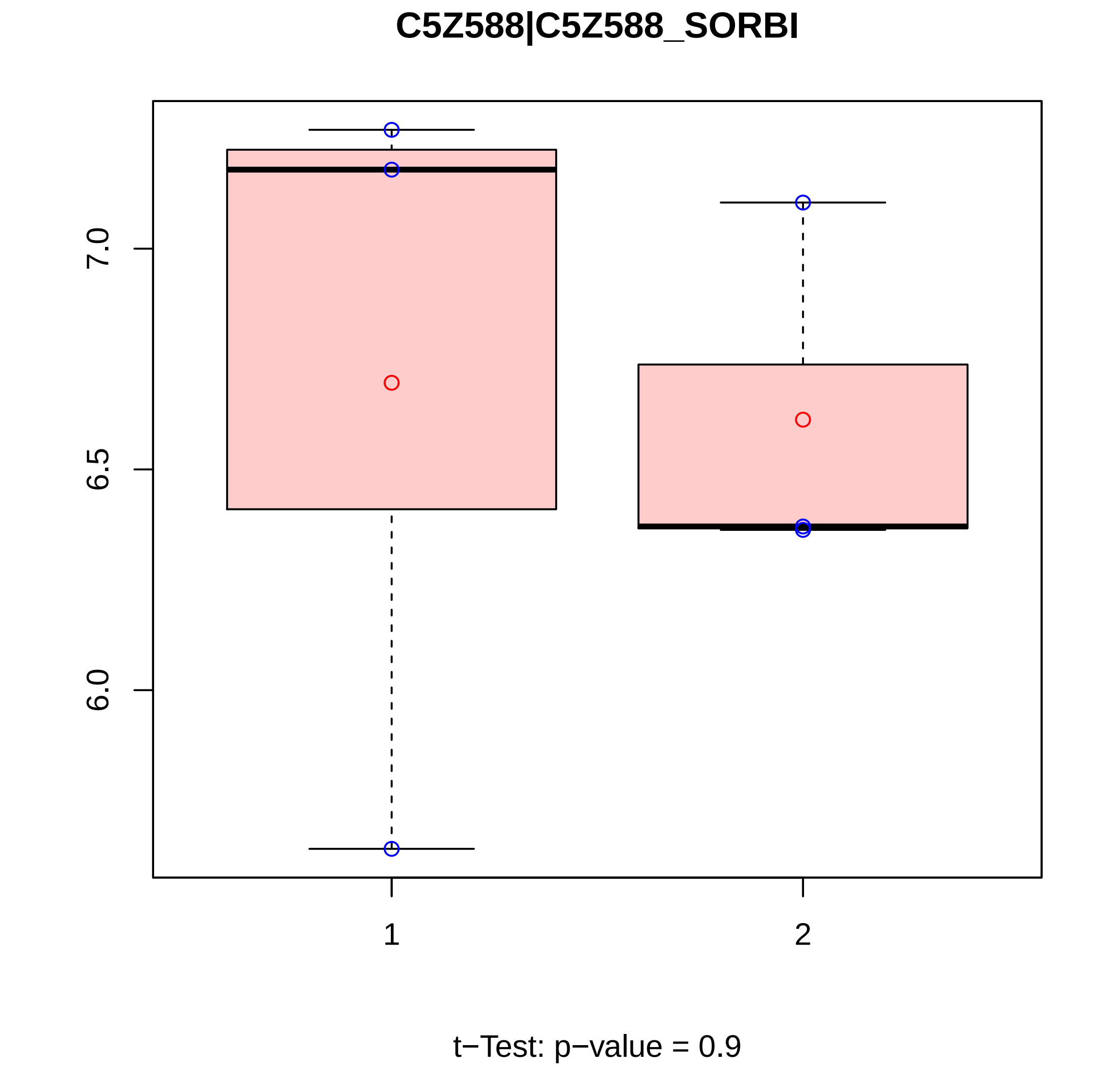
C5Z588|C5Z588_SORBI
7.0
6.5
6.0
1
2
t−Test: p−value = 0.9

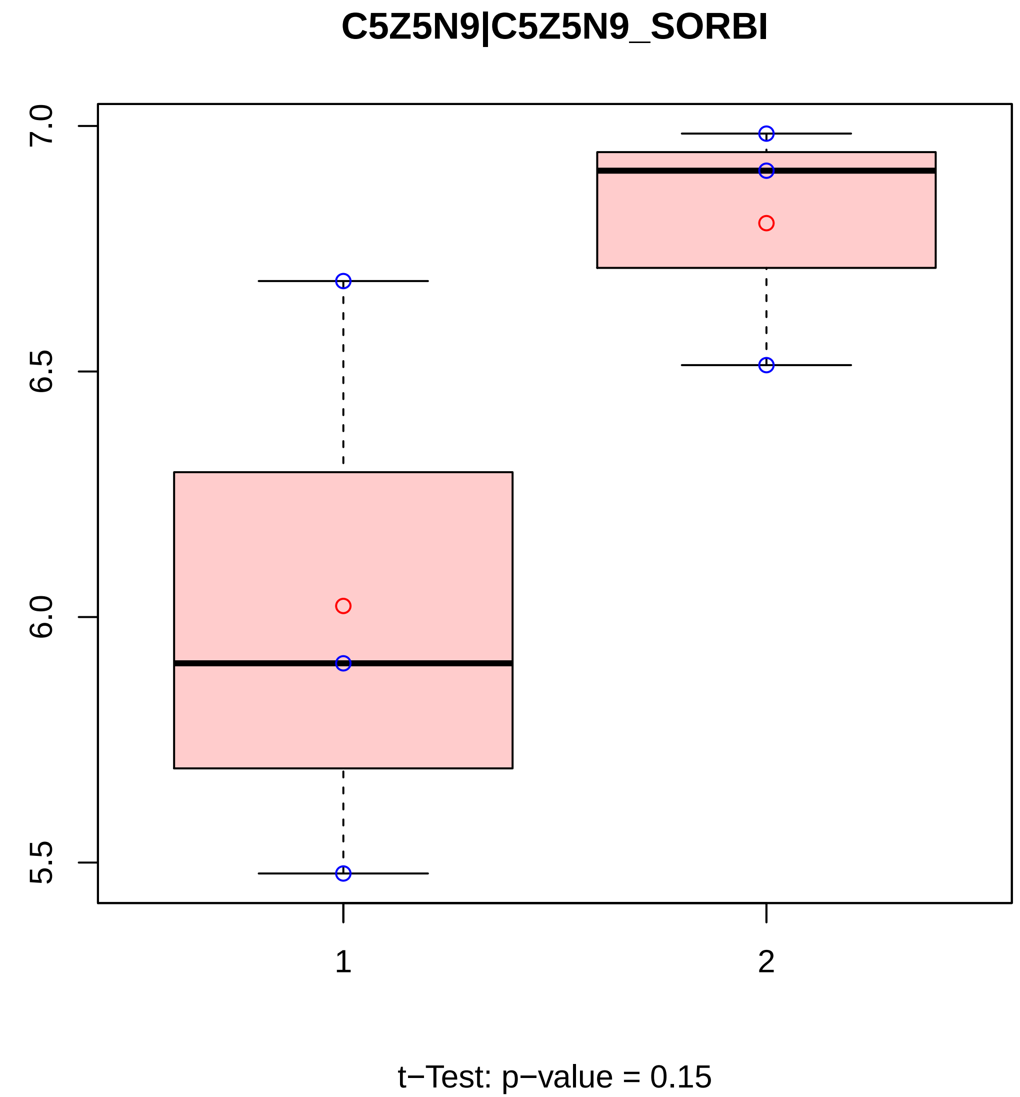
C5Z5N9|C5Z5N9_SORBI
7.0
6.5
6.0
5.5
1
2
t−Test: p−value = 0.15

C5Z5R1|C5Z5R1_SORBI

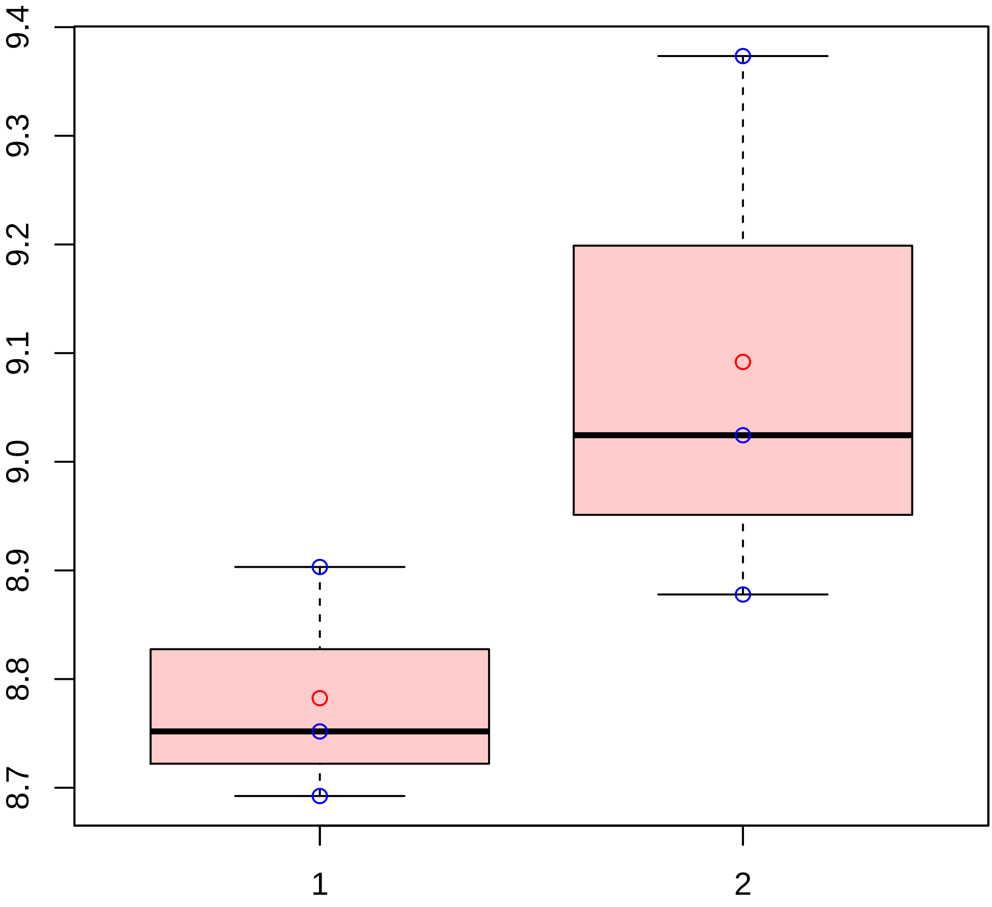

t−Test: p−value = 0.16

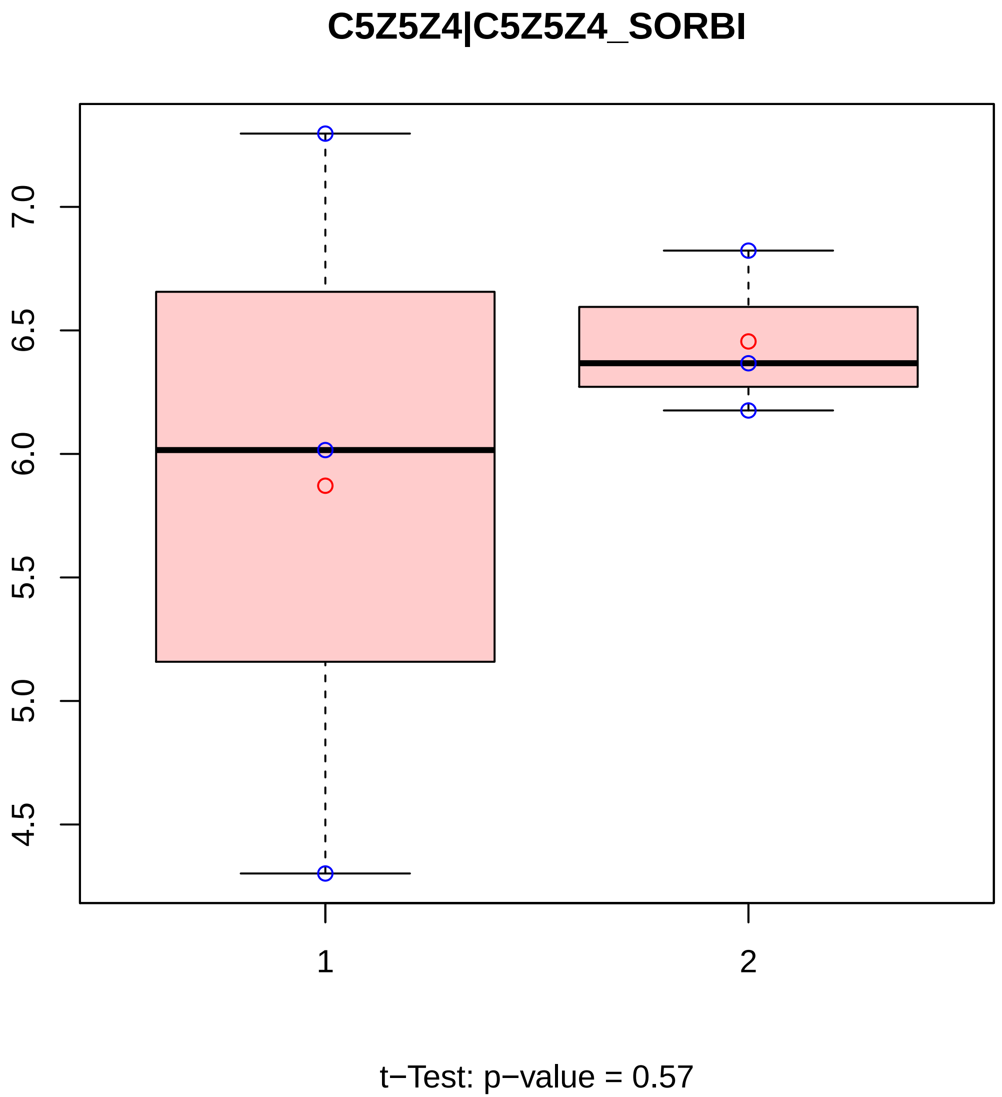
C5Z5Z4|C5Z5Z4_SORBI
7.0
6.5
6.0
5.5
5.0
4.5
1
2
t−Test: p−value = 0.57

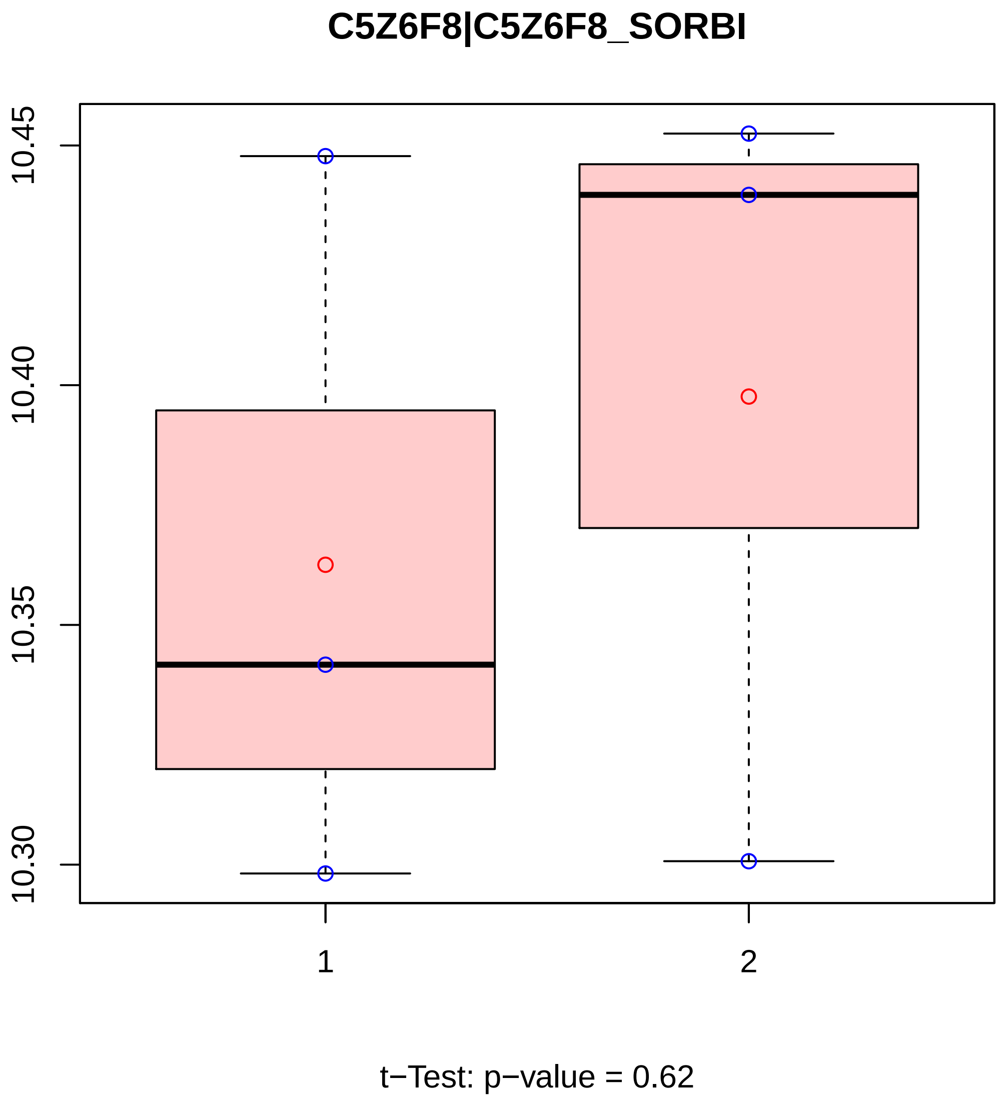
C5Z6F8|C5Z6F8_SORBI
10.45
10.40
10.35
10.30
1
2
t−Test: p−value = 0.62

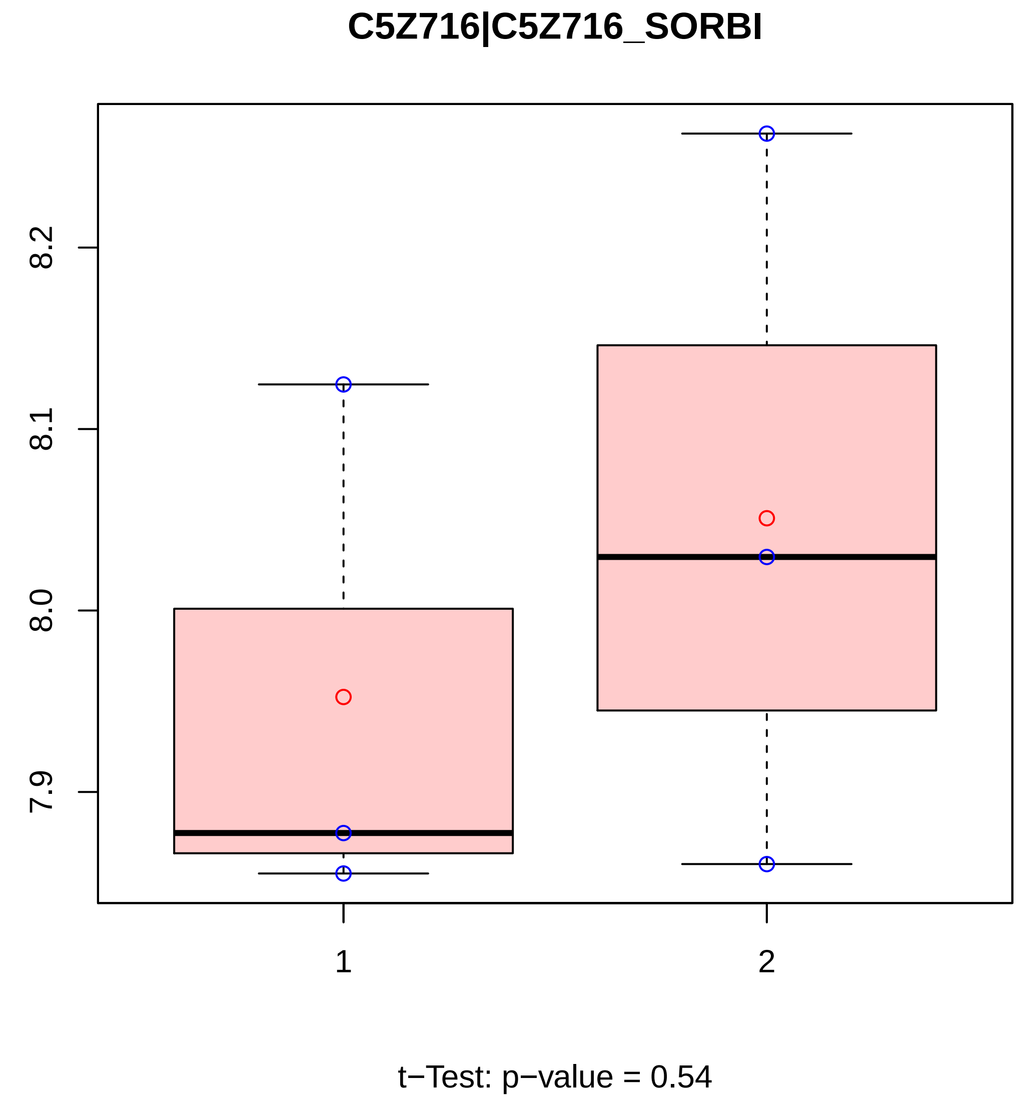

C5Z716|C5Z716_SORBI
8.2
8.1
8.0
7.9
1
2
t−Test: p−value = 0.54

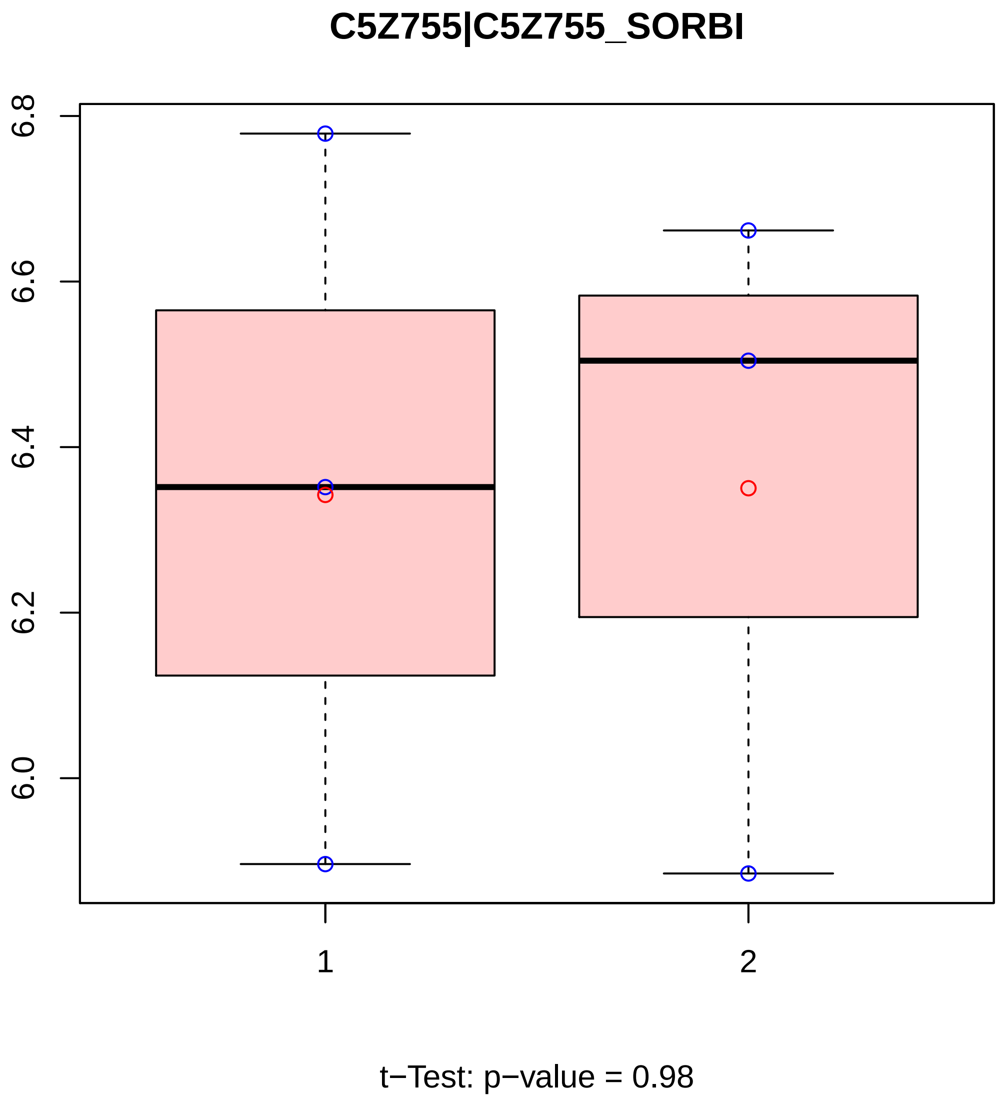
C5Z755|C5Z755_SORBI
6.0
6.2
6.4
6.6
6.8
1
2
t−Test: p−value = 0.98

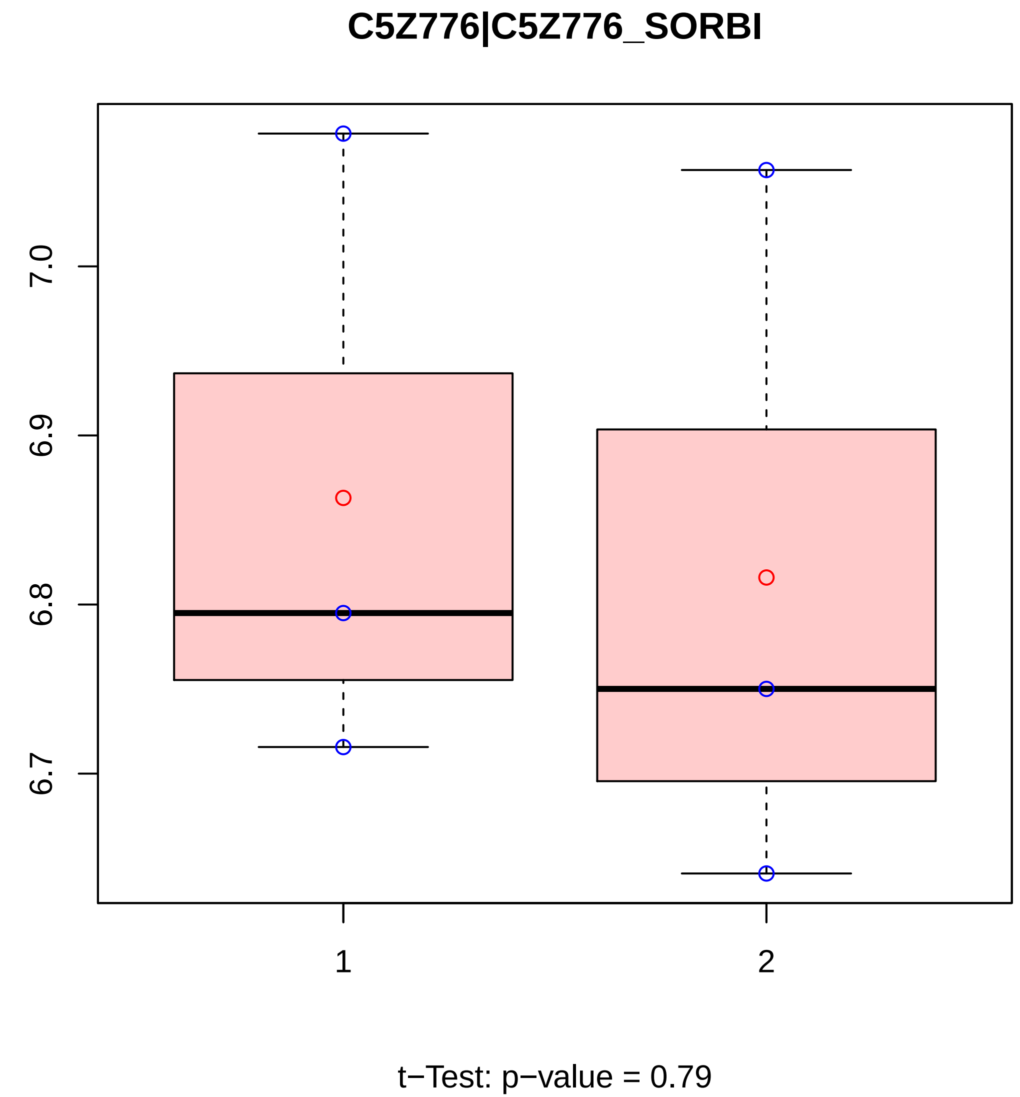
C5Z776|C5Z776_SORBI
7.0
6.9
6.8
6.7
1
2
t−Test: p−value = 0.79

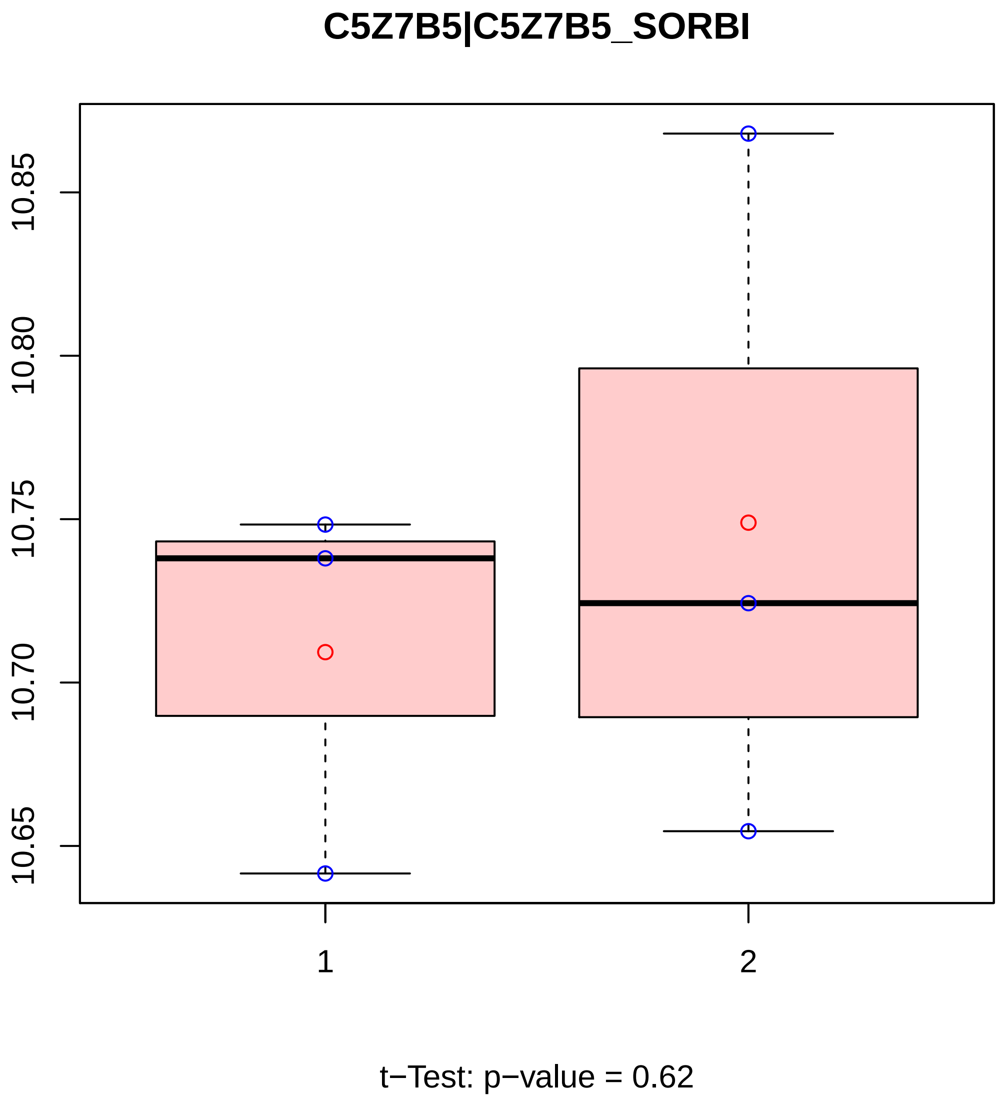
C5Z7B5|C5Z7B5_SORBI
10.65
10.70
10.75
10.80
10.85
1
2
t−Test: p−value = 0.62

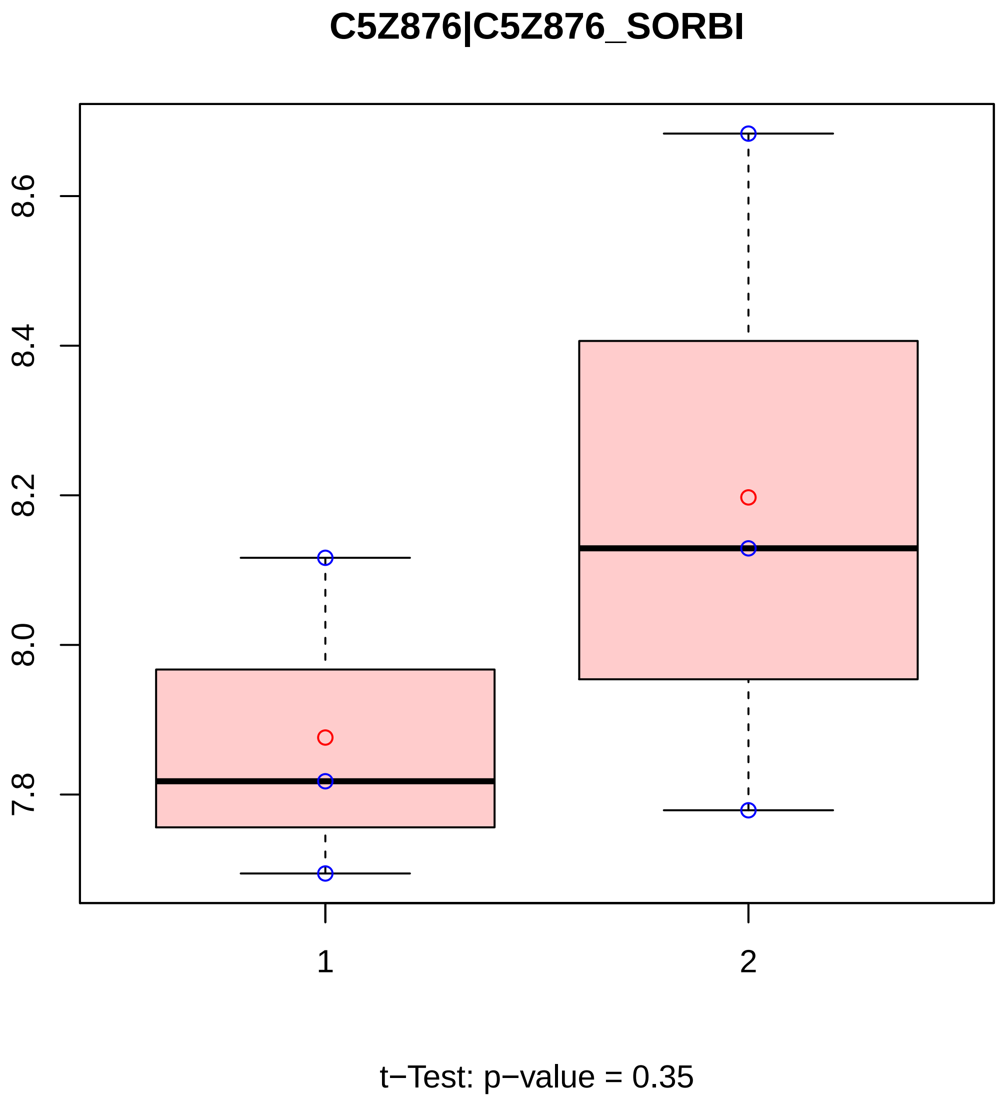
C5Z876|C5Z876_SORBI
8.6
8.4
8.2
8.0
7.8
1
2
t−Test: p−value = 0.35

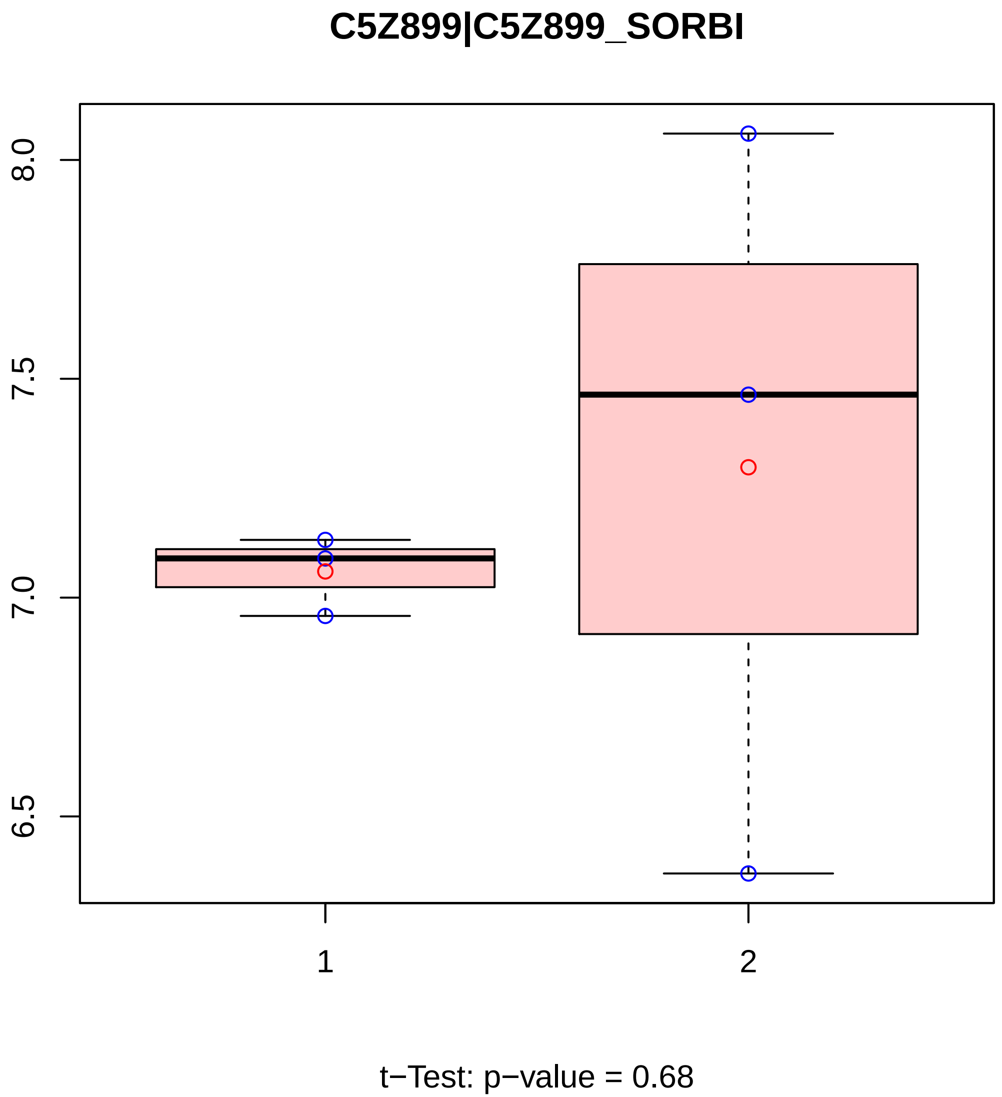
C5Z899|C5Z899_SORBI
8.0
7.5
7.0
6.5
1
2
t−Test: p−value = 0.68

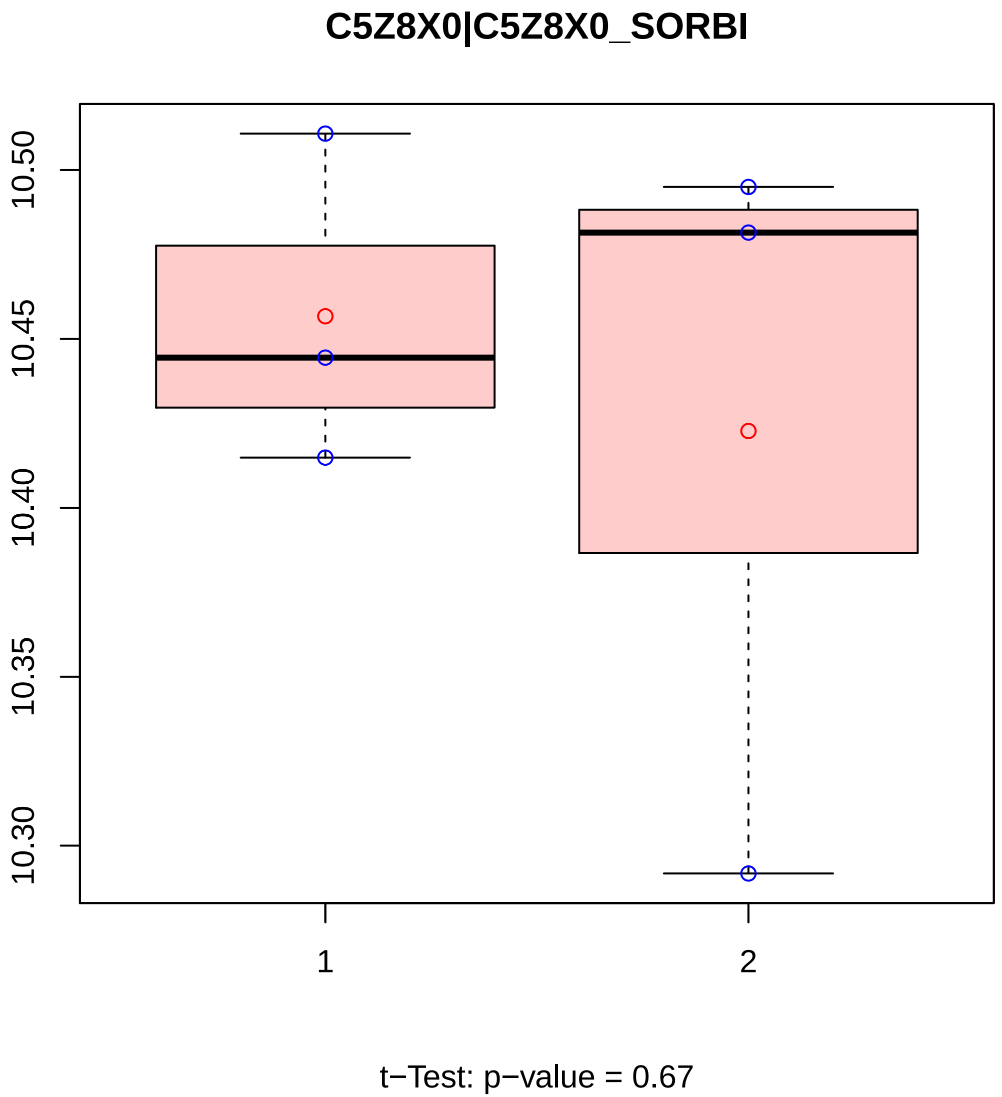

C5Z8X0|C5Z8X0_SORBI
10.50
10.45
10.40
10.35
10.30
1
2
t−Test: p−value = 0.67

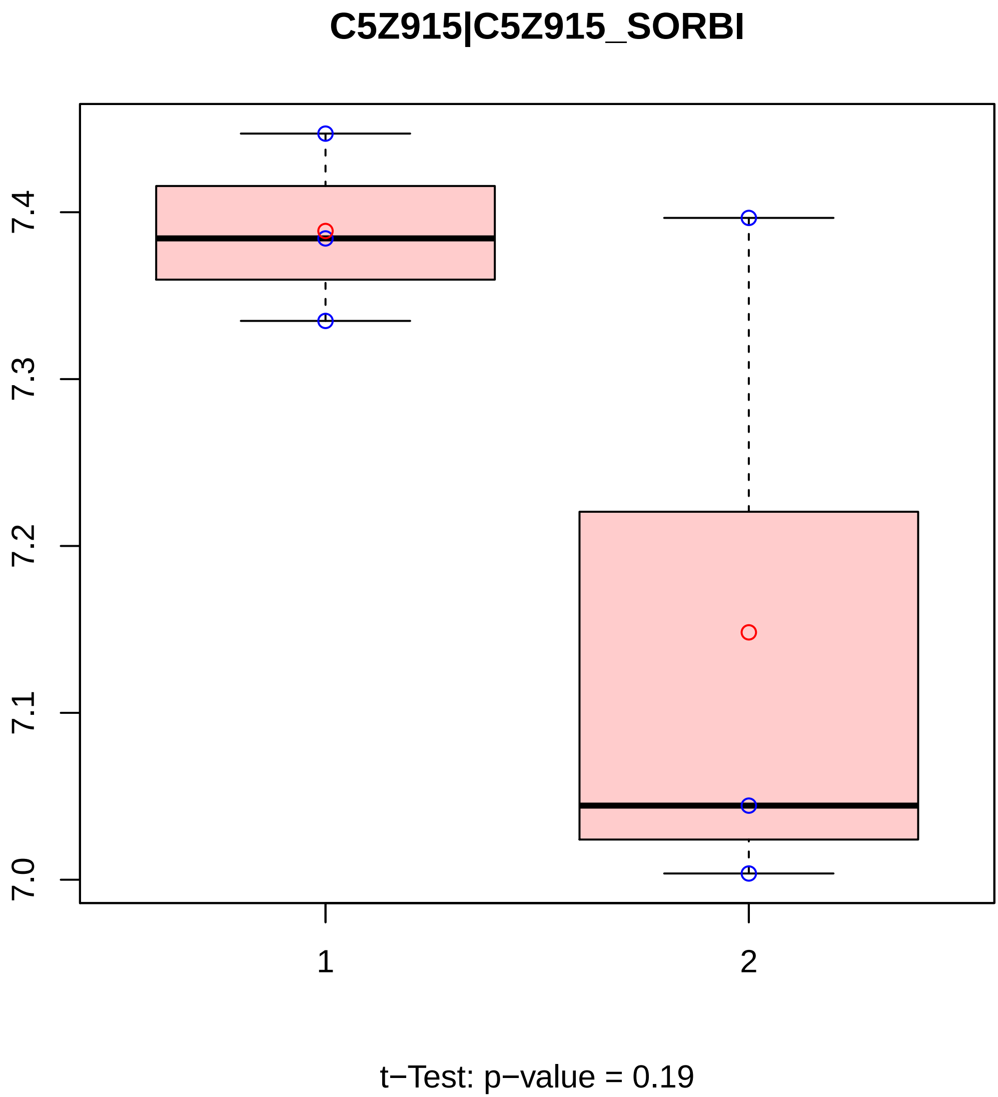
C5Z915|C5Z915_SORBI
7.0
7.1
7.2
7.3
7.4
1
2
t−Test: p−value = 0.19

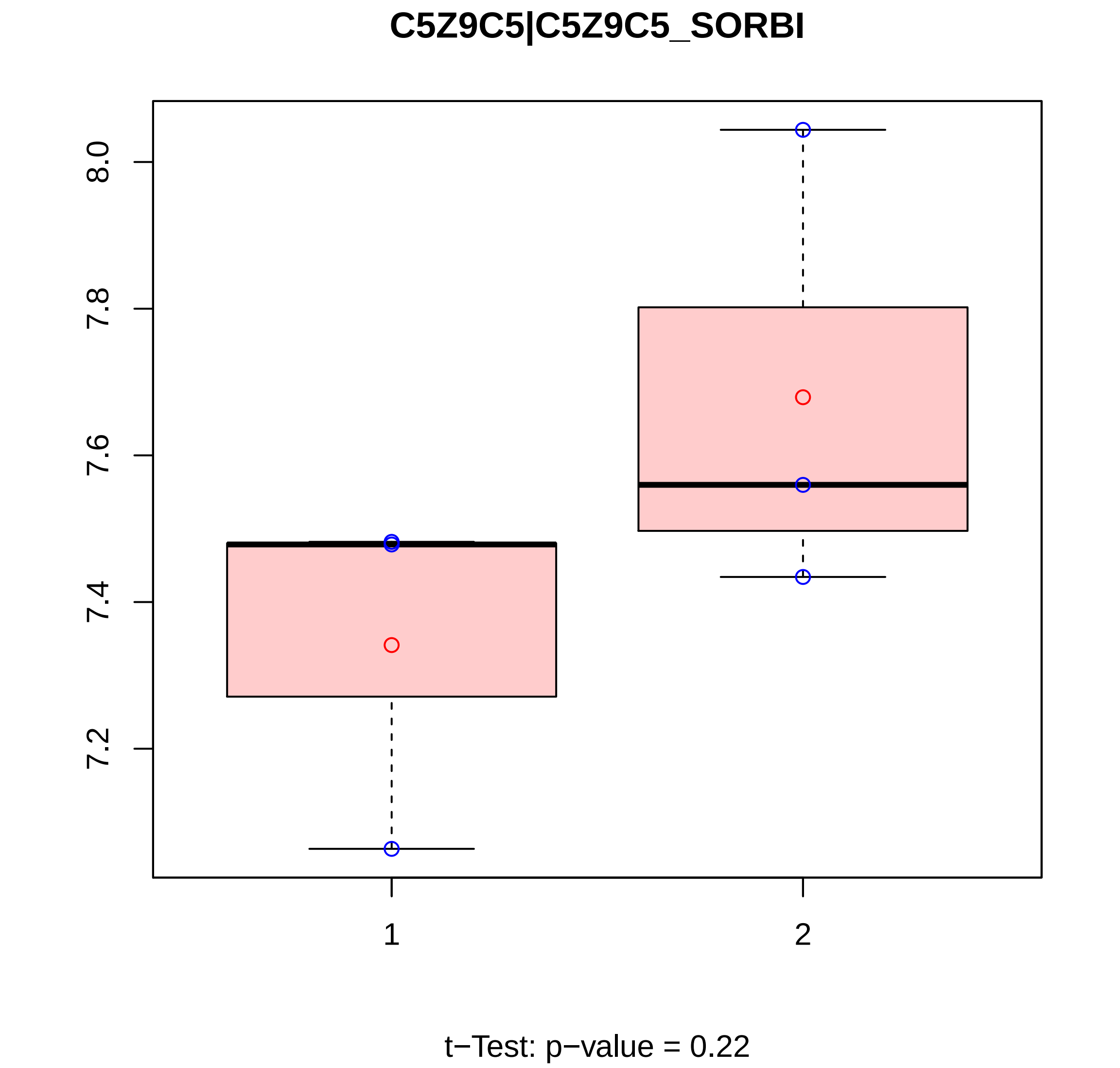
C5Z9C5|C5Z9C5_SORBI
8.0
7.8
7.6
7.4
7.2
1
2
t−Test: p−value = 0.22

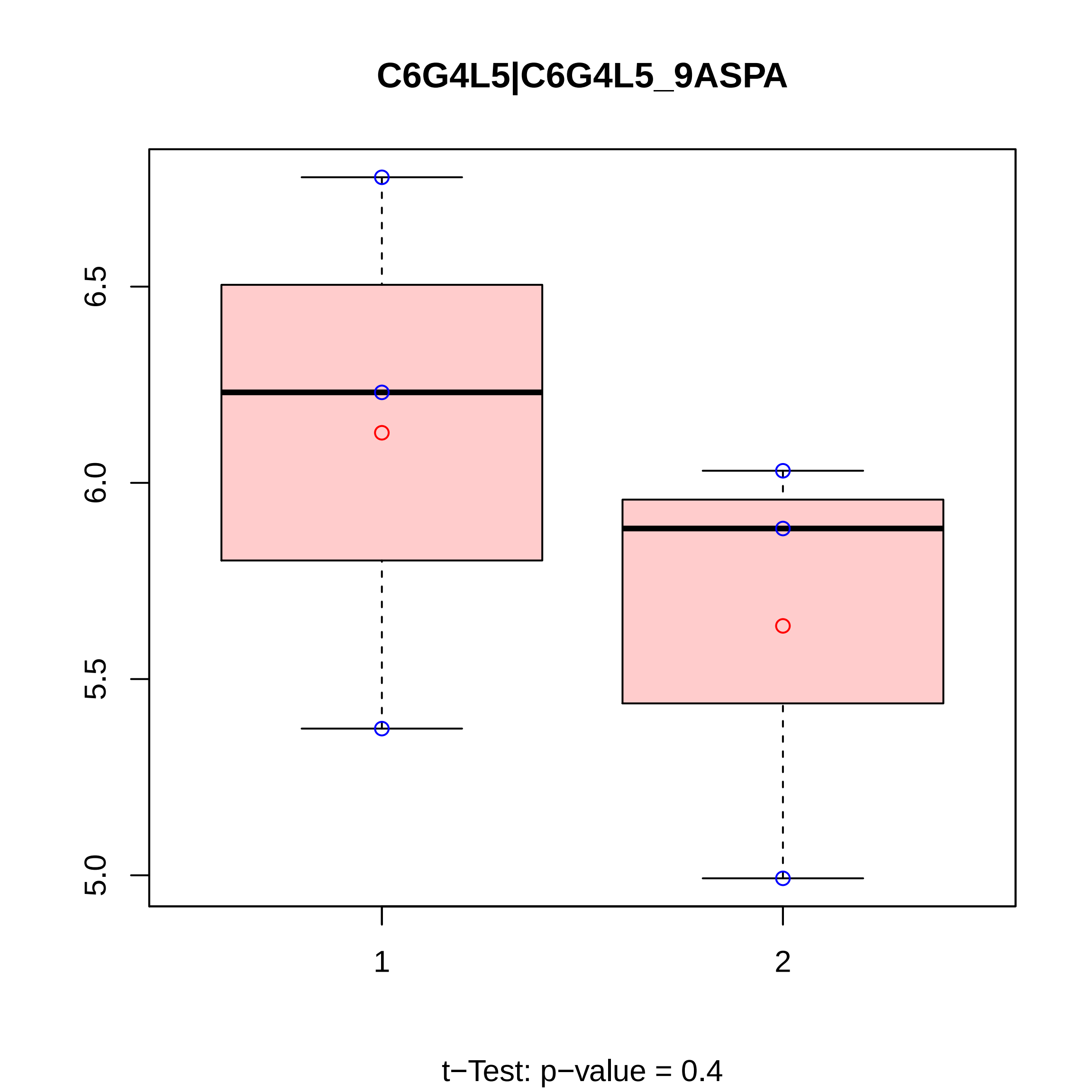
C6G4L5|C6G4L5_9ASPA
6.5
6.0
5.5
5.0
1
2
t−Test: p−value = 0.4

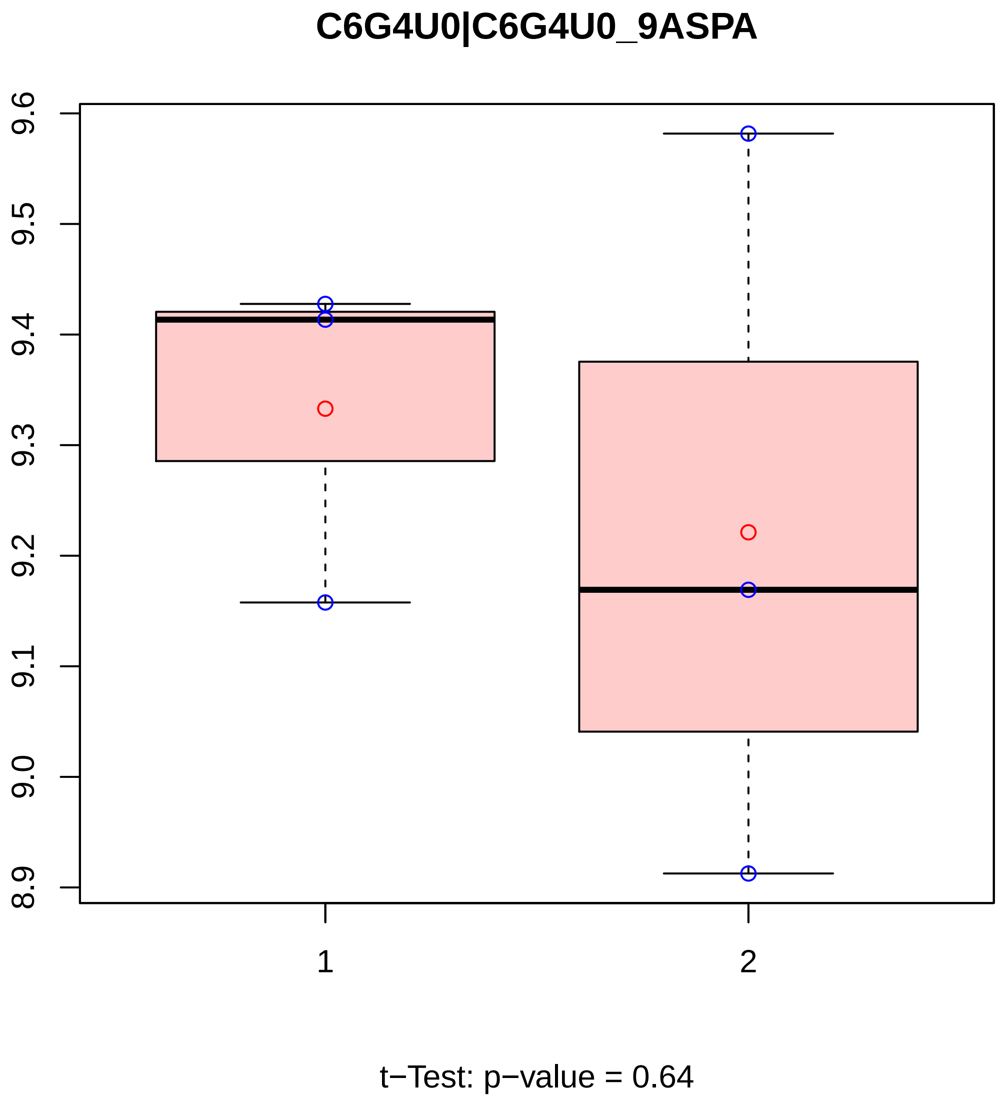
C6G4U0|C6G4U0_9ASPA
9.6
9.5
9.4
9.3
9.2
9.1
9.0
8.9
1
2
t−Test: p−value = 0.64

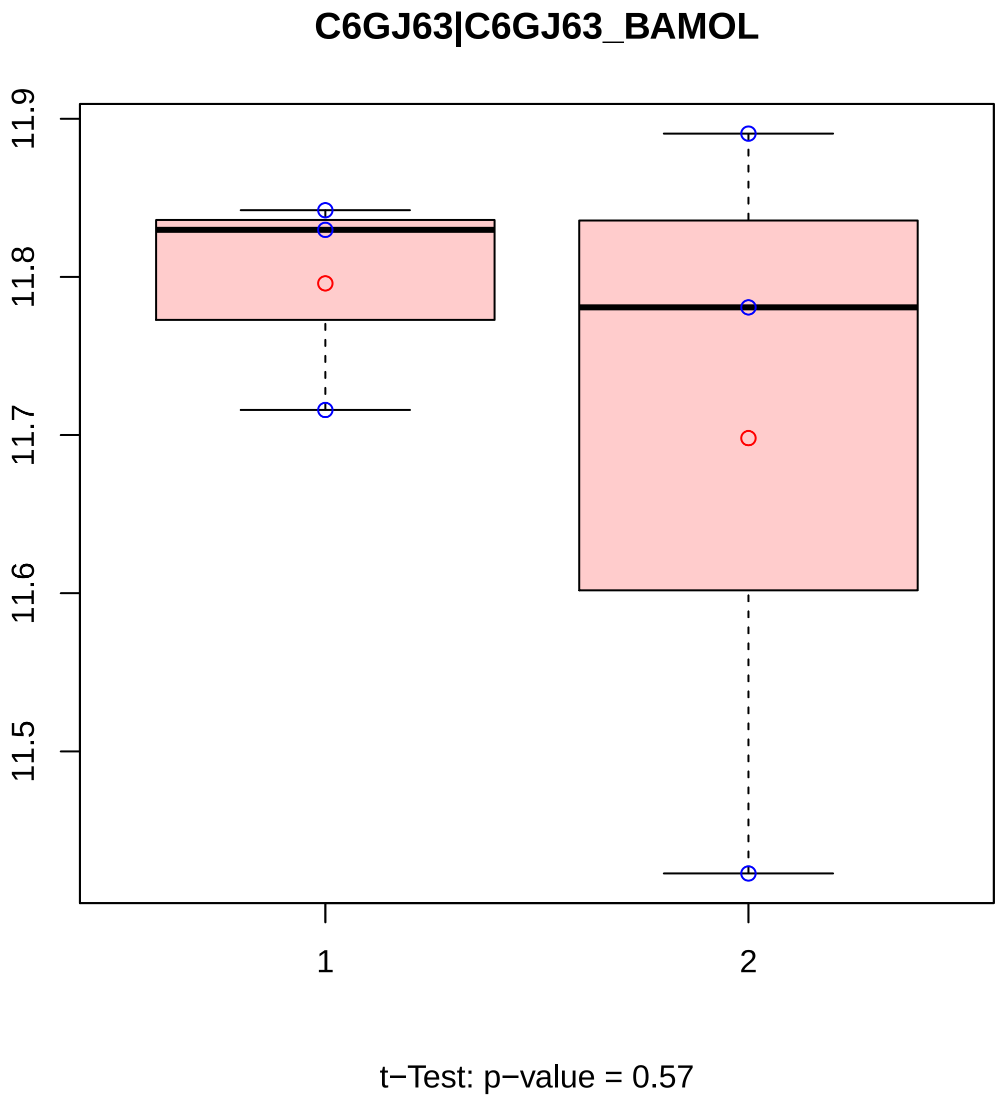
C6GJ63|C6GJ63_BAMOL
11.9
11.8
11.7
11.6
11.5
1
2
t−Test: p−value = 0.57

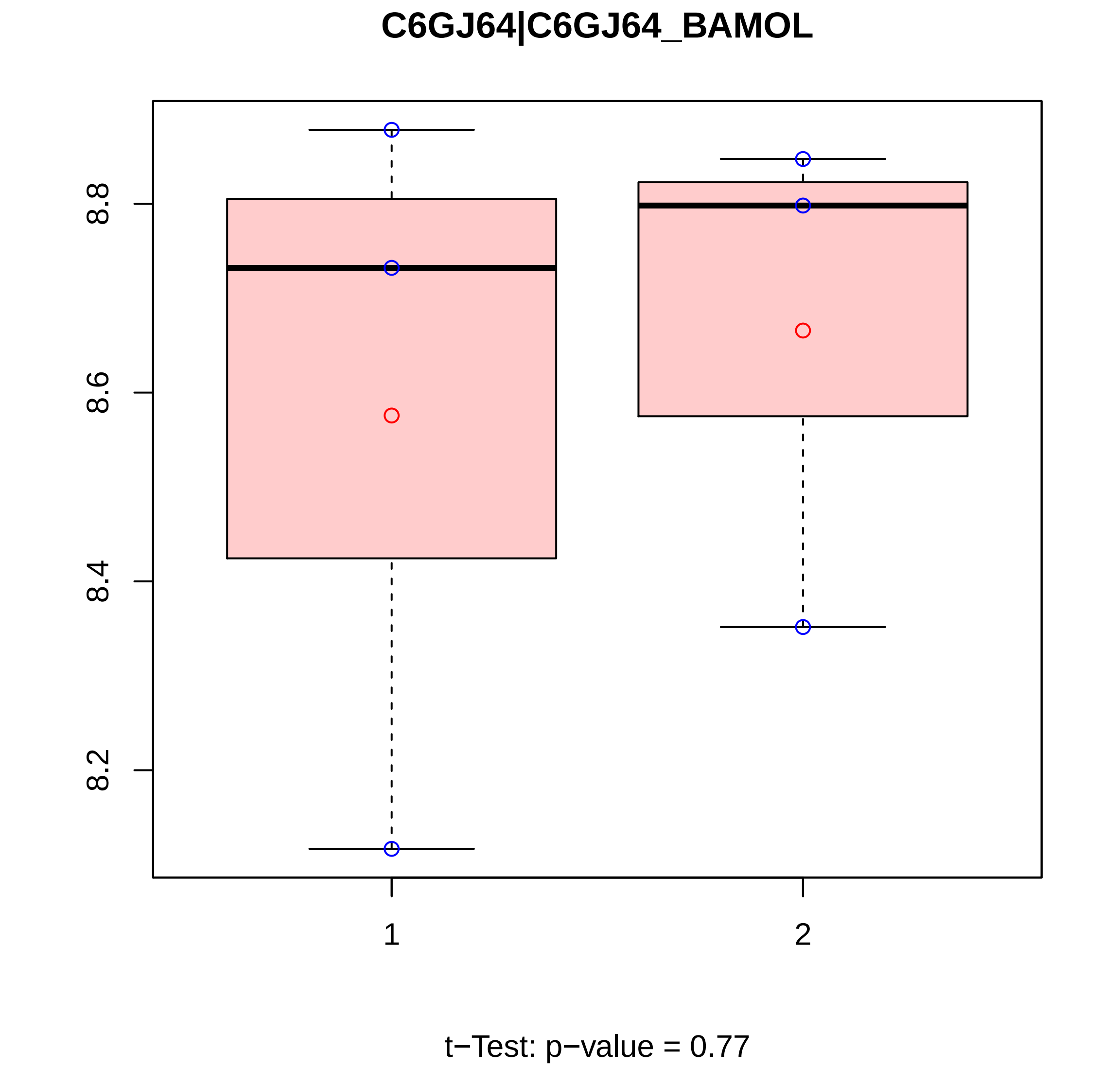

C6GJ64|C6GJ64_BAMOL
8.2
8.4
8.6
8.8
1
2
t−Test: p−value = 0.77

C6JRU8|C6JRU8_SORBI

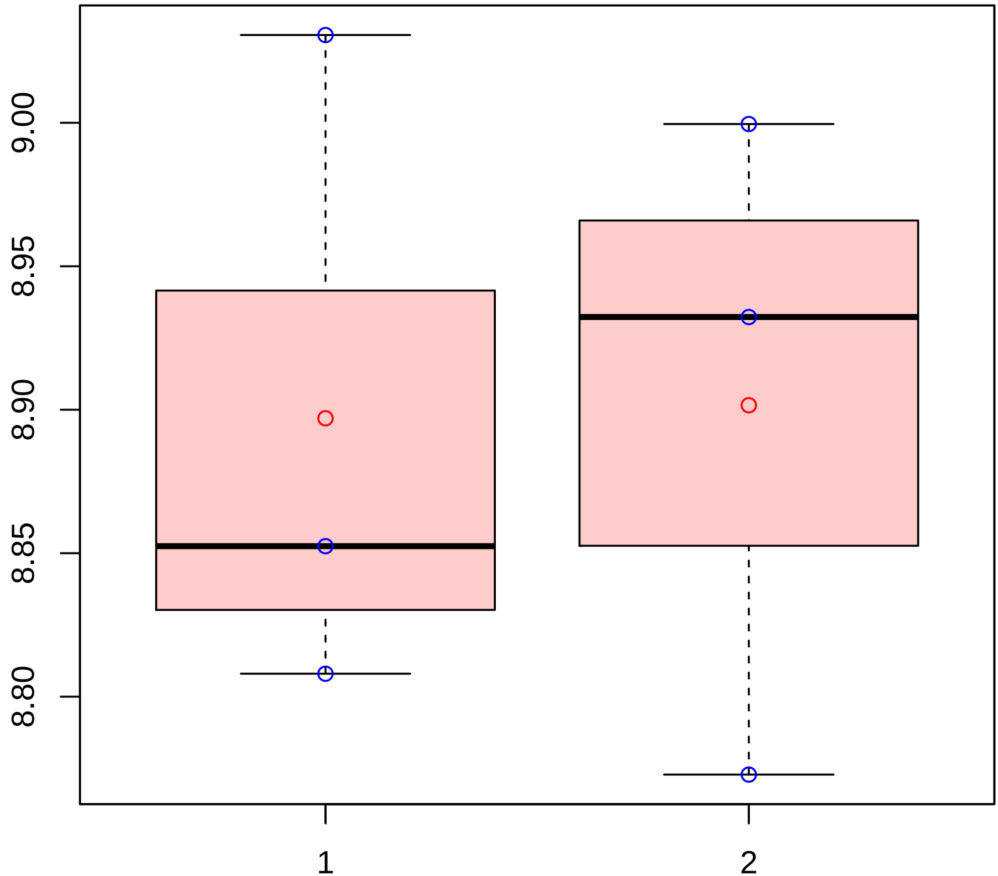

t−Test: p−value = 0.96

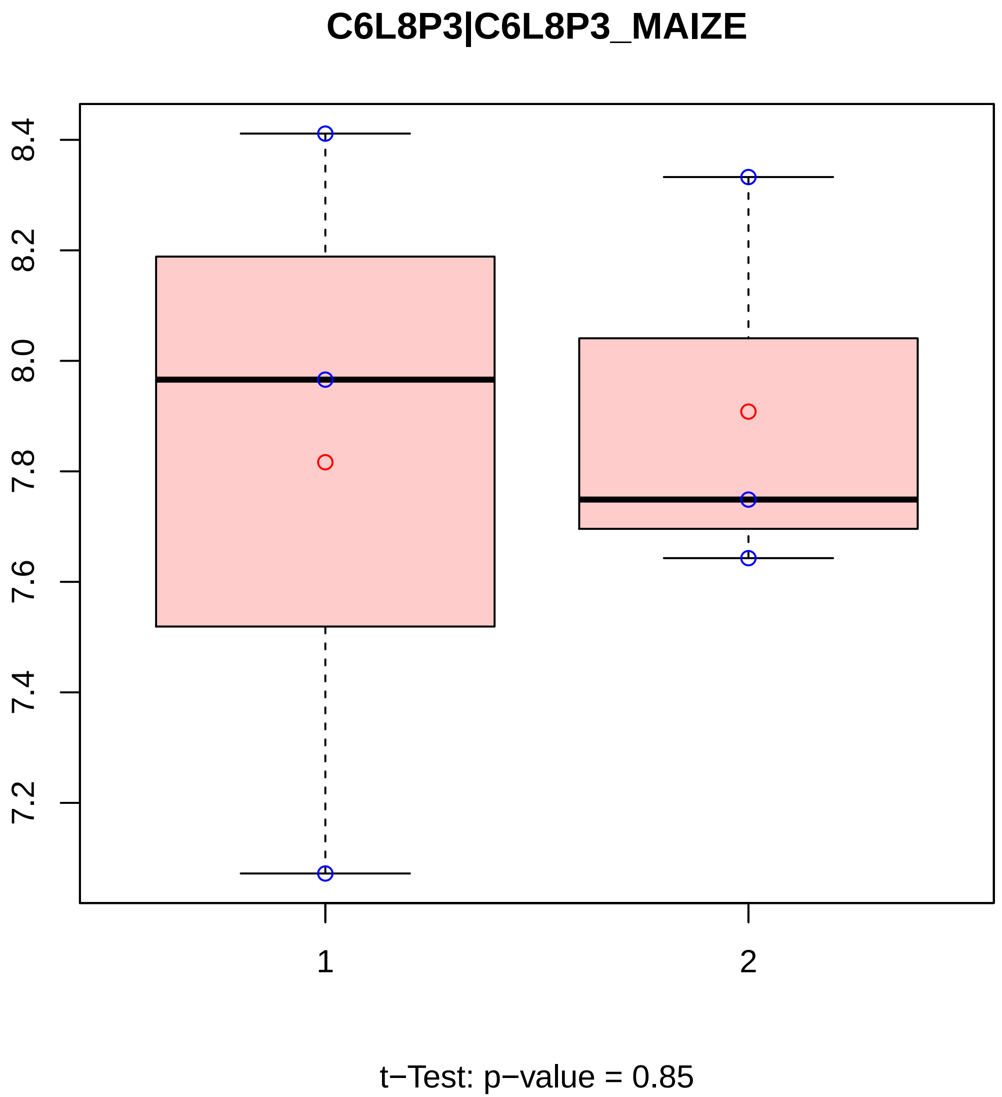
C6L8P3|C6L8P3_MAIZE
8.4
8.2
8.0
7.8
7.6
7.4
7.2
1
2
t−Test: p−value = 0.85

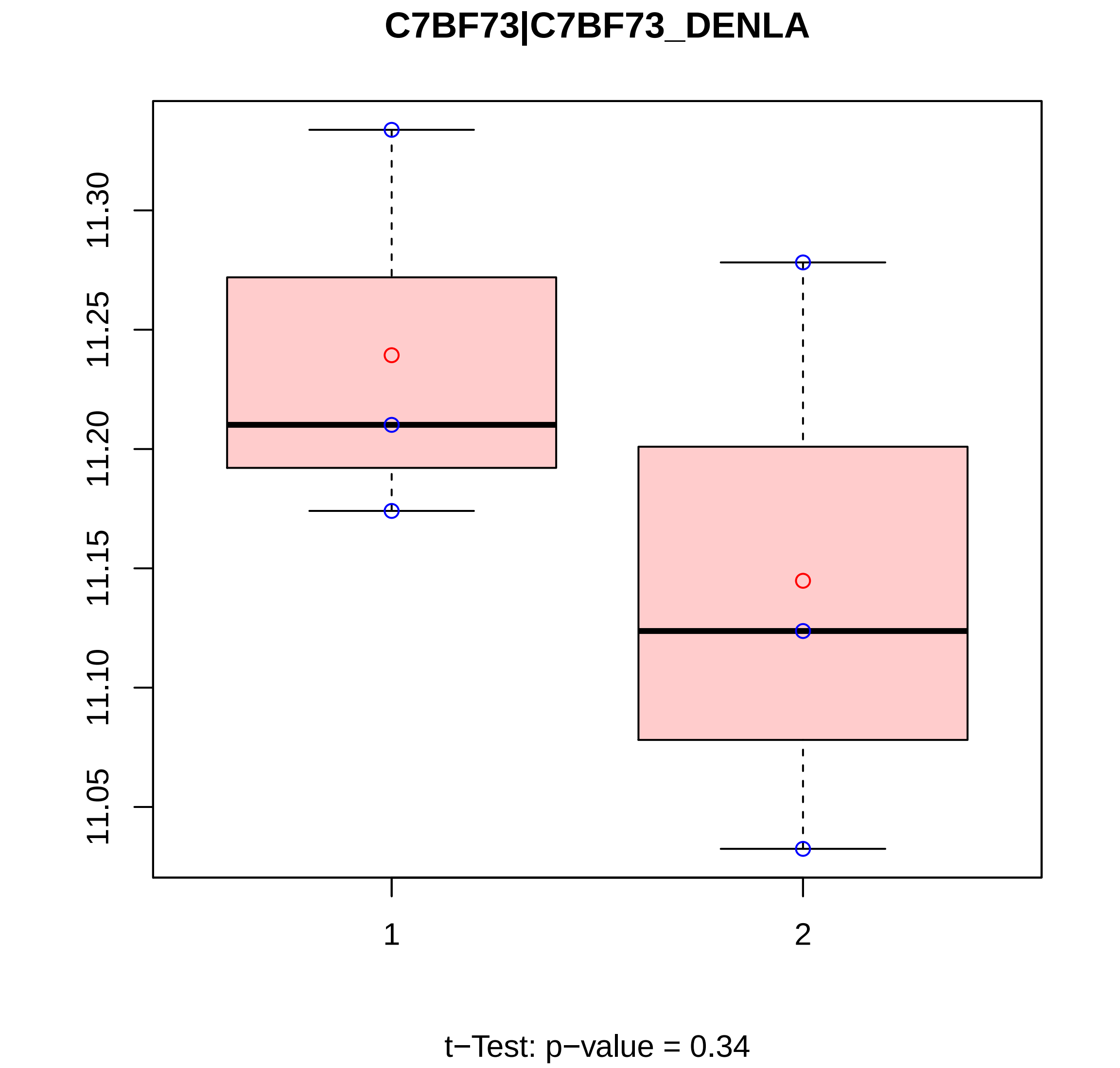
C7BF73|C7BF73_DENLA
11.05
11.10
11.15
11.20
11.25
11.30
1
2
t−Test: p−value = 0.34

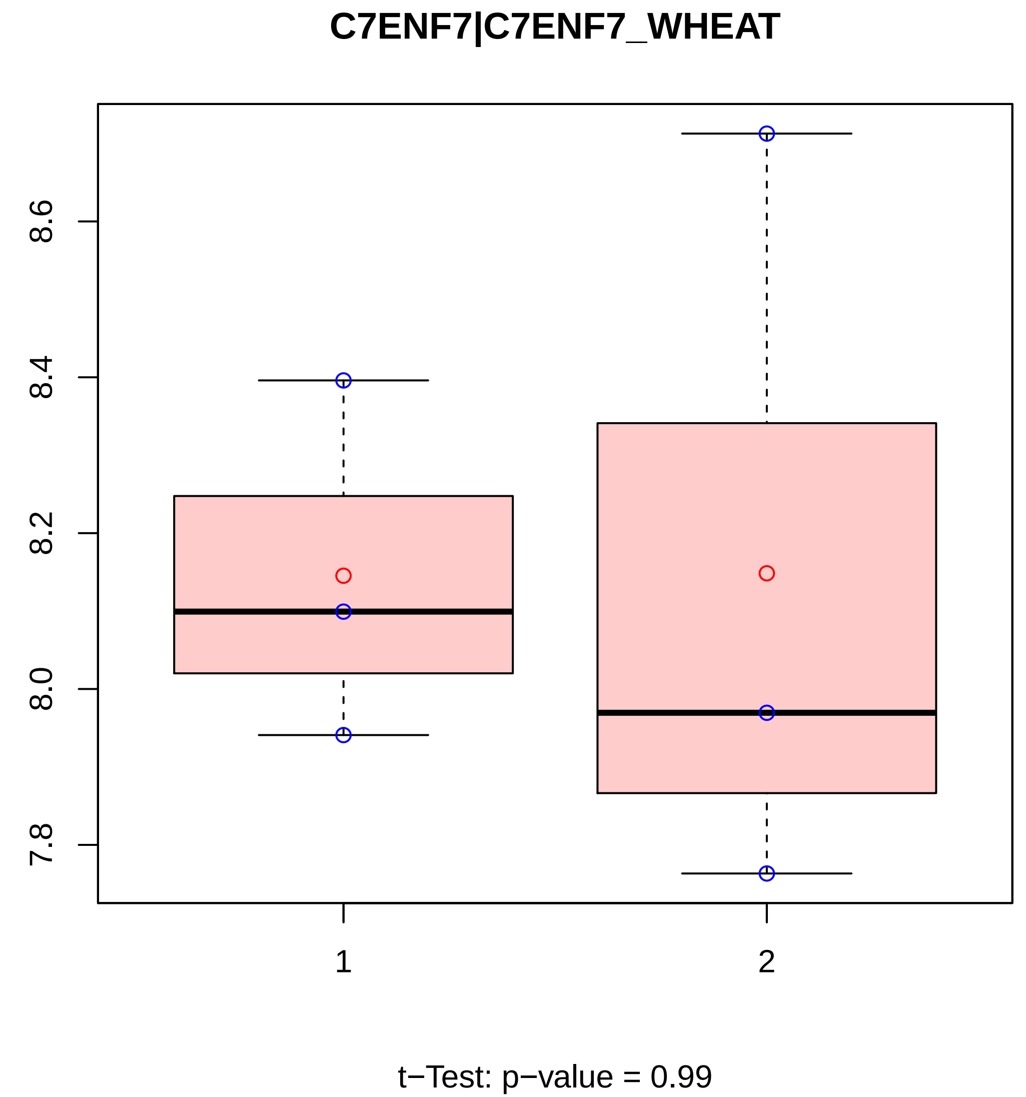
C7ENF7|C7ENF7_WHEAT
8.6
8.4
8.2
8.0
7.8
1
2
t−Test: p−value = 0.99

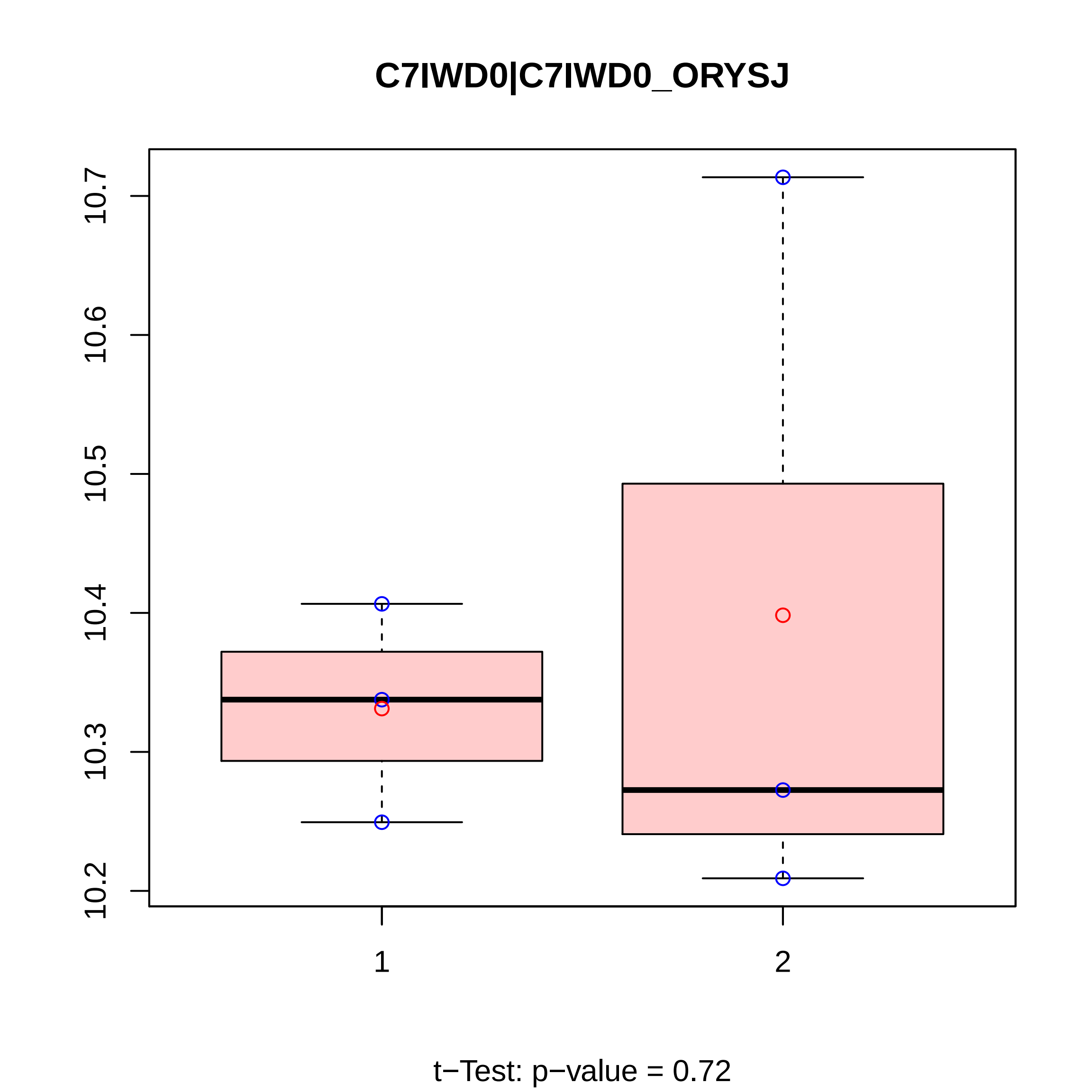

C7IWD0|C7IWD0_ORYSJ
10.2
10.3
10.4
10.5
10.6
10.7
1
2
t−Test: p−value = 0.72

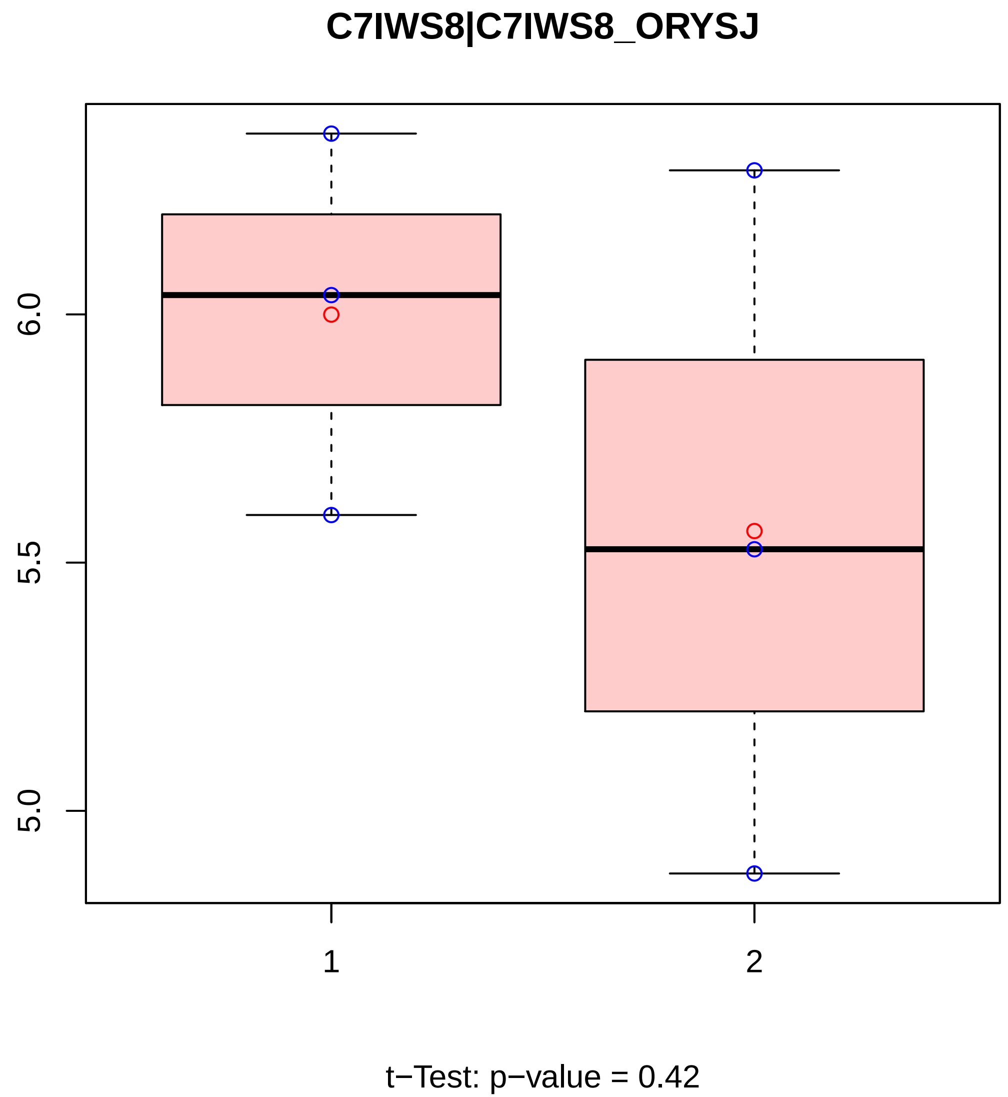

C7IWS8|C7IWS8_ORYSJ
6.0
5.5
5.0
1
2
t−Test: p−value = 0.42

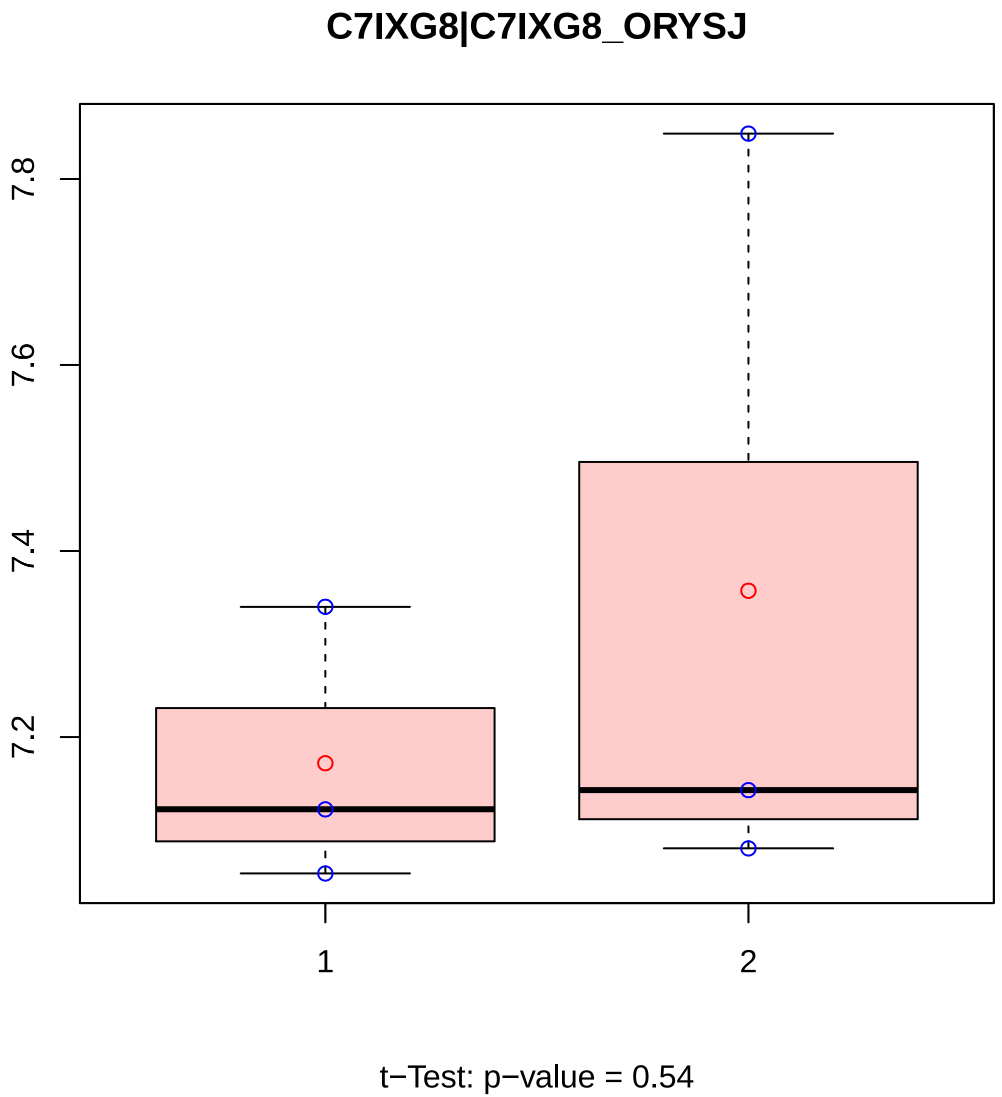

C7IXG8|C7IXG8_ORYSJ
7.2
7.4
7.6
7.8
1
2
t−Test: p−value = 0.54

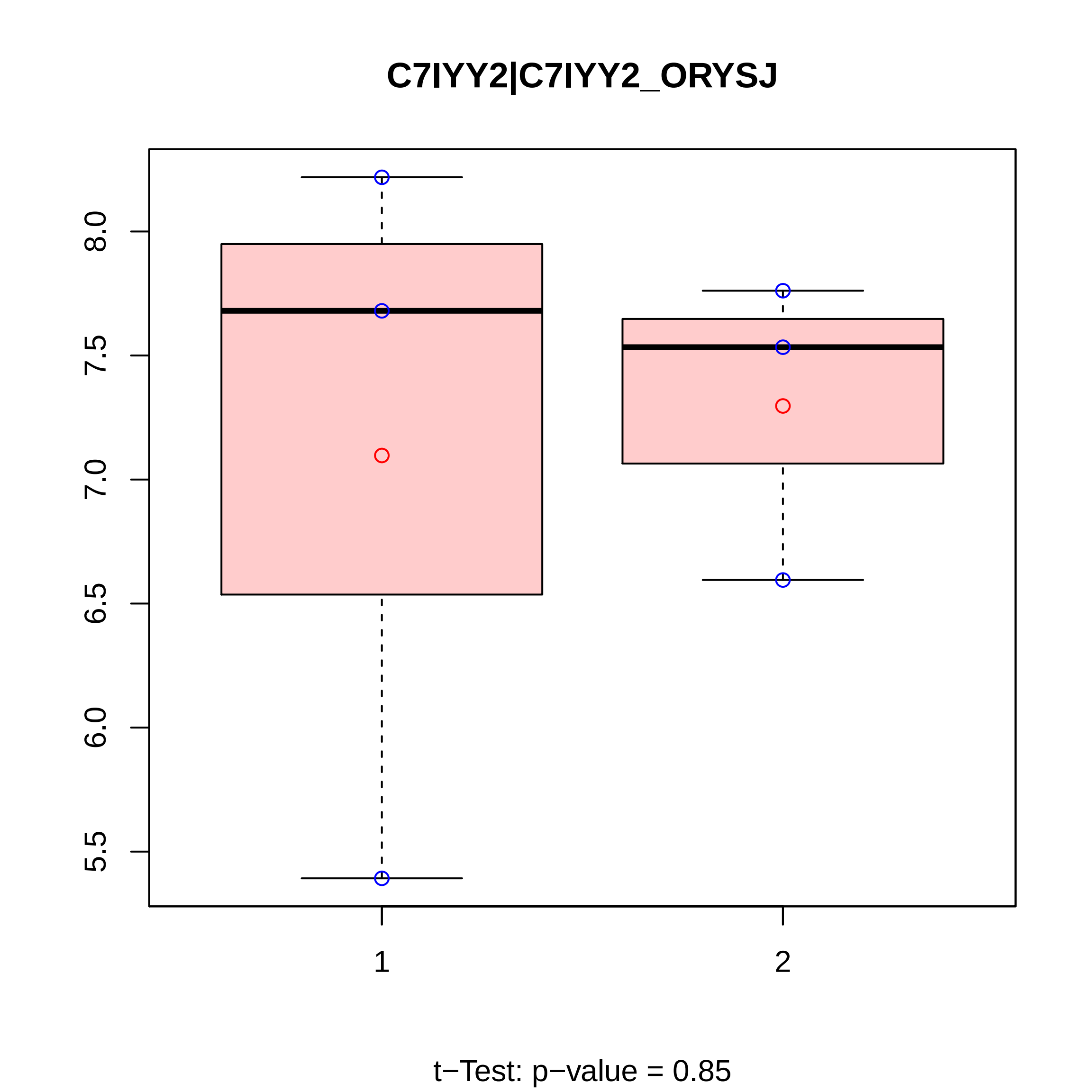

C7IYY2|C7IYY2_ORYSJ
8.0
7.5
7.0
6.5
6.0
5.5
1
2
t−Test: p−value = 0.85

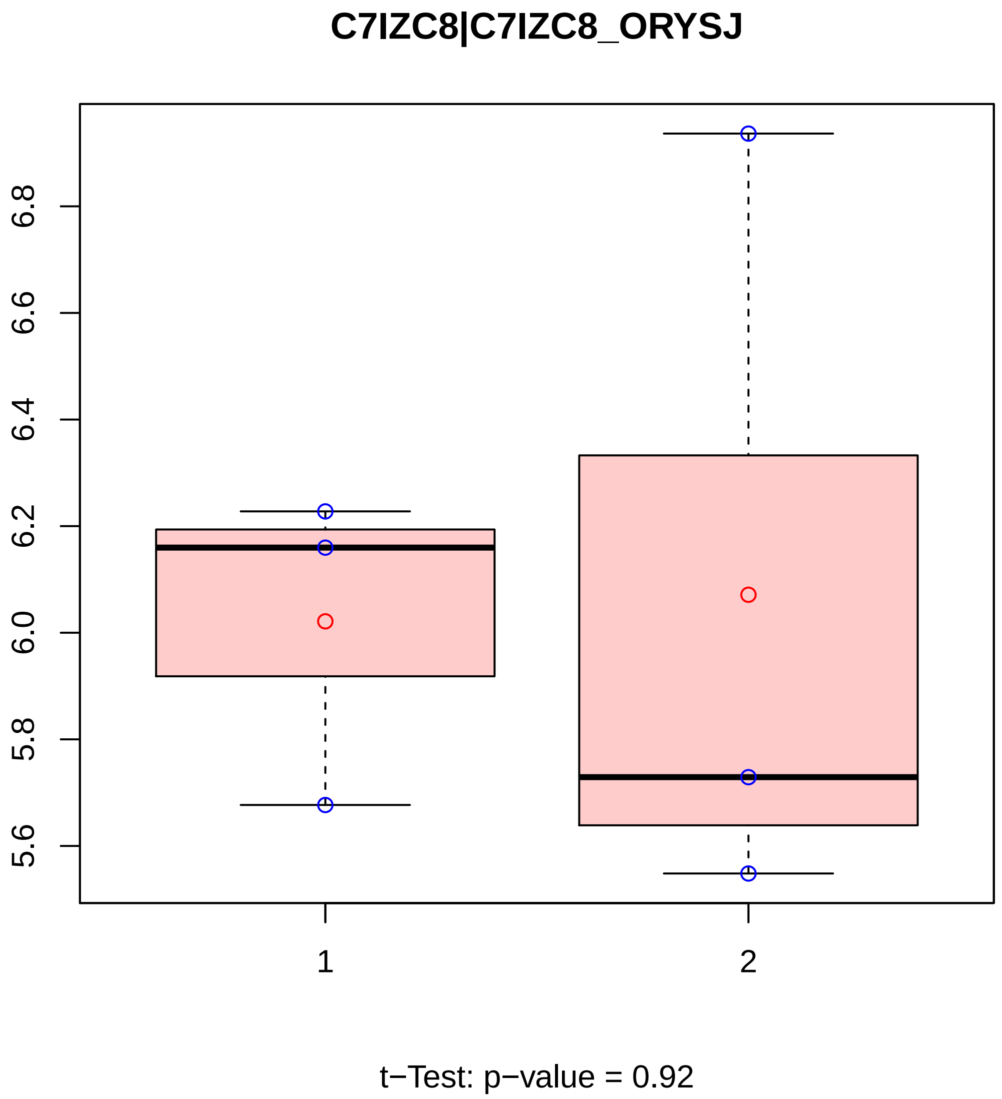
C7IZC8|C7IZC8_ORYSJ
6.8
6.6
6.4
6.2
6.0
5.8
5.6
1
2
t−Test: p−value = 0.92

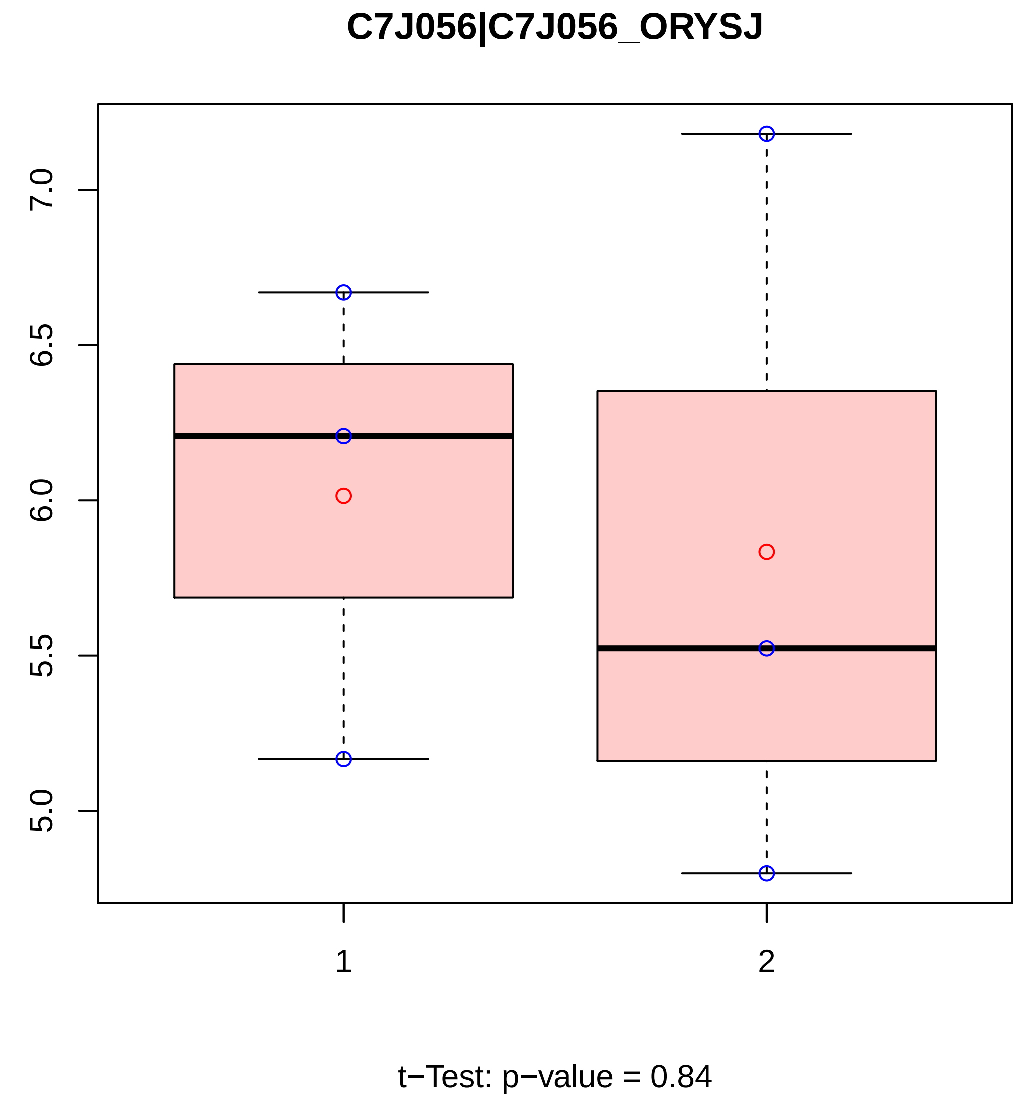

C7J056|C7J056_ORYSJ
7.0
6.5
6.0
5.5
5.0
1
2
t−Test: p−value = 0.84

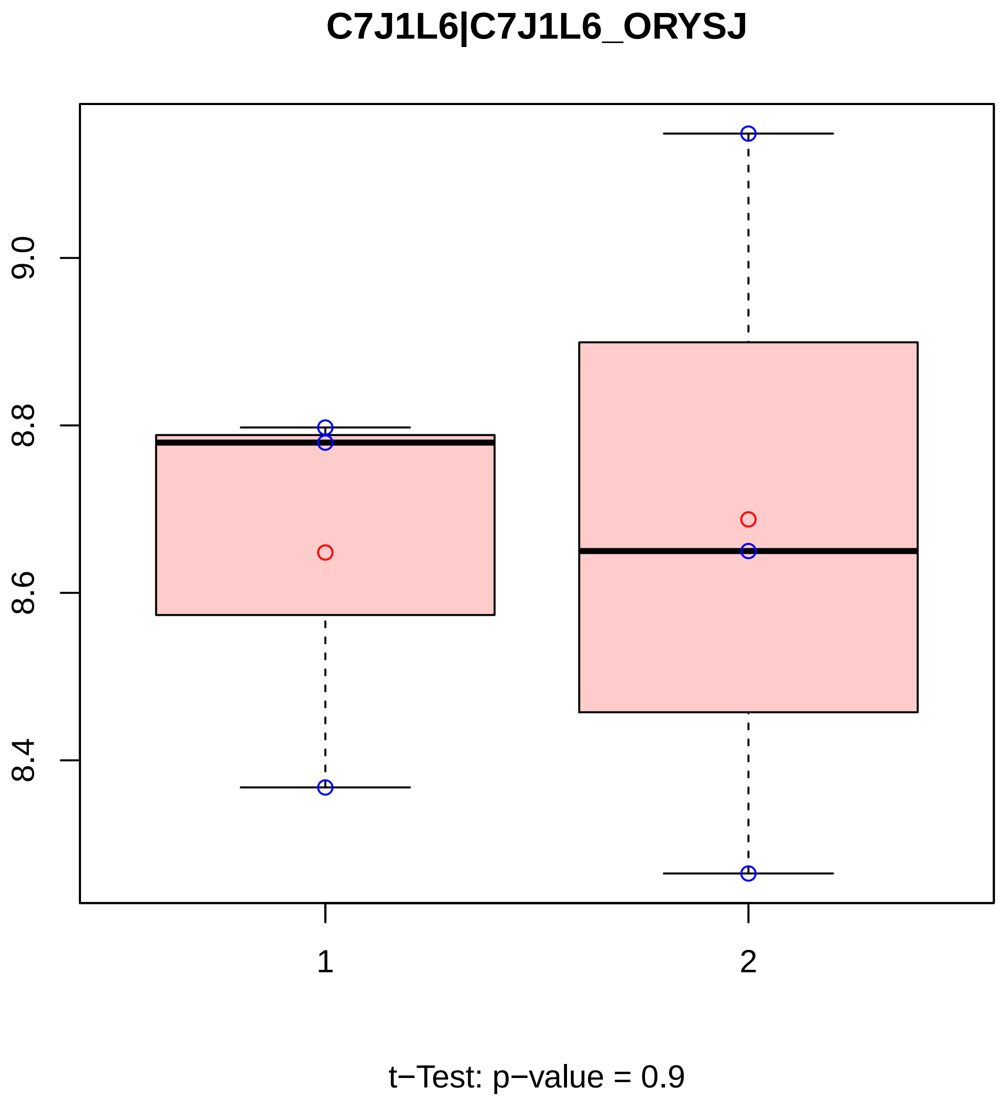

C7J1L6|C7J1L6_ORYSJ
9.0
8.8
8.6
8.4
1
2
t−Test: p−value = 0.9

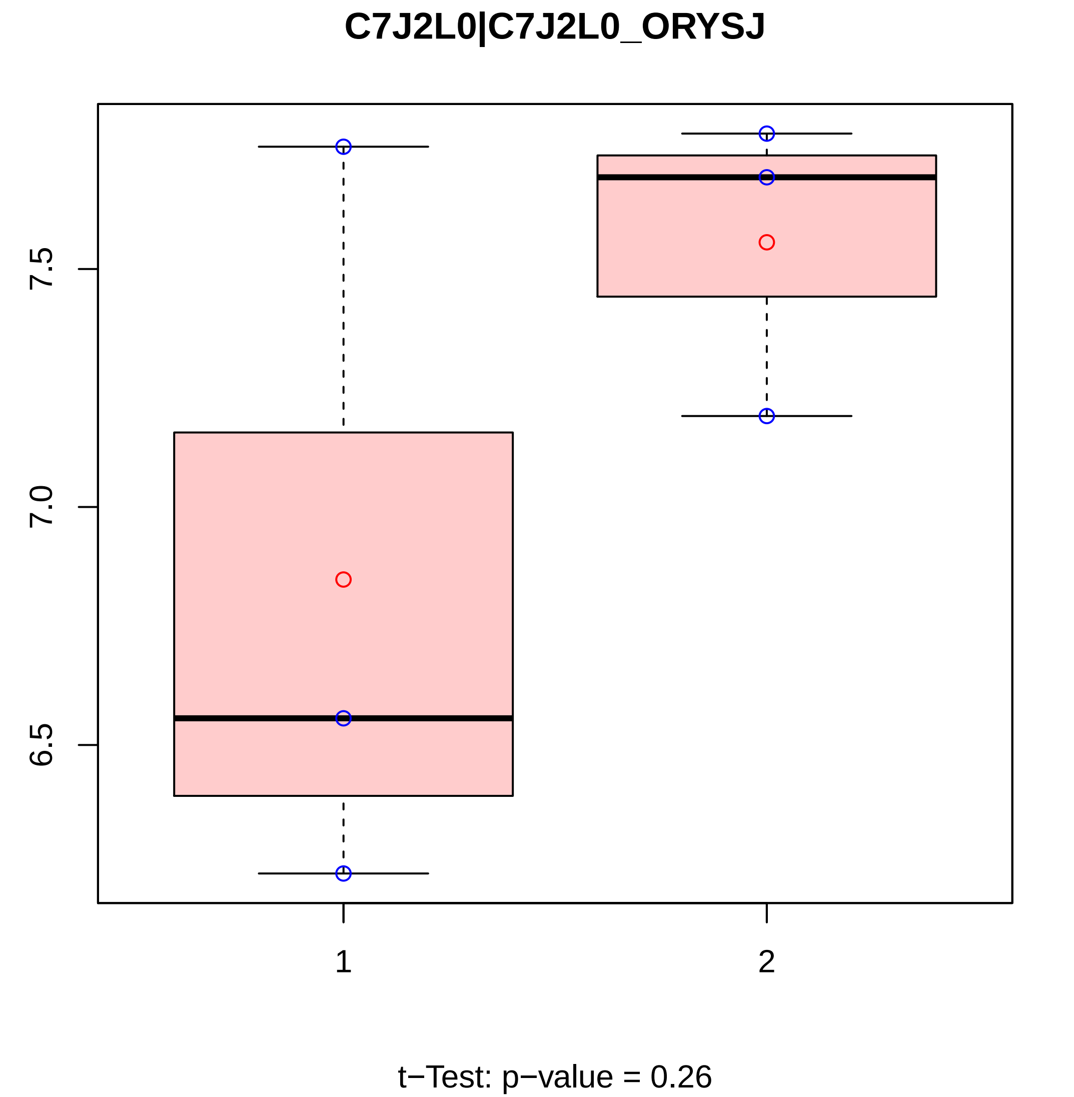
C7J2L0|C7J2L0_ORYSJ
7.5
7.0
6.5
1
2
t−Test: p−value = 0.26

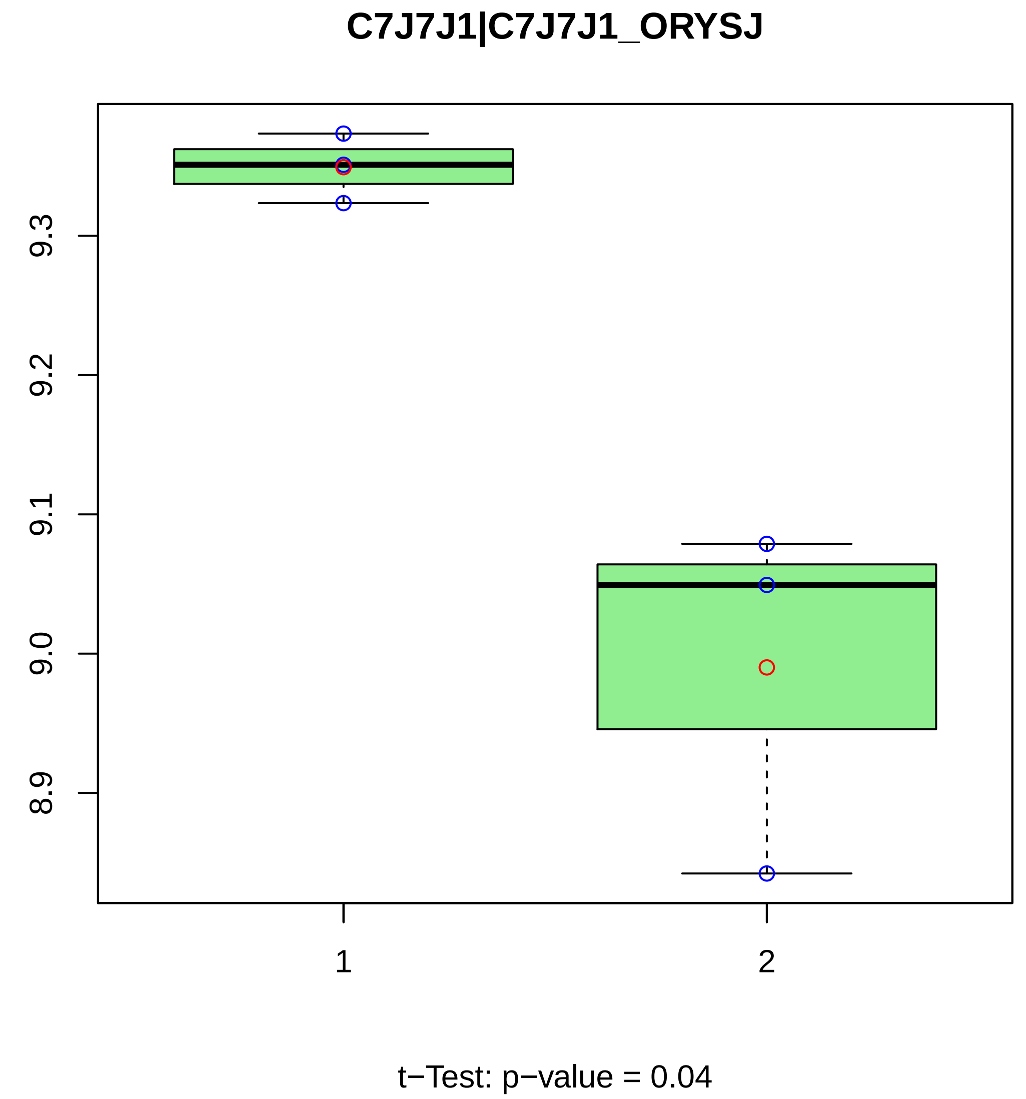
C7J7J1|C7J7J1_ORYSJ
9.3
9.2
9.1
9.0
8.9
1
2
t−Test: p−value = 0.04

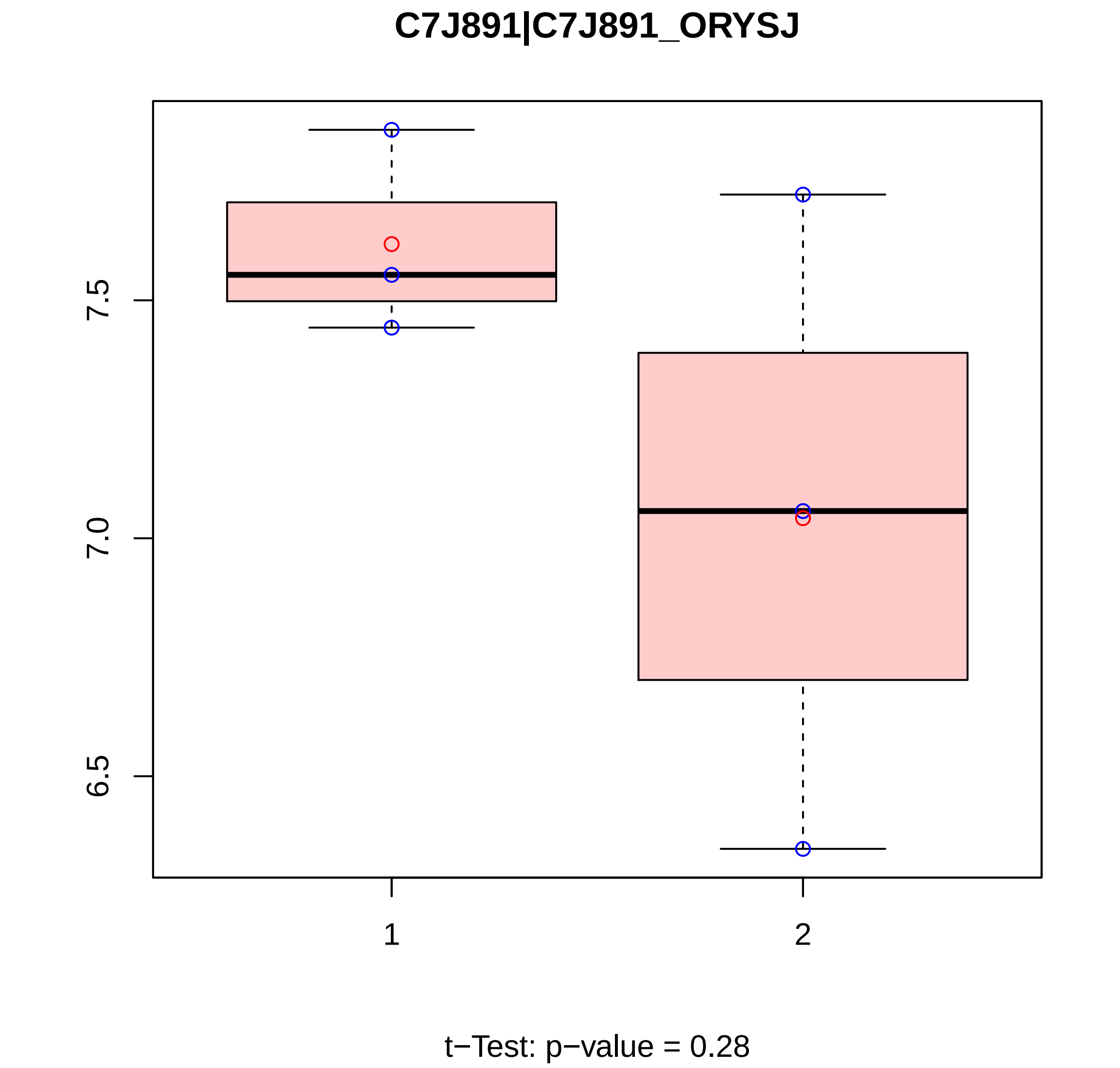
C7J891|C7J891_ORYSJ
7.5
7.0
6.5
1
2
t−Test: p−value = 0.28

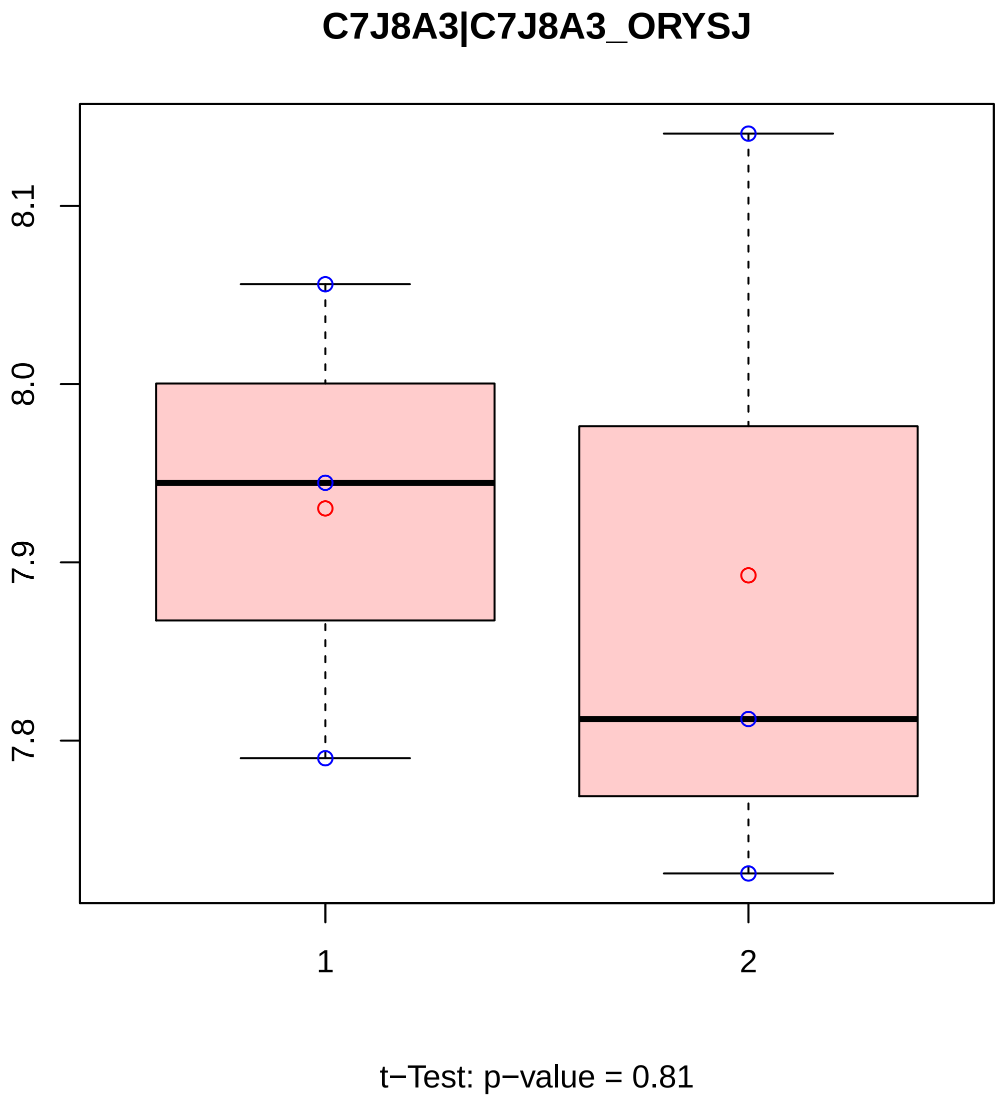
C7J8A3|C7J8A3_ORYSJ
8.1
8.0
7.9
7.8
1
2
t−Test: p−value = 0.81

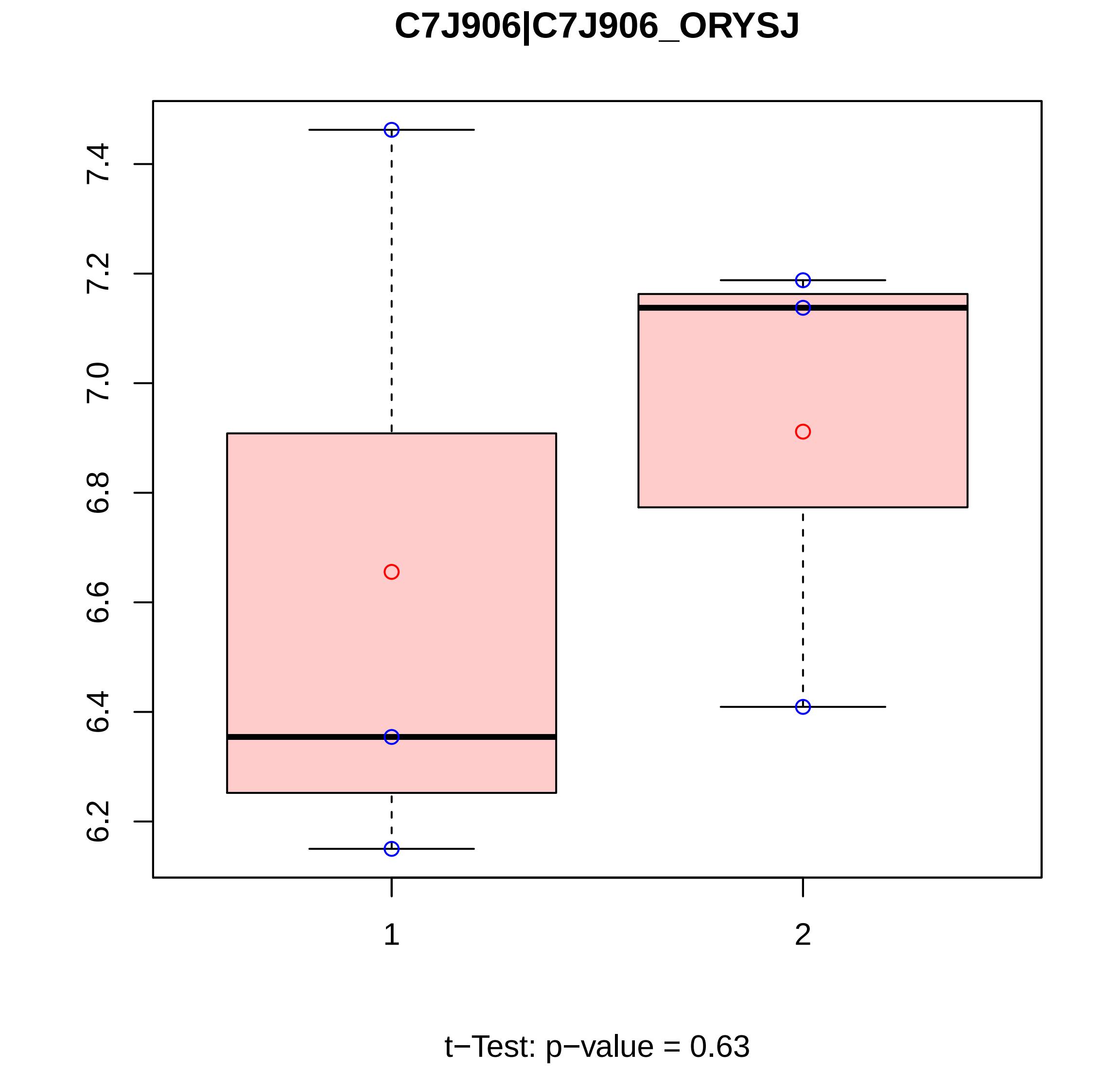
C7J906|C7J906_ORYSJ
7.4
7.2
7.0
6.8
6.6
6.4
6.2
1
2
t−Test: p−value = 0.63

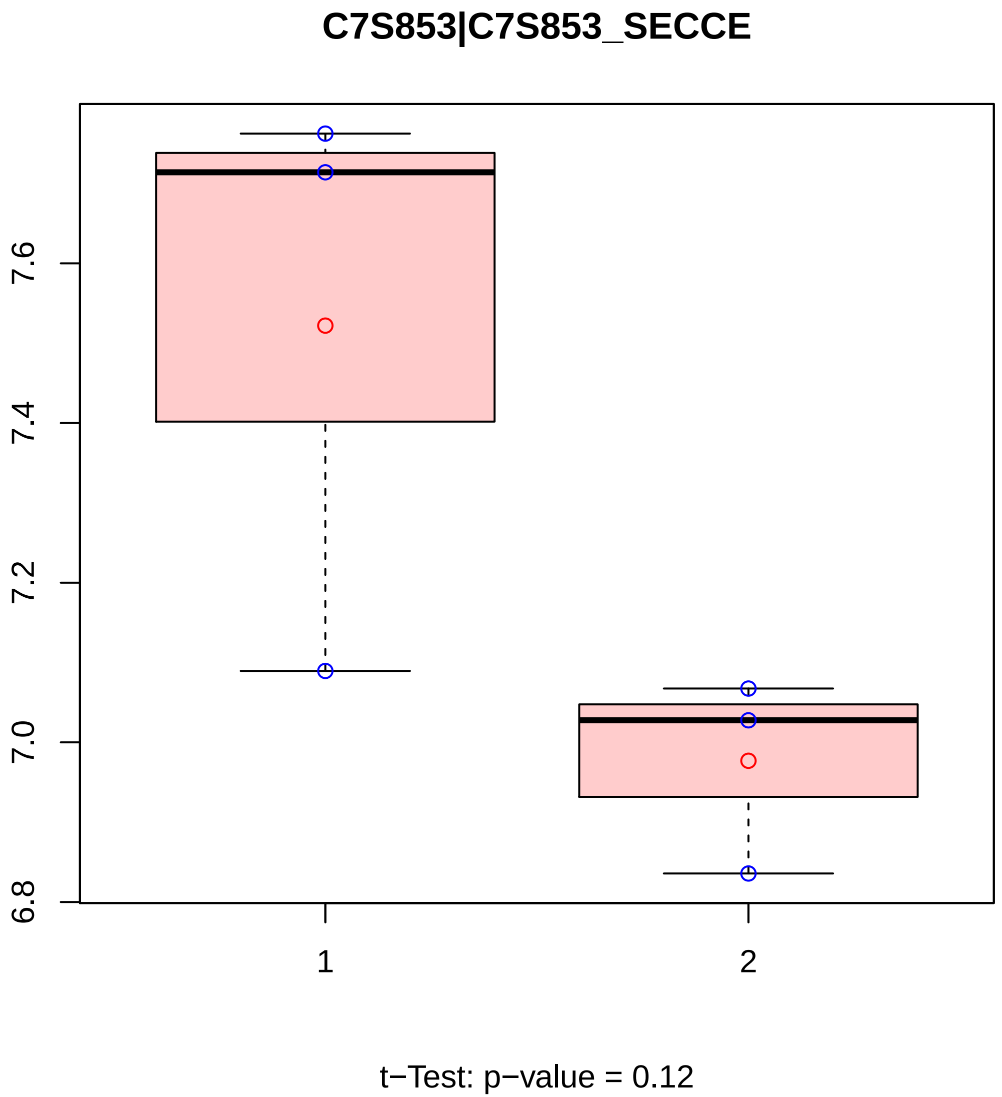
C7S853|C7S853_SECCE
7.6
7.4
7.2
7.0
6.8
1
2
t−Test: p−value = 0.12

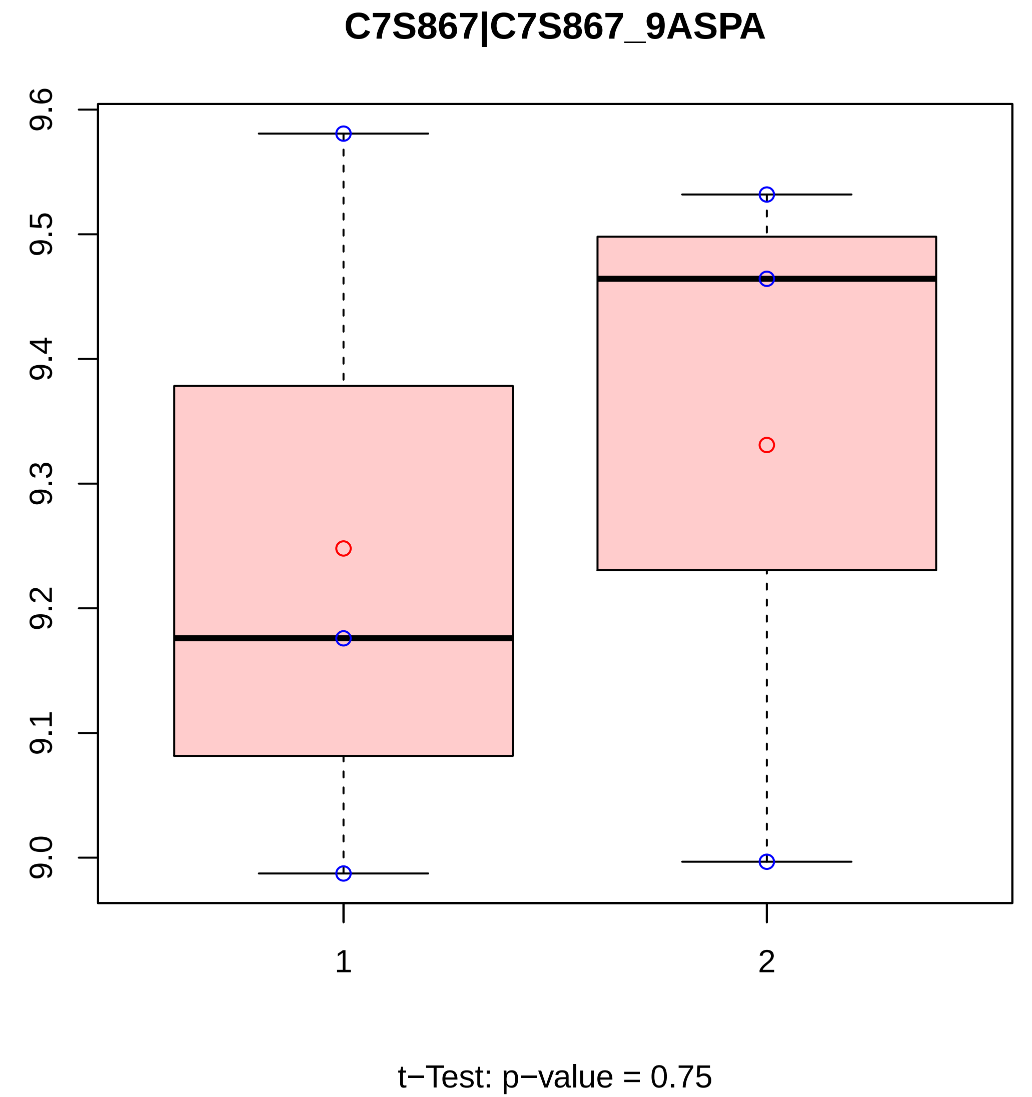
C7S867|C7S867_9ASPA
9.0
9.1
9.2
9.3
9.4
9.5
9.6
1
2
t−Test: p−value = 0.75

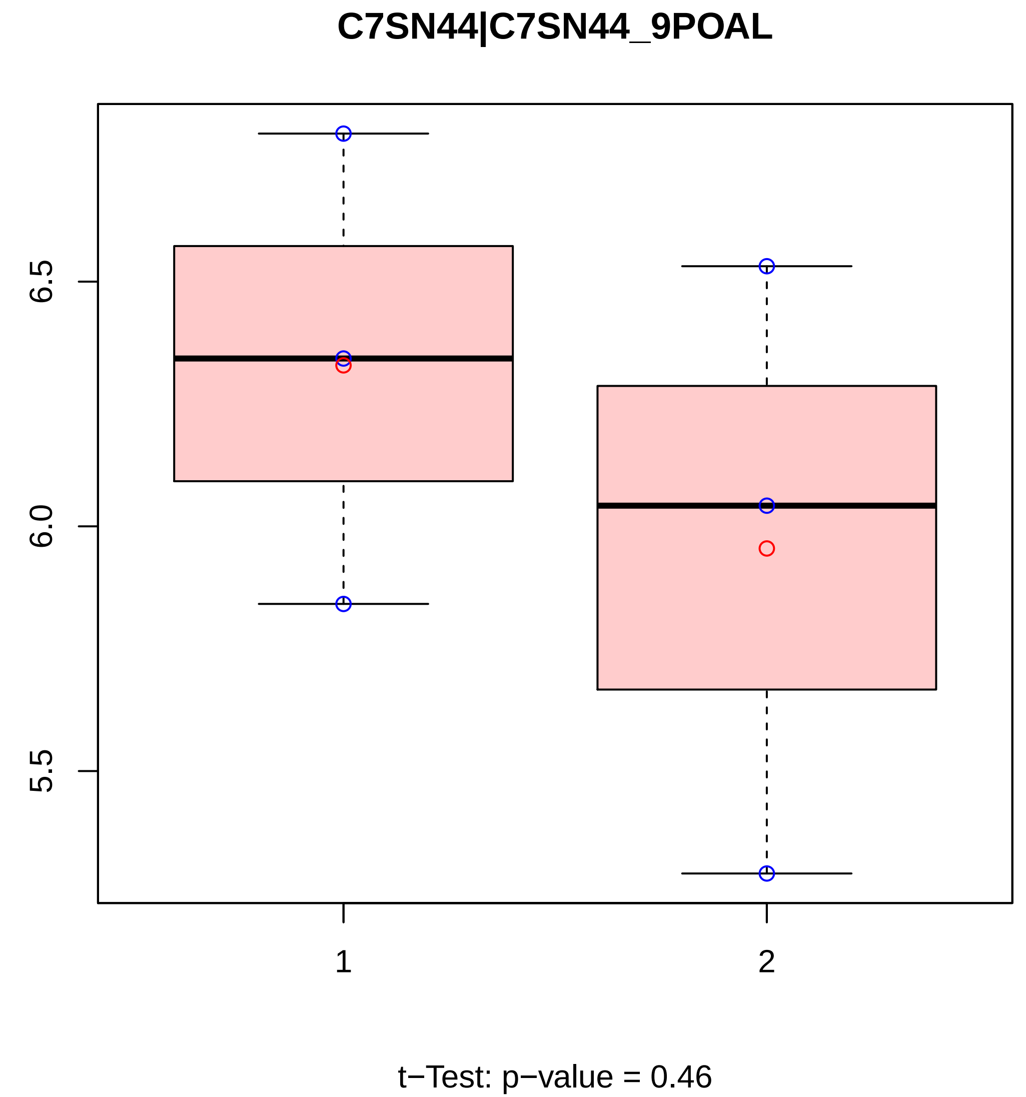
C7SN44|C7SN44_9POAL
6.5
6.0
5.5
1
2
t−Test: p−value = 0.46

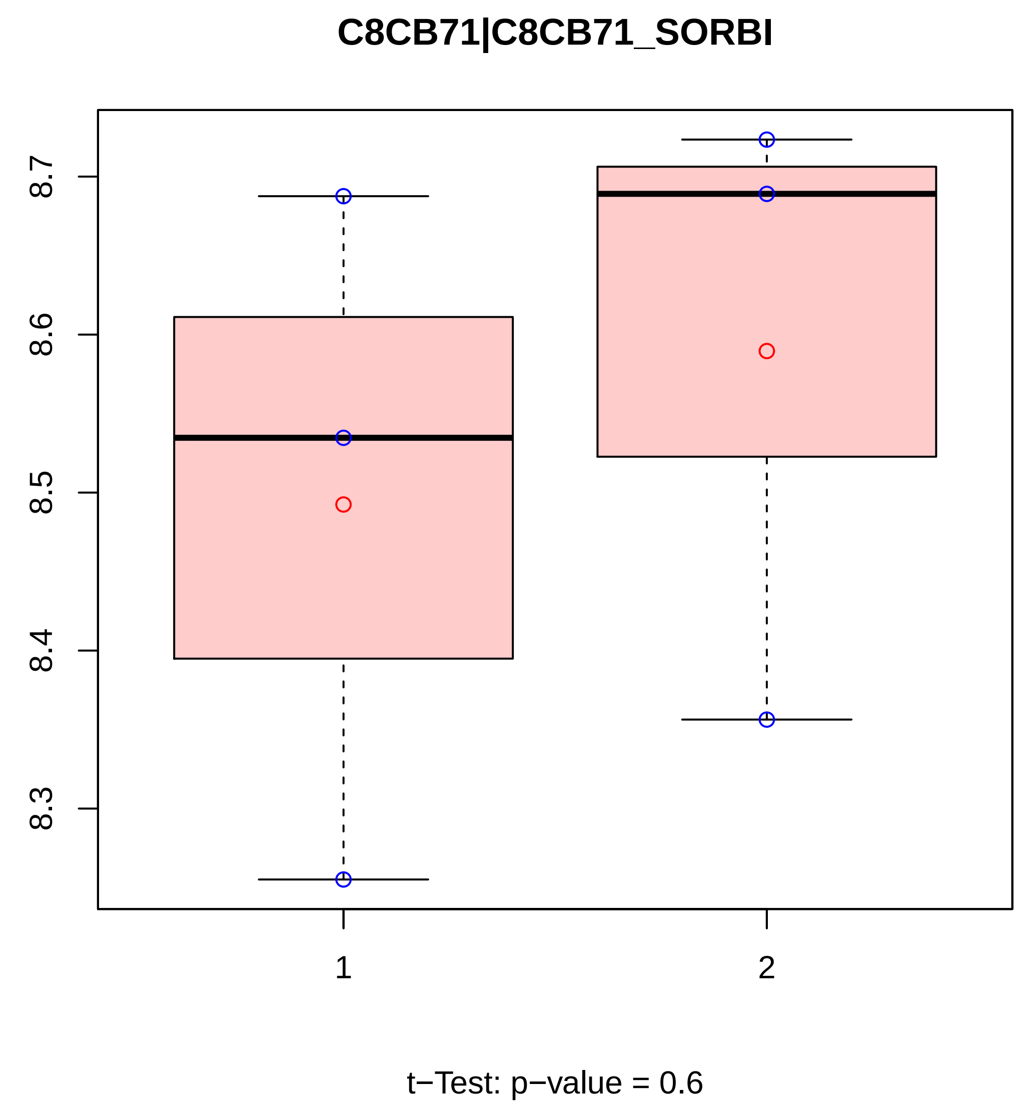
C8CB71|C8CB71_SORBI
8.3
8.4
8.5
8.6
8.7
1
2
t−Test: p−value = 0.6

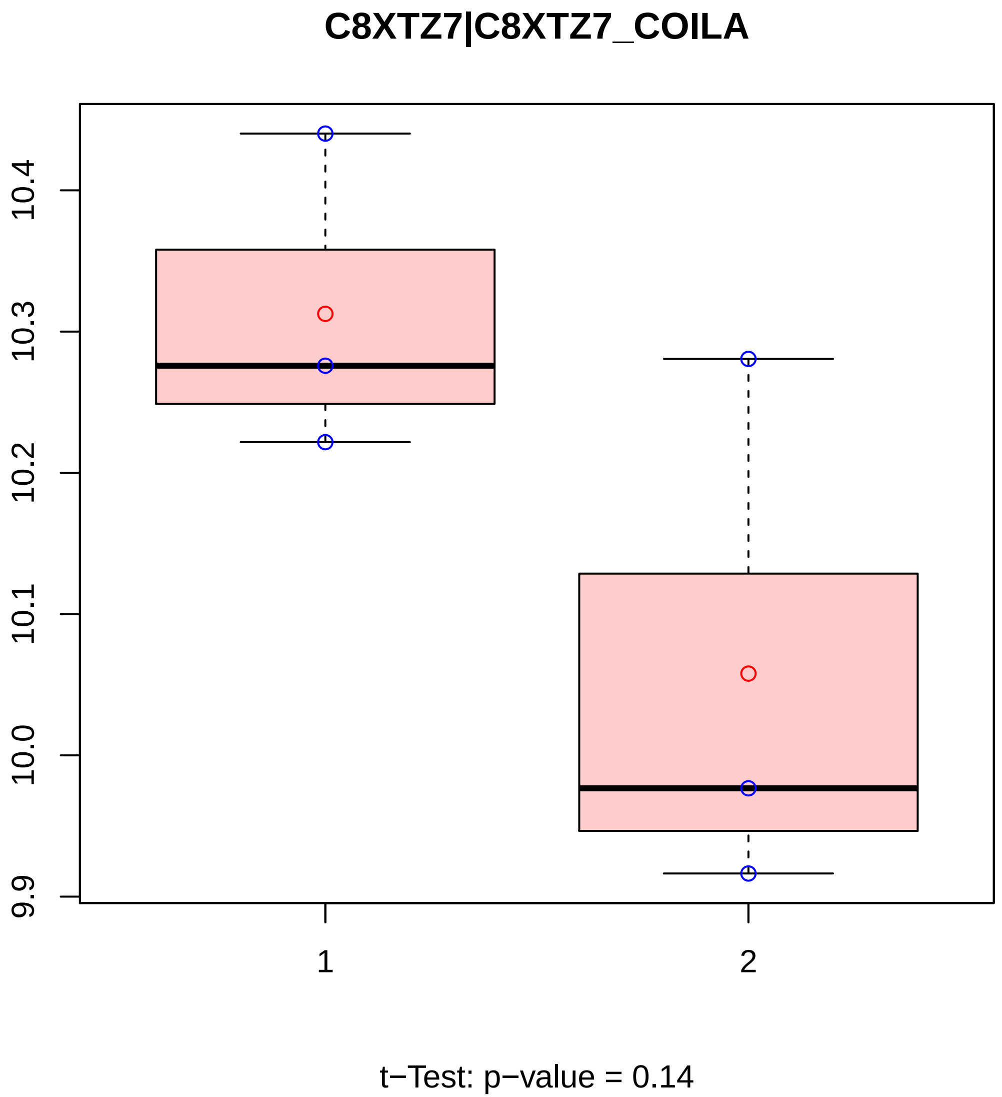

C8XTZ7|C8XTZ7_COILA
10.4
10.3
10.2
10.1
10.0
9.9
1
2
t−Test: p−value = 0.14

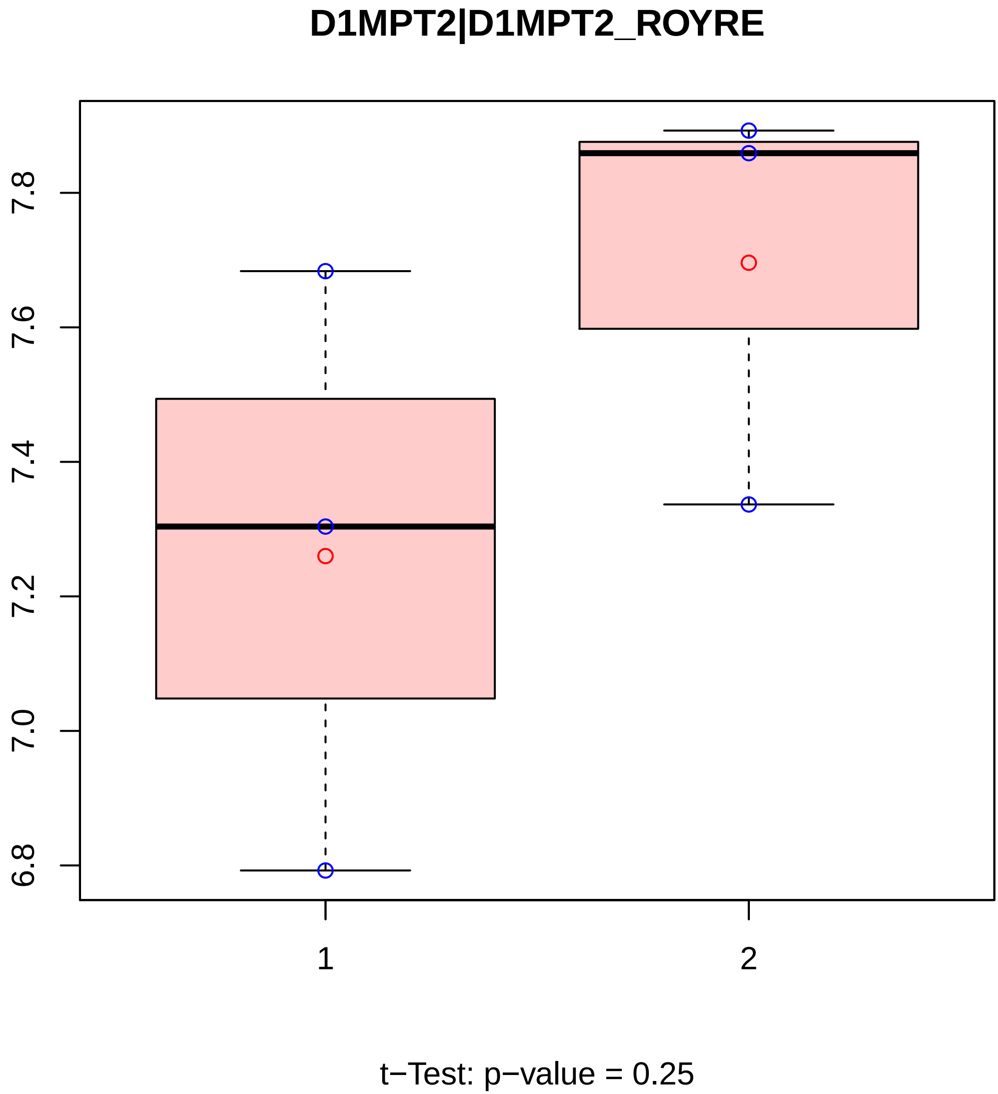
D1MPT2|D1MPT2_ROYRE
7.8
7.6
7.4
7.2
7.0
6.8
1
2
t−Test: p−value = 0.25

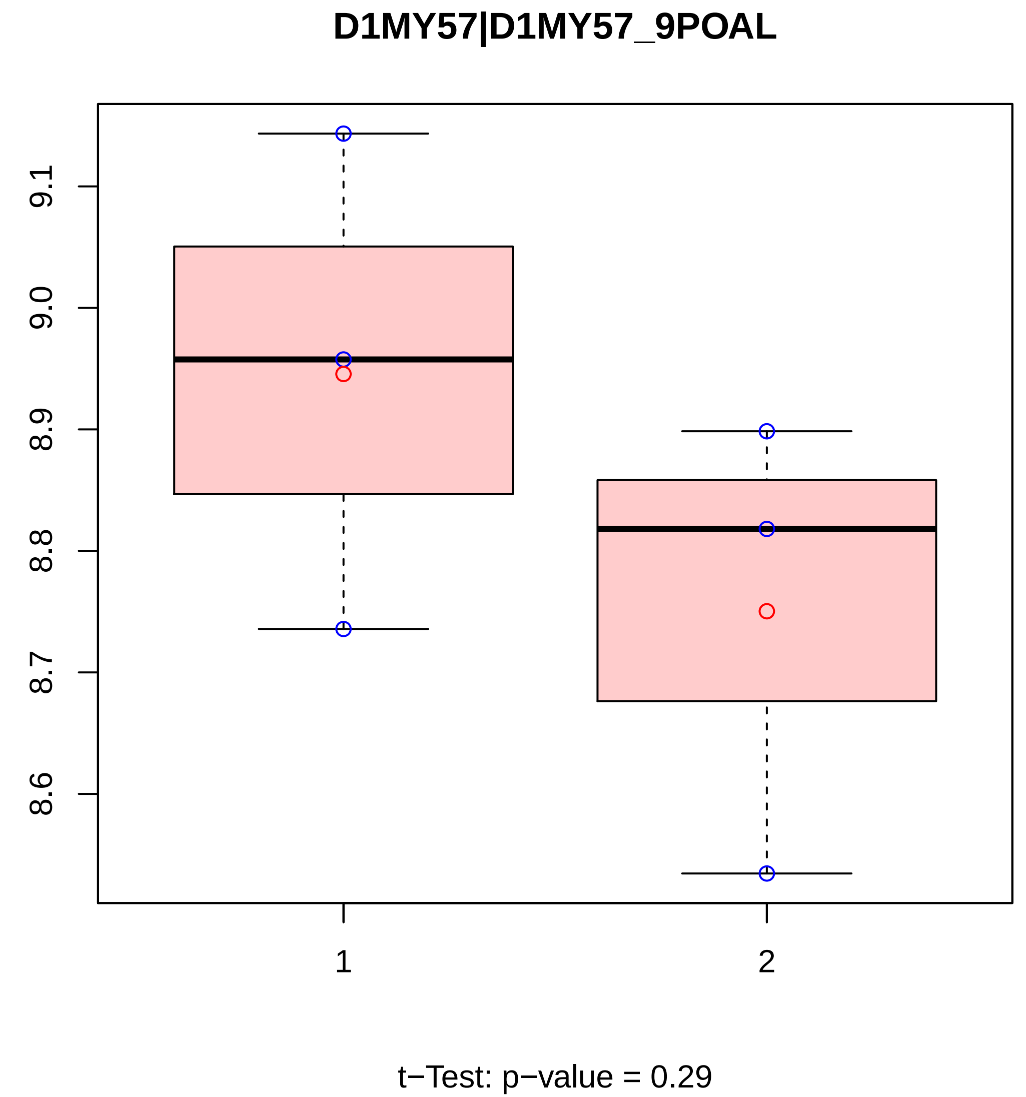

D1MY57|D1MY57_9POAL
9.1
9.0
8.9
8.8
8.7
8.6
1
2
t−Test: p−value = 0.29

D2IX41|D2IX41_PANVG

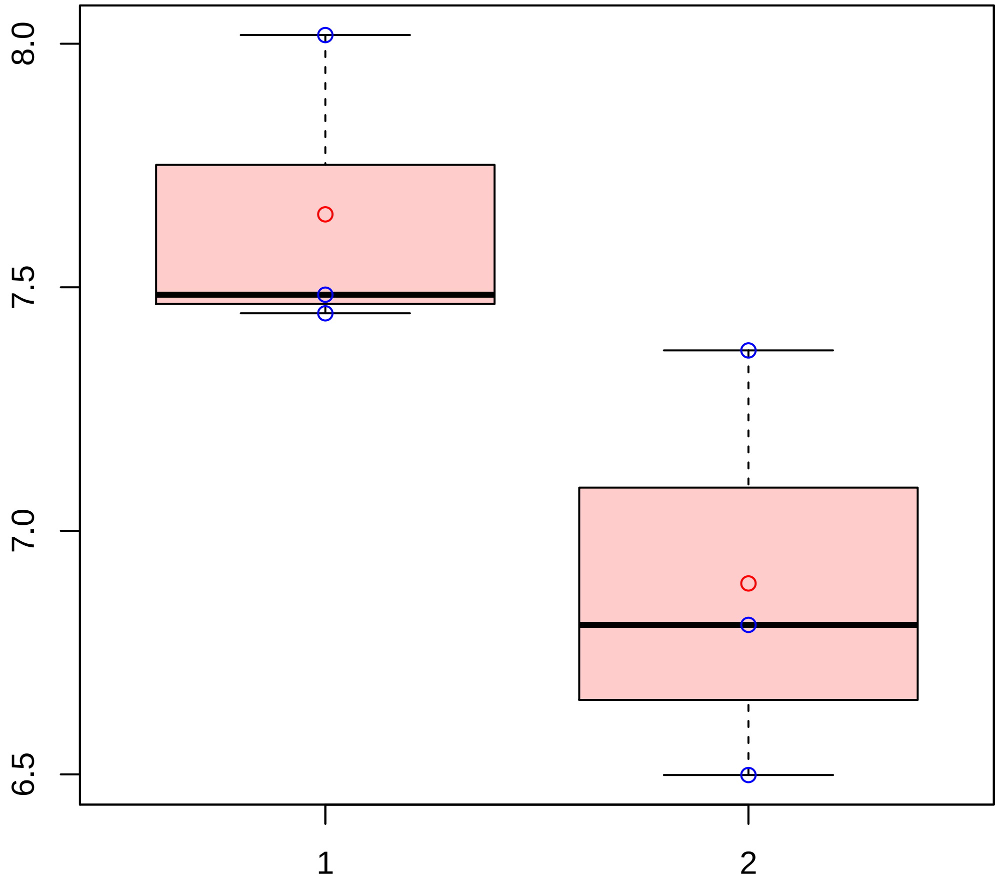

t−Test: p−value = 0.08

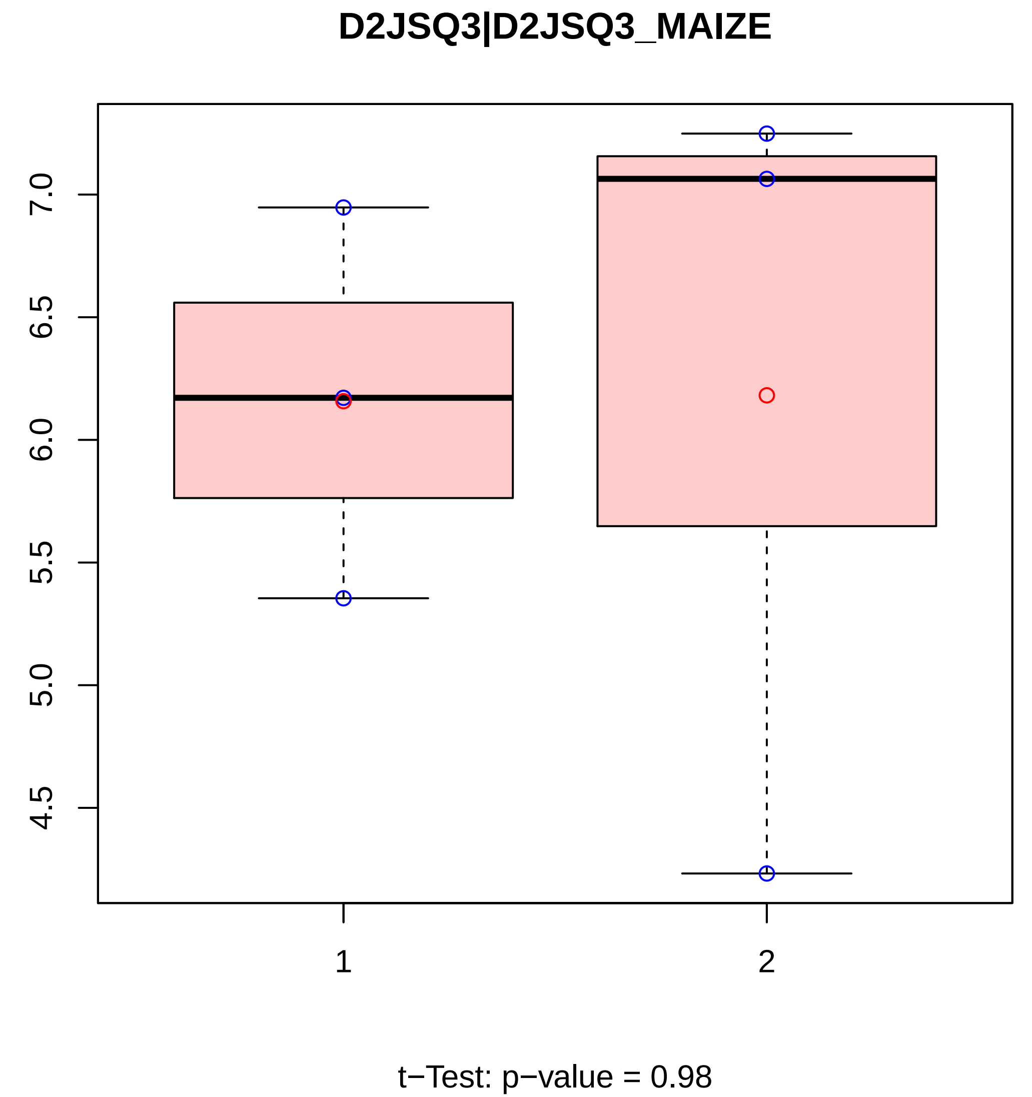
D2JSQ3|D2JSQ3_MAIZE
7.0
6.5
6.0
5.5
5.0
4.5
1
2
t−Test: p−value = 0.98

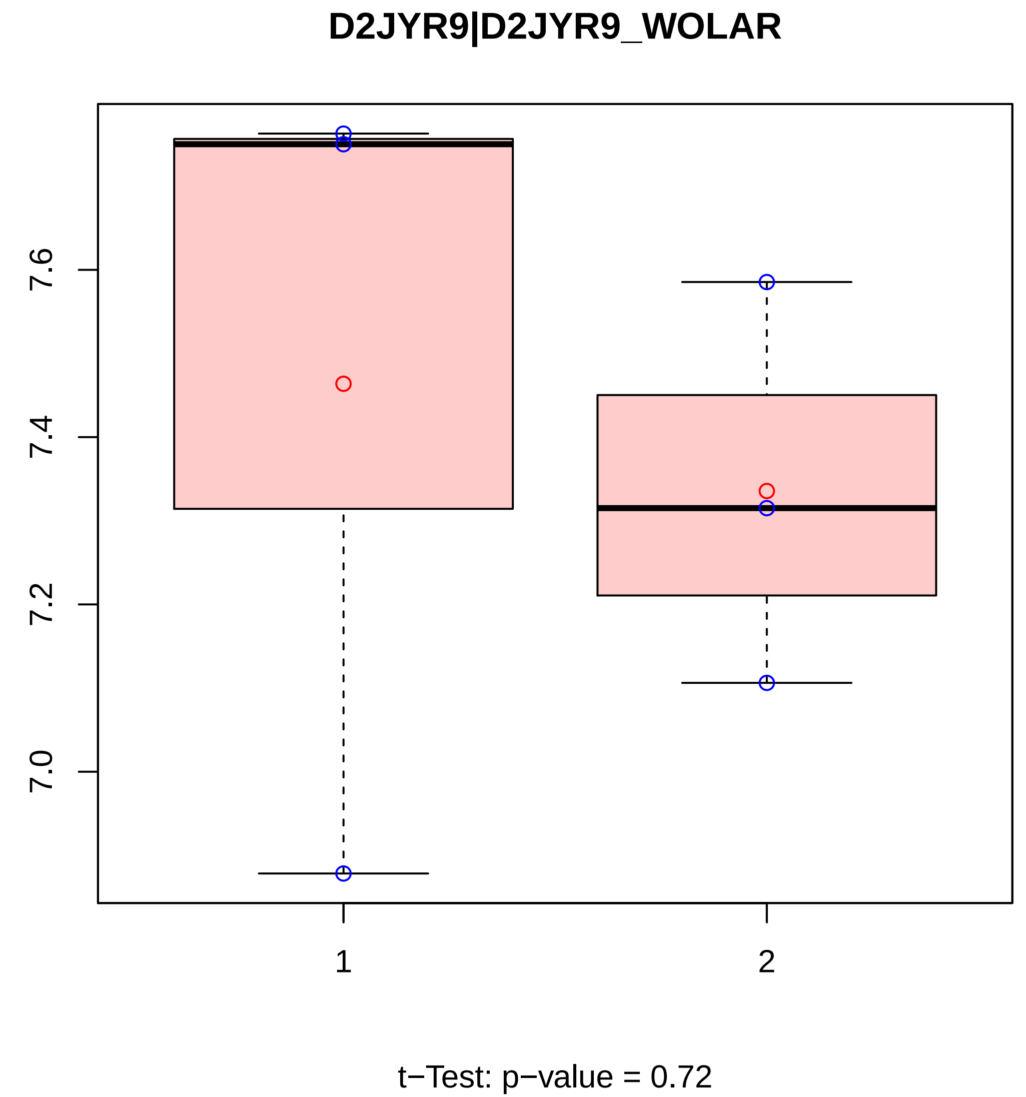
D2JYR9|D2JYR9_WOLAR
7.0
7.2
7.4
7.6
1
2
t−Test: p−value = 0.72

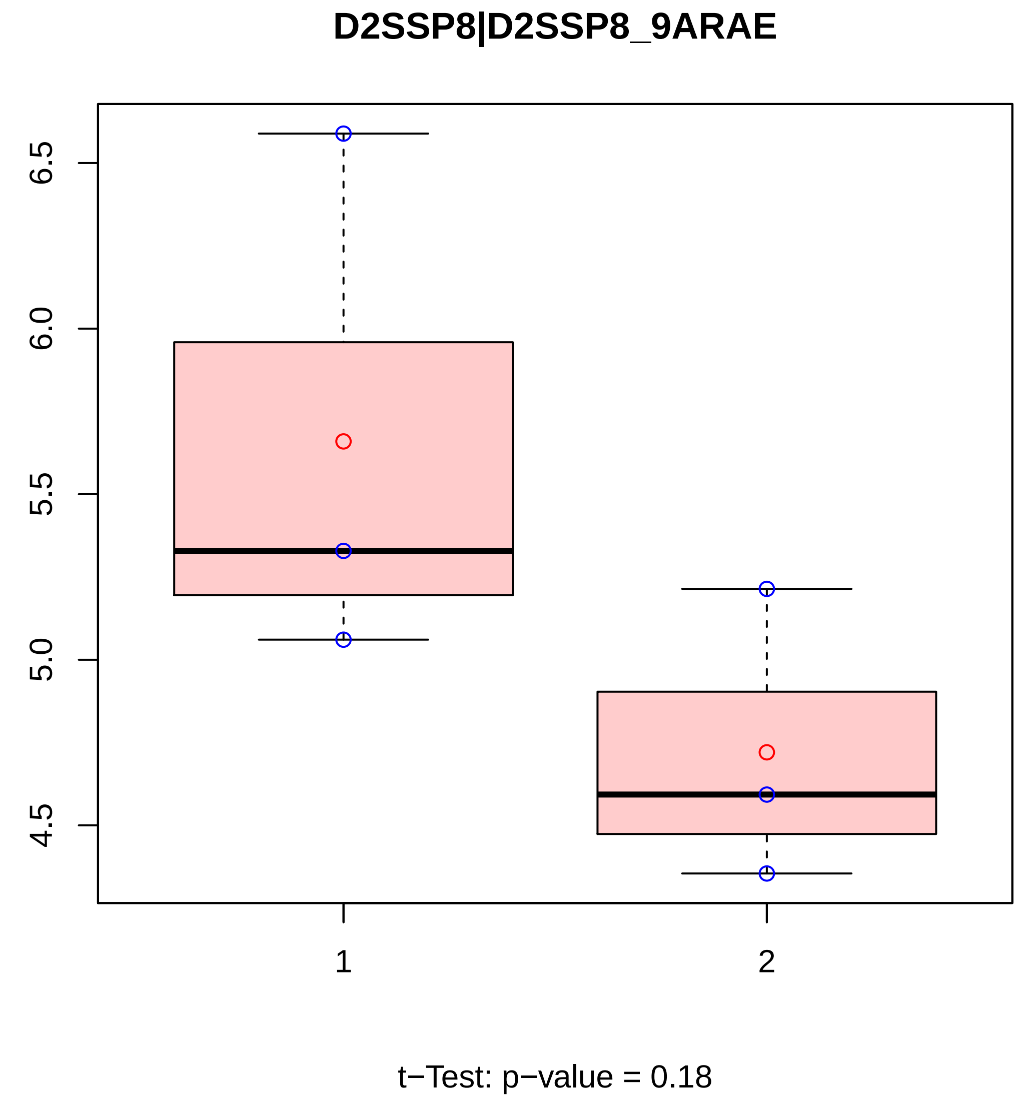
D2SSP8|D2SSP8_9ARAE
6.5
6.0
5.5
5.0
4.5
1
2
t−Test: p−value = 0.18

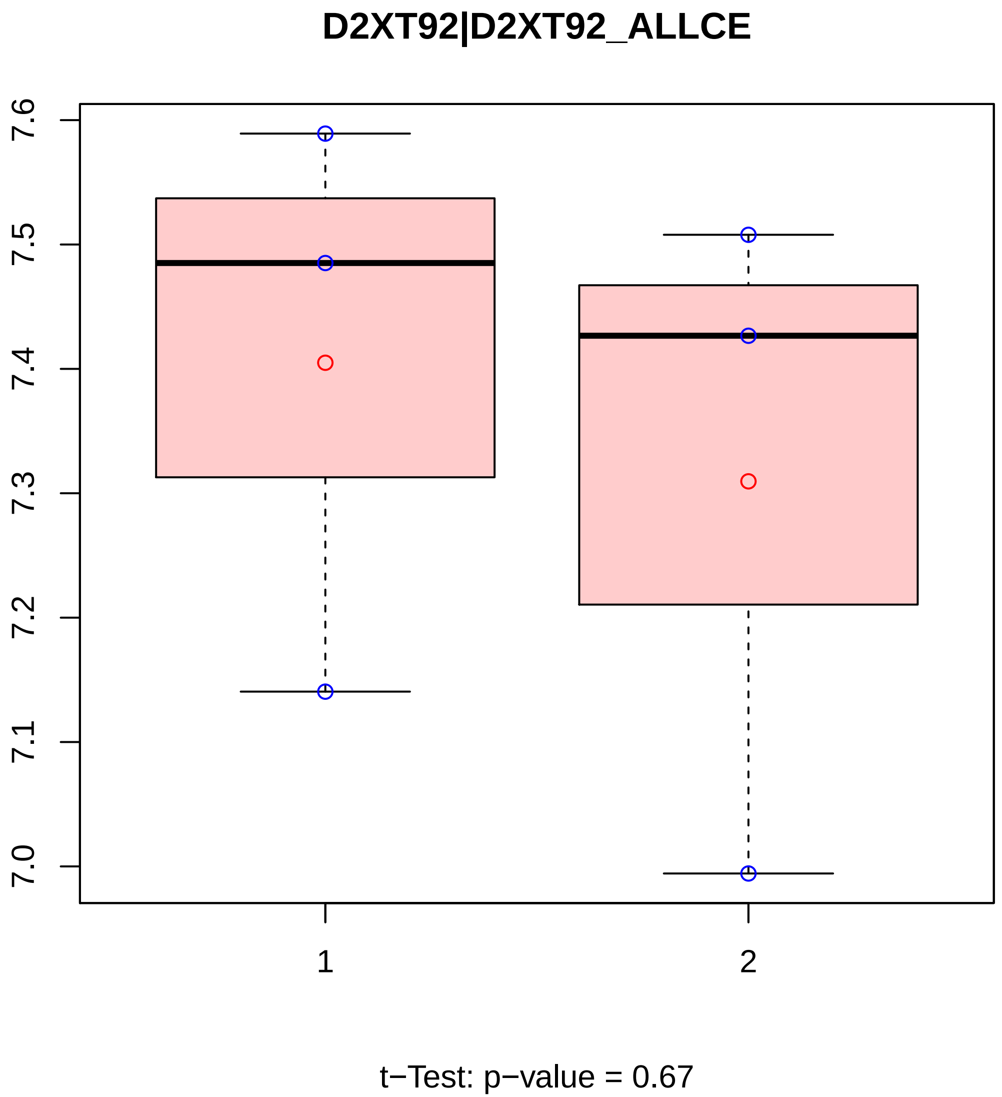
D2XT92|D2XT92_ALLCE
7.0
7.1
7.2
7.3
7.4
7.5
7.6
1
2
t−Test: p−value = 0.67

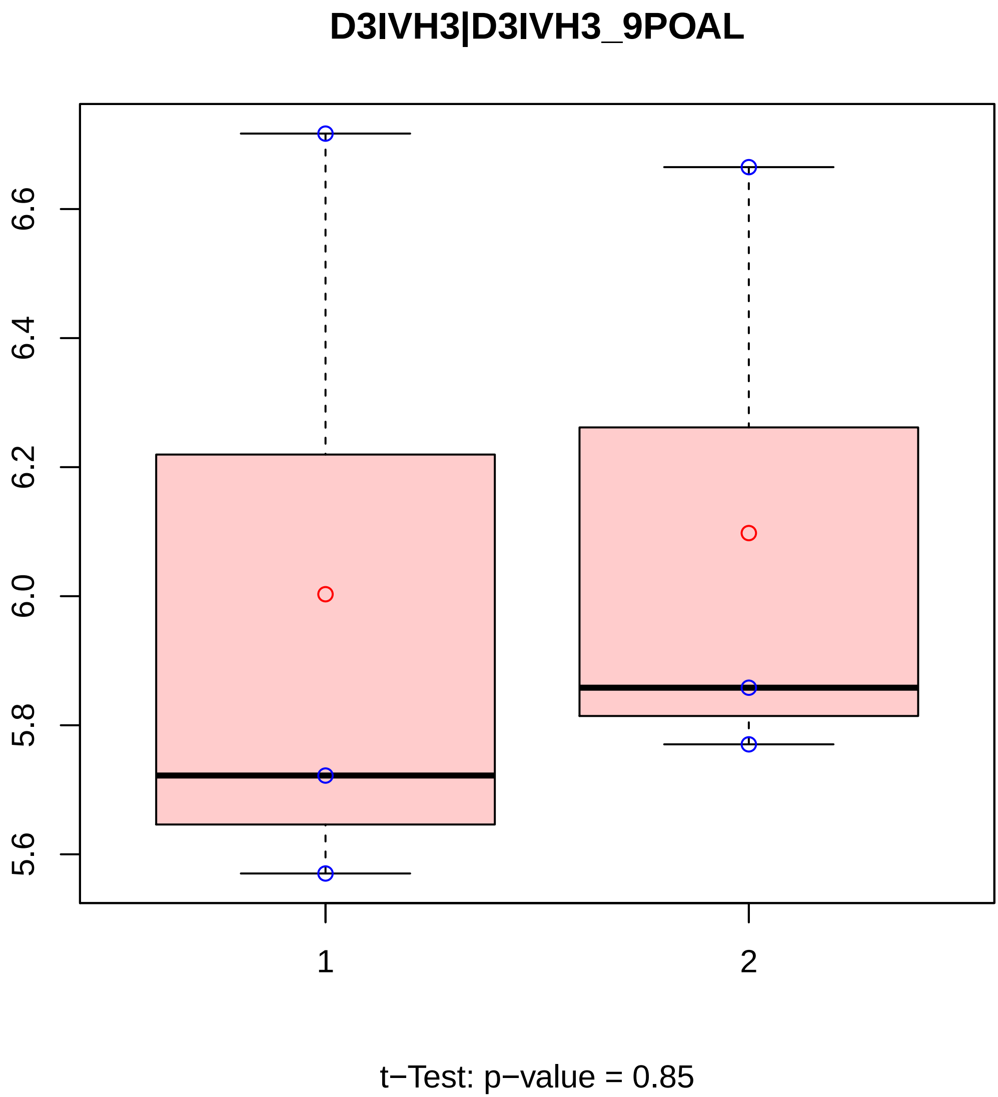
D3IVH3|D3IVH3_9POAL
6.6
6.4
6.2
6.0
5.8
5.6
1
2
t−Test: p−value = 0.85

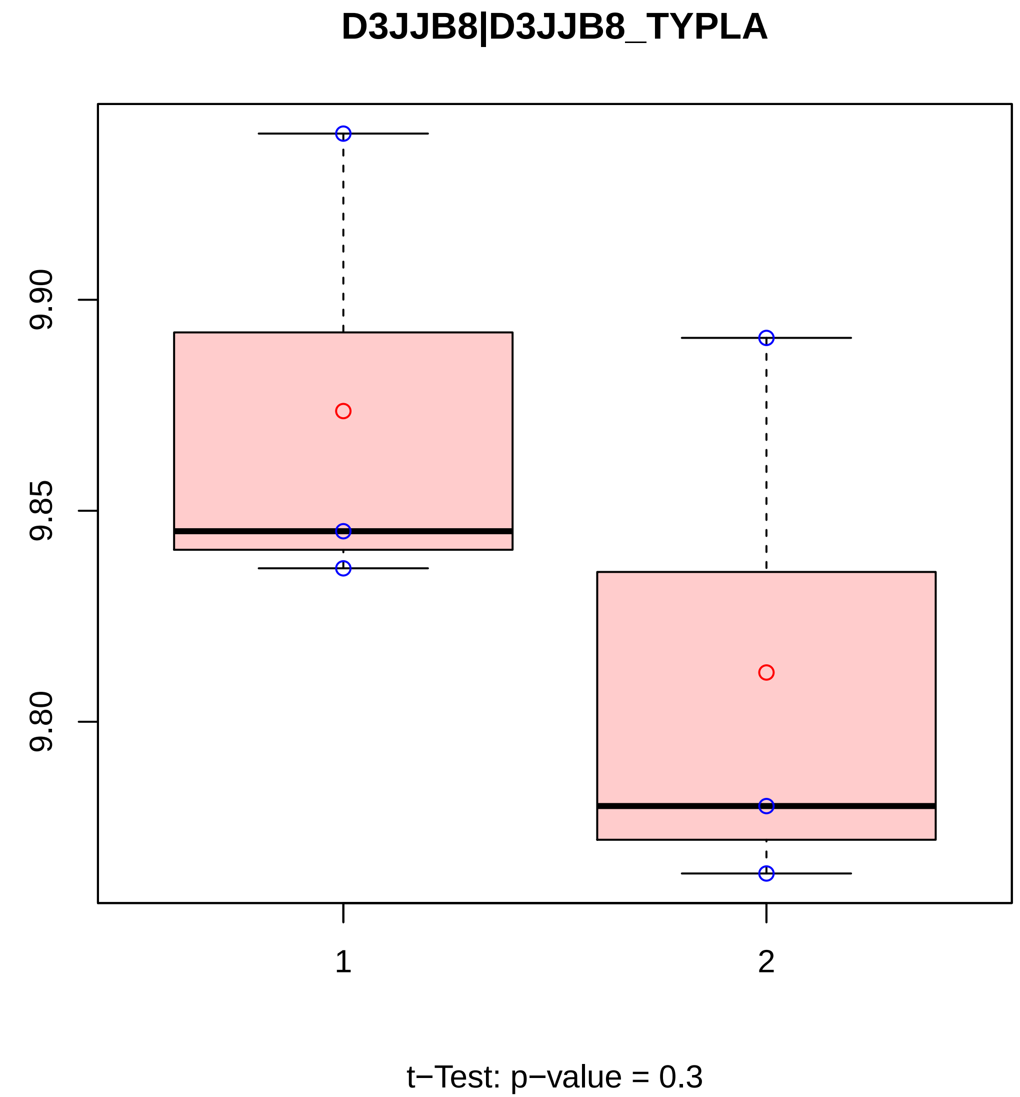
D3JJB8|D3JJB8_TYPLA
9.90
9.85
9.80
1
2
t−Test: p−value = 0.3

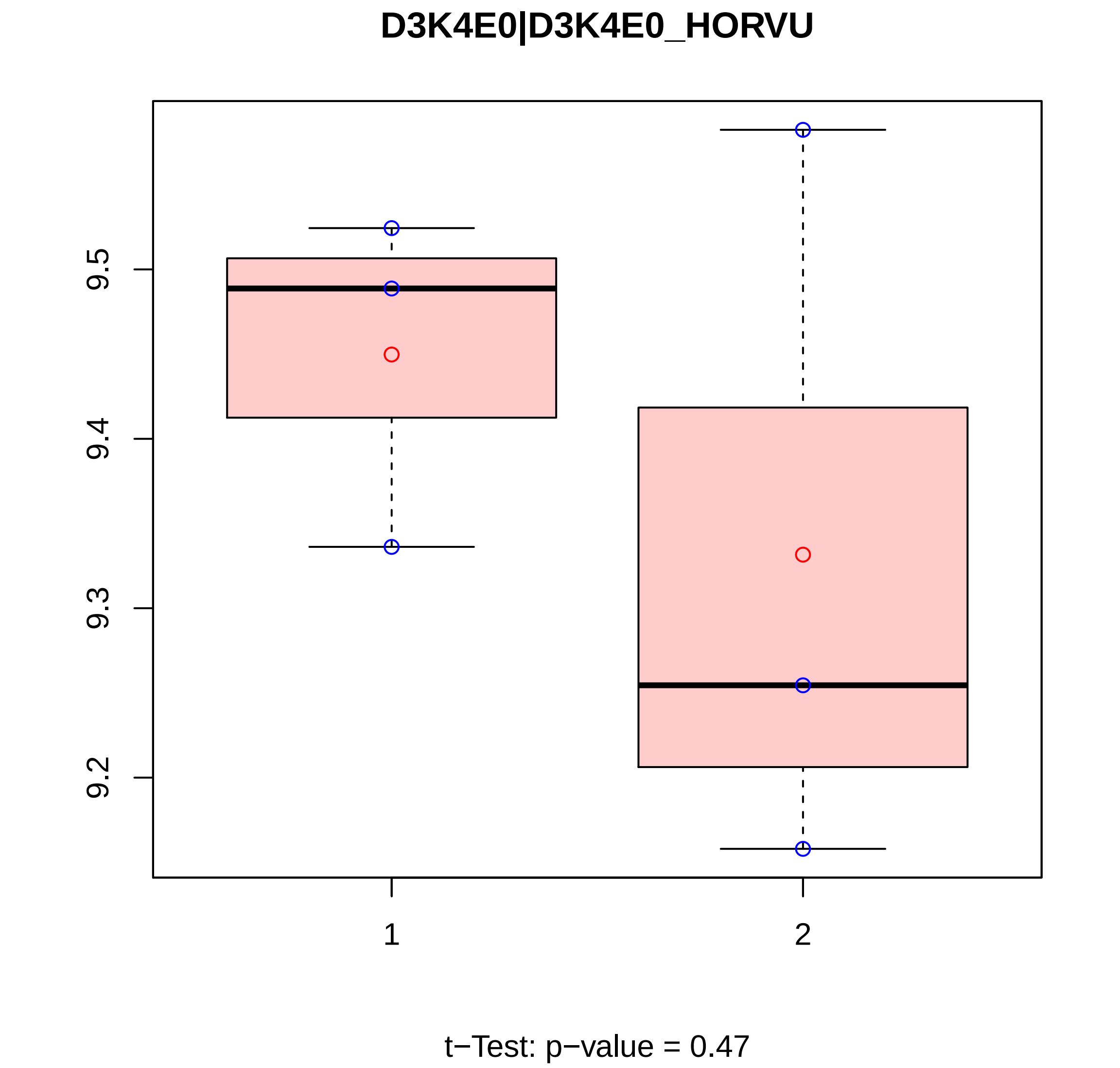
D3K4E0|D3K4E0_HORVU
9.2
9.3
9.4
9.5
1
2
t−Test: p−value = 0.47

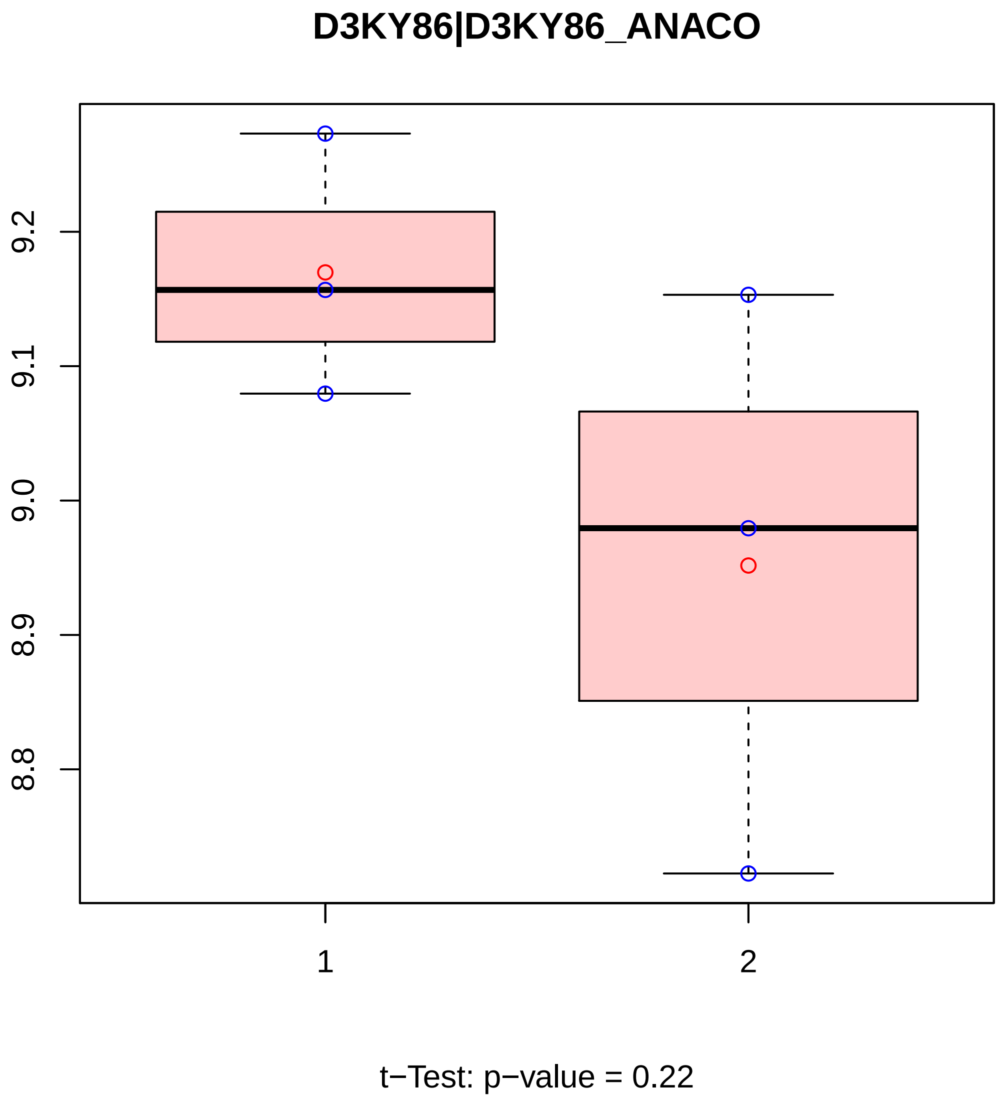
D3KY86|D3KY86_ANACO
9.2
9.1
9.0
8.9
8.8
1
2
t−Test: p−value = 0.22

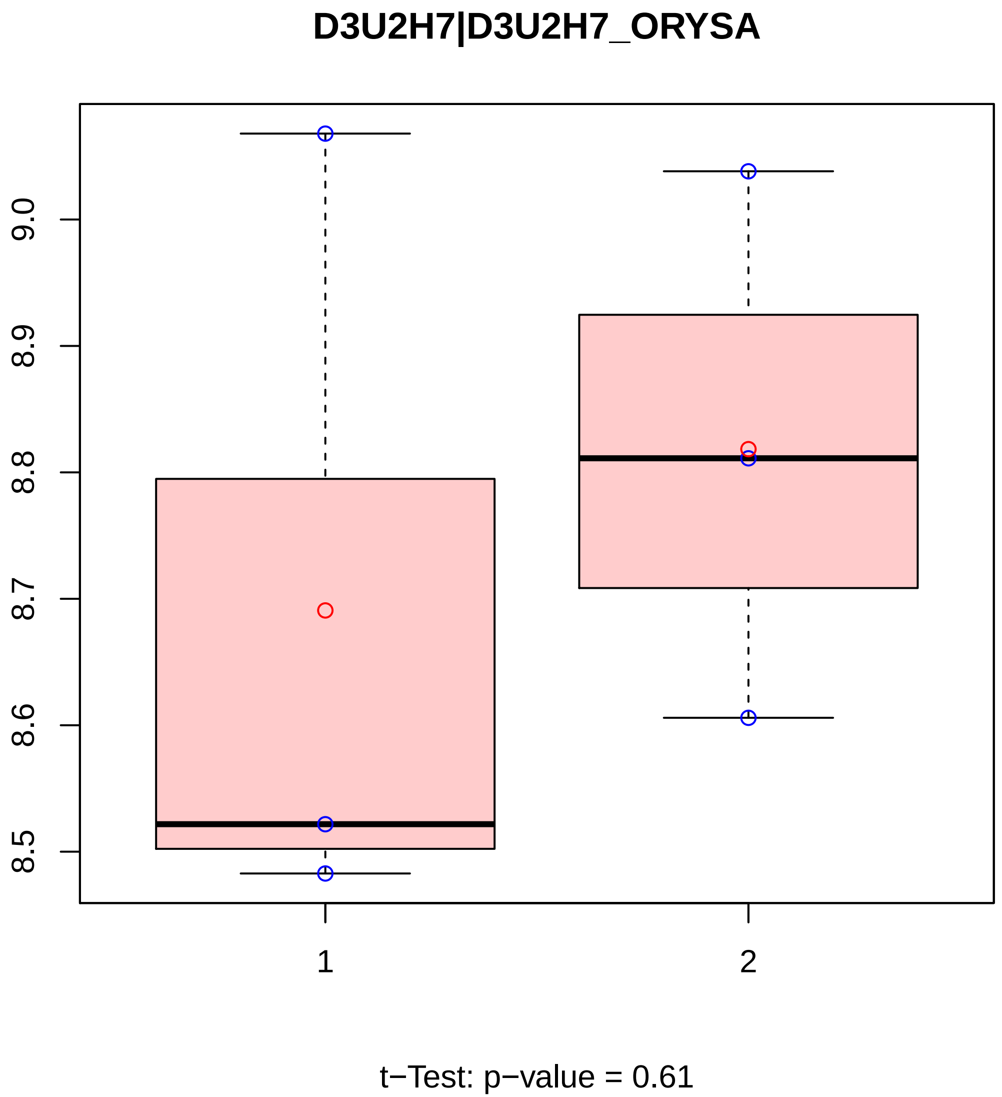
D3U2H7|D3U2H7_ORYSA
9.0
8.9
8.8
8.7
8.6
8.5
1
2
t−Test: p−value = 0.61

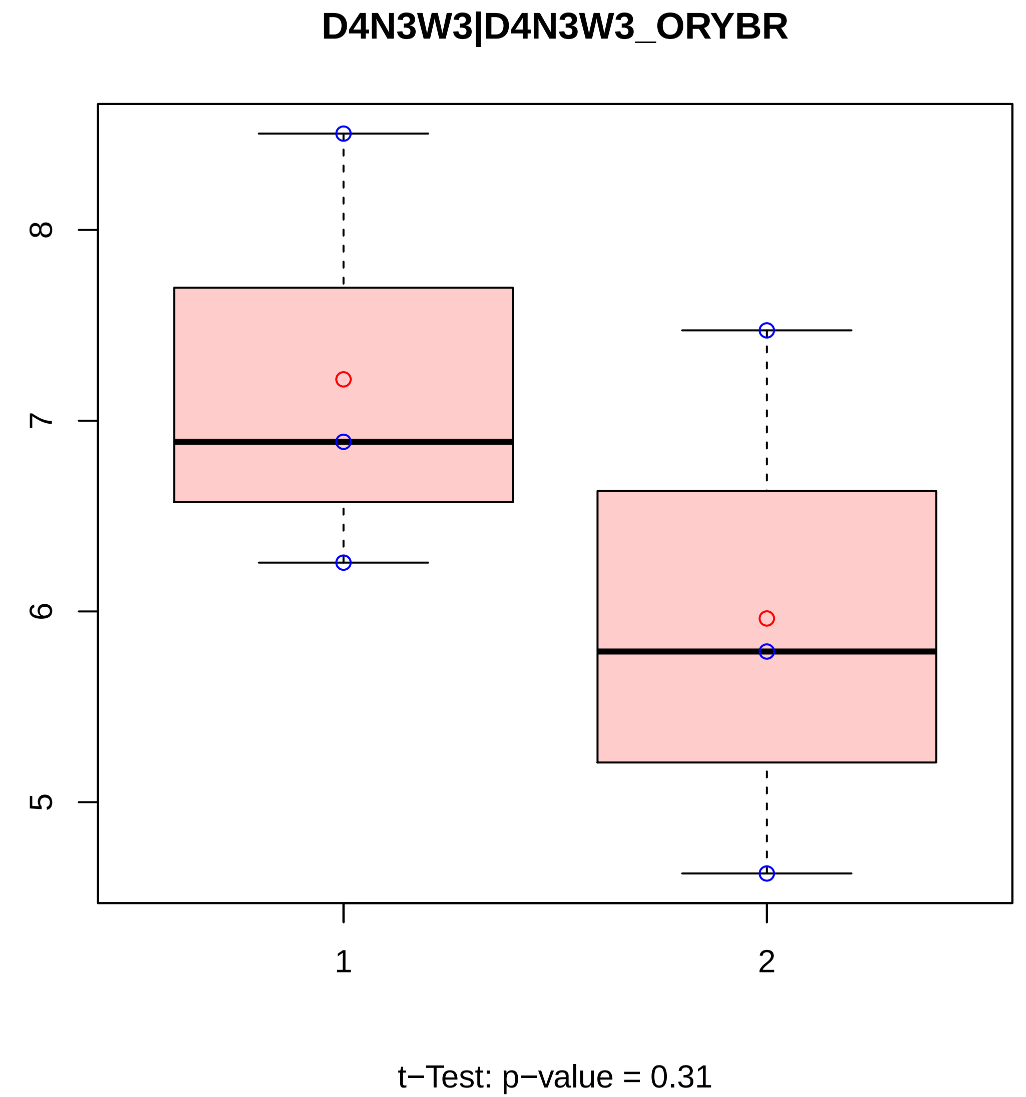

D4N3W3|D4N3W3_ORYBR
8
7
6
5
1
2
t−Test: p−value = 0.31

D5HQ36|D5HQ36_ONCHC

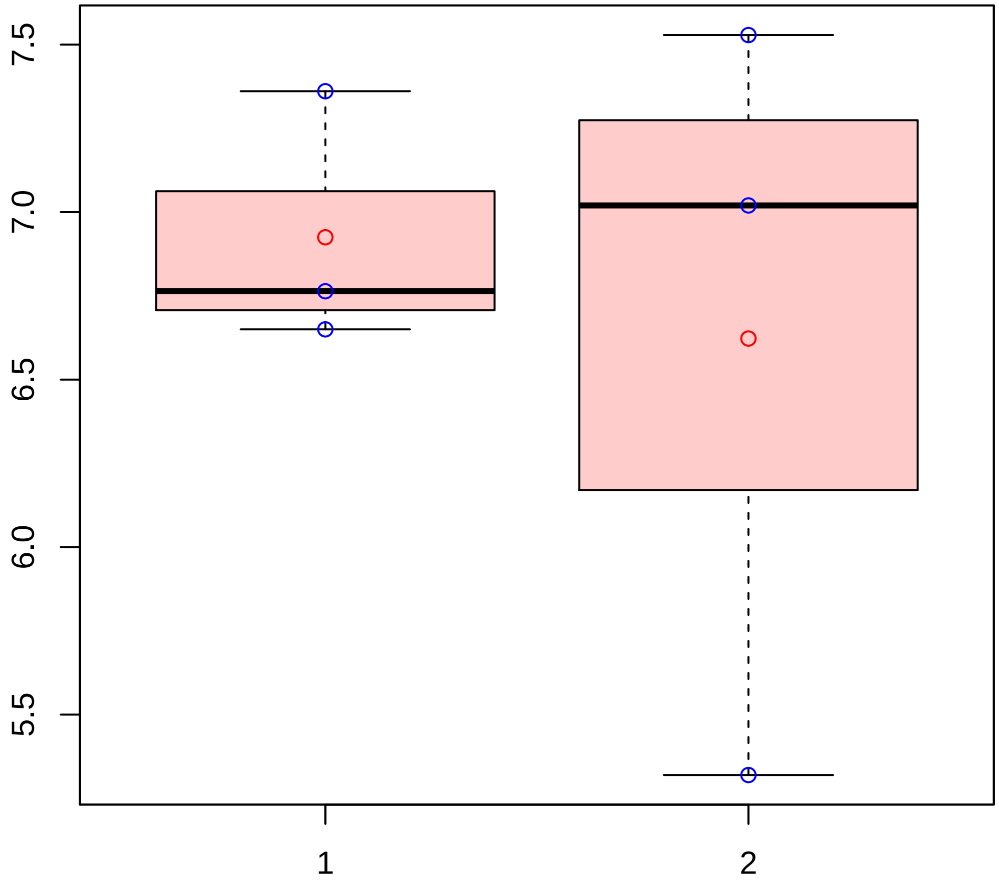

t−Test: p−value = 0.7

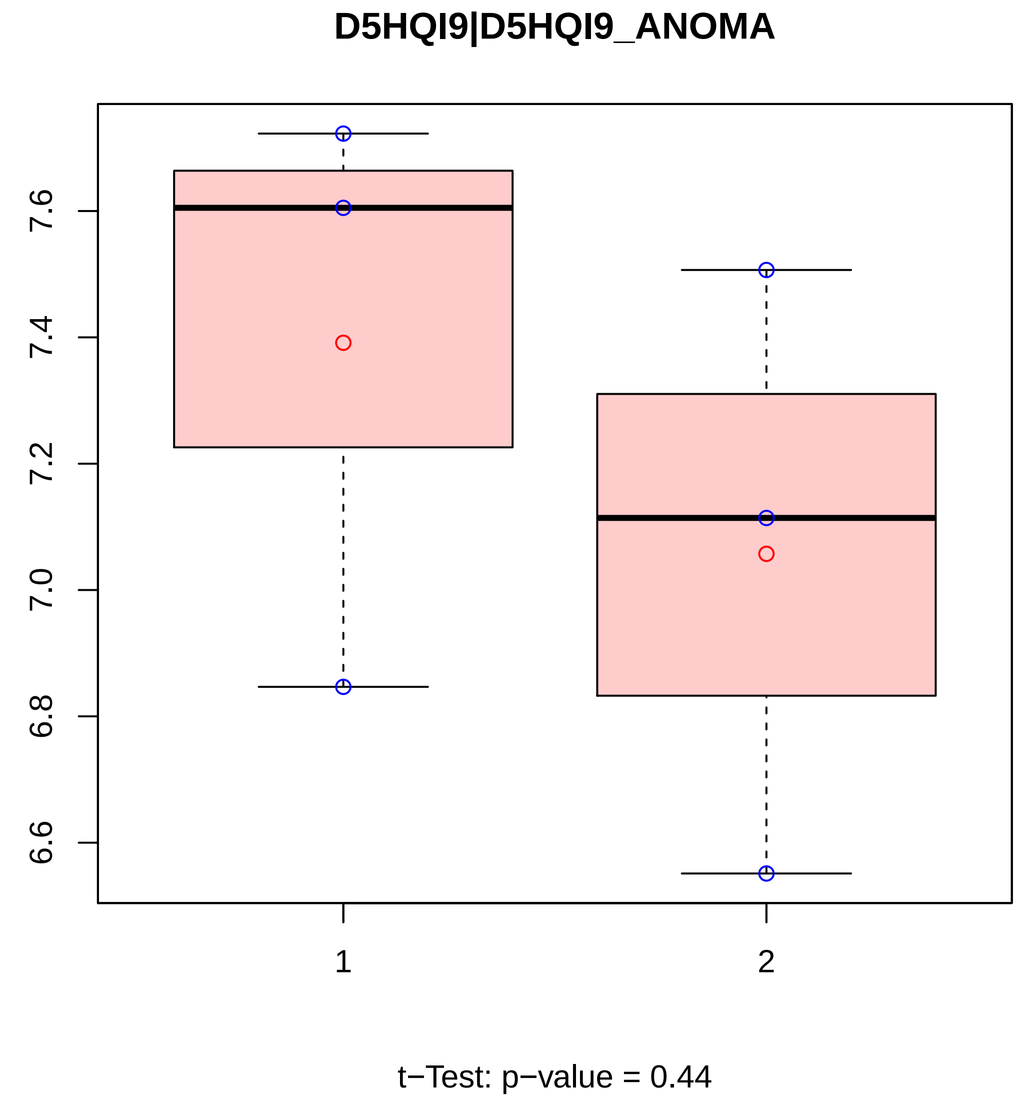
D5HQI9|D5HQI9_ANOMA
7.6
7.4
7.2
7.0
6.8
6.6
1
2
t−Test: p−value = 0.44

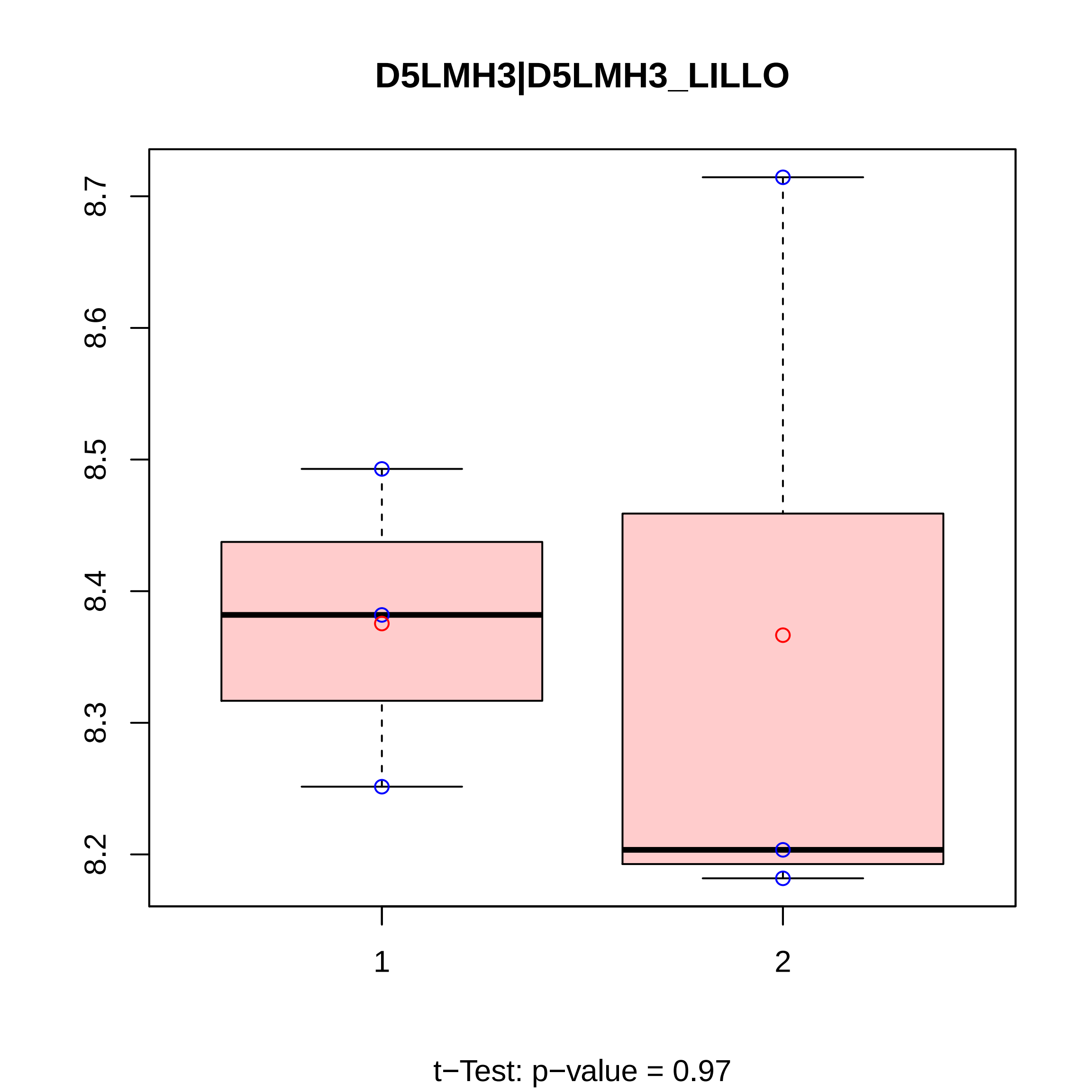

D5LMH3|D5LMH3_LILLO
8.2
8.3
8.4
8.5
8.6
8.7
1
2
t−Test: p−value = 0.97

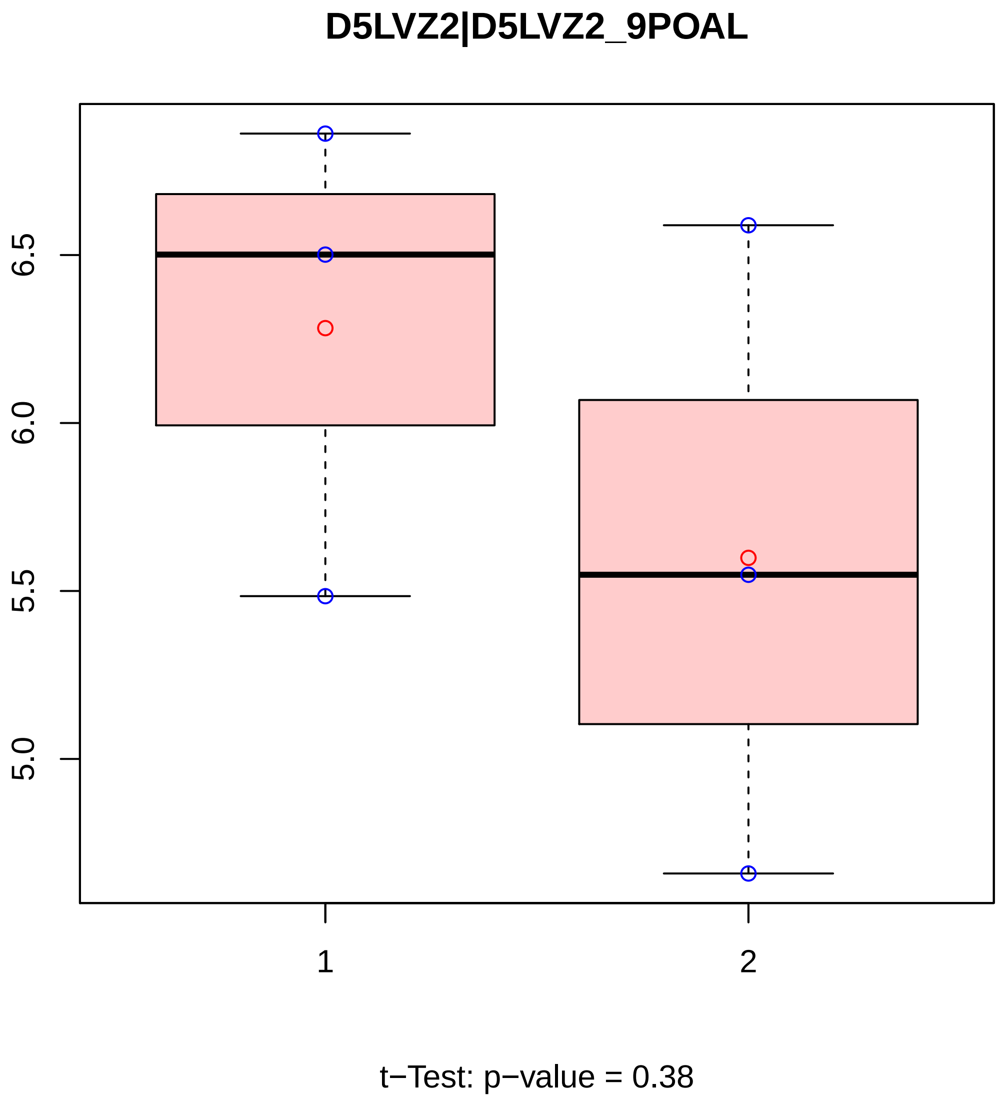
D5LVZ2|D5LVZ2_9POAL
6.5
6.0
5.5
5.0
1
2
t−Test: p−value = 0.38

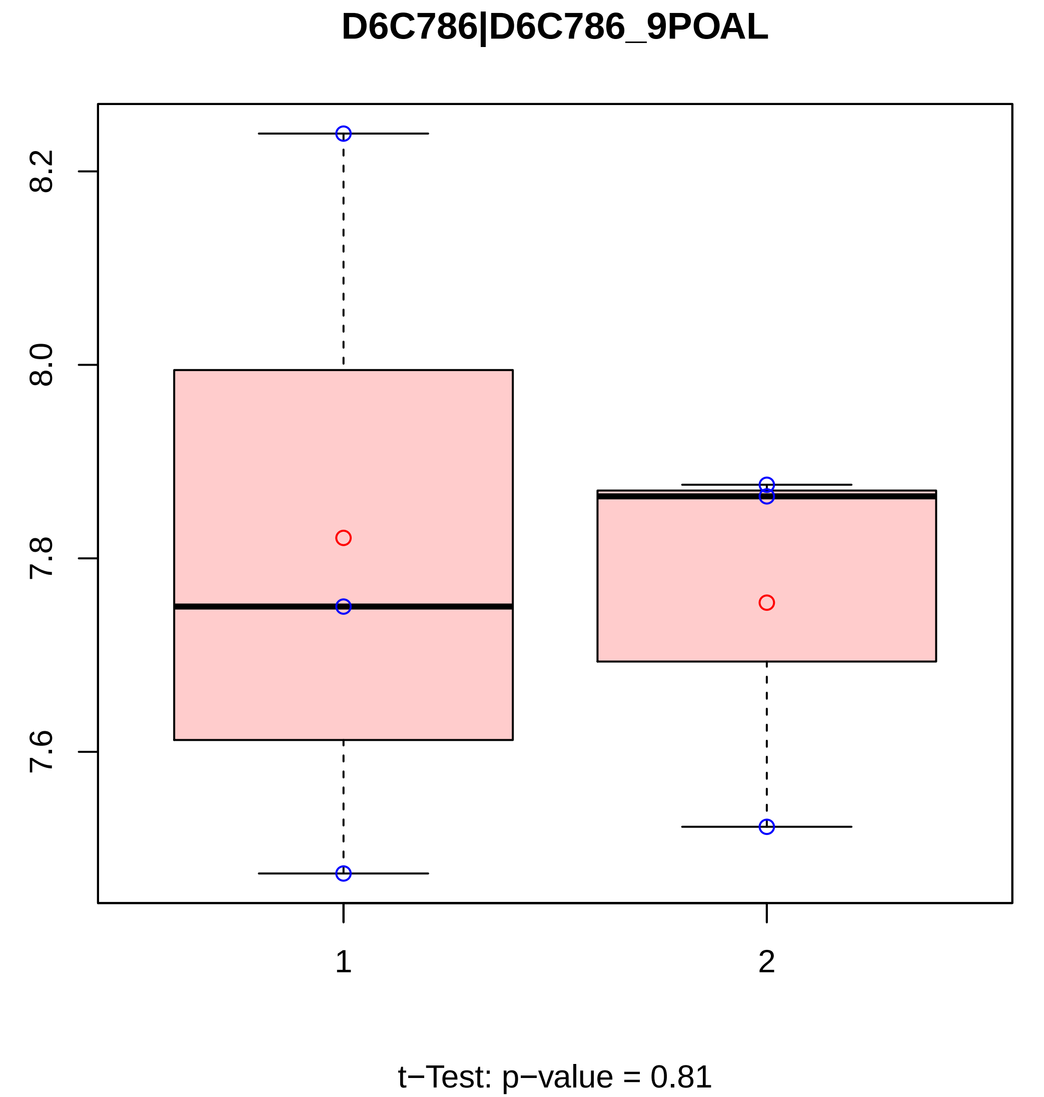
D6C786|D6C786_9POAL
8.2
8.0
7.8
7.6
1
2
t−Test: p−value = 0.81

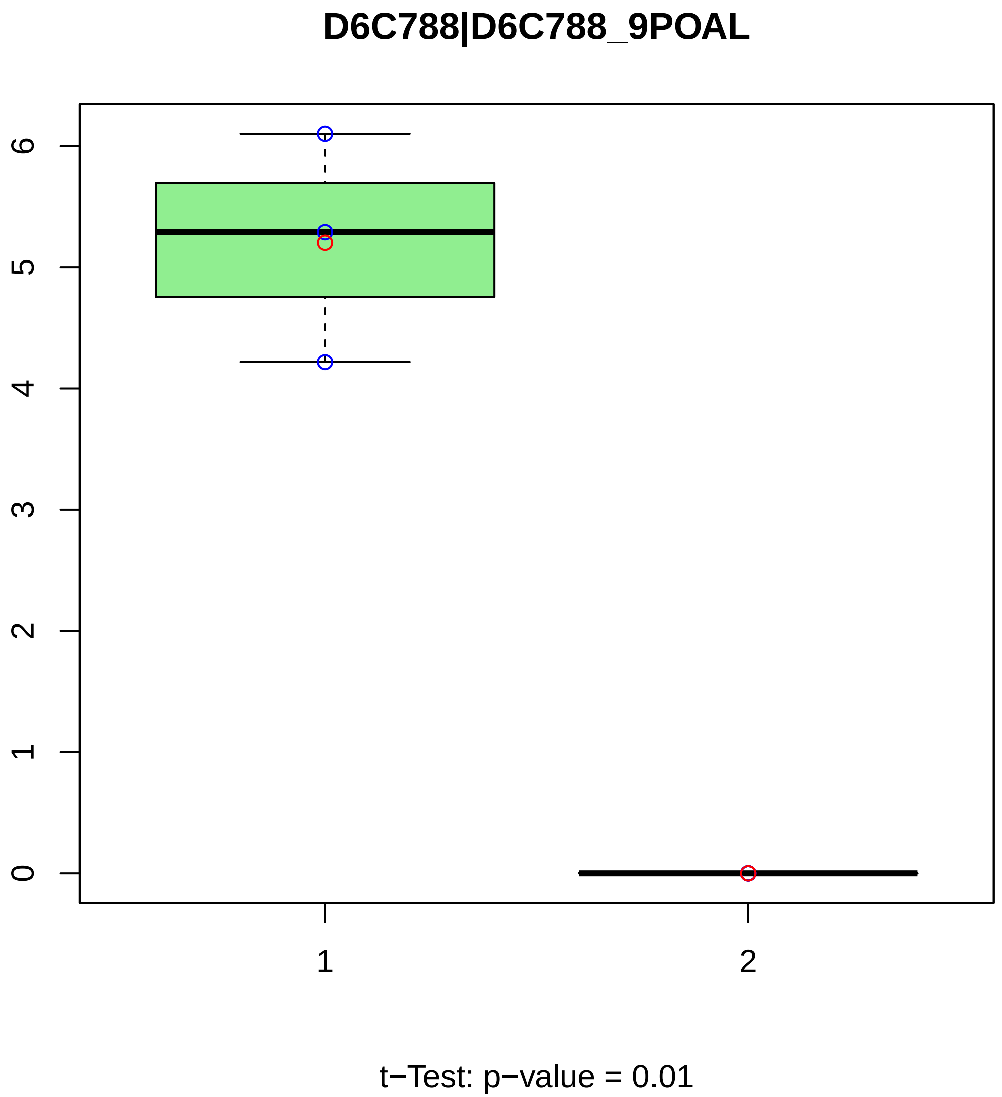

D6C788|D6C788_9POAL
0
1
2
3
4
5
6
1
2
t−Test: p−value = 0.01

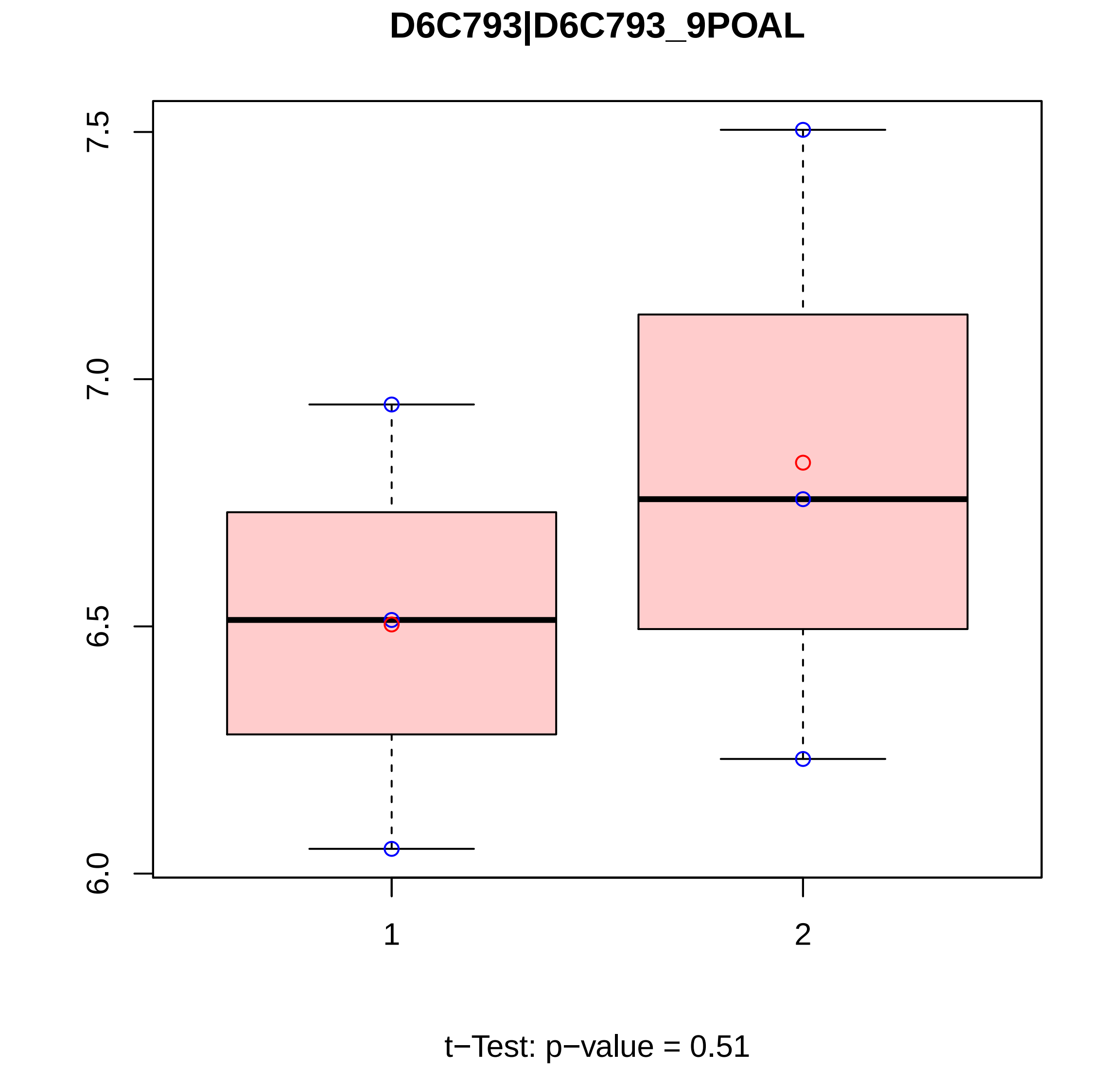
D6C793|D6C793_9POAL
6.0
6.5
7.0
7.5
1
2
t−Test: p−value = 0.51

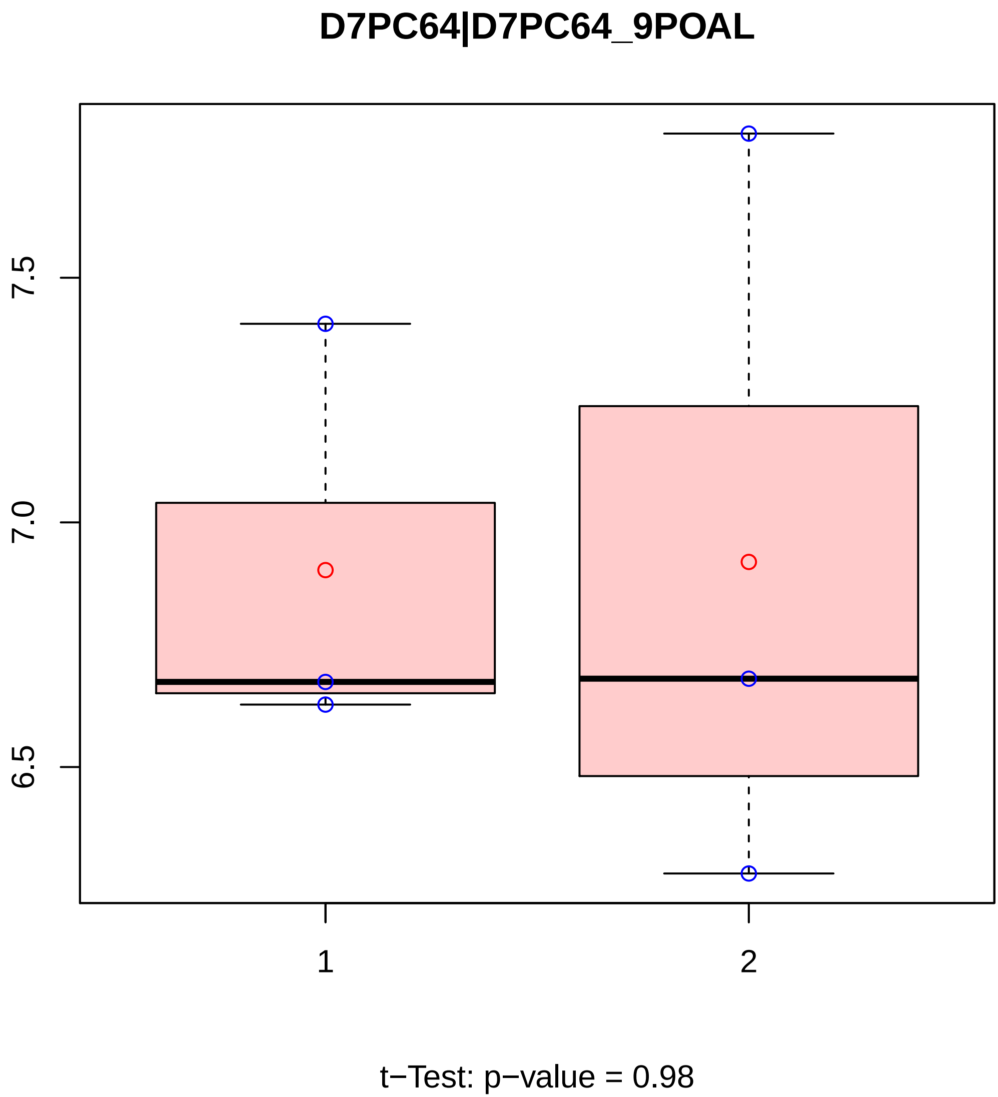
D7PC64|D7PC64_9POAL
7.5
7.0
6.5
1
2
t−Test: p−value = 0.98

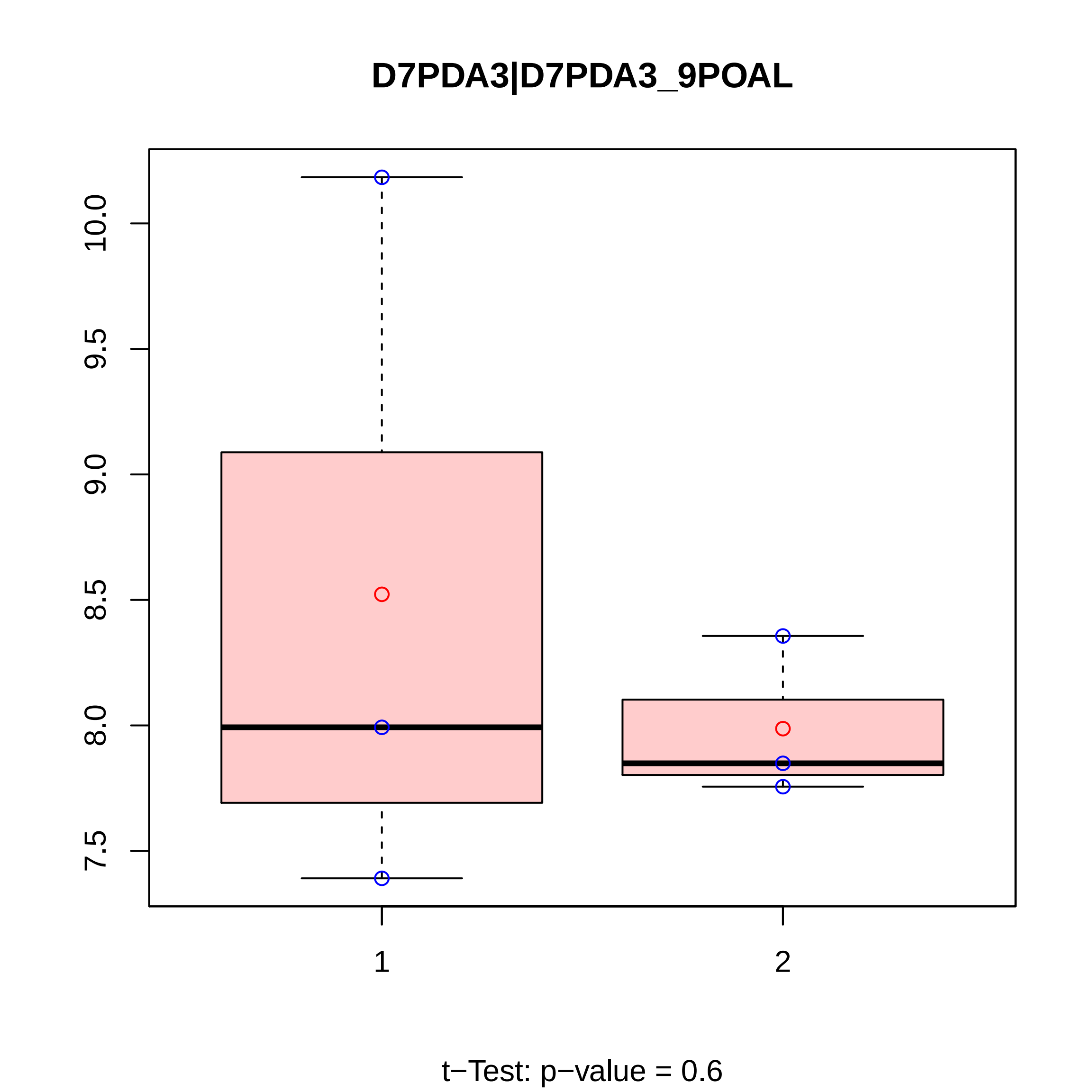

D7PDA3|D7PDA3_9POAL
10.0
9.5
9.0
8.5
8.0
7.5
1
2
t−Test: p−value = 0.6

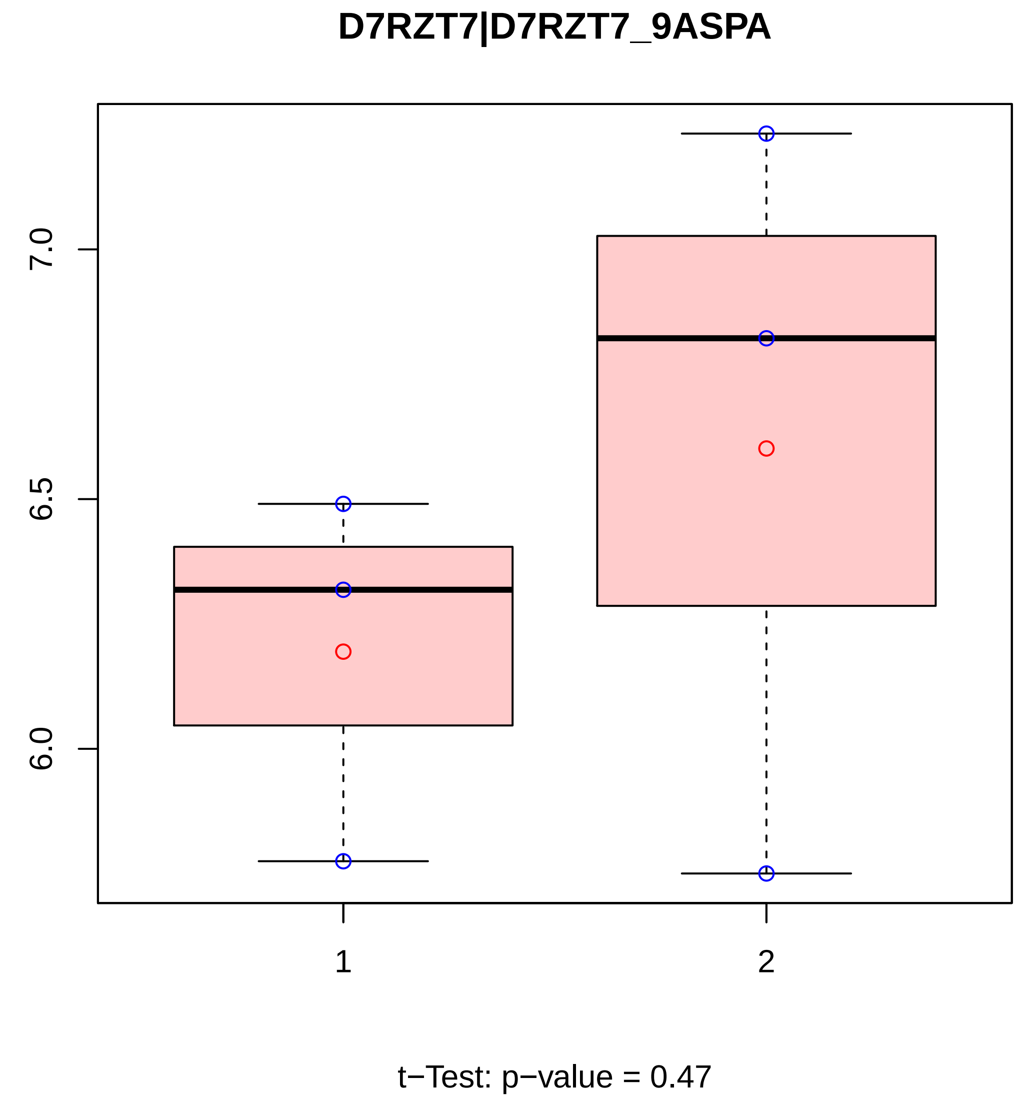
D7RZT7|D7RZT7_9ASPA
7.0
6.5
6.0
1
2
t−Test: p−value = 0.47

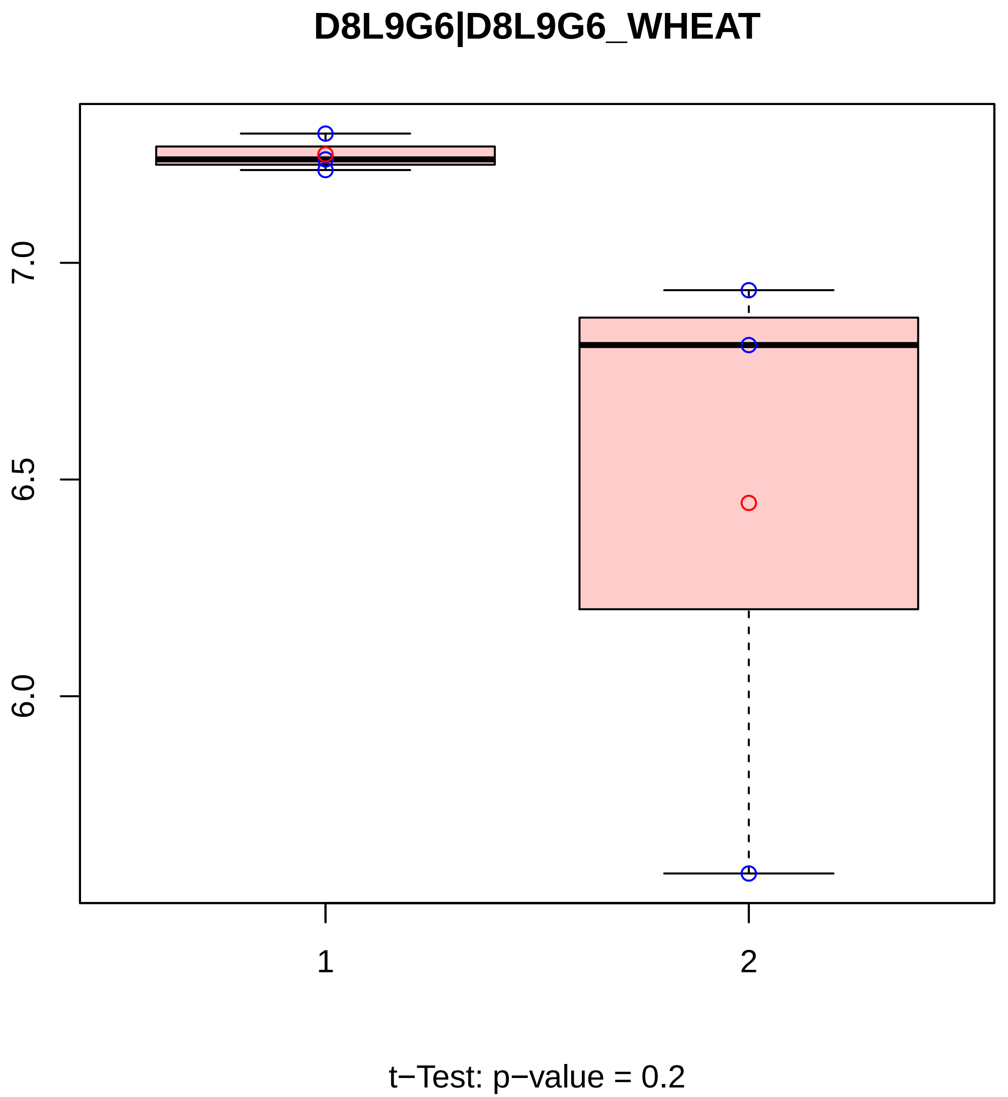
D8L9G6|D8L9G6_WHEAT
7.0
6.5
6.0
1
2
t−Test: p−value = 0.2

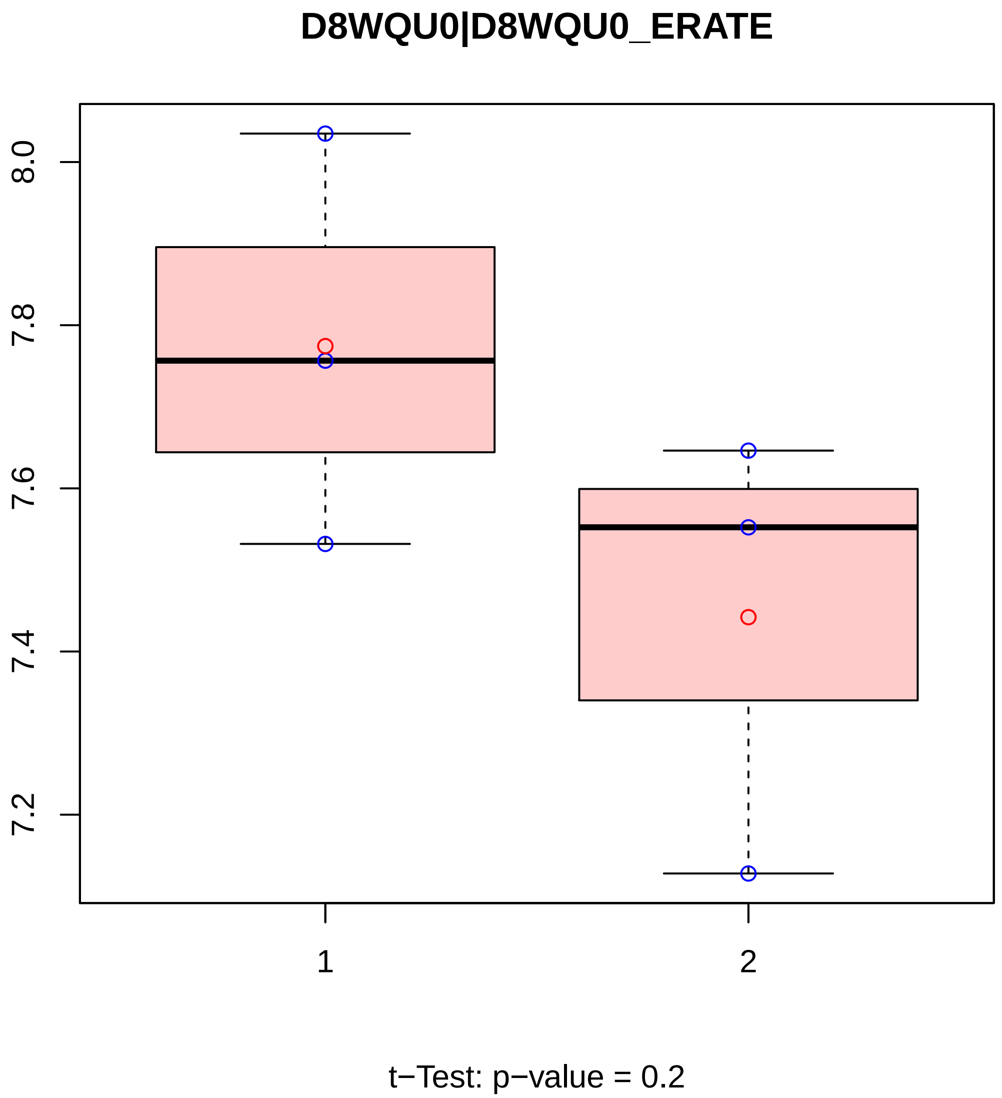
D8WQU0|D8WQU0_ERATE
8.0
7.8
7.6
7.4
7.2
1
2
t−Test: p−value = 0.2

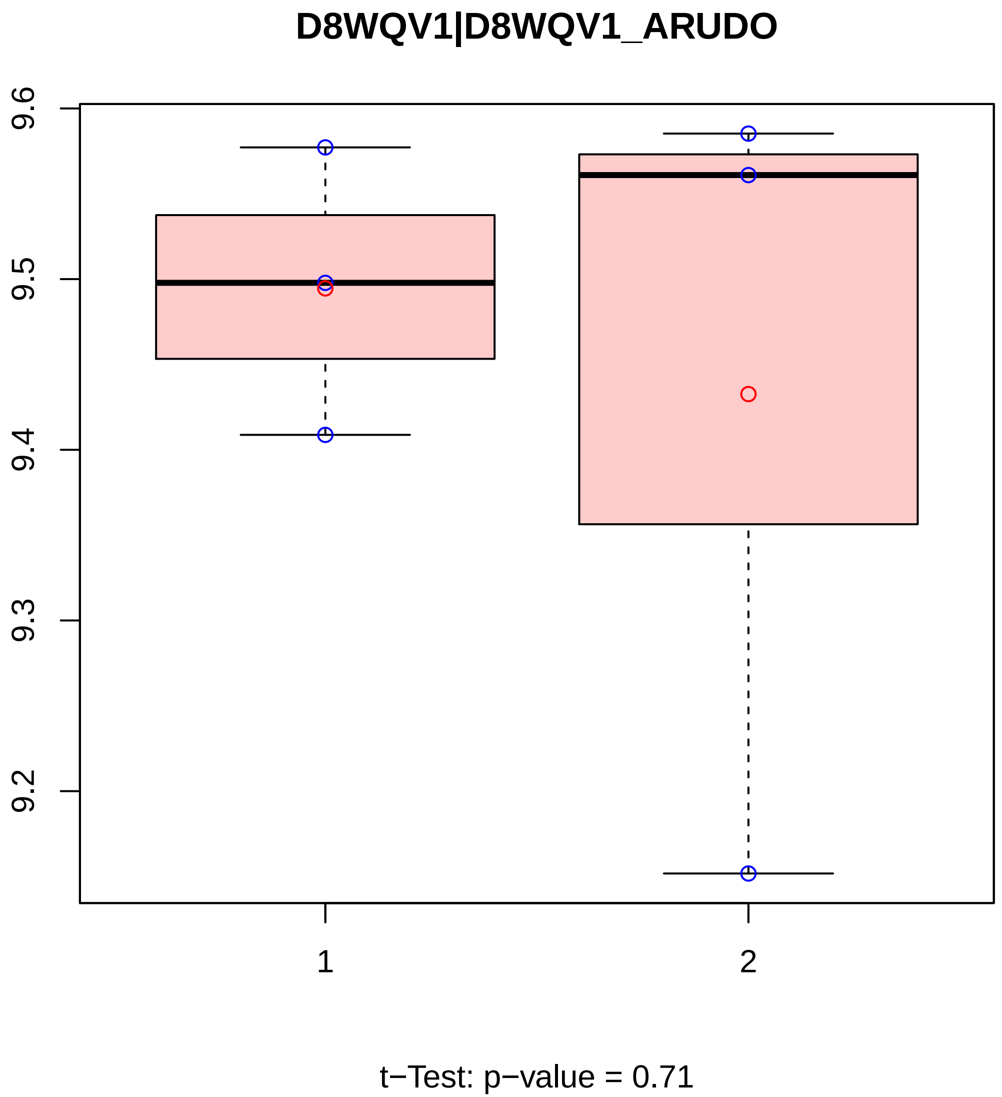
D8WQV1|D8WQV1_ARUDO
9.6
9.5
9.4
9.3
9.2
1
2
t−Test: p−value = 0.71

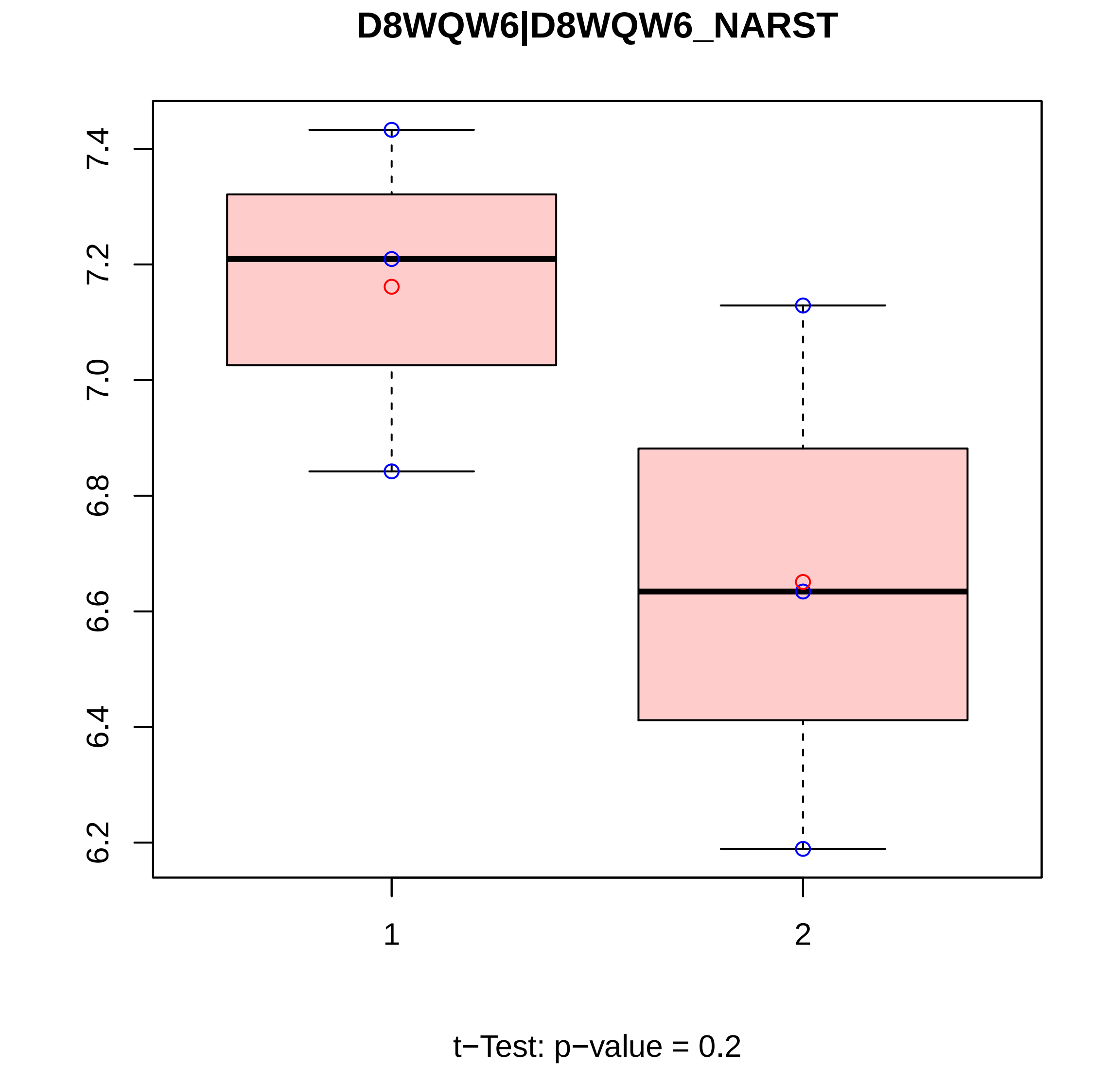
D8WQW6|D8WQW6_NARST
7.4
7.2
7.0
6.8
6.6
6.4
6.2
1
2
t−Test: p−value = 0.2

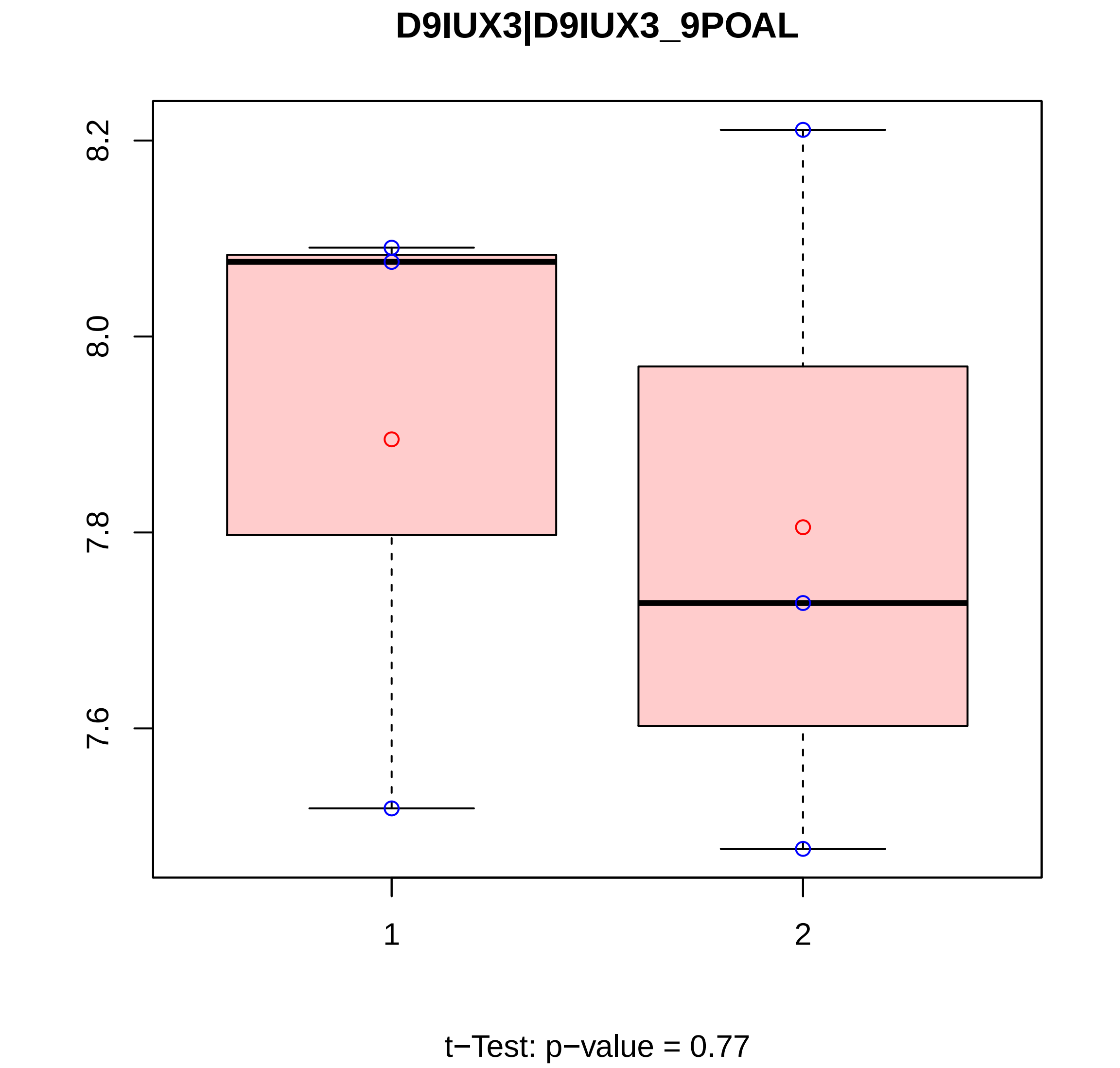
D9IUX3|D9IUX3_9POAL
8.2
8.0
7.8
7.6
1
2
t−Test: p−value = 0.77

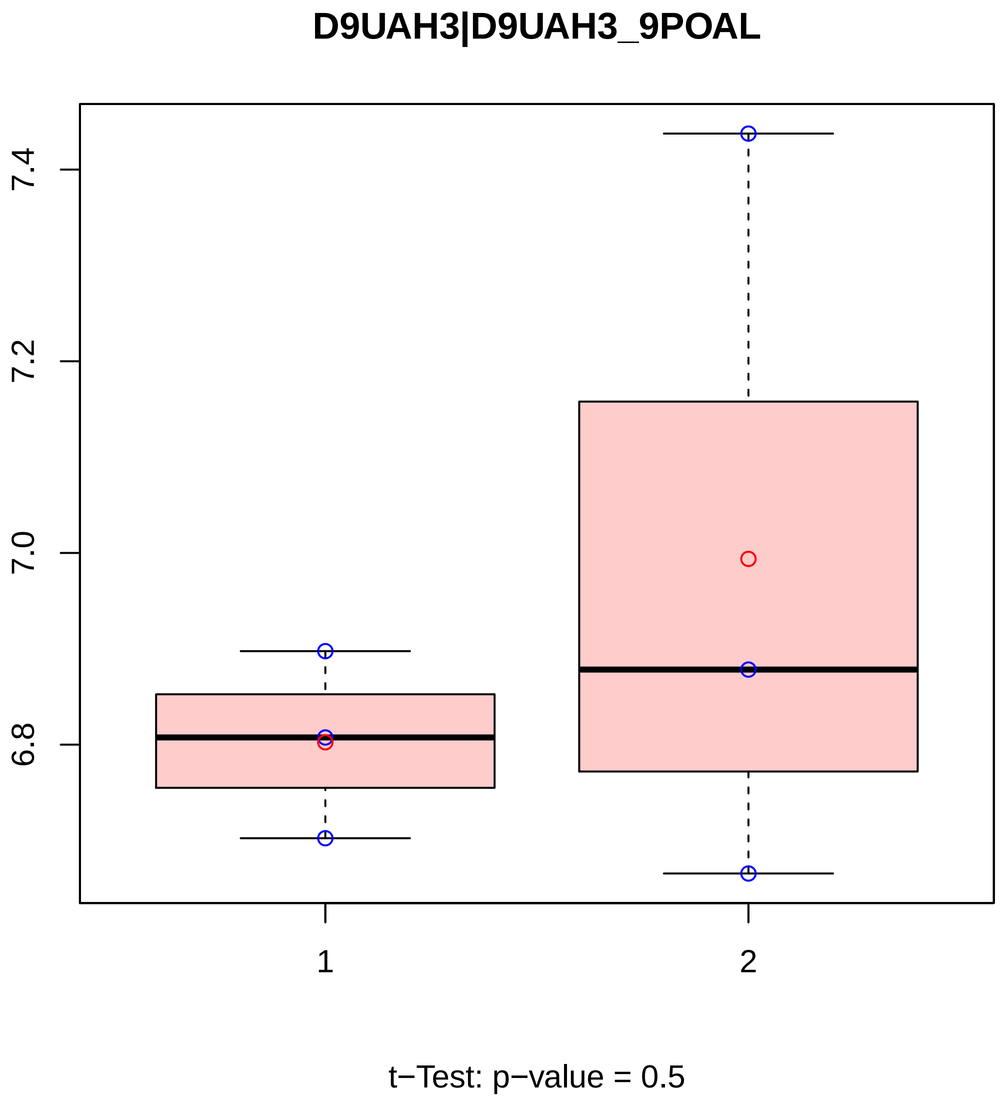
D9UAH3|D9UAH3_9POAL
7.4
7.2
7.0
6.8
1
2
t−Test: p−value = 0.5

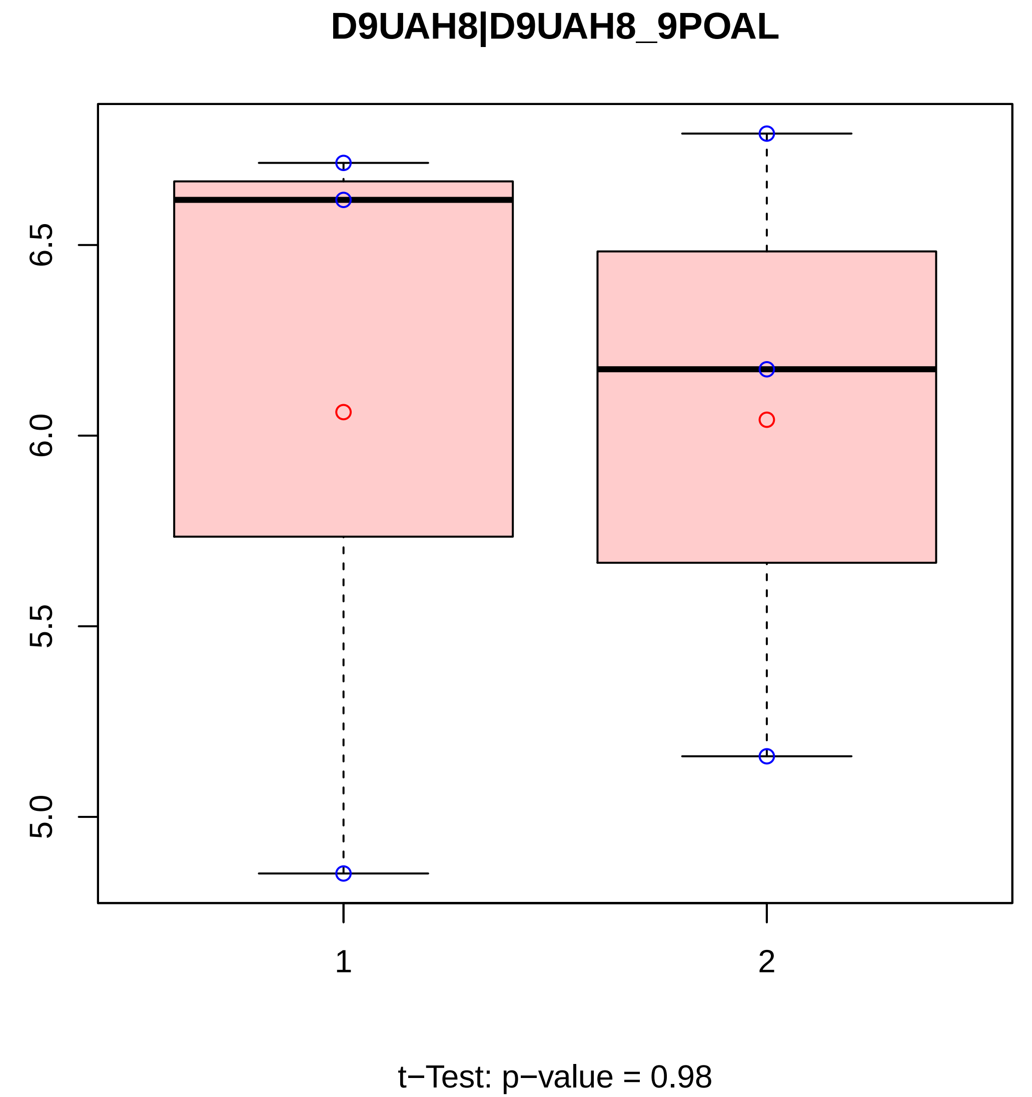
D9UAH8|D9UAH8_9POAL
6.5
6.0
5.5
5.0
1
2
t−Test: p−value = 0.98

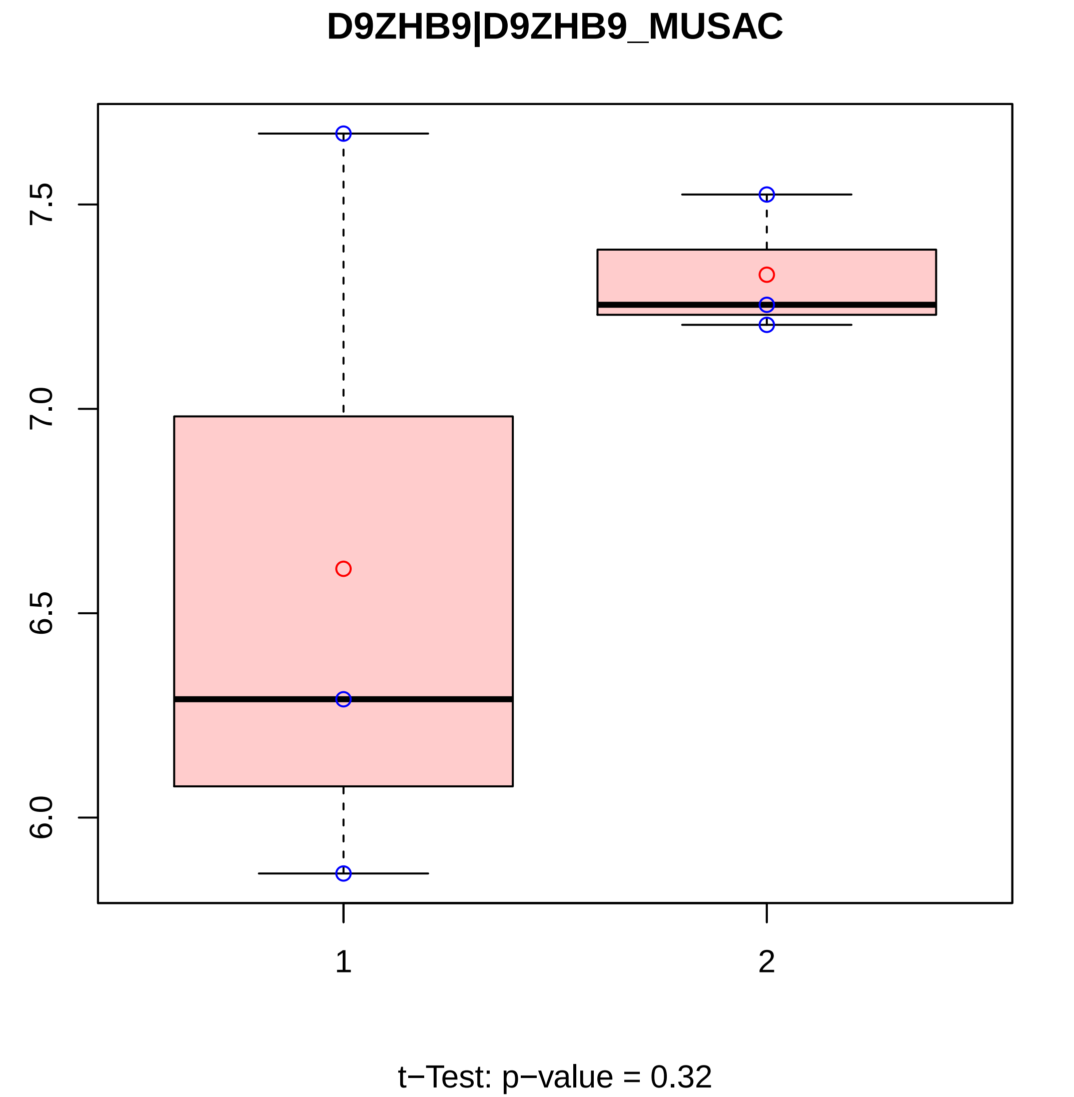
D9ZHB9|D9ZHB9_MUSAC
7.5
7.0
6.5
6.0
1
2
t−Test: p−value = 0.32

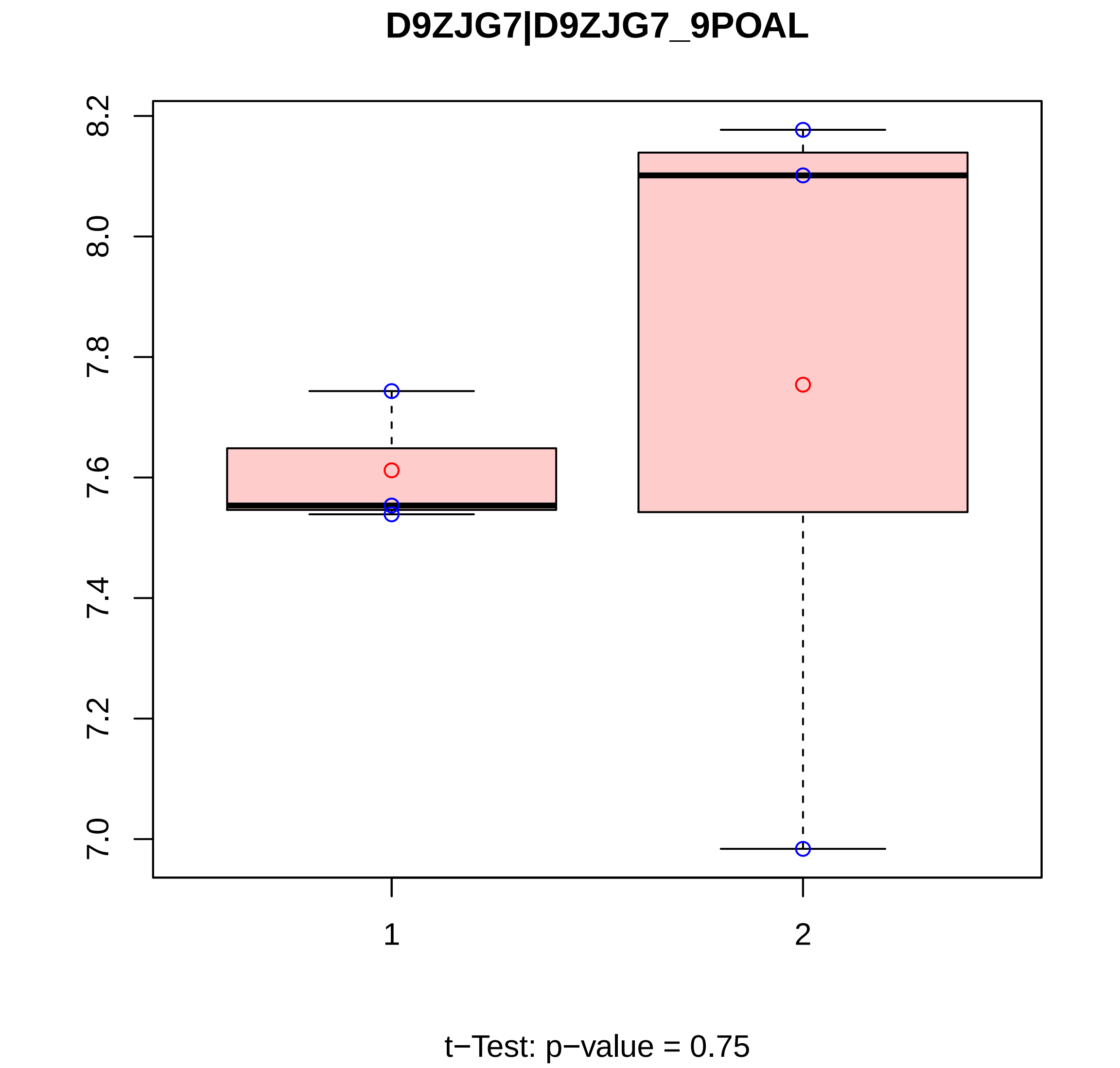
D9ZJG7|D9ZJG7_9POAL
8.2
8.0
7.8
7.6
7.4
7.2
7.0
1
2
t−Test: p−value = 0.75

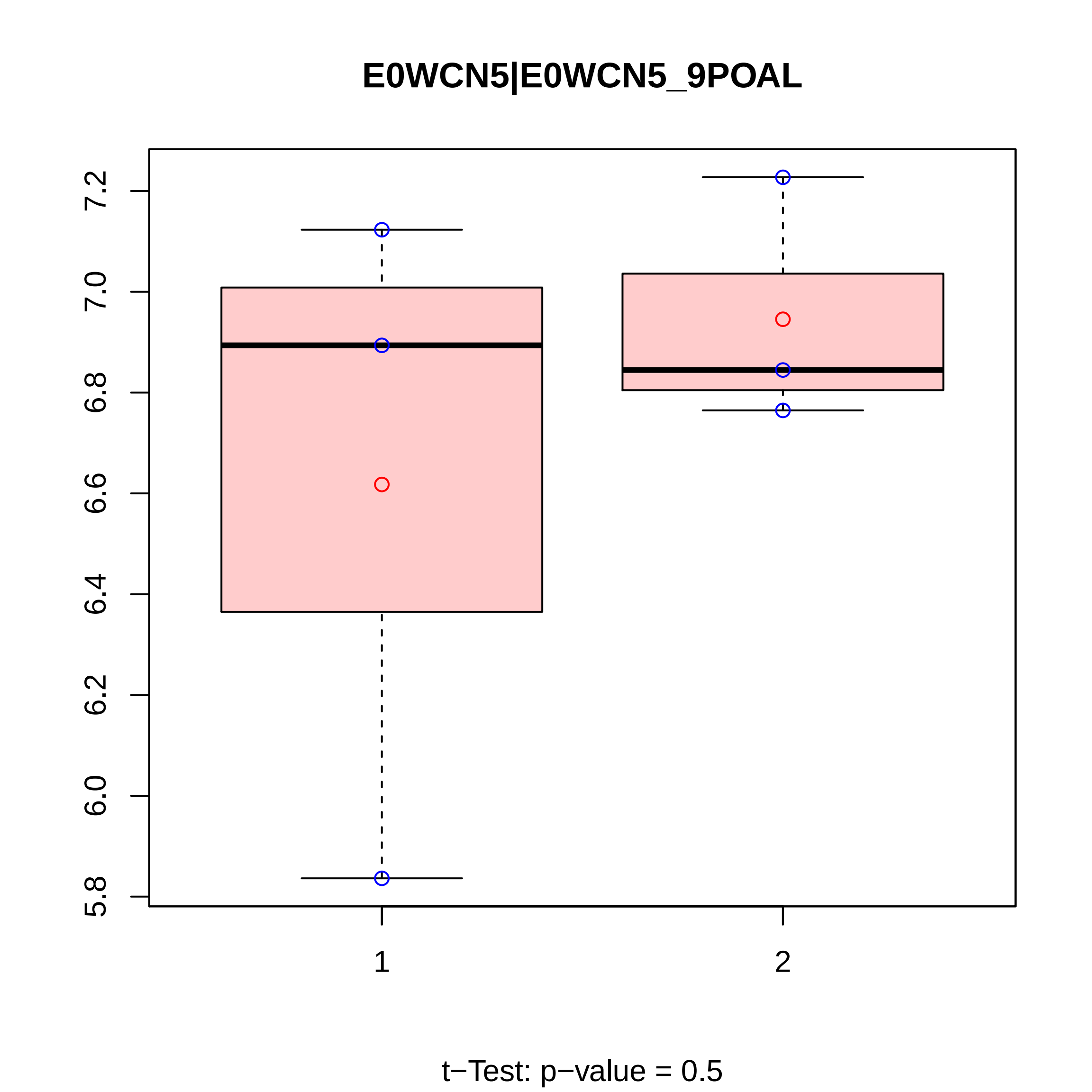

E0WCN5|E0WCN5_9POAL
7.2
7.0
6.8
6.6
6.4
6.2
6.0
5.8
1
2
t−Test: p−value = 0.5

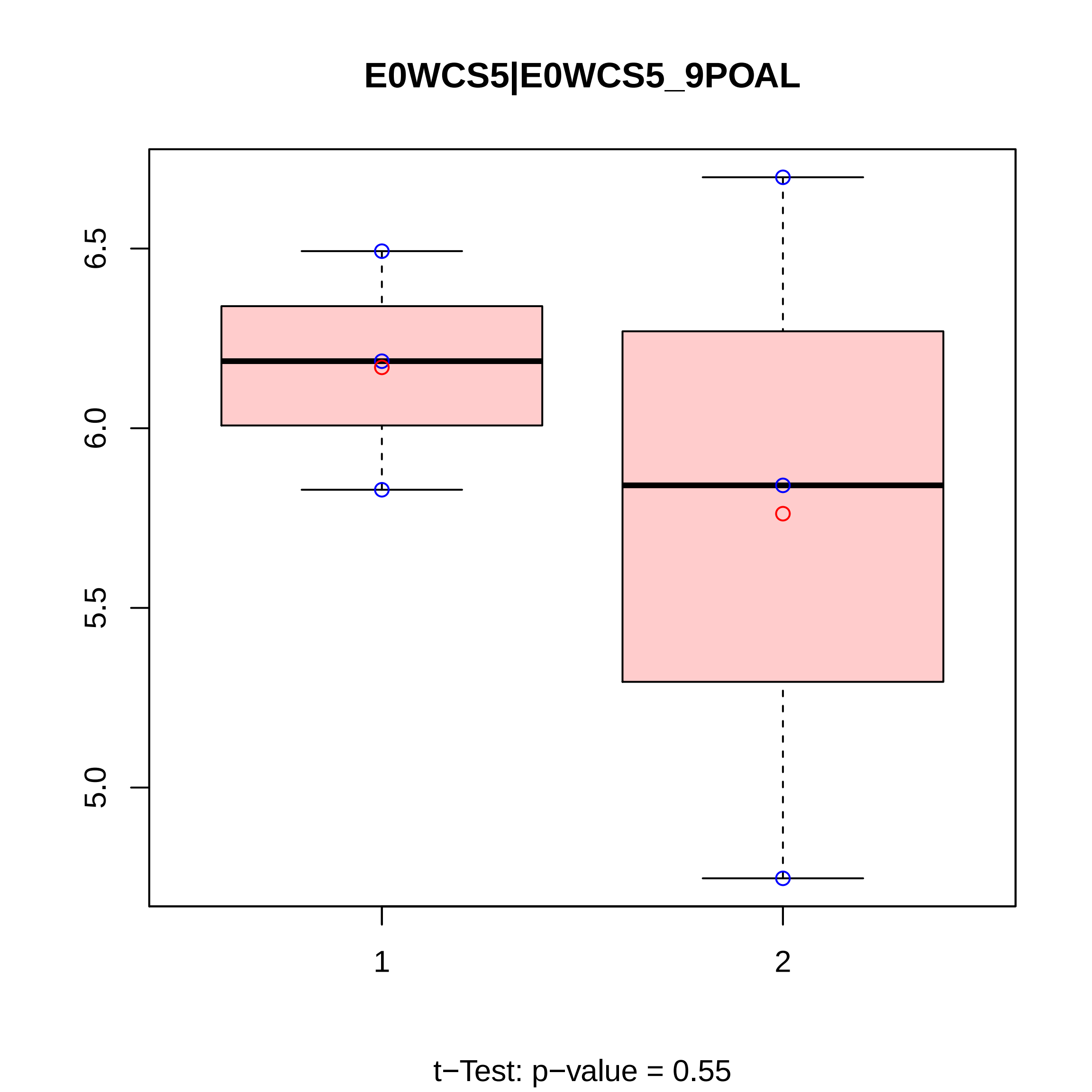
E0WCS5|E0WCS5_9POAL
6.5
6.0
5.5
5.0
1
2
t−Test: p−value = 0.55

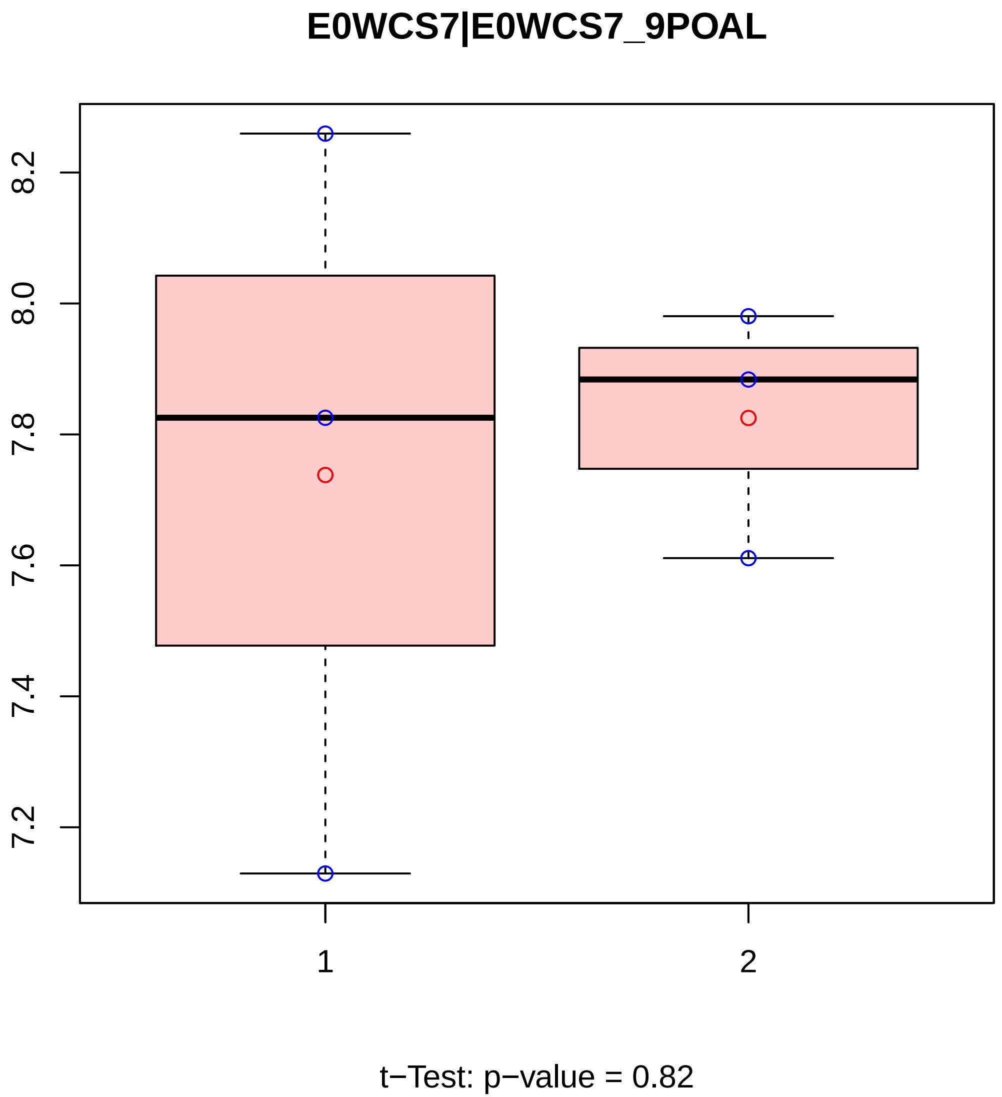
E0WCS7|E0WCS7_9POAL
8.2
8.0
7.8
7.6
7.4
7.2
1
2
t−Test: p−value = 0.82

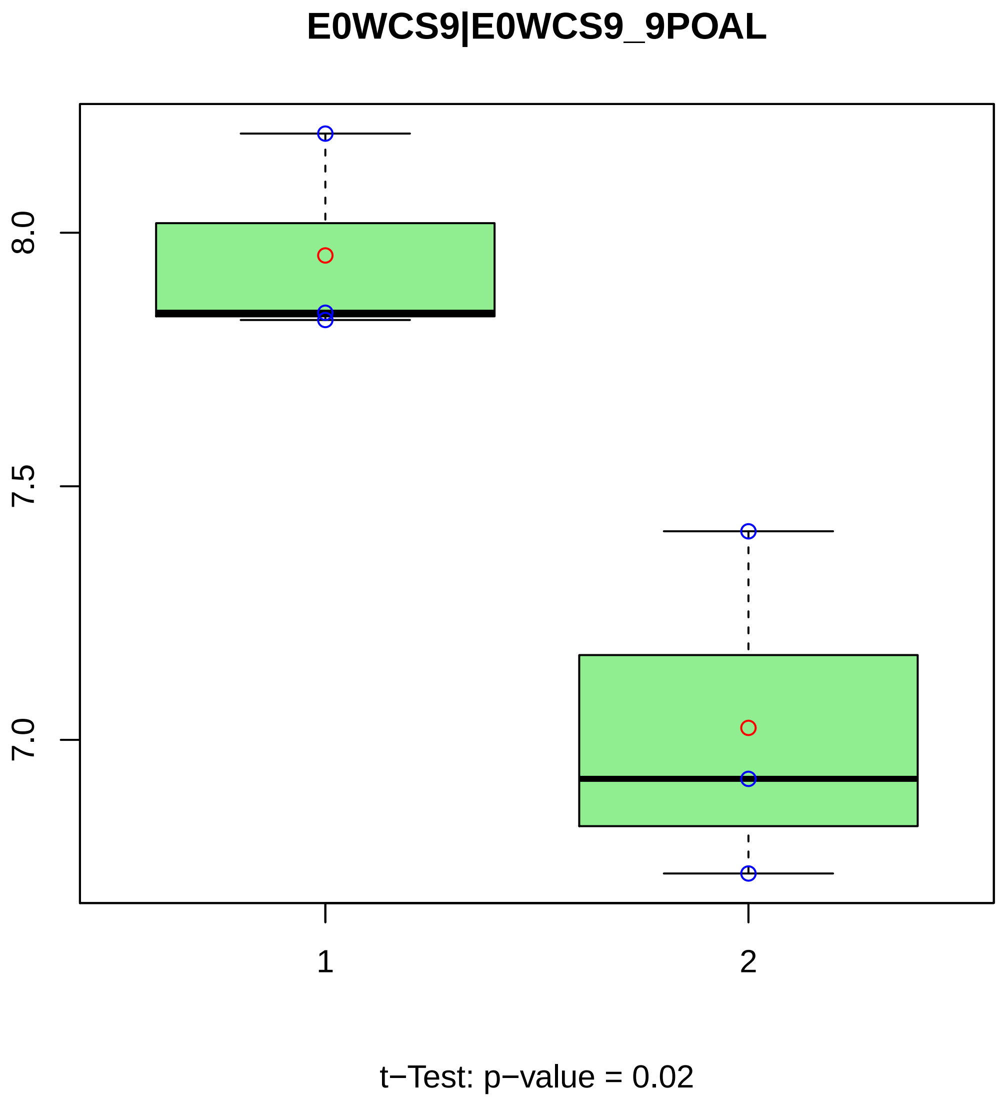

E0WCS9|E0WCS9_9POAL
8.0
7.5
7.0
1
2
t−Test: p−value = 0.02

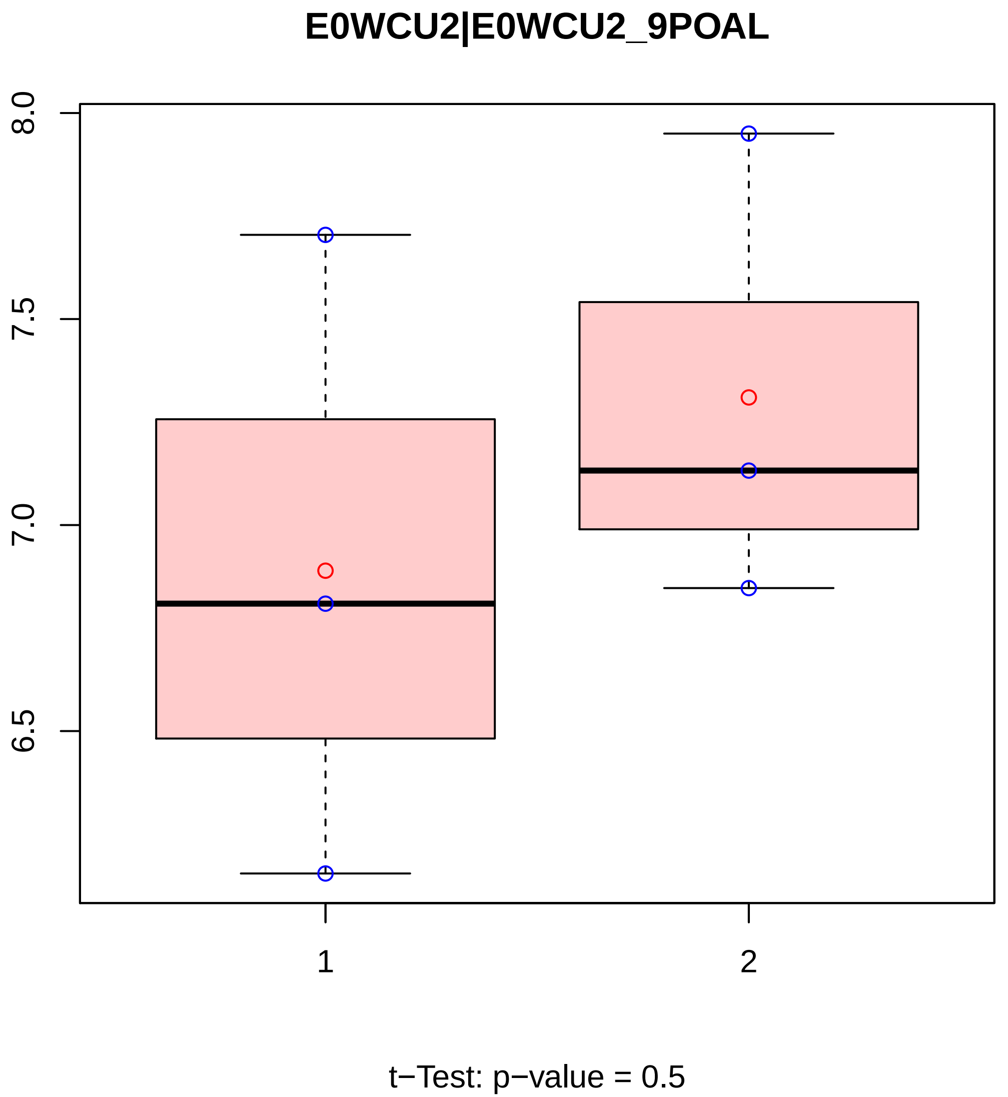
E0WCU2|E0WCU2_9POAL
8.0
7.5
7.0
6.5
1
2
t−Test: p−value = 0.5

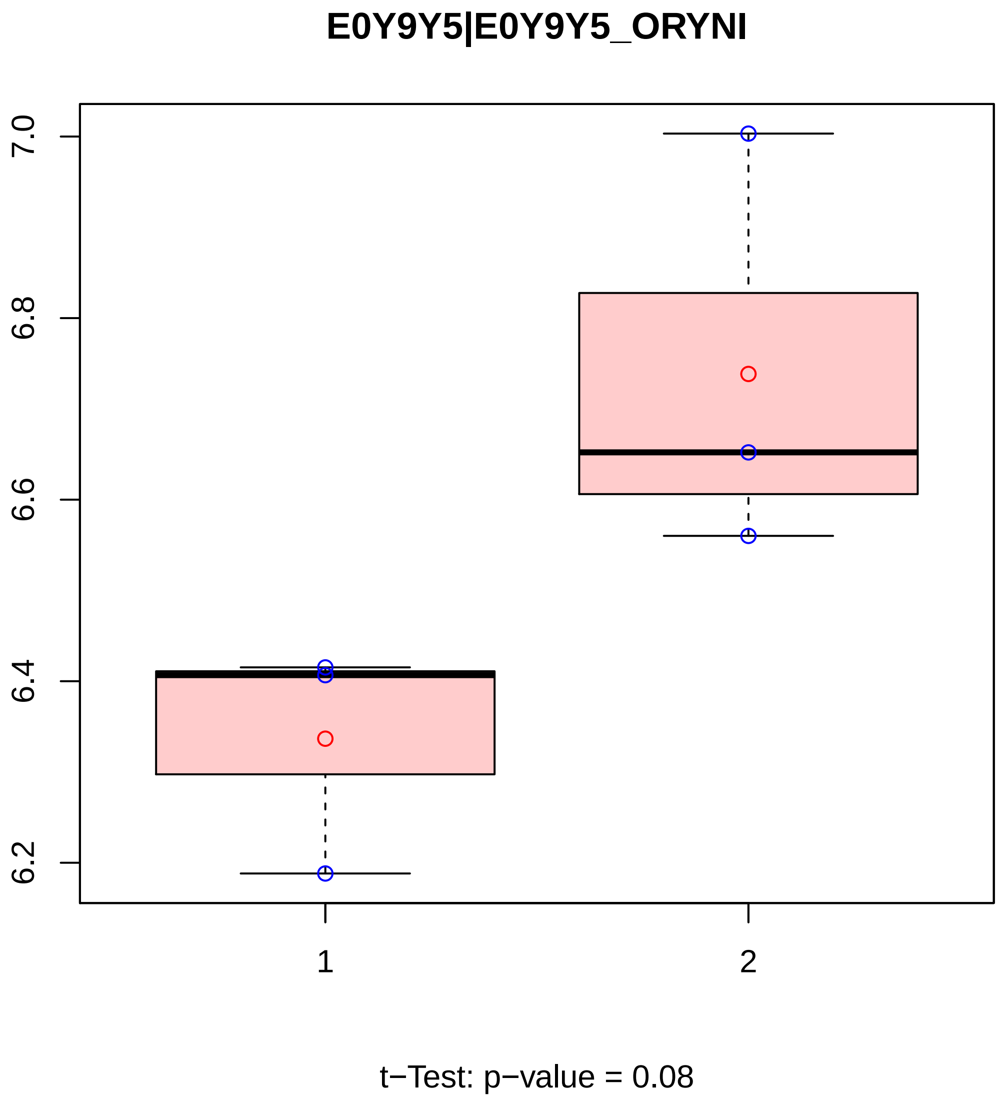
E0Y9Y5|E0Y9Y5_ORYNI
7.0
6.8
6.6
6.4
6.2
1
2
t−Test: p−value = 0.08

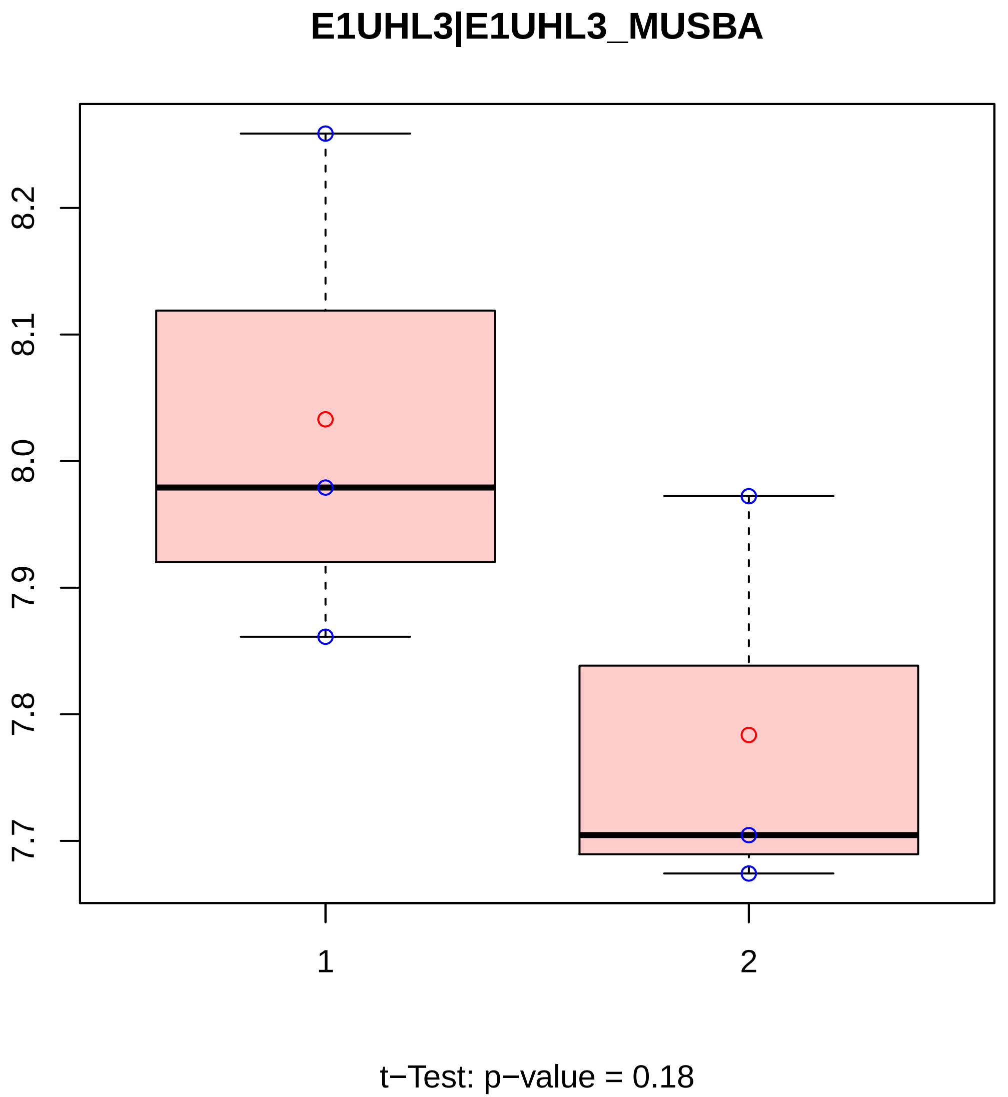

E1UHL3|E1UHL3_MUSBA
8.2
8.1
8.0
7.9
7.8
7.7
1
2
t−Test: p−value = 0.18

E1XUD2|E1XUD2_9POAL

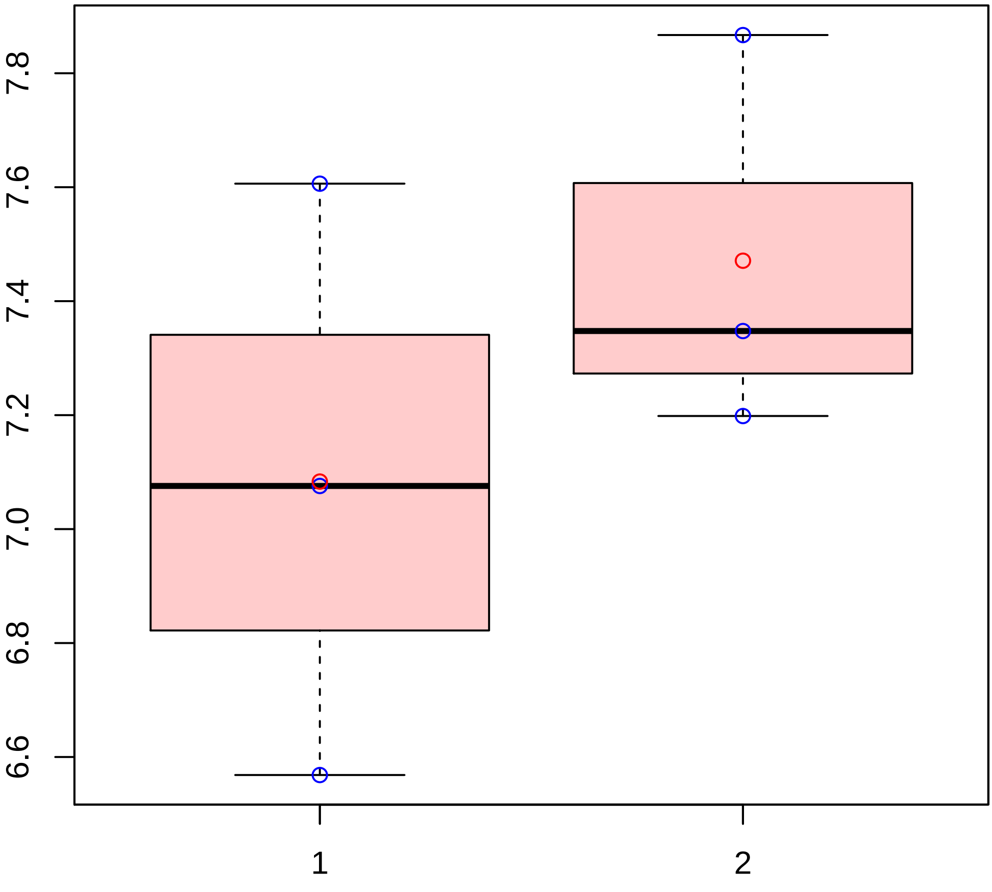

t−Test: p−value = 0.35

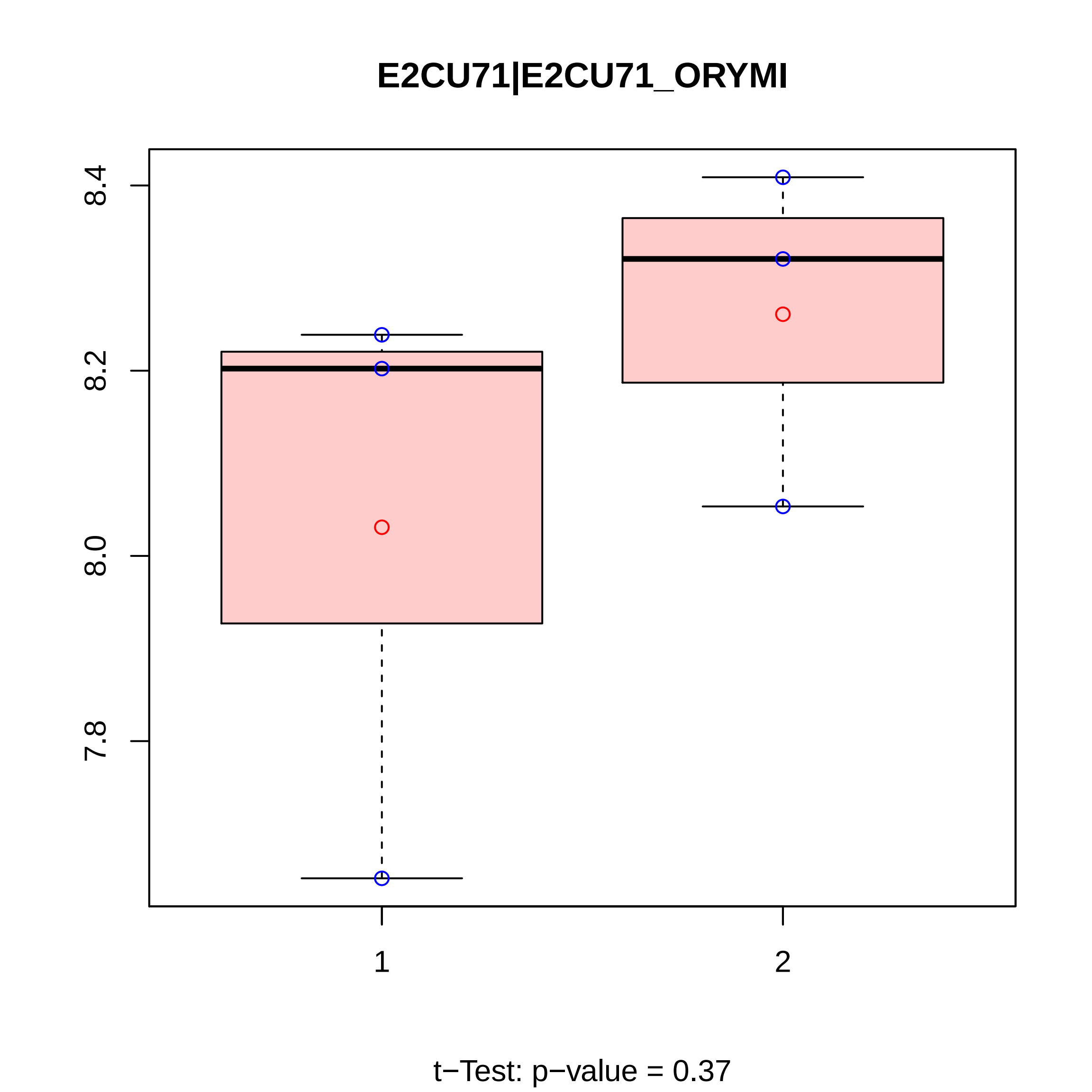

E2CU71|E2CU71_ORYMI
8.4
8.2
8.0
7.8
1
2
t−Test: p−value = 0.37

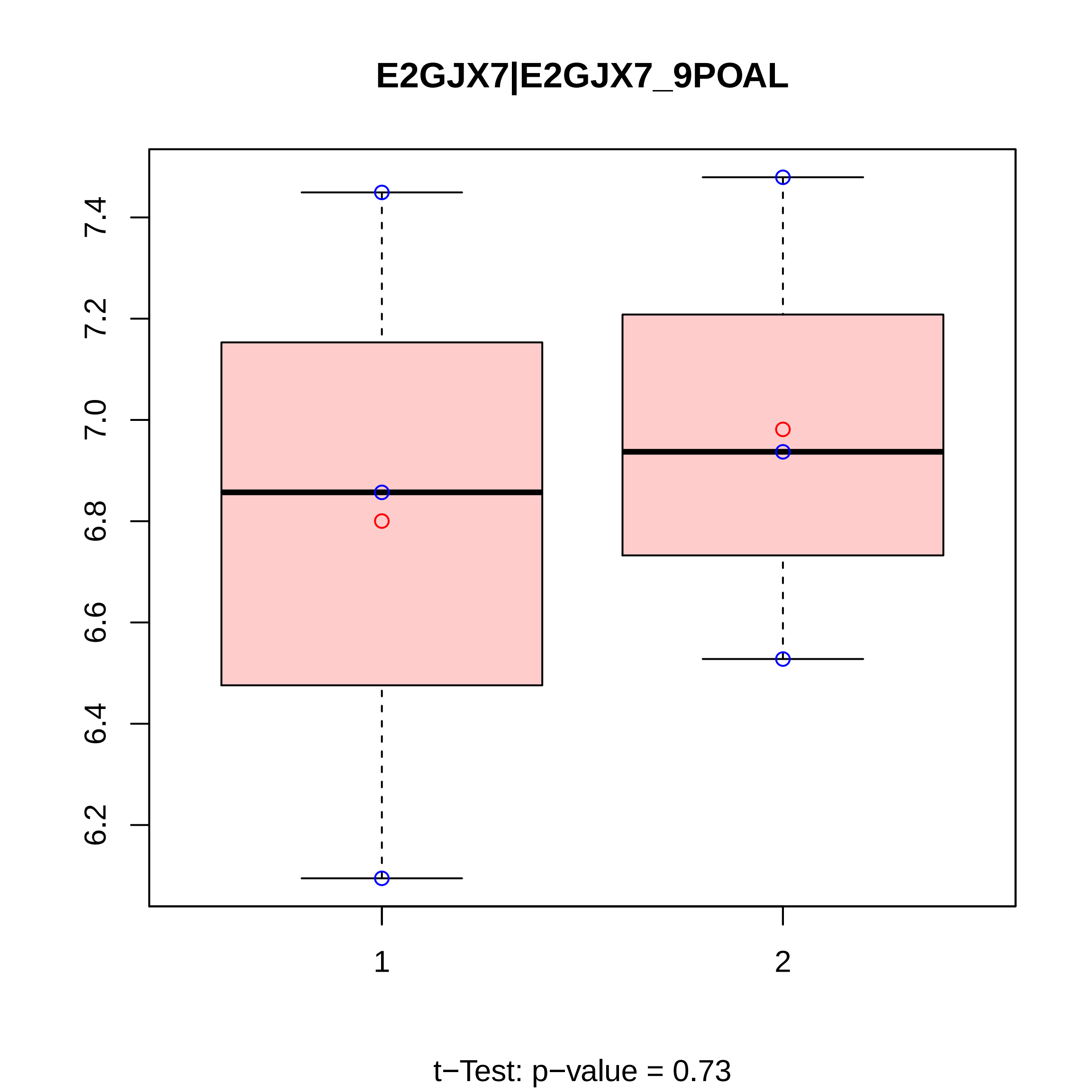
E2GJX7|E2GJX7_9POAL
7.4
7.2
7.0
6.8
6.6
6.4
6.2
1
2
t−Test: p−value = 0.73

E3W9C5|E3W9C5_ZINZE

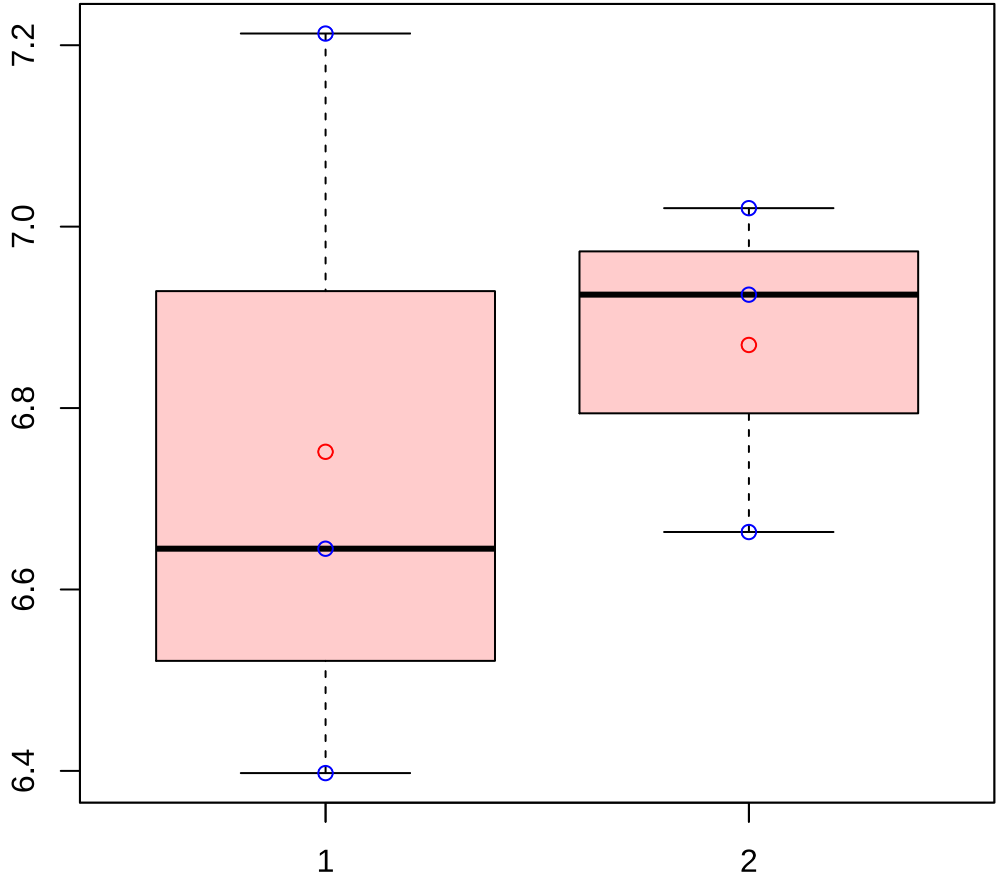

t−Test: p−value = 0.69

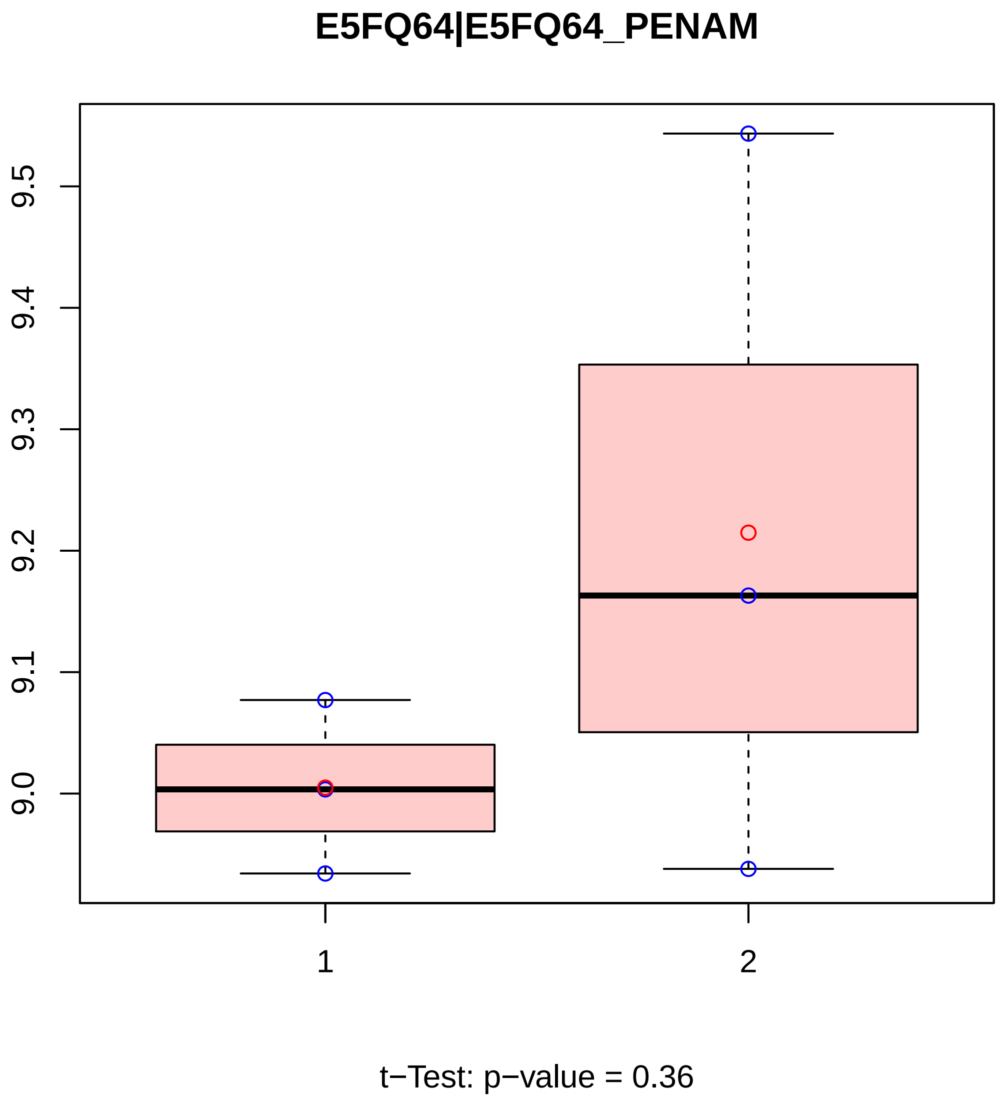

E5FQ64|E5FQ64_PENAM
9.0
9.1
9.2
9.3
9.4
9.5
1
2
t−Test: p−value = 0.36

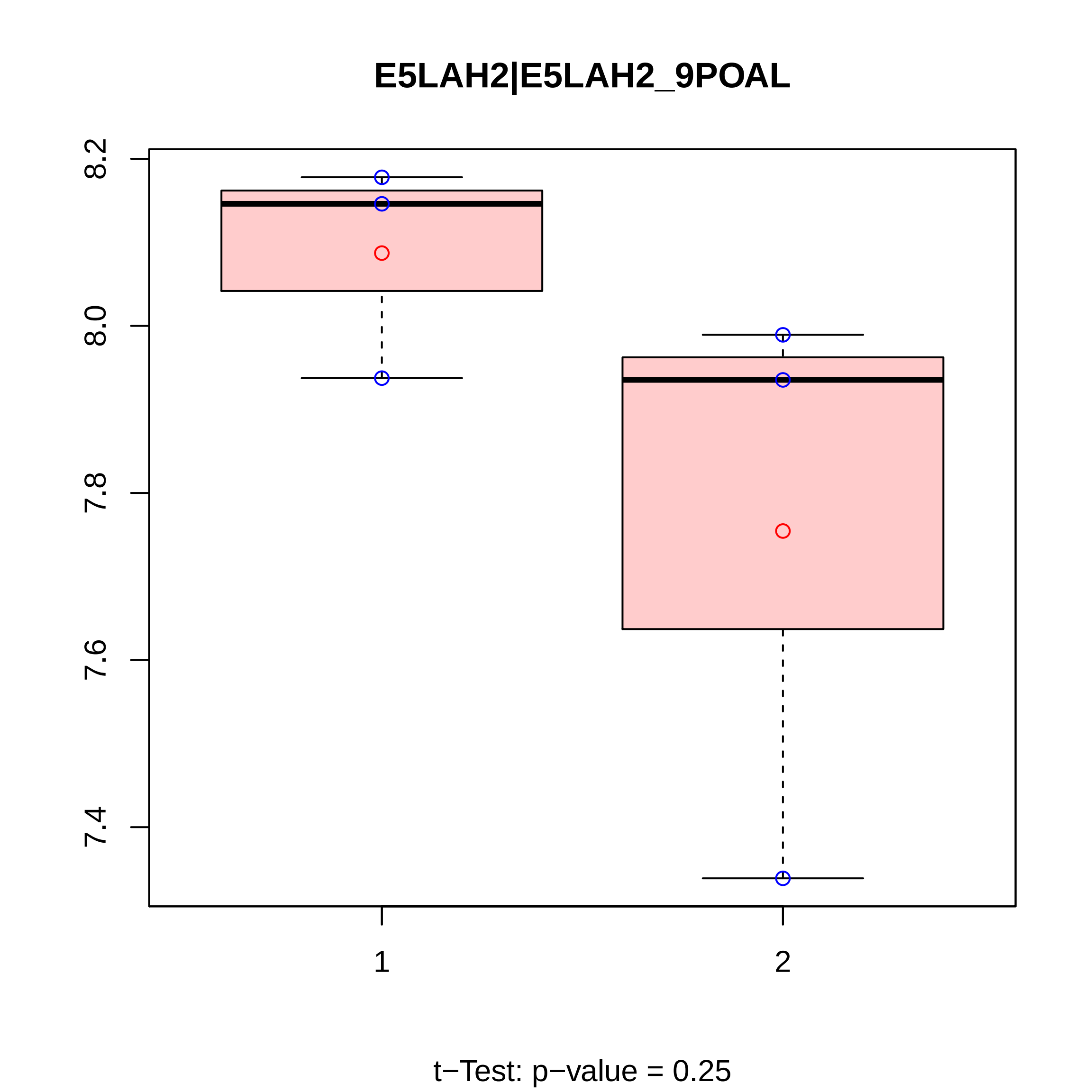
E5LAH2|E5LAH2_9POAL
8.2
8.0
7.8
7.6
7.4
1
2
t−Test: p−value = 0.25

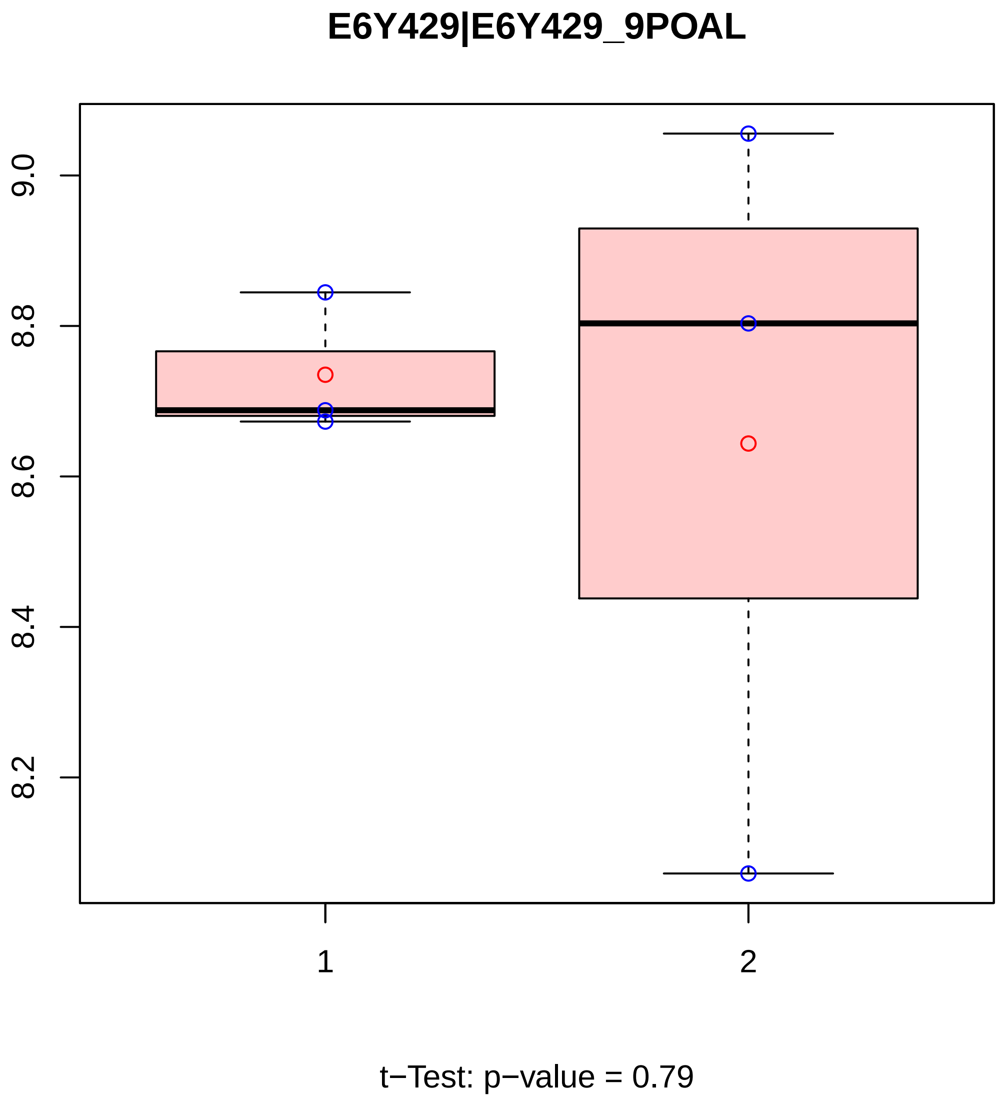
E6Y429|E6Y429_9POAL
9.0
8.8
8.6
8.4
8.2
1
2
t−Test: p−value = 0.79

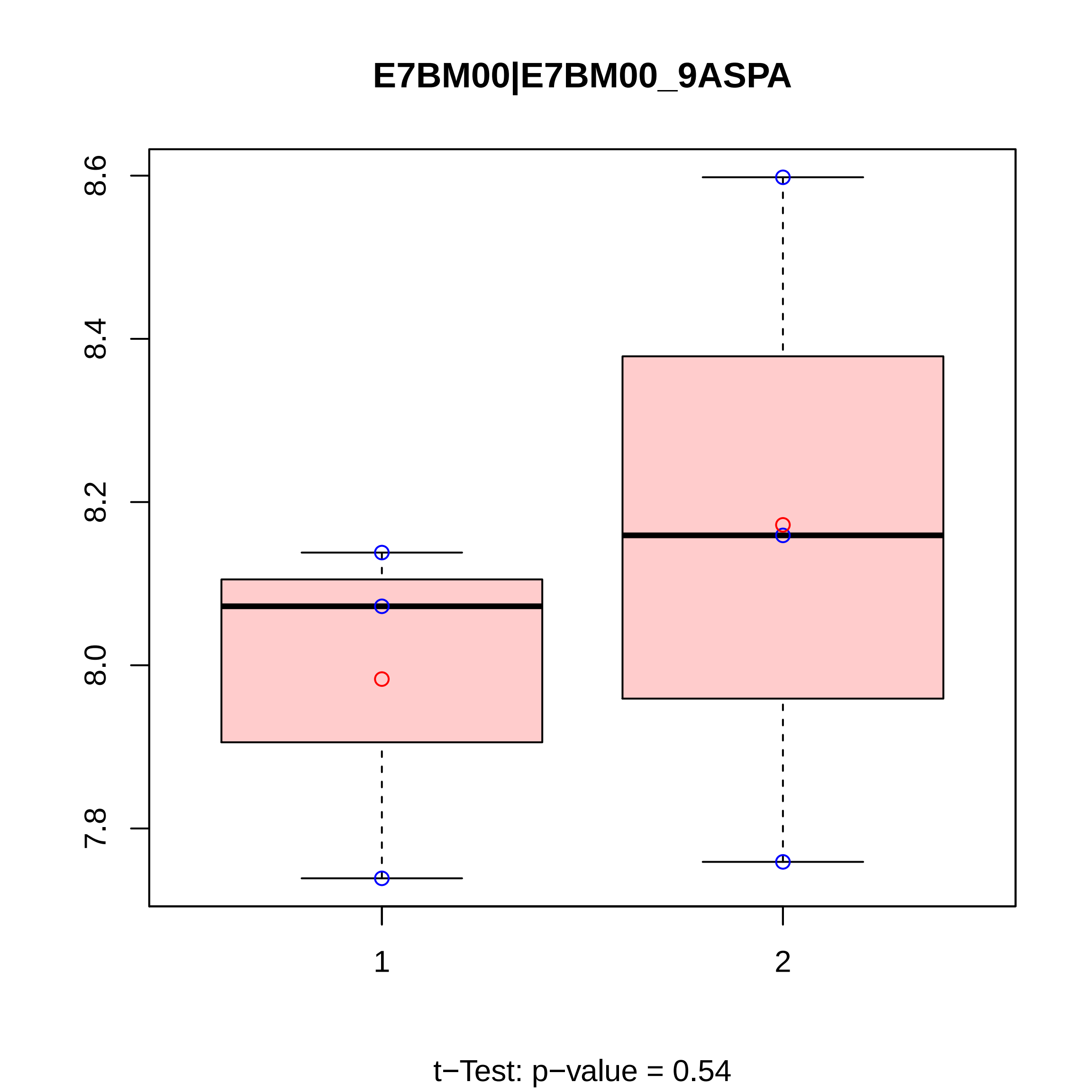
E7BM00|E7BM00_9ASPA
8.6
8.4
8.2
8.0
7.8
1
2
t−Test: p−value = 0.54

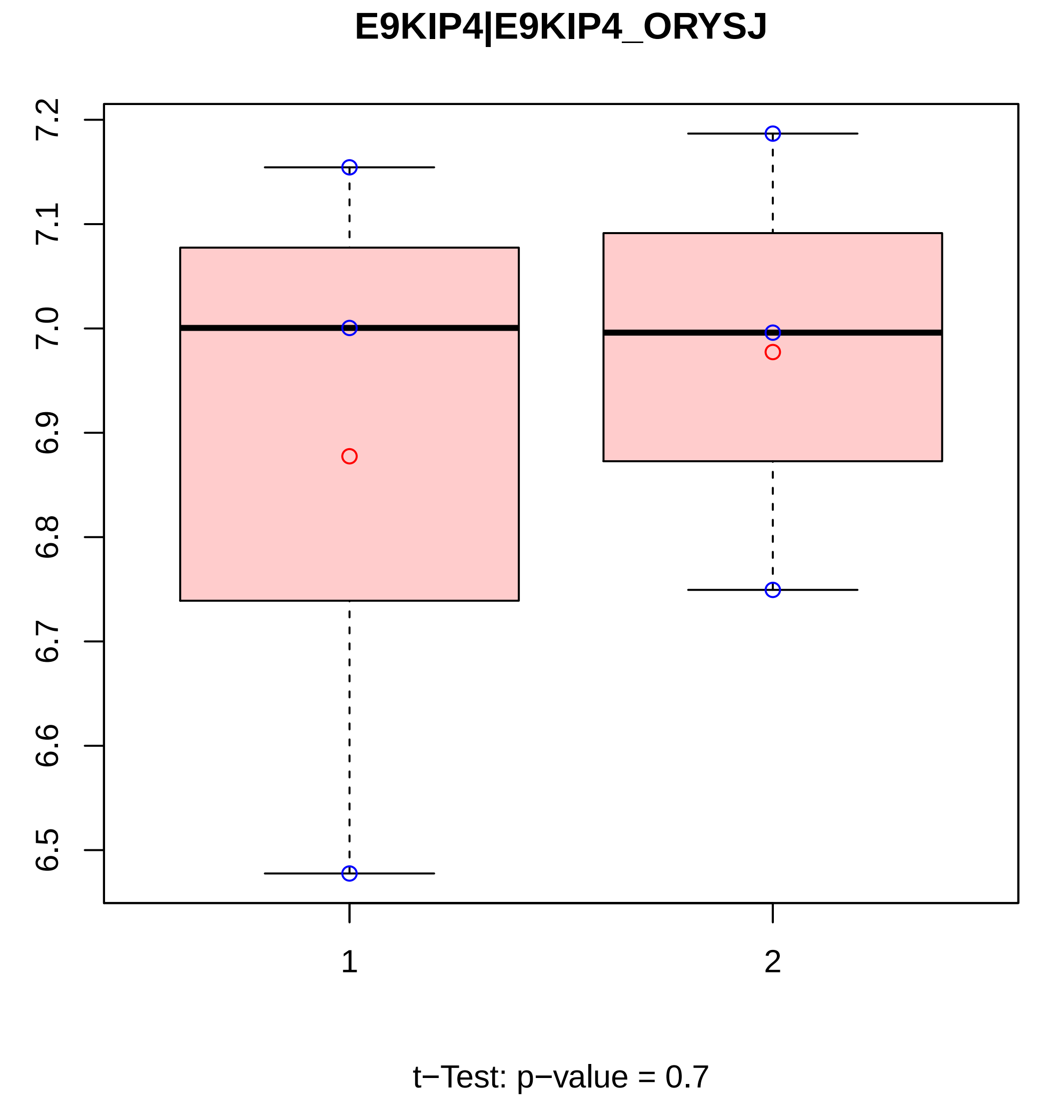
E9KIP4|E9KIP4_ORYSJ
7.2
7.1
7.0
6.9
6.8
6.7
6.6
6.5
1
2
t−Test: p−value = 0.7

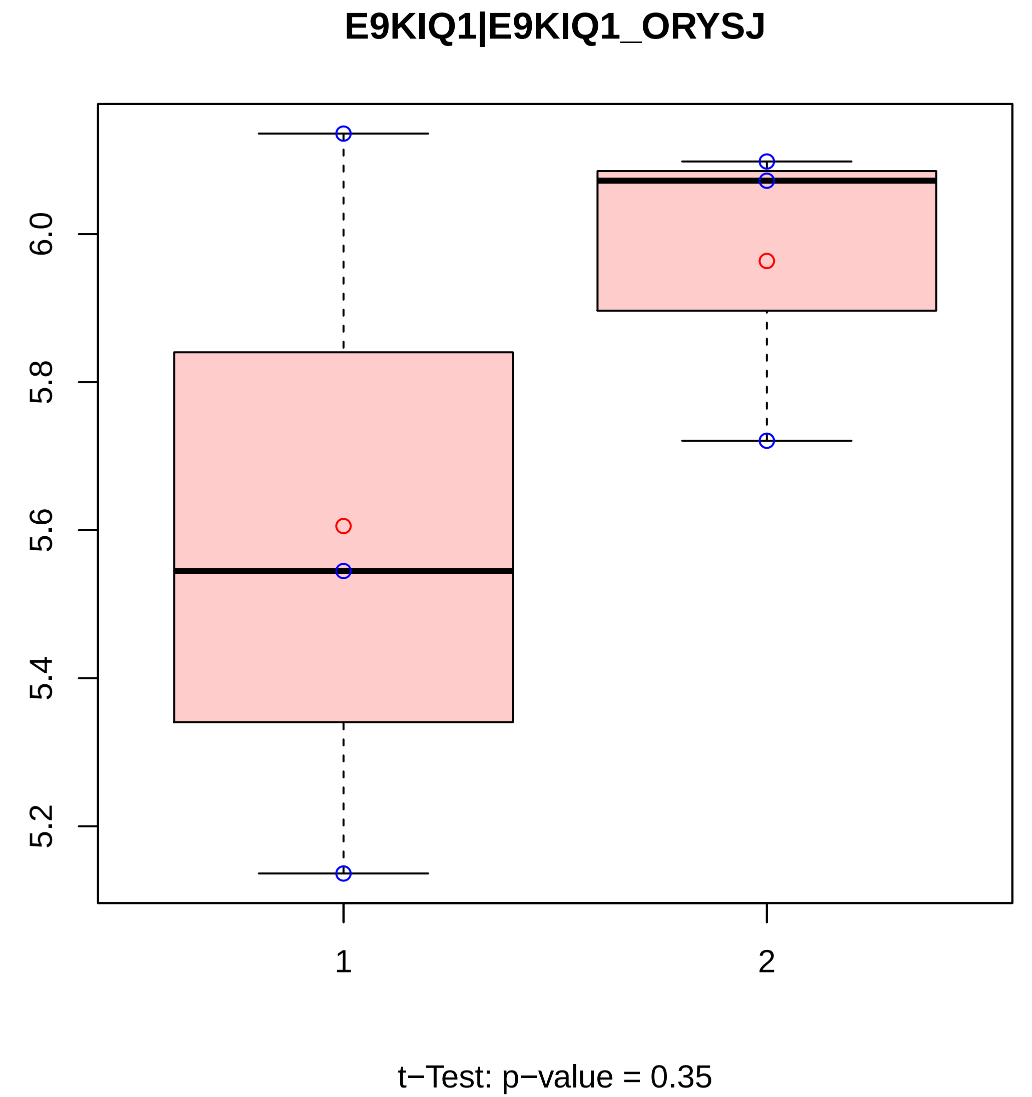
E9KIQ1|E9KIQ1_ORYSJ
5.2
5.4
5.6
5.8
6.0
1
2
t−Test: p−value = 0.35

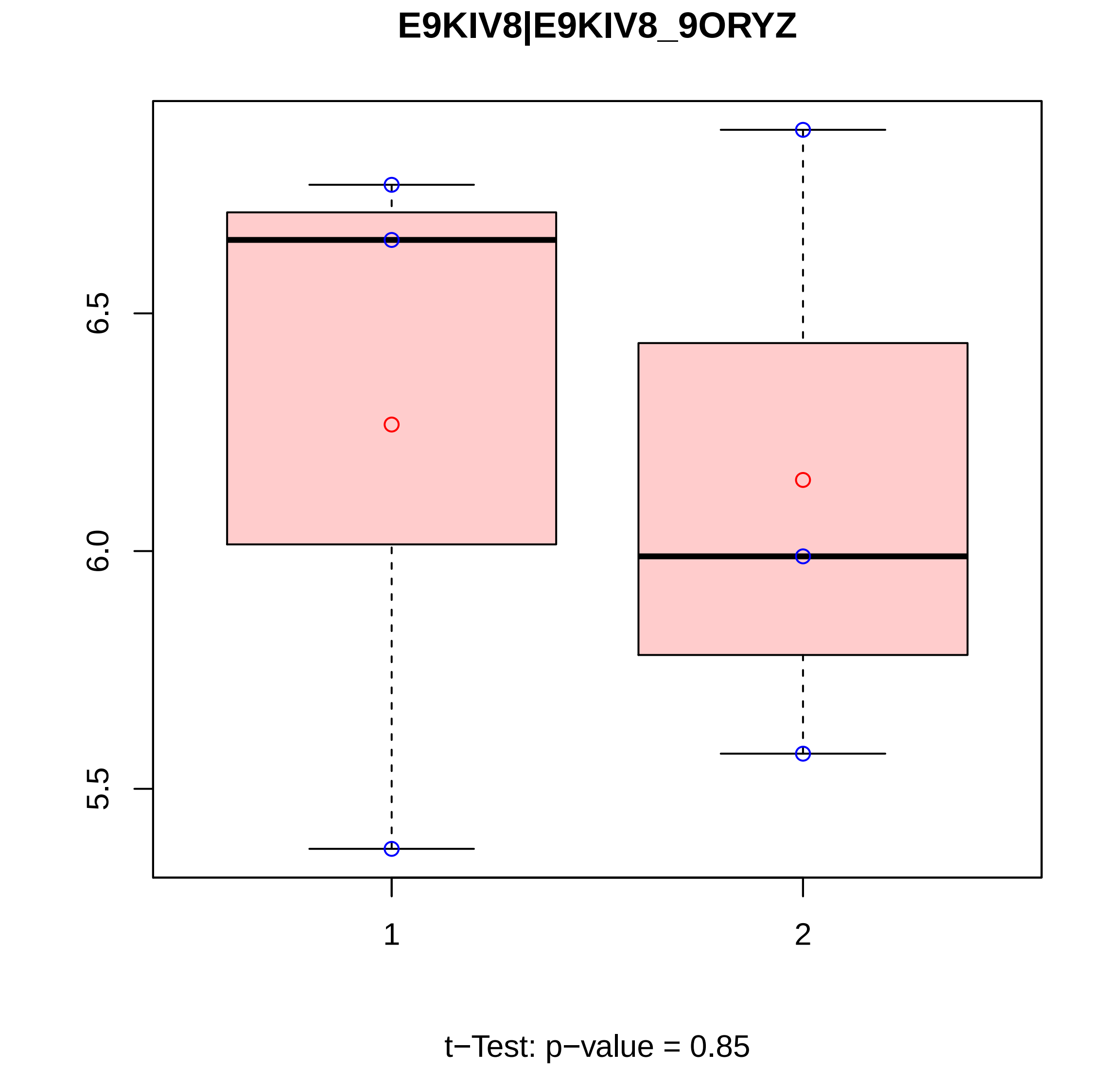
E9KIV8|E9KIV8_9ORYZ
6.5
6.0
5.5
1
2
t−Test: p−value = 0.85

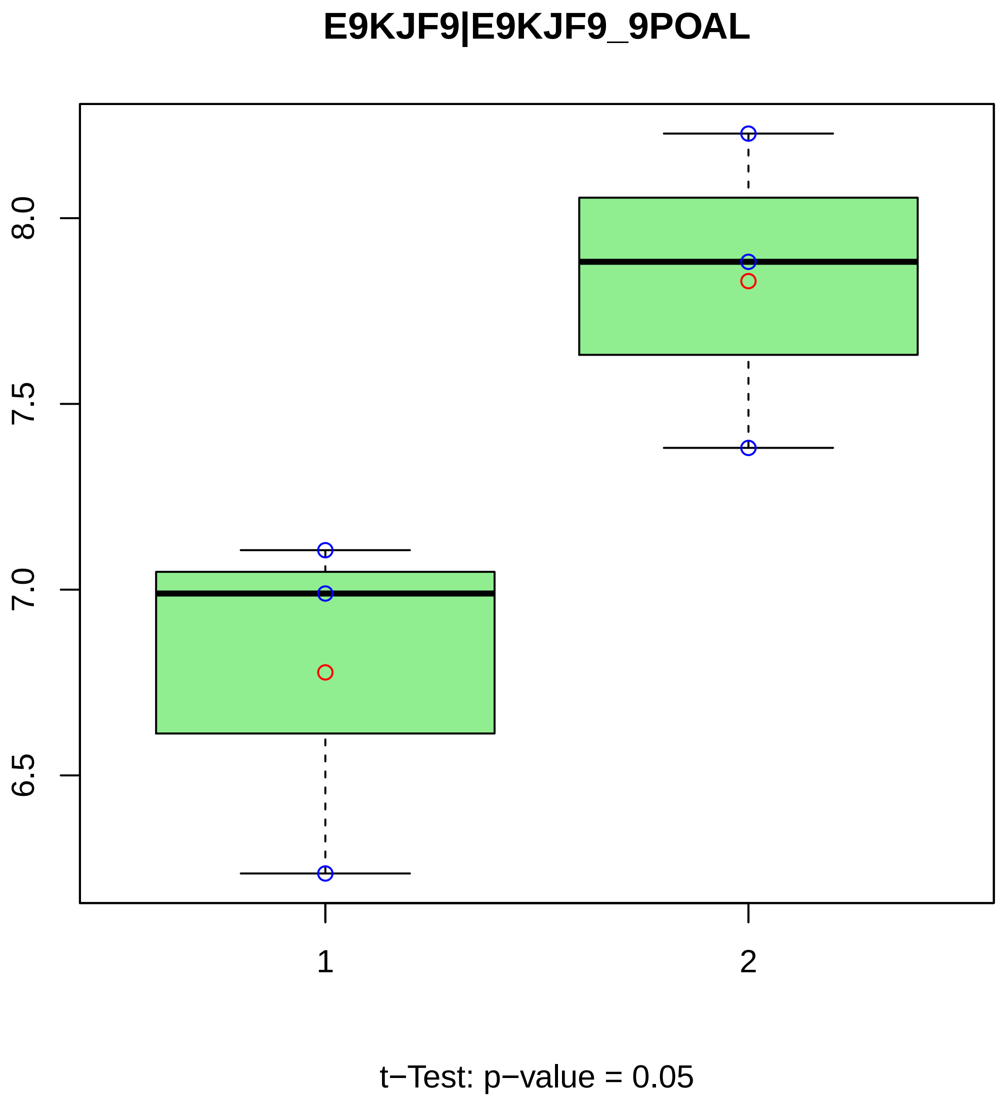
E9KJF9|E9KJF9_9POAL
8.0
7.5
7.0
6.5
1
2
t−Test: p−value = 0.05

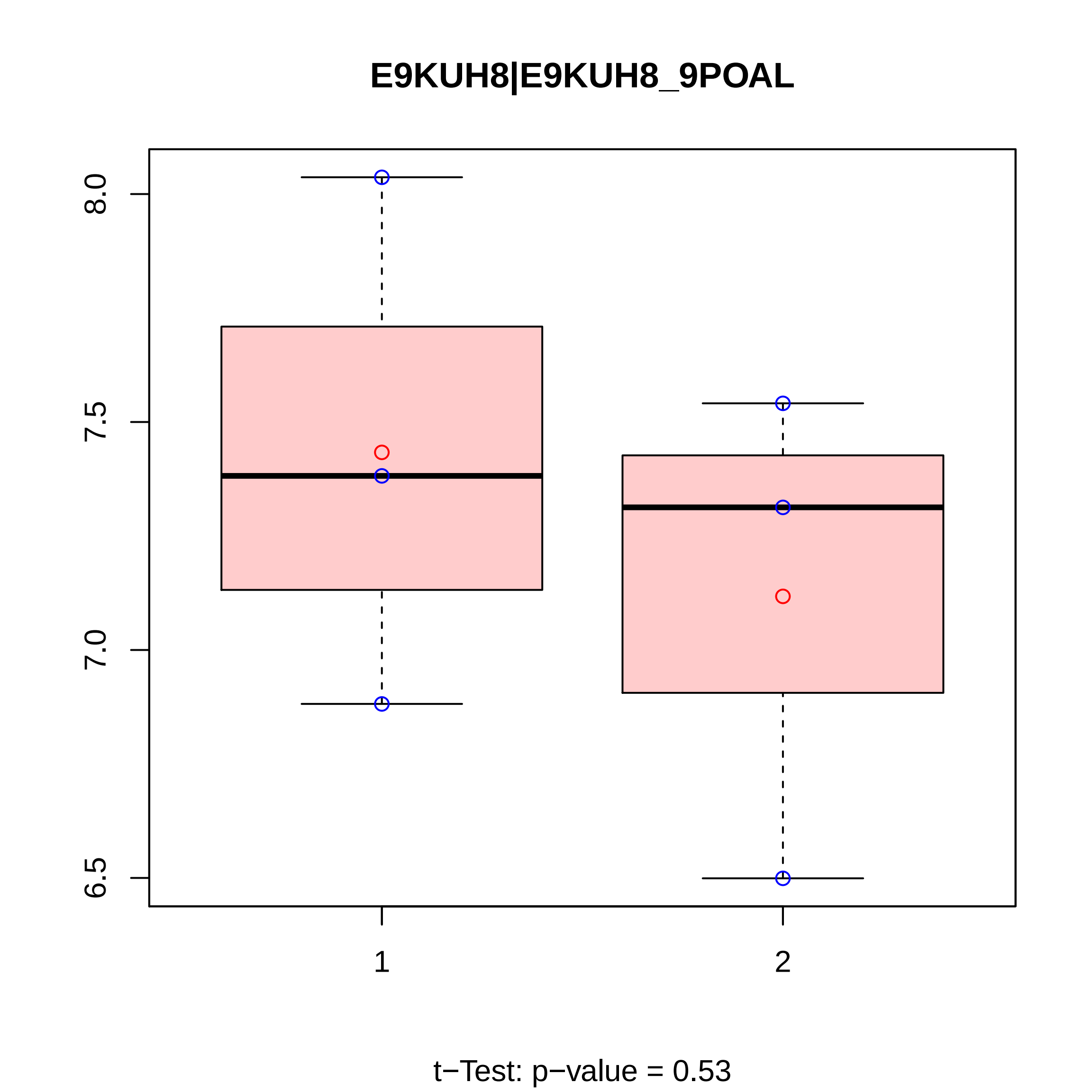
E9KUH8|E9KUH8_9POAL
8.0
7.5
7.0
6.5
1
2
t−Test: p−value = 0.53

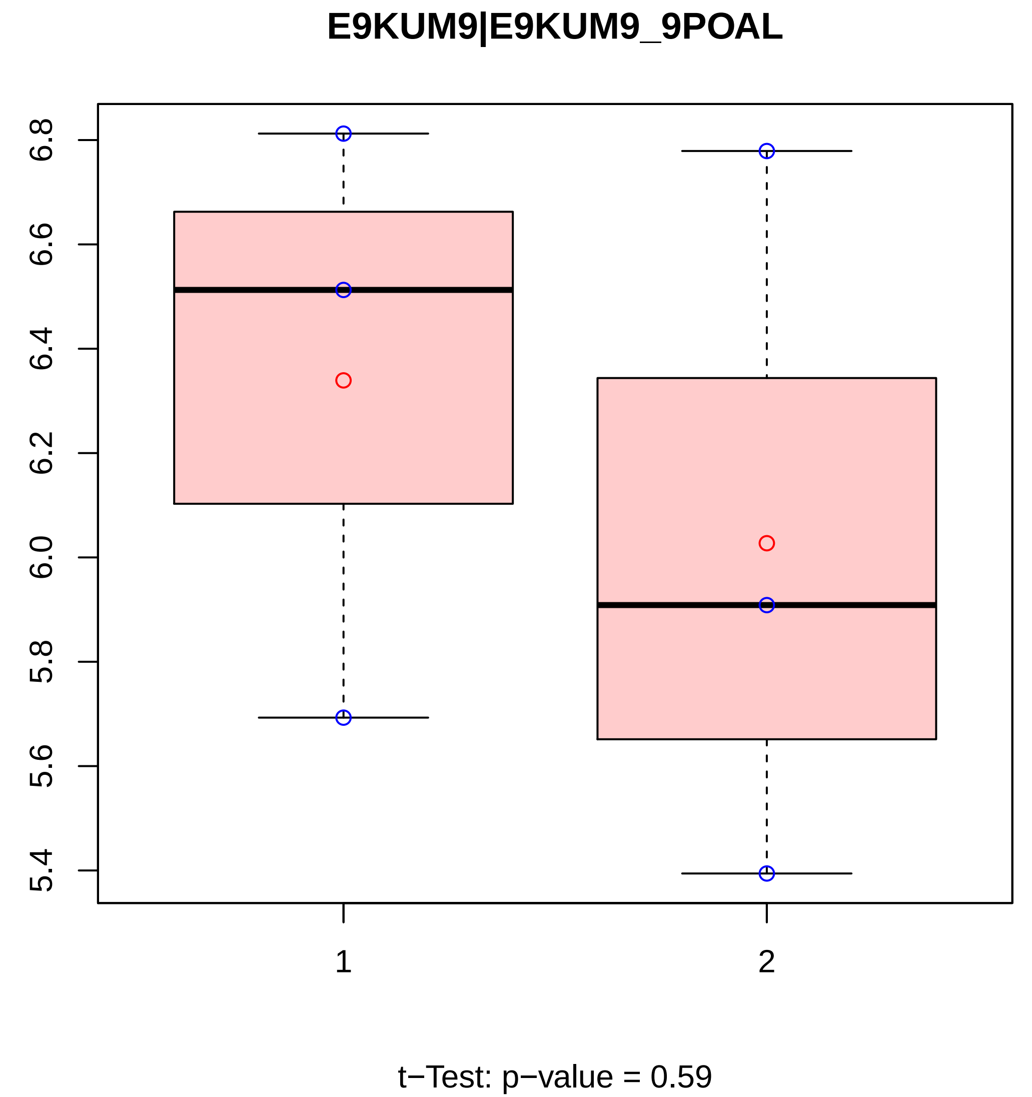
E9KUM9|E9KUM9_9POAL
6.8
6.6
6.4
6.2
6.0
5.8
5.6
5.4
1
2
t−Test: p−value = 0.59

E9NM57|E9NM57_MUSAC

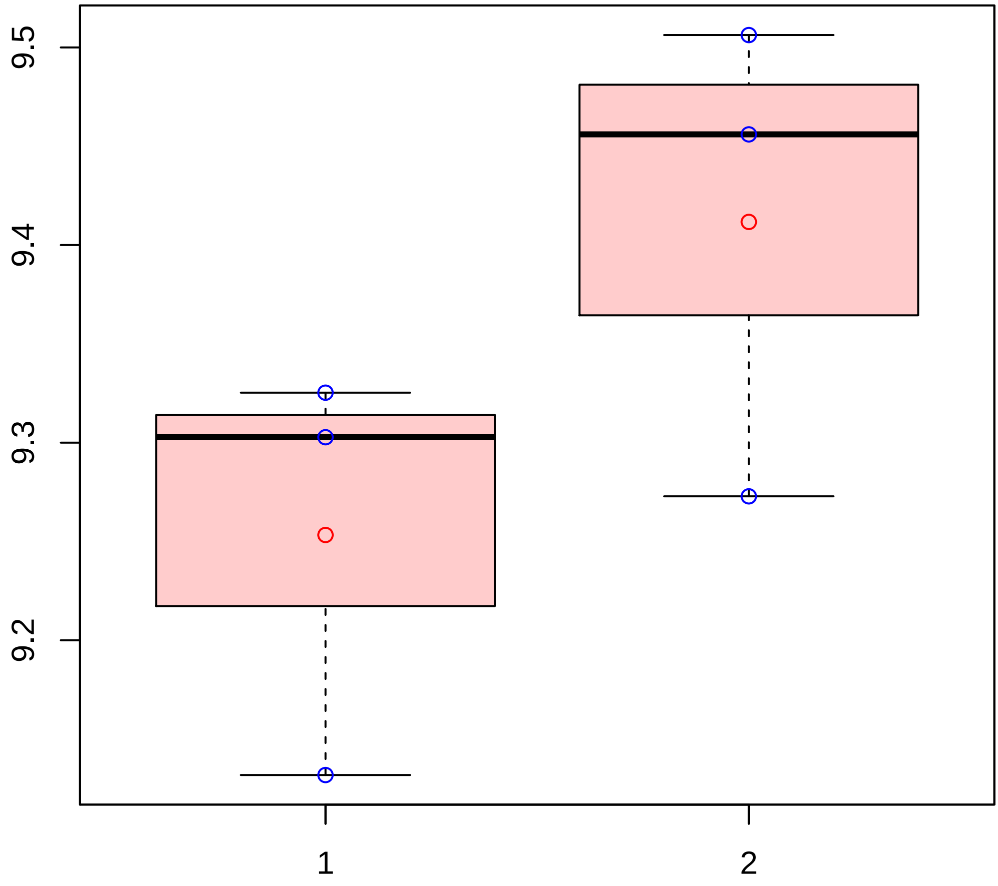

t−Test: p−value = 0.17

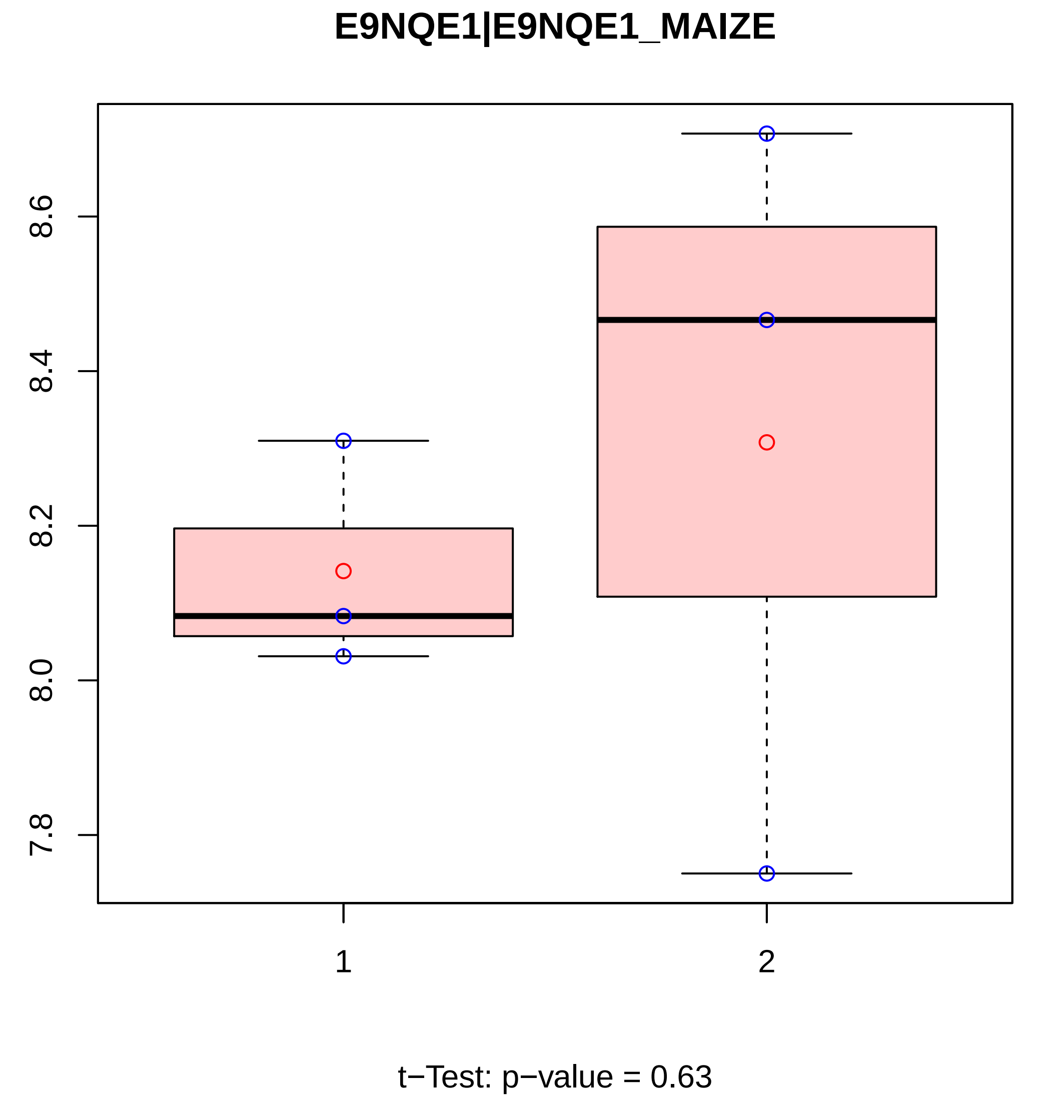

E9NQE1|E9NQE1_MAIZE
7.8
8.0
8.2
8.4
8.6
1
2
t−Test: p−value = 0.63

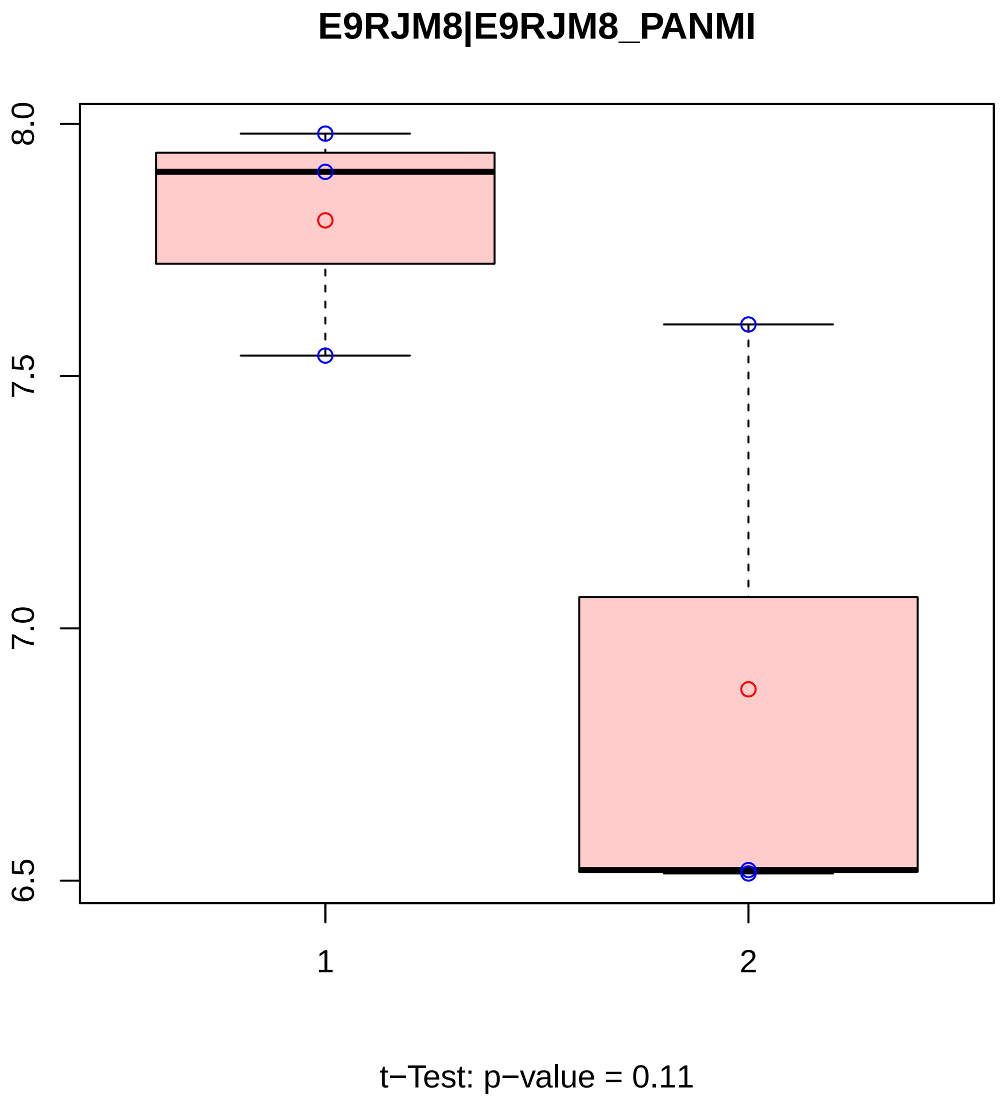
E9RJM8|E9RJM8_PANMI
8.0
7.5
7.0
6.5
1
2
t−Test: p−value = 0.11

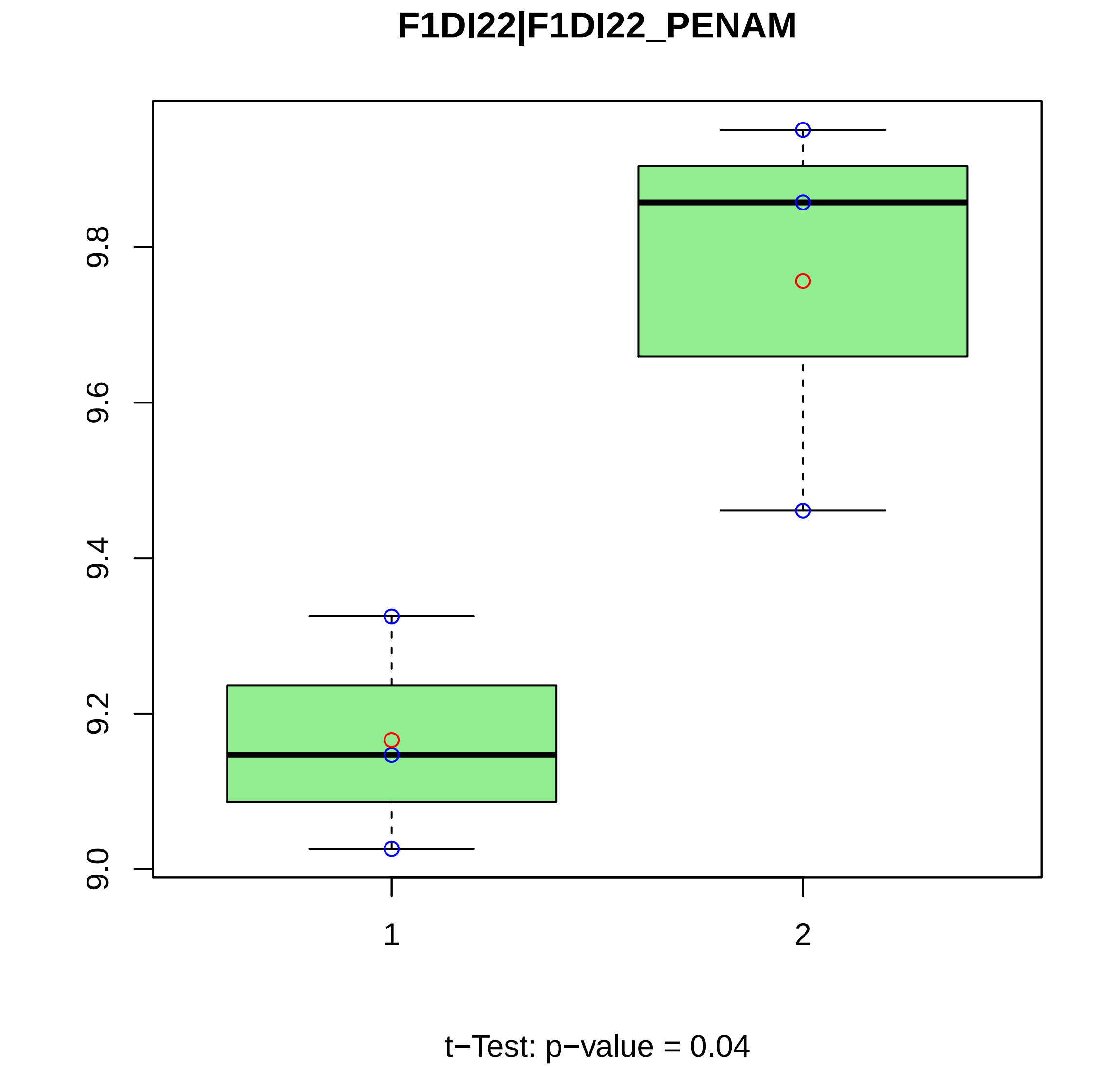
F1DI22|F1DI22_PENAM
9.0
9.2
9.4
9.6
9.8
1
2
t−Test: p−value = 0.04

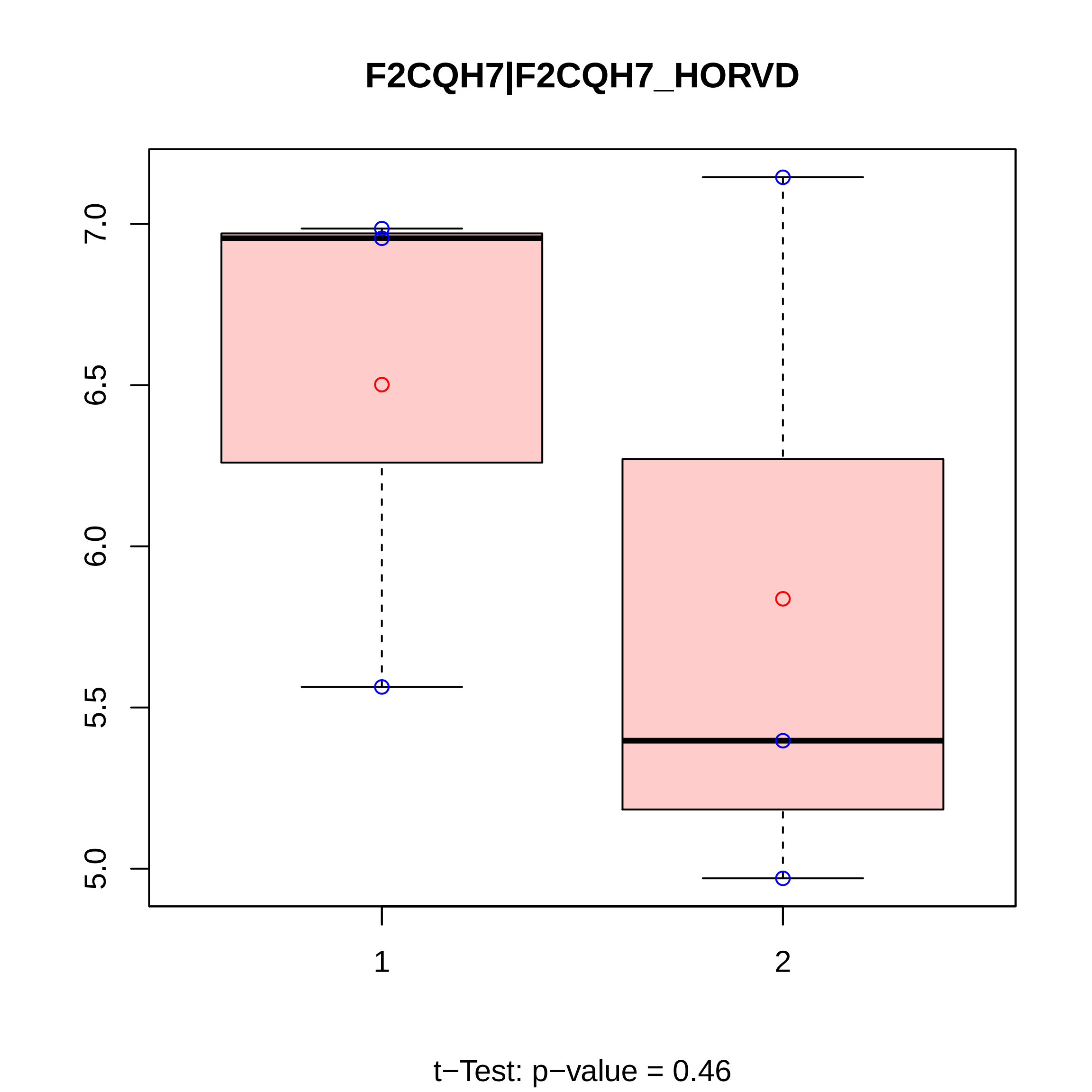
F2CQH7|F2CQH7_HORVD
7.0
6.5
6.0
5.5
5.0
1
2
t−Test: p−value = 0.46

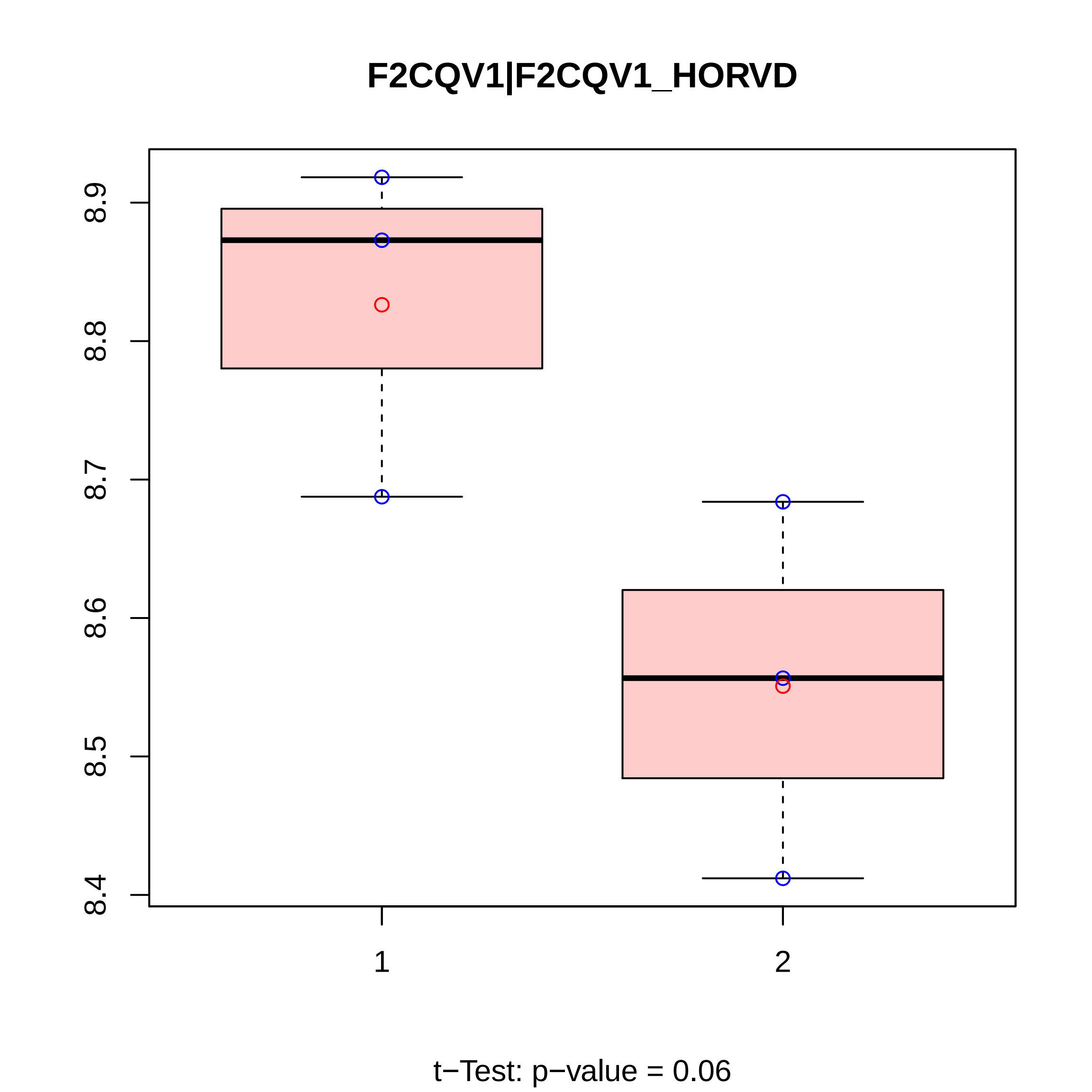
F2CQV1|F2CQV1_HORVD
8.4
8.5
8.6
8.7
8.8
8.9
1
2
t−Test: p−value = 0.06

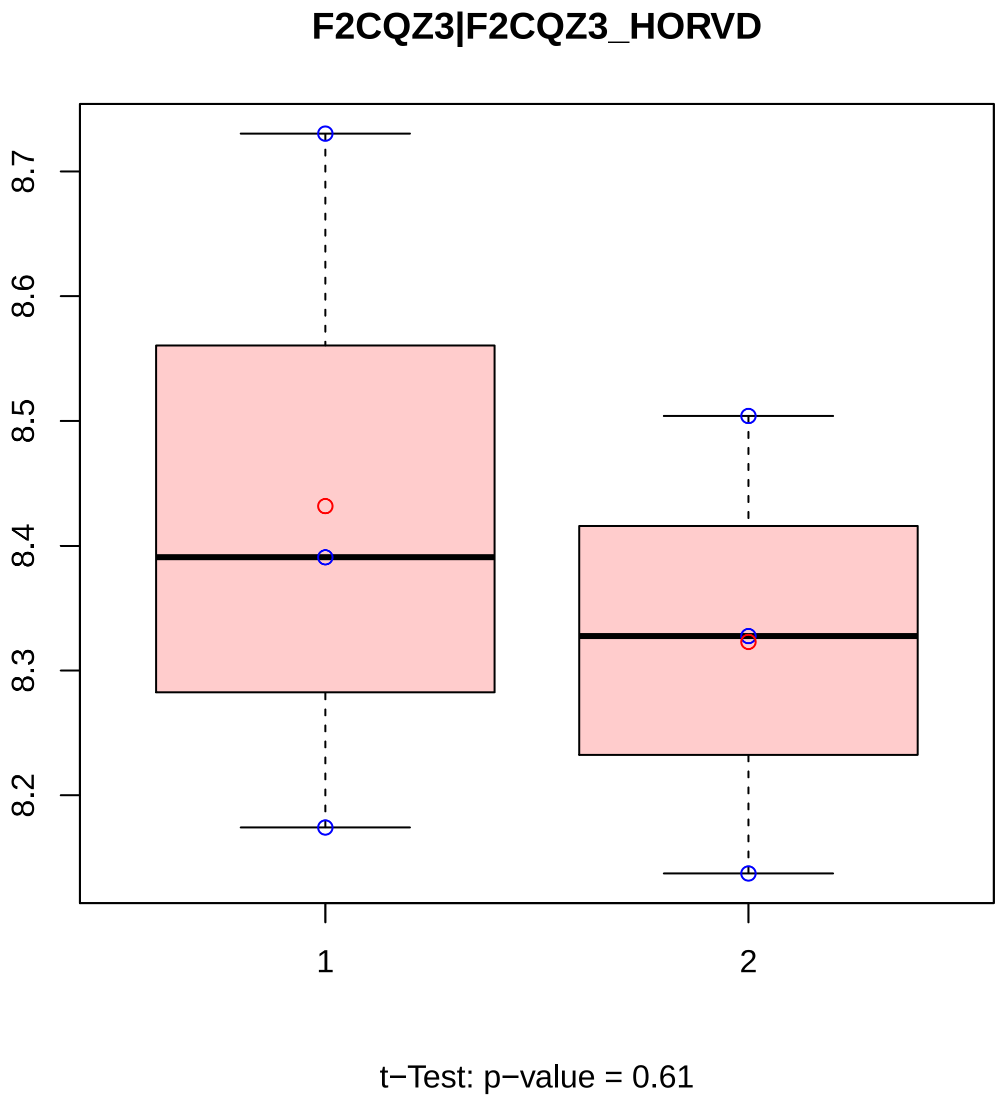
F2CQZ3|F2CQZ3_HORVD
8.2
8.3
8.4
8.5
8.6
8.7
1
2
t−Test: p−value = 0.61

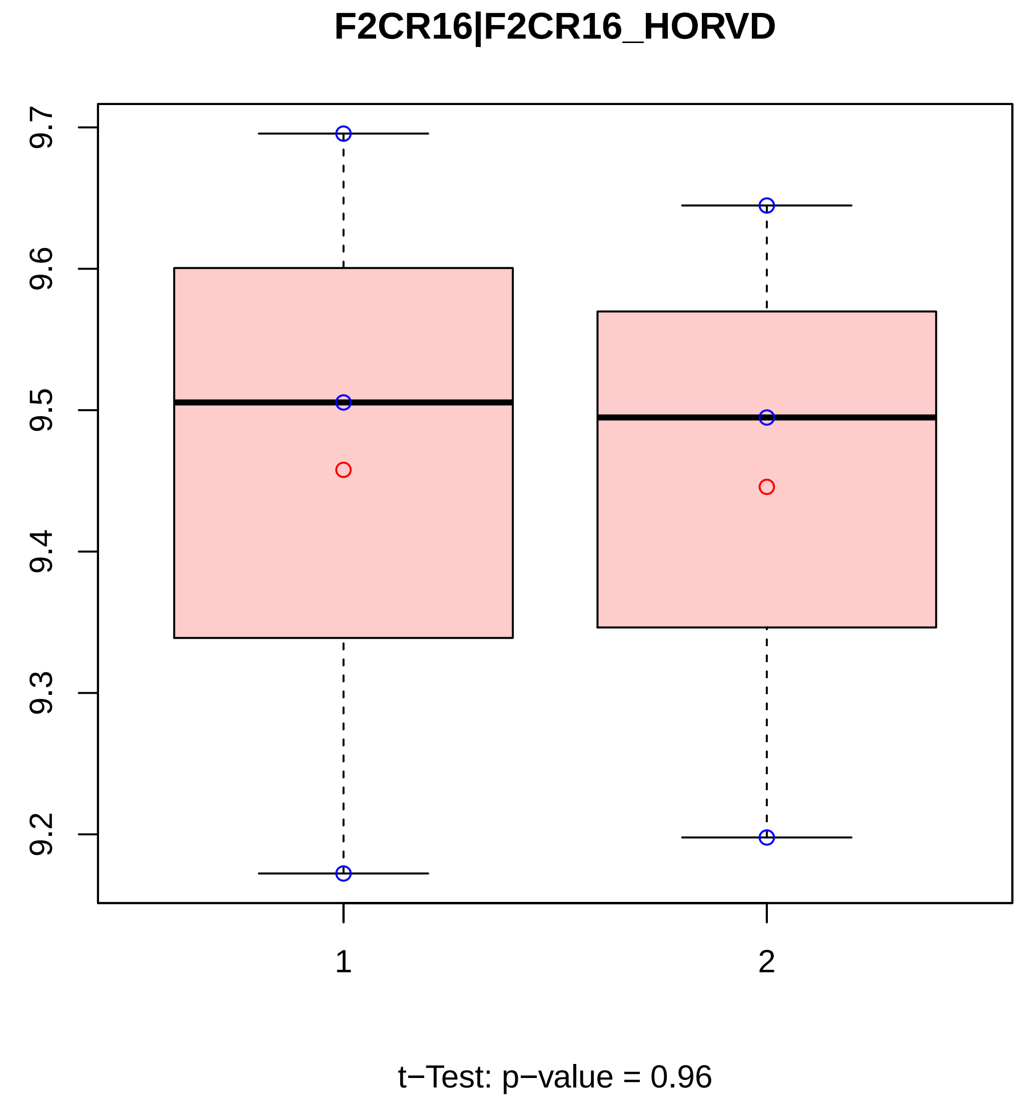

F2CR16|F2CR16_HORVD
9.2
9.3
9.4
9.5
9.6
9.7
1
2
t−Test: p−value = 0.96

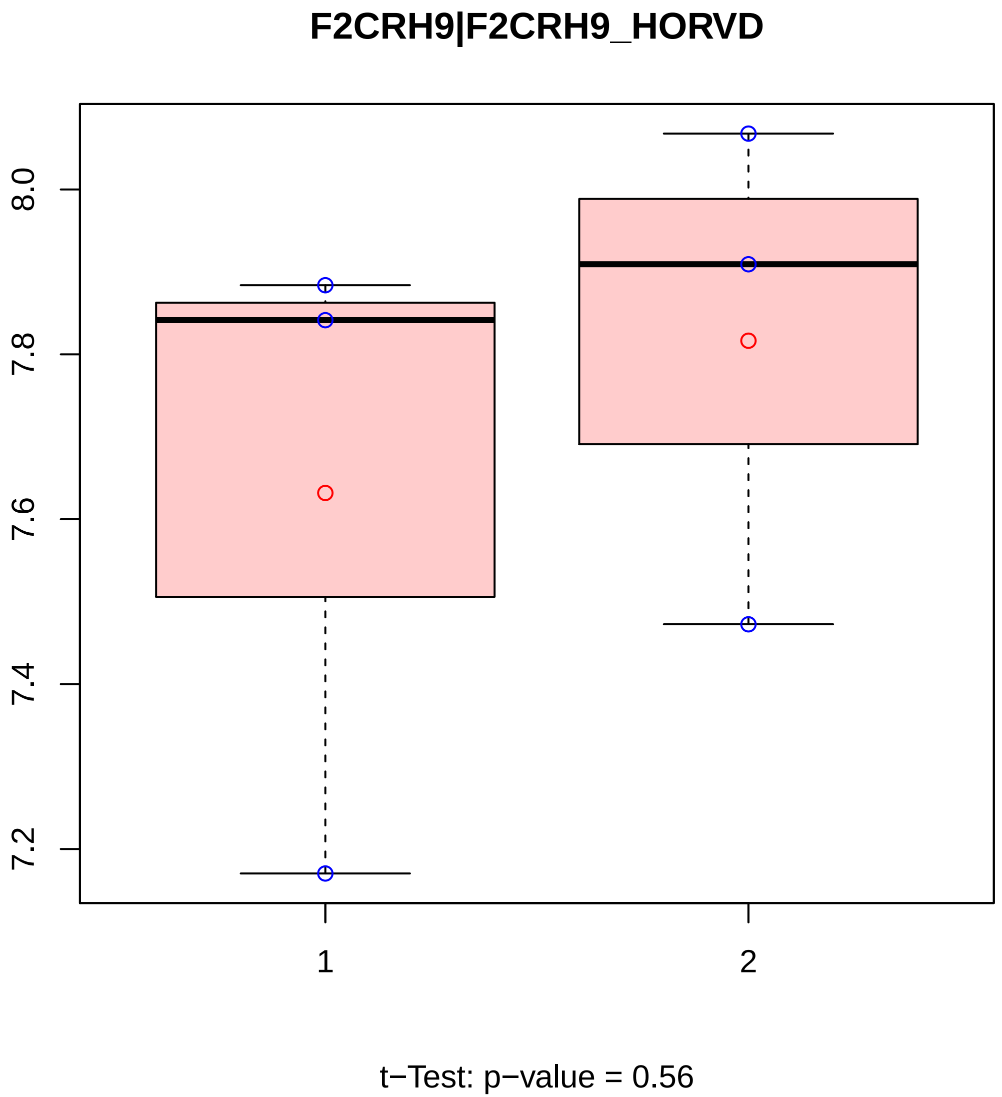

F2CRH9|F2CRH9_HORVD
8.0
7.8
7.6
7.4
7.2
1
2
t−Test: p−value = 0.56

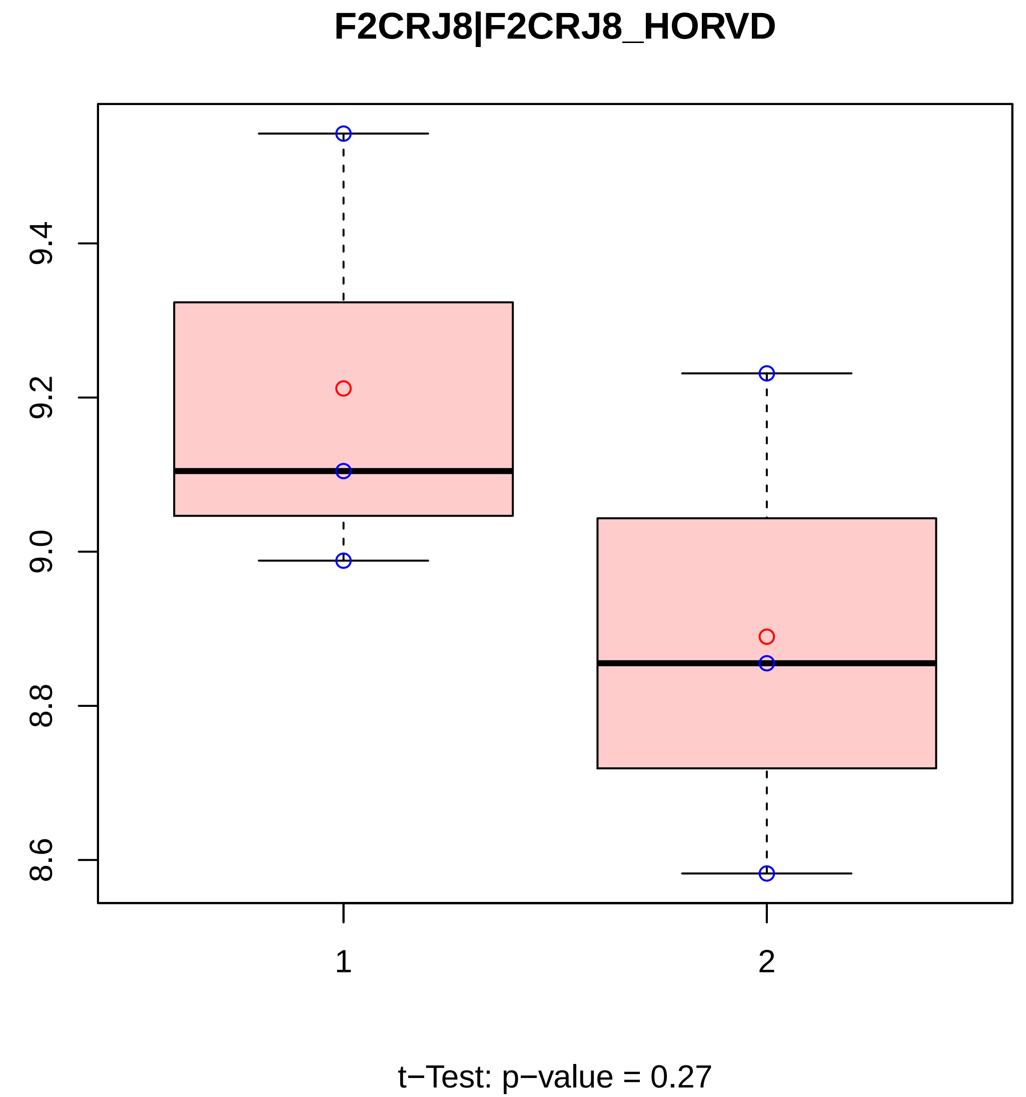
F2CRJ8|F2CRJ8_HORVD
9.4
9.2
9.0
8.8
8.6
1
2
t−Test: p−value = 0.27

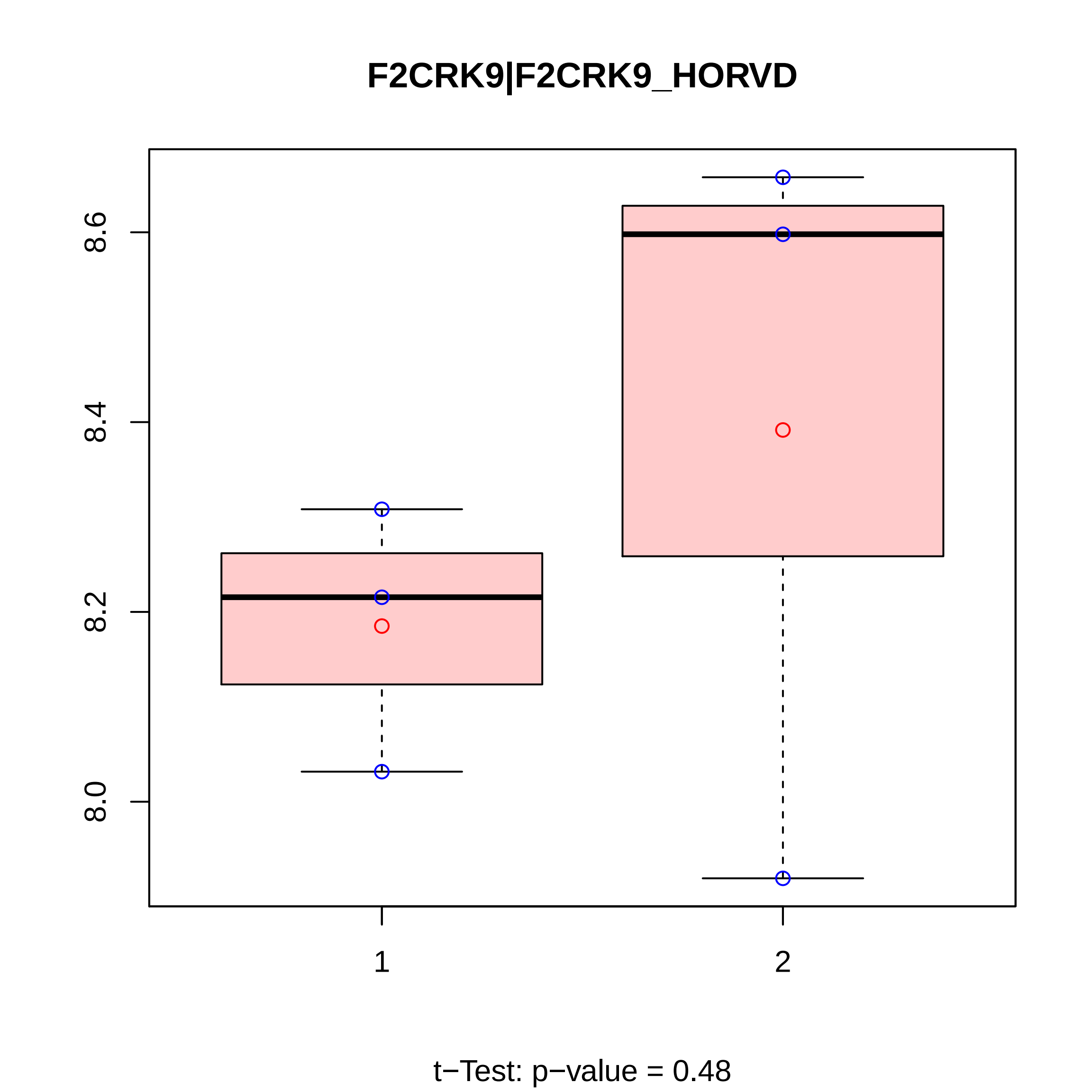
F2CRK9|F2CRK9_HORVD
8.0
8.2
8.4
8.6
1
2
t−Test: p−value = 0.48

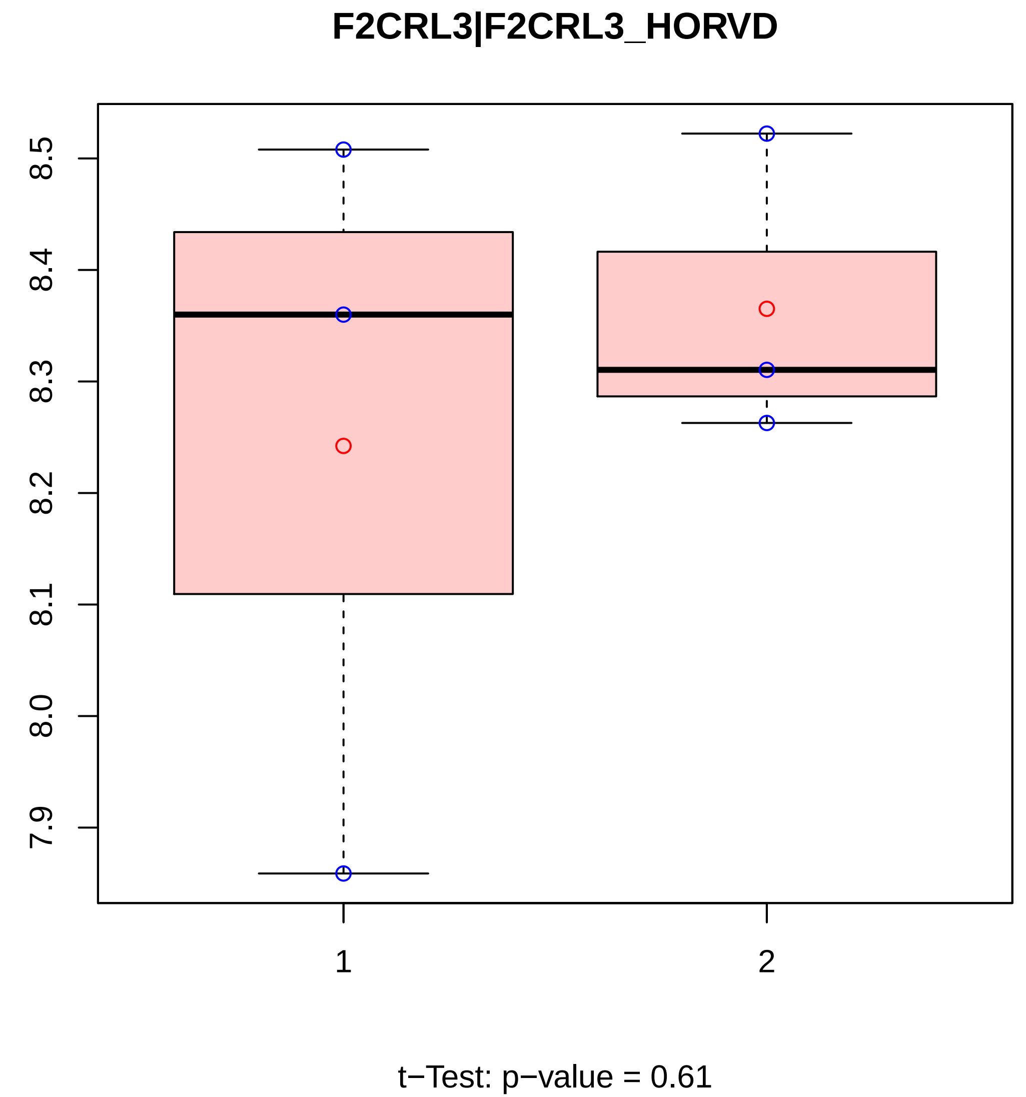
F2CRL3|F2CRL3_HORVD
8.5
8.4
8.3
8.2
8.1
8.0
7.9
1
2
t−Test: p−value = 0.61

F2CRM3|F2CRM3_HORVD

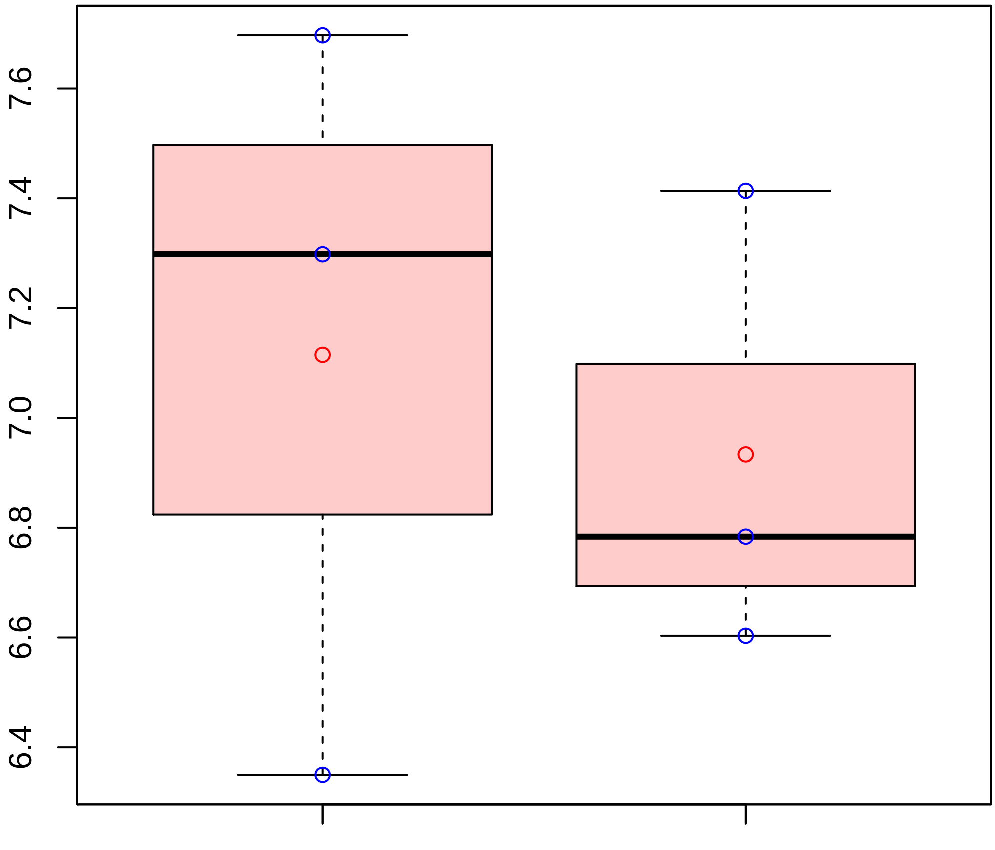

t−Test: p−value = 0.72

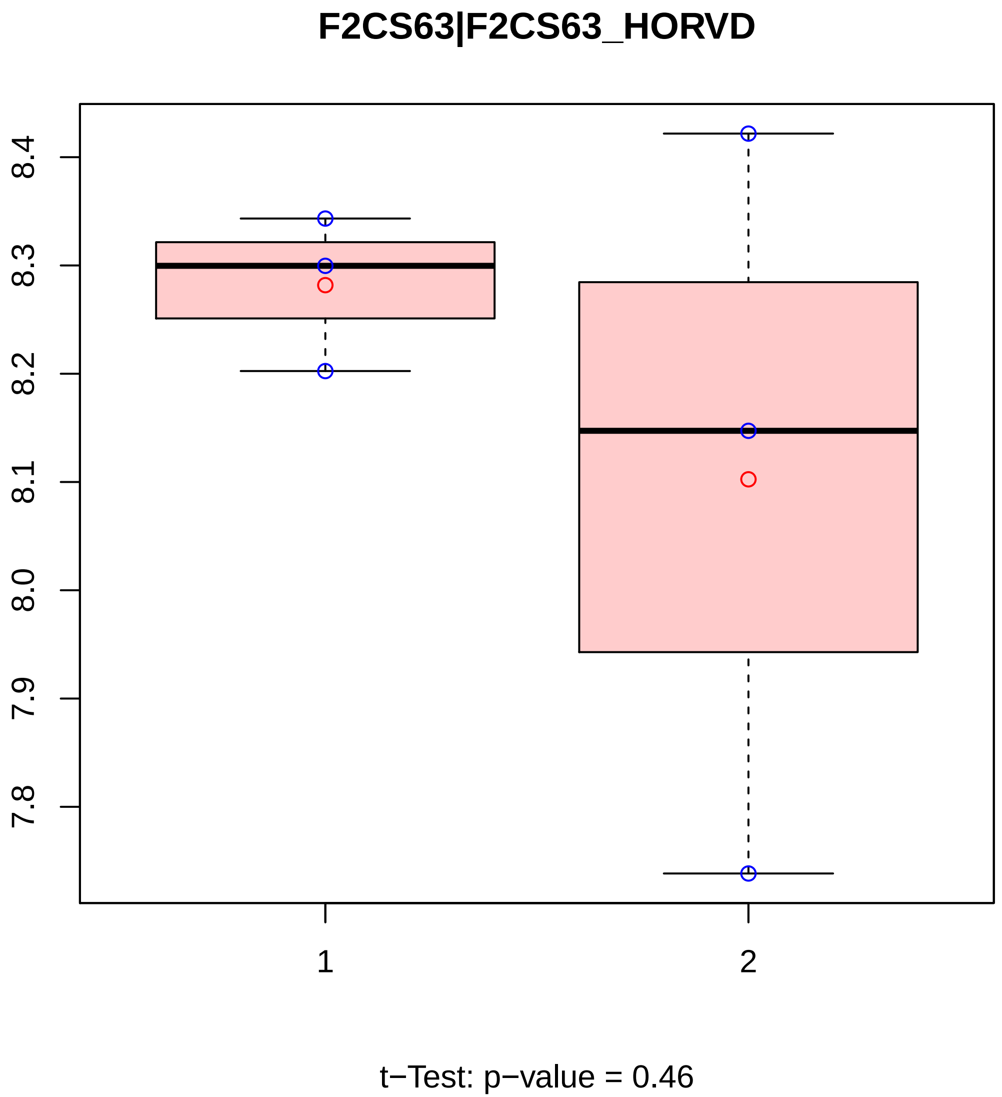

F2CS63|F2CS63_HORVD
8.4
8.3
8.2
8.1
8.0
7.9
7.8
1
2
t−Test: p−value = 0.46

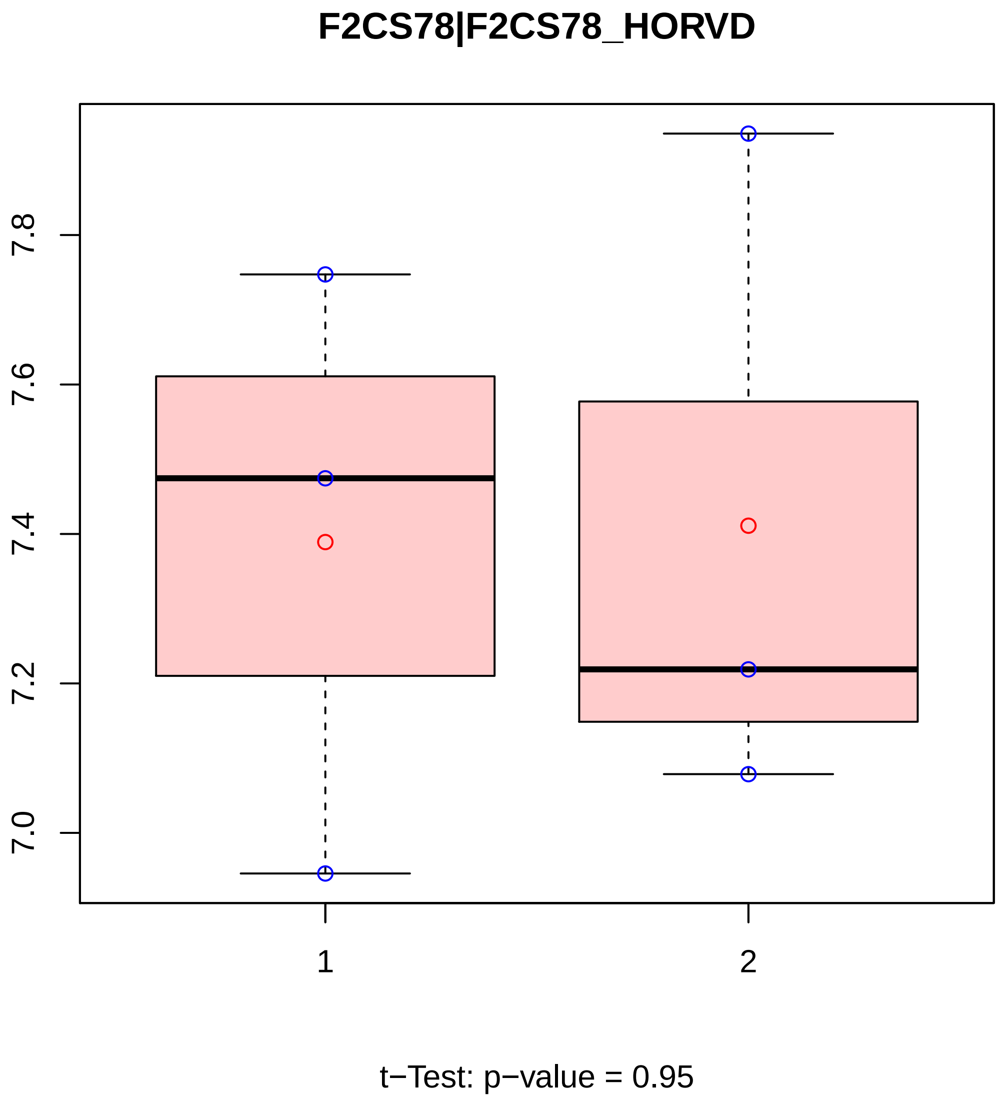

F2CS78|F2CS78_HORVD
7.0
7.2
7.4
7.6
7.8
1
2
t−Test: p−value = 0.95

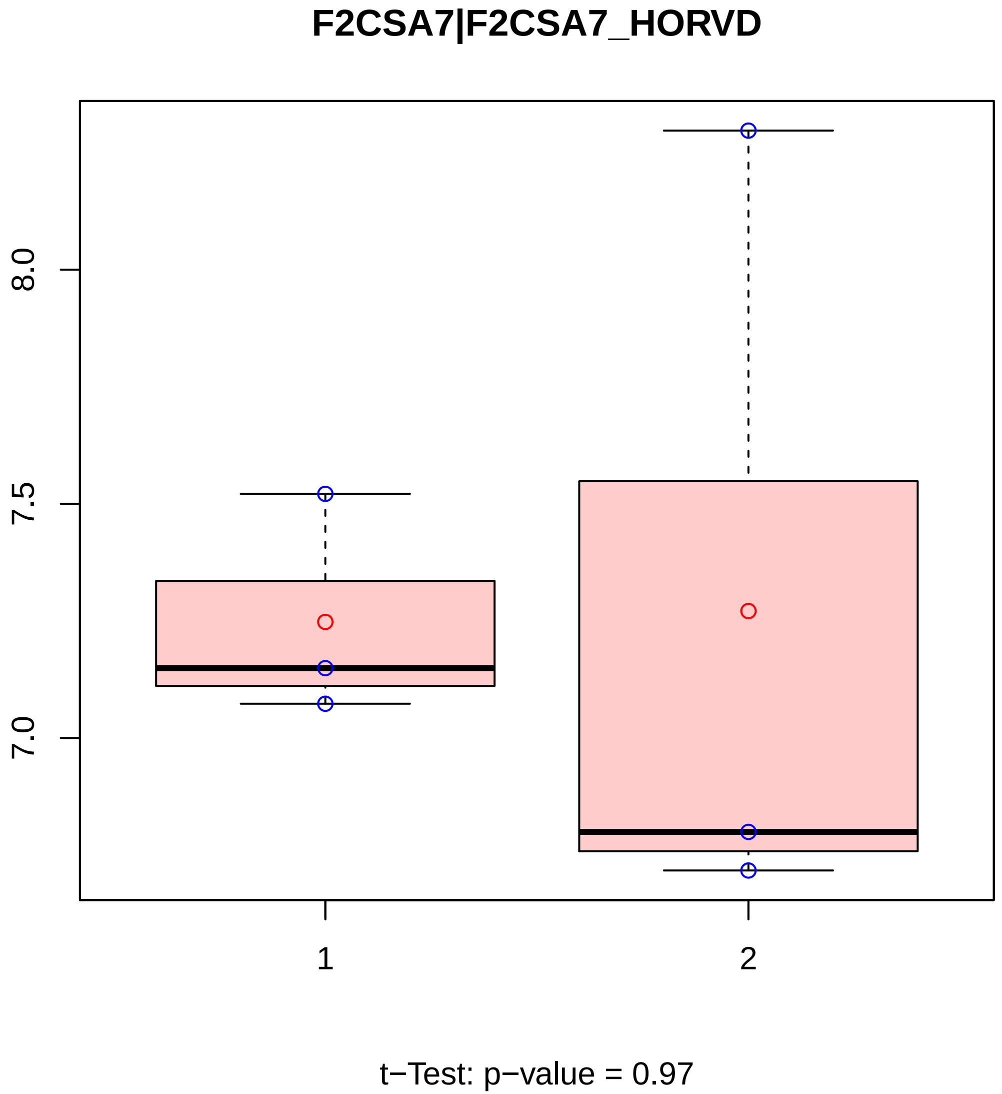
F2CSA7|F2CSA7_HORVD
8.0
7.5
7.0
1
2
t−Test: p−value = 0.97

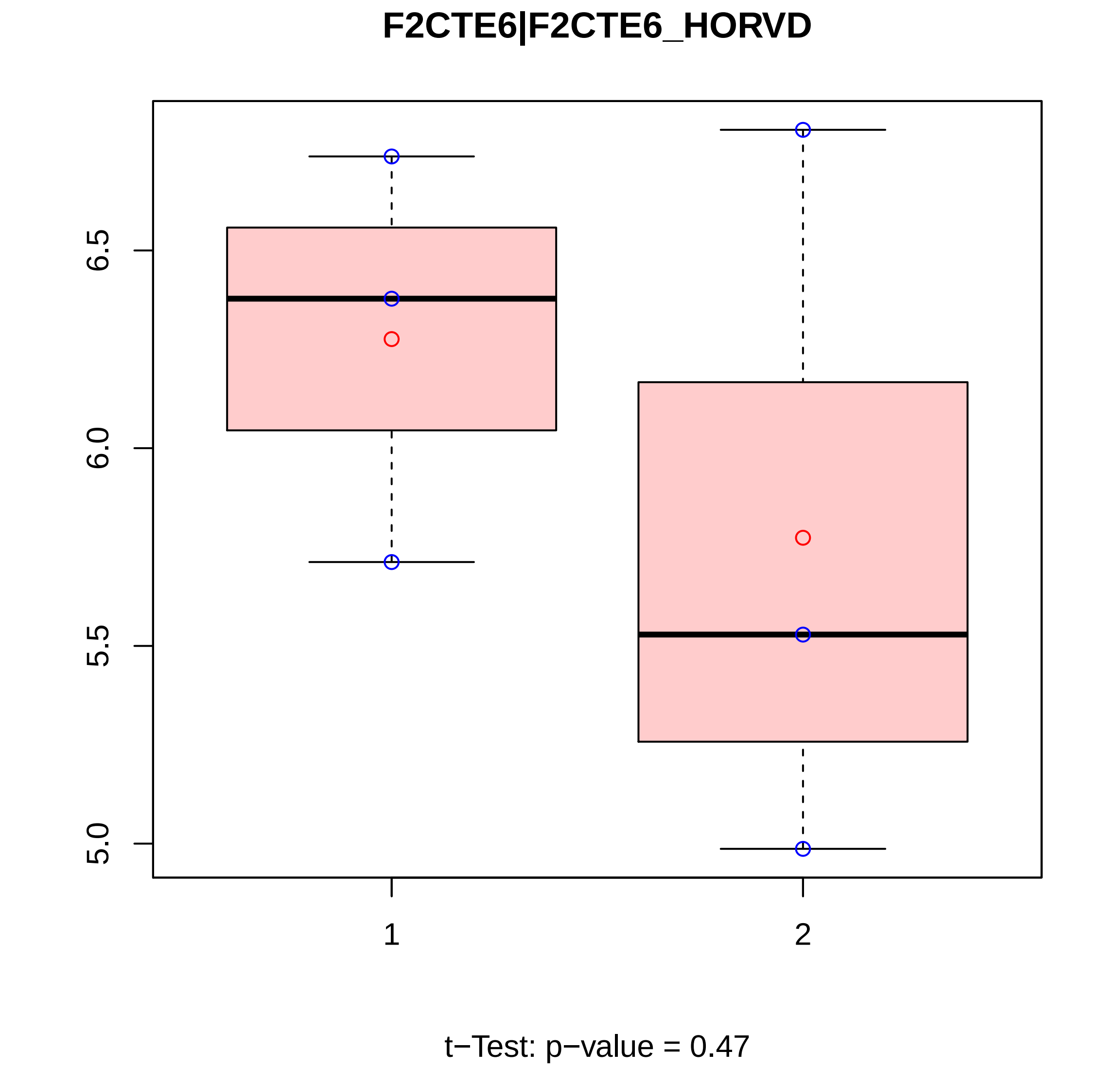
F2CTE6|F2CTE6_HORVD
6.5
6.0
5.5
5.0
1
2
t−Test: p−value = 0.47

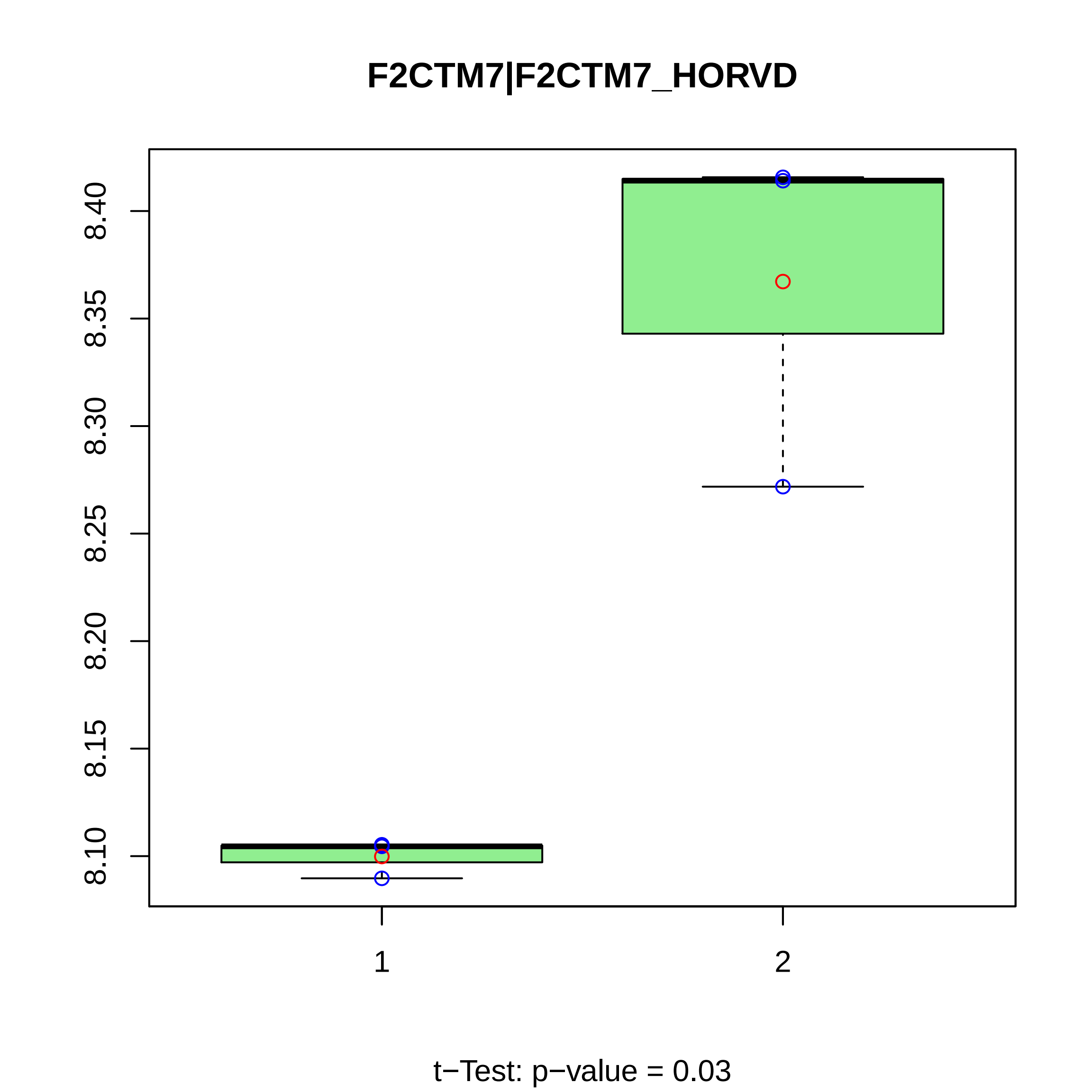

F2CTM7|F2CTM7_HORVD
8.10
8.15
8.20
8.25
8.30
8.35
8.40
1
2
t−Test: p−value = 0.03

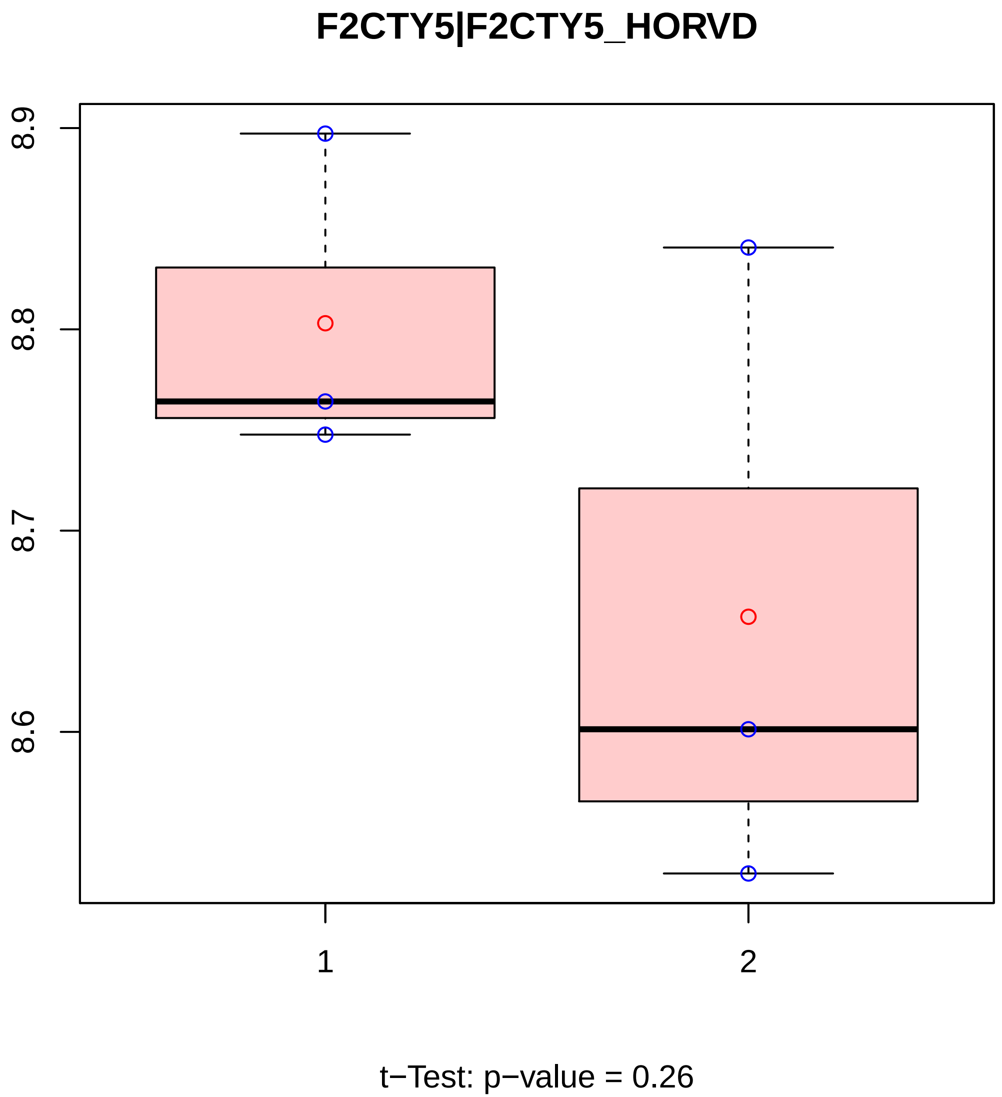
F2CTY5|F2CTY5_HORVD
8.9
8.8
8.7
8.6
1
2
t−Test: p−value = 0.26

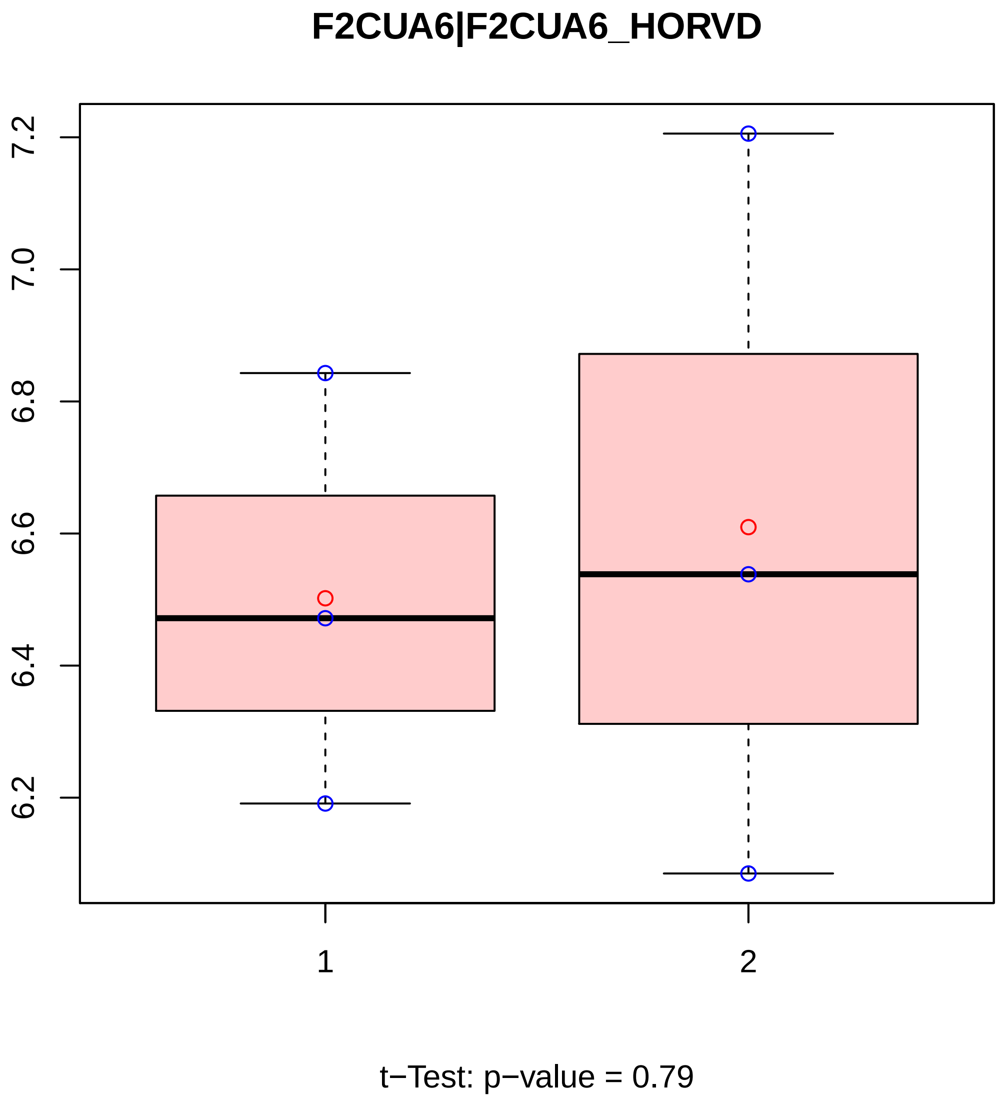
F2CUA6|F2CUA6_HORVD
7.2
7.0
6.8
6.6
6.4
6.2
1
2
t−Test: p−value = 0.79

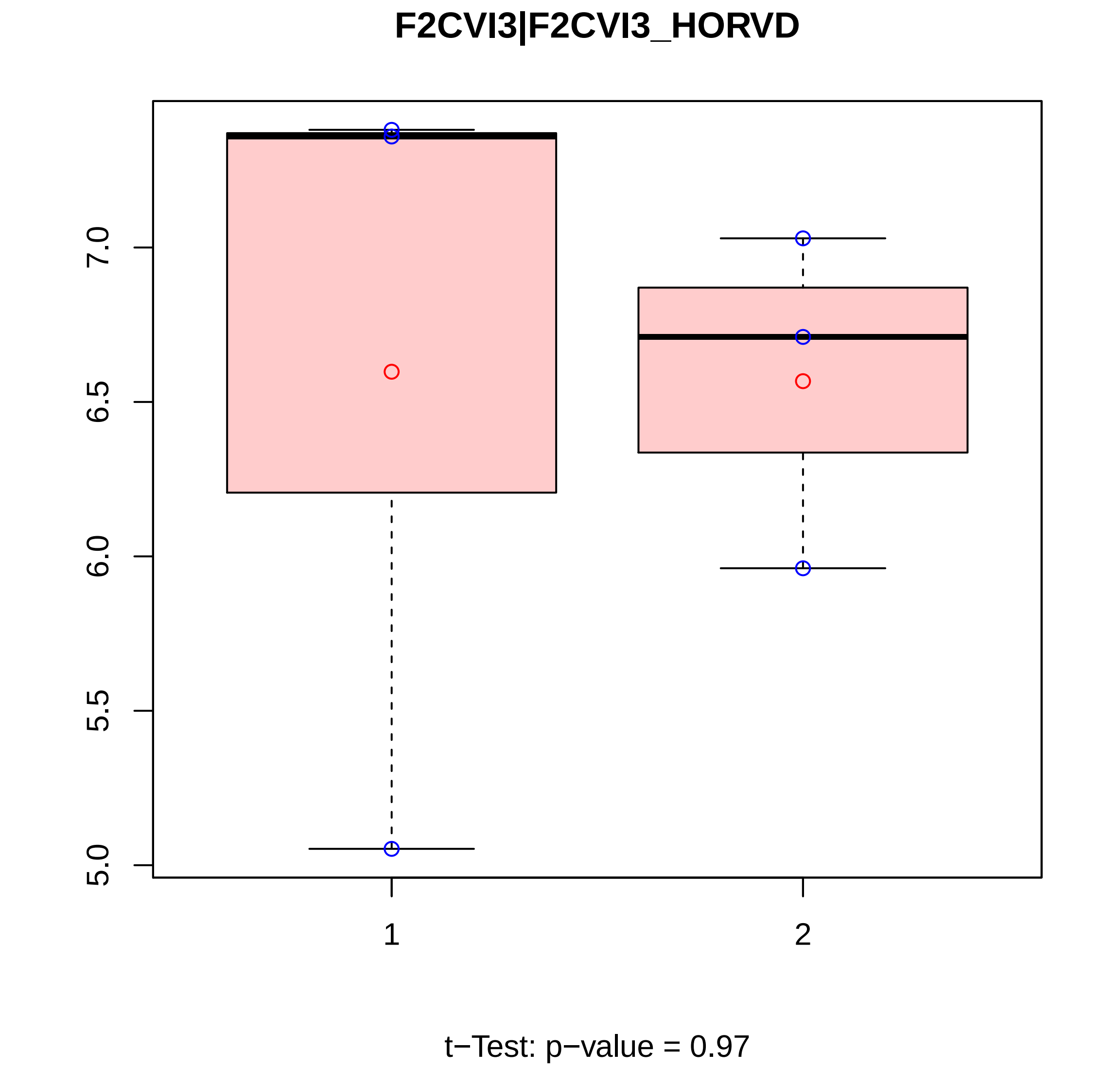
F2CVI3|F2CVI3_HORVD
7.0
6.5
6.0
5.5
5.0
1
2
t−Test: p−value = 0.97

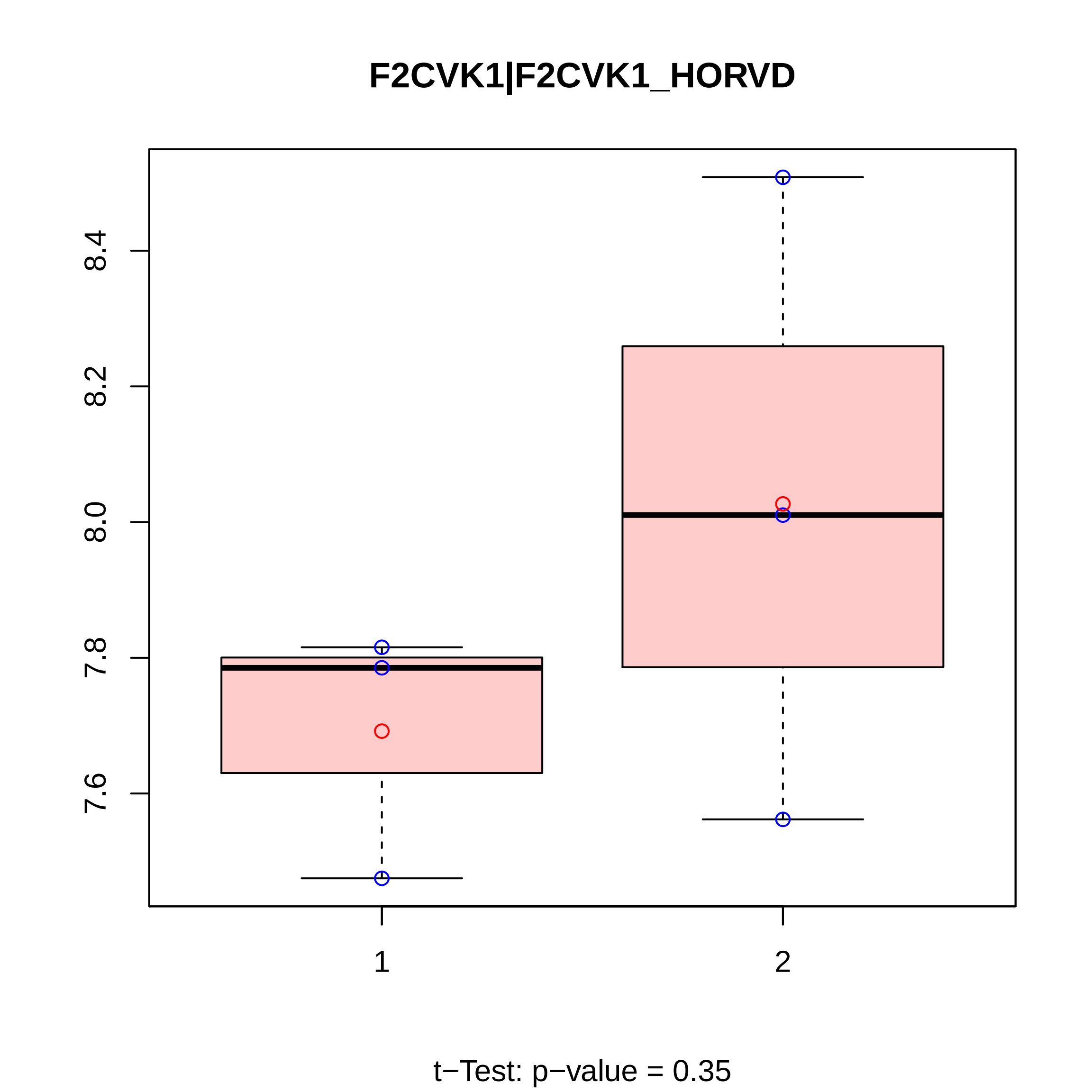
F2CVK1|F2CVK1_HORVD
8.4
8.2
8.0
7.8
7.6
1
2
t−Test: p−value = 0.35

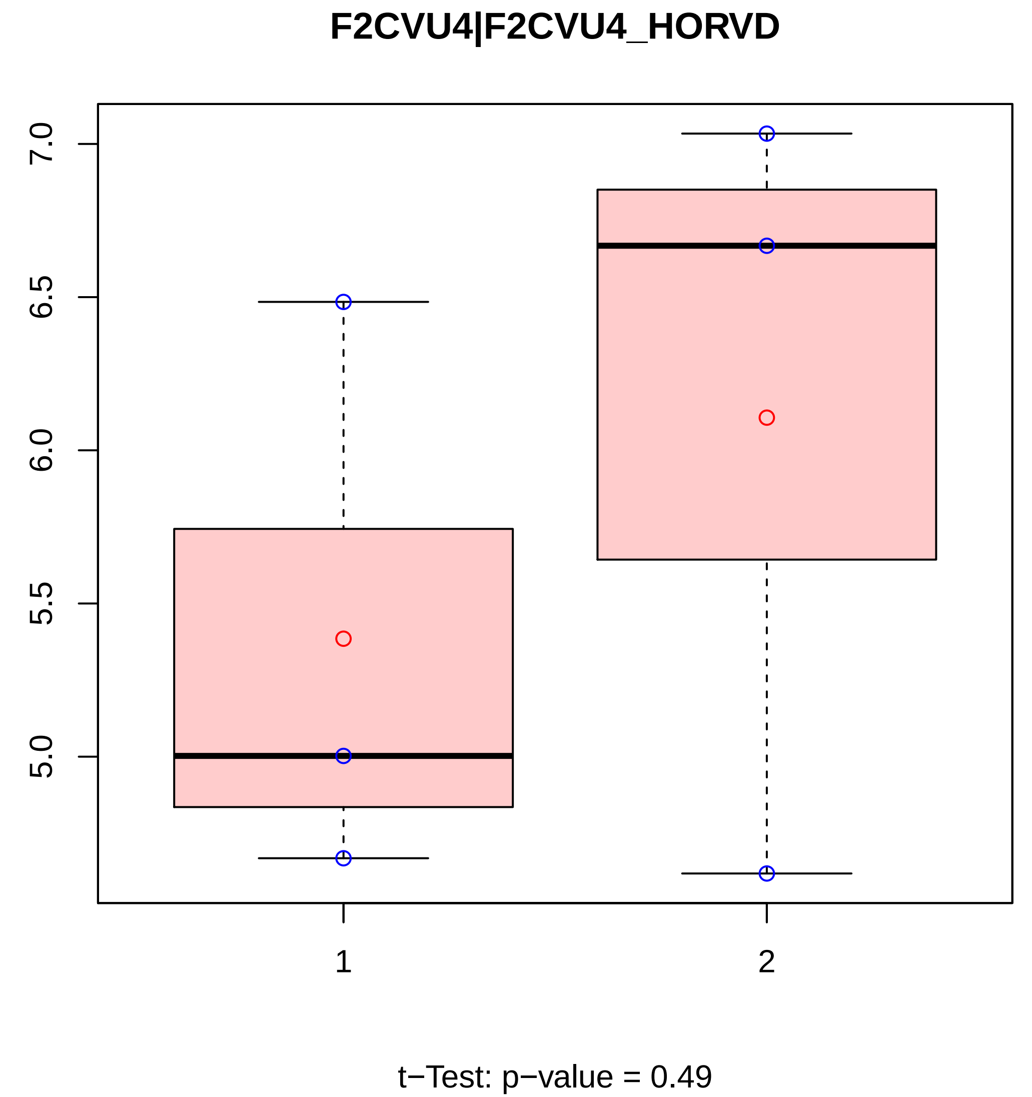
F2CVU4|F2CVU4_HORVD
7.0
6.5
6.0
5.5
5.0
1
2
t−Test: p−value = 0.49

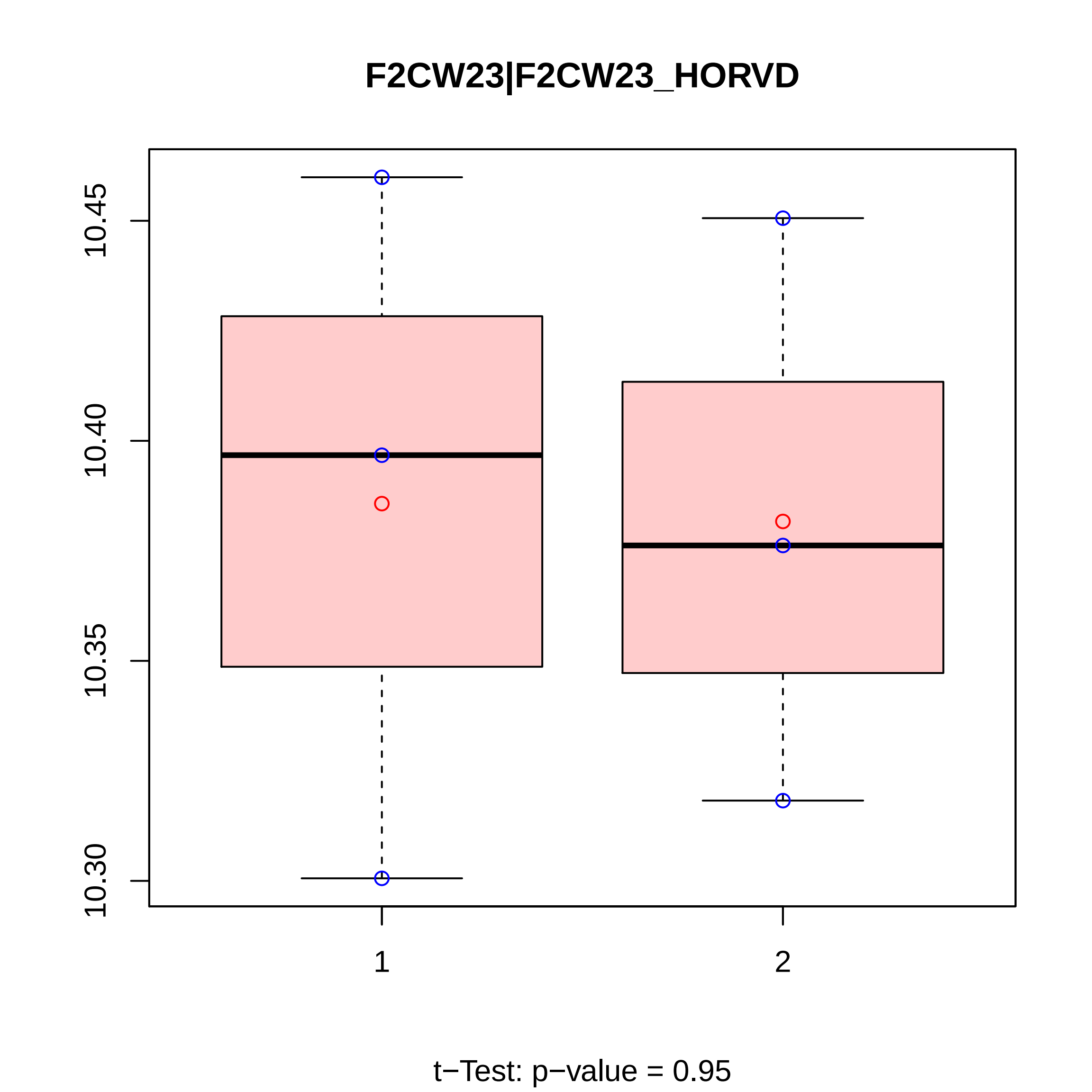
F2CW23|F2CW23_HORVD
10.30
10.35
10.40
10.45
1
2
t−Test: p−value = 0.95

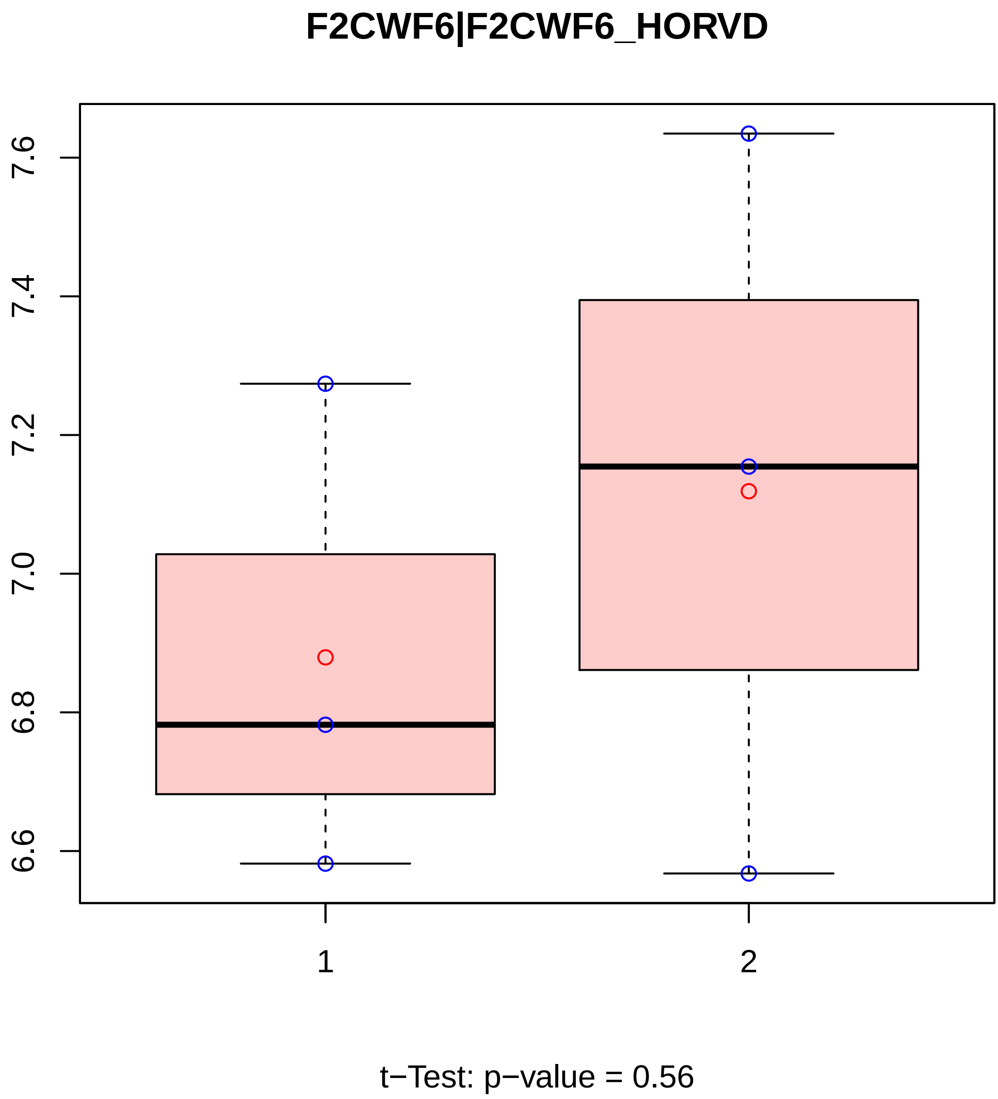

F2CWF6|F2CWF6_HORVD
7.6
7.4
7.2
7.0
6.8
6.6
1
2
t−Test: p−value = 0.56

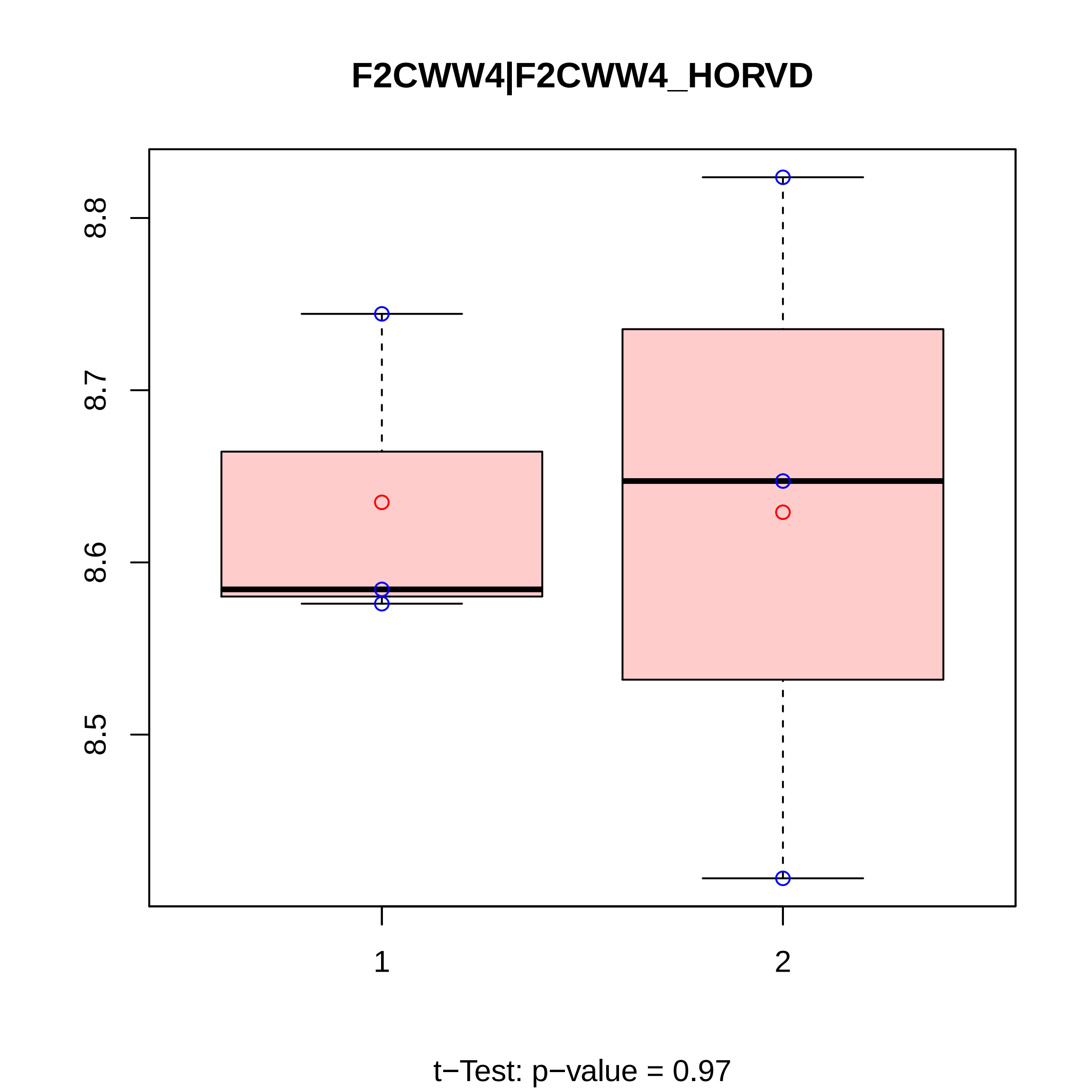
F2CWW4|F2CWW4_HORVD
8.8
8.7
8.6
8.5
1
2
t−Test: p−value = 0.97

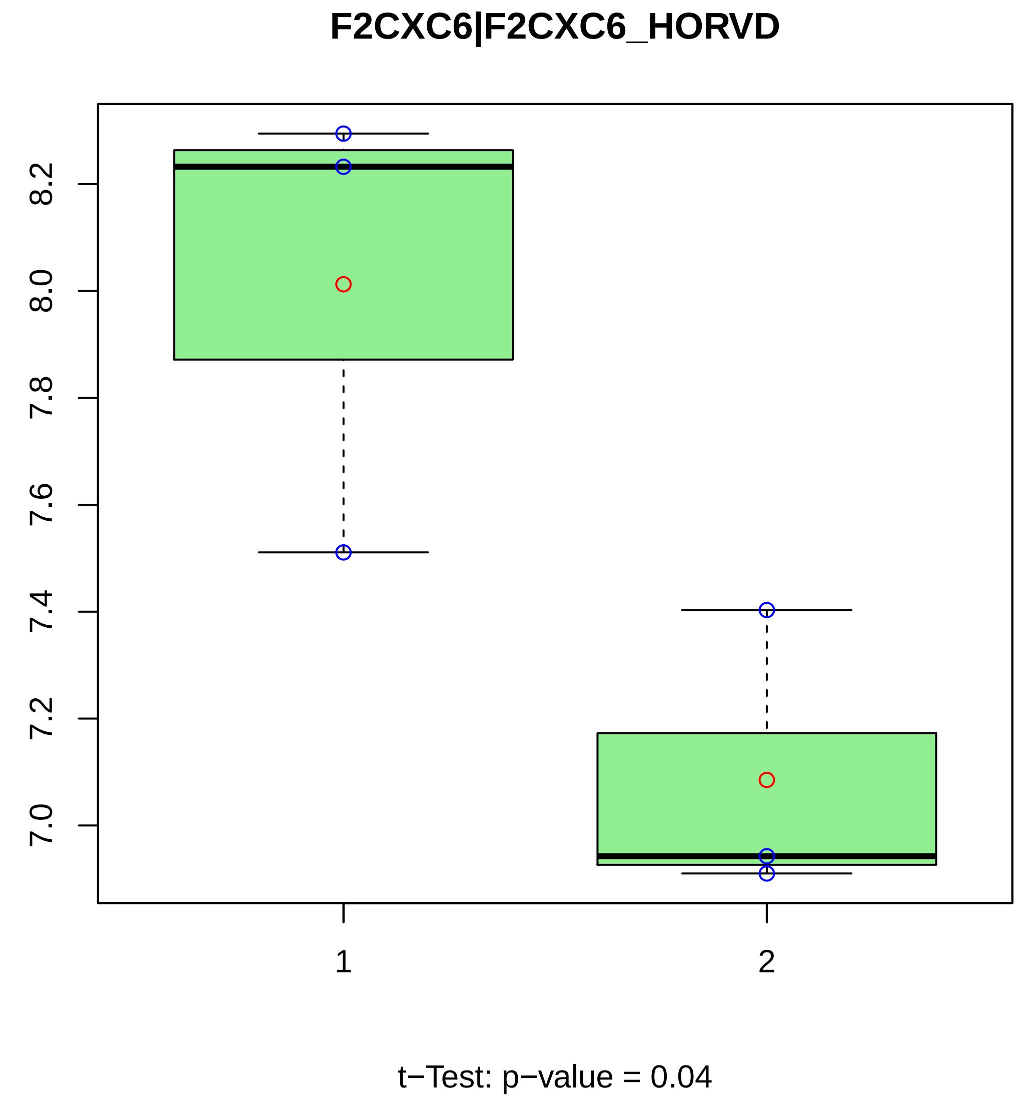

F2CXC6|F2CXC6_HORVD
8.2
8.0
7.8
7.6
7.4
7.2
7.0
1
2
t−Test: p−value = 0.04

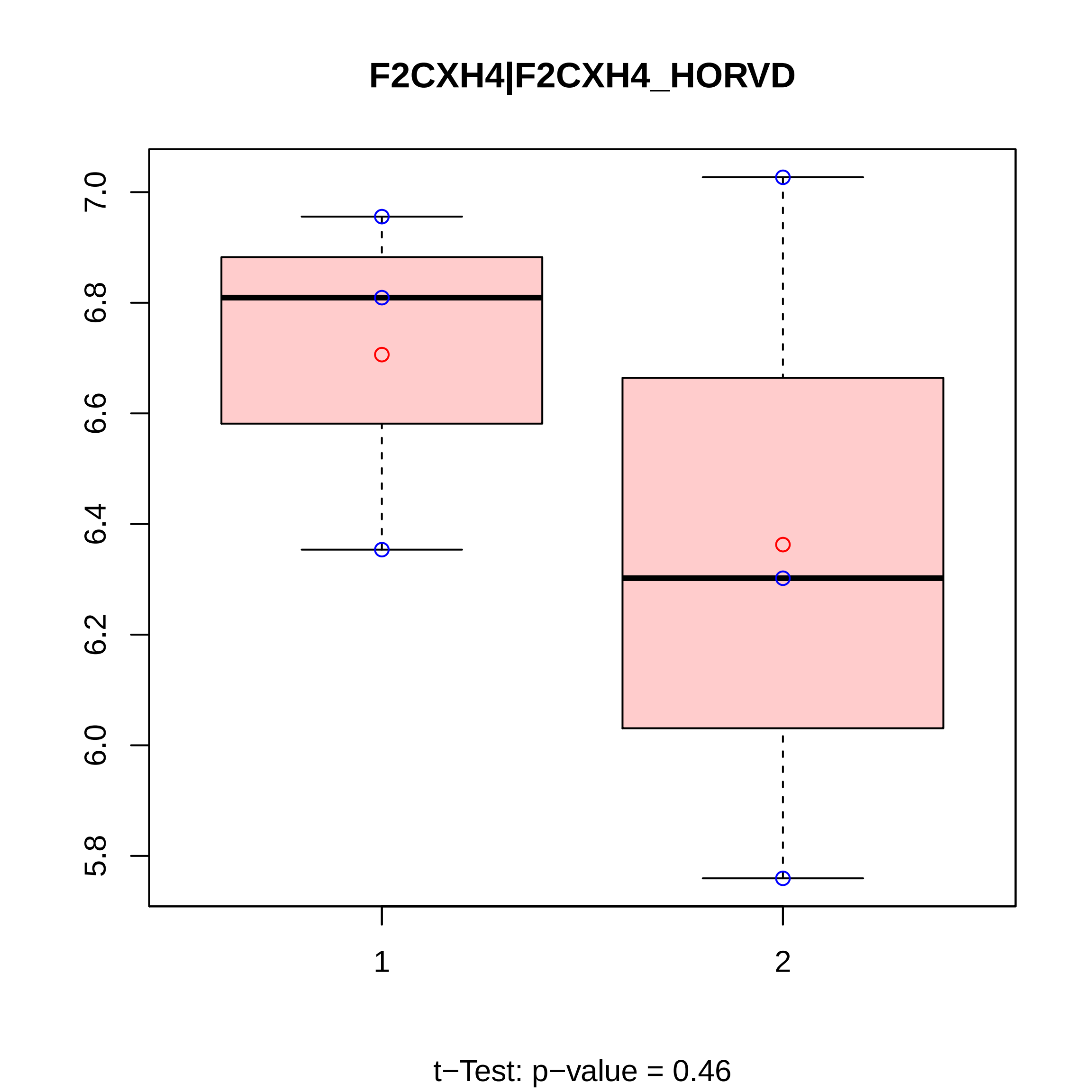

F2CXH4|F2CXH4_HORVD
7.0
6.8
6.6
6.4
6.2
6.0
5.8
1
2
t−Test: p−value = 0.46

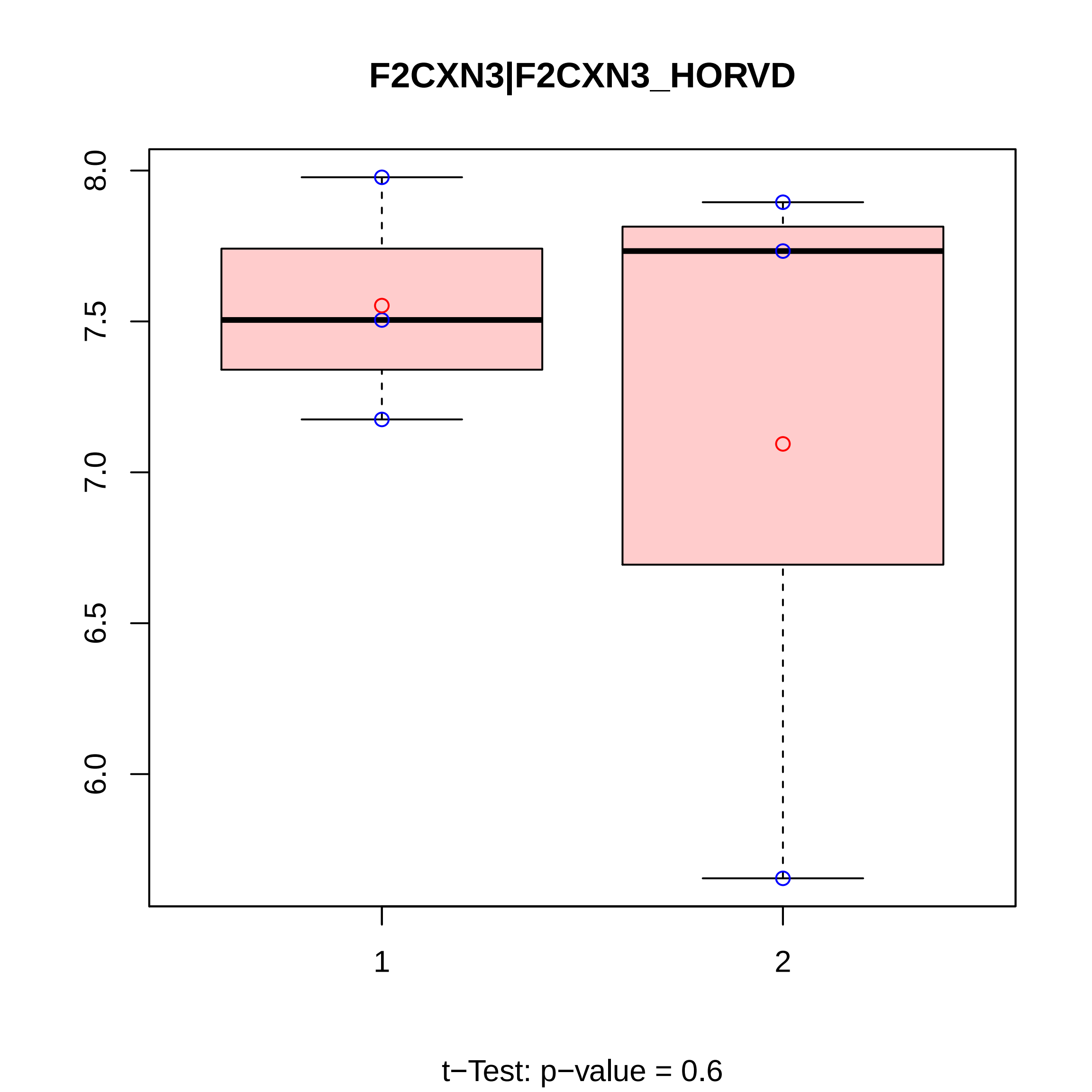
F2CXN3|F2CXN3_HORVD
8.0
7.5
7.0
6.5
6.0
1
2
t−Test: p−value = 0.6

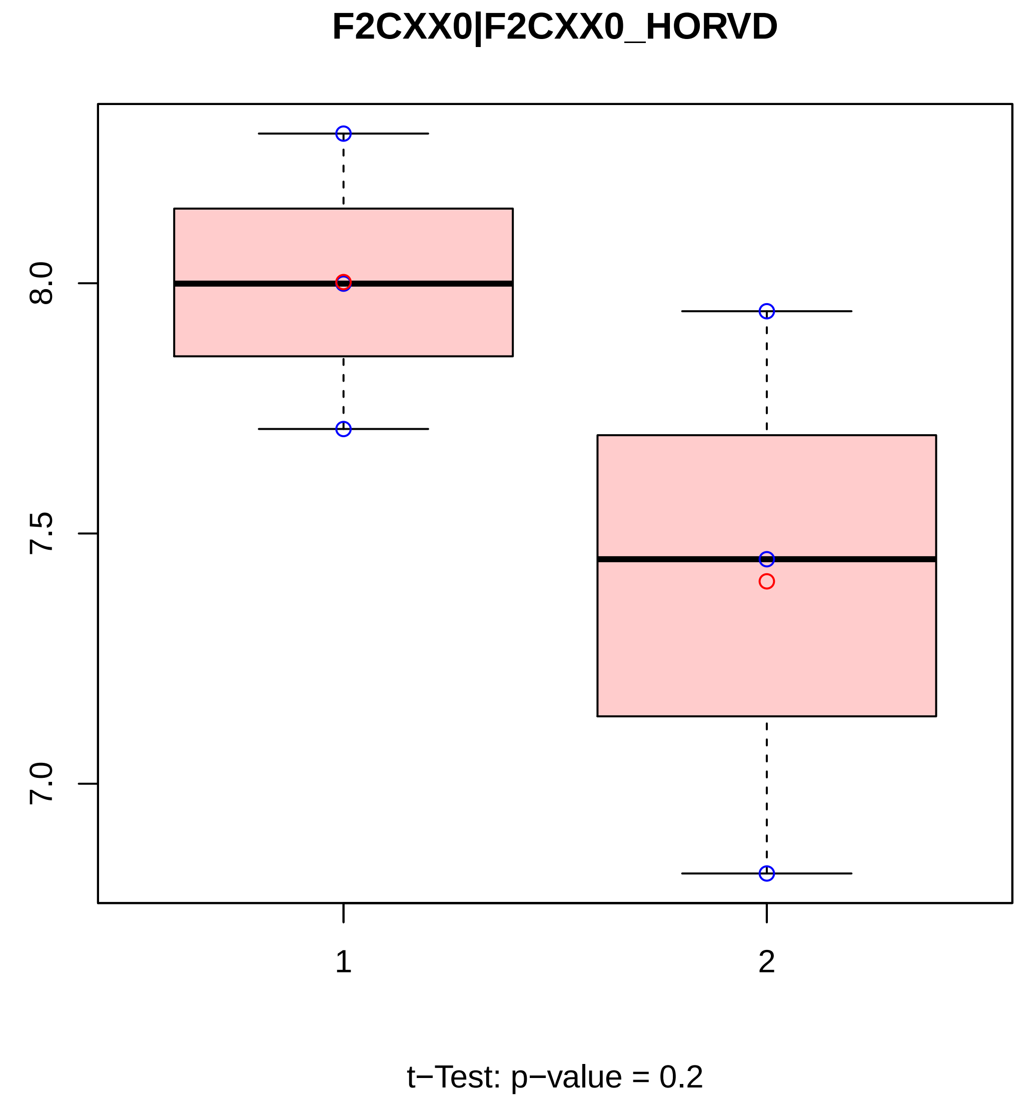

F2CXX0|F2CXX0_HORVD
8.0
7.5
7.0
1
2
t−Test: p−value = 0.2

F2CY15|F2CY15_HORVD

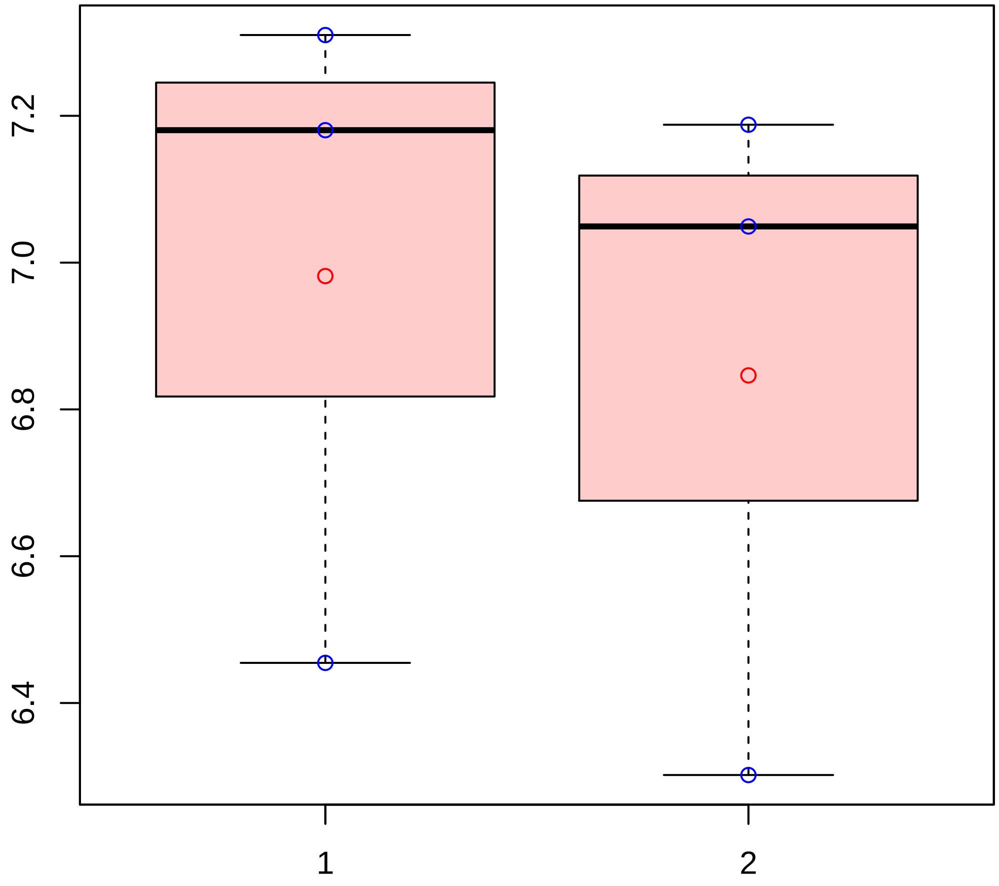

t−Test: p−value = 0.74

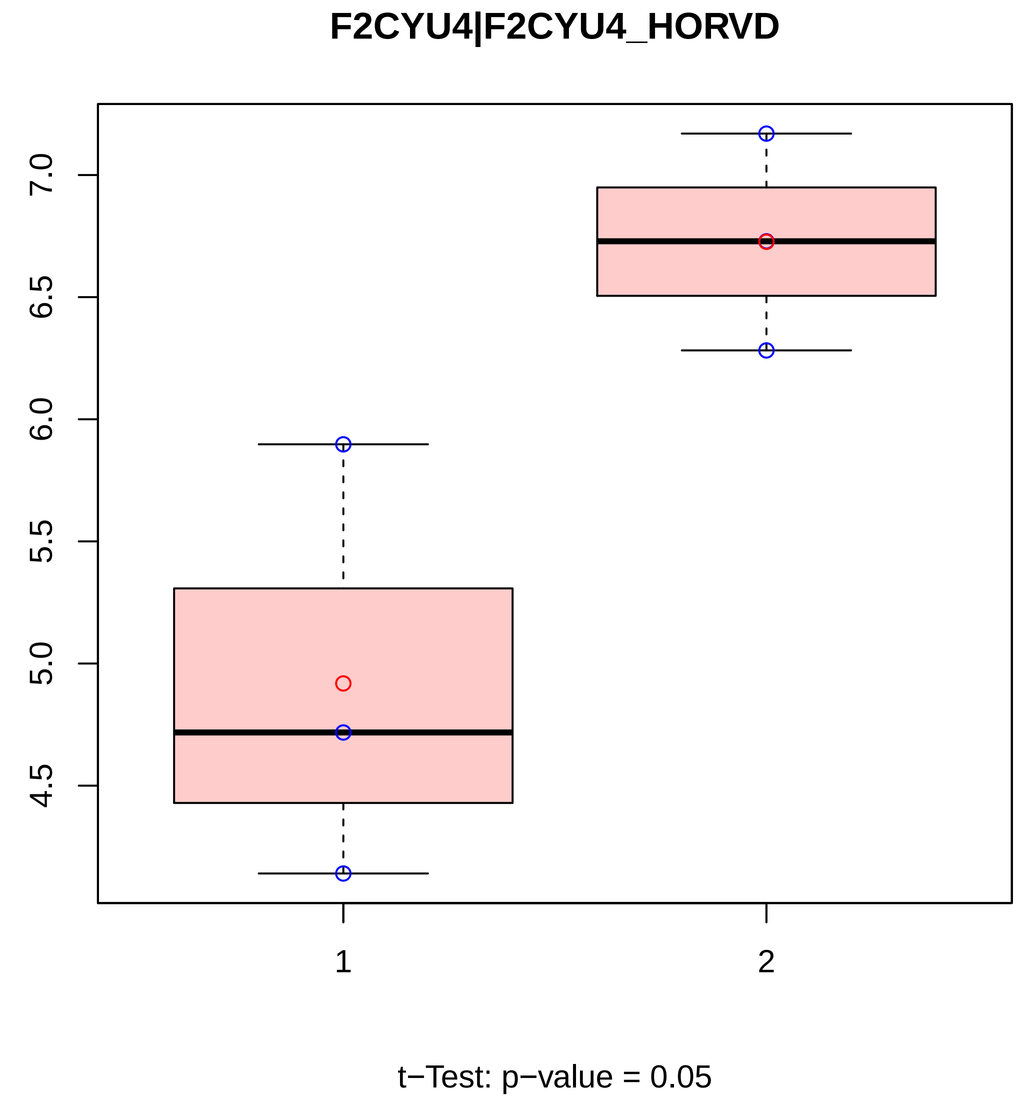

F2CYU4|F2CYU4_HORVD
7.0
6.5
6.0
5.5
5.0
4.5
1
2
t−Test: p−value = 0.05

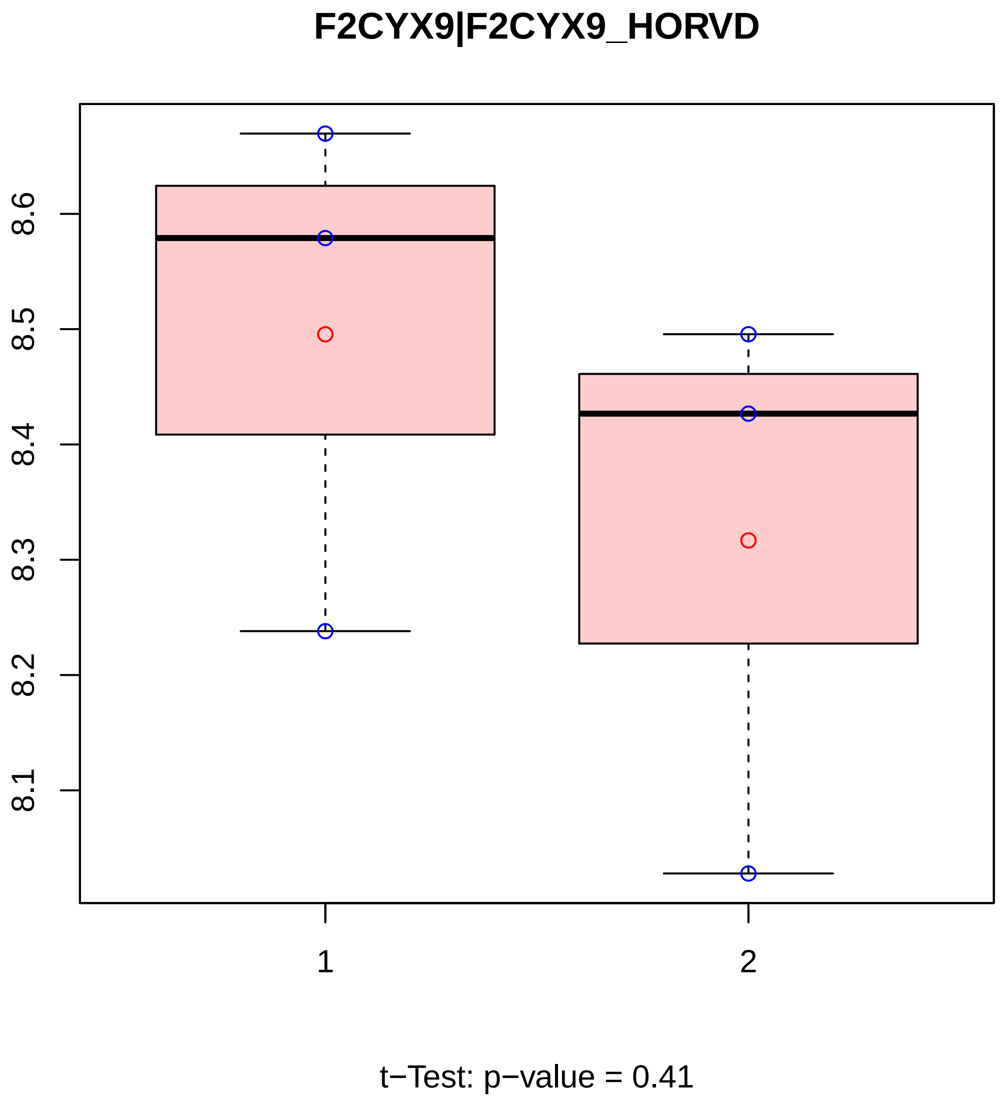
F2CYX9|F2CYX9_HORVD
8.1
8.2
8.3
8.4
8.5
8.6
1
2
t−Test: p−value = 0.41

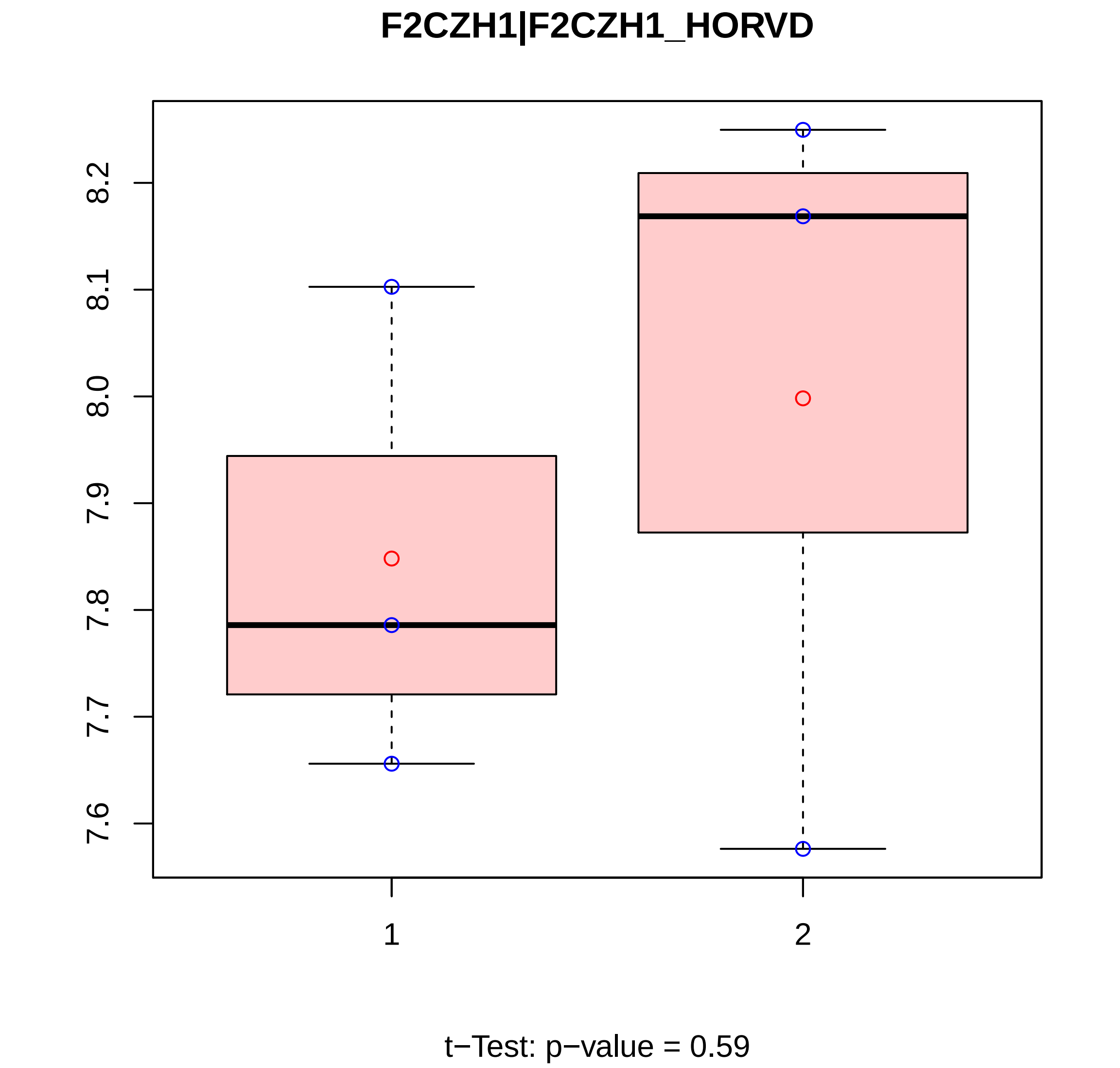

F2CZH1|F2CZH1_HORVD
8.2
8.1
8.0
7.9
7.8
7.7
7.6
1
2
t−Test: p−value = 0.59

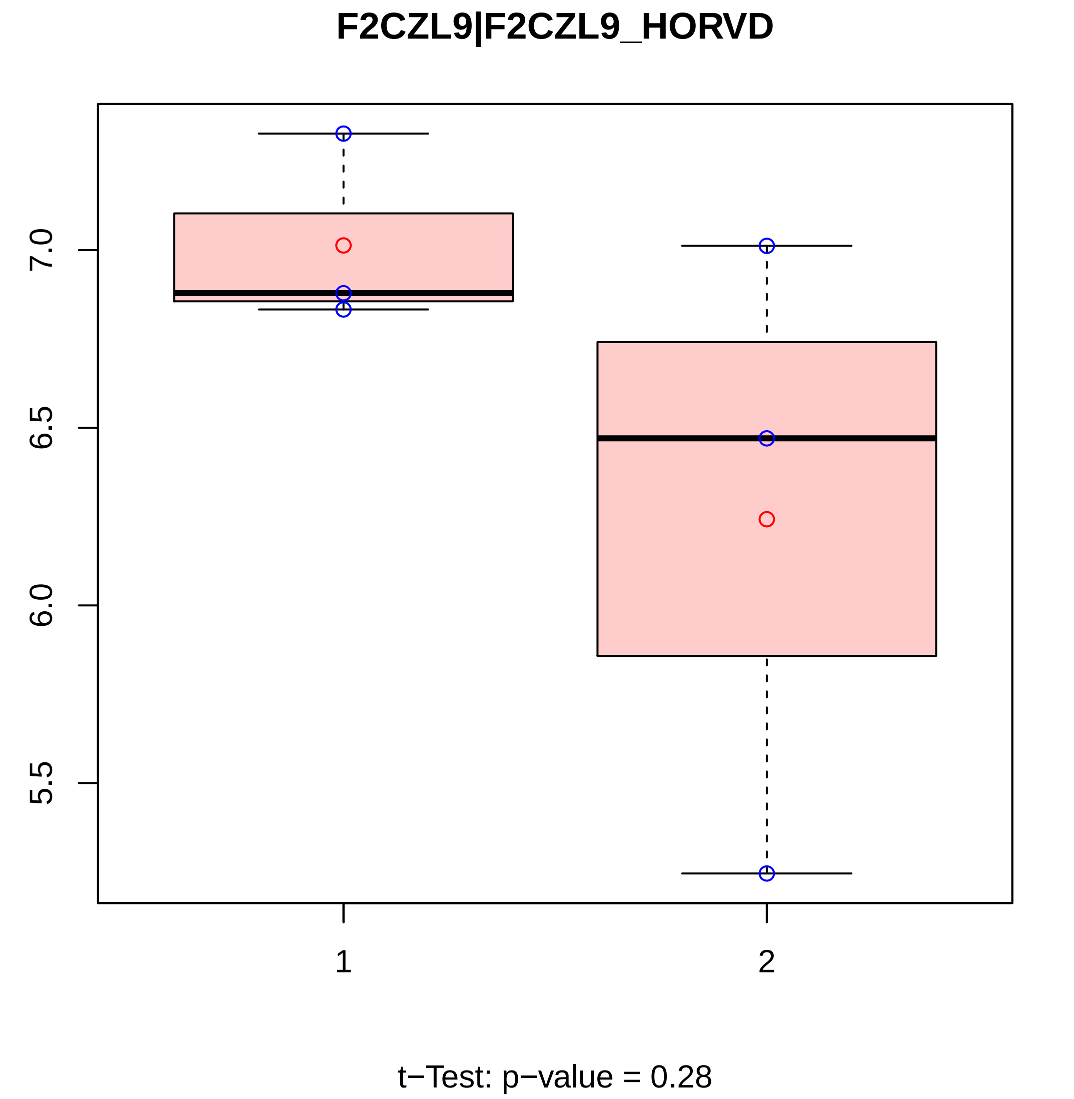
F2CZL9|F2CZL9_HORVD
7.0
6.5
6.0
5.5
1
2
t−Test: p−value = 0.28

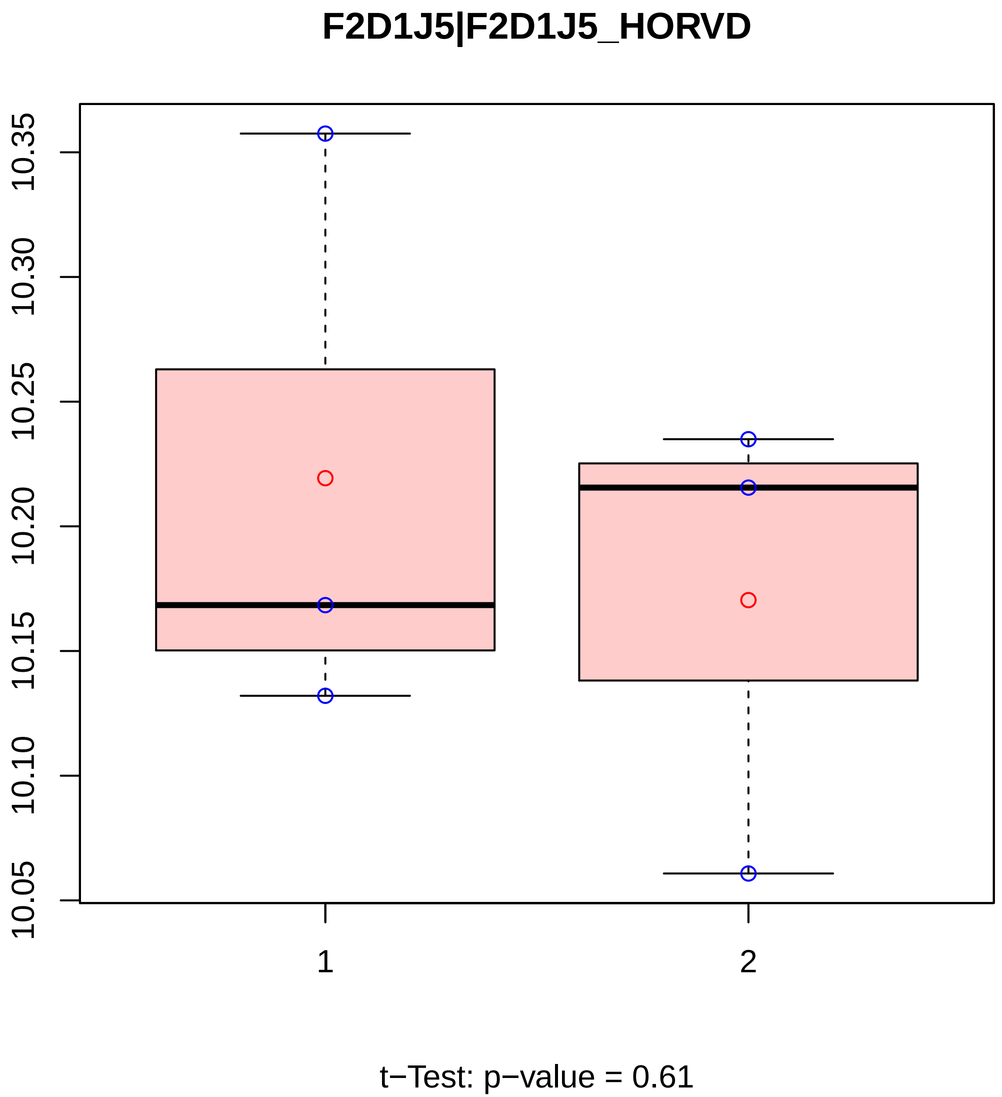
F2D1J5|F2D1J5_HORVD
10.05
10.10
10.15
10.20
10.25
10.30
10.35
1
2
t−Test: p−value = 0.61

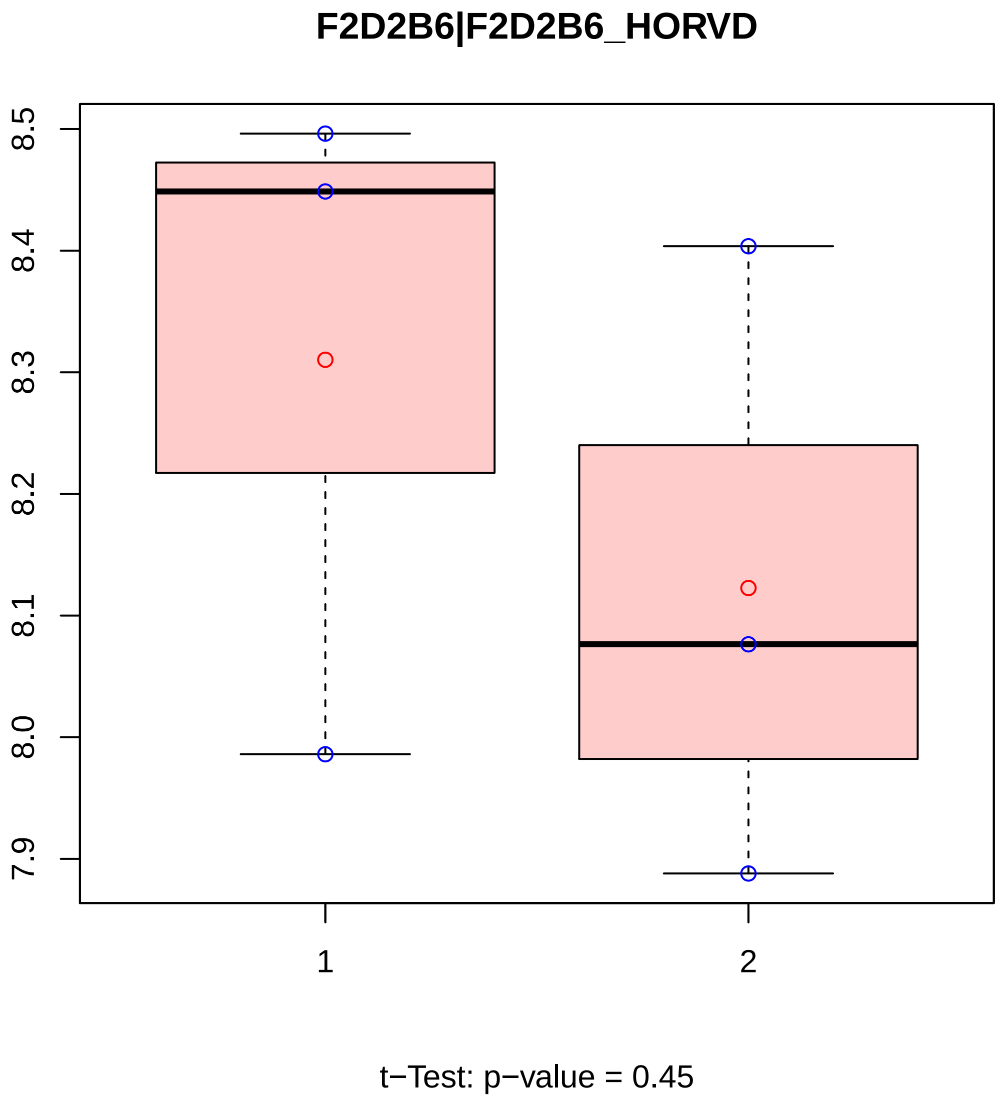
F2D2B6|F2D2B6_HORVD
8.5
8.4
8.3
8.2
8.1
8.0
7.9
1
2
t−Test: p−value = 0.45

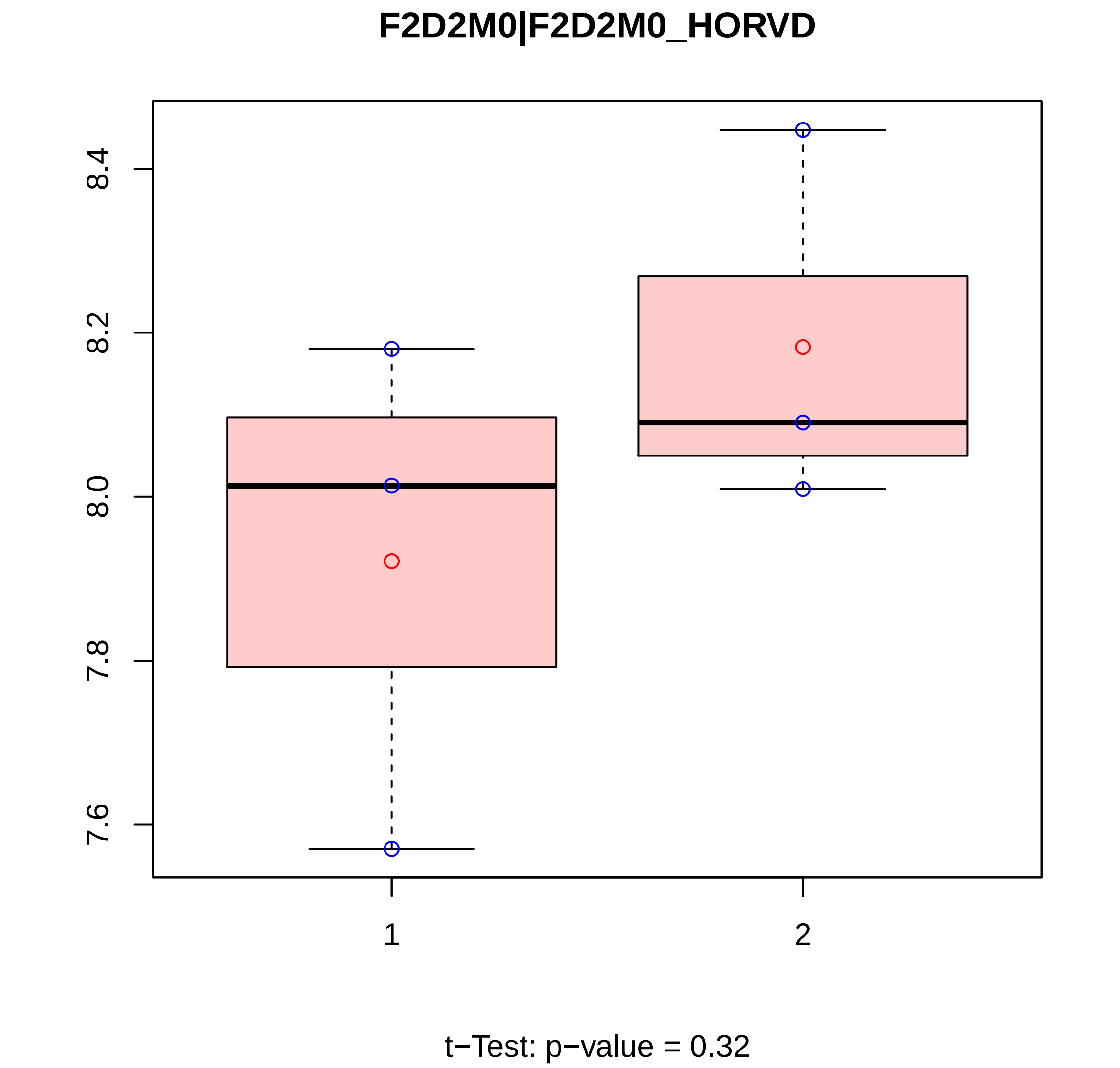
F2D2M0|F2D2M0_HORVD
8.4
8.2
8.0
7.8
7.6
1
2
t−Test: p−value = 0.32

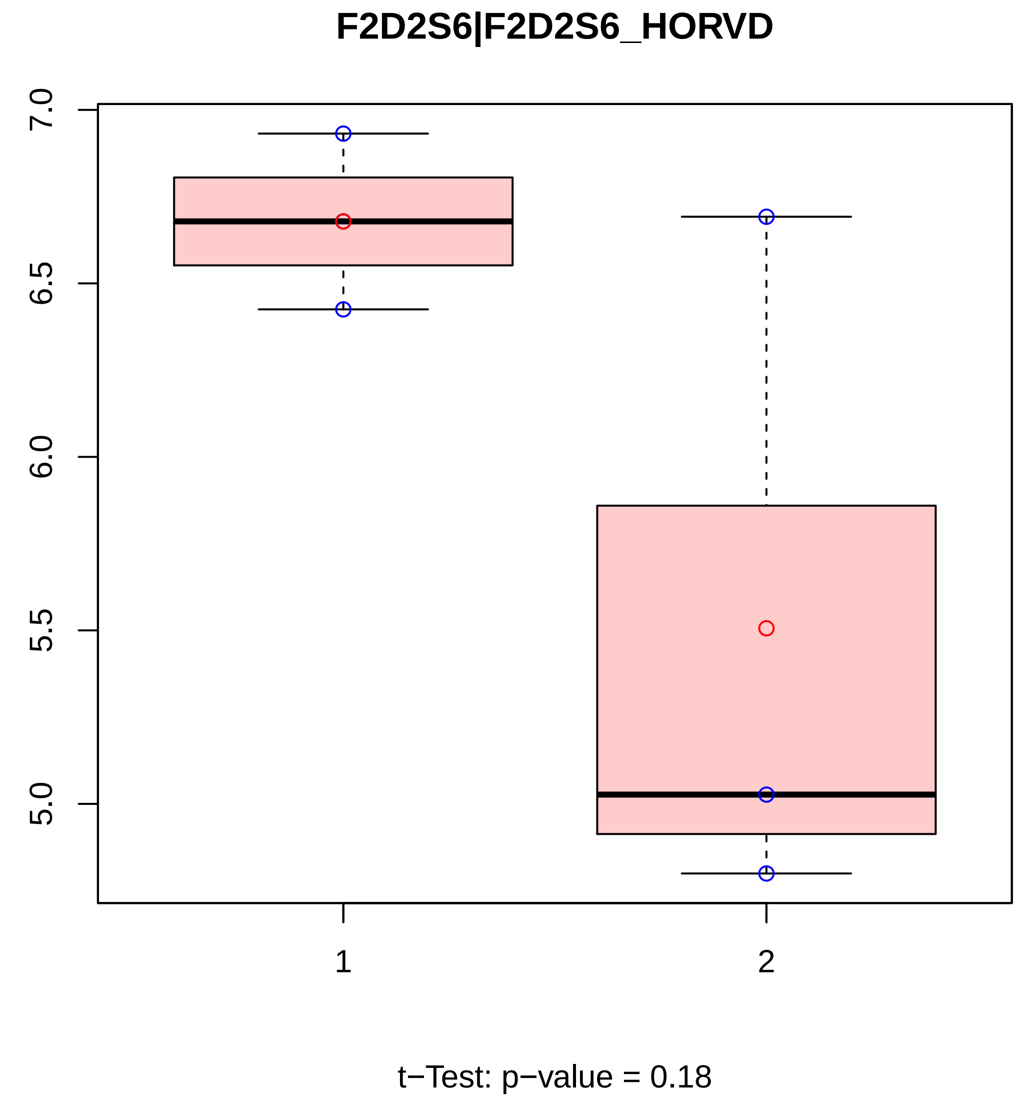
F2D2S6|F2D2S6_HORVD
7.0
6.5
6.0
5.5
5.0
1
2
t−Test: p−value = 0.18

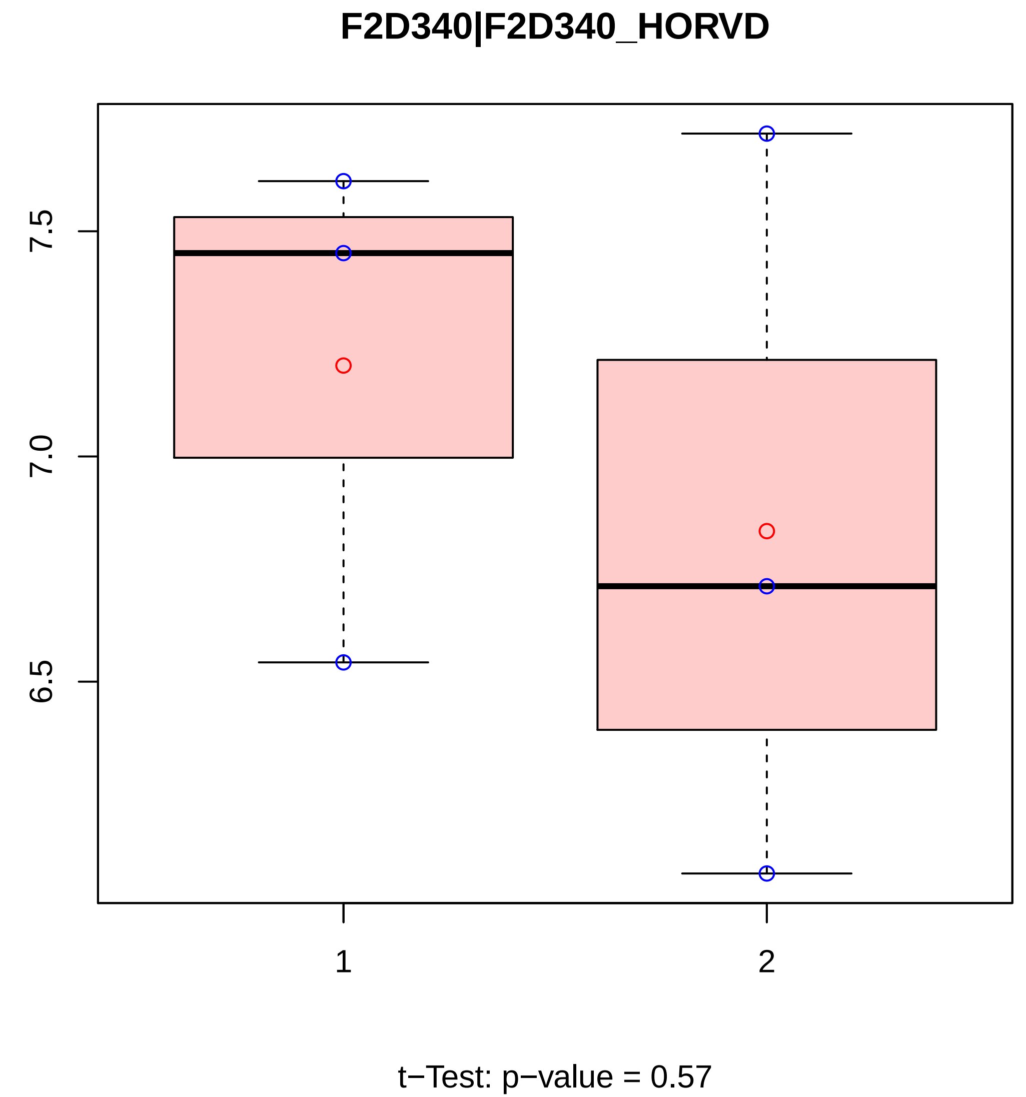

F2D340|F2D340_HORVD
7.5
7.0
6.5
1
2
t−Test: p−value = 0.57

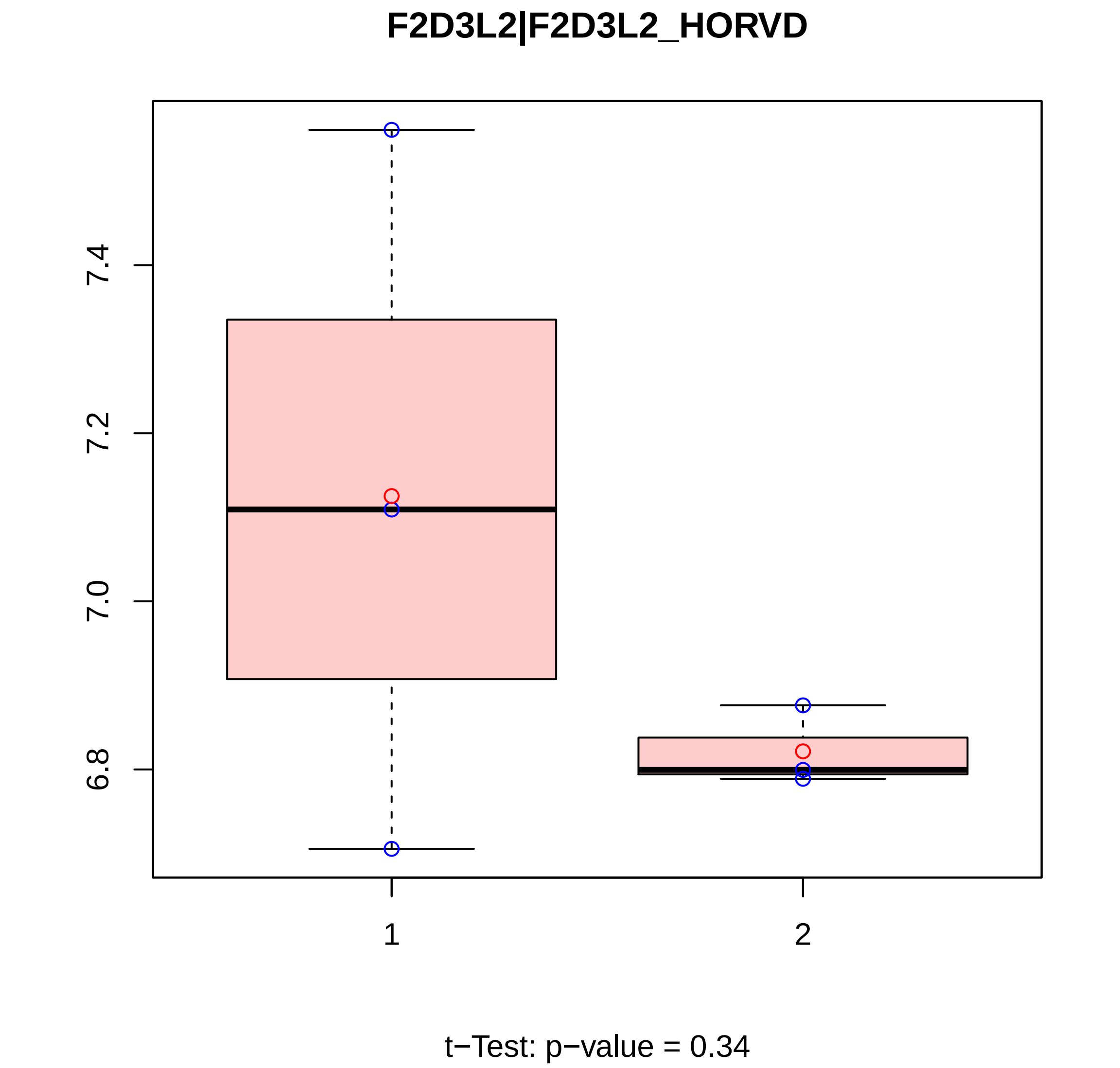
F2D3L2|F2D3L2_HORVD
7.4
7.2
7.0
6.8
1
2
t−Test: p−value = 0.34

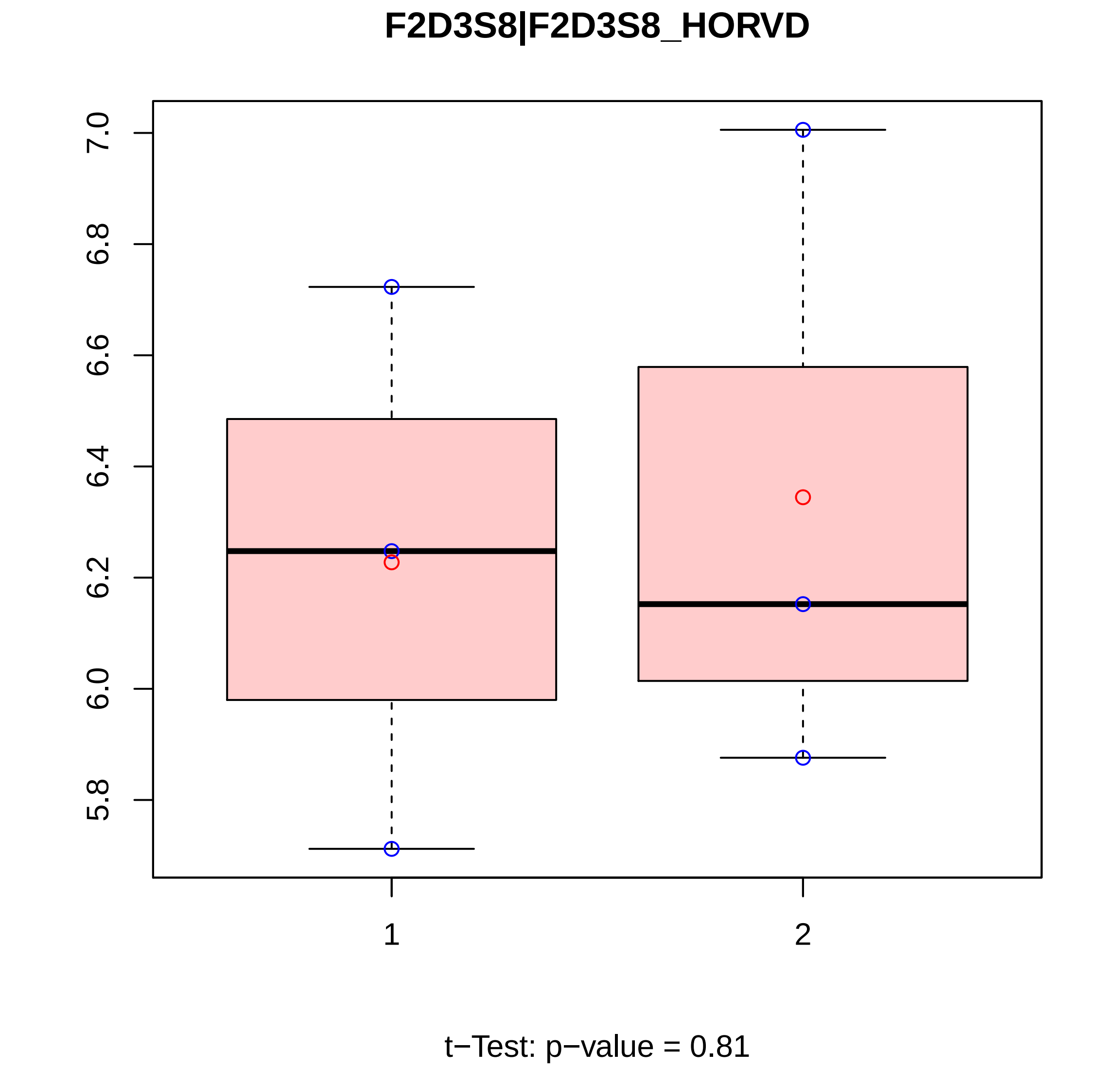
F2D3S8|F2D3S8_HORVD
7.0
6.8
6.6
6.4
6.2
6.0
5.8
1
2
t−Test: p−value = 0.81

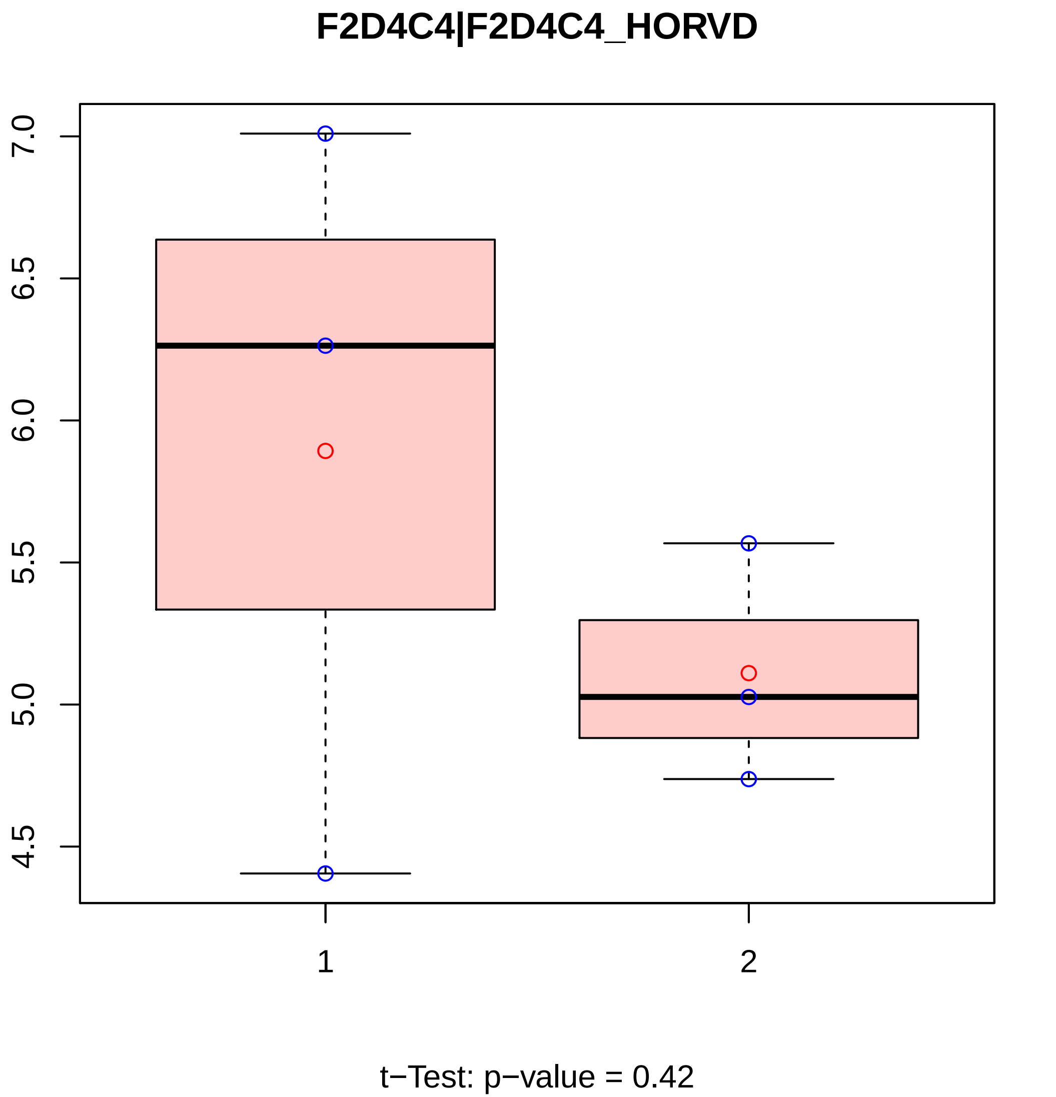
F2D4C4|F2D4C4_HORVD
7.0
6.5
6.0
5.5
5.0
4.5
1
2
t−Test: p−value = 0.42

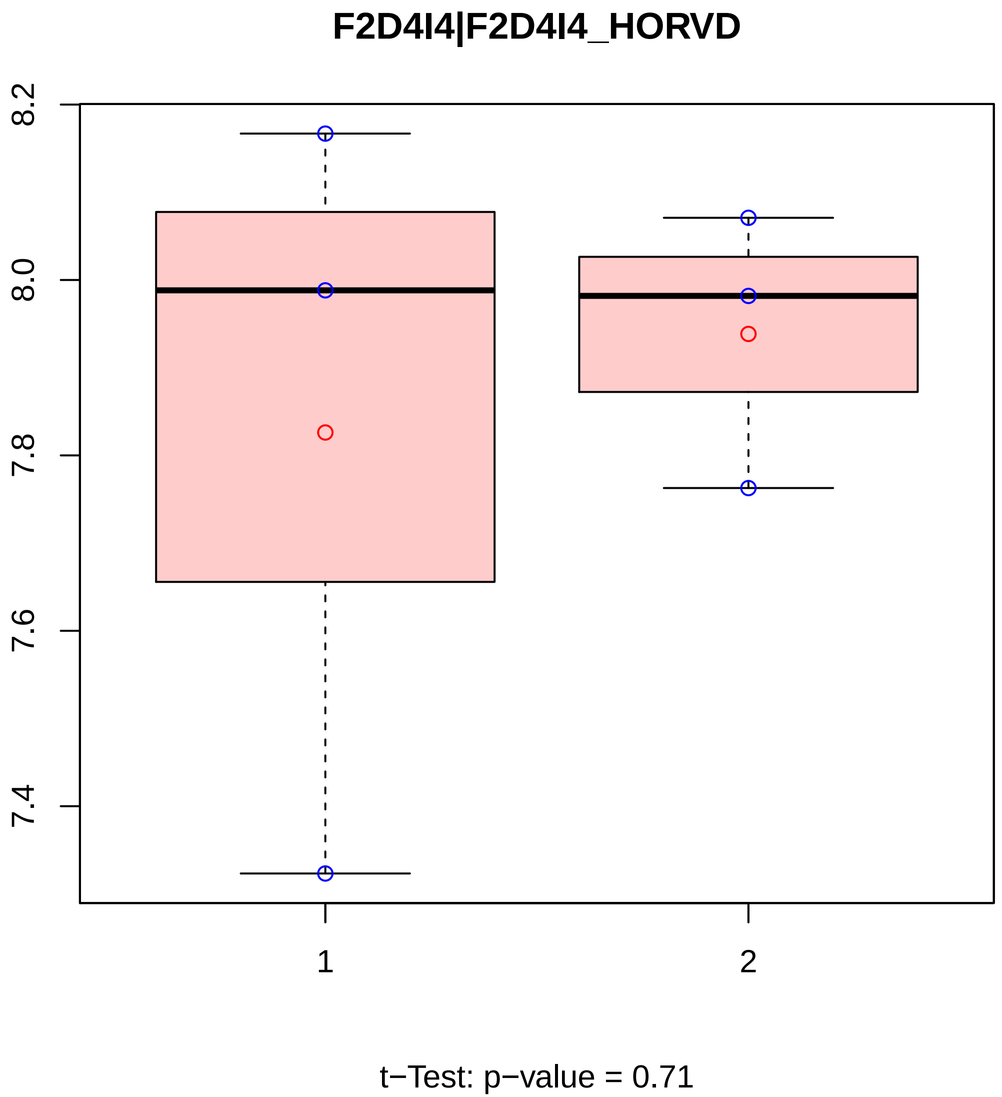
F2D4I4|F2D4I4_HORVD
8.2
8.0
7.8
7.6
7.4
1
2
t−Test: p−value = 0.71

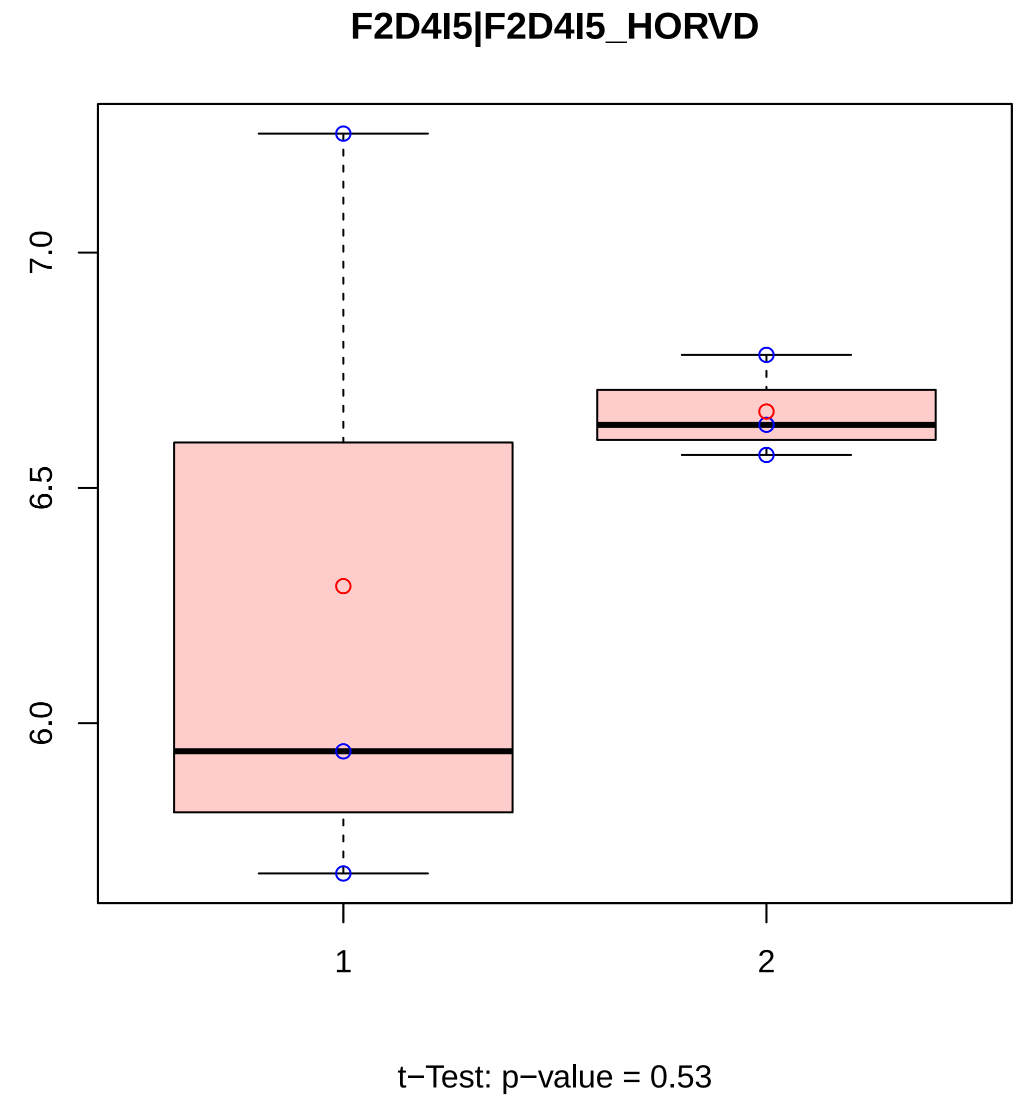

F2D4I5|F2D4I5_HORVD
7.0
6.5
6.0
1
2
t−Test: p−value = 0.53

F2D4I8|F2D4I8_HORVD

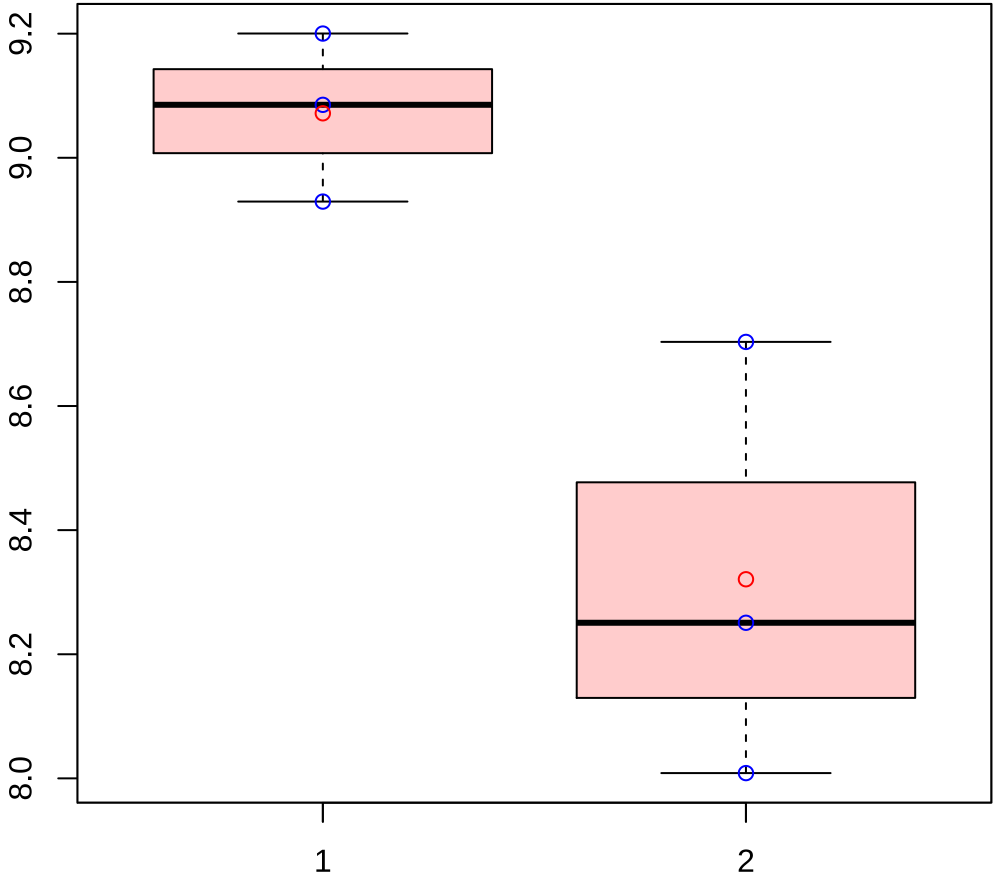

t−Test: p−value = 0.05

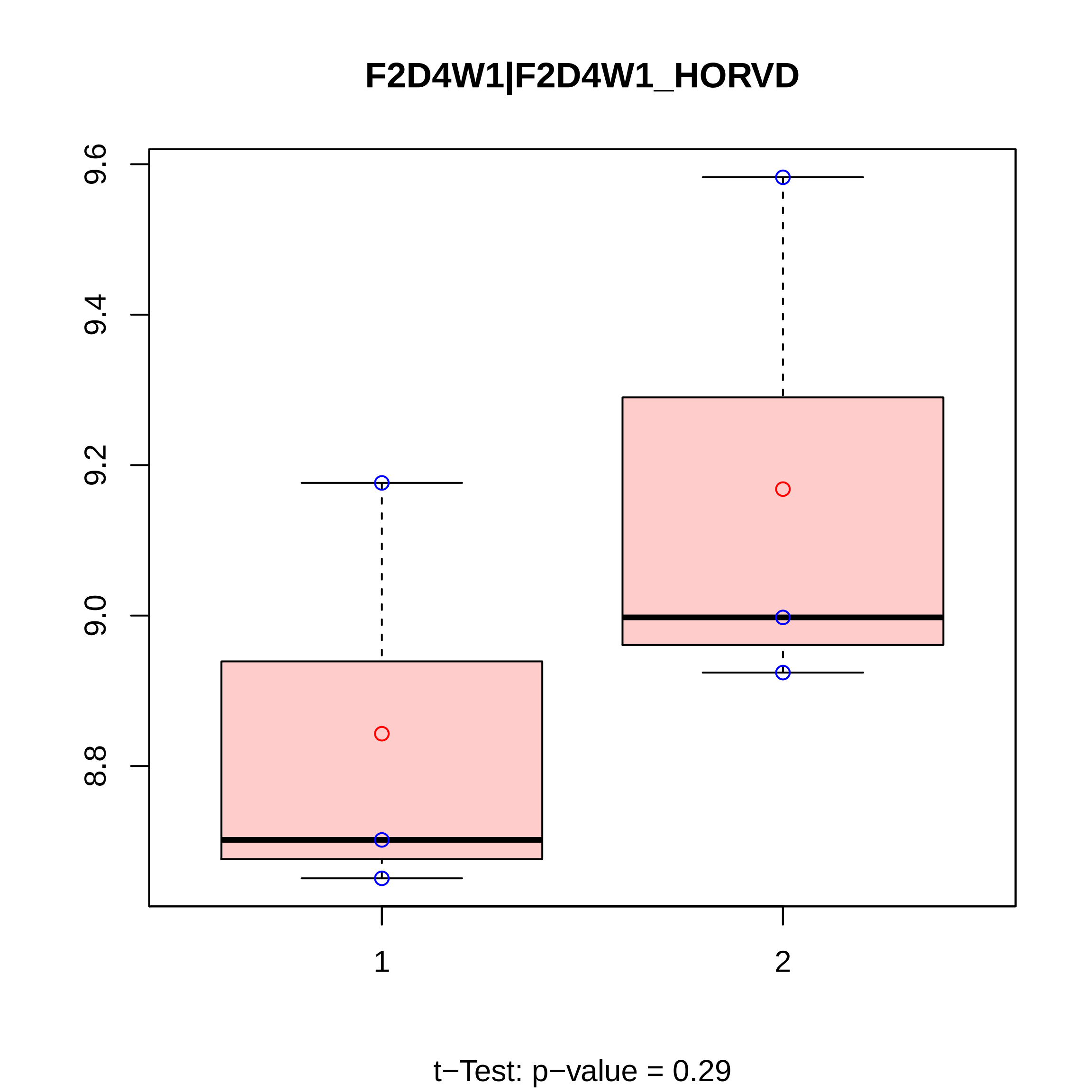
F2D4W1|F2D4W1_HORVD
9.6
9.4
9.2
9.0
8.8
1
2
t−Test: p−value = 0.29

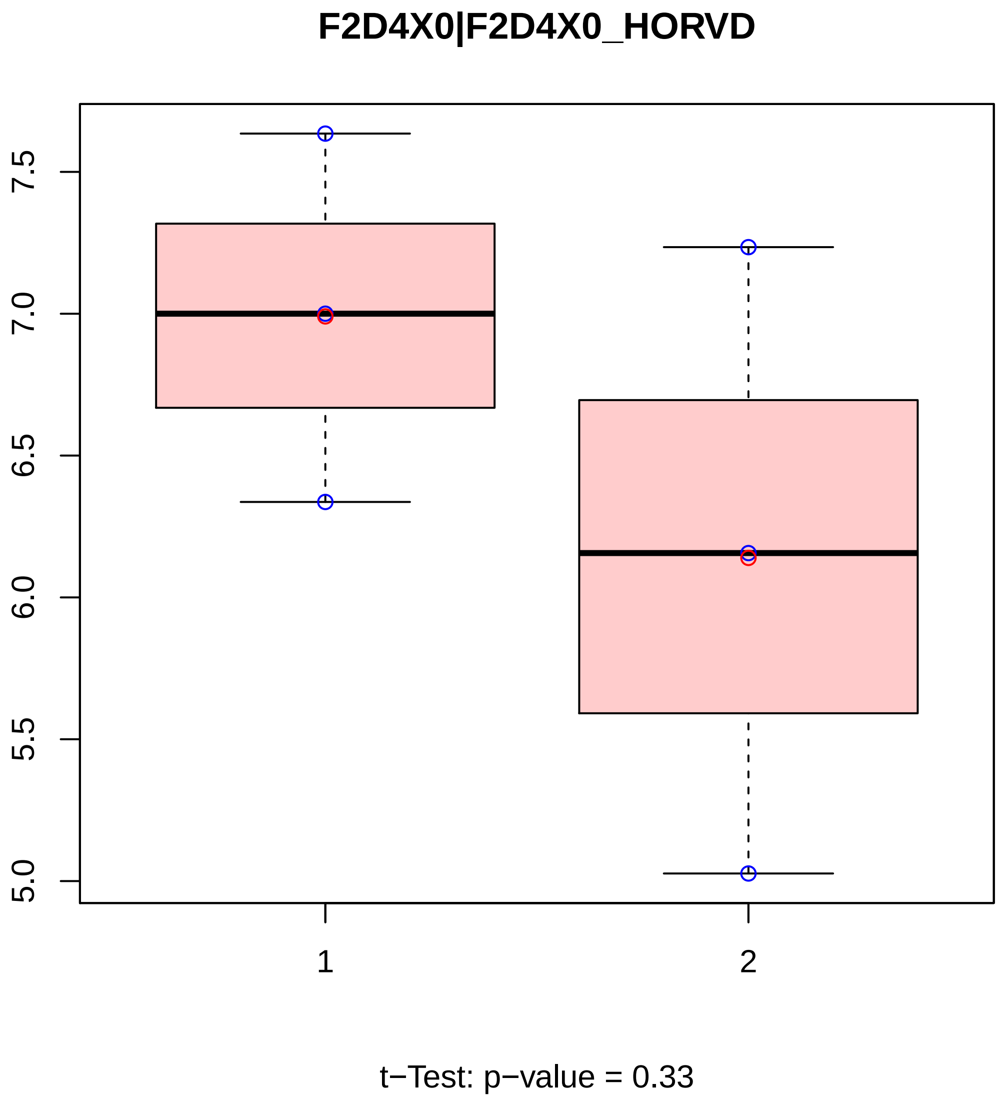
F2D4X0|F2D4X0_HORVD
7.5
7.0
6.5
6.0
5.5
5.0
1
2
t−Test: p−value = 0.33

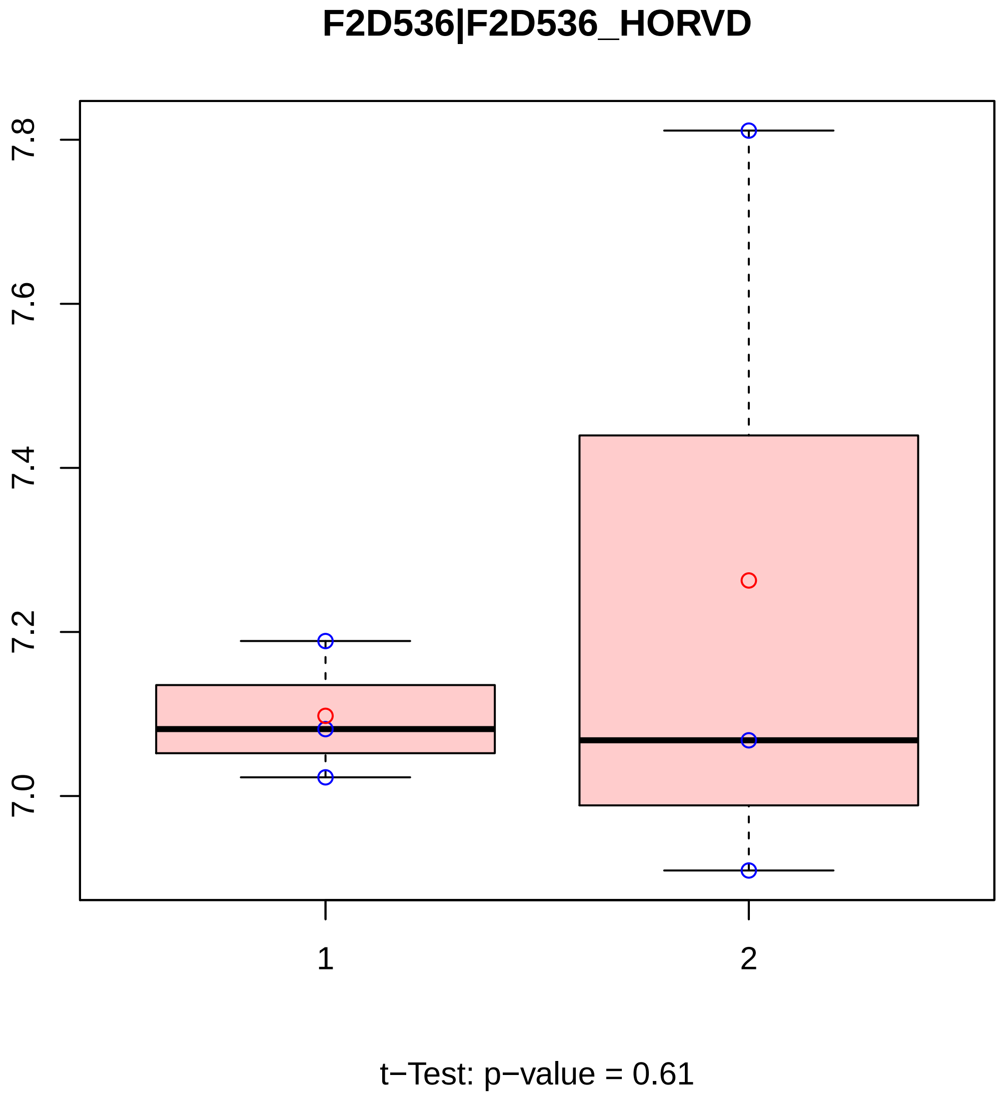

F2D536|F2D536_HORVD
7.0
7.2
7.4
7.6
7.8
1
2
t−Test: p−value = 0.61

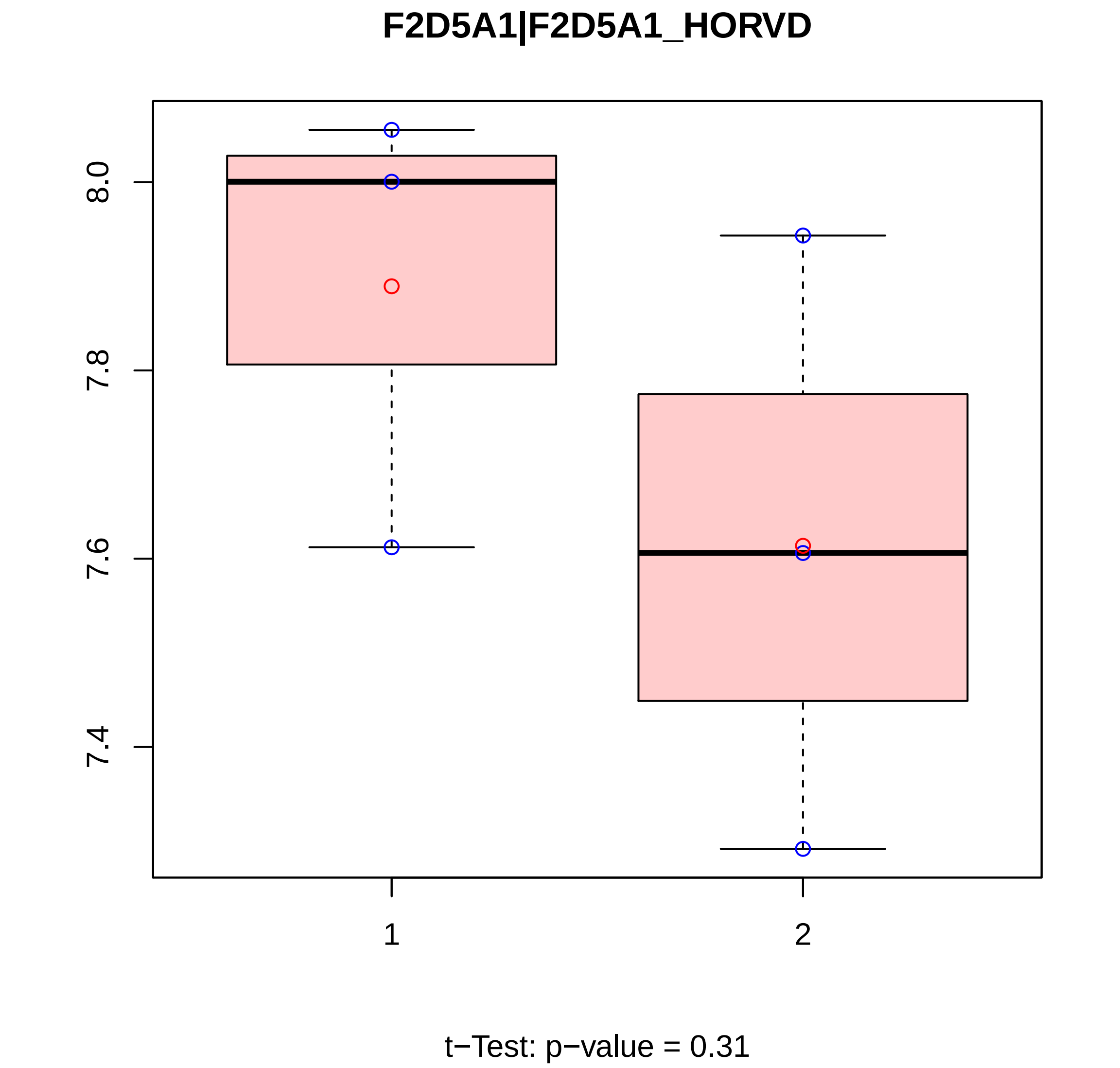
F2D5A1|F2D5A1_HORVD
8.0
7.8
7.6
7.4
1
2
t−Test: p−value = 0.31

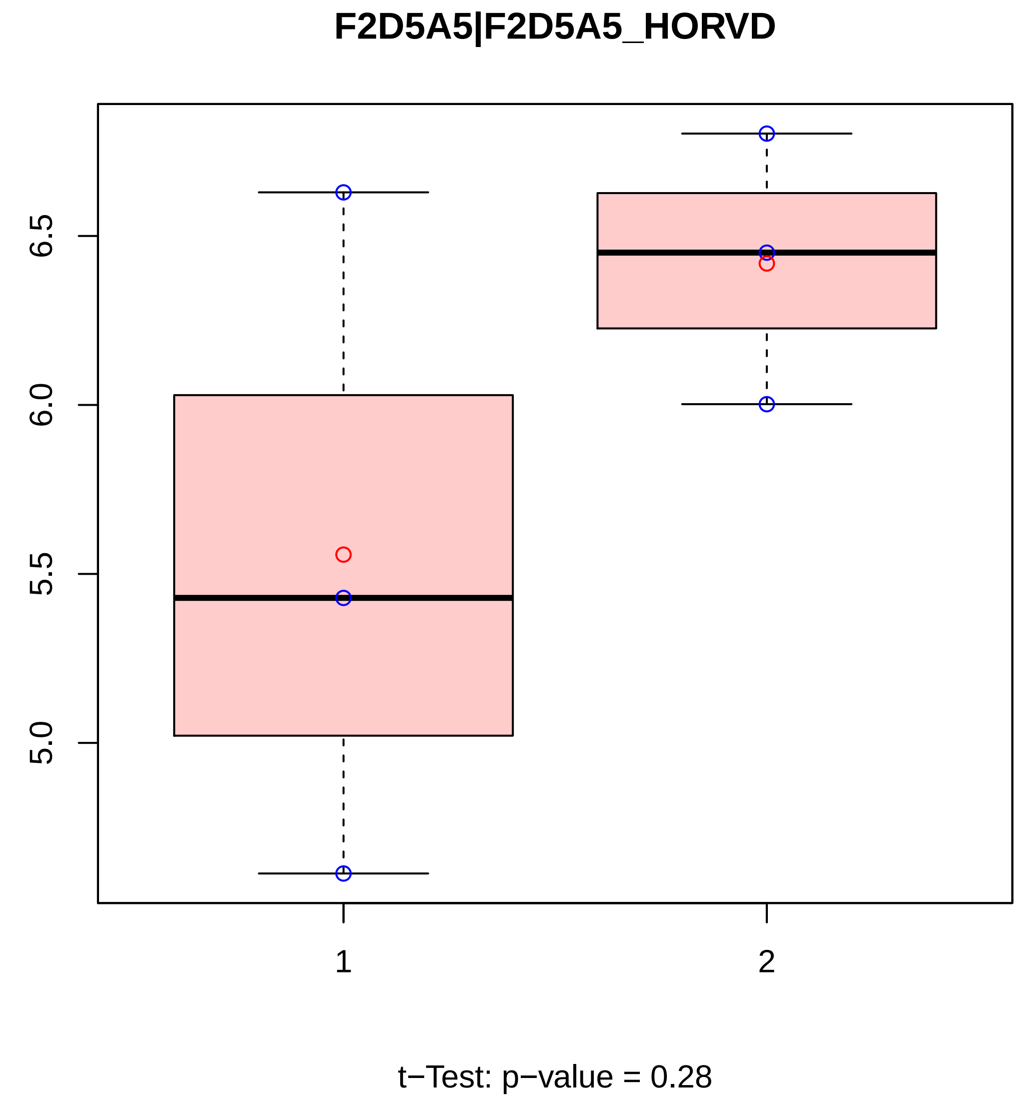

F2D5A5|F2D5A5_HORVD
6.5
6.0
5.5
5.0
1
2
t−Test: p−value = 0.28

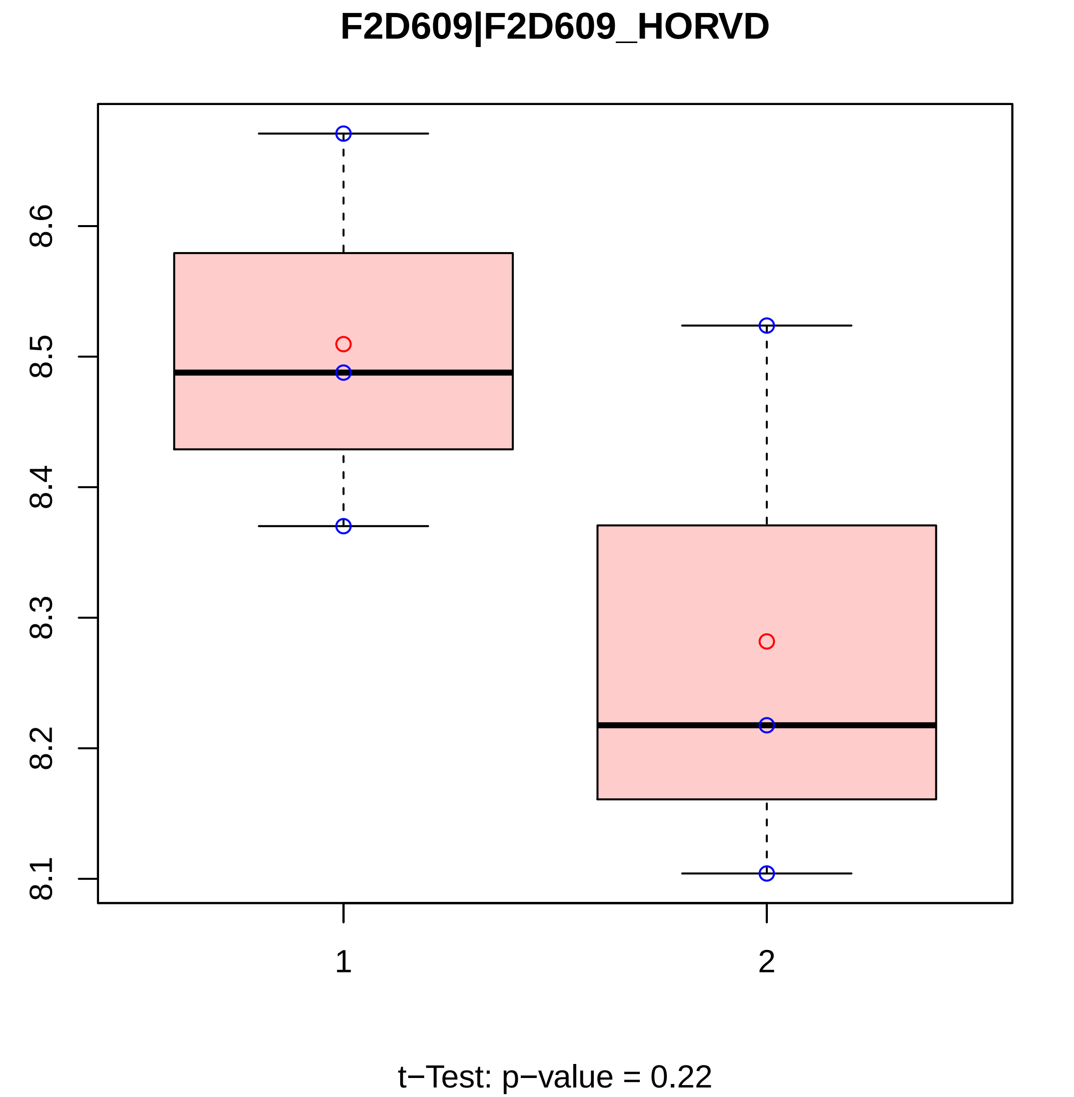

F2D609|F2D609_HORVD
8.1
8.2
8.3
8.4
8.5
8.6
1
2
t−Test: p−value = 0.22

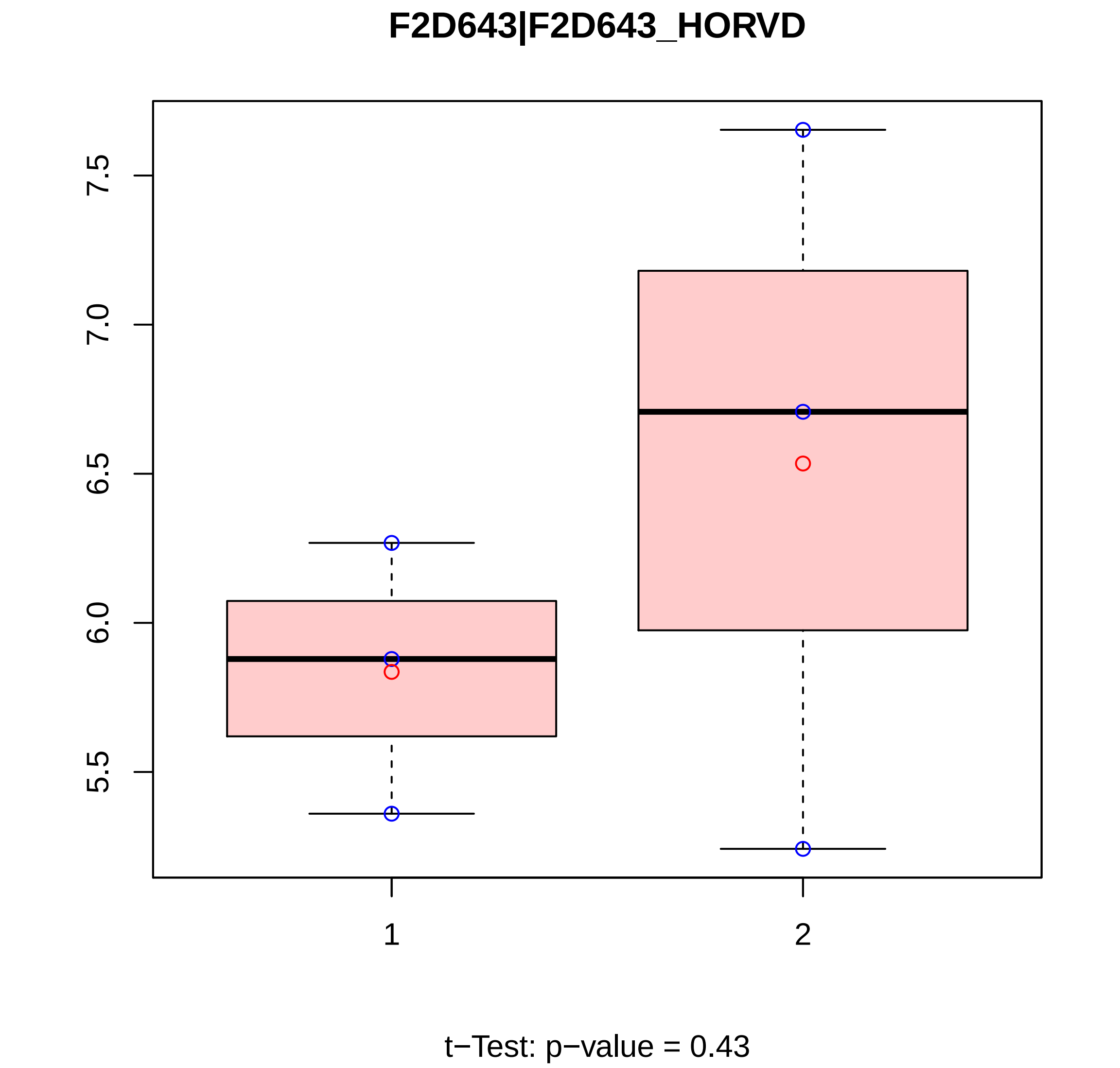
F2D643|F2D643_HORVD
7.5
7.0
6.5
6.0
5.5
1
2
t−Test: p−value = 0.43

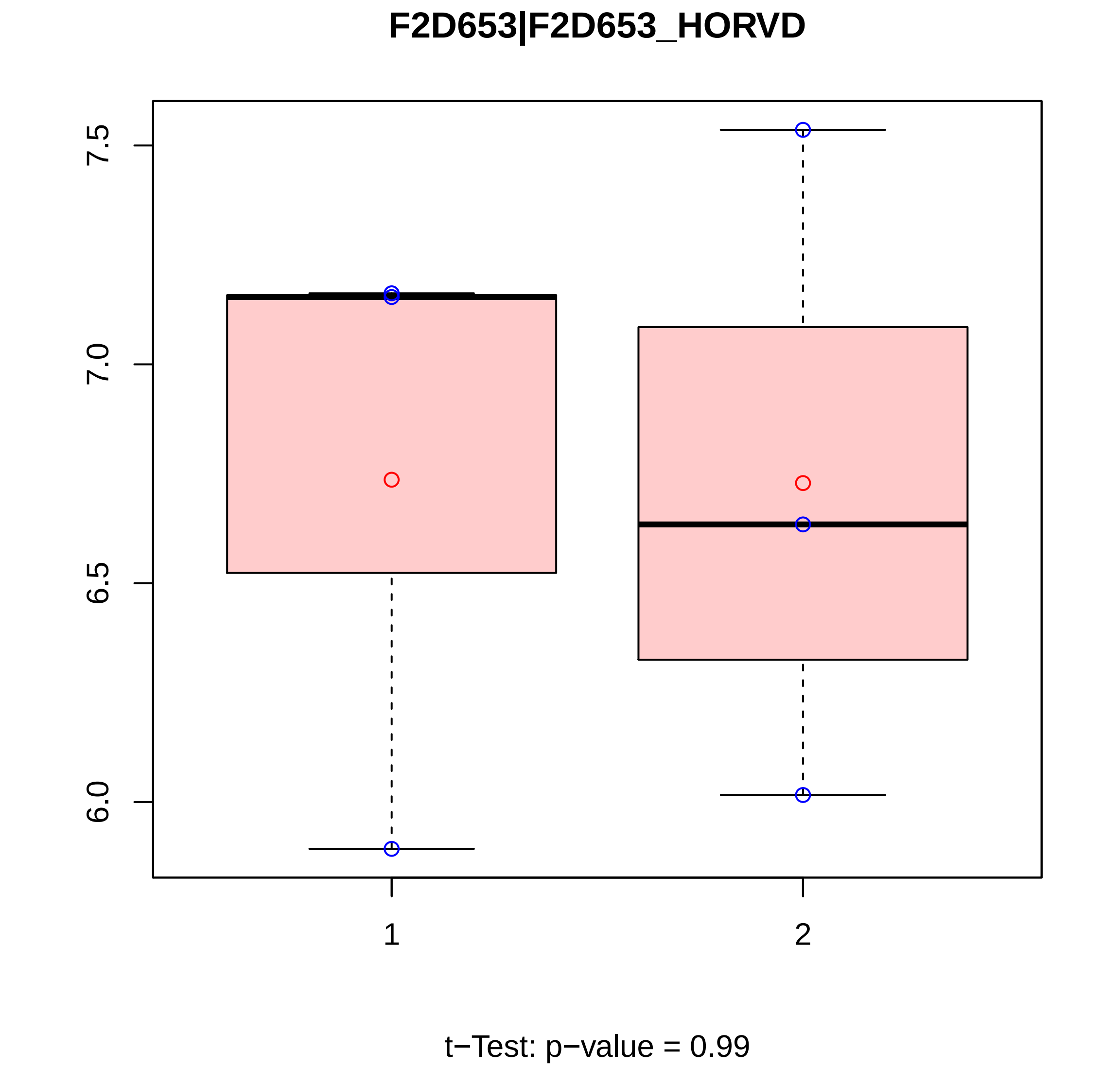
F2D653|F2D653_HORVD
7.5
7.0
6.5
6.0
1
2
t−Test: p−value = 0.99

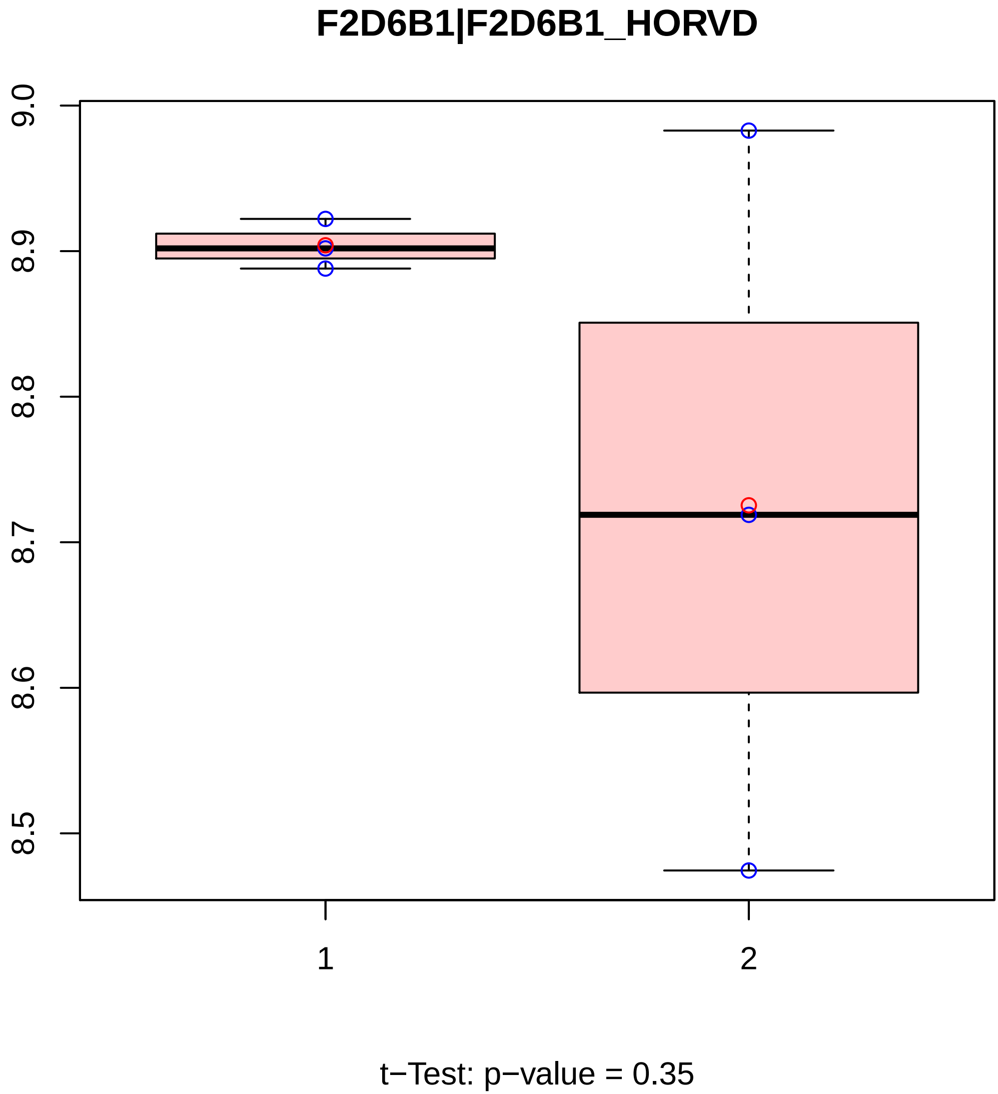

F2D6B1|F2D6B1_HORVD
9.0
8.9
8.8
8.7
8.6
8.5
1
2
t−Test: p−value = 0.35

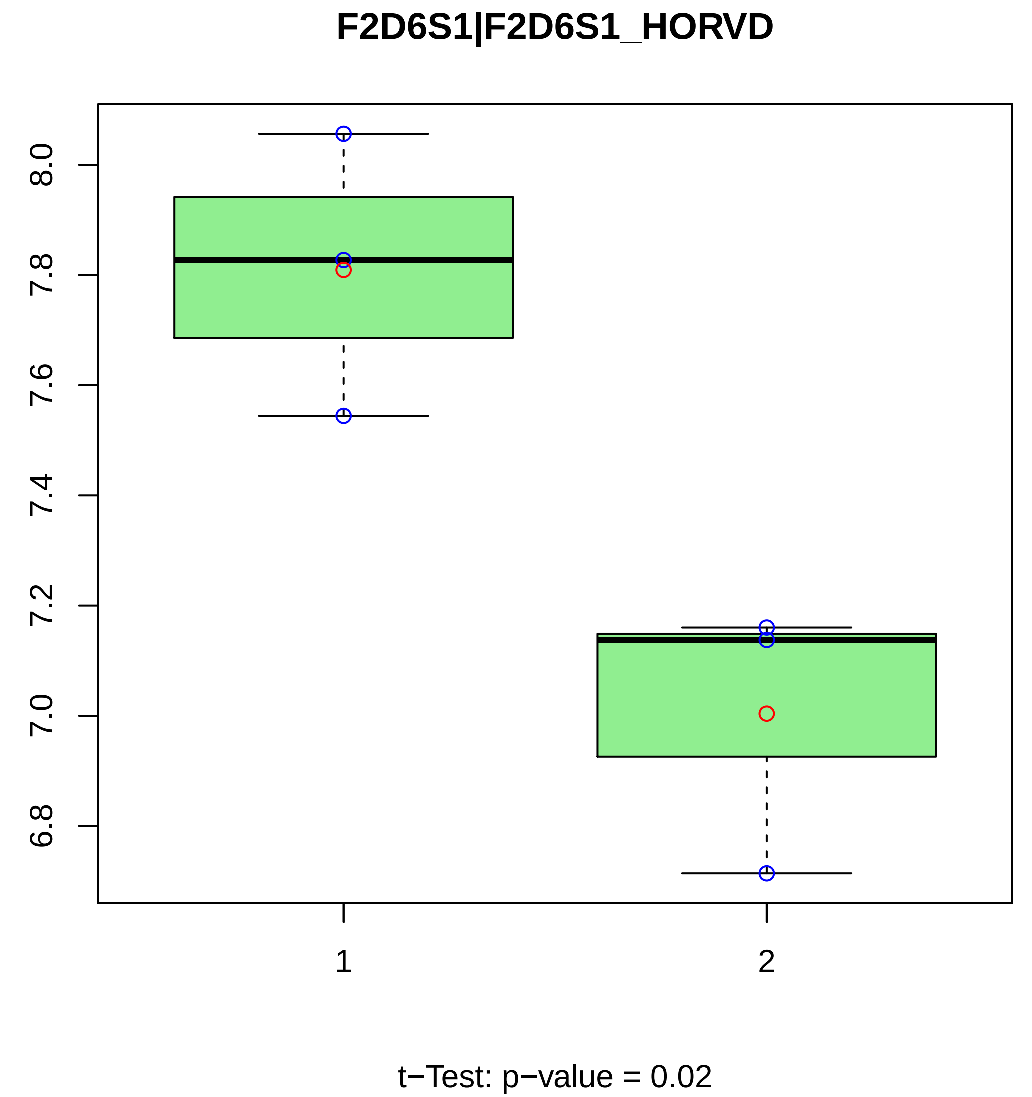
F2D6S1|F2D6S1_HORVD
8.0
7.8
7.6
7.4
7.2
7.0
6.8
1
2
t−Test: p−value = 0.02

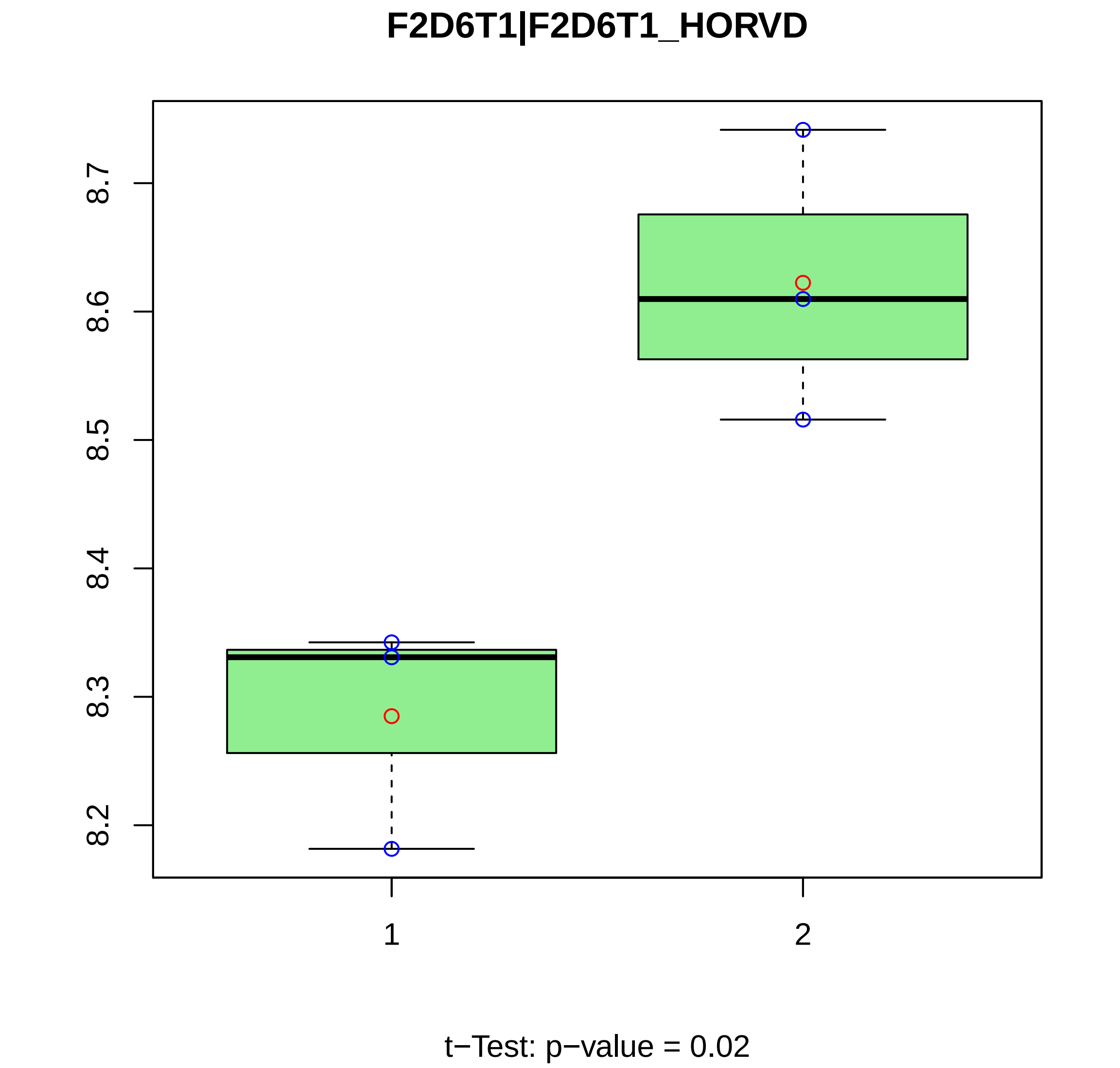
F2D6T1|F2D6T1_HORVD
8.2
8.3
8.4
8.5
8.6
8.7
1
2
t−Test: p−value = 0.02

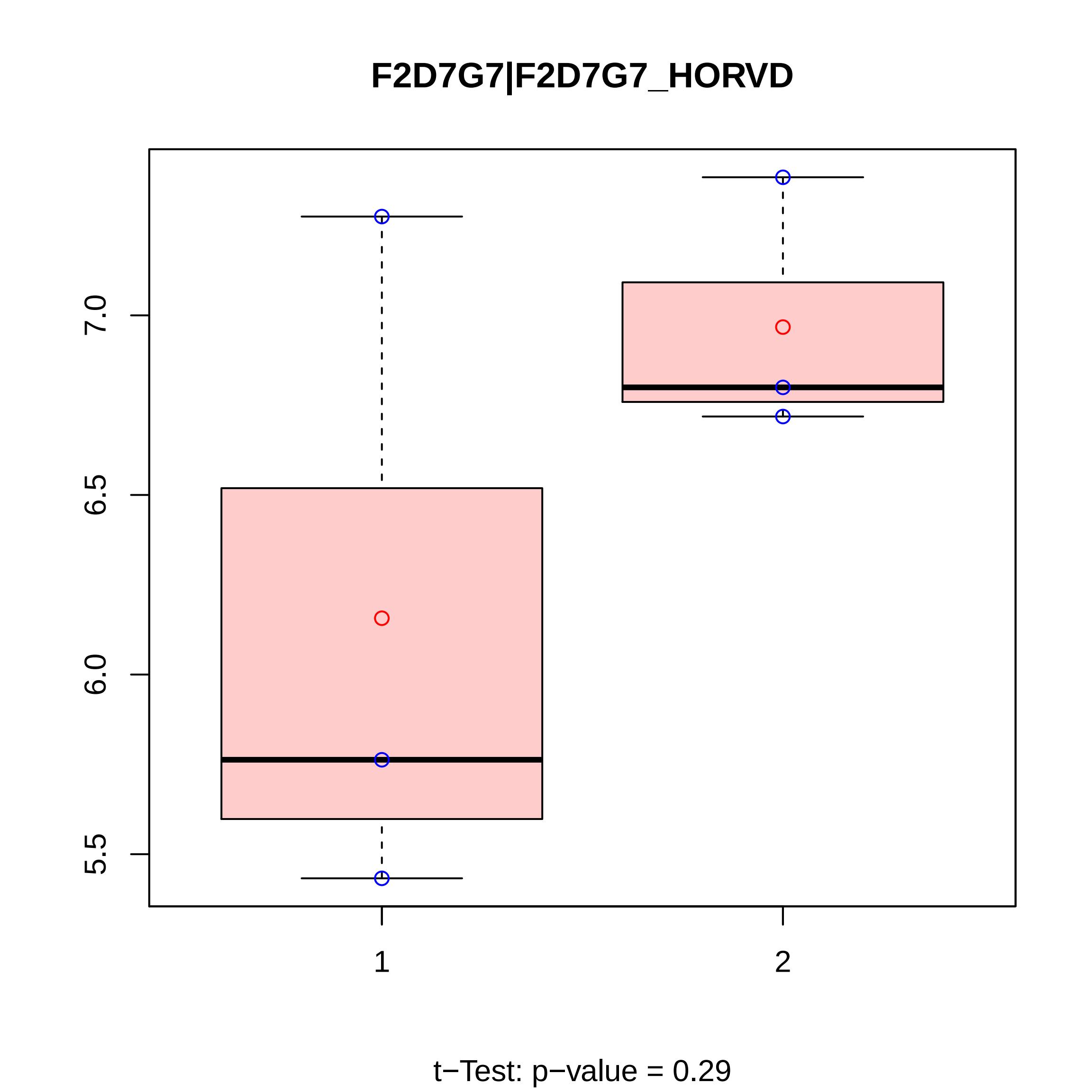
F2D7G7|F2D7G7_HORVD
5.5
6.0
6.5
7.0
1
2
t−Test: p−value = 0.29

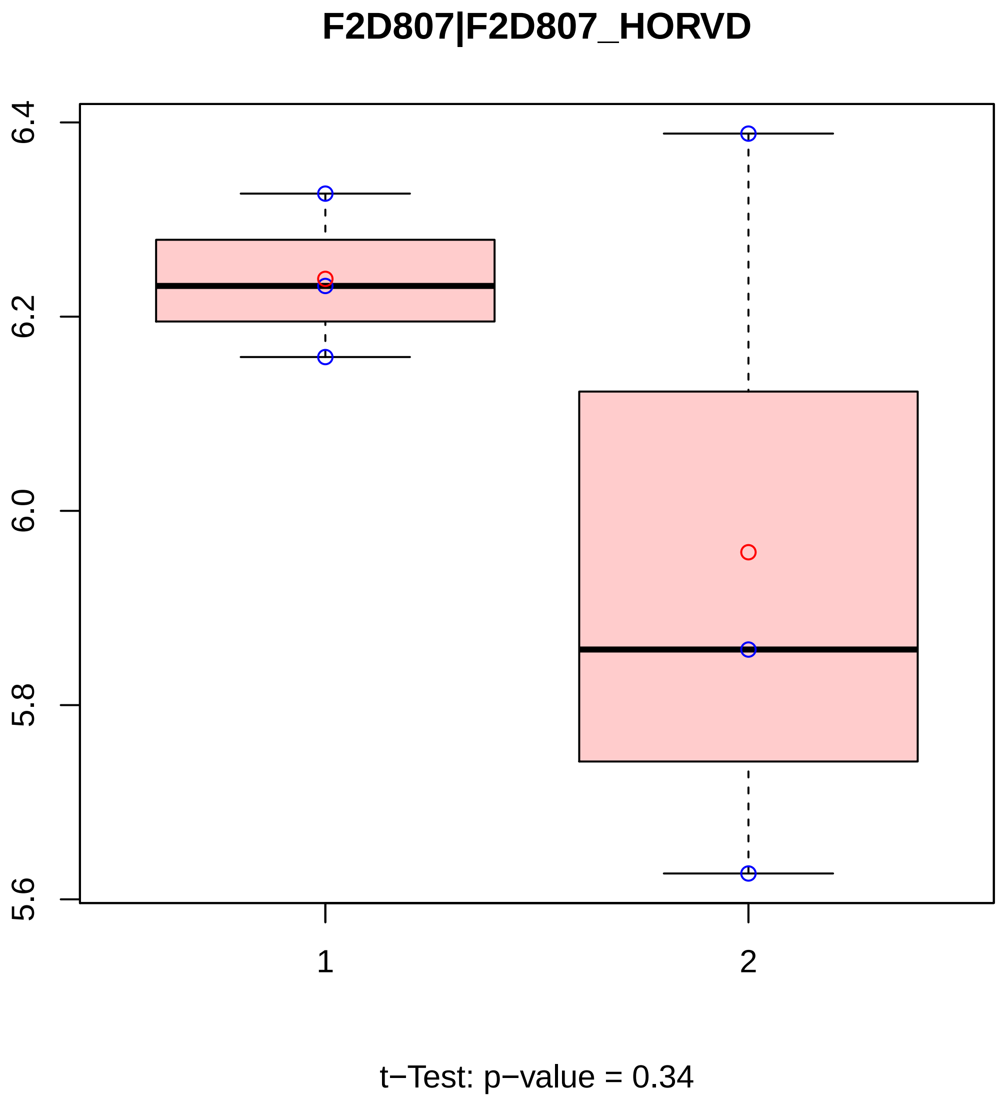
F2D807|F2D807_HORVD
6.4
6.2
6.0
5.8
5.6
1
2
t−Test: p−value = 0.34

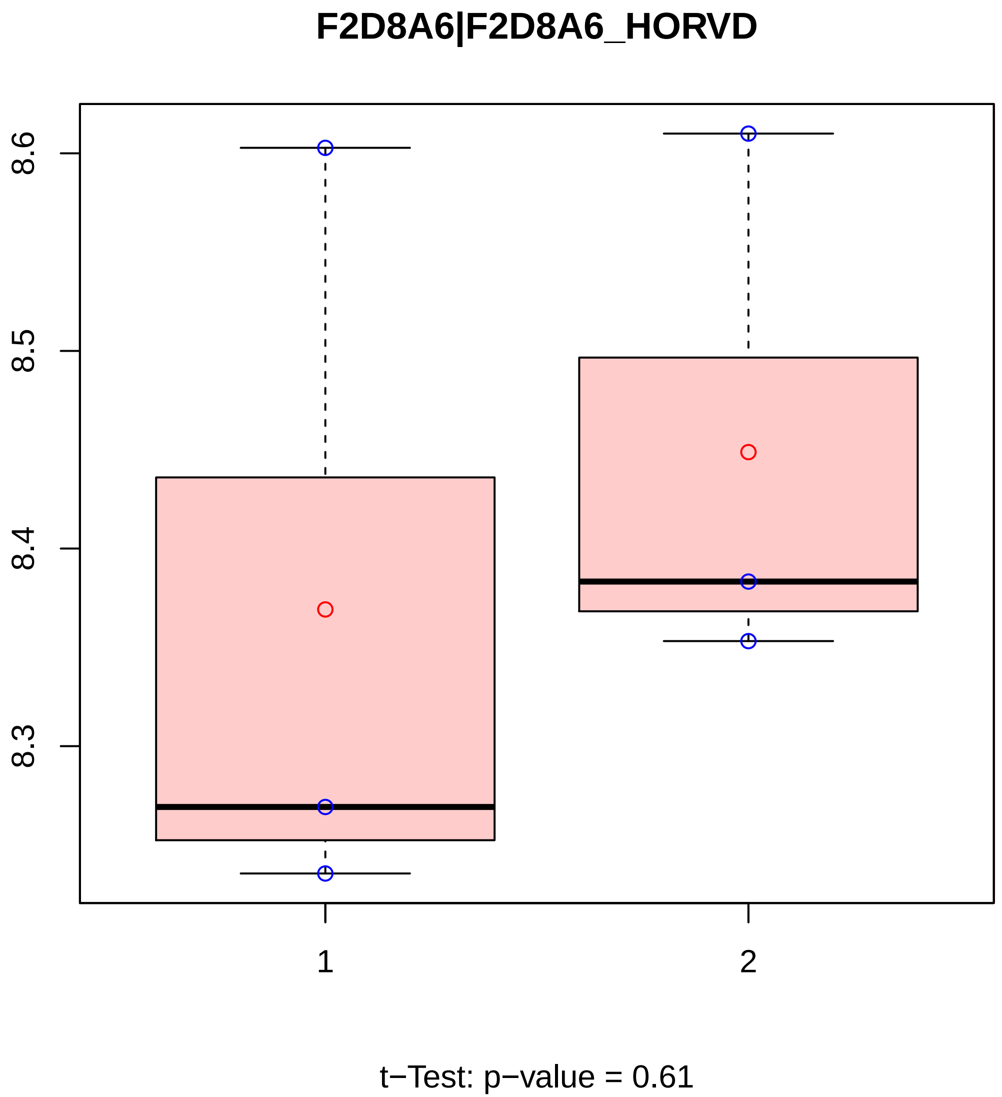

F2D8A6|F2D8A6_HORVD
8.6
8.5
8.4
8.3
1
2
t−Test: p−value = 0.61

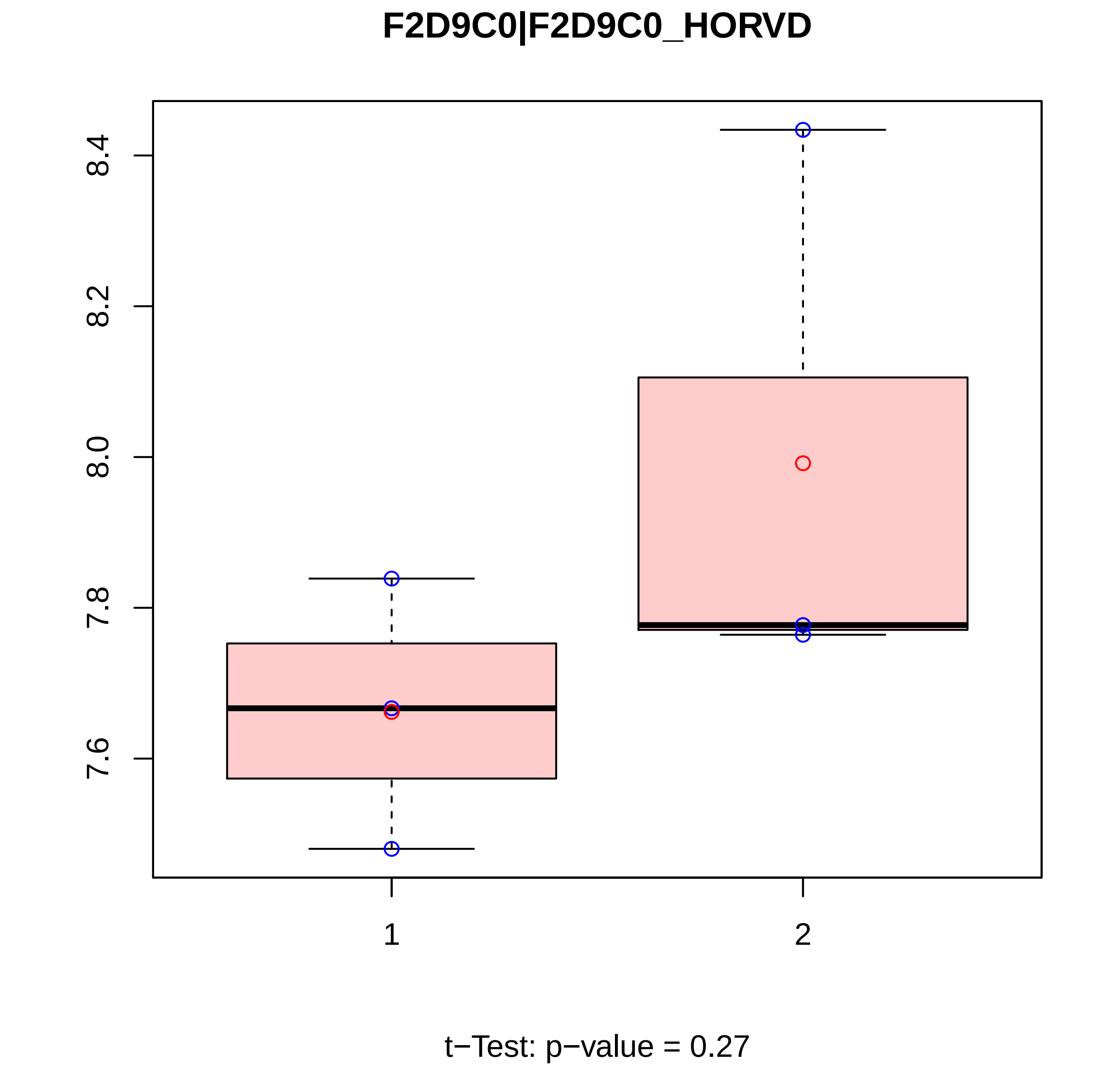
F2D9C0|F2D9C0_HORVD
8.4
8.2
8.0
7.8
7.6
1
2
t−Test: p−value = 0.27

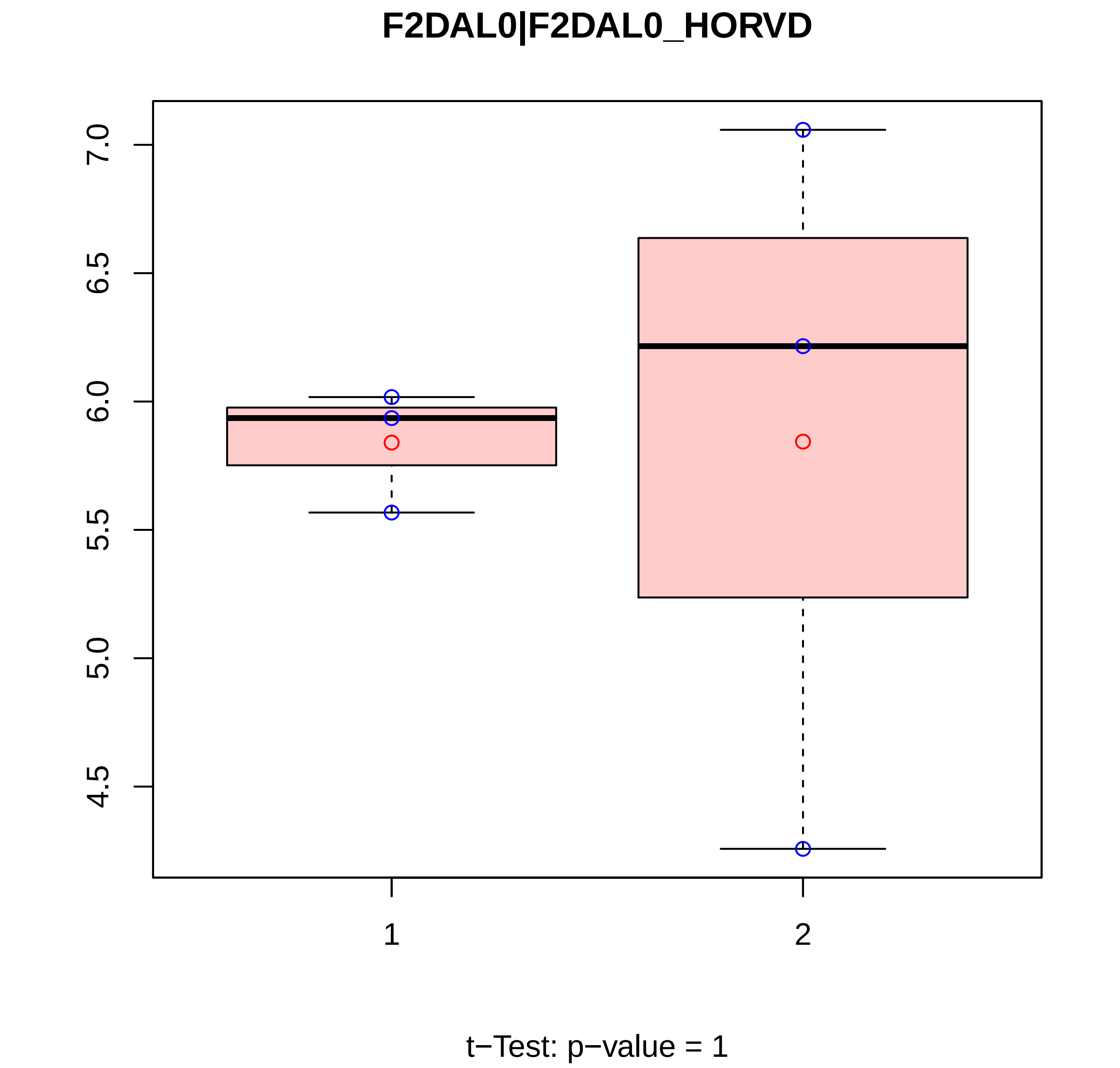
F2DAL0|F2DAL0_HORVD
7.0
6.5
6.0
5.5
5.0
4.5
1
2
t−Test: p−value = 1

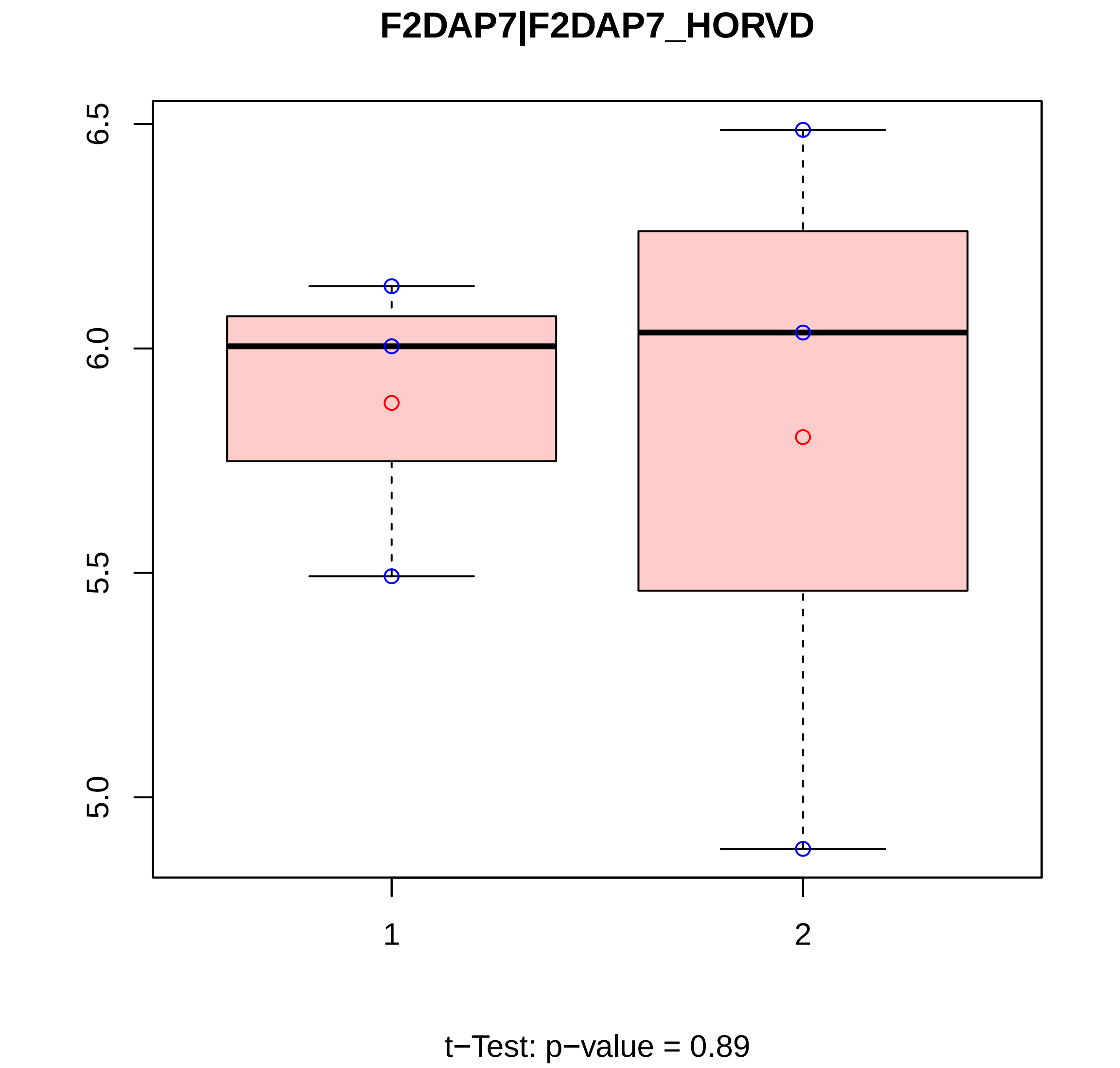
F2DAP7|F2DAP7_HORVD
6.5
6.0
5.5
5.0
1
2
t−Test: p−value = 0.89

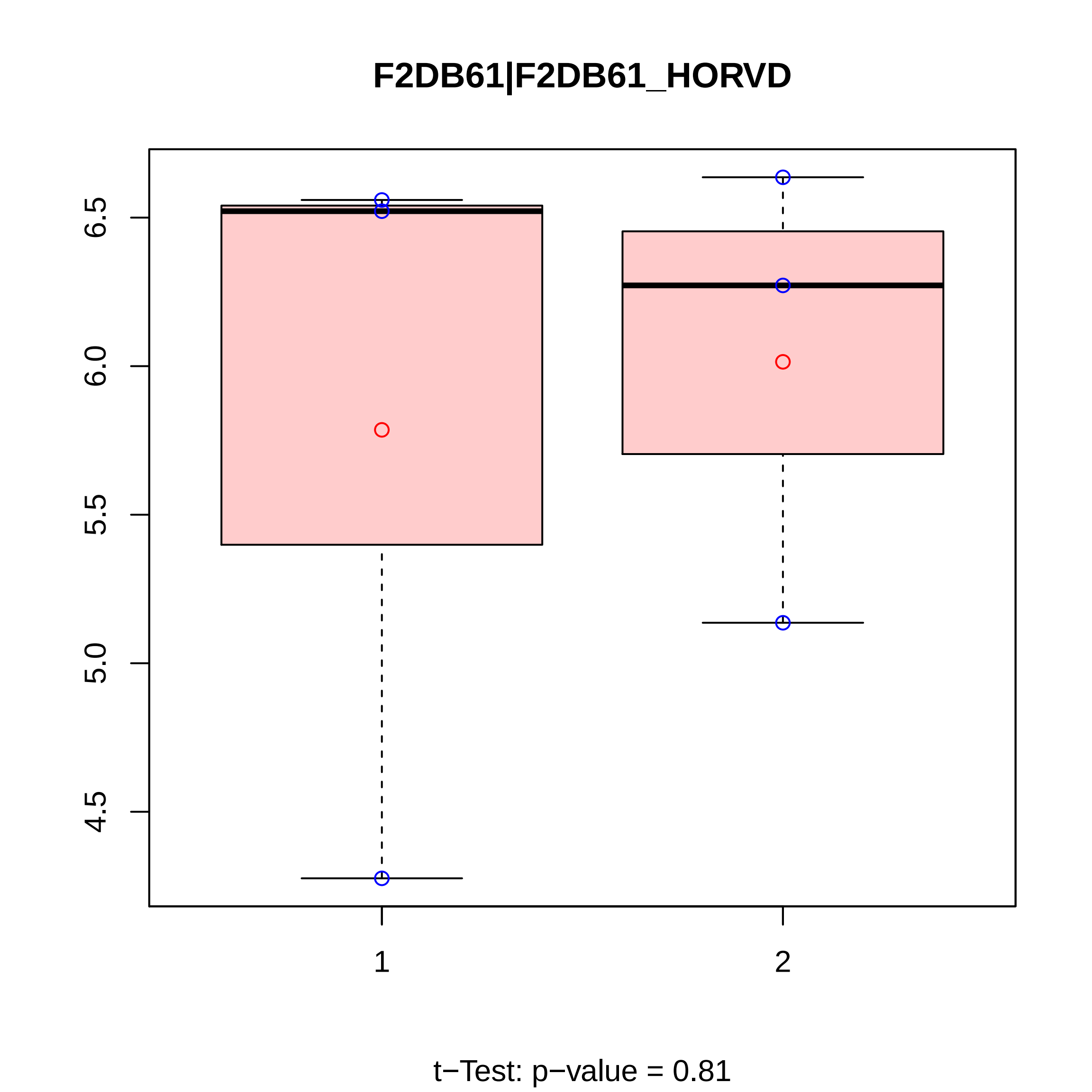
F2DB61|F2DB61_HORVD
4.5
5.0
5.5
6.0
6.5
1
2
t−Test: p−value = 0.81

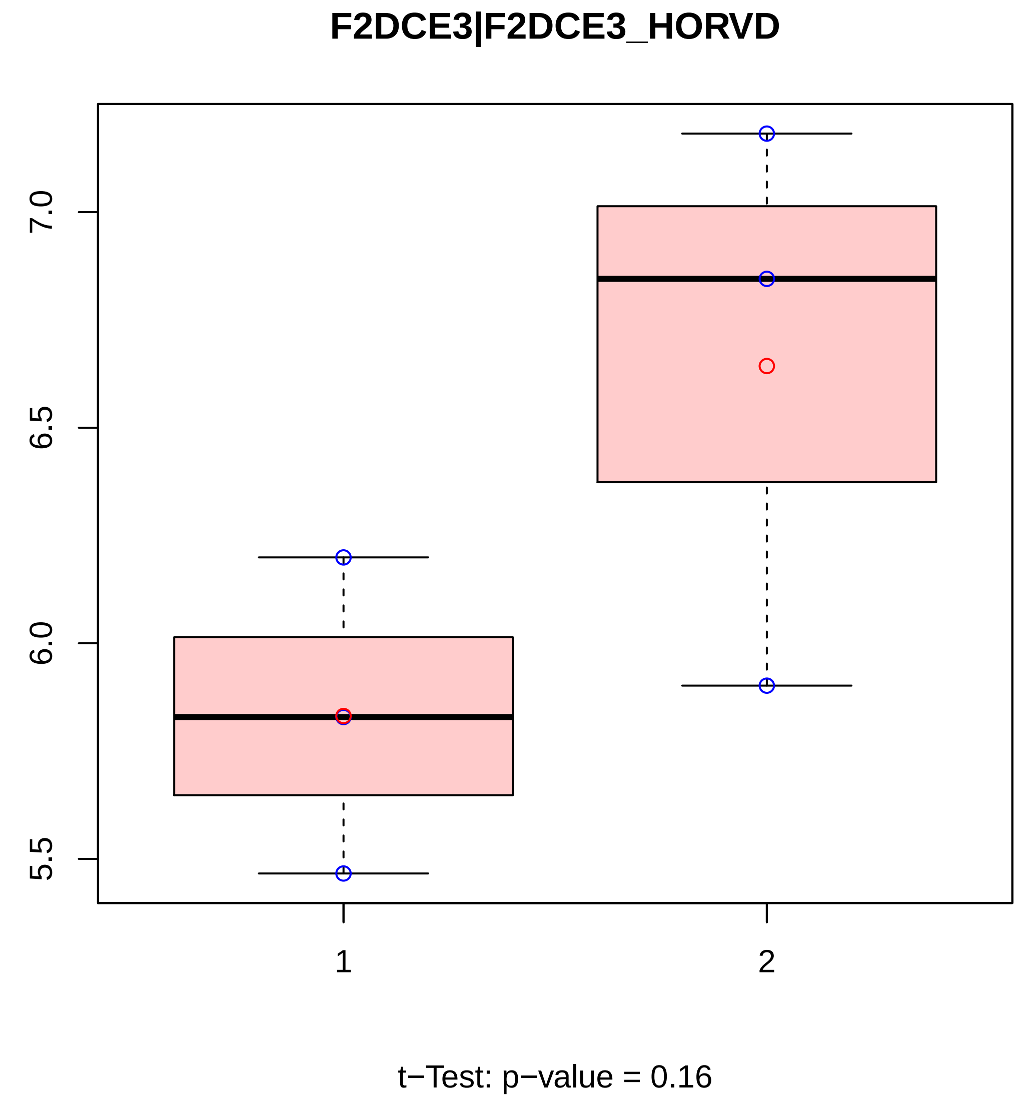
F2DCE3|F2DCE3_HORVD
7.0
6.5
6.0
5.5
1
2
t−Test: p−value = 0.16

F2DCJ3|F2DCJ3_HORVD

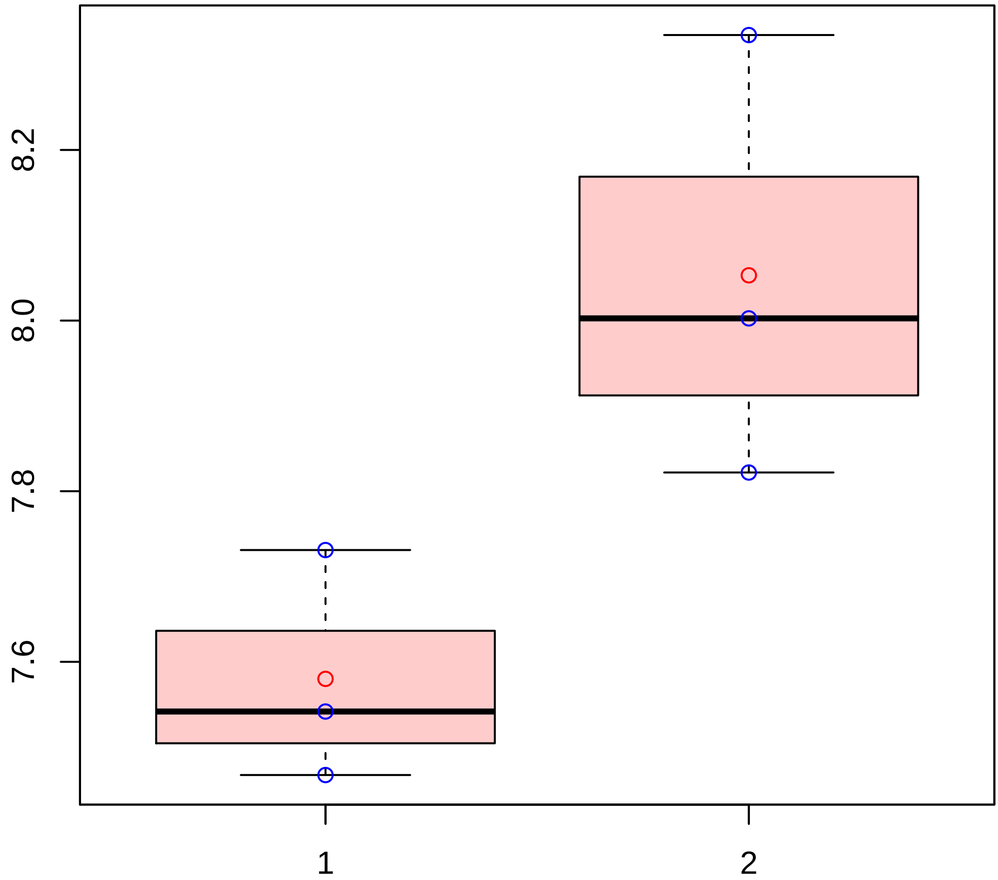

t−Test: p−value = 0.07

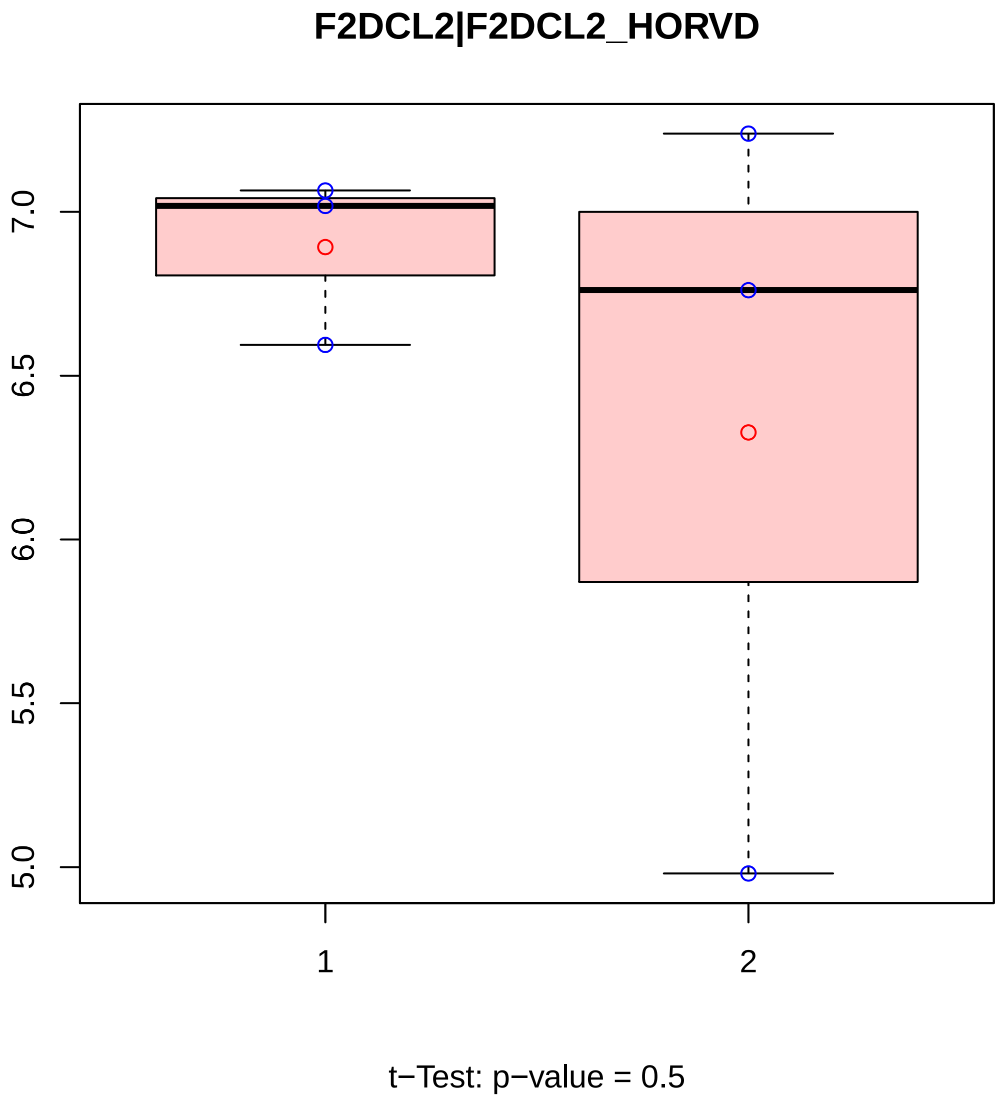

F2DCL2|F2DCL2_HORVD
7.0
6.5
6.0
5.5
5.0
1
2
t−Test: p−value = 0.5

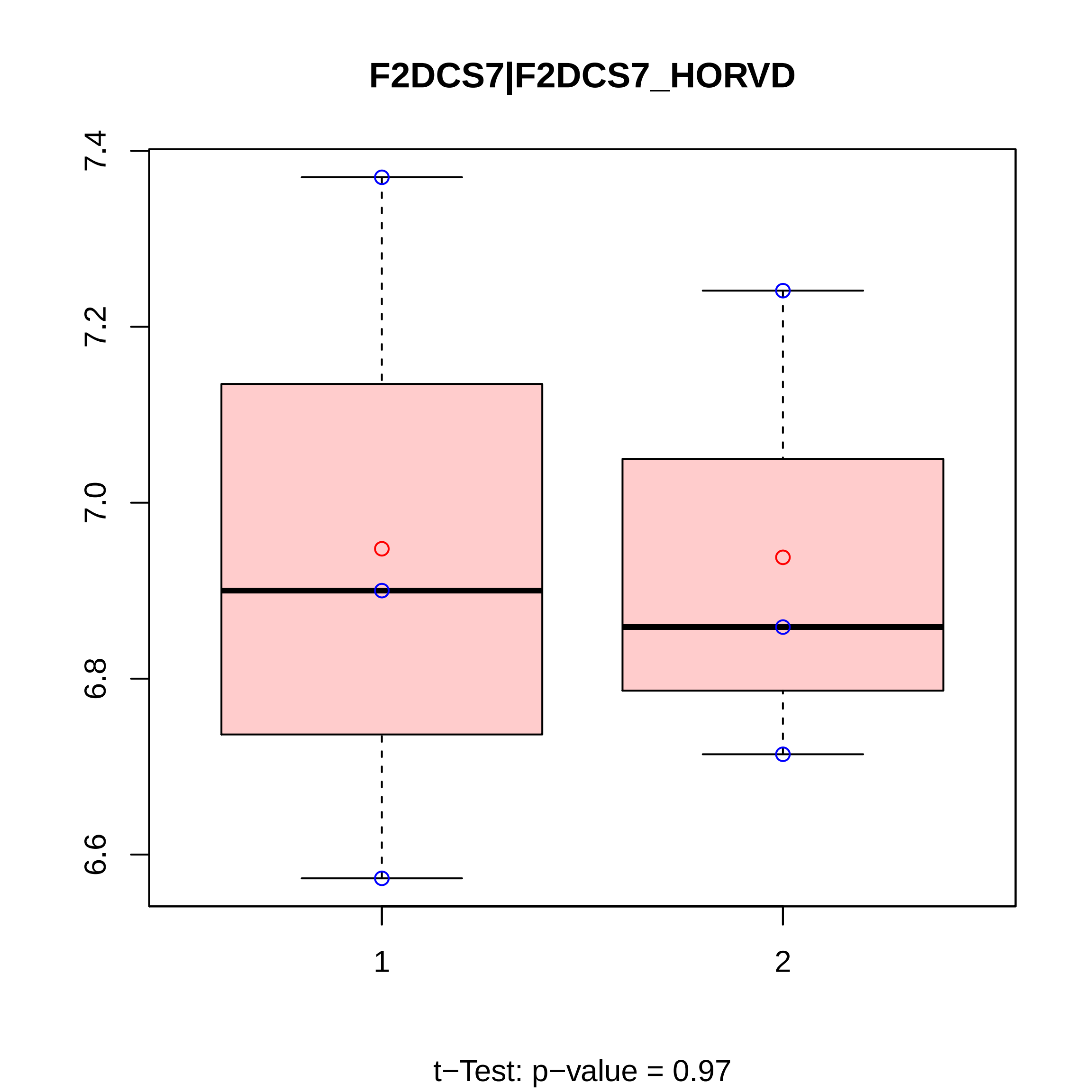

F2DCS7|F2DCS7_HORVD
7.4
7.2
7.0
6.8
6.6
1
2
t−Test: p−value = 0.97

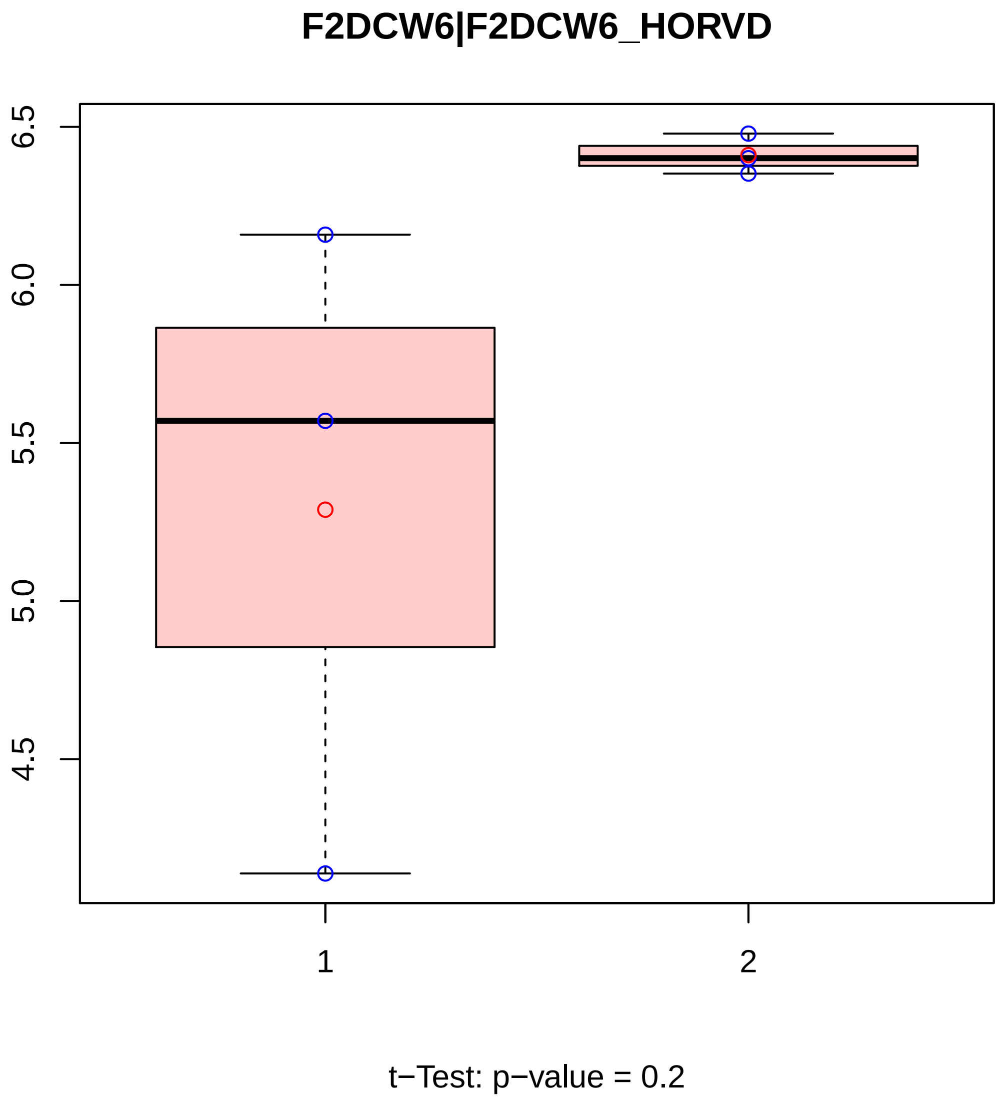
F2DCW6|F2DCW6_HORVD
6.5
6.0
5.5
5.0
4.5
1
2
t−Test: p−value = 0.2

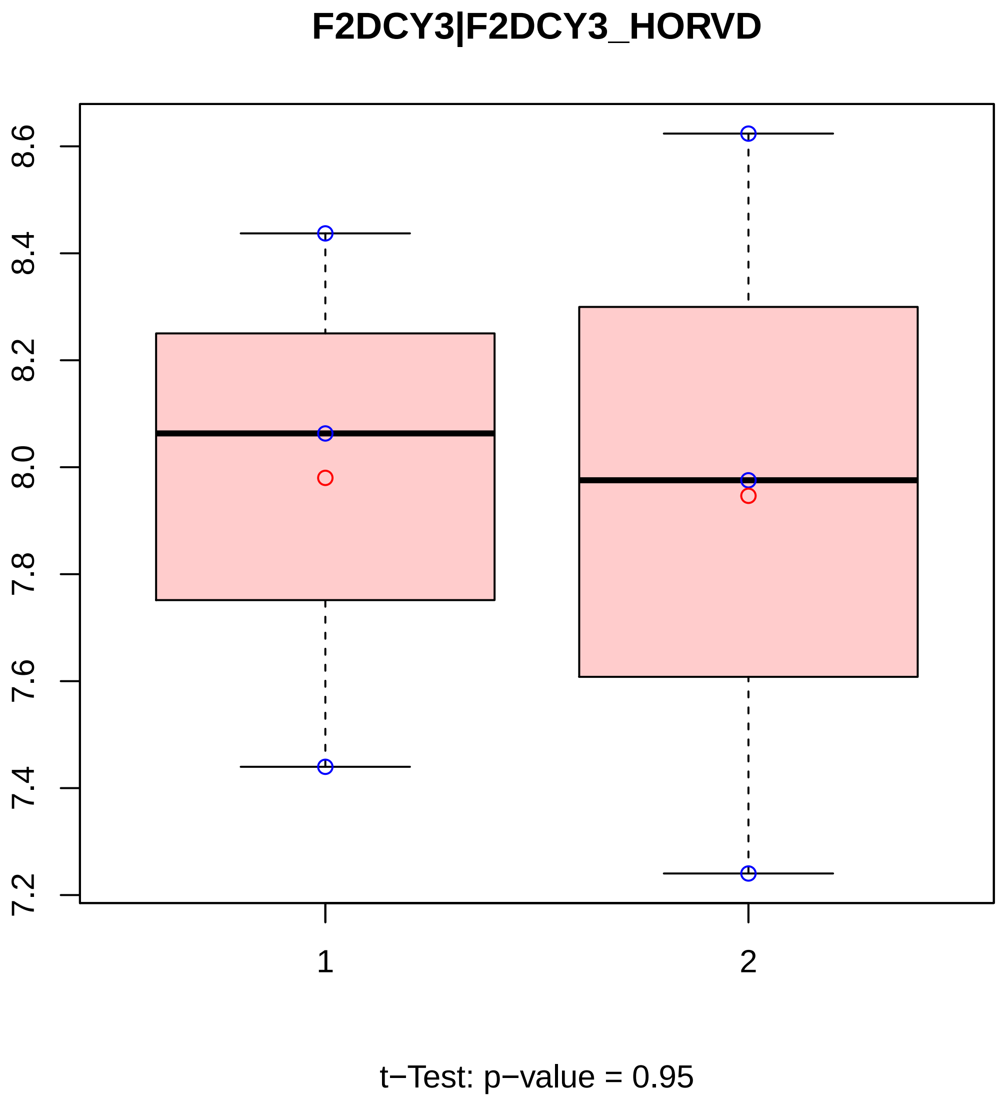
F2DCY3|F2DCY3_HORVD
8.6
8.4
8.2
8.0
7.8
7.6
7.4
7.2
1
2
t−Test: p−value = 0.95

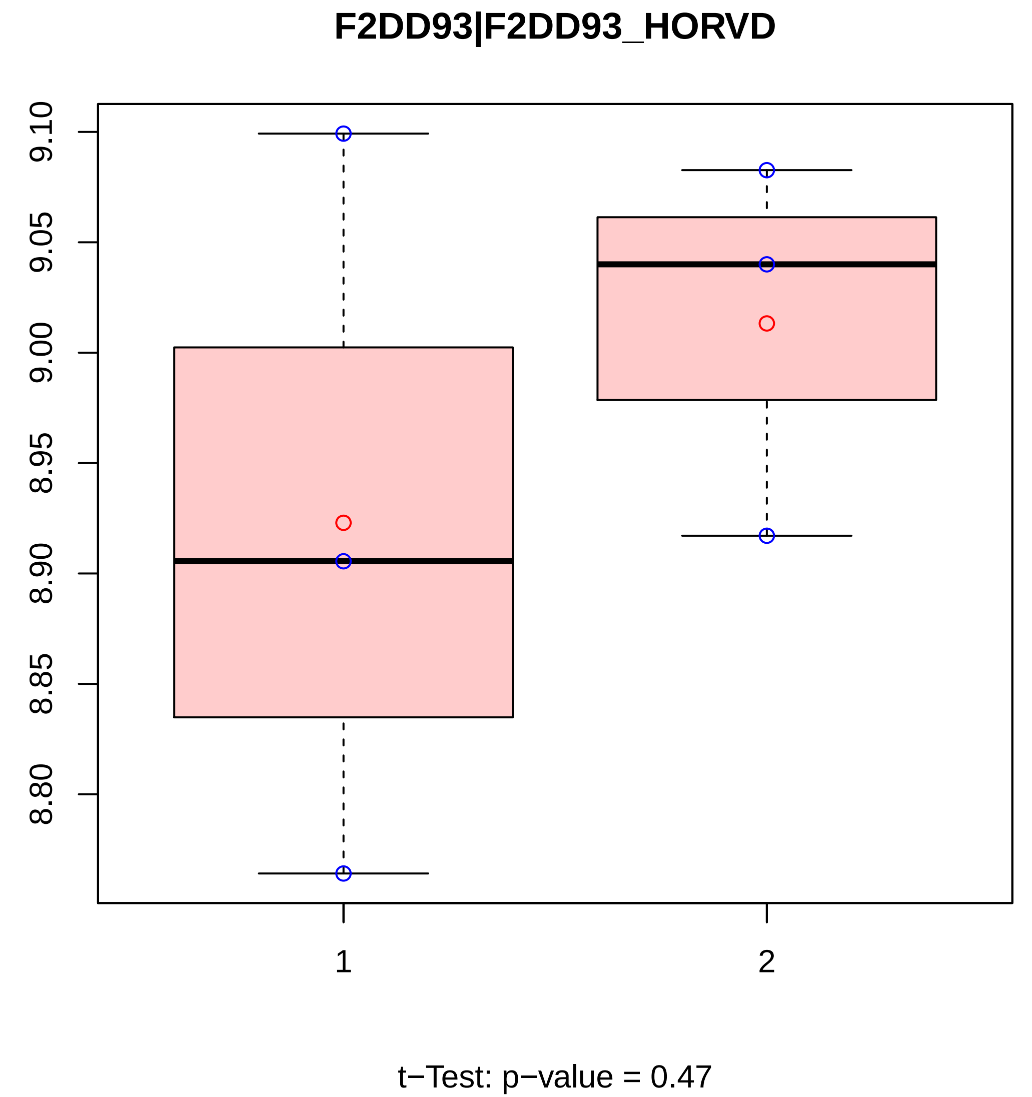
F2DD93|F2DD93_HORVD
9.10
9.05
9.00
8.95
8.90
8.85
8.80
1
2
t−Test: p−value = 0.47

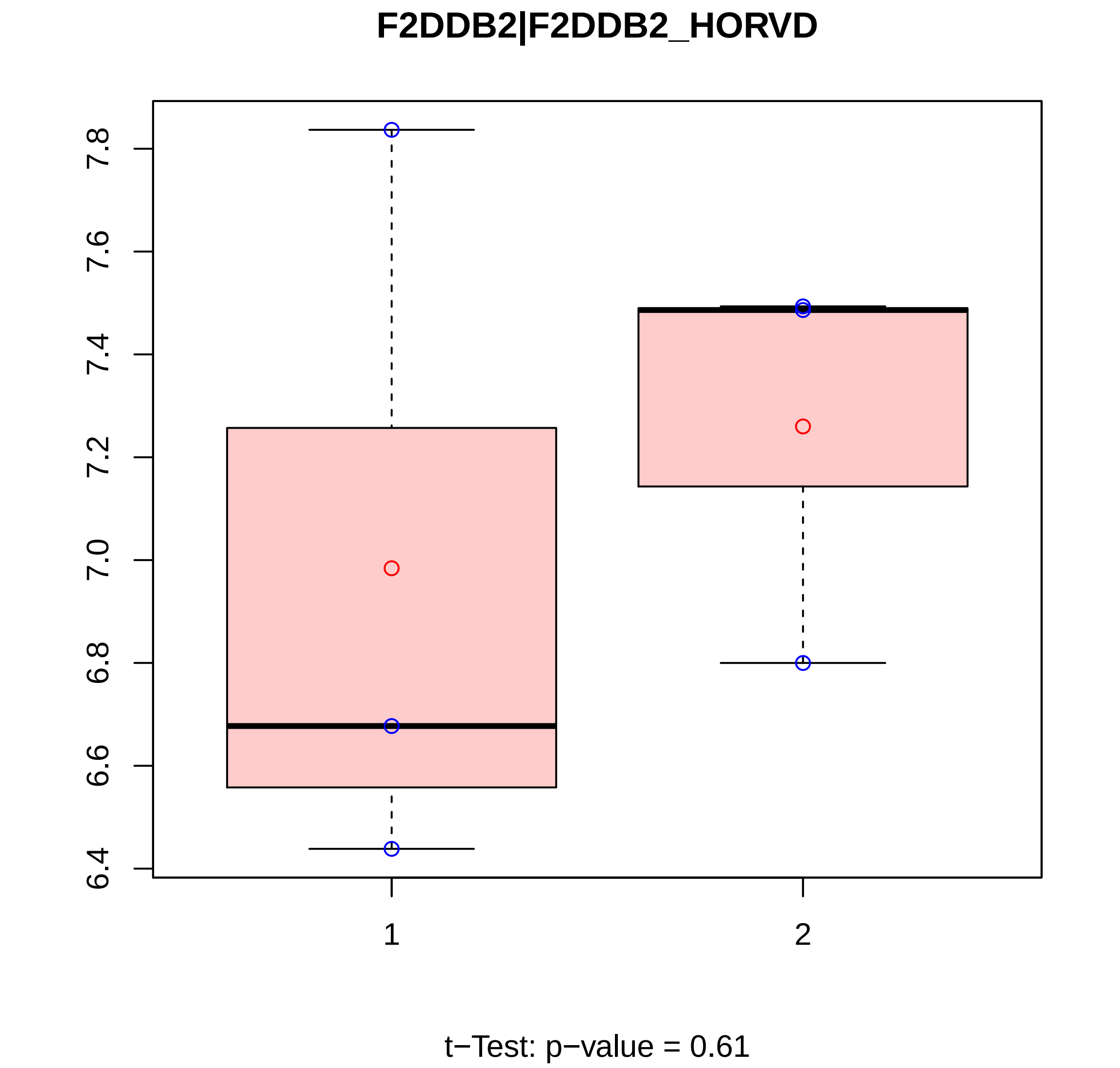
F2DDB2|F2DDB2_HORVD
7.8
7.6
7.4
7.2
7.0
6.8
6.6
6.4
1
2
t−Test: p−value = 0.61

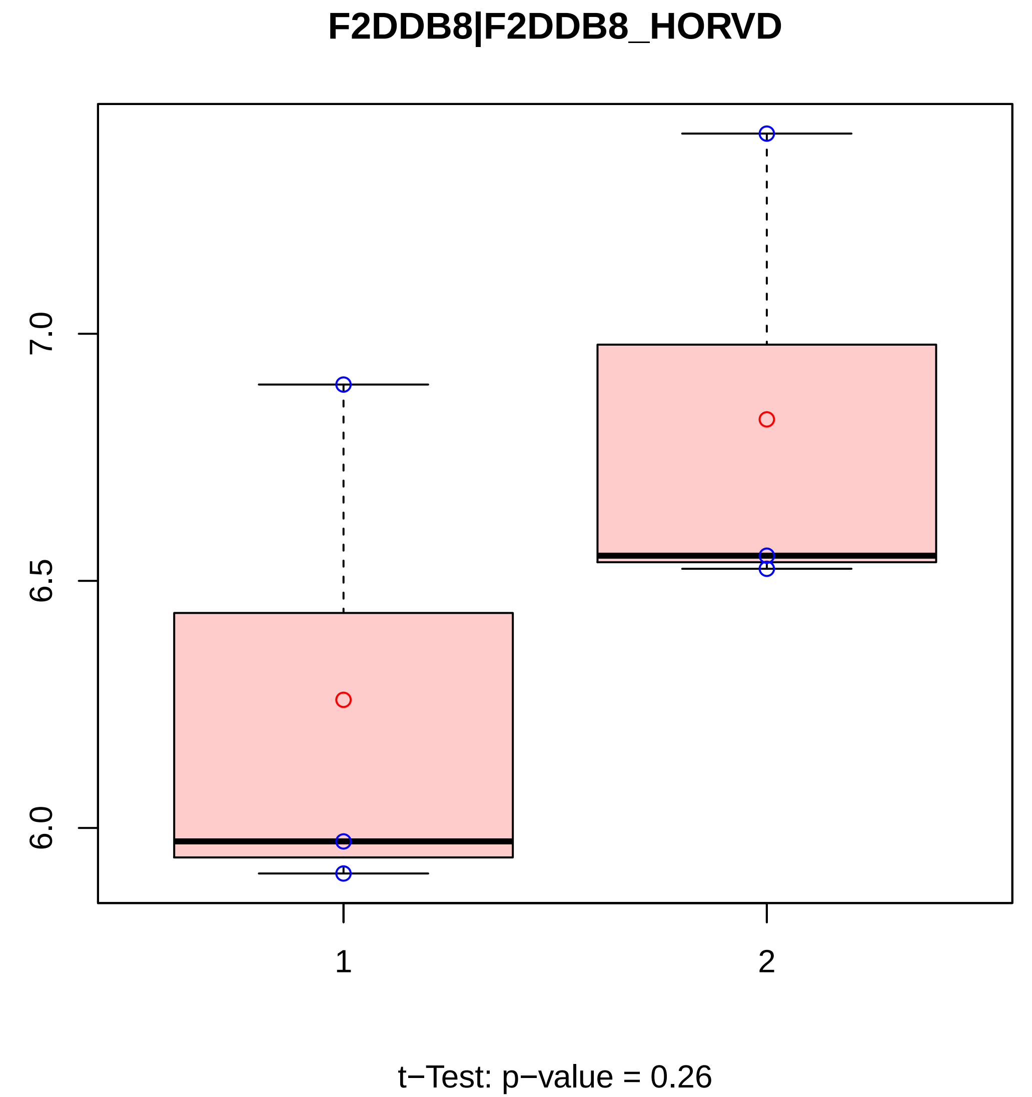
F2DDB8|F2DDB8_HORVD
7.0
6.5
6.0
1
2
t−Test: p−value = 0.26

F2DDE9|F2DDE9_HORVD

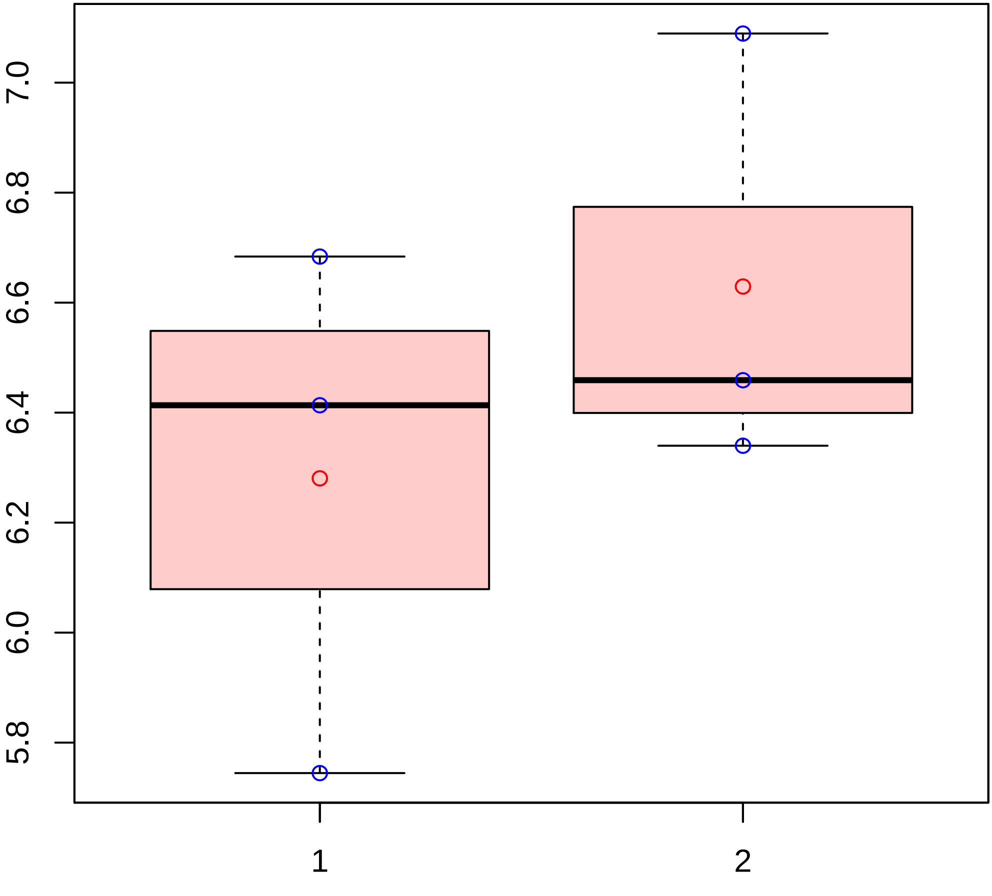

t−Test: p−value = 0.39

F2DDK1|F2DDK1_HORVD

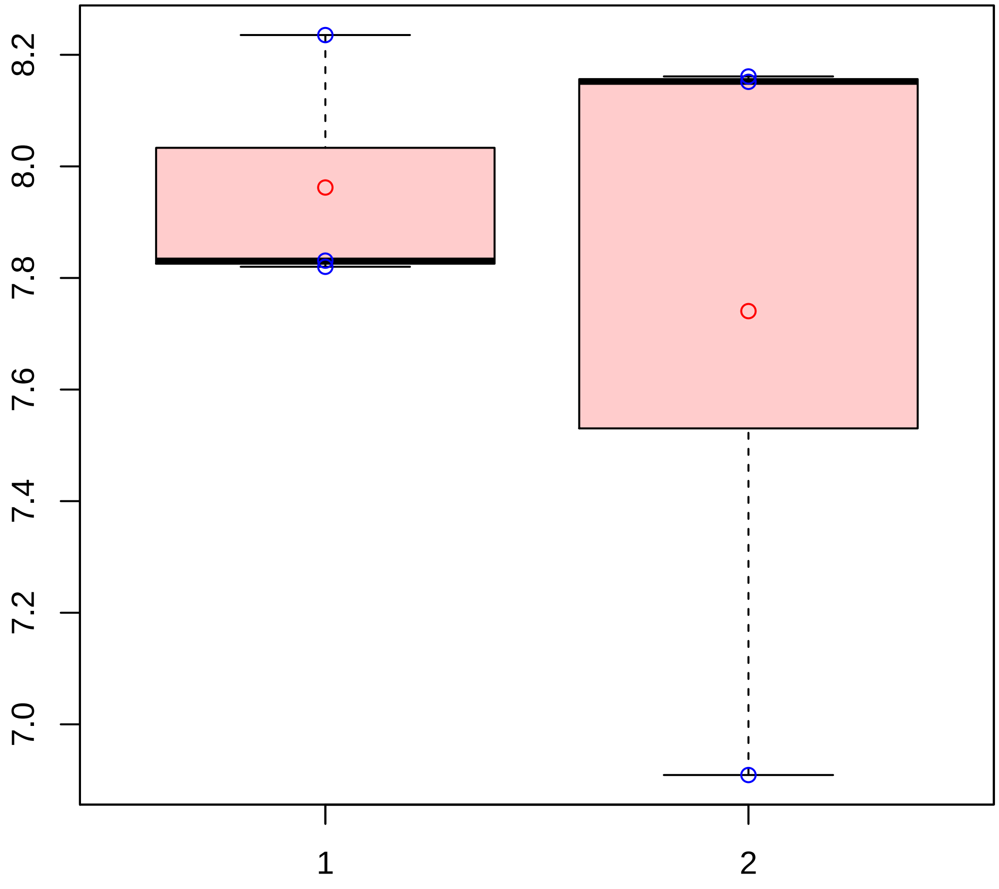

t−Test: p−value = 0.66

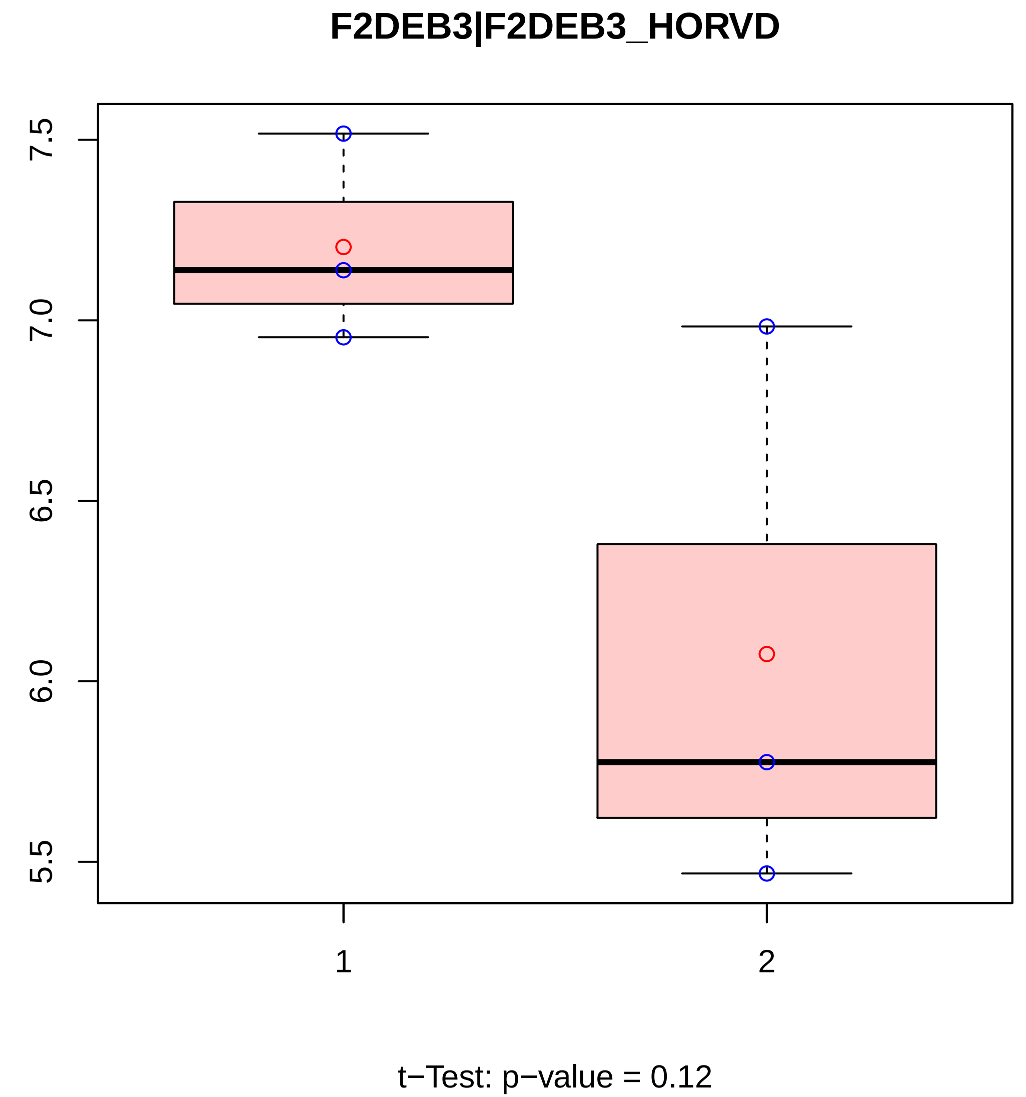
F2DEB3|F2DEB3_HORVD
7.5
7.0
6.5
6.0
5.5
1
2
t−Test: p−value = 0.12

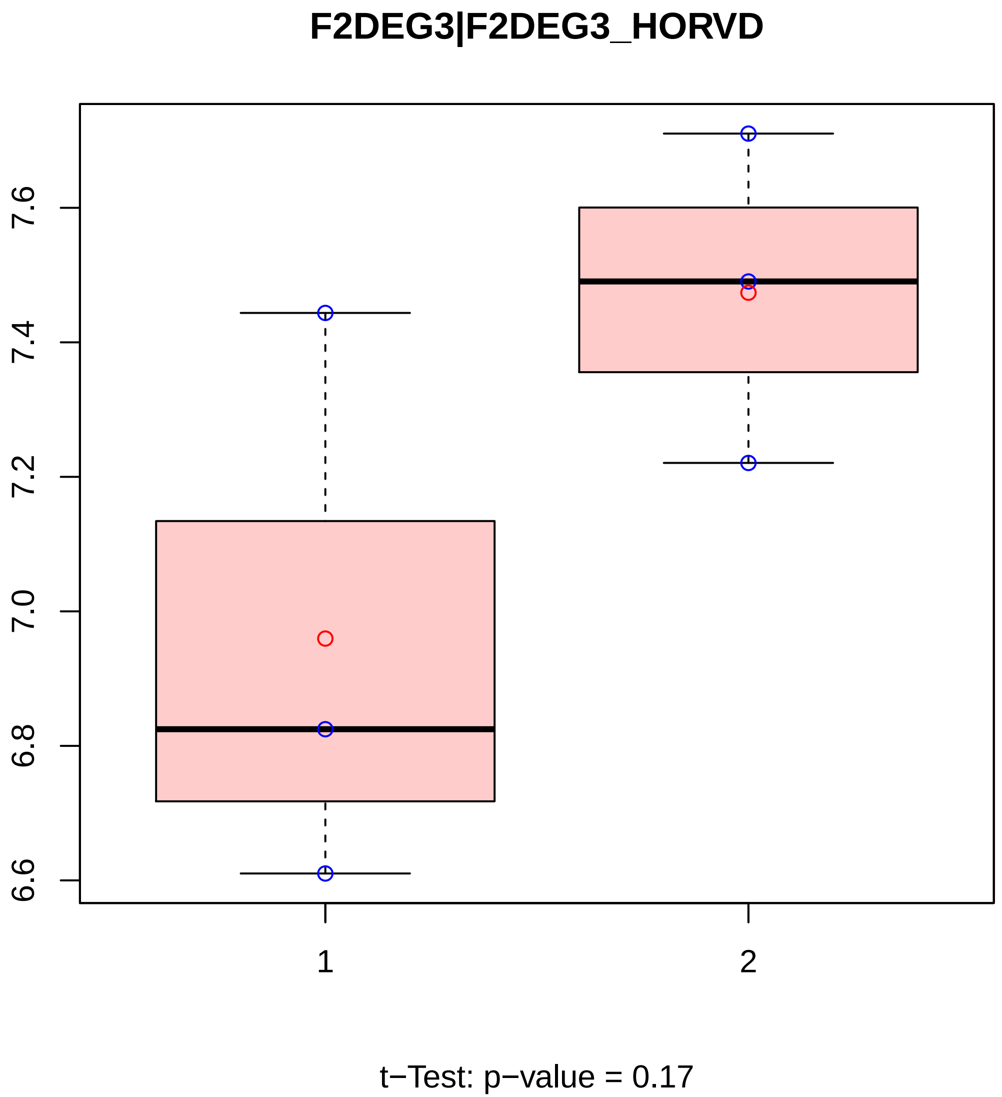
F2DEG3|F2DEG3_HORVD
7.6
7.4
7.2
7.0
6.8
6.6
1
2
t−Test: p−value = 0.17

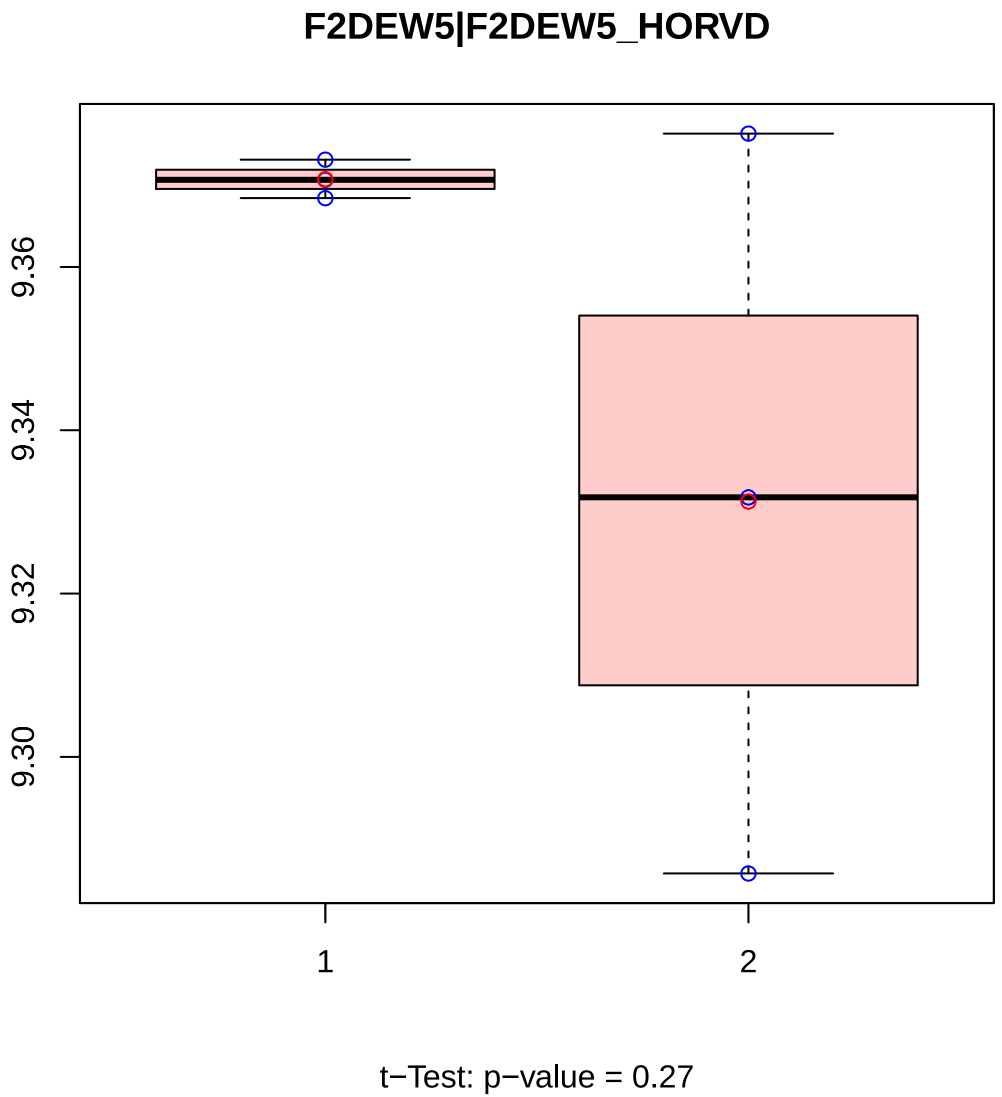

F2DEW5|F2DEW5_HORVD
9.30
9.32
9.34
9.36
1
2
t−Test: p−value = 0.27

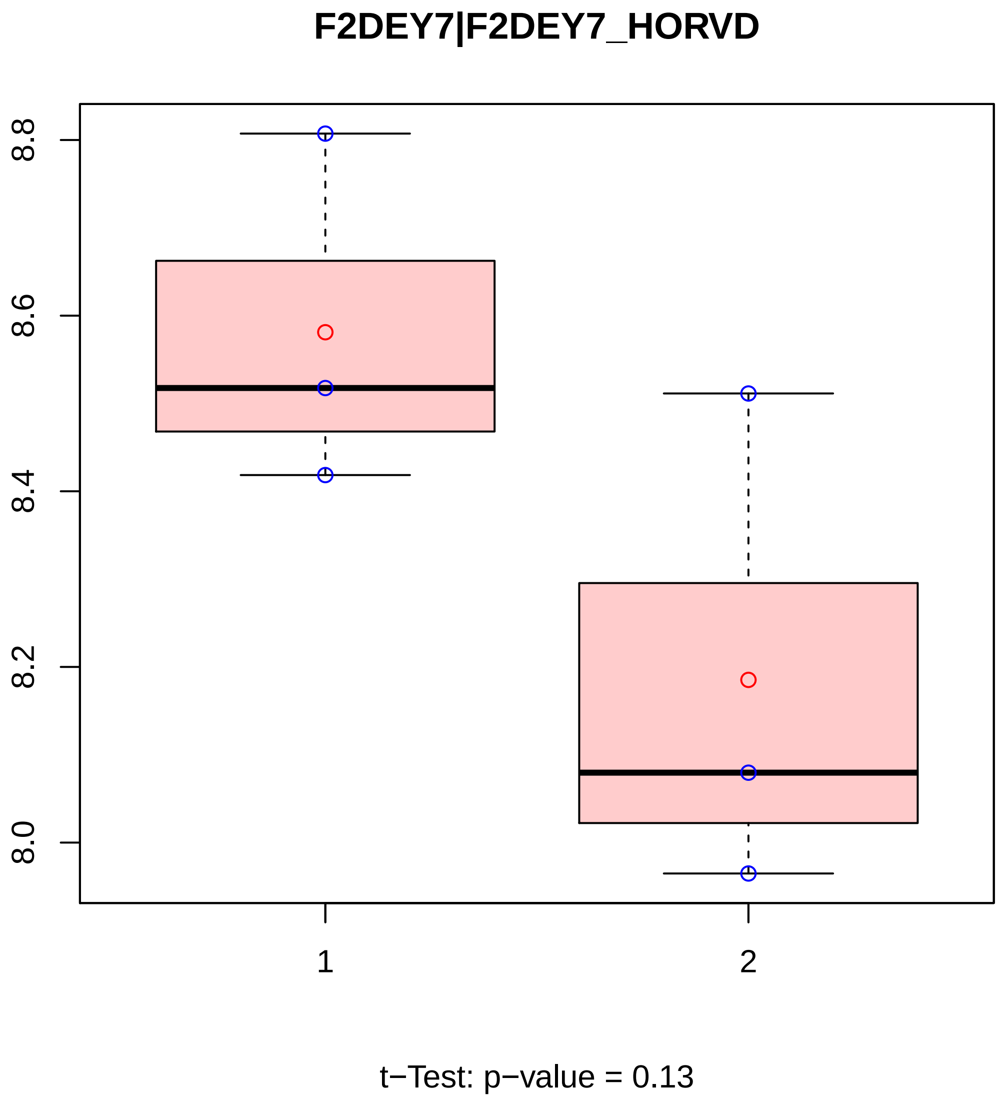
F2DEY7|F2DEY7_HORVD
8.0
8.2
8.4
8.6
8.8
1
2
t−Test: p−value = 0.13

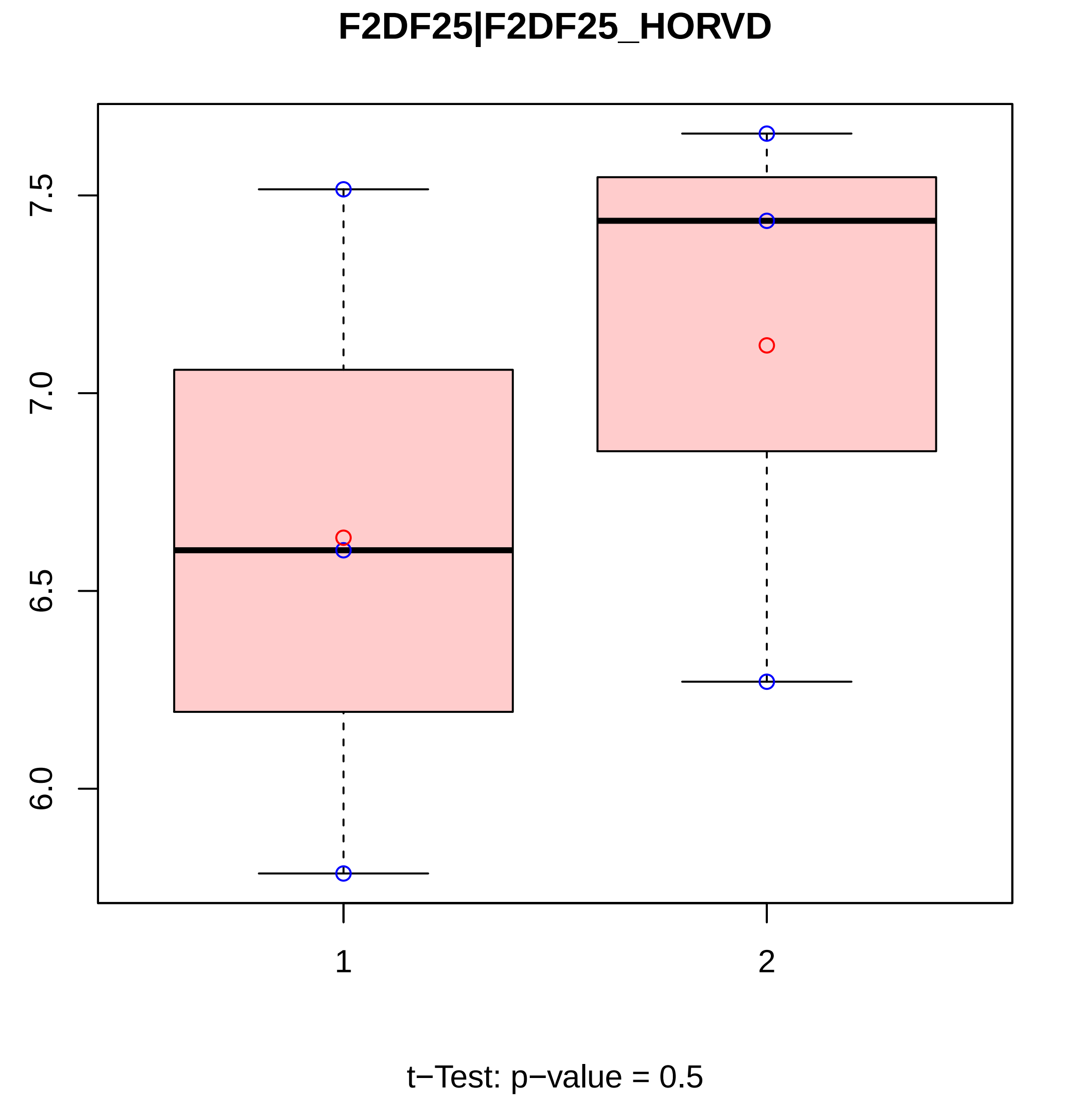

F2DF25|F2DF25_HORVD
7.5
7.0
6.5
6.0
1
2
t−Test: p−value = 0.5

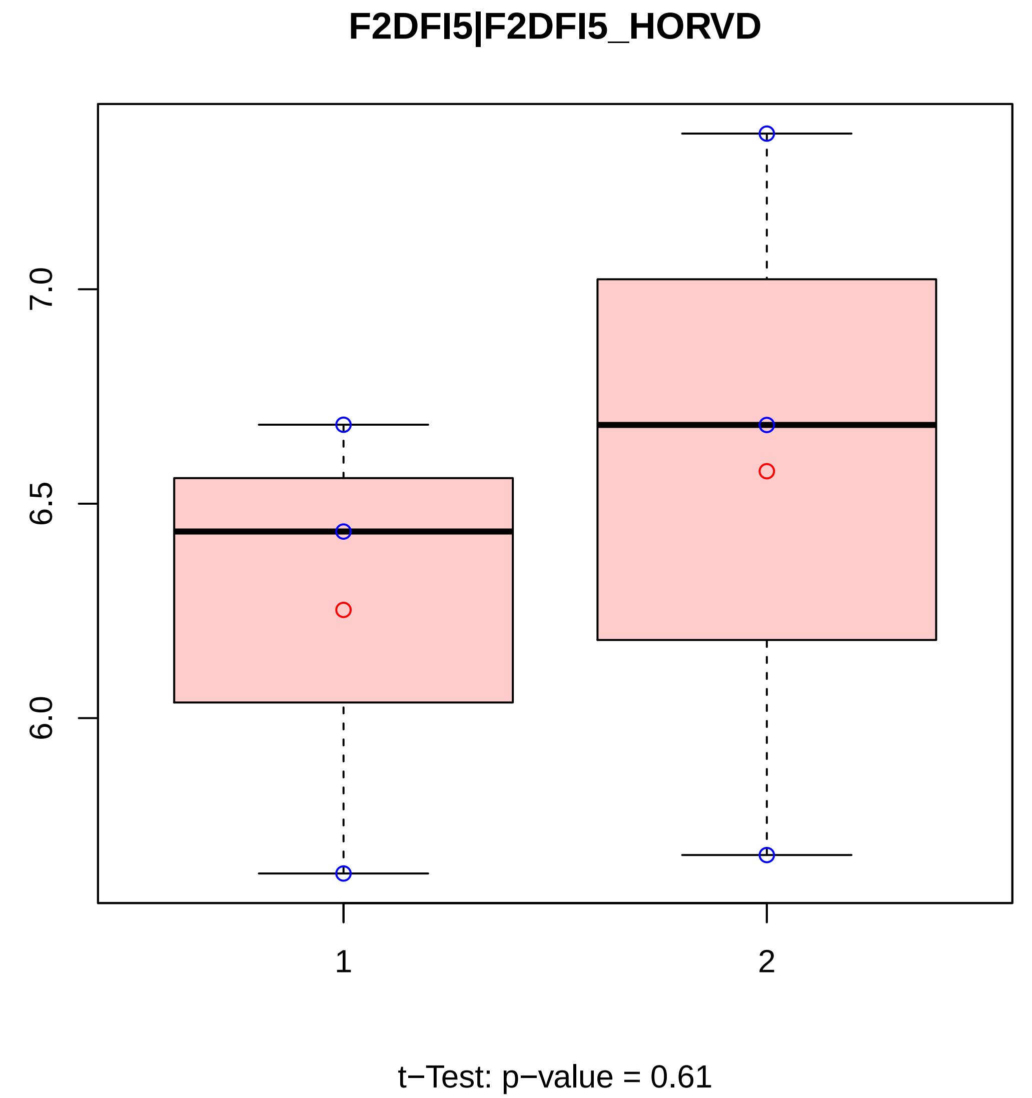

F2DFI5|F2DFI5_HORVD
7.0
6.5
6.0
1
2
t−Test: p−value = 0.61

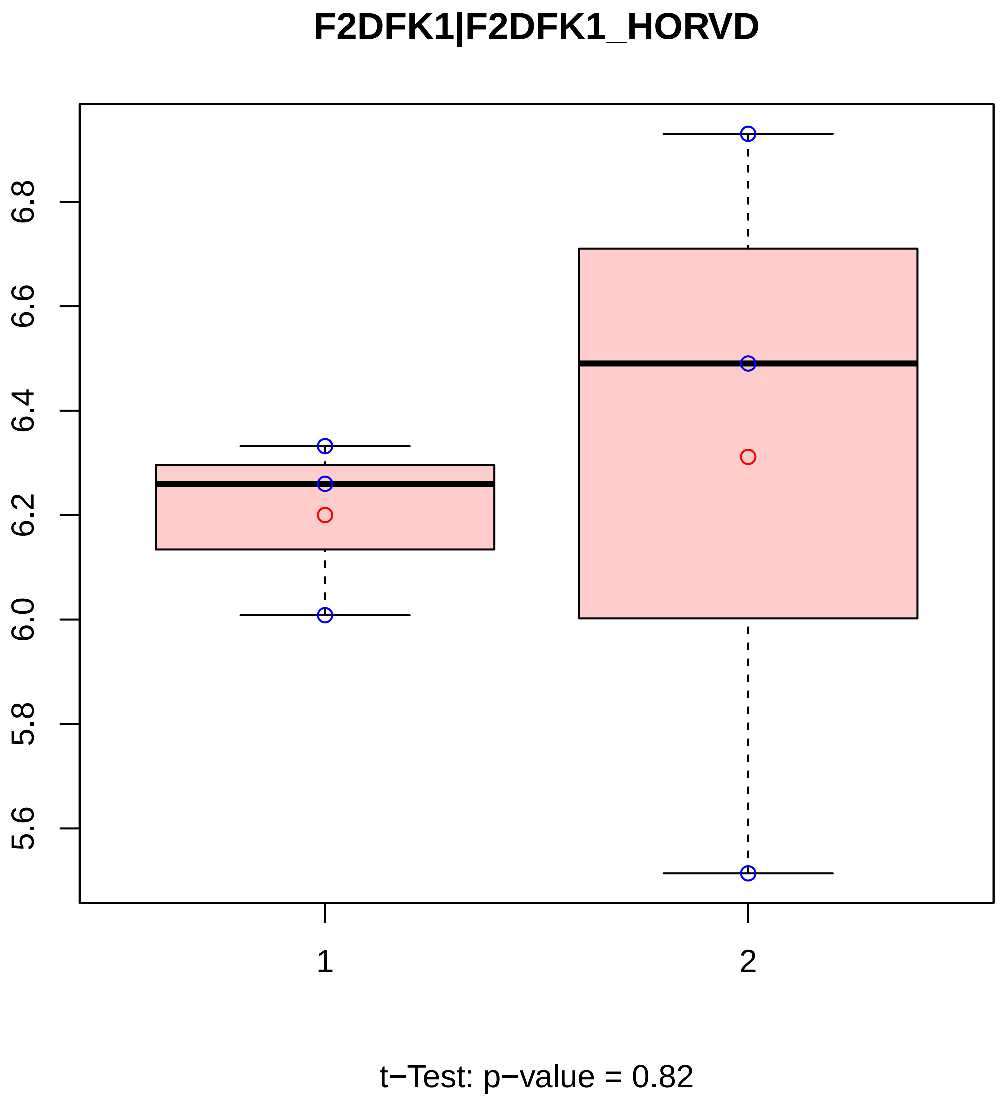
F2DFK1|F2DFK1_HORVD
6.8
6.6
6.4
6.2
6.0
5.8
5.6
1
2
t−Test: p−value = 0.82

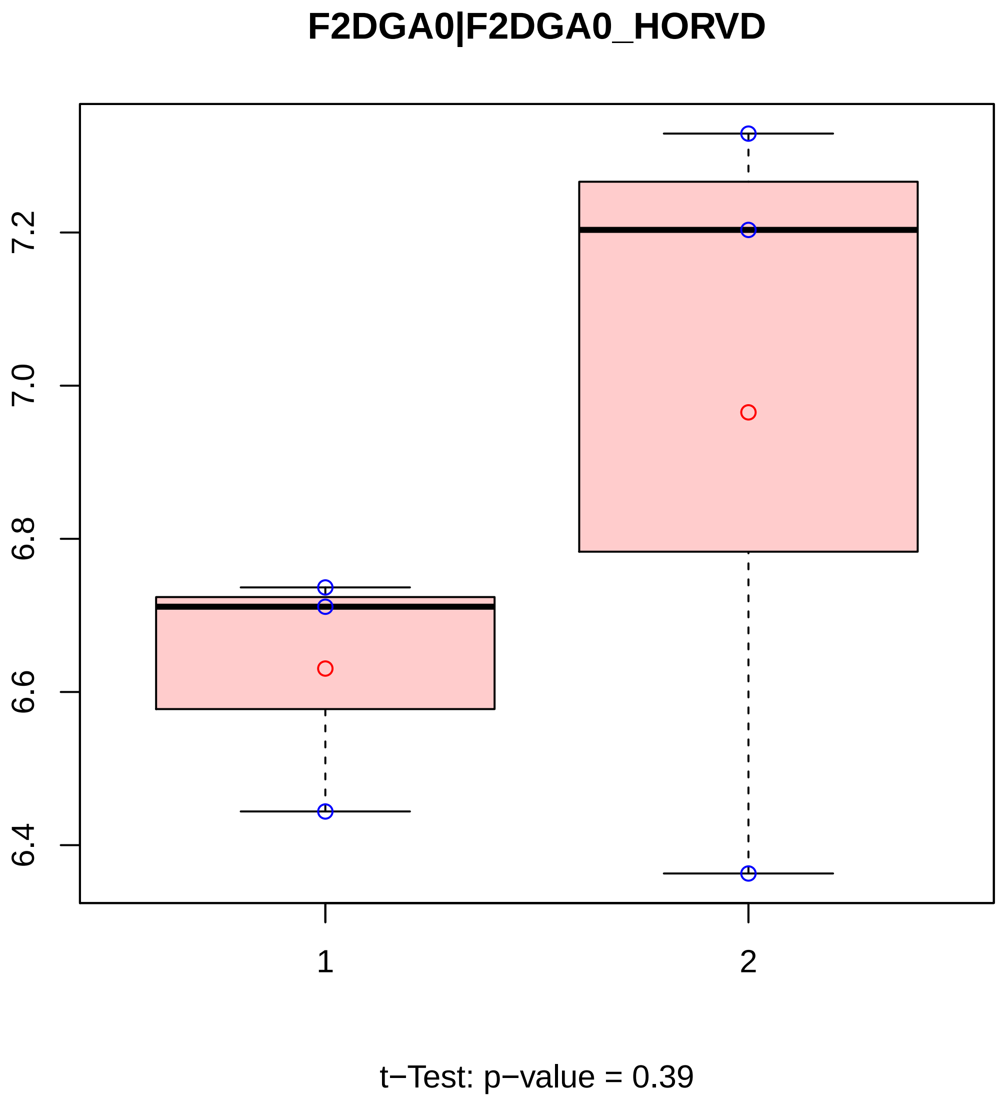
F2DGA0|F2DGA0_HORVD
6.4
6.6
6.8
7.0
7.2
1
2
t−Test: p−value = 0.39

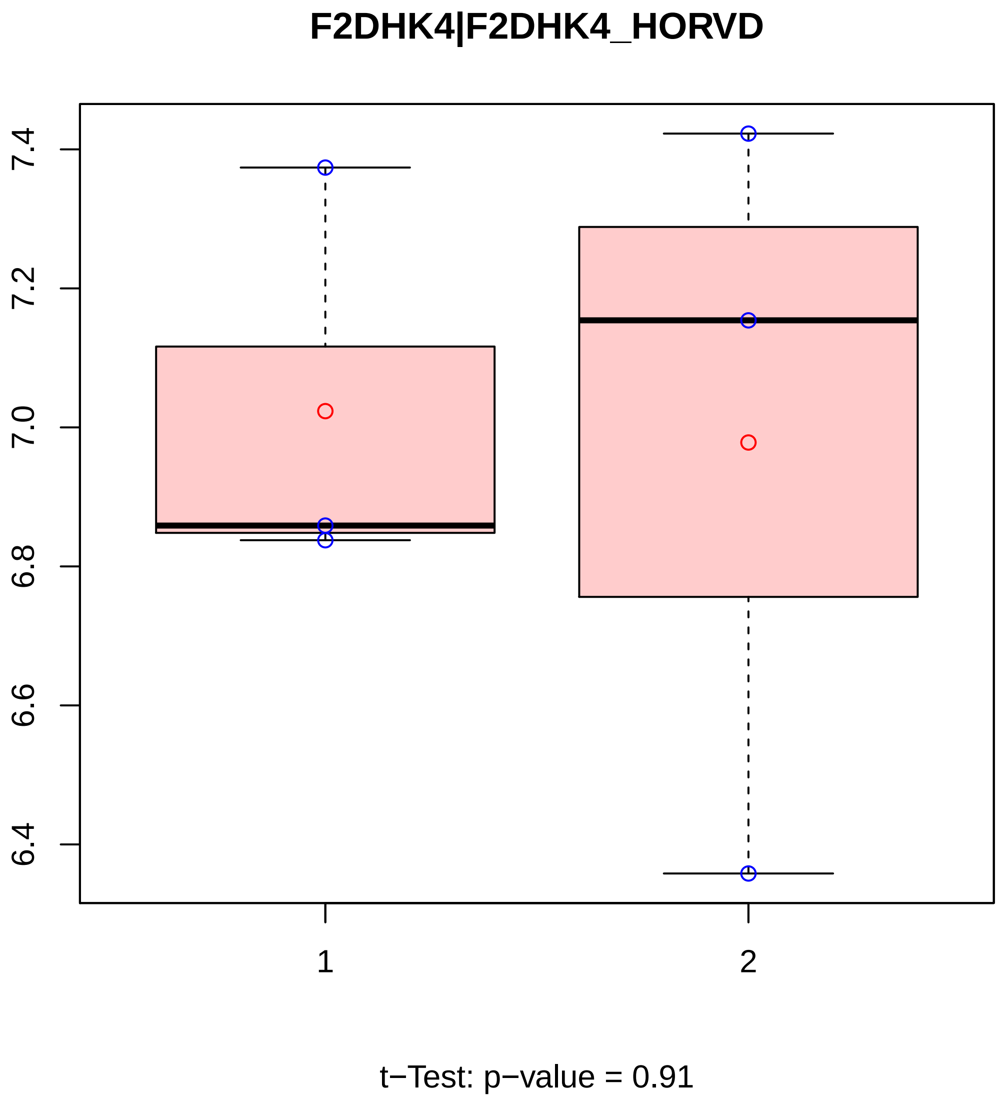

F2DHK4|F2DHK4_HORVD
7.4
7.2
7.0
6.8
6.6
6.4
1
2
t−Test: p−value = 0.91

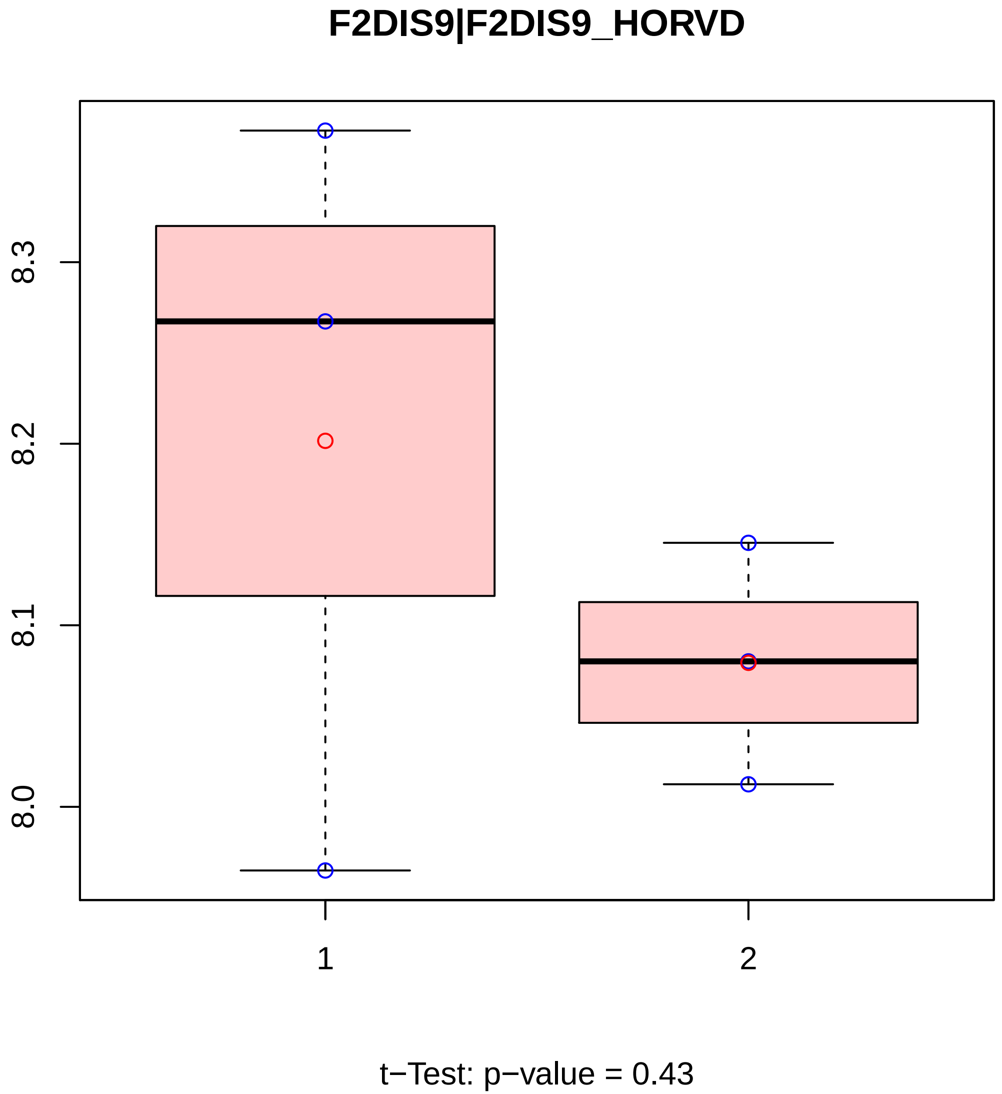

F2DIS9|F2DIS9_HORVD
8.0
8.1
8.2
8.3
1
2
t−Test: p−value = 0.43

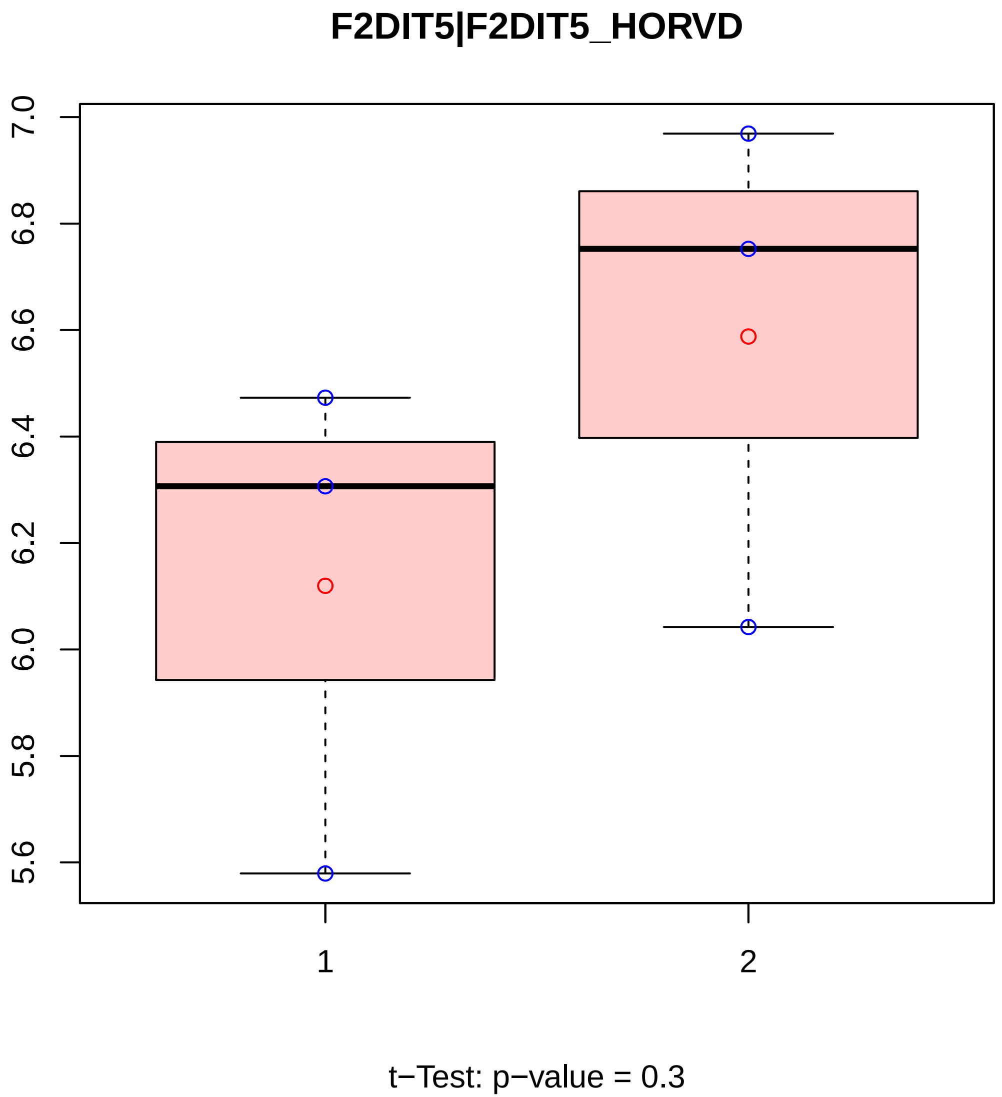
F2DIT5|F2DIT5_HORVD
7.0
6.8
6.6
6.4
6.2
6.0
5.8
5.6
1
2
t−Test: p−value = 0.3

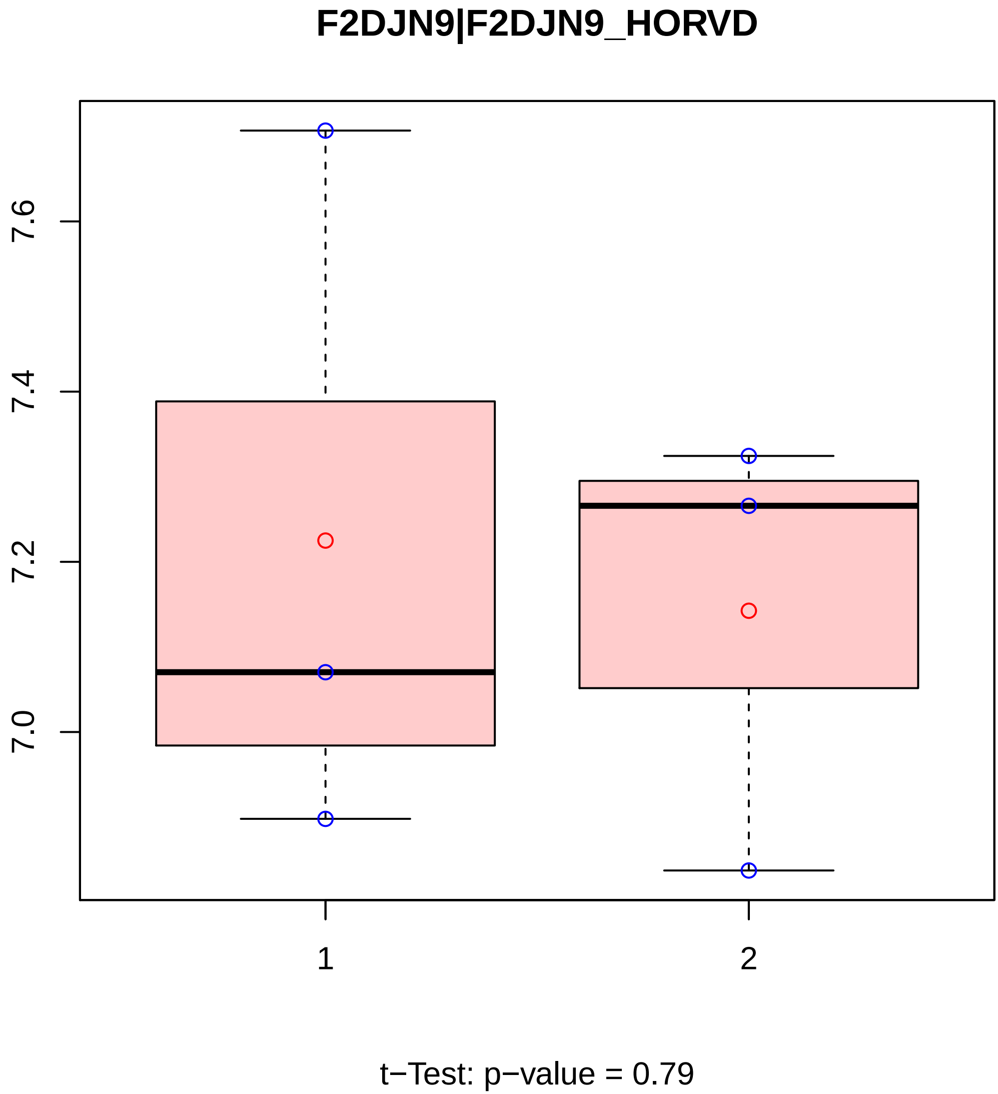

F2DJN9|F2DJN9_HORVD
7.6
7.4
7.2
7.0
1
2
t−Test: p−value = 0.79

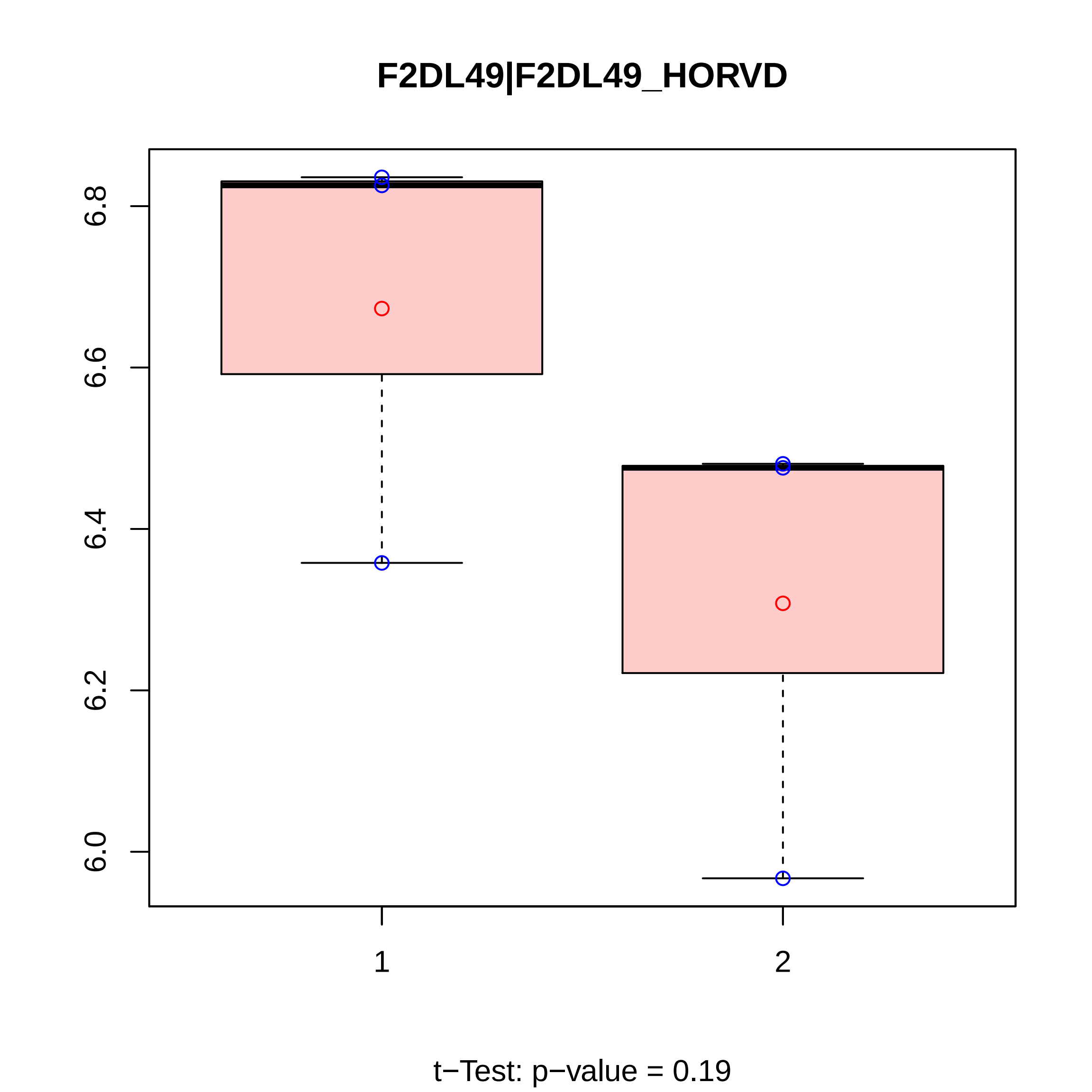
F2DL49|F2DL49_HORVD
6.0
6.2
6.4
6.6
6.8
1
2
t−Test: p−value = 0.19

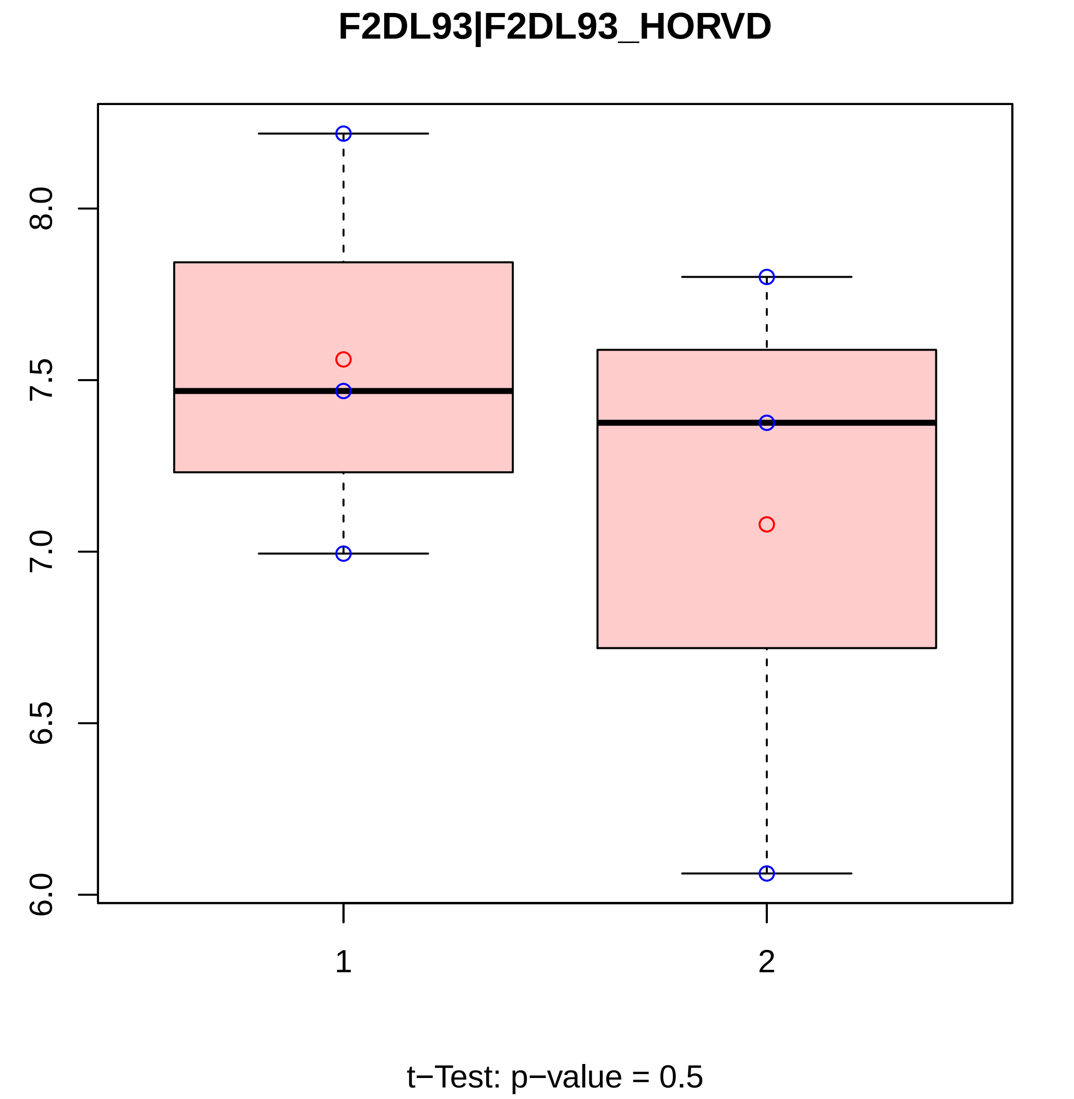
F2DL93|F2DL93_HORVD
8.0
7.5
7.0
6.5
6.0
1
2
t−Test: p−value = 0.5

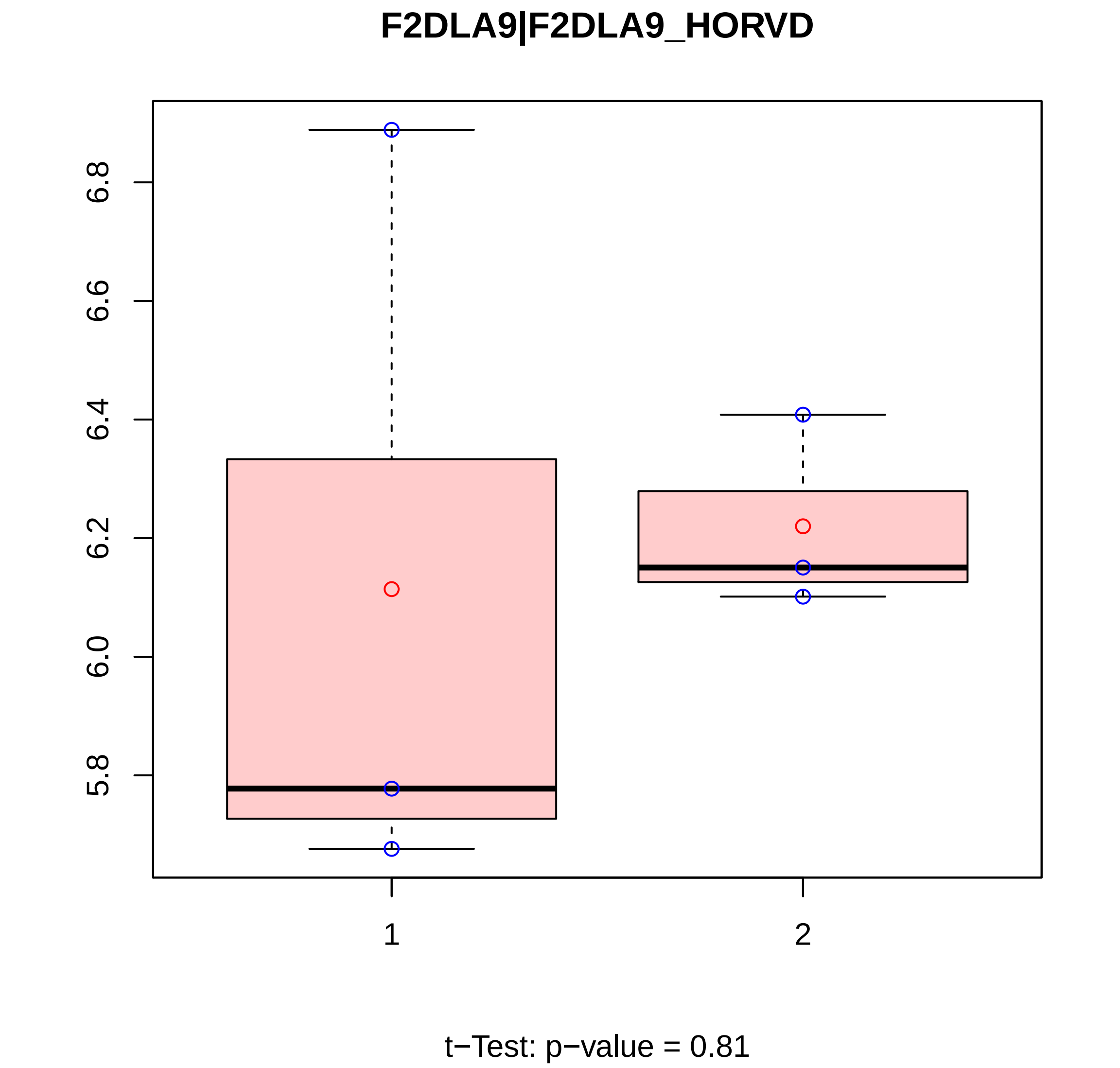
F2DLA9|F2DLA9_HORVD
5.8
6.0
6.2
6.4
6.6
6.8
1
2
t−Test: p−value = 0.81

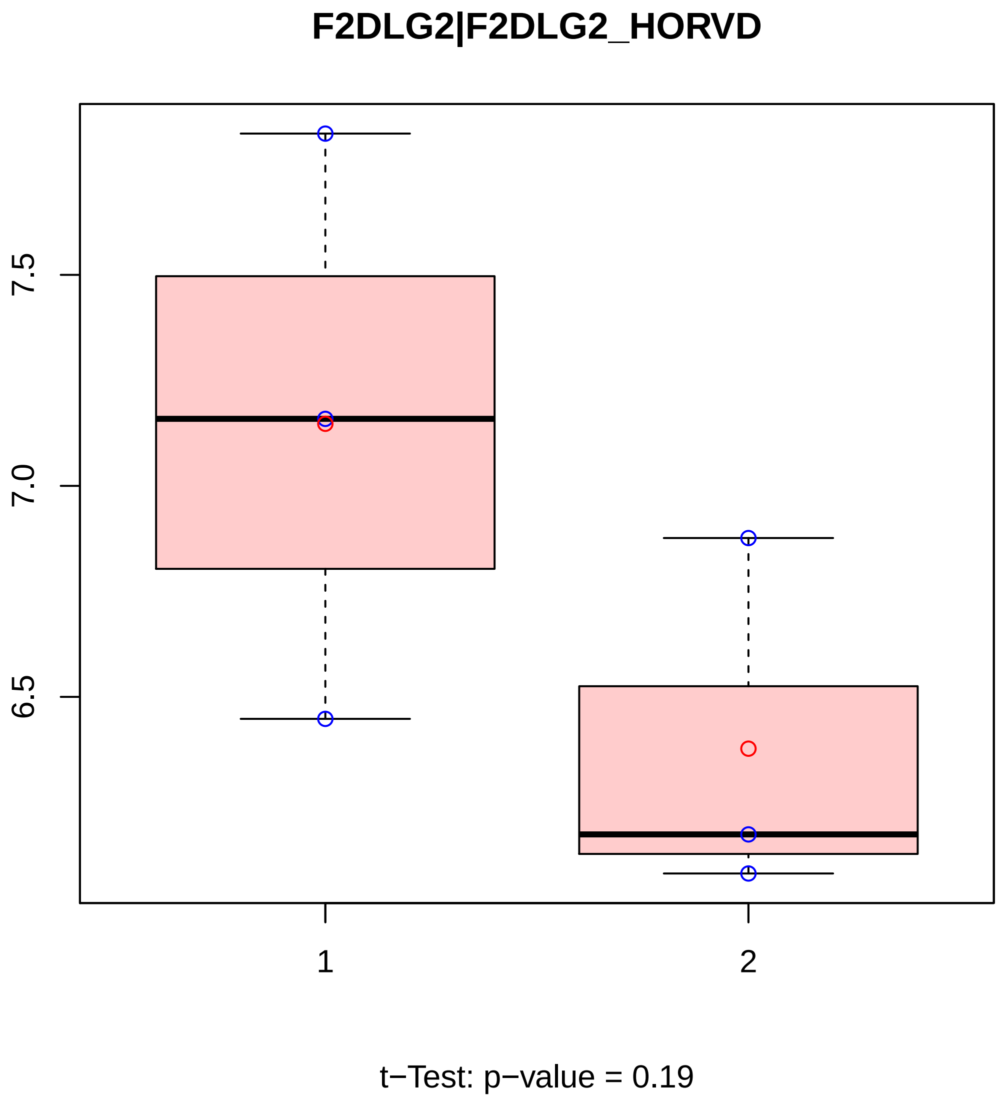
F2DLG2|F2DLG2_HORVD
7.5
7.0
6.5
1
2
t−Test: p−value = 0.19

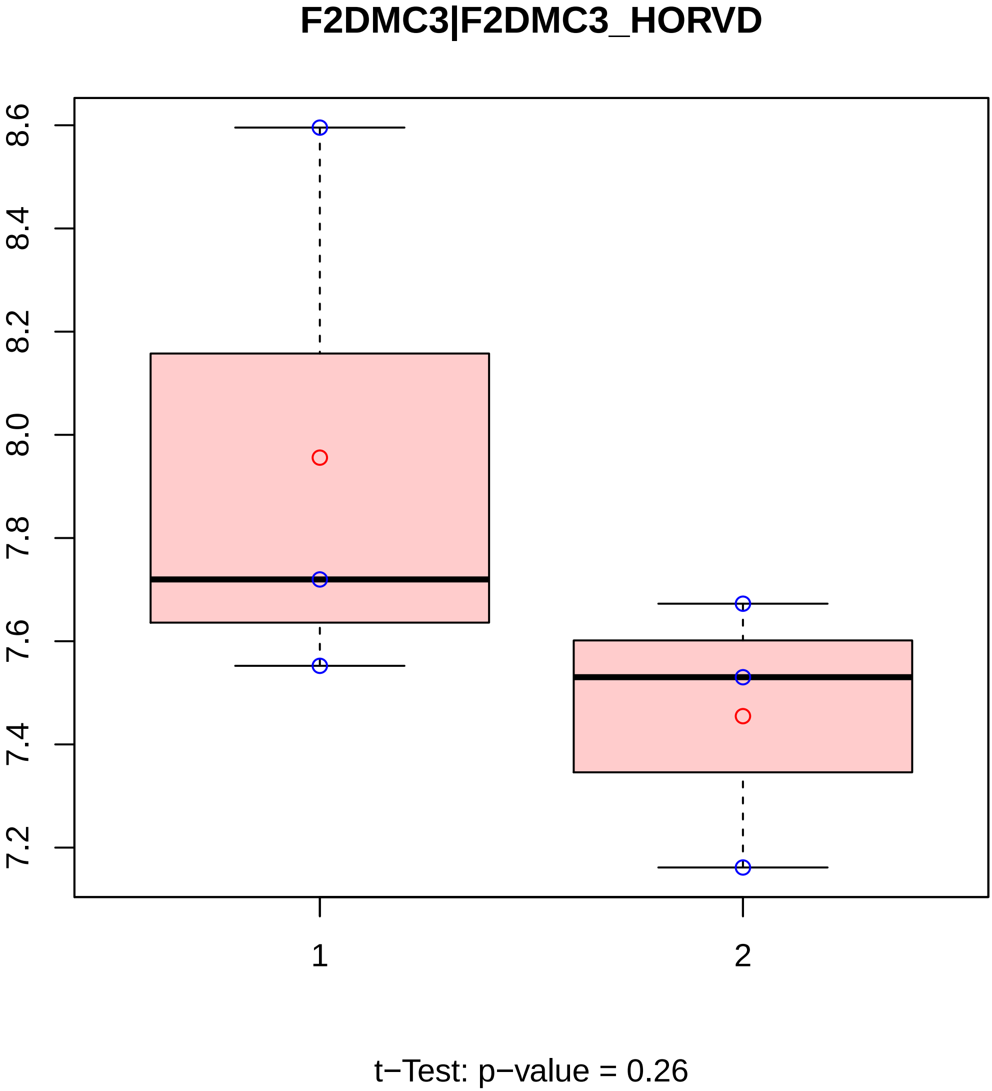

F2DMC3|F2DMC3_HORVD
8.6
8.4
8.2
8.0
7.8
7.6
7.4
7.2
1
2
t−Test: p−value = 0.26

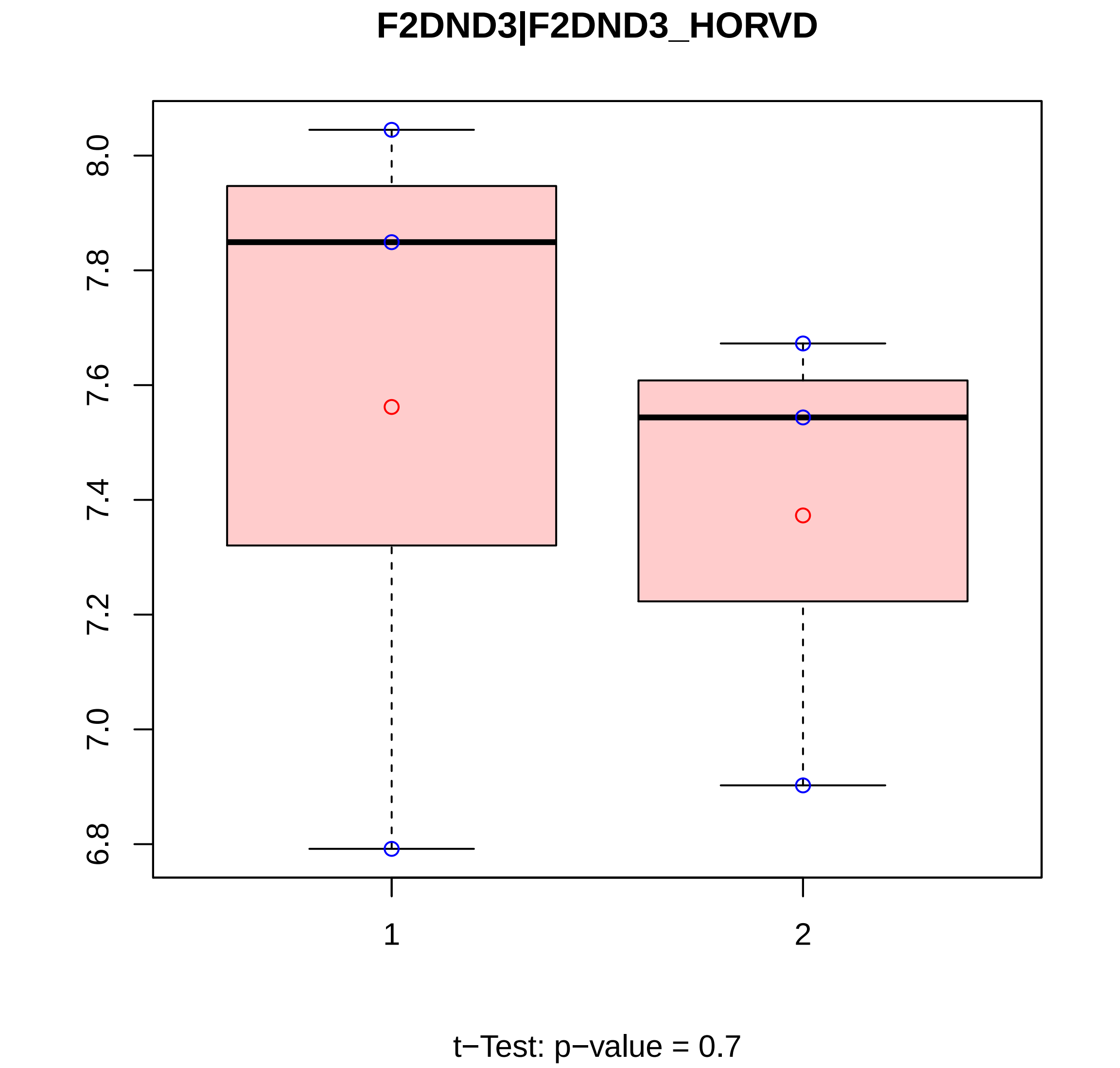
F2DND3|F2DND3_HORVD
8.0
7.8
7.6
7.4
7.2
7.0
6.8
1
2
t−Test: p−value = 0.7

F2DNQ6|F2DNQ6_HORVD

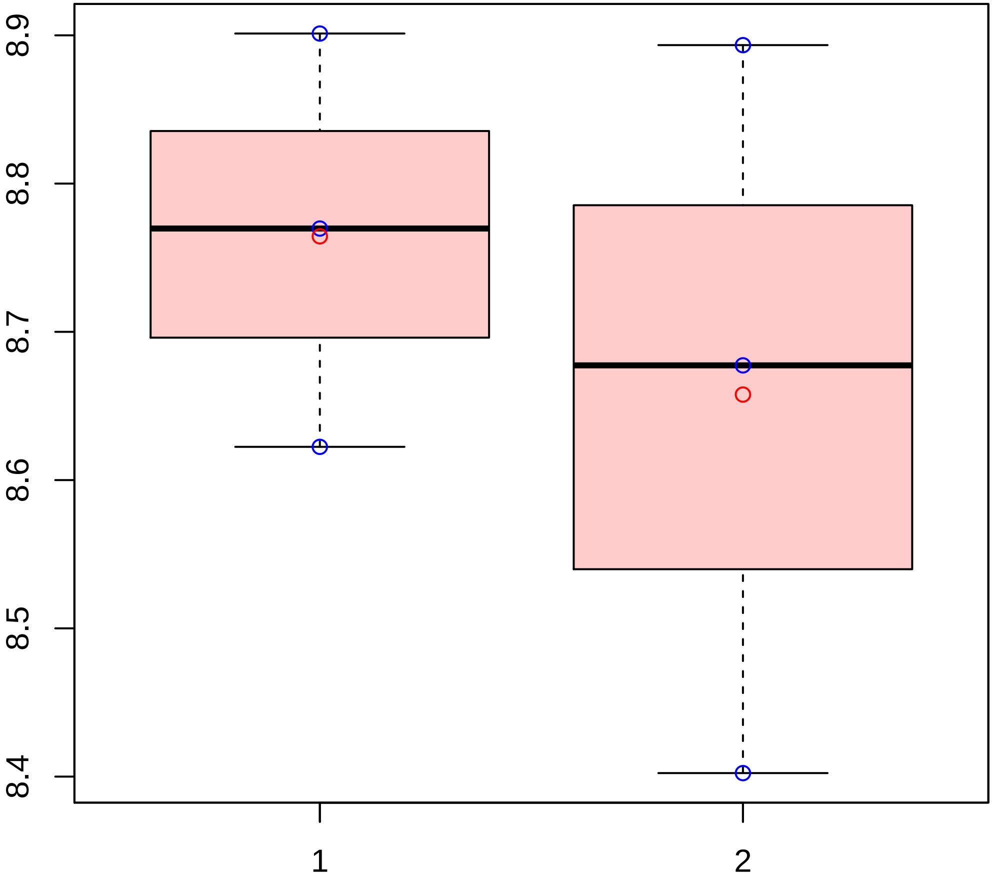

t−Test: p−value = 0.56

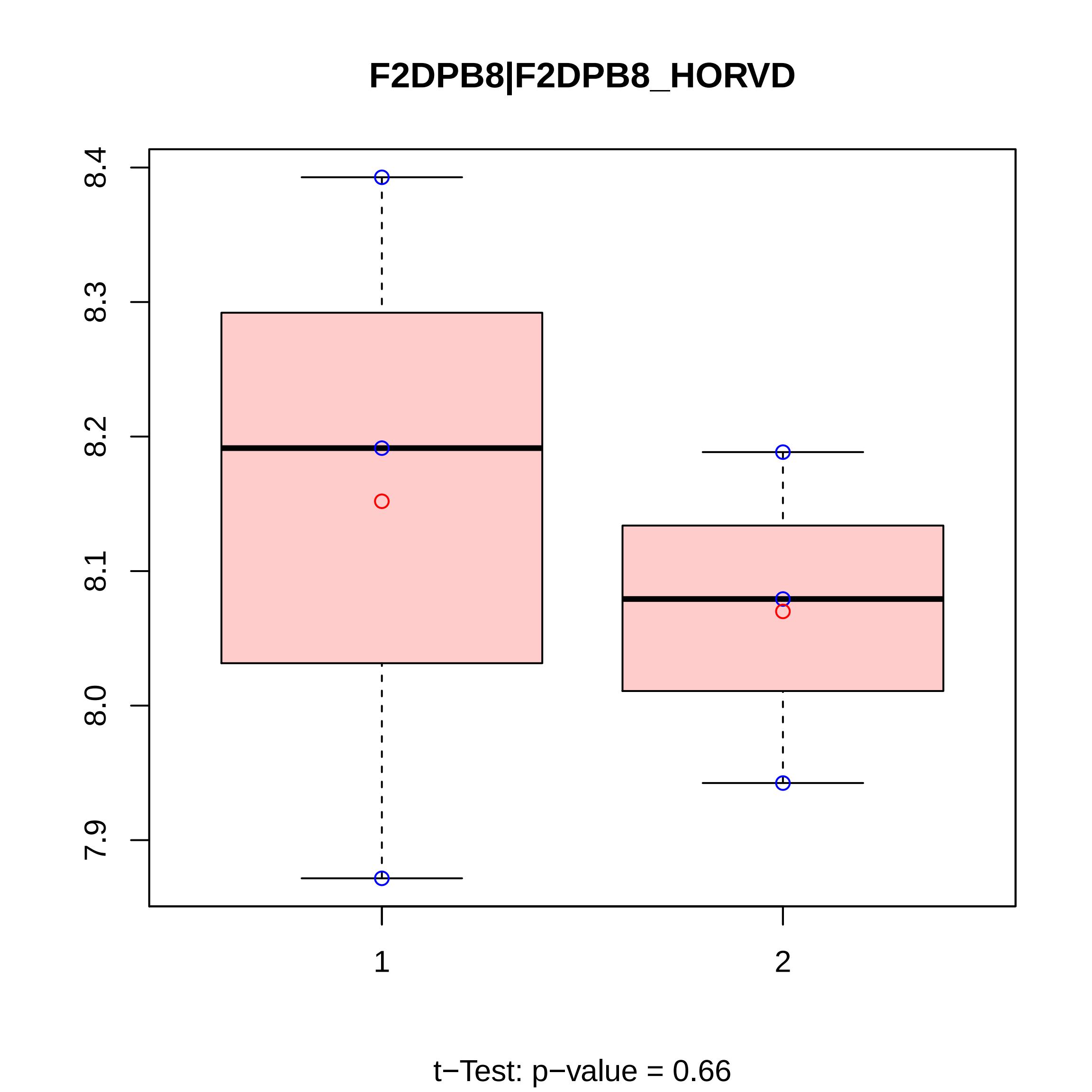

F2DPB8|F2DPB8_HORVD
8.4
8.3
8.2
8.1
8.0
7.9
1
2
t−Test: p−value = 0.66

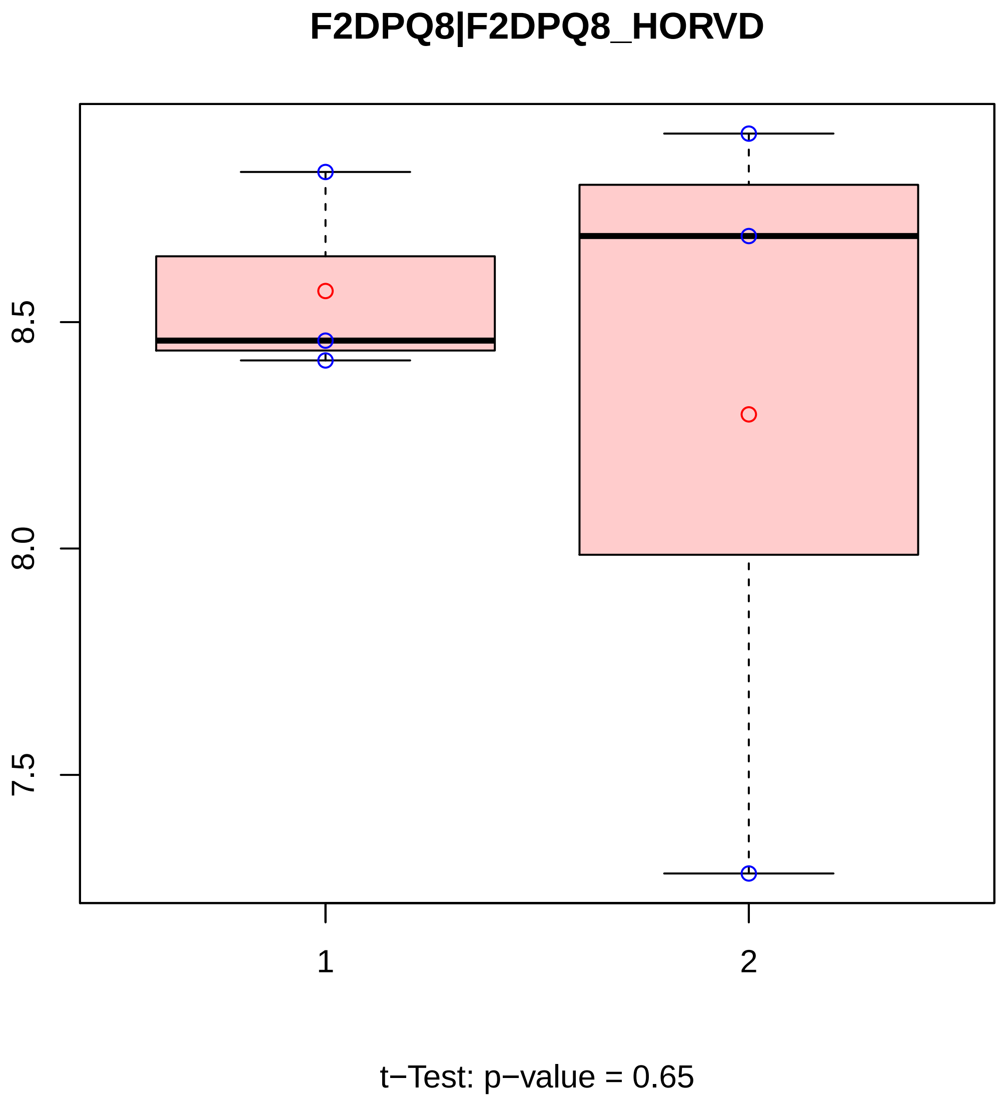
F2DPQ8|F2DPQ8_HORVD
8.5
8.0
7.5
1
2
t−Test: p−value = 0.65

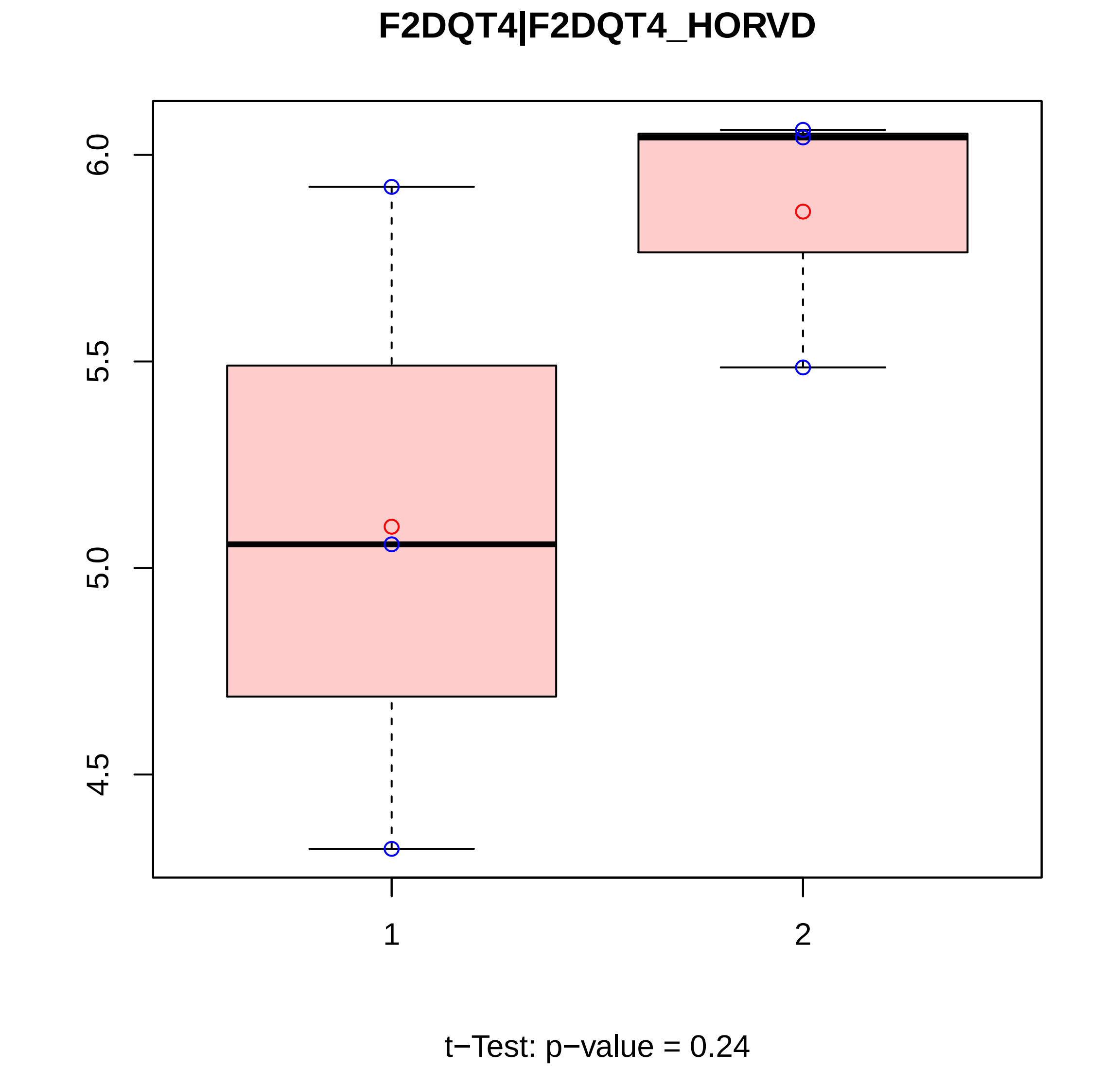
F2DQT4|F2DQT4_HORVD
6.0
5.5
5.0
4.5
1
2
t−Test: p−value = 0.24

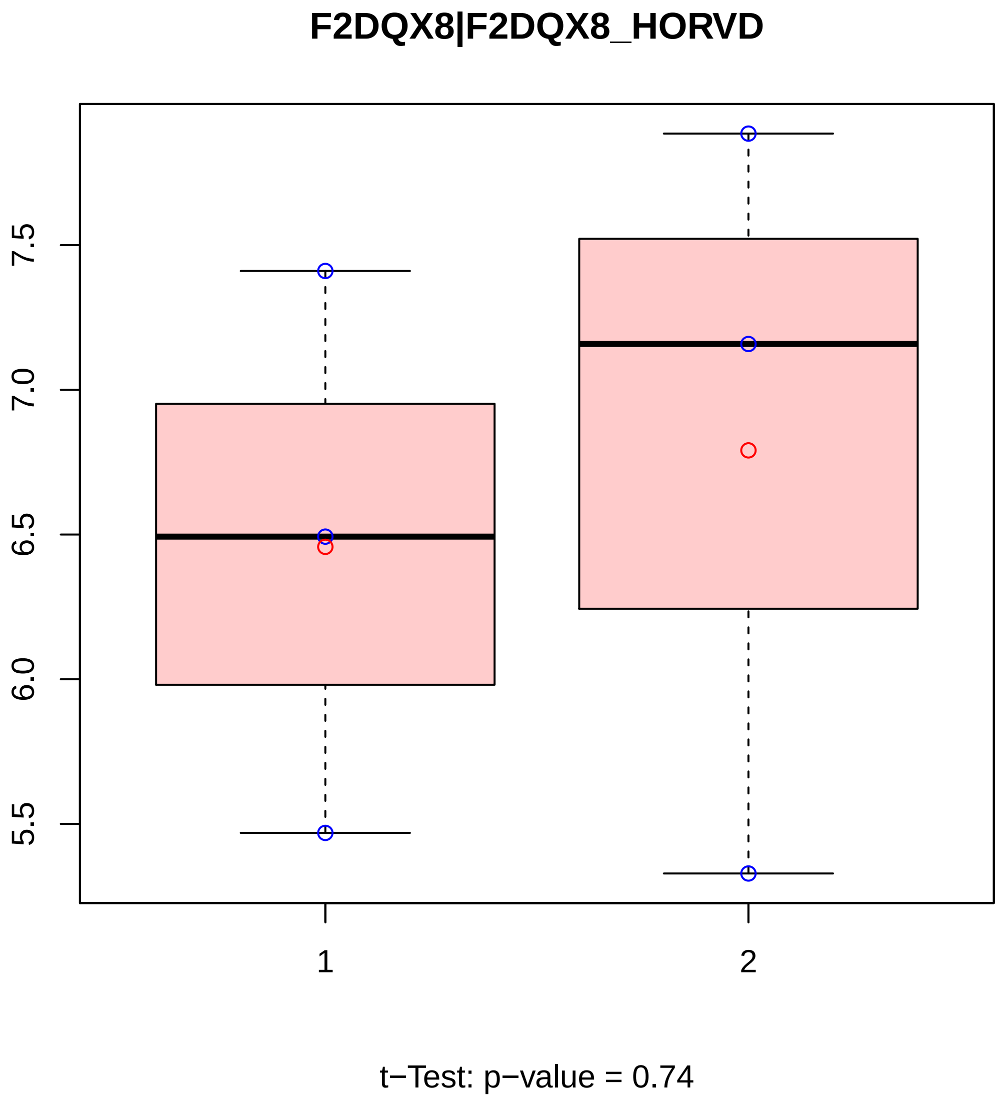
F2DQX8|F2DQX8_HORVD
7.5
7.0
6.5
6.0
5.5
1
2
t−Test: p−value = 0.74

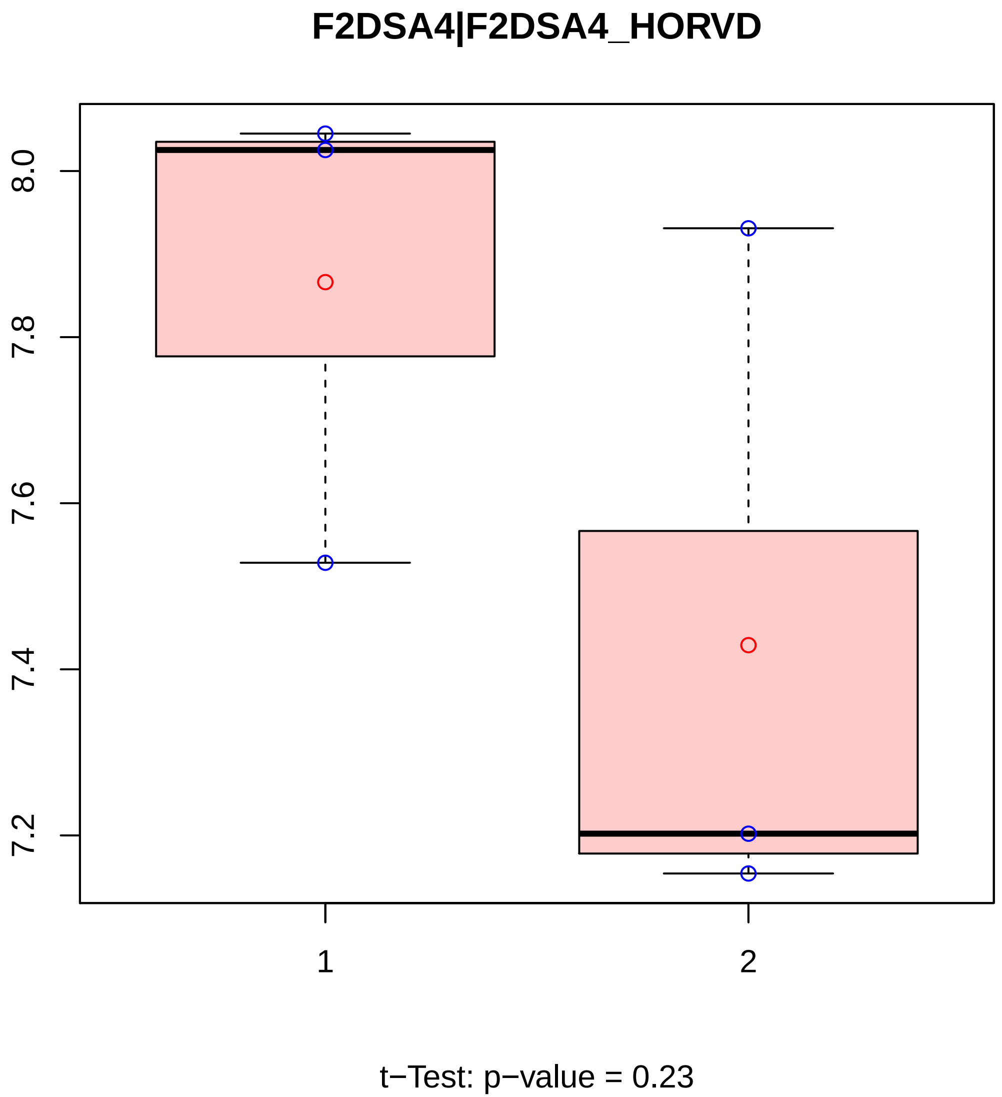

F2DSA4|F2DSA4_HORVD
8.0
7.8
7.6
7.4
7.2
1
2
t−Test: p−value = 0.23

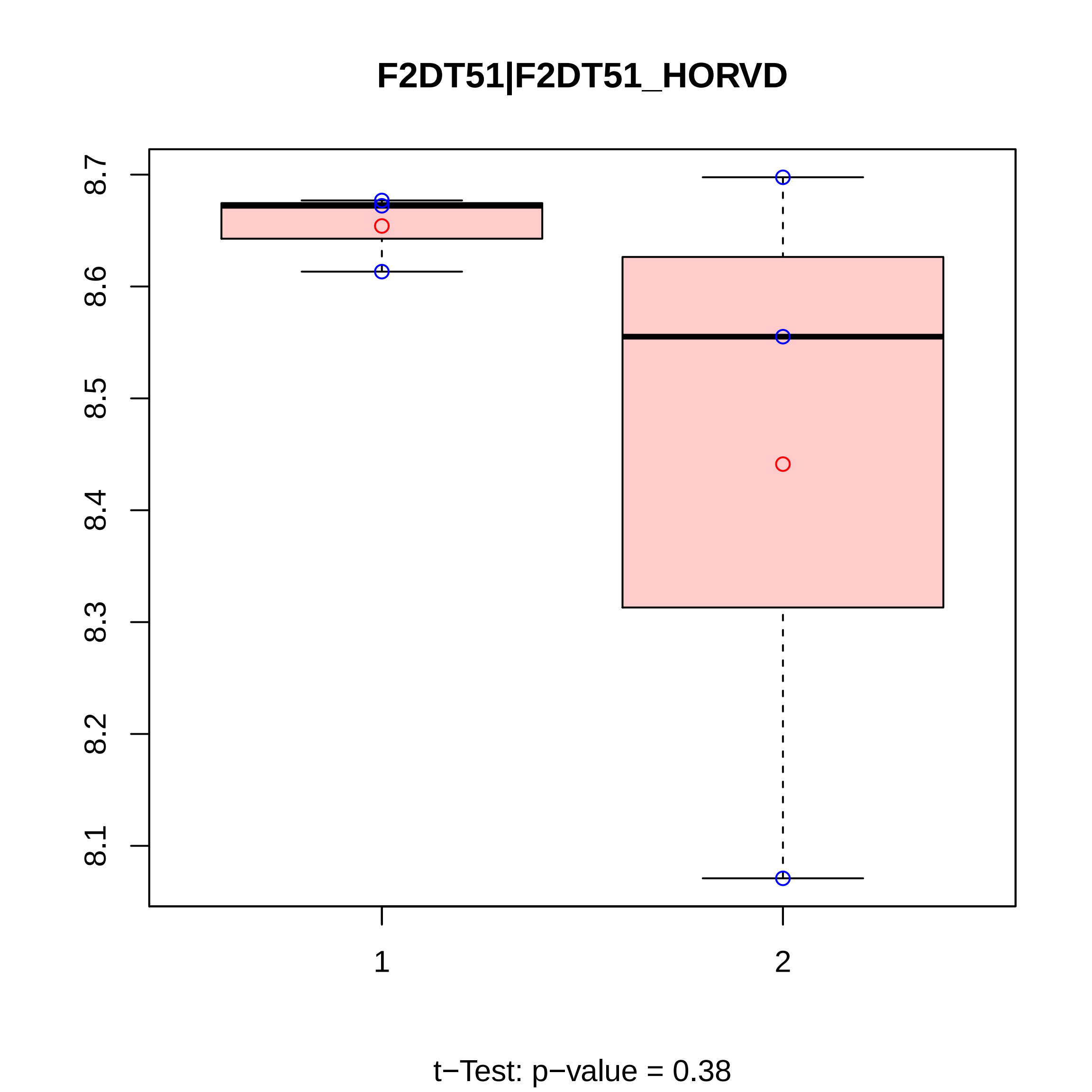

F2DT51|F2DT51_HORVD
8.1
8.2
8.3
8.4
8.5
8.6
8.7
1
2
t−Test: p−value = 0.38

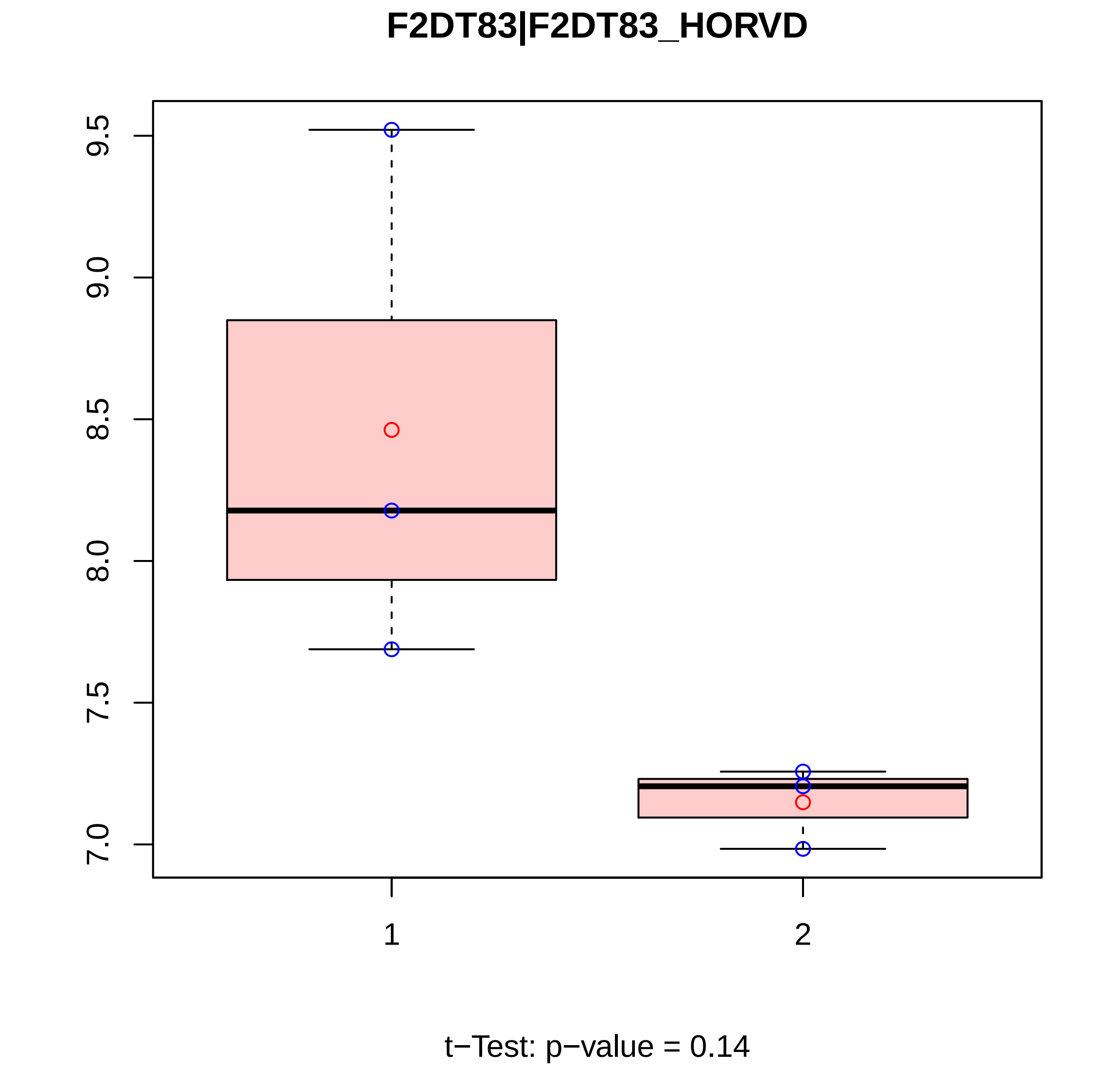
F2DT83|F2DT83_HORVD
9.5
9.0
8.5
8.0
7.5
7.0
1
2
t−Test: p−value = 0.14

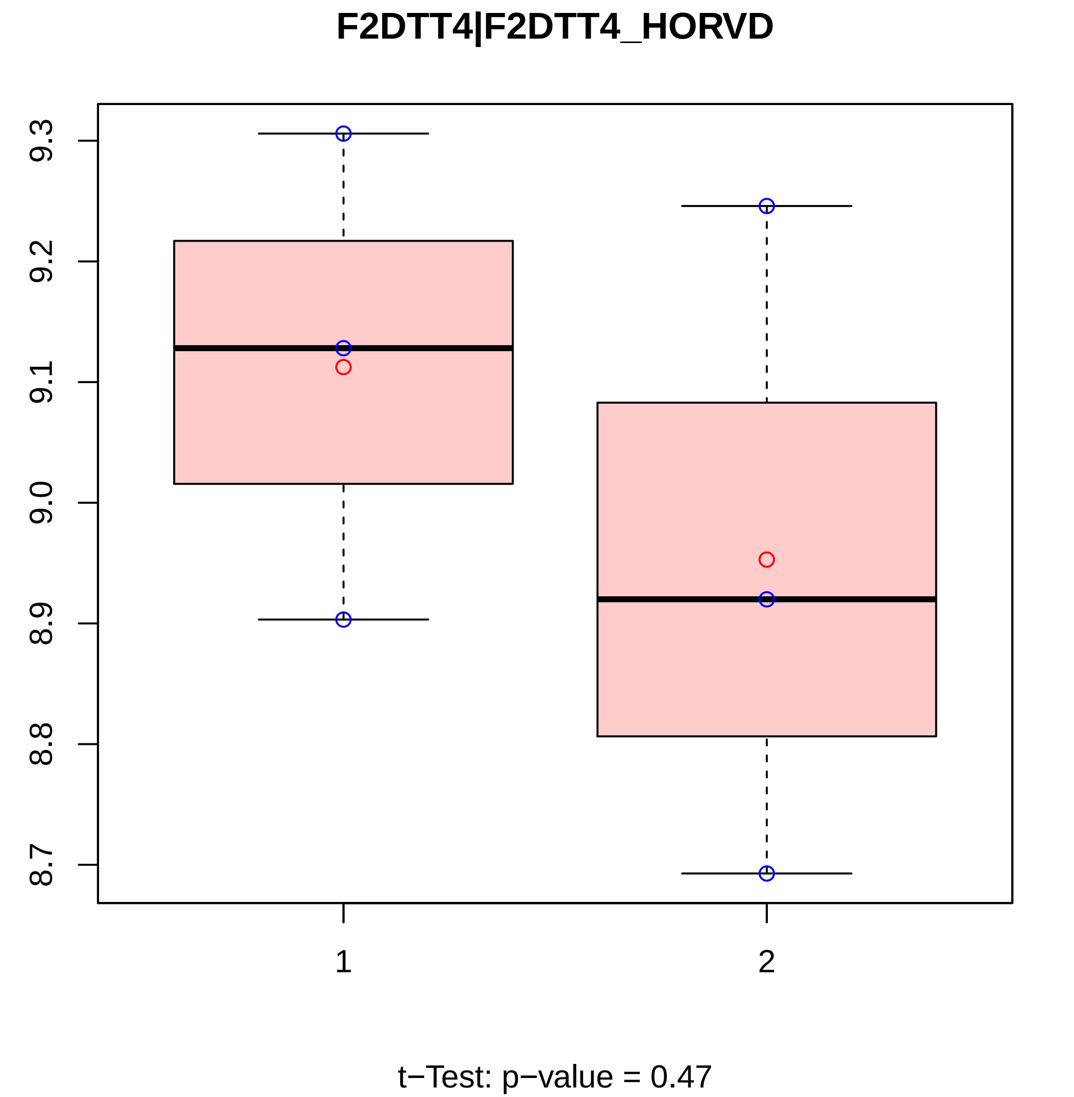
F2DTT4|F2DTT4_HORVD
9.3
9.2
9.1
9.0
8.9
8.8
8.7
1
2
t−Test: p−value = 0.47

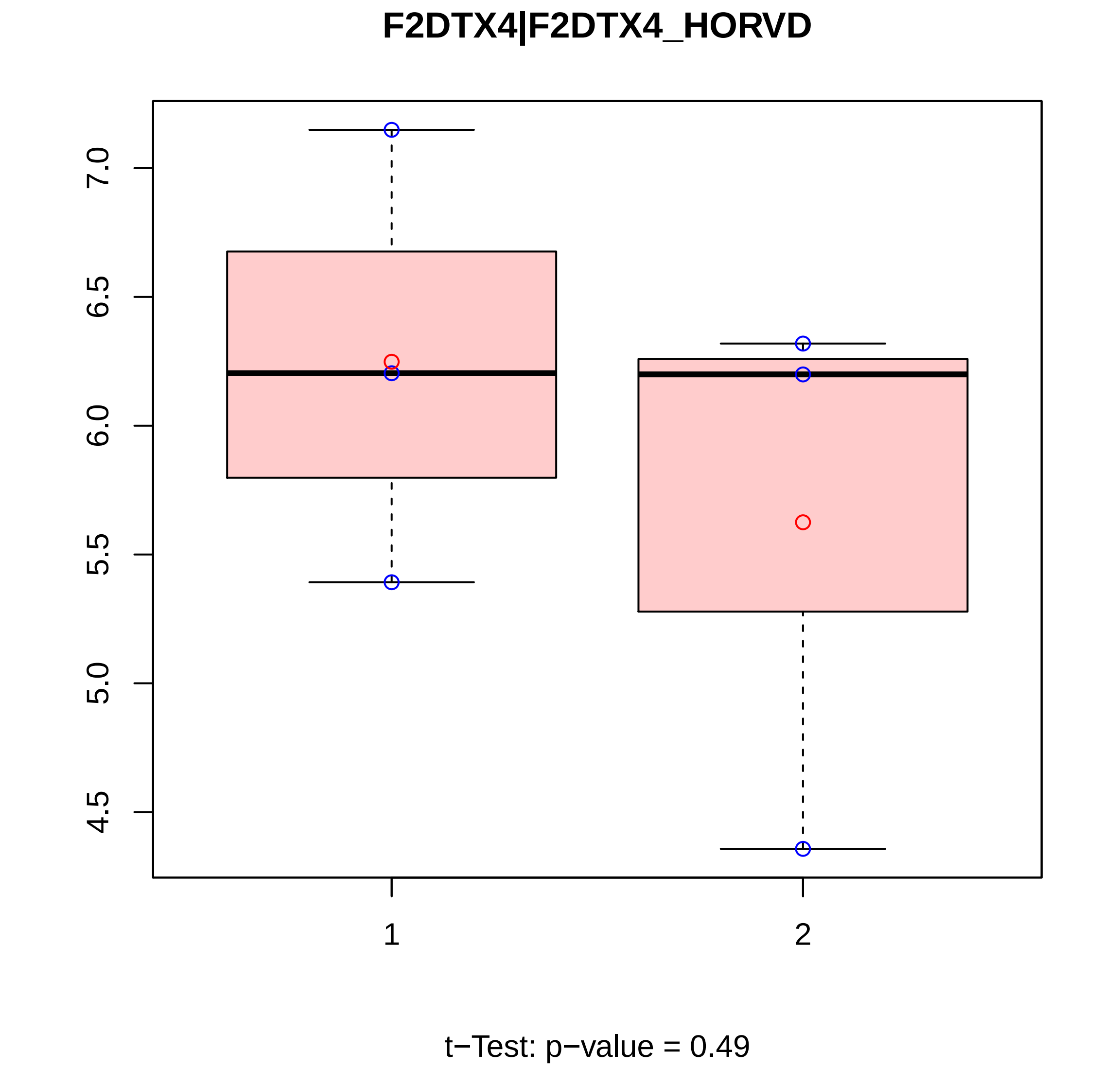
F2DTX4|F2DTX4_HORVD
7.0
6.5
6.0
5.5
5.0
4.5
1
2
t−Test: p−value = 0.49

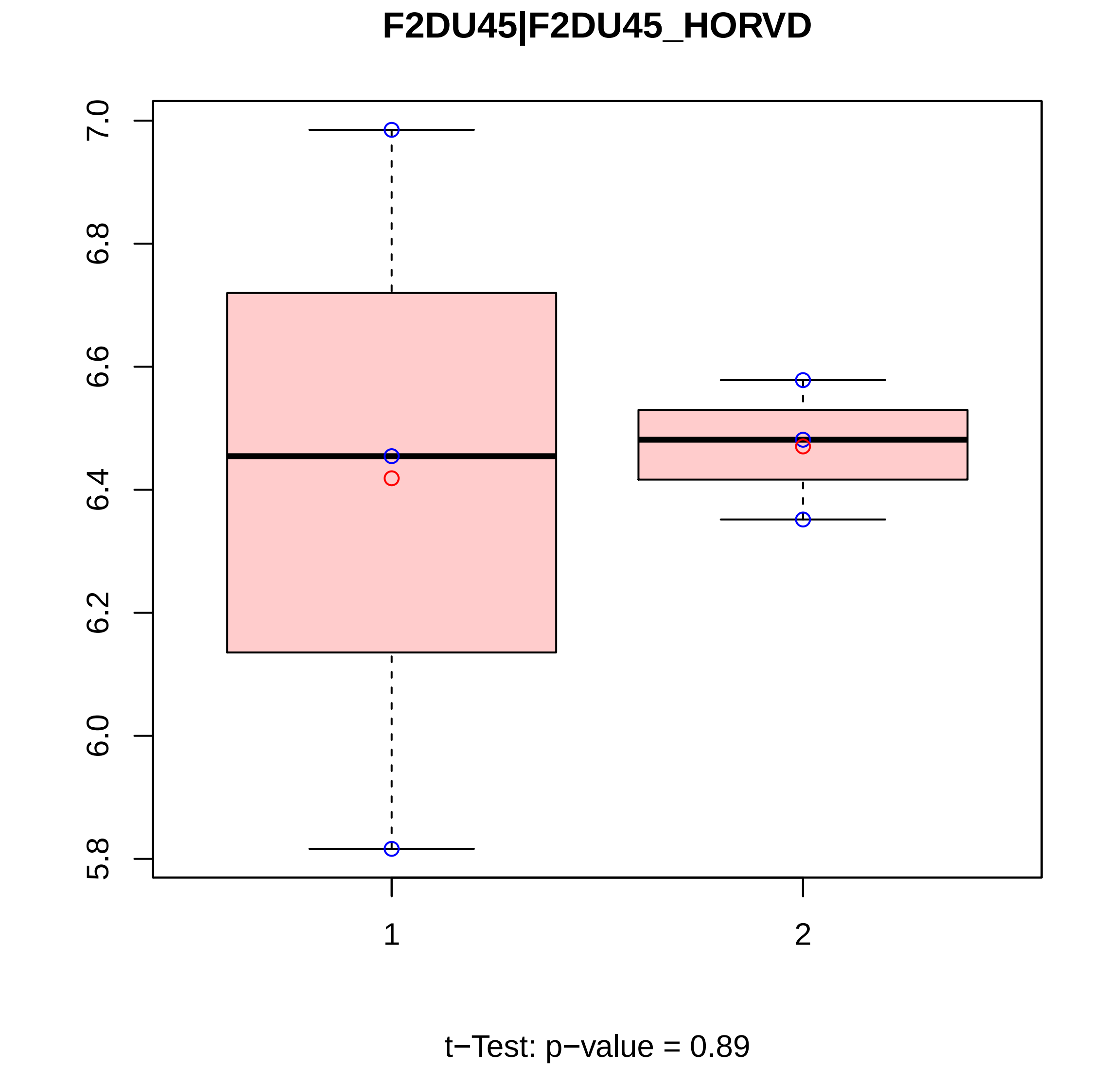
F2DU45|F2DU45_HORVD
7.0
6.8
6.6
6.4
6.2
6.0
5.8
1
2
t−Test: p−value = 0.89

F2DUB8|F2DUB8_HORVD

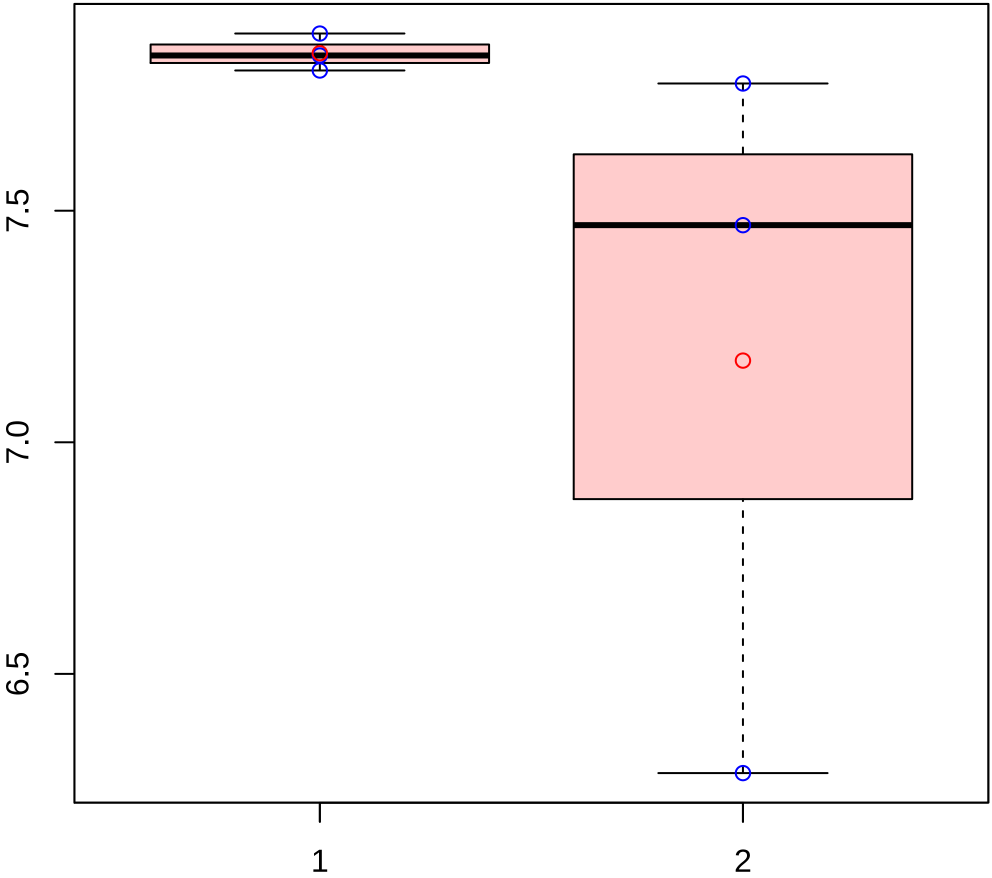

t−Test: p−value = 0.28

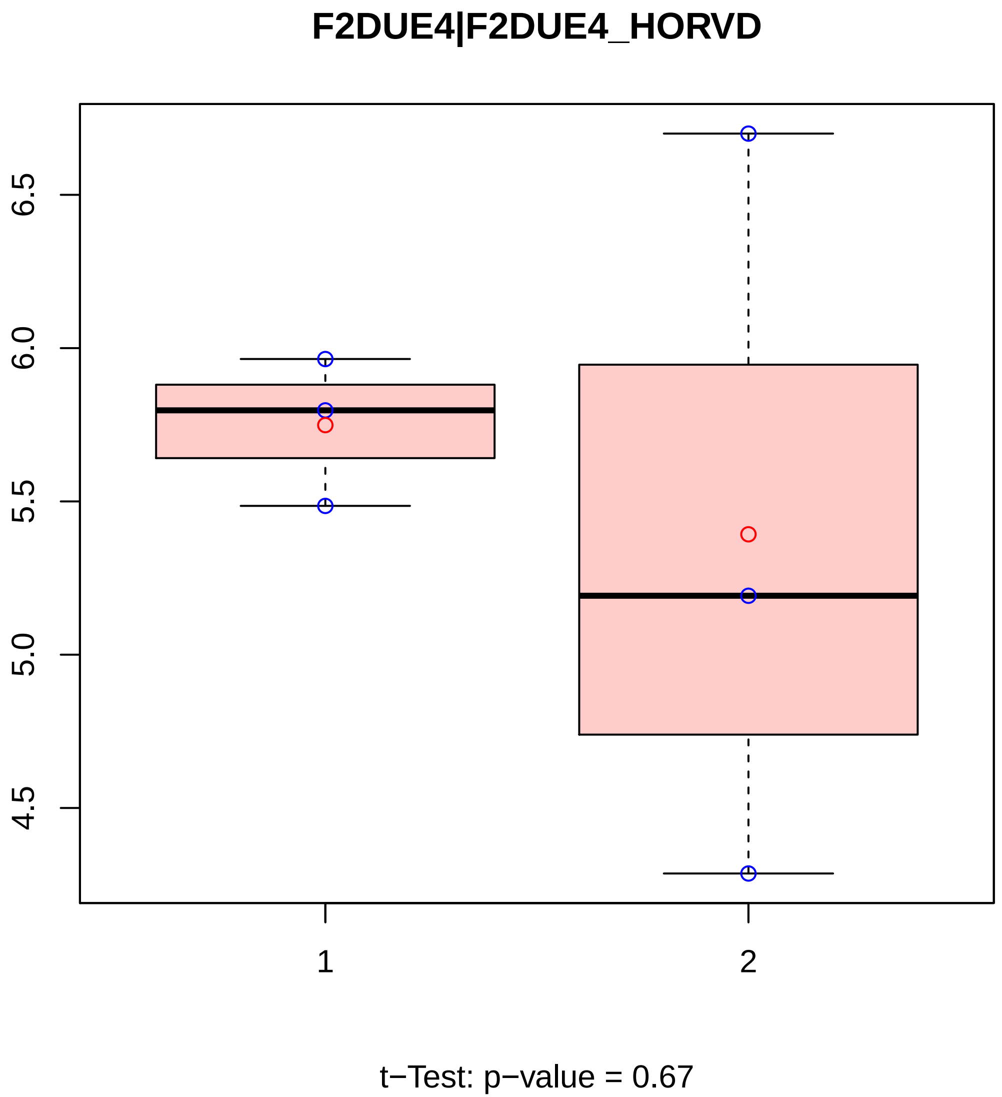
F2DUE4|F2DUE4_HORVD
6.5
6.0
5.5
5.0
4.5
1
2
t−Test: p−value = 0.67

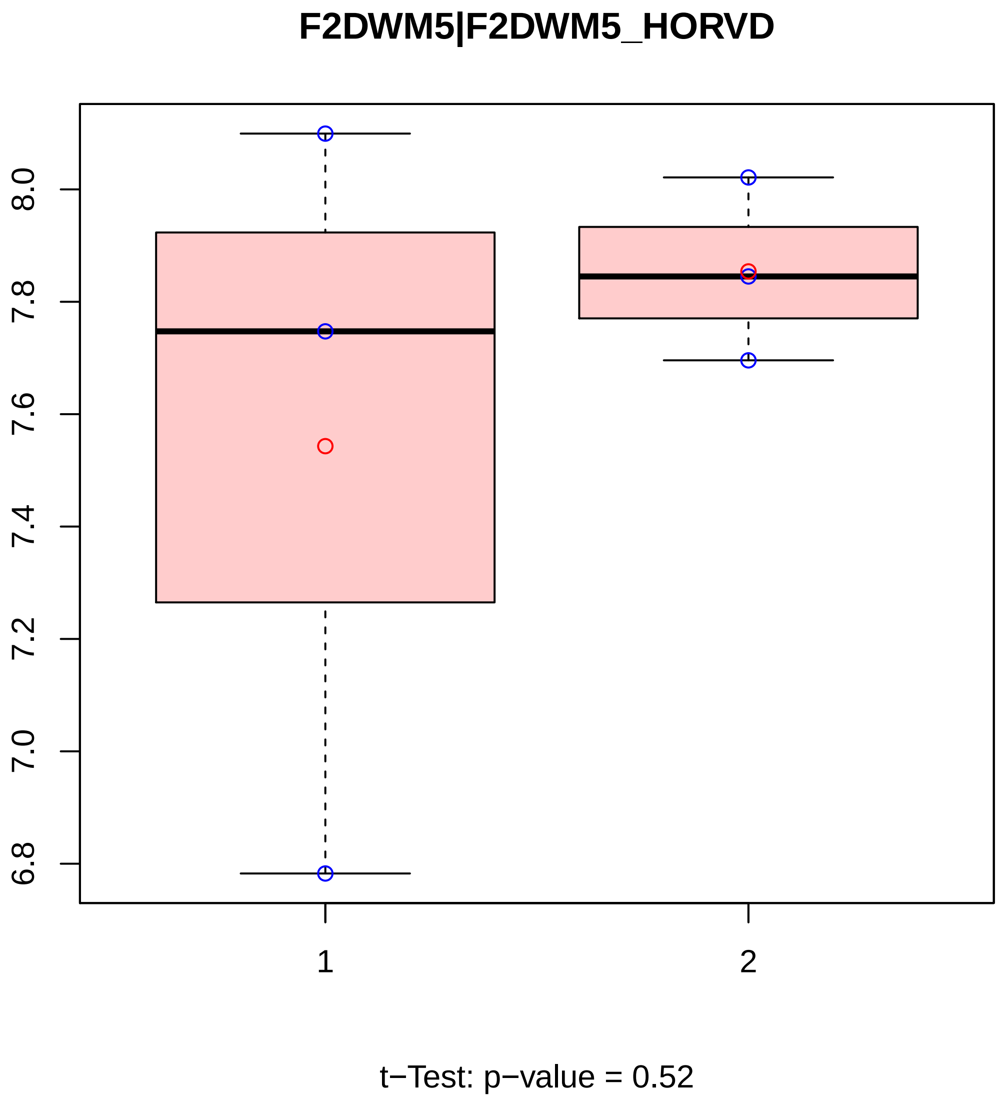
F2DWM5|F2DWM5_HORVD
8.0
7.8
7.6
7.4
7.2
7.0
6.8
1
2
t−Test: p−value = 0.52

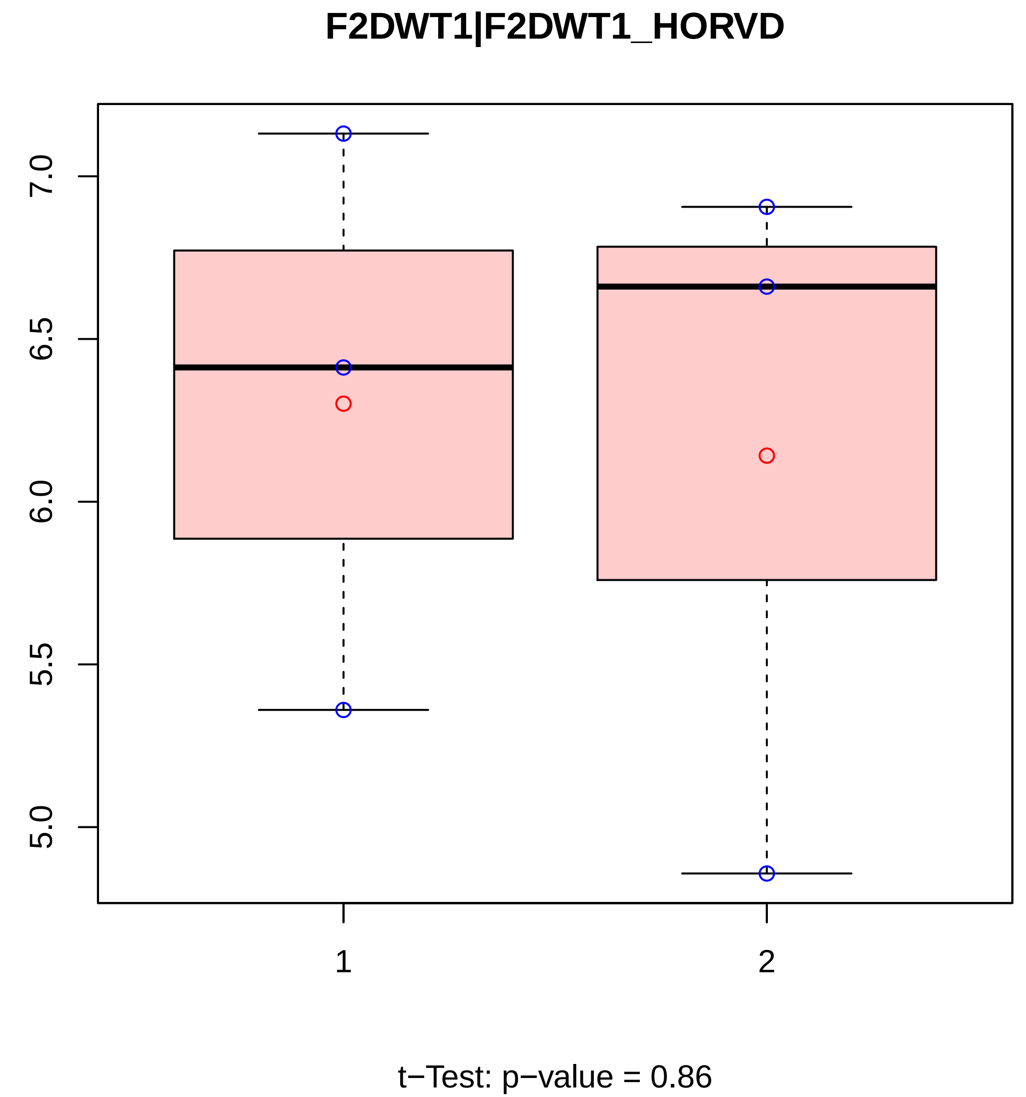
F2DWT1|F2DWT1_HORVD
7.0
6.5
6.0
5.5
5.0
1
2
t−Test: p−value = 0.86

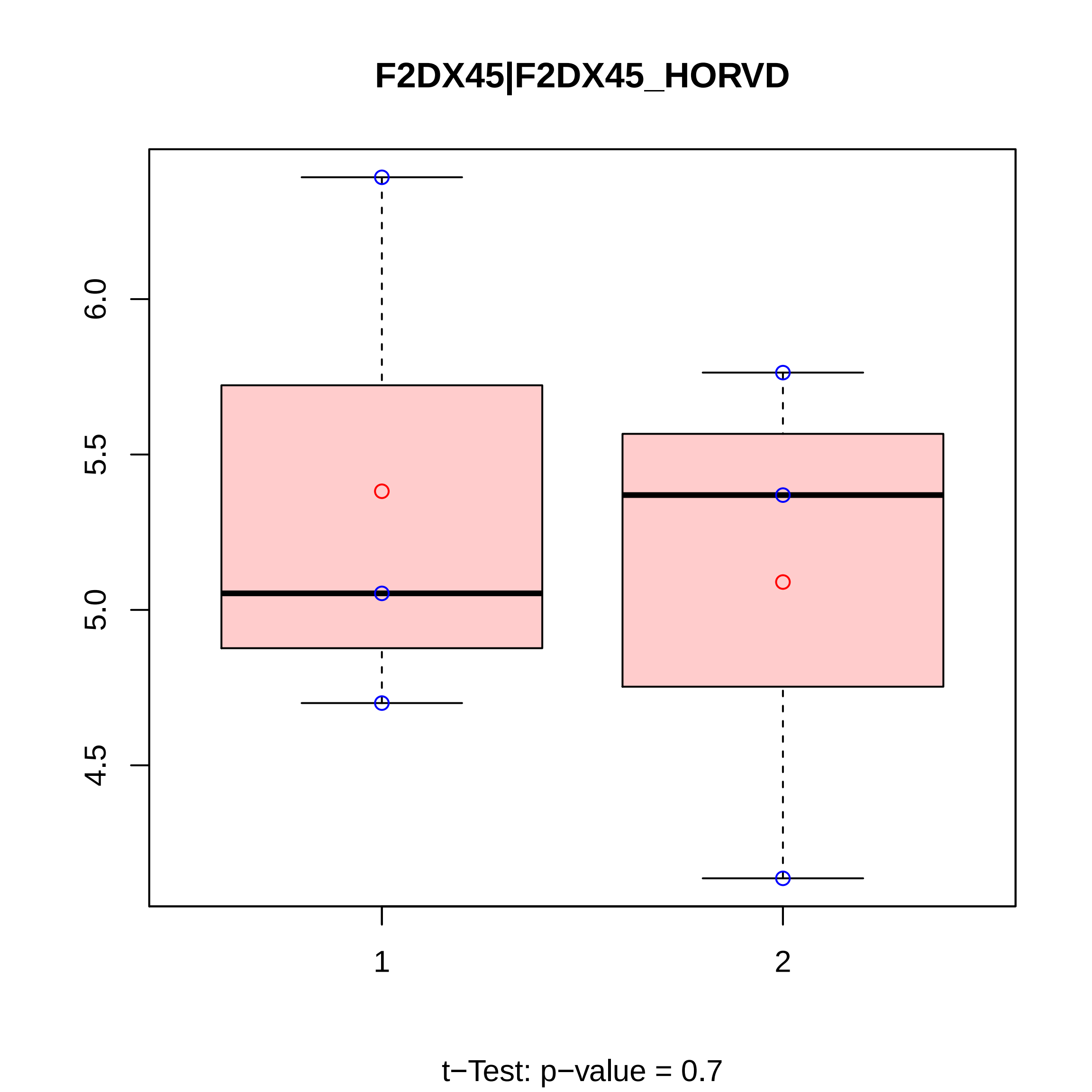
F2DX45|F2DX45_HORVD
6.0
5.5
5.0
4.5
1
2
t−Test: p−value = 0.7

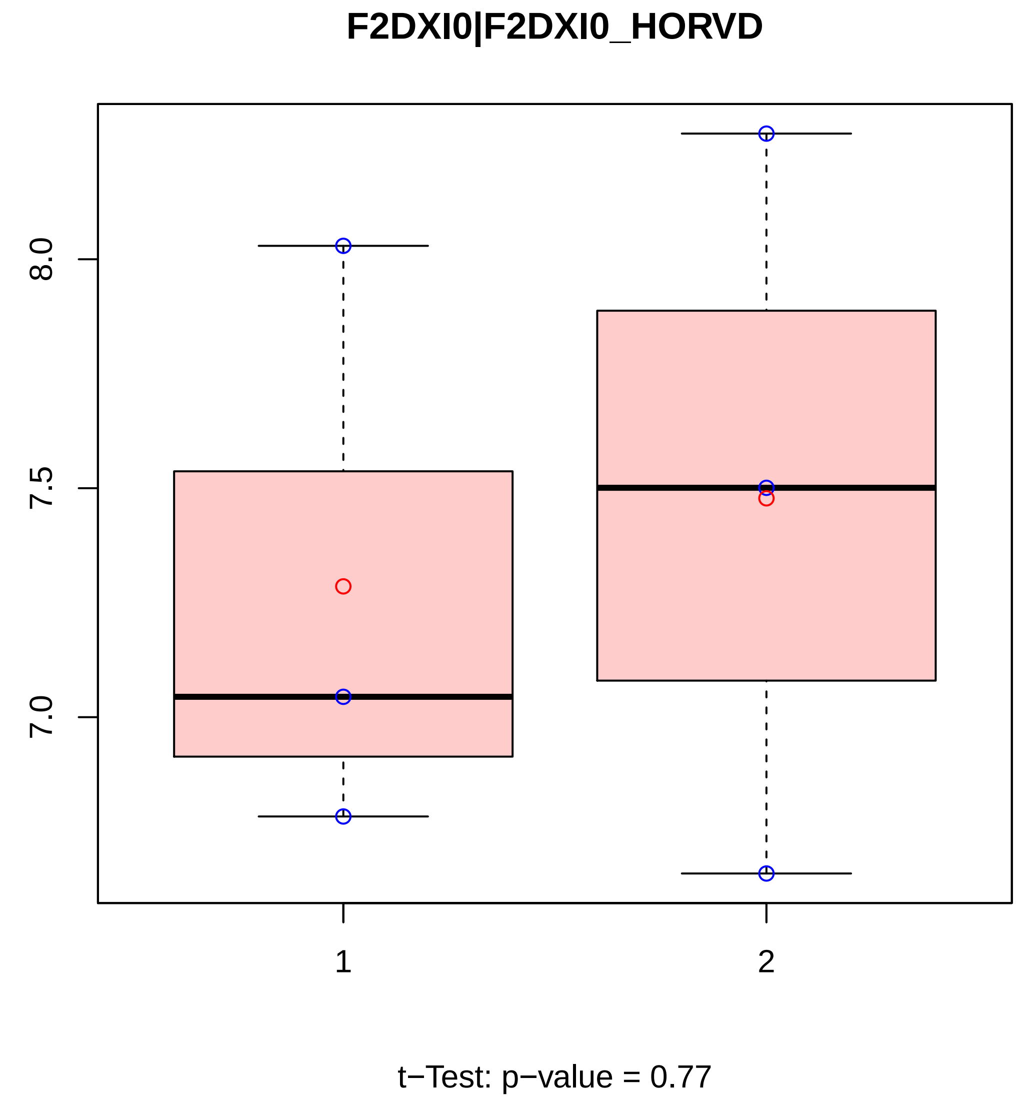
F2DXI0|F2DXI0_HORVD
8.0
7.5
7.0
1
2
t−Test: p−value = 0.77

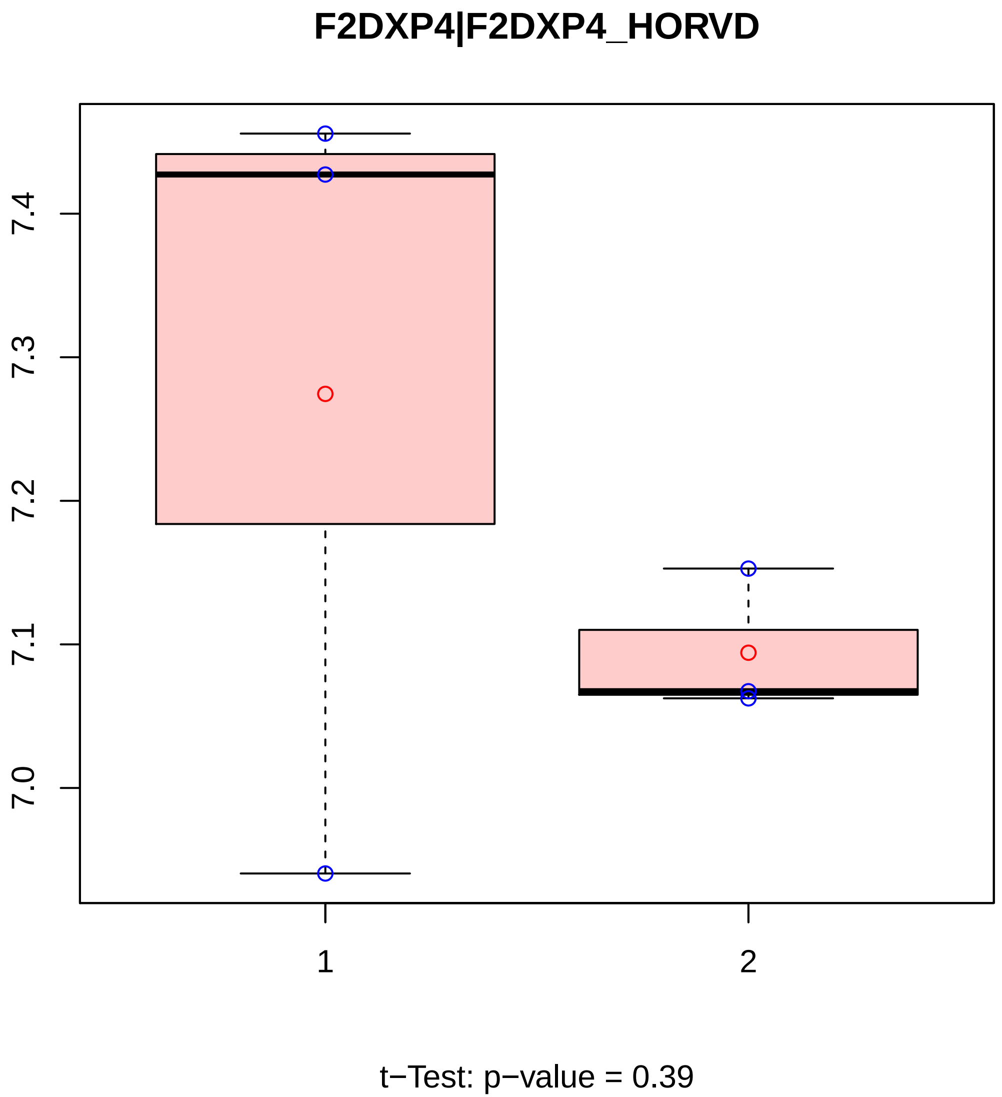
F2DXP4|F2DXP4_HORVD
7.0
7.1
7.2
7.3
7.4
1
2
t−Test: p−value = 0.39

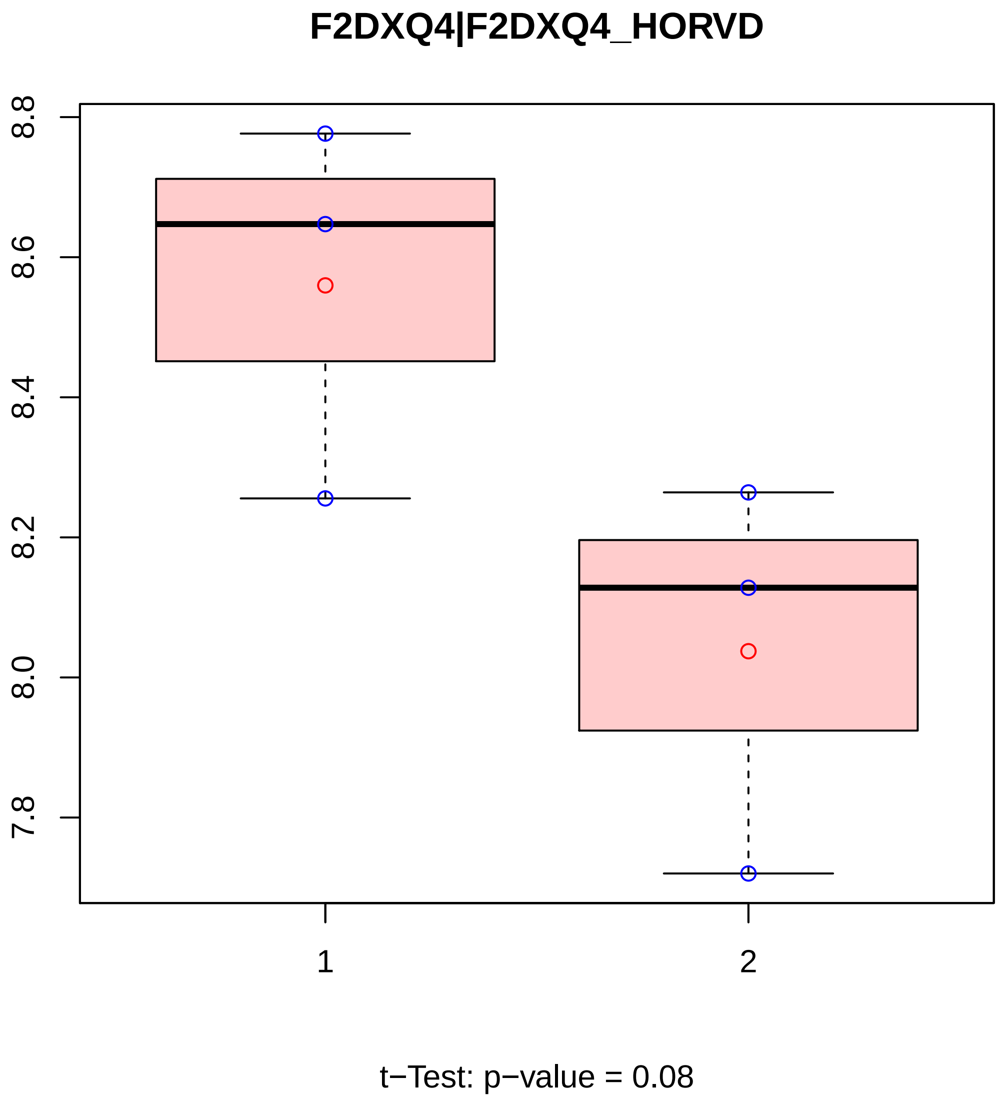

F2DXQ4|F2DXQ4_HORVD
8.8
8.6
8.4
8.2
8.0
7.8
1
2
t−Test: p−value = 0.08

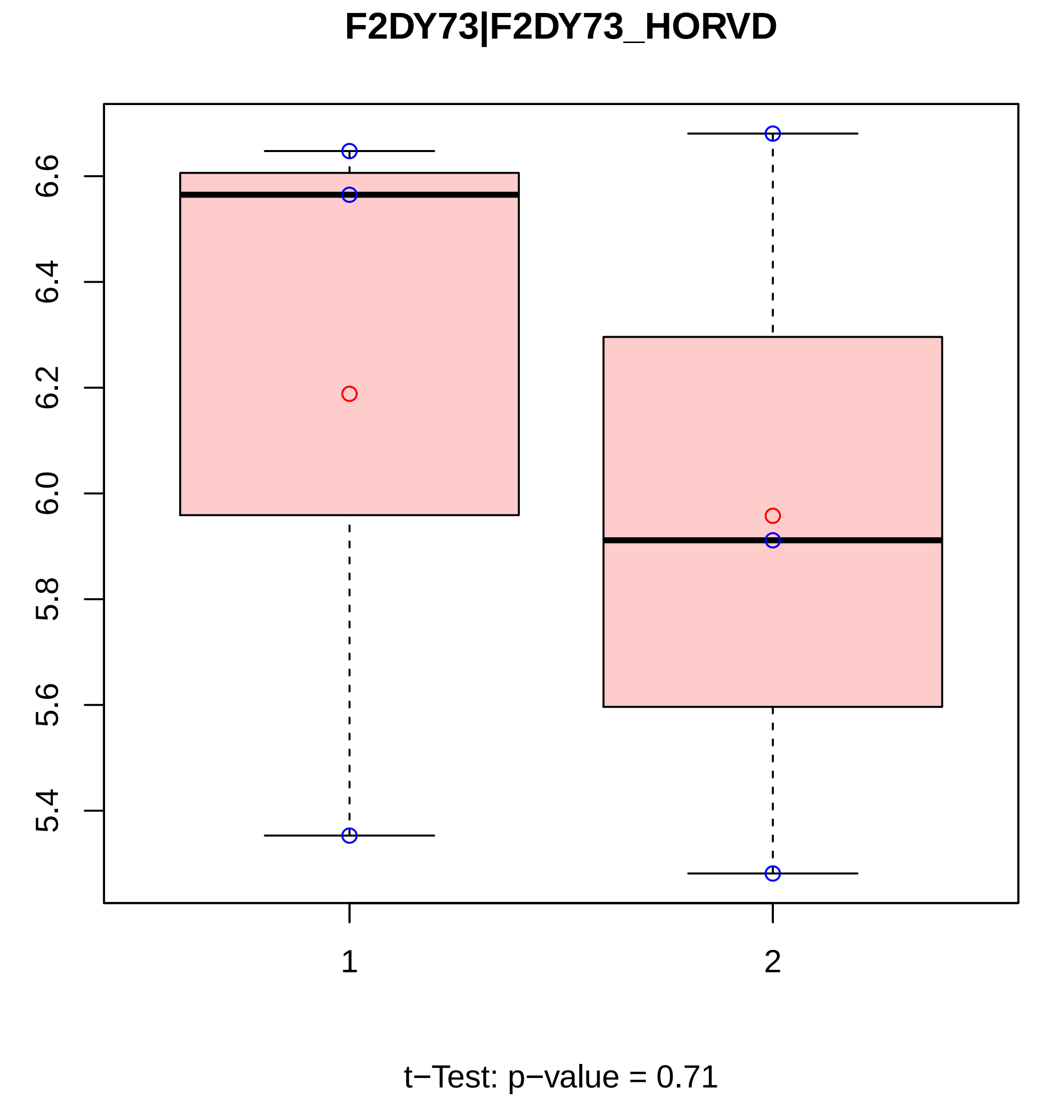

F2DY73|F2DY73_HORVD
6.6
6.4
6.2
6.0
5.8
5.6
5.4
1
2
t−Test: p−value = 0.71

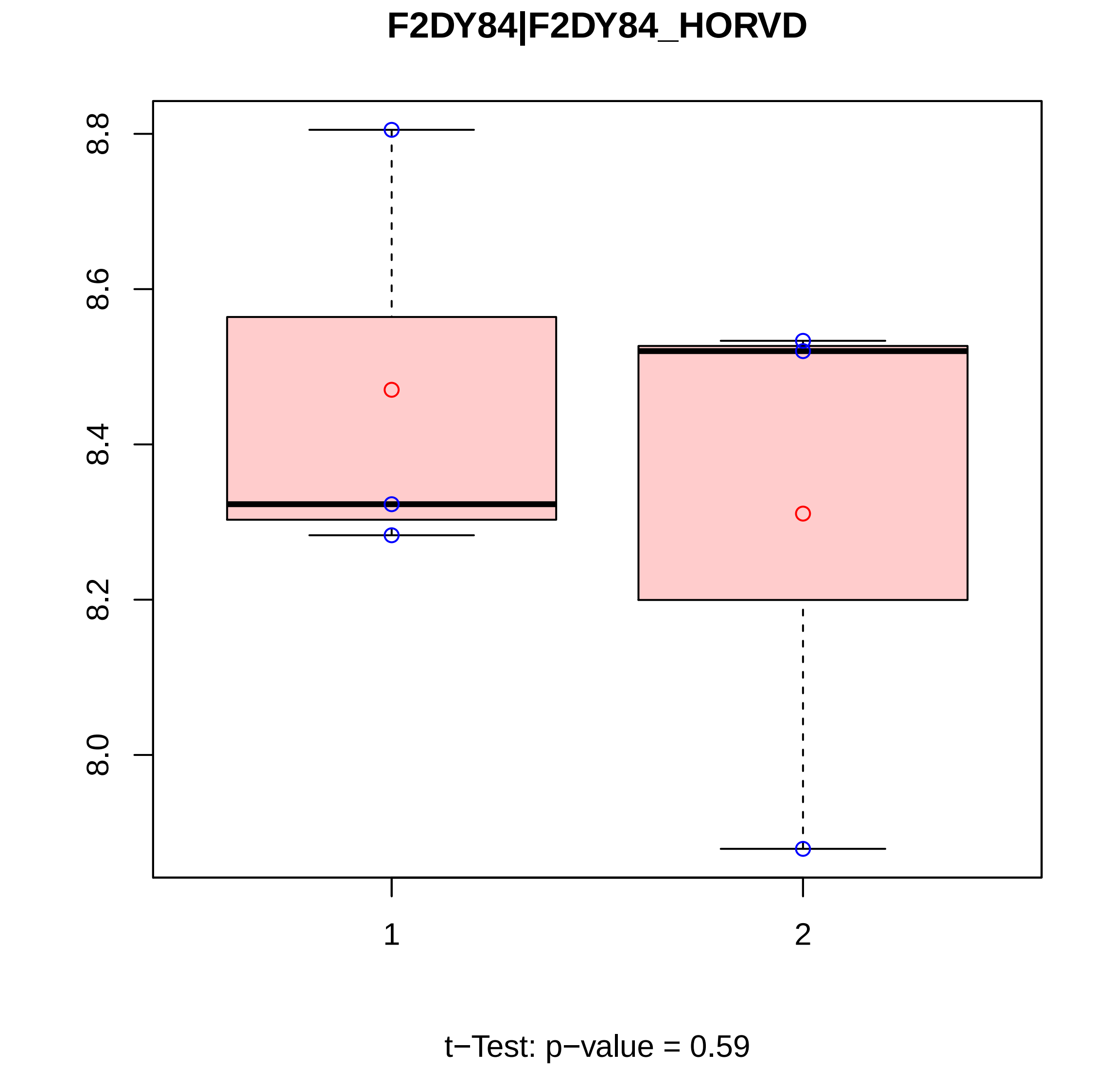

F2DY84|F2DY84_HORVD
8.8
8.6
8.4
8.2
8.0
1
2
t−Test: p−value = 0.59

F2DYG3|F2DYG3_HORVD

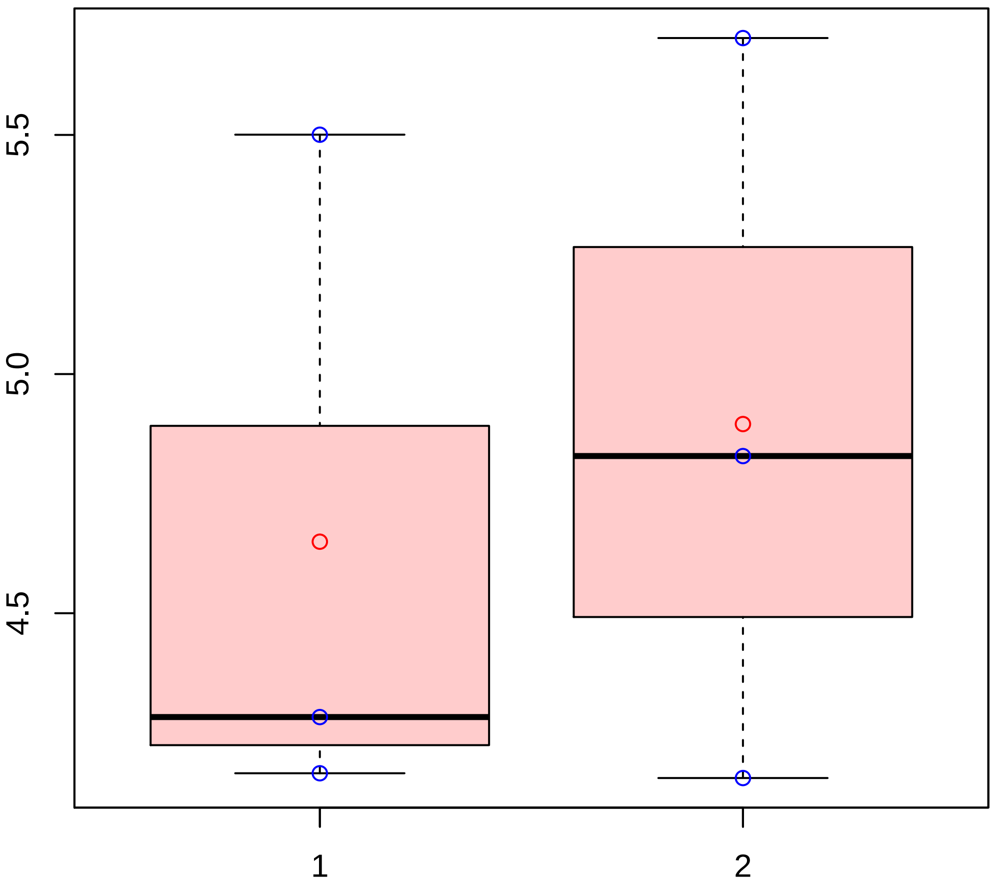

t−Test: p−value = 0.71

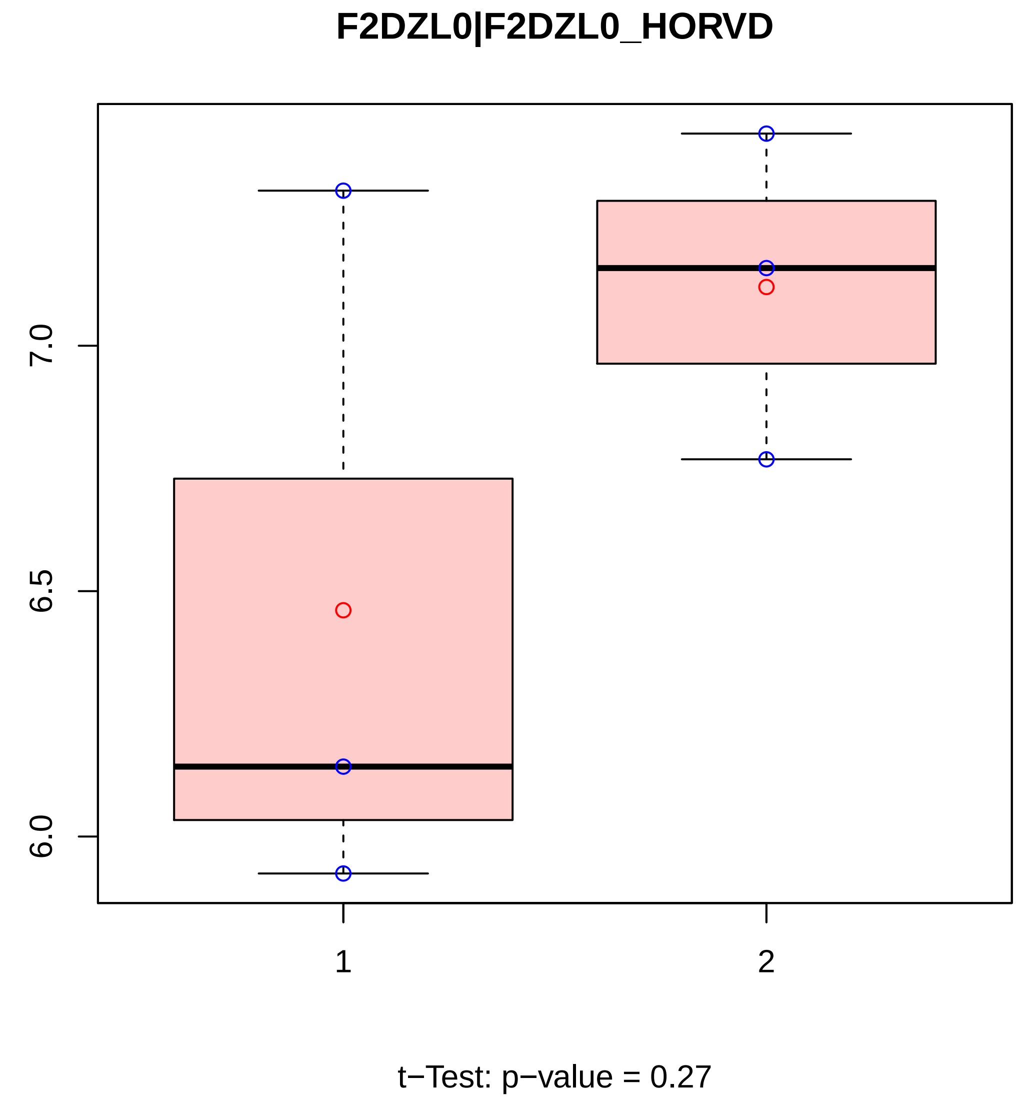
F2DZL0|F2DZL0_HORVD
7.0
6.5
6.0
1
2
t−Test: p−value = 0.27

F2E035|F2E035_HORVD

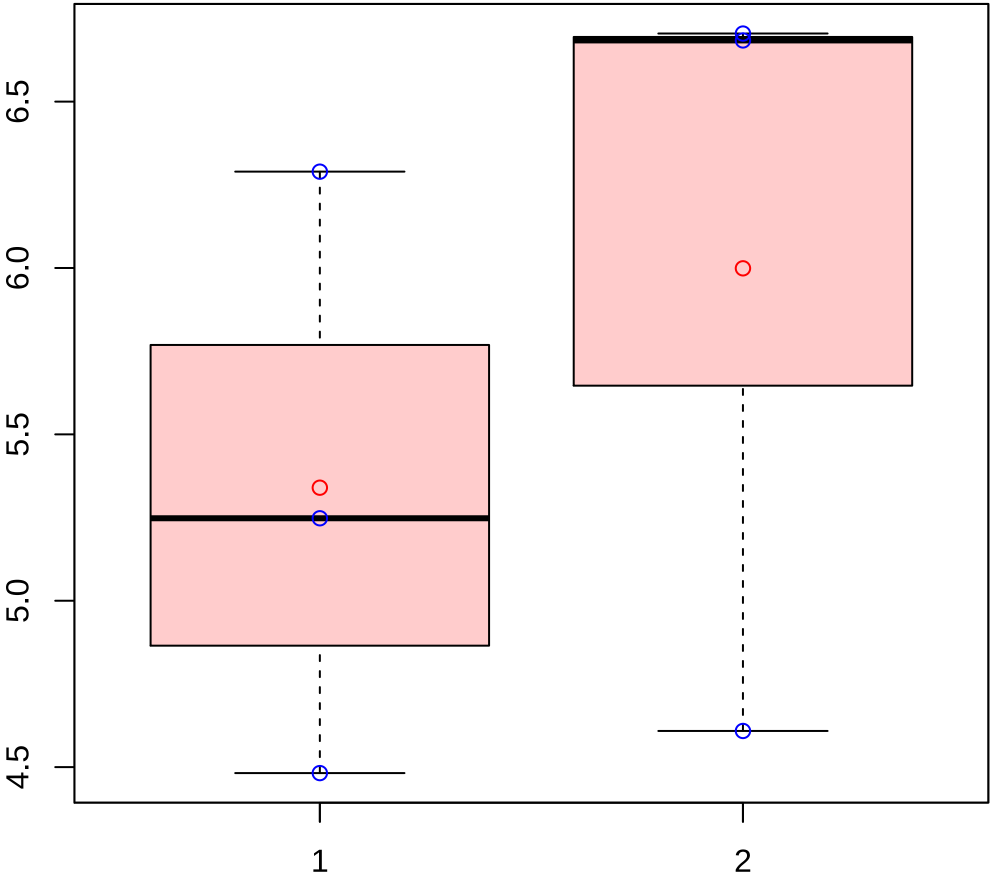

t−Test: p−value = 0.49

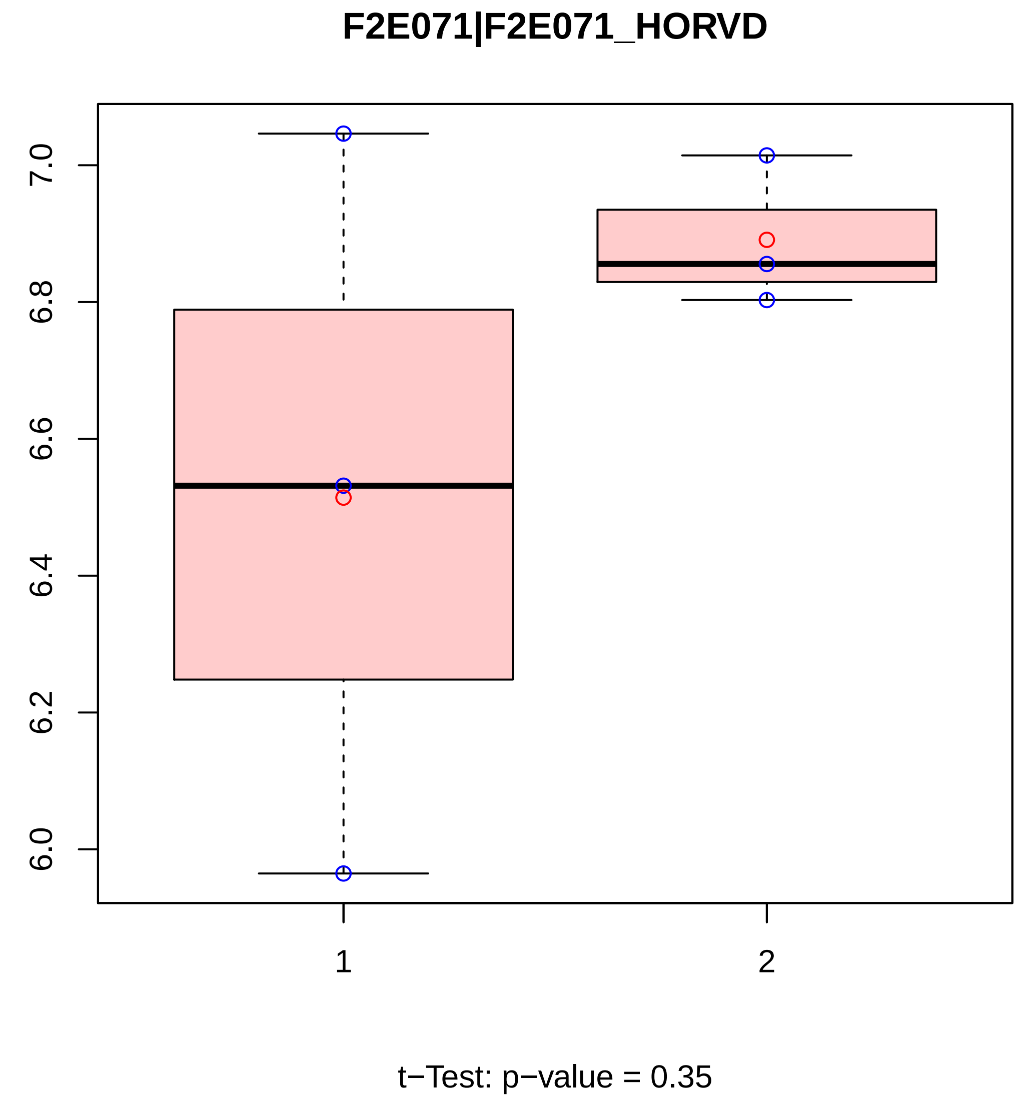
F2E071|F2E071_HORVD
7.0
6.8
6.6
6.4
6.2
6.0
1
2
t−Test: p−value = 0.35

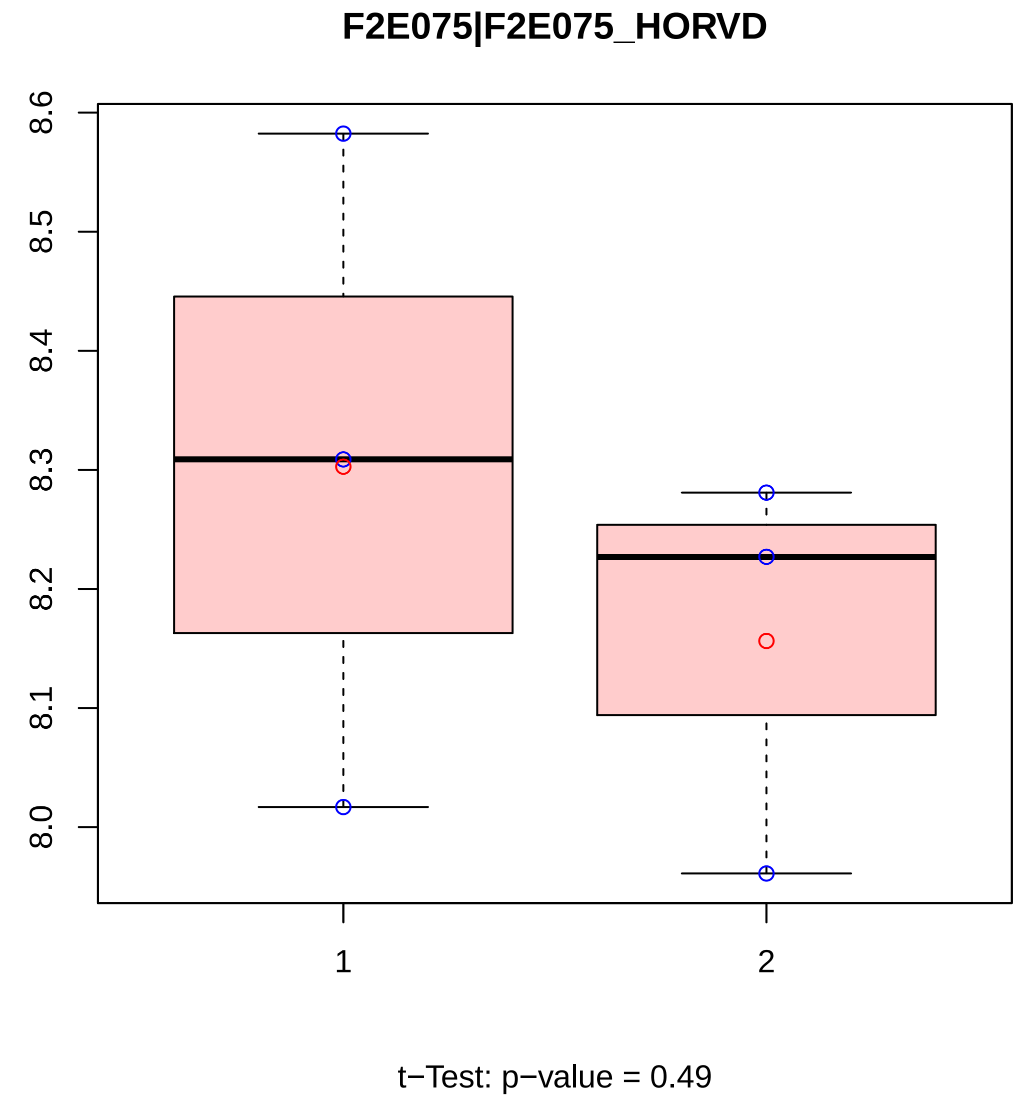

F2E075|F2E075_HORVD
8.0
8.1
8.2
8.3
8.4
8.5
8.6
1
2
t−Test: p−value = 0.49

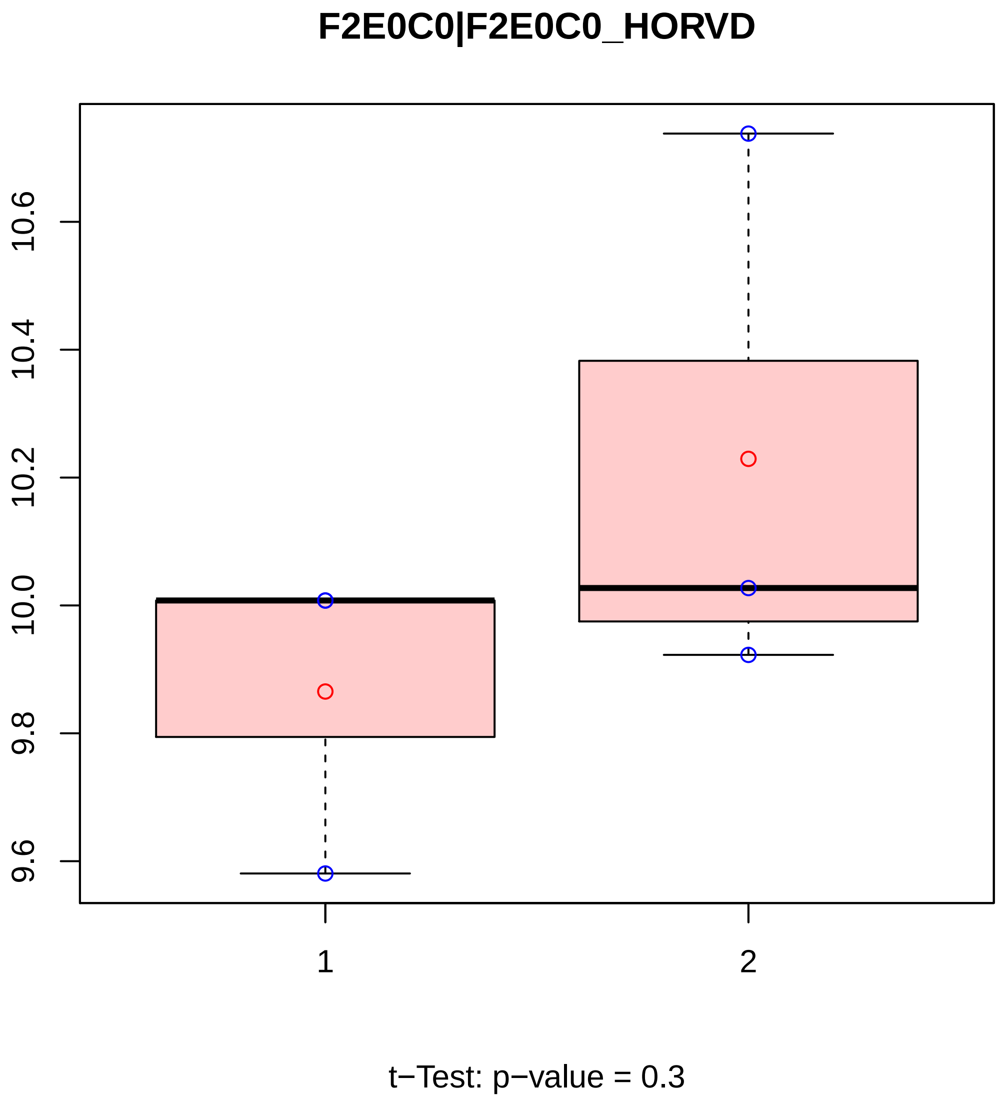

F2E0C0|F2E0C0_HORVD
10.6
10.4
10.2
10.0
9.8
9.6
1
2
t−Test: p−value = 0.3

F2E1A4|F2E1A4_HORVD

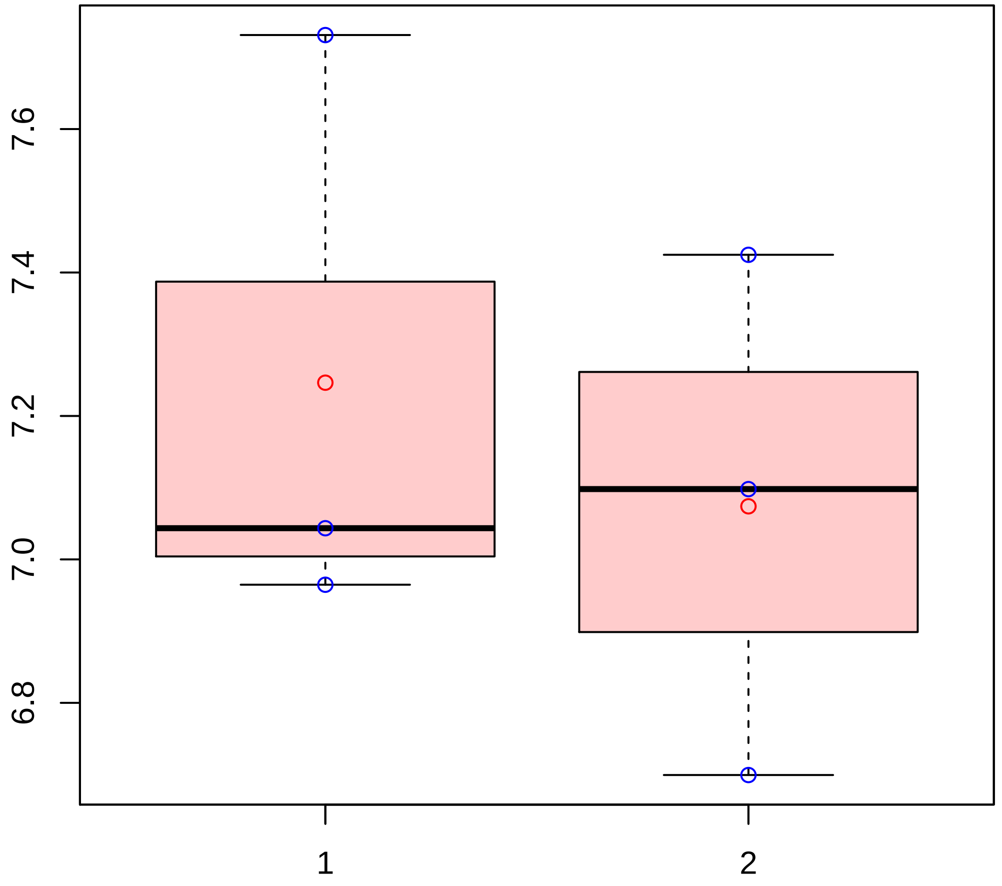

t−Test: p−value = 0.62

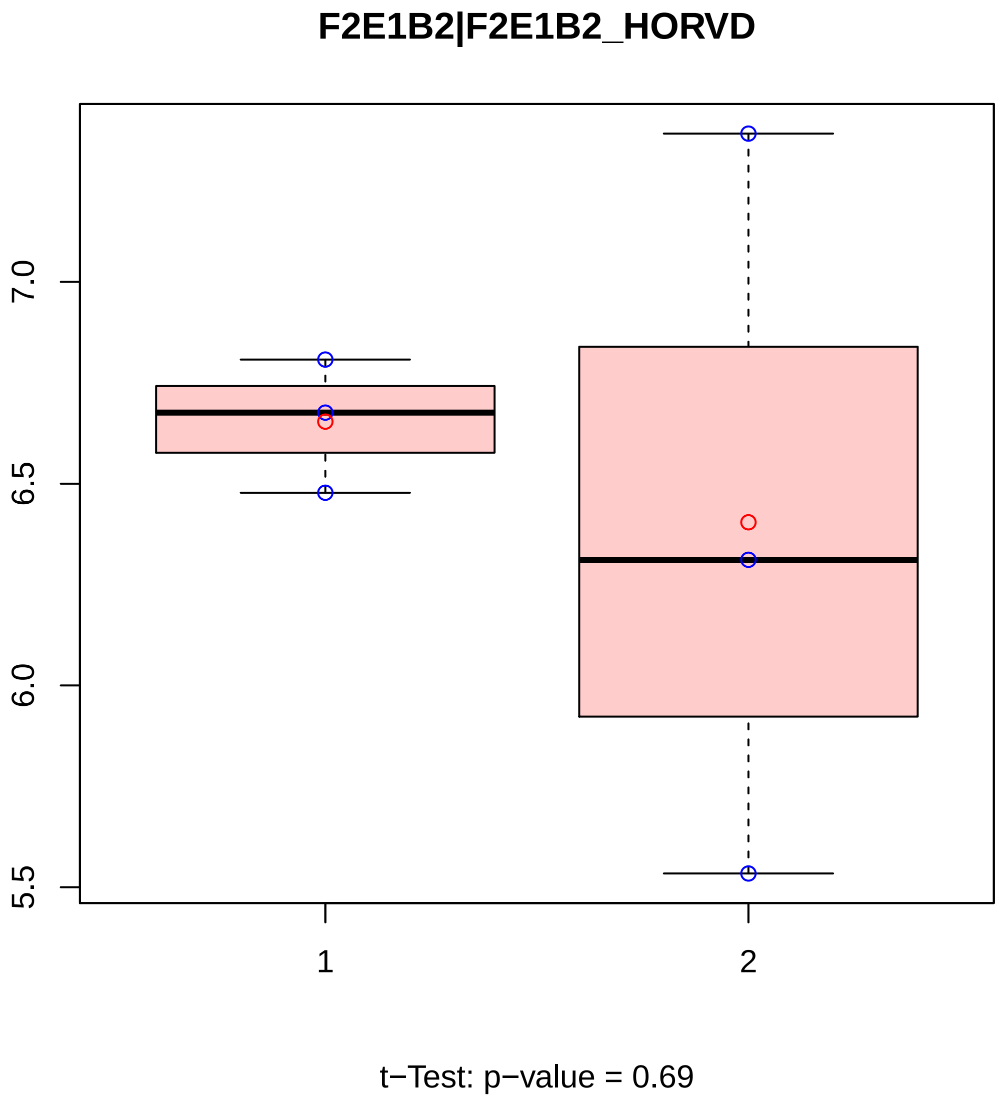
F2E1B2|F2E1B2_HORVD
7.0
6.5
6.0
5.5
1
2
t−Test: p−value = 0.69

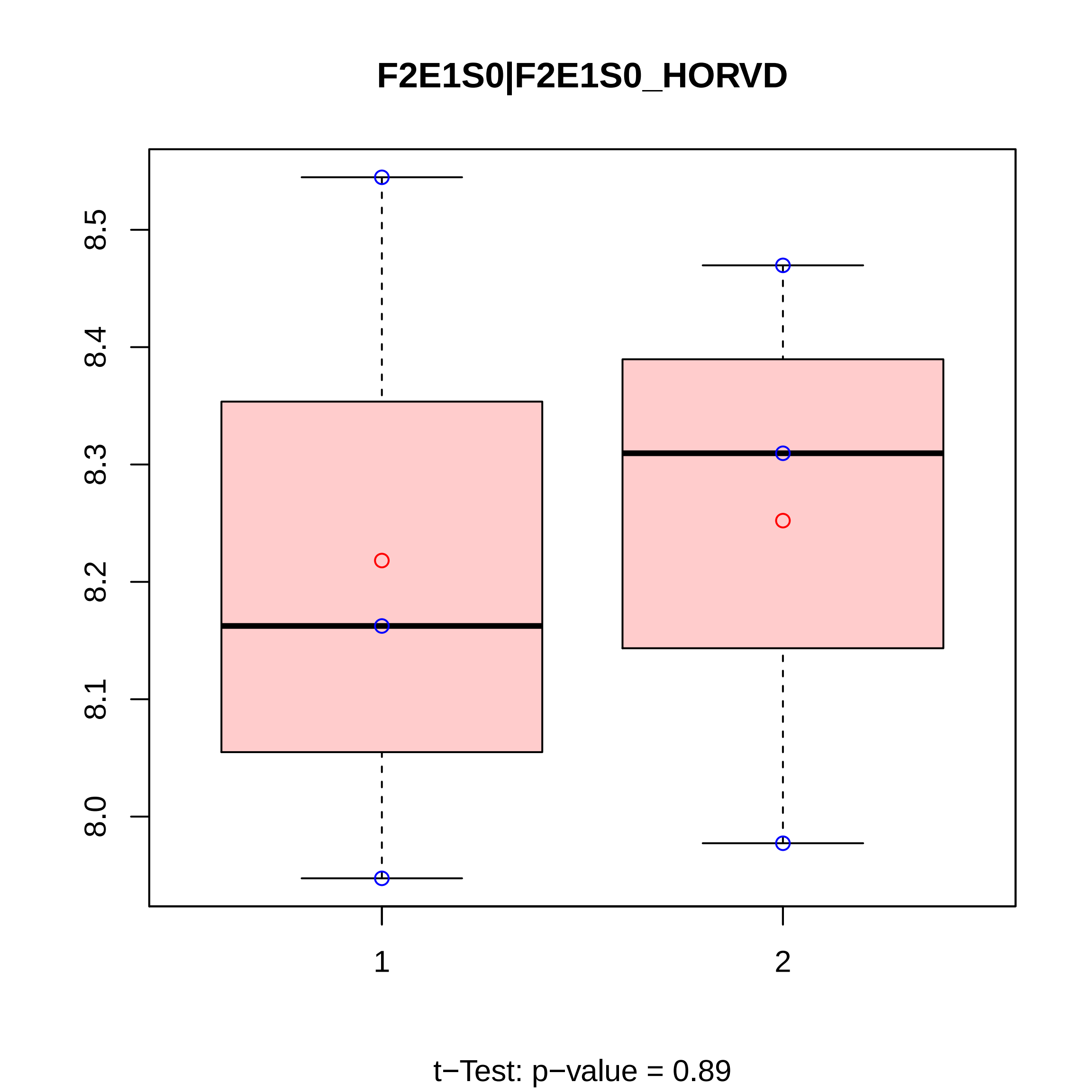
F2E1S0|F2E1S0_HORVD
8.0
8.1
8.2
8.3
8.4
8.5
1
2
t−Test: p−value = 0.89

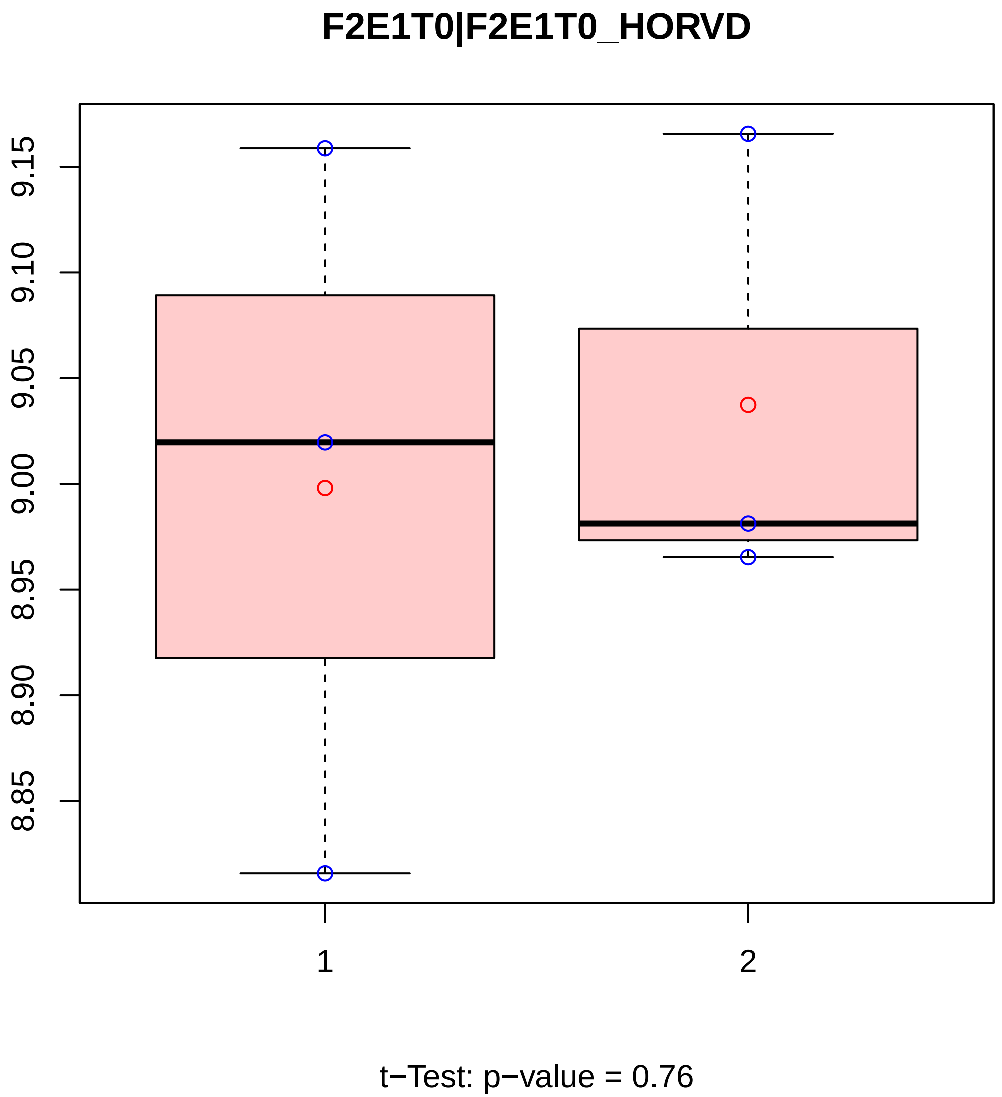
F2E1T0|F2E1T0_HORVD
9.15
9.10
9.05
9.00
8.95
8.90
8.85
1
2
t−Test: p−value = 0.76

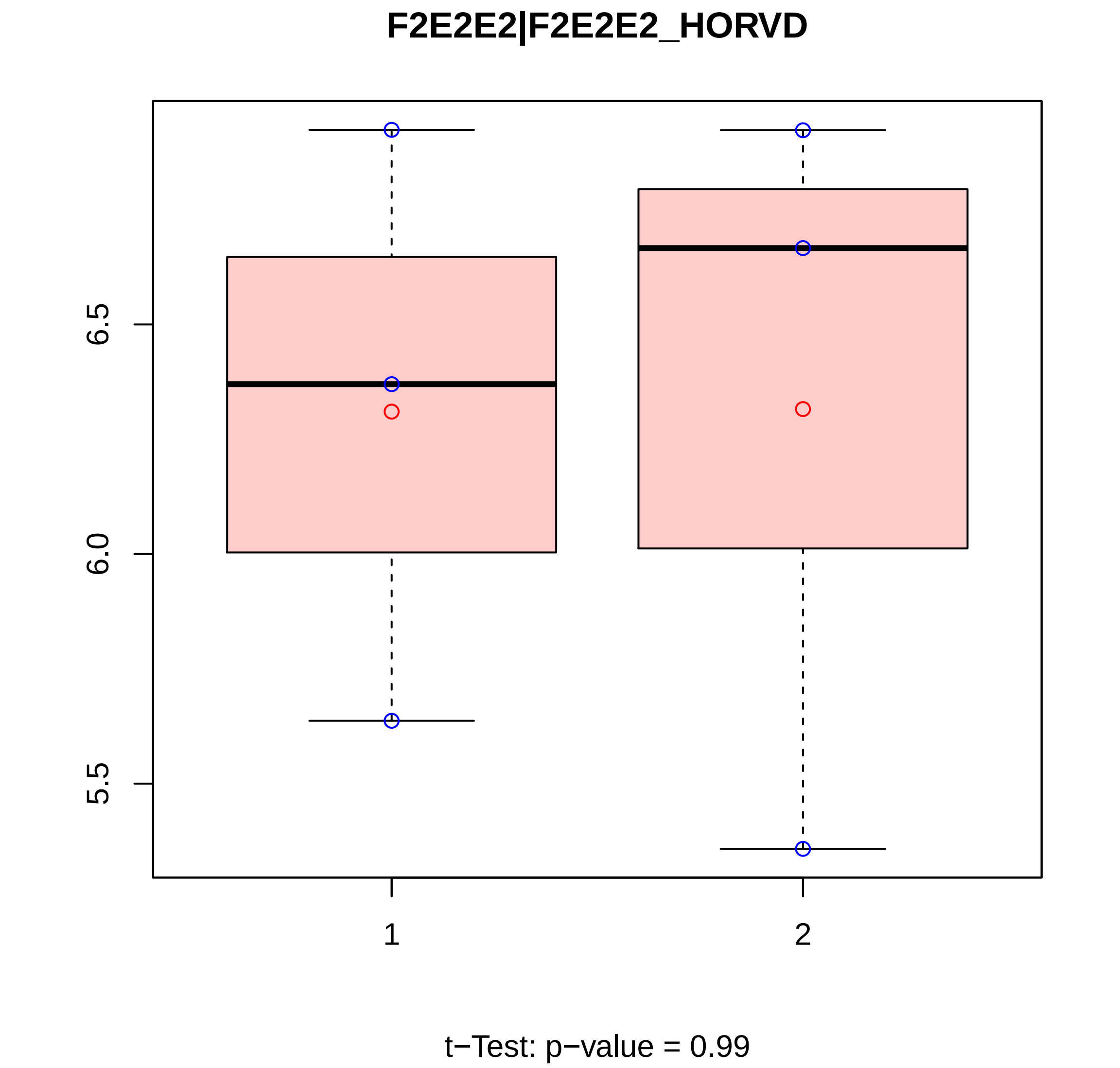
F2E2E2|F2E2E2_HORVD
5.5
6.0
6.5
1
2
t−Test: p−value = 0.99

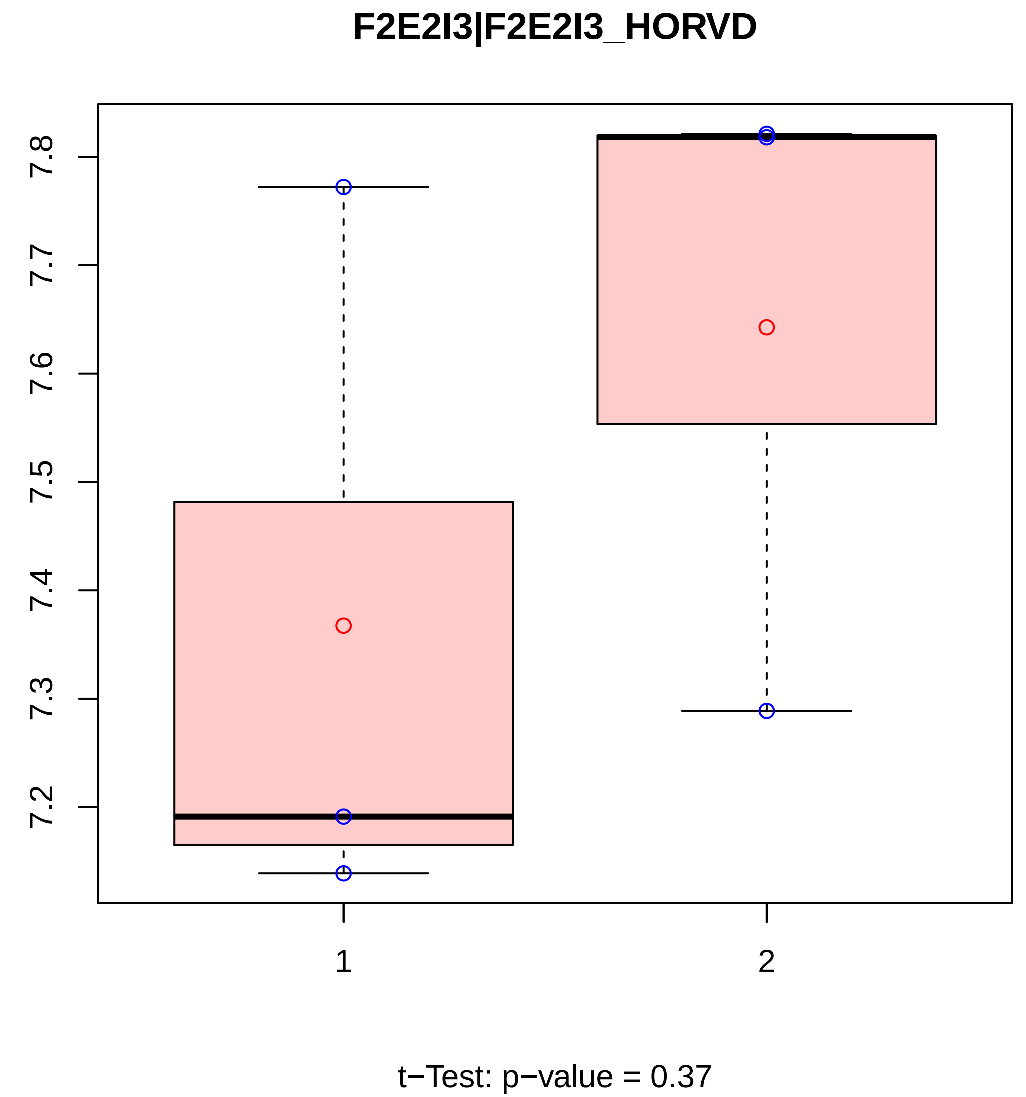
F2E2I3|F2E2I3_HORVD
7.2
7.3
7.4
7.5
7.6
7.7
7.8
1
2
t−Test: p−value = 0.37

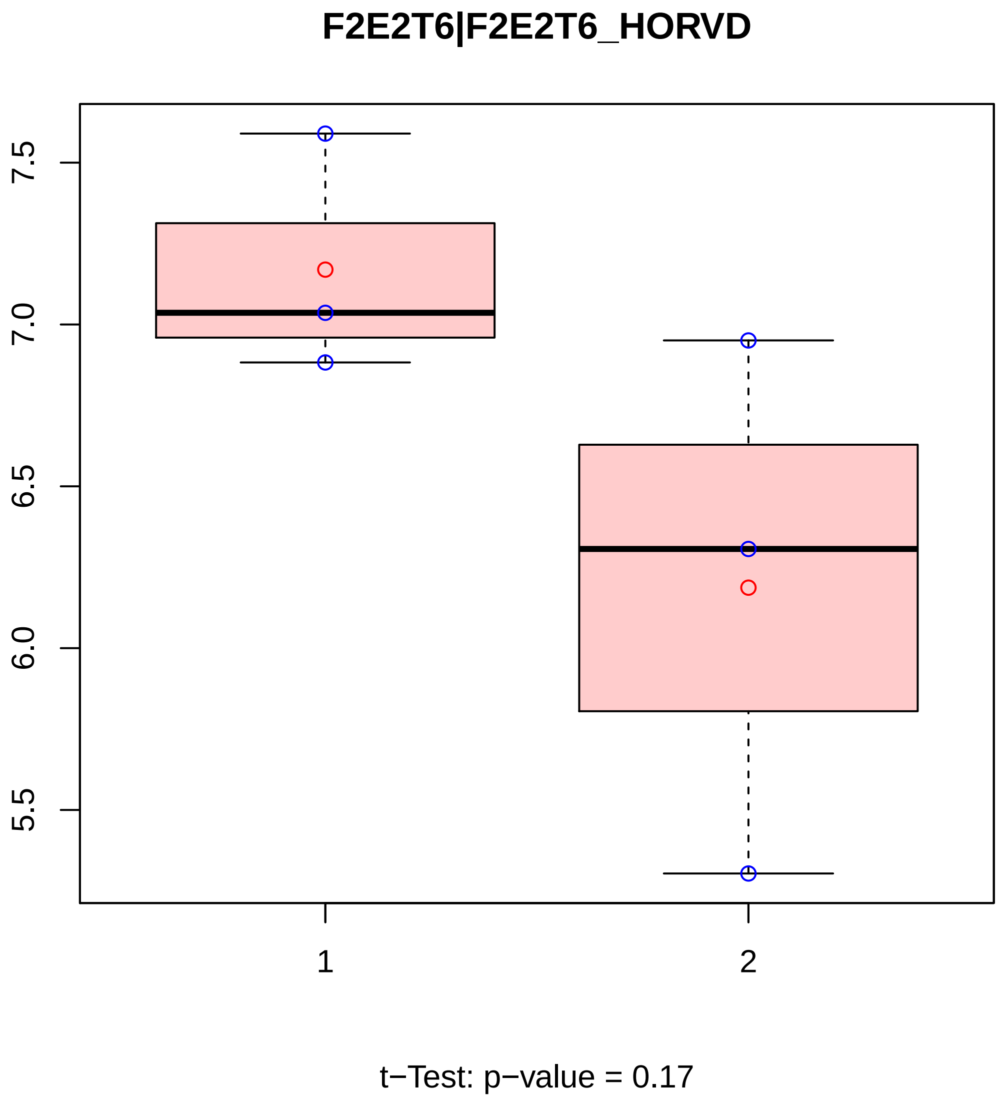
F2E2T6|F2E2T6_HORVD
7.5
7.0
6.5
6.0
5.5
1
2
t−Test: p−value = 0.17

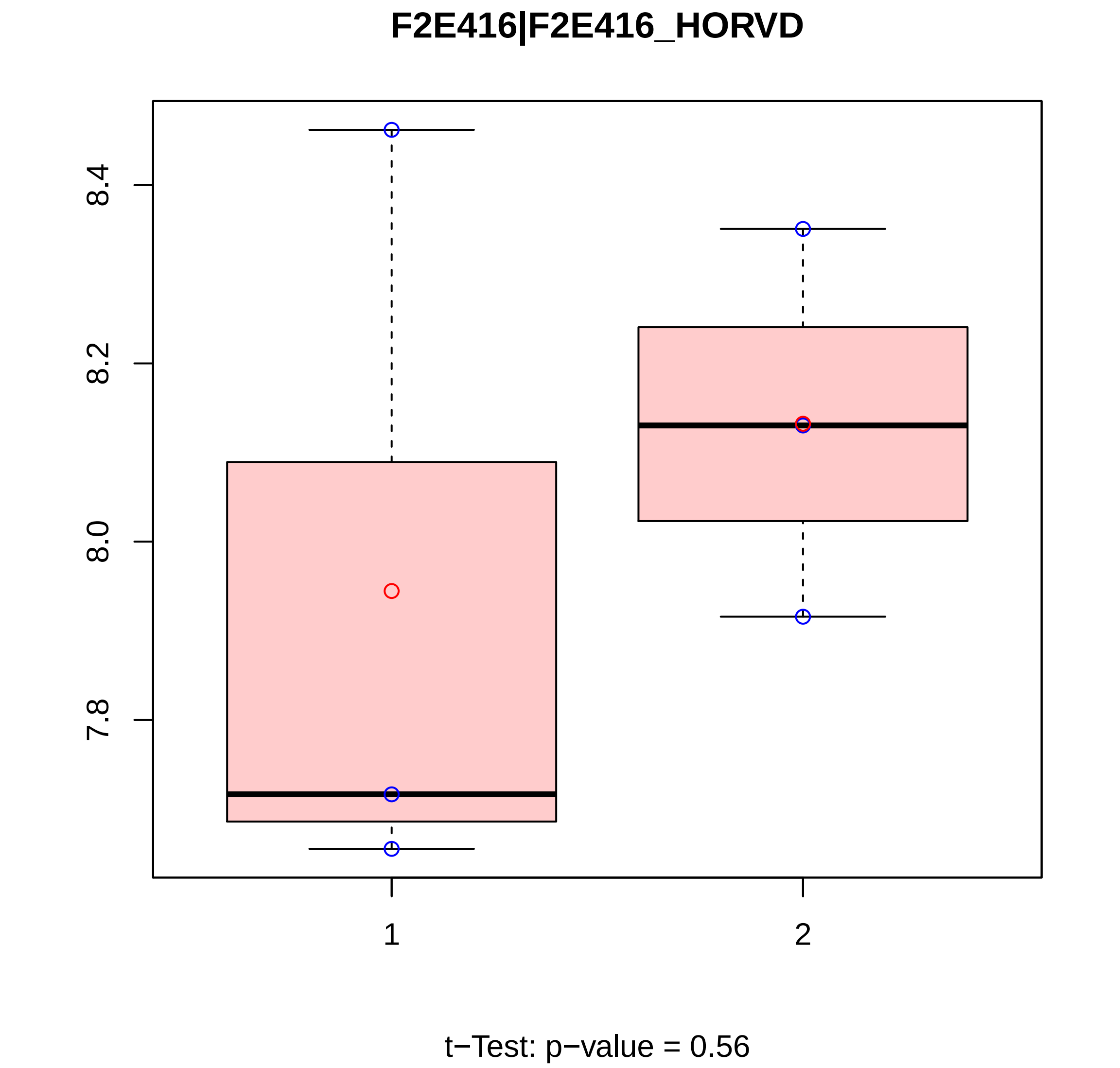
F2E416|F2E416_HORVD
8.4
8.2
8.0
7.8
1
2
t−Test: p−value = 0.56

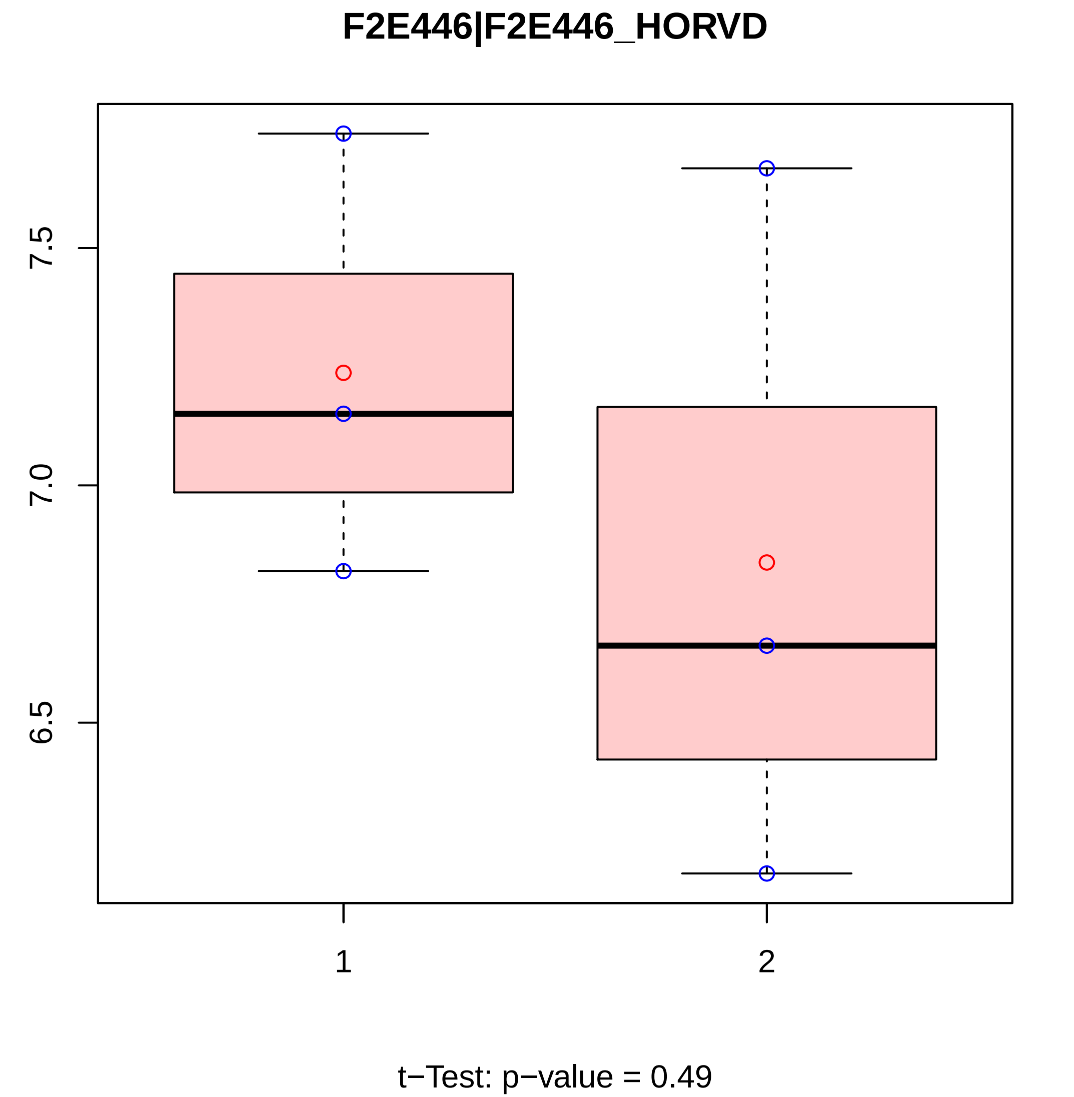
F2E446|F2E446_HORVD
7.5
7.0
6.5
1
2
t−Test: p−value = 0.49

F2E4B4|F2E4B4_HORVD

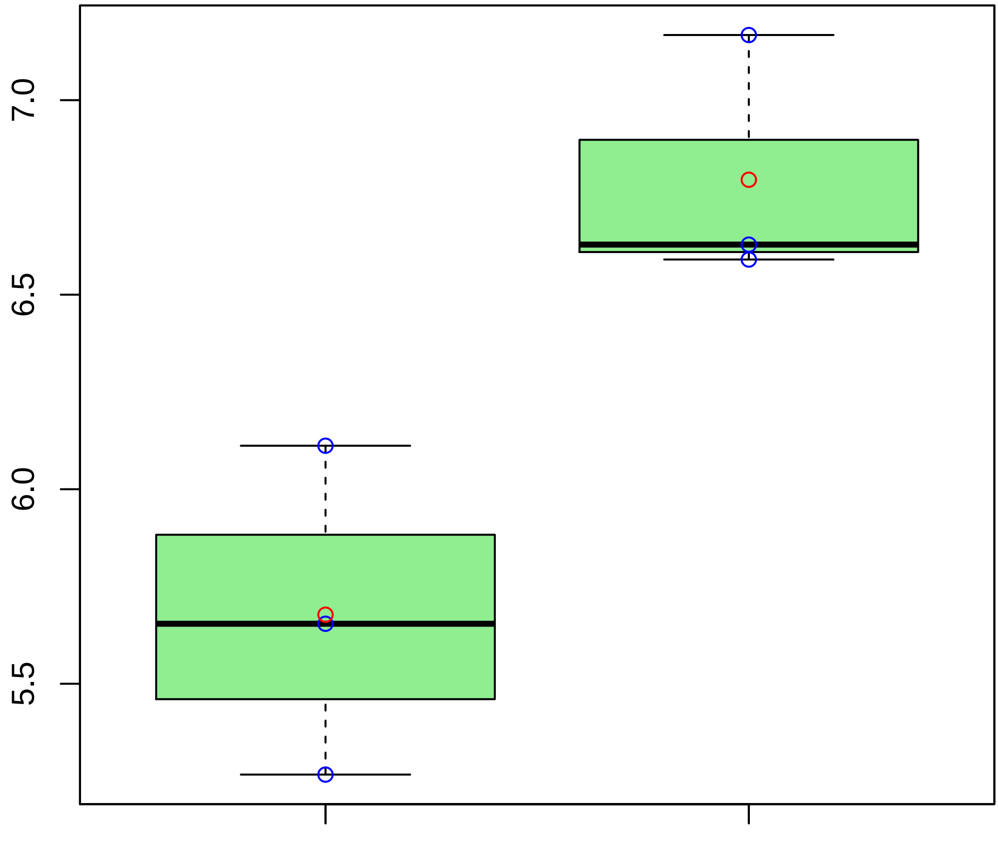

t−Test: p−value = 0.02

F2E4I2|F2E4I2_HORVD

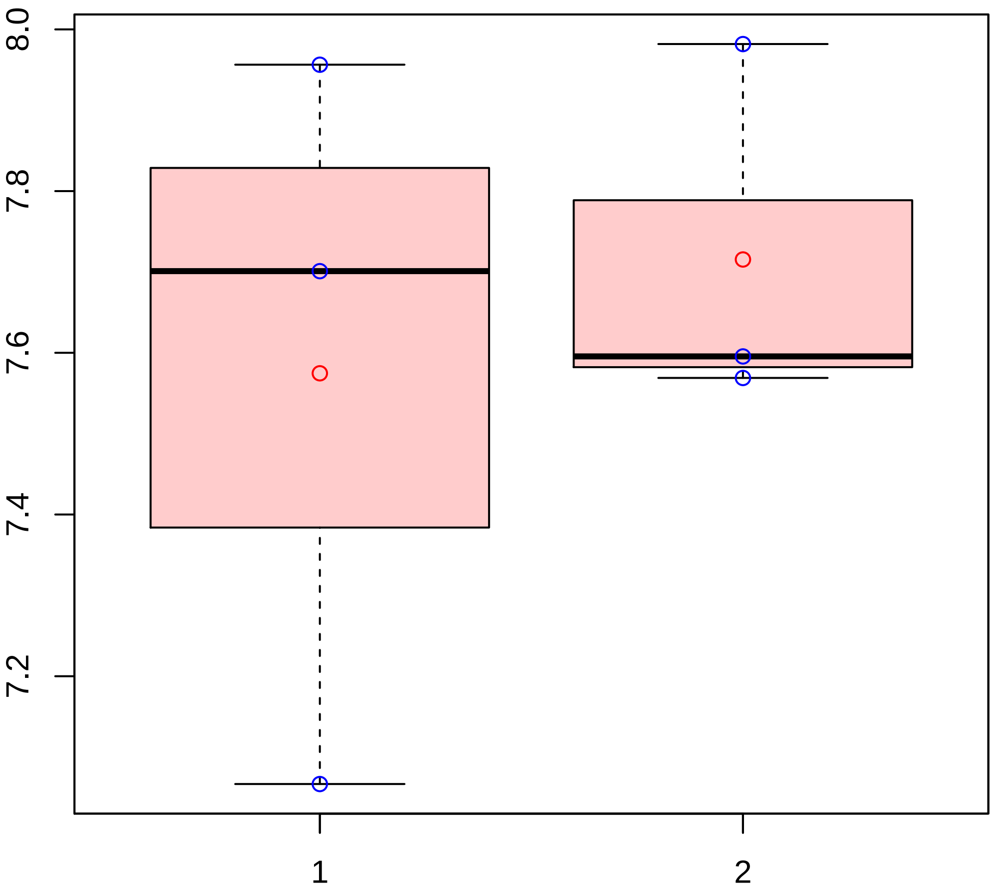

t−Test: p−value = 0.67

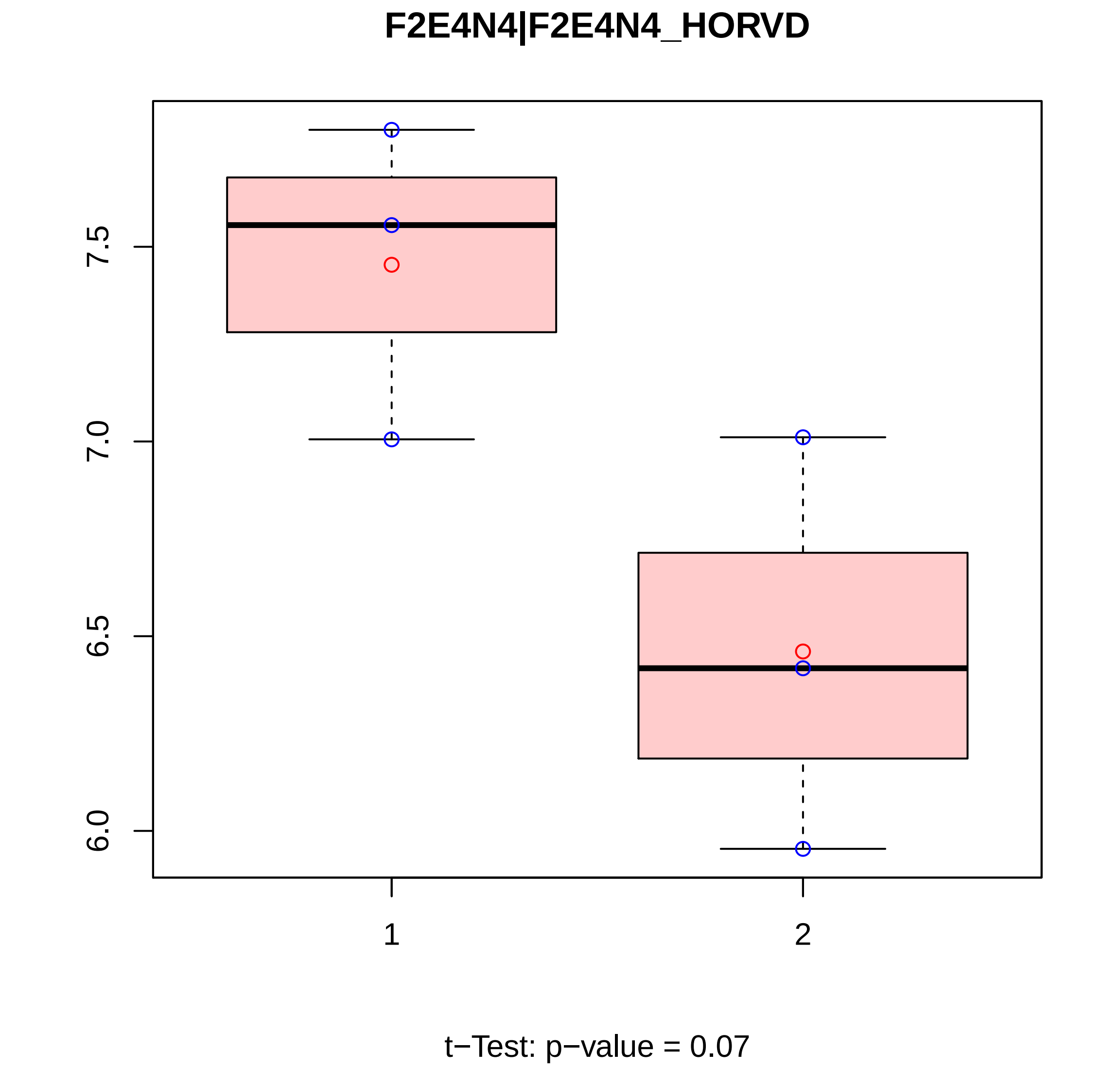
F2E4N4|F2E4N4_HORVD
7.5
7.0
6.5
6.0
1
2
t−Test: p−value = 0.07

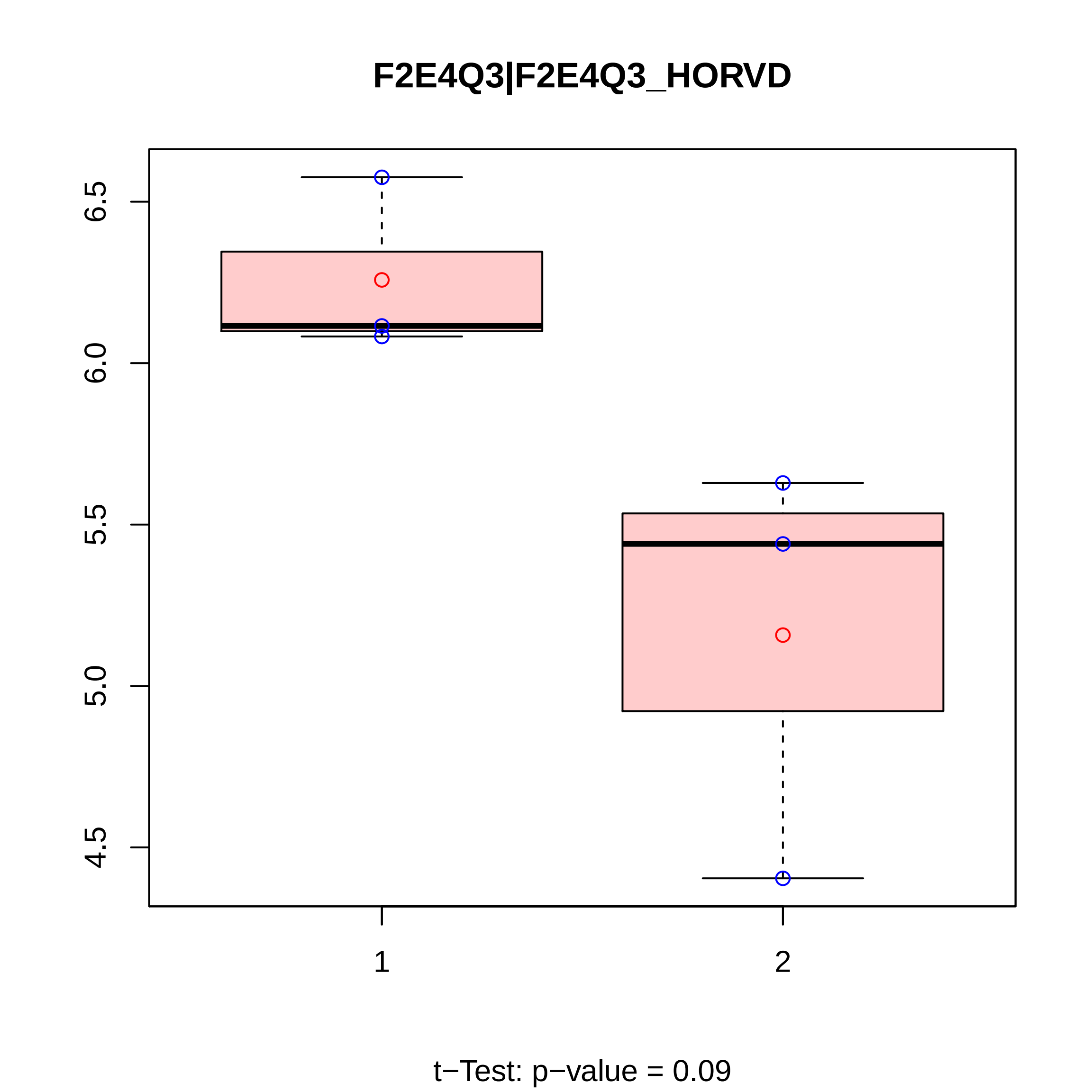

F2E4Q3|F2E4Q3_HORVD
6.5
6.0
5.5
5.0
4.5
1
2
t−Test: p−value = 0.09

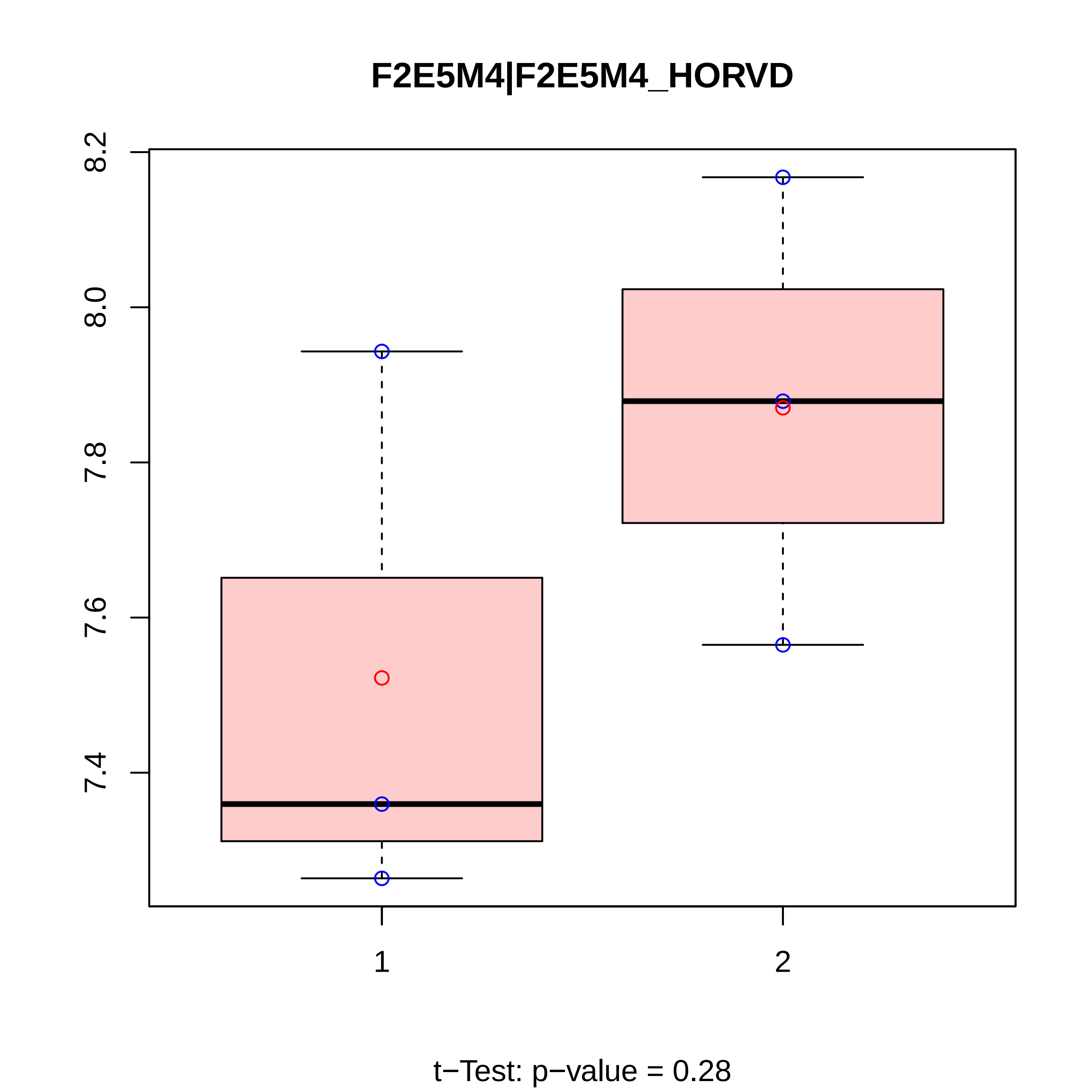
F2E5M4|F2E5M4_HORVD
8.2
8.0
7.8
7.6
7.4
1
2
t−Test: p−value = 0.28

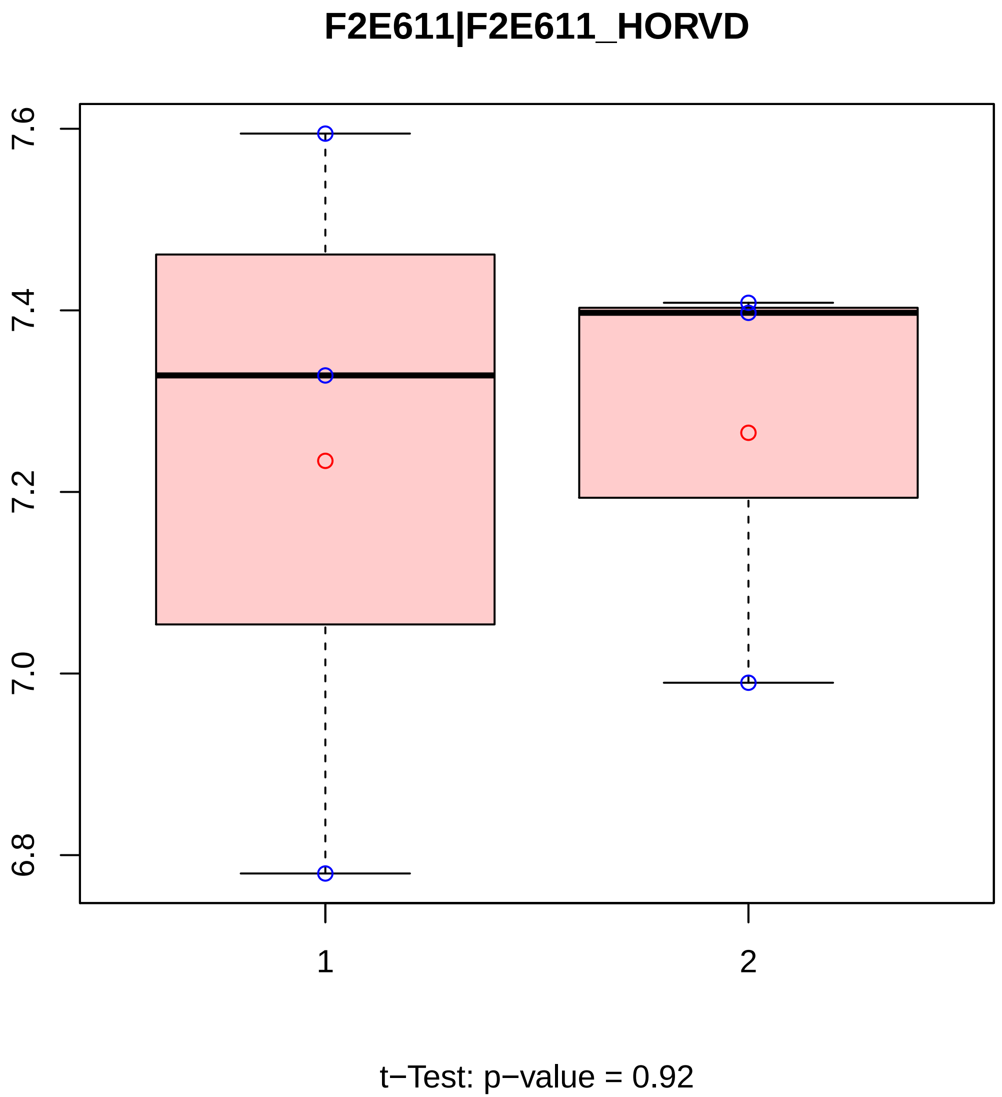
F2E611|F2E611_HORVD
7.6
7.4
7.2
7.0
6.8
1
2
t−Test: p−value = 0.92

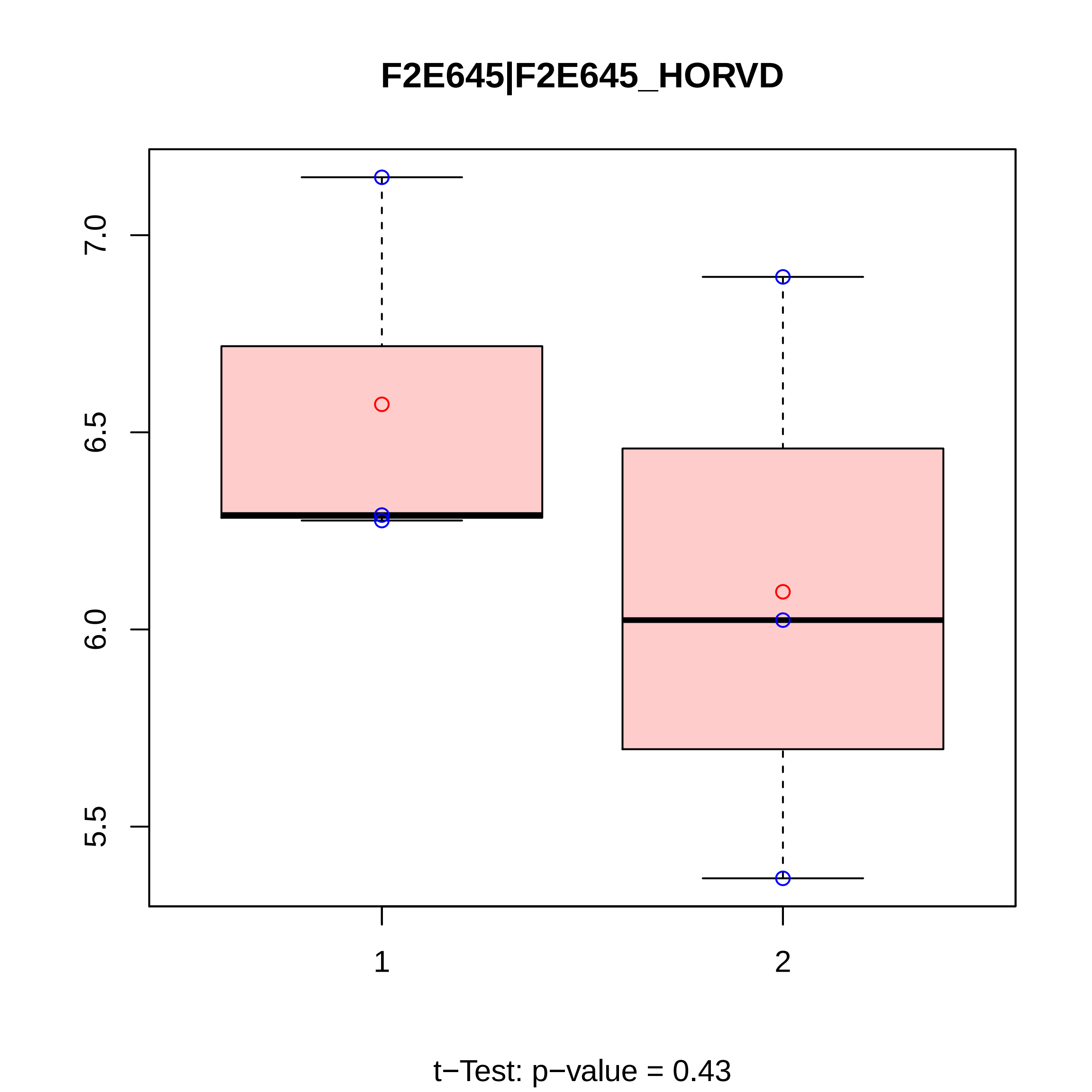
F2E645|F2E645_HORVD
7.0
6.5
6.0
5.5
1
2
t−Test: p−value = 0.43

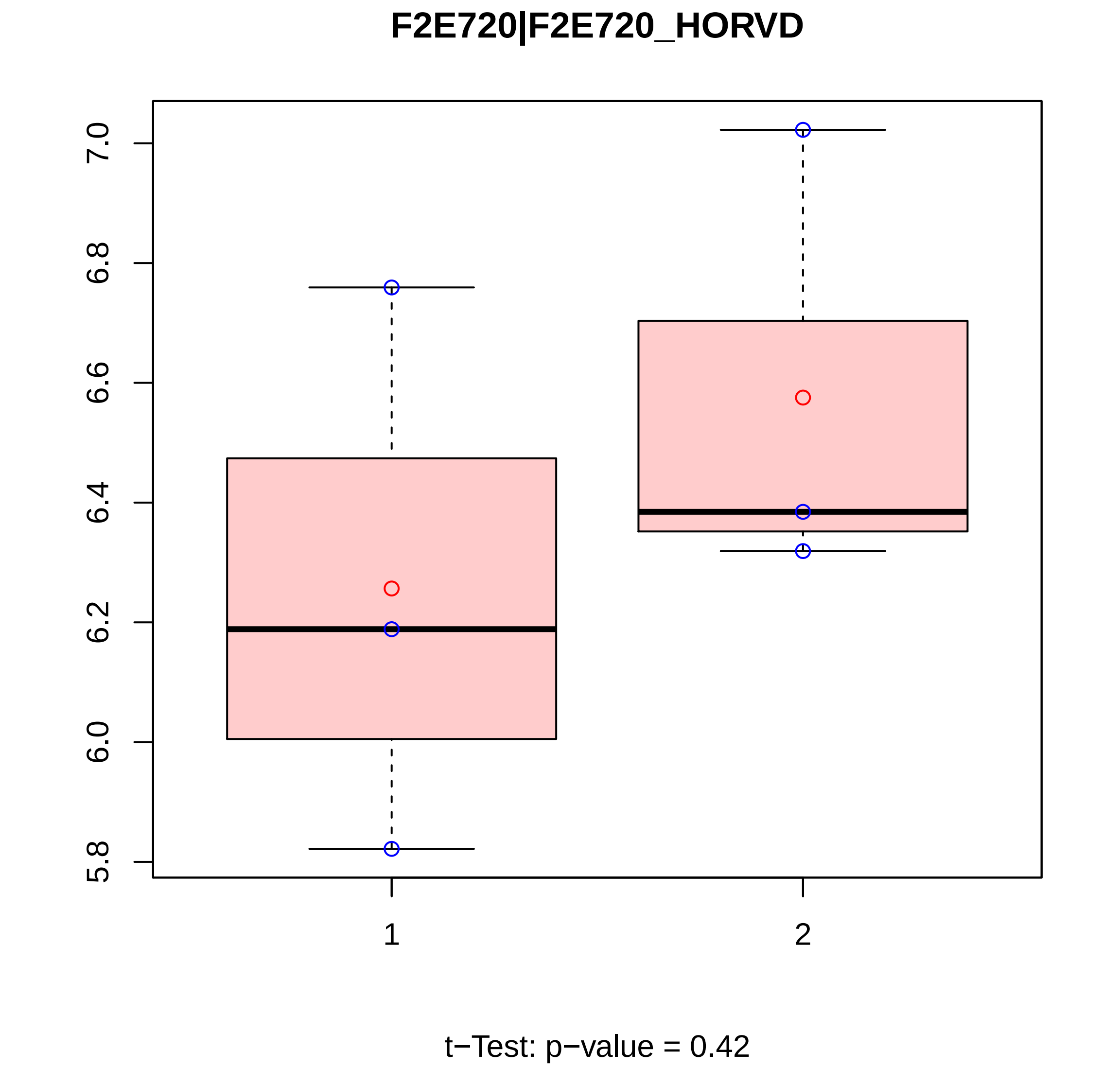
F2E720|F2E720_HORVD
7.0
6.8
6.6
6.4
6.2
6.0
5.8
1
2
t−Test: p−value = 0.42

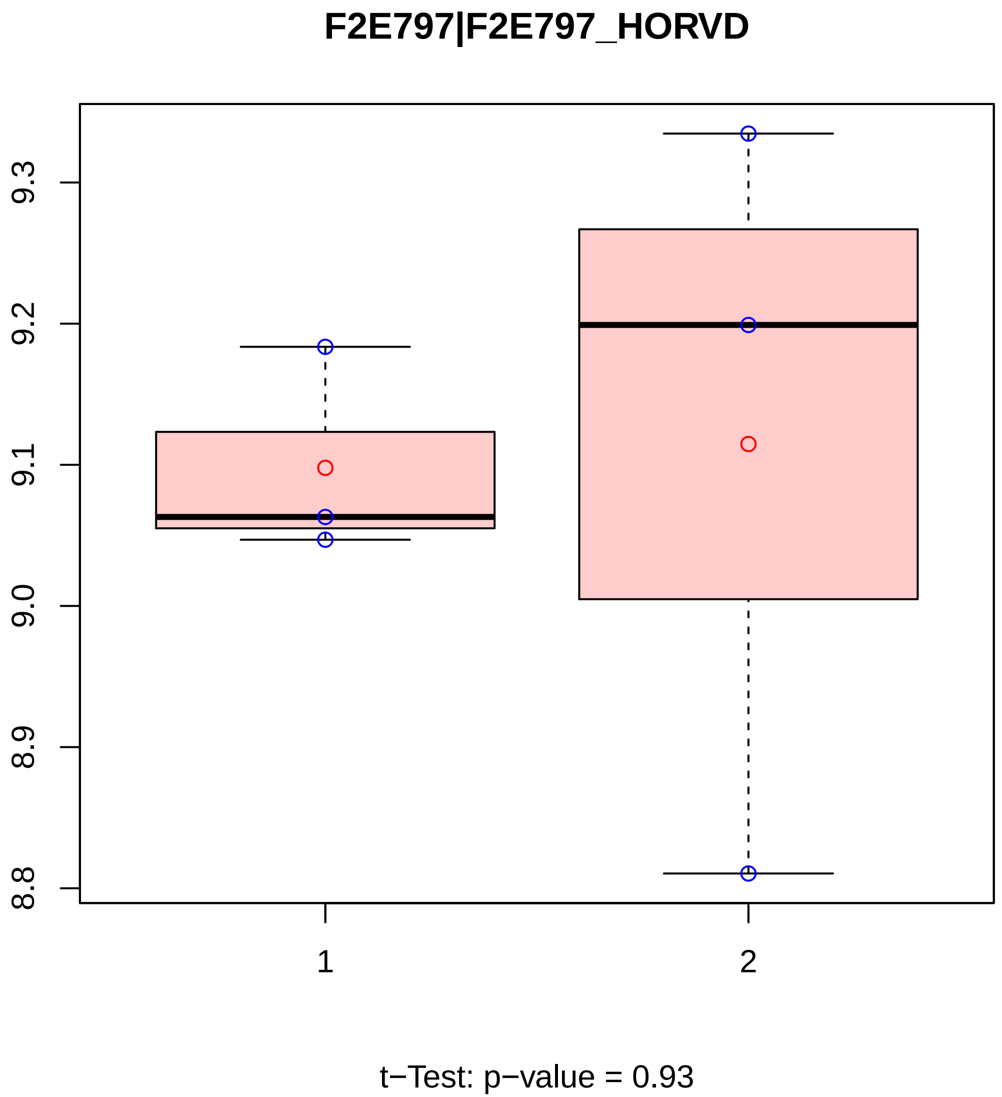

F2E797|F2E797_HORVD
9.3
9.2
9.1
9.0
8.9
8.8
1
2
t−Test: p−value = 0.93

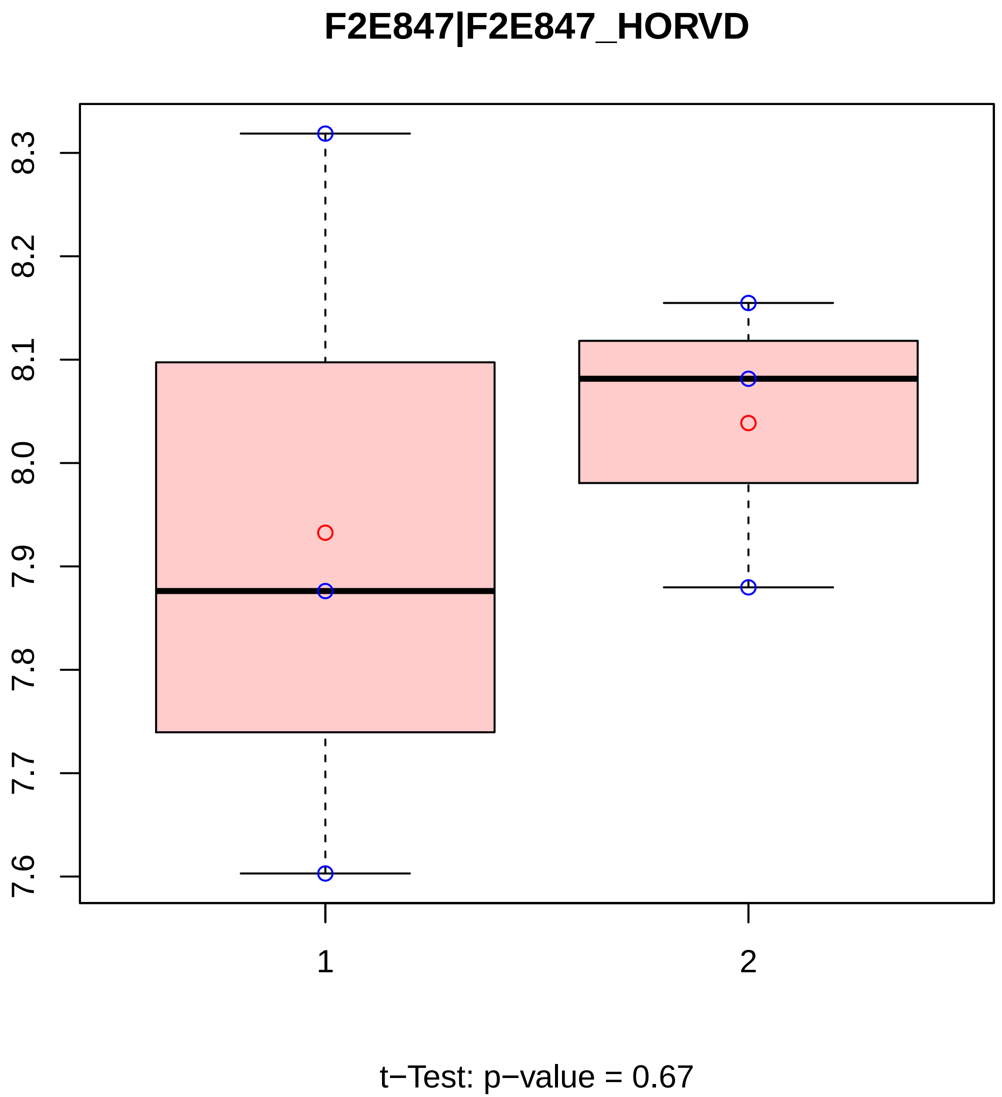
F2E847|F2E847_HORVD
8.3
8.2
8.1
8.0
7.9
7.8
7.7
7.6
1
2
t−Test: p−value = 0.67

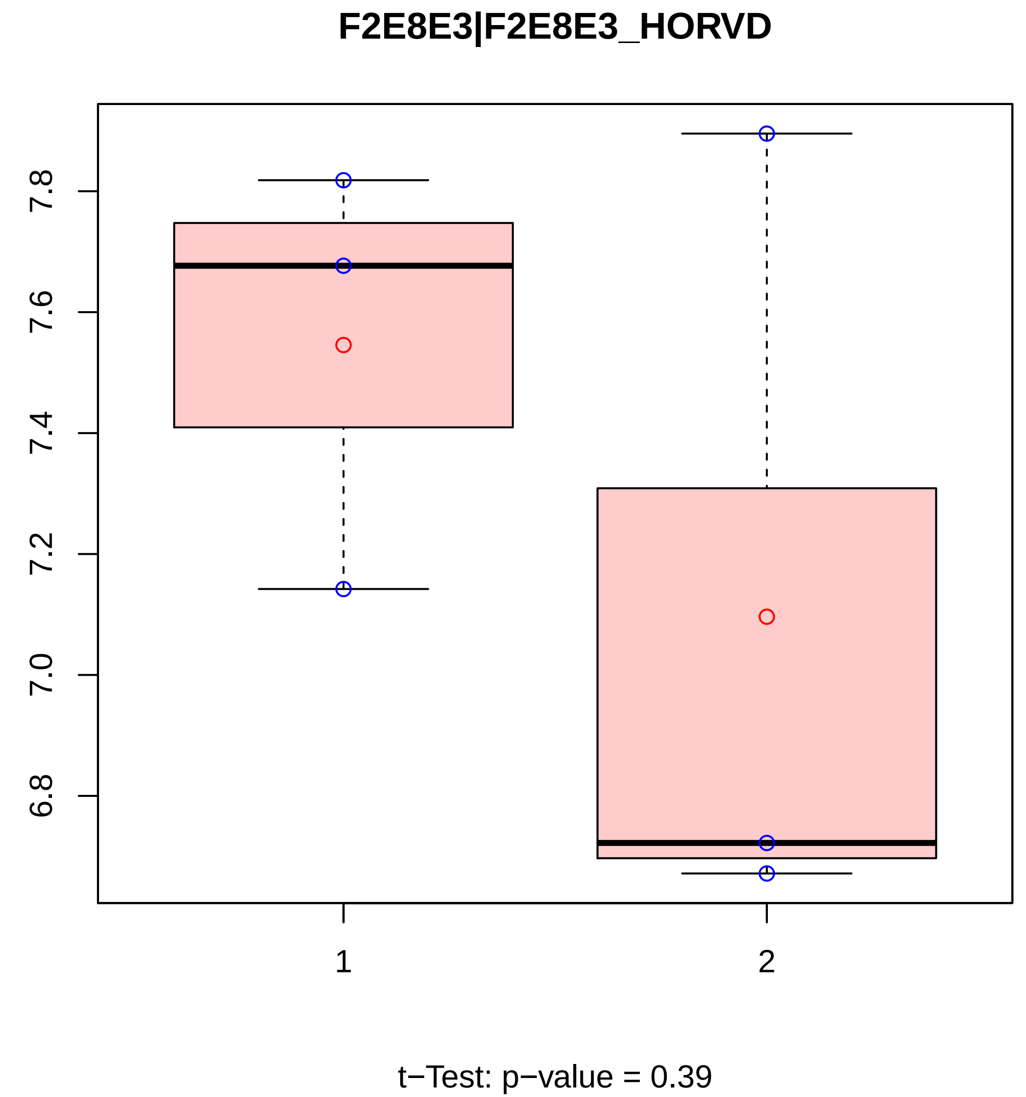
F2E8E3|F2E8E3_HORVD
7.8
7.6
7.4
7.2
7.0
6.8
1
2
t−Test: p−value = 0.39

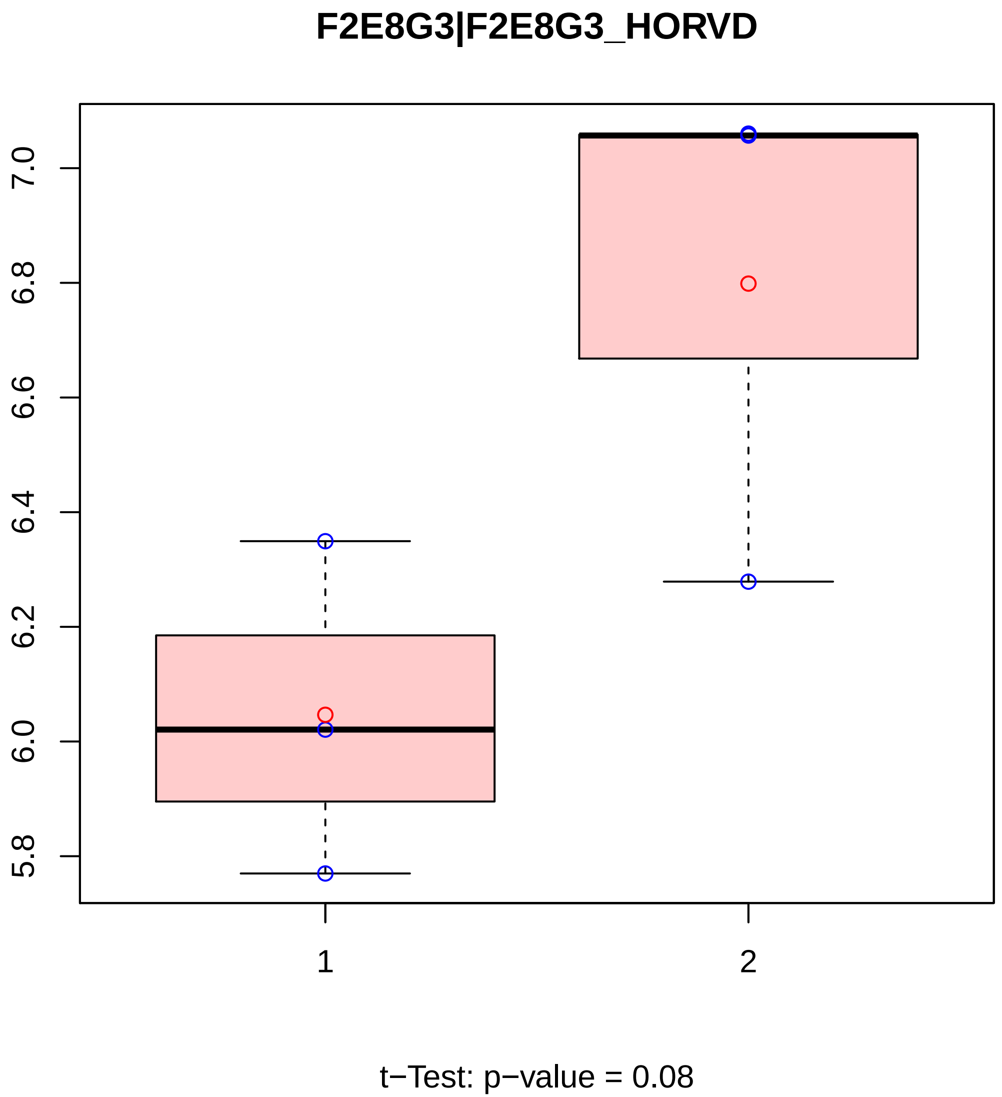
F2E8G3|F2E8G3_HORVD
7.0
6.8
6.6
6.4
6.2
6.0
5.8
1
2
t−Test: p−value = 0.08

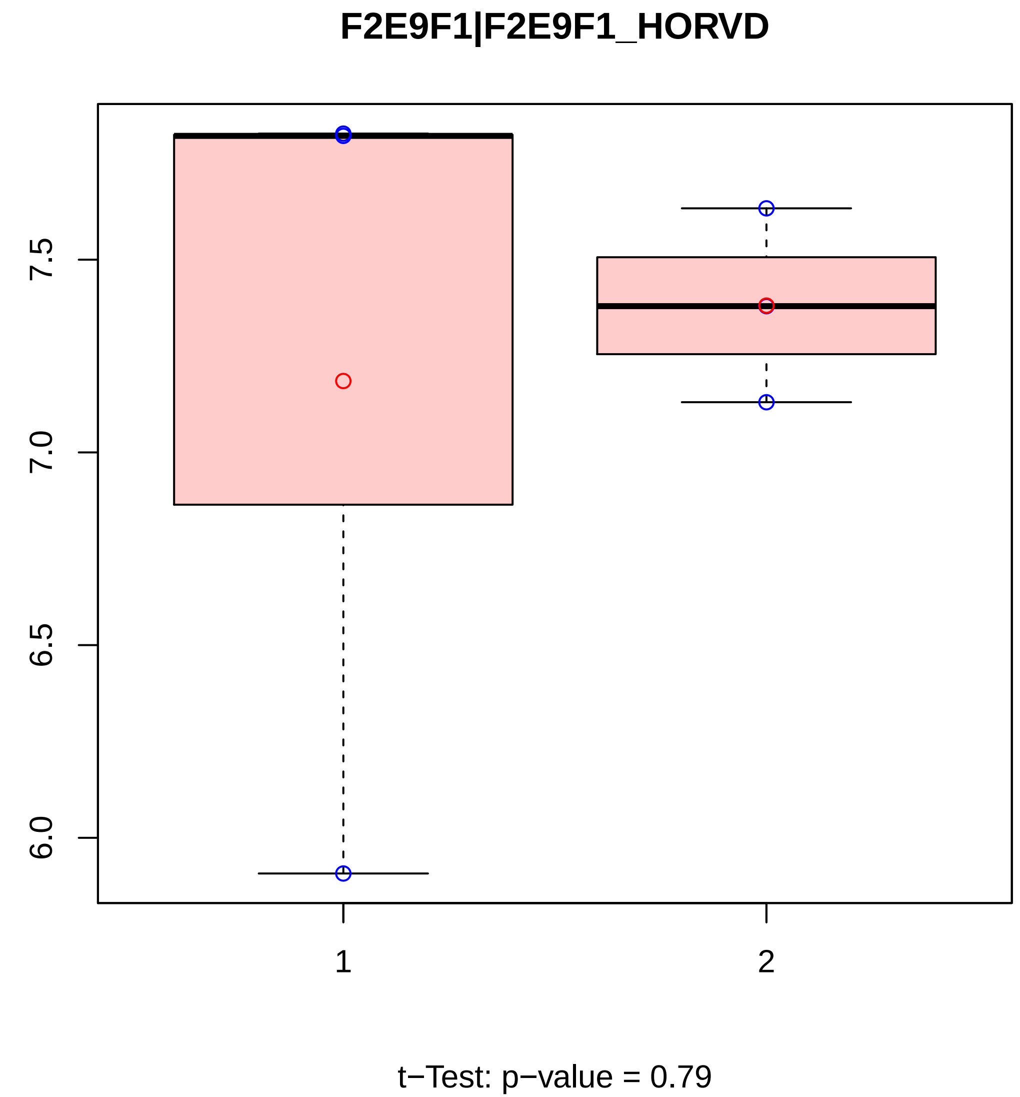
F2E9F1|F2E9F1_HORVD
7.5
7.0
6.5
6.0
1
2
t−Test: p−value = 0.79

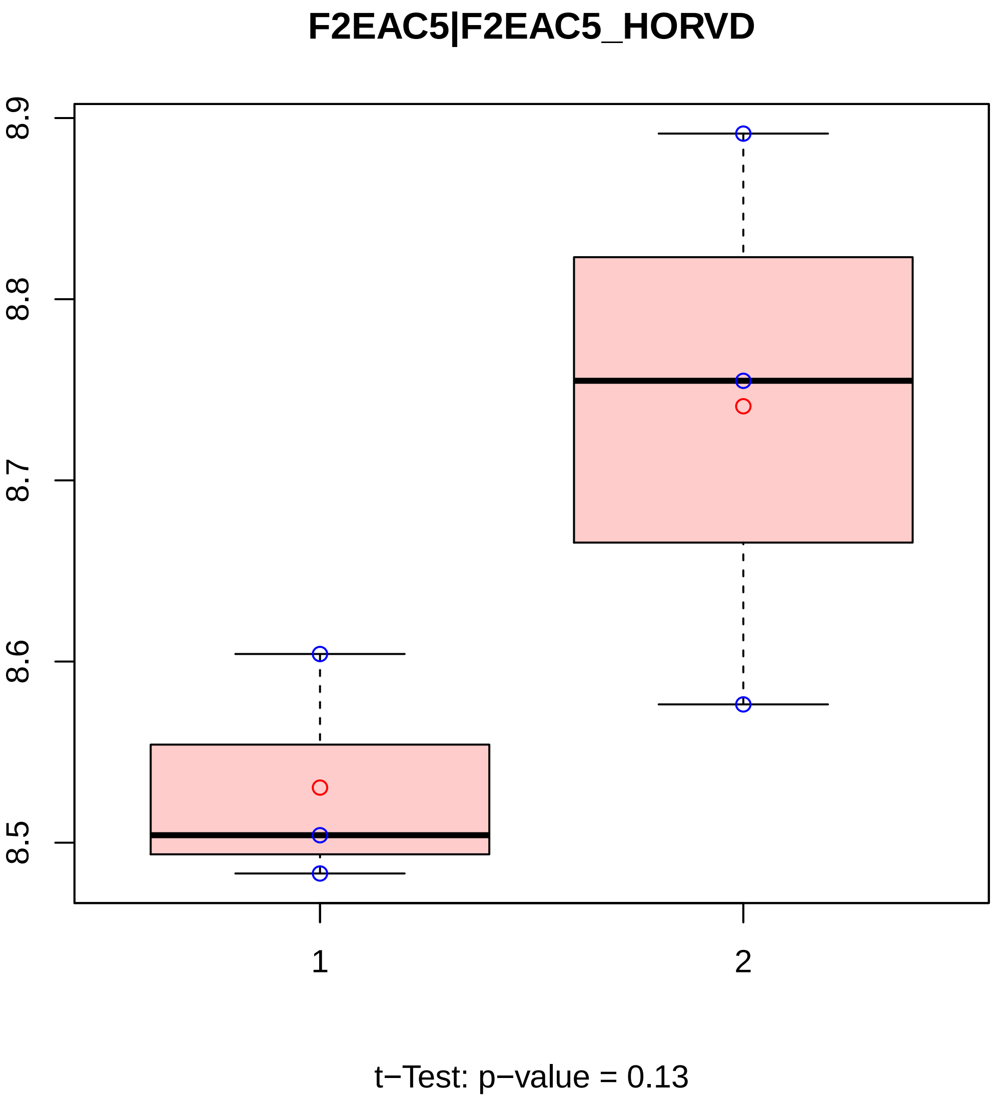
F2EAC5|F2EAC5_HORVD
8.5
8.6
8.7
8.8
8.9
1
2
t−Test: p−value = 0.13

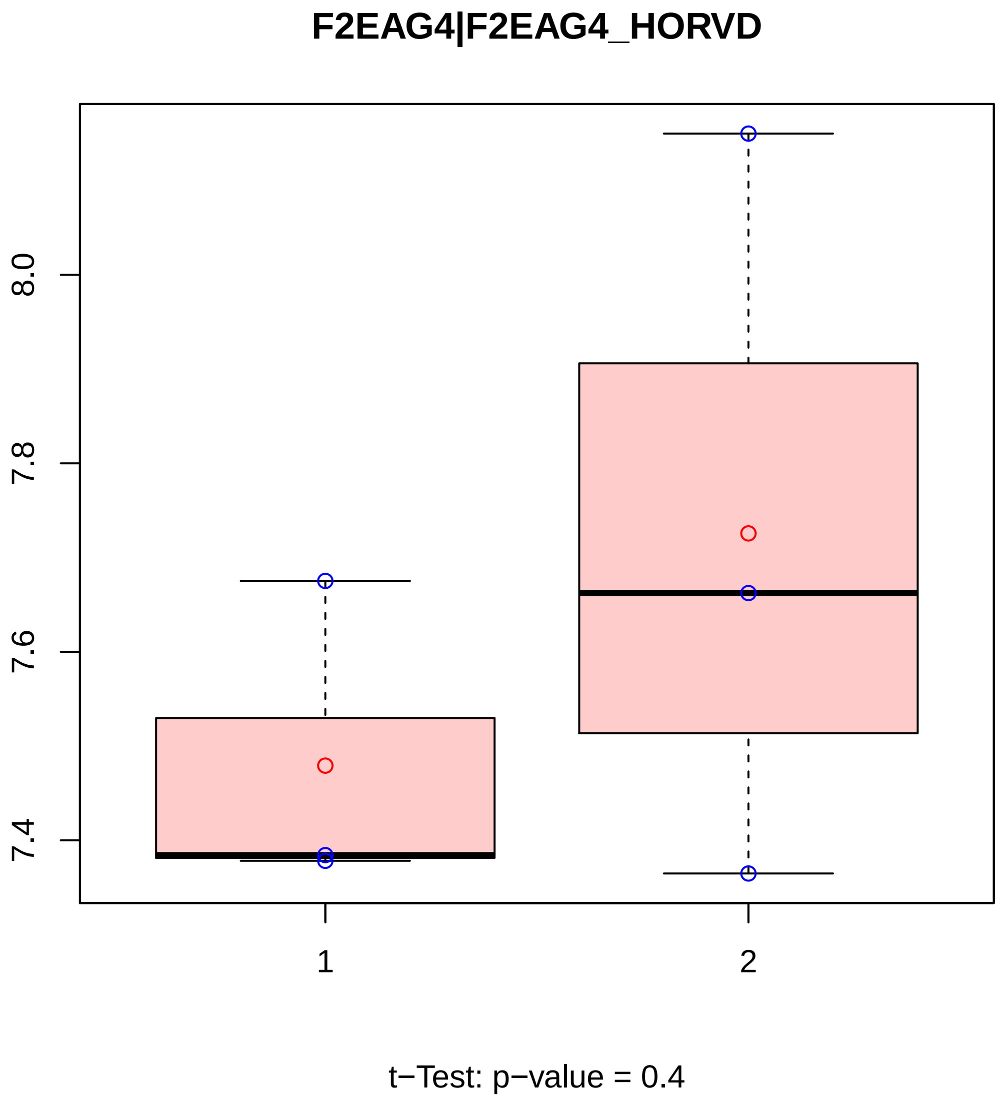
F2EAG4|F2EAG4_HORVD
8.0
7.8
7.6
7.4
1
2
t−Test: p−value = 0.4

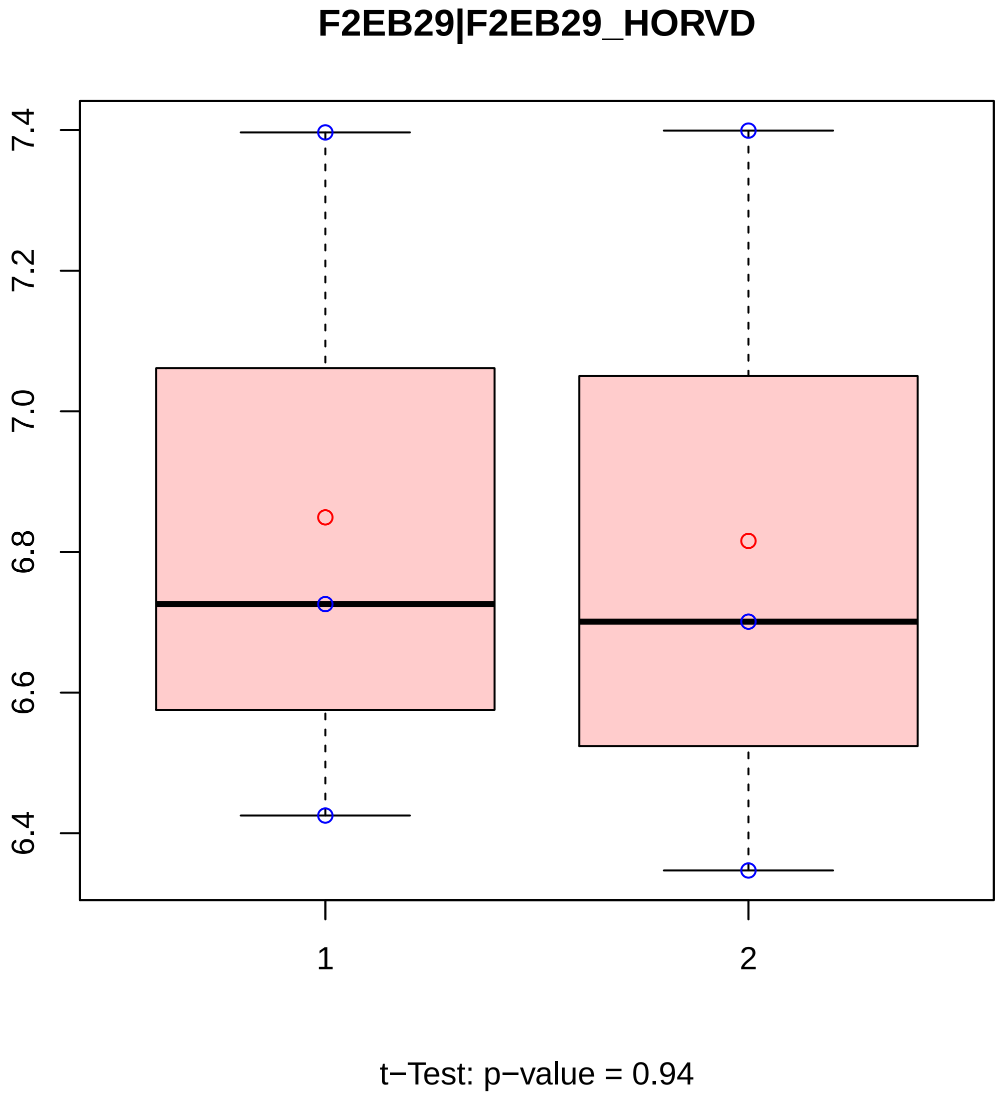
F2EB29|F2EB29_HORVD
7.4
7.2
7.0
6.8
6.6
6.4
1
2
t−Test: p−value = 0.94

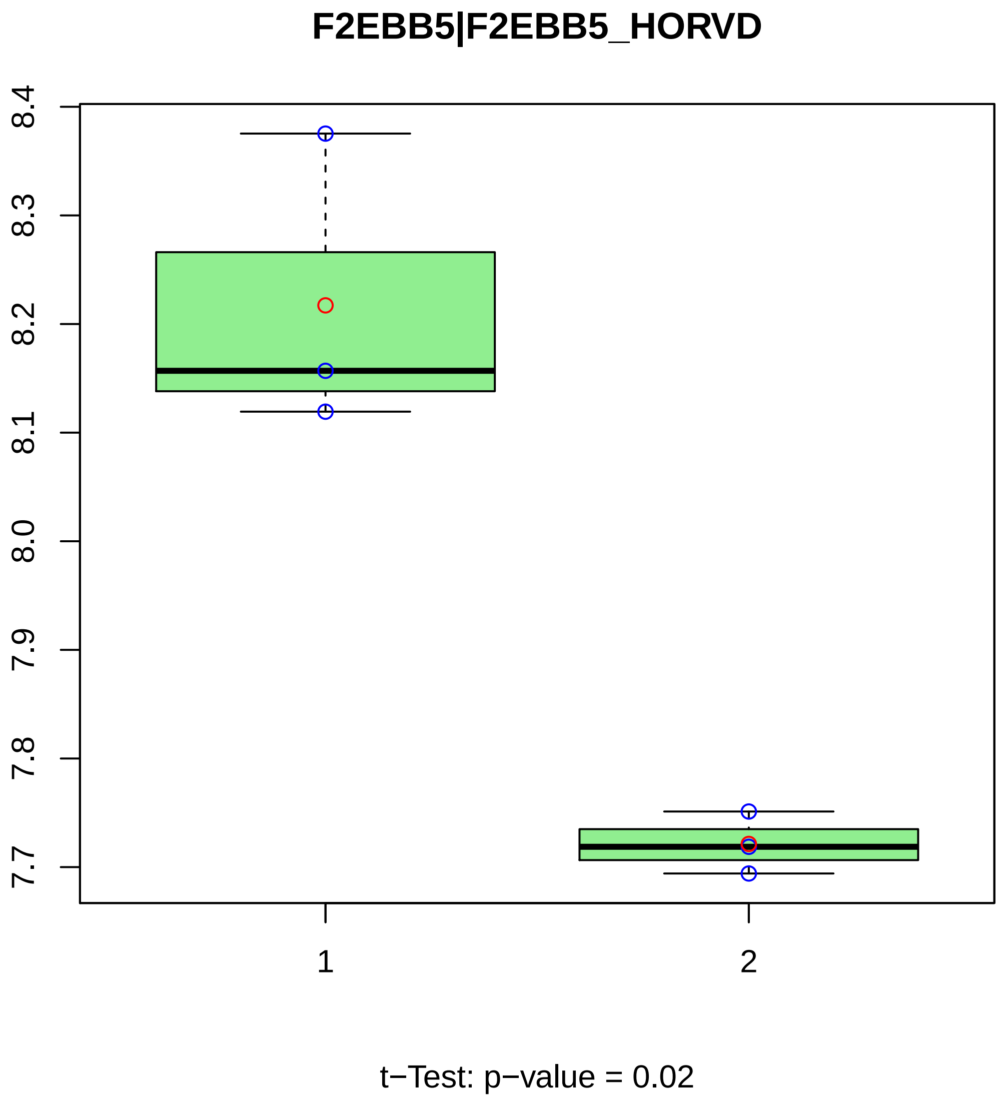

F2EBB5|F2EBB5_HORVD
8.4
8.3
8.2
8.1
8.0
7.9
7.8
7.7
1
2
t−Test: p−value = 0.02

F2EBI3|F2EBI3_HORVD

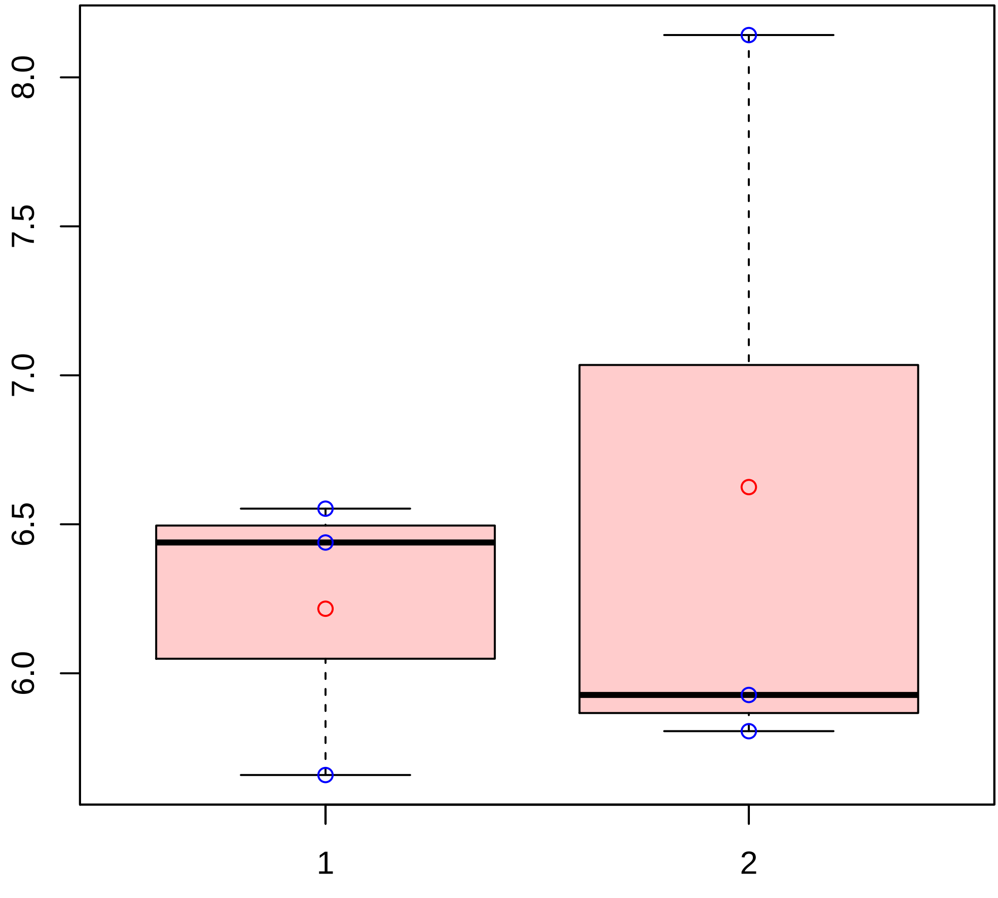

t−Test: p−value = 0.65

F2ECA4|F2ECA4_HORVD

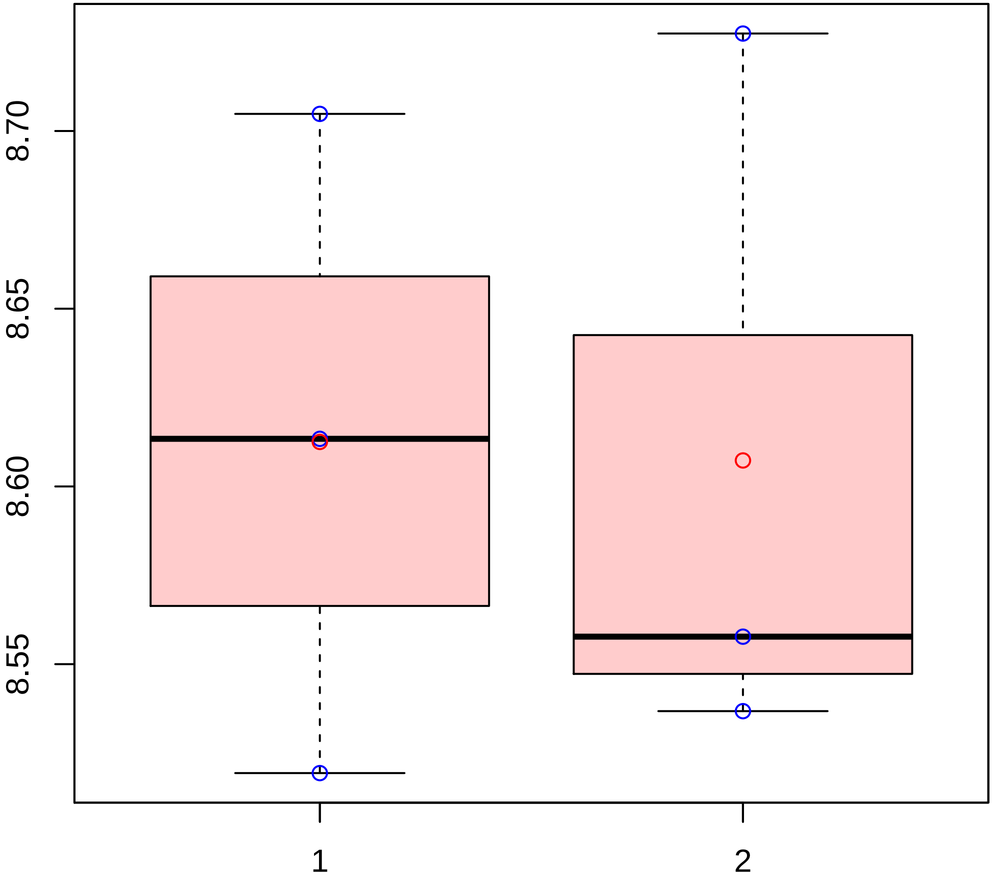

t−Test: p−value = 0.95

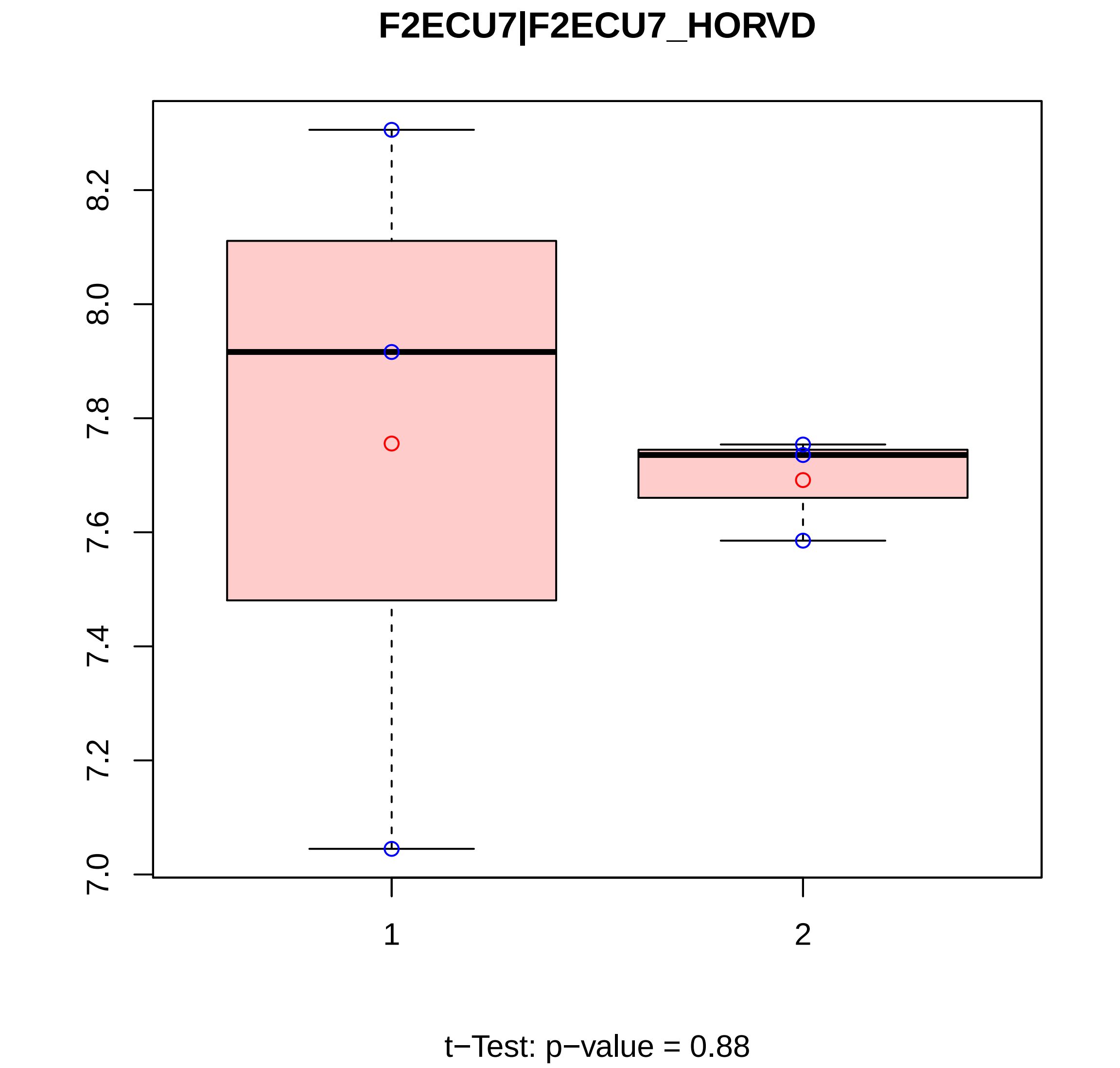
F2ECU7|F2ECU7_HORVD
8.2
8.0
7.8
7.6
7.4
7.2
7.0
1
2
t−Test: p−value = 0.88

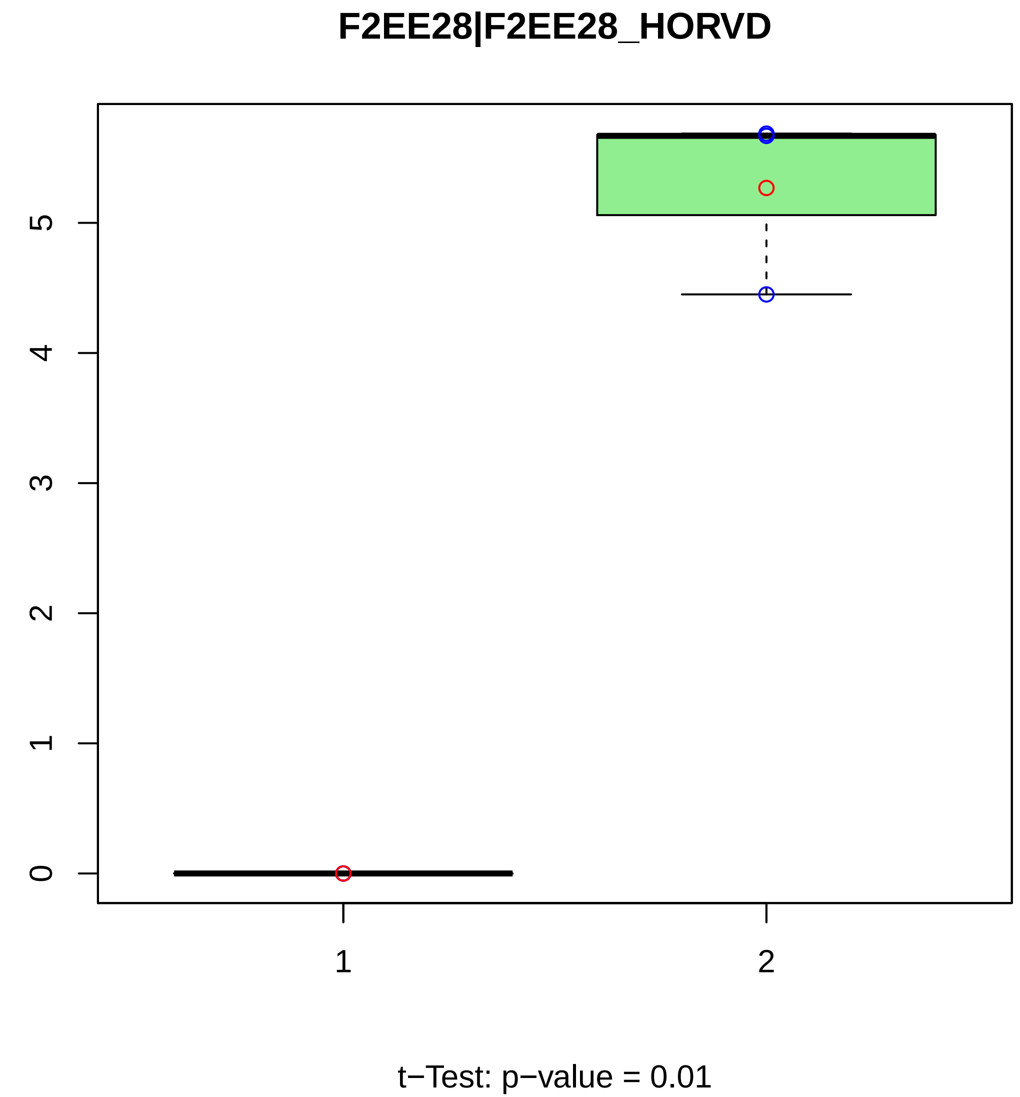

F2EE28|F2EE28_HORVD
0
1
2
3
4
5
1
2
t−Test: p−value = 0.01

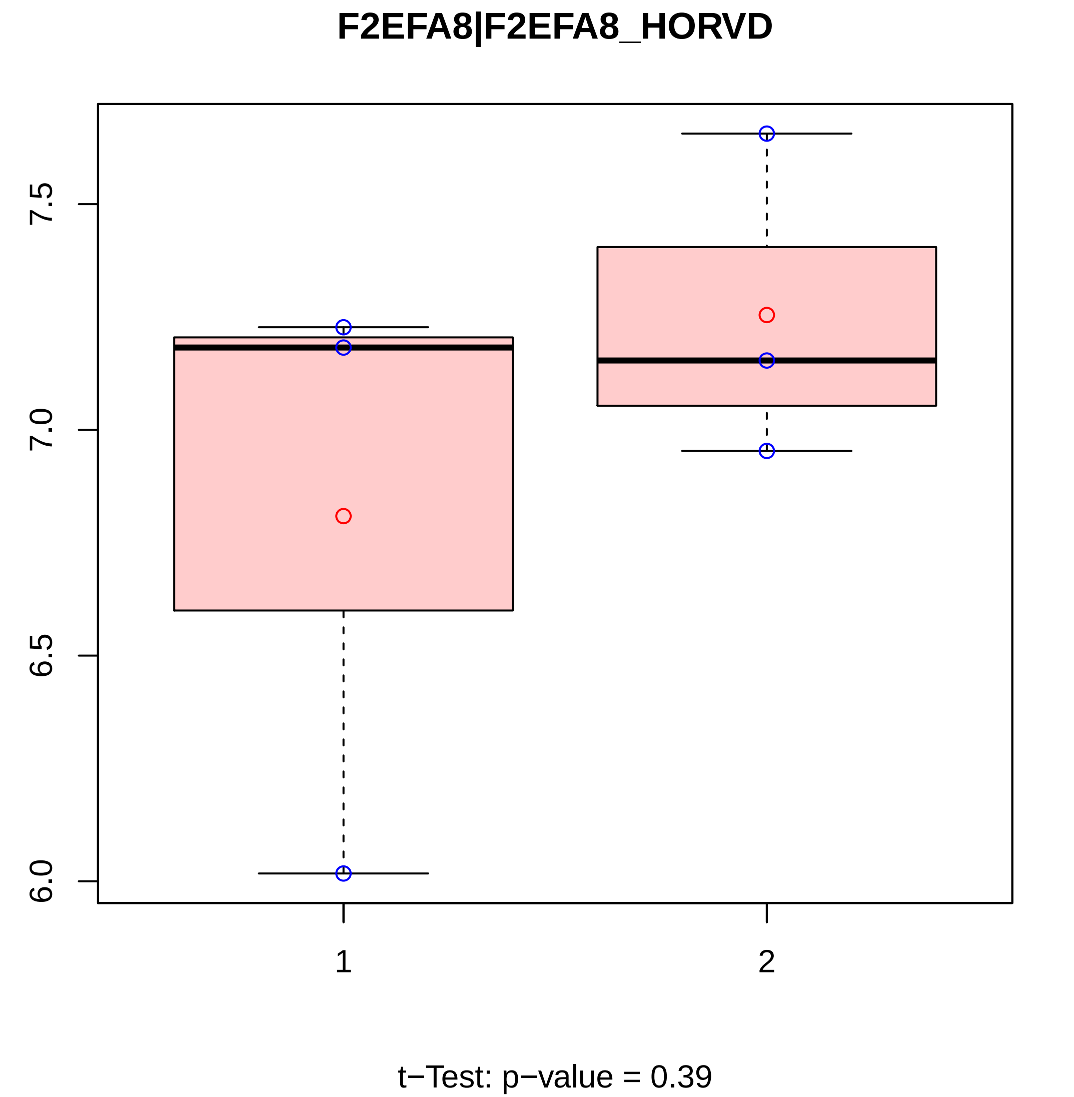
F2EFA8|F2EFA8_HORVD
7.5
7.0
6.5
6.0
1
2
t−Test: p−value = 0.39

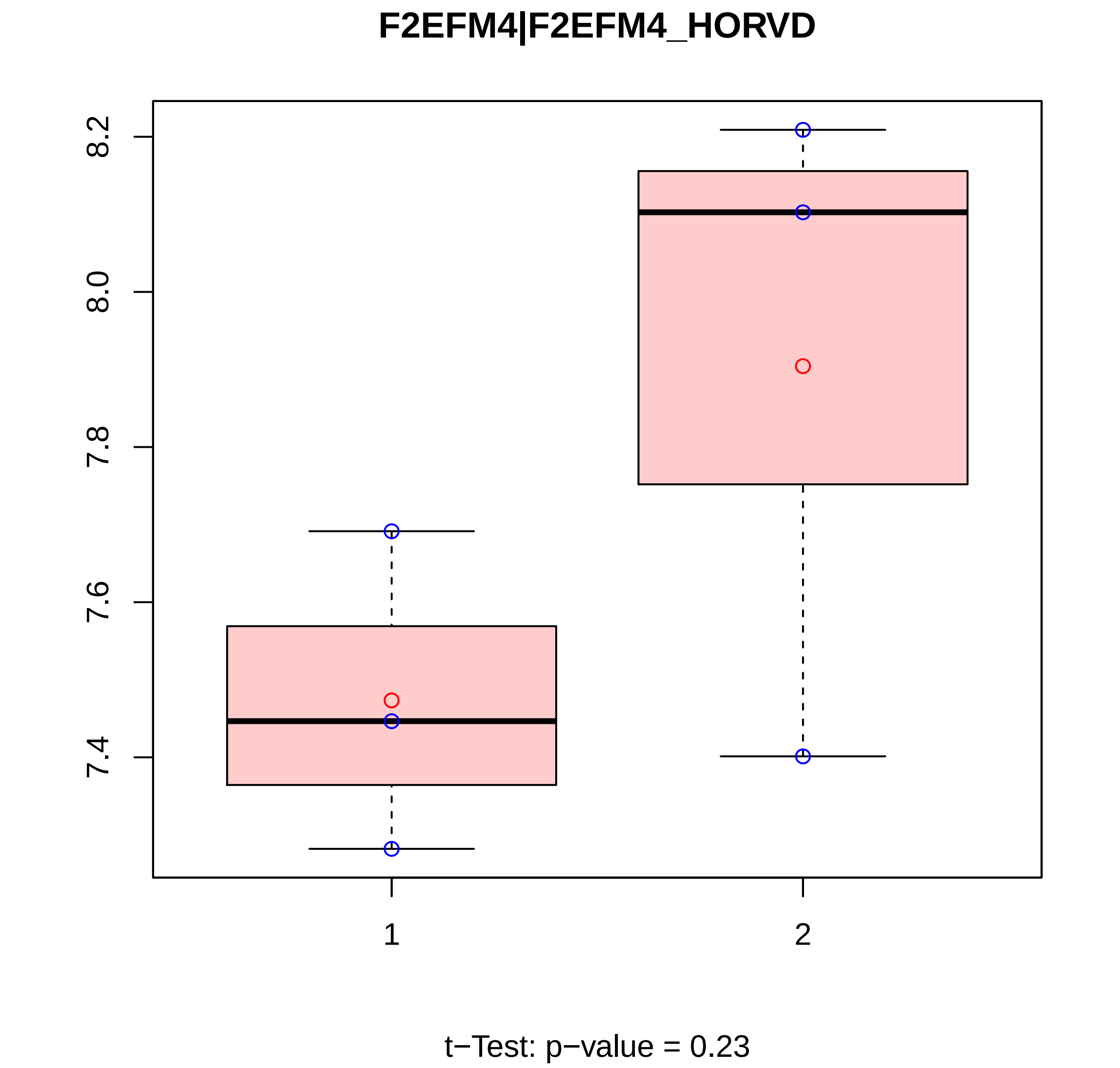
F2EFM4|F2EFM4_HORVD
8.2
8.0
7.8
7.6
7.4
1
2
t−Test: p−value = 0.23

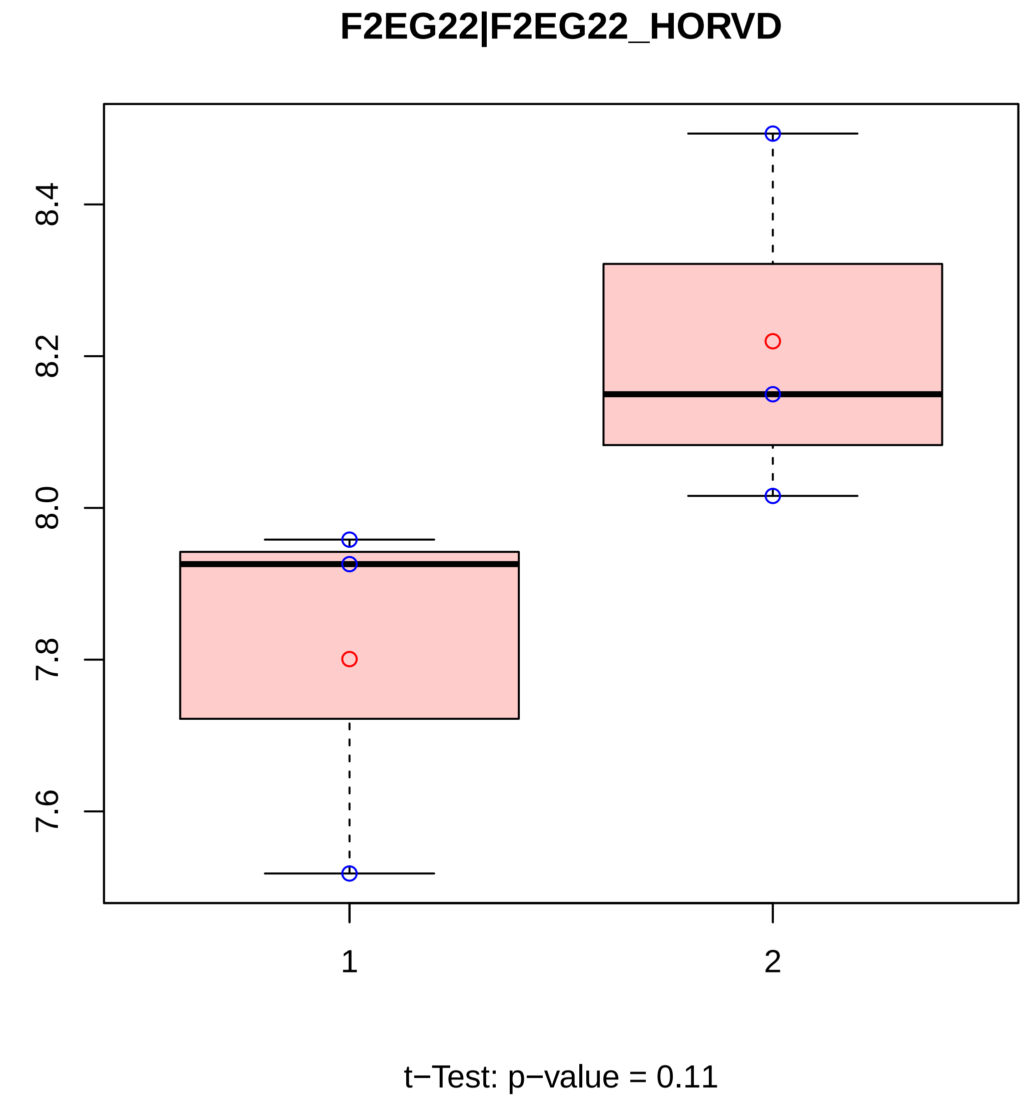
F2EG22|F2EG22_HORVD
7.6
7.8
8.0
8.2
8.4
1
2
t−Test: p−value = 0.11

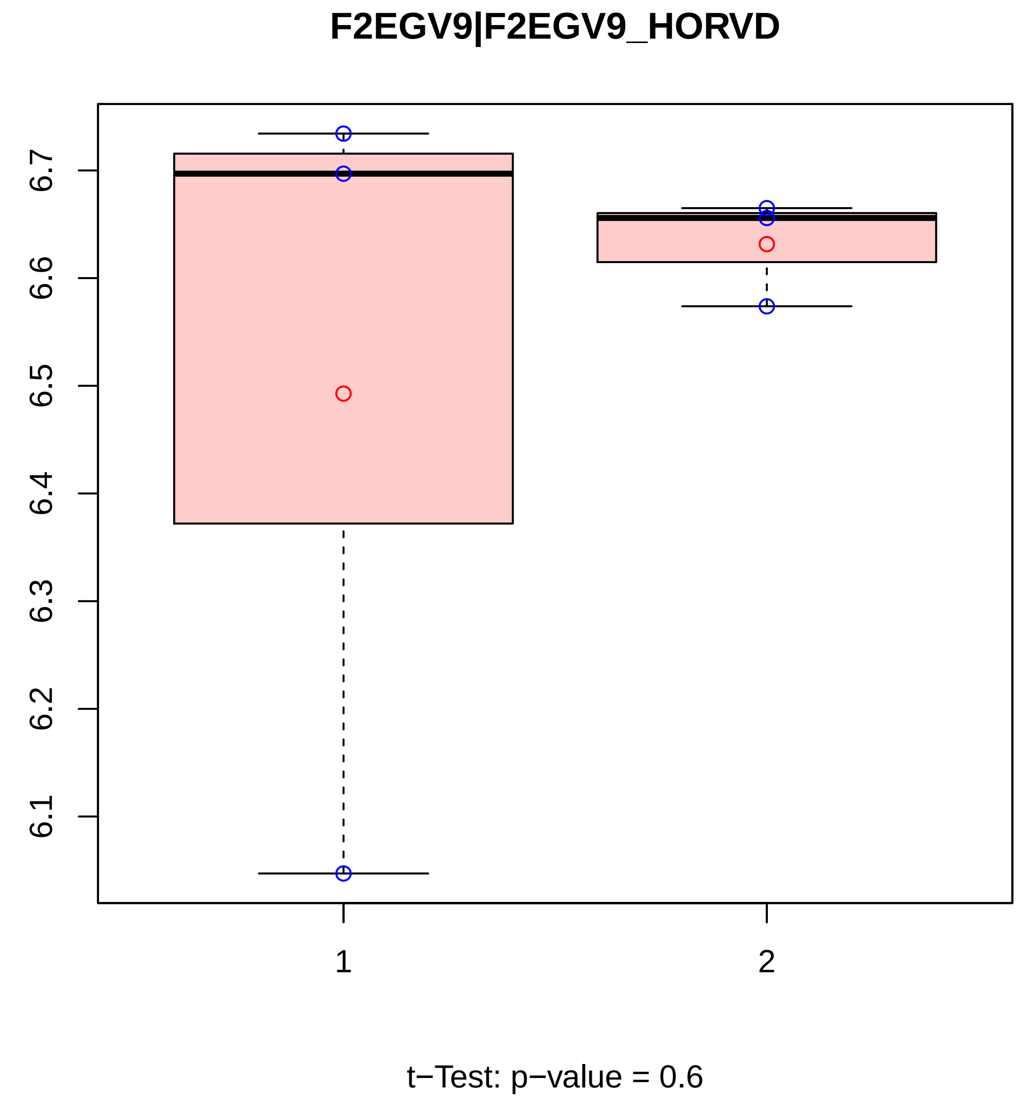
F2EGV9|F2EGV9_HORVD
6.1
6.2
6.3
6.4
6.5
6.6
6.7
1
2
t−Test: p−value = 0.6

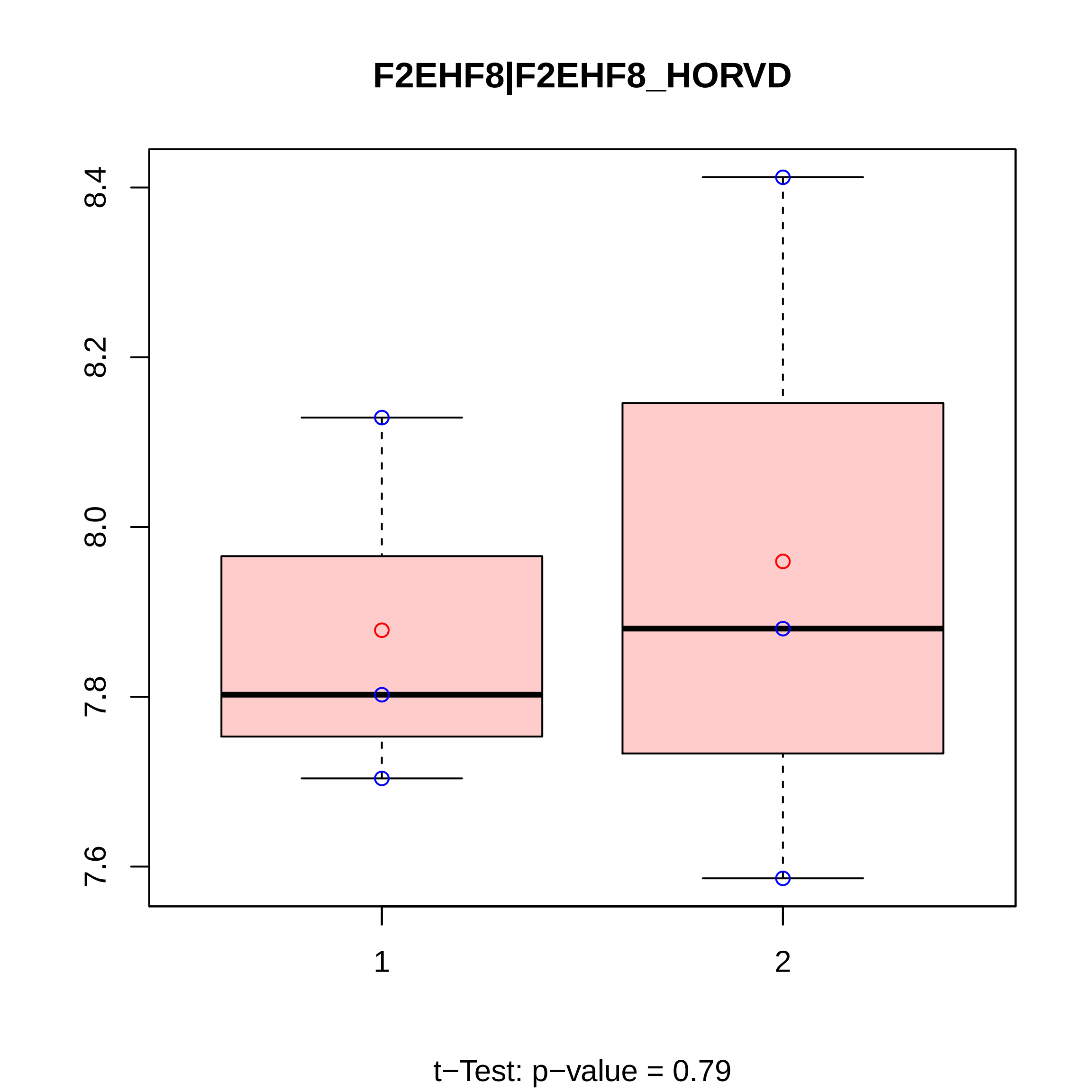

F2EHF8|F2EHF8_HORVD
8.4
8.2
8.0
7.8
7.6
1
2
t−Test: p−value = 0.79

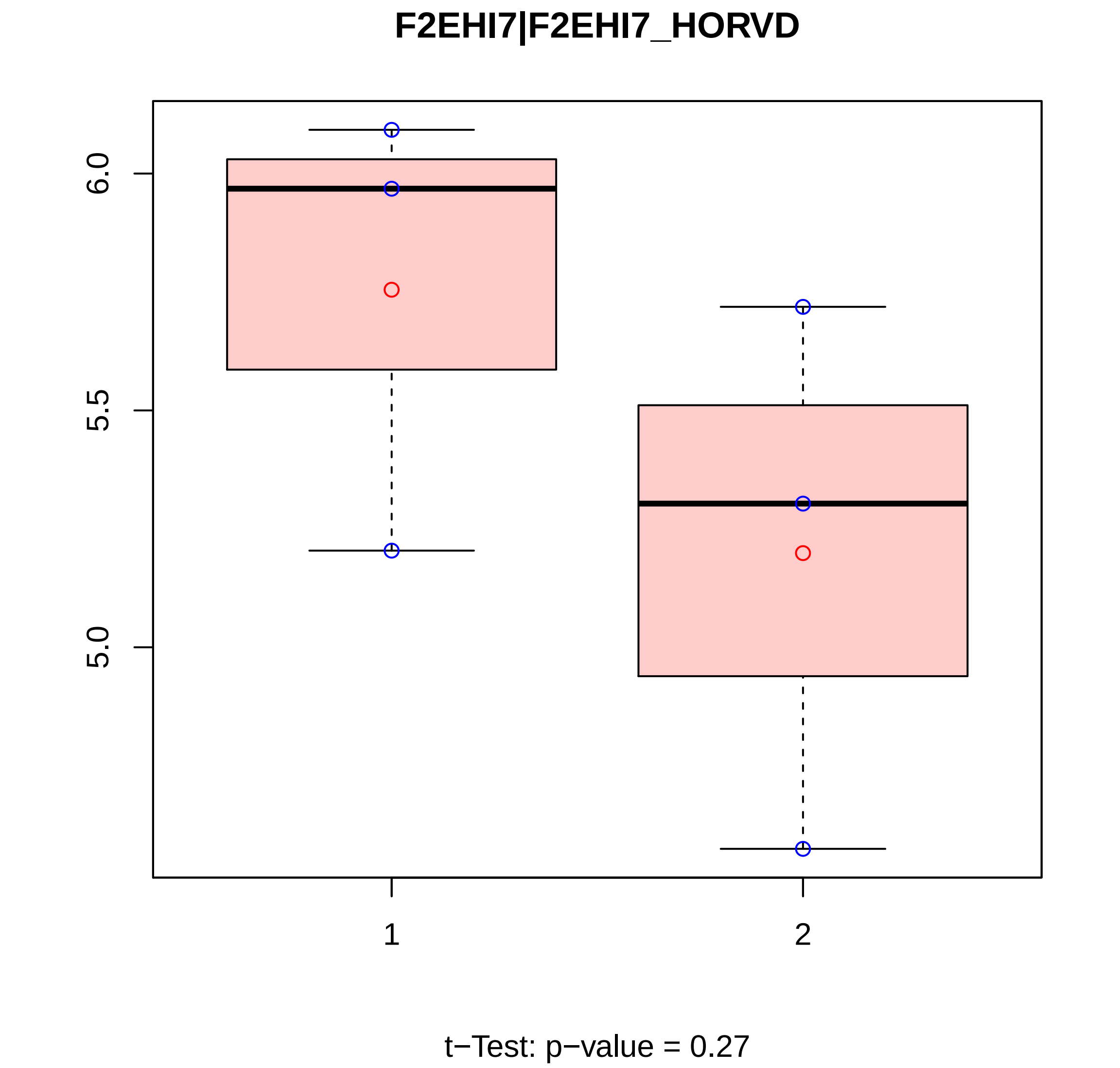
F2EHI7|F2EHI7_HORVD
6.0
5.5
5.0
1
2
t−Test: p−value = 0.27

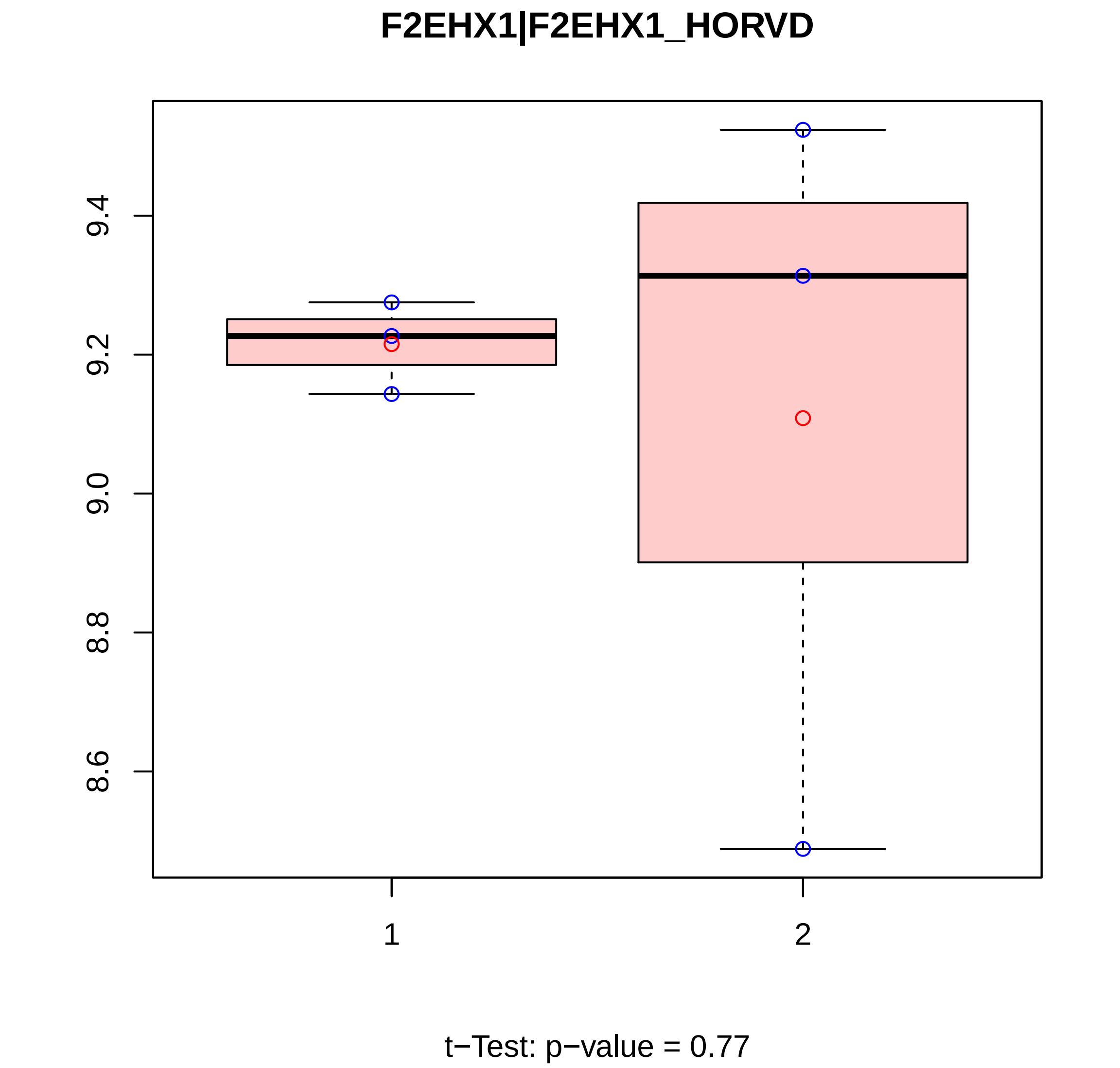
F2EHX1|F2EHX1_HORVD
9.4
9.2
9.0
8.8
8.6
1
2
t−Test: p−value = 0.77

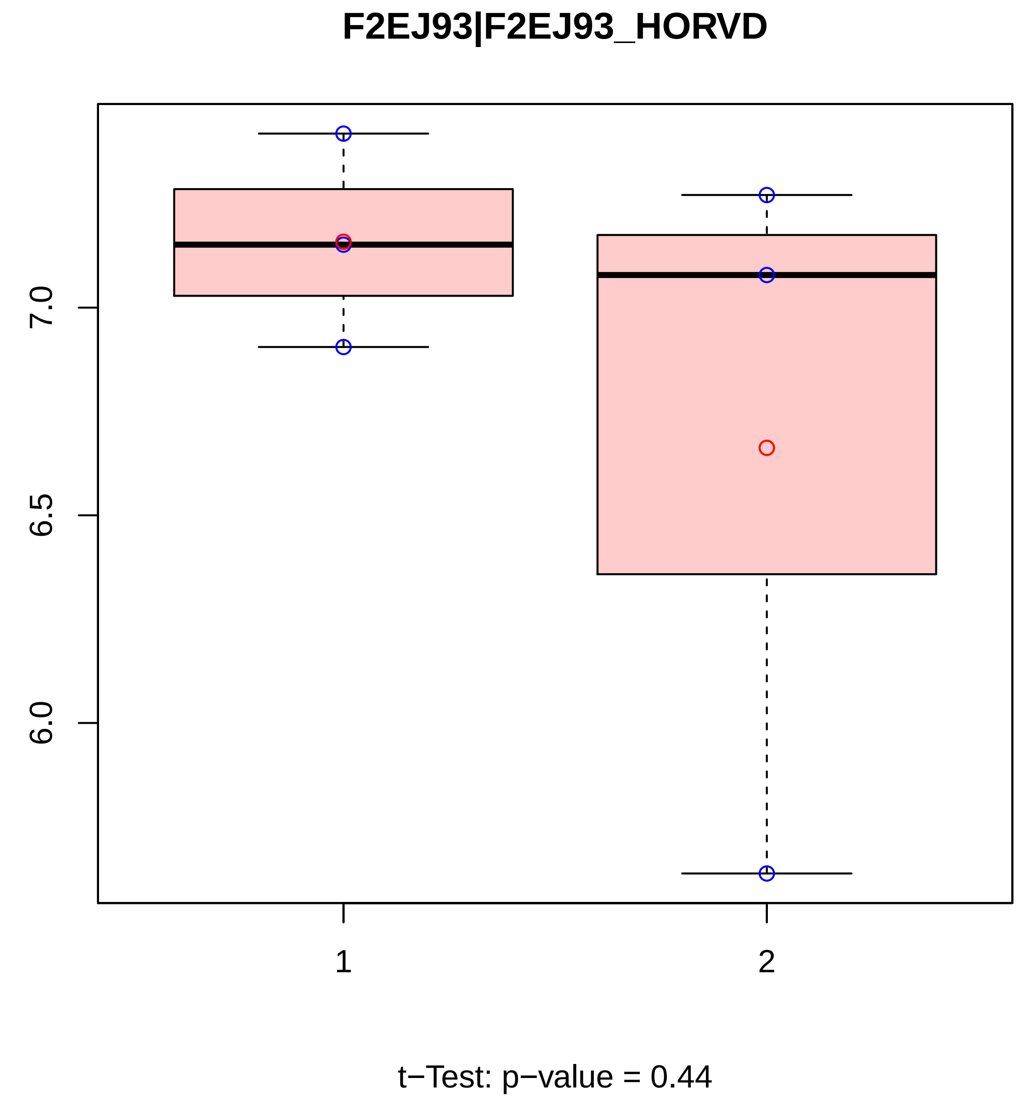
F2EJ93|F2EJ93_HORVD
7.0
6.5
6.0
1
2
t−Test: p−value = 0.44

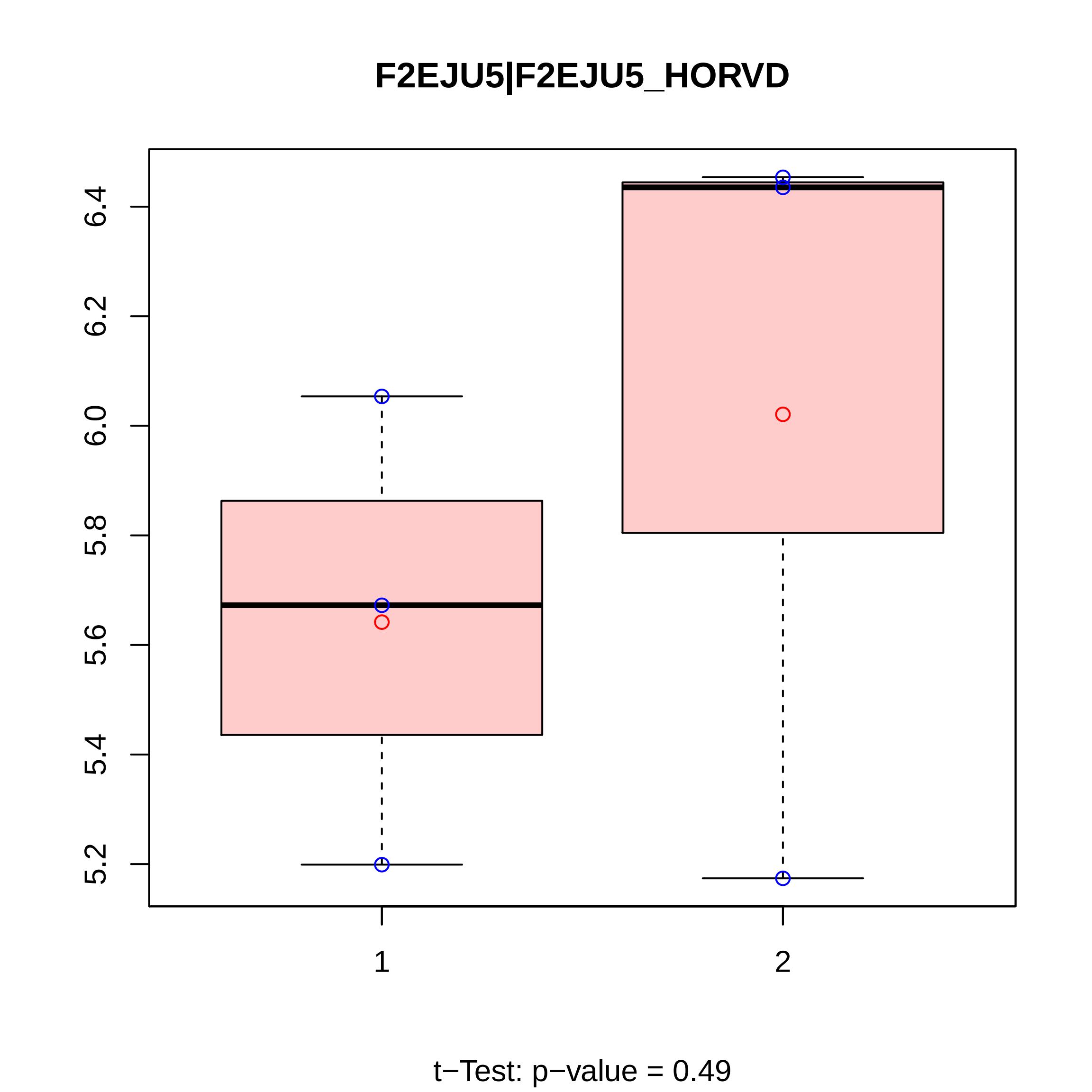

F2EJU5|F2EJU5_HORVD
6.4
6.2
6.0
5.8
5.6
5.4
5.2
1
2
t−Test: p−value = 0.49

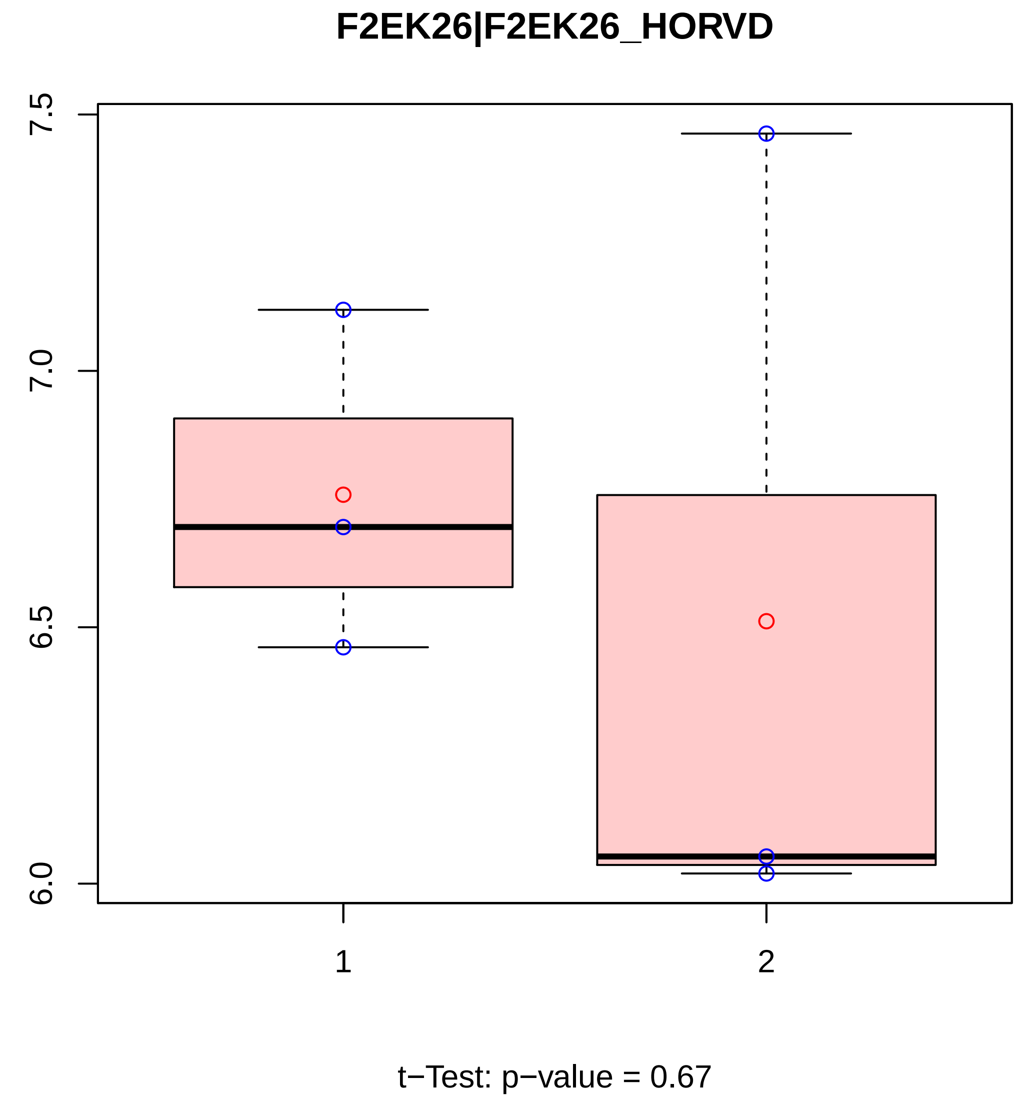
F2EK26|F2EK26_HORVD
7.5
7.0
6.5
6.0
1
2
t−Test: p−value = 0.67

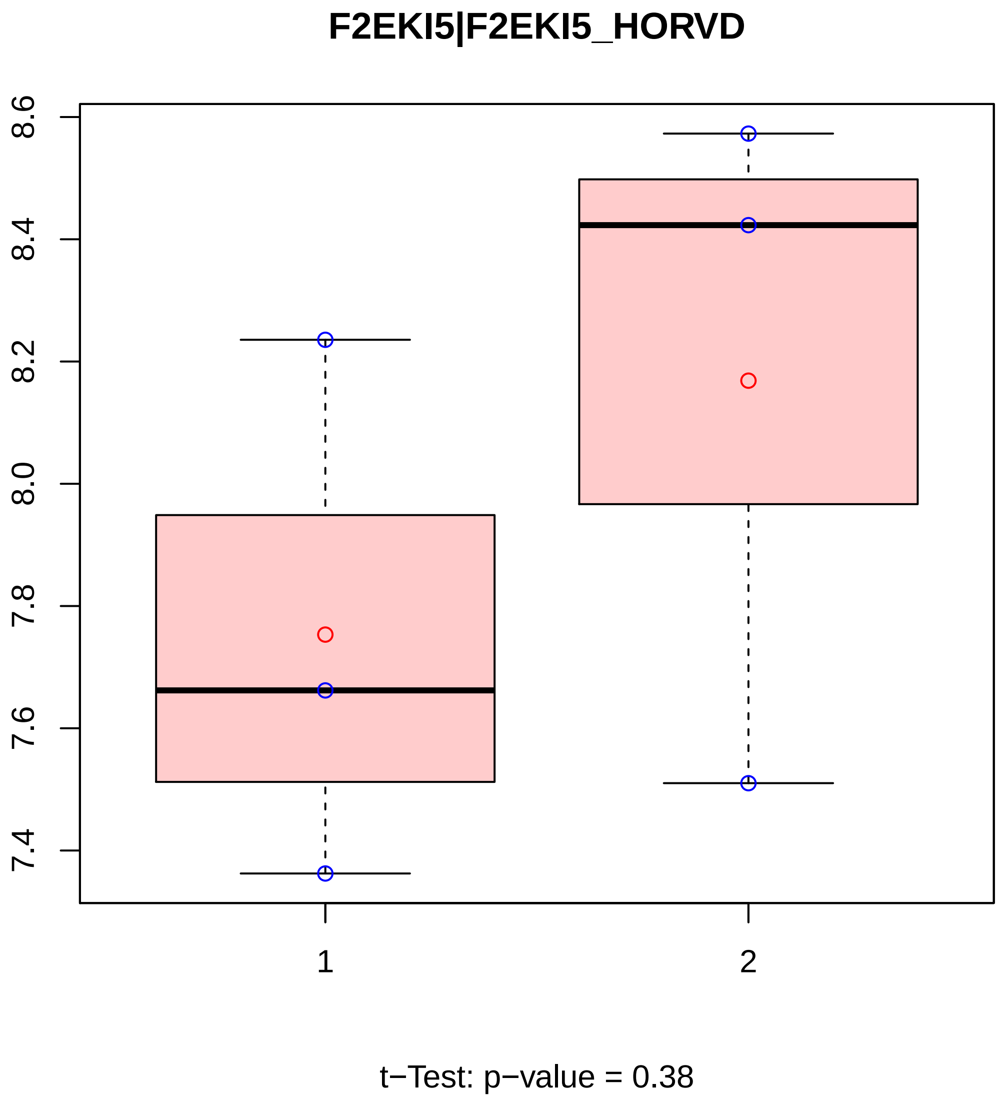
F2EKI5|F2EKI5_HORVD
8.6
8.4
8.2
8.0
7.8
7.6
7.4
1
2
t−Test: p−value = 0.38

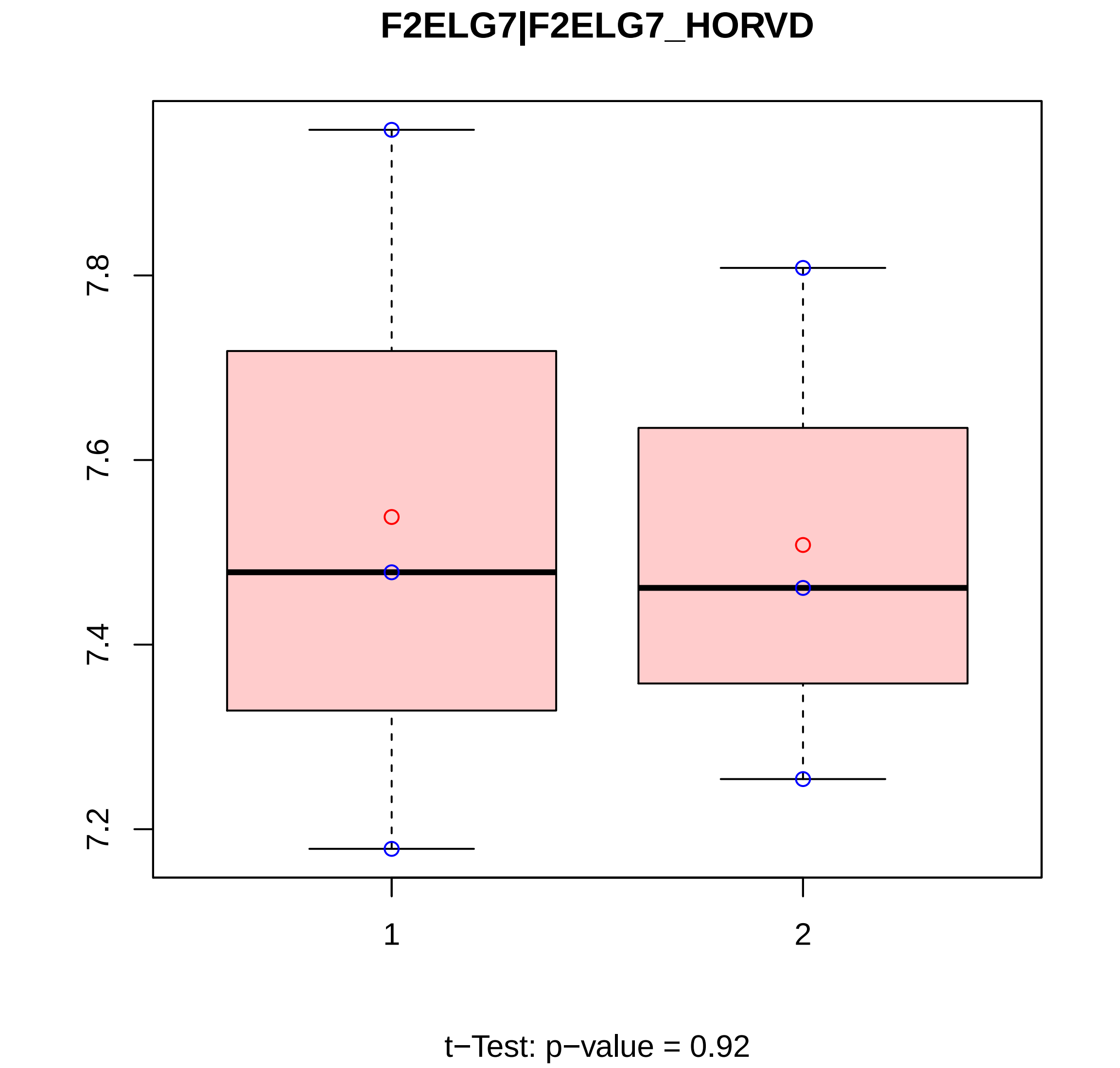

F2ELG7|F2ELG7_HORVD
7.2
7.4
7.6
7.8
1
2
t−Test: p−value = 0.92

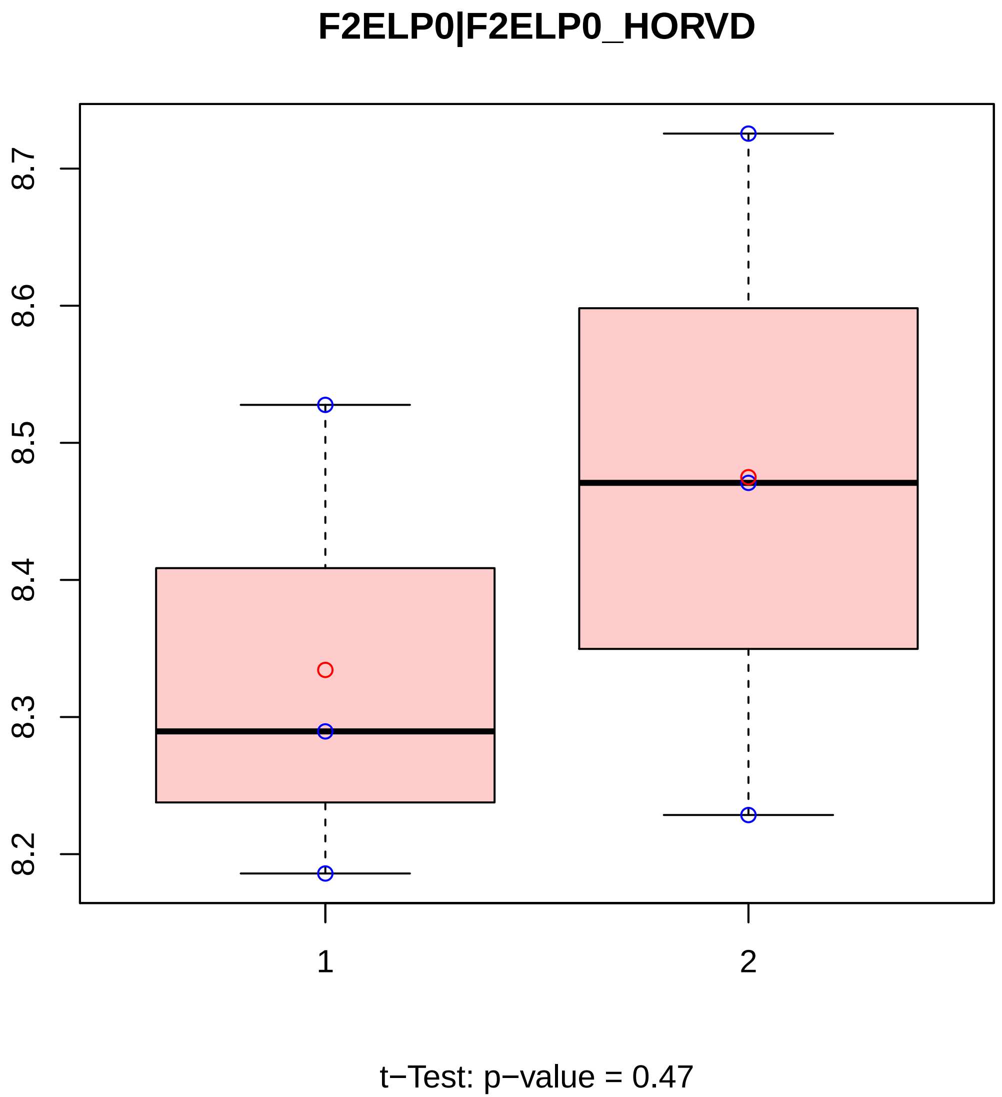
F2ELP0|F2ELP0_HORVD
8.2
8.3
8.4
8.5
8.6
8.7
1
2
t−Test: p−value = 0.47

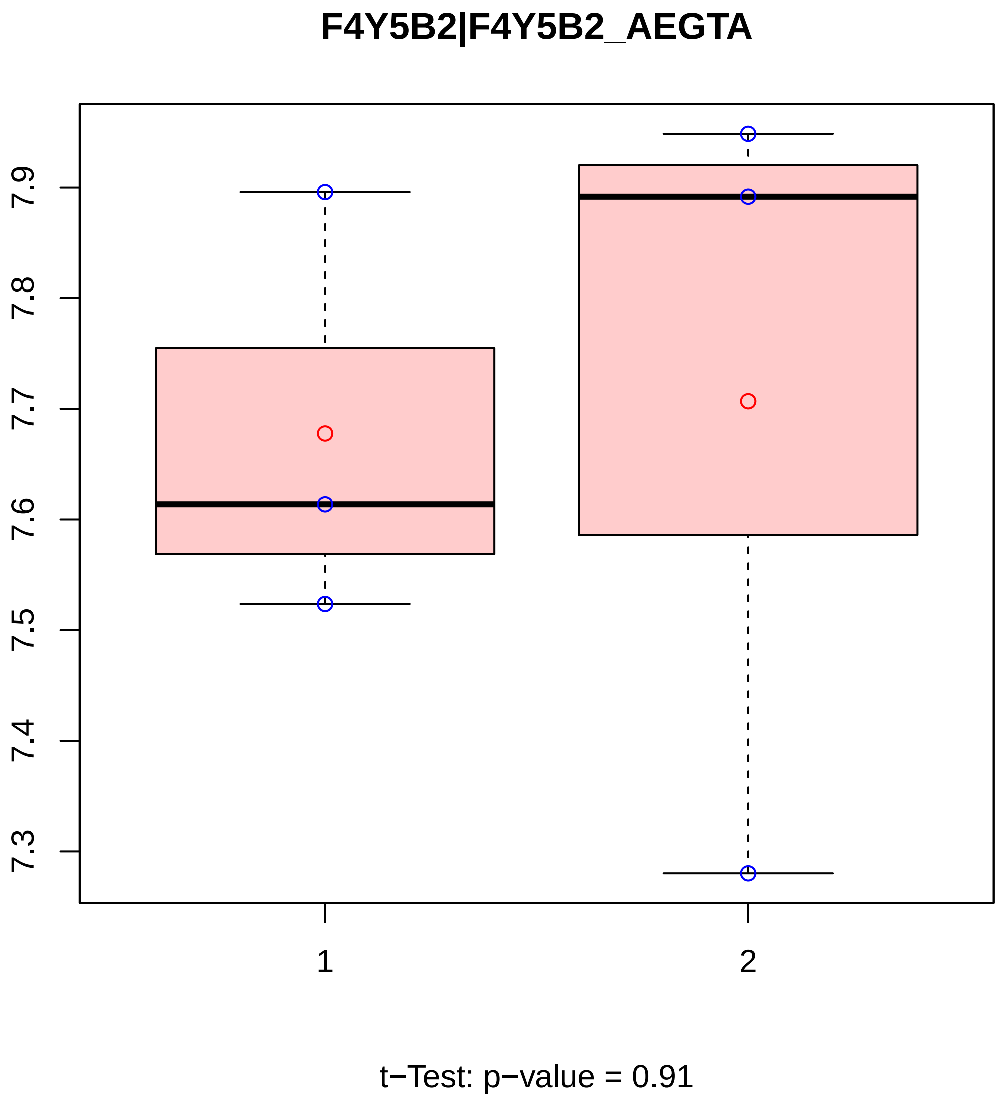

F4Y5B2|F4Y5B2_AEGTA
7.3
7.4
7.5
7.6
7.7
7.8
7.9
1
2
t−Test: p−value = 0.91

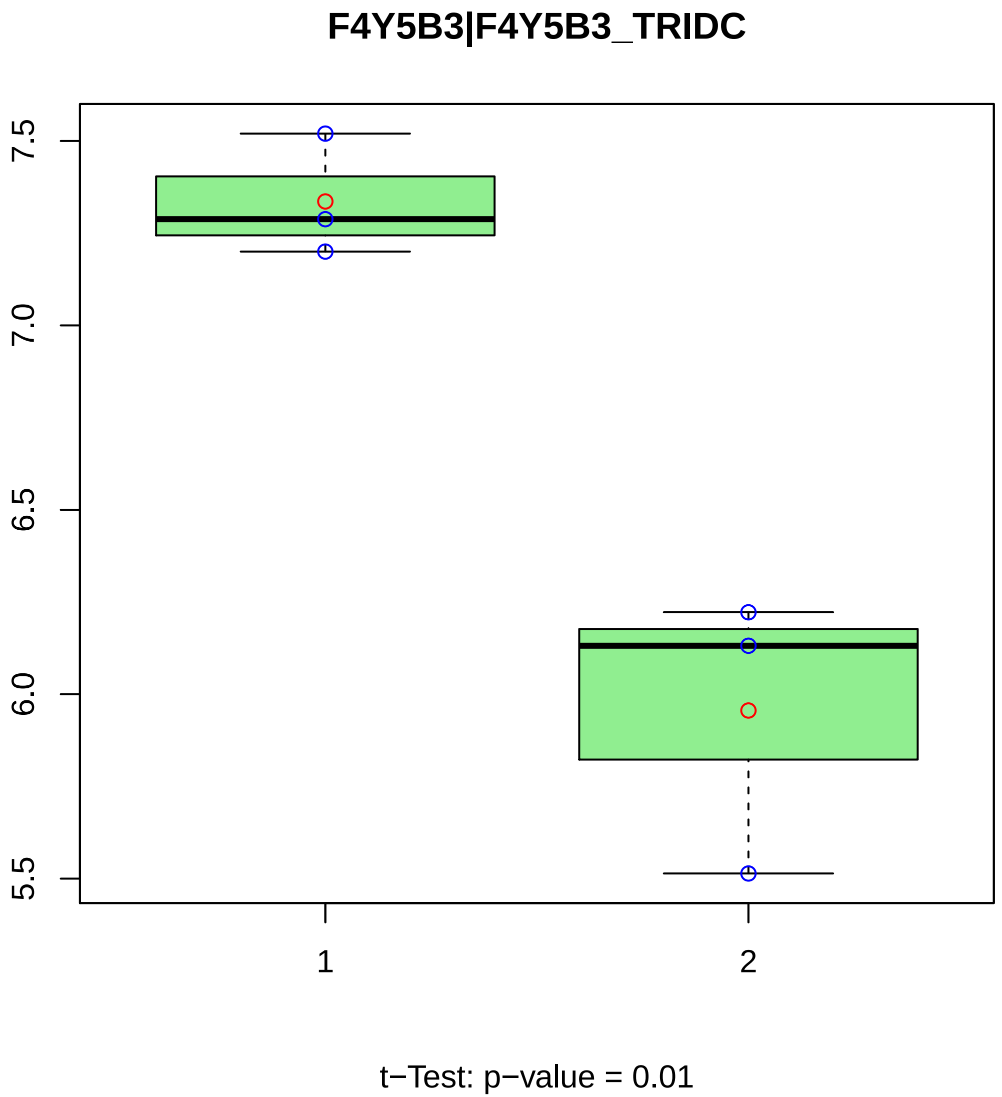
F4Y5B3|F4Y5B3_TRIDC
7.5
7.0
6.5
6.0
5.5
1
2
t−Test: p−value = 0.01

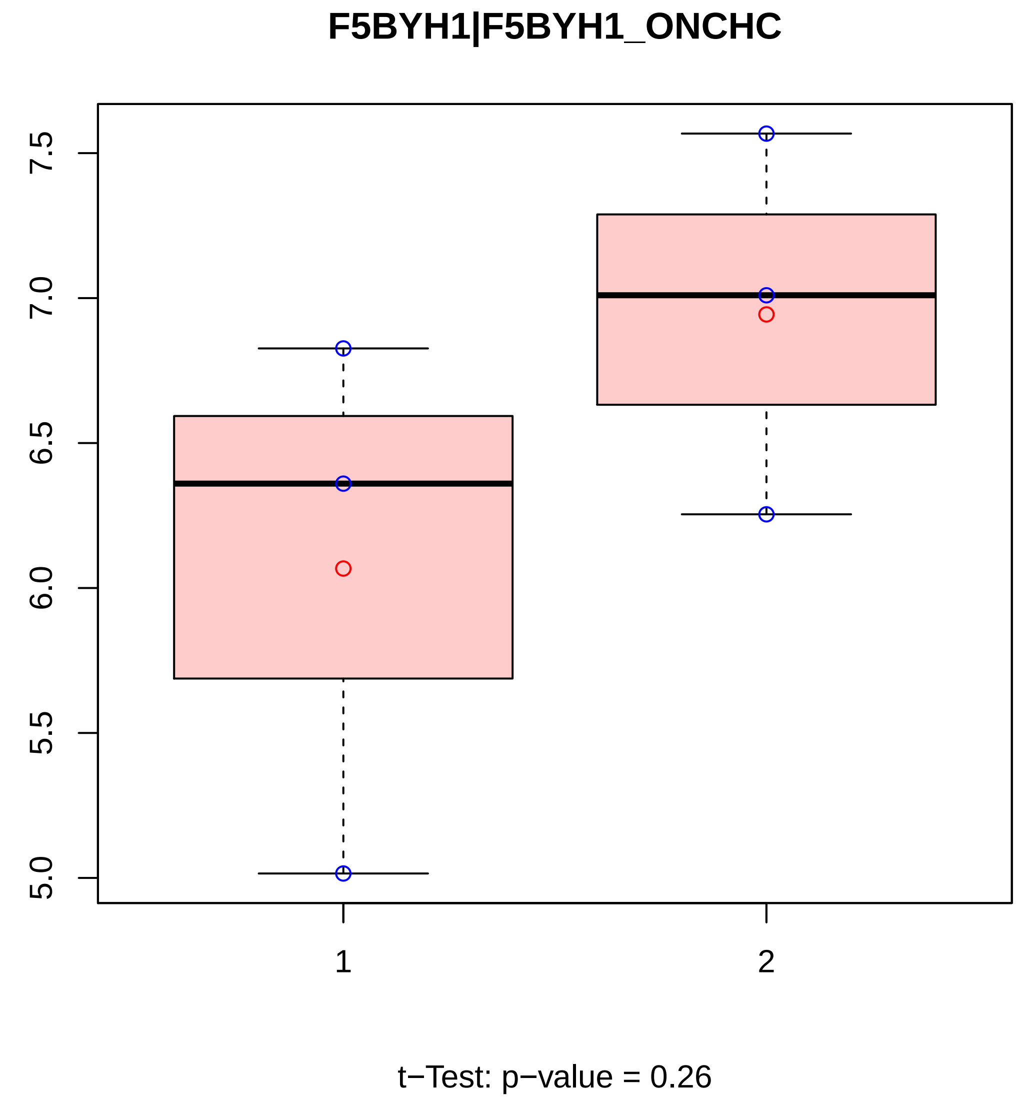

F5BYH1|F5BYH1_ONCHC
7.5
7.0
6.5
6.0
5.5
5.0
1
2
t−Test: p−value = 0.26

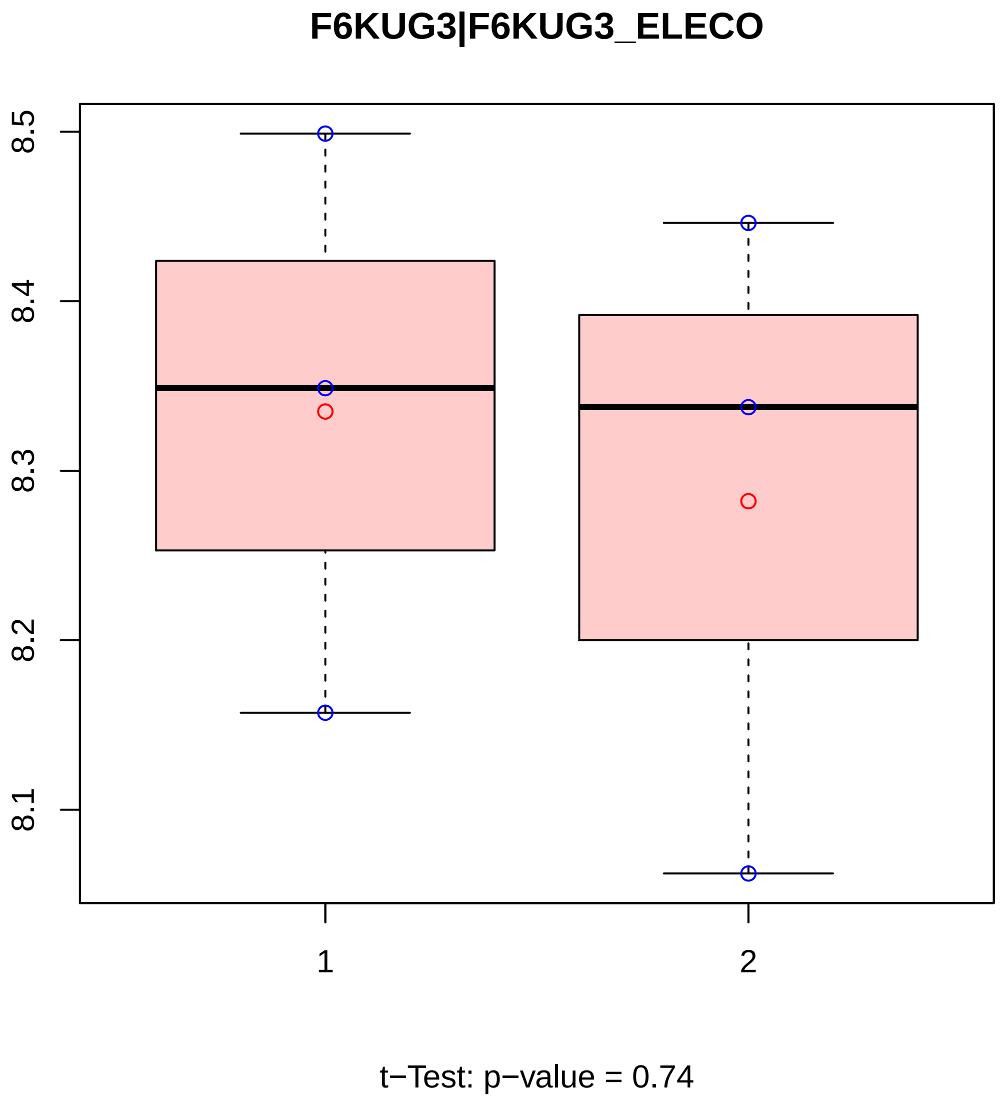

F6KUG3|F6KUG3_ELECO
8.5
8.4
8.3
8.2
8.1
1
2
t−Test: p−value = 0.74

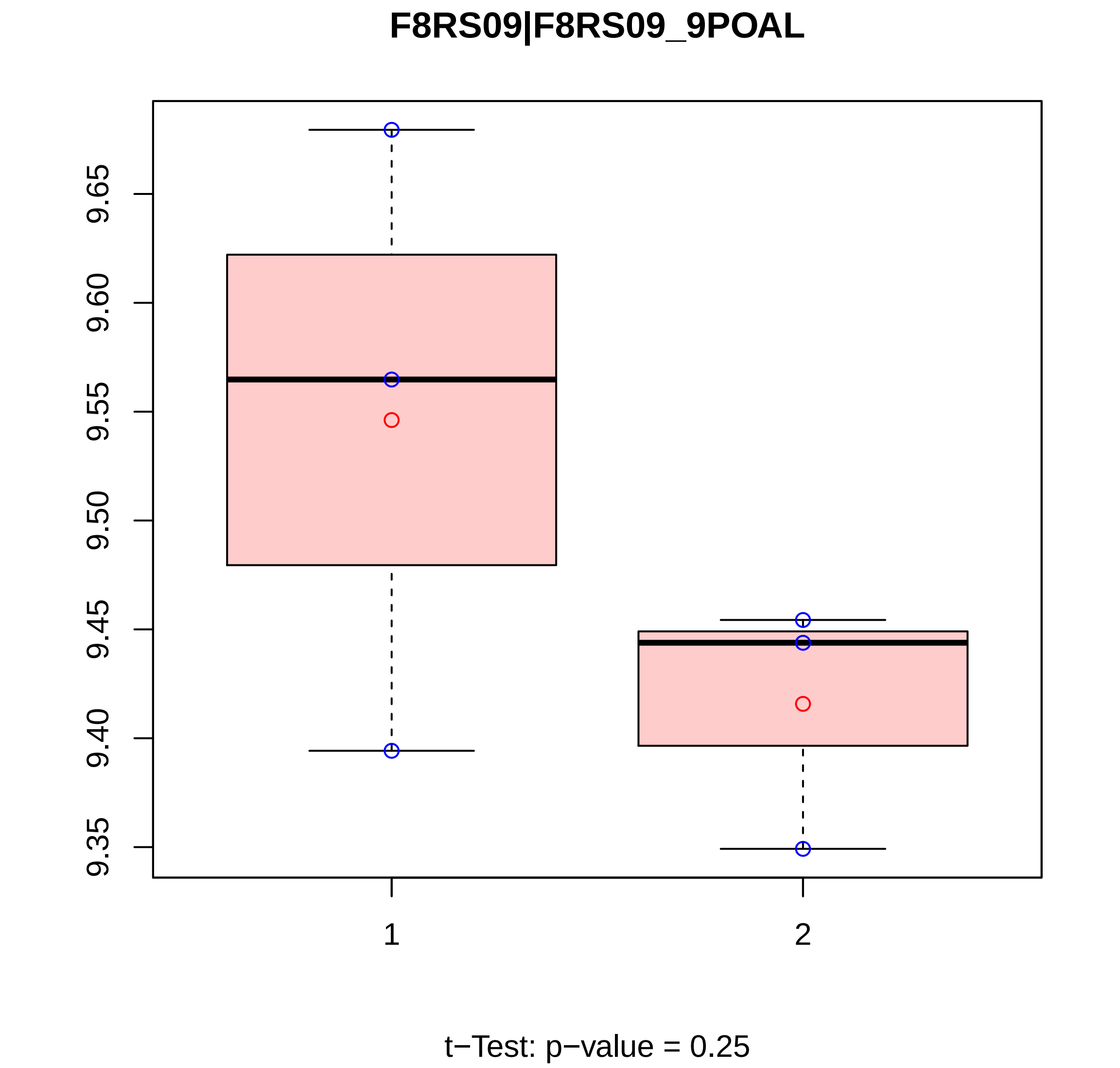
F8RS09|F8RS09_9POAL
9.35
9.40
9.45
9.50
9.55
9.60
9.65
1
2
t−Test: p−value = 0.25

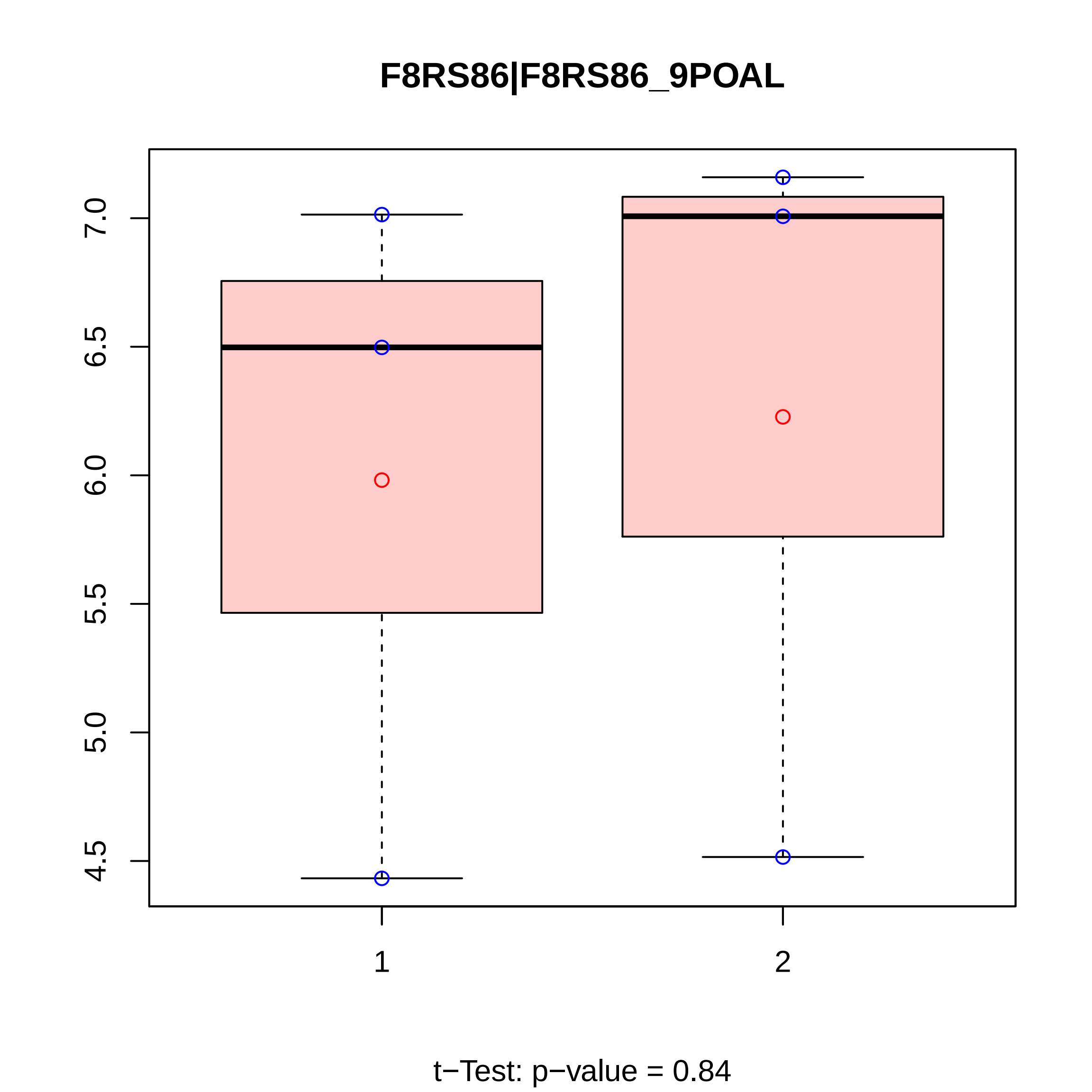

F8RS86|F8RS86_9POAL
7.0
6.5
6.0
5.5
5.0
4.5
1
2
t−Test: p−value = 0.84

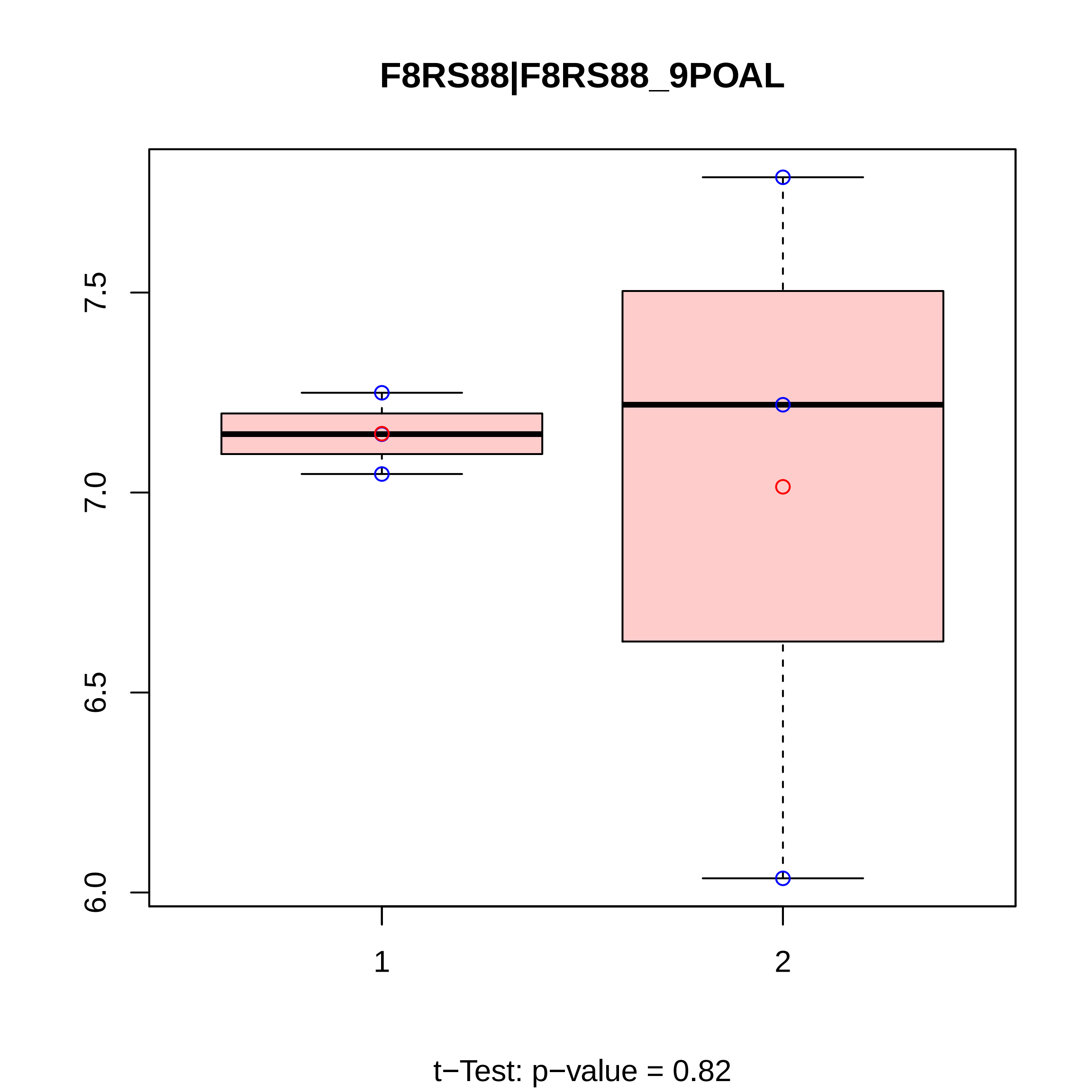
F8RS88|F8RS88_9POAL
7.5
7.0
6.5
6.0
1
2
t−Test: p−value = 0.82

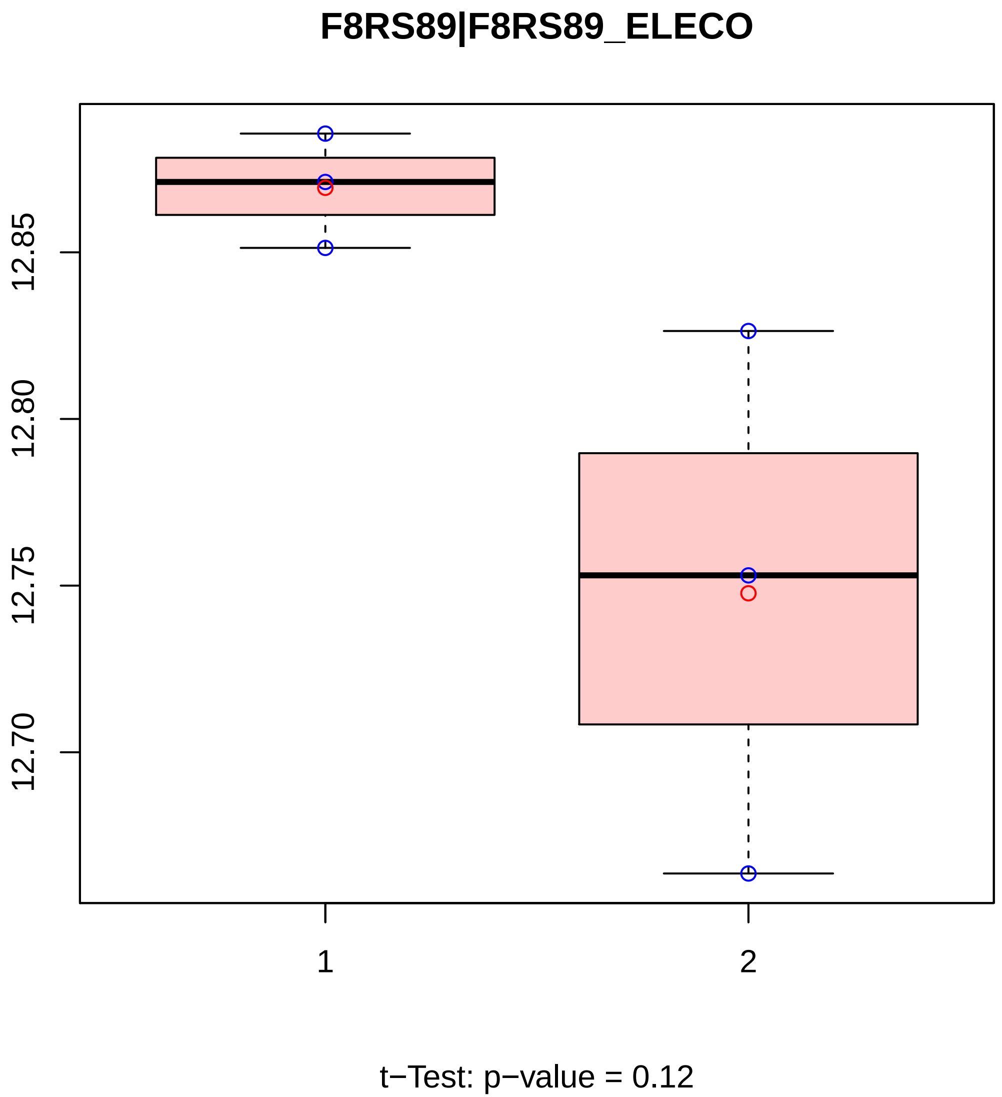
F8RS89|F8RS89_ELECO
12.70
12.75
12.80
12.85
1
2
t−Test: p−value = 0.12

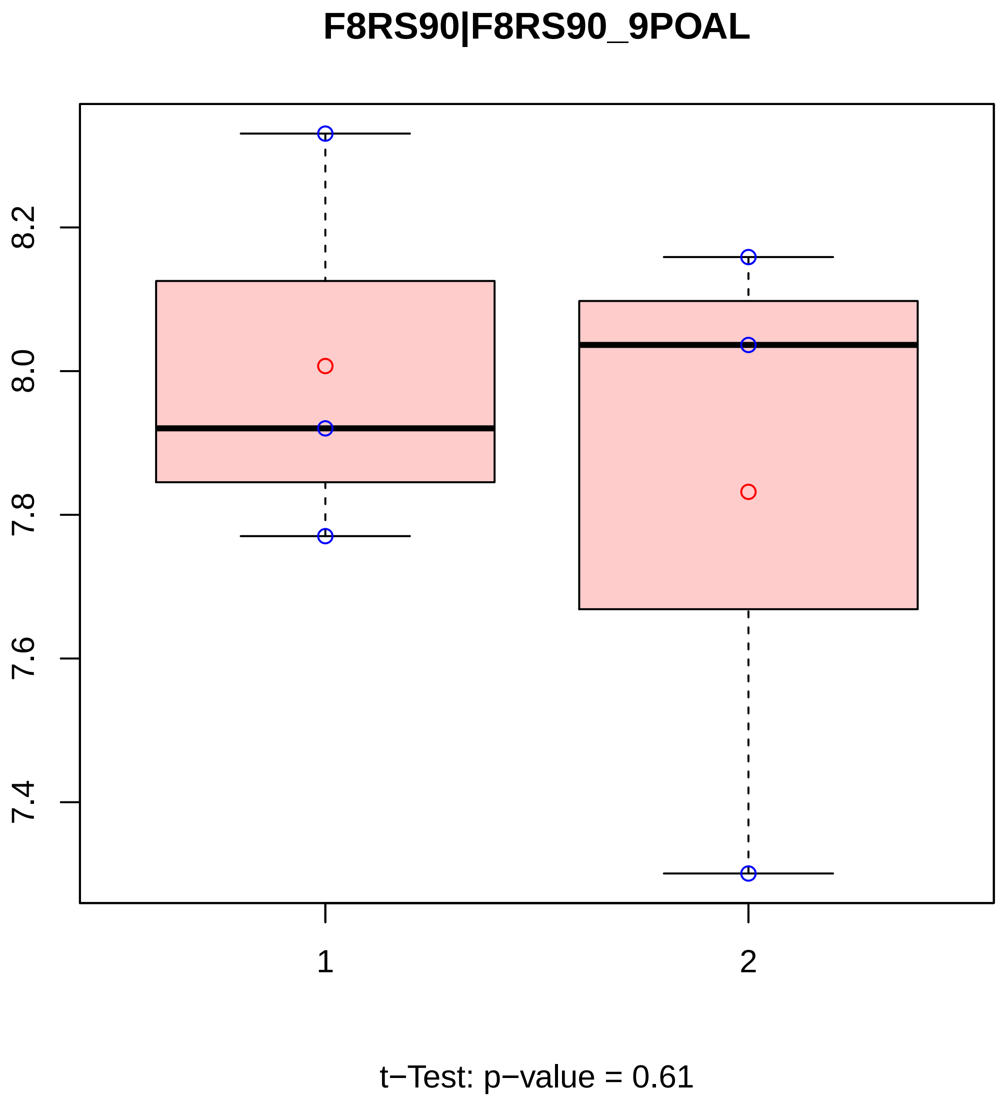
F8RS90|F8RS90_9POAL
8.2
8.0
7.8
7.6
7.4
1
2
t−Test: p−value = 0.61

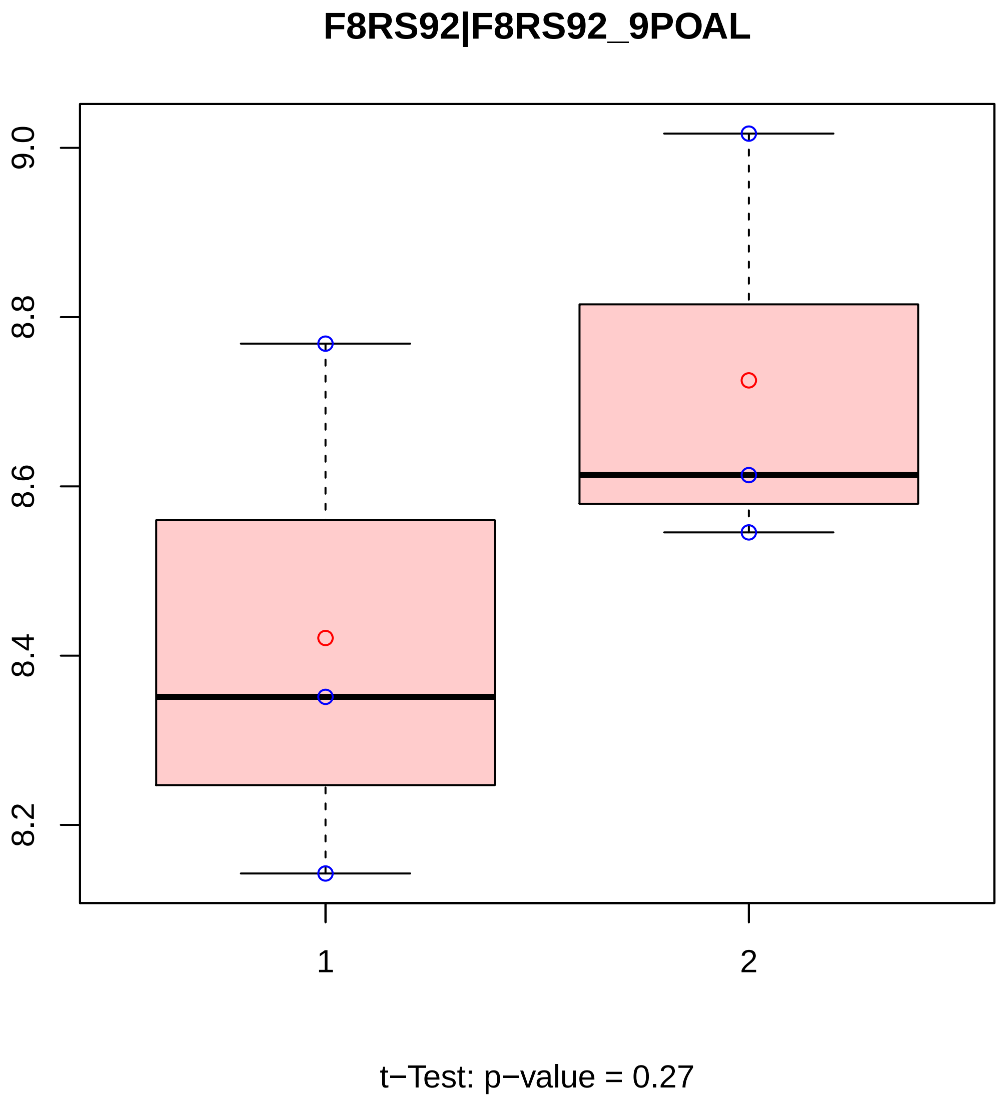
F8RS92|F8RS92_9POAL
9.0
8.8
8.6
8.4
8.2
1
2
t−Test: p−value = 0.27

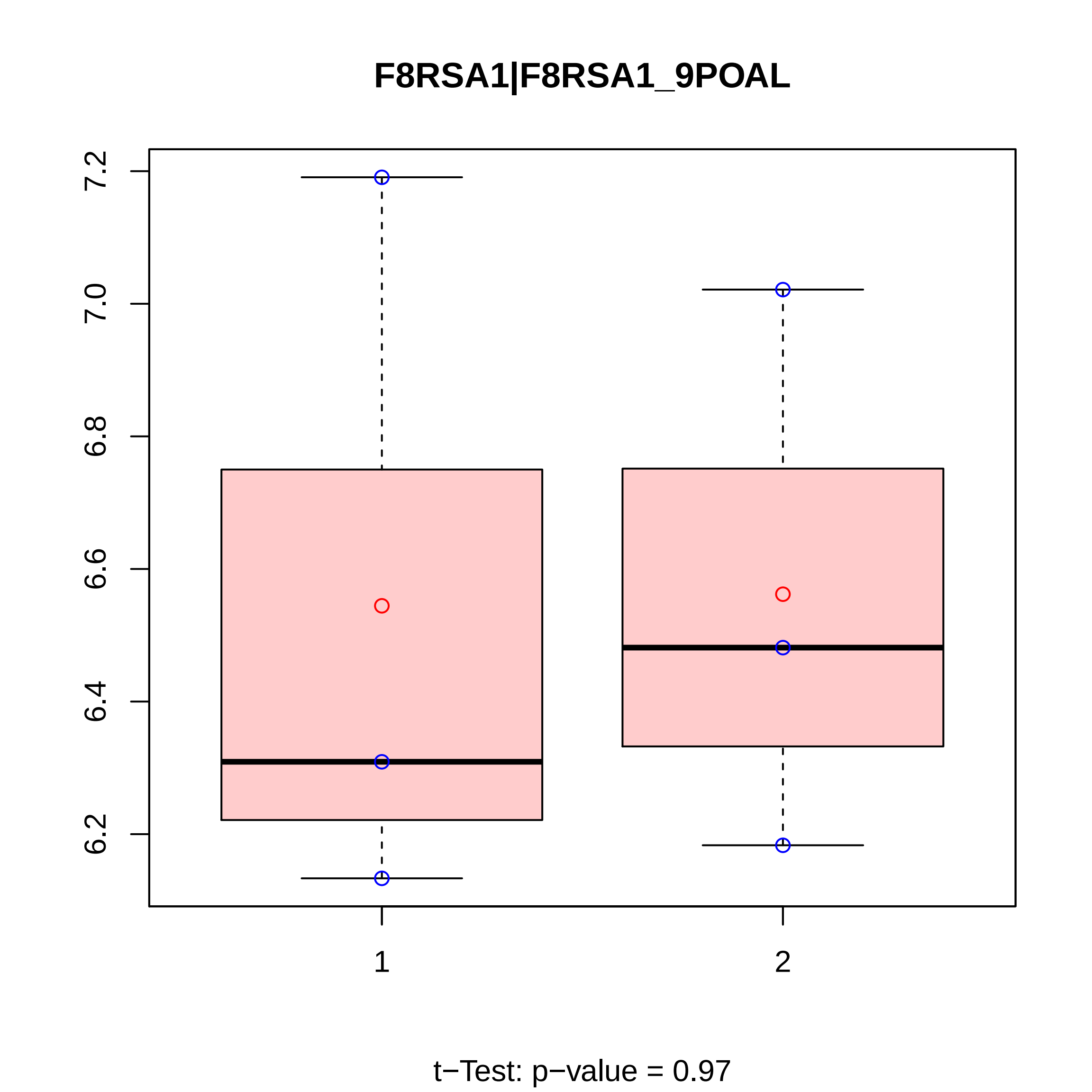

F8RSA1|F8RSA1_9POAL
7.2
7.0
6.8
6.6
6.4
6.2
1
2
t−Test: p−value = 0.97

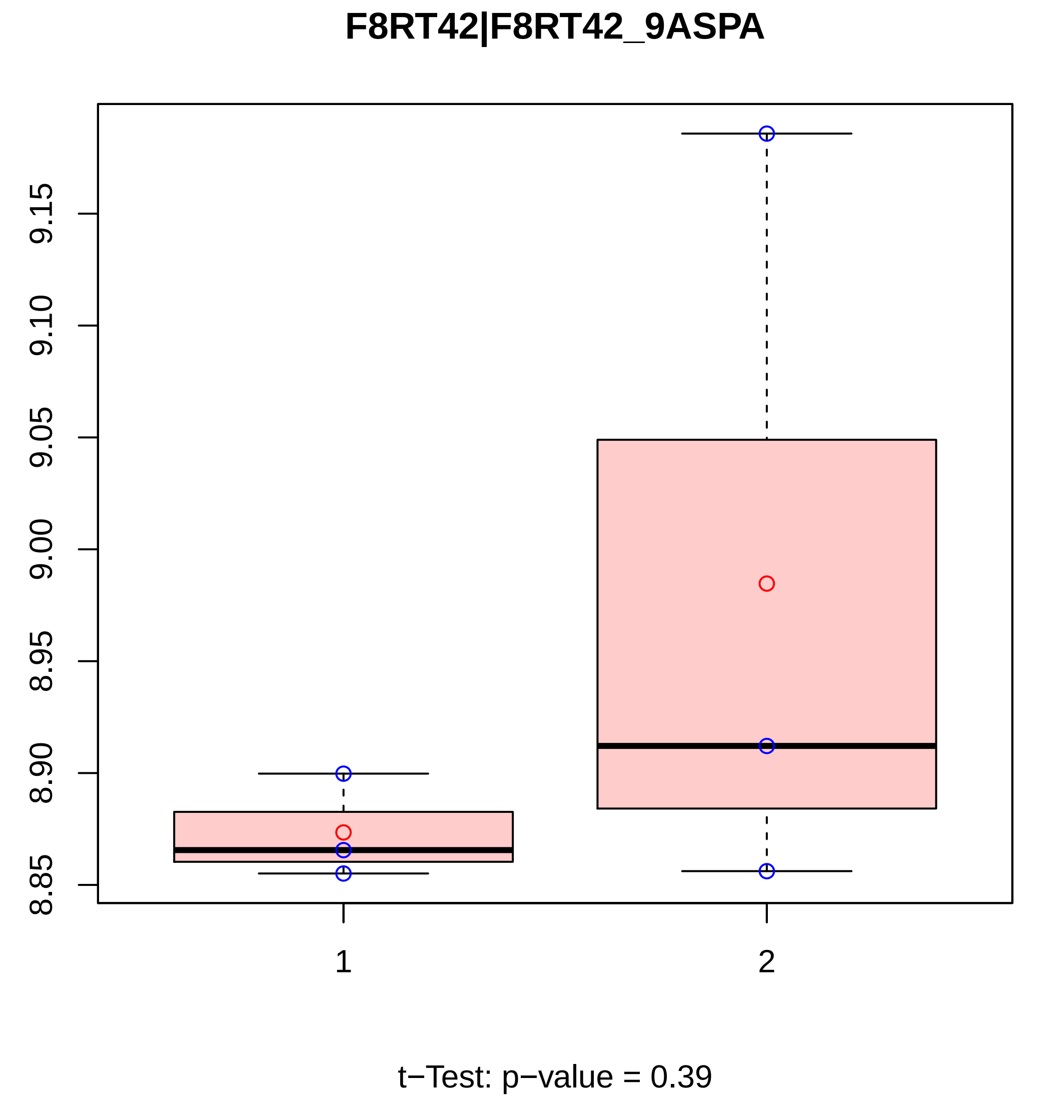
F8RT42|F8RT42_9ASPA
8.85
8.90
8.95
9.00
9.05
9.10
9.15
1
2
t−Test: p−value = 0.39

F8RT59|F8RT59_9LILI

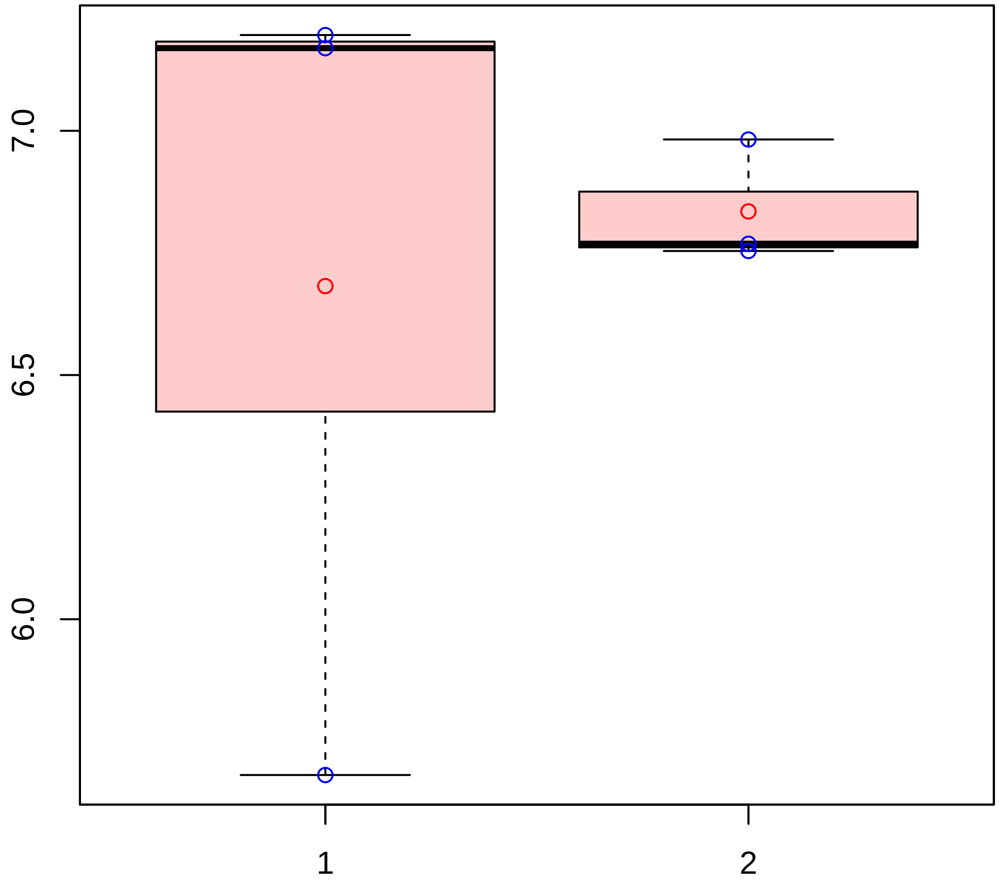

t−Test: p−value = 0.79

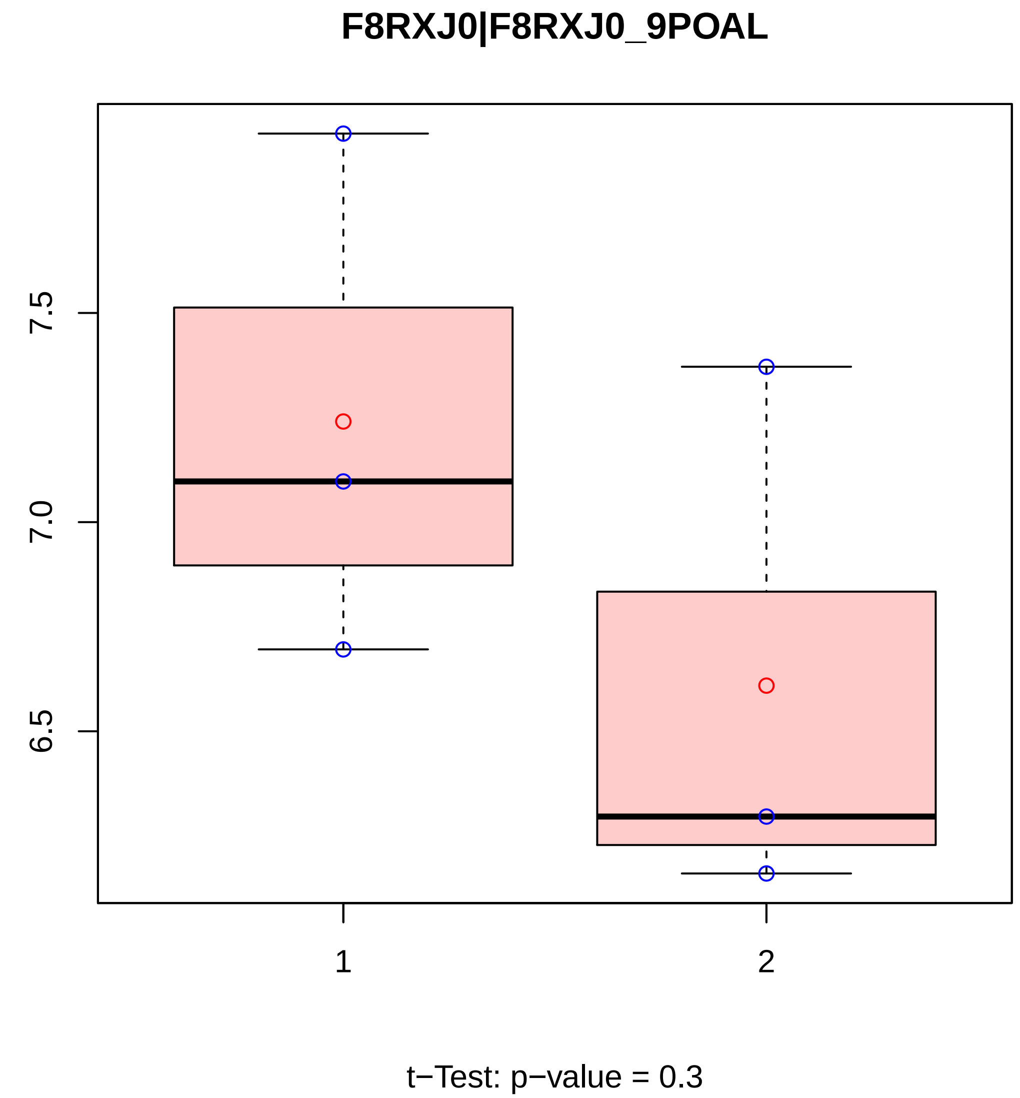
F8RXJ0|F8RXJ0_9POAL
7.5
7.0
6.5
1
2
t−Test: p−value = 0.3

F8RYE6|F8RYE6_9POAL

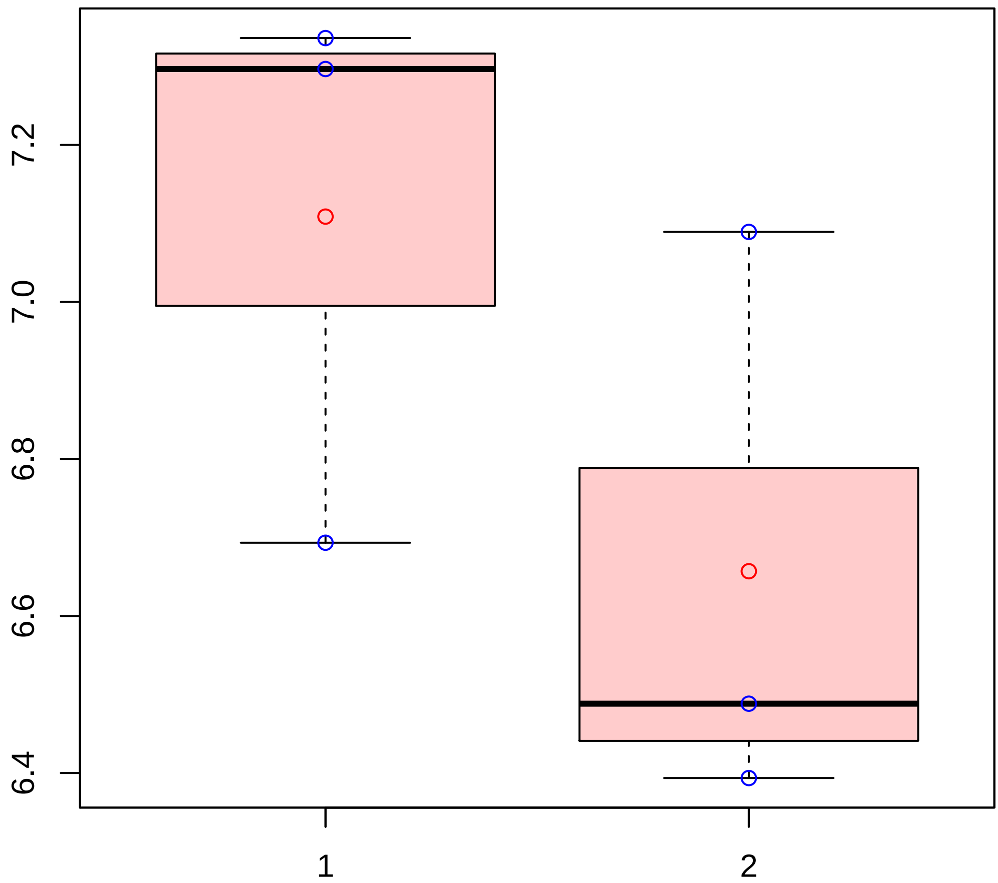

t−Test: p−value = 0.21

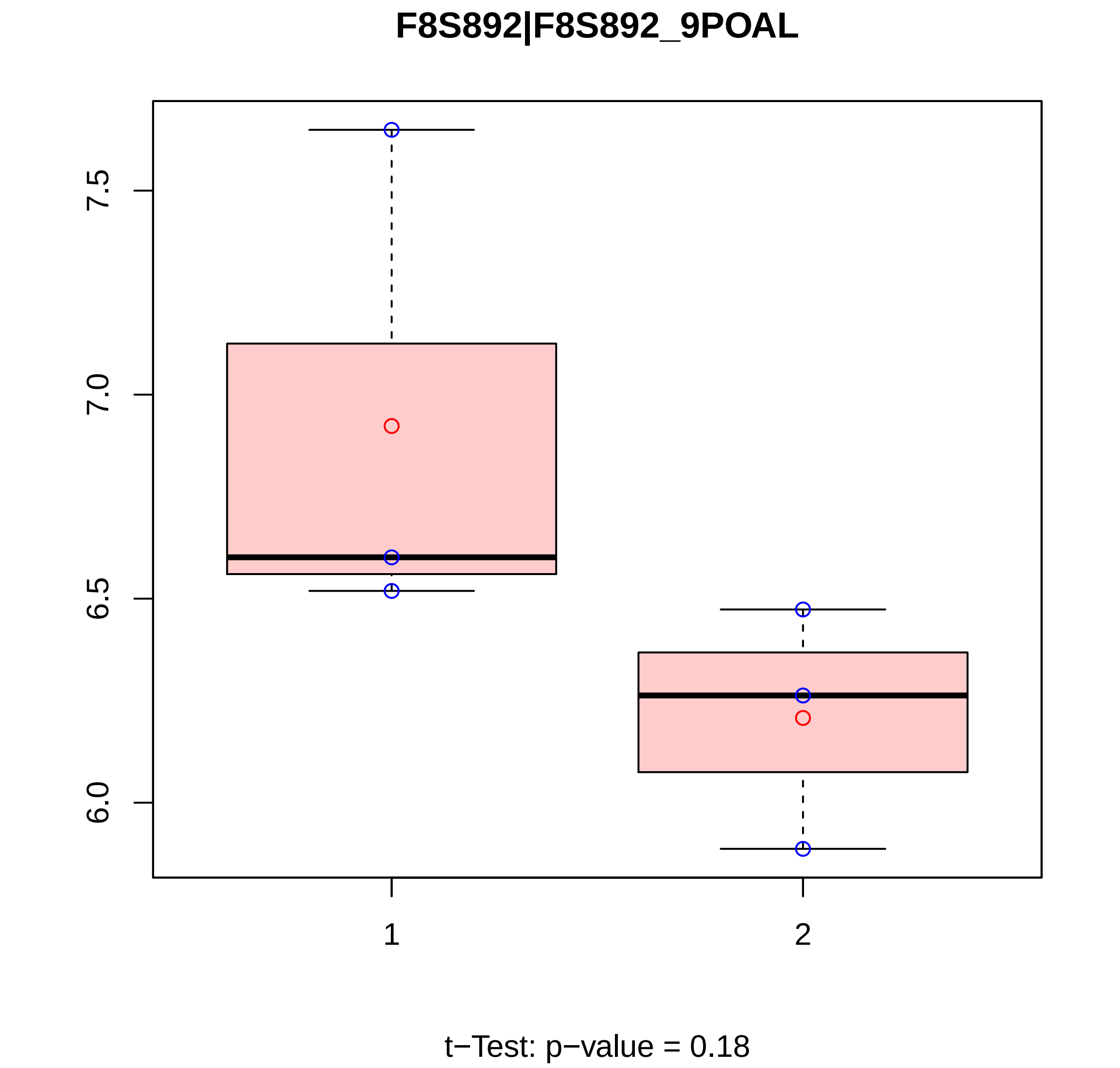
F8S892|F8S892_9POAL
6.0
6.5
7.0
7.5
1
2
t−Test: p−value = 0.18

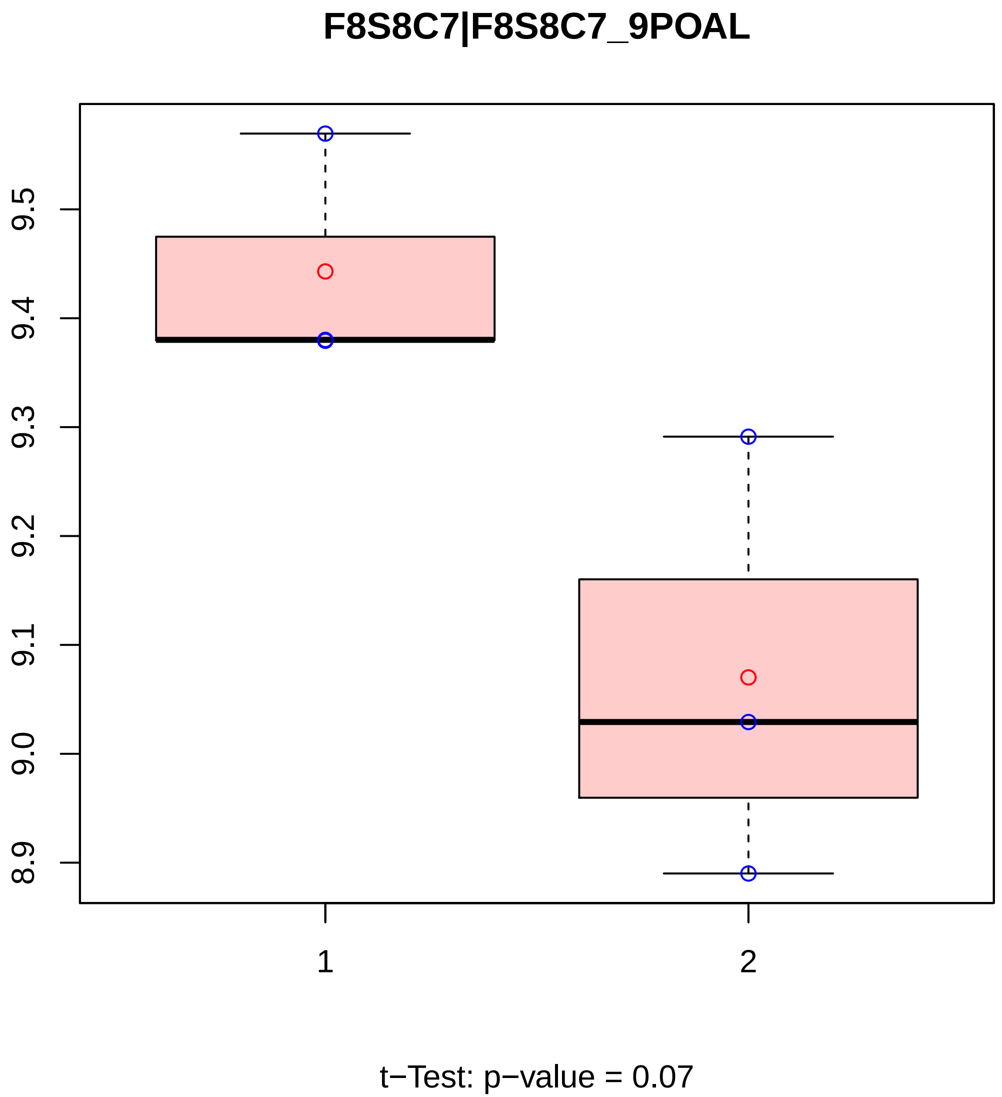
F8S8C7|F8S8C7_9POAL
9.5
9.4
9.3
9.2
9.1
9.0
8.9
1
2
t−Test: p−value = 0.07

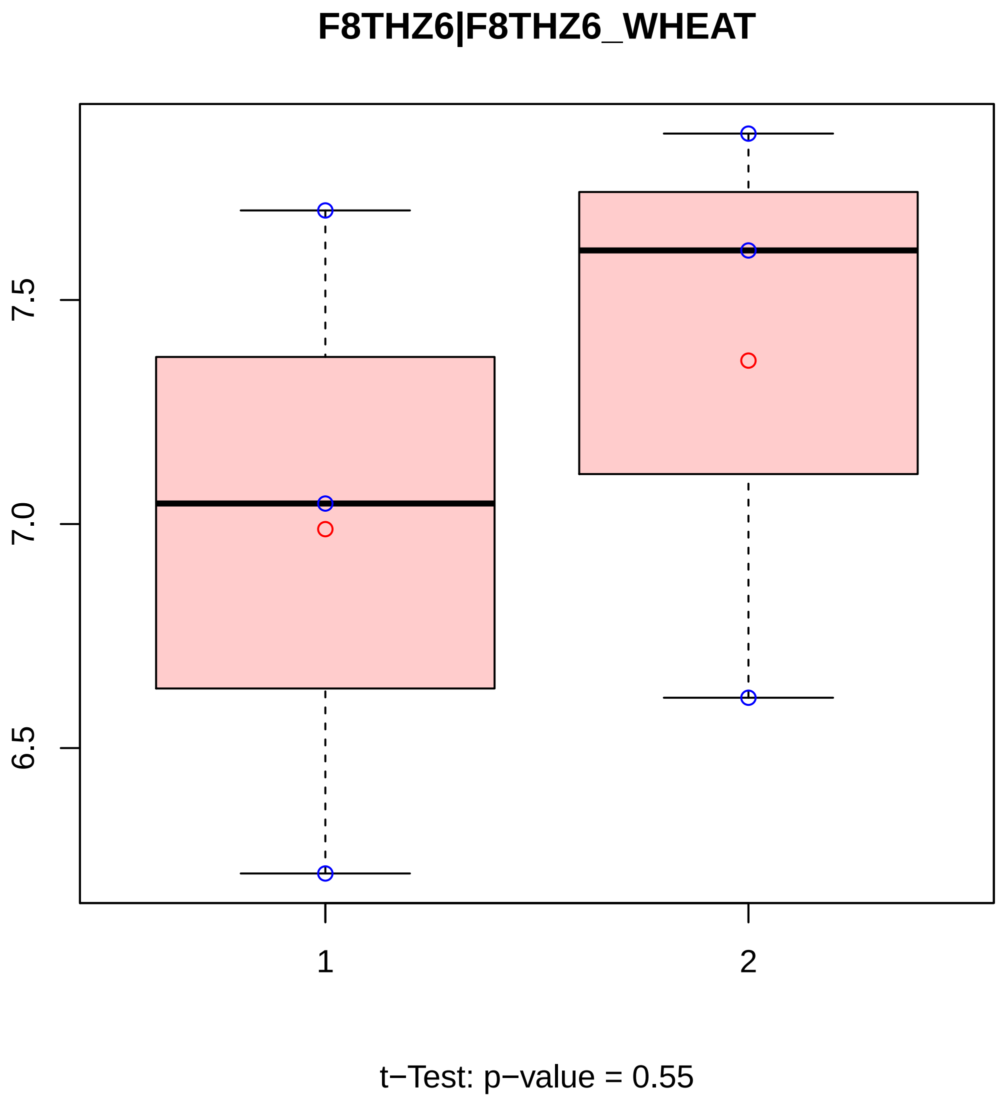
F8THZ6|F8THZ6_WHEAT
7.5
7.0
6.5
1
2
t−Test: p−value = 0.55

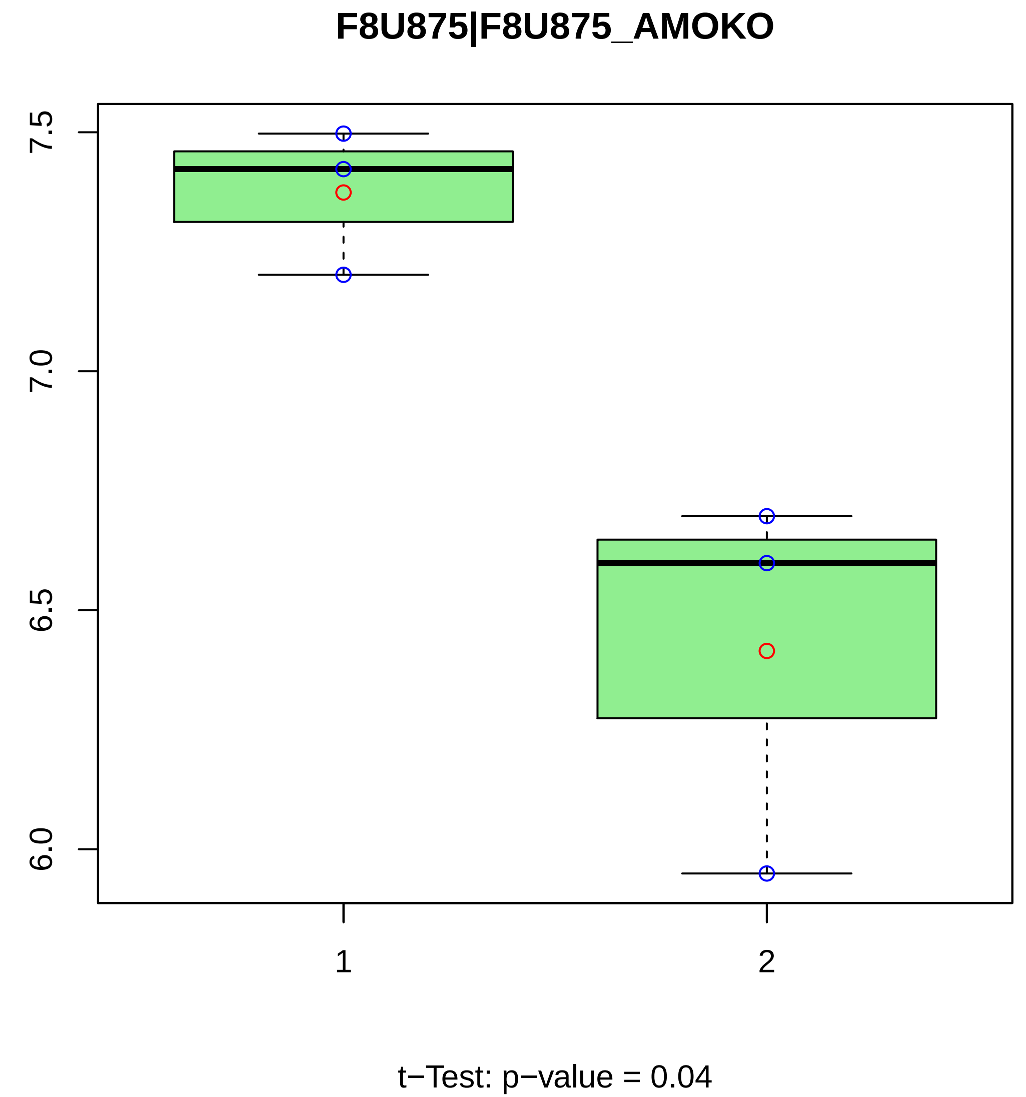
F8U875|F8U875_AMOKO
7.5
7.0
6.5
6.0
1
2
t−Test: p−value = 0.04

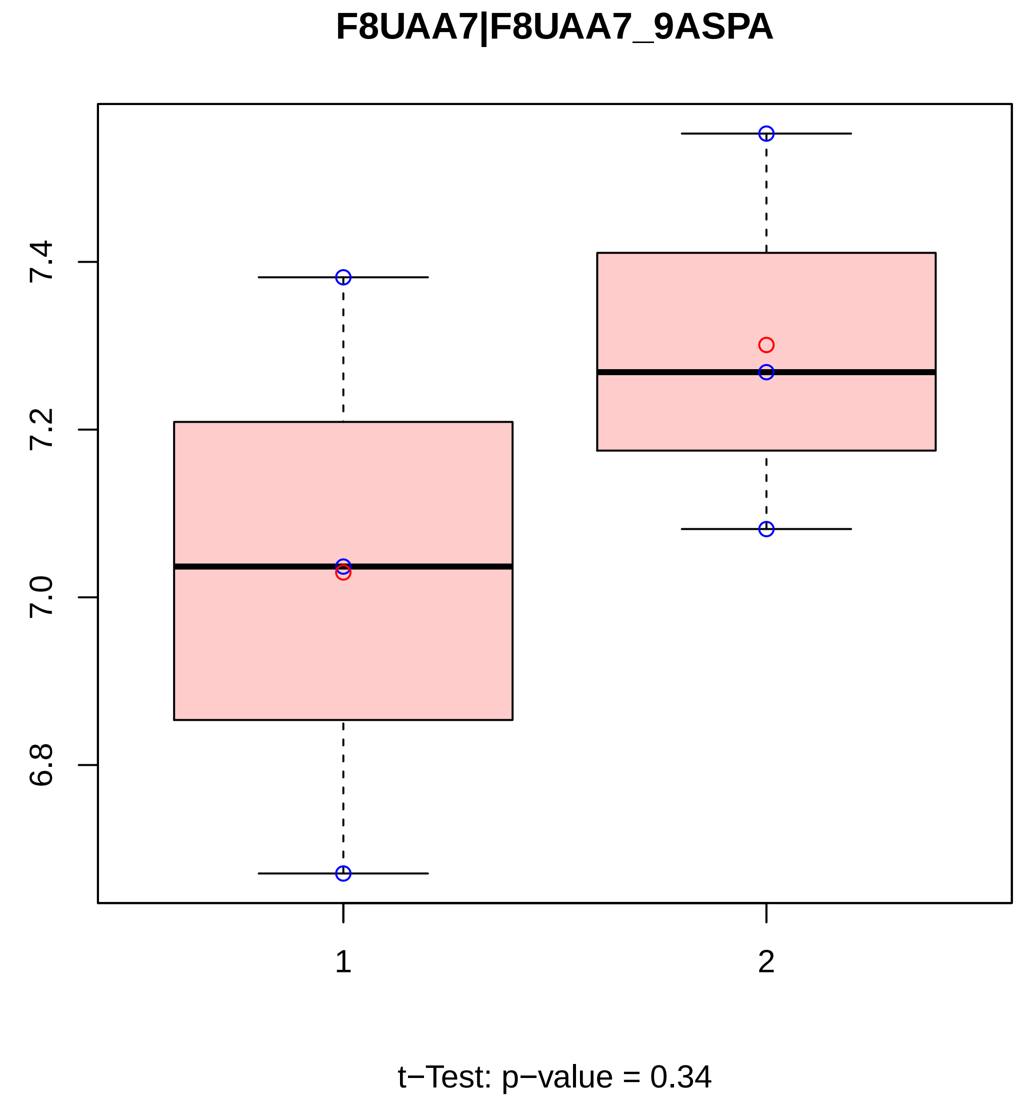
F8UAA7|F8UAA7_9ASPA
7.4
7.2
7.0
6.8
1
2
t−Test: p−value = 0.34

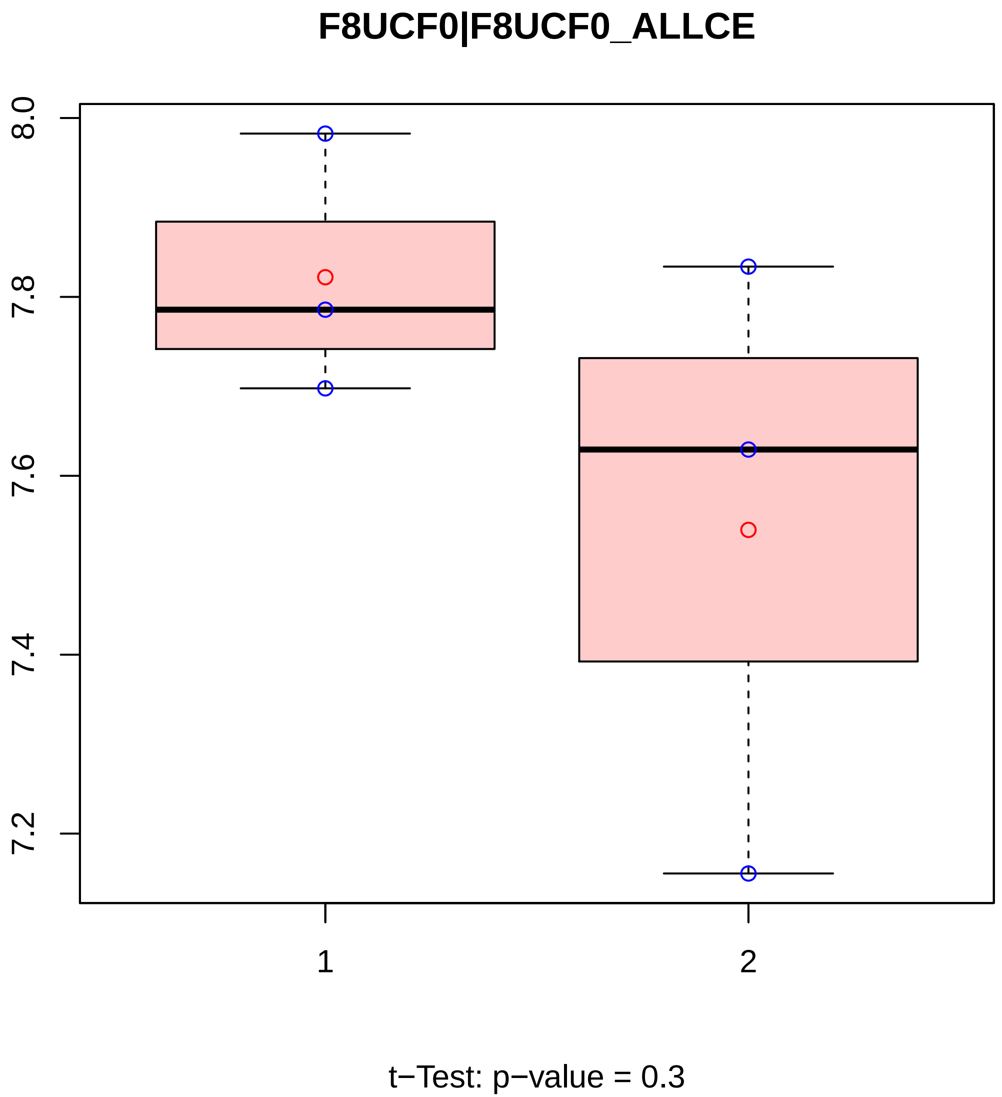
F8UCF0|F8UCF0_ALLCE
8.0
7.8
7.6
7.4
7.2
1
2
t−Test: p−value = 0.3

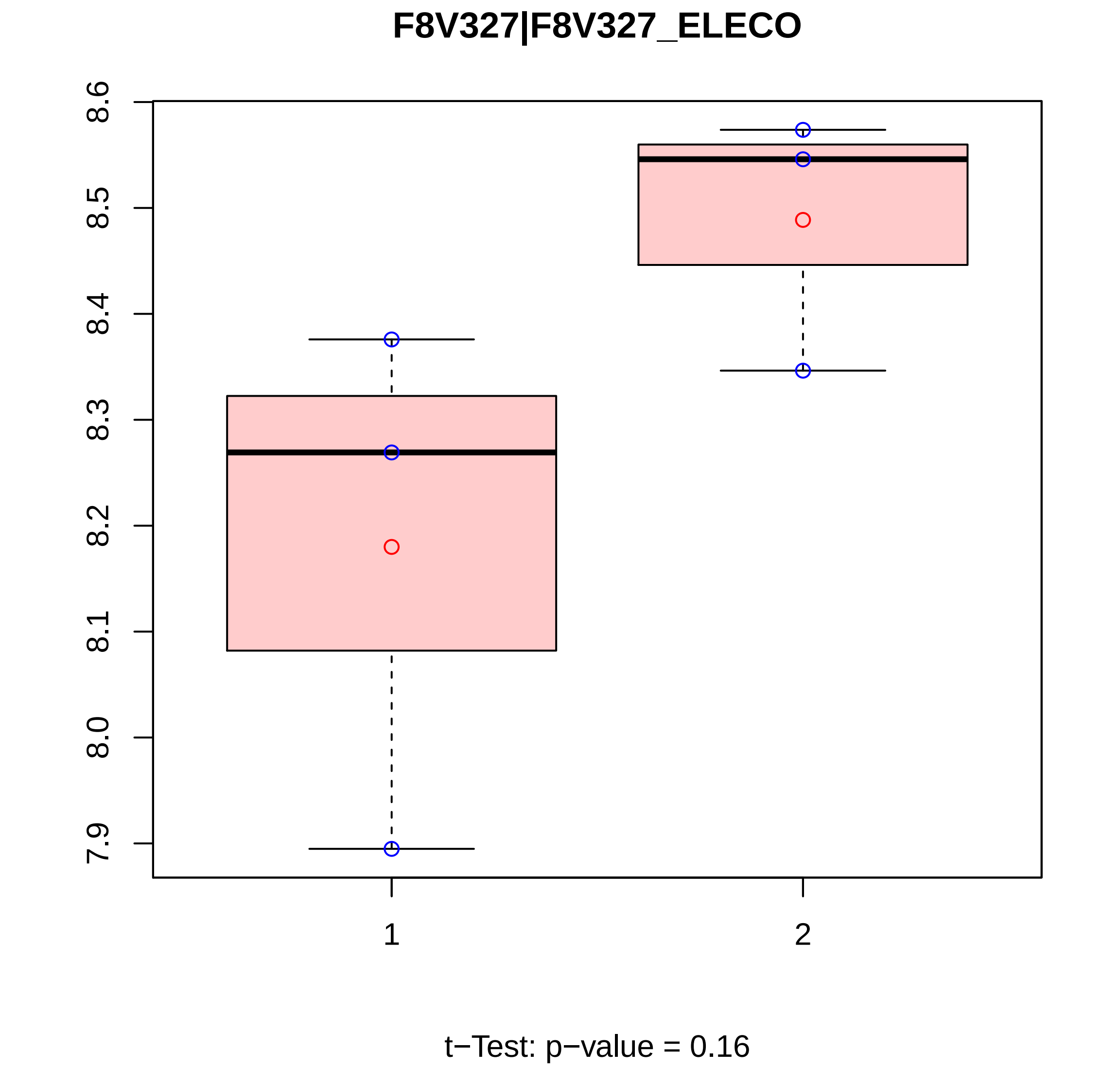

F8V327|F8V327_ELECO
8.6
8.5
8.4
8.3
8.2
8.1
8.0
7.9
1
2
t−Test: p−value = 0.16

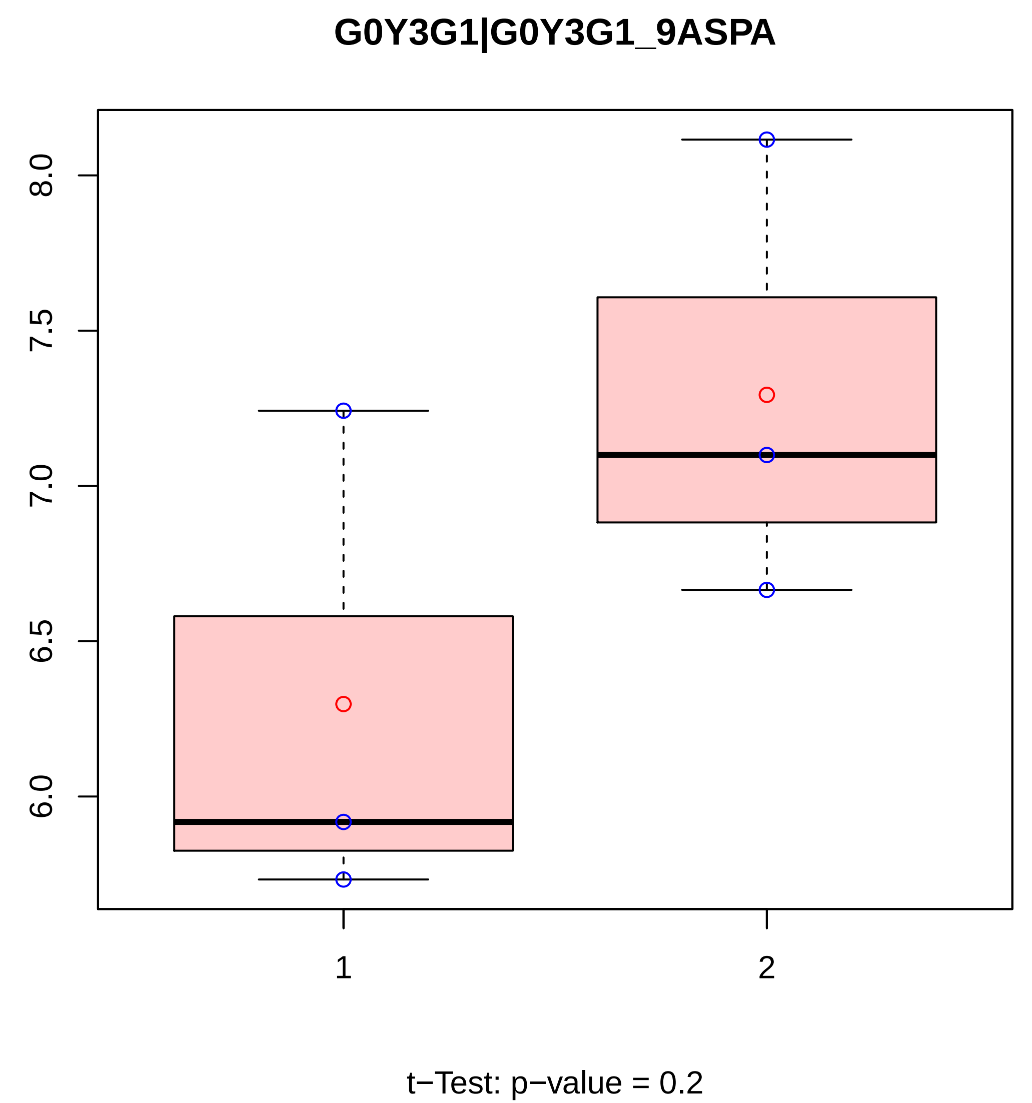
G0Y3G1|G0Y3G1_9ASPA
8.0
7.5
7.0
6.5
6.0
1
2
t−Test: p−value = 0.2

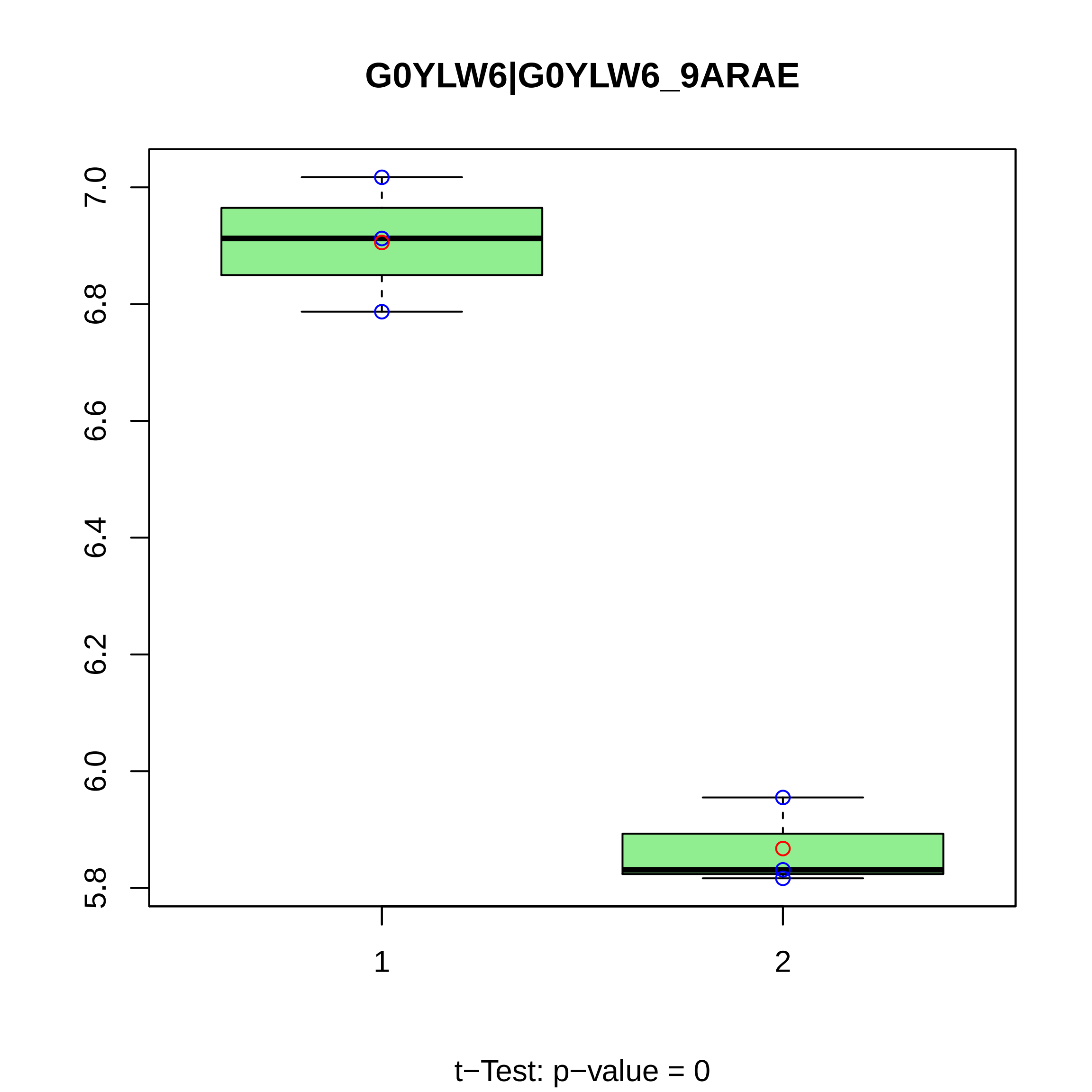
G0YLW6|G0YLW6_9ARAE
7.0
6.8
6.6
6.4
6.2
6.0
5.8
1
2
t−Test: p−value = 0

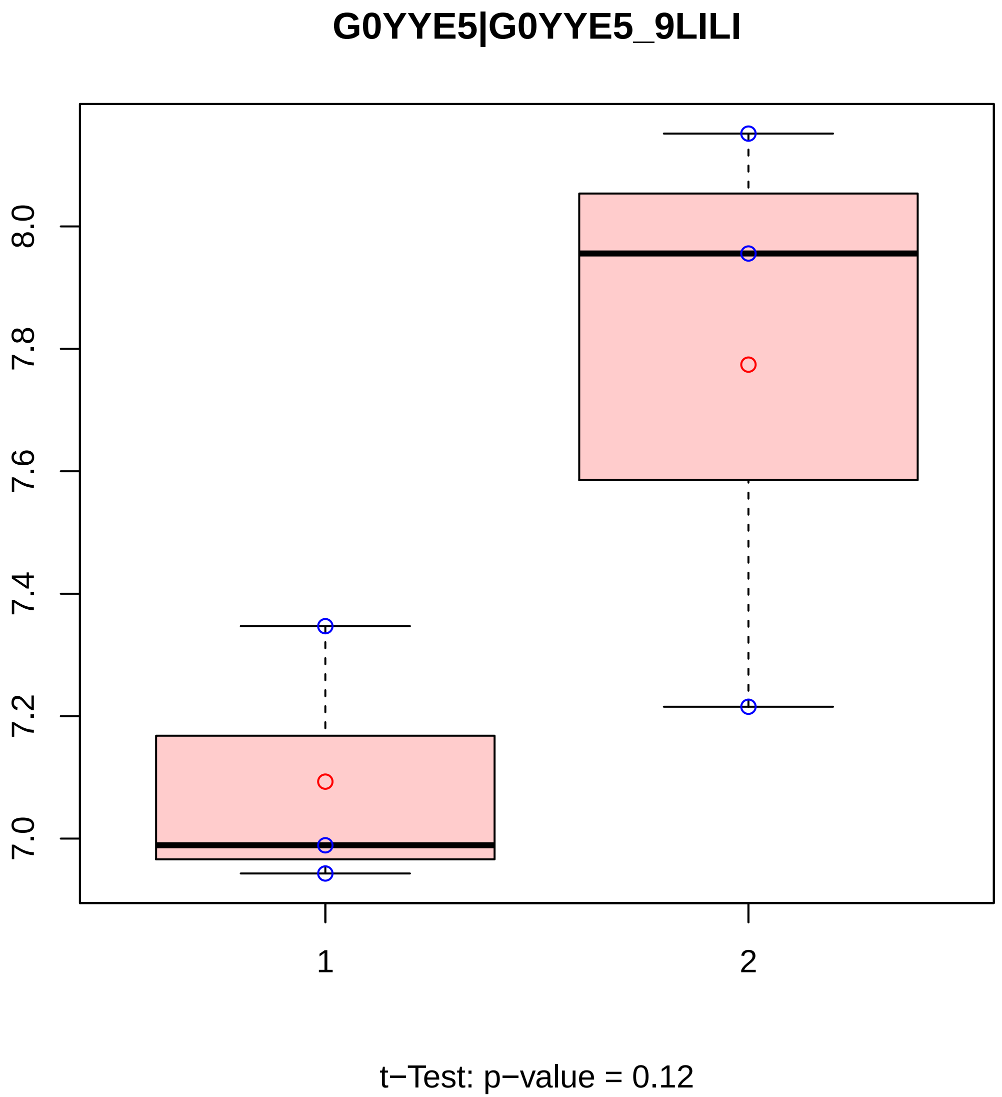
G0YYE5|G0YYE5_9LILI
8.0
7.8
7.6
7.4
7.2
7.0
1
2
t−Test: p−value = 0.12

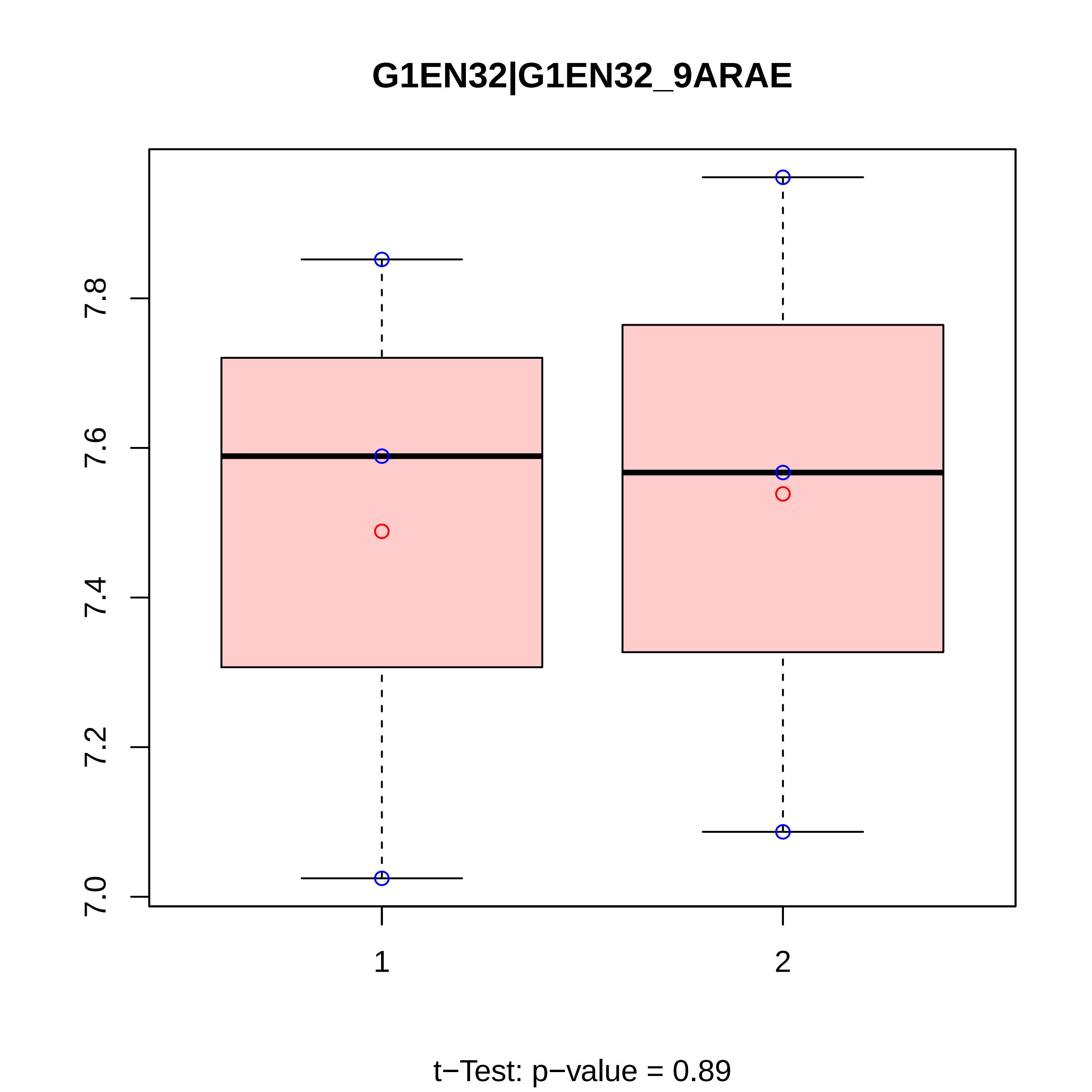
G1EN32|G1EN32_9ARAE
7.0
7.2
7.4
7.6
7.8
1
2
t−Test: p−value = 0.89

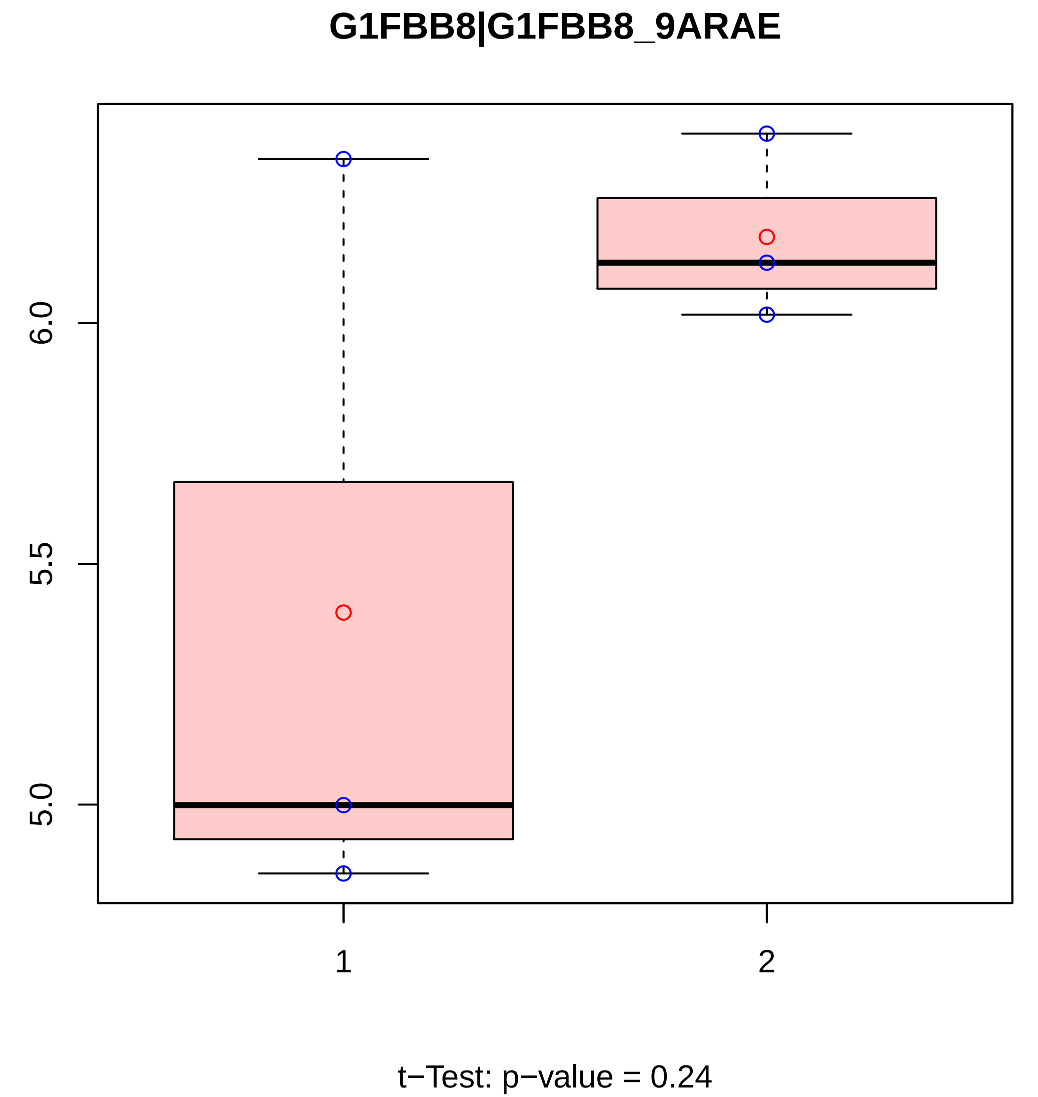
G1FBB8|G1FBB8_9ARAE
6.0
5.5
5.0
1
2
t−Test: p−value = 0.24

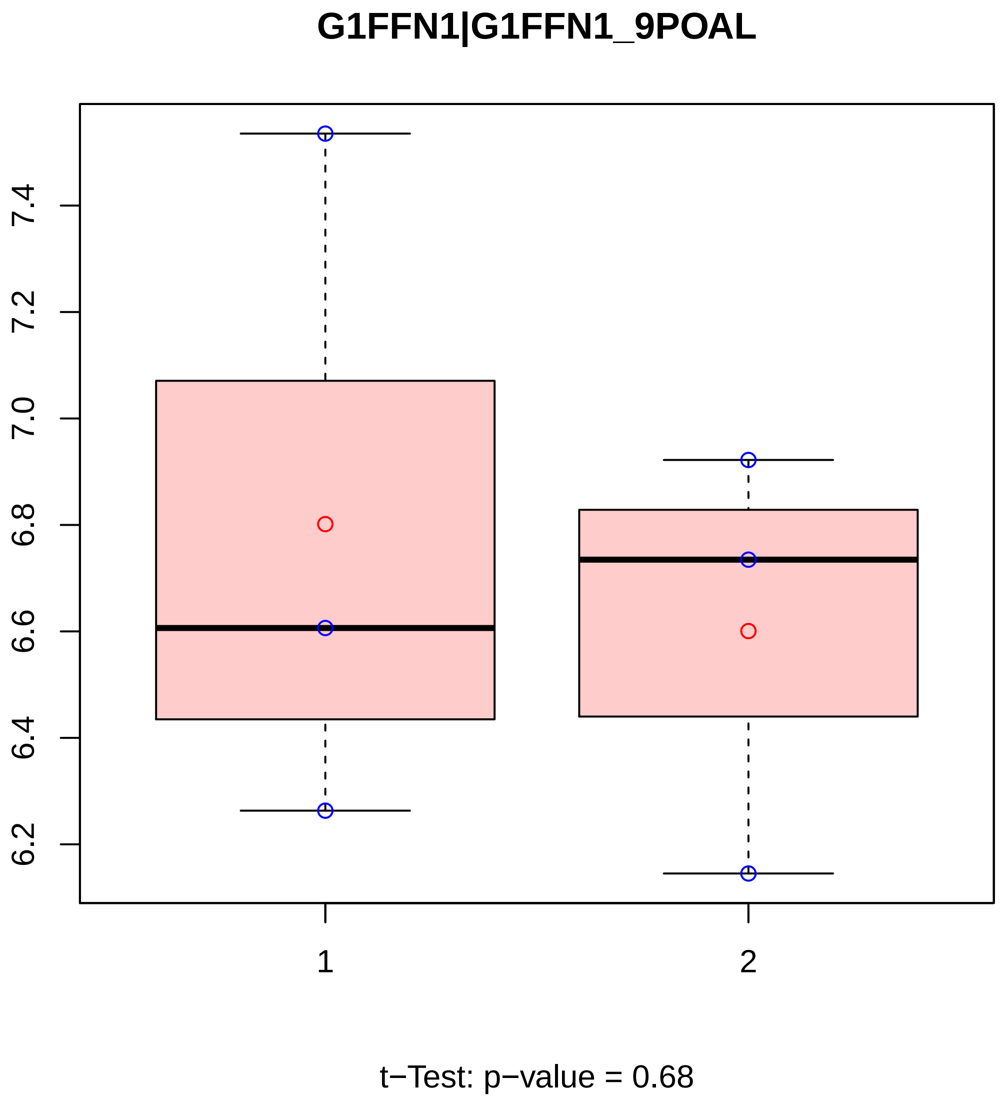
G1FFN1|G1FFN1_9POAL
7.4
7.2
7.0
6.8
6.6
6.4
6.2
1
2
t−Test: p−value = 0.68

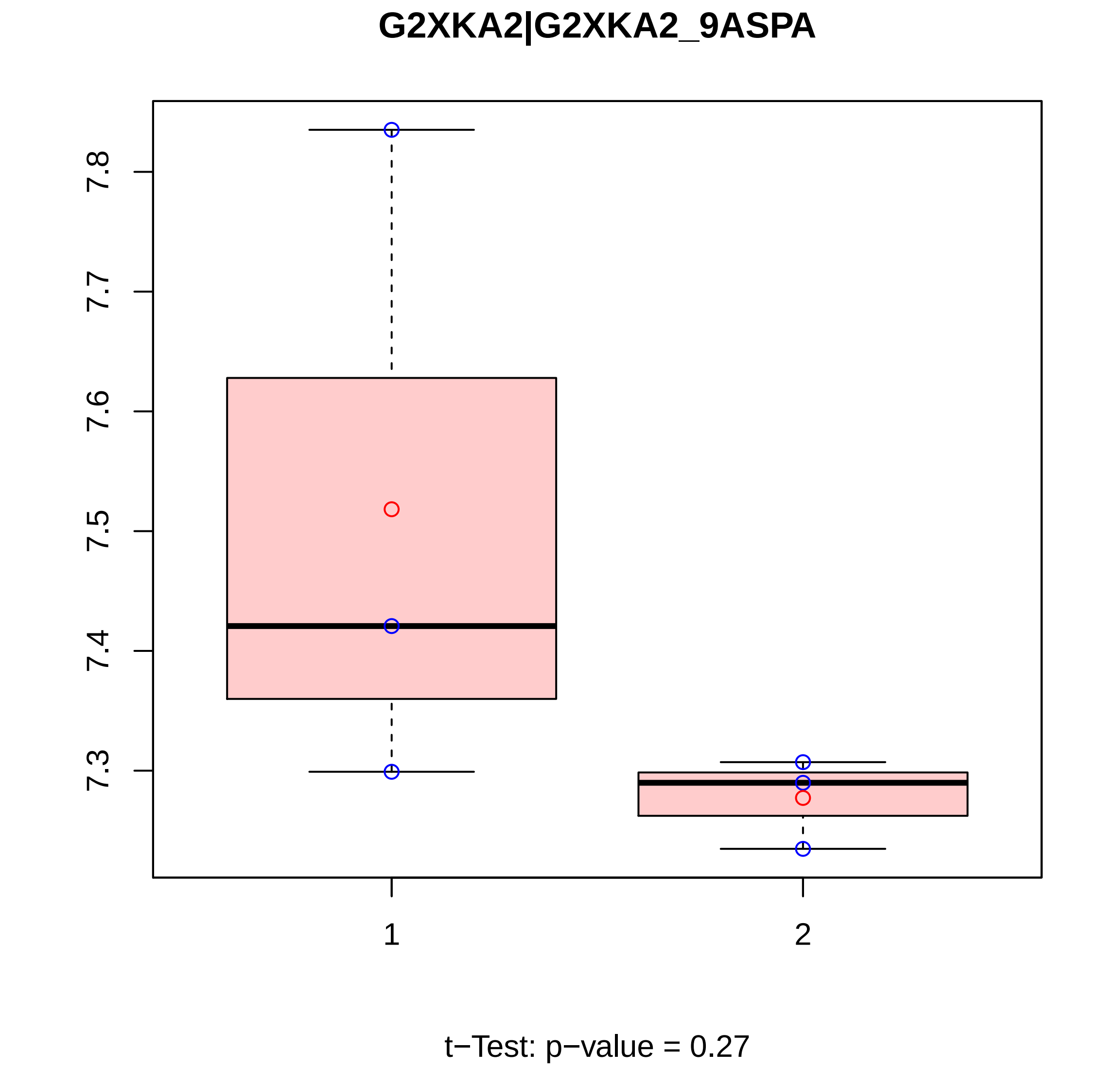
G2XKA2|G2XKA2_9ASPA
7.3
7.4
7.5
7.6
7.7
7.8
1
2
t−Test: p−value = 0.27

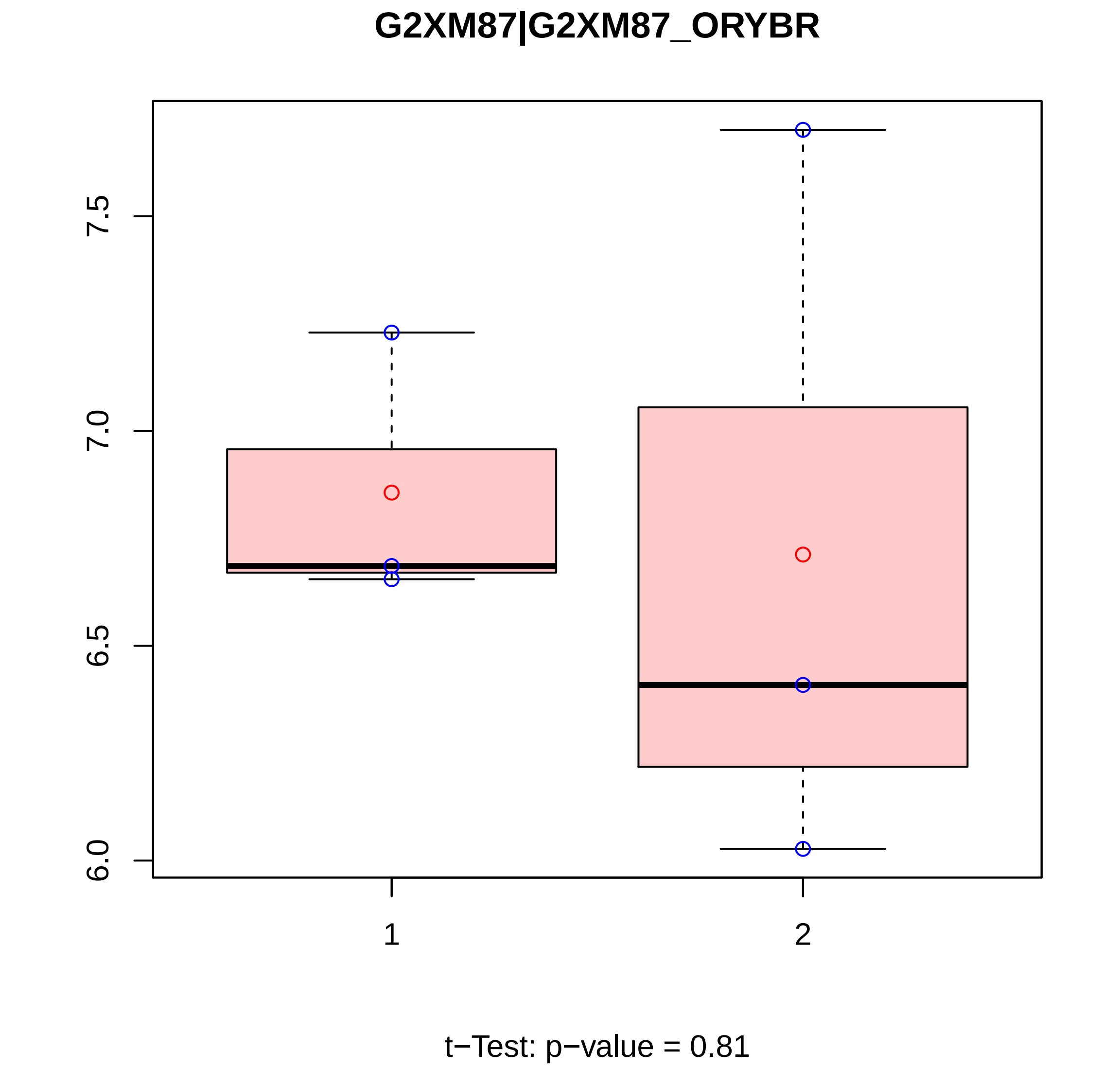
G2XM87|G2XM87_ORYBR
7.5
7.0
6.5
6.0
1
2
t−Test: p−value = 0.81

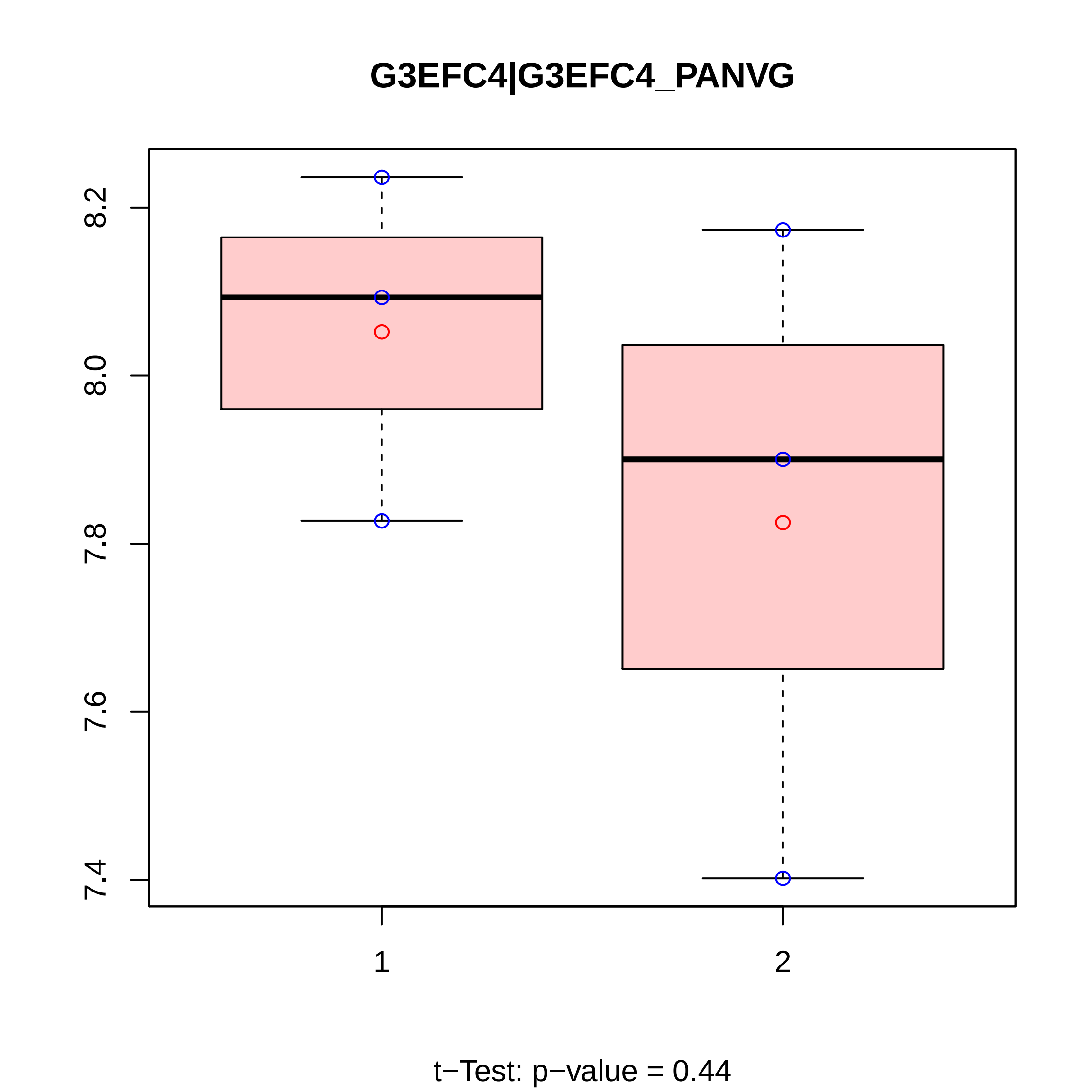
G3EFC4|G3EFC4_PANVG
8.2
8.0
7.8
7.6
7.4
1
2
t−Test: p−value = 0.44

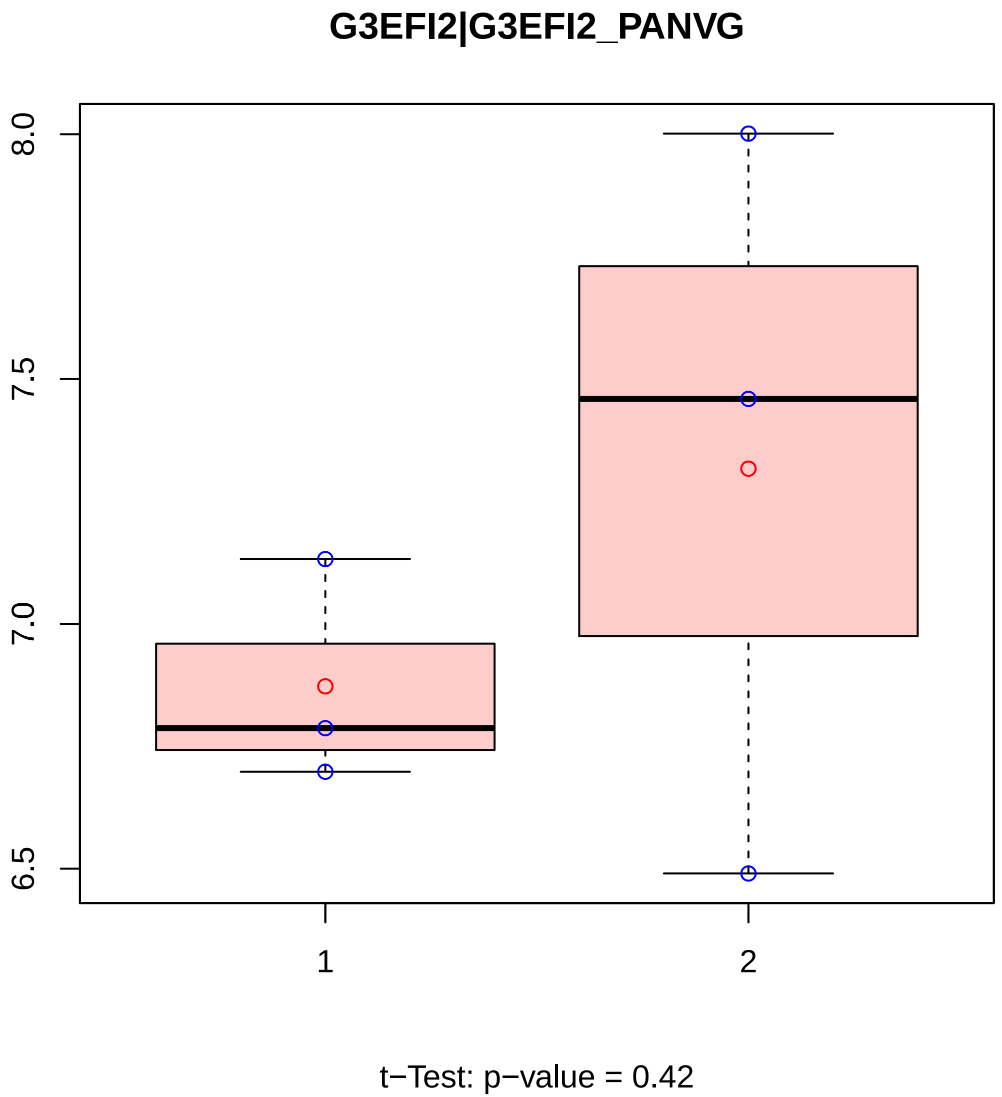
G3EFI2|G3EFI2_PANVG
8.0
7.5
7.0
6.5
1
2
t−Test: p−value = 0.42

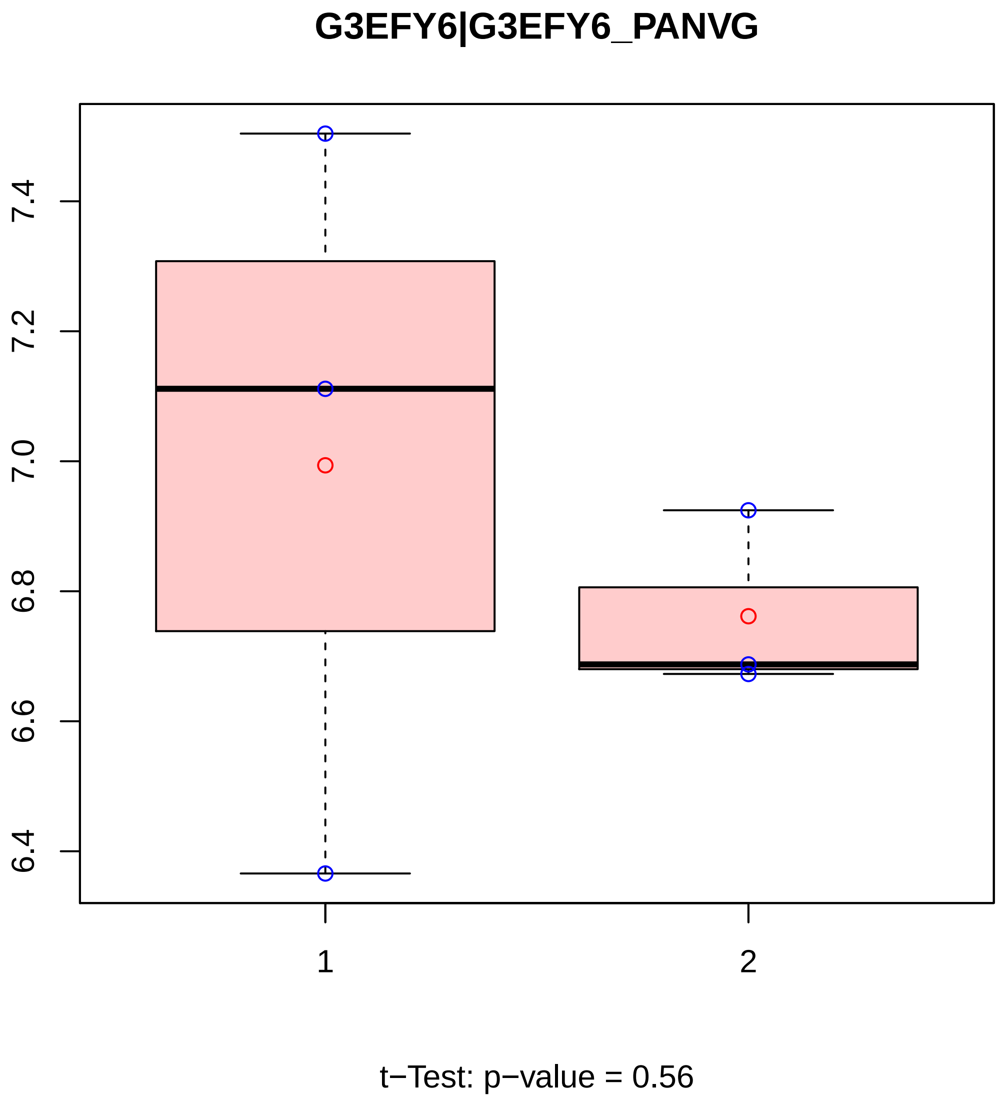
G3EFY6|G3EFY6_PANVG
7.4
7.2
7.0
6.8
6.6
6.4
1
2
t−Test: p−value = 0.56

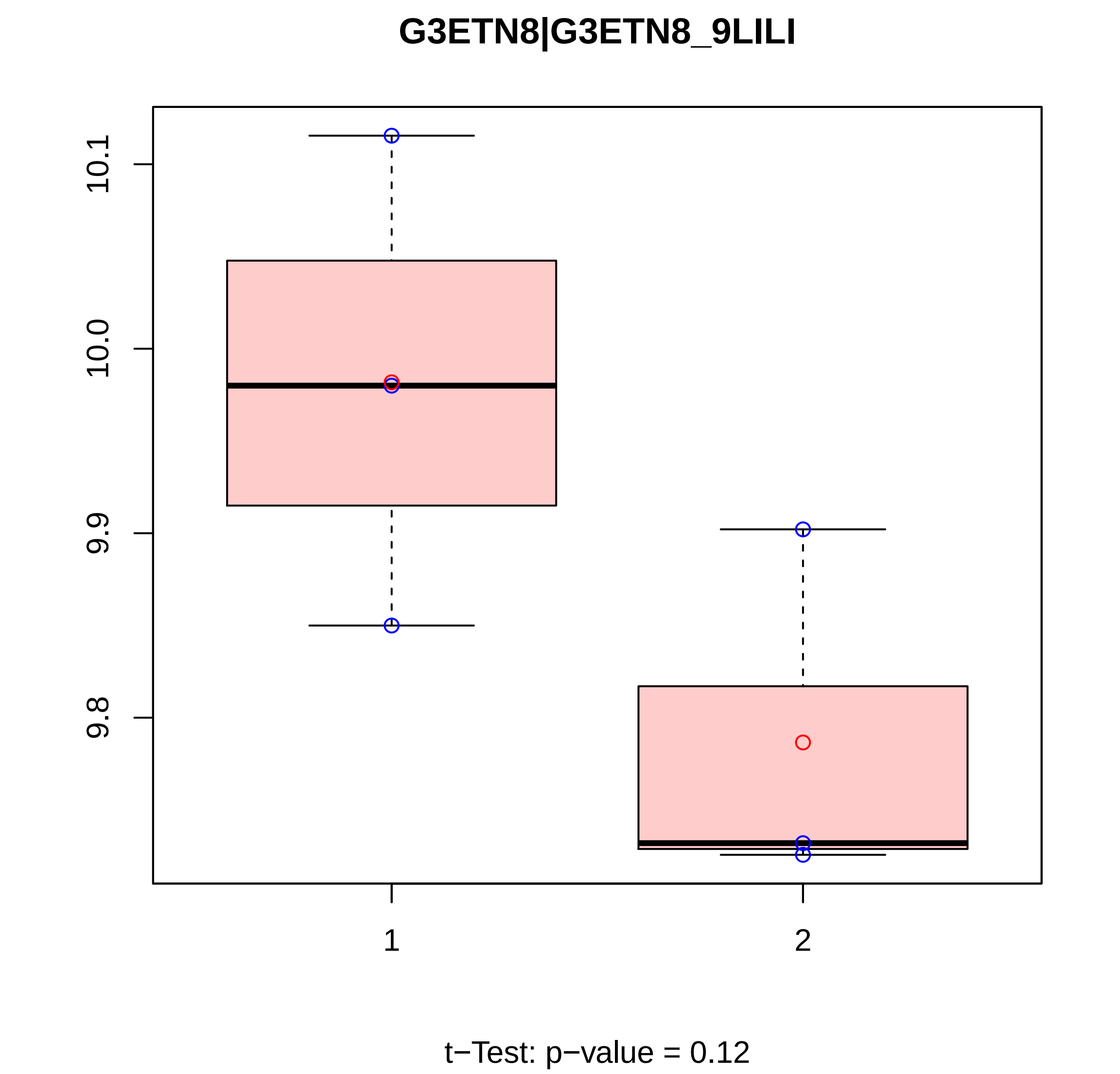

G3ETN8|G3ETN8_9LILI
10.1
10.0
9.9
9.8
1
2
t−Test: p−value = 0.12

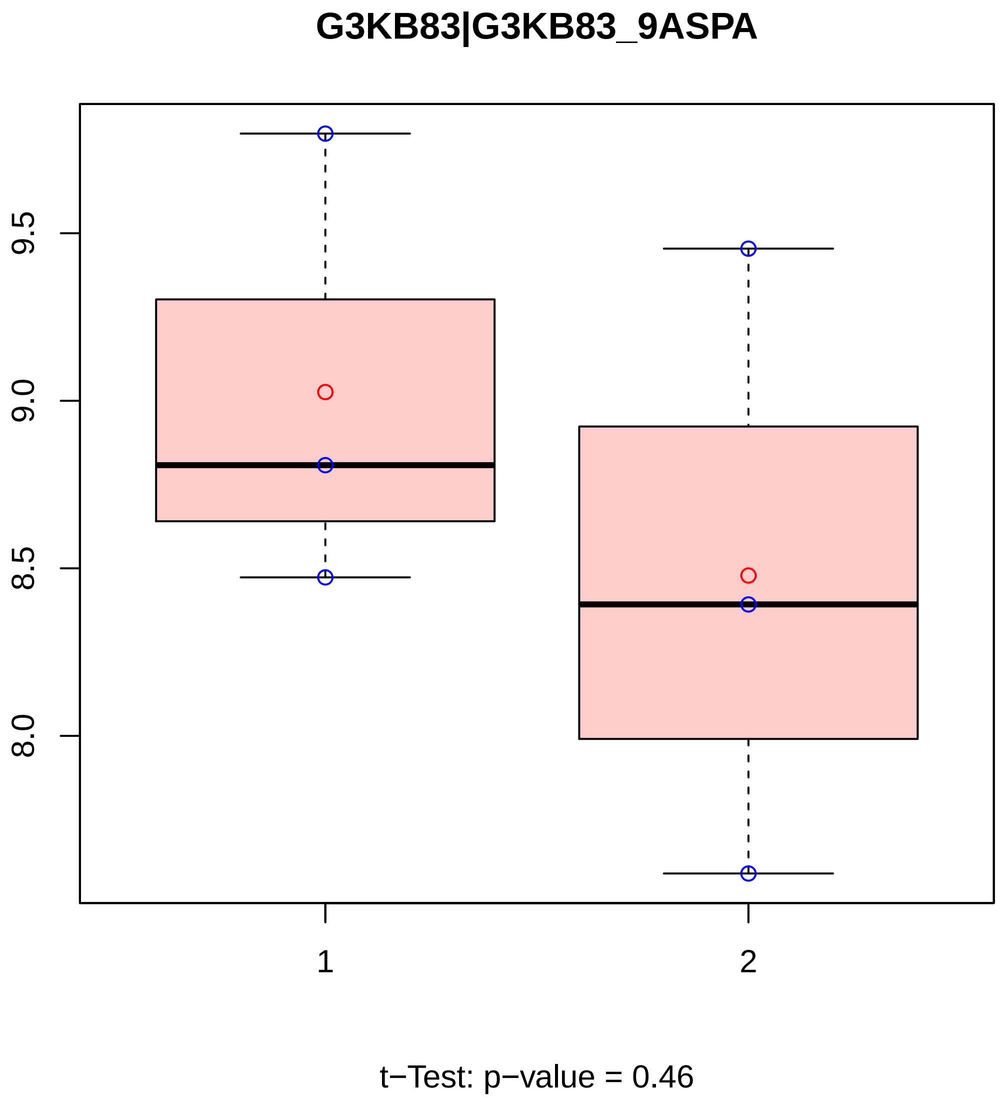

G3KB83|G3KB83_9ASPA
9.5
9.0
8.5
8.0
1
2
t−Test: p−value = 0.46

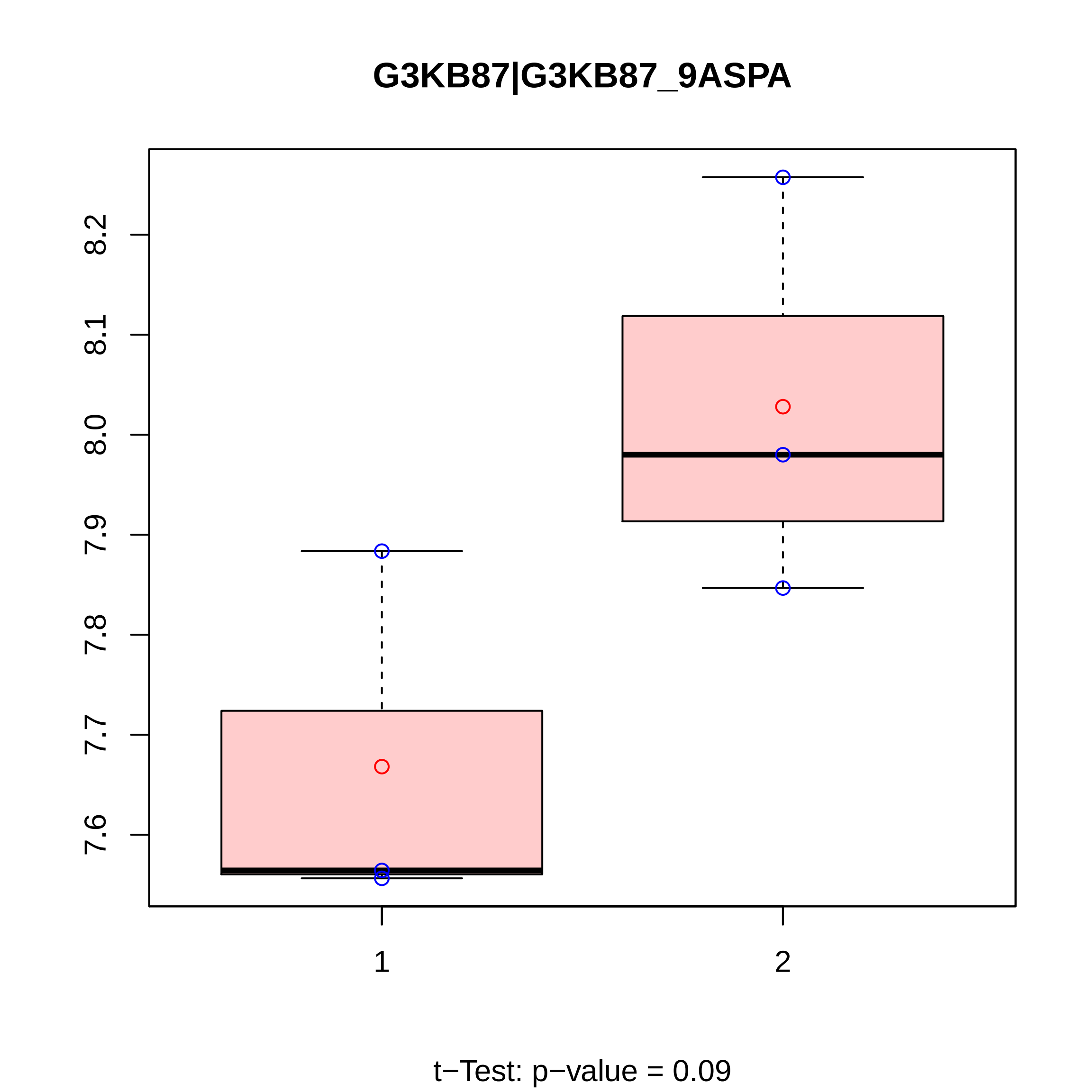
G3KB87|G3KB87_9ASPA
8.2
8.1
8.0
7.9
7.8
7.7
7.6
1
2
t−Test: p−value = 0.09

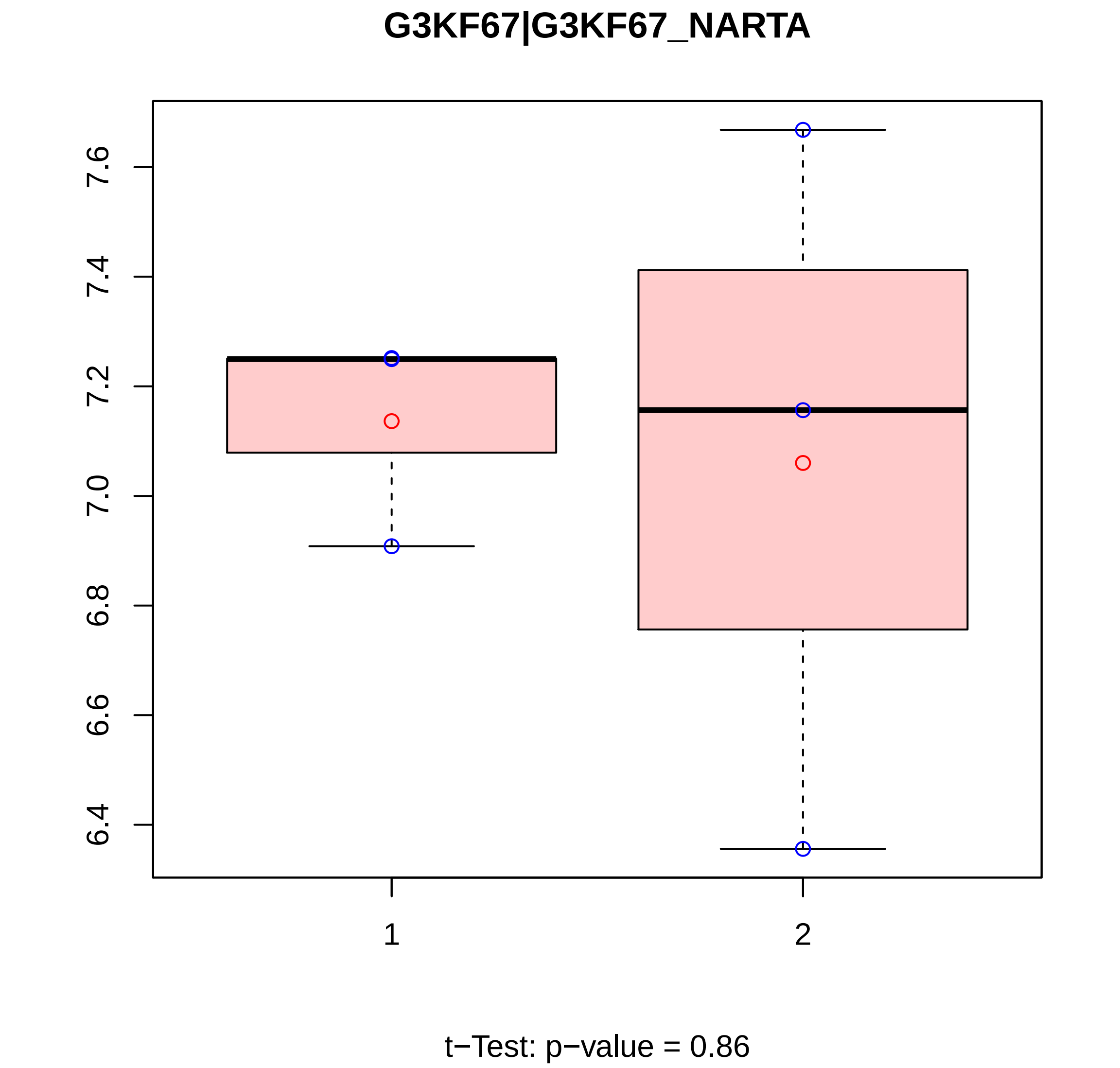
G3KF67|G3KF67_NARTA
7.6
7.4
7.2
7.0
6.8
6.6
6.4
1
2
t−Test: p−value = 0.86

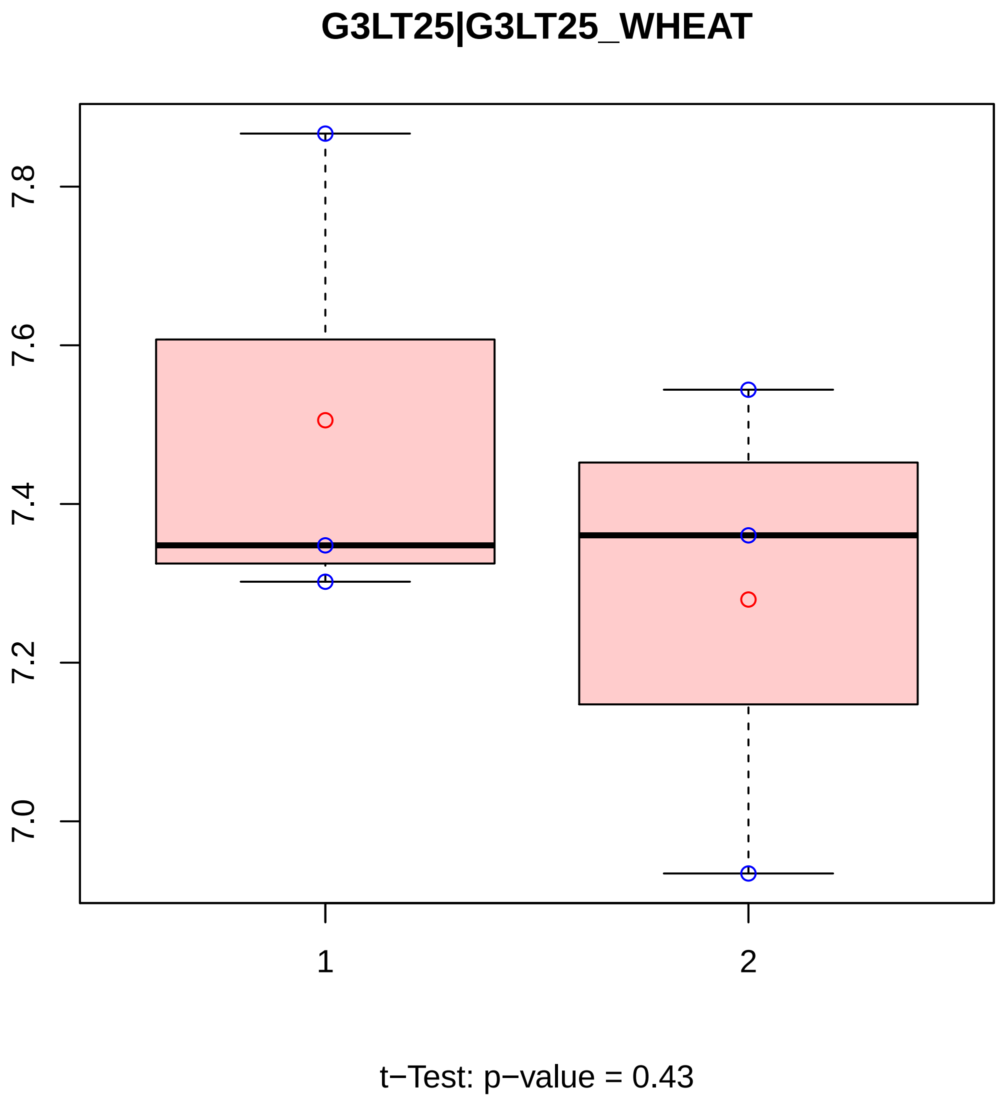
G3LT25|G3LT25_WHEAT
7.0
7.2
7.4
7.6
7.8
1
2
t−Test: p−value = 0.43

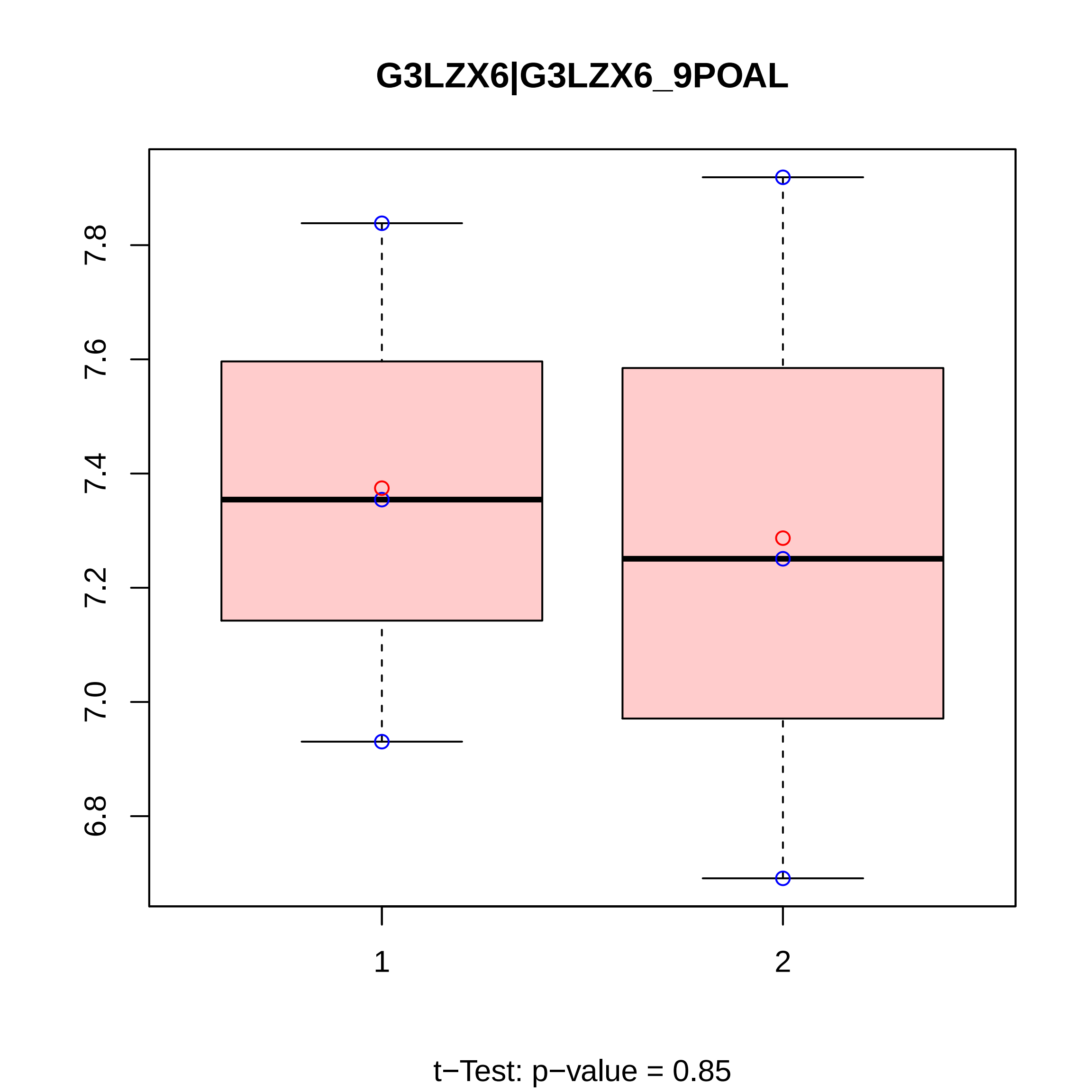

G3LZX6|G3LZX6_9POAL
7.8
7.6
7.4
7.2
7.0
6.8
1
2
t−Test: p−value = 0.85

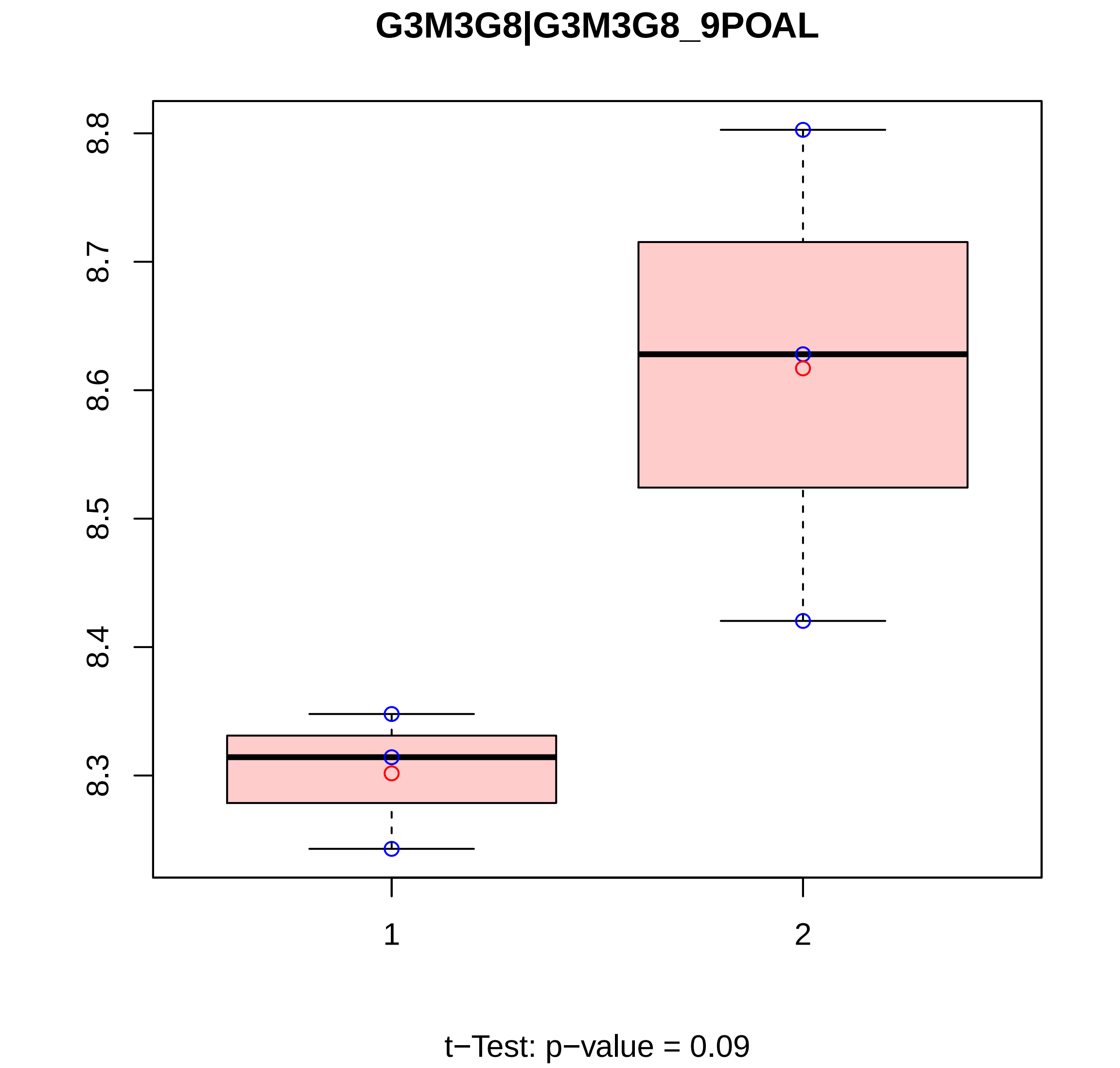
G3M3G8|G3M3G8_9POAL
8.8
8.7
8.6
8.5
8.4
8.3
1
2
t−Test: p−value = 0.09

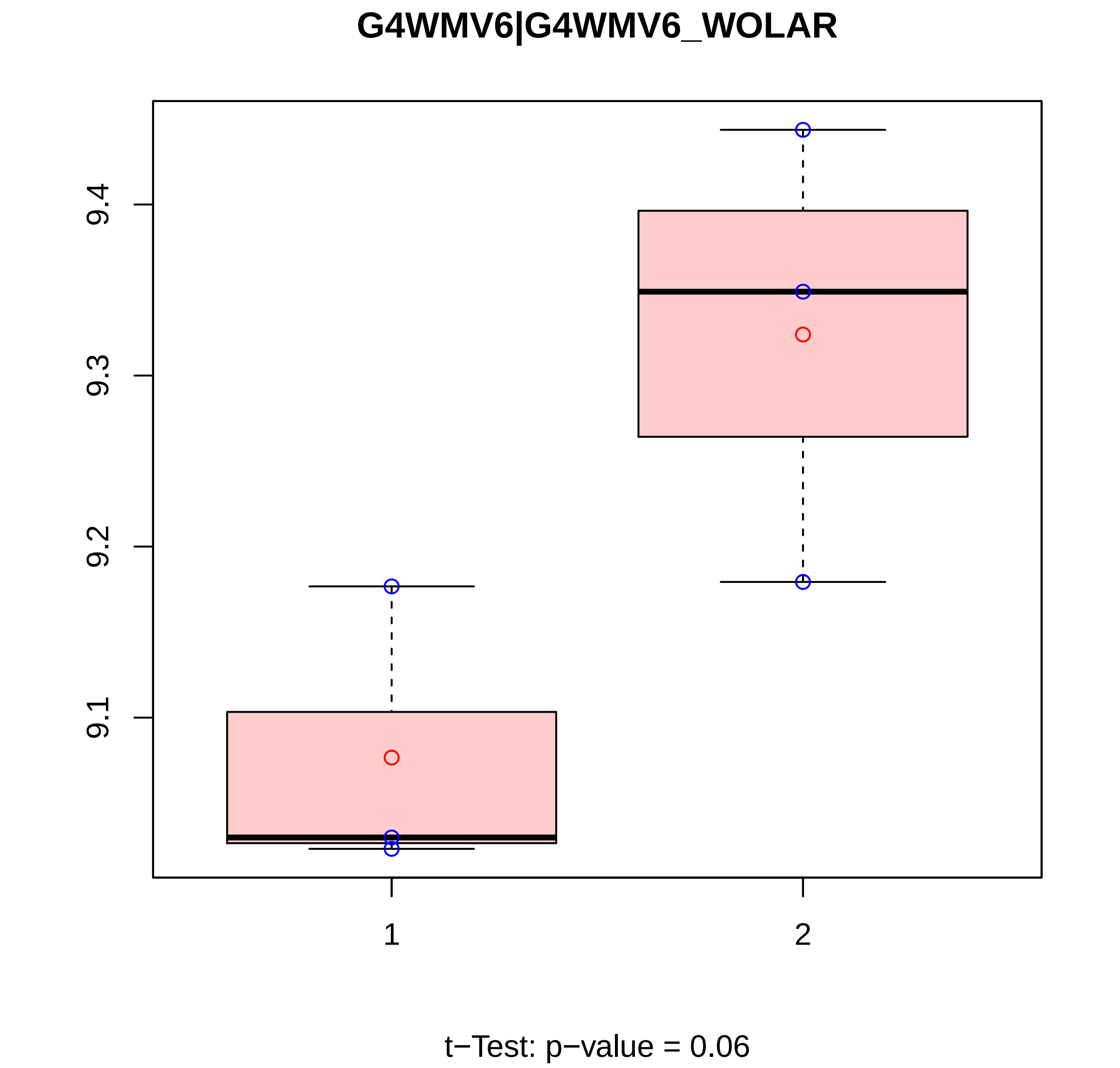
G4WMV6|G4WMV6_WOLAR
9.1
9.2
9.3
9.4
1
2
t−Test: p−value = 0.06

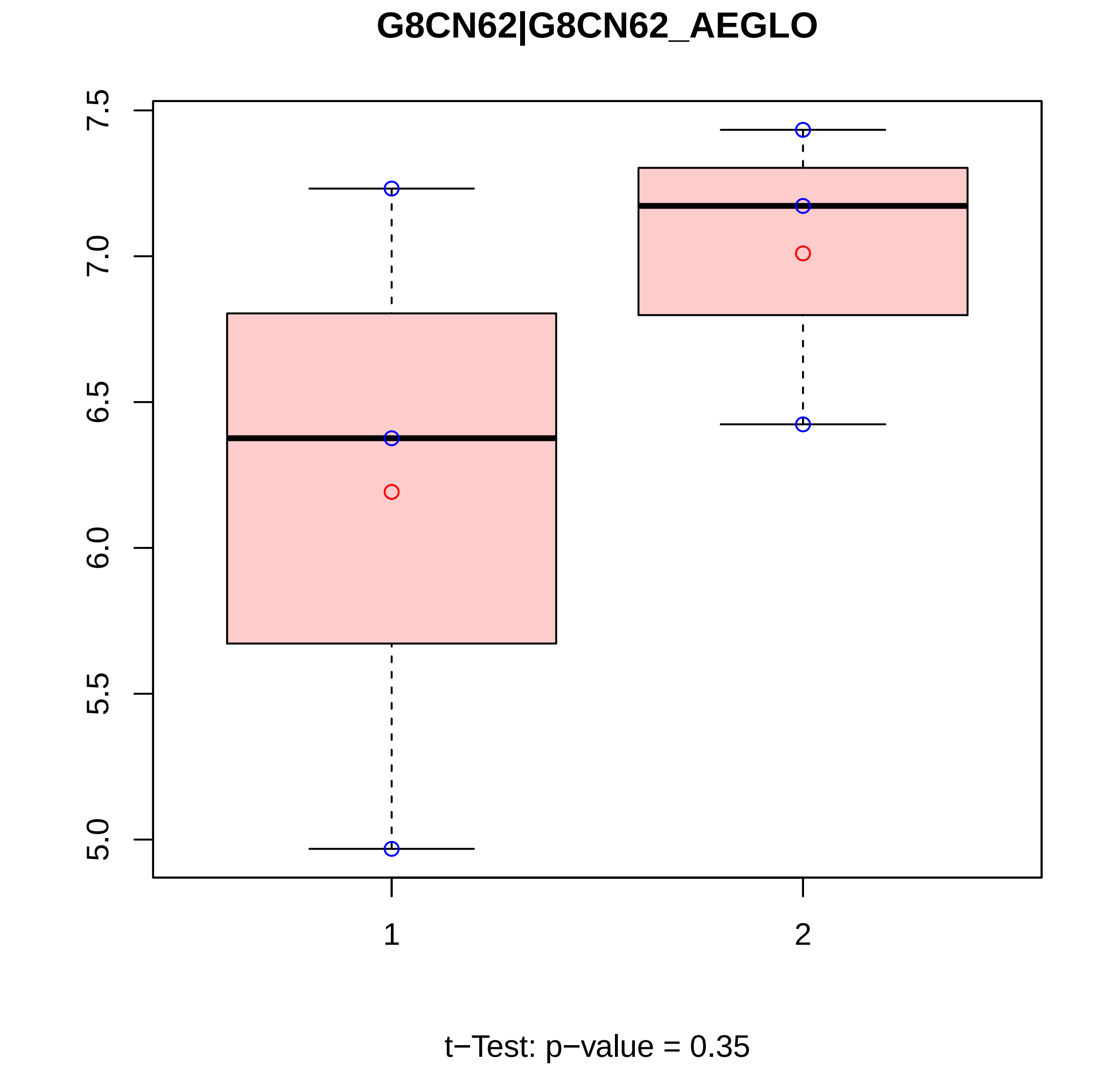

G8CN62|G8CN62_AEGLO
7.5
7.0
6.5
6.0
5.5
5.0
1
2
t−Test: p−value = 0.35

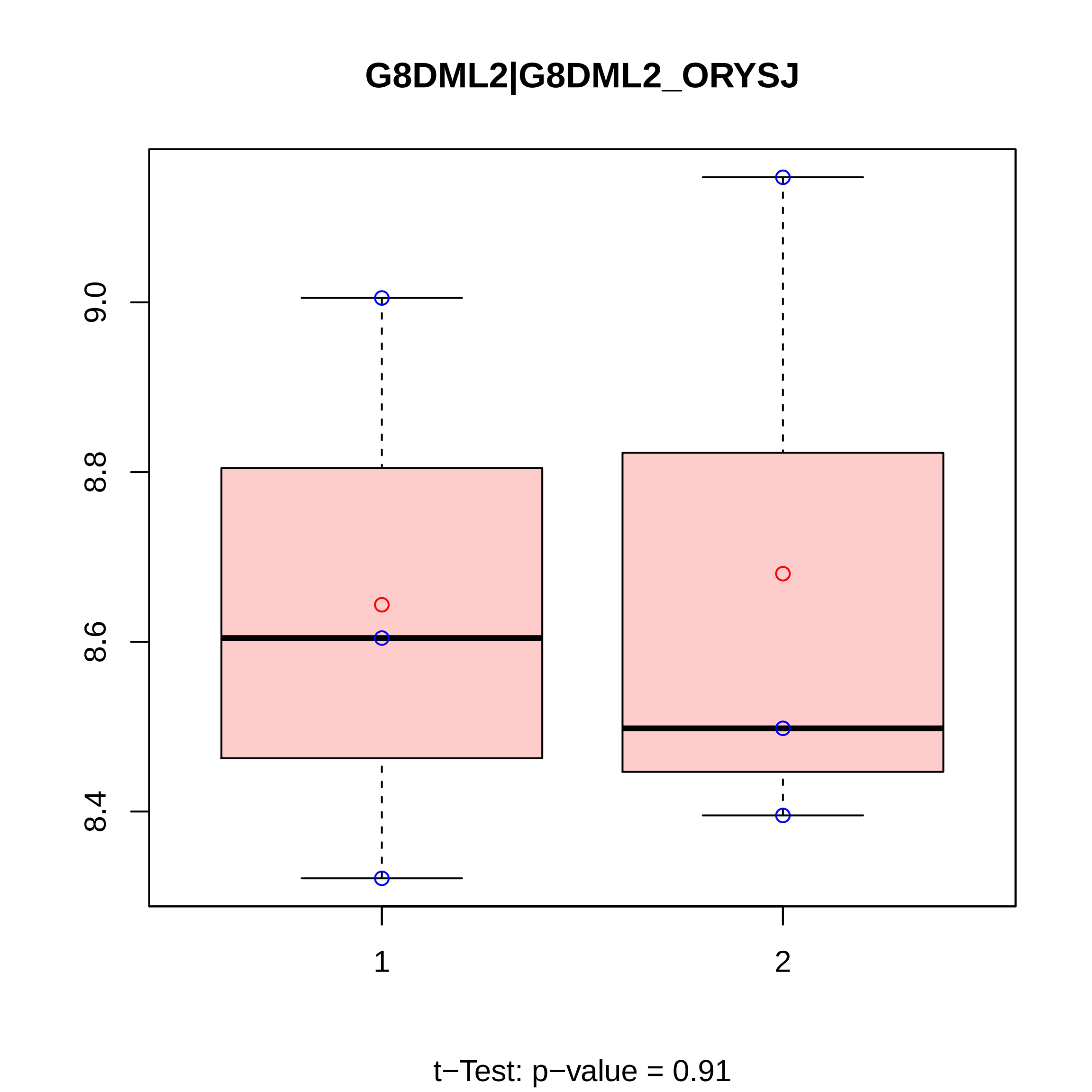
G8DML2|G8DML2_ORYSJ
9.0
8.8
8.6
8.4
1
2
t−Test: p−value = 0.91

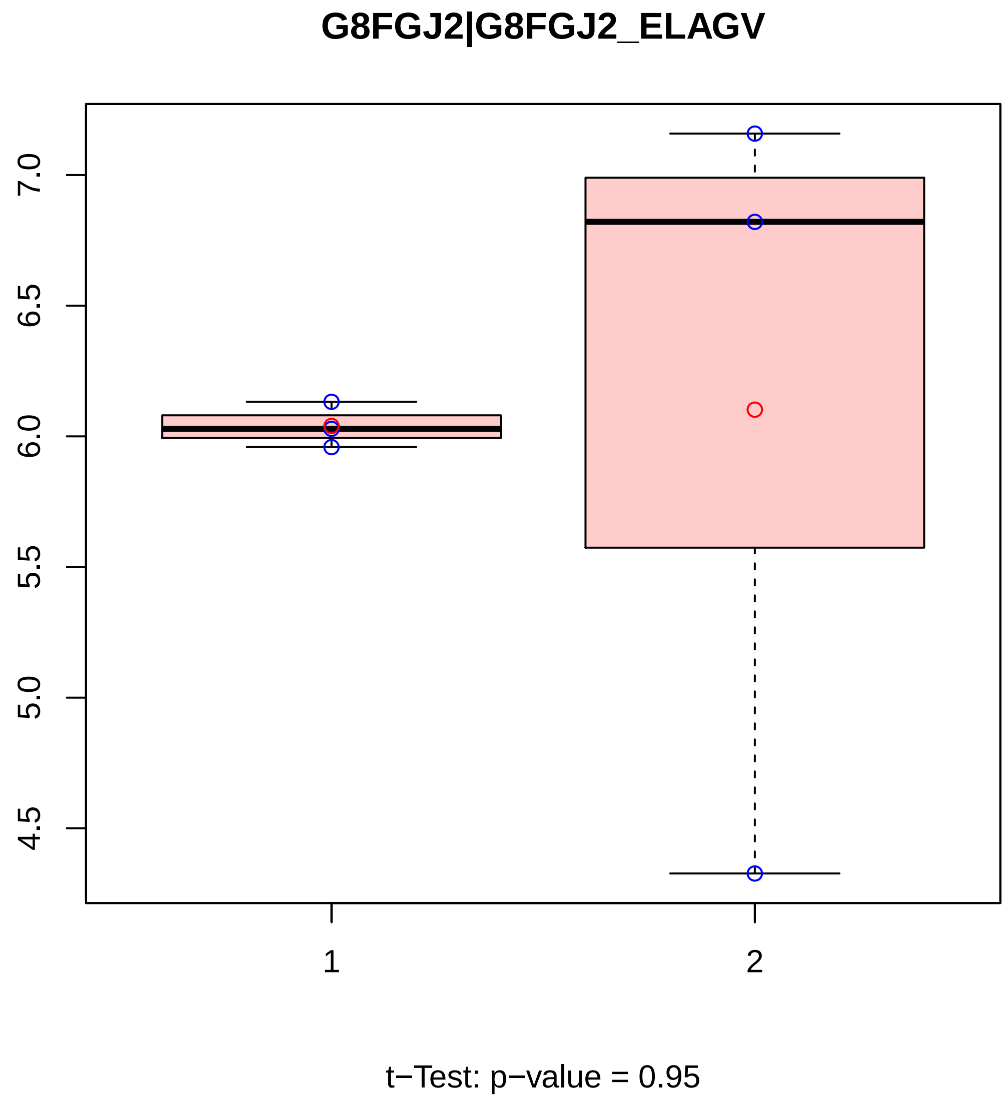
G8FGJ2|G8FGJ2_ELAGV
7.0
6.5
6.0
5.5
5.0
4.5
1
2
t−Test: p−value = 0.95

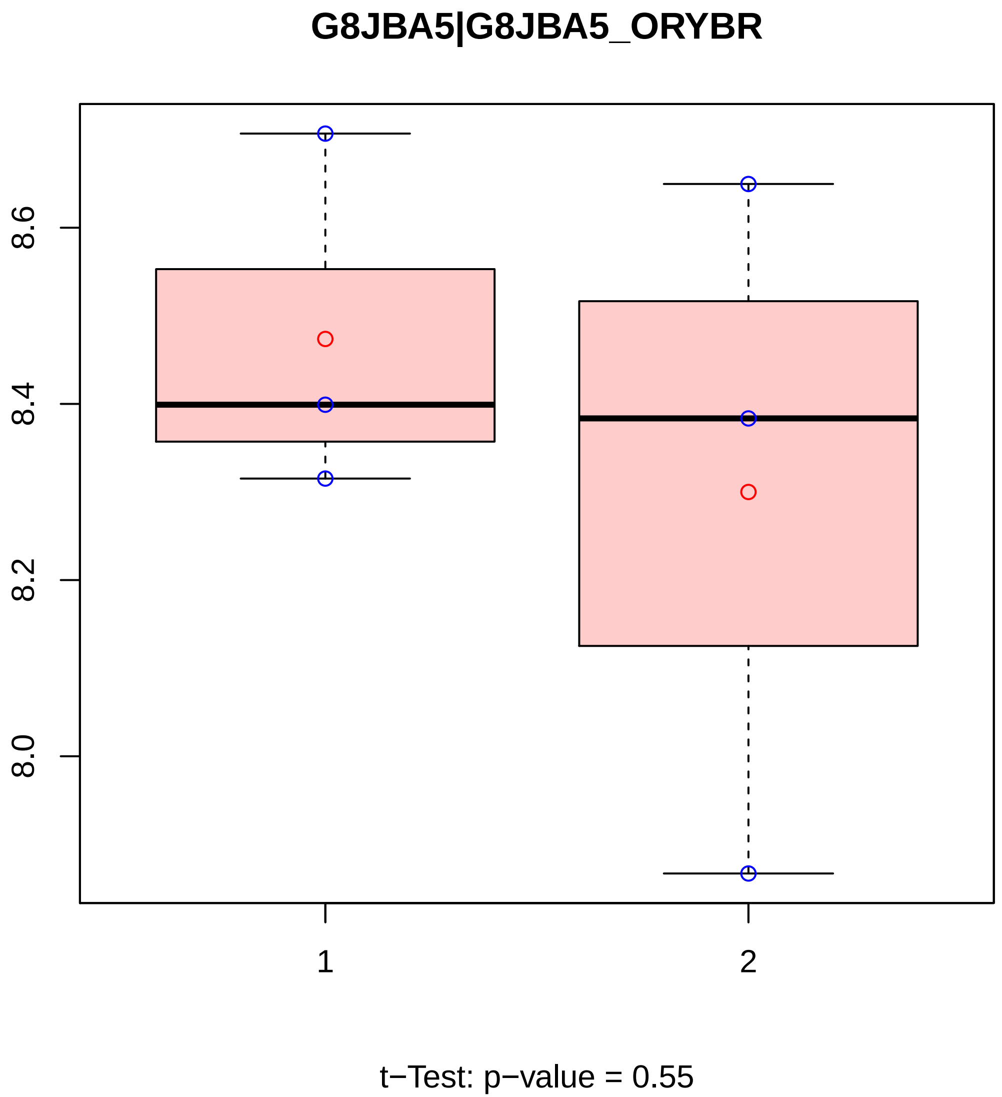
G8JBA5|G8JBA5_ORYBR
8.0
8.2
8.4
8.6
1
2
t−Test: p−value = 0.55

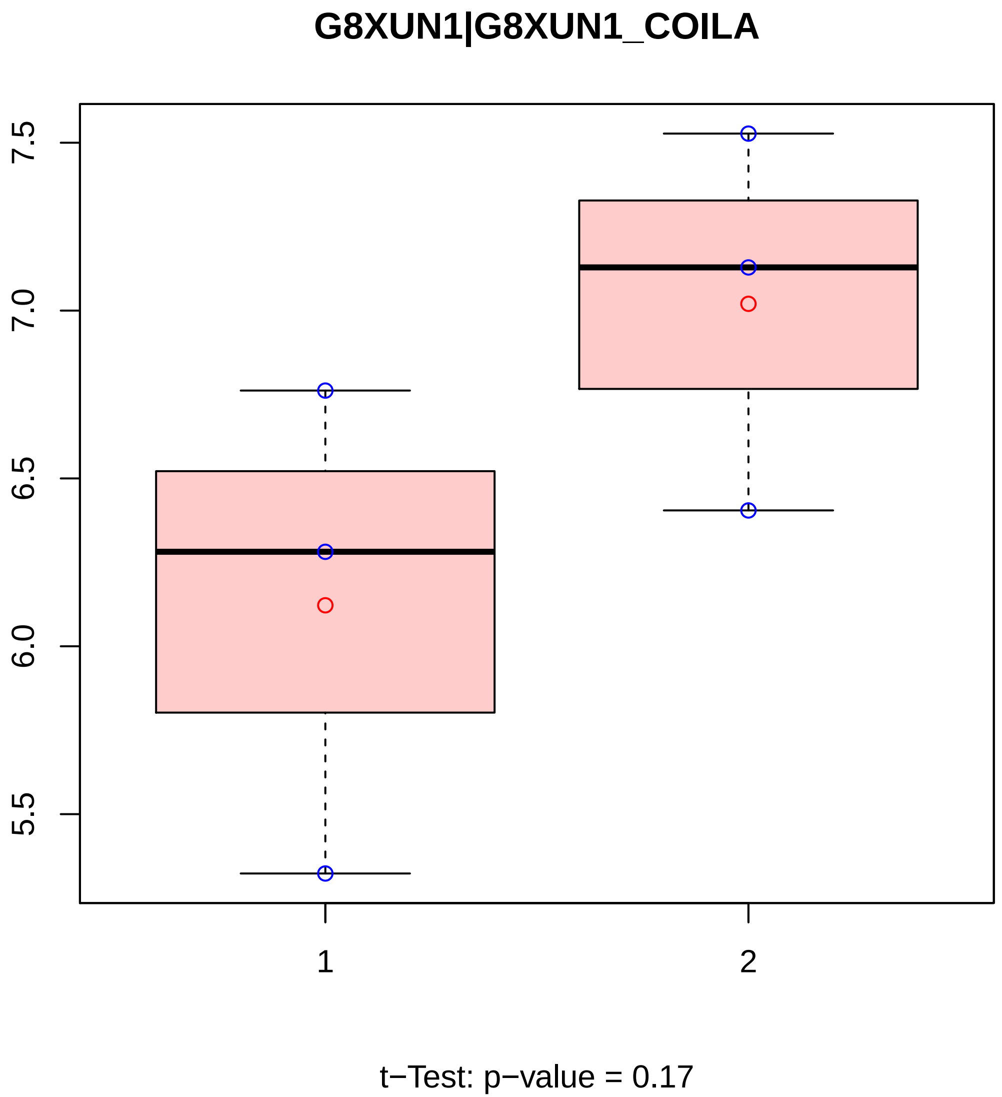
G8XUN1|G8XUN1_COILA
7.5
7.0
6.5
6.0
5.5
1
2
t−Test: p−value = 0.17

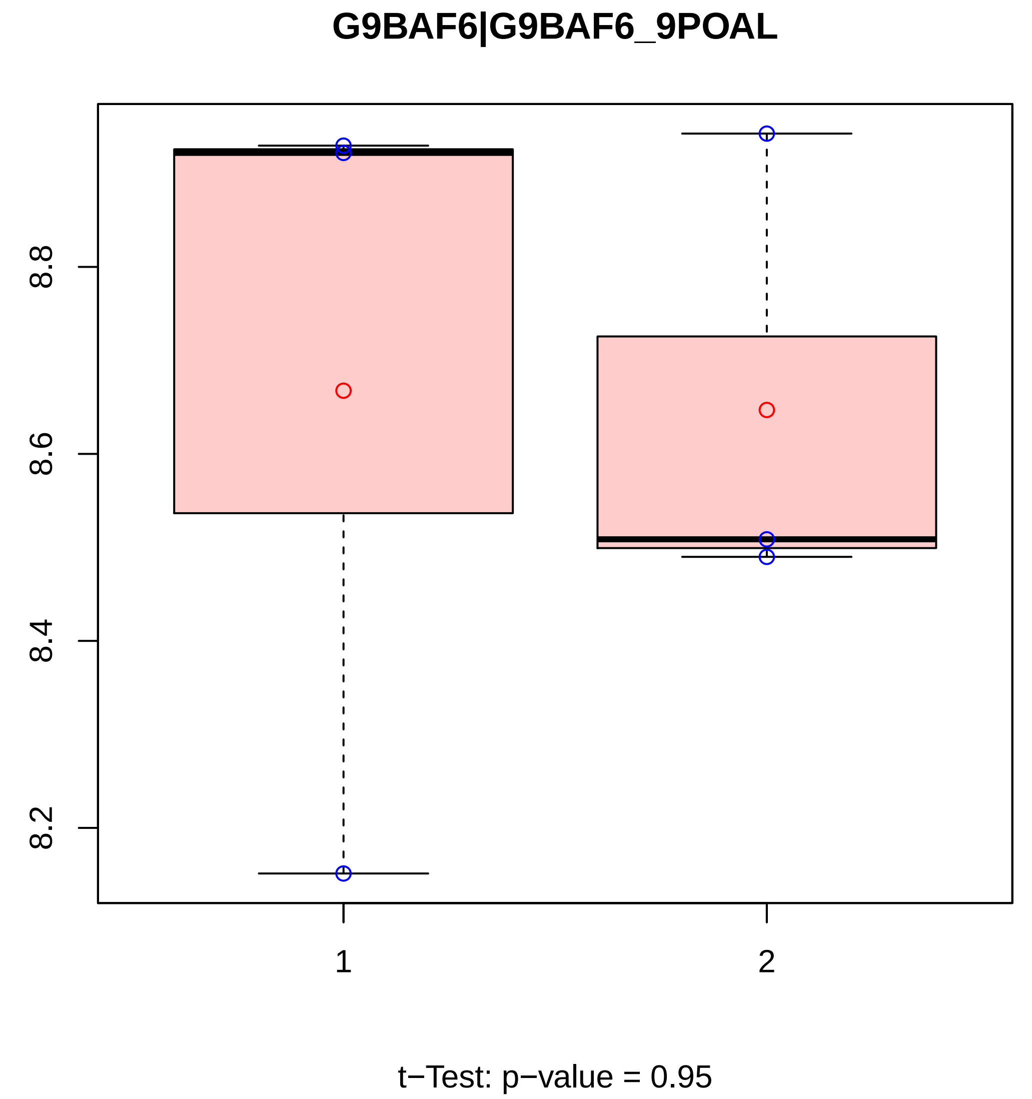
G9BAF6|G9BAF6_9POAL
8.2
8.4
8.6
8.8
1
2
t−Test: p−value = 0.95

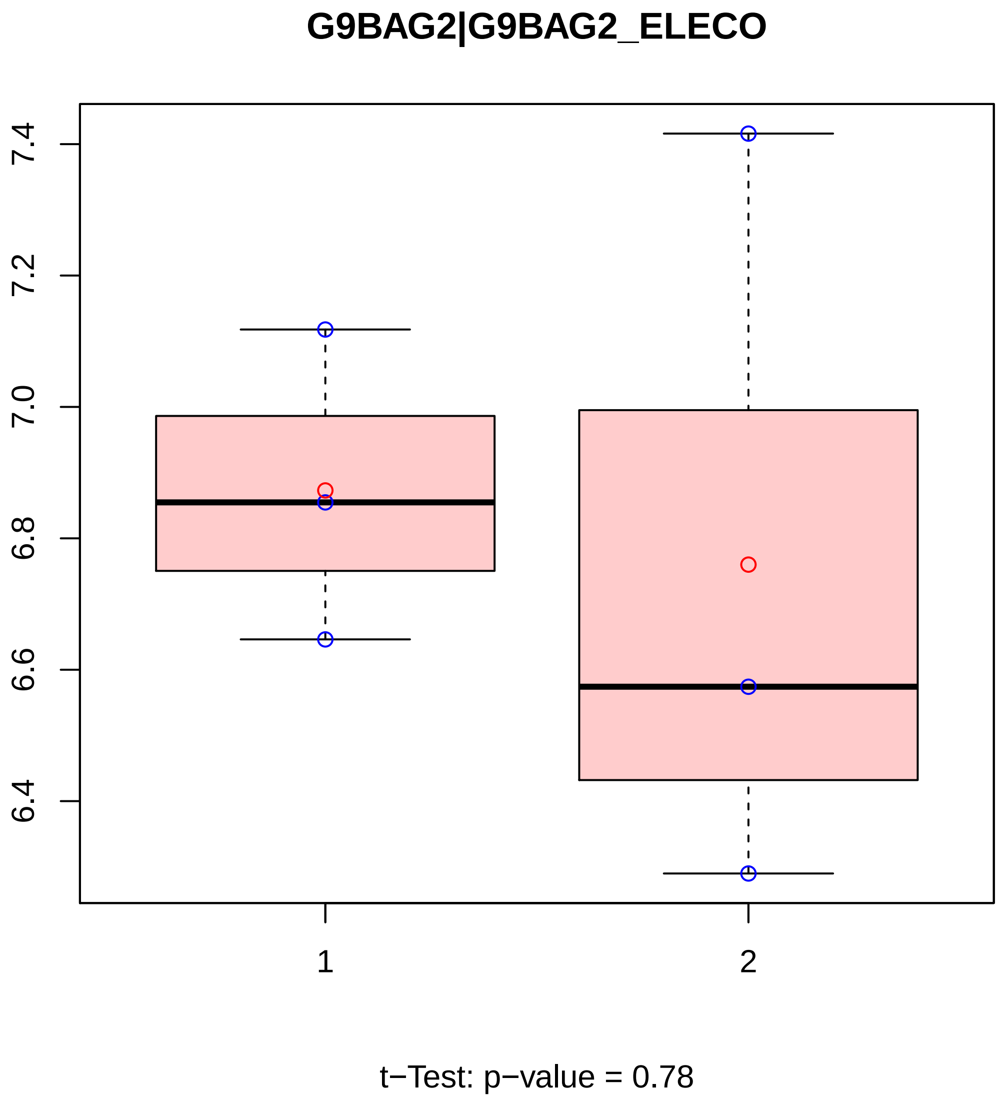

G9BAG2|G9BAG2_ELECO
7.4
7.2
7.0
6.8
6.6
6.4
1
2
t−Test: p−value = 0.78

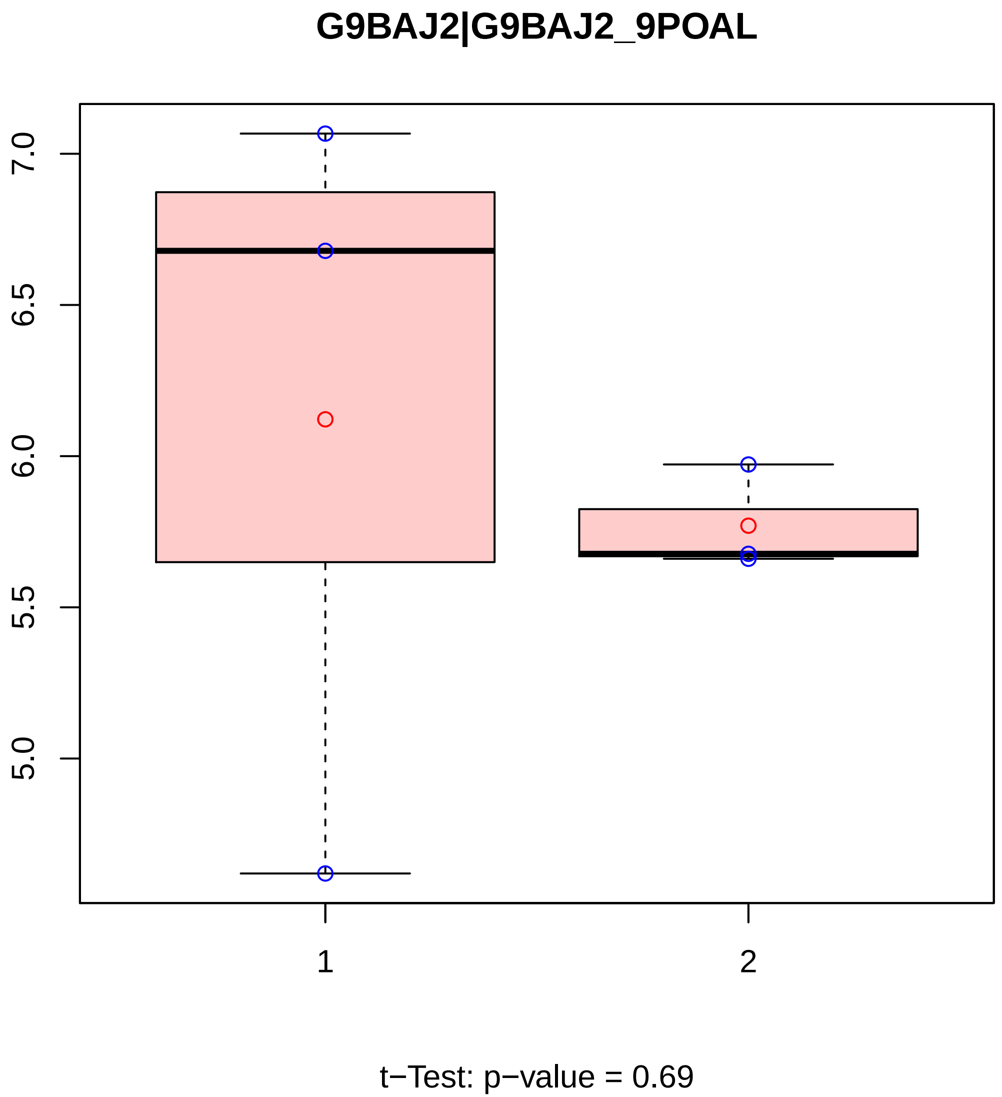
G9BAJ2|G9BAJ2_9POAL
7.0
6.5
6.0
5.5
5.0
1
2
t−Test: p−value = 0.69

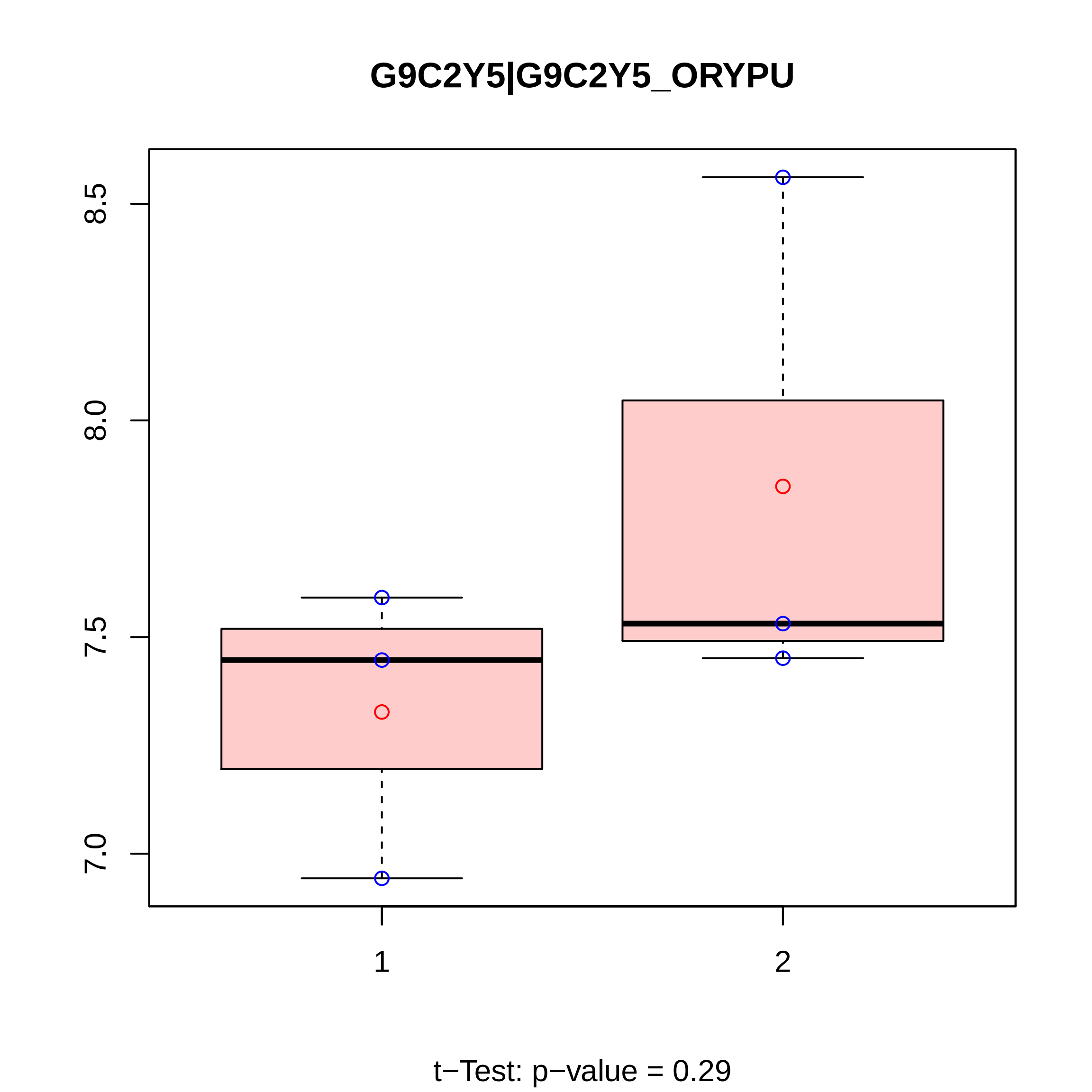
G9C2Y5|G9C2Y5_ORYPU
8.5
8.0
7.5
7.0
1
2
t−Test: p−value = 0.29

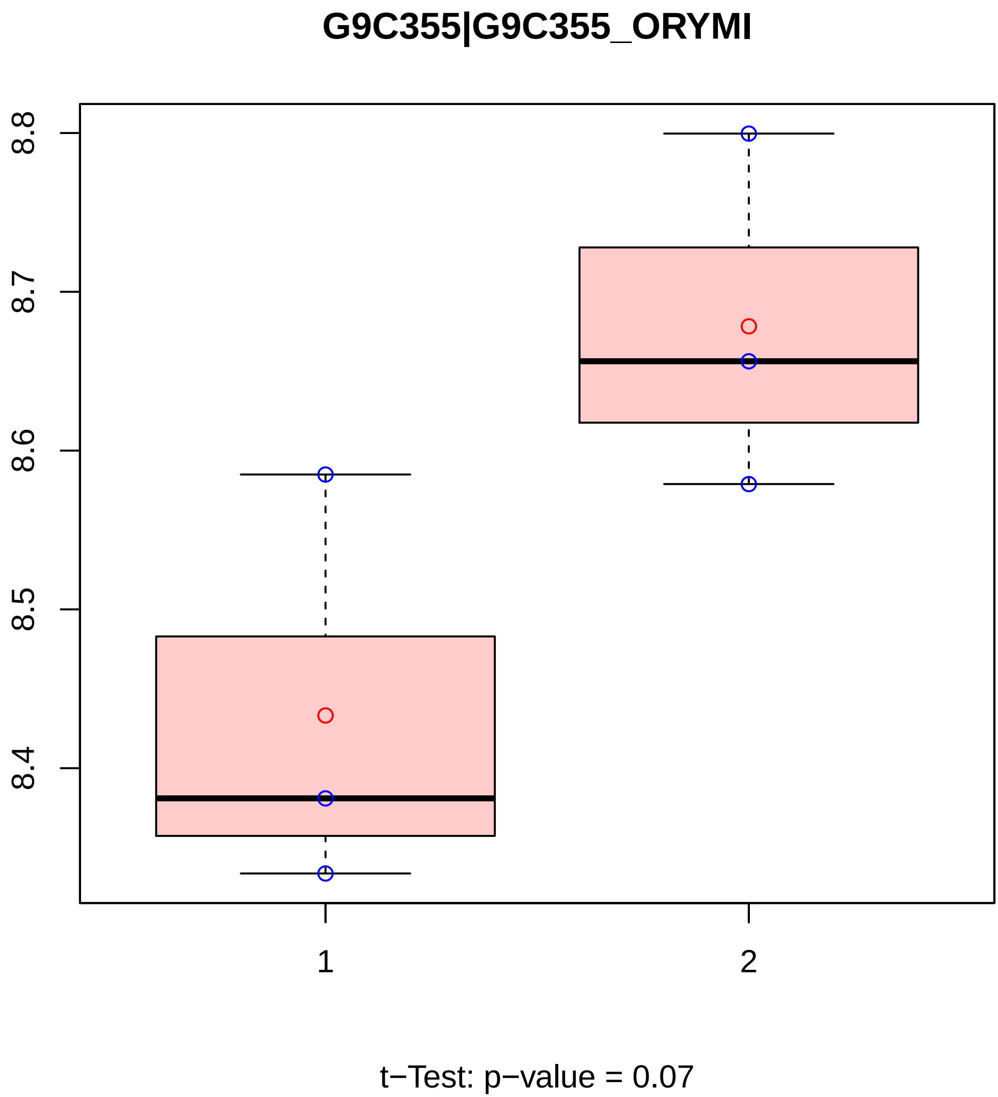
G9C355|G9C355_ORYMI
8.4
8.5
8.6
8.7
8.8
1
2
t−Test: p−value = 0.07

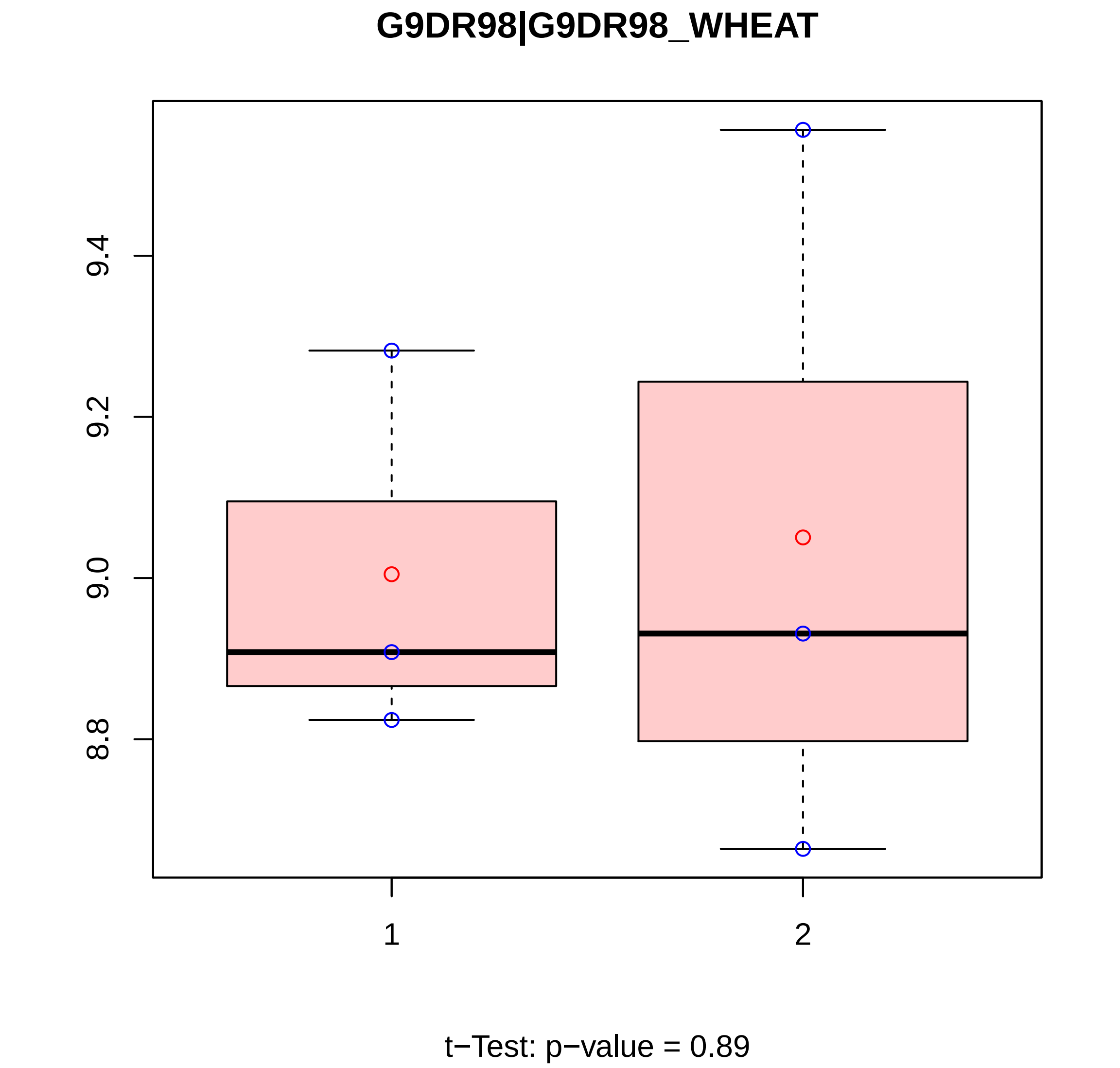

G9DR98|G9DR98_WHEAT
9.4
9.2
9.0
8.8
1
2
t−Test: p−value = 0.89

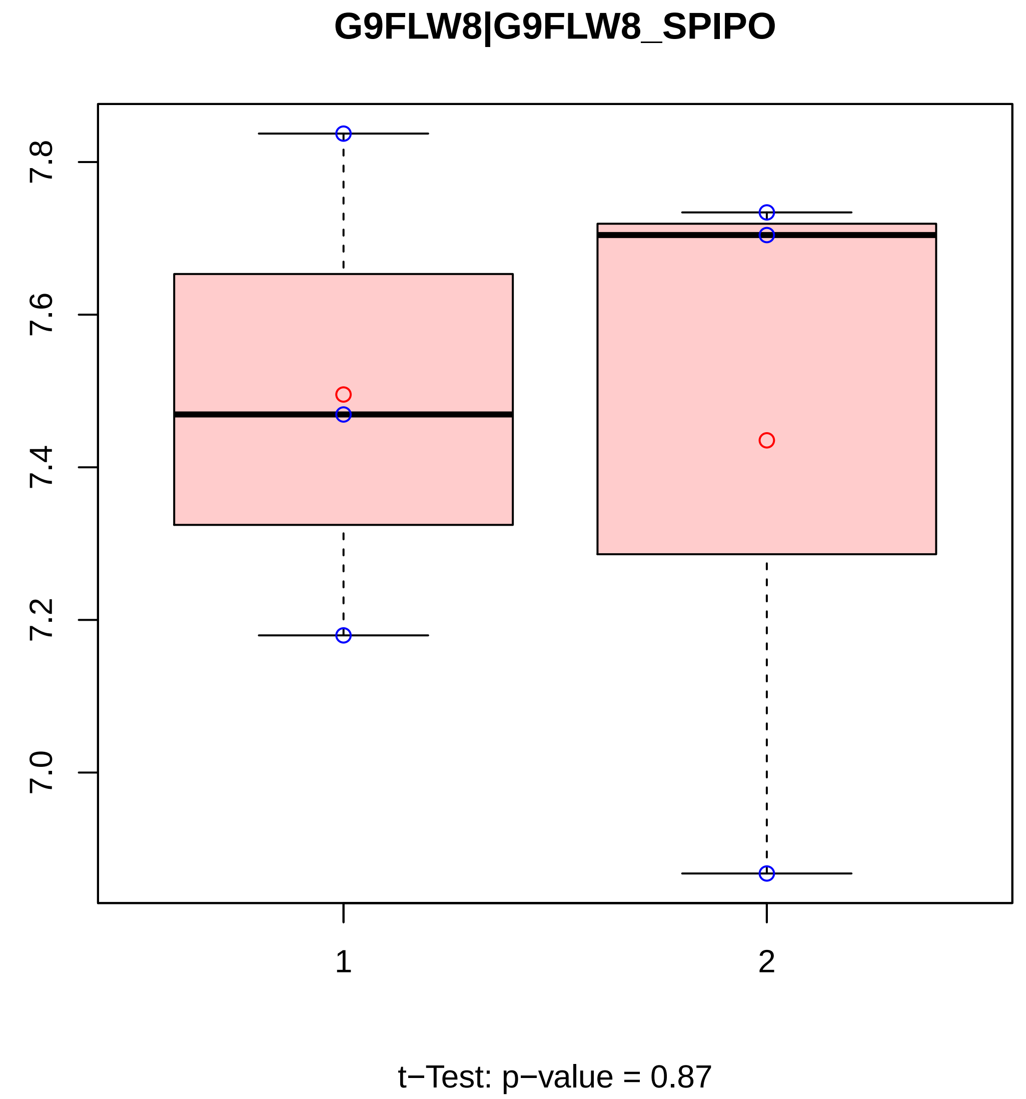
G9FLW8|G9FLW8_SPIPO
7.8
7.6
7.4
7.2
7.0
1
2
t−Test: p−value = 0.87

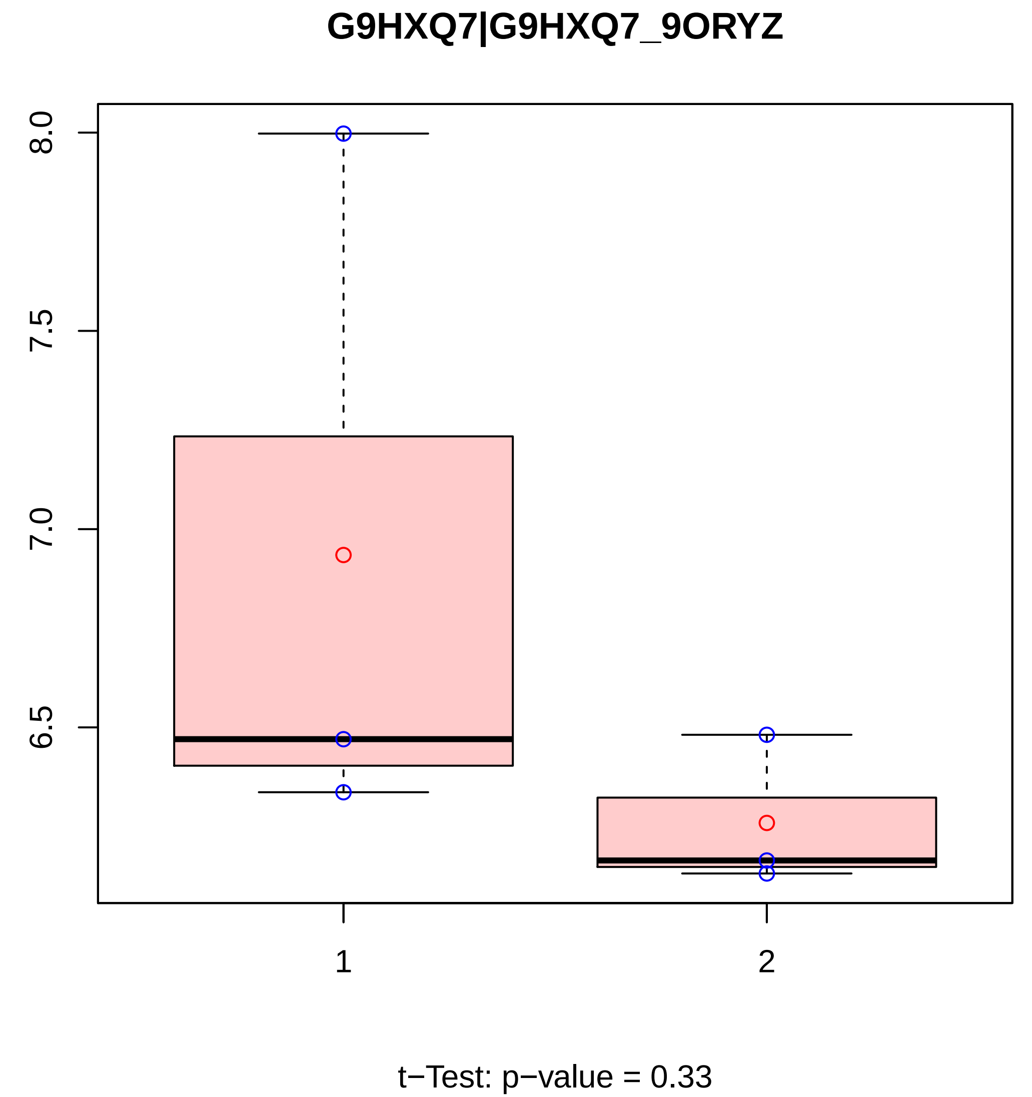
G9HXQ7|G9HXQ7_9ORYZ
8.0
7.5
7.0
6.5
1
2
t−Test: p−value = 0.33

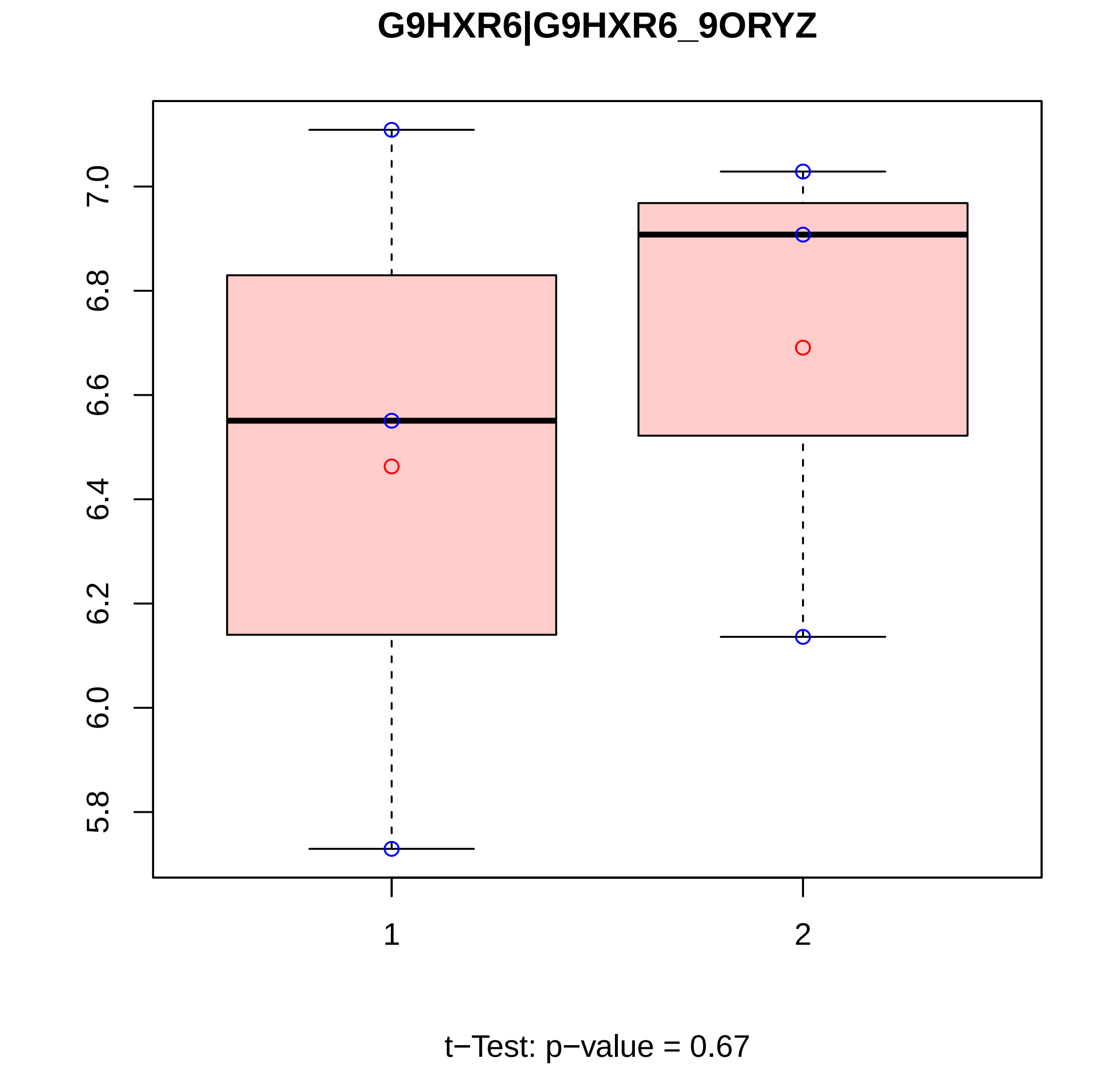
G9HXR6|G9HXR6_9ORYZ
7.0
6.8
6.6
6.4
6.2
6.0
5.8
1
2
t−Test: p−value = 0.67

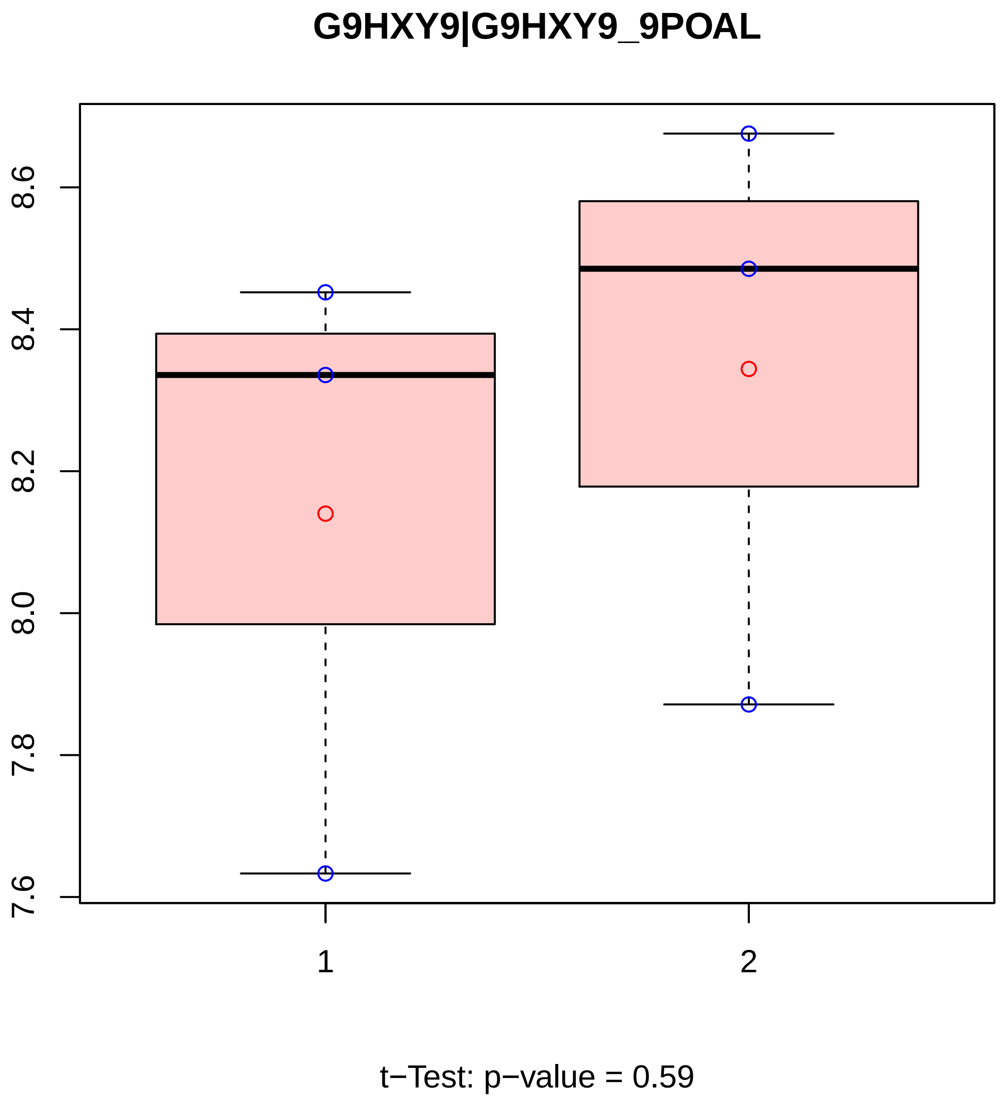
G9HXY9|G9HXY9_9POAL
8.6
8.4
8.2
8.0
7.8
7.6
1
2
t−Test: p−value = 0.59

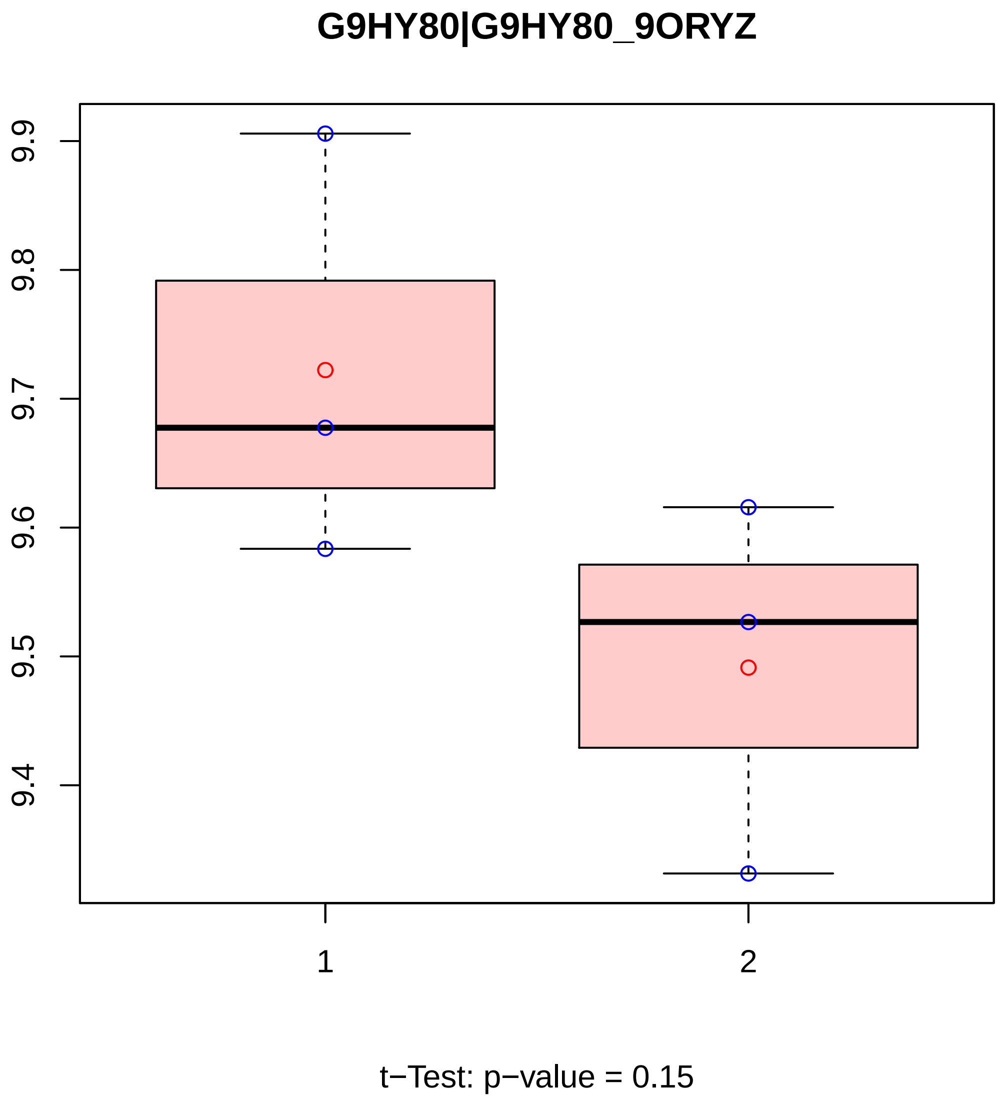
G9HY80|G9HY80_9ORYZ
9.9
9.8
9.7
9.6
9.5
9.4
1
2
t−Test: p−value = 0.15

G9JKH7|G9JKH7_9LILI

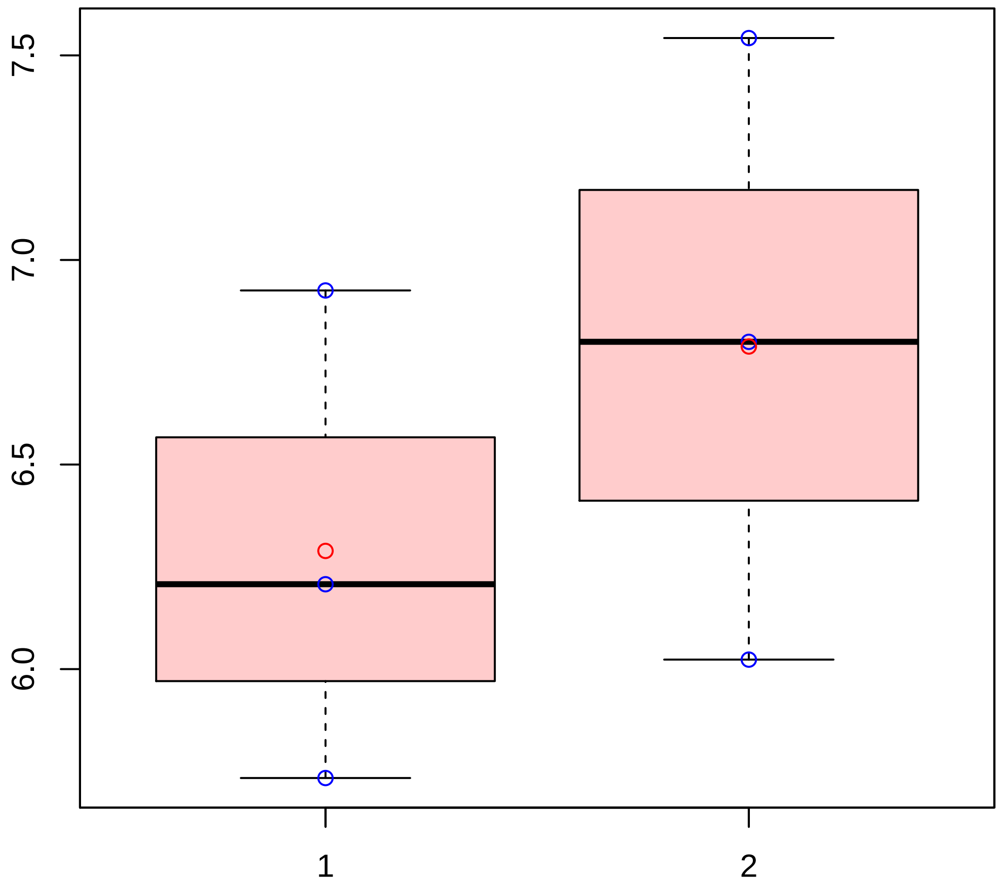

t−Test: p−value = 0.42

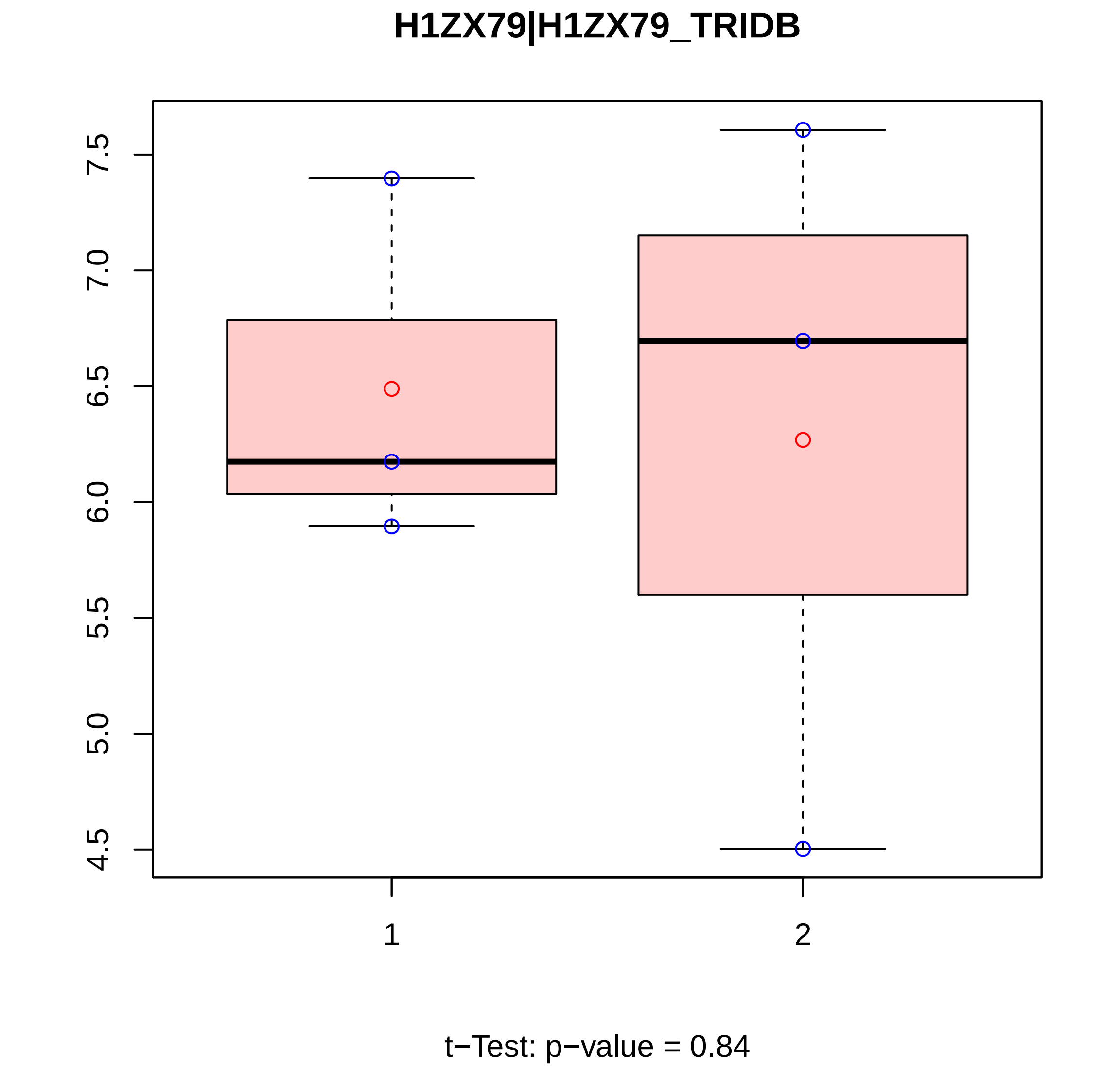
H1ZX79|H1ZX79_TRIDB
7.5
7.0
6.5
6.0
5.5
5.0
4.5
1
2
t−Test: p−value = 0.84

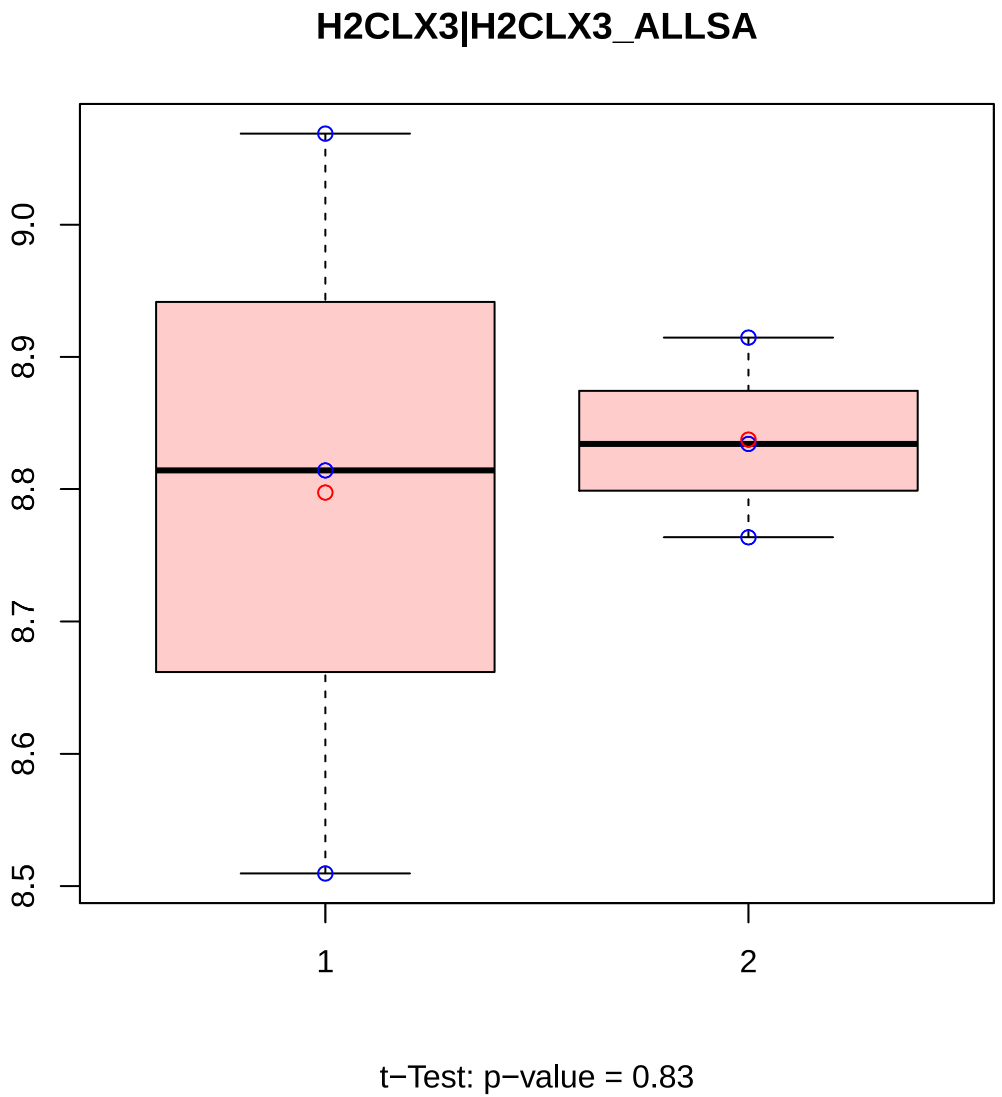
H2CLX3|H2CLX3_ALLSA
9.0
8.9
8.8
8.7
8.6
8.5
1
2
t−Test: p−value = 0.83

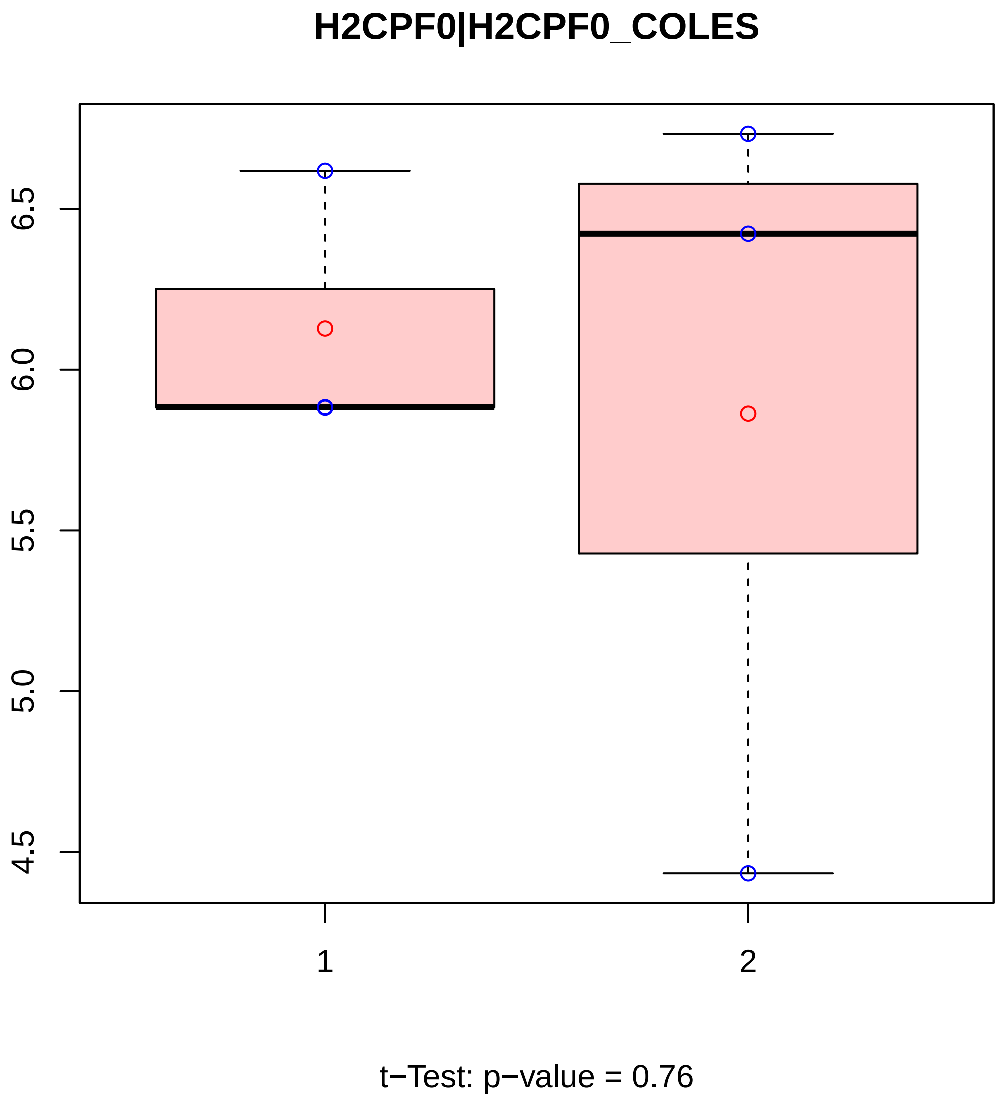
H2CPF0|H2CPF0_COLES
6.5
6.0
5.5
5.0
4.5
1
2
t−Test: p−value = 0.76

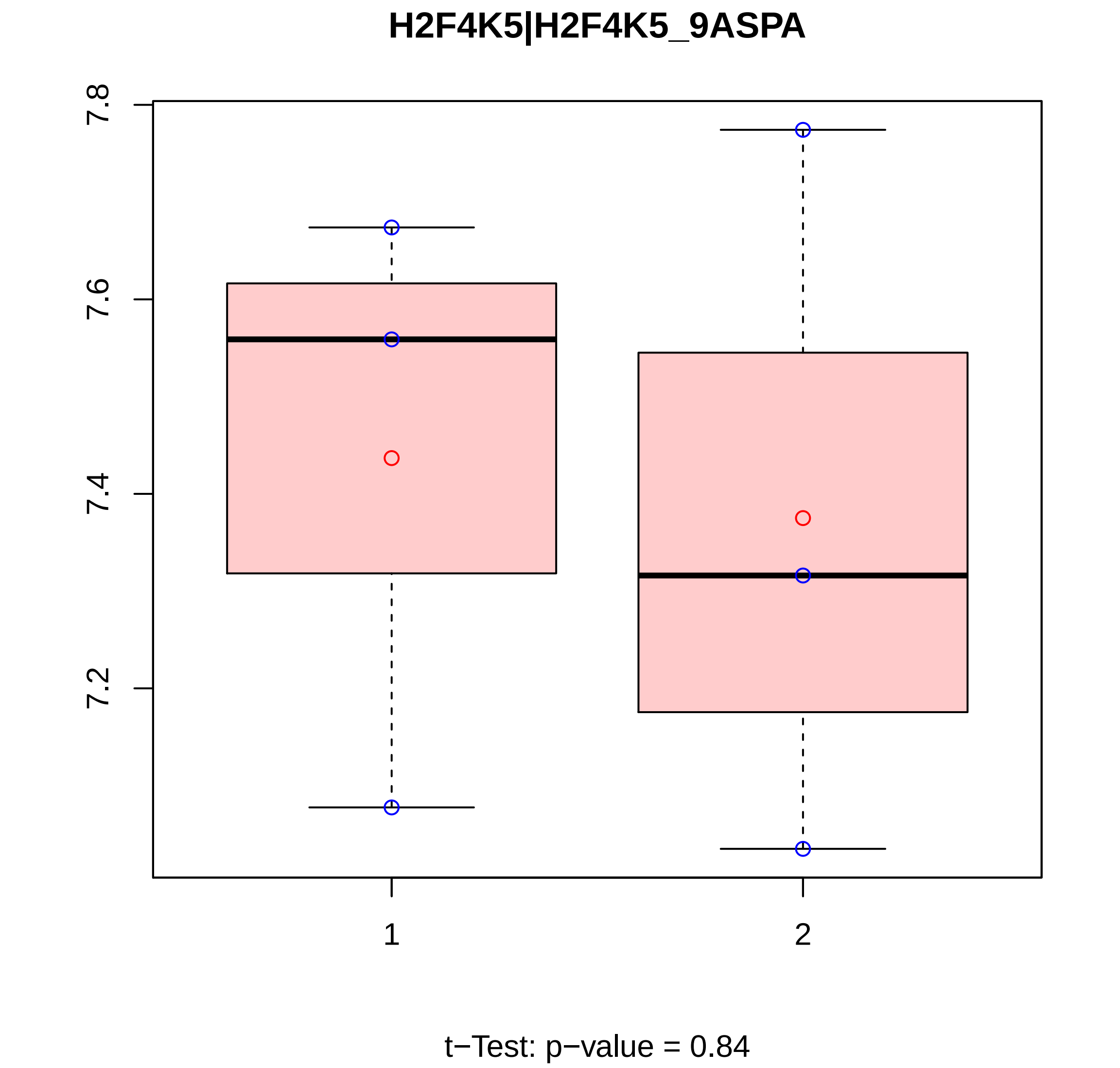
H2F4K5|H2F4K5_9ASPA
7.8
7.6
7.4
7.2
1
2
t−Test: p−value = 0.84

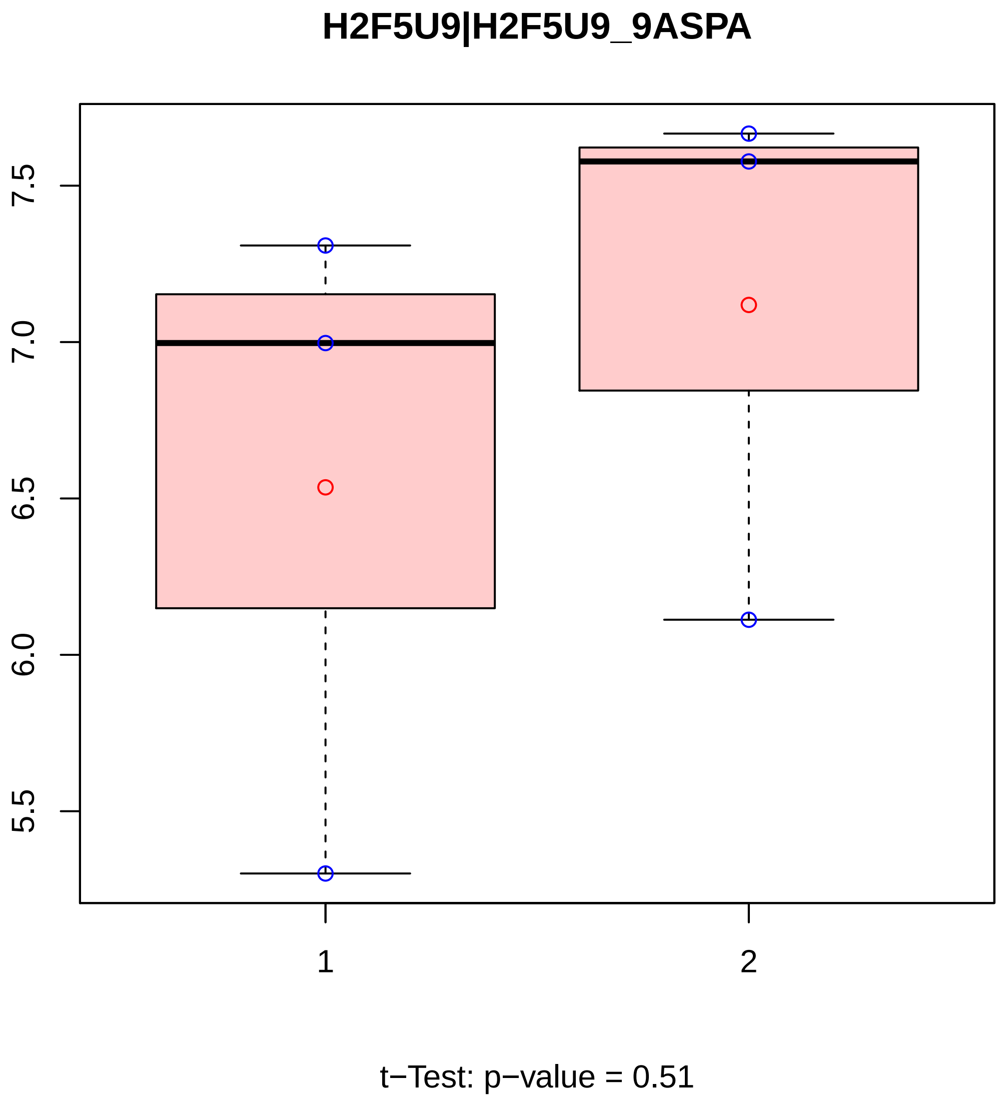
H2F5U9|H2F5U9_9ASPA
7.5
7.0
6.5
6.0
5.5
1
2
t−Test: p−value = 0.51

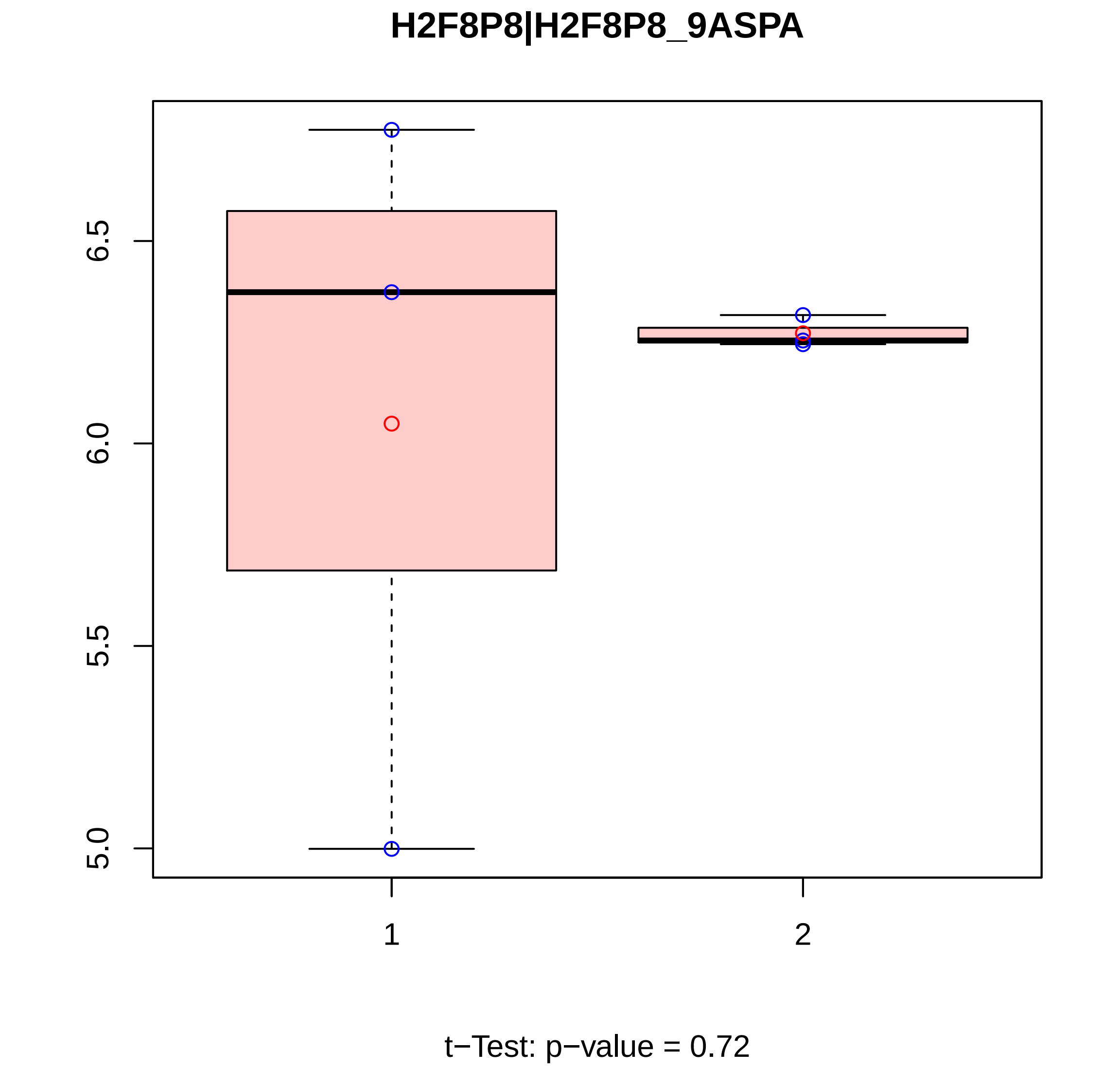
H2F8P8|H2F8P8_9ASPA
6.5
6.0
5.5
5.0
1
2
t−Test: p−value = 0.72

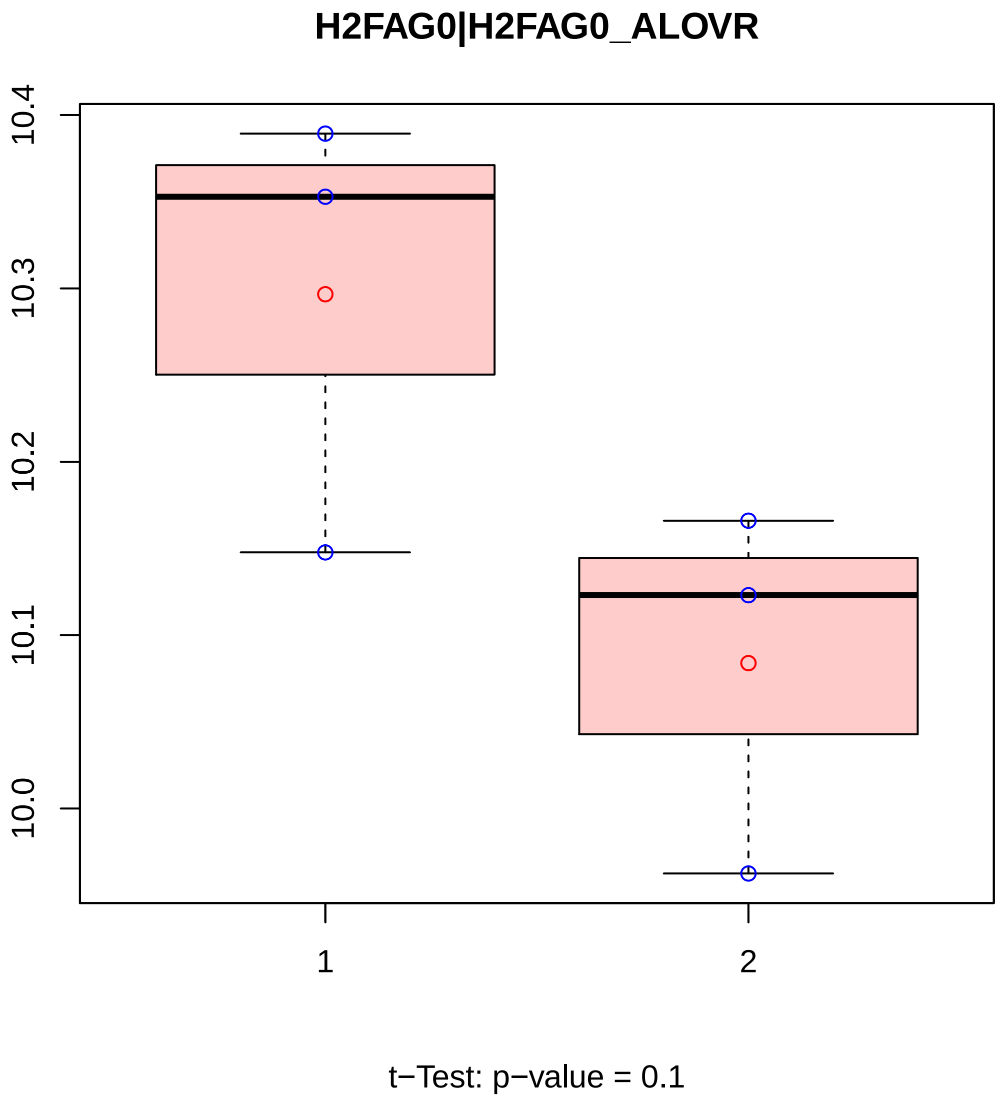

H2FAG0|H2FAG0_ALOVR
10.4
10.3
10.2
10.1
10.0
1
2
t−Test: p−value = 0.1

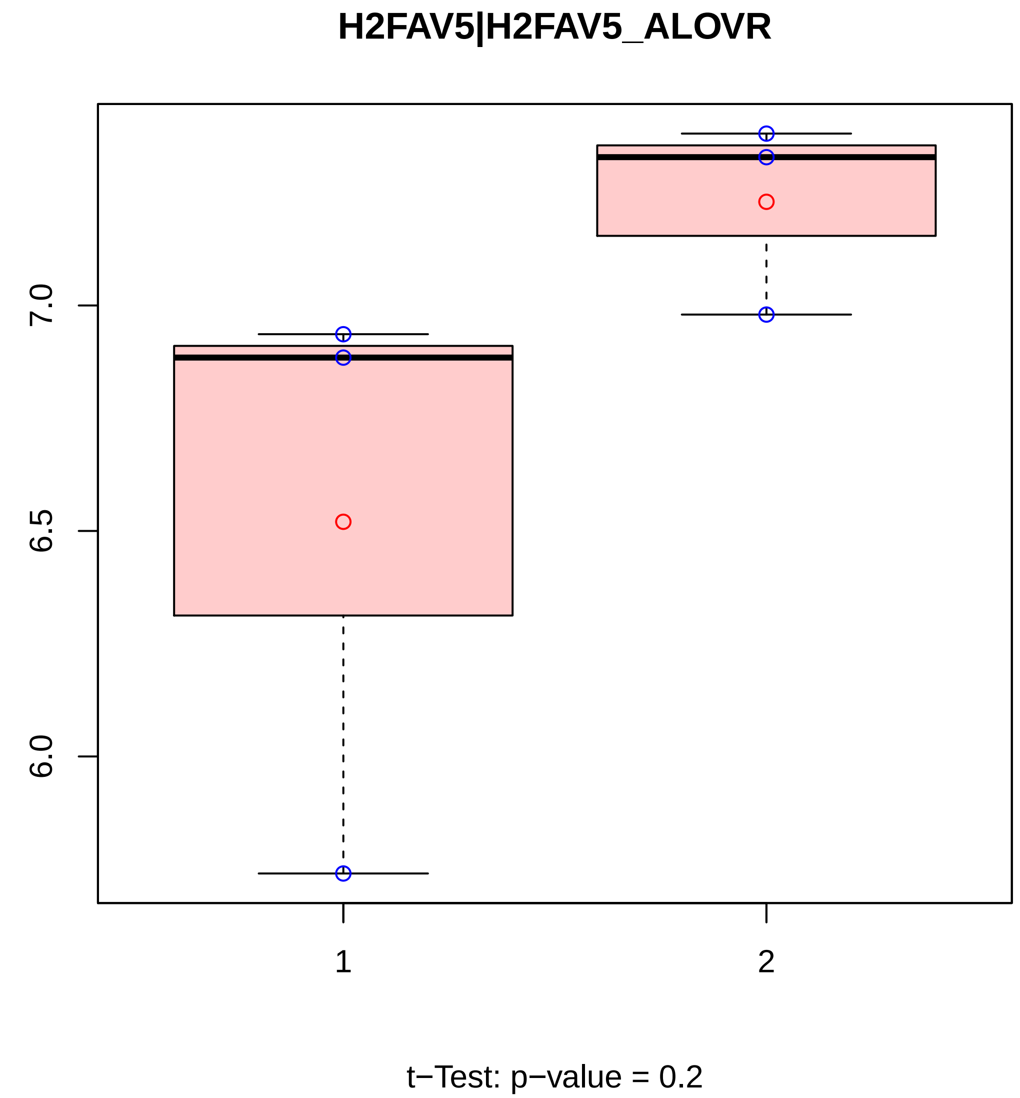
H2FAV5|H2FAV5_ALOVR
7.0
6.5
6.0
1
2
t−Test: p−value = 0.2

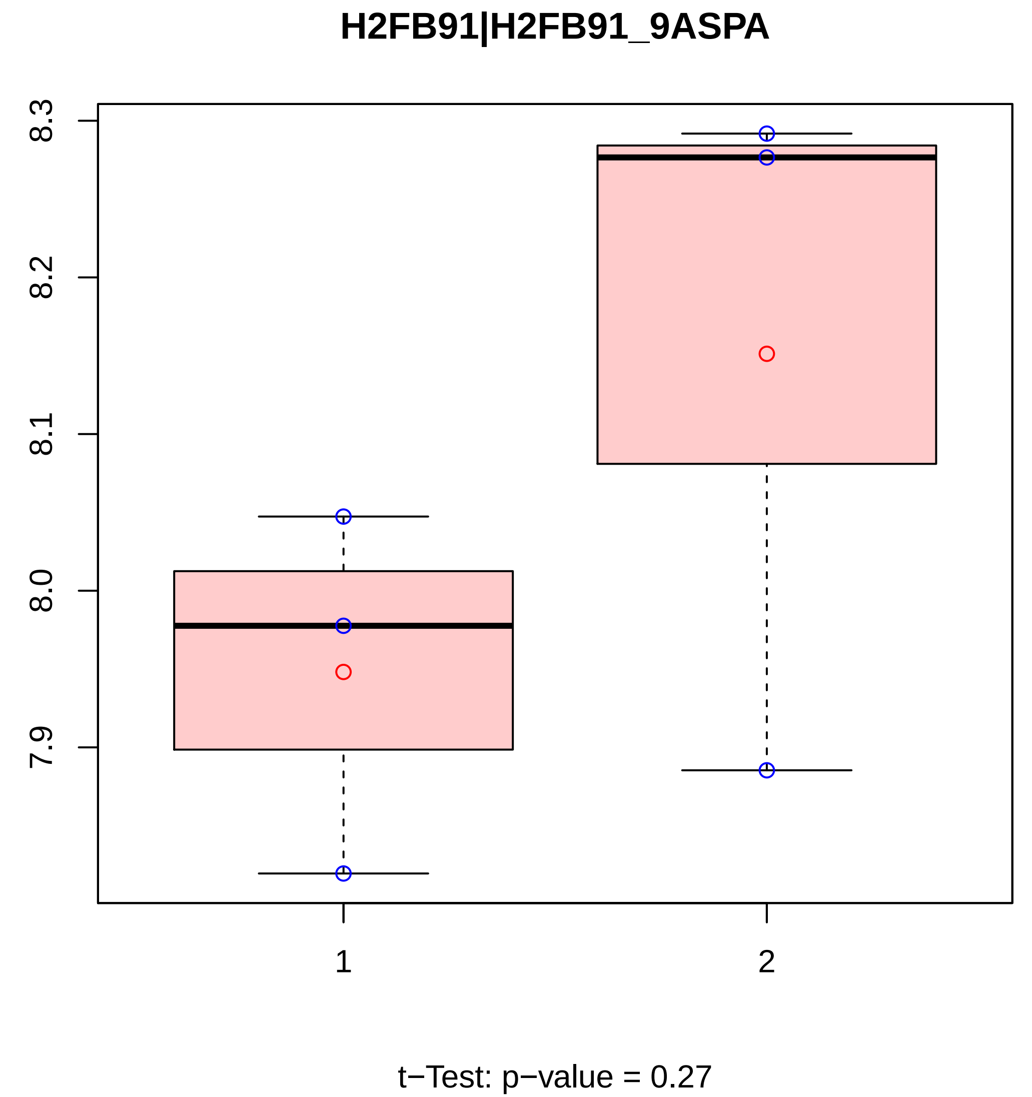
H2FB91|H2FB91_9ASPA
8.3
8.2
8.1
8.0
7.9
1
2
t−Test: p−value = 0.27

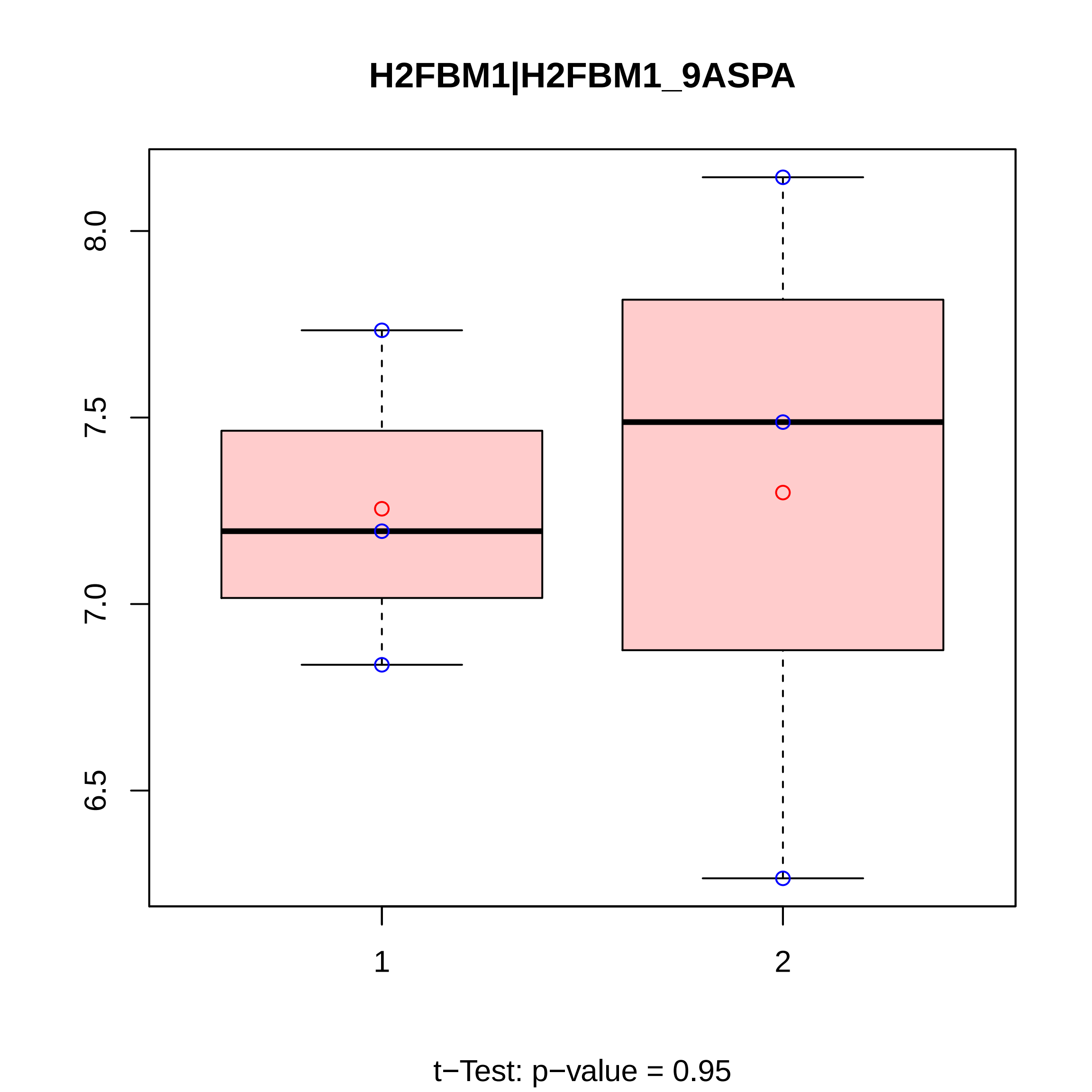

H2FBM1|H2FBM1_9ASPA
8.0
7.5
7.0
6.5
1
2
t−Test: p−value = 0.95

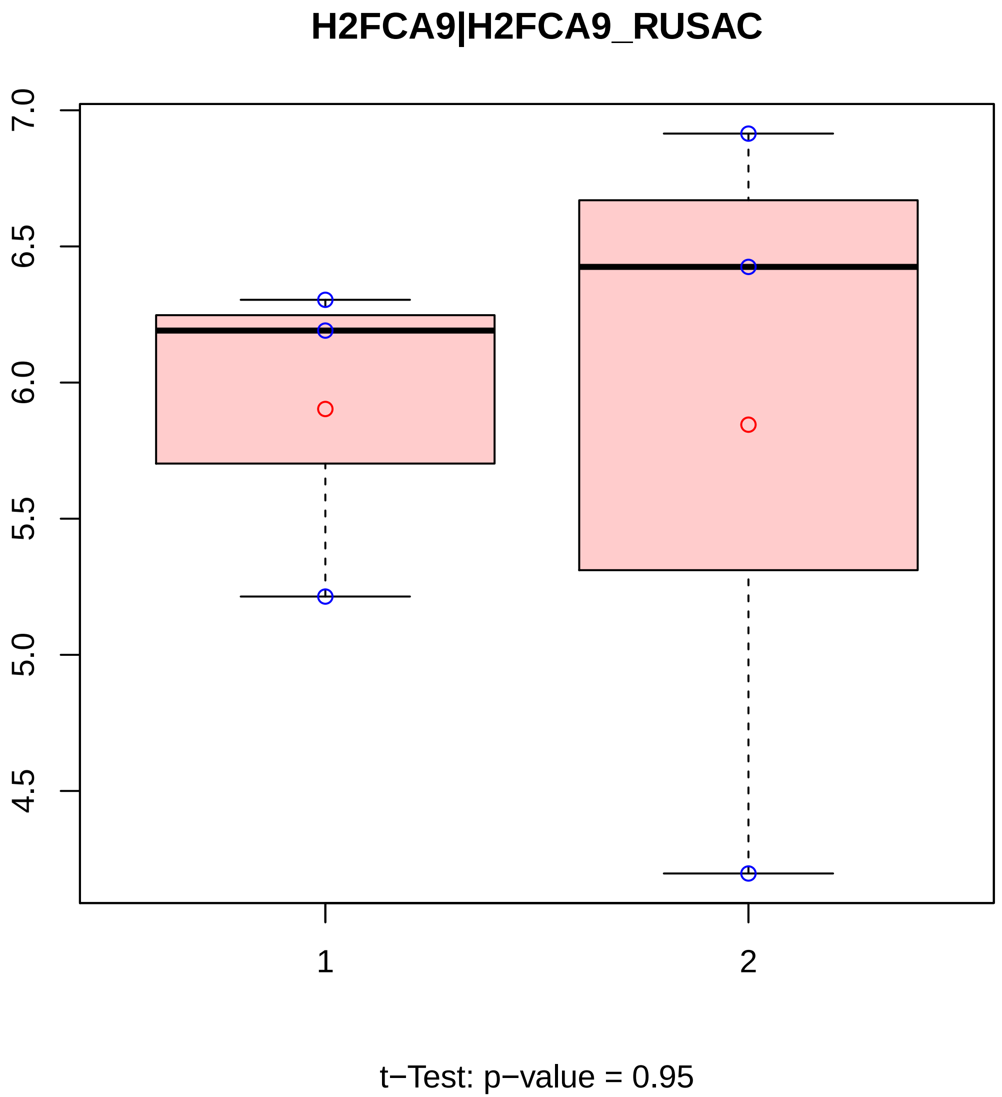
H2FCA9|H2FCA9_RUSAC
7.0
6.5
6.0
5.5
5.0
4.5
1
2
t−Test: p−value = 0.95

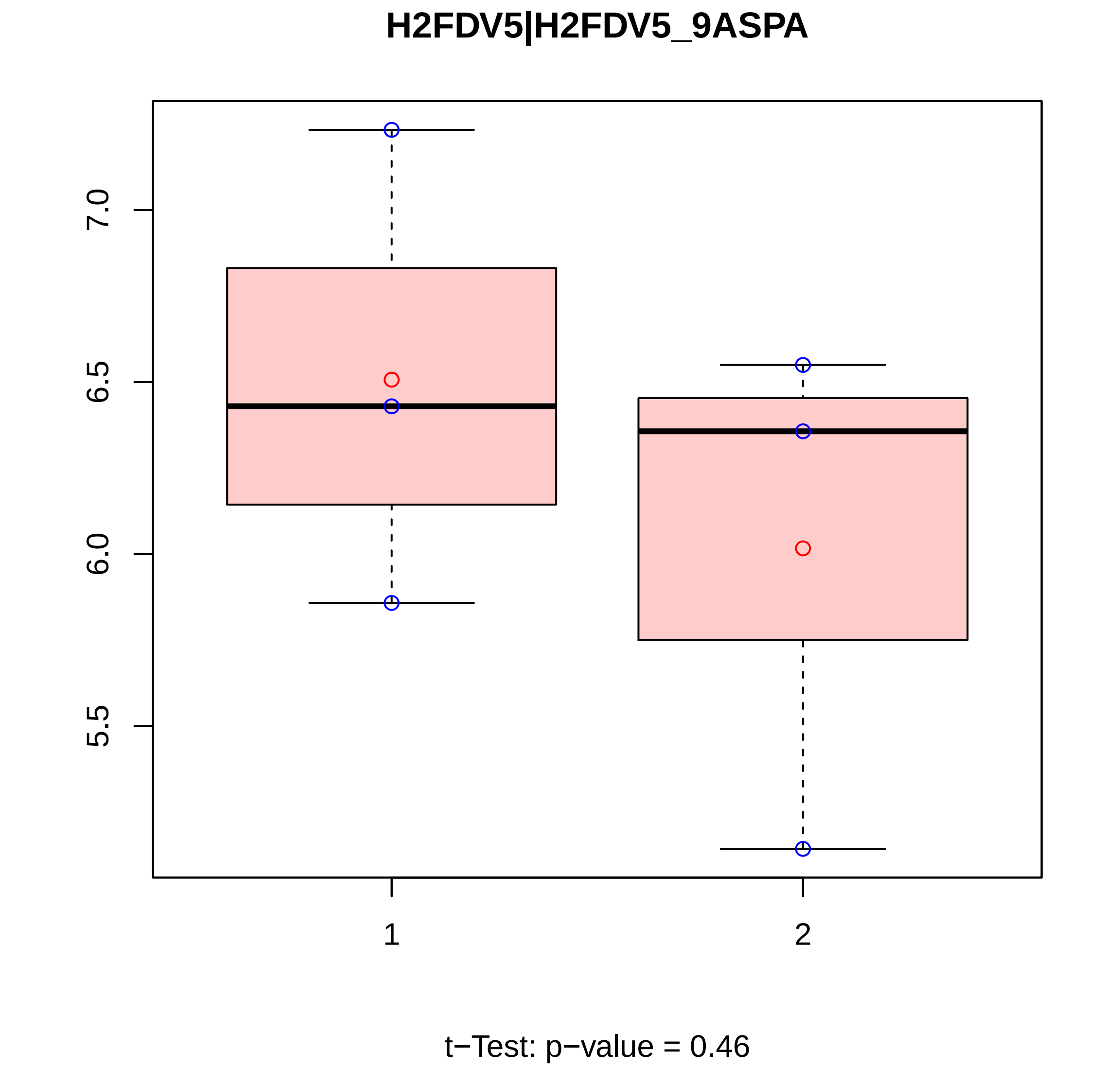
H2FDV5|H2FDV5_9ASPA
7.0
6.5
6.0
5.5
1
2
t−Test: p−value = 0.46

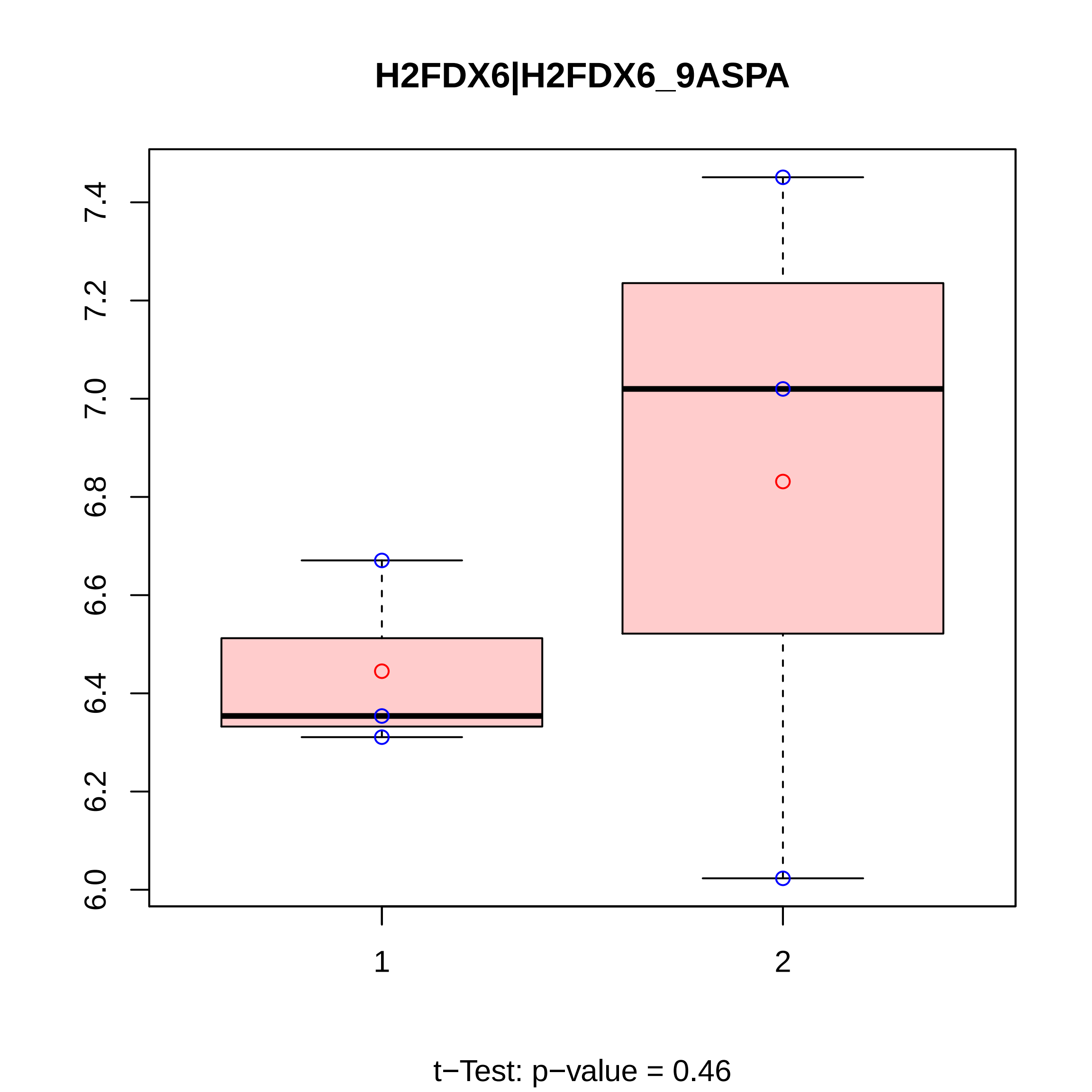
H2FDX6|H2FDX6_9ASPA
7.4
7.2
7.0
6.8
6.6
6.4
6.2
6.0
1
2
t−Test: p−value = 0.46

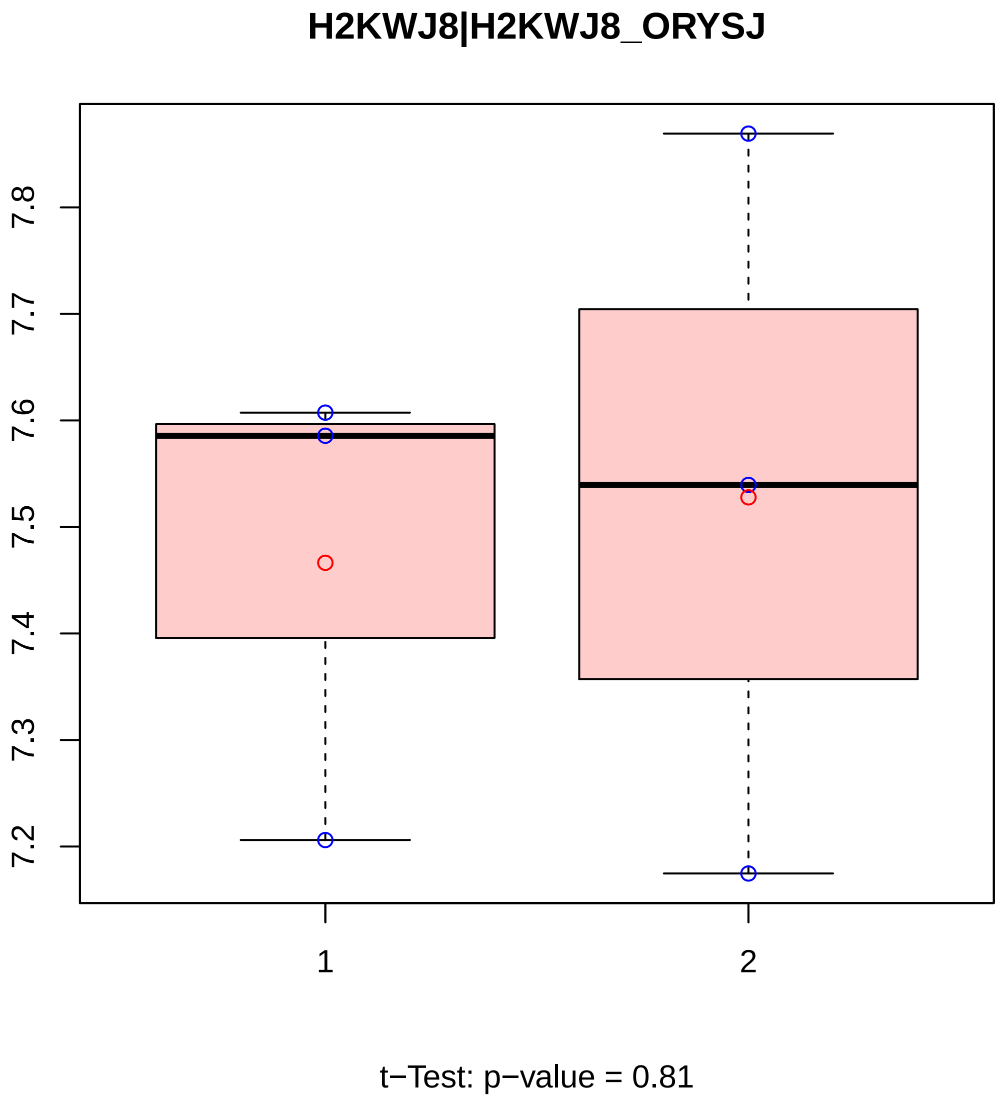
H2KWJ8|H2KWJ8_ORYSJ
7.2
7.3
7.4
7.5
7.6
7.7
7.8
1
2
t−Test: p−value = 0.81

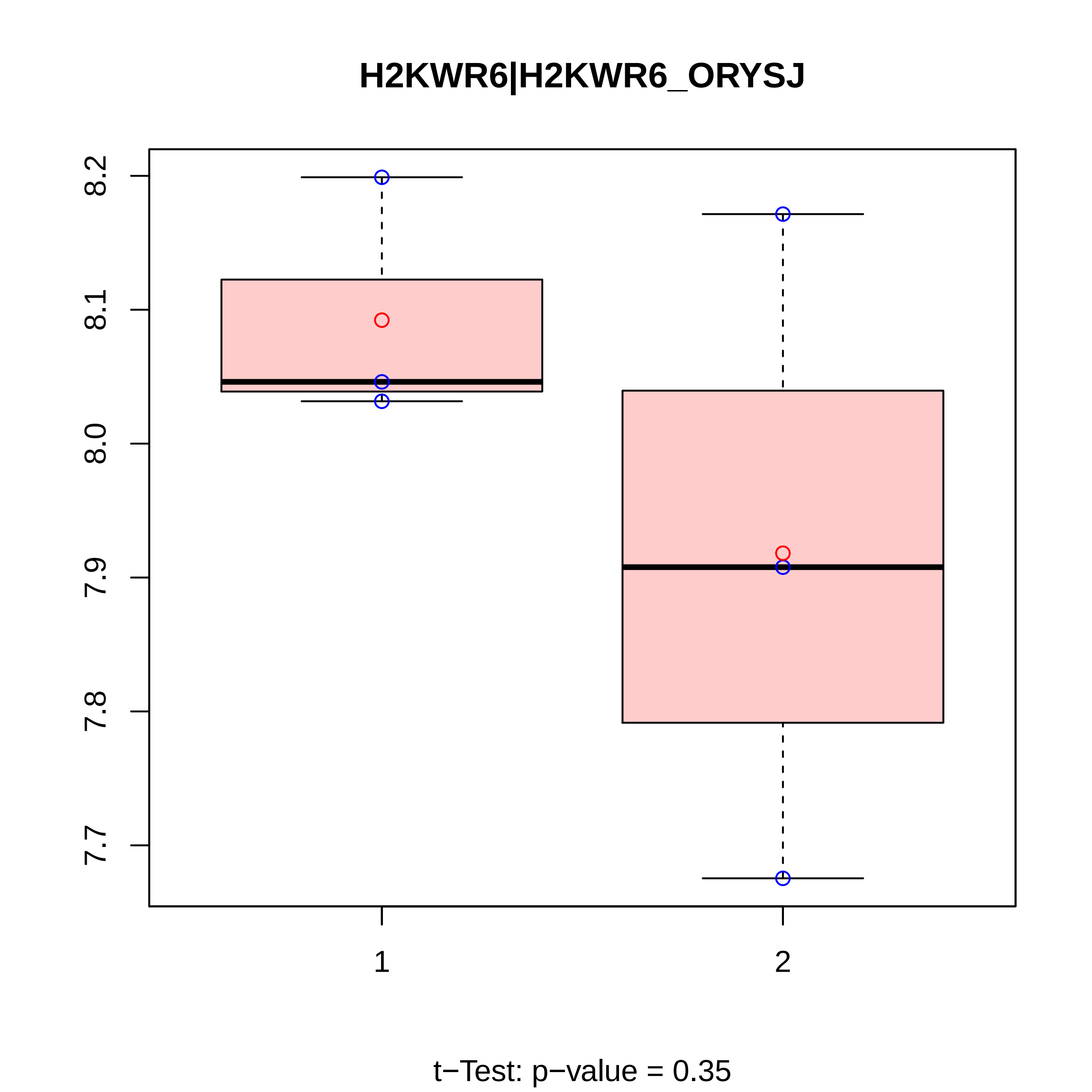
H2KWR6|H2KWR6_ORYSJ
8.2
8.1
8.0
7.9
7.8
7.7
1
2
t−Test: p−value = 0.35

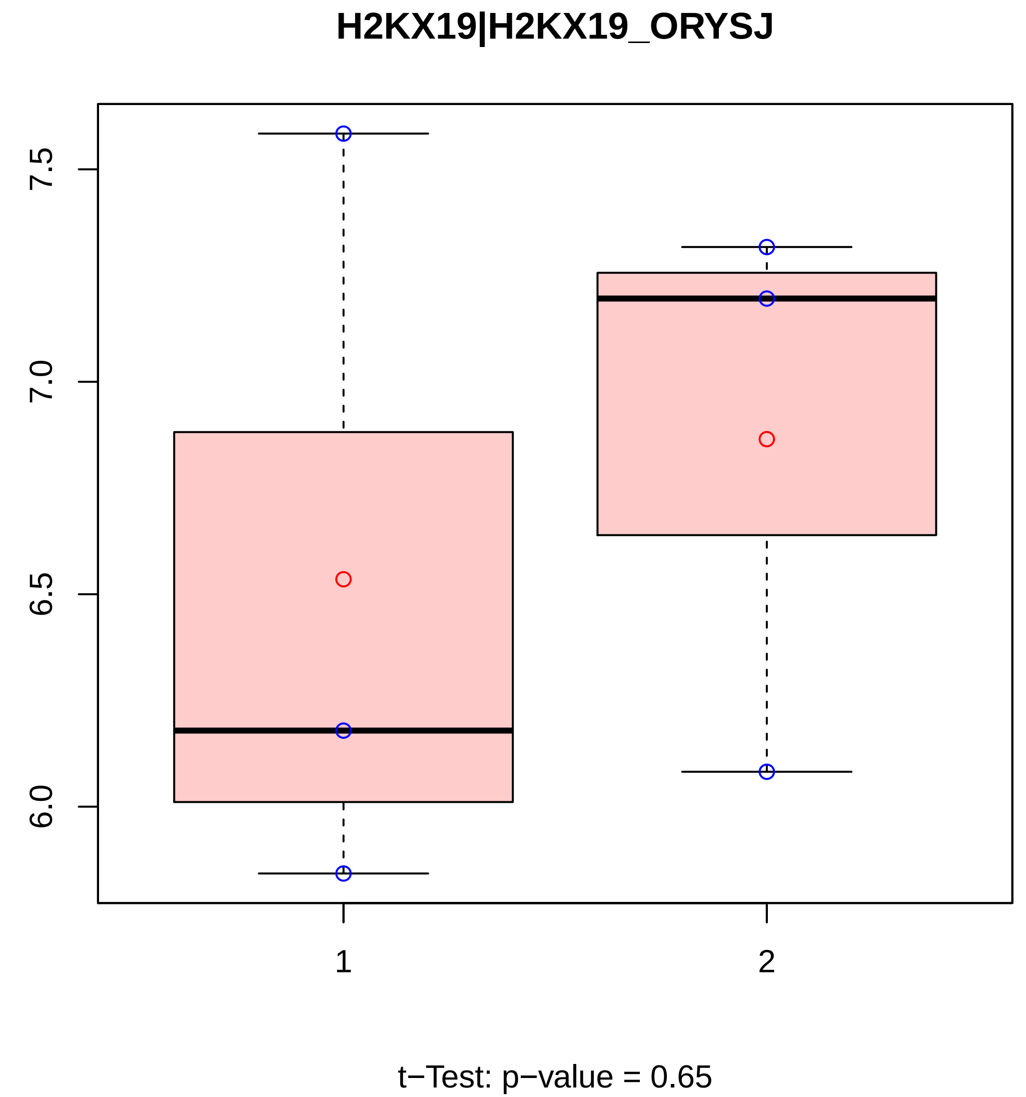
H2KX19|H2KX19_ORYSJ
7.5
7.0
6.5
6.0
1
2
t−Test: p−value = 0.65

H2KX75|H2KX75_ORYSJ

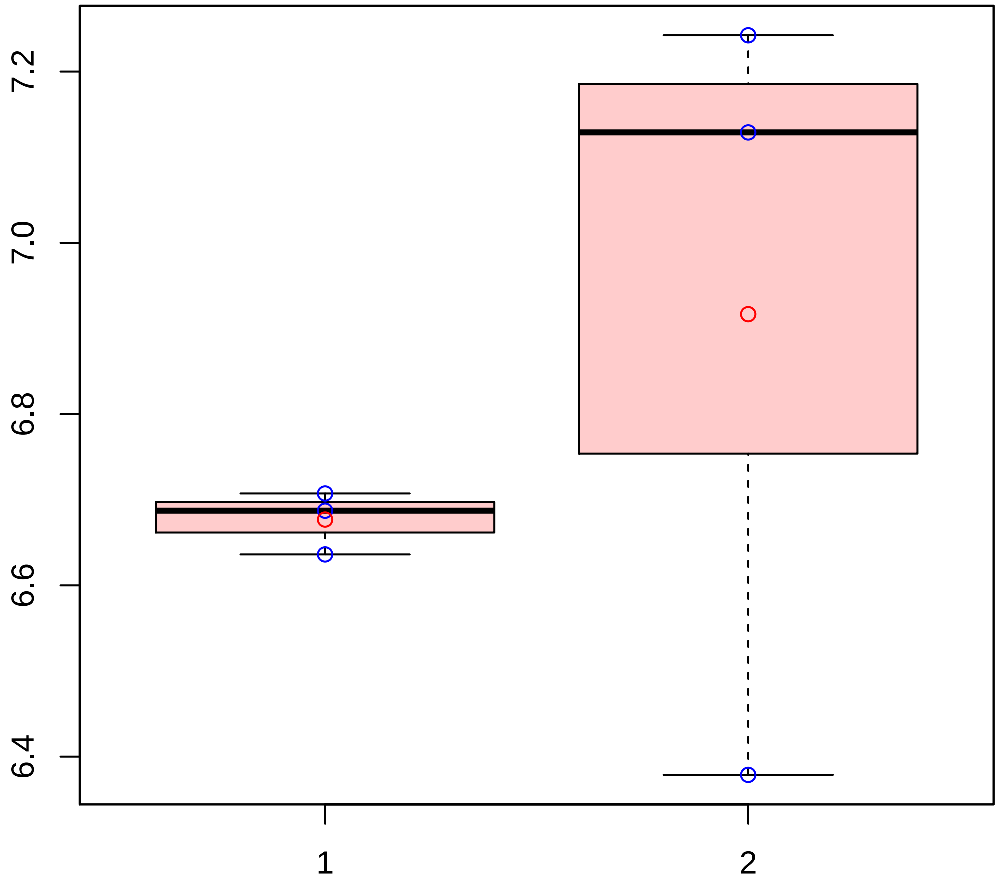

t−Test: p−value = 0.47

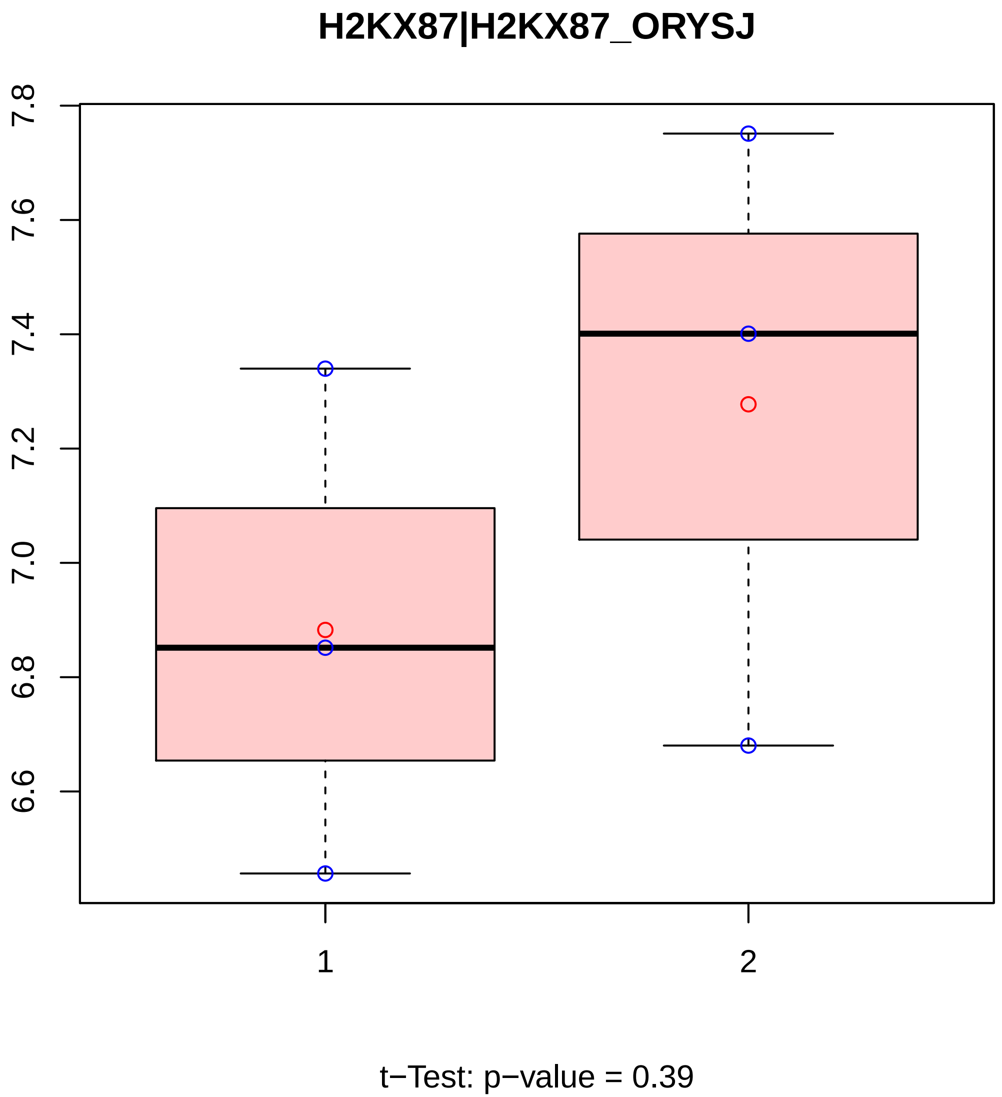
H2KX87|H2KX87_ORYSJ
7.8
7.6
7.4
7.2
7.0
6.8
6.6
1
2
t−Test: p−value = 0.39

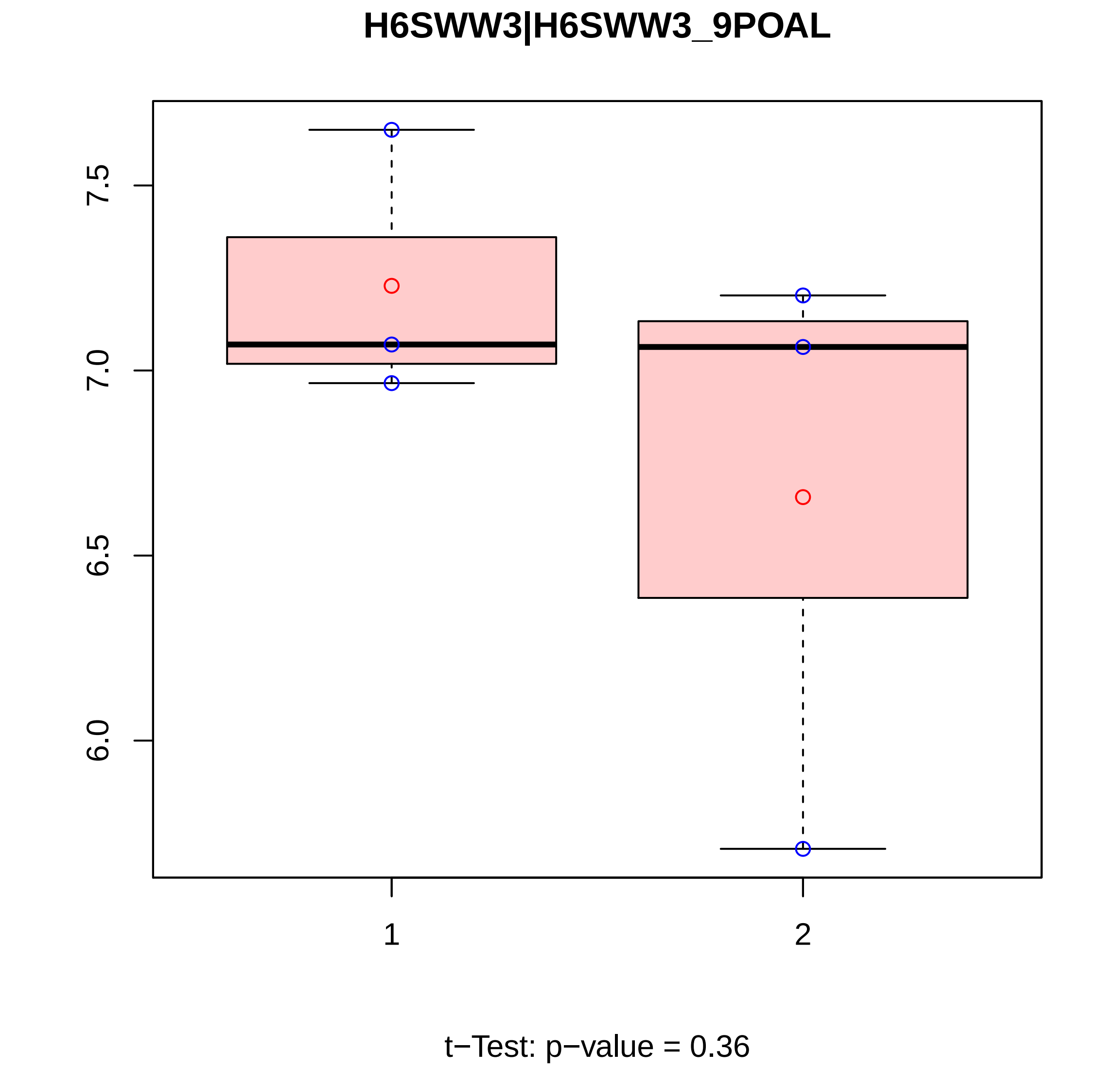
H6SWW3|H6SWW3_9POAL
7.5
7.0
6.5
6.0
1
2
t−Test: p−value = 0.36

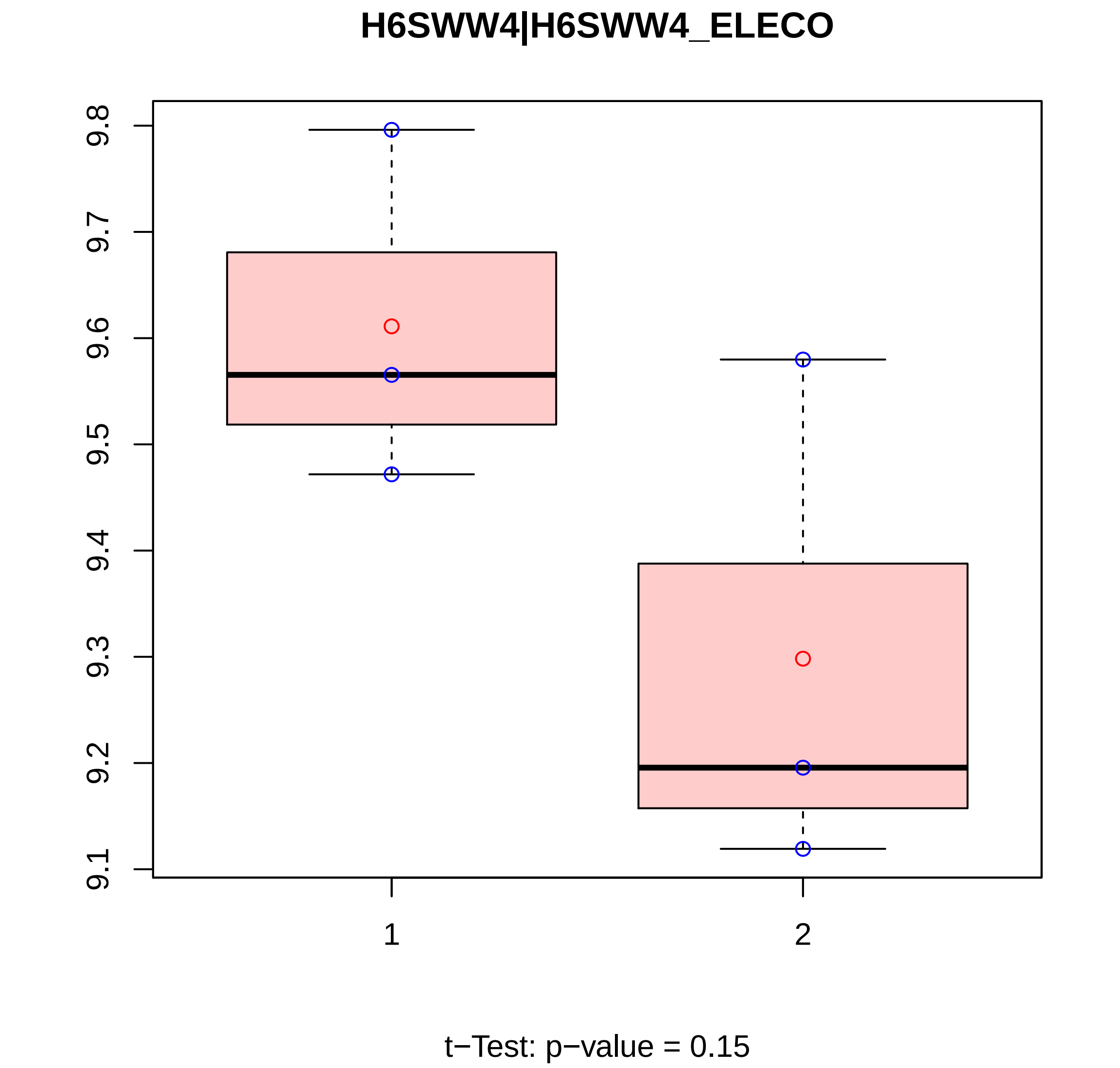

H6SWW4|H6SWW4_ELECO
9.1
9.2
9.3
9.4
9.5
9.6
9.7
9.8
1
2
t−Test: p−value = 0.15

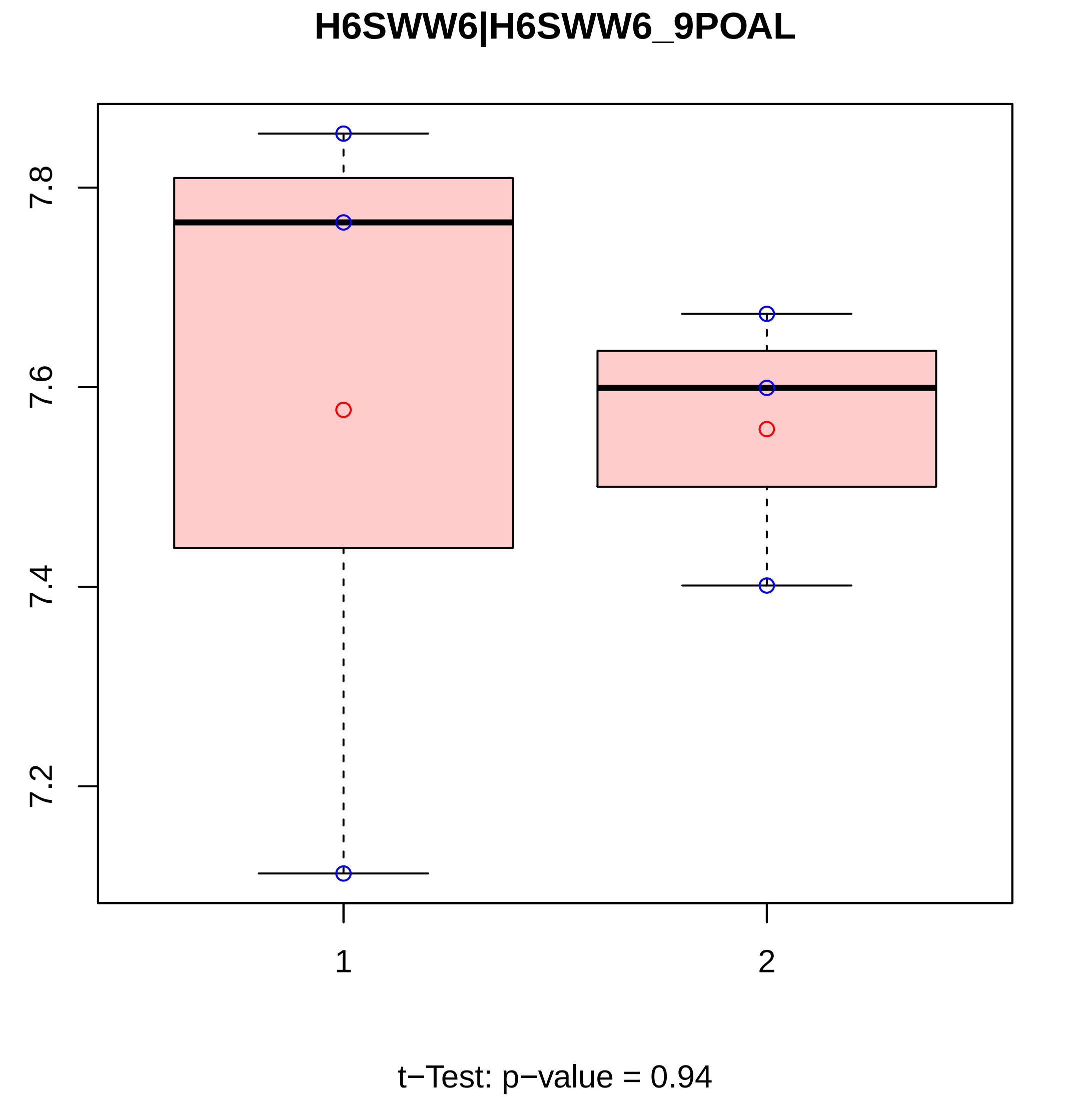
H6SWW6|H6SWW6_9POAL
7.2
7.4
7.6
7.8
1
2
t−Test: p−value = 0.94

H6SX50|H6SX50_9ASPA

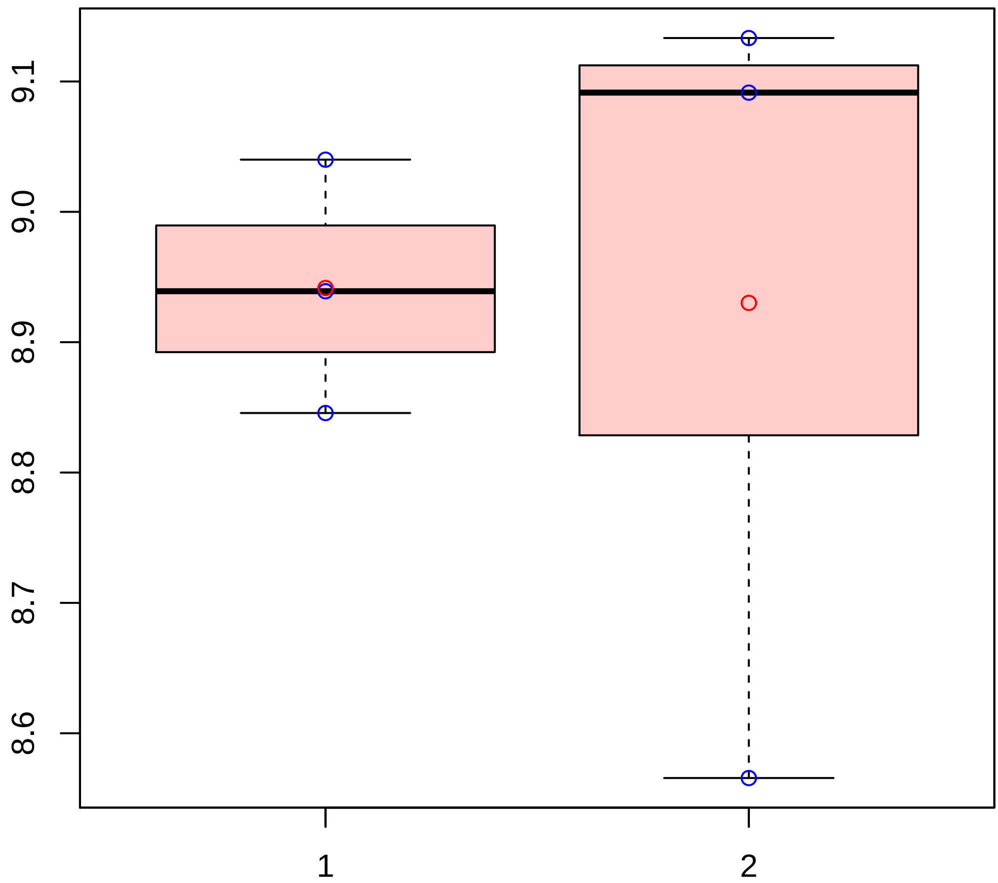

t−Test: p−value = 0.96

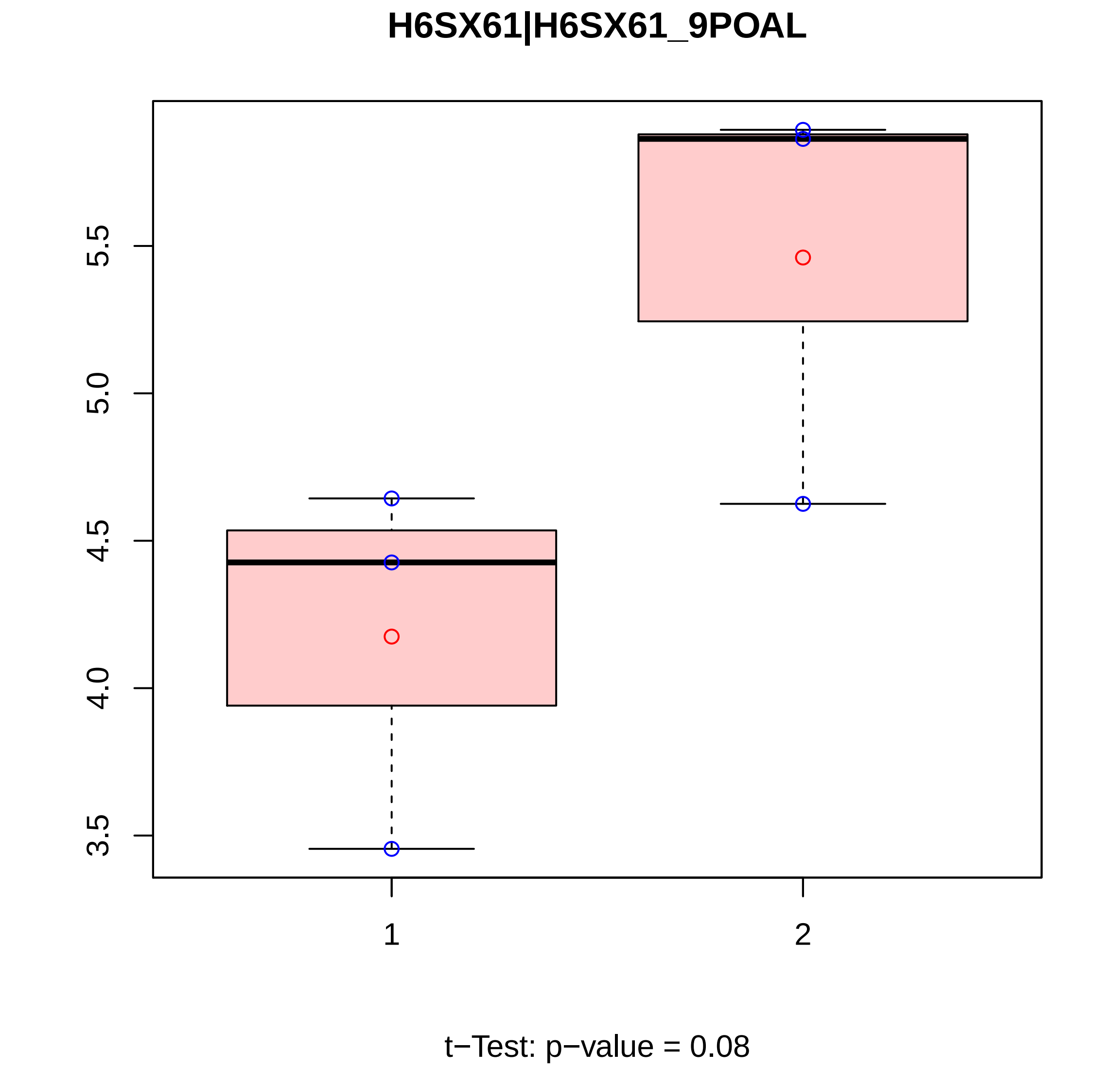
H6SX61|H6SX61_9POAL
5.5
5.0
4.5
4.0
3.5
1
2
t−Test: p−value = 0.08

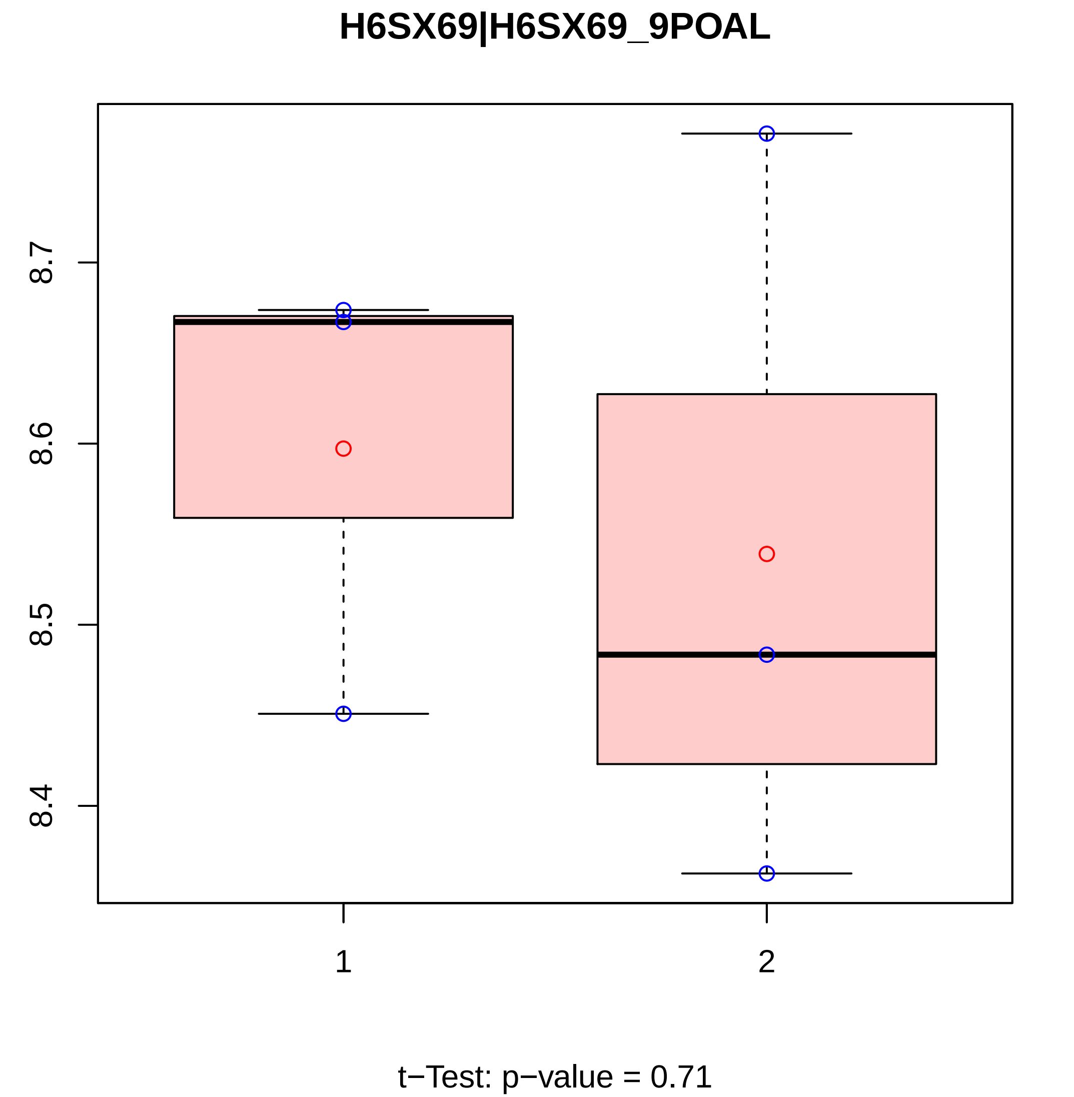

H6SX69|H6SX69_9POAL
8.4
8.5
8.6
8.7
1
2
t−Test: p−value = 0.71

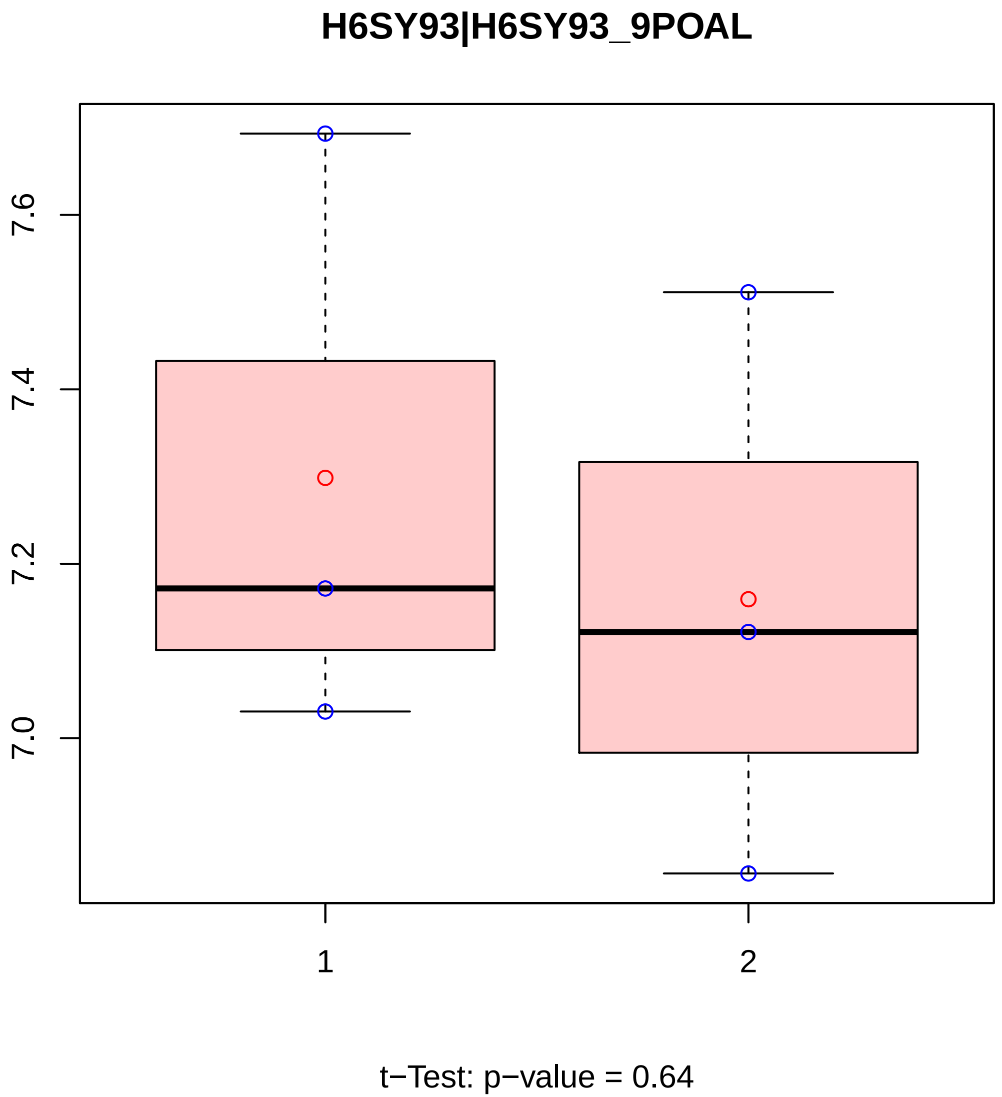

H6SY93|H6SY93_9POAL
7.0
7.2
7.4
7.6
1
2
t−Test: p−value = 0.64

H6SYE8|H6SYE8_ELECO

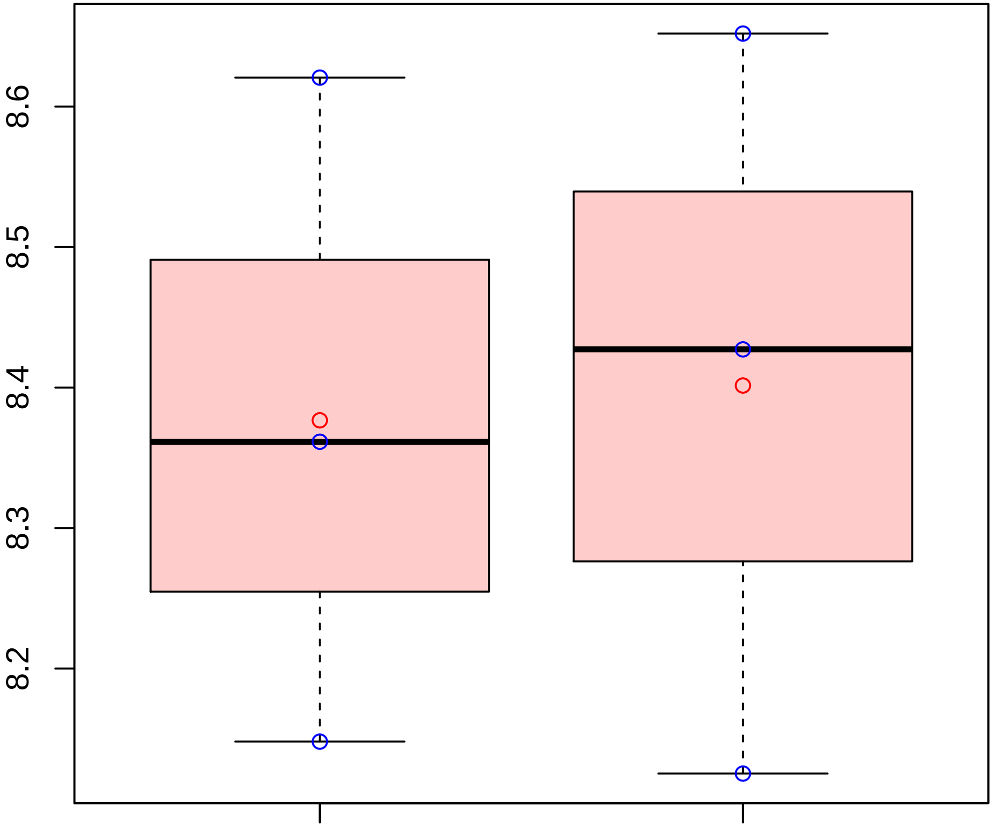

t−Test: p−value = 0.91

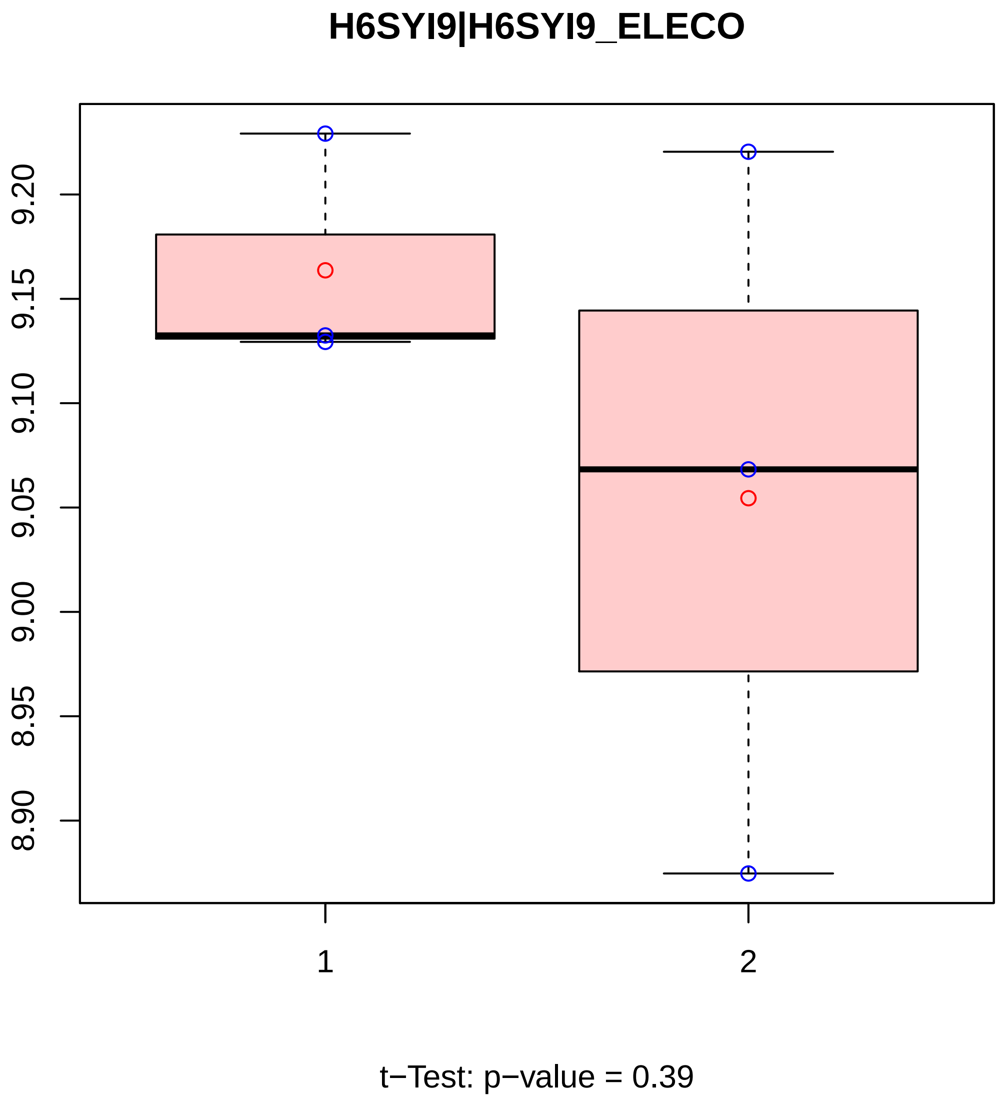
H6SYI9|H6SYI9_ELECO
9.20
9.15
9.10
9.05
9.00
8.95
8.90
1
2
t−Test: p−value = 0.39

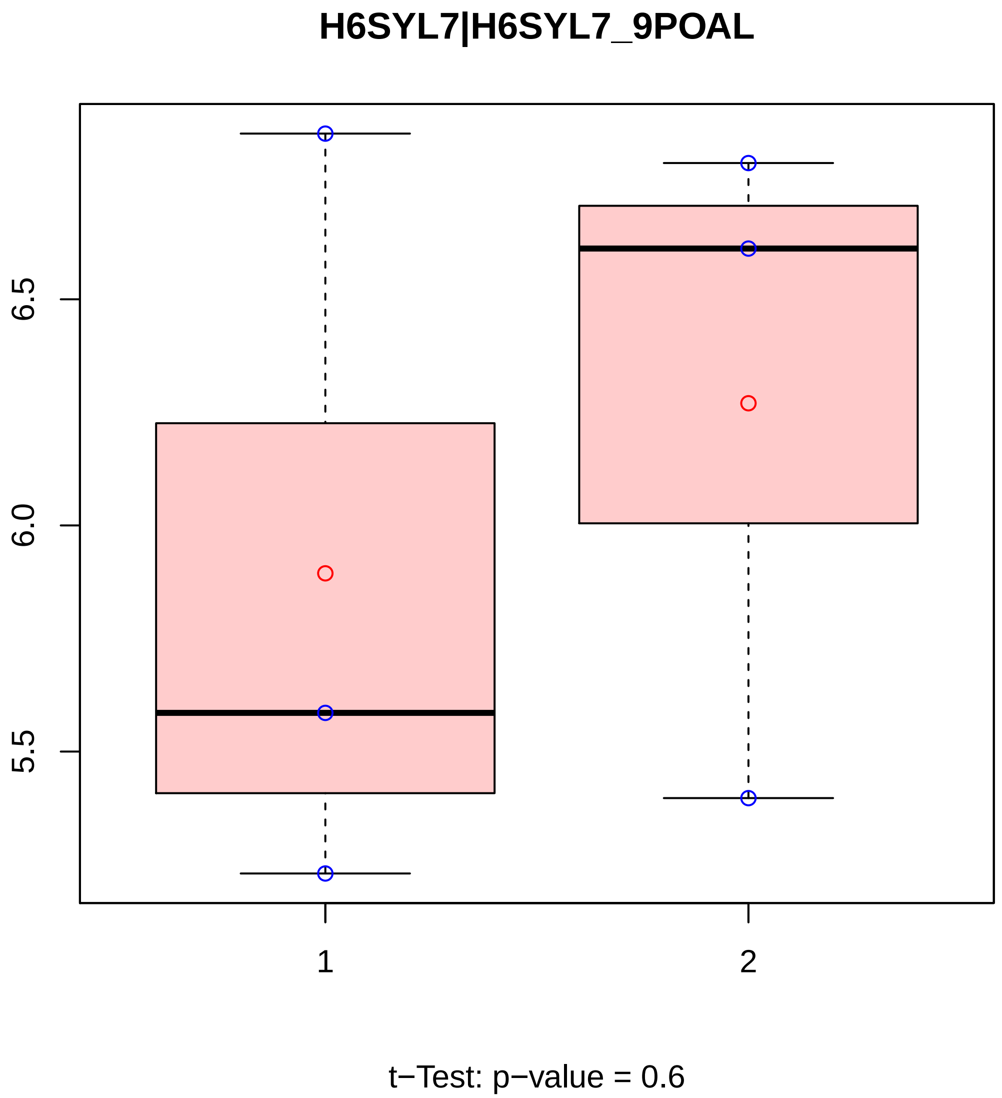

H6SYL7|H6SYL7_9POAL
6.5
6.0
5.5
1
2
t−Test: p−value = 0.6

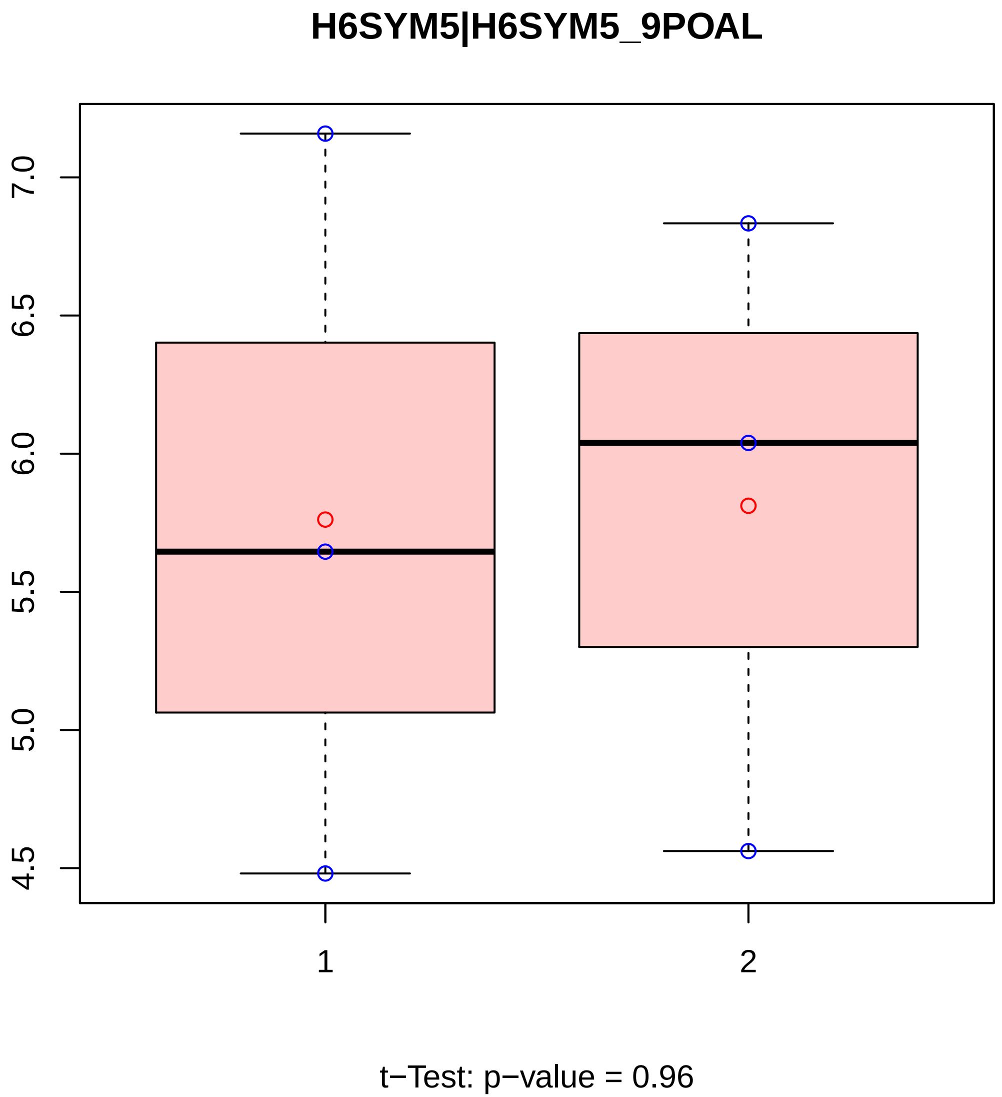

H6SYM5|H6SYM5_9POAL
7.0
6.5
6.0
5.5
5.0
4.5
1
2
t−Test: p−value = 0.96

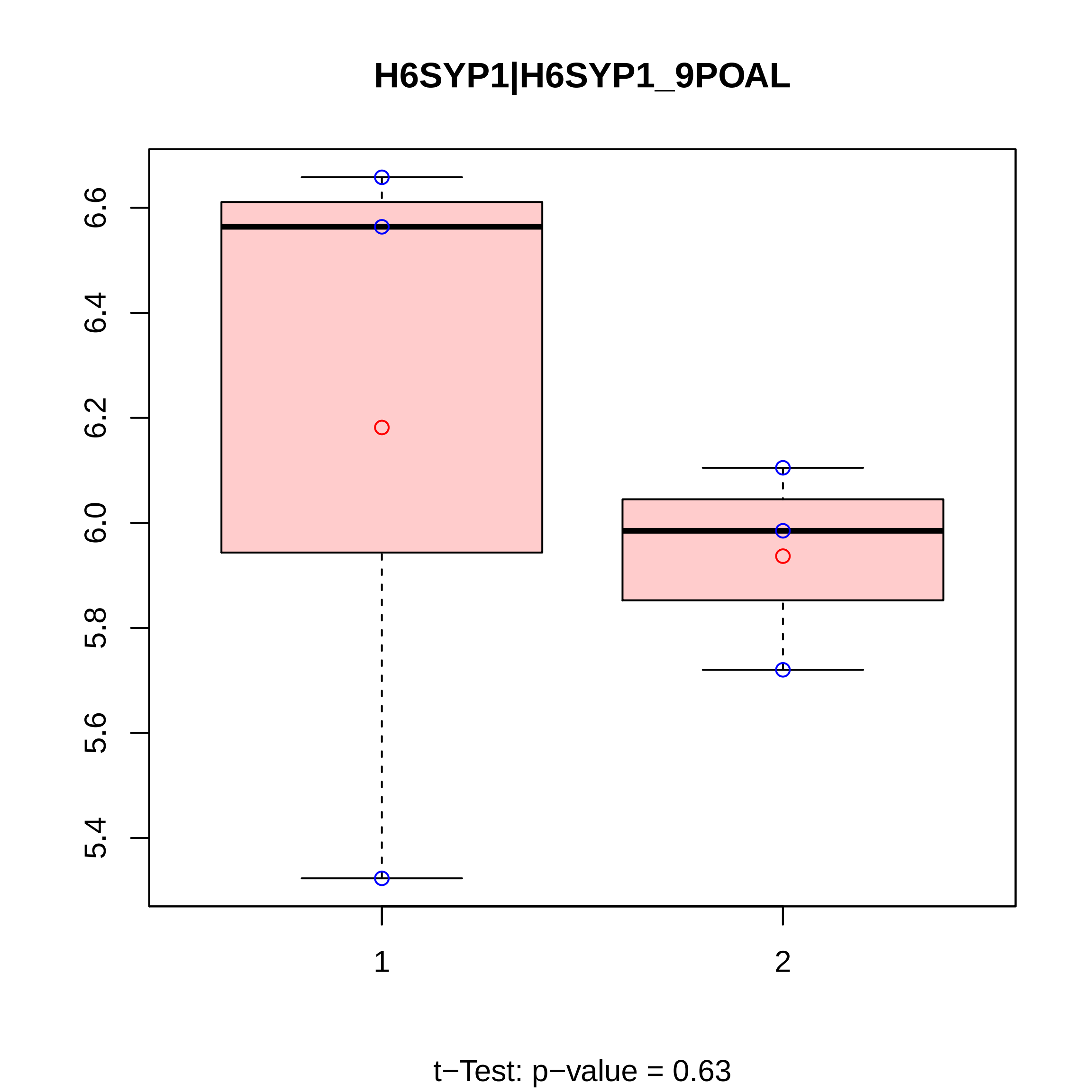

H6SYP1|H6SYP1_9POAL
6.6
6.4
6.2
6.0
5.8
5.6
5.4
1
2
t−Test: p−value = 0.63

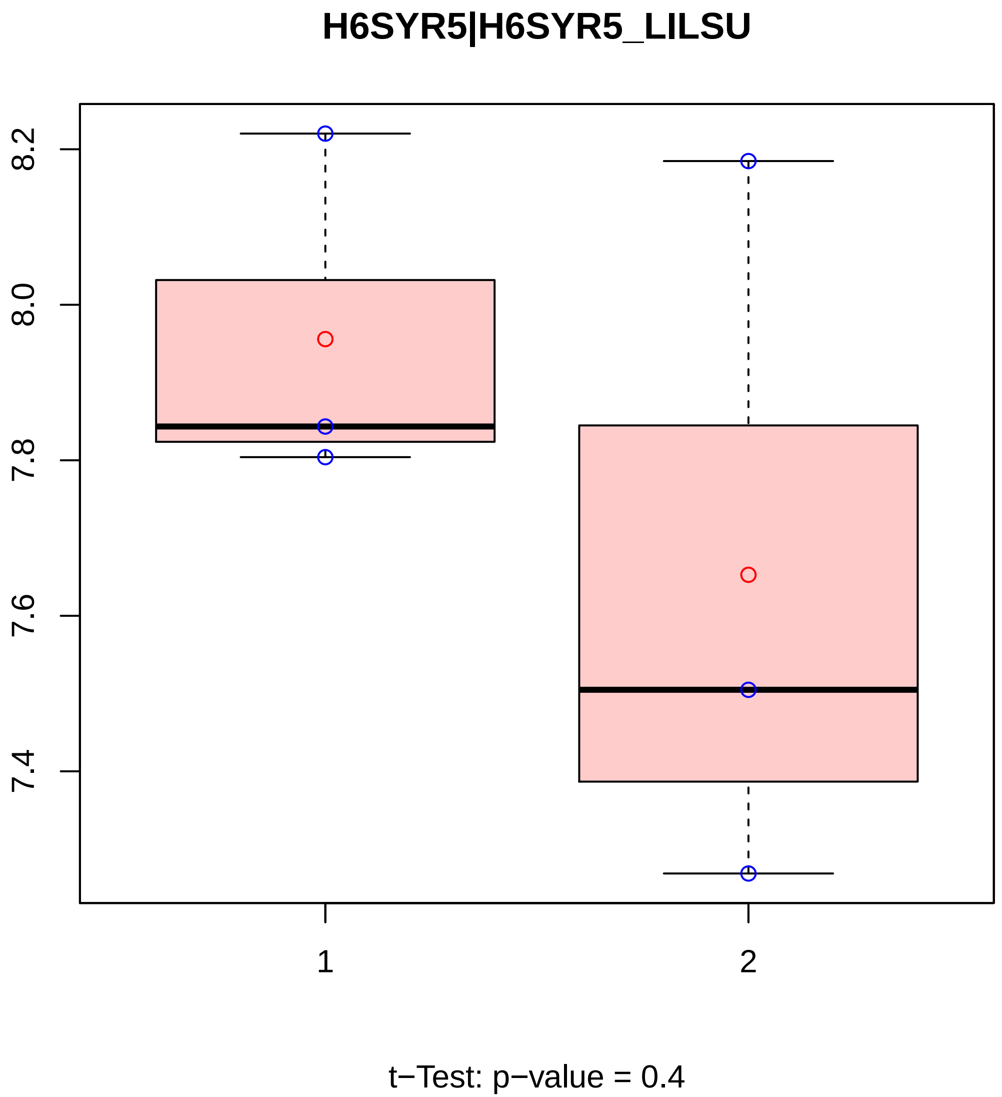
H6SYR5|H6SYR5_LILSU
8.2
8.0
7.8
7.6
7.4
1
2
t−Test: p−value = 0.4

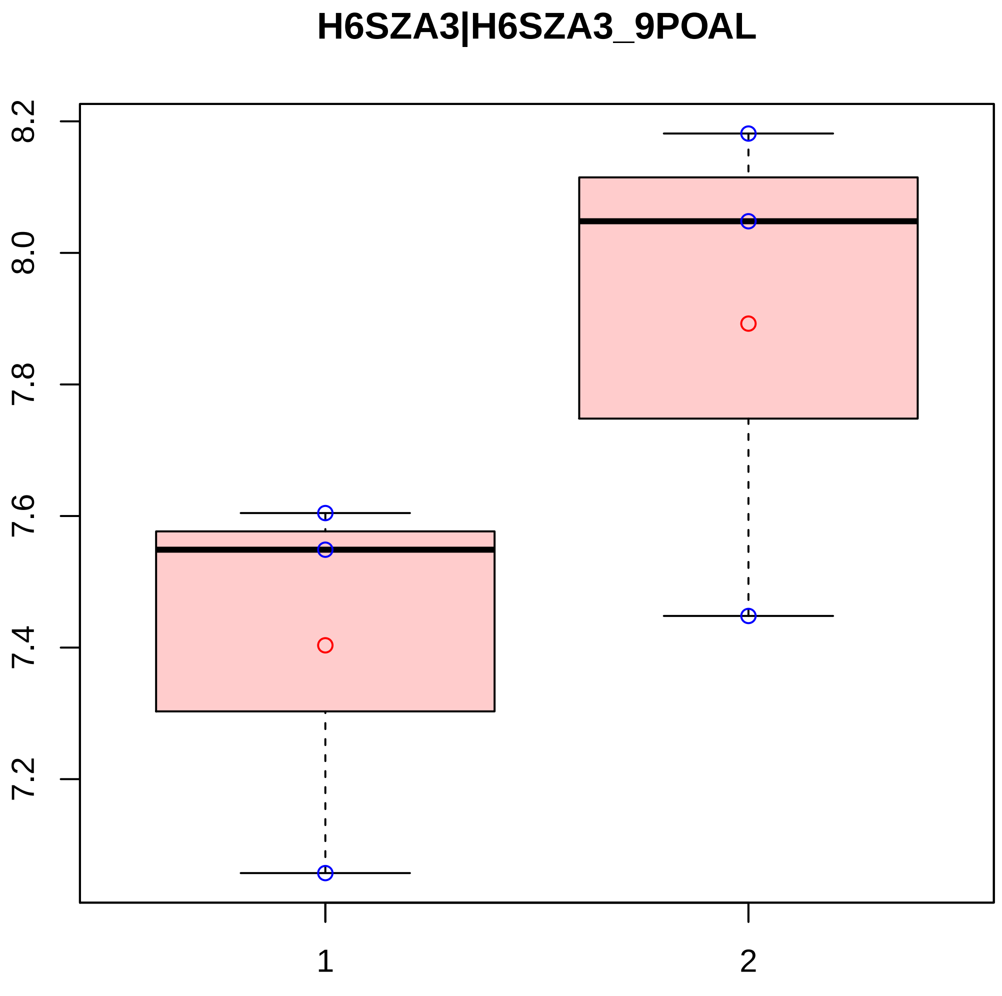

t−Test: p−value = 0.17

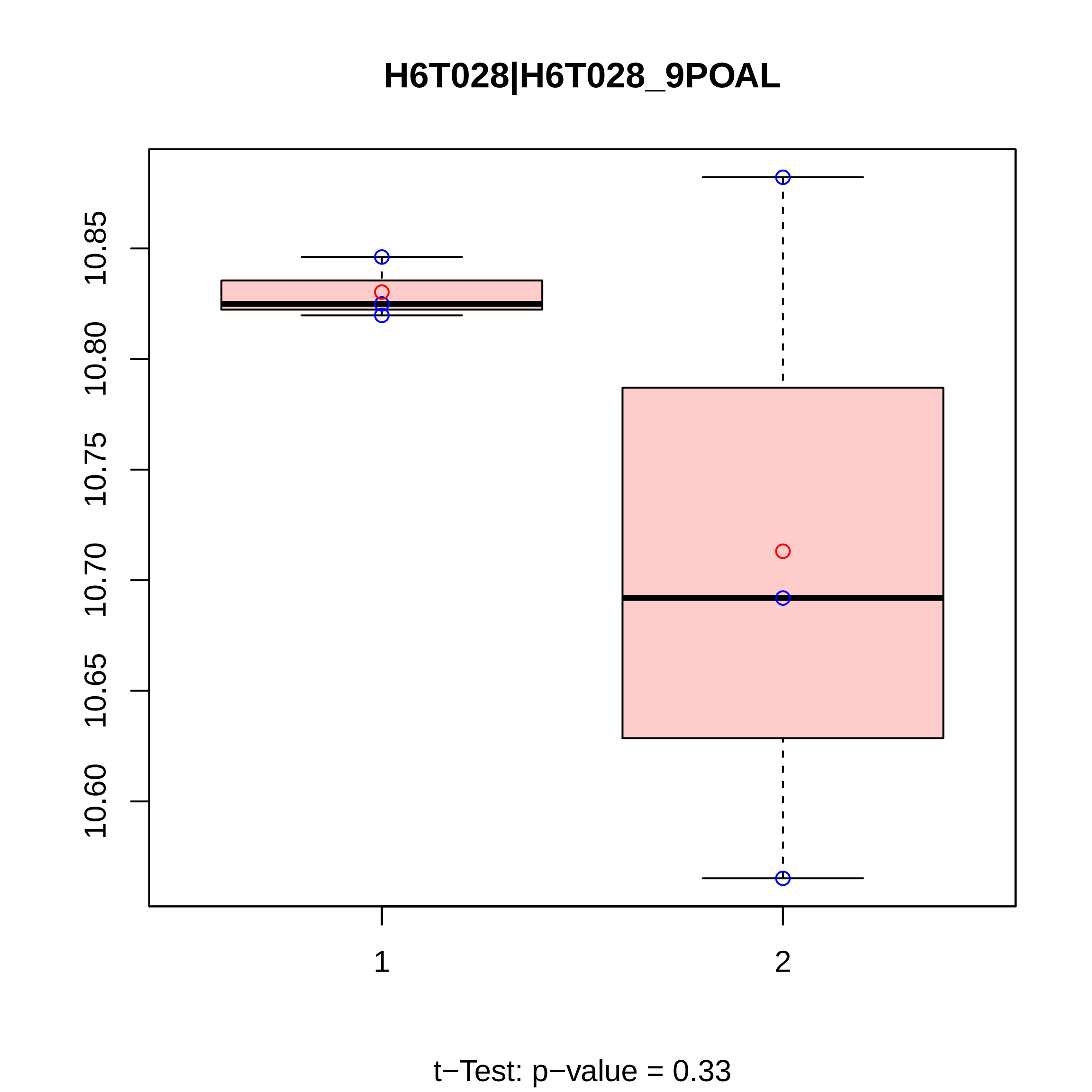
H6T028|H6T028_9POAL
10.60
10.65
10.70
10.75
10.80
10.85
1
2
t−Test: p−value = 0.33

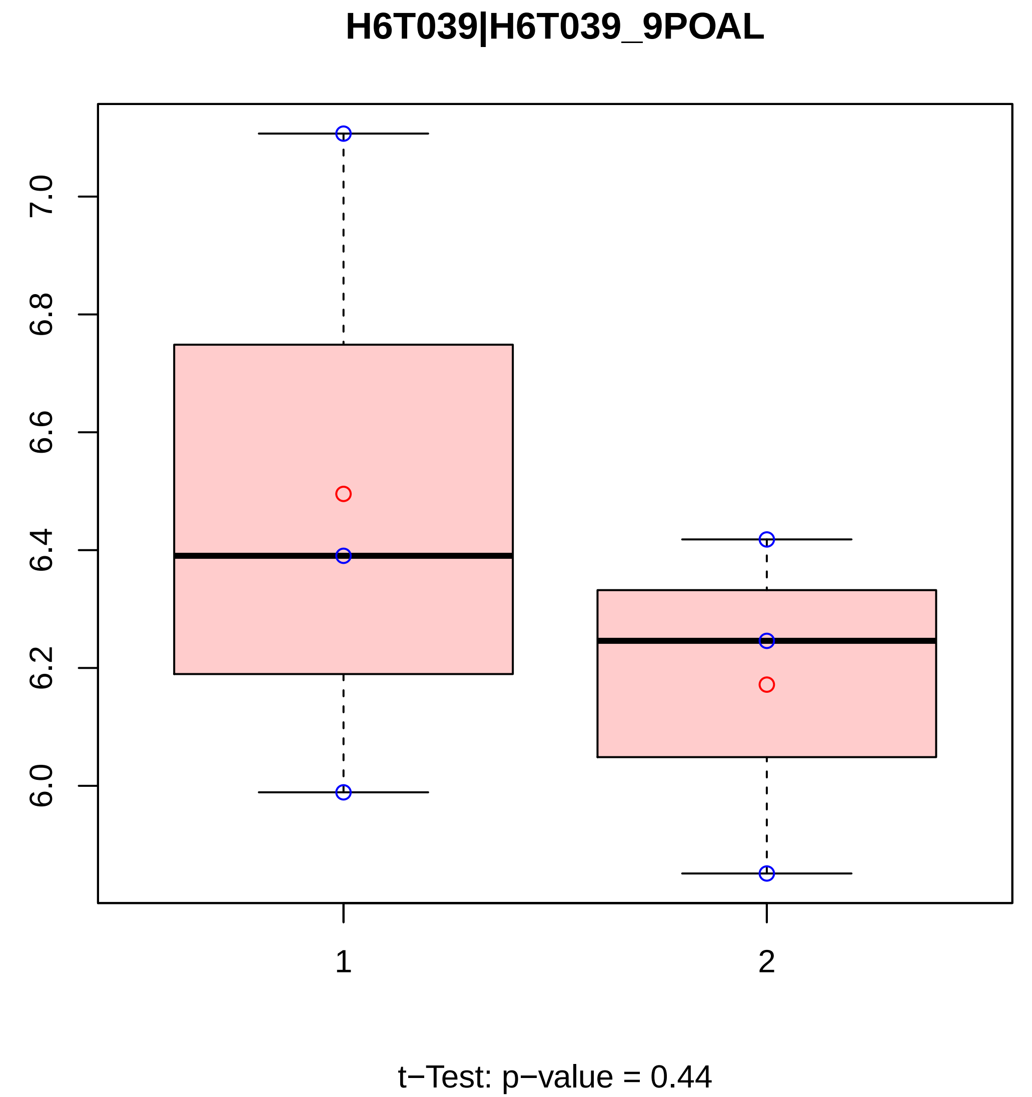
H6T039|H6T039_9POAL
7.0
6.8
6.6
6.4
6.2
6.0
1
2
t−Test: p−value = 0.44

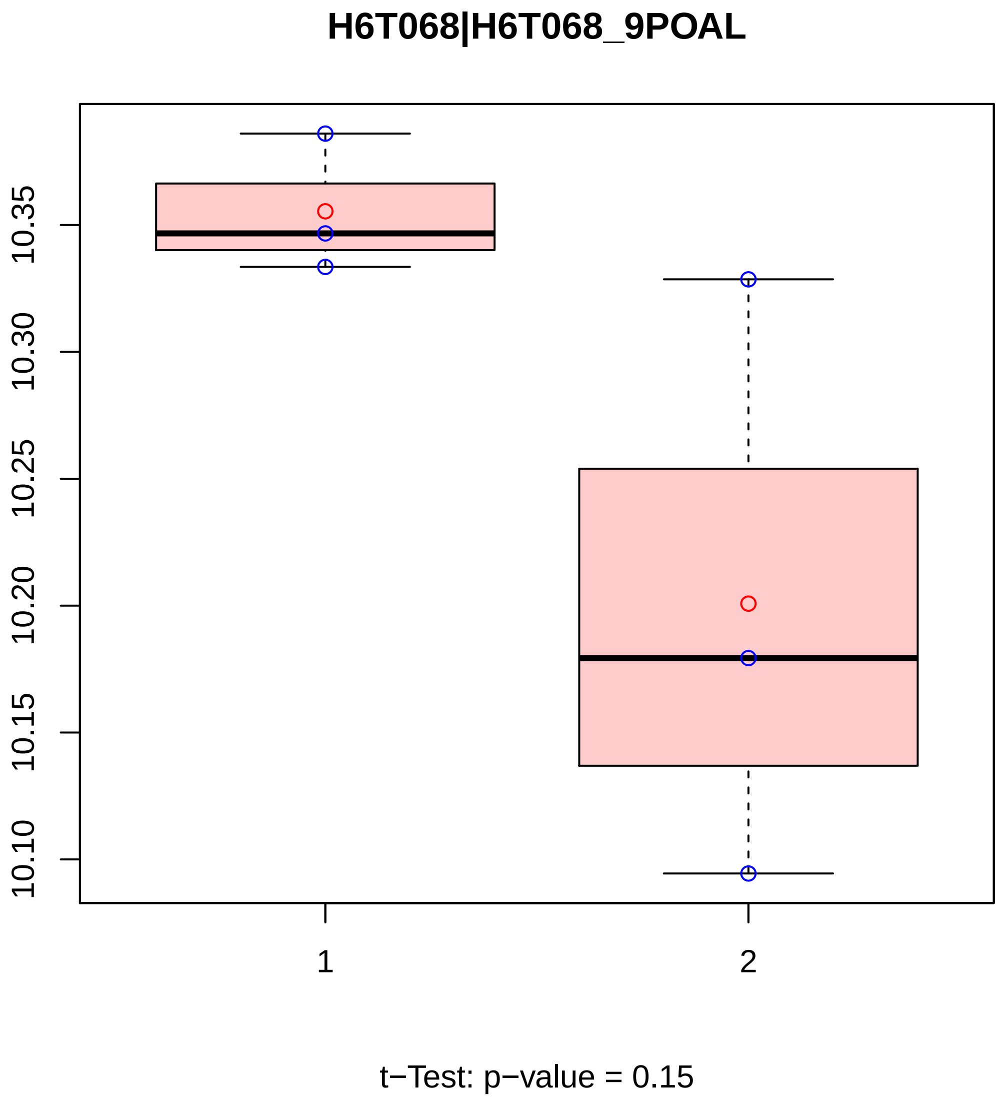
H6T068|H6T068_9POAL
10.10
10.15
10.20
10.25
10.30
10.35
1
2
t−Test: p−value = 0.15

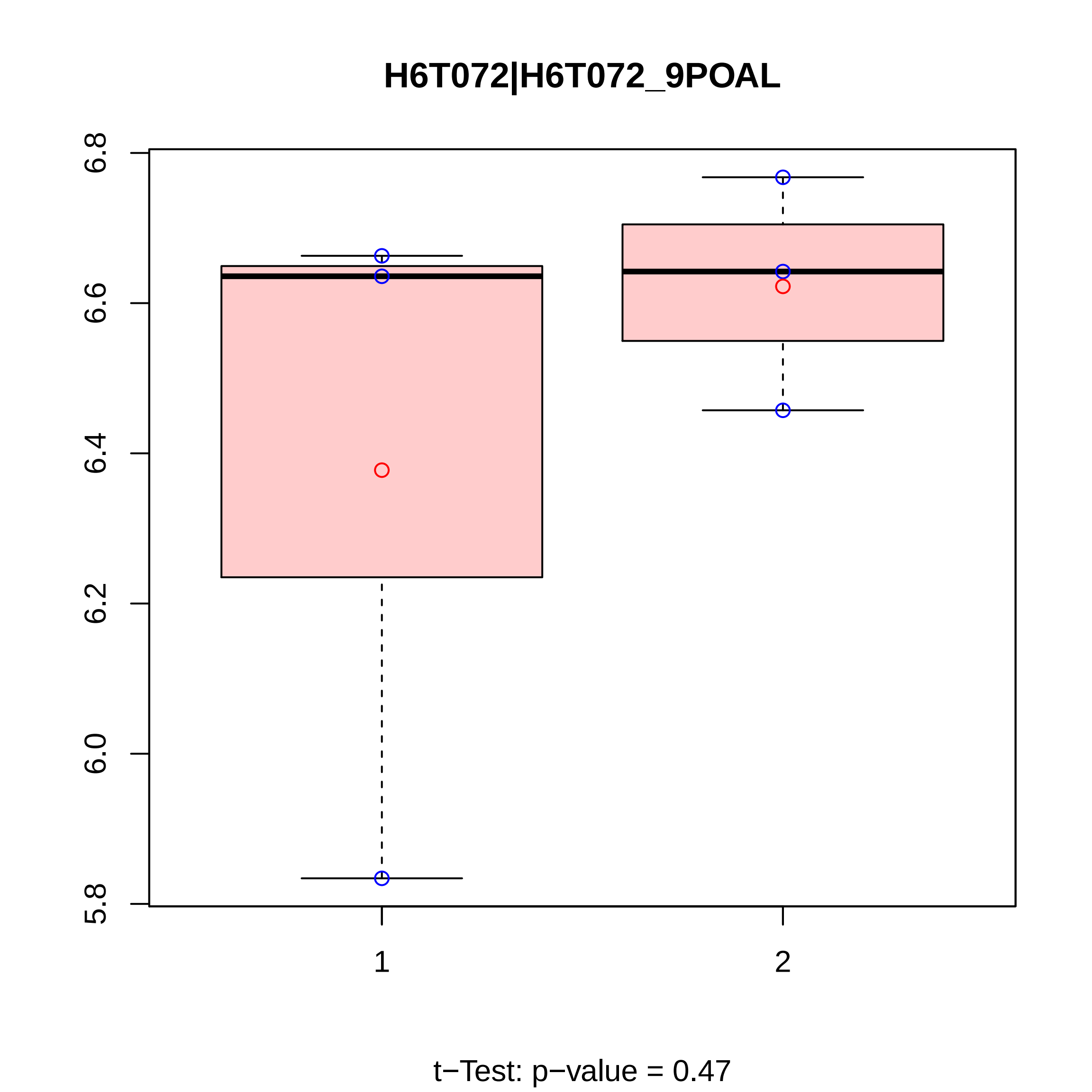
H6T072|H6T072_9POAL
6.8
6.6
6.4
6.2
6.0
5.8
1
2
t−Test: p−value = 0.47

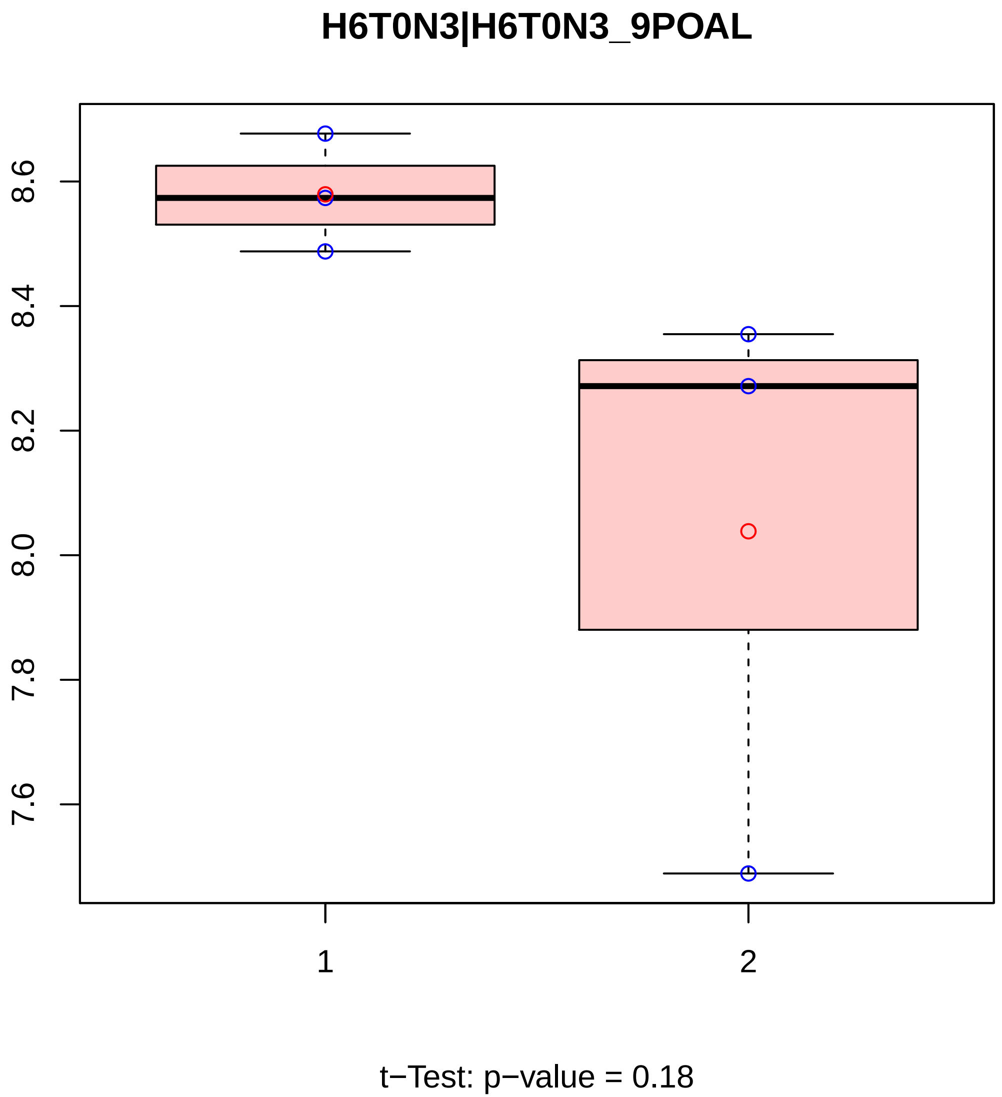
H6T0N3|H6T0N3_9POAL
8.6
8.4
8.2
8.0
7.8
7.6
1
2
t−Test: p−value = 0.18

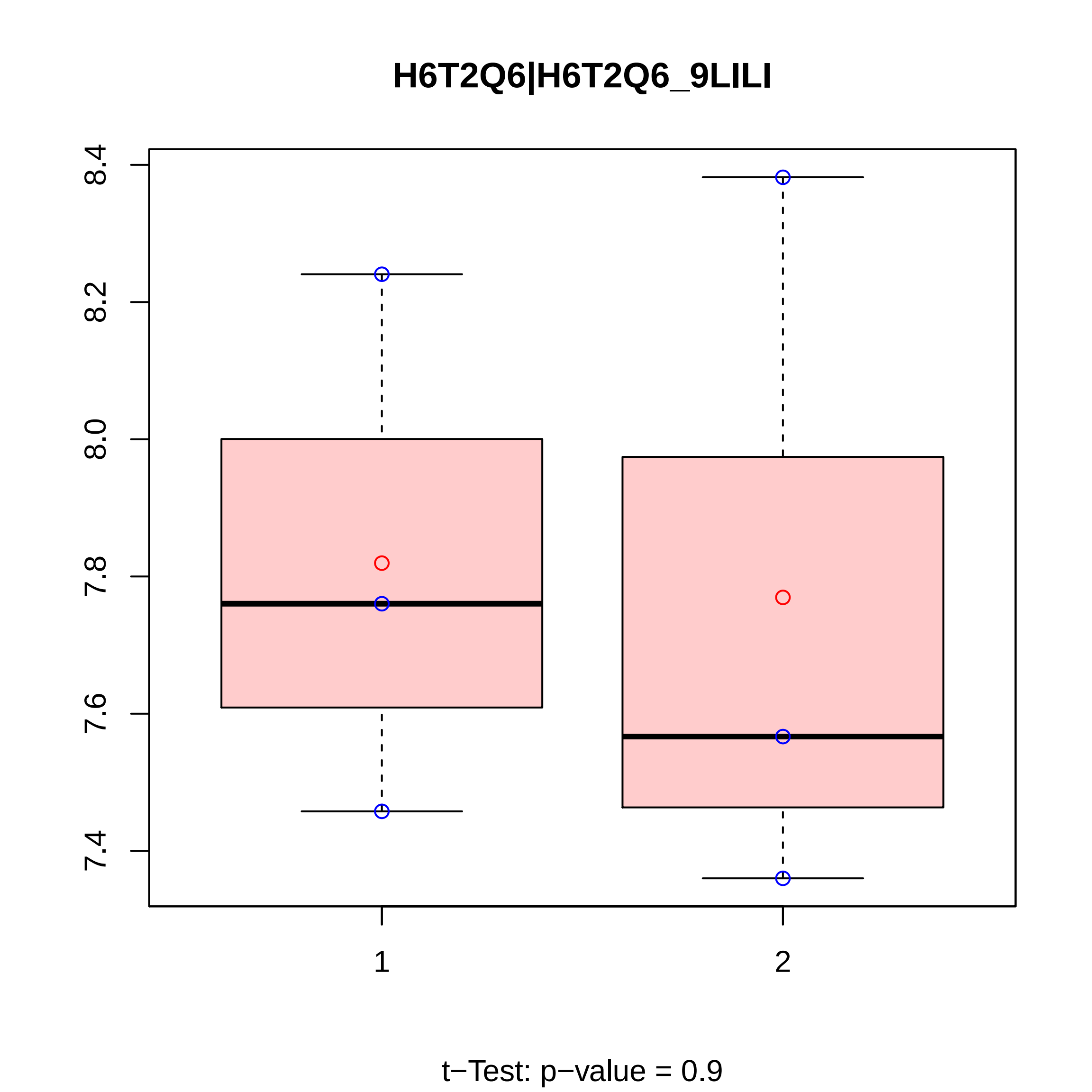

H6T2Q6|H6T2Q6_9LILI
8.4
8.2
8.0
7.8
7.6
7.4
1
2
t−Test: p−value = 0.9

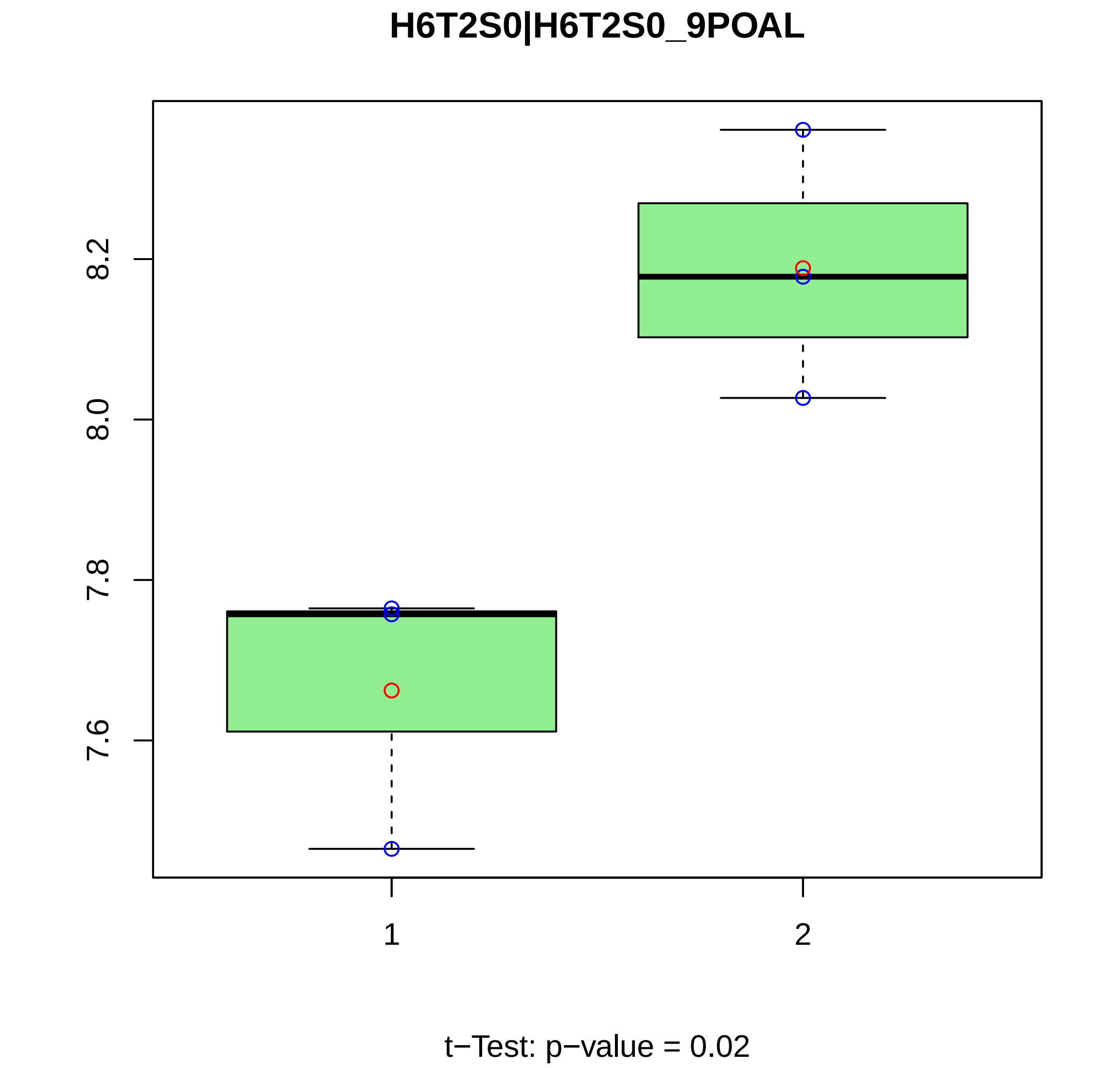
H6T2S0|H6T2S0_9POAL
7.6
7.8
8.0
8.2
1
2
t−Test: p−value = 0.02

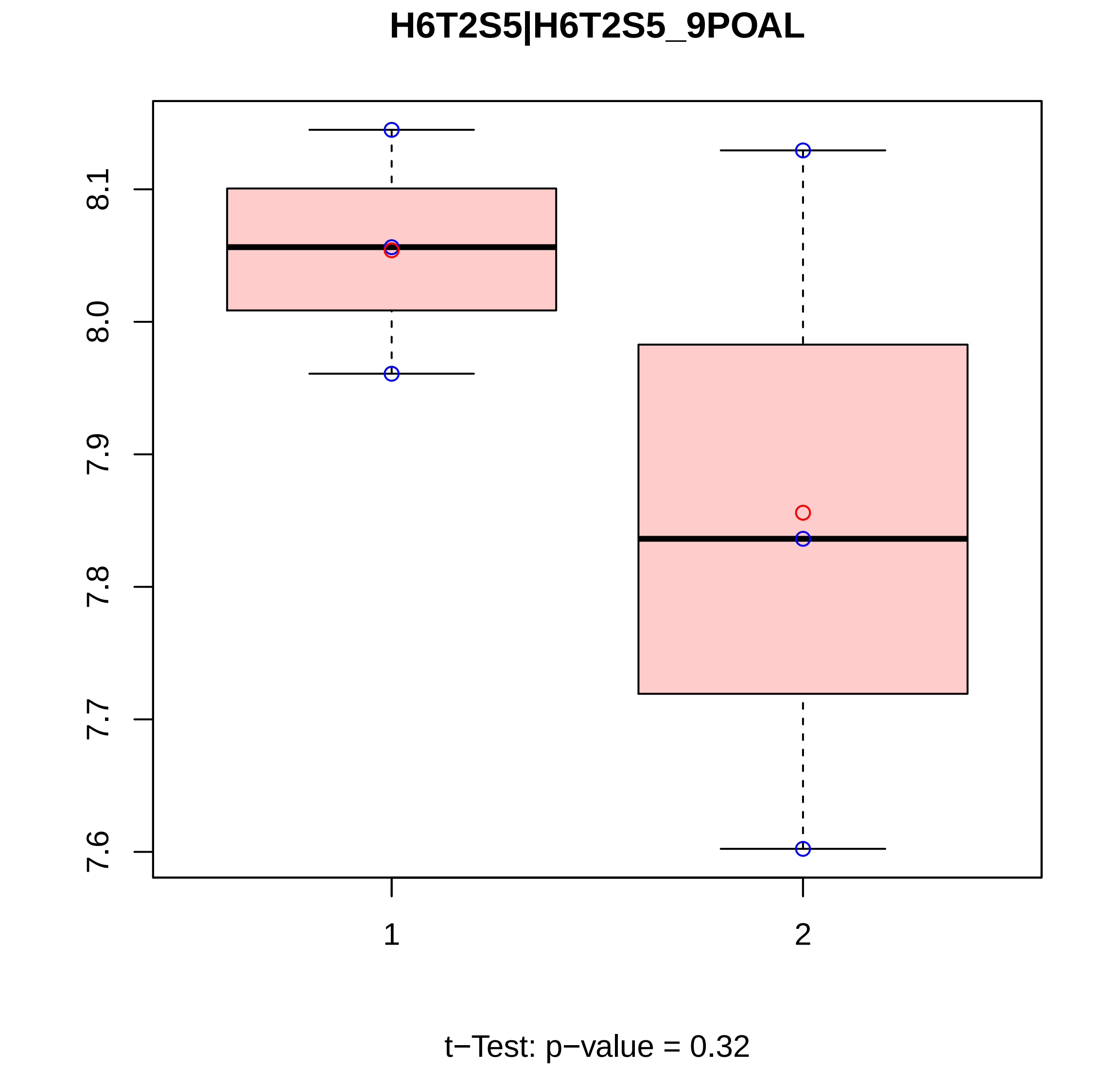
H6T2S5|H6T2S5_9POAL
8.1
8.0
7.9
7.8
7.7
7.6
1
2
t−Test: p−value = 0.32

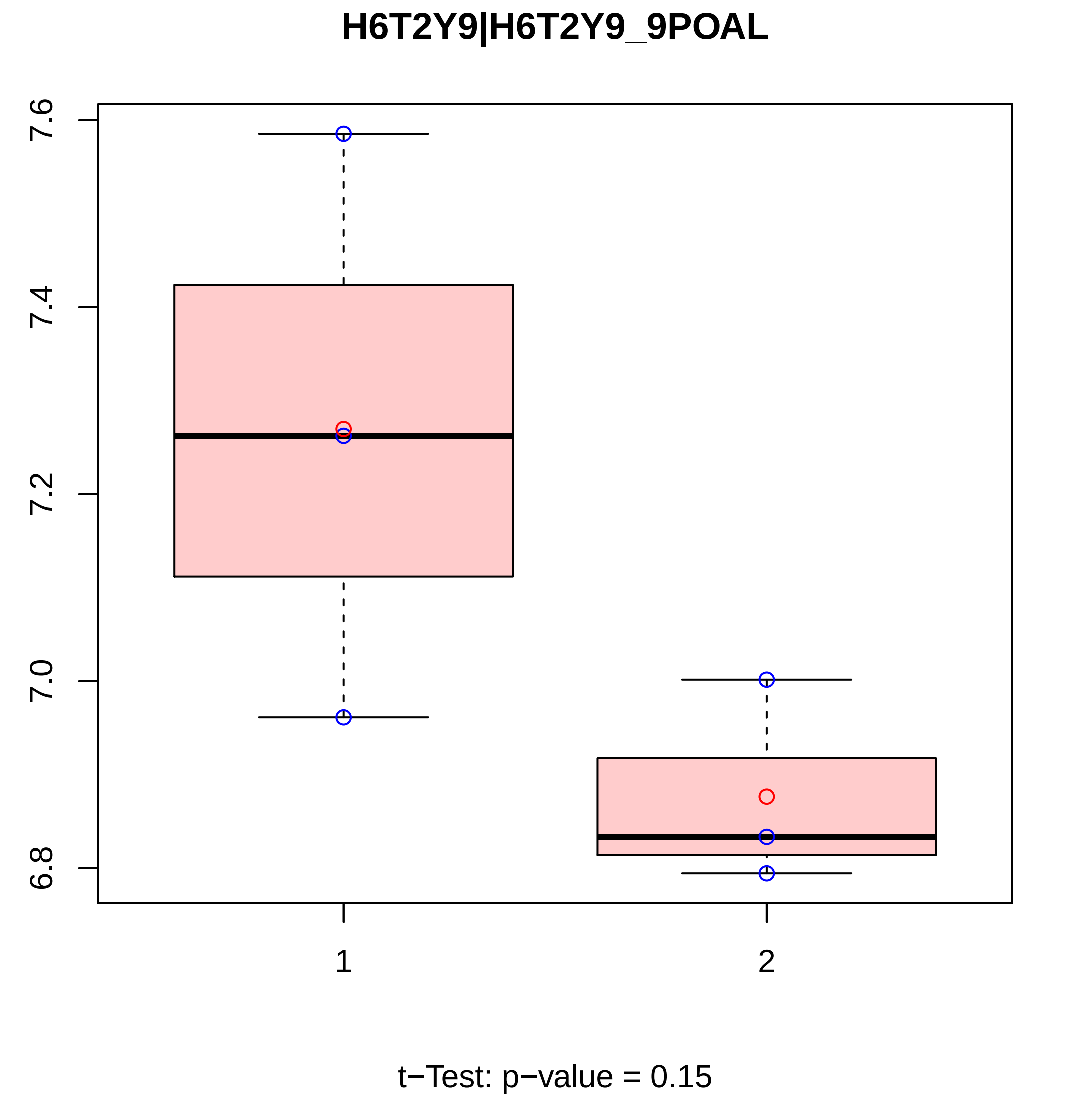
H6T2Y9|H6T2Y9_9POAL
7.6
7.4
7.2
7.0
6.8
1
2
t−Test: p−value = 0.15

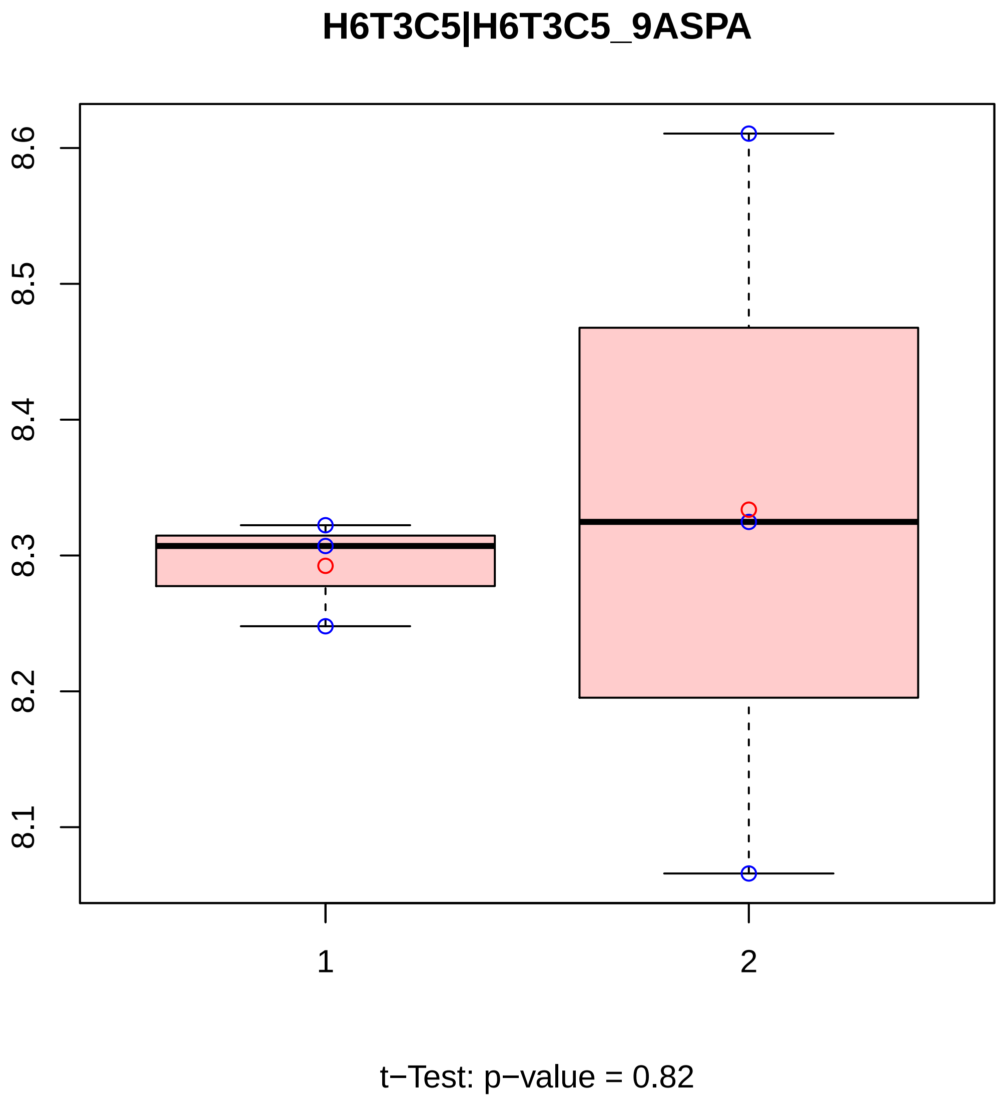
H6T3C5|H6T3C5_9ASPA
8.1
8.2
8.3
8.4
8.5
8.6
1
2
t−Test: p−value = 0.82

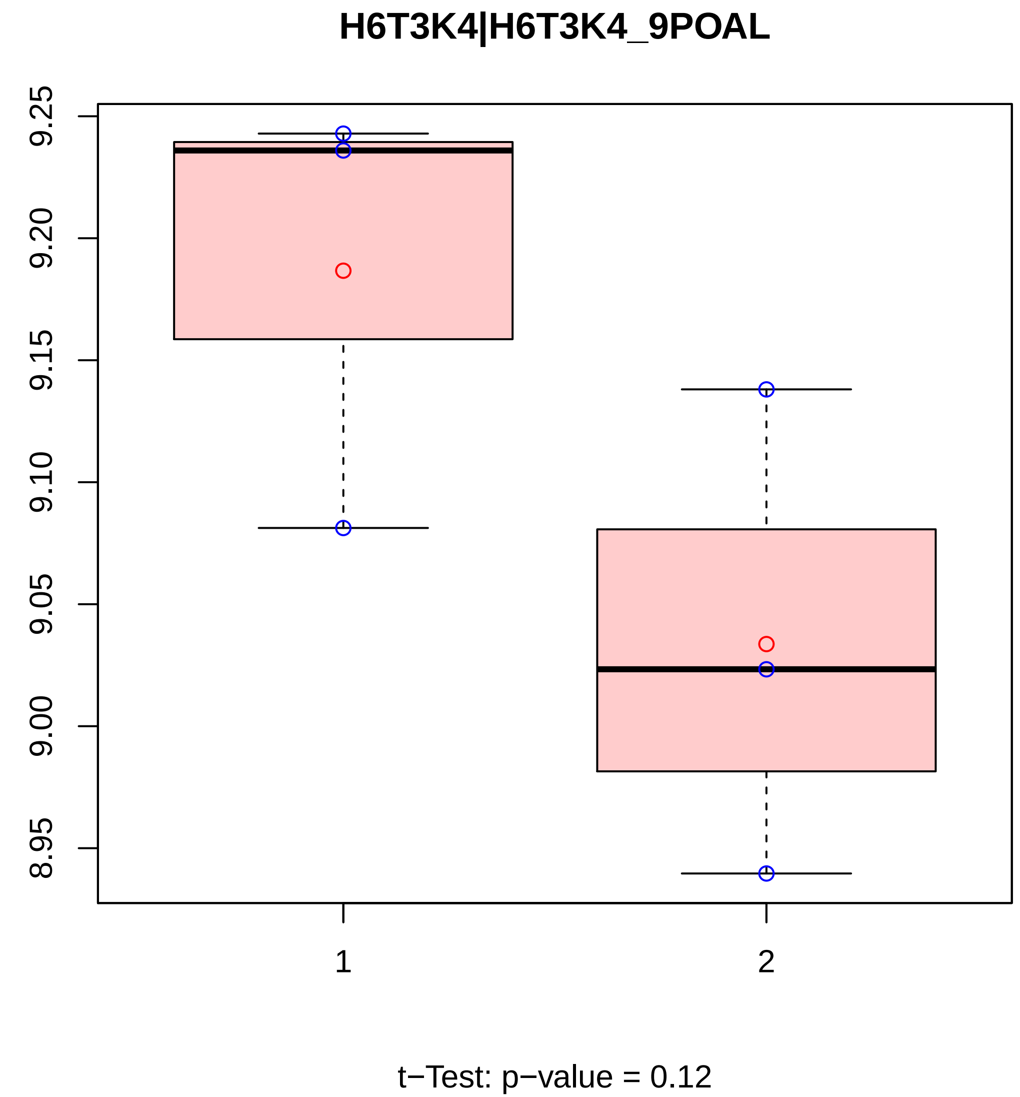
H6T3K4|H6T3K4_9POAL
9.25
9.20
9.15
9.10
9.05
9.00
8.95
1
2
t−Test: p−value = 0.12

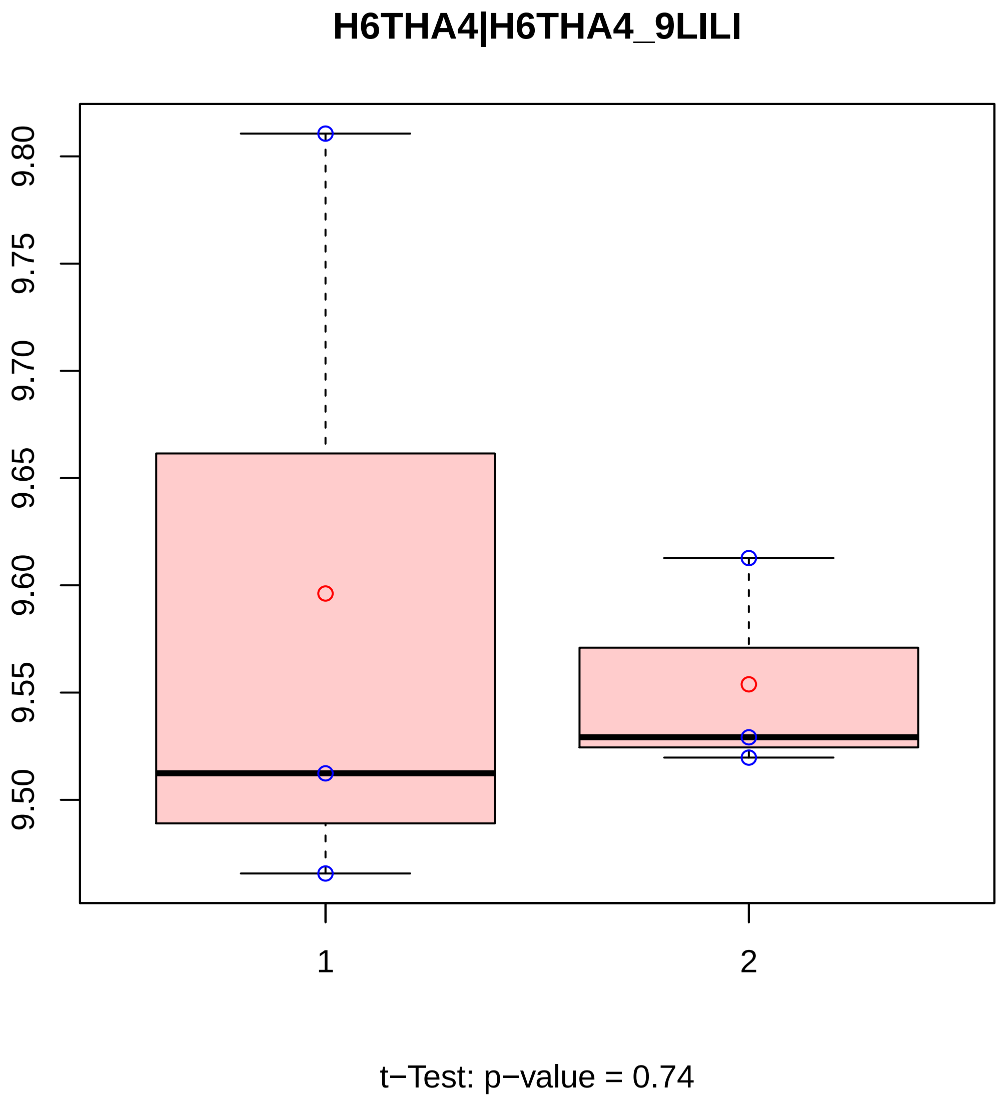
H6THA4|H6THA4_9LILI
9.50
9.55
9.60
9.65
9.70
9.75
9.80
1
2
t−Test: p−value = 0.74

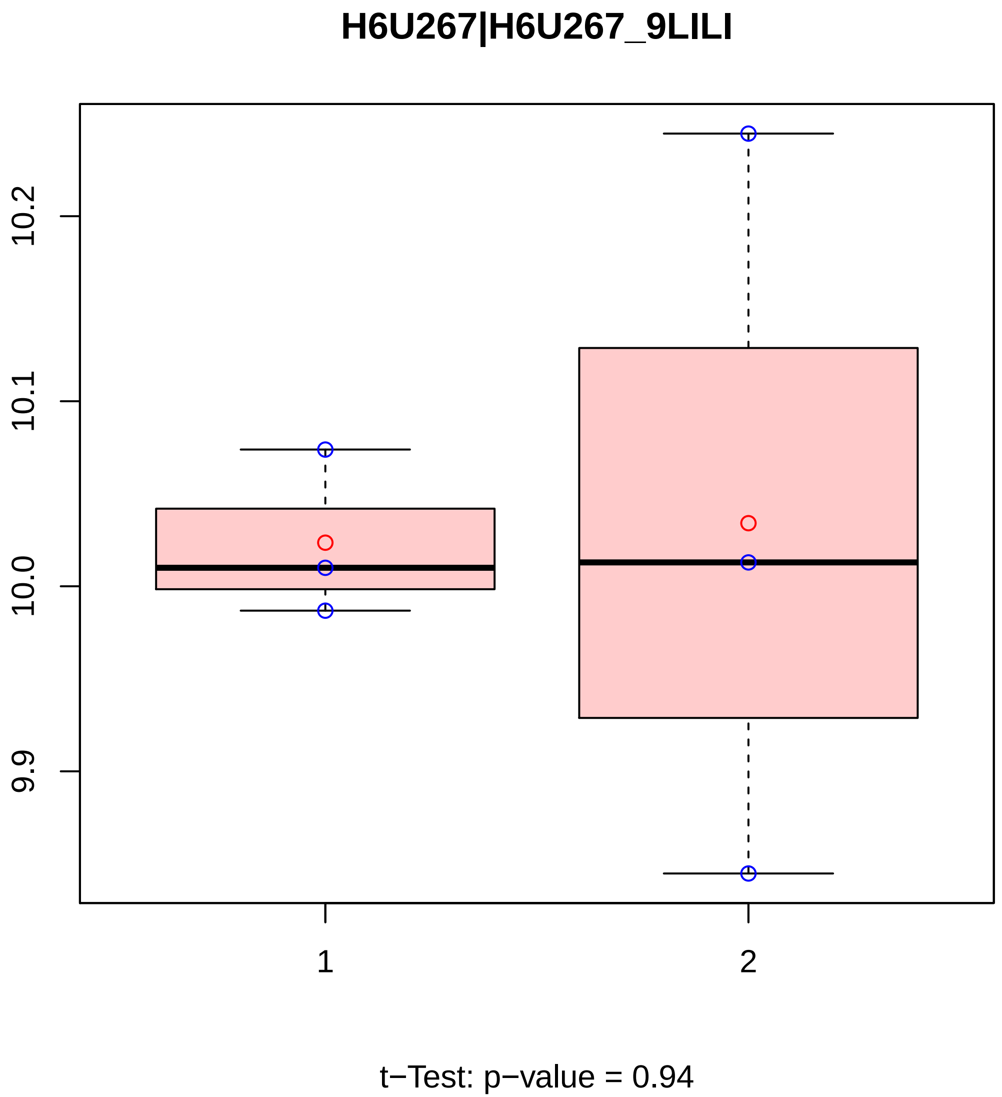
H6U267|H6U267_9LILI
10.2
10.1
10.0
9.9
1
2
t−Test: p−value = 0.94

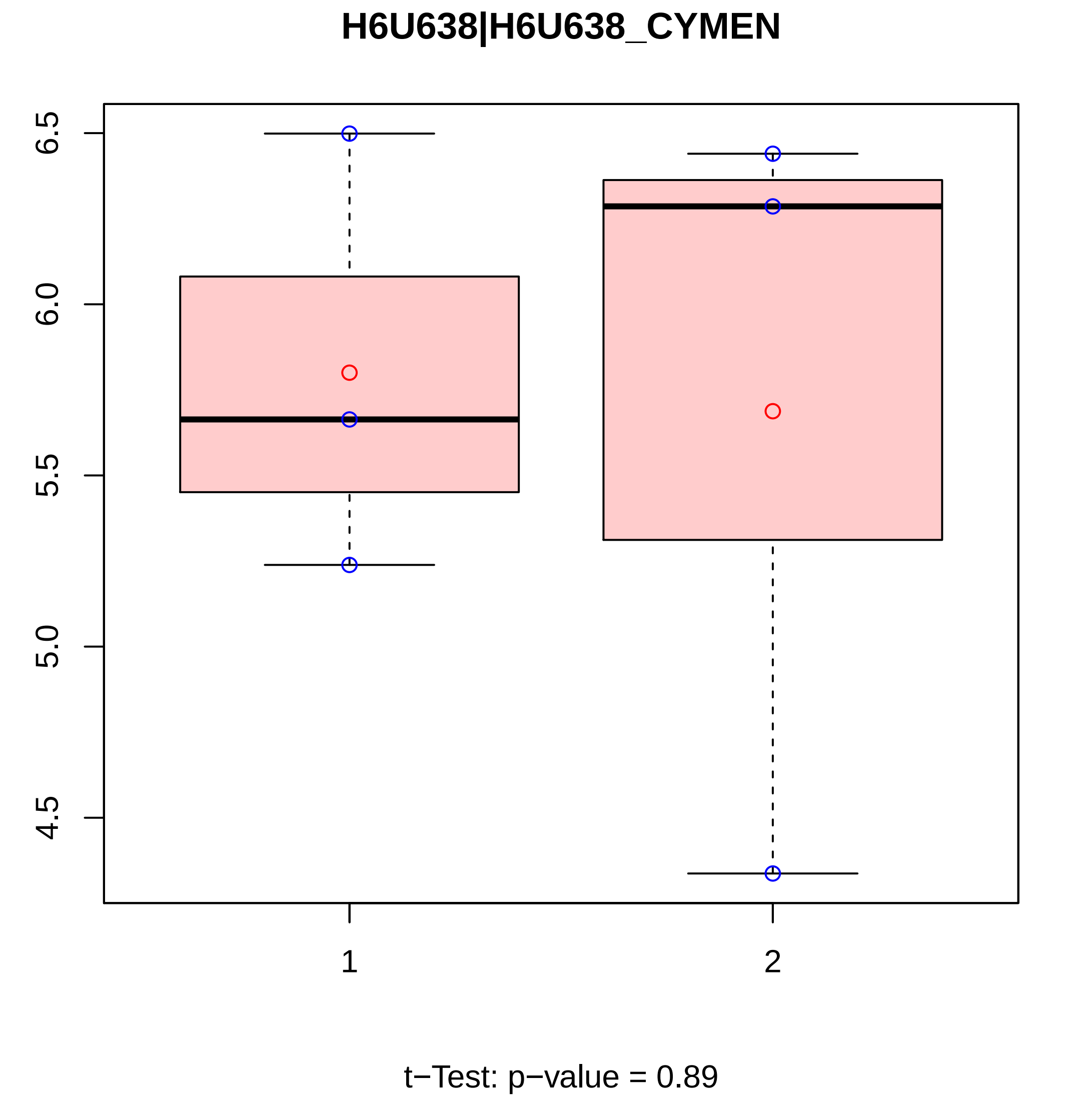
H6U638|H6U638_CYMEN
6.5
6.0
5.5
5.0
4.5
1
2
t−Test: p−value = 0.89

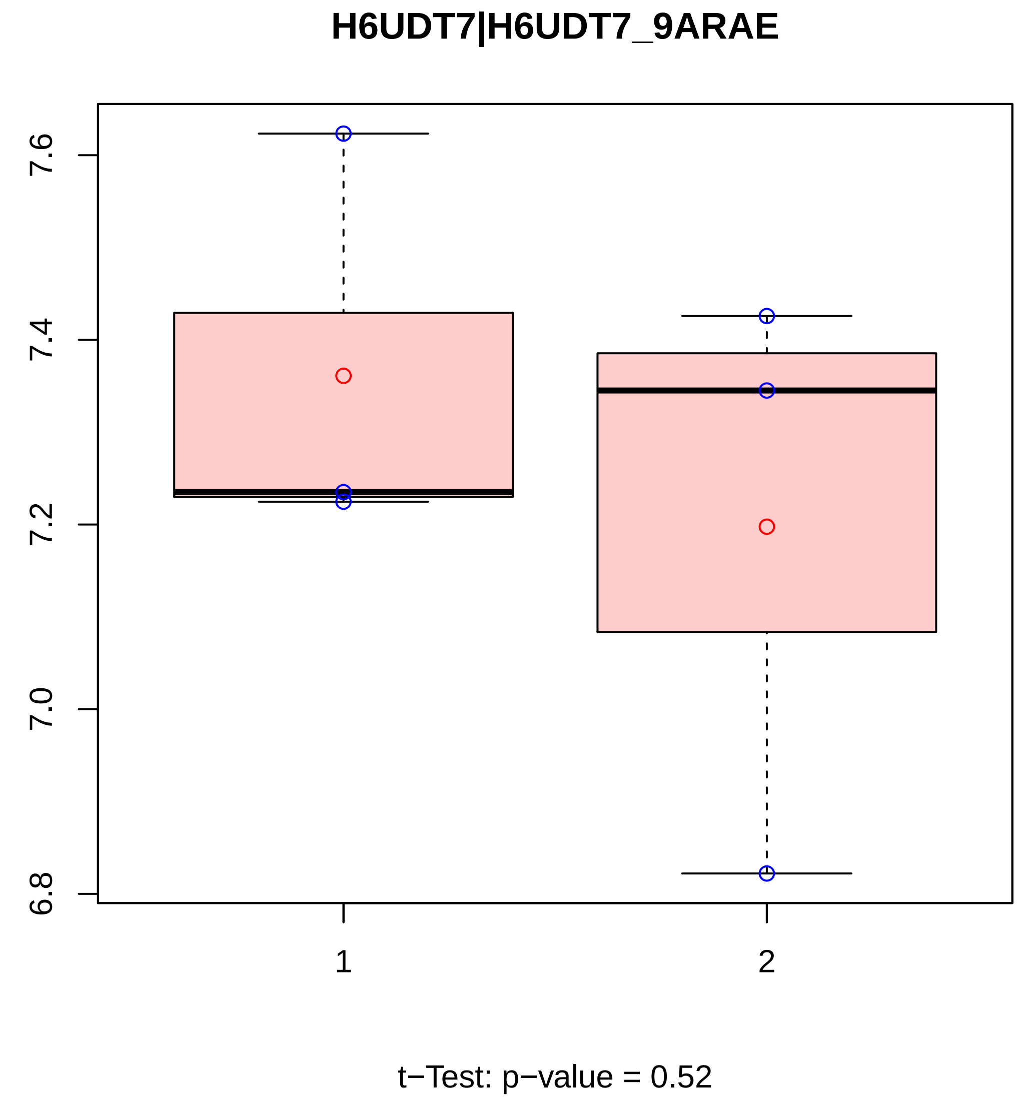
H6UDT7|H6UDT7_9ARAE
7.6
7.4
7.2
7.0
6.8
1
2
t−Test: p−value = 0.52

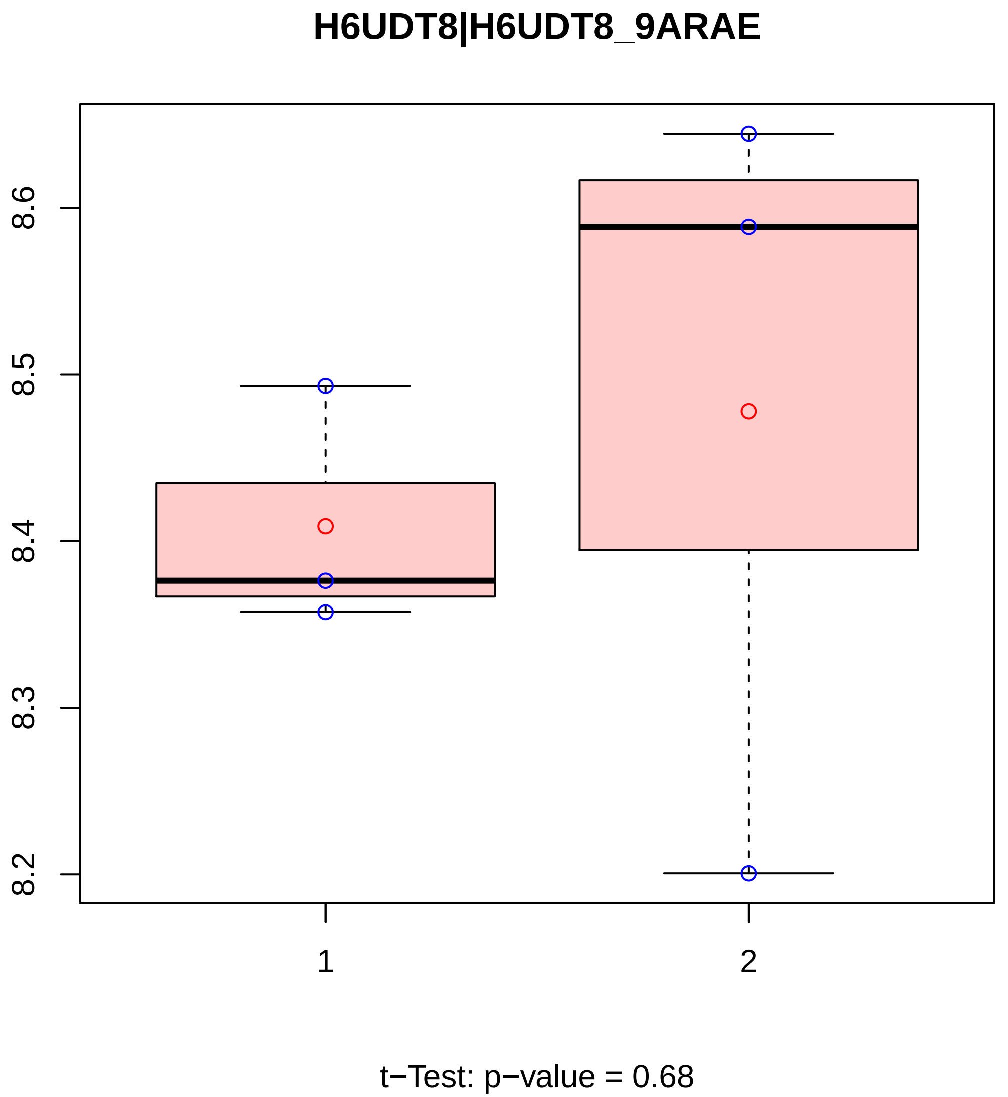
H6UDT8|H6UDT8_9ARAE
8.2
8.3
8.4
8.5
8.6
1
2
t−Test: p−value = 0.68

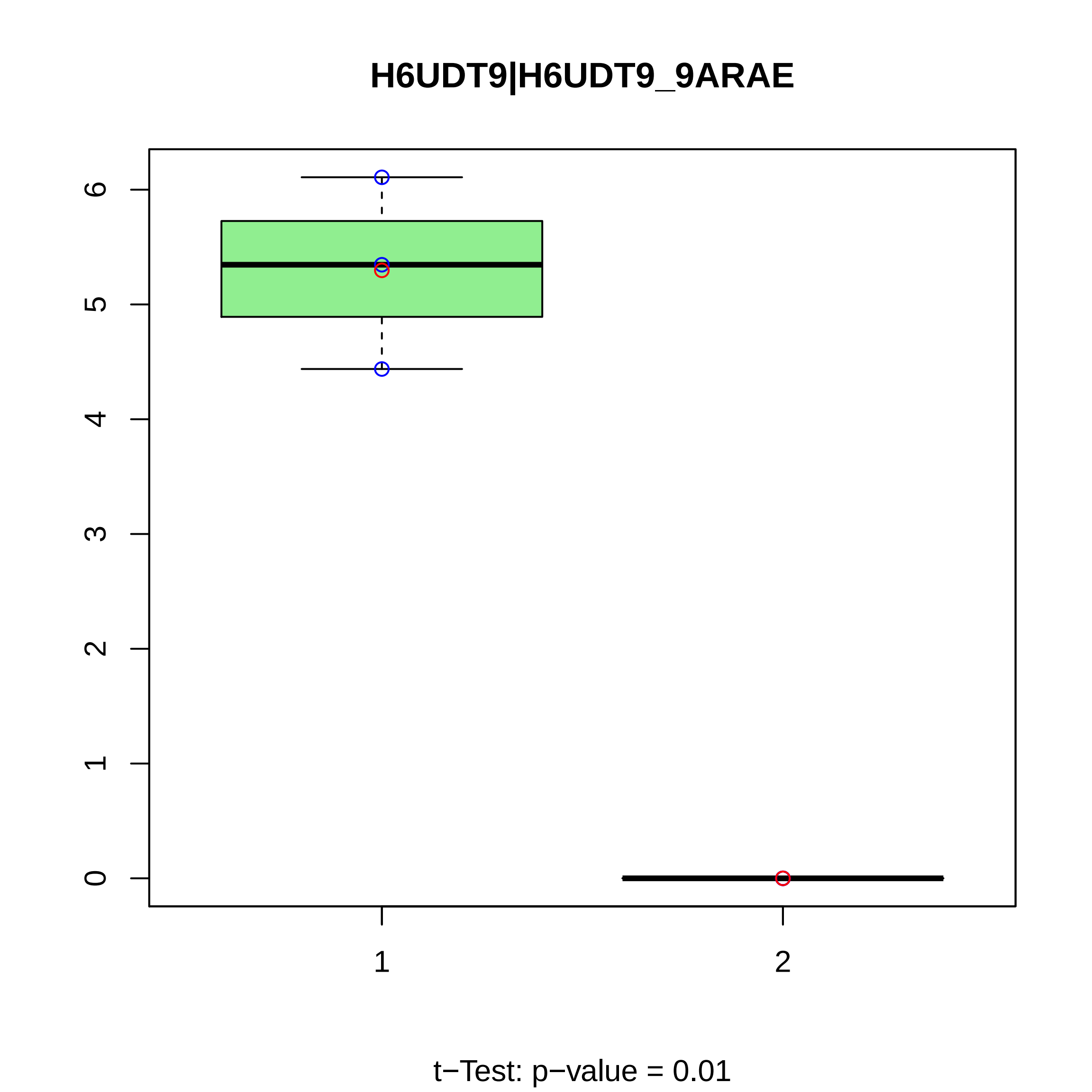

H6UDT9|H6UDT9_9ARAE
0
1
2
3
4
5
6
1
2
t−Test: p−value = 0.01

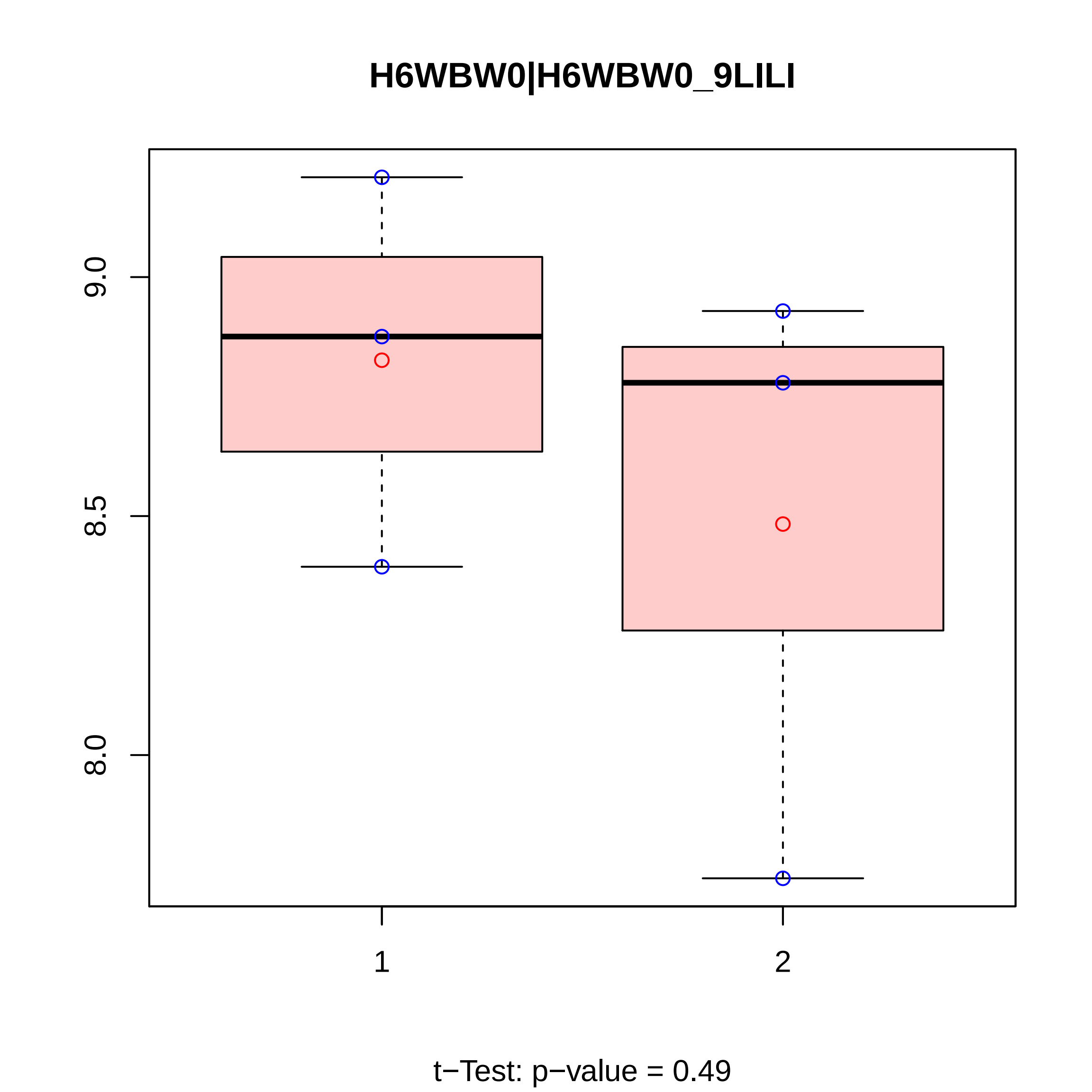
H6WBW0|H6WBW0_9LILI
9.0
8.5
8.0
1
2
t−Test: p−value = 0.49

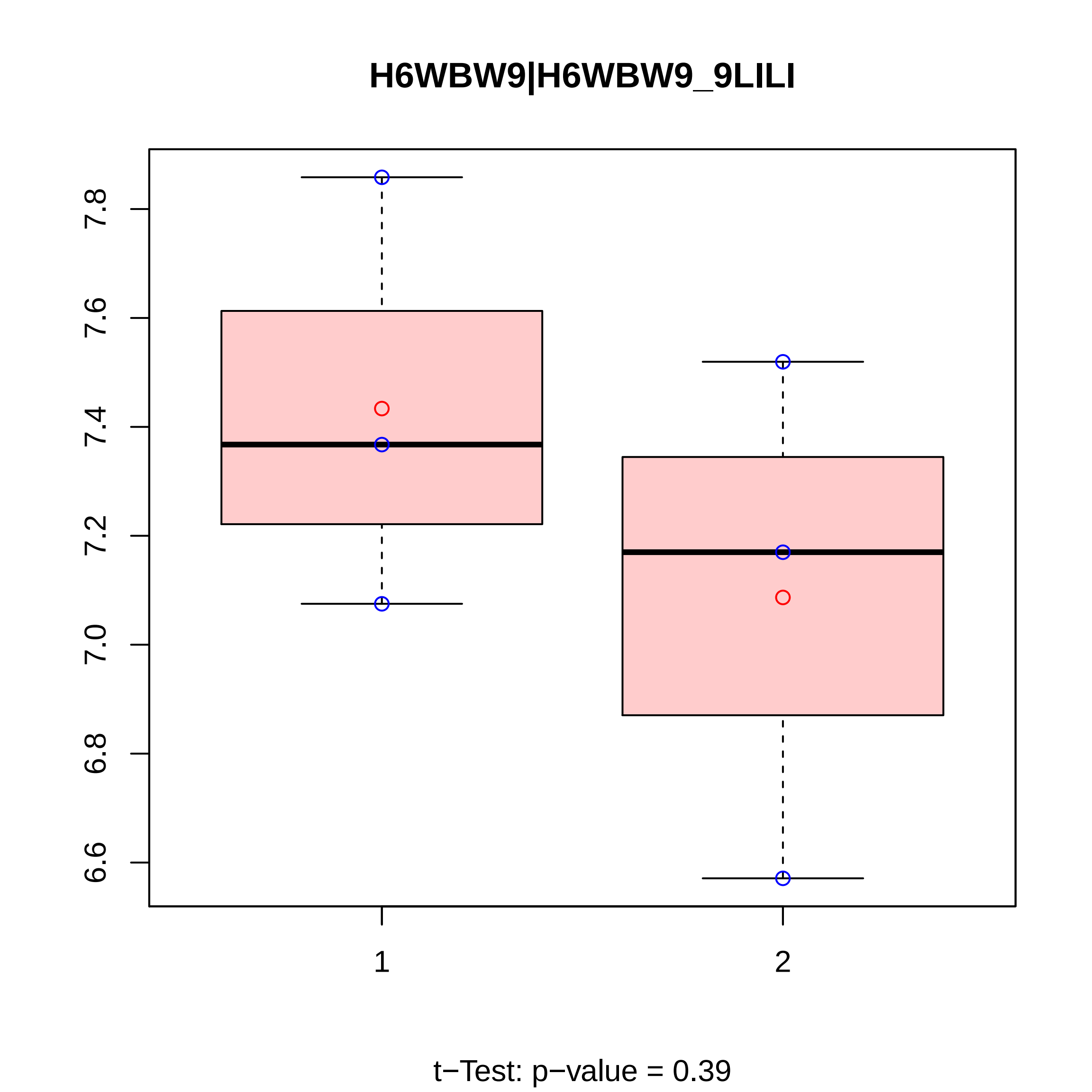

H6WBW9|H6WBW9_9LILI
7.8
7.6
7.4
7.2
7.0
6.8
6.6
1
2
t−Test: p−value = 0.39

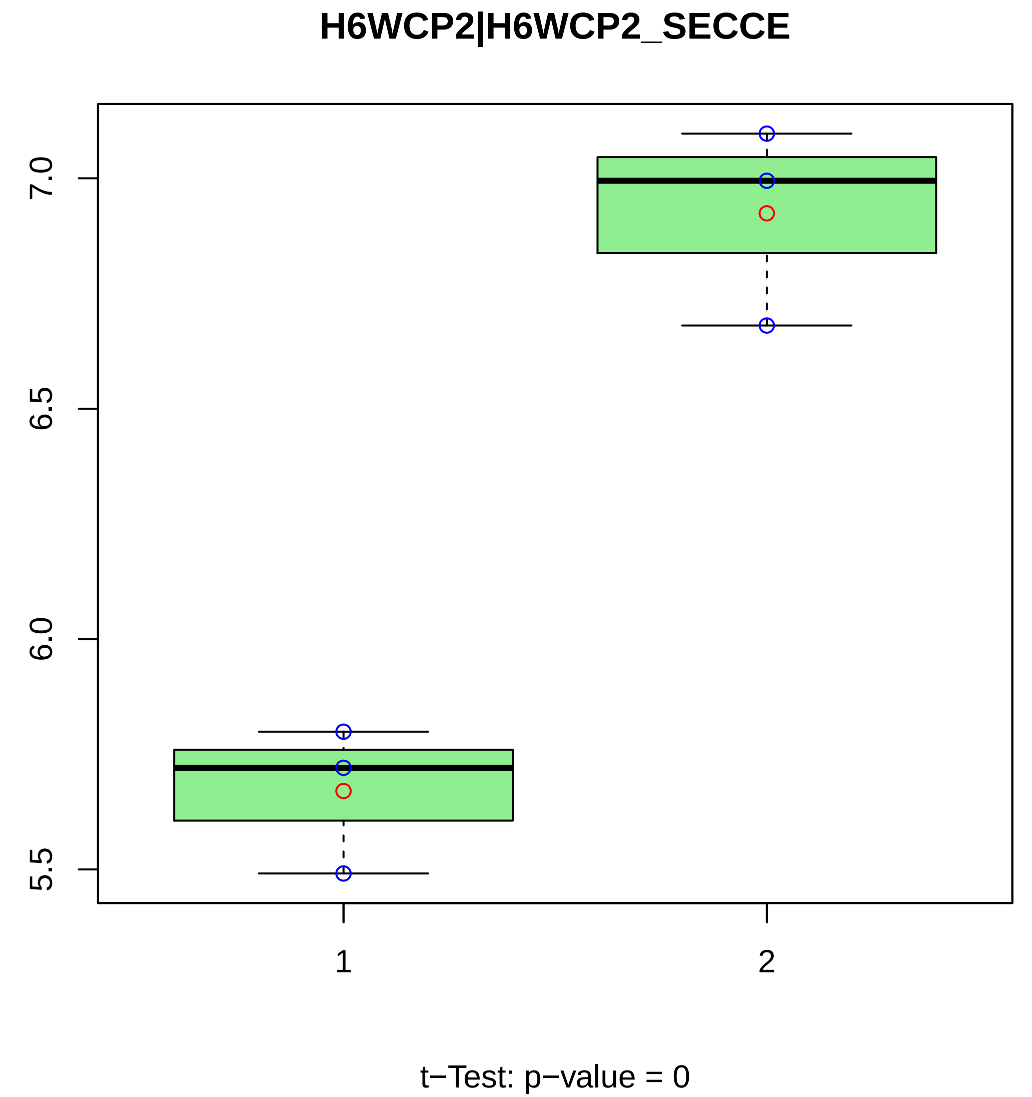
H6WCP2|H6WCP2_SECCE
7.0
6.5
6.0
5.5
1
2
t−Test: p−value = 0

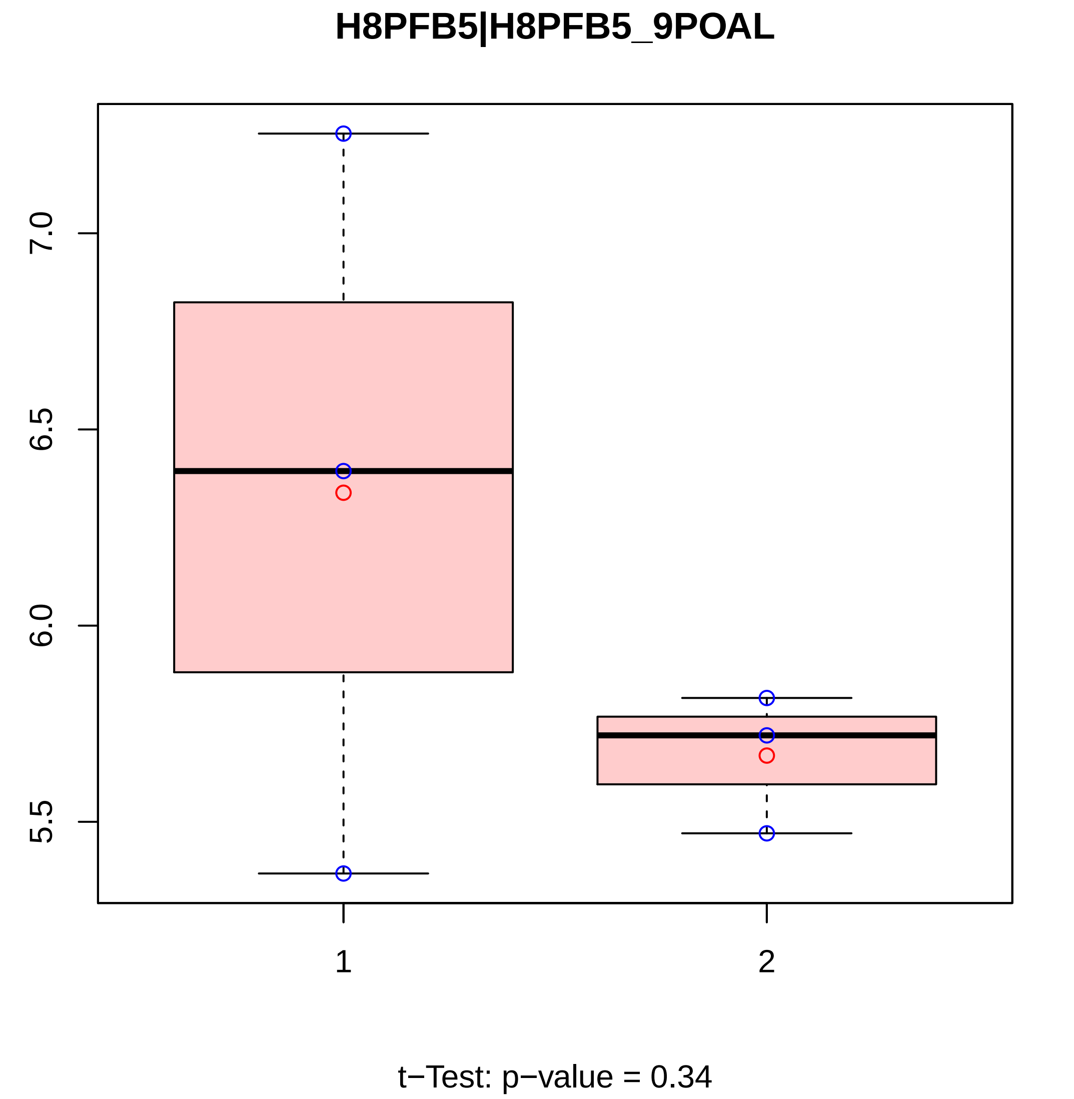

H8PFB5|H8PFB5_9POAL
7.0
6.5
6.0
5.5
1
2
t−Test: p−value = 0.34

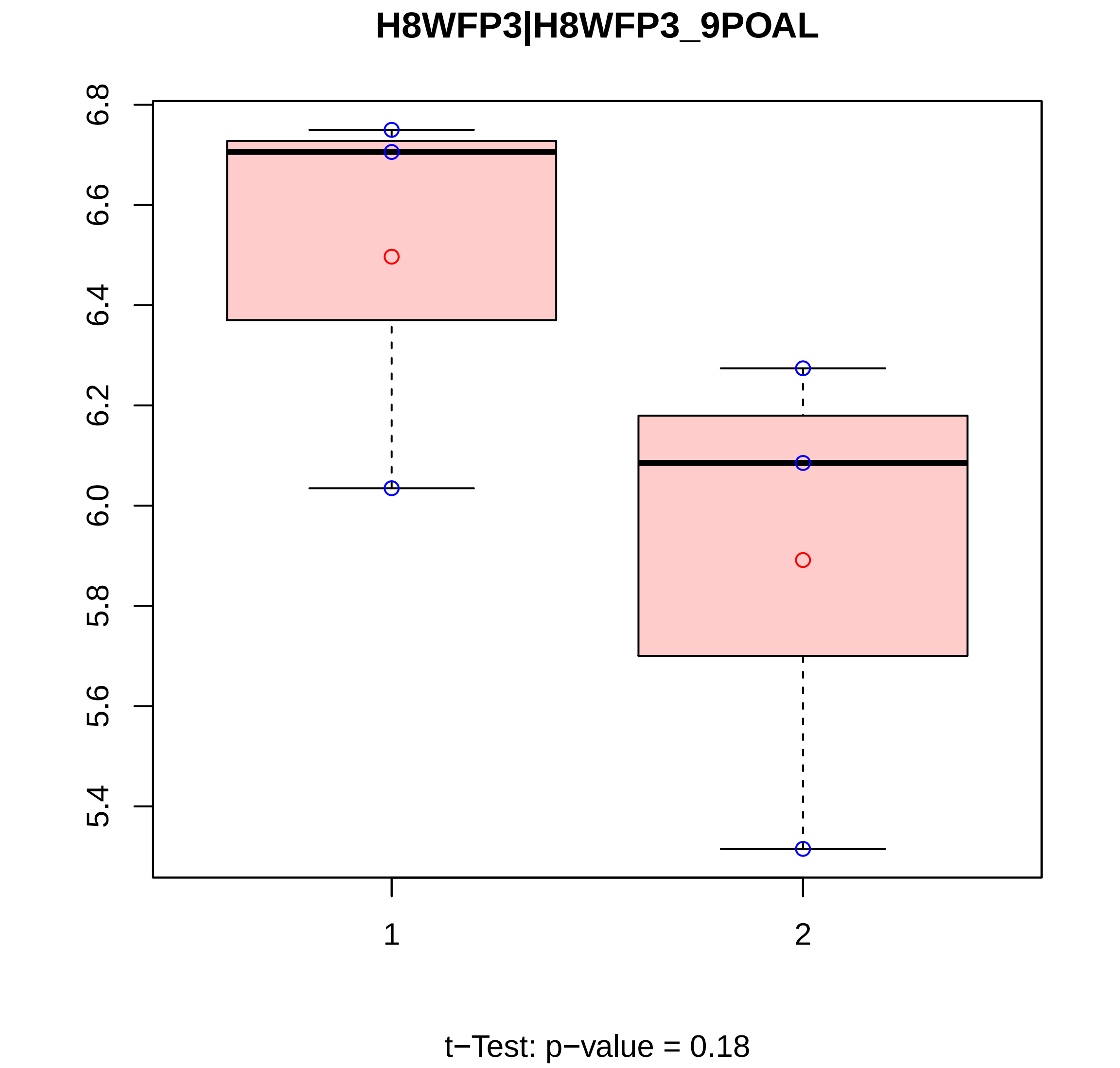
H8WFP3|H8WFP3_9POAL
6.8
6.6
6.4
6.2
6.0
5.8
5.6
5.4
1
2
t−Test: p−value = 0.18

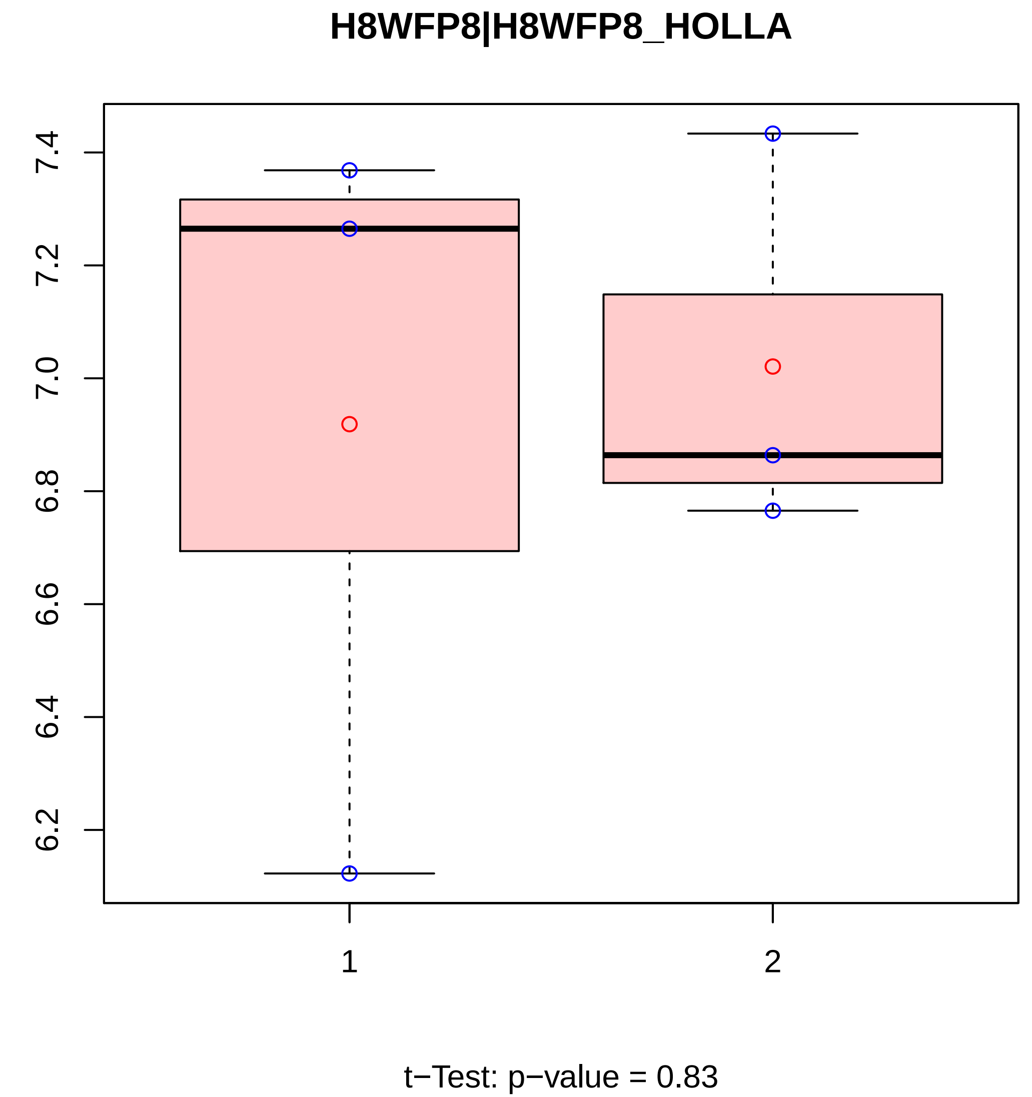
H8WFP8|H8WFP8_HOLLA
7.4
7.2
7.0
6.8
6.6
6.4
6.2
1
2
t−Test: p−value = 0.83

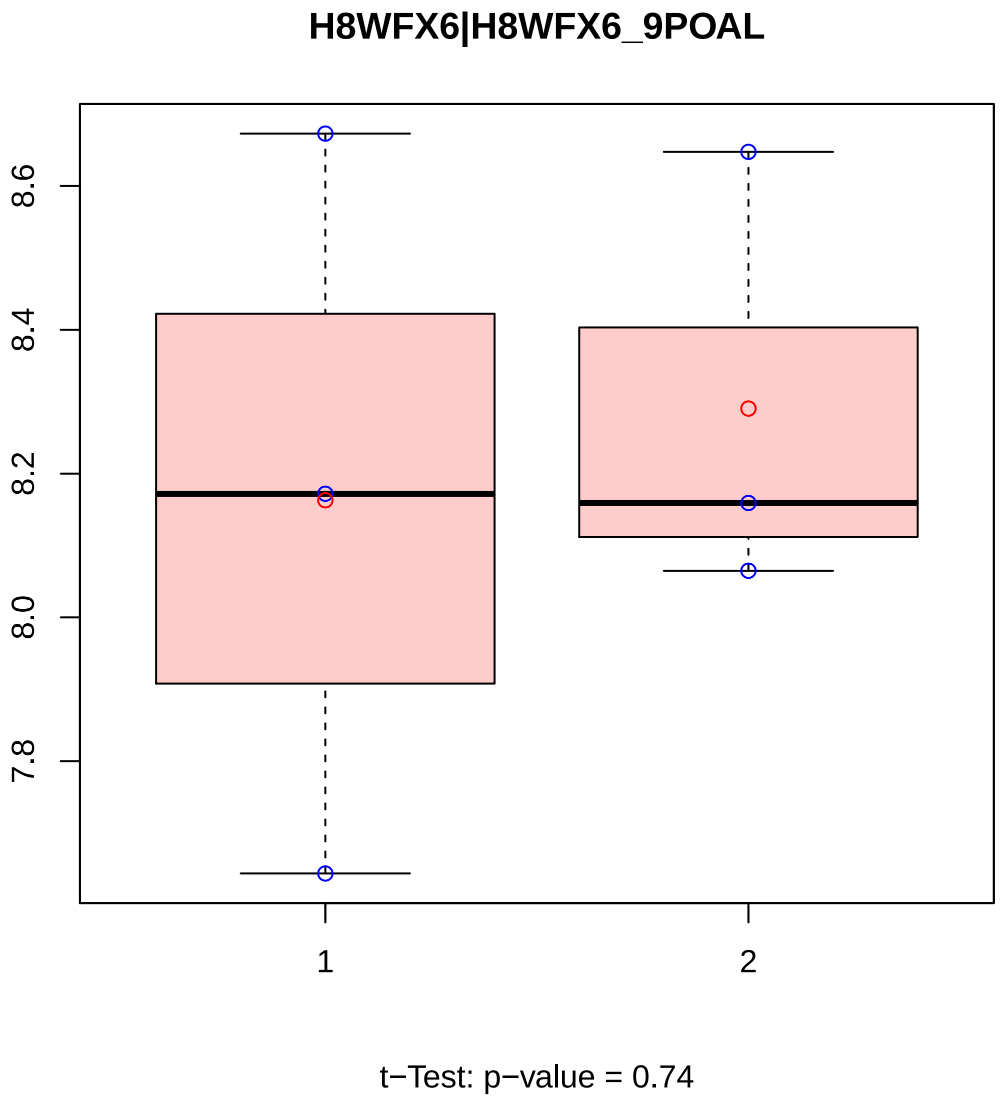
H8WFX6|H8WFX6_9POAL
8.6
8.4
8.2
8.0
7.8
1
2
t−Test: p−value = 0.74

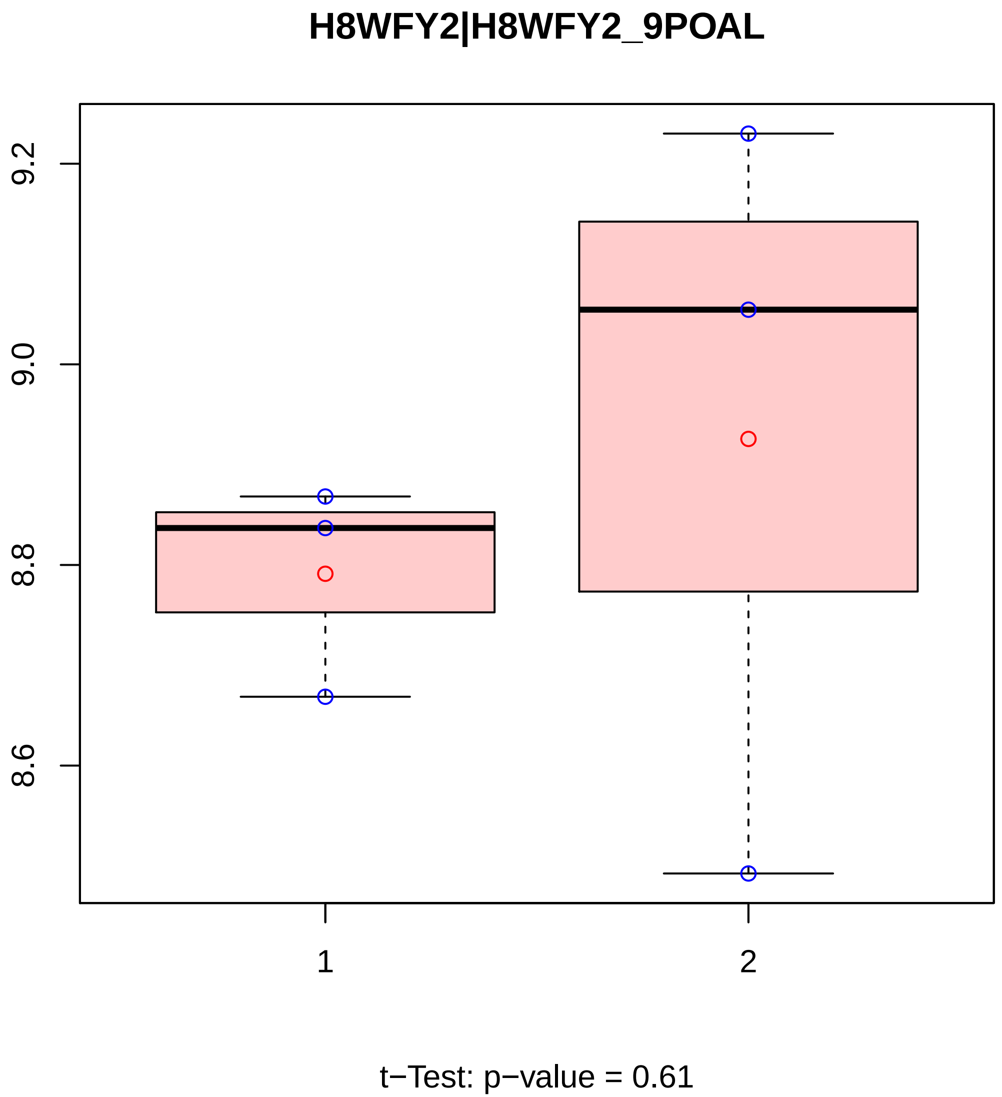

H8WFY2|H8WFY2_9POAL
9.2
9.0
8.8
8.6
1
2
t−Test: p−value = 0.61

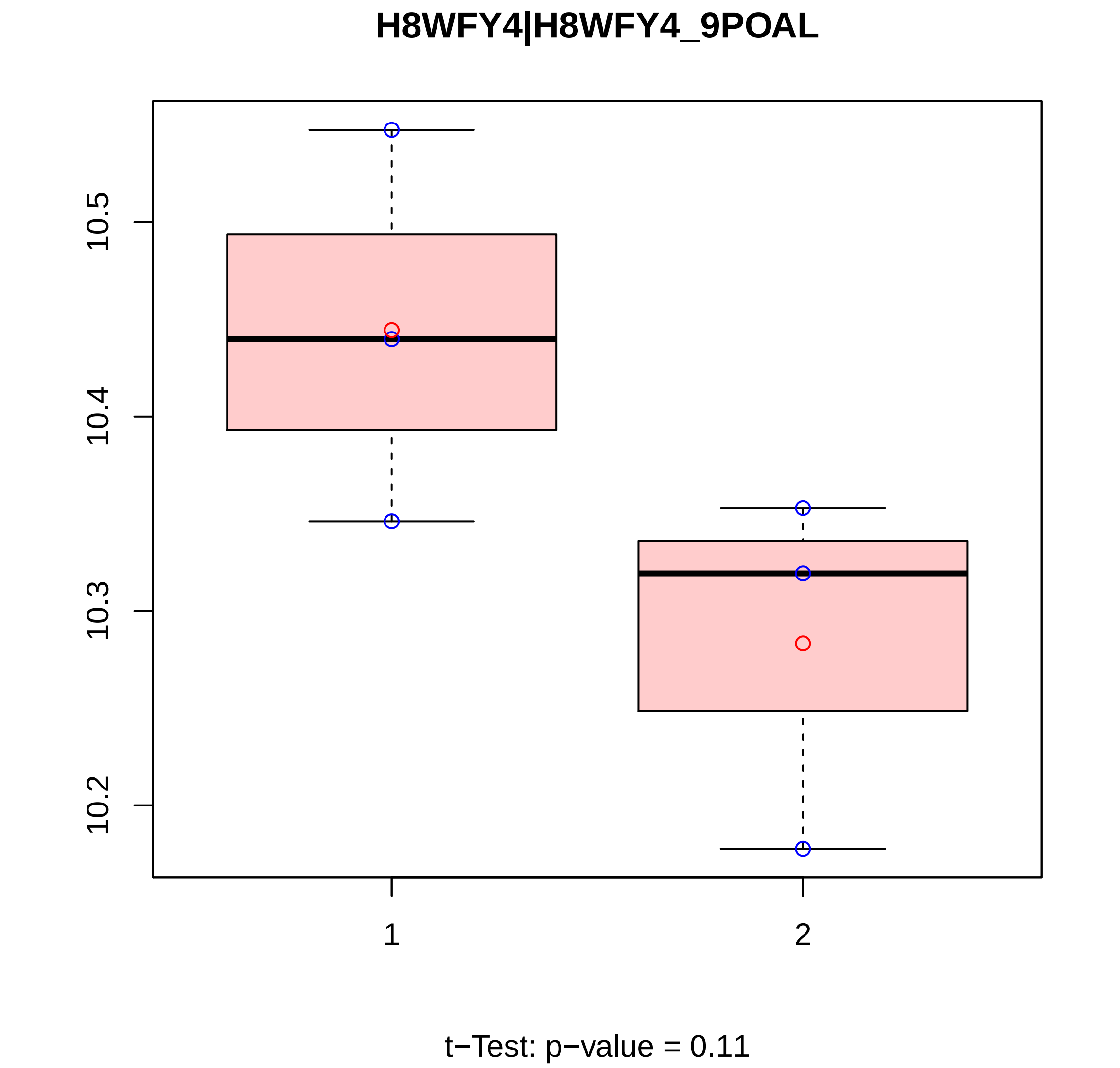
H8WFY4|H8WFY4_9POAL
10.5
10.4
10.3
10.2
1
2
t−Test: p−value = 0.11

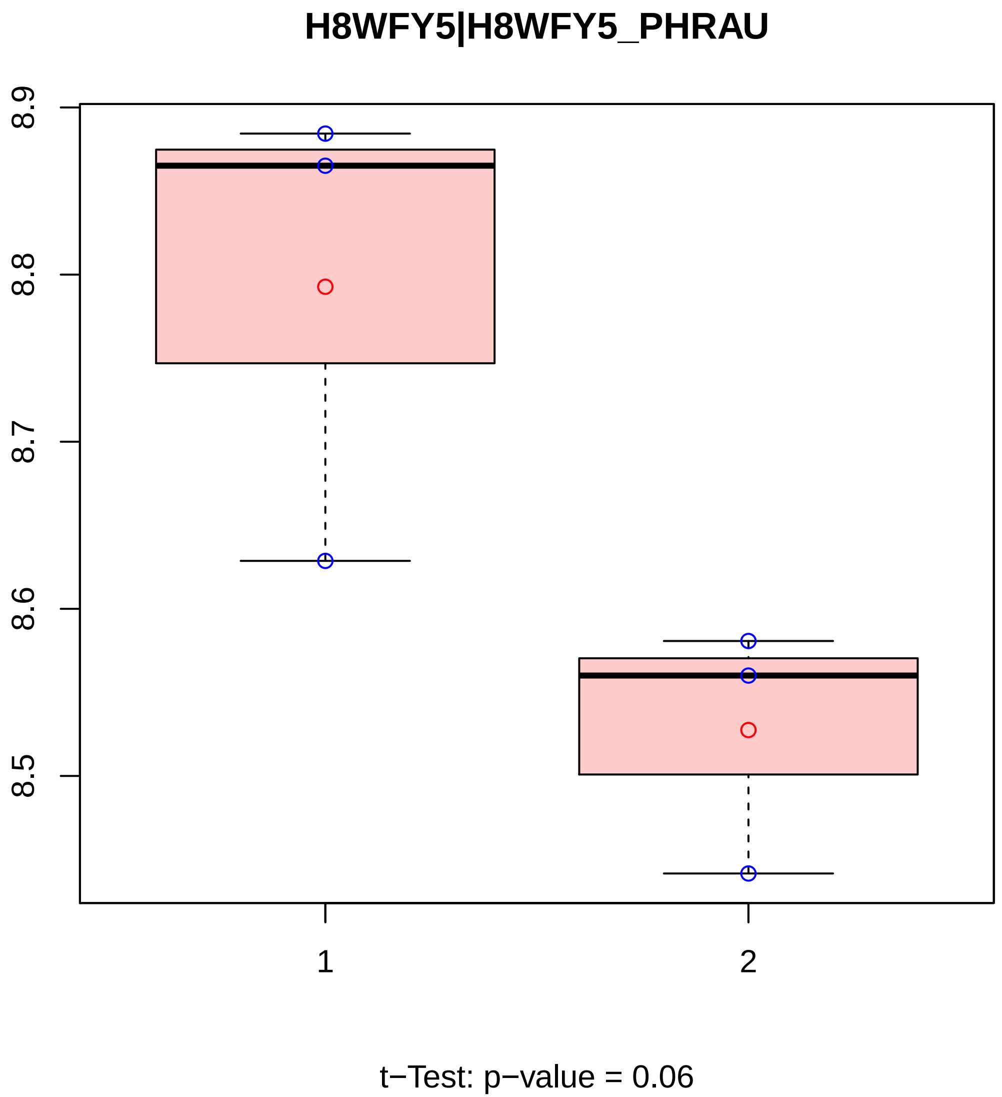
H8WFY5|H8WFY5_PHRAU
8.9
8.8
8.7
8.6
8.5
1
2
t−Test: p−value = 0.06

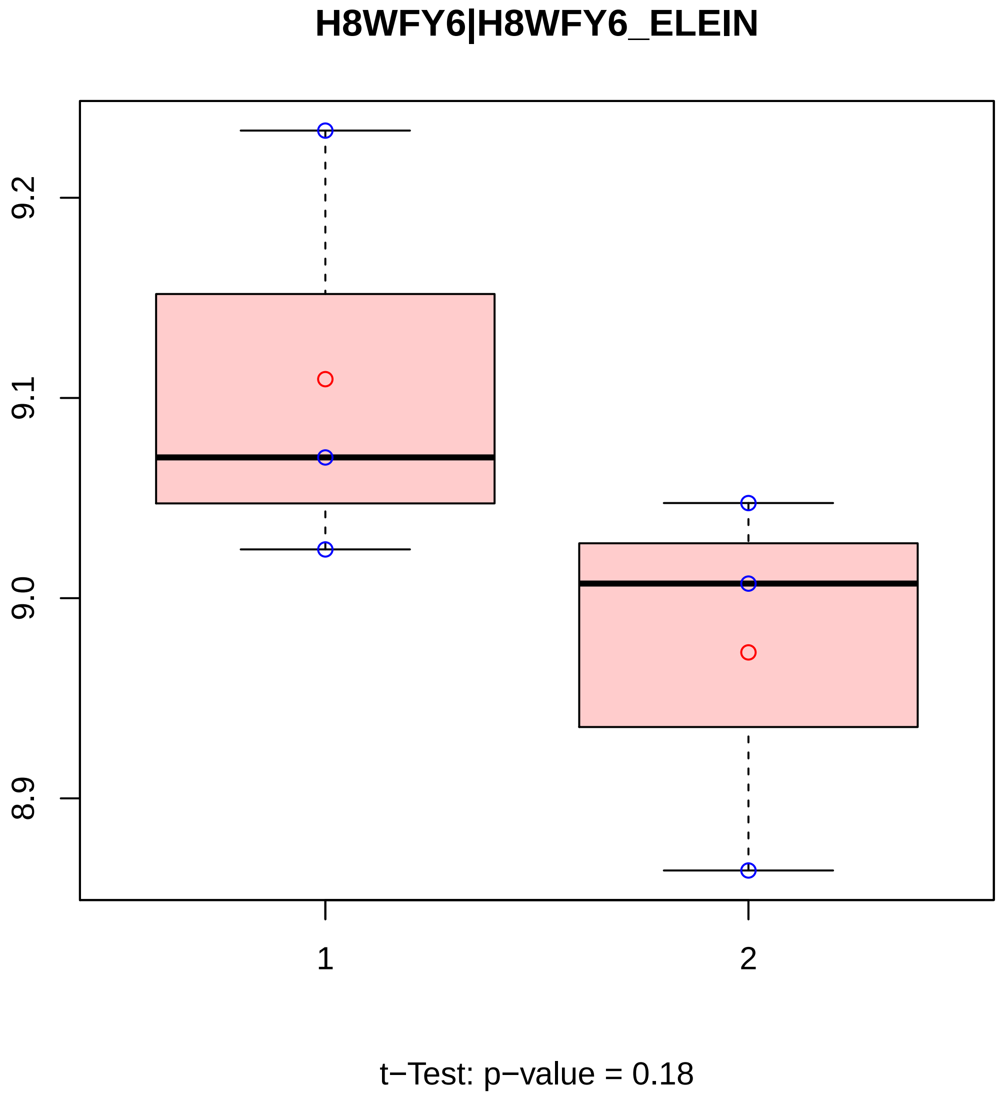
H8WFY6|H8WFY6_ELEIN
9.2
9.1
9.0
8.9
1
2
t−Test: p−value = 0.18

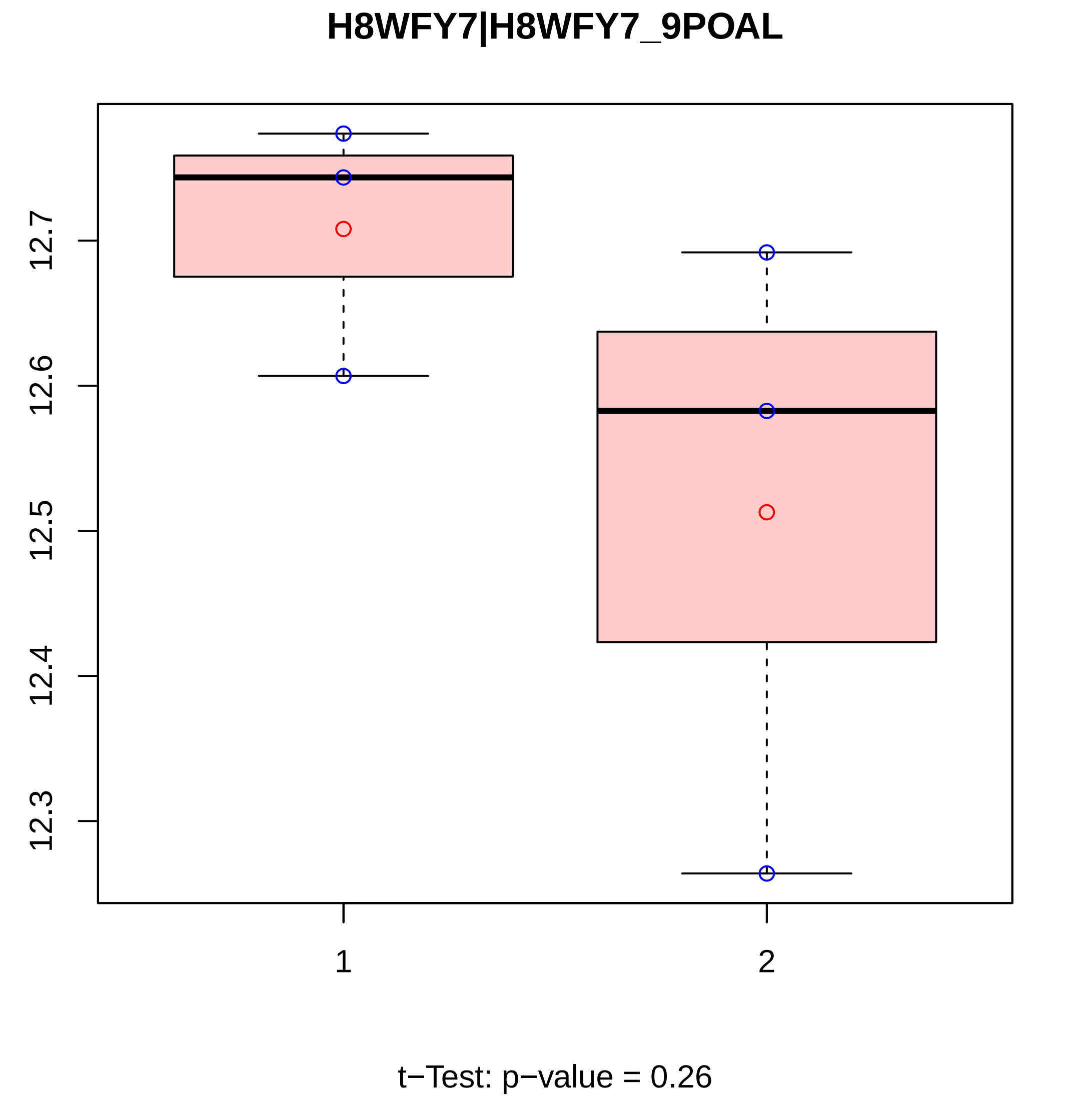

H8WFY7|H8WFY7_9POAL
12.3
12.4
12.5
12.6
12.7
1
2
t−Test: p−value = 0.26

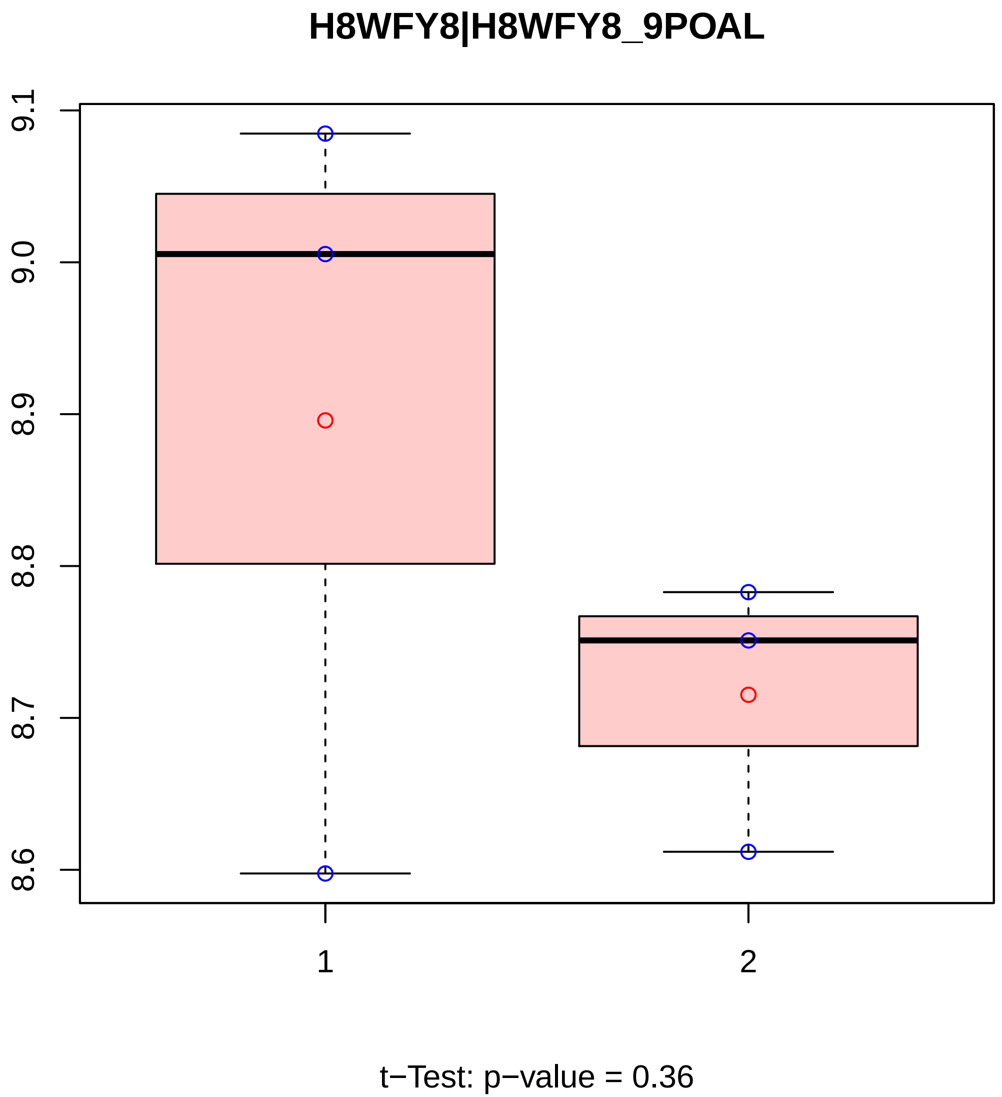

H8WFY8|H8WFY8_9POAL
9.1
9.0
8.9
8.8
8.7
8.6
1
2
t−Test: p−value = 0.36

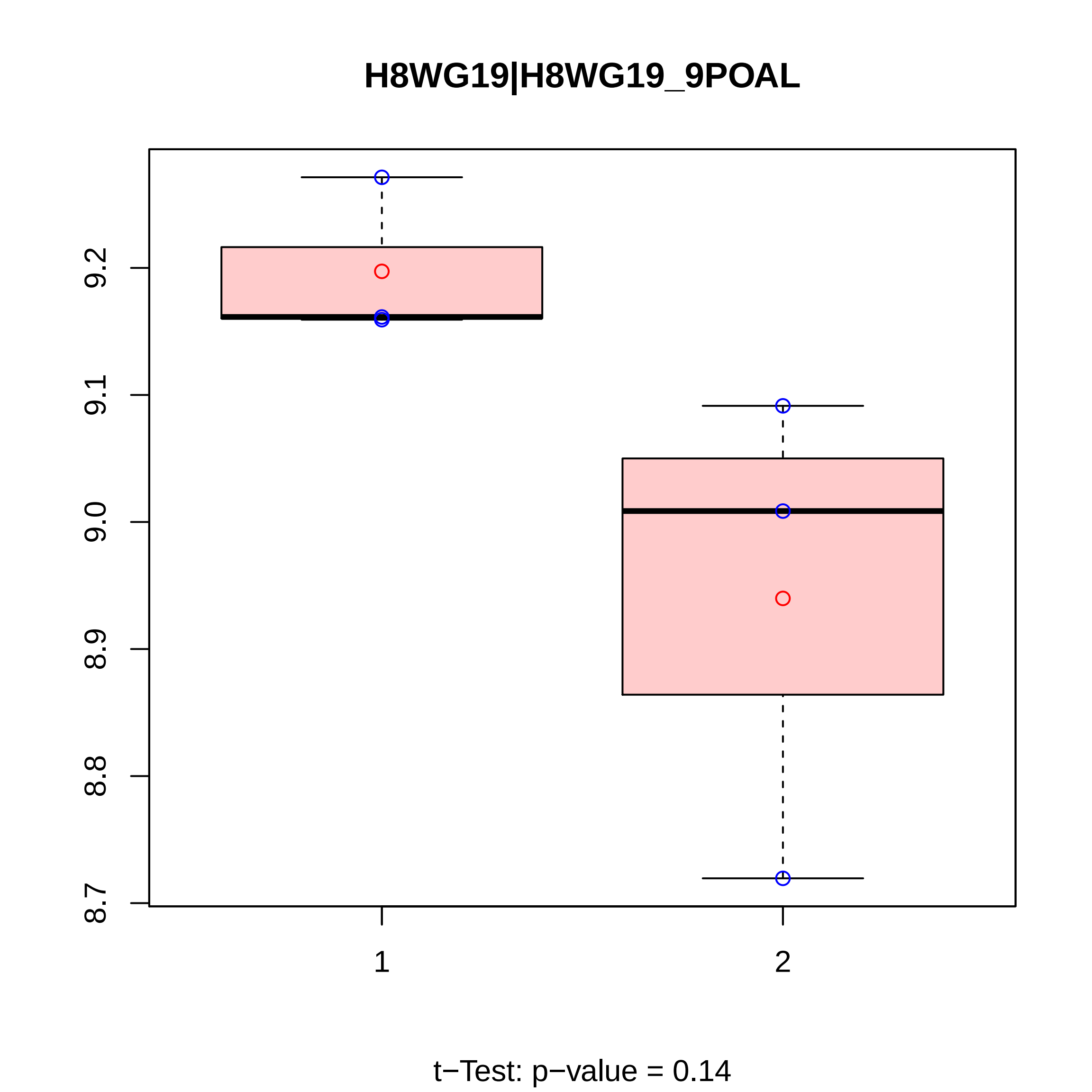
H8WG19|H8WG19_9POAL
9.2
9.1
9.0
8.9
8.8
8.7
1
2
t−Test: p−value = 0.14

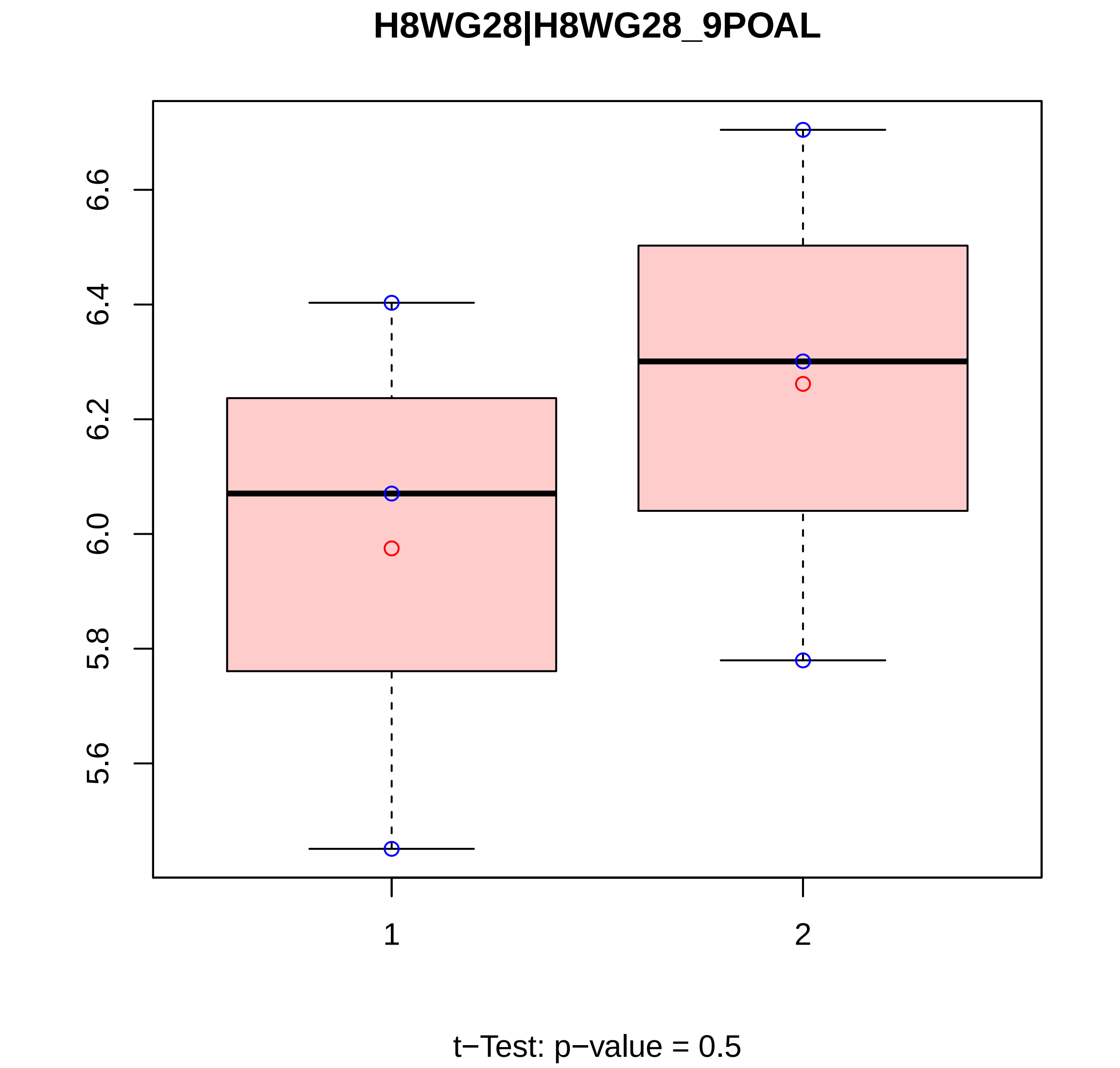
H8WG28|H8WG28_9POAL
6.6
6.4
6.2
6.0
5.8
5.6
1
2
t−Test: p−value = 0.5

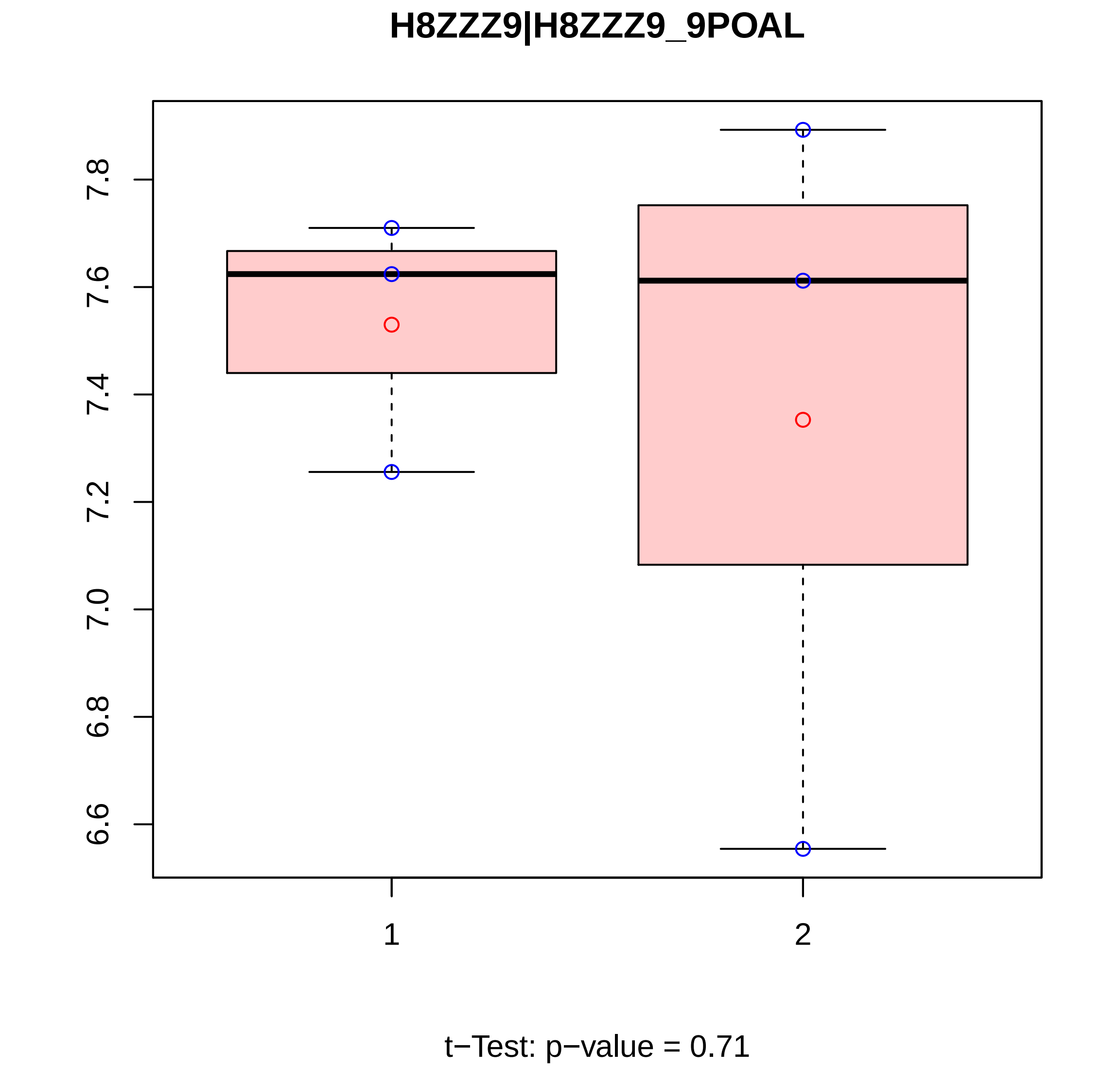
H8ZZZ9|H8ZZZ9_9POAL
7.8
7.6
7.4
7.2
7.0
6.8
6.6
1
2
t−Test: p−value = 0.71

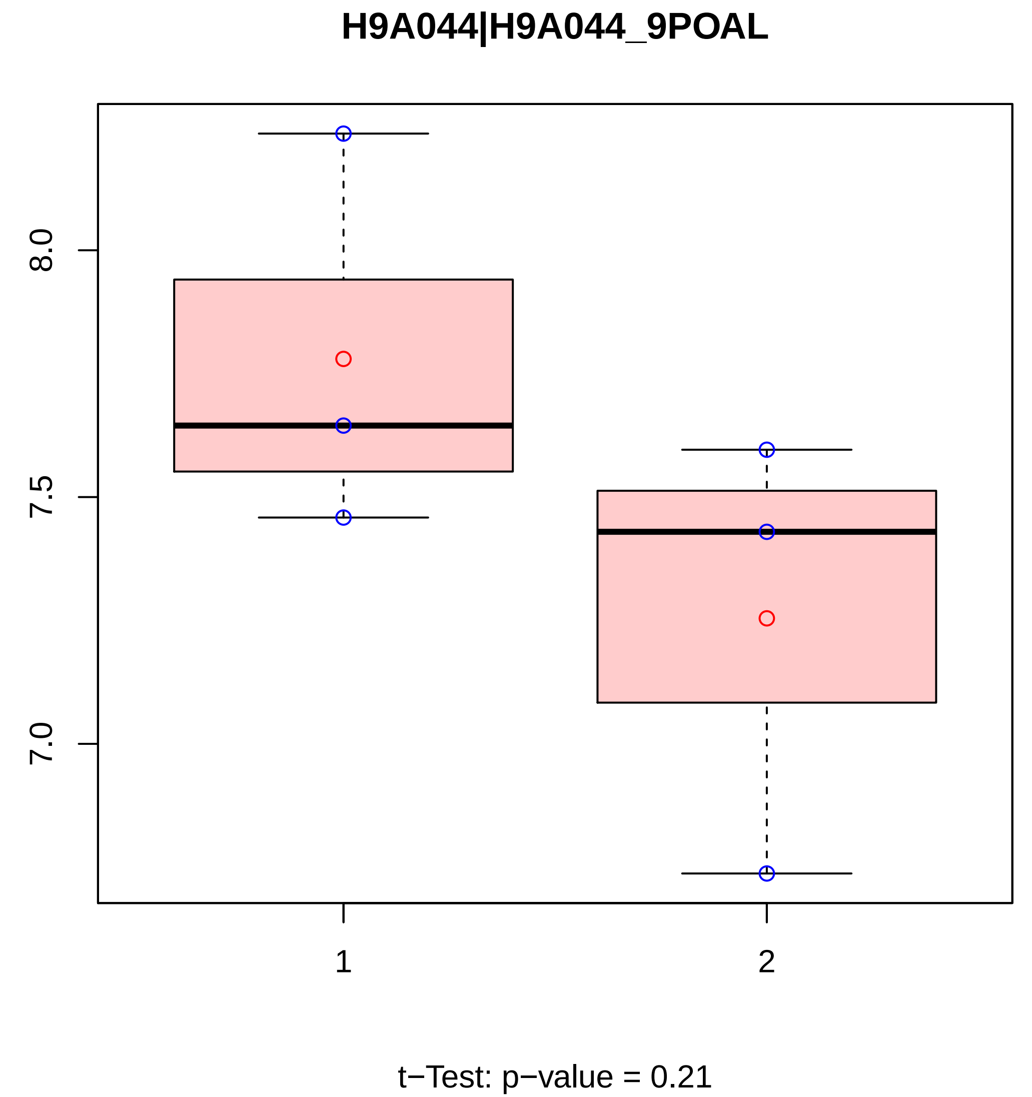
H9A044|H9A044_9POAL
8.0
7.5
7.0
1
2
t−Test: p−value = 0.21

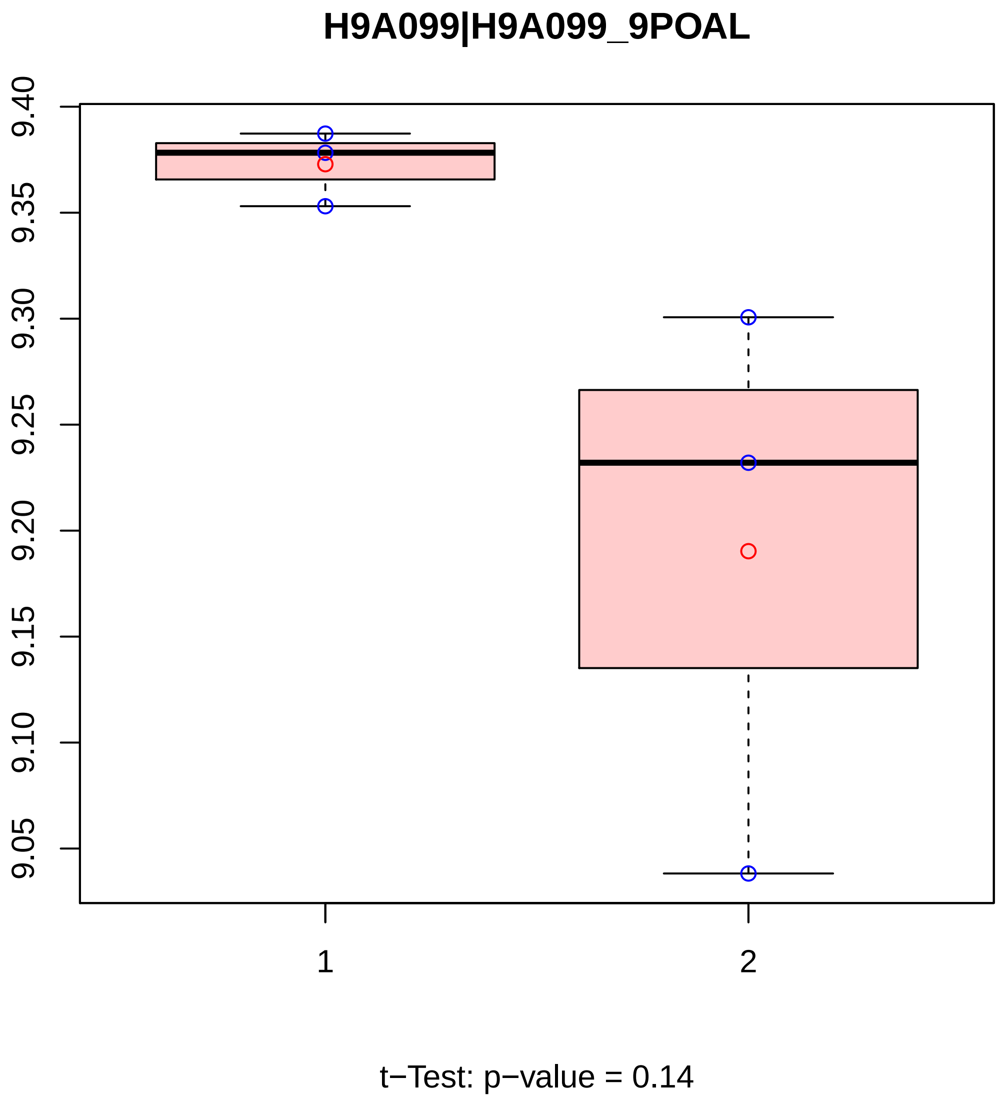

H9A099|H9A099_9POAL
9.40
9.35
9.30
9.25
9.20
9.15
9.10
9.05
1
2
t−Test: p−value = 0.14

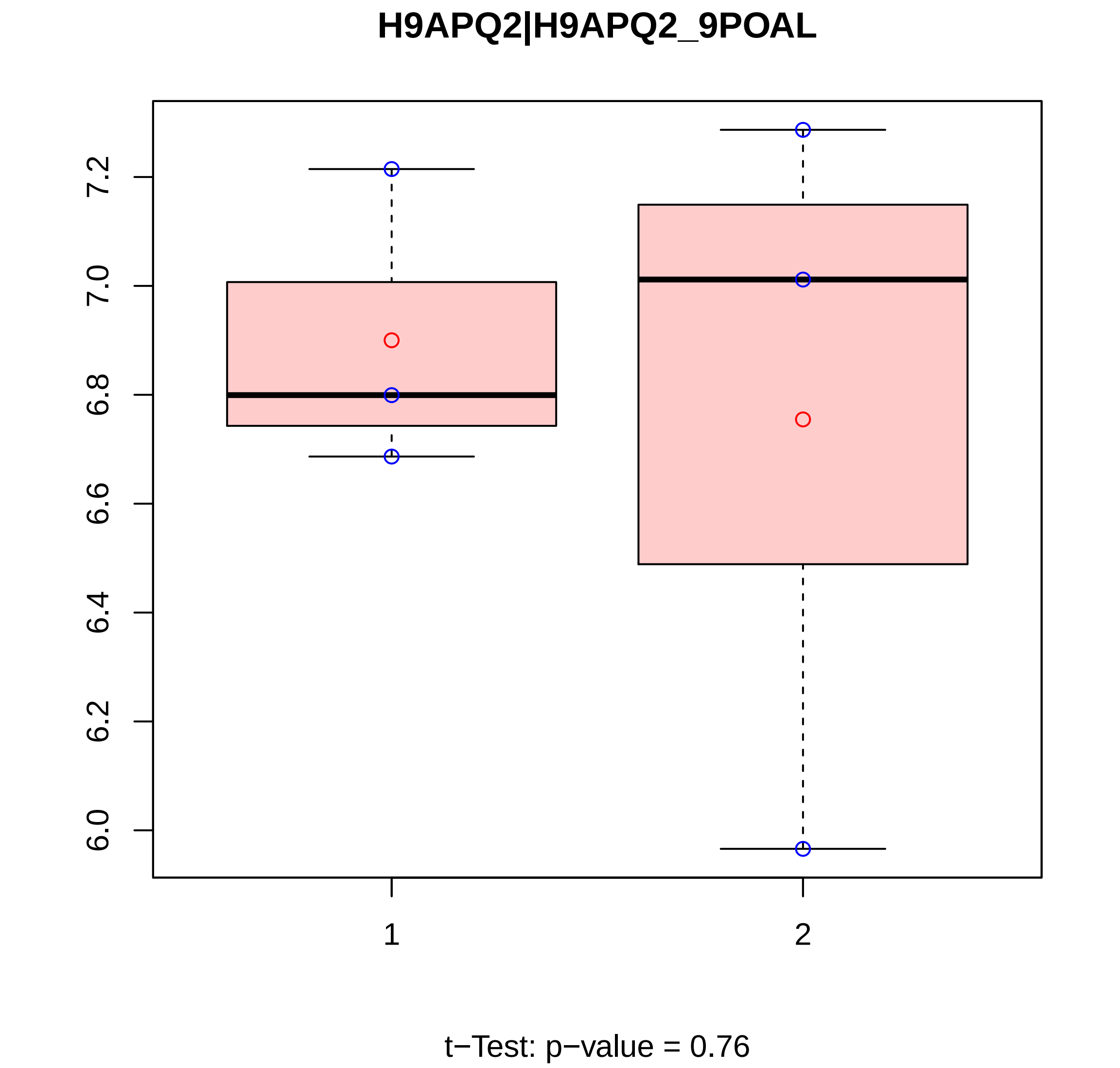
H9APQ2|H9APQ2_9POAL
7.2
7.0
6.8
6.6
6.4
6.2
6.0
1
2
t−Test: p−value = 0.76

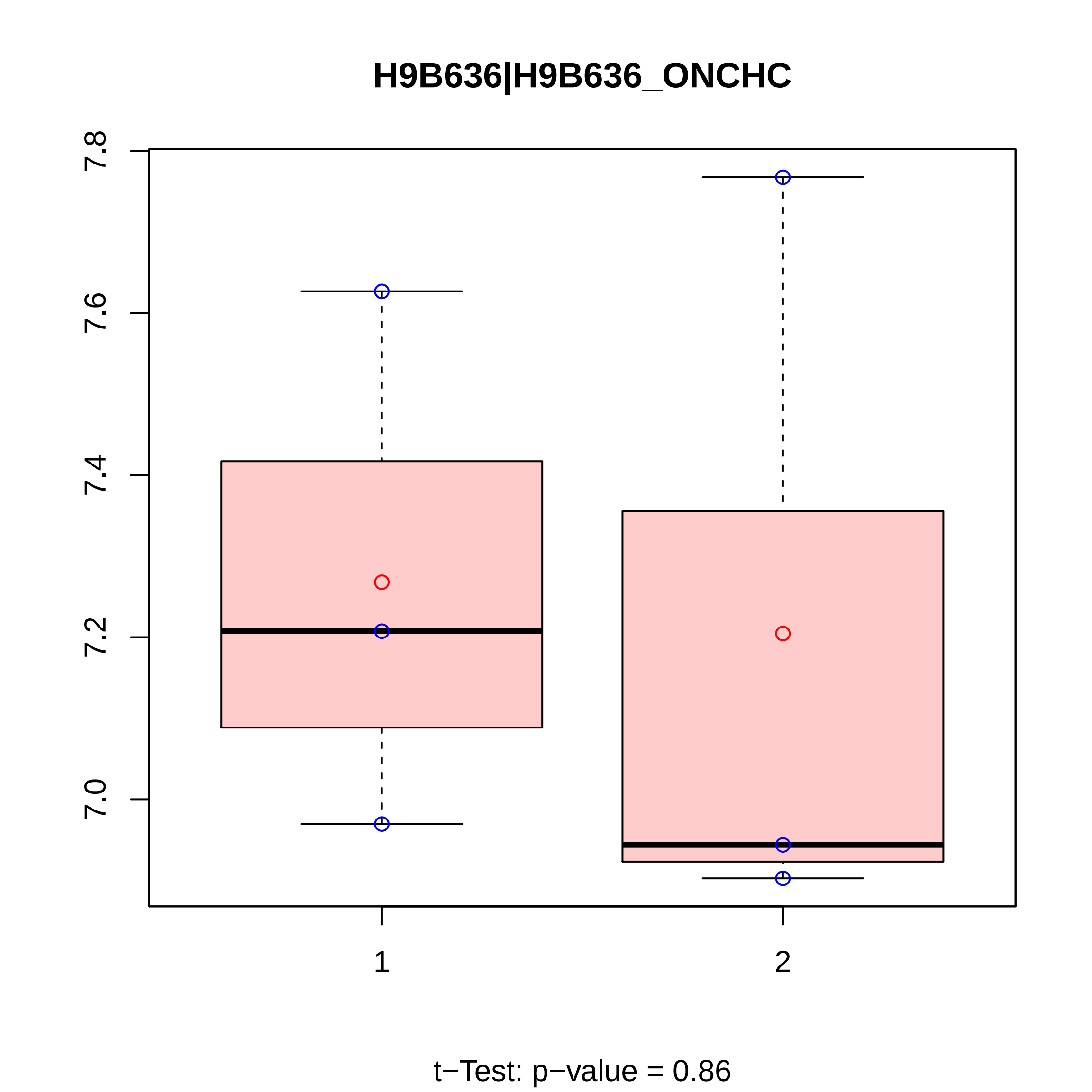
H9B636|H9B636_ONCHC
7.8
7.6
7.4
7.2
7.0
1
2
t−Test: p−value = 0.86

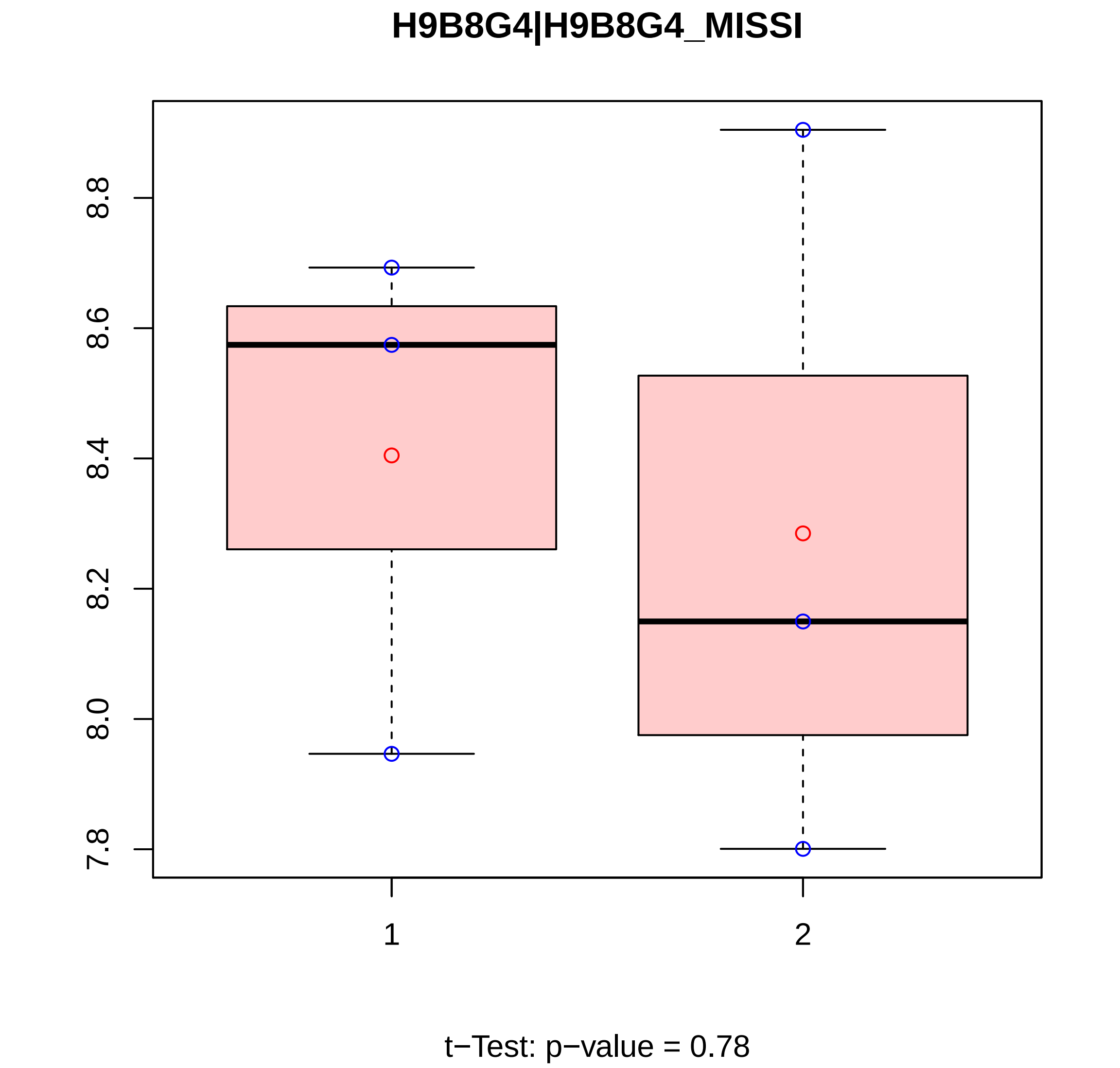
H9B8G4|H9B8G4_MISSI
8.8
8.6
8.4
8.2
8.0
7.8
1
2
t−Test: p−value = 0.78

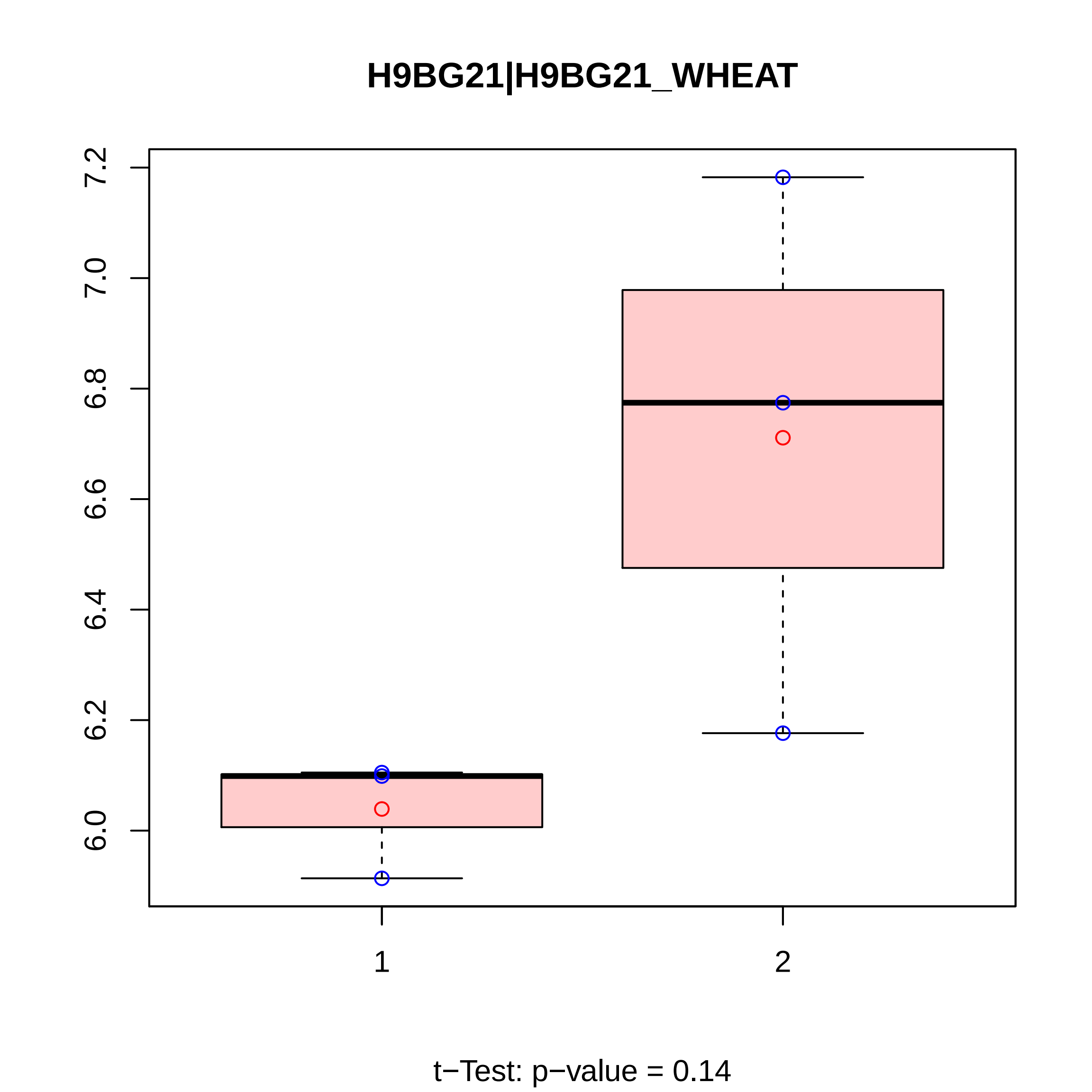
H9BG21|H9BG21_WHEAT
7.2
7.0
6.8
6.6
6.4
6.2
6.0
1
2
t−Test: p−value = 0.14

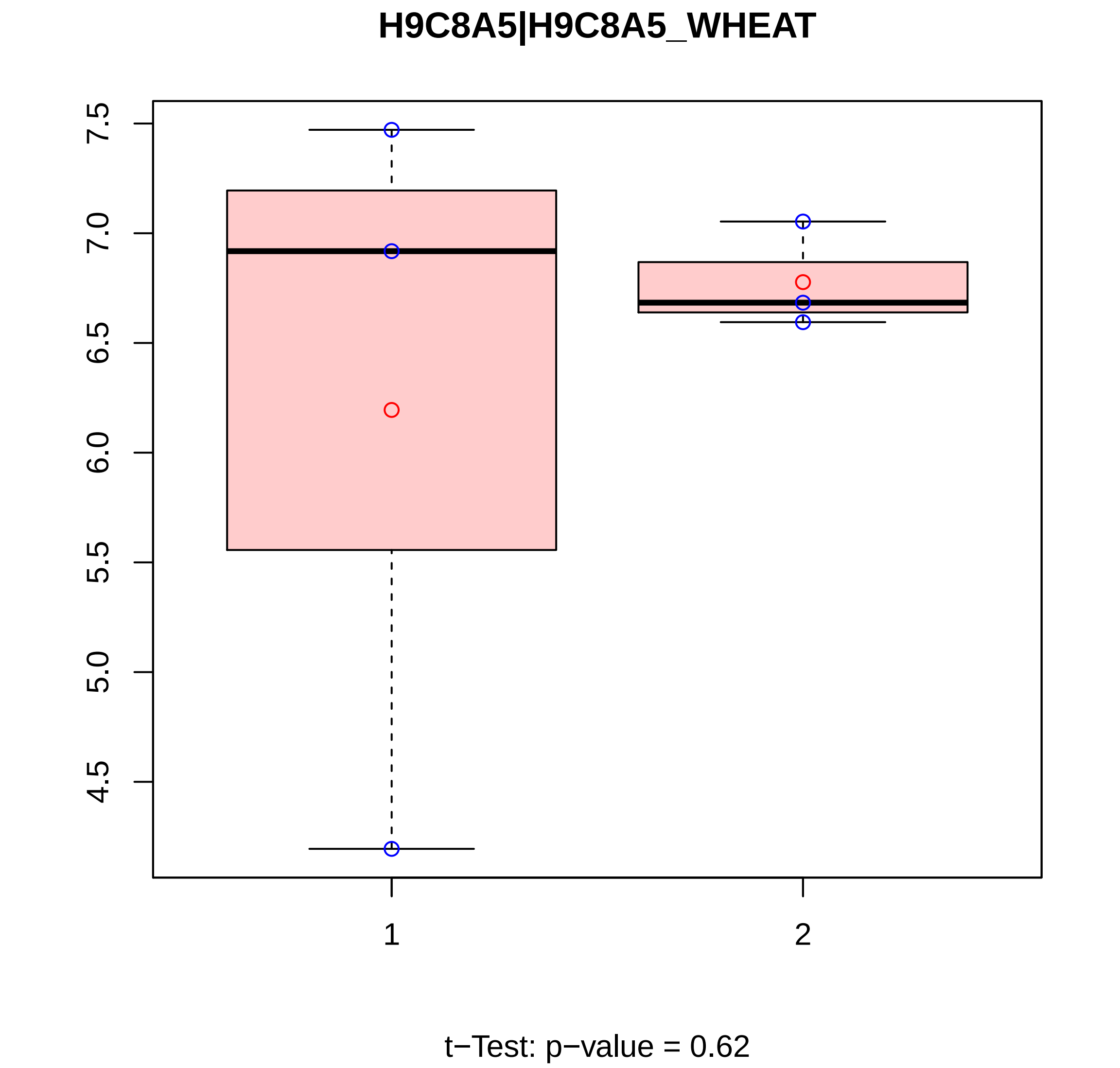

H9C8A5|H9C8A5_WHEAT
7.5
7.0
6.5
6.0
5.5
5.0
4.5
1
2
t−Test: p−value = 0.62

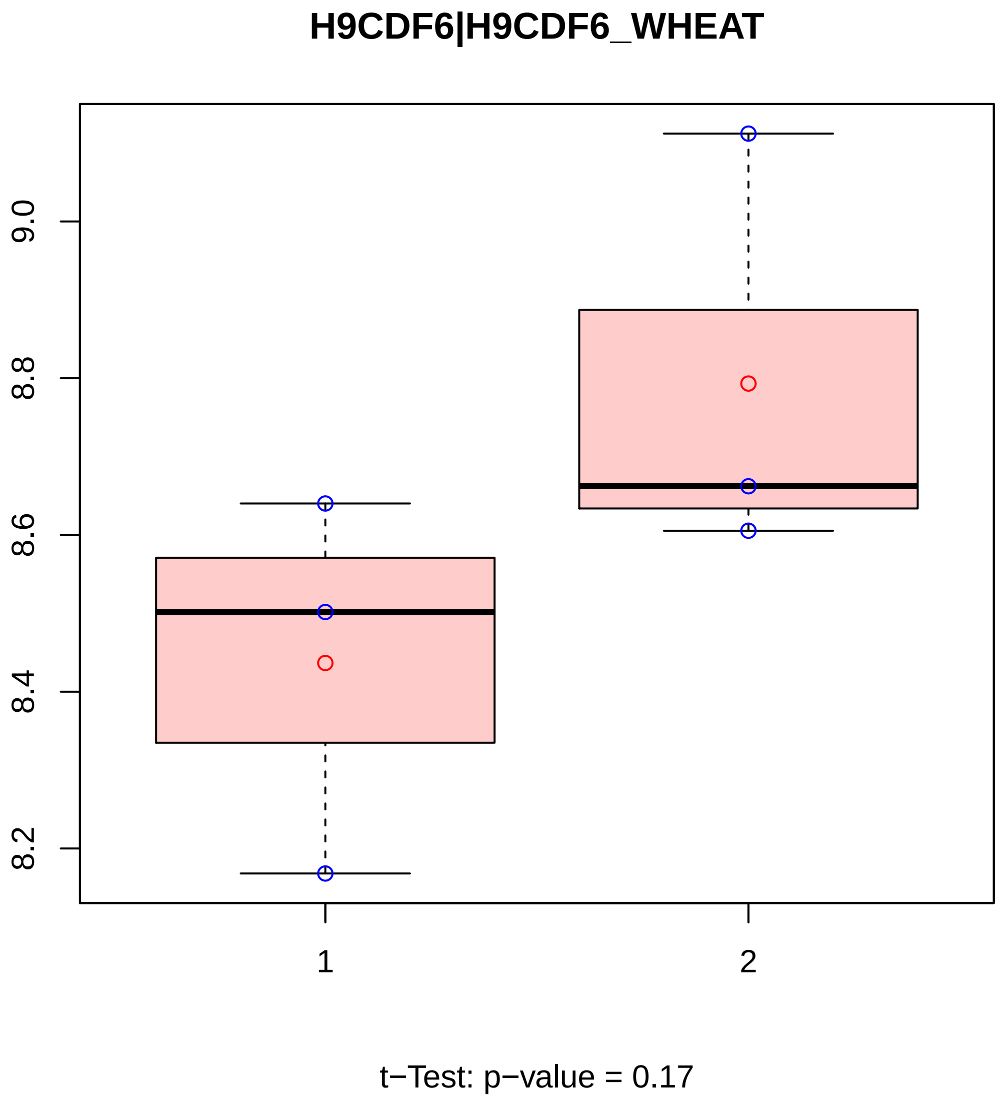

H9CDF6|H9CDF6_WHEAT
9.0
8.8
8.6
8.4
8.2
1
2
t−Test: p−value = 0.17

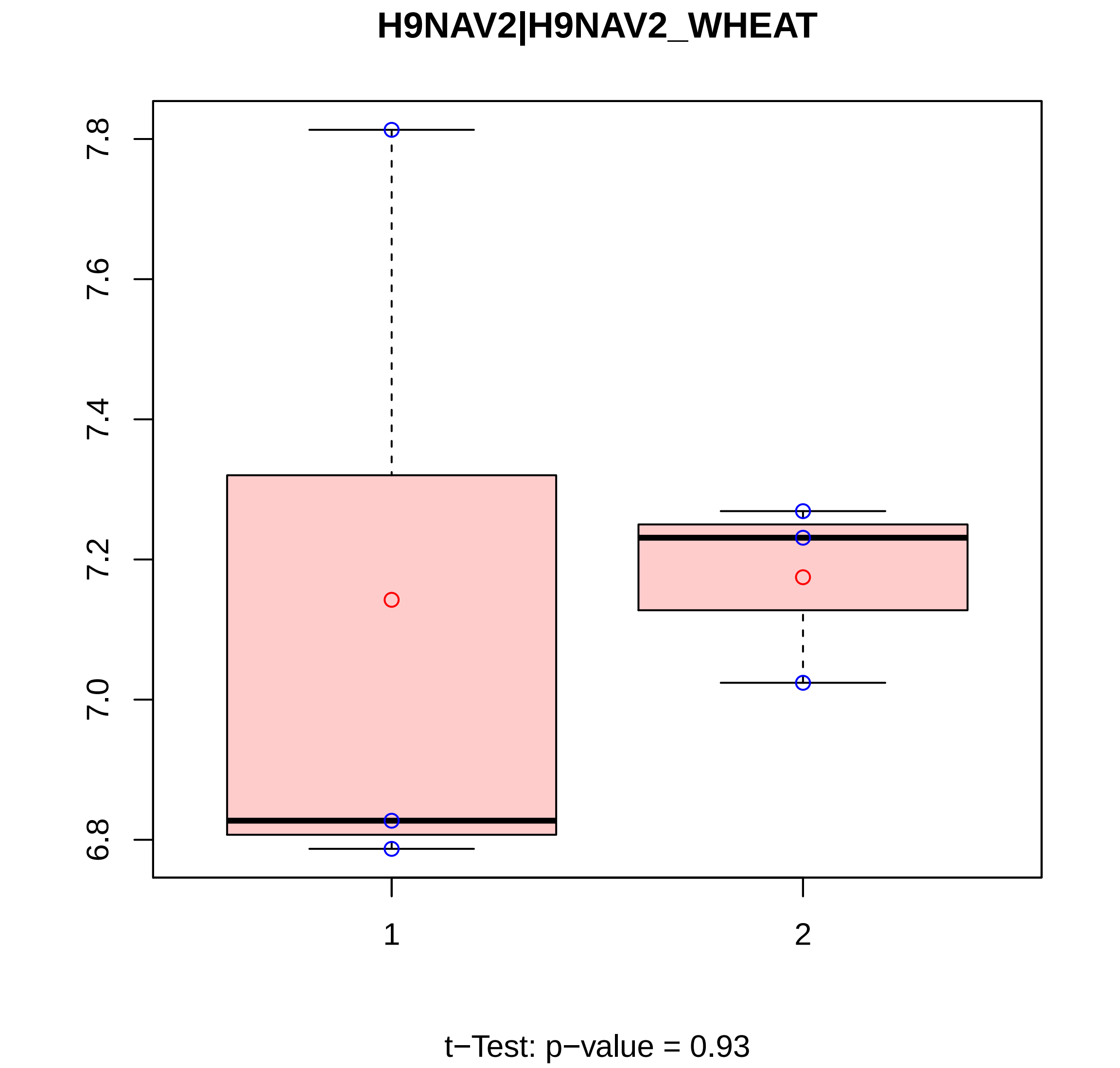
H9NAV2|H9NAV2_WHEAT
7.8
7.6
7.4
7.2
7.0
6.8
1
2
t−Test: p−value = 0.93

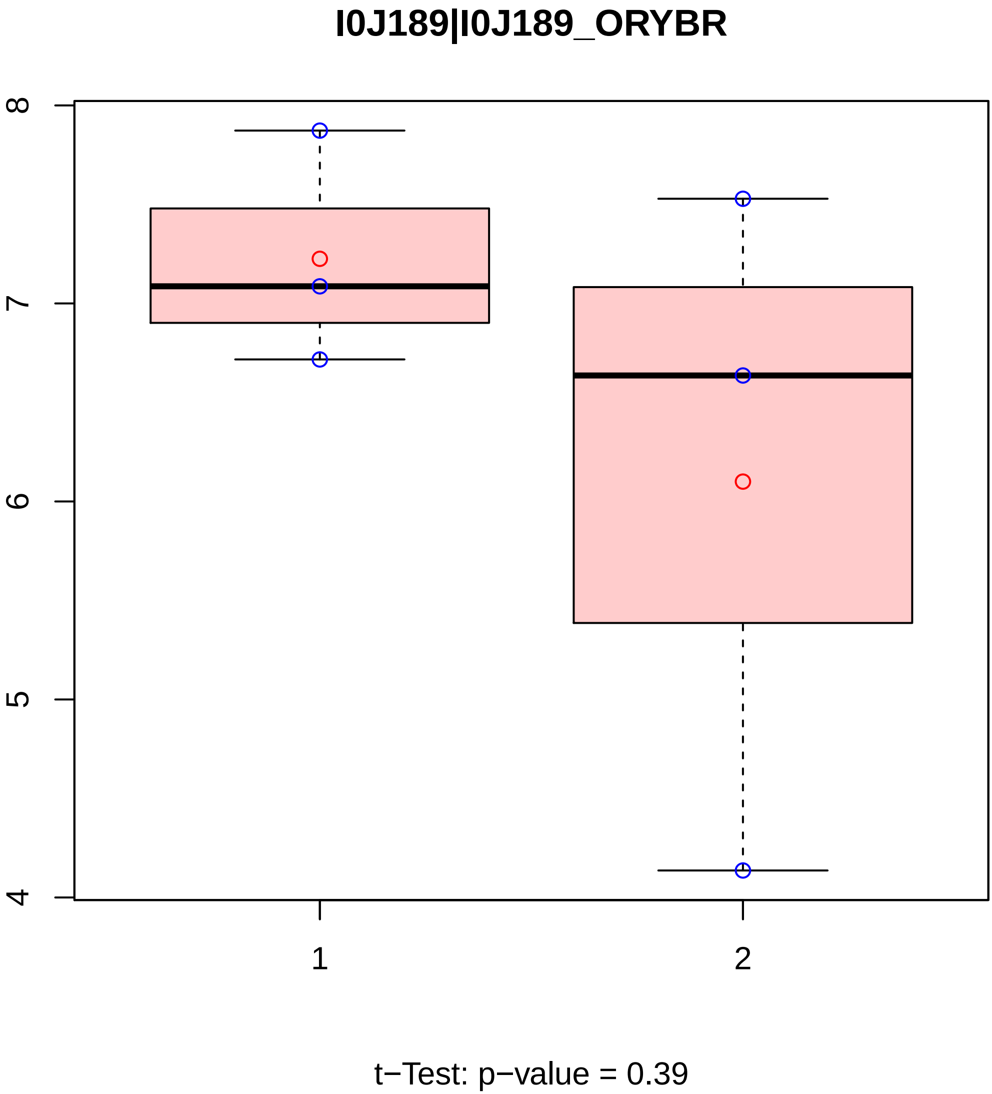

I0J189|I0J189_ORYBR
8
7
6
5
4
1
2
t−Test: p−value = 0.39

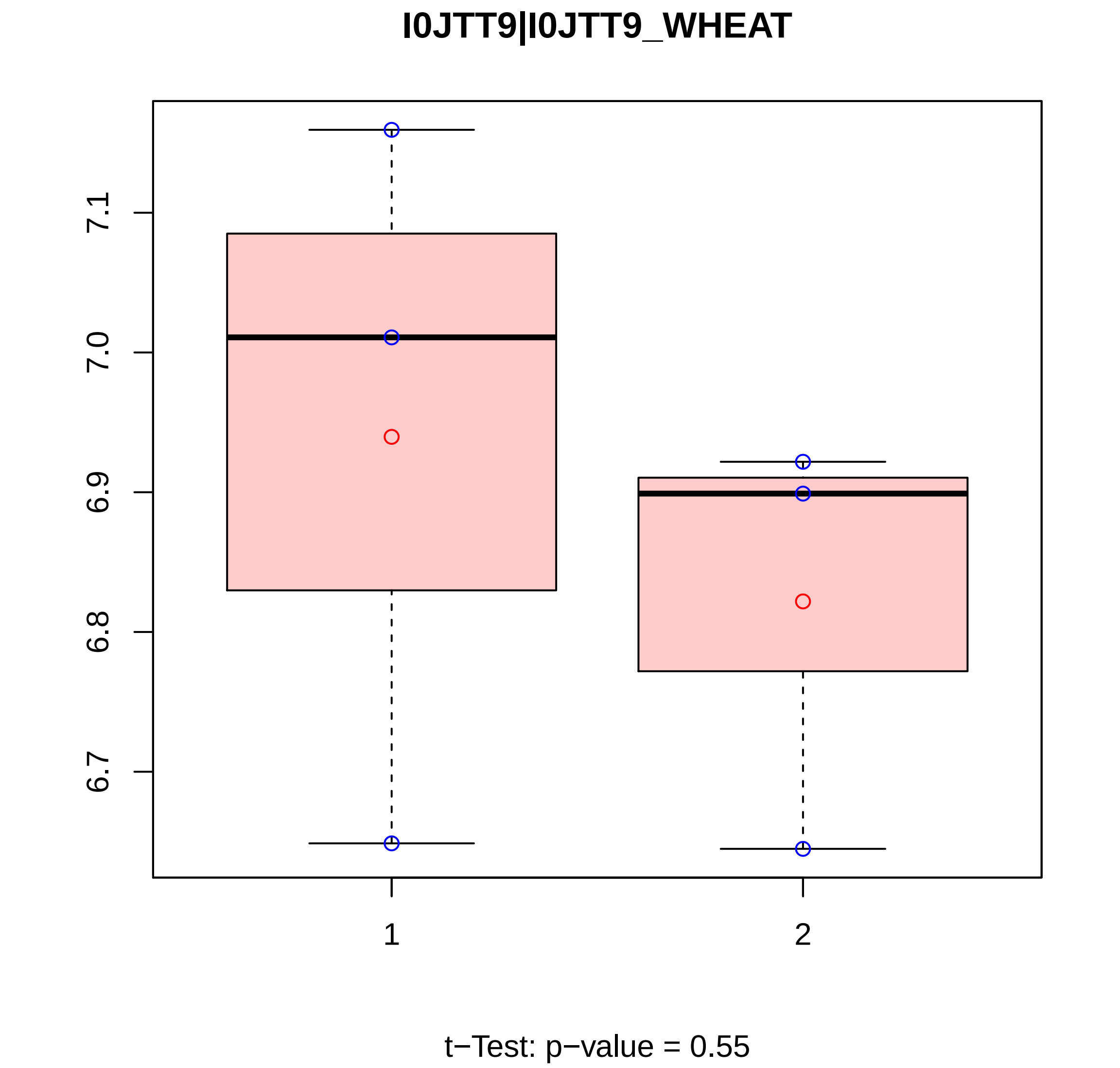
I0JTT9|I0JTT9_WHEAT
7.1
7.0
6.9
6.8
6.7
1
2
t−Test: p−value = 0.55

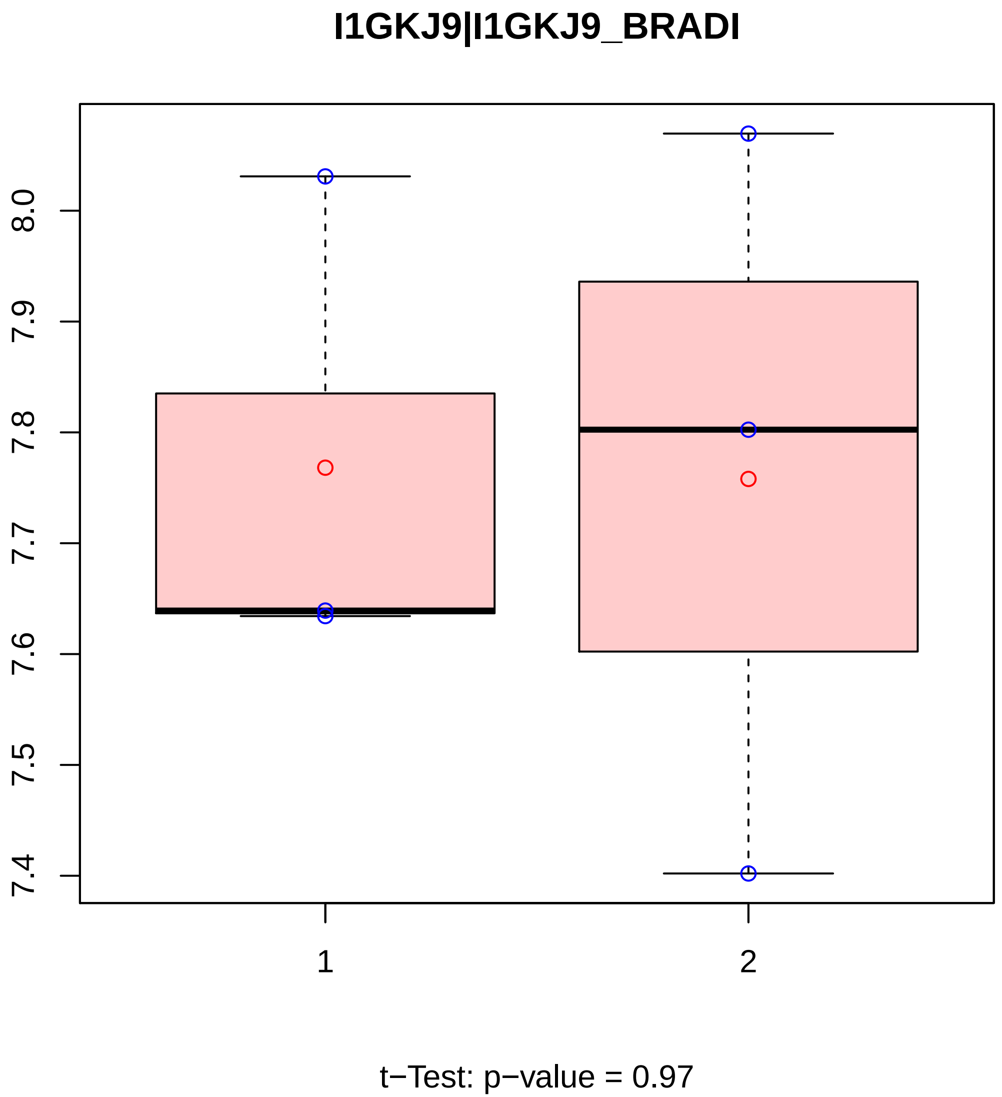
I1GKJ9|I1GKJ9_BRADI
8.0
7.9
7.8
7.7
7.6
7.5
7.4
1
2
t−Test: p−value = 0.97

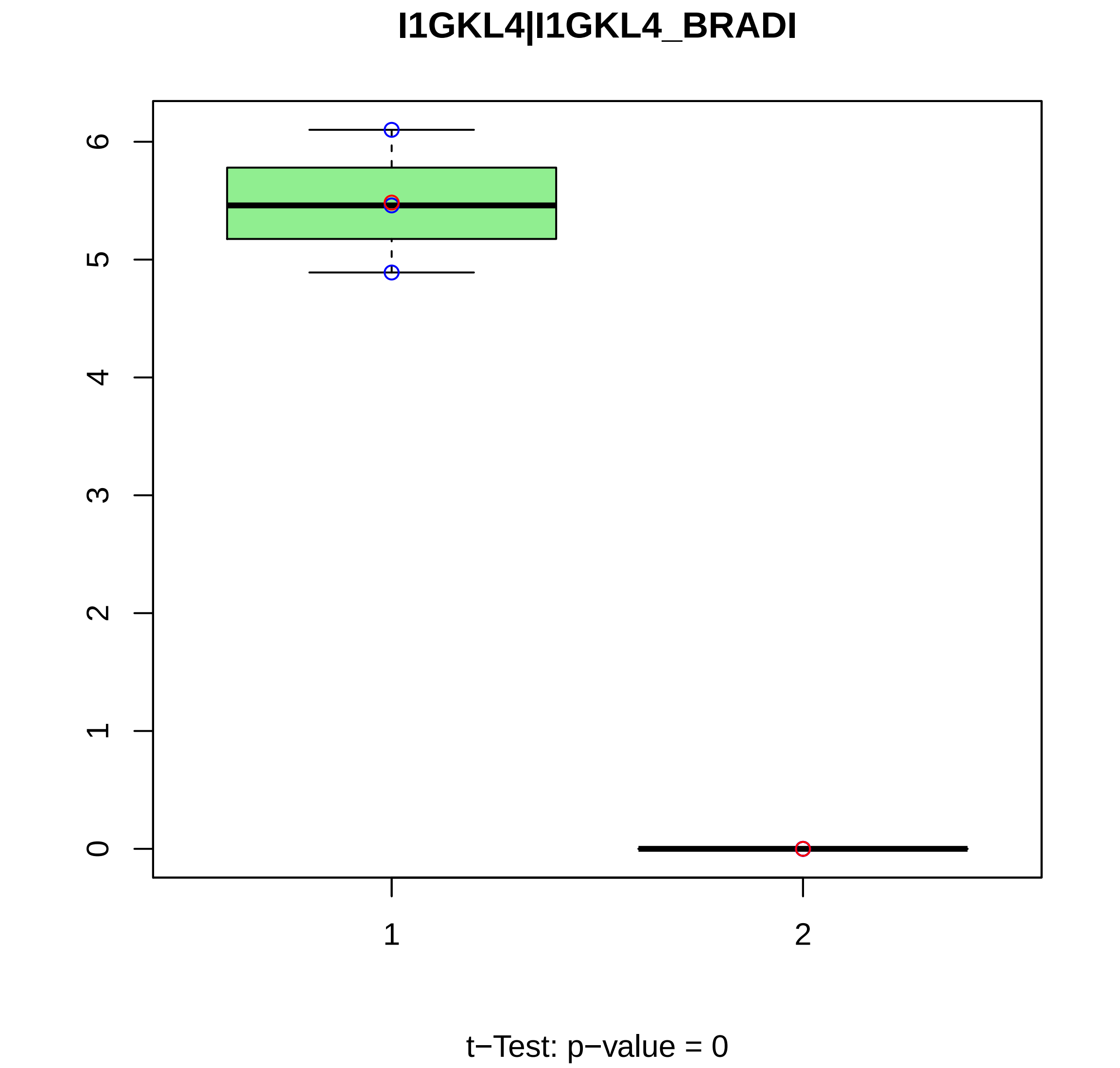

I1GKL4|I1GKL4_BRADI
0
1
2
3
4
5
6
1
2
t−Test: p−value = 0

I1GL11|I1GL11_BRADI

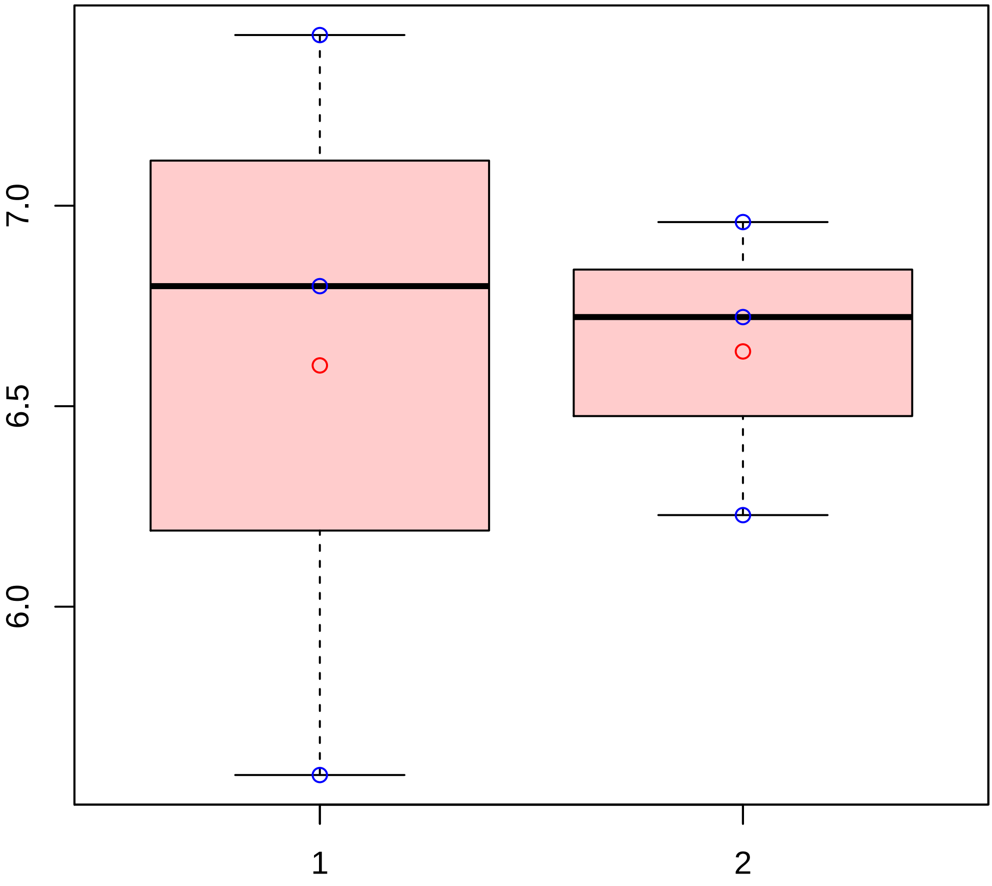

t−Test: p−value = 0.96

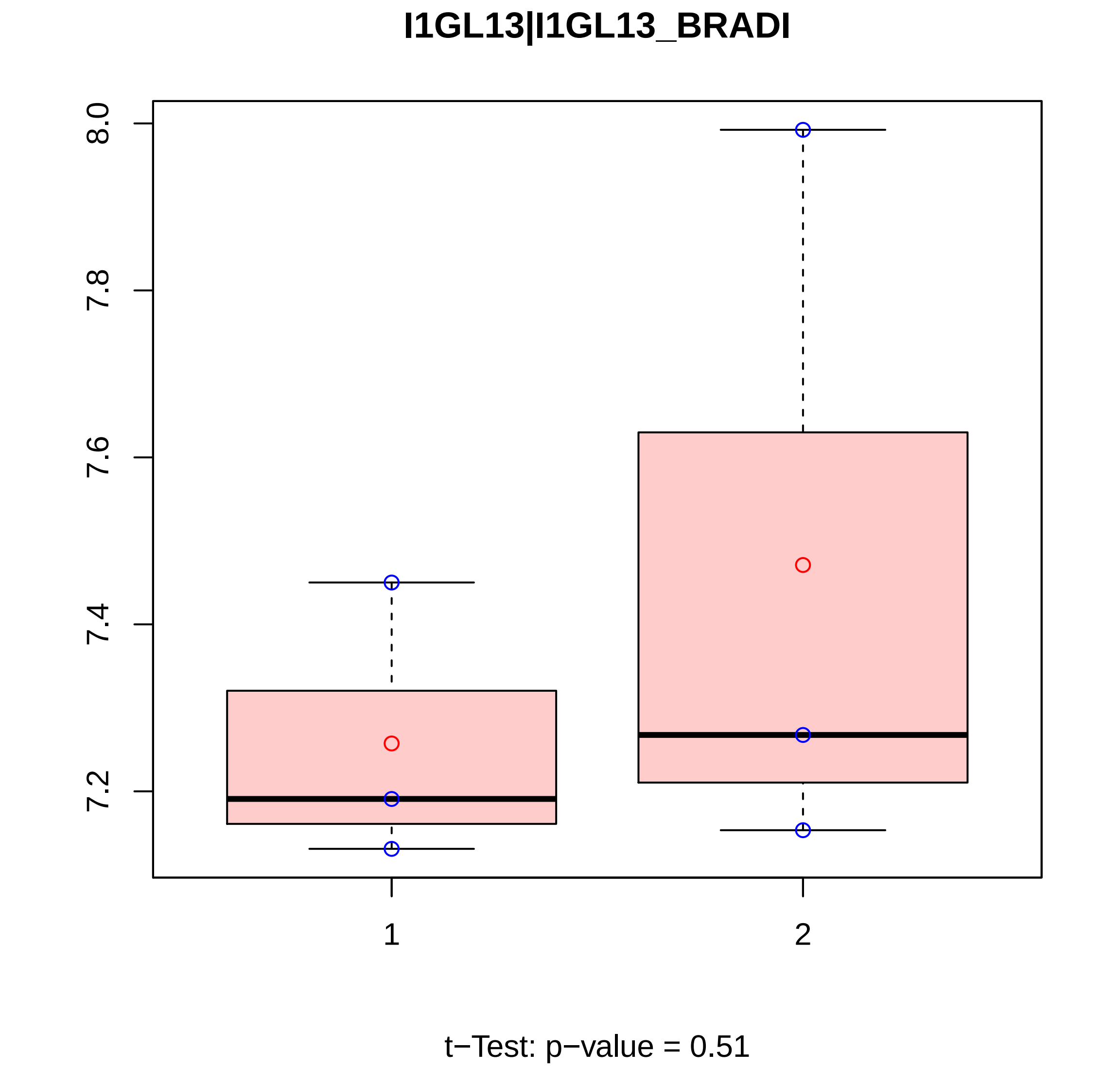
I1GL13|I1GL13_BRADI
8.0
7.8
7.6
7.4
7.2
1
2
t−Test: p−value = 0.51

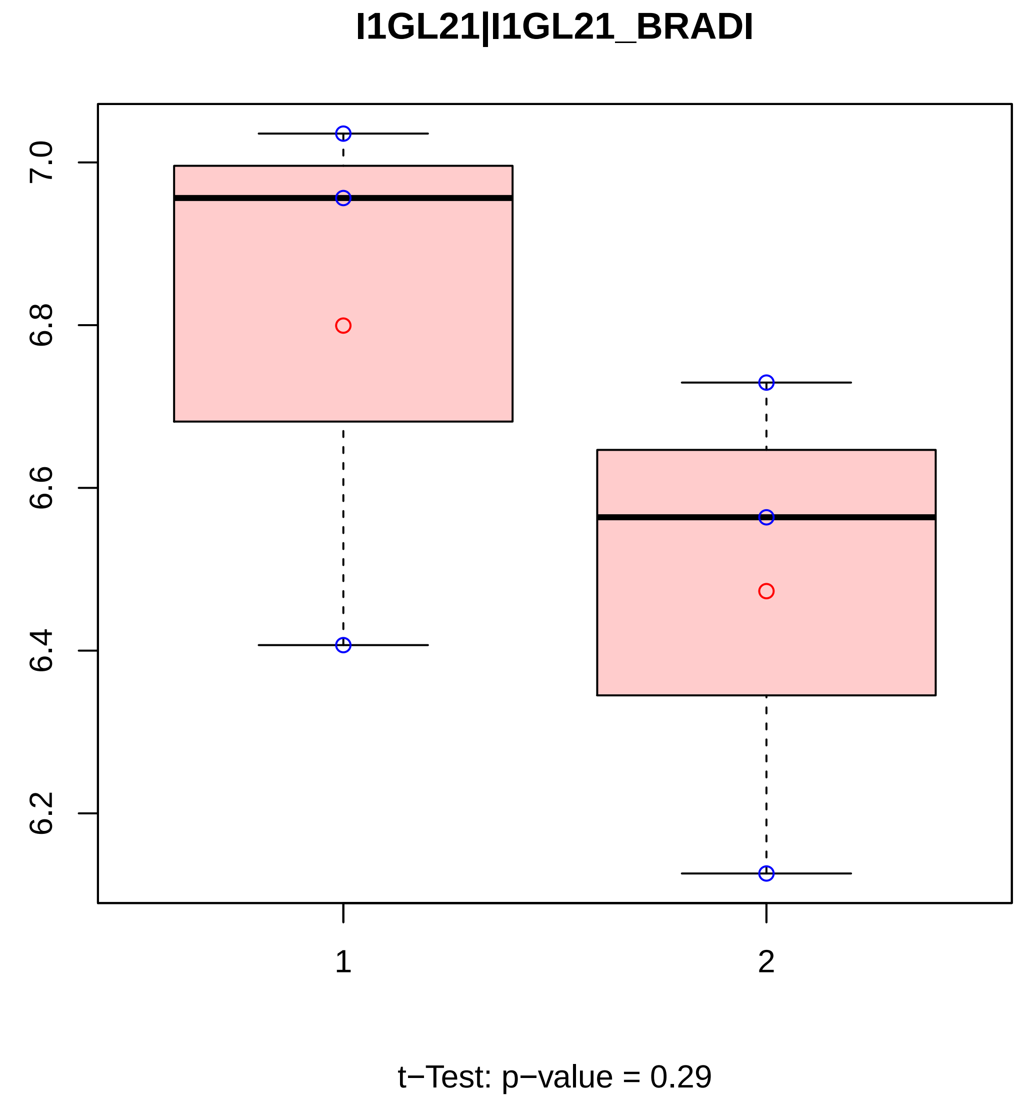
I1GL21|I1GL21_BRADI
7.0
6.8
6.6
6.4
6.2
1
2
t−Test: p−value = 0.29

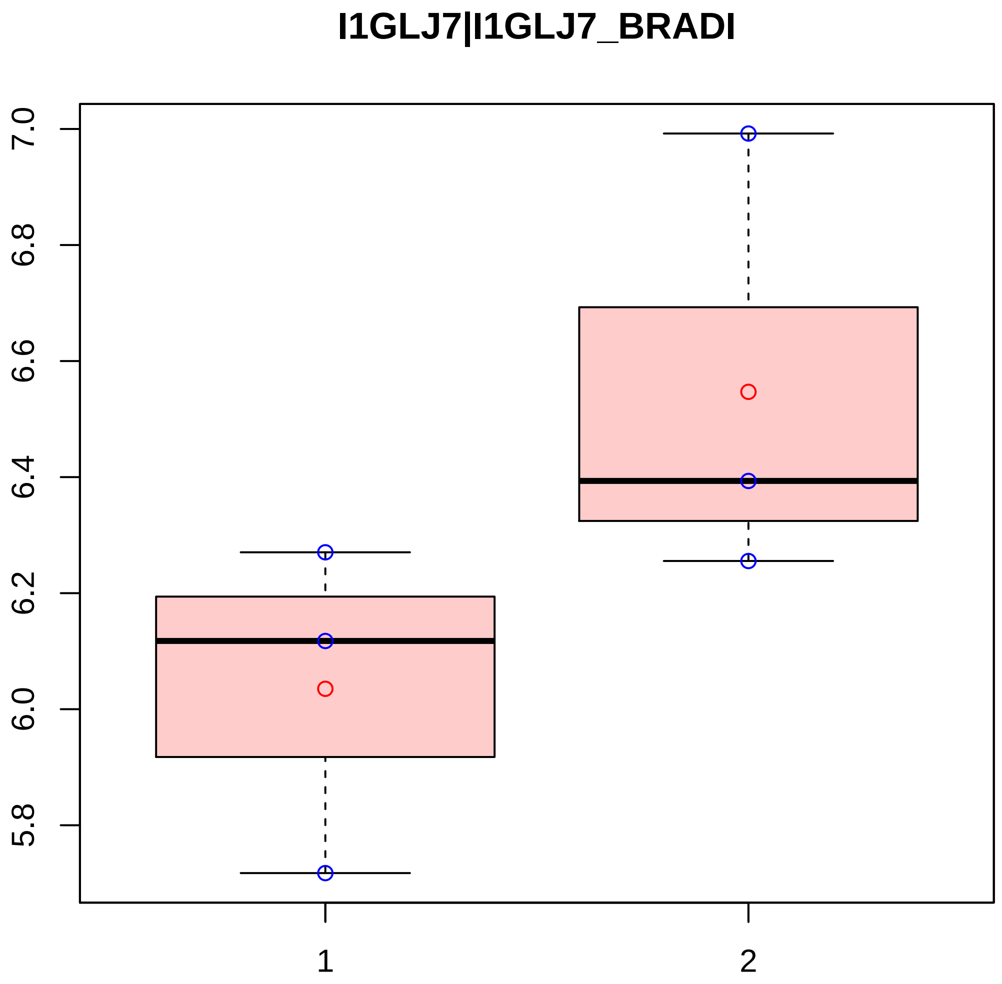

t−Test: p−value = 0.15

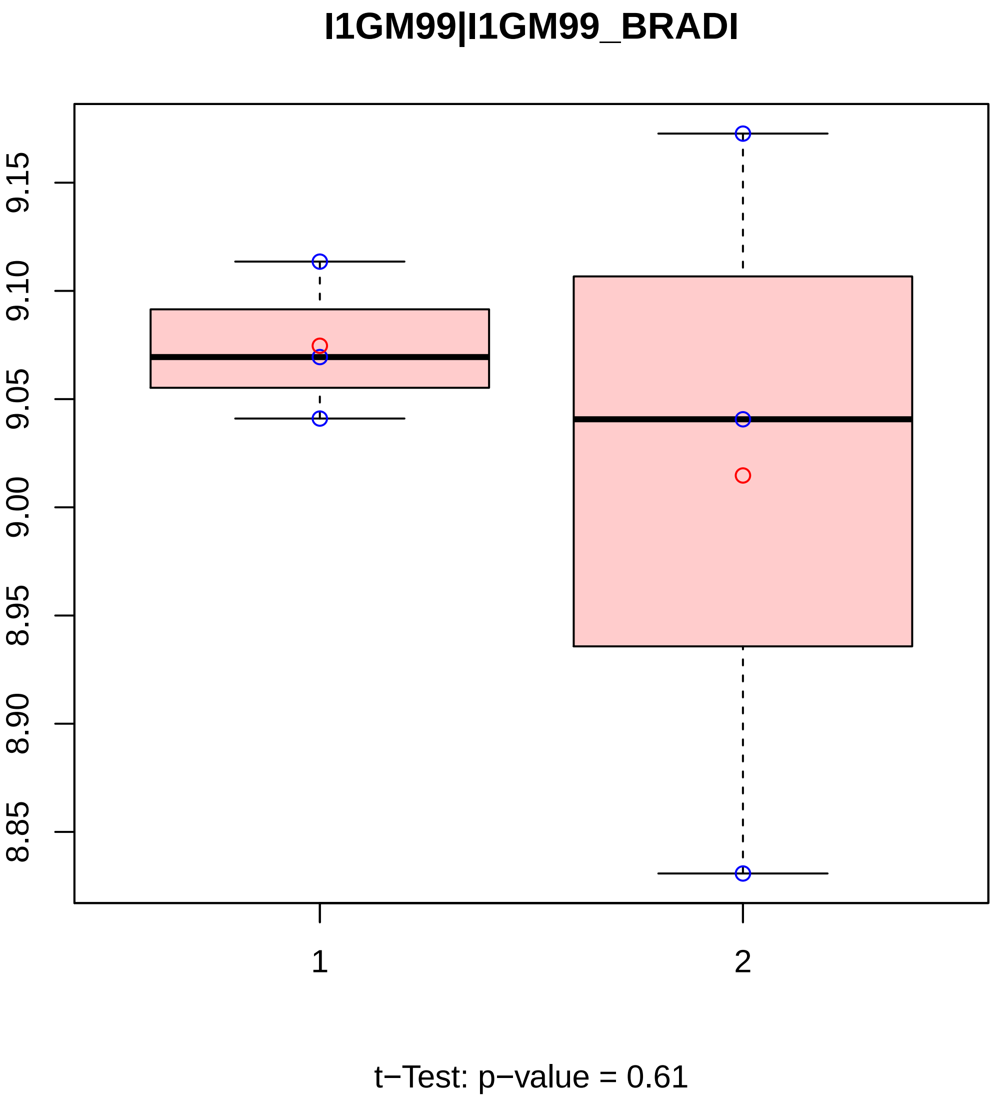
I1GM99|I1GM99_BRADI
9.15
9.10
9.05
9.00
8.95
8.90
8.85
1
2
t−Test: p−value = 0.61

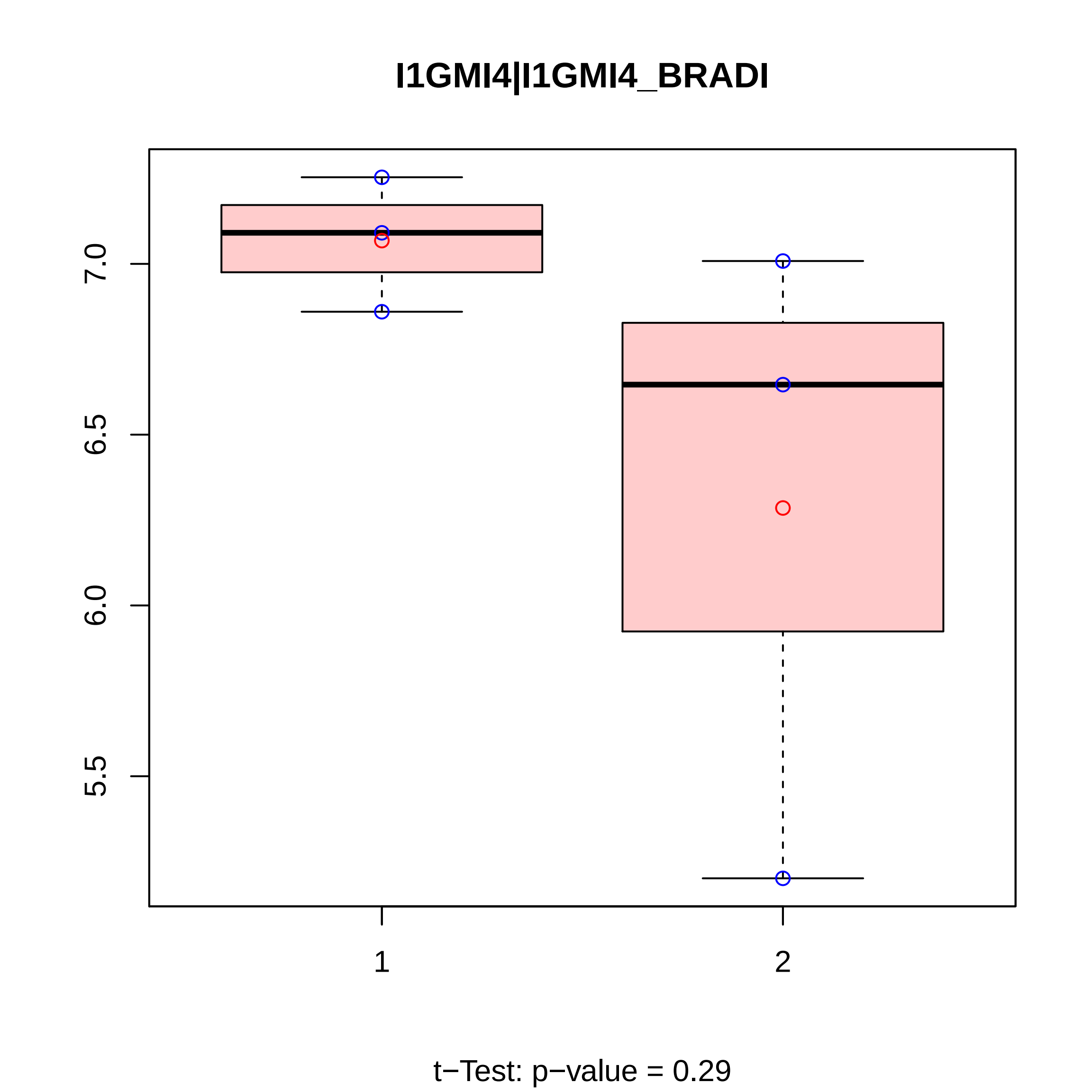
I1GMI4|I1GMI4_BRADI
7.0
6.5
6.0
5.5
1
2
t−Test: p−value = 0.29

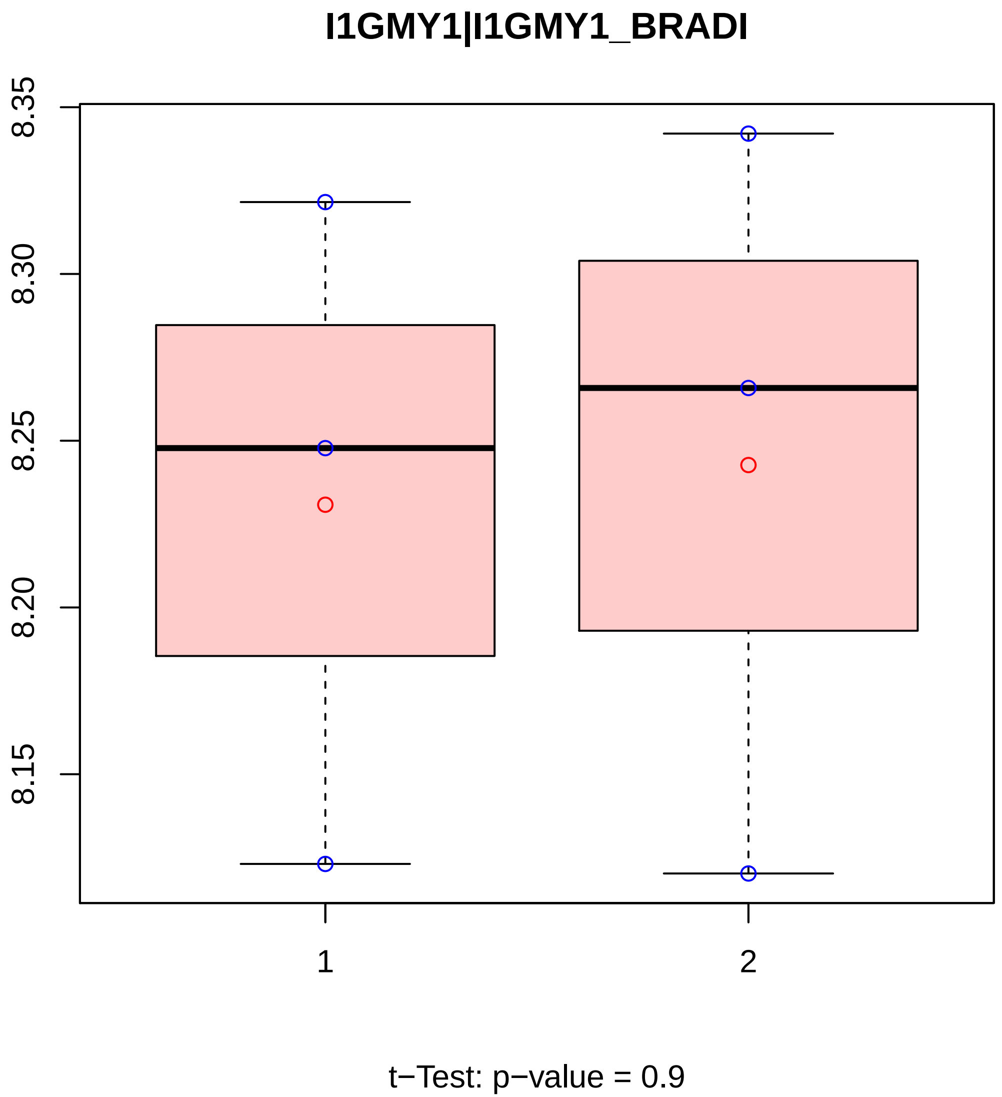
I1GMY1|I1GMY1_BRADI
8.35
8.30
8.25
8.20
8.15
1
2
t−Test: p−value = 0.9

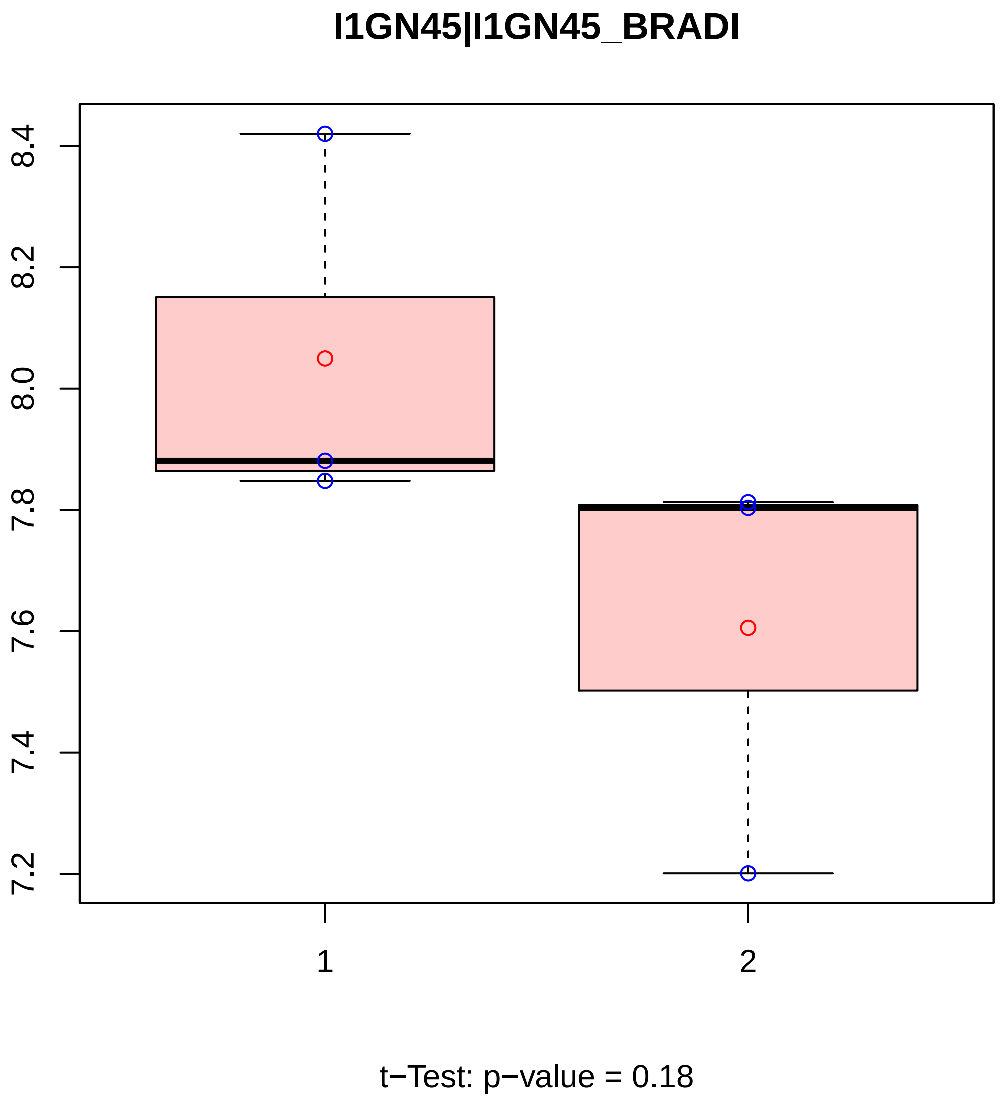
I1GN45|I1GN45_BRADI
8.4
8.2
8.0
7.8
7.6
7.4
7.2
1
2
t−Test: p−value = 0.18

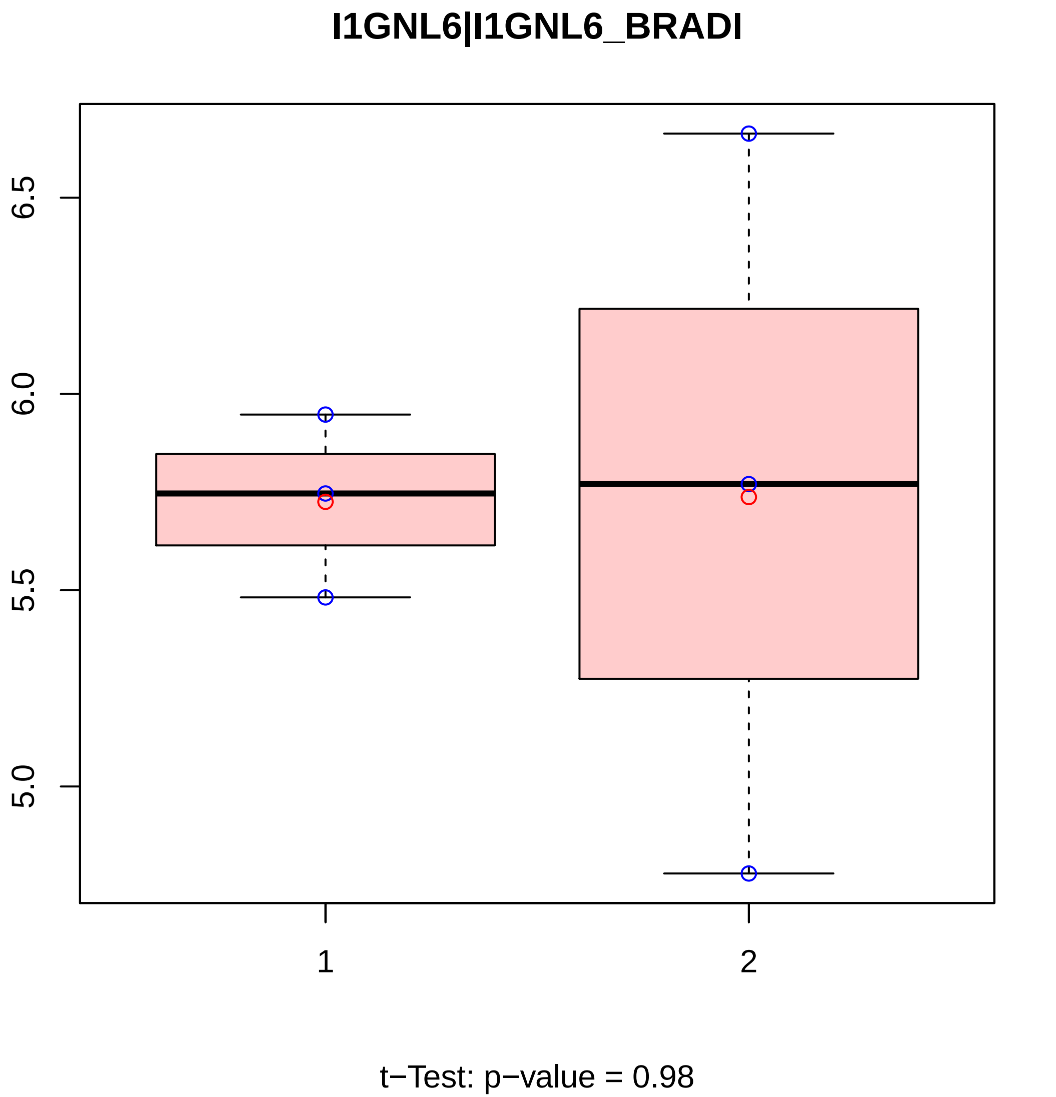
I1GNL6|I1GNL6_BRADI
6.5
6.0
5.5
5.0
1
2
t−Test: p−value = 0.98

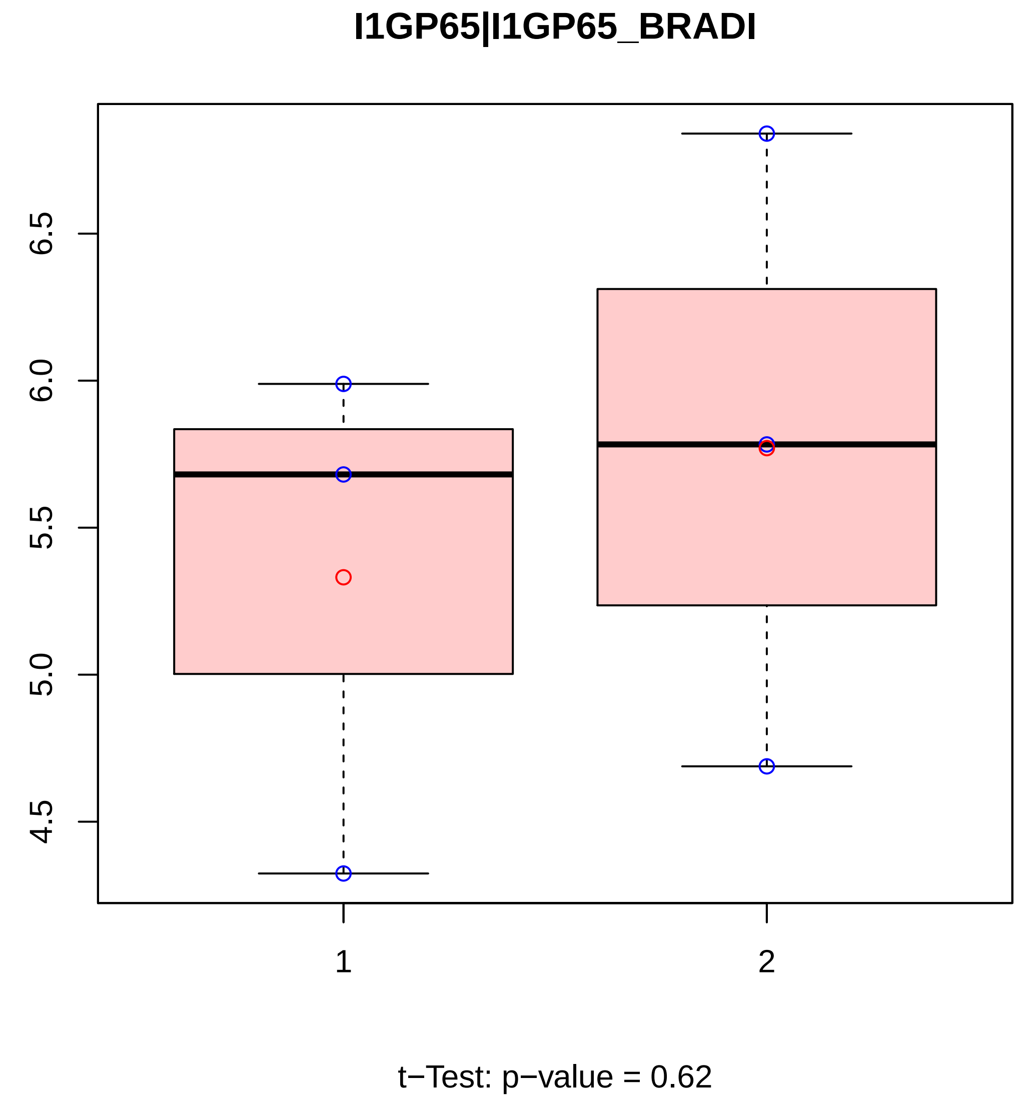
I1GP65|I1GP65_BRADI
4.5
5.0
5.5
6.0
6.5
1
2
t−Test: p−value = 0.62

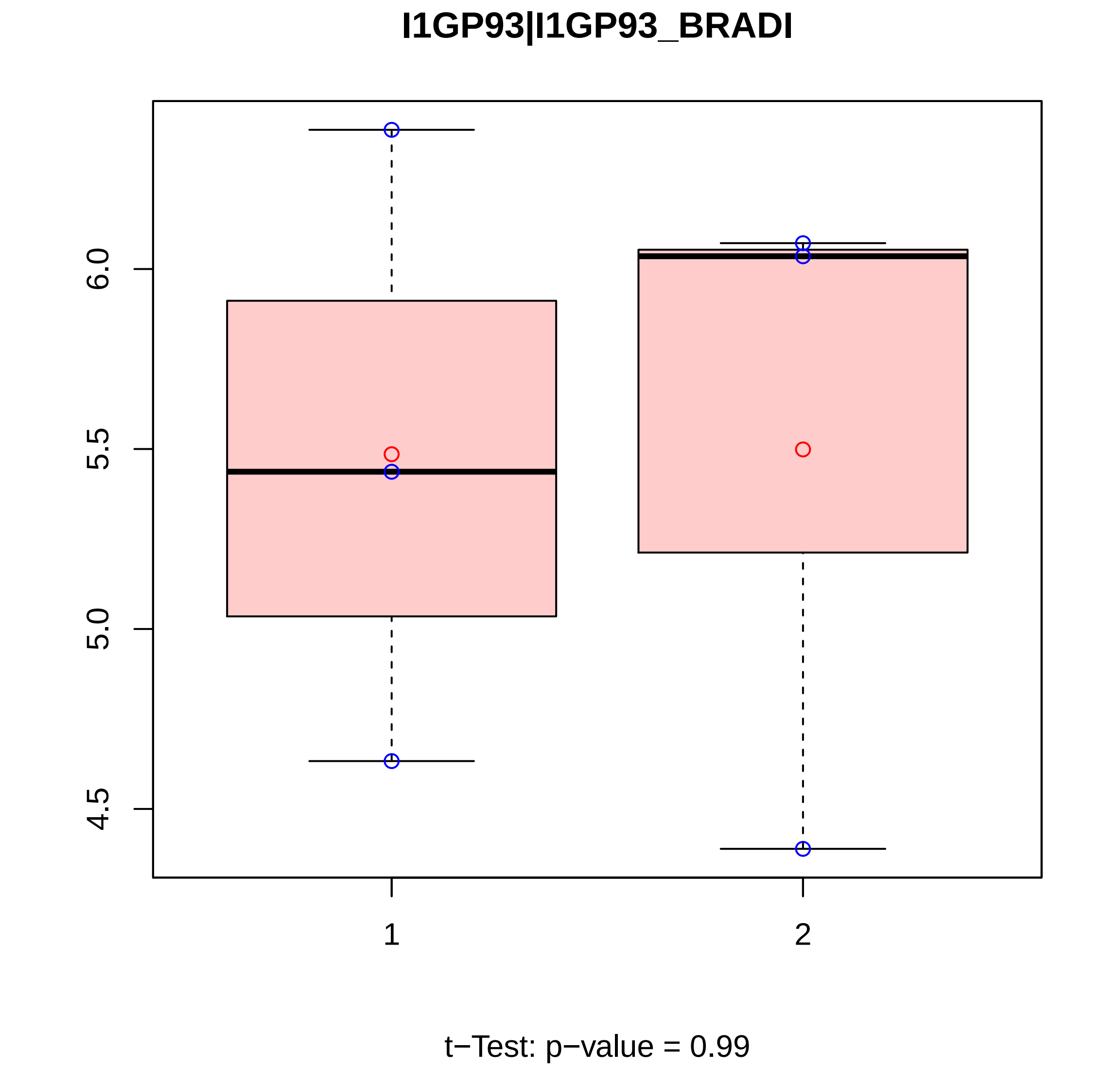
I1GP93|I1GP93_BRADI
6.0
5.5
5.0
4.5
1
2
t−Test: p−value = 0.99

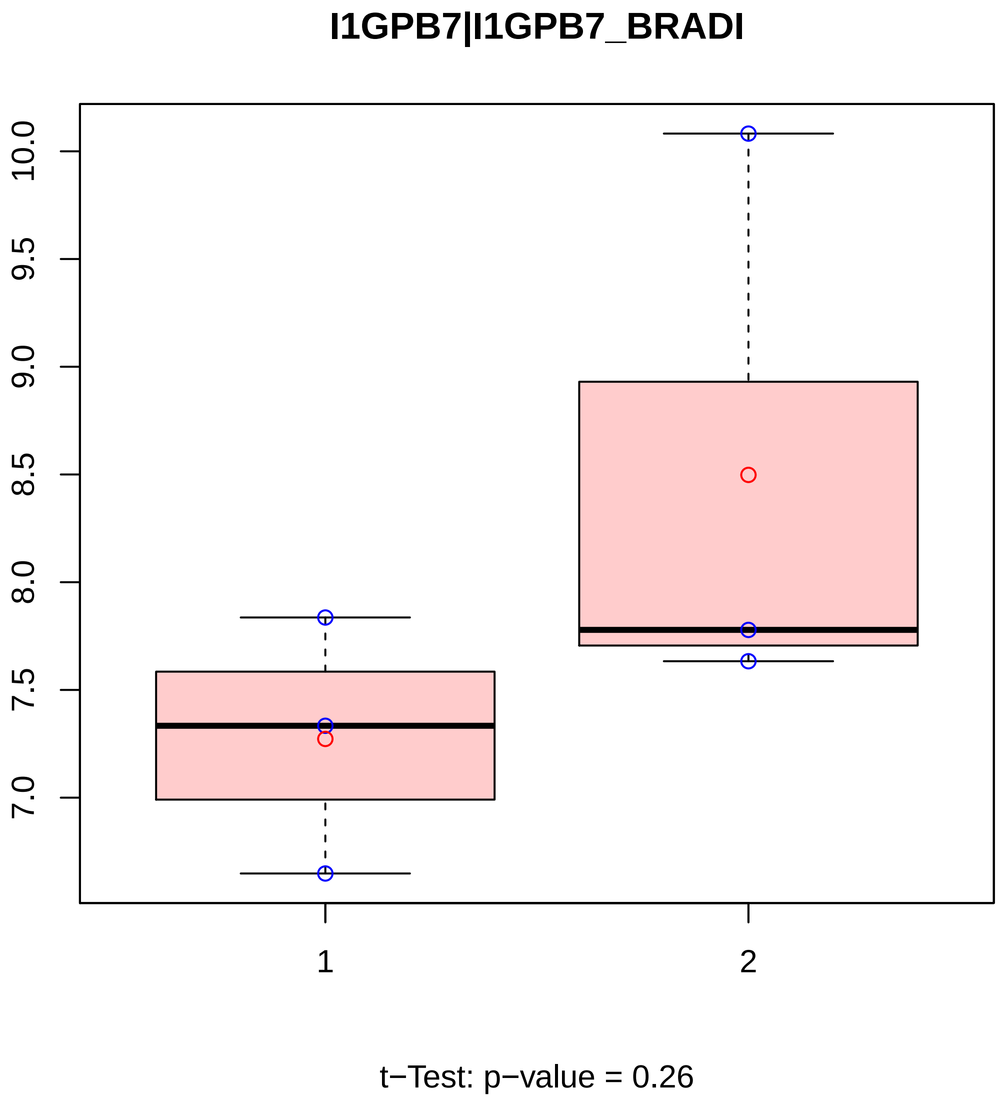
I1GPB7|I1GPB7_BRADI
10.0
9.5
9.0
8.5
8.0
7.5
7.0
1
2
t−Test: p−value = 0.26

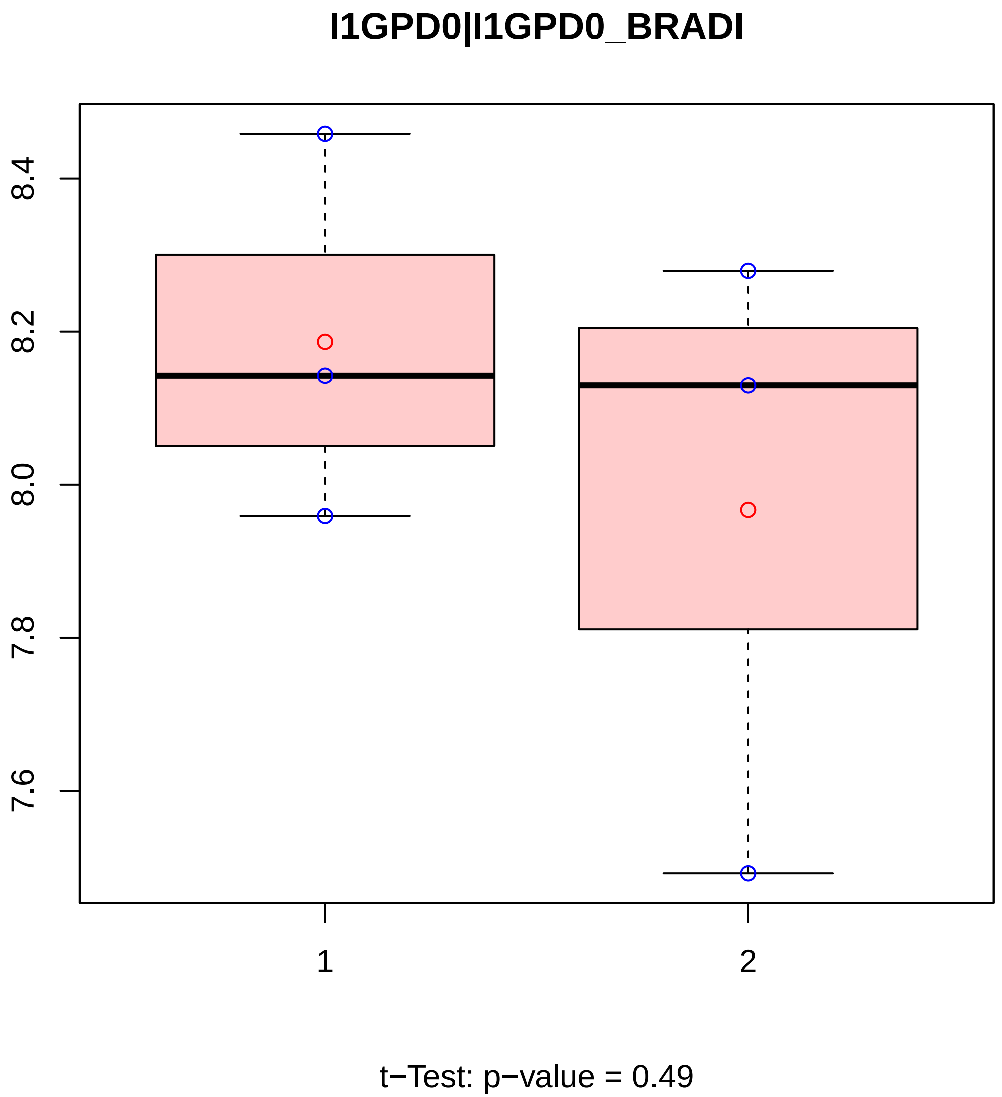
I1GPD0|I1GPD0_BRADI
8.4
8.2
8.0
7.8
7.6
1
2
t−Test: p−value = 0.49

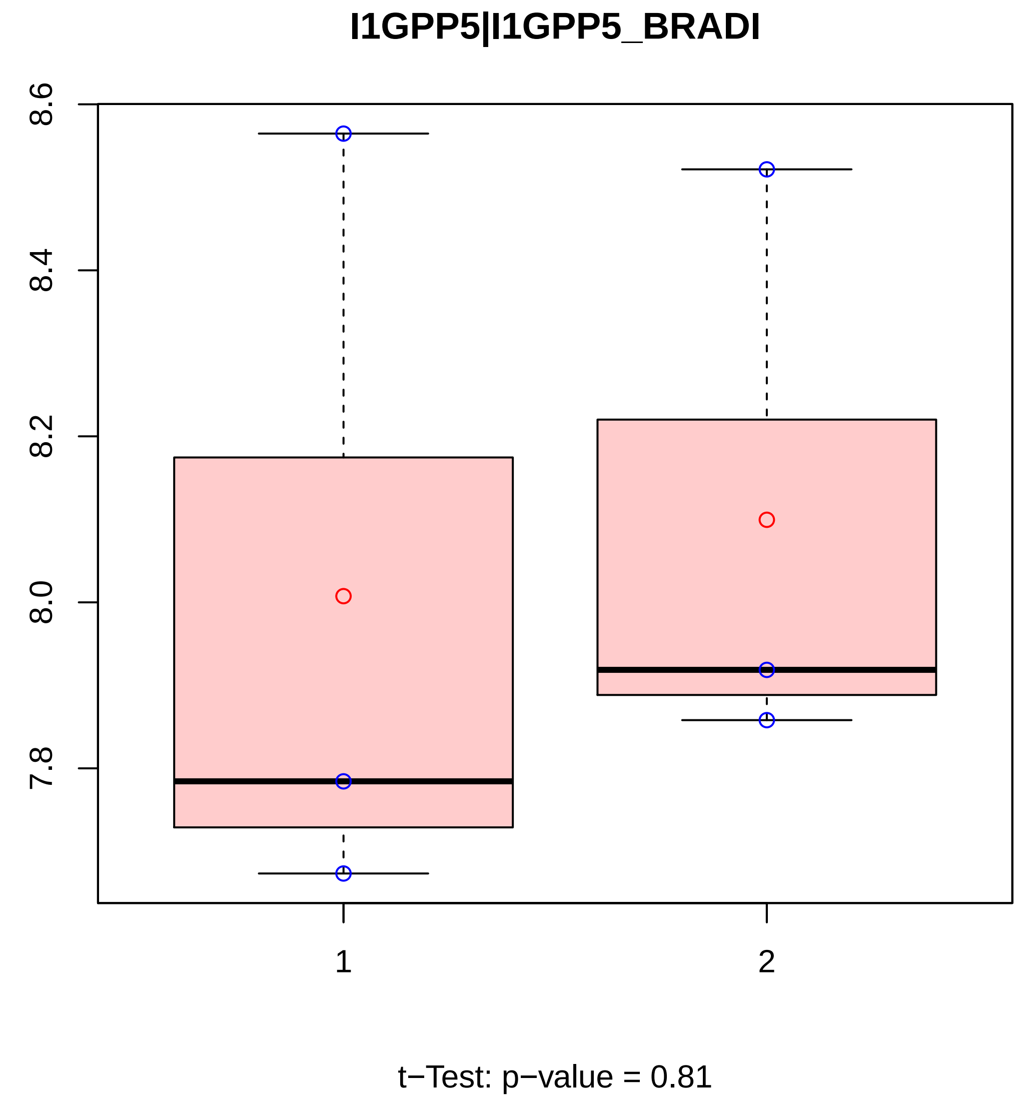
I1GPP5|I1GPP5_BRADI
8.6
8.4
8.2
8.0
7.8
1
2
t−Test: p−value = 0.81

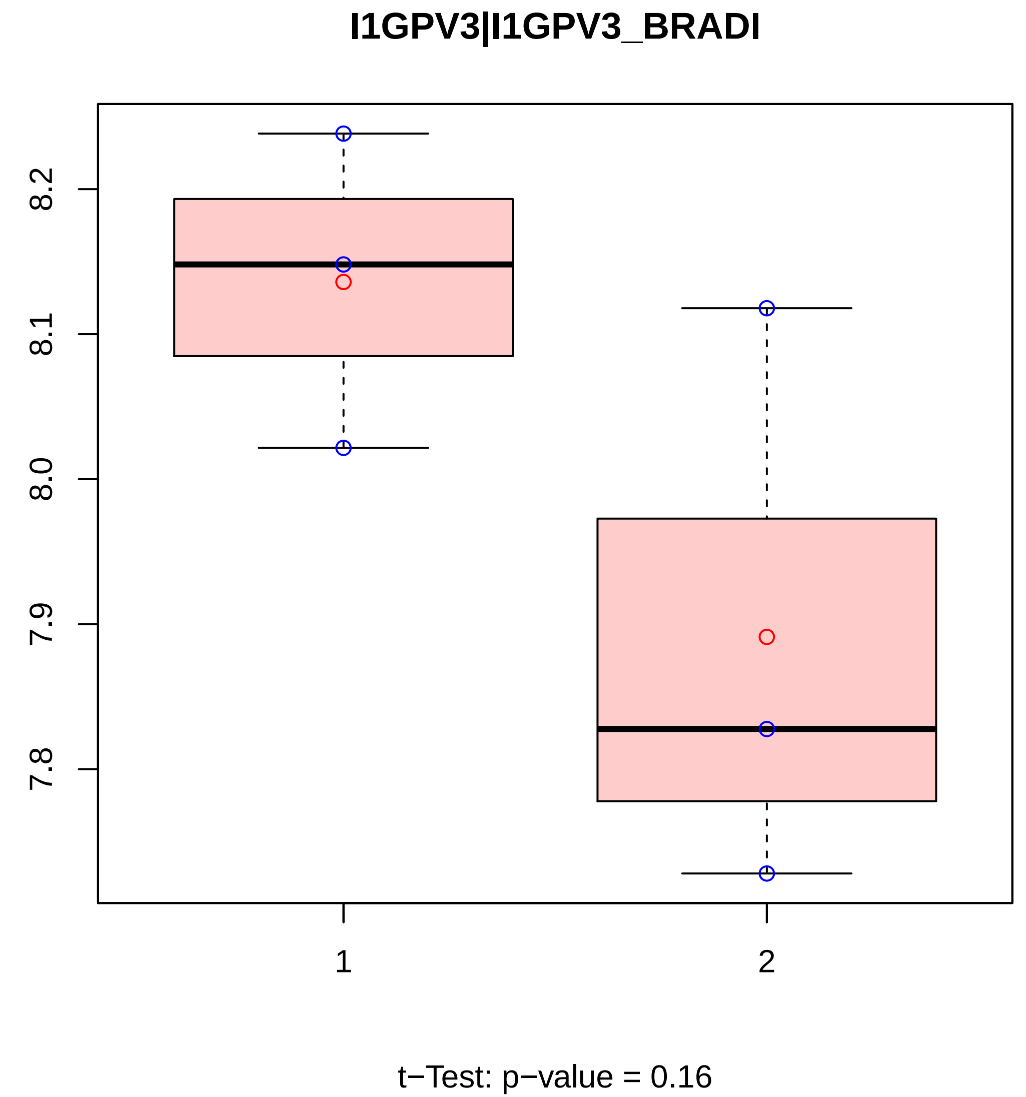
I1GPV3|I1GPV3_BRADI
8.2
8.1
8.0
7.9
7.8
1
2
t−Test: p−value = 0.16

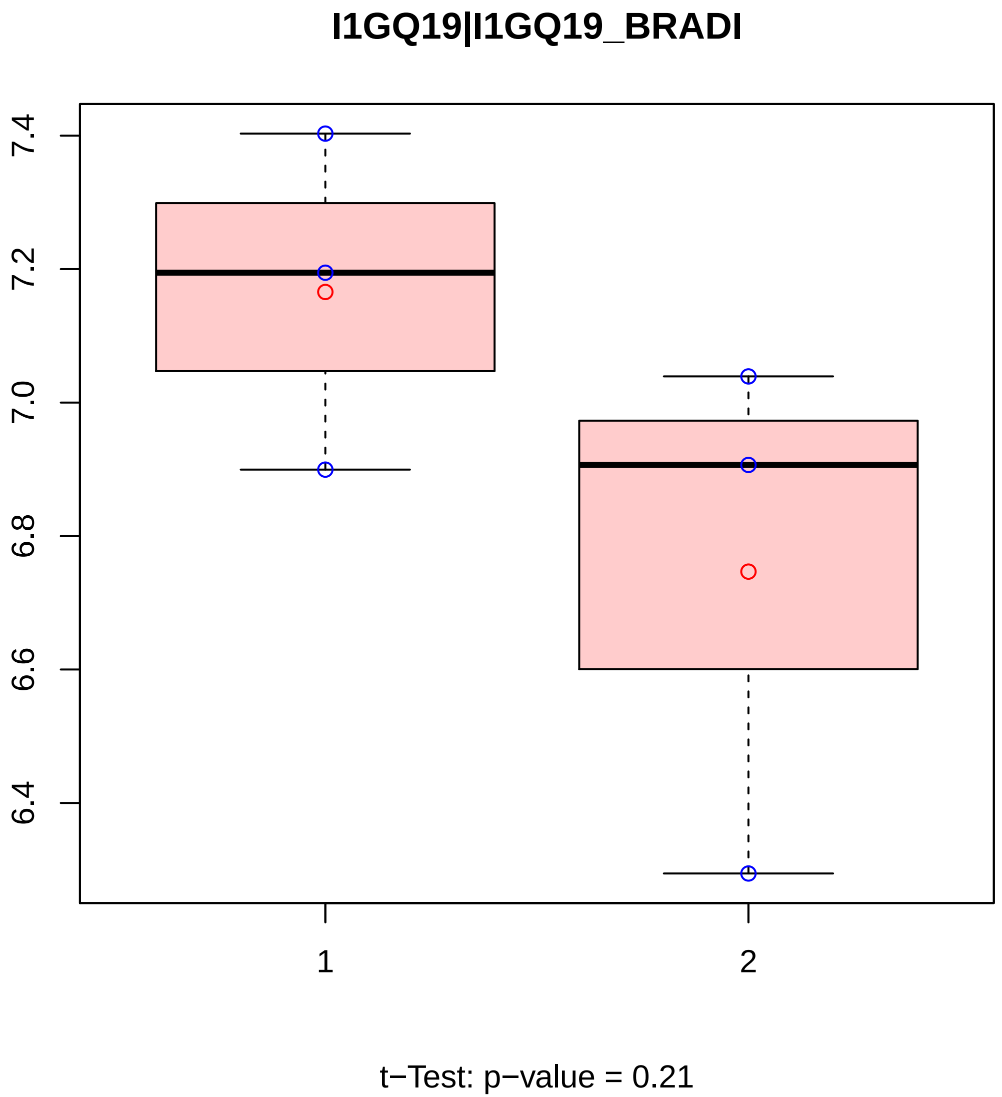
I1GQ19|I1GQ19_BRADI
7.4
7.2
7.0
6.8
6.6
6.4
1
2
t−Test: p−value = 0.21

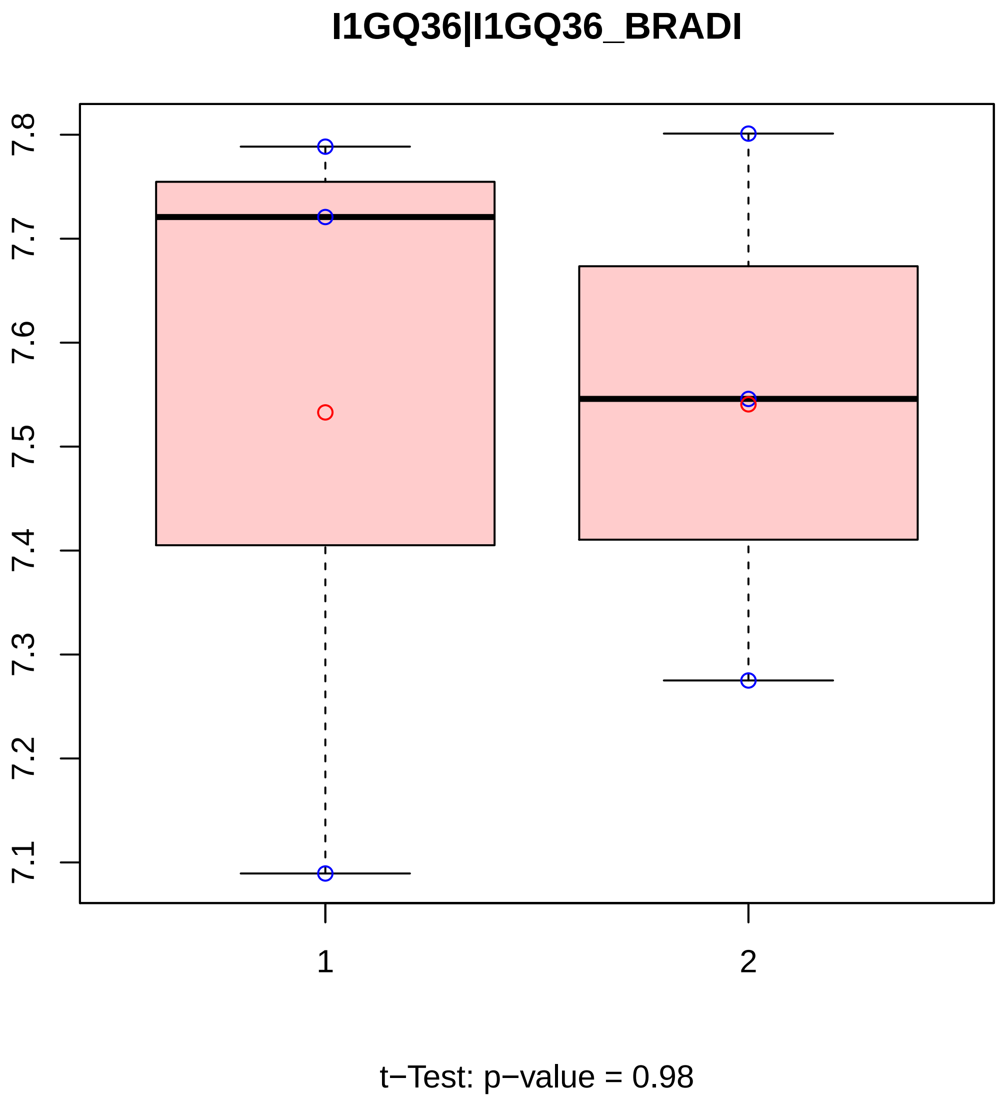

I1GQ36|I1GQ36_BRADI
7.1
7.2
7.3
7.4
7.5
7.6
7.7
7.8
1
2
t−Test: p−value = 0.98

I1GQ59|I1GQ59_BRADI

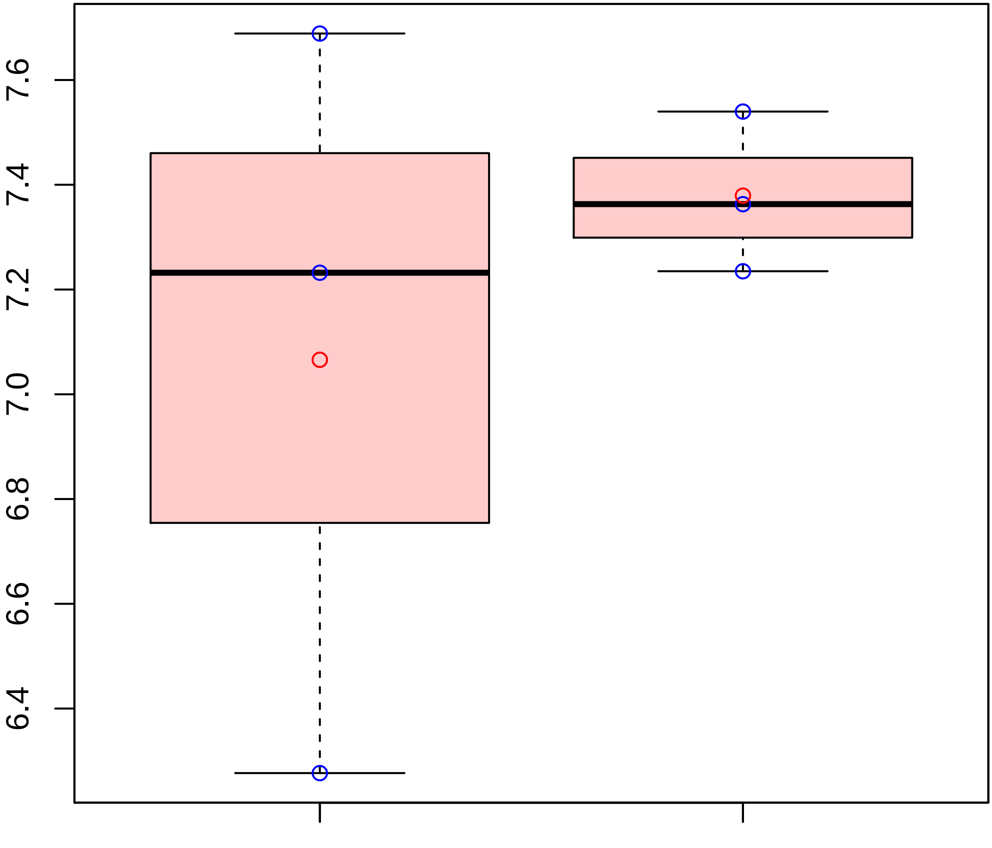

t−Test: p−value = 0.53

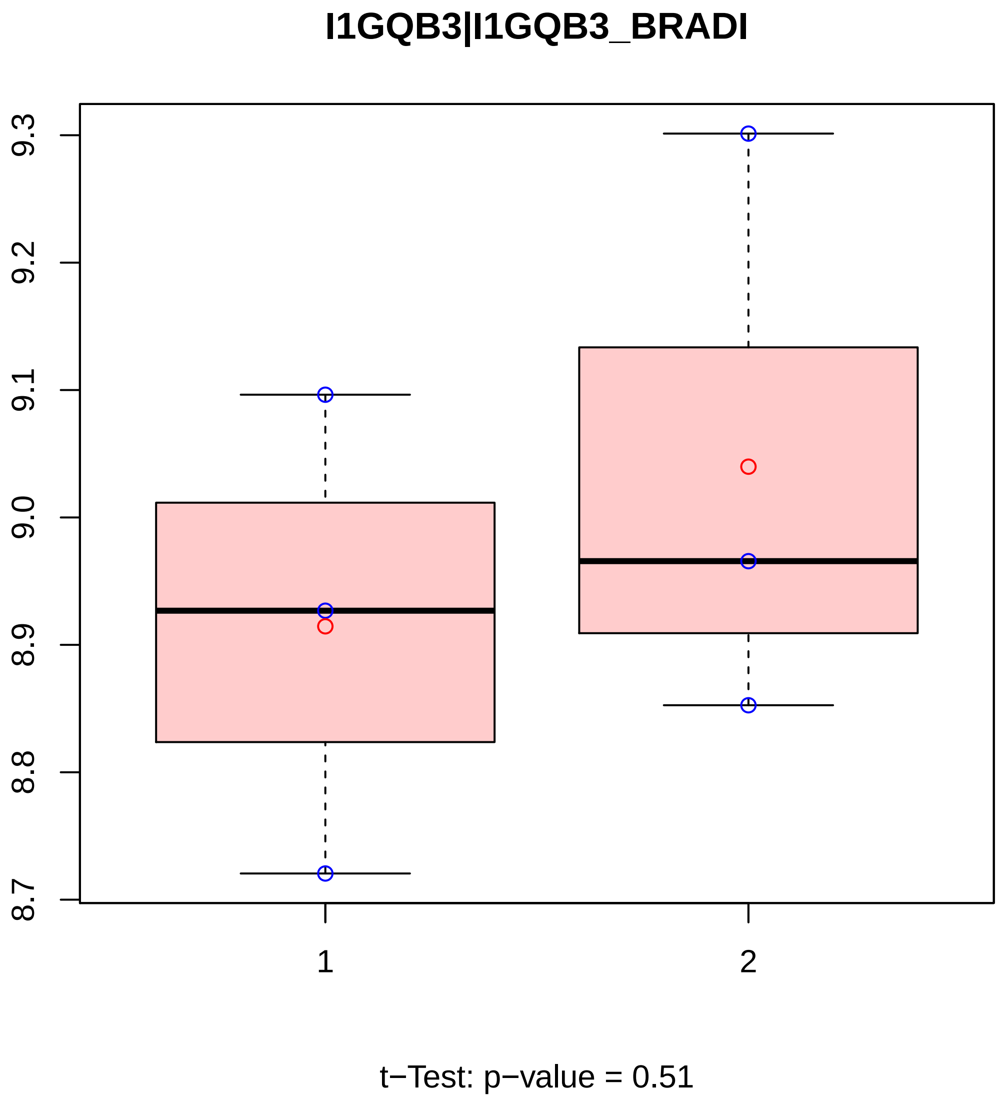
I1GQB3|I1GQB3_BRADI
9.3
9.2
9.1
9.0
8.9
8.8
8.7
1
2
t−Test: p−value = 0.51

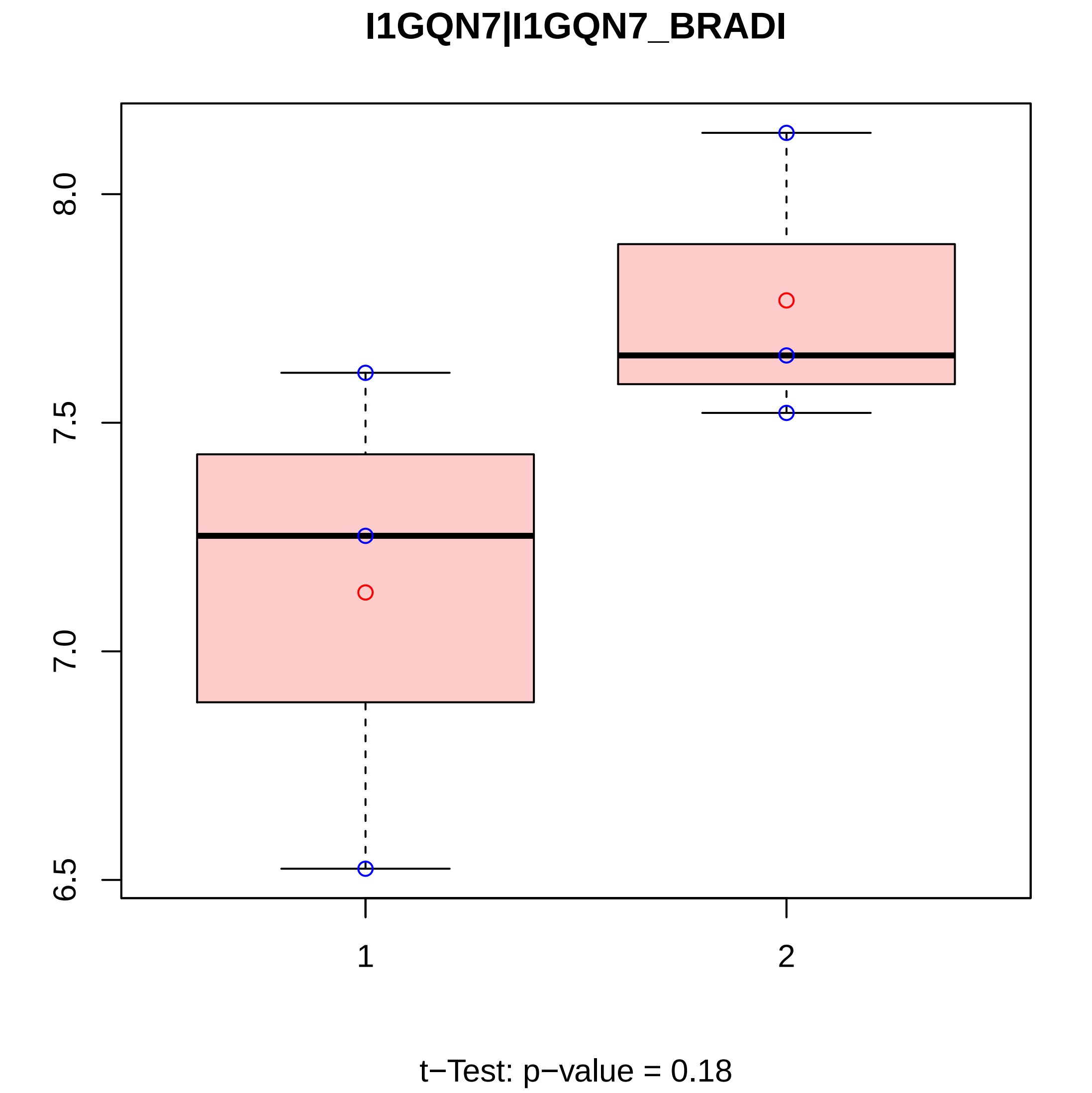
I1GQN7|I1GQN7_BRADI
8.0
7.5
7.0
6.5
1
2
t−Test: p−value = 0.18

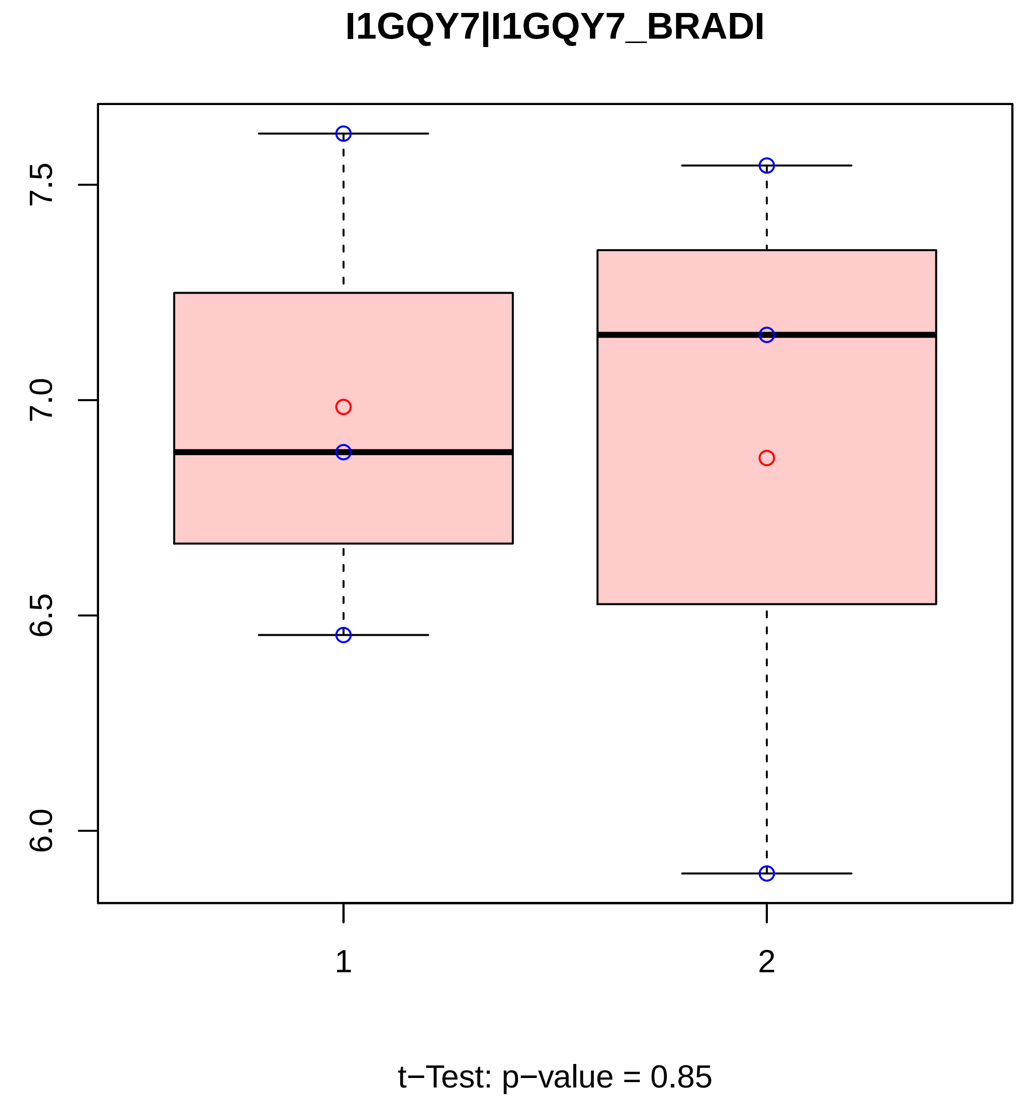
I1GQY7|I1GQY7_BRADI
7.5
7.0
6.5
6.0
1
2
t−Test: p−value = 0.85

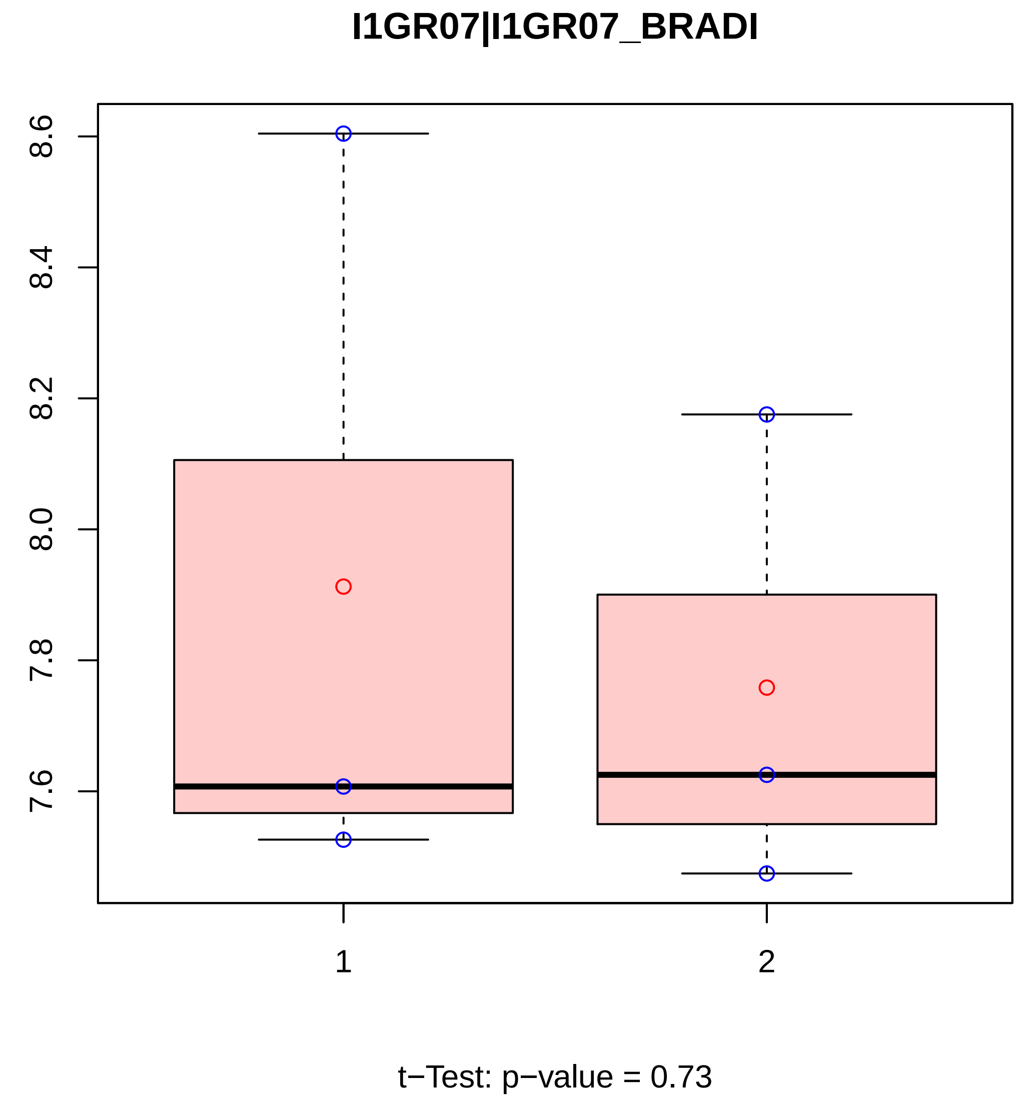
I1GR07|I1GR07_BRADI
8.6
8.4
8.2
8.0
7.8
7.6
1
2
t−Test: p−value = 0.73

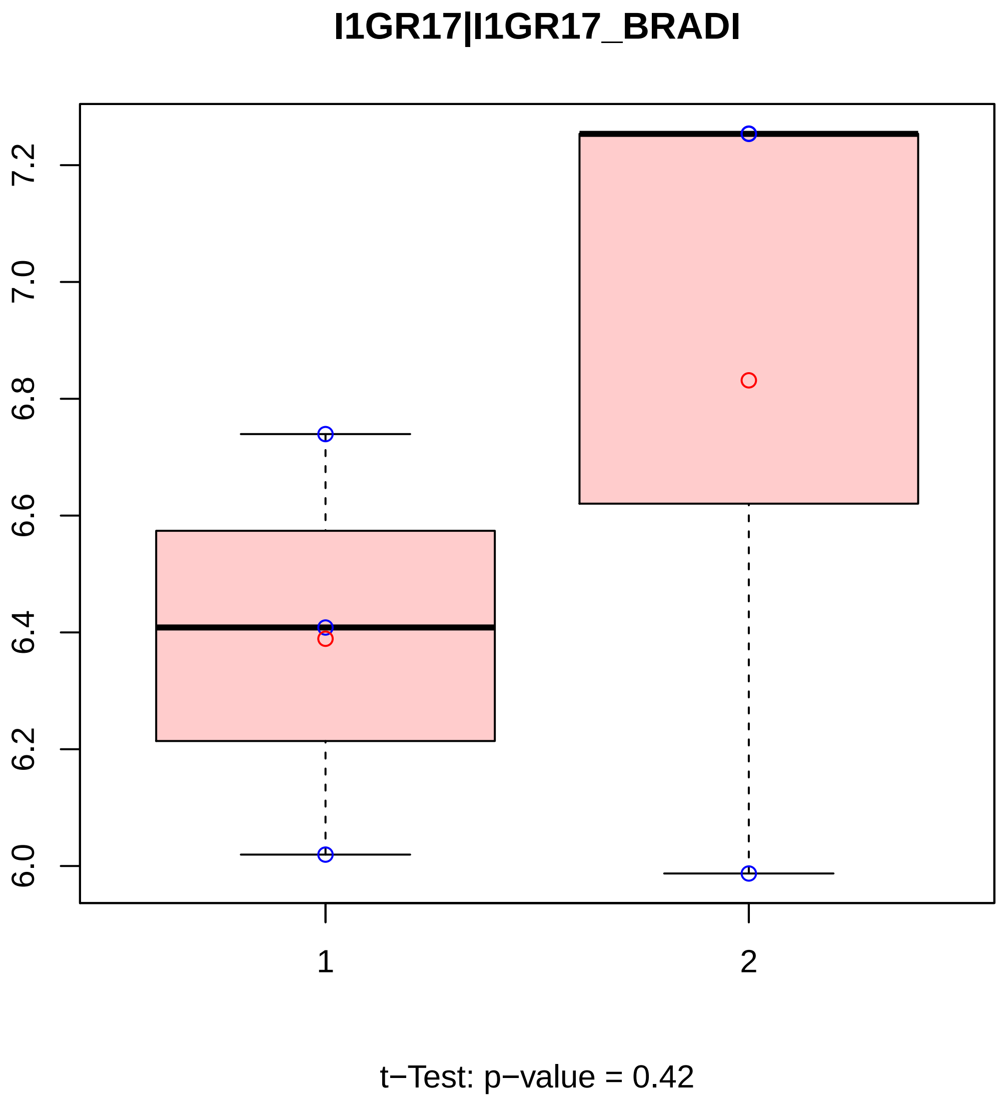

I1GR17|I1GR17_BRADI
7.2
7.0
6.8
6.6
6.4
6.2
6.0
1
2
t−Test: p−value = 0.42

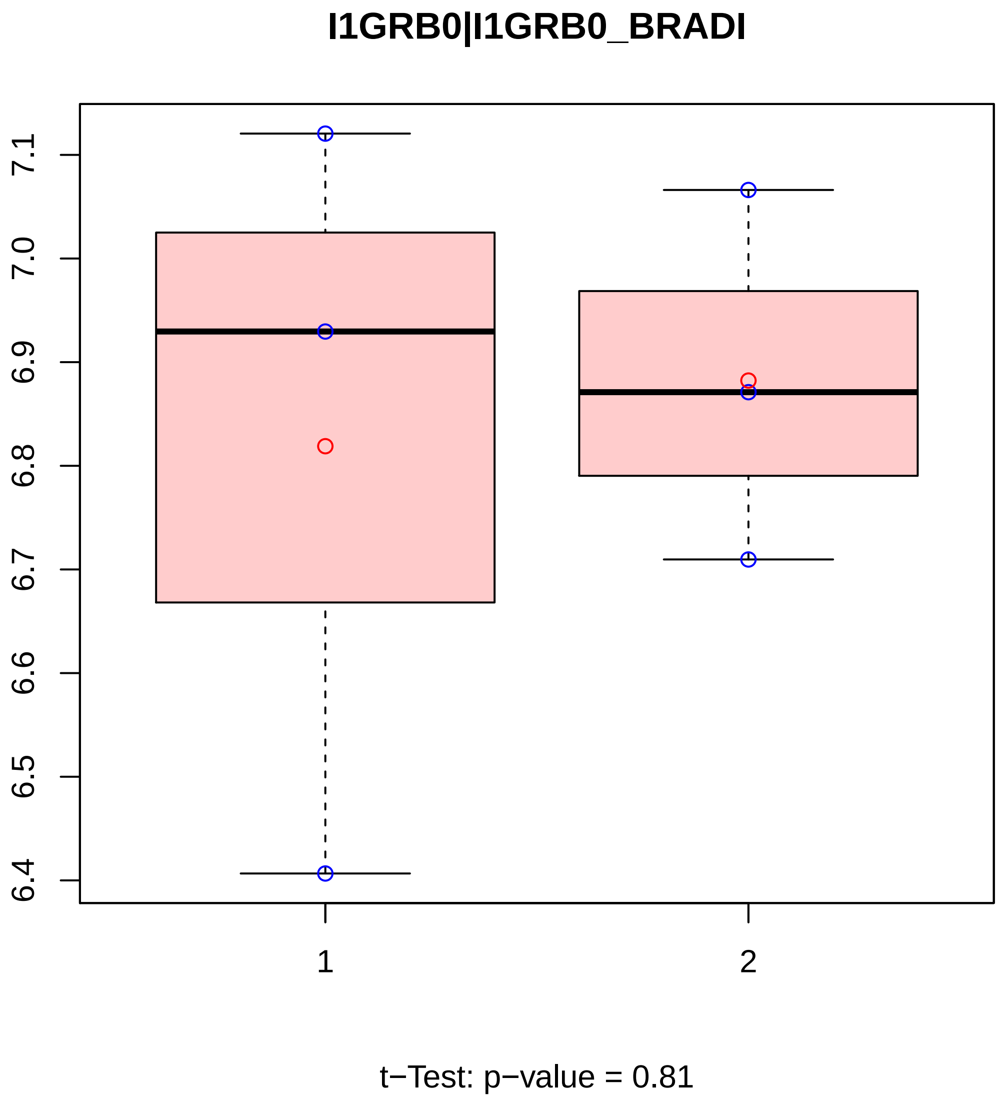

I1GRB0|I1GRB0_BRADI
7.1
7.0
6.9
6.8
6.7
6.6
6.5
6.4
1
2
t−Test: p−value = 0.81

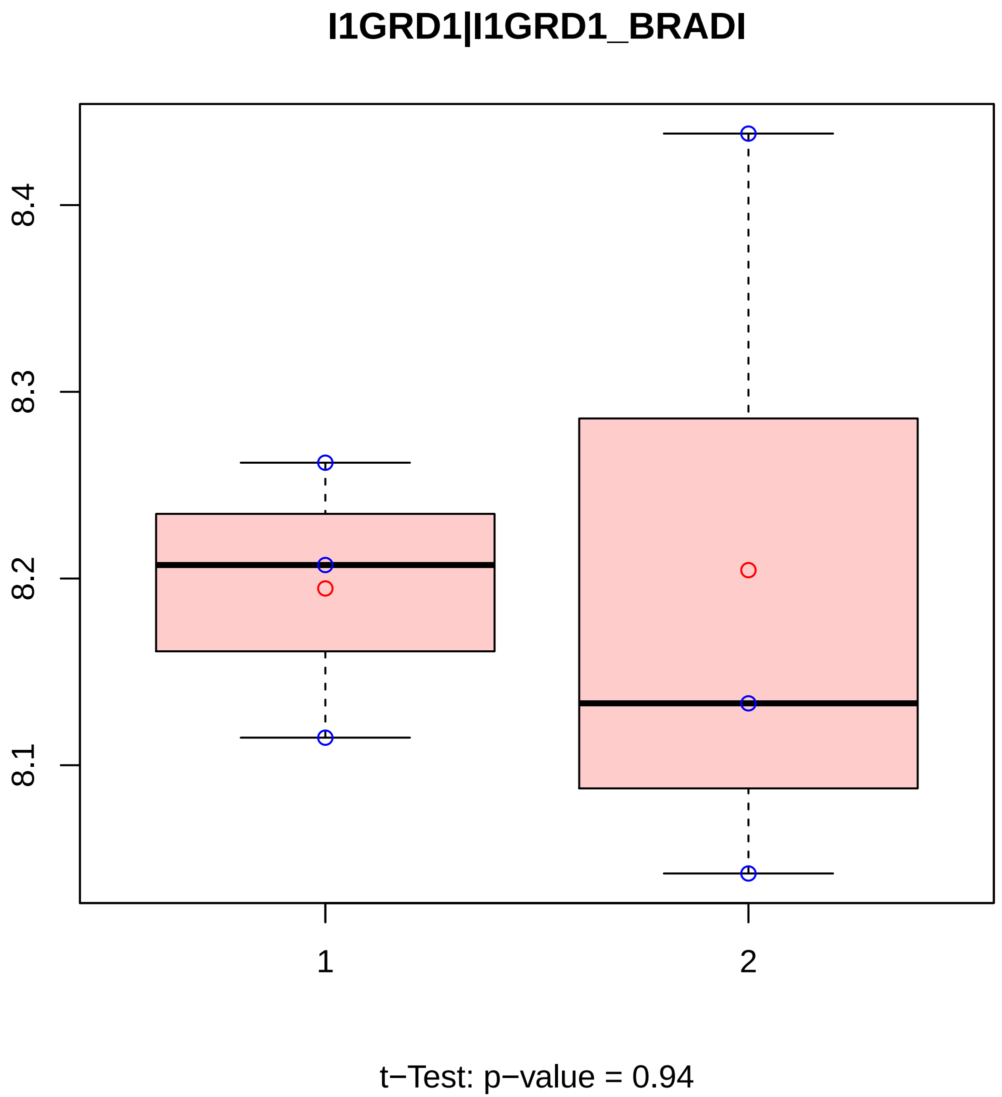
I1GRD1|I1GRD1_BRADI
8.1
8.2
8.3
8.4
1
2
t−Test: p−value = 0.94

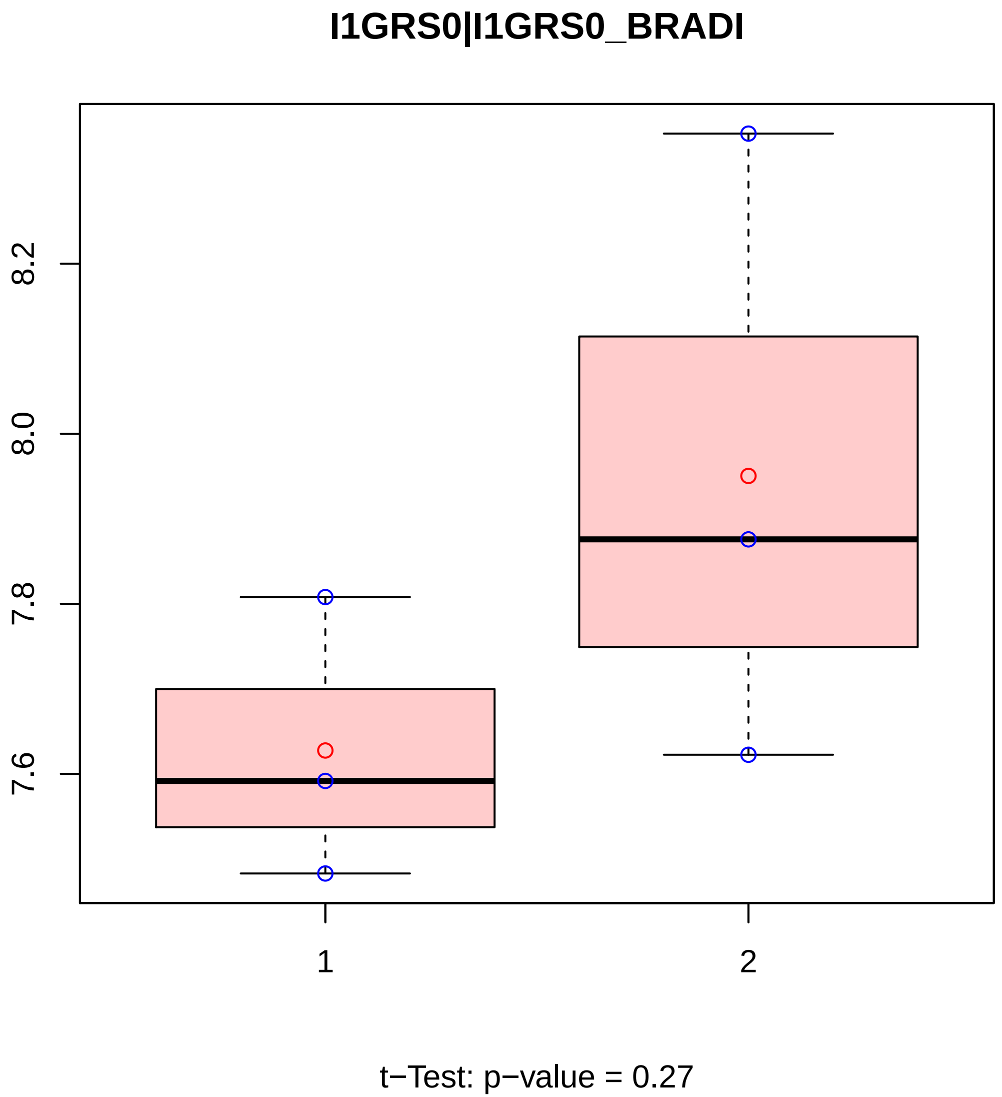
I1GRS0|I1GRS0_BRADI
8.2
8.0
7.8
7.6
1
2
t−Test: p−value = 0.27

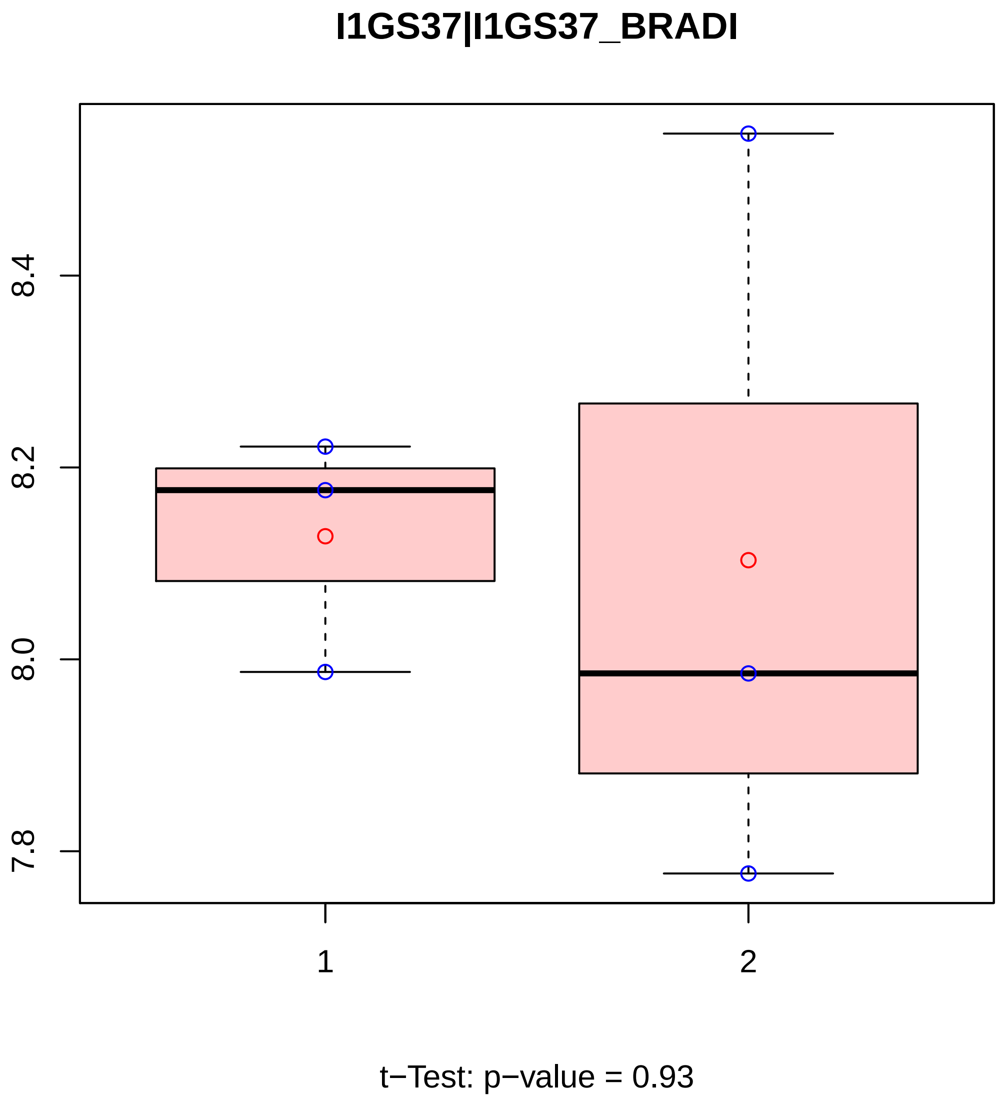
I1GS37|I1GS37_BRADI
8.4
8.2
8.0
7.8
1
2
t−Test: p−value = 0.93

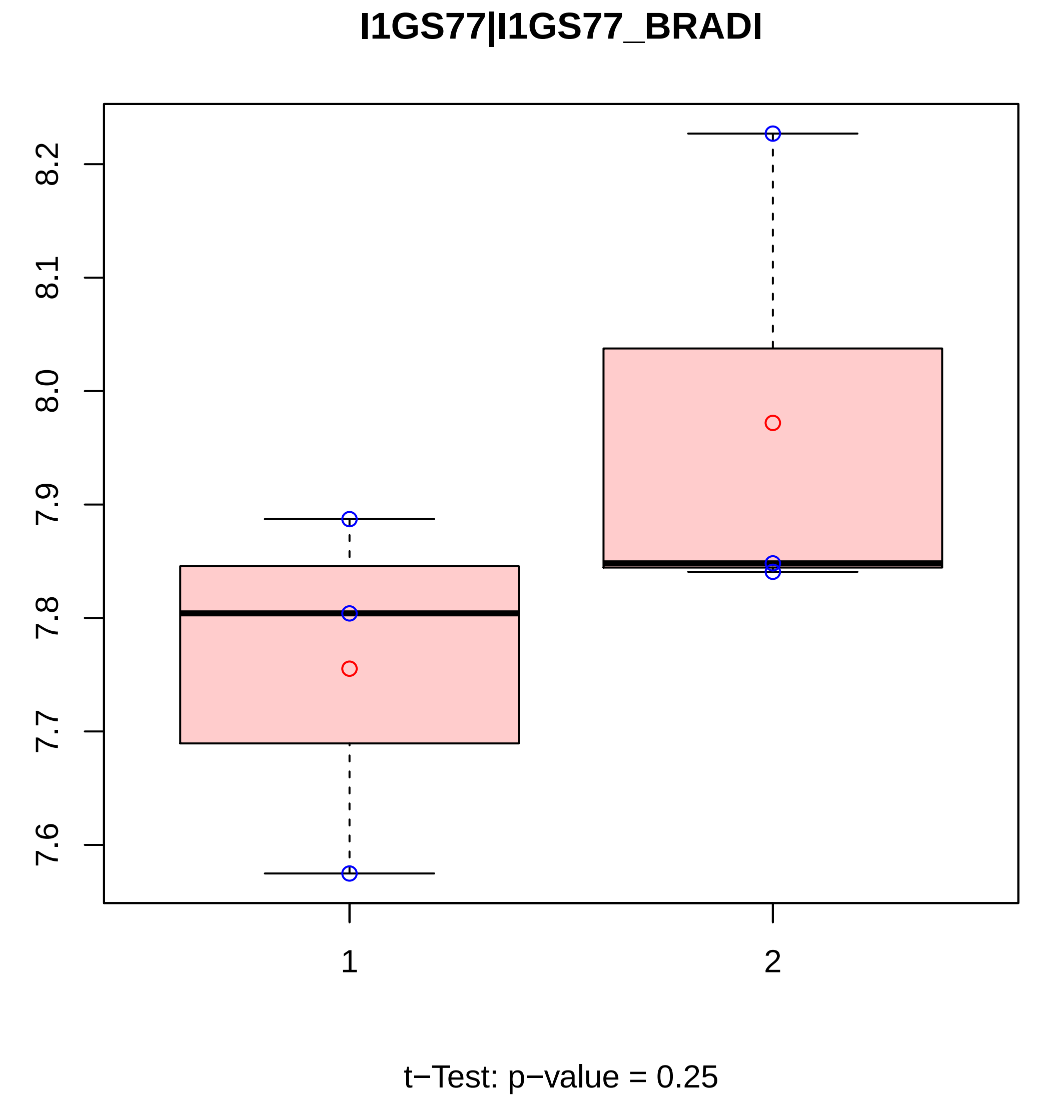
I1GS77|I1GS77_BRADI
8.2
8.1
8.0
7.9
7.8
7.7
7.6
1
2
t−Test: p−value = 0.25

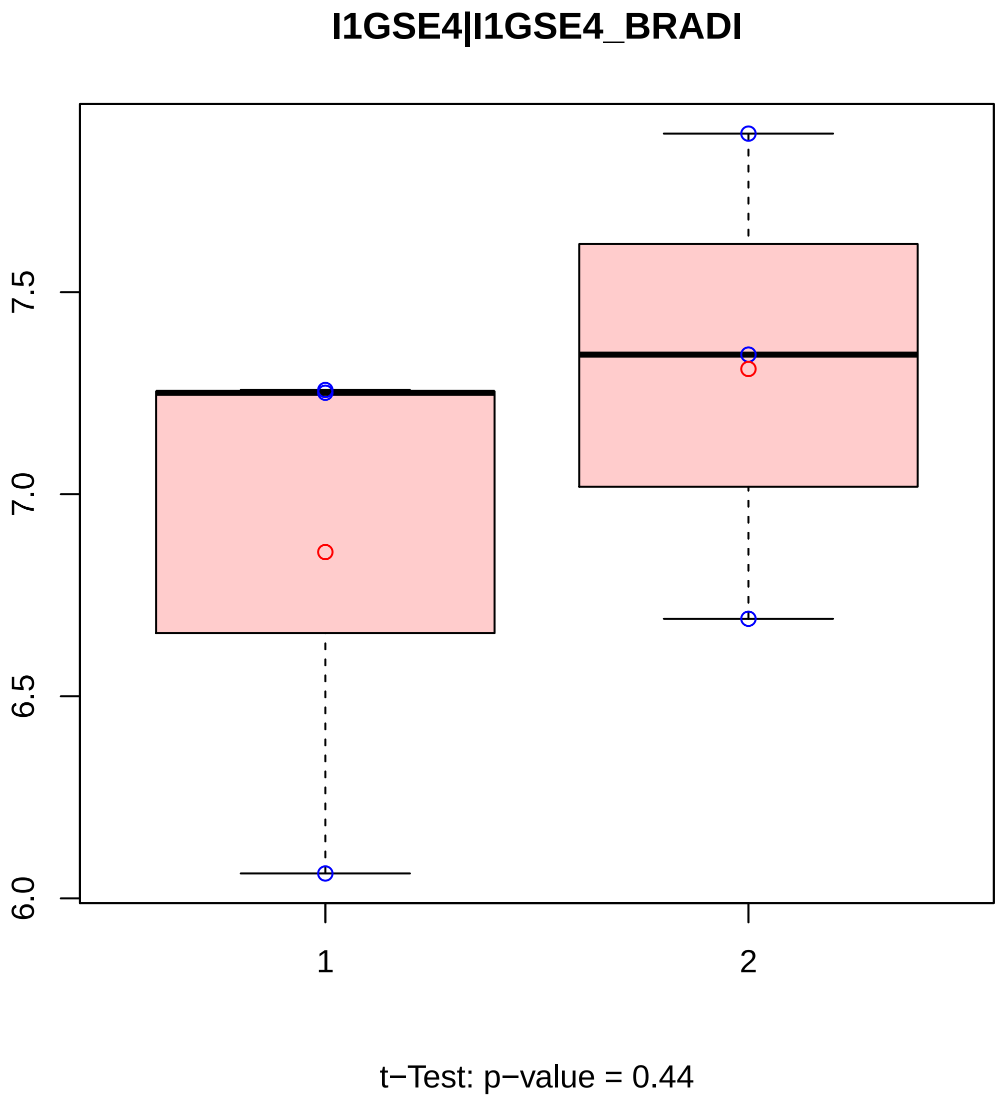
I1GSE4|I1GSE4_BRADI
6.0
6.5
7.0
7.5
1
2
t−Test: p−value = 0.44

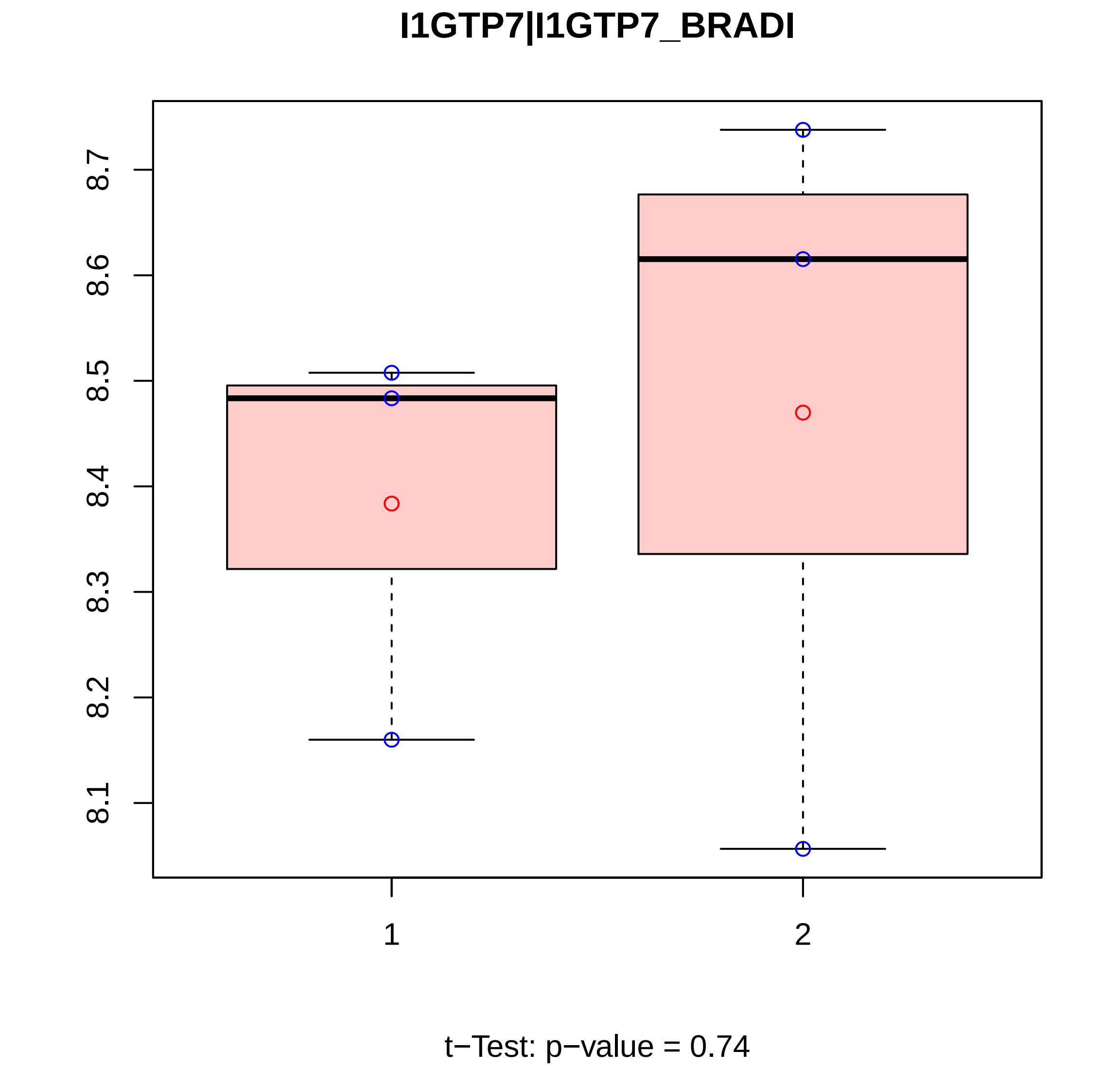
I1GTP7|I1GTP7_BRADI
8.1
8.2
8.3
8.4
8.5
8.6
8.7
1
2
t−Test: p−value = 0.74

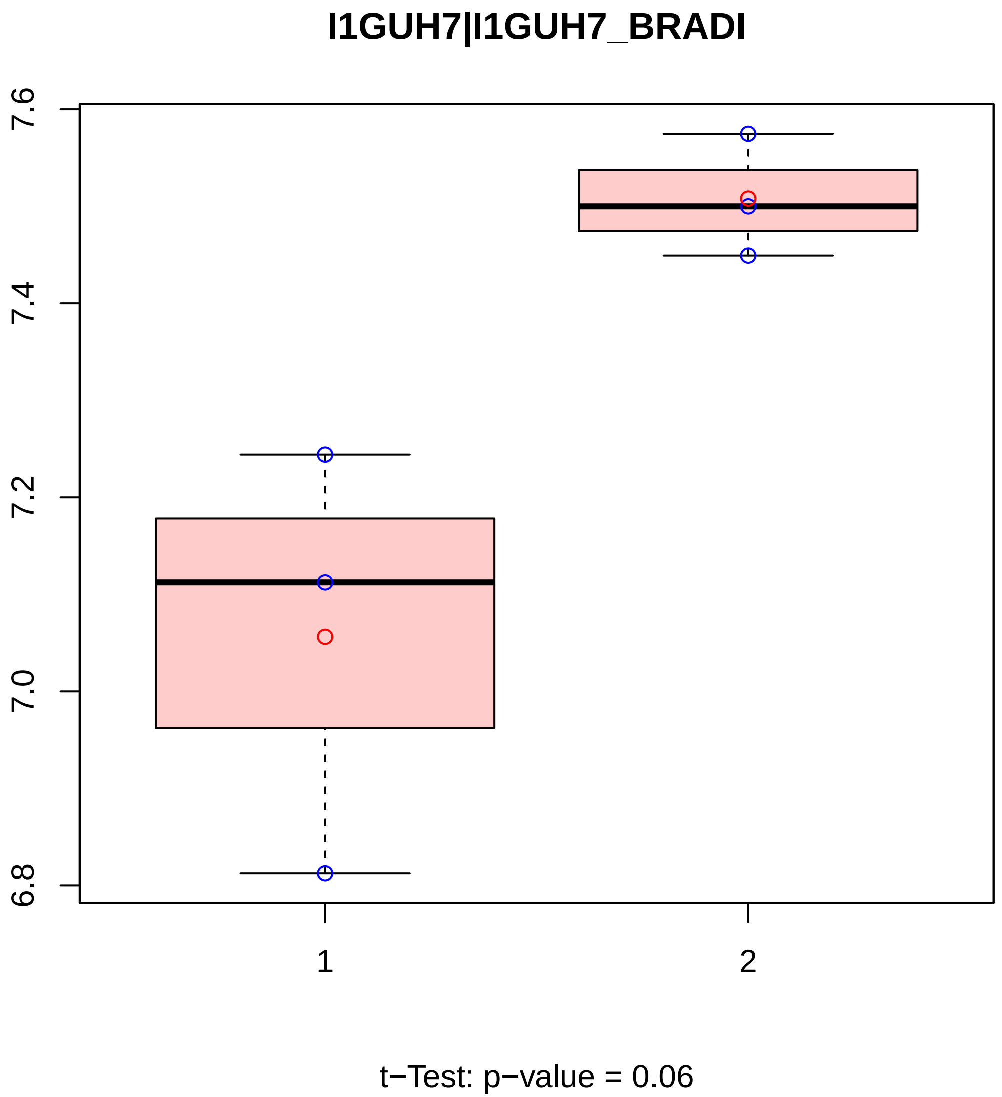
I1GUH7|I1GUH7_BRADI
7.6
7.4
7.2
7.0
6.8
1
2
t−Test: p−value = 0.06

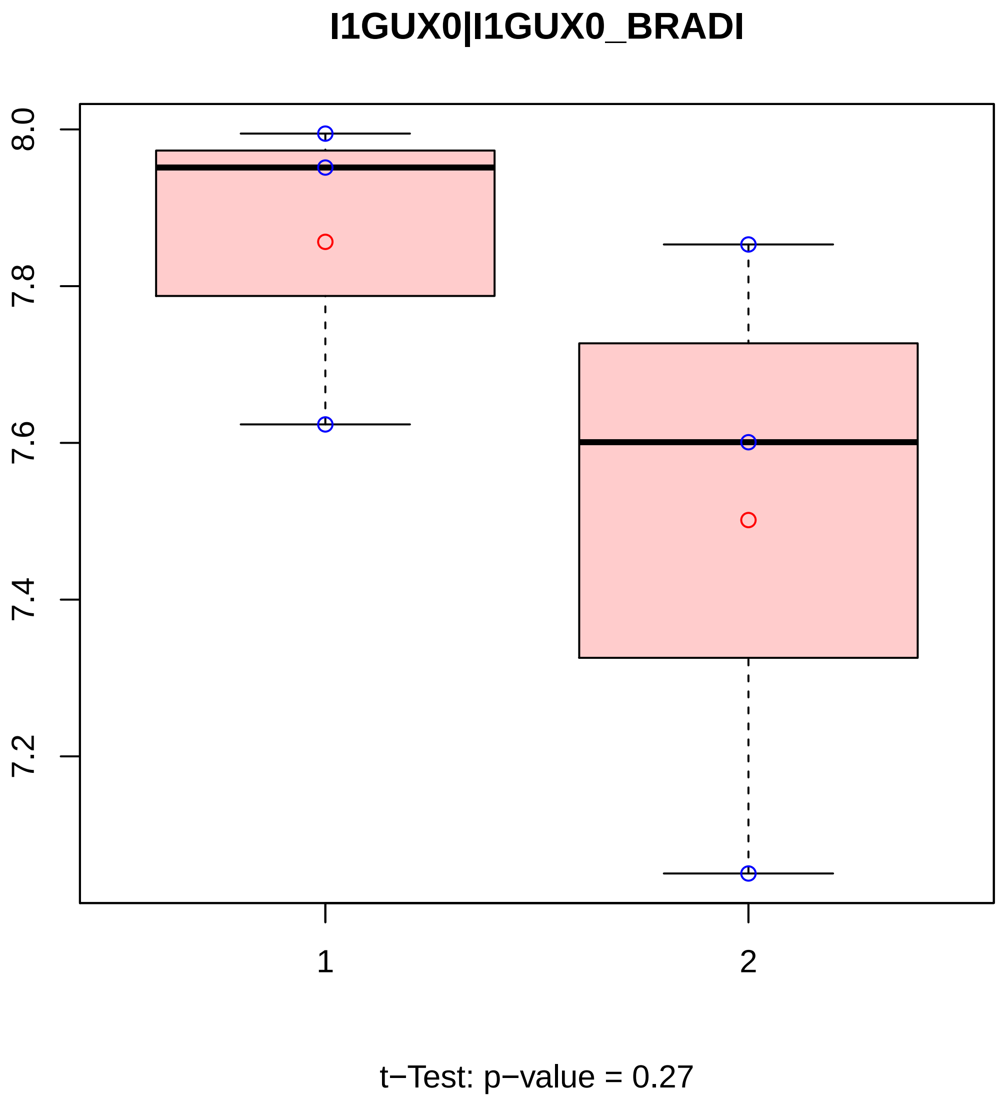

I1GUX0|I1GUX0_BRADI
8.0
7.8
7.6
7.4
7.2
1
2
t−Test: p−value = 0.27

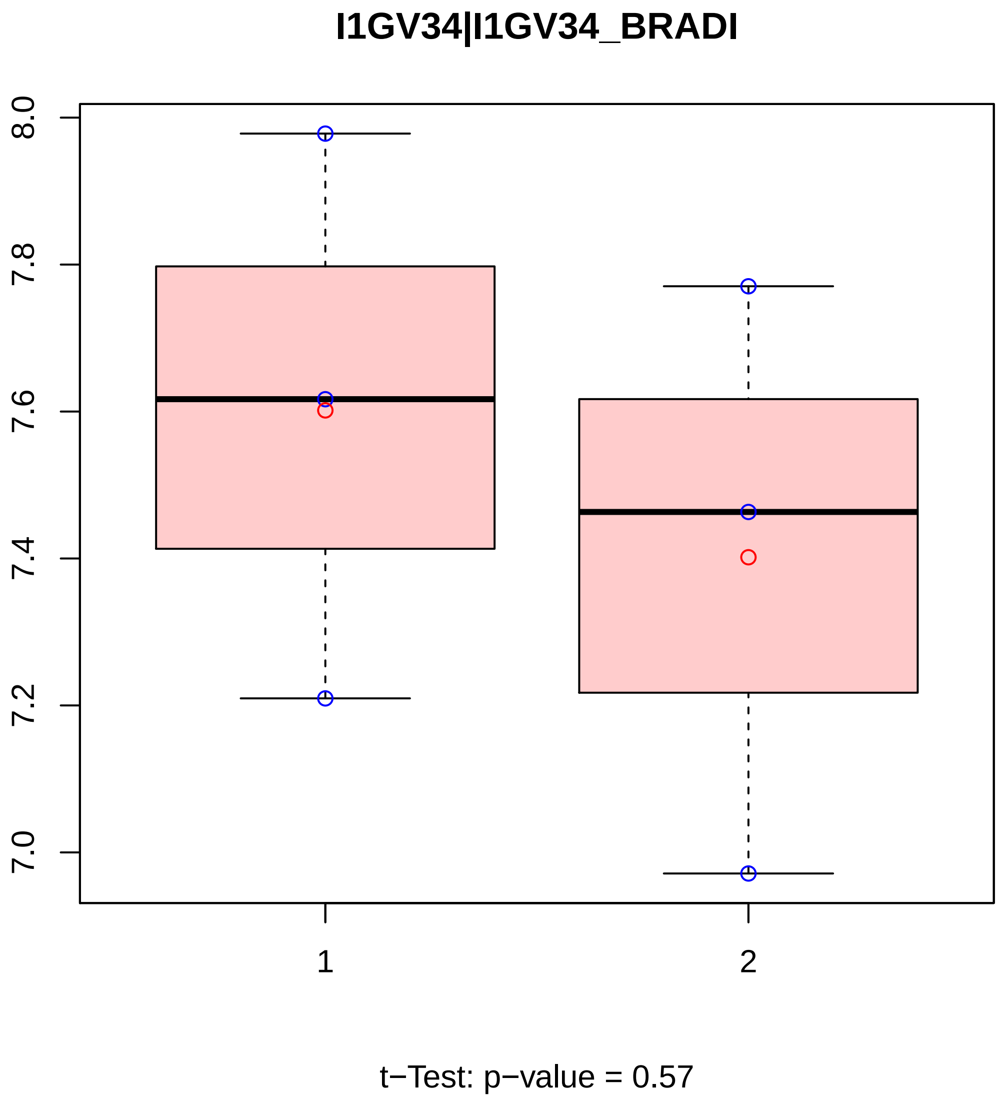
I1GV34|I1GV34_BRADI
8.0
7.8
7.6
7.4
7.2
7.0
1
2
t−Test: p−value = 0.57

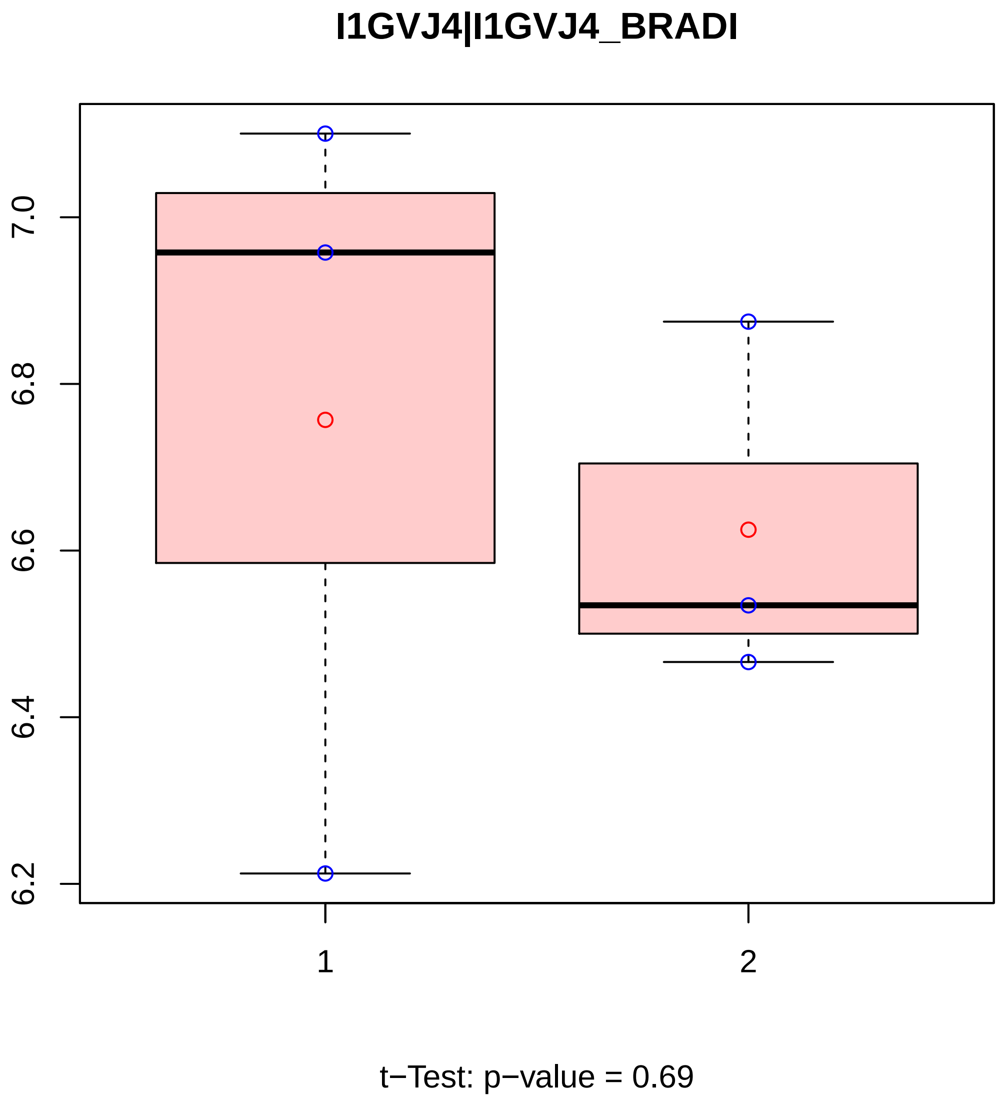
I1GVJ4|I1GVJ4_BRADI
7.0
6.8
6.6
6.4
6.2
1
2
t−Test: p−value = 0.69

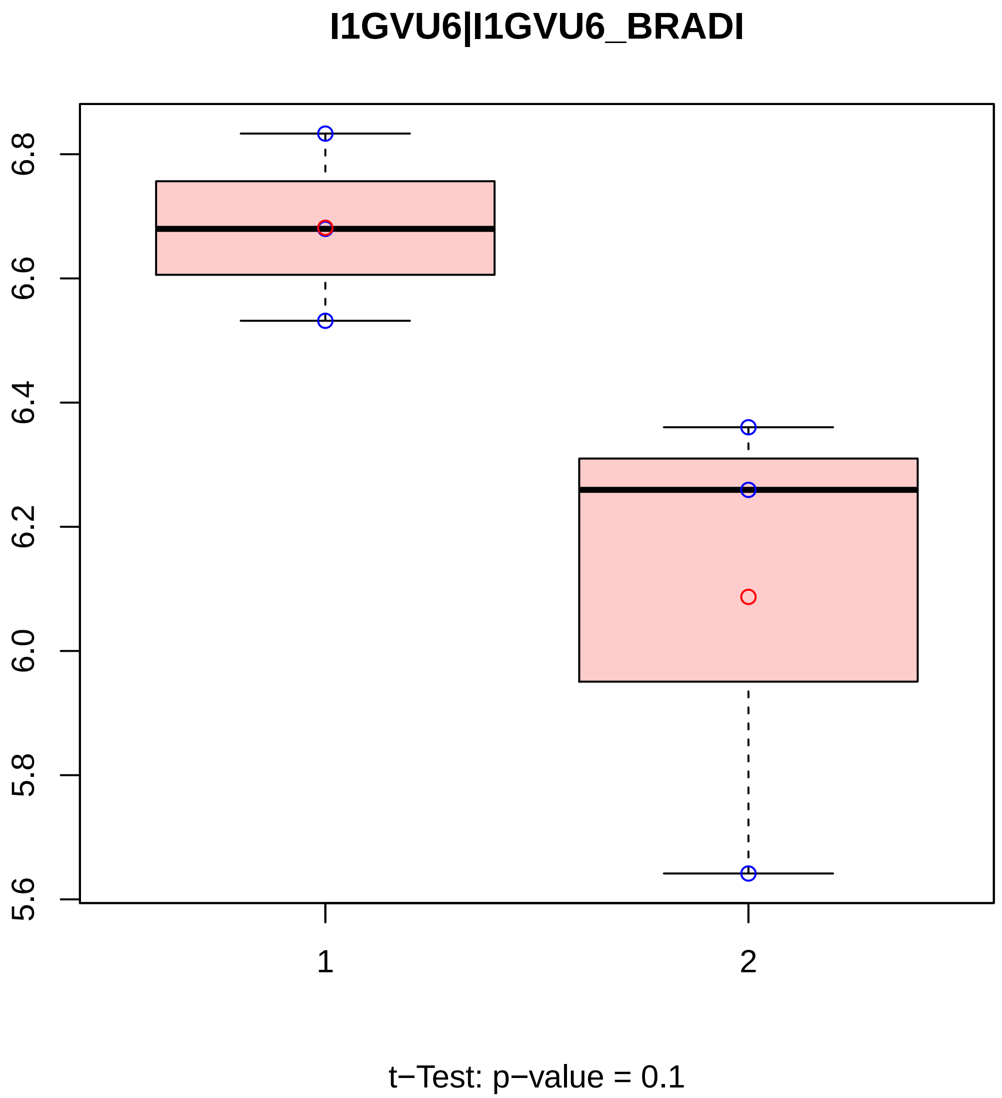
I1GVU6|I1GVU6_BRADI
6.8
6.6
6.4
6.2
6.0
5.8
5.6
1
2
t−Test: p−value = 0.1

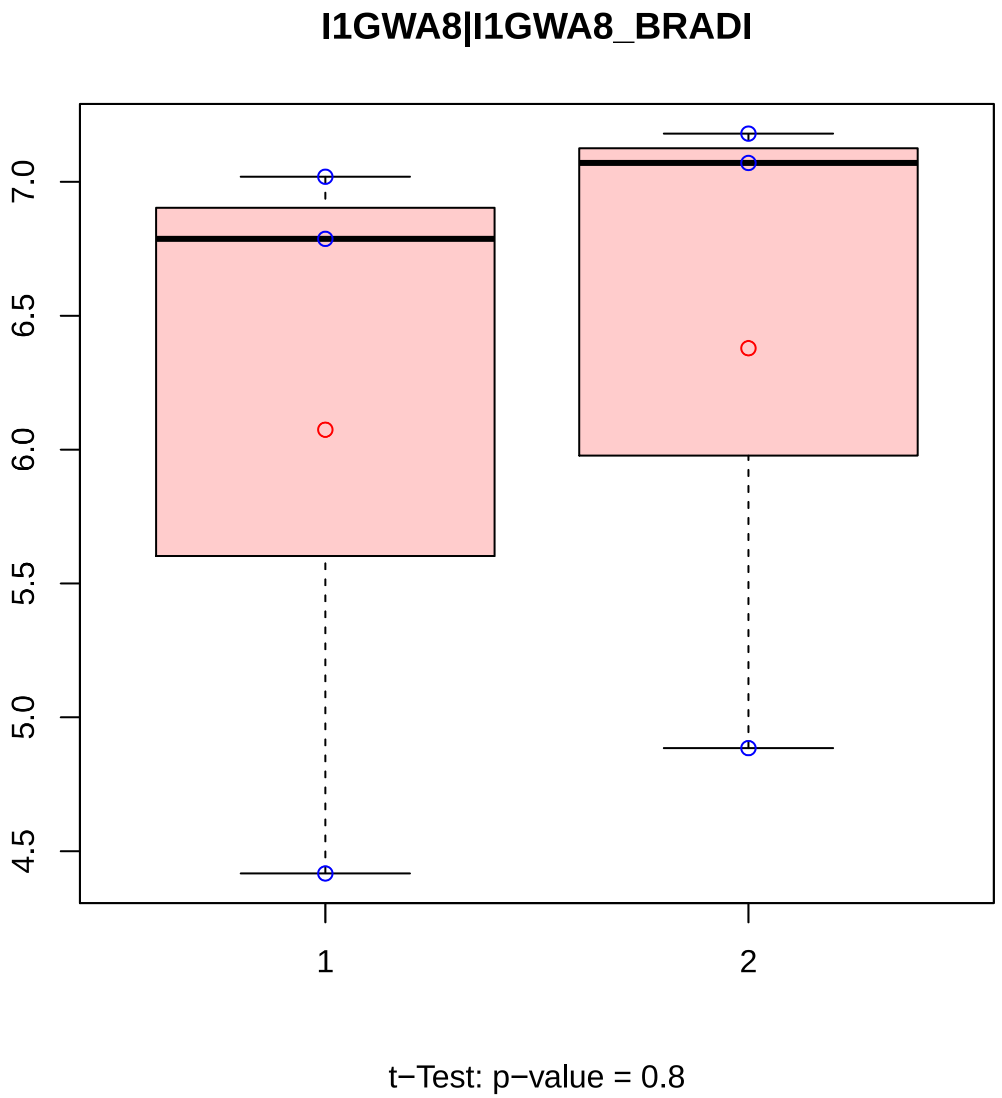

I1GWA8|I1GWA8_BRADI
7.0
6.5
6.0
5.5
5.0
4.5
1
2
t−Test: p−value = 0.8

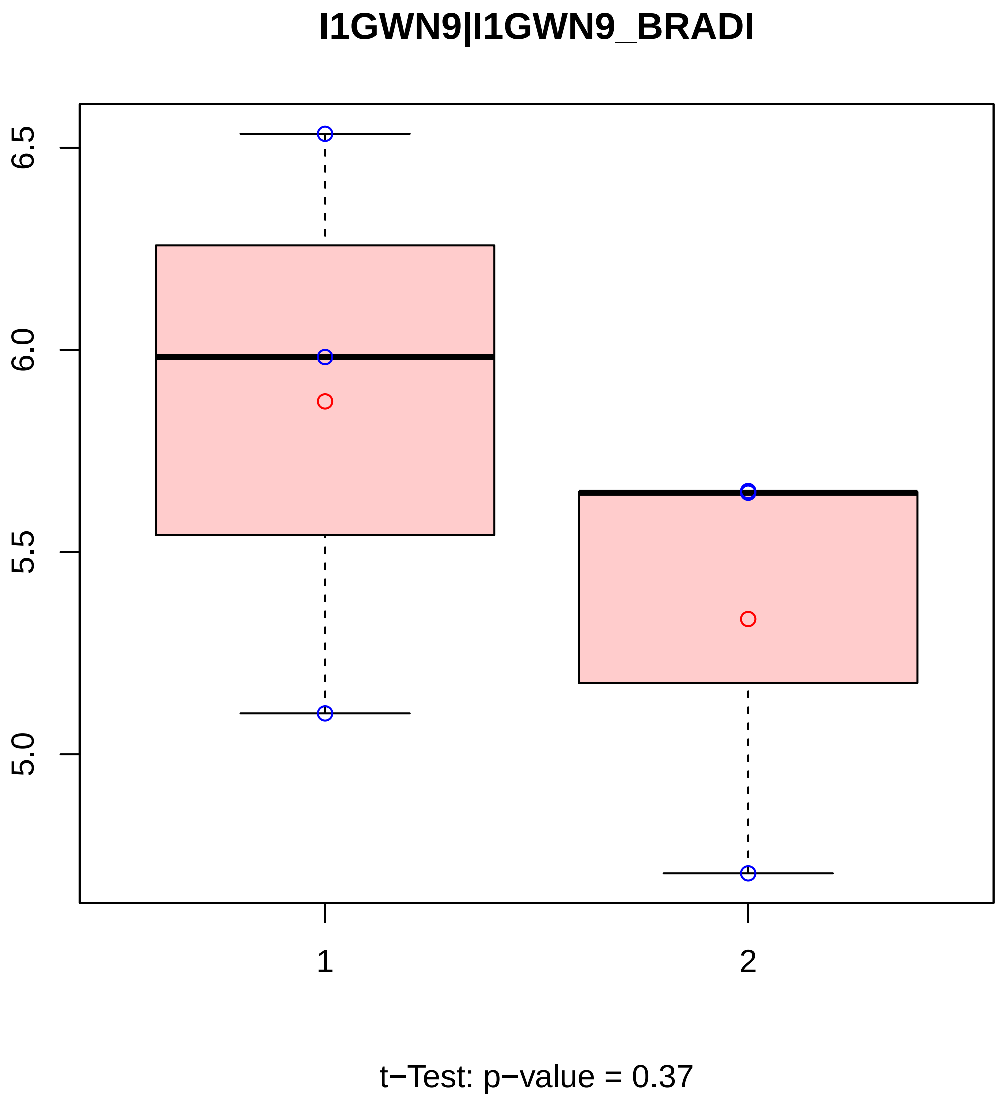

I1GWN9|I1GWN9_BRADI
6.5
6.0
5.5
5.0
1
2
t−Test: p−value = 0.37

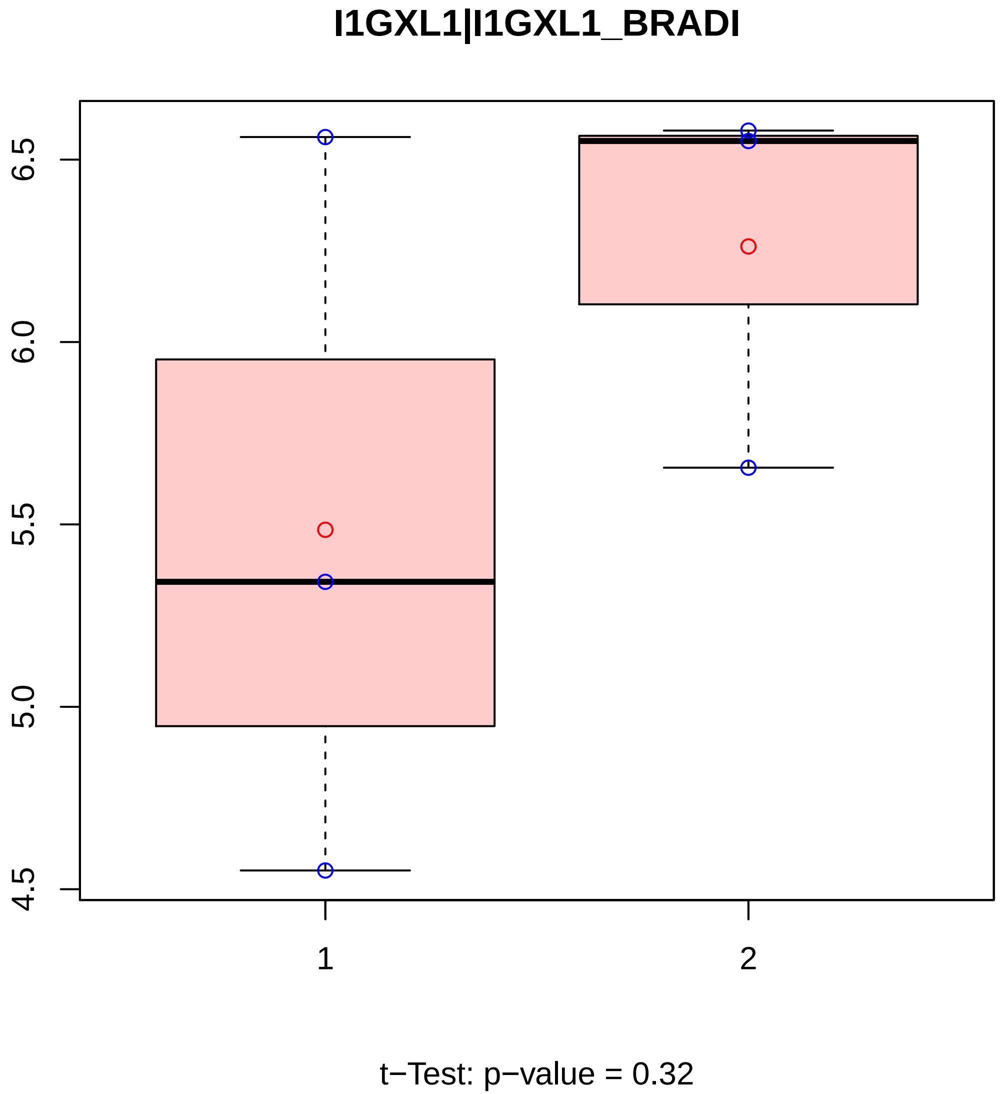
I1GXL1|I1GXL1_BRADI
6.5
6.0
5.5
5.0
4.5
1
2
t−Test: p−value = 0.32

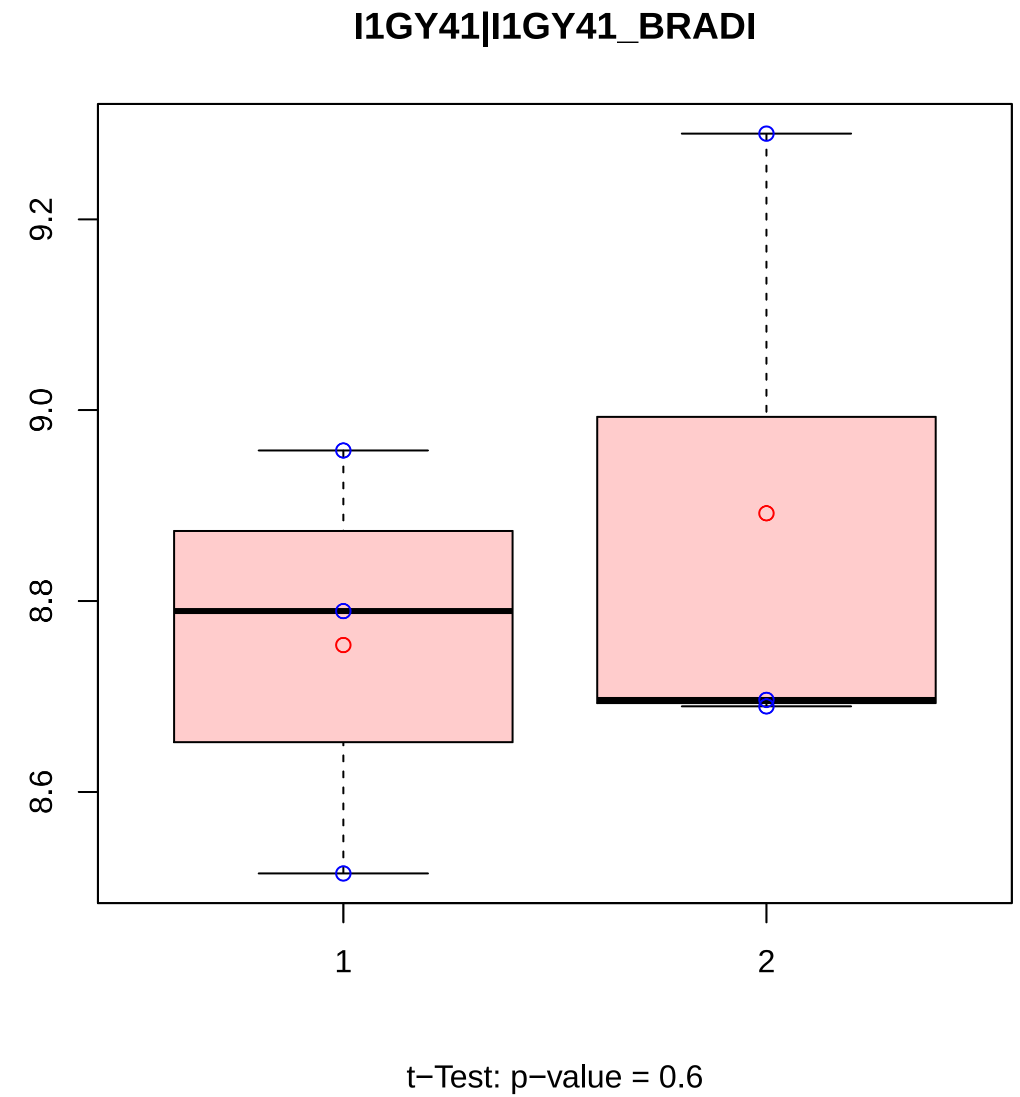
I1GY41|I1GY41_BRADI
9.2
9.0
8.8
8.6
1
2
t−Test: p−value = 0.6

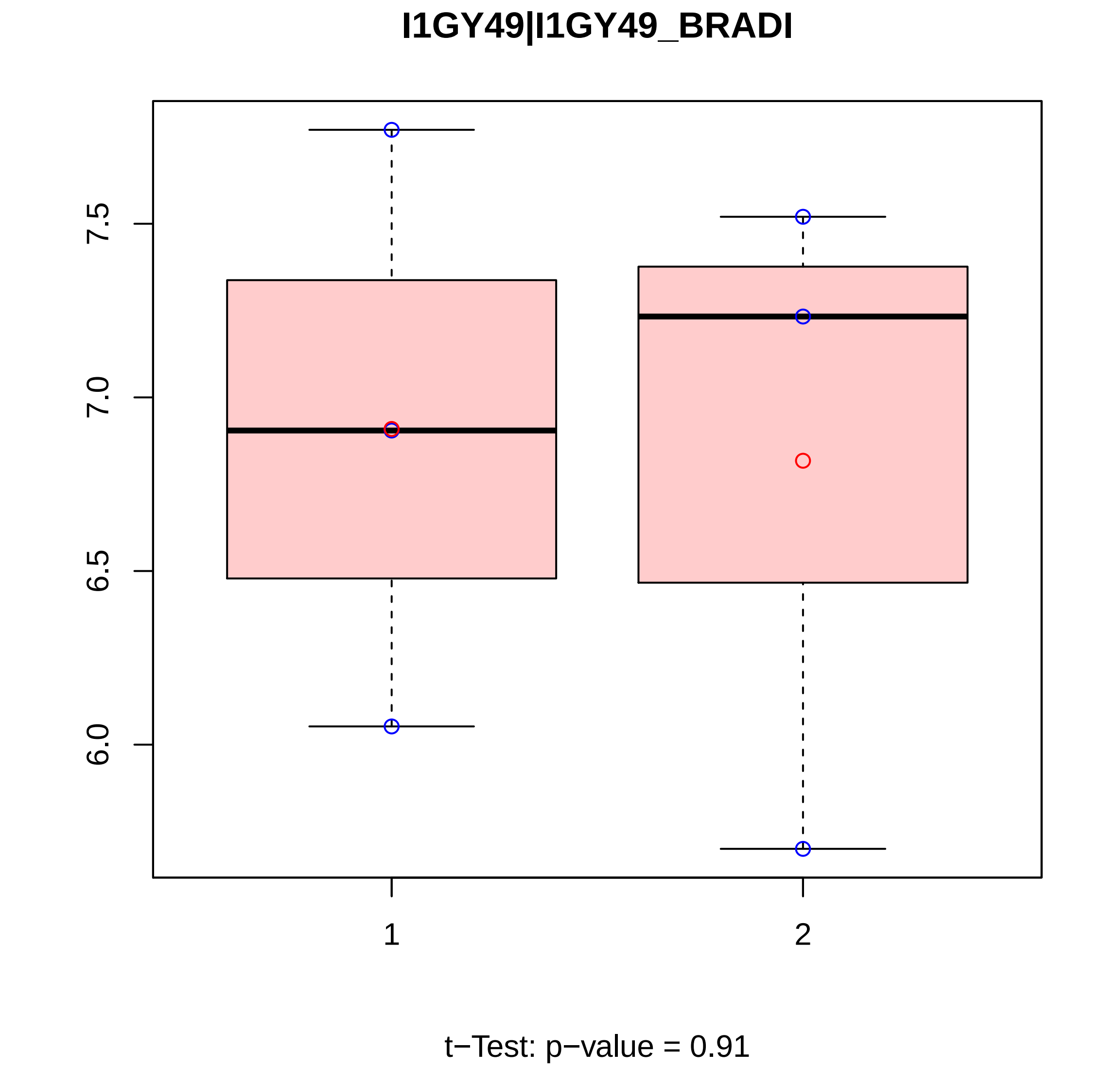
I1GY49|I1GY49_BRADI
7.5
7.0
6.5
6.0
1
2
t−Test: p−value = 0.91

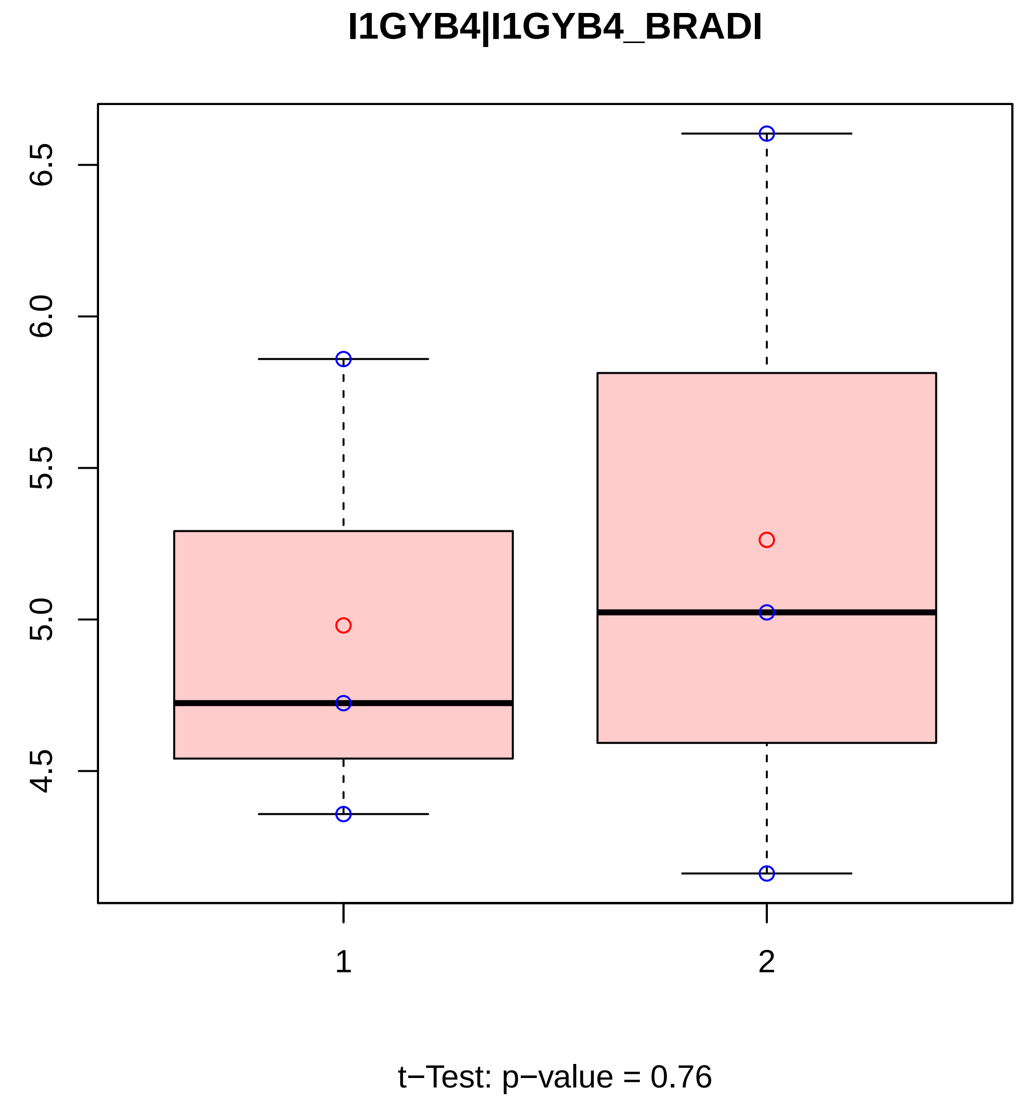
I1GYB4|I1GYB4_BRADI
6.5
6.0
5.5
5.0
4.5
1
2
t−Test: p−value = 0.76

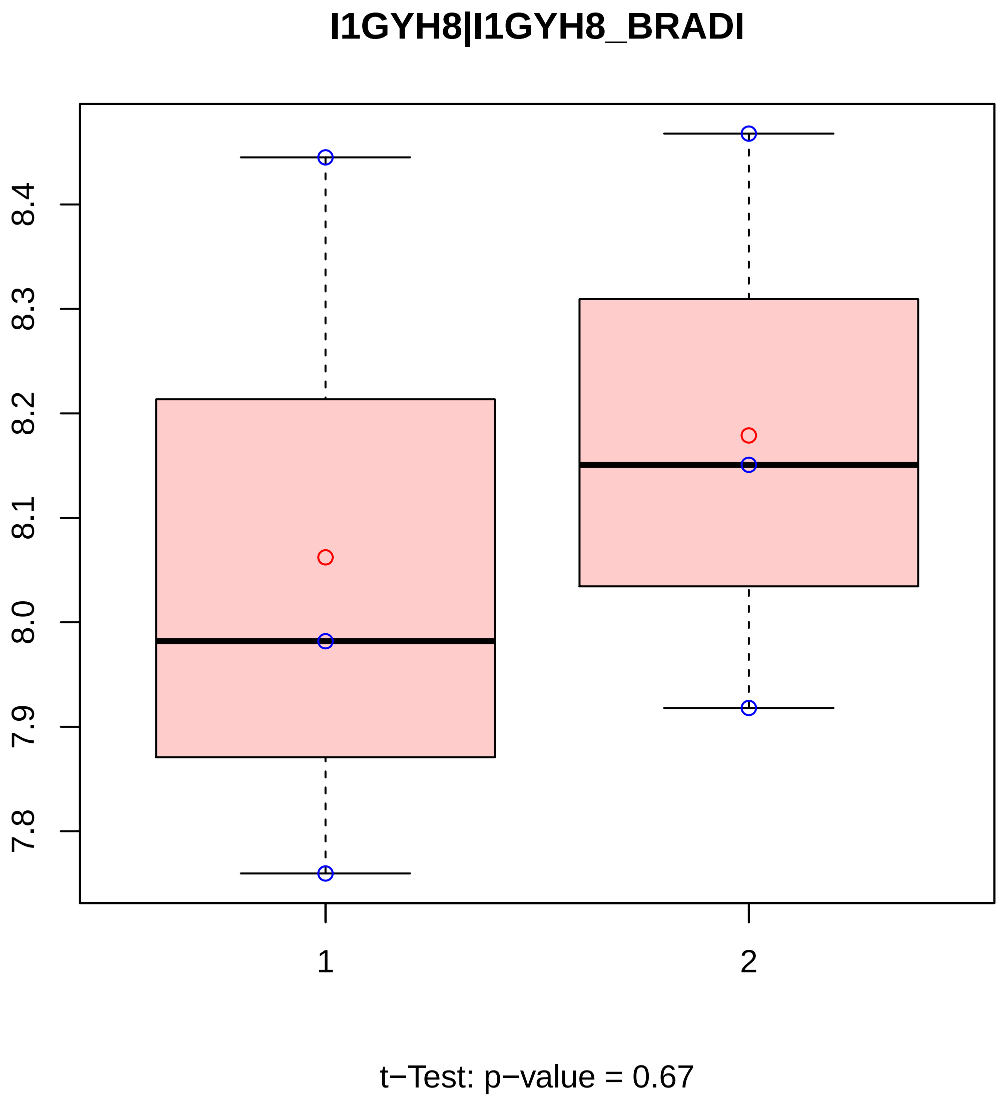
I1GYH8|I1GYH8_BRADI
8.4
8.3
8.2
8.1
8.0
7.9
7.8
1
2
t−Test: p−value = 0.67

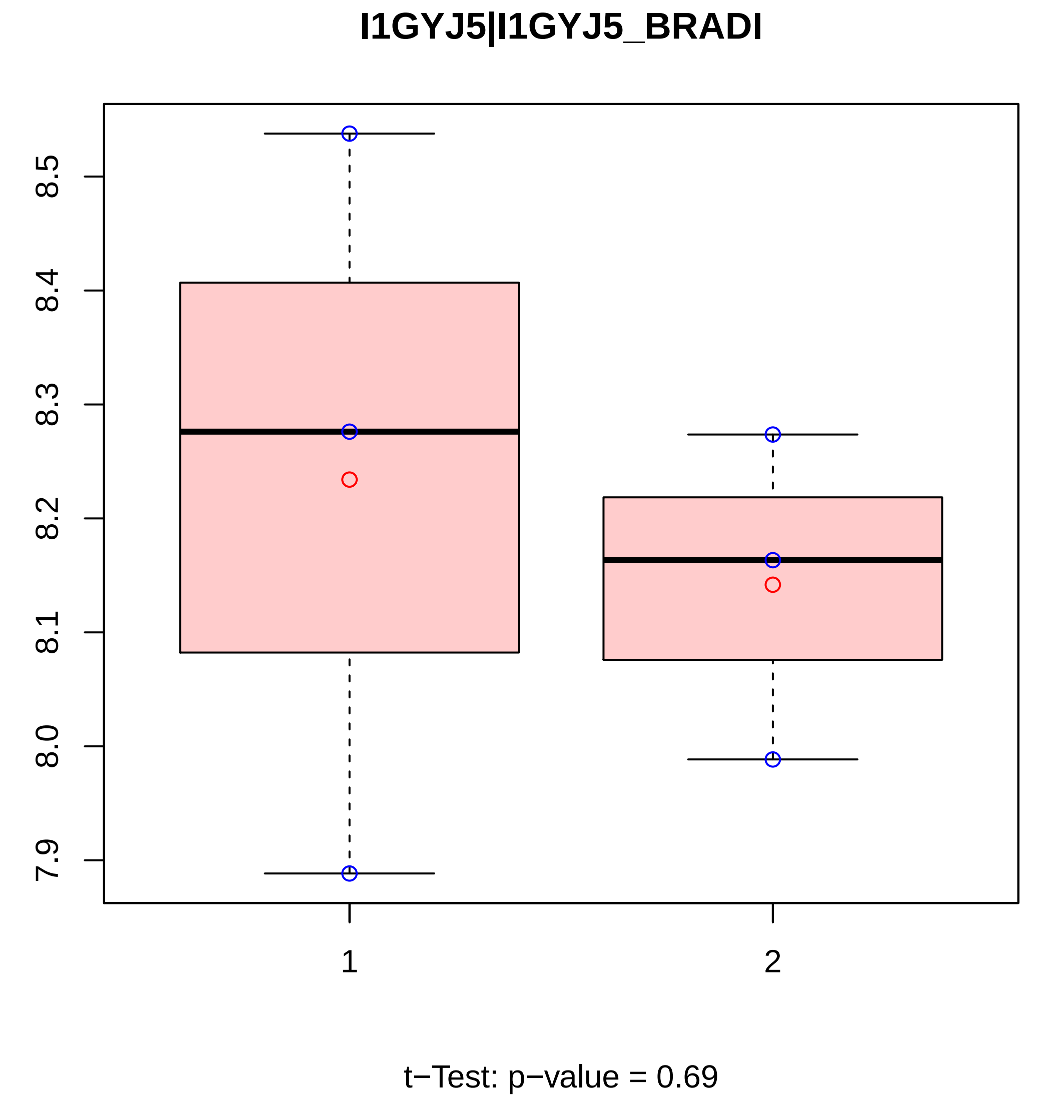
I1GYJ5|I1GYJ5_BRADI
8.5
8.4
8.3
8.2
8.1
8.0
7.9
1
2
t−Test: p−value = 0.69

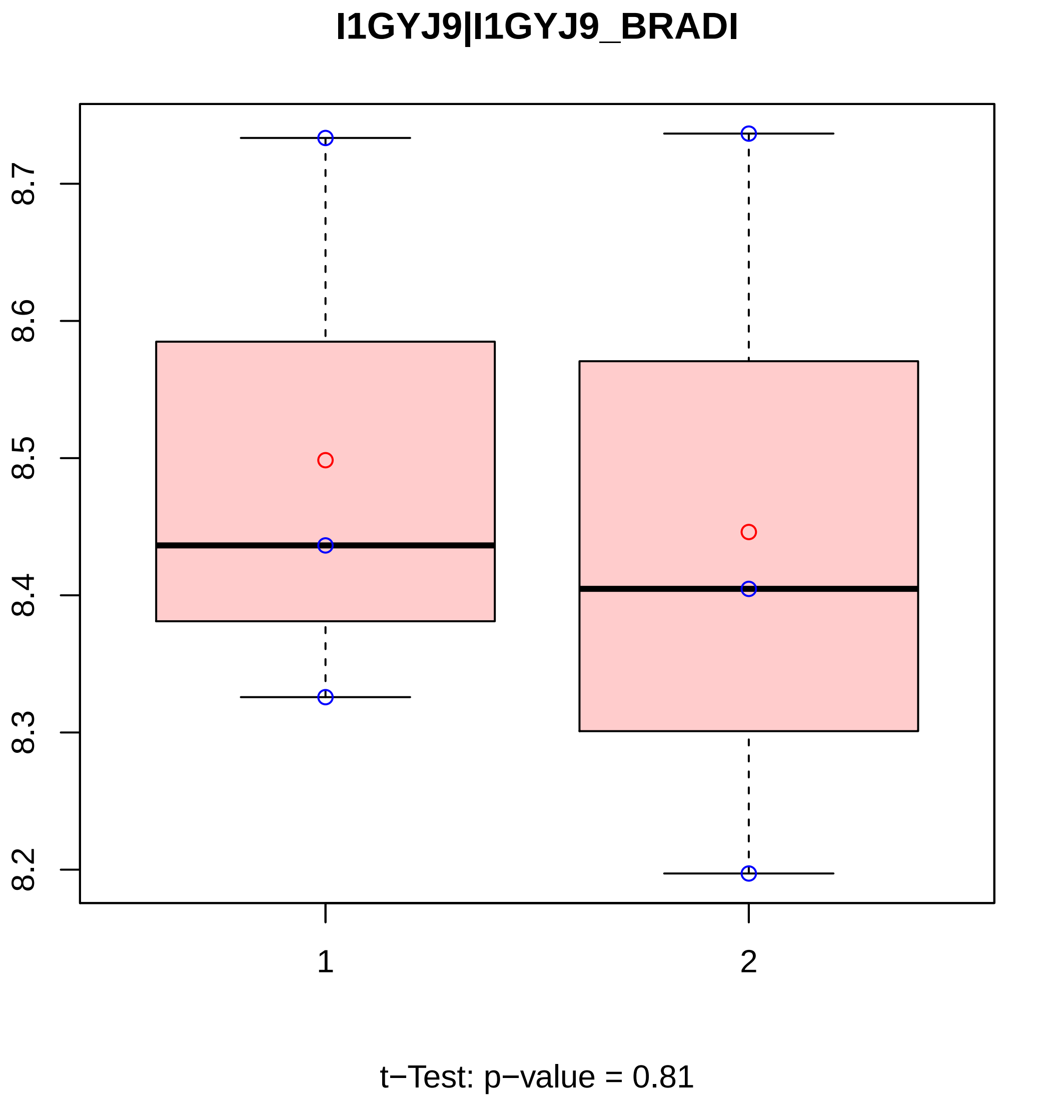
I1GYJ9|I1GYJ9_BRADI
8.2
8.3
8.4
8.5
8.6
8.7
1
2
t−Test: p−value = 0.81

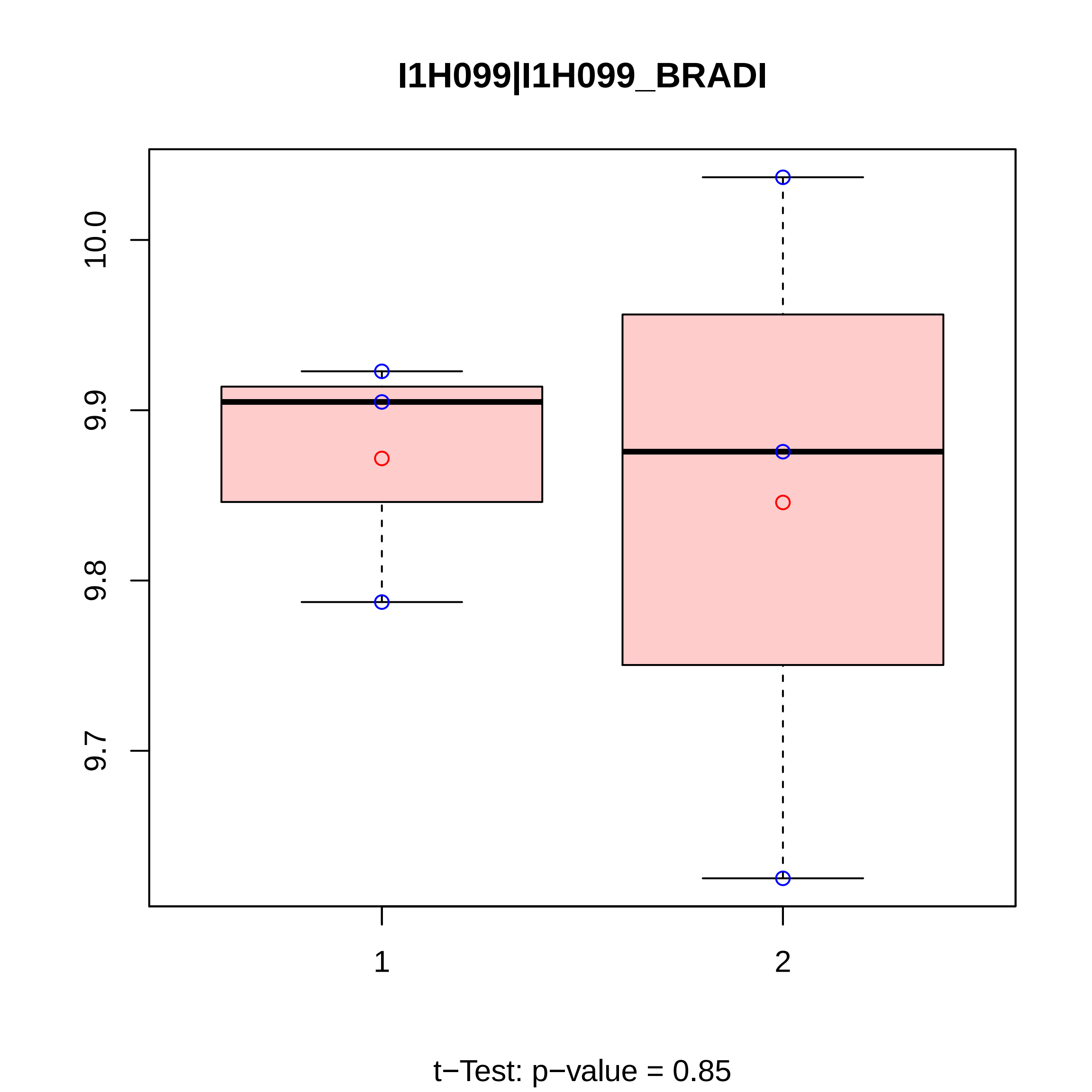
I1H099|I1H099_BRADI
10.0
9.9
9.8
9.7
1
2
t−Test: p−value = 0.85

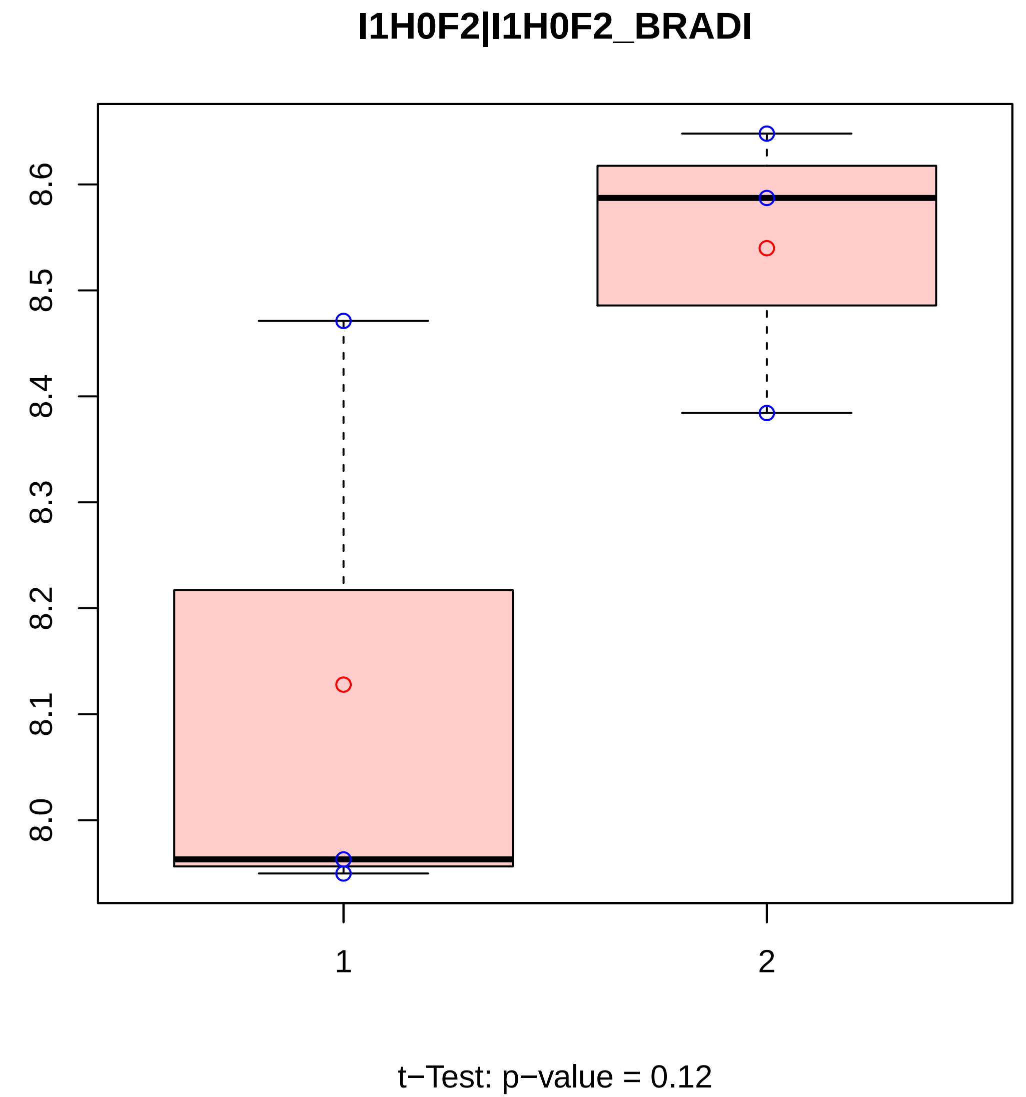
I1H0F2|I1H0F2_BRADI
8.0
8.1
8.2
8.3
8.4
8.5
8.6
1
2
t−Test: p−value = 0.12

I1H0F4|I1H0F4_BRADI

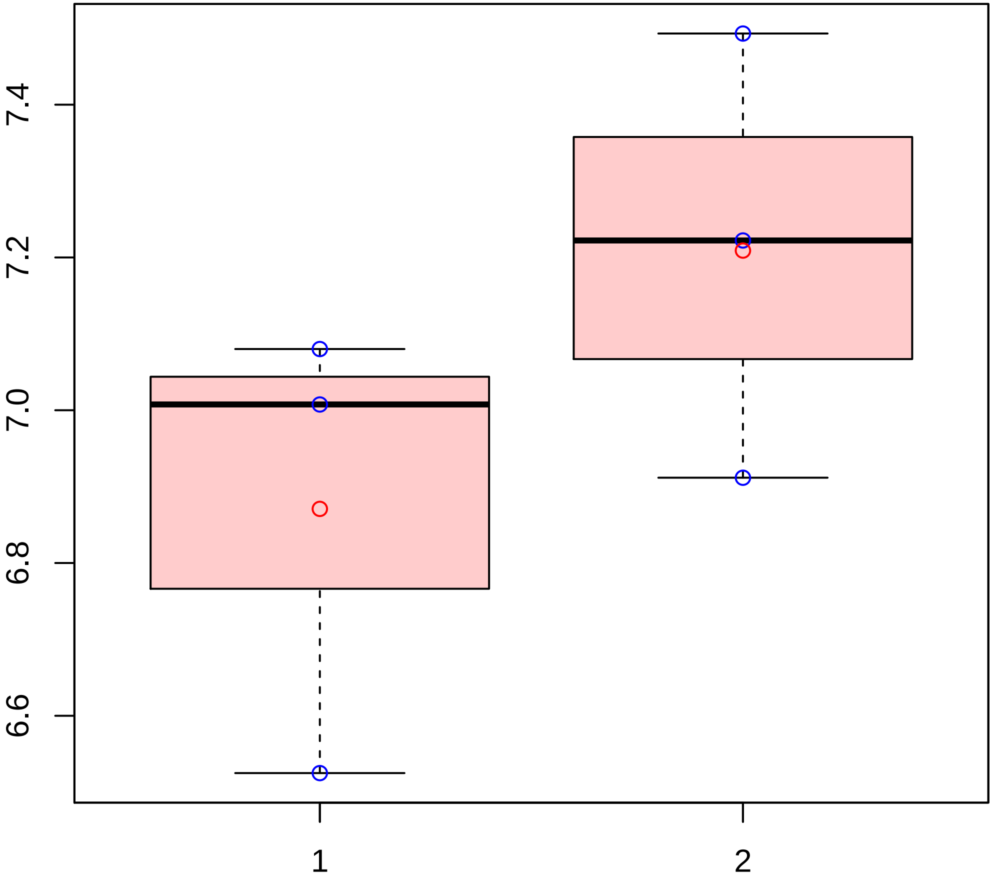

t−Test: p−value = 0.23

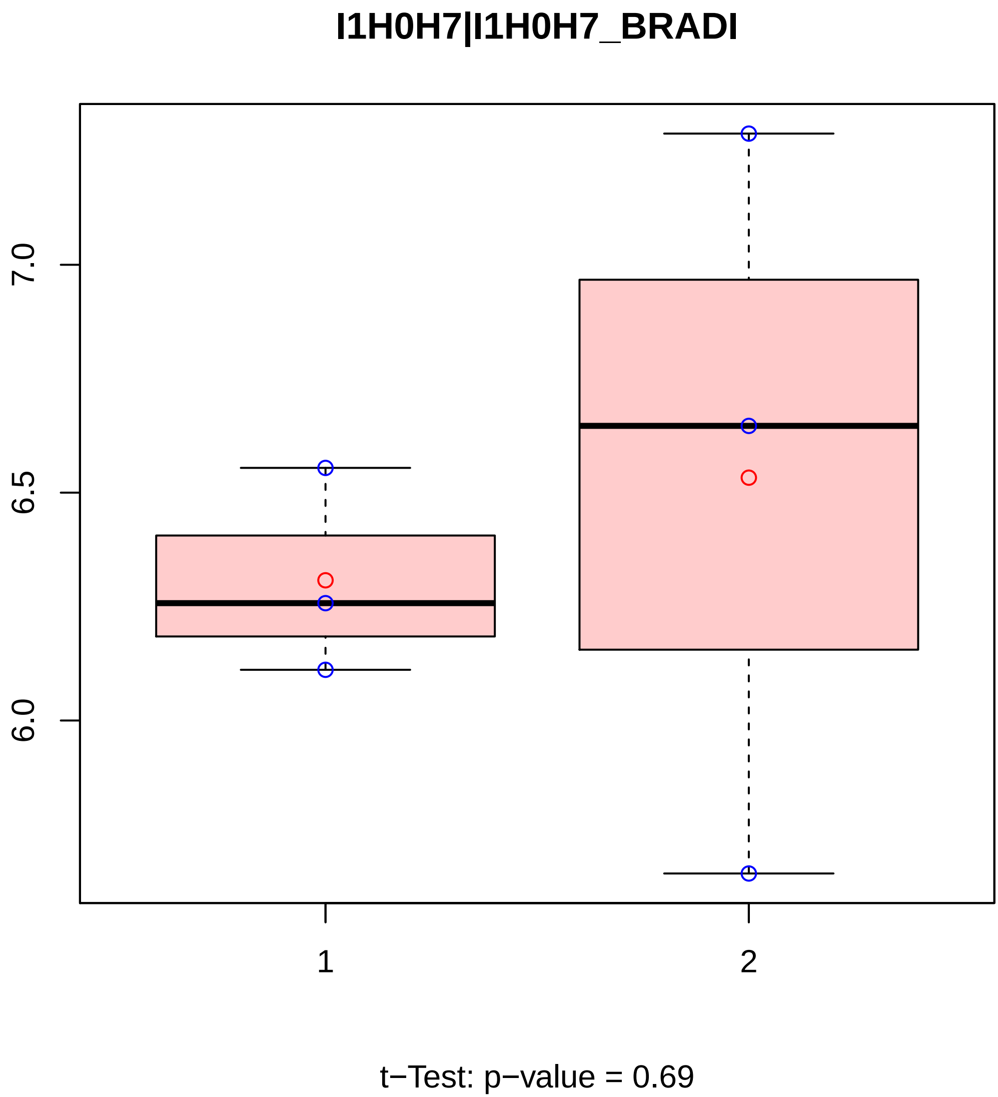
I1H0H7|I1H0H7_BRADI
7.0
6.5
6.0
1
2
t−Test: p−value = 0.69

I1H0K5|I1H0K5_BRADI

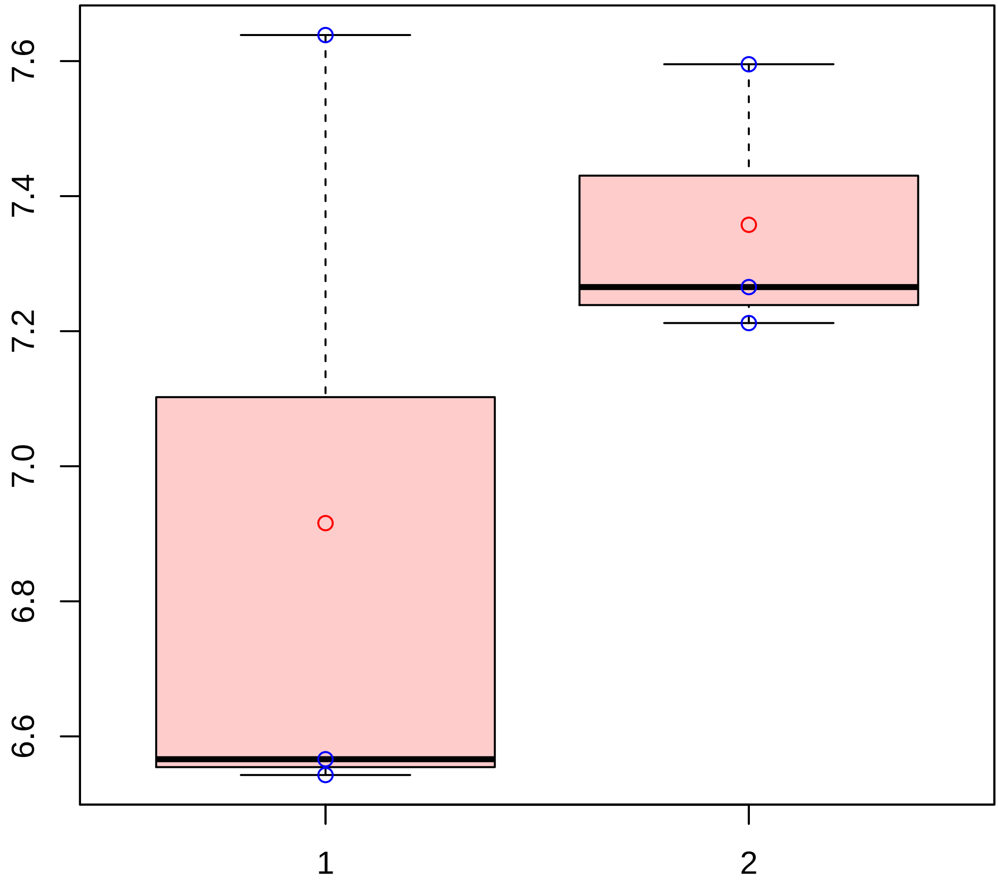

t−Test: p−value = 0.35

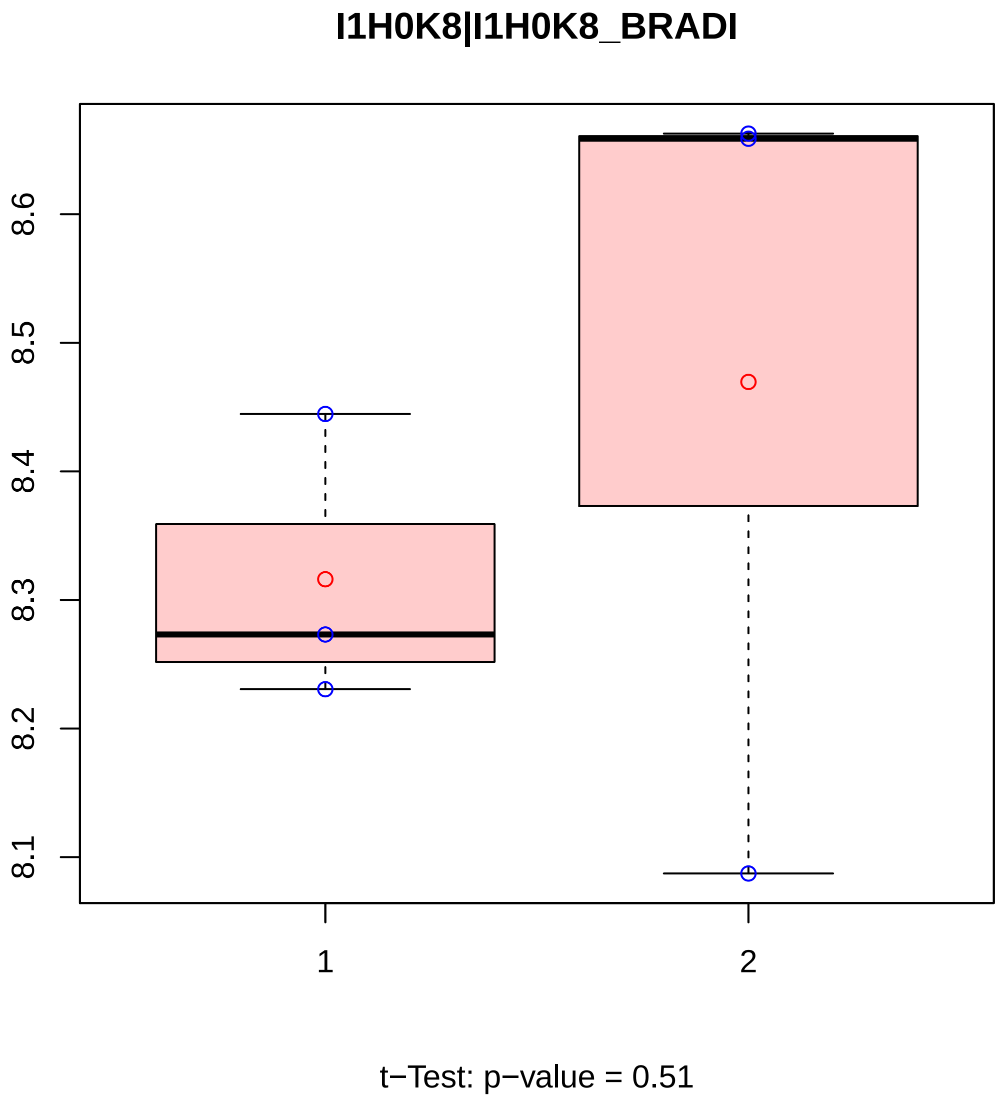

I1H0K8|I1H0K8_BRADI
8.1
8.2
8.3
8.4
8.5
8.6
1
2
t−Test: p−value = 0.51

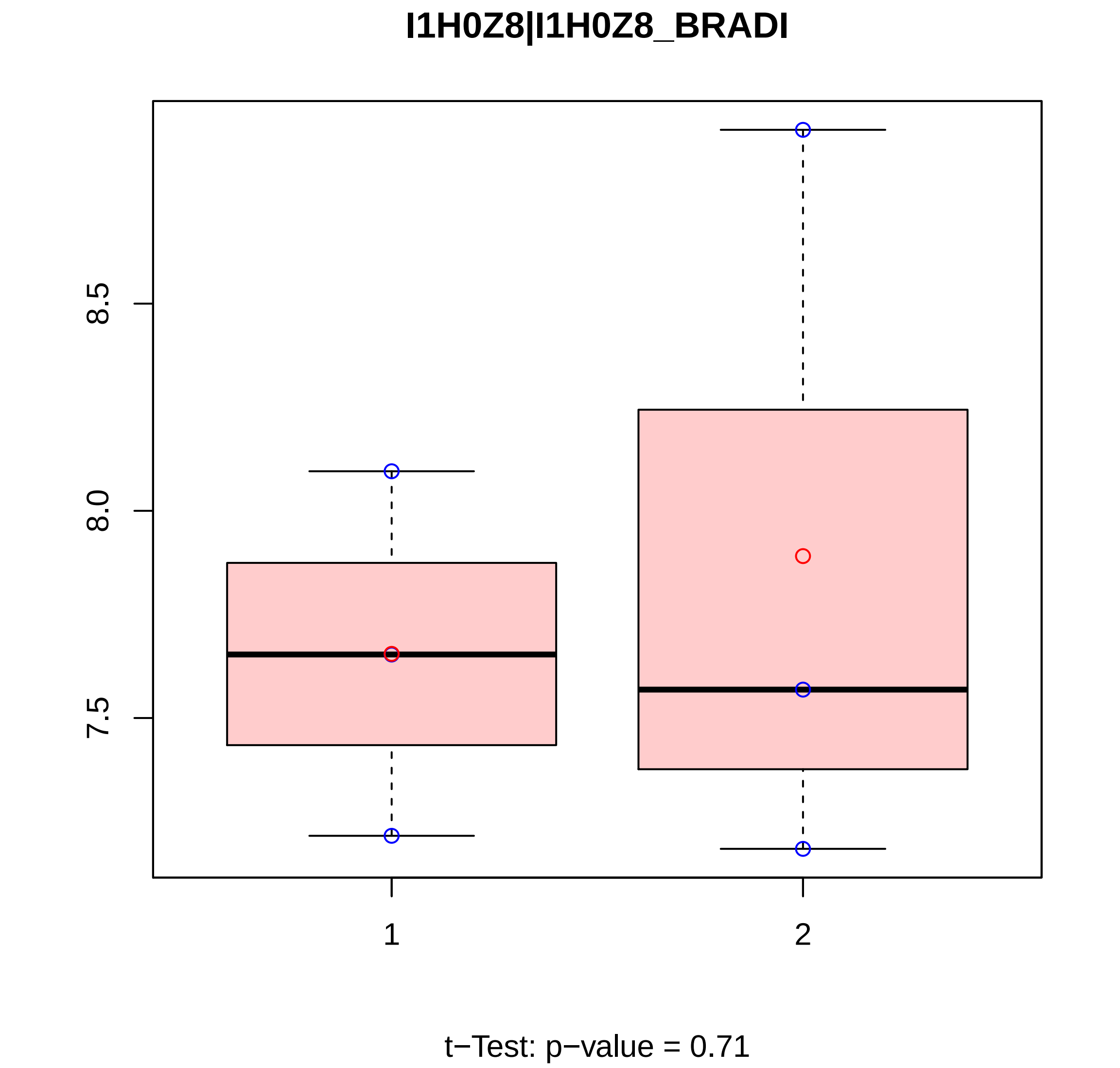
I1H0Z8|I1H0Z8_BRADI
8.5
8.0
7.5
1
2
t−Test: p−value = 0.71

I1H117|I1H117_BRADI

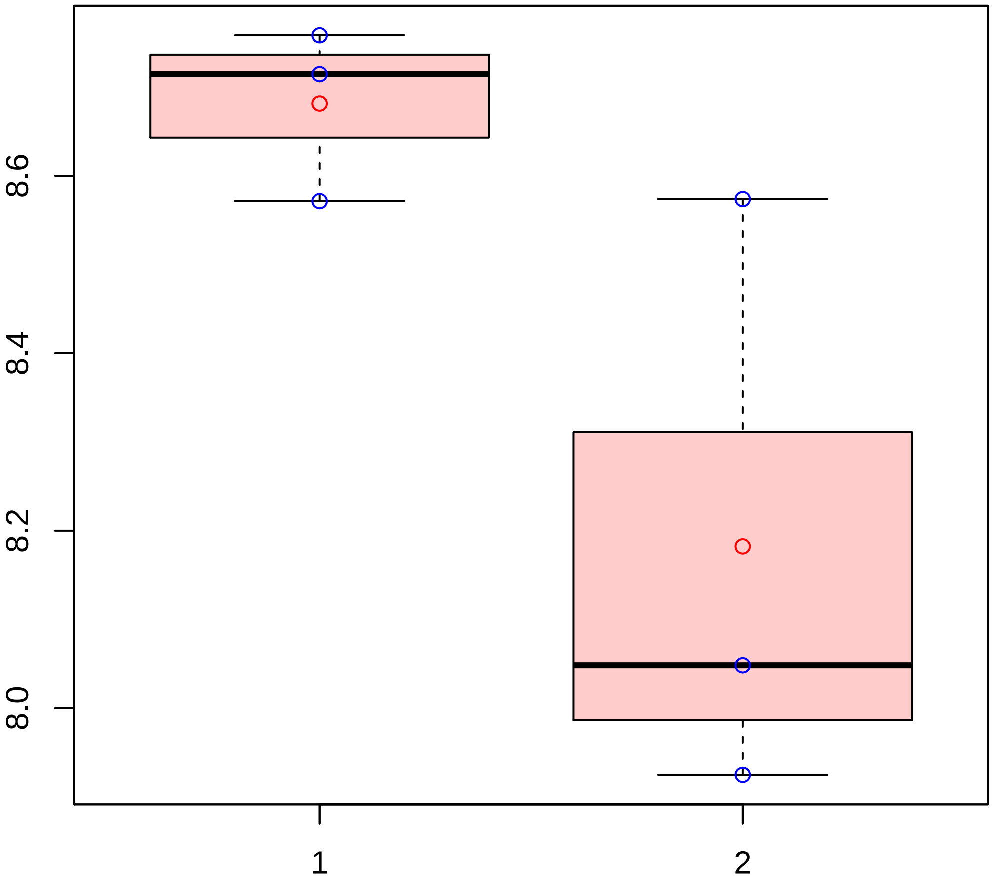

t−Test: p−value = 0.12

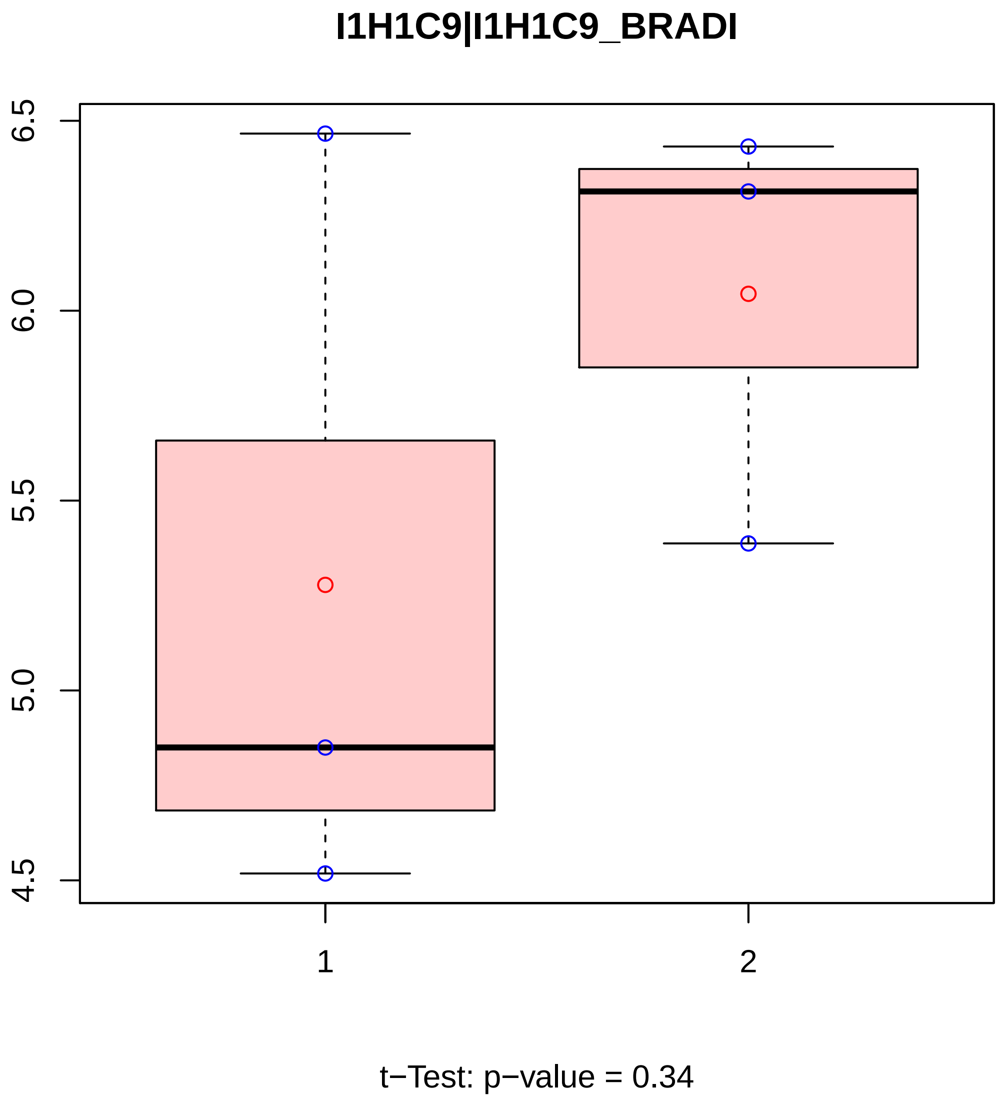
I1H1C9|I1H1C9_BRADI
6.5
6.0
5.5
5.0
4.5
1
2
t−Test: p−value = 0.34

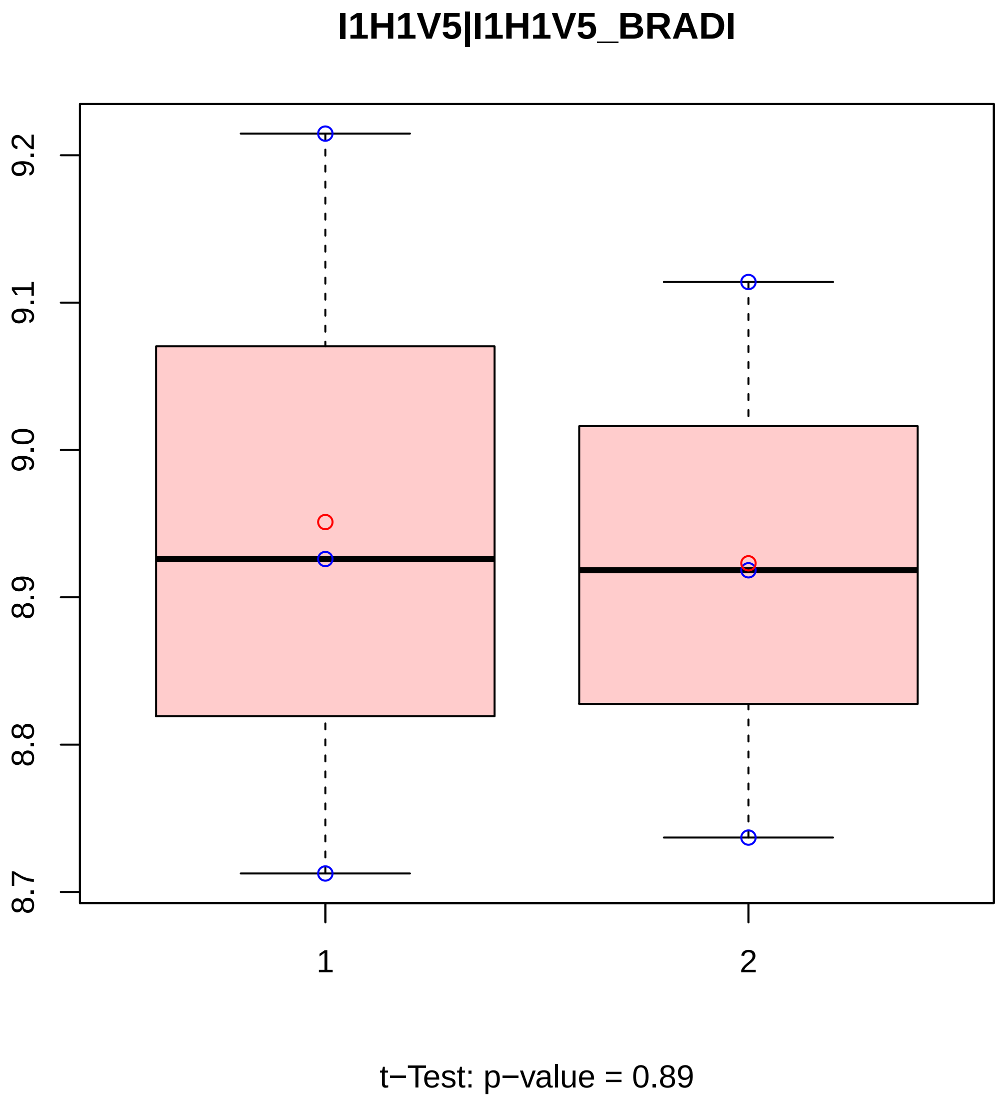
I1H1V5|I1H1V5_BRADI
9.2
9.1
9.0
8.9
8.8
8.7
1
2
t−Test: p−value = 0.89

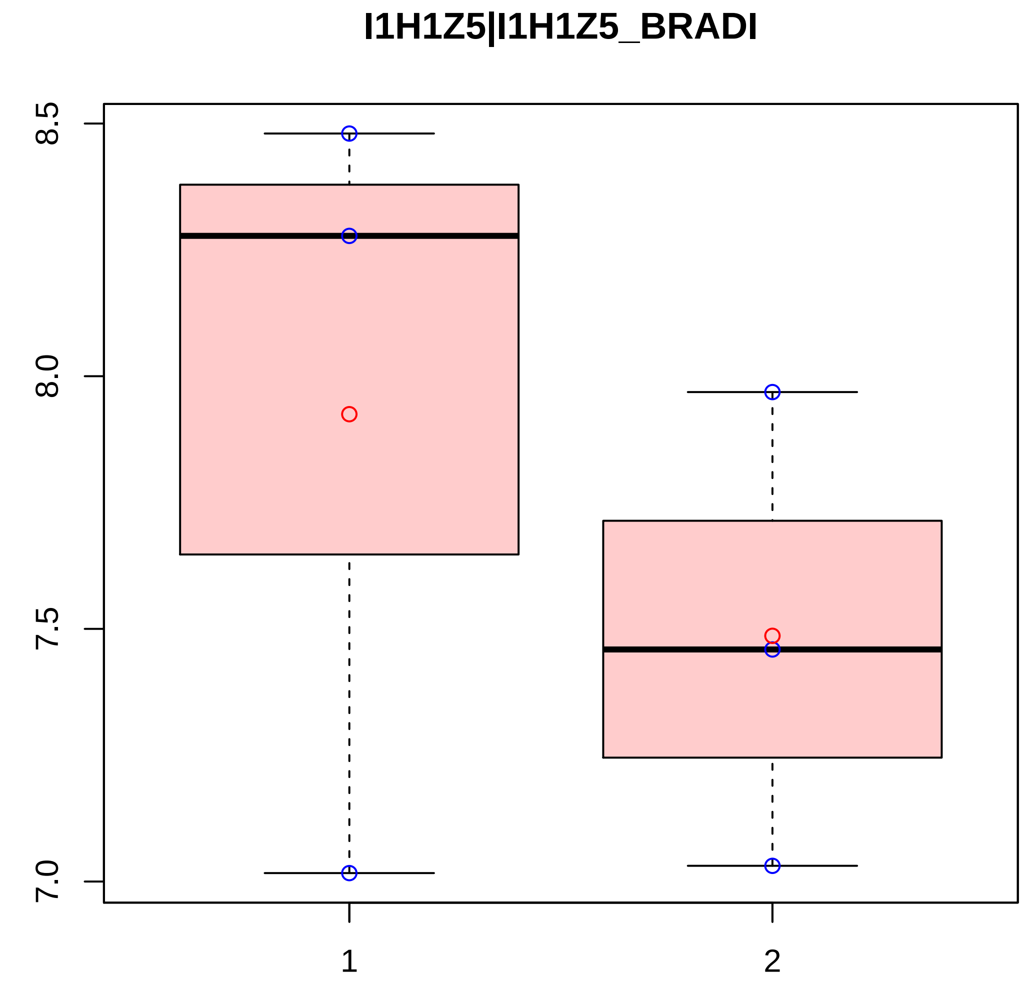

t−Test: p−value = 0.47

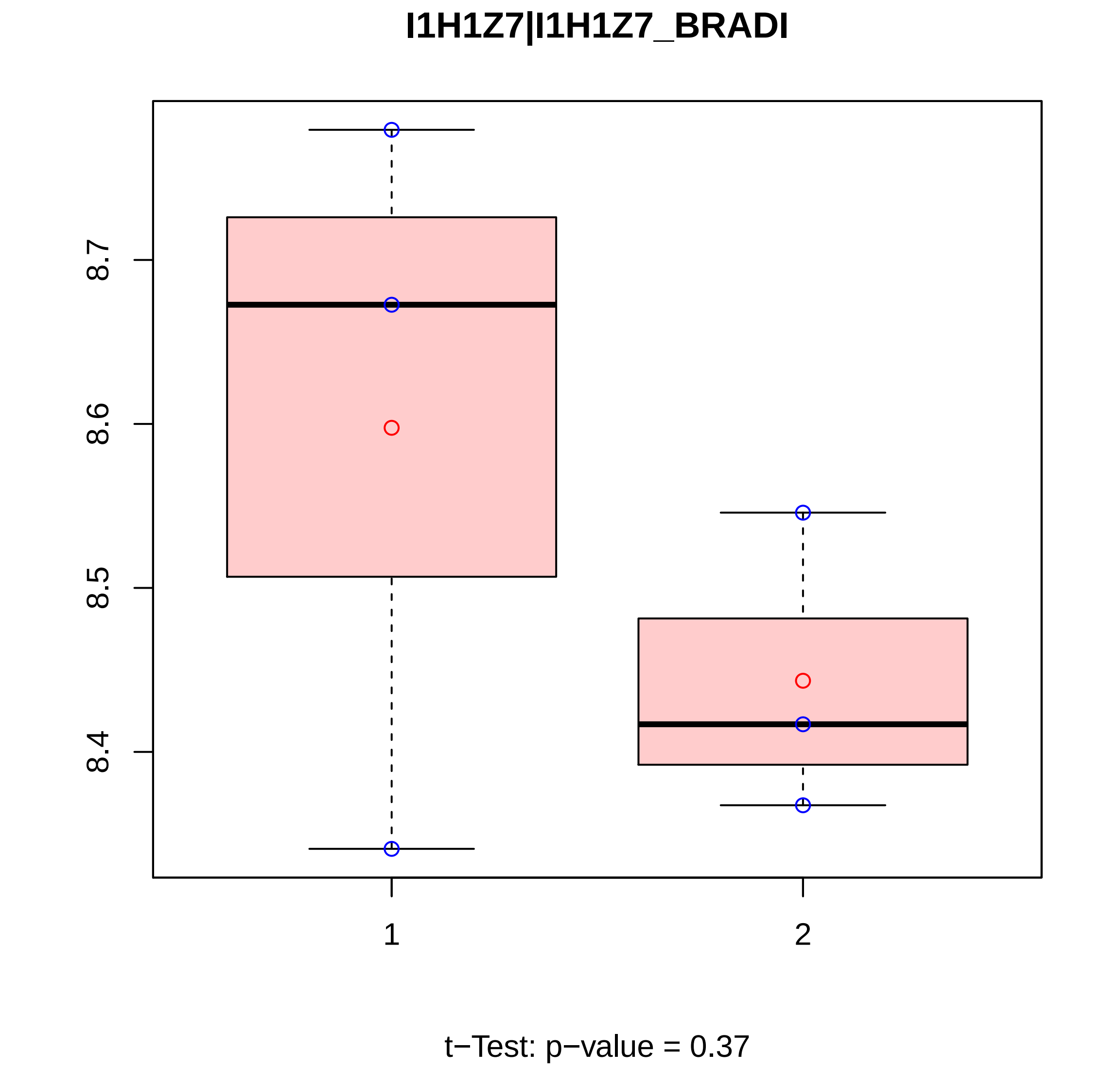
I1H1Z7|I1H1Z7_BRADI
8.4
8.5
8.6
8.7
1
2
t−Test: p−value = 0.37

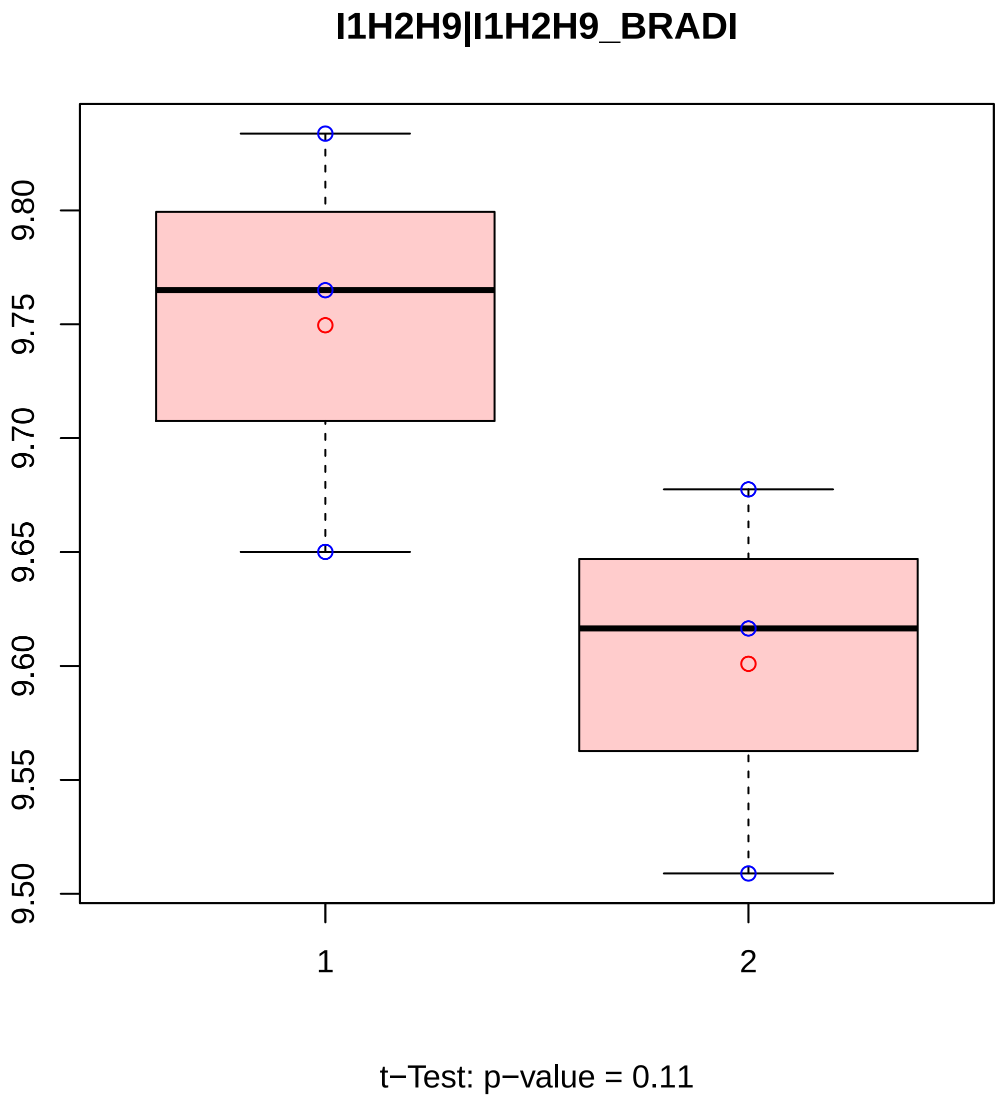
I1H2H9|I1H2H9_BRADI
9.50
9.55
9.60
9.65
9.70
9.75
9.80
1
2
t−Test: p−value = 0.11

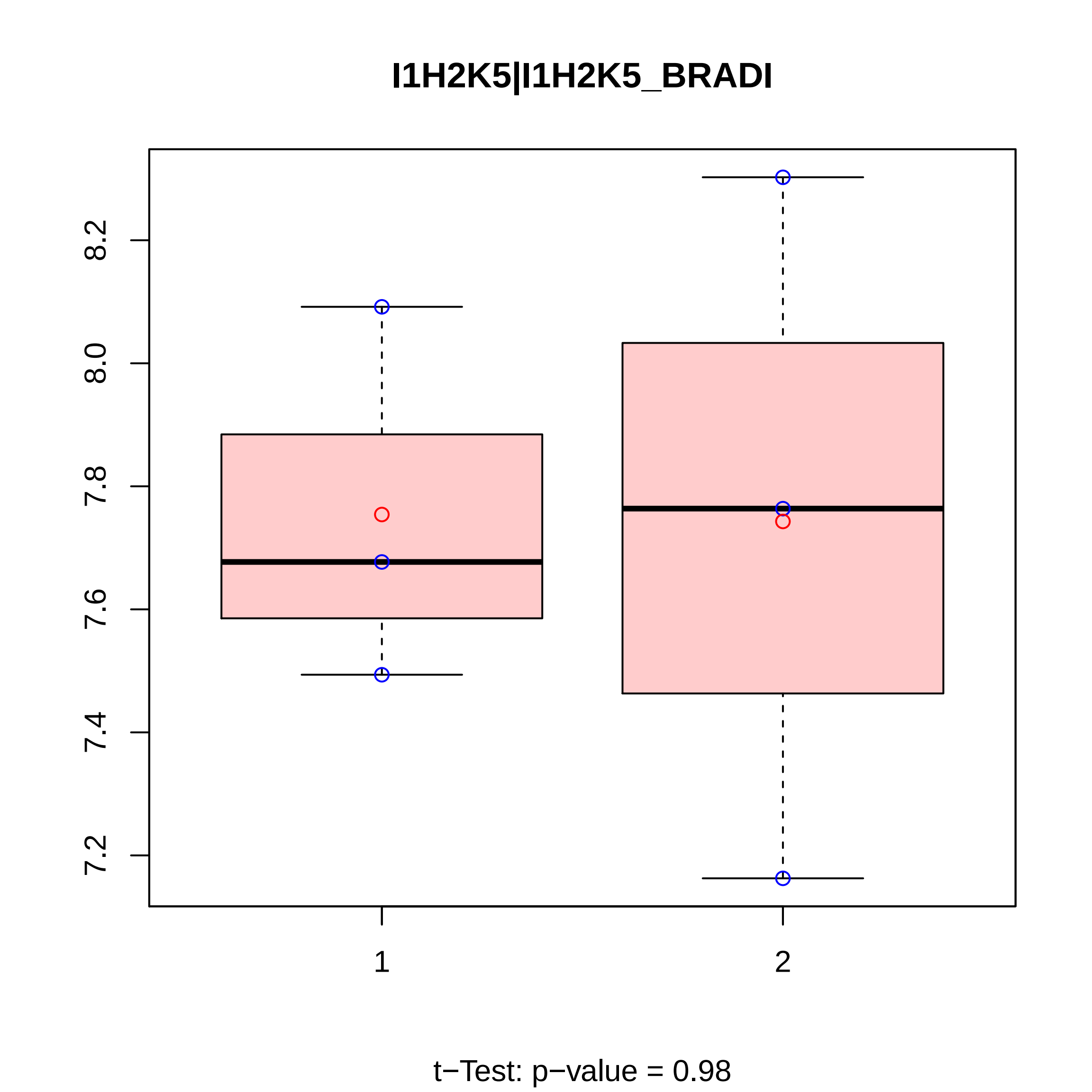
I1H2K5|I1H2K5_BRADI
8.2
8.0
7.8
7.6
7.4
7.2
1
2
t−Test: p−value = 0.98

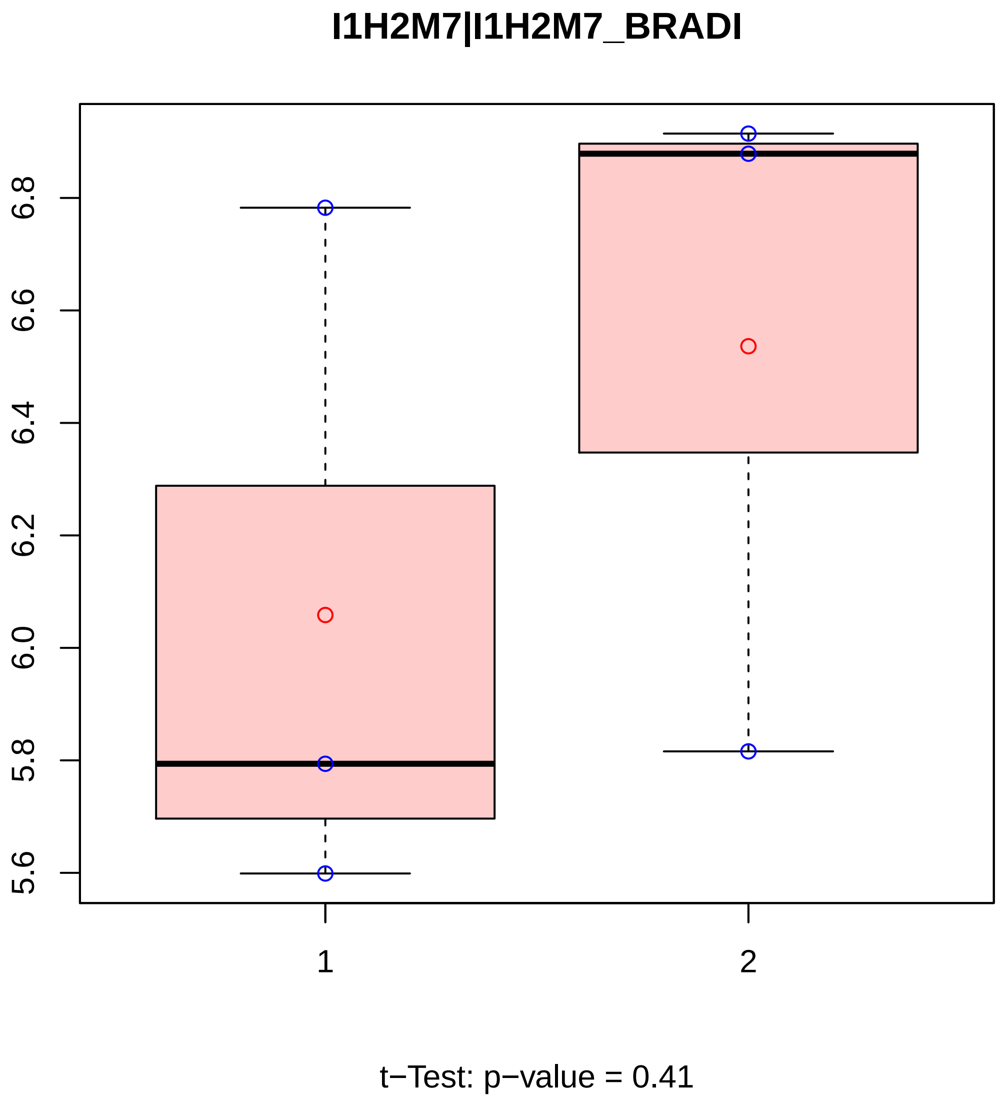
I1H2M7|I1H2M7_BRADI
6.8
6.6
6.4
6.2
6.0
5.8
5.6
1
2
t−Test: p−value = 0.41

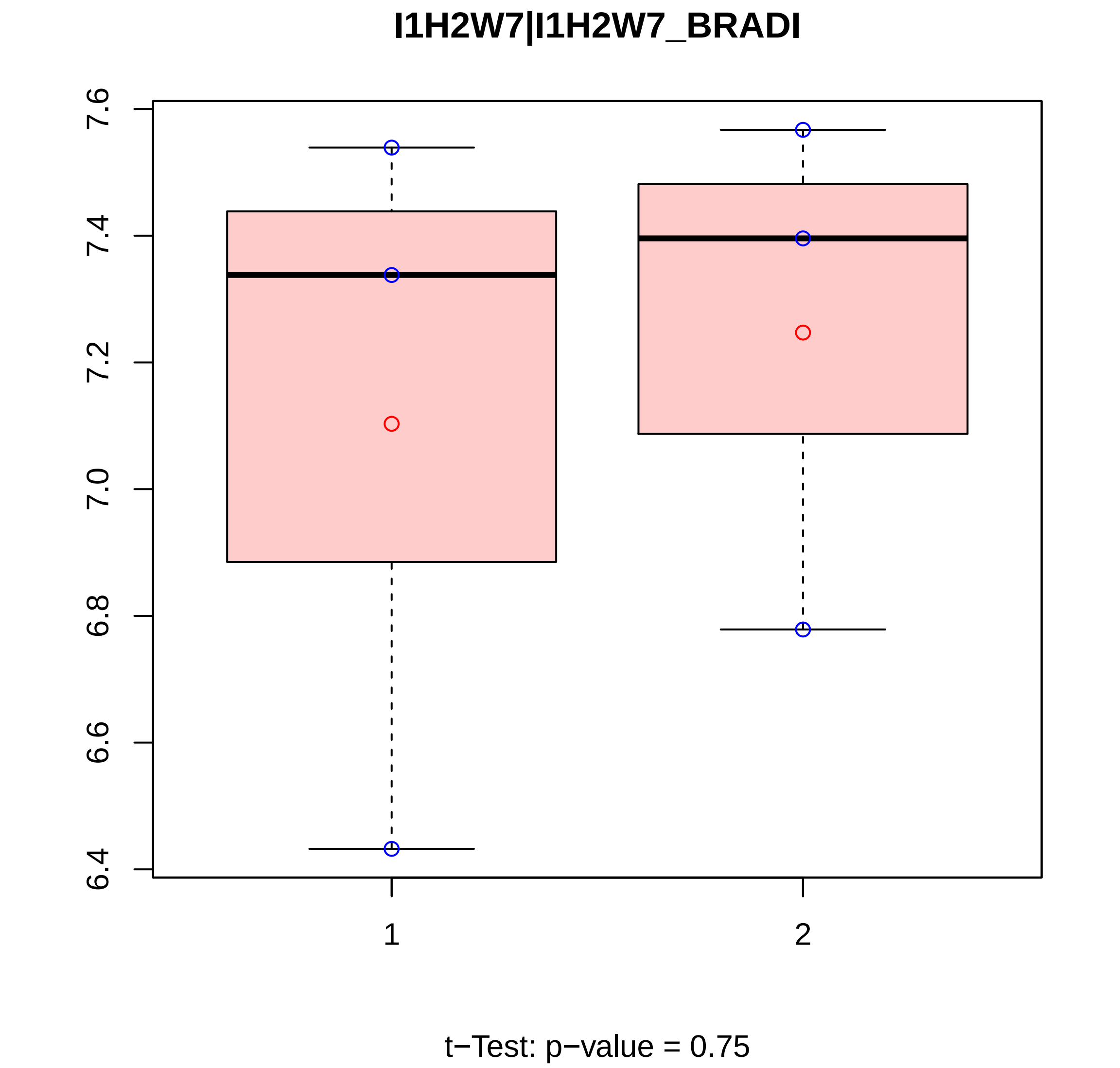
I1H2W7|I1H2W7_BRADI
7.6
7.4
7.2
7.0
6.8
6.6
6.4
1
2
t−Test: p−value = 0.75

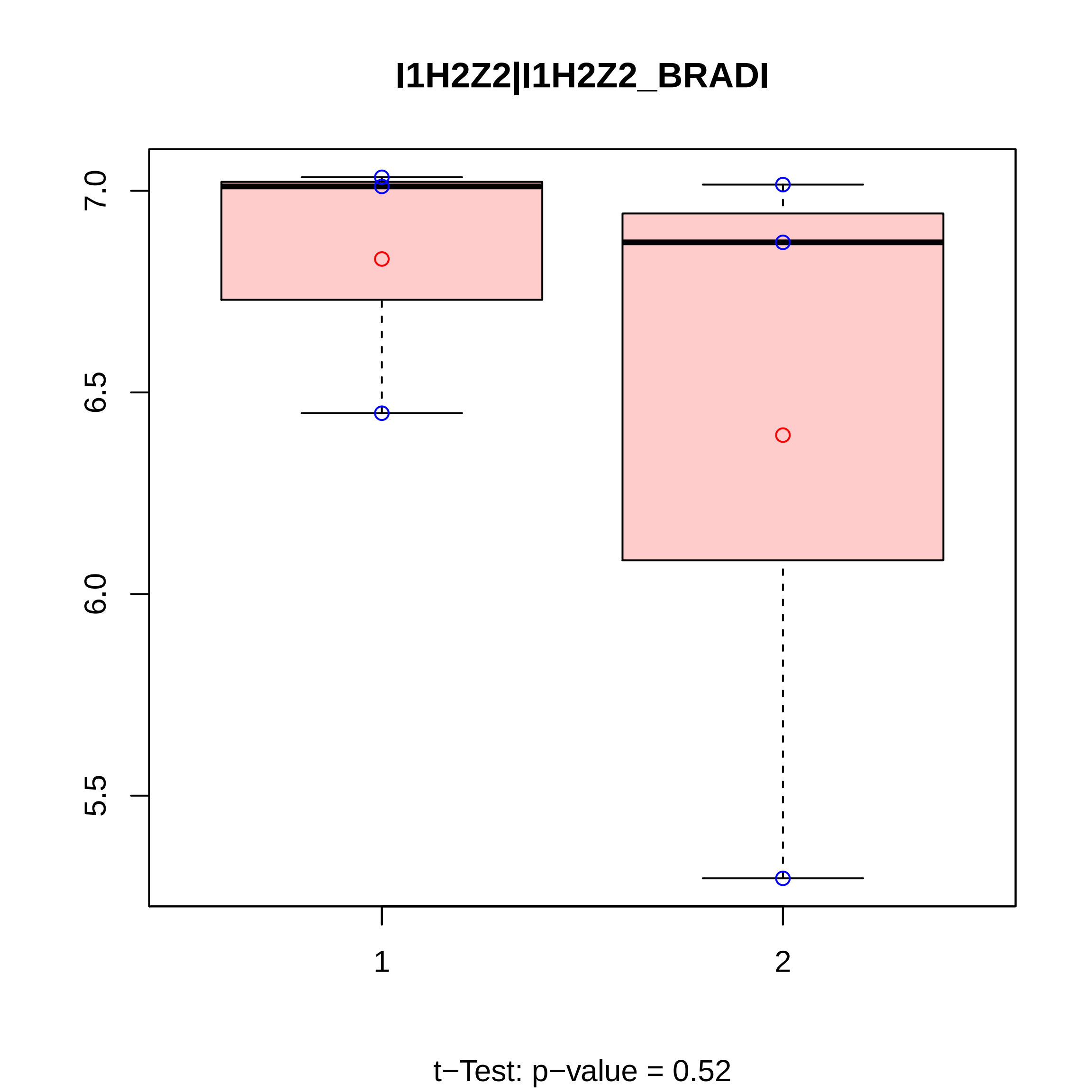
I1H2Z2|I1H2Z2_BRADI
7.0
6.5
6.0
5.5
1
2
t−Test: p−value = 0.52

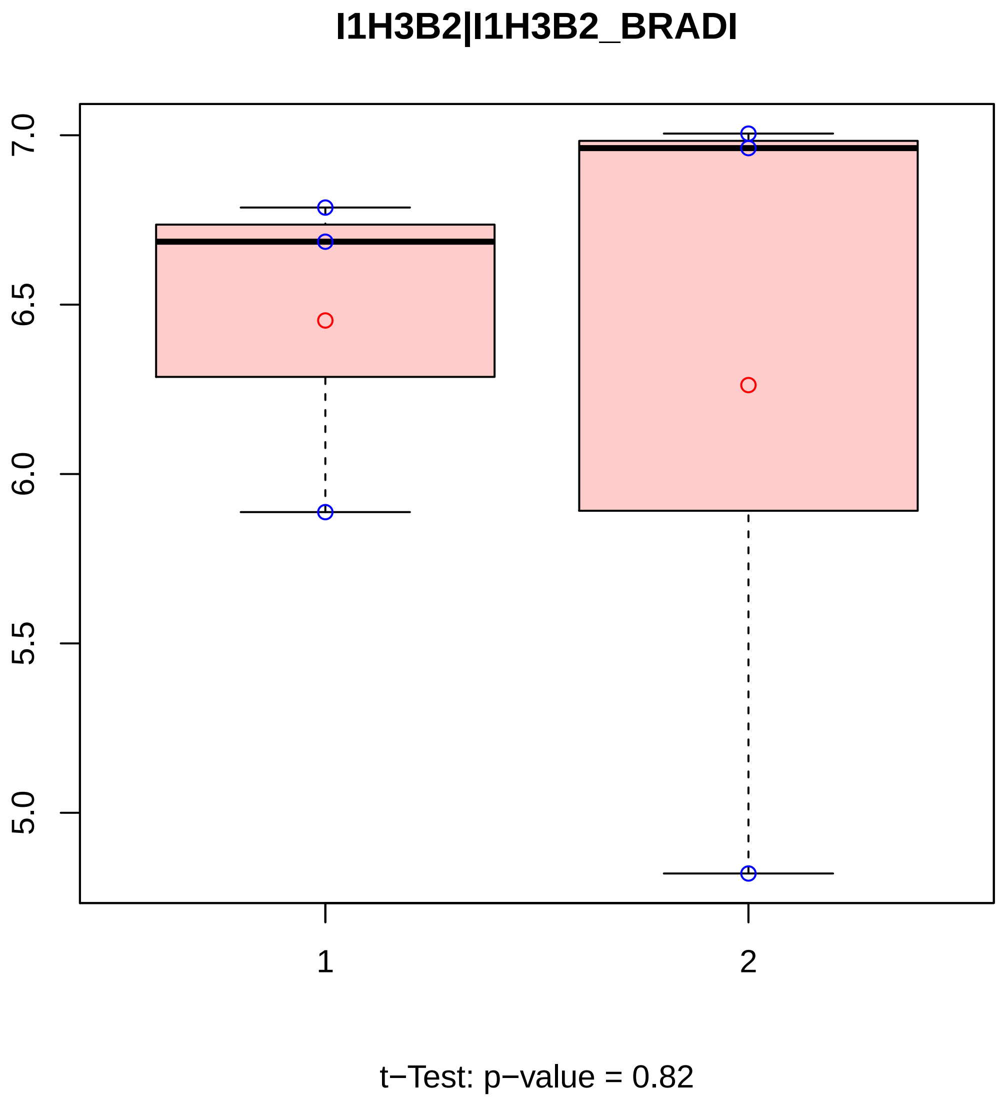
I1H3B2|I1H3B2_BRADI
7.0
6.5
6.0
5.5
5.0
1
2
t−Test: p−value = 0.82

I1H3F3|I1H3F3_BRADI

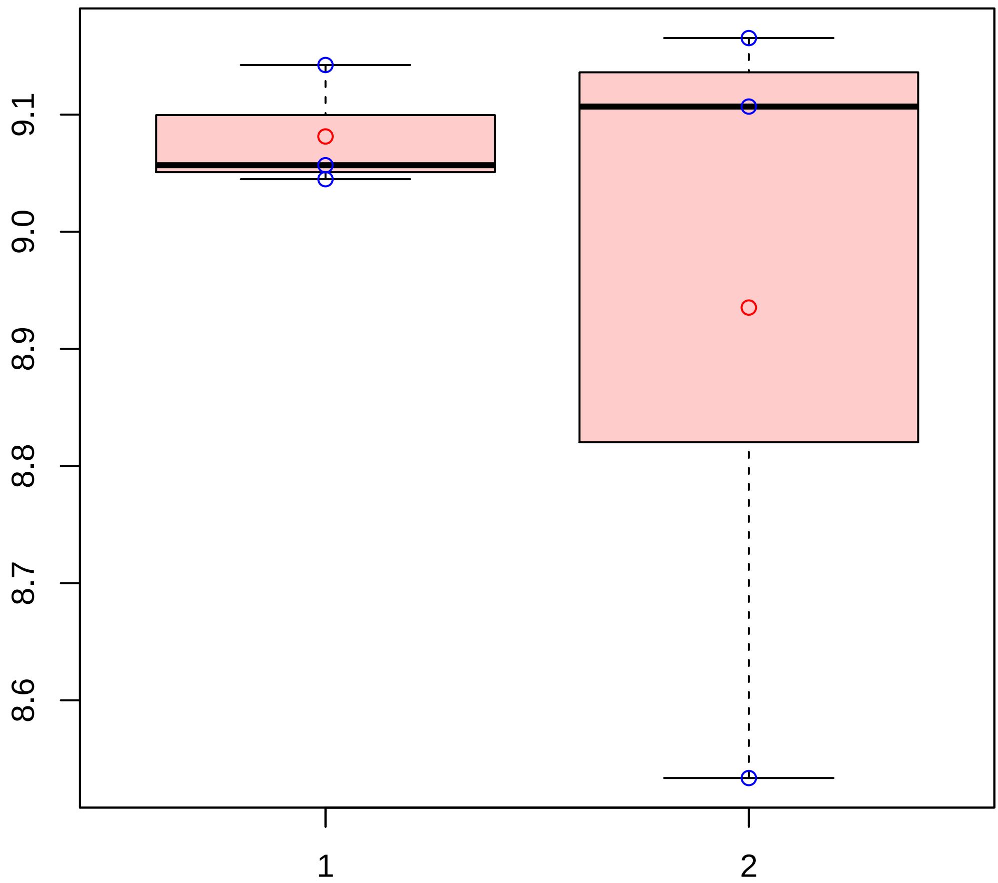

t−Test: p−value = 0.55

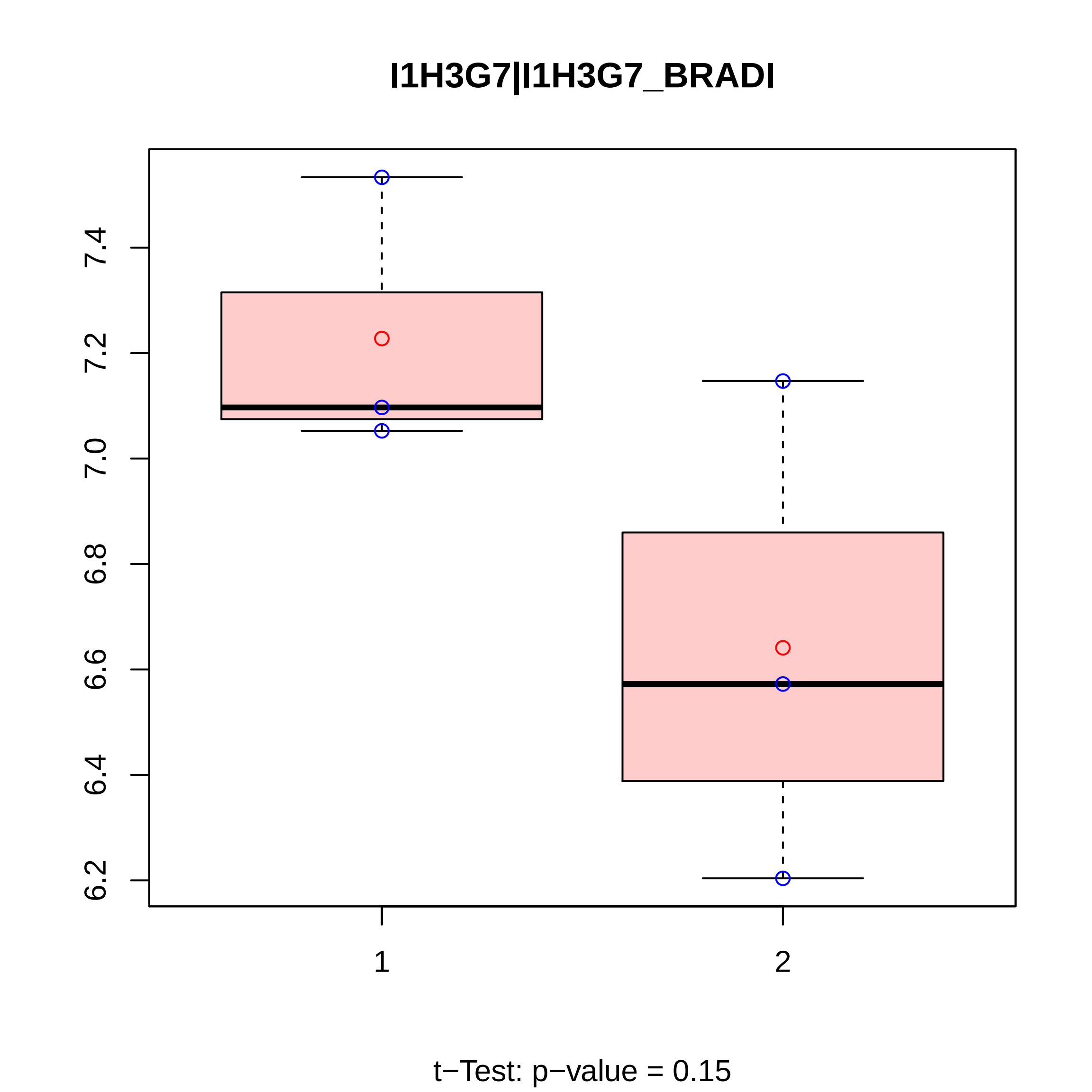
I1H3G7|I1H3G7_BRADI
7.4
7.2
7.0
6.8
6.6
6.4
6.2
1
2
t−Test: p−value = 0.15

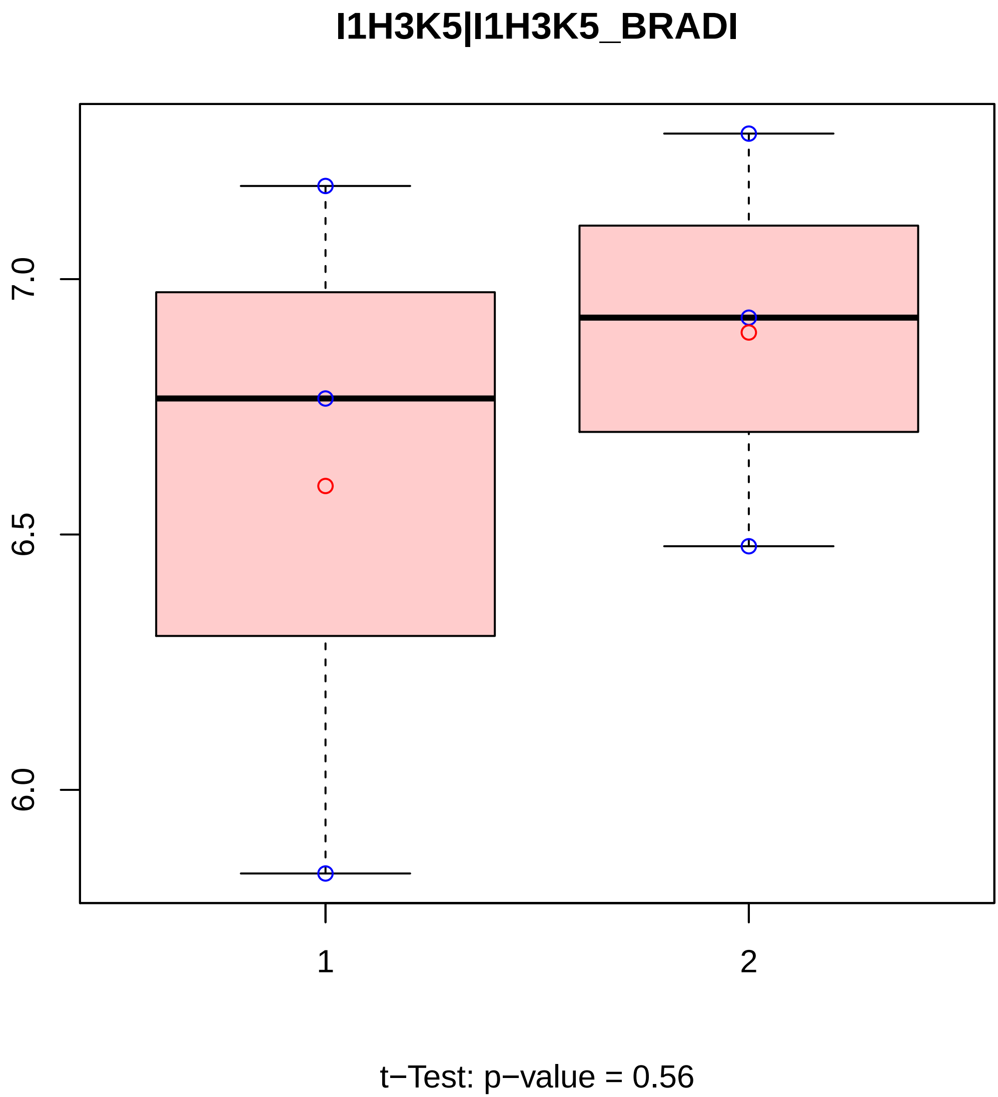
I1H3K5|I1H3K5_BRADI
7.0
6.5
6.0
1
2
t−Test: p−value = 0.56

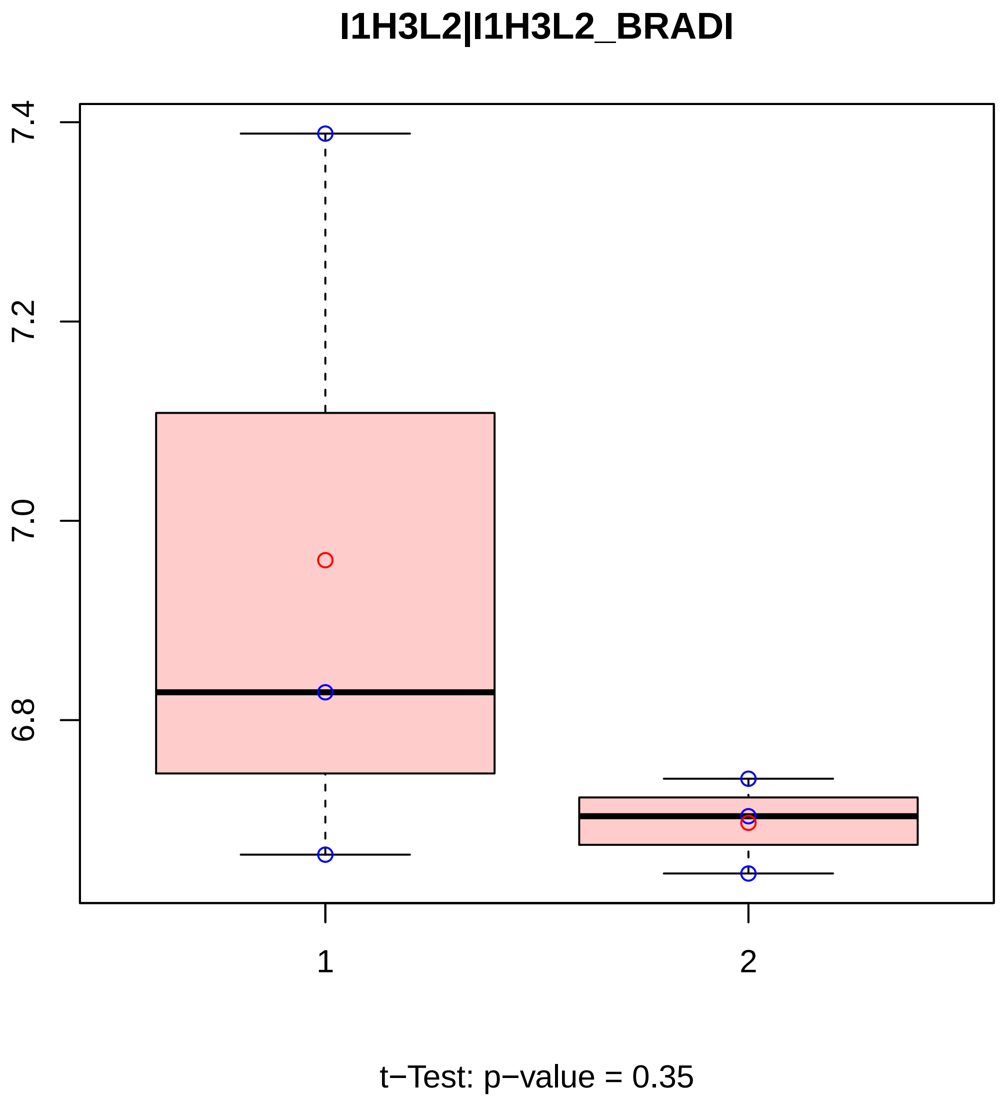
I1H3L2|I1H3L2_BRADI
7.4
7.2
7.0
6.8
1
2
t−Test: p−value = 0.35

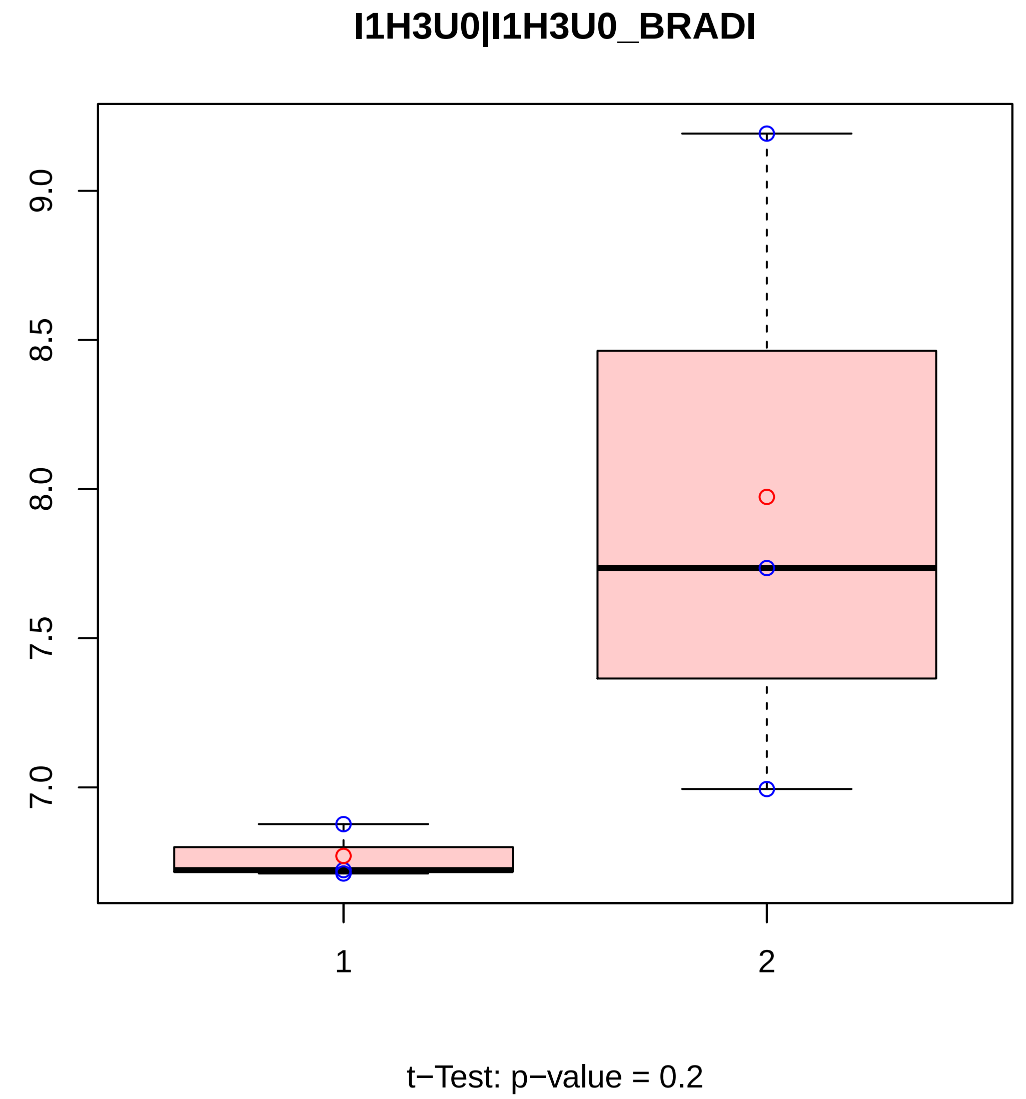
I1H3U0|I1H3U0_BRADI
9.0
8.5
8.0
7.5
7.0
1
2
t−Test: p−value = 0.2

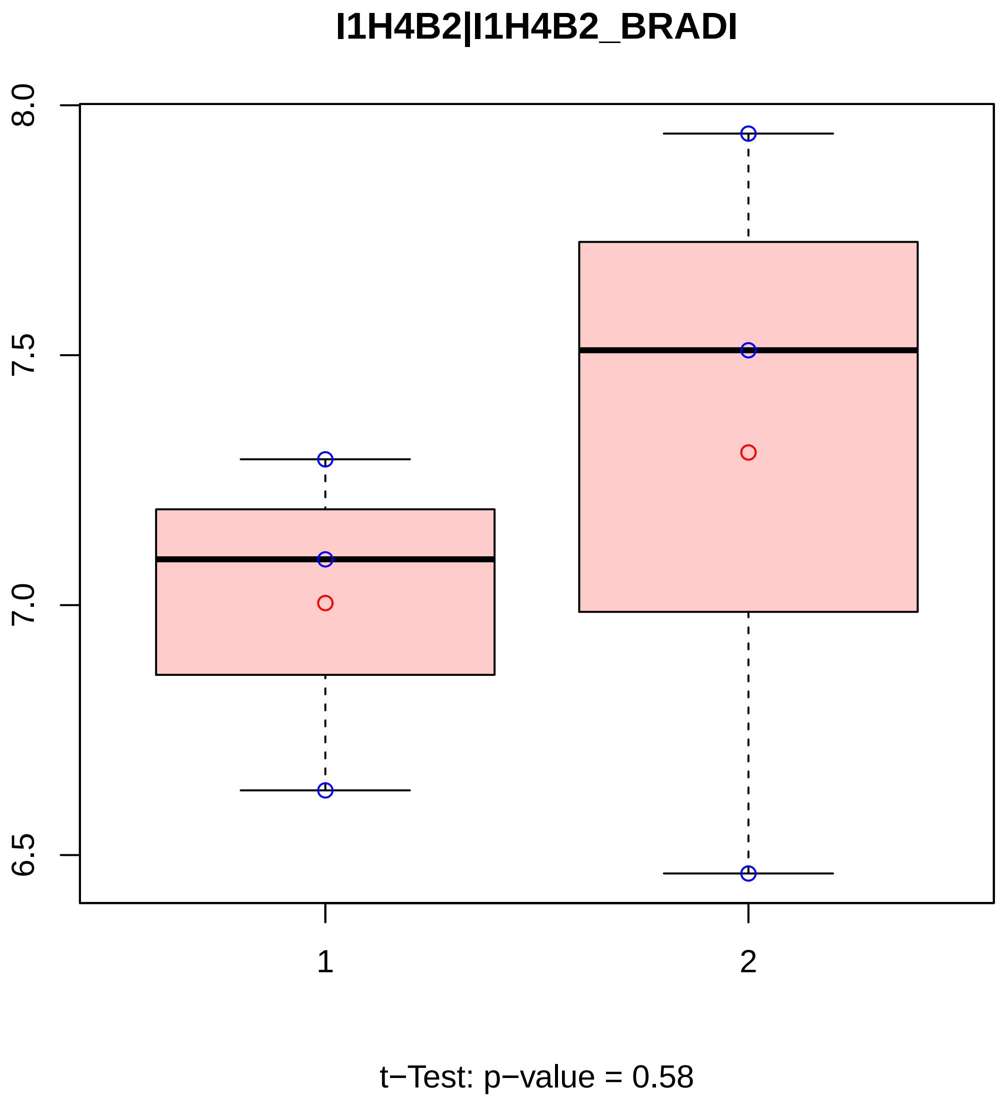

I1H4B2|I1H4B2_BRADI
8.0
7.5
7.0
6.5
1
2
t−Test: p−value = 0.58

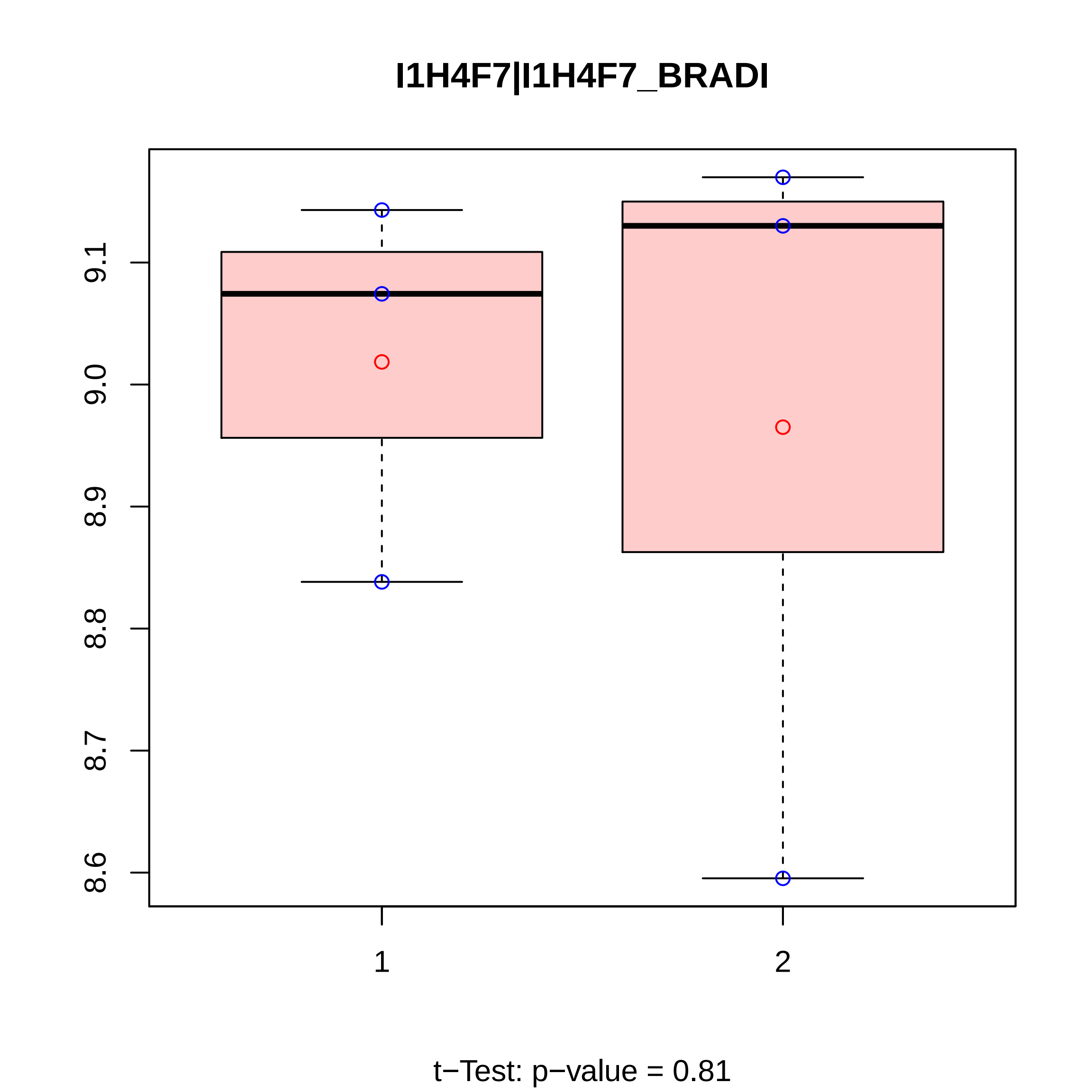
I1H4F7|I1H4F7_BRADI
9.1
9.0
8.9
8.8
8.7
8.6
1
2
t−Test: p−value = 0.81

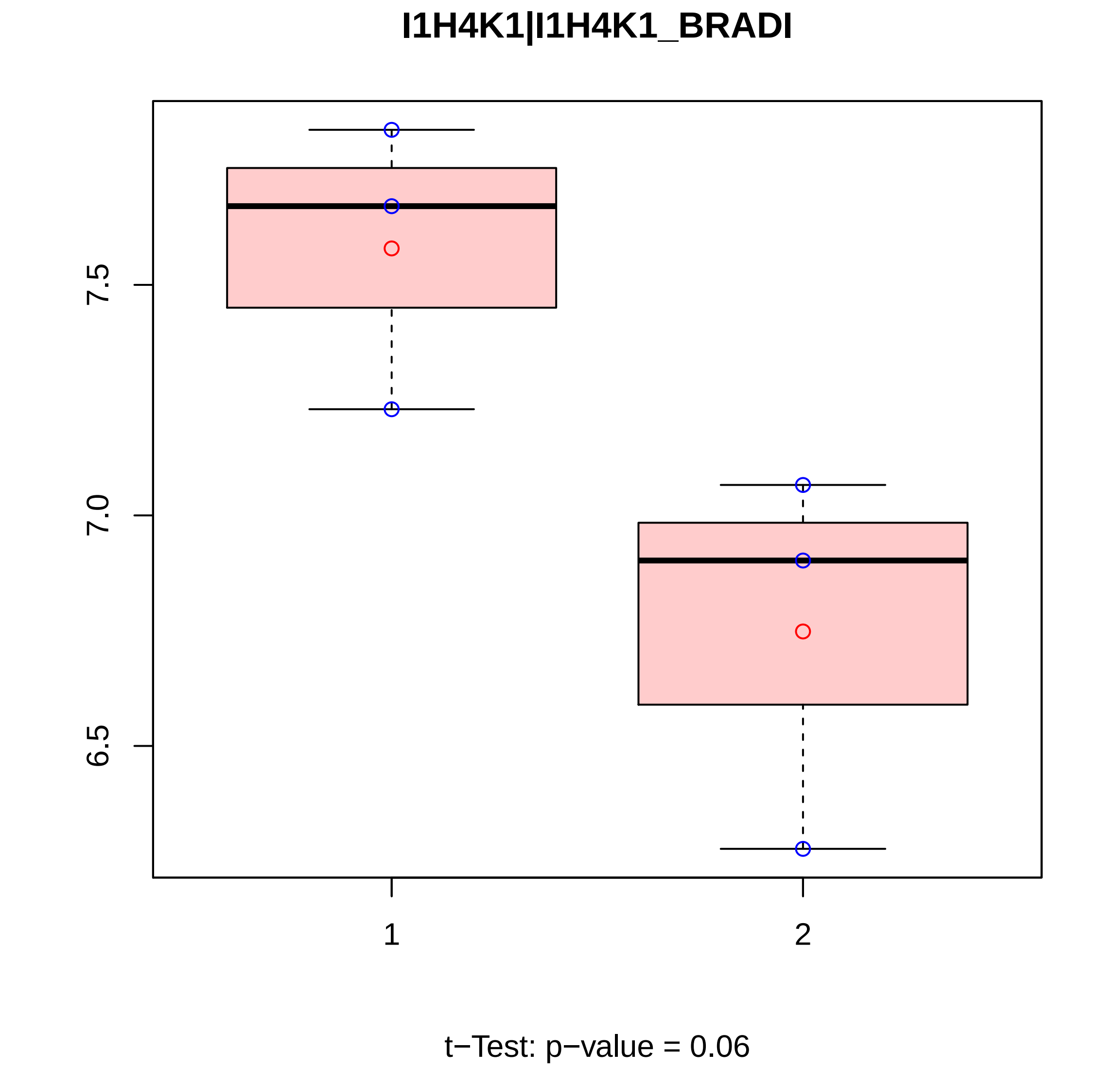
I1H4K1|I1H4K1_BRADI
7.5
7.0
6.5
1
2
t−Test: p−value = 0.06

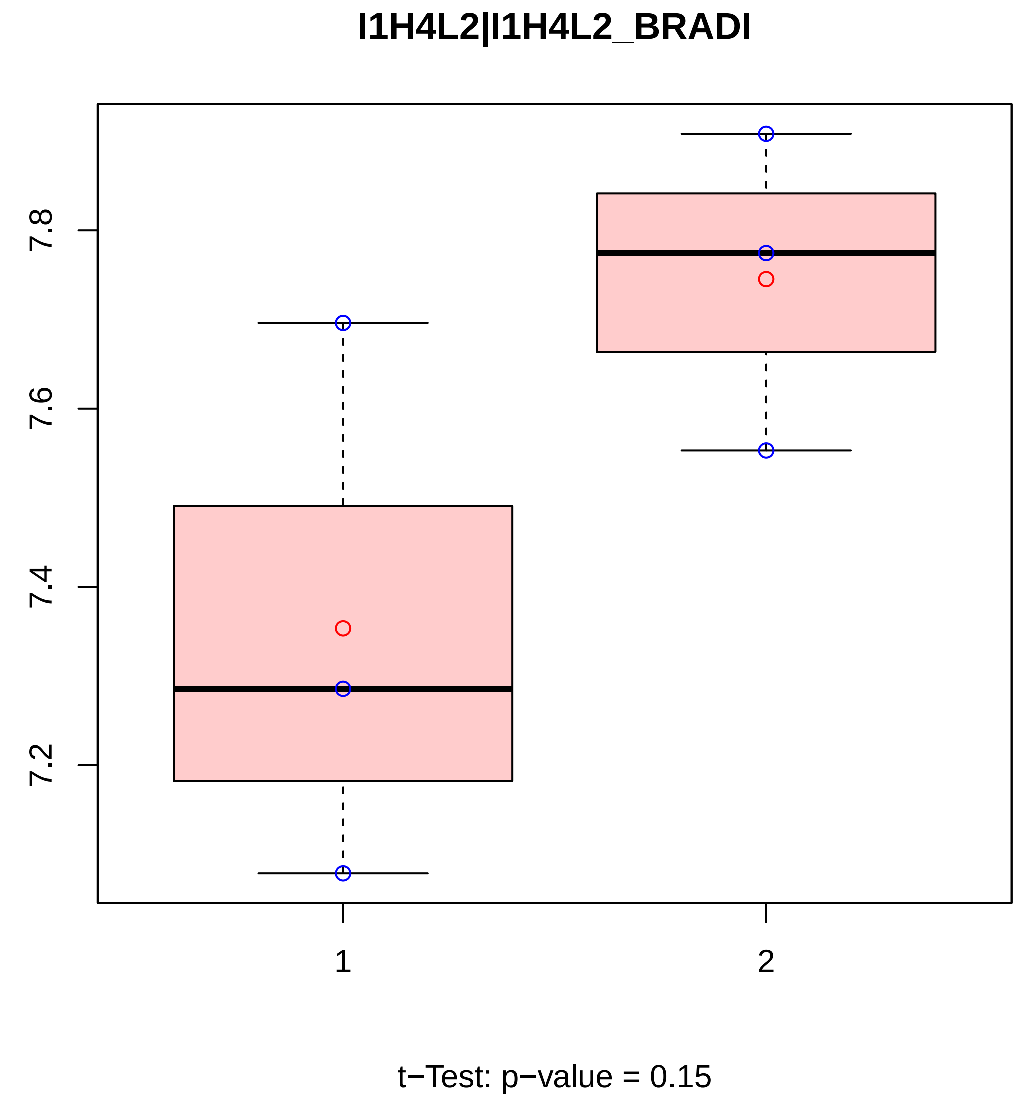

I1H4L2|I1H4L2_BRADI
7.2
7.4
7.6
7.8
1
2
t−Test: p−value = 0.15

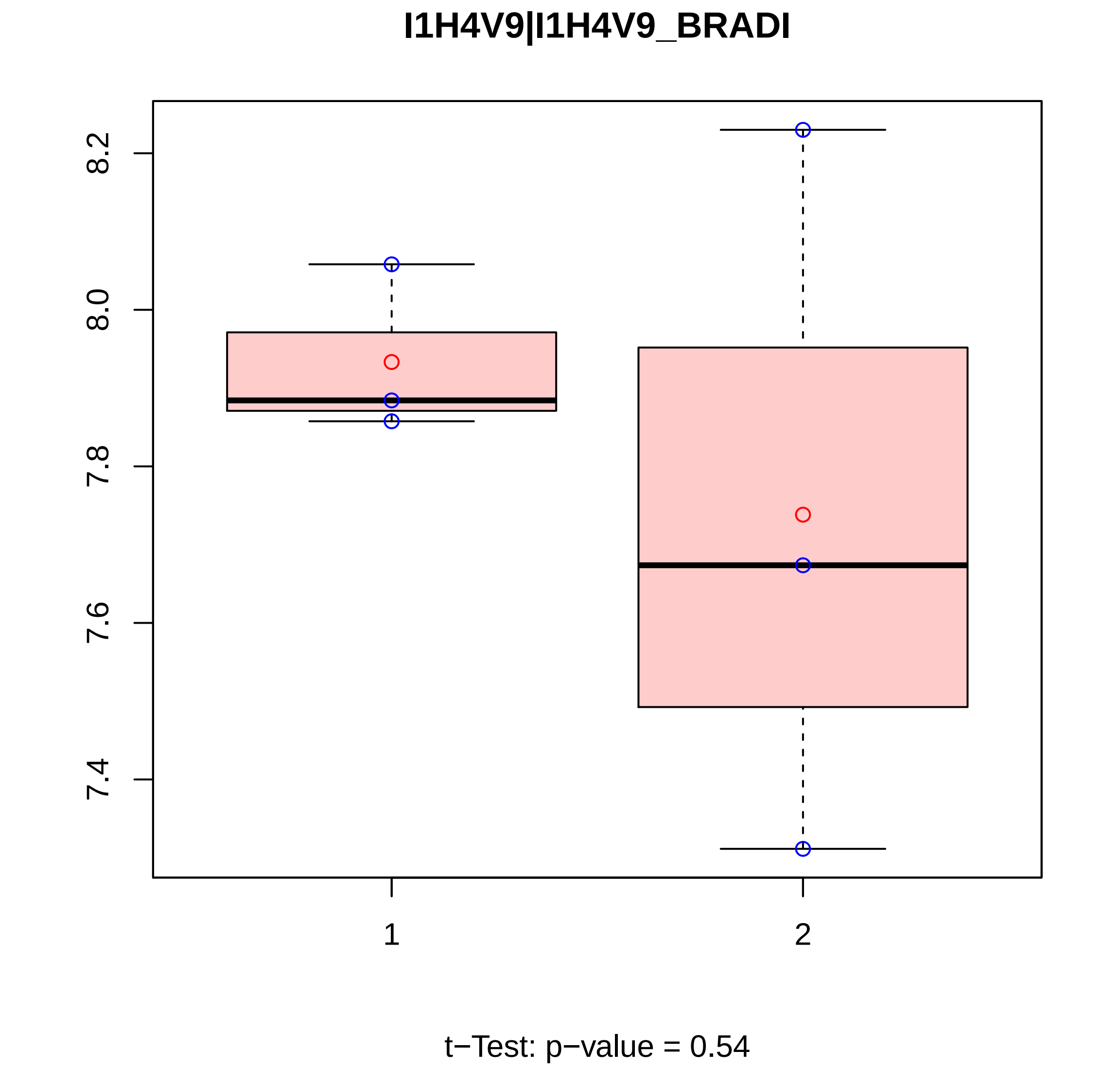
I1H4V9|I1H4V9_BRADI
8.2
8.0
7.8
7.6
7.4
1
2
t−Test: p−value = 0.54

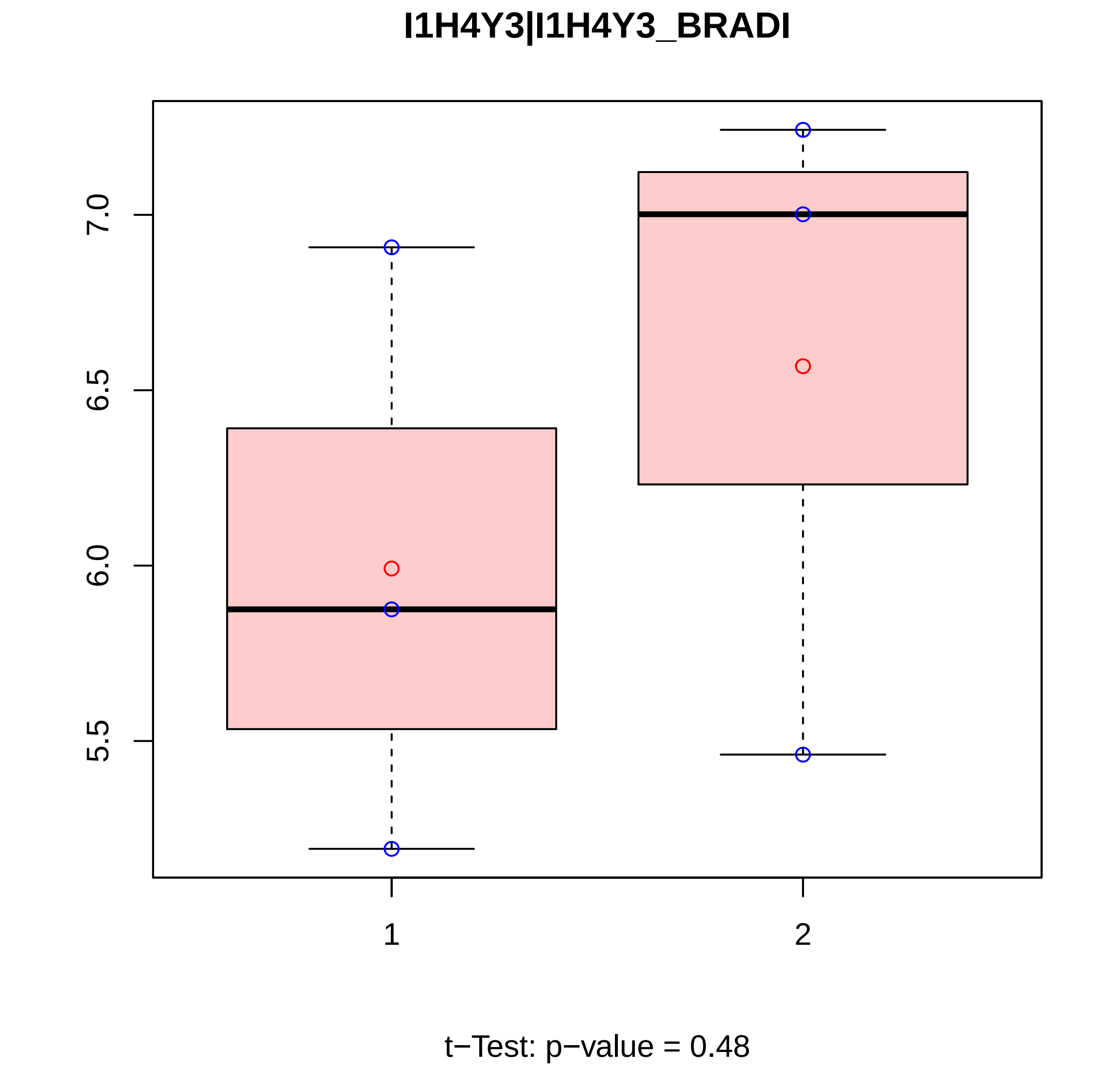

I1H4Y3|I1H4Y3_BRADI
7.0
6.5
6.0
5.5
1
2
t−Test: p−value = 0.48

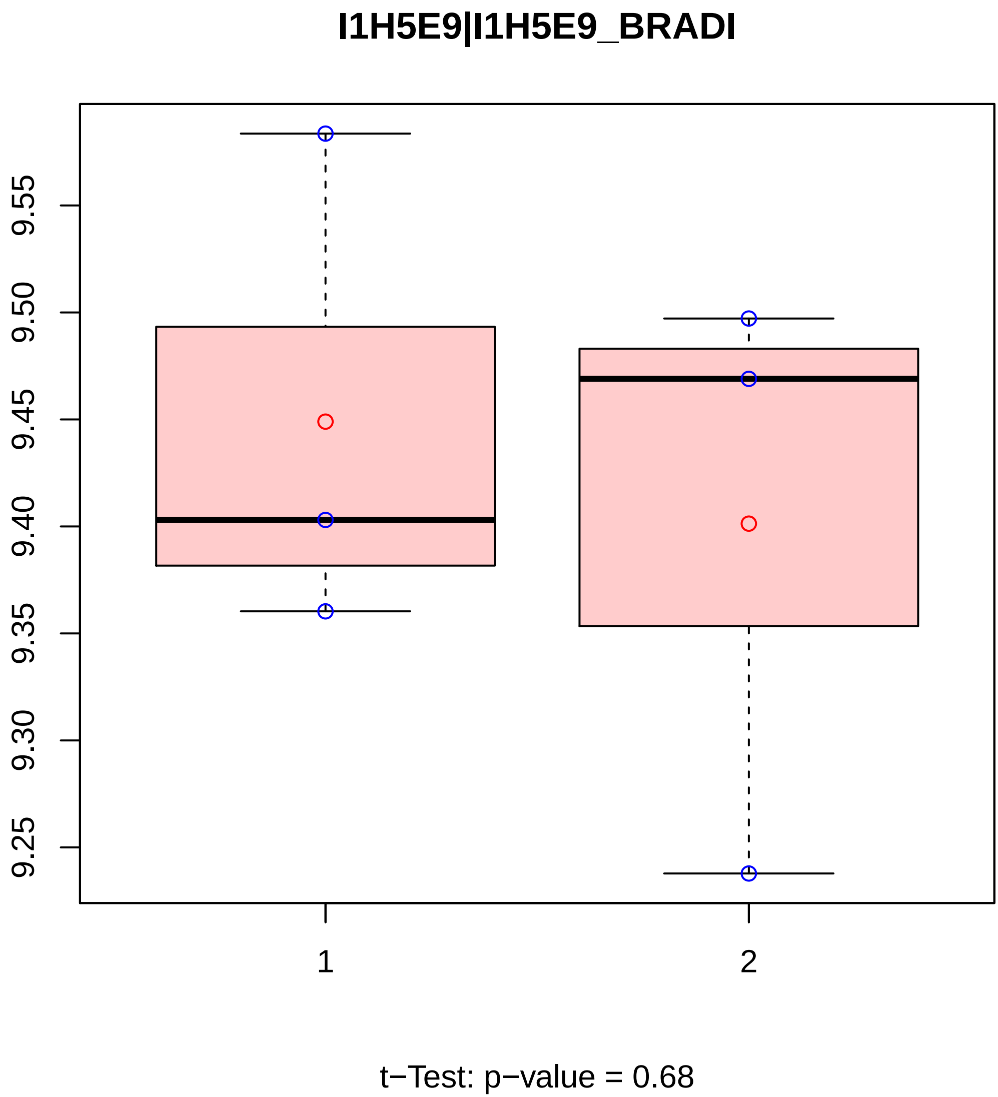
I1H5E9|I1H5E9_BRADI
9.25
9.30
9.35
9.40
9.45
9.50
9.55
1
2
t−Test: p−value = 0.68

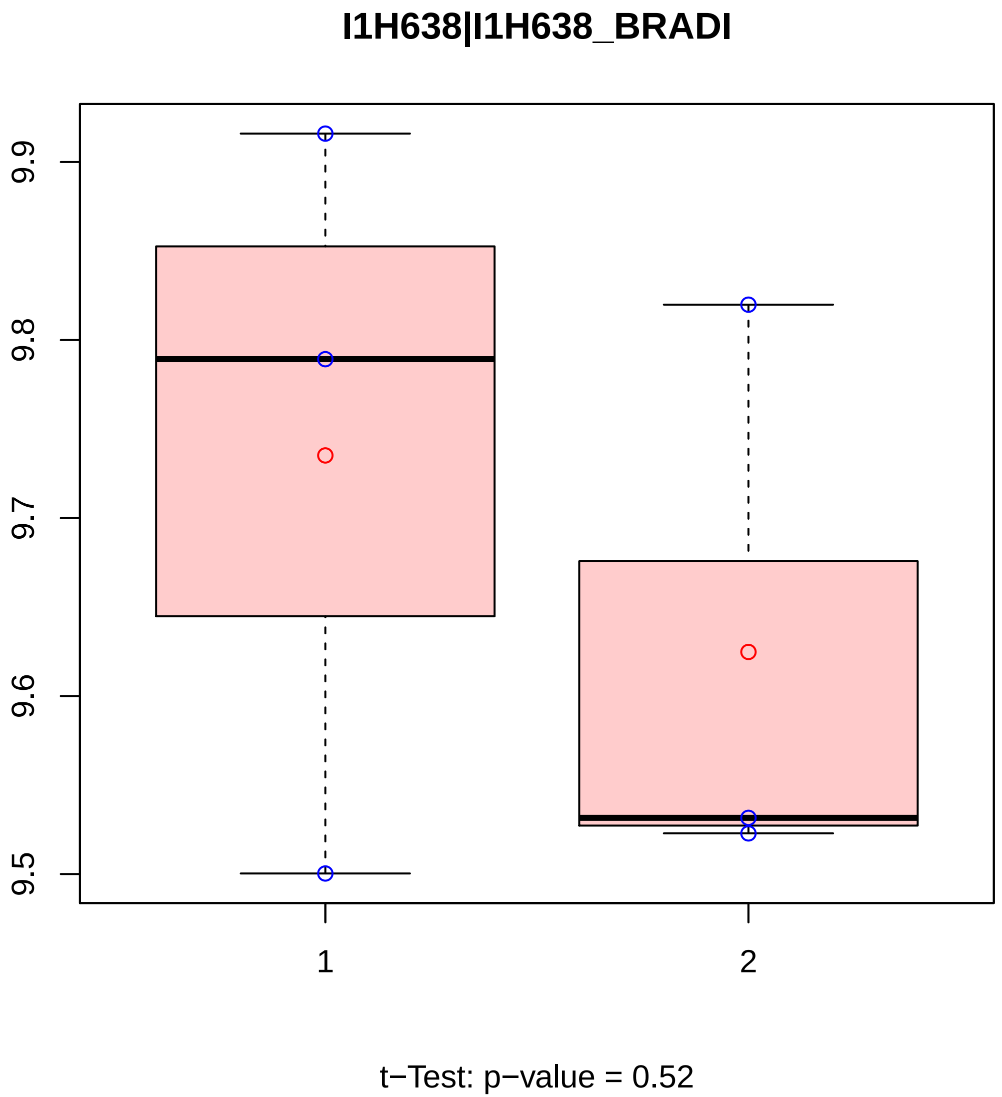

I1H638|I1H638_BRADI
9.5
9.6
9.7
9.8
9.9
1
2
t−Test: p−value = 0.52

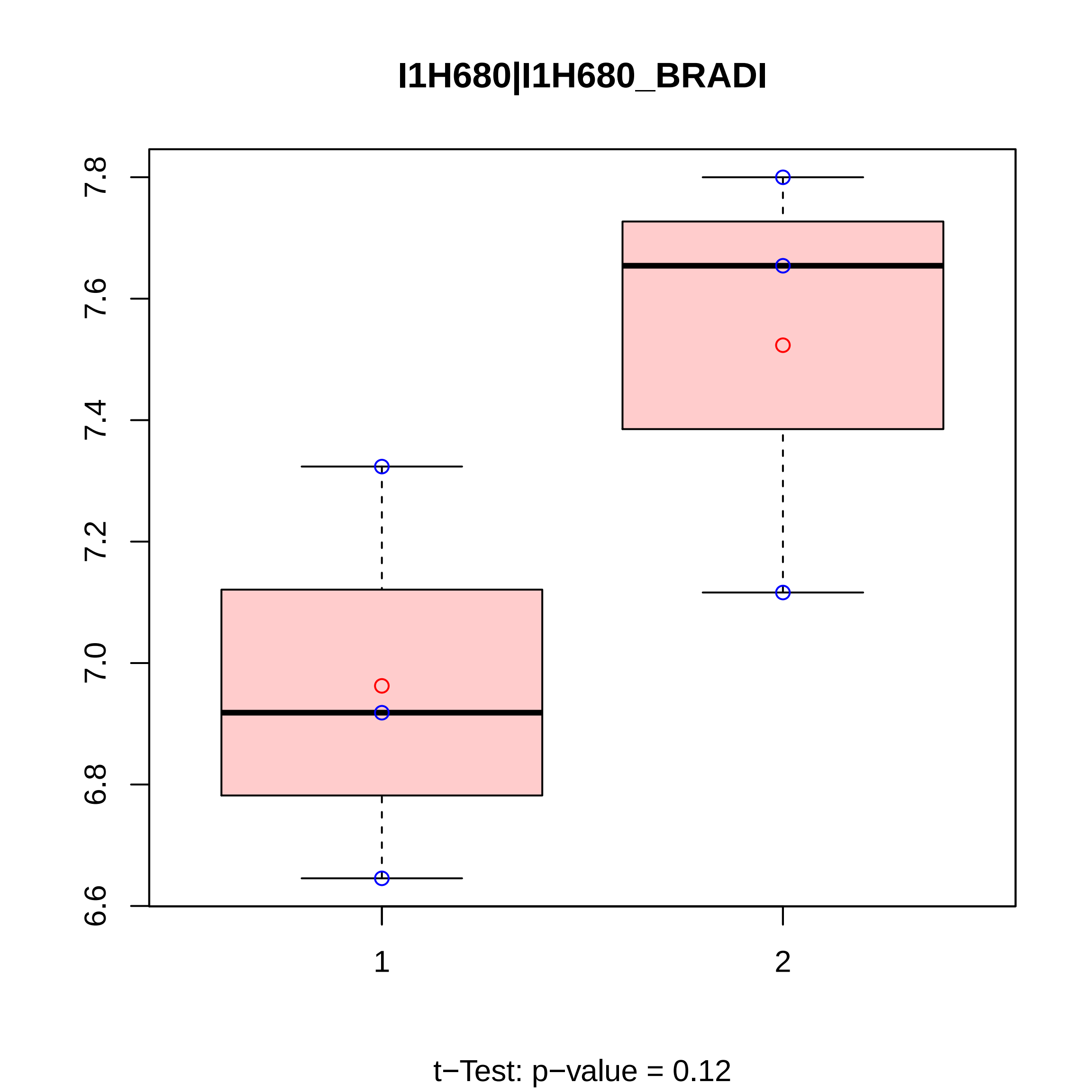

I1H680|I1H680_BRADI
7.8
7.6
7.4
7.2
7.0
6.8
6.6
1
2
t−Test: p−value = 0.12

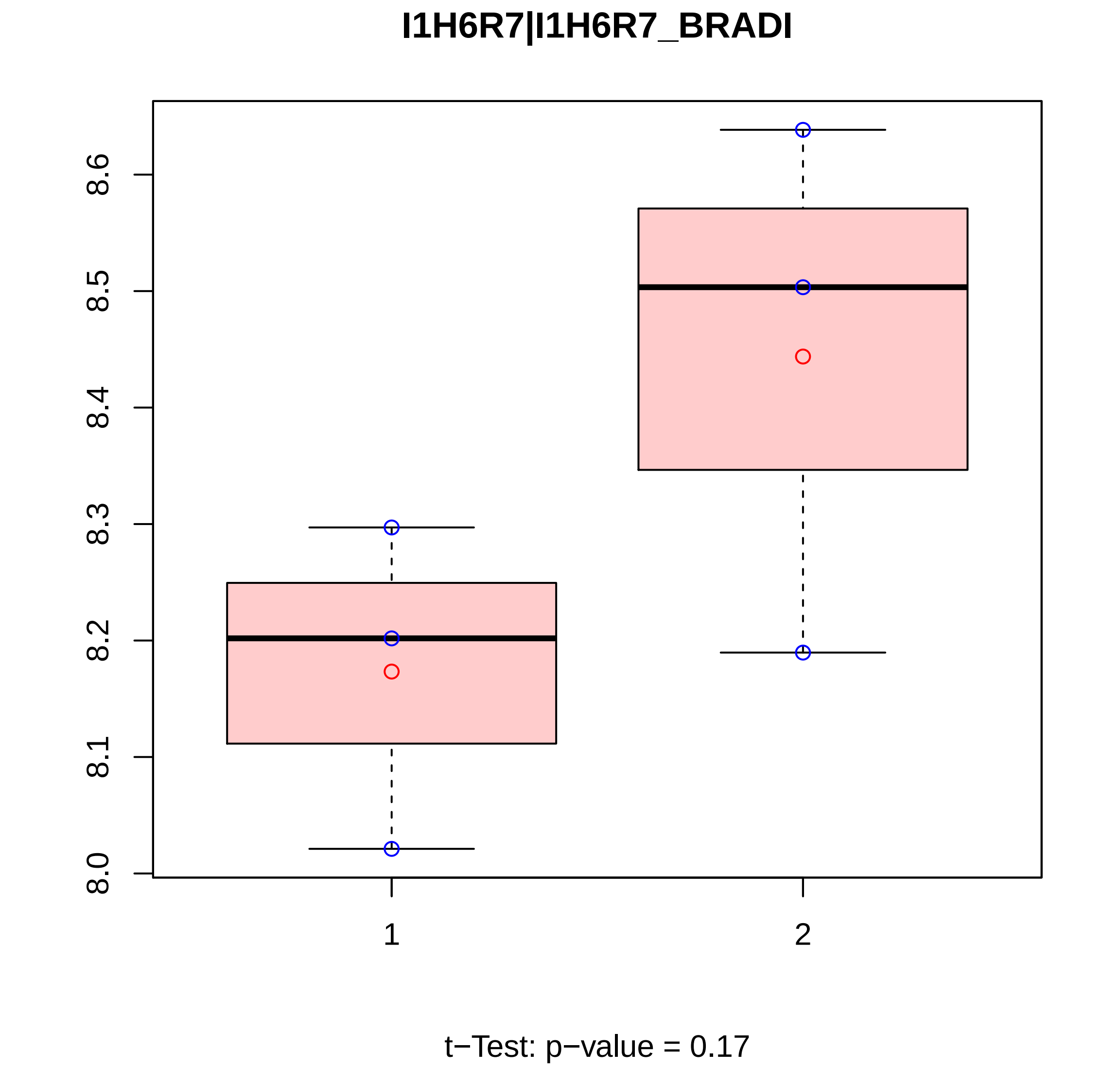
I1H6R7|I1H6R7_BRADI
8.0
8.1
8.2
8.3
8.4
8.5
8.6
1
2
t−Test: p−value = 0.17

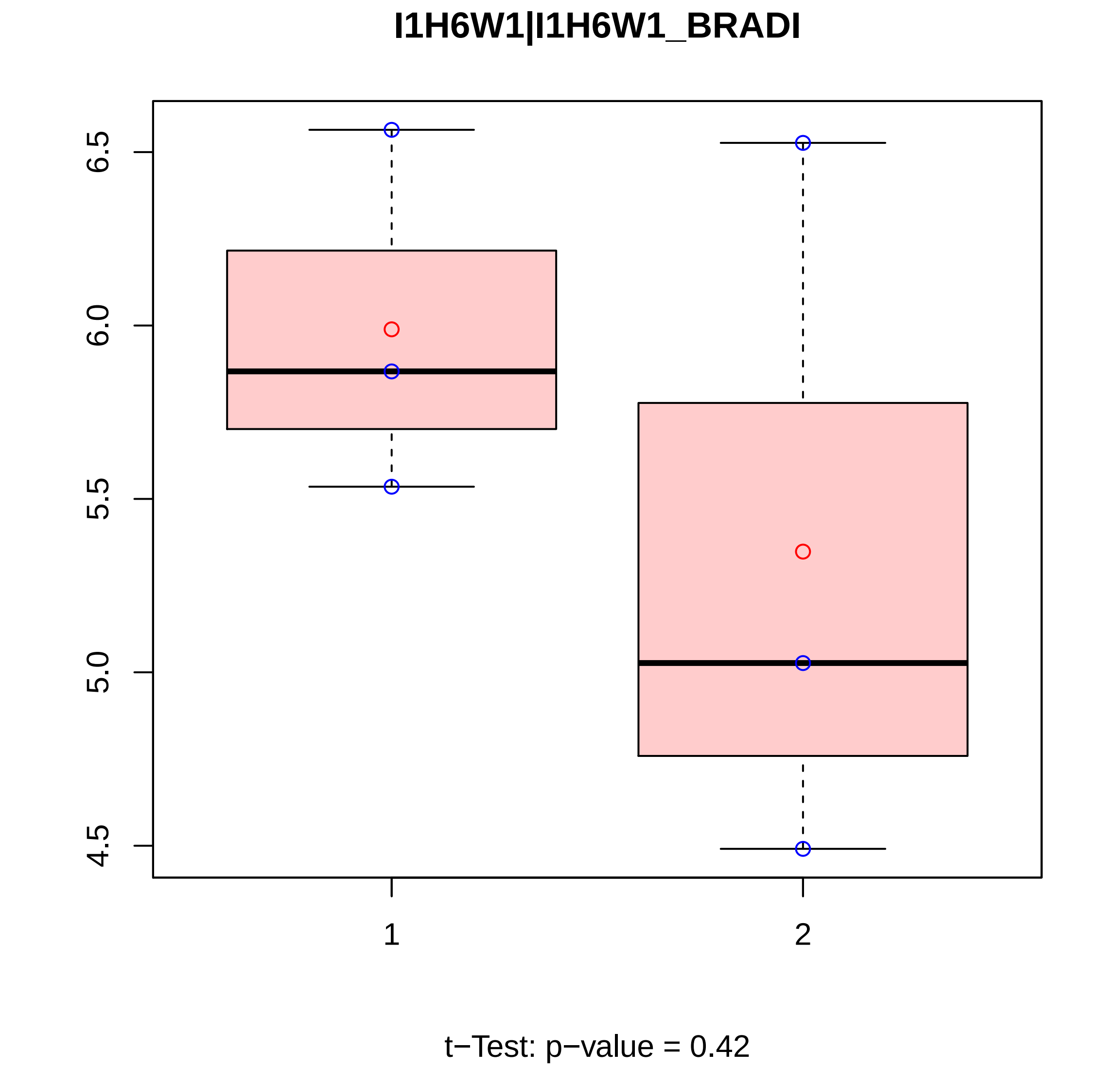
I1H6W1|I1H6W1_BRADI
6.5
6.0
5.5
5.0
4.5
1
2
t−Test: p−value = 0.42

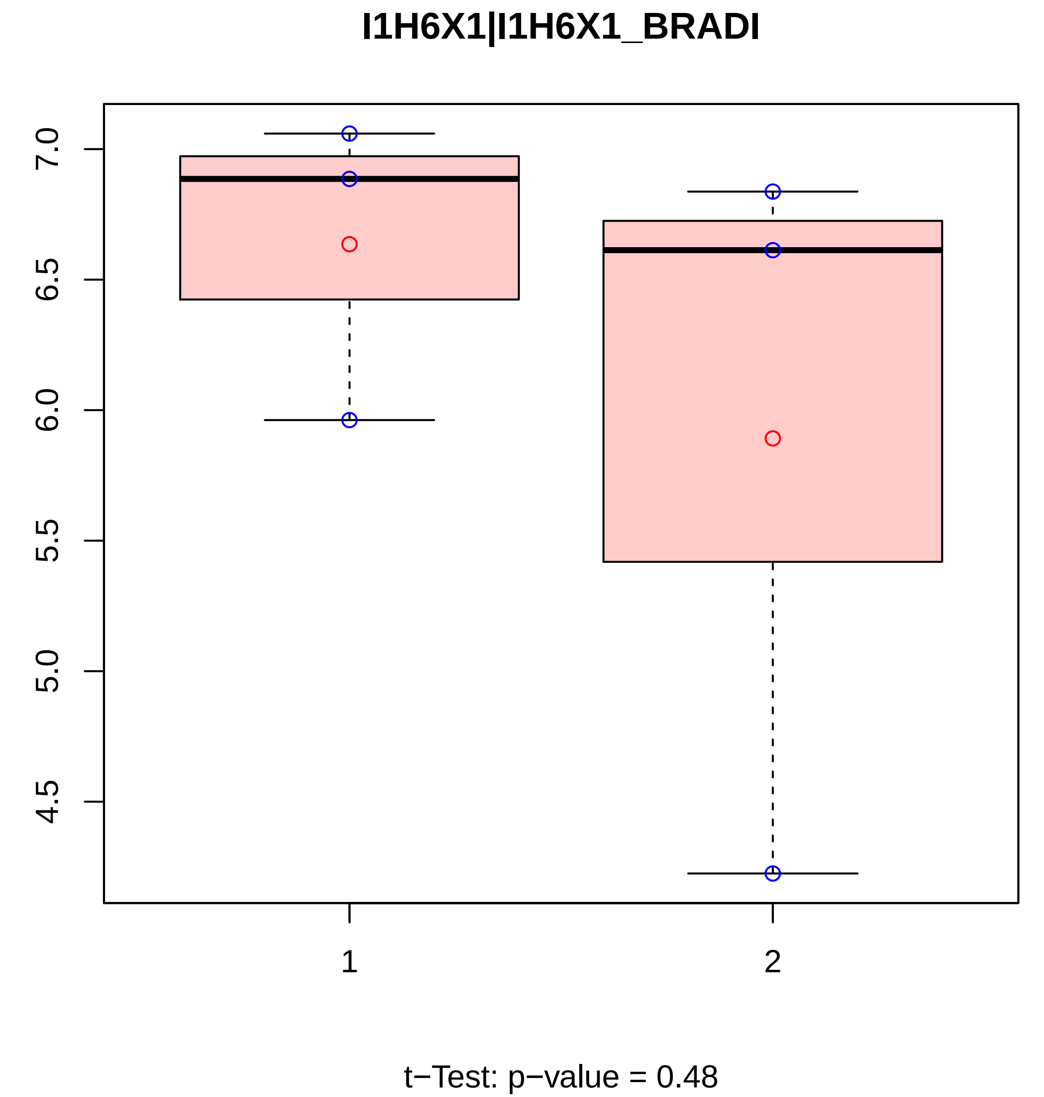
I1H6X1|I1H6X1_BRADI
7.0
6.5
6.0
5.5
5.0
4.5
1
2
t−Test: p−value = 0.48

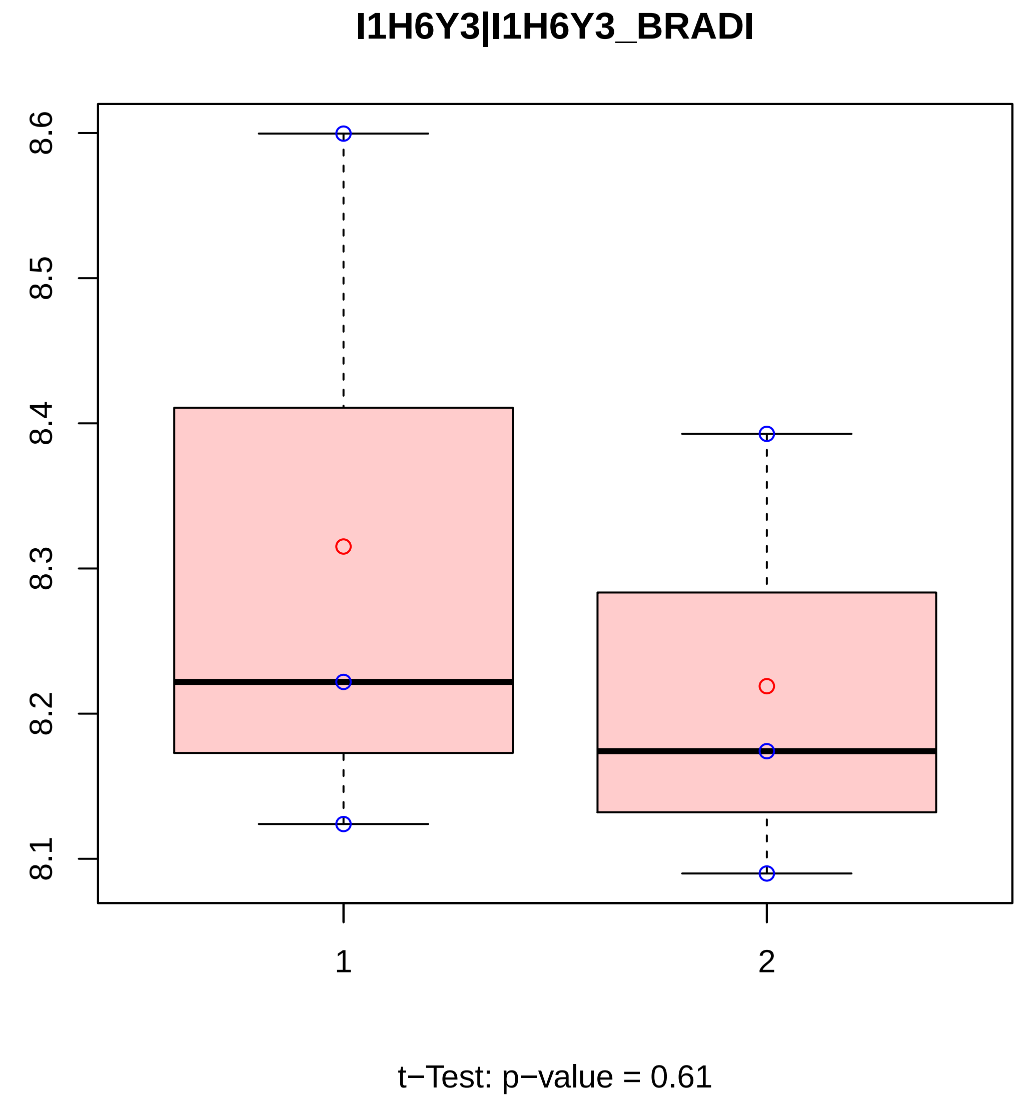
I1H6Y3|I1H6Y3_BRADI
8.1
8.2
8.3
8.4
8.5
8.6
1
2
t−Test: p−value = 0.61

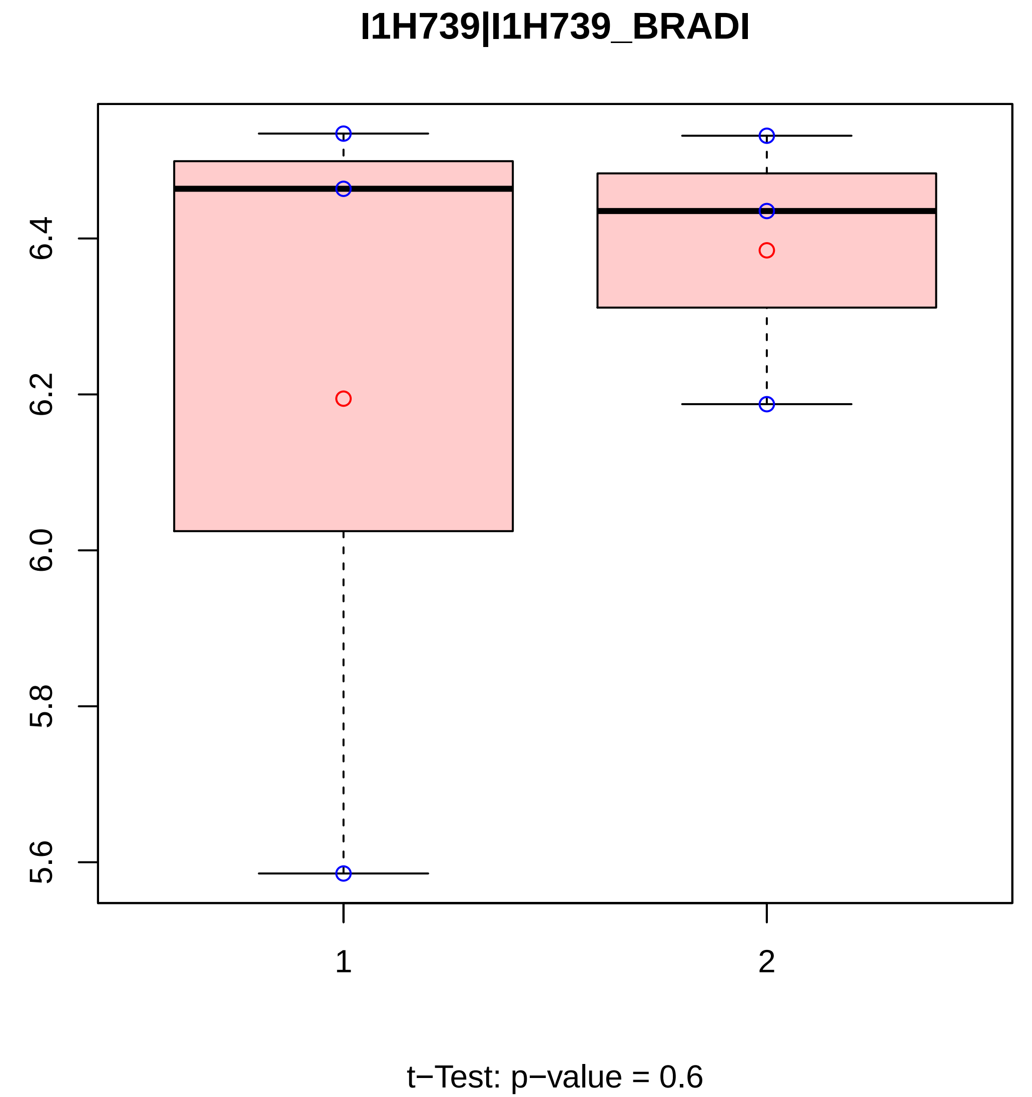
I1H739|I1H739_BRADI
6.4
6.2
6.0
5.8
5.6
1
2
t−Test: p−value = 0.6

I1H755|I1H755_BRADI

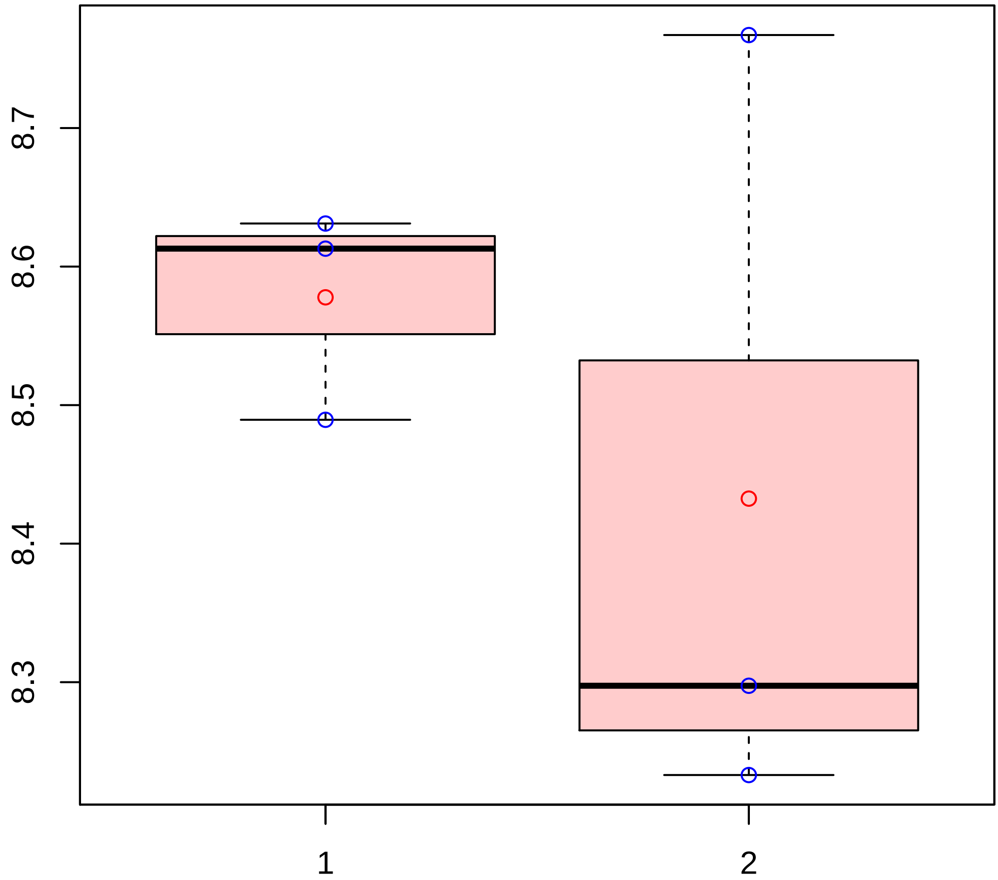

t−Test: p−value = 0.48

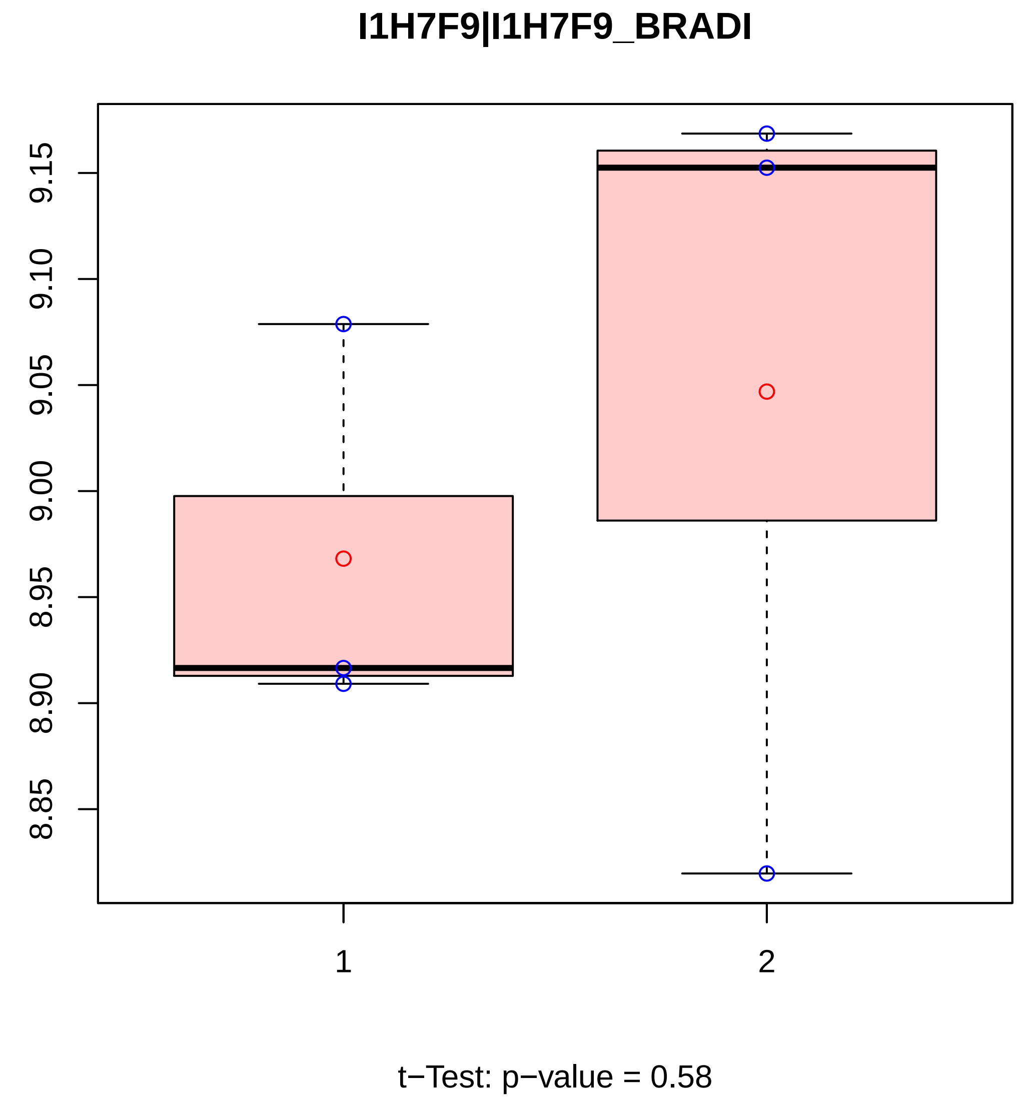
I1H7F9|I1H7F9_BRADI
9.15
9.10
9.05
9.00
8.95
8.90
8.85
1
2
t−Test: p−value = 0.58

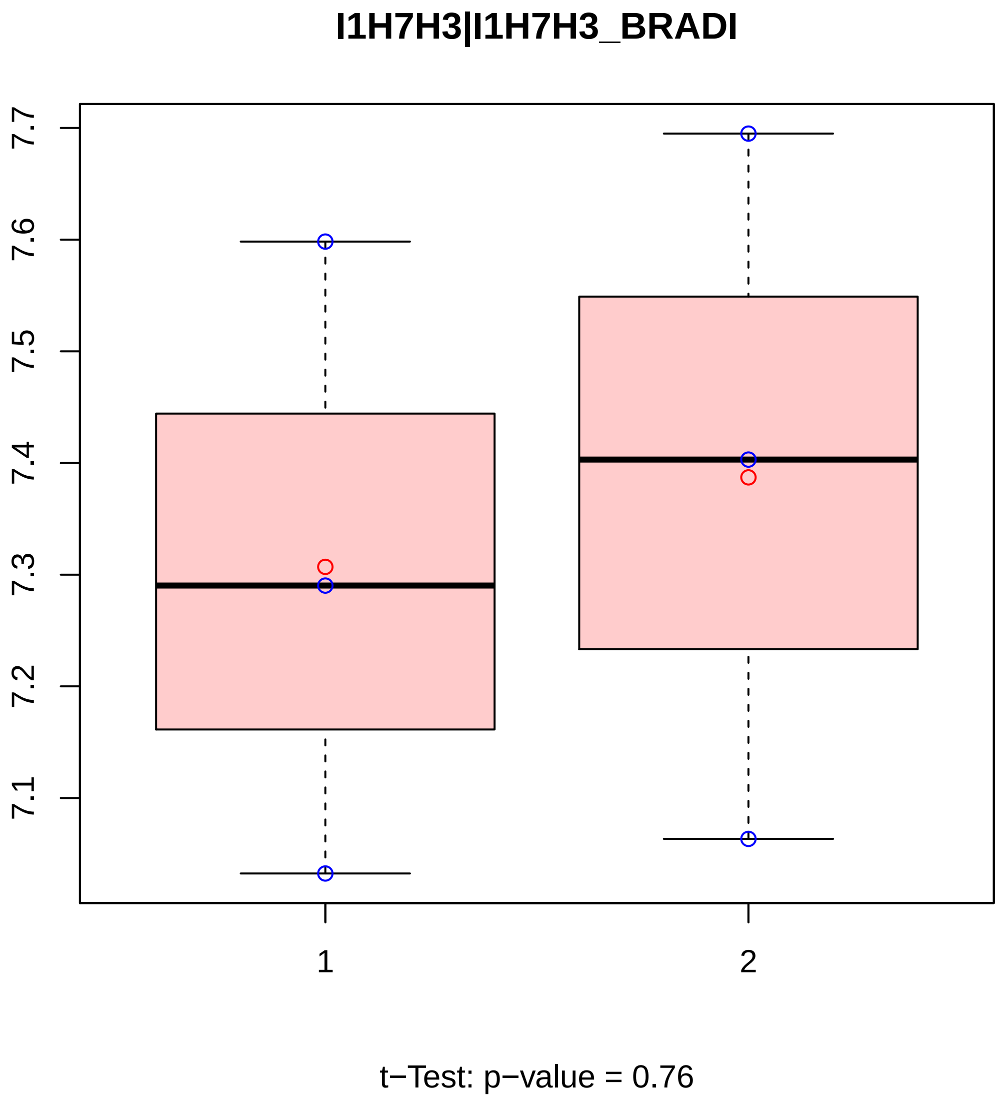
I1H7H3|I1H7H3_BRADI
7.1
7.2
7.3
7.4
7.5
7.6
7.7
1
2
t−Test: p−value = 0.76

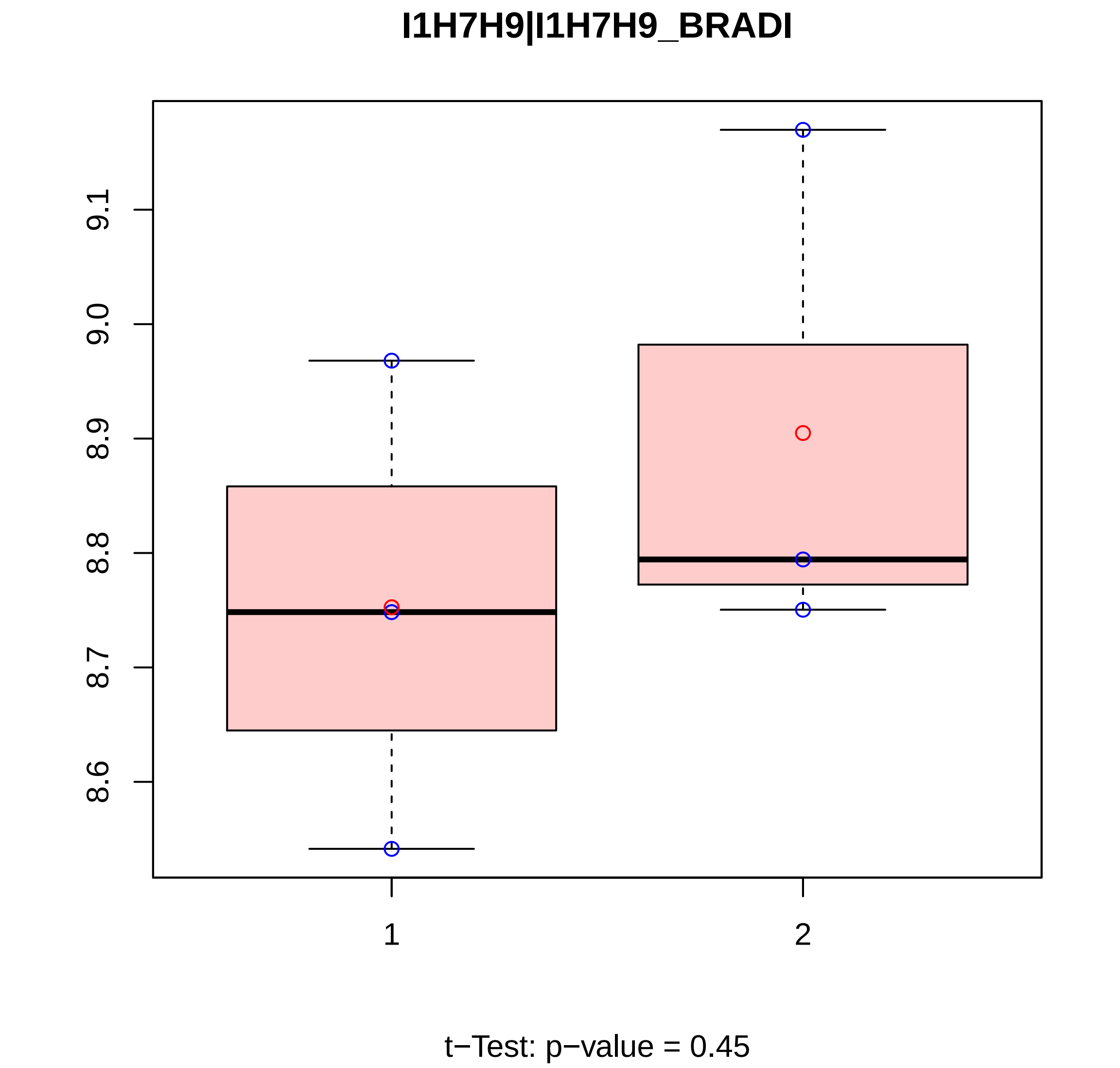

I1H7H9|I1H7H9_BRADI
9.1
9.0
8.9
8.8
8.7
8.6
1
2
t−Test: p−value = 0.45

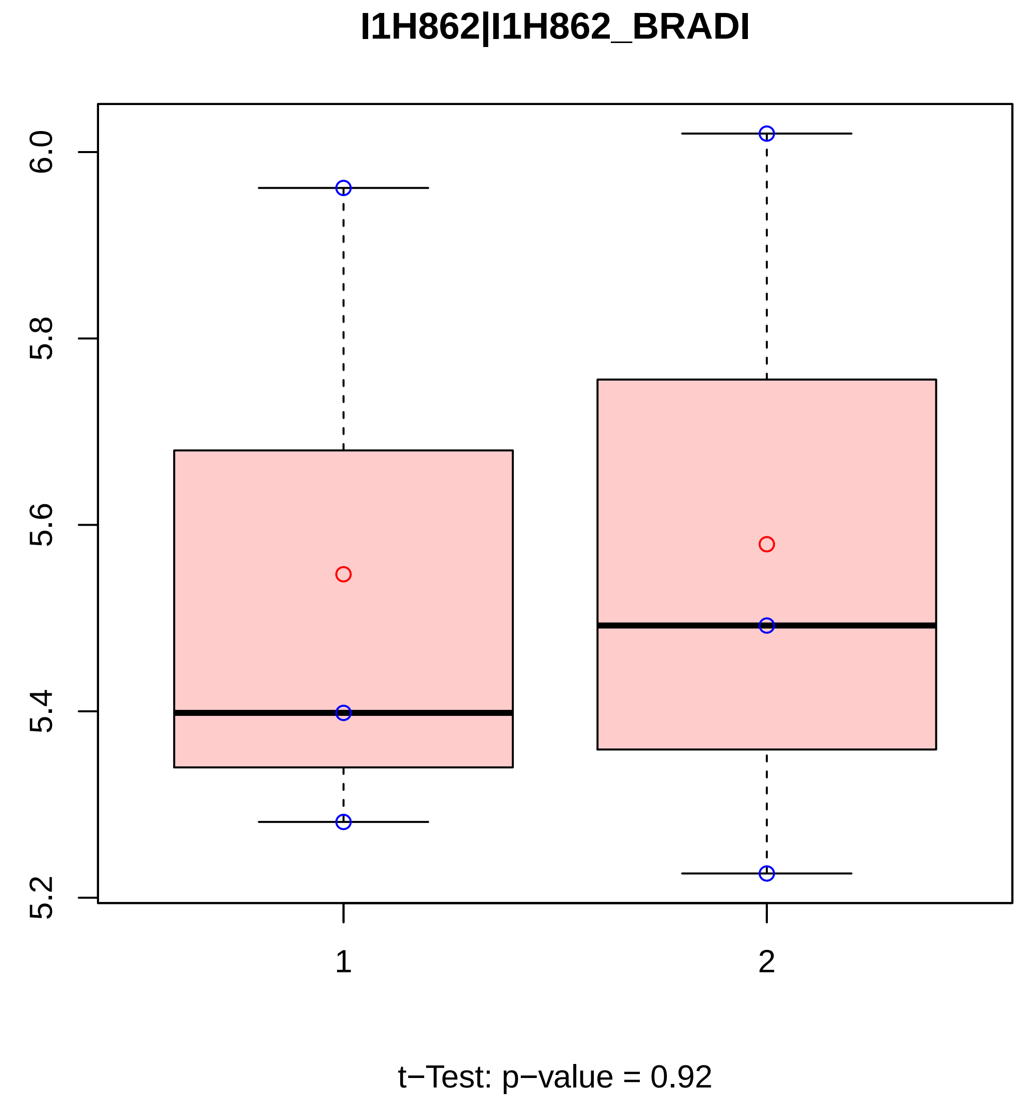

I1H862|I1H862_BRADI
6.0
5.8
5.6
5.4
5.2
1
2
t−Test: p−value = 0.92

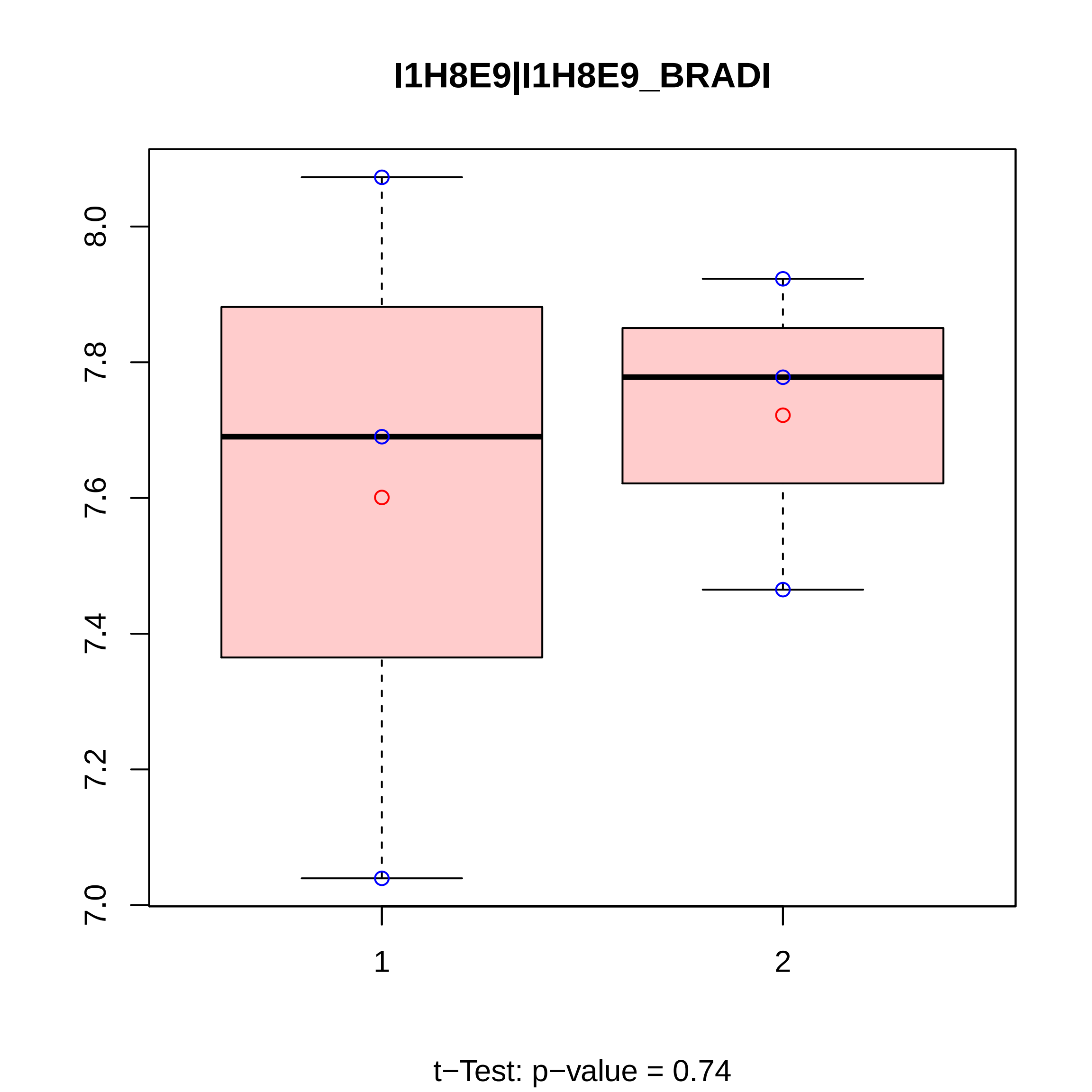
I1H8E9|I1H8E9_BRADI
8.0
7.8
7.6
7.4
7.2
7.0
1
2
t−Test: p−value = 0.74

I1H8G4|I1H8G4_BRADI

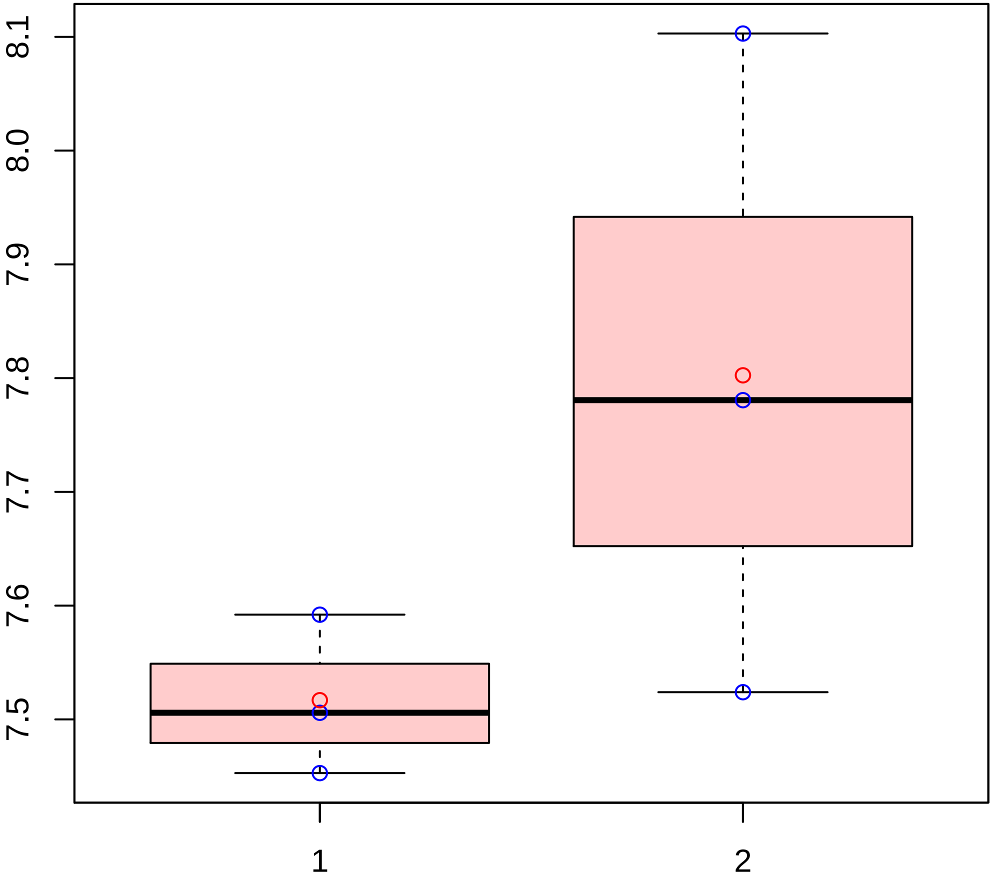

t−Test: p−value = 0.23

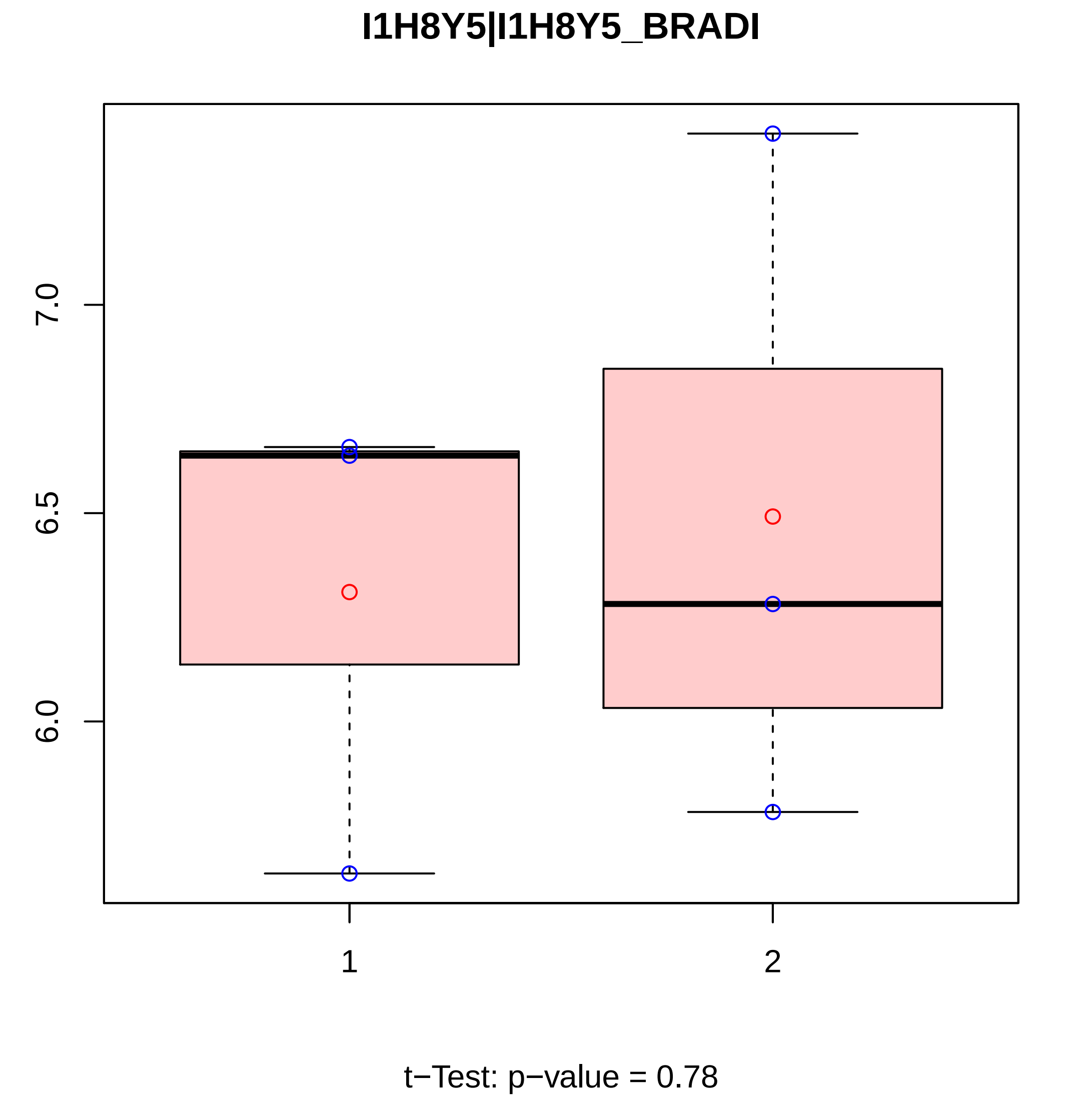
I1H8Y5|I1H8Y5_BRADI
7.0
6.5
6.0
1
2
t−Test: p−value = 0.78

I1H940|I1H940_BRADI

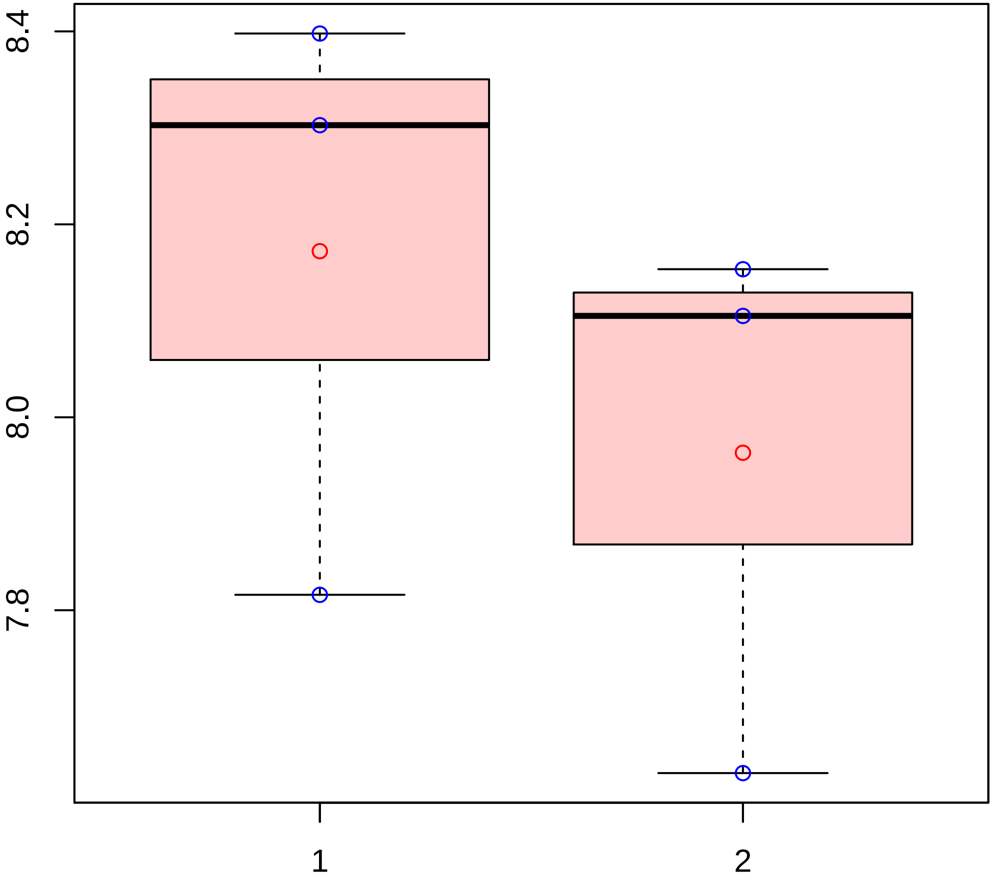

t−Test: p−value = 0.44

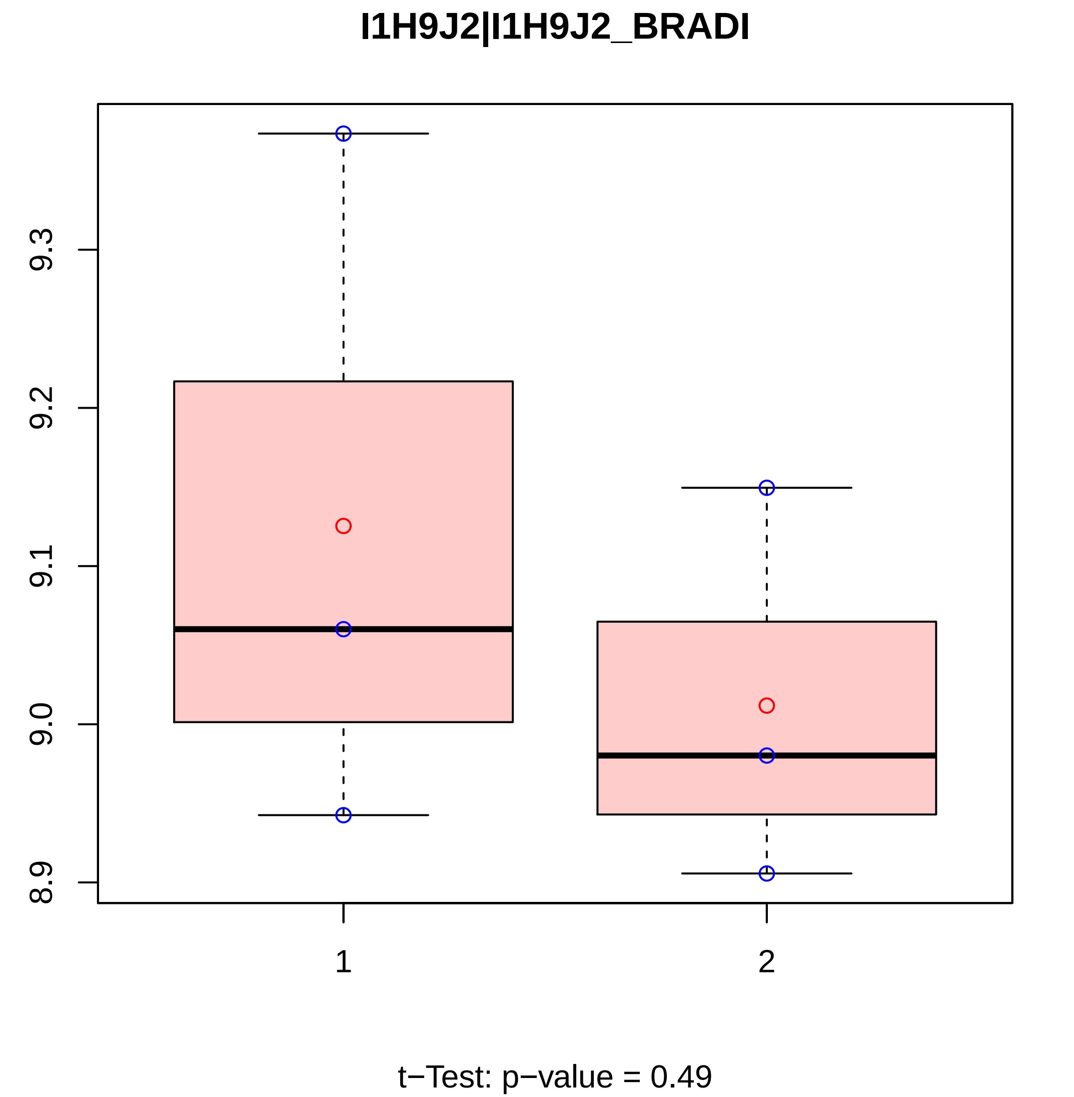

I1H9J2|I1H9J2_BRADI
9.3
9.2
9.1
9.0
8.9
1
2
t−Test: p−value = 0.49

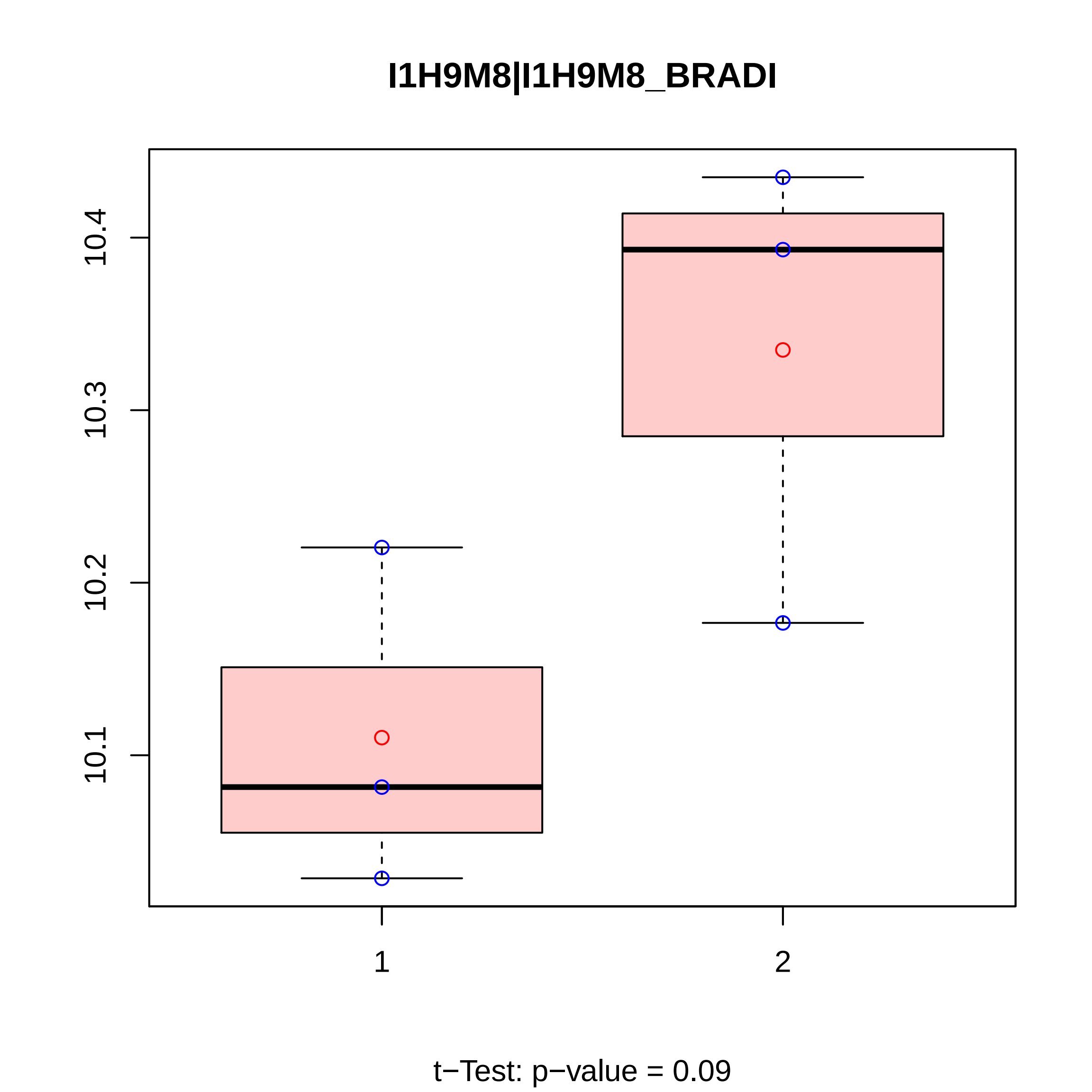
I1H9M8|I1H9M8_BRADI
10.1
10.2
10.3
10.4
1
2
t−Test: p−value = 0.09

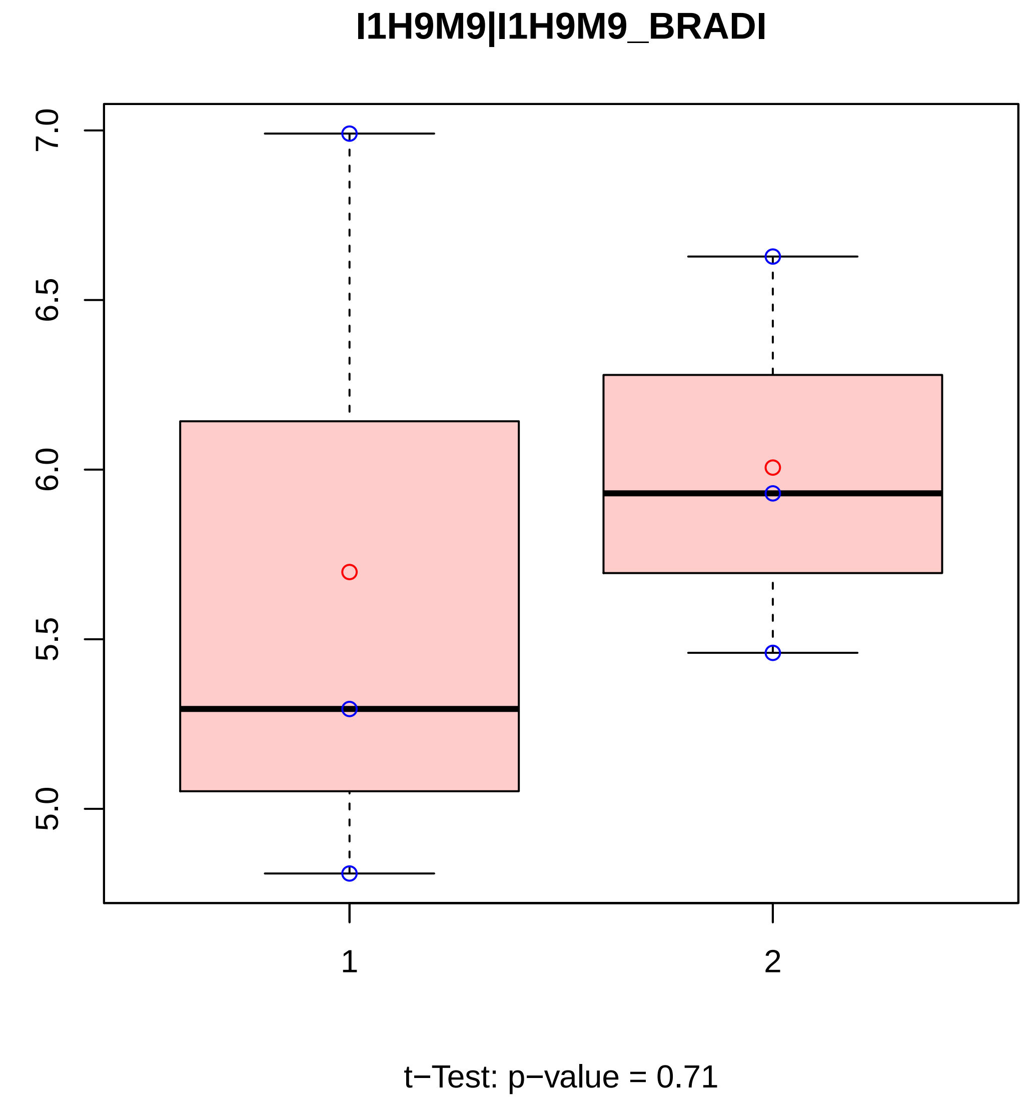
I1H9M9|I1H9M9_BRADI
7.0
6.5
6.0
5.5
5.0
1
2
t−Test: p−value = 0.71

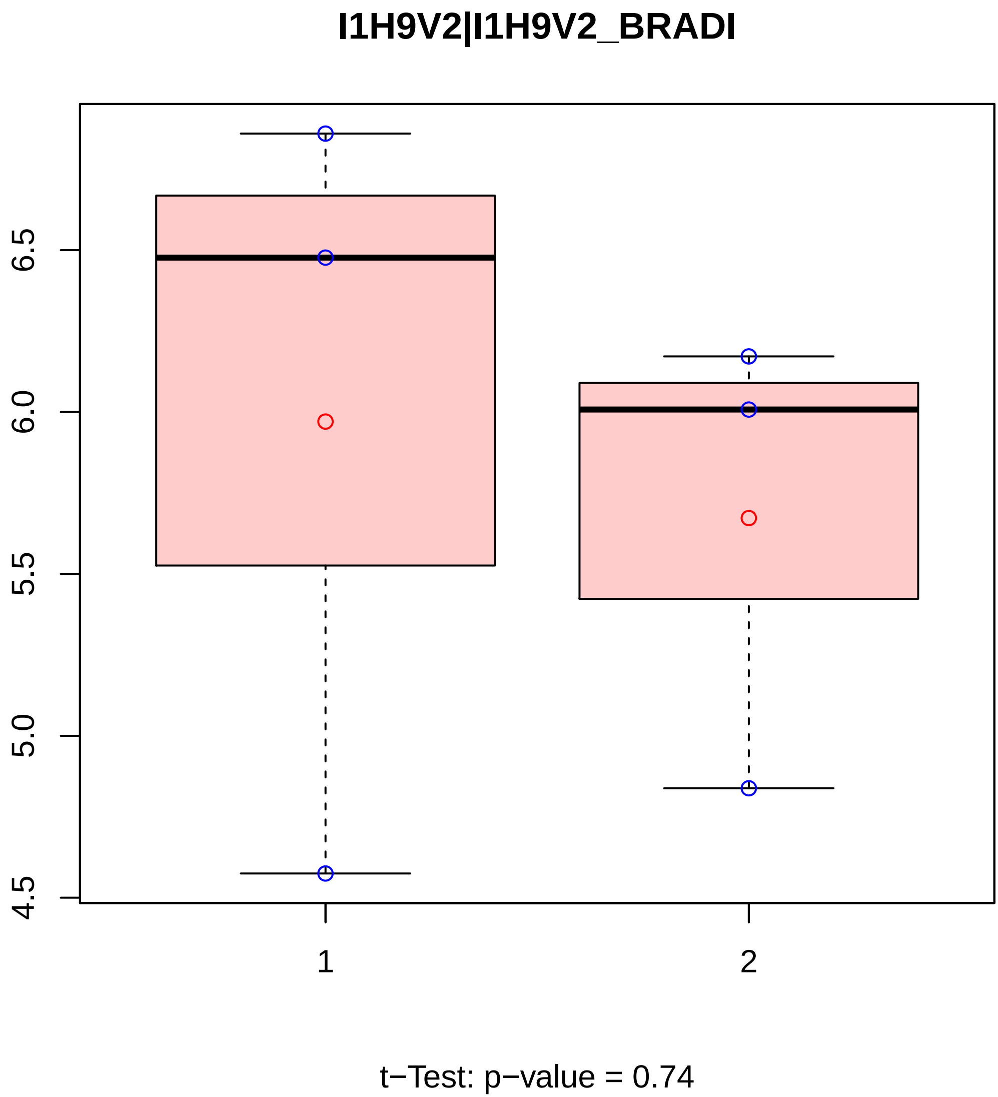
I1H9V2|I1H9V2_BRADI
6.5
6.0
5.5
5.0
4.5
1
2
t−Test: p−value = 0.74

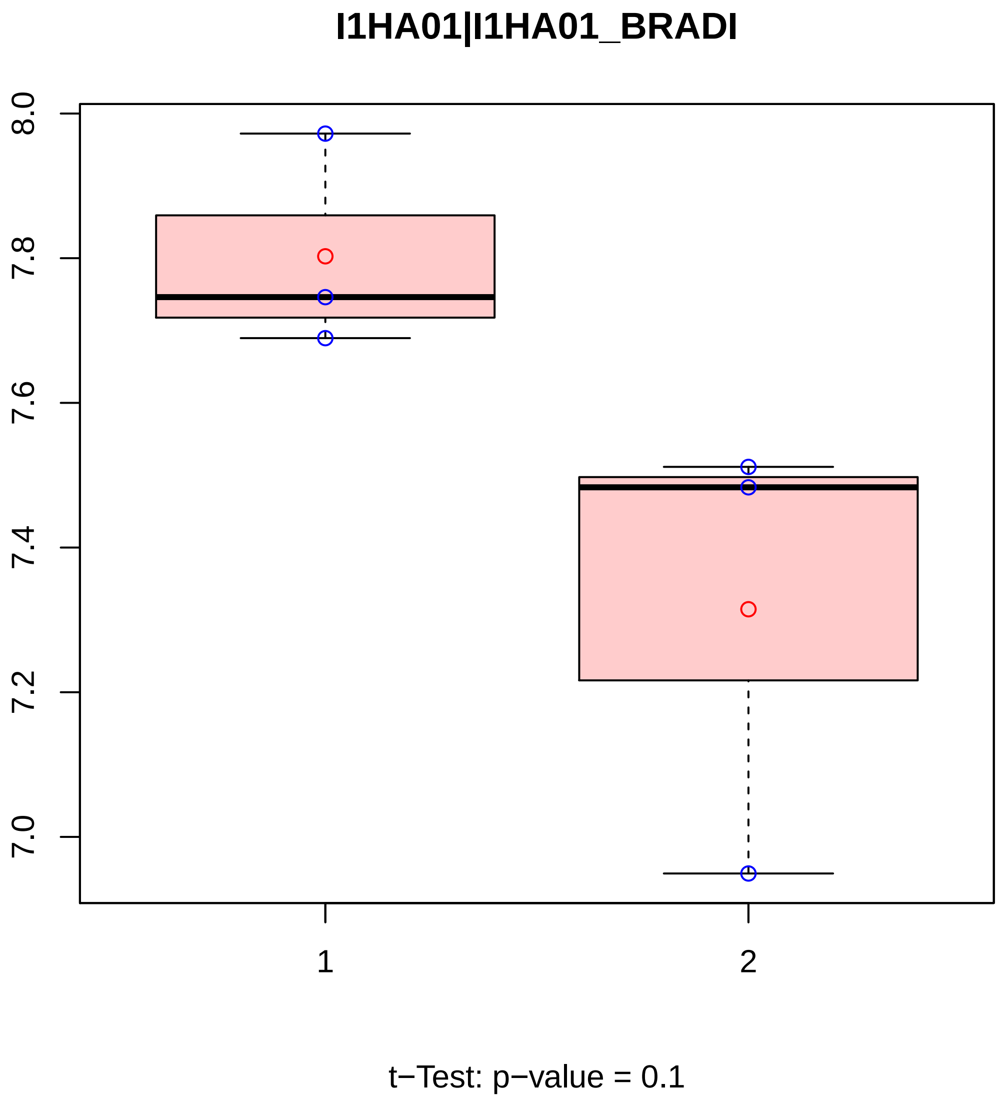
I1HA01|I1HA01_BRADI
8.0
7.8
7.6
7.4
7.2
7.0
1
2
t−Test: p−value = 0.1

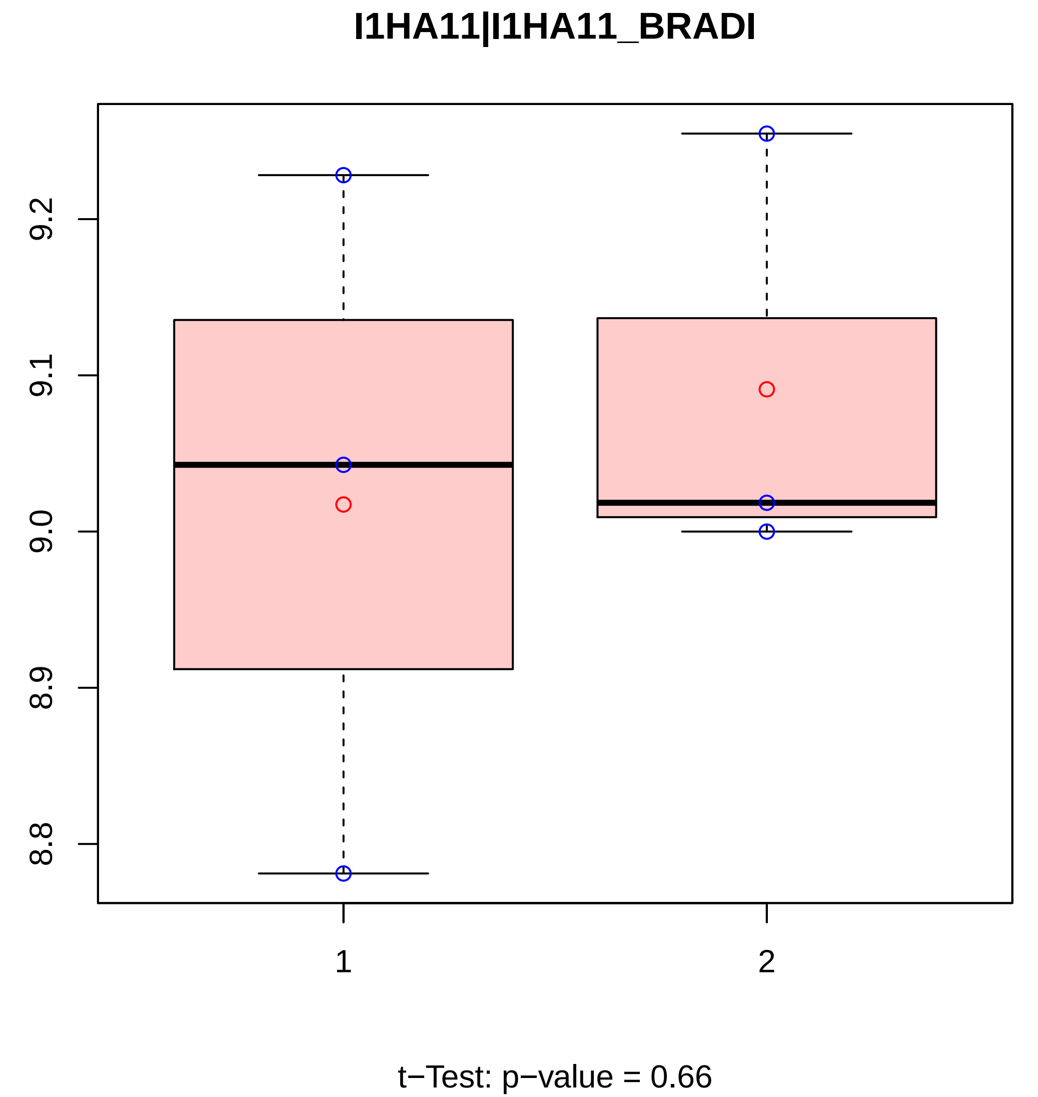

I1HA11|I1HA11_BRADI
9.2
9.1
9.0
8.9
8.8
1
2
t−Test: p−value = 0.66

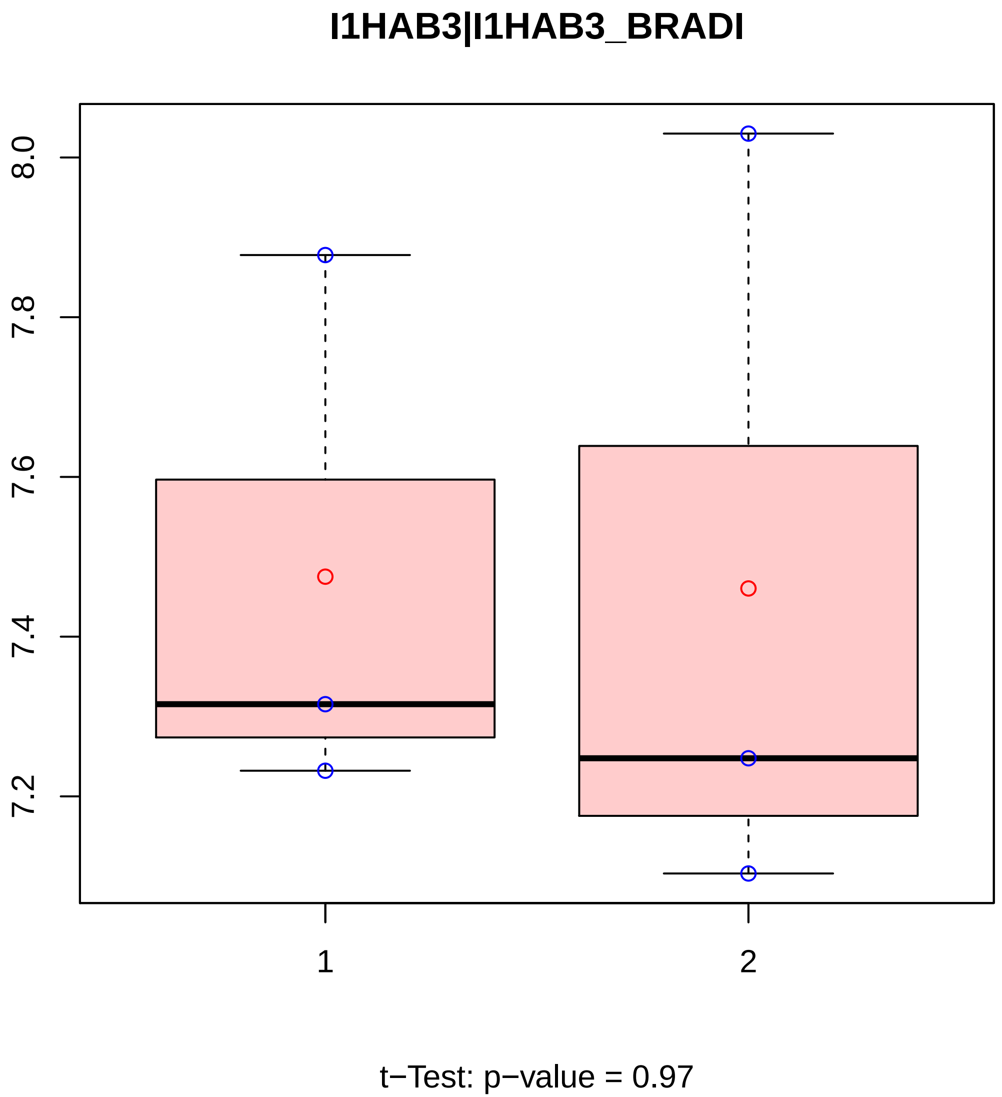
I1HAB3|I1HAB3_BRADI
8.0
7.8
7.6
7.4
7.2
1
2
t−Test: p−value = 0.97

I1HAK6|I1HAK6_BRADI

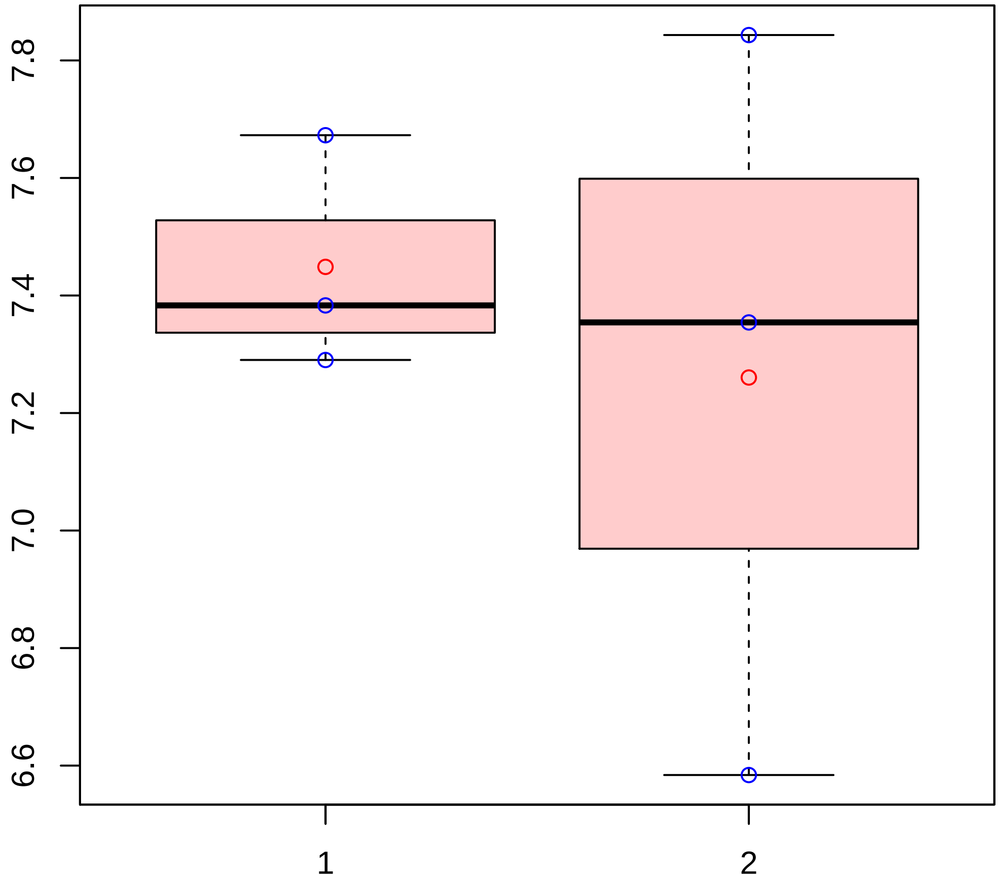

t−Test: p−value = 0.67

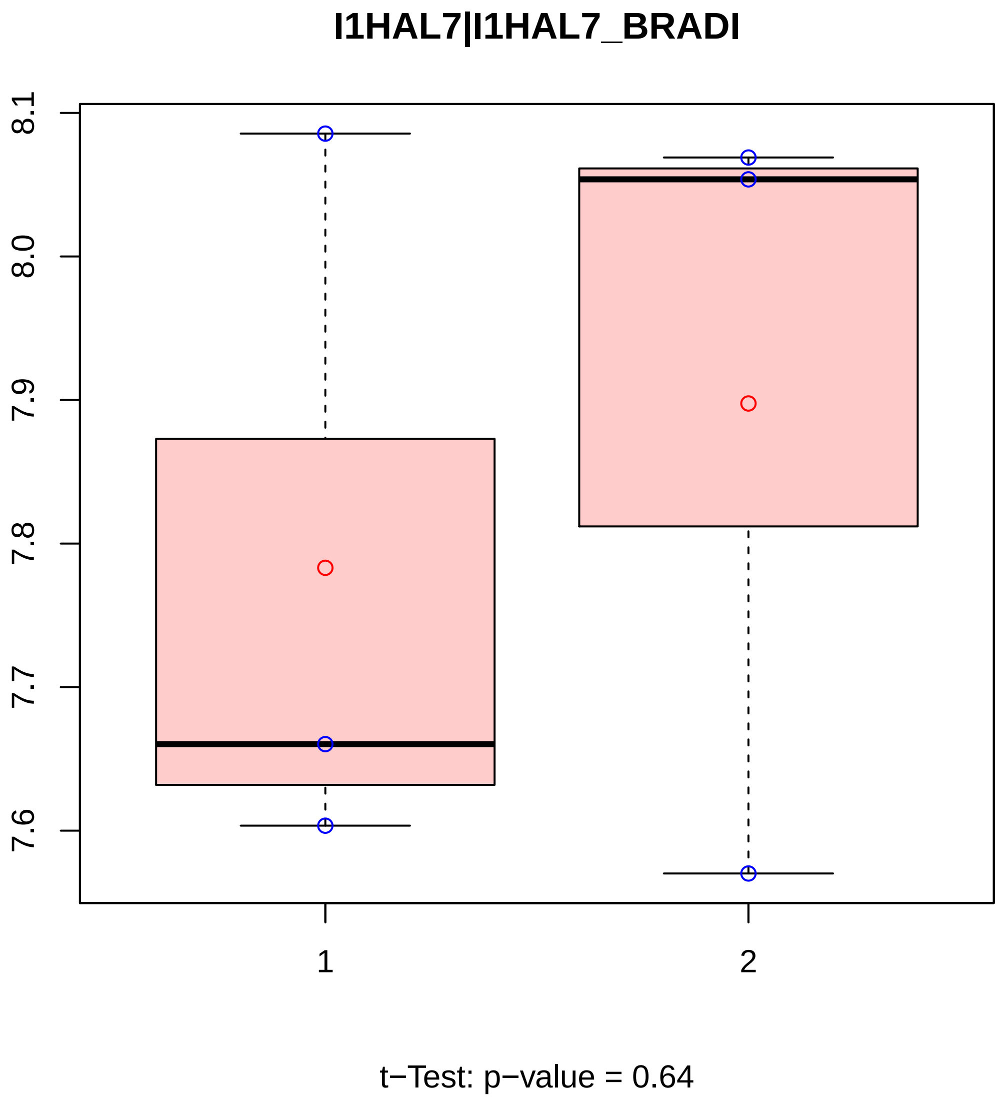
I1HAL7|I1HAL7_BRADI
8.1
8.0
7.9
7.8
7.7
7.6
1
2
t−Test: p−value = 0.64

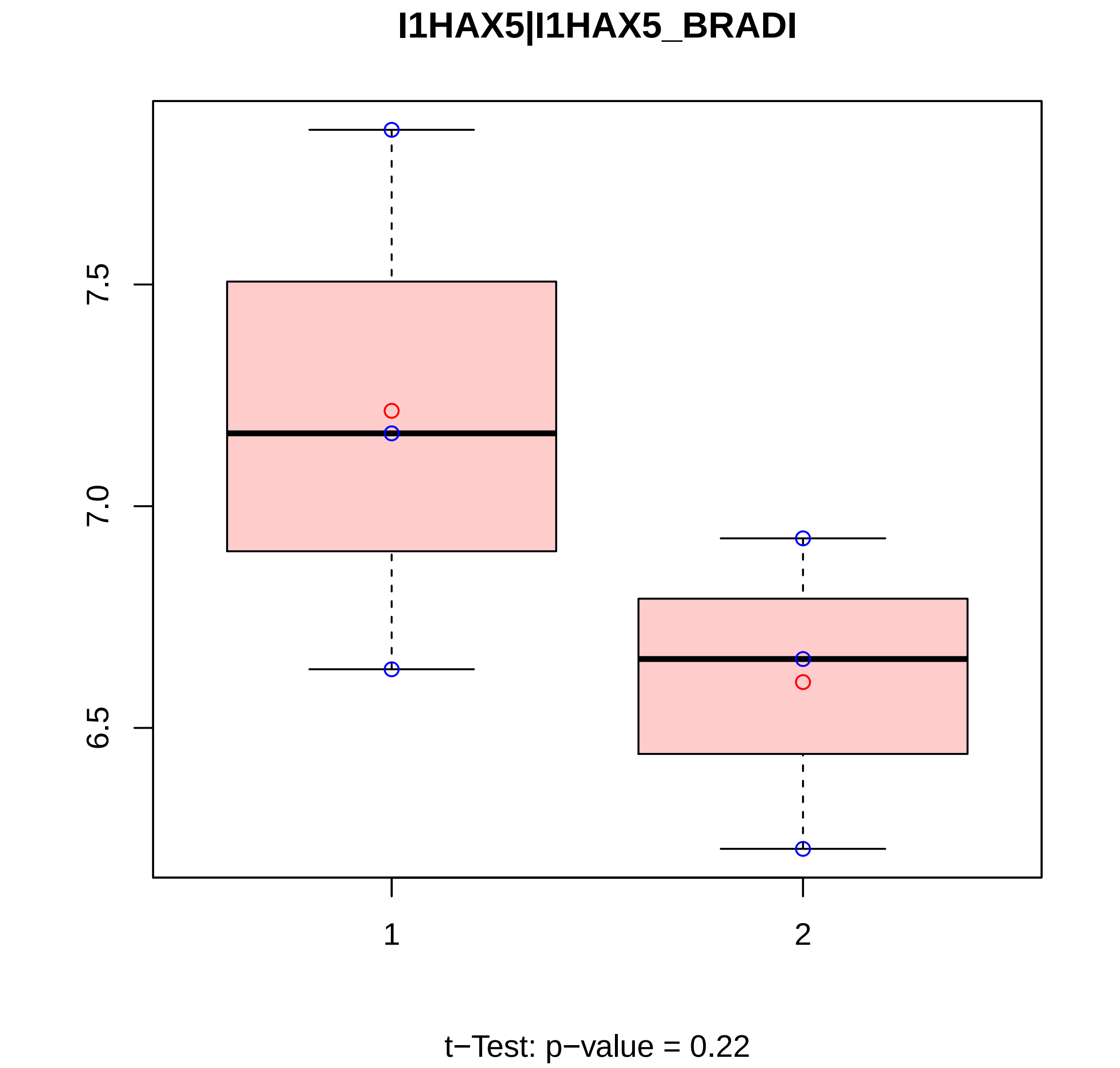
I1HAX5|I1HAX5_BRADI
7.5
7.0
6.5
1
2
t−Test: p−value = 0.22

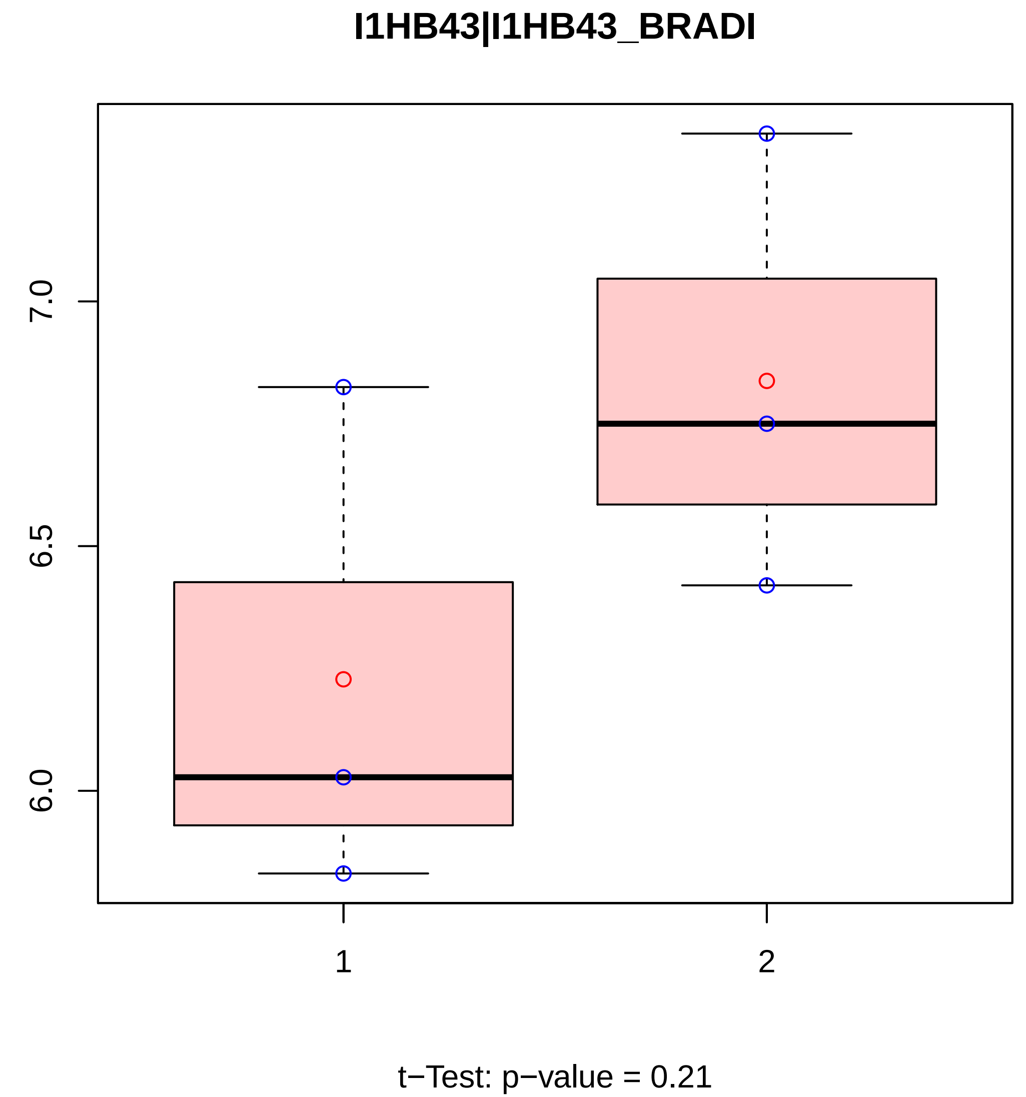
I1HB43|I1HB43_BRADI
7.0
6.5
6.0
1
2
t−Test: p−value = 0.21

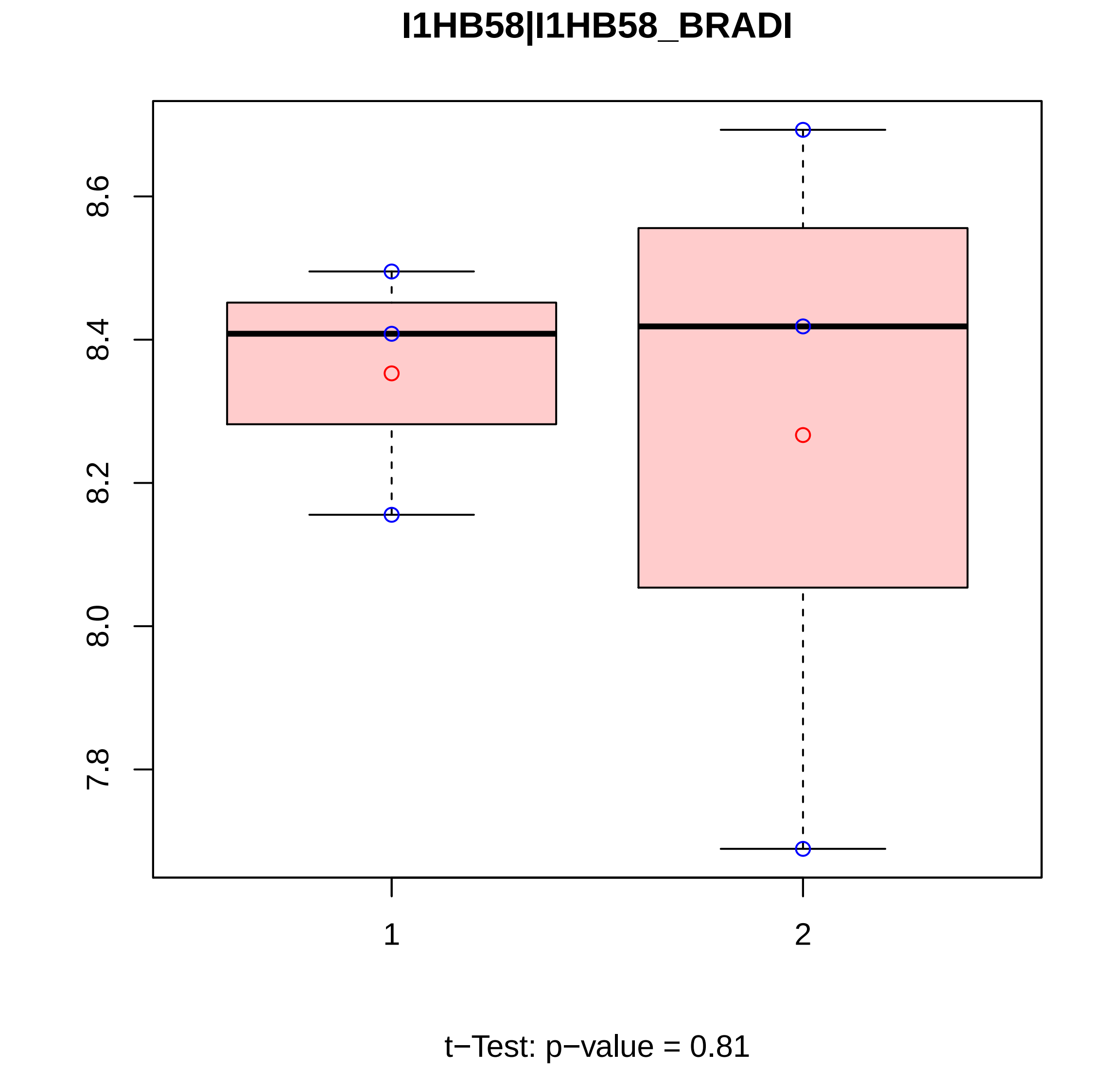

I1HB58|I1HB58_BRADI
8.6
8.4
8.2
8.0
7.8
1
2
t−Test: p−value = 0.81

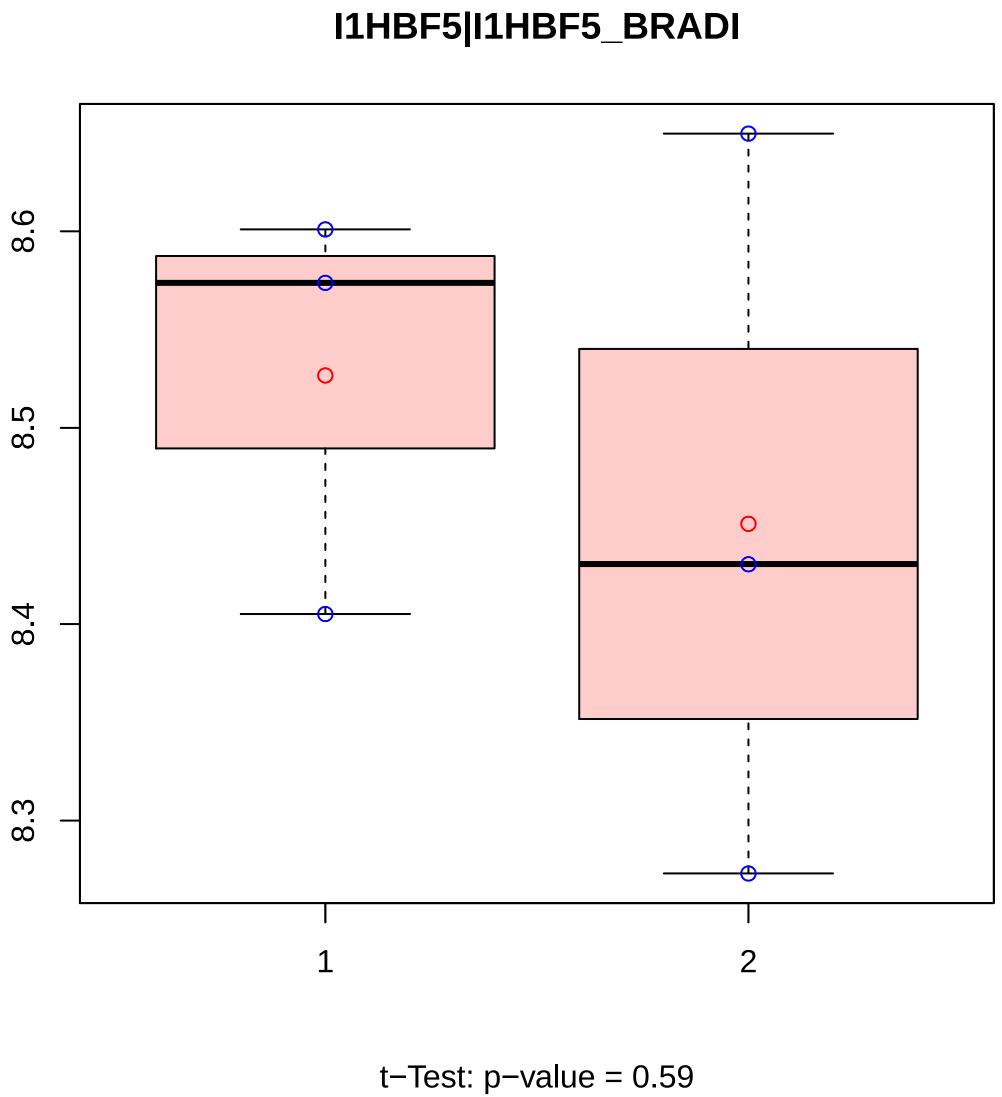
I1HBF5|I1HBF5_BRADI
8.3
8.4
8.5
8.6
1
2
t−Test: p−value = 0.59

I1HCH2|I1HCH2_BRADI

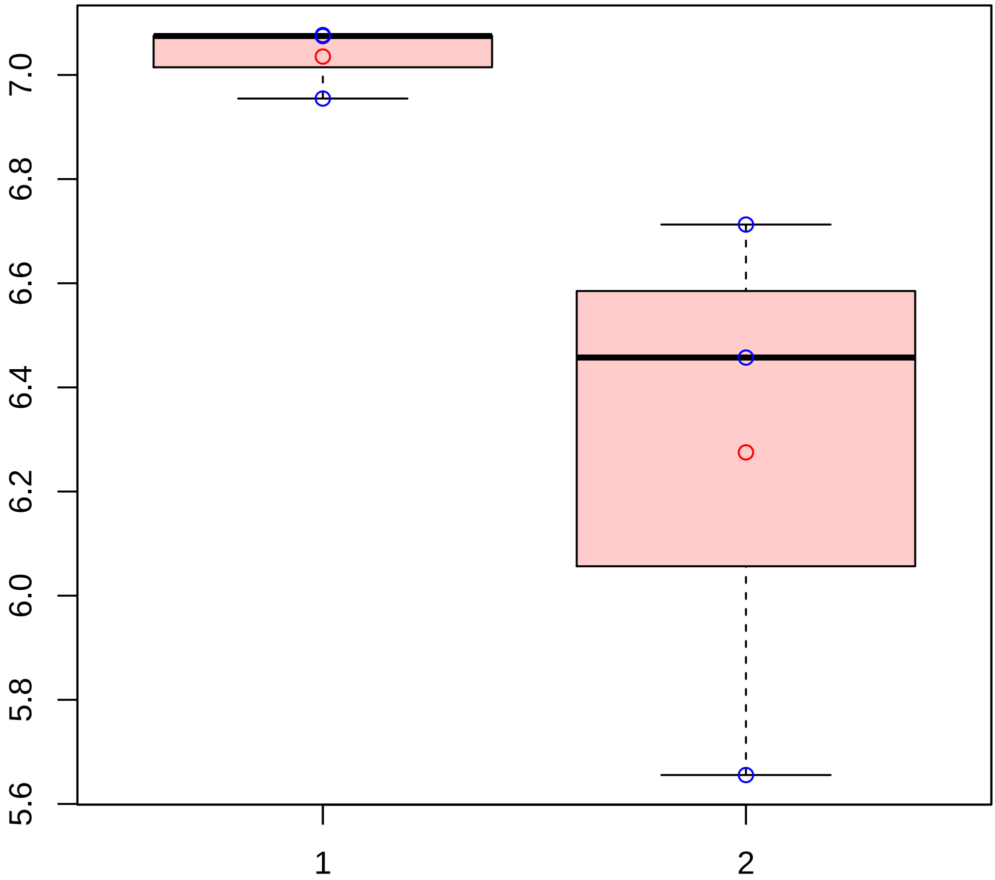

t−Test: p−value = 0.14

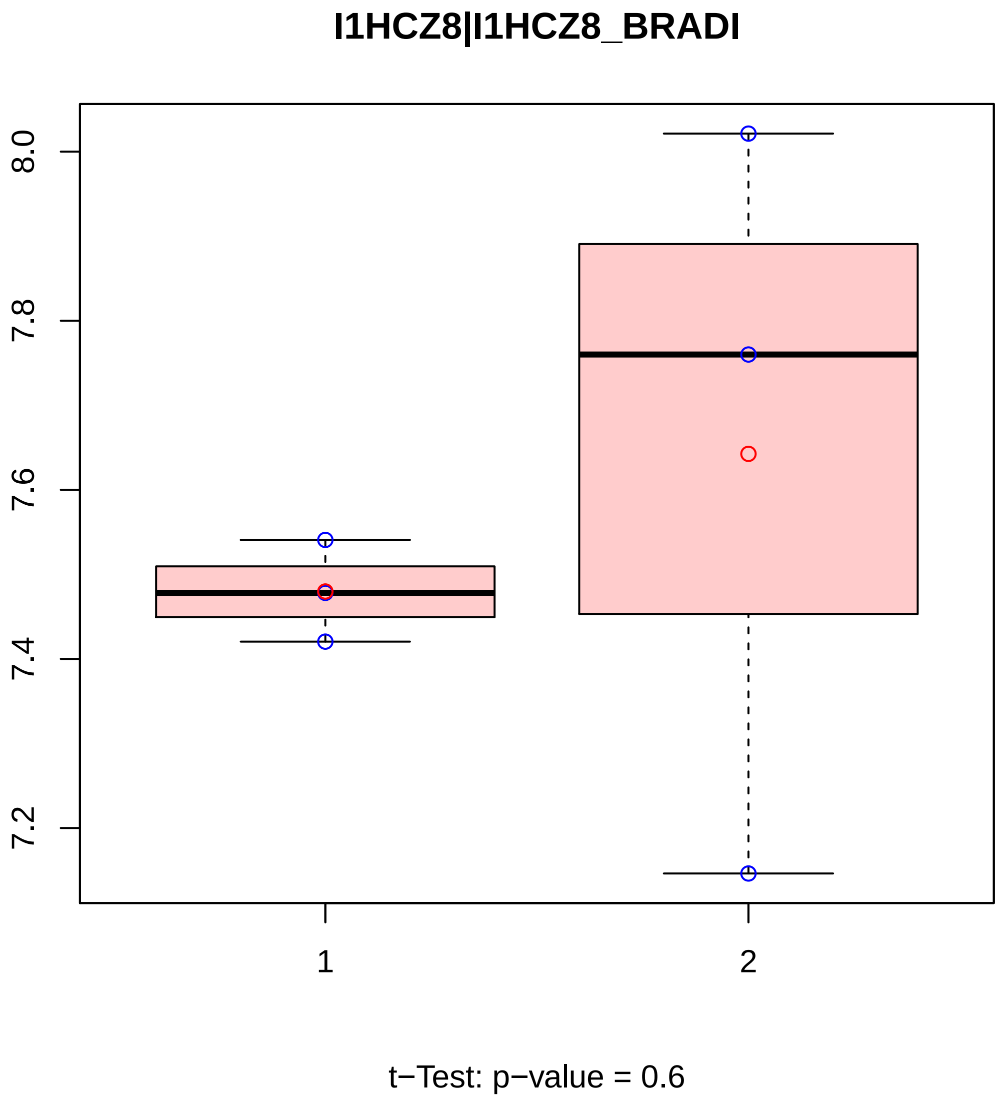

I1HCZ8|I1HCZ8_BRADI
8.0
7.8
7.6
7.4
7.2
1
2
t−Test: p−value = 0.6

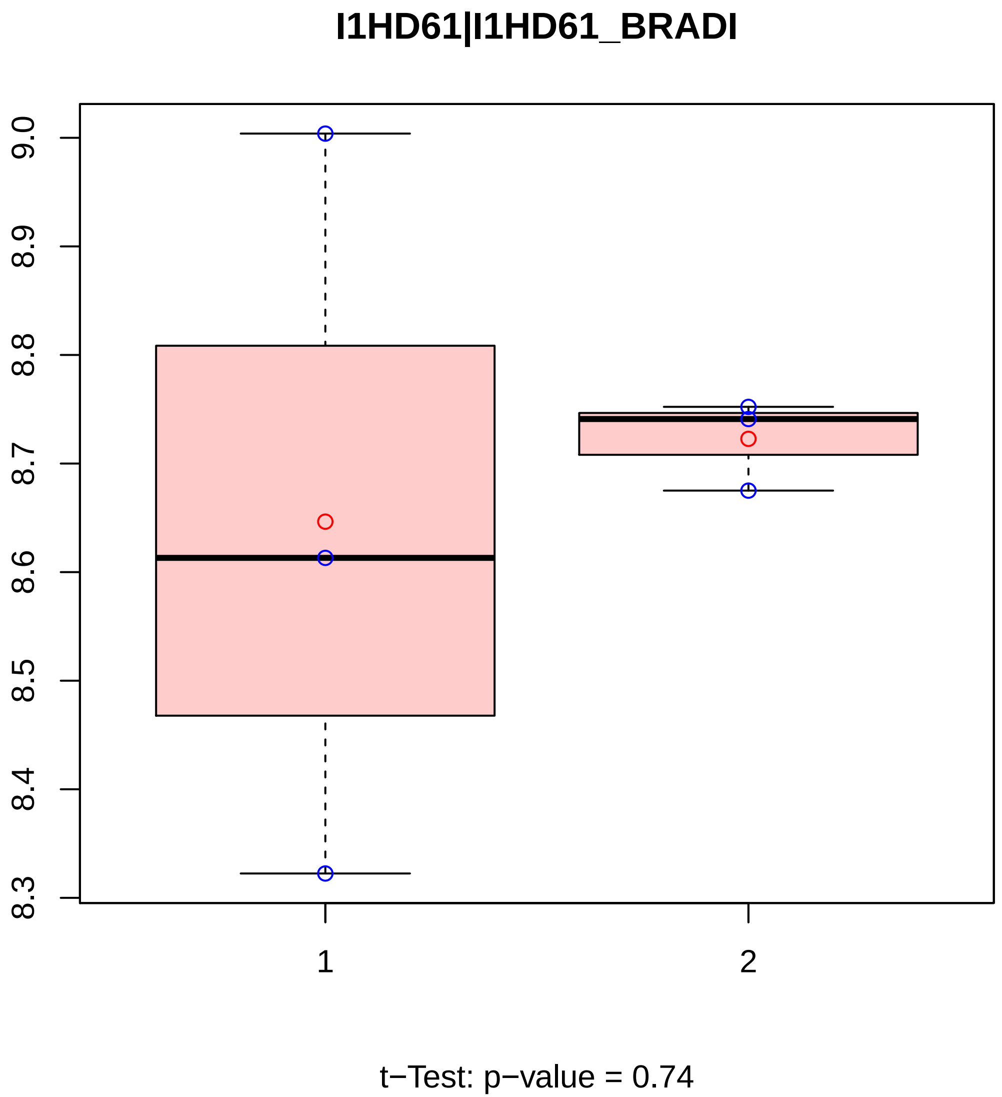
I1HD61|I1HD61_BRADI
9.0
8.9
8.8
8.7
8.6
8.5
8.4
8.3
1
2
t−Test: p−value = 0.74

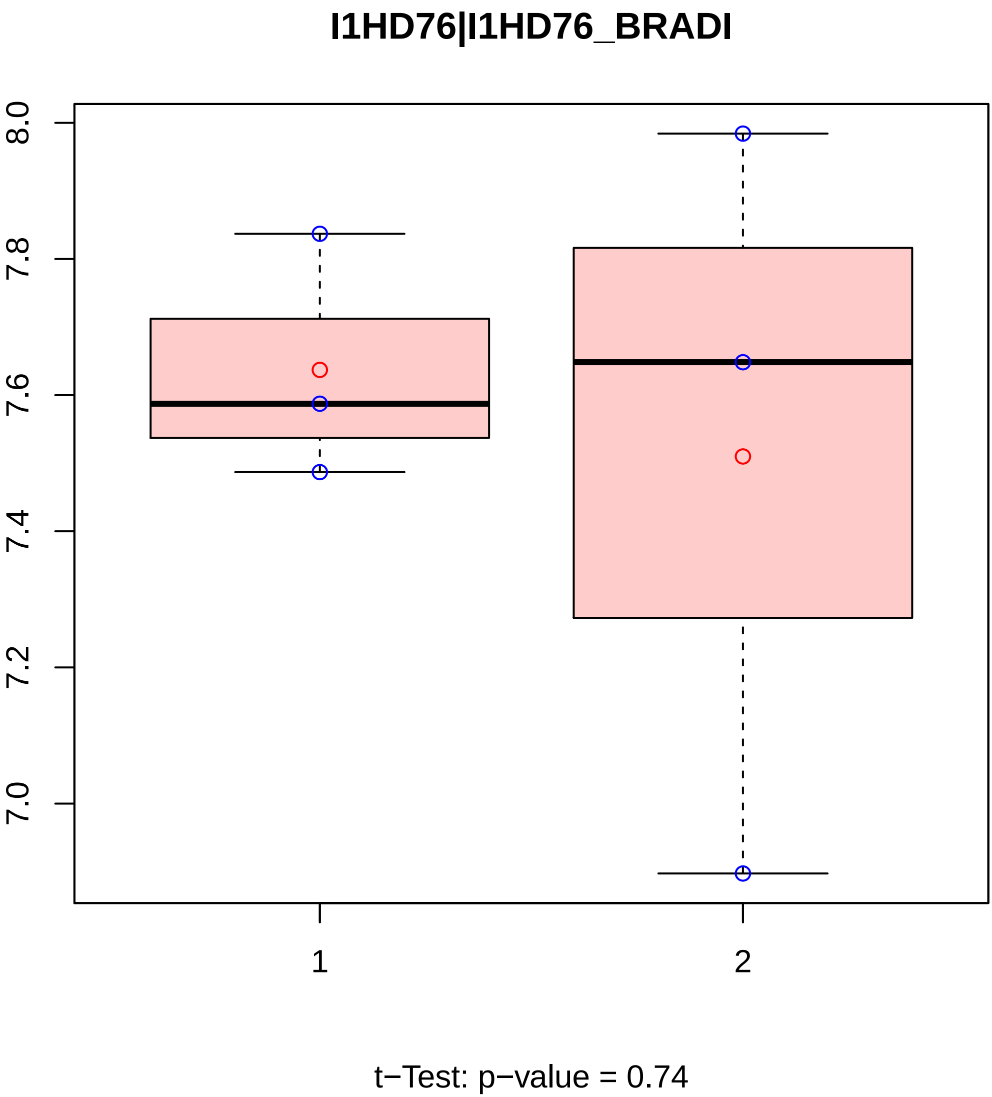
I1HD76|I1HD76_BRADI
8.0
7.8
7.6
7.4
7.2
7.0
1
2
t−Test: p−value = 0.74

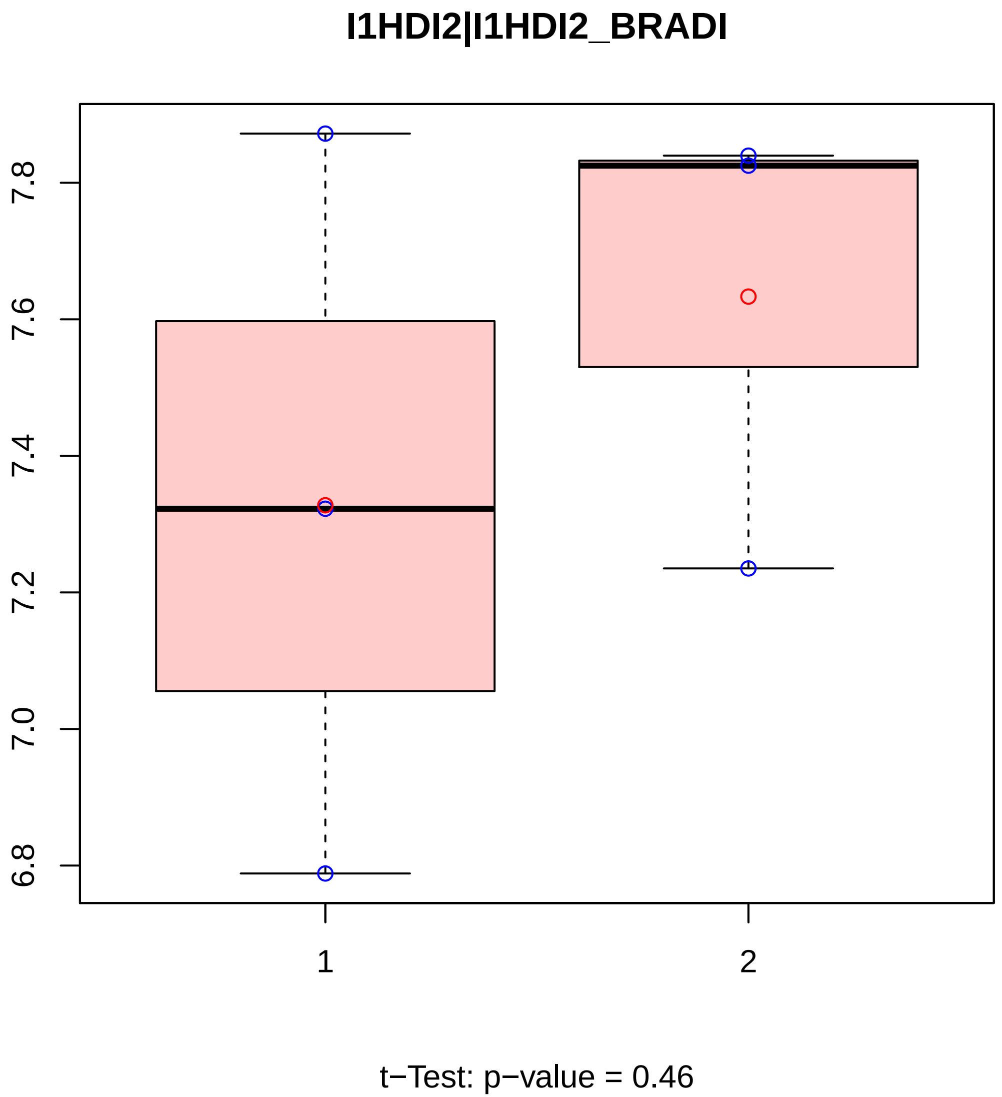
I1HDI2|I1HDI2_BRADI
7.8
7.6
7.4
7.2
7.0
6.8
1
2
t−Test: p−value = 0.46

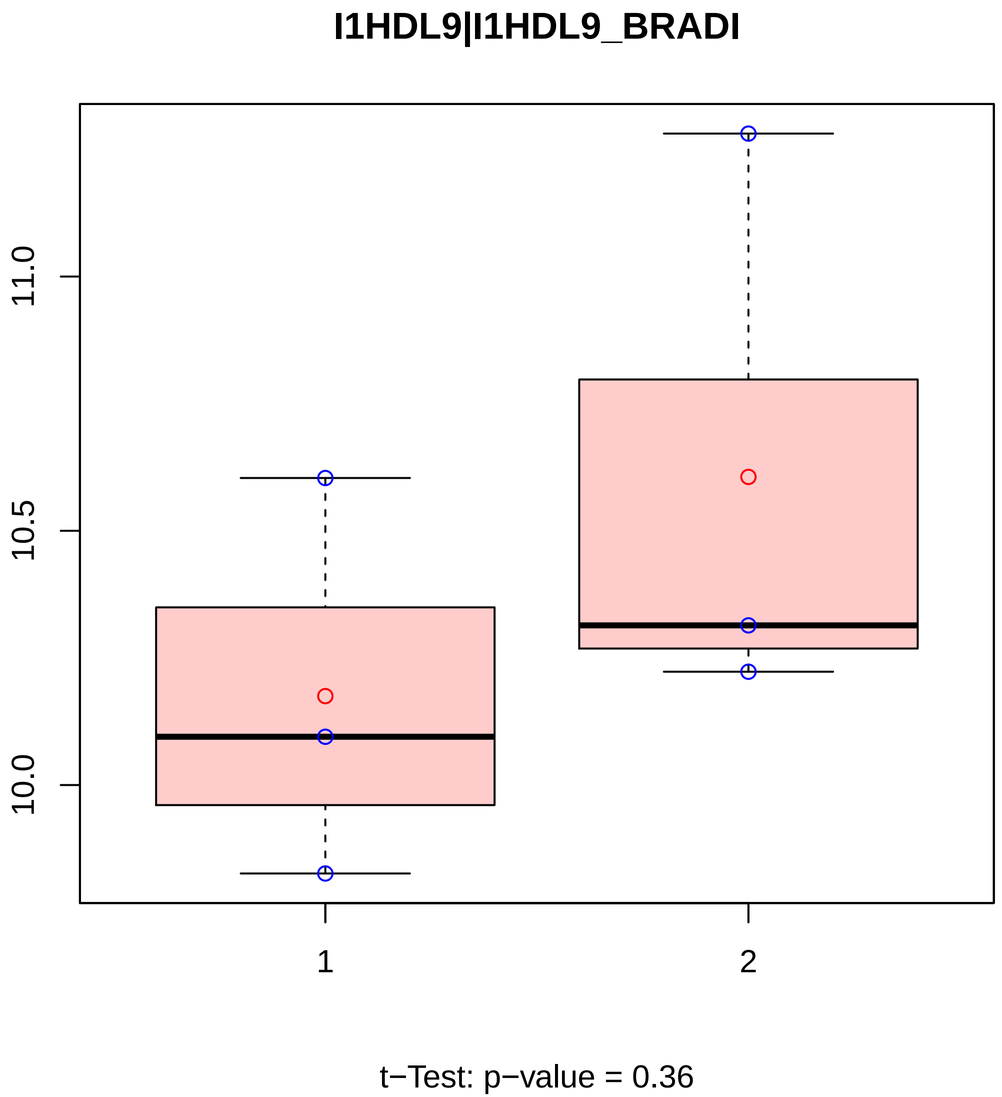
I1HDL9|I1HDL9_BRADI
11.0
10.5
10.0
1
2
t−Test: p−value = 0.36

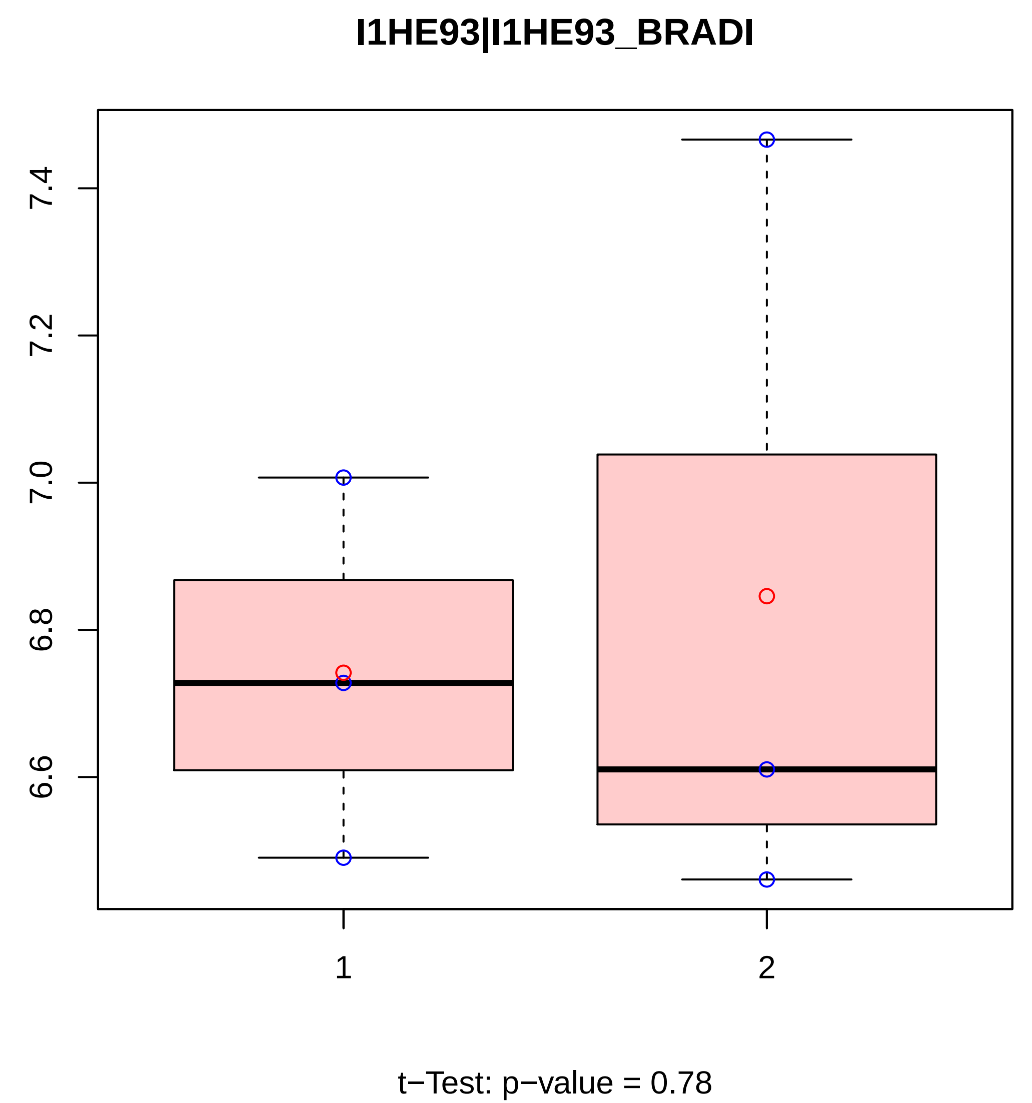
I1HE93|I1HE93_BRADI
7.4
7.2
7.0
6.8
6.6
1
2
t−Test: p−value = 0.78

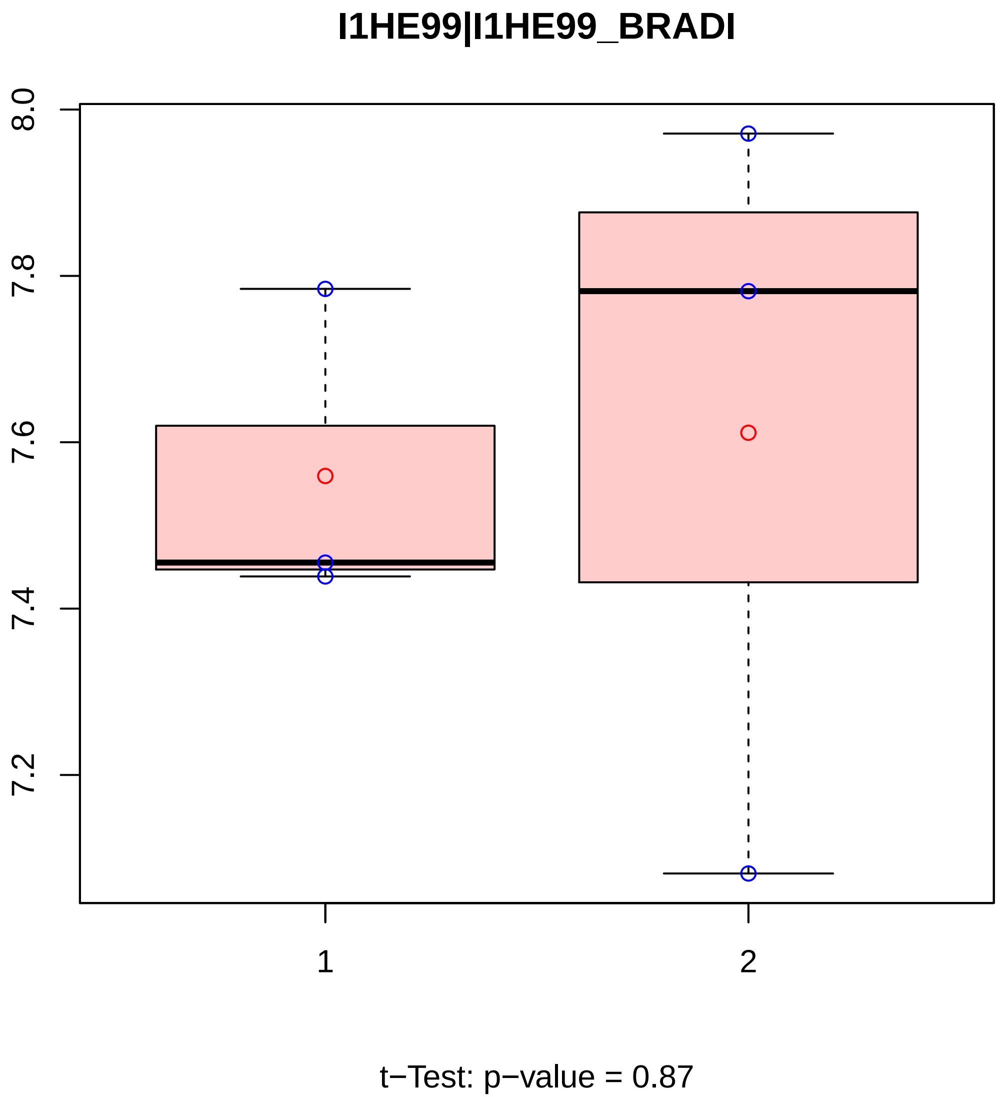
I1HE99|I1HE99_BRADI
8.0
7.8
7.6
7.4
7.2
1
2
t−Test: p−value = 0.87

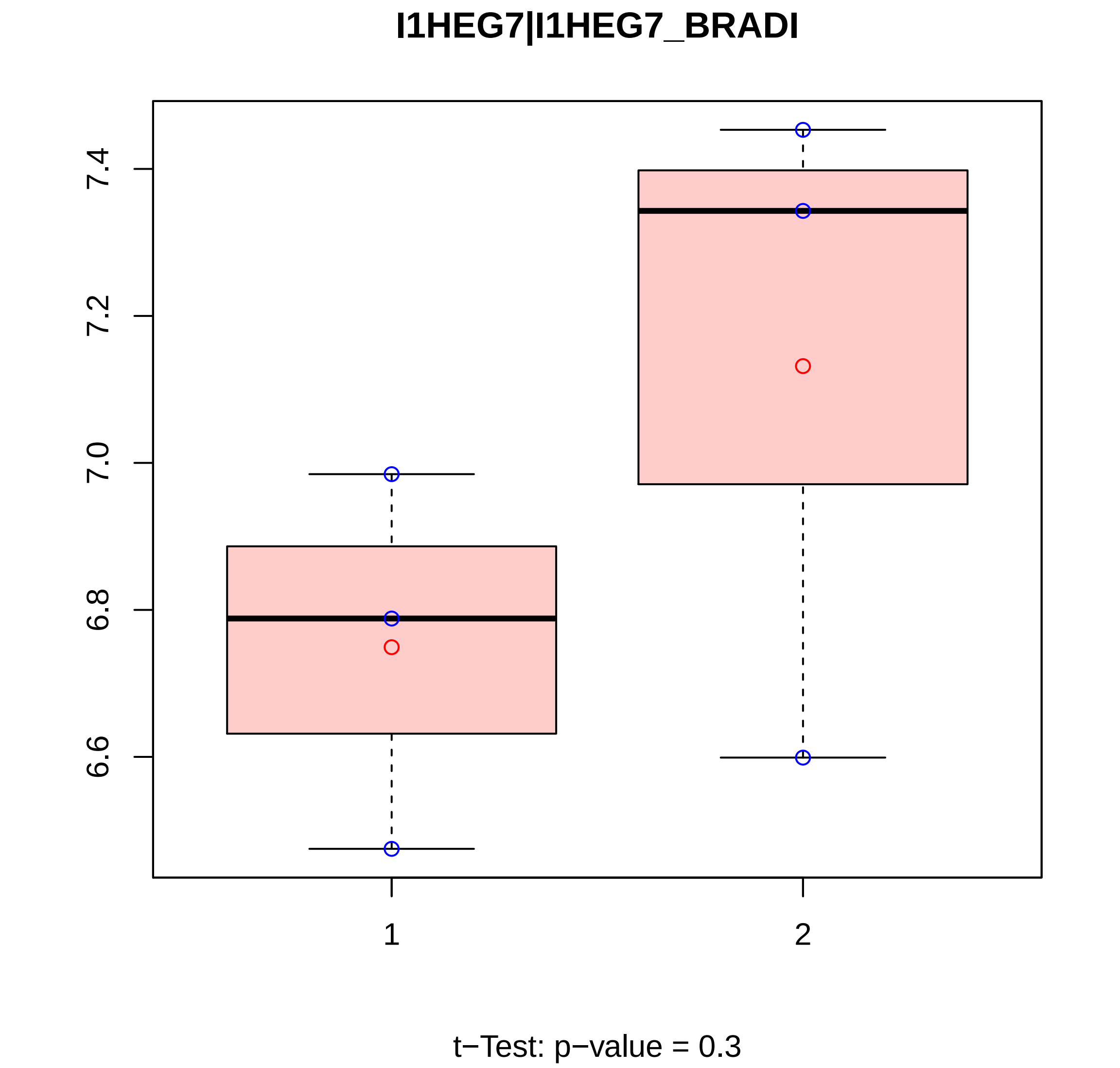
I1HEG7|I1HEG7_BRADI
7.4
7.2
7.0
6.8
6.6
1
2
t−Test: p−value = 0.3

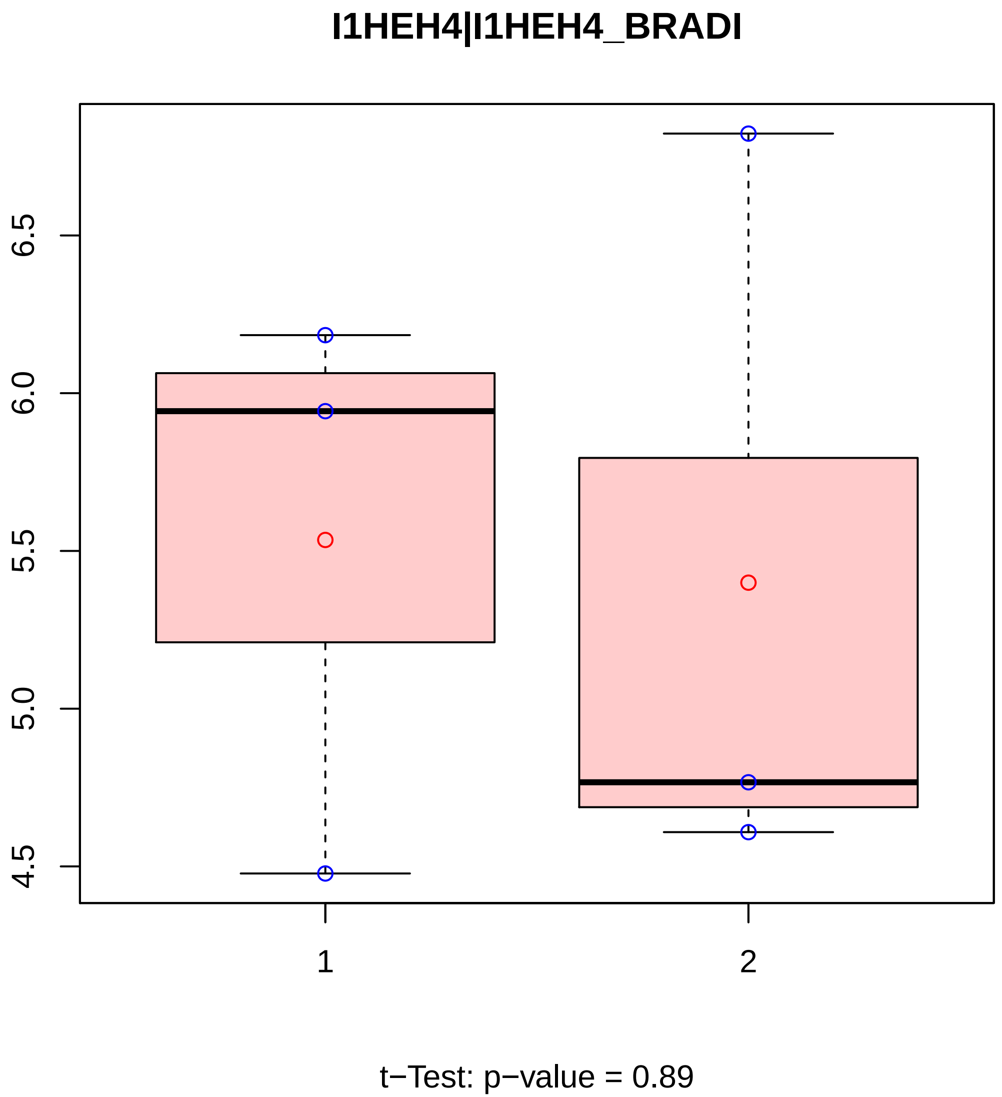
I1HEH4|I1HEH4_BRADI
6.5
6.0
5.5
5.0
4.5
1
2
t−Test: p−value = 0.89

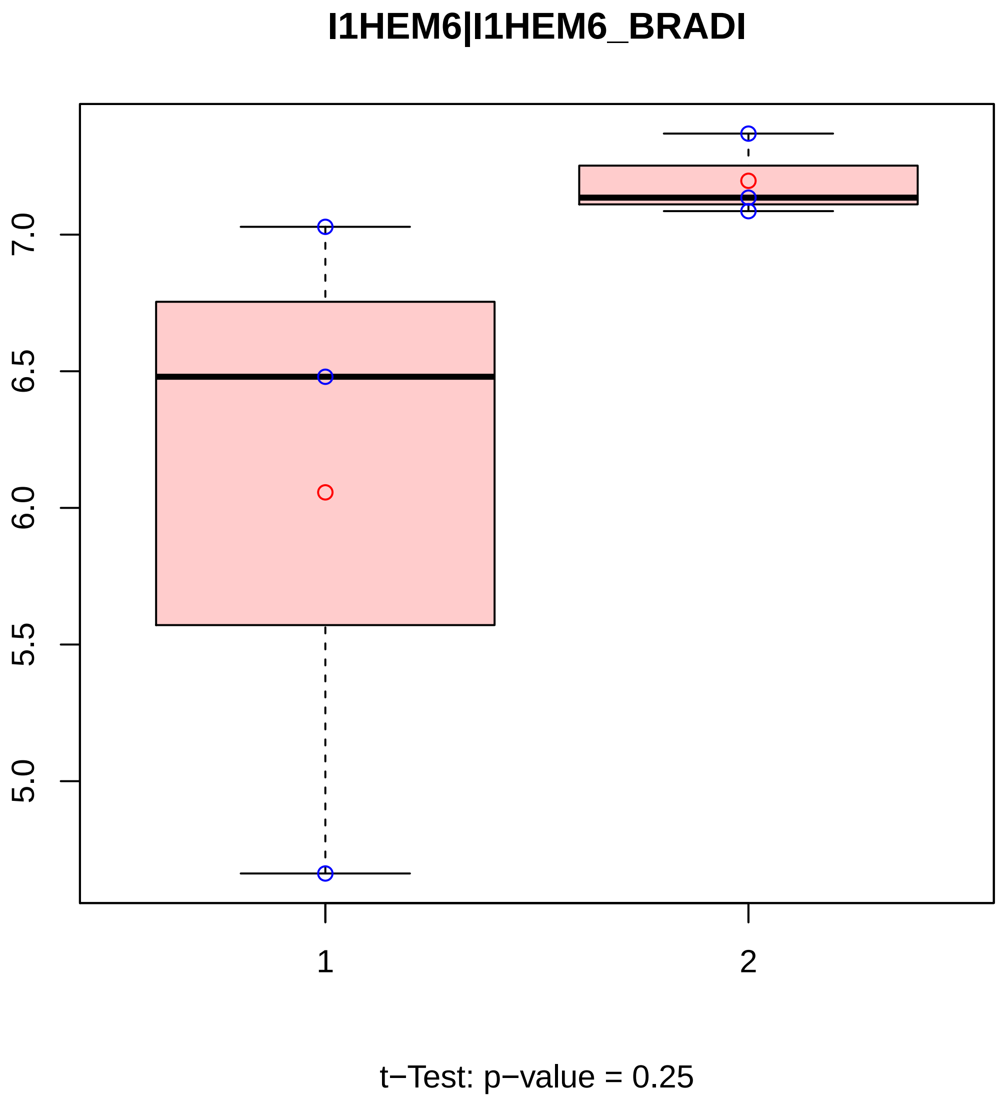

I1HEM6|I1HEM6_BRADI
5.0
5.5
6.0
6.5
7.0
1
2
t−Test: p−value = 0.25

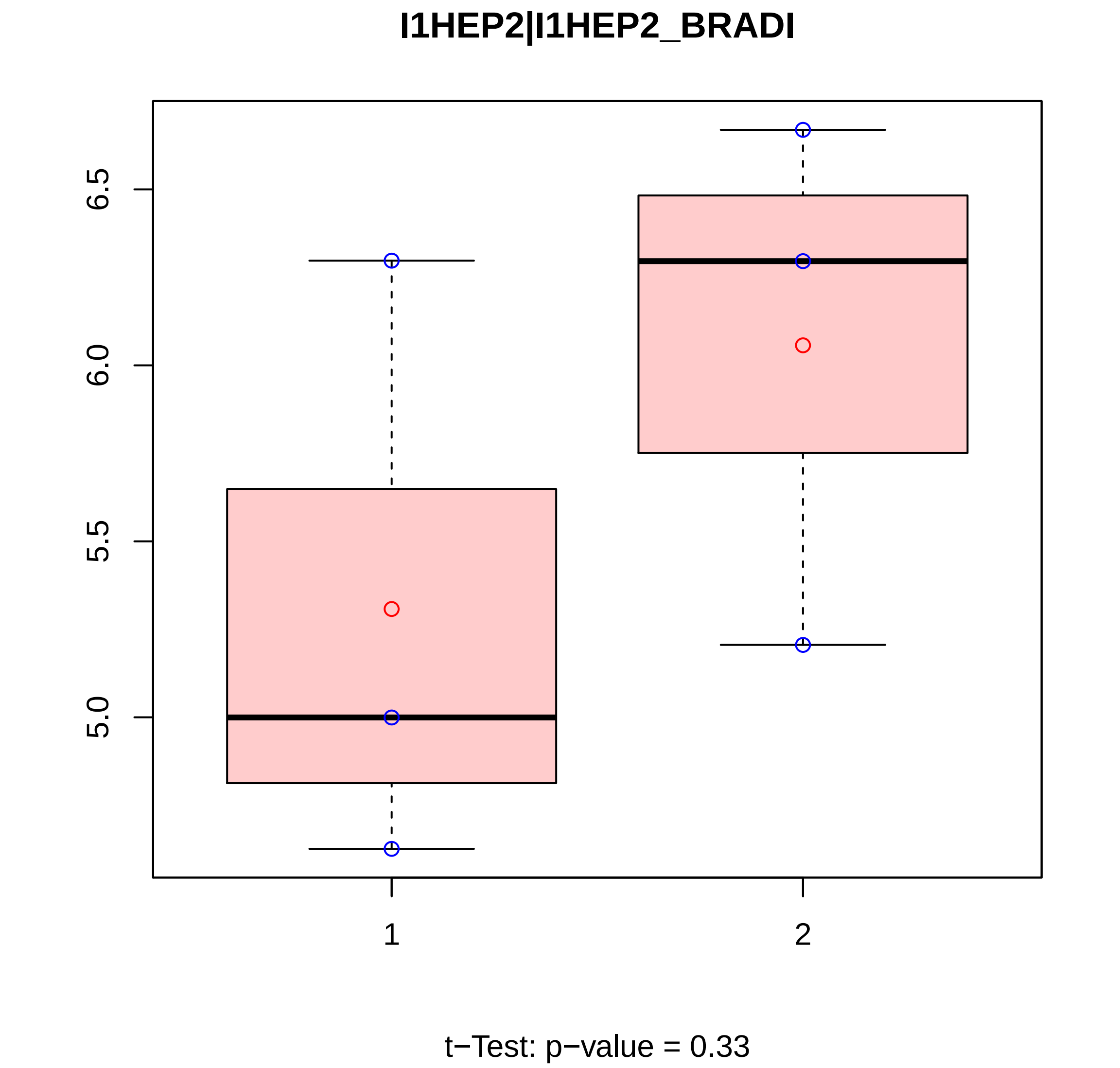
I1HEP2|I1HEP2_BRADI
6.5
6.0
5.5
5.0
1
2
t−Test: p−value = 0.33

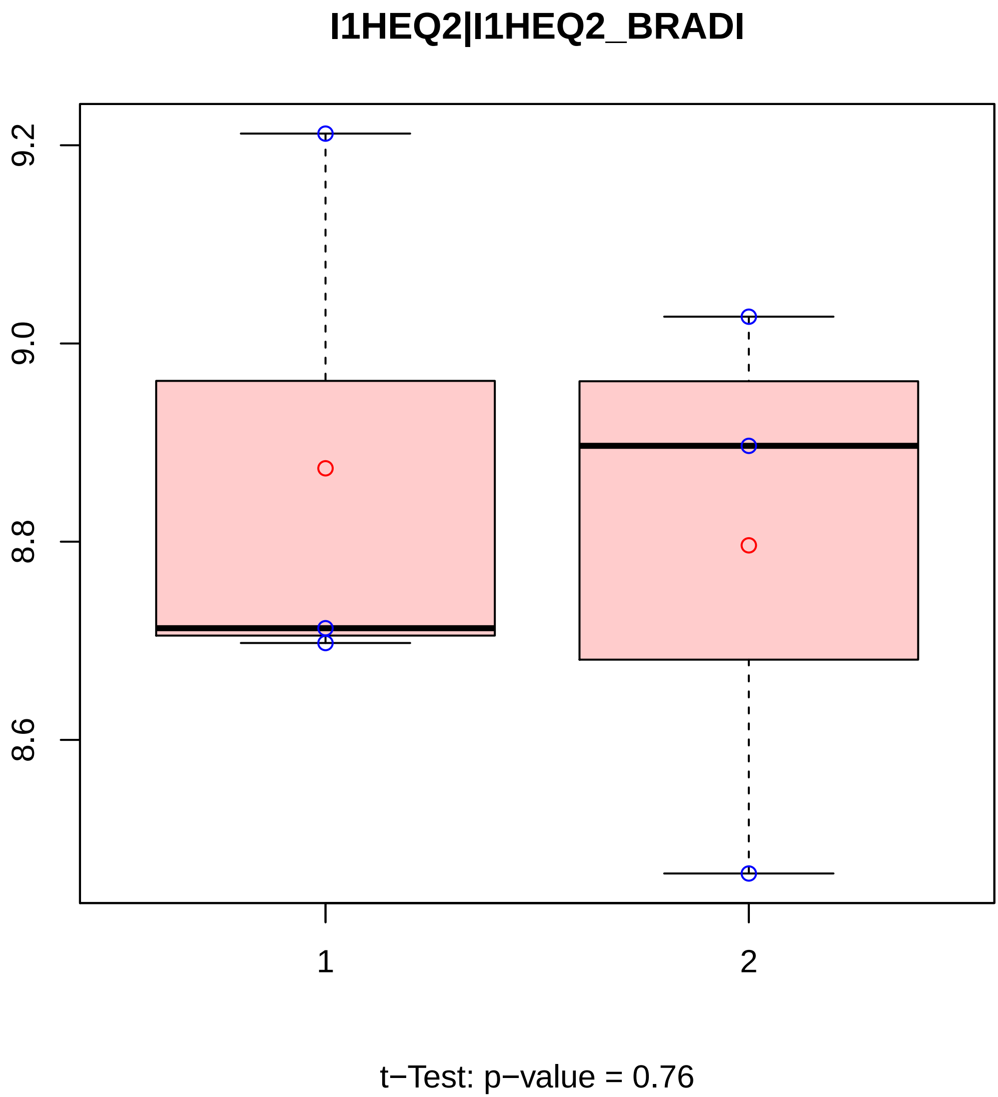
I1HEQ2|I1HEQ2_BRADI
9.2
9.0
8.8
8.6
1
2
t−Test: p−value = 0.76

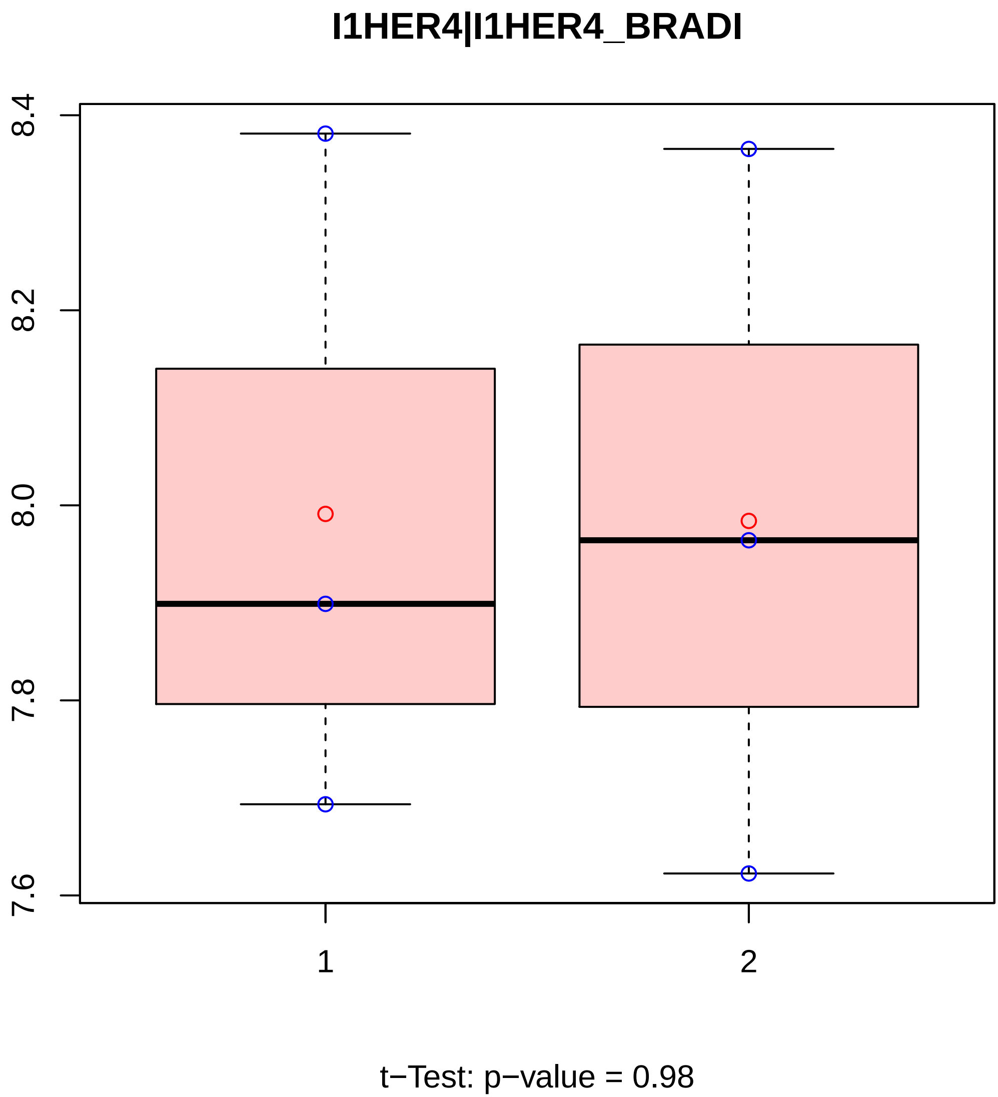

I1HER4|I1HER4_BRADI
8.4
8.2
8.0
7.8
7.6
1
2
t−Test: p−value = 0.98

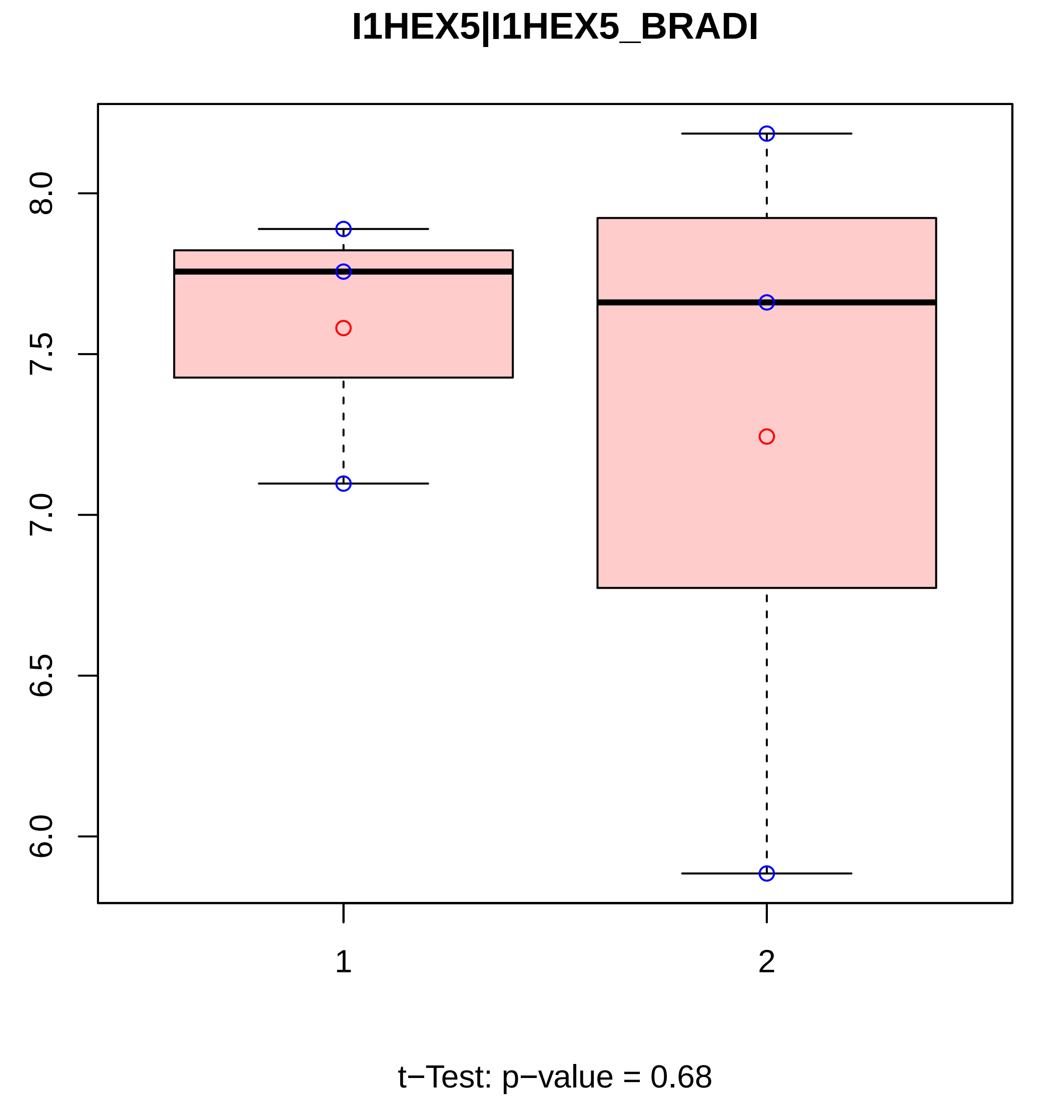
I1HEX5|I1HEX5_BRADI
8.0
7.5
7.0
6.5
6.0
1
2
t−Test: p−value = 0.68

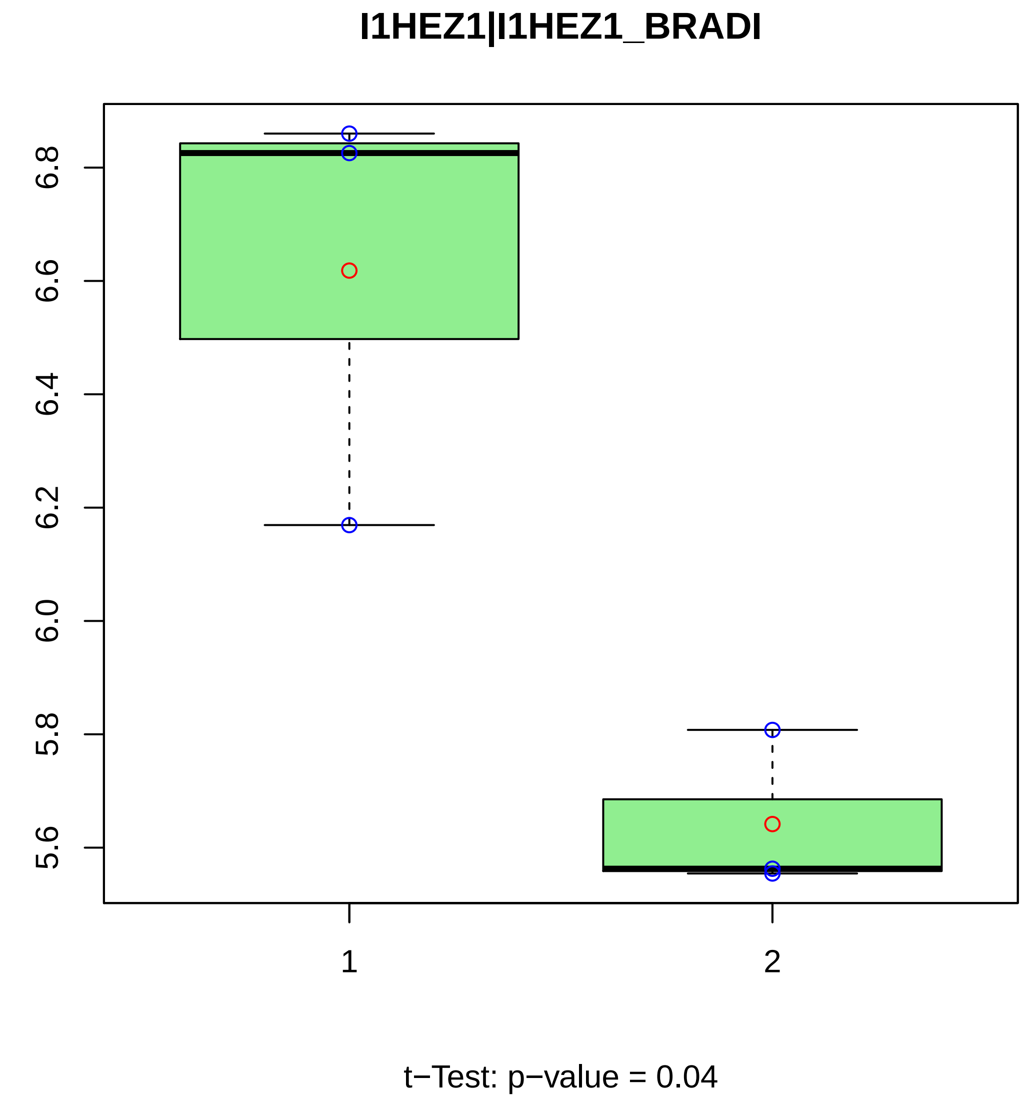
I1HEZ1|I1HEZ1_BRADI
6.8
6.6
6.4
6.2
6.0
5.8
5.6
1
2
t−Test: p−value = 0.04

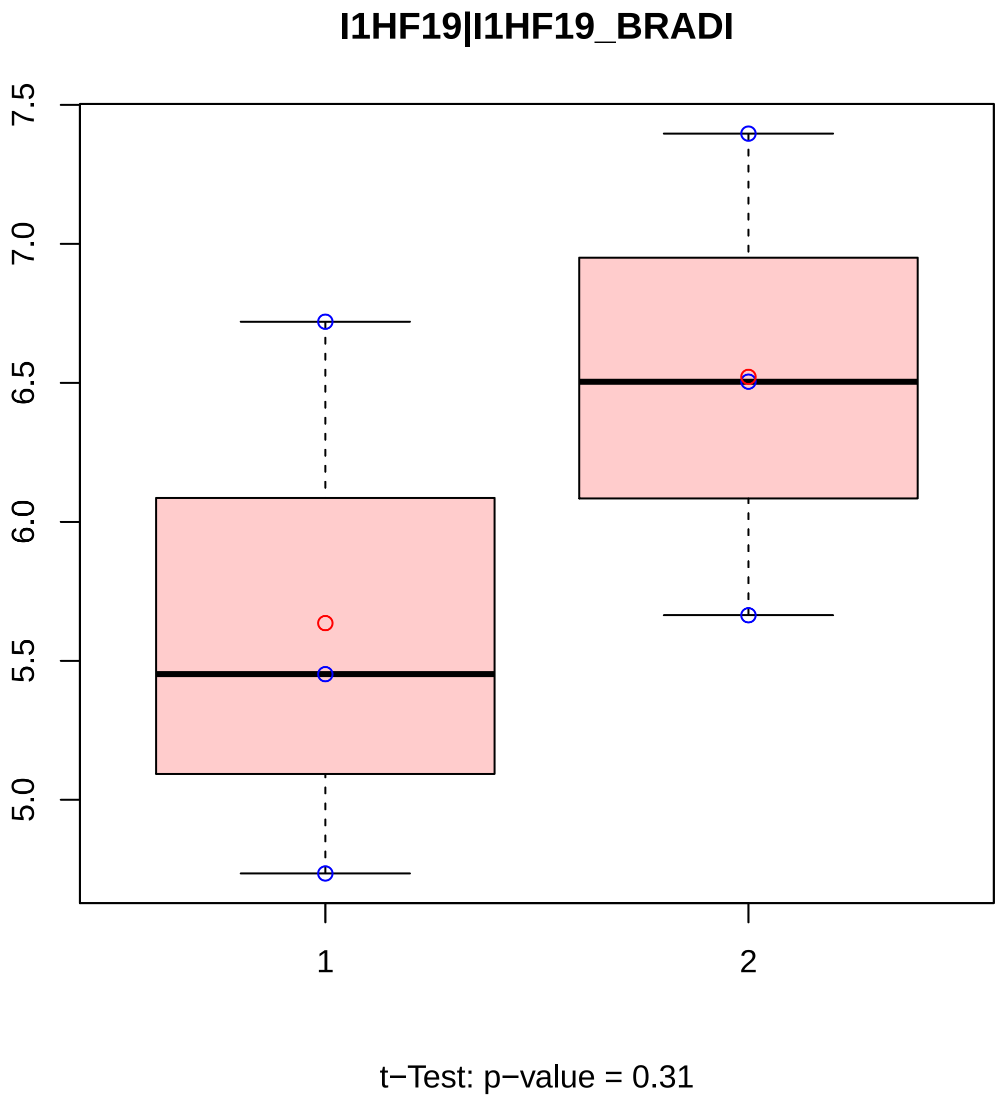
I1HF19|I1HF19_BRADI
7.5
7.0
6.5
6.0
5.5
5.0
1
2
t−Test: p−value = 0.31

I1HF20|I1HF20_BRADI

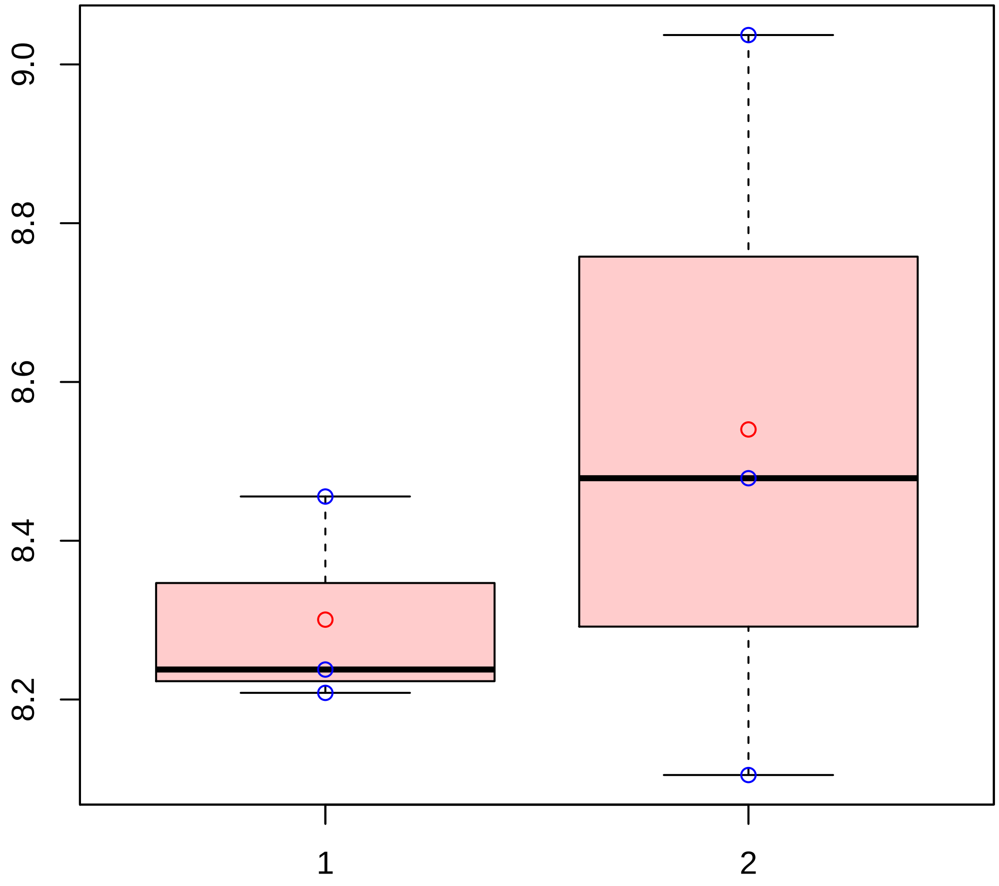

t−Test: p−value = 0.47

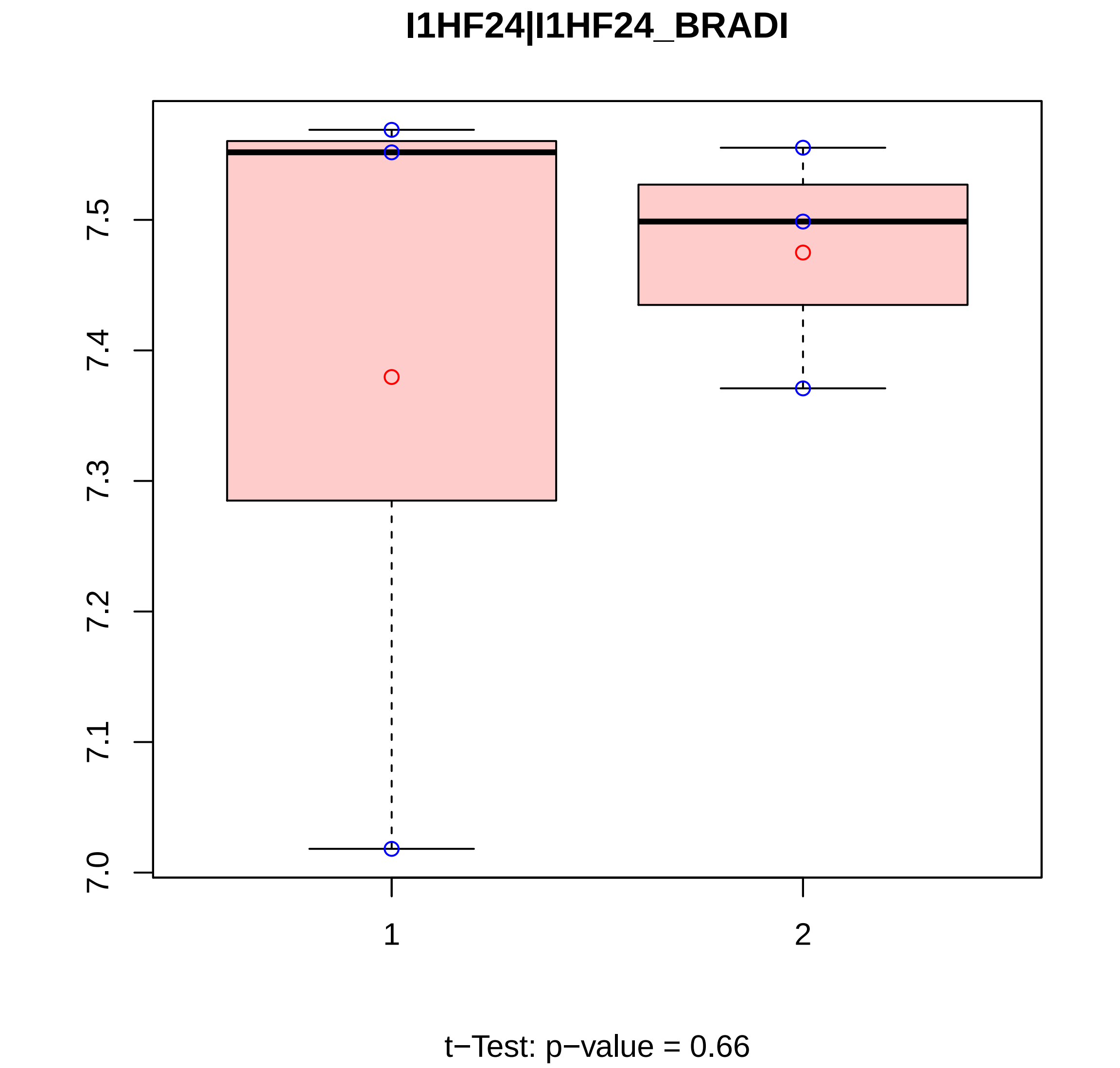
I1HF24|I1HF24_BRADI
7.0
7.1
7.2
7.3
7.4
7.5
1
2
t−Test: p−value = 0.66

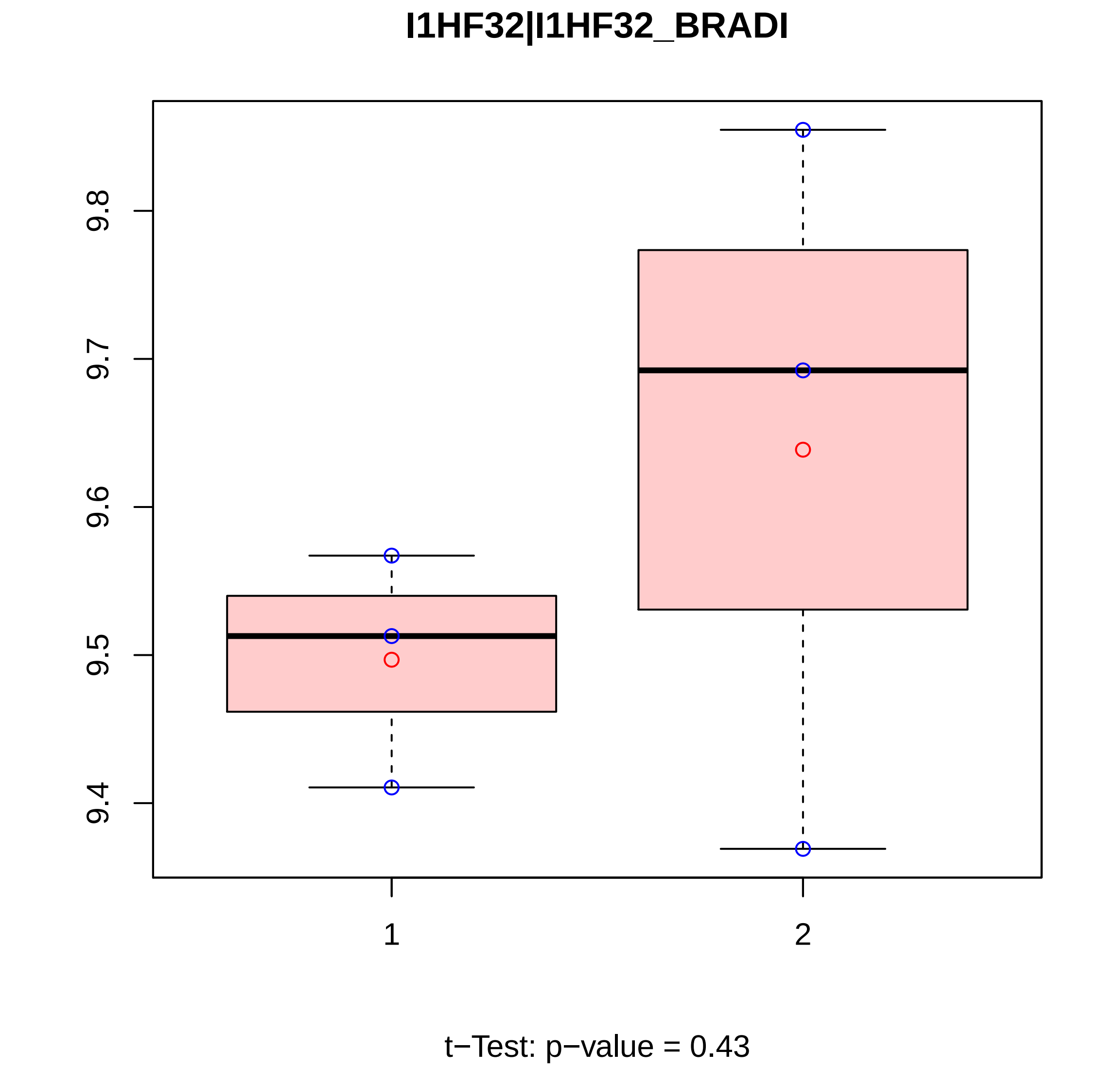
I1HF32|I1HF32_BRADI
9.4
9.5
9.6
9.7
9.8
1
2
t−Test: p−value = 0.43

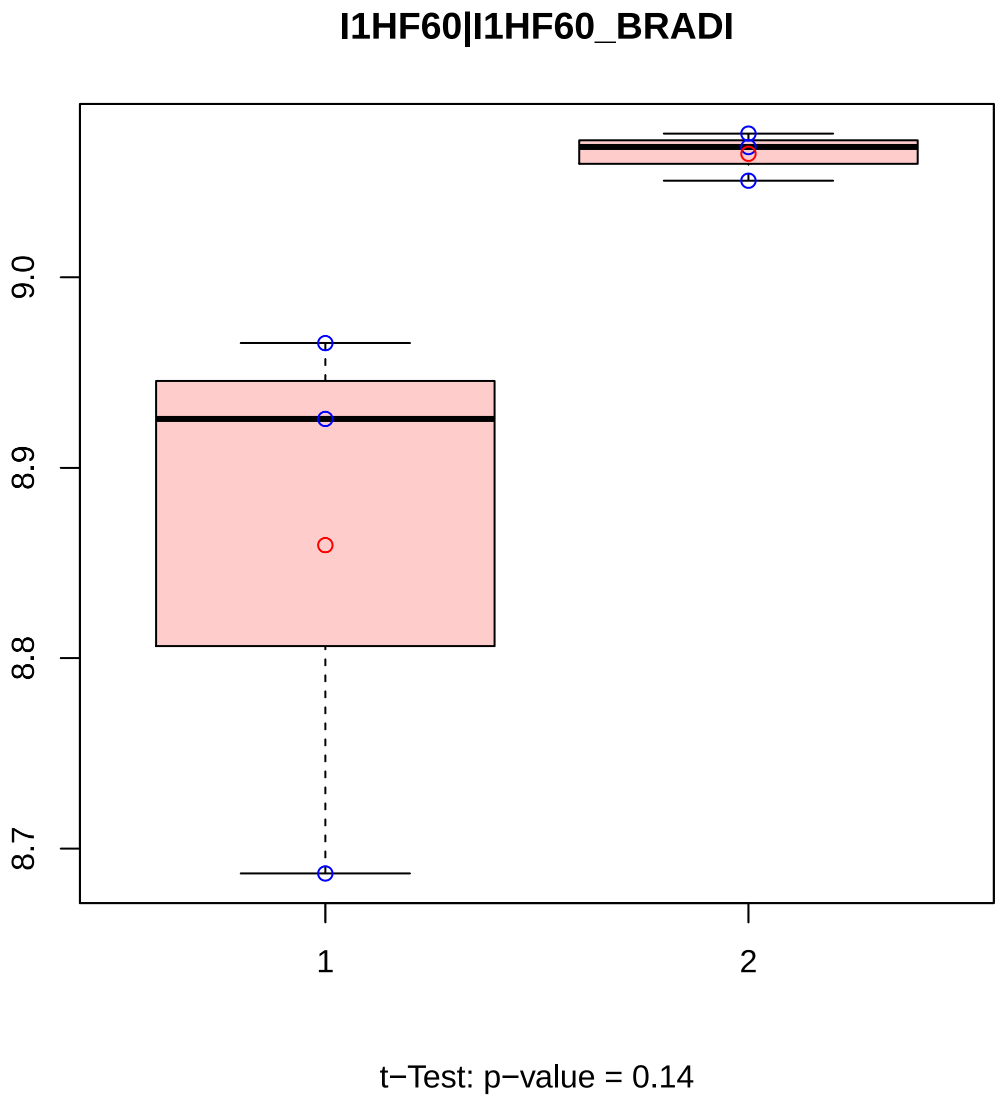
I1HF60|I1HF60_BRADI
9.0
8.9
8.8
8.7
1
2
t−Test: p−value = 0.14

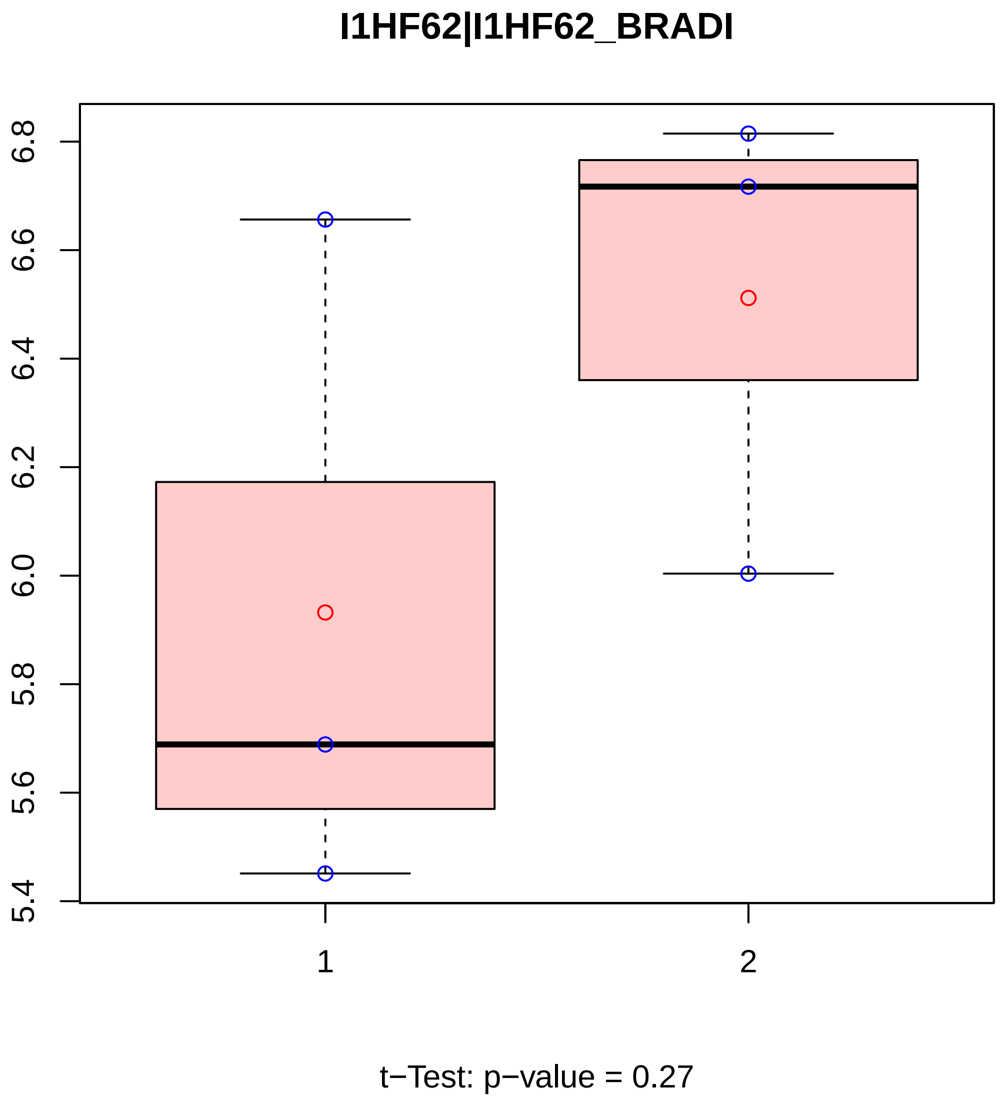
I1HF62|I1HF62_BRADI
6.8
6.6
6.4
6.2
6.0
5.8
5.6
5.4
1
2
t−Test: p−value = 0.27

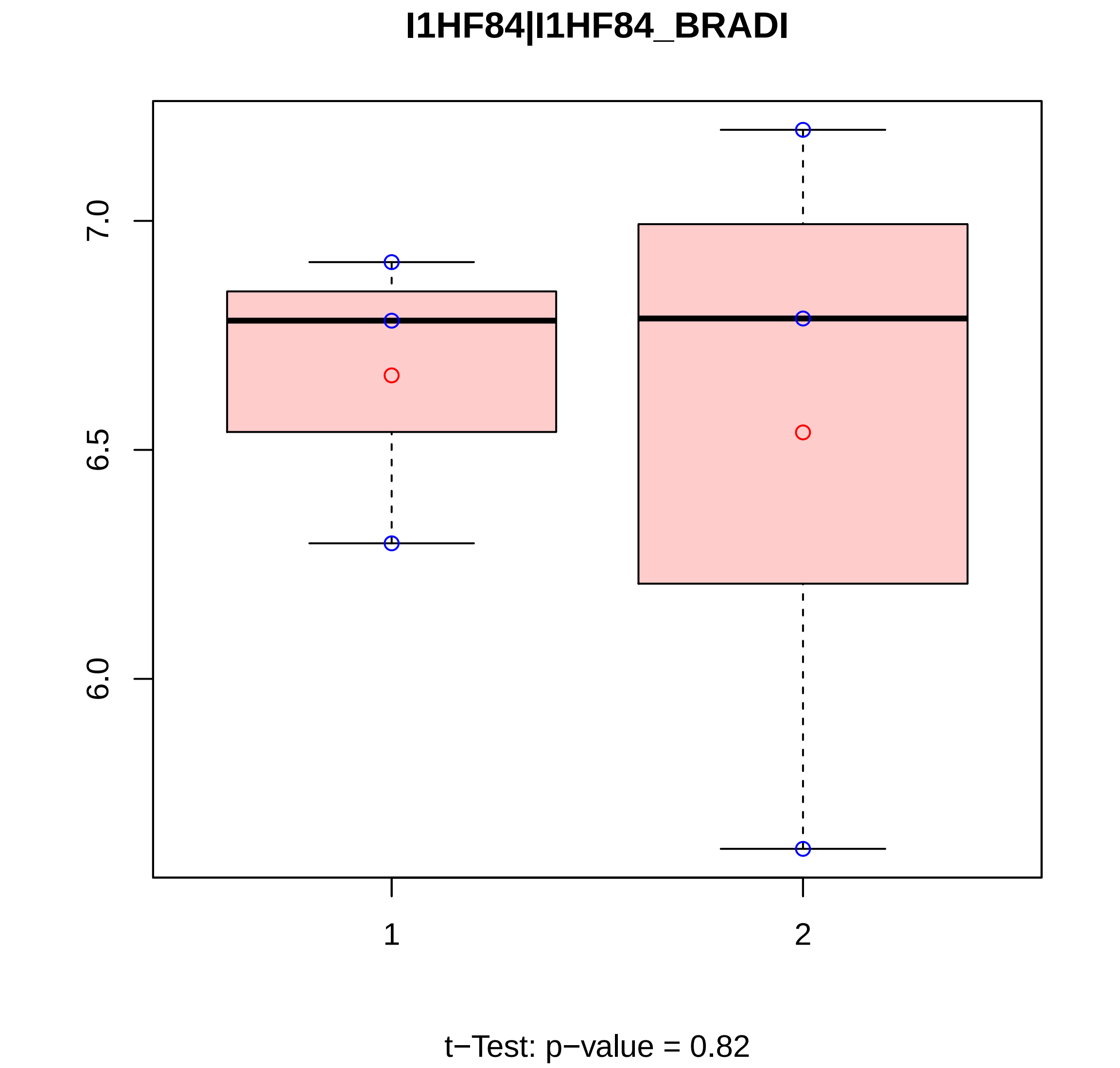
I1HF84|I1HF84_BRADI
7.0
6.5
6.0
1
2
t−Test: p−value = 0.82

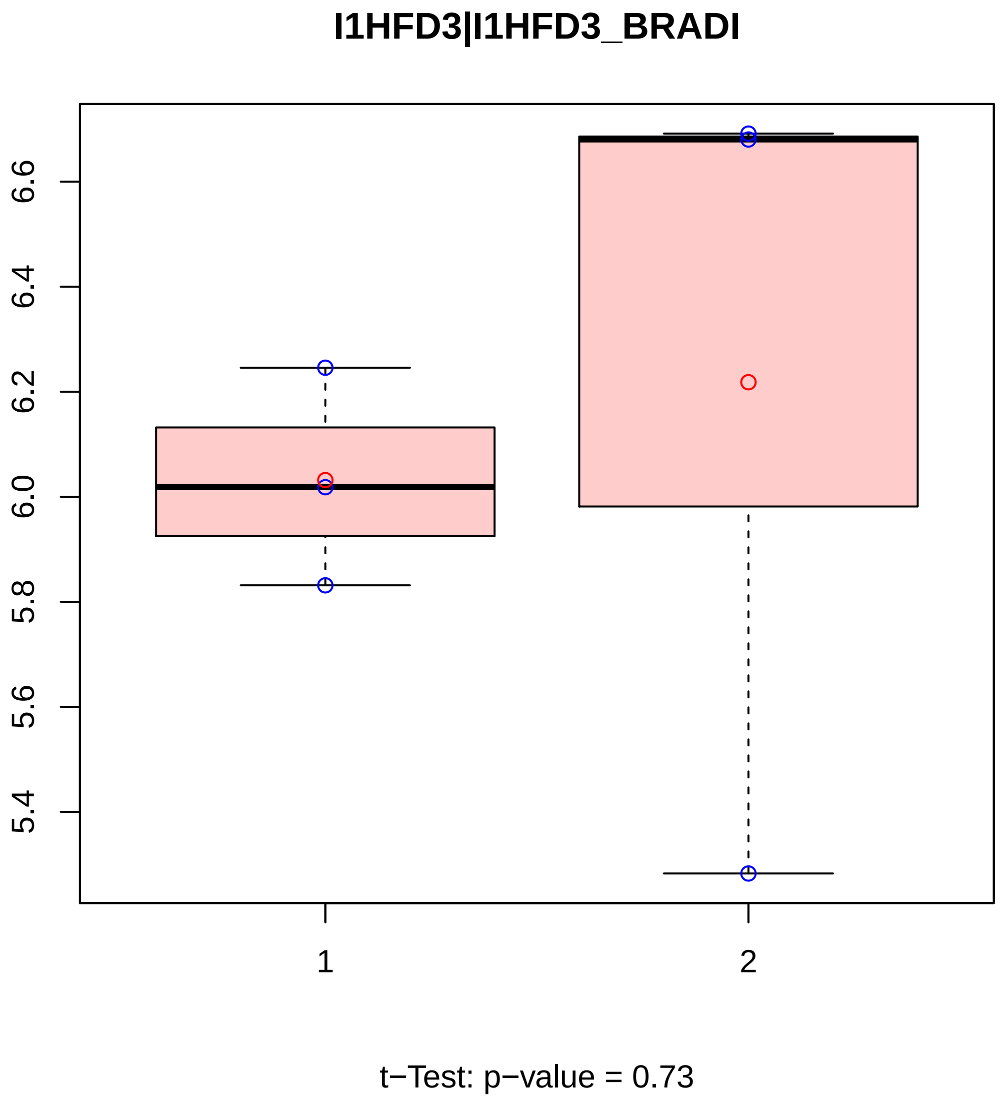
I1HFD3|I1HFD3_BRADI
6.6
6.4
6.2
6.0
5.8
5.6
5.4
1
2
t−Test: p−value = 0.73

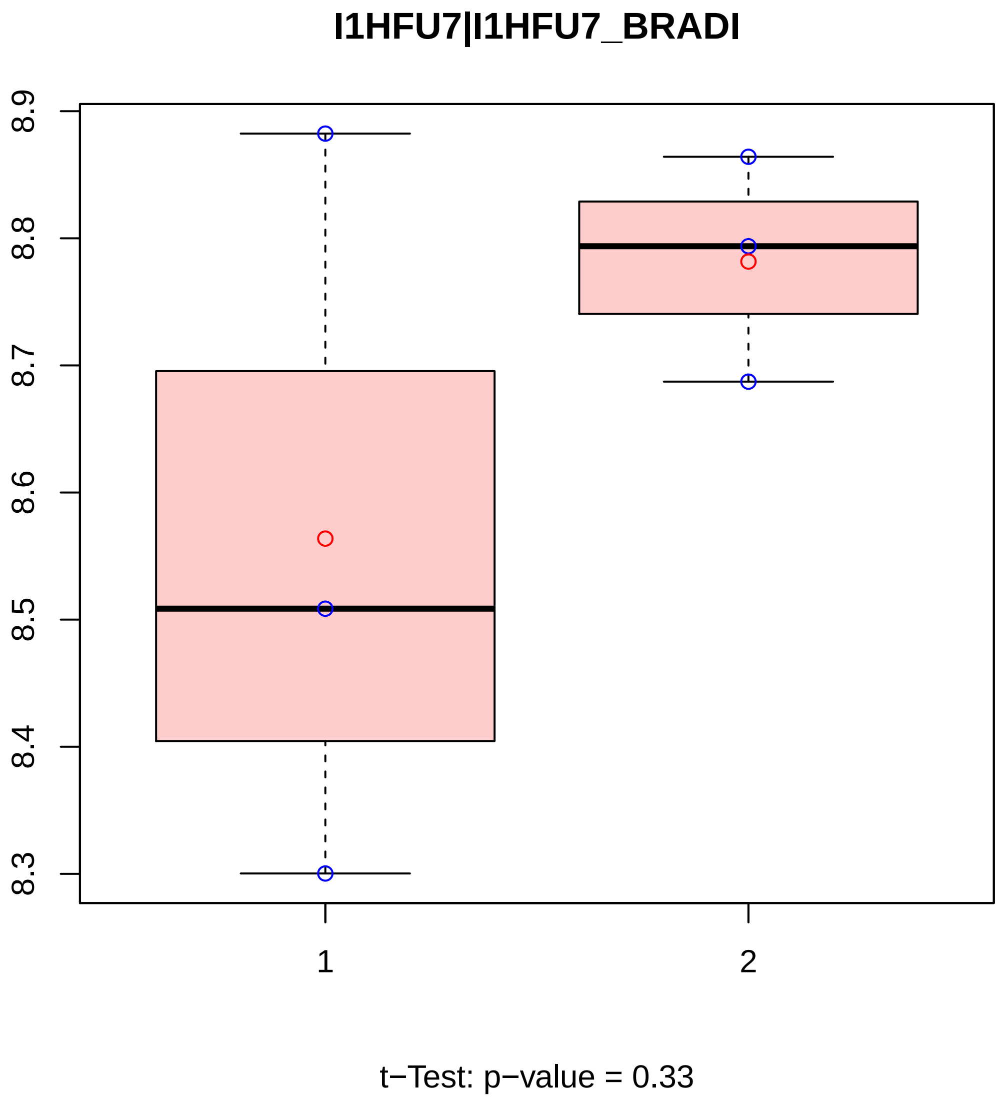
I1HFU7|I1HFU7_BRADI
8.3
8.4
8.5
8.6
8.7
8.8
8.9
1
2
t−Test: p−value = 0.33

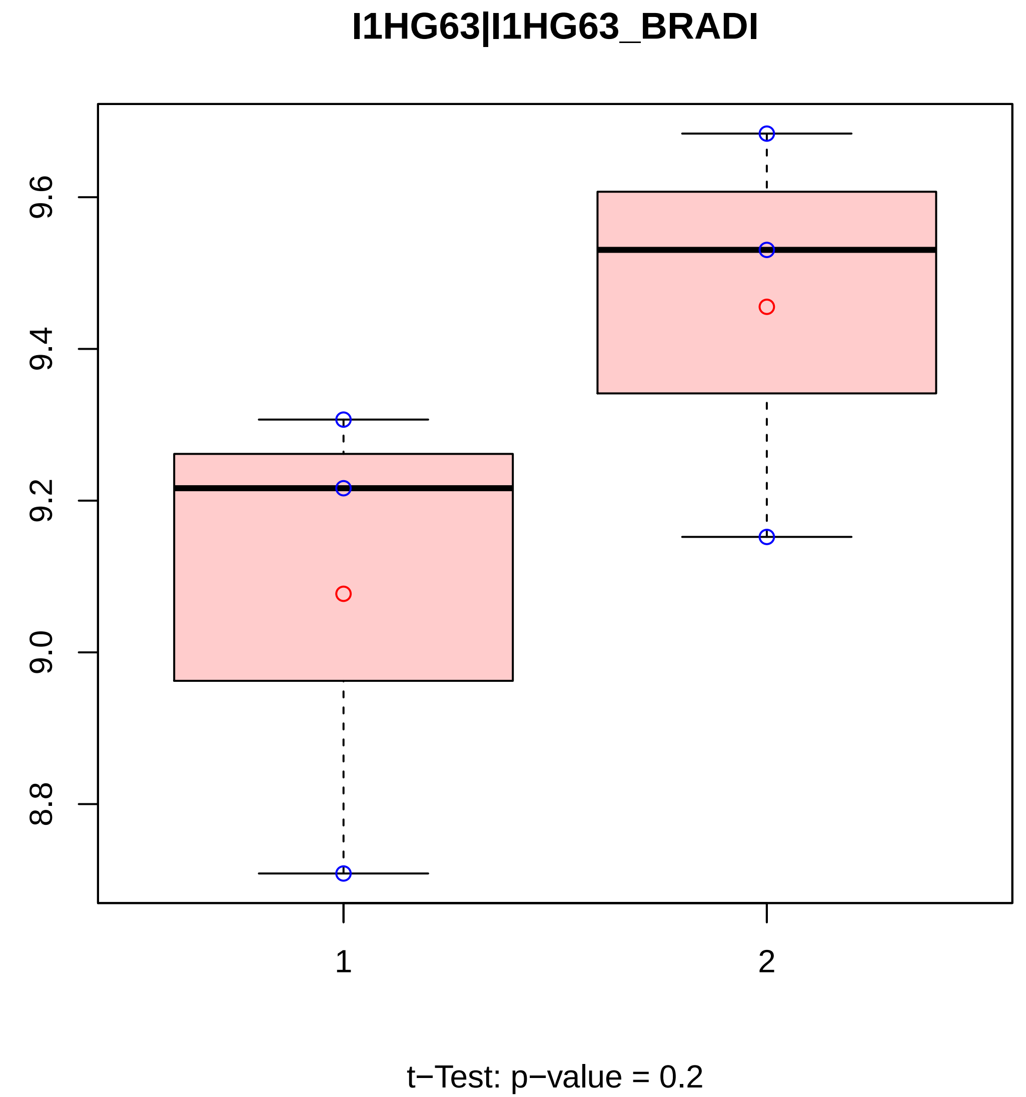
I1HG63|I1HG63_BRADI
9.6
9.4
9.2
9.0
8.8
1
2
t−Test: p−value = 0.2

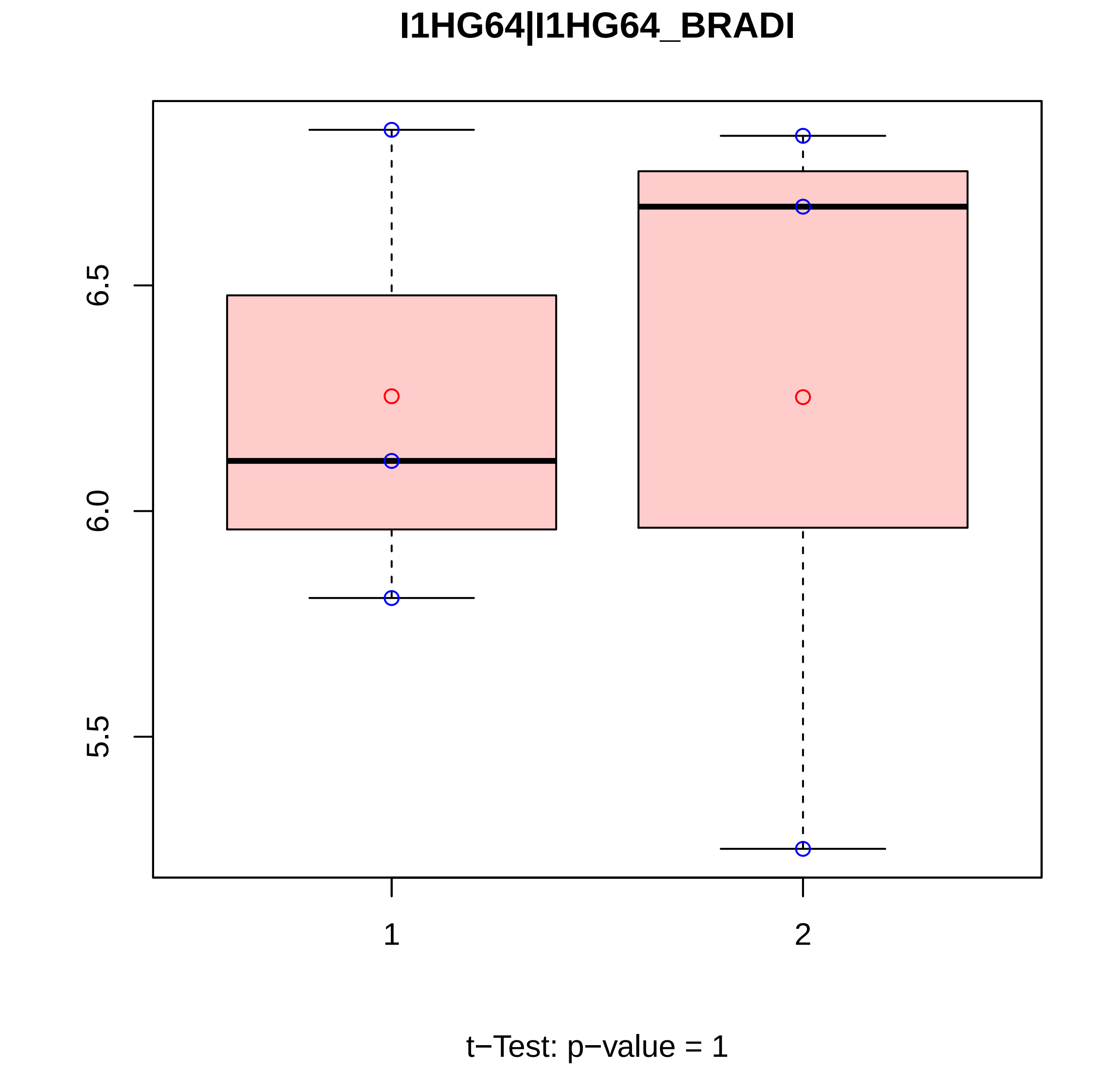

I1HG64|I1HG64_BRADI
6.5
6.0
5.5
1
2
t−Test: p−value = 1

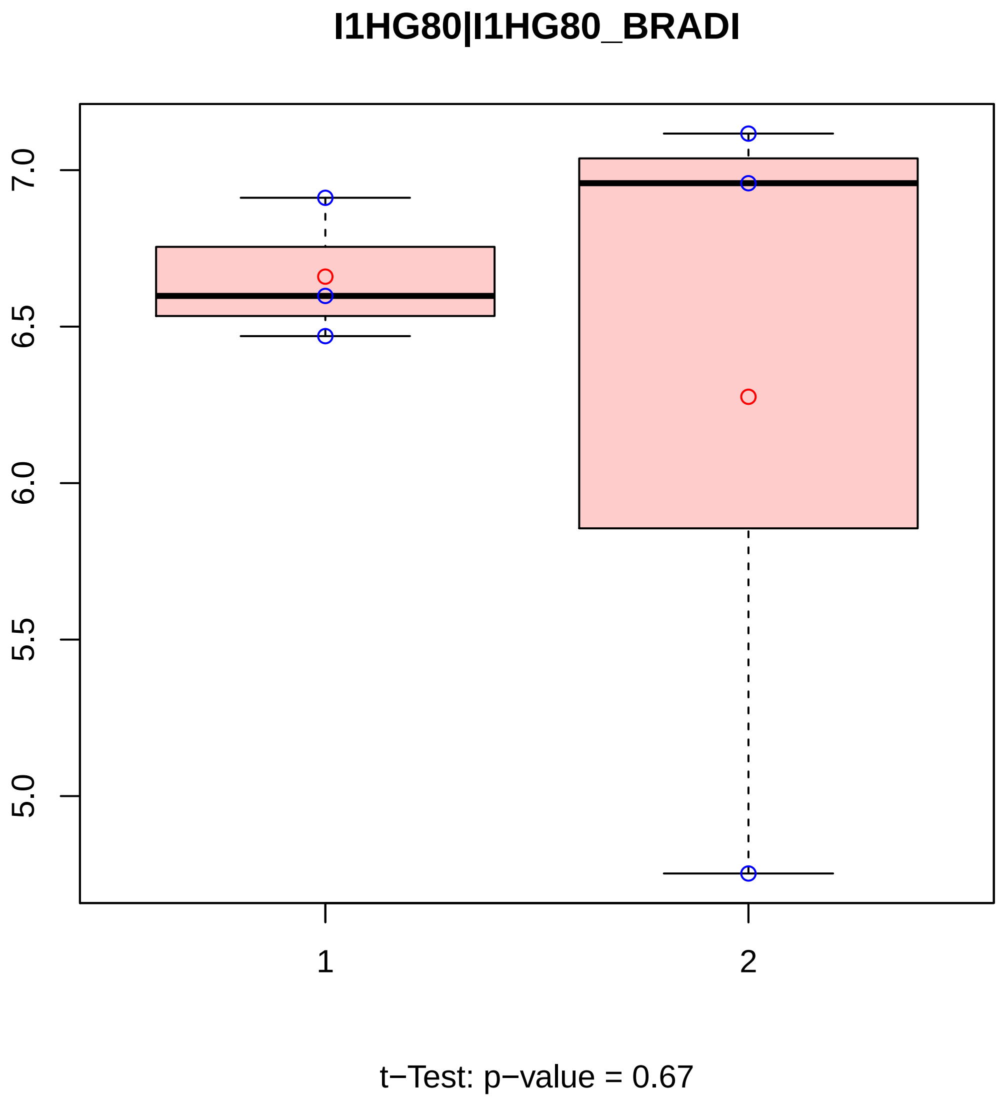
I1HG80|I1HG80_BRADI
7.0
6.5
6.0
5.5
5.0
1
2
t−Test: p−value = 0.67

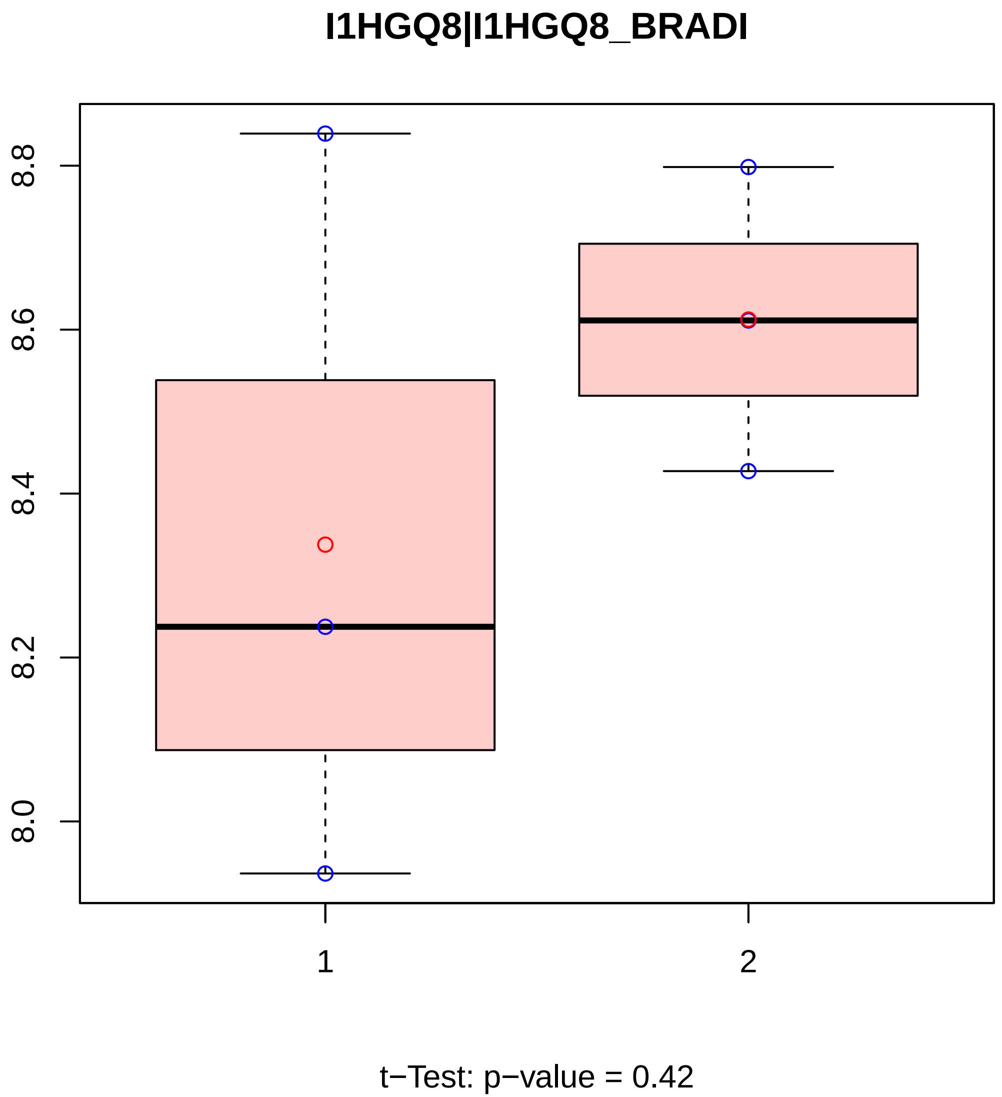
I1HGQ8|I1HGQ8_BRADI
8.0
8.2
8.4
8.6
8.8
1
2
t−Test: p−value = 0.42

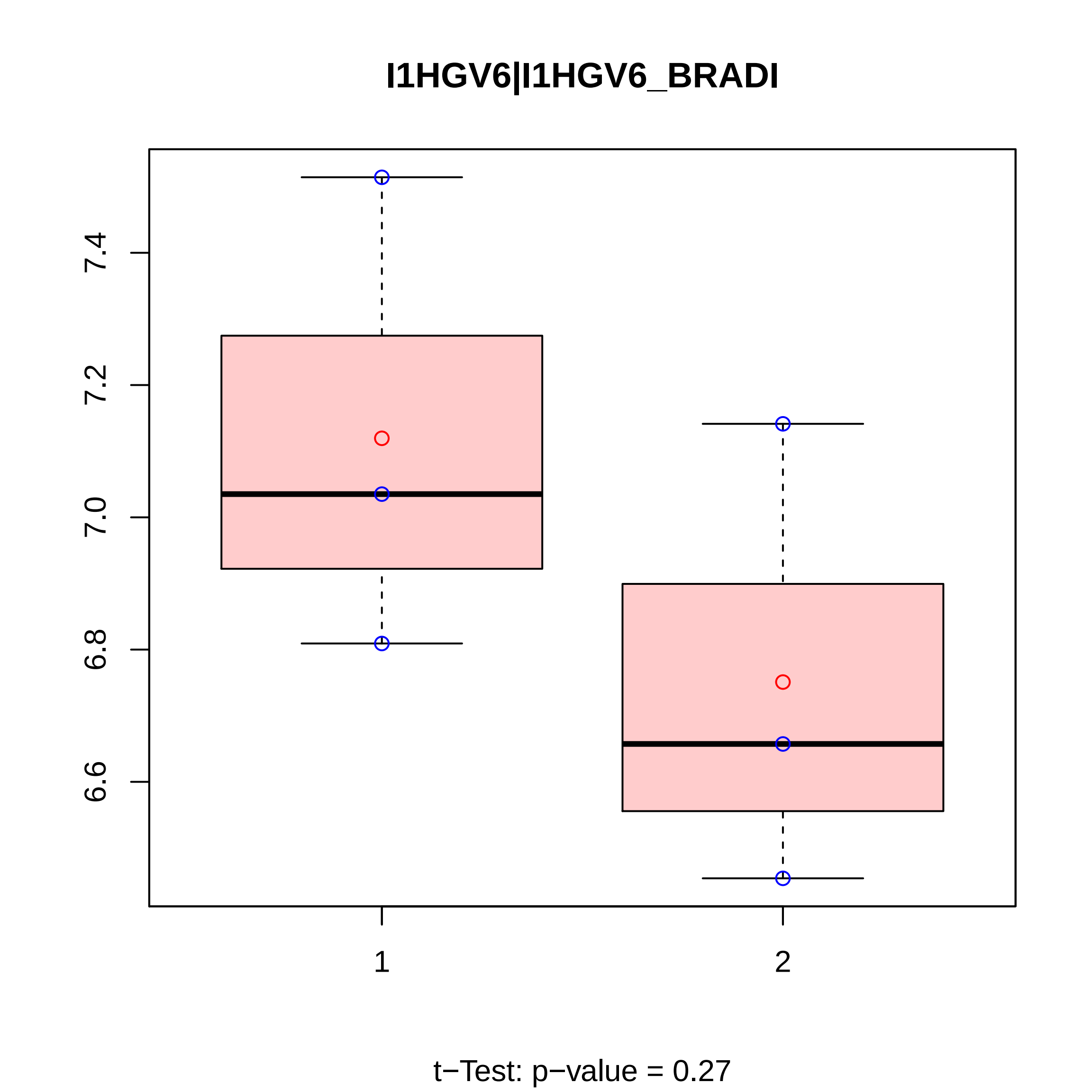

I1HGV6|I1HGV6_BRADI
7.4
7.2
7.0
6.8
6.6
1
2
t−Test: p−value = 0.27

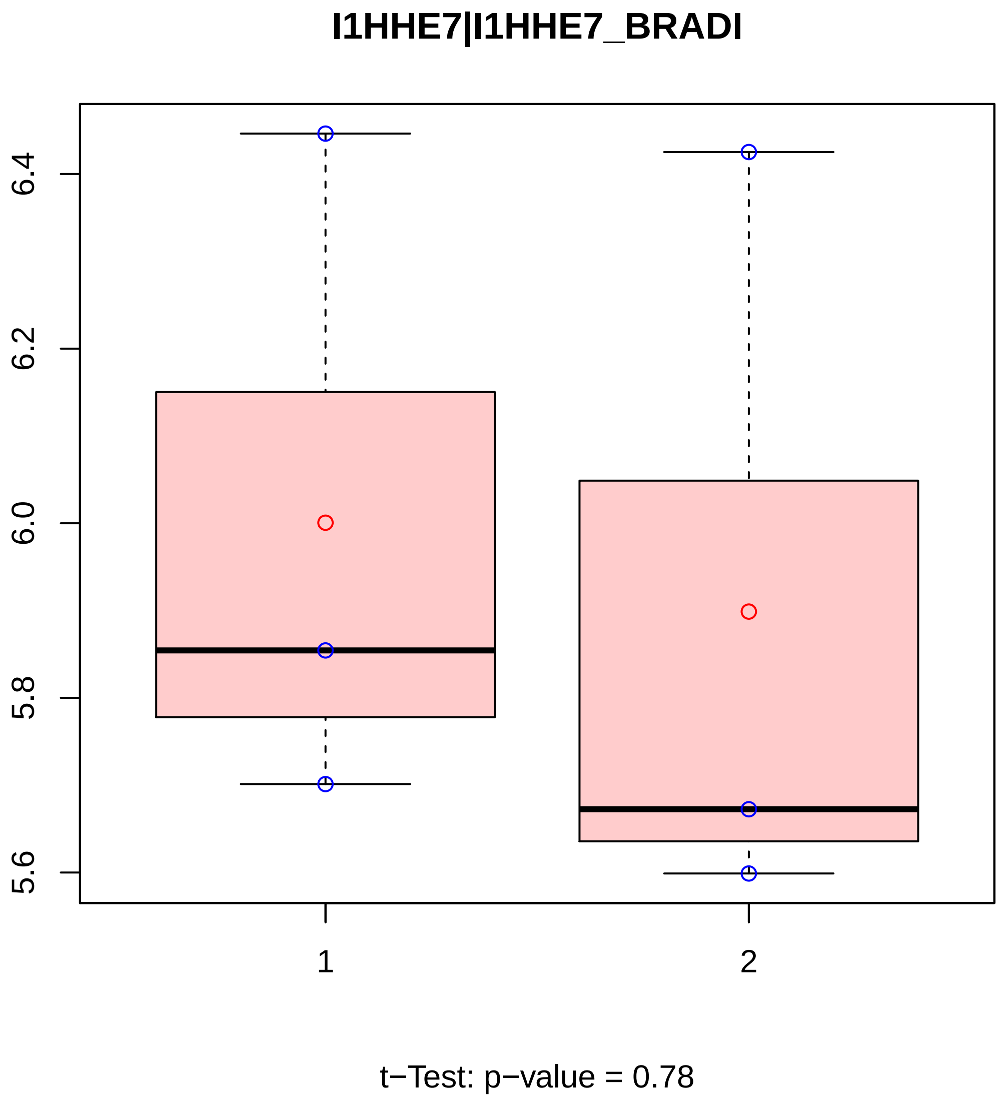
I1HHE7|I1HHE7_BRADI
6.4
6.2
6.0
5.8
5.6
1
2
t−Test: p−value = 0.78

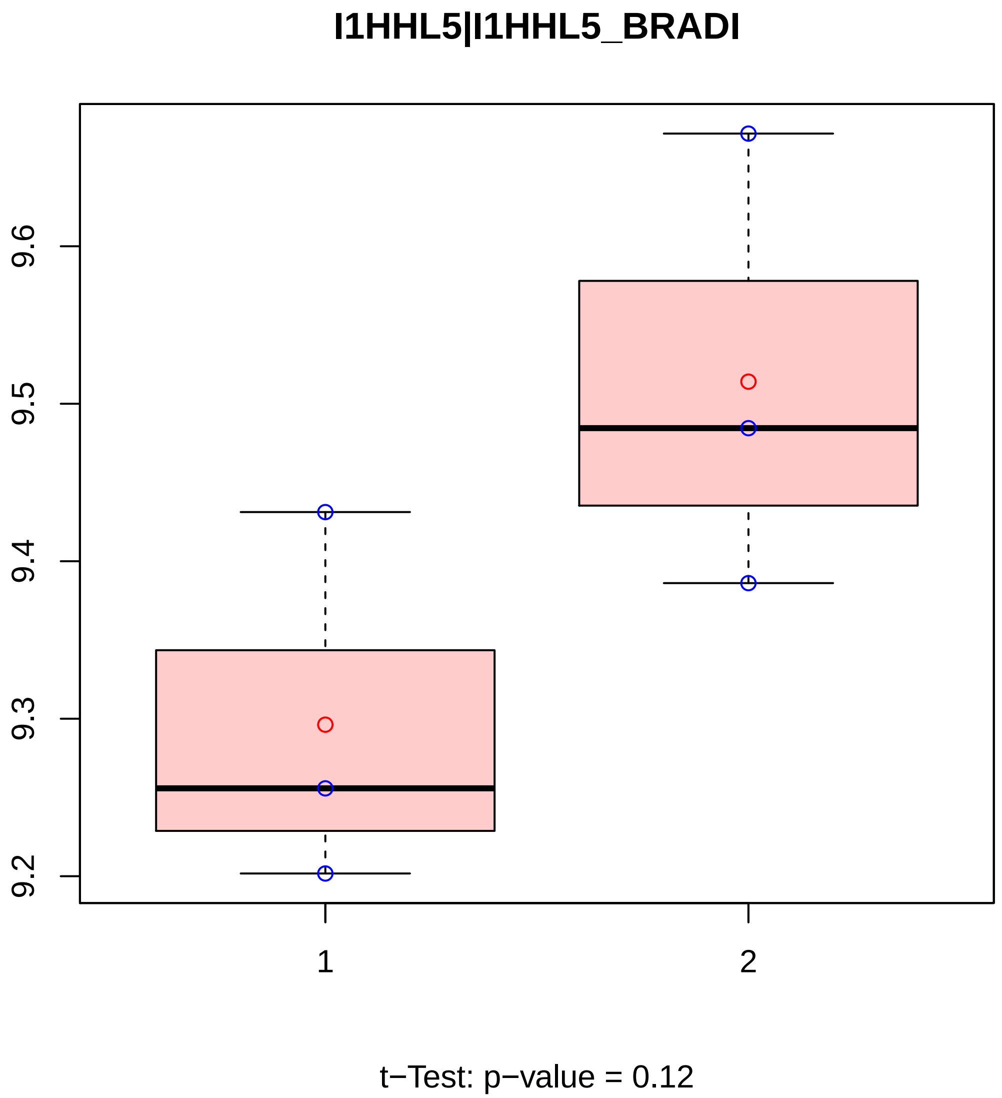

I1HHL5|I1HHL5_BRADI
9.2
9.3
9.4
9.5
9.6
1
2
t−Test: p−value = 0.12

I1HI06|I1HI06_BRADI

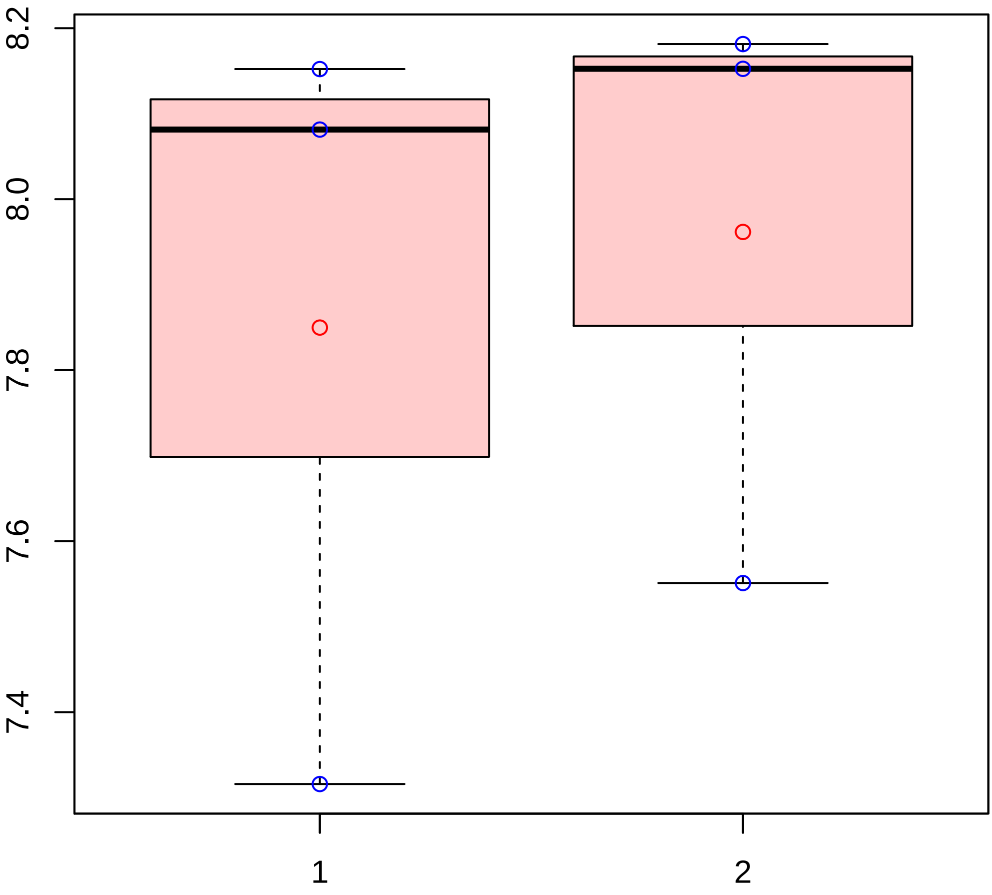

t−Test: p−value = 0.76

I1HI26|I1HI26_BRADI

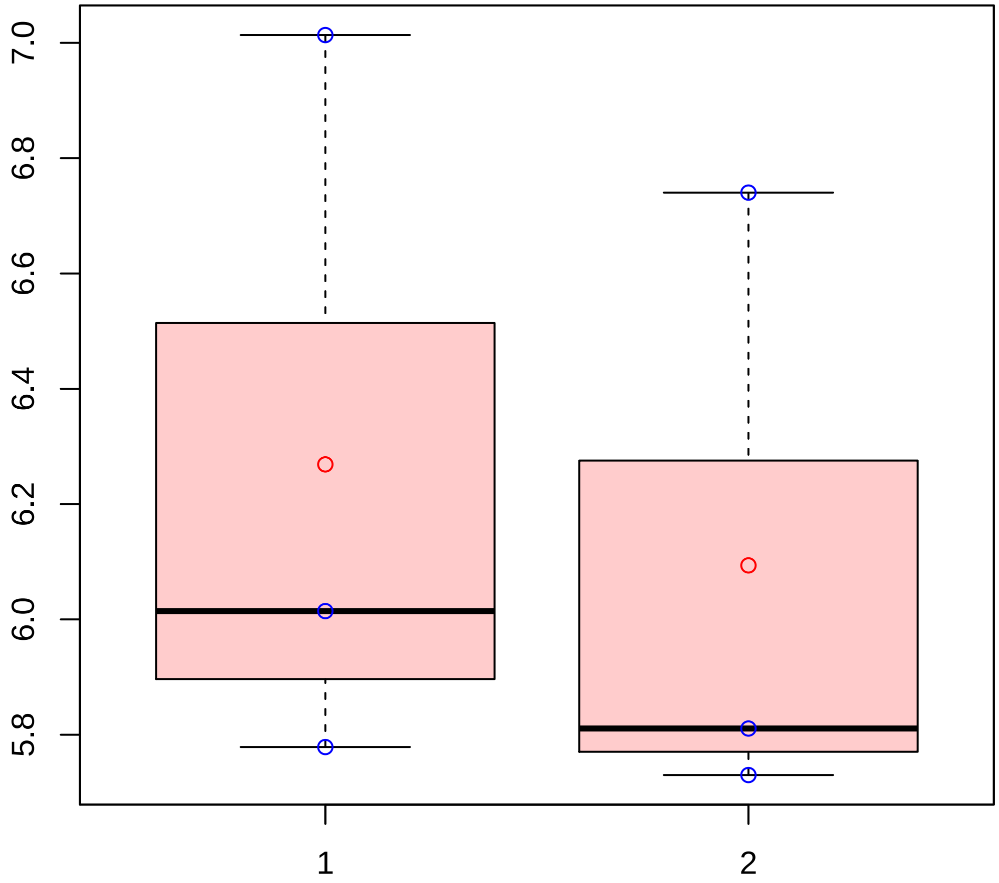

t−Test: p−value = 0.74

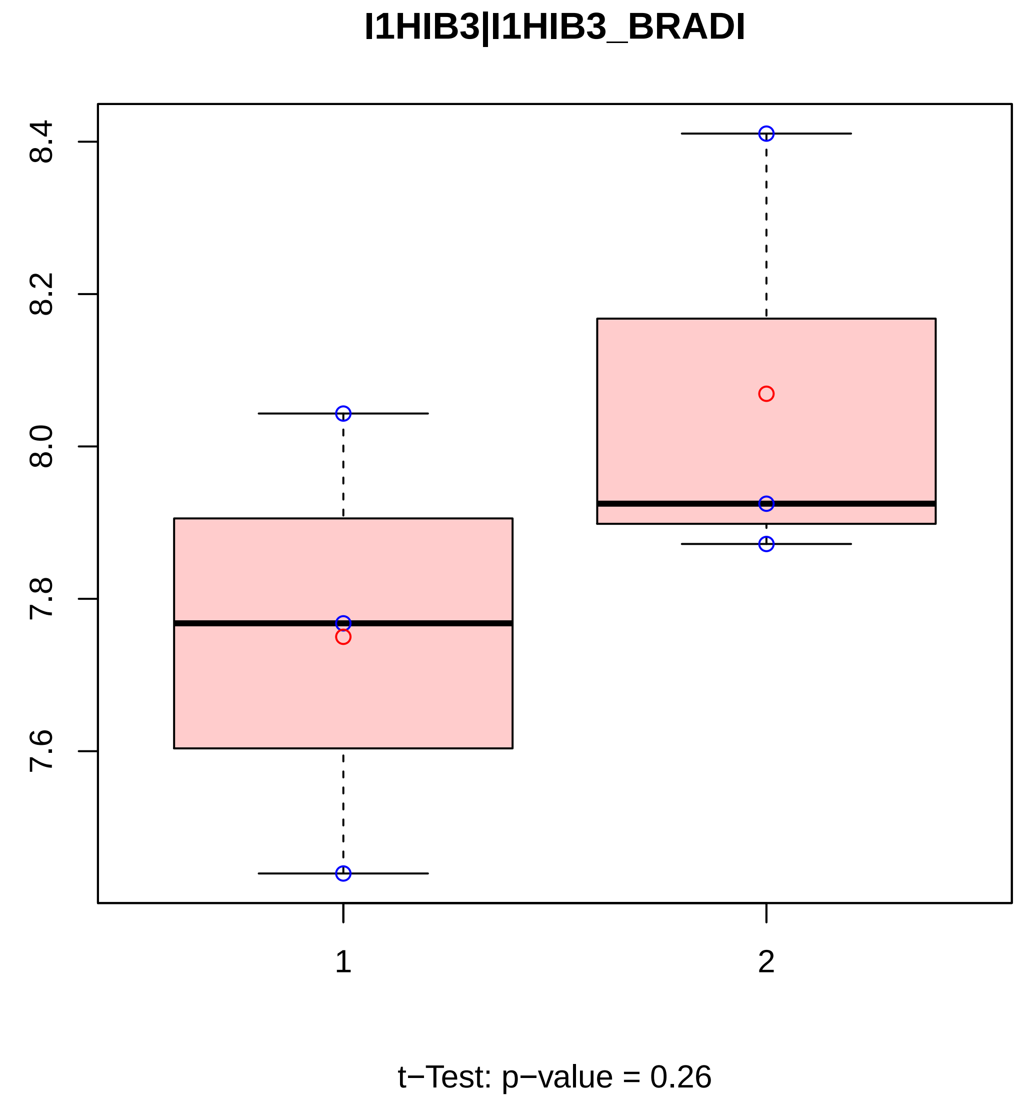
I1HIB3|I1HIB3_BRADI
8.4
8.2
8.0
7.8
7.6
1
2
t−Test: p−value = 0.26

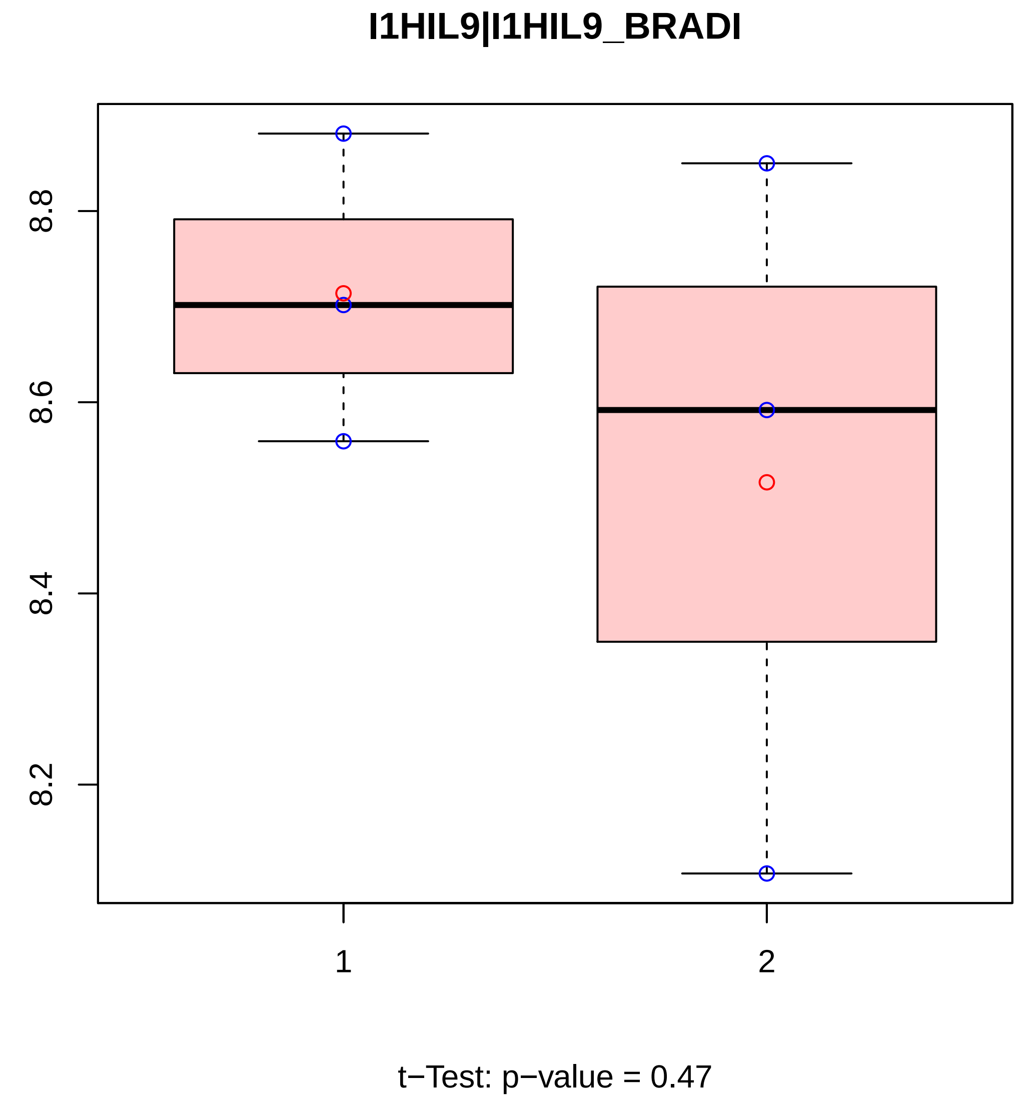
I1HIL9|I1HIL9_BRADI
8.2
8.4
8.6
8.8
1
2
t−Test: p−value = 0.47

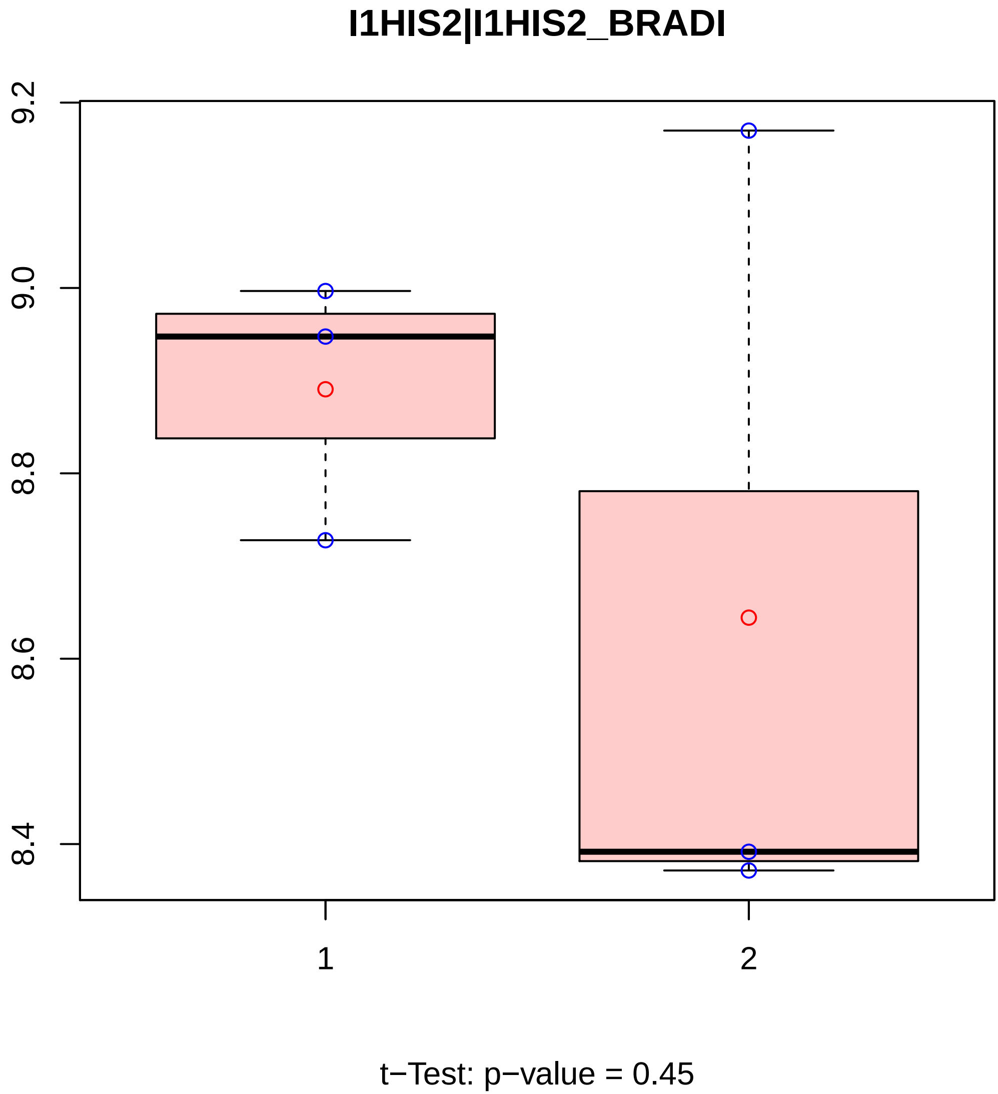
I1HIS2|I1HIS2_BRADI
9.2
9.0
8.8
8.6
8.4
1
2
t−Test: p−value = 0.45

I1HJ07|I1HJ07_BRADI

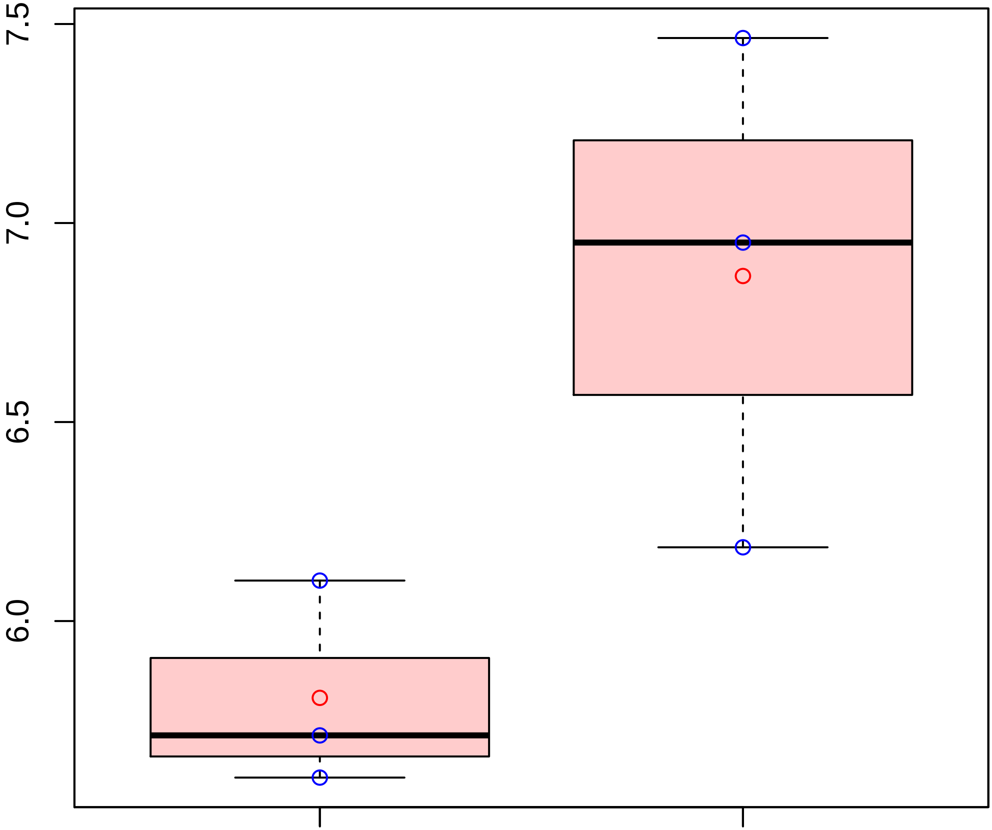

t−Test: p−value = 0.09

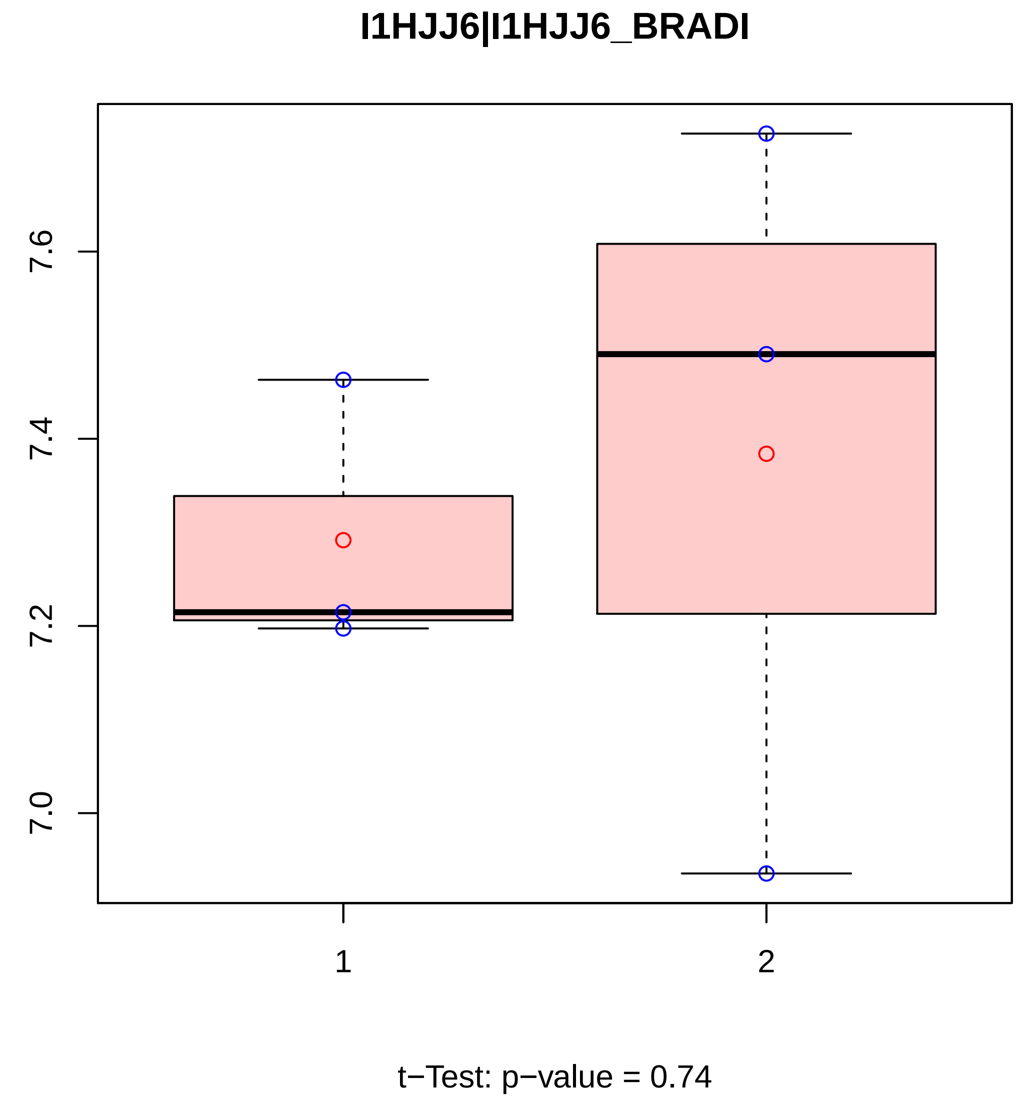
I1HJJ6|I1HJJ6_BRADI
7.0
7.2
7.4
7.6
1
2
t−Test: p−value = 0.74

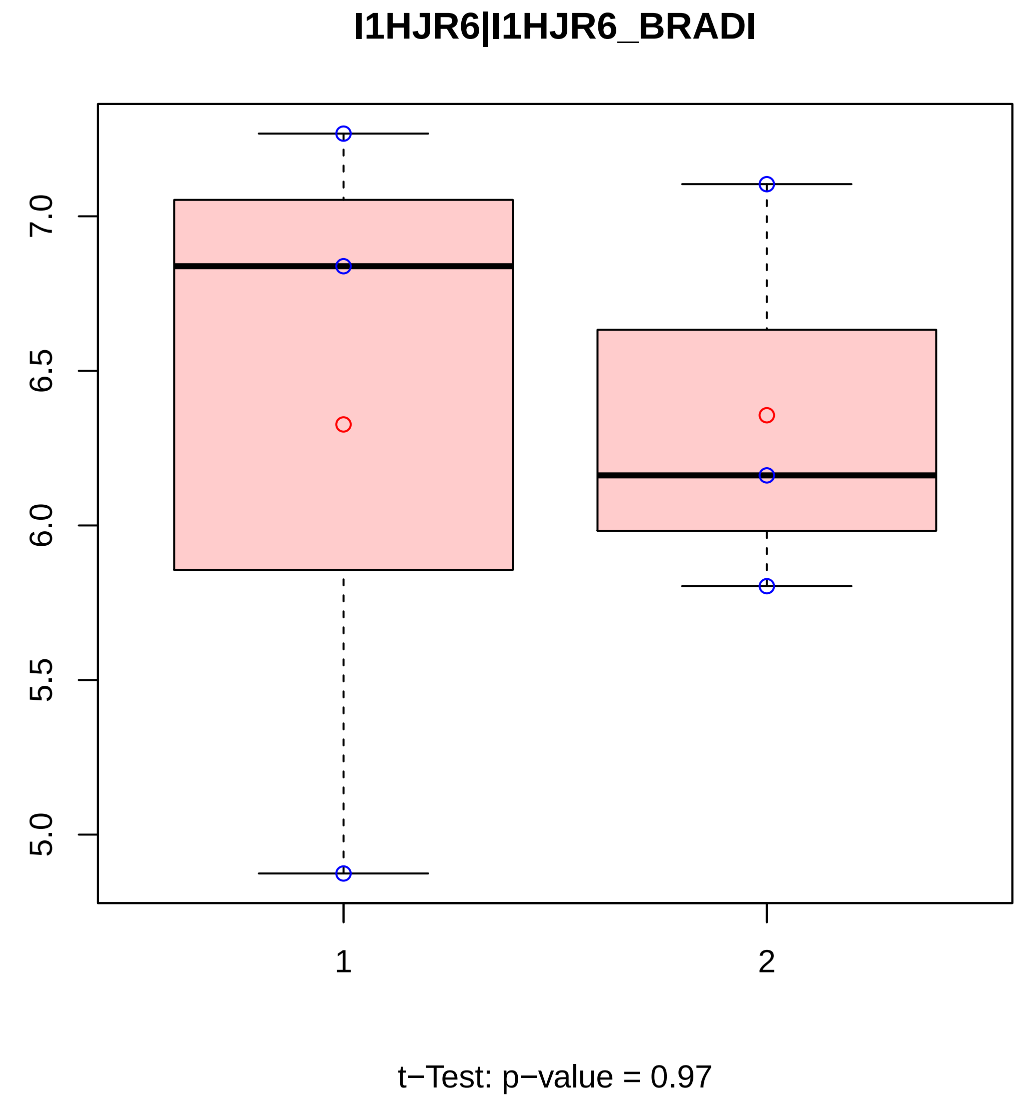
I1HJR6|I1HJR6_BRADI
7.0
6.5
6.0
5.5
5.0
1
2
t−Test: p−value = 0.97

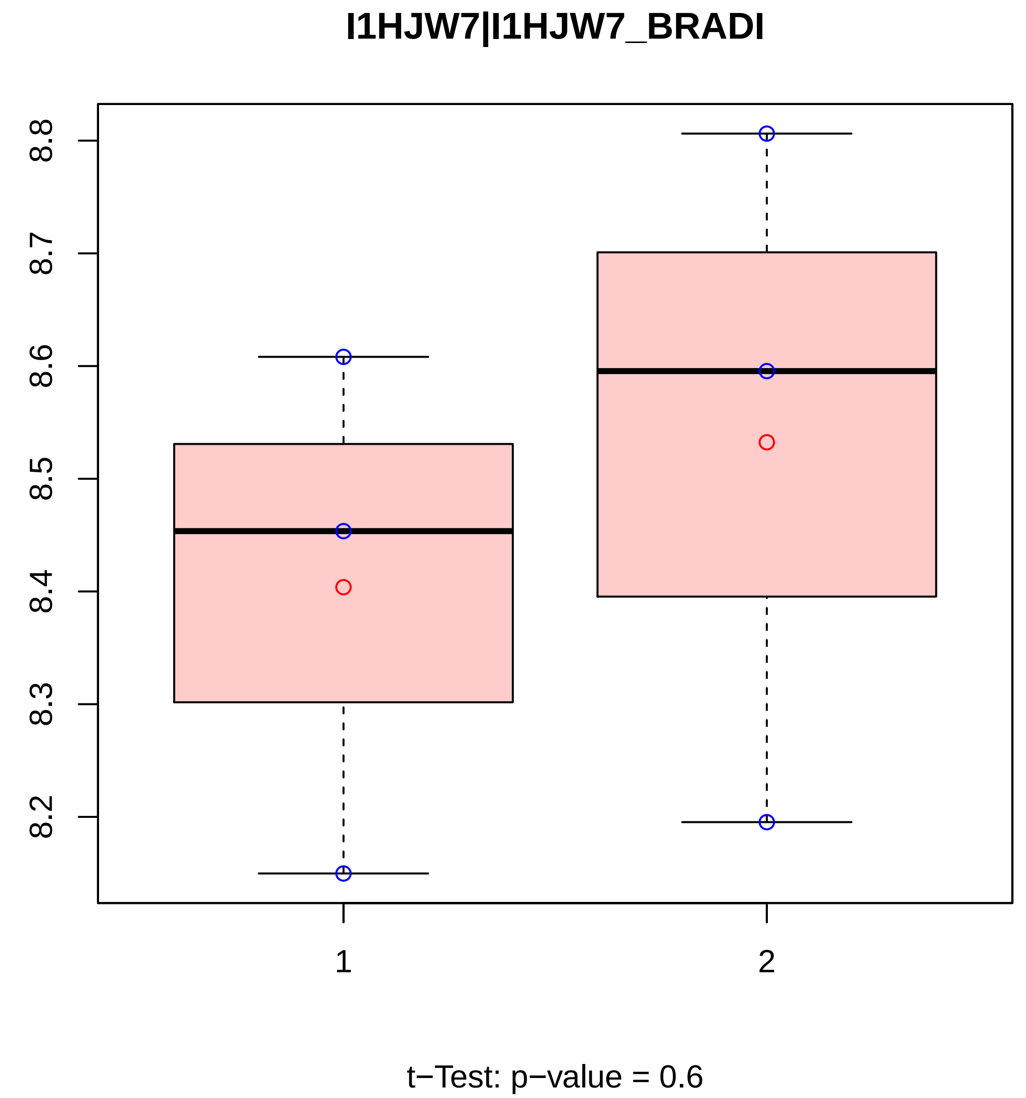
I1HJW7|I1HJW7_BRADI
8.2
8.3
8.4
8.5
8.6
8.7
8.8
1
2
t−Test: p−value = 0.6

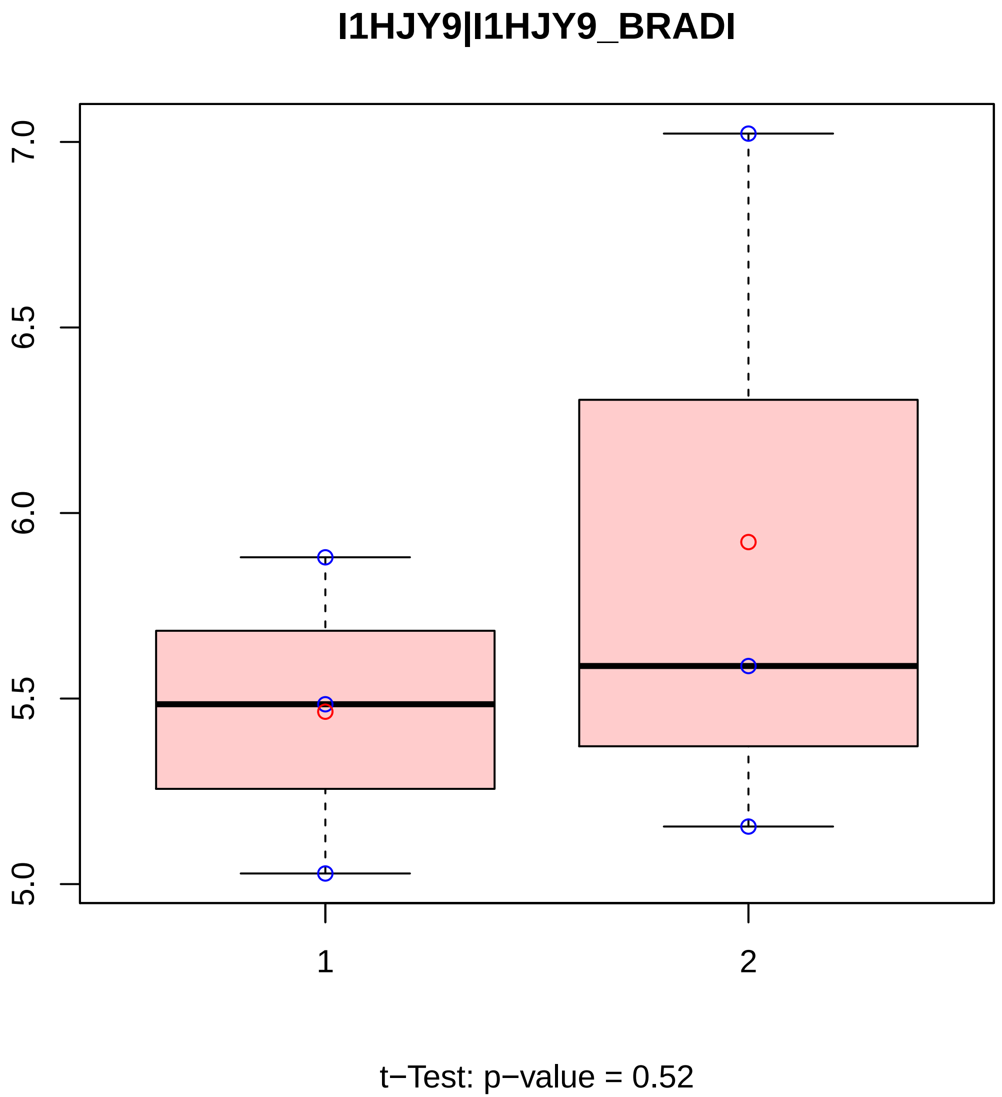
I1HJY9|I1HJY9_BRADI
7.0
6.5
6.0
5.5
5.0
1
2
t−Test: p−value = 0.52

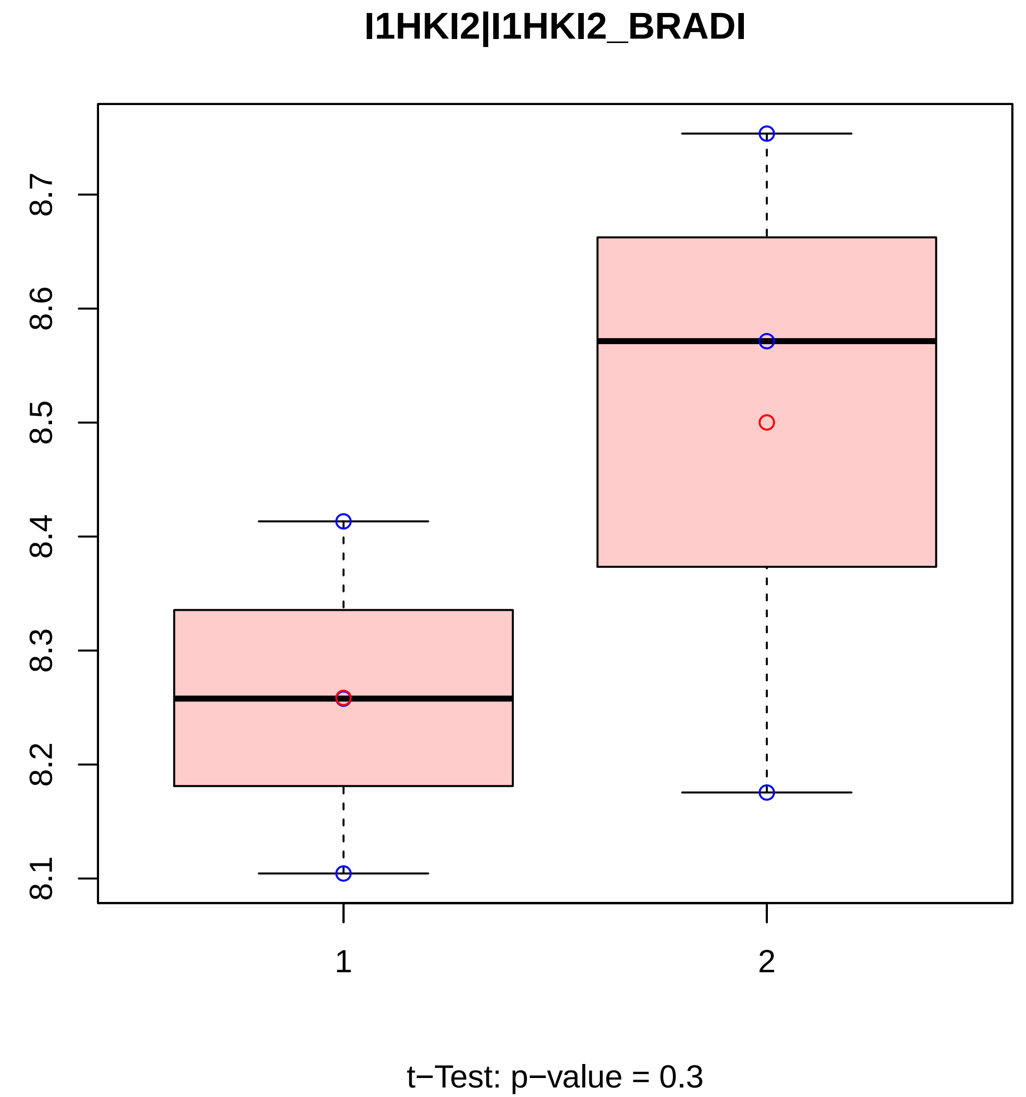
I1HKI2|I1HKI2_BRADI
8.1
8.2
8.3
8.4
8.5
8.6
8.7
1
2
t−Test: p−value = 0.3

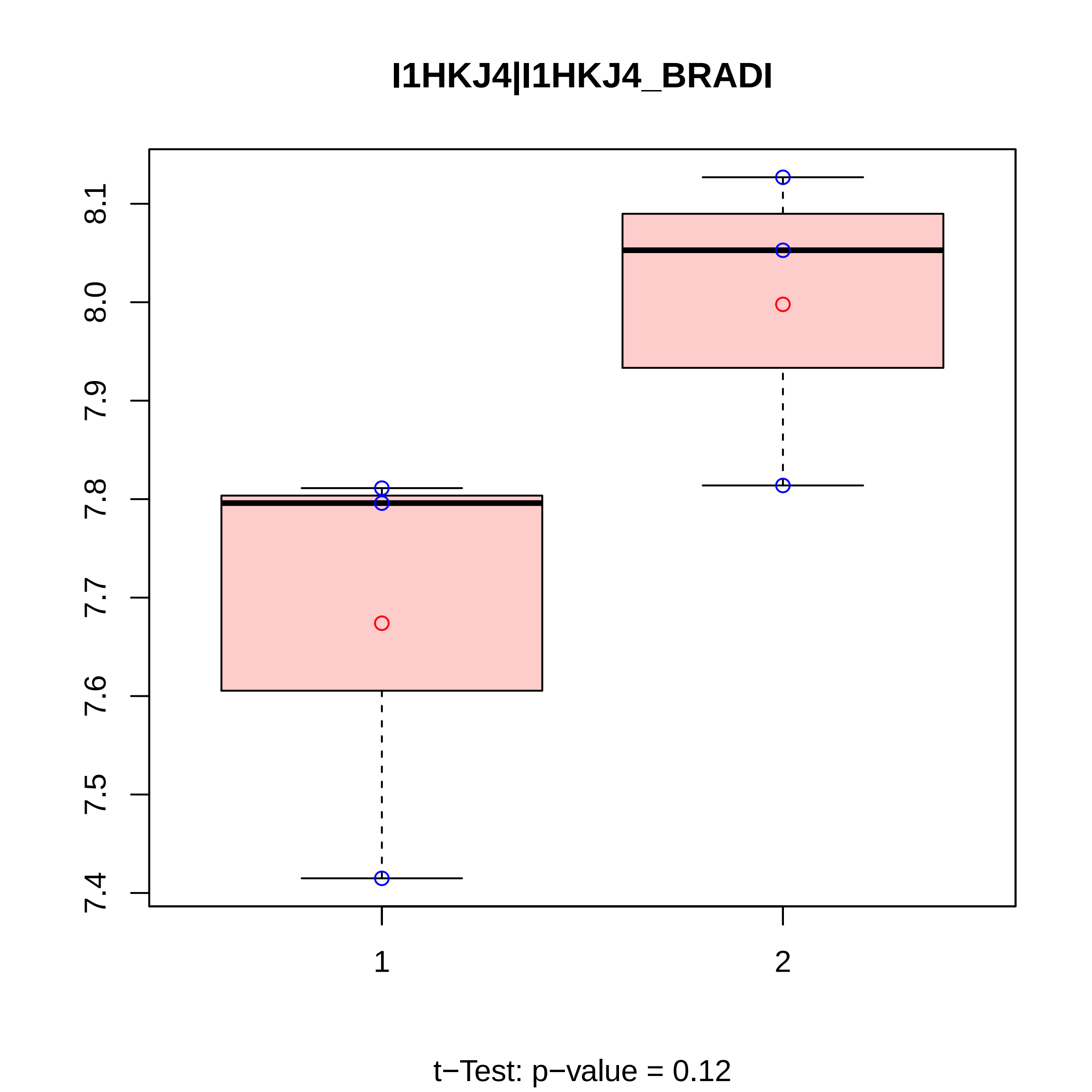
I1HKJ4|I1HKJ4_BRADI
8.1
8.0
7.9
7.8
7.7
7.6
7.5
7.4
1
2
t−Test: p−value = 0.12

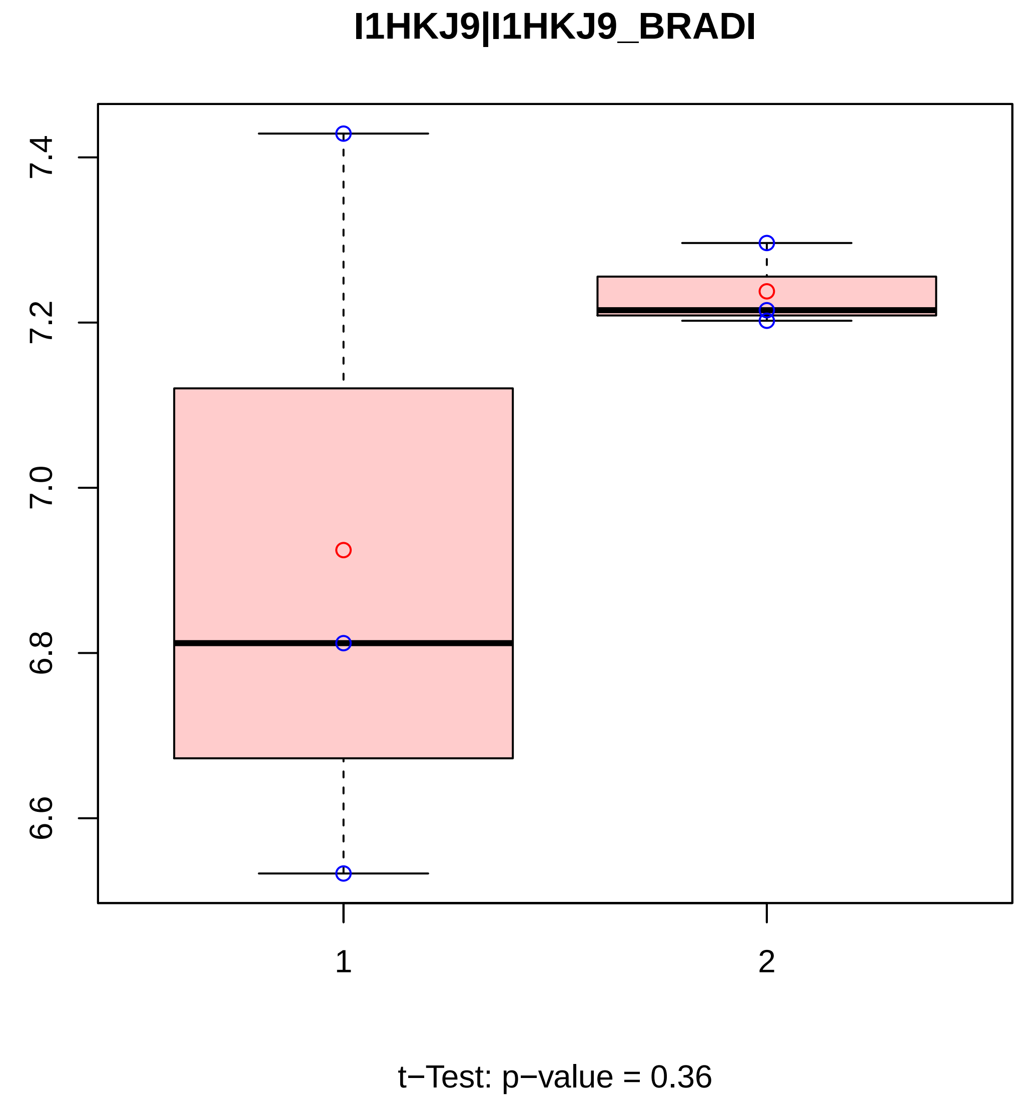
I1HKJ9|I1HKJ9_BRADI
7.4
7.2
7.0
6.8
6.6
1
2
t−Test: p−value = 0.36

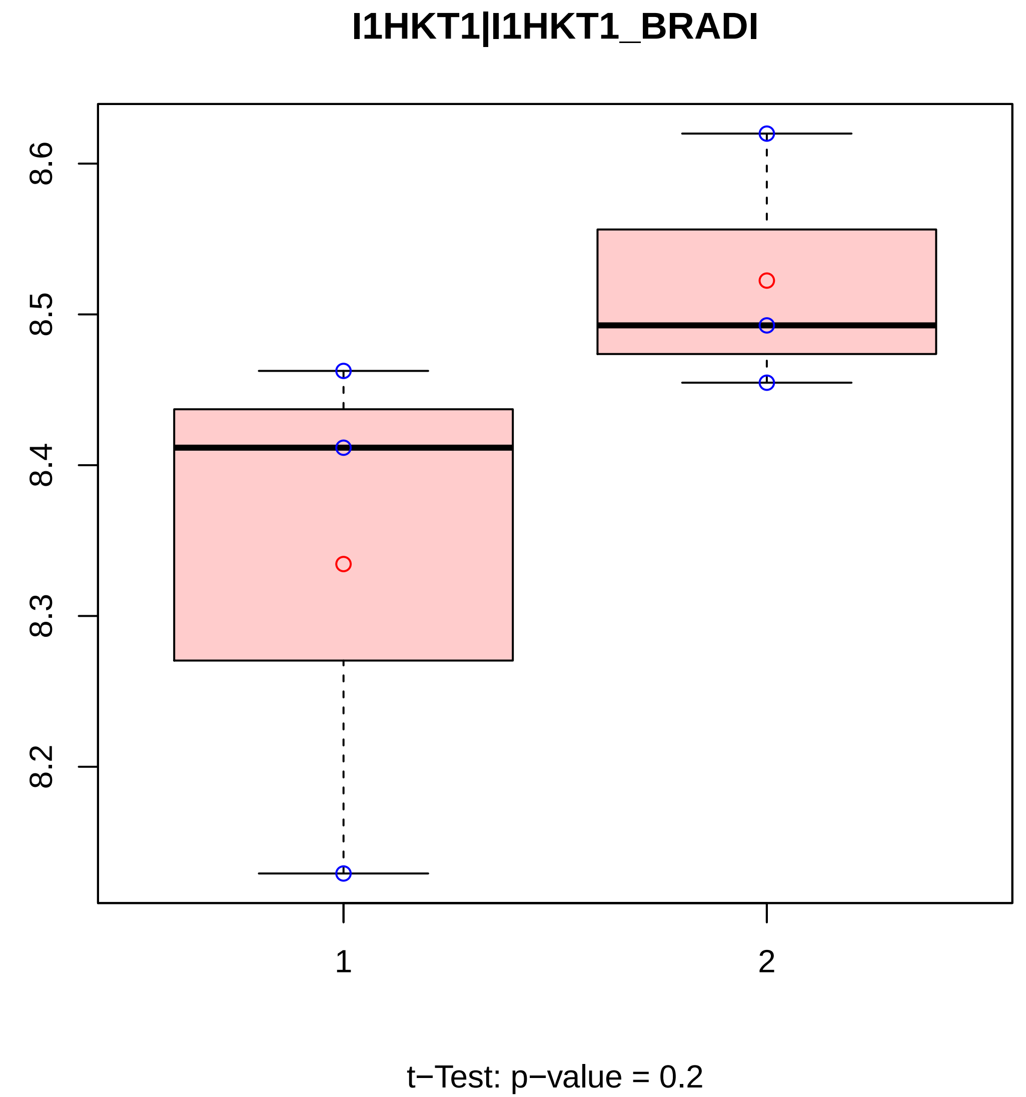
I1HKT1|I1HKT1_BRADI
8.6
8.5
8.4
8.3
8.2
1
2
t−Test: p−value = 0.2

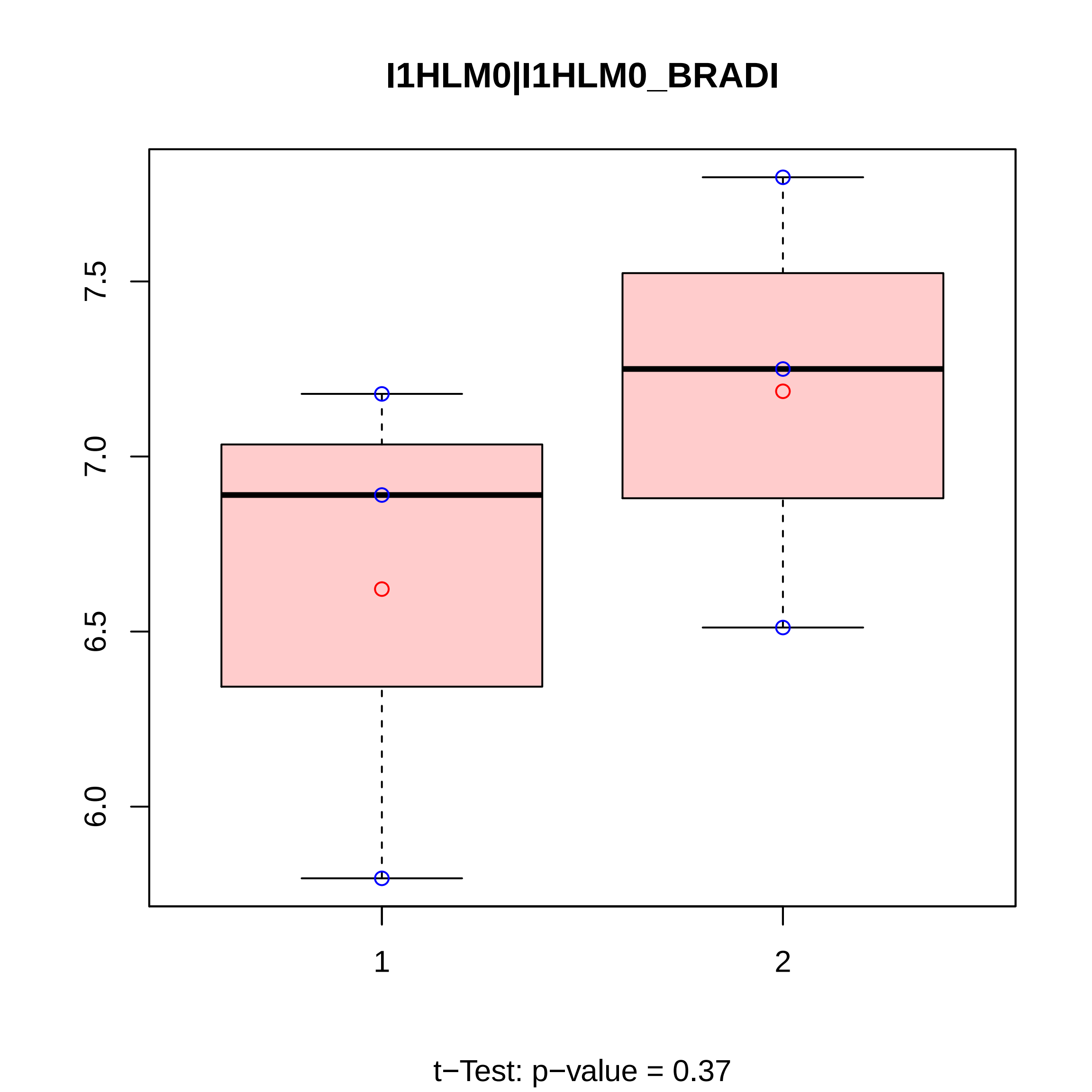

I1HLM0|I1HLM0_BRADI
6.0
6.5
7.0
7.5
1
2
t−Test: p−value = 0.37

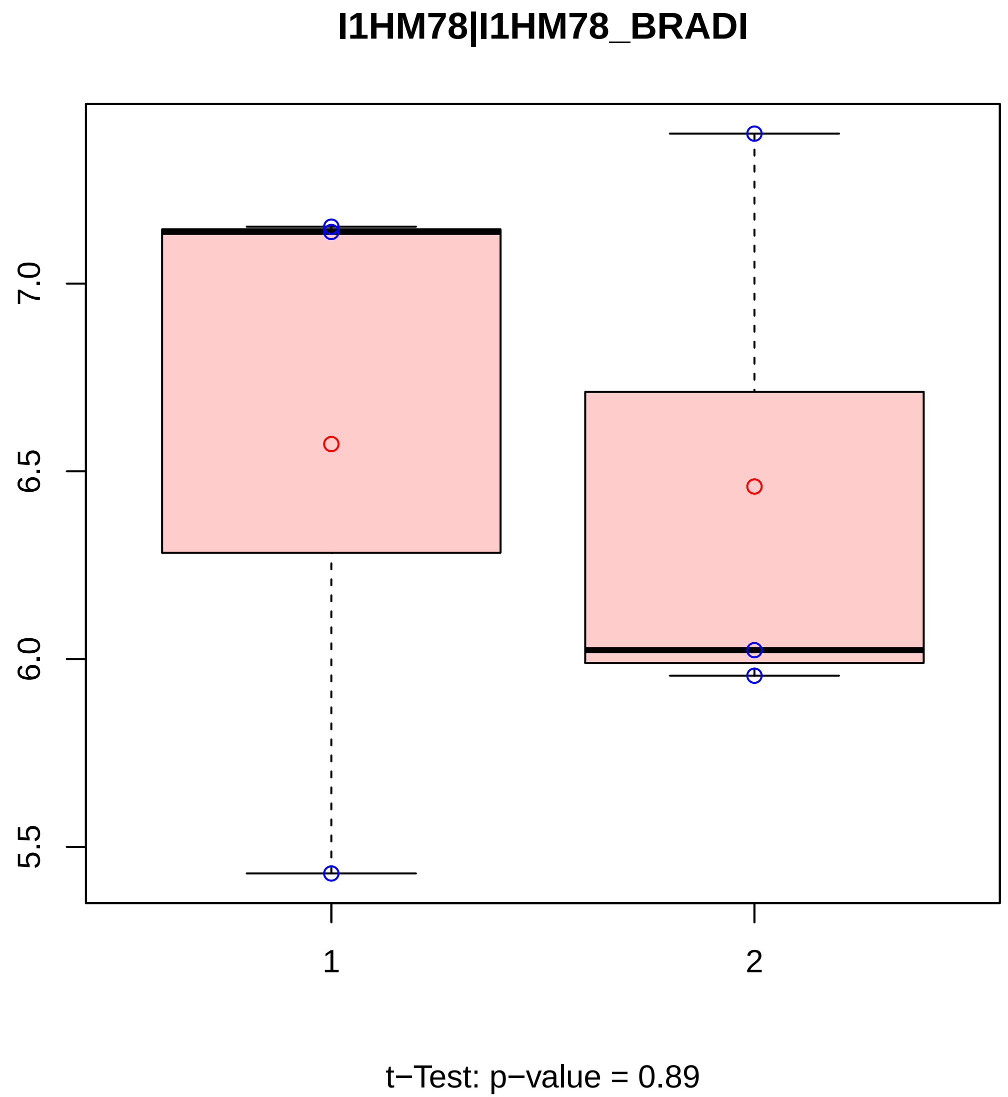
I1HM78|I1HM78_BRADI
7.0
6.5
6.0
5.5
1
2
t−Test: p−value = 0.89

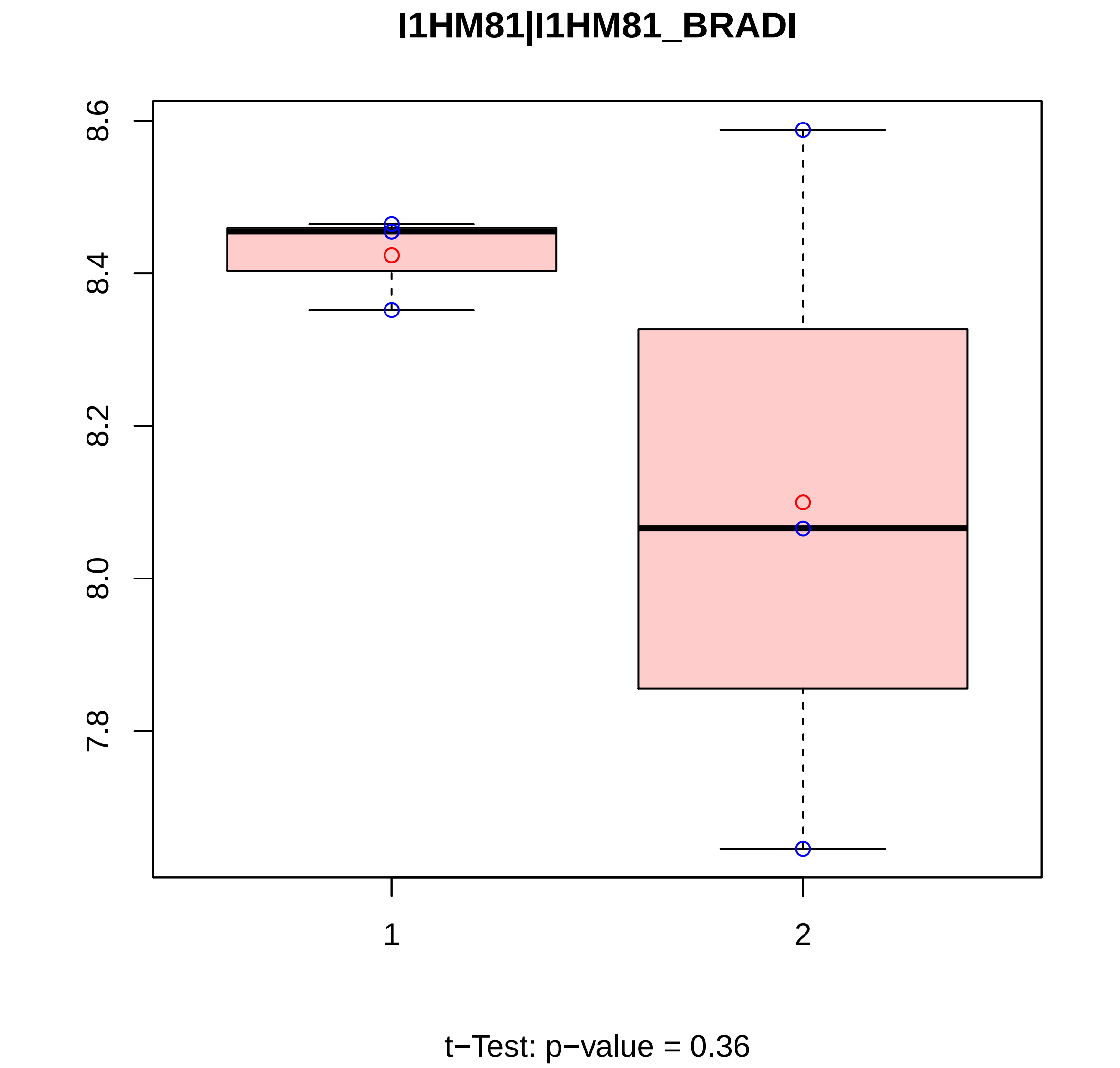

I1HM81|I1HM81_BRADI
8.6
8.4
8.2
8.0
7.8
1
2
t−Test: p−value = 0.36

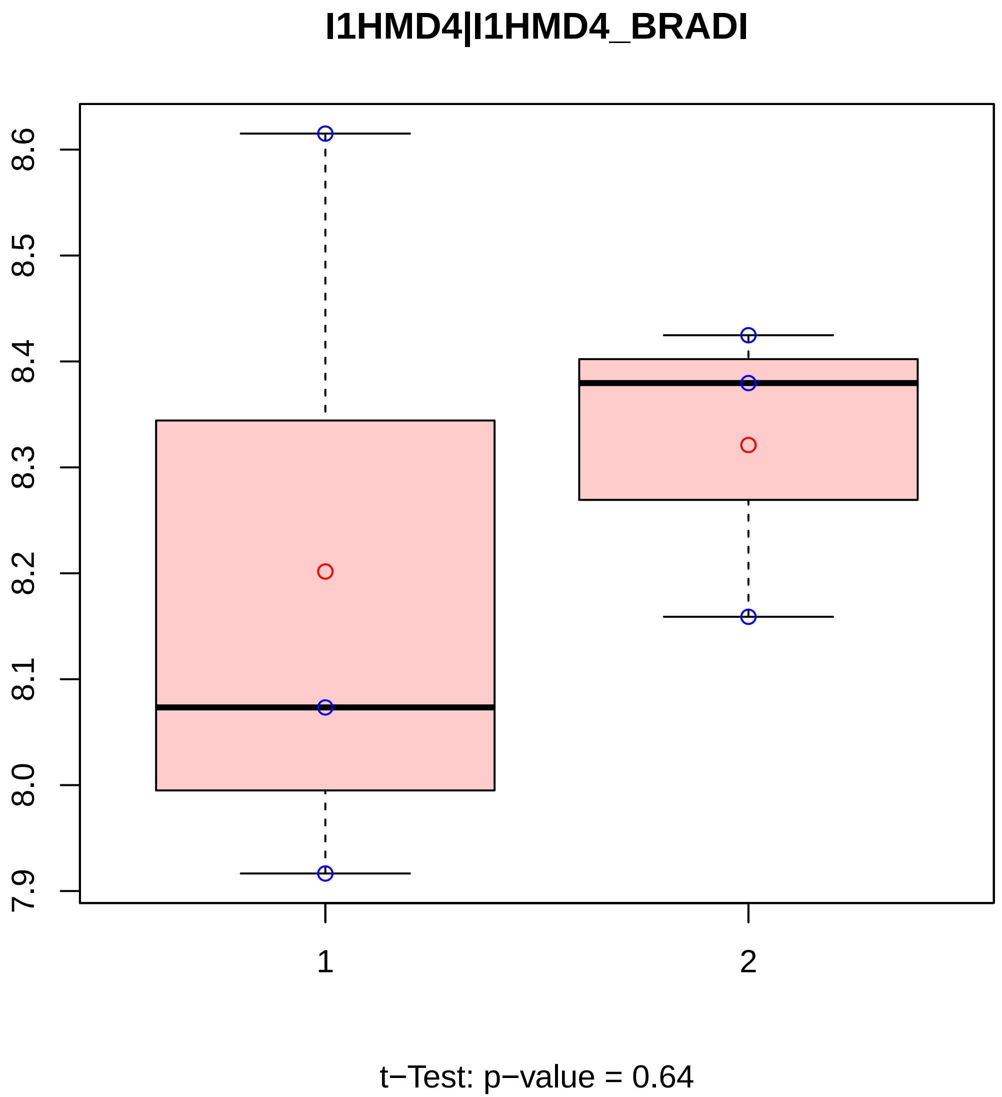
I1HMD4|I1HMD4_BRADI
8.6
8.5
8.4
8.3
8.2
8.1
8.0
7.9
1
2
t−Test: p−value = 0.64

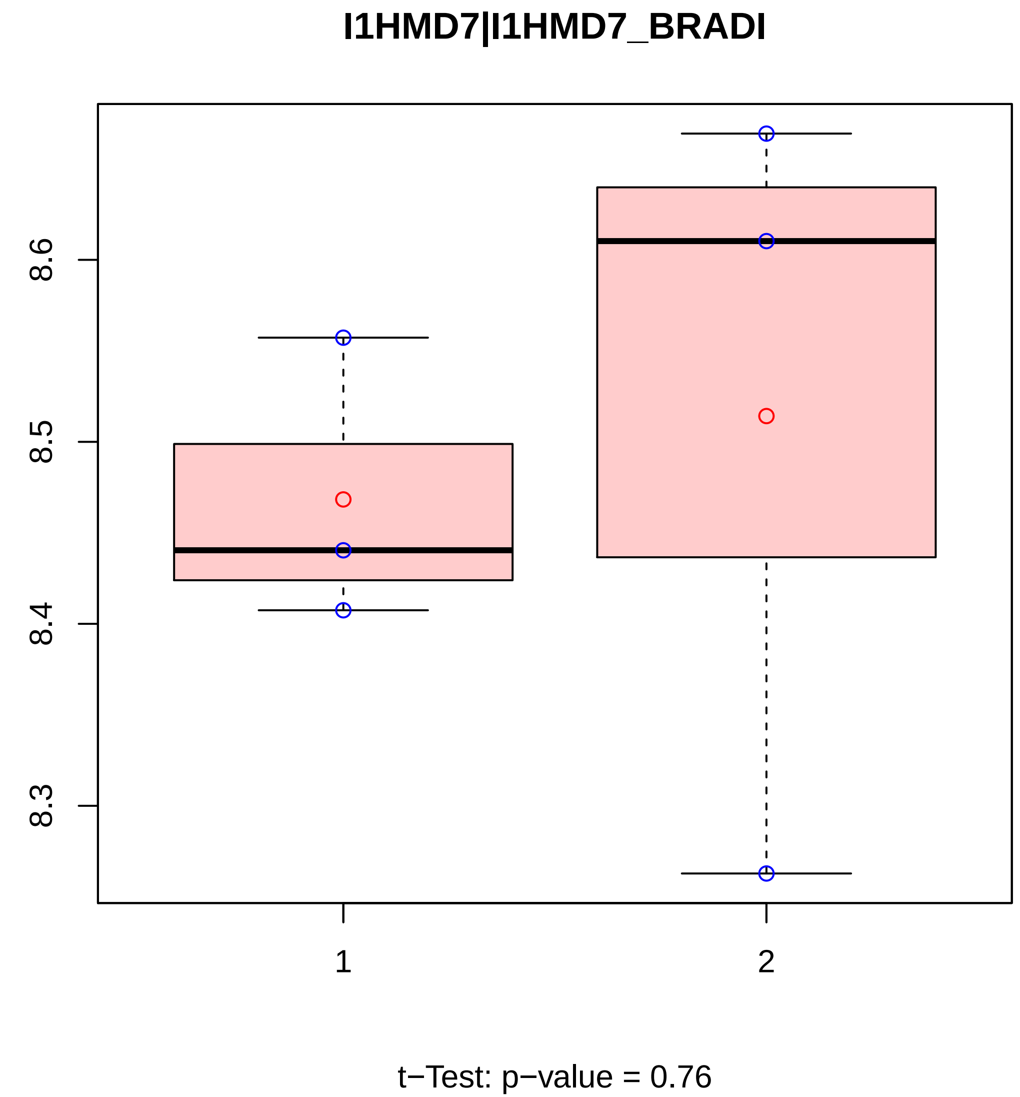
I1HMD7|I1HMD7_BRADI
8.6
8.5
8.4
8.3
1
2
t−Test: p−value = 0.76

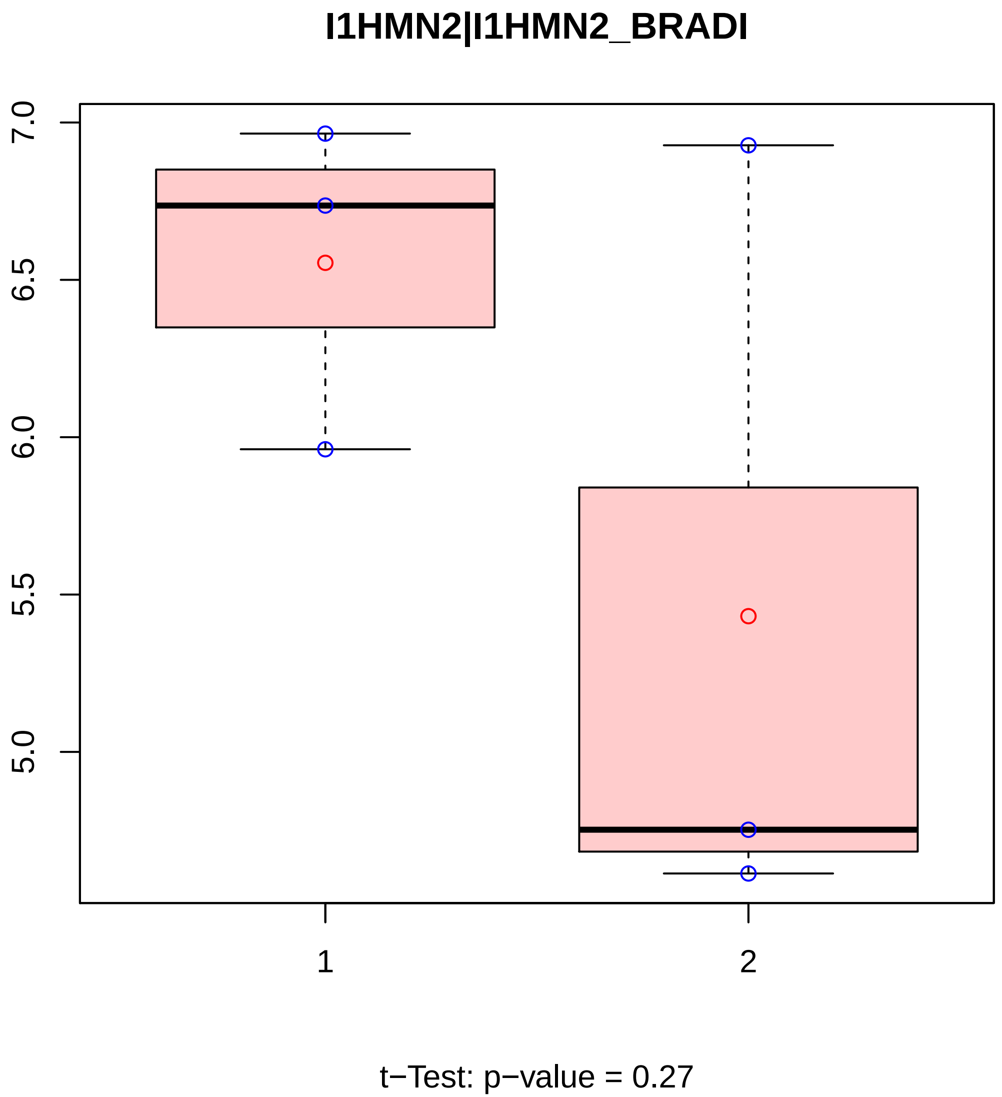
I1HMN2|I1HMN2_BRADI
7.0
6.5
6.0
5.5
5.0
1
2
t−Test: p−value = 0.27

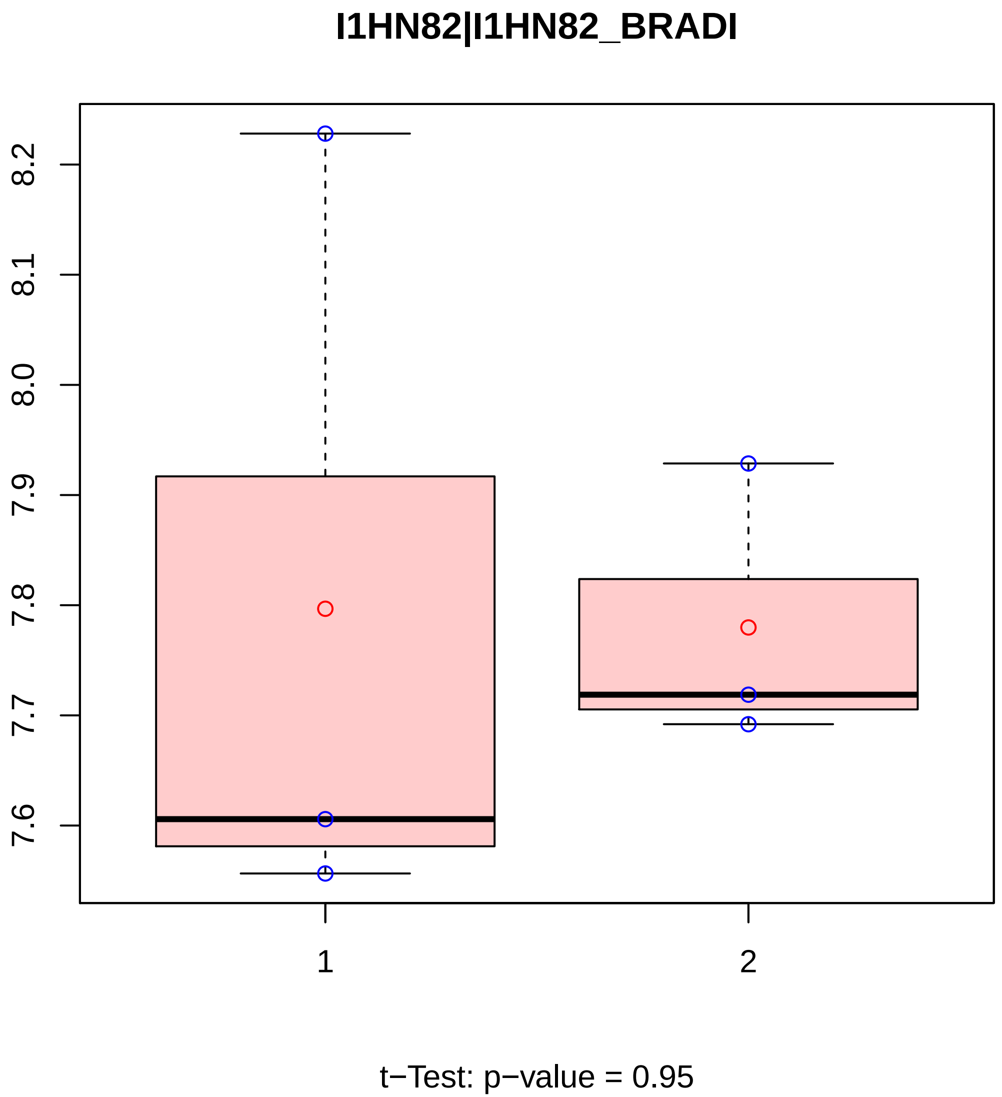
I1HN82|I1HN82_BRADI
8.2
8.1
8.0
7.9
7.8
7.7
7.6
1
2
t−Test: p−value = 0.95

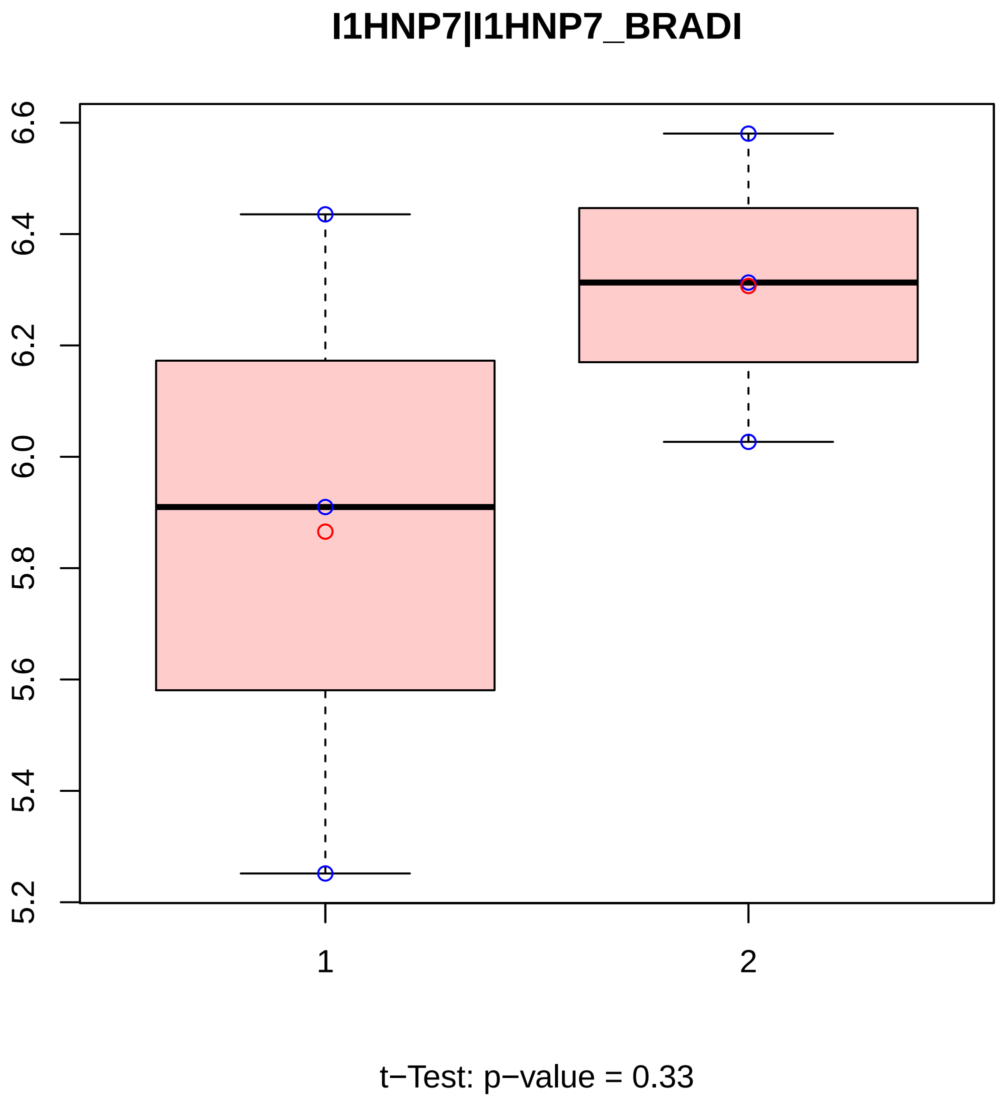

I1HNP7|I1HNP7_BRADI
6.6
6.4
6.2
6.0
5.8
5.6
5.4
5.2
1
2
t−Test: p−value = 0.33

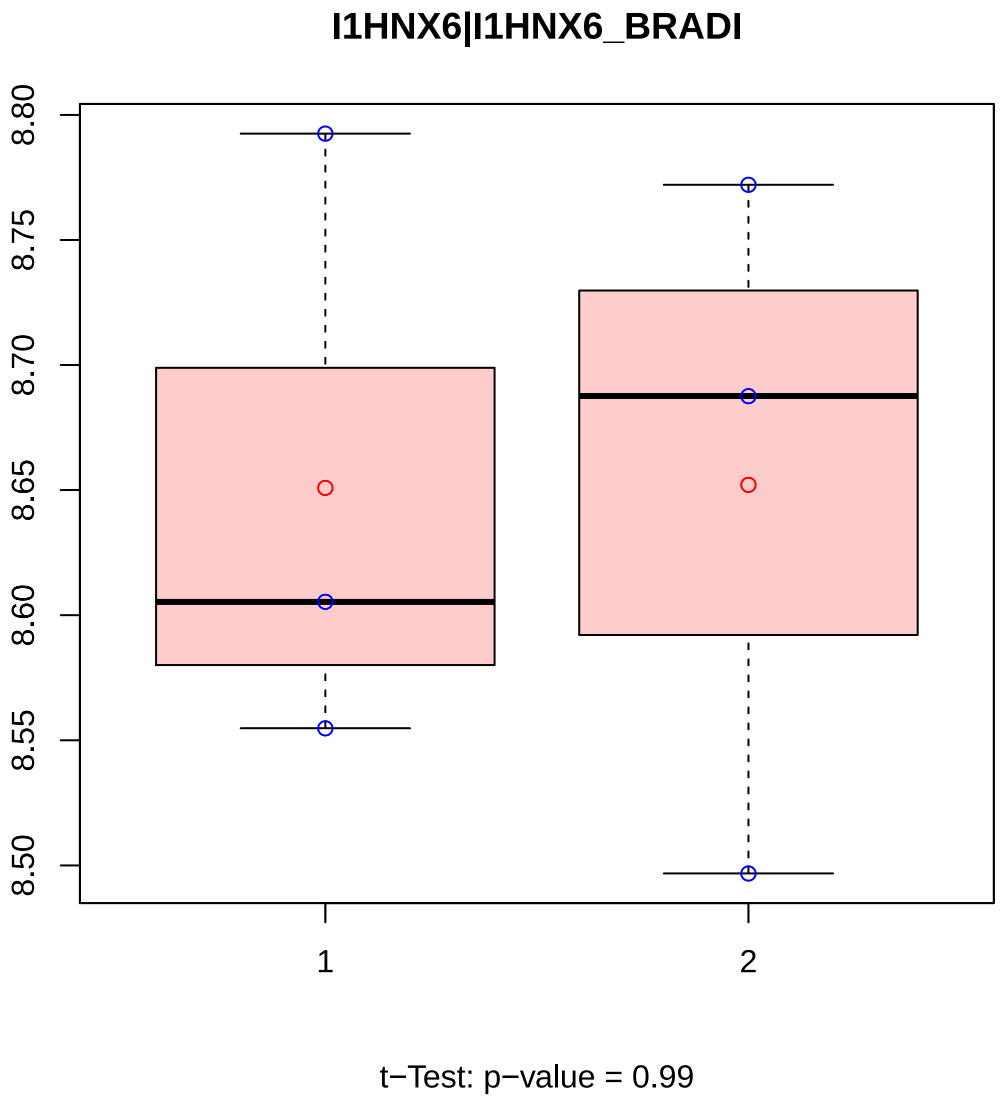
I1HNX6|I1HNX6_BRADI
8.80
8.75
8.70
8.65
8.60
8.55
8.50
1
2
t−Test: p−value = 0.99

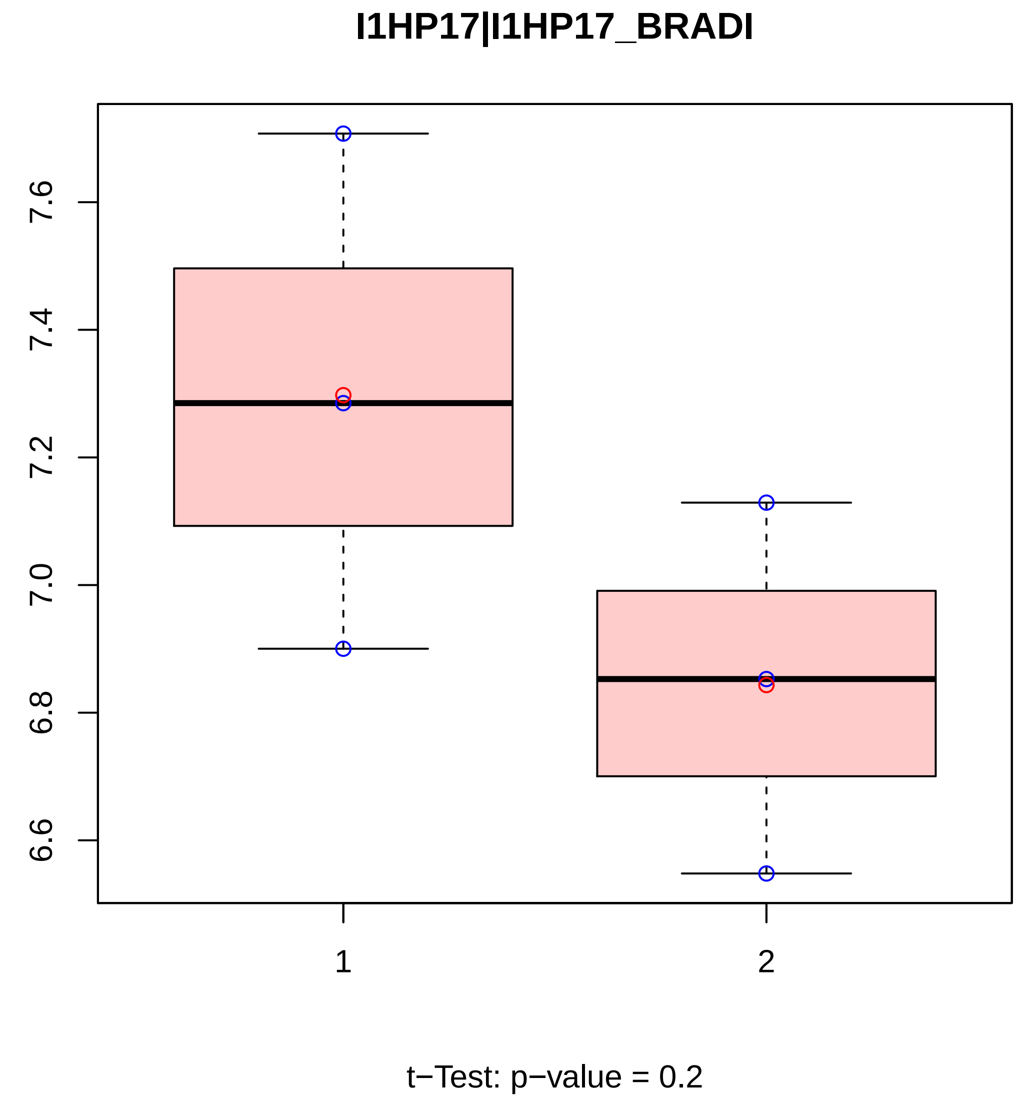
I1HP17|I1HP17_BRADI
7.6
7.4
7.2
7.0
6.8
6.6
1
2
t−Test: p−value = 0.2

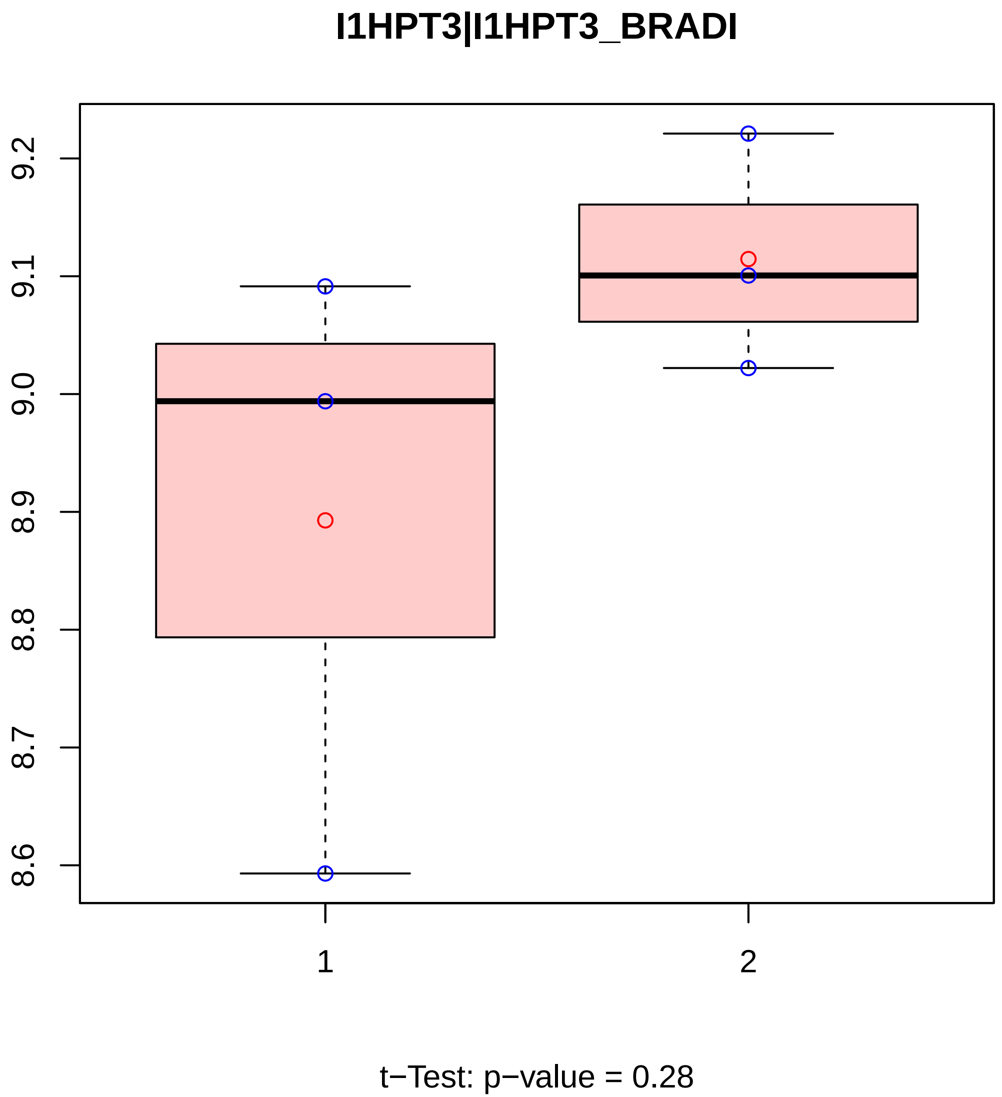

I1HPT3|I1HPT3_BRADI
9.2
9.1
9.0
8.9
8.8
8.7
8.6
1
2
t−Test: p−value = 0.28

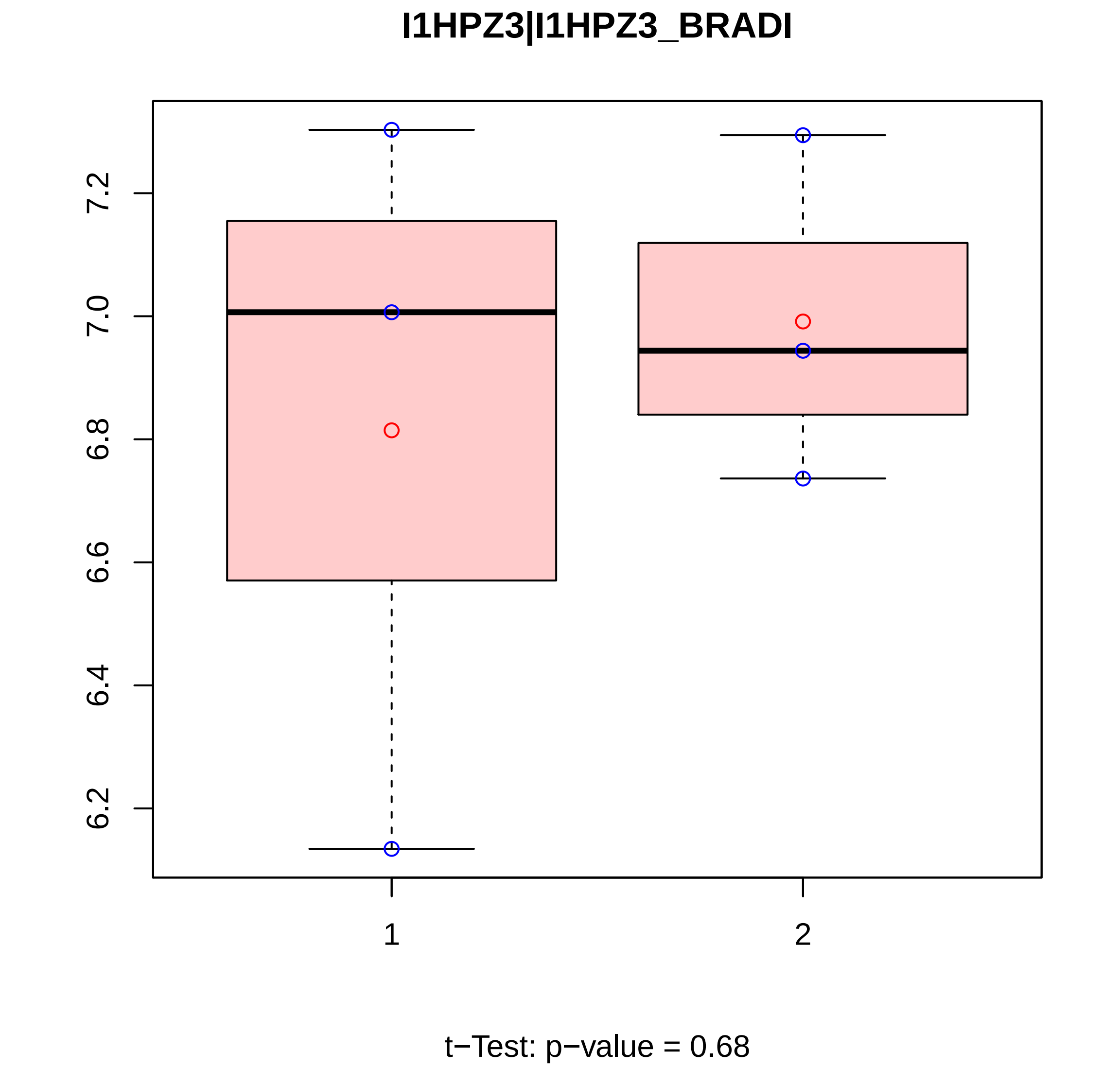

I1HPZ3|I1HPZ3_BRADI
7.2
7.0
6.8
6.6
6.4
6.2
1
2
t−Test: p−value = 0.68

I1HPZ8|I1HPZ8_BRADI

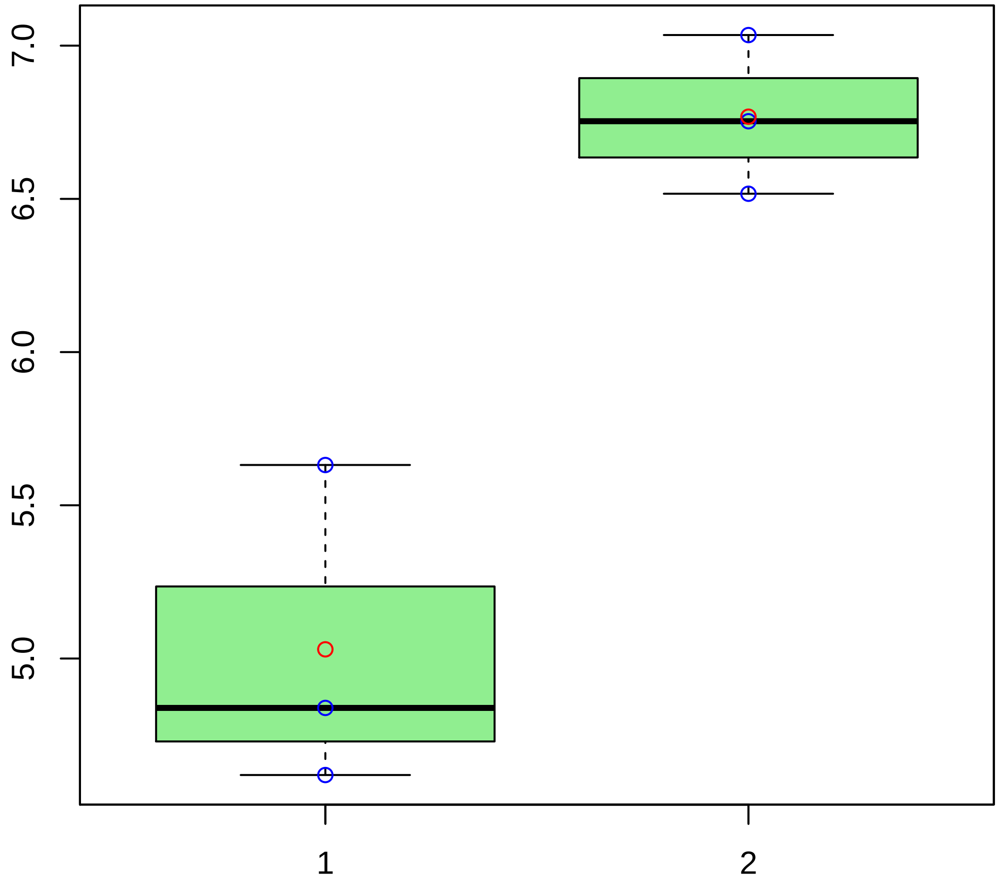

t−Test: p−value = 0.02

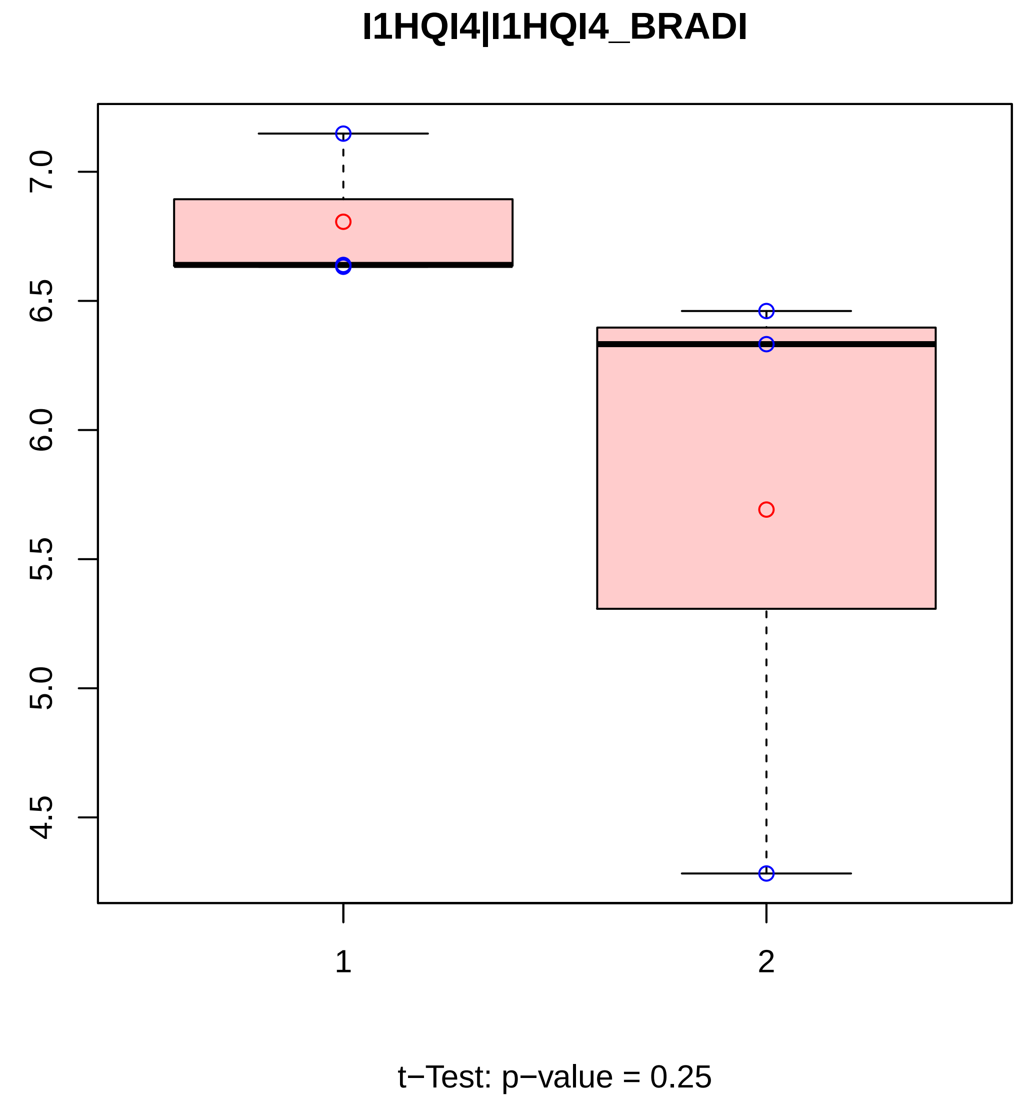
I1HQI4|I1HQI4_BRADI
7.0
6.5
6.0
5.5
5.0
4.5
1
2
t−Test: p−value = 0.25

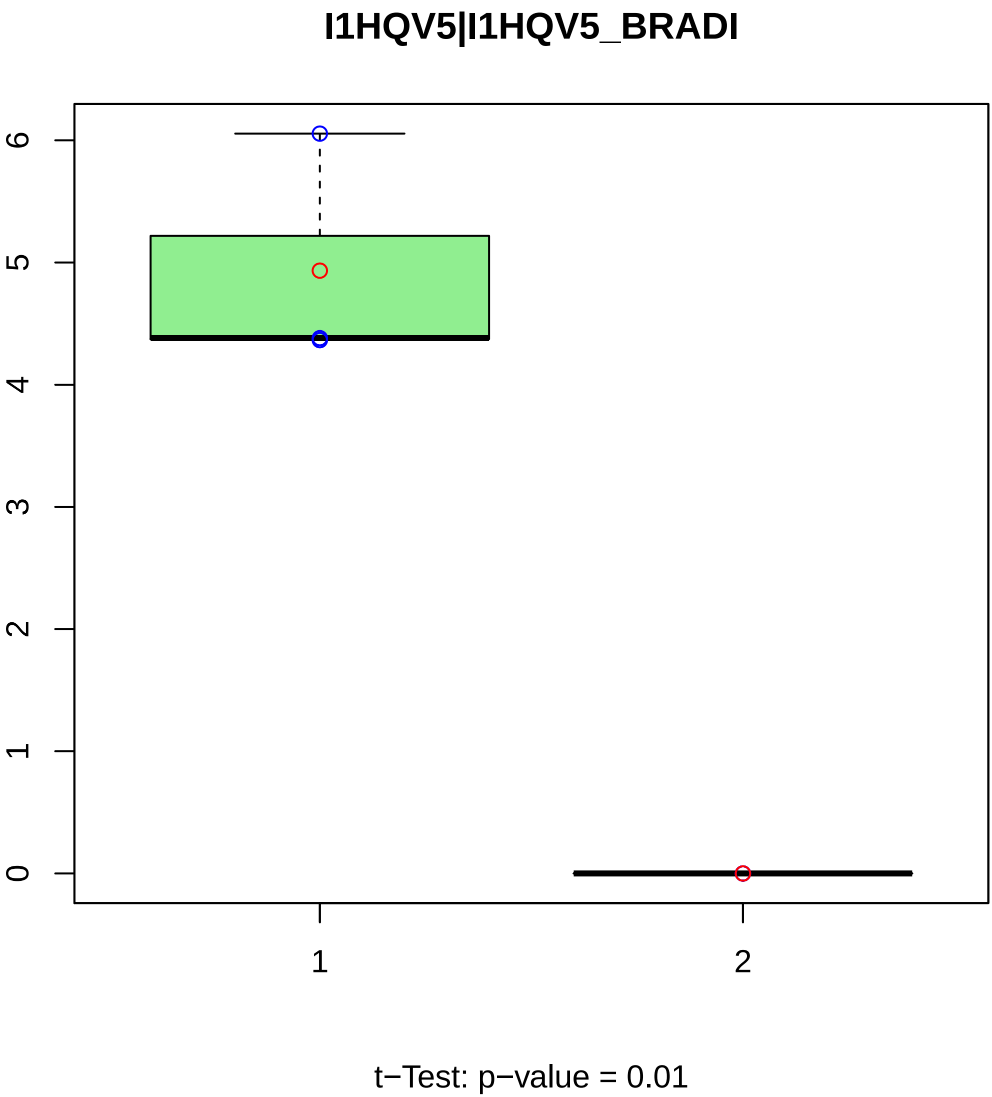
I1HQV5|I1HQV5_BRADI
0
1
2
3
4
5
6
1
2
t−Test: p−value = 0.01

I1HR90|I1HR90_BRADI

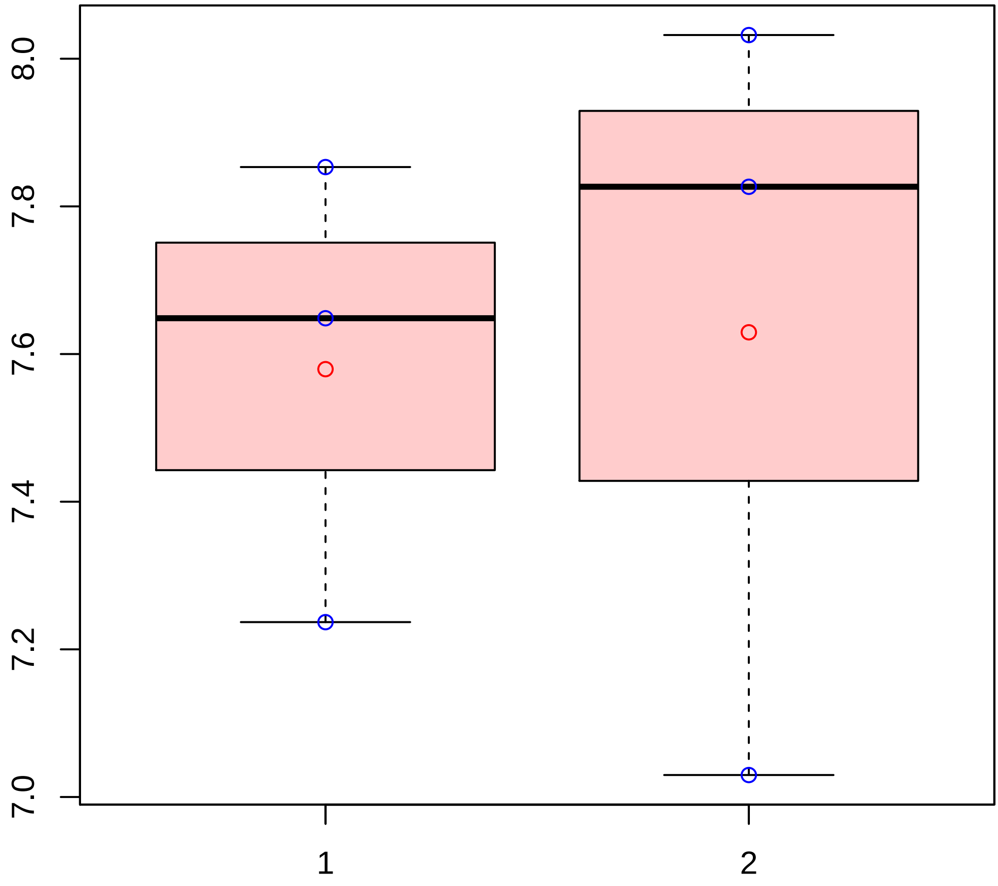

t−Test: p−value = 0.9

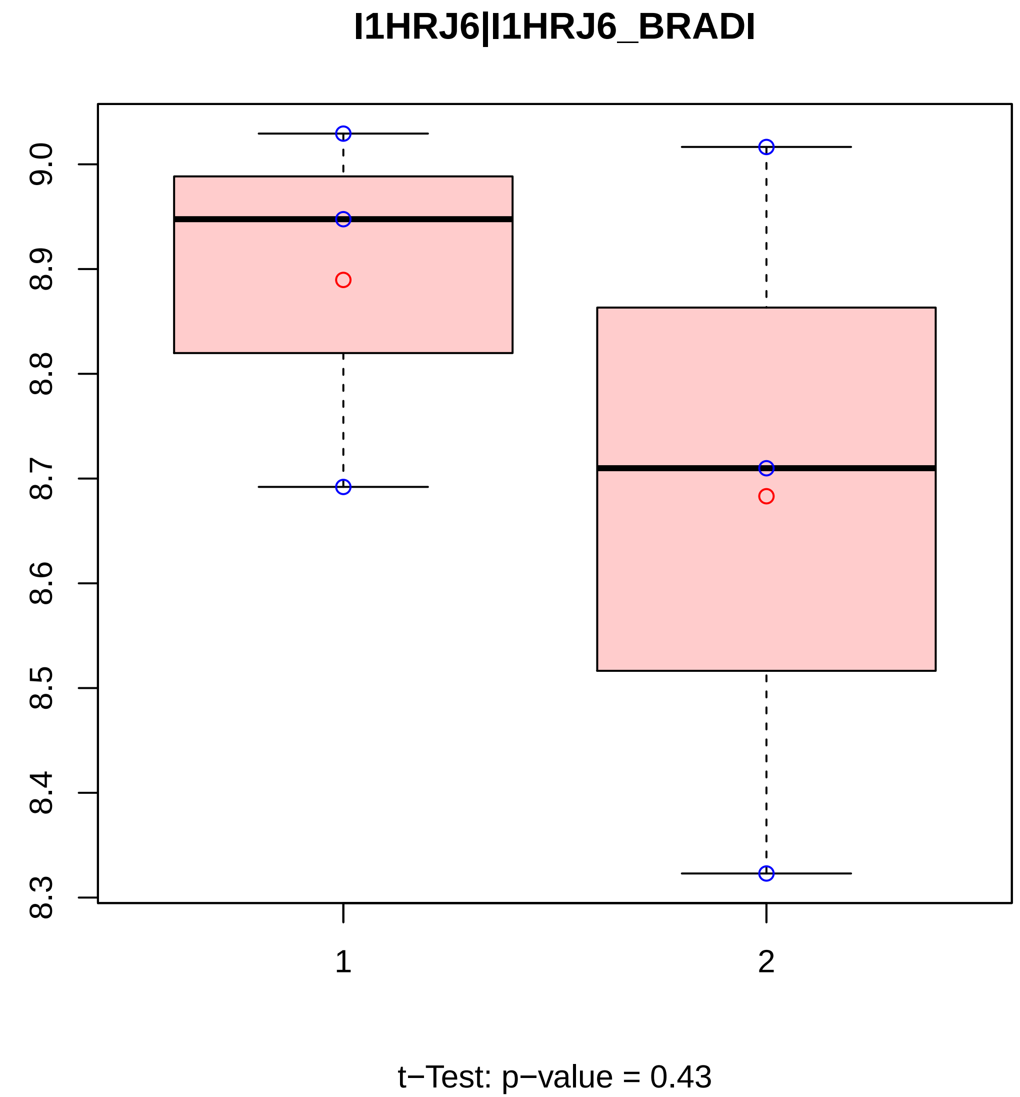
I1HRJ6|I1HRJ6_BRADI
9.0
8.9
8.8
8.7
8.6
8.5
8.4
8.3
1
2
t−Test: p−value = 0.43

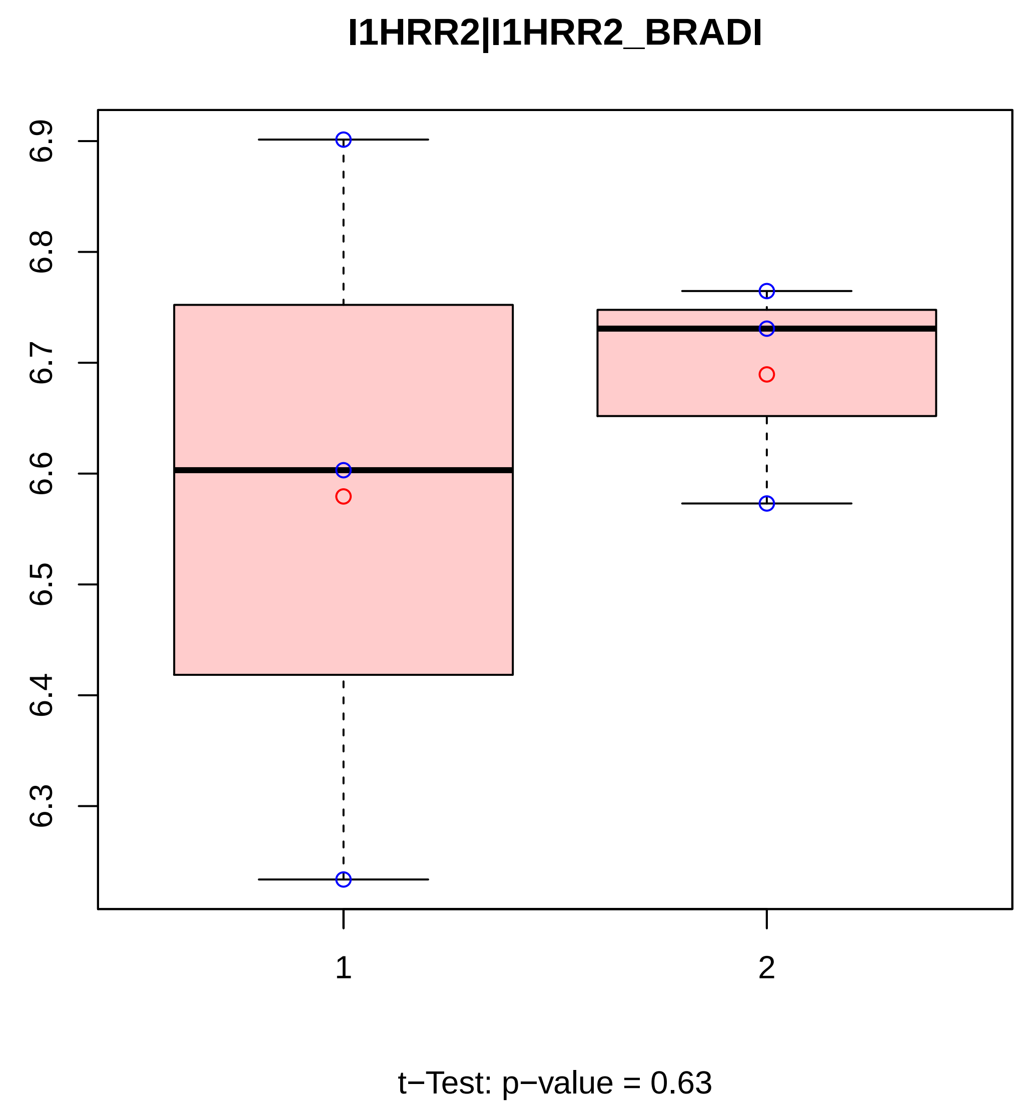
I1HRR2|I1HRR2_BRADI
6.3
6.4
6.5
6.6
6.7
6.8
6.9
1
2
t−Test: p−value = 0.63

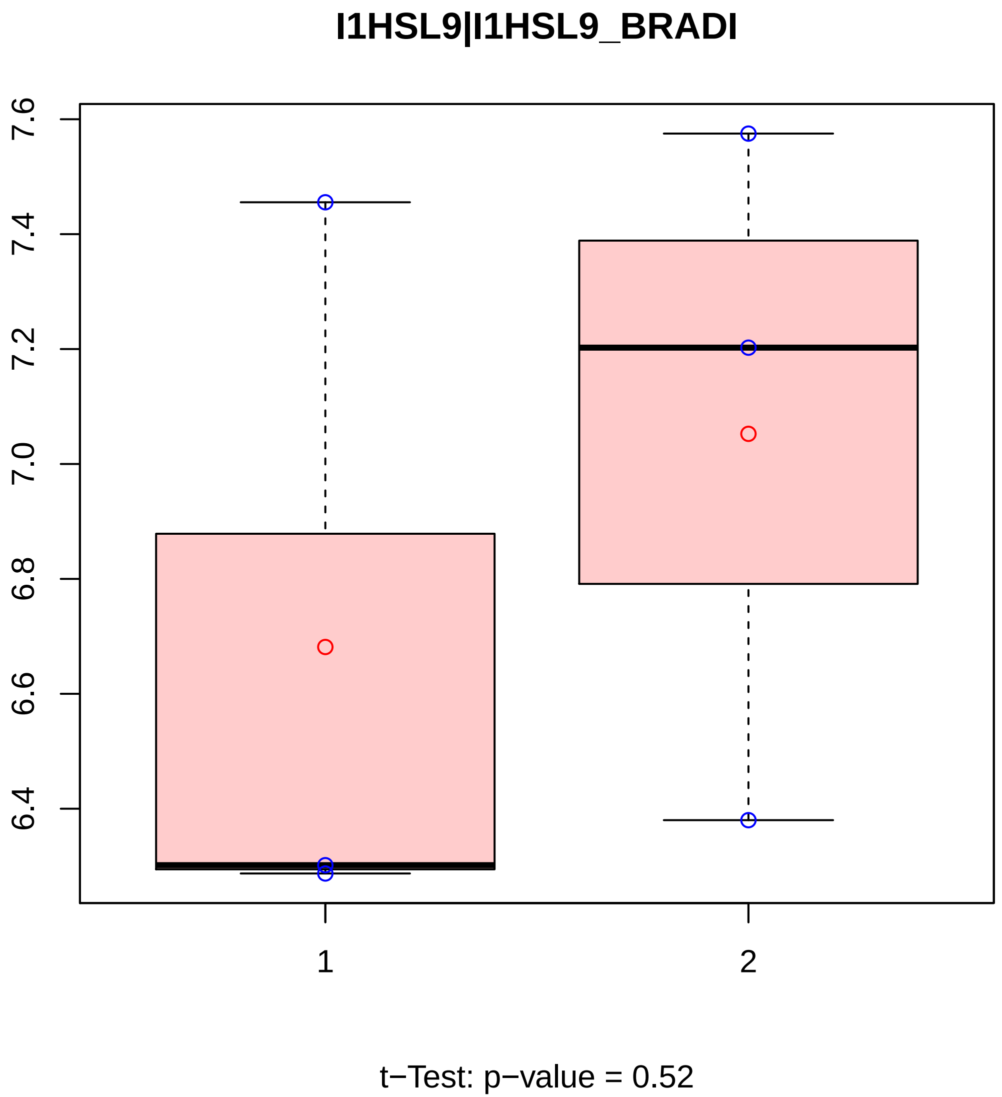

I1HSL9|I1HSL9_BRADI
7.6
7.4
7.2
7.0
6.8
6.6
6.4
1
2
t−Test: p−value = 0.52

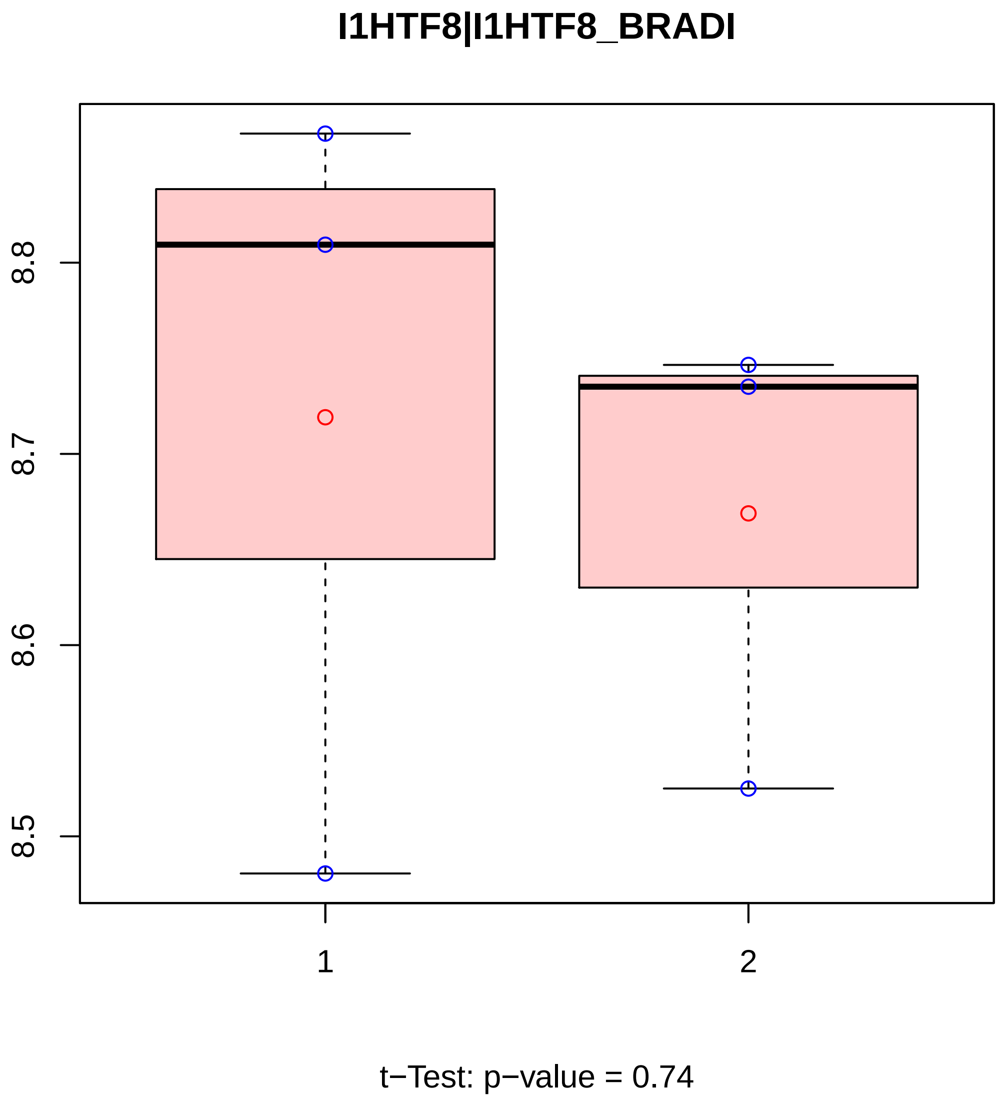
I1HTF8|I1HTF8_BRADI
8.5
8.6
8.7
8.8
1
2
t−Test: p−value = 0.74

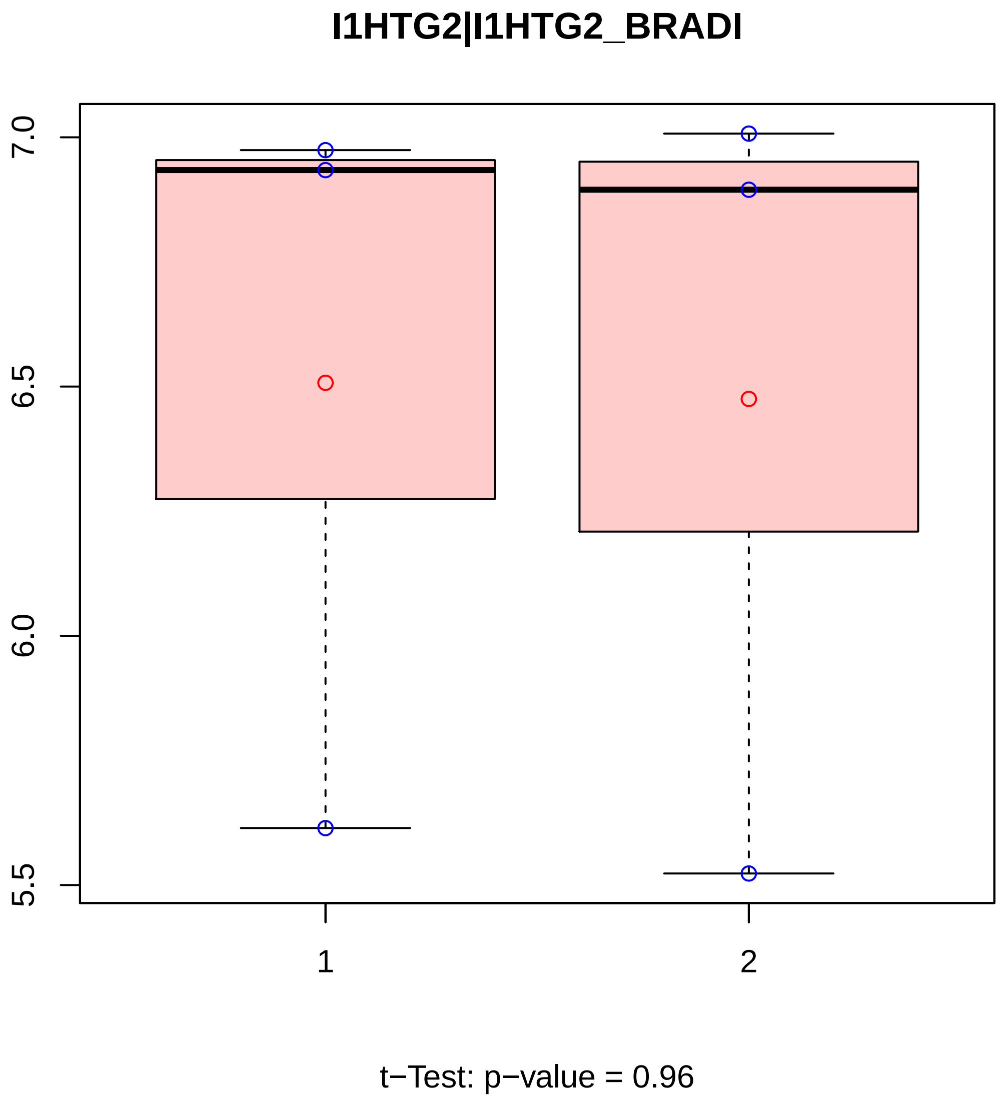
I1HTG2|I1HTG2_BRADI
7.0
6.5
6.0
5.5
1
2
t−Test: p−value = 0.96

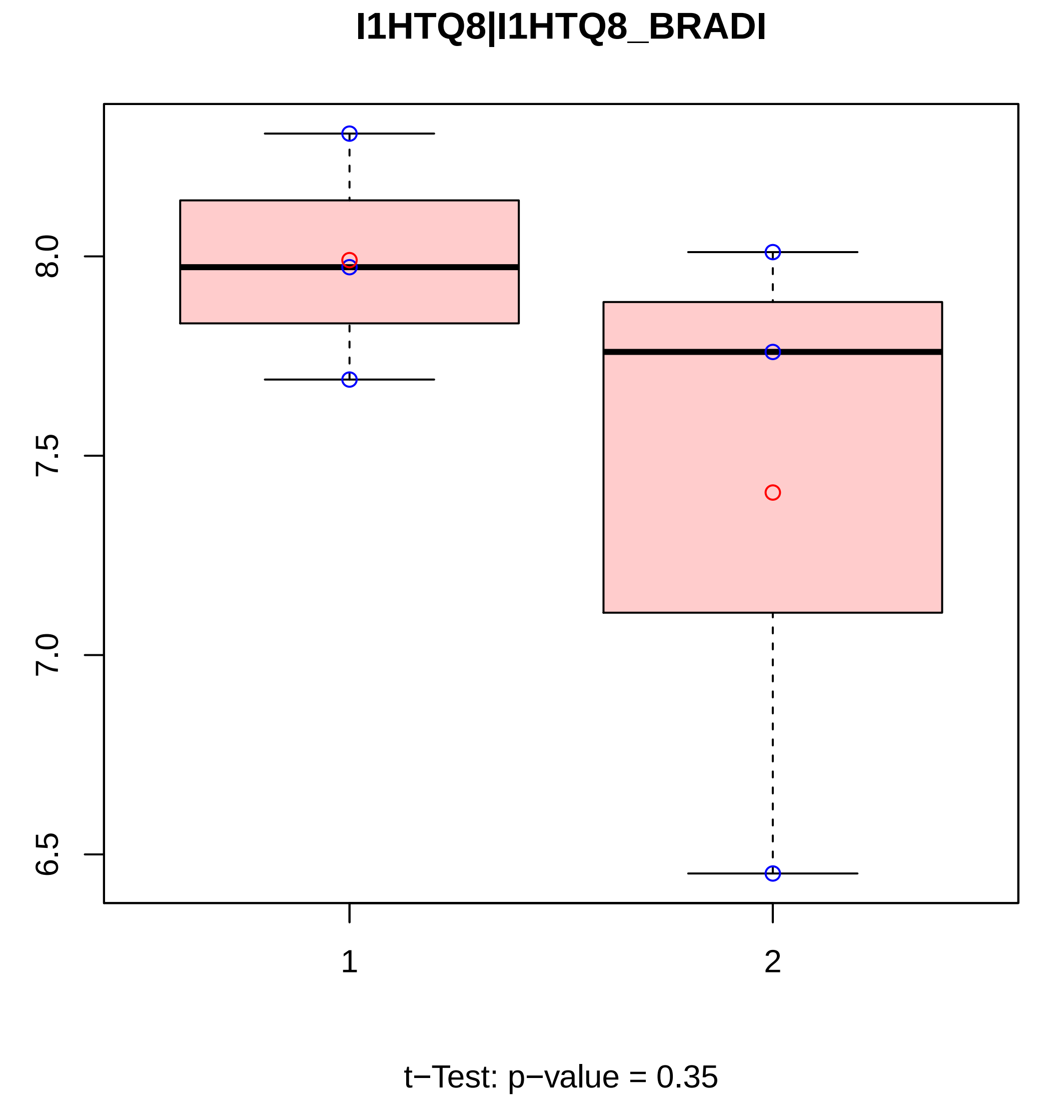

I1HTQ8|I1HTQ8_BRADI
8.0
7.5
7.0
6.5
1
2
t−Test: p−value = 0.35

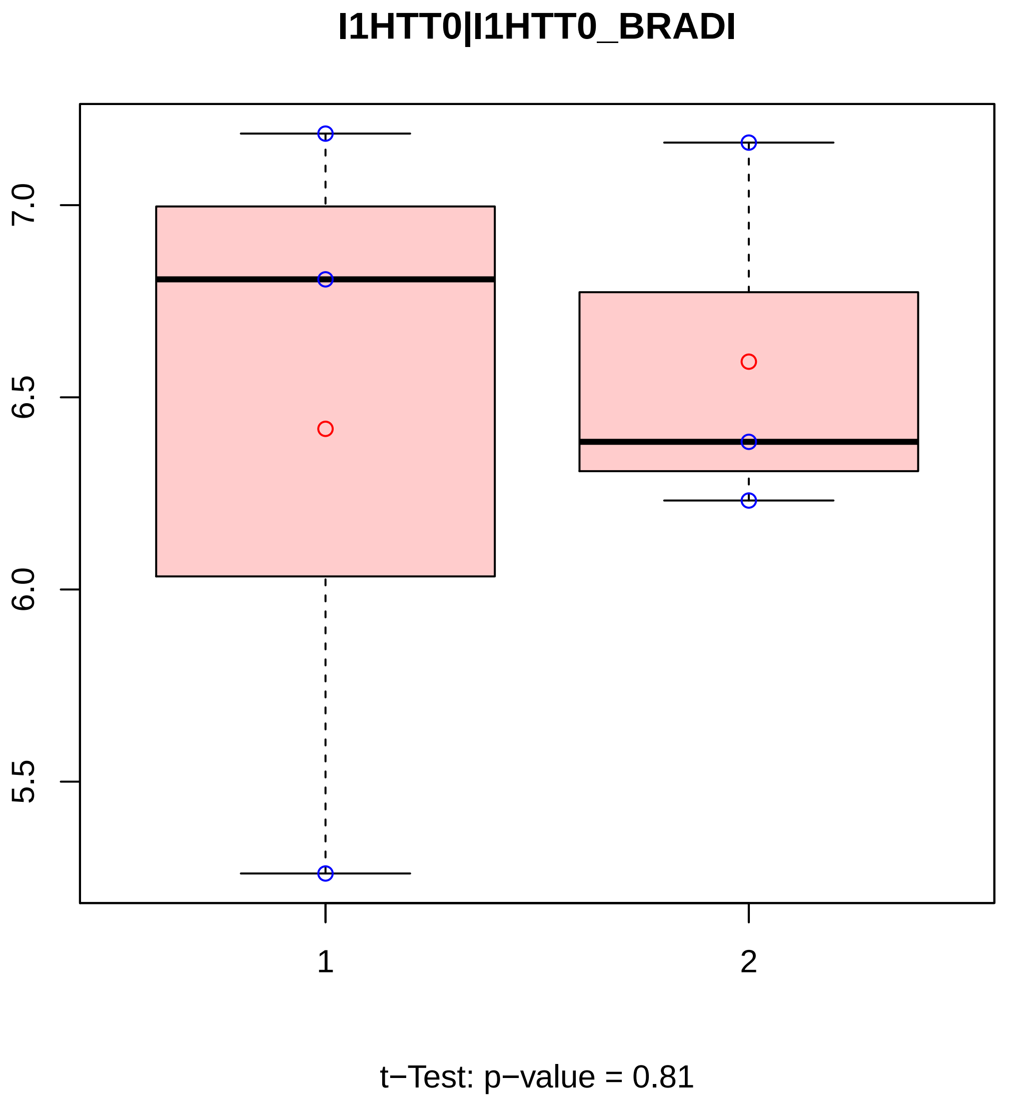
I1HTT0|I1HTT0_BRADI
7.0
6.5
6.0
5.5
1
2
t−Test: p−value = 0.81

I1HTW6|I1HTW6_BRADI

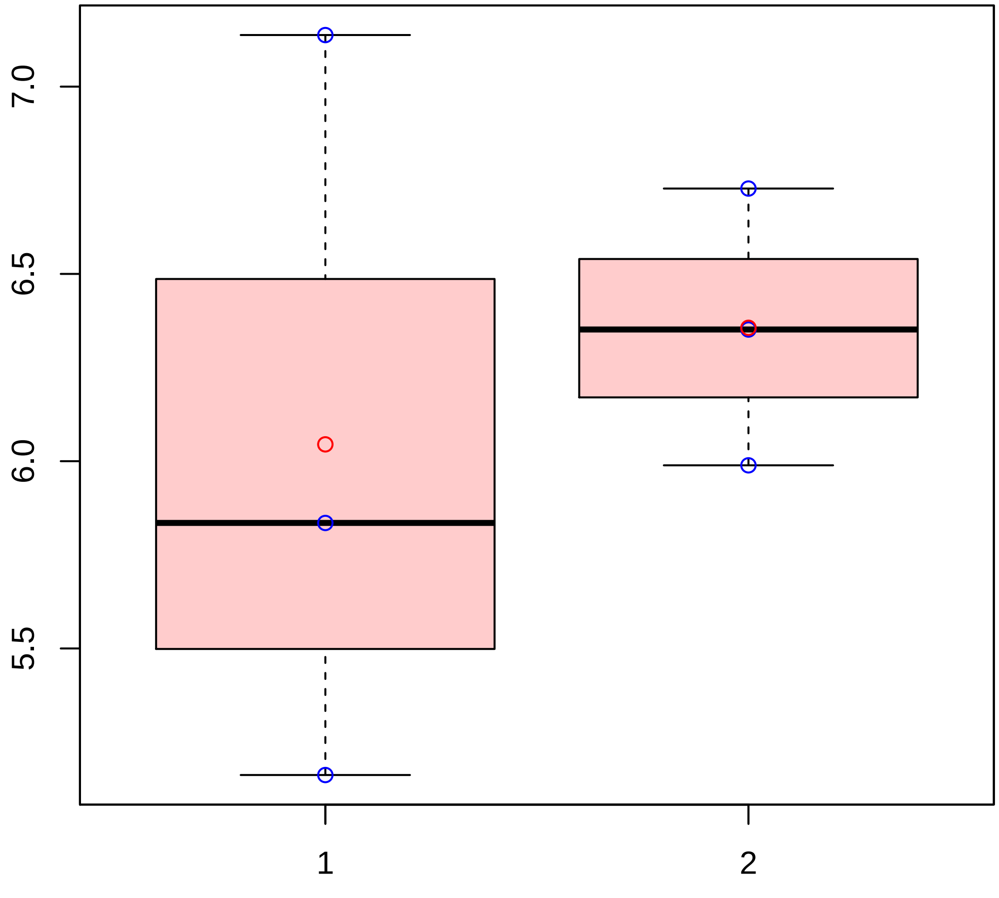

t−Test: p−value = 0.66

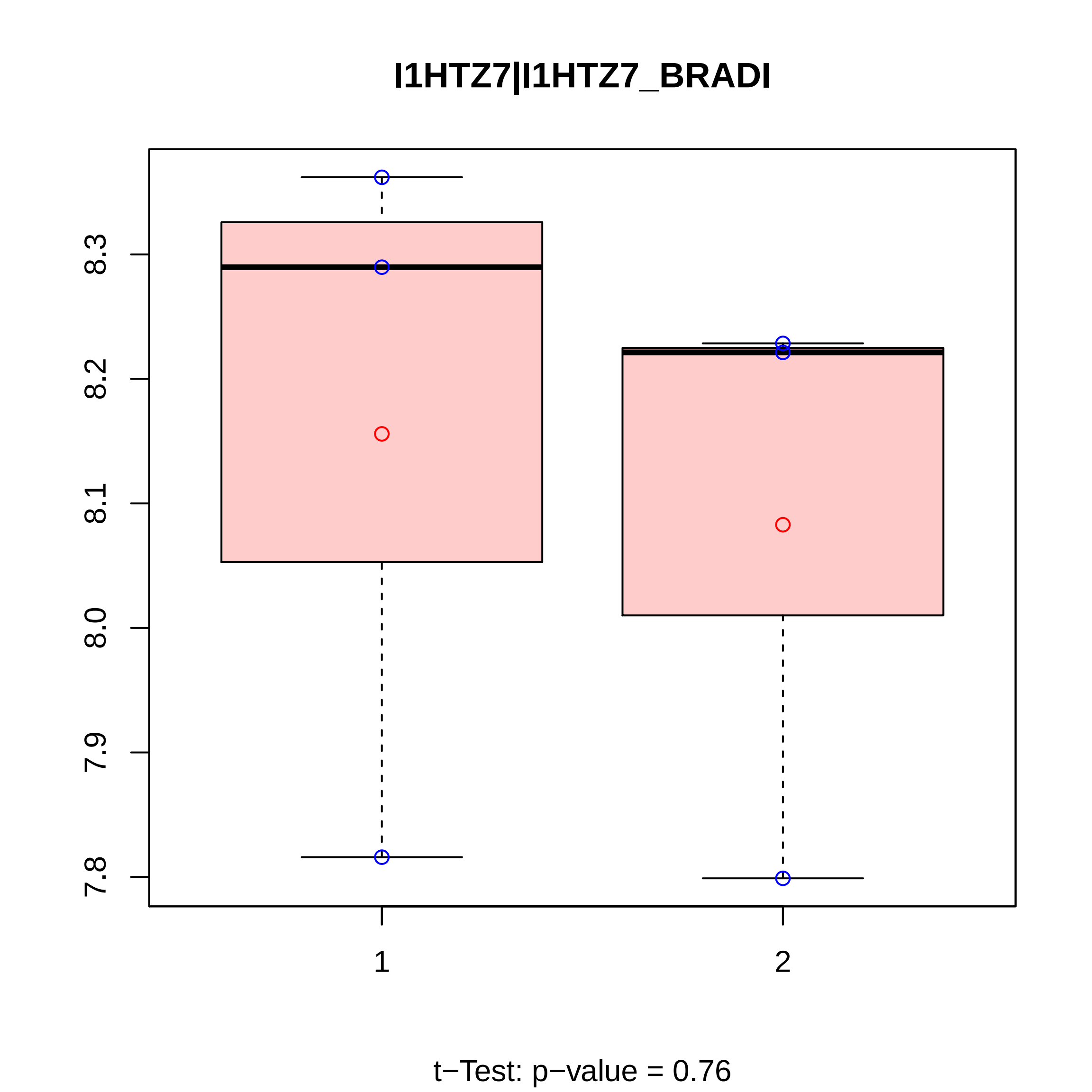
I1HTZ7|I1HTZ7_BRADI
8.3
8.2
8.1
8.0
7.9
7.8
1
2
t−Test: p−value = 0.76

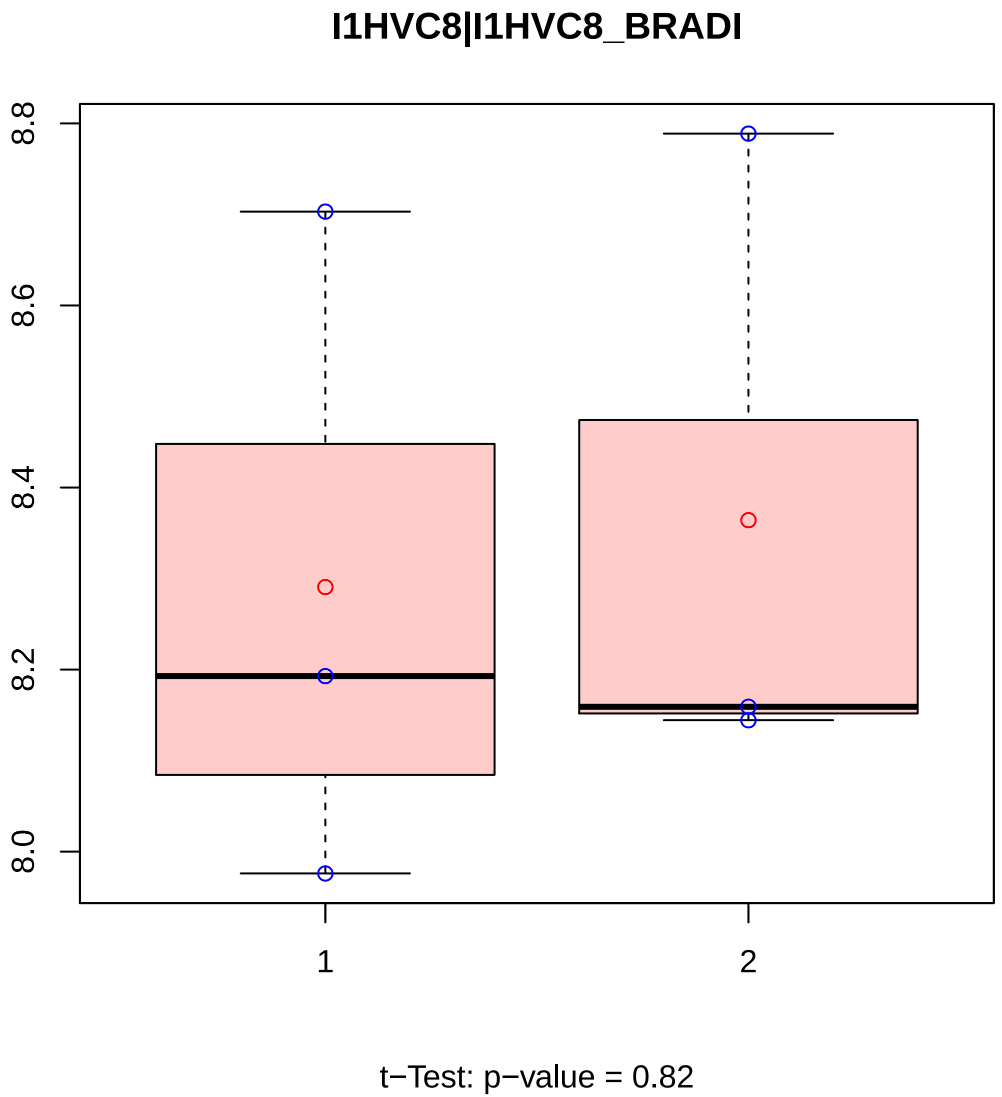

I1HVC8|I1HVC8_BRADI
8.0
8.2
8.4
8.6
8.8
1
2
t−Test: p−value = 0.82

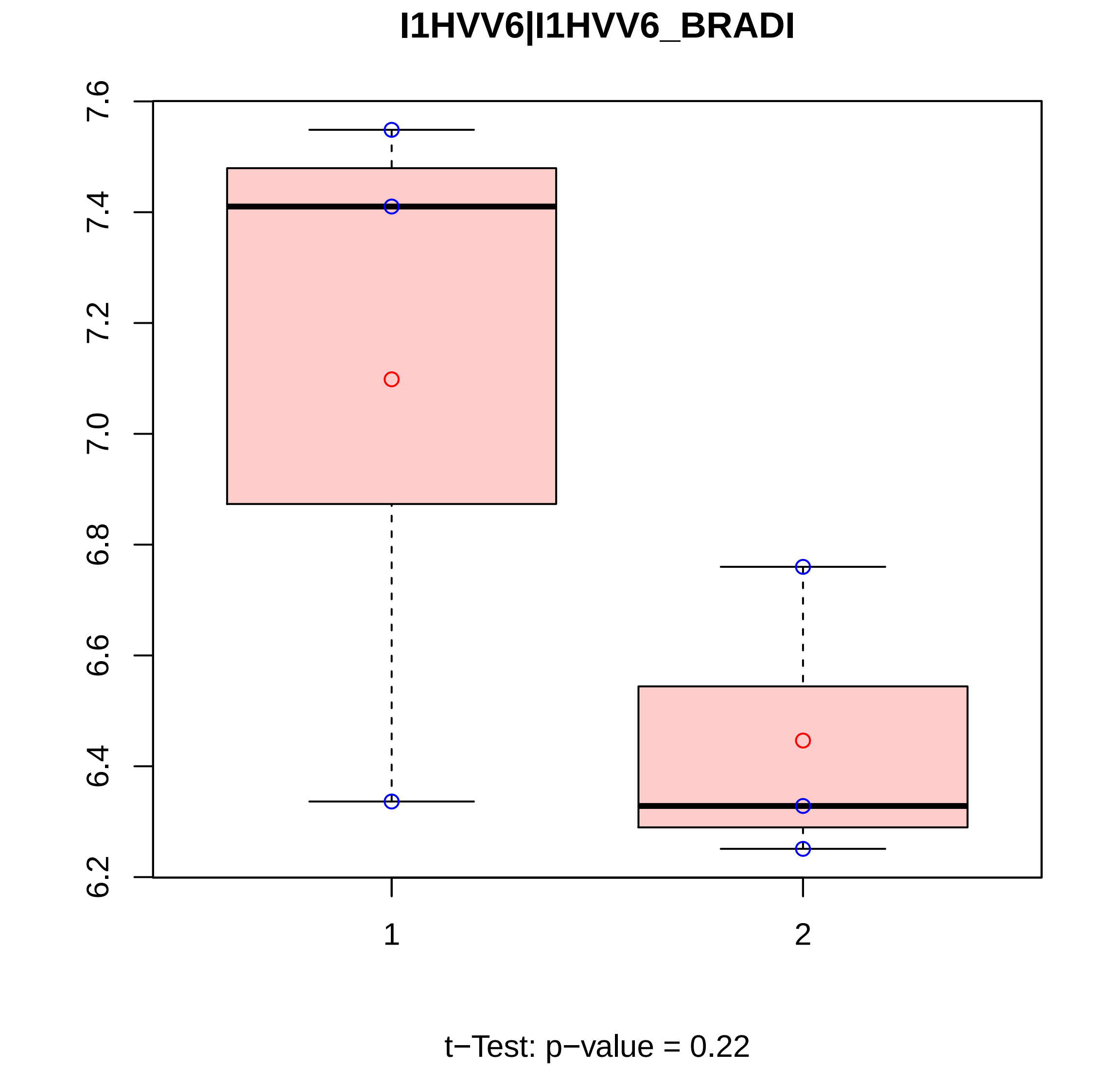
I1HVV6|I1HVV6_BRADI
7.6
7.4
7.2
7.0
6.8
6.6
6.4
6.2
1
2
t−Test: p−value = 0.22

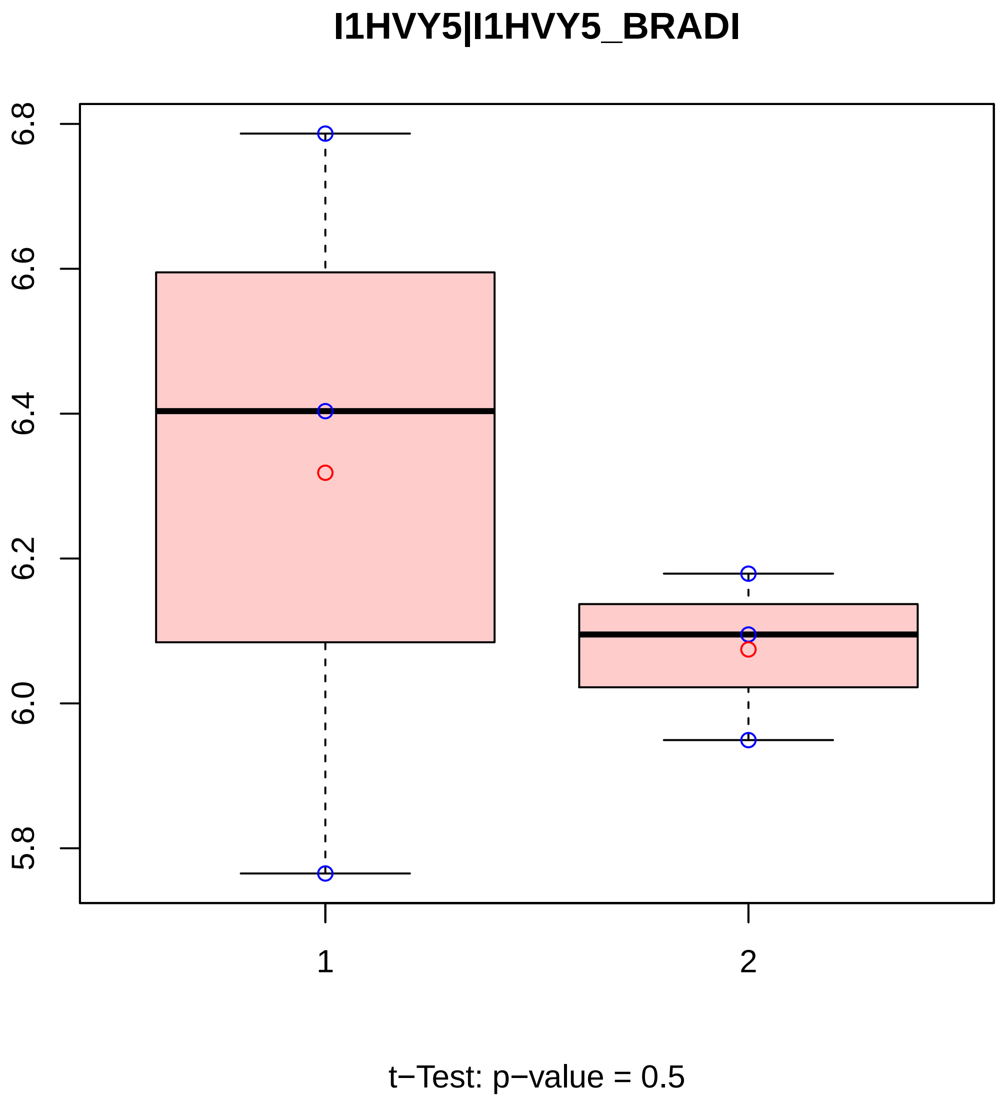

I1HVY5|I1HVY5_BRADI
6.8
6.6
6.4
6.2
6.0
5.8
1
2
t−Test: p−value = 0.5

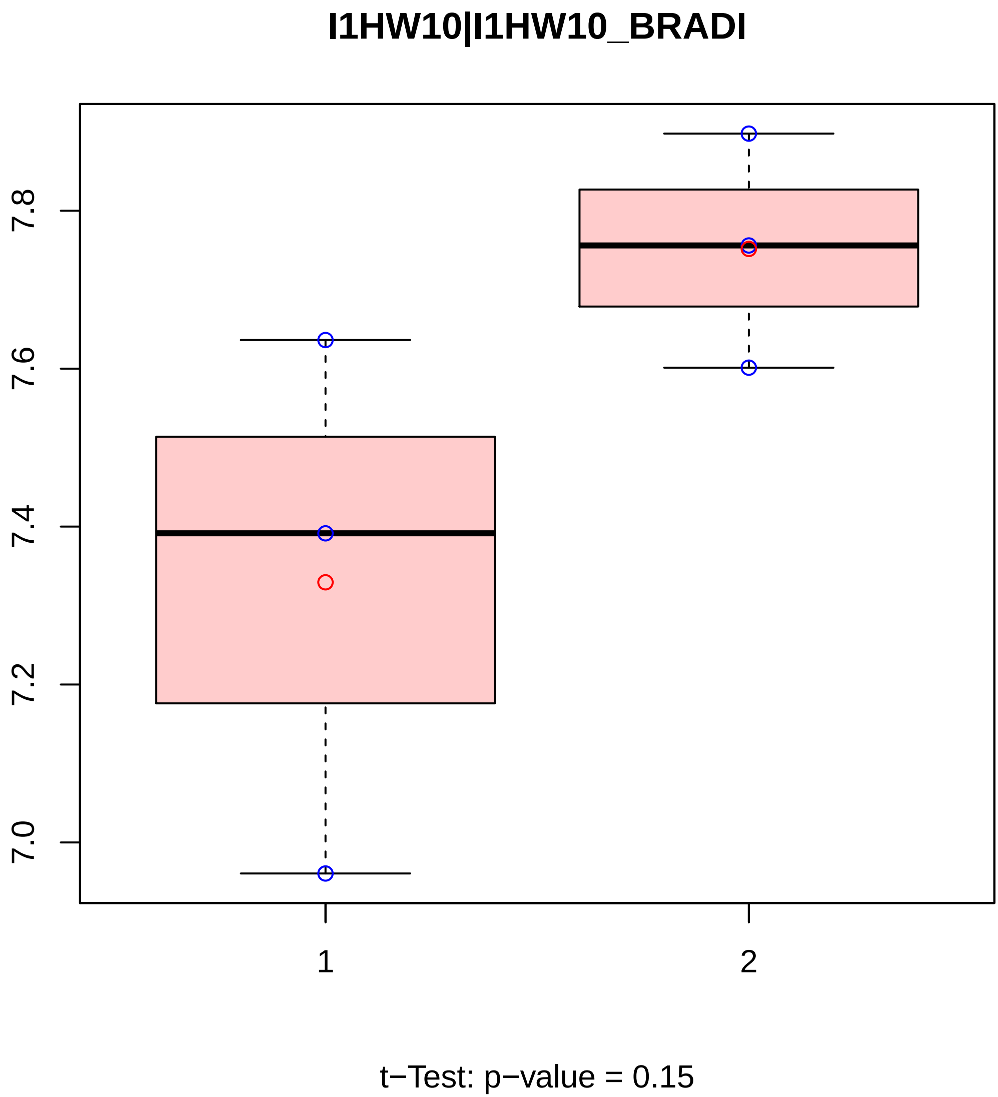
I1HW10|I1HW10_BRADI
7.0
7.2
7.4
7.6
7.8
1
2
t−Test: p−value = 0.15

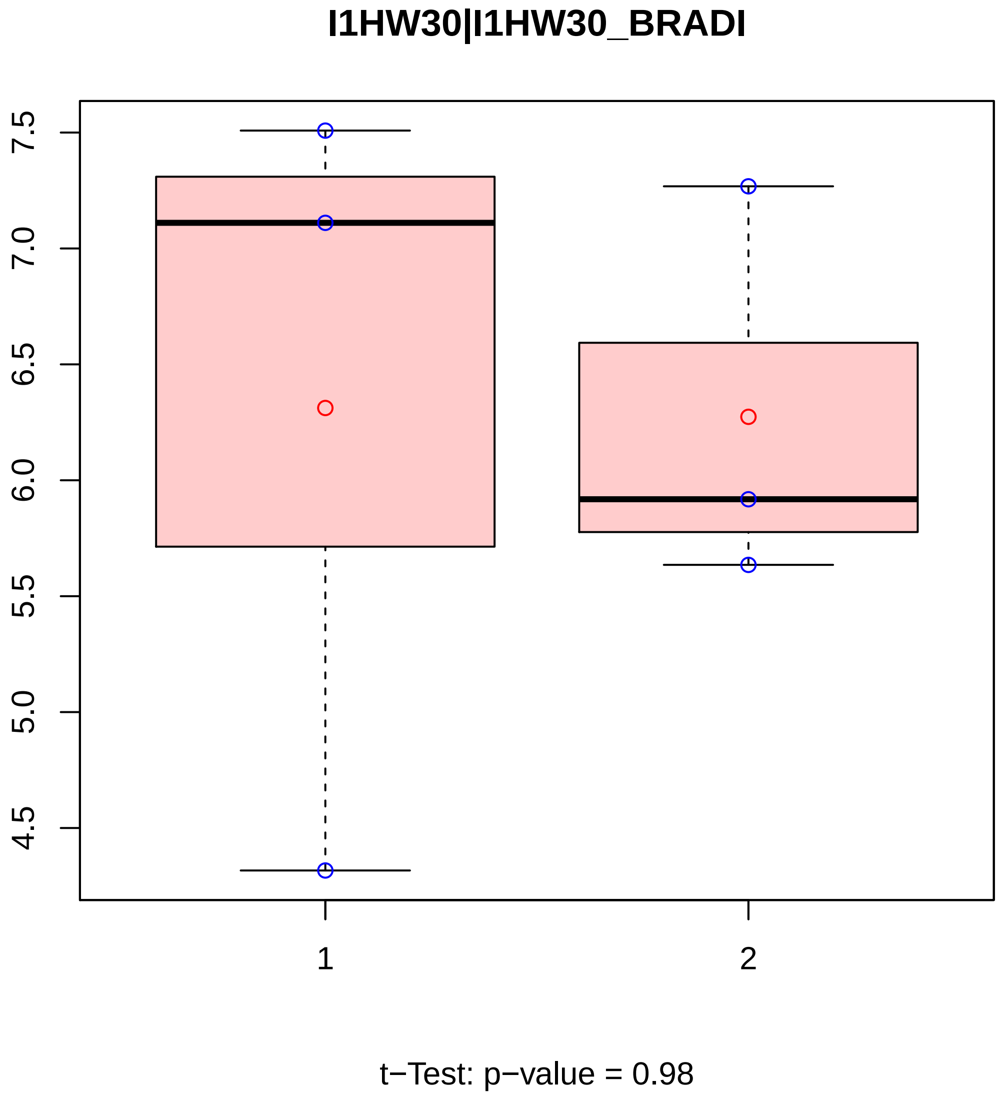
I1HW30|I1HW30_BRADI
7.5
7.0
6.5
6.0
5.5
5.0
4.5
1
2
t−Test: p−value = 0.98

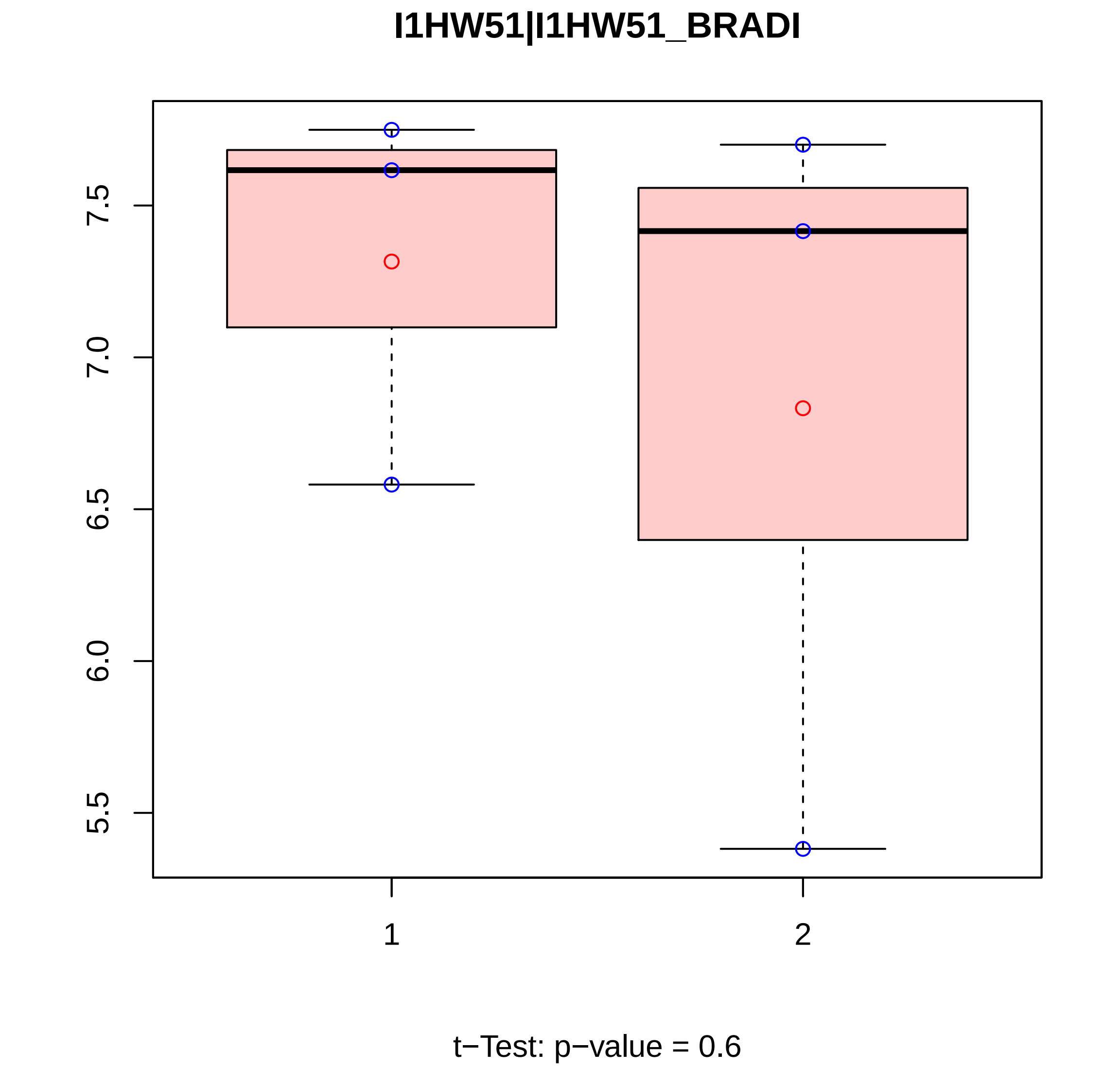
I1HW51|I1HW51_BRADI
7.5
7.0
6.5
6.0
5.5
1
2
t−Test: p−value = 0.6

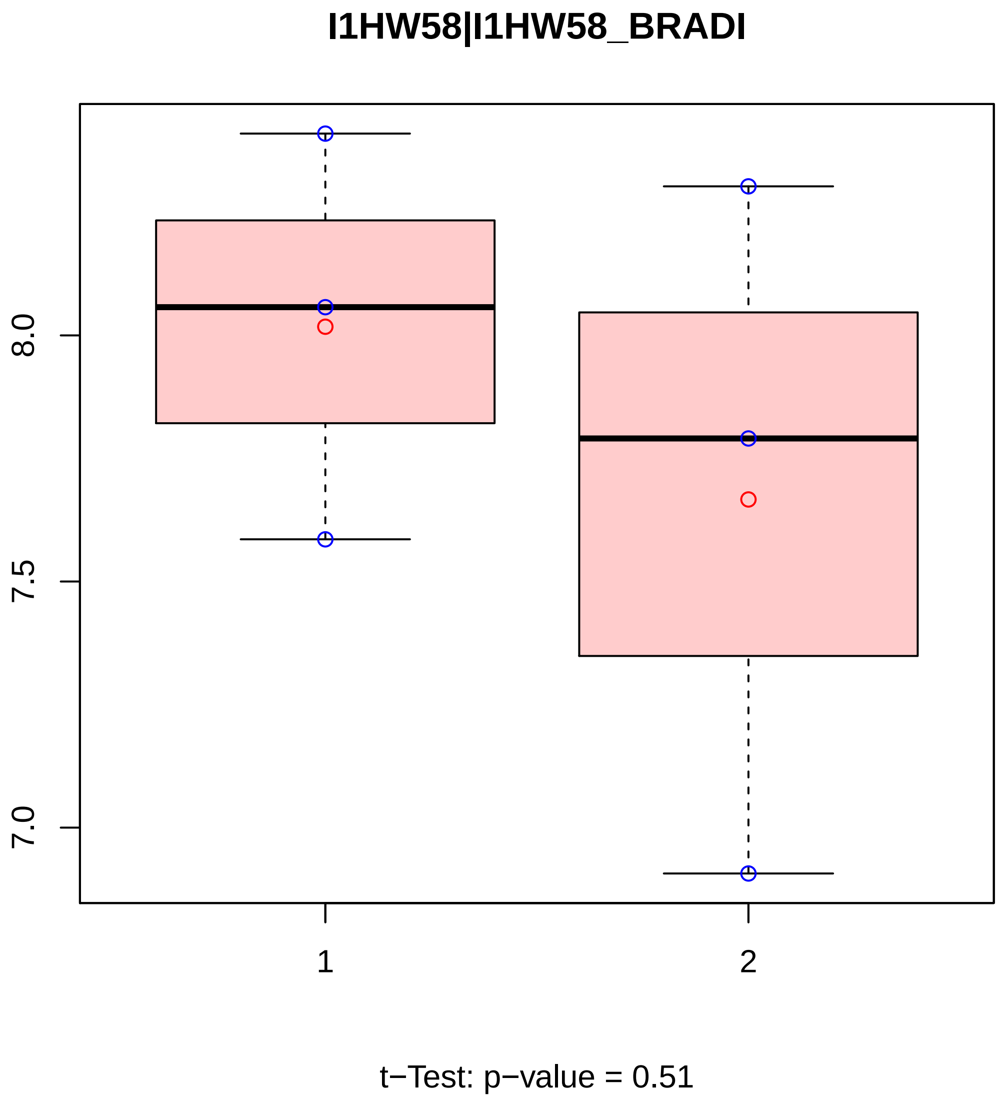
I1HW58|I1HW58_BRADI
8.0
7.5
7.0
1
2
t−Test: p−value = 0.51

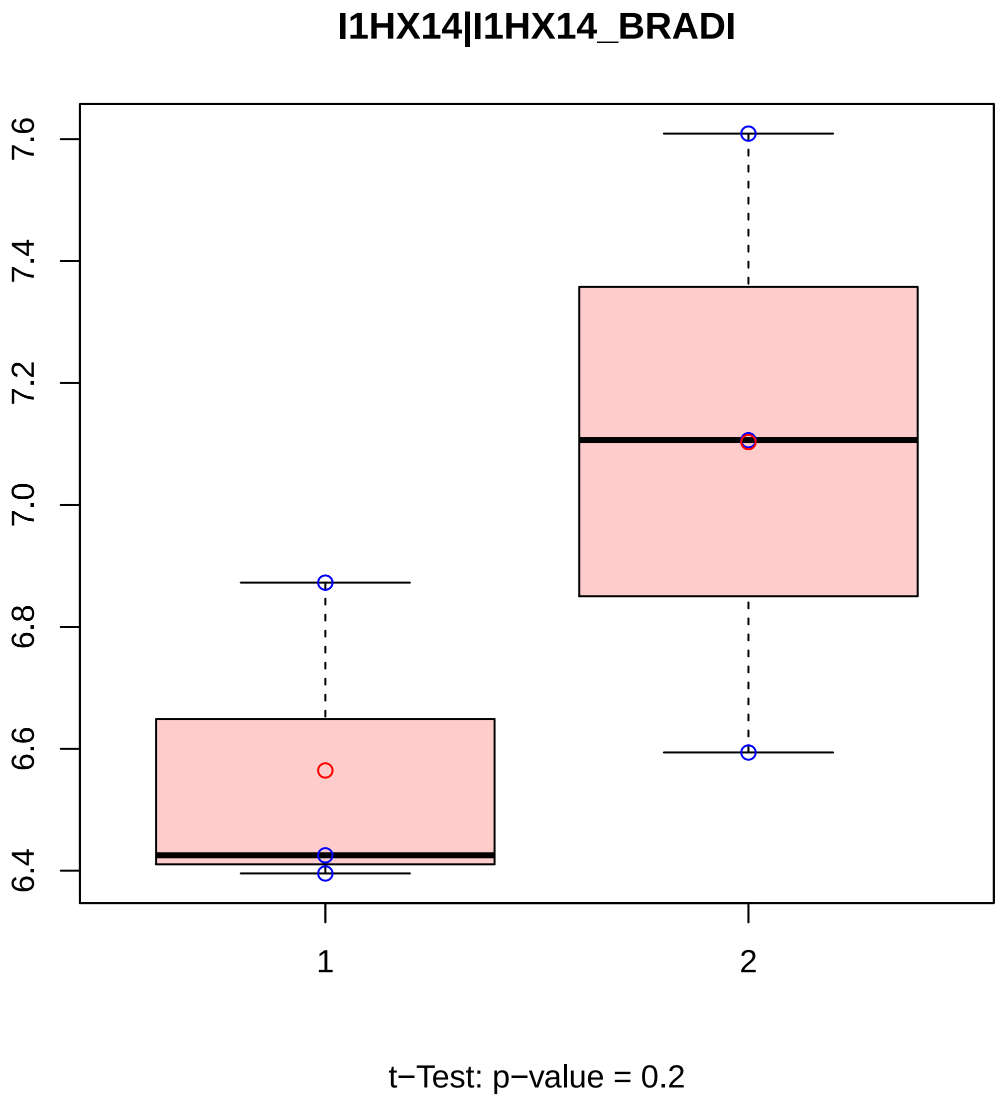
I1HX14|I1HX14_BRADI
7.6
7.4
7.2
7.0
6.8
6.6
6.4
1
2
t−Test: p−value = 0.2

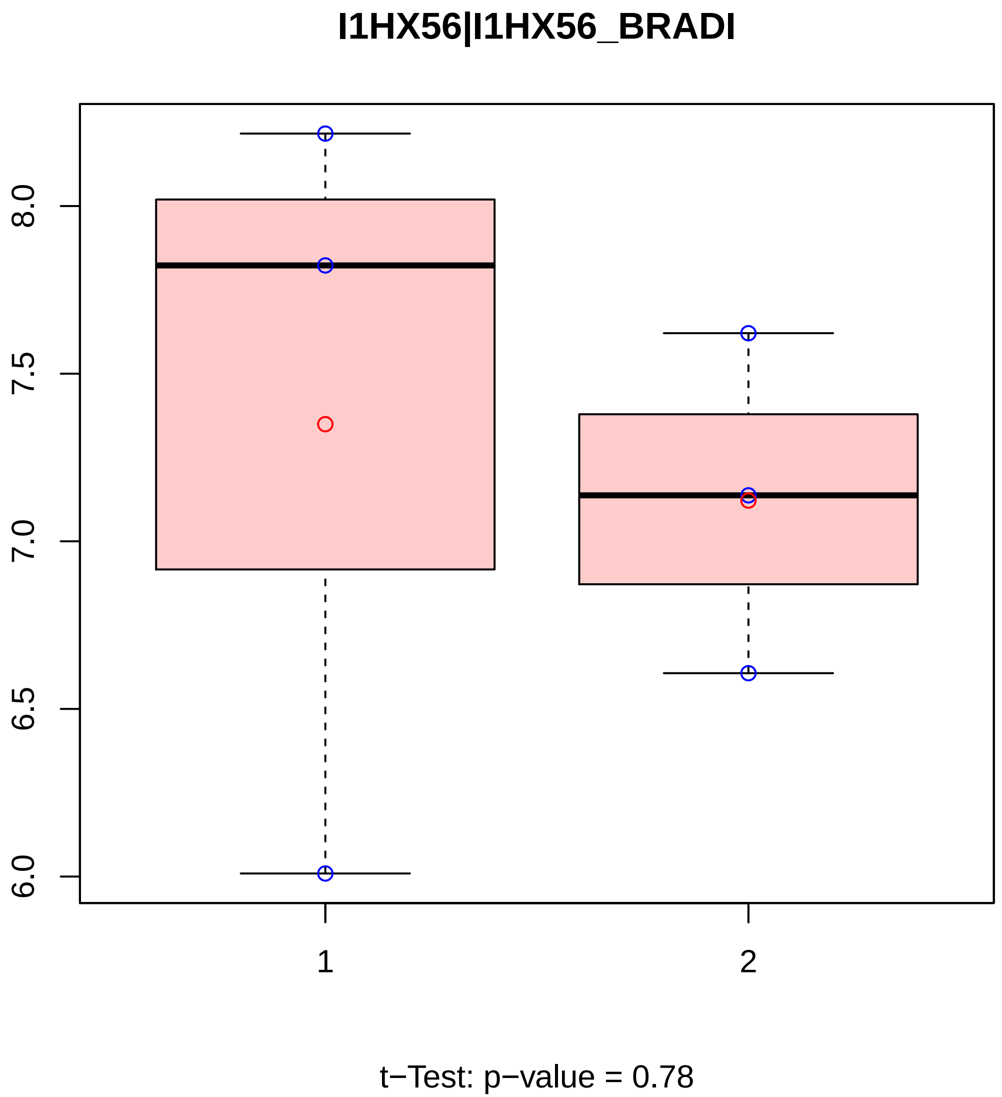
I1HX56|I1HX56_BRADI
8.0
7.5
7.0
6.5
6.0
1
2
t−Test: p−value = 0.78

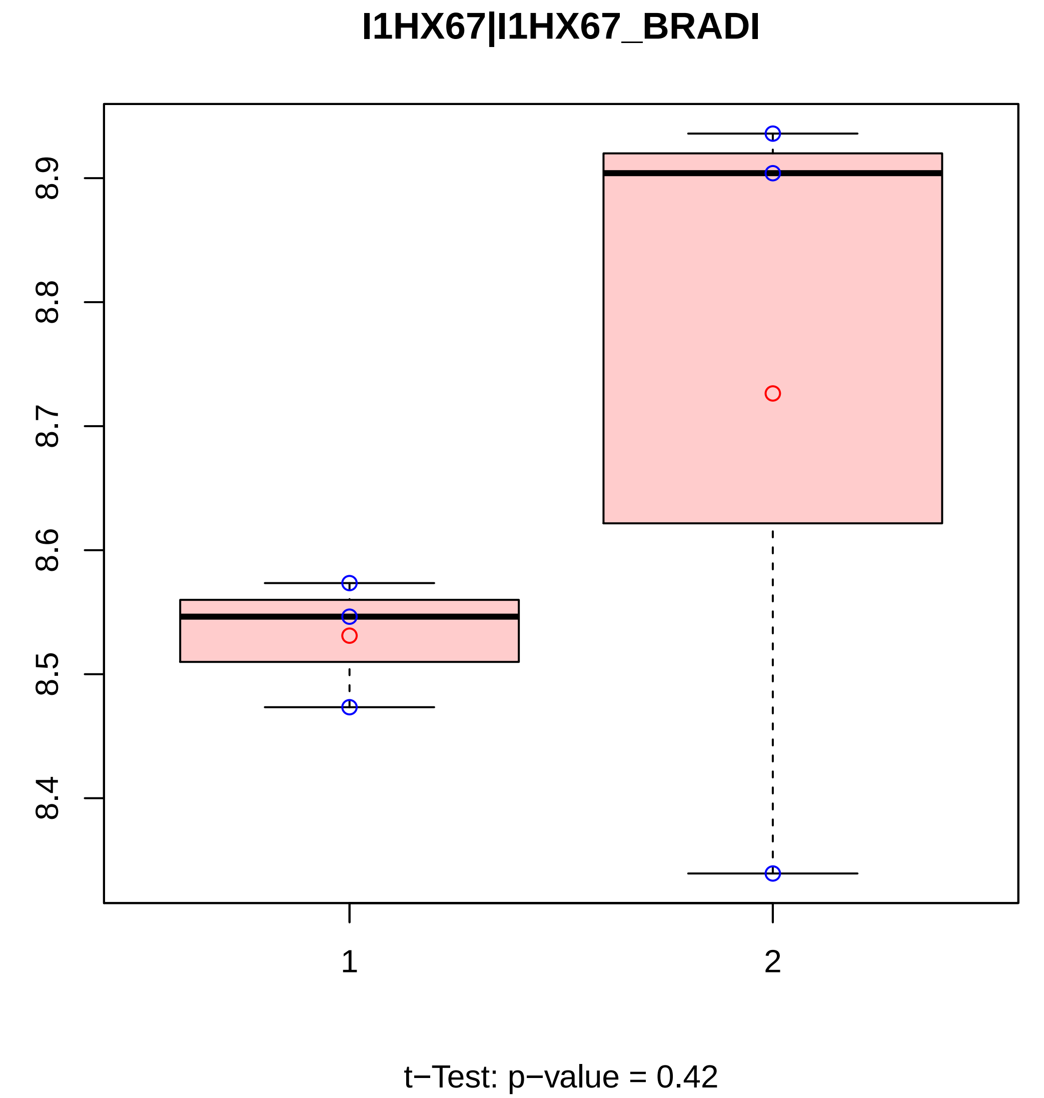
I1HX67|I1HX67_BRADI
8.4
8.5
8.6
8.7
8.8
8.9
1
2
t−Test: p−value = 0.42

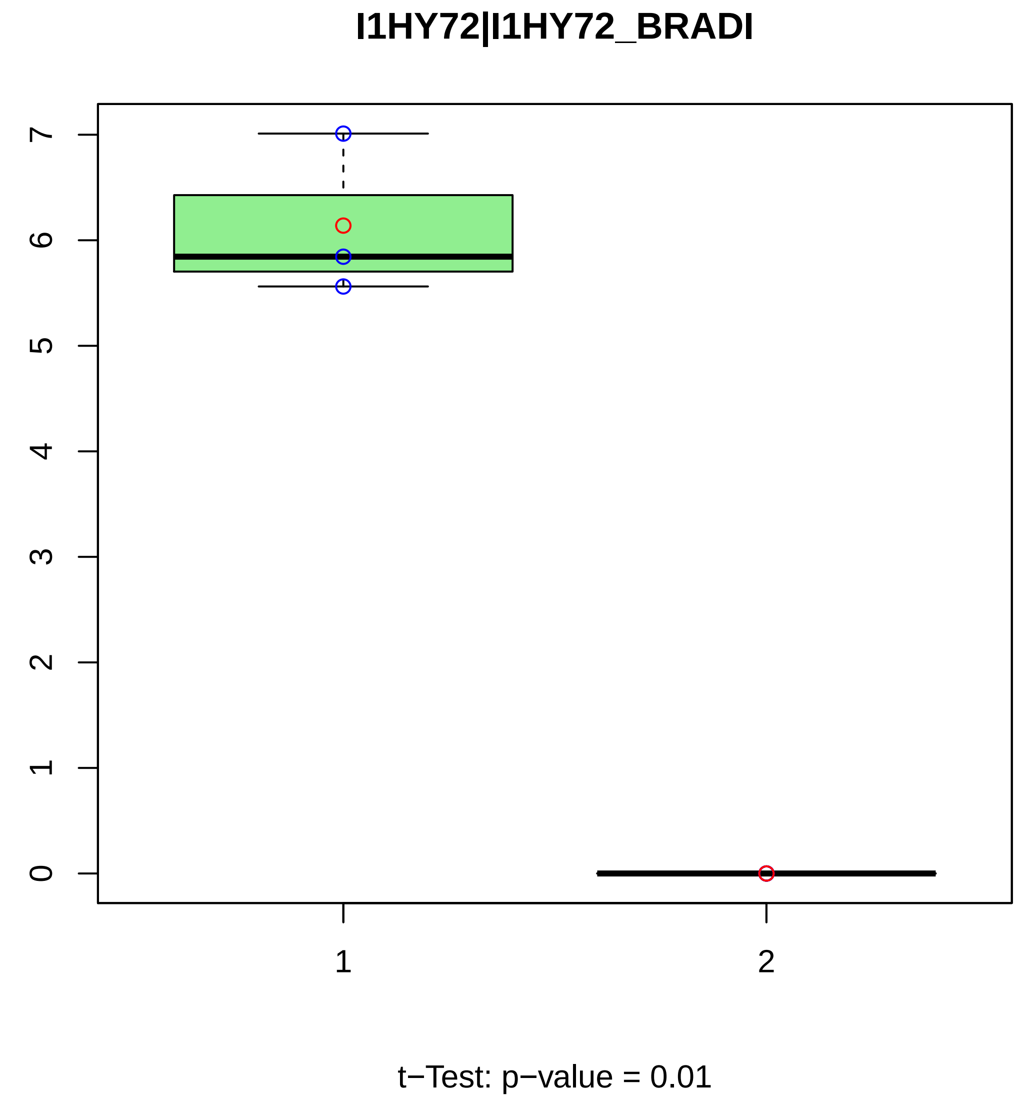
I1HY72|I1HY72_BRADI
0
1
2
3
4
5
6
7
1
2
t−Test: p−value = 0.01

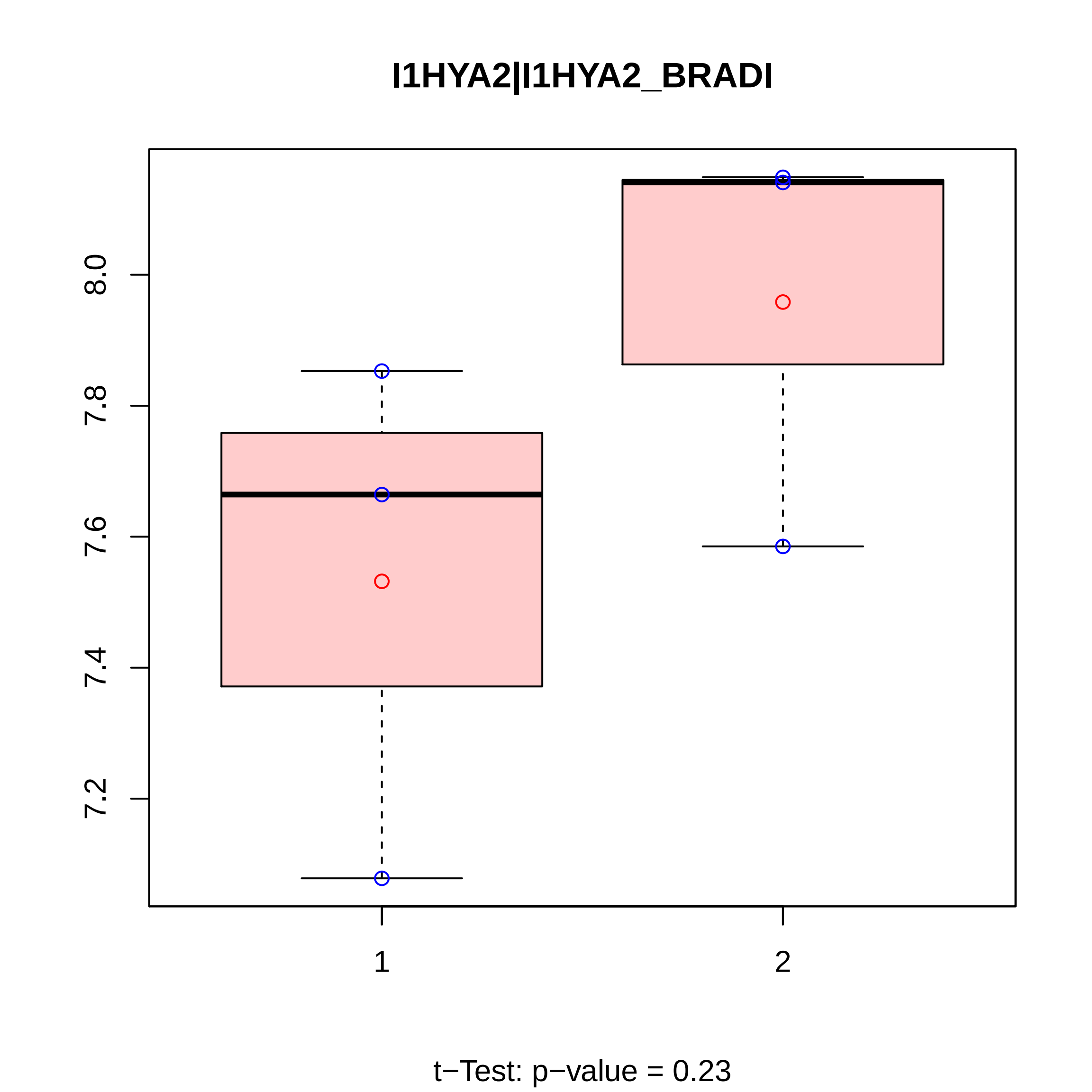
I1HYA2|I1HYA2_BRADI
8.0
7.8
7.6
7.4
7.2
1
2
t−Test: p−value = 0.23

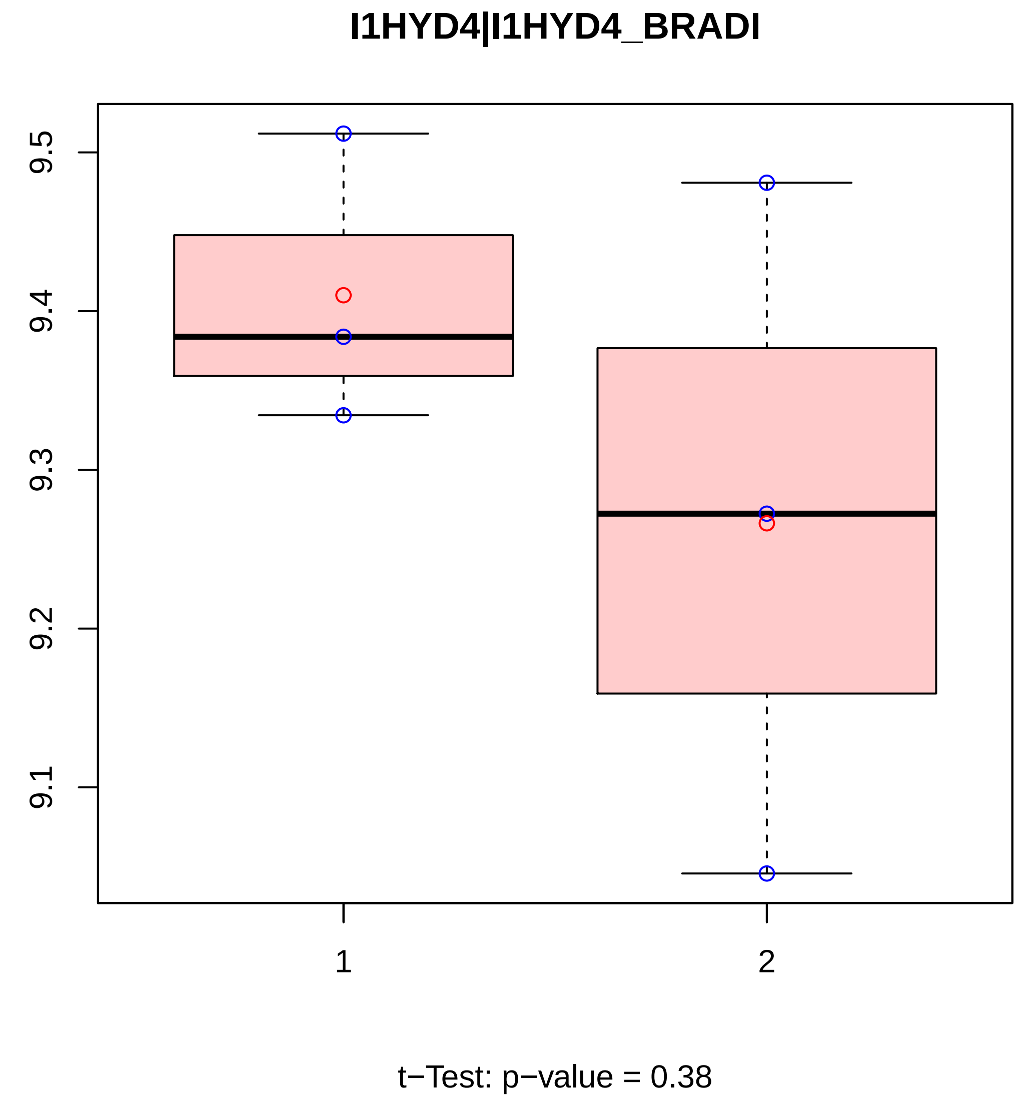
I1HYD4|I1HYD4_BRADI
9.1
9.2
9.3
9.4
9.5
1
2
t−Test: p−value = 0.38

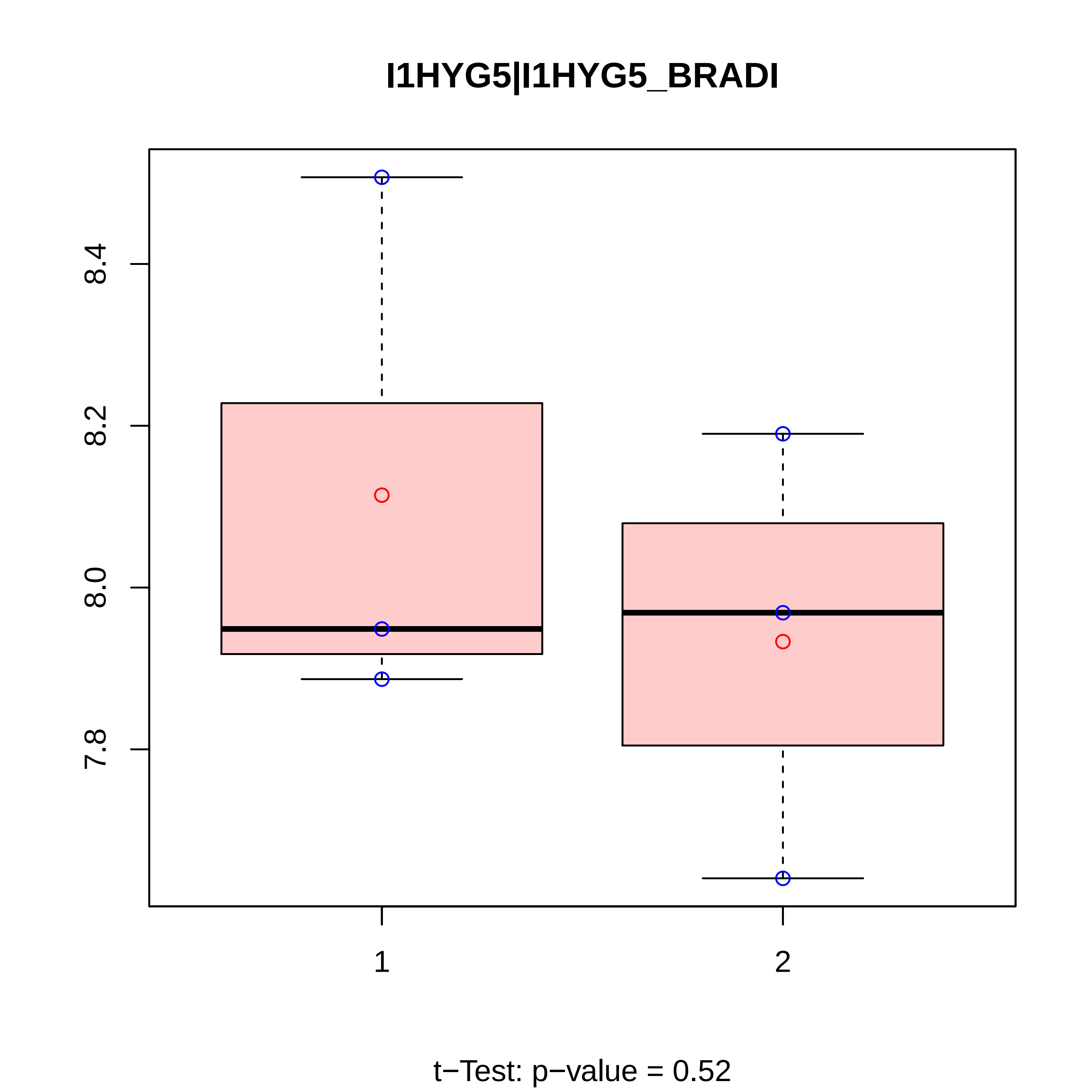

I1HYG5|I1HYG5_BRADI
8.4
8.2
8.0
7.8
1
2
t−Test: p−value = 0.52

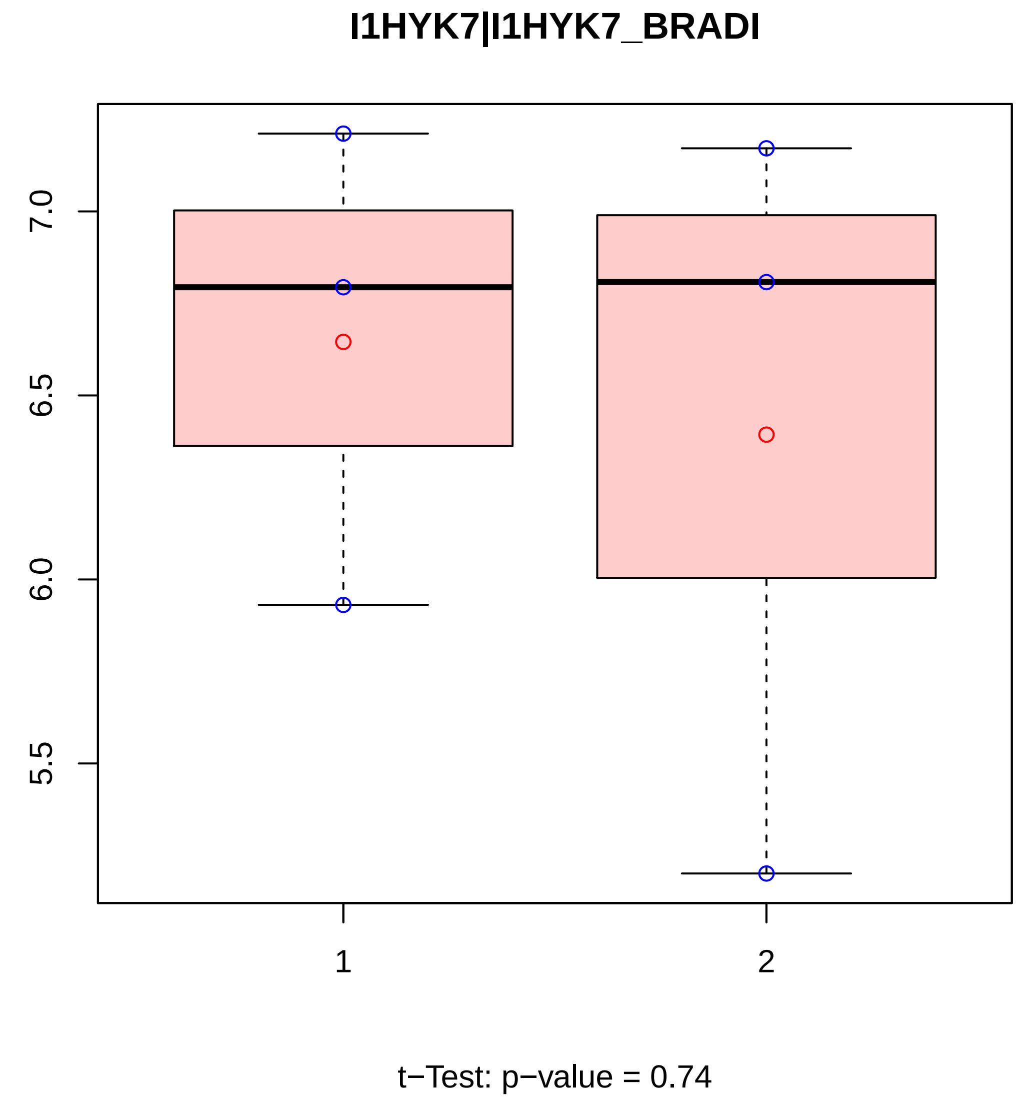
I1HYK7|I1HYK7_BRADI
7.0
6.5
6.0
5.5
1
2
t−Test: p−value = 0.74

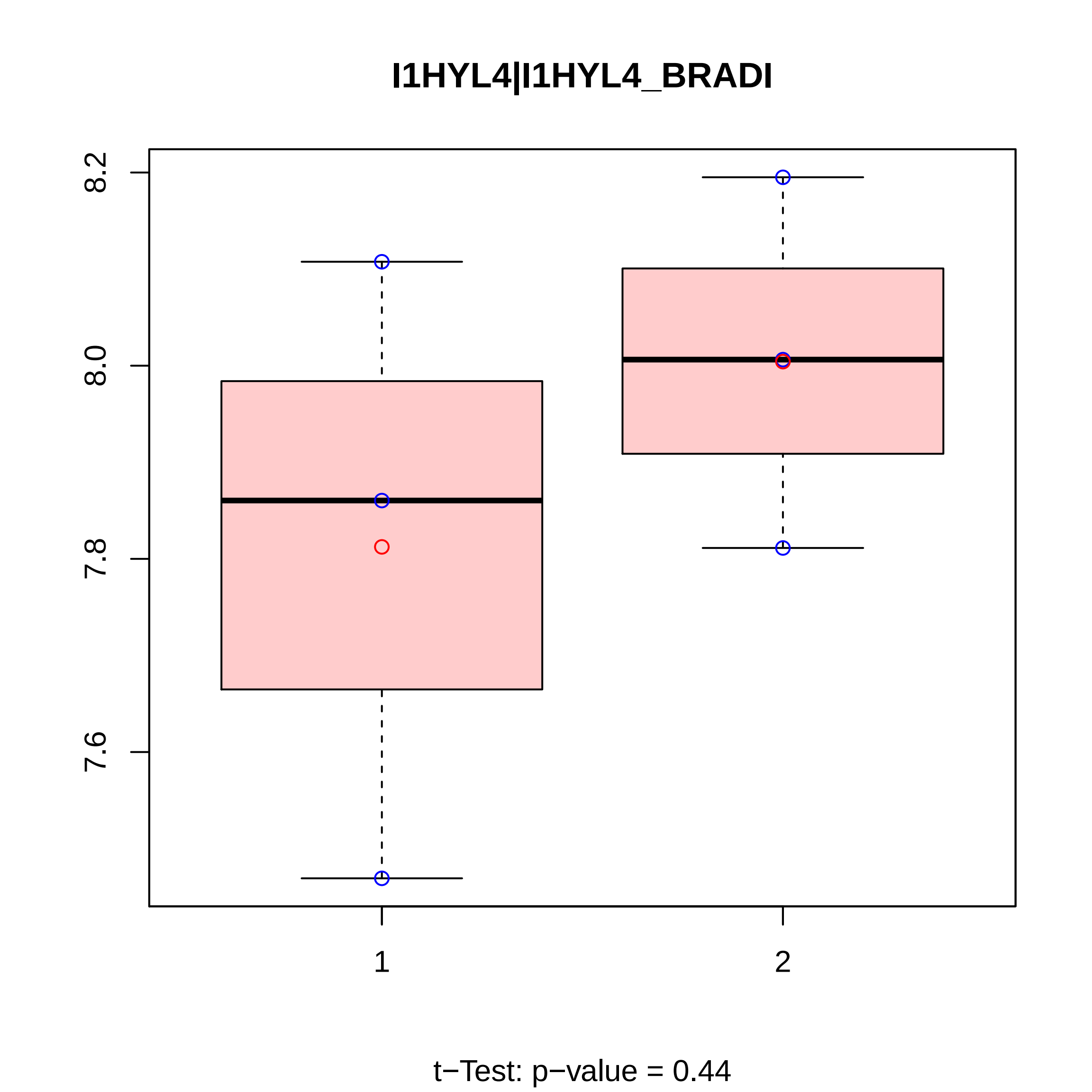
I1HYL4|I1HYL4_BRADI
8.2
8.0
7.8
7.6
1
2
t−Test: p−value = 0.44

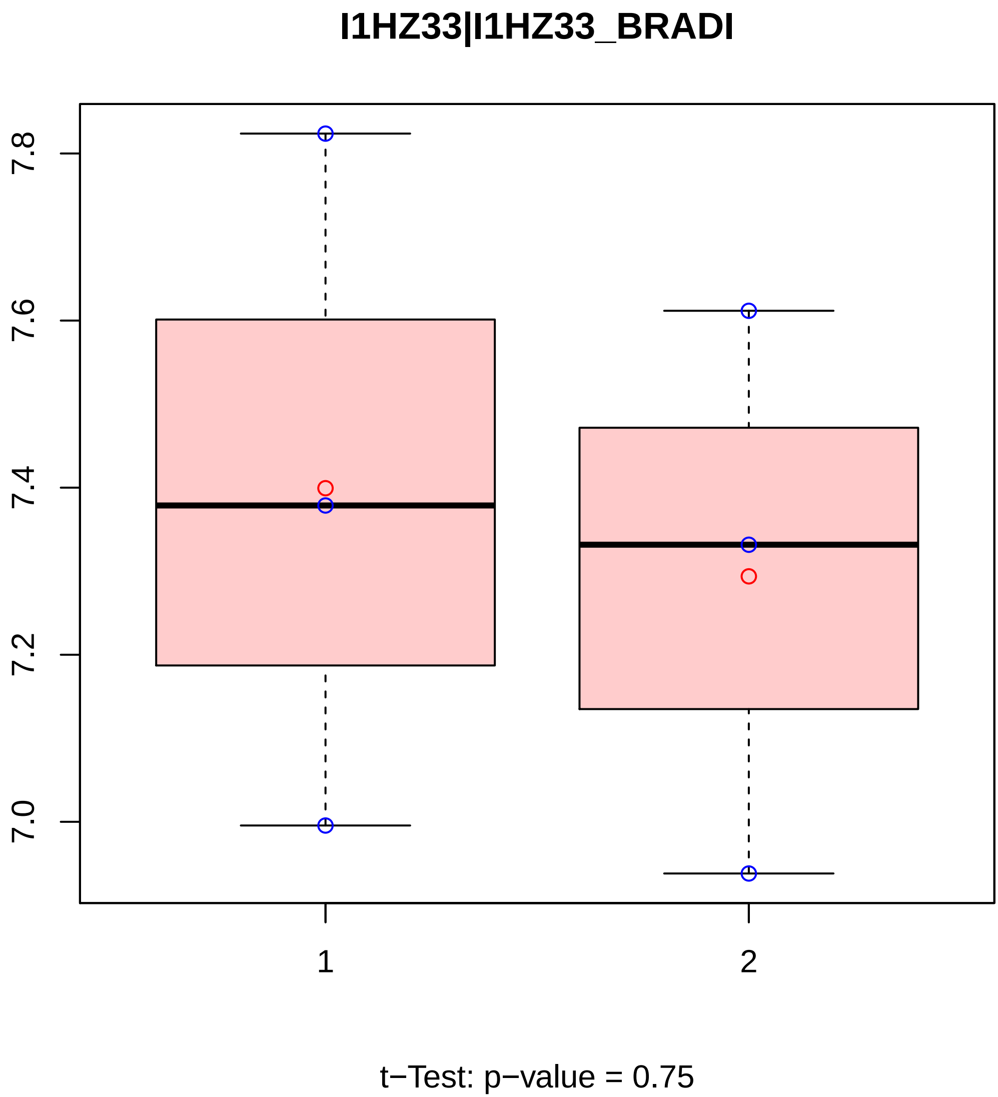
I1HZ33|I1HZ33_BRADI
7.0
7.2
7.4
7.6
7.8
1
2
t−Test: p−value = 0.75

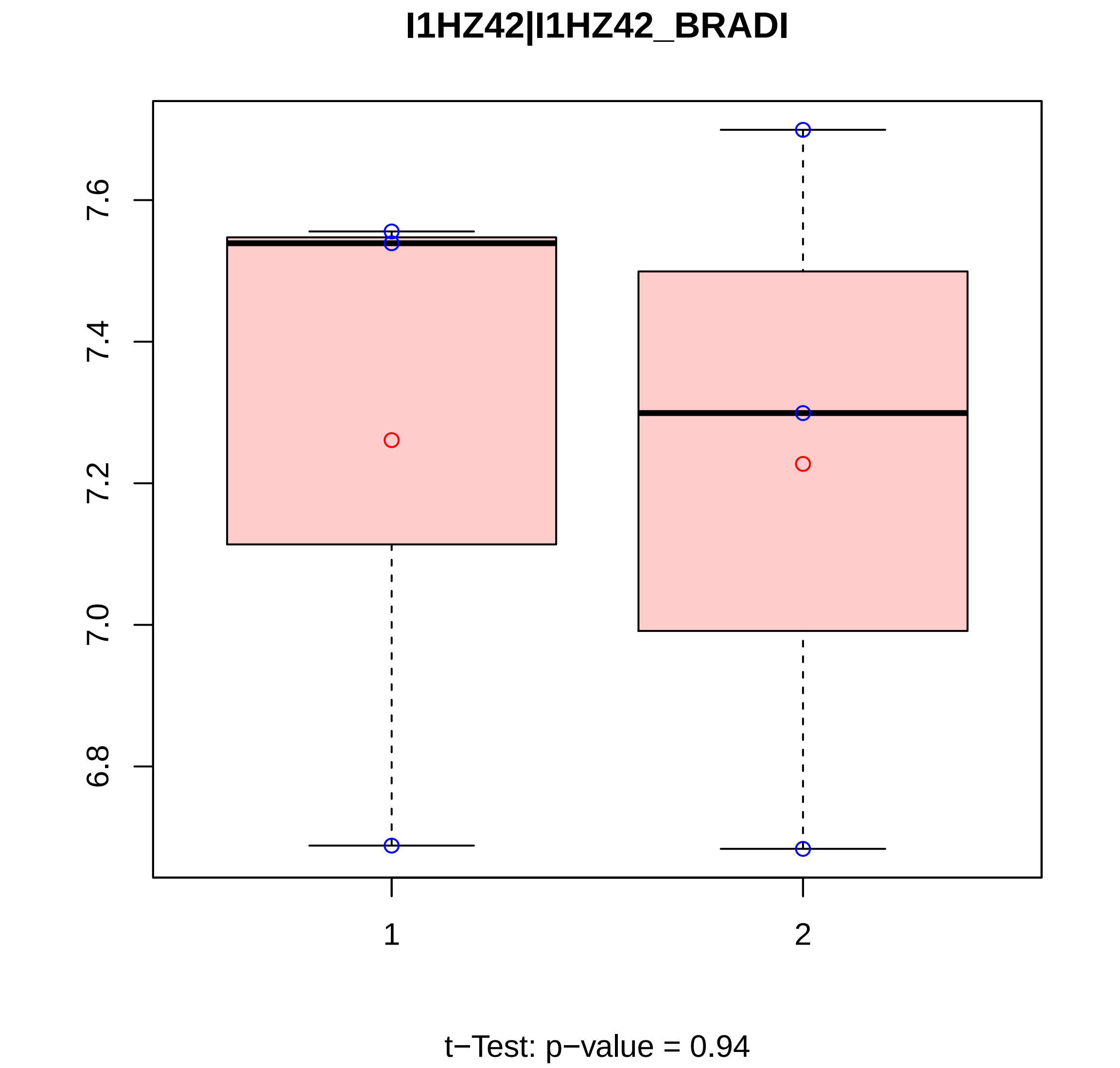
I1HZ42|I1HZ42_BRADI
7.6
7.4
7.2
7.0
6.8
1
2
t−Test: p−value = 0.94

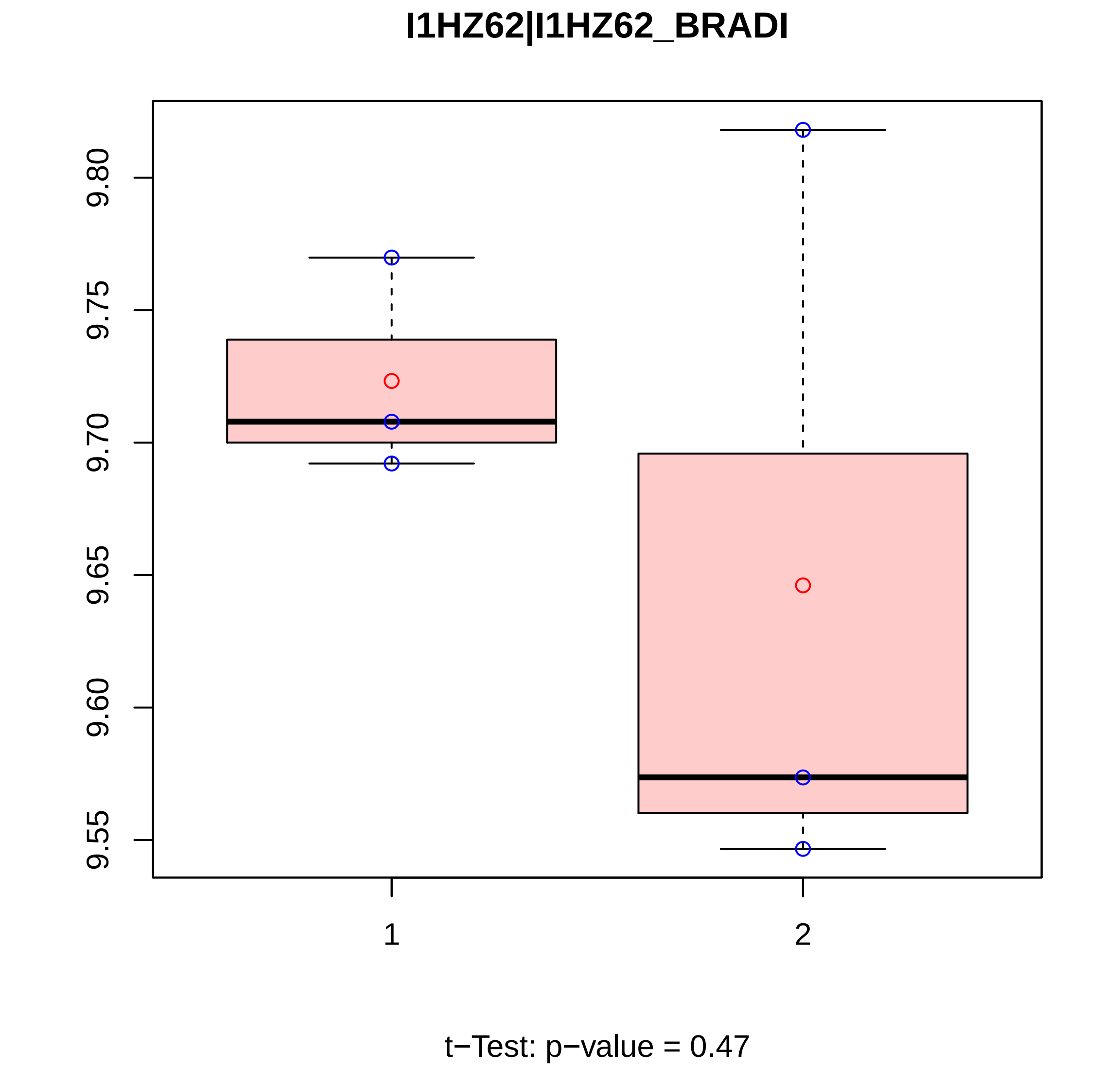

I1HZ62|I1HZ62_BRADI
9.55
9.60
9.65
9.70
9.75
9.80
1
2
t−Test: p−value = 0.47

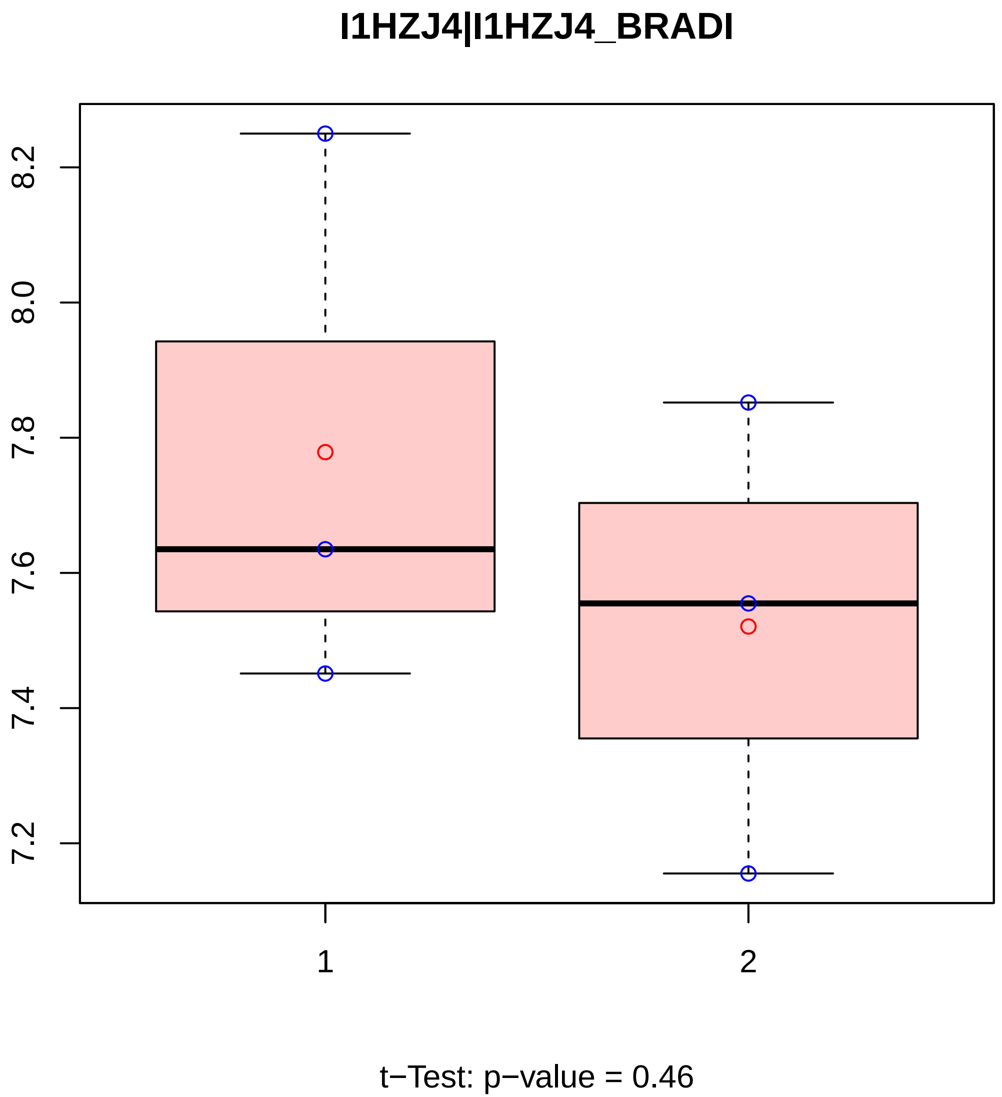
I1HZJ4|I1HZJ4_BRADI
8.2
8.0
7.8
7.6
7.4
7.2
1
2
t−Test: p−value = 0.46

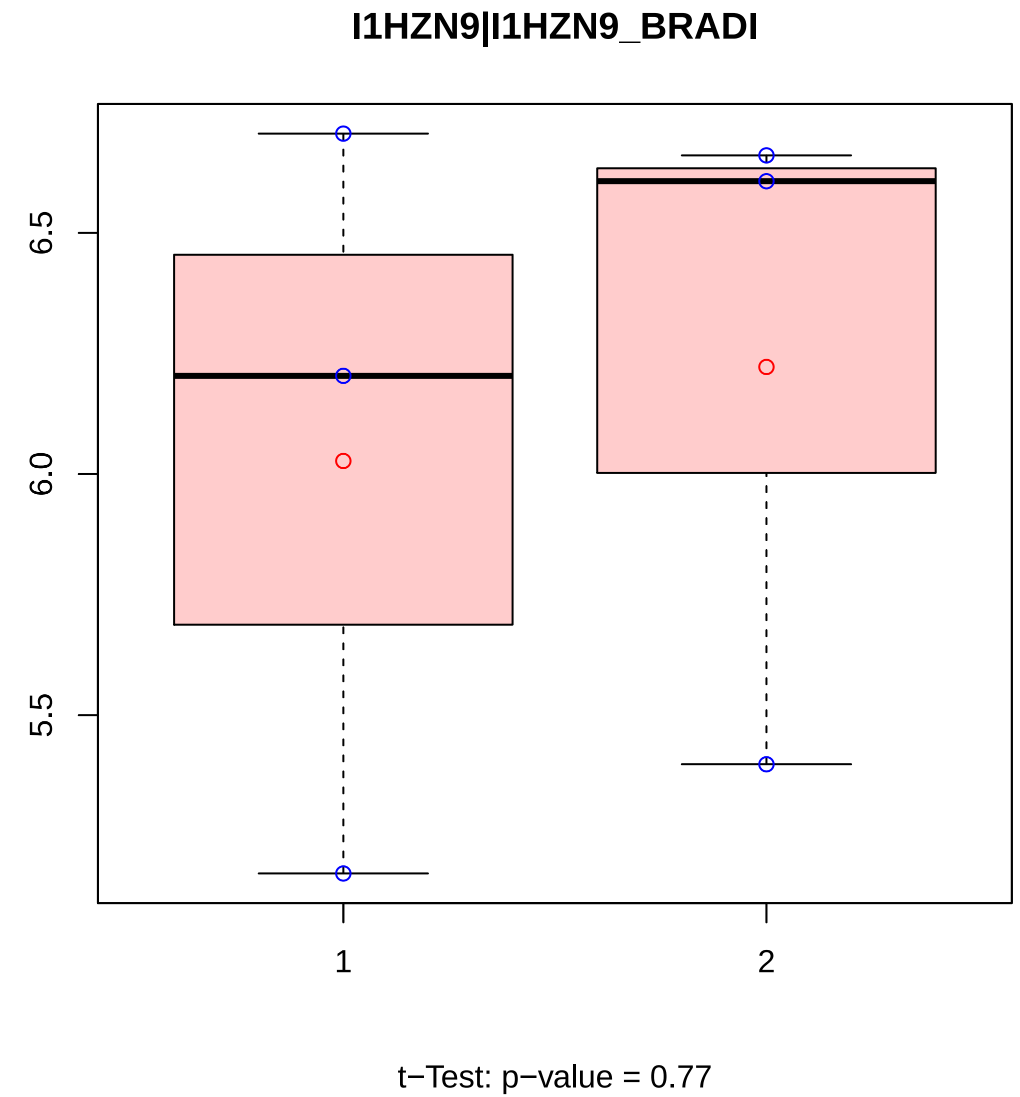
I1HZN9|I1HZN9_BRADI
6.5
6.0
5.5
1
2
t−Test: p−value = 0.77

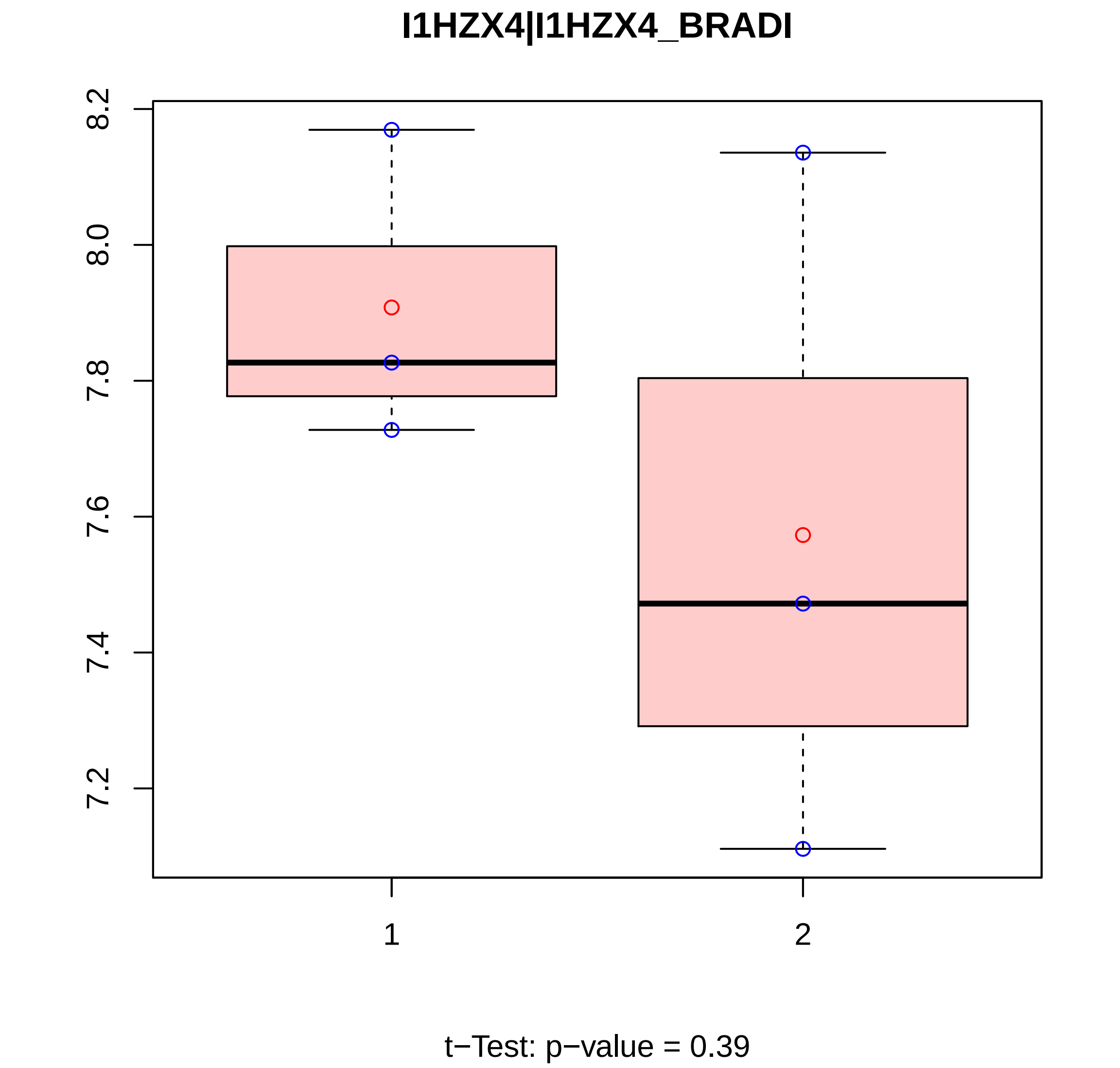
I1HZX4|I1HZX4_BRADI
8.2
8.0
7.8
7.6
7.4
7.2
1
2
t−Test: p−value = 0.39

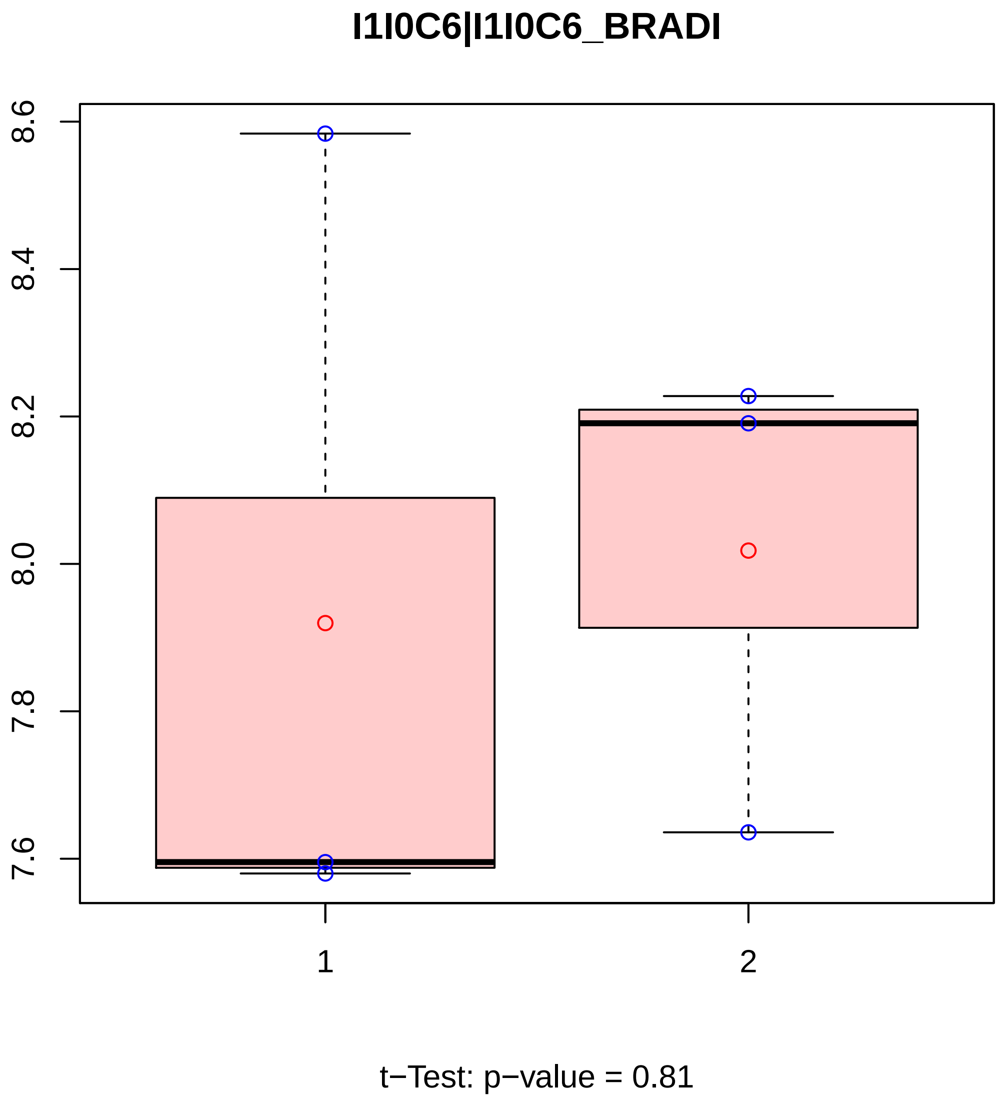
I1I0C6|I1I0C6_BRADI
8.6
8.4
8.2
8.0
7.8
7.6
1
2
t−Test: p−value = 0.81

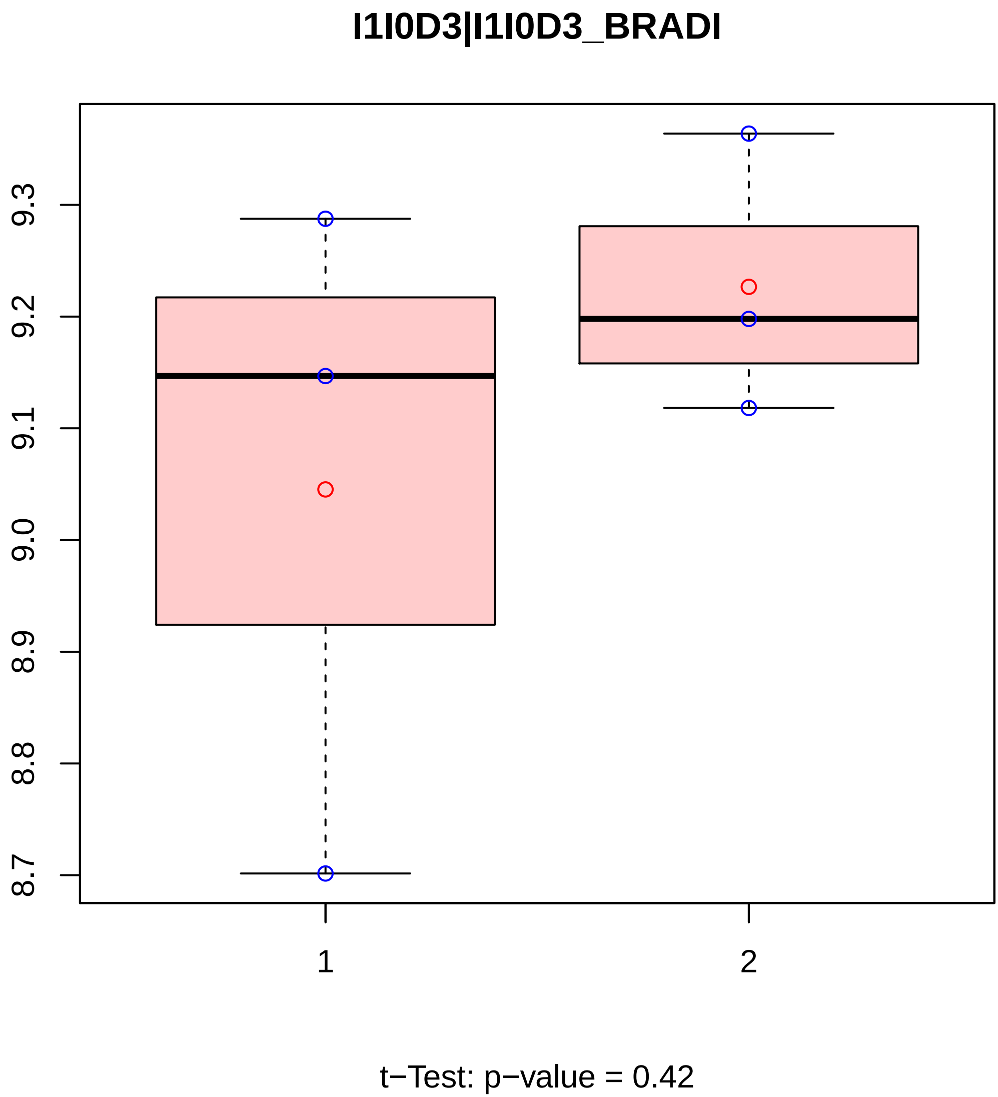
I1I0D3|I1I0D3_BRADI
9.3
9.2
9.1
9.0
8.9
8.8
8.7
1
2
t−Test: p−value = 0.42

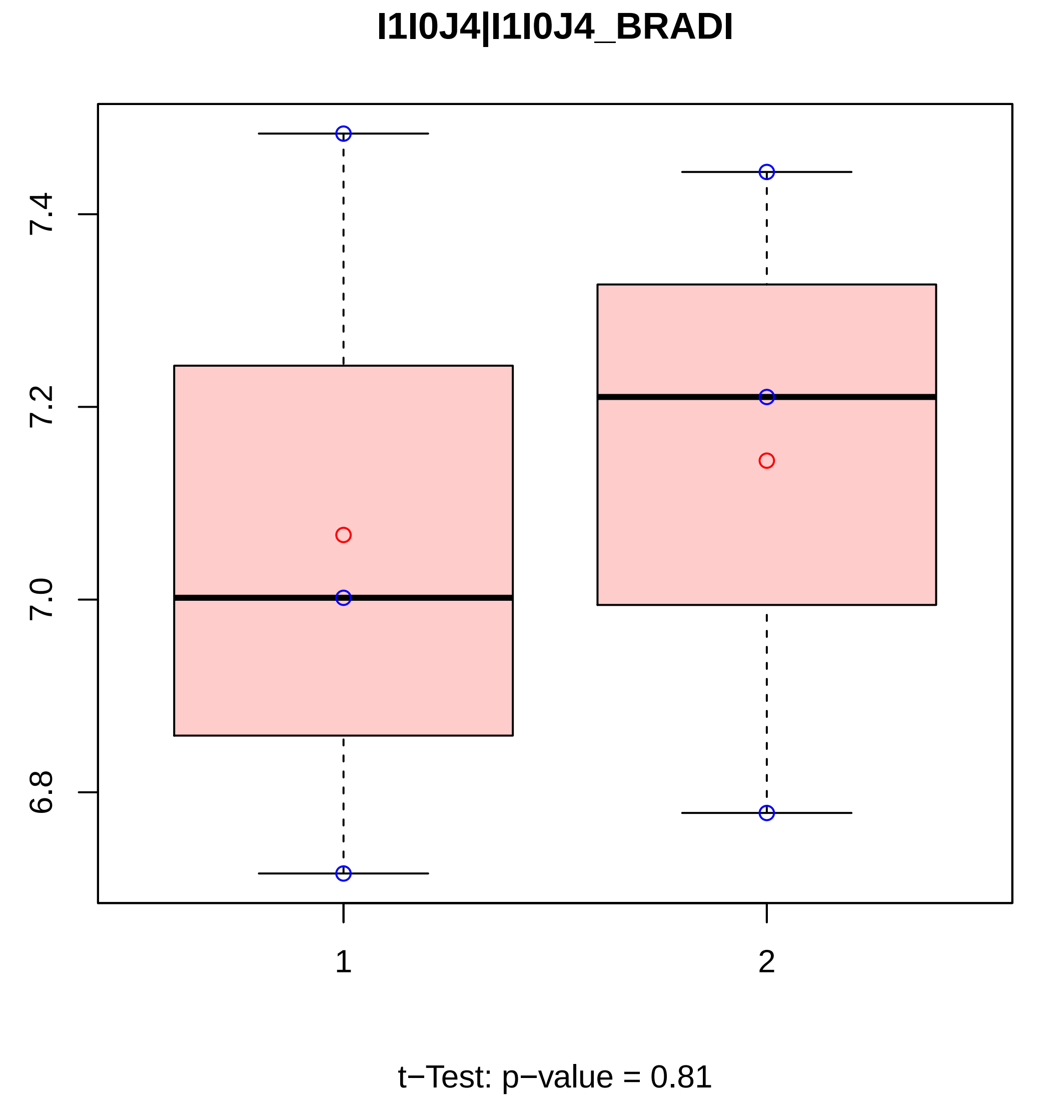
I1I0J4|I1I0J4_BRADI
7.4
7.2
7.0
6.8
1
2
t−Test: p−value = 0.81

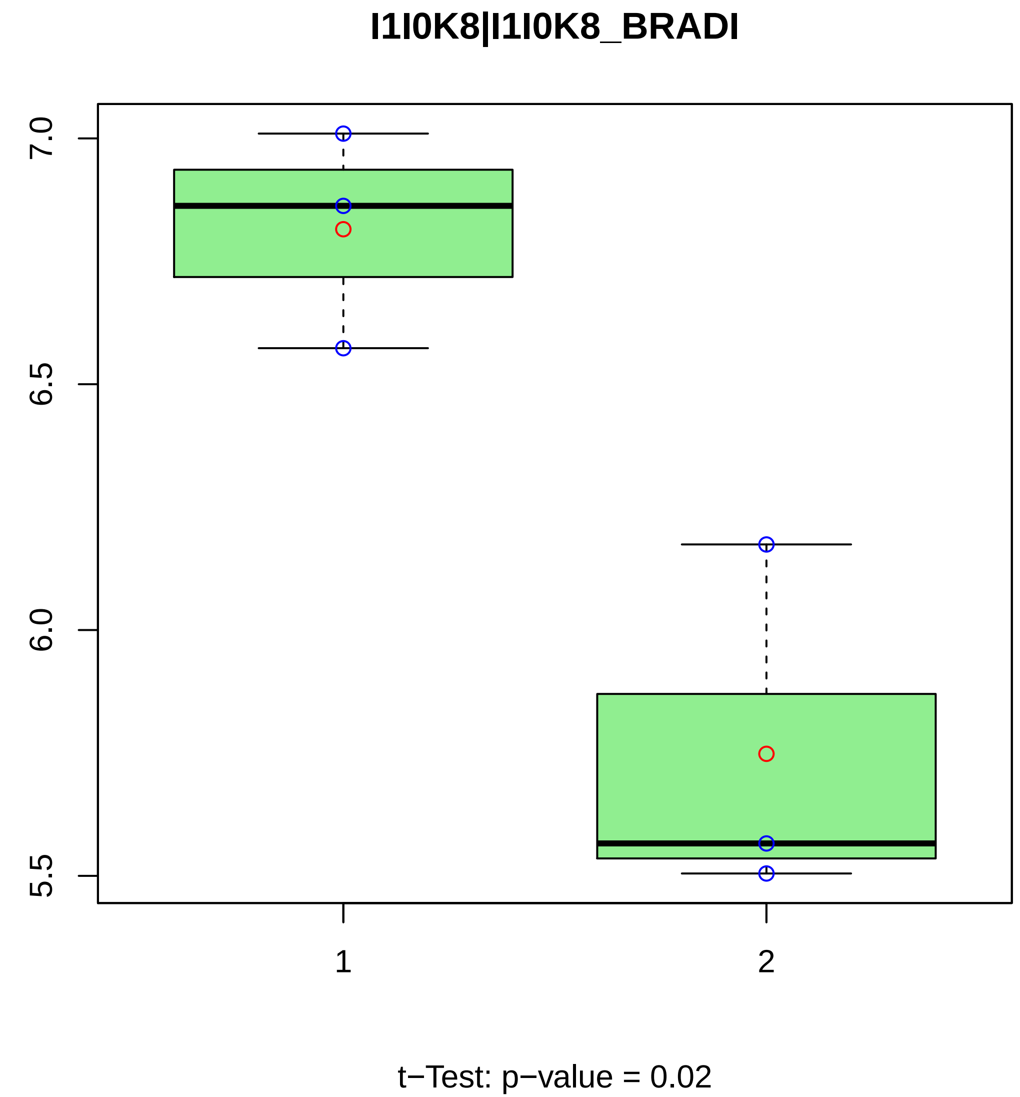
I1I0K8|I1I0K8_BRADI
7.0
6.5
6.0
5.5
1
2
t−Test: p−value = 0.02

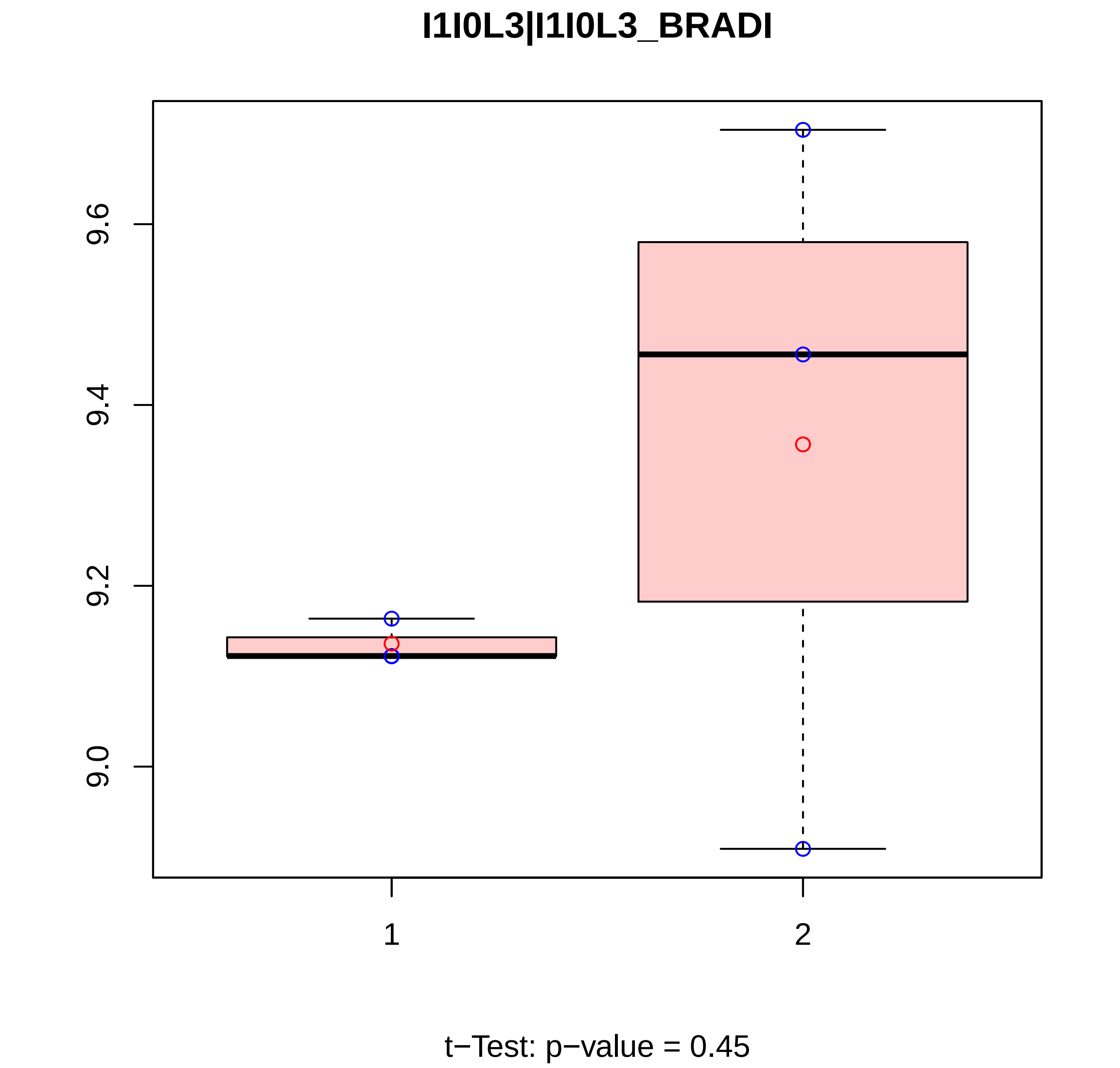
I1I0L3|I1I0L3_BRADI
9.0
9.2
9.4
9.6
1
2
t−Test: p−value = 0.45

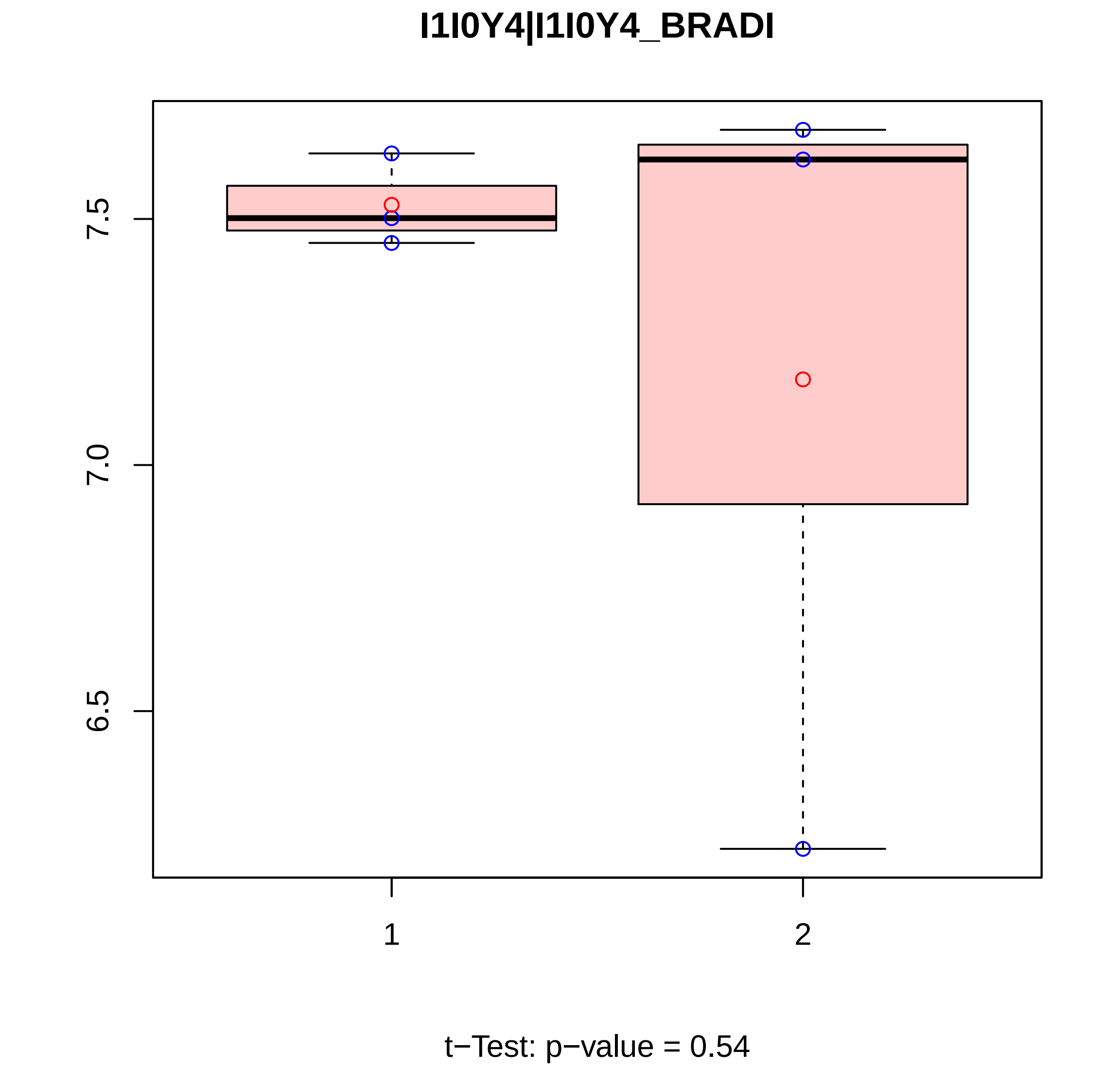
I1I0Y4|I1I0Y4_BRADI
7.5
7.0
6.5
1
2
t−Test: p−value = 0.54

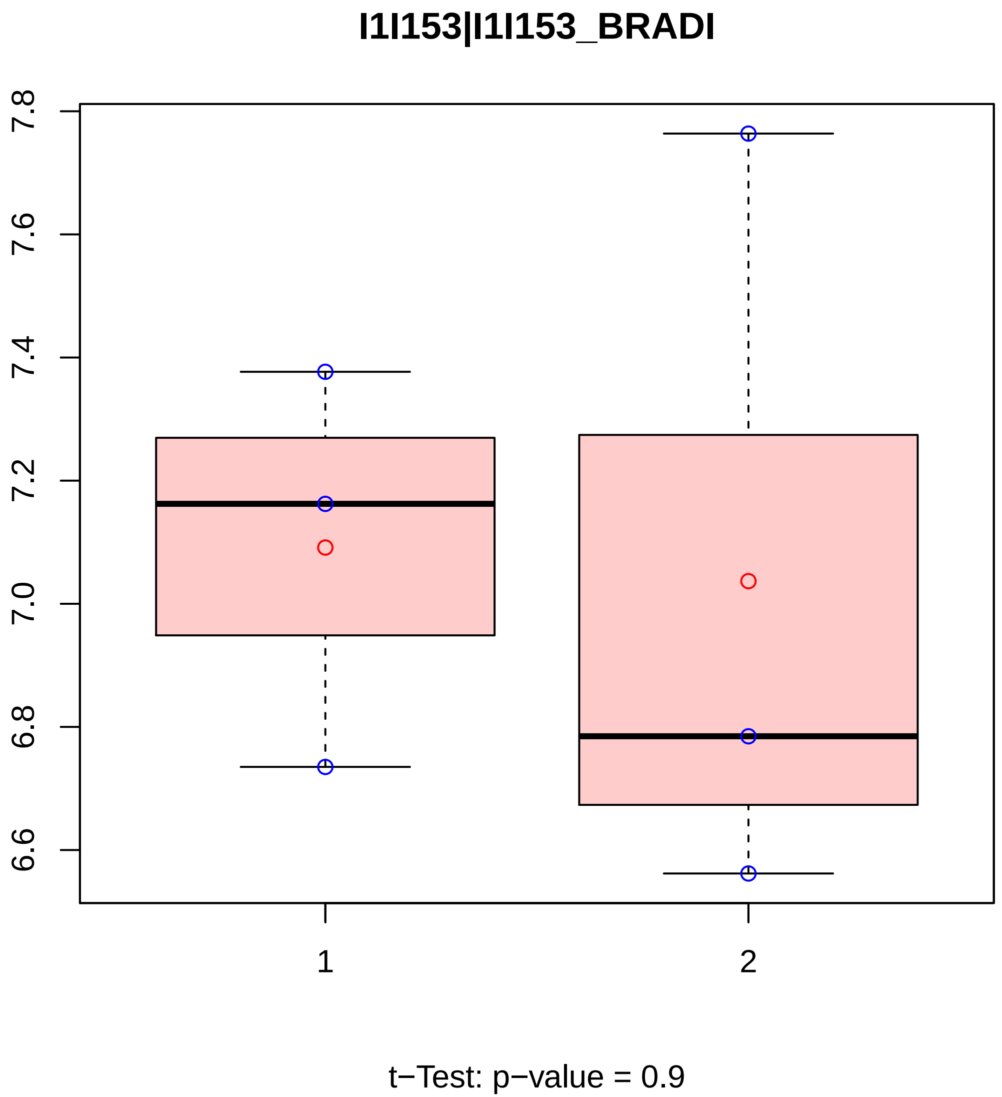
I1I153|I1I153_BRADI
7.8
7.6
7.4
7.2
7.0
6.8
6.6
1
2
t−Test: p−value = 0.9

I1I1M4|I1I1M4_BRADI

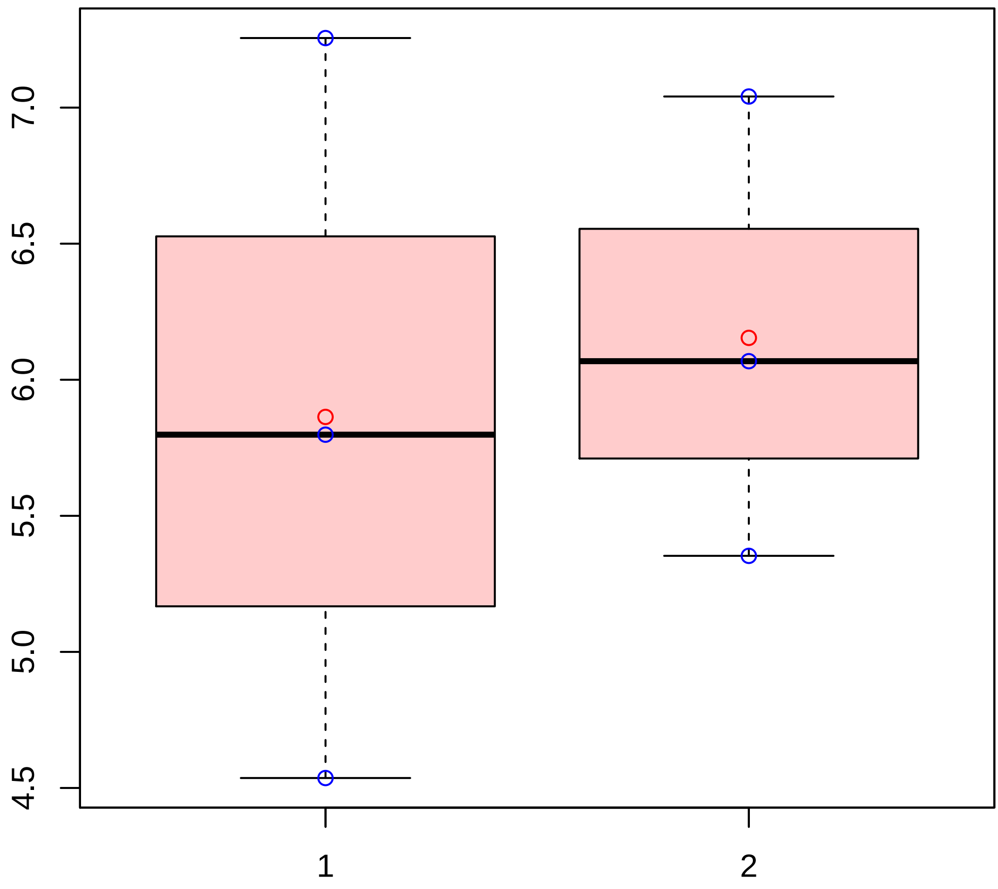

t−Test: p−value = 0.77

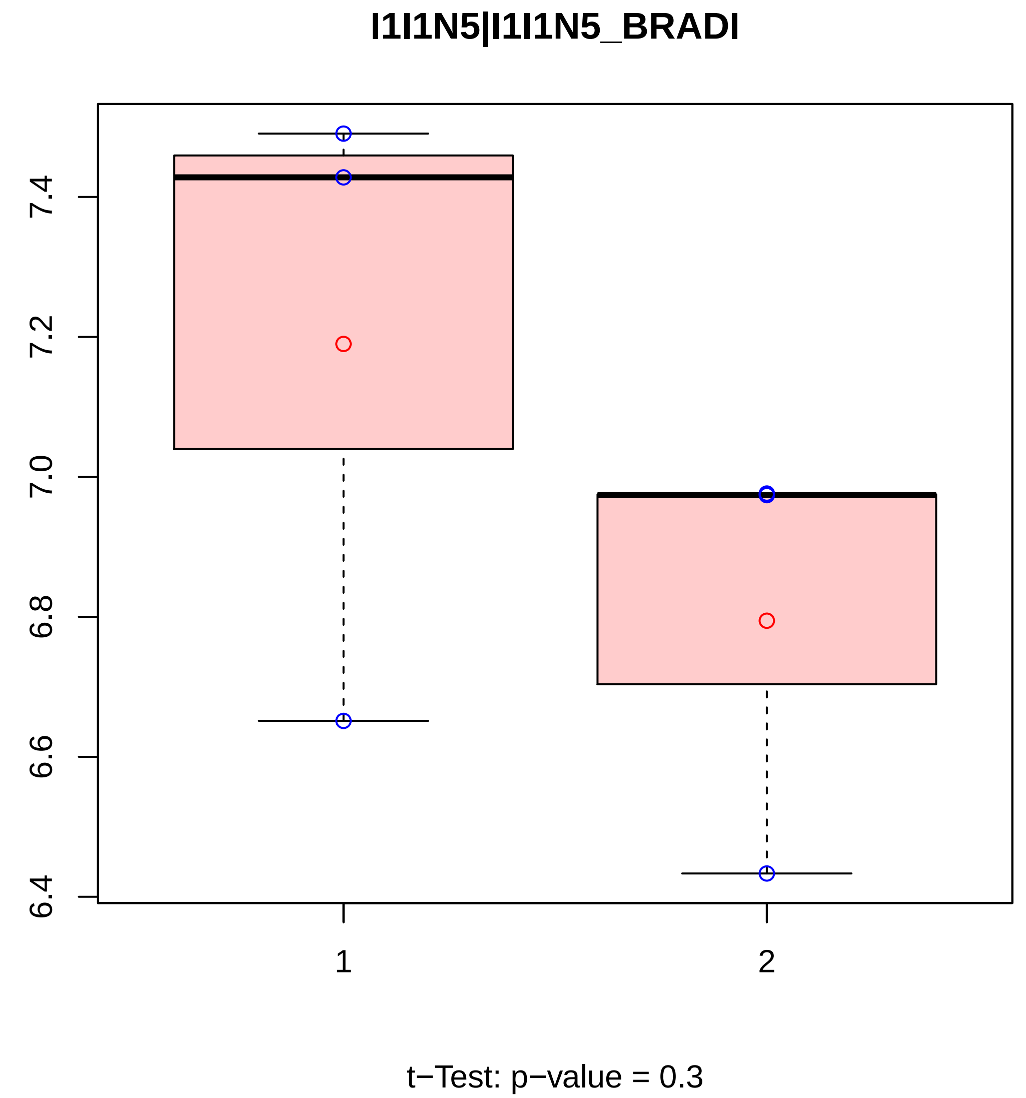
I1I1N5|I1I1N5_BRADI
7.4
7.2
7.0
6.8
6.6
6.4
1
2
t−Test: p−value = 0.3

I1I1U7|I1I1U7_BRADI

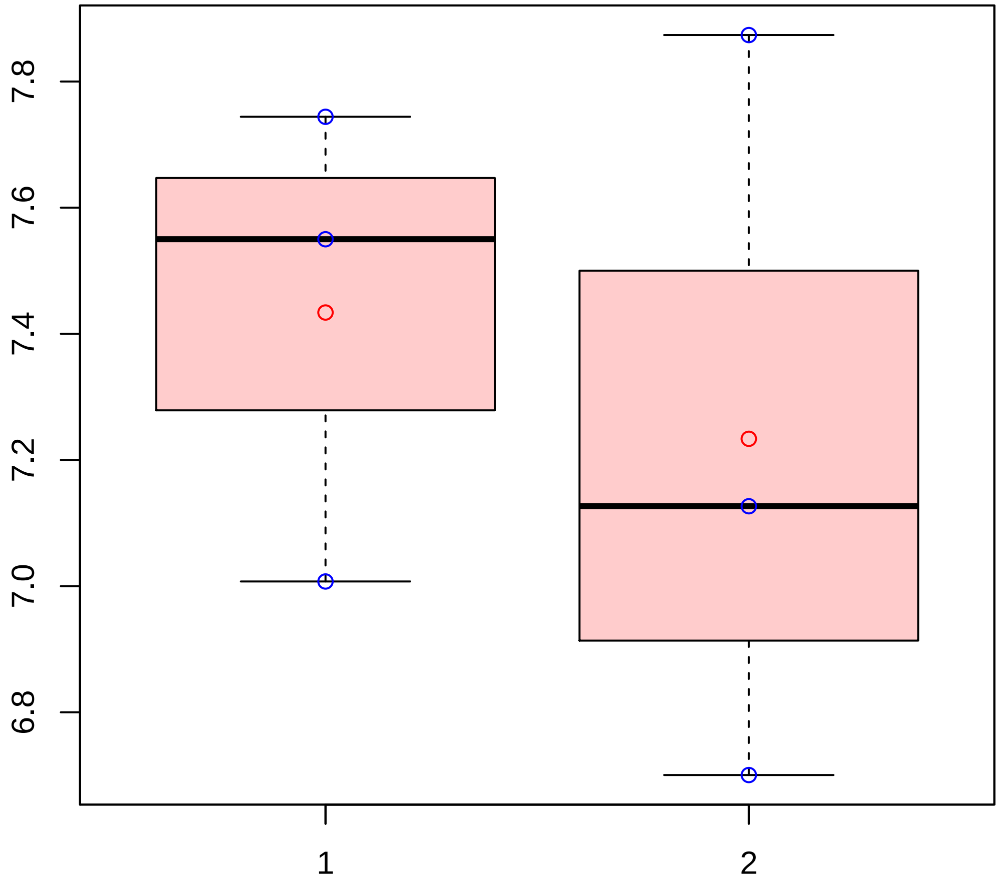

t−Test: p−value = 0.65

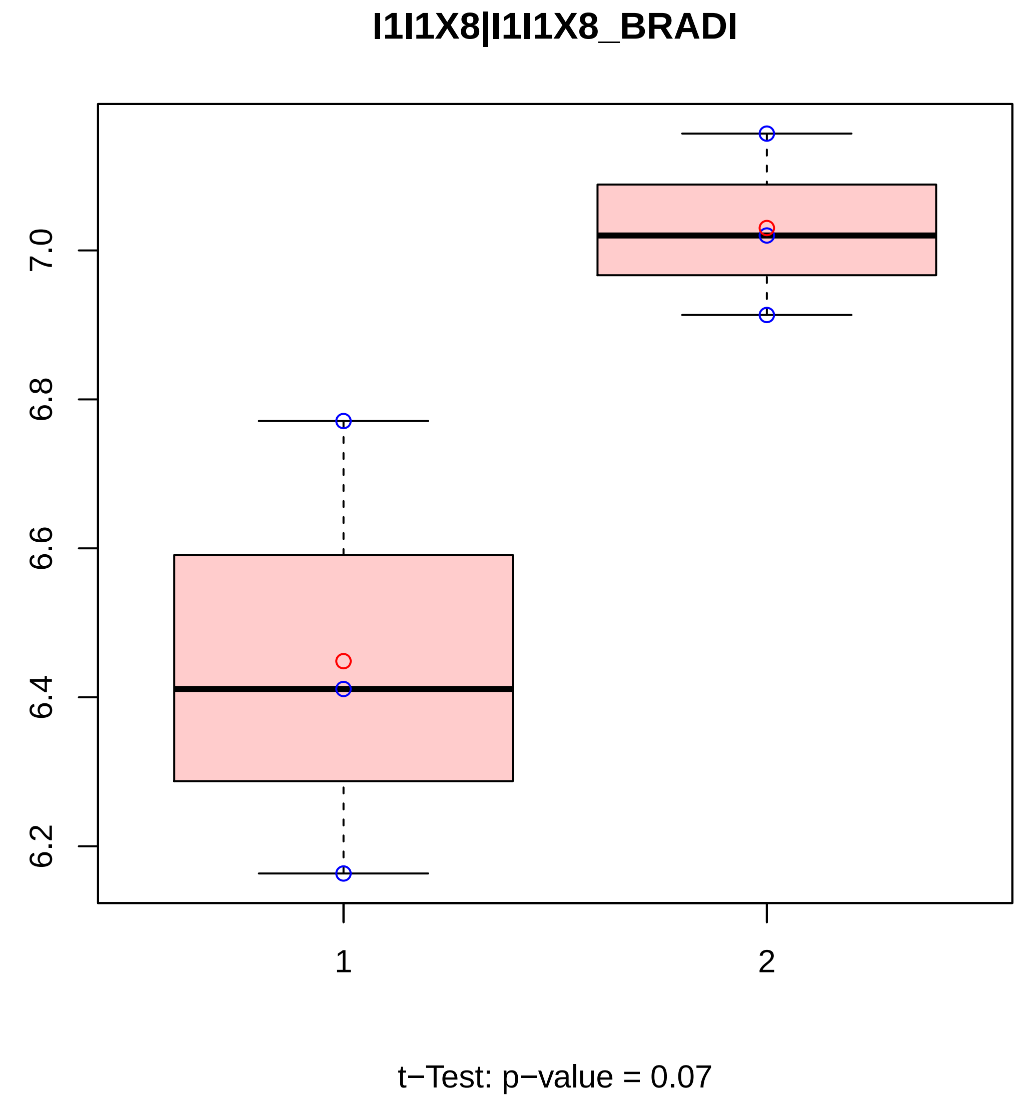

I1I1X8|I1I1X8_BRADI
7.0
6.8
6.6
6.4
6.2
1
2
t−Test: p−value = 0.07

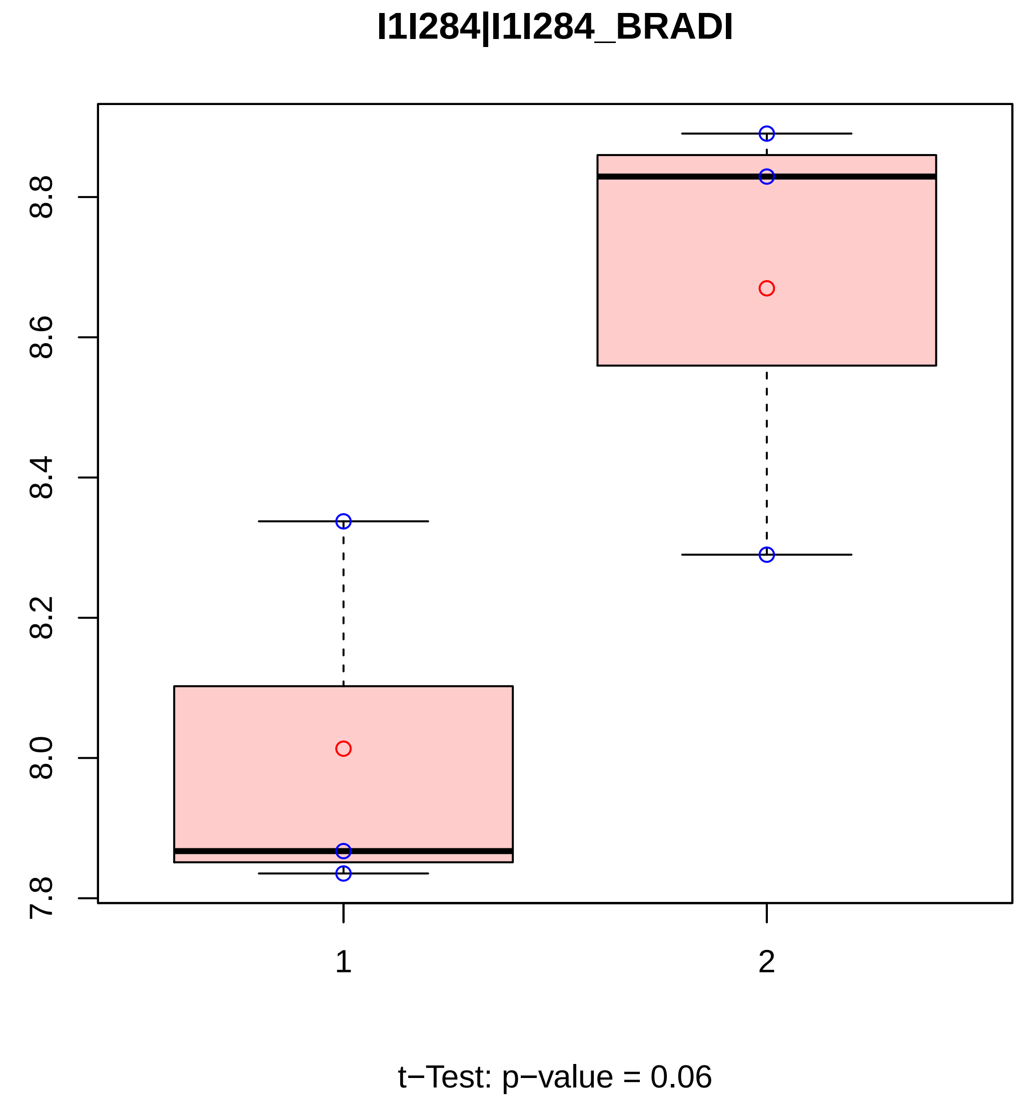
I1I284|I1I284_BRADI
8.8
8.6
8.4
8.2
8.0
7.8
1
2
t−Test: p−value = 0.06

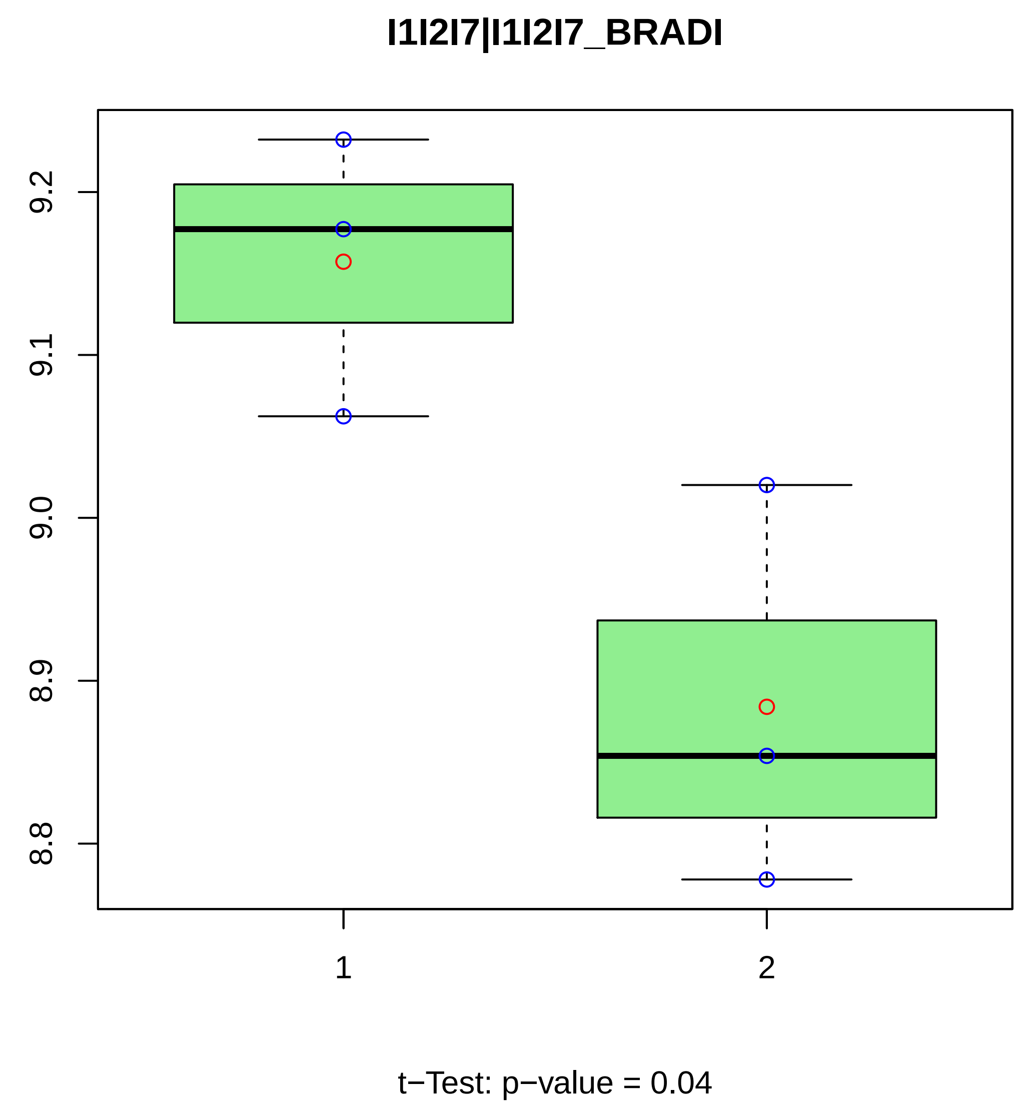

I1I2I7|I1I2I7_BRADI
9.2
9.1
9.0
8.9
8.8
1
2
t−Test: p−value = 0.04

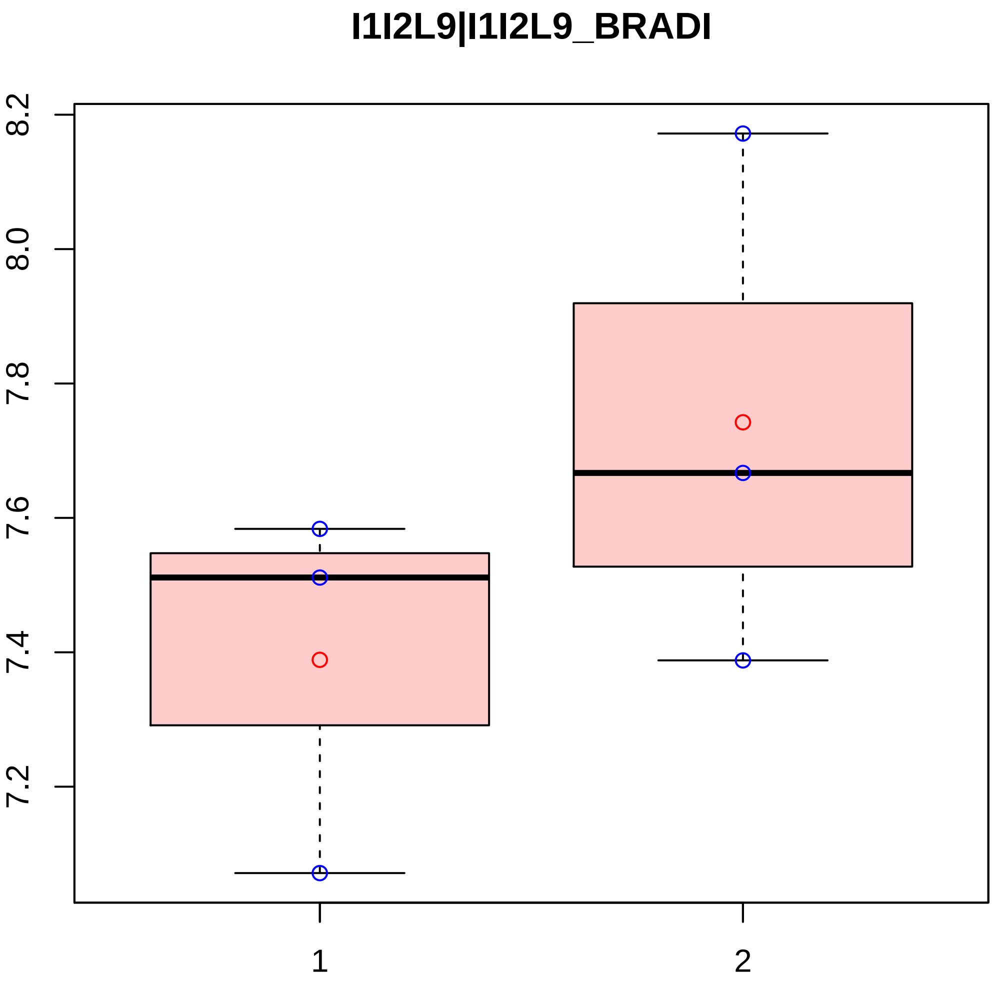

t−Test: p−value = 0.28

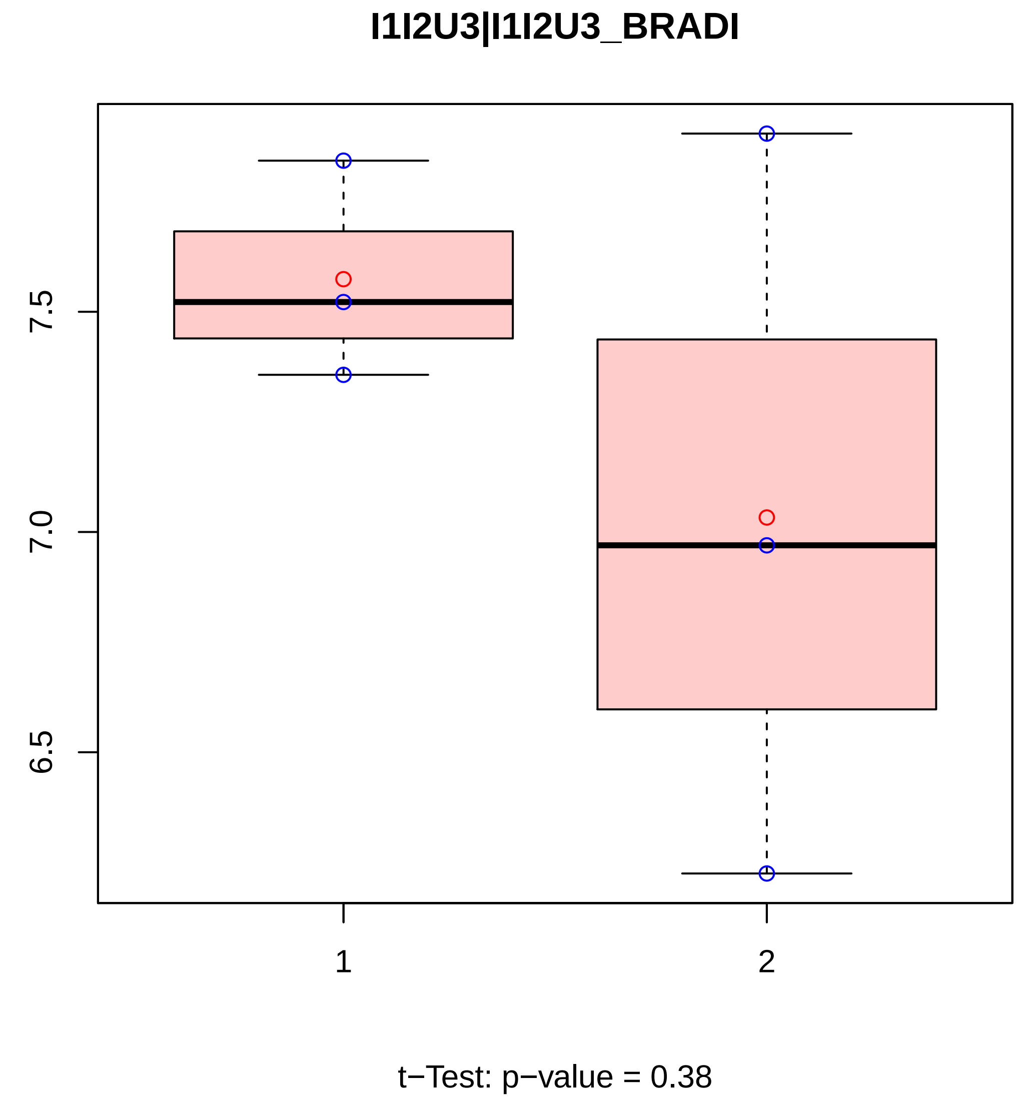
I1I2U3|I1I2U3_BRADI
7.5
7.0
6.5
1
2
t−Test: p−value = 0.38

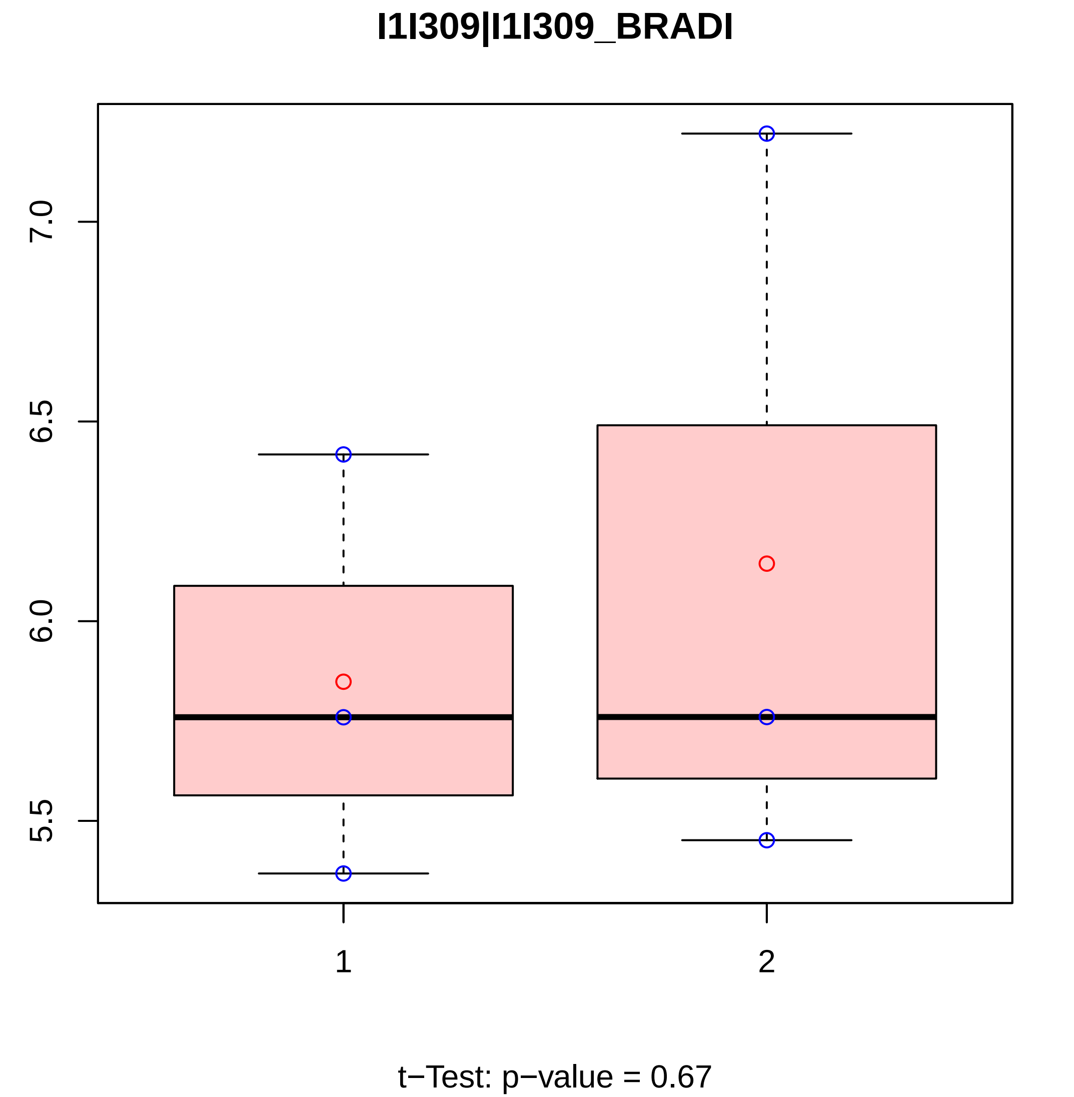
I1I309|I1I309_BRADI
7.0
6.5
6.0
5.5
1
2
t−Test: p−value = 0.67

I1I399|I1I399_BRADI

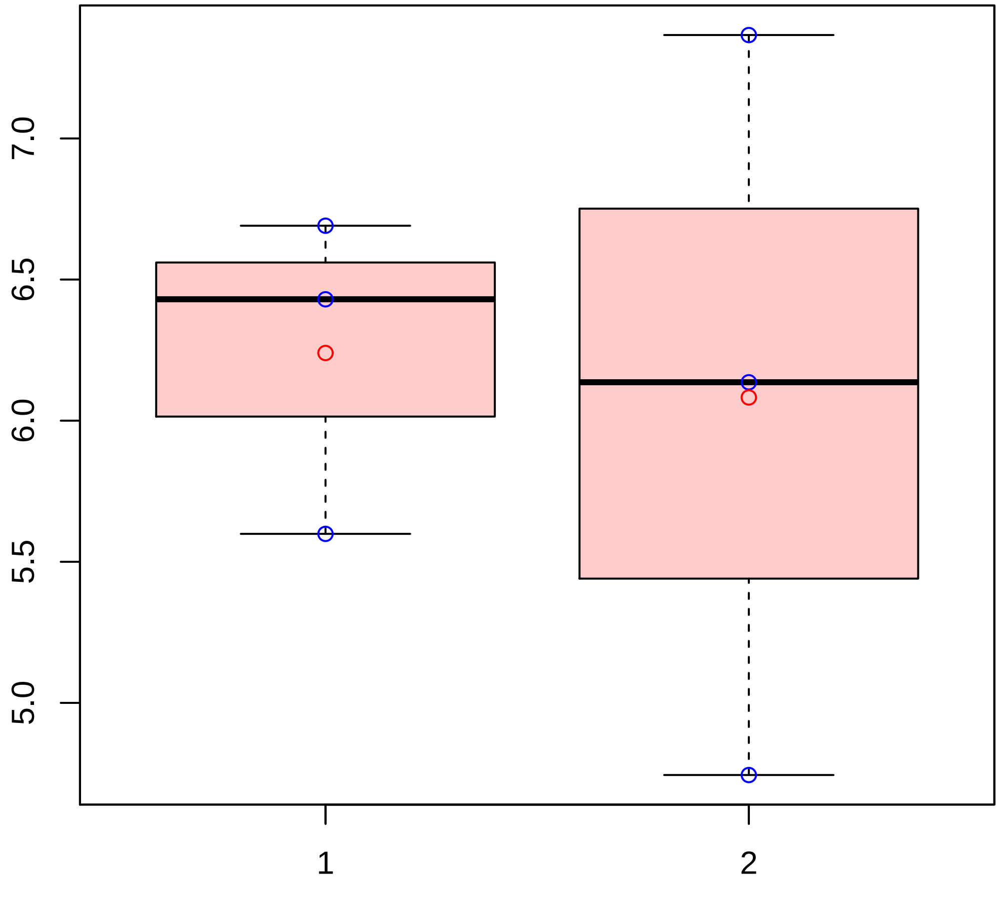

t−Test: p−value = 0.86

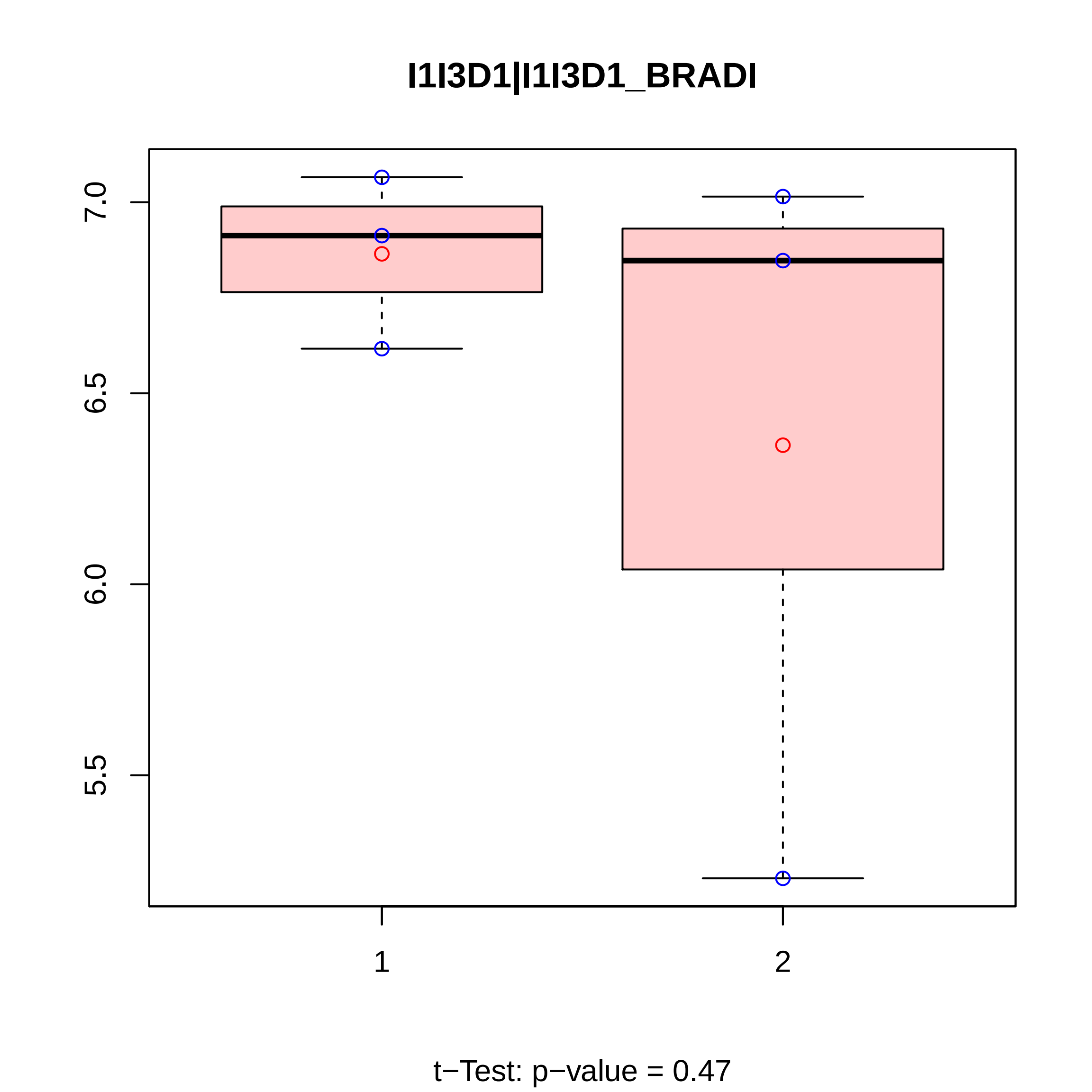
I1I3D1|I1I3D1_BRADI
7.0
6.5
6.0
5.5
1
2
t−Test: p−value = 0.47

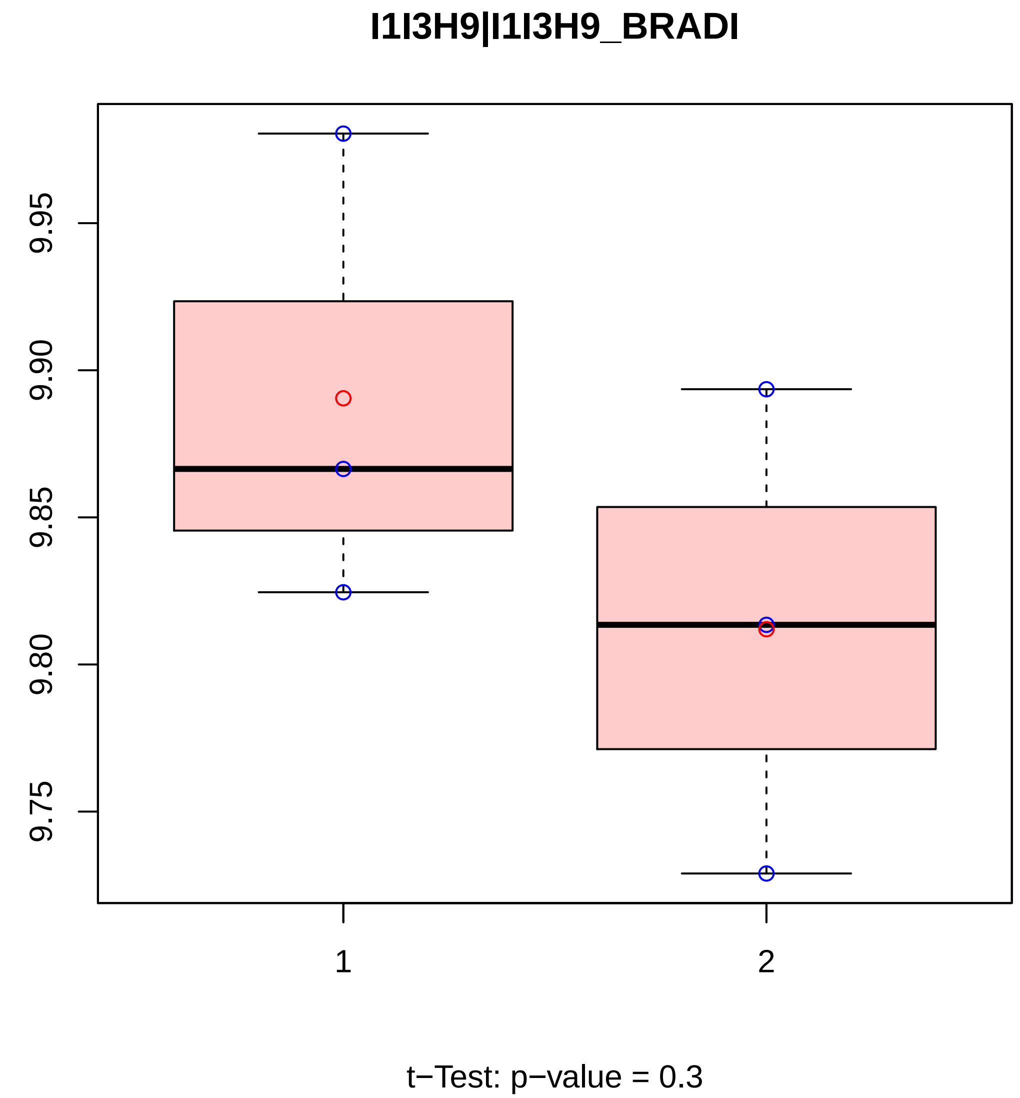
I1I3H9|I1I3H9_BRADI
9.75
9.80
9.85
9.90
9.95
1
2
t−Test: p−value = 0.3

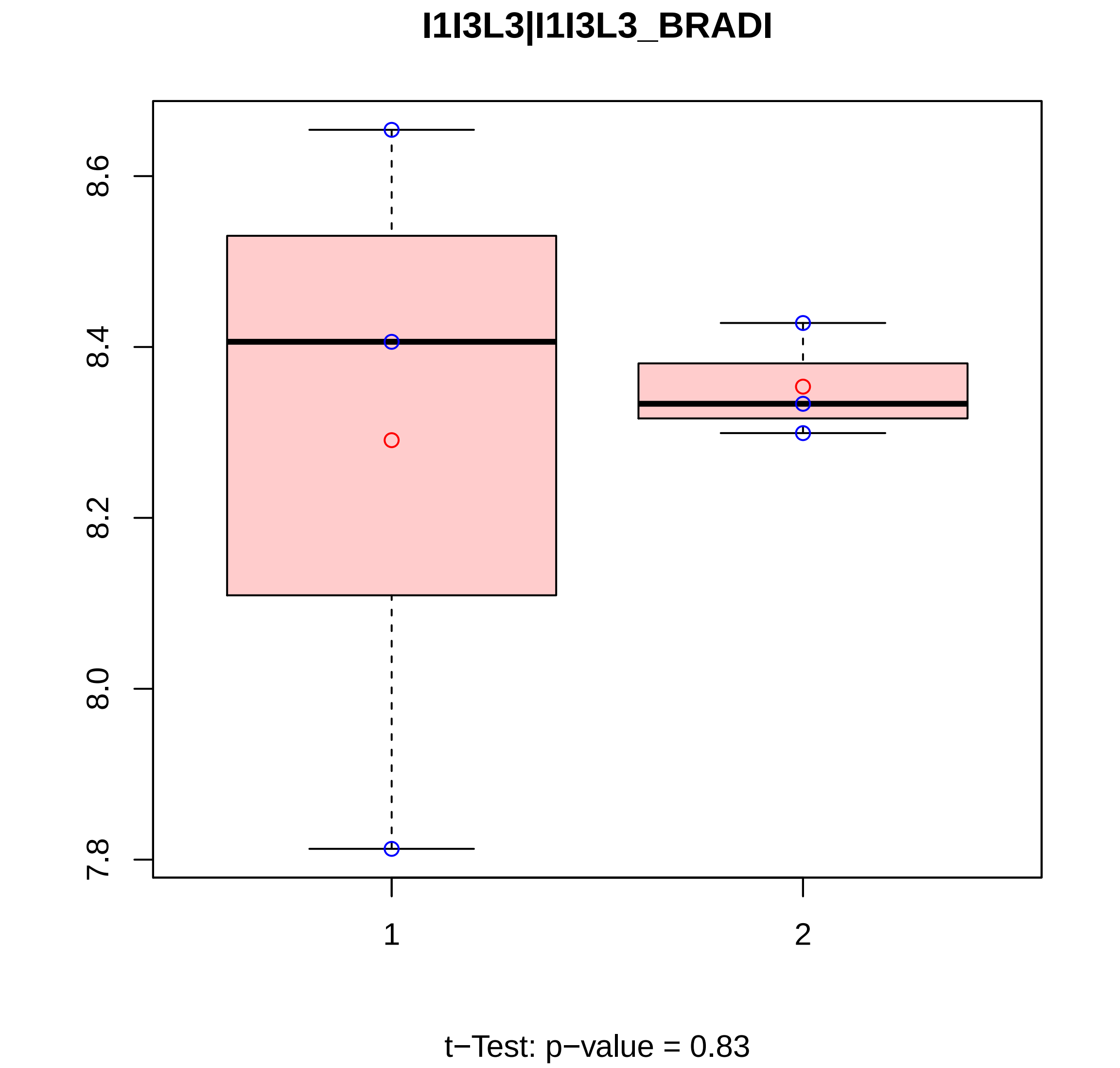
I1I3L3|I1I3L3_BRADI
8.6
8.4
8.2
8.0
7.8
1
2
t−Test: p−value = 0.83

I1I3T4|I1I3T4_BRADI

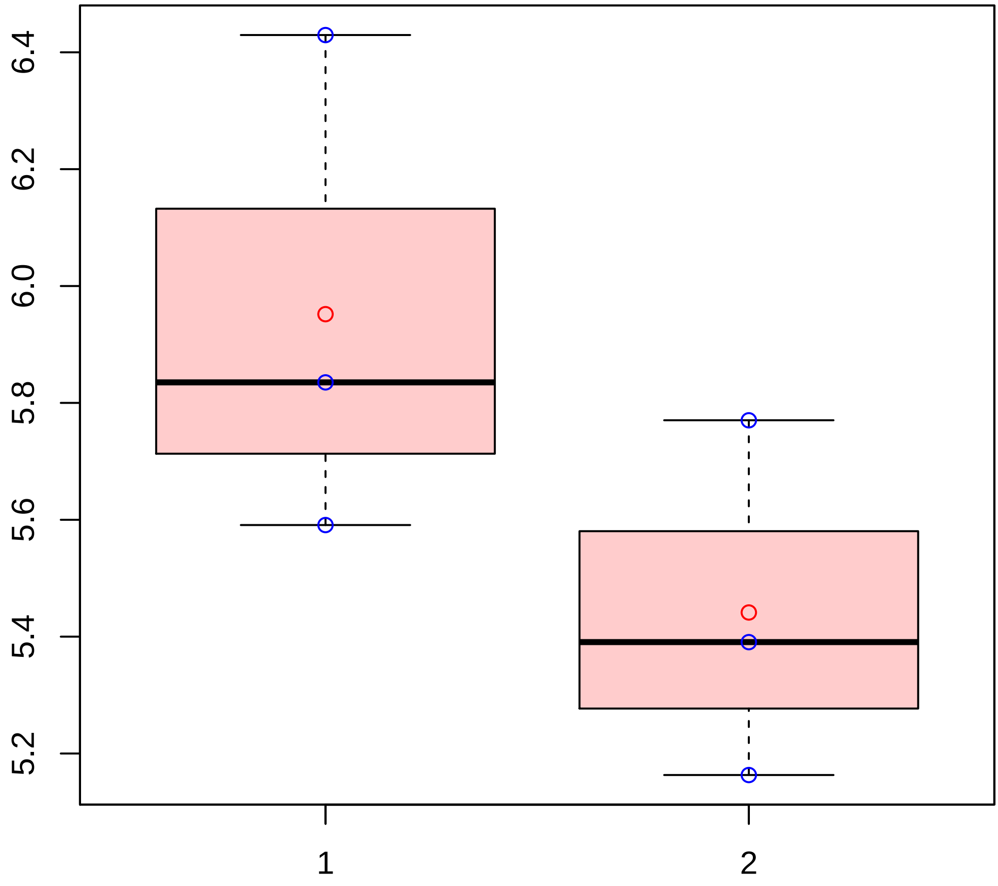

t−Test: p−value = 0.18

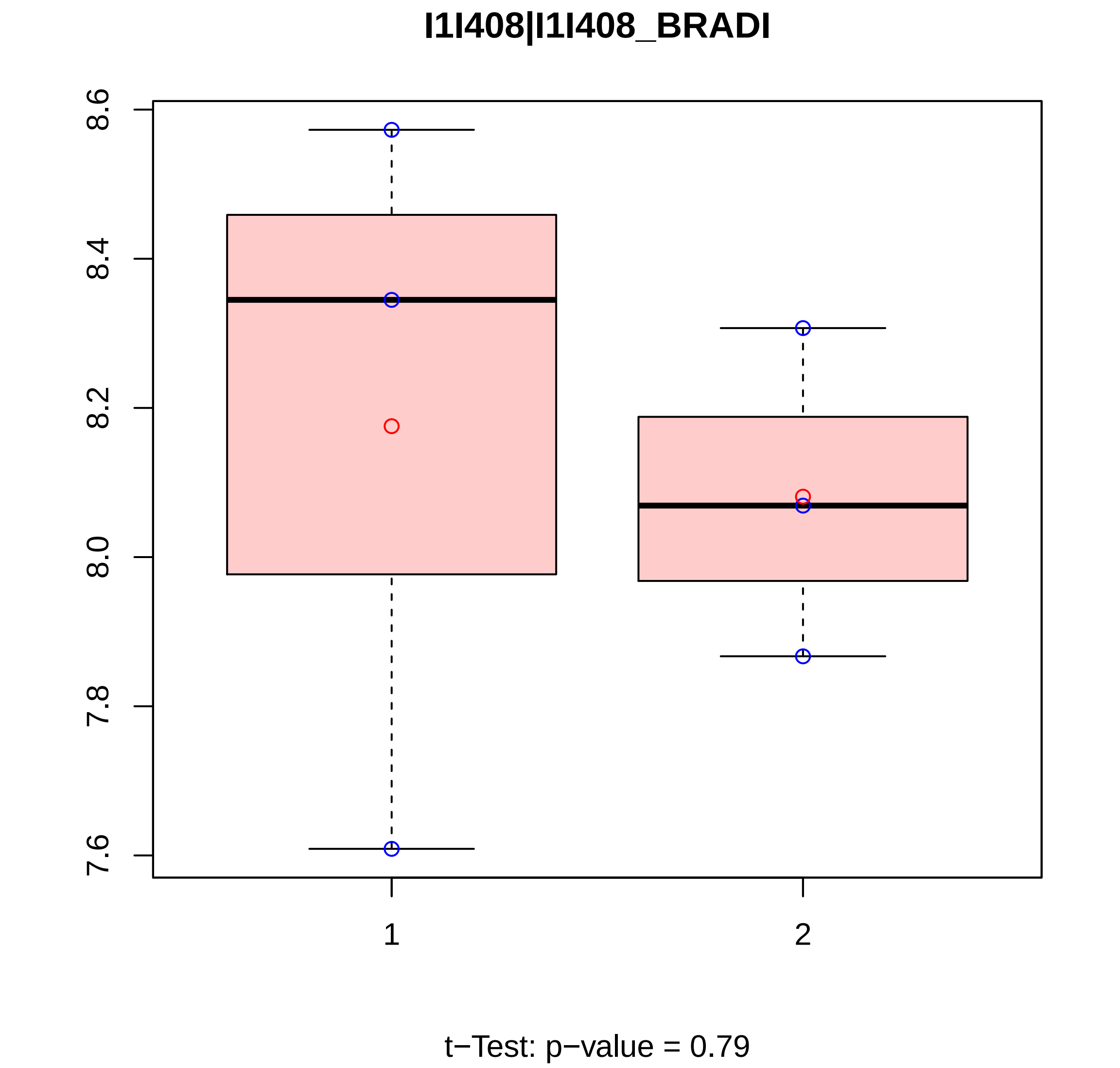

I1I408|I1I408_BRADI
8.6
8.4
8.2
8.0
7.8
7.6
1
2
t−Test: p−value = 0.79

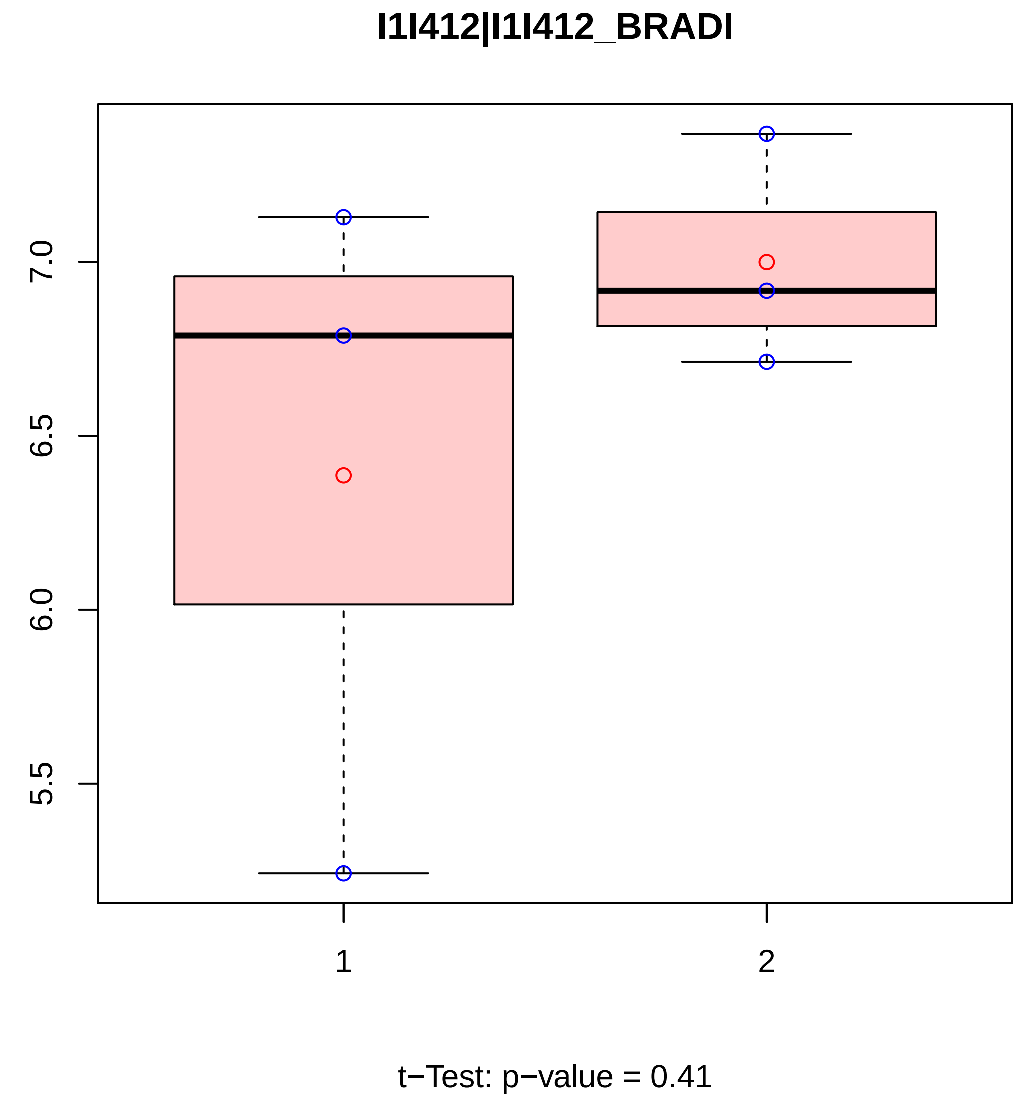
I1I412|I1I412_BRADI
7.0
6.5
6.0
5.5
1
2
t−Test: p−value = 0.41

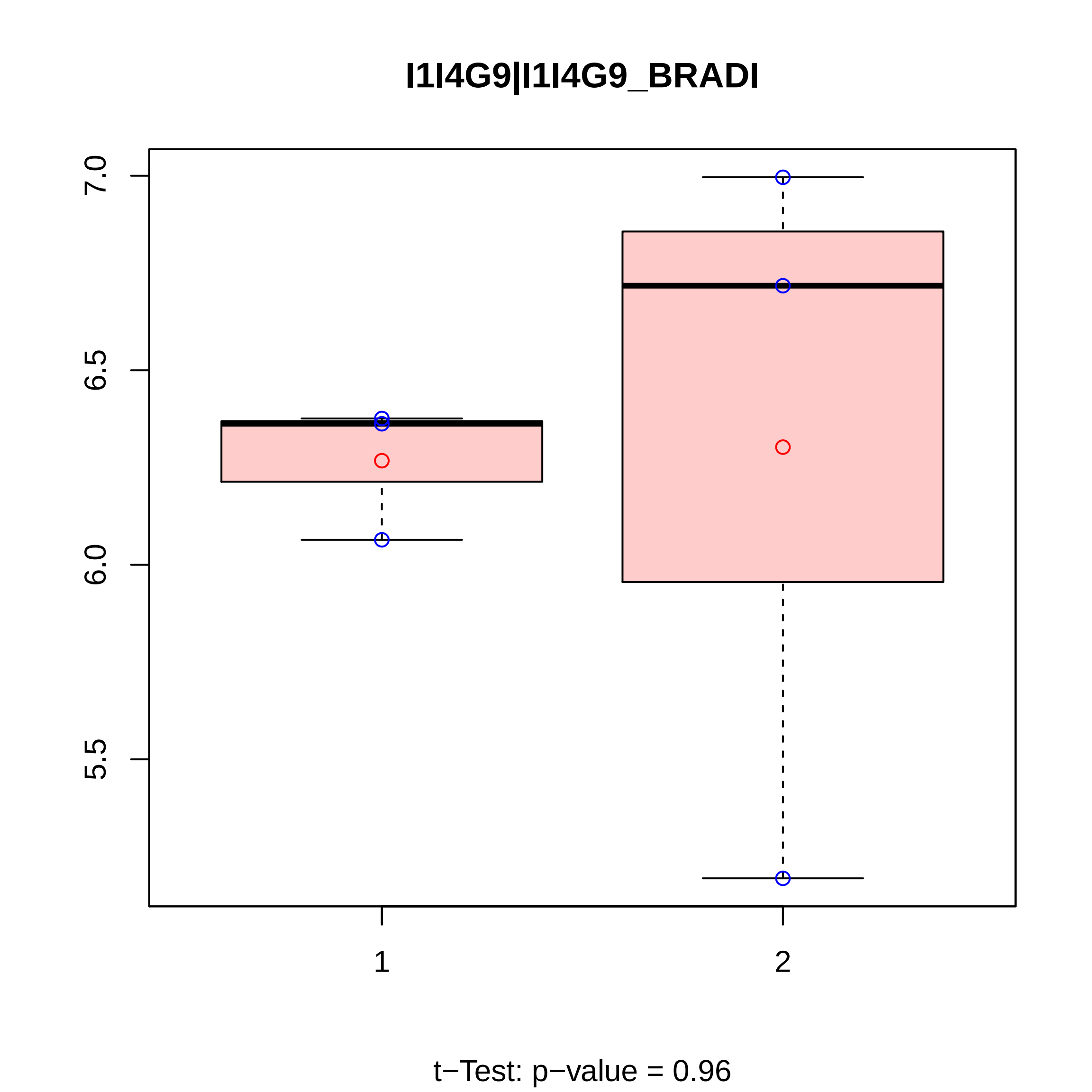
I1I4G9|I1I4G9_BRADI
7.0
6.5
6.0
5.5
1
2
t−Test: p−value = 0.96

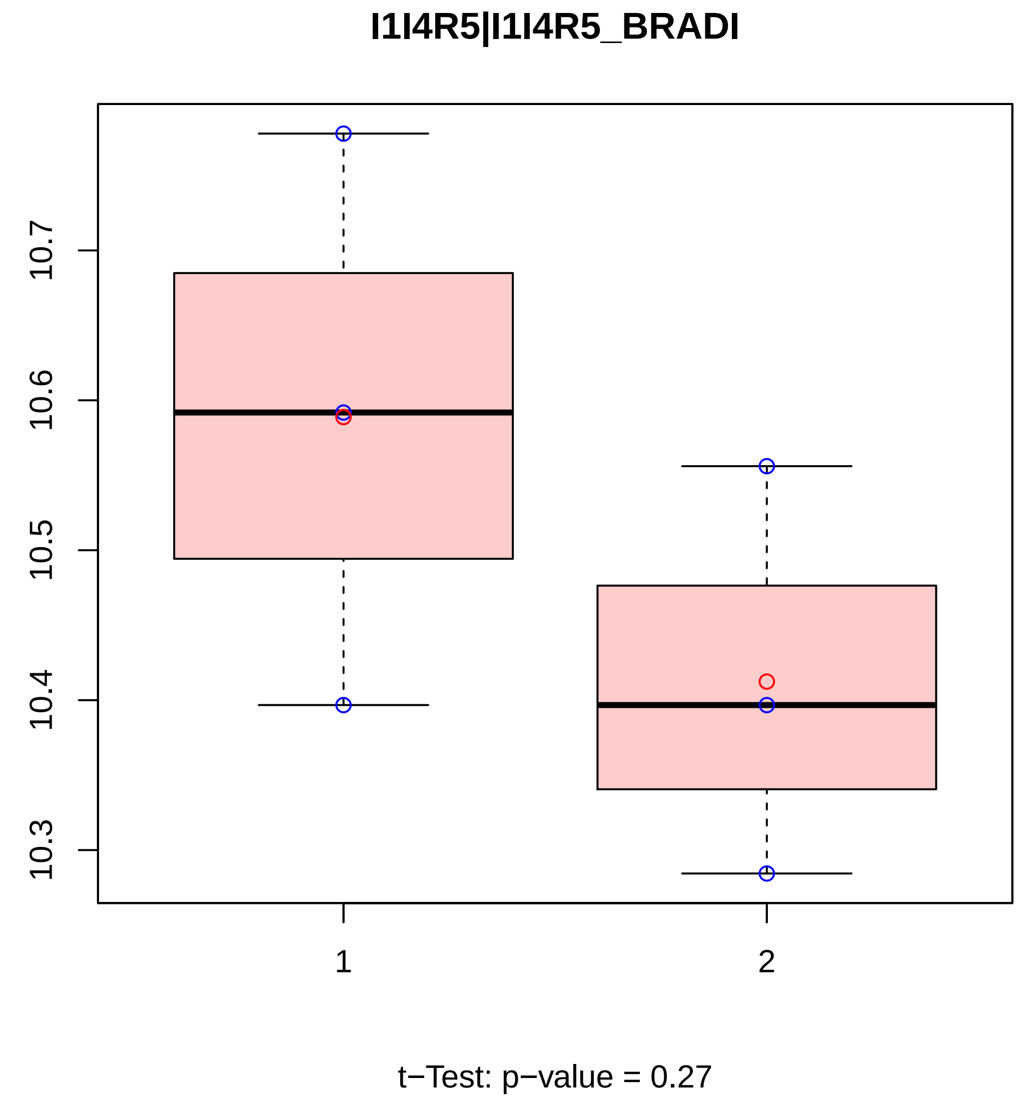
I1I4R5|I1I4R5_BRADI
10.3
10.4
10.5
10.6
10.7
1
2
t−Test: p−value = 0.27

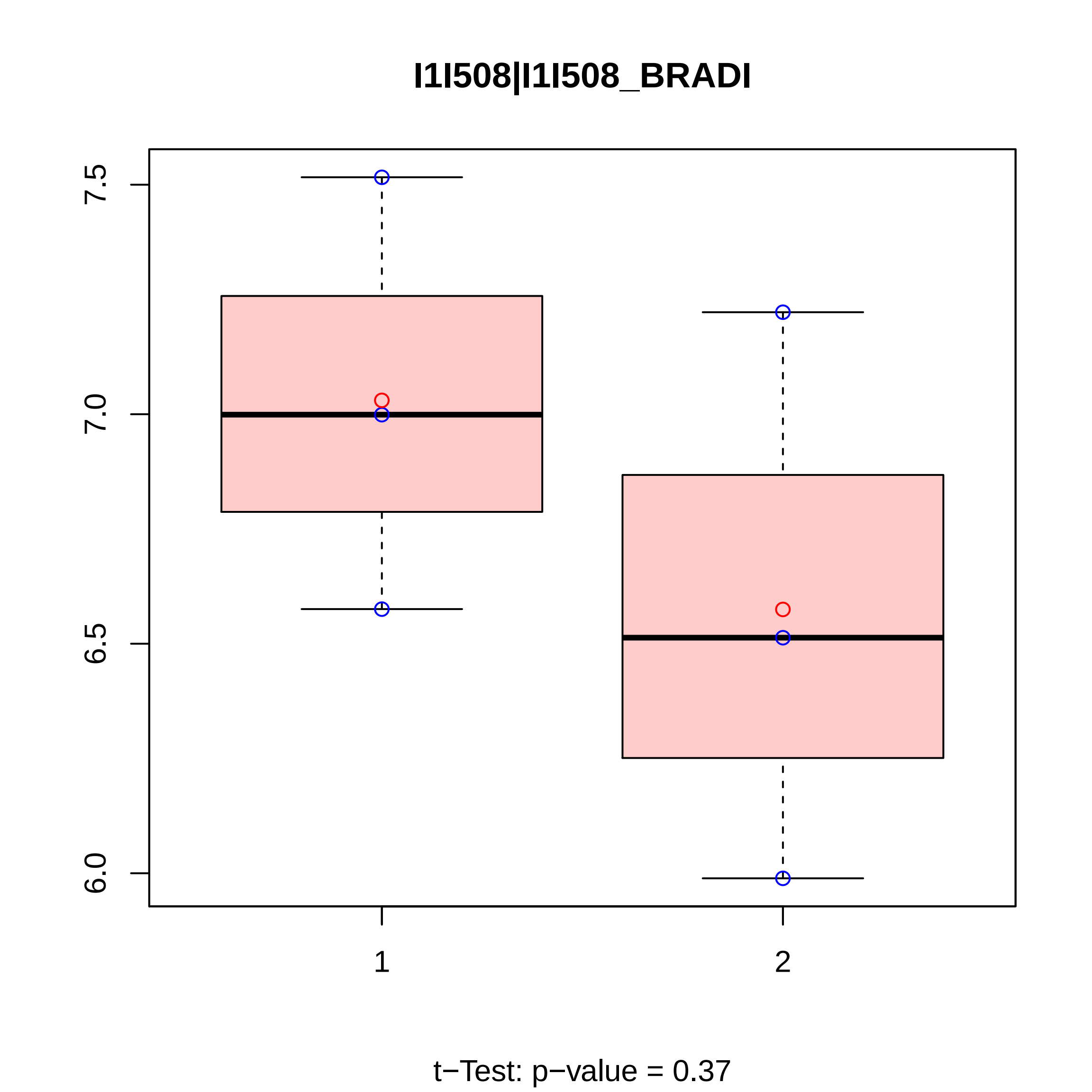
I1I508|I1I508_BRADI
7.5
7.0
6.5
6.0
1
2
t−Test: p−value = 0.37

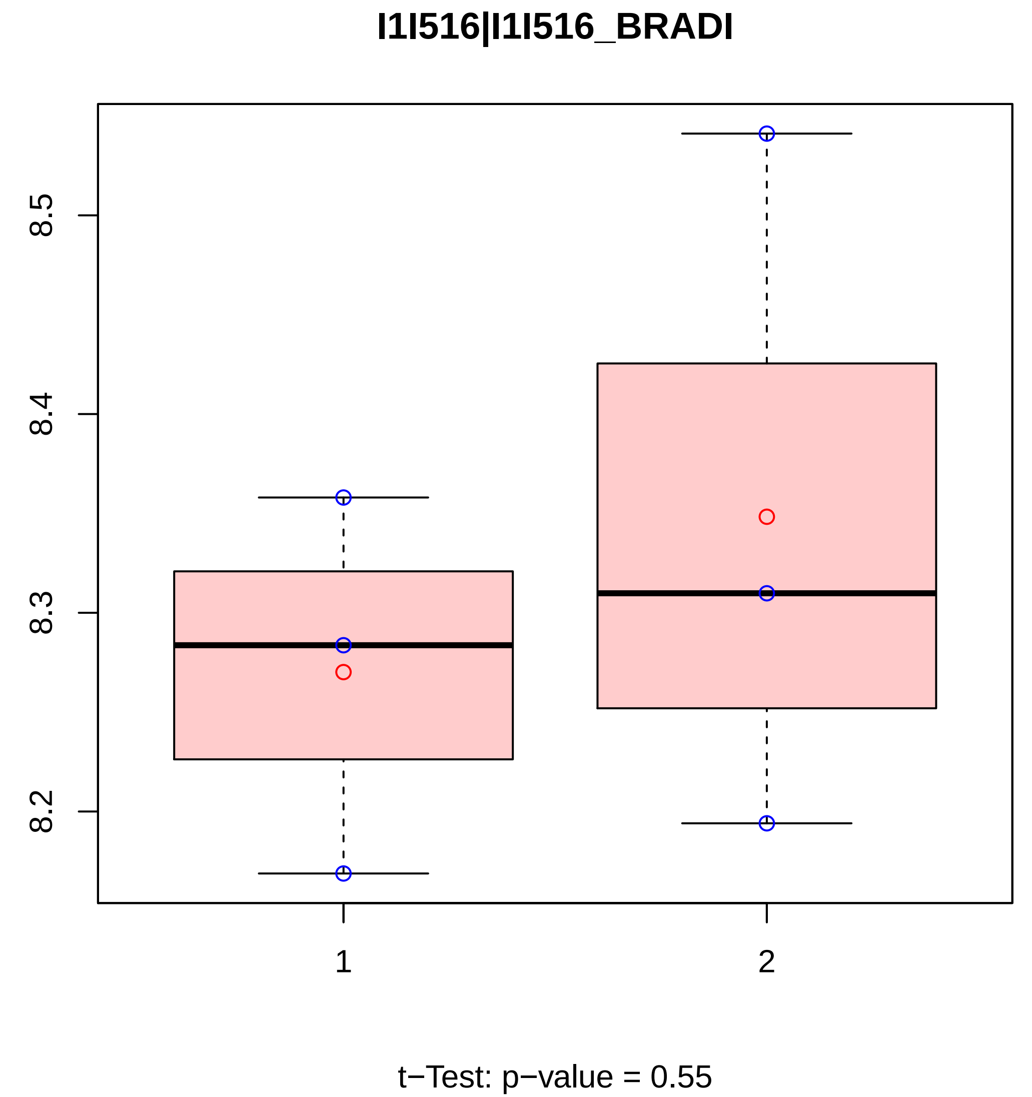

I1I516|I1I516_BRADI
8.2
8.3
8.4
8.5
1
2
t−Test: p−value = 0.55

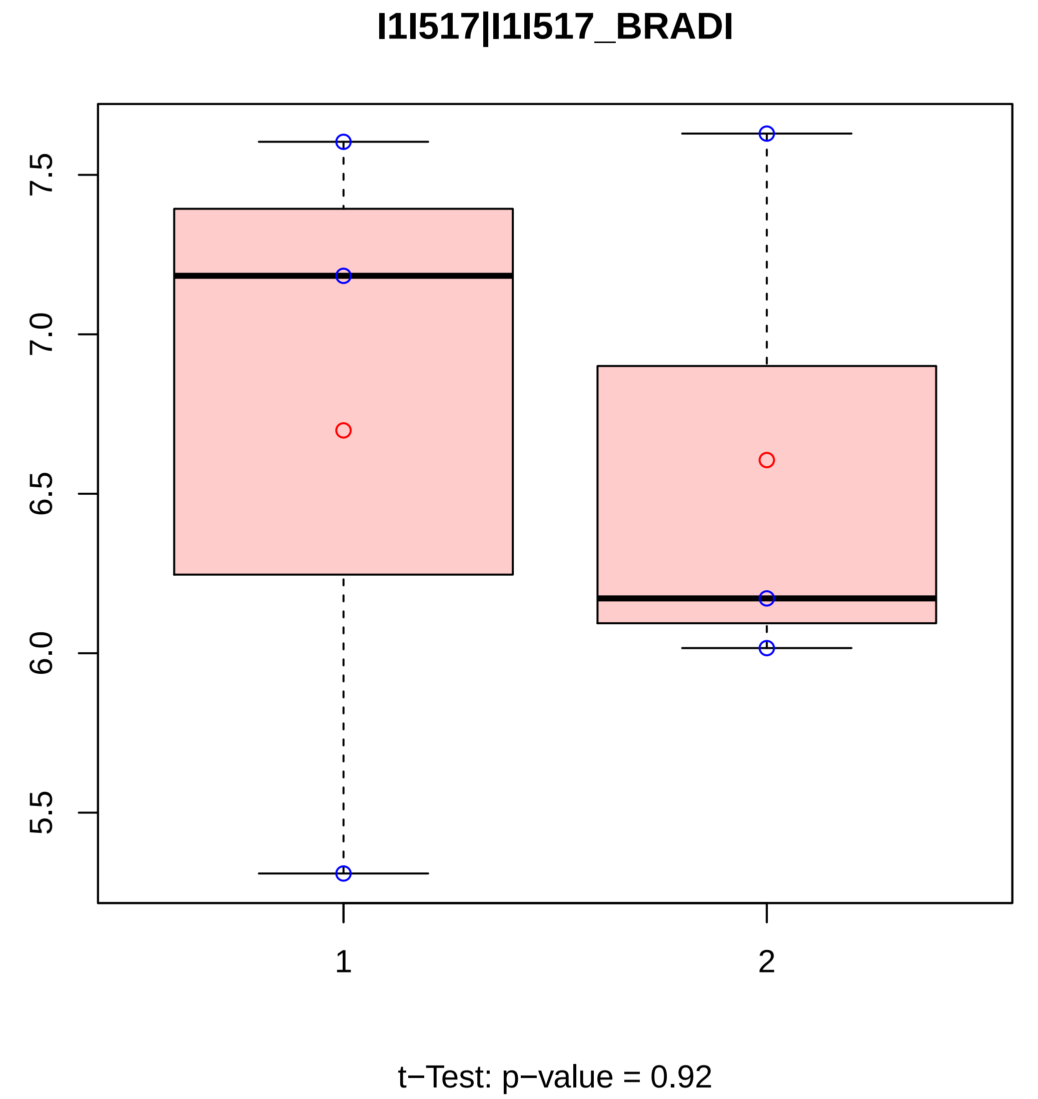
I1I517|I1I517_BRADI
7.5
7.0
6.5
6.0
5.5
1
2
t−Test: p−value = 0.92

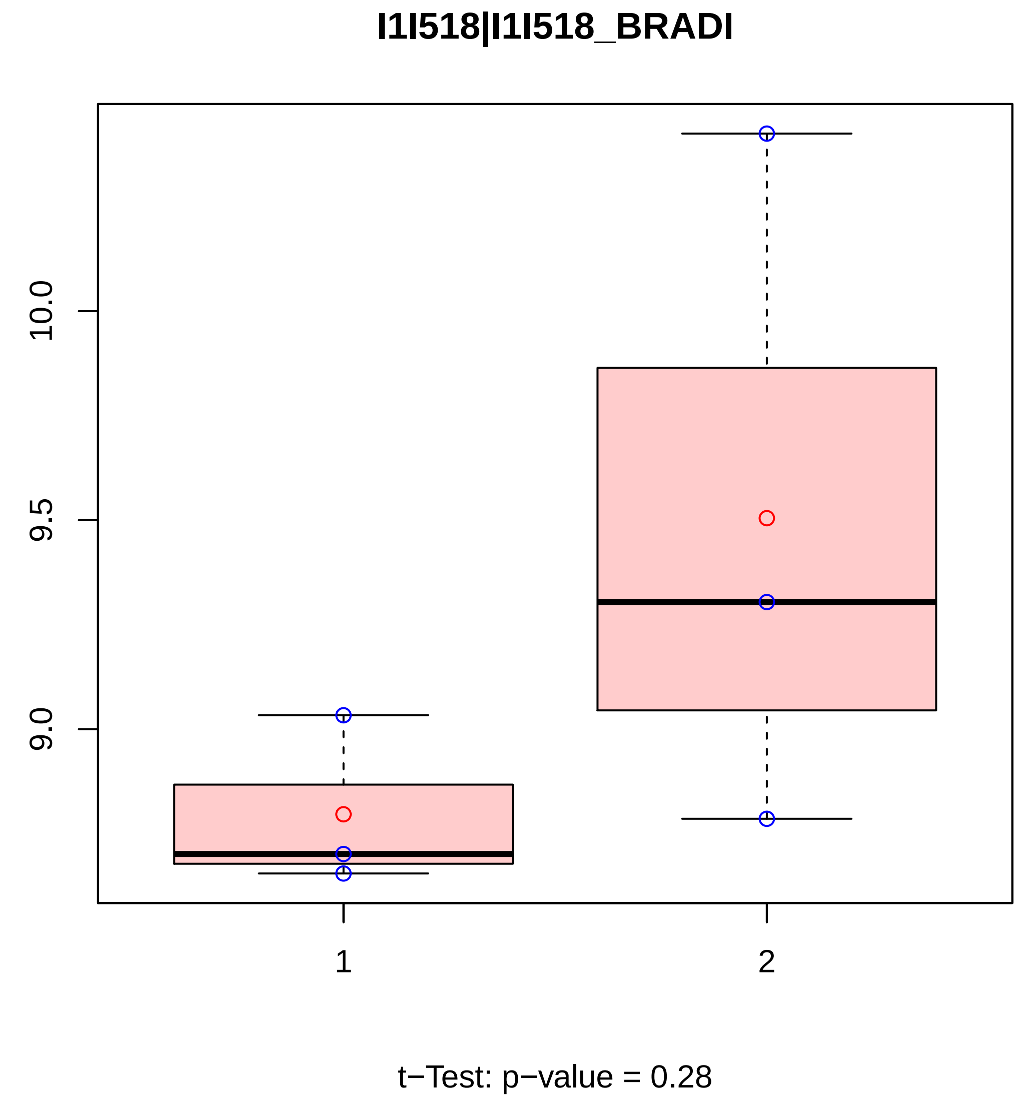
I1I518|I1I518_BRADI
10.0
9.5
9.0
1
2
t−Test: p−value = 0.28

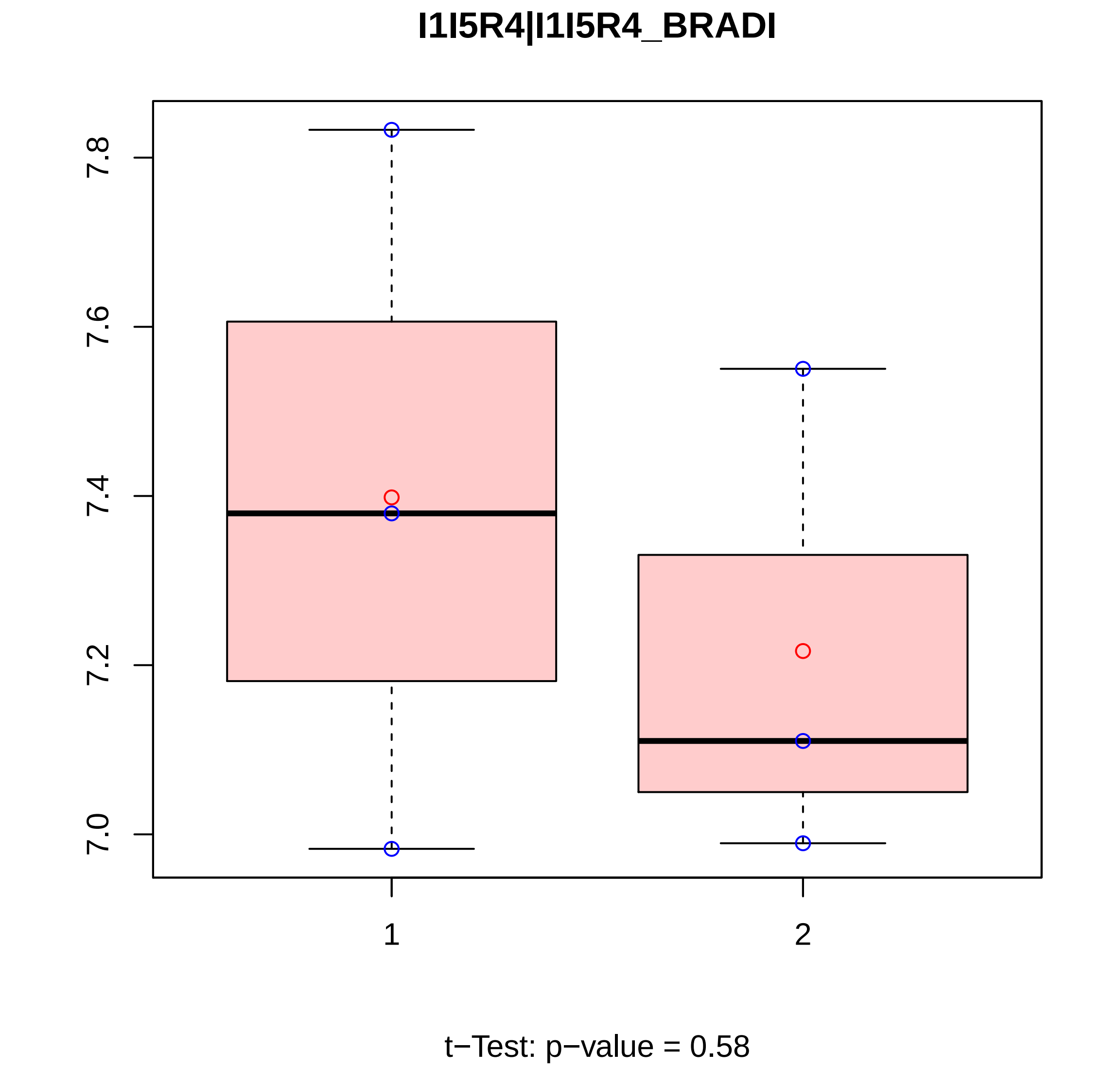
I1I5R4|I1I5R4_BRADI
7.8
7.6
7.4
7.2
7.0
1
2
t−Test: p−value = 0.58

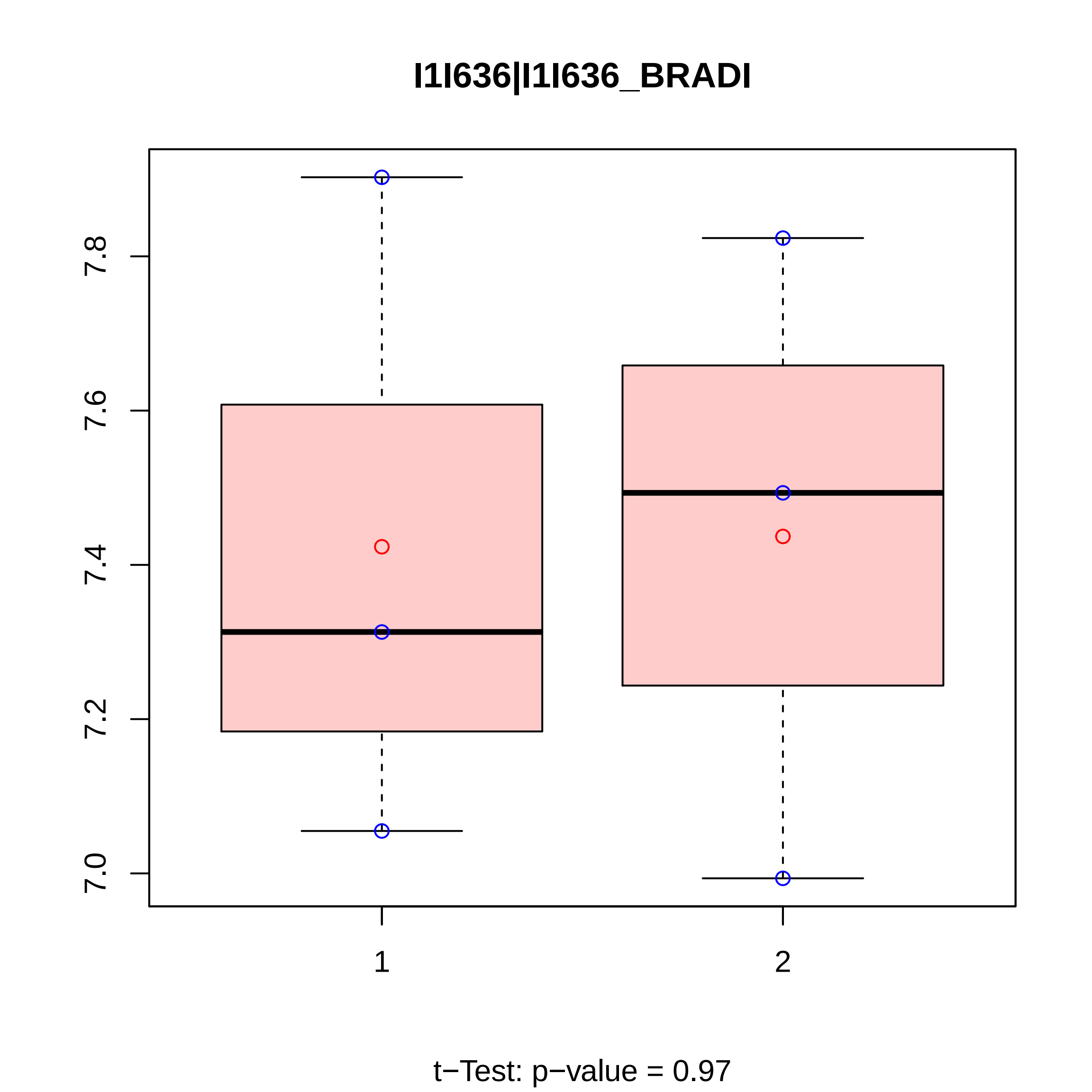

I1I636|I1I636_BRADI
7.0
7.2
7.4
7.6
7.8
1
2
t−Test: p−value = 0.97

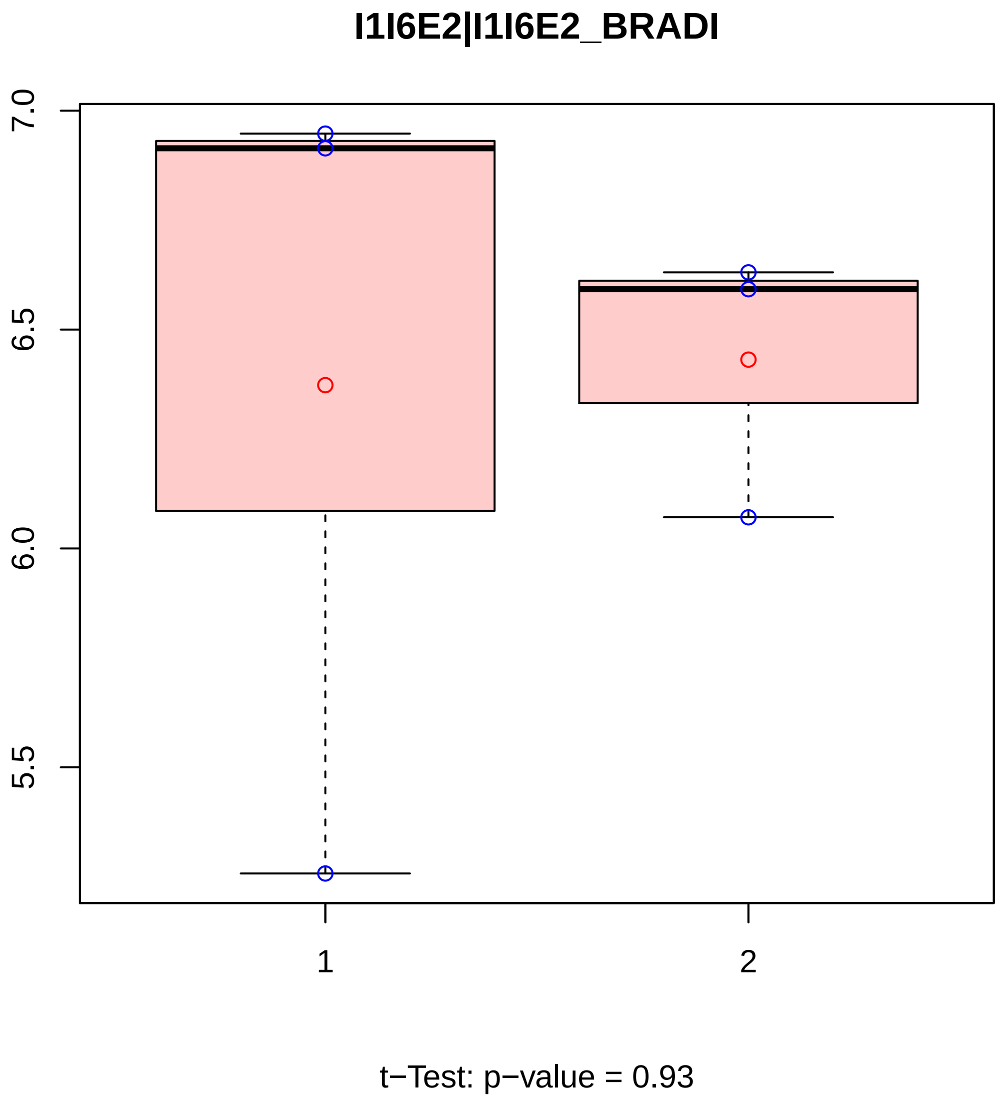
I1I6E2|I1I6E2_BRADI
7.0
6.5
6.0
5.5
1
2
t−Test: p−value = 0.93

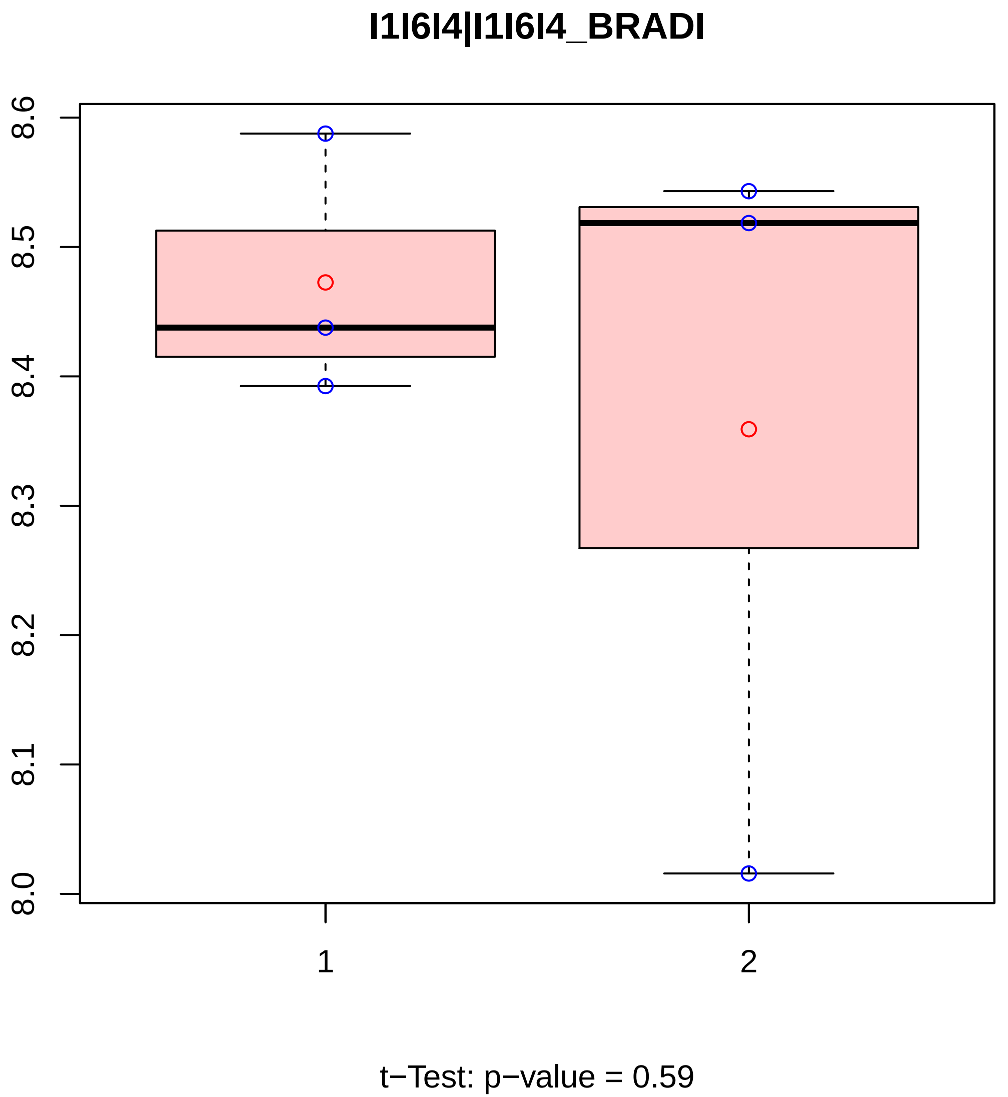

I1I6I4|I1I6I4_BRADI
8.0
8.1
8.2
8.3
8.4
8.5
8.6
1
2
t−Test: p−value = 0.59

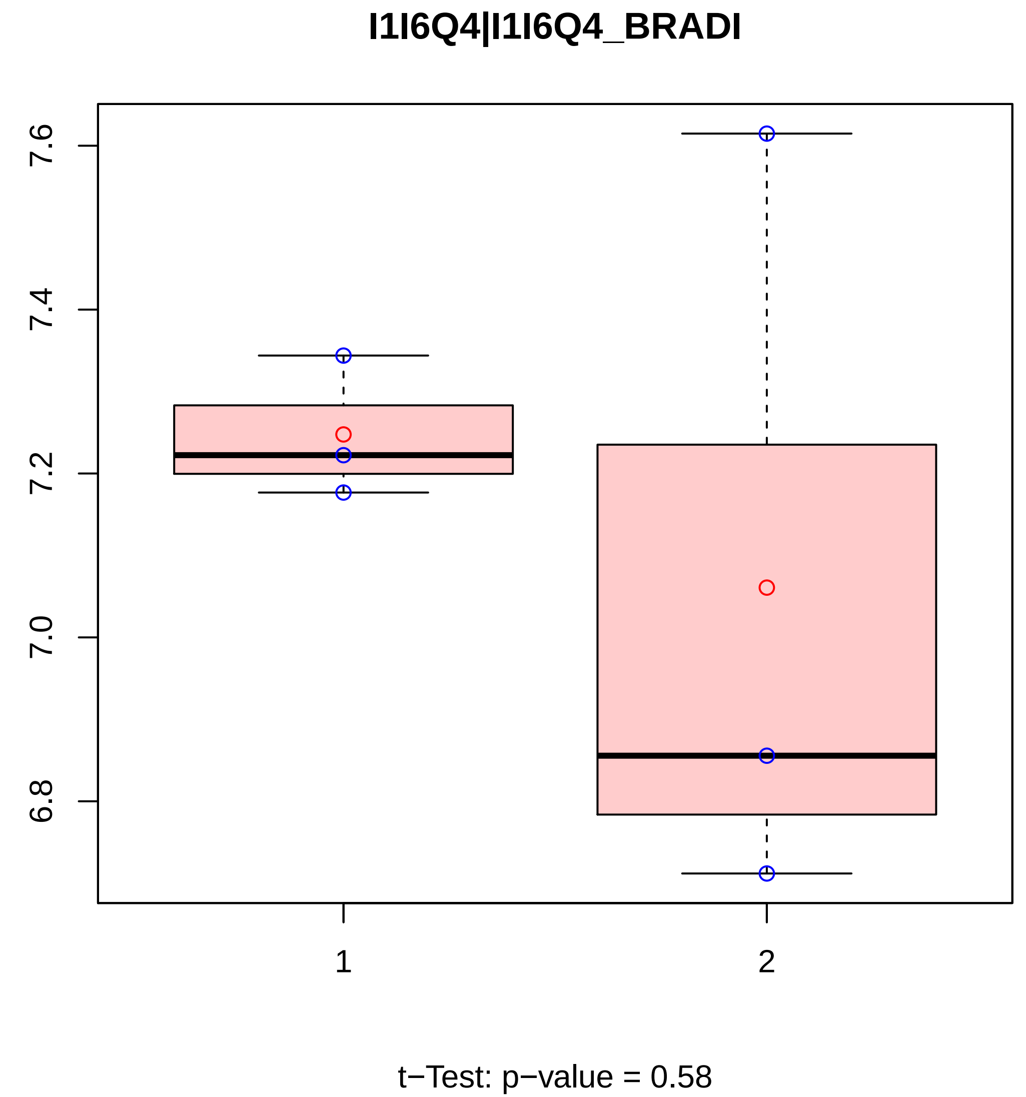
I1I6Q4|I1I6Q4_BRADI
7.6
7.4
7.2
7.0
6.8
1
2
t−Test: p−value = 0.58

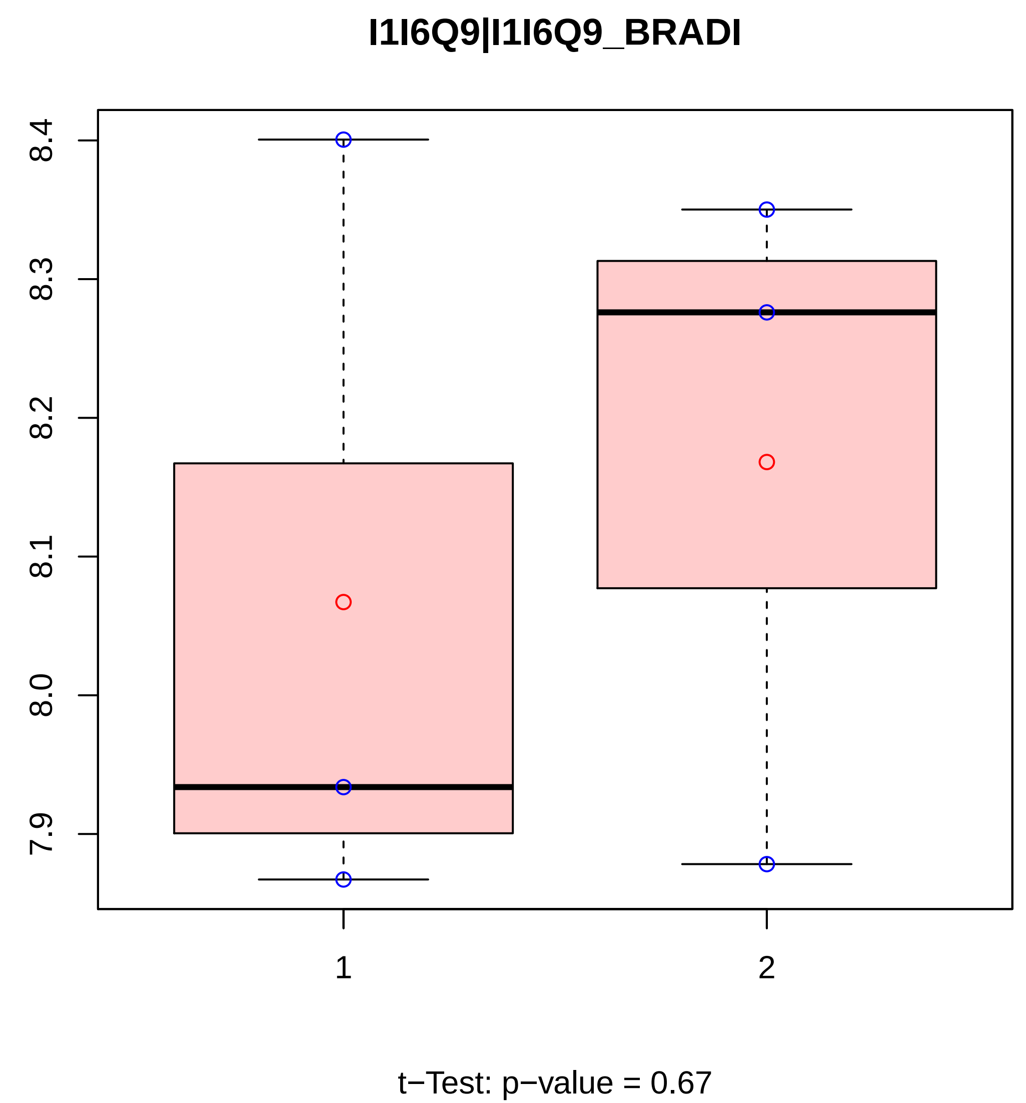
I1I6Q9|I1I6Q9_BRADI
8.4
8.3
8.2
8.1
8.0
7.9
1
2
t−Test: p−value = 0.67

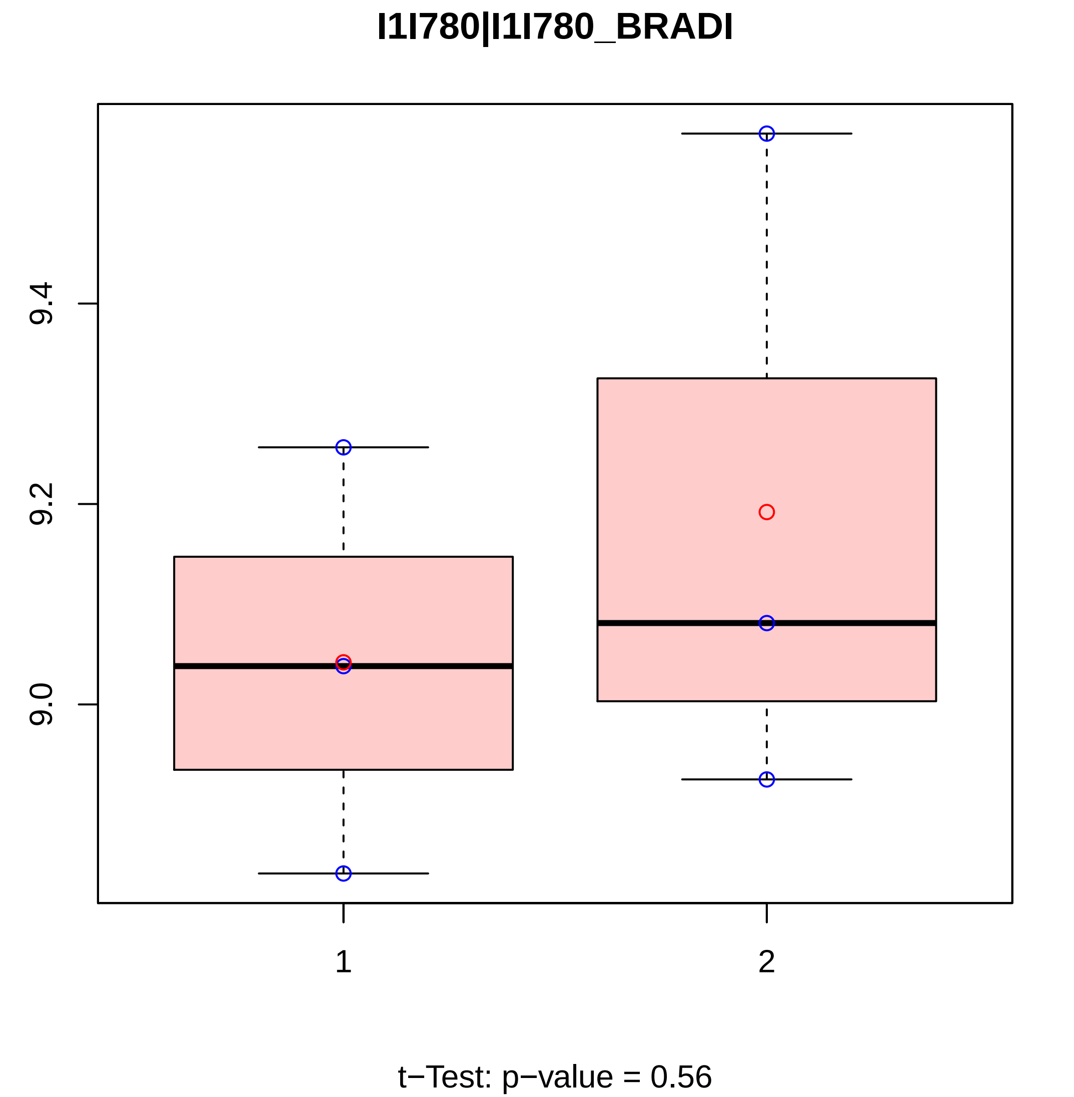
I1I780|I1I780_BRADI
9.0
9.2
9.4
1
2
t−Test: p−value = 0.56

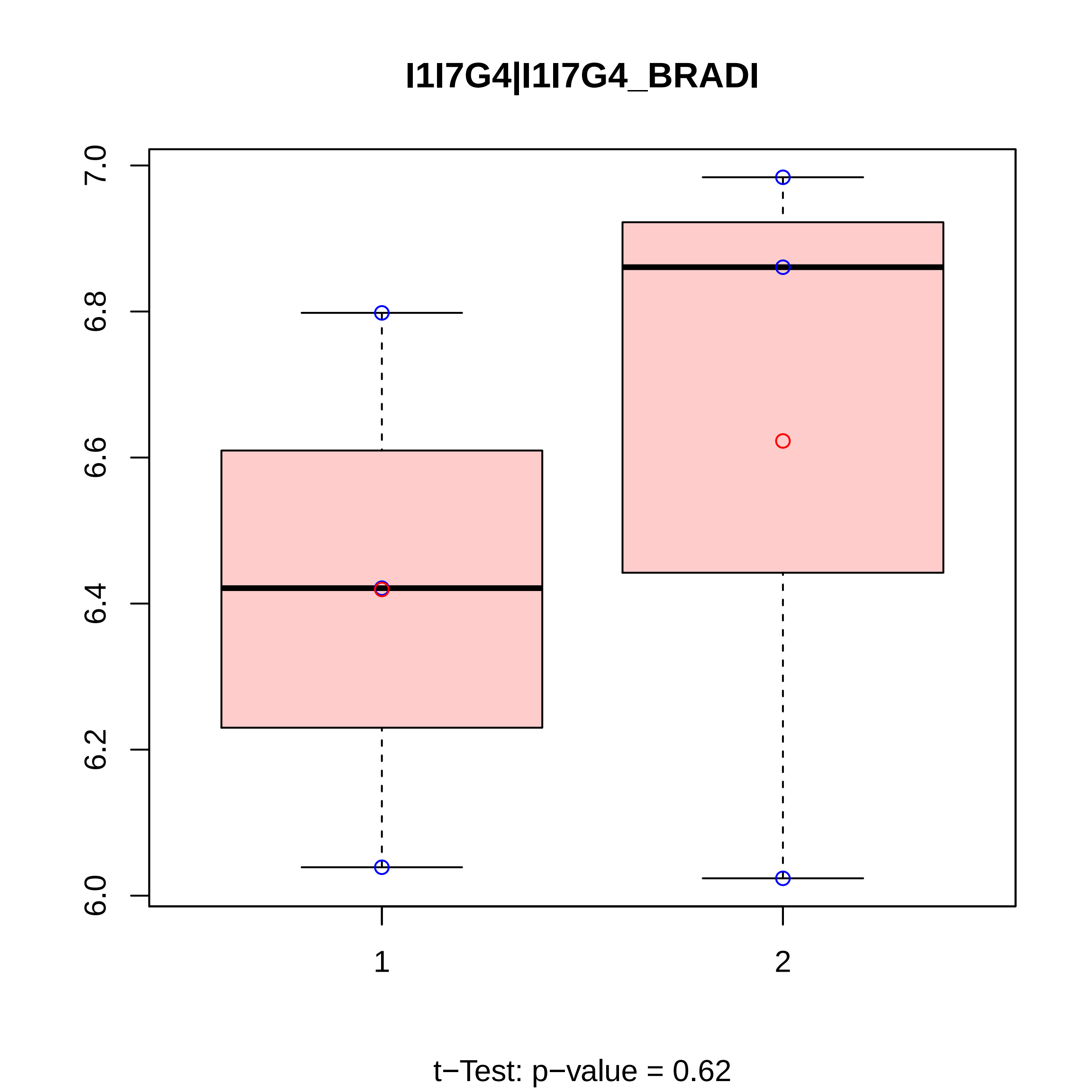
I1I7G4|I1I7G4_BRADI
7.0
6.8
6.6
6.4
6.2
6.0
1
2
t−Test: p−value = 0.62

I1I7K9|I1I7K9_BRADI

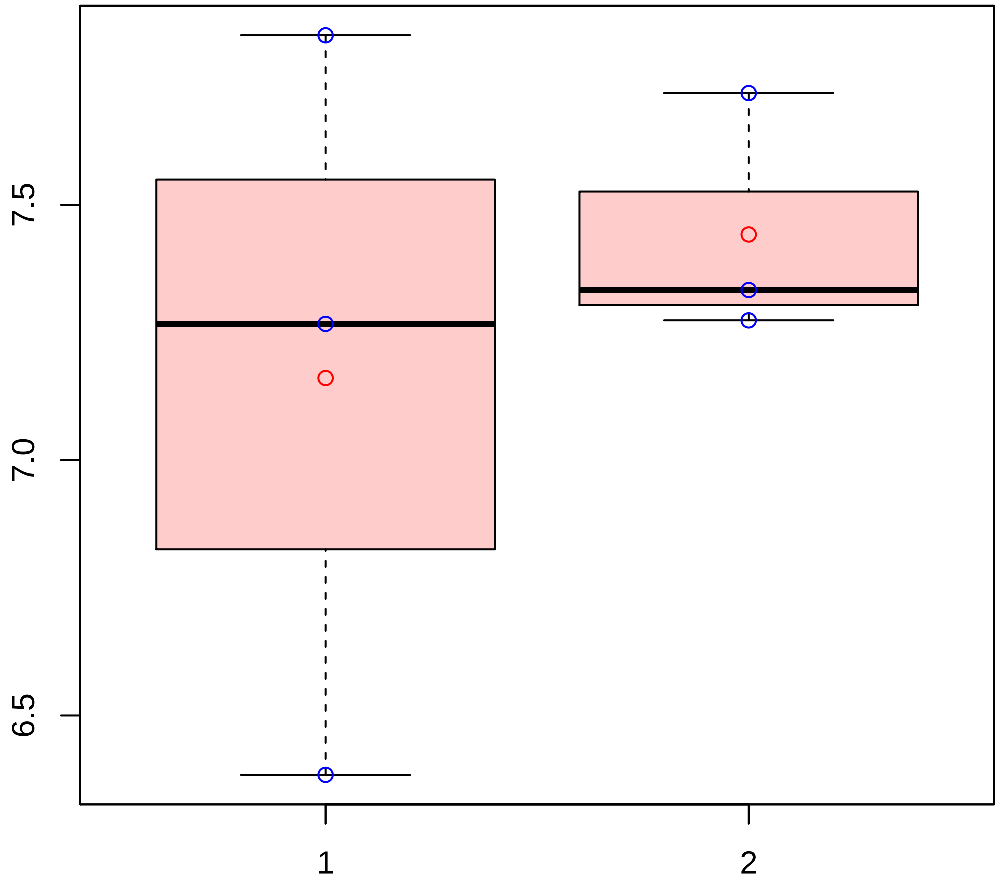

t−Test: p−value = 0.58

I1I7T6|I1I7T6_BRADI

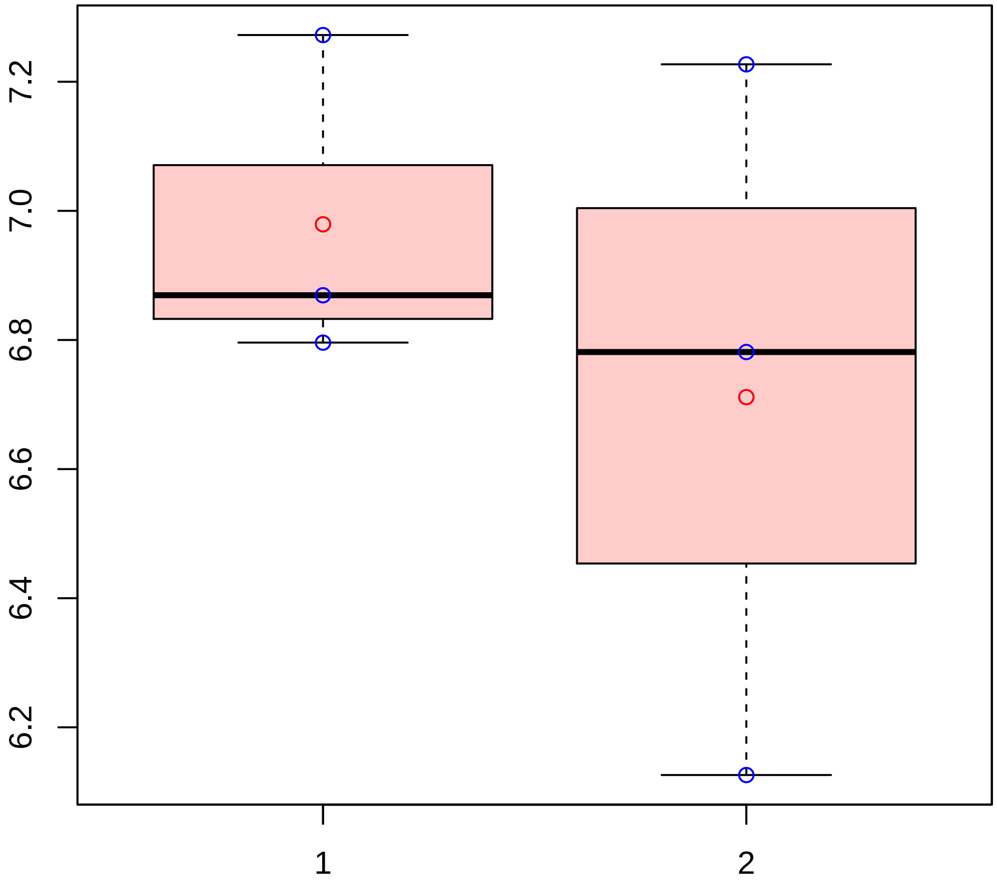

t−Test: p−value = 0.51

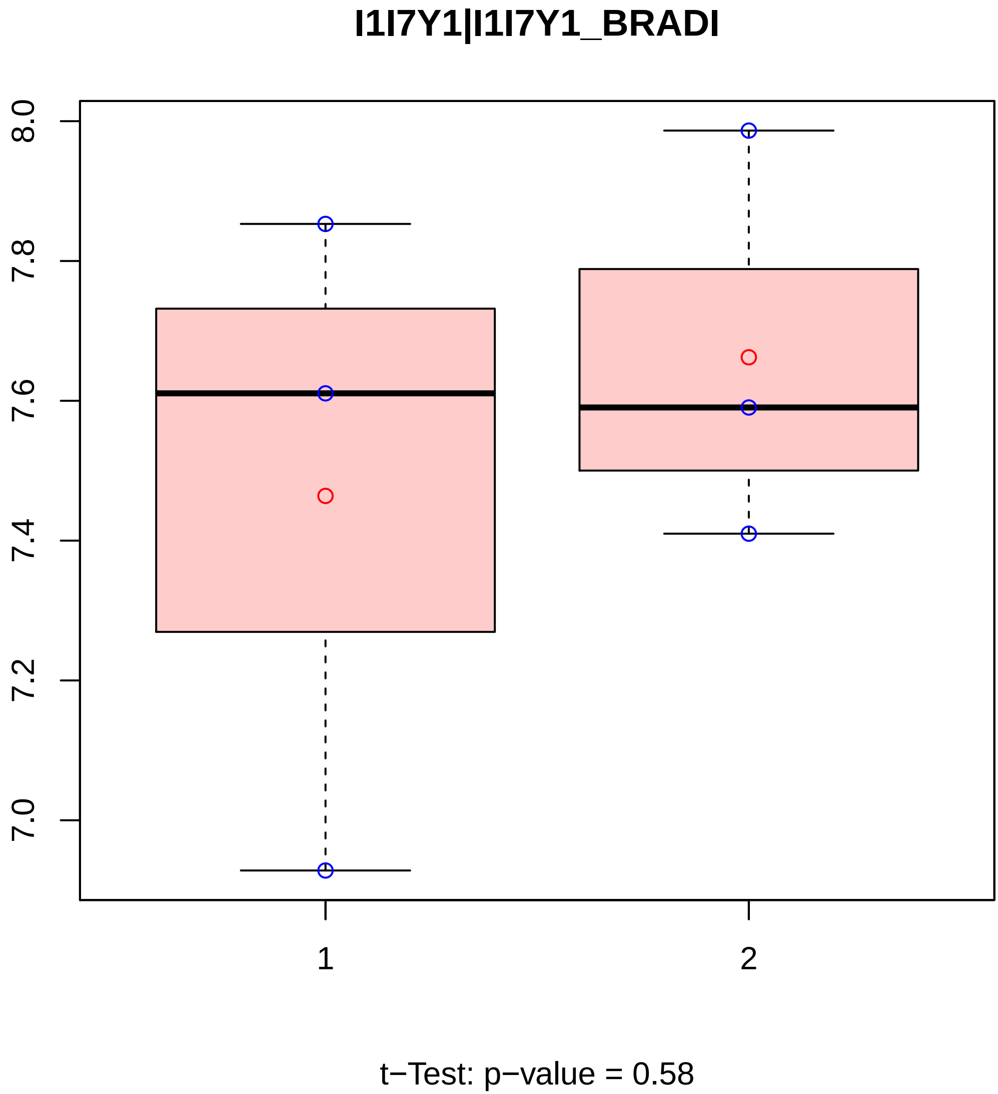

I1I7Y1|I1I7Y1_BRADI
8.0
7.8
7.6
7.4
7.2
7.0
1
2
t−Test: p−value = 0.58

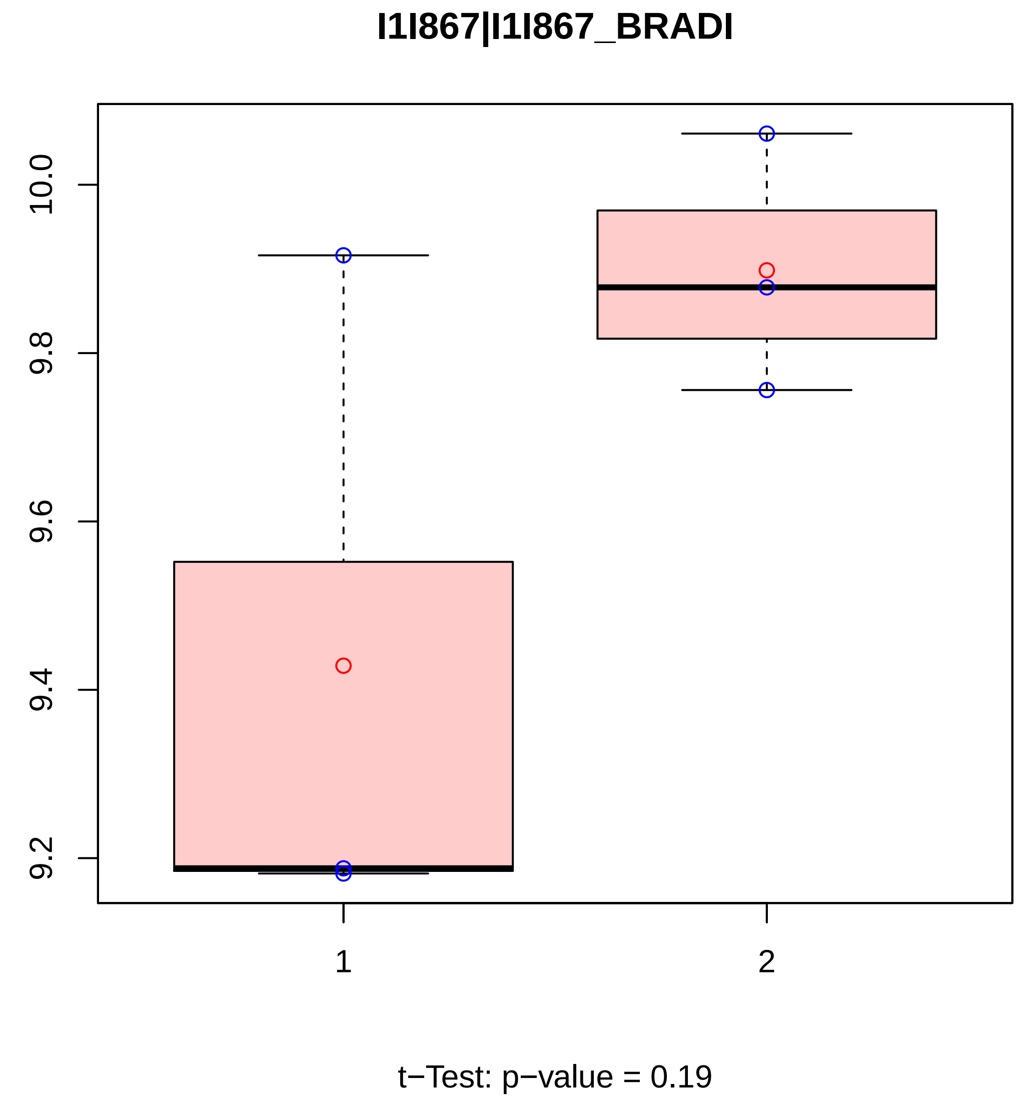
I1I867|I1I867_BRADI
10.0
9.8
9.6
9.4
9.2
1
2
t−Test: p−value = 0.19

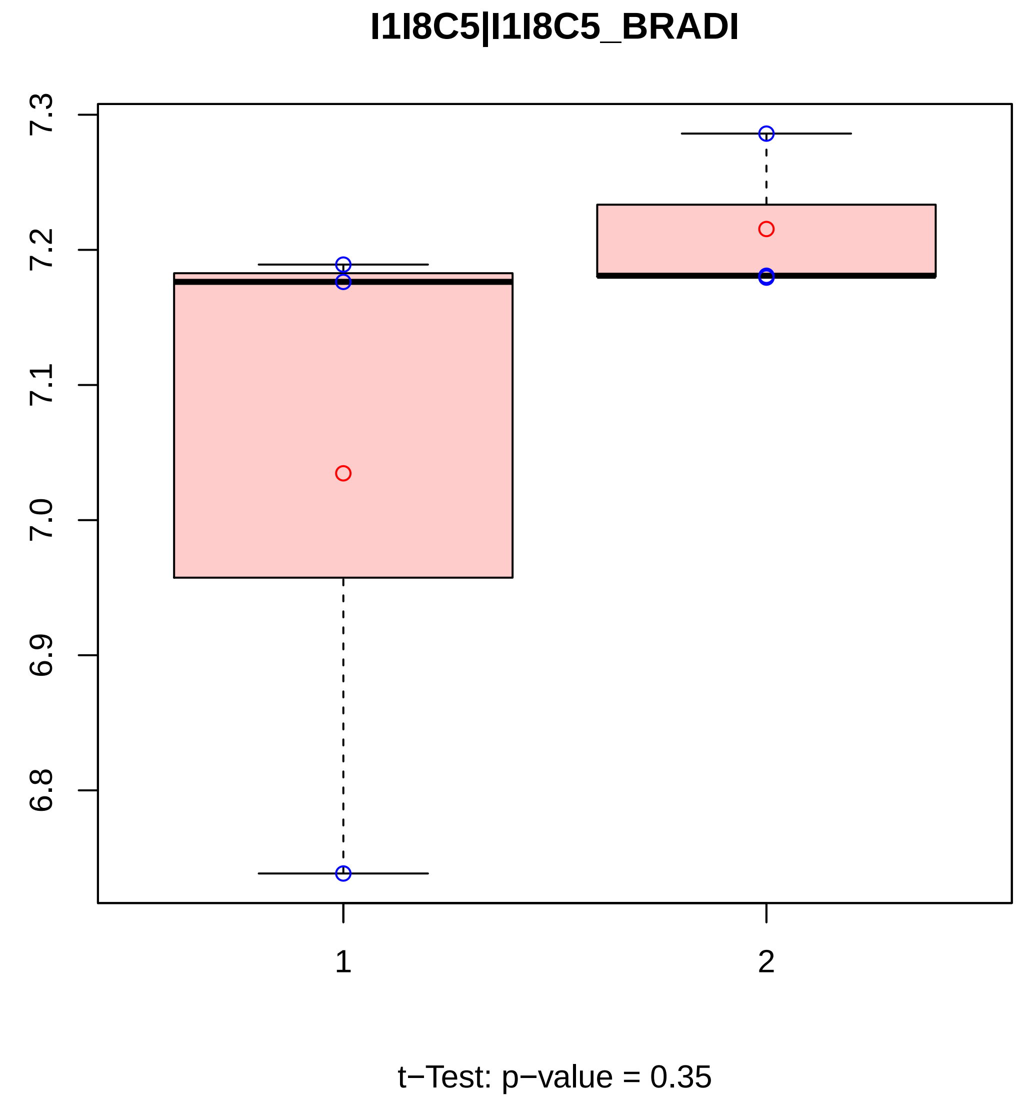
I1I8C5|I1I8C5_BRADI
7.3
7.2
7.1
7.0
6.9
6.8
1
2
t−Test: p−value = 0.35

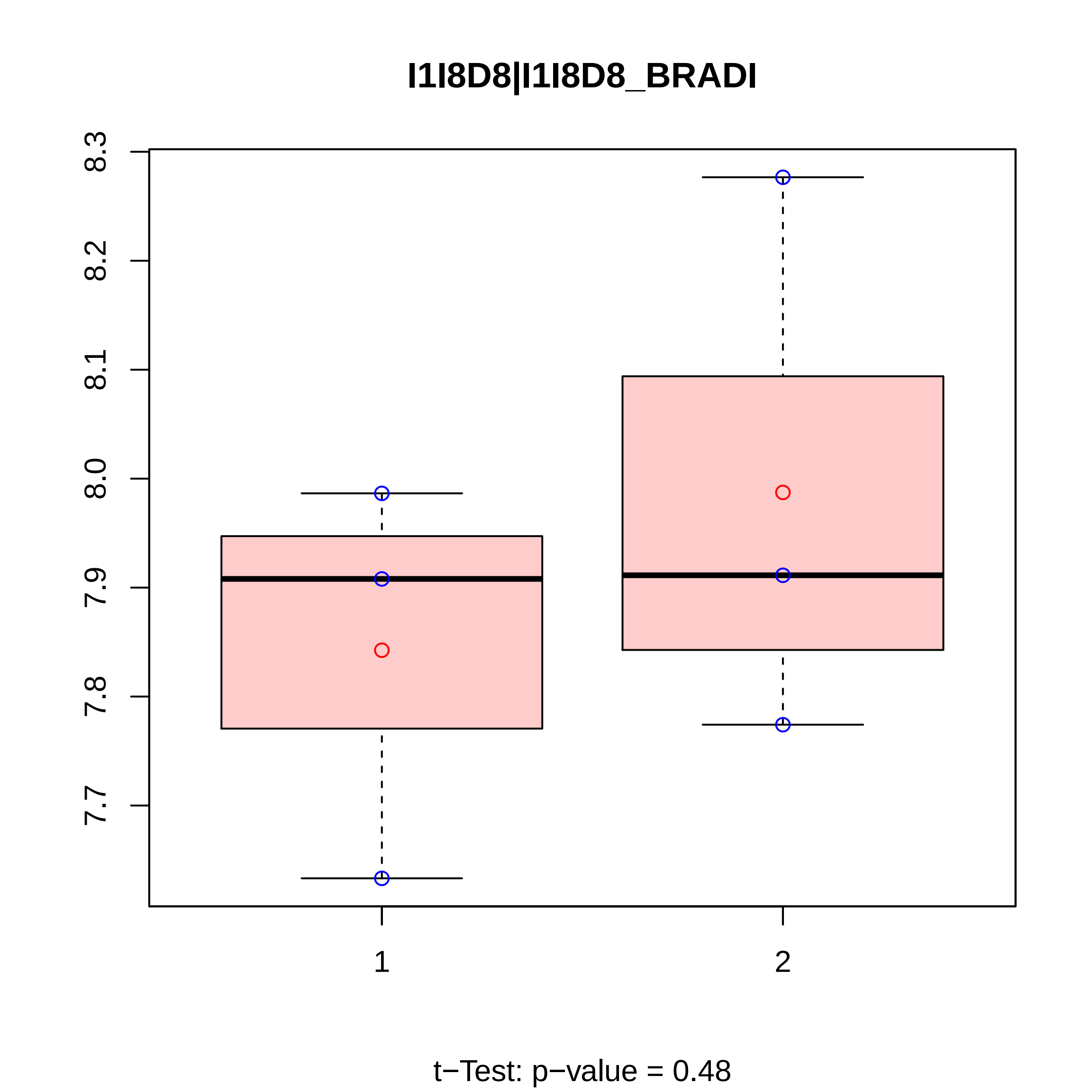
I1I8D8|I1I8D8_BRADI
8.3
8.2
8.1
8.0
7.9
7.8
7.7
1
2
t−Test: p−value = 0.48

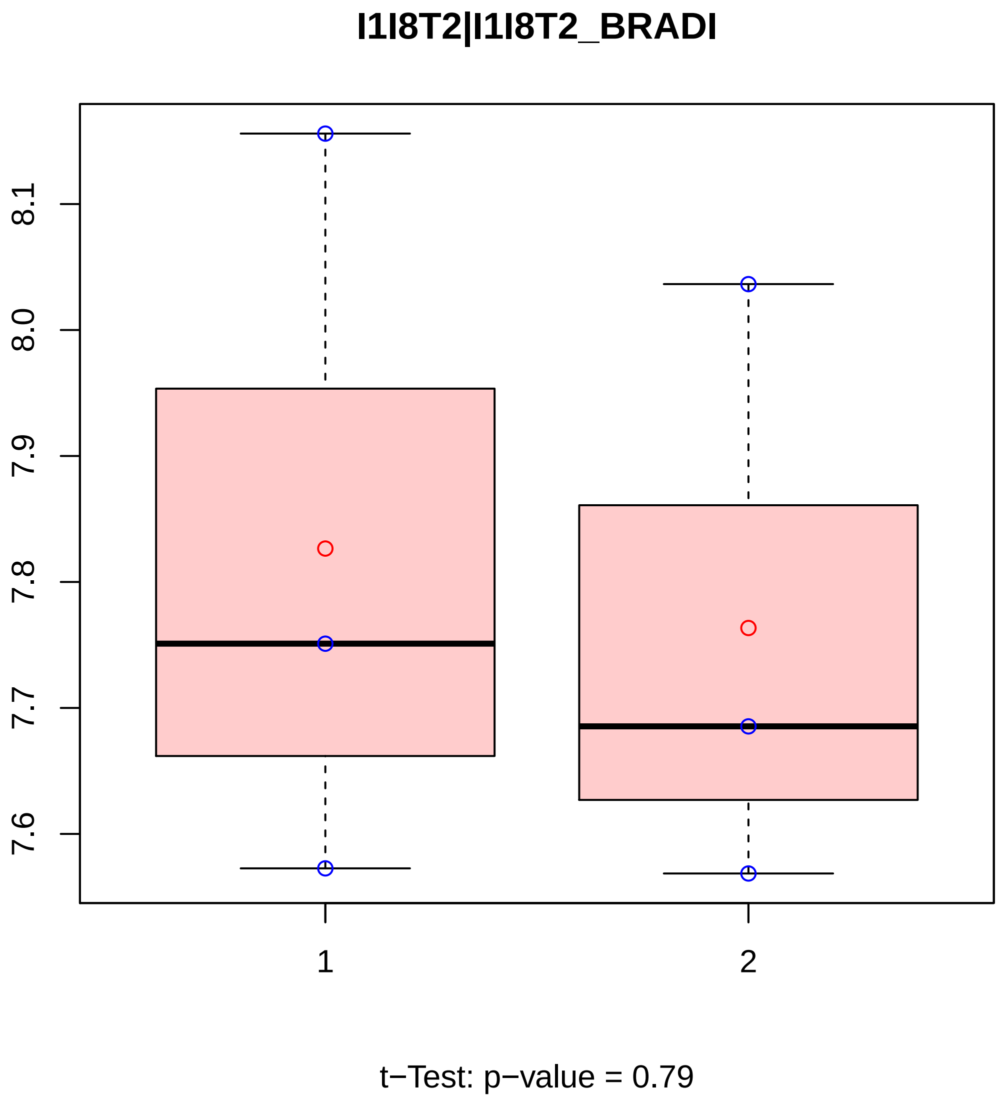
I1I8T2|I1I8T2_BRADI
8.1
8.0
7.9
7.8
7.7
7.6
1
2
t−Test: p−value = 0.79

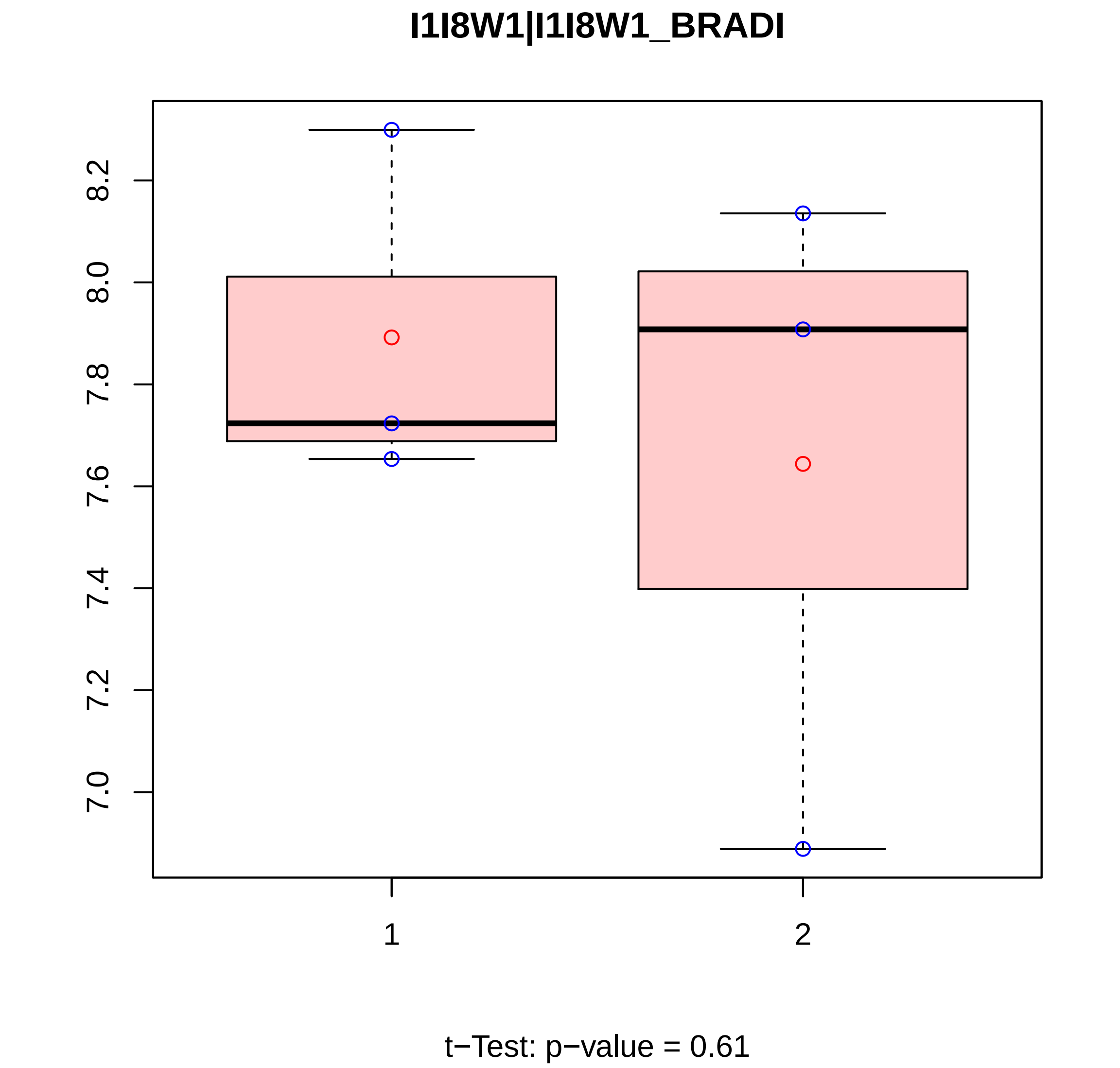
I1I8W1|I1I8W1_BRADI
8.2
8.0
7.8
7.6
7.4
7.2
7.0
1
2
t−Test: p−value = 0.61

I1I8X4|I1I8X4_BRADI

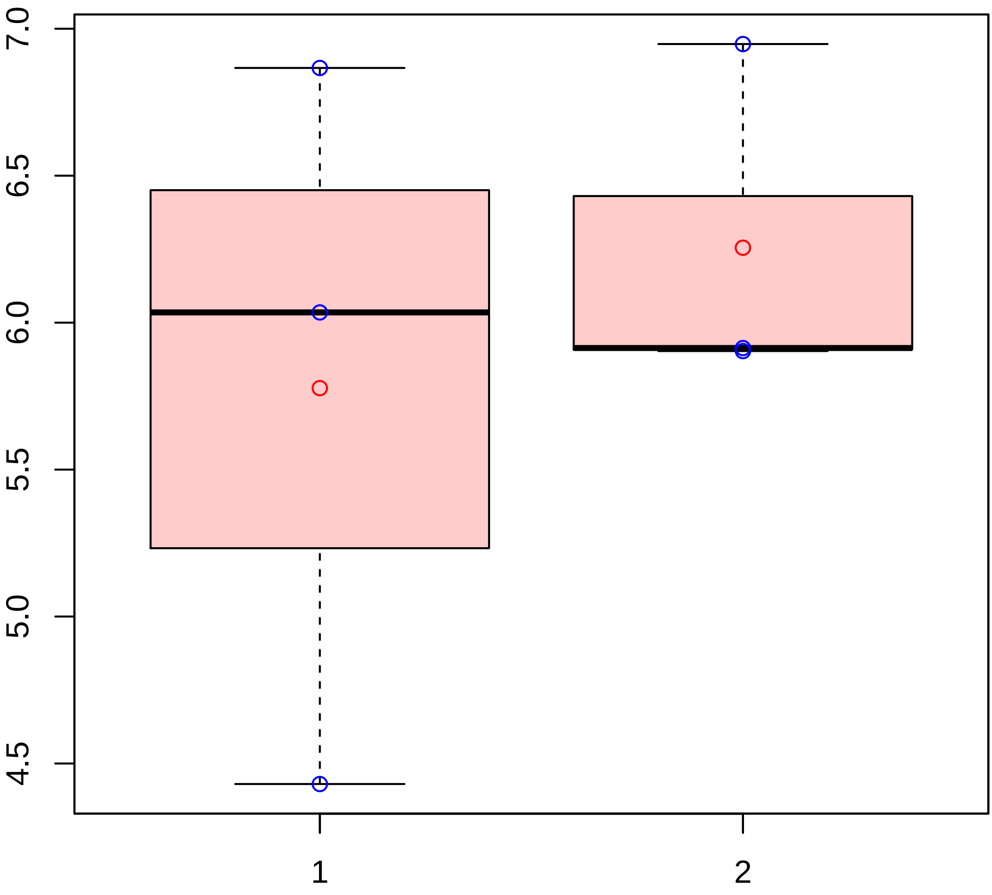

t−Test: p−value = 0.59

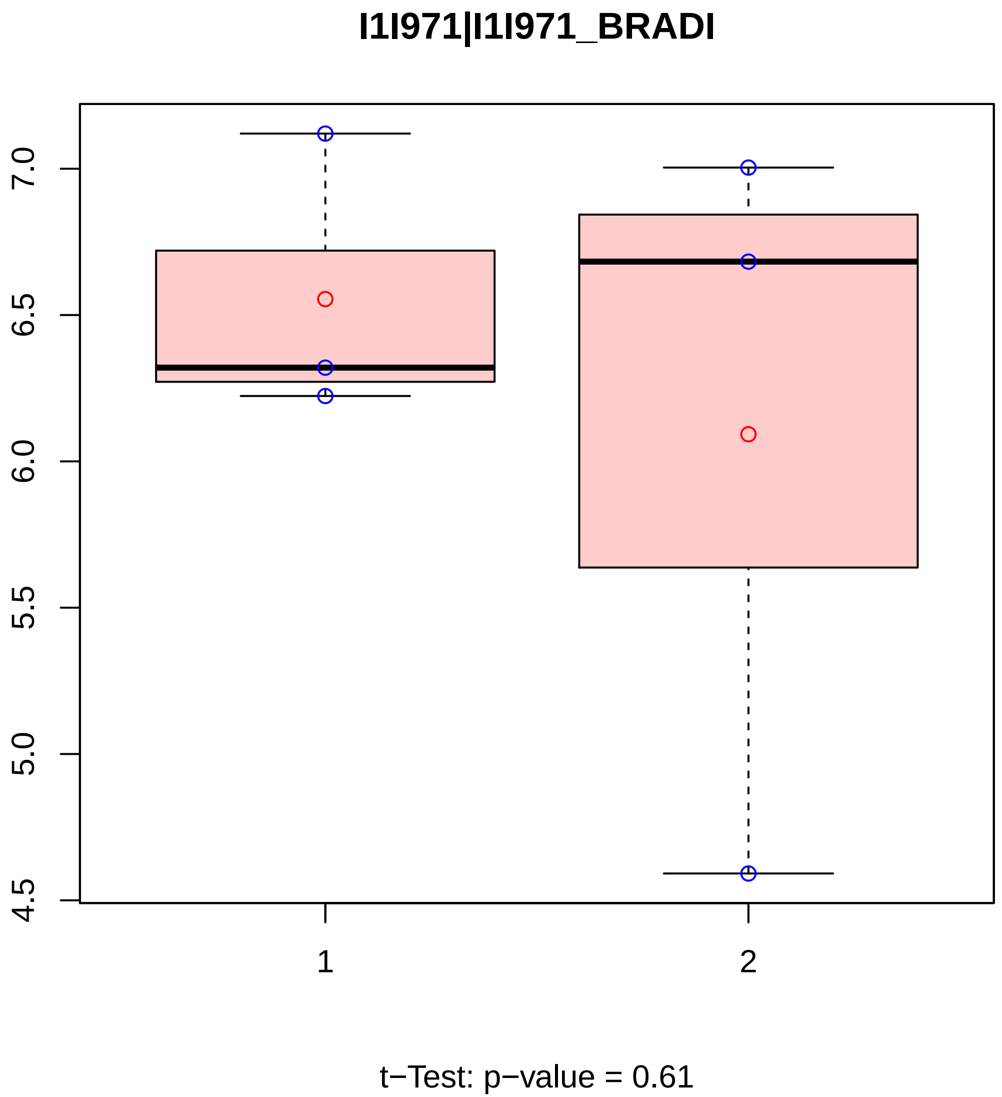
I1I971|I1I971_BRADI
7.0
6.5
6.0
5.5
5.0
4.5
1
2
t−Test: p−value = 0.61

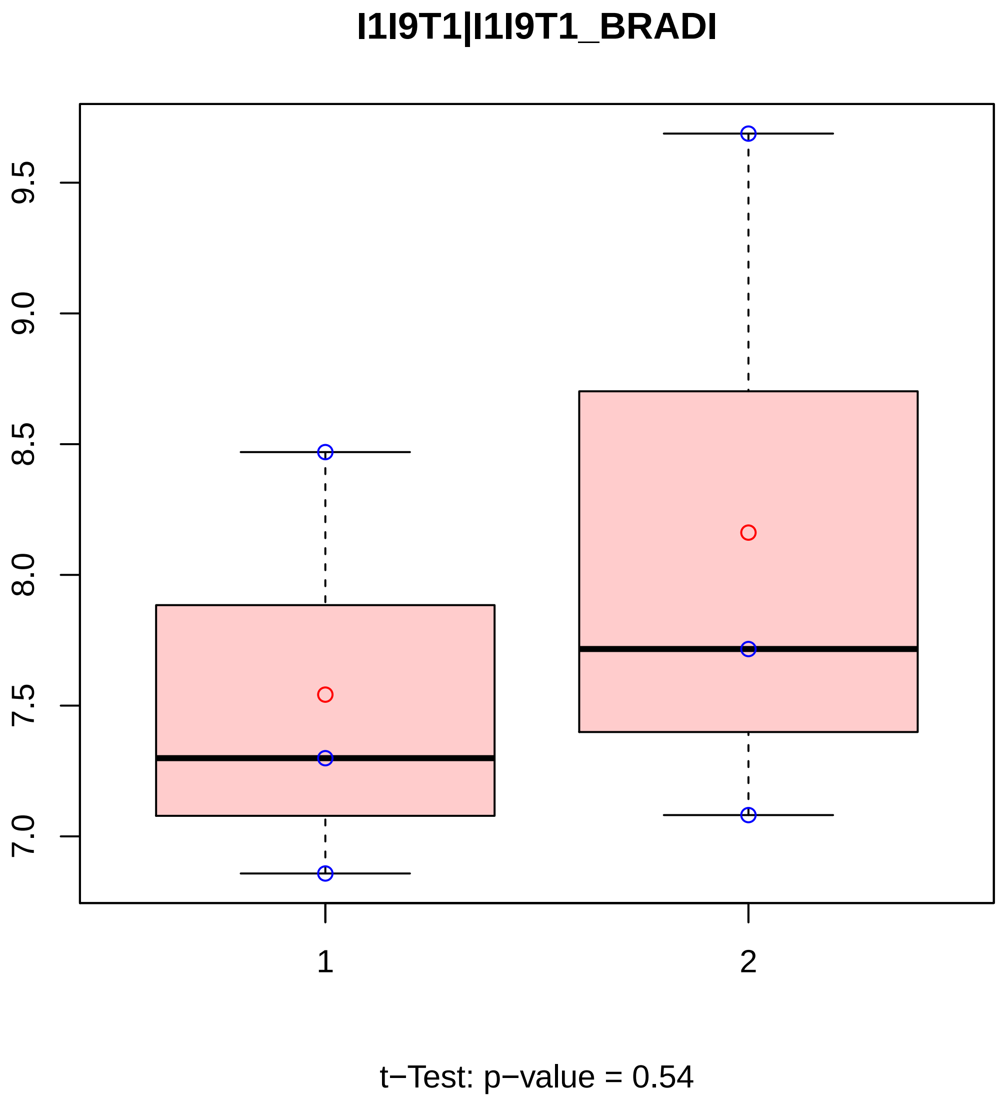
I1I9T1|I1I9T1_BRADI
7.0
7.5
8.0
8.5
9.0
9.5
1
2
t−Test: p−value = 0.54

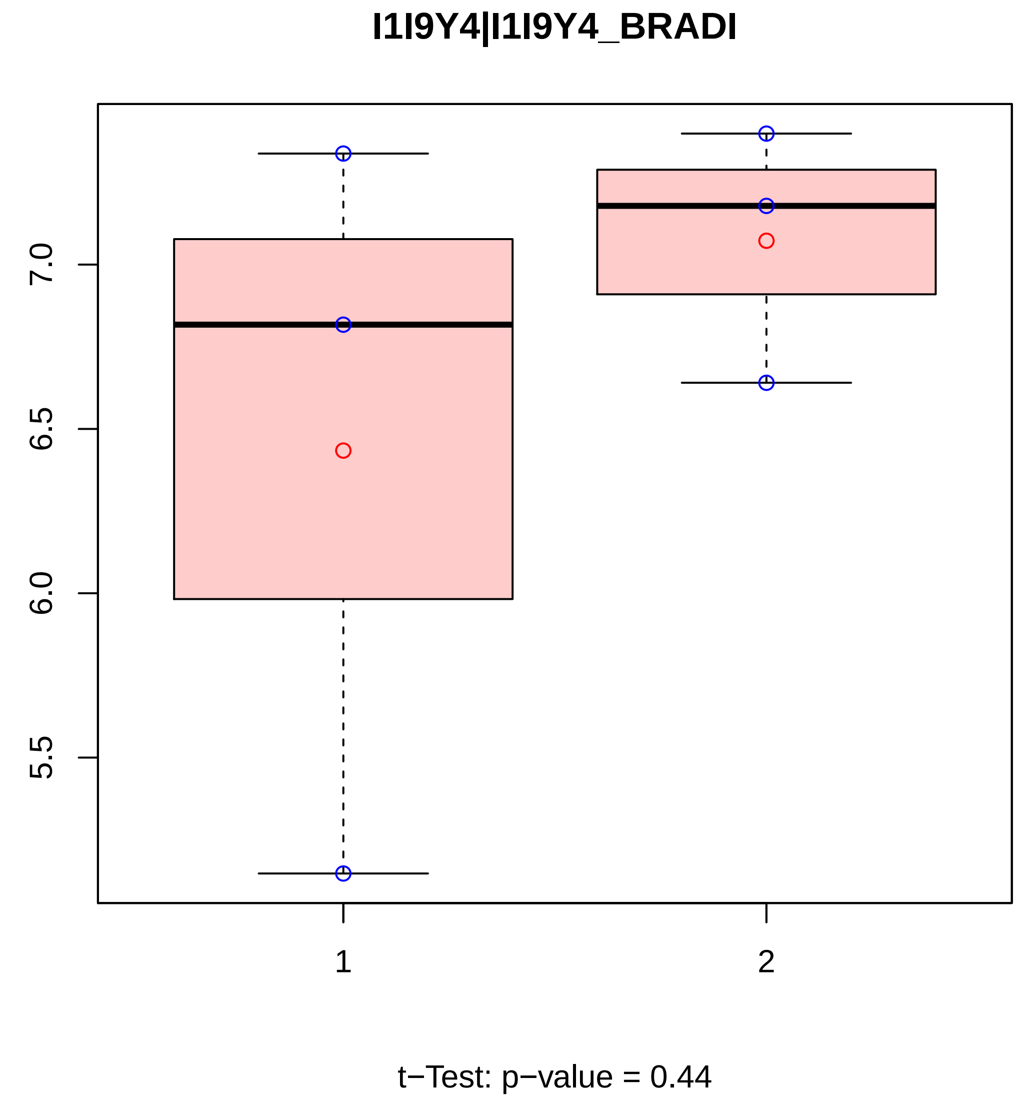

I1I9Y4|I1I9Y4_BRADI
7.0
6.5
6.0
5.5
1
2
t−Test: p−value = 0.44

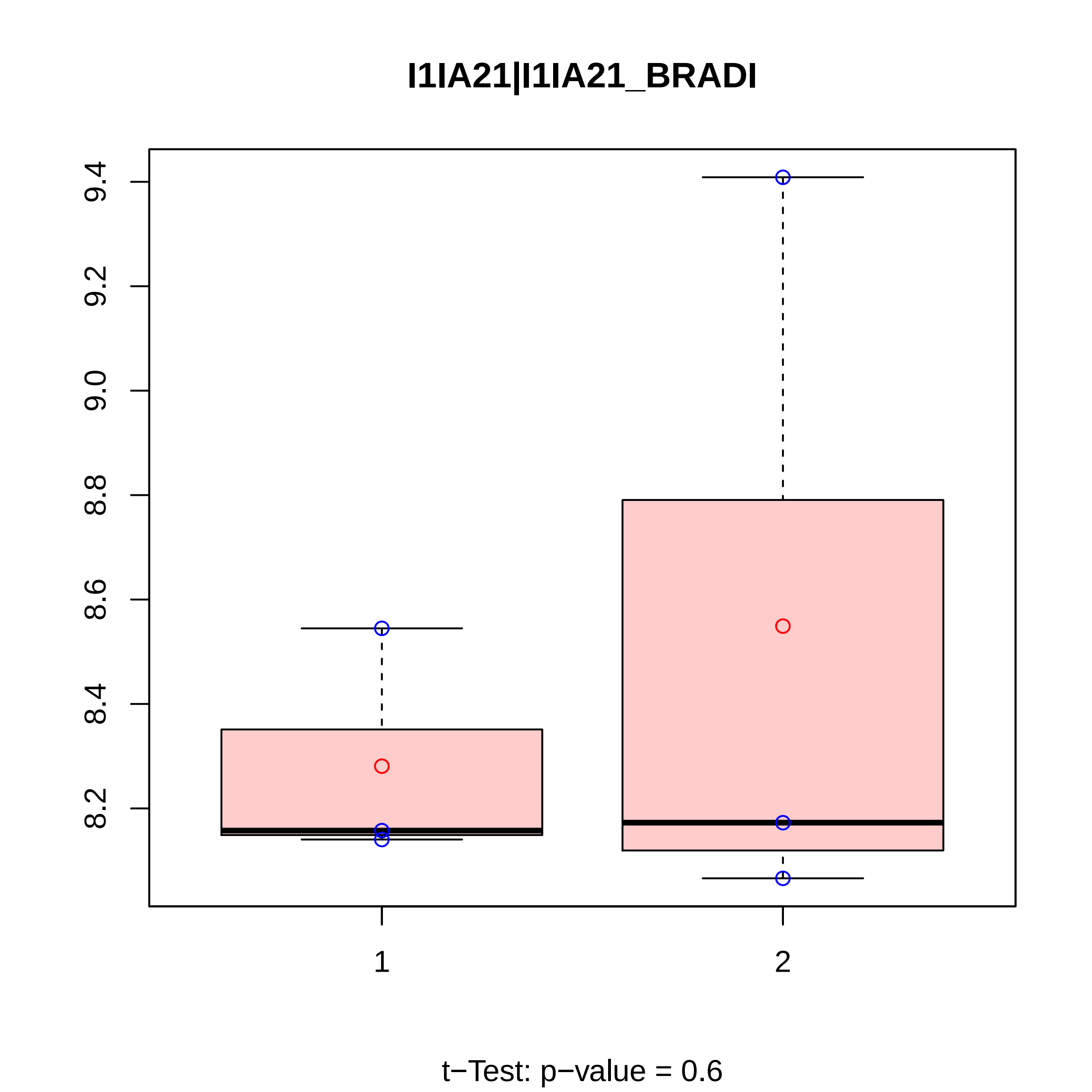

I1IA21|I1IA21_BRADI
9.4
9.2
9.0
8.8
8.6
8.4
8.2
1
2
t−Test: p−value = 0.6

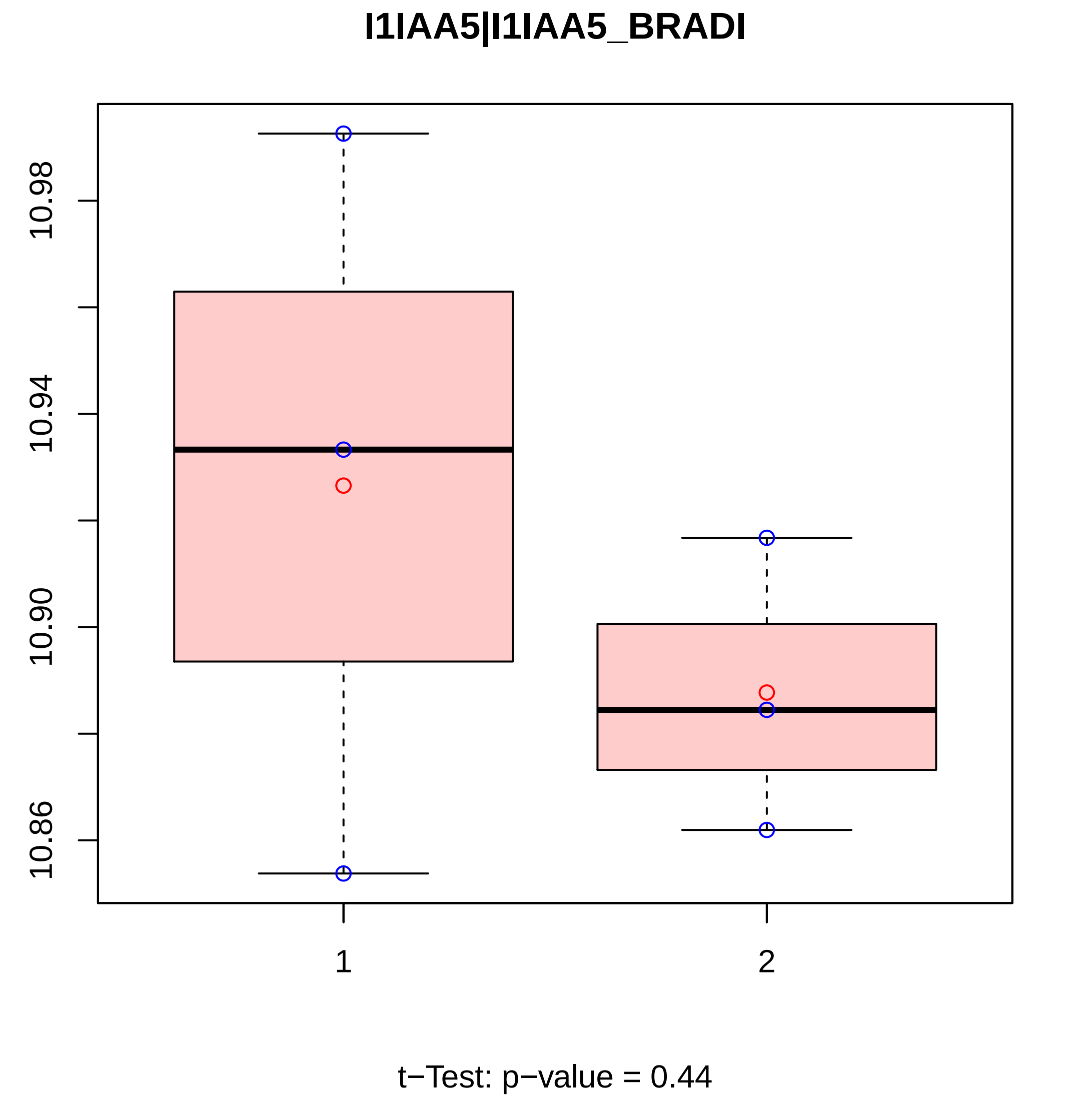
I1IAA5|I1IAA5_BRADI
10.86
10.90
10.94
10.98
1
2
t−Test: p−value = 0.44

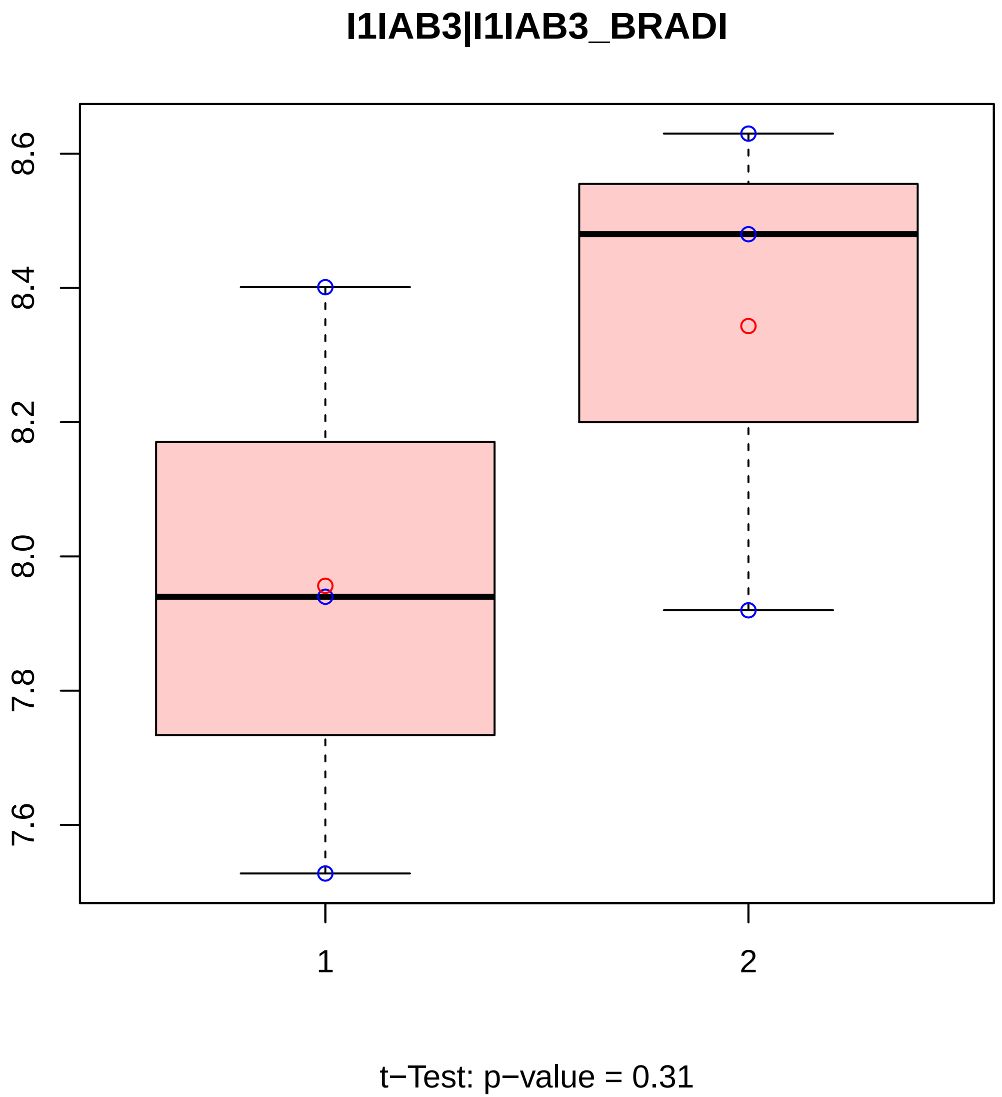
I1IAB3|I1IAB3_BRADI
8.6
8.4
8.2
8.0
7.8
7.6
1
2
t−Test: p−value = 0.31

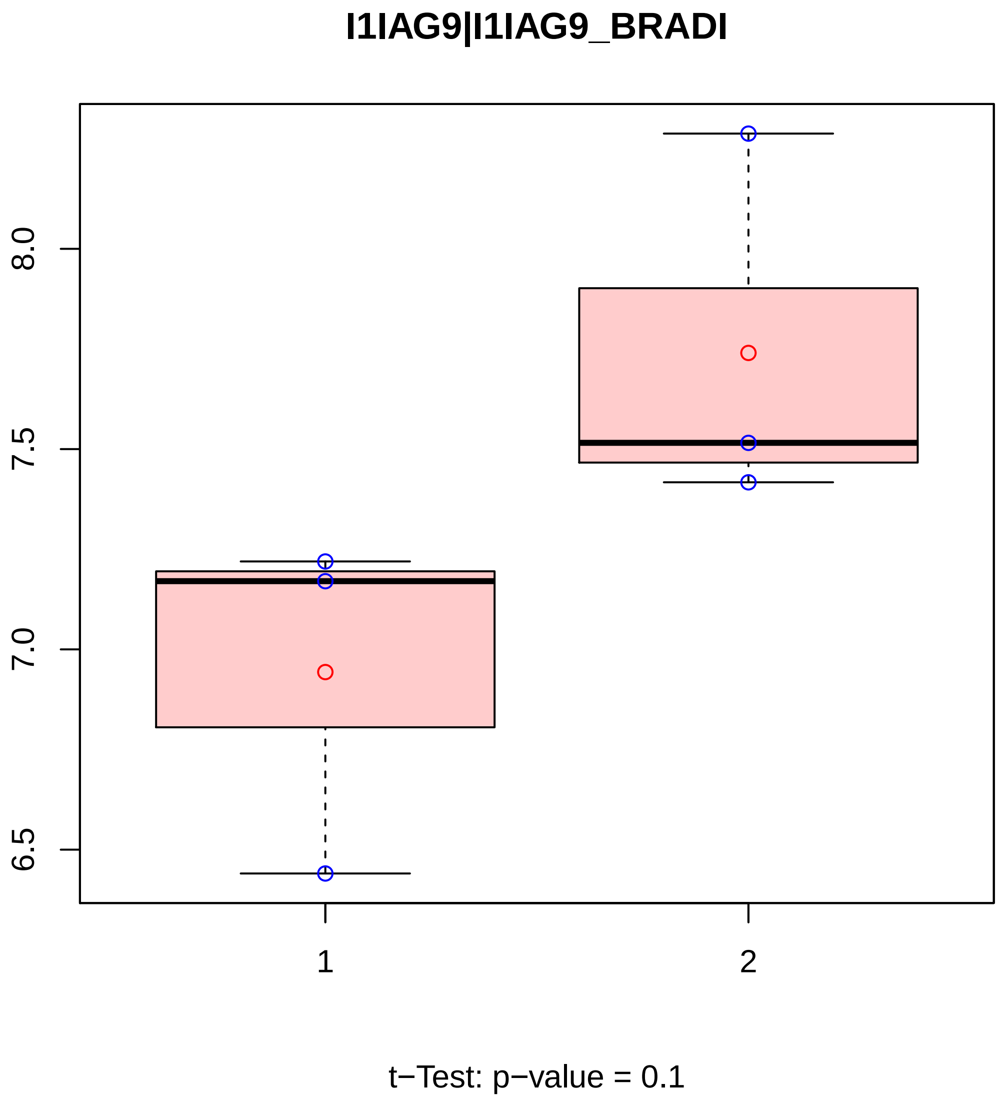
I1IAG9|I1IAG9_BRADI
8.0
7.5
7.0
6.5
1
2
t−Test: p−value = 0.1

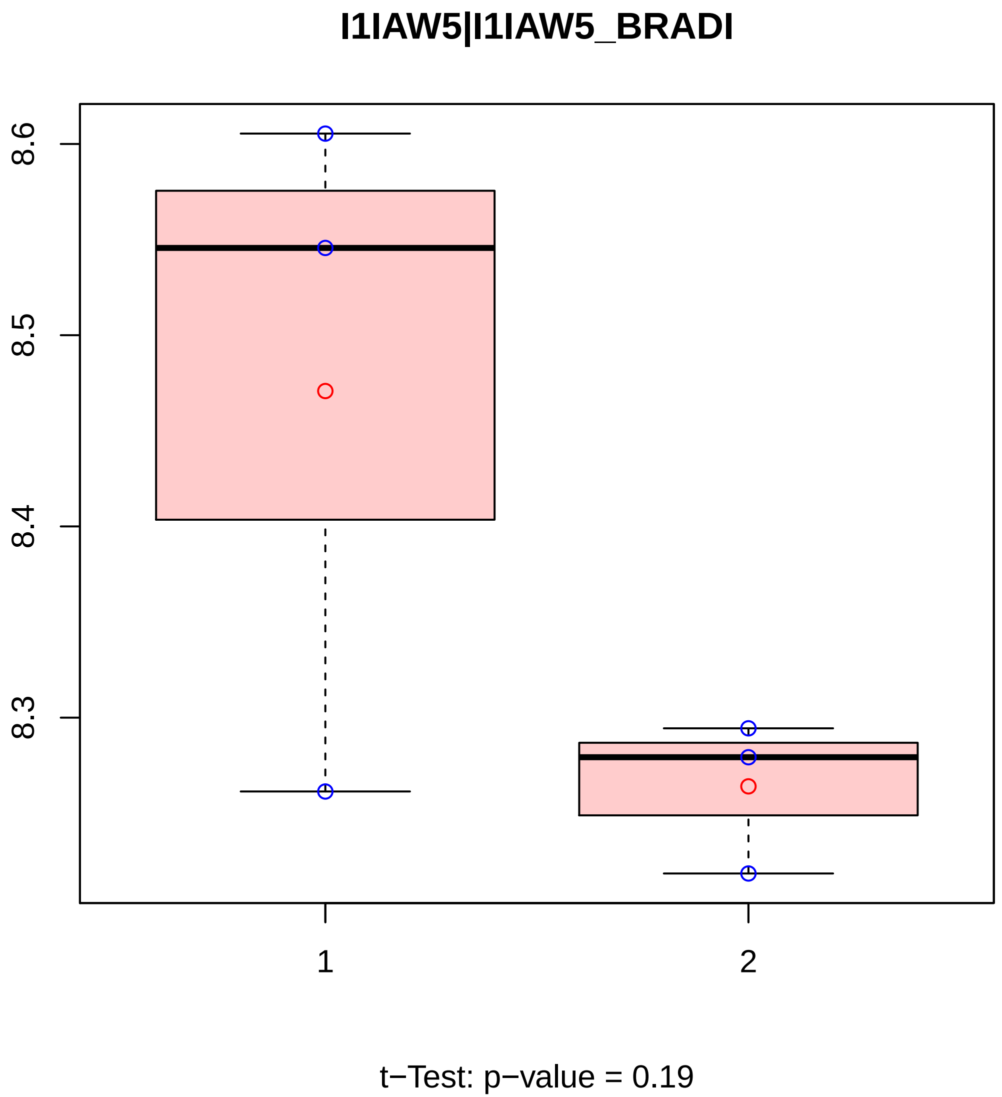
I1IAW5|I1IAW5_BRADI
8.6
8.5
8.4
8.3
1
2
t−Test: p−value = 0.19

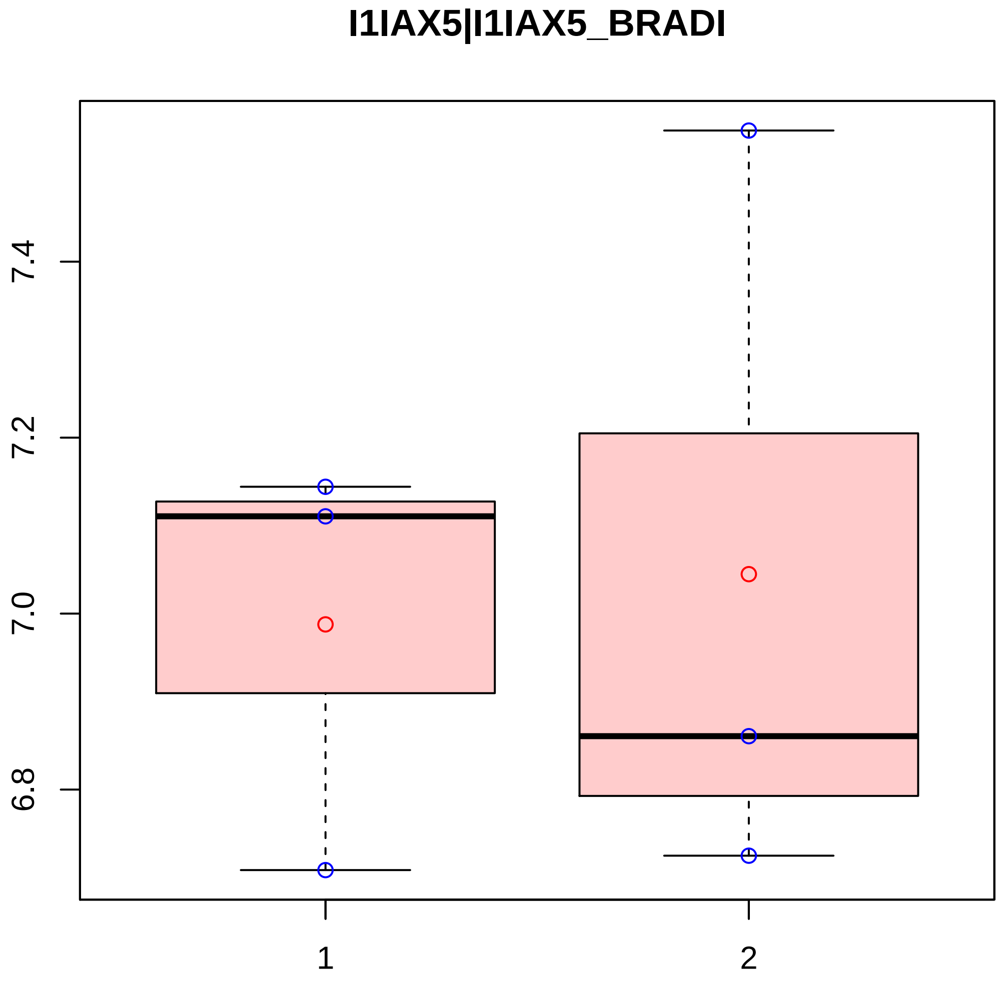

t−Test: p−value = 0.86

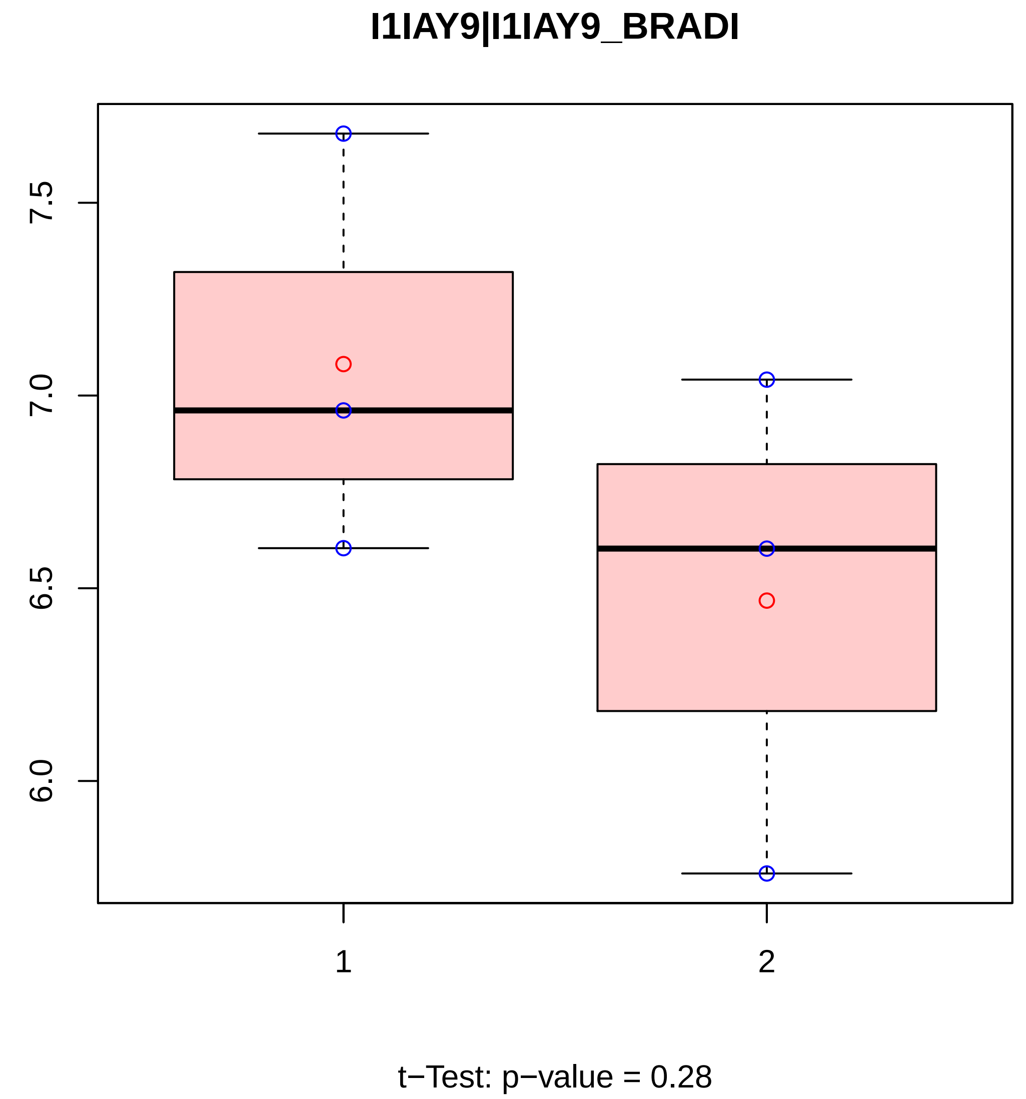

I1IAY9|I1IAY9_BRADI
7.5
7.0
6.5
6.0
1
2
t−Test: p−value = 0.28

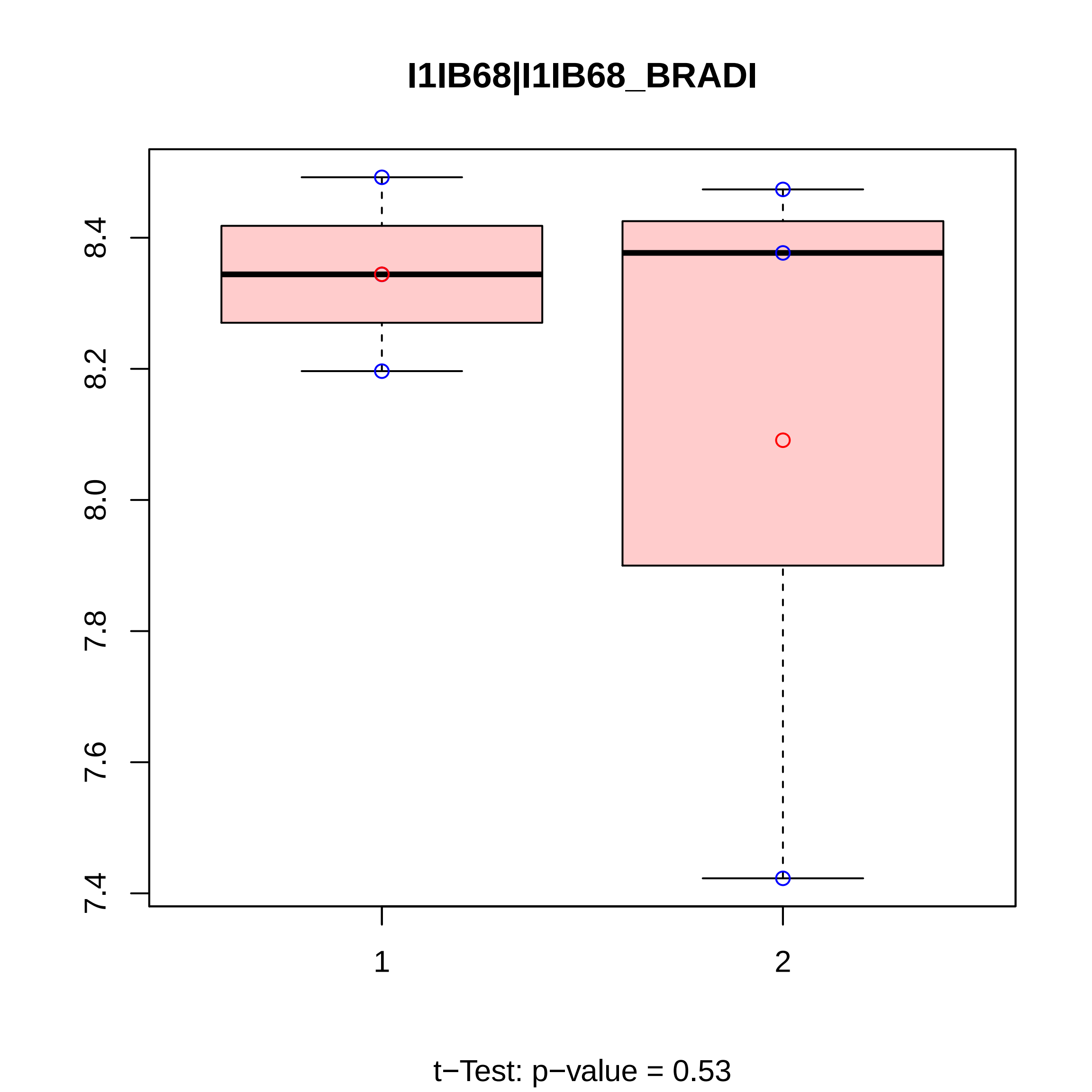

I1IB68|I1IB68_BRADI
8.4
8.2
8.0
7.8
7.6
7.4
1
2
t−Test: p−value = 0.53

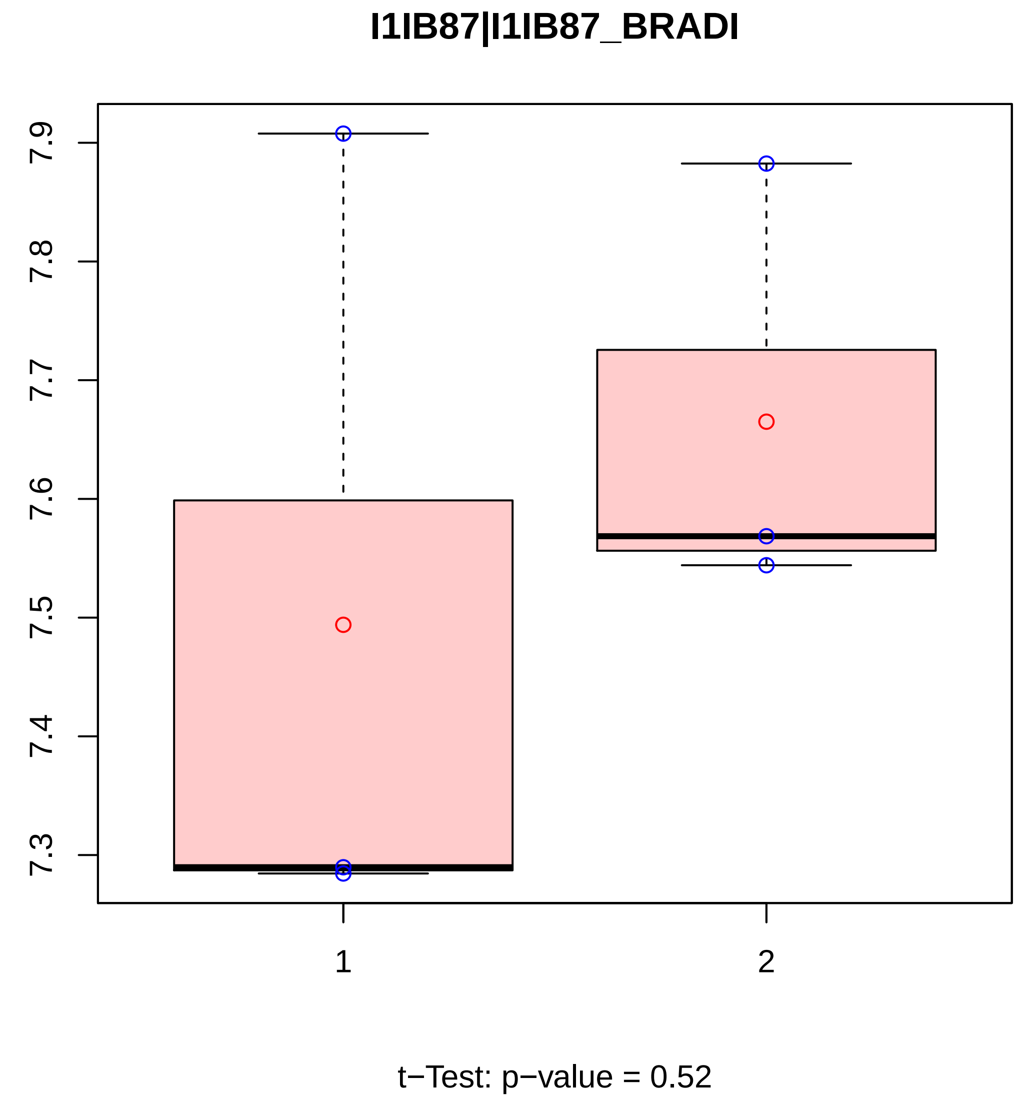

I1IB87|I1IB87_BRADI
7.3
7.4
7.5
7.6
7.7
7.8
7.9
1
2
t−Test: p−value = 0.52

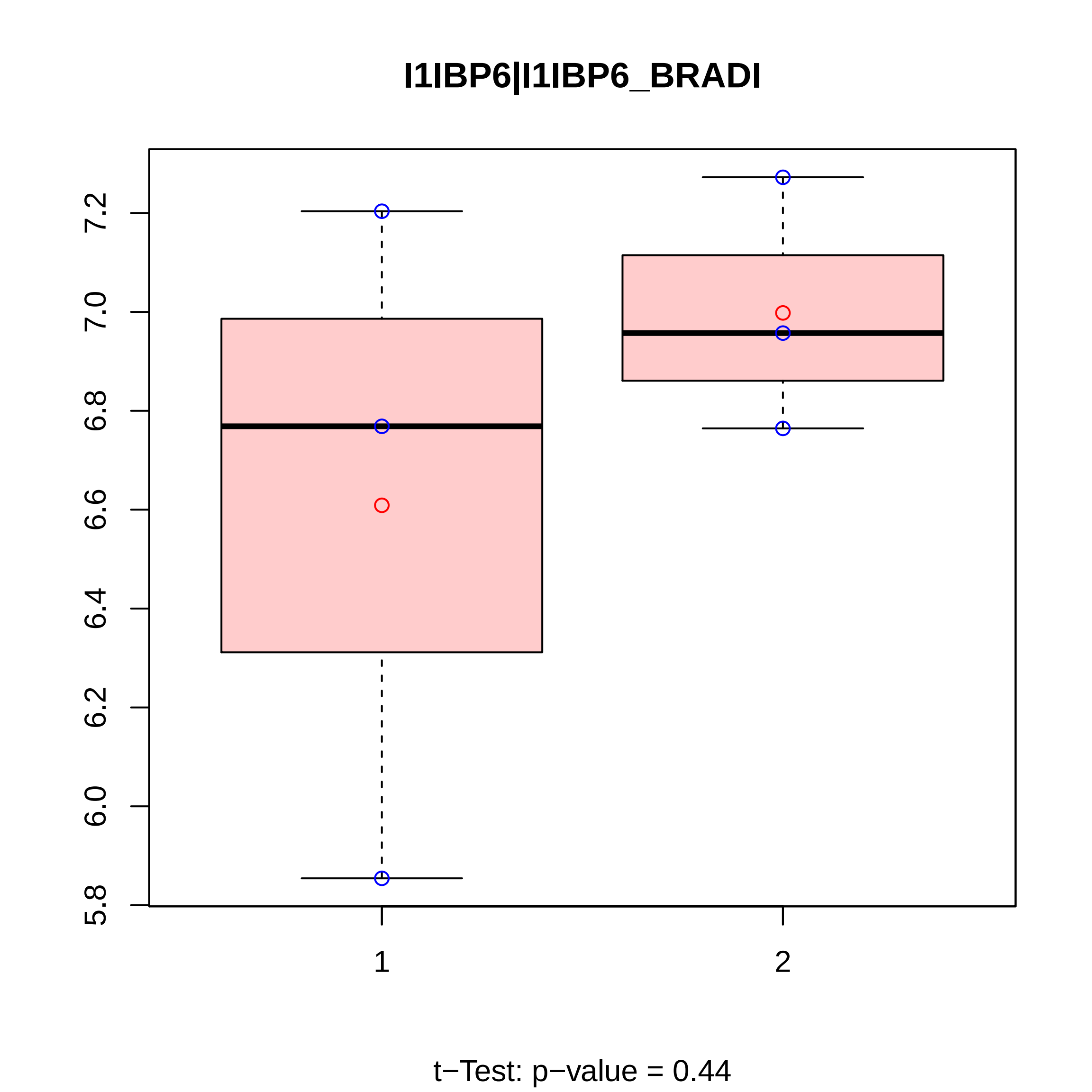
I1IBP6|I1IBP6_BRADI
7.2
7.0
6.8
6.6
6.4
6.2
6.0
5.8
1
2
t−Test: p−value = 0.44

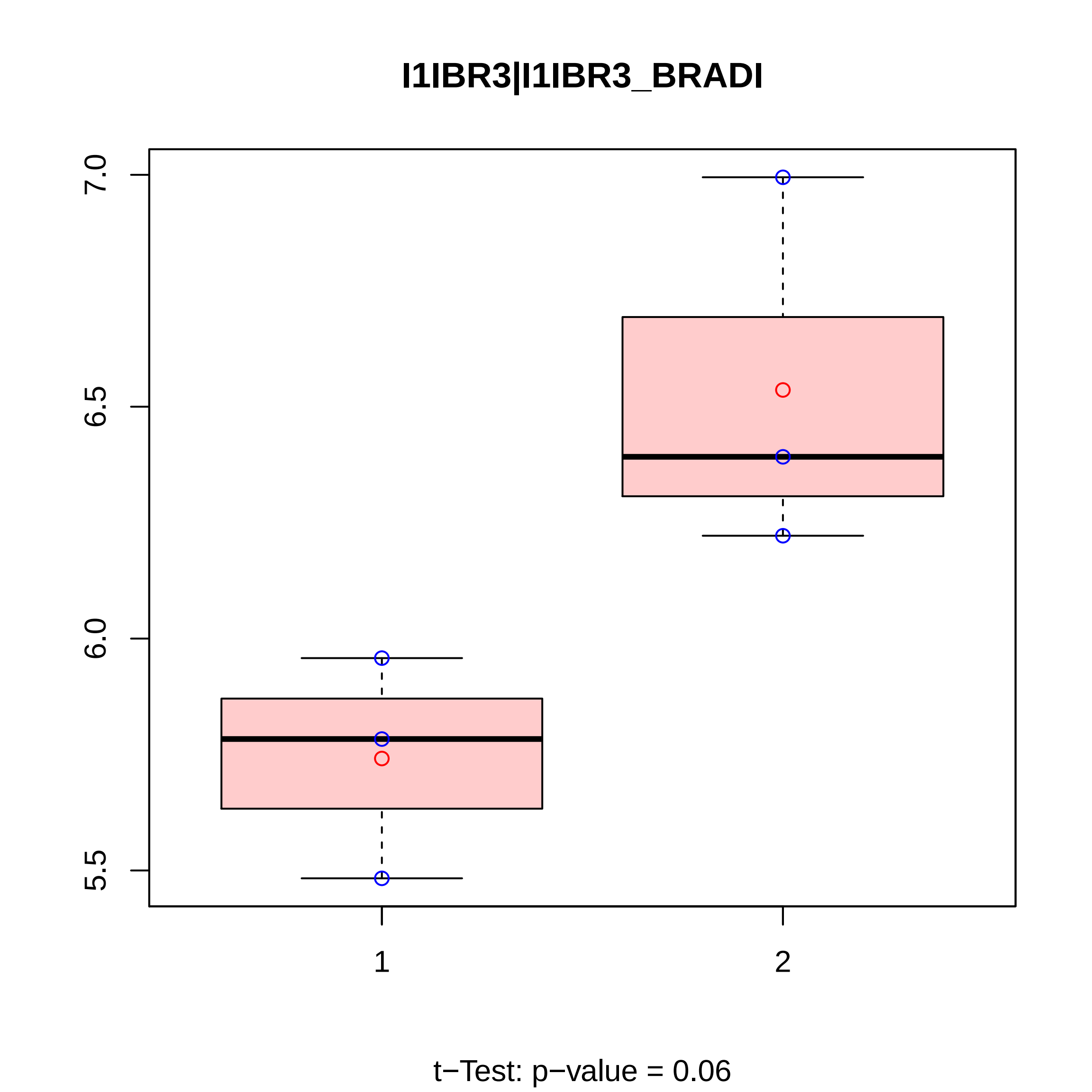
I1IBR3|I1IBR3_BRADI
7.0
6.5
6.0
5.5
1
2
t−Test: p−value = 0.06

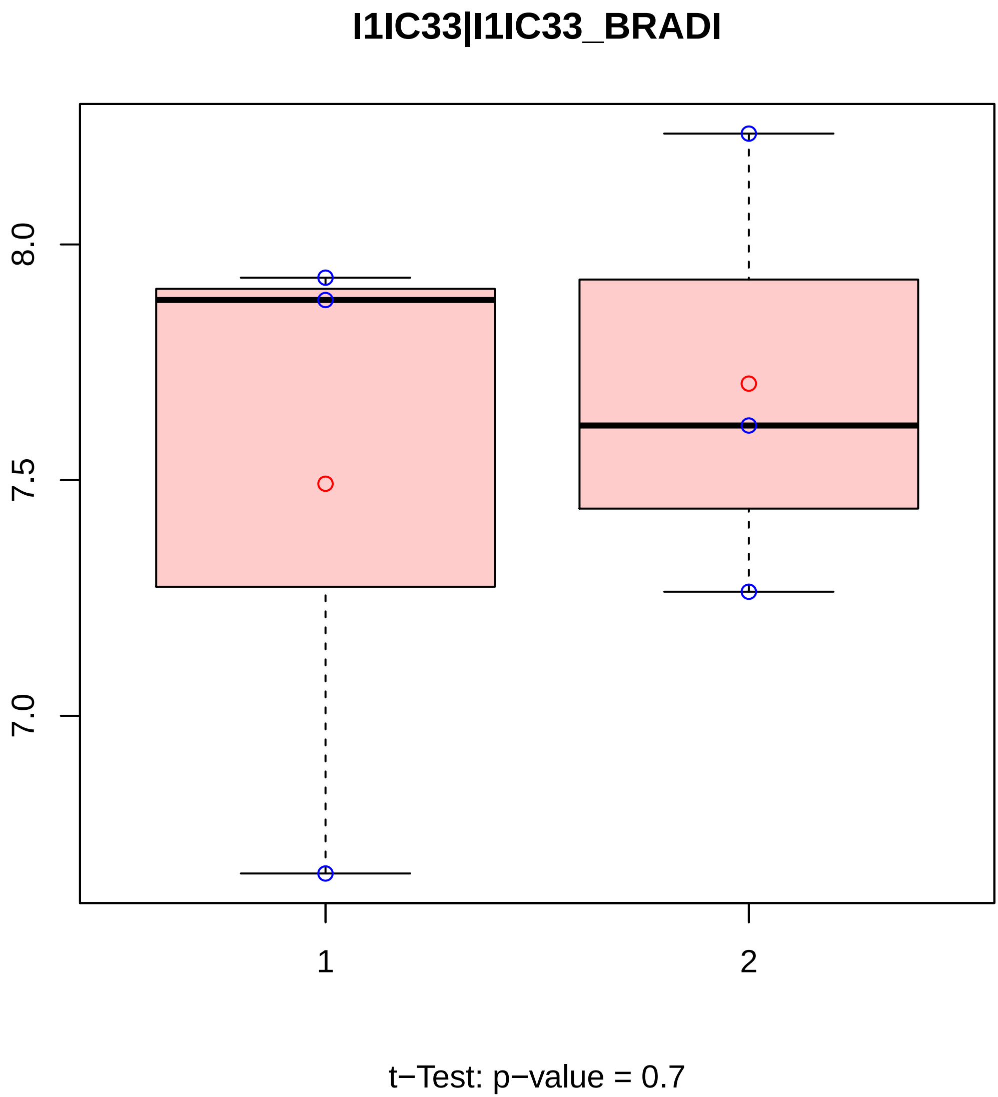
I1IC33|I1IC33_BRADI
8.0
7.5
7.0
1
2
t−Test: p−value = 0.7

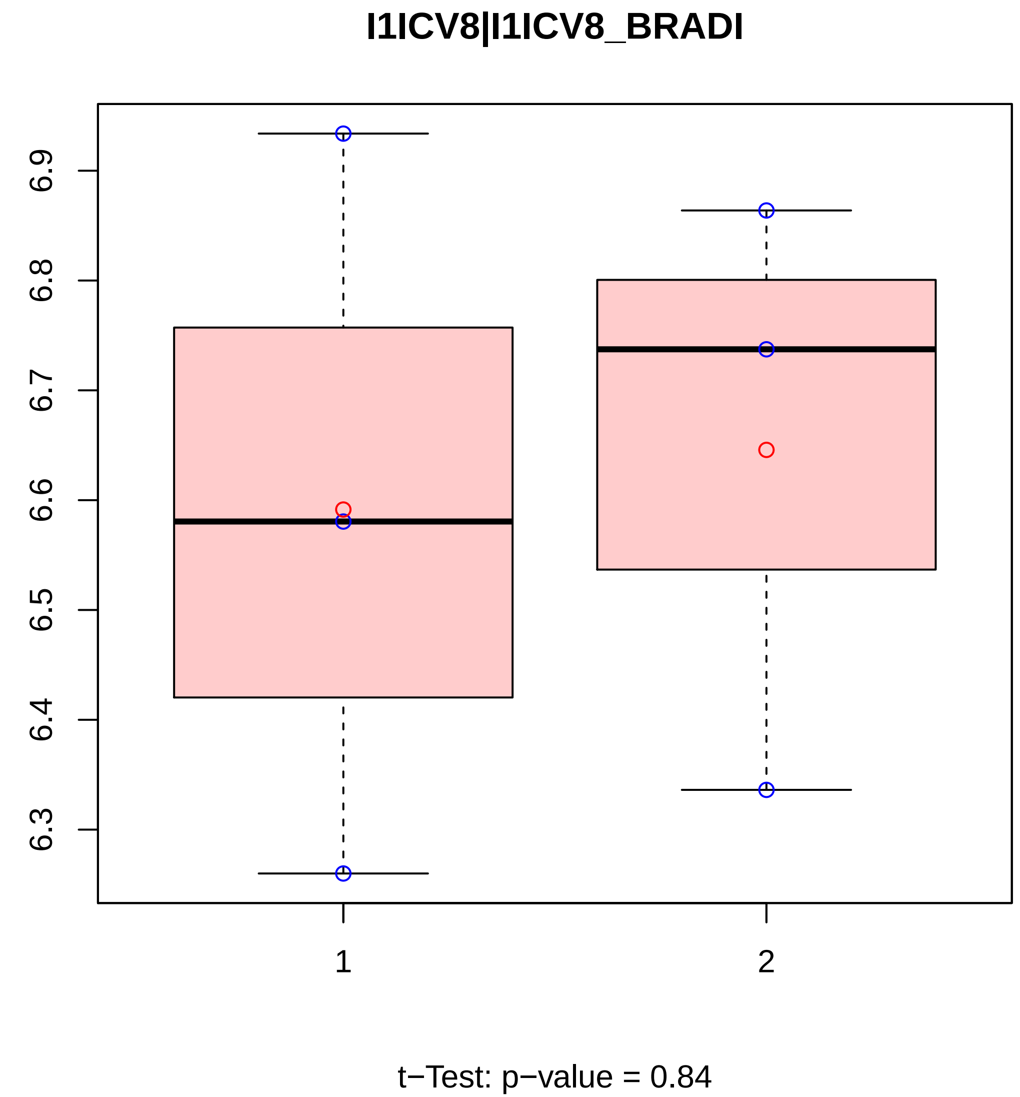
I1ICV8|I1ICV8_BRADI
6.3
6.4
6.5
6.6
6.7
6.8
6.9
1
2
t−Test: p−value = 0.84

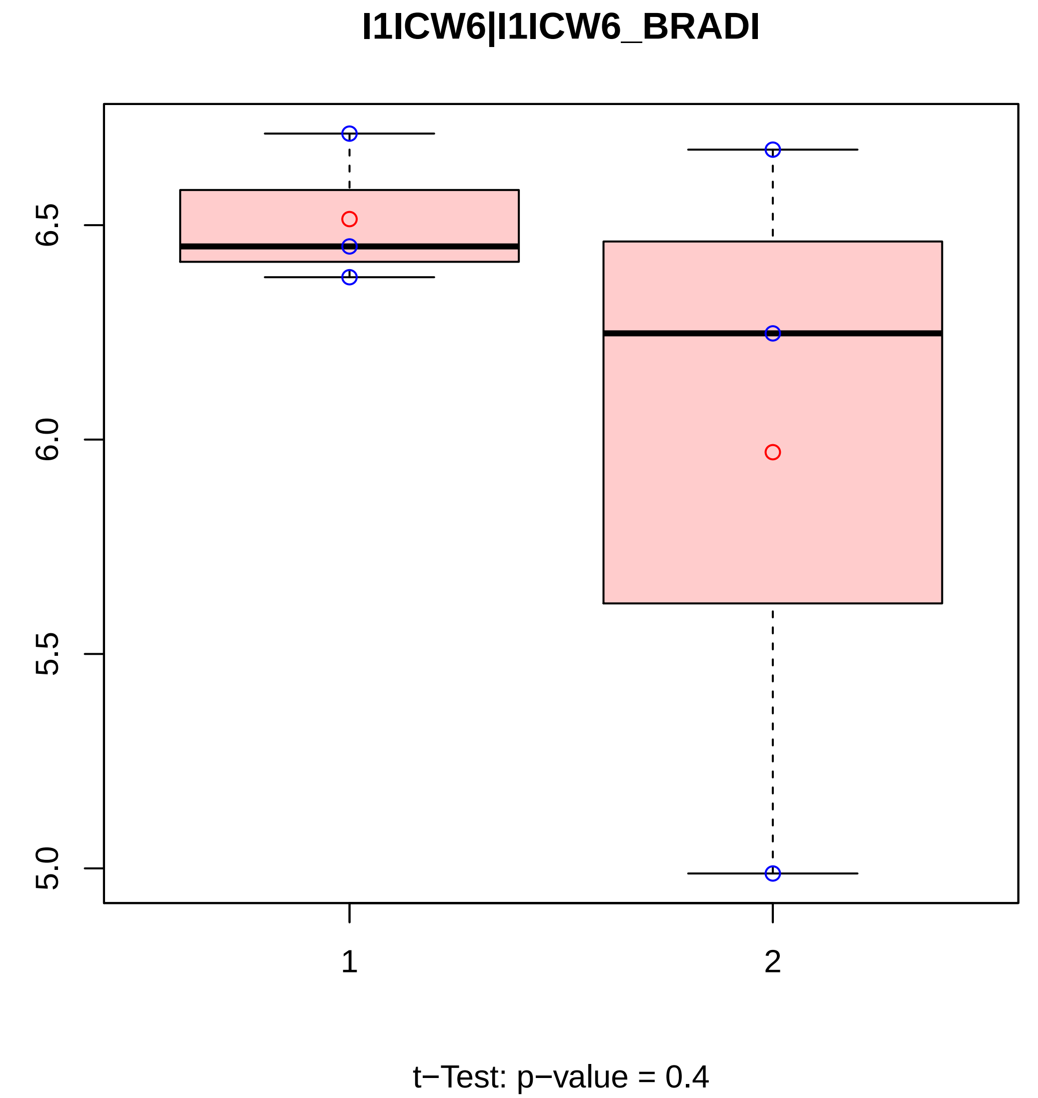
I1ICW6|I1ICW6_BRADI
6.5
6.0
5.5
5.0
1
2
t−Test: p−value = 0.4

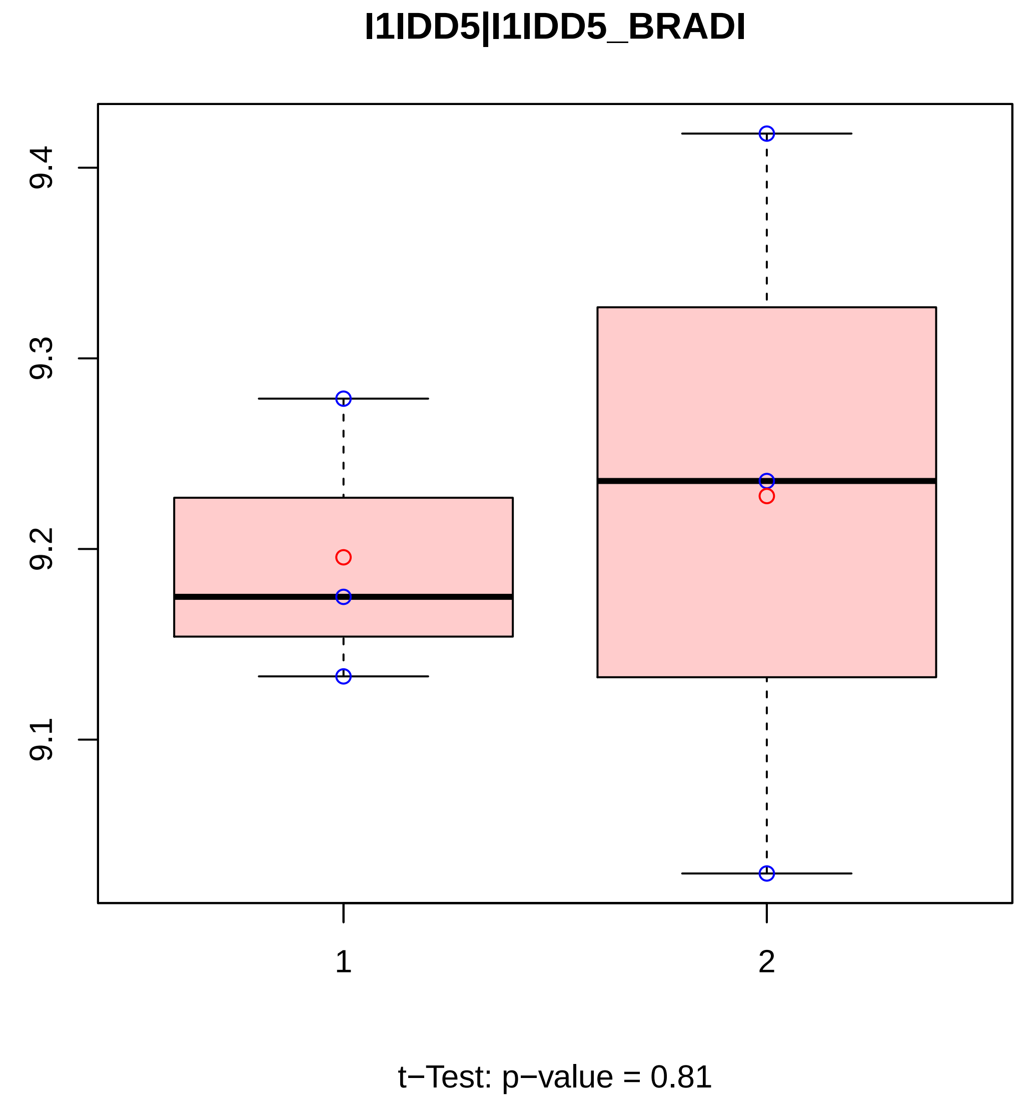
I1IDD5|I1IDD5_BRADI
9.1
9.2
9.3
9.4
1
2
t−Test: p−value = 0.81

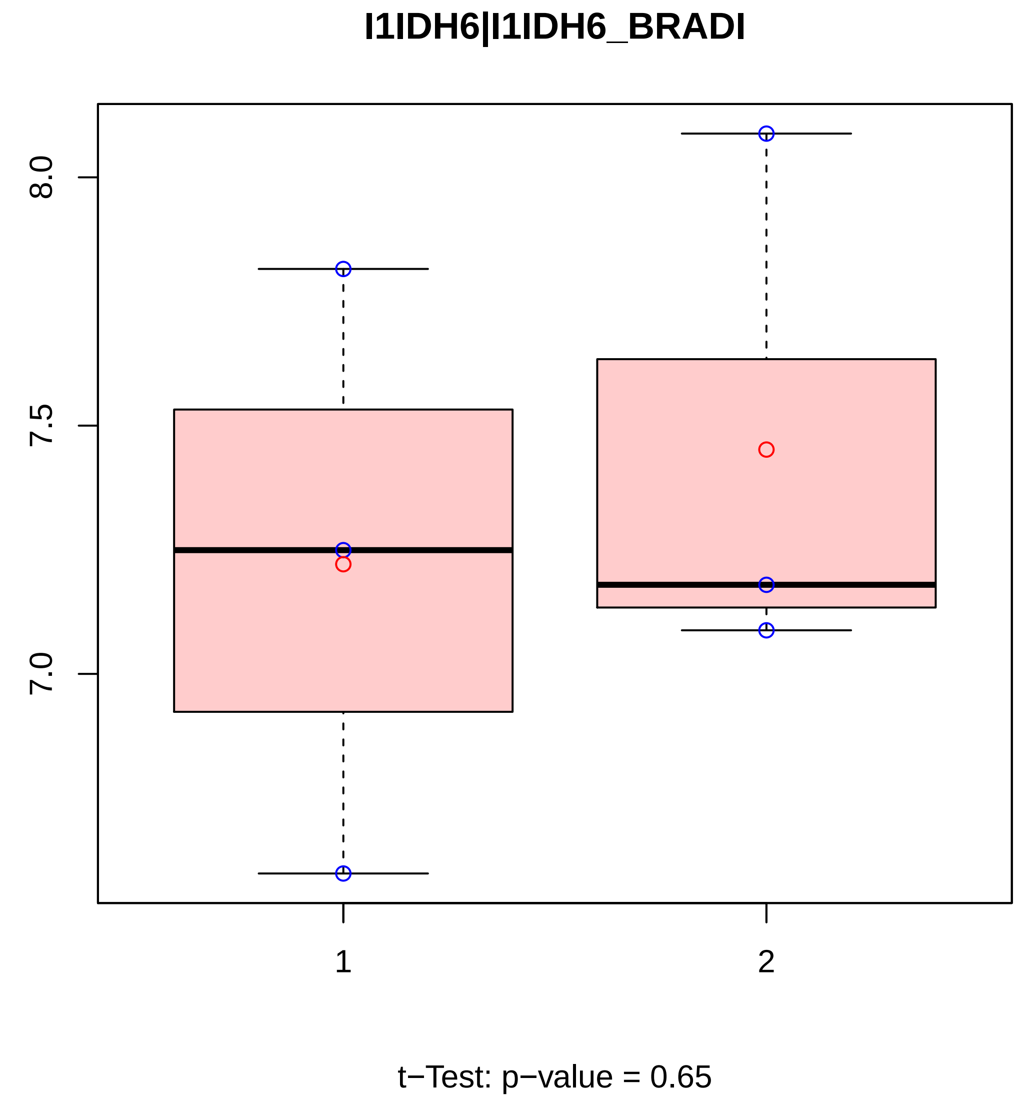
I1IDH6|I1IDH6_BRADI
8.0
7.5
7.0
1
2
t−Test: p−value = 0.65

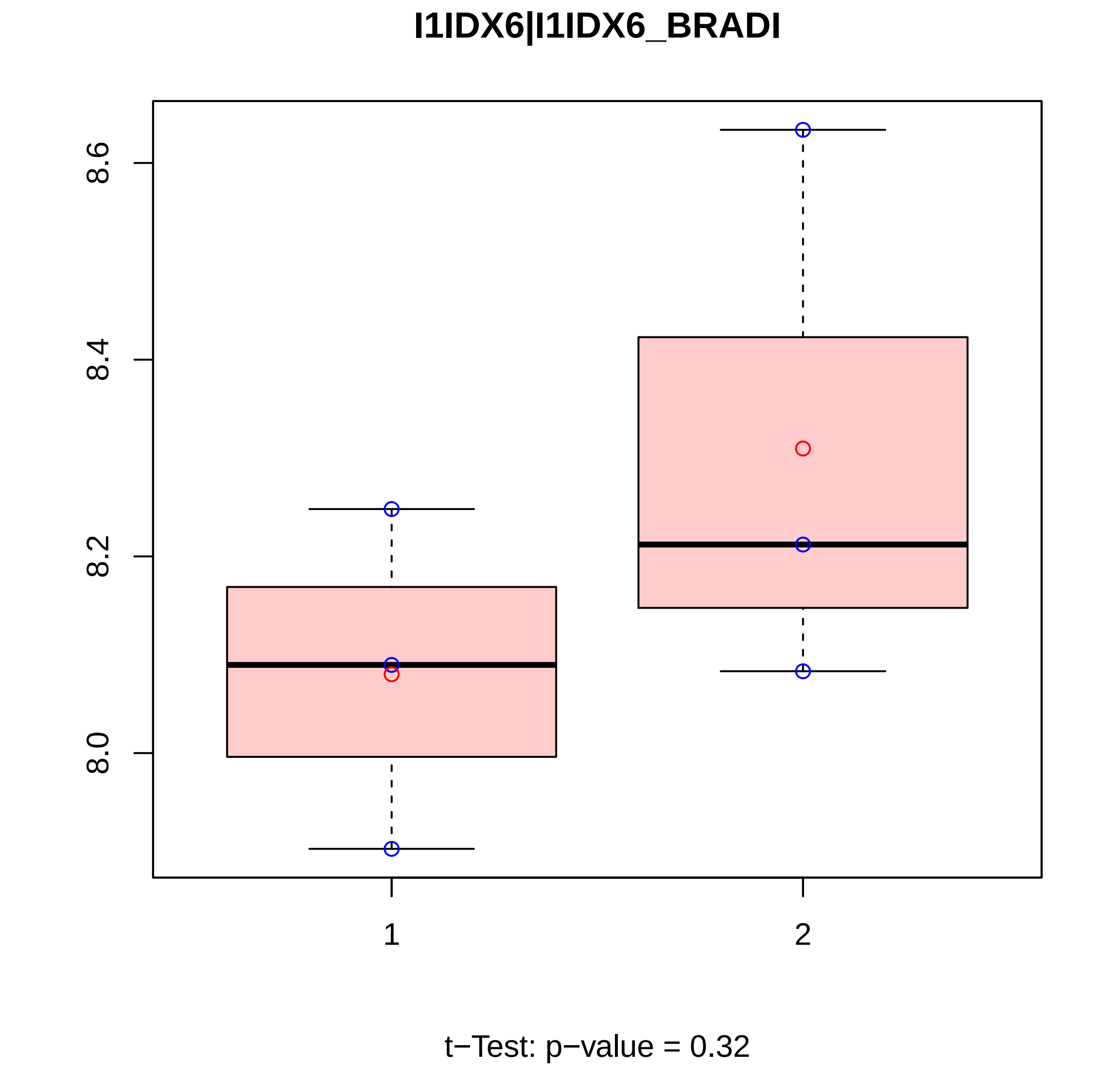
I1IDX6|I1IDX6_BRADI
8.0
8.2
8.4
8.6
1
2
t−Test: p−value = 0.32

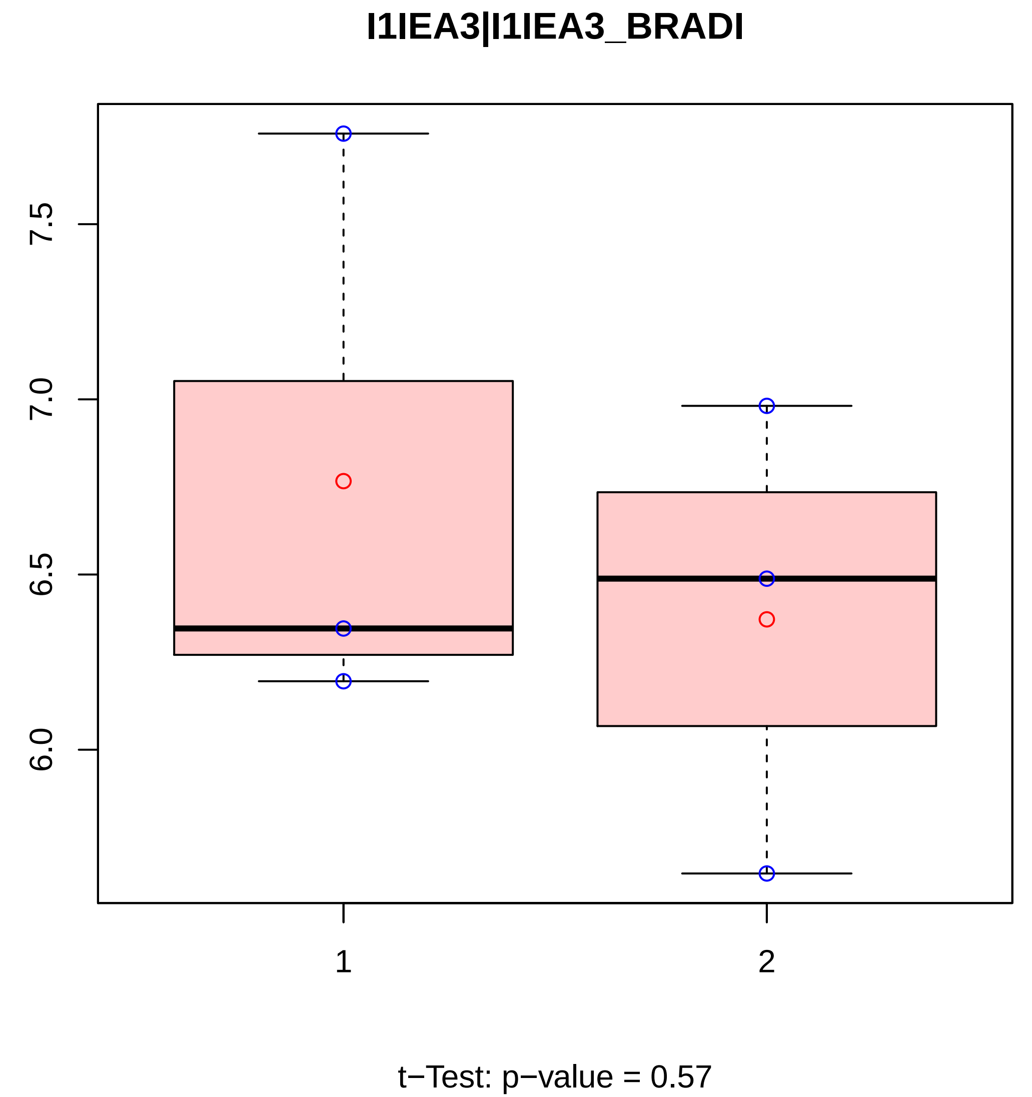
I1IEA3|I1IEA3_BRADI
7.5
7.0
6.5
6.0
1
2
t−Test: p−value = 0.57

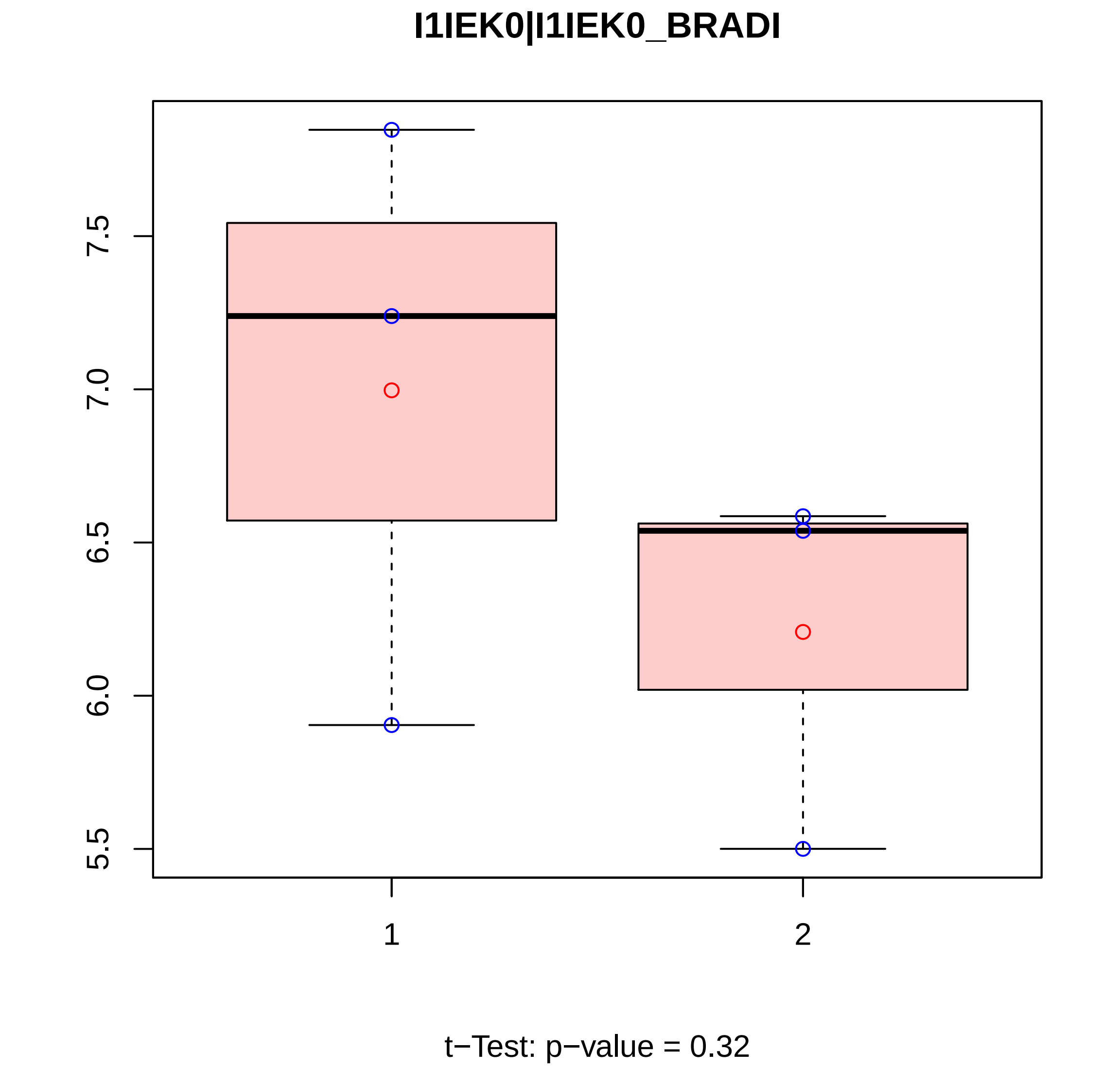
I1IEK0|I1IEK0_BRADI
7.5
7.0
6.5
6.0
5.5
1
2
t−Test: p−value = 0.32

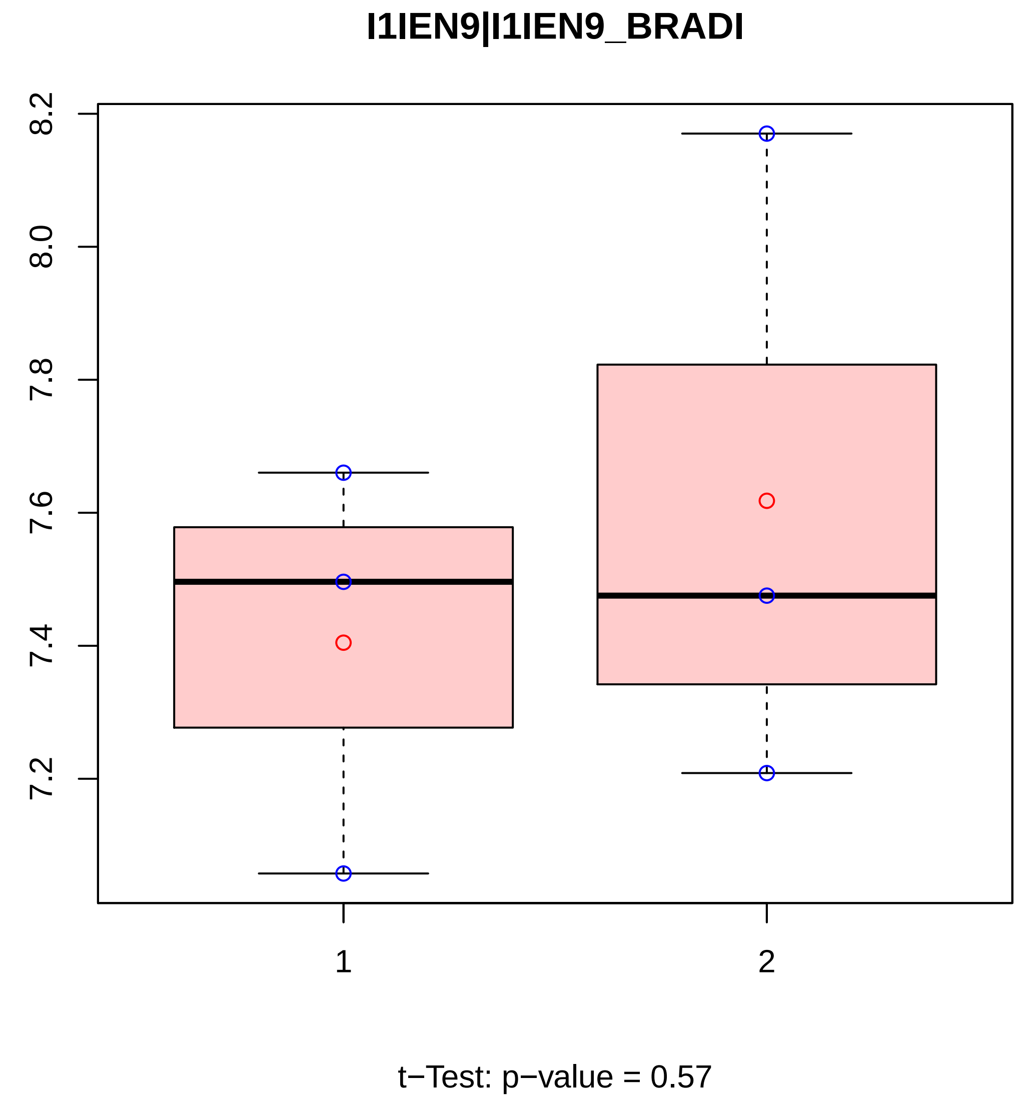
I1IEN9|I1IEN9_BRADI
8.2
8.0
7.8
7.6
7.4
7.2
1
2
t−Test: p−value = 0.57

I1IES0|I1IES0_BRADI

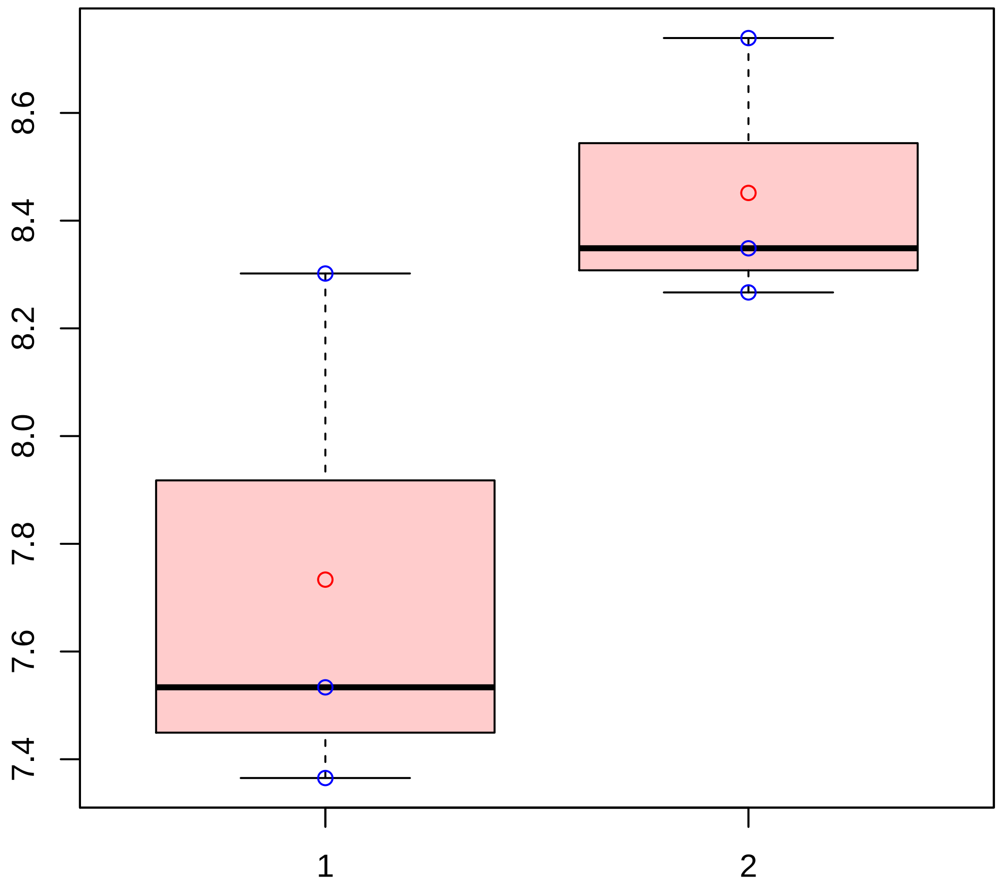

t−Test: p−value = 0.11

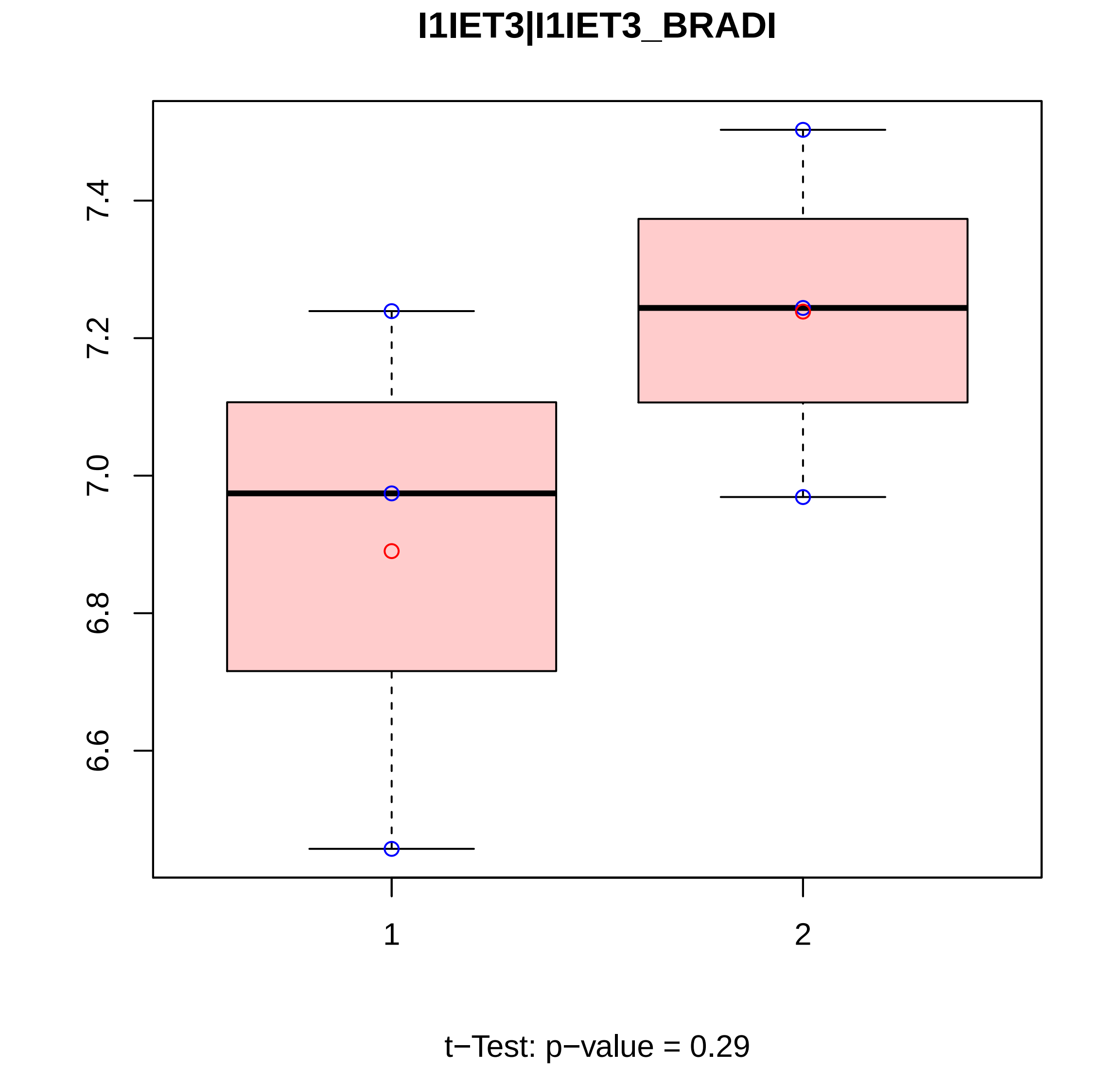
I1IET3|I1IET3_BRADI
7.4
7.2
7.0
6.8
6.6
1
2
t−Test: p−value = 0.29

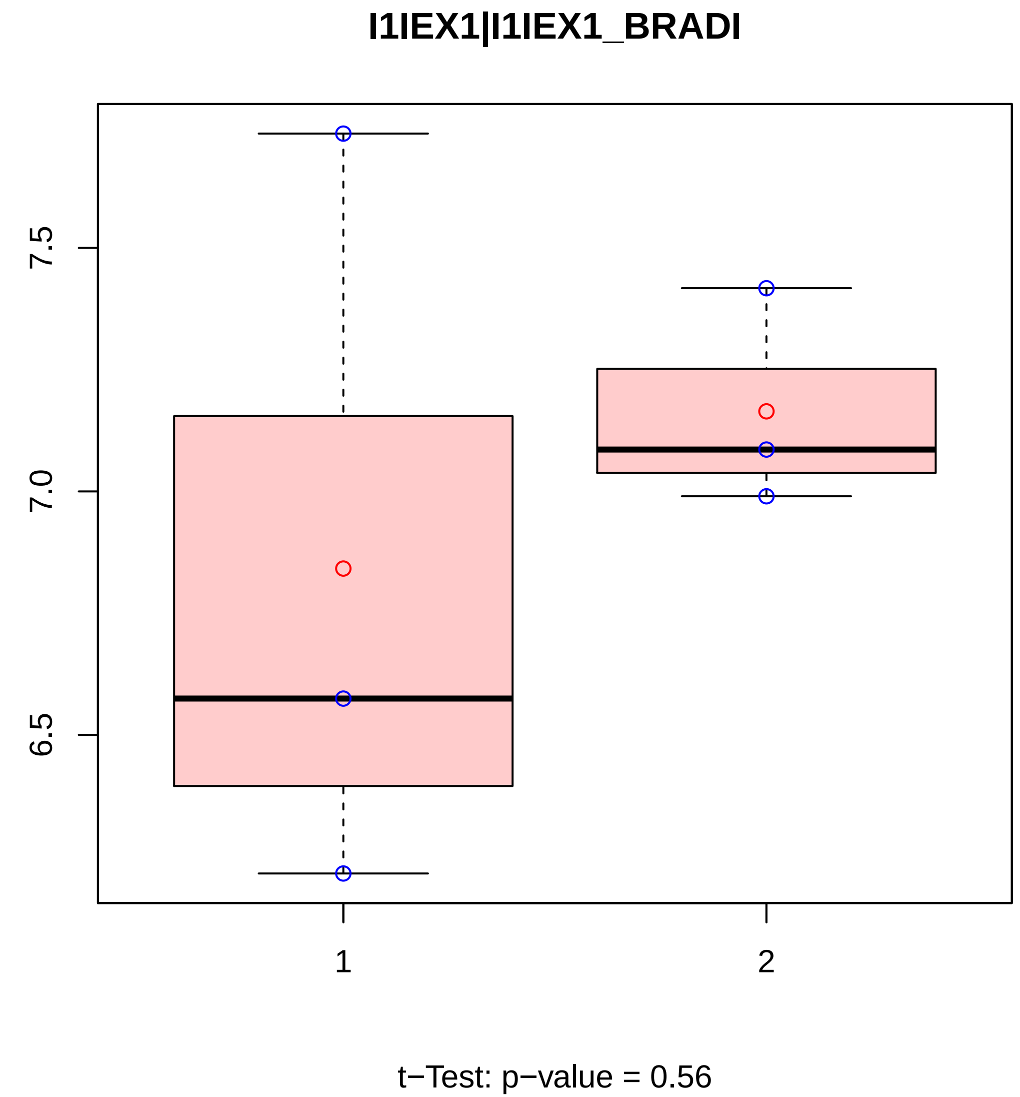
I1IEX1|I1IEX1_BRADI
7.5
7.0
6.5
1
2
t−Test: p−value = 0.56

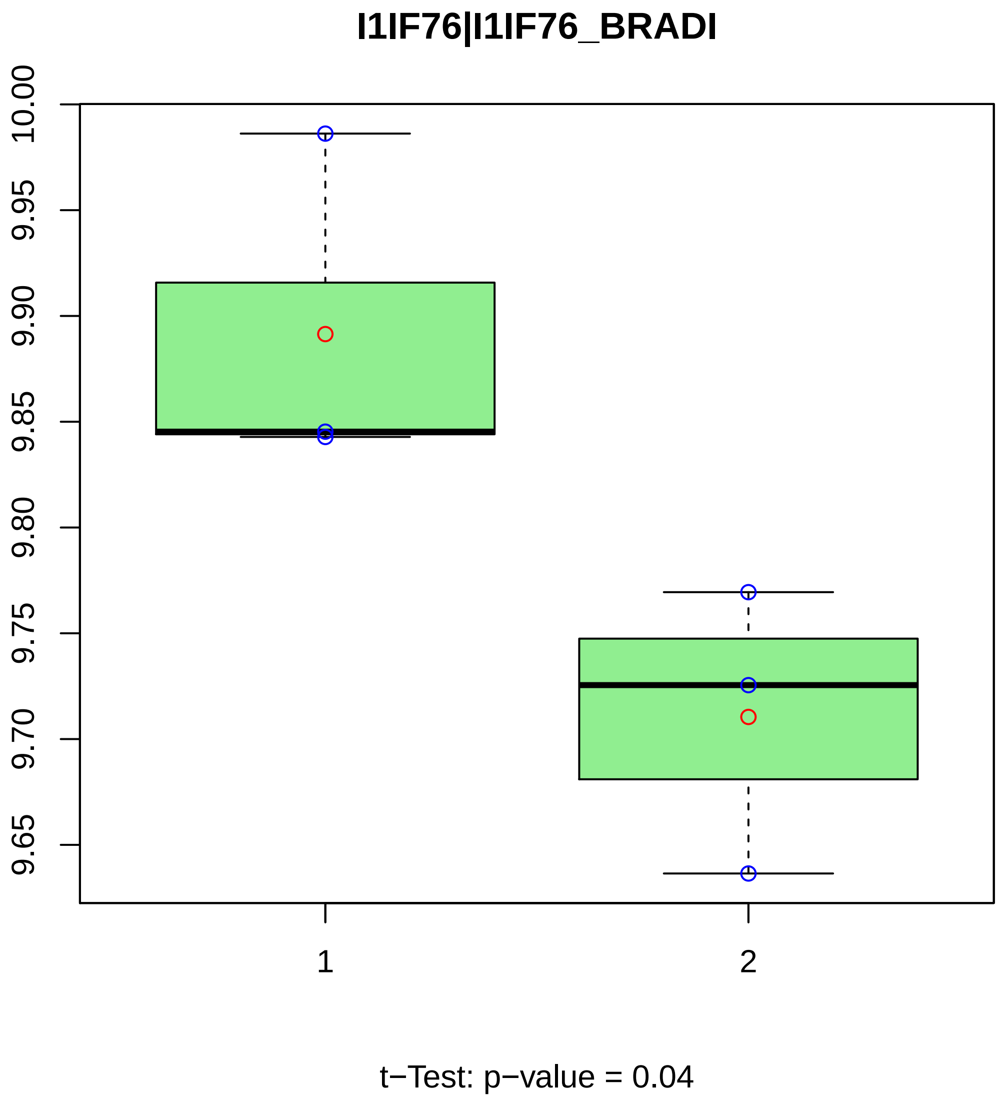

I1IF76|I1IF76_BRADI
10.00
9.95
9.90
9.85
9.80
9.75
9.70
9.65
1
2
t−Test: p−value = 0.04

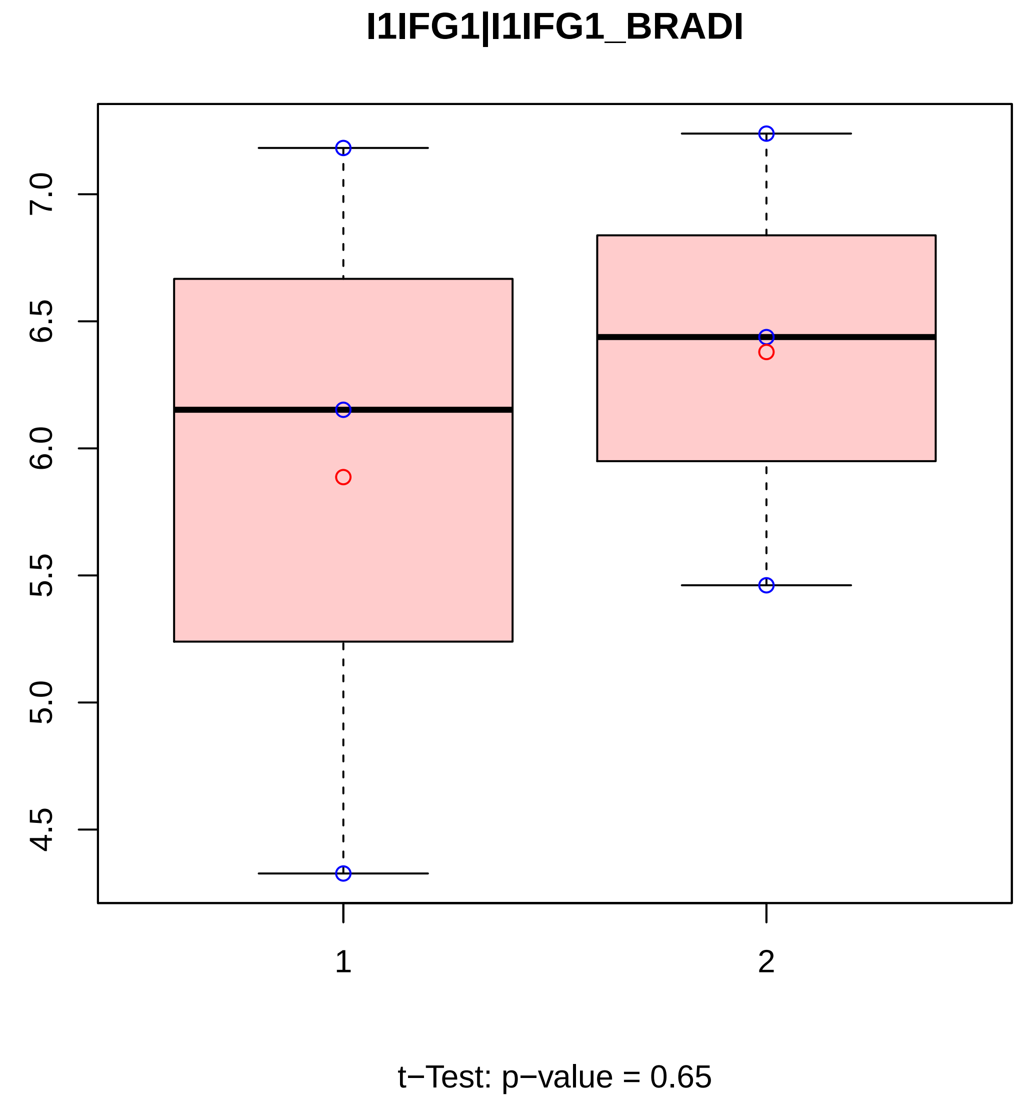
I1IFG1|I1IFG1_BRADI
7.0
6.5
6.0
5.5
5.0
4.5
1
2
t−Test: p−value = 0.65

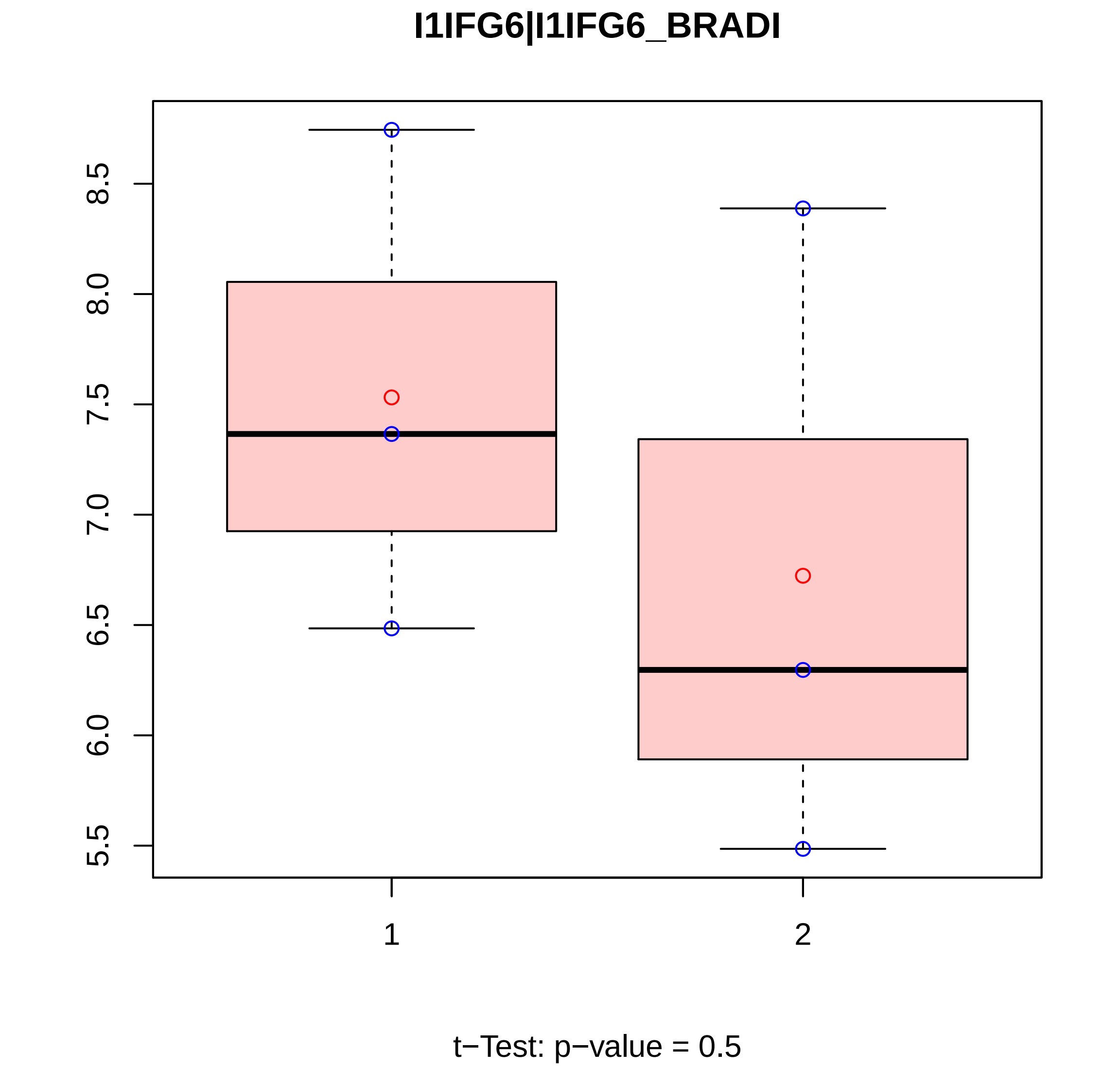
I1IFG6|I1IFG6_BRADI
8.5
8.0
7.5
7.0
6.5
6.0
5.5
1
2
t−Test: p−value = 0.5

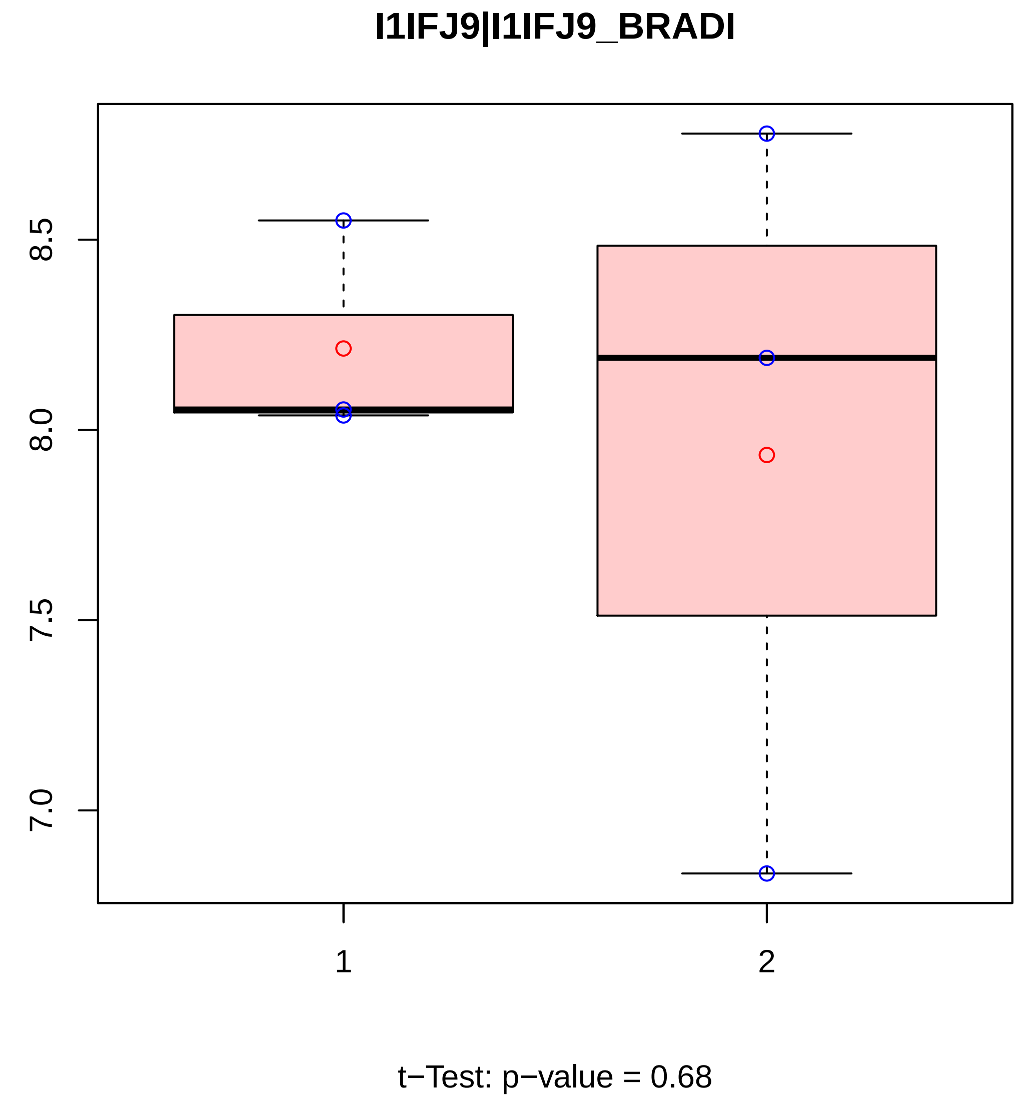
I1IFJ9|I1IFJ9_BRADI
8.5
8.0
7.5
7.0
1
2
t−Test: p−value = 0.68

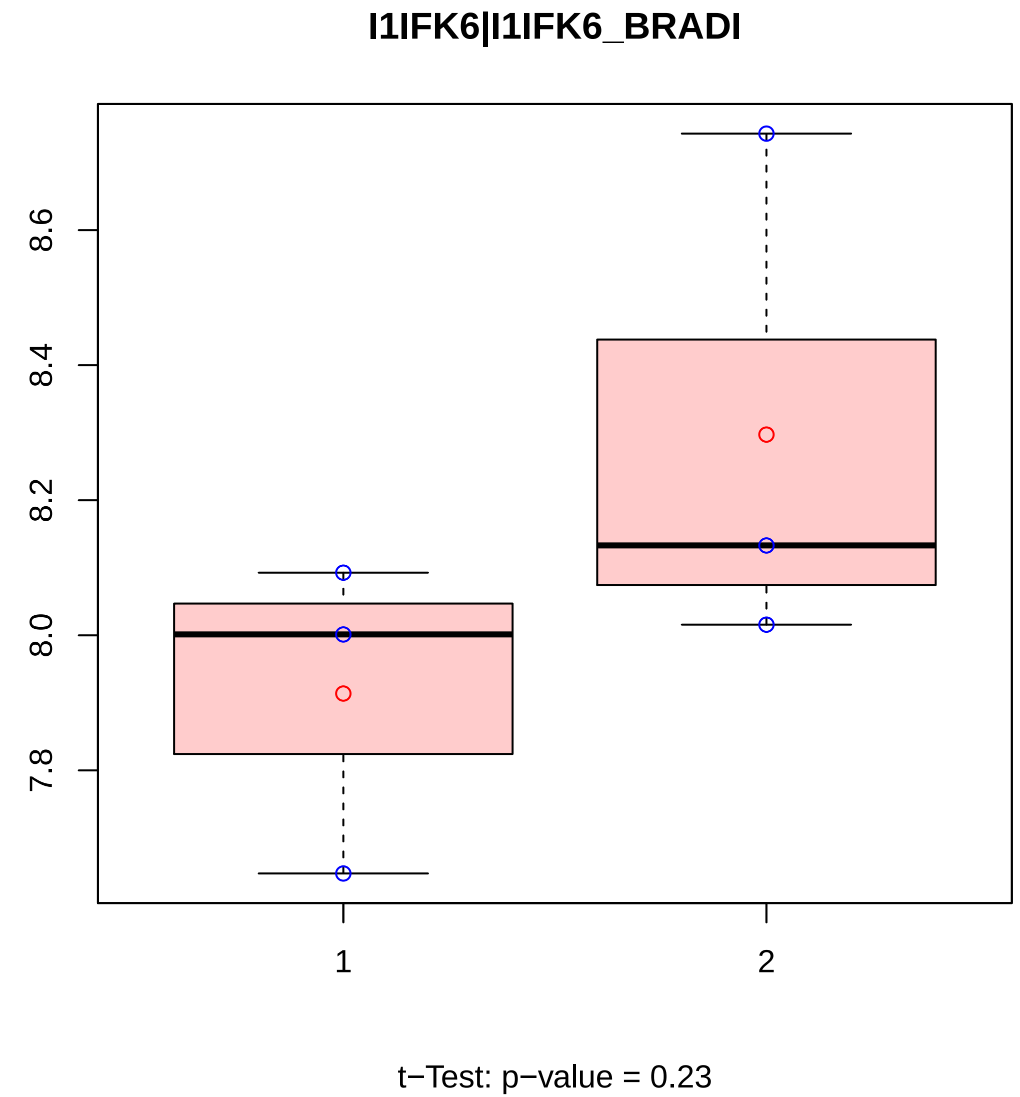
I1IFK6|I1IFK6_BRADI
8.6
8.4
8.2
8.0
7.8
1
2
t−Test: p−value = 0.23

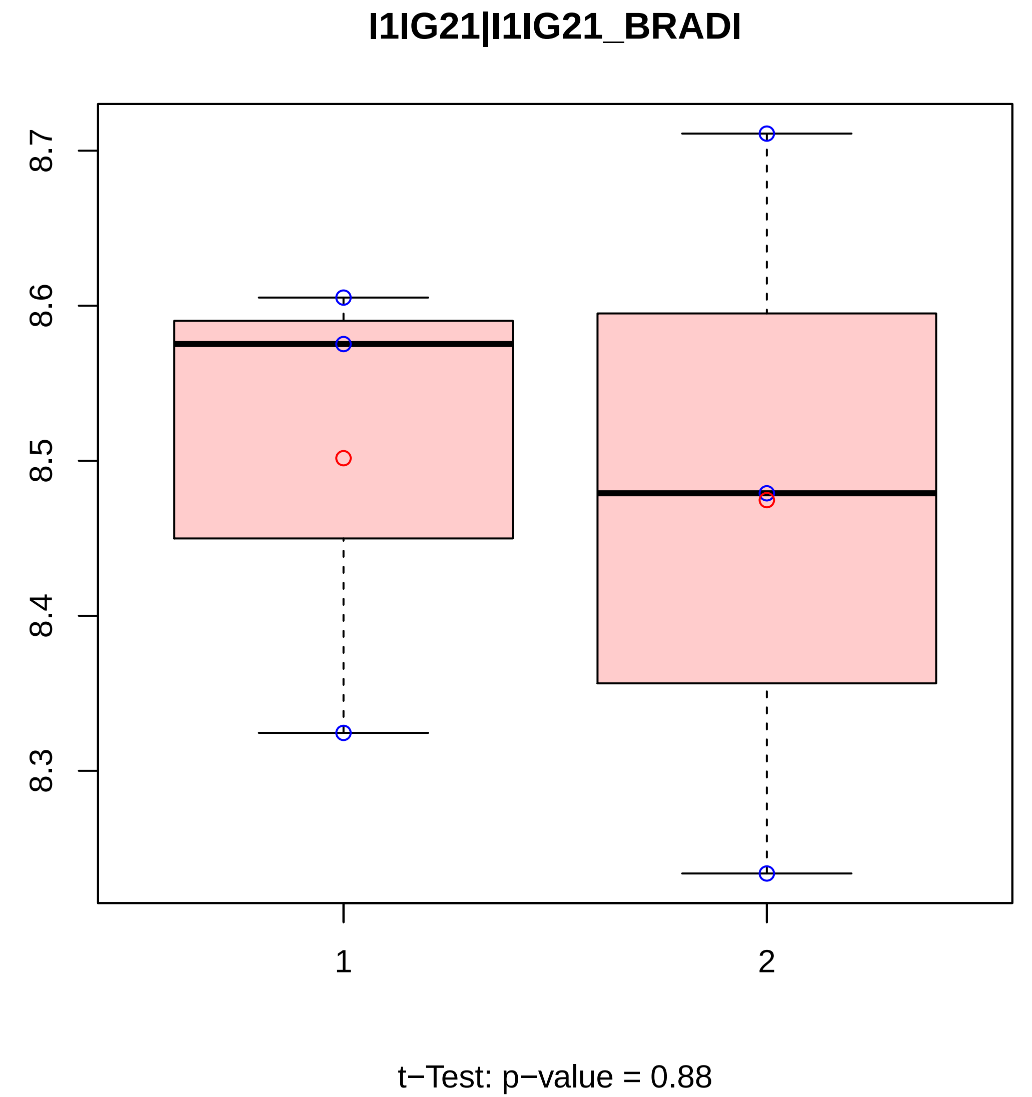
I1IG21|I1IG21_BRADI
8.7
8.6
8.5
8.4
8.3
1
2
t−Test: p−value = 0.88

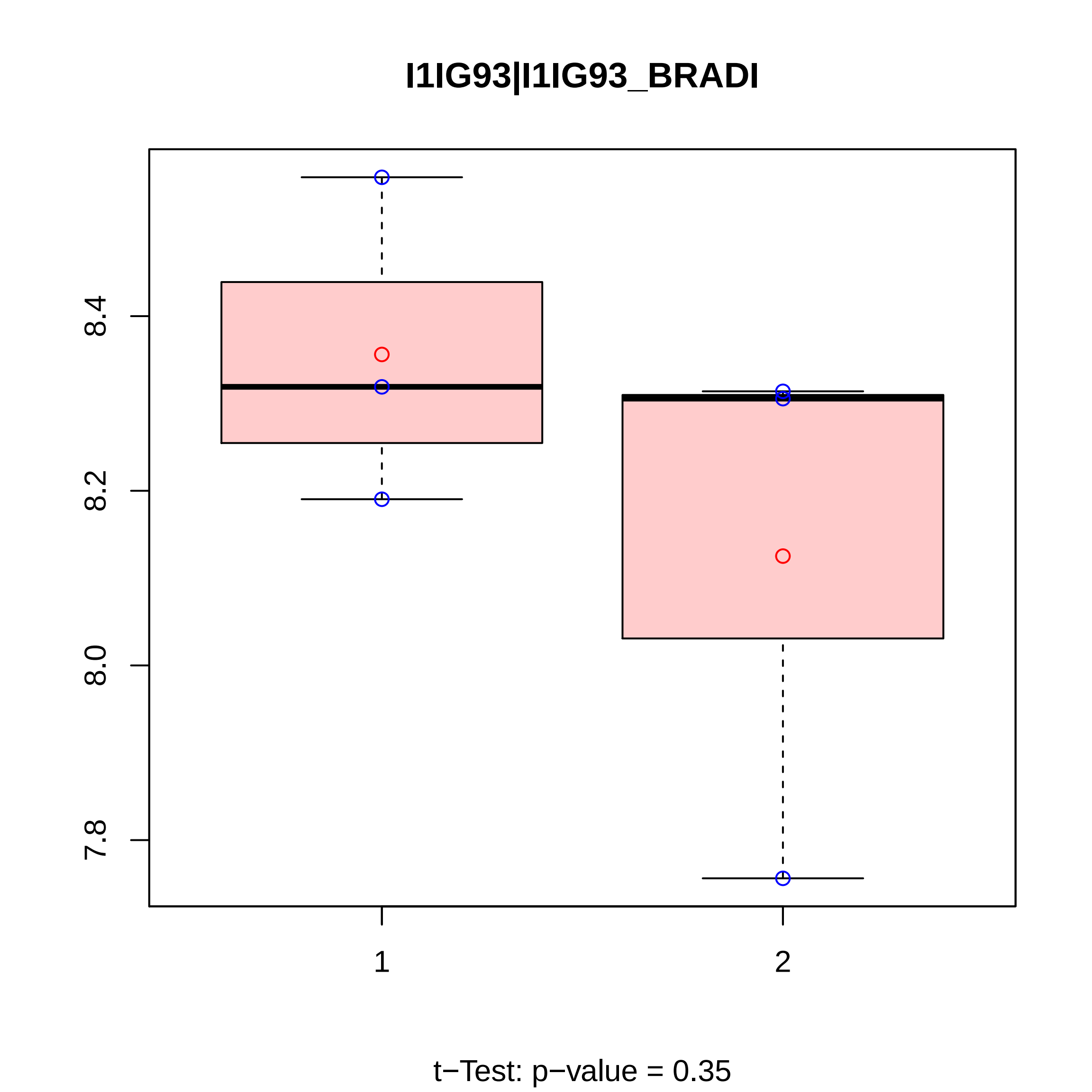
I1IG93|I1IG93_BRADI
8.4
8.2
8.0
7.8
1
2
t−Test: p−value = 0.35

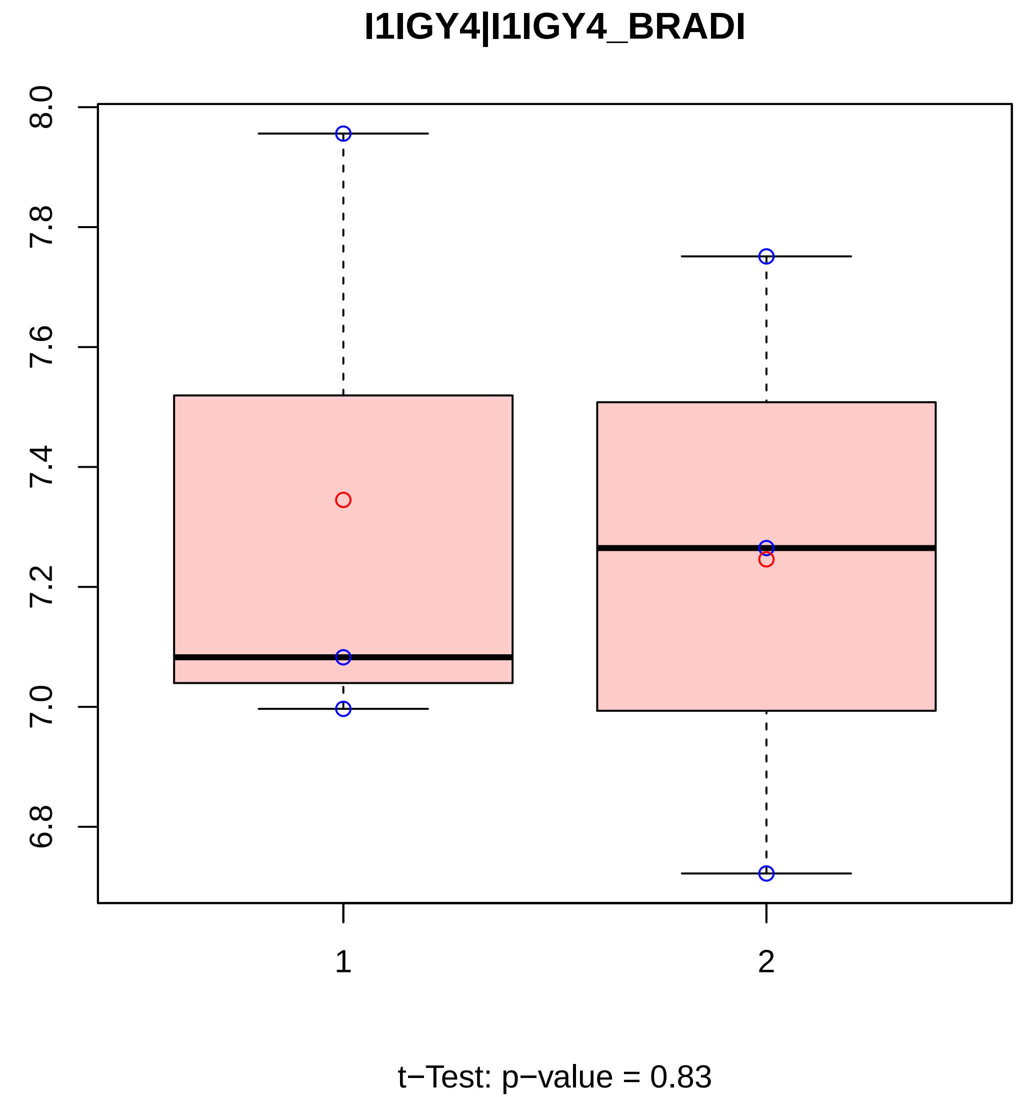
I1IGY4|I1IGY4_BRADI
8.0
7.8
7.6
7.4
7.2
7.0
6.8
1
2
t−Test: p−value = 0.83

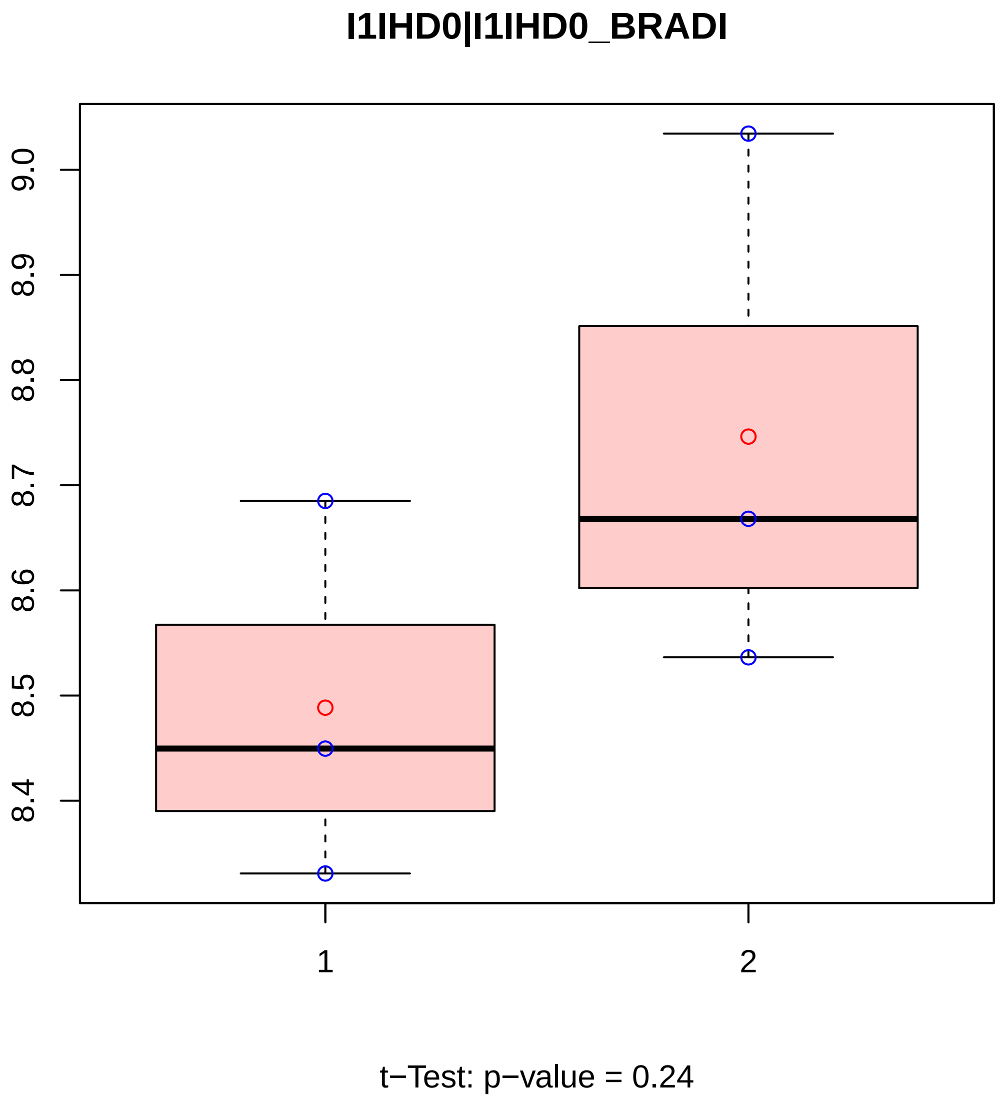
I1IHD0|I1IHD0_BRADI
9.0
8.9
8.8
8.7
8.6
8.5
8.4
1
2
t−Test: p−value = 0.24

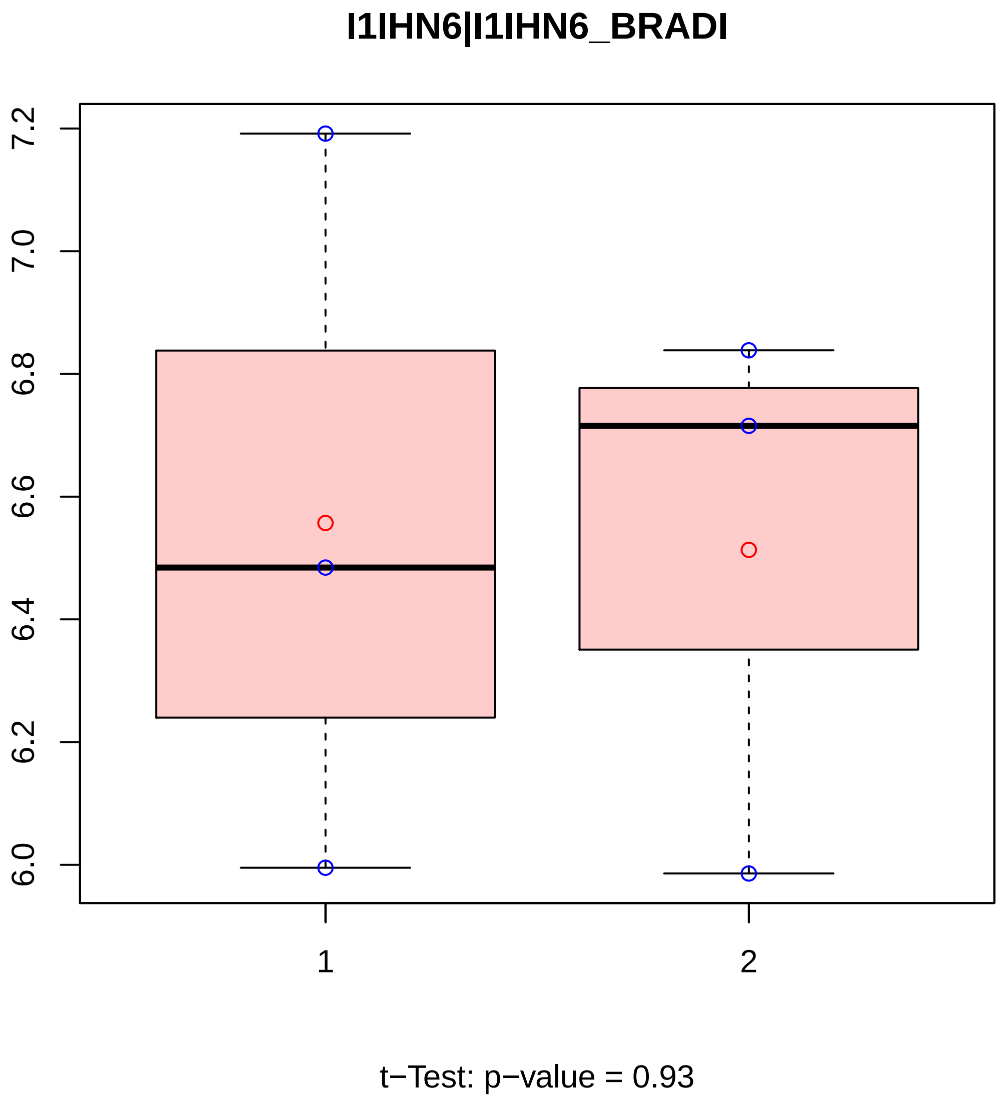
I1IHN6|I1IHN6_BRADI
7.2
7.0
6.8
6.6
6.4
6.2
6.0
1
2
t−Test: p−value = 0.93

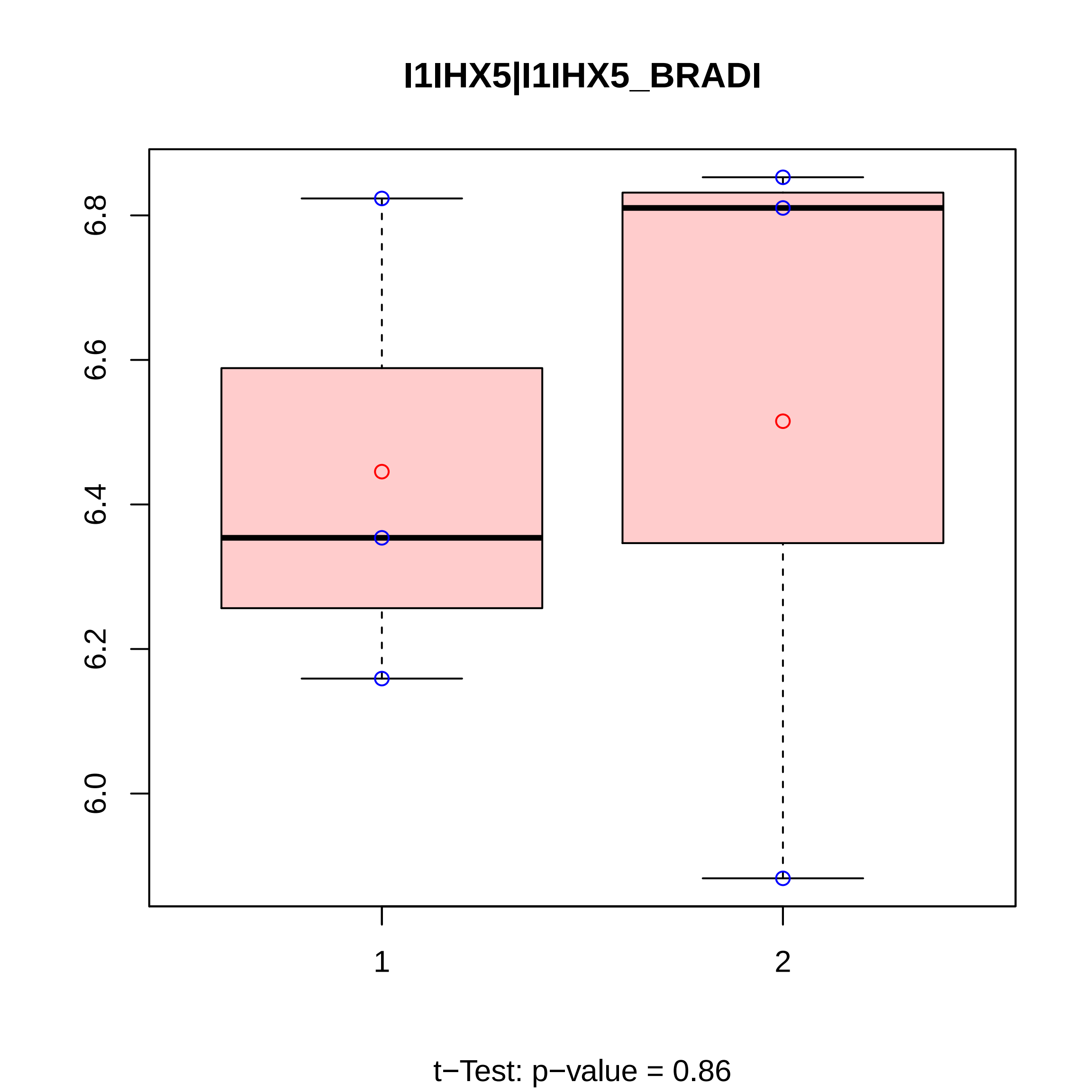
I1IHX5|I1IHX5_BRADI
6.0
6.2
6.4
6.6
6.8
1
2
t−Test: p−value = 0.86

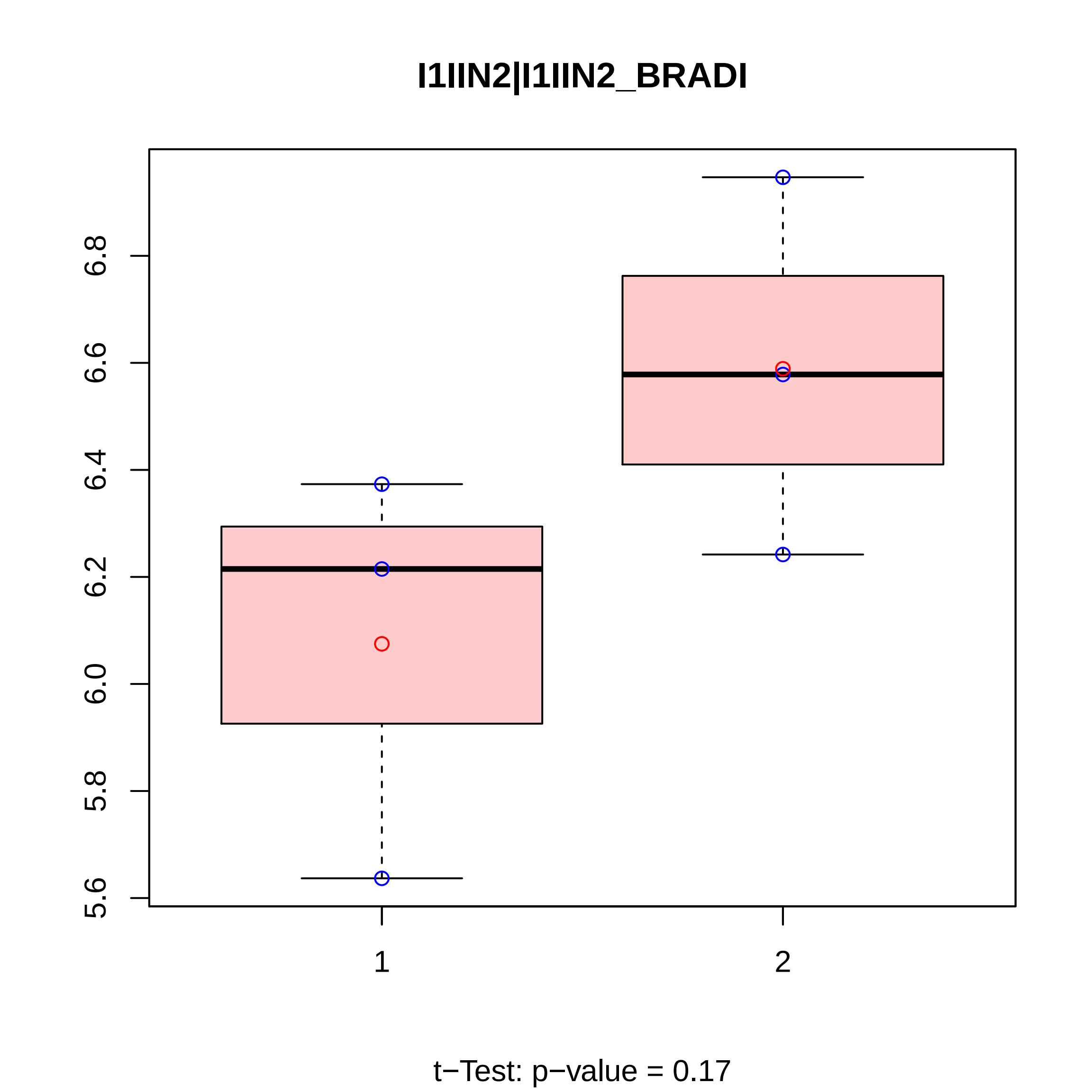
I1IIN2|I1IIN2_BRADI
5.6
5.8
6.0
6.2
6.4
6.6
6.8
1
2
t−Test: p−value = 0.17

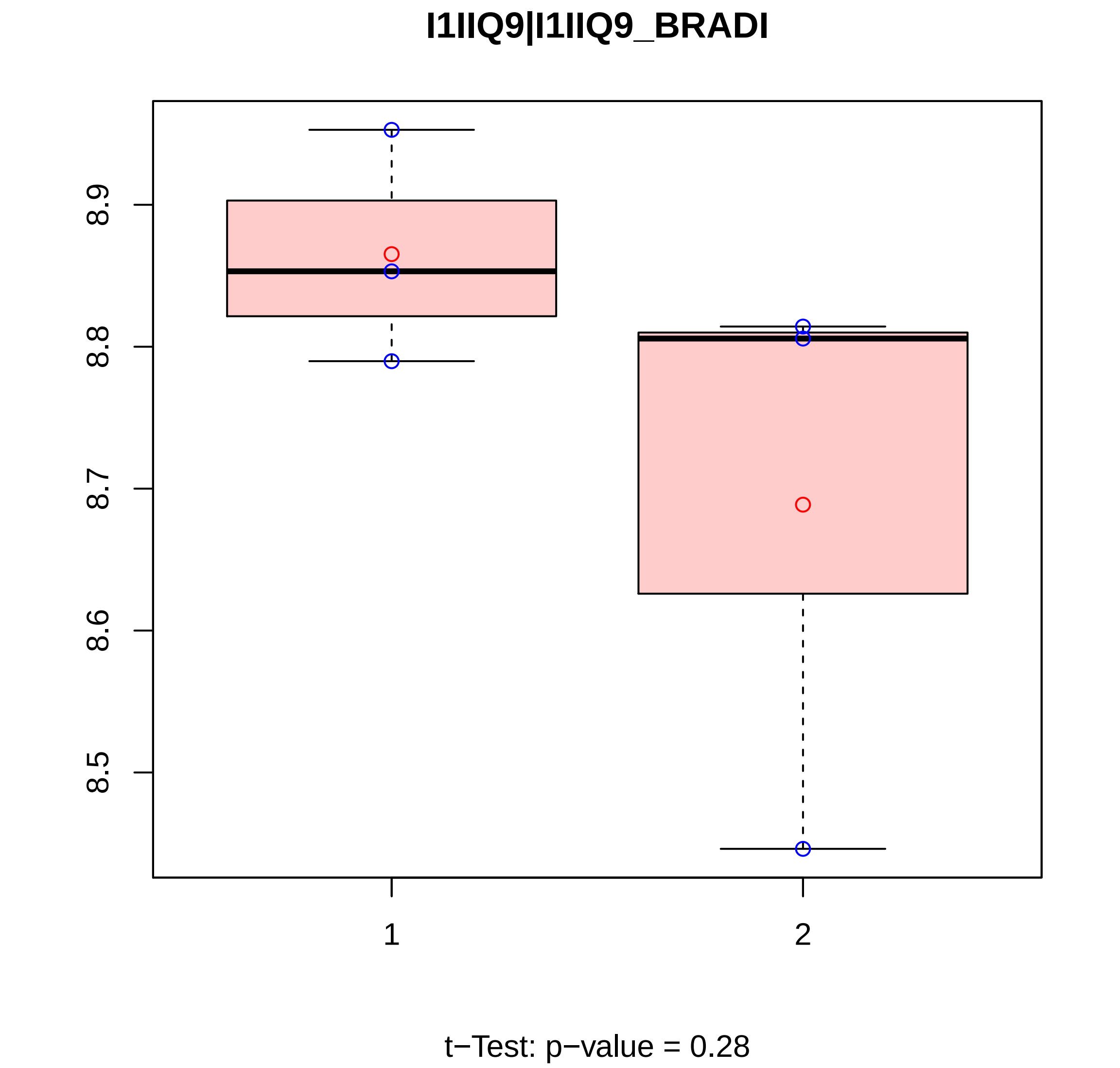
I1IIQ9|I1IIQ9_BRADI
8.5
8.6
8.7
8.8
8.9
1
2
t−Test: p−value = 0.28

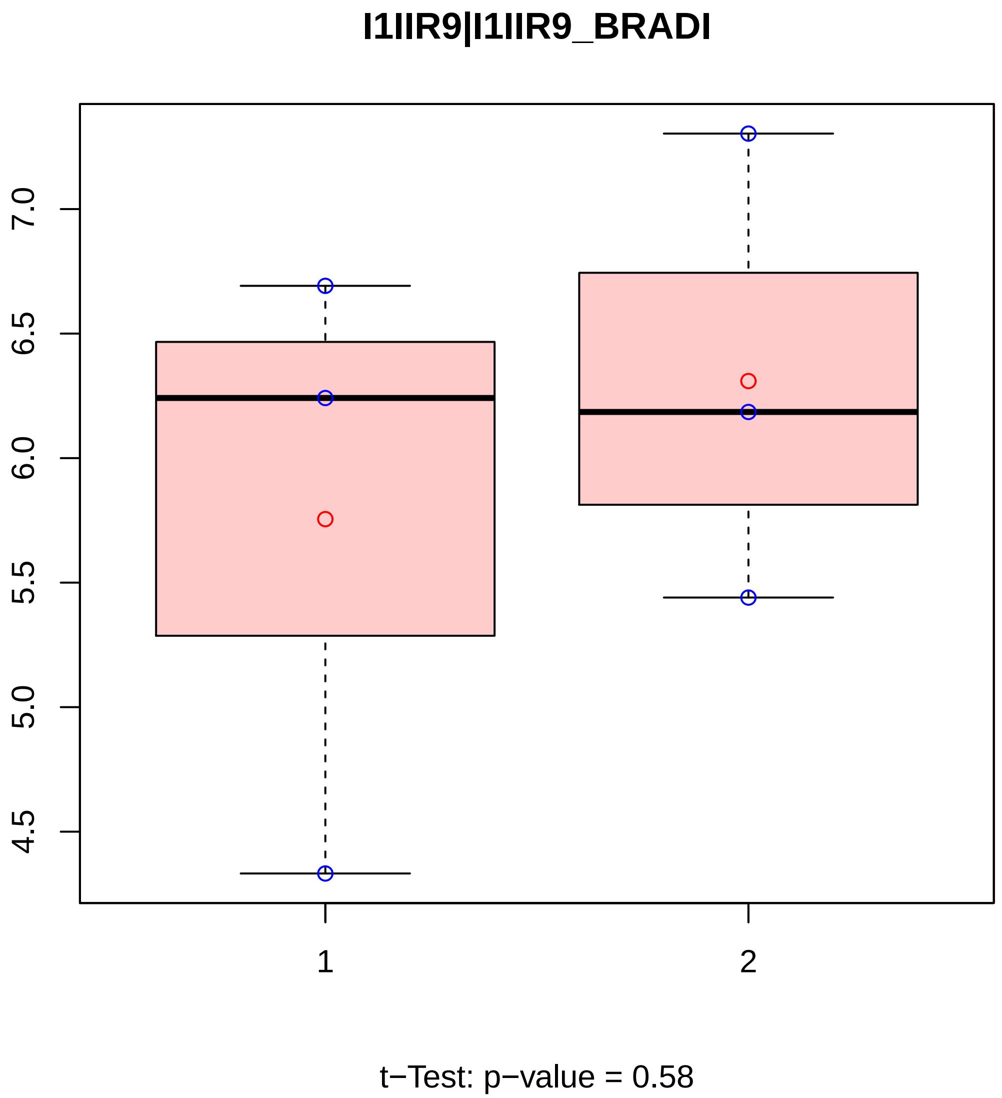
I1IIR9|I1IIR9_BRADI
7.0
6.5
6.0
5.5
5.0
4.5
1
2
t−Test: p−value = 0.58

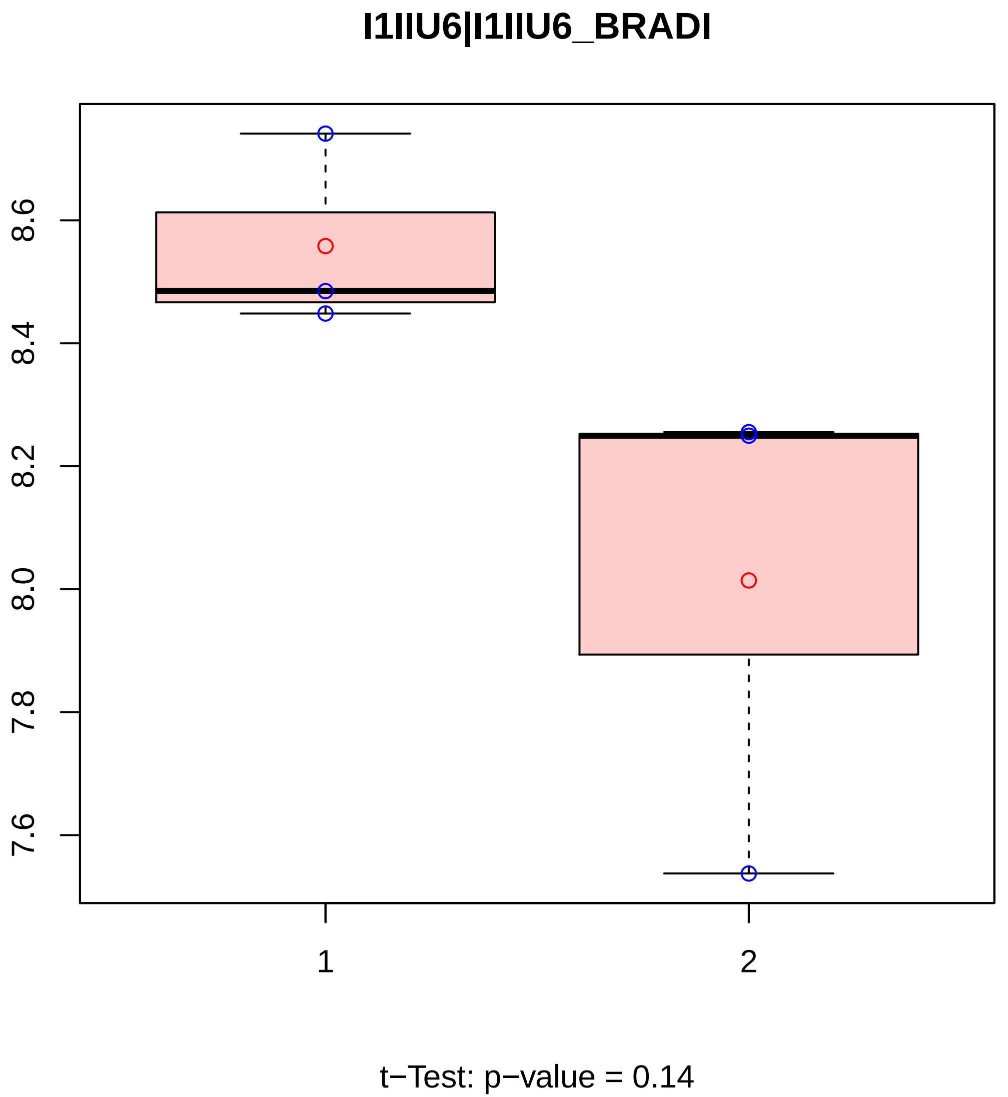
I1IIU6|I1IIU6_BRADI
8.6
8.4
8.2
8.0
7.8
7.6
1
2
t−Test: p−value = 0.14

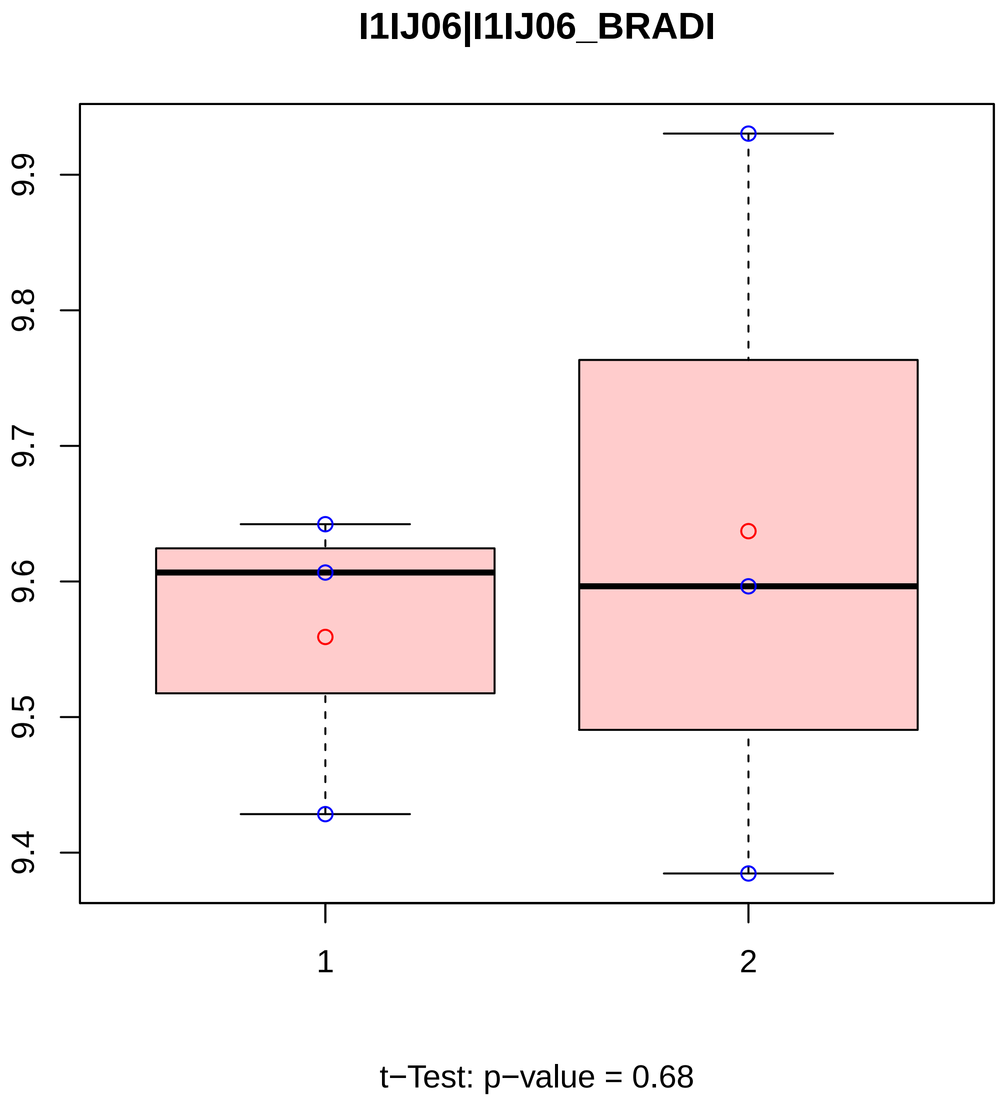
I1IJ06|I1IJ06_BRADI
9.9
9.8
9.7
9.6
9.5
9.4
1
2
t−Test: p−value = 0.68

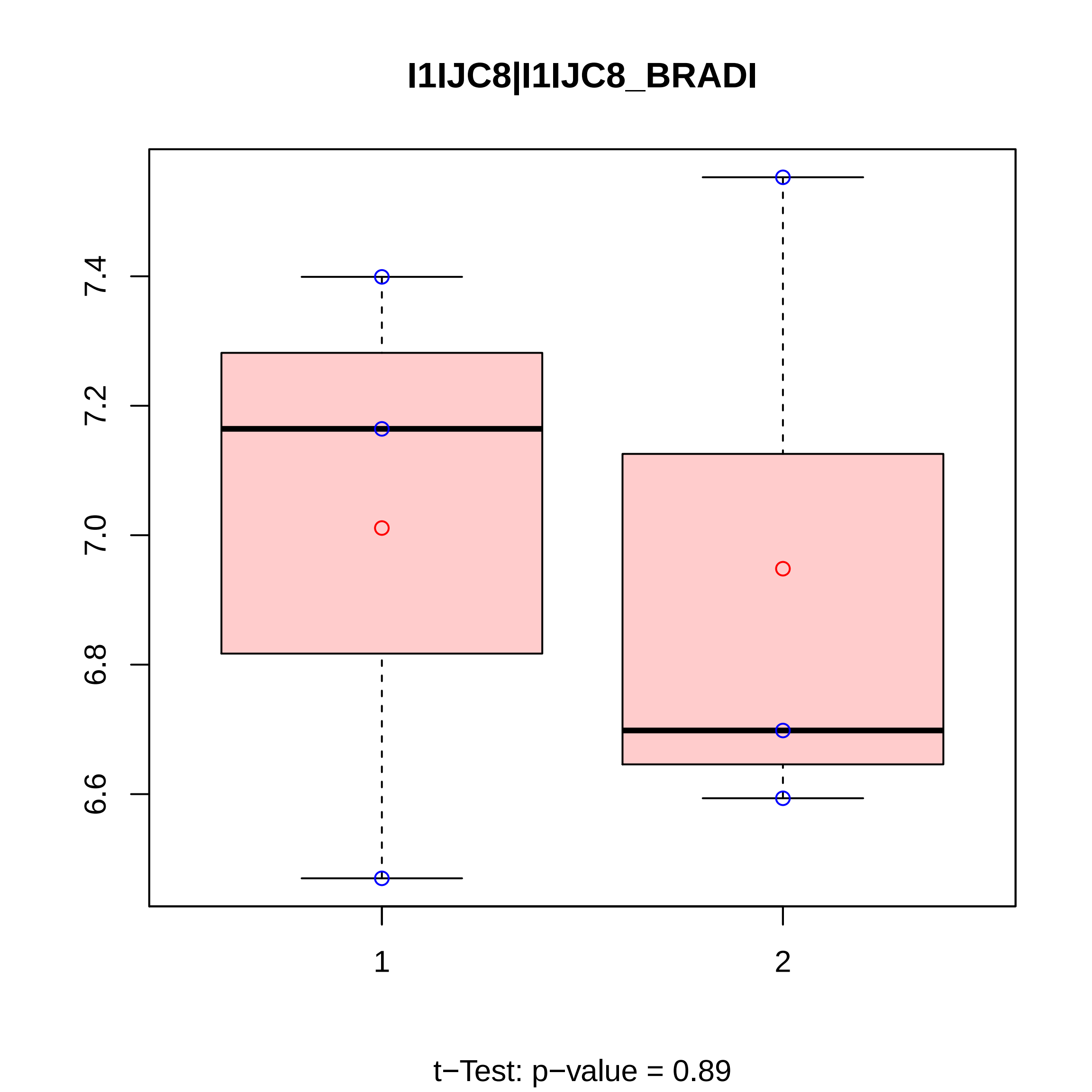
I1IJC8|I1IJC8_BRADI
7.4
7.2
7.0
6.8
6.6
1
2
t−Test: p−value = 0.89

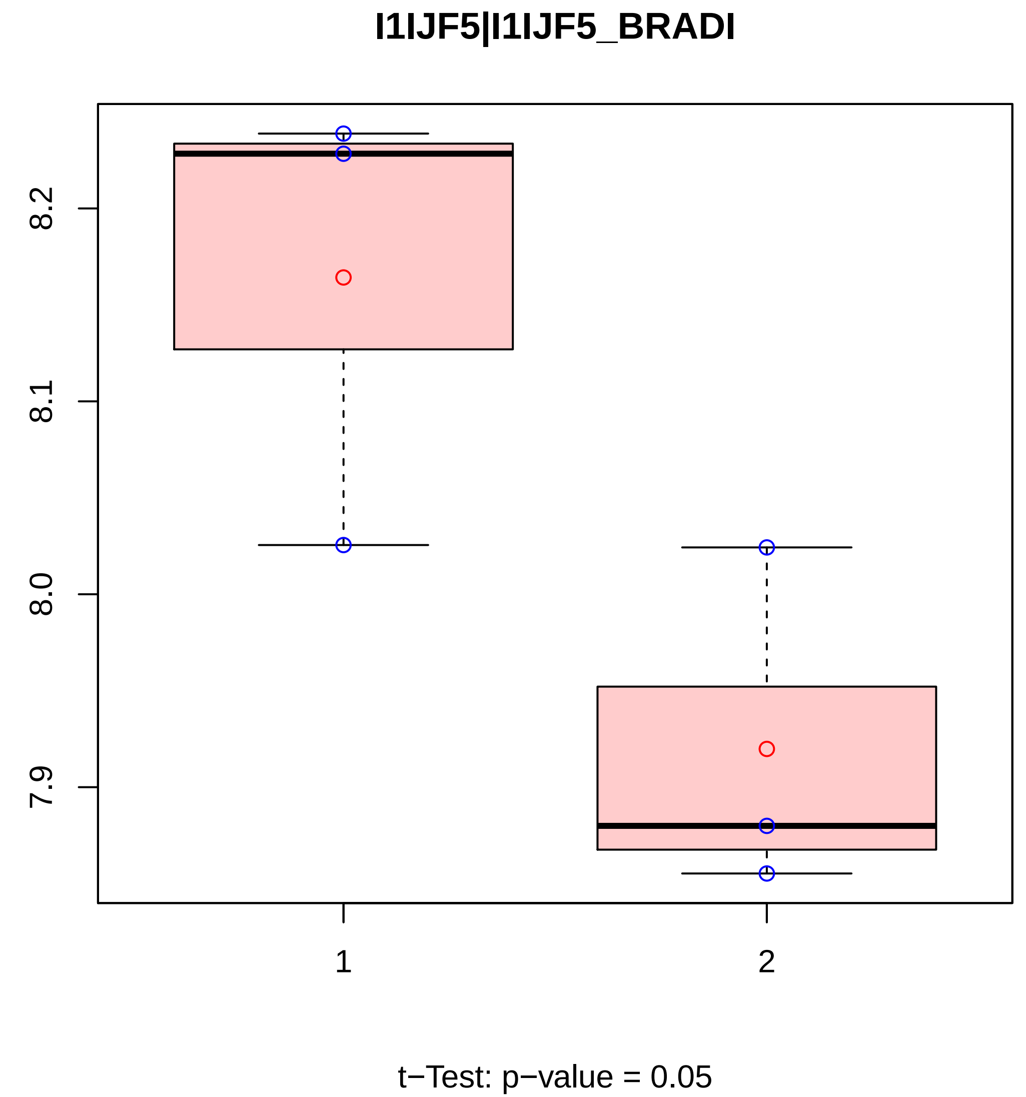
I1IJF5|I1IJF5_BRADI
8.2
8.1
8.0
7.9
1
2
t−Test: p−value = 0.05

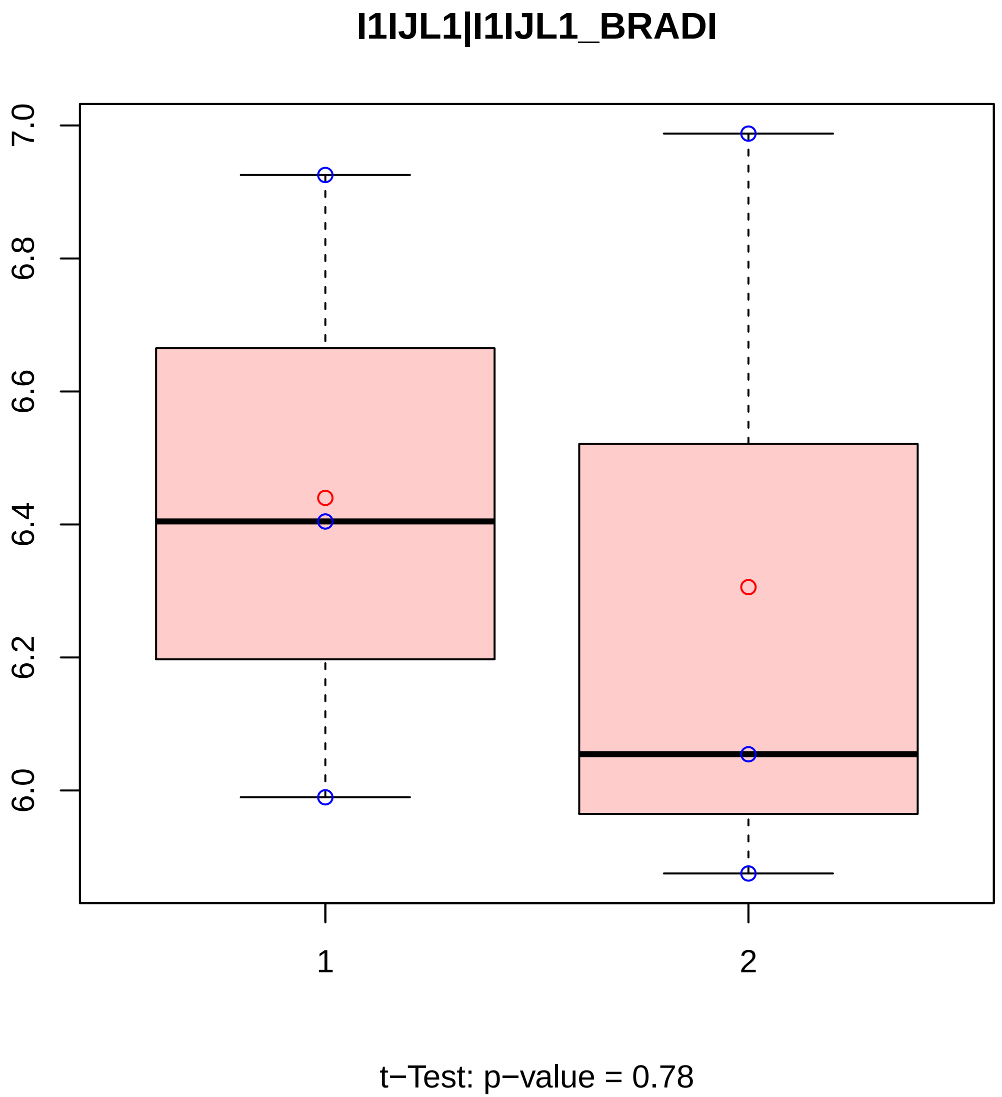

I1IJL1|I1IJL1_BRADI
7.0
6.8
6.6
6.4
6.2
6.0
1
2
t−Test: p−value = 0.78

I1IJW0|I1IJW0_BRADI

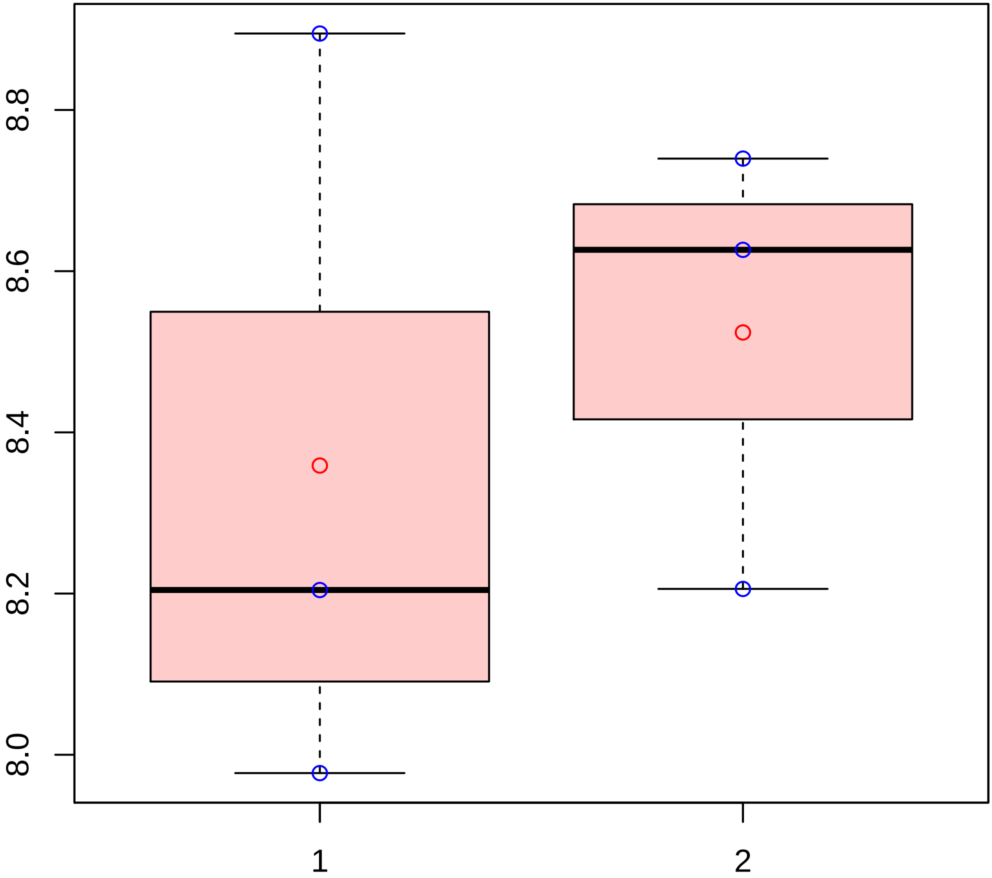

t−Test: p−value = 0.64

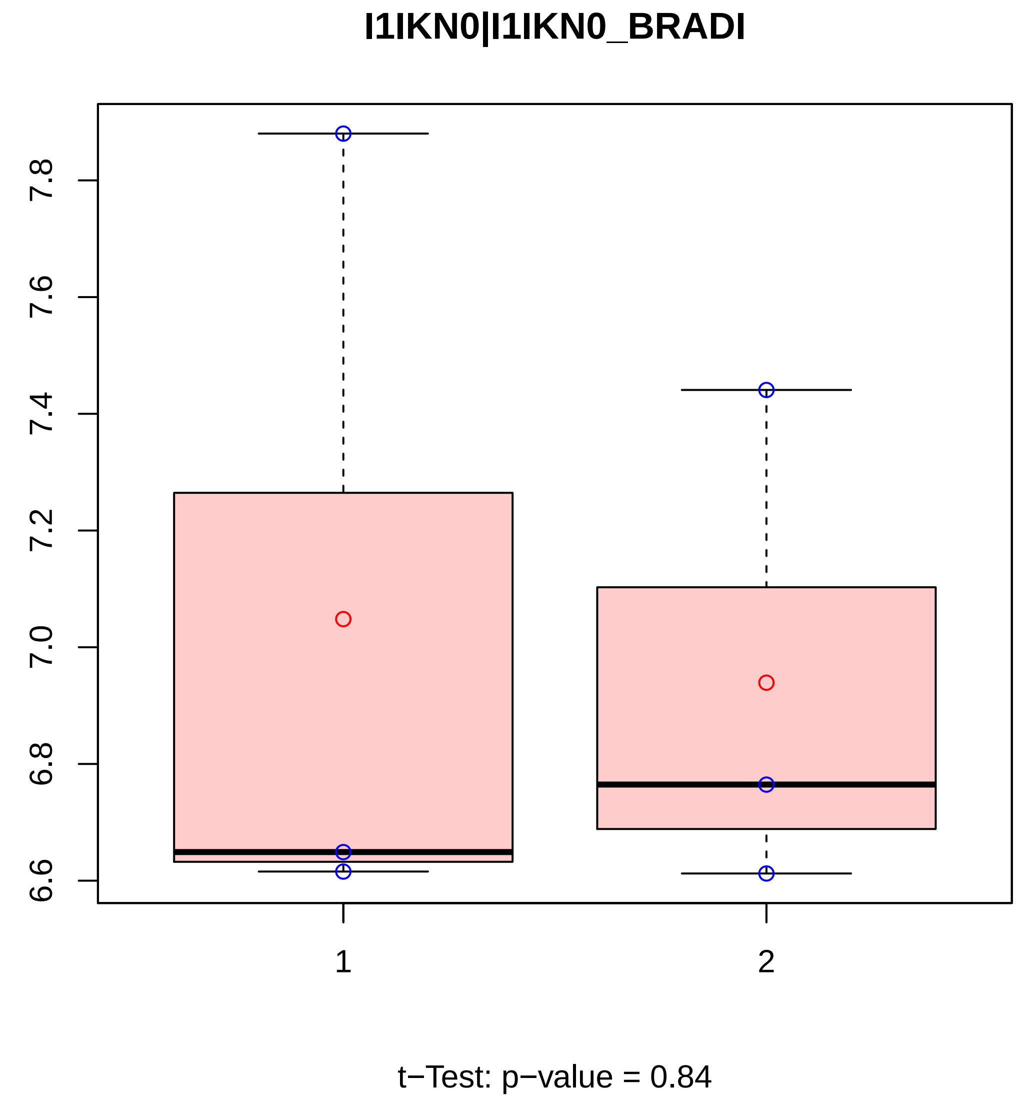
I1IKN0|I1IKN0_BRADI
7.8
7.6
7.4
7.2
7.0
6.8
6.6
1
2
t−Test: p−value = 0.84

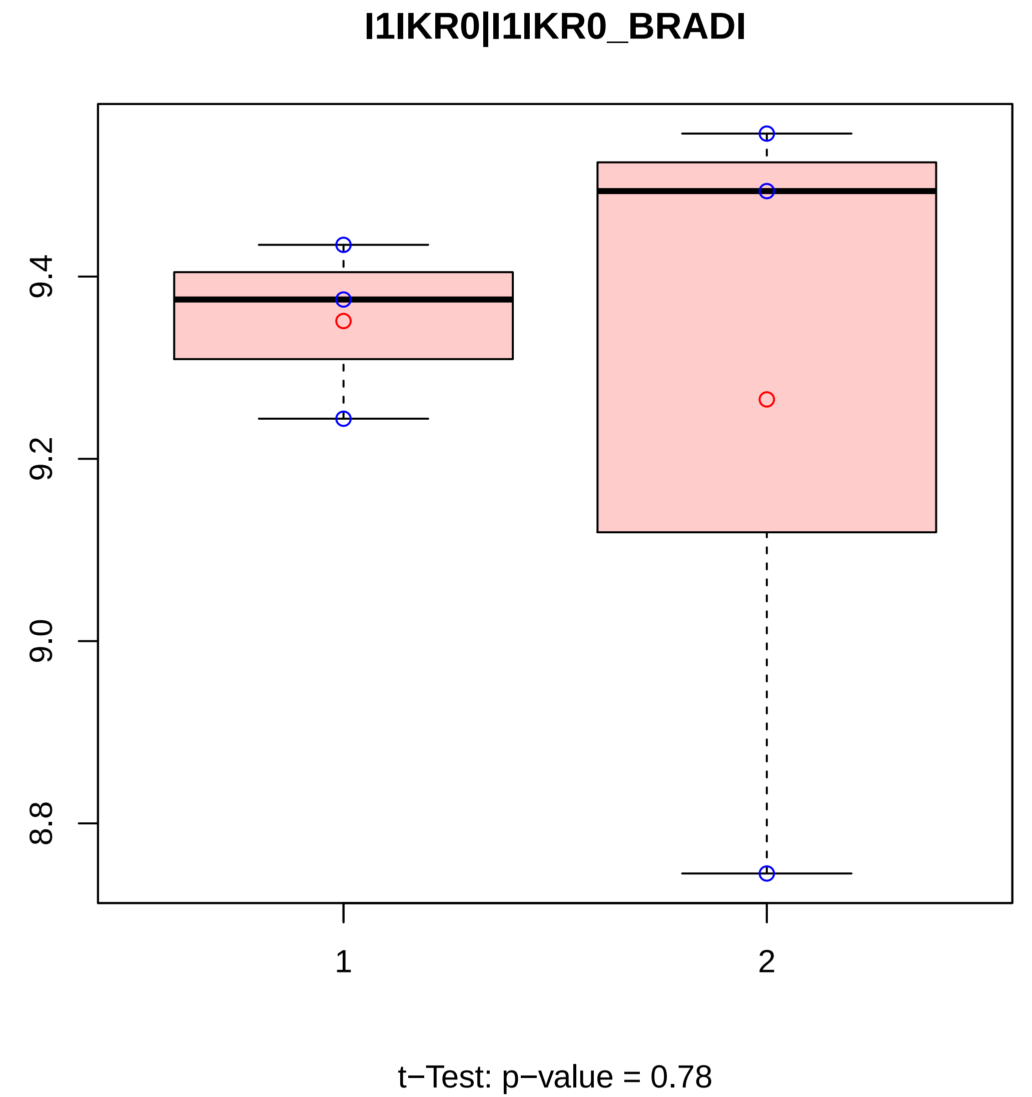
I1IKR0|I1IKR0_BRADI
9.4
9.2
9.0
8.8
1
2
t−Test: p−value = 0.78

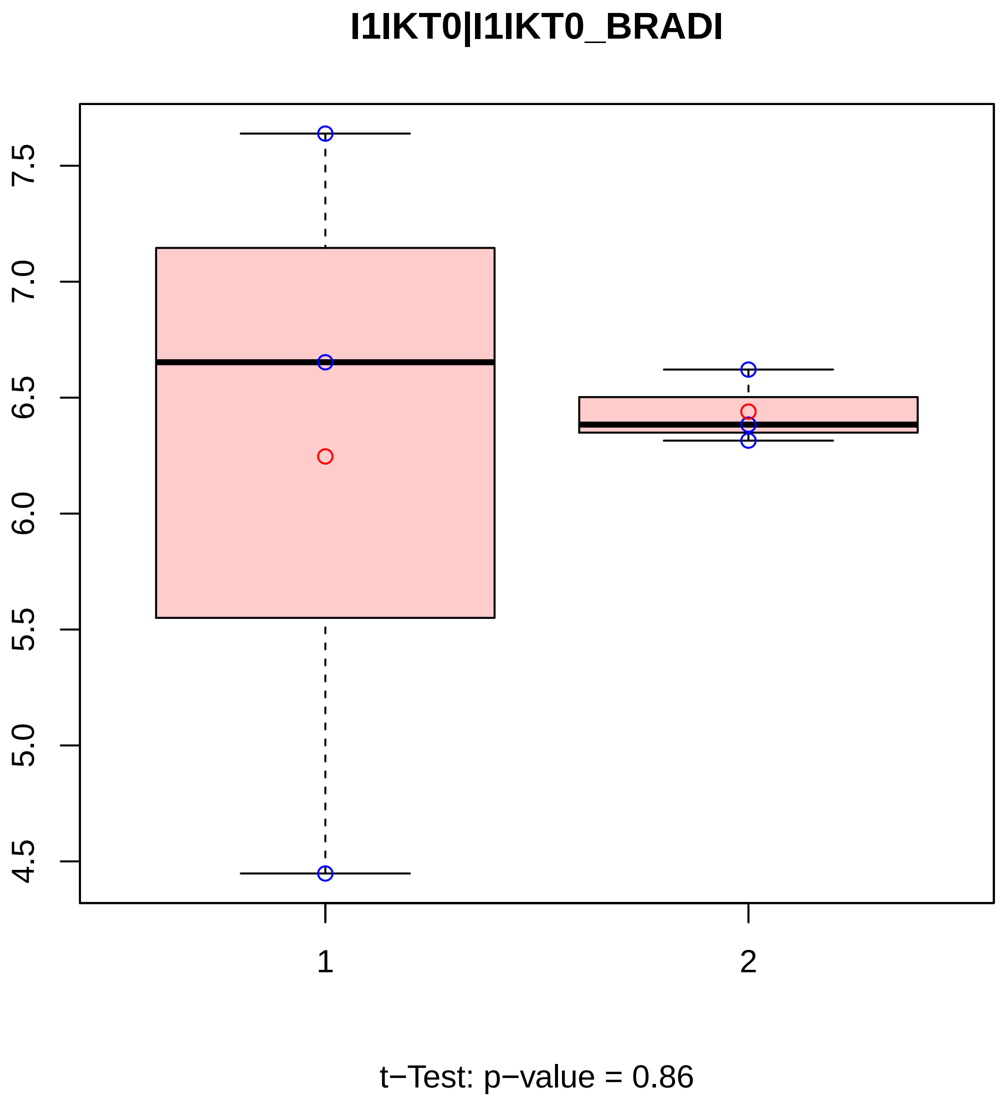

I1IKT0|I1IKT0_BRADI
7.5
7.0
6.5
6.0
5.5
5.0
4.5
1
2
t−Test: p−value = 0.86

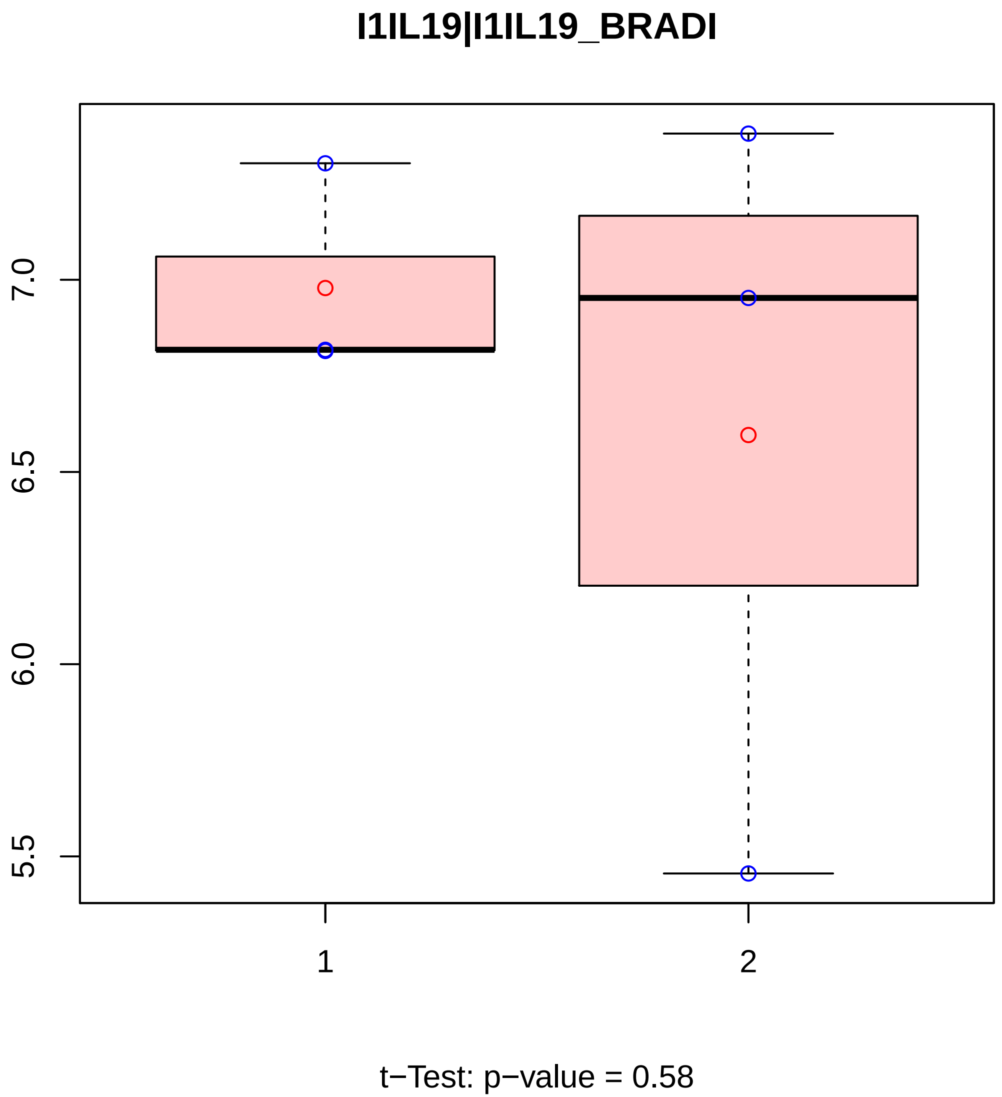
I1IL19|I1IL19_BRADI
7.0
6.5
6.0
5.5
1
2
t−Test: p−value = 0.58

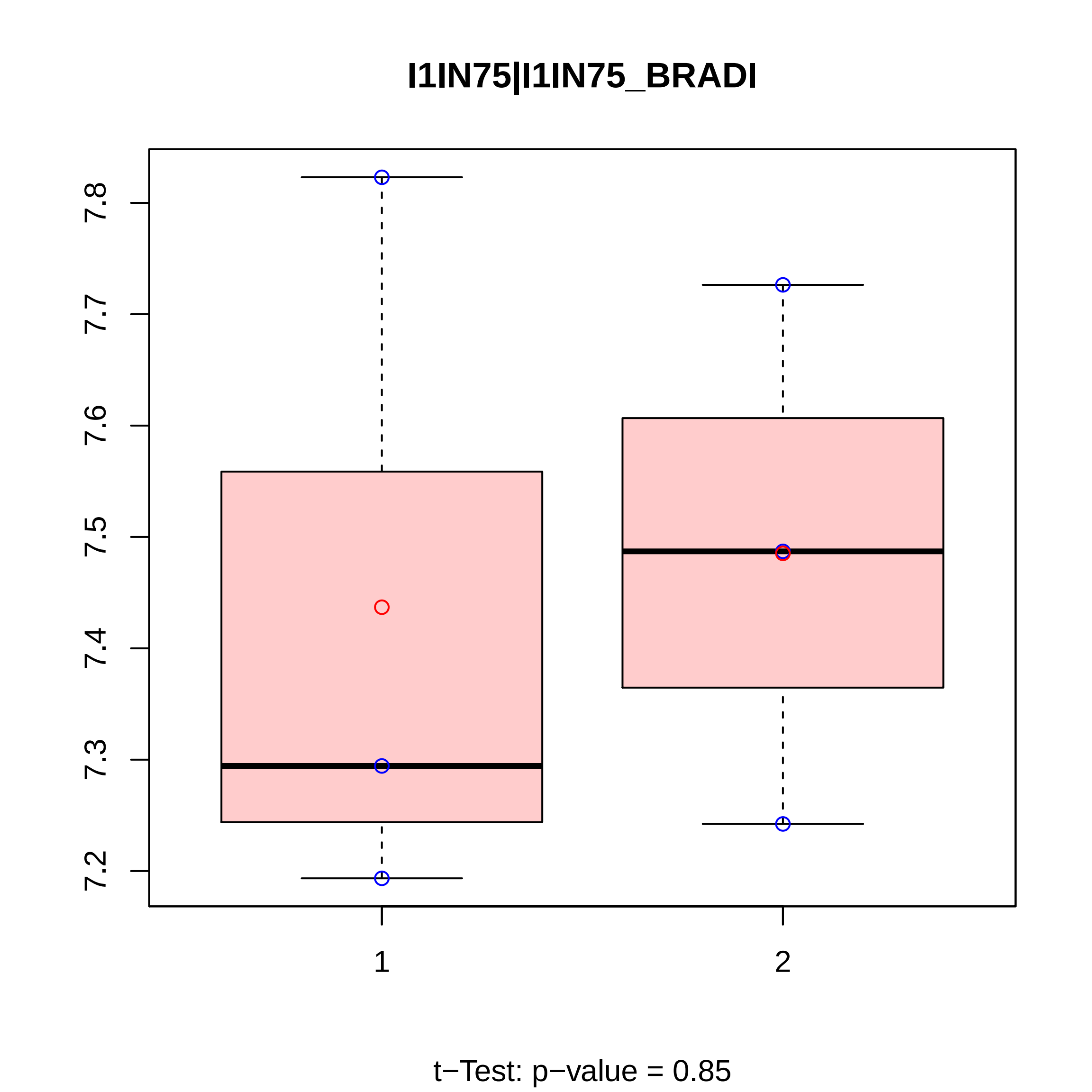
I1IN75|I1IN75_BRADI
7.2
7.3
7.4
7.5
7.6
7.7
7.8
1
2
t−Test: p−value = 0.85

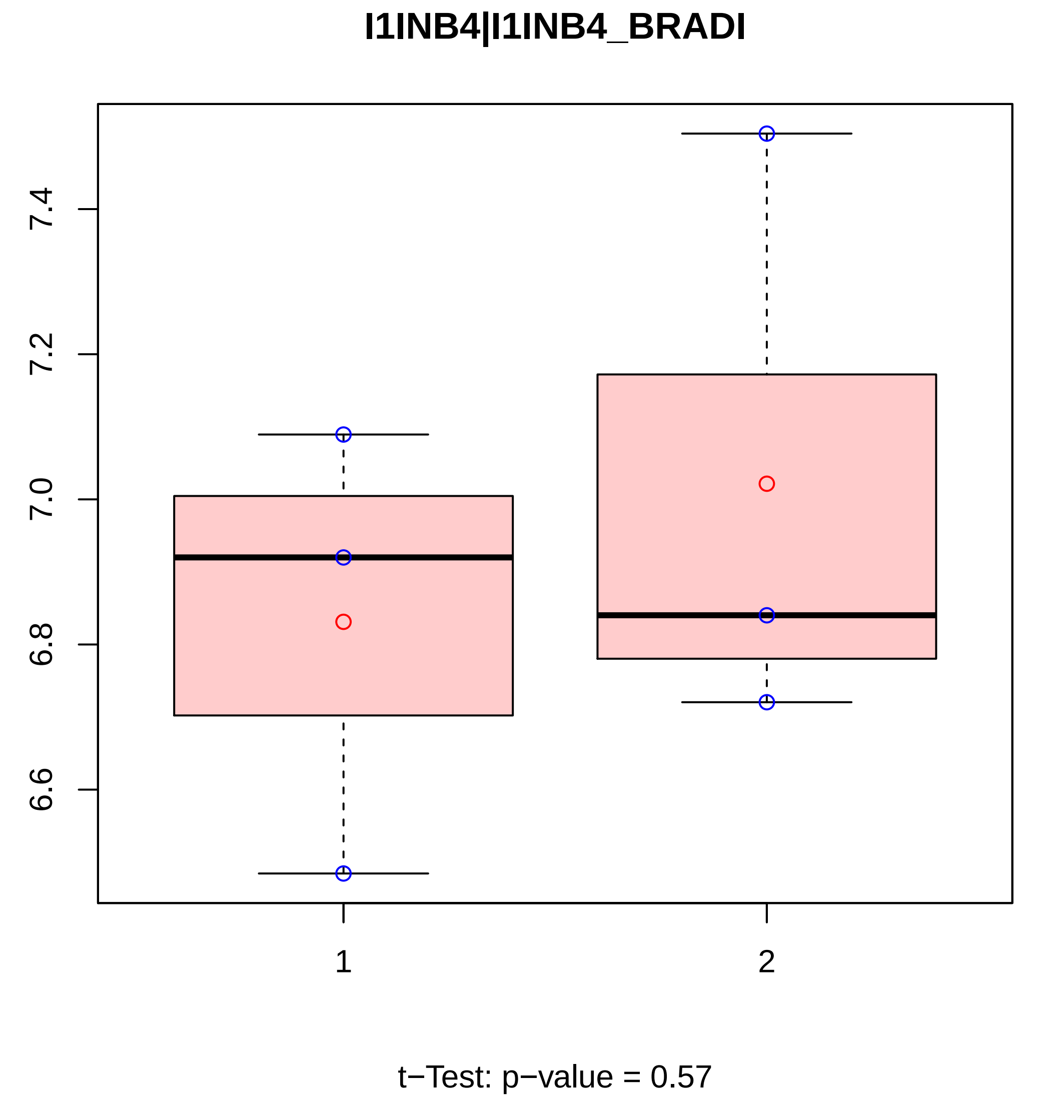
I1INB4|I1INB4_BRADI
7.4
7.2
7.0
6.8
6.6
1
2
t−Test: p−value = 0.57

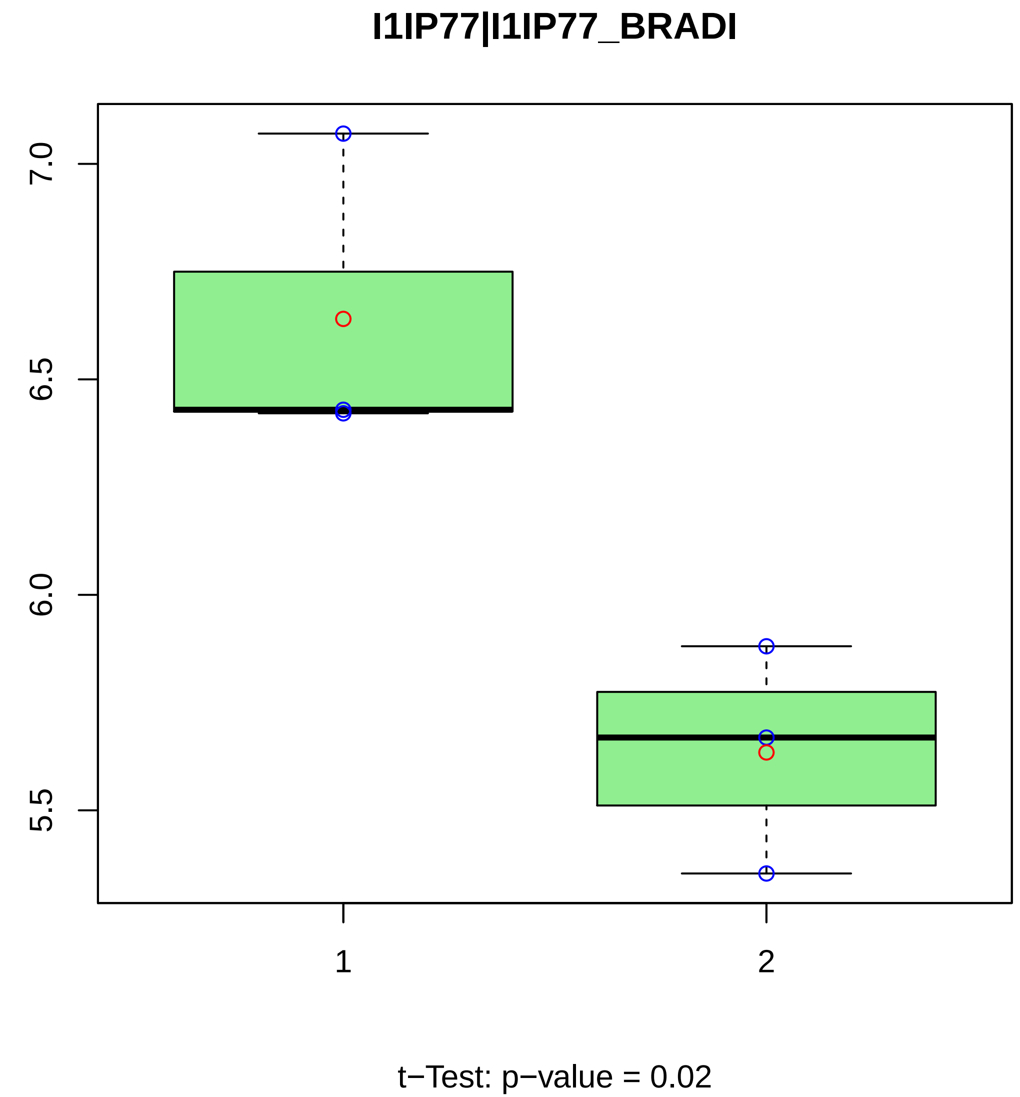
I1IP77|I1IP77_BRADI
7.0
6.5
6.0
5.5
1
2
t−Test: p−value = 0.02

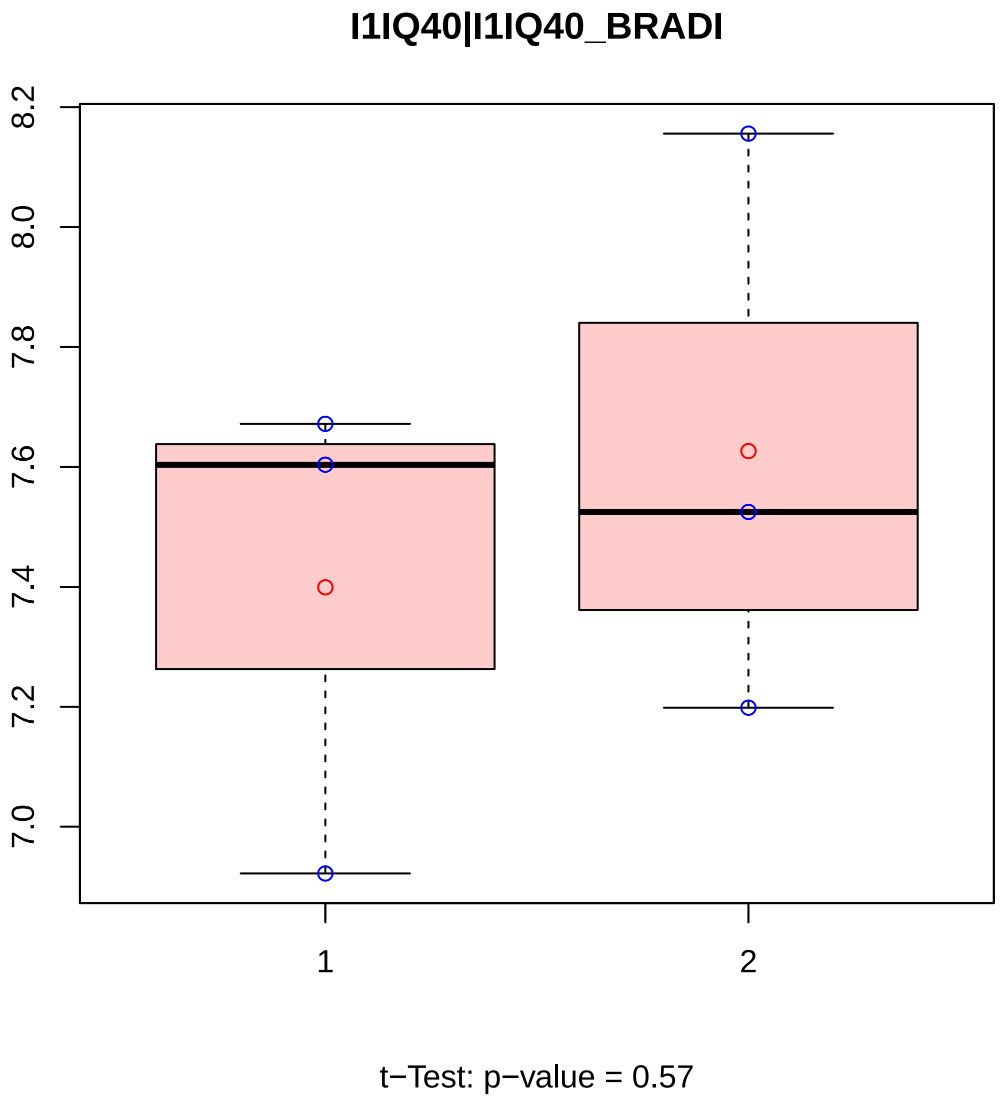
I1IQ40|I1IQ40_BRADI
8.2
8.0
7.8
7.6
7.4
7.2
7.0
1
2
t−Test: p−value = 0.57

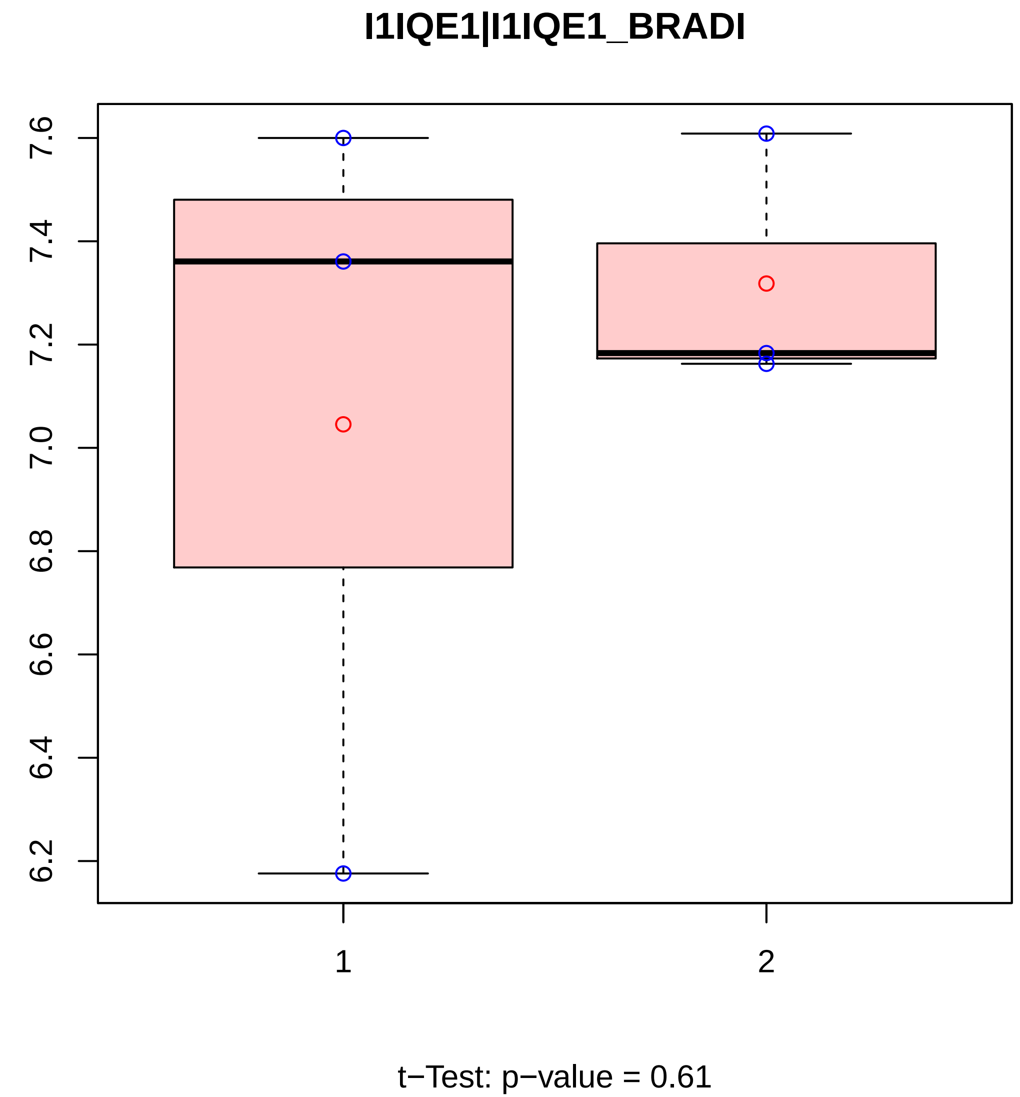
I1IQE1|I1IQE1_BRADI
7.6
7.4
7.2
7.0
6.8
6.6
6.4
6.2
1
2
t−Test: p−value = 0.61

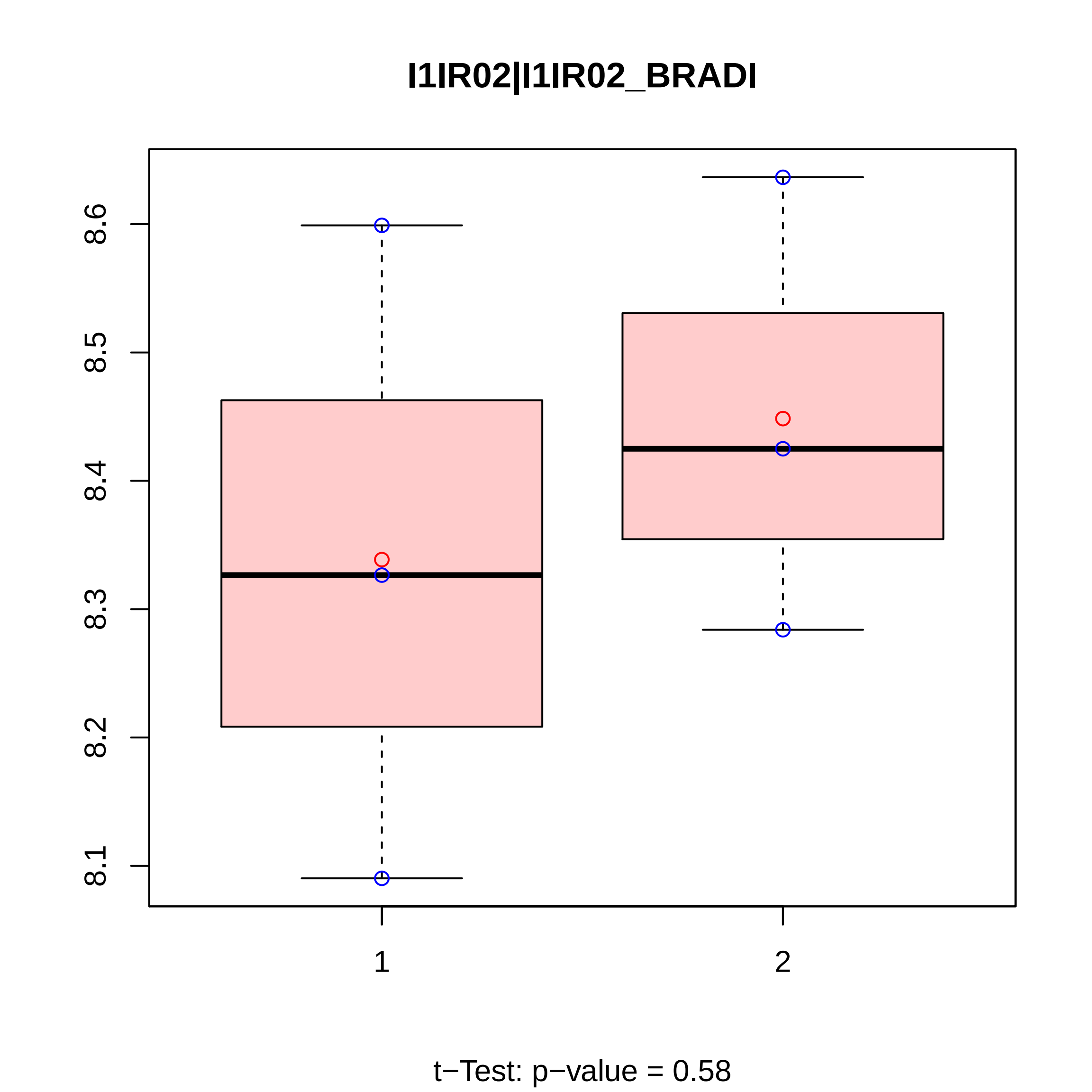
I1IR02|I1IR02_BRADI
8.1
8.2
8.3
8.4
8.5
8.6
1
2
t−Test: p−value = 0.58

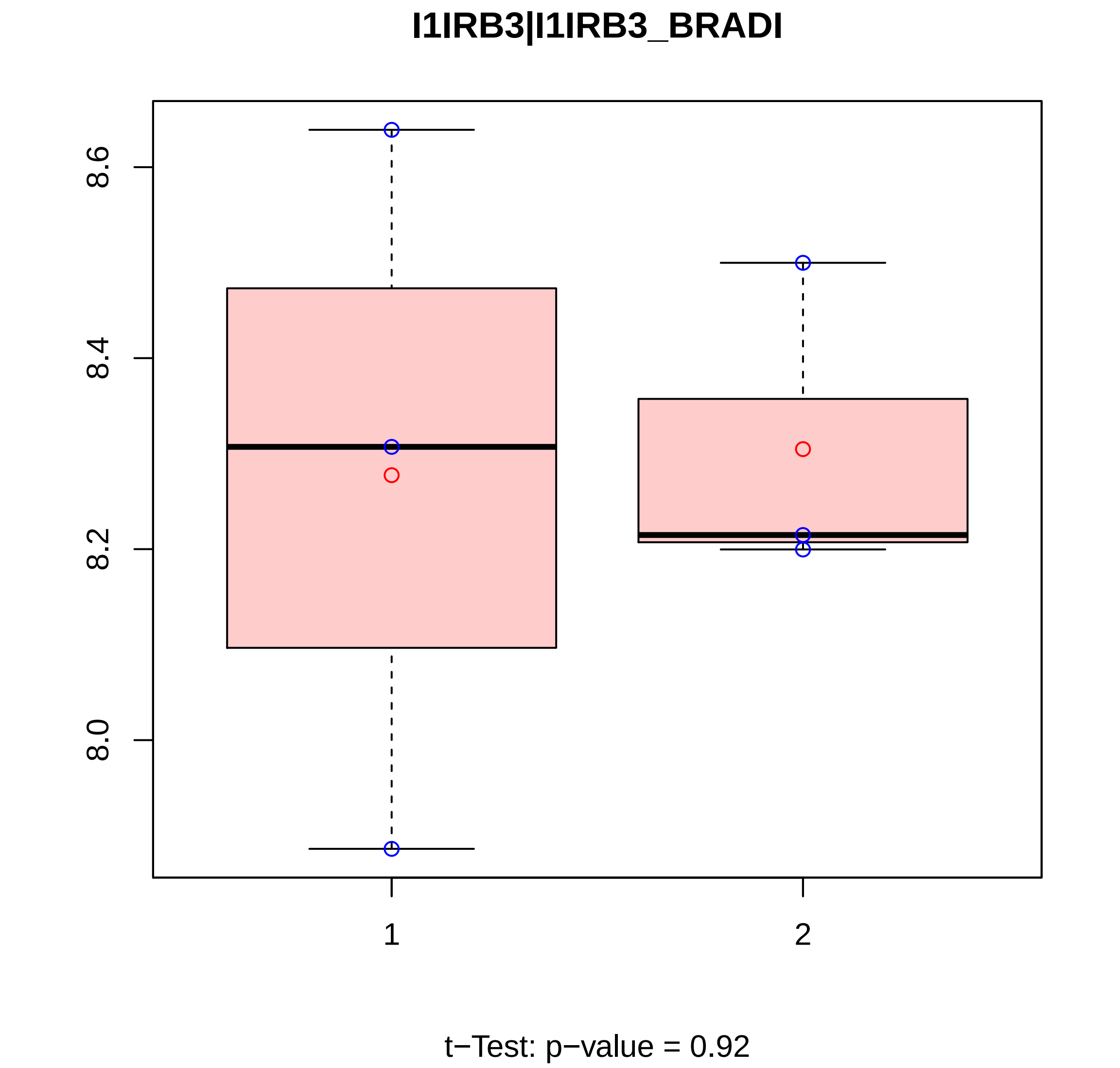
I1IRB3|I1IRB3_BRADI
8.0
8.2
8.4
8.6
1
2
t−Test: p−value = 0.92

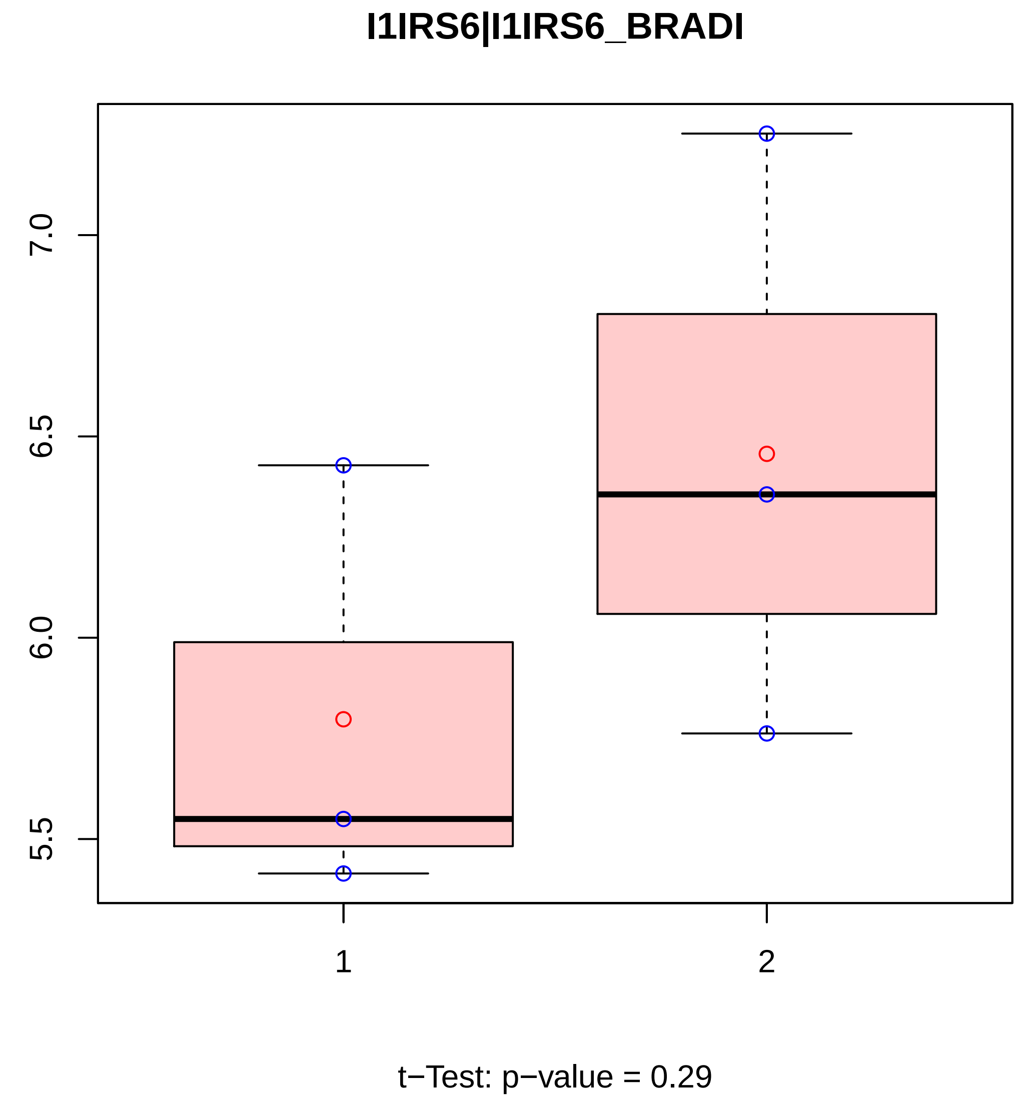
I1IRS6|I1IRS6_BRADI
7.0
6.5
6.0
5.5
1
2
t−Test: p−value = 0.29

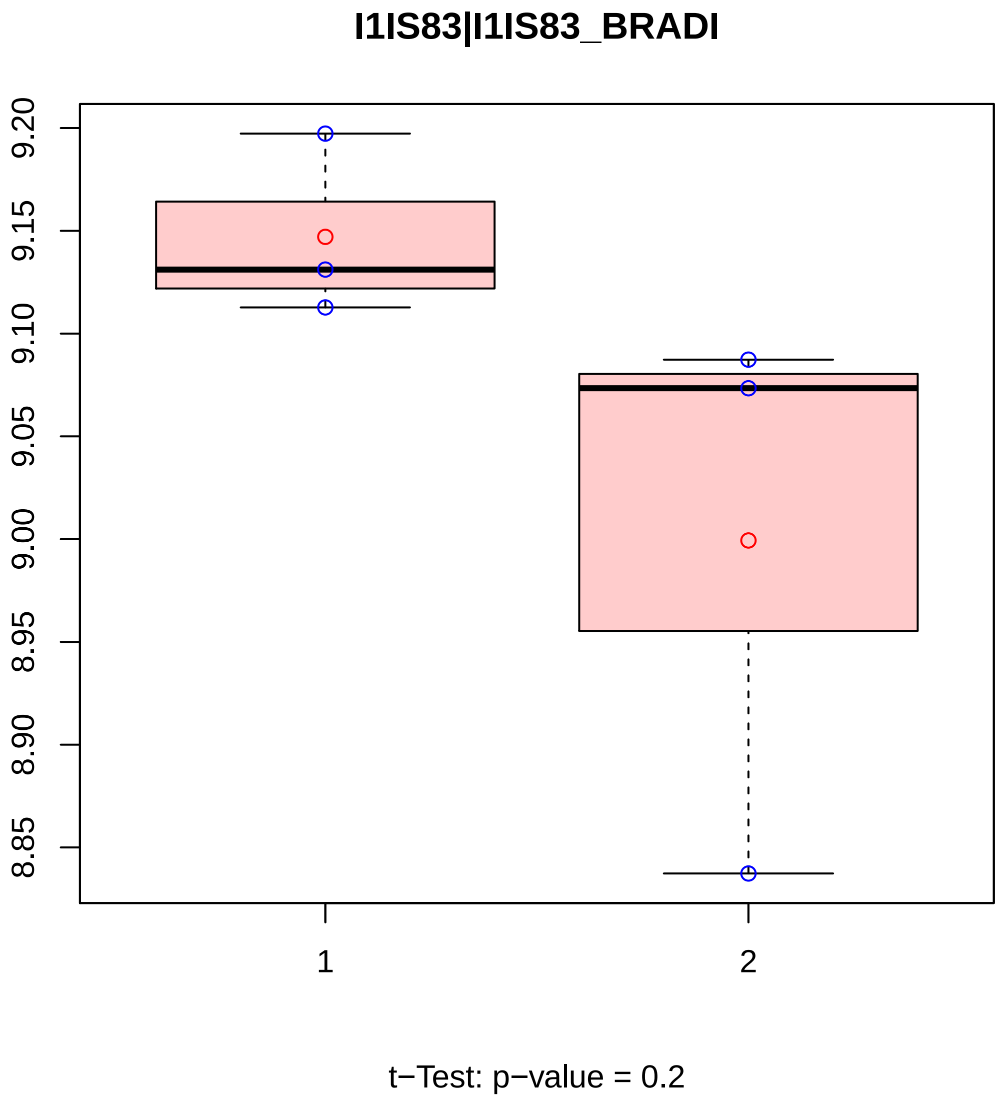
I1IS83|I1IS83_BRADI
9.20
9.15
9.10
9.05
9.00
8.95
8.90
8.85
1
2
t−Test: p−value = 0.2

I1ISA6|I1ISA6_BRADI

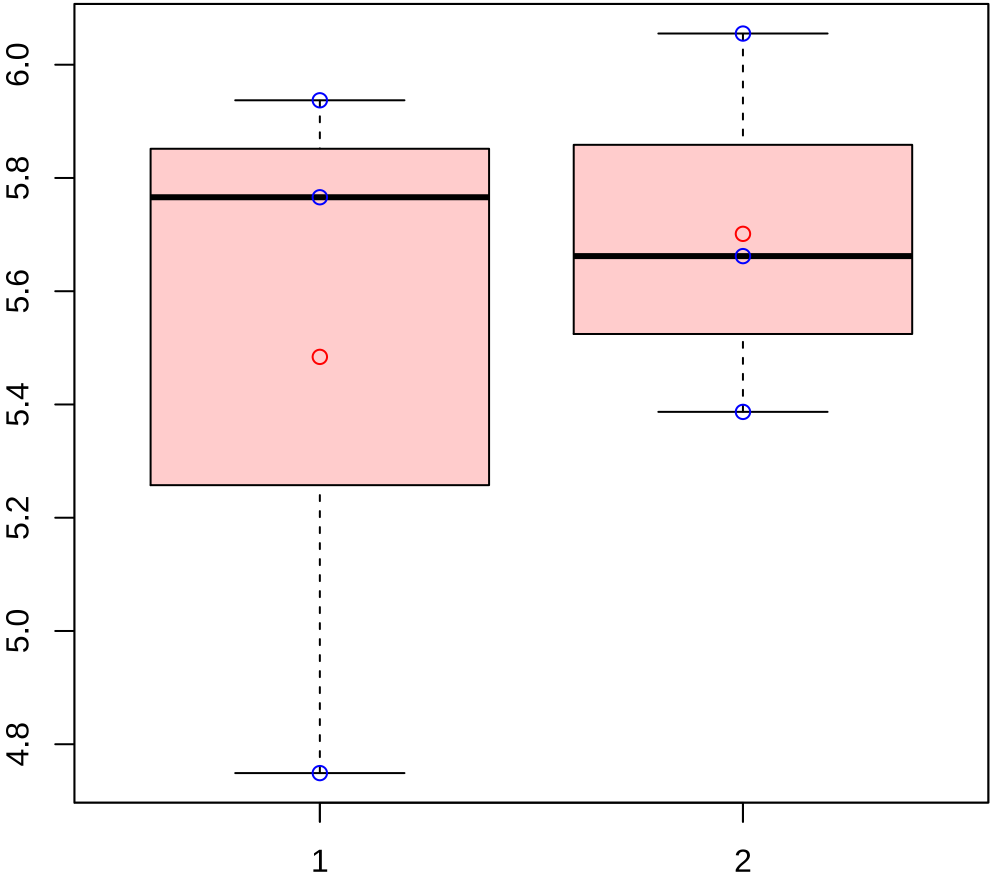

t−Test: p−value = 0.64

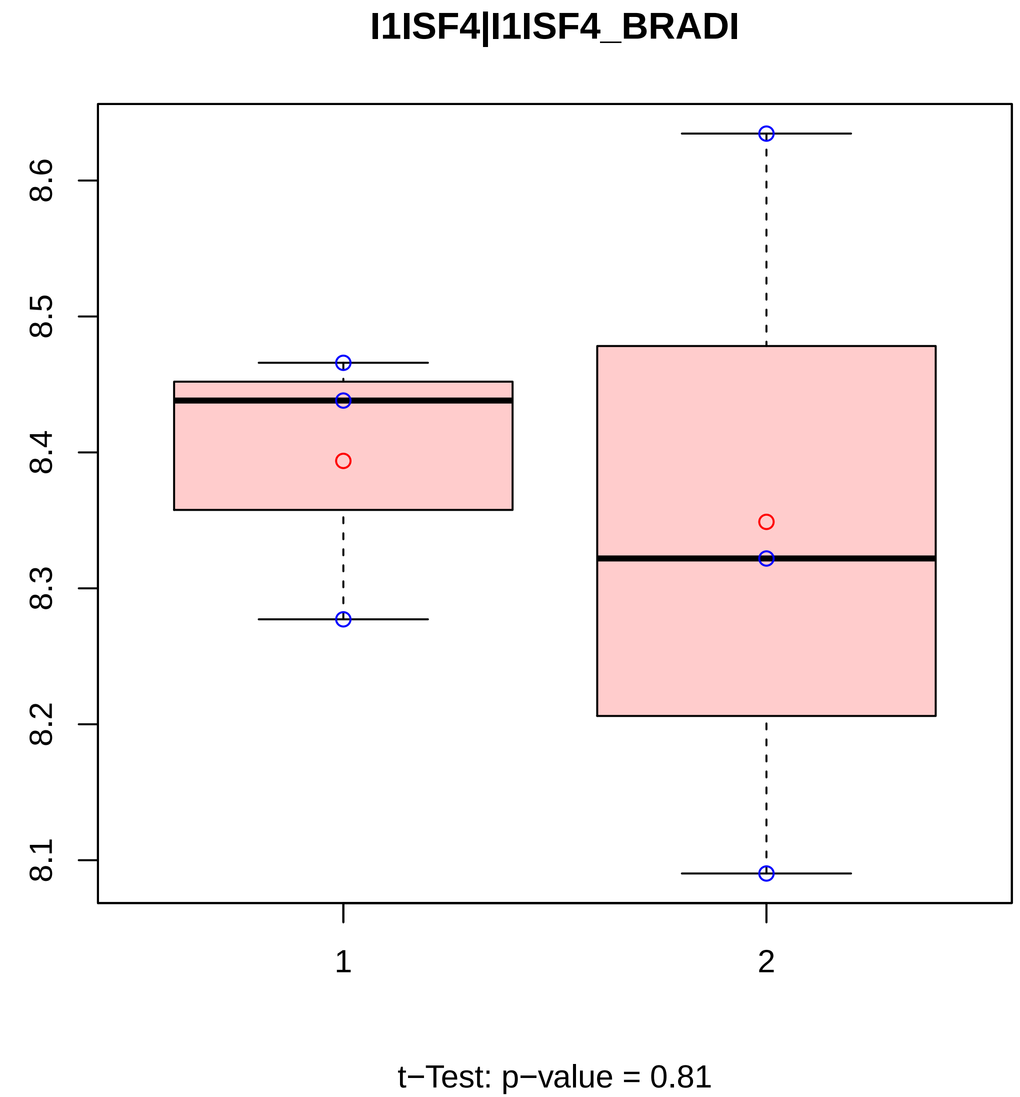
I1ISF4|I1ISF4_BRADI
8.1
8.2
8.3
8.4
8.5
8.6
1
2
t−Test: p−value = 0.81

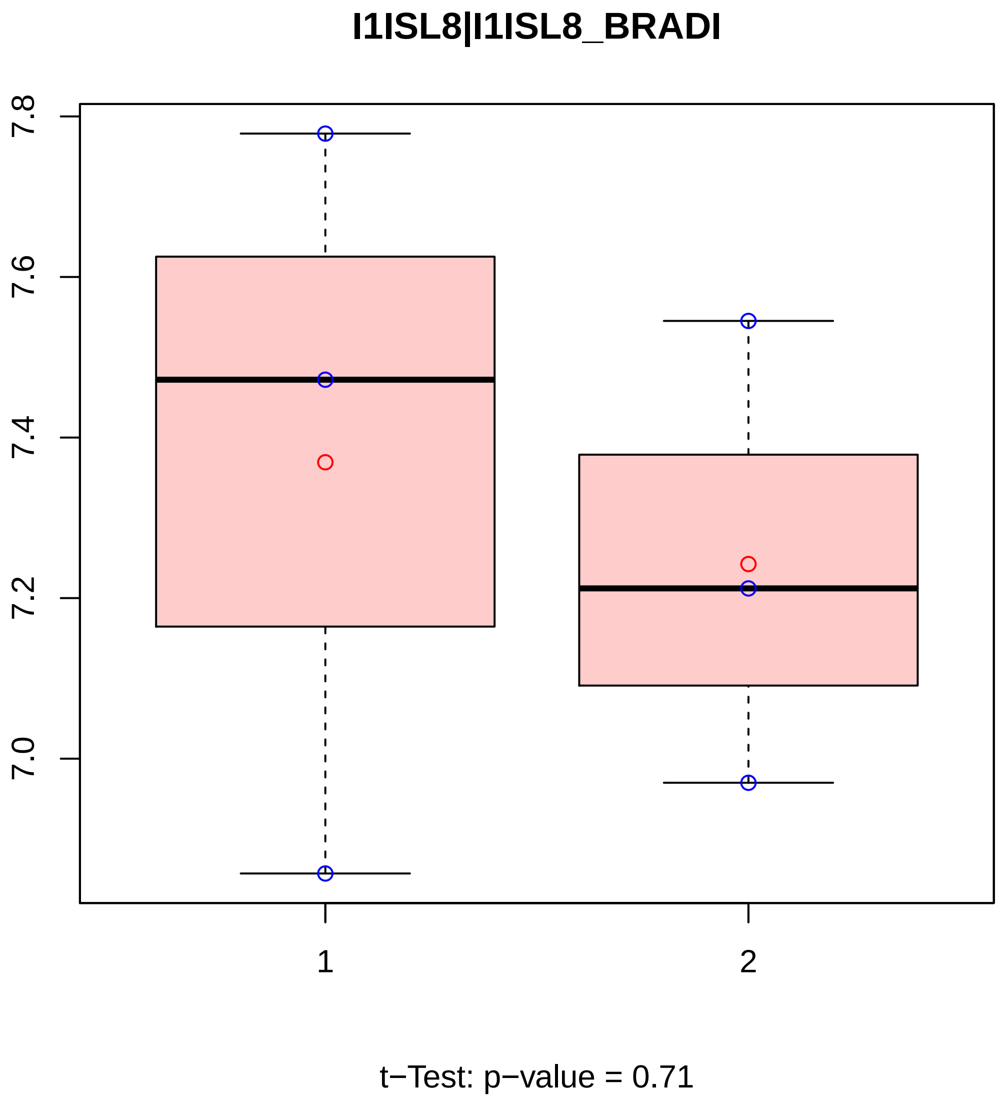

I1ISL8|I1ISL8_BRADI
7.8
7.6
7.4
7.2
7.0
1
2
t−Test: p−value = 0.71

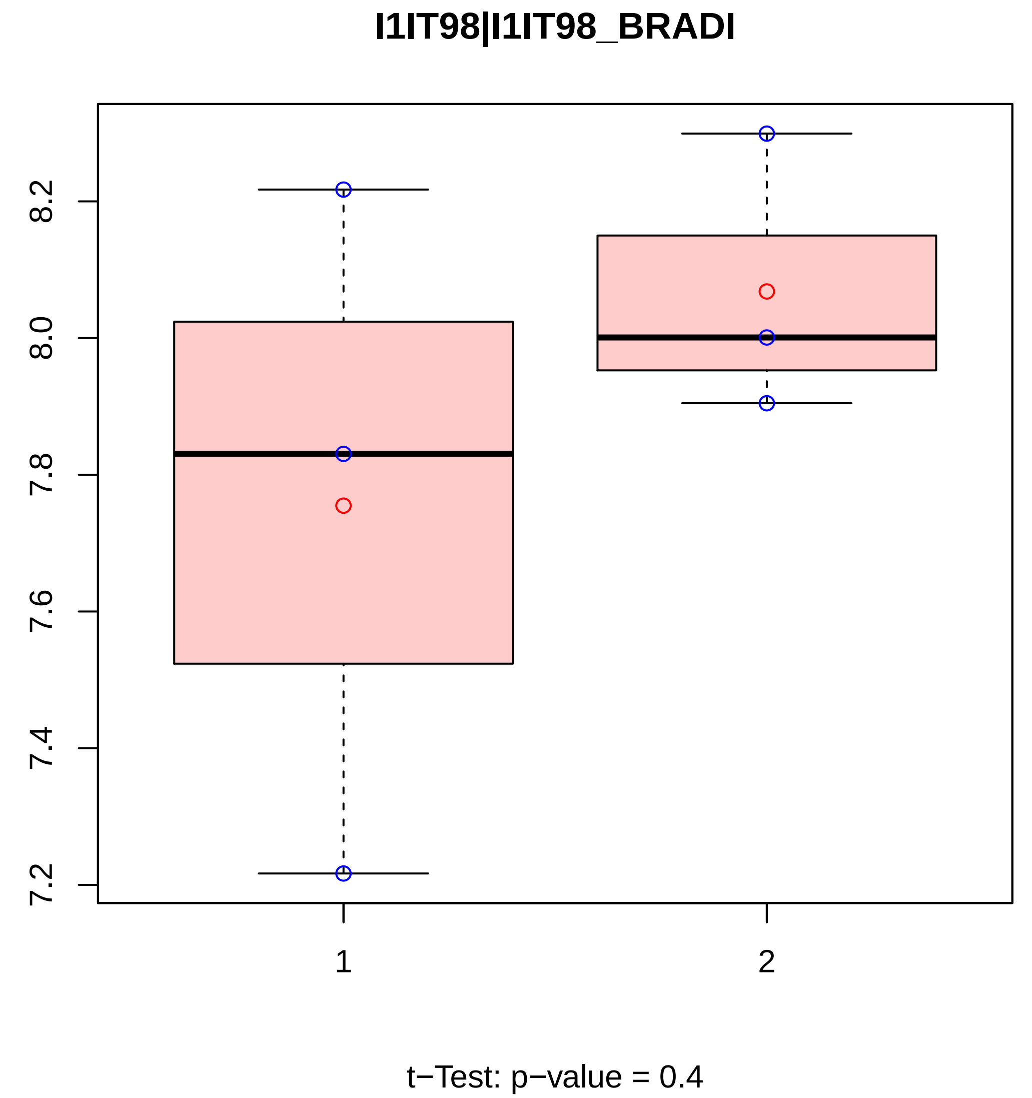
I1IT98|I1IT98_BRADI
8.2
8.0
7.8
7.6
7.4
7.2
1
2
t−Test: p−value = 0.4

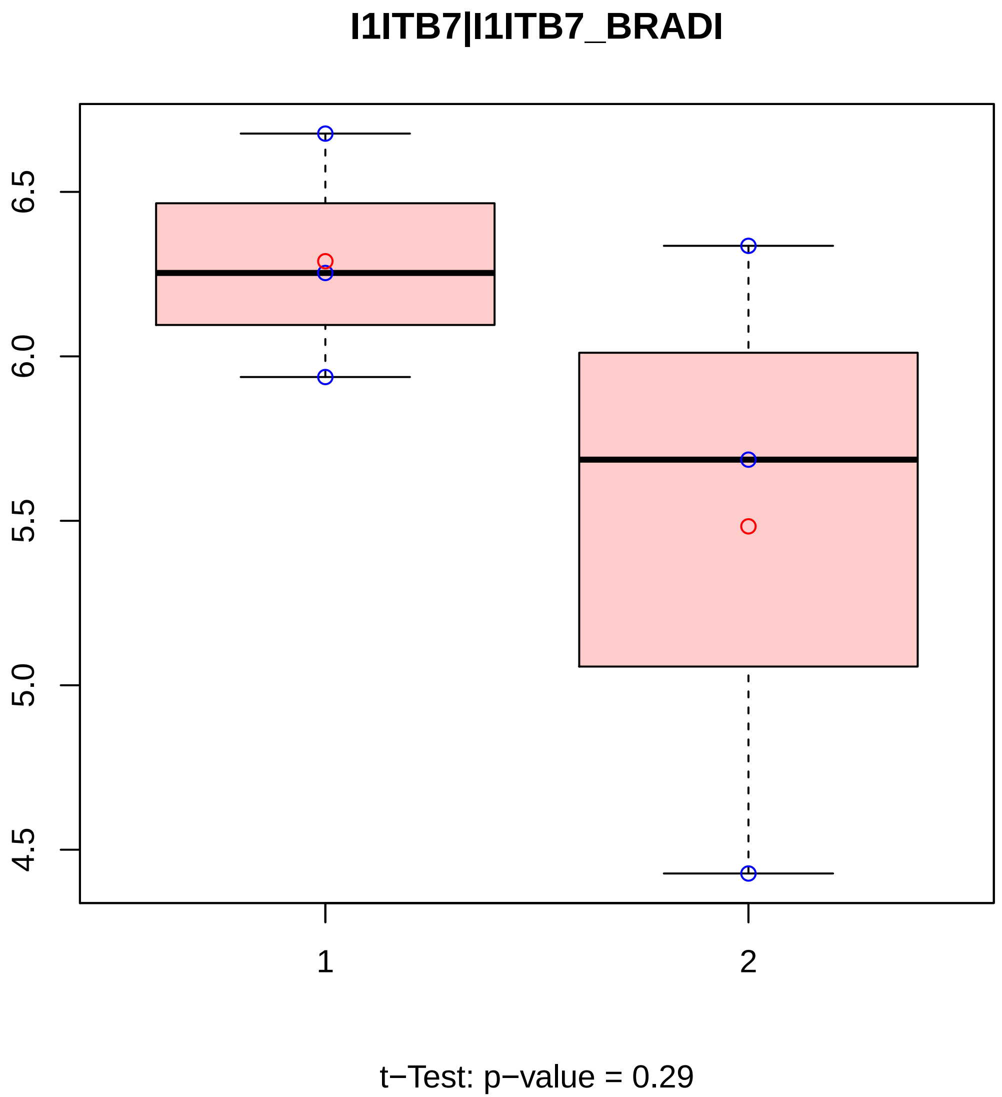
I1ITB7|I1ITB7_BRADI
6.5
6.0
5.5
5.0
4.5
1
2
t−Test: p−value = 0.29

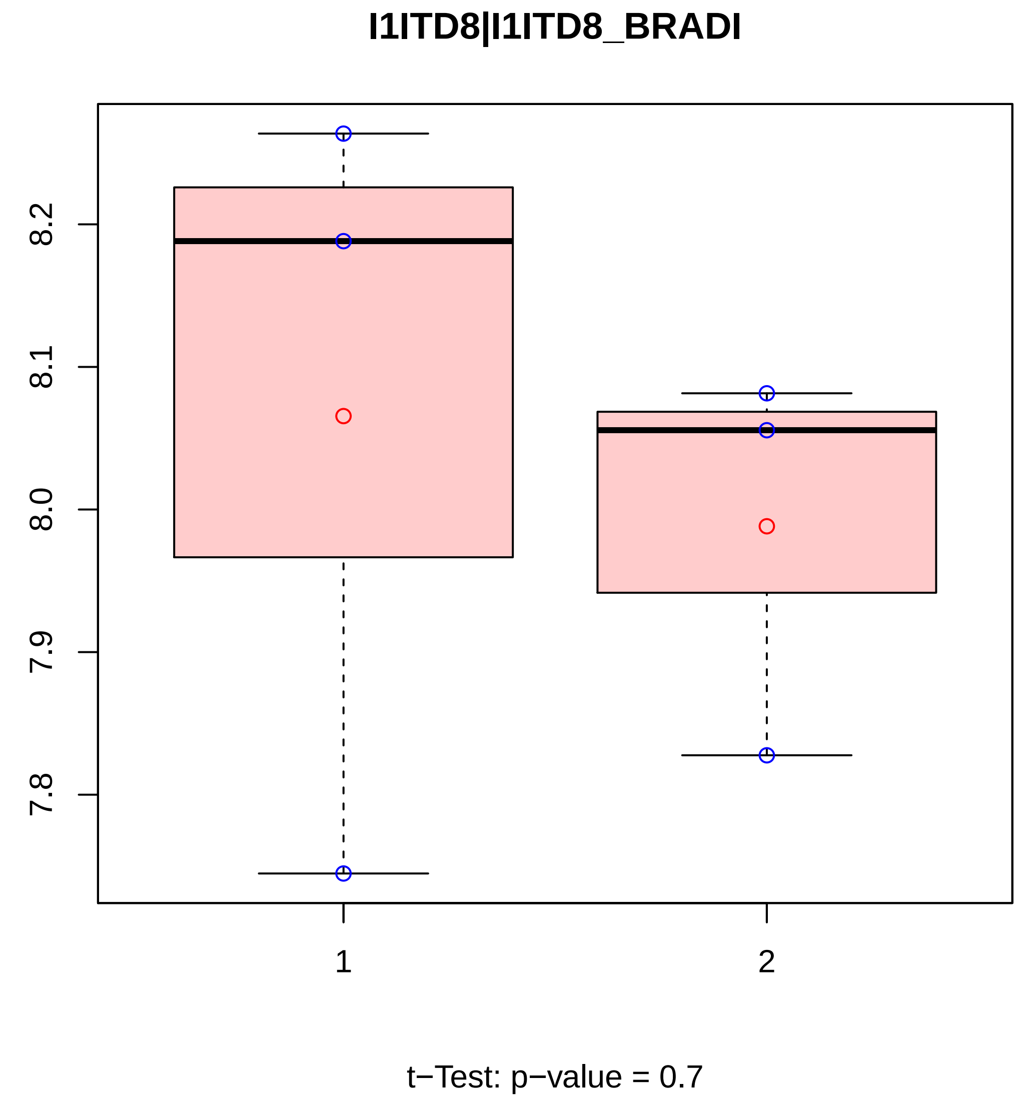
I1ITD8|I1ITD8_BRADI
8.2
8.1
8.0
7.9
7.8
1
2
t−Test: p−value = 0.7

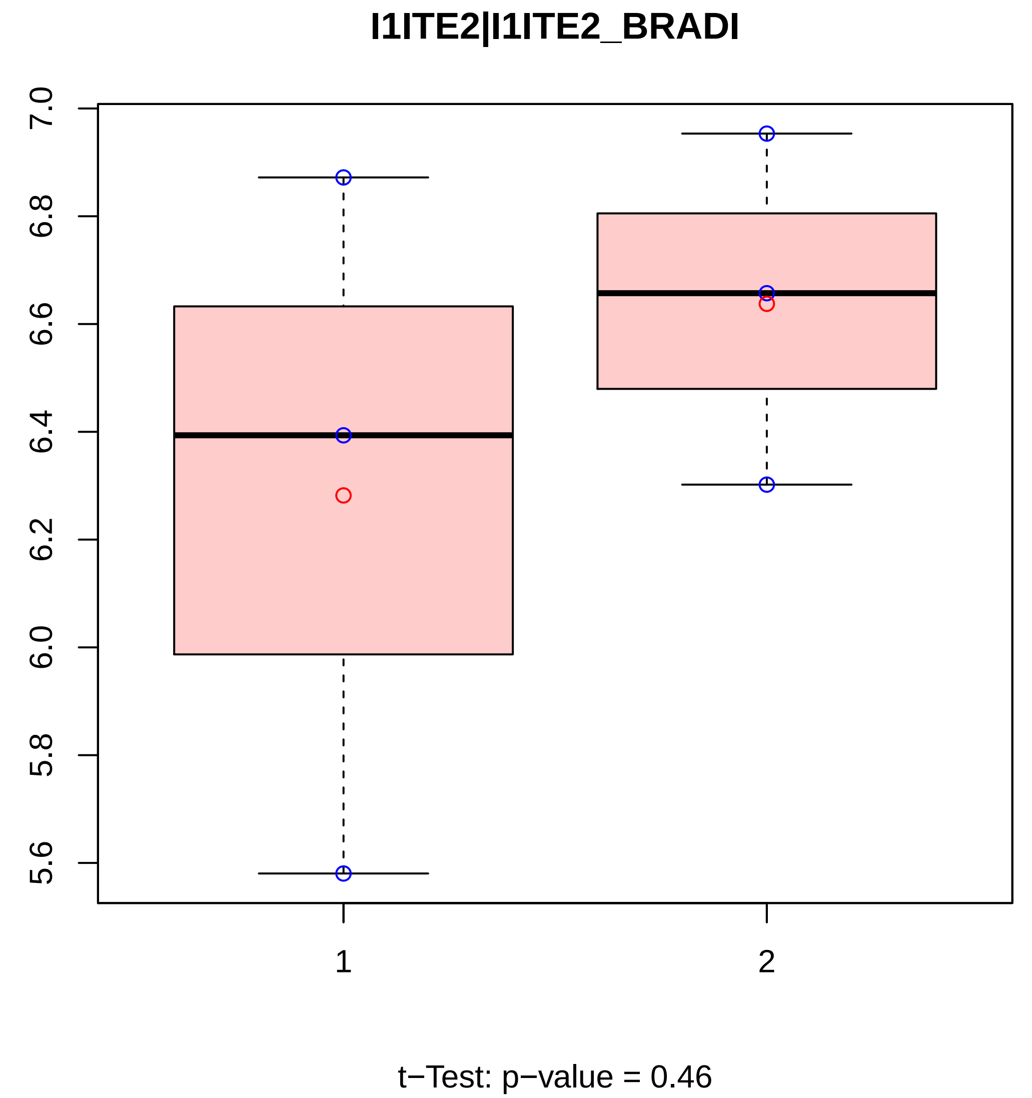
I1ITE2|I1ITE2_BRADI
7.0
6.8
6.6
6.4
6.2
6.0
5.8
5.6
1
2
t−Test: p−value = 0.46

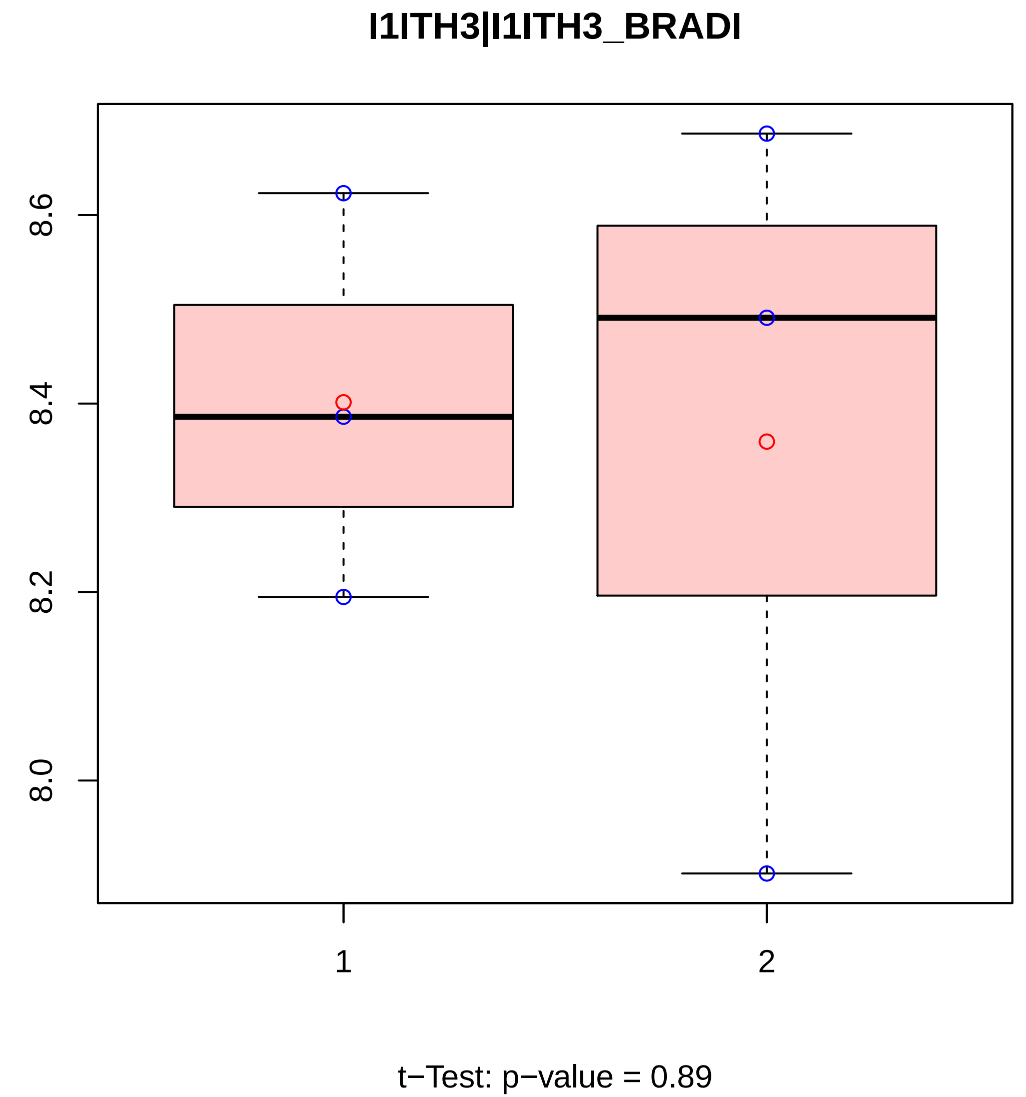
I1ITH3|I1ITH3_BRADI
8.0
8.2
8.4
8.6
1
2
t−Test: p−value = 0.89

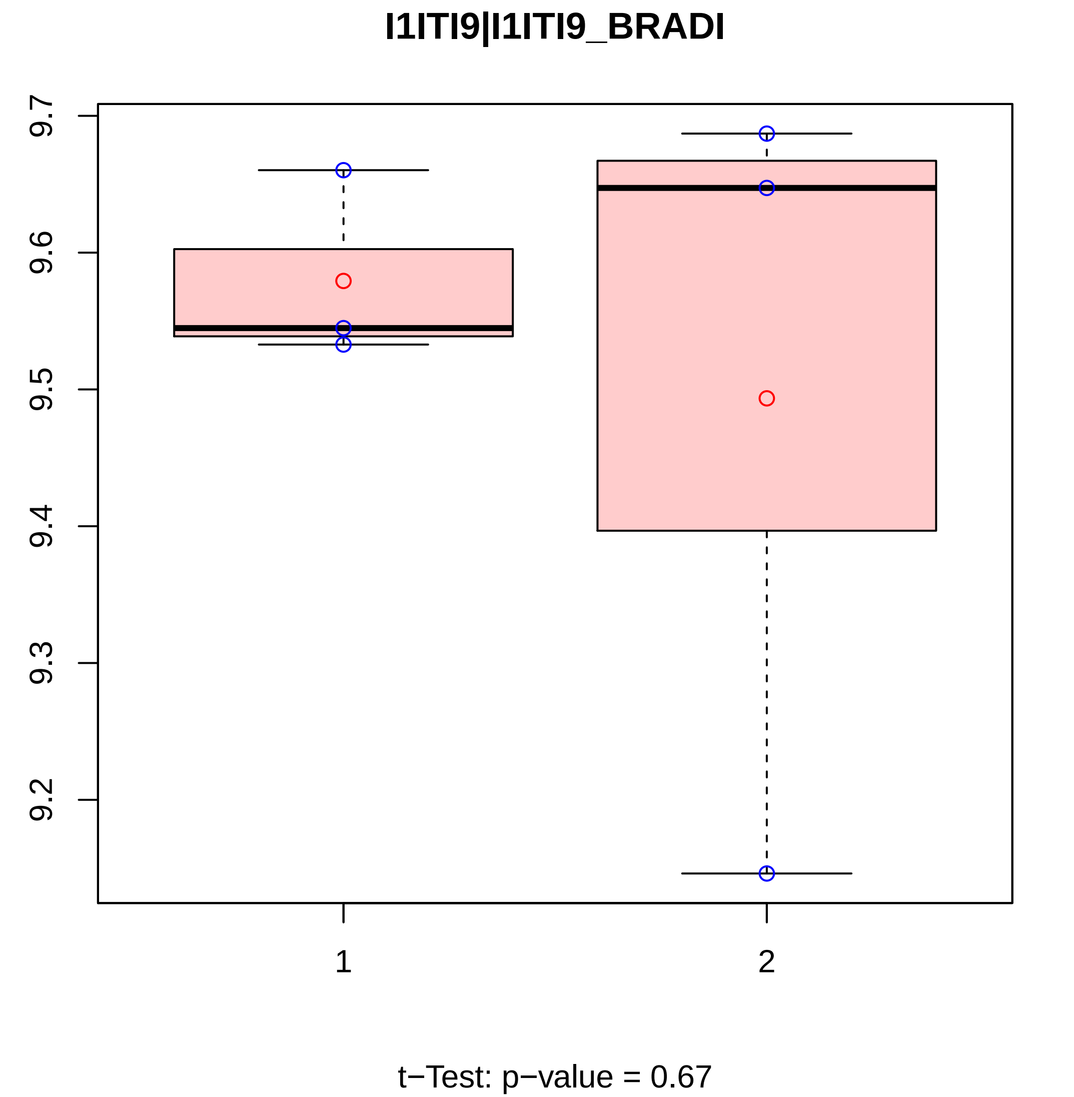

I1ITI9|I1ITI9_BRADI
9.7
9.6
9.5
9.4
9.3
9.2
1
2
t−Test: p−value = 0.67

I1ITJ3|I1ITJ3_BRADI

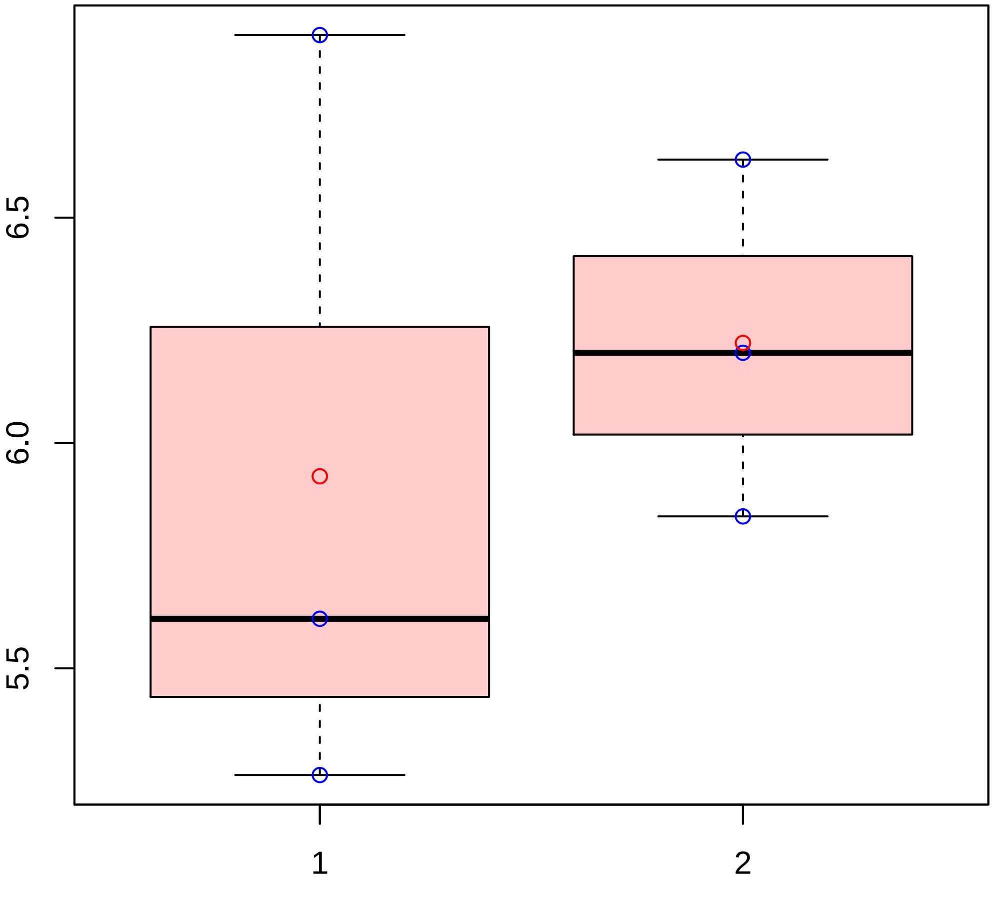

t−Test: p−value = 0.63

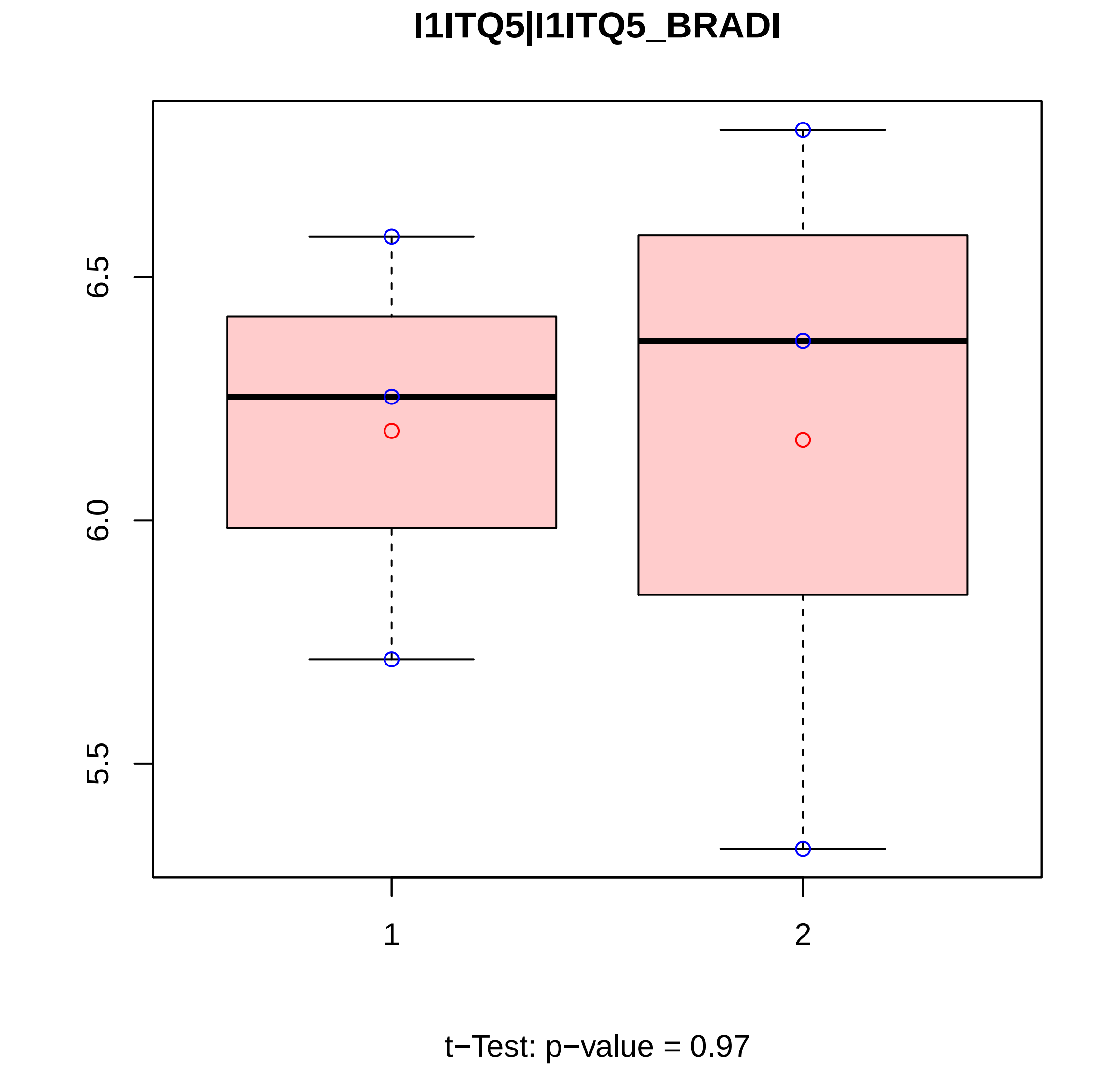
I1ITQ5|I1ITQ5_BRADI
6.5
6.0
5.5
1
2
t−Test: p−value = 0.97

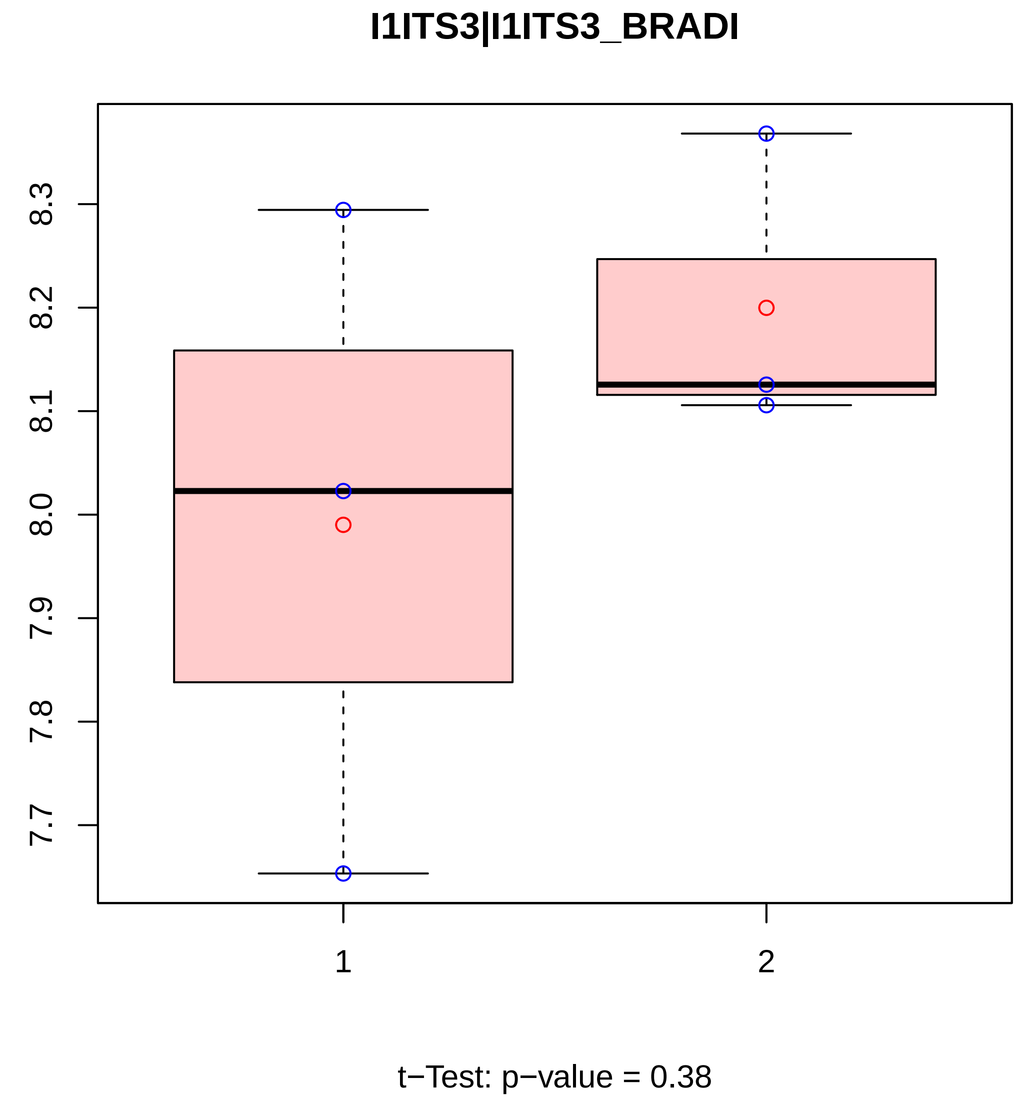

I1ITS3|I1ITS3_BRADI
8.3
8.2
8.1
8.0
7.9
7.8
7.7
1
2
t−Test: p−value = 0.38

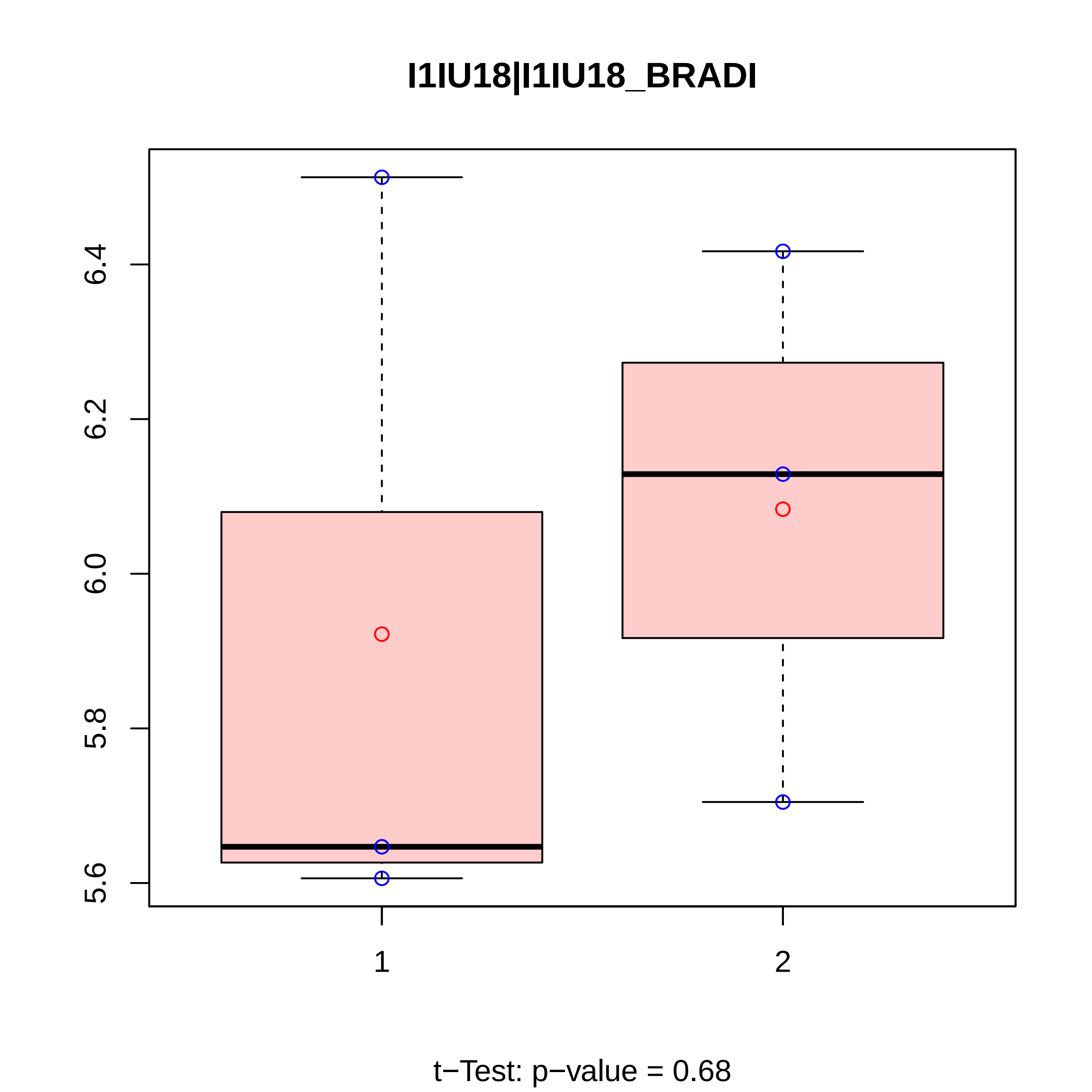
I1IU18|I1IU18_BRADI
6.4
6.2
6.0
5.8
5.6
1
2
t−Test: p−value = 0.68

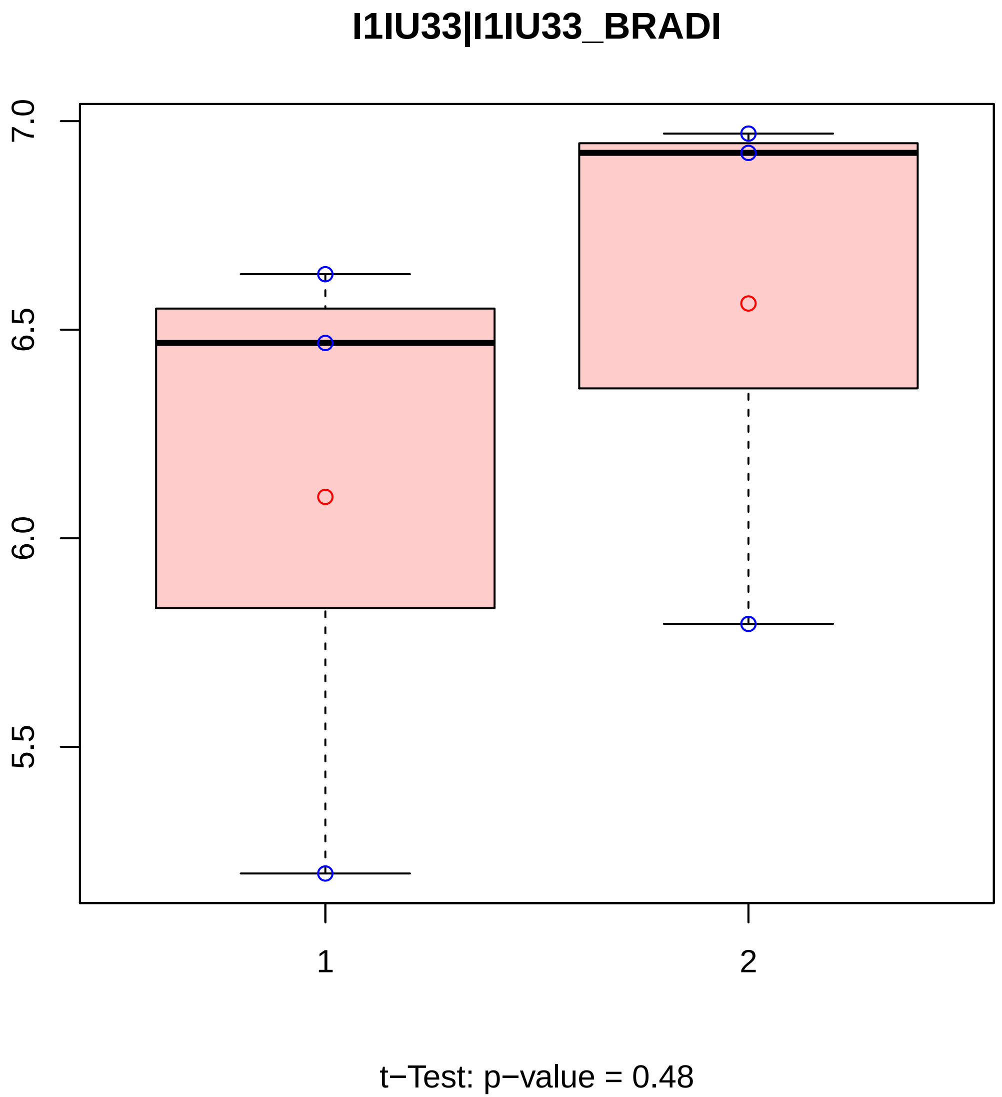
I1IU33|I1IU33_BRADI
7.0
6.5
6.0
5.5
1
2
t−Test: p−value = 0.48

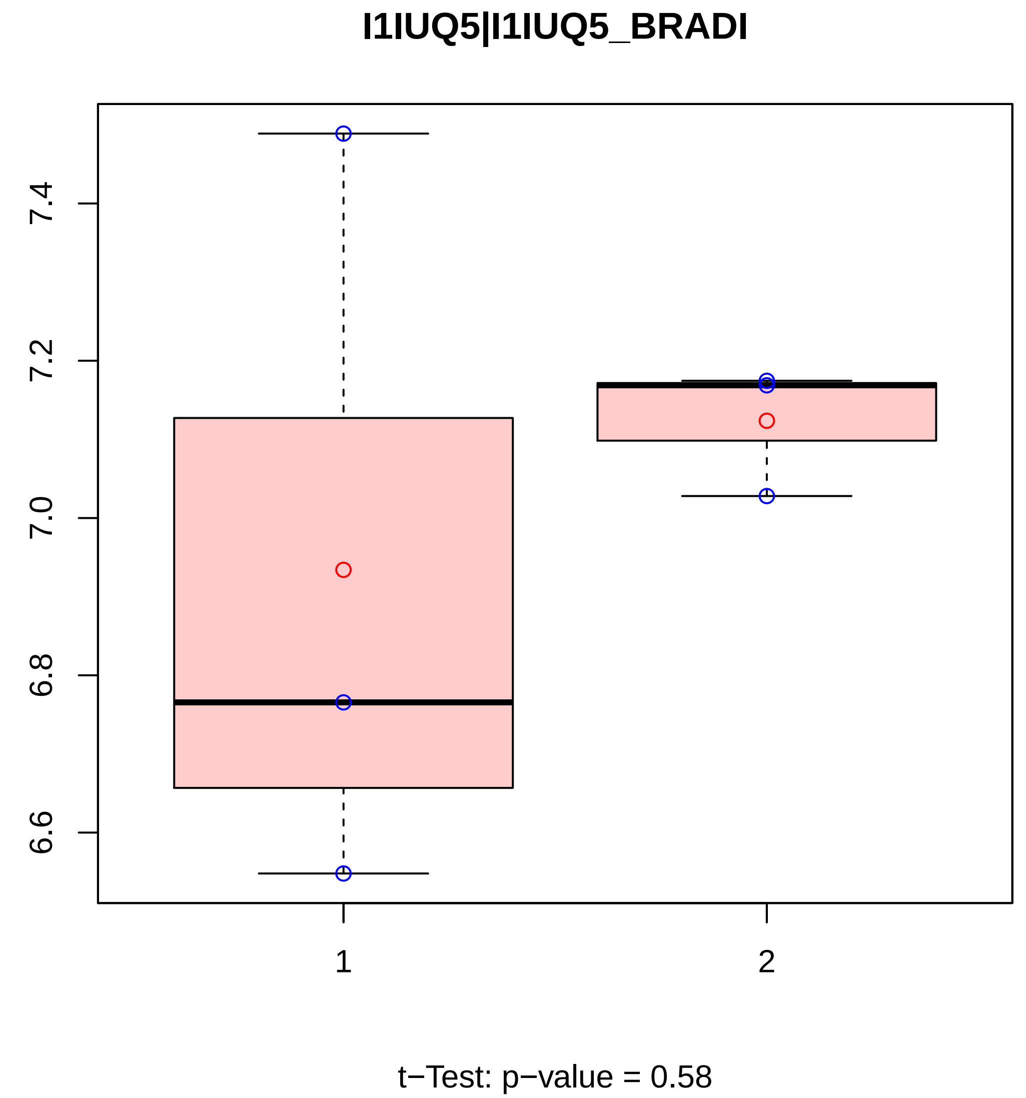
I1IUQ5|I1IUQ5_BRADI
6.6
6.8
7.0
7.2
7.4
1
2
t−Test: p−value = 0.58

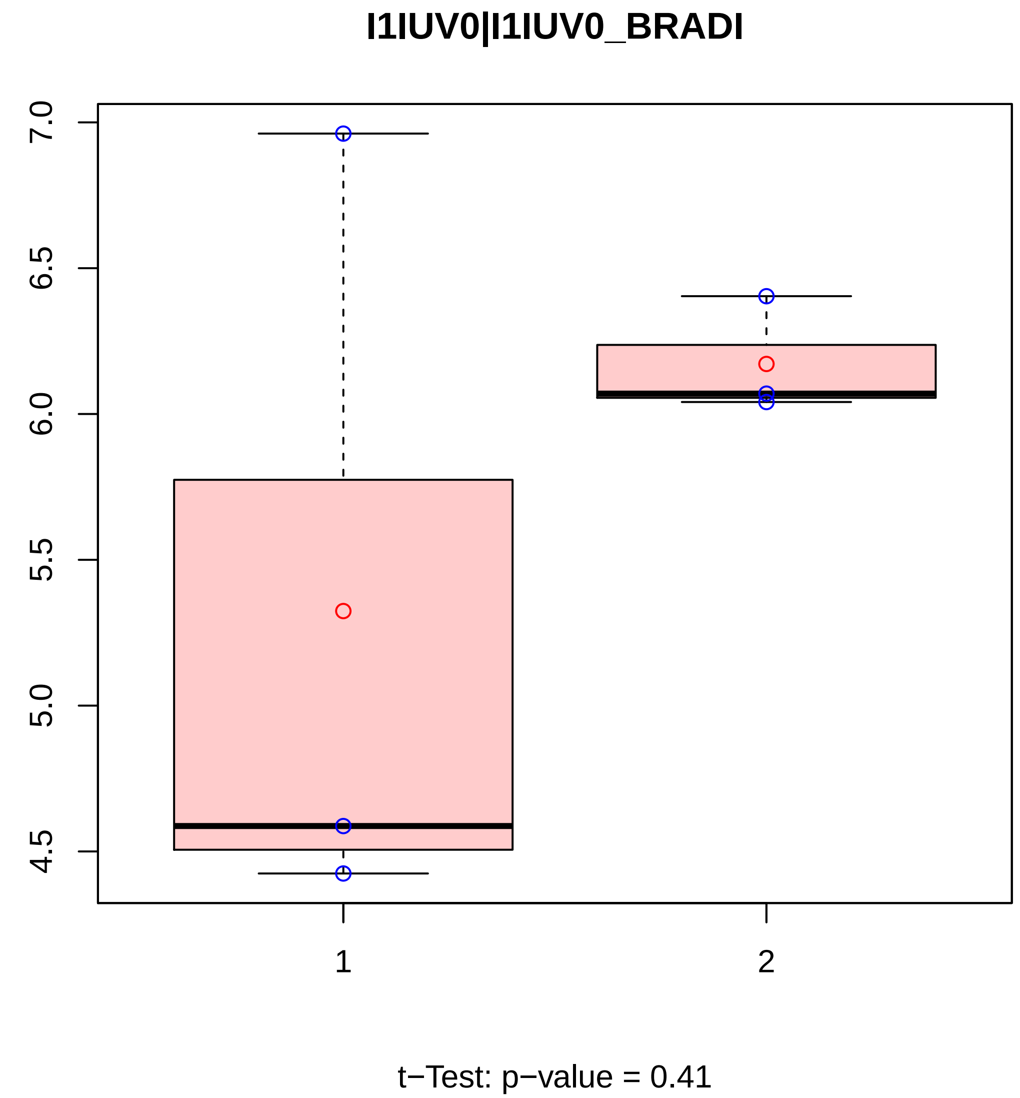
I1IUV0|I1IUV0_BRADI
7.0
6.5
6.0
5.5
5.0
4.5
1
2
t−Test: p−value = 0.41

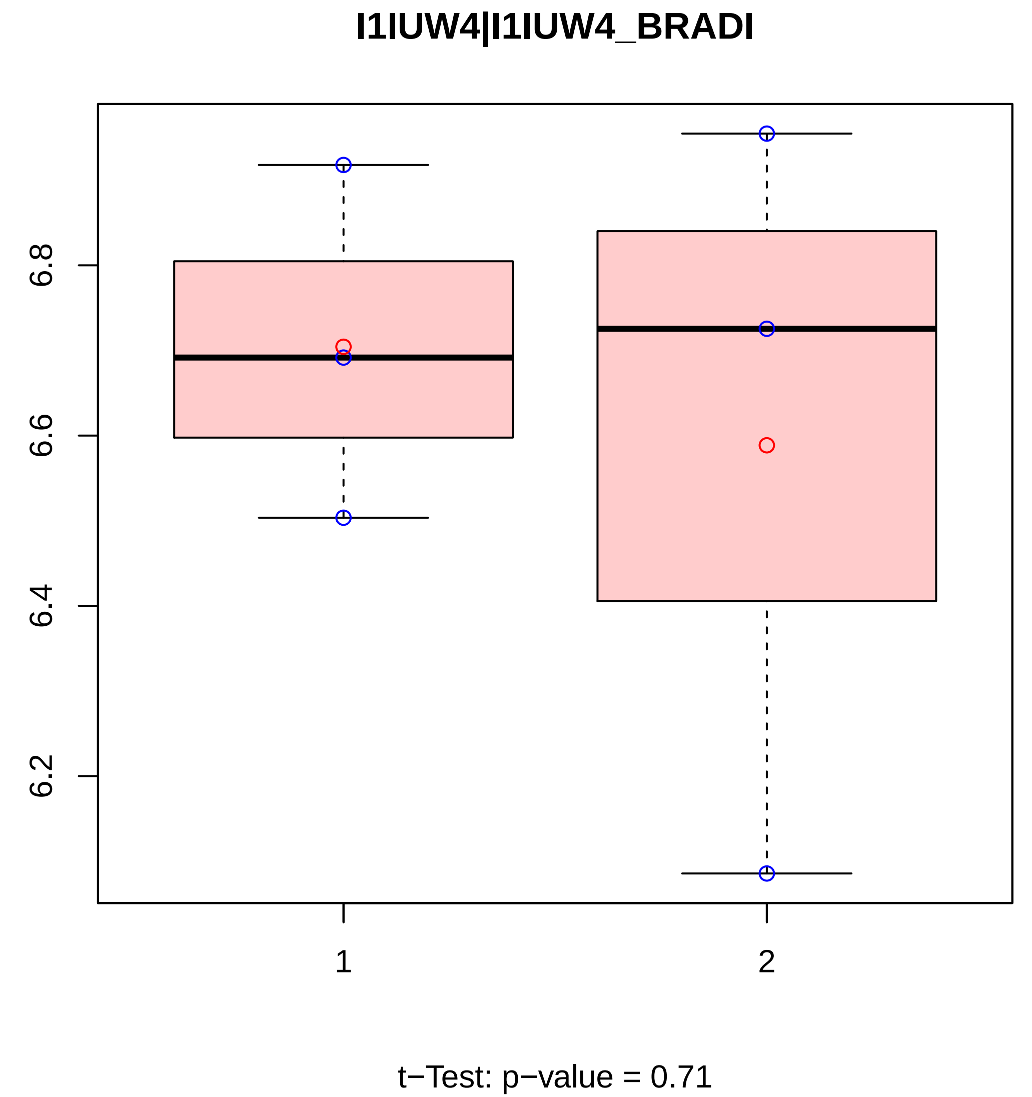

I1IUW4|I1IUW4_BRADI
6.2
6.4
6.6
6.8
1
2
t−Test: p−value = 0.71

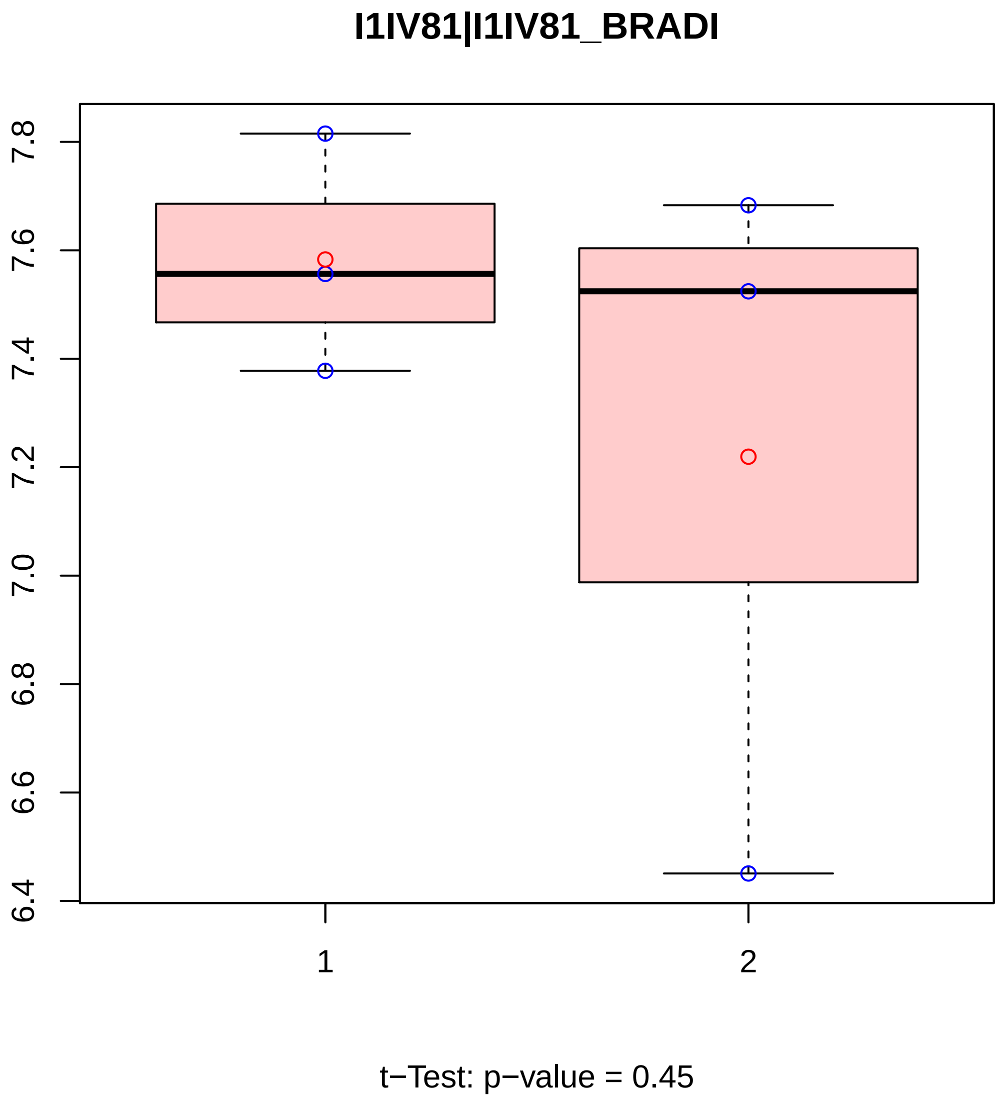
I1IV81|I1IV81_BRADI
7.8
7.6
7.4
7.2
7.0
6.8
6.6
6.4
1
2
t−Test: p−value = 0.45

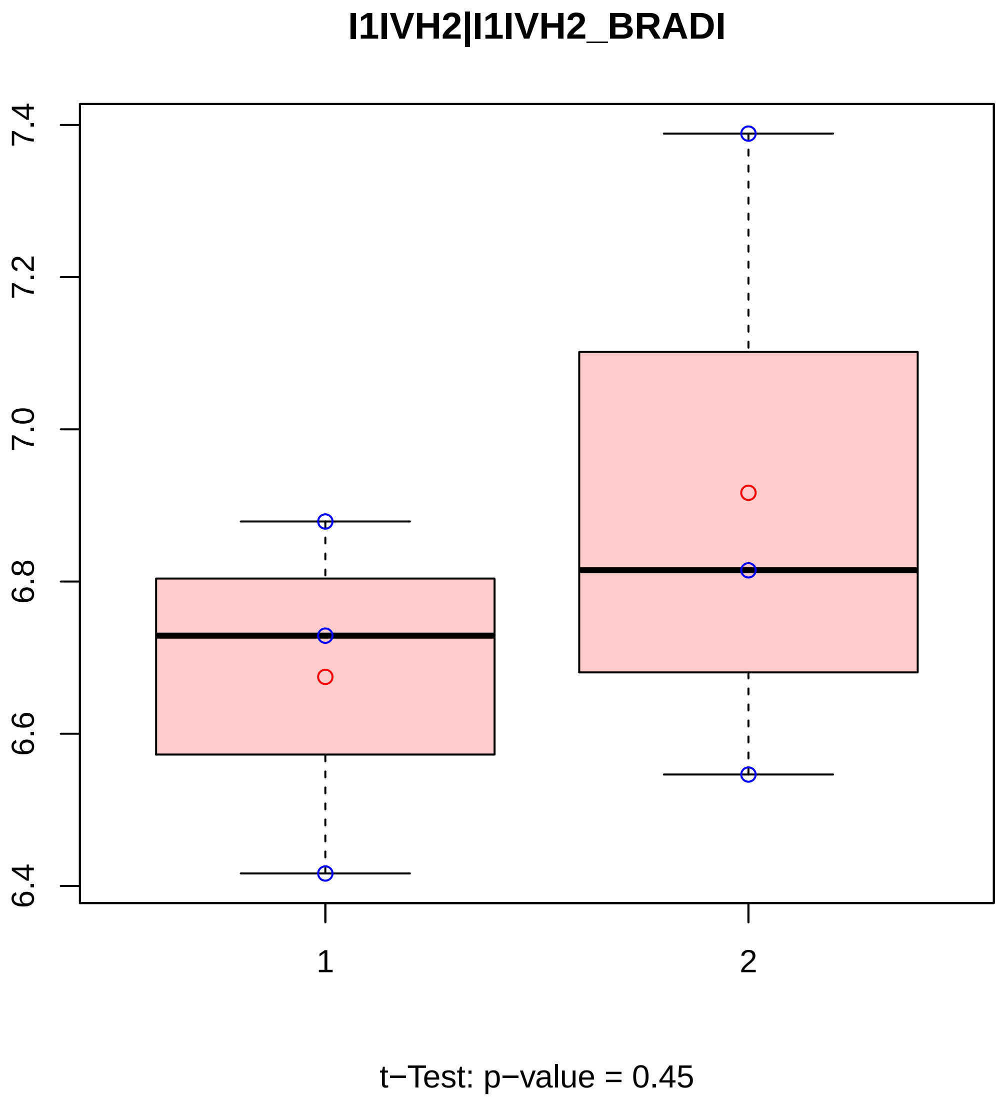
I1IVH2|I1IVH2_BRADI
7.4
7.2
7.0
6.8
6.6
6.4
1
2
t−Test: p−value = 0.45

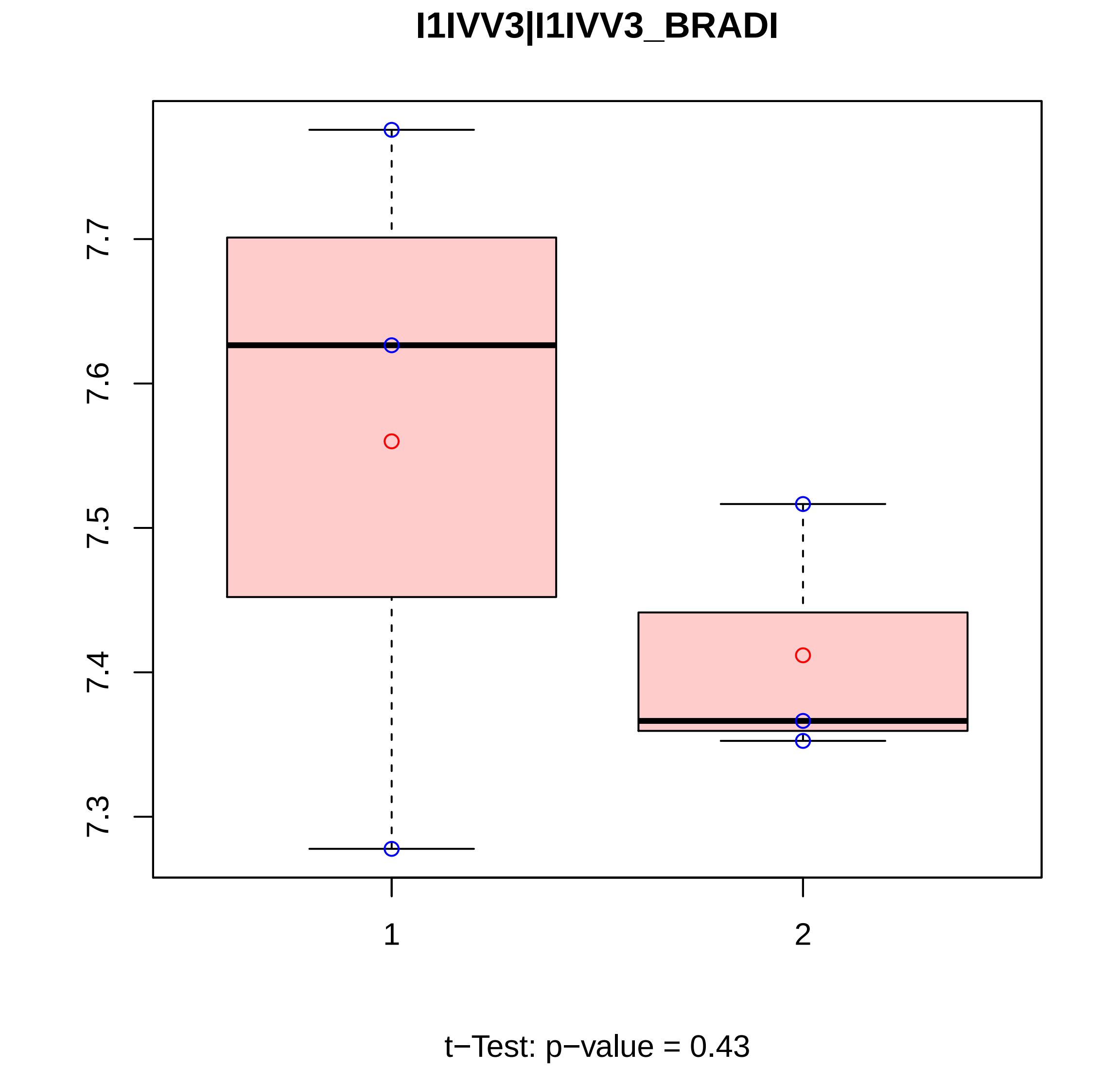

I1IVV3|I1IVV3_BRADI
7.3
7.4
7.5
7.6
7.7
1
2
t−Test: p−value = 0.43

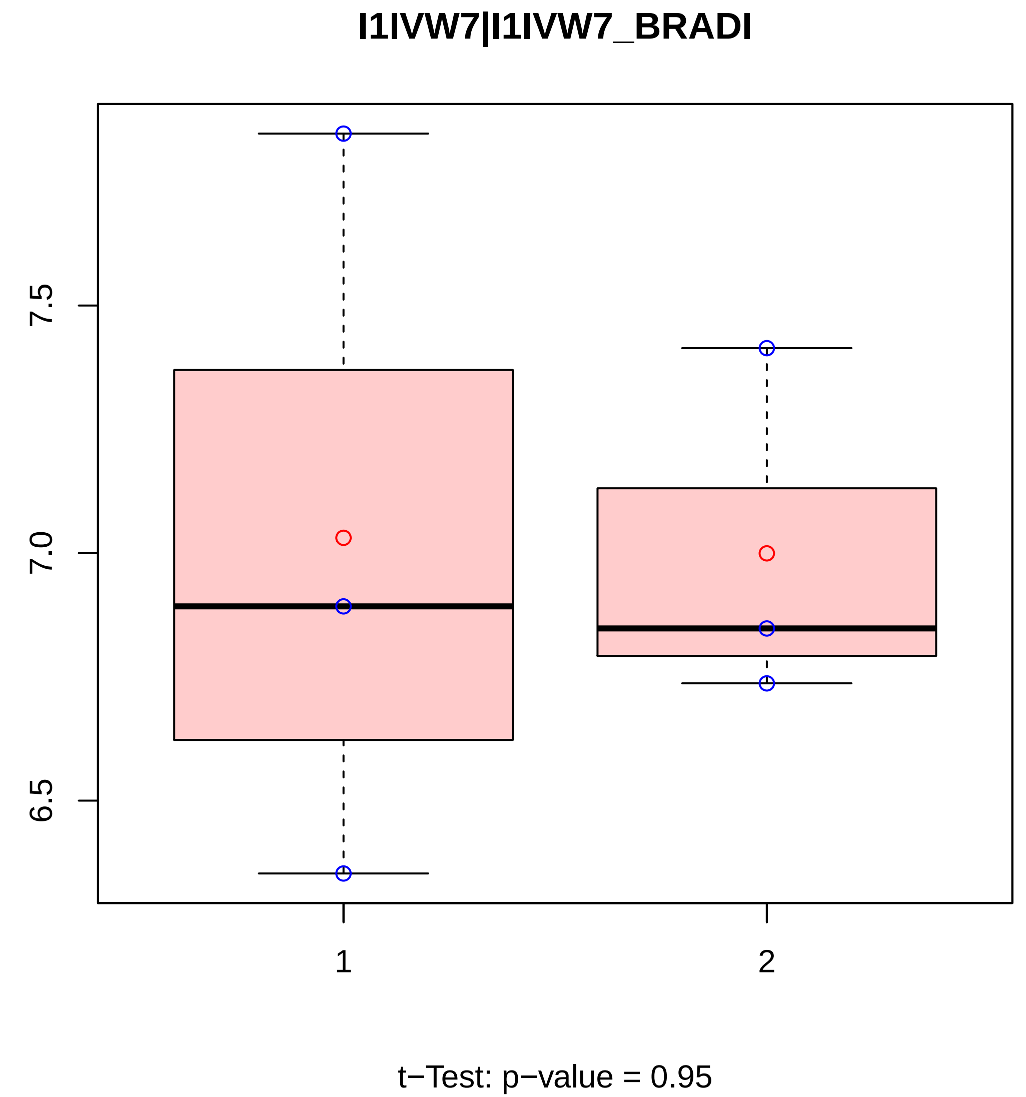
I1IVW7|I1IVW7_BRADI
7.5
7.0
6.5
1
2
t−Test: p−value = 0.95

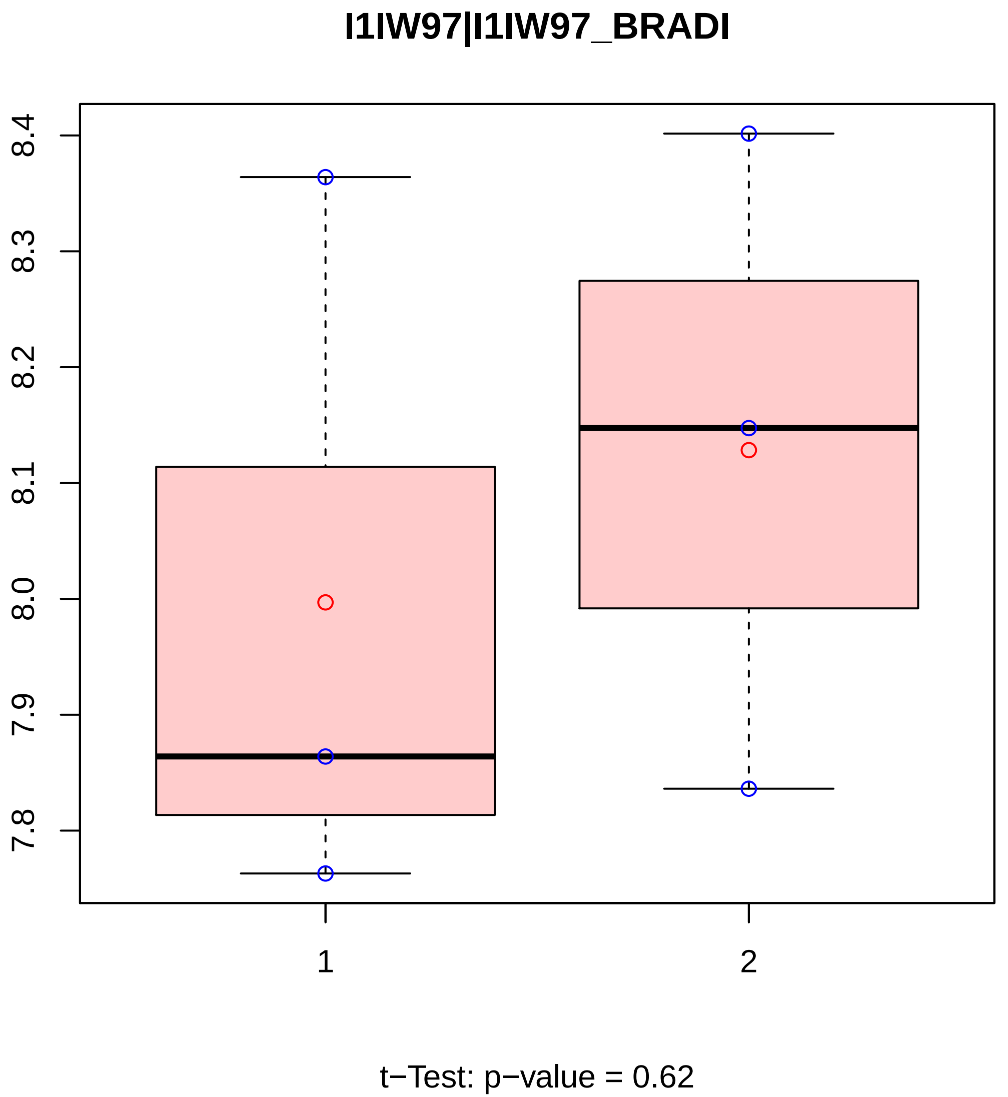
I1IW97|I1IW97_BRADI
8.4
8.3
8.2
8.1
8.0
7.9
7.8
1
2
t−Test: p−value = 0.62

I1IWF2|I1IWF2_BRADI

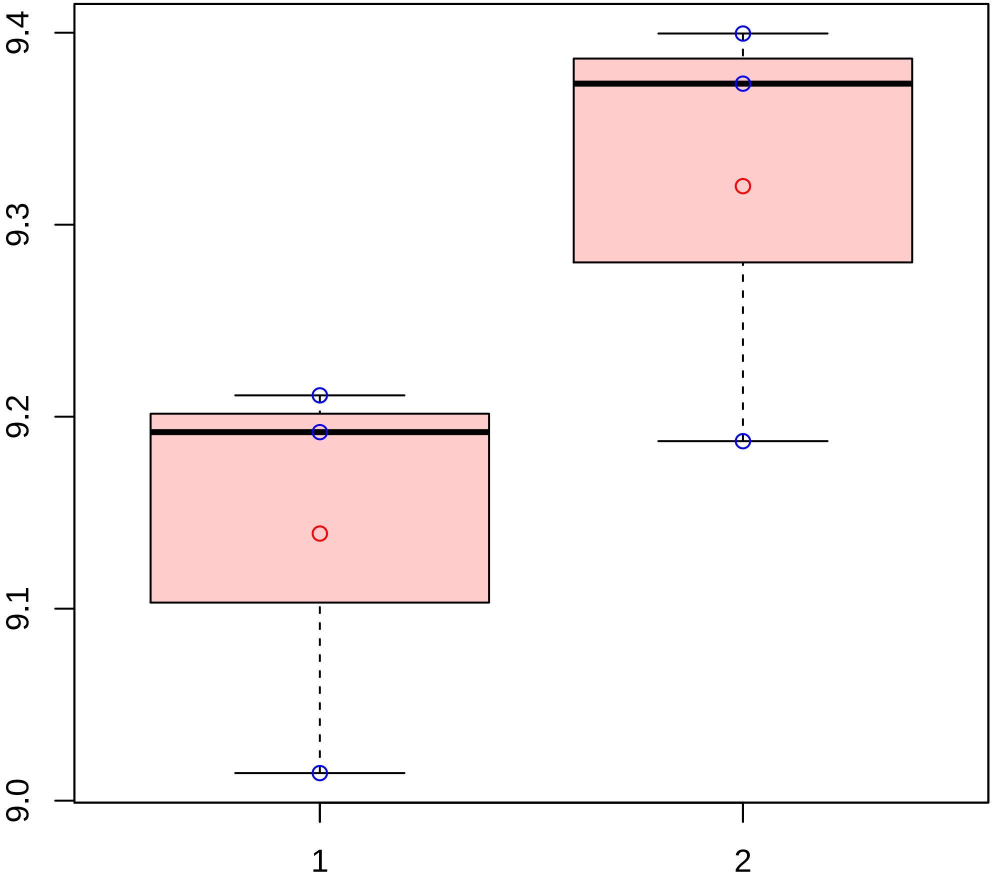

t−Test: p−value = 0.12

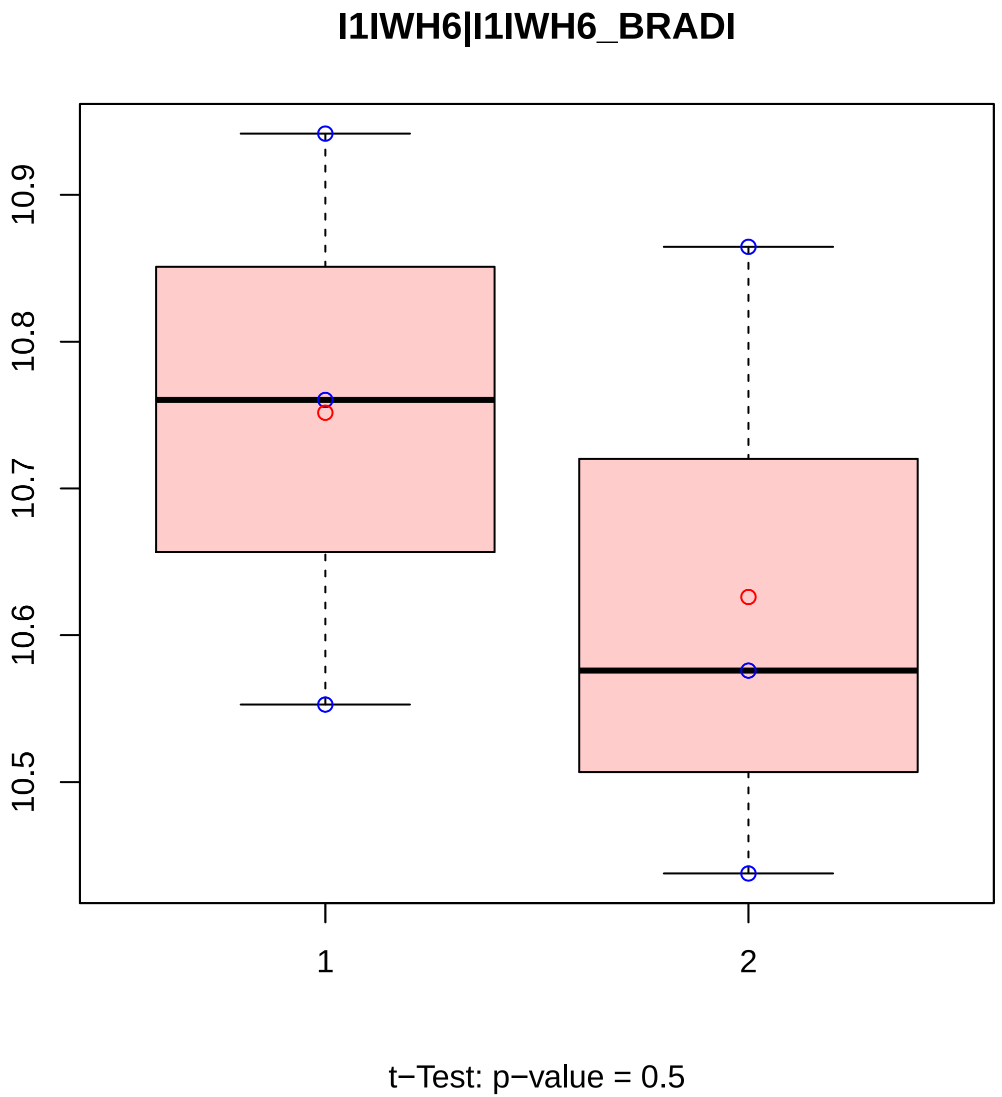
I1IWH6|I1IWH6_BRADI
10.9
10.8
10.7
10.6
10.5
1
2
t−Test: p−value = 0.5

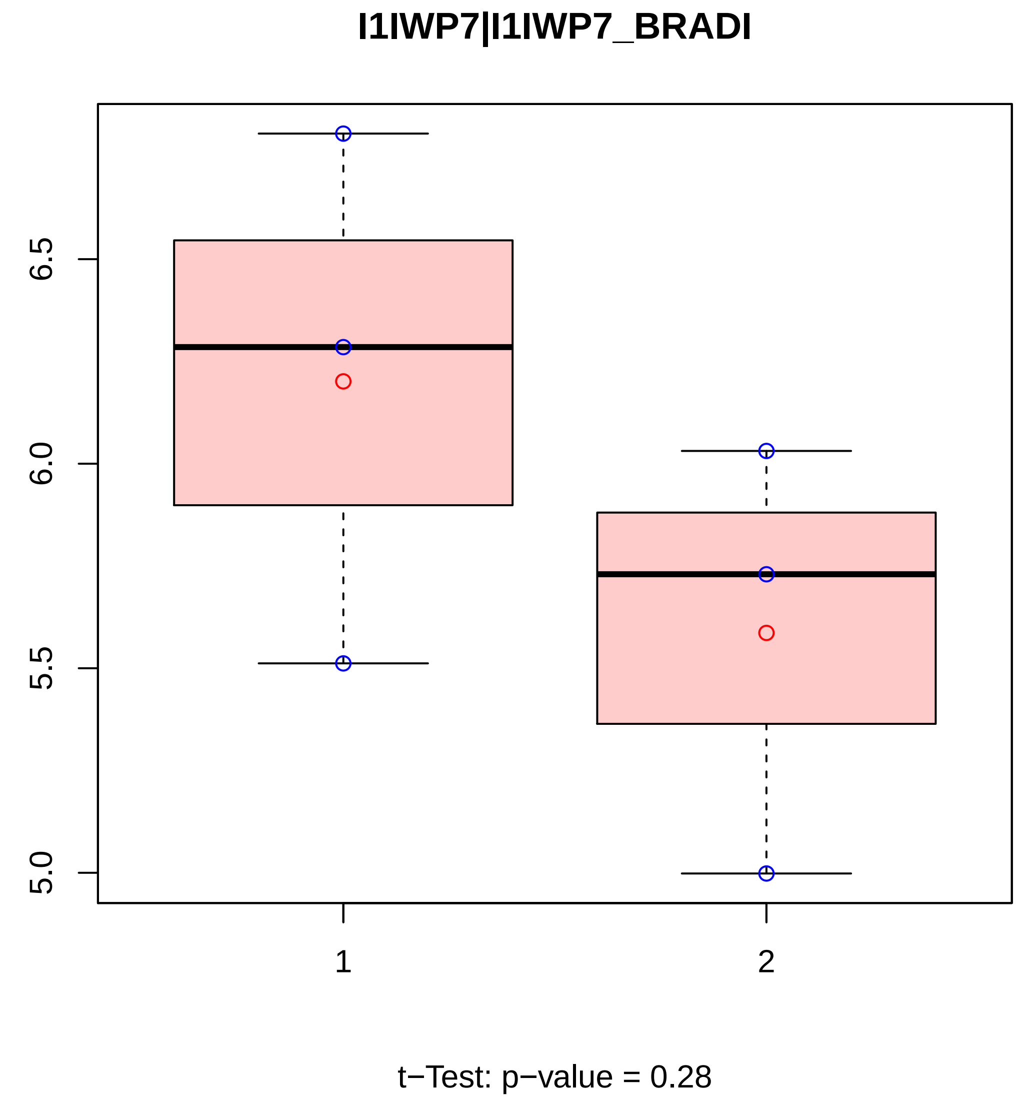
I1IWP7|I1IWP7_BRADI
5.0
5.5
6.0
6.5
1
2
t−Test: p−value = 0.28

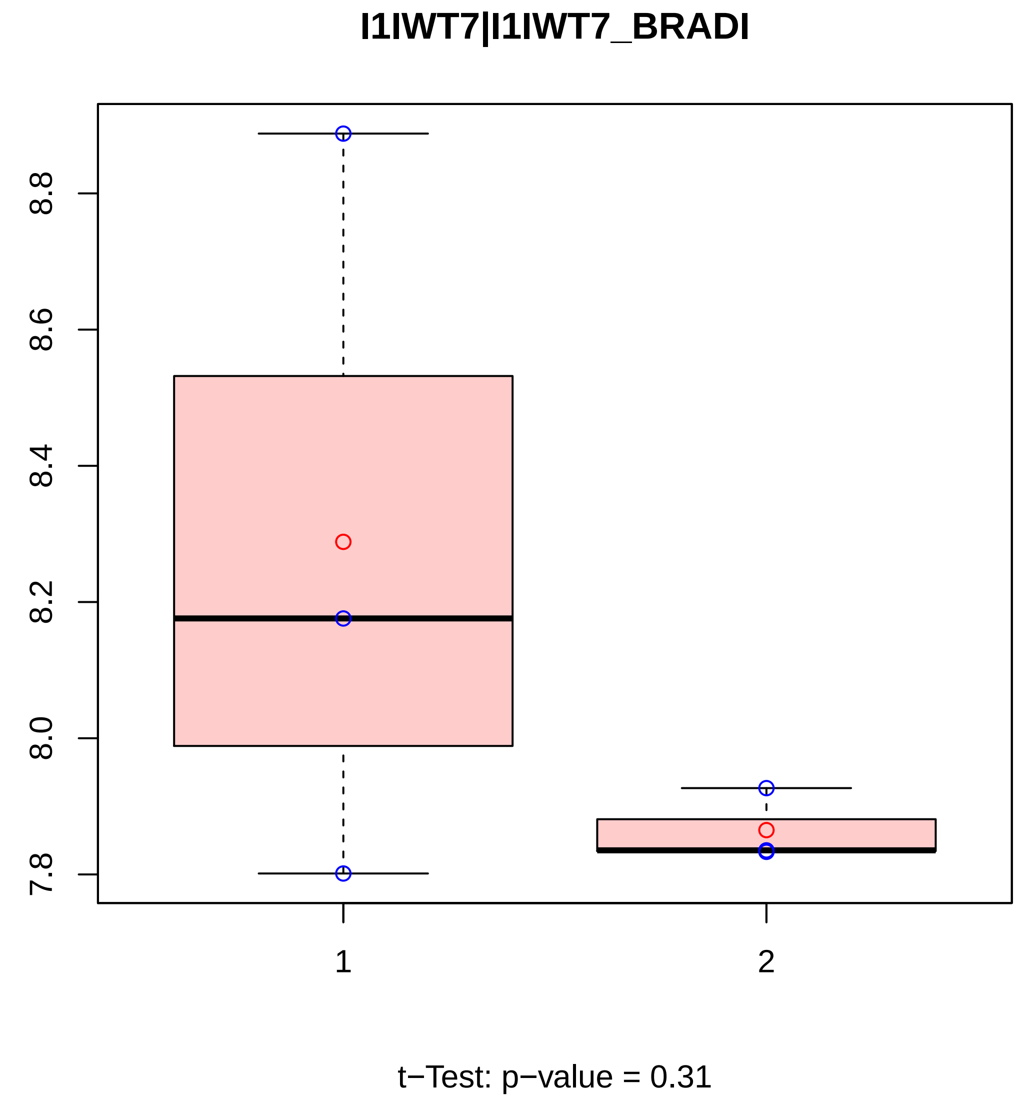
I1IWT7|I1IWT7_BRADI
8.8
8.6
8.4
8.2
8.0
7.8
1
2
t−Test: p−value = 0.31

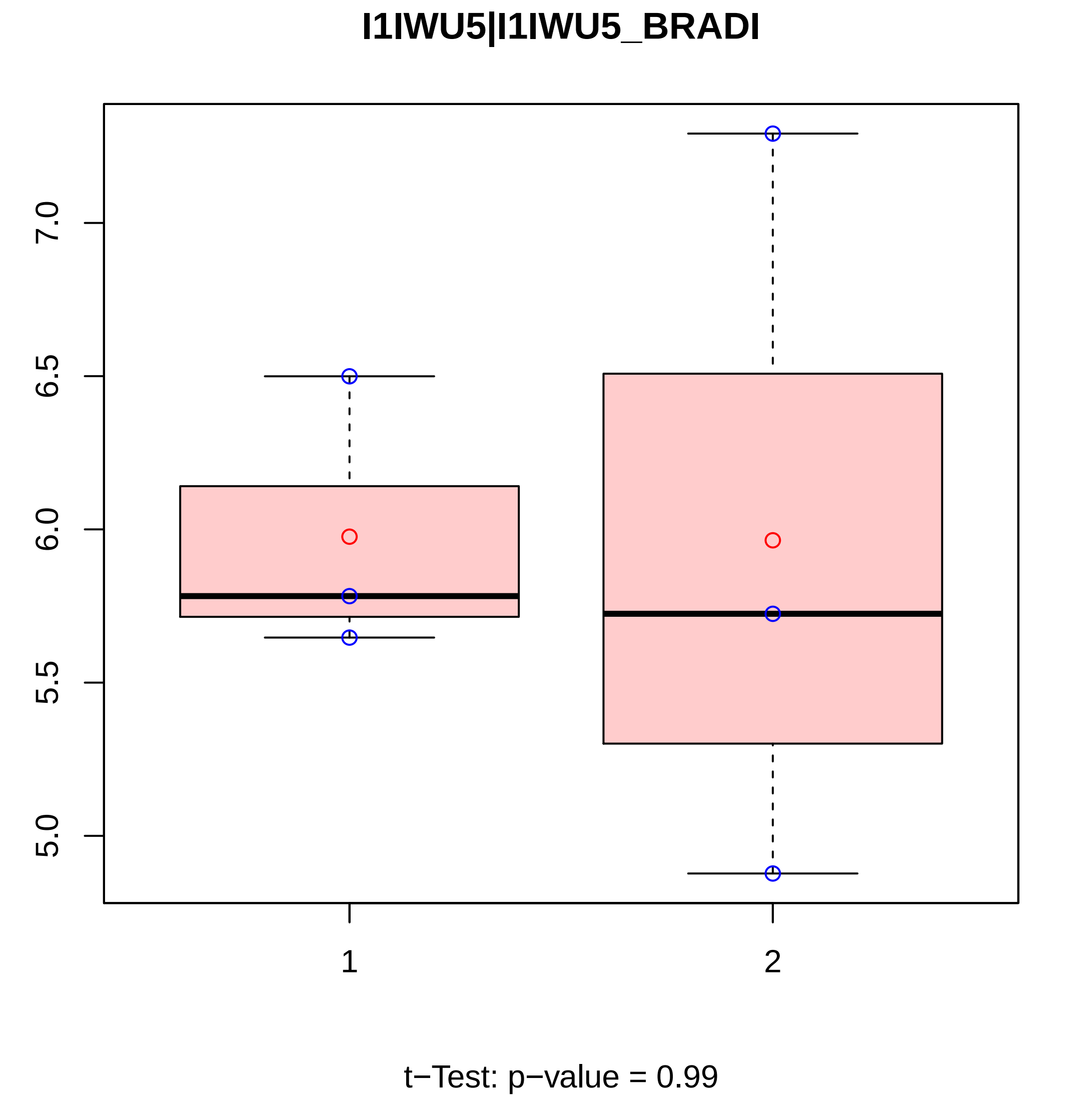
I1IWU5|I1IWU5_BRADI
7.0
6.5
6.0
5.5
5.0
1
2
t−Test: p−value = 0.99

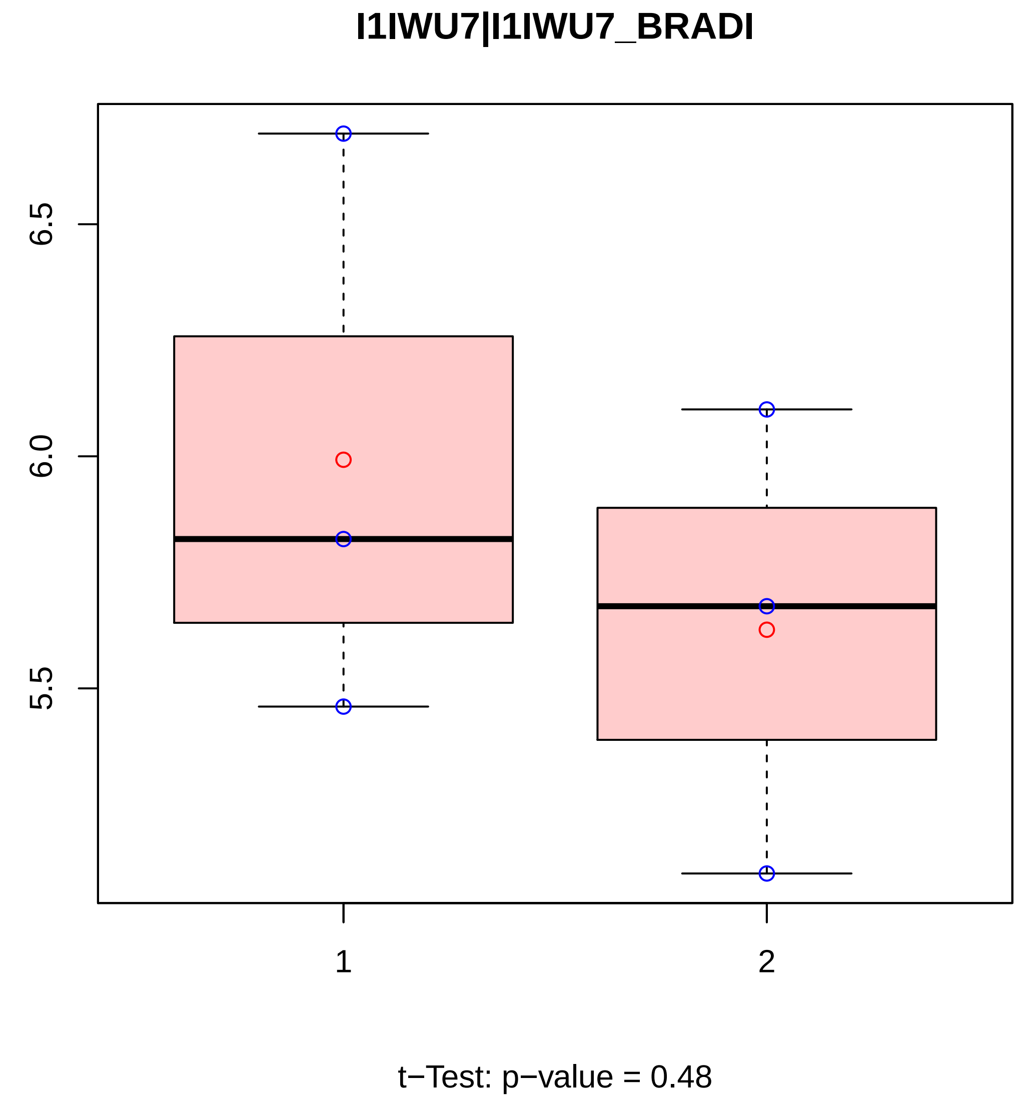
I1IWU7|I1IWU7_BRADI
6.5
6.0
5.5
1
2
t−Test: p−value = 0.48

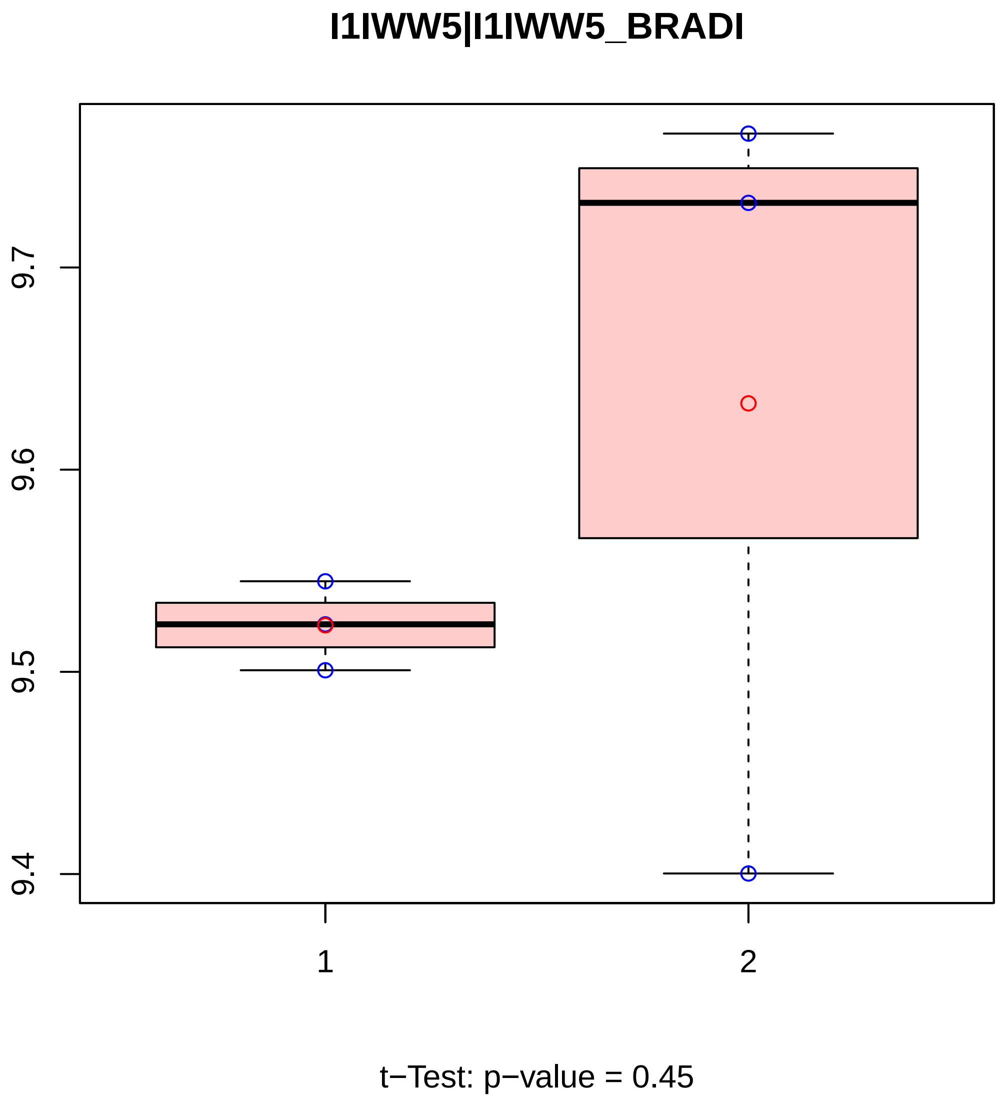
I1IWW5|I1IWW5_BRADI
9.4
9.5
9.6
9.7
1
2
t−Test: p−value = 0.45

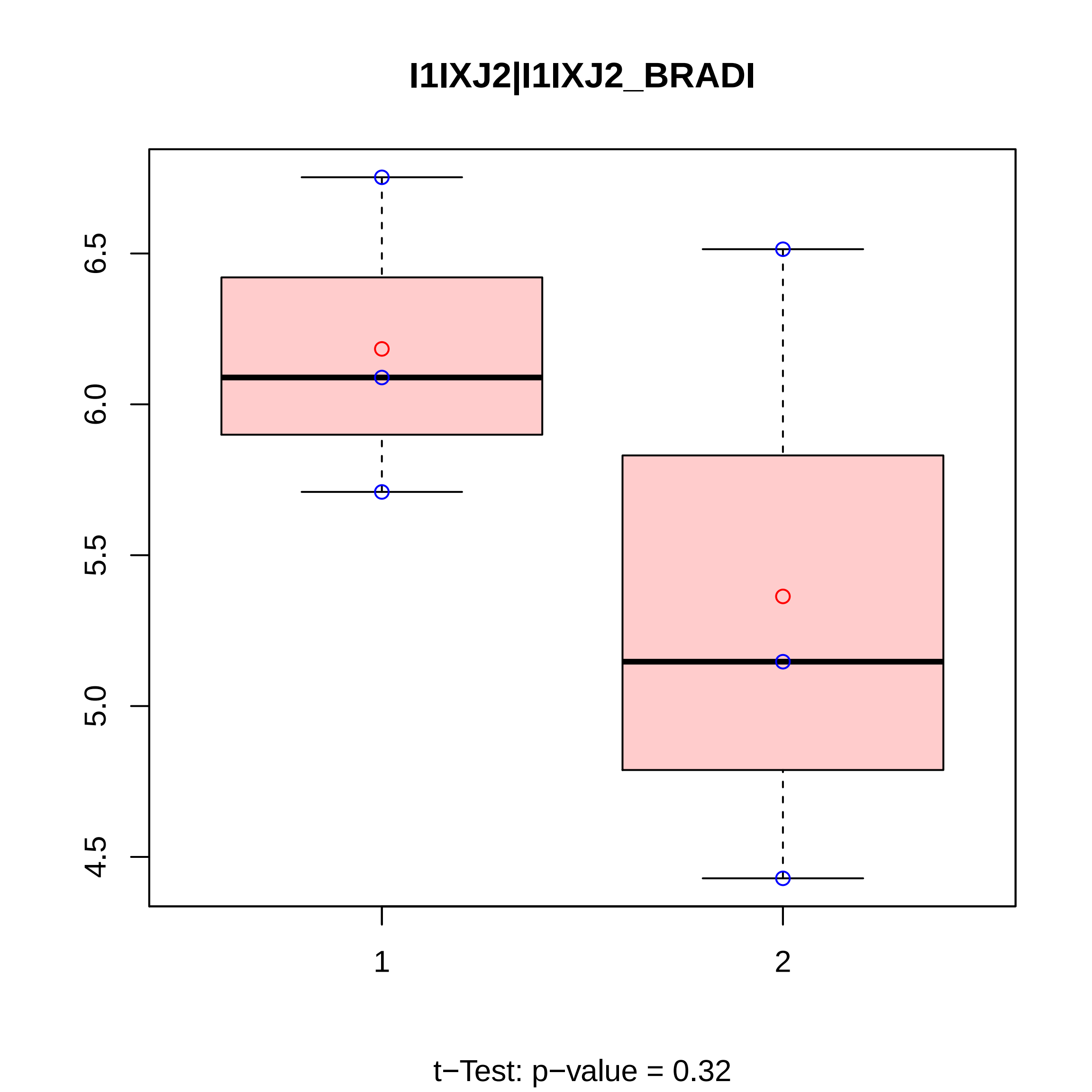
I1IXJ2|I1IXJ2_BRADI
6.5
6.0
5.5
5.0
4.5
1
2
t−Test: p−value = 0.32

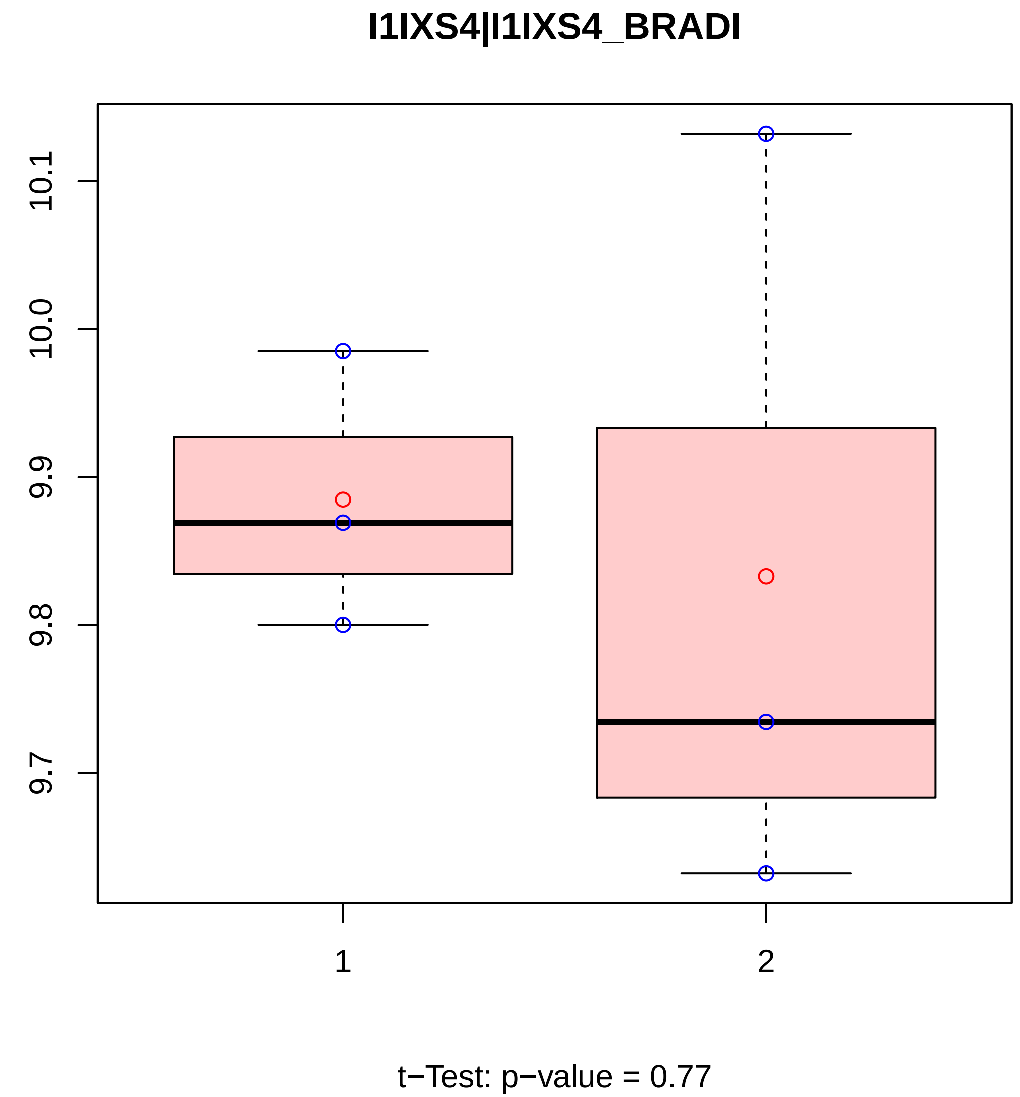
I1IXS4|I1IXS4_BRADI
10.1
10.0
9.9
9.8
9.7
1
2
t−Test: p−value = 0.77

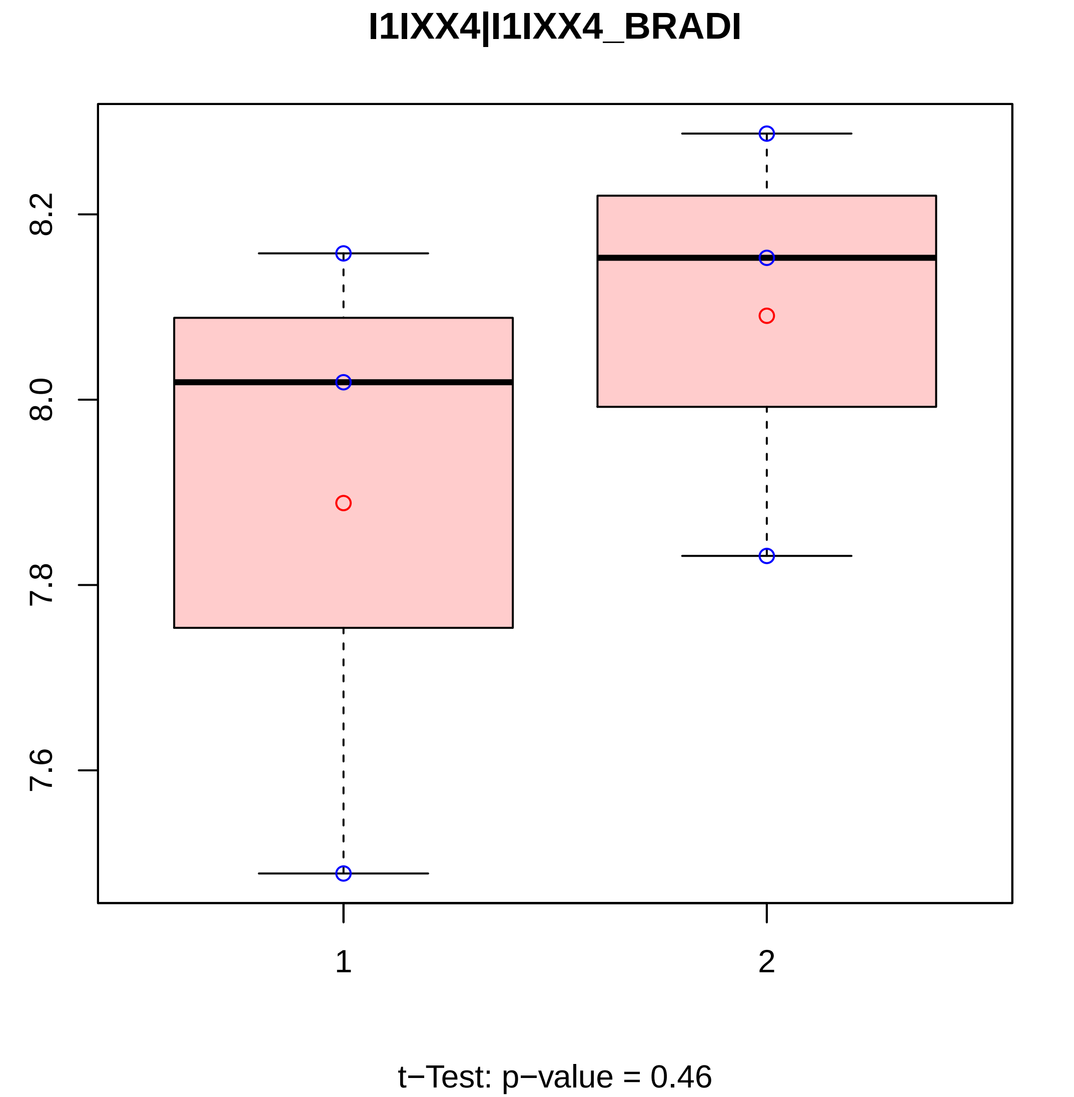
I1IXX4|I1IXX4_BRADI
8.2
8.0
7.8
7.6
1
2
t−Test: p−value = 0.46

I1IY80|I1IY80_BRADI

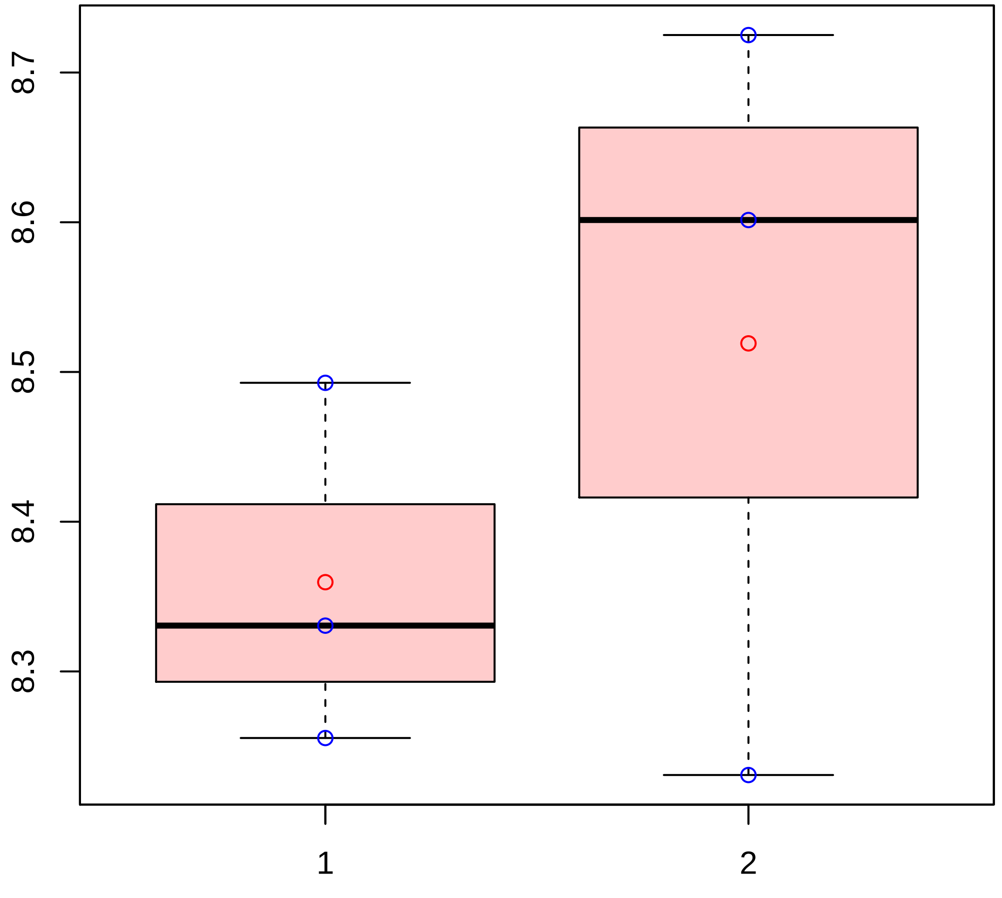

t−Test: p−value = 0.41

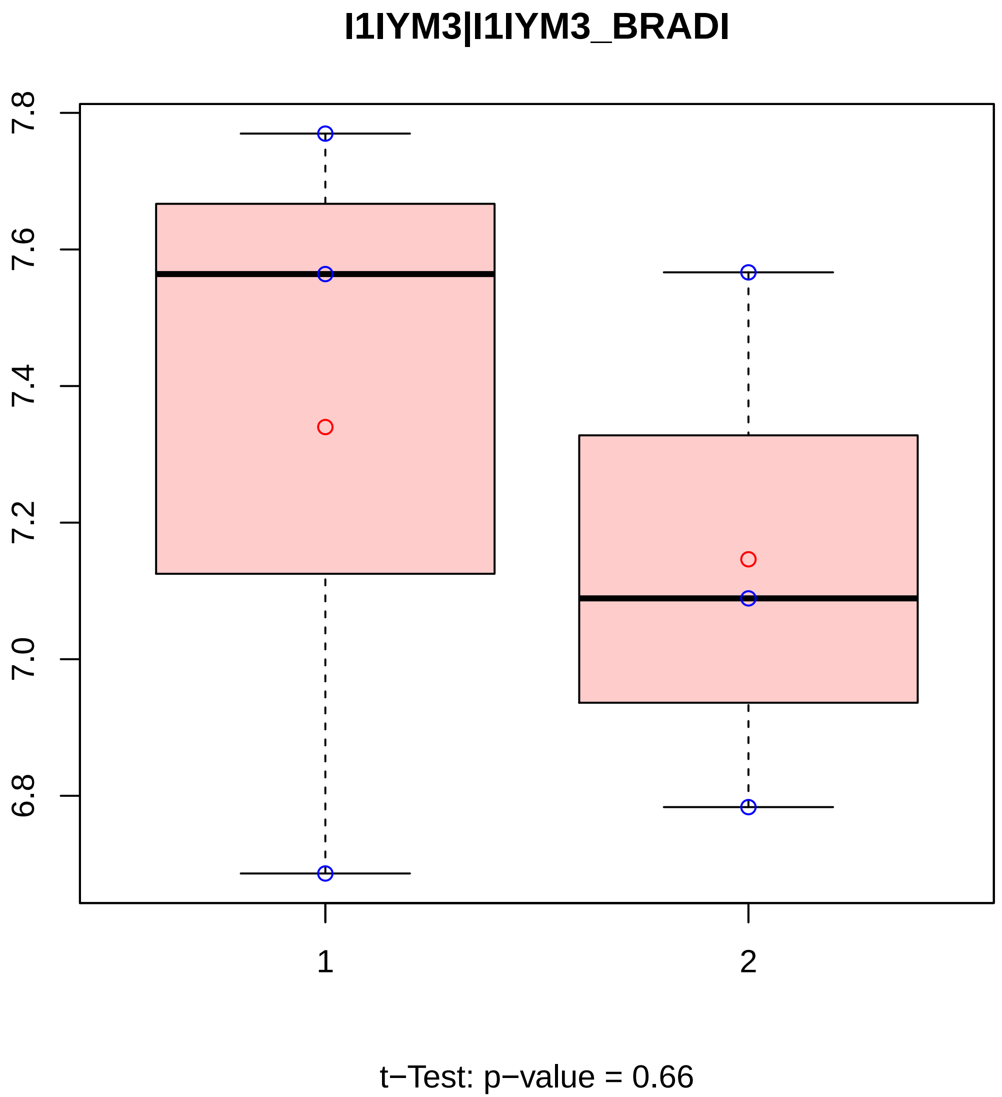
I1IYM3|I1IYM3_BRADI
7.8
7.6
7.4
7.2
7.0
6.8
1
2
t−Test: p−value = 0.66

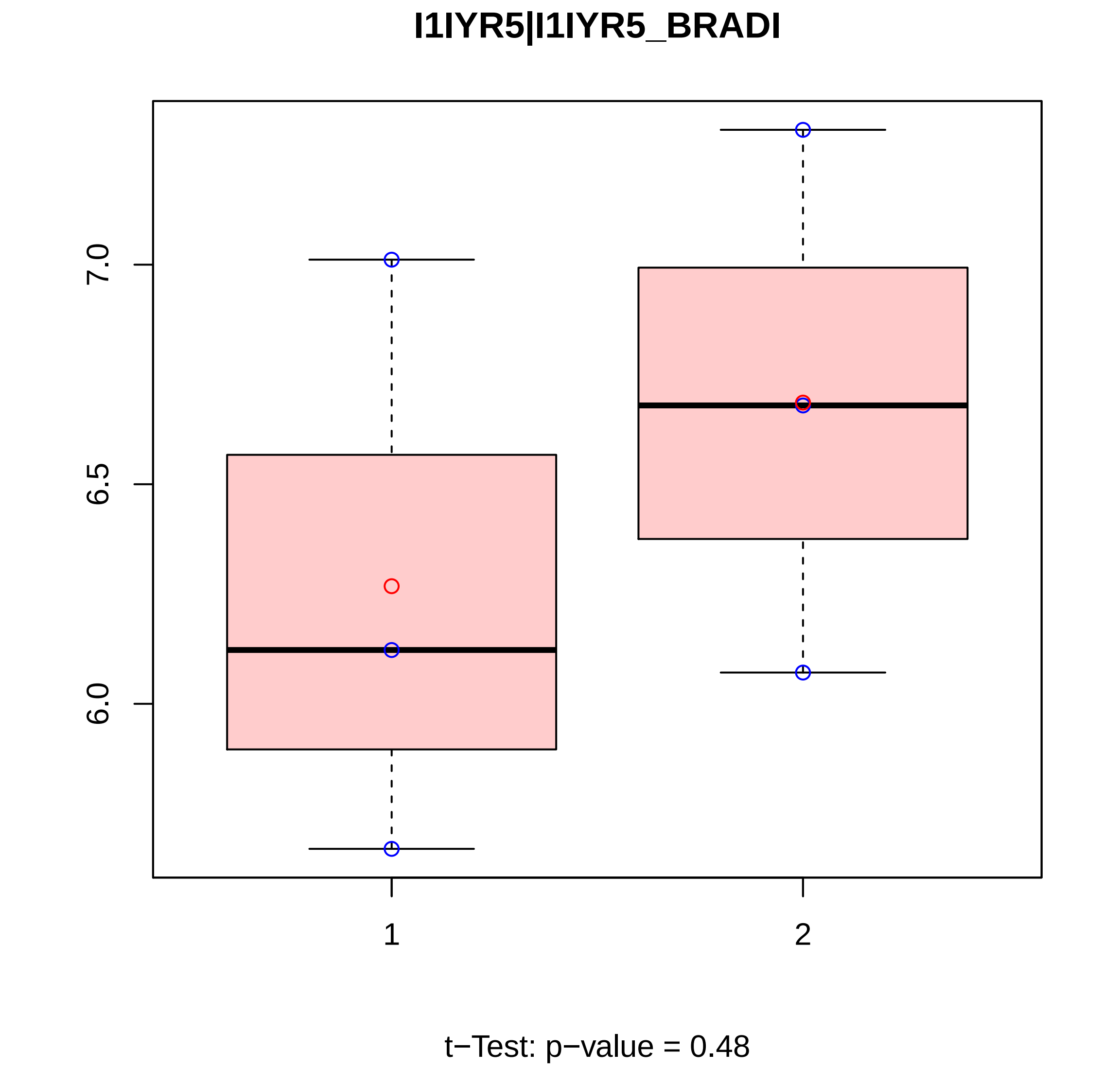

I1IYR5|I1IYR5_BRADI
7.0
6.5
6.0
1
2
t−Test: p−value = 0.48

I1IZ44|I1IZ44_BRADI

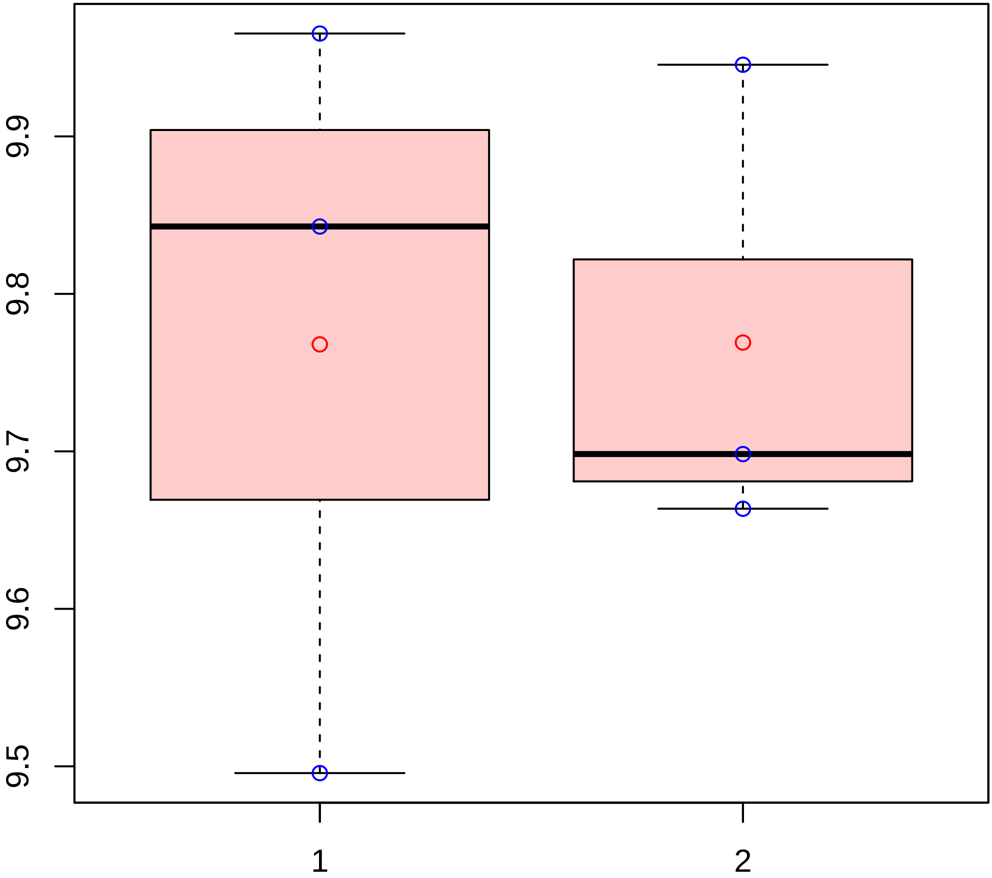

t−Test: p−value = 0.99

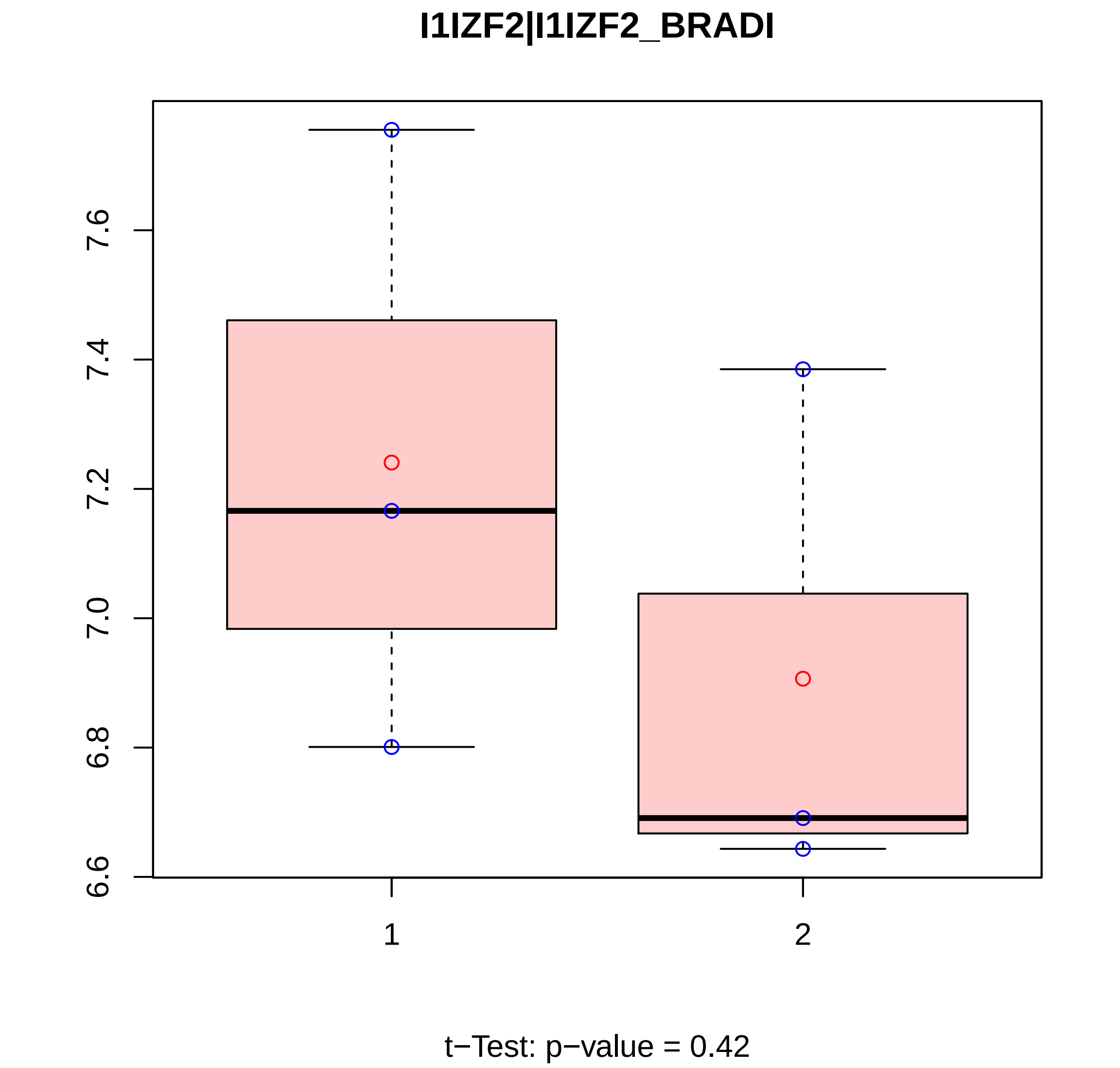
I1IZF2|I1IZF2_BRADI
7.6
7.4
7.2
7.0
6.8
6.6
1
2
t−Test: p−value = 0.42

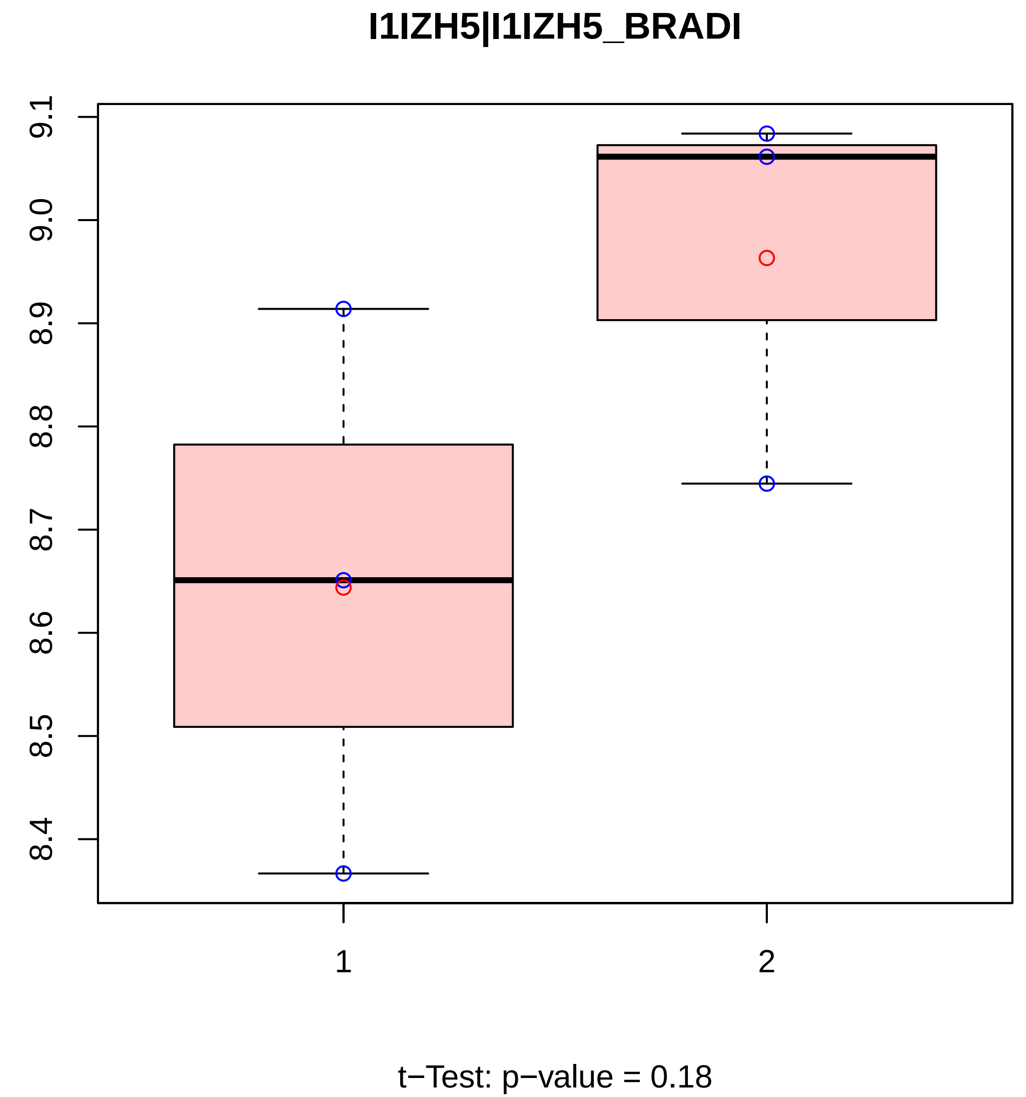
I1IZH5|I1IZH5_BRADI
9.1
9.0
8.9
8.8
8.7
8.6
8.5
8.4
1
2
t−Test: p−value = 0.18

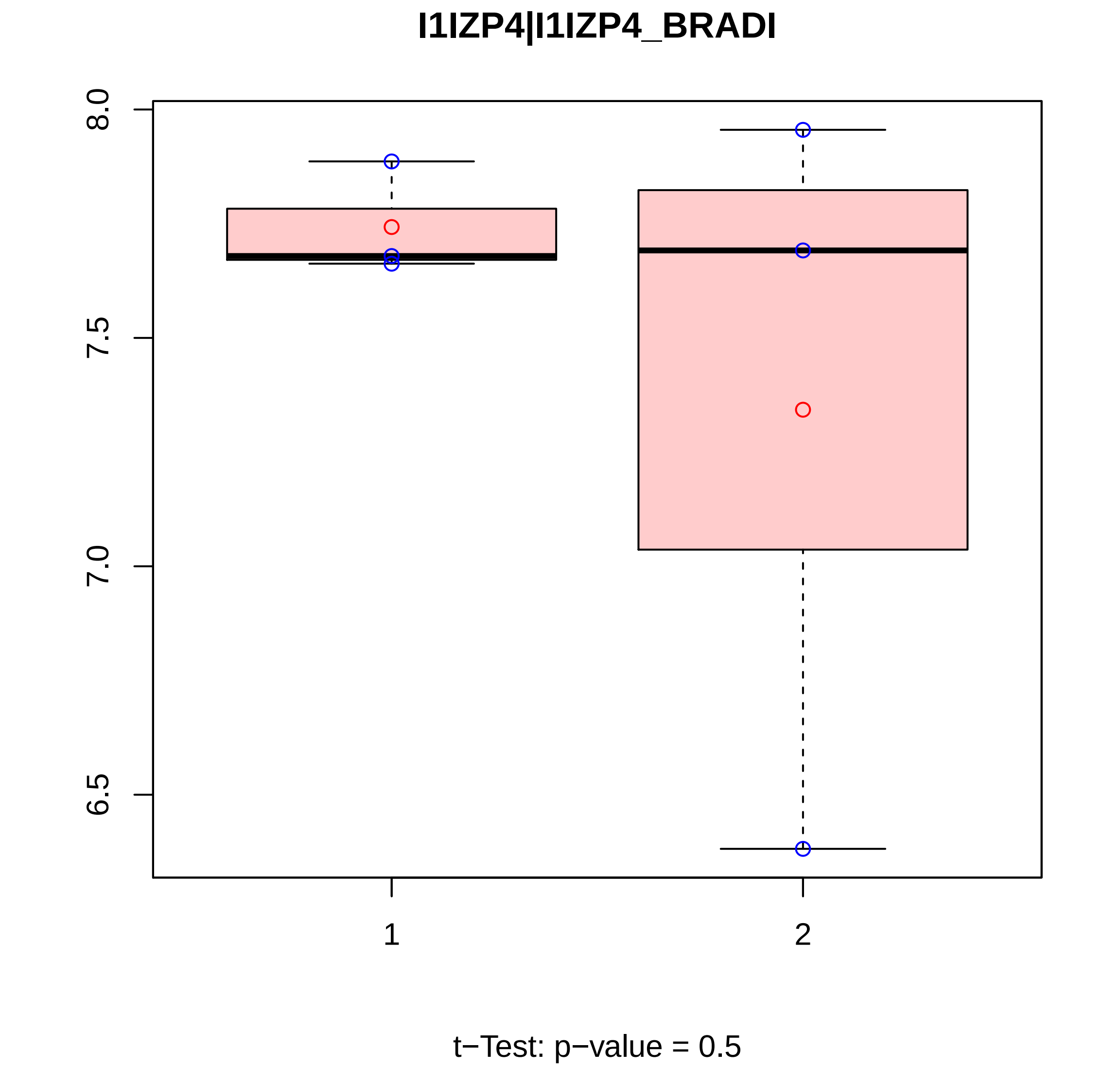
I1IZP4|I1IZP4_BRADI
8.0
7.5
7.0
6.5
1
2
t−Test: p−value = 0.5

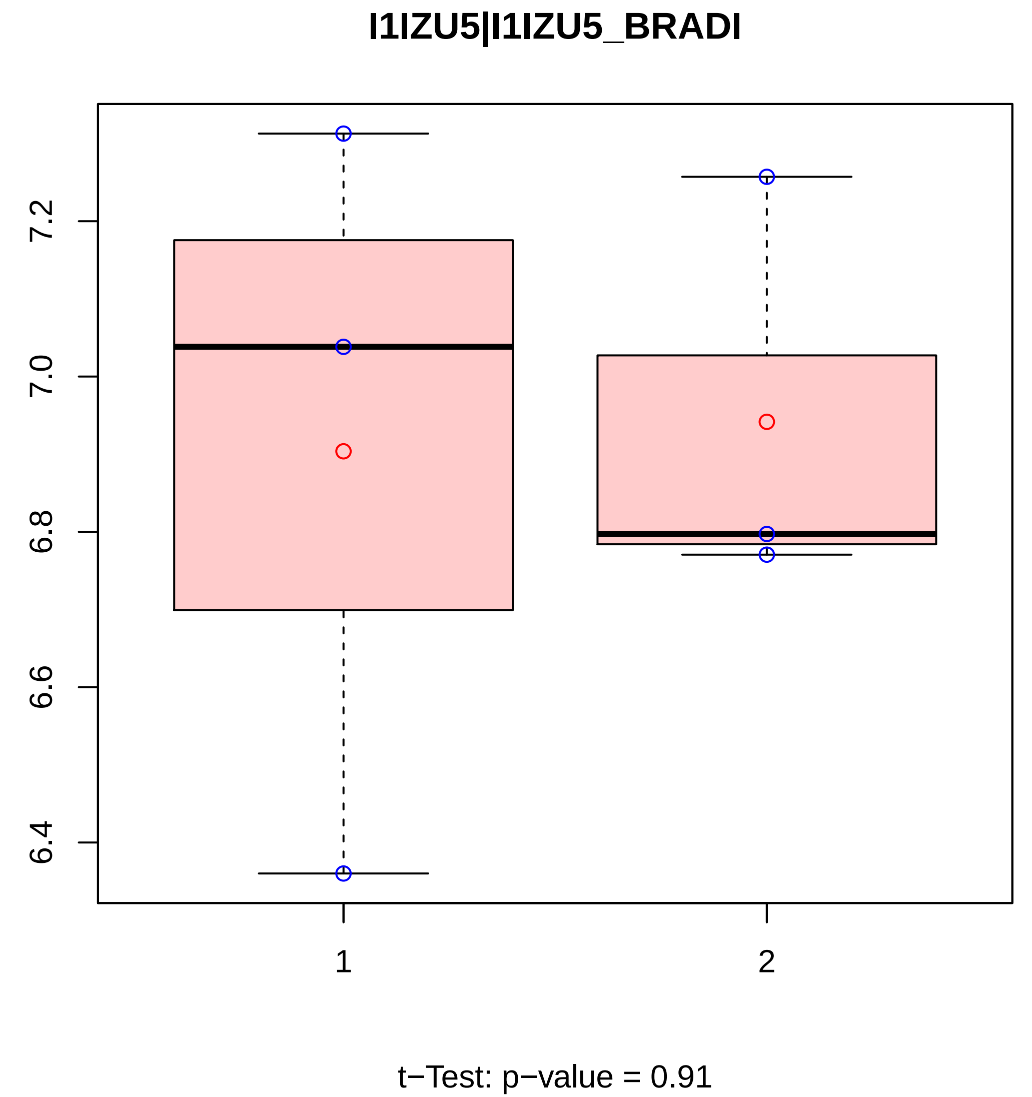
I1IZU5|I1IZU5_BRADI
7.2
7.0
6.8
6.6
6.4
1
2
t−Test: p−value = 0.91

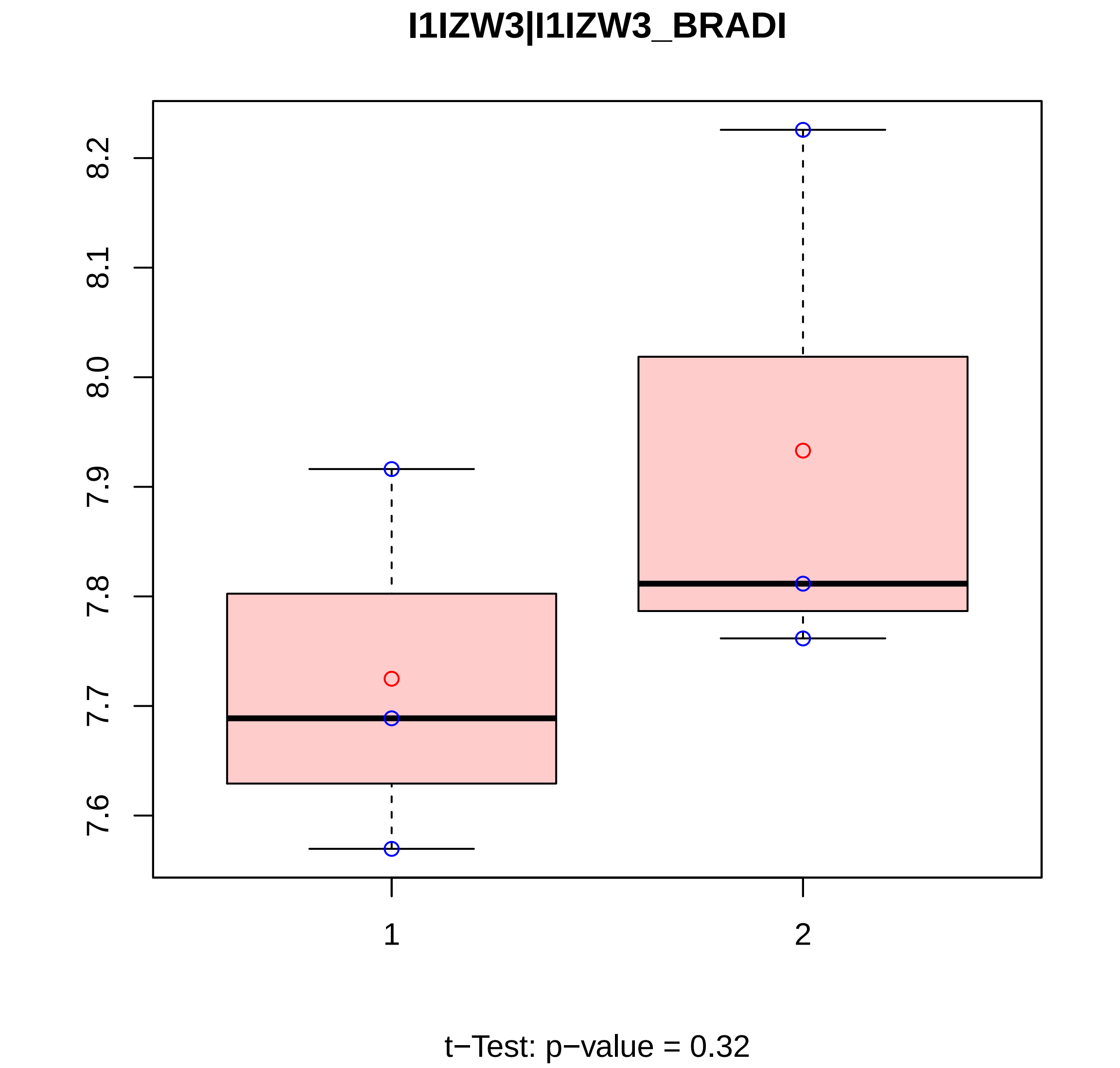
I1IZW3|I1IZW3_BRADI
8.2
8.1
8.0
7.9
7.8
7.7
7.6
1
2
t−Test: p−value = 0.32

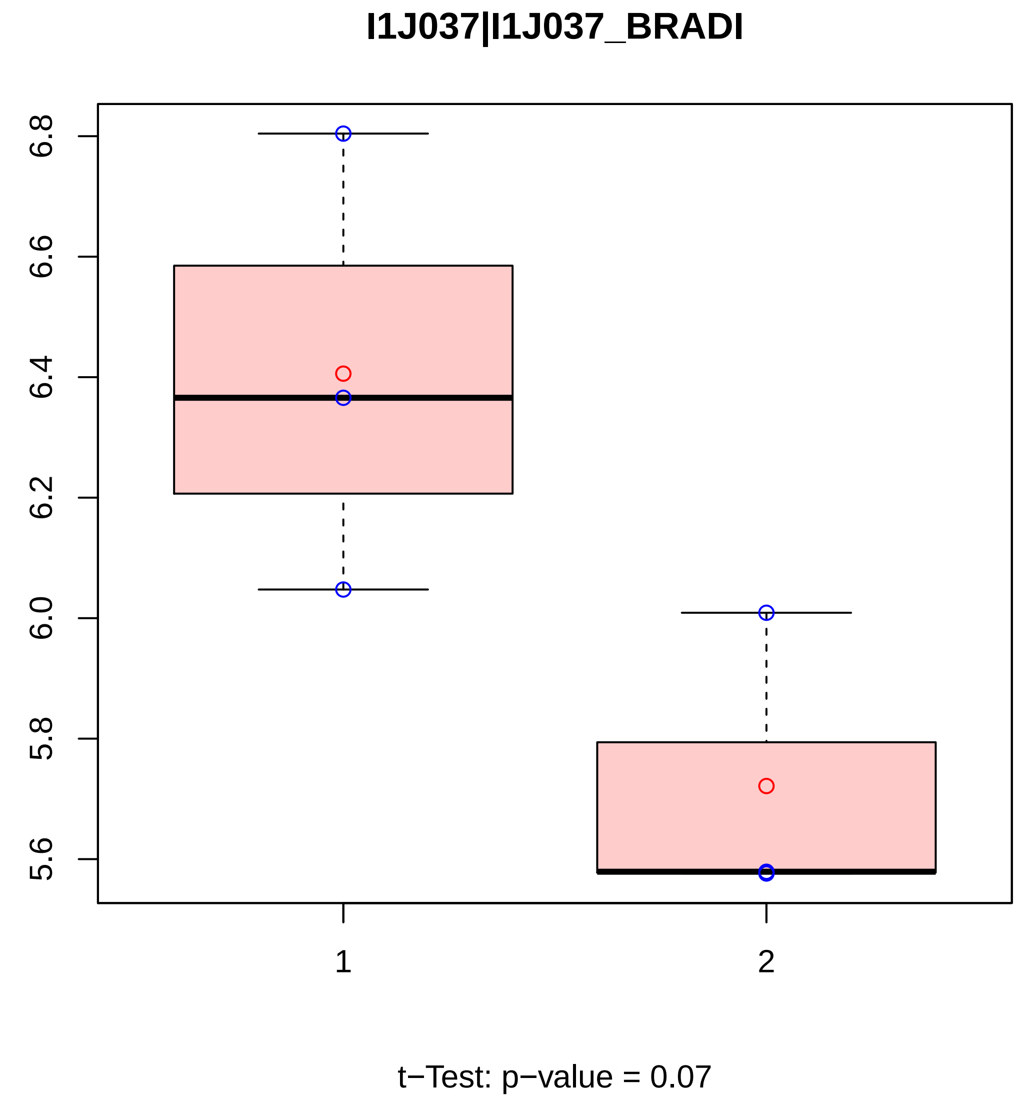

I1J037|I1J037_BRADI
6.8
6.6
6.4
6.2
6.0
5.8
5.6
1
2
t−Test: p−value = 0.07

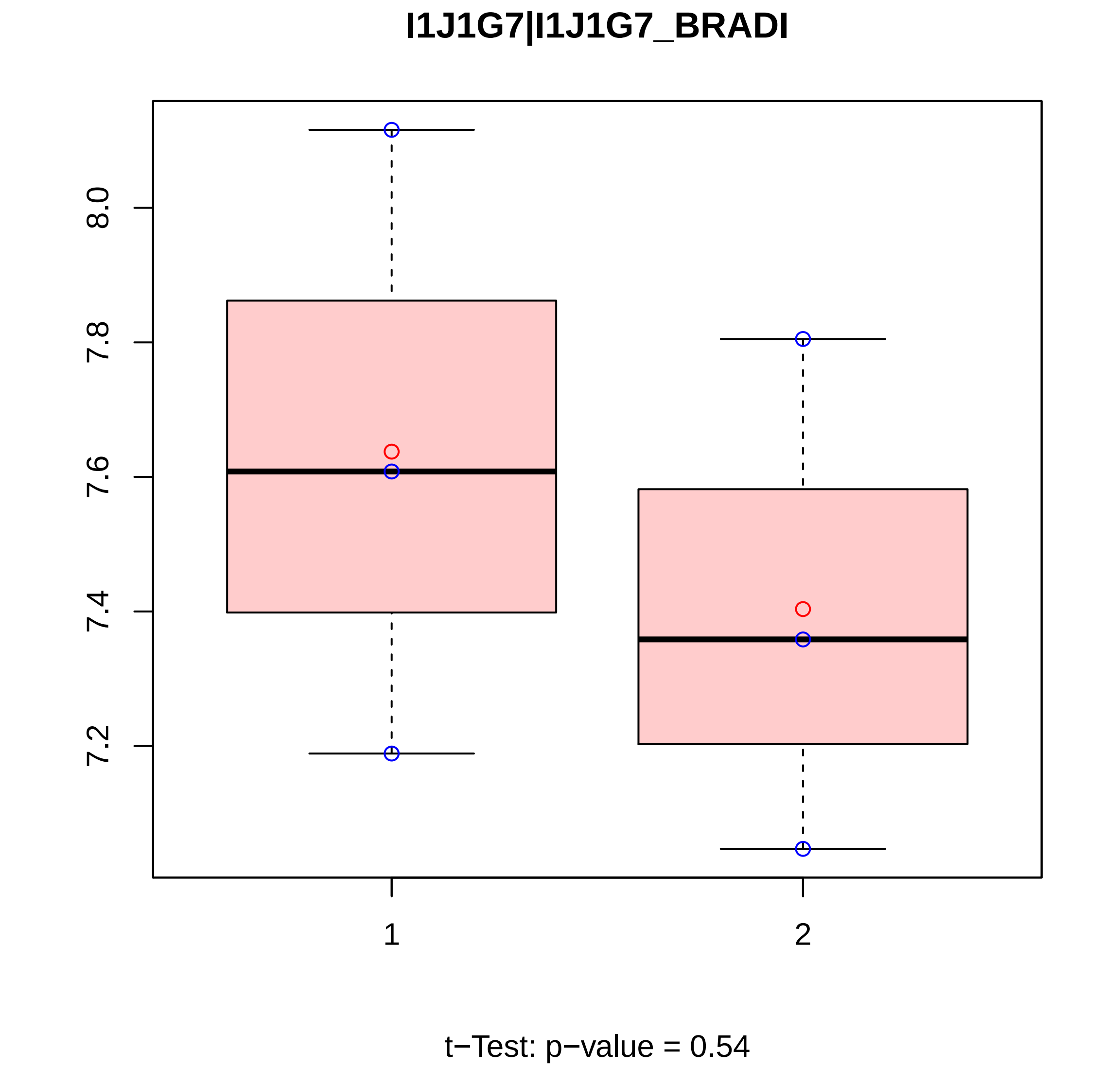
I1J1G7|I1J1G7_BRADI
8.0
7.8
7.6
7.4
7.2
1
2
t−Test: p−value = 0.54

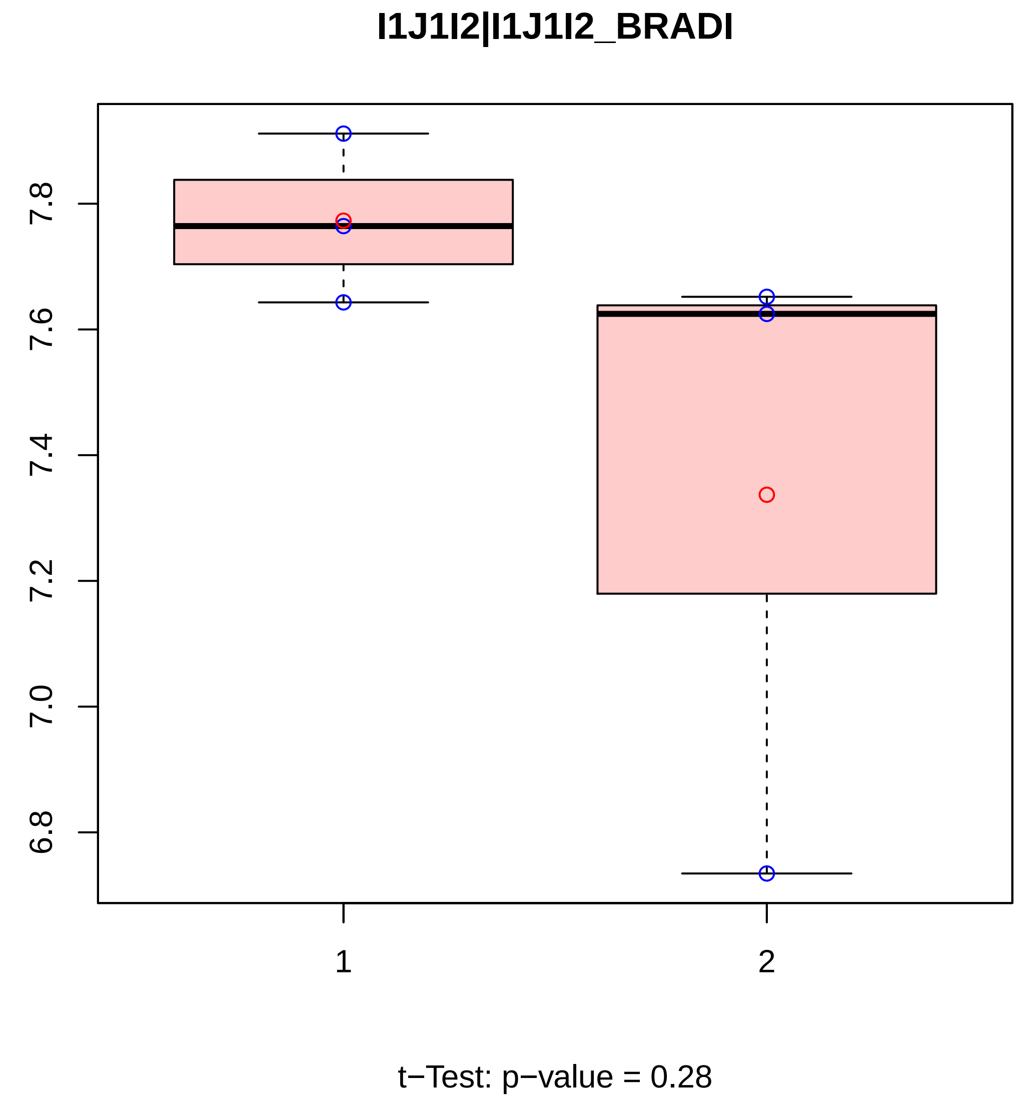
I1J1I2|I1J1I2_BRADI
7.8
7.6
7.4
7.2
7.0
6.8
1
2
t−Test: p−value = 0.28

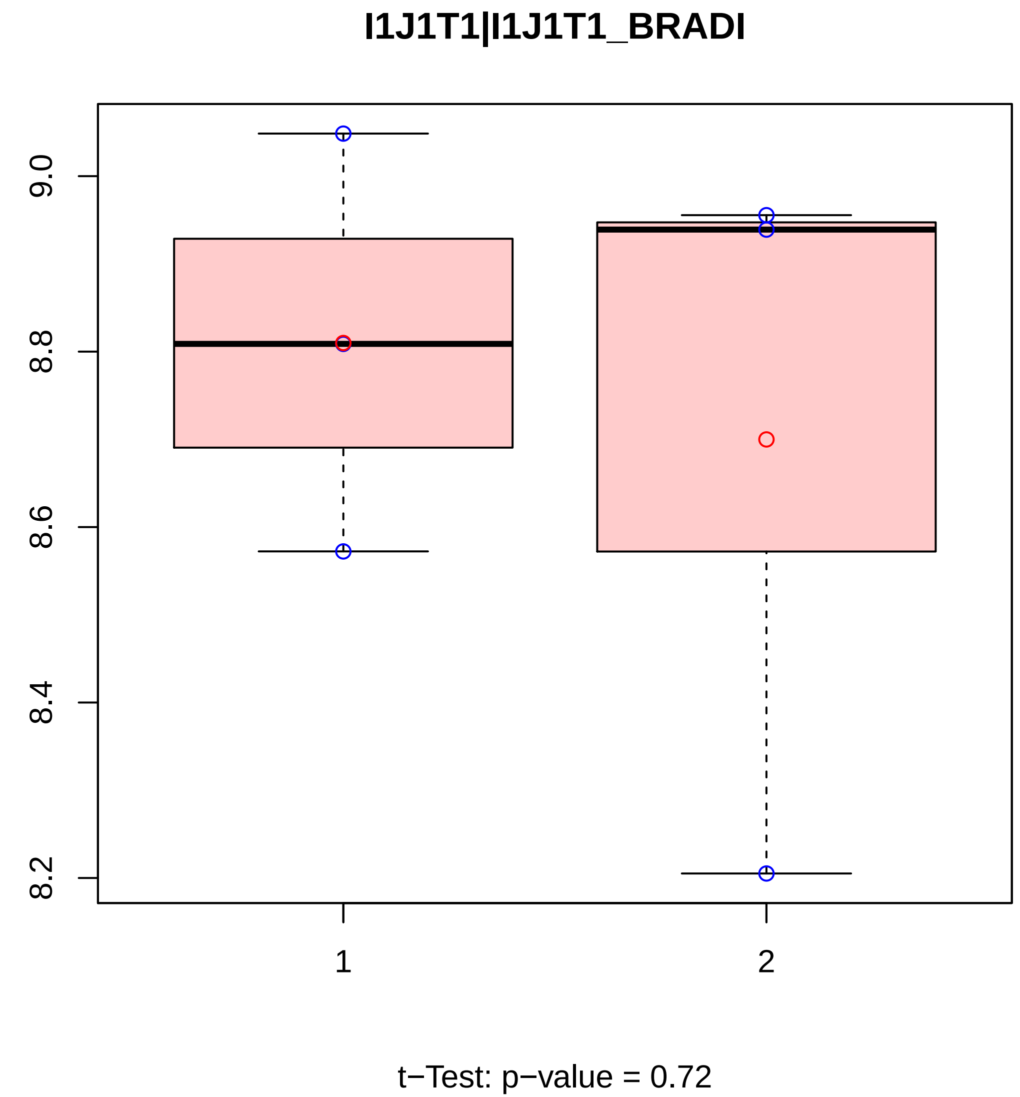
I1J1T1|I1J1T1_BRADI
9.0
8.8
8.6
8.4
8.2
1
2
t−Test: p−value = 0.72

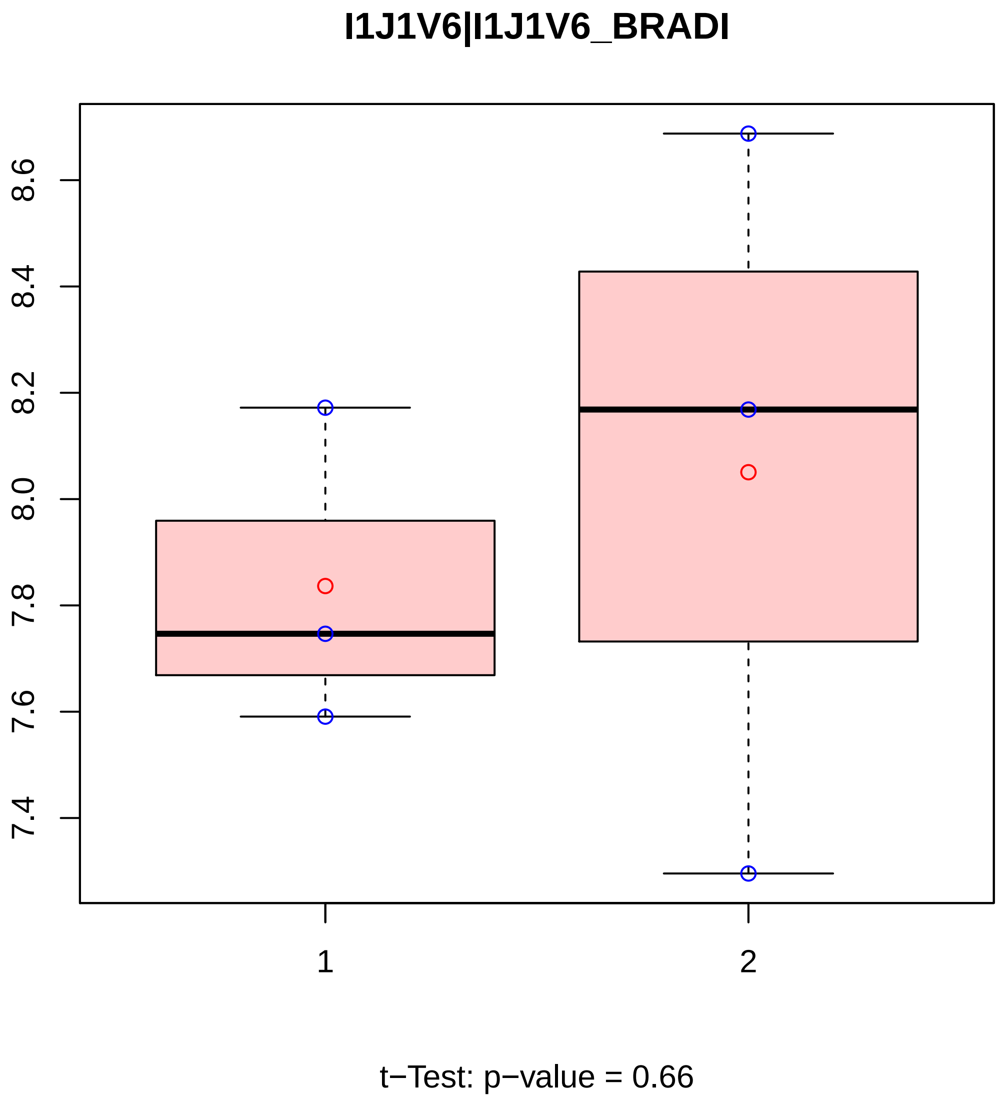
I1J1V6|I1J1V6_BRADI
8.6
8.4
8.2
8.0
7.8
7.6
7.4
1
2
t−Test: p−value = 0.66

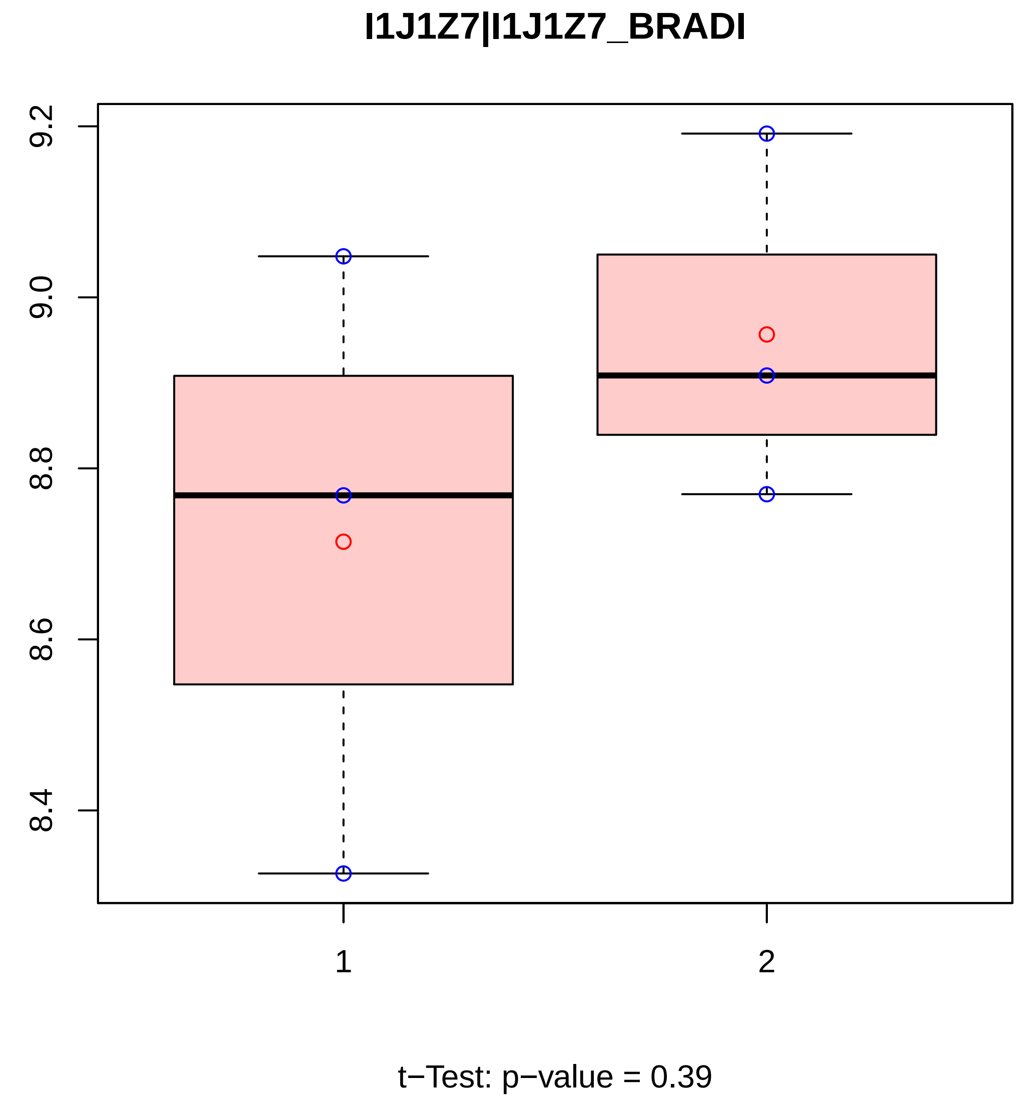
I1J1Z7|I1J1Z7_BRADI
9.2
9.0
8.8
8.6
8.4
1
2
t−Test: p−value = 0.39

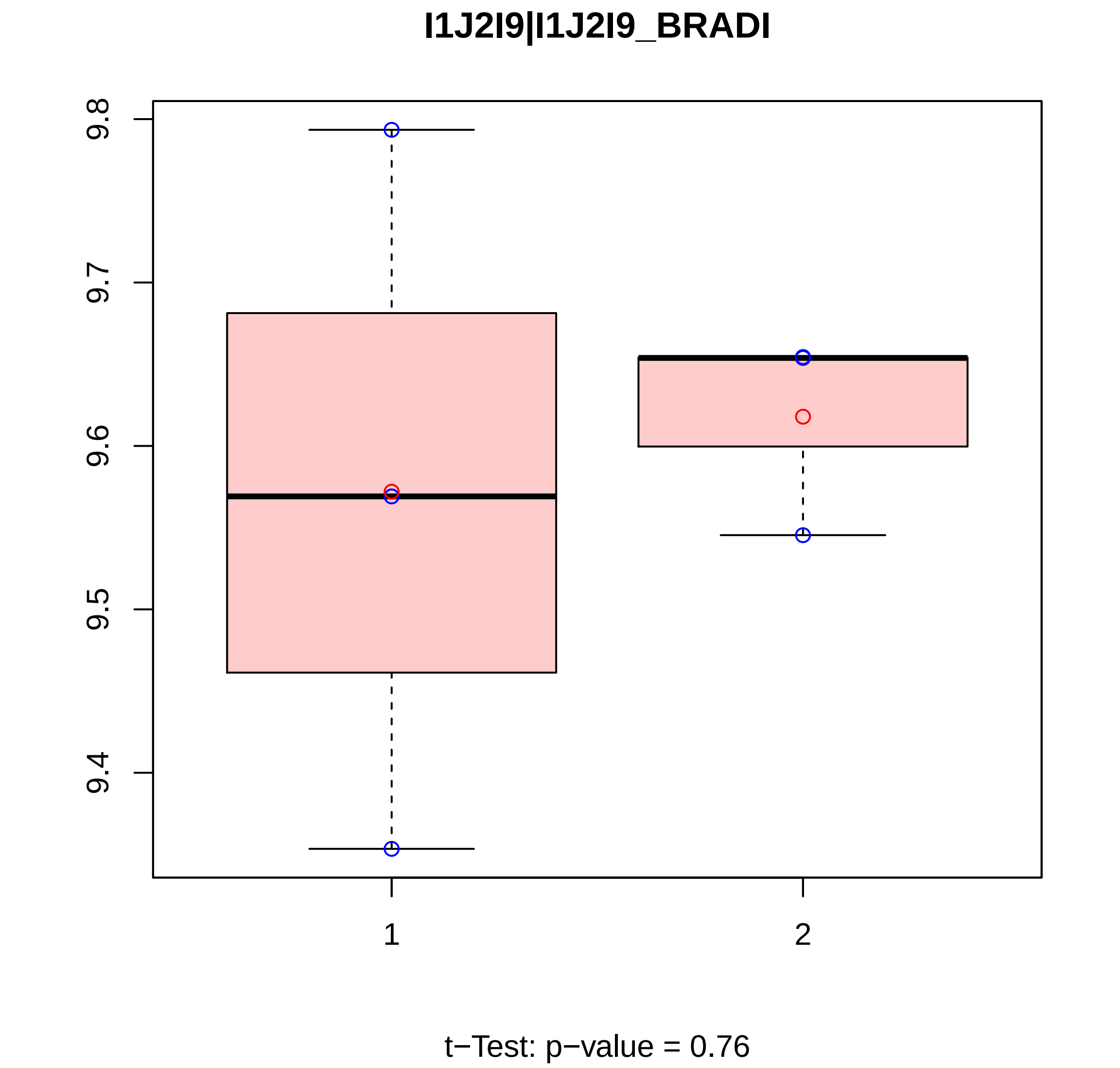
I1J2I9|I1J2I9_BRADI
9.4
9.5
9.6
9.7
9.8
1
2
t−Test: p−value = 0.76

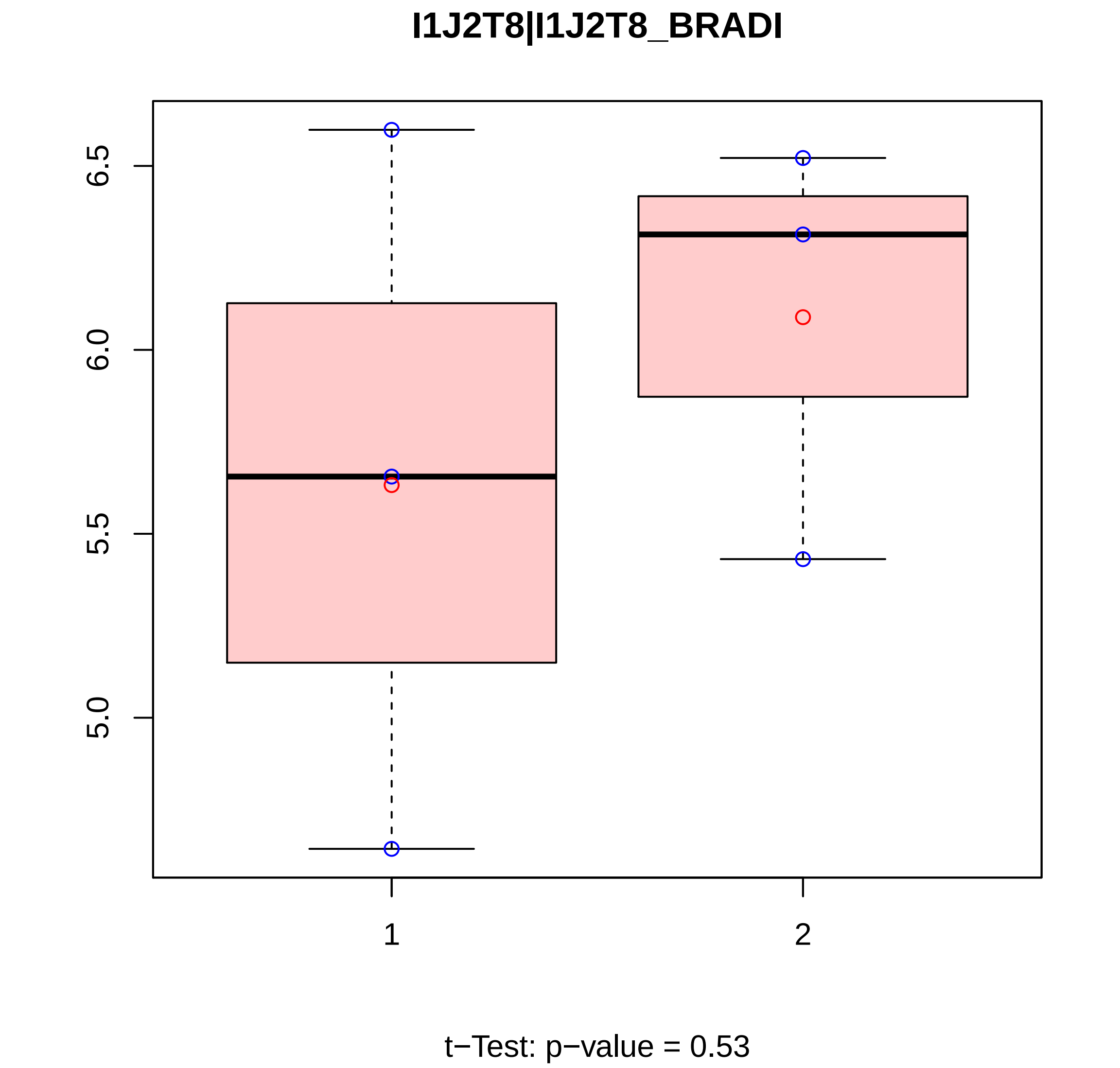
I1J2T8|I1J2T8_BRADI
6.5
6.0
5.5
5.0
1
2
t−Test: p−value = 0.53

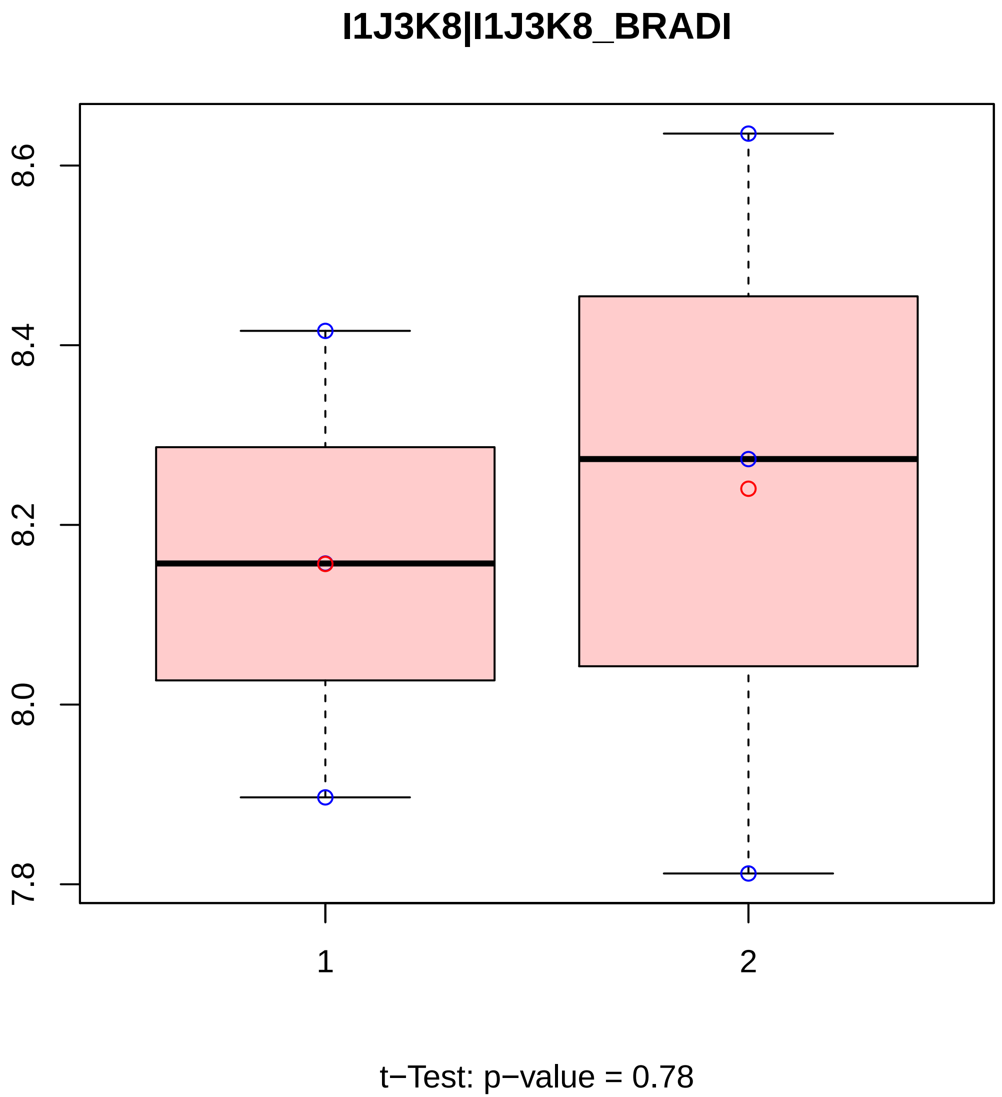
I1J3K8|I1J3K8_BRADI
8.6
8.4
8.2
8.0
7.8
1
2
t−Test: p−value = 0.78

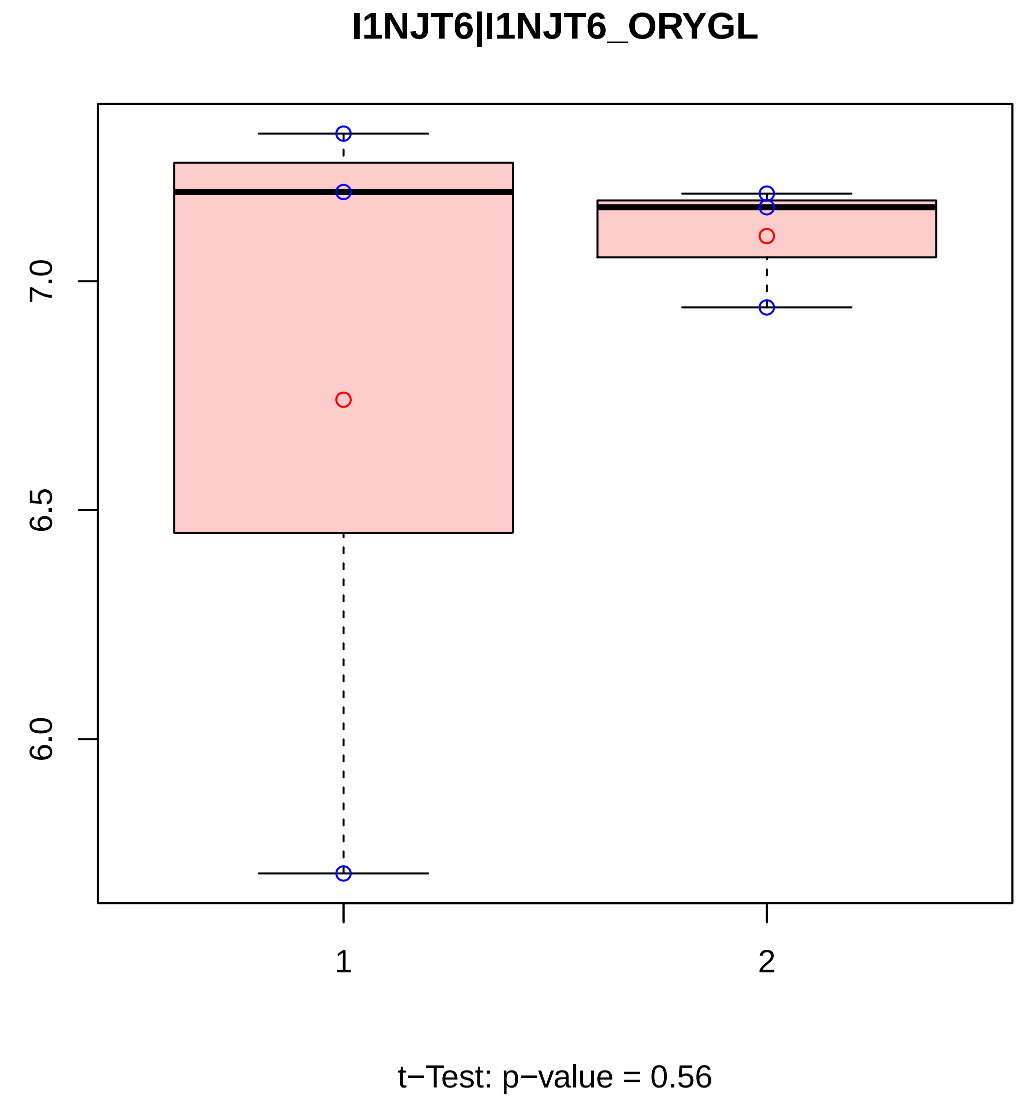
I1NJT6|I1NJT6_ORYGL
7.0
6.5
6.0
1
2
t−Test: p−value = 0.56

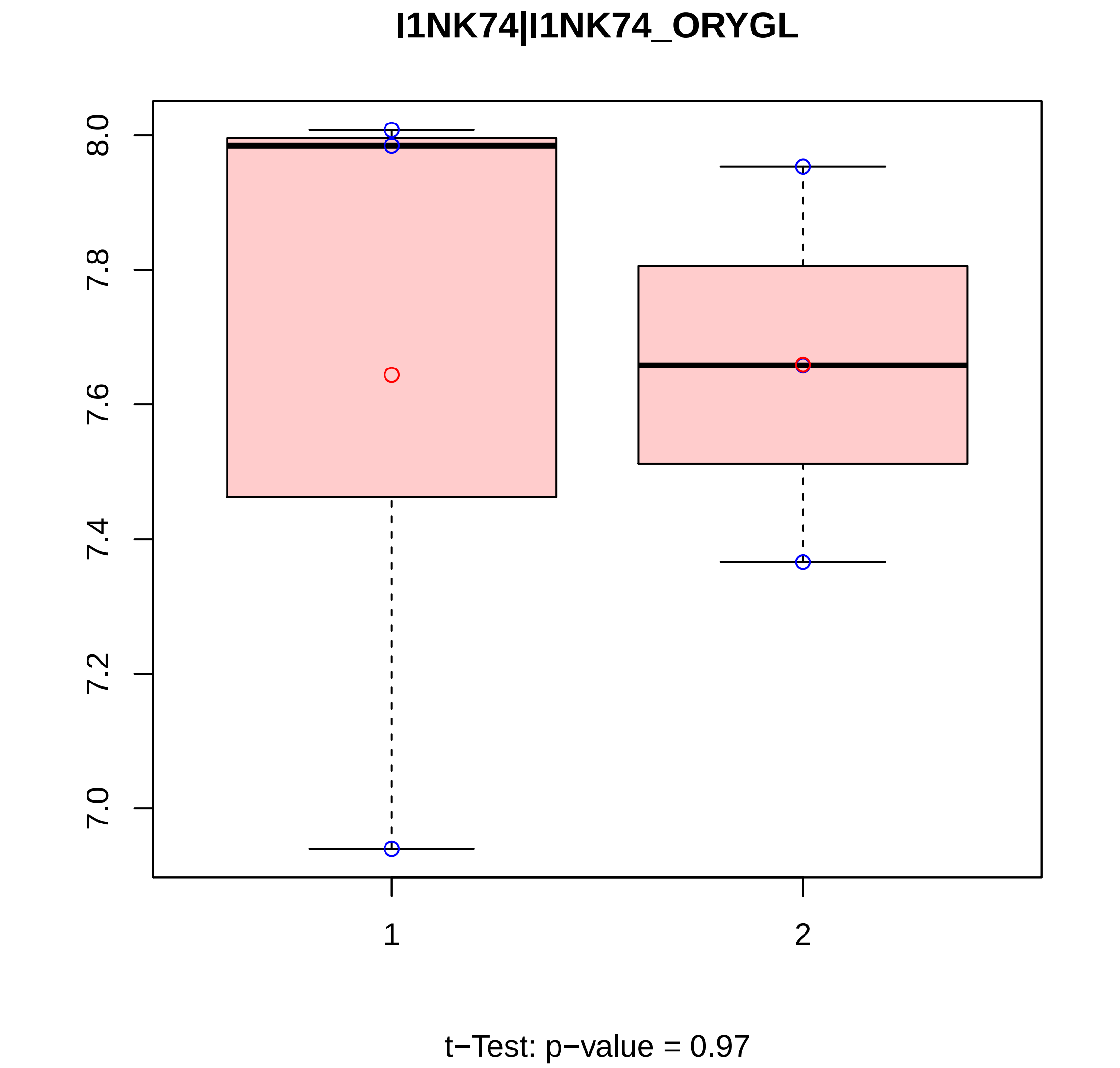

I1NK74|I1NK74_ORYGL
7.0
7.2
7.4
7.6
7.8
8.0
1
2
t−Test: p−value = 0.97

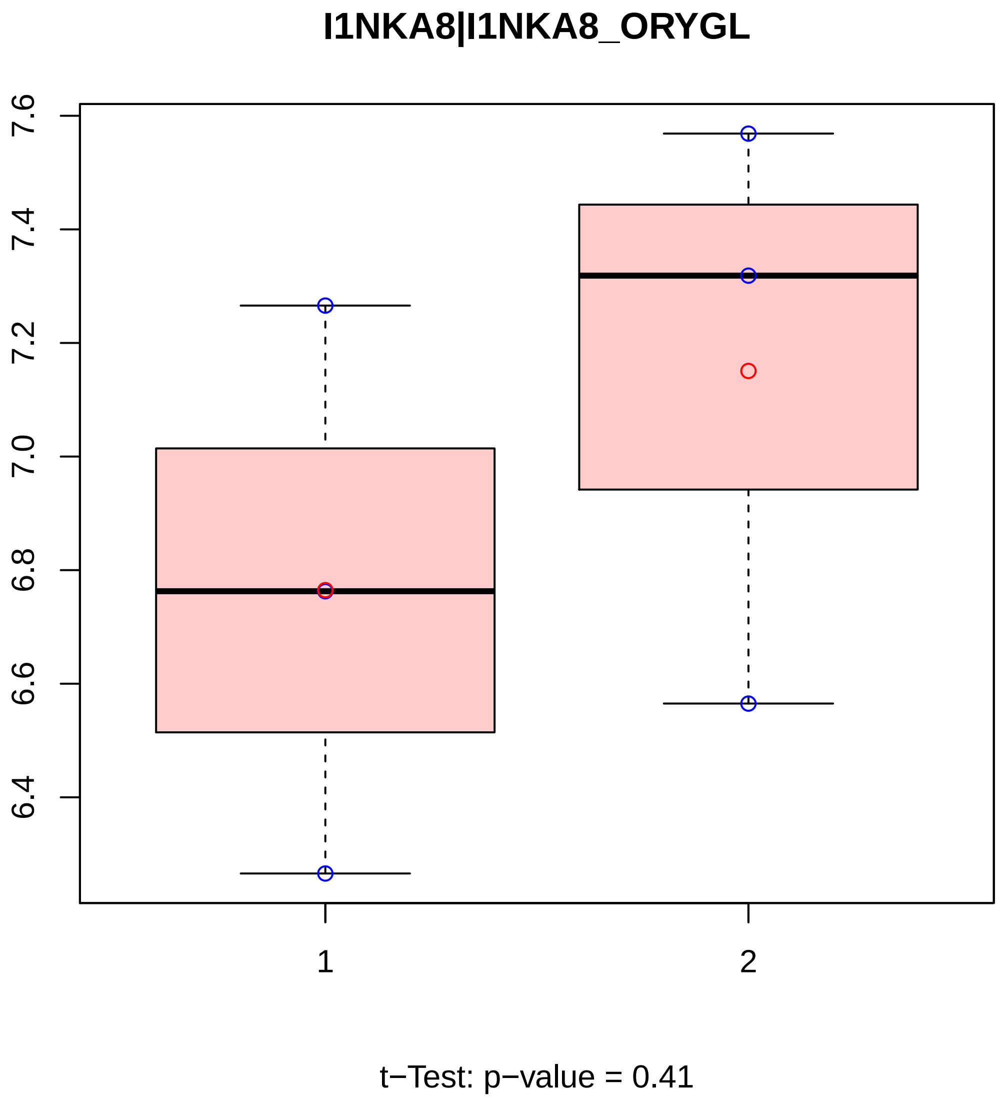

I1NKA8|I1NKA8_ORYGL
7.6
7.4
7.2
7.0
6.8
6.6
6.4
1
2
t−Test: p−value = 0.41

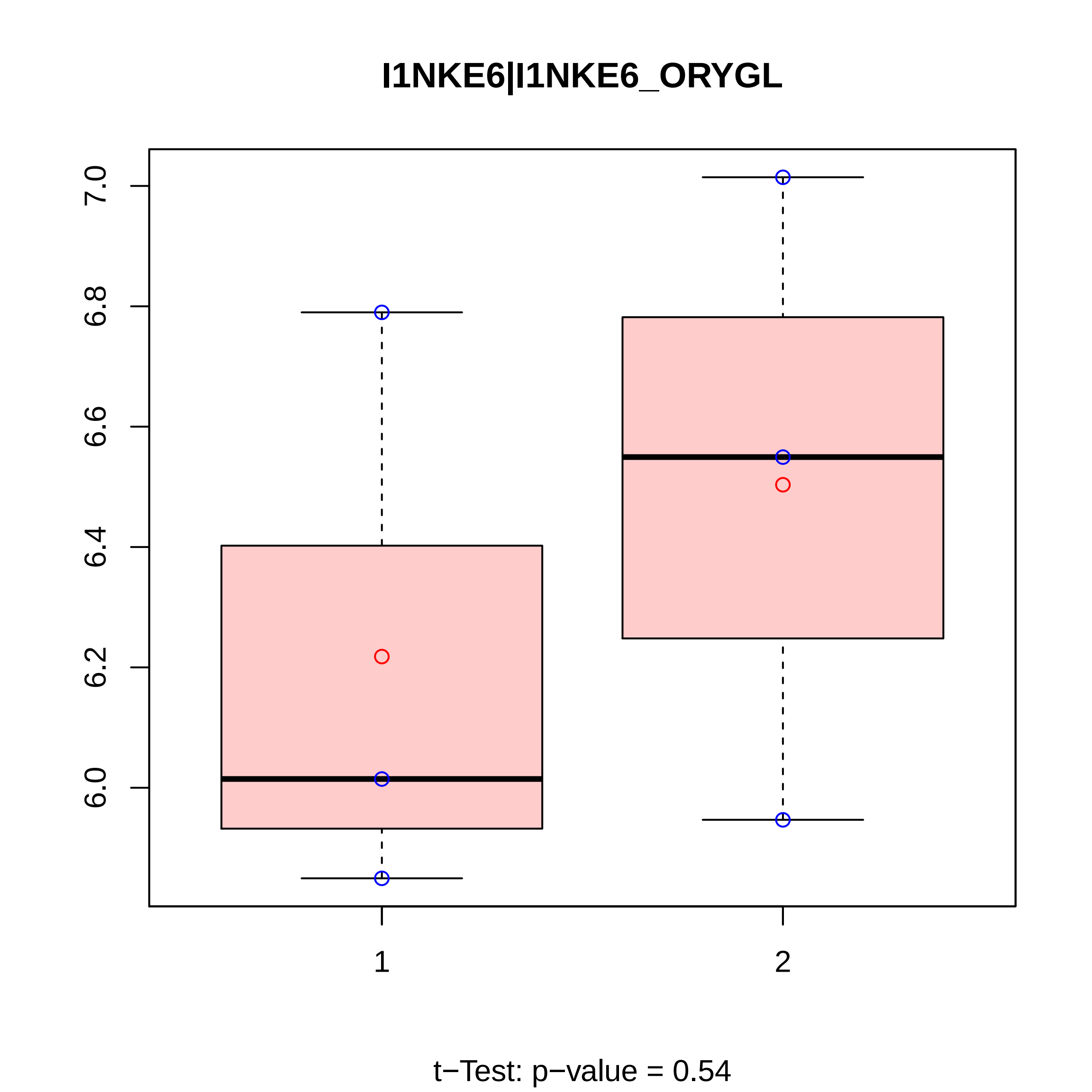

I1NKE6|I1NKE6_ORYGL
7.0
6.8
6.6
6.4
6.2
6.0
1
2
t−Test: p−value = 0.54

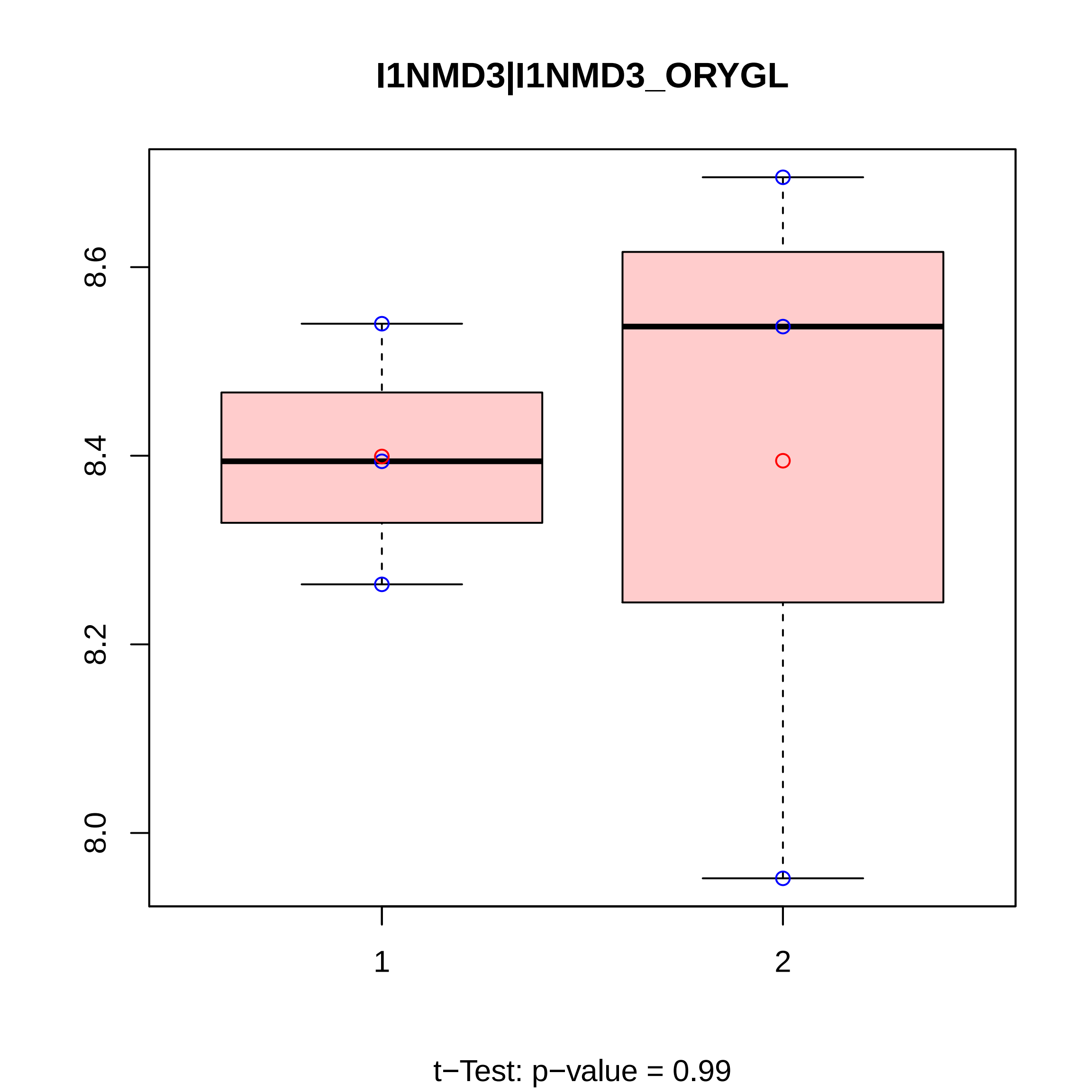
I1NMD3|I1NMD3_ORYGL
8.6
8.4
8.2
8.0
1
2
t−Test: p−value = 0.99

I1NMG9|I1NMG9_ORYGL

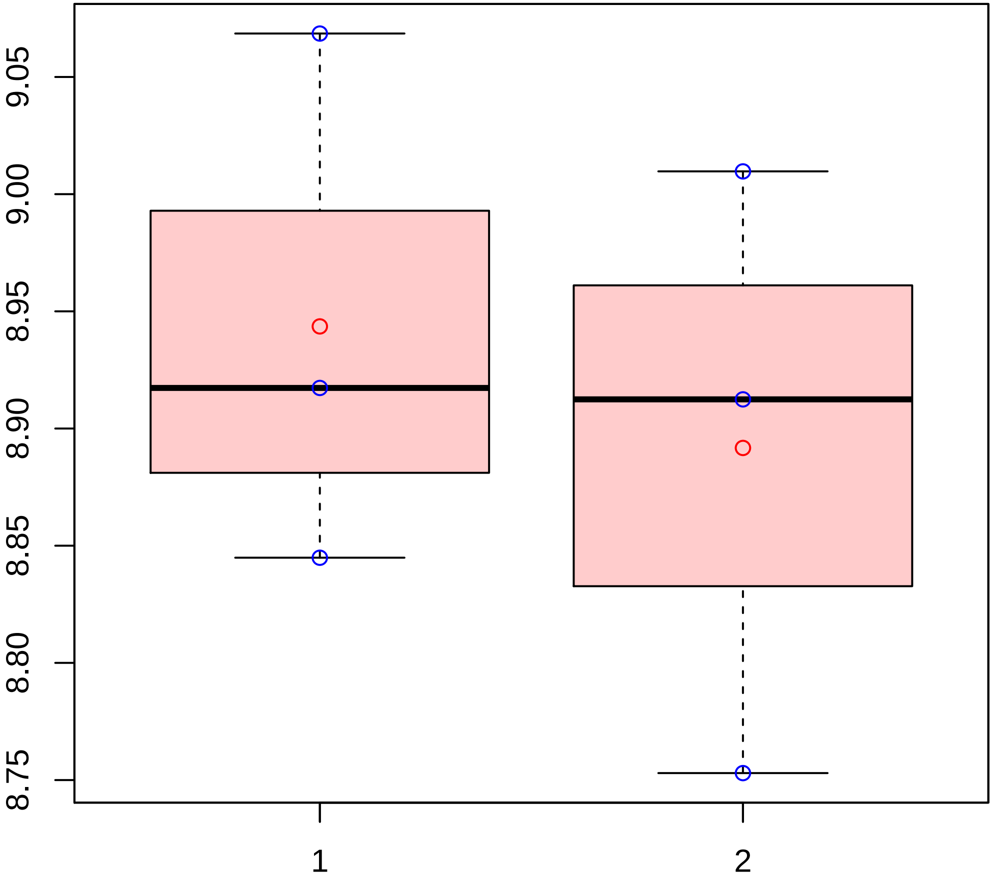

t−Test: p−value = 0.63

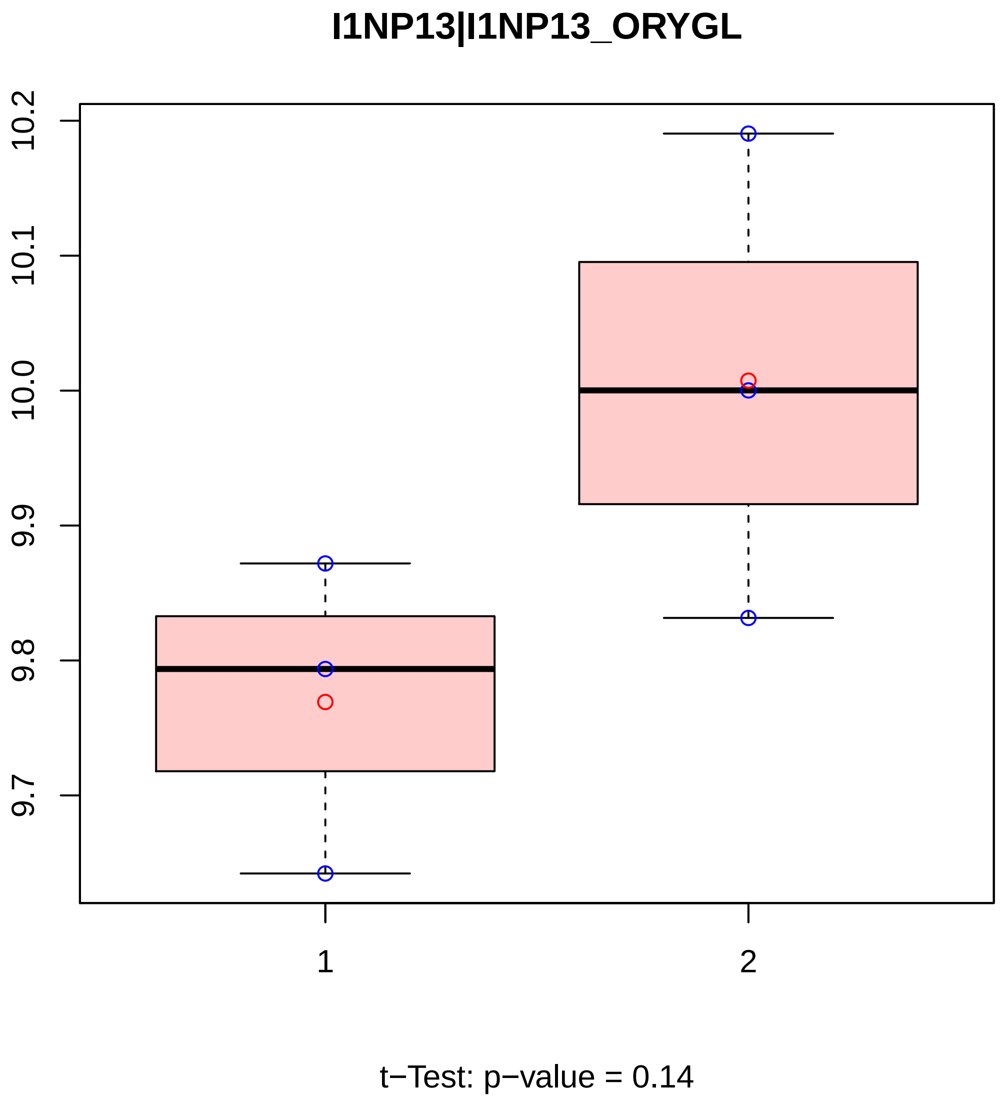
I1NP13|I1NP13_ORYGL
10.2
10.1
10.0
9.9
9.8
9.7
1
2
t−Test: p−value = 0.14

I1NP22|I1NP22_ORYGL

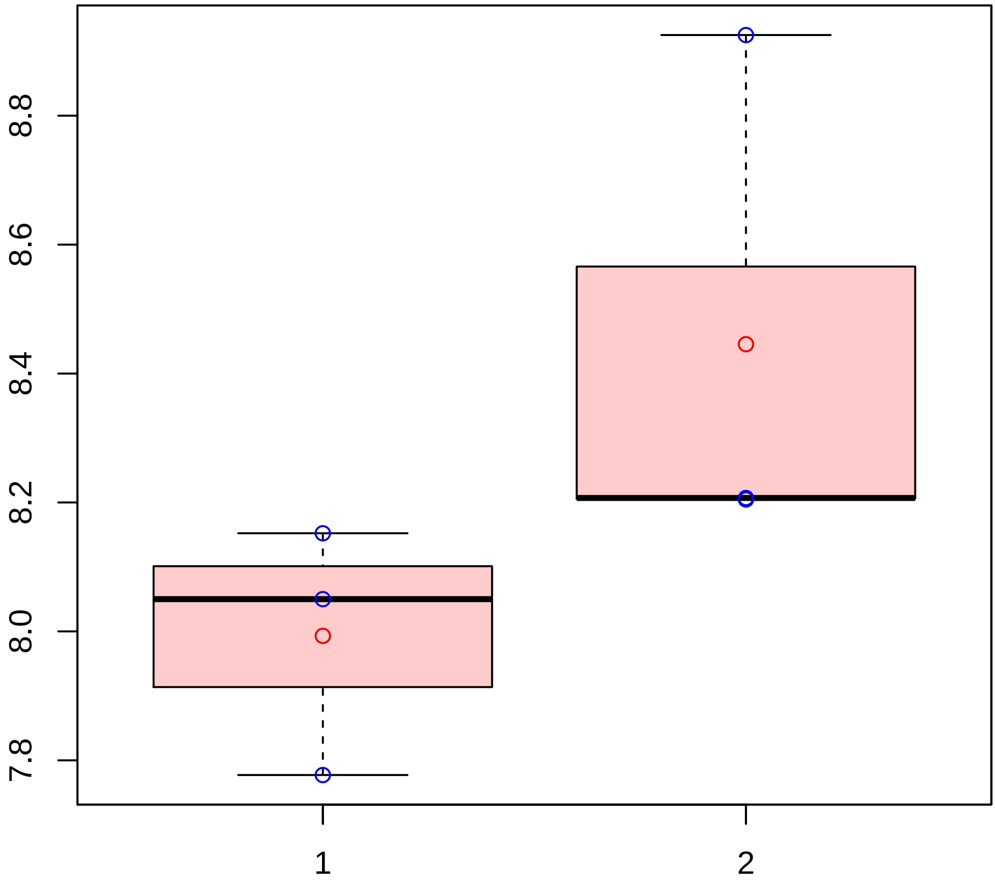

t−Test: p−value = 0.19

I1NQX1|I1NQX1_ORYGL

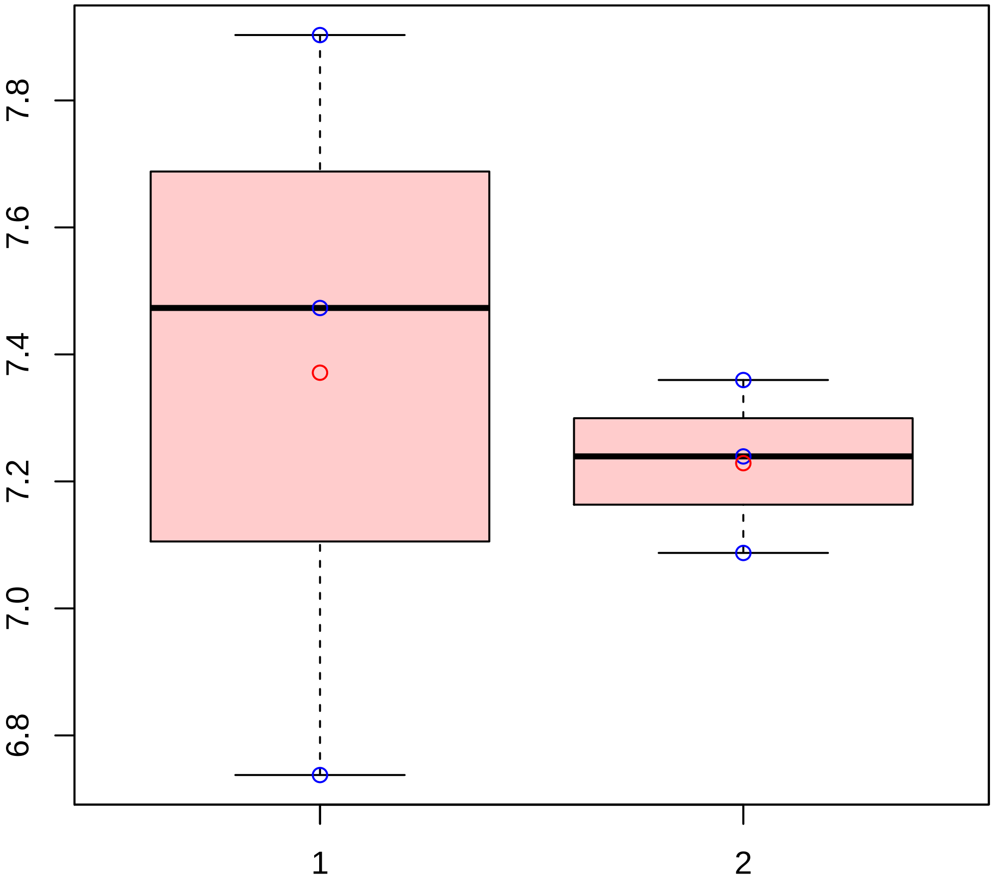

t−Test: p−value = 0.72

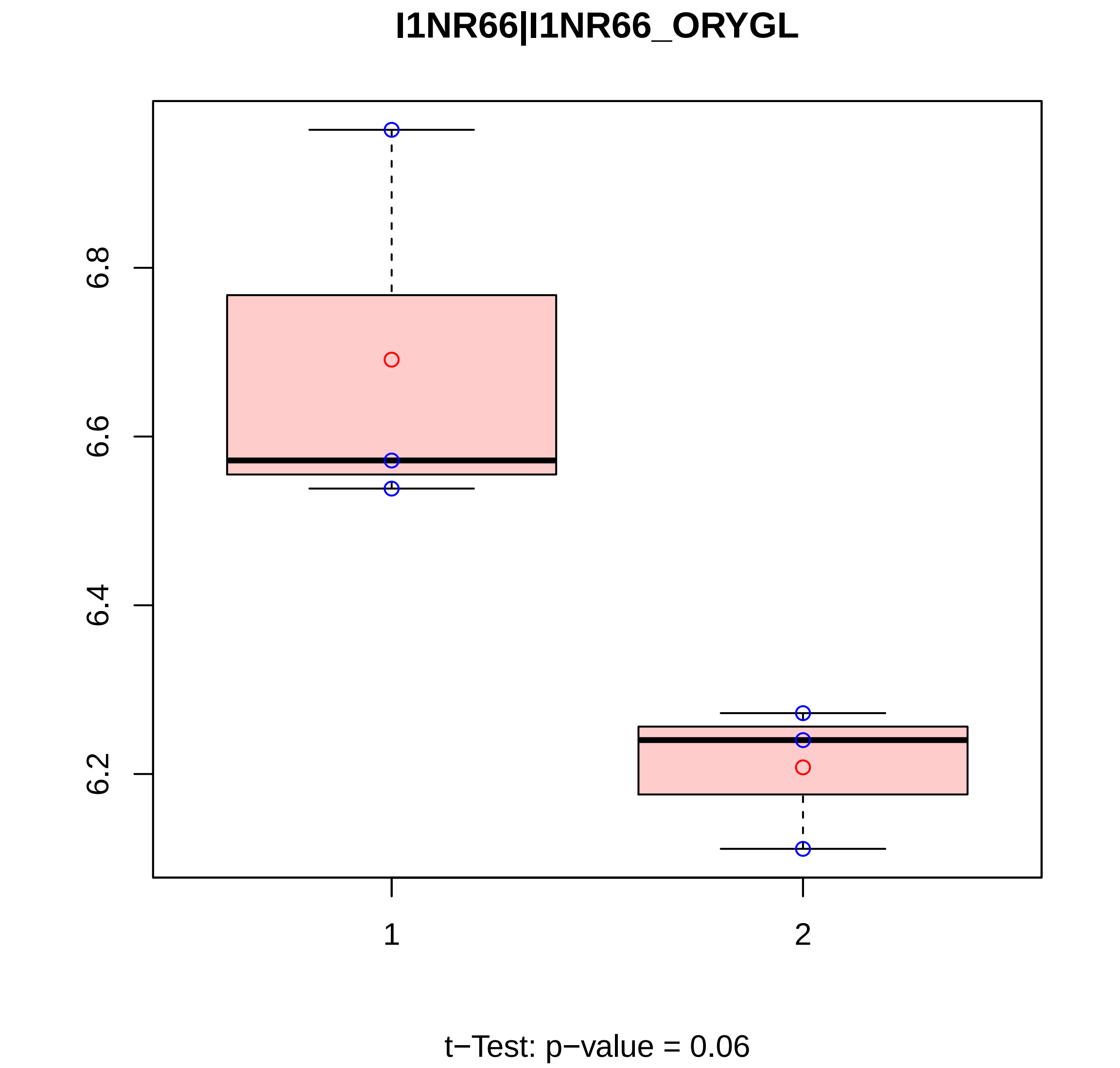
I1NR66|I1NR66_ORYGL
6.2
6.4
6.6
6.8
1
2
t−Test: p−value = 0.06

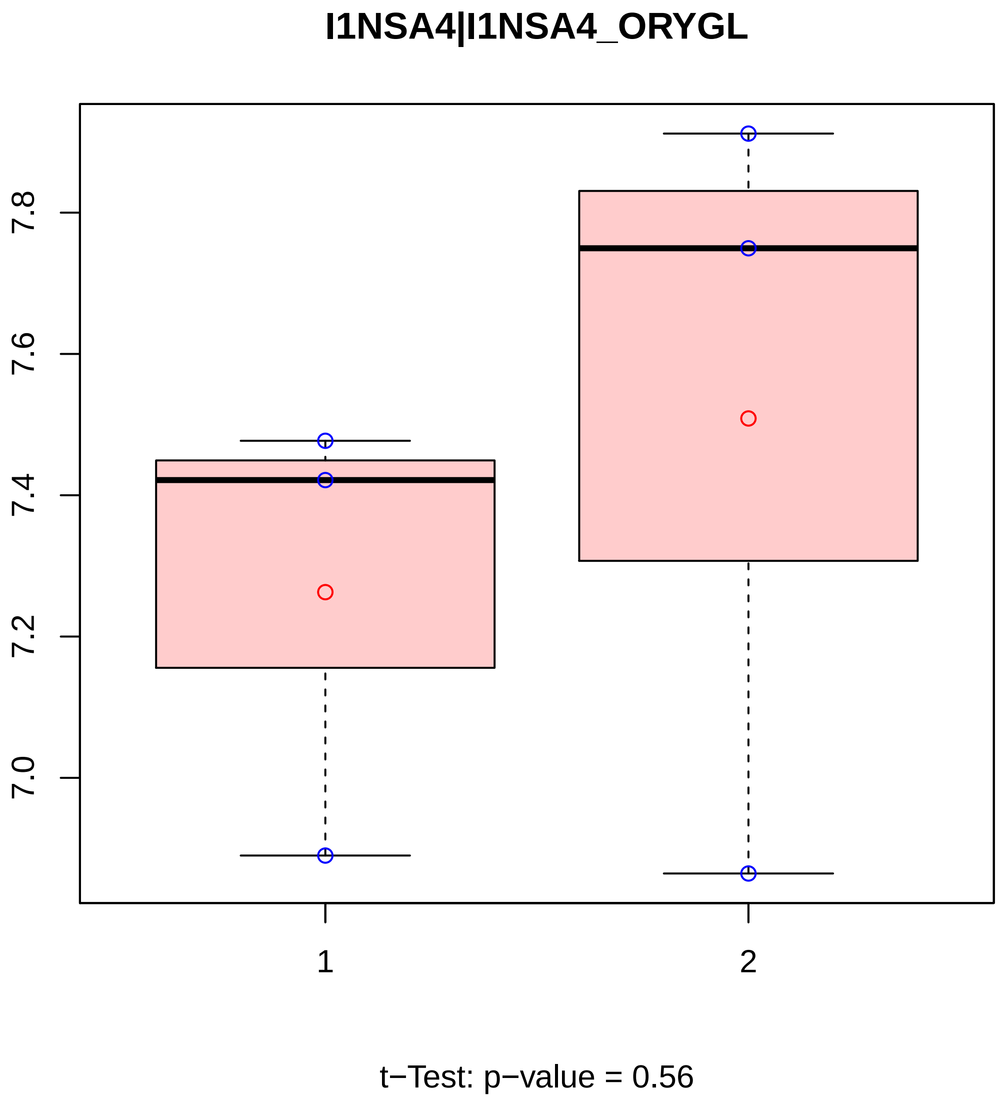
I1NSA4|I1NSA4_ORYGL
7.0
7.2
7.4
7.6
7.8
1
2
t−Test: p−value = 0.56

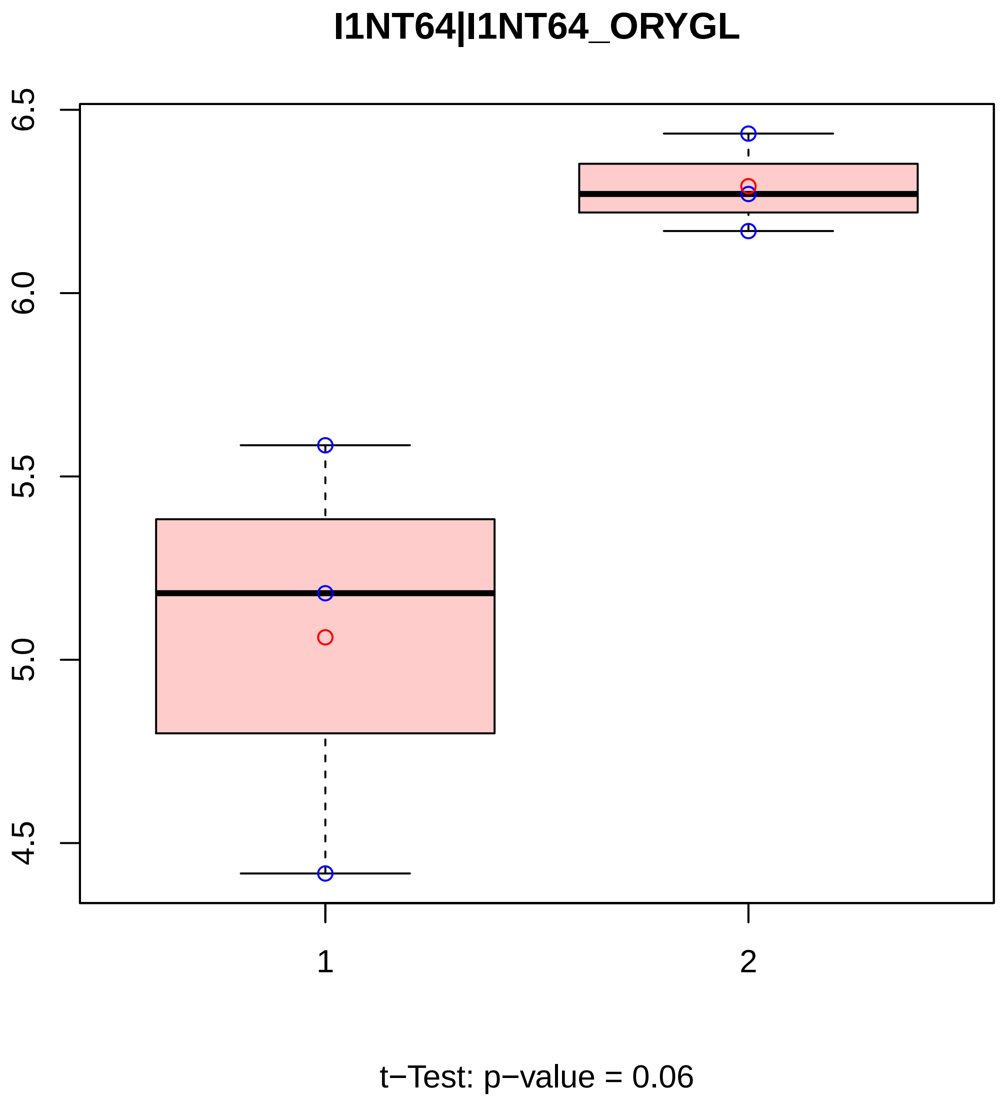
I1NT64|I1NT64_ORYGL
6.5
6.0
5.5
5.0
4.5
1
2
t−Test: p−value = 0.06

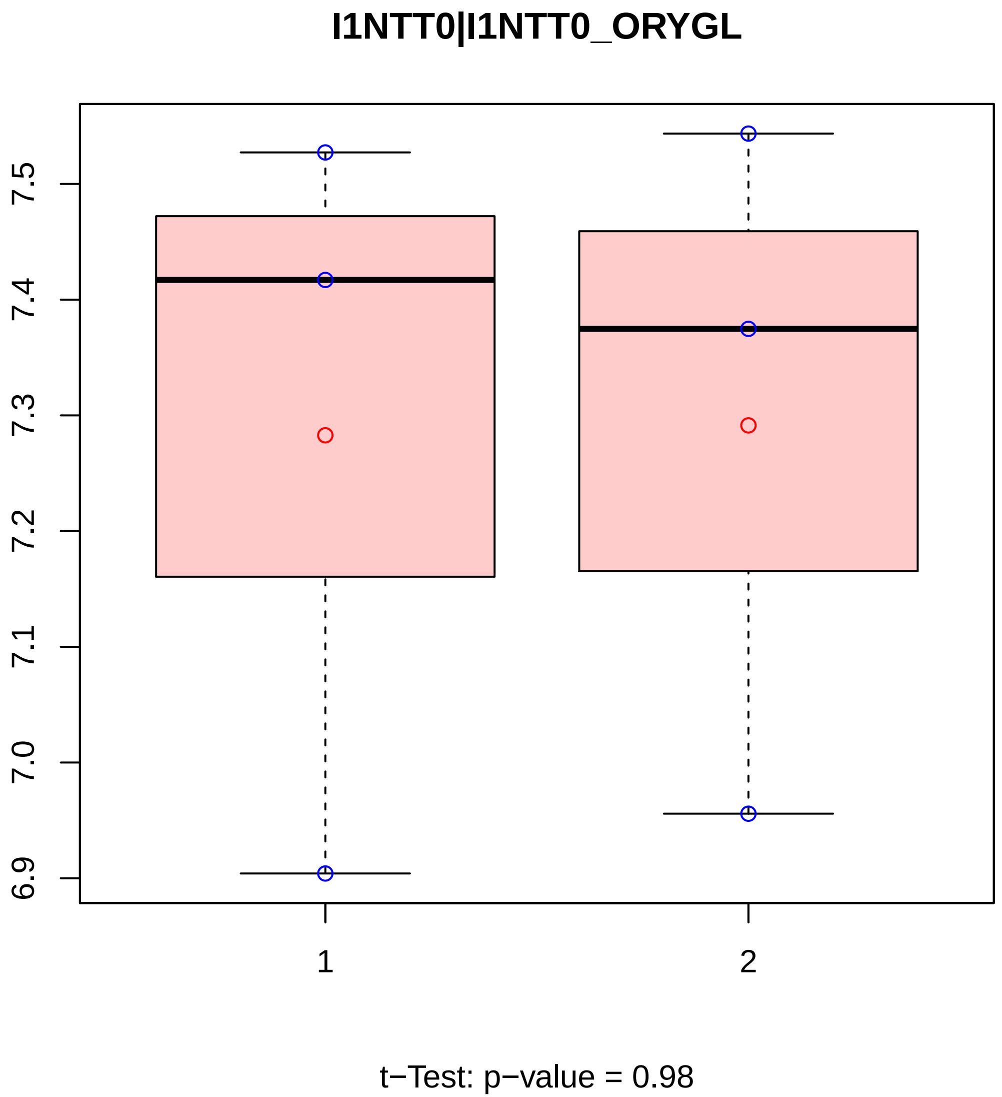
I1NTT0|I1NTT0_ORYGL
7.5
7.4
7.3
7.2
7.1
7.0
6.9
1
2
t−Test: p−value = 0.98

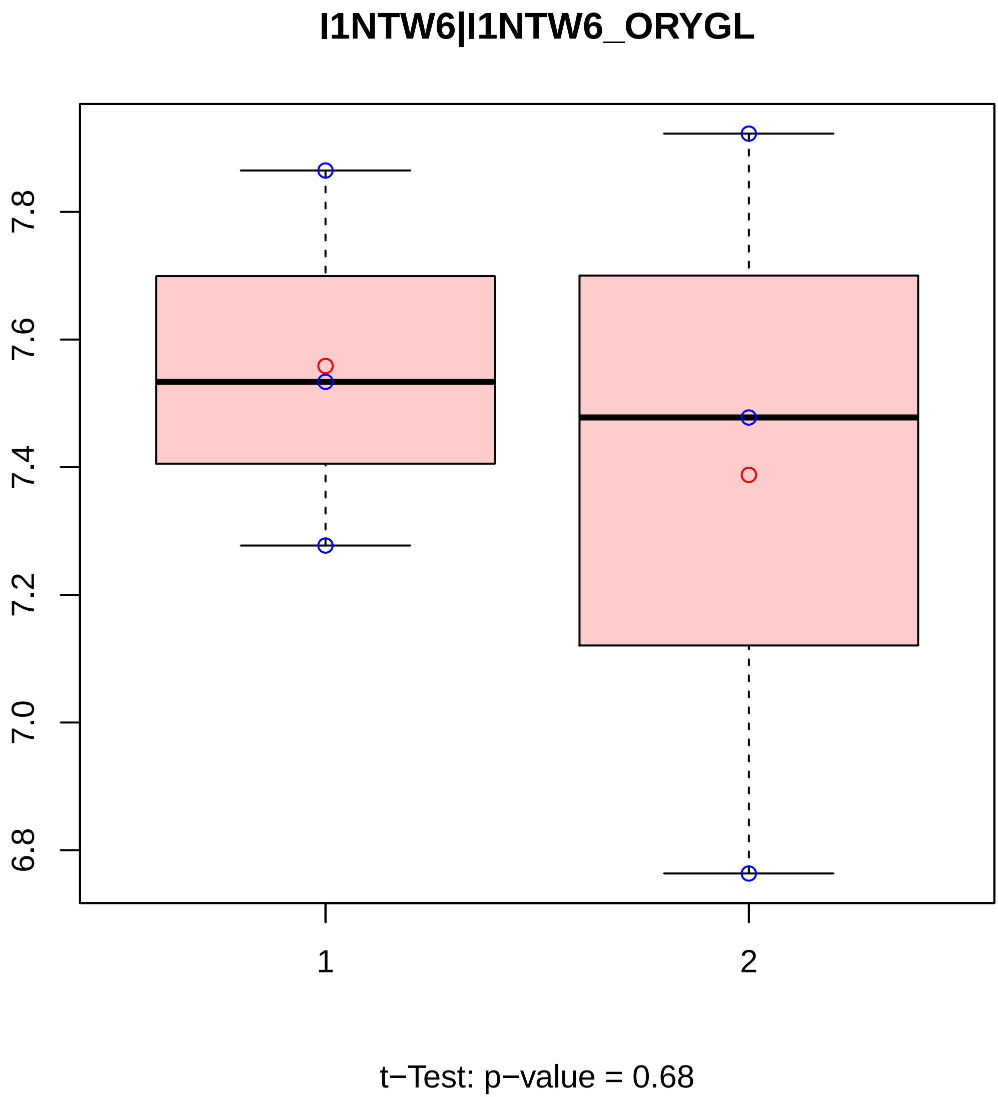

I1NTW6|I1NTW6_ORYGL
7.8
7.6
7.4
7.2
7.0
6.8
1
2
t−Test: p−value = 0.68

I1NU41|I1NU41_ORYGL

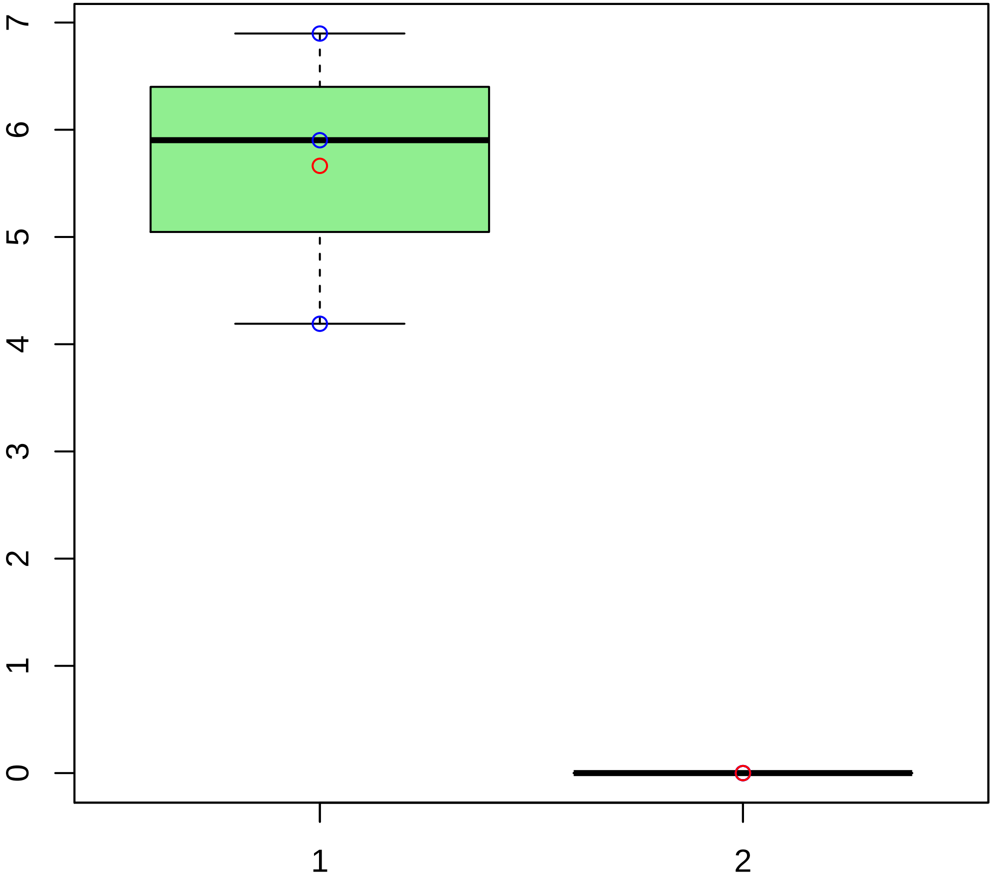

t−Test: p−value = 0.02

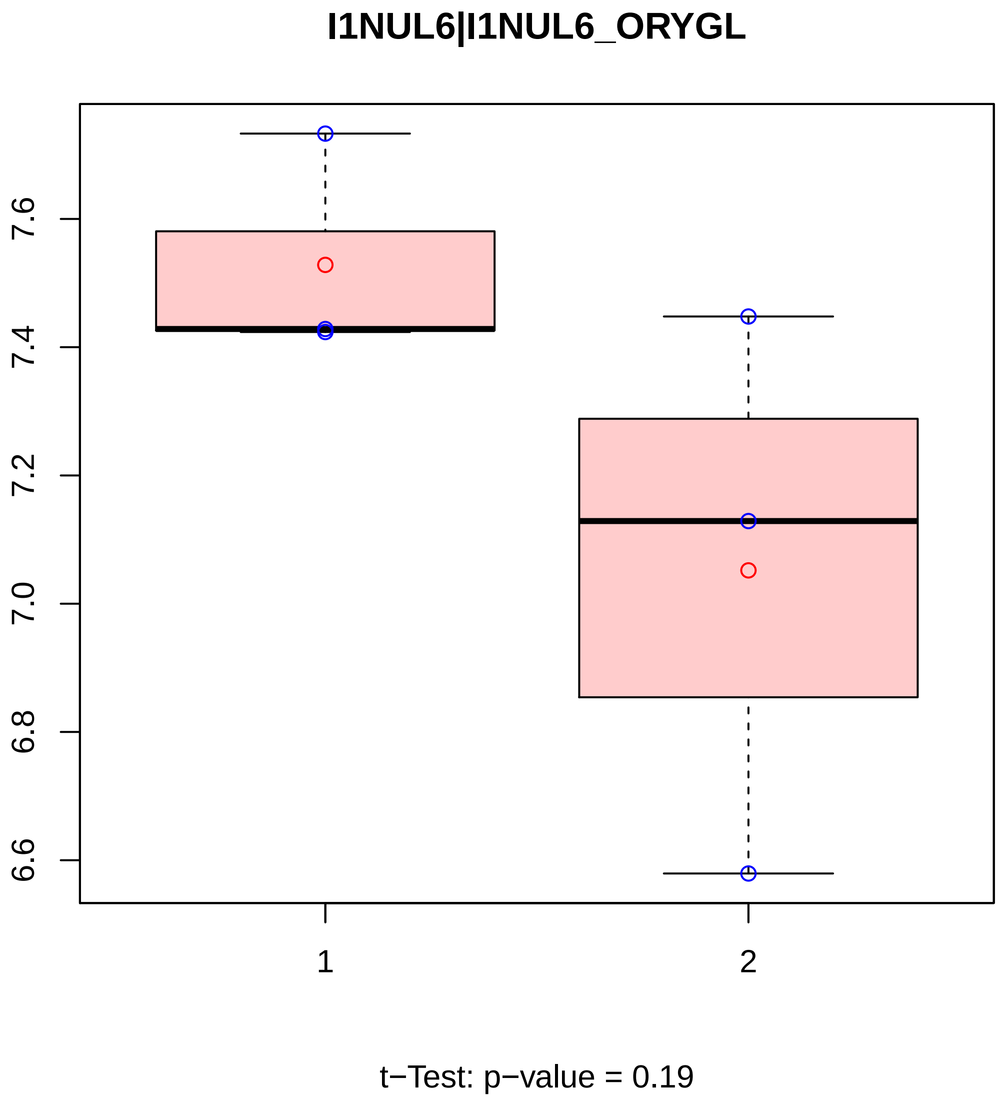
I1NUL6|I1NUL6_ORYGL
7.6
7.4
7.2
7.0
6.8
6.6
1
2
t−Test: p−value = 0.19

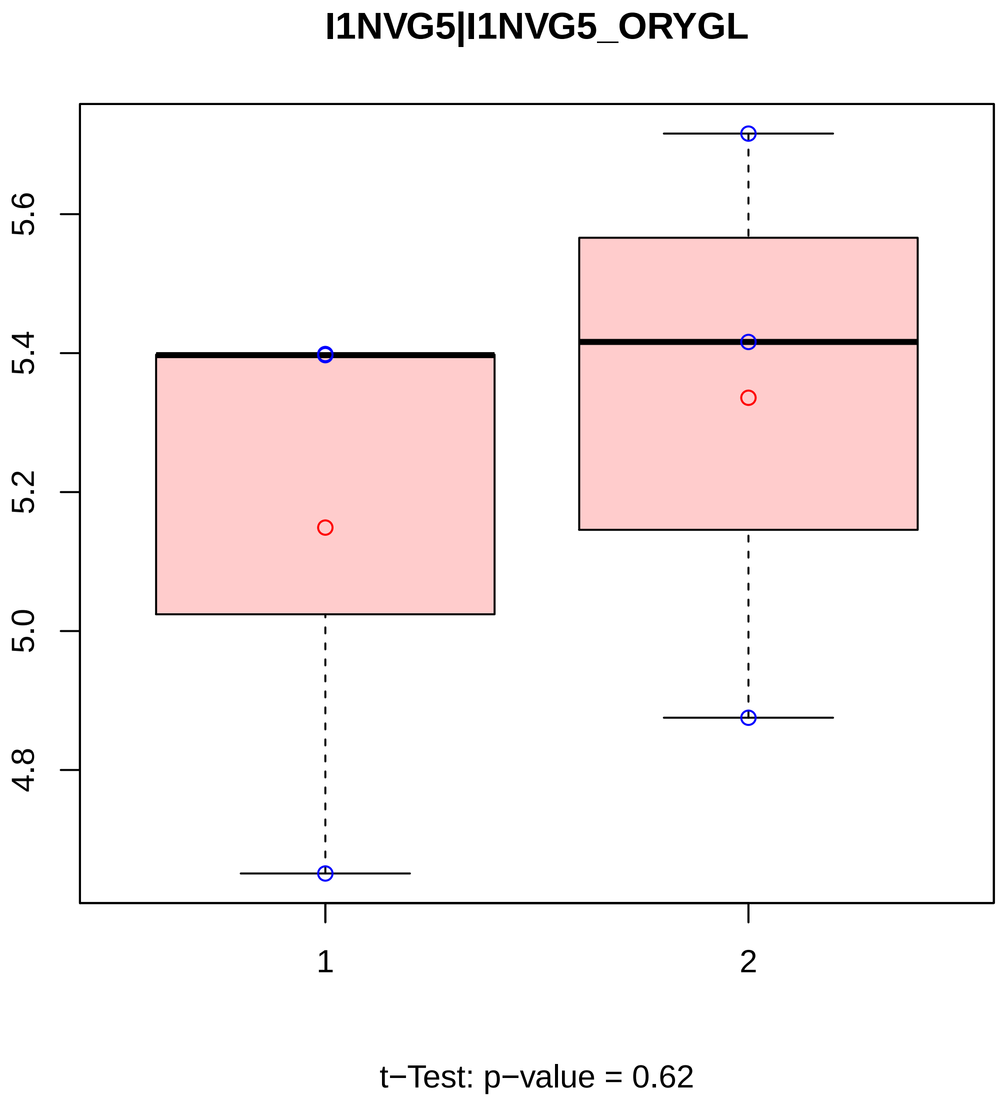
I1NVG5|I1NVG5_ORYGL
5.6
5.4
5.2
5.0
4.8
1
2
t−Test: p−value = 0.62

I1NWA5|I1NWA5_ORYGL

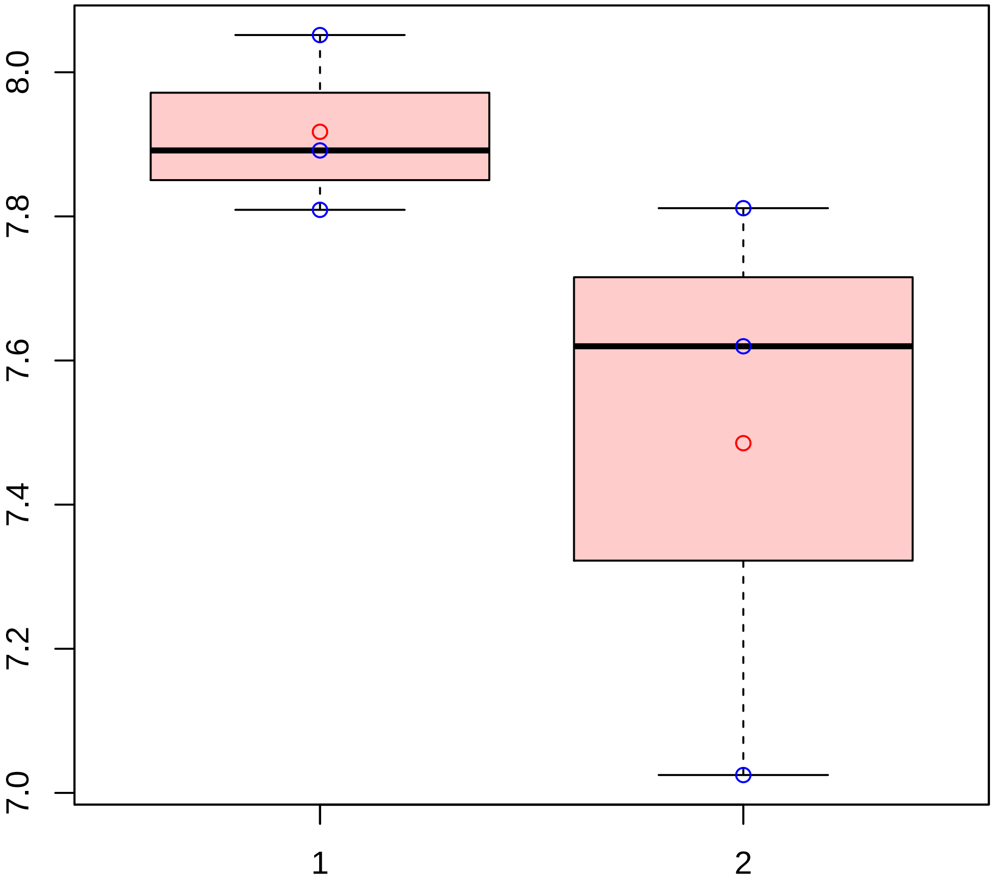

t−Test: p−value = 0.2

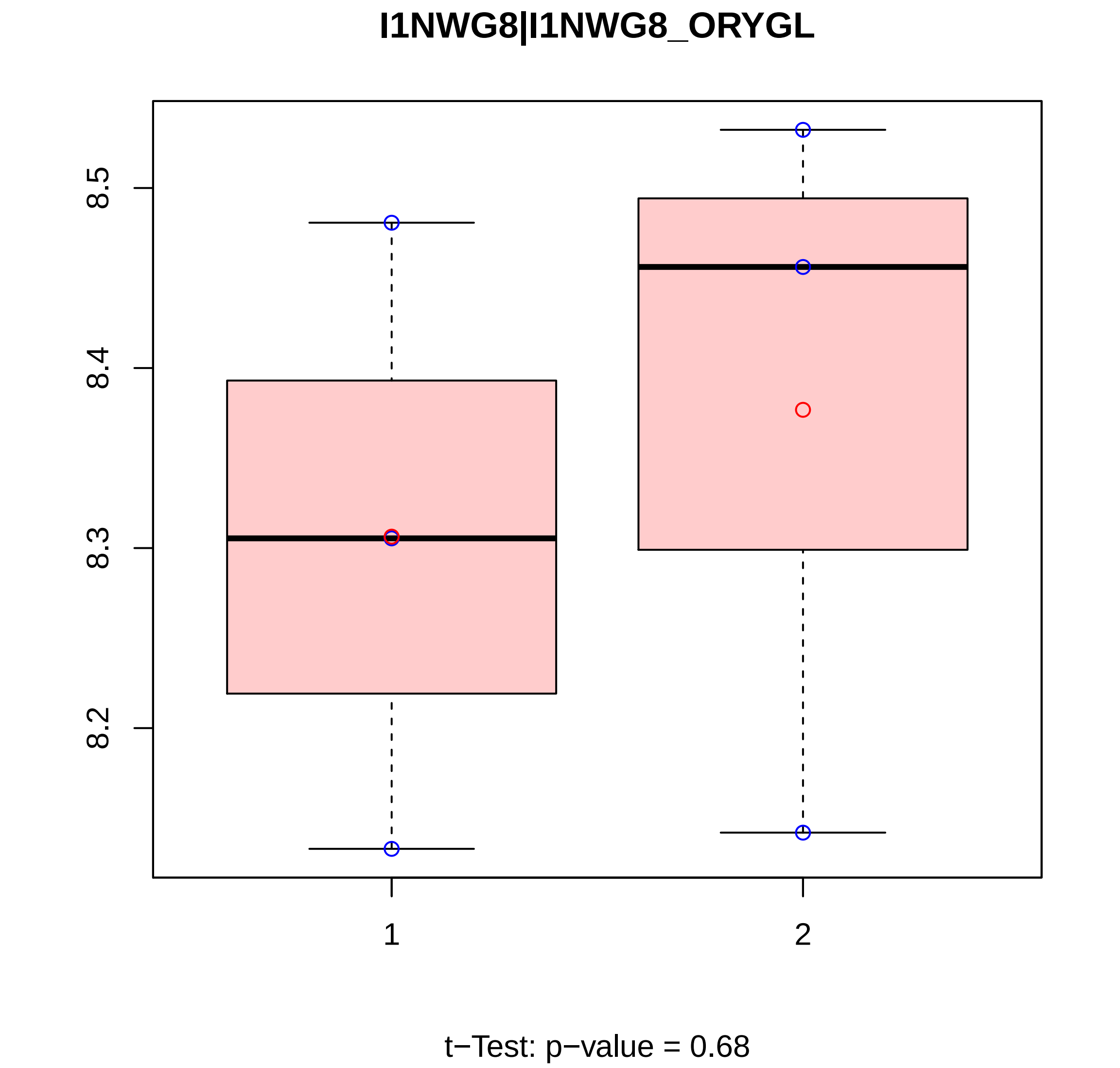
I1NWG8|I1NWG8_ORYGL
8.2
8.3
8.4
8.5
1
2
t−Test: p−value = 0.68

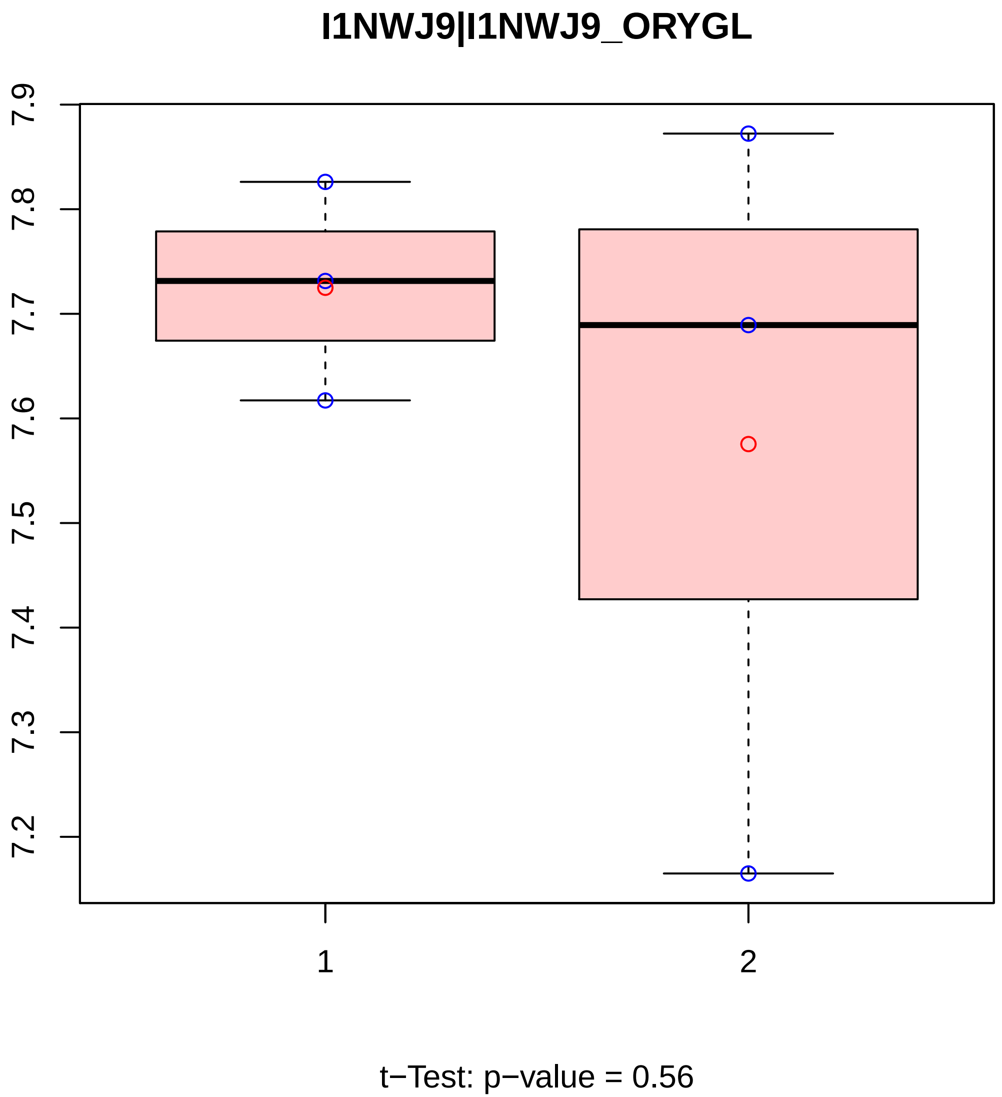
I1NWJ9|I1NWJ9_ORYGL
7.9
7.8
7.7
7.6
7.5
7.4
7.3
7.2
1
2
t−Test: p−value = 0.56

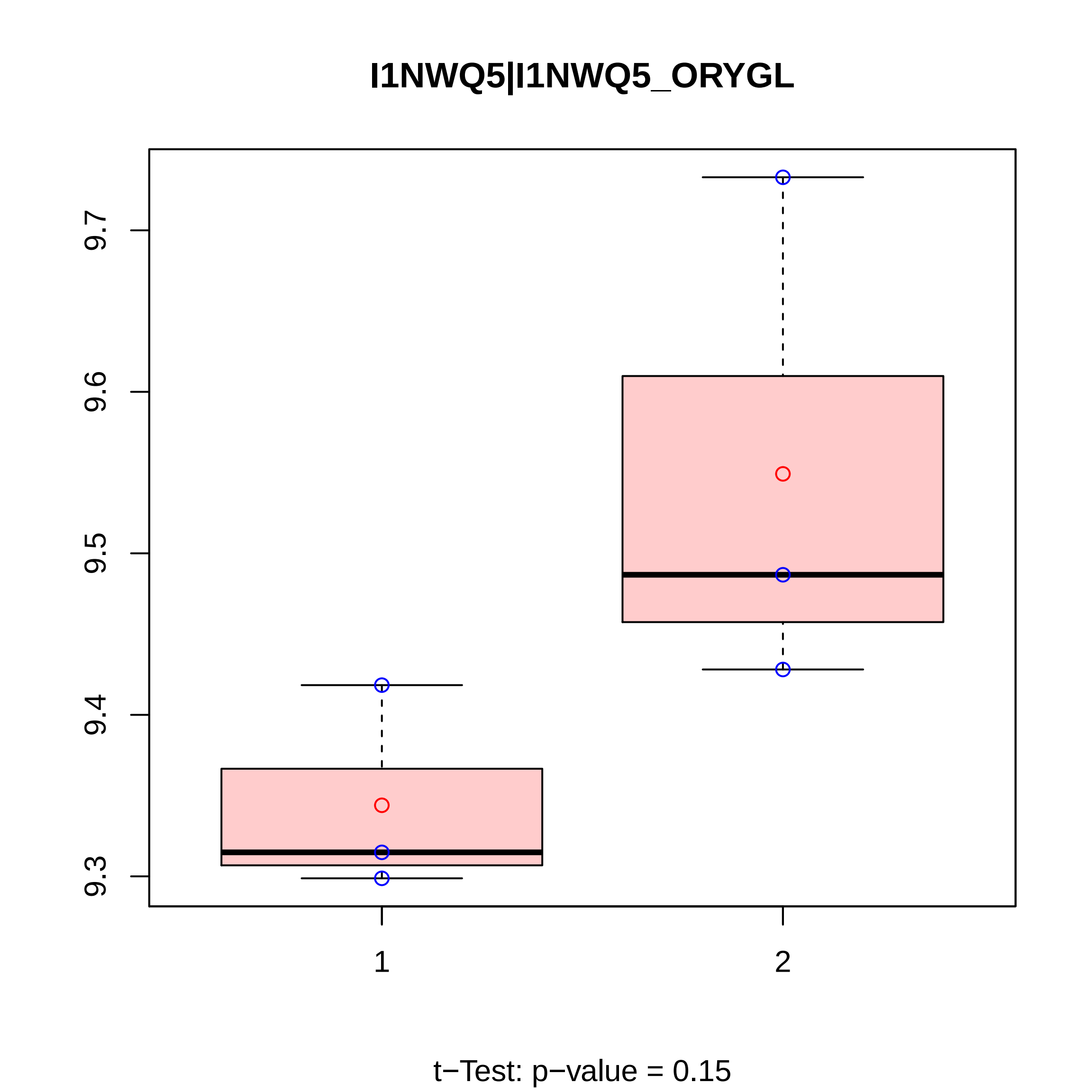
I1NWQ5|I1NWQ5_ORYGL
9.3
9.4
9.5
9.6
9.7
1
2
t−Test: p−value = 0.15

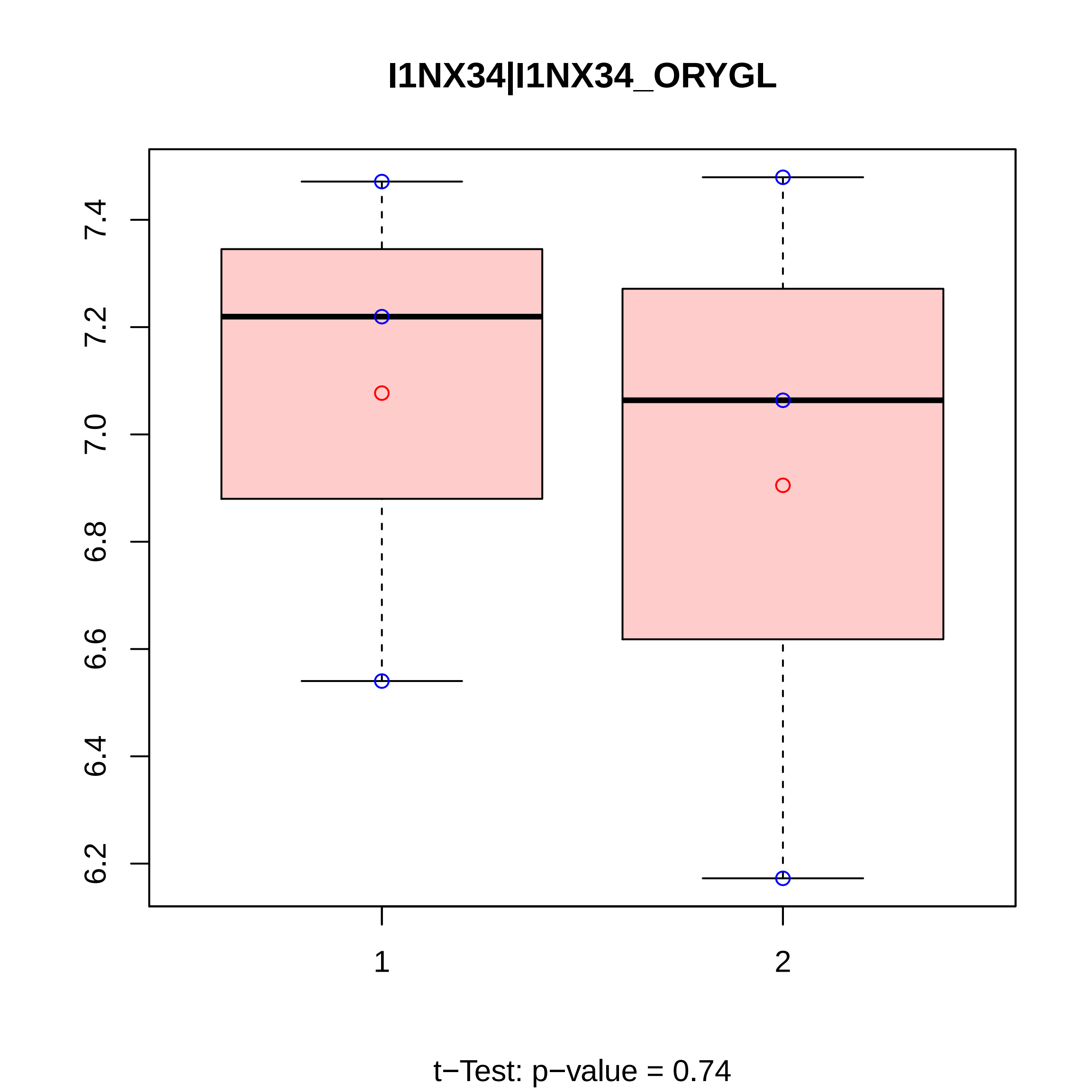
I1NX34|I1NX34_ORYGL
7.4
7.2
7.0
6.8
6.6
6.4
6.2
1
2
t−Test: p−value = 0.74

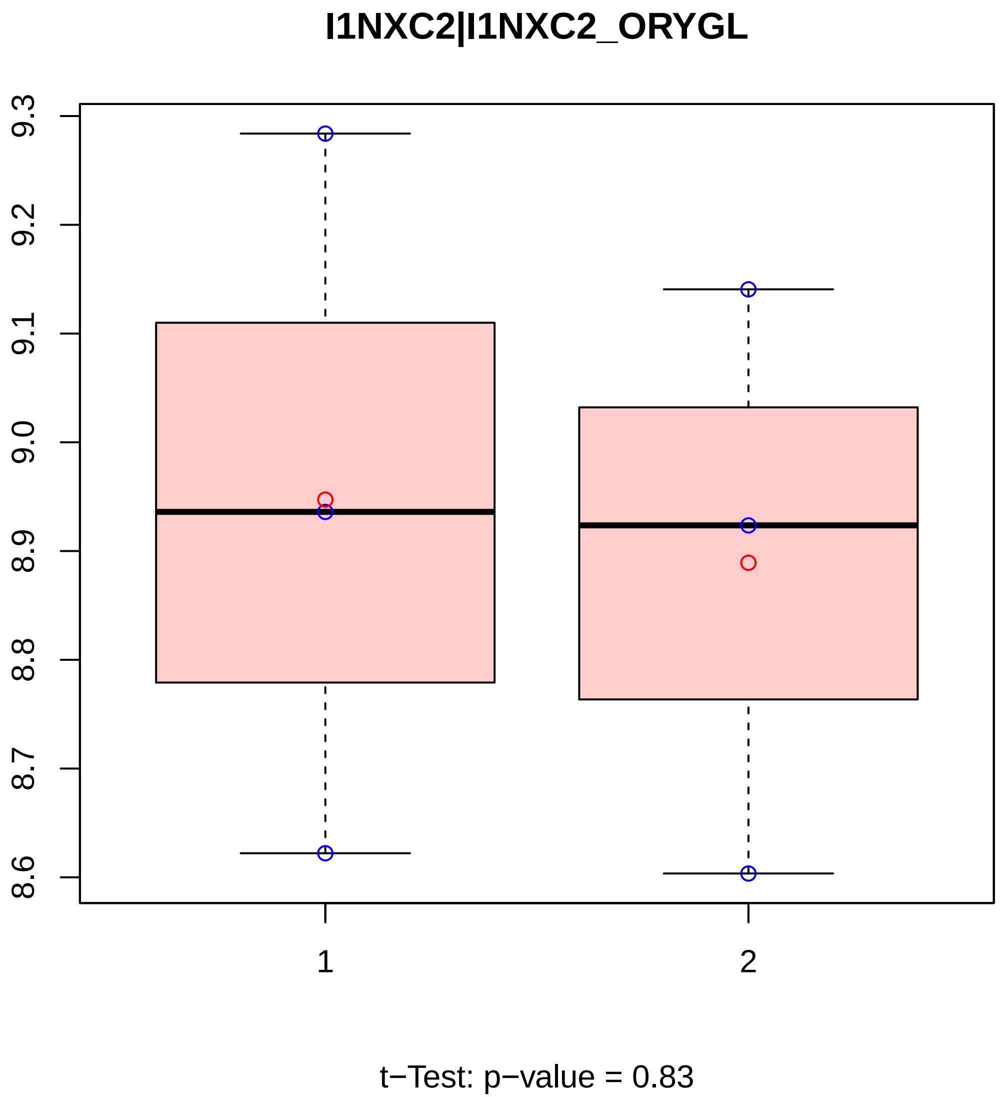
I1NXC2|I1NXC2_ORYGL
9.3
9.2
9.1
9.0
8.9
8.8
8.7
8.6
1
2
t−Test: p−value = 0.83

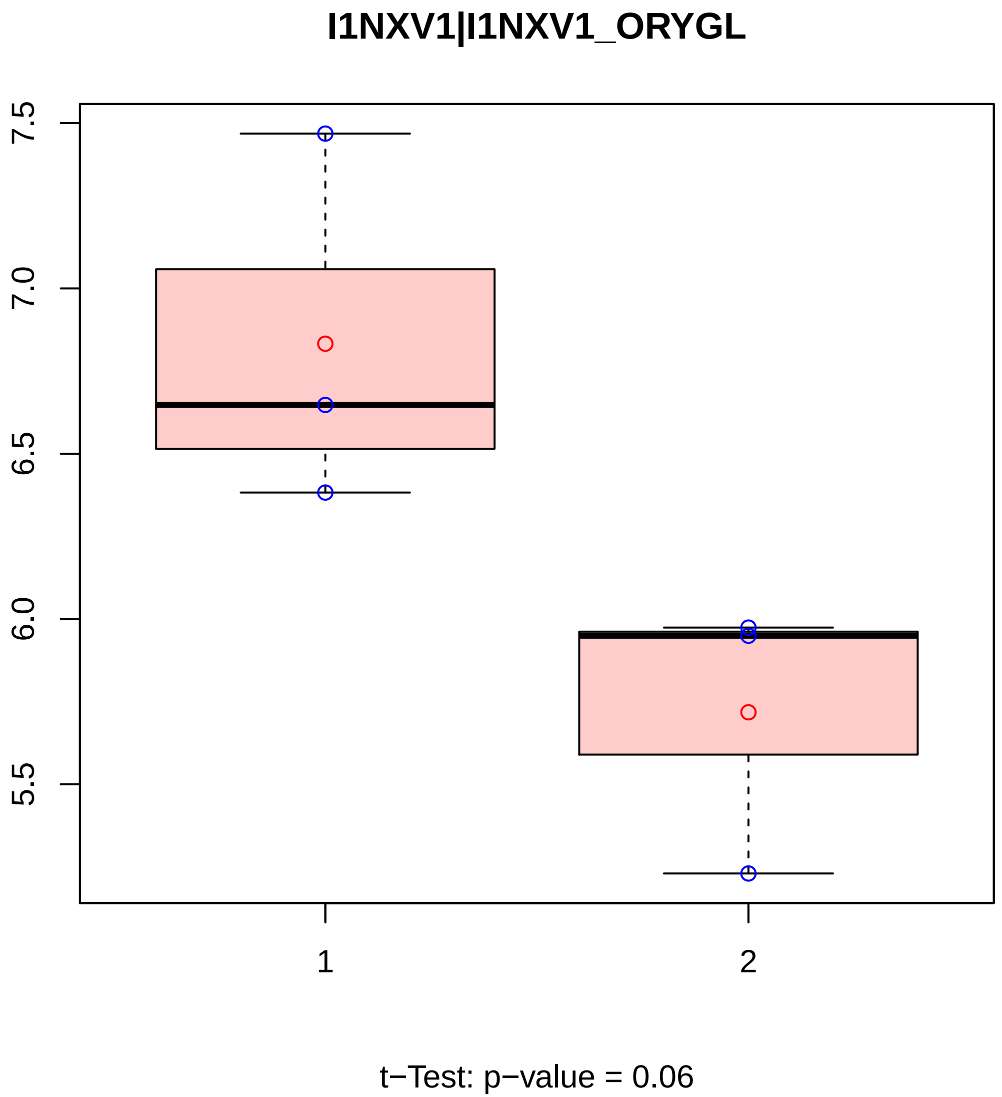
I1NXV1|I1NXV1_ORYGL
7.5
7.0
6.5
6.0
5.5
1
2
t−Test: p−value = 0.06

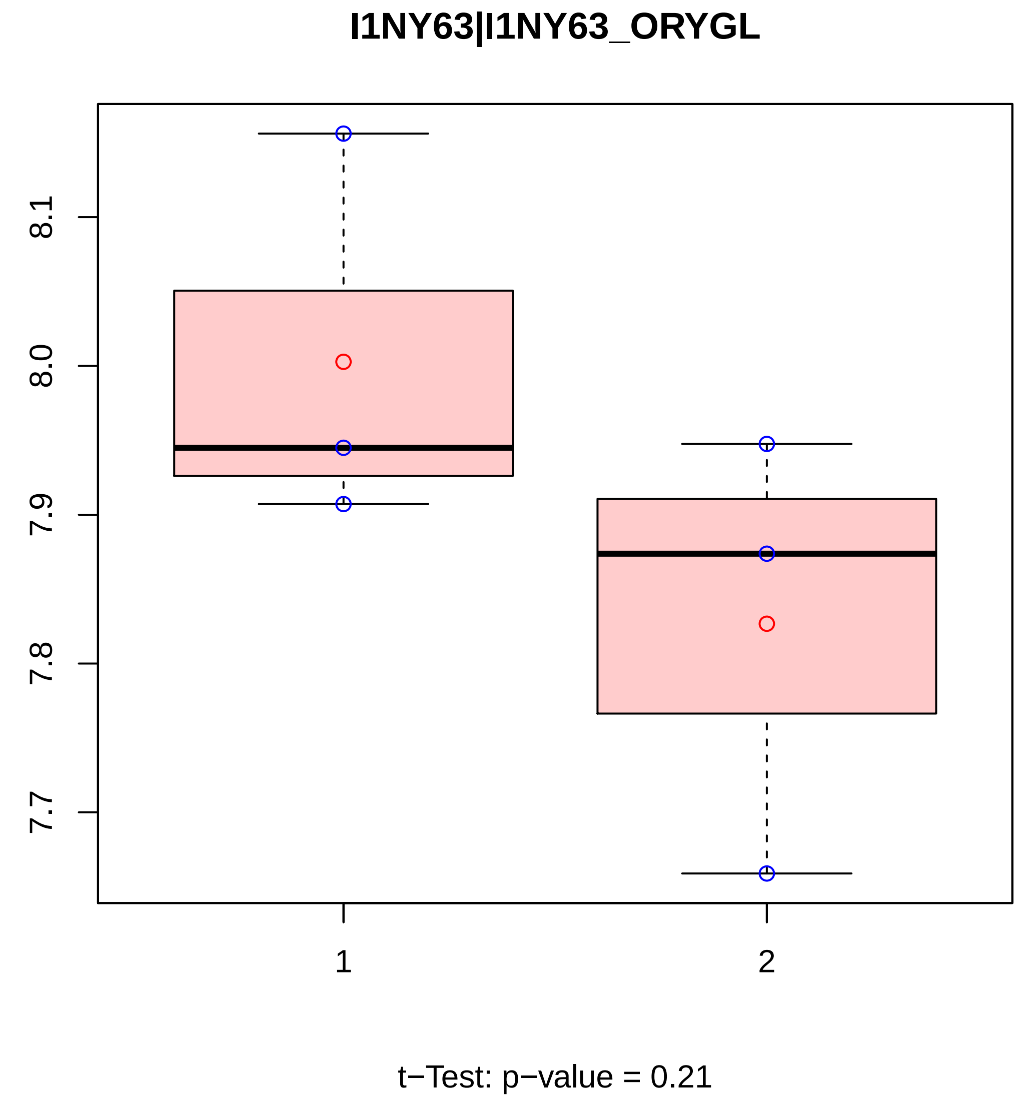
I1NY63|I1NY63_ORYGL
8.1
8.0
7.9
7.8
7.7
1
2
t−Test: p−value = 0.21

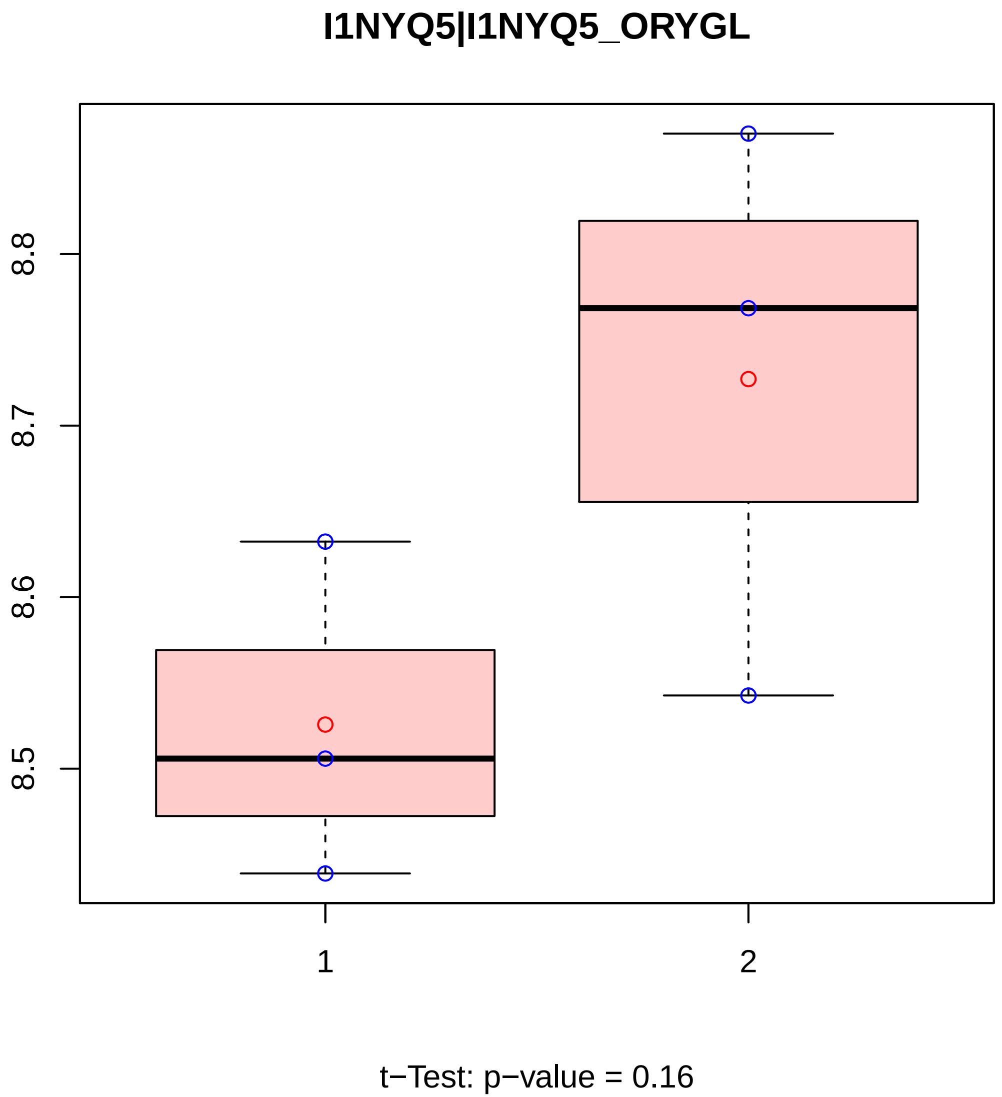
I1NYQ5|I1NYQ5_ORYGL
8.5
8.6
8.7
8.8
1
2
t−Test: p−value = 0.16

I1NZ48|I1NZ48_ORYGL

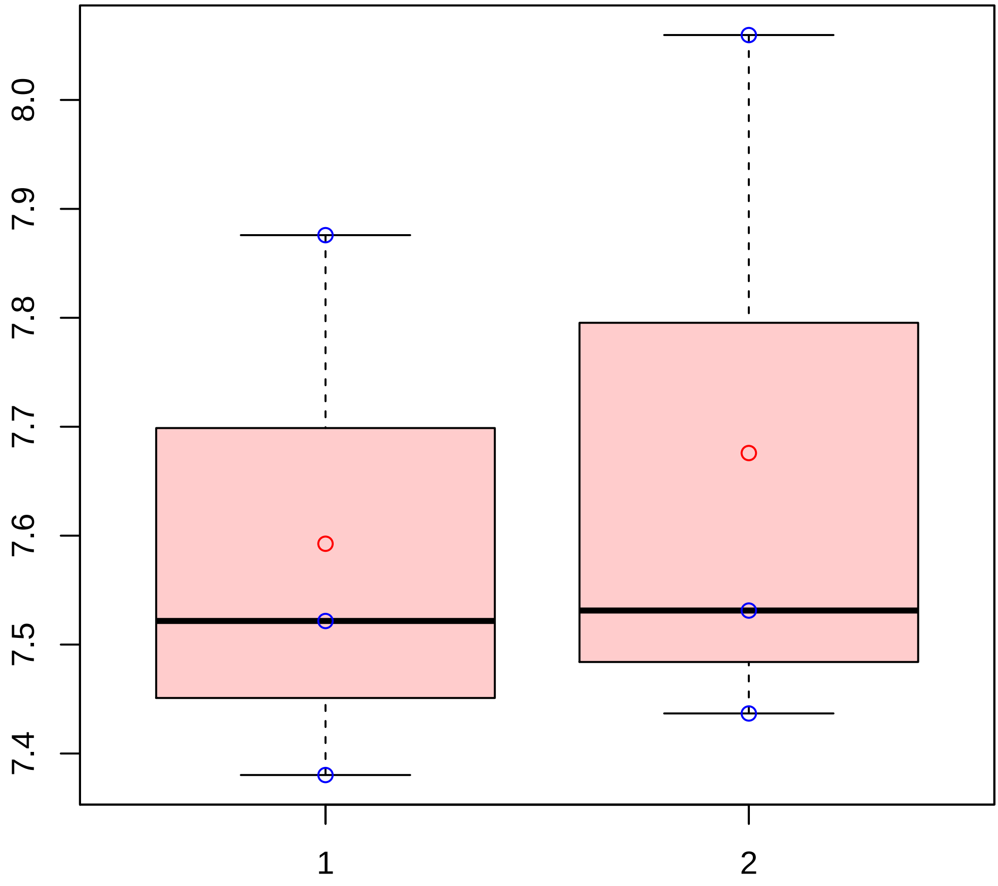

t−Test: p−value = 0.75

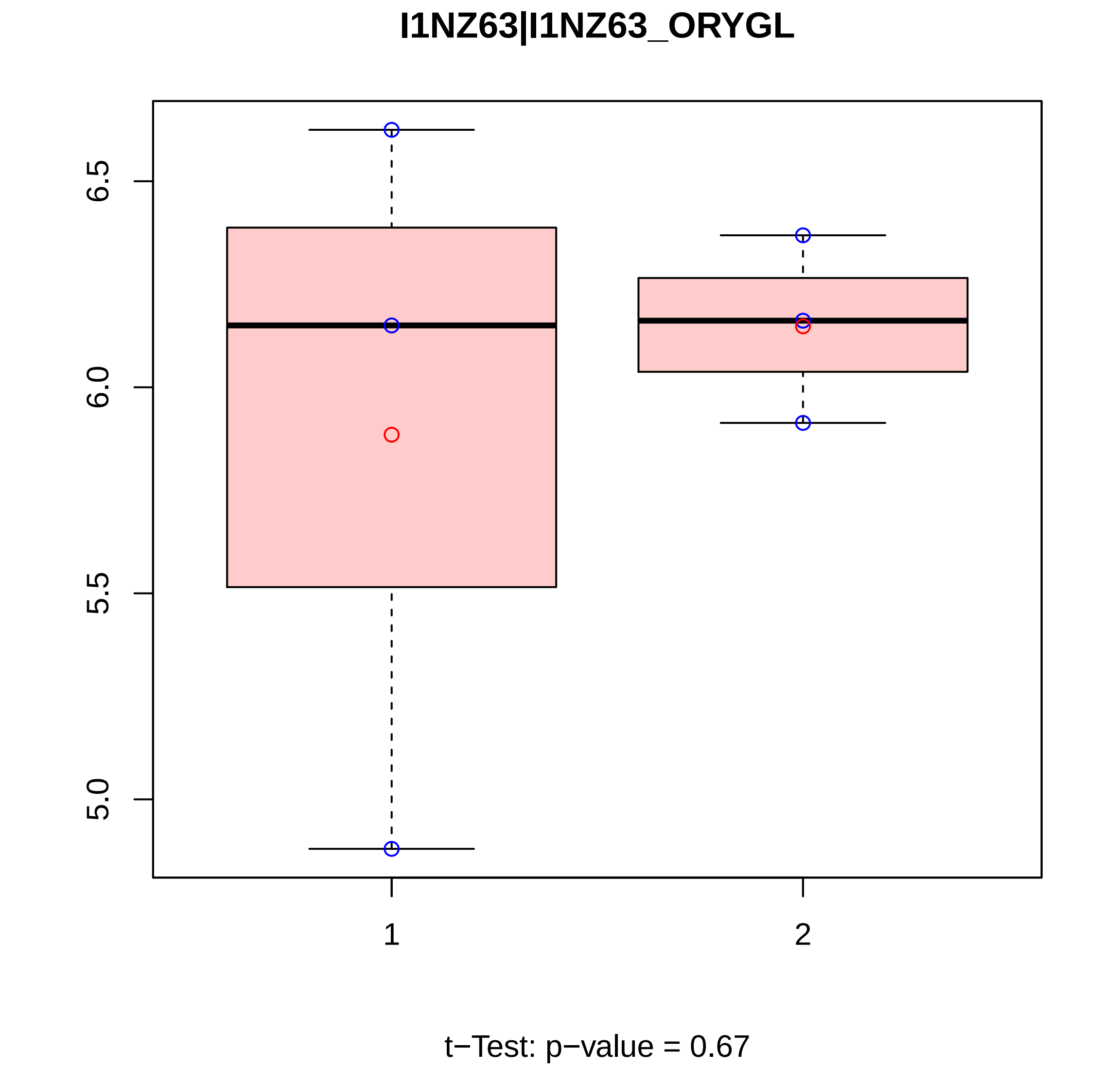

I1NZ63|I1NZ63_ORYGL
6.5
6.0
5.5
5.0
1
2
t−Test: p−value = 0.67

I1NZI0|I1NZI0_ORYGL

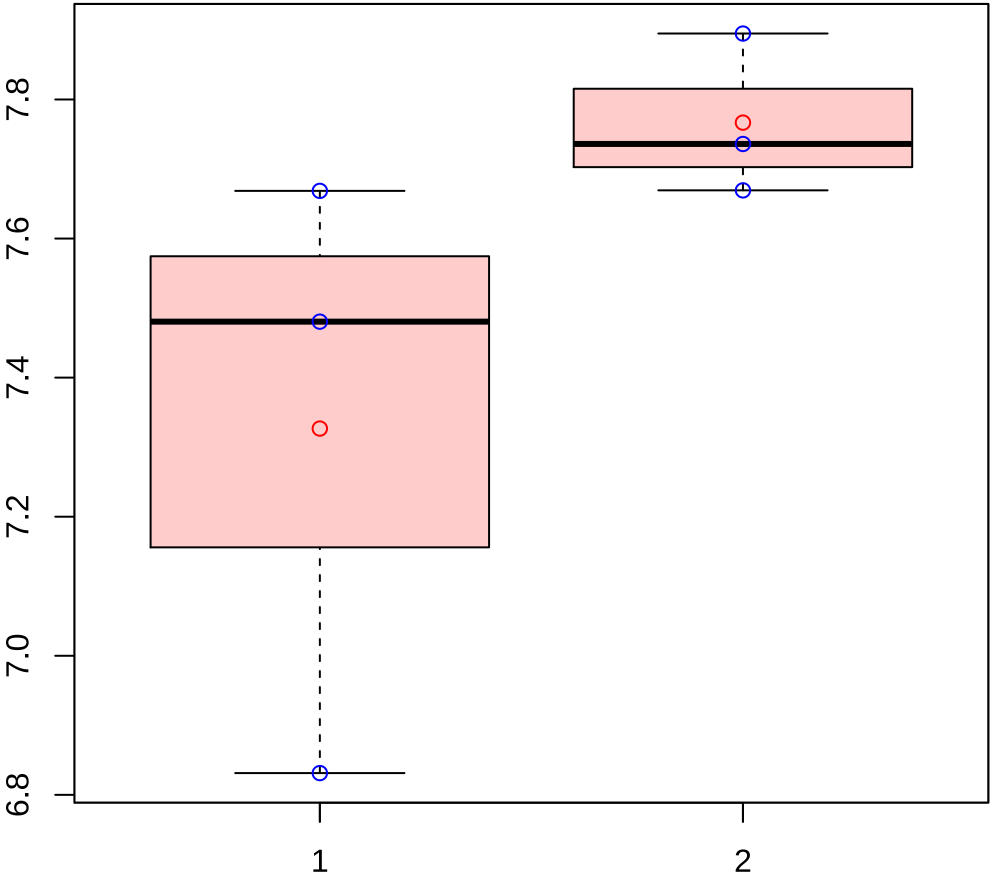

t−Test: p−value = 0.22

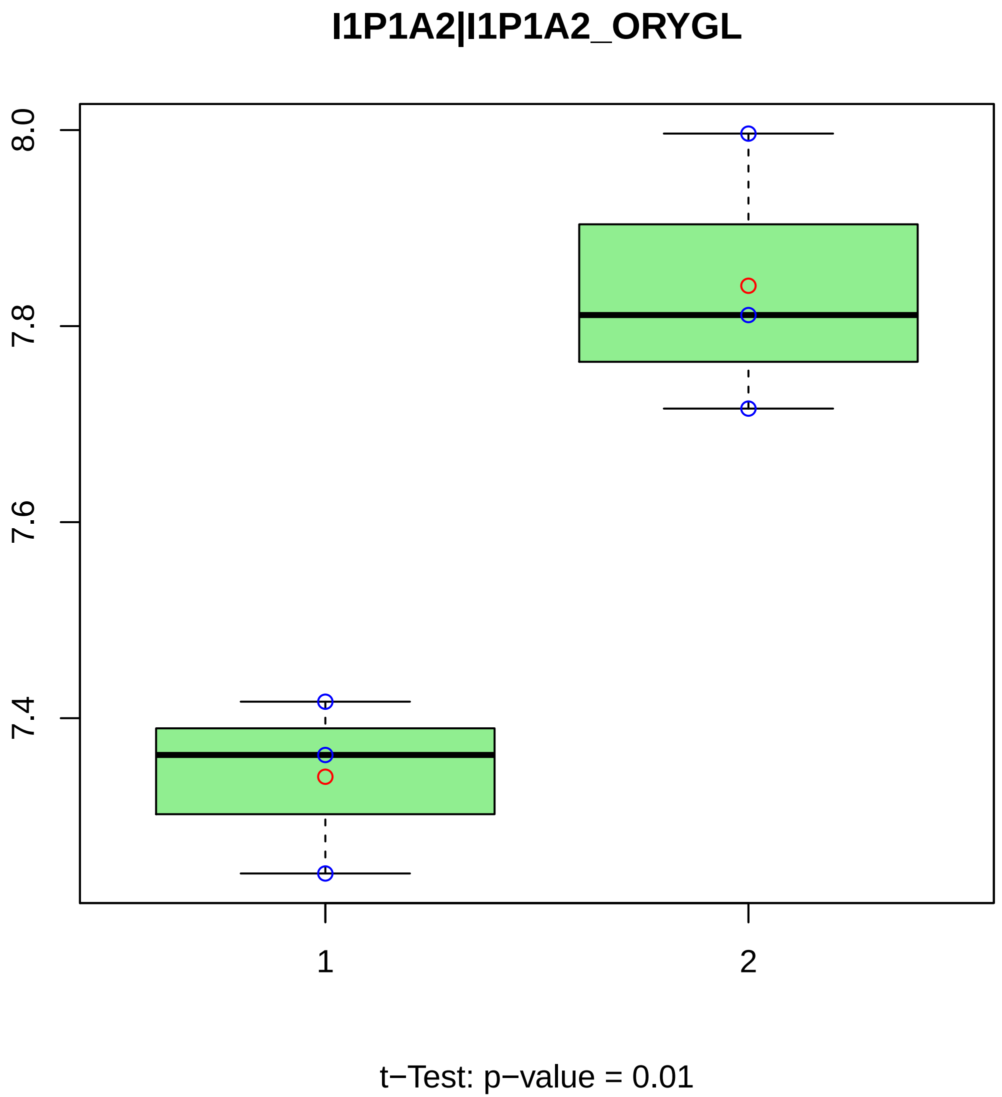
I1P1A2|I1P1A2_ORYGL
8.0
7.8
7.6
7.4
1
2
t−Test: p−value = 0.01

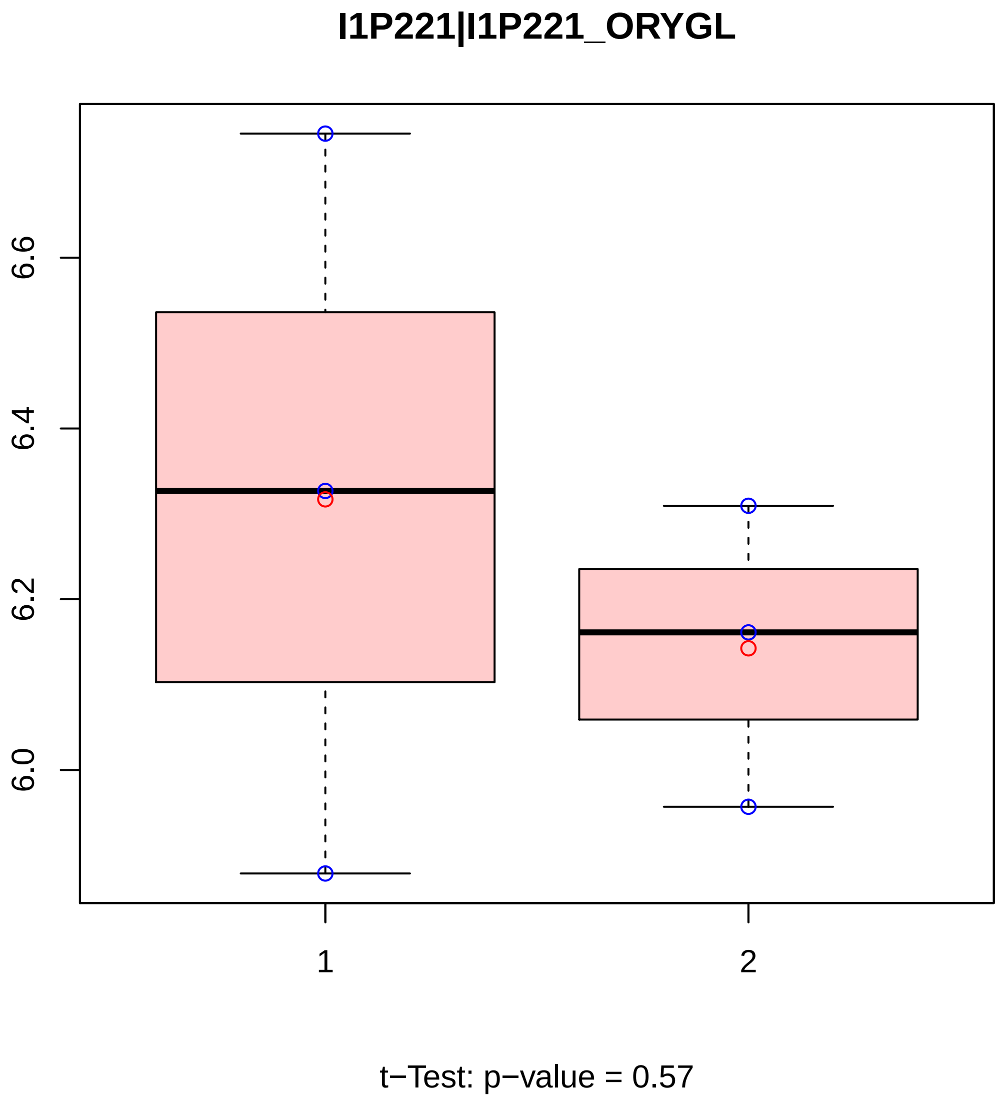
I1P221|I1P221_ORYGL
6.0
6.2
6.4
6.6
1
2
t−Test: p−value = 0.57

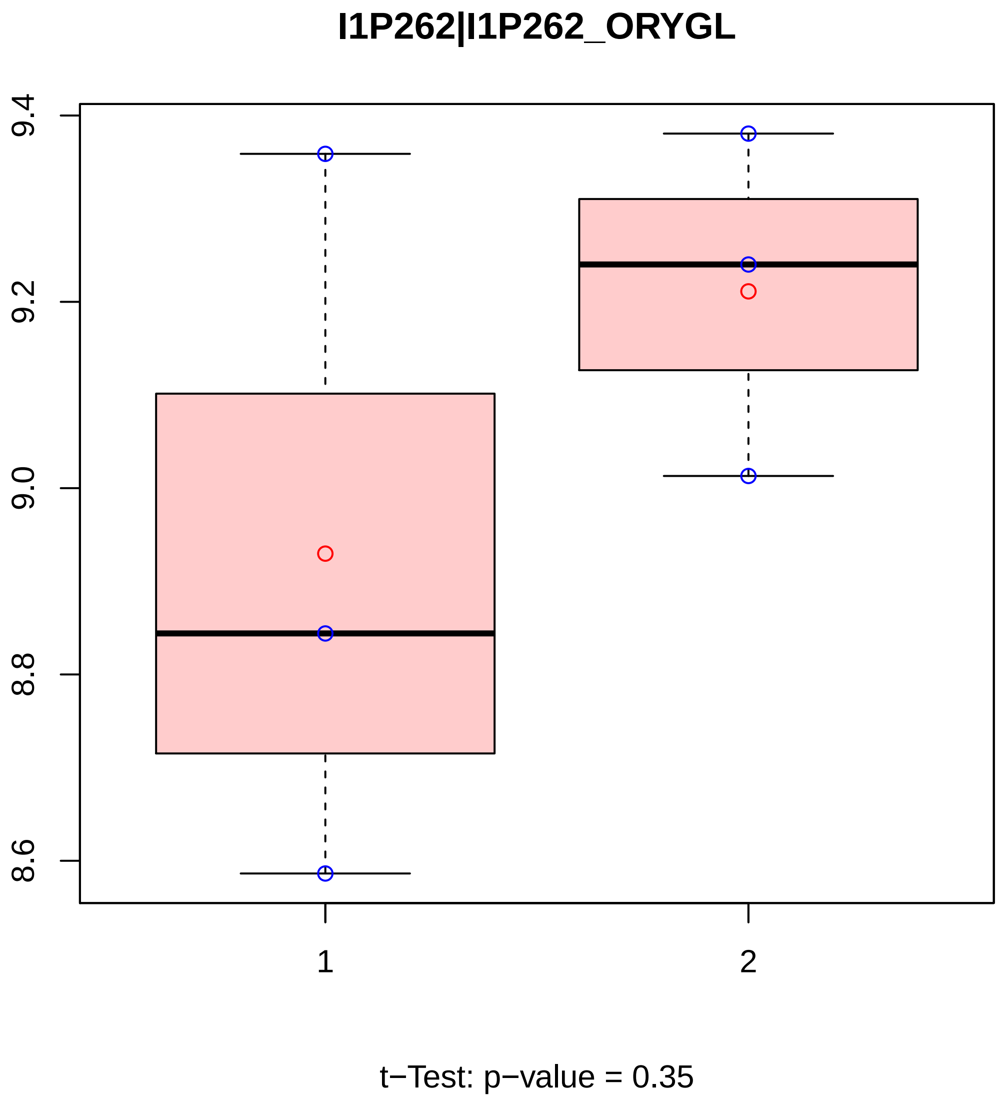
I1P262|I1P262_ORYGL
9.4
9.2
9.0
8.8
8.6
1
2
t−Test: p−value = 0.35

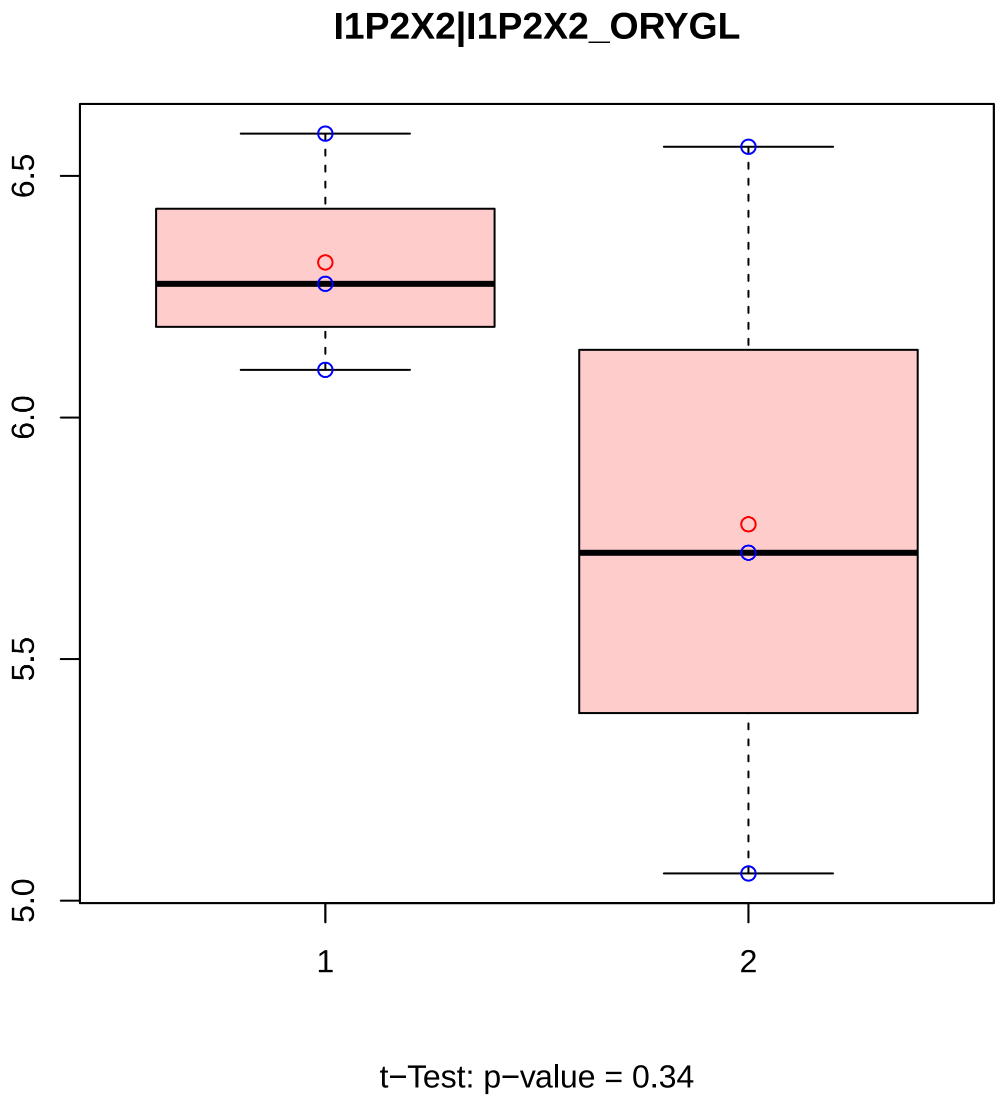

I1P2X2|I1P2X2_ORYGL
6.5
6.0
5.5
5.0
1
2
t−Test: p−value = 0.34

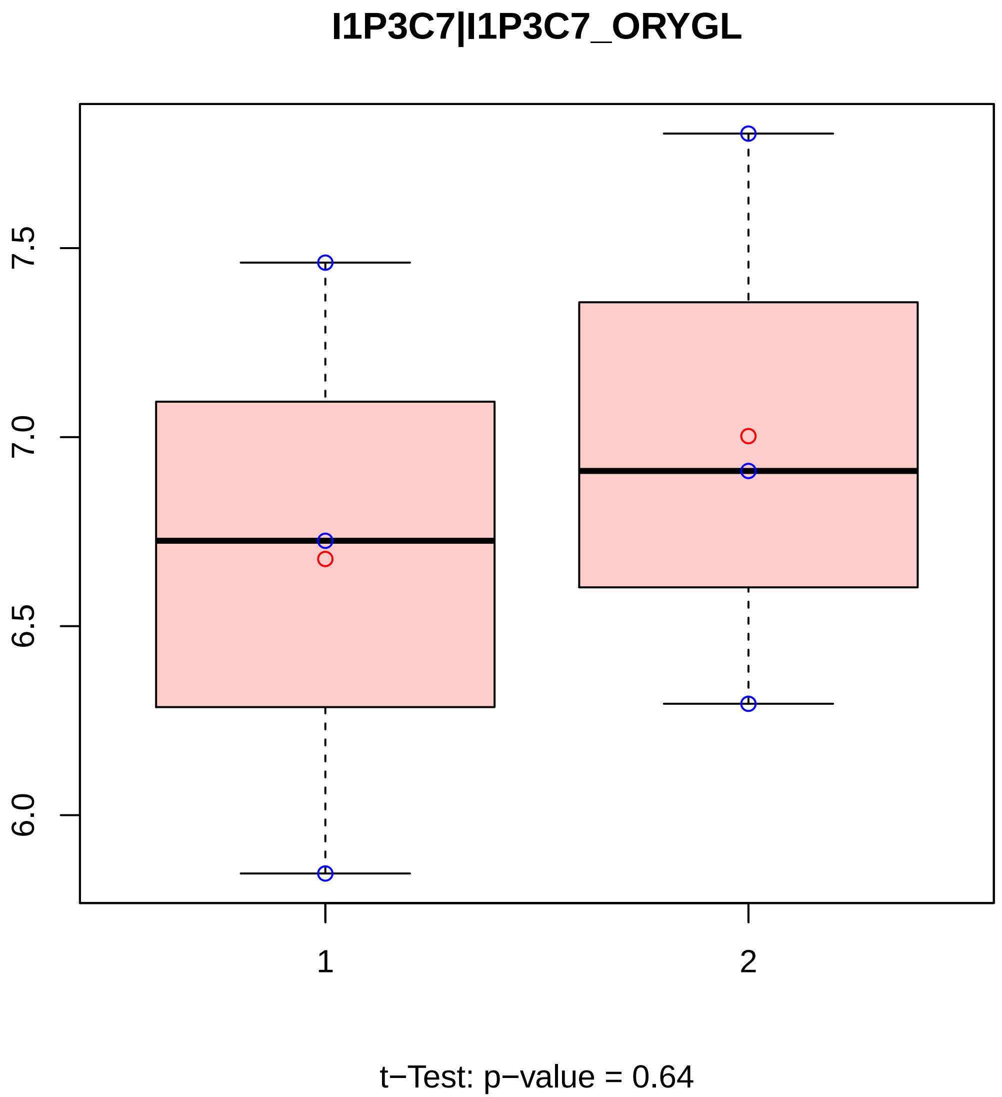
I1P3C7|I1P3C7_ORYGL
6.0
6.5
7.0
7.5
1
2
t−Test: p−value = 0.64

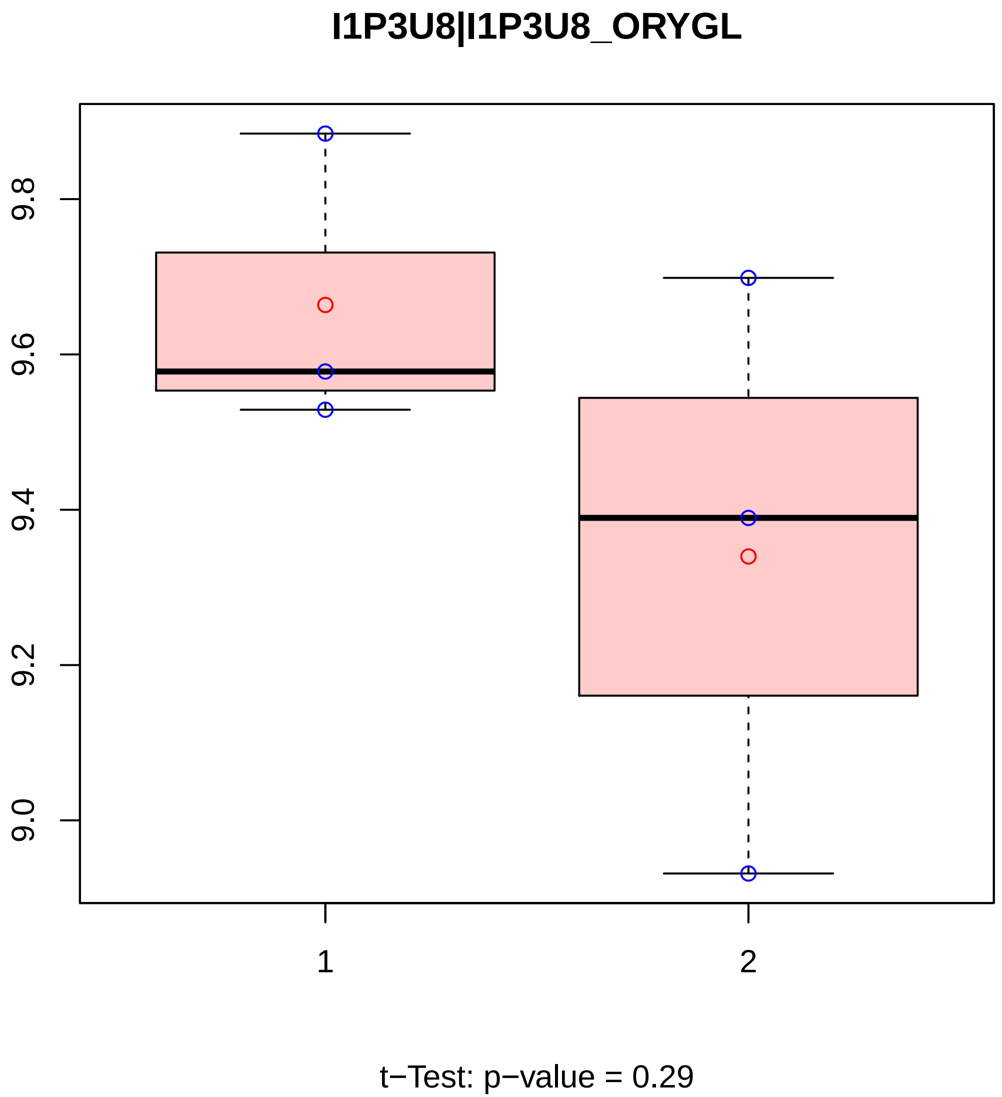
I1P3U8|I1P3U8_ORYGL
9.0
9.2
9.4
9.6
9.8
1
2
t−Test: p−value = 0.29

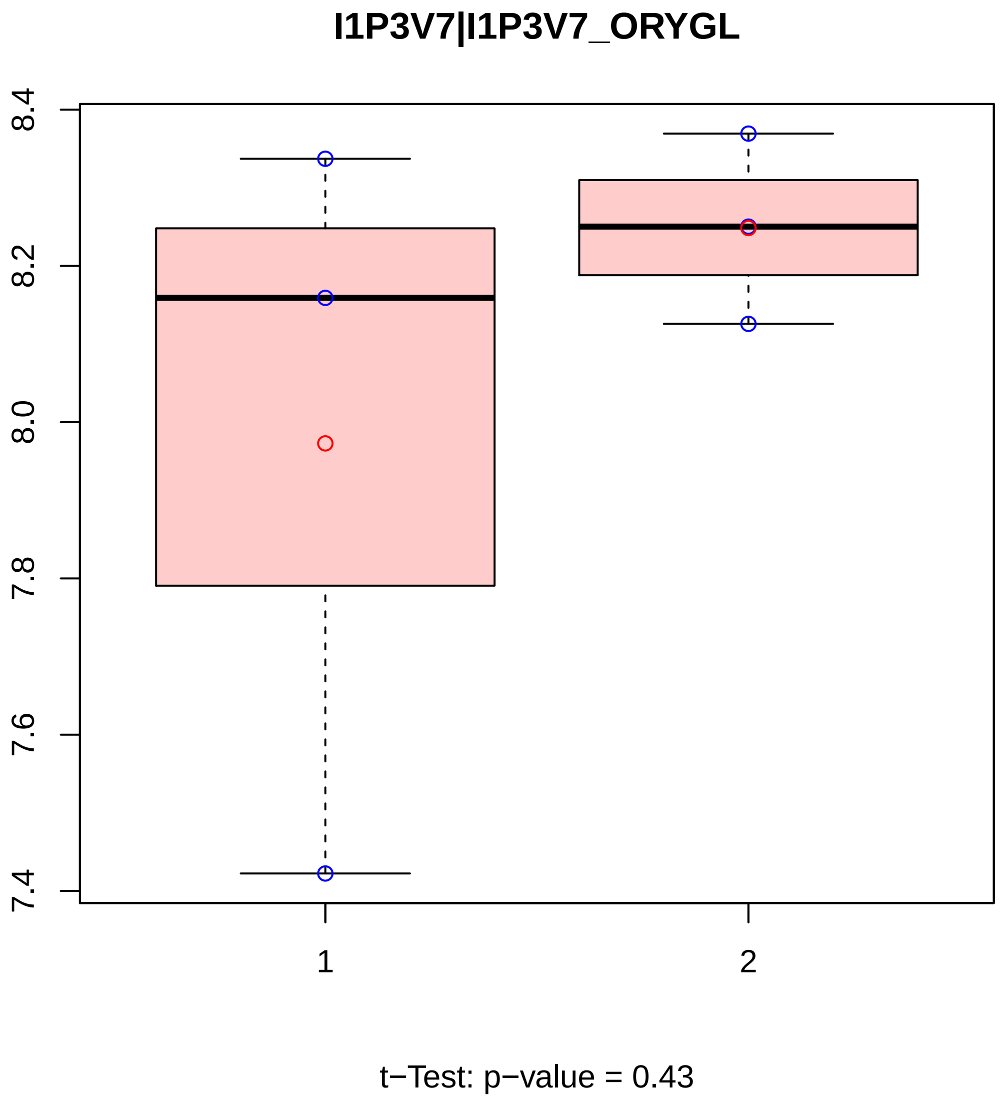
I1P3V7|I1P3V7_ORYGL
8.4
8.2
8.0
7.8
7.6
7.4
1
2
t−Test: p−value = 0.43

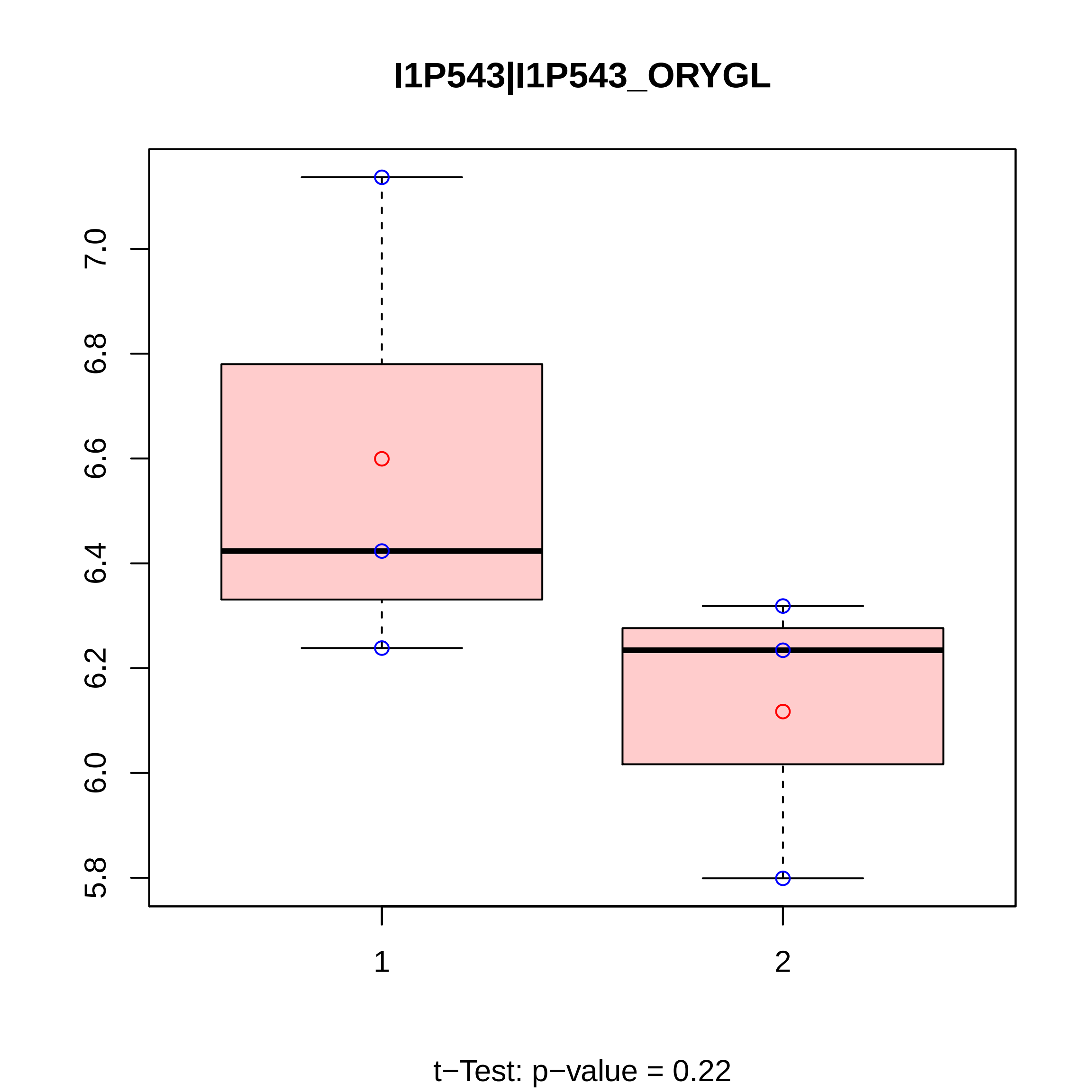
I1P543|I1P543_ORYGL
7.0
6.8
6.6
6.4
6.2
6.0
5.8
1
2
t−Test: p−value = 0.22

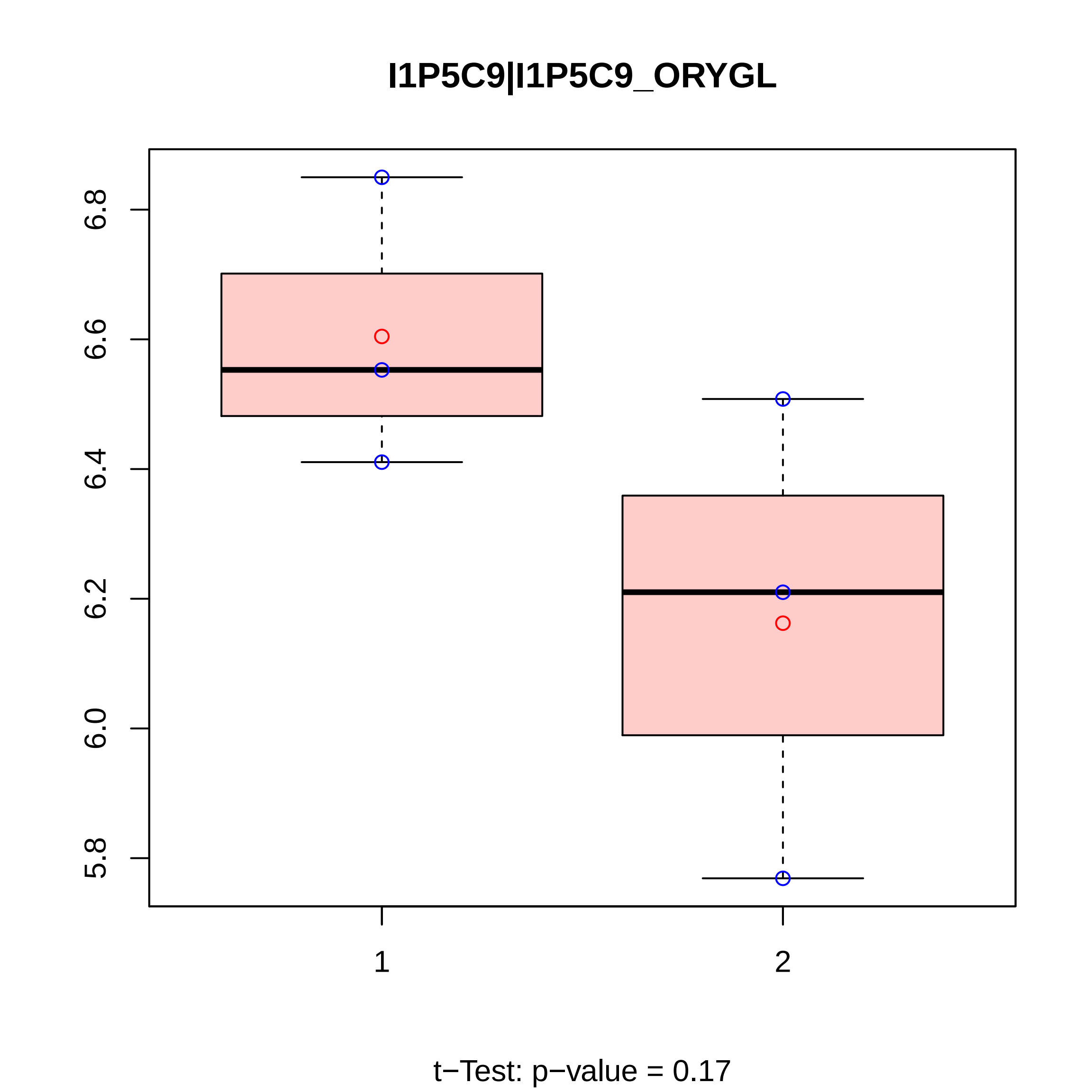
I1P5C9|I1P5C9_ORYGL
6.8
6.6
6.4
6.2
6.0
5.8
1
2
t−Test: p−value = 0.17

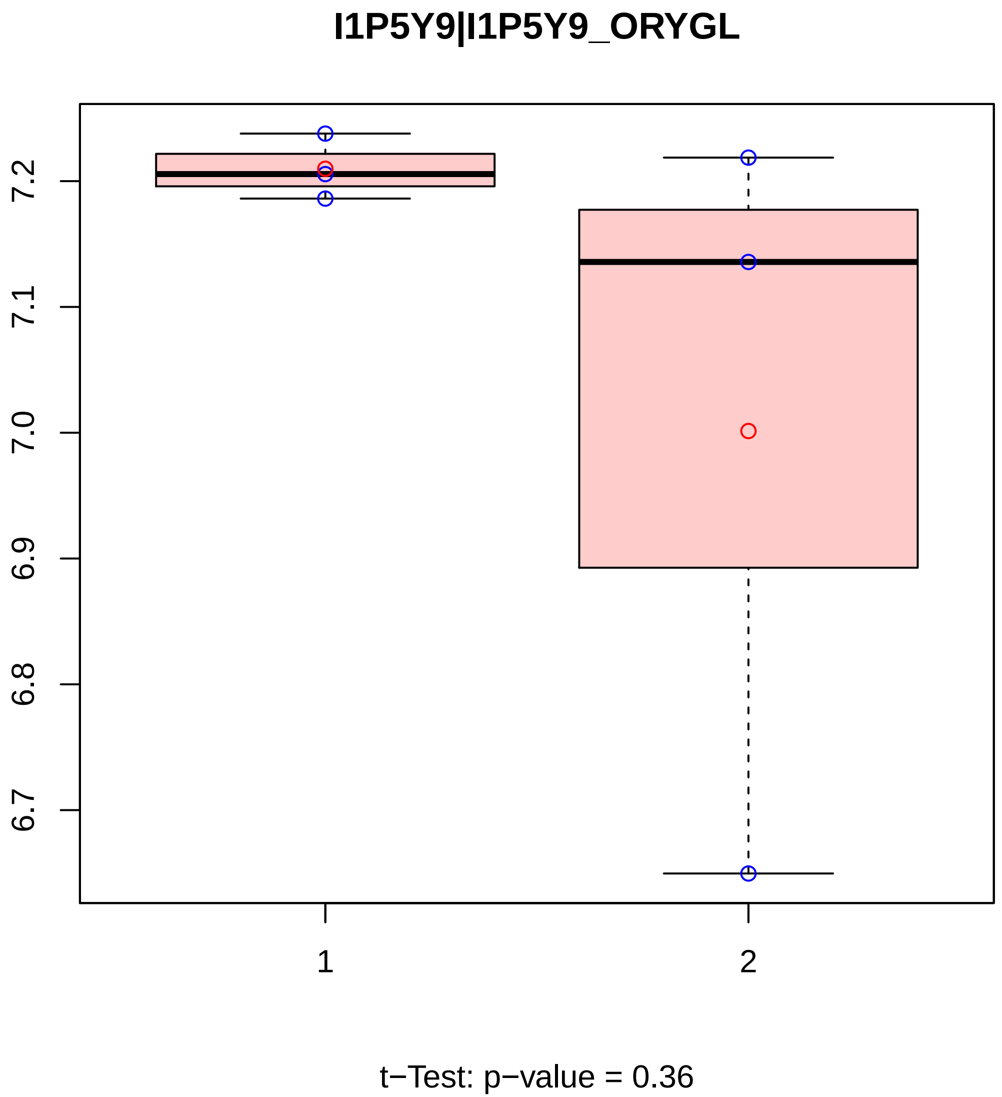
I1P5Y9|I1P5Y9_ORYGL
7.2
7.1
7.0
6.9
6.8
6.7
1
2
t−Test: p−value = 0.36

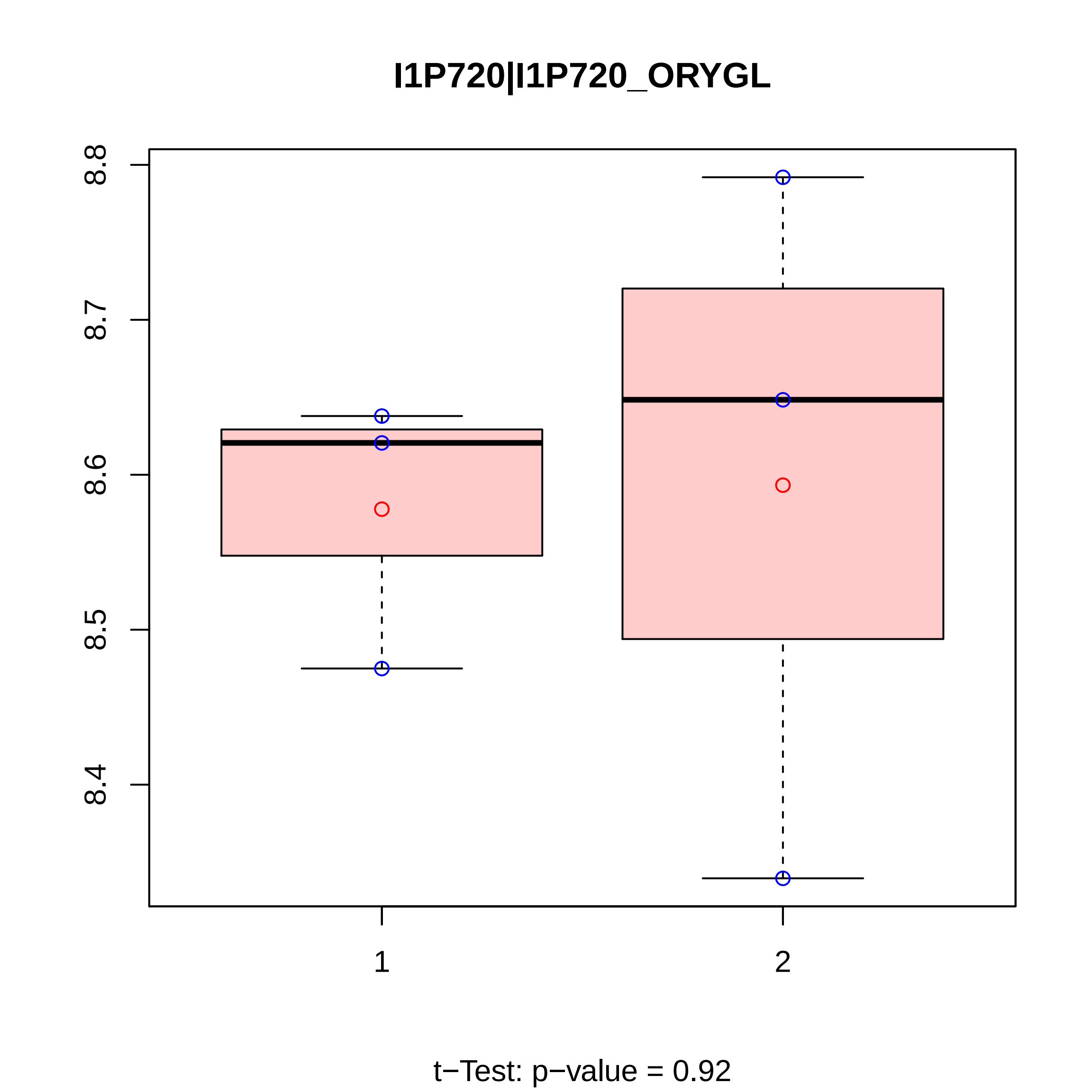
I1P720|I1P720_ORYGL
8.4
8.5
8.6
8.7
8.8
1
2
t−Test: p−value = 0.92

I1P7F9|I1P7F9_ORYGL

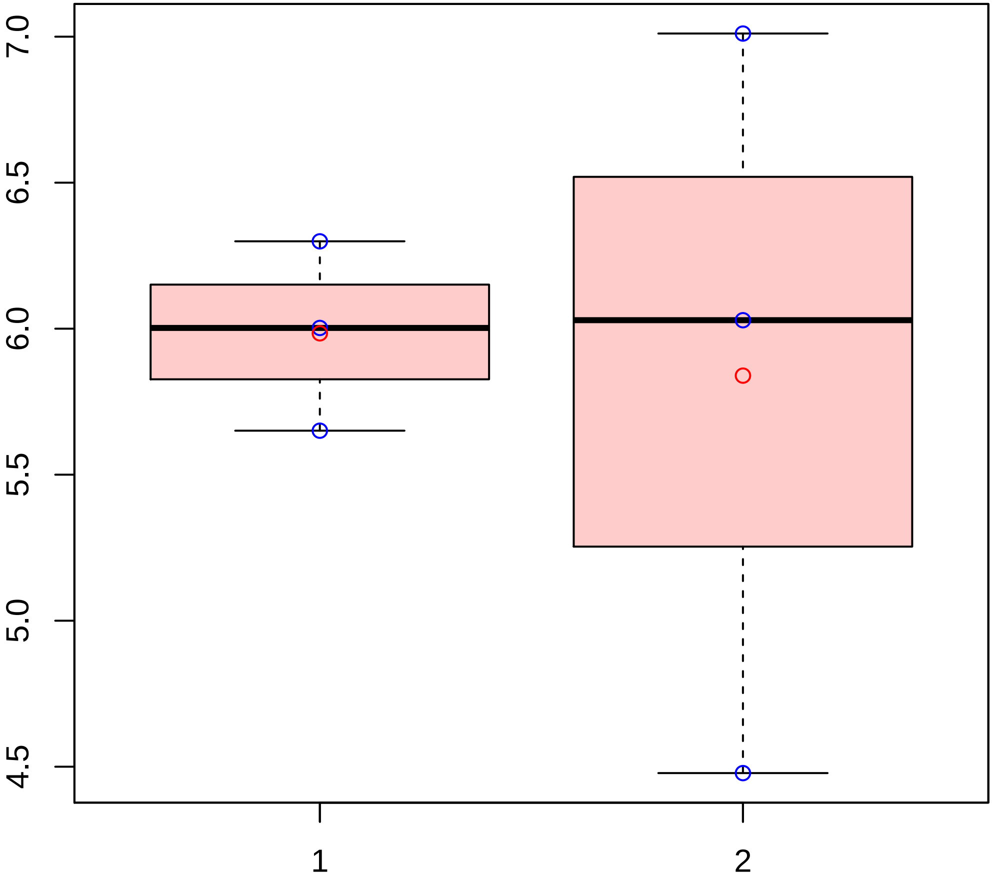

t−Test: p−value = 0.86

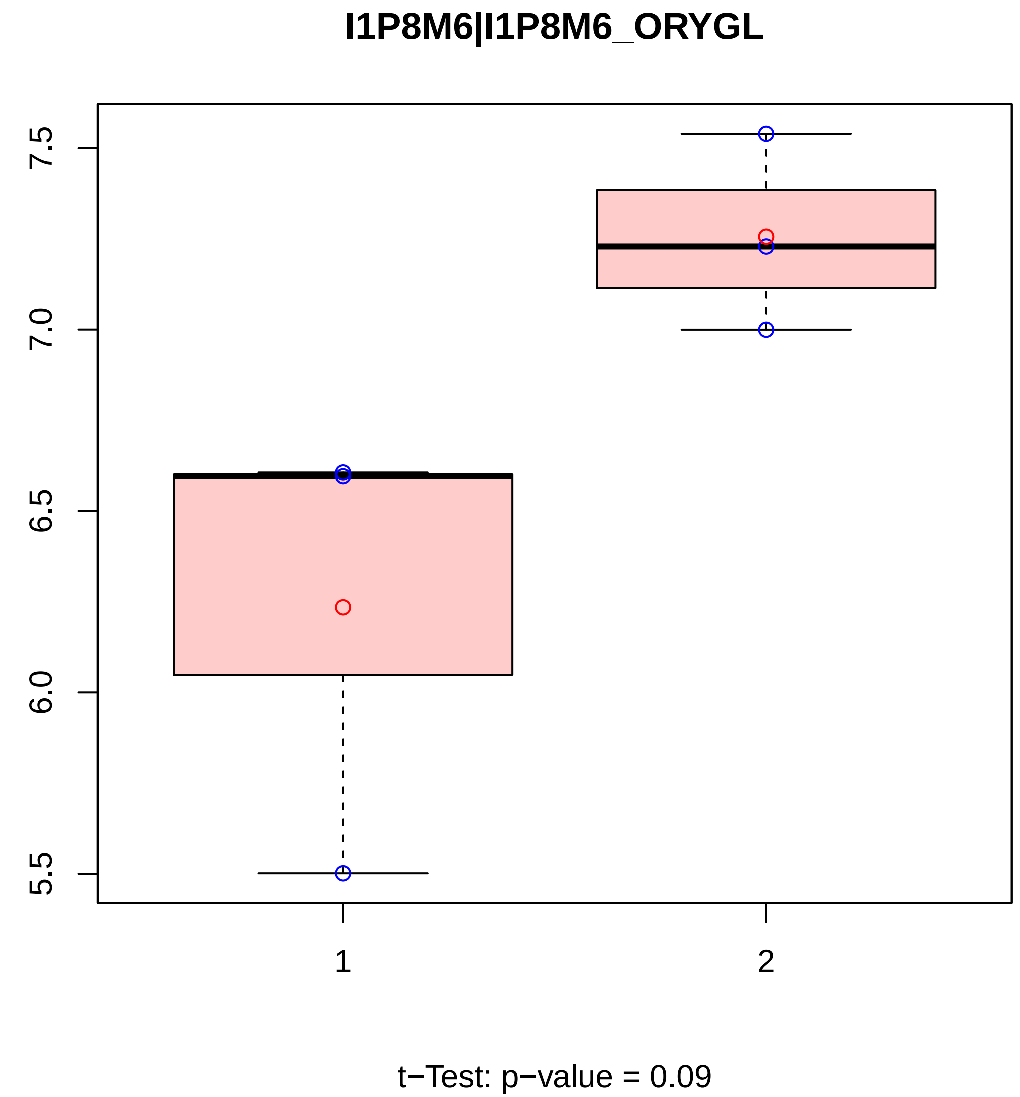
I1P8M6|I1P8M6_ORYGL
7.5
7.0
6.5
6.0
5.5
1
2
t−Test: p−value = 0.09

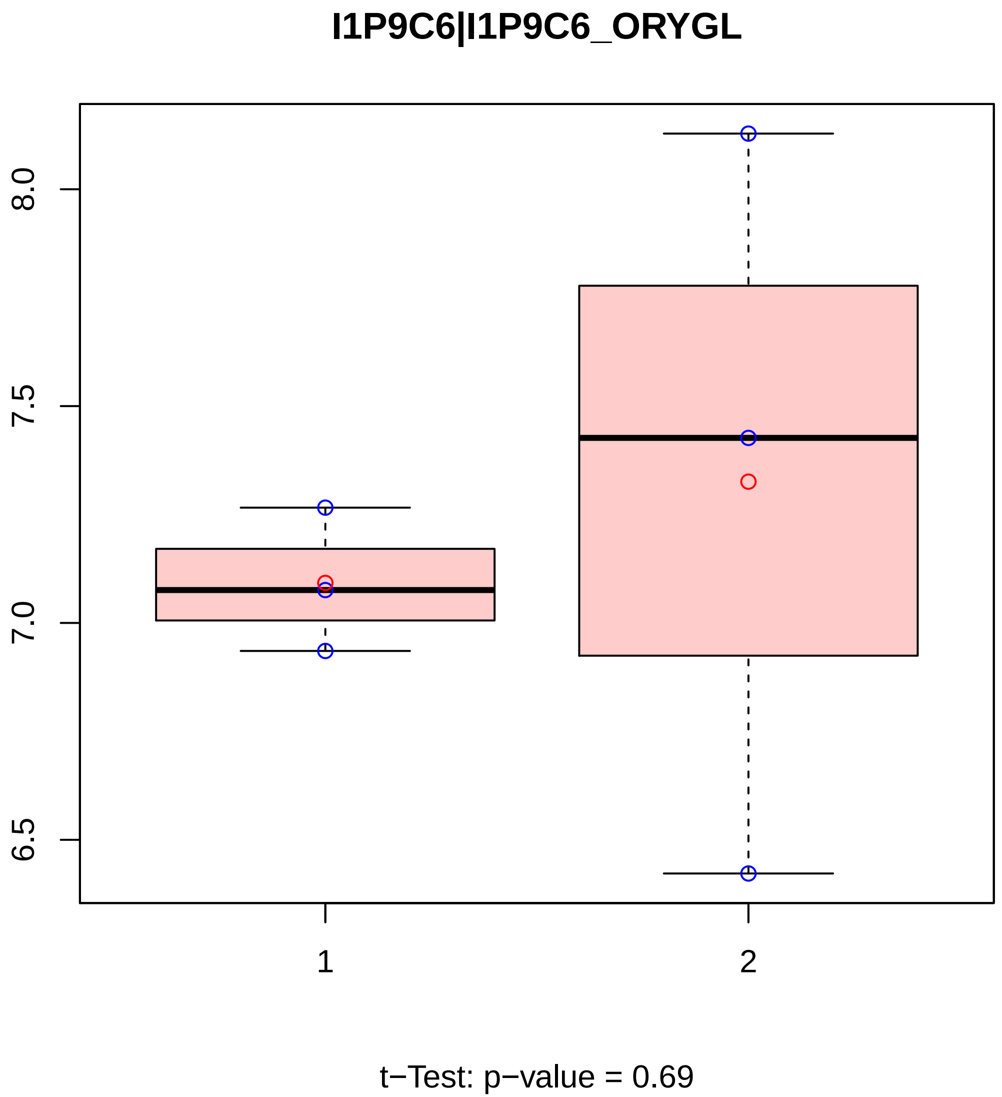

I1P9C6|I1P9C6_ORYGL
8.0
7.5
7.0
6.5
1
2
t−Test: p−value = 0.69

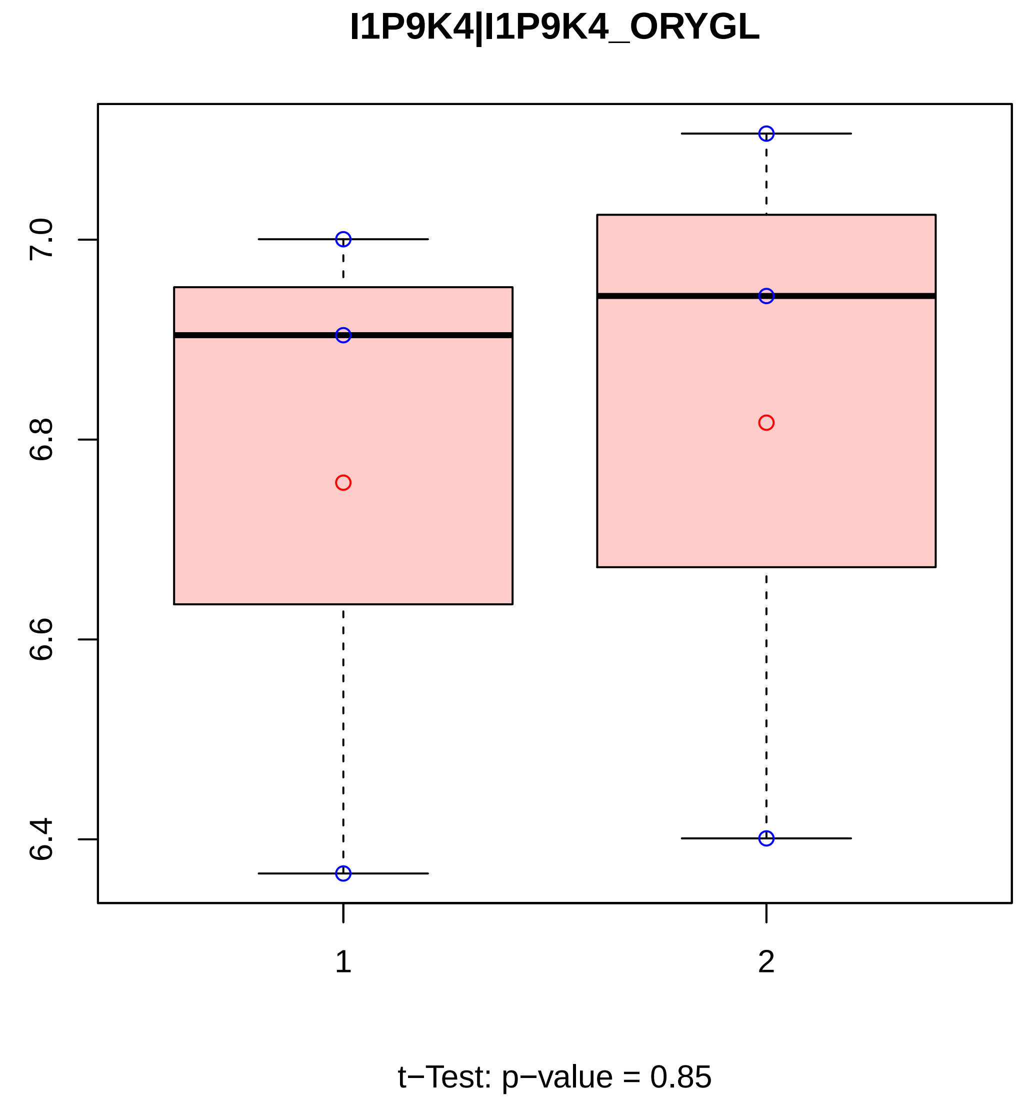
I1P9K4|I1P9K4_ORYGL
7.0
6.8
6.6
6.4
1
2
t−Test: p−value = 0.85

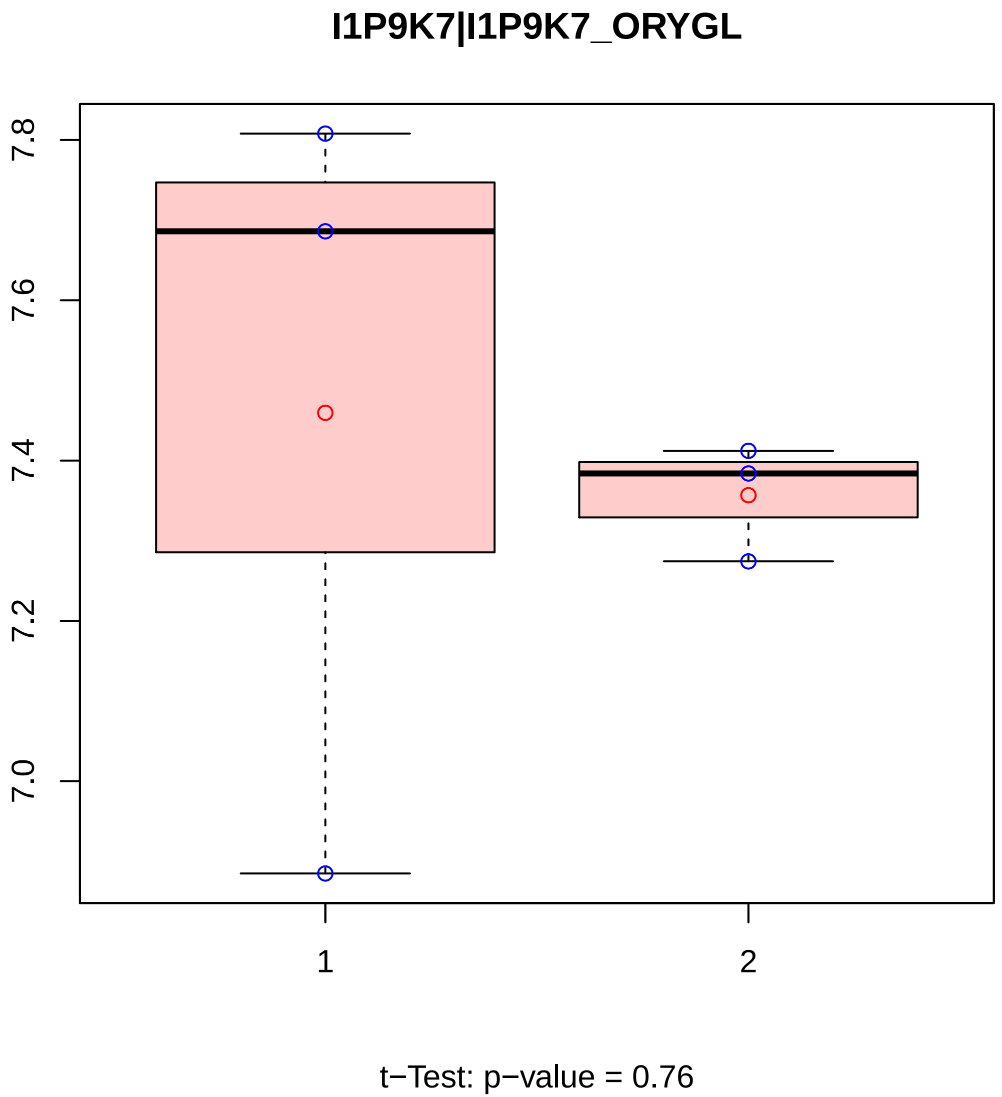
I1P9K7|I1P9K7_ORYGL
7.8
7.6
7.4
7.2
7.0
1
2
t−Test: p−value = 0.76

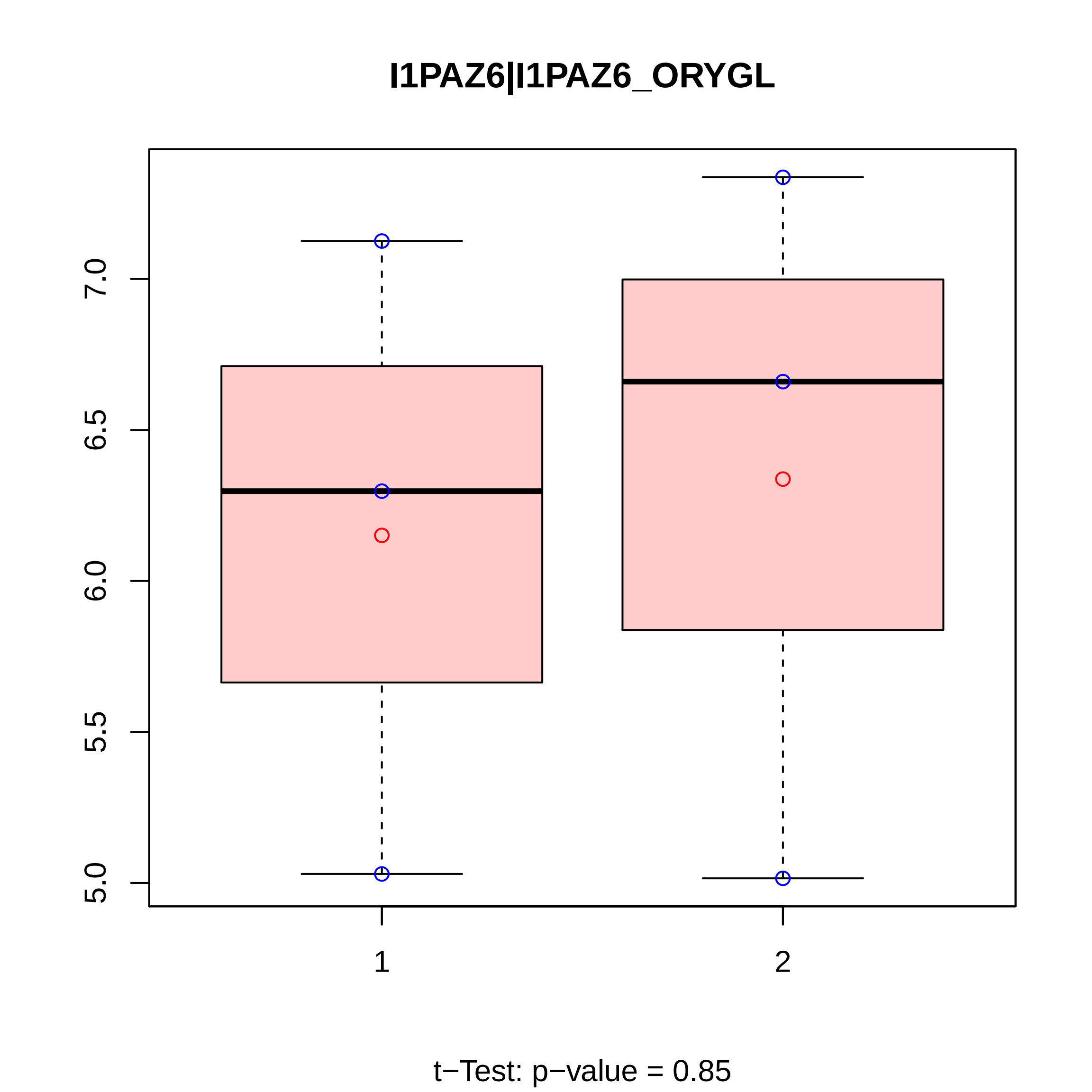
I1PAZ6|I1PAZ6_ORYGL
5.0
5.5
6.0
6.5
7.0
1
2
t−Test: p−value = 0.85

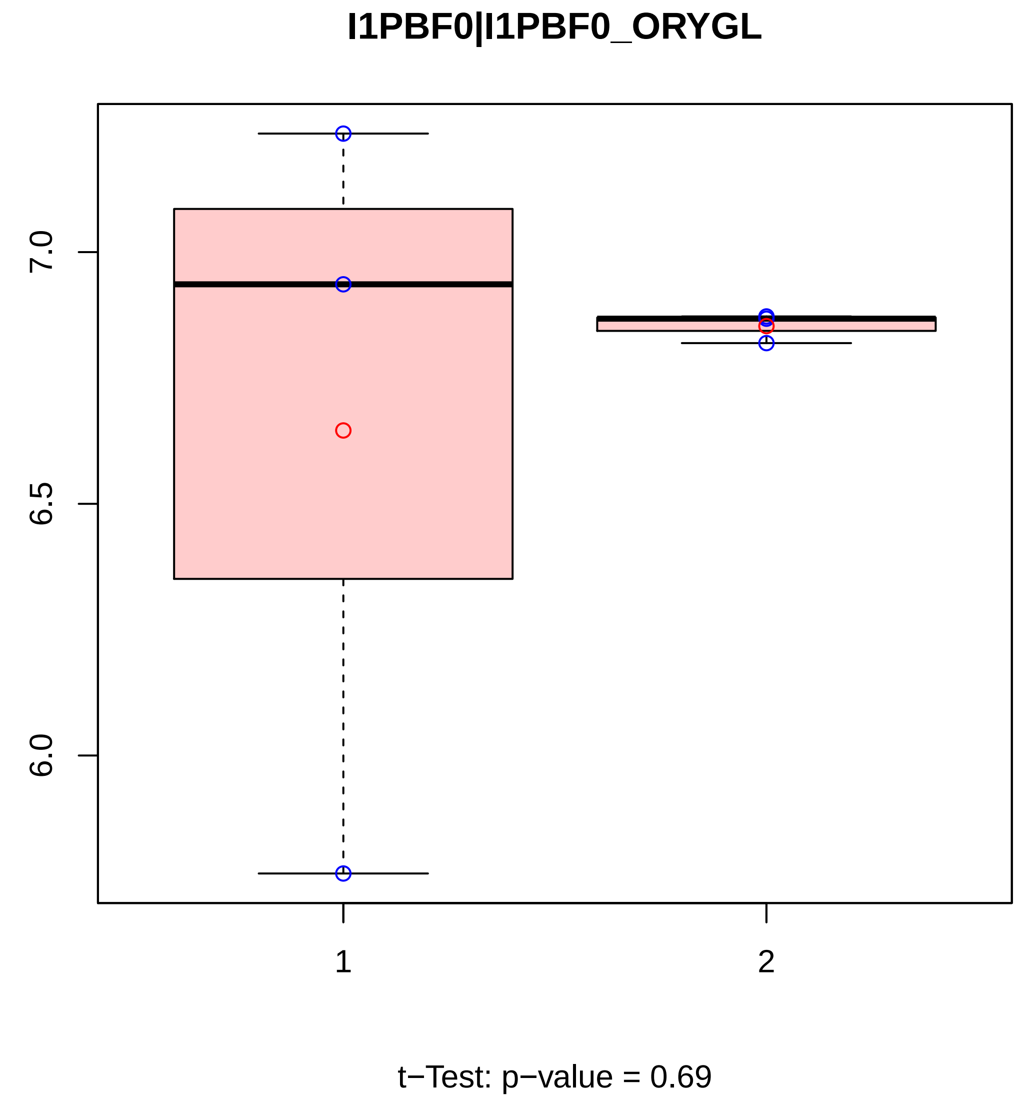
I1PBF0|I1PBF0_ORYGL
7.0
6.5
6.0
1
2
t−Test: p−value = 0.69

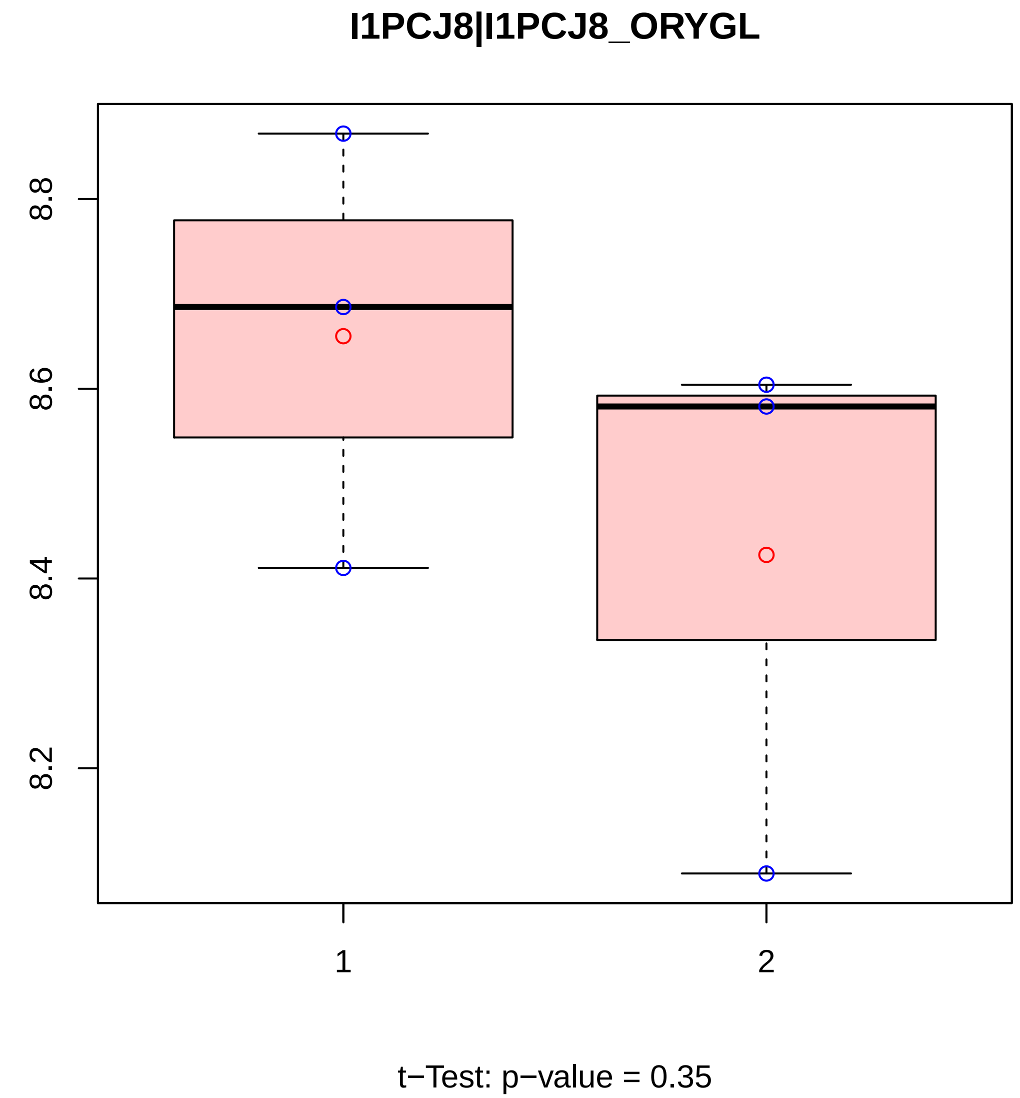

I1PCJ8|I1PCJ8_ORYGL
8.2
8.4
8.6
8.8
1
2
t−Test: p−value = 0.35

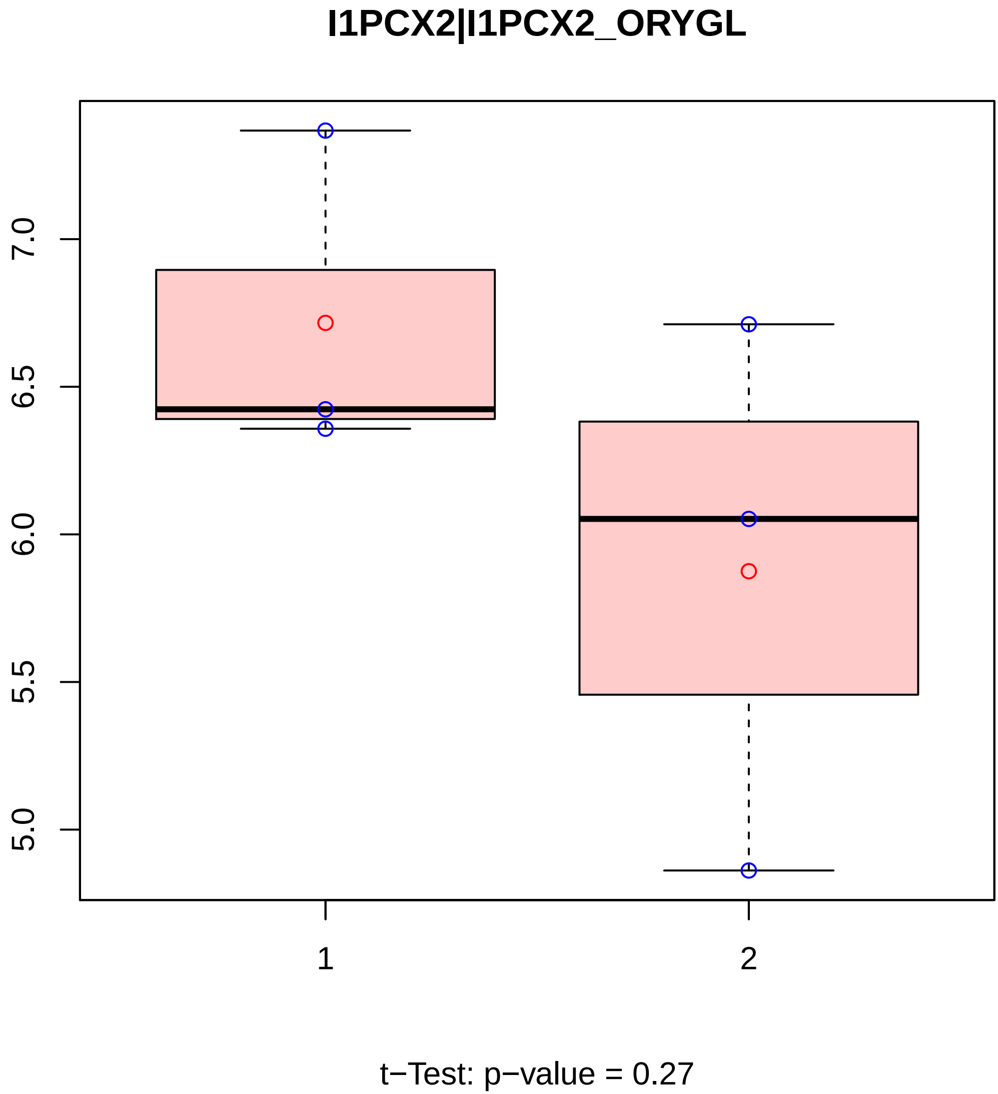
I1PCX2|I1PCX2_ORYGL
7.0
6.5
6.0
5.5
5.0
1
2
t−Test: p−value = 0.27

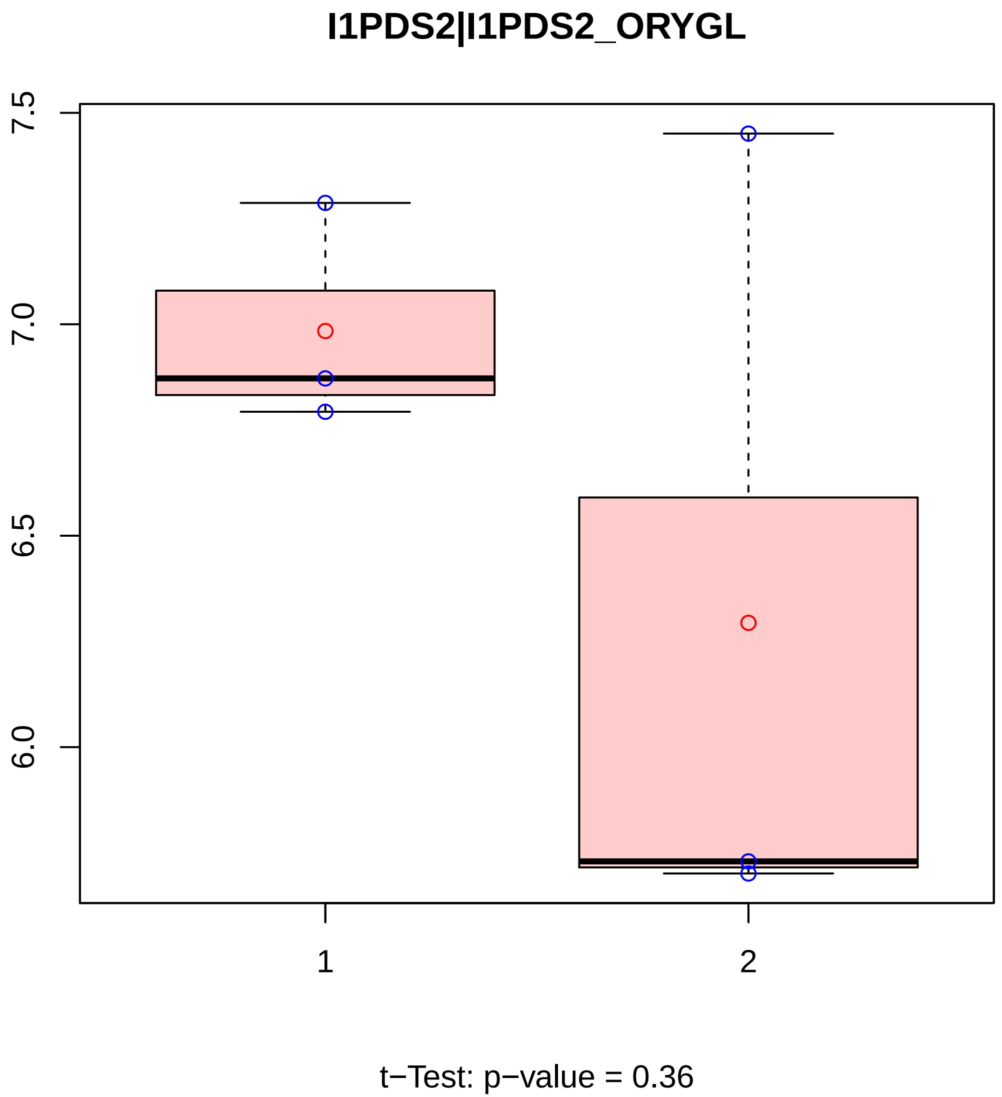
I1PDS2|I1PDS2_ORYGL
7.5
7.0
6.5
6.0
1
2
t−Test: p−value = 0.36

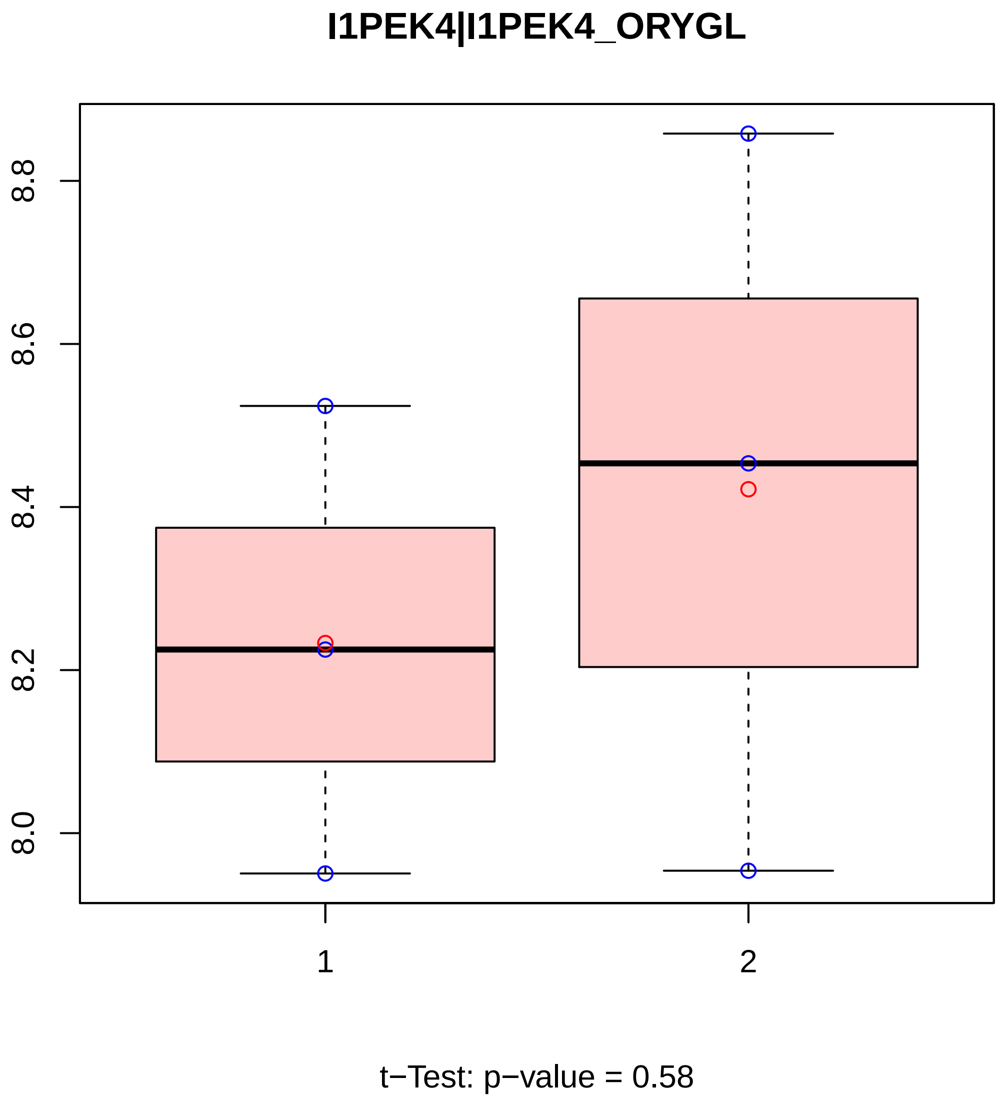
I1PEK4|I1PEK4_ORYGL
8.0
8.2
8.4
8.6
8.8
1
2
t−Test: p−value = 0.58

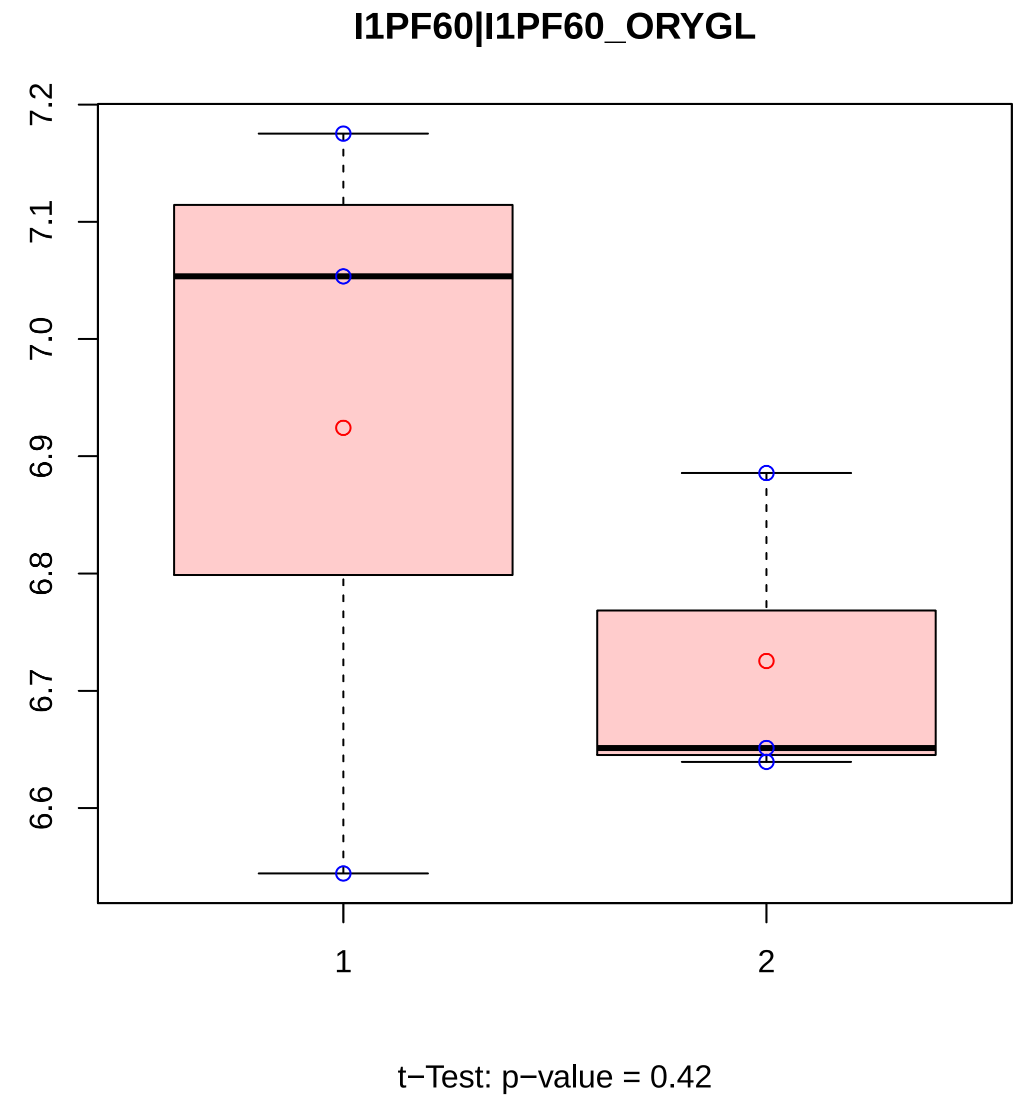
I1PF60|I1PF60_ORYGL
7.2
7.1
7.0
6.9
6.8
6.7
6.6
1
2
t−Test: p−value = 0.42

I1PFC8|I1PFC8_ORYGL

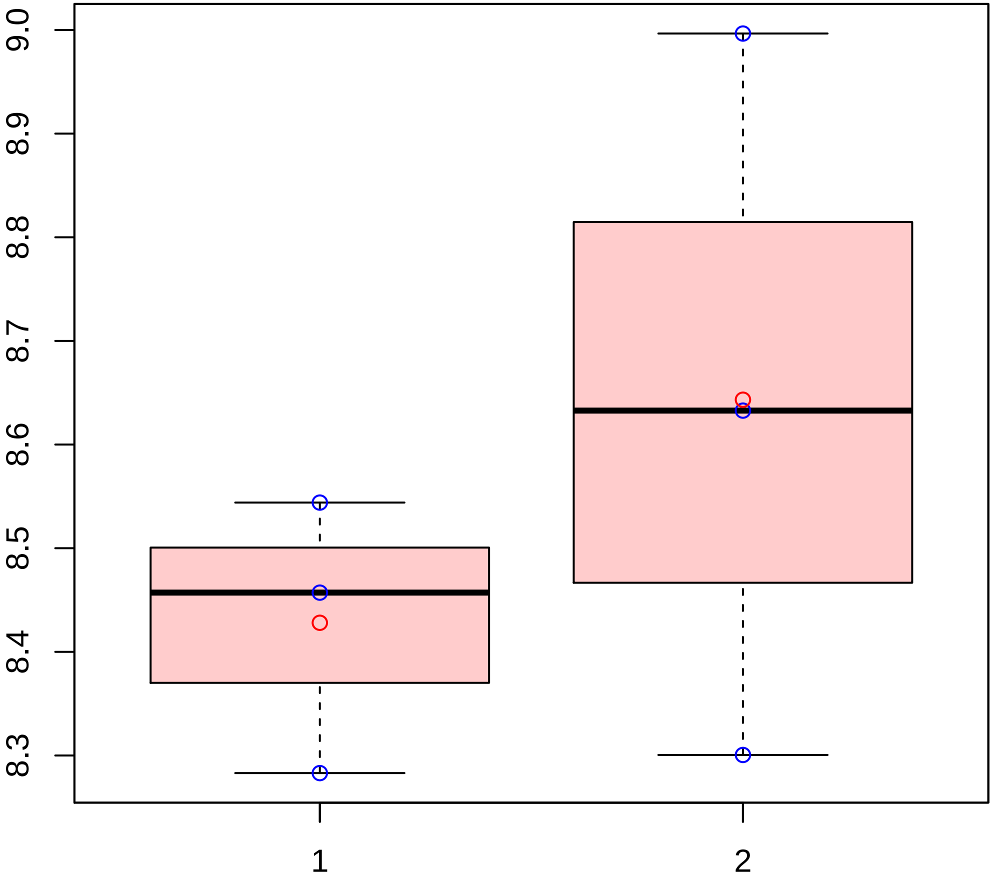

t−Test: p−value = 0.4

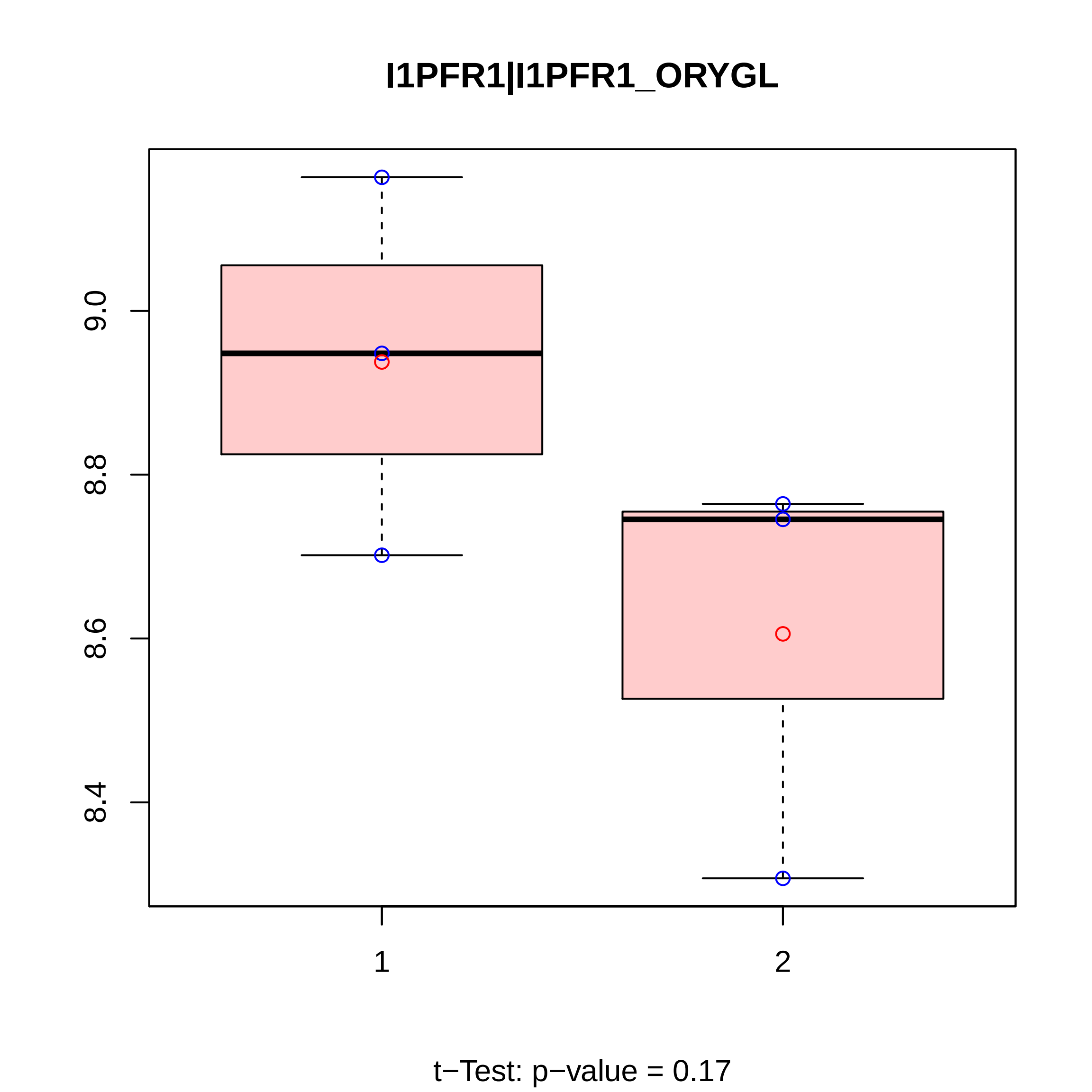

I1PFR1|I1PFR1_ORYGL
9.0
8.8
8.6
8.4
1
2
t−Test: p−value = 0.17

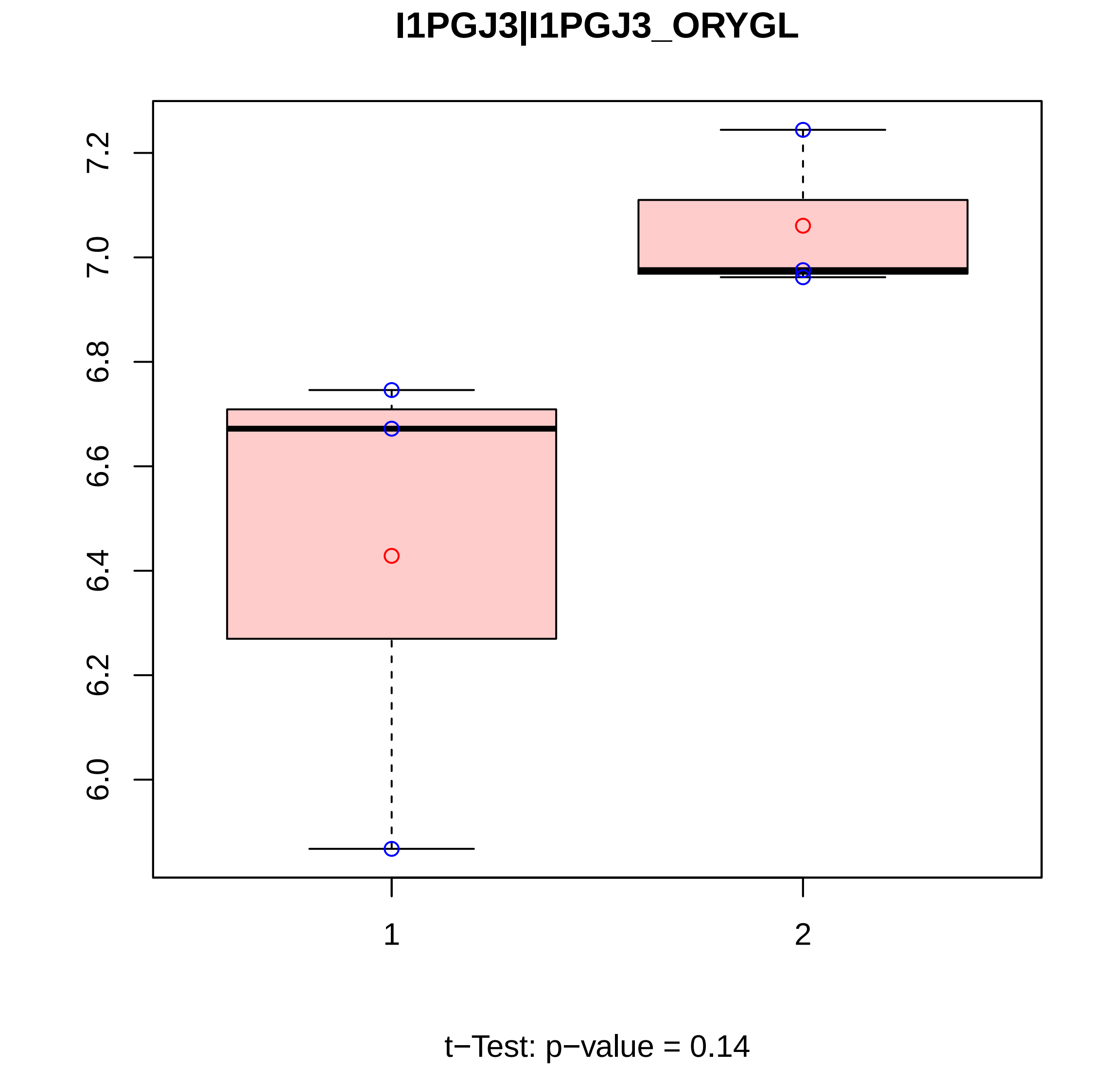
I1PGJ3|I1PGJ3_ORYGL
7.2
7.0
6.8
6.6
6.4
6.2
6.0
1
2
t−Test: p−value = 0.14

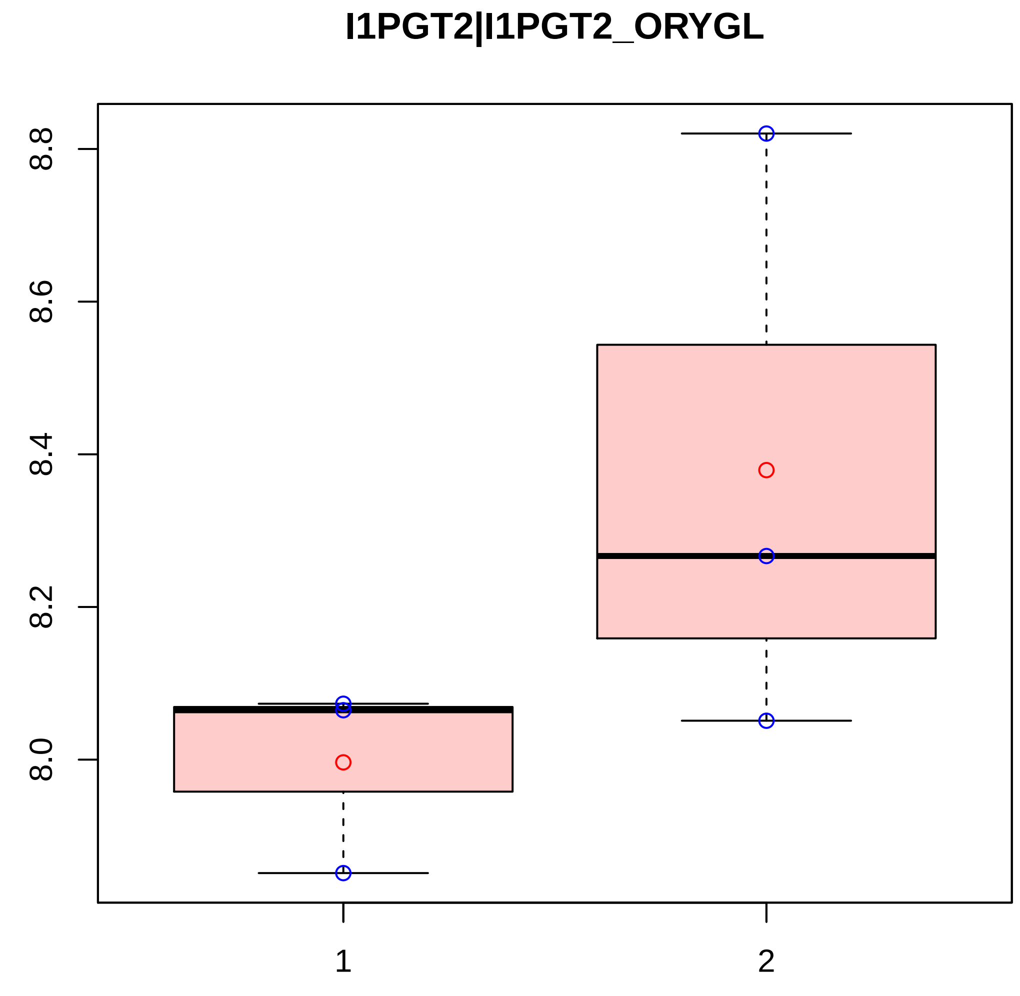

t−Test: p−value = 0.23

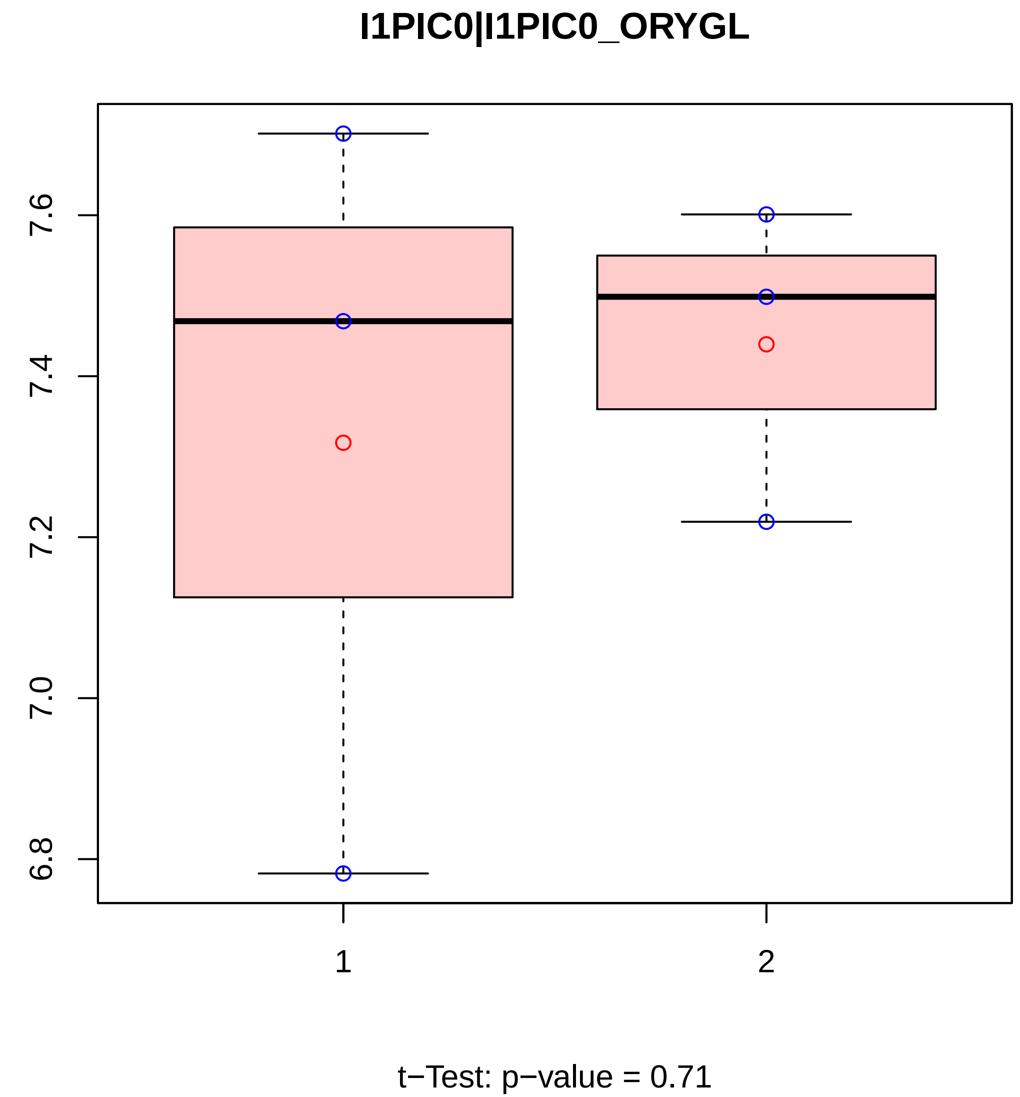
I1PIC0|I1PIC0_ORYGL
7.6
7.4
7.2
7.0
6.8
1
2
t−Test: p−value = 0.71

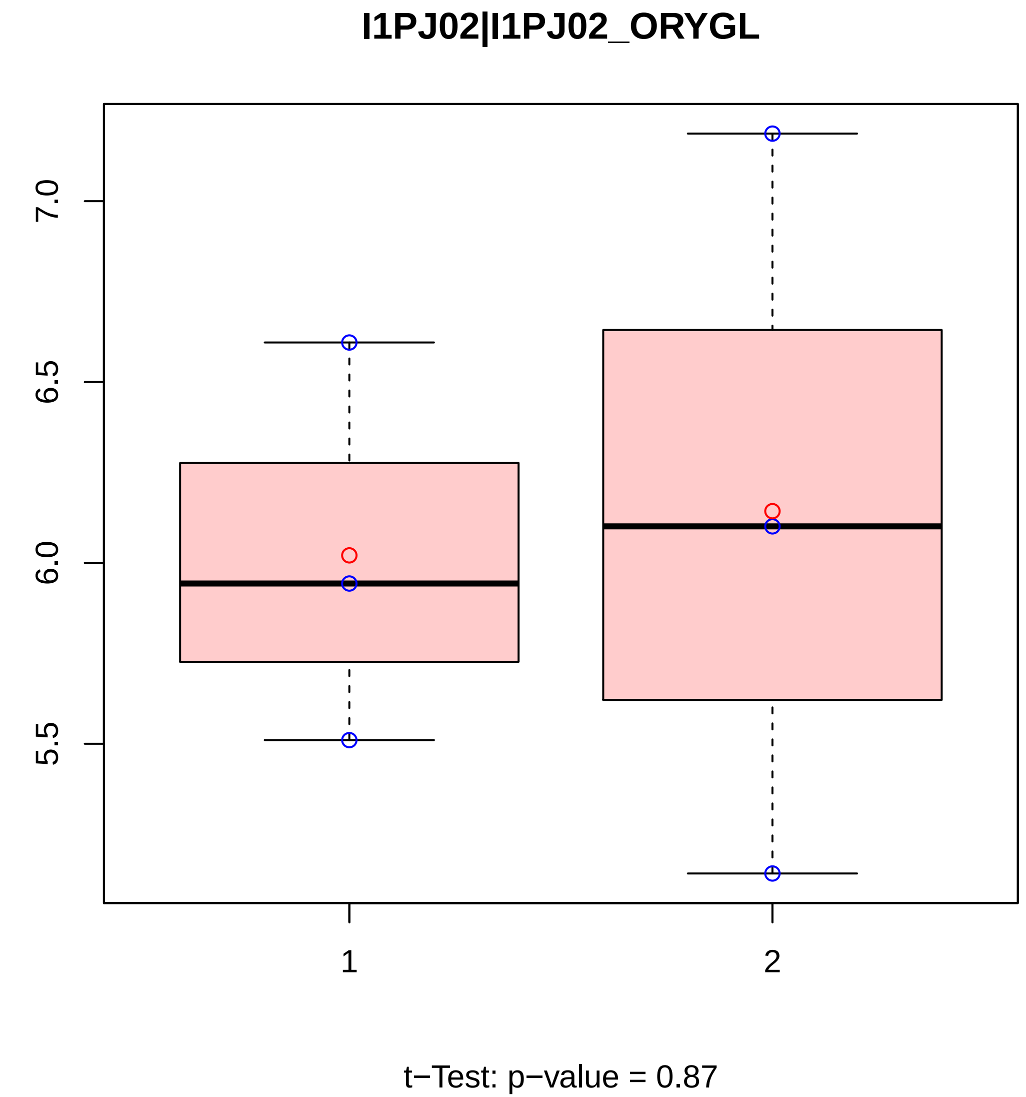

I1PJ02|I1PJ02_ORYGL
7.0
6.5
6.0
5.5
1
2
t−Test: p−value = 0.87

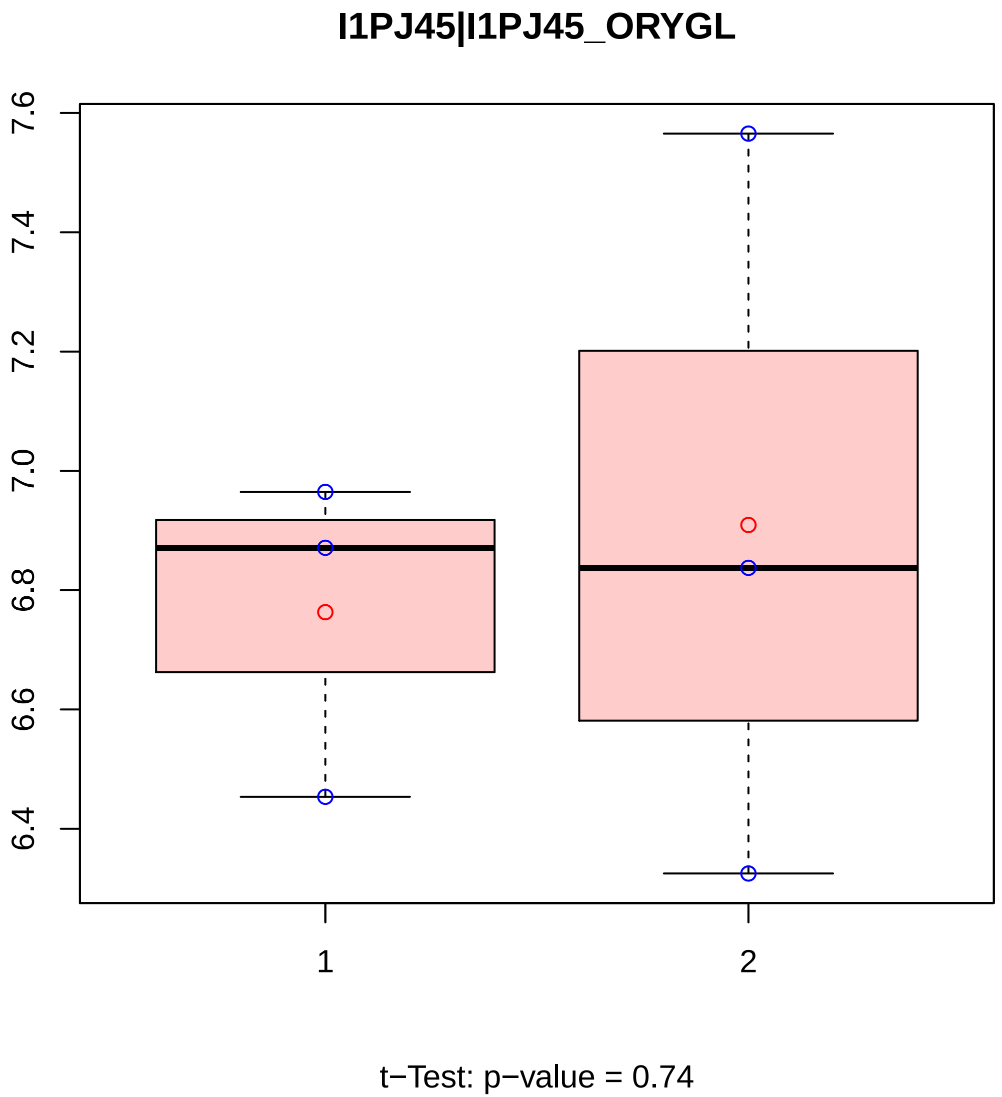

I1PJ45|I1PJ45_ORYGL
7.6
7.4
7.2
7.0
6.8
6.6
6.4
1
2
t−Test: p−value = 0.74

I1PK82|I1PK82_ORYGL

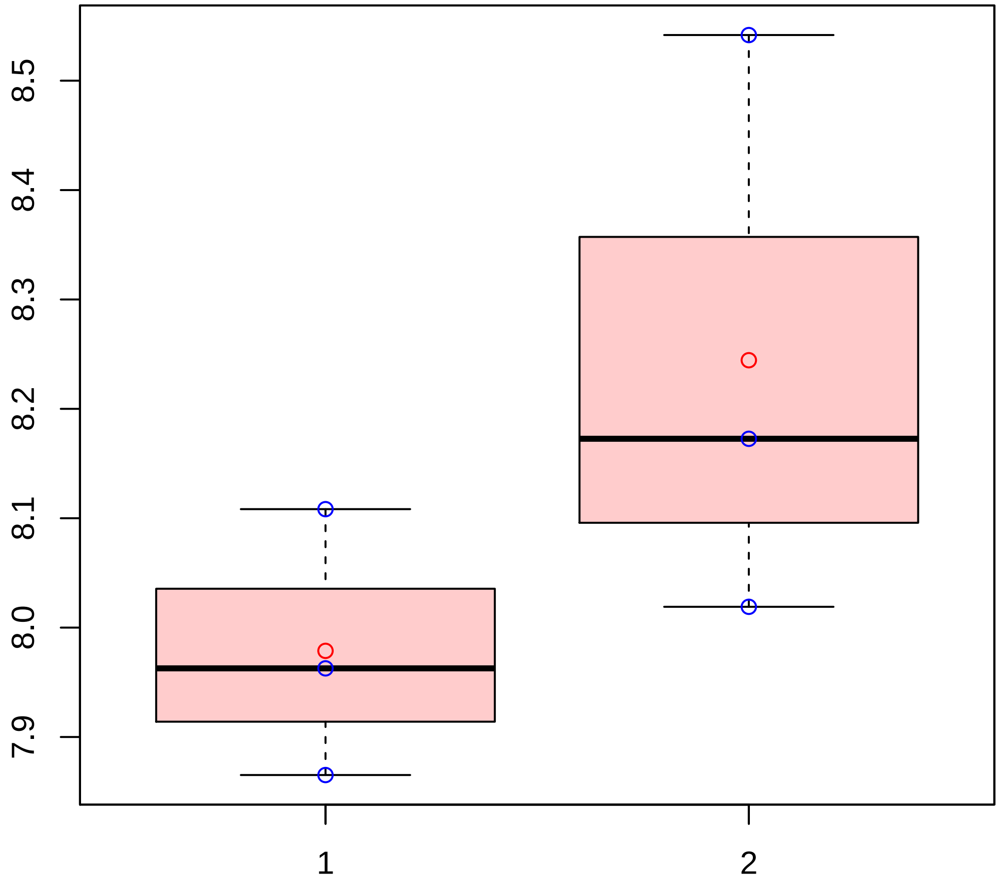

t−Test: p−value = 0.22

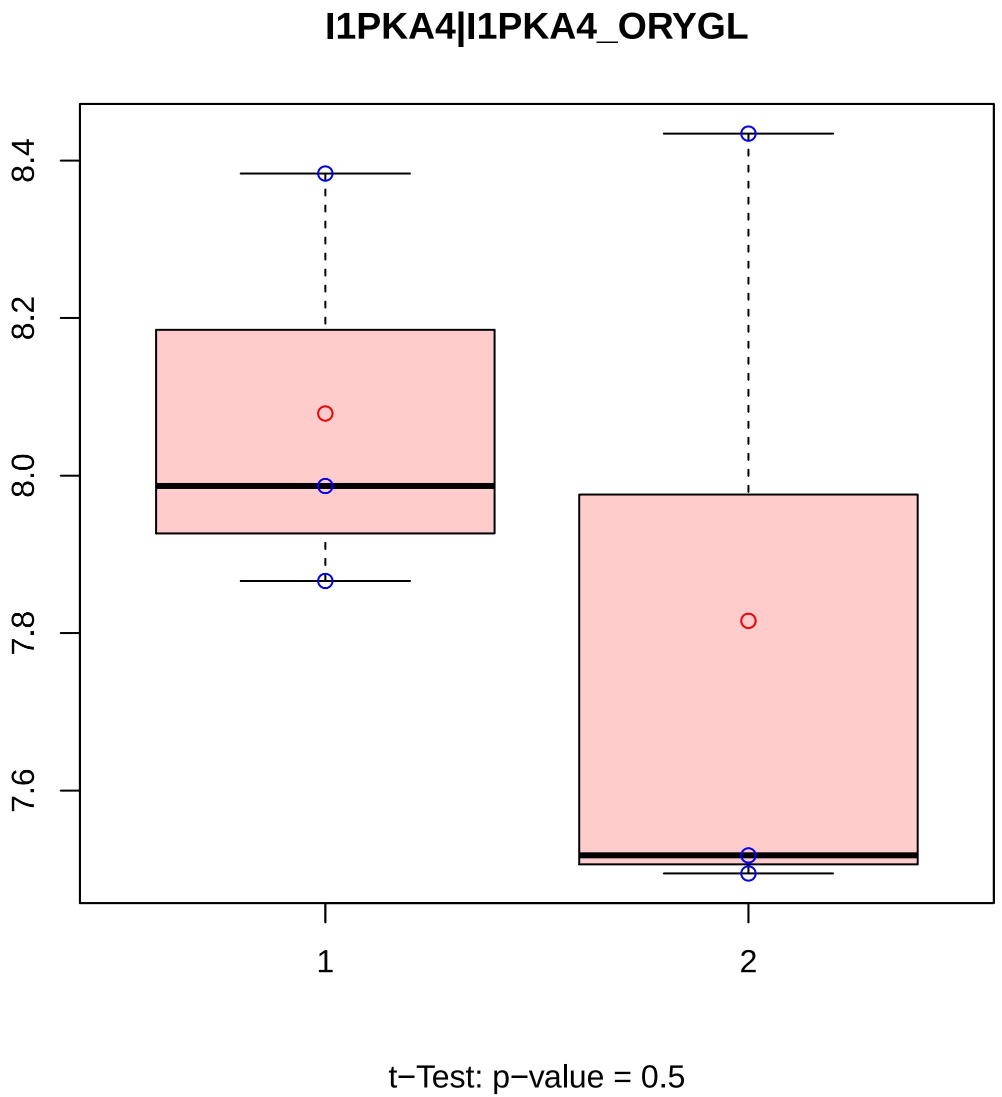

I1PKA4|I1PKA4_ORYGL
8.4
8.2
8.0
7.8
7.6
1
2
t−Test: p−value = 0.5

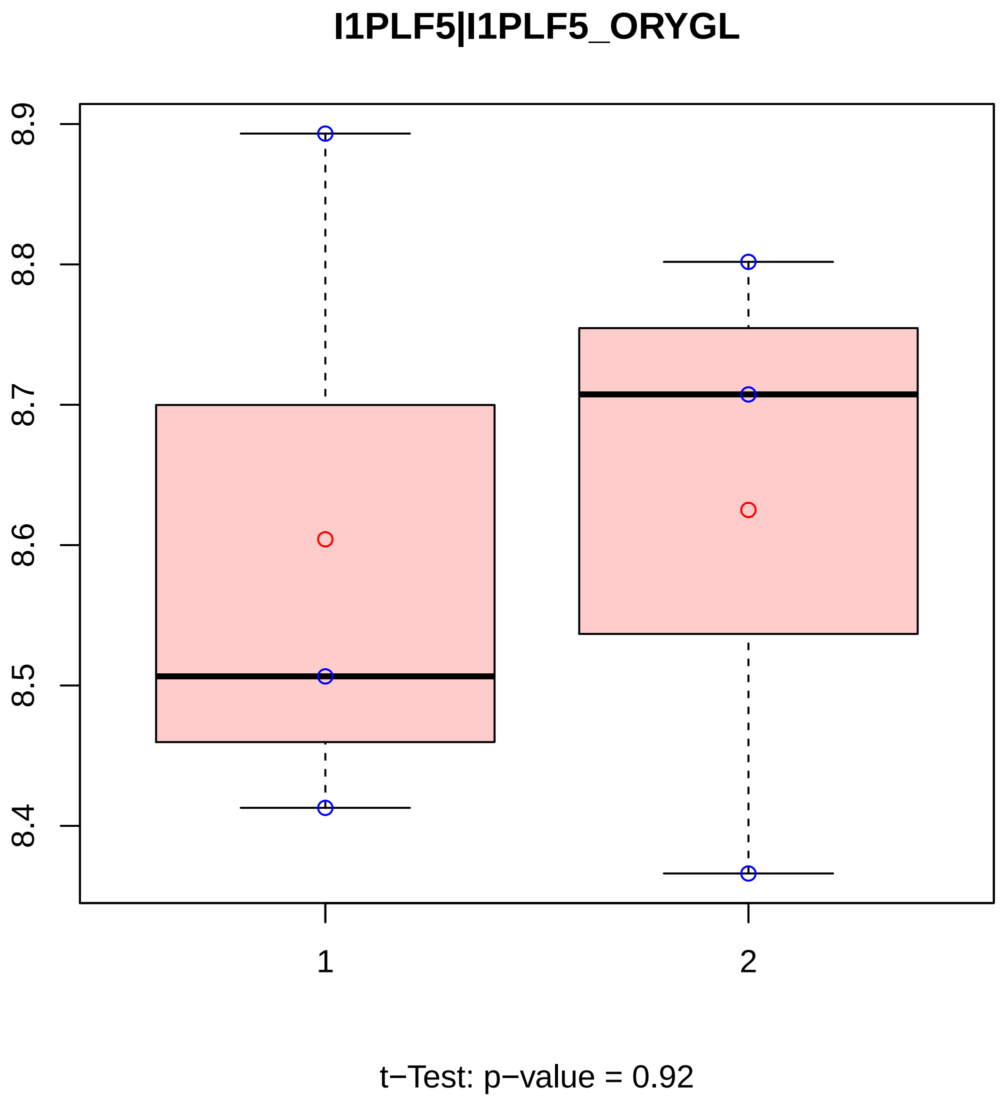
I1PLF5|I1PLF5_ORYGL
8.4
8.5
8.6
8.7
8.8
8.9
1
2
t−Test: p−value = 0.92

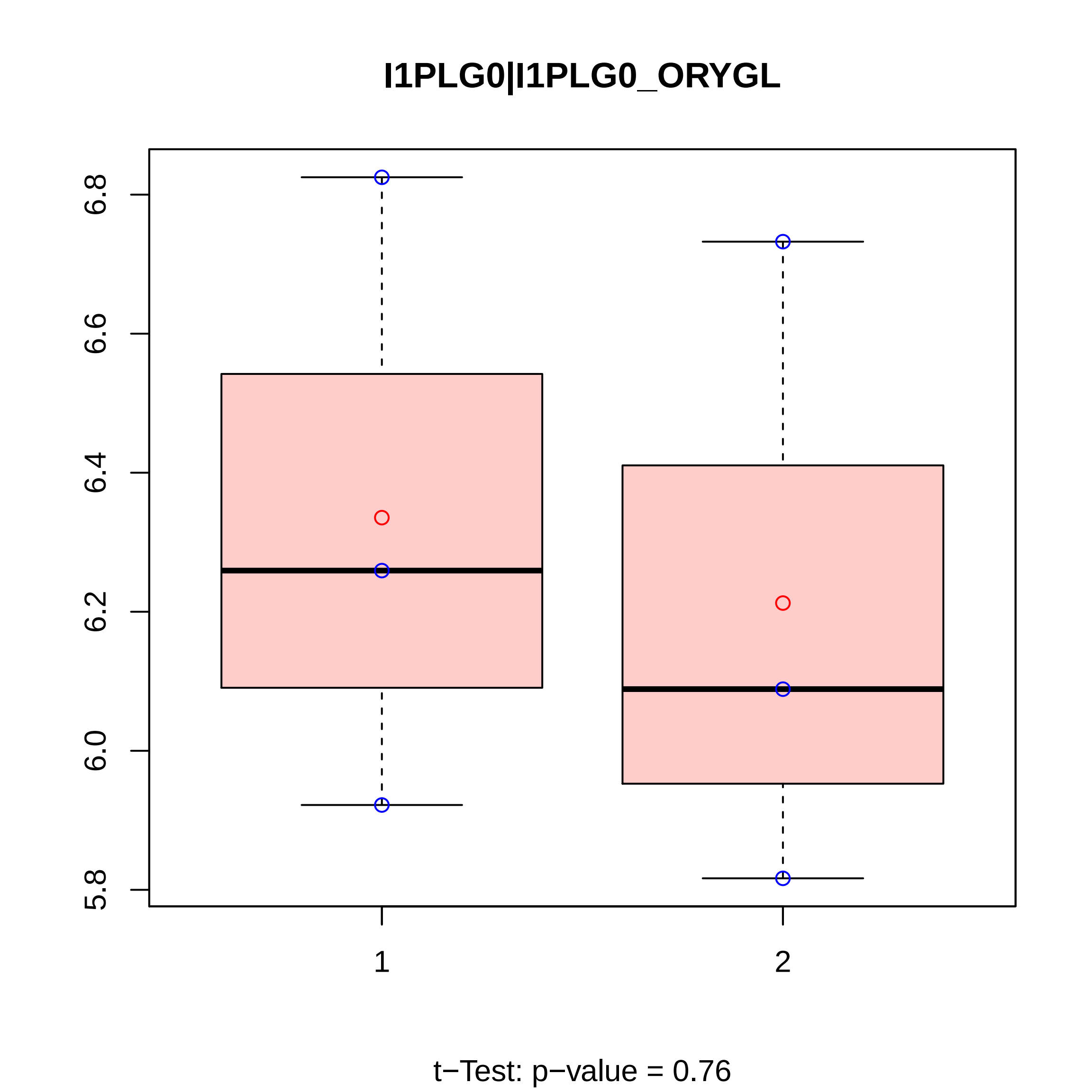
I1PLG0|I1PLG0_ORYGL
6.8
6.6
6.4
6.2
6.0
5.8
1
2
t−Test: p−value = 0.76

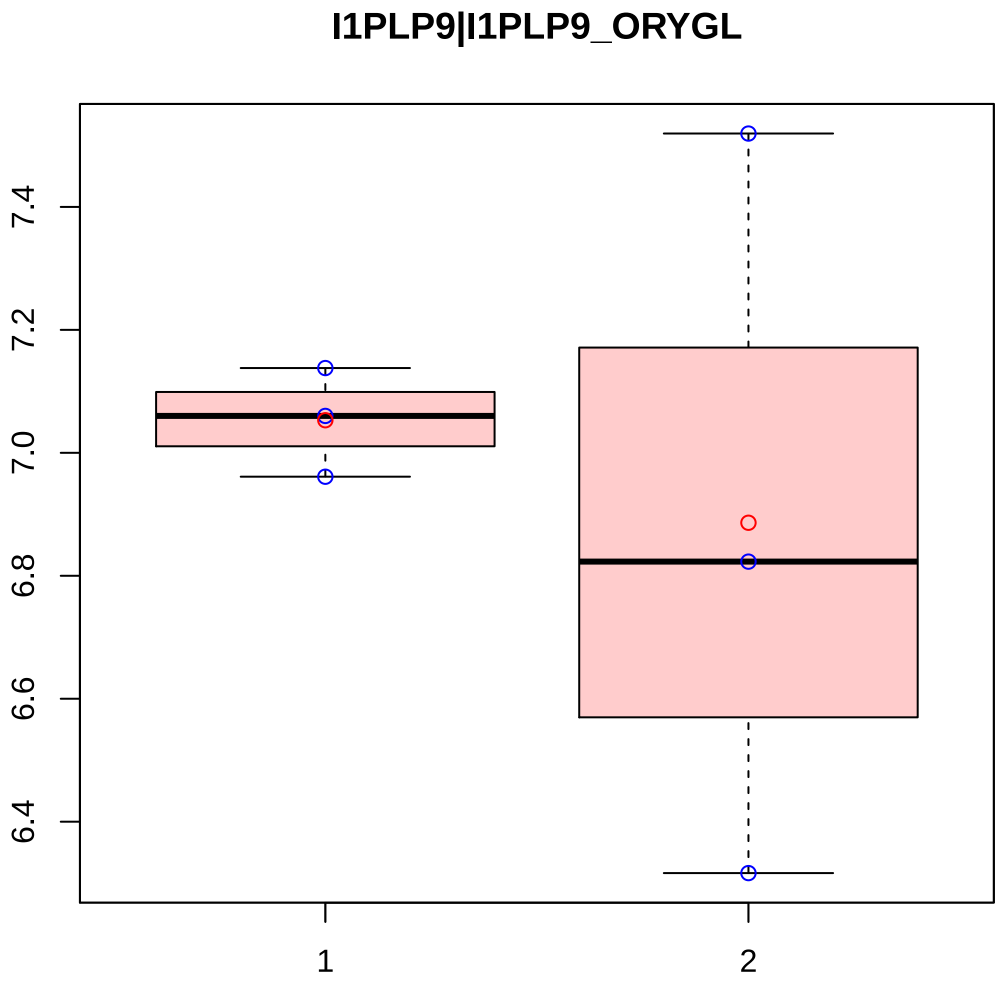

t−Test: p−value = 0.68

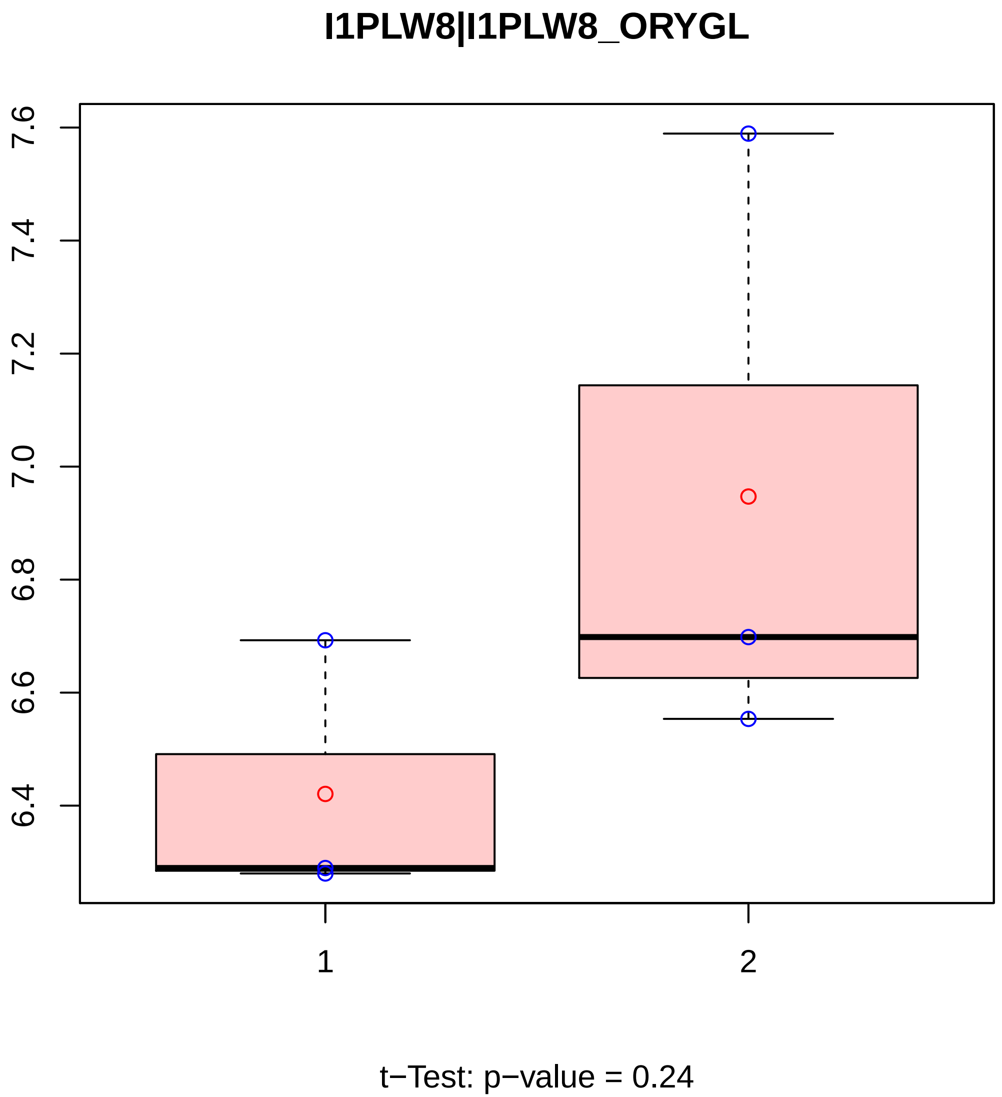
I1PLW8|I1PLW8_ORYGL
7.6
7.4
7.2
7.0
6.8
6.6
6.4
1
2
t−Test: p−value = 0.24

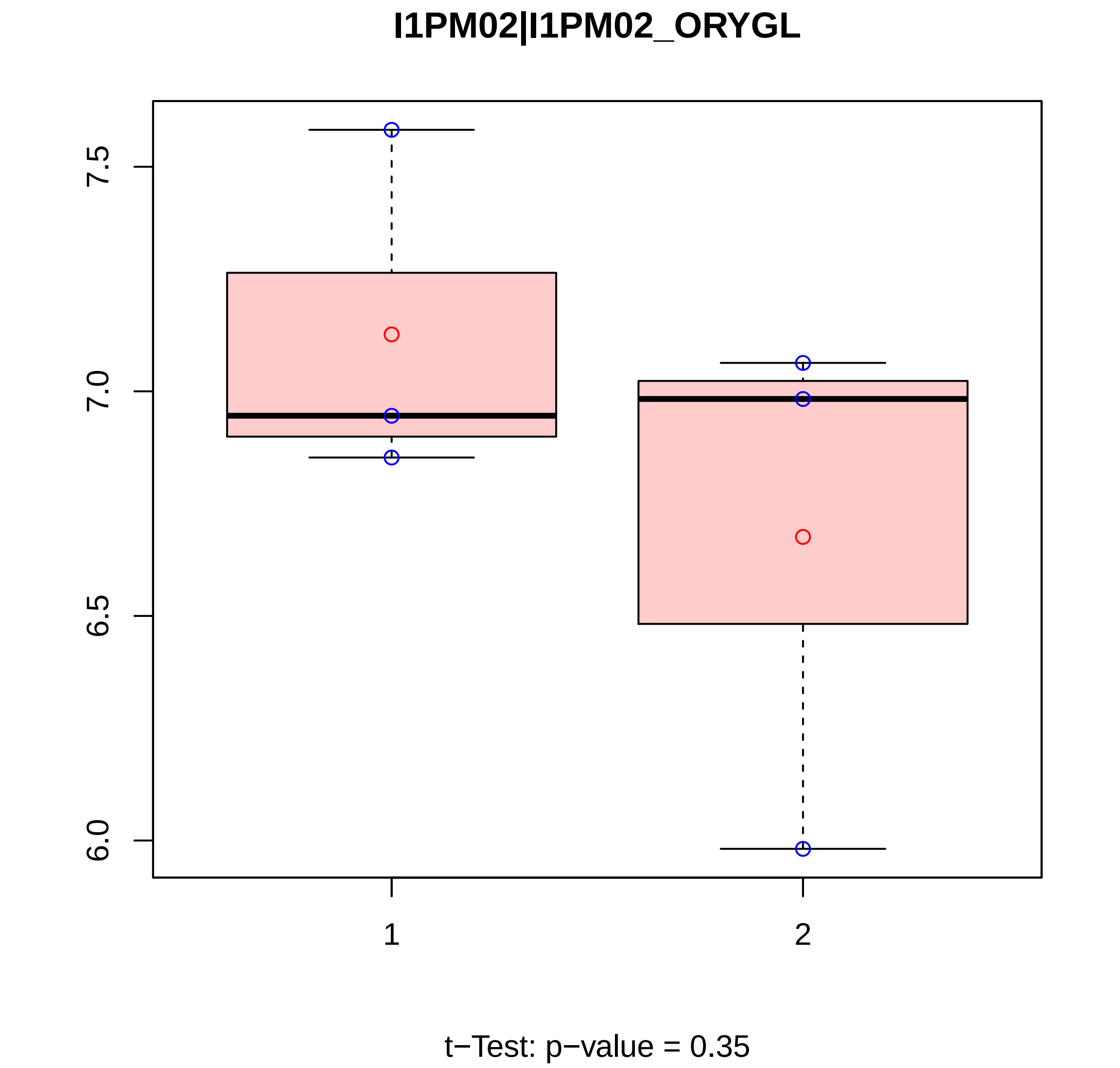

I1PM02|I1PM02_ORYGL
7.5
7.0
6.5
6.0
1
2
t−Test: p−value = 0.35

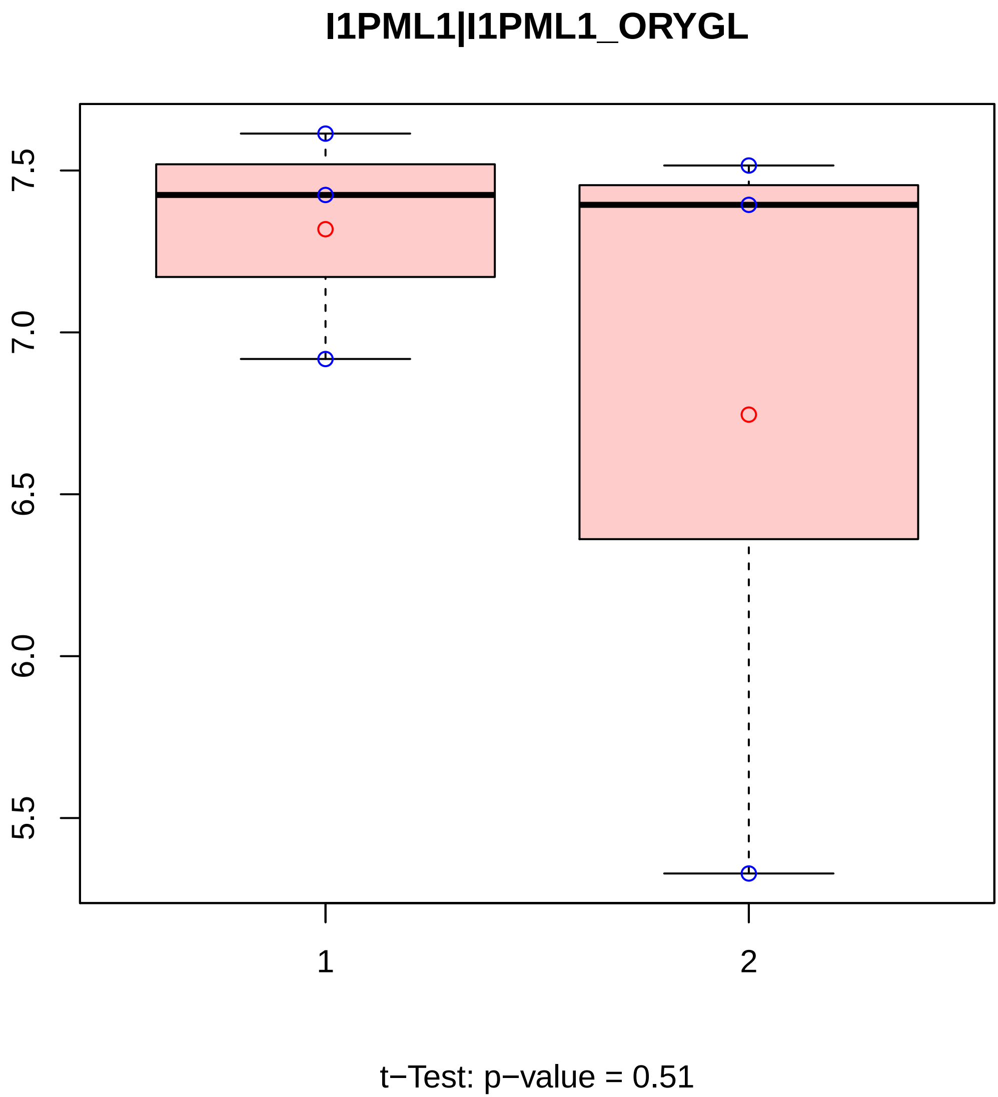

I1PML1|I1PML1_ORYGL
7.5
7.0
6.5
6.0
5.5
1
2
t−Test: p−value = 0.51

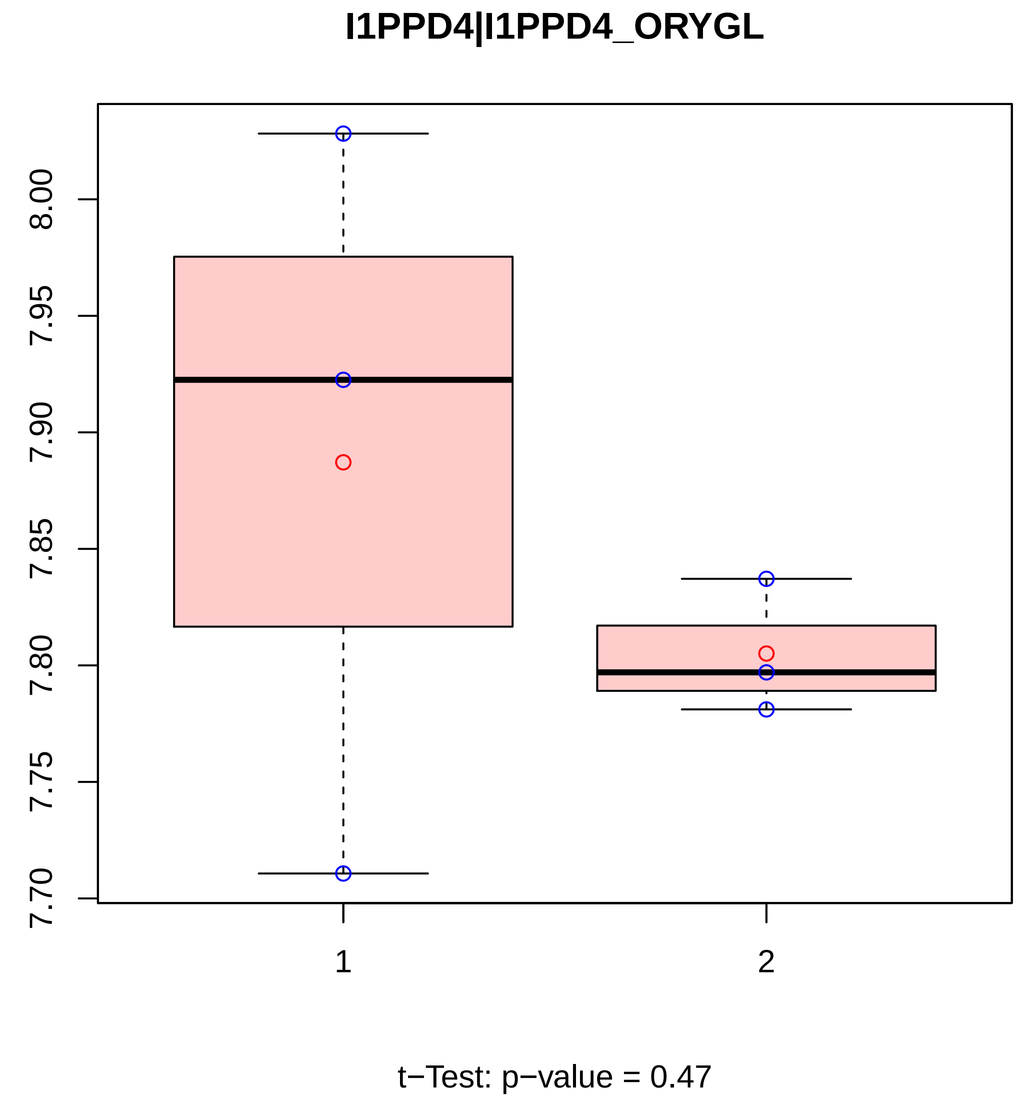
I1PPD4|I1PPD4_ORYGL
8.00
7.95
7.90
7.85
7.80
7.75
7.70
1
2
t−Test: p−value = 0.47

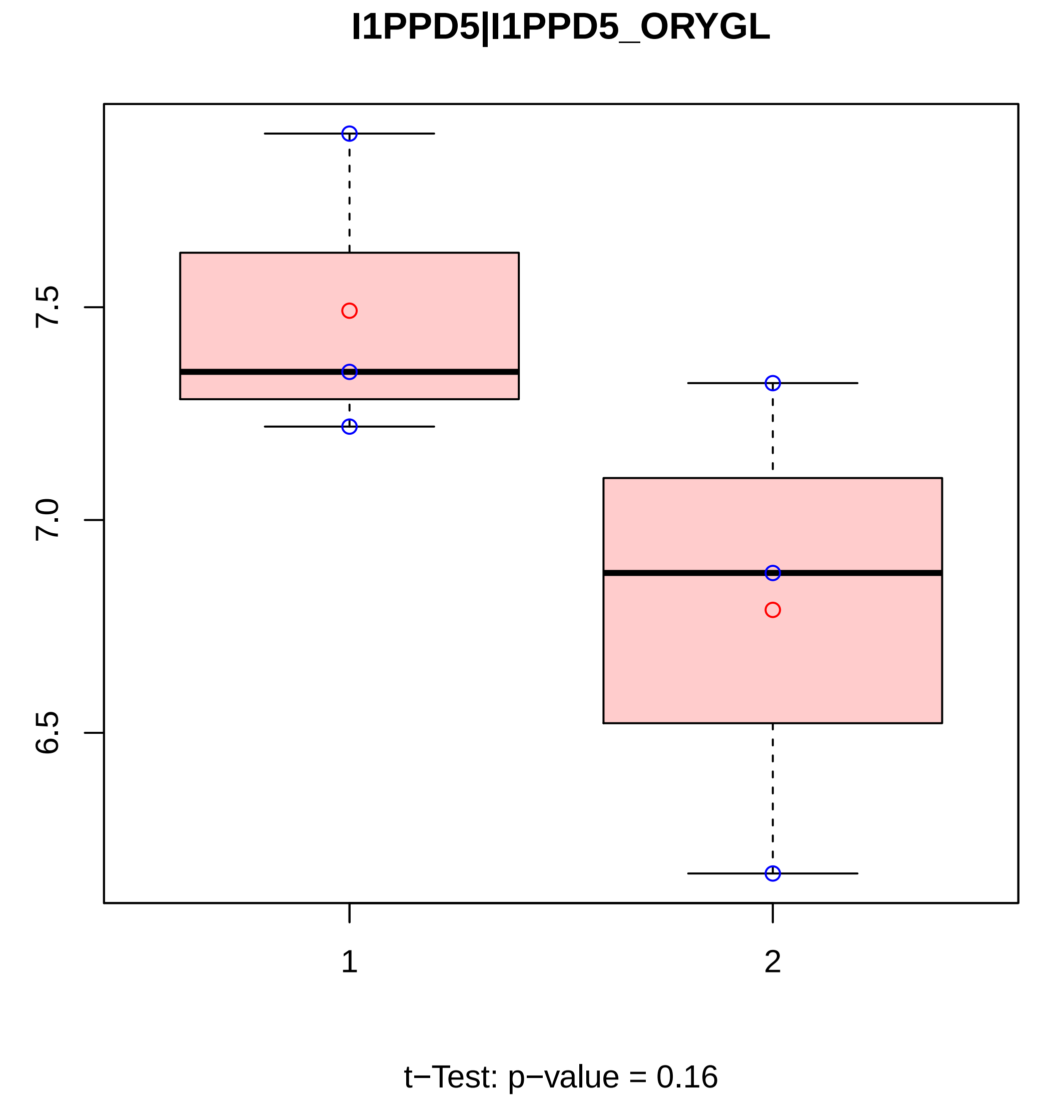
I1PPD5|I1PPD5_ORYGL
7.5
7.0
6.5
1
2
t−Test: p−value = 0.16

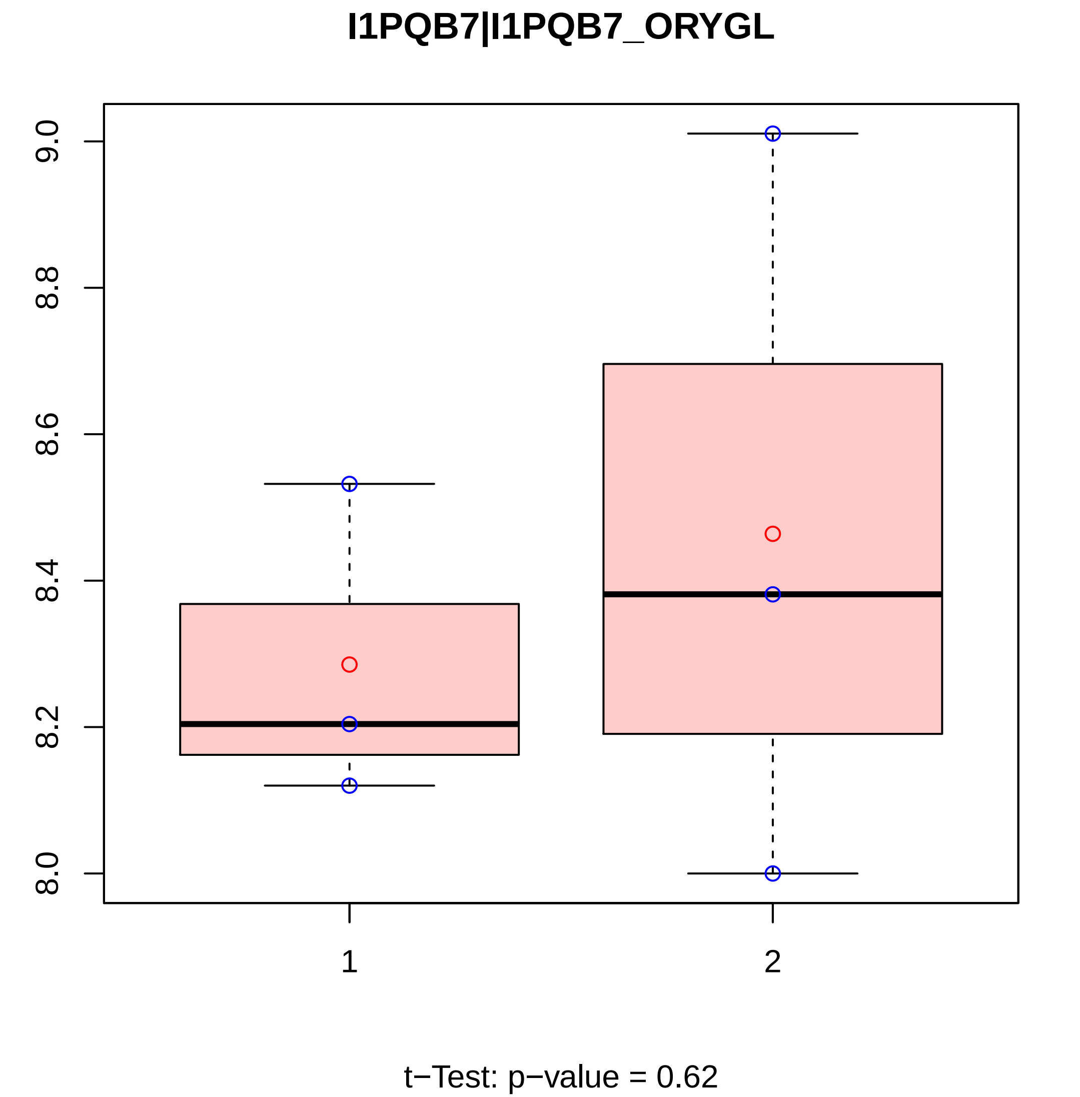
I1PQB7|I1PQB7_ORYGL
9.0
8.8
8.6
8.4
8.2
8.0
1
2
t−Test: p−value = 0.62

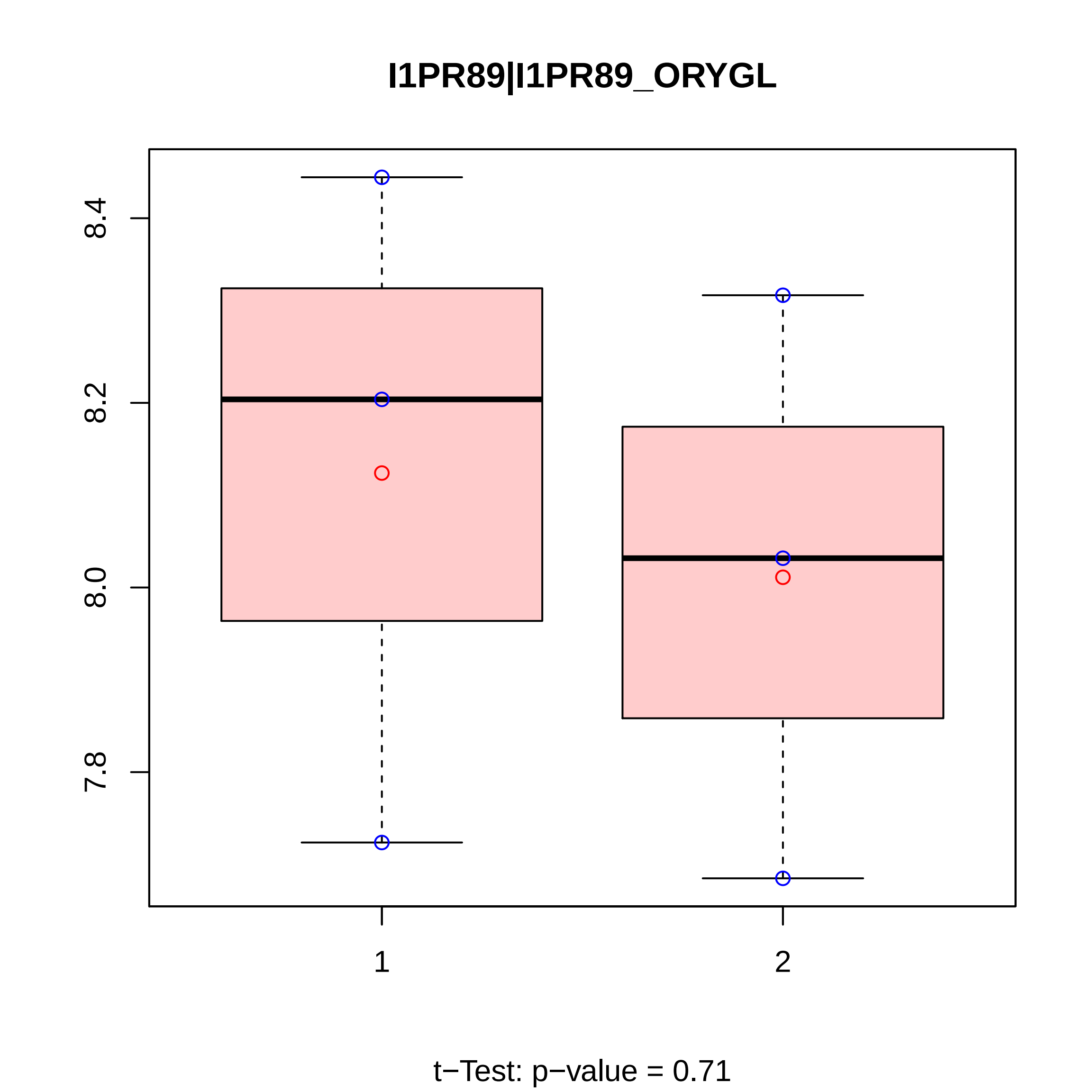
I1PR89|I1PR89_ORYGL
8.4
8.2
8.0
7.8
1
2
t−Test: p−value = 0.71

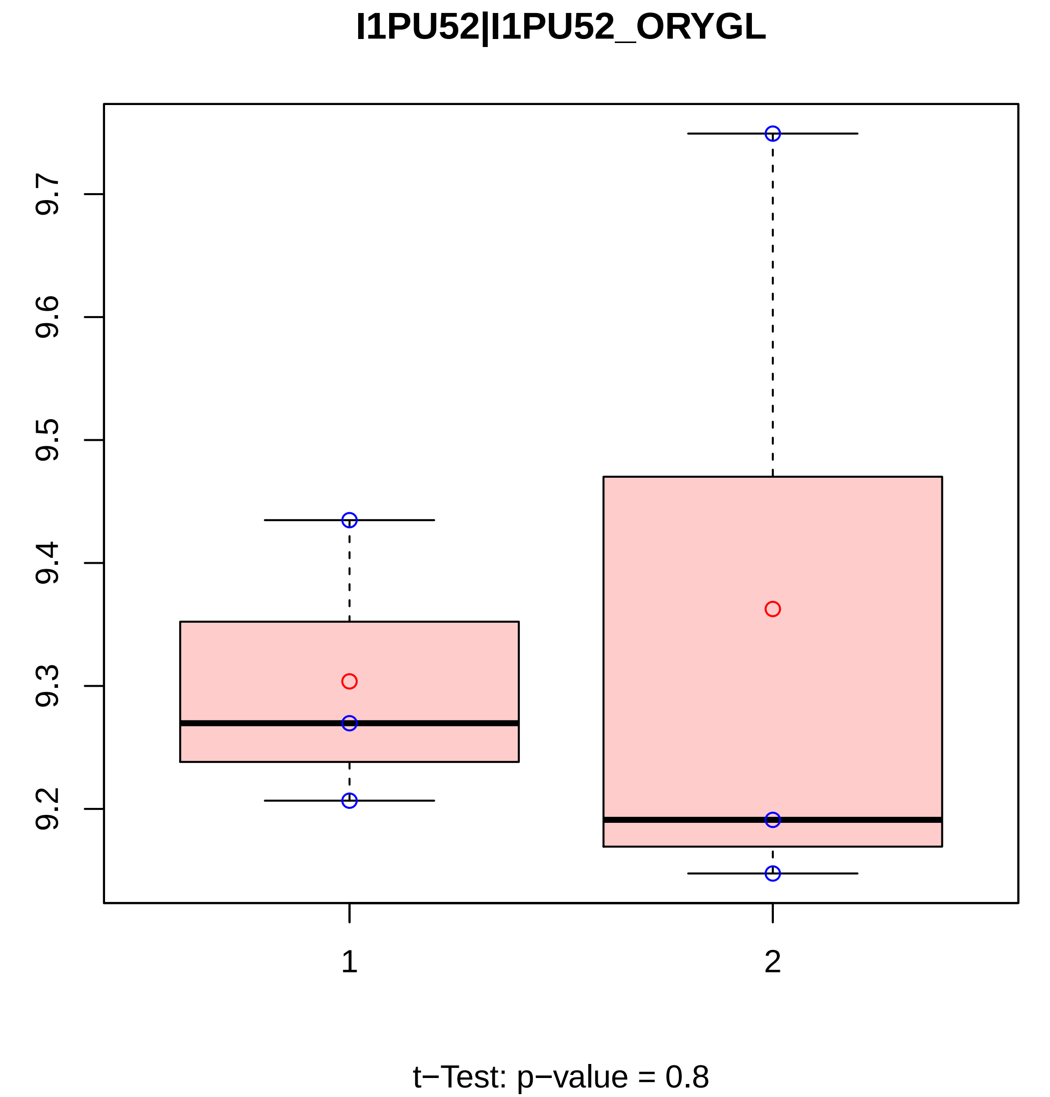
I1PU52|I1PU52_ORYGL
9.2
9.3
9.4
9.5
9.6
9.7
1
2
t−Test: p−value = 0.8

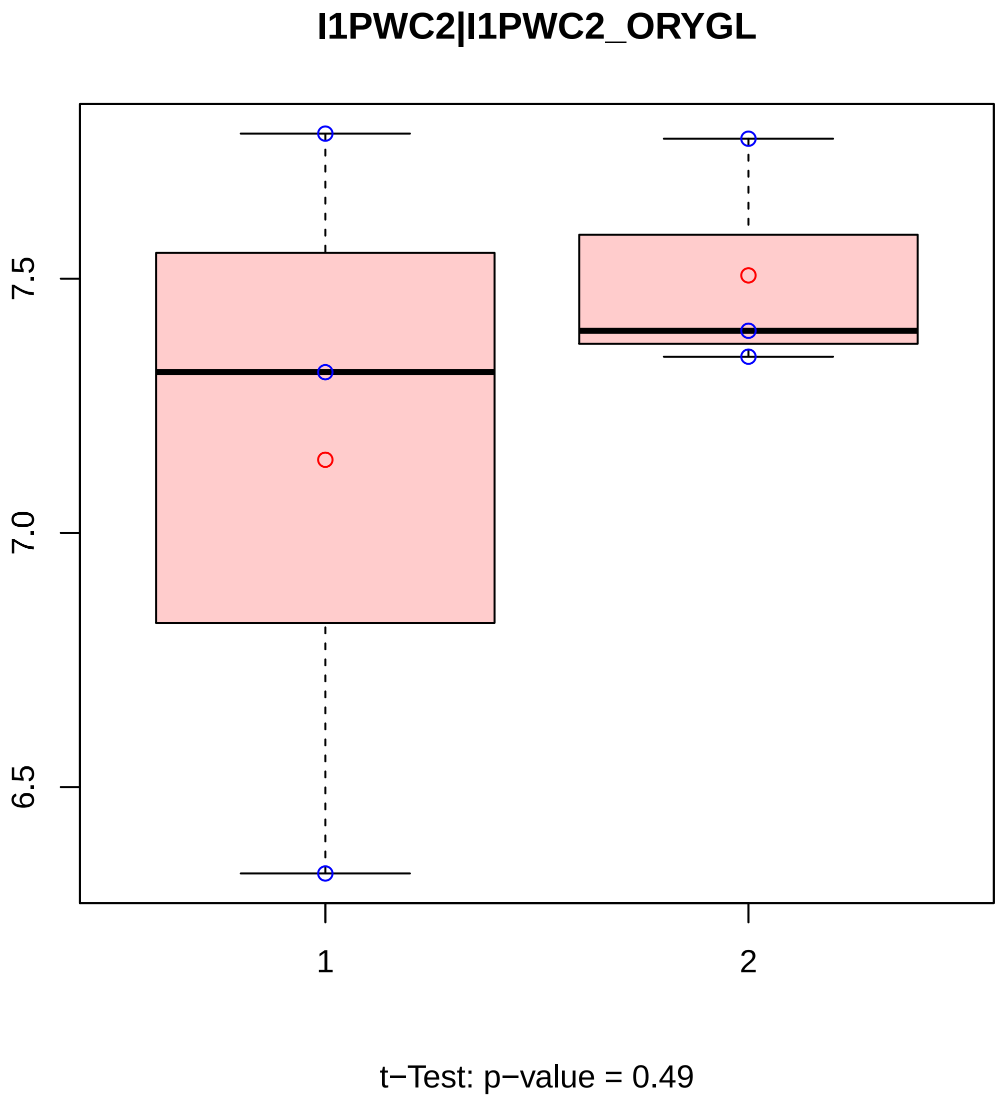
I1PWC2|I1PWC2_ORYGL
7.5
7.0
6.5
1
2
t−Test: p−value = 0.49

I1PWN0|I1PWN0_ORYGL

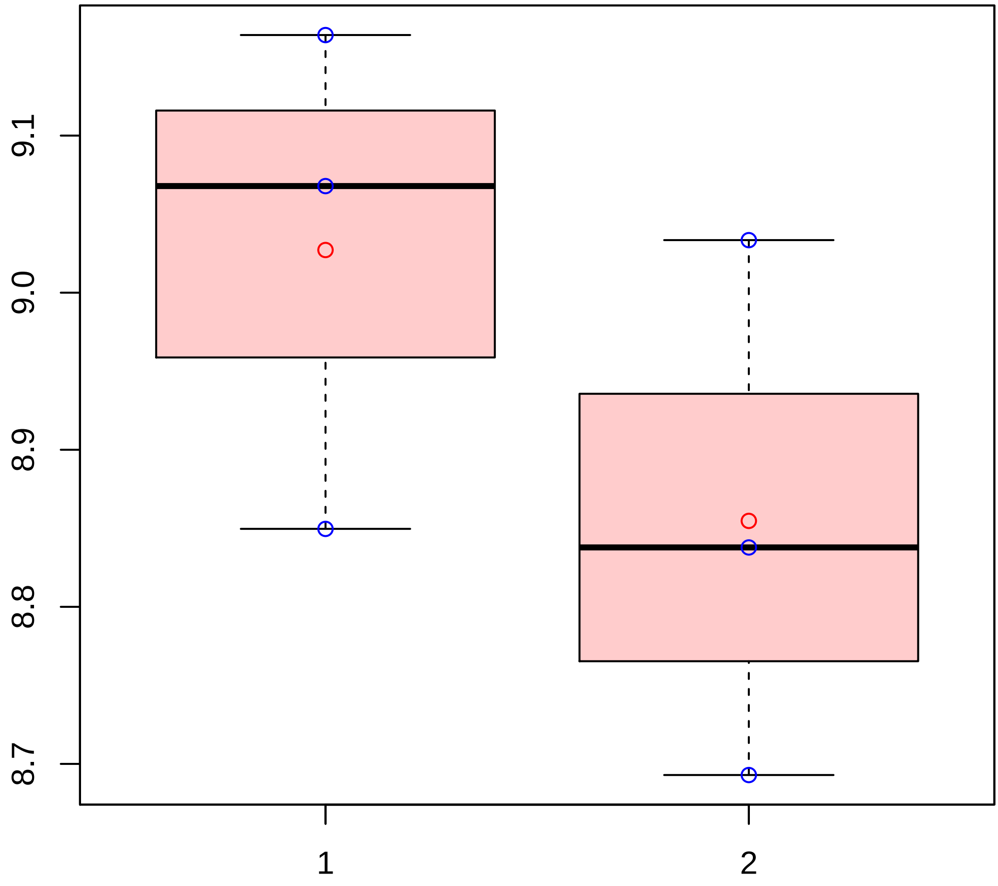

t−Test: p−value = 0.27

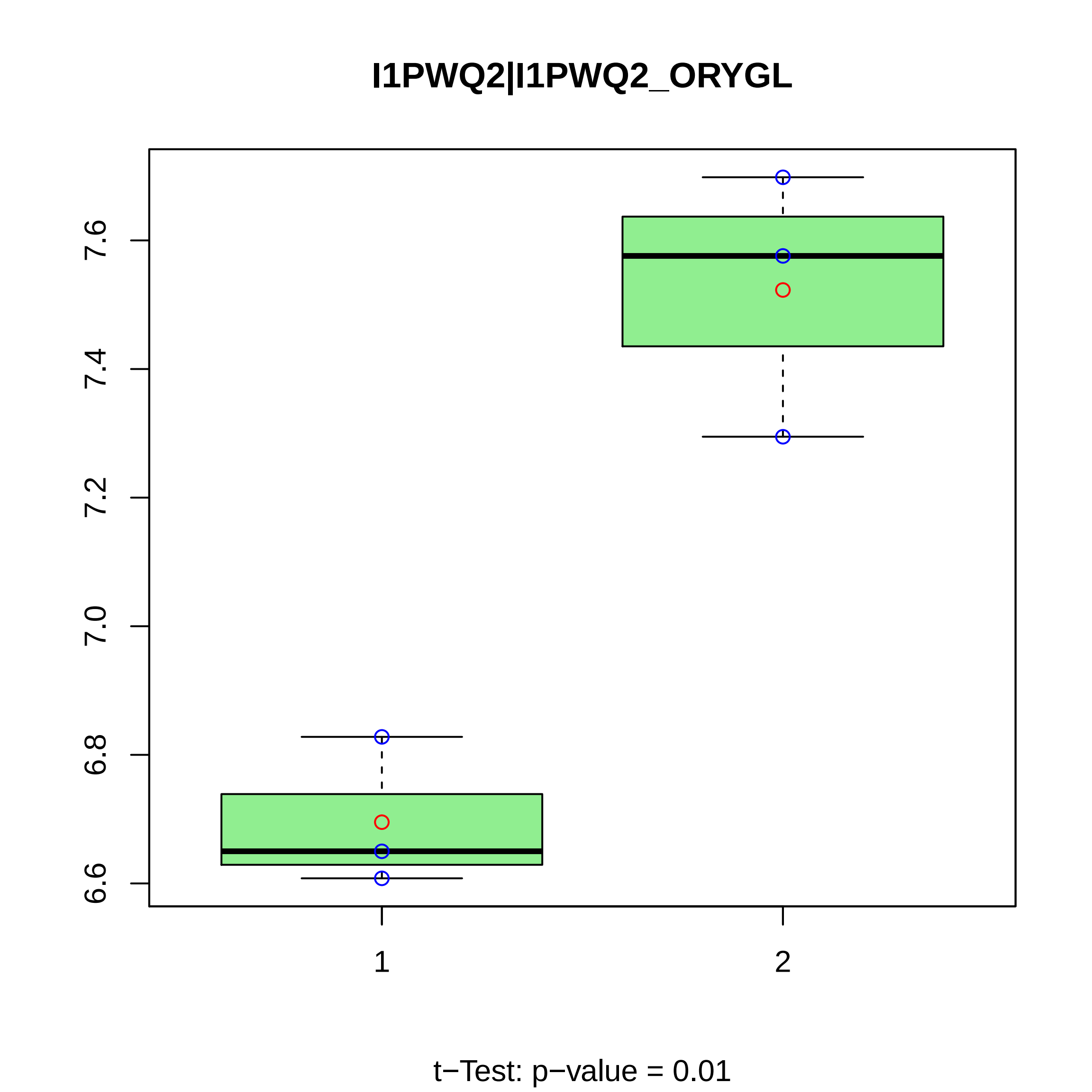

I1PWQ2|I1PWQ2_ORYGL
7.6
7.4
7.2
7.0
6.8
6.6
1
2
t−Test: p−value = 0.01

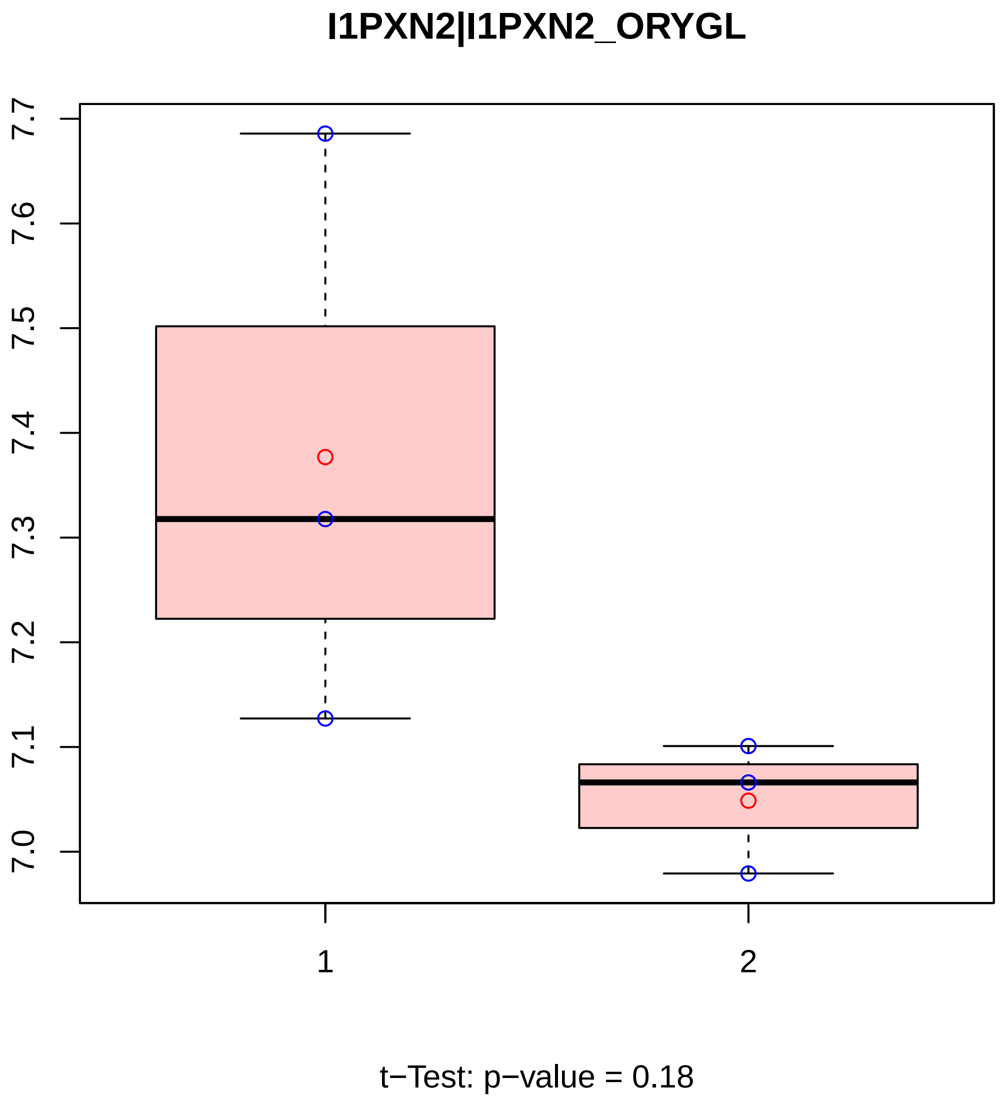
I1PXN2|I1PXN2_ORYGL
7.0
7.1
7.2
7.3
7.4
7.5
7.6
7.7
1
2
t−Test: p−value = 0.18

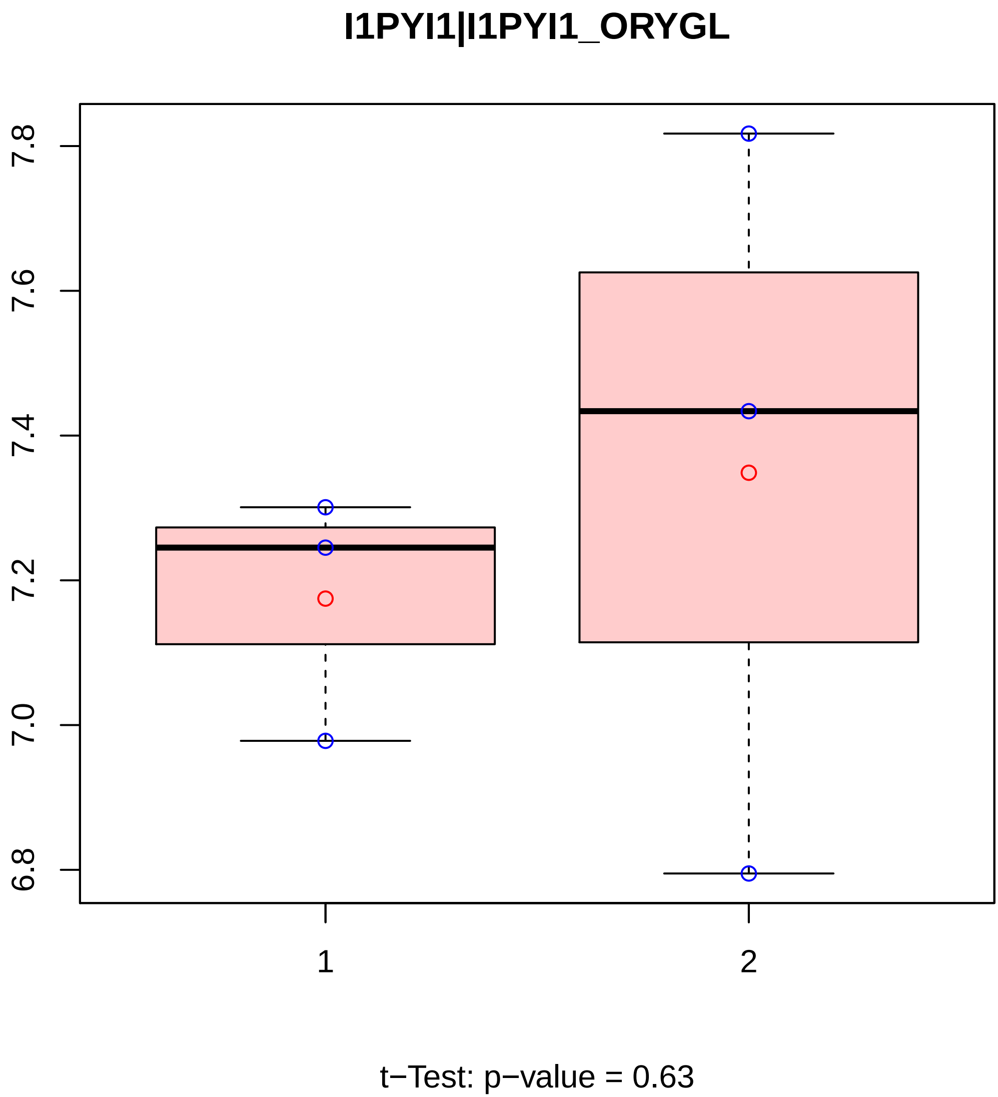
I1PYI1|I1PYI1_ORYGL
7.8
7.6
7.4
7.2
7.0
6.8
1
2
t−Test: p−value = 0.63

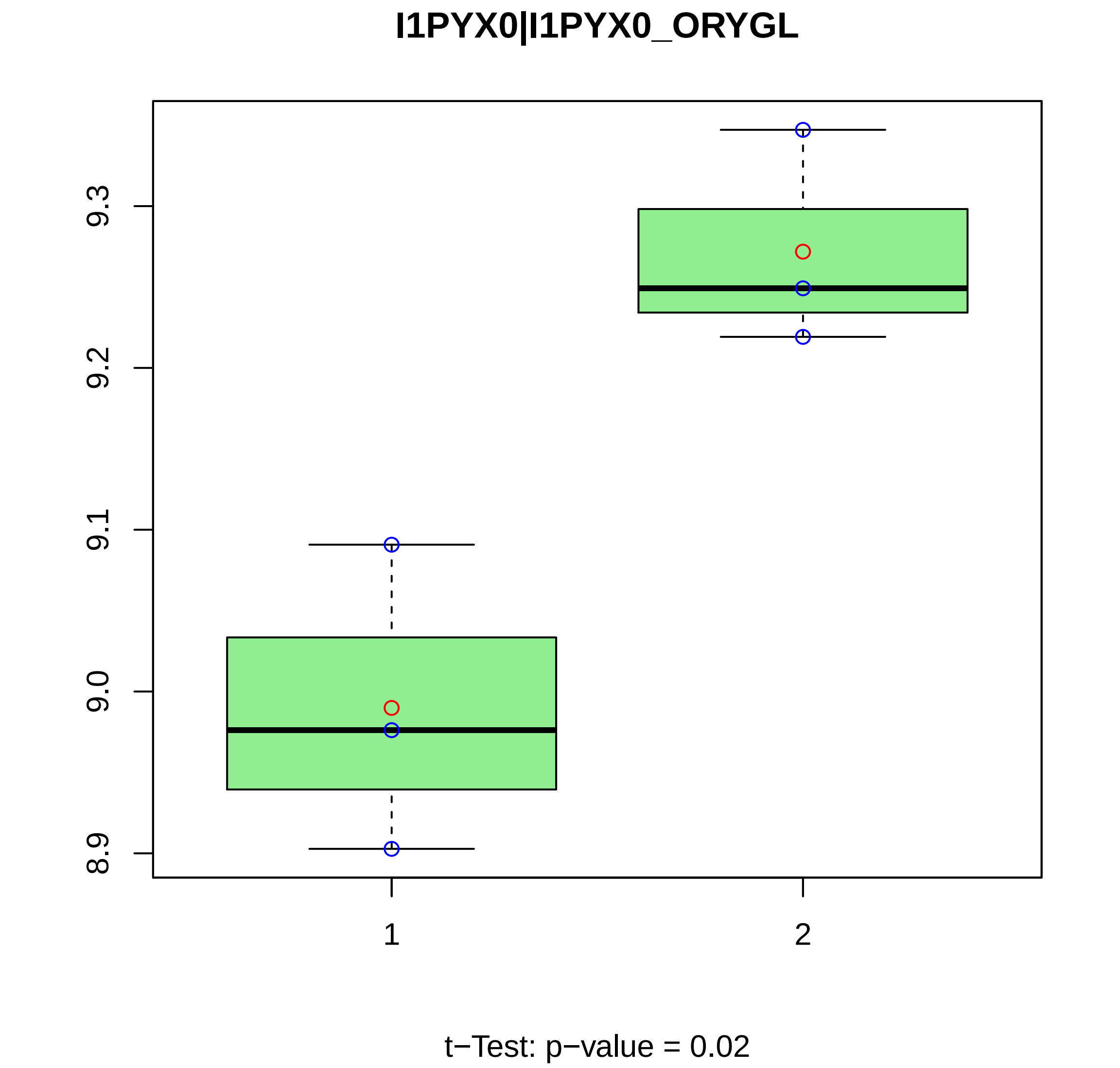
I1PYX0|I1PYX0_ORYGL
9.3
9.2
9.1
9.0
8.9
1
2
t−Test: p−value = 0.02

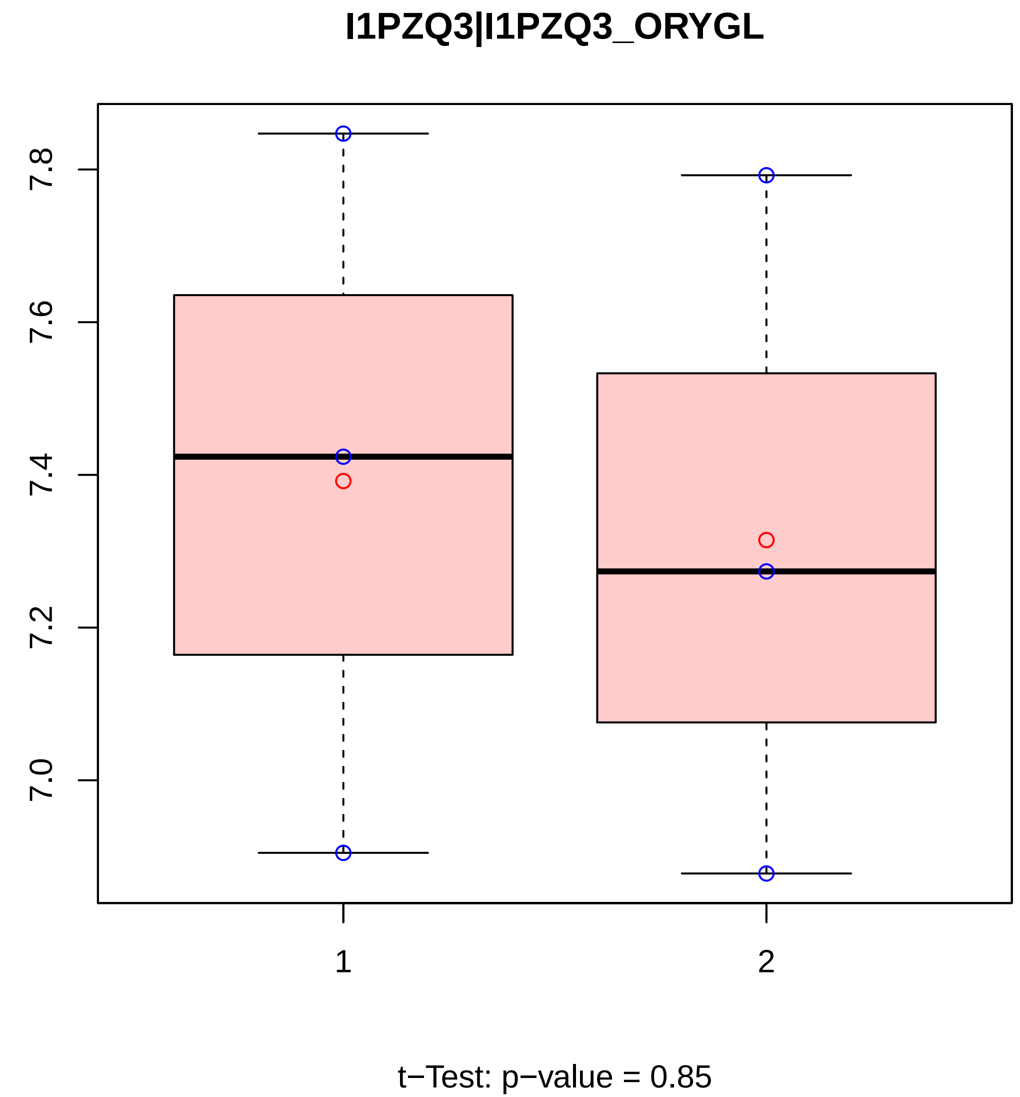
I1PZQ3|I1PZQ3_ORYGL
7.0
7.2
7.4
7.6
7.8
1
2
t−Test: p−value = 0.85

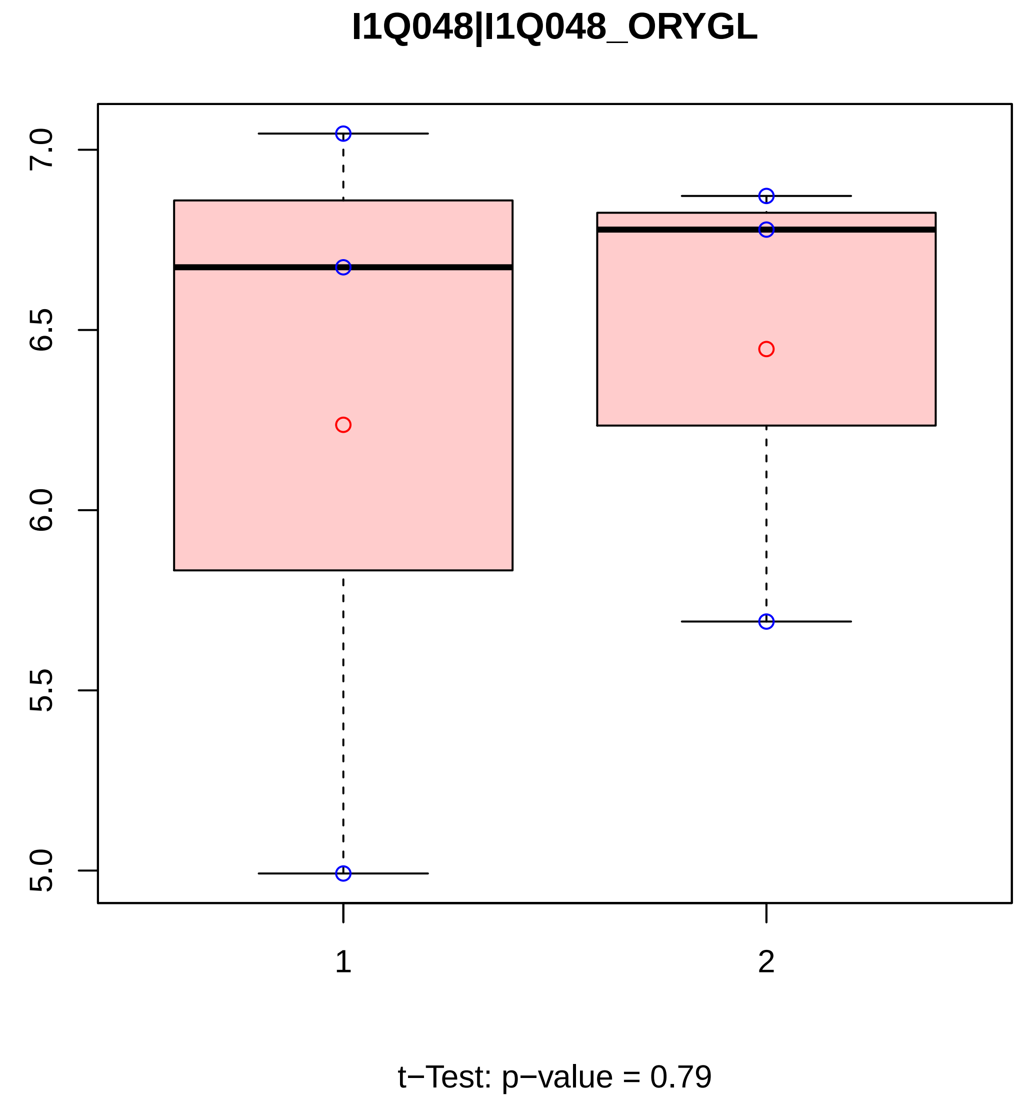
I1Q048|I1Q048_ORYGL
7.0
6.5
6.0
5.5
5.0
1
2
t−Test: p−value = 0.79

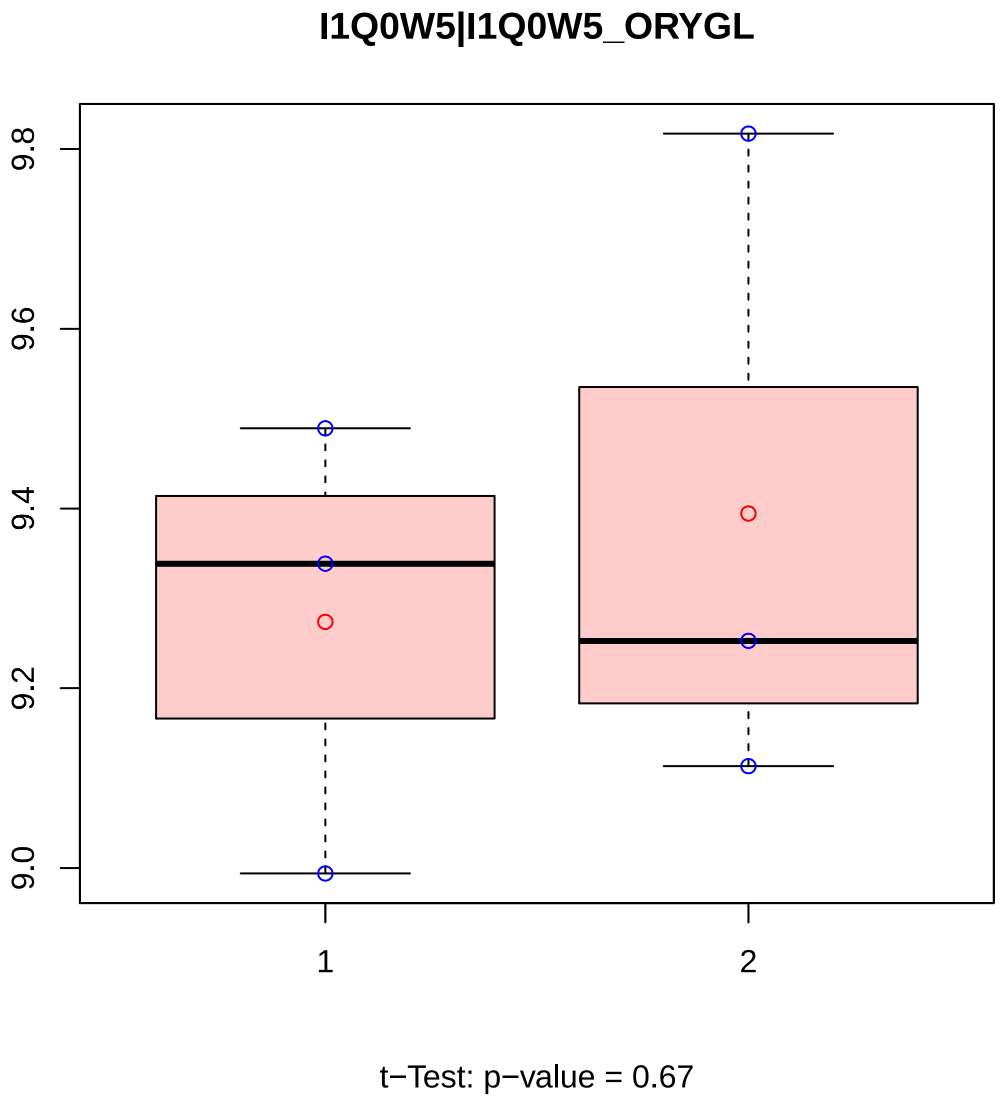
I1Q0W5|I1Q0W5_ORYGL
9.0
9.2
9.4
9.6
9.8
1
2
t−Test: p−value = 0.67

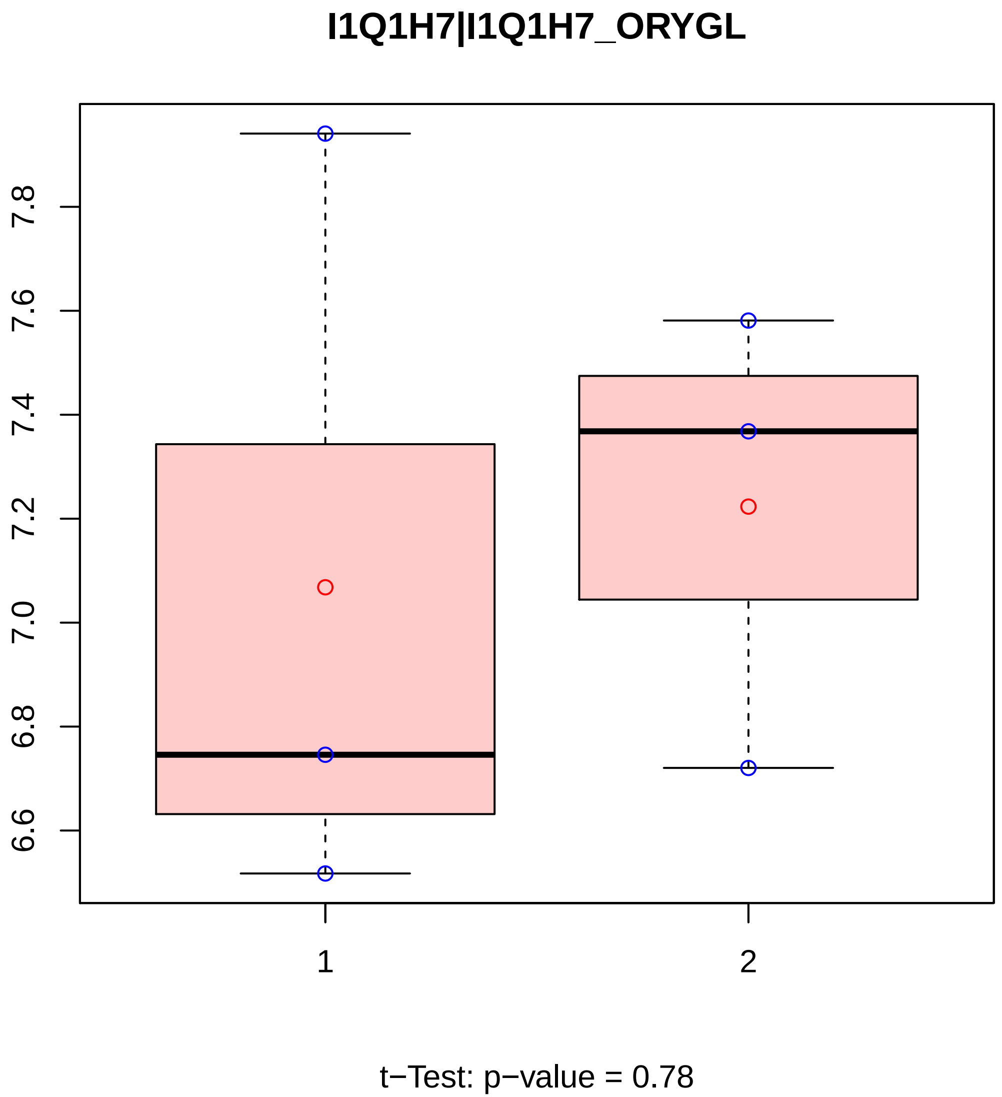
I1Q1H7|I1Q1H7_ORYGL
6.6
6.8
7.0
7.2
7.4
7.6
7.8
1
2
t−Test: p−value = 0.78

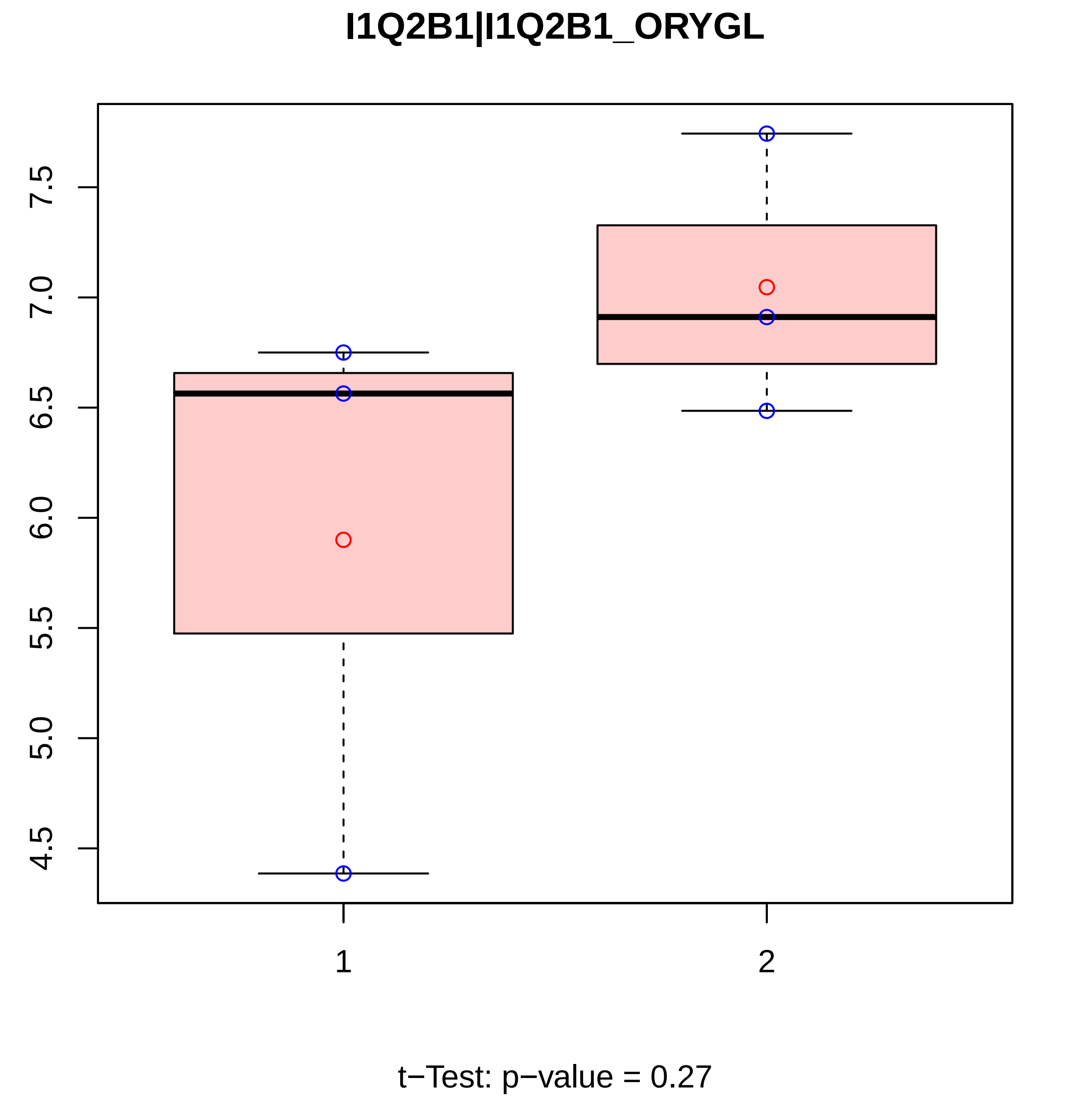
I1Q2B1|I1Q2B1_ORYGL
7.5
7.0
6.5
6.0
5.5
5.0
4.5
1
2
t−Test: p−value = 0.27

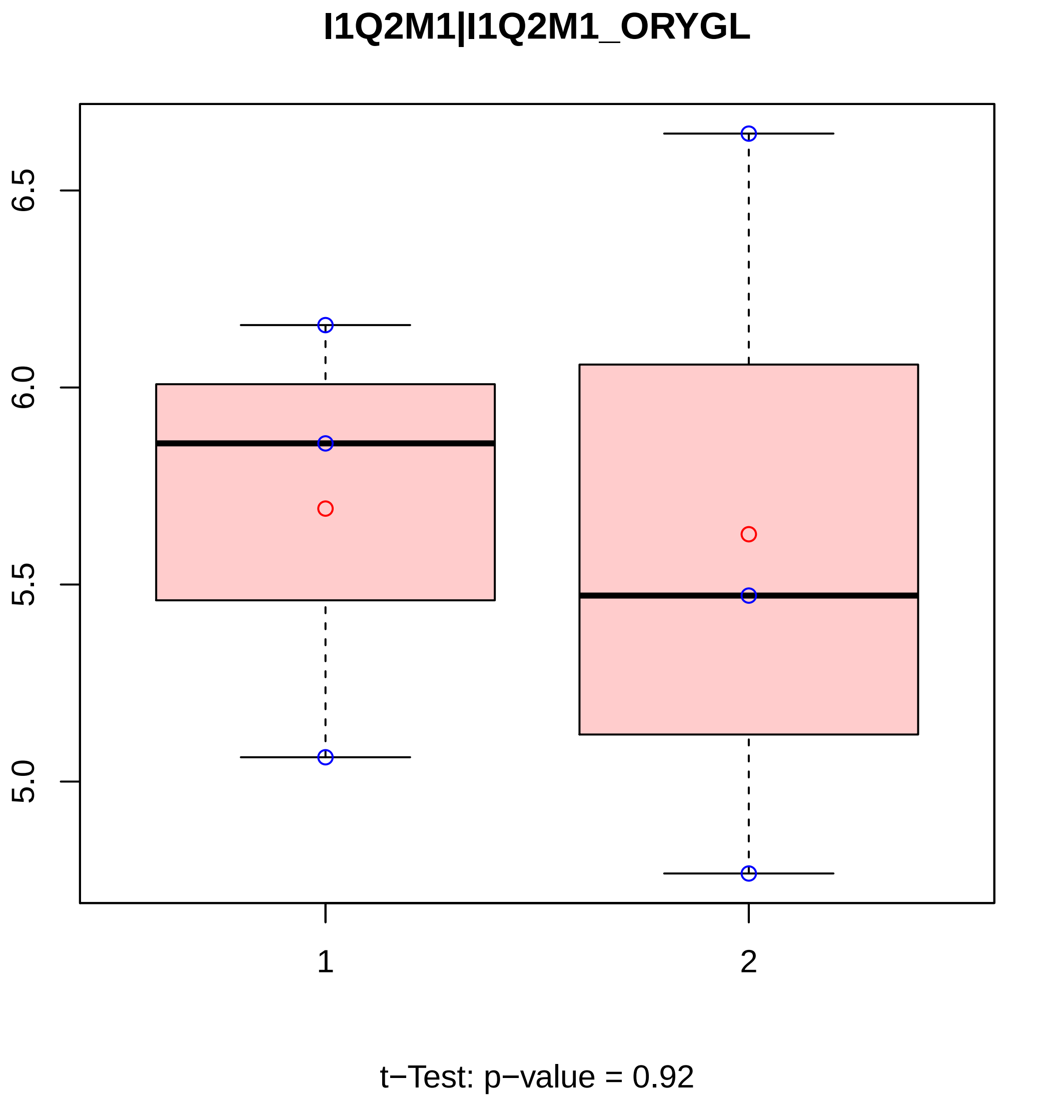
I1Q2M1|I1Q2M1_ORYGL
6.5
6.0
5.5
5.0
1
2
t−Test: p−value = 0.92

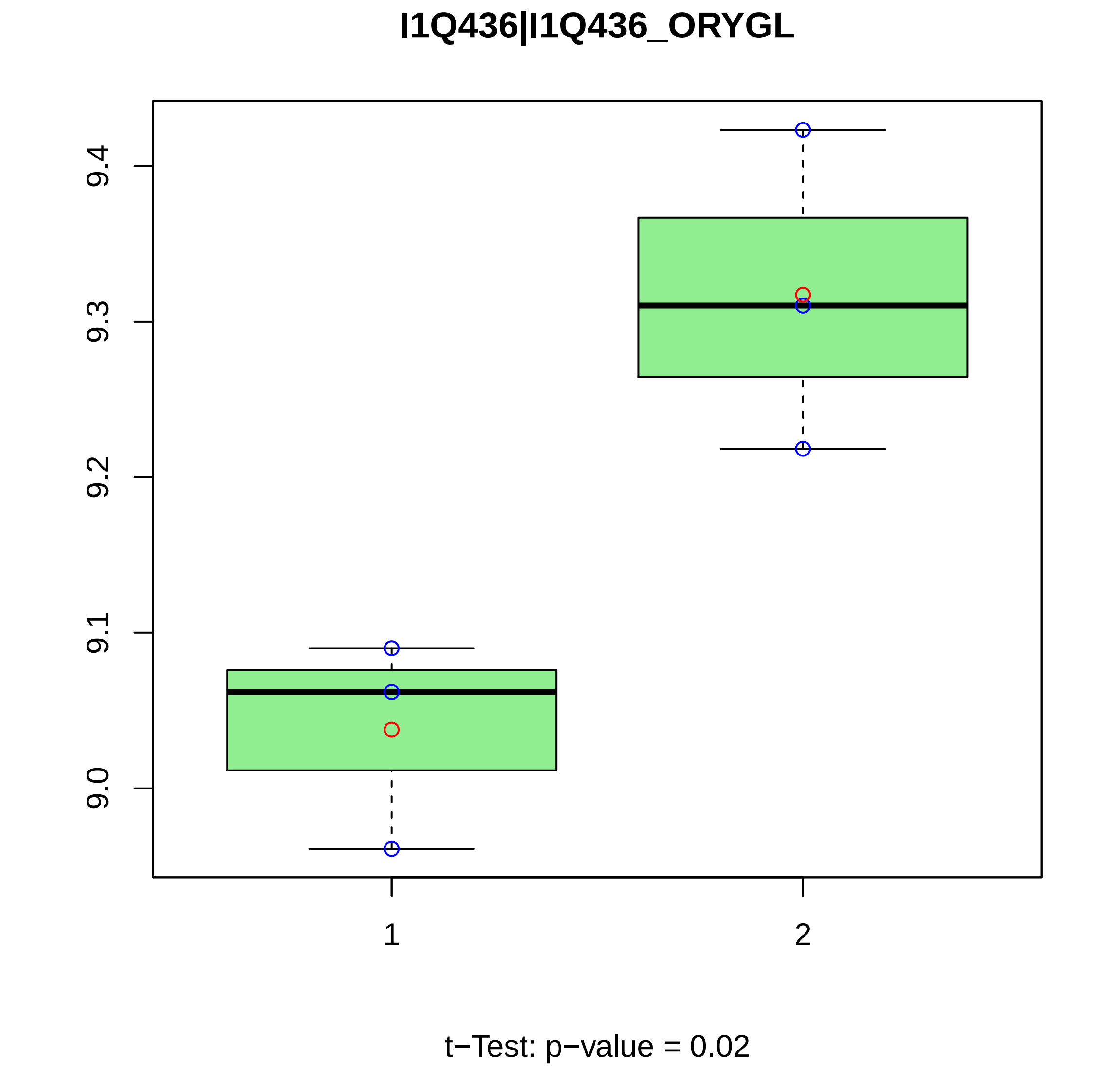
I1Q436|I1Q436_ORYGL
9.0
9.1
9.2
9.3
9.4
1
2
t−Test: p−value = 0.02

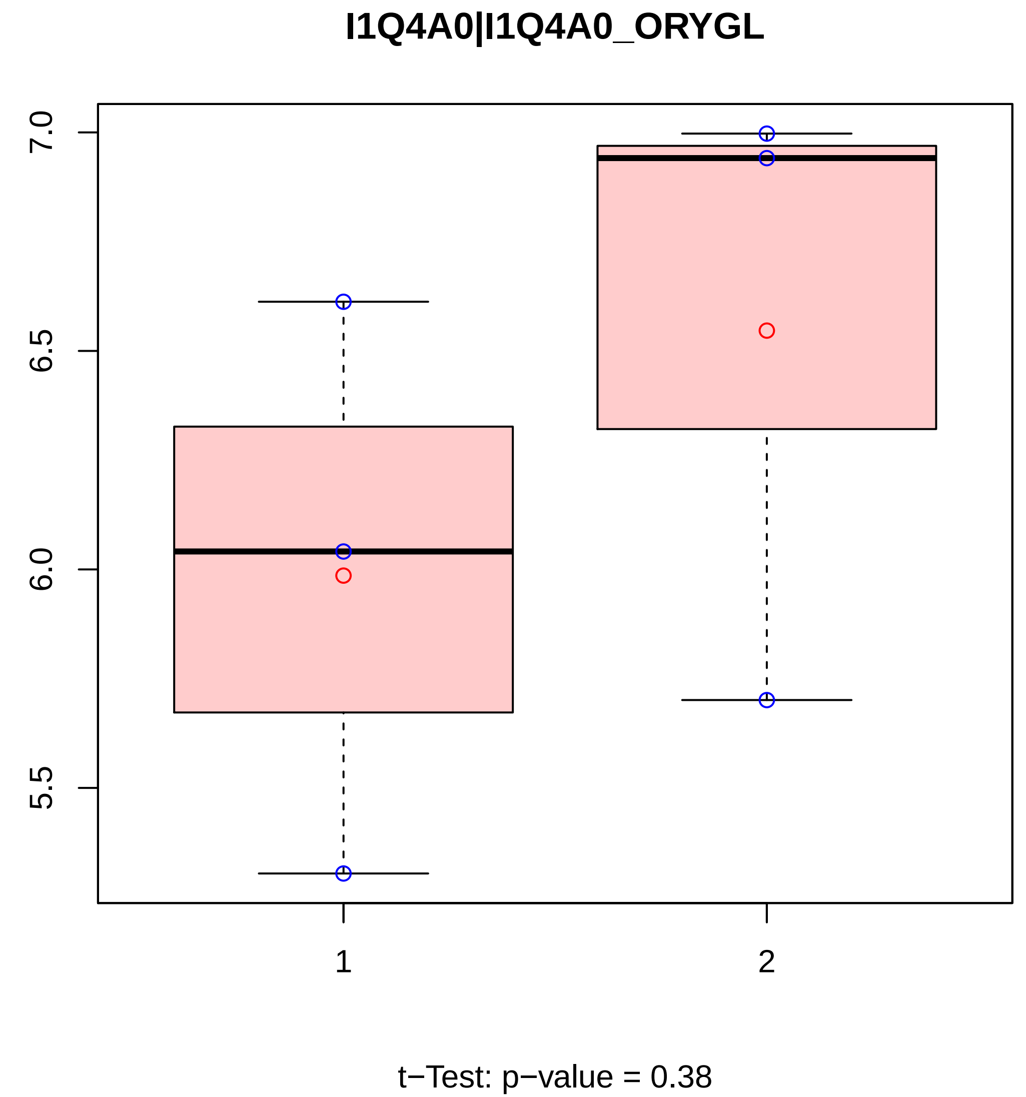
I1Q4A0|I1Q4A0_ORYGL
7.0
6.5
6.0
5.5
1
2
t−Test: p−value = 0.38

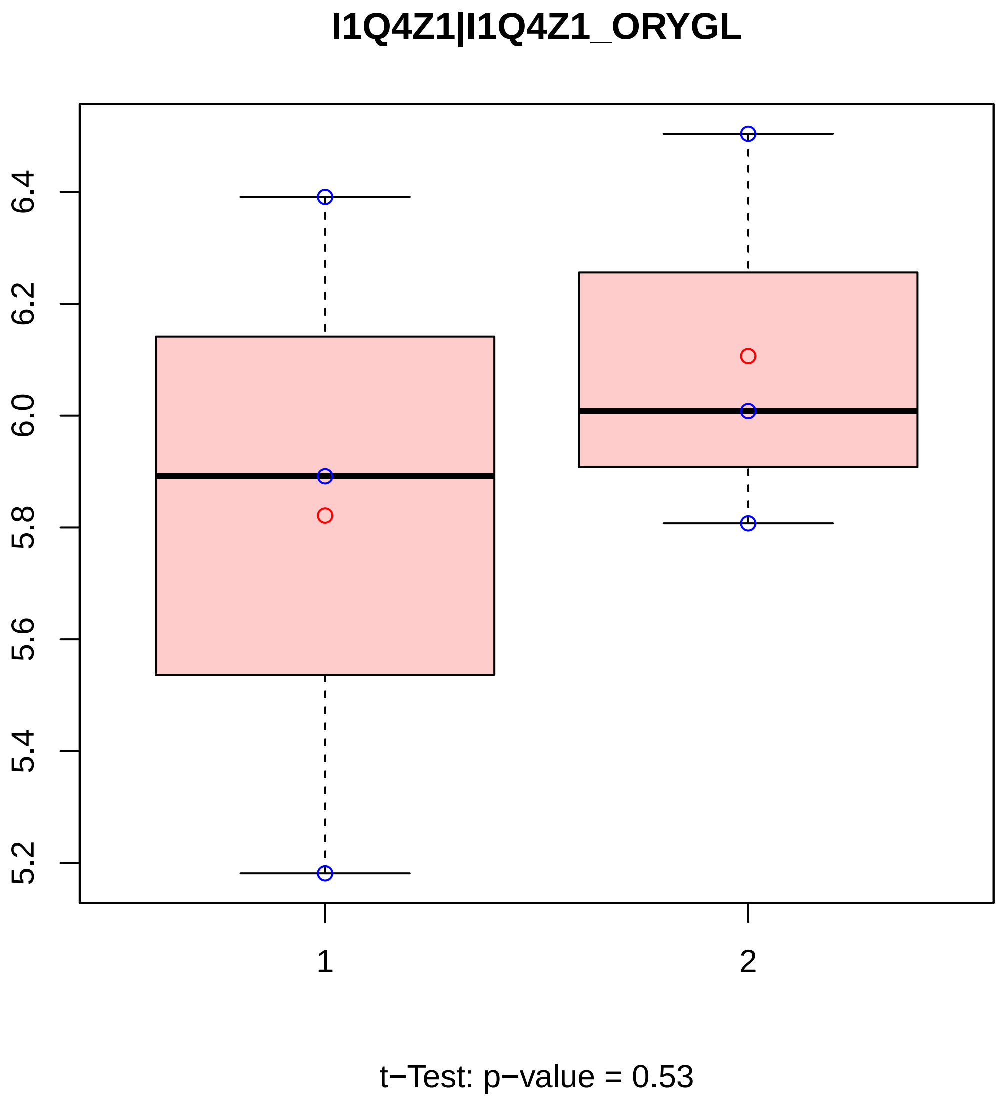
I1Q4Z1|I1Q4Z1_ORYGL
6.4
6.2
6.0
5.8
5.6
5.4
5.2
1
2
t−Test: p−value = 0.53

I1Q526|I1Q526_ORYGL

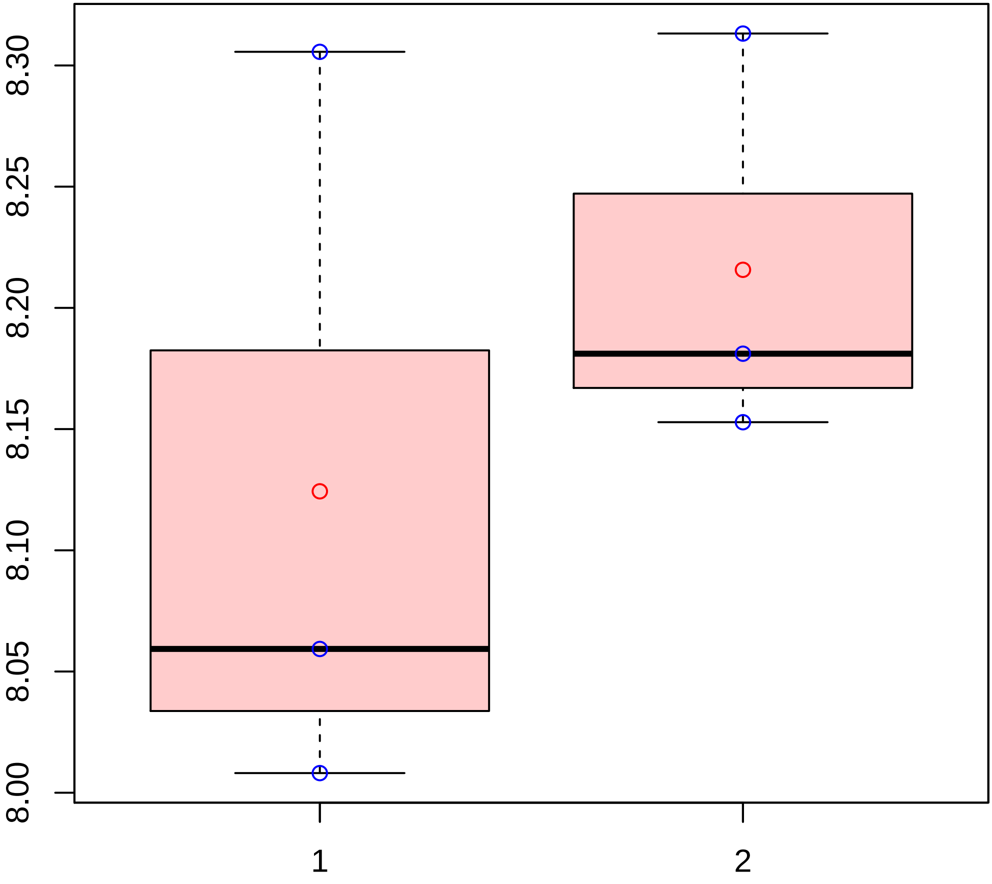

t−Test: p−value = 0.44

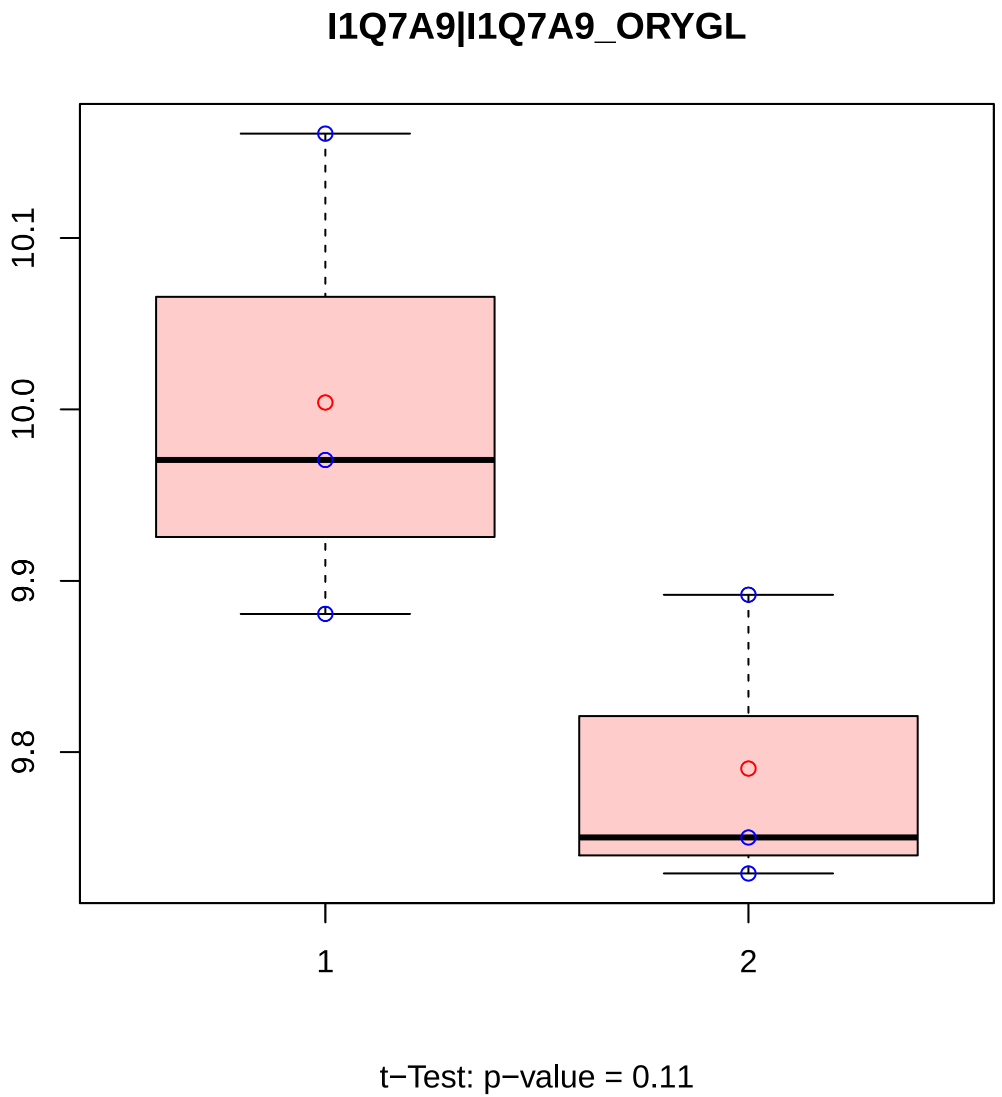
I1Q7A9|I1Q7A9_ORYGL
10.1
10.0
9.9
9.8
1
2
t−Test: p−value = 0.11

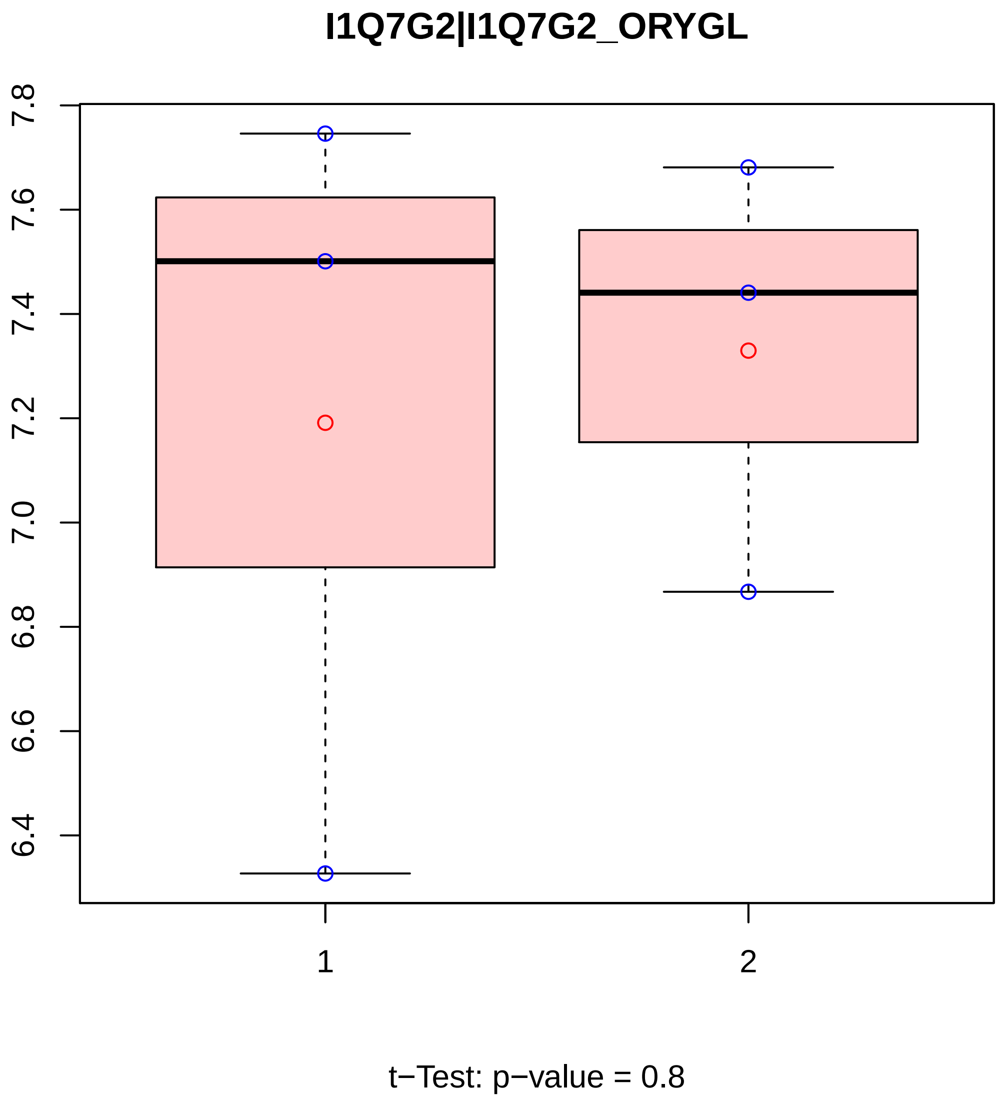
I1Q7G2|I1Q7G2_ORYGL
7.8
7.6
7.4
7.2
7.0
6.8
6.6
6.4
1
2
t−Test: p−value = 0.8

I1Q7V9|I1Q7V9_ORYGL

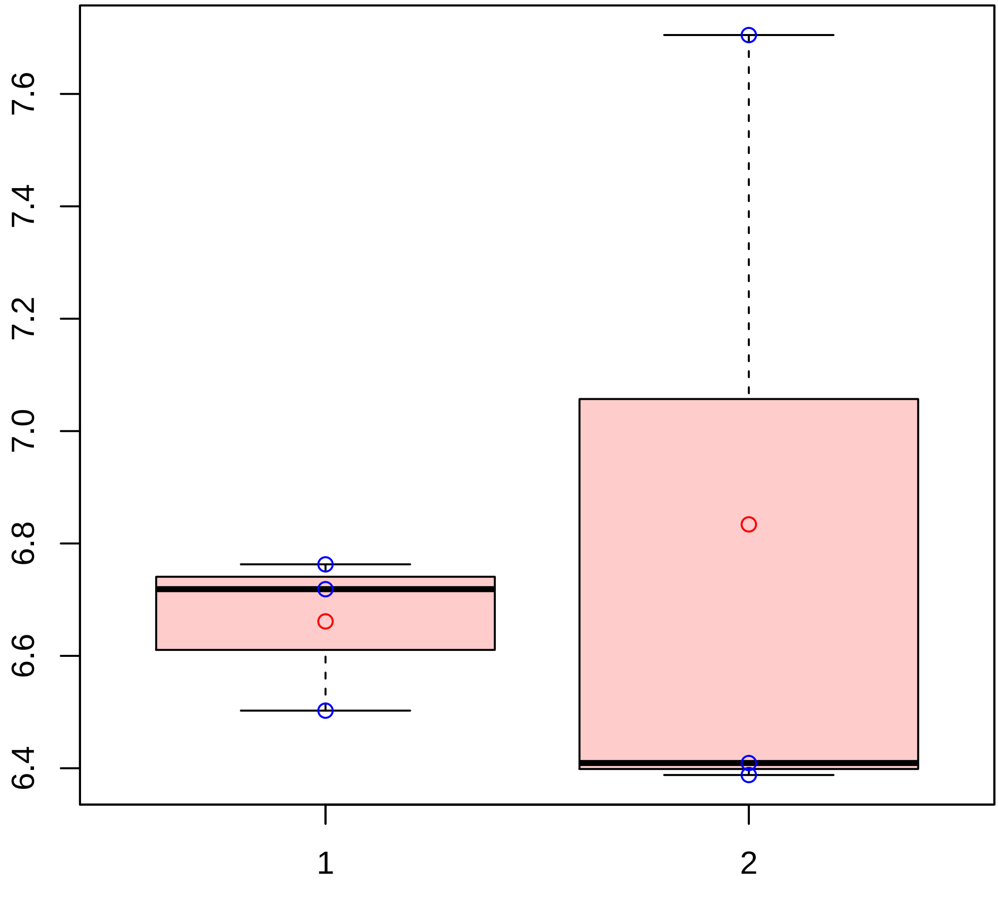

t−Test: p−value = 0.73

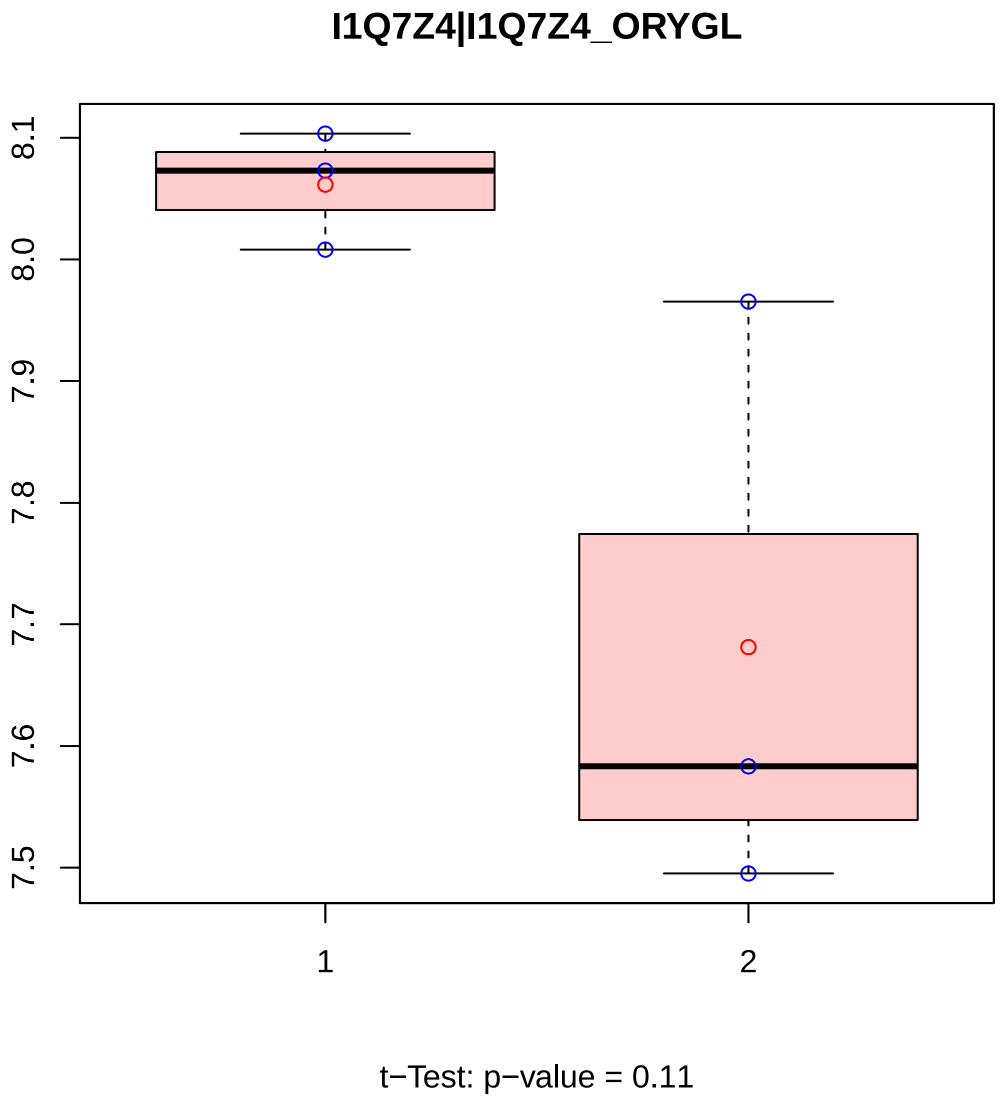
I1Q7Z4|I1Q7Z4_ORYGL
8.1
8.0
7.9
7.8
7.7
7.6
7.5
1
2
t−Test: p−value = 0.11

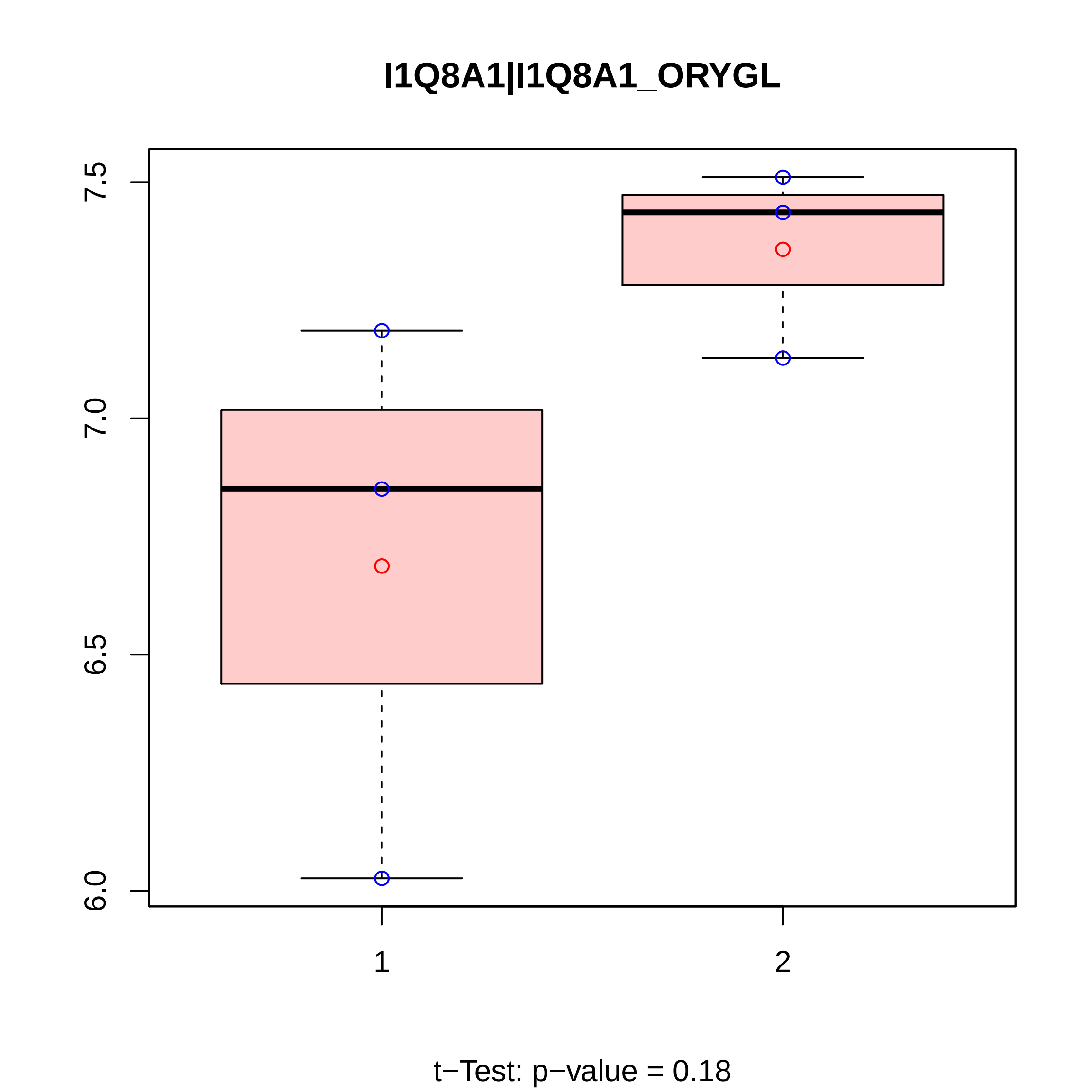
I1Q8A1|I1Q8A1_ORYGL
7.5
7.0
6.5
6.0
1
2
t−Test: p−value = 0.18

I1Q8A3|I1Q8A3_ORYGL

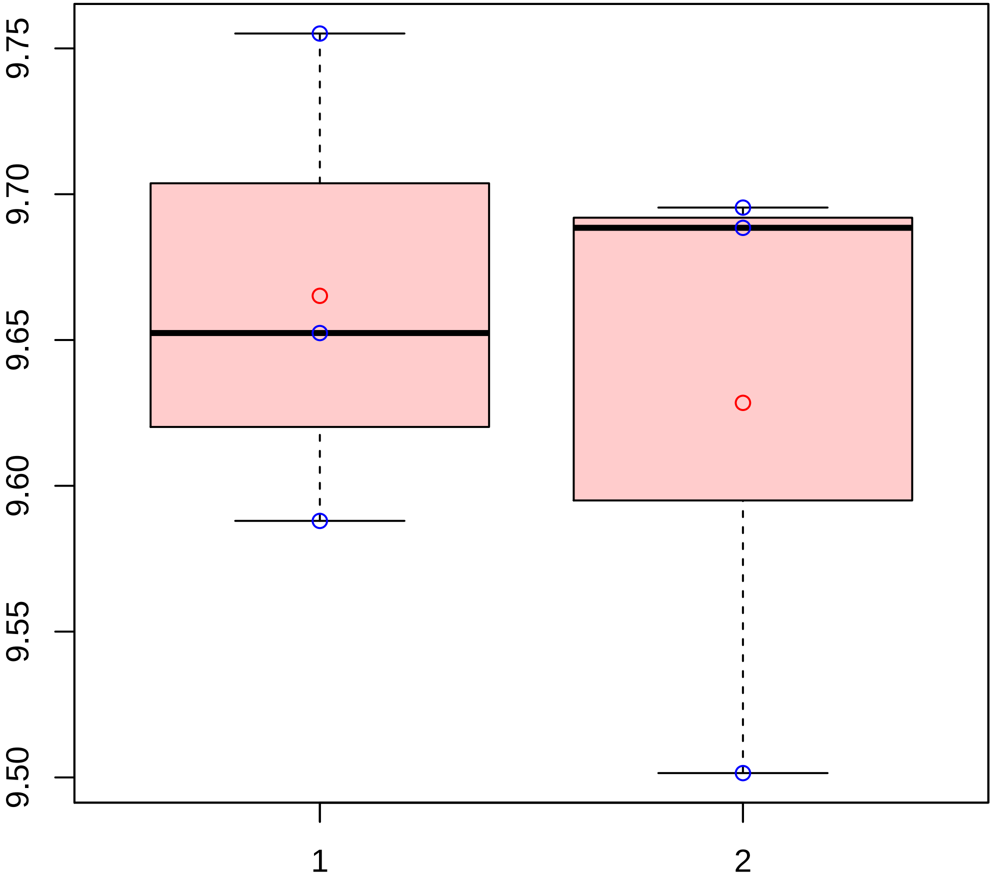

t−Test: p−value = 0.67

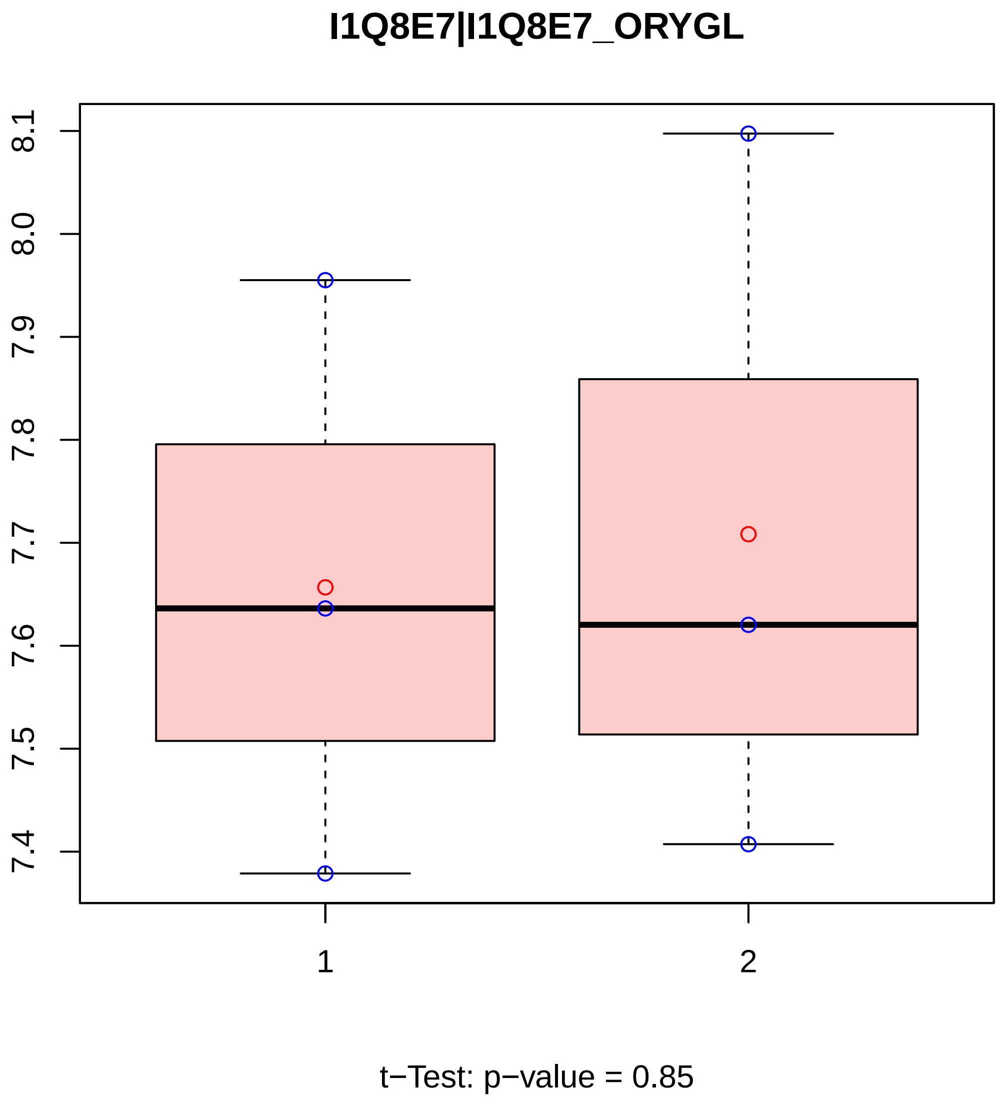
I1Q8E7|I1Q8E7_ORYGL
8.1
8.0
7.9
7.8
7.7
7.6
7.5
7.4
1
2
t−Test: p−value = 0.85

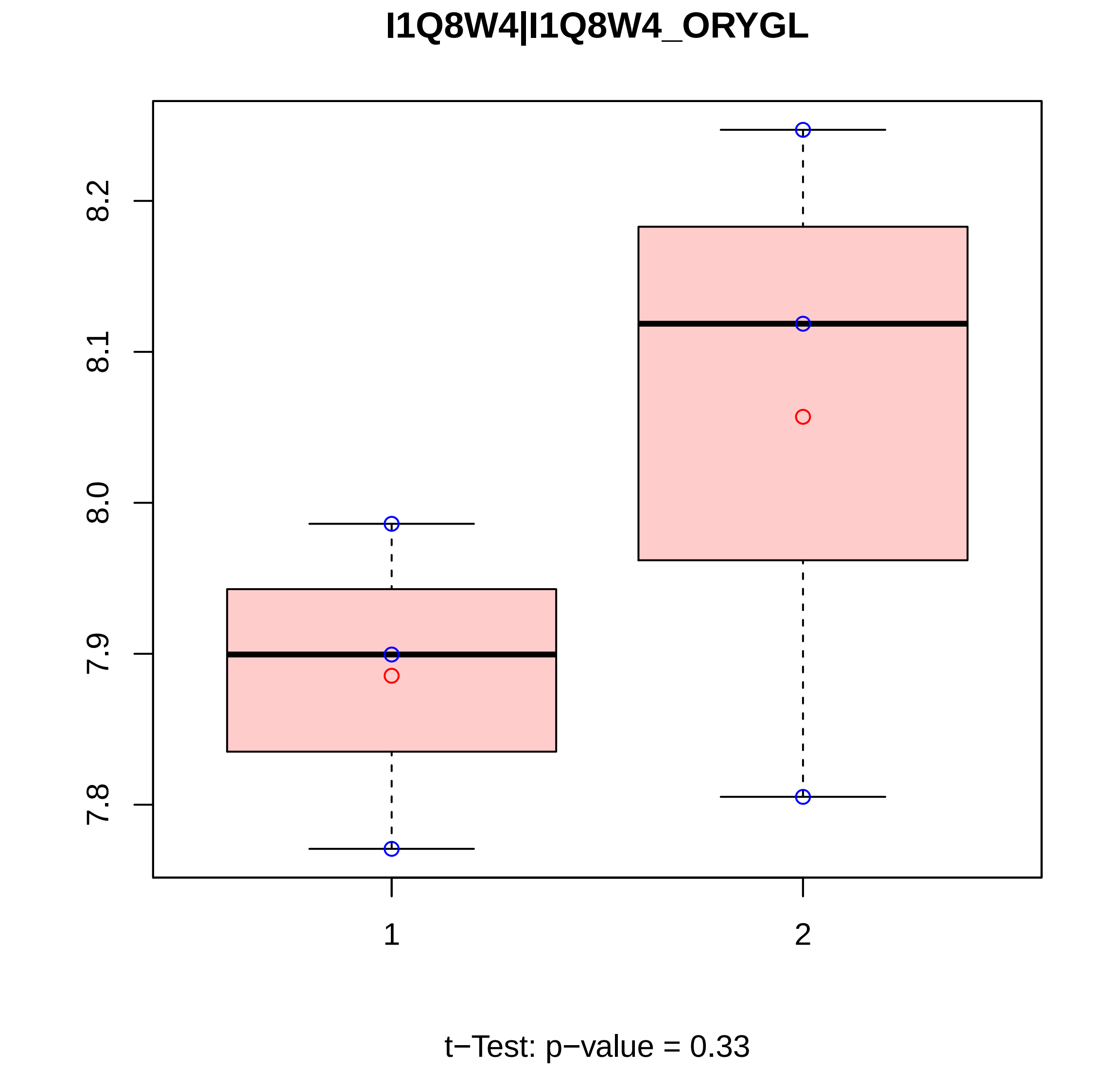
I1Q8W4|I1Q8W4_ORYGL
8.2
8.1
8.0
7.9
7.8
1
2
t−Test: p−value = 0.33

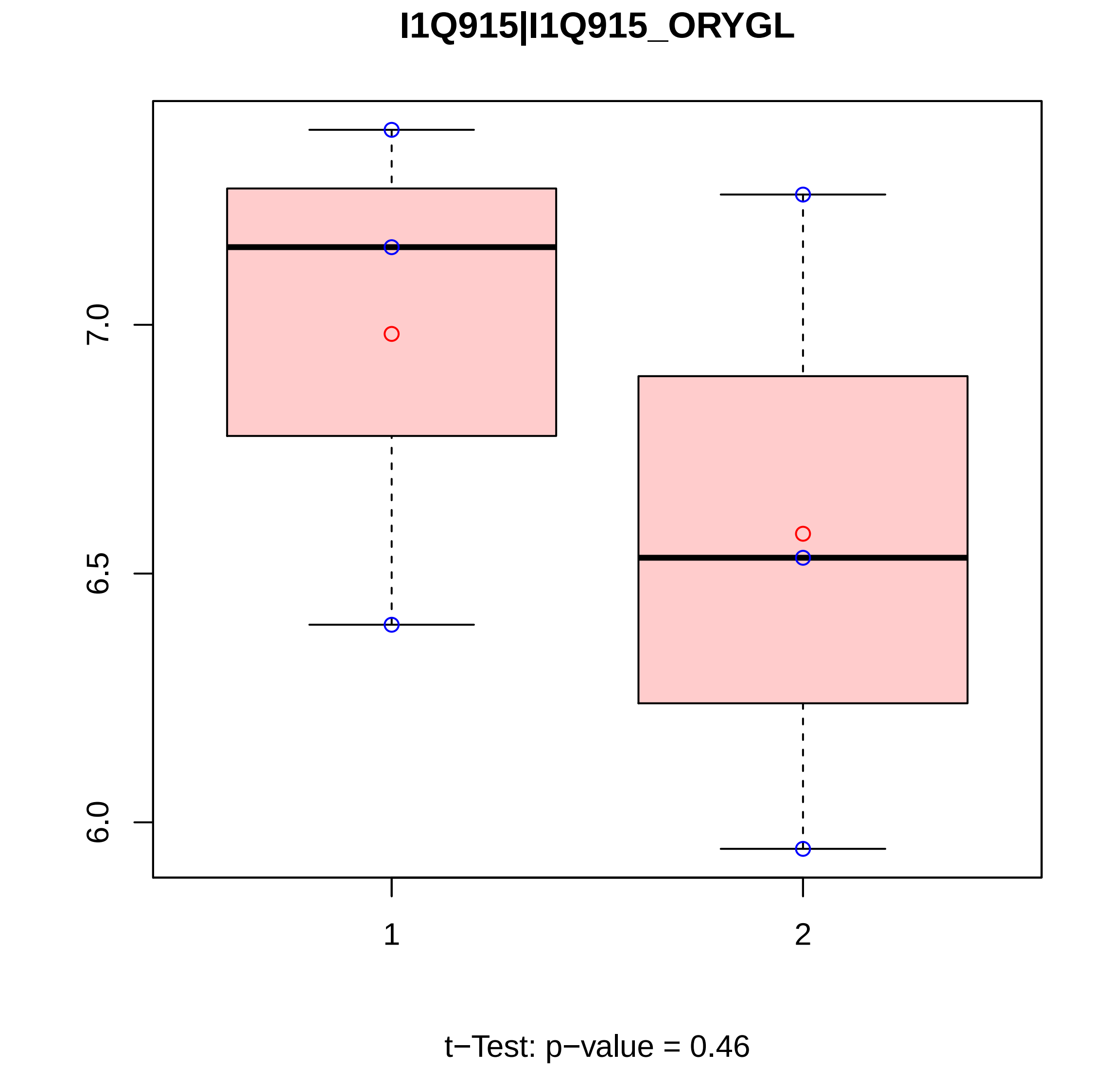
I1Q915|I1Q915_ORYGL
7.0
6.5
6.0
1
2
t−Test: p−value = 0.46

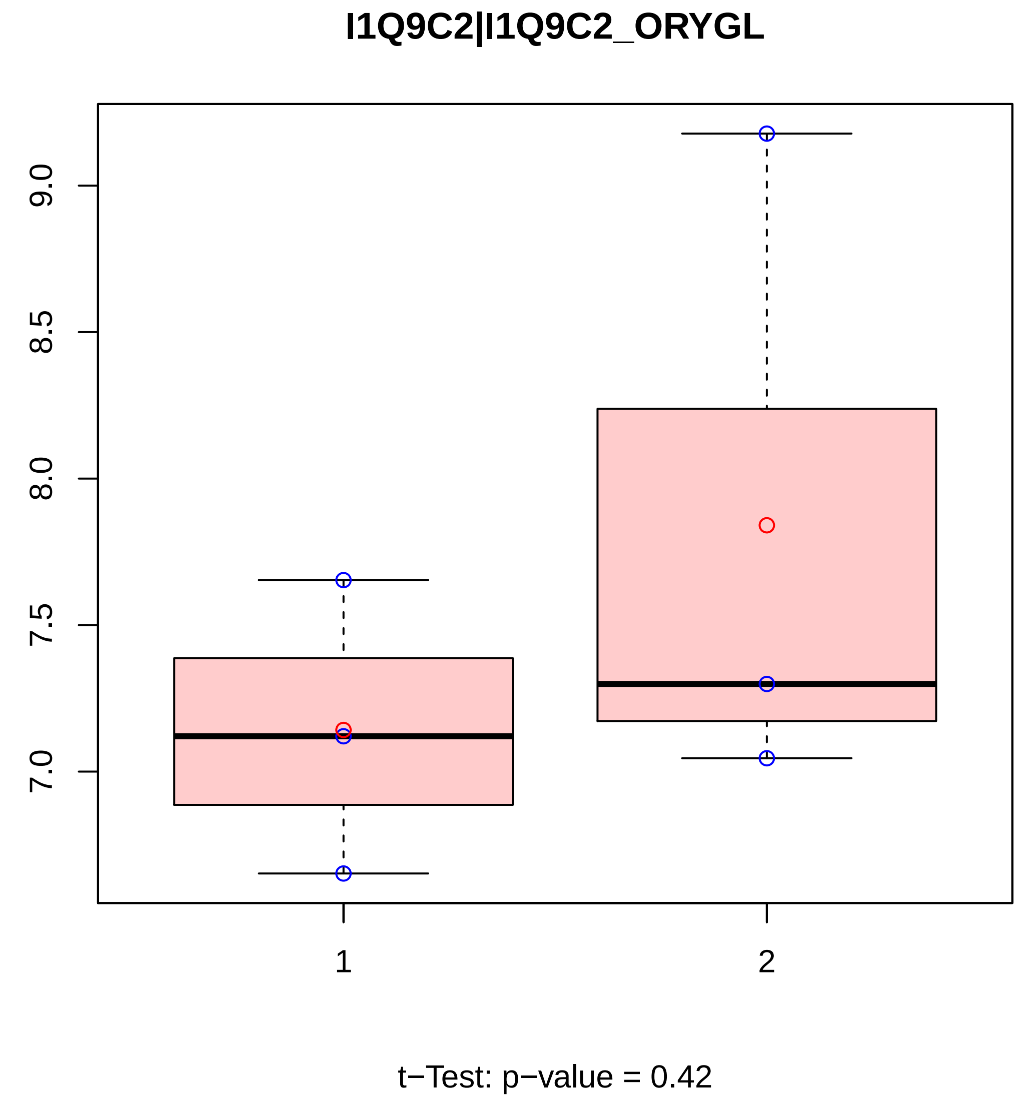
I1Q9C2|I1Q9C2_ORYGL
9.0
8.5
8.0
7.5
7.0
1
2
t−Test: p−value = 0.42

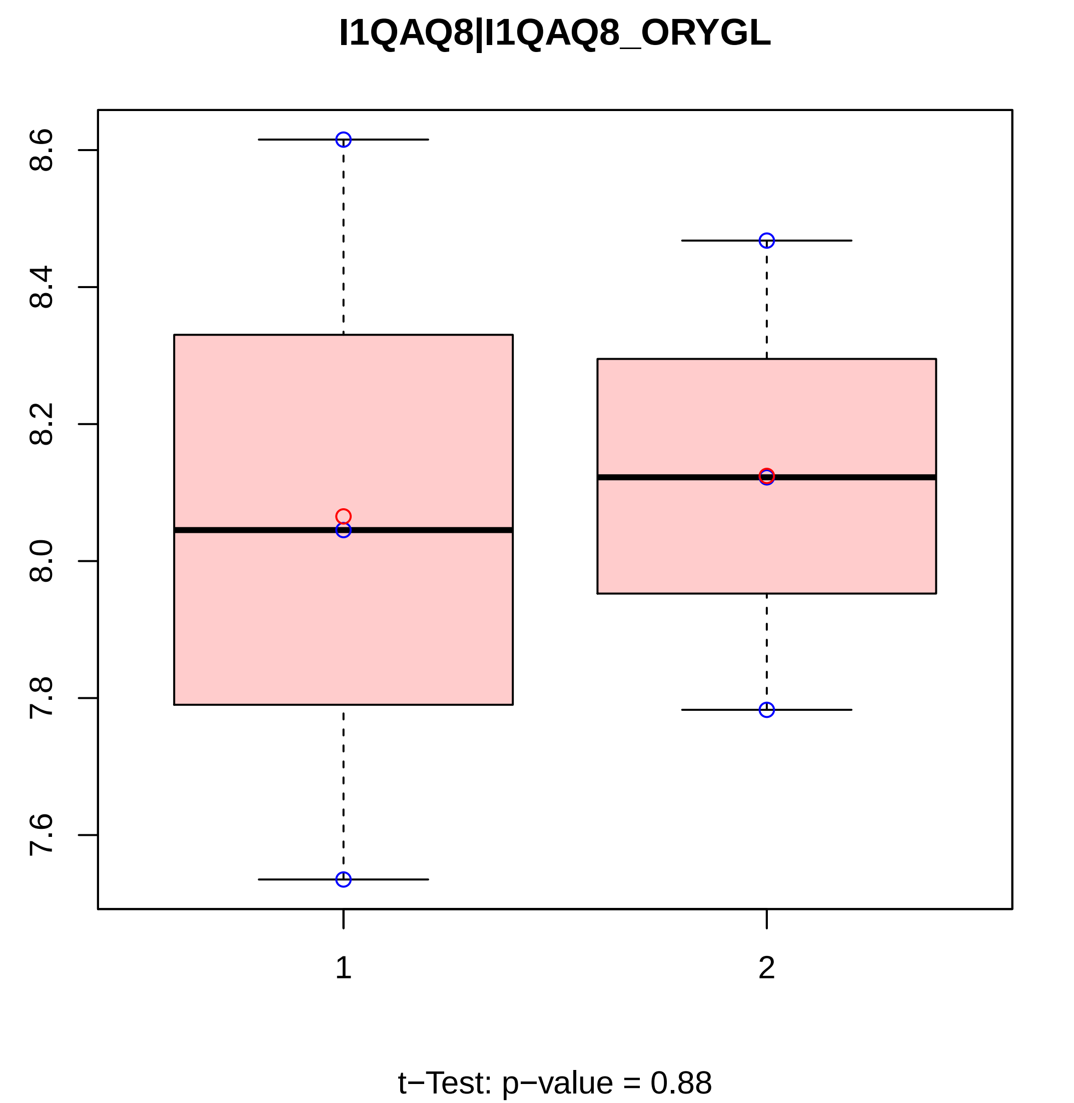
I1QAQ8|I1QAQ8_ORYGL
8.6
8.4
8.2
8.0
7.8
7.6
1
2
t−Test: p−value = 0.88

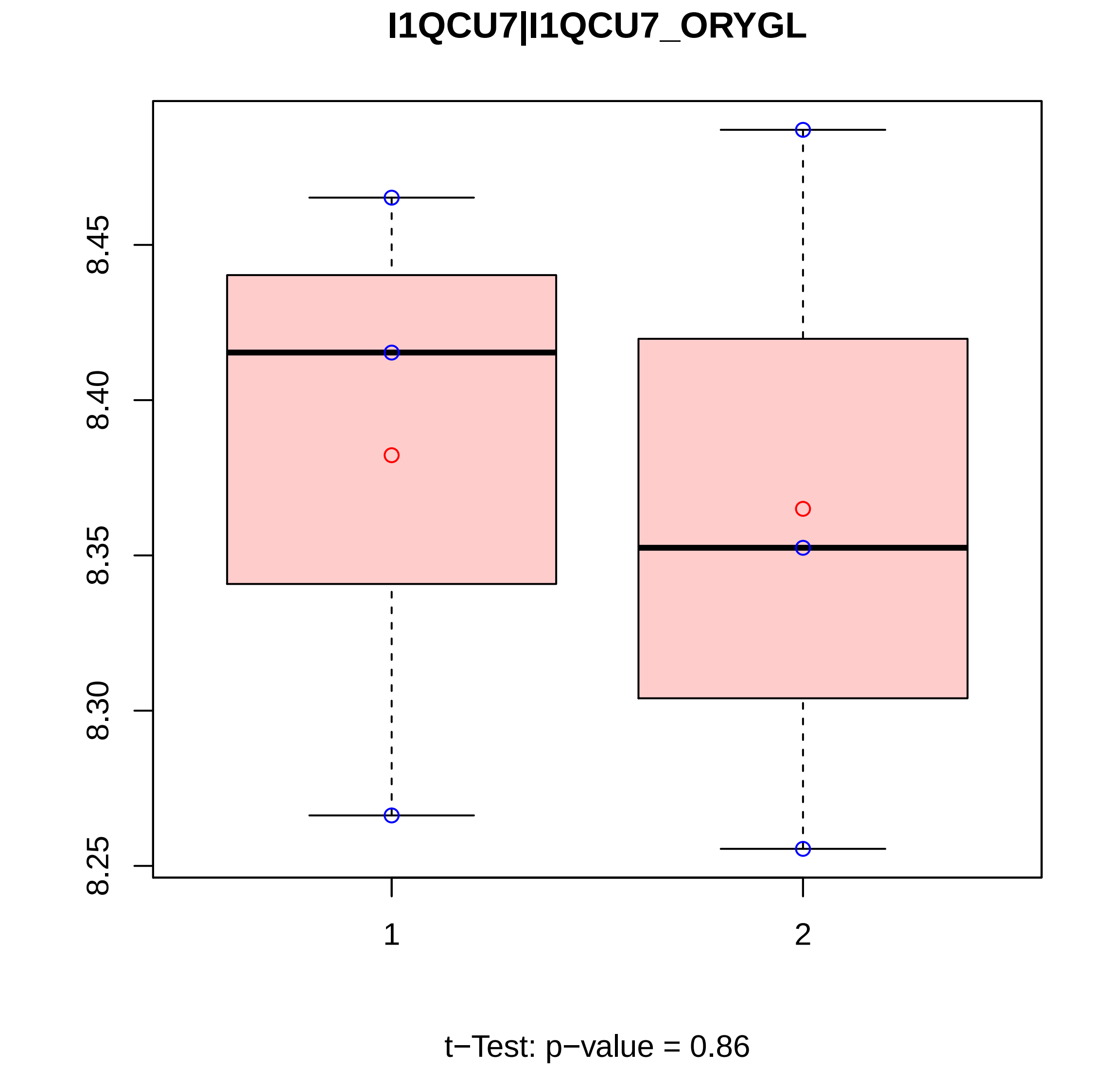
I1QCU7|I1QCU7_ORYGL
8.25
8.30
8.35
8.40
8.45
1
2
t−Test: p−value = 0.86

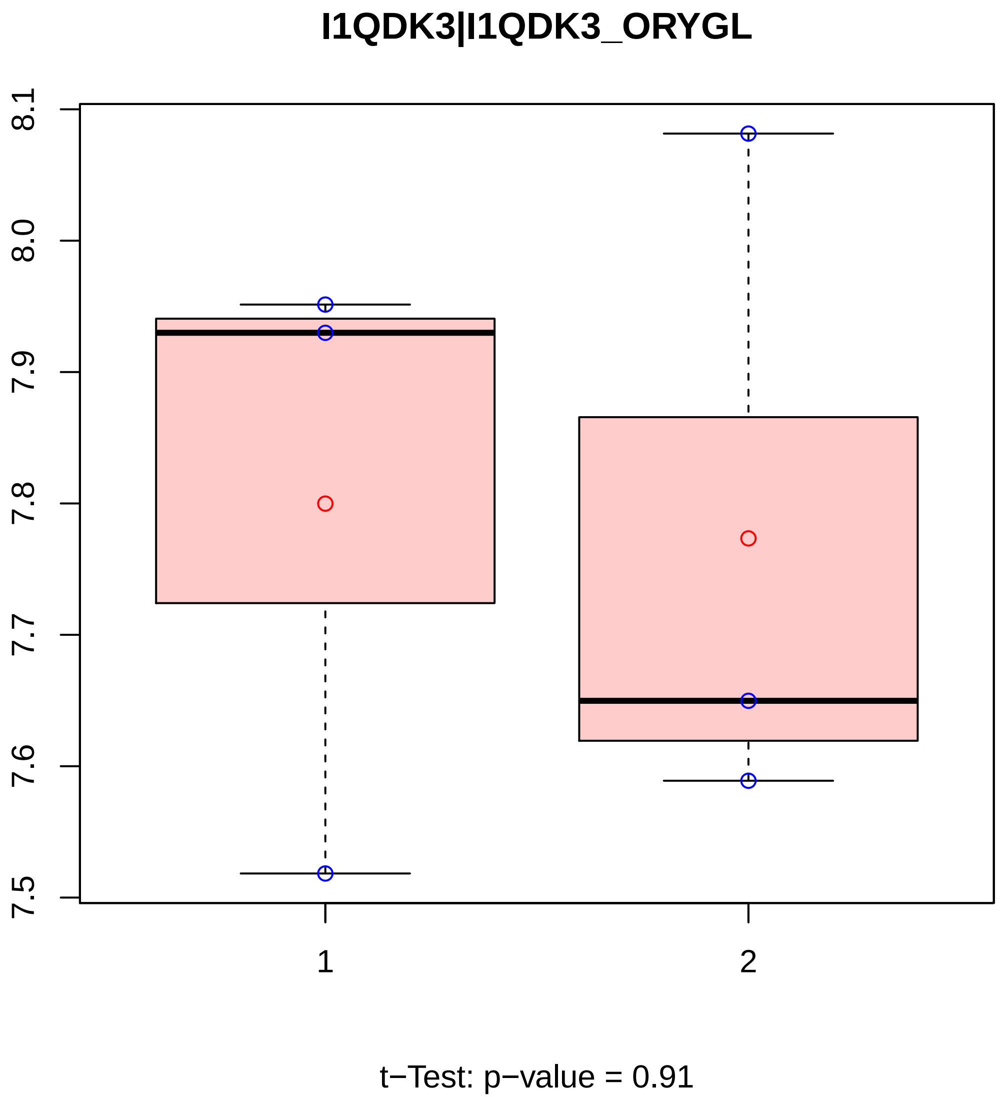
I1QDK3|I1QDK3_ORYGL
8.1
8.0
7.9
7.8
7.7
7.6
7.5
1
2
t−Test: p−value = 0.91

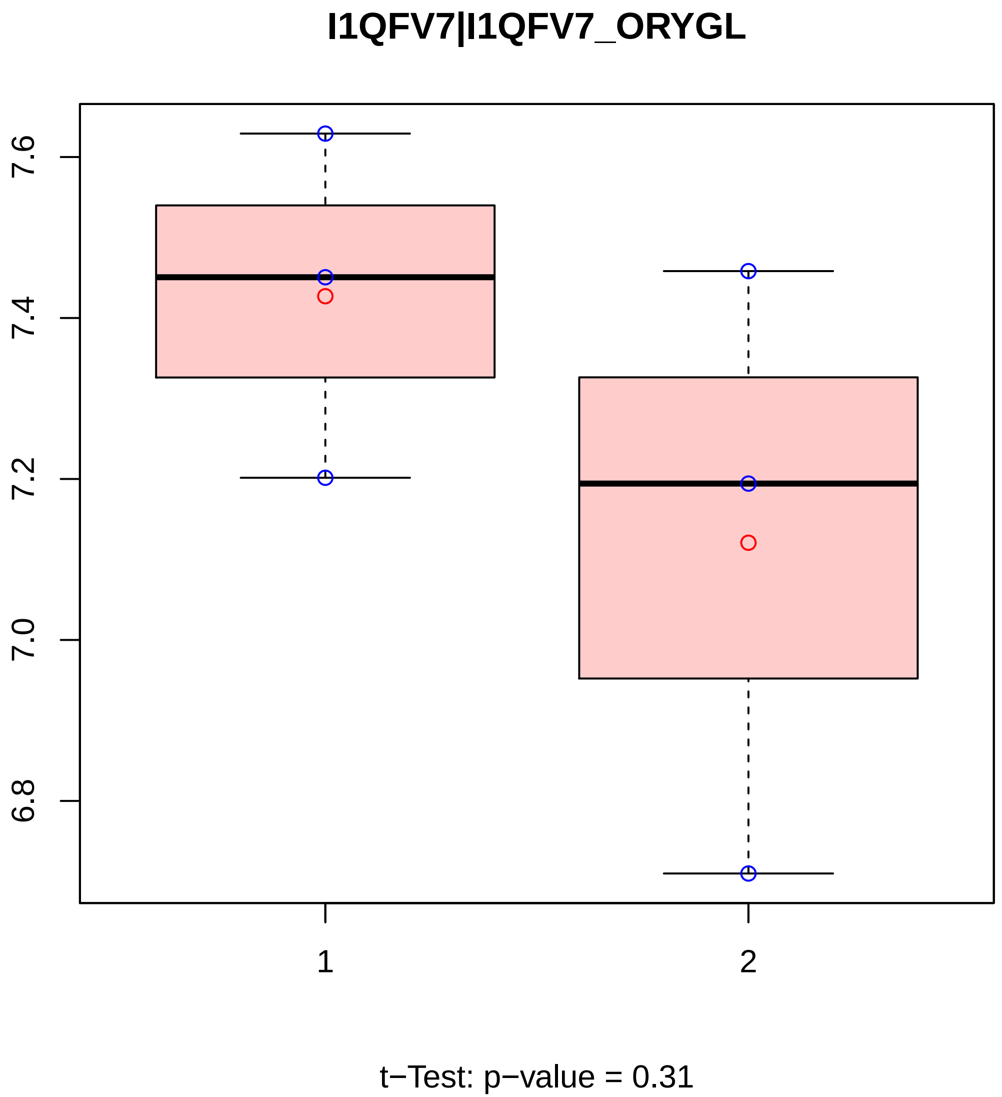
I1QFV7|I1QFV7_ORYGL
7.6
7.4
7.2
7.0
6.8
1
2
t−Test: p−value = 0.31

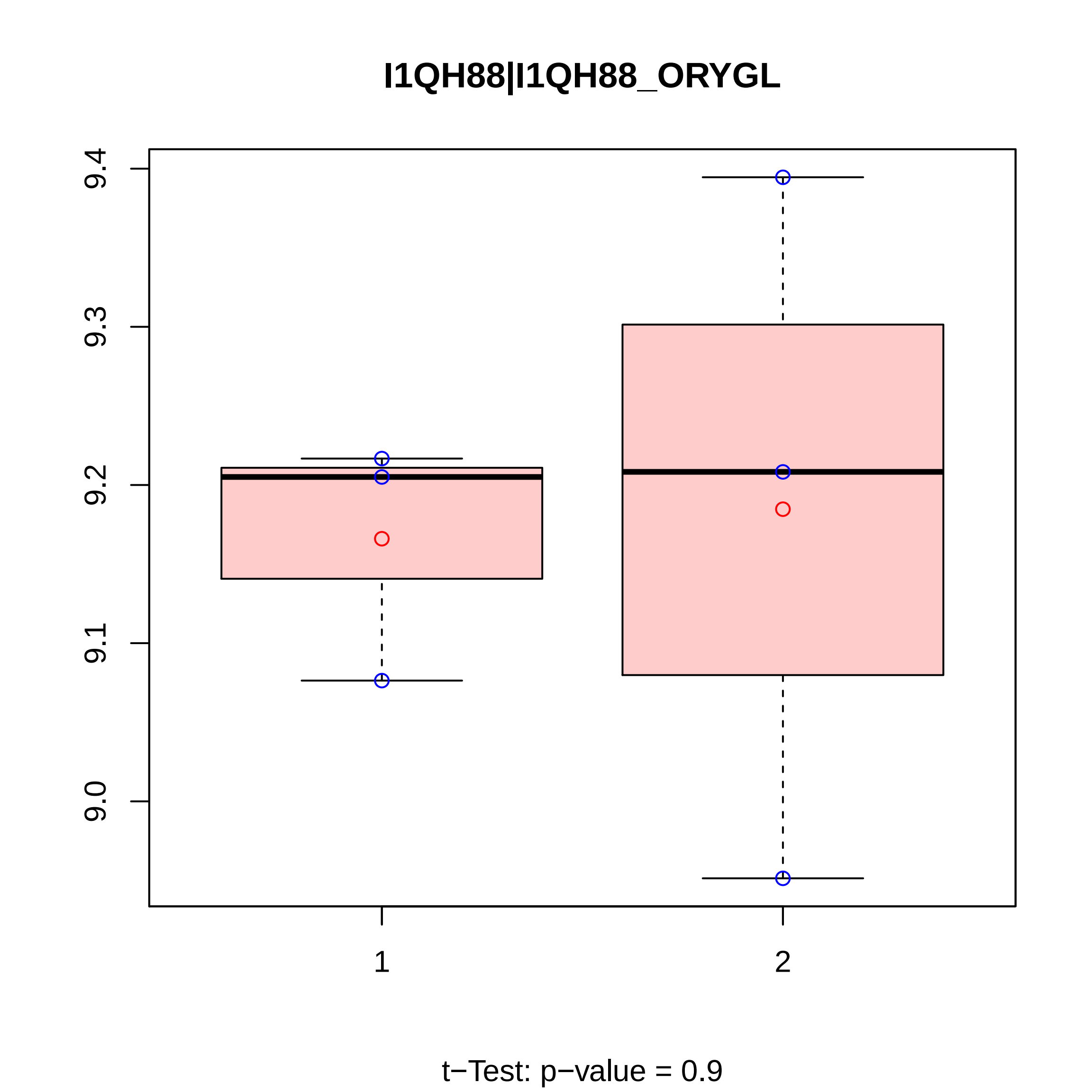

I1QH88|I1QH88_ORYGL
9.0
9.1
9.2
9.3
9.4
1
2
t−Test: p−value = 0.9

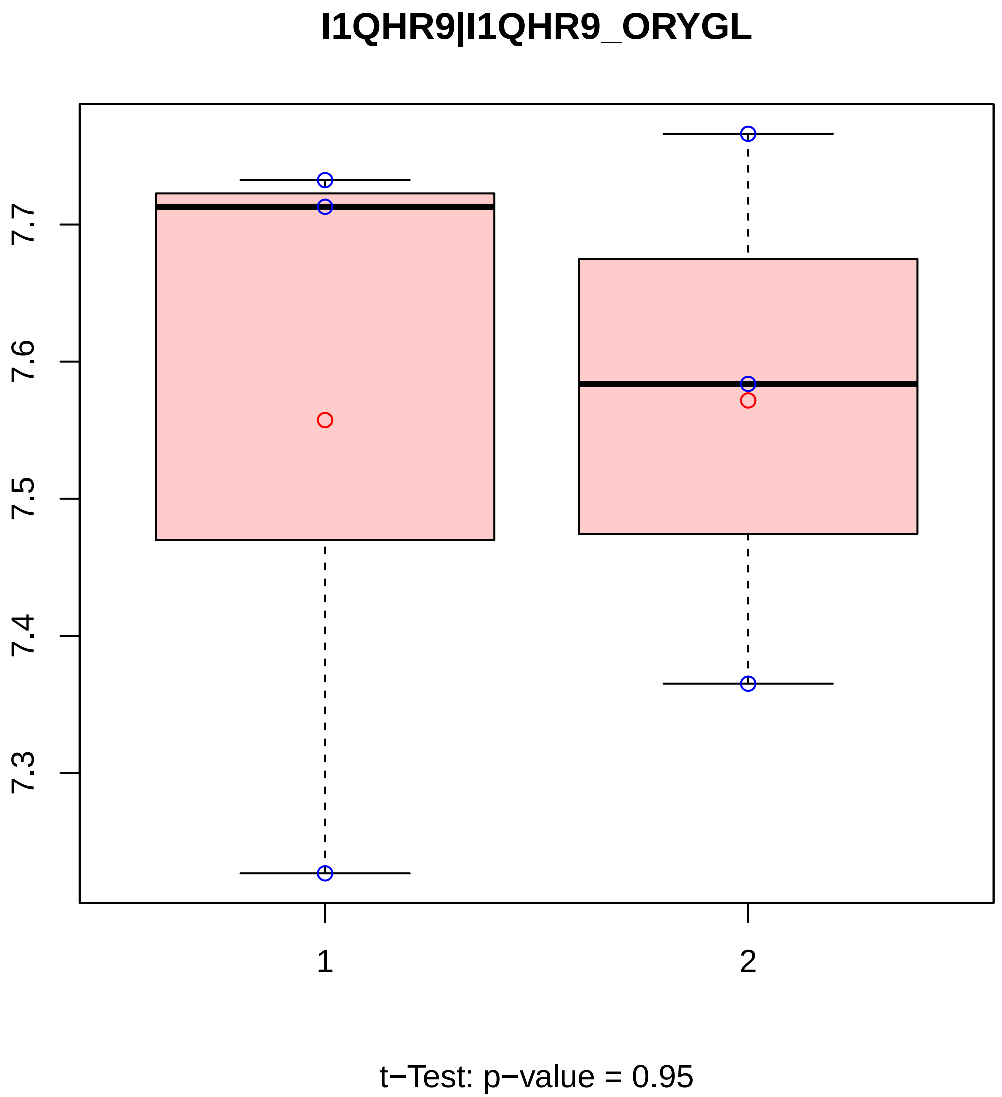
I1QHR9|I1QHR9_ORYGL
7.3
7.4
7.5
7.6
7.7
1
2
t−Test: p−value = 0.95

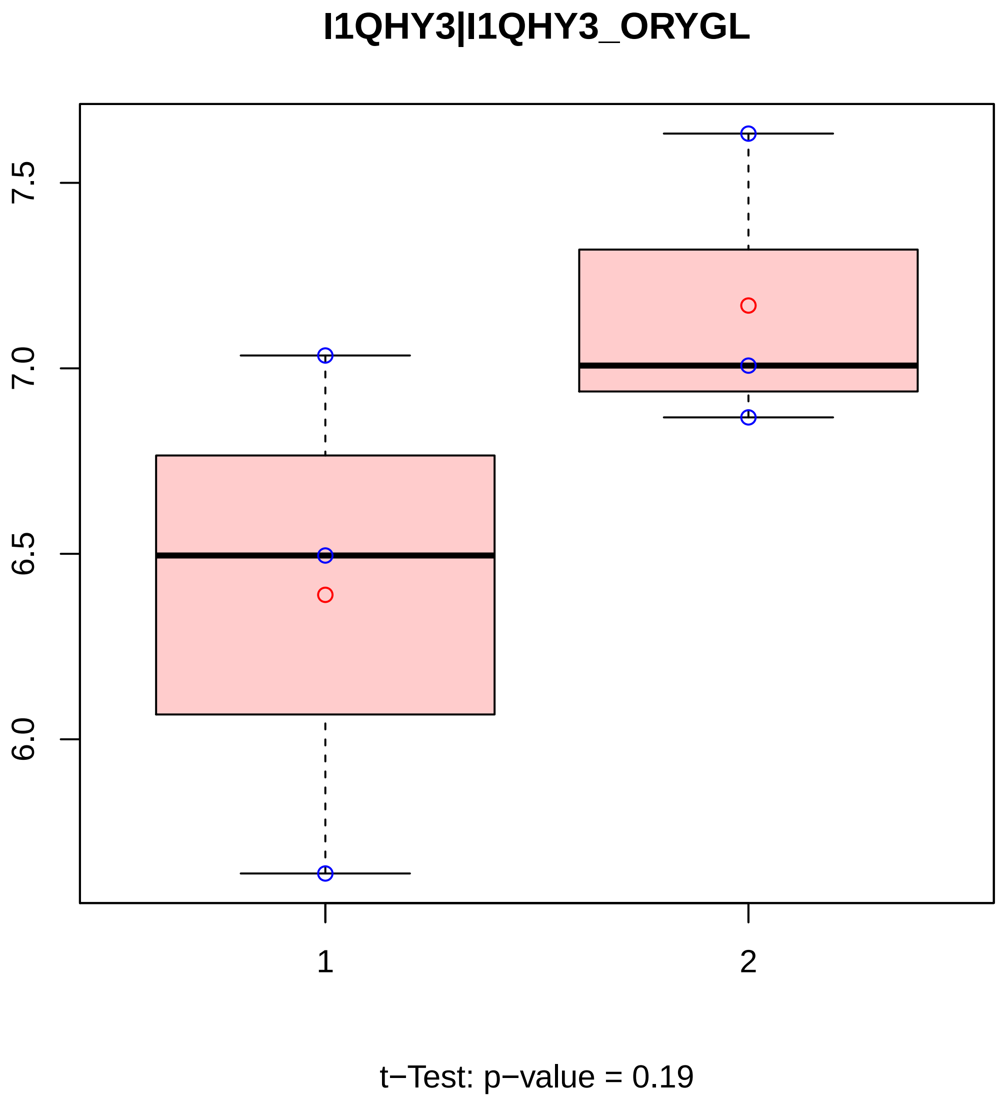

I1QHY3|I1QHY3_ORYGL
7.5
7.0
6.5
6.0
1
2
t−Test: p−value = 0.19

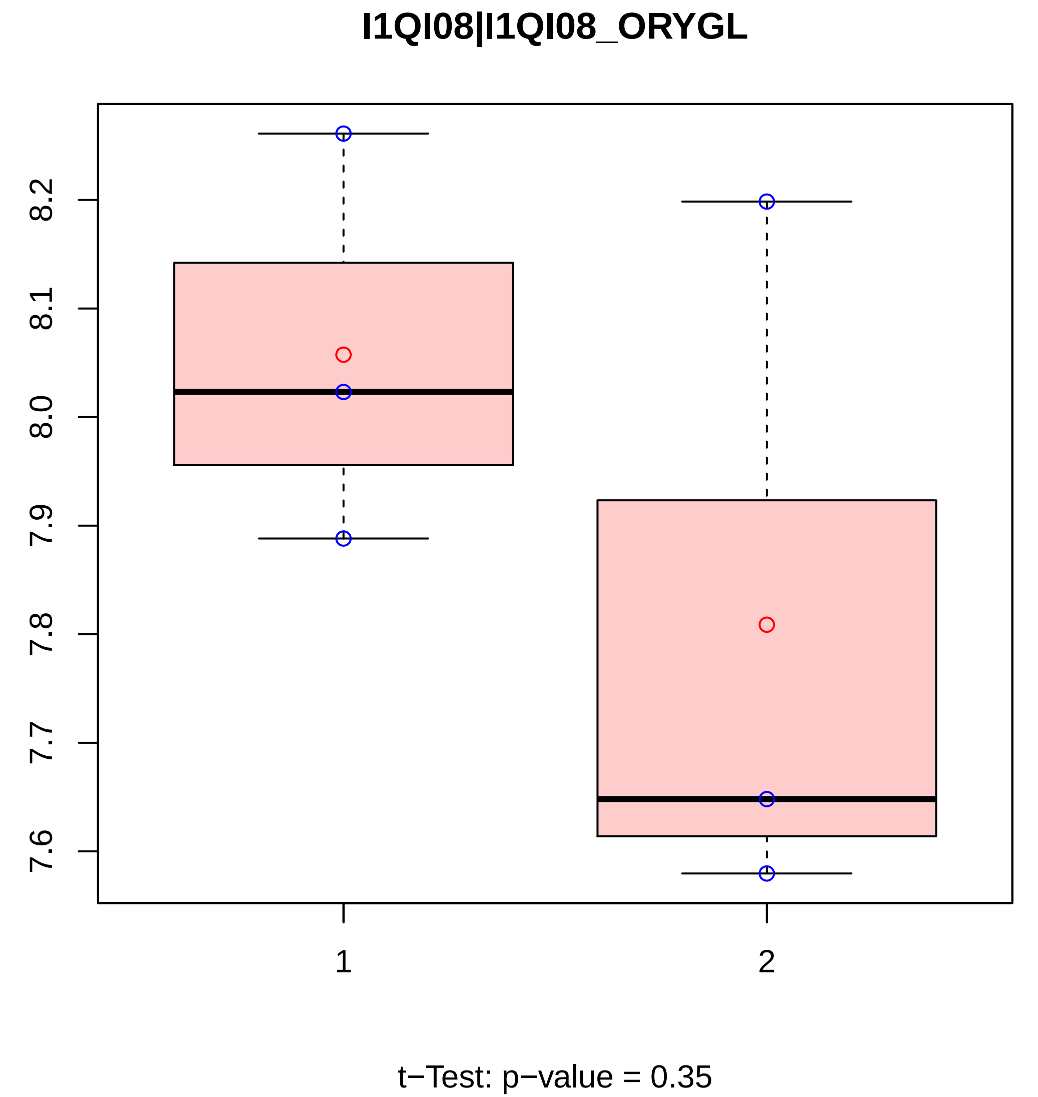
I1QI08|I1QI08_ORYGL
8.2
8.1
8.0
7.9
7.8
7.7
7.6
1
2
t−Test: p−value = 0.35

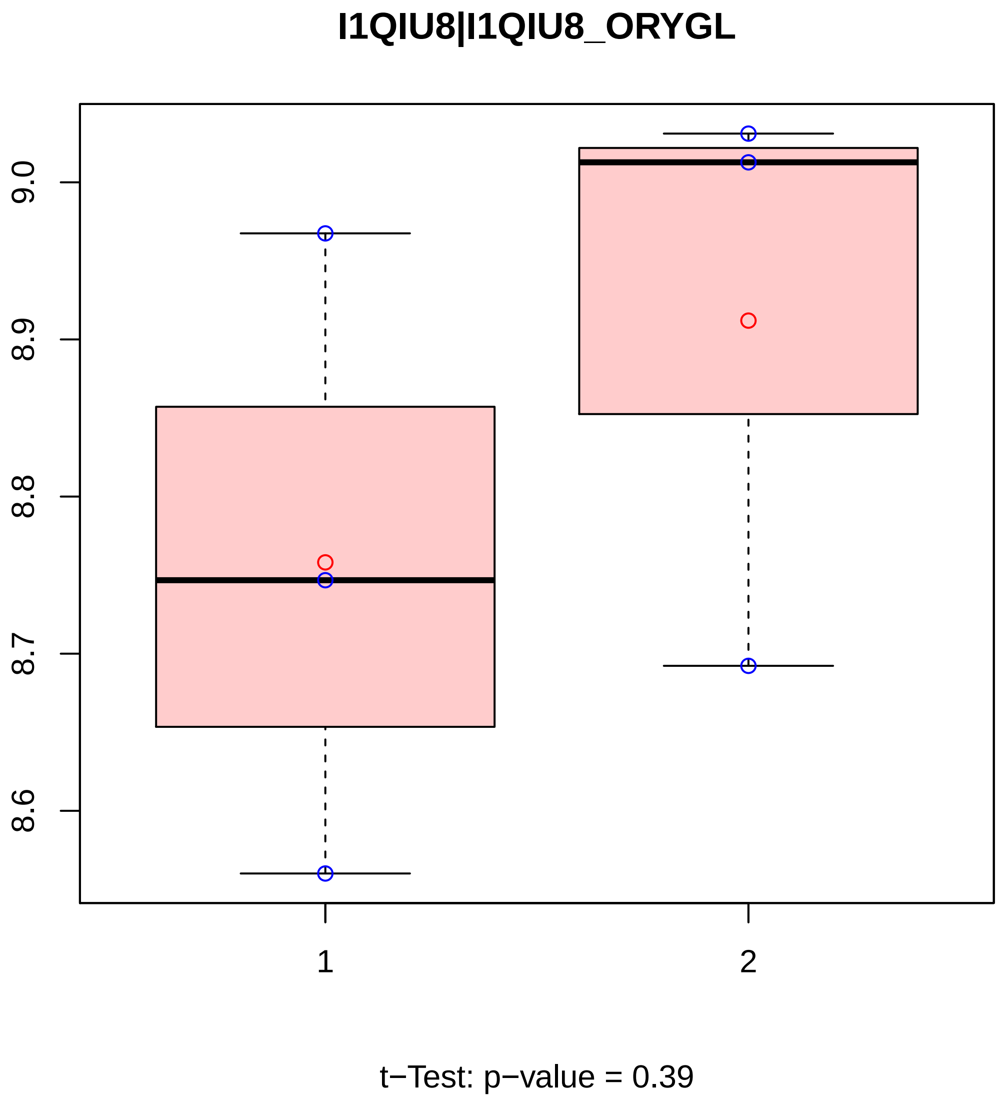

I1QIU8|I1QIU8_ORYGL
9.0
8.9
8.8
8.7
8.6
1
2
t−Test: p−value = 0.39

I1QJH7|I1QJH7_ORYGL

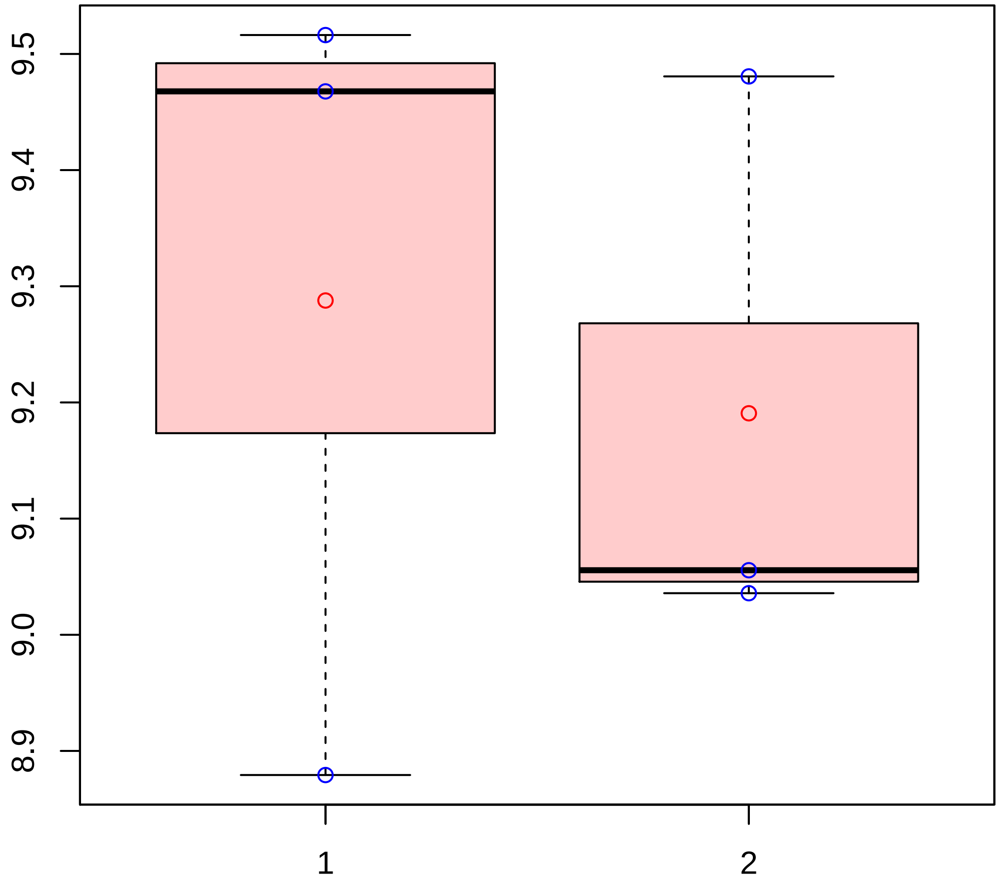

t−Test: p−value = 0.72

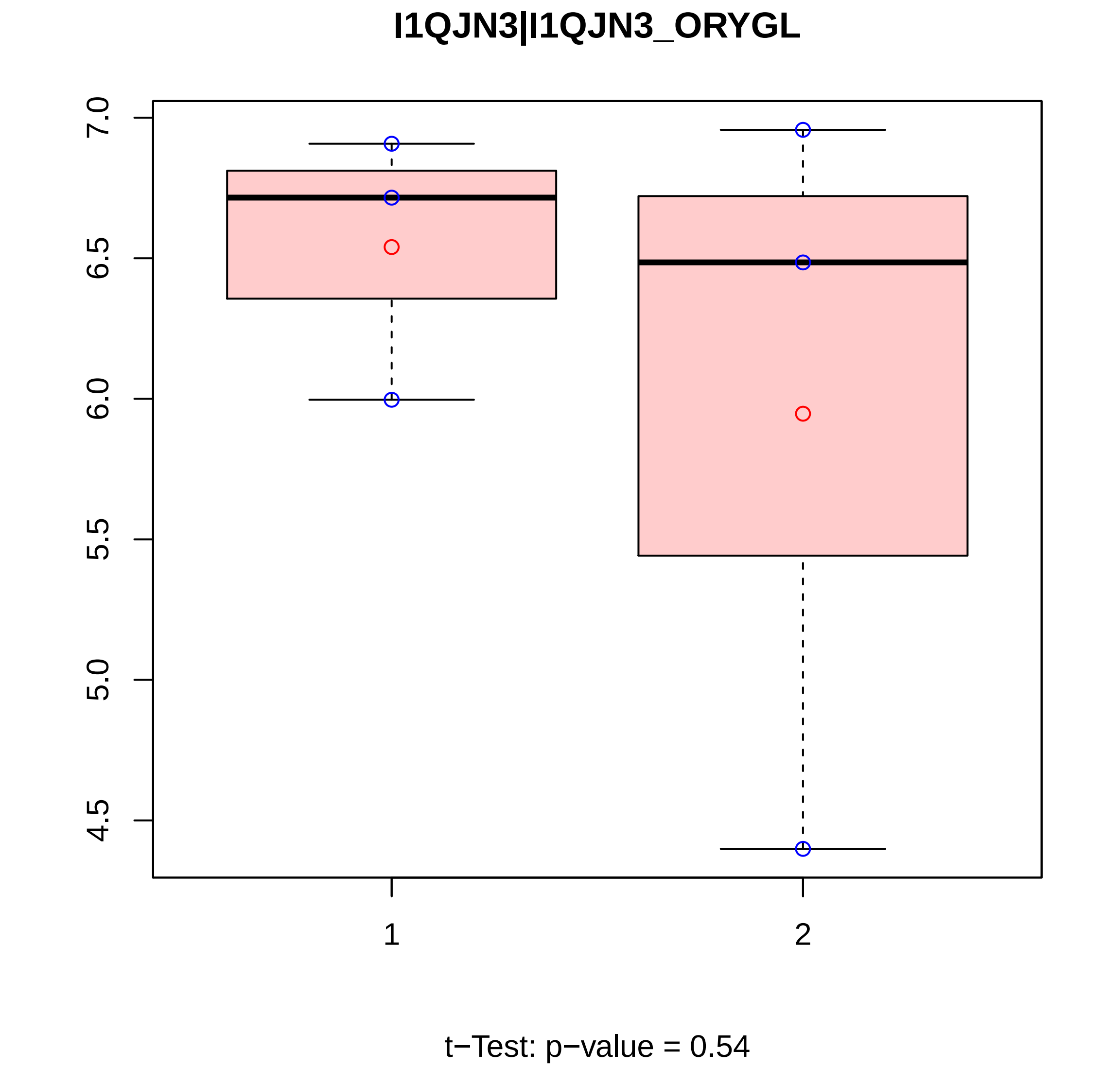
I1QJN3|I1QJN3_ORYGL
7.0
6.5
6.0
5.5
5.0
4.5
1
2
t−Test: p−value = 0.54

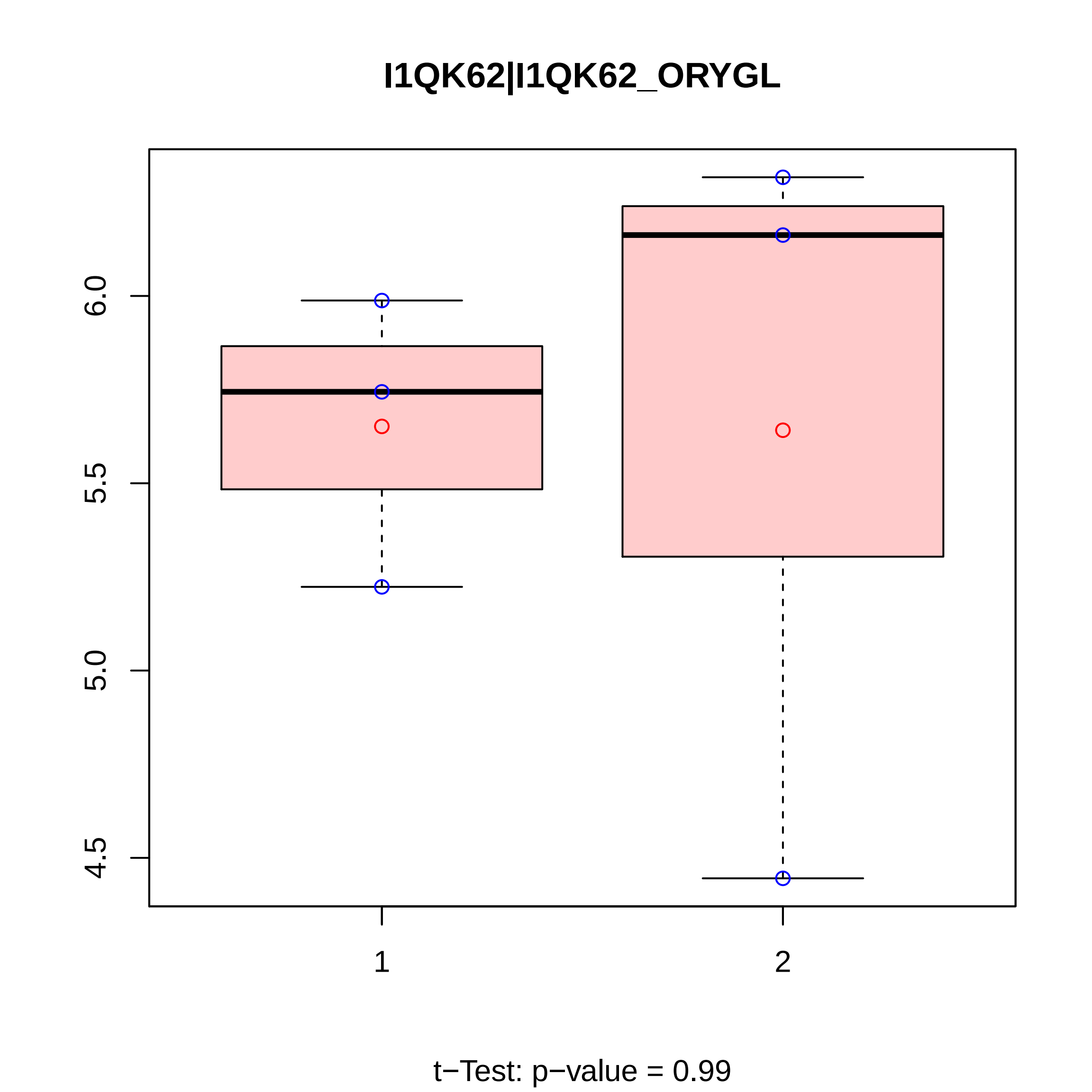
I1QK62|I1QK62_ORYGL
6.0
5.5
5.0
4.5
1
2
t−Test: p−value = 0.99

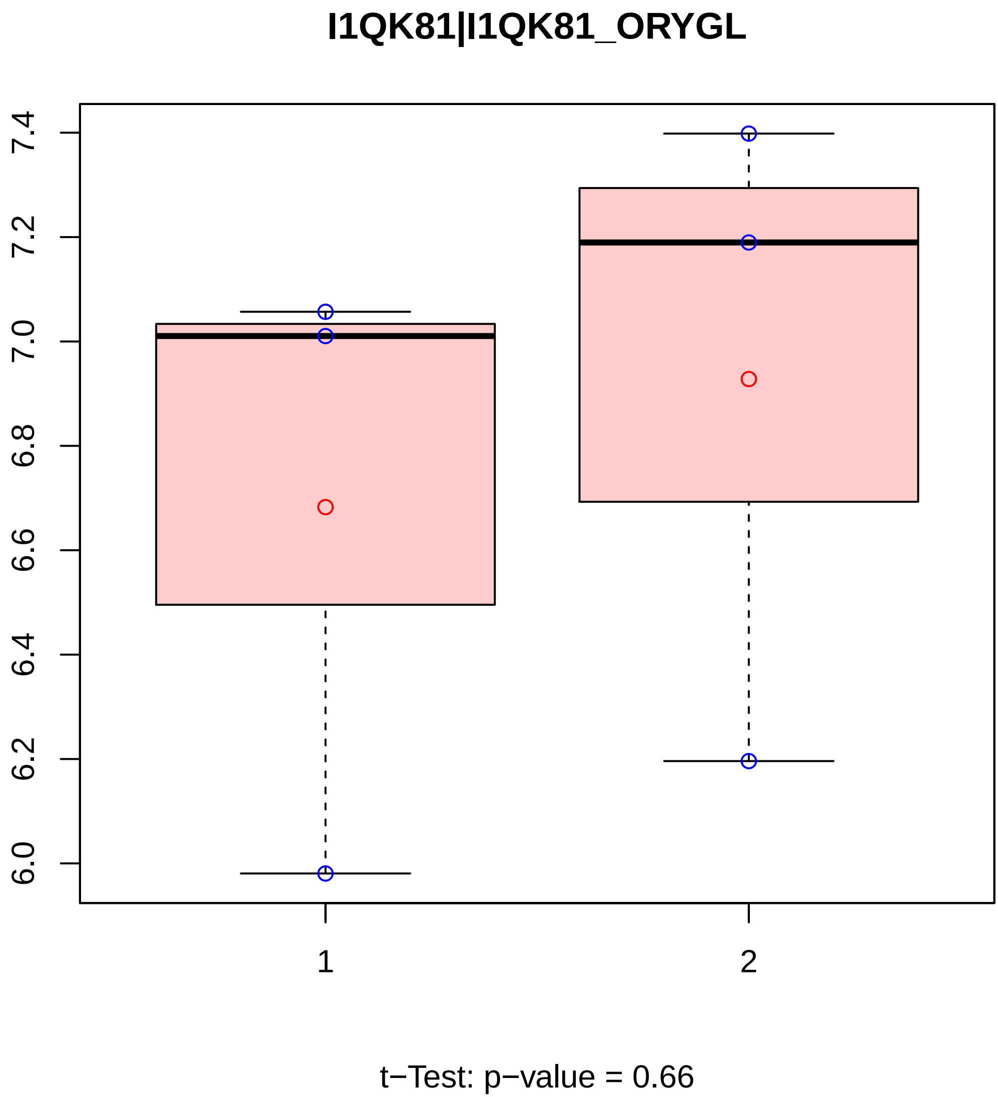
I1QK81|I1QK81_ORYGL
7.4
7.2
7.0
6.8
6.6
6.4
6.2
6.0
1
2
t−Test: p−value = 0.66

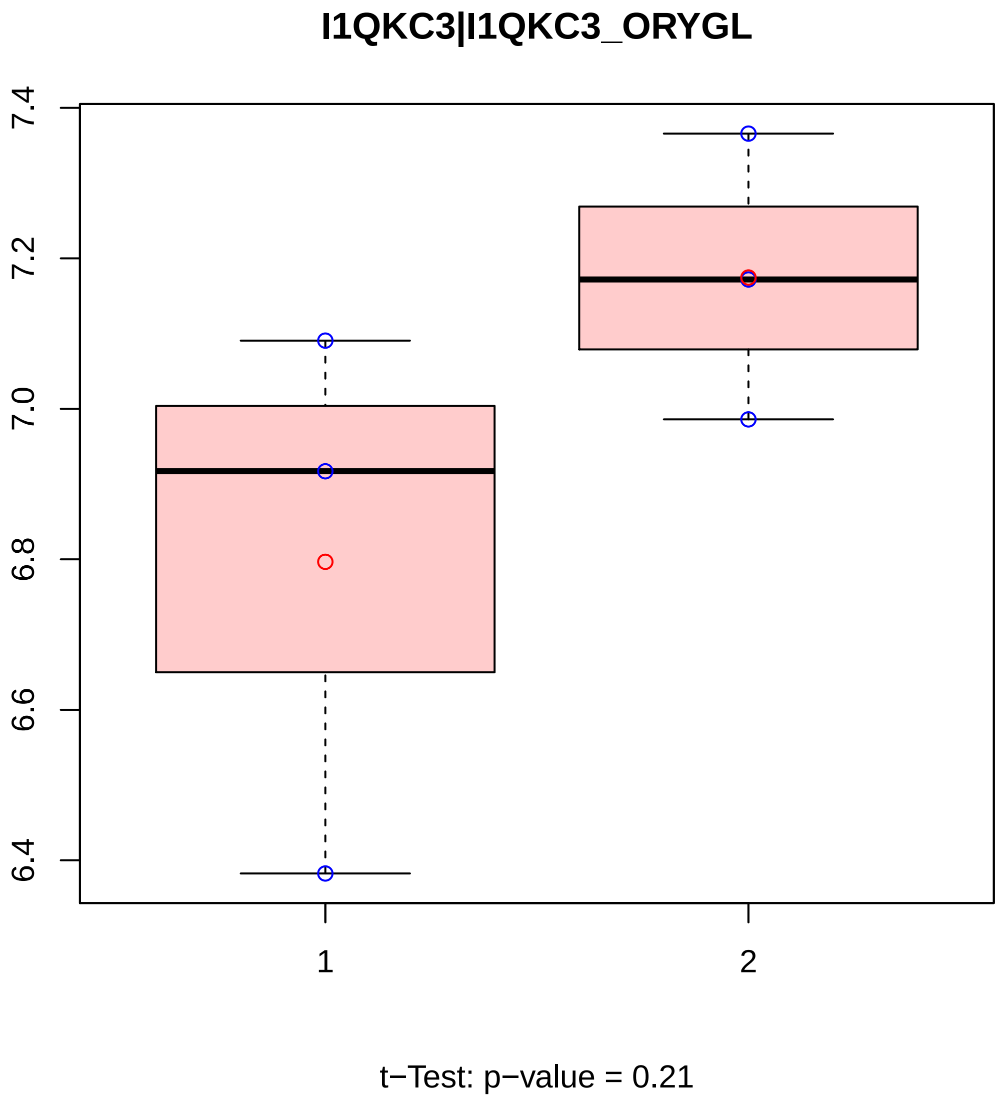
I1QKC3|I1QKC3_ORYGL
7.4
7.2
7.0
6.8
6.6
6.4
1
2
t−Test: p−value = 0.21

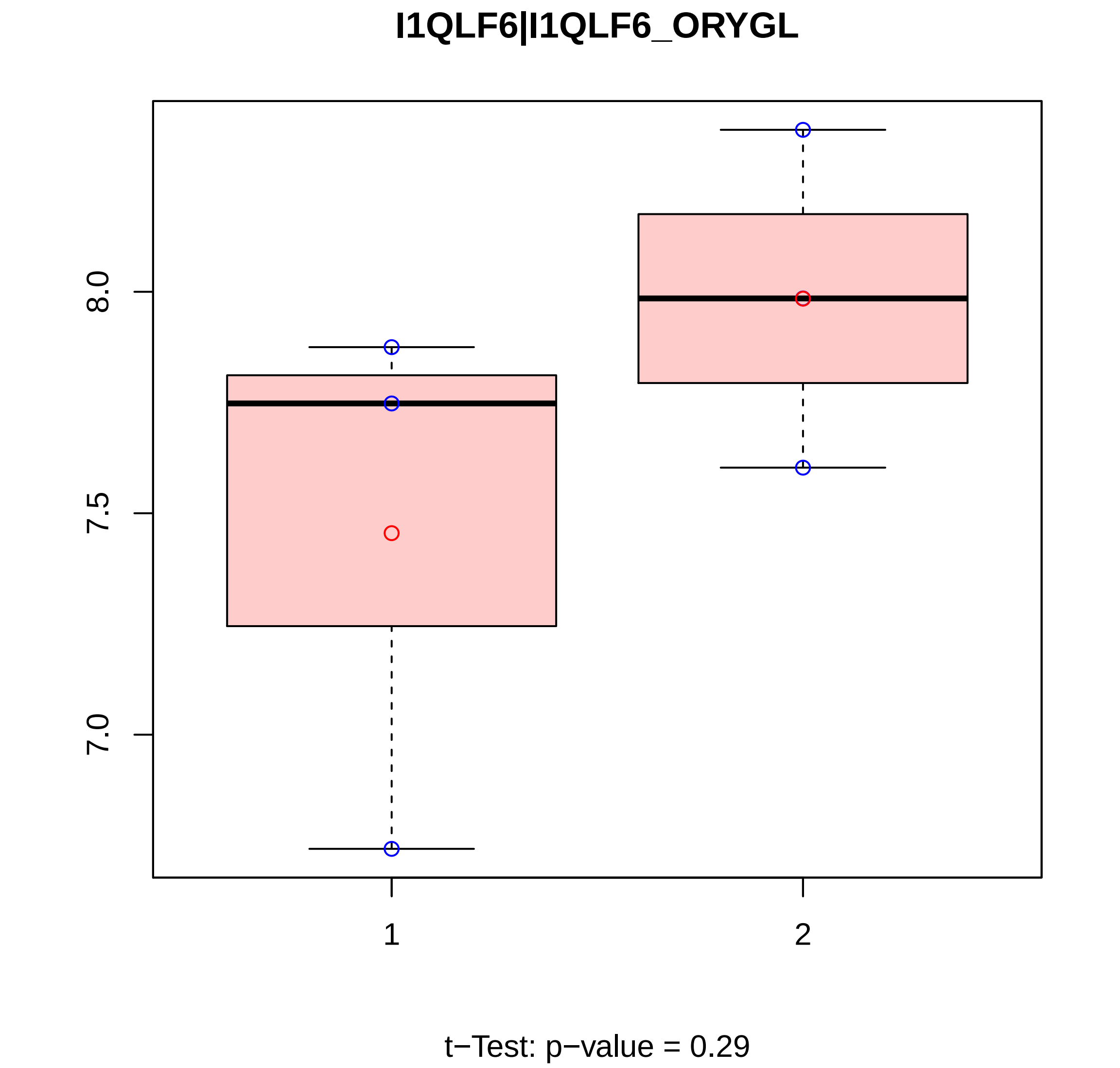
I1QLF6|I1QLF6_ORYGL
8.0
7.5
7.0
1
2
t−Test: p−value = 0.29

I1QLJ6|I1QLJ6_ORYGL

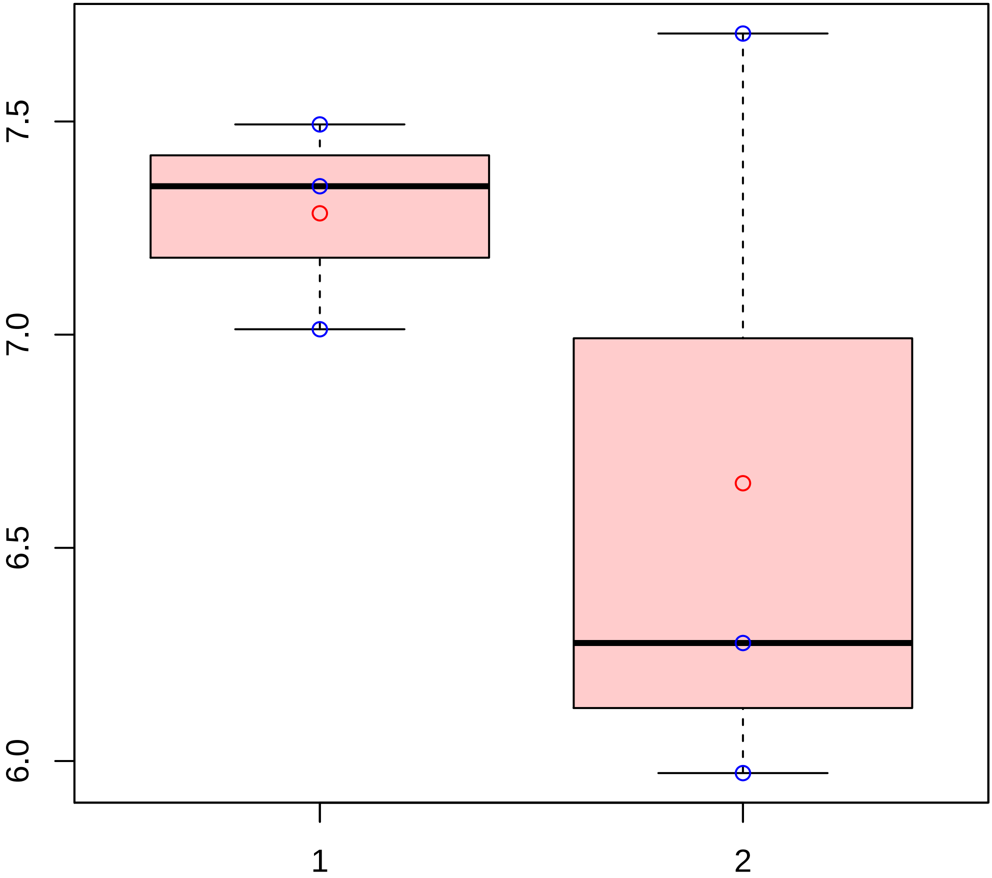

t−Test: p−value = 0.36

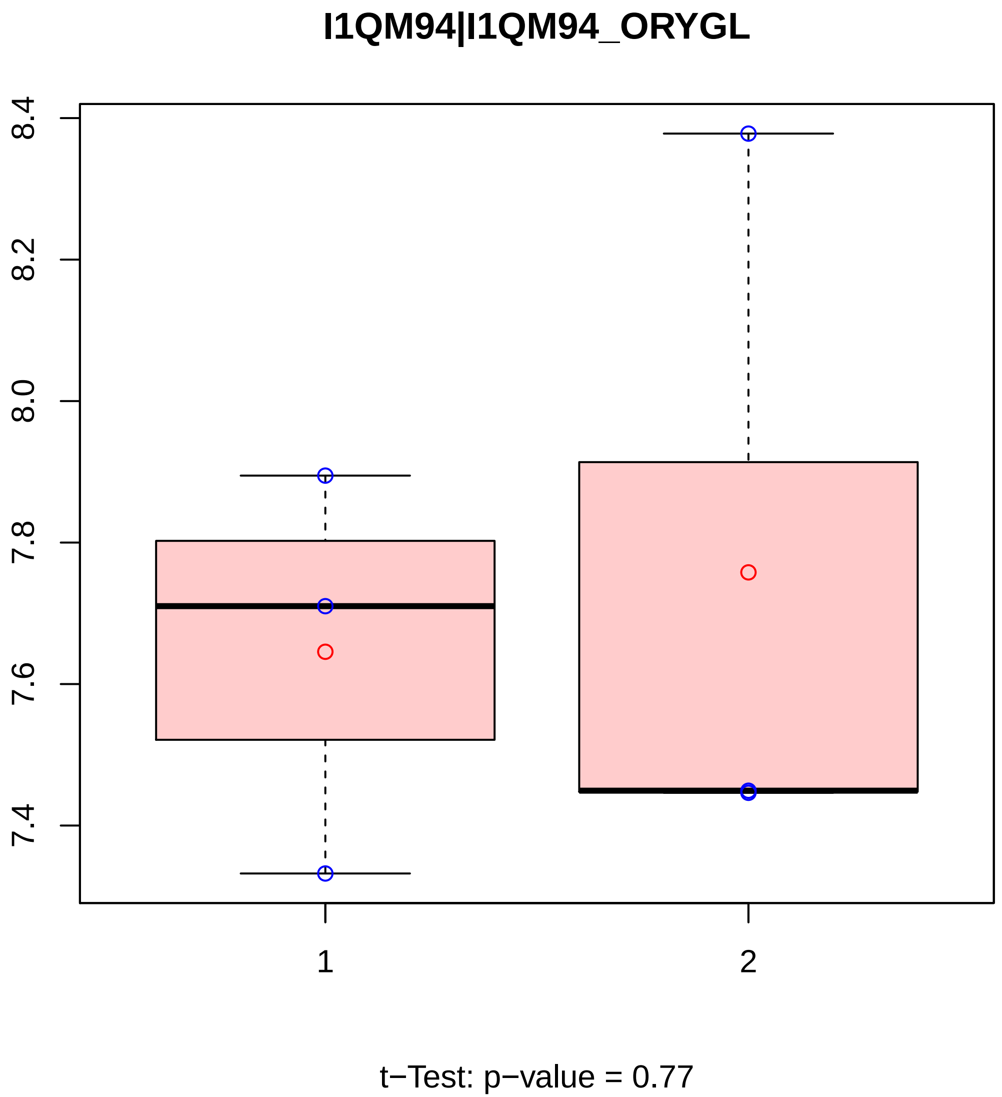

I1QM94|I1QM94_ORYGL
8.4
8.2
8.0
7.8
7.6
7.4
1
2
t−Test: p−value = 0.77

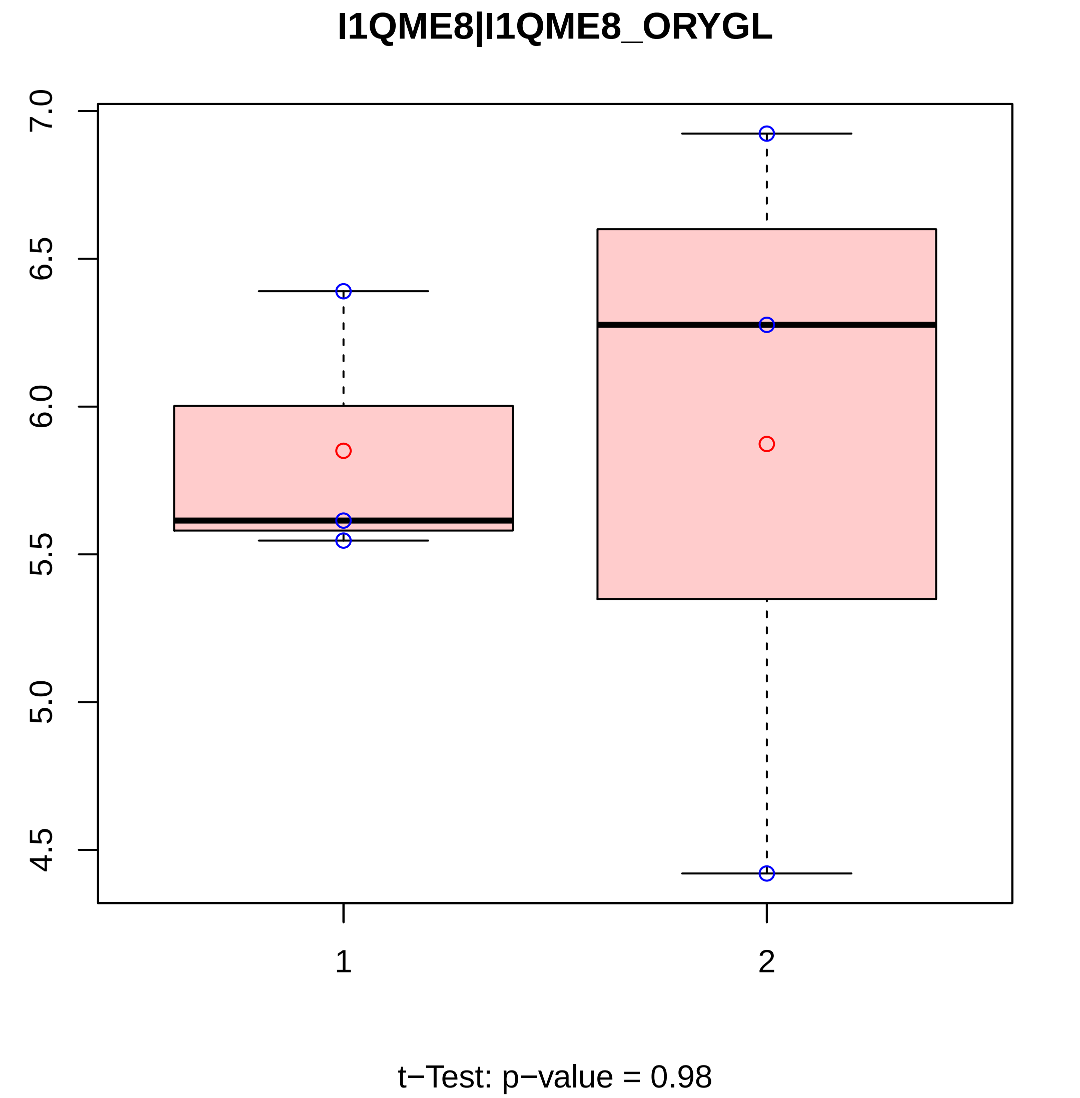
I1QME8|I1QME8_ORYGL
7.0
6.5
6.0
5.5
5.0
4.5
1
2
t−Test: p−value = 0.98

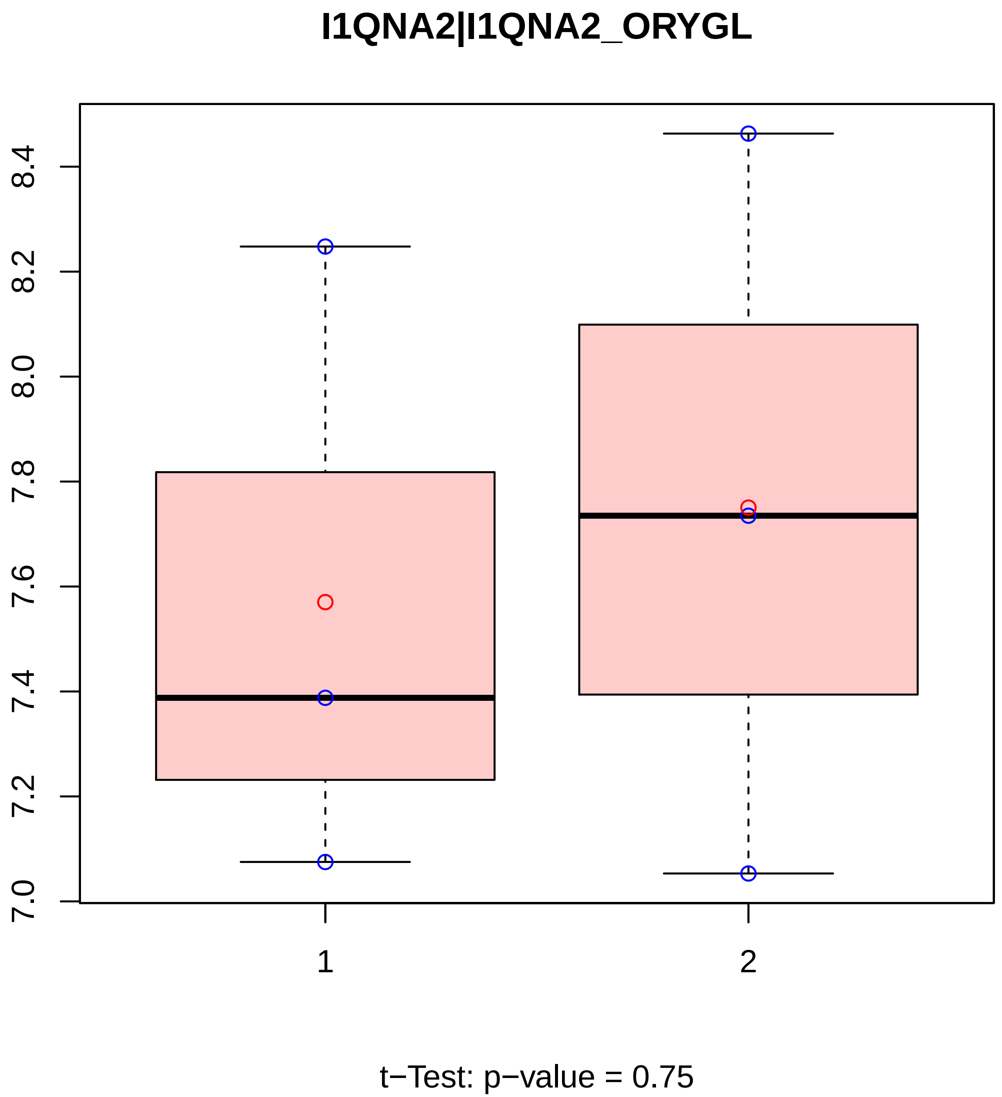
I1QNA2|I1QNA2_ORYGL
8.4
8.2
8.0
7.8
7.6
7.4
7.2
7.0
1
2
t−Test: p−value = 0.75

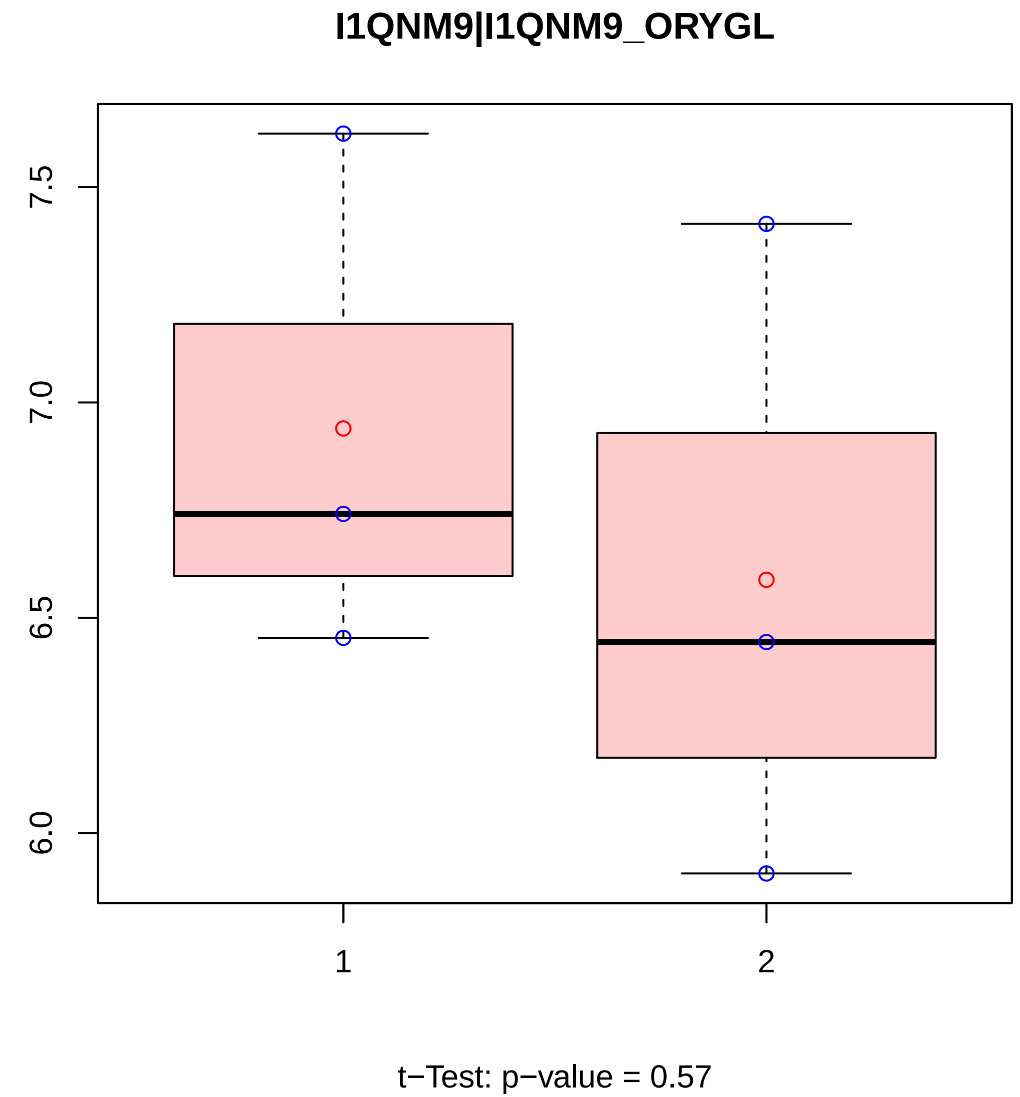
I1QNM9|I1QNM9_ORYGL
7.5
7.0
6.5
6.0
1
2
t−Test: p−value = 0.57

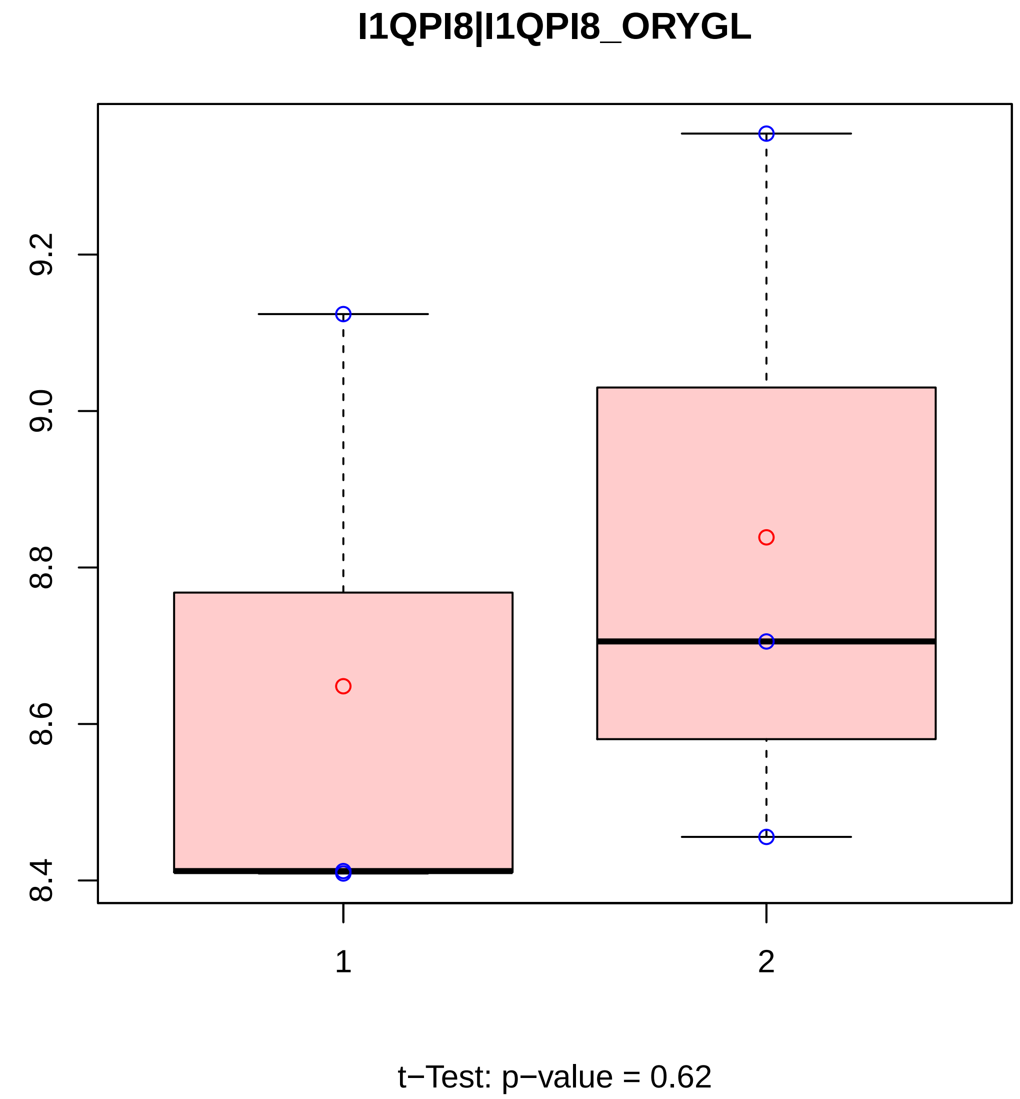
I1QPI8|I1QPI8_ORYGL
8.4
8.6
8.8
9.0
9.2
1
2
t−Test: p−value = 0.62

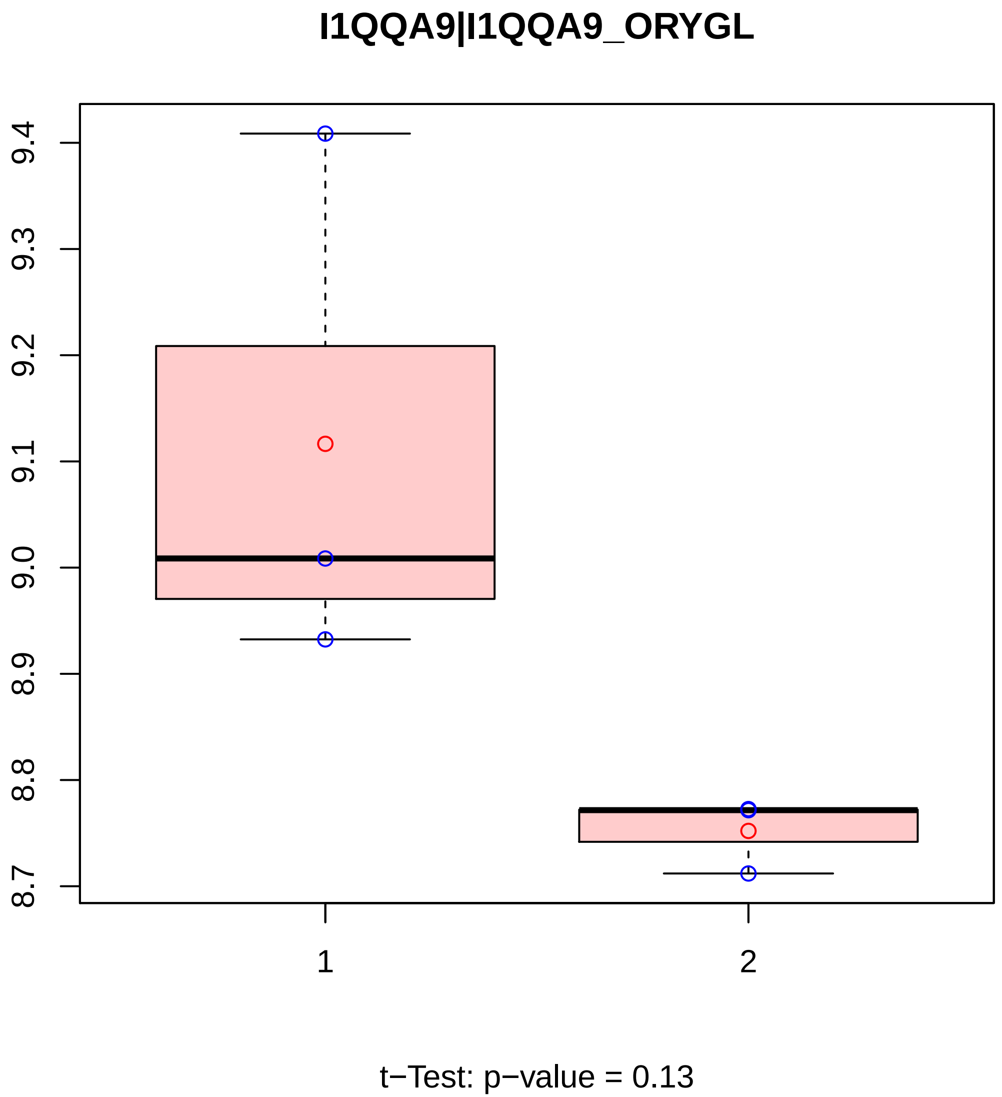

I1QQA9|I1QQA9_ORYGL
9.4
9.3
9.2
9.1
9.0
8.9
8.8
8.7
1
2
t−Test: p−value = 0.13

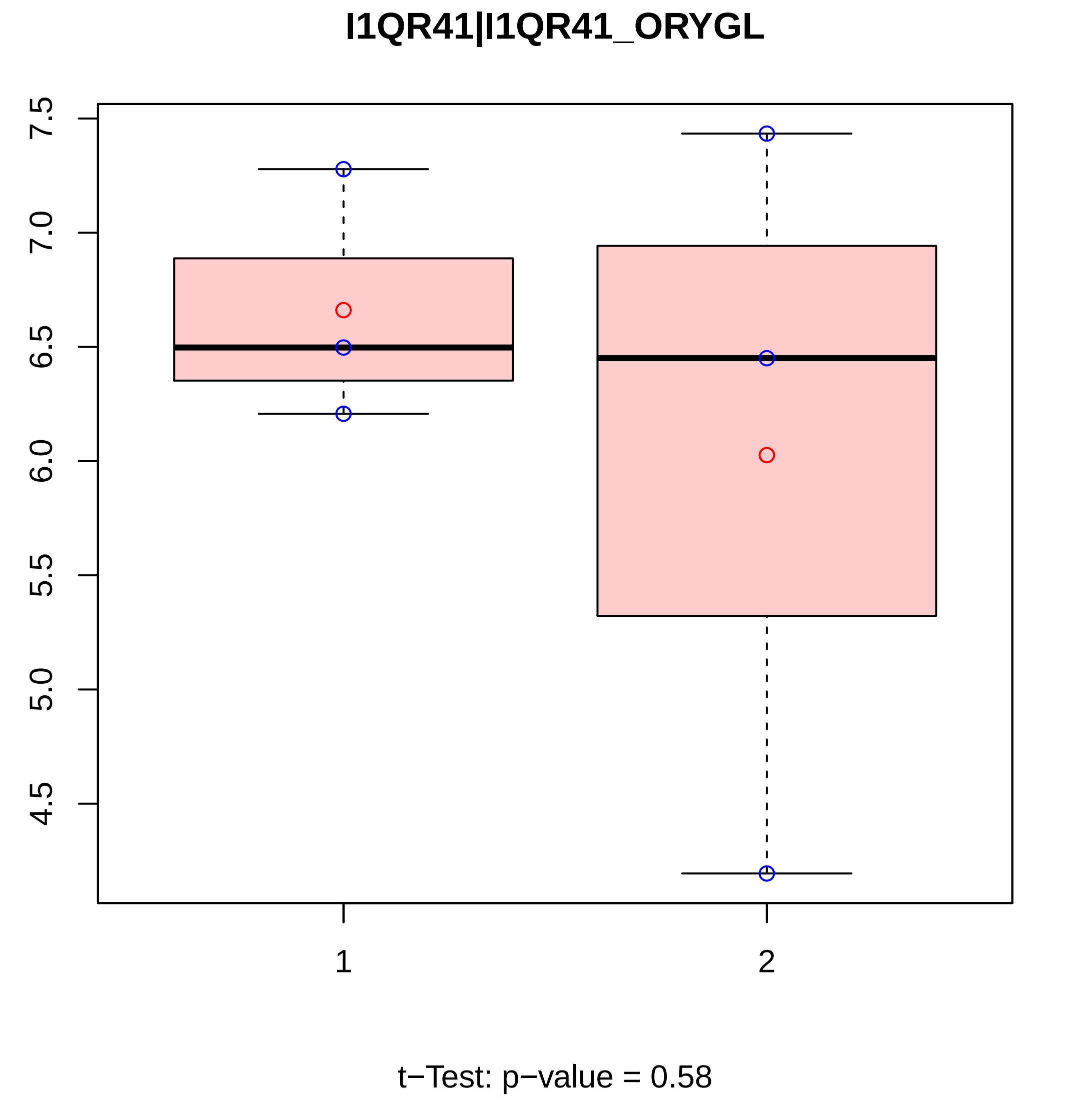
I1QR41|I1QR41_ORYGL
7.5
7.0
6.5
6.0
5.5
5.0
4.5
1
2
t−Test: p−value = 0.58

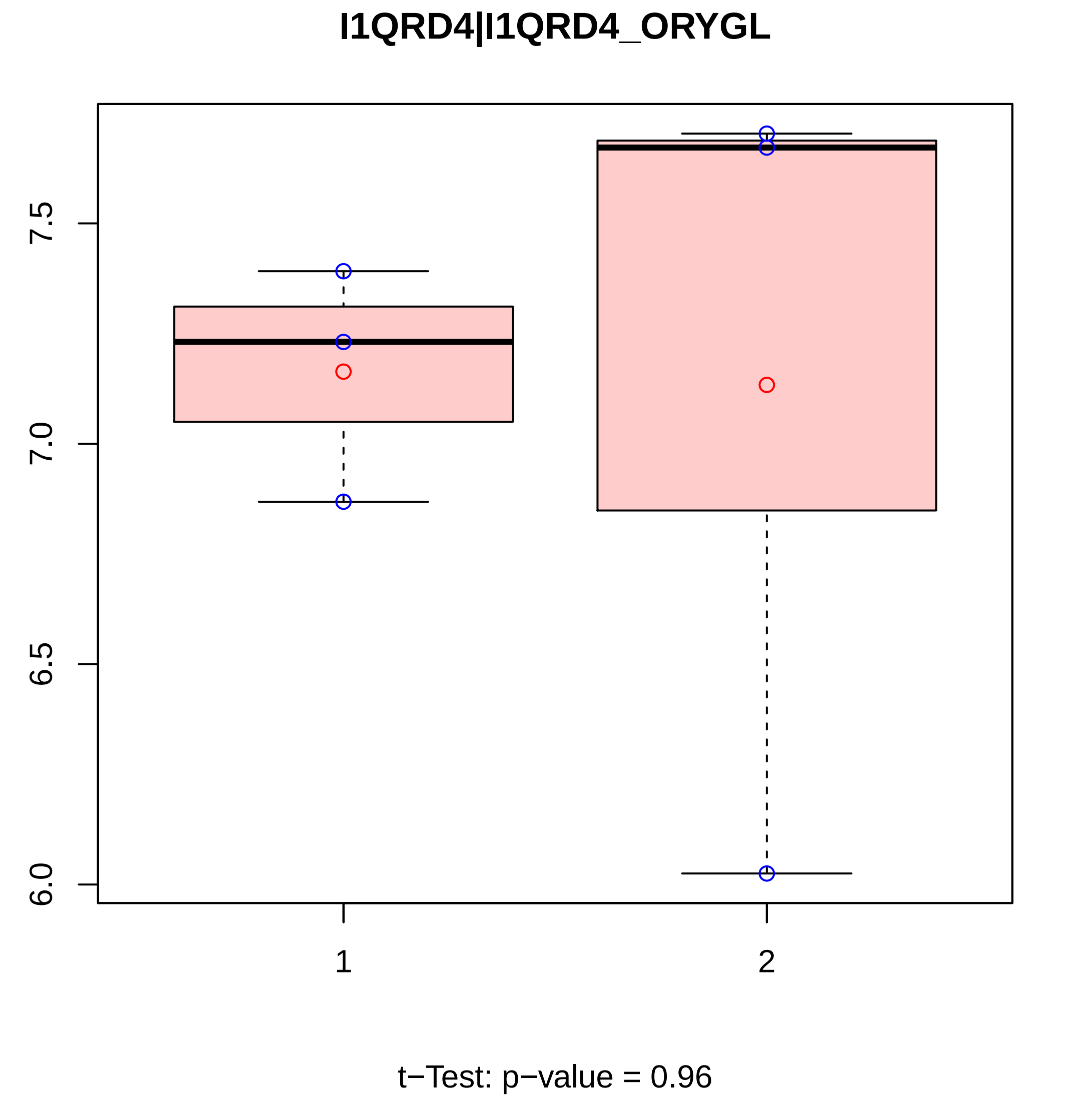
I1QRD4|I1QRD4_ORYGL
7.5
7.0
6.5
6.0
1
2
t−Test: p−value = 0.96

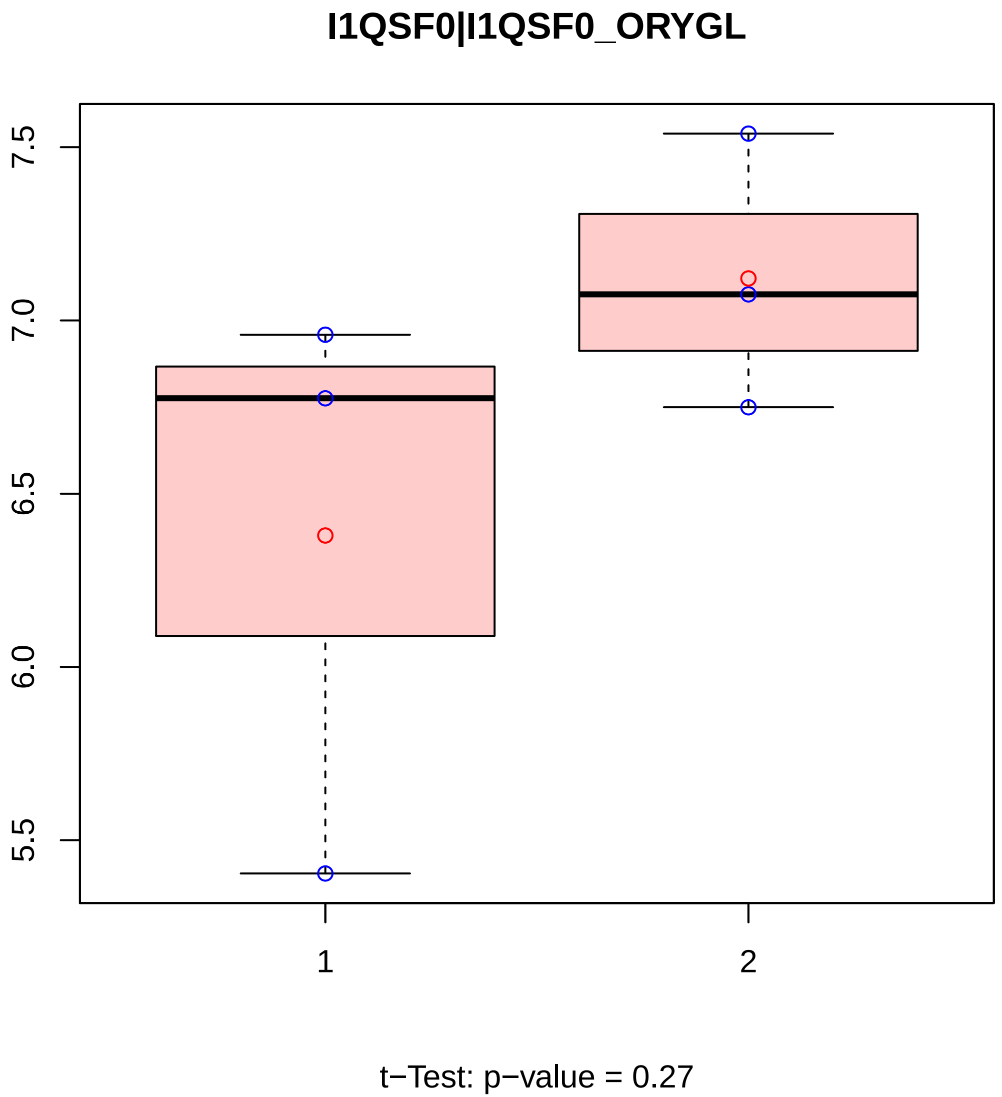

I1QSF0|I1QSF0_ORYGL
7.5
7.0
6.5
6.0
5.5
1
2
t−Test: p−value = 0.27

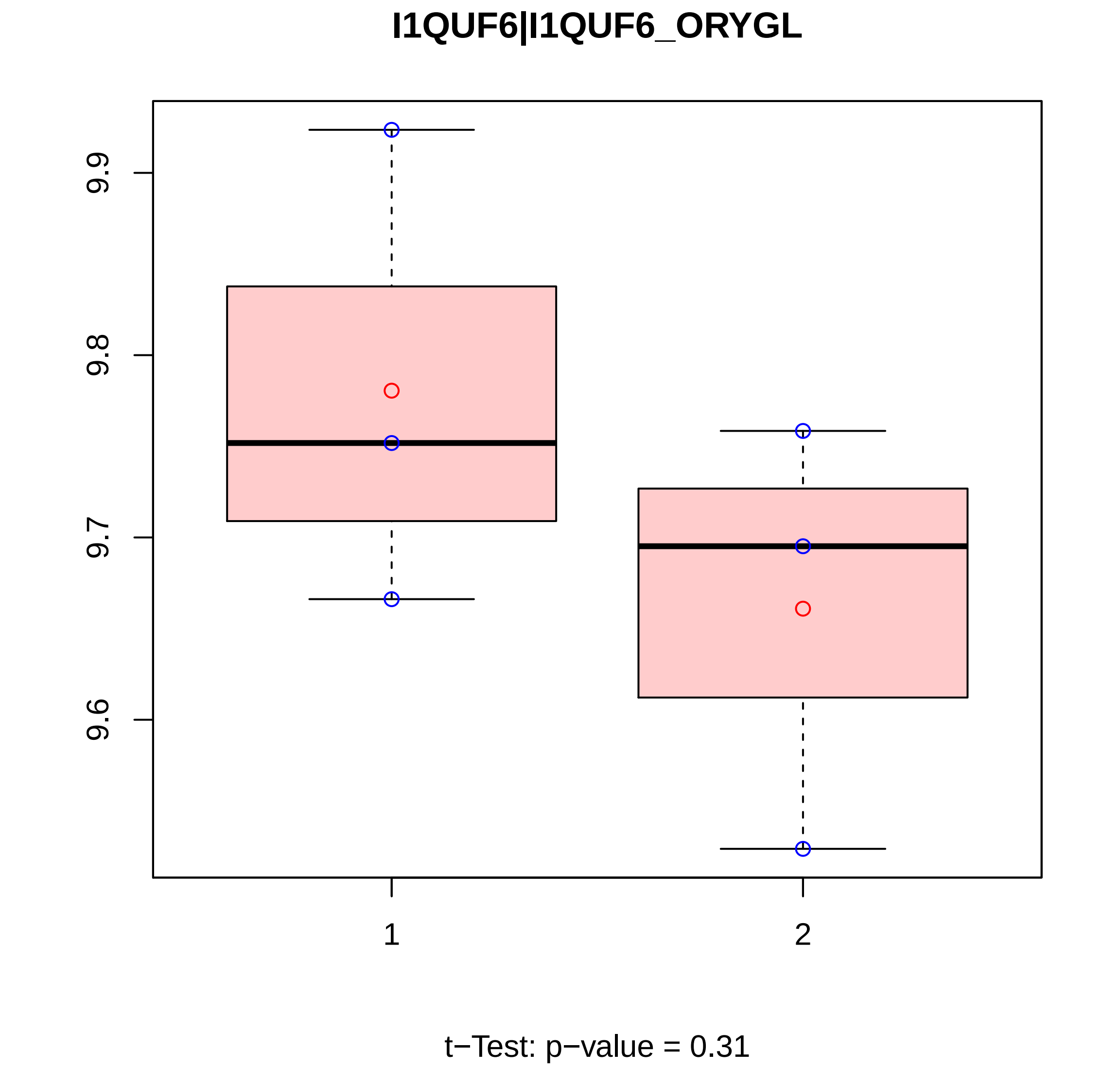
I1QUF6|I1QUF6_ORYGL
9.9
9.8
9.7
9.6
1
2
t−Test: p−value = 0.31

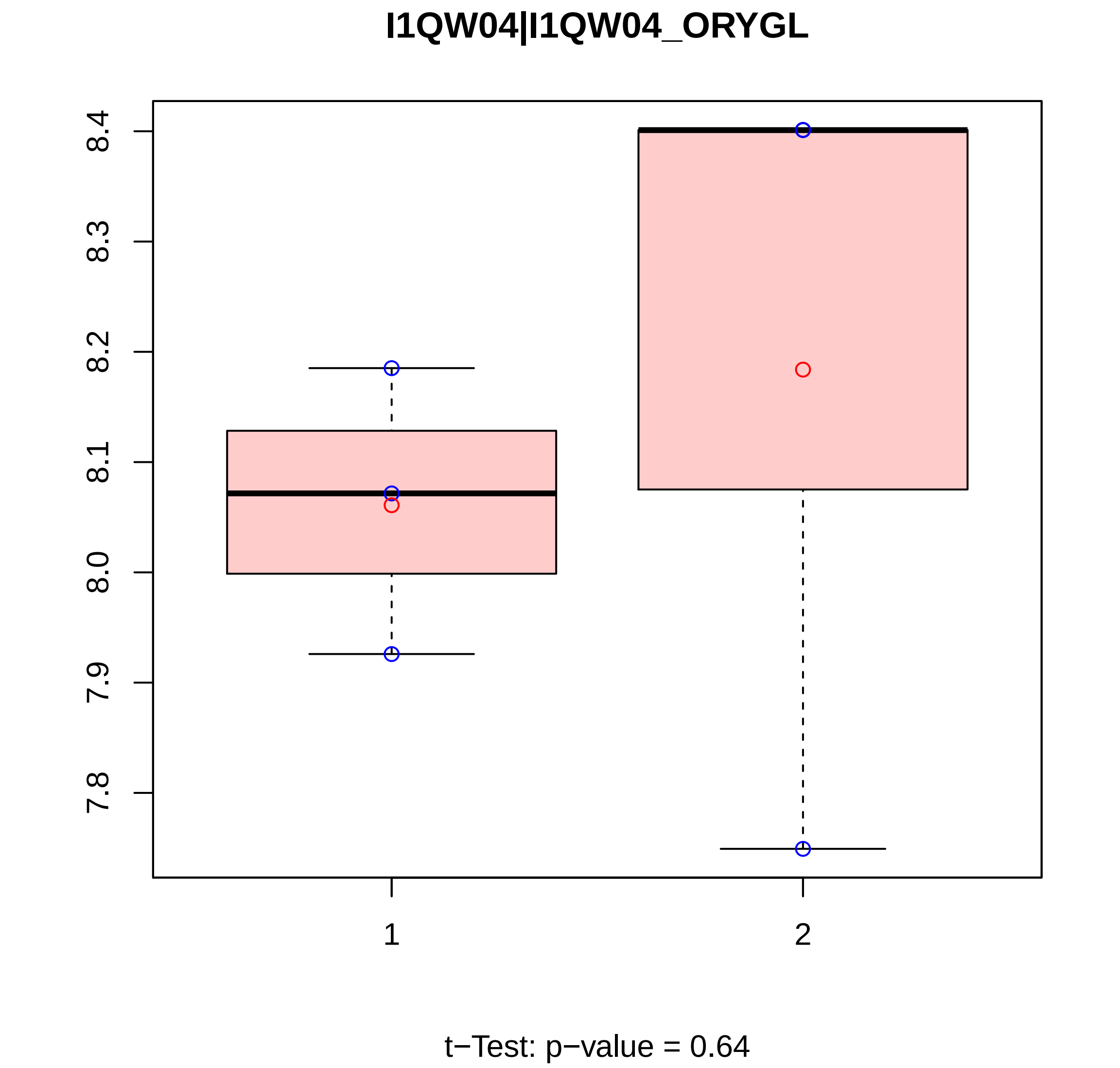
I1QW04|I1QW04_ORYGL
8.4
8.3
8.2
8.1
8.0
7.9
7.8
1
2
t−Test: p−value = 0.64

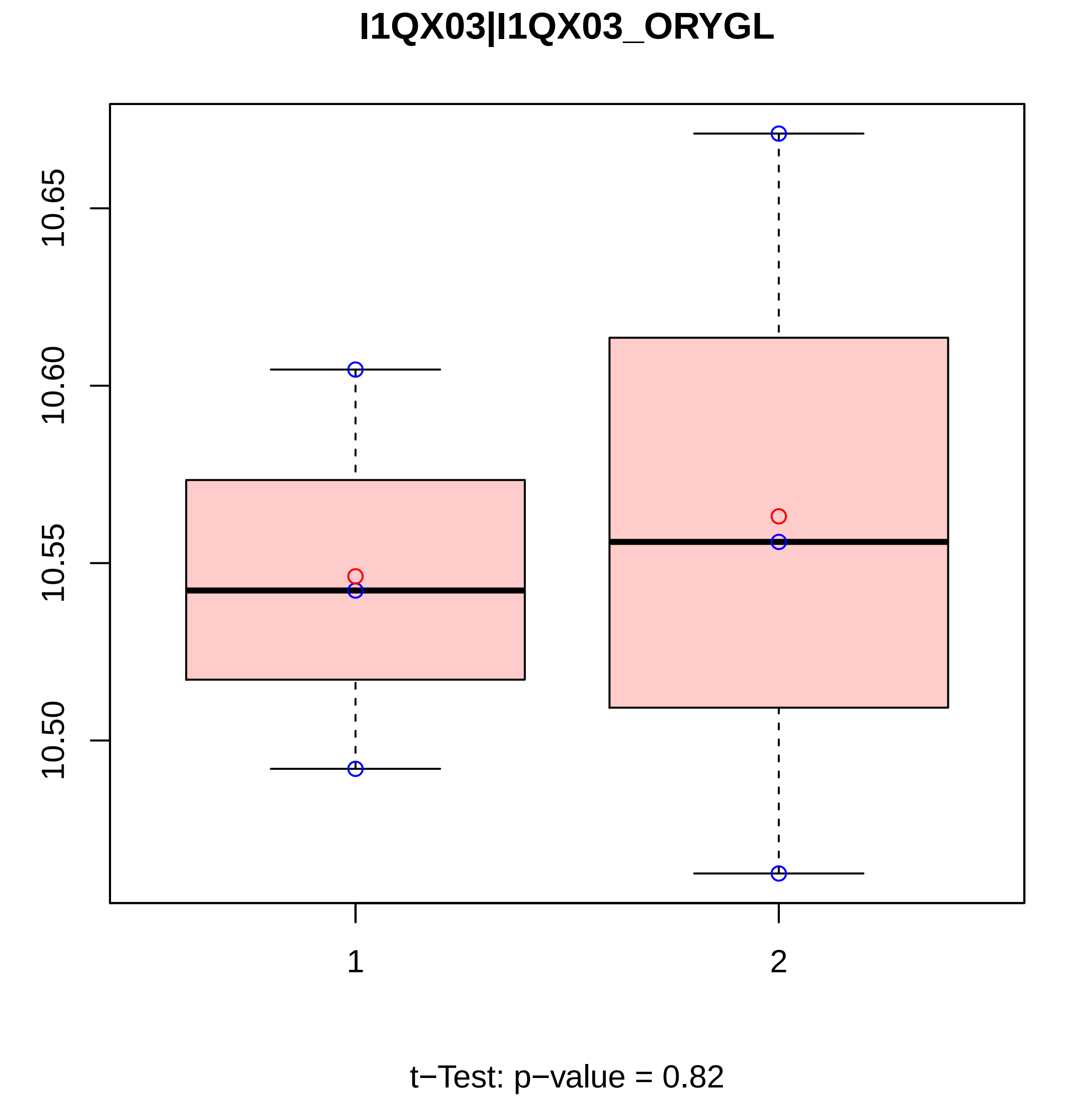
I1QX03|I1QX03_ORYGL
10.50
10.55
10.60
10.65
1
2
t−Test: p−value = 0.82

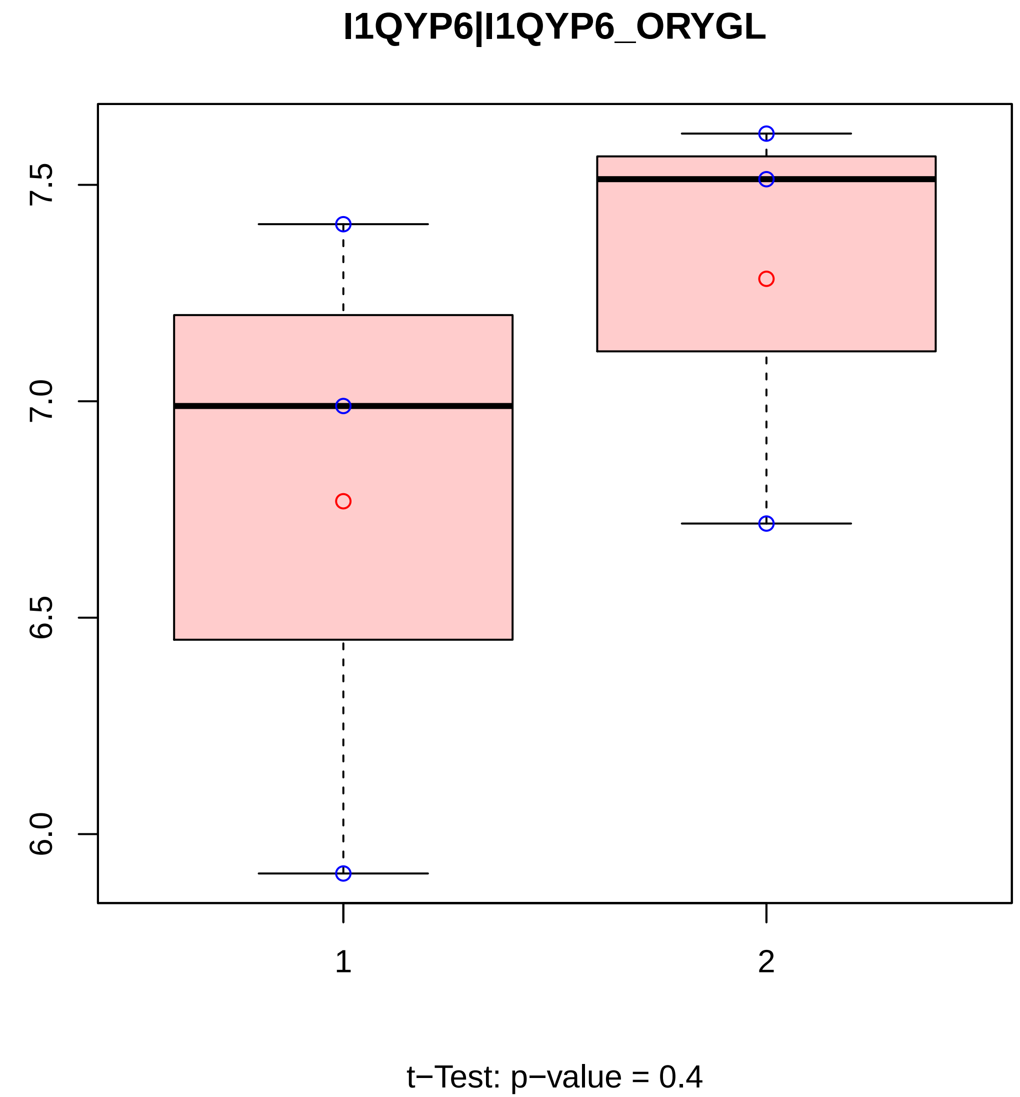
I1QYP6|I1QYP6_ORYGL
7.5
7.0
6.5
6.0
1
2
t−Test: p−value = 0.4

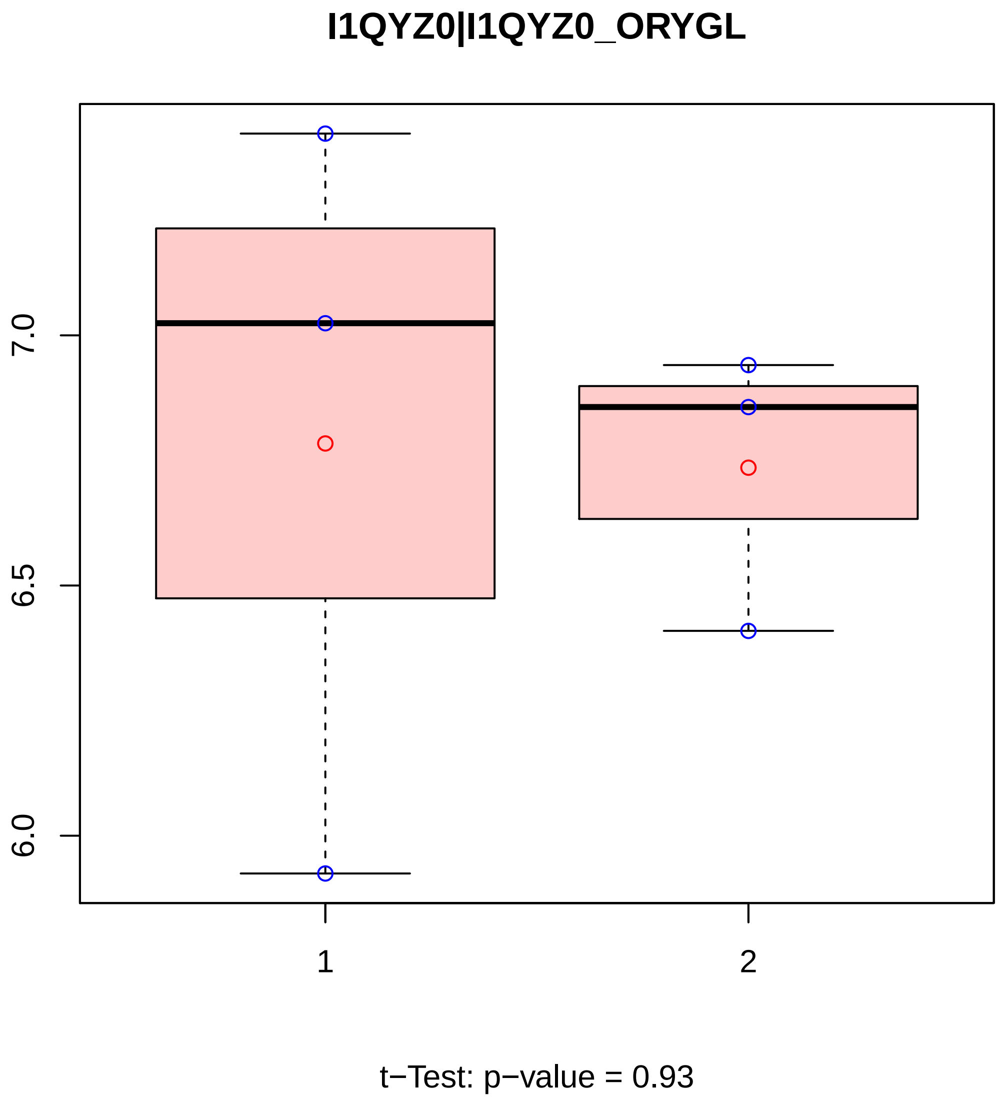
I1QYZ0|I1QYZ0_ORYGL
7.0
6.5
6.0
1
2
t−Test: p−value = 0.93

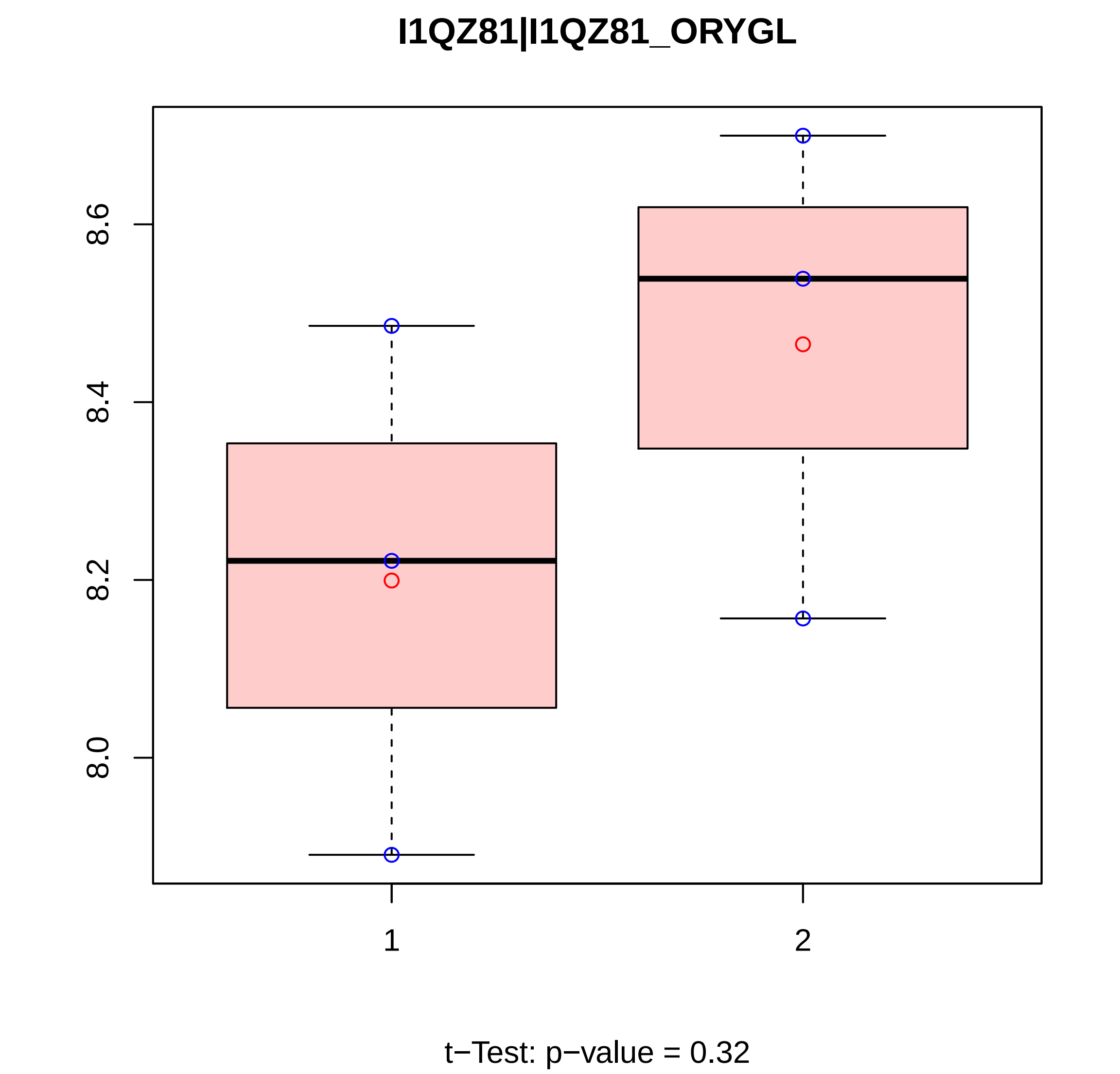
I1QZ81|I1QZ81_ORYGL
8.0
8.2
8.4
8.6
1
2
t−Test: p−value = 0.32

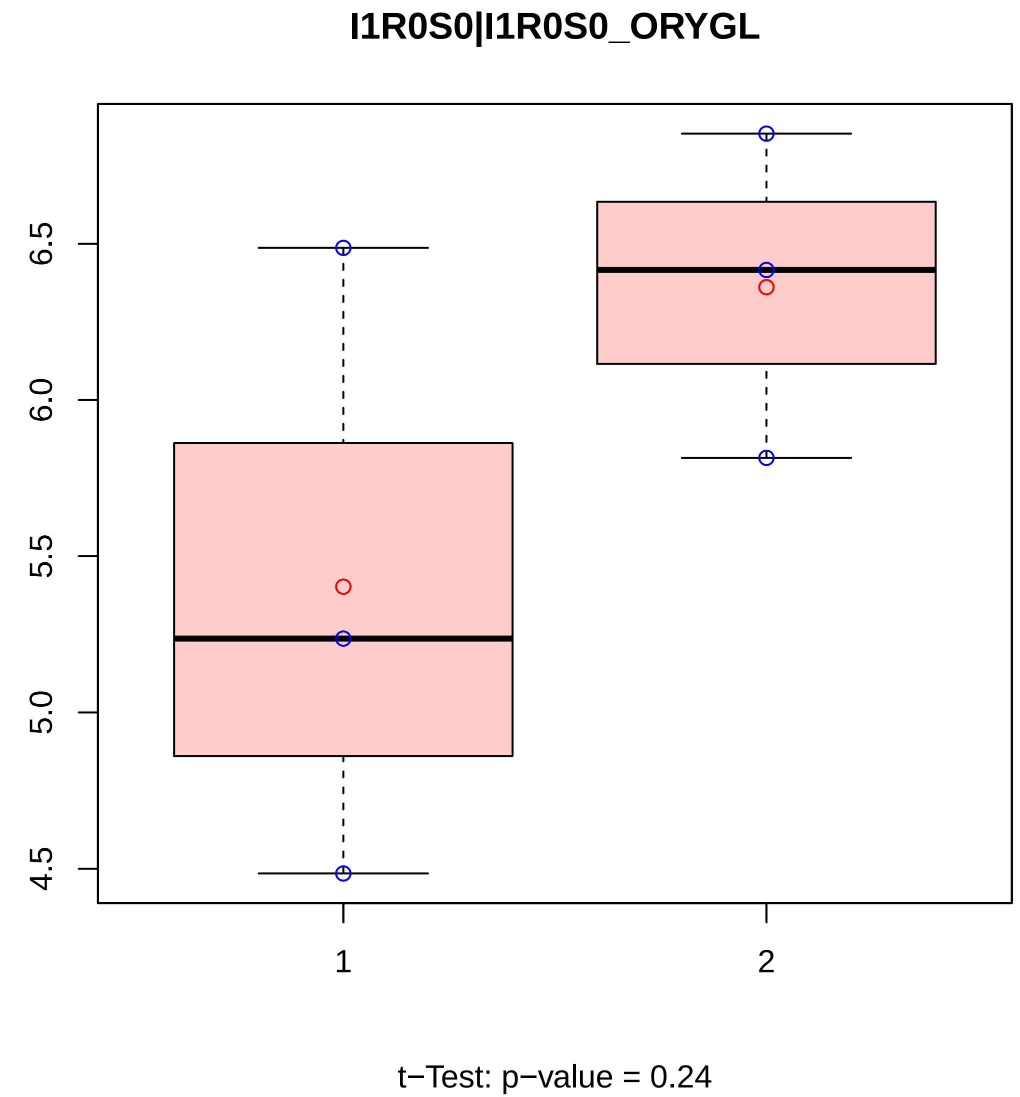
I1R0S0|I1R0S0_ORYGL
6.5
6.0
5.5
5.0
4.5
1
2
t−Test: p−value = 0.24

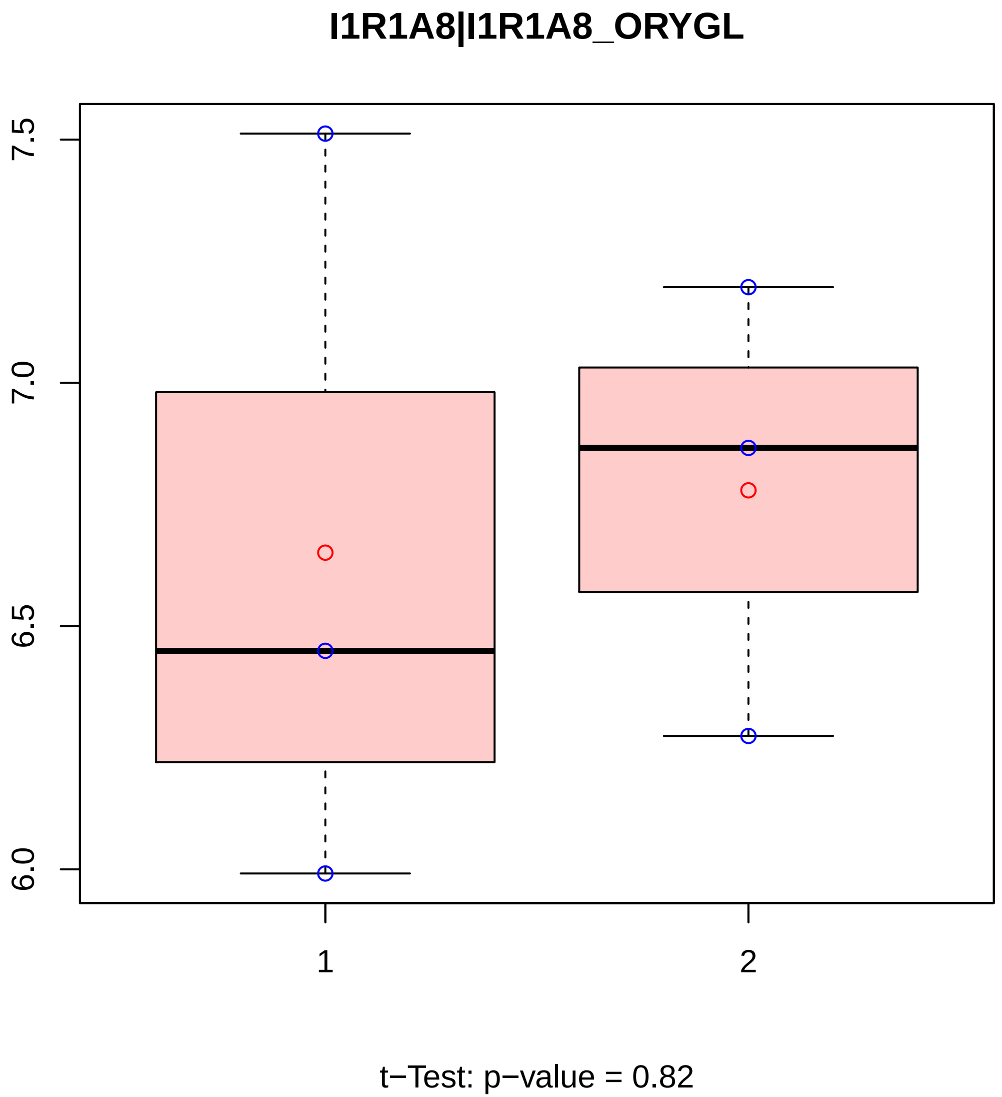

I1R1A8|I1R1A8_ORYGL
7.5
7.0
6.5
6.0
1
2
t−Test: p−value = 0.82

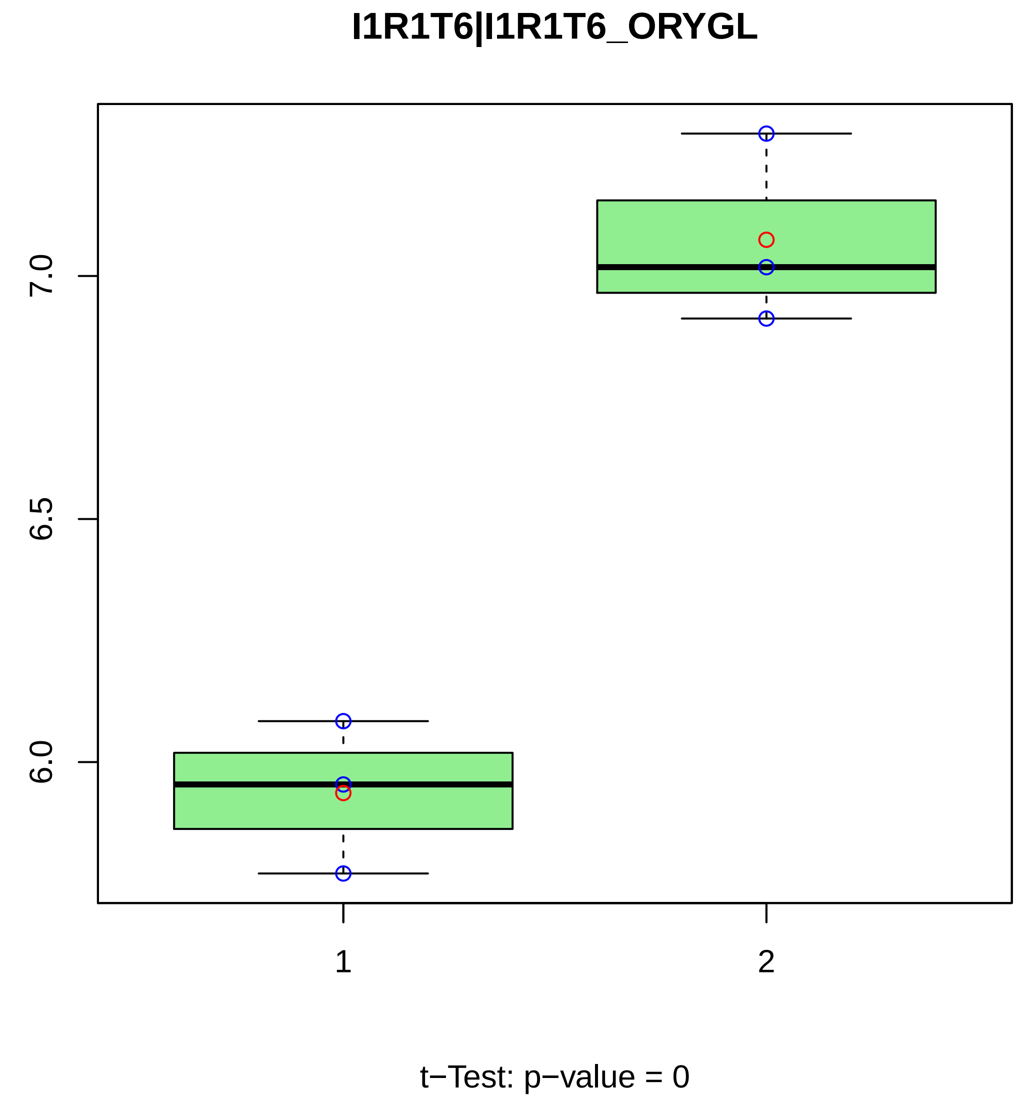

I1R1T6|I1R1T6_ORYGL
7.0
6.5
6.0
1
2
t−Test: p−value = 0

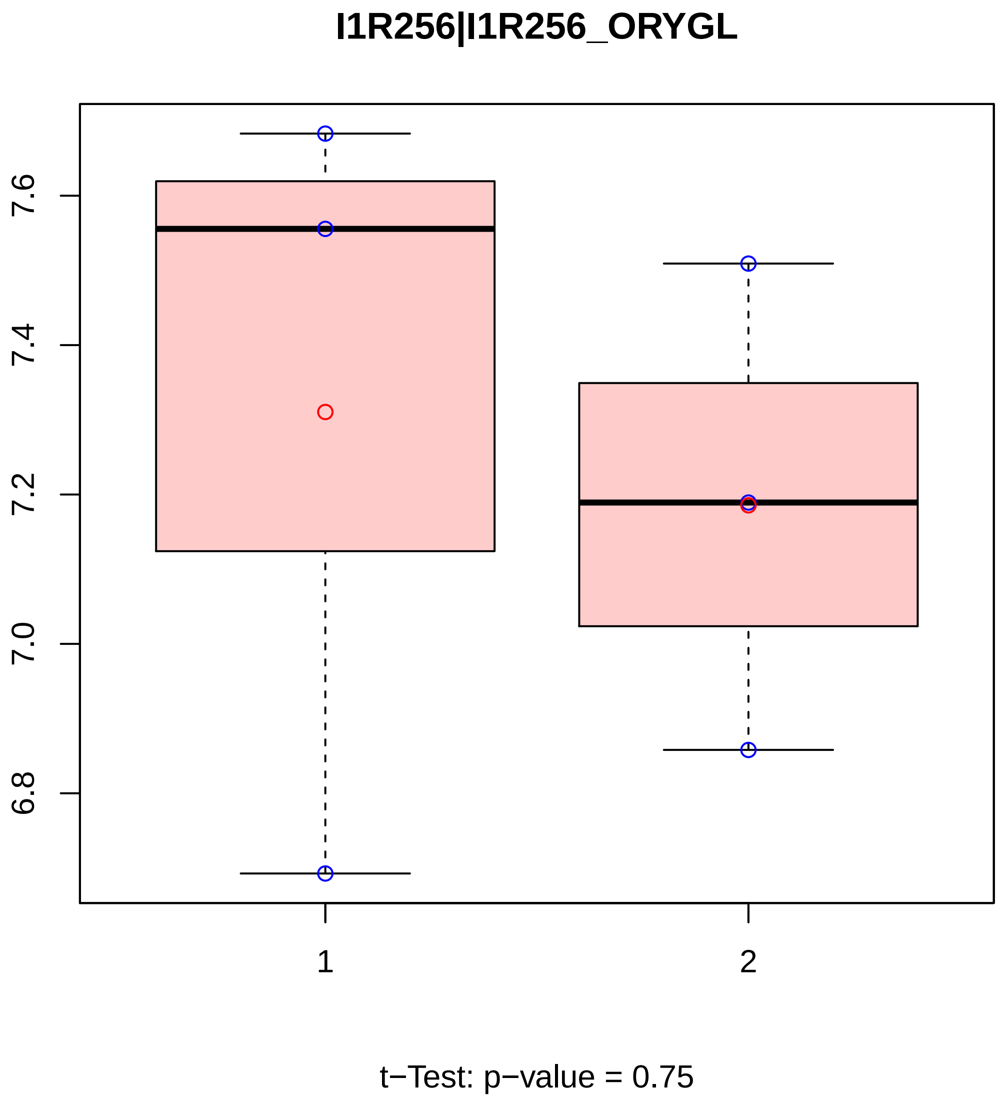
I1R256|I1R256_ORYGL
6.8
7.0
7.2
7.4
7.6
1
2
t−Test: p−value = 0.75

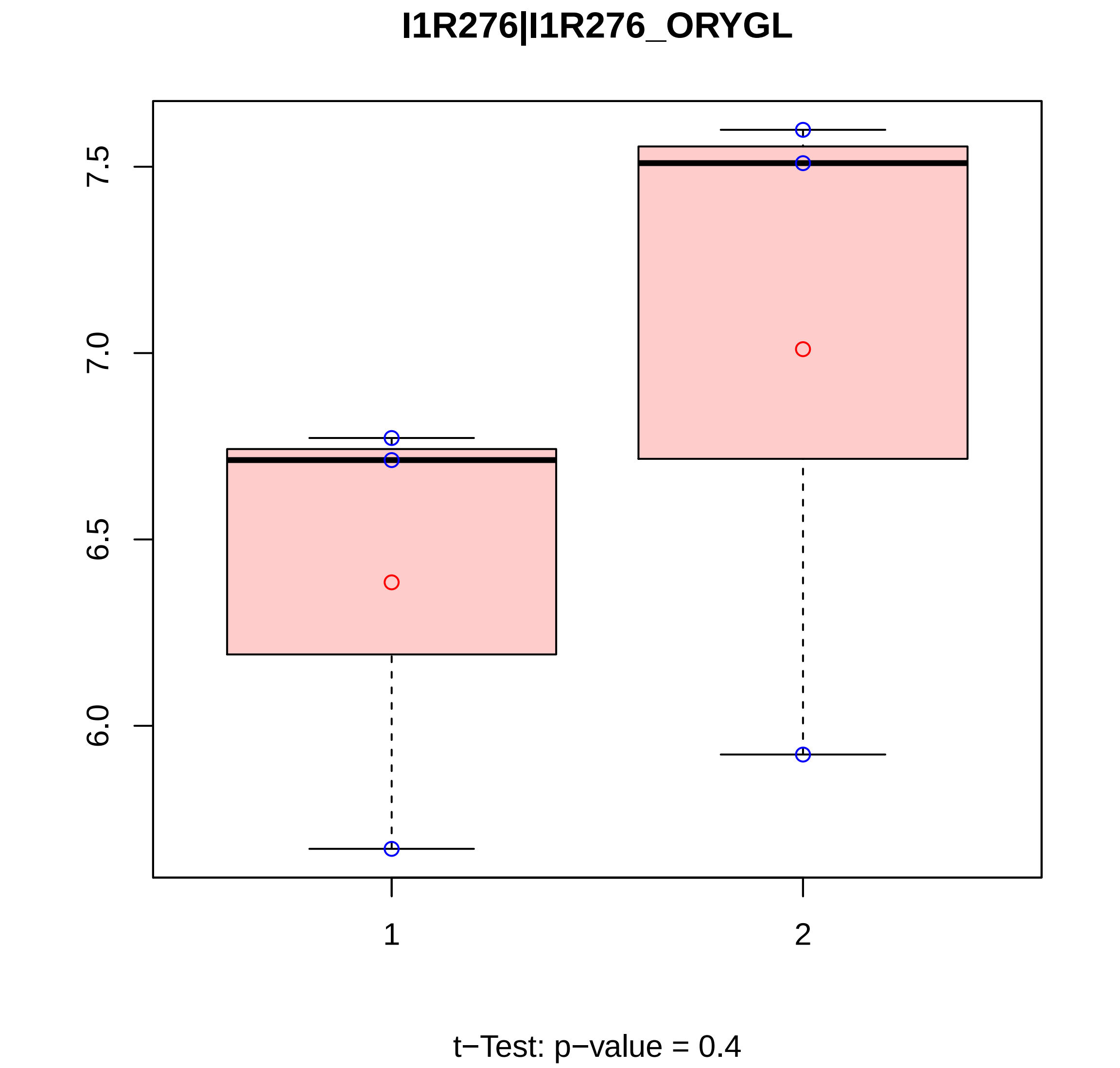
I1R276|I1R276_ORYGL
7.5
7.0
6.5
6.0
1
2
t−Test: p−value = 0.4

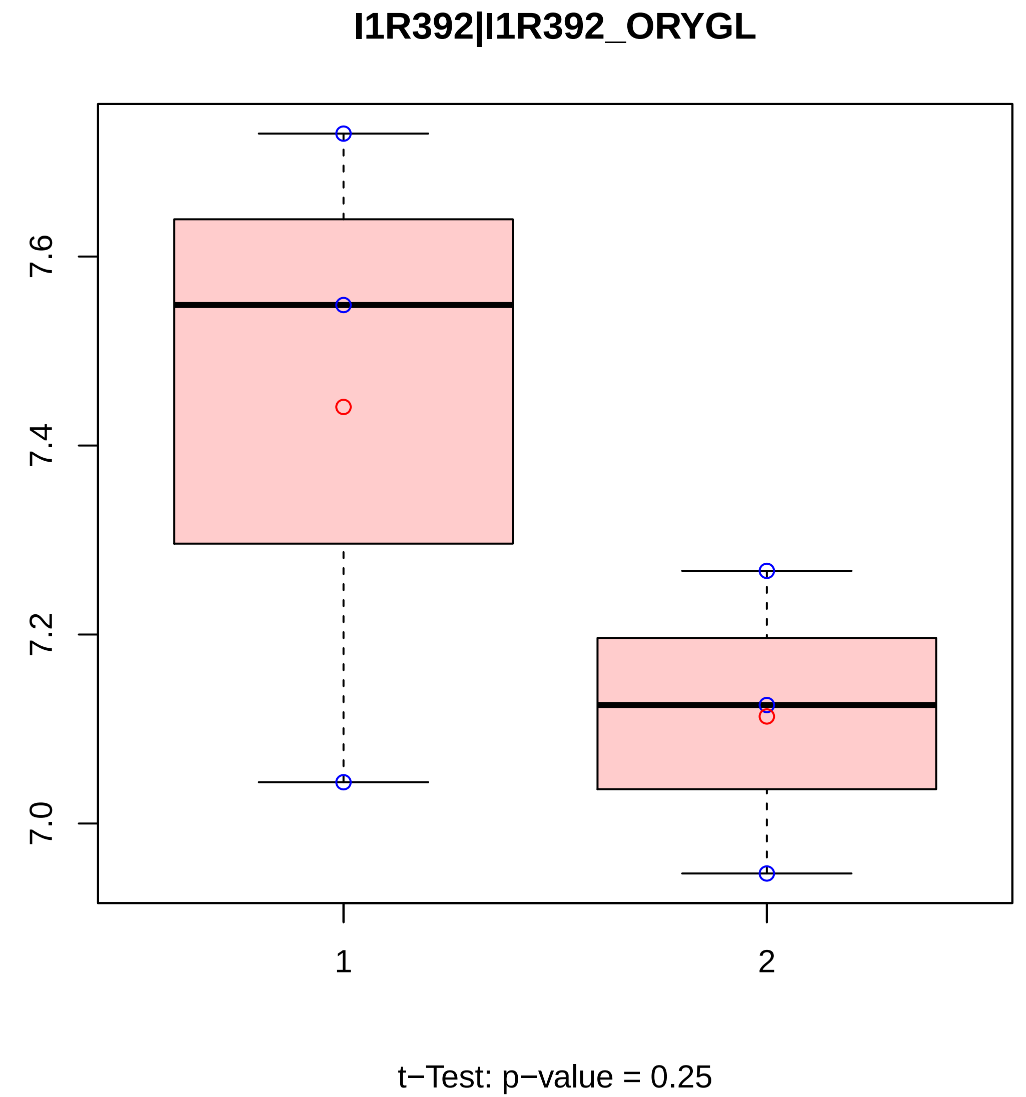
I1R392|I1R392_ORYGL
7.6
7.4
7.2
7.0
1
2
t−Test: p−value = 0.25

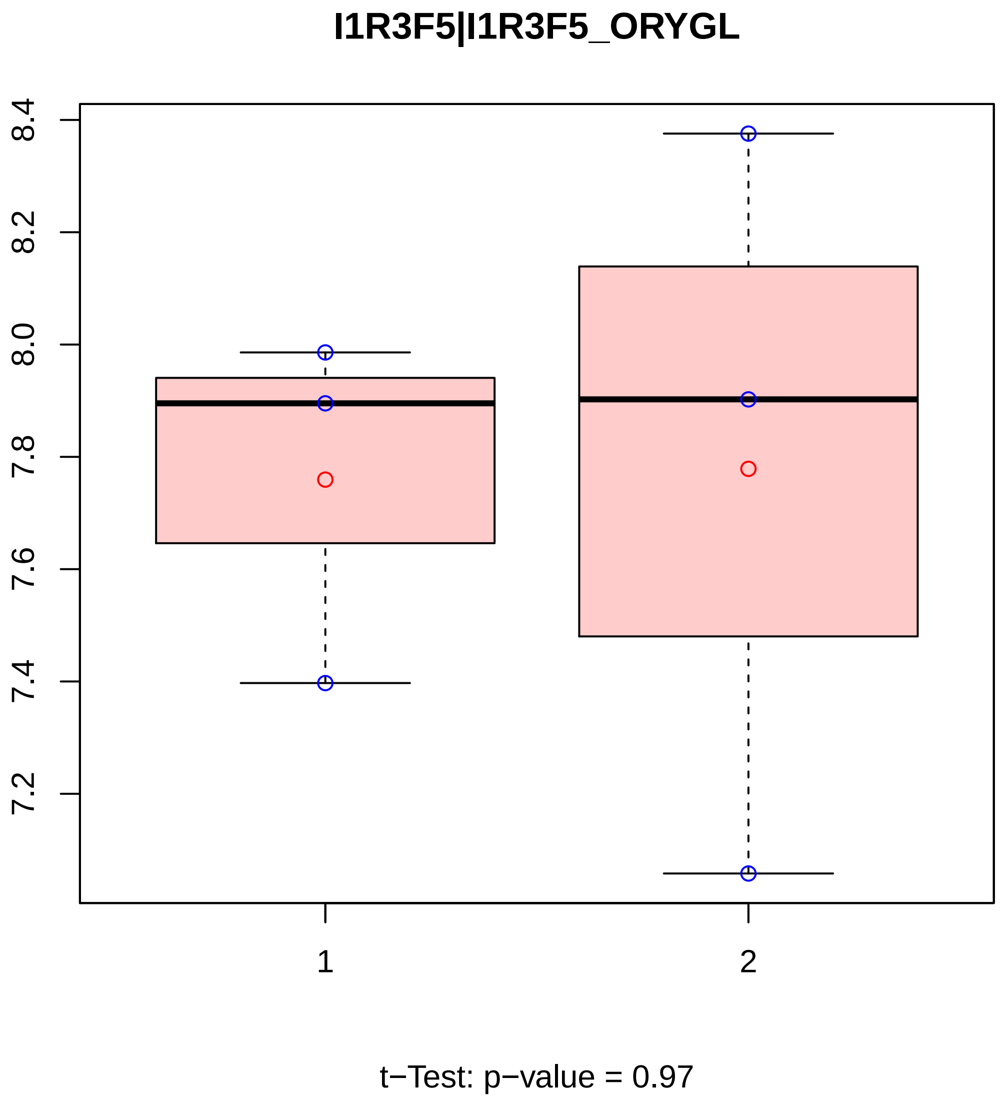
I1R3F5|I1R3F5_ORYGL
8.4
8.2
8.0
7.8
7.6
7.4
7.2
1
2
t−Test: p−value = 0.97

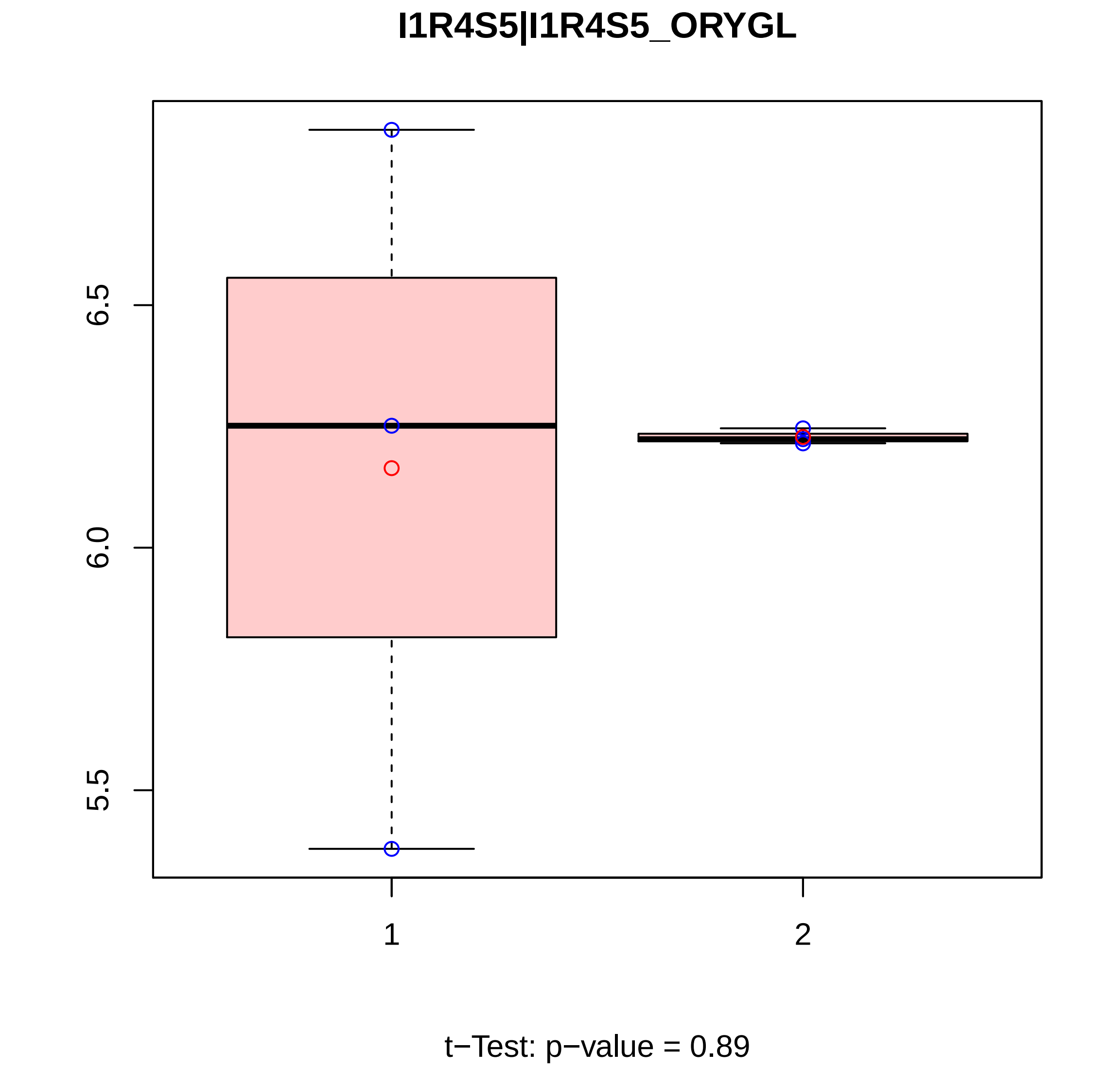
I1R4S5|I1R4S5_ORYGL
6.5
6.0
5.5
1
2
t−Test: p−value = 0.89

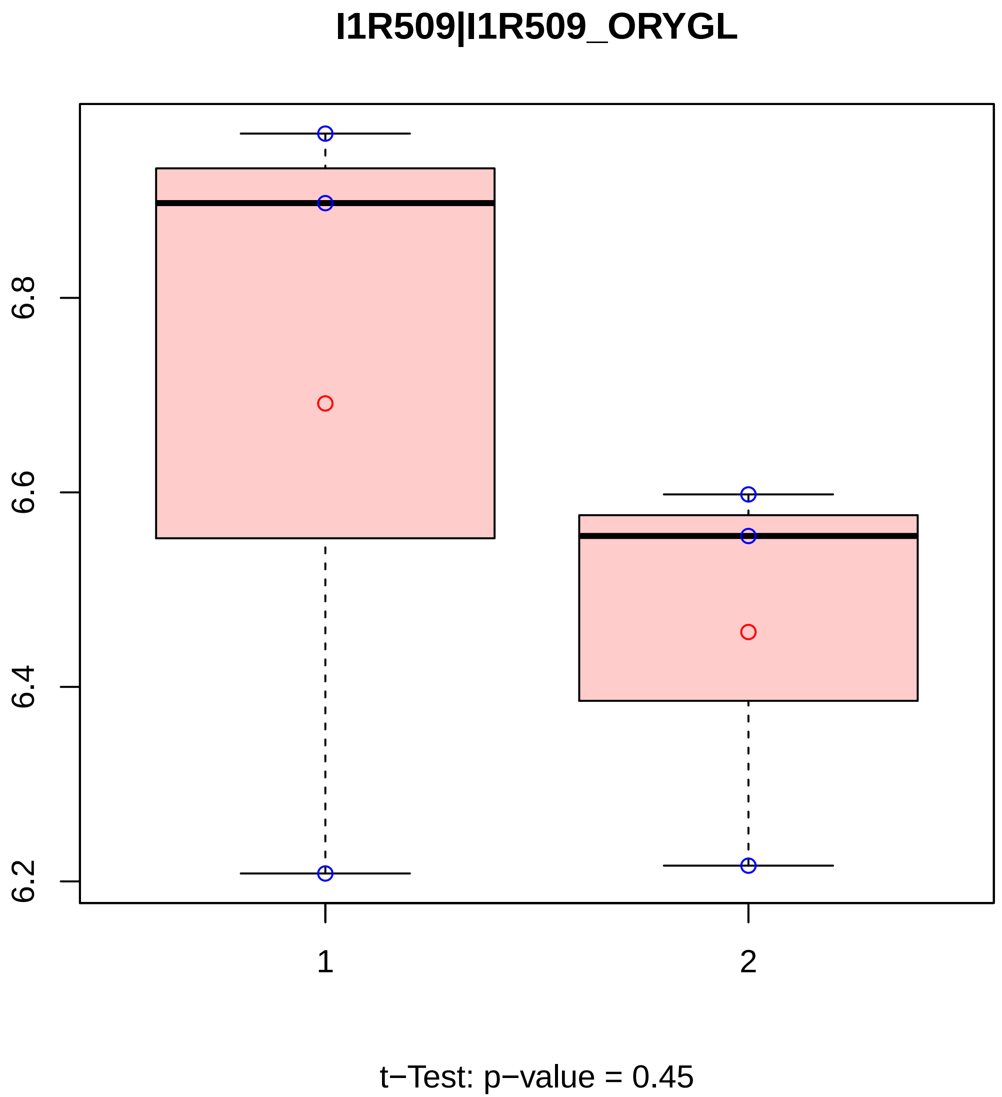
I1R509|I1R509_ORYGL
6.2
6.4
6.6
6.8
1
2
t−Test: p−value = 0.45

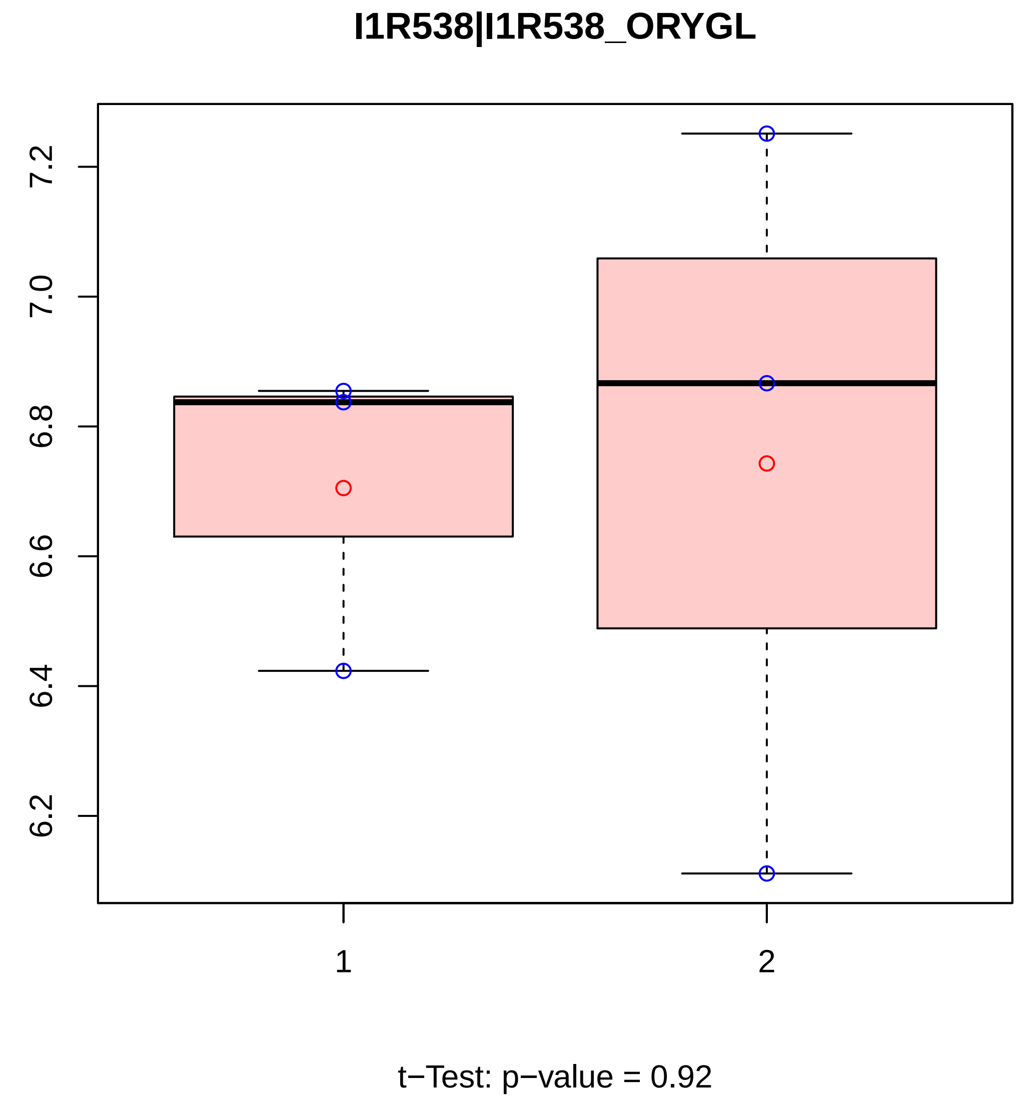
I1R538|I1R538_ORYGL
7.2
7.0
6.8
6.6
6.4
6.2
1
2
t−Test: p−value = 0.92

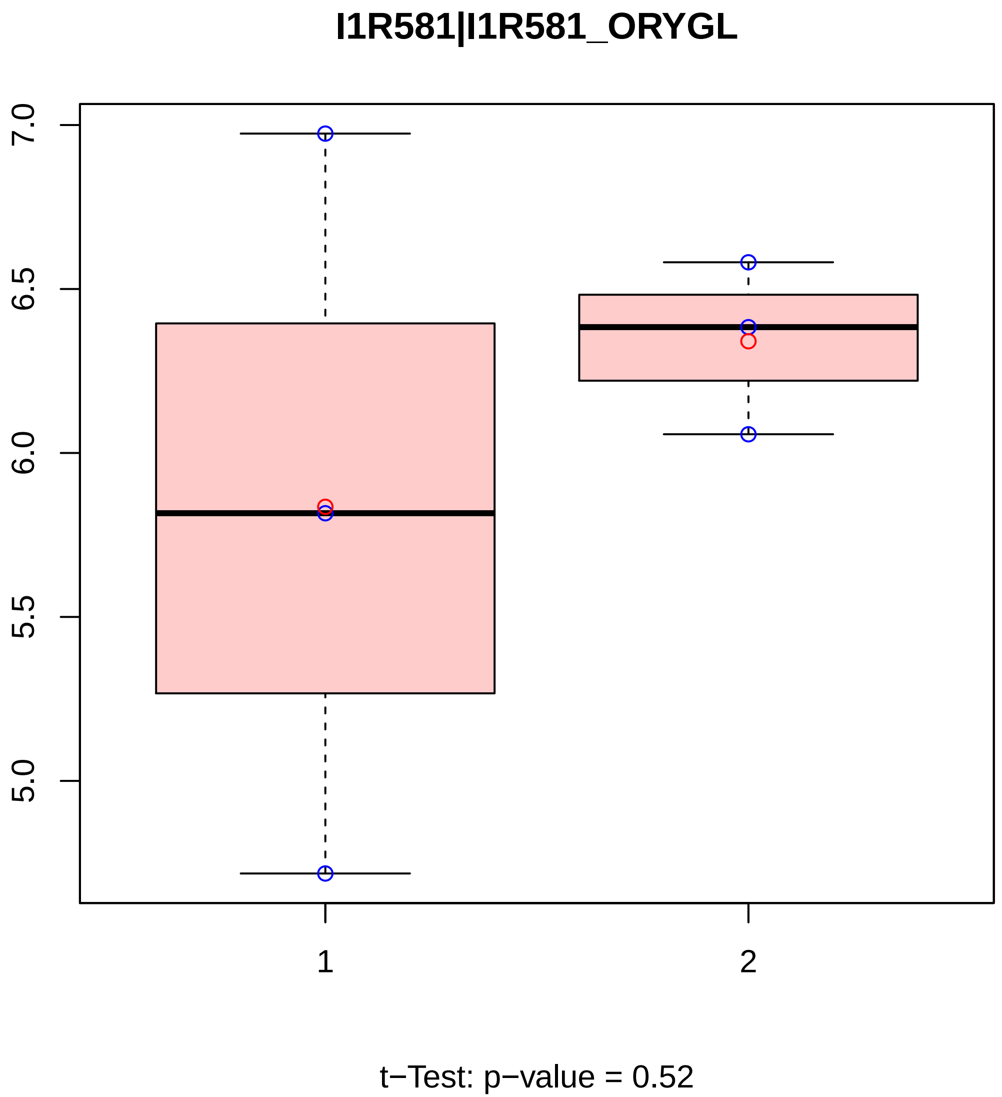

I1R581|I1R581_ORYGL
7.0
6.5
6.0
5.5
5.0
1
2
t−Test: p−value = 0.52

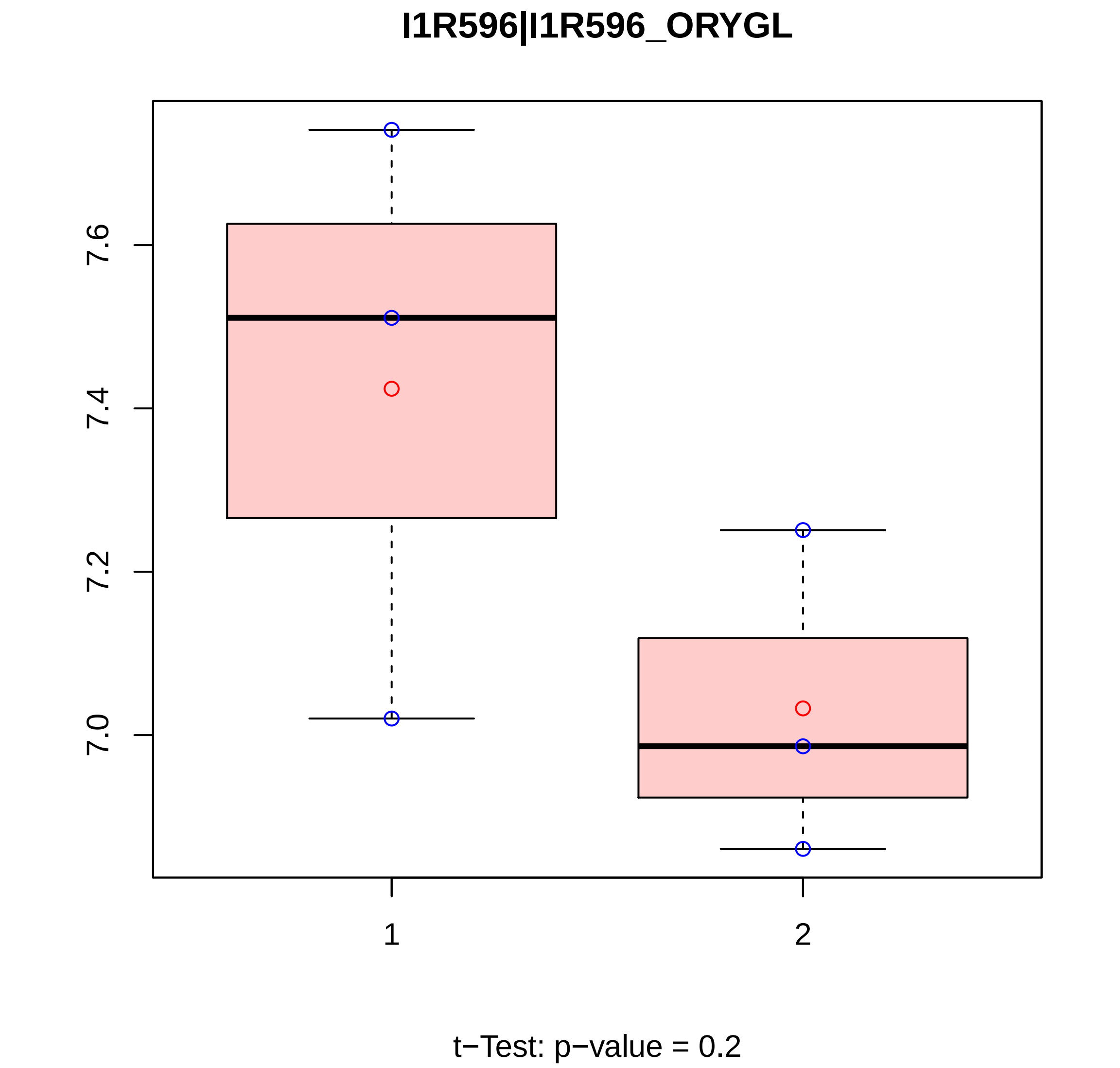
I1R596|I1R596_ORYGL
7.0
7.2
7.4
7.6
1
2
t−Test: p−value = 0.2

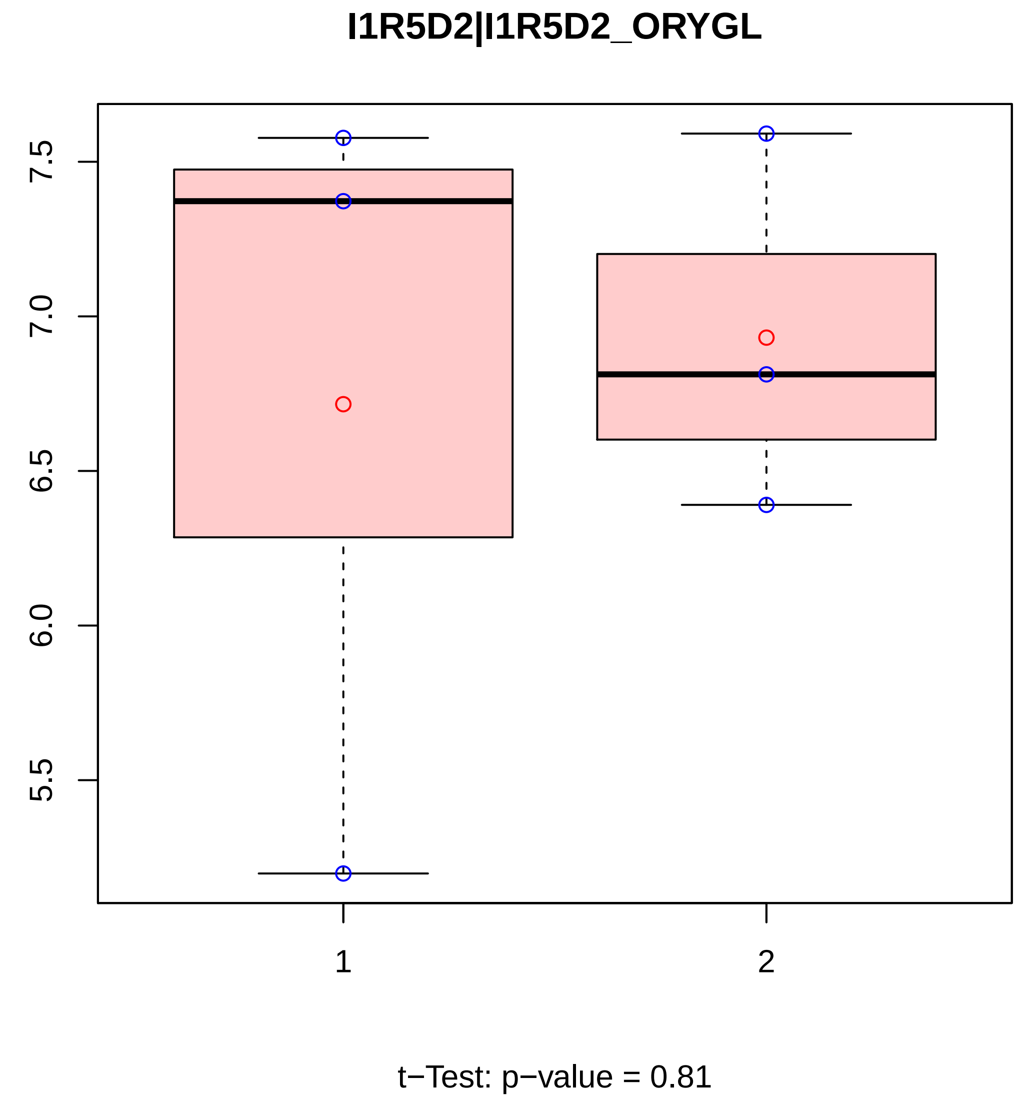

I1R5D2|I1R5D2_ORYGL
7.5
7.0
6.5
6.0
5.5
1
2
t−Test: p−value = 0.81

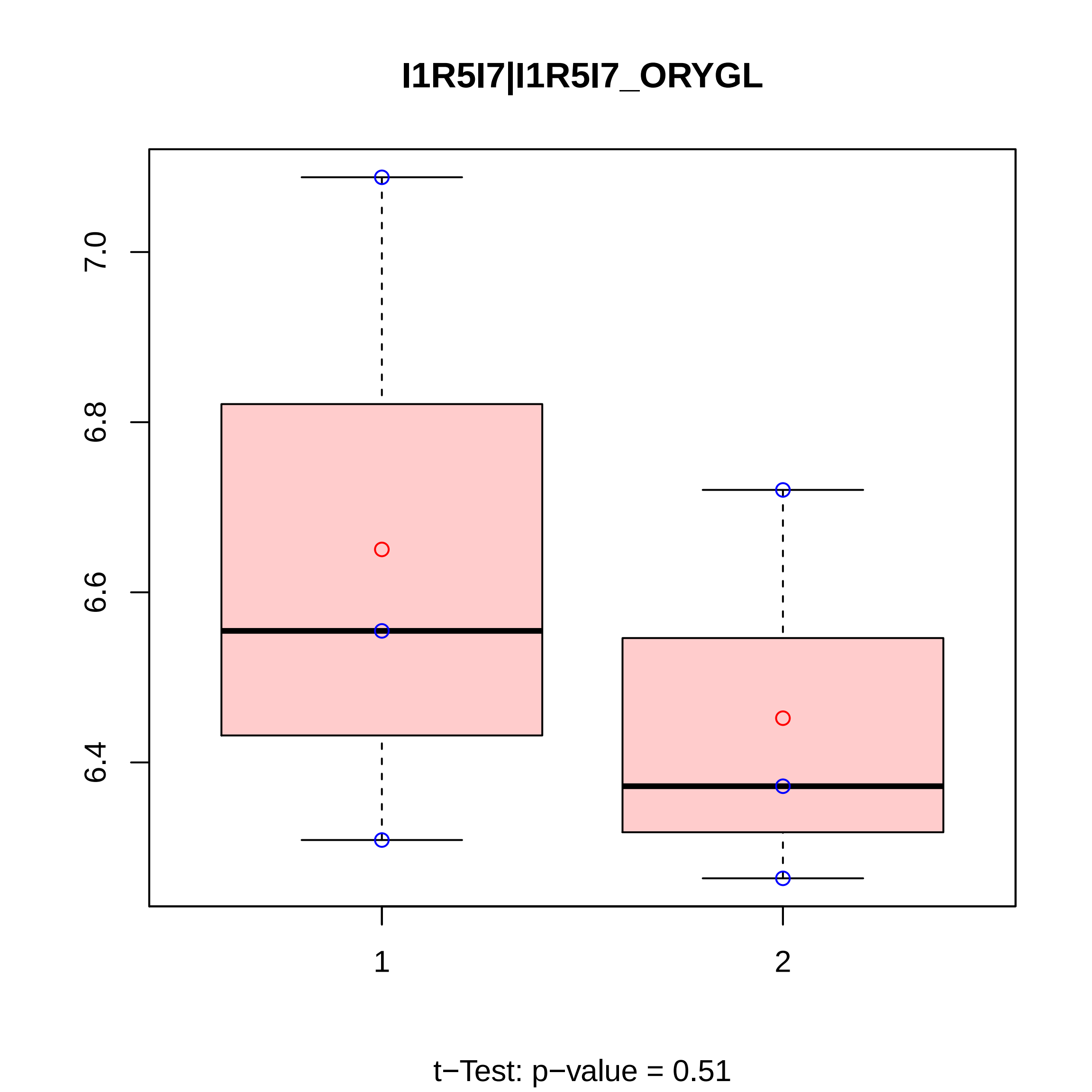
I1R5I7|I1R5I7_ORYGL
7.0
6.8
6.6
6.4
1
2
t−Test: p−value = 0.51

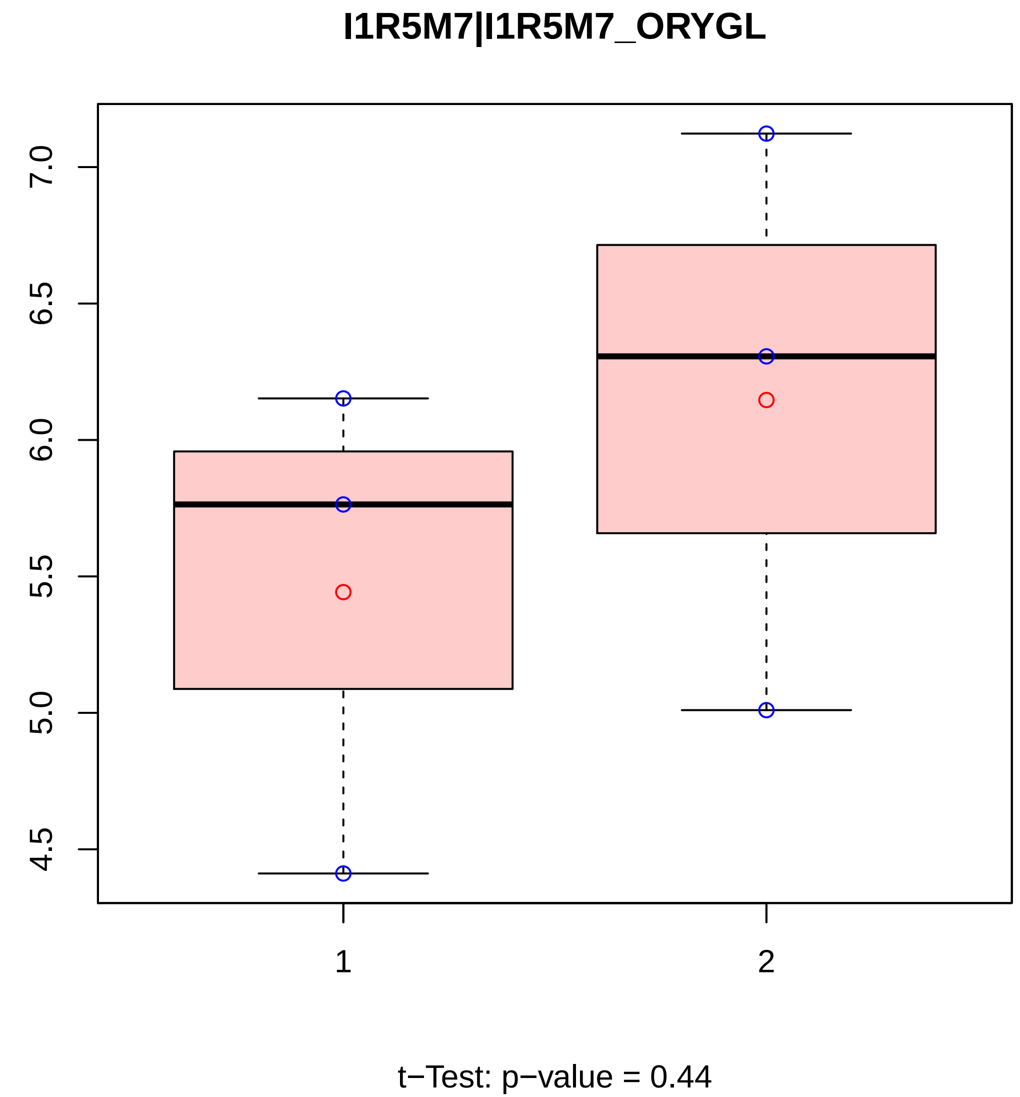
I1R5M7|I1R5M7_ORYGL
7.0
6.5
6.0
5.5
5.0
4.5
1
2
t−Test: p−value = 0.44

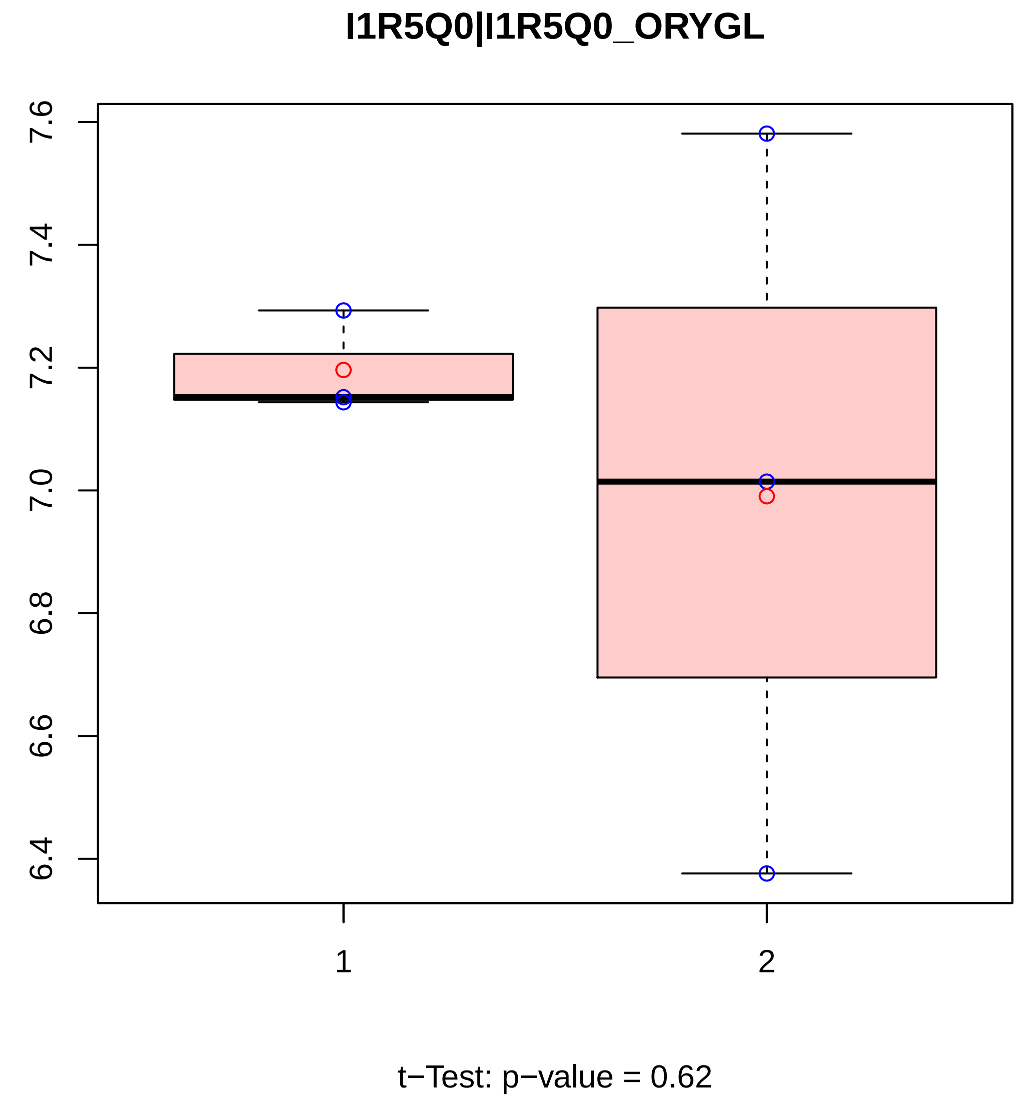

I1R5Q0|I1R5Q0_ORYGL
7.6
7.4
7.2
7.0
6.8
6.6
6.4
1
2
t−Test: p−value = 0.62

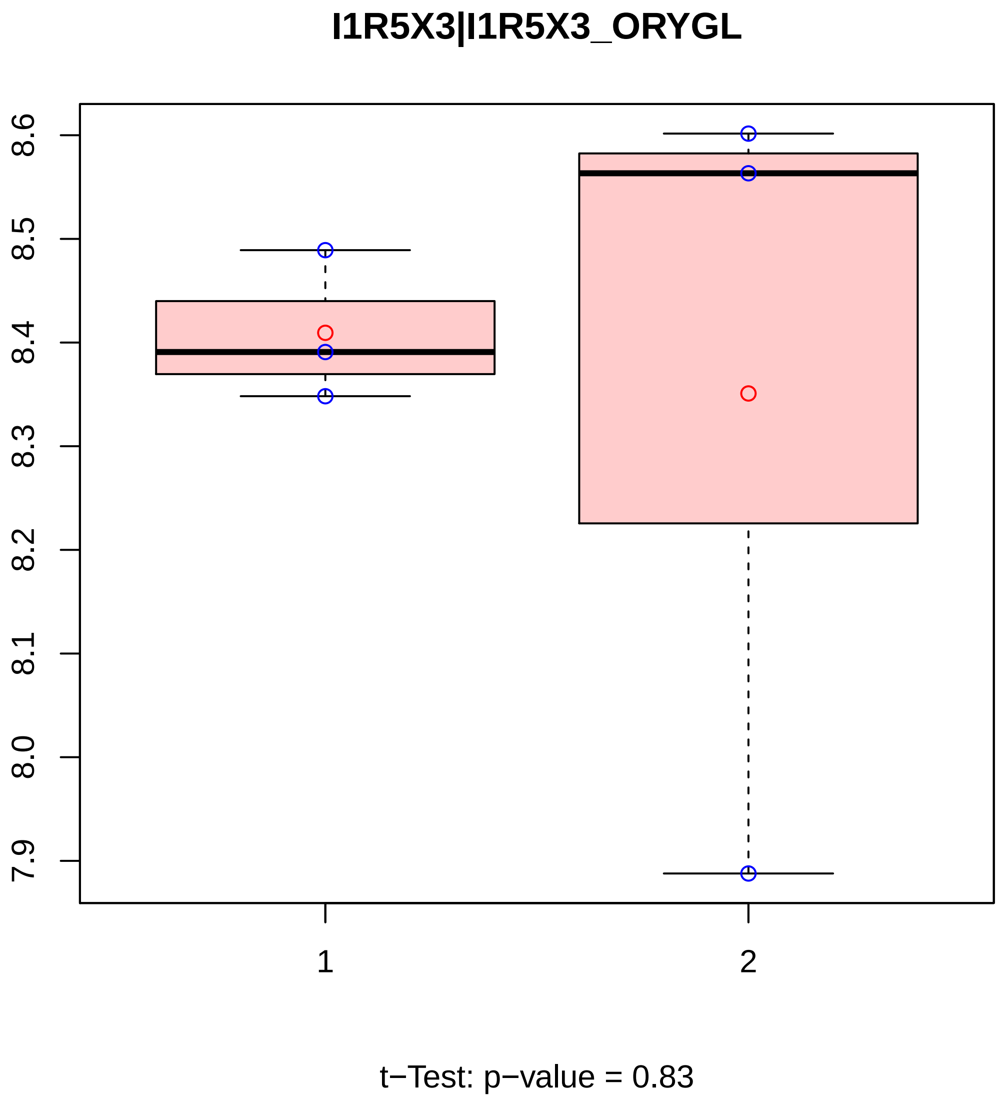
I1R5X3|I1R5X3_ORYGL
8.6
8.5
8.4
8.3
8.2
8.1
8.0
7.9
1
2
t−Test: p−value = 0.83

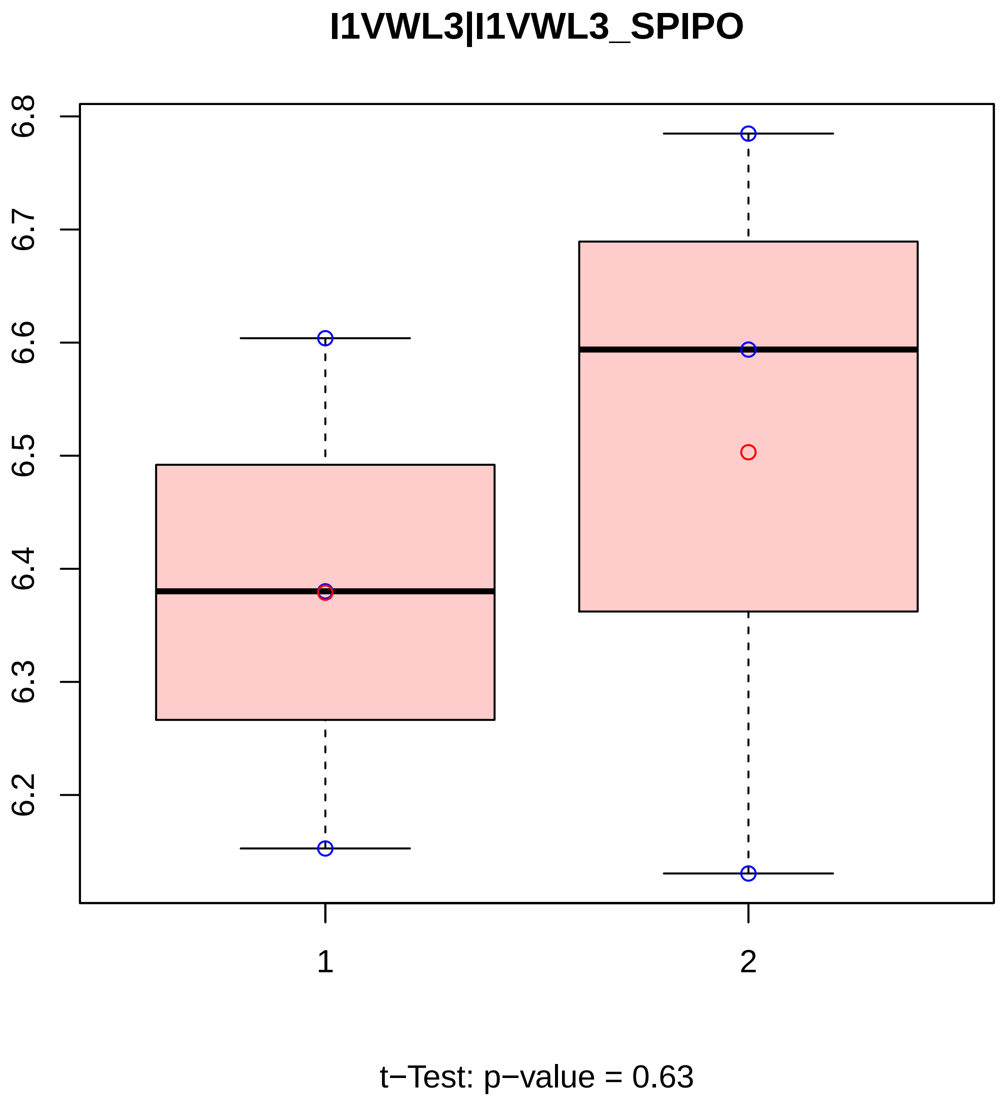

I1VWL3|I1VWL3_SPIPO
6.2
6.3
6.4
6.5
6.6
6.7
6.8
1
2
t−Test: p−value = 0.63

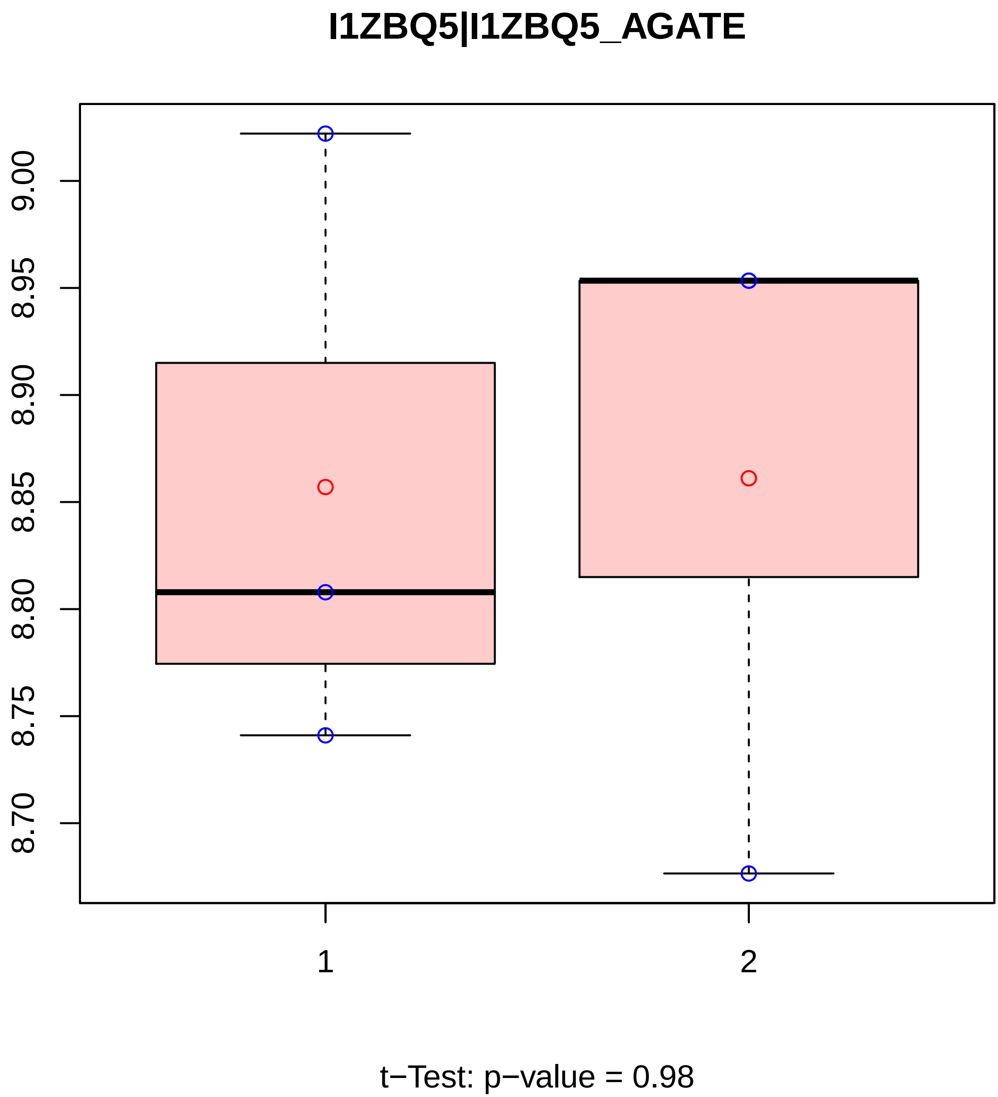
I1ZBQ5|I1ZBQ5_AGATE
9.00
8.95
8.90
8.85
8.80
8.75
8.70
1
2
t−Test: p−value = 0.98

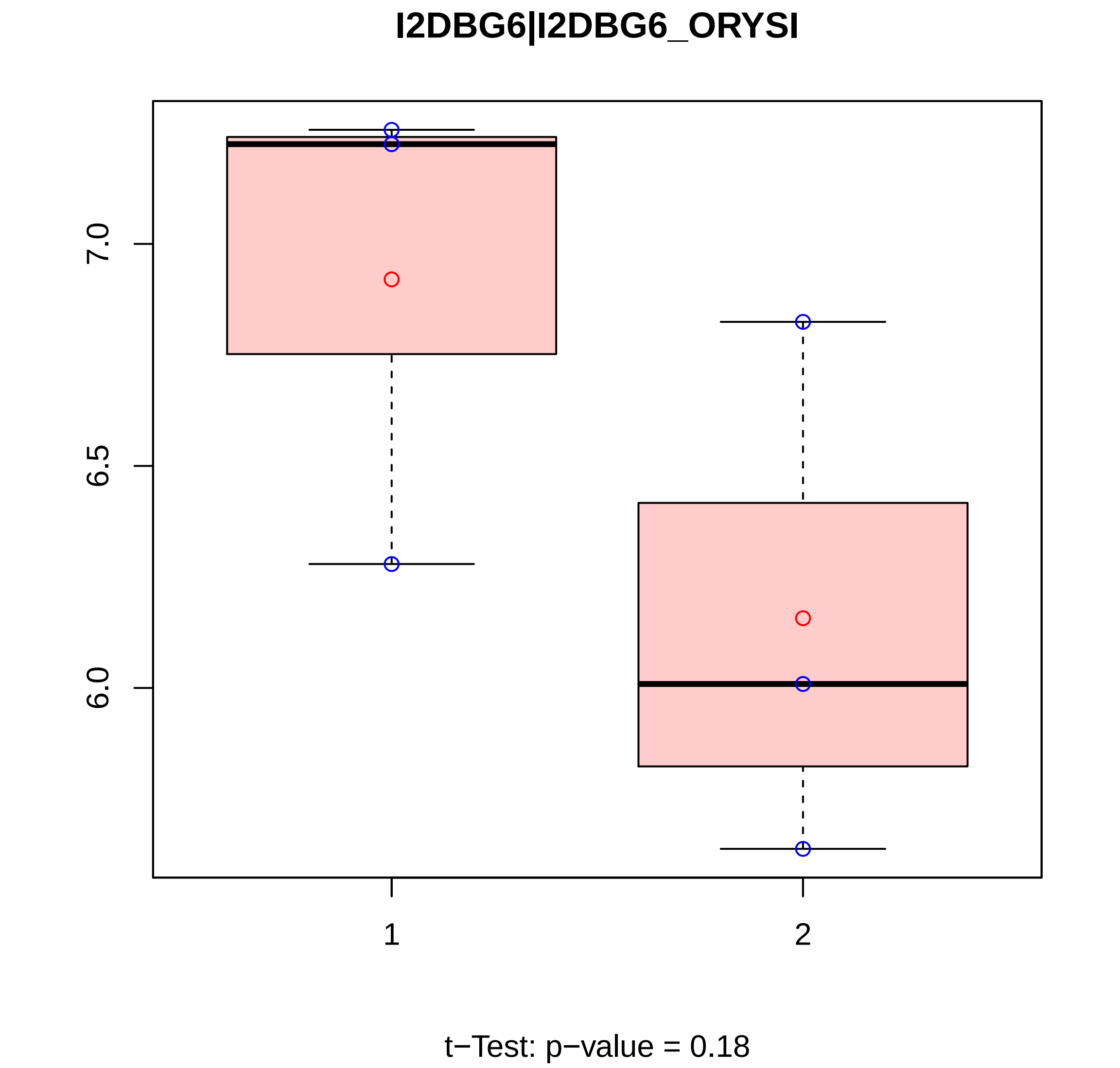
I2DBG6|I2DBG6_ORYSI
7.0
6.5
6.0
1
2
t−Test: p−value = 0.18

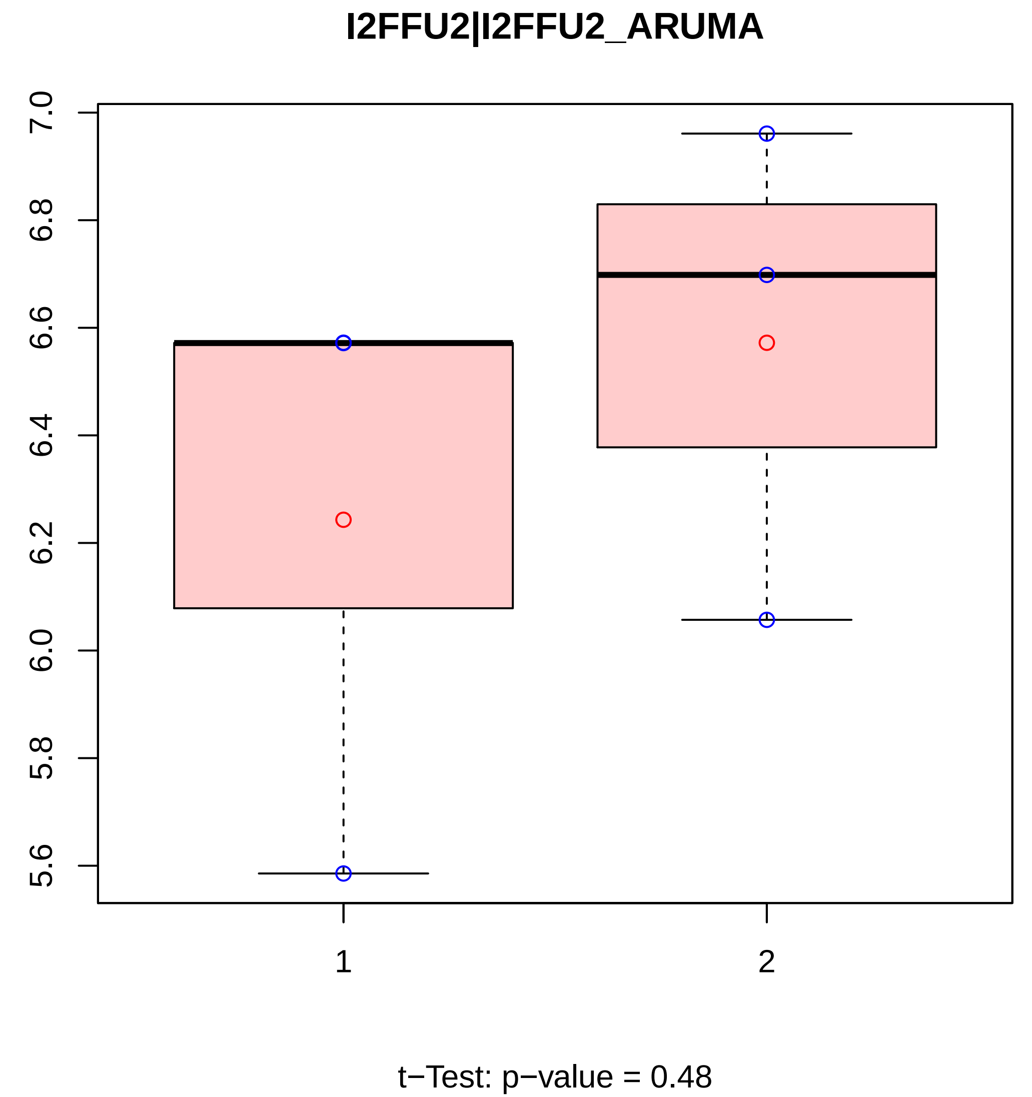
I2FFU2|I2FFU2_ARUMA
7.0
6.8
6.6
6.4
6.2
6.0
5.8
5.6
1
2
t−Test: p−value = 0.48

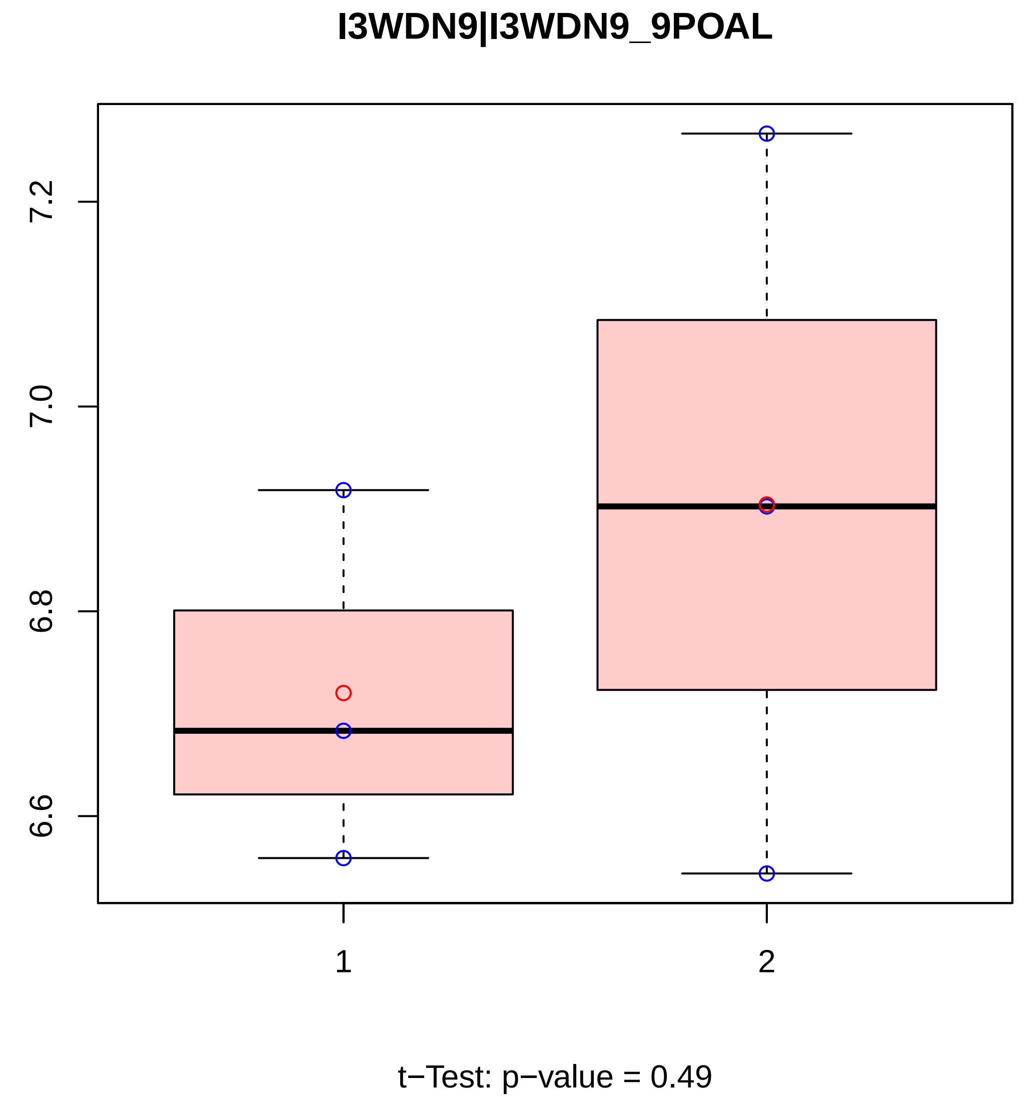

I3WDN9|I3WDN9_9POAL
7.2
7.0
6.8
6.6
1
2
t−Test: p−value = 0.49

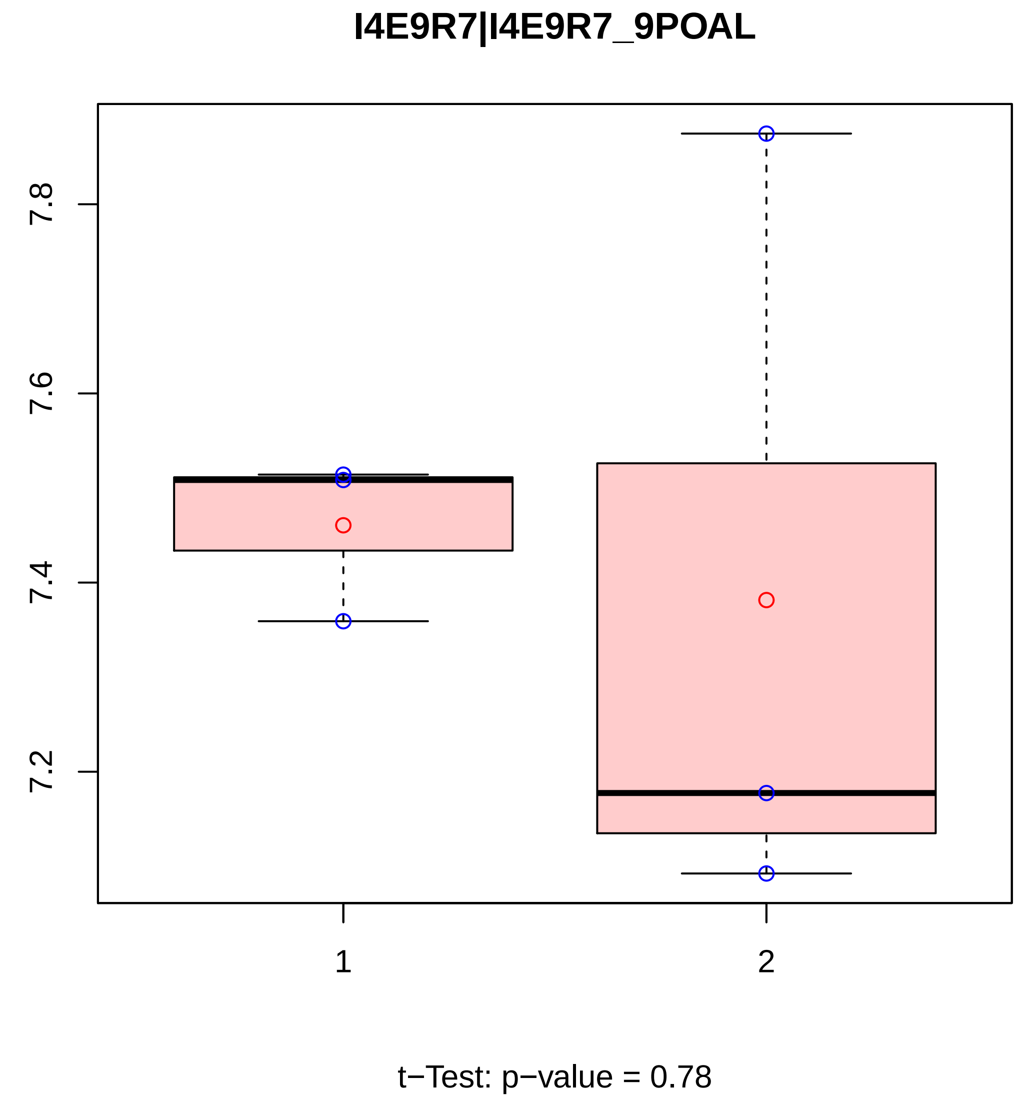

I4E9R7|I4E9R7_9POAL
7.2
7.4
7.6
7.8
1
2
t−Test: p−value = 0.78

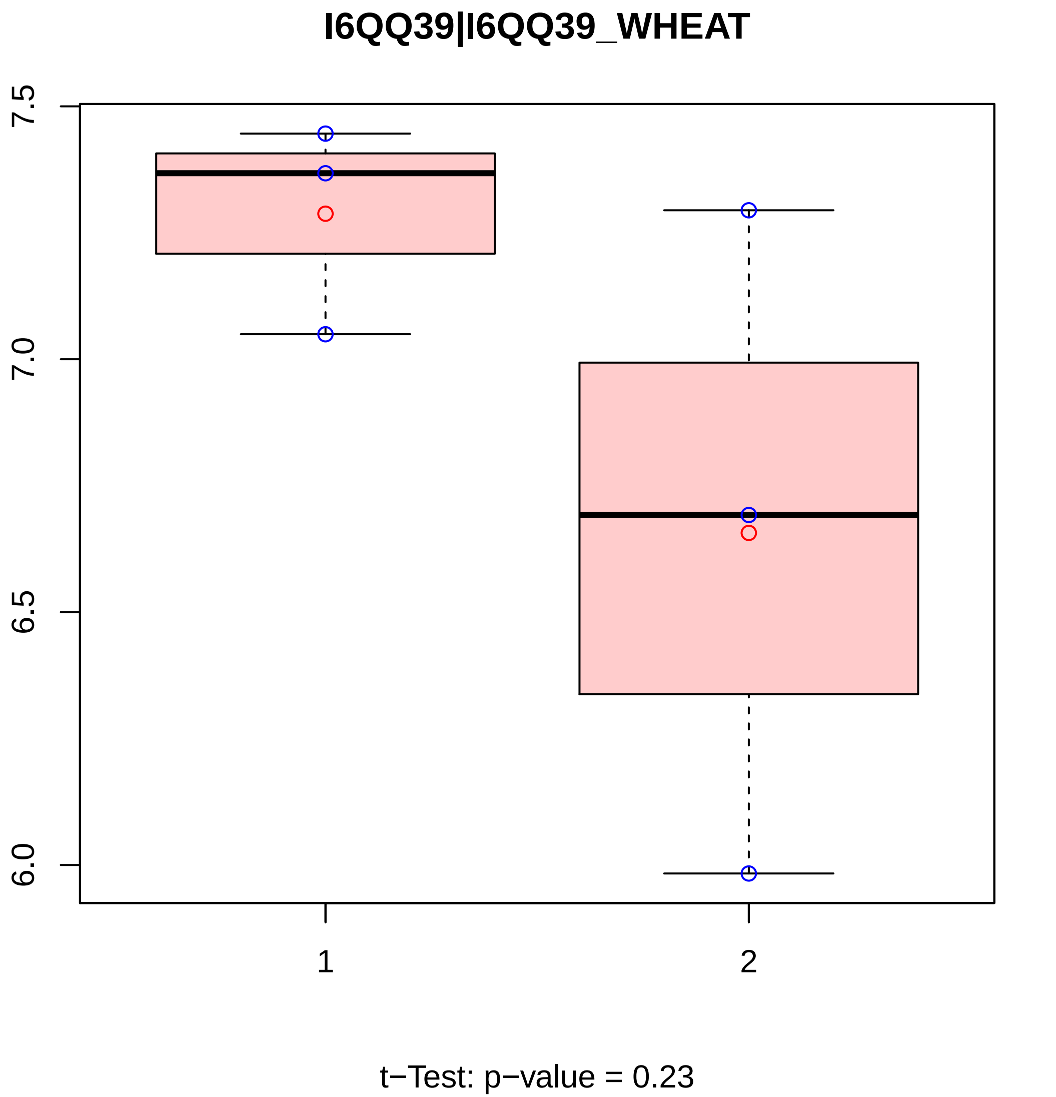

I6QQ39|I6QQ39_WHEAT
7.5
7.0
6.5
6.0
1
2
t−Test: p−value = 0.23

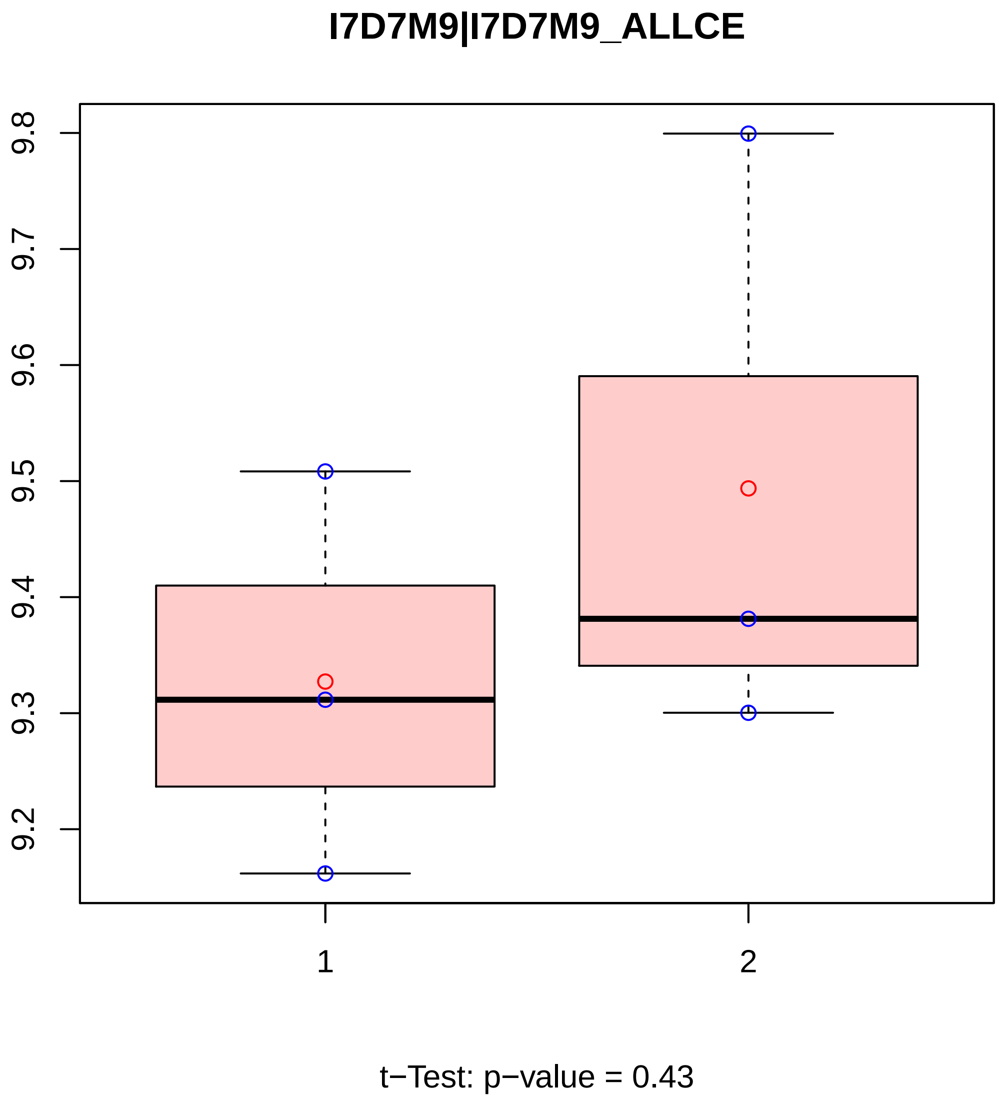
I7D7M9|I7D7M9_ALLCE
9.2
9.3
9.4
9.5
9.6
9.7
9.8
1
2
t−Test: p−value = 0.43

I7HB64|I7HB64_9LILI

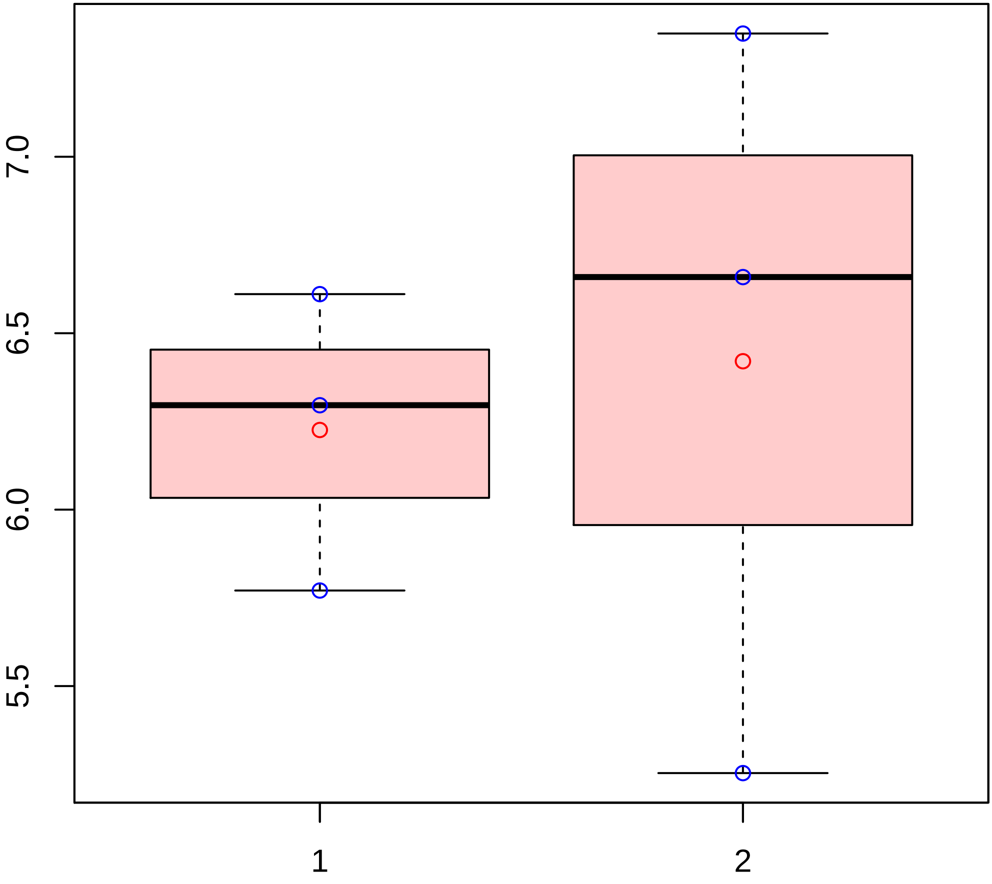

t−Test: p−value = 0.79

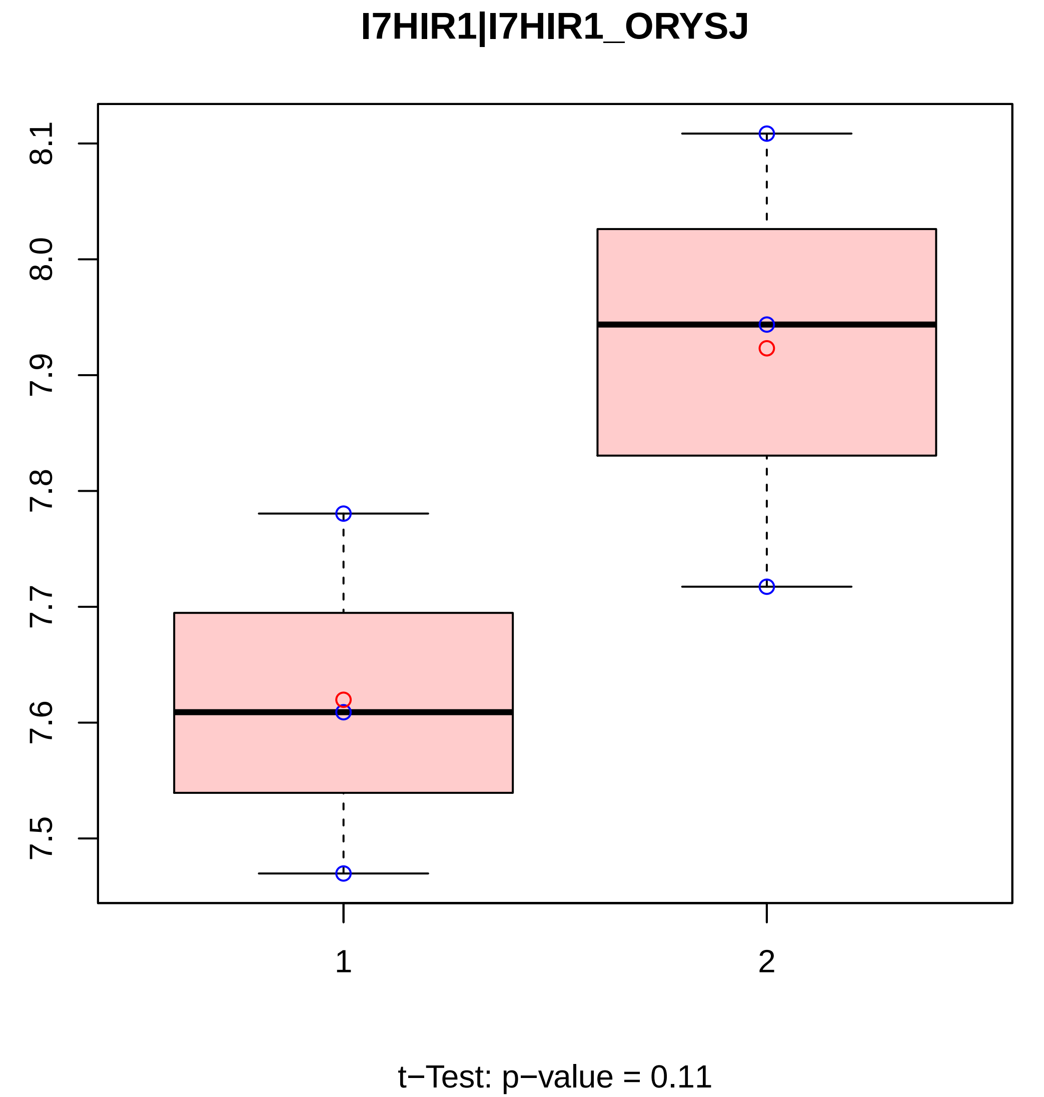
I7HIR1|I7HIR1_ORYSJ
8.1
8.0
7.9
7.8
7.7
7.6
7.5
1
2
t−Test: p−value = 0.11

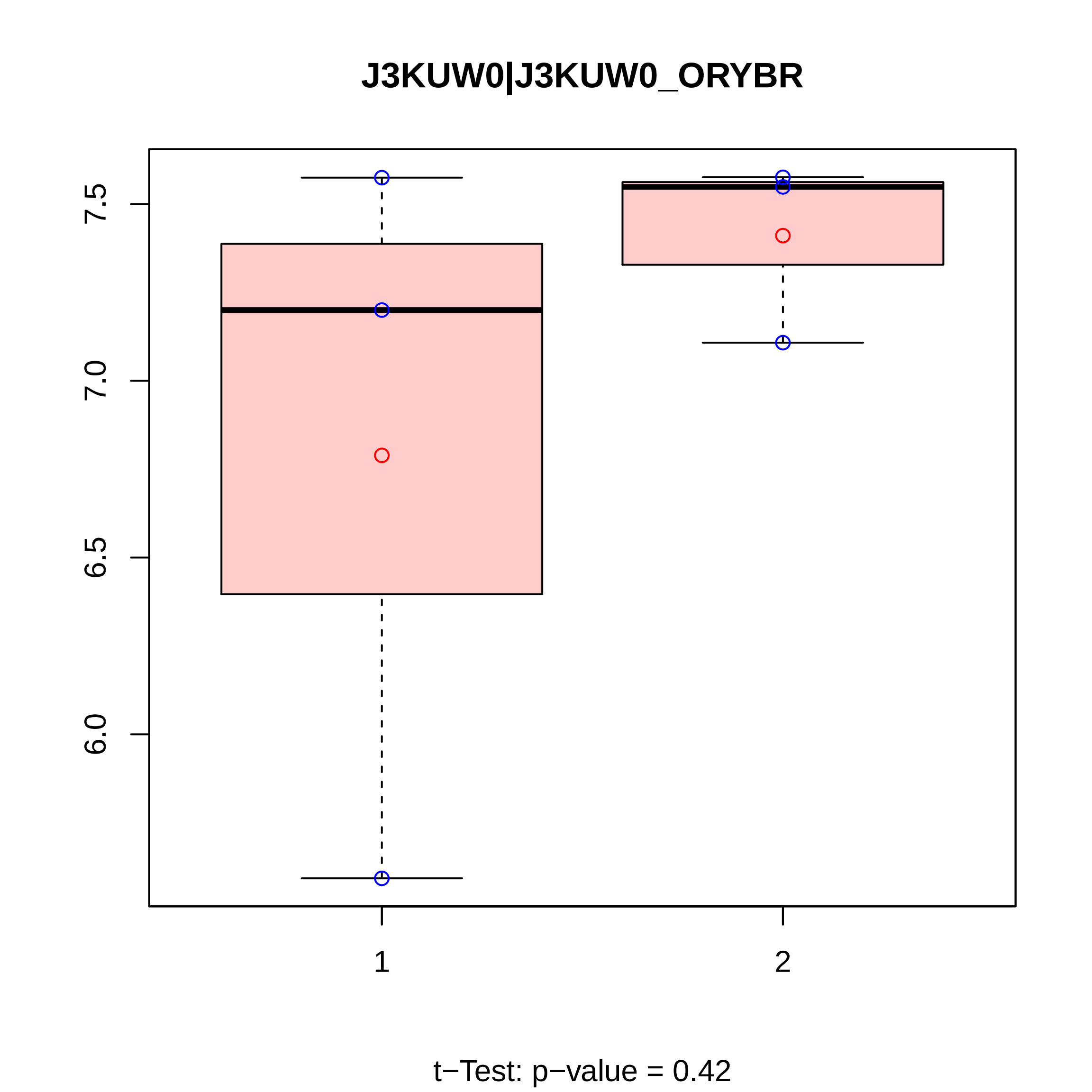
J3KUW0|J3KUW0_ORYBR
7.5
7.0
6.5
6.0
1
2
t−Test: p−value = 0.42

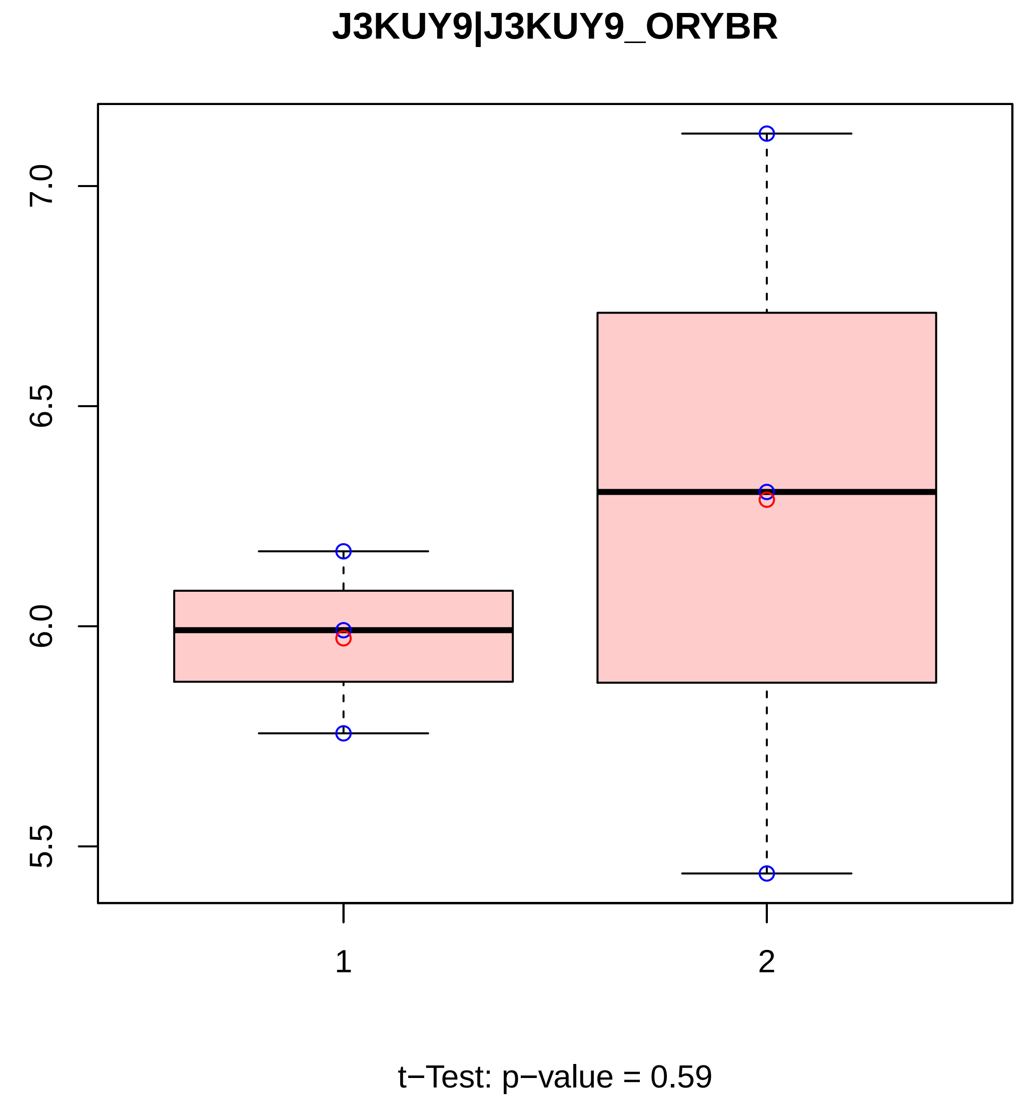

J3KUY9|J3KUY9_ORYBR
7.0
6.5
6.0
5.5
1
2
t−Test: p−value = 0.59

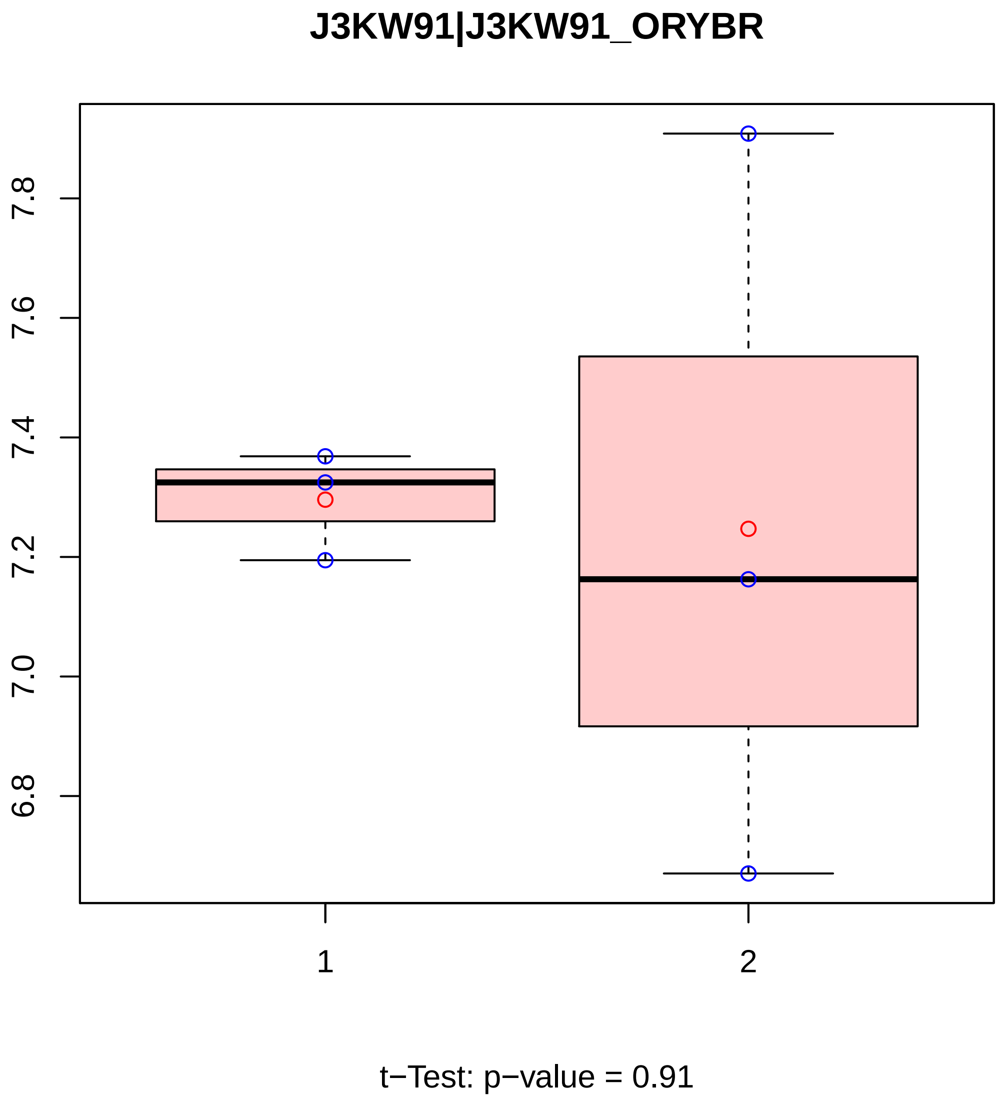

J3KW91|J3KW91_ORYBR
7.8
7.6
7.4
7.2
7.0
6.8
1
2
t−Test: p−value = 0.91

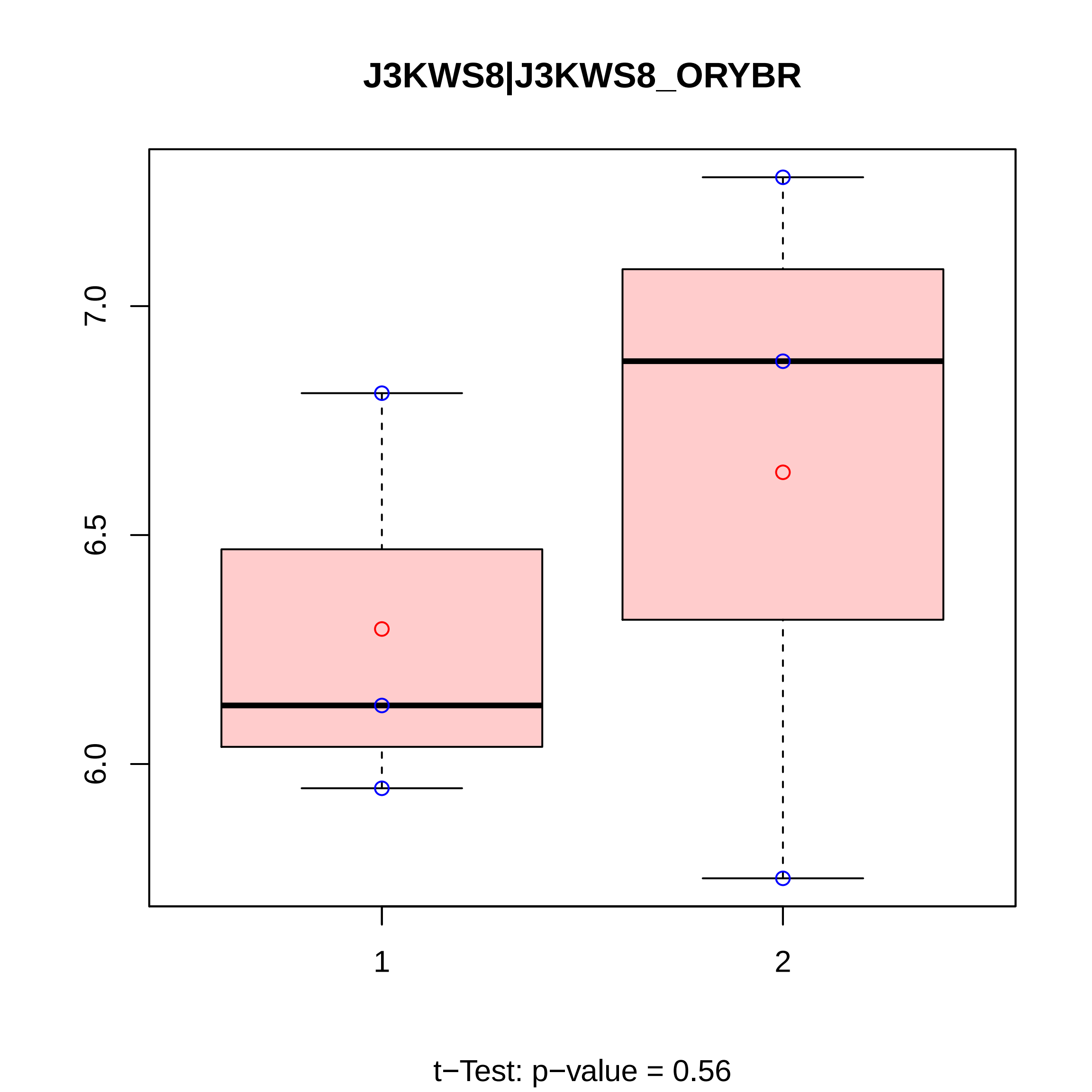
J3KWS8|J3KWS8_ORYBR
7.0
6.5
6.0
1
2
t−Test: p−value = 0.56

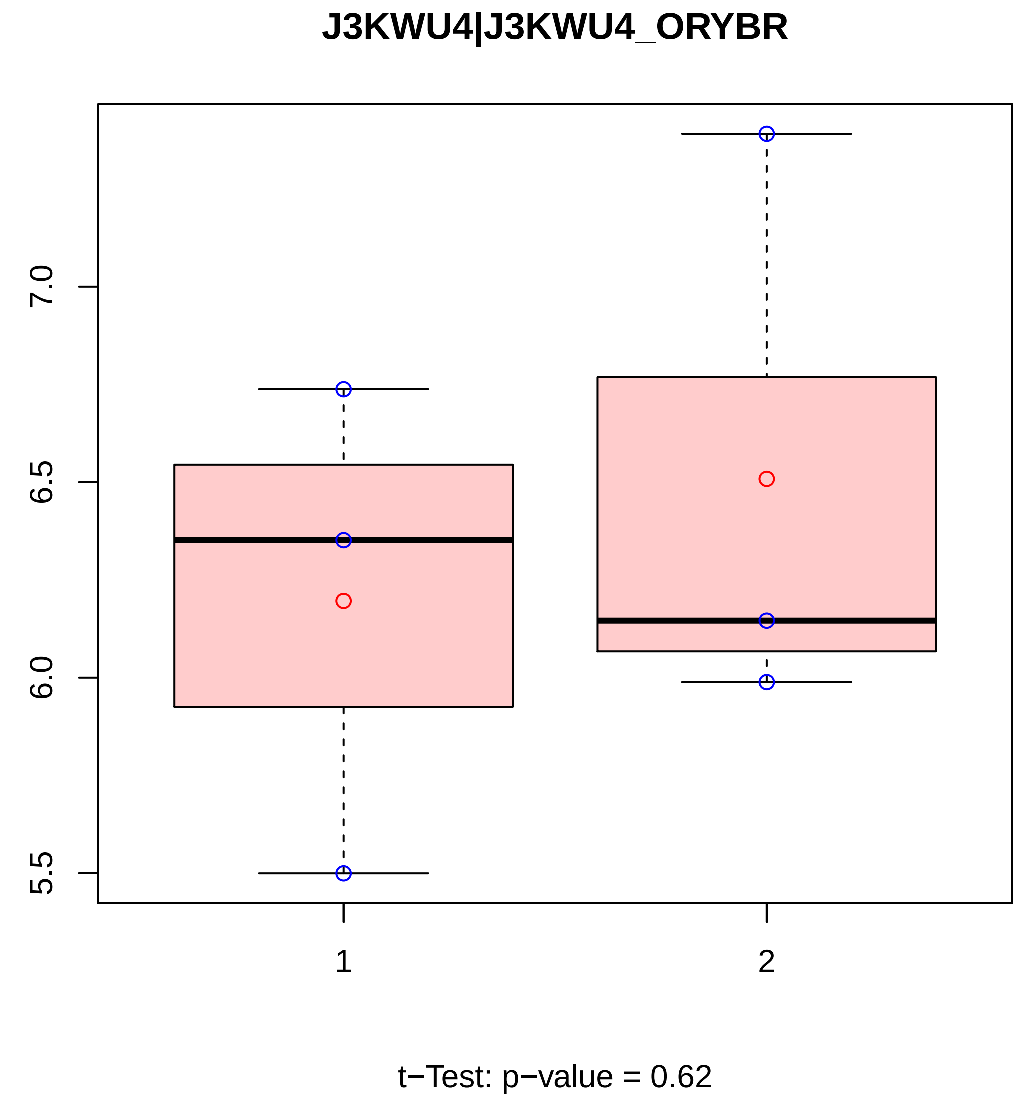

J3KWU4|J3KWU4_ORYBR
7.0
6.5
6.0
5.5
1
2
t−Test: p−value = 0.62

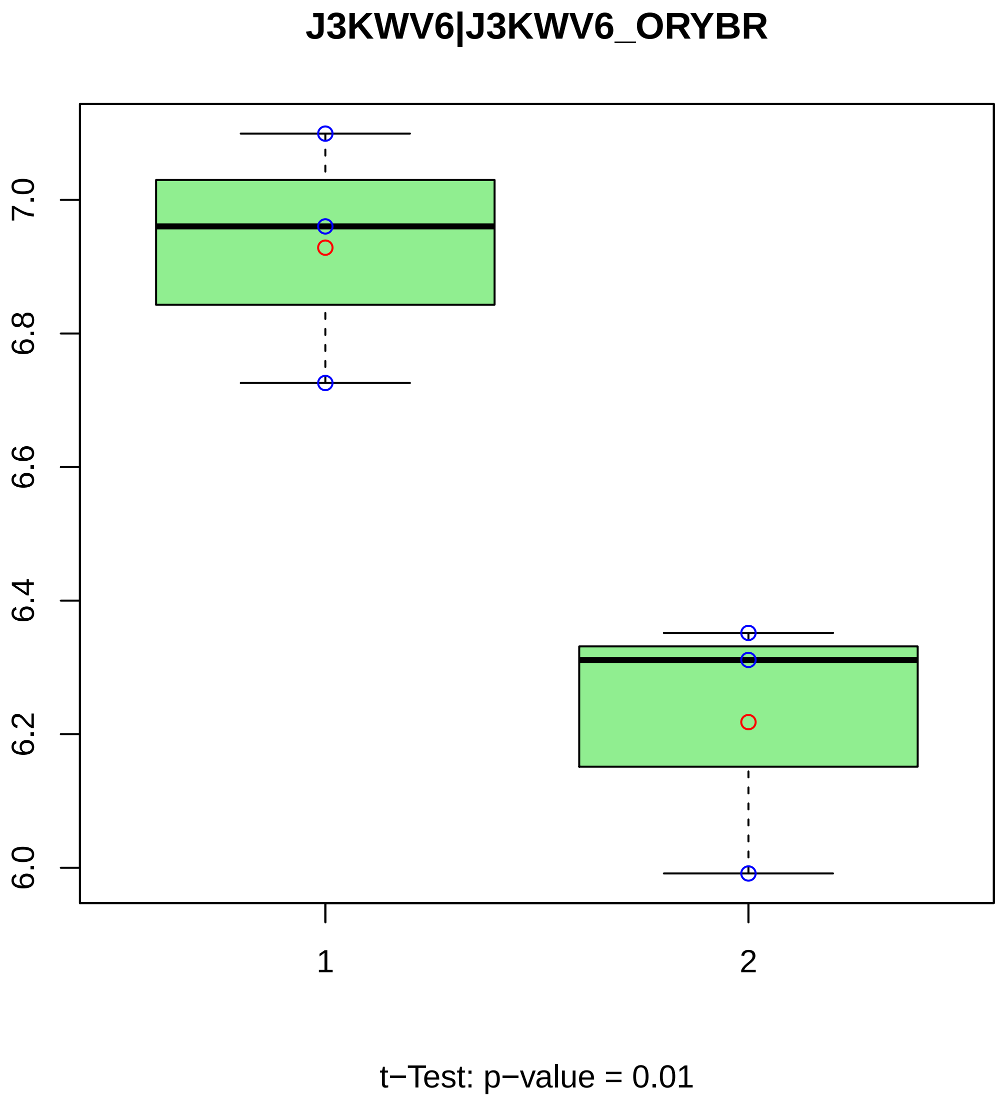

J3KWV6|J3KWV6_ORYBR
7.0
6.8
6.6
6.4
6.2
6.0
1
2
t−Test: p−value = 0.01

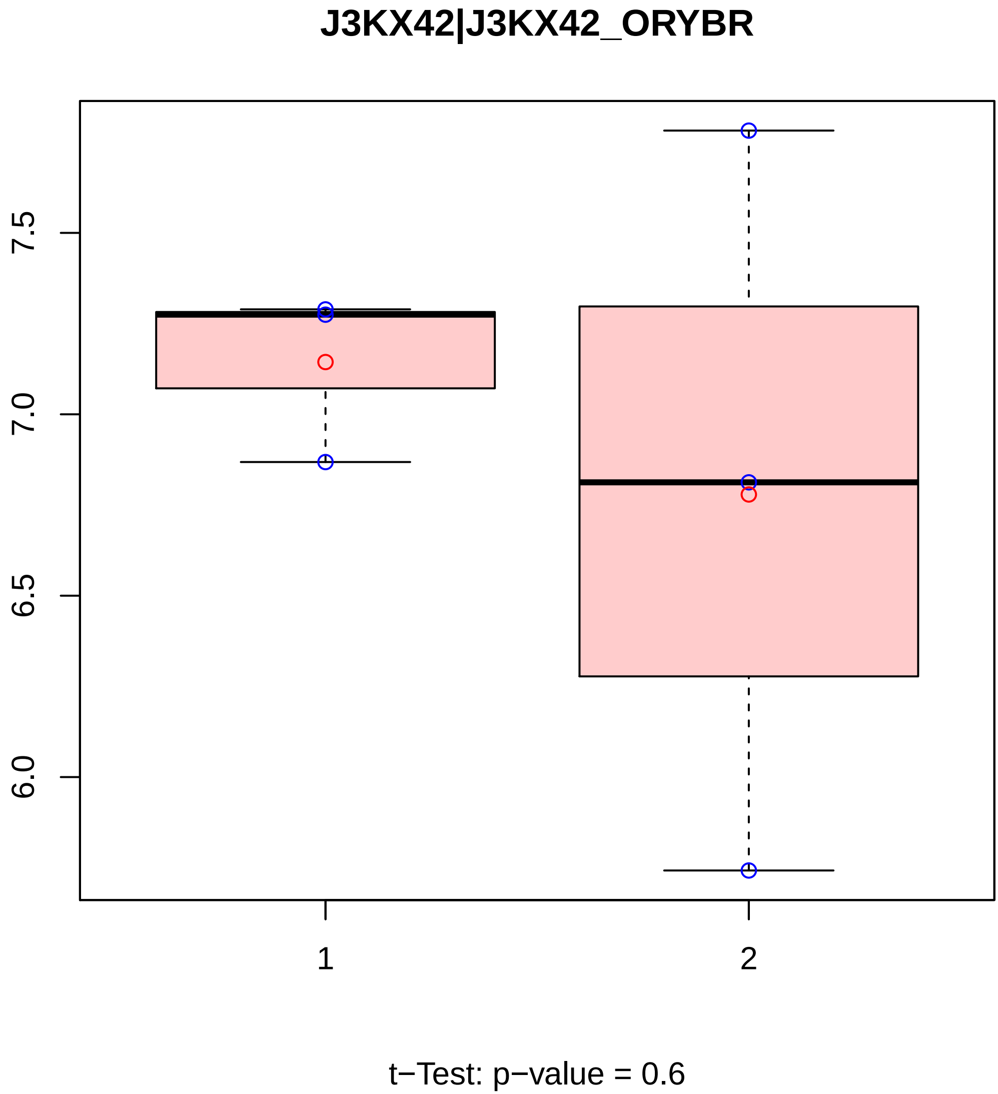
J3KX42|J3KX42_ORYBR
7.5
7.0
6.5
6.0
1
2
t−Test: p−value = 0.6

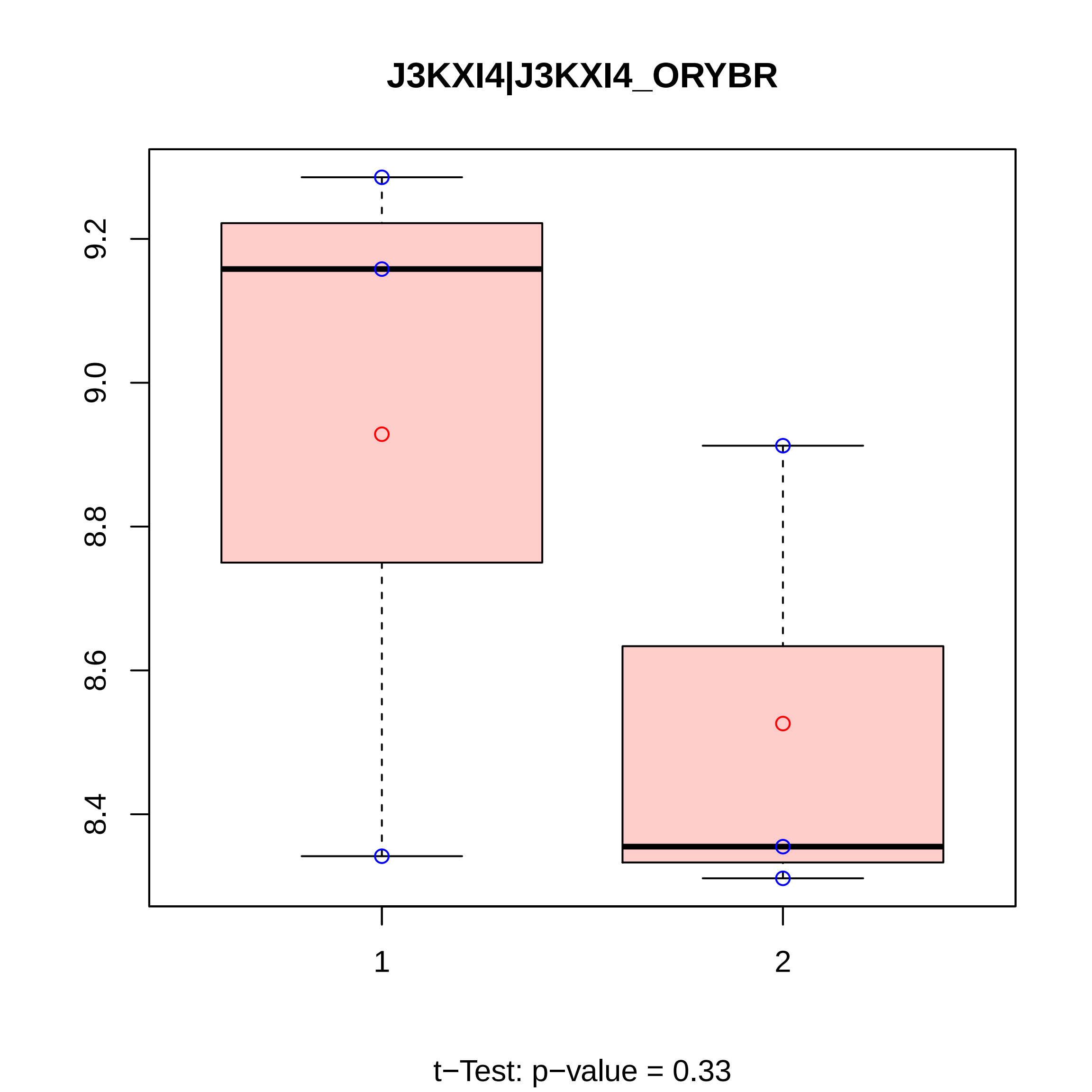
J3KXI4|J3KXI4_ORYBR
9.2
9.0
8.8
8.6
8.4
1
2
t−Test: p−value = 0.33

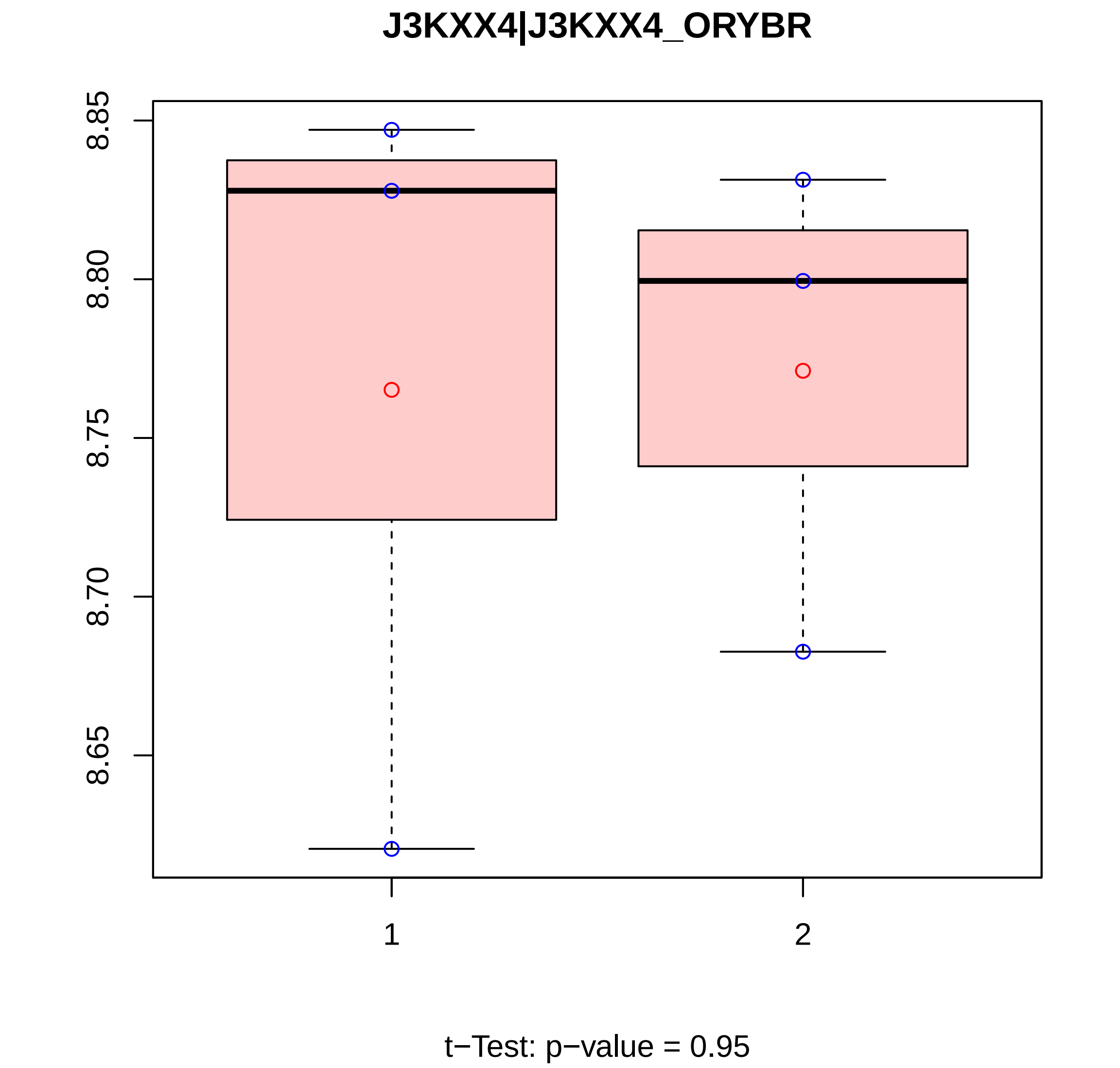

J3KXX4|J3KXX4_ORYBR
8.65
8.70
8.75
8.80
8.85
1
2
t−Test: p−value = 0.95

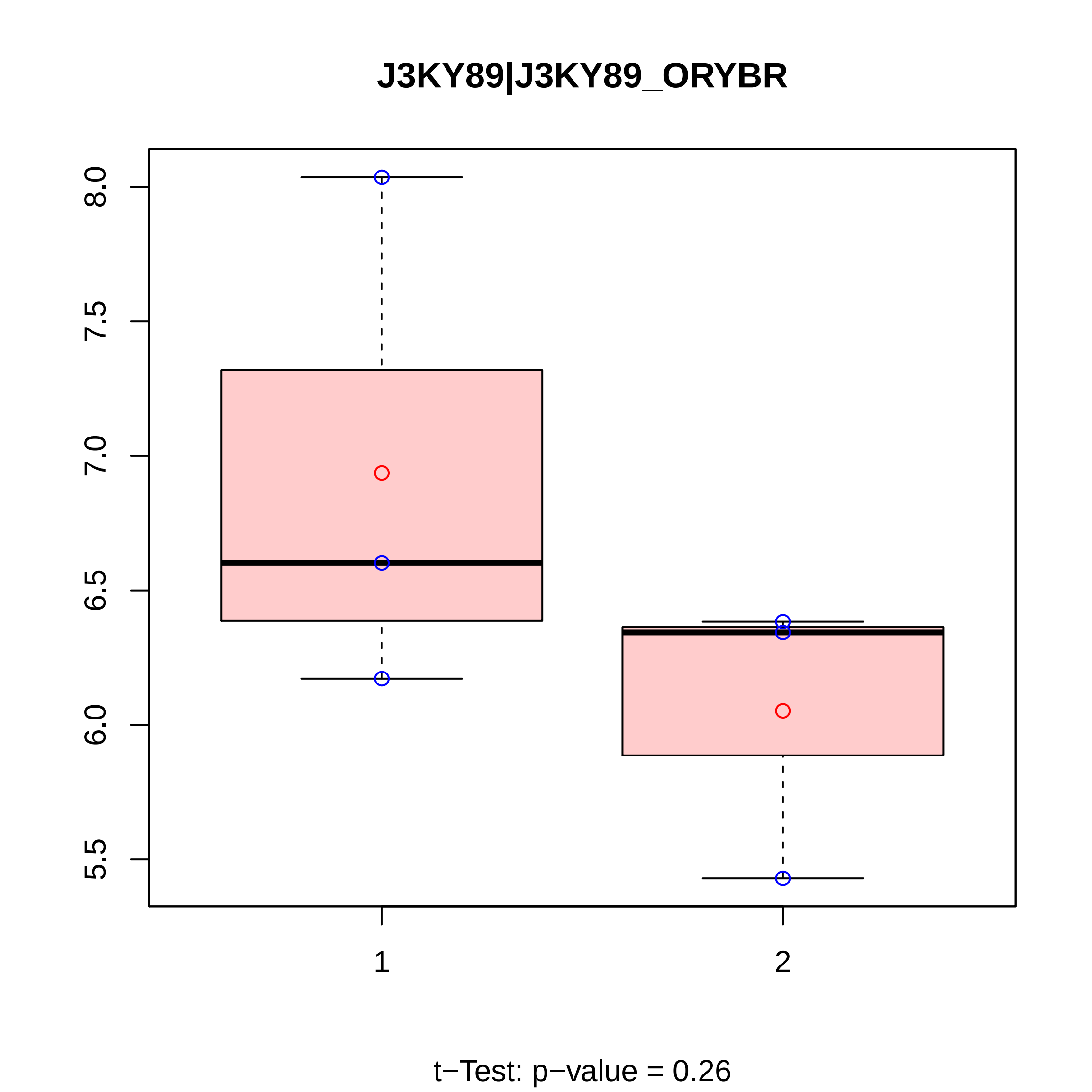

J3KY89|J3KY89_ORYBR
8.0
7.5
7.0
6.5
6.0
5.5
1
2
t−Test: p−value = 0.26

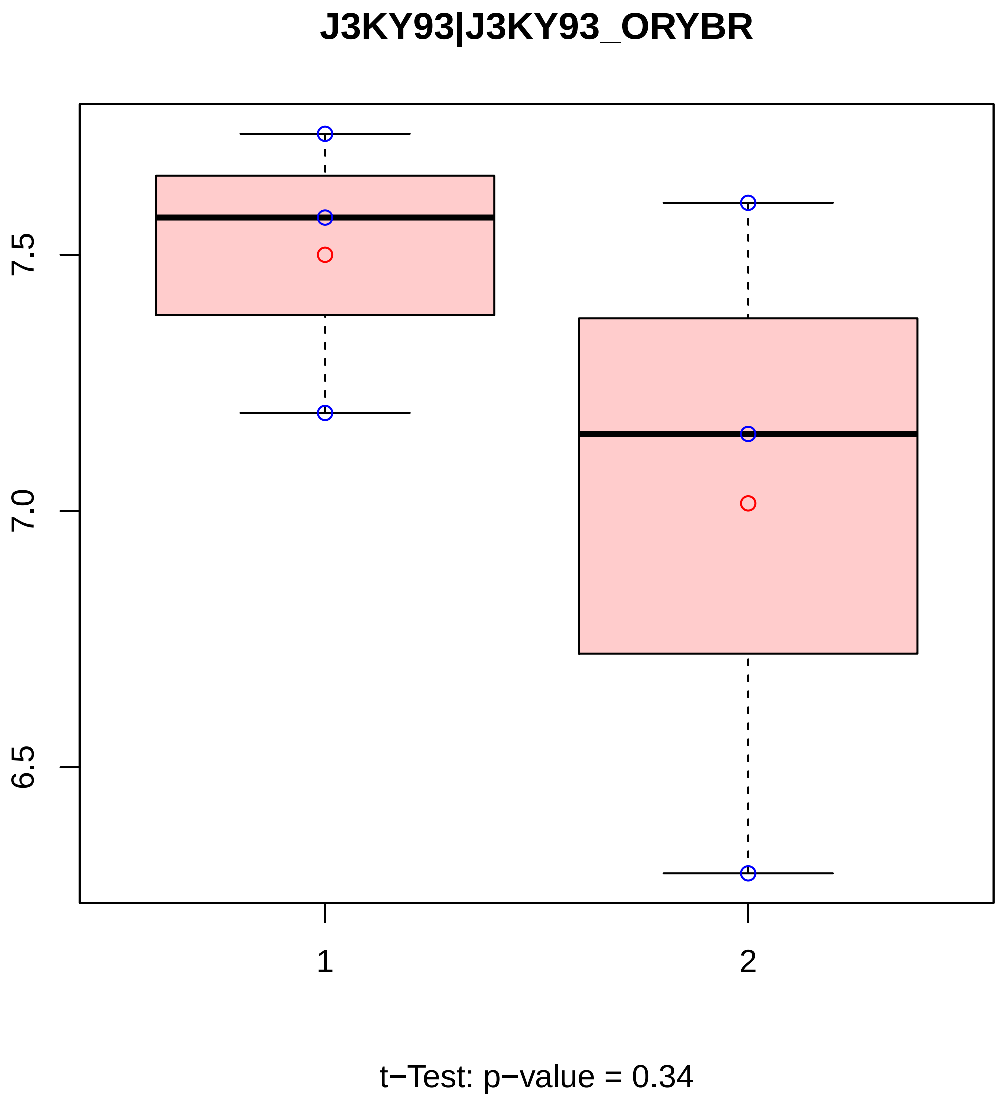

J3KY93|J3KY93_ORYBR
7.5
7.0
6.5
1
2
t−Test: p−value = 0.34

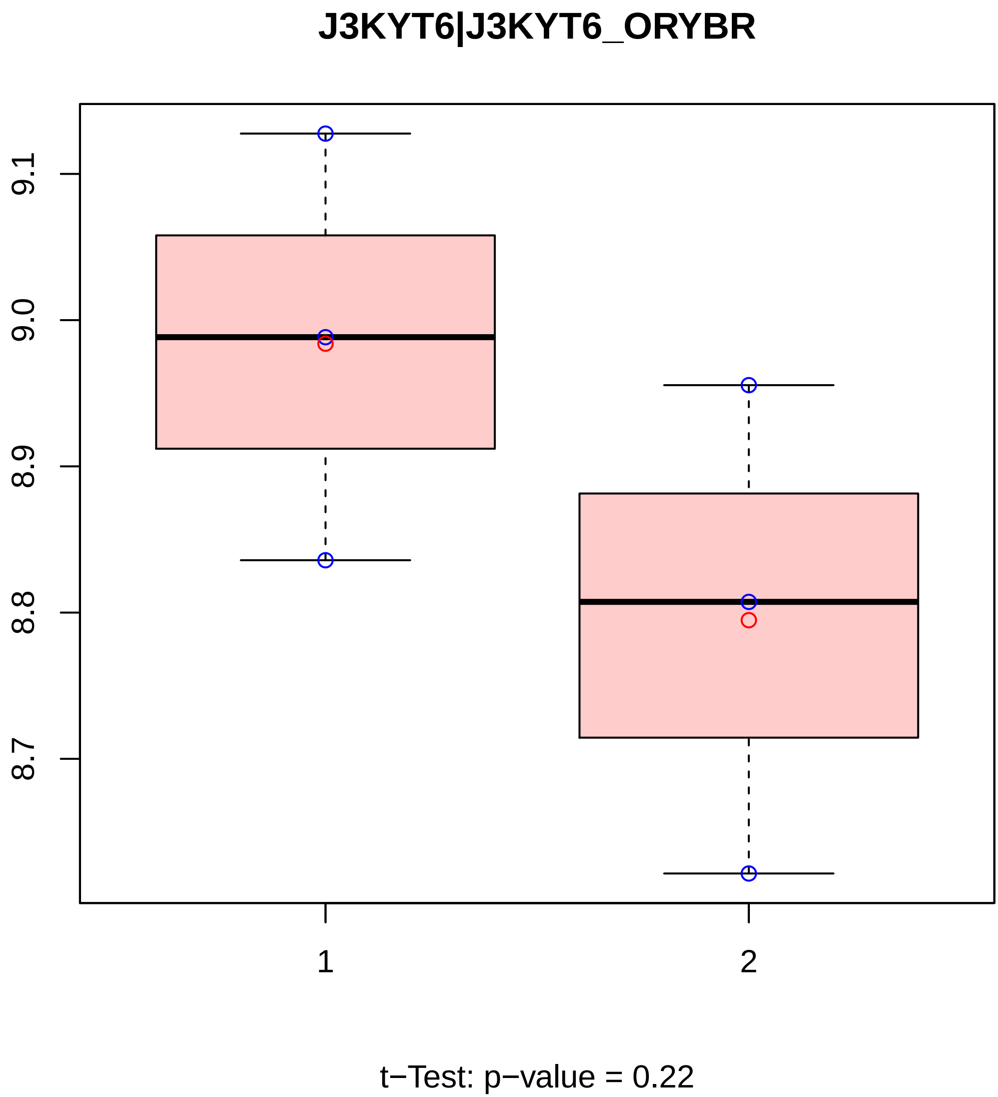
J3KYT6|J3KYT6_ORYBR
9.1
9.0
8.9
8.8
8.7
1
2
t−Test: p−value = 0.22

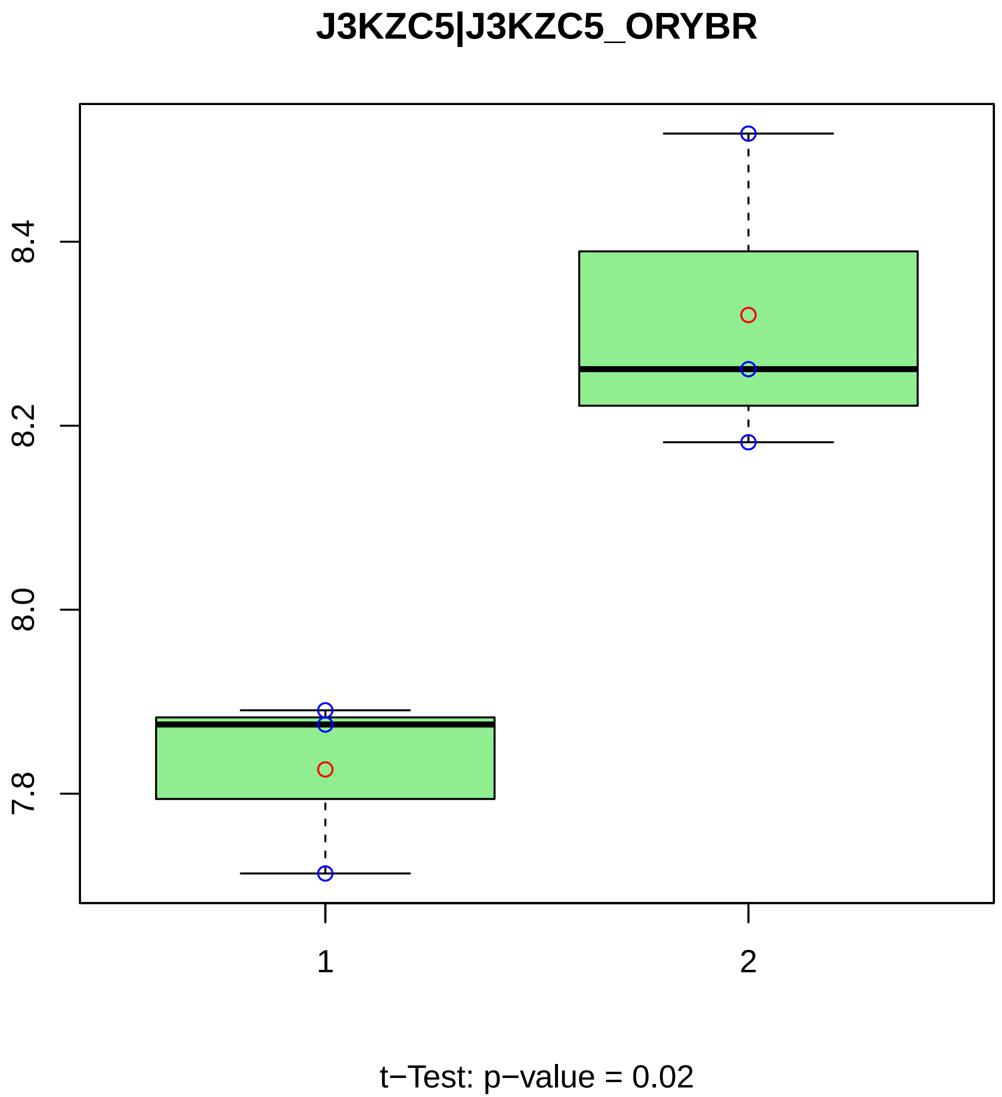
J3KZC5|J3KZC5_ORYBR
8.4
8.2
8.0
7.8
1
2
t−Test: p−value = 0.02

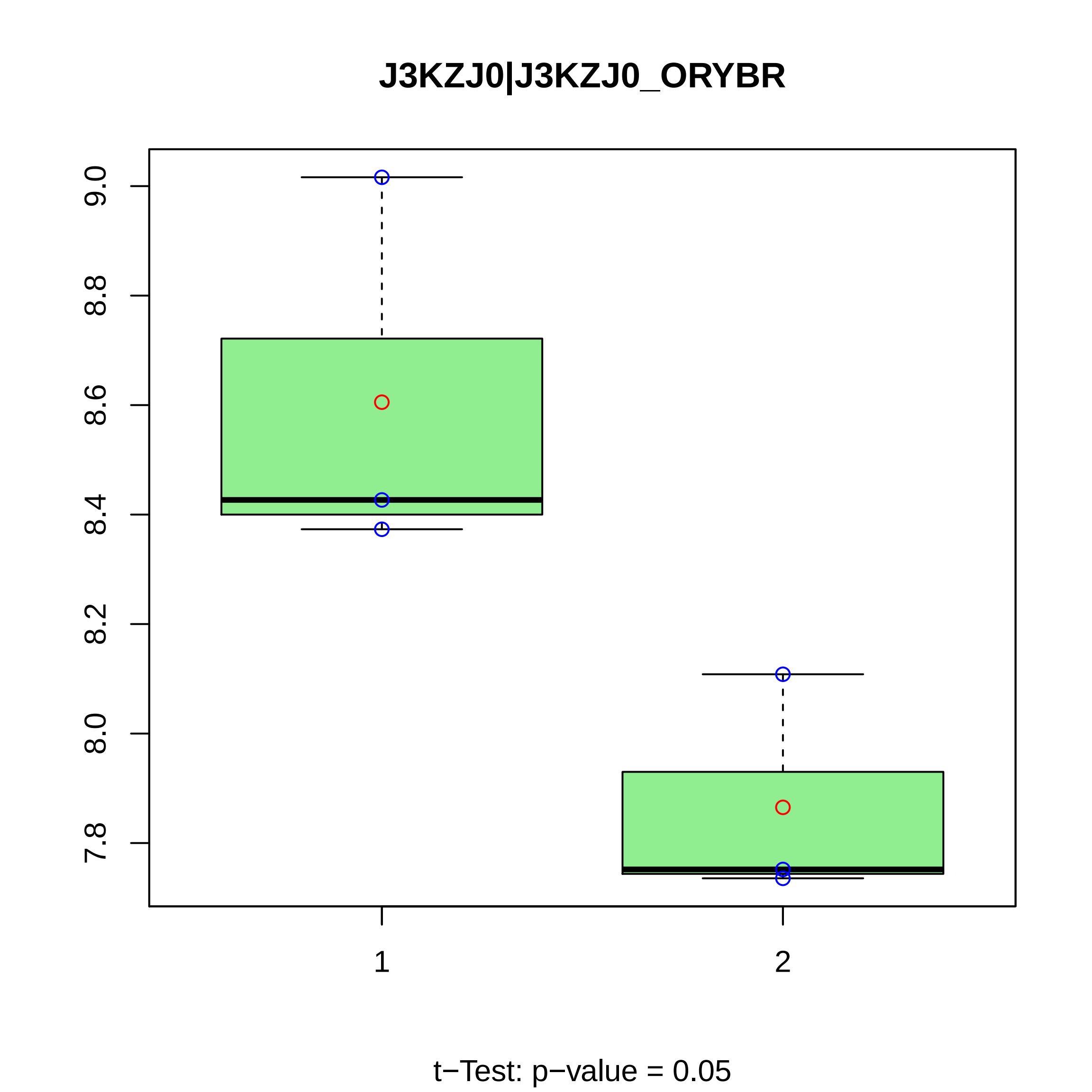
J3KZJ0|J3KZJ0_ORYBR
9.0
8.8
8.6
8.4
8.2
8.0
7.8
1
2
t−Test: p−value = 0.05

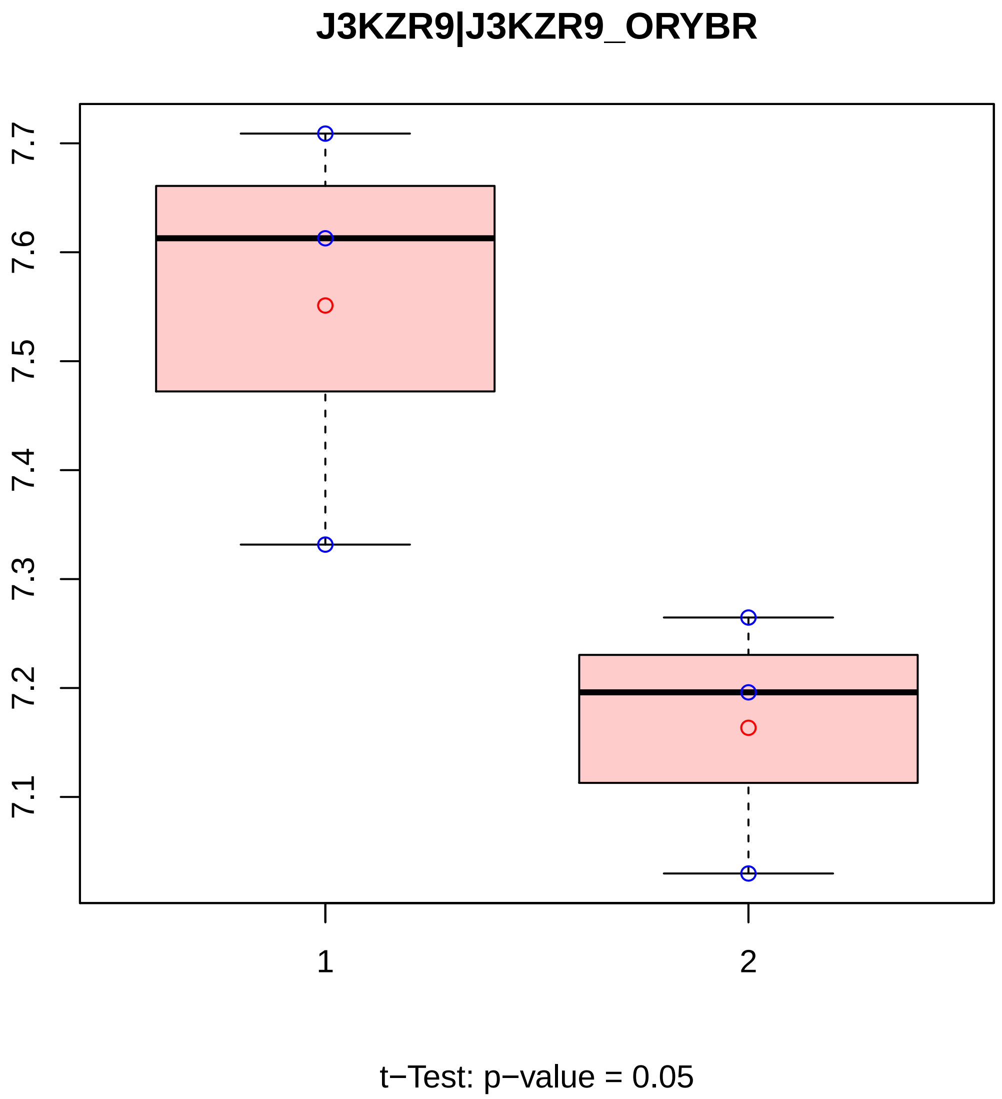
J3KZR9|J3KZR9_ORYBR
7.7
7.6
7.5
7.4
7.3
7.2
7.1
1
2
t−Test: p−value = 0.05

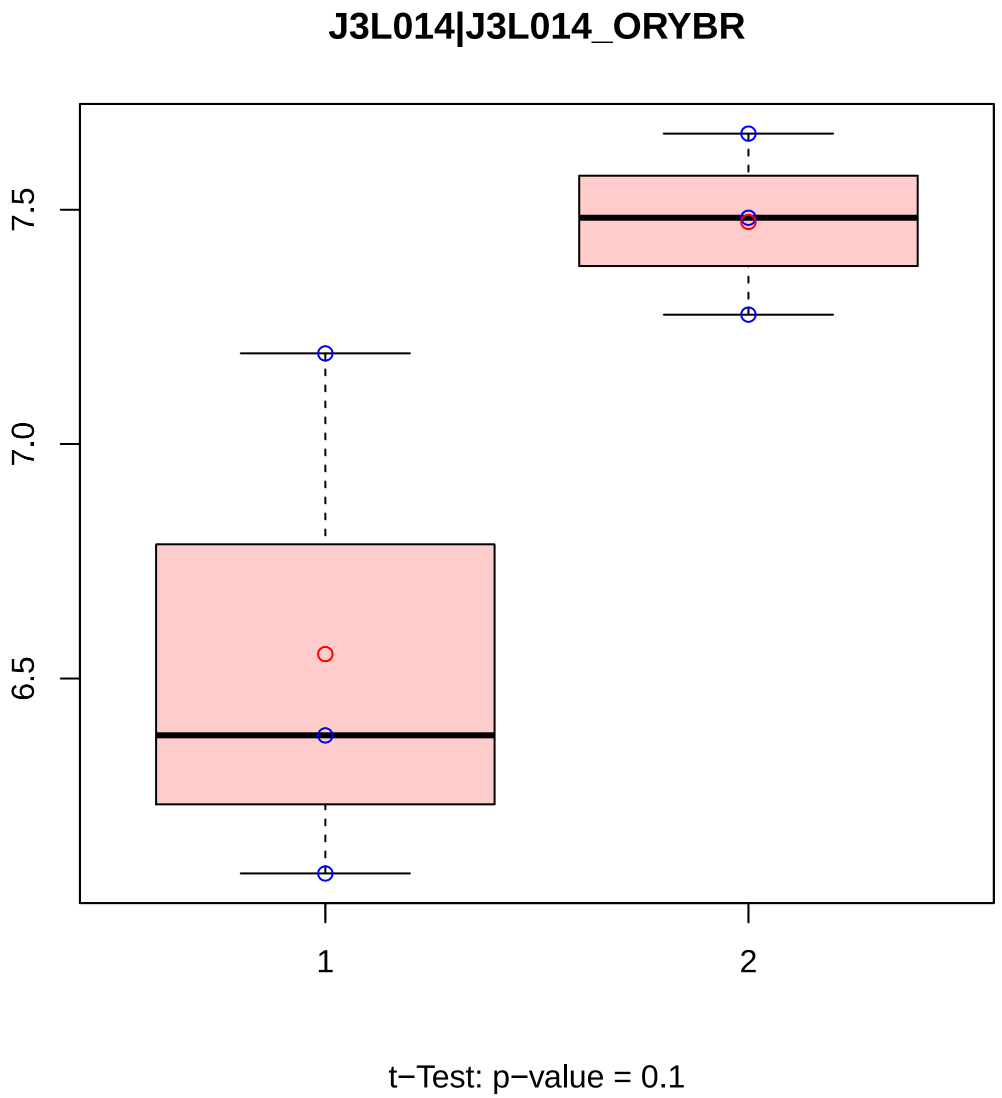
J3L014|J3L014_ORYBR
7.5
7.0
6.5
1
2
t−Test: p−value = 0.1

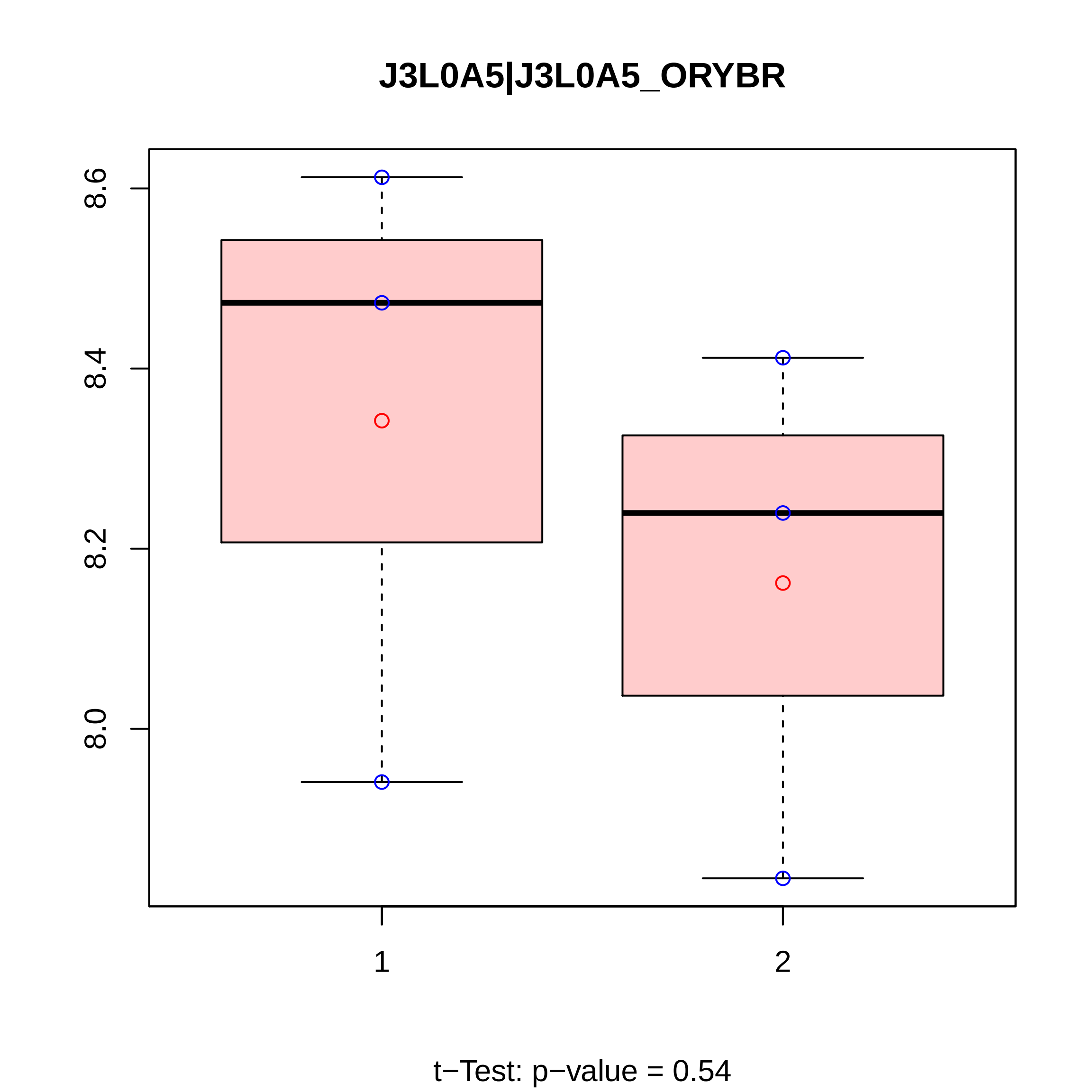
J3L0A5|J3L0A5_ORYBR
8.0
8.2
8.4
8.6
1
2
t−Test: p−value = 0.54

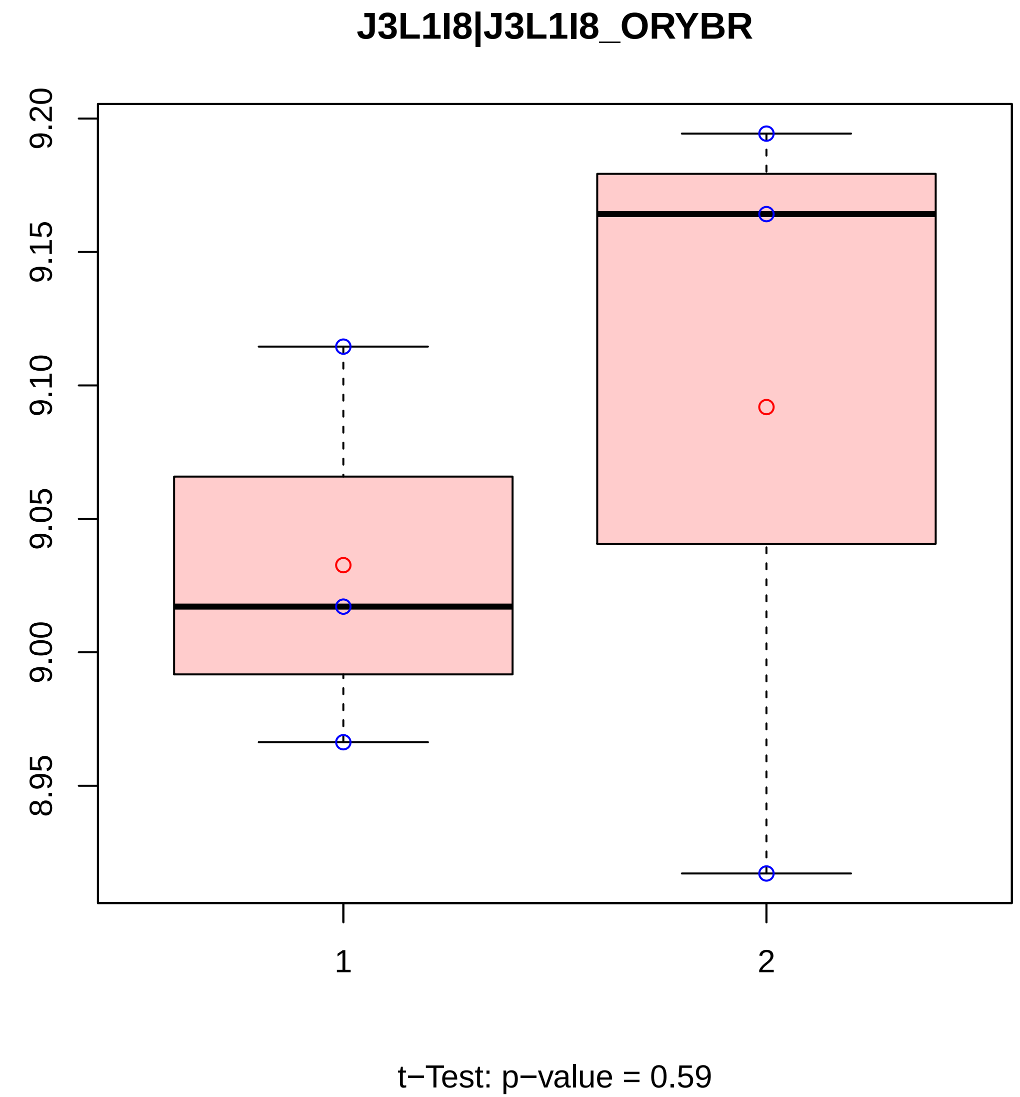
J3L1I8|J3L1I8_ORYBR
9.20
9.15
9.10
9.05
9.00
8.95
1
2
t−Test: p−value = 0.59

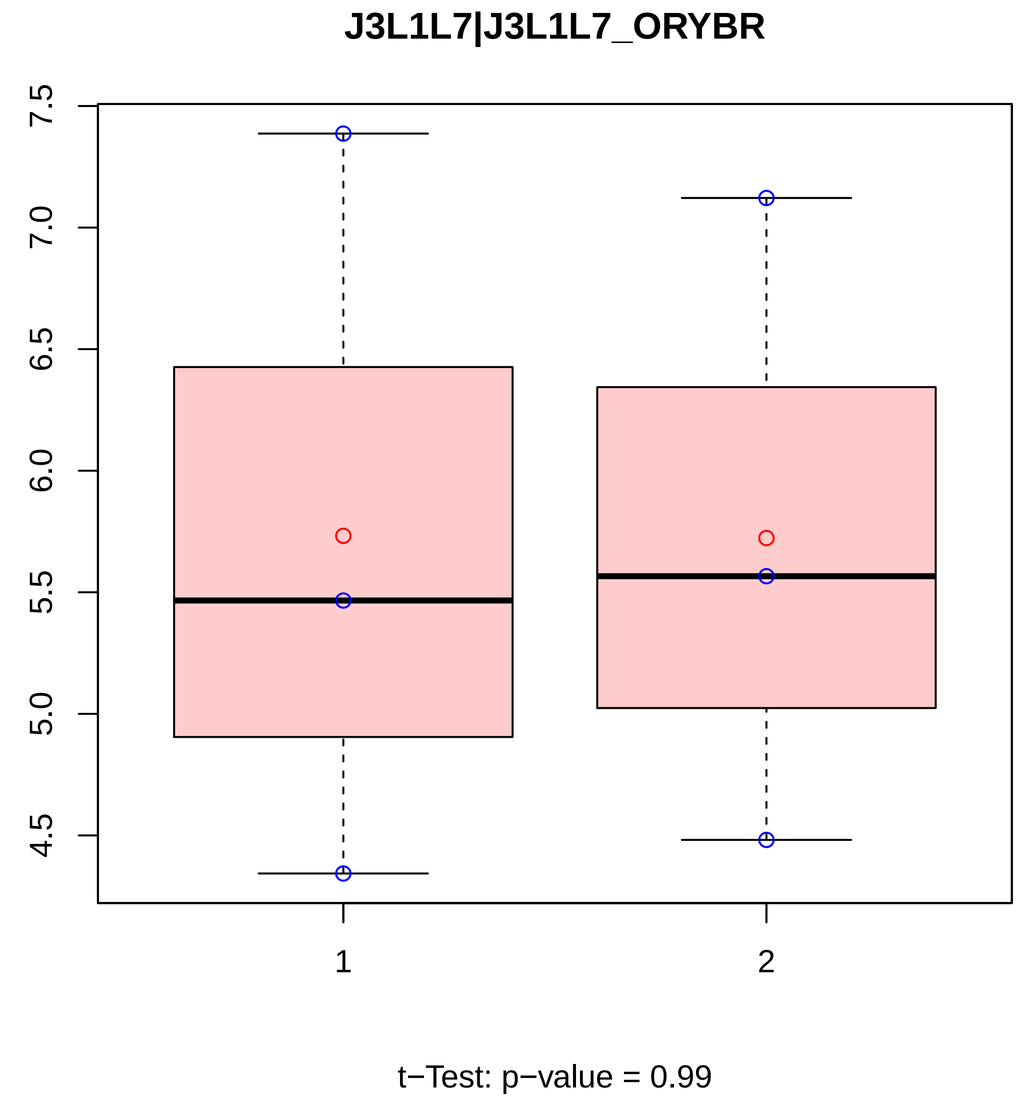
J3L1L7|J3L1L7_ORYBR
7.5
7.0
6.5
6.0
5.5
5.0
4.5
1
2
t−Test: p−value = 0.99

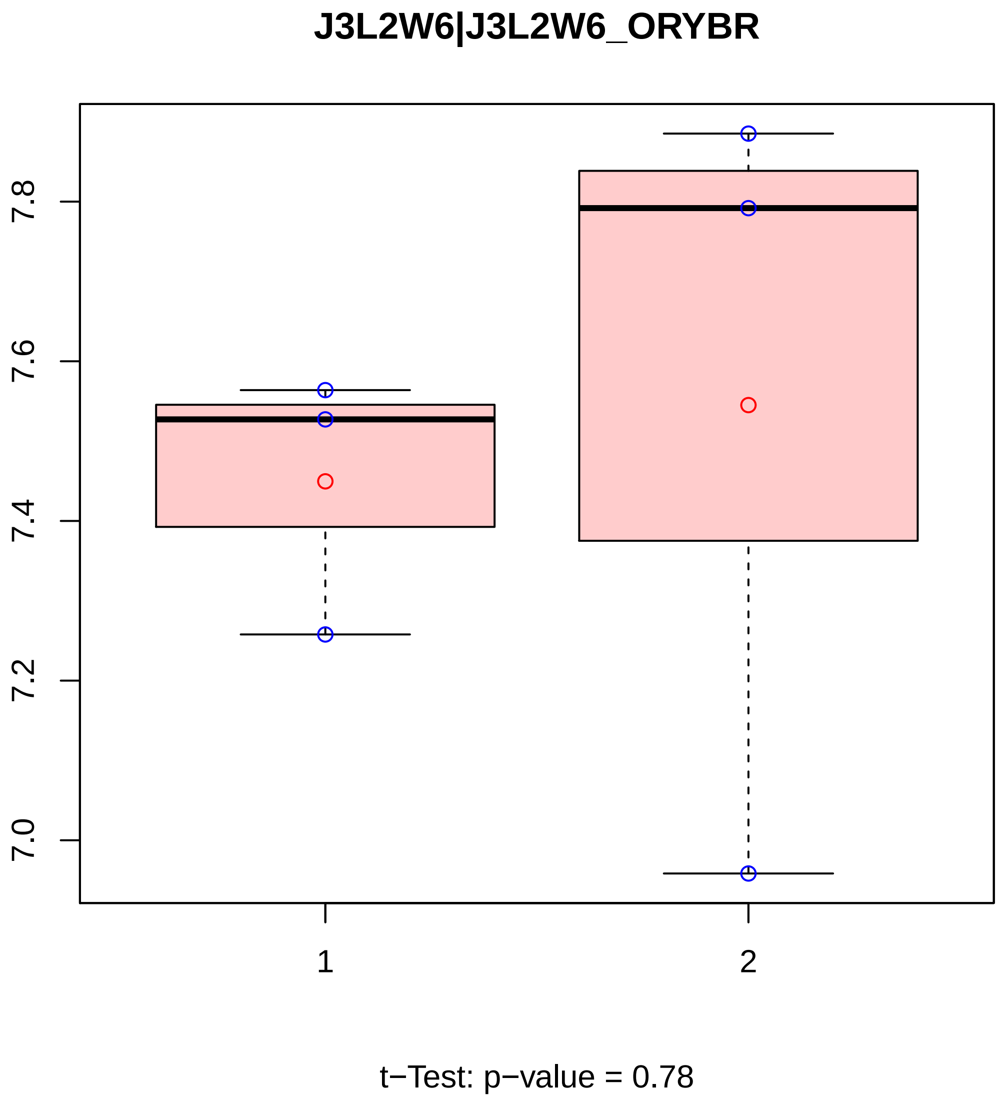
J3L2W6|J3L2W6_ORYBR
7.0
7.2
7.4
7.6
7.8
1
2
t−Test: p−value = 0.78

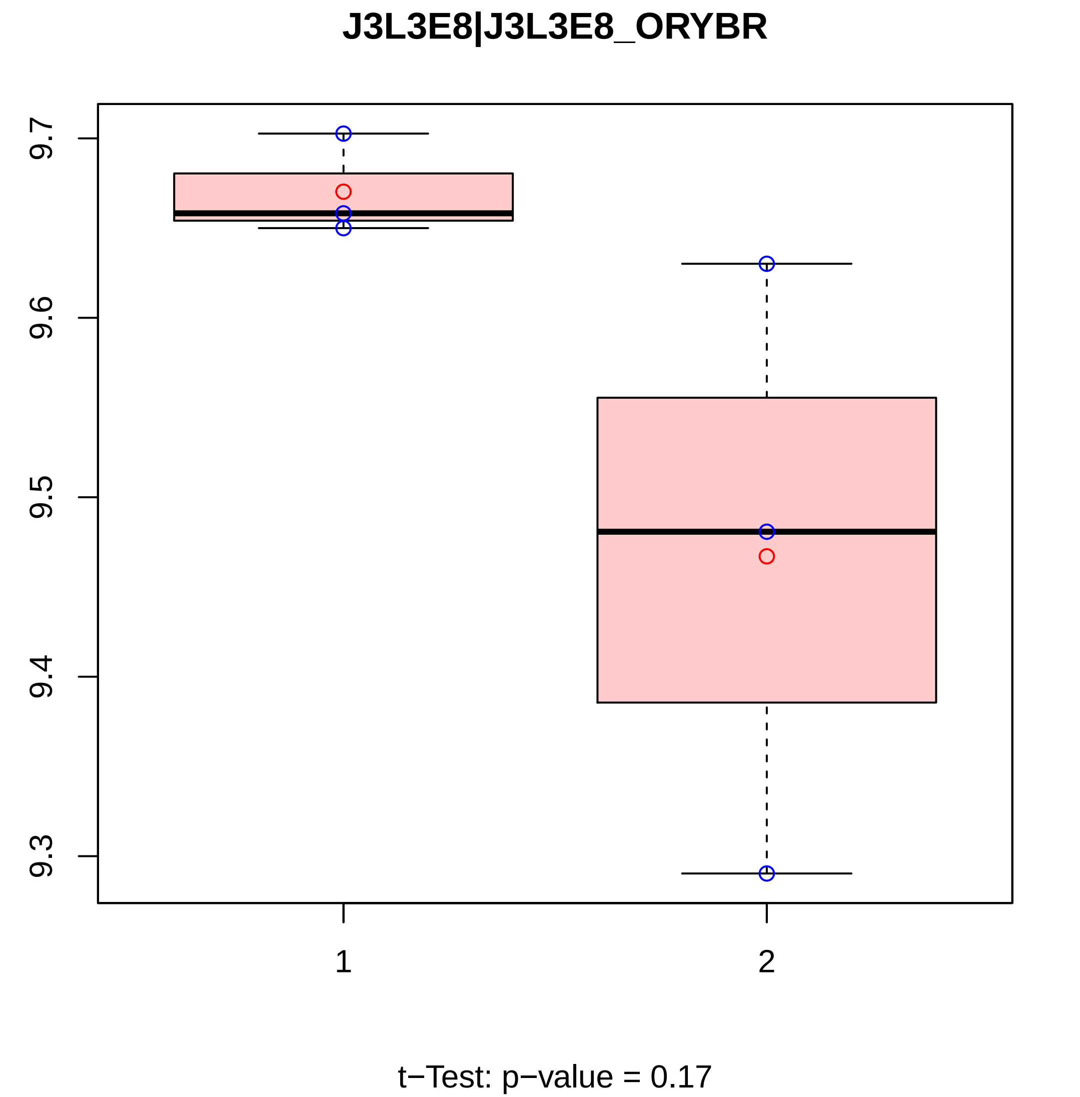
J3L3E8|J3L3E8_ORYBR
9.7
9.6
9.5
9.4
9.3
1
2
t−Test: p−value = 0.17

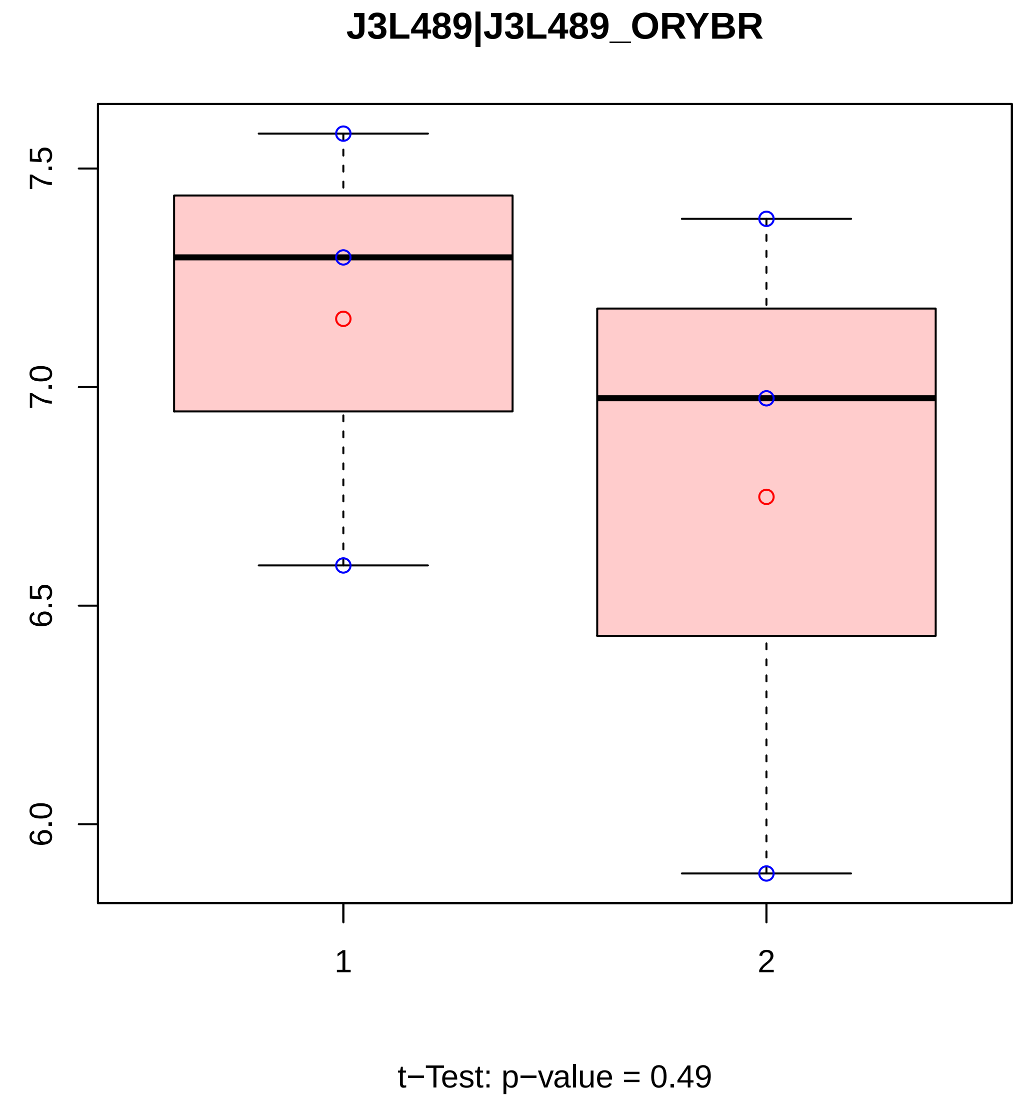
J3L489|J3L489_ORYBR
7.5
7.0
6.5
6.0
1
2
t−Test: p−value = 0.49

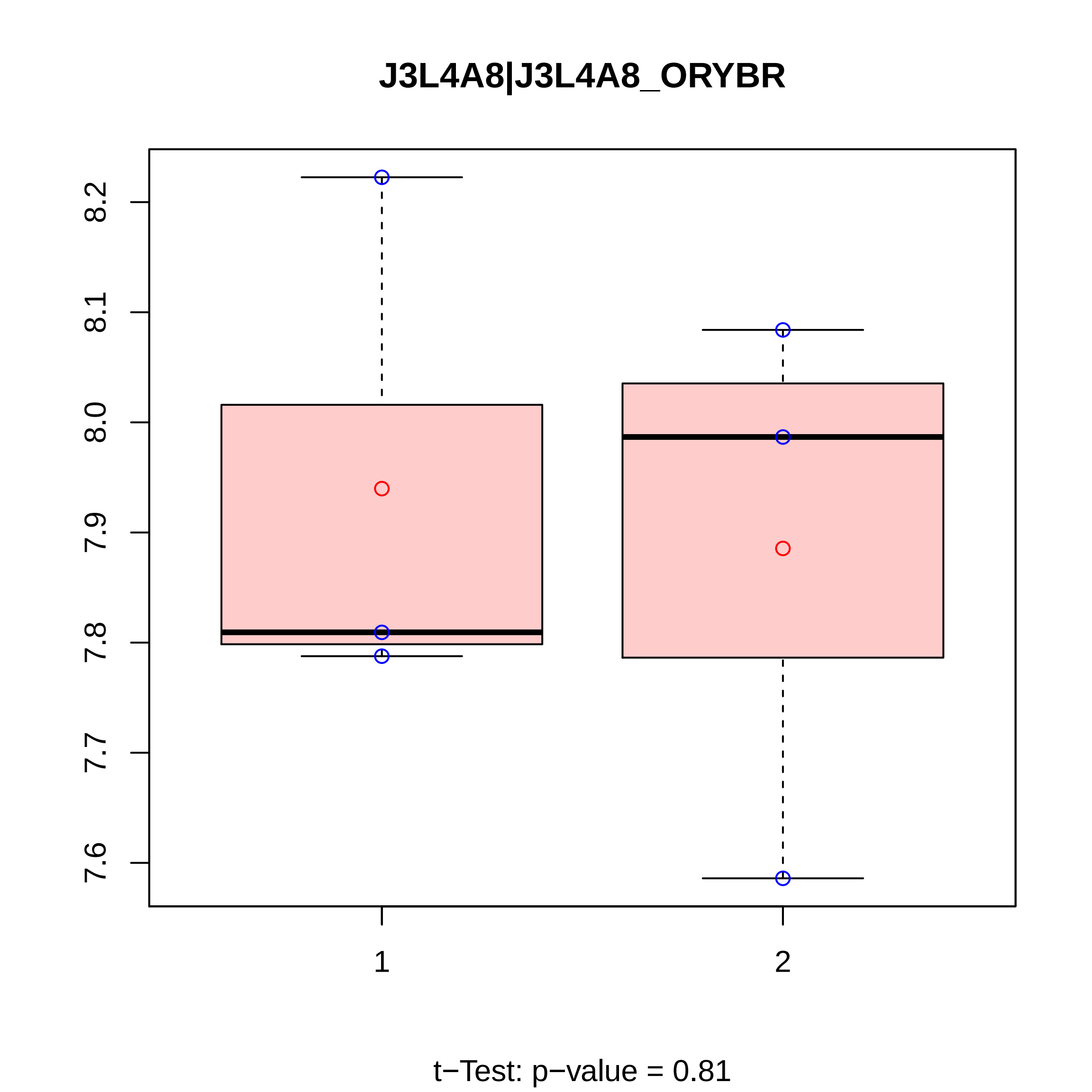

J3L4A8|J3L4A8_ORYBR
8.2
8.1
8.0
7.9
7.8
7.7
7.6
1
2
t−Test: p−value = 0.81

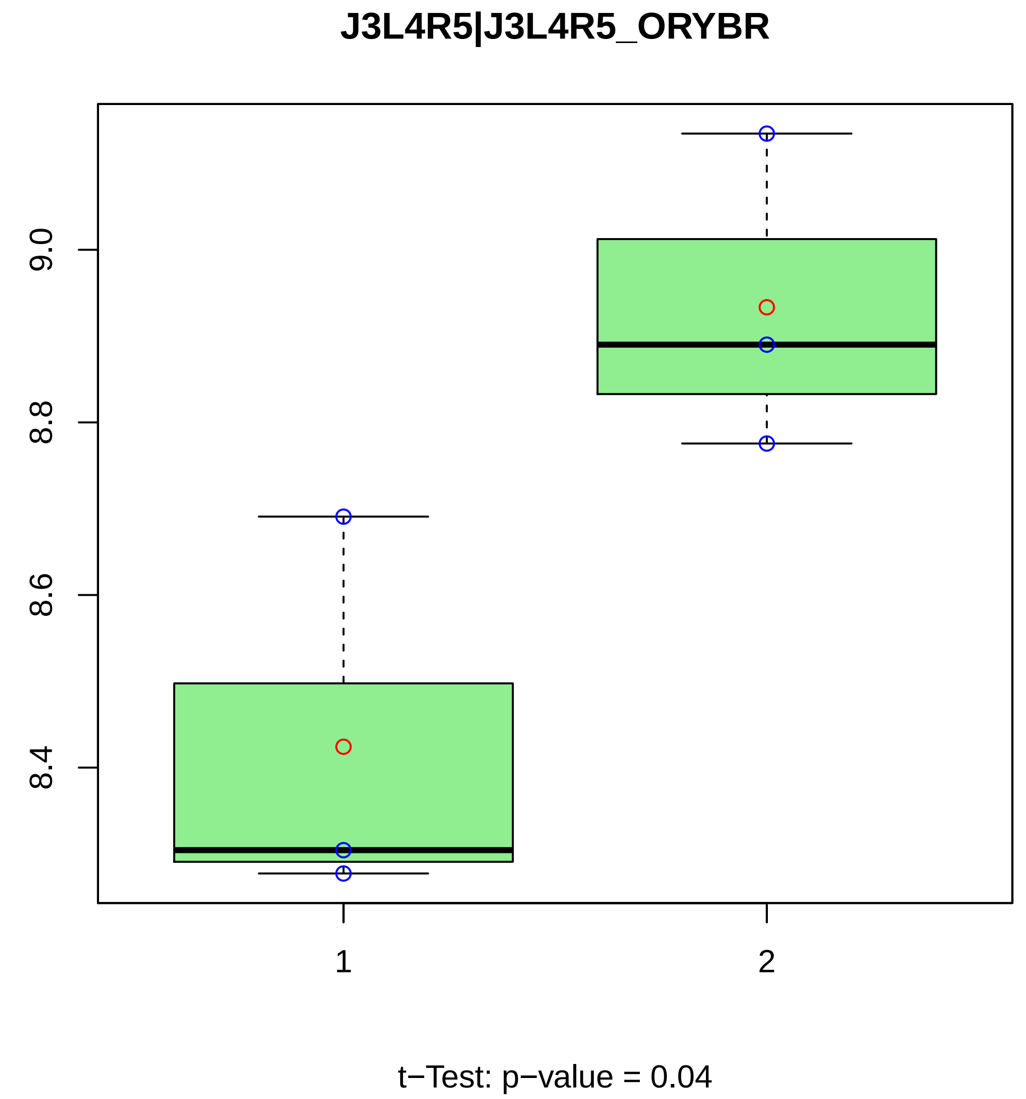
J3L4R5|J3L4R5_ORYBR
9.0
8.8
8.6
8.4
1
2
t−Test: p−value = 0.04

J3L4S8|J3L4S8_ORYBR

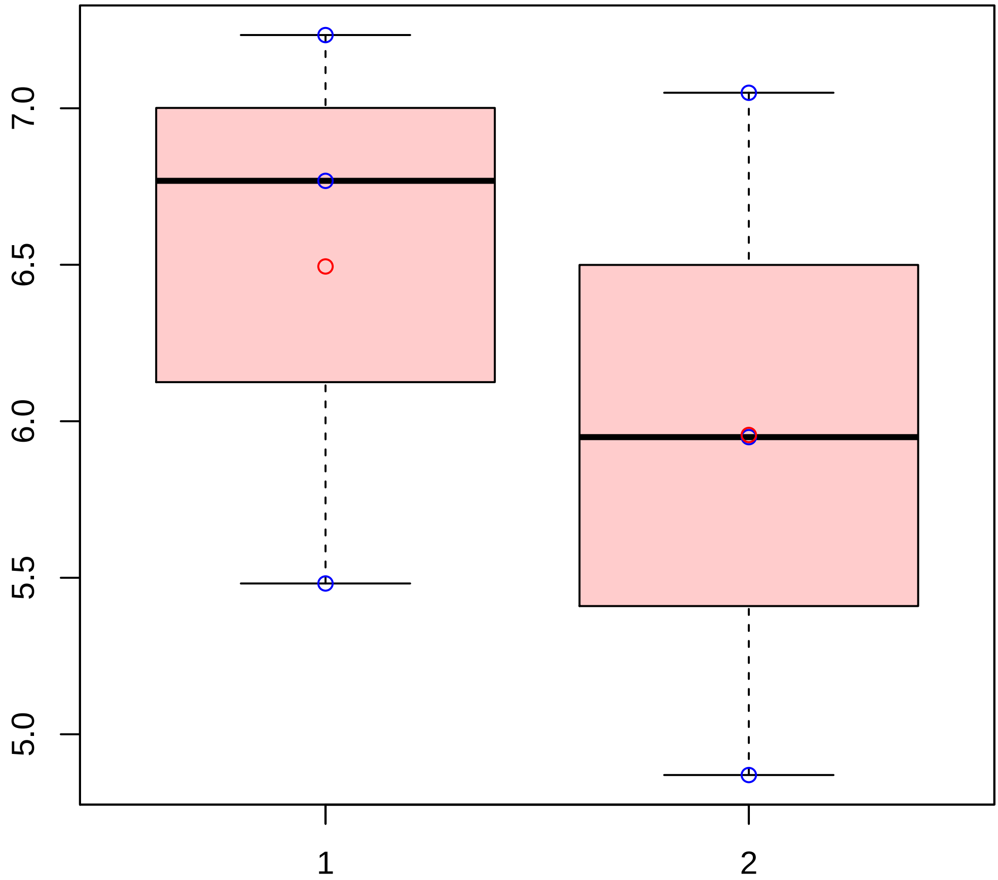

t−Test: p−value = 0.55

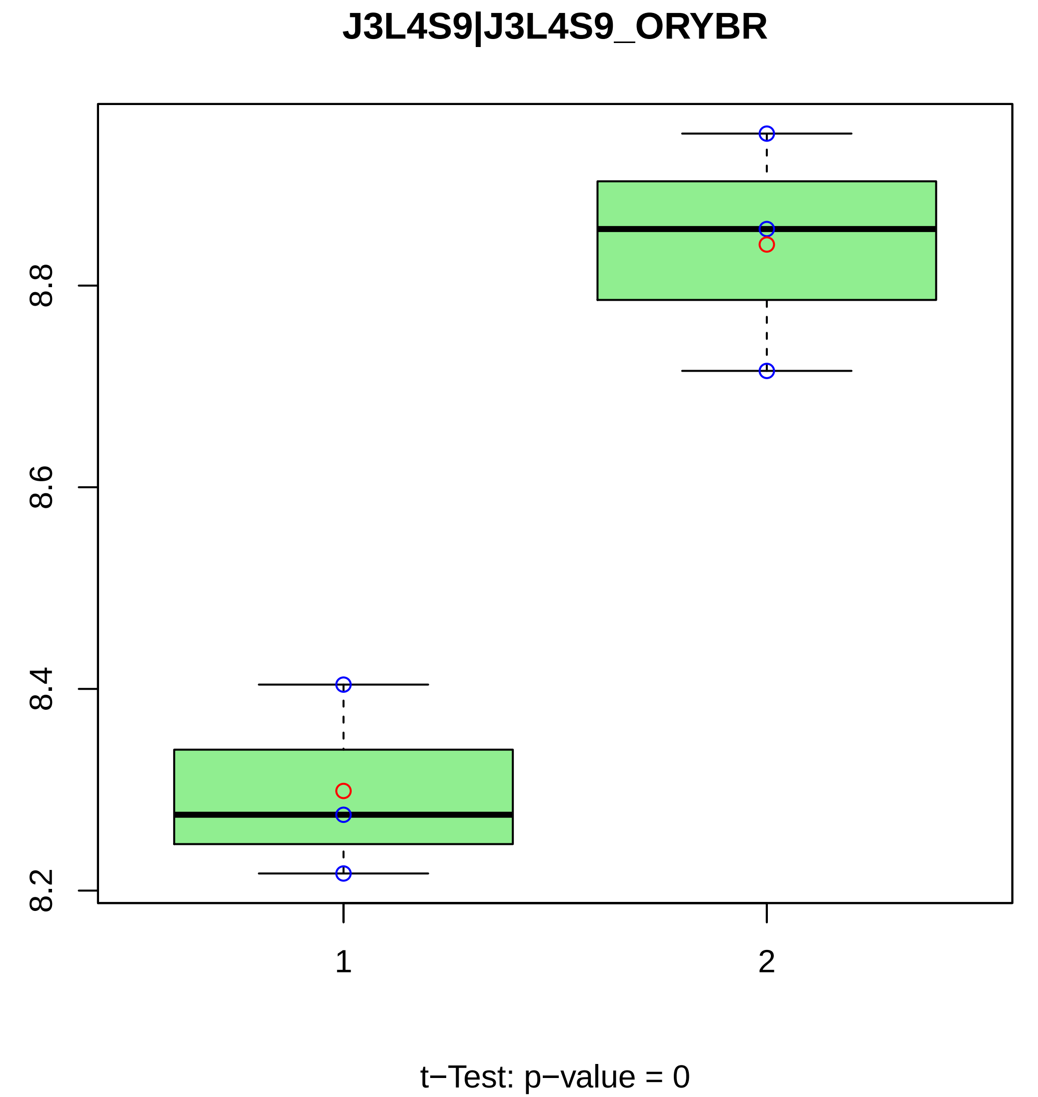
J3L4S9|J3L4S9_ORYBR
8.2
8.4
8.6
8.8
1
2
t−Test: p−value = 0

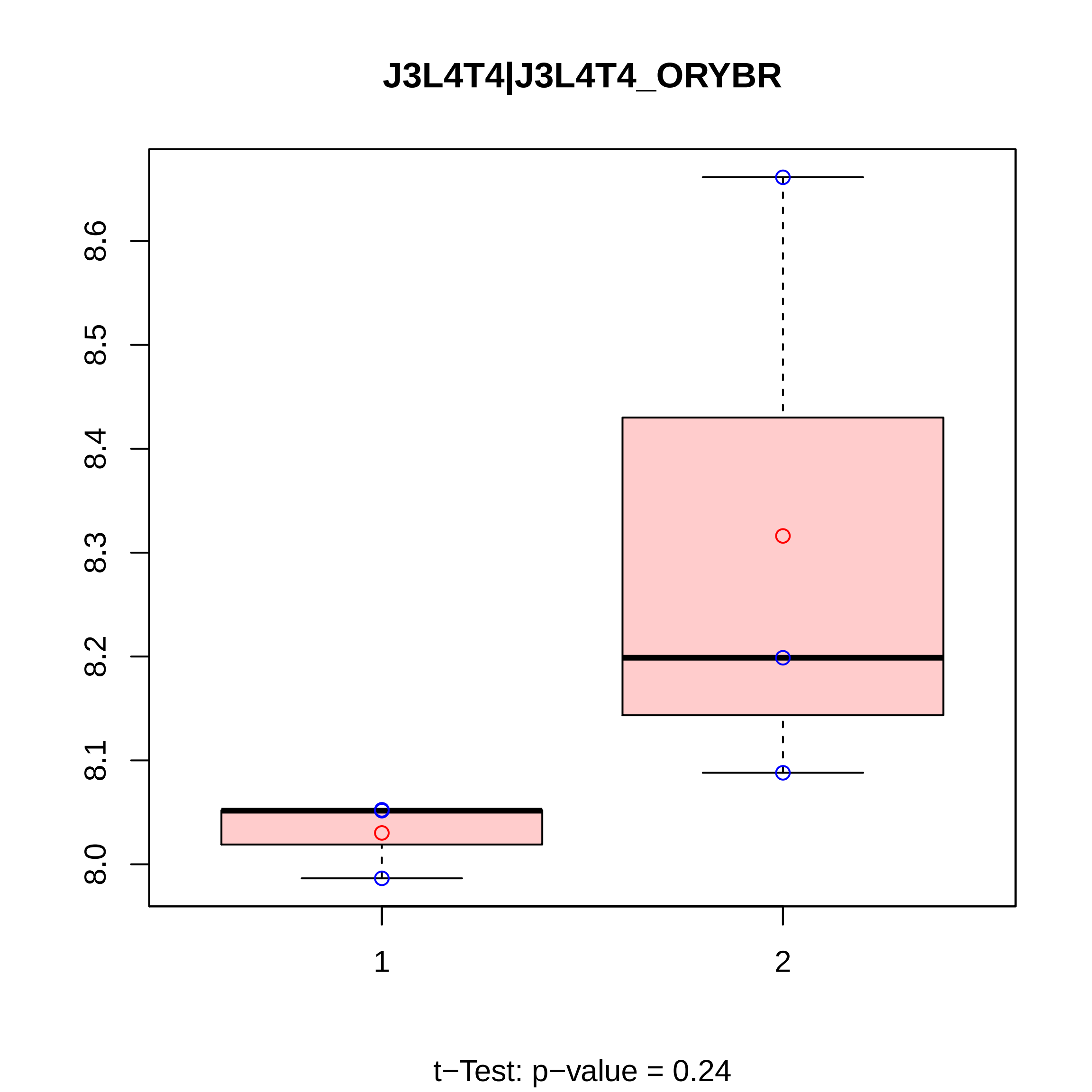
J3L4T4|J3L4T4_ORYBR
8.0
8.1
8.2
8.3
8.4
8.5
8.6
1
2
t−Test: p−value = 0.24

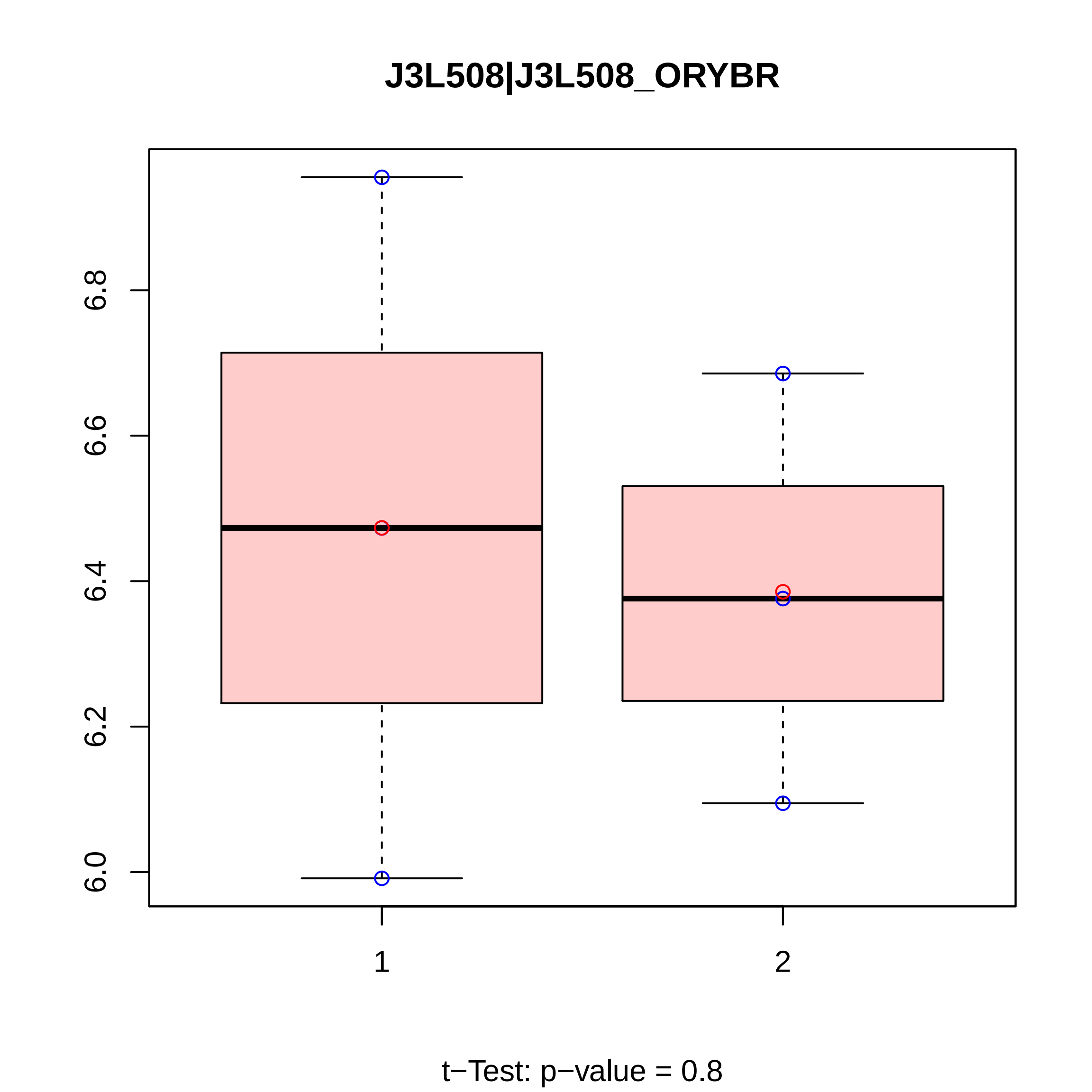
J3L508|J3L508_ORYBR
6.0
6.2
6.4
6.6
6.8
1
2
t−Test: p−value = 0.8

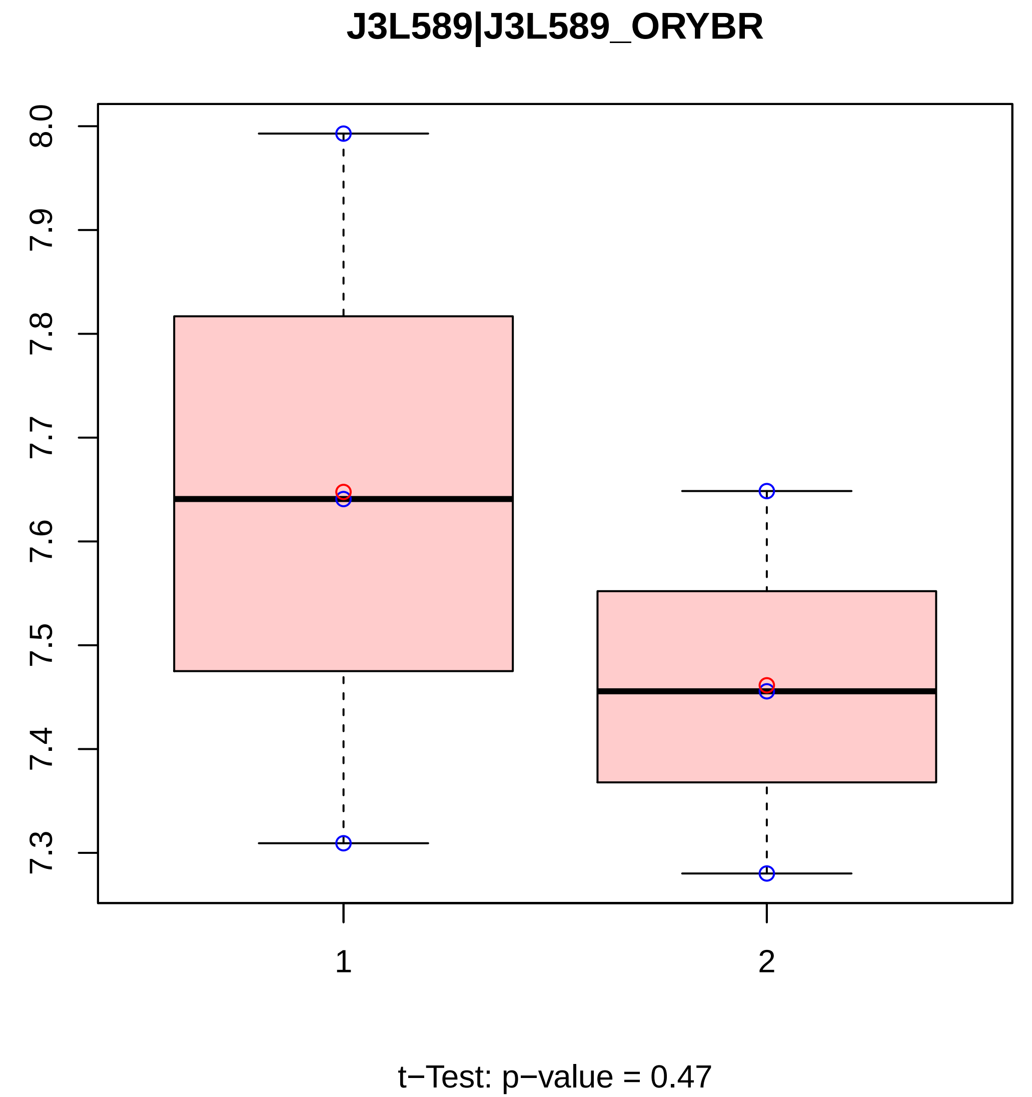
J3L589|J3L589_ORYBR
8.0
7.9
7.8
7.7
7.6
7.5
7.4
7.3
1
2
t−Test: p−value = 0.47

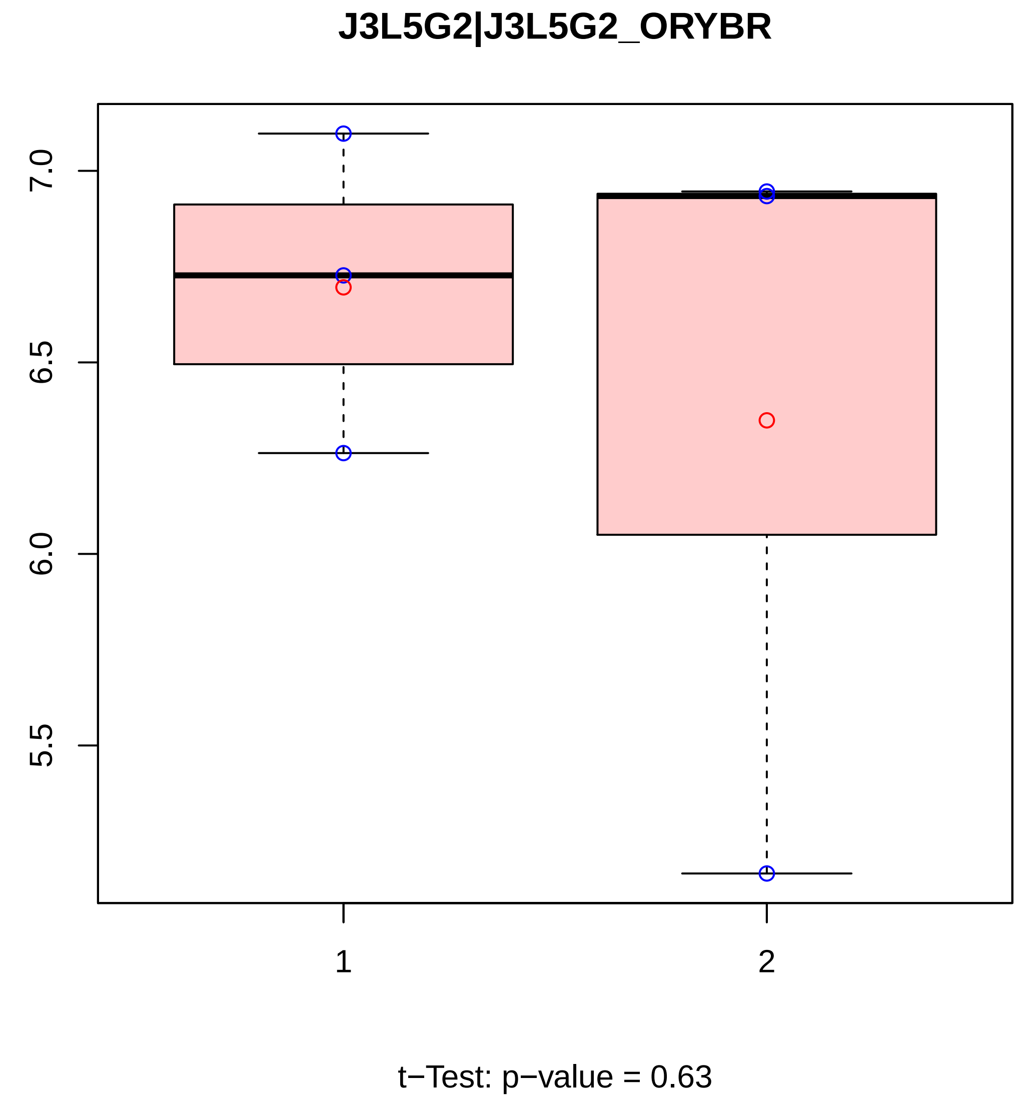
J3L5G2|J3L5G2_ORYBR
7.0
6.5
6.0
5.5
1
2
t−Test: p−value = 0.63

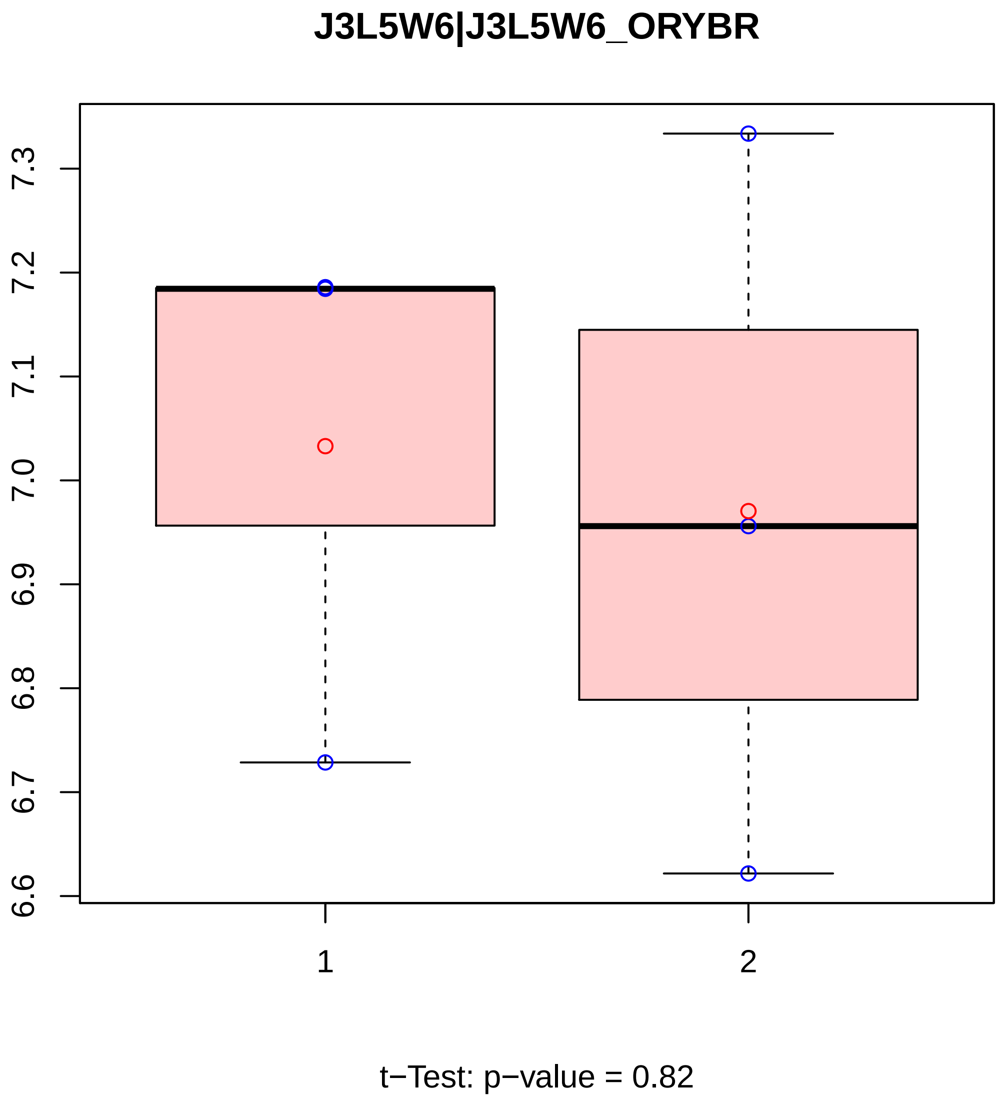

J3L5W6|J3L5W6_ORYBR
7.3
7.2
7.1
7.0
6.9
6.8
6.7
6.6
1
2
t−Test: p−value = 0.82

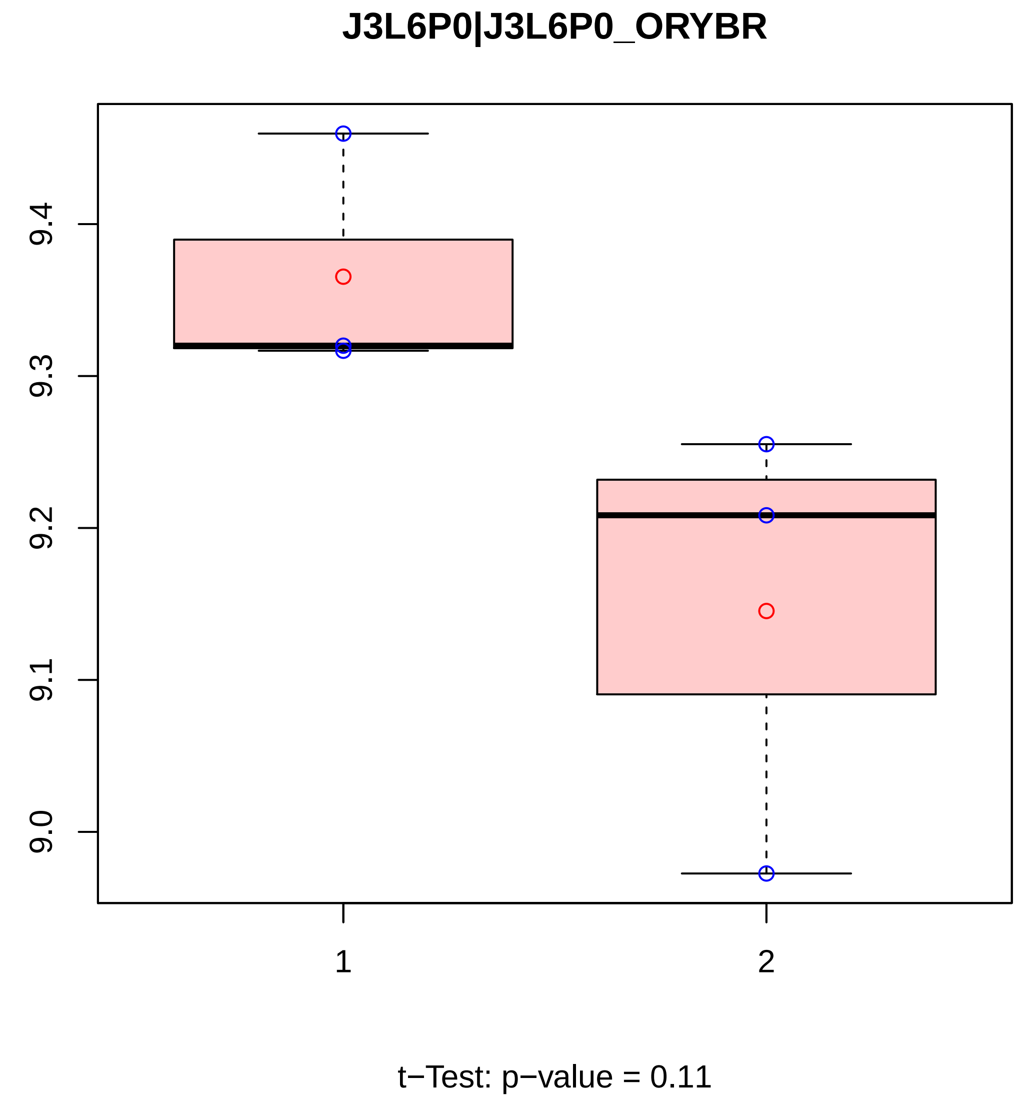
J3L6P0|J3L6P0_ORYBR
9.0
9.1
9.2
9.3
9.4
1
2
t−Test: p−value = 0.11

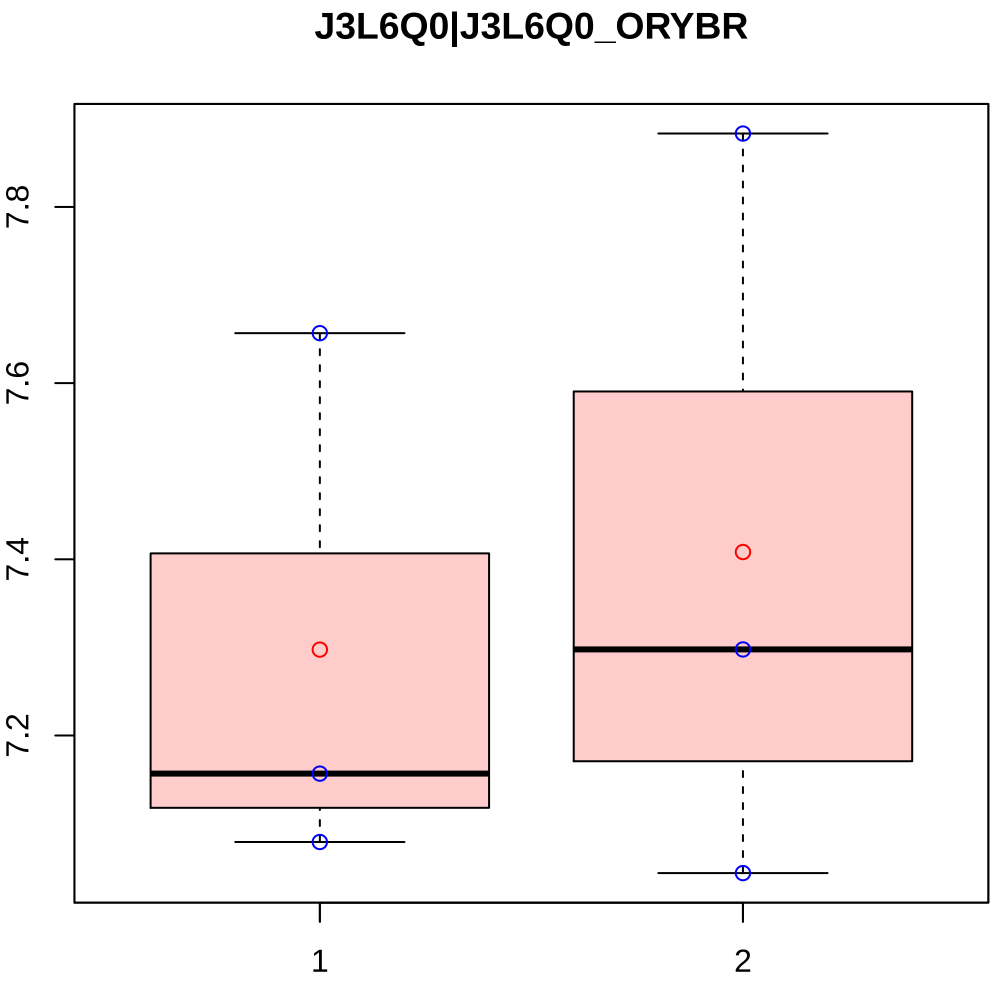

t−Test: p−value = 0.74

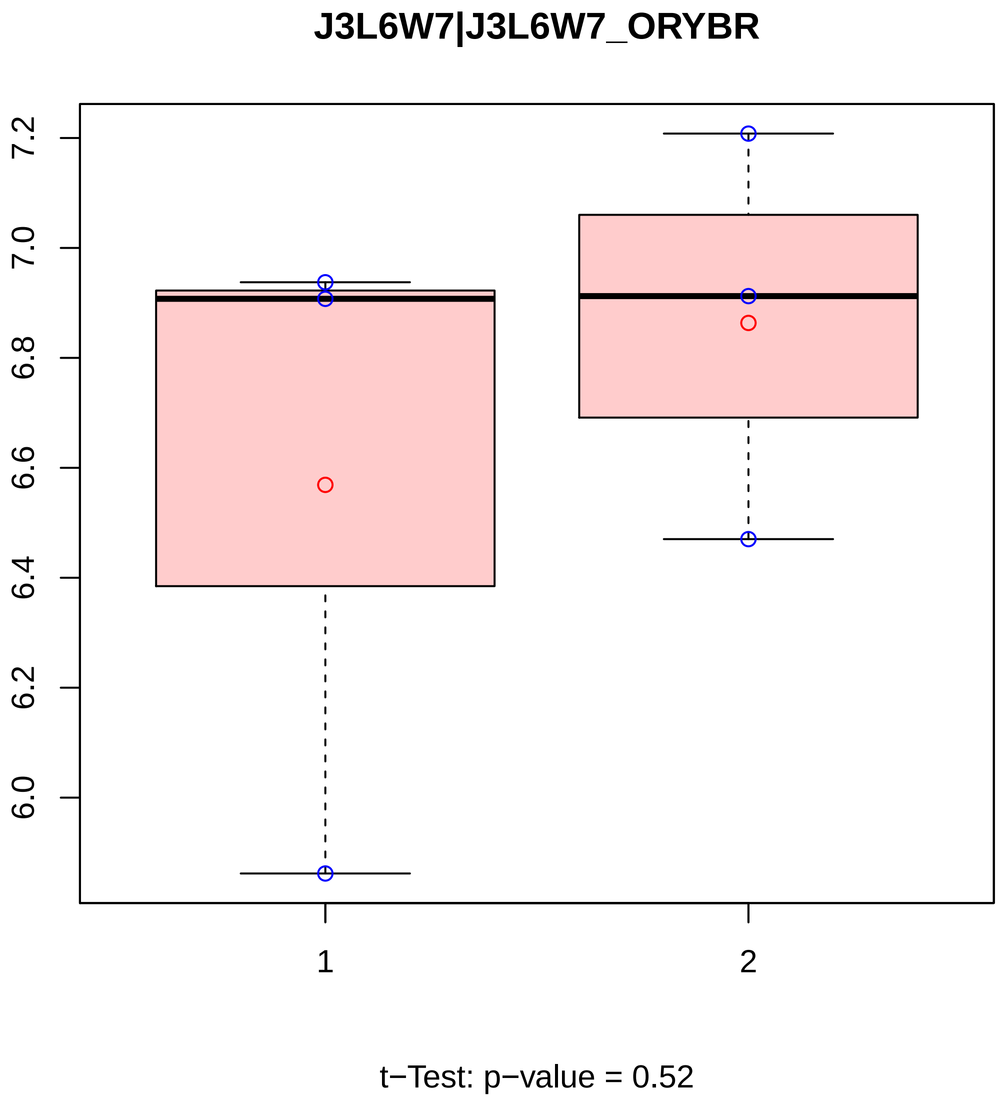

J3L6W7|J3L6W7_ORYBR
7.2
7.0
6.8
6.6
6.4
6.2
6.0
1
2
t−Test: p−value = 0.52

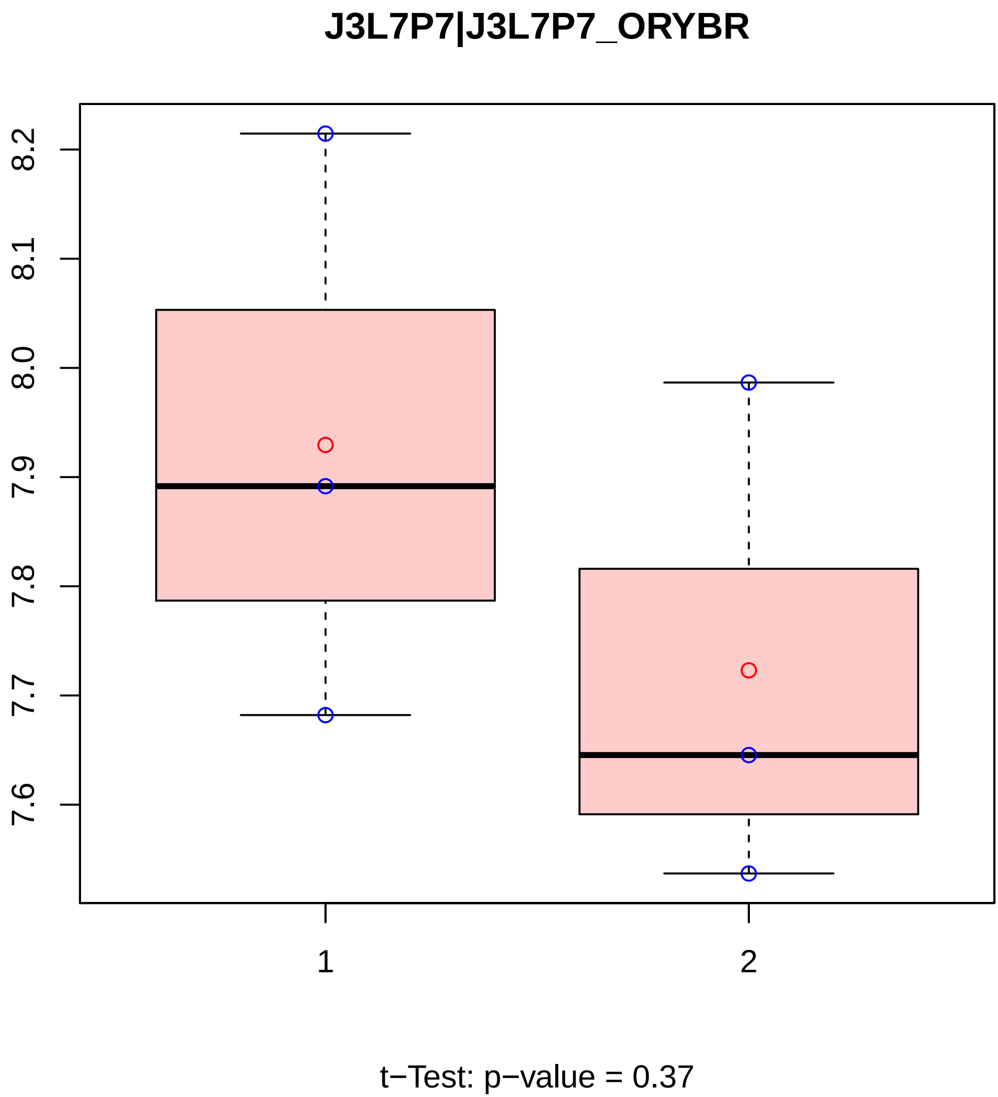
J3L7P7|J3L7P7_ORYBR
8.2
8.1
8.0
7.9
7.8
7.7
7.6
1
2
t−Test: p−value = 0.37

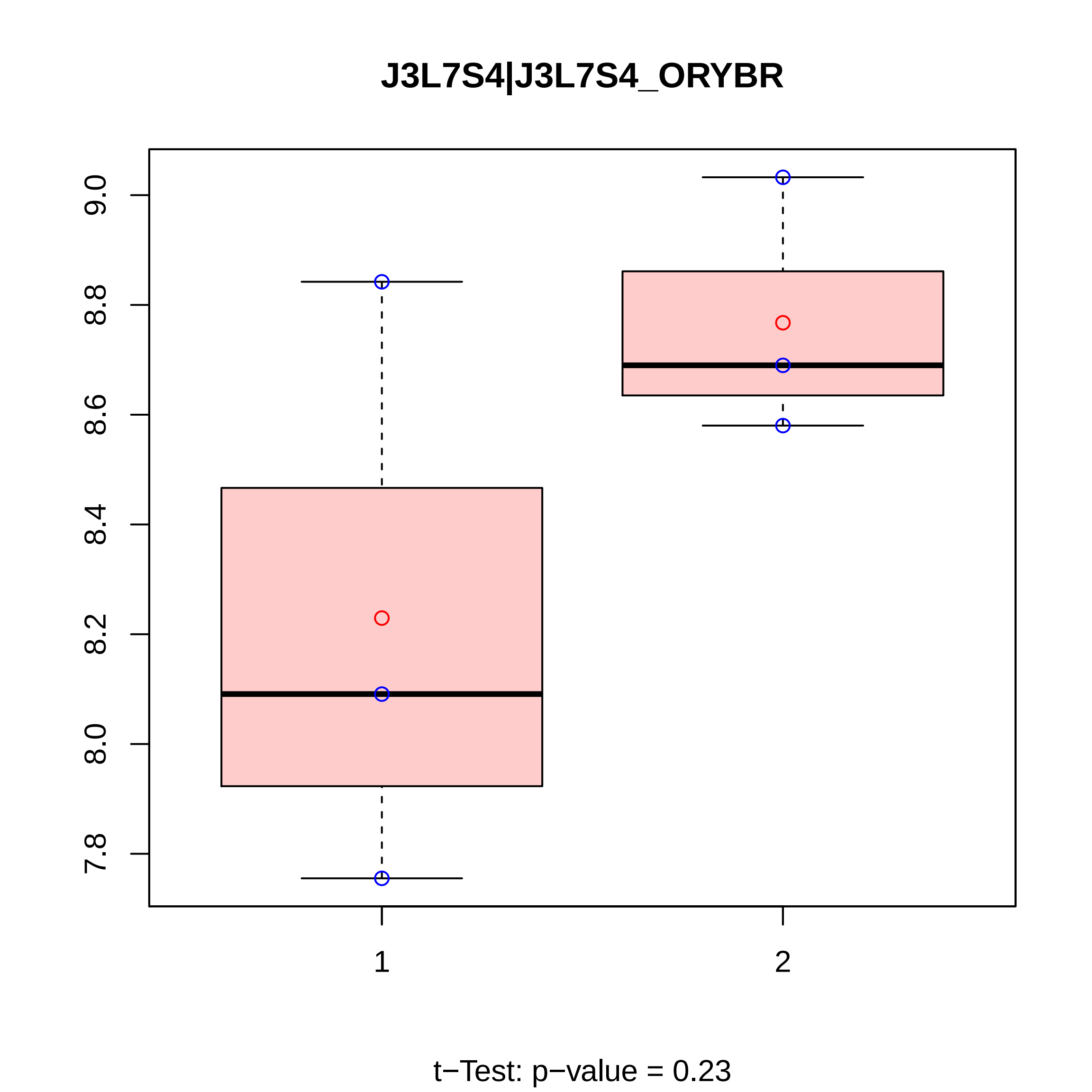
J3L7S4|J3L7S4_ORYBR
9.0
8.8
8.6
8.4
8.2
8.0
7.8
1
2
t−Test: p−value = 0.23

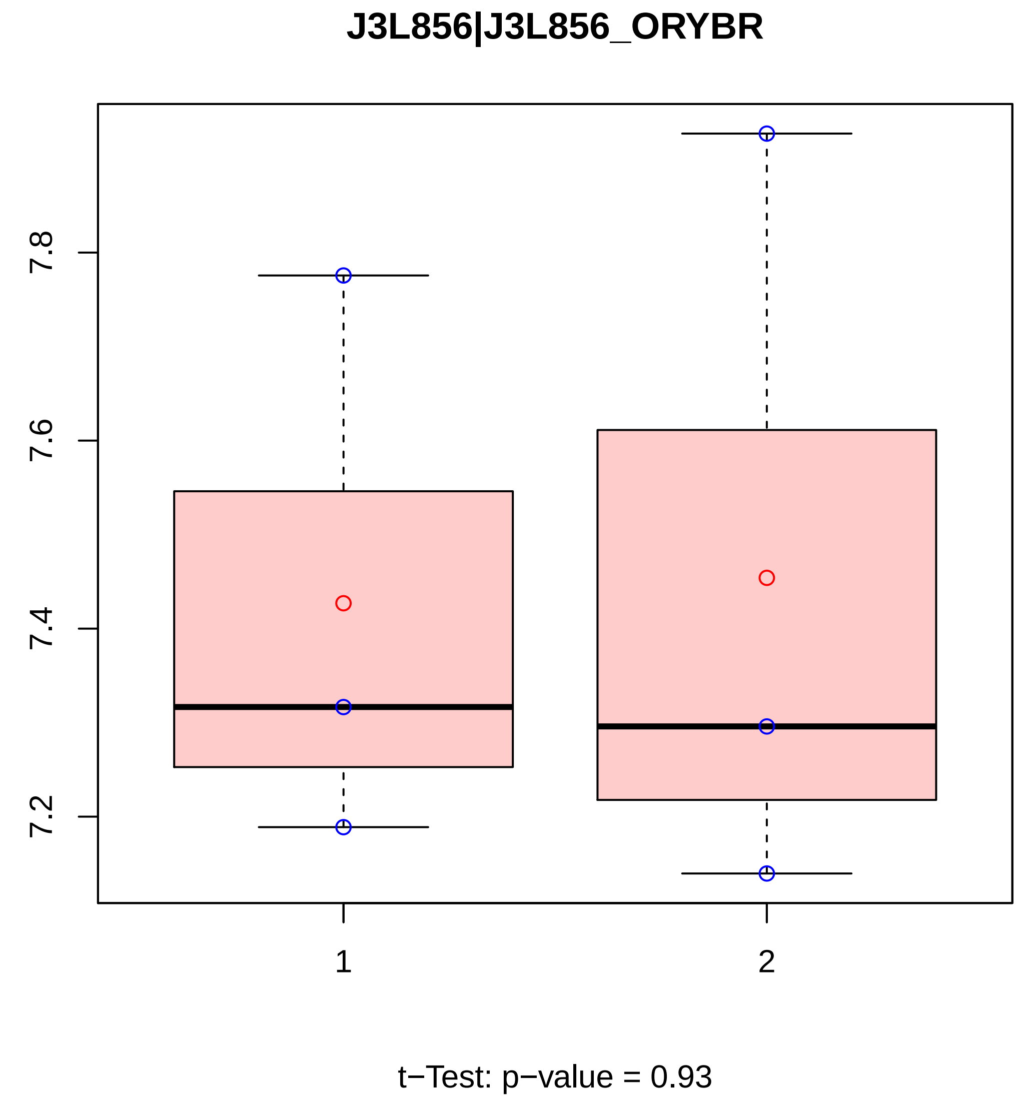

J3L856|J3L856_ORYBR
7.2
7.4
7.6
7.8
1
2
t−Test: p−value = 0.93

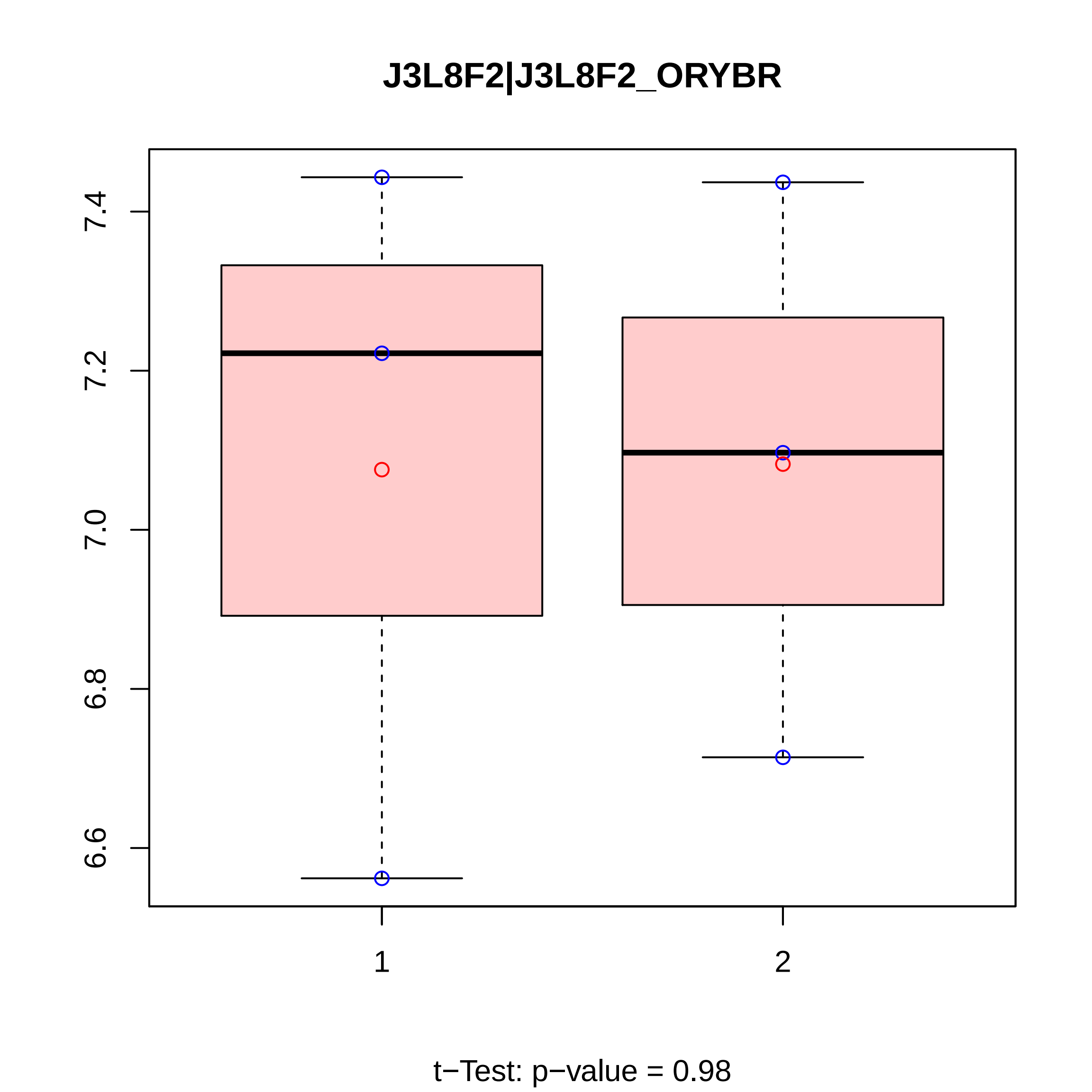

J3L8F2|J3L8F2_ORYBR
7.4
7.2
7.0
6.8
6.6
1
2
t−Test: p−value = 0.98

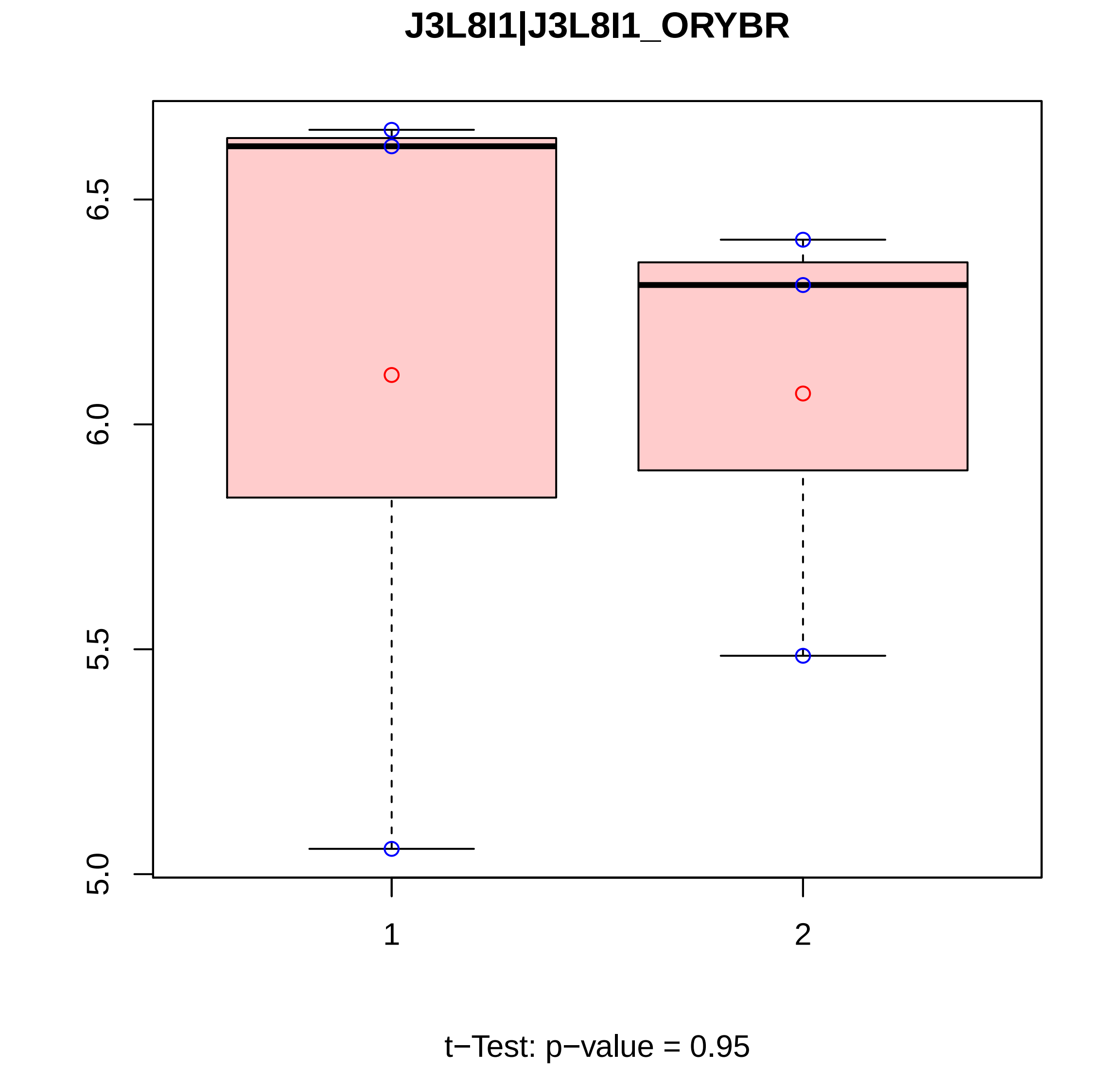
J3L8I1|J3L8I1_ORYBR
5.0
5.5
6.0
6.5
1
2
t−Test: p−value = 0.95

J3L8Y0|J3L8Y0_ORYBR

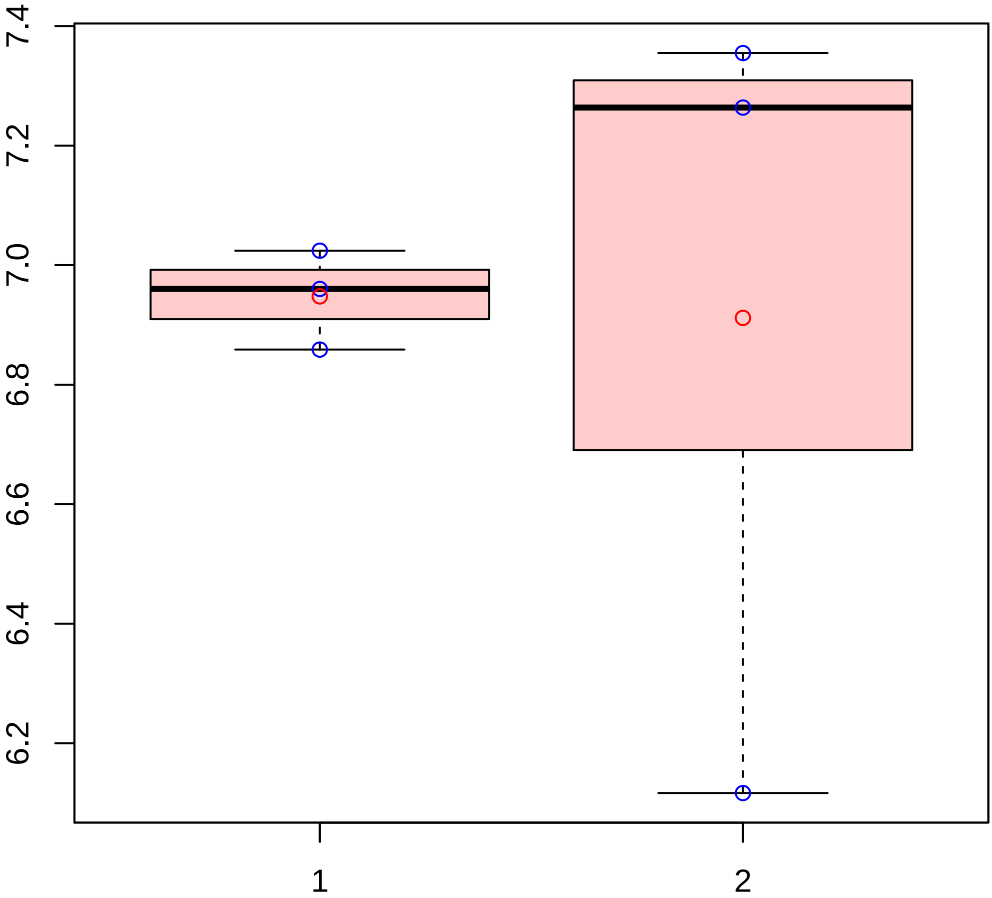

t−Test: p−value = 0.94

J3L9E8|J3L9E8_ORYBR

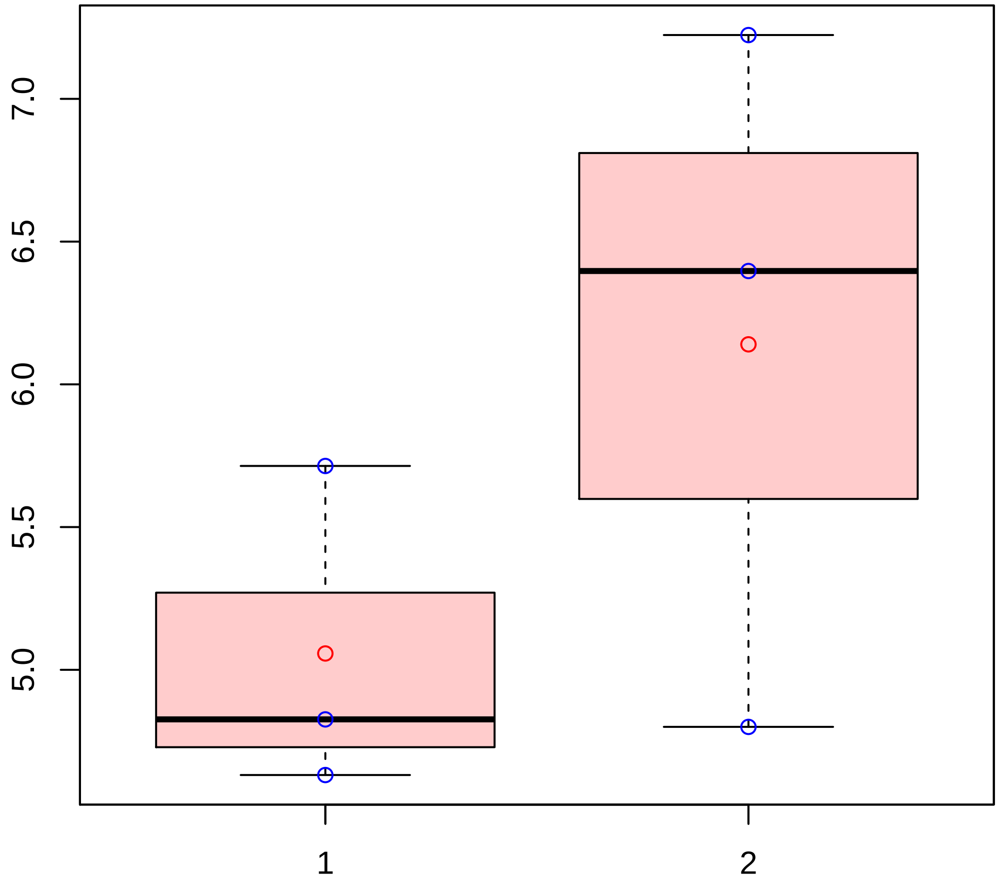

t−Test: p−value = 0.27

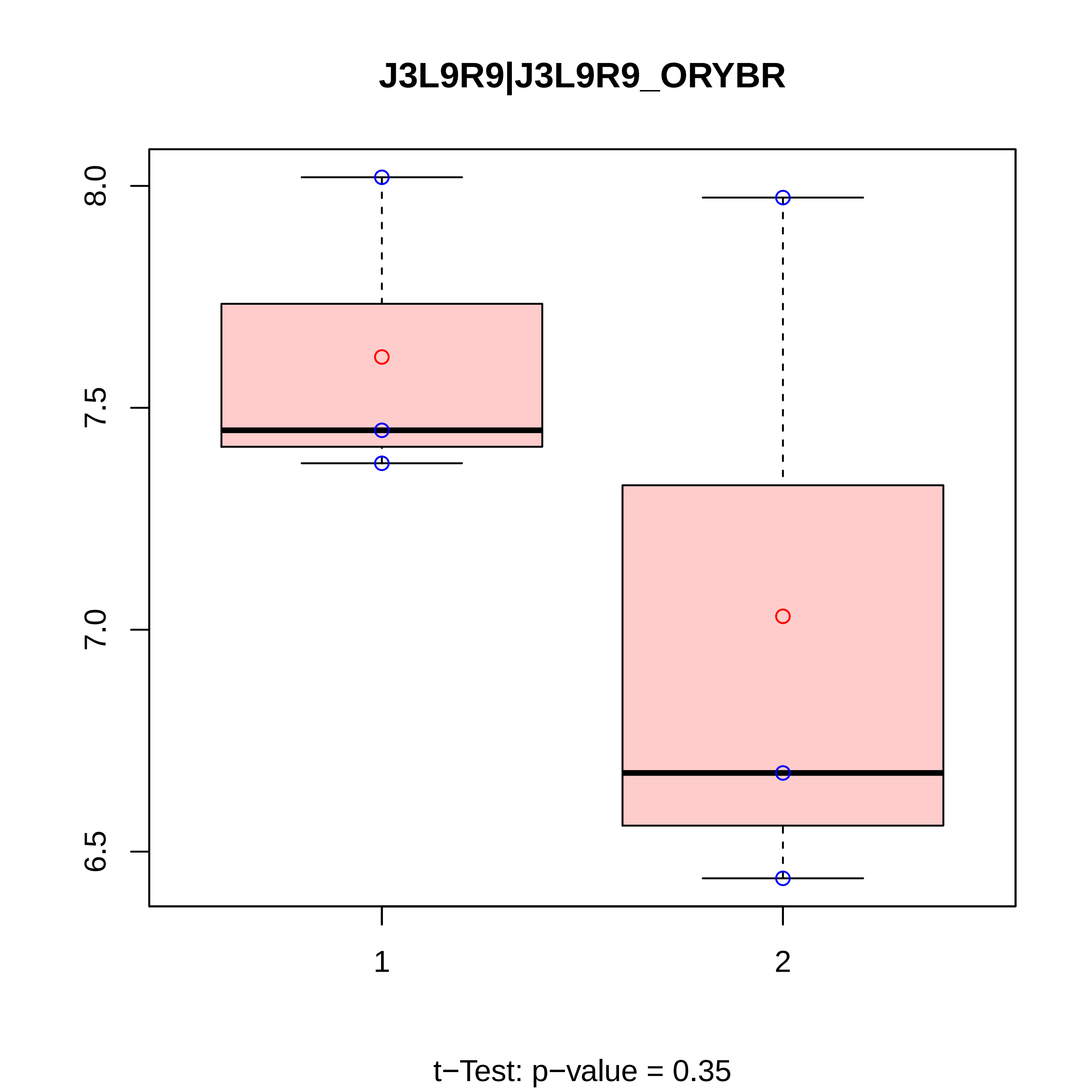
J3L9R9|J3L9R9_ORYBR
8.0
7.5
7.0
6.5
1
2
t−Test: p−value = 0.35

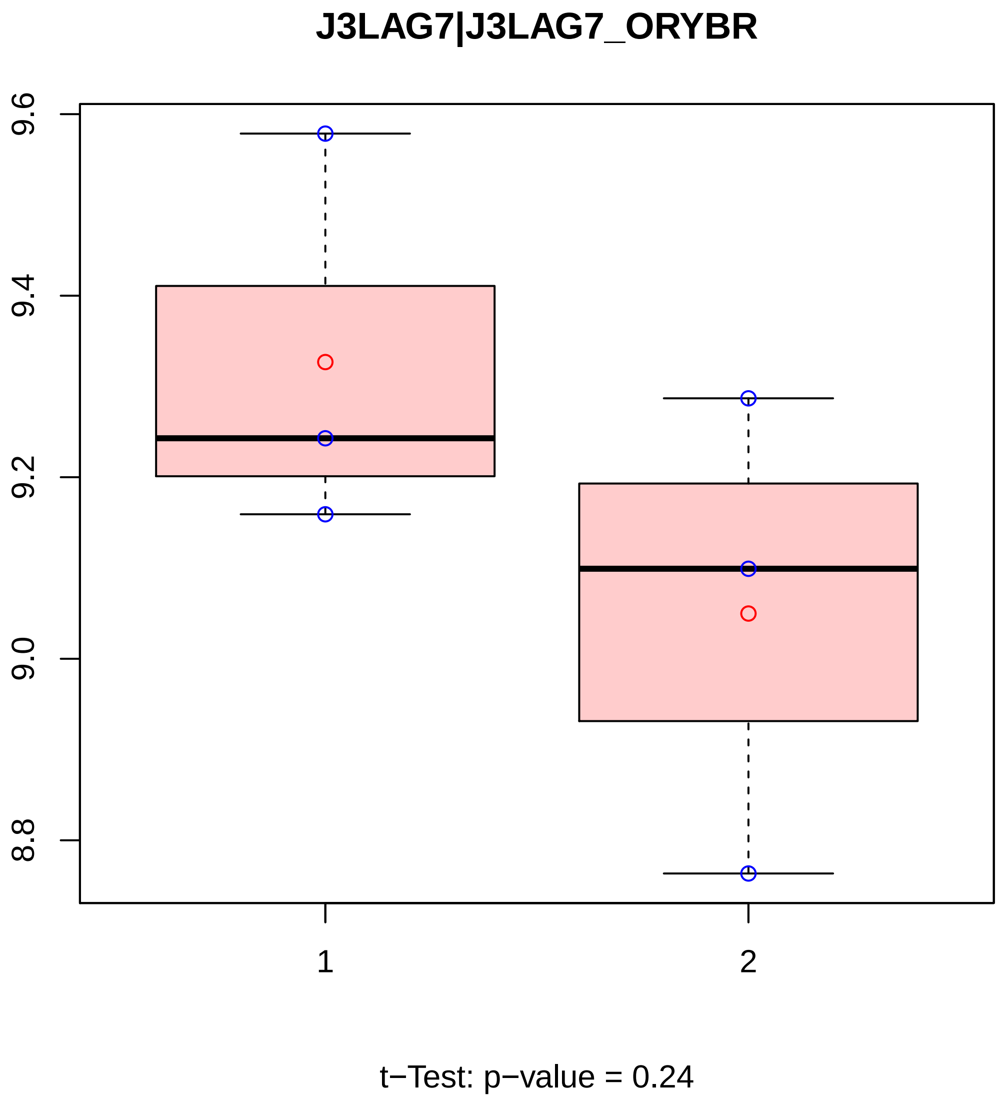
J3LAG7|J3LAG7_ORYBR
9.6
9.4
9.2
9.0
8.8
1
2
t−Test: p−value = 0.24

J3LAH9|J3LAH9_ORYBR

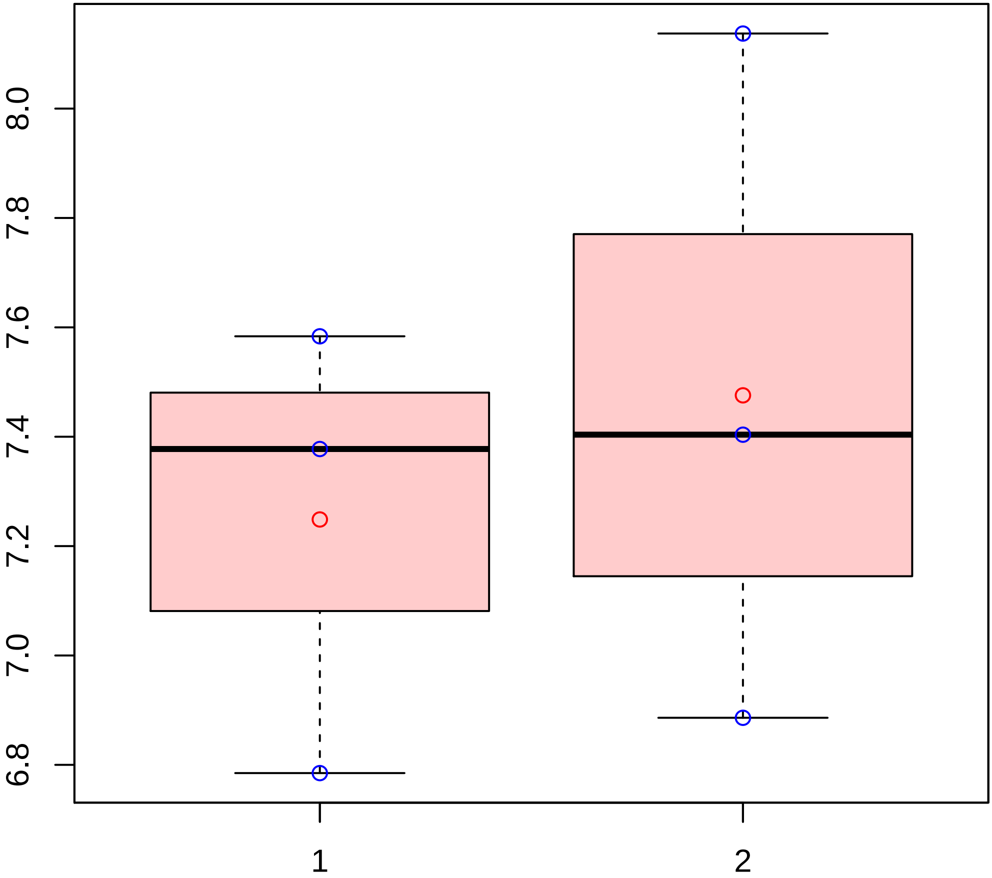

t−Test: p−value = 0.63

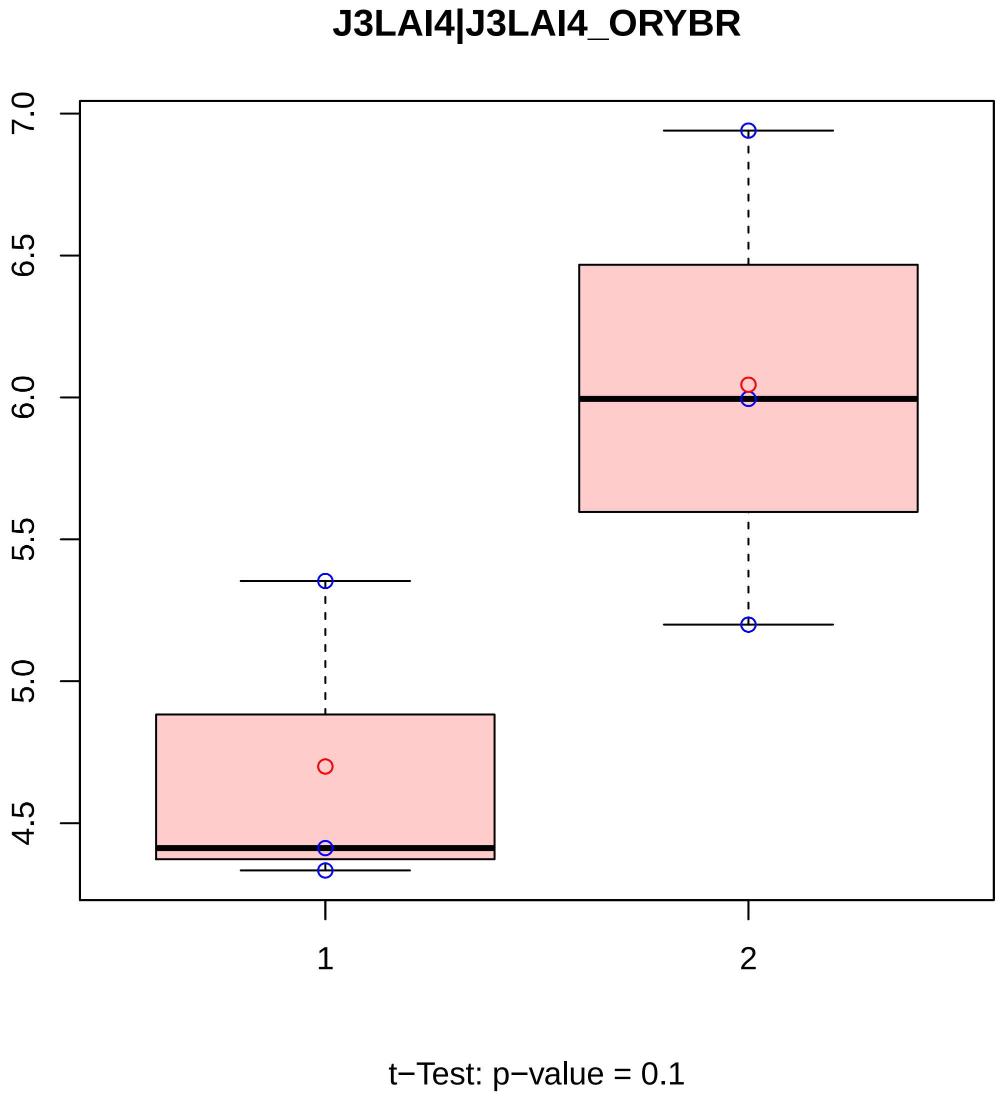
J3LAI4|J3LAI4_ORYBR
7.0
6.5
6.0
5.5
5.0
4.5
1
2
t−Test: p−value = 0.1

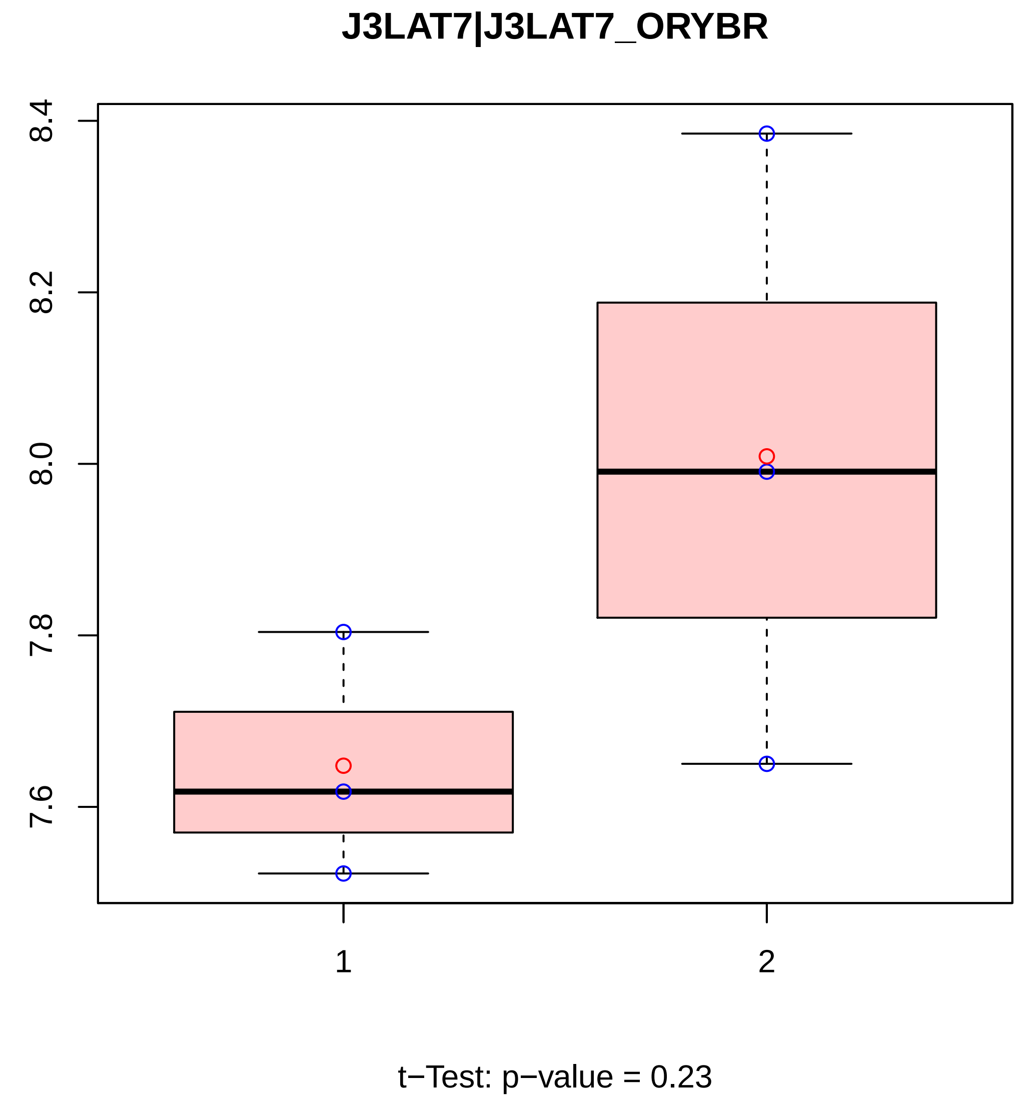
J3LAT7|J3LAT7_ORYBR
8.4
8.2
8.0
7.8
7.6
1
2
t−Test: p−value = 0.23

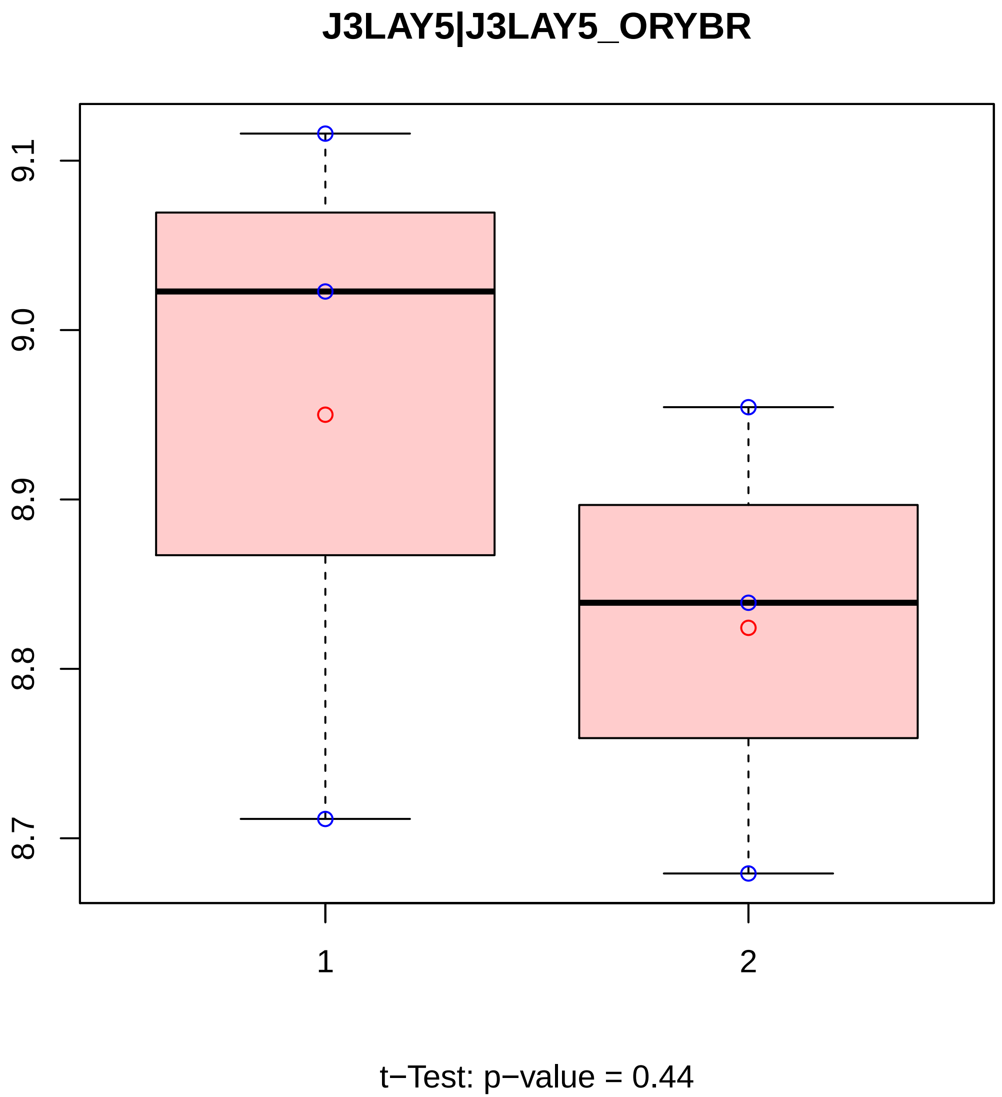
J3LAY5|J3LAY5_ORYBR
9.1
9.0
8.9
8.8
8.7
1
2
t−Test: p−value = 0.44

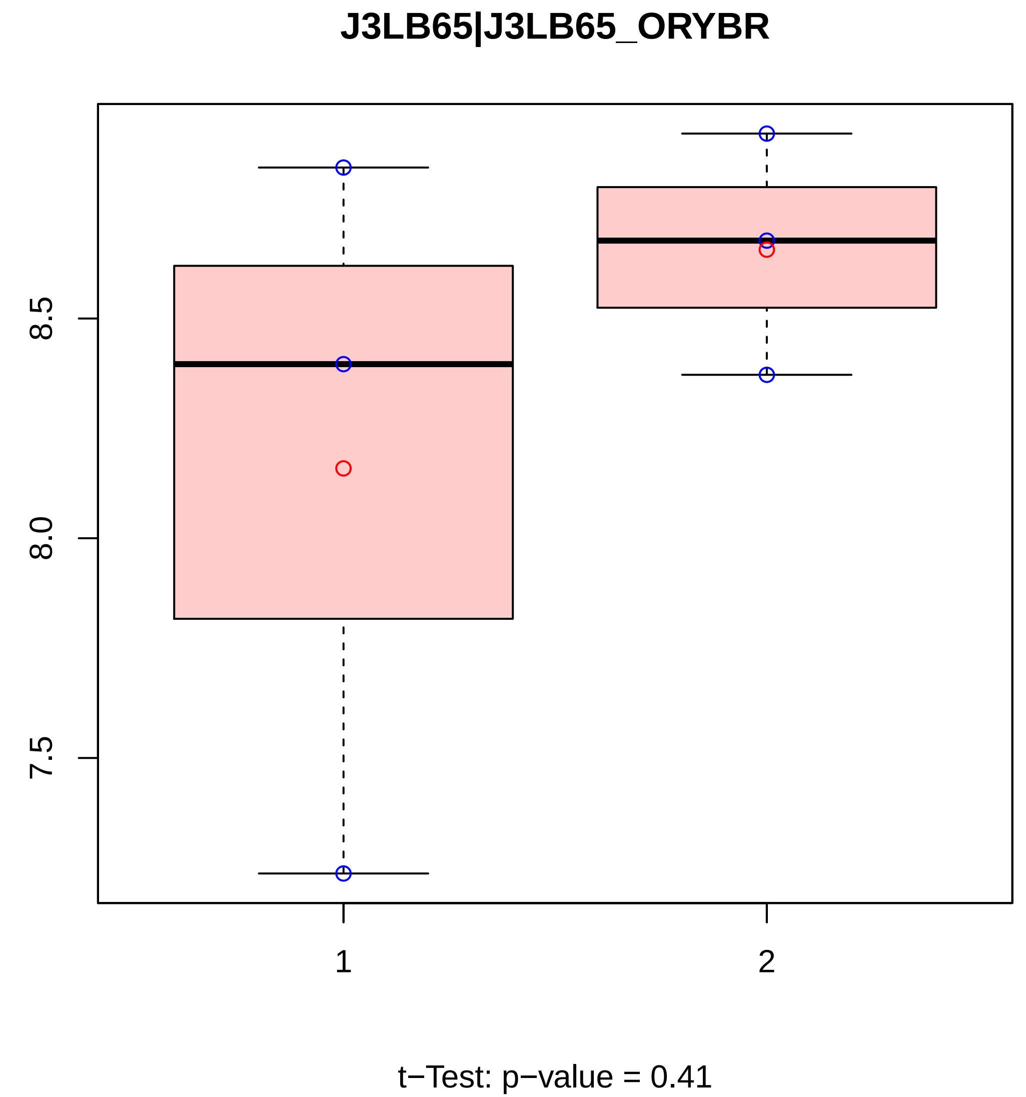

J3LB65|J3LB65_ORYBR
8.5
8.0
7.5
1
2
t−Test: p−value = 0.41

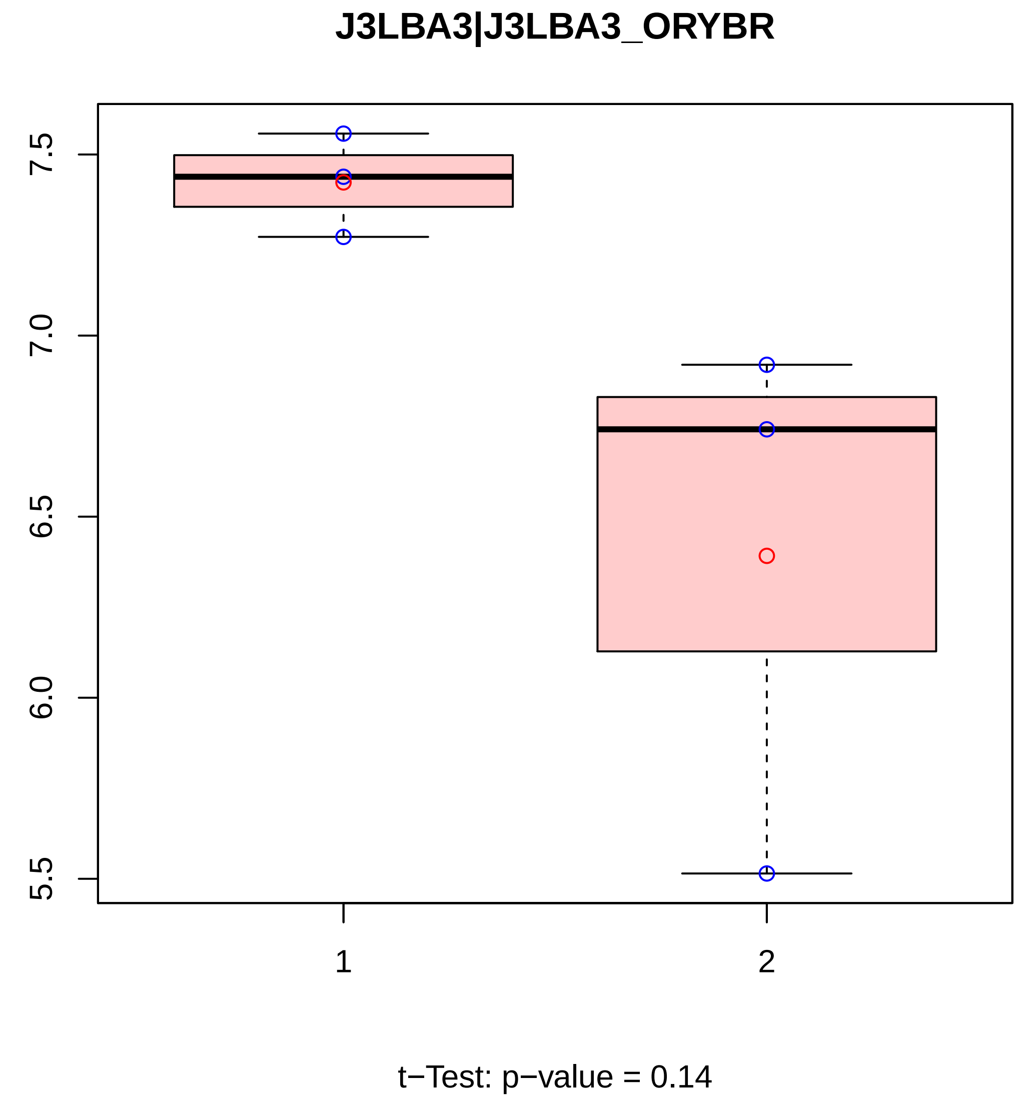
J3LBA3|J3LBA3_ORYBR
7.5
7.0
6.5
6.0
5.5
1
2
t−Test: p−value = 0.14

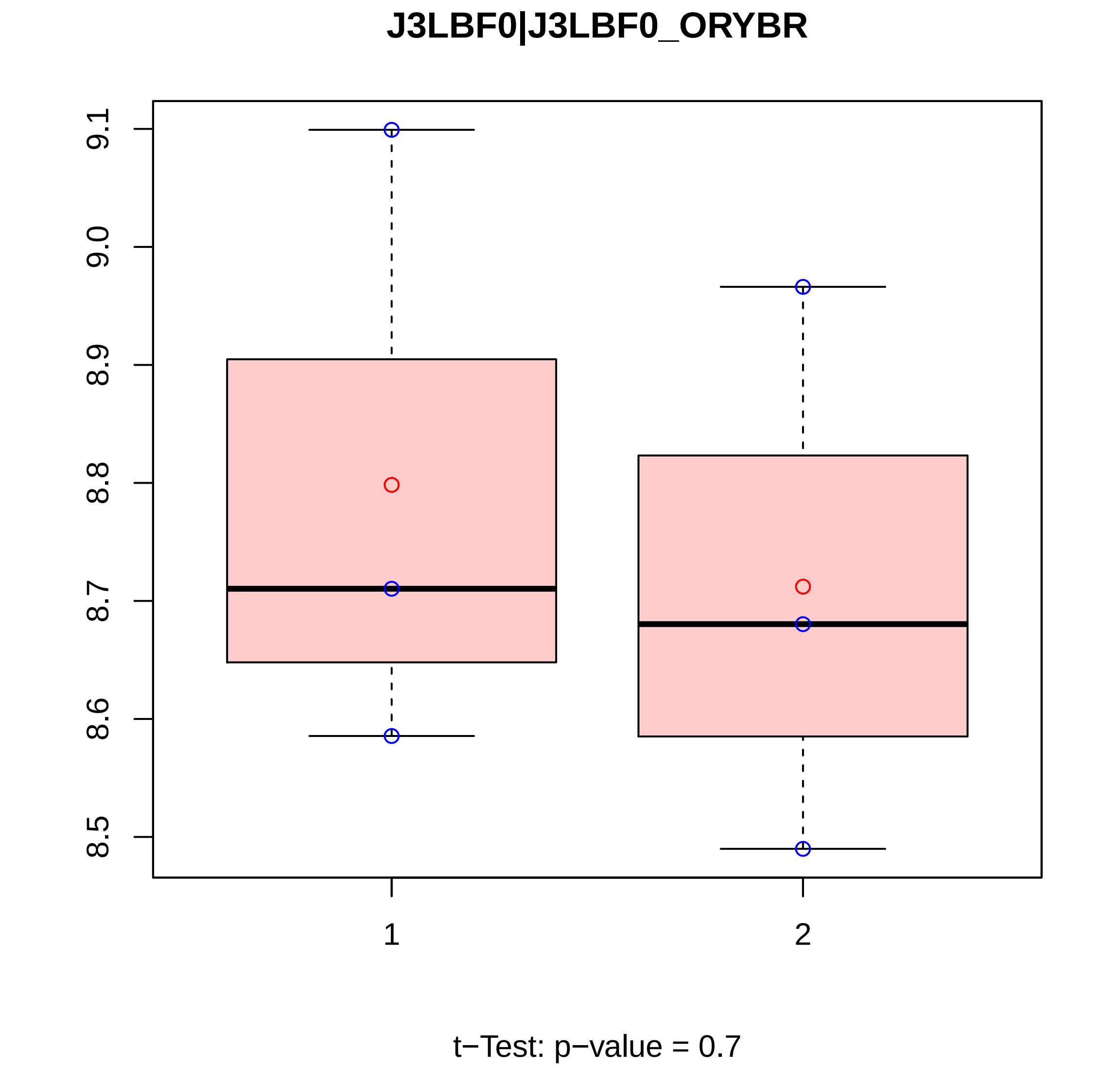
J3LBF0|J3LBF0_ORYBR
9.1
9.0
8.9
8.8
8.7
8.6
8.5
1
2
t−Test: p−value = 0.7

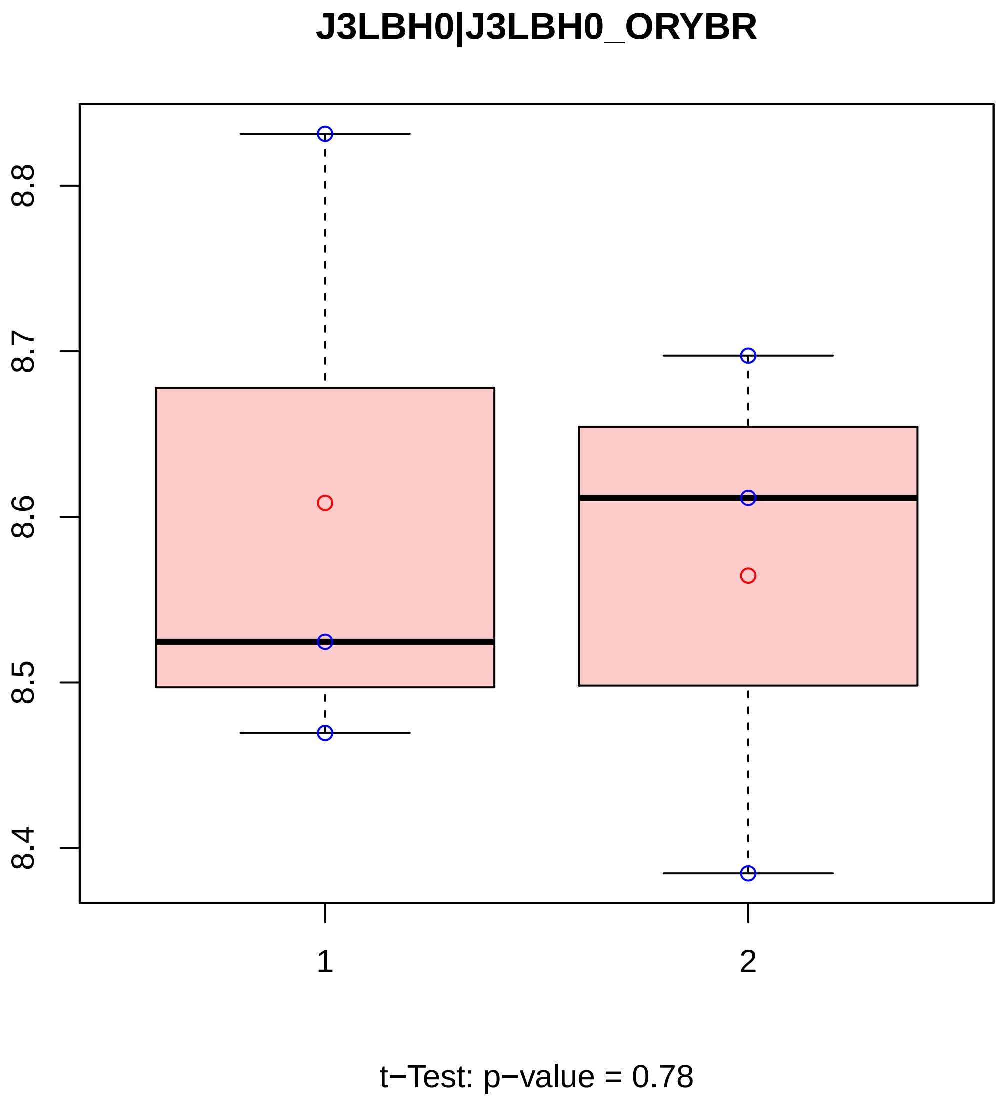

J3LBH0|J3LBH0_ORYBR
8.4
8.5
8.6
8.7
8.8
1
2
t−Test: p−value = 0.78

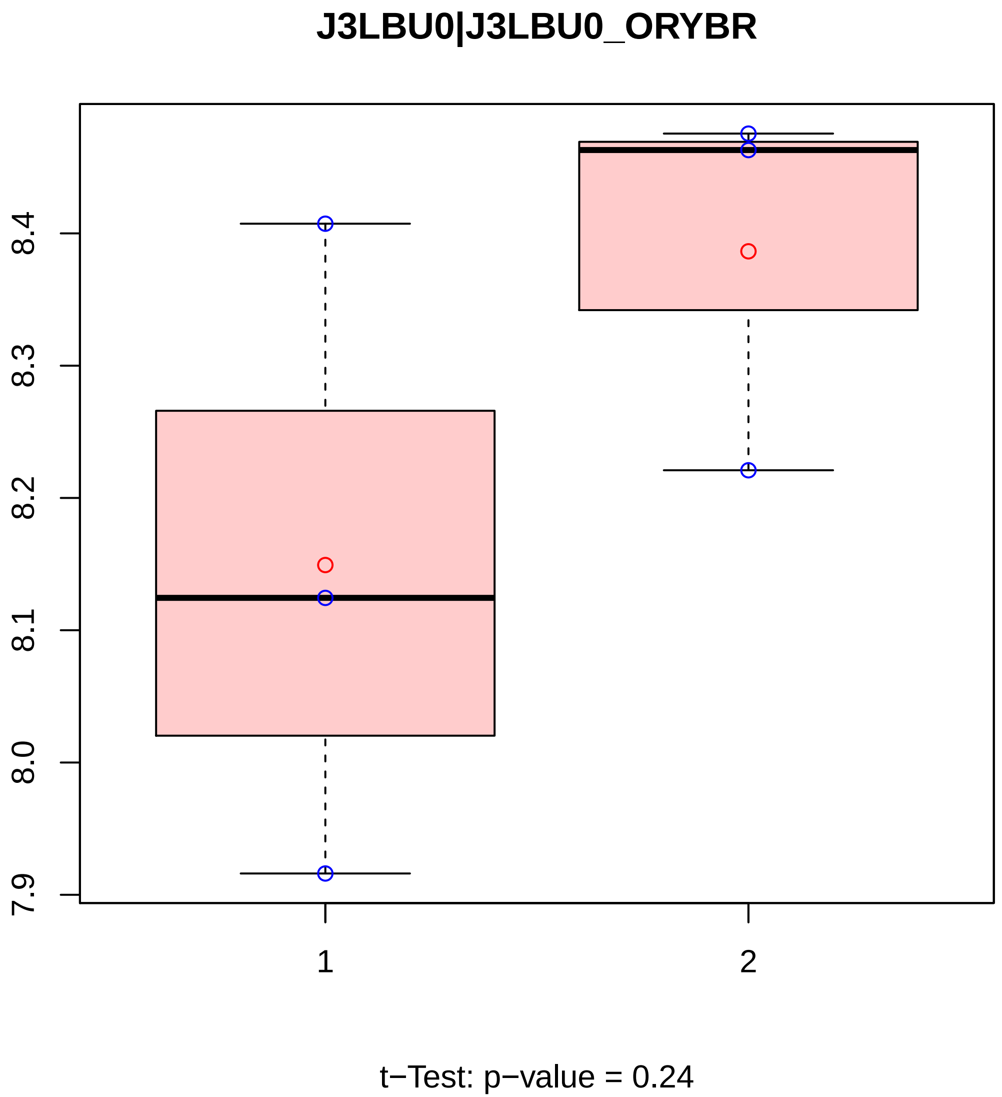
J3LBU0|J3LBU0_ORYBR
7.9
8.0
8.1
8.2
8.3
8.4
1
2
t−Test: p−value = 0.24

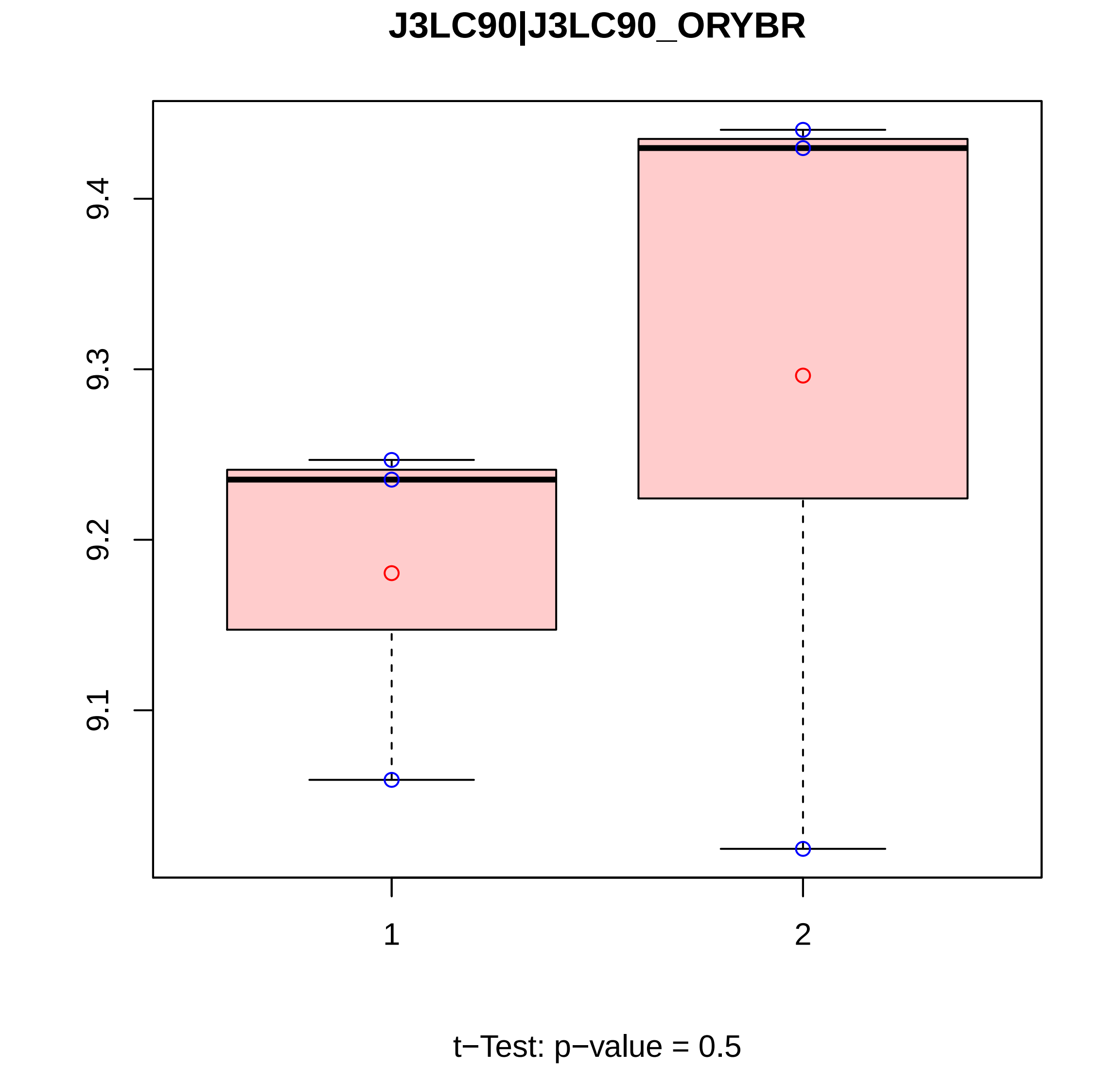
J3LC90|J3LC90_ORYBR
9.1
9.2
9.3
9.4
1
2
t−Test: p−value = 0.5

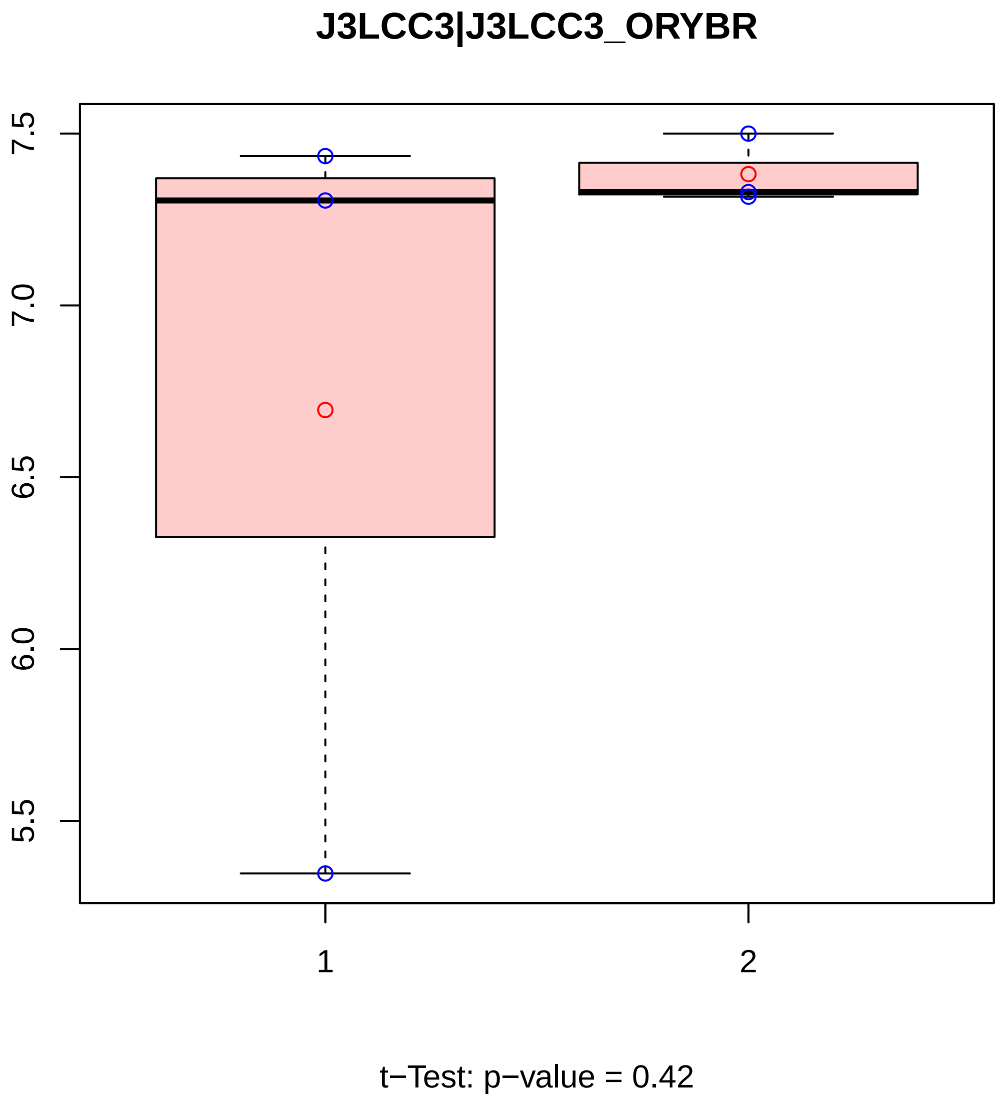
J3LCC3|J3LCC3_ORYBR
7.5
7.0
6.5
6.0
5.5
1
2
t−Test: p−value = 0.42

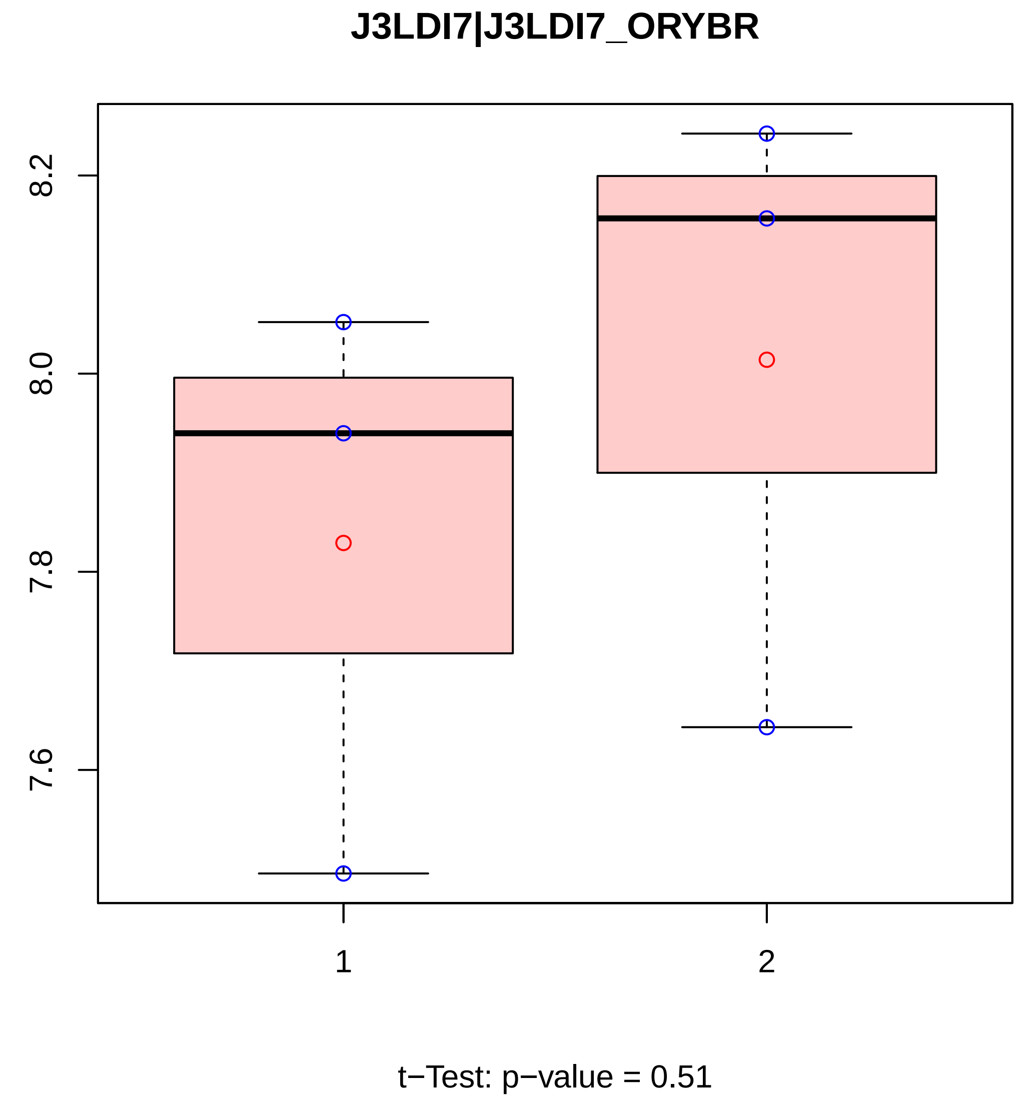
J3LDI7|J3LDI7_ORYBR
8.2
8.0
7.8
7.6
1
2
t−Test: p−value = 0.51

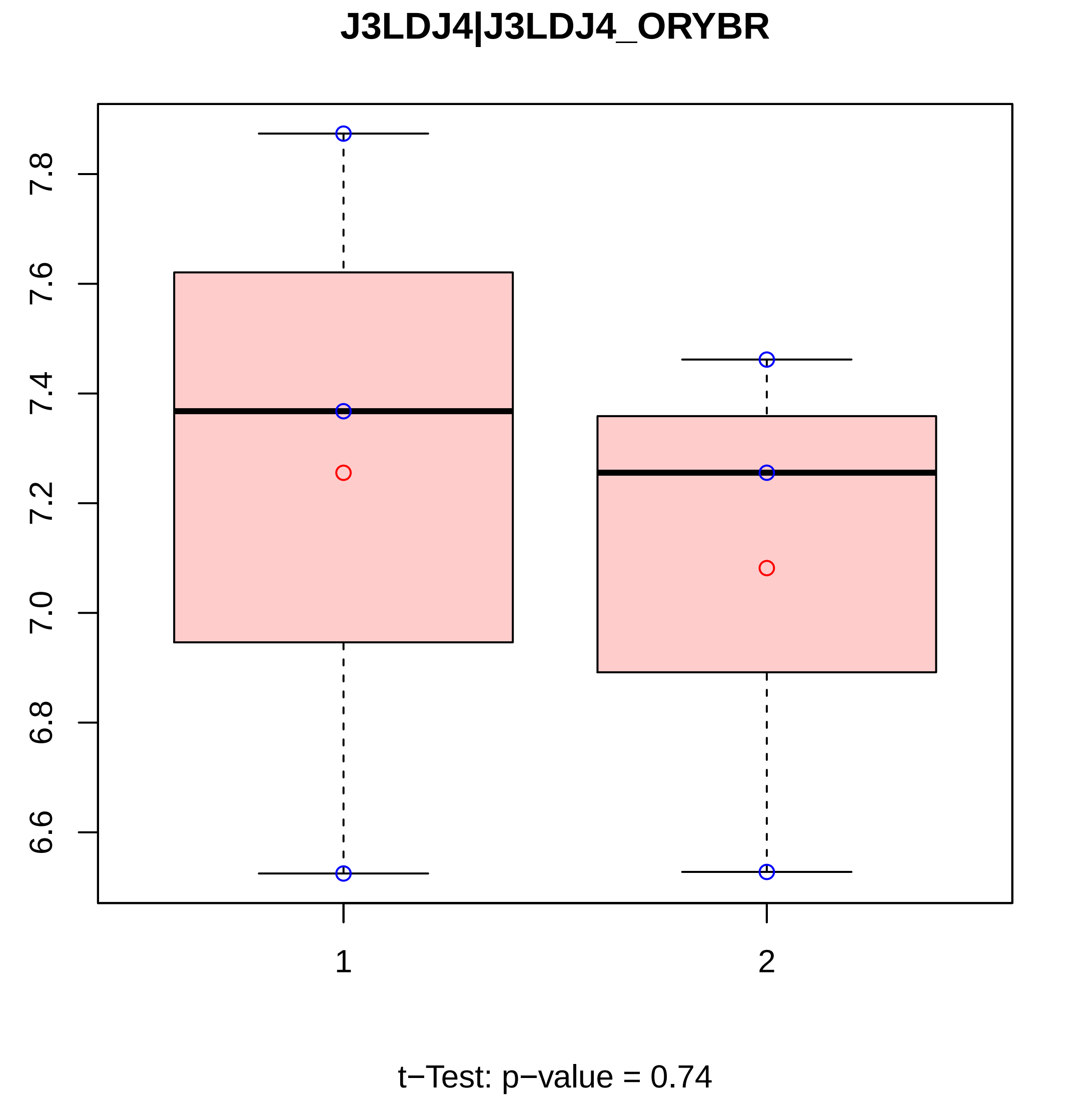
J3LDJ4|J3LDJ4_ORYBR
7.8
7.6
7.4
7.2
7.0
6.8
6.6
1
2
t−Test: p−value = 0.74

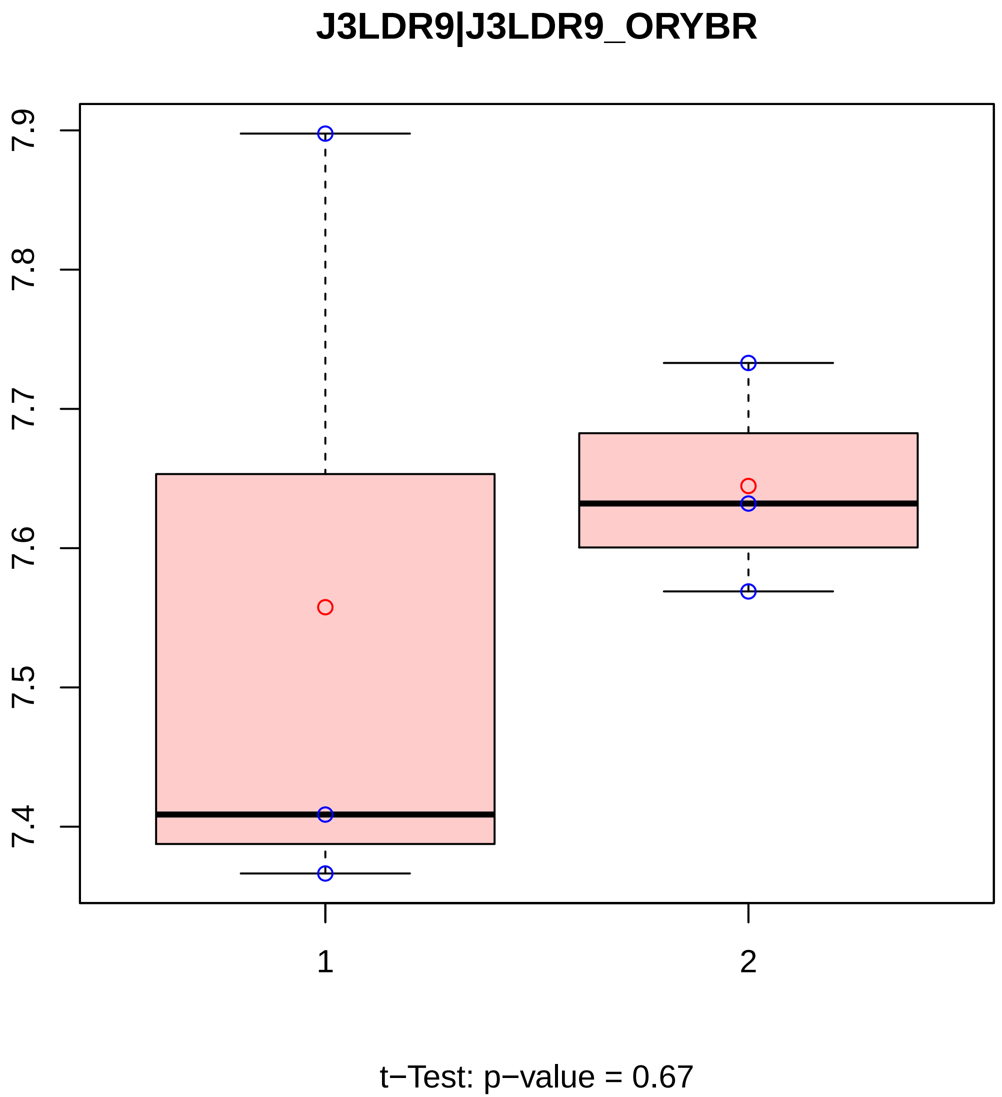
J3LDR9|J3LDR9_ORYBR
7.9
7.8
7.7
7.6
7.5
7.4
1
2
t−Test: p−value = 0.67

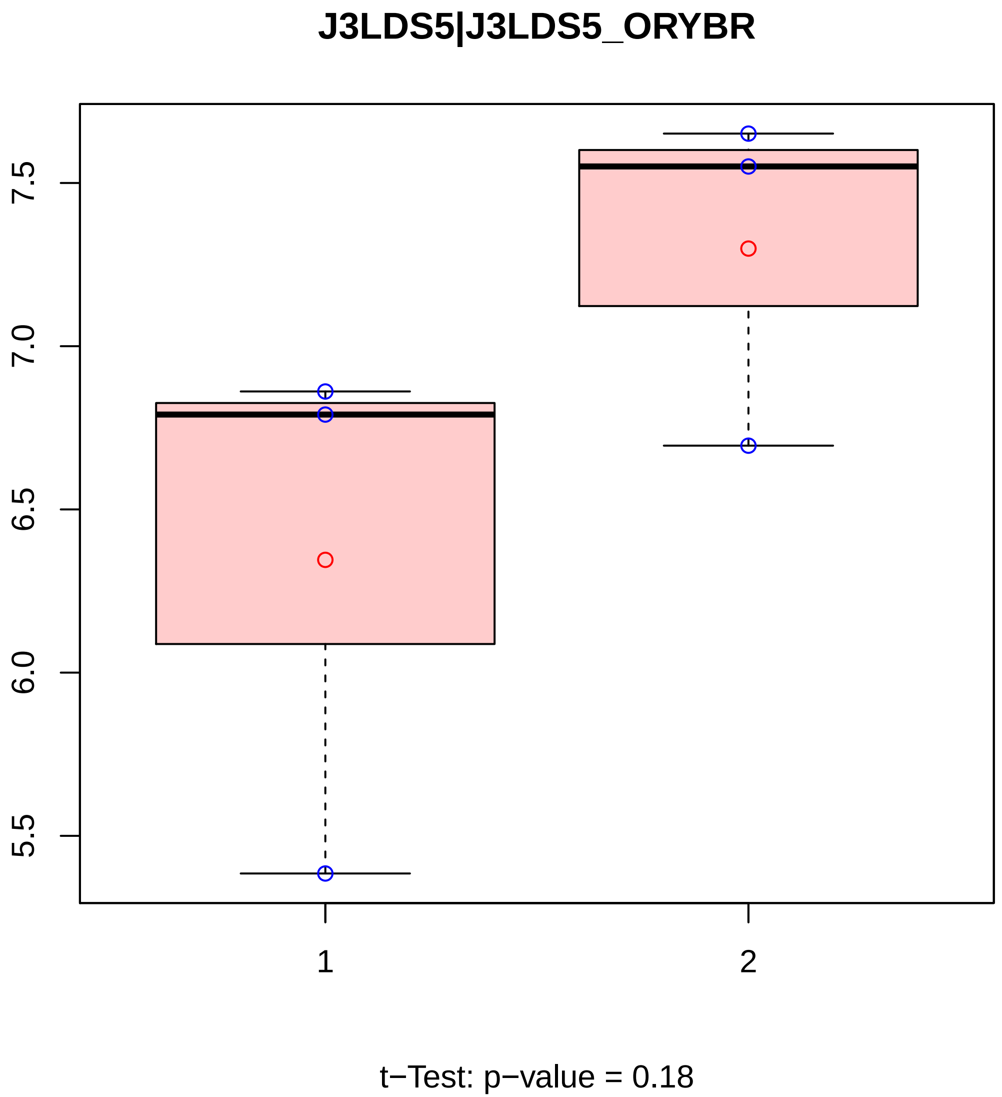
J3LDS5|J3LDS5_ORYBR
7.5
7.0
6.5
6.0
5.5
1
2
t−Test: p−value = 0.18

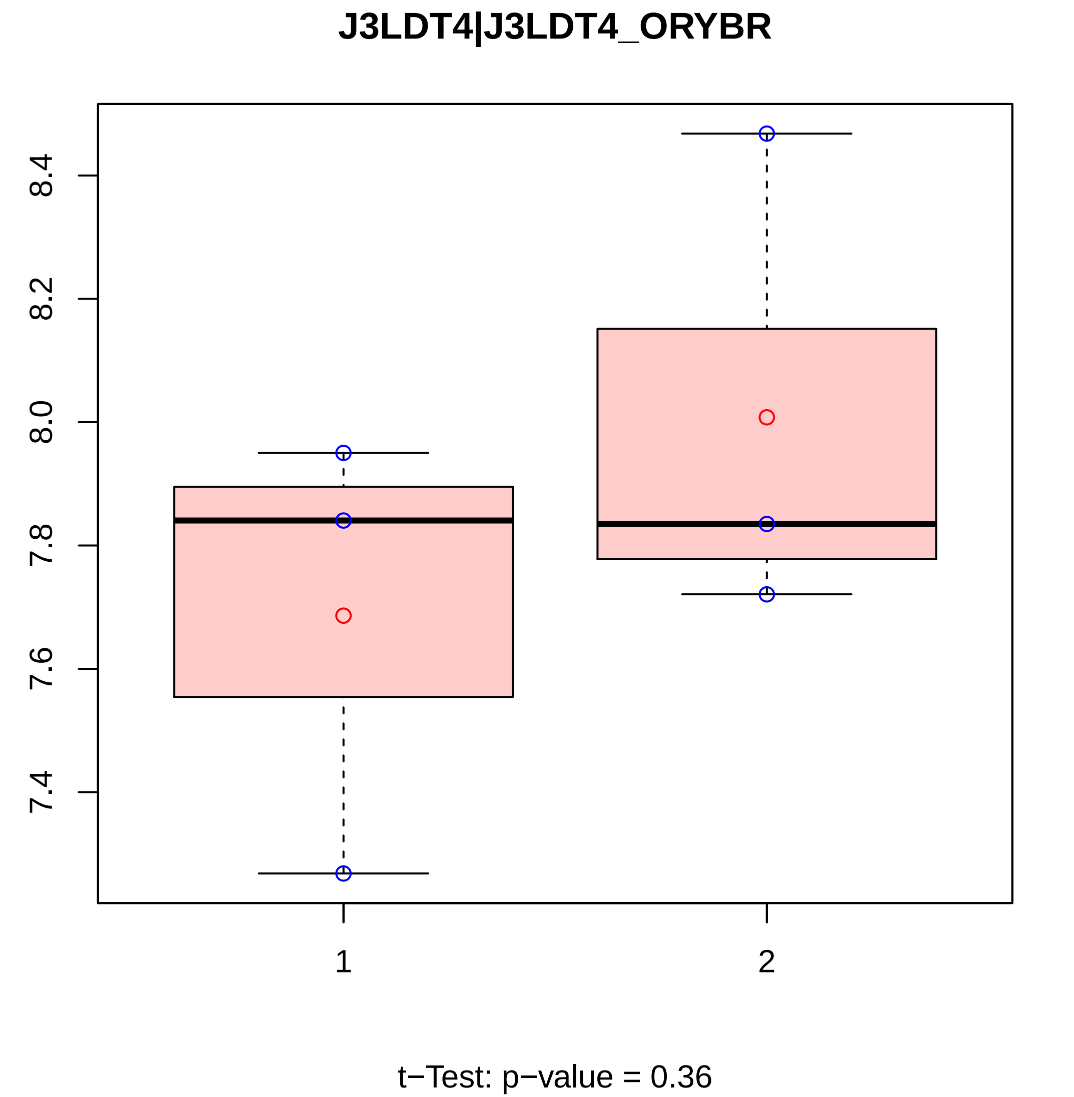
J3LDT4|J3LDT4_ORYBR
8.4
8.2
8.0
7.8
7.6
7.4
1
2
t−Test: p−value = 0.36

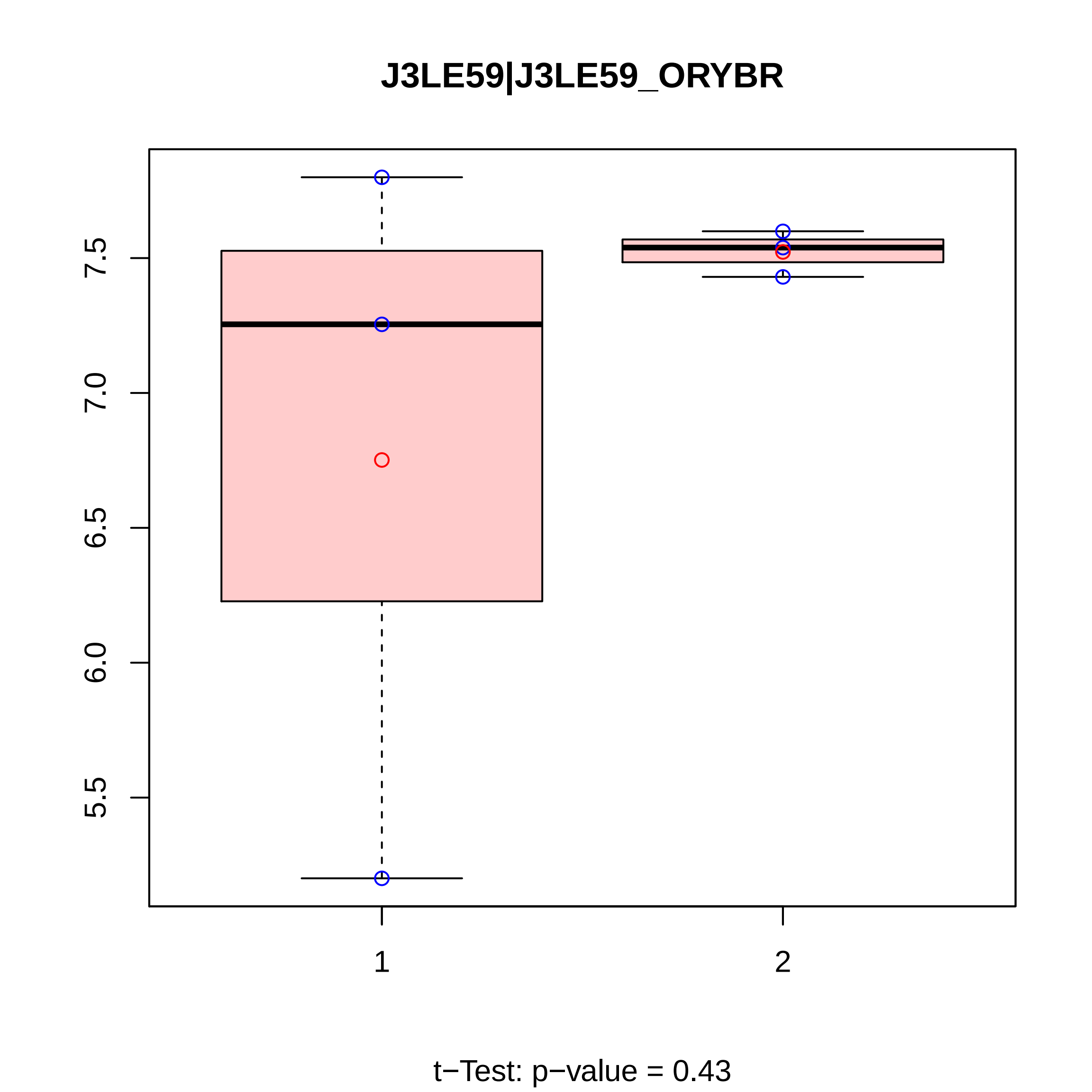
J3LE59|J3LE59_ORYBR
7.5
7.0
6.5
6.0
5.5
1
2
t−Test: p−value = 0.43

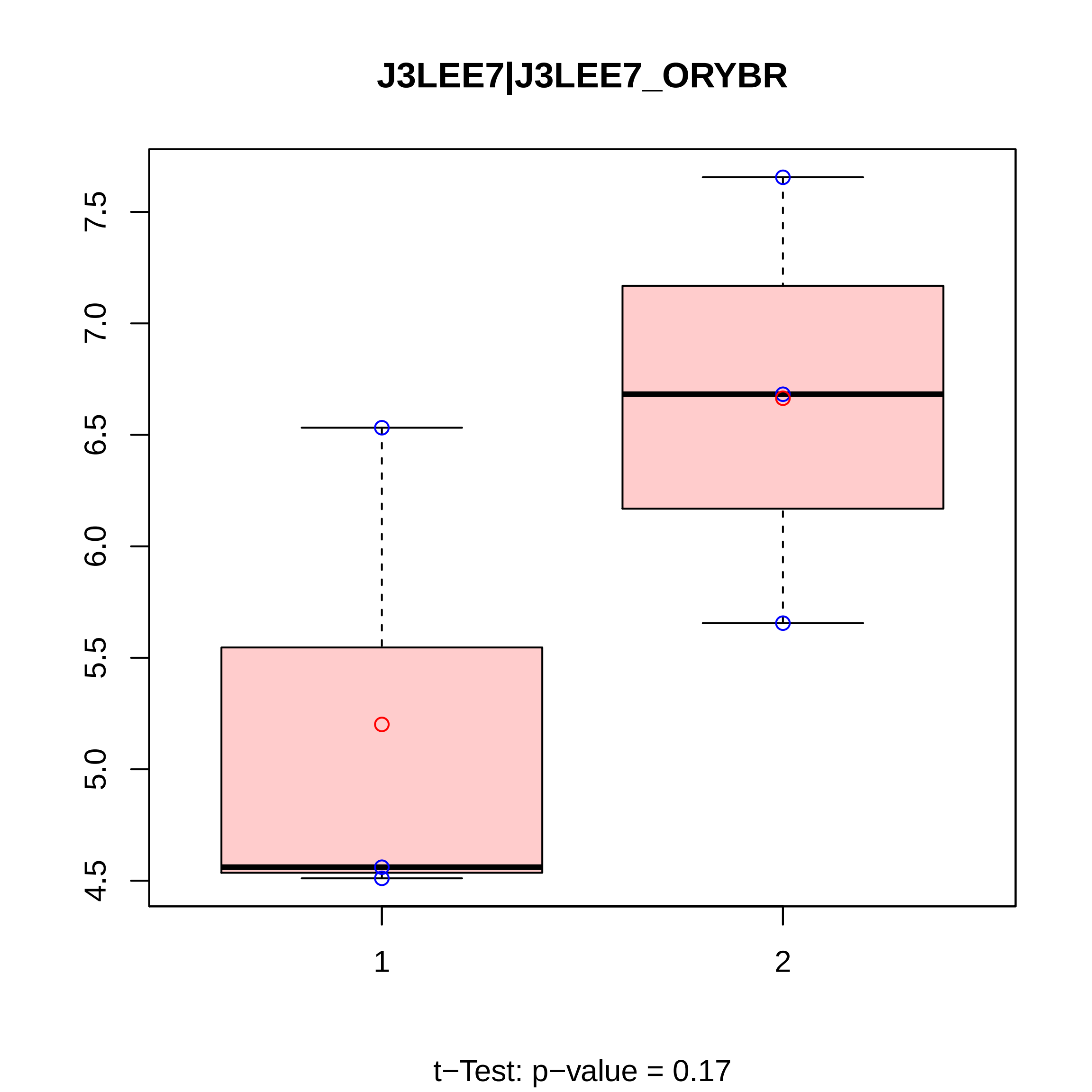
J3LEE7|J3LEE7_ORYBR
7.5
7.0
6.5
6.0
5.5
5.0
4.5
1
2
t−Test: p−value = 0.17

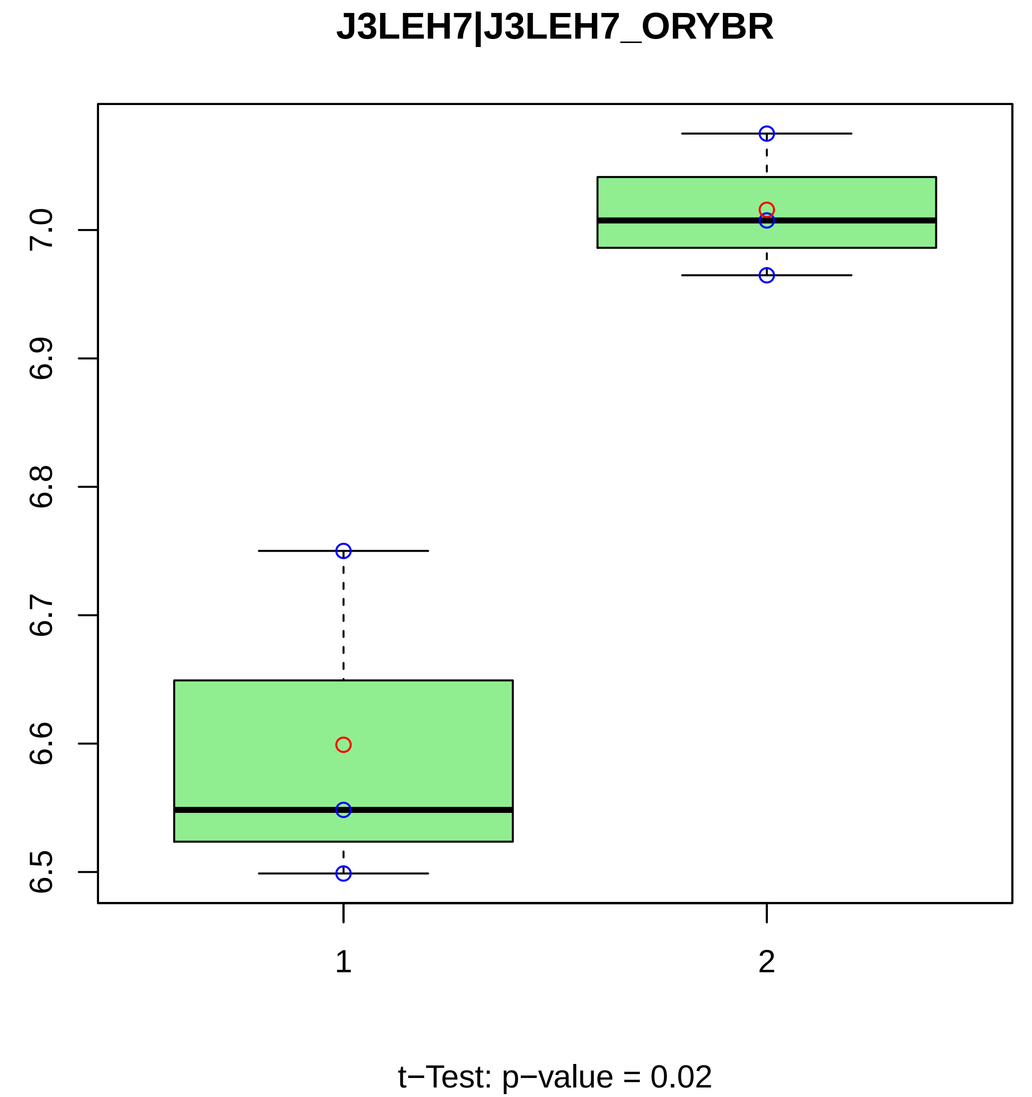

J3LEH7|J3LEH7_ORYBR
7.0
6.9
6.8
6.7
6.6
6.5
1
2
t−Test: p−value = 0.02

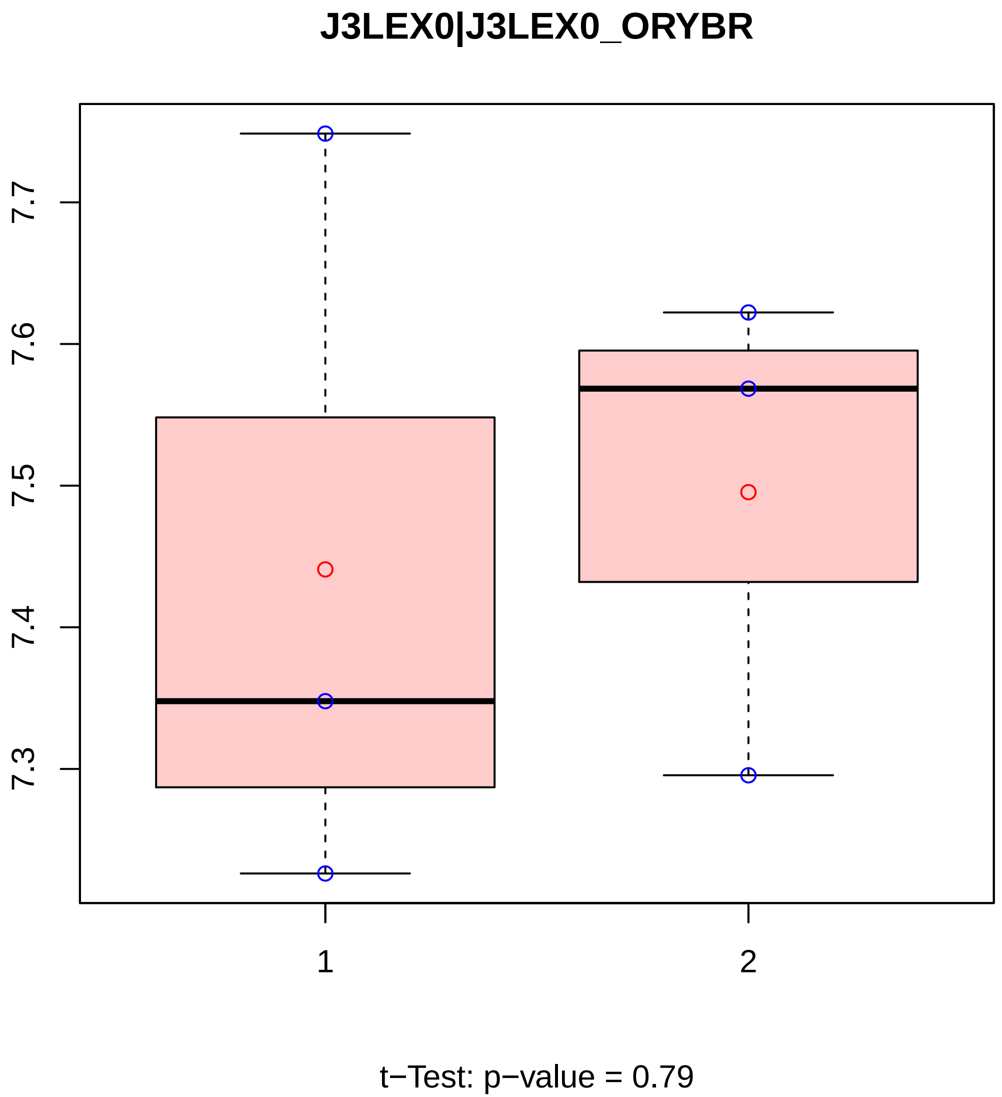
J3LEX0|J3LEX0_ORYBR
7.3
7.4
7.5
7.6
7.7
1
2
t−Test: p−value = 0.79

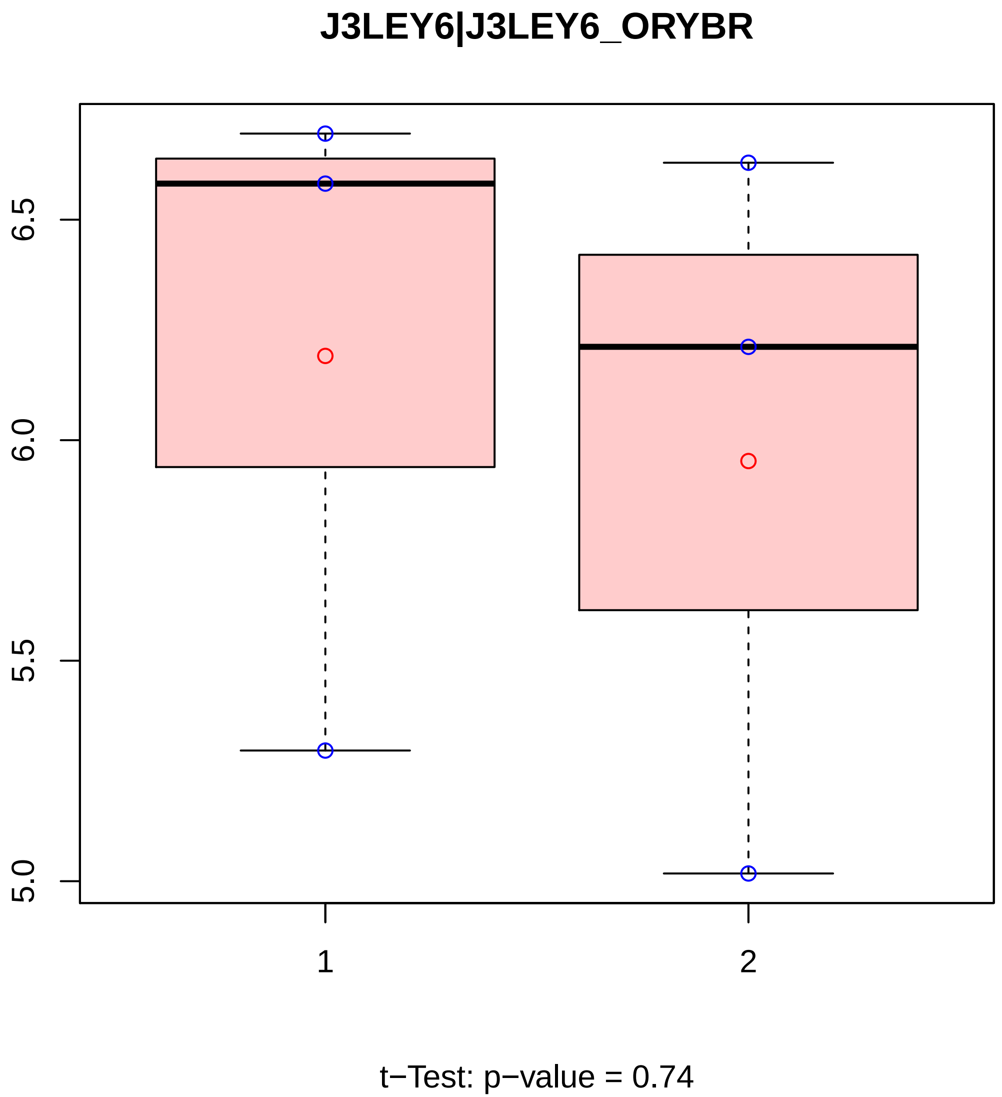
J3LEY6|J3LEY6_ORYBR
6.5
6.0
5.5
5.0
1
2
t−Test: p−value = 0.74

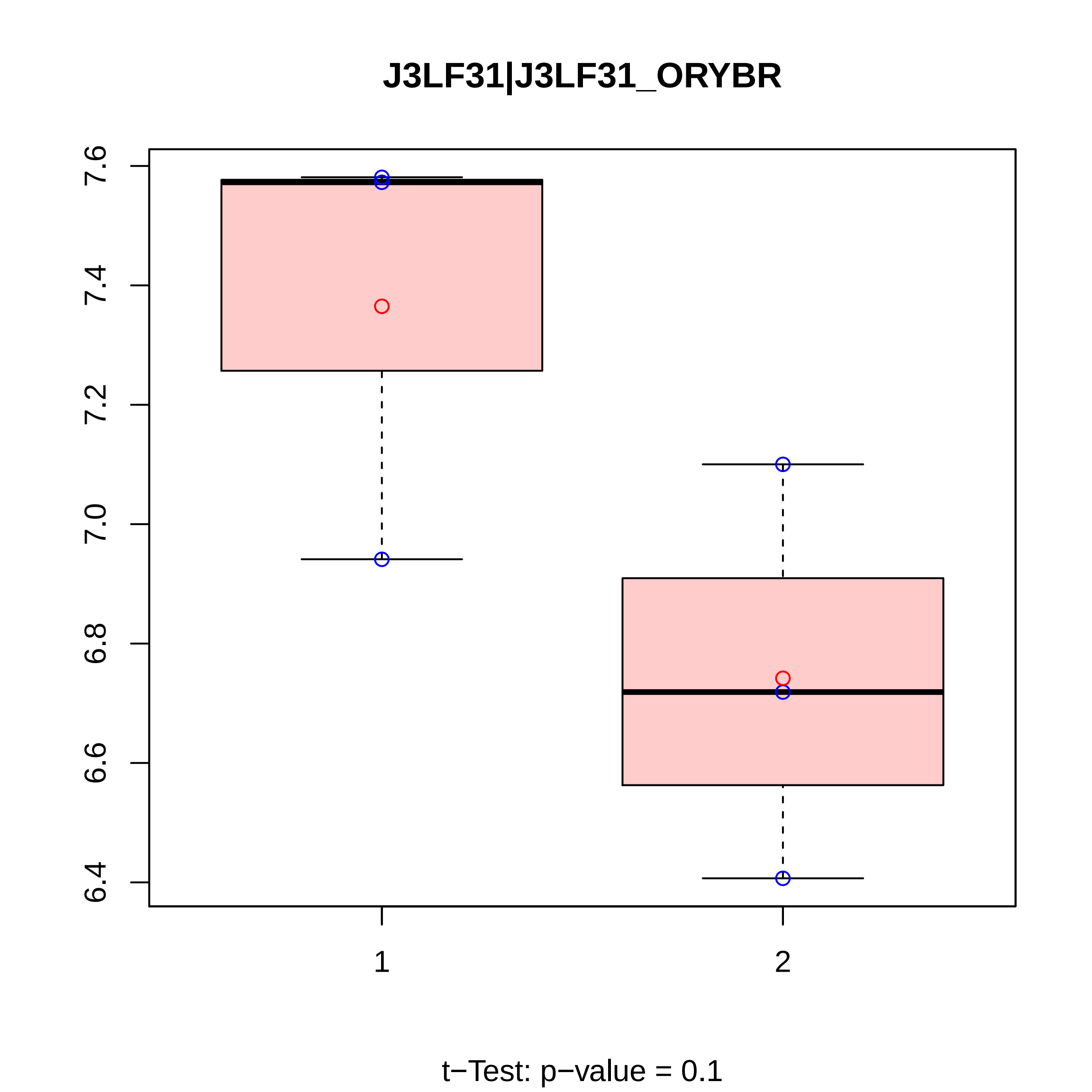
J3LF31|J3LF31_ORYBR
7.6
7.4
7.2
7.0
6.8
6.6
6.4
1
2
t−Test: p−value = 0.1

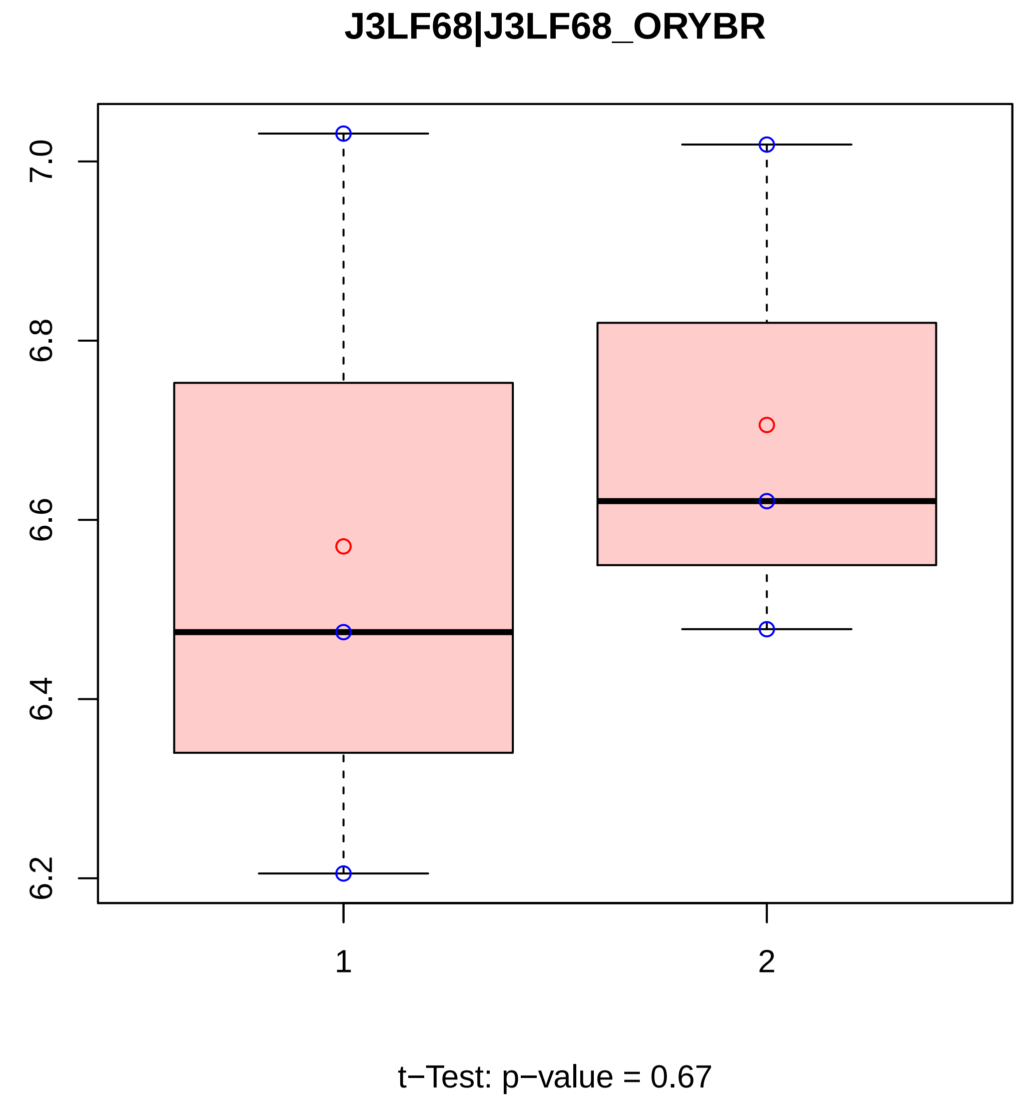
J3LF68|J3LF68_ORYBR
7.0
6.8
6.6
6.4
6.2
1
2
t−Test: p−value = 0.67

J3LF89|J3LF89_ORYBR

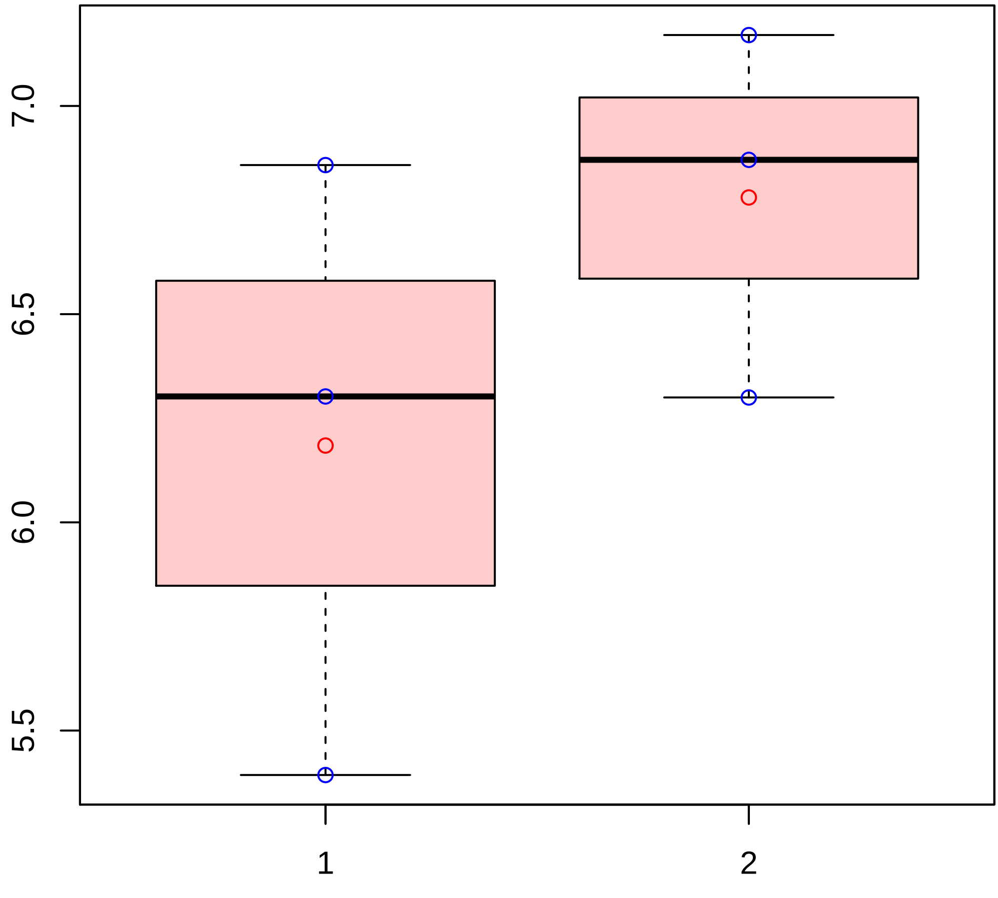

t−Test: p−value = 0.31

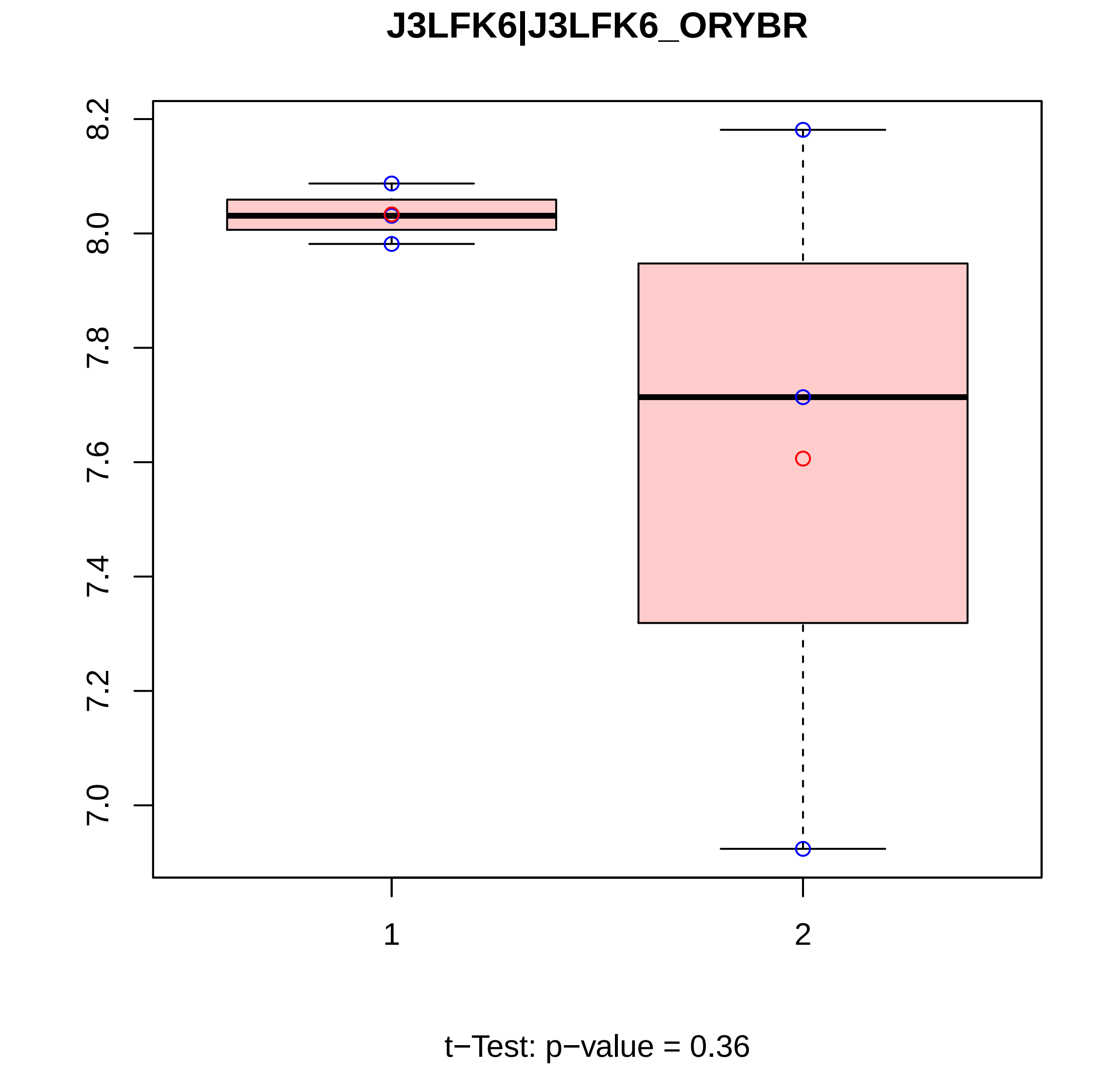
J3LFK6|J3LFK6_ORYBR
8.2
8.0
7.8
7.6
7.4
7.2
7.0
1
2
t−Test: p−value = 0.36

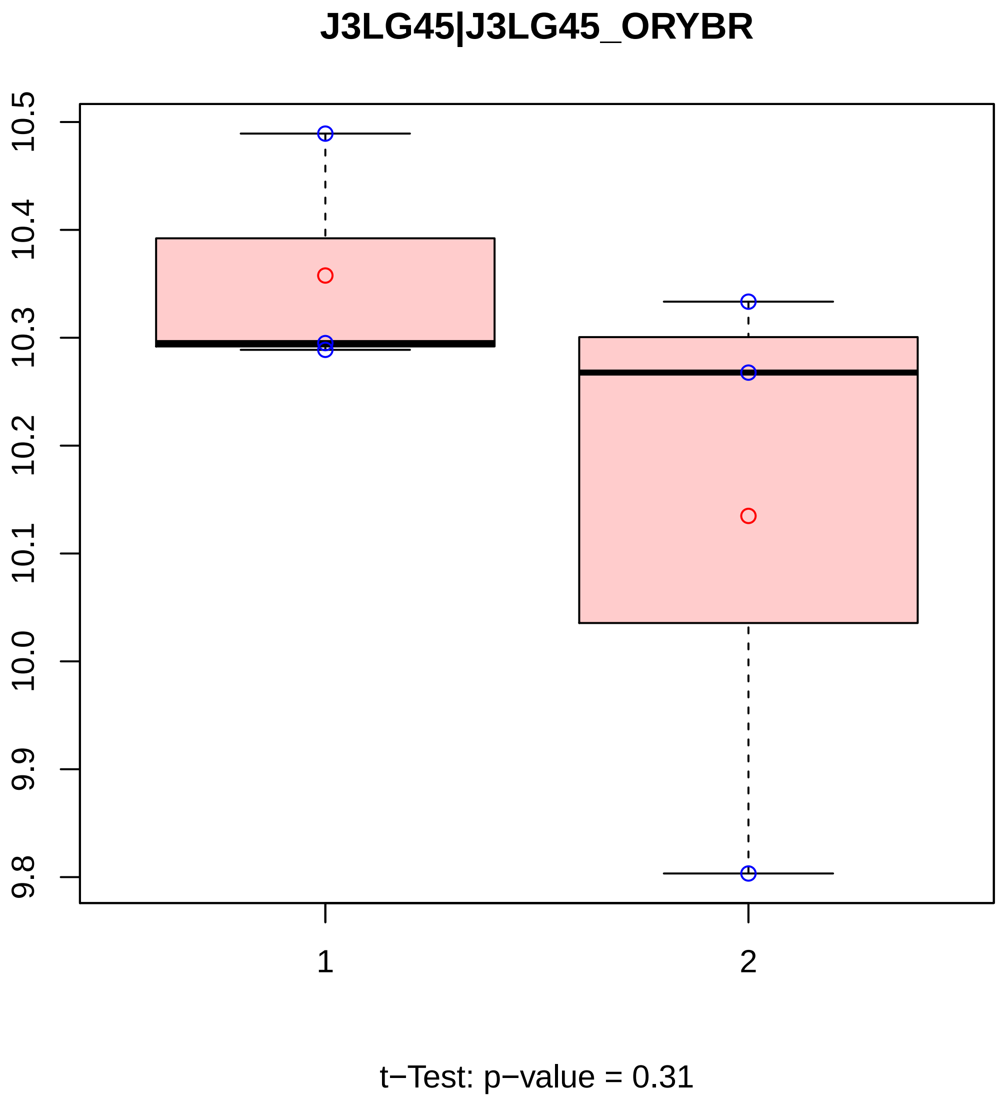
J3LG45|J3LG45_ORYBR
10.5
10.4
10.3
10.2
10.1
10.0
9.9
9.8
1
2
t−Test: p−value = 0.31

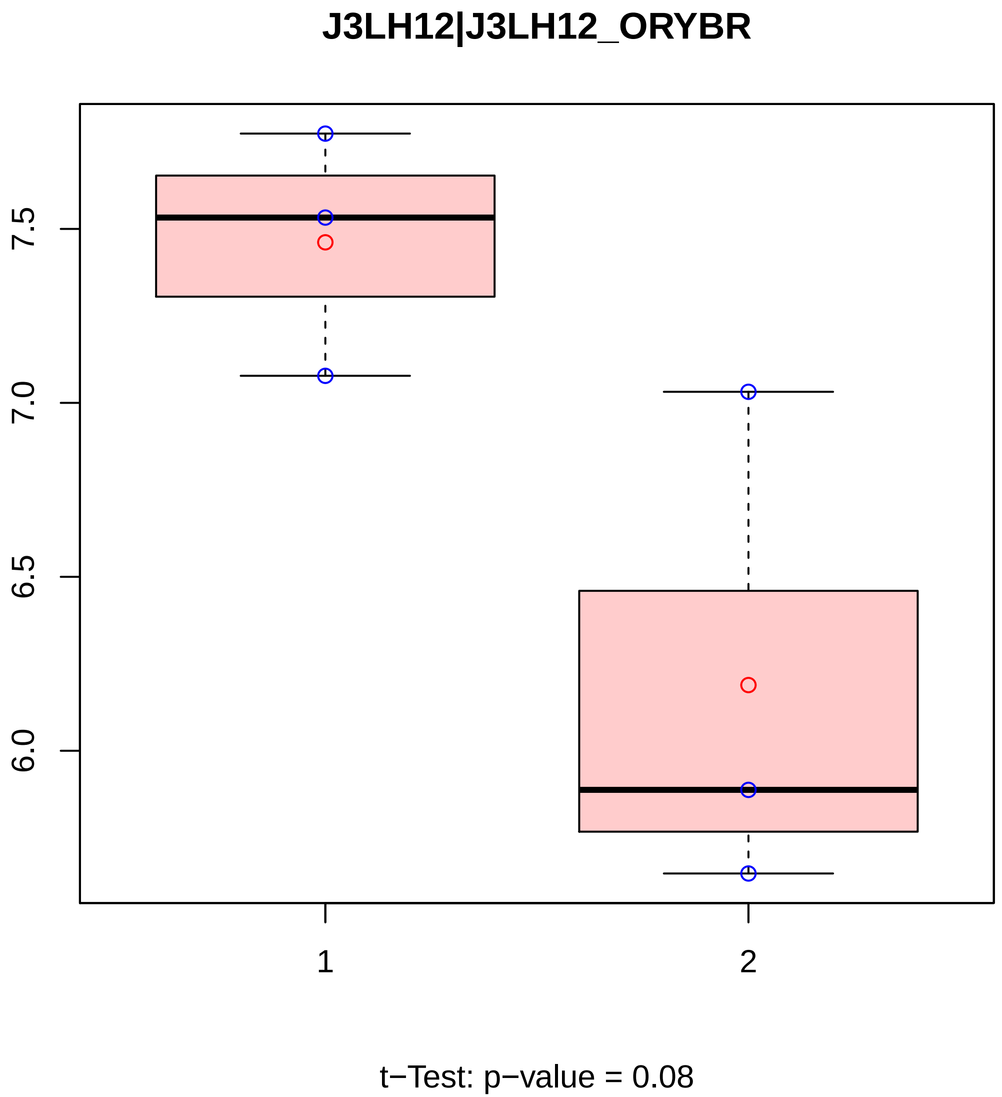

J3LH12|J3LH12_ORYBR
7.5
7.0
6.5
6.0
1
2
t−Test: p−value = 0.08

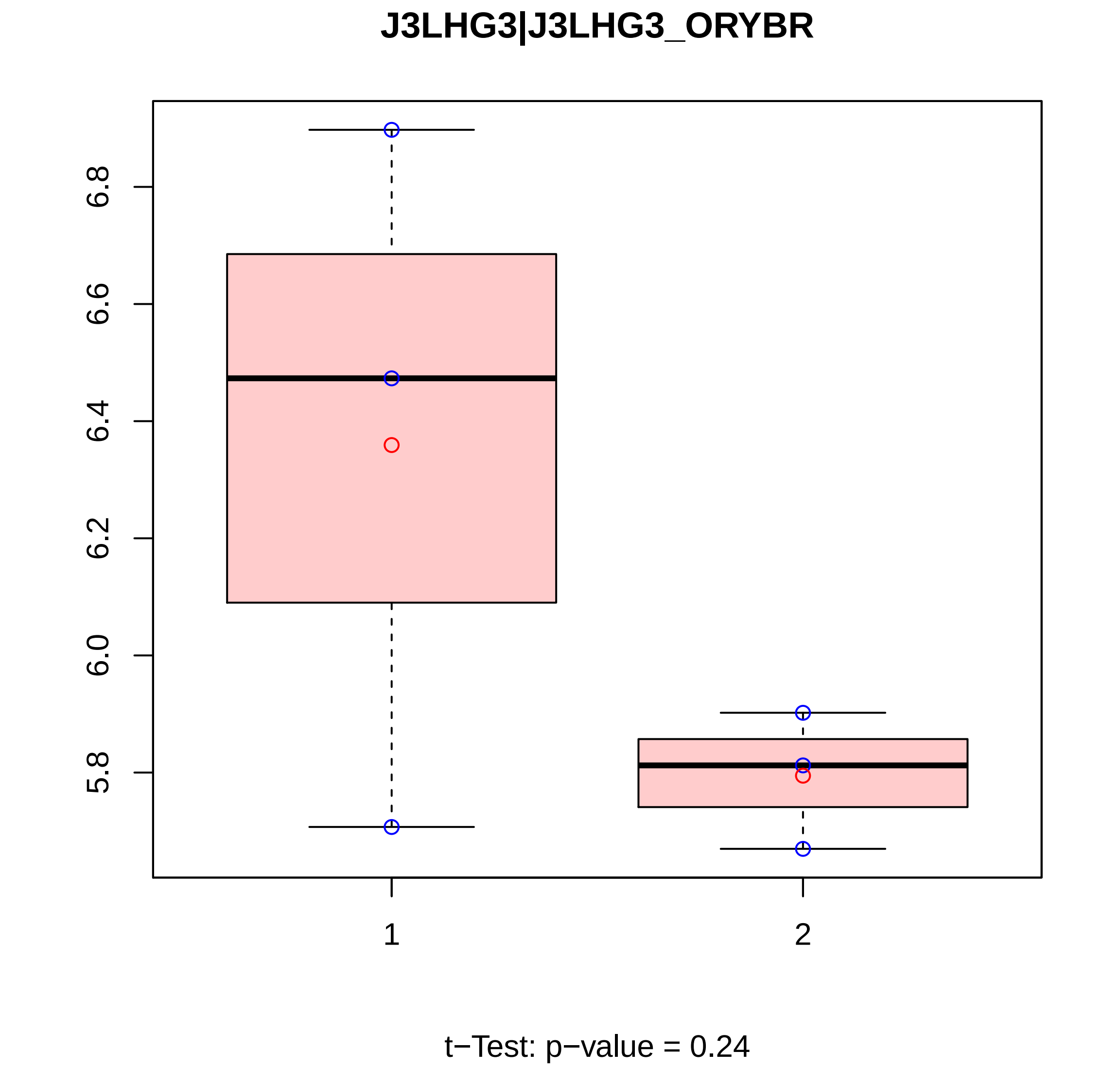
J3LHG3|J3LHG3_ORYBR
6.8
6.6
6.4
6.2
6.0
5.8
1
2
t−Test: p−value = 0.24

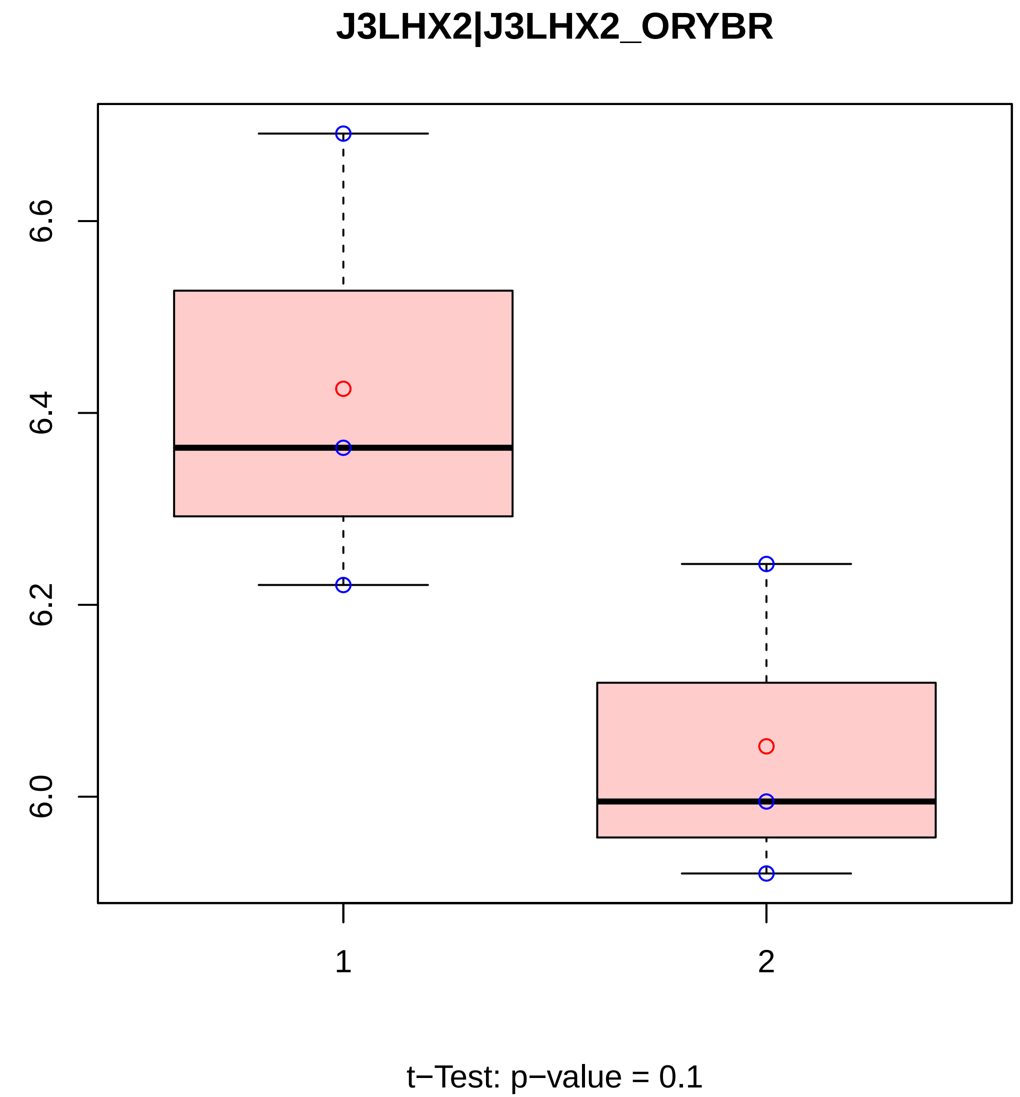

J3LHX2|J3LHX2_ORYBR
6.0
6.2
6.4
6.6
1
2
t−Test: p−value = 0.1

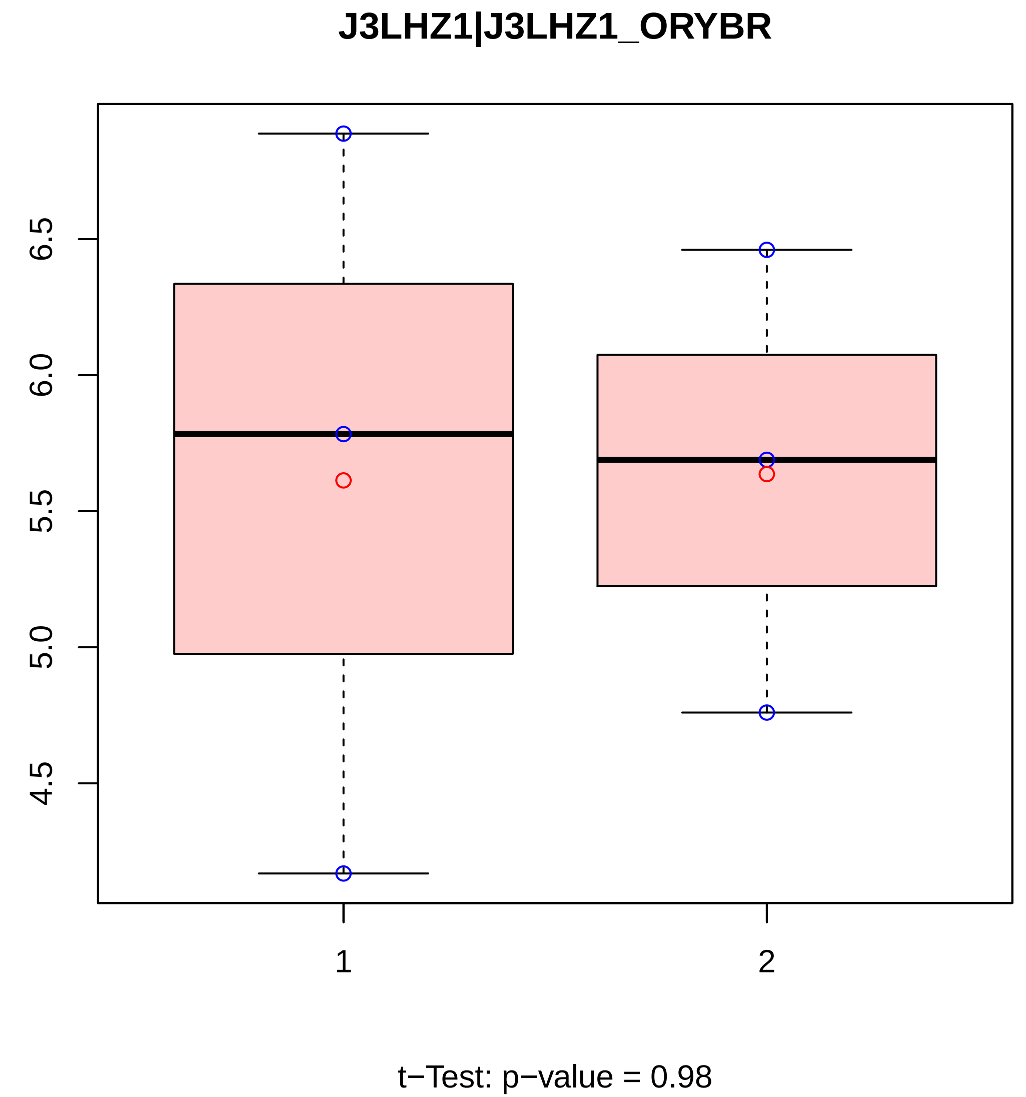
J3LHZ1|J3LHZ1_ORYBR
6.5
6.0
5.5
5.0
4.5
1
2
t−Test: p−value = 0.98

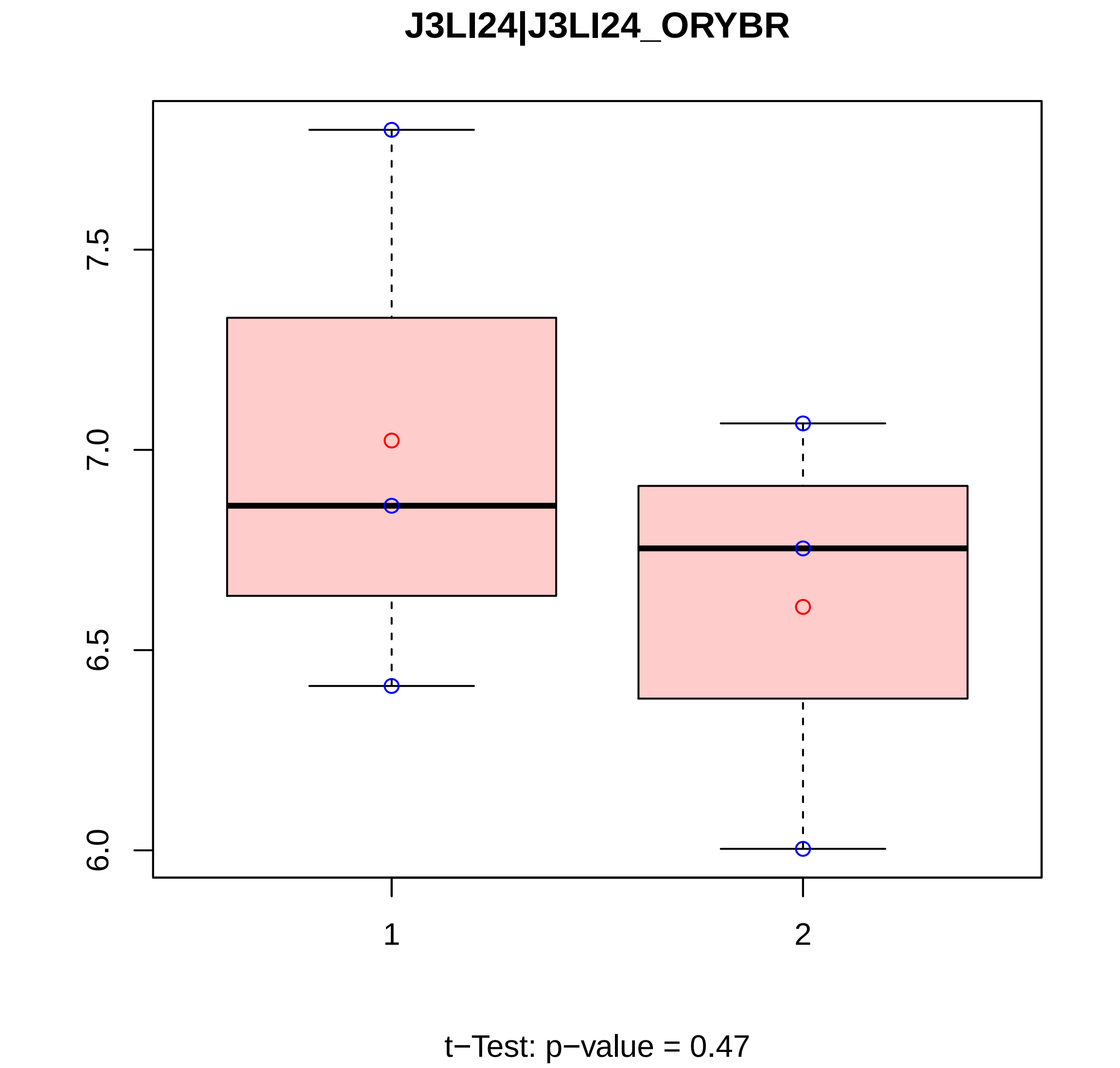
J3LI24|J3LI24_ORYBR
7.5
7.0
6.5
6.0
1
2
t−Test: p−value = 0.47

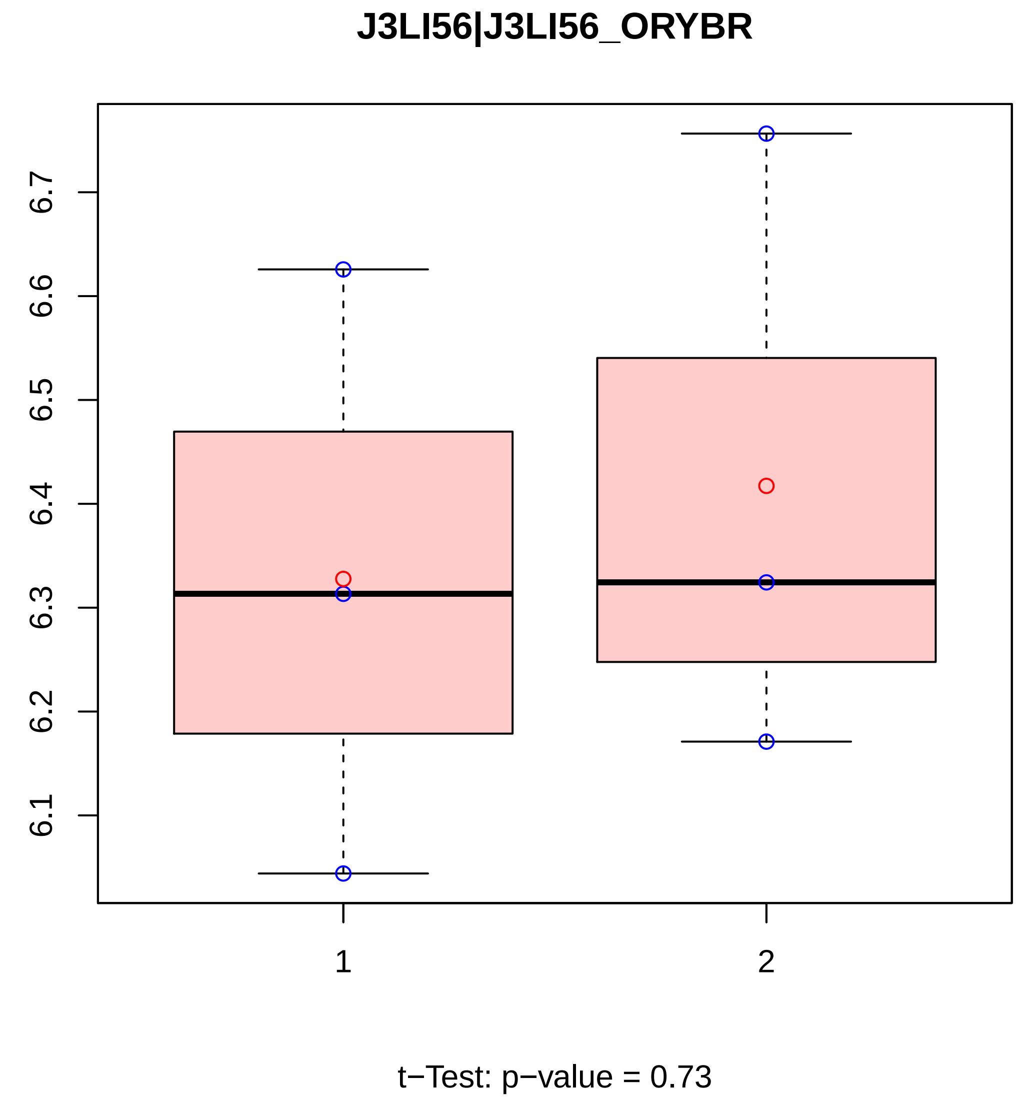
J3LI56|J3LI56_ORYBR
6.1
6.2
6.3
6.4
6.5
6.6
6.7
1
2
t−Test: p−value = 0.73

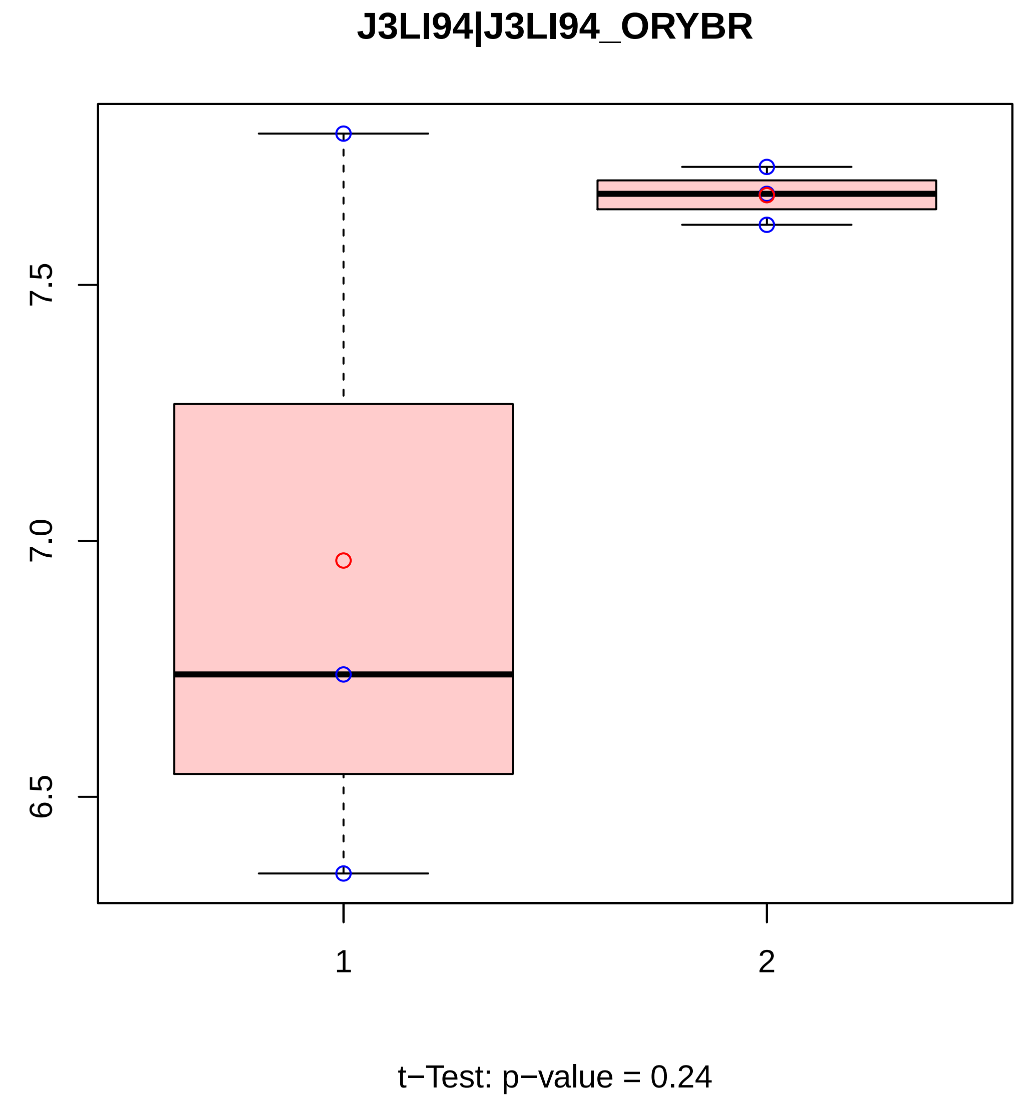
J3LI94|J3LI94_ORYBR
7.5
7.0
6.5
1
2
t−Test: p−value = 0.24

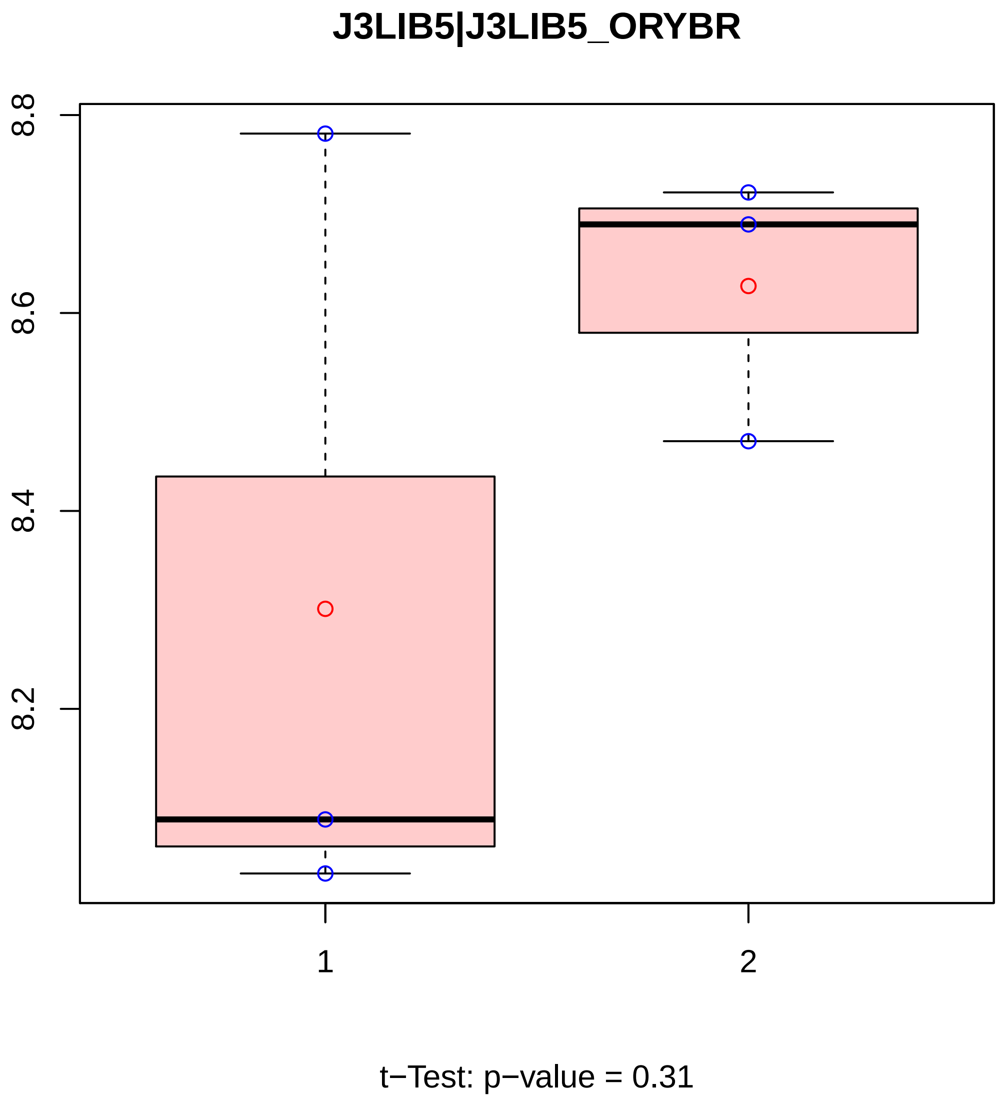
J3LIB5|J3LIB5_ORYBR
8.8
8.6
8.4
8.2
1
2
t−Test: p−value = 0.31

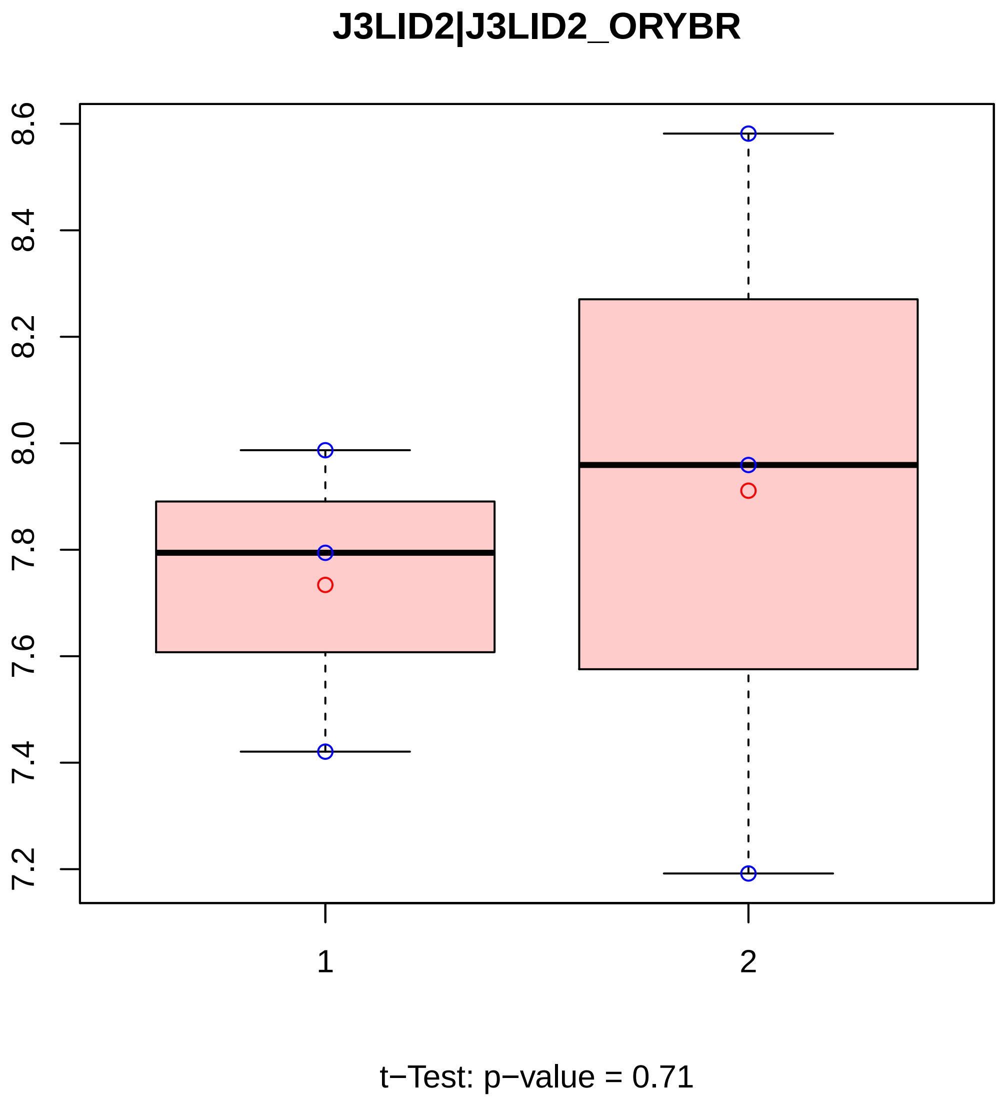
J3LID2|J3LID2_ORYBR
8.6
8.4
8.2
8.0
7.8
7.6
7.4
7.2
1
2
t−Test: p−value = 0.71

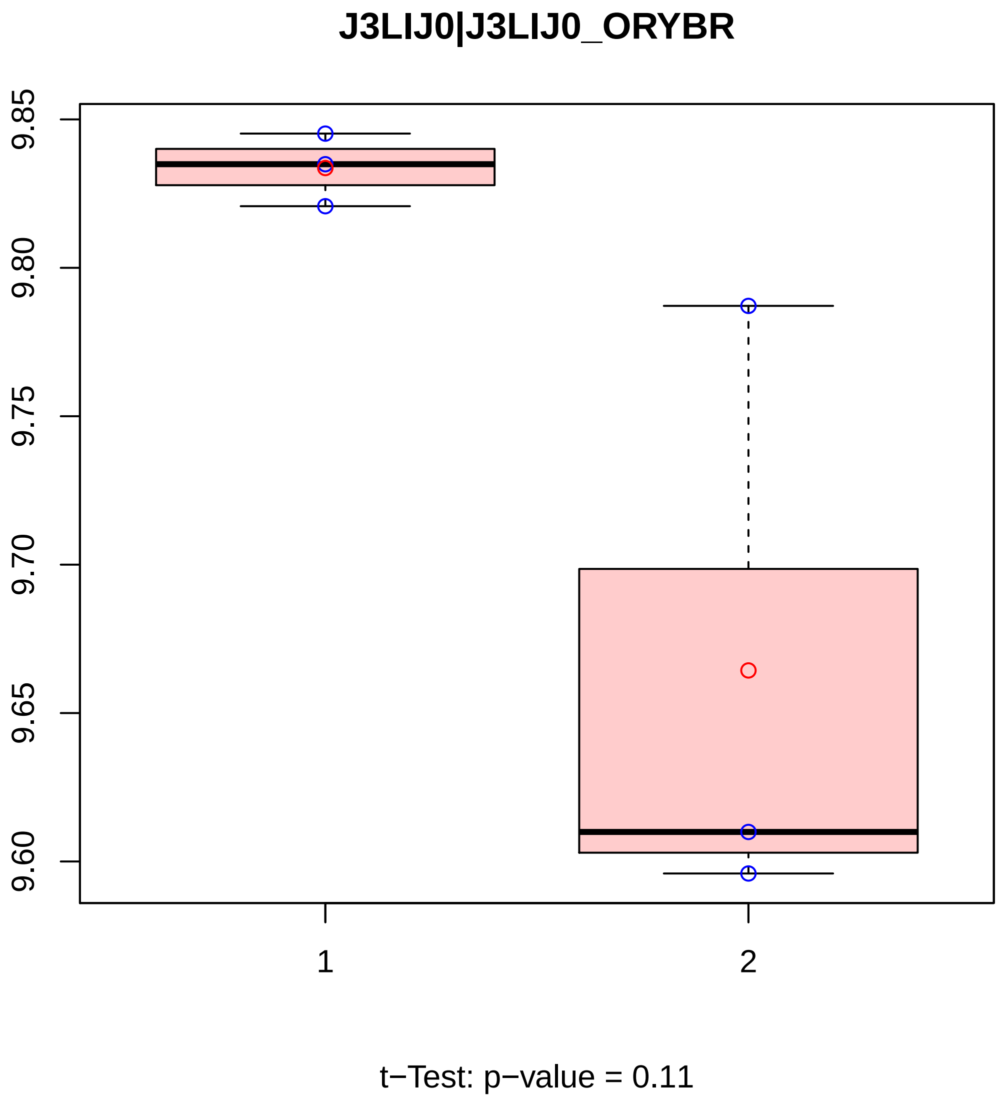
J3LIJ0|J3LIJ0_ORYBR
9.60
9.65
9.70
9.75
9.80
9.85
1
2
t−Test: p−value = 0.11

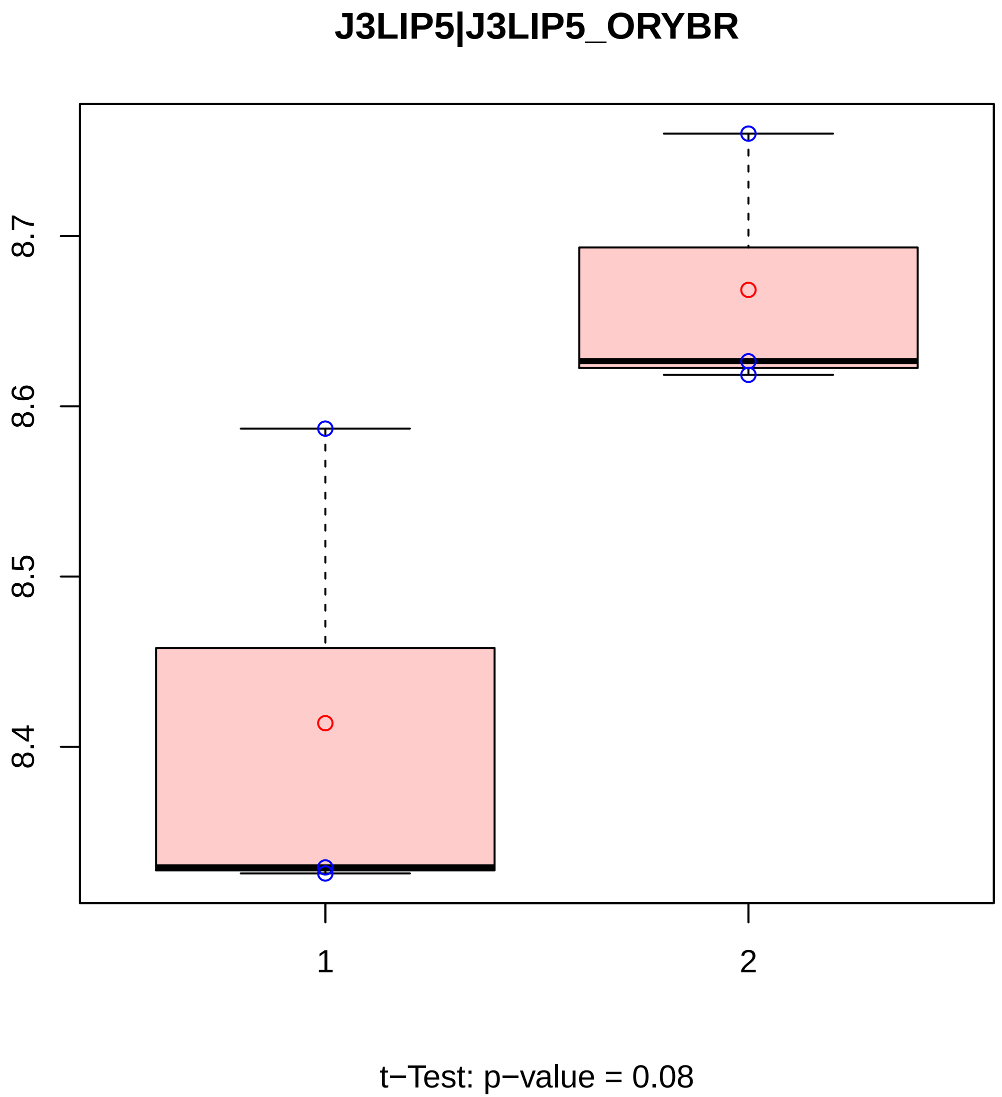
J3LIP5|J3LIP5_ORYBR
8.4
8.5
8.6
8.7
1
2
t−Test: p−value = 0.08

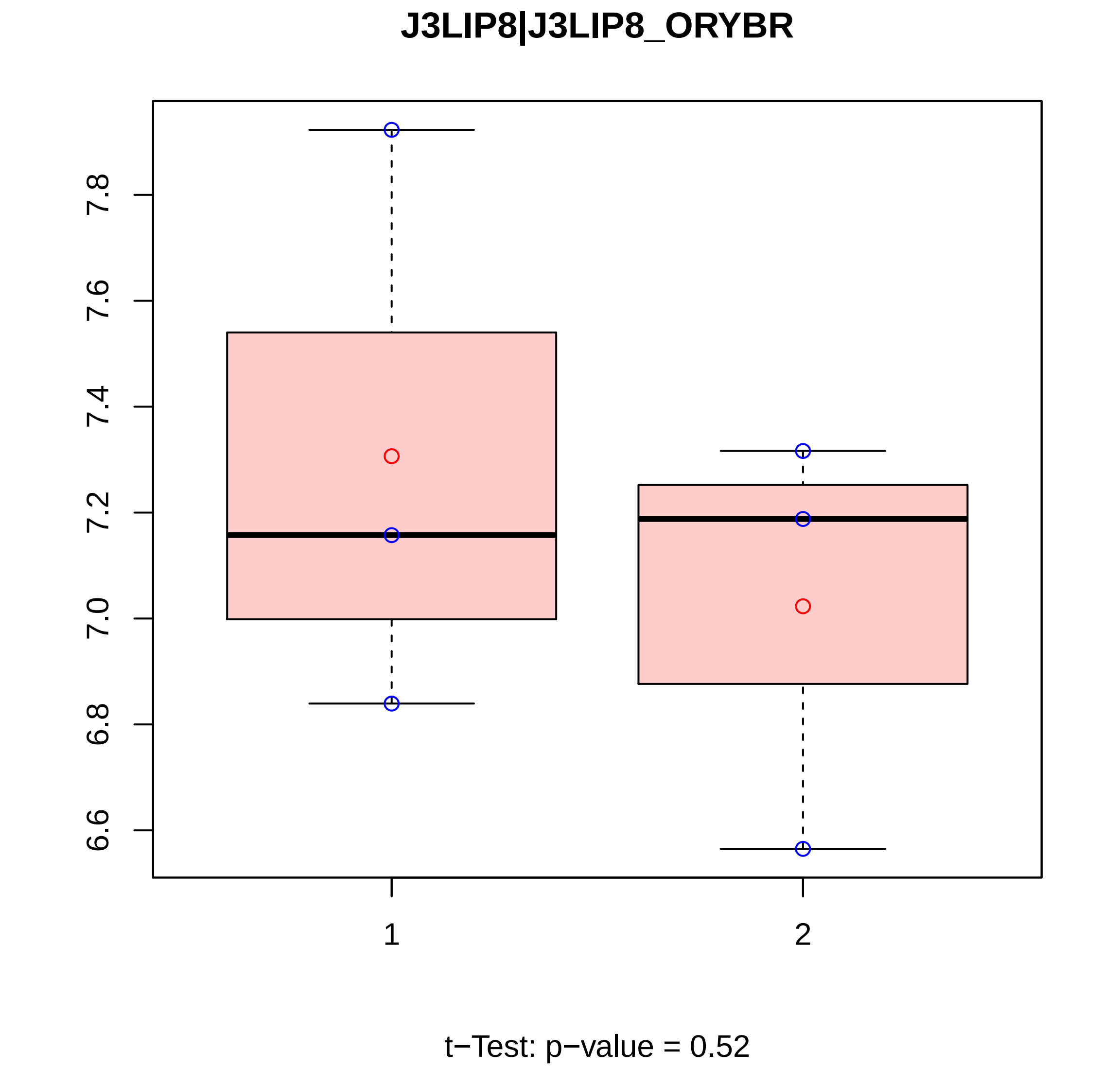
J3LIP8|J3LIP8_ORYBR
6.6
6.8
7.0
7.2
7.4
7.6
7.8
1
2
t−Test: p−value = 0.52

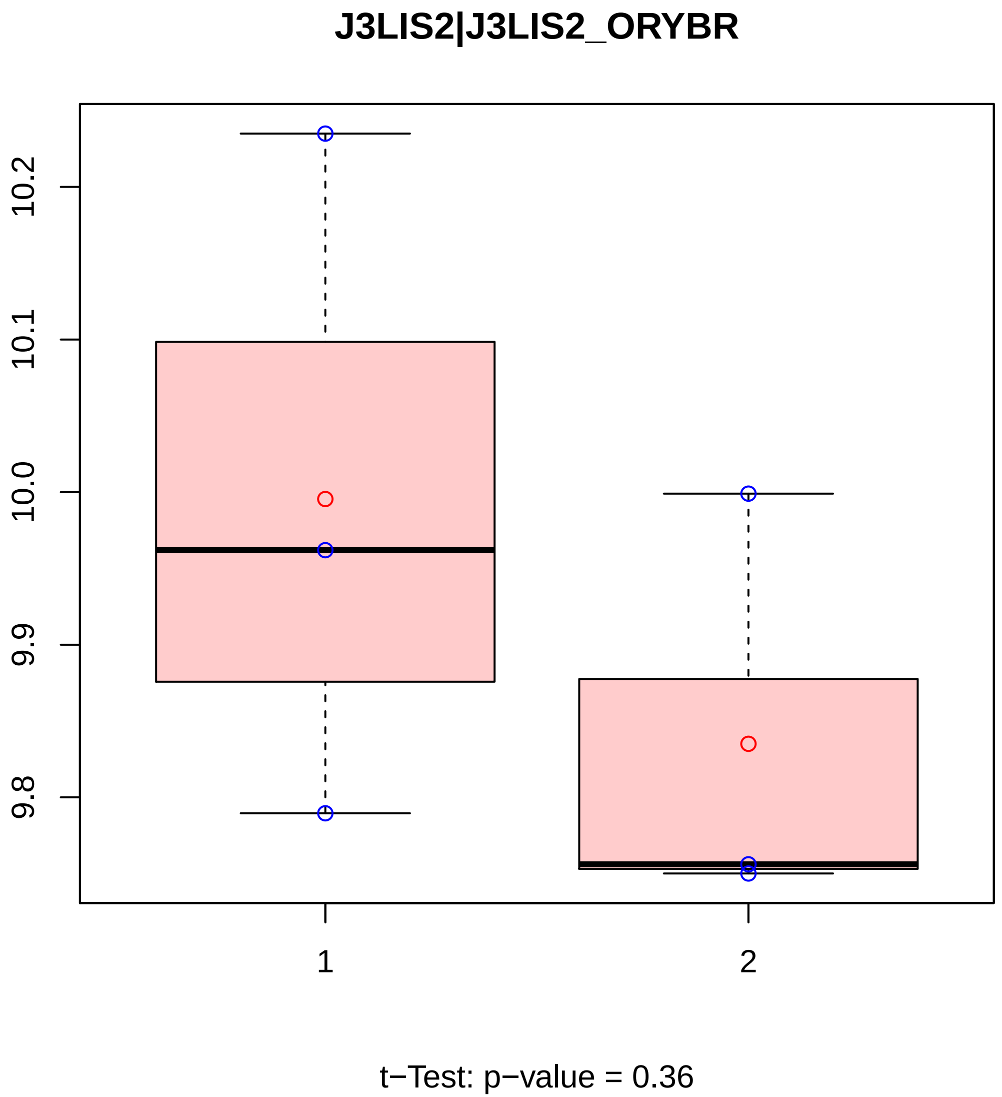
J3LIS2|J3LIS2_ORYBR
10.2
10.1
10.0
9.9
9.8
1
2
t−Test: p−value = 0.36

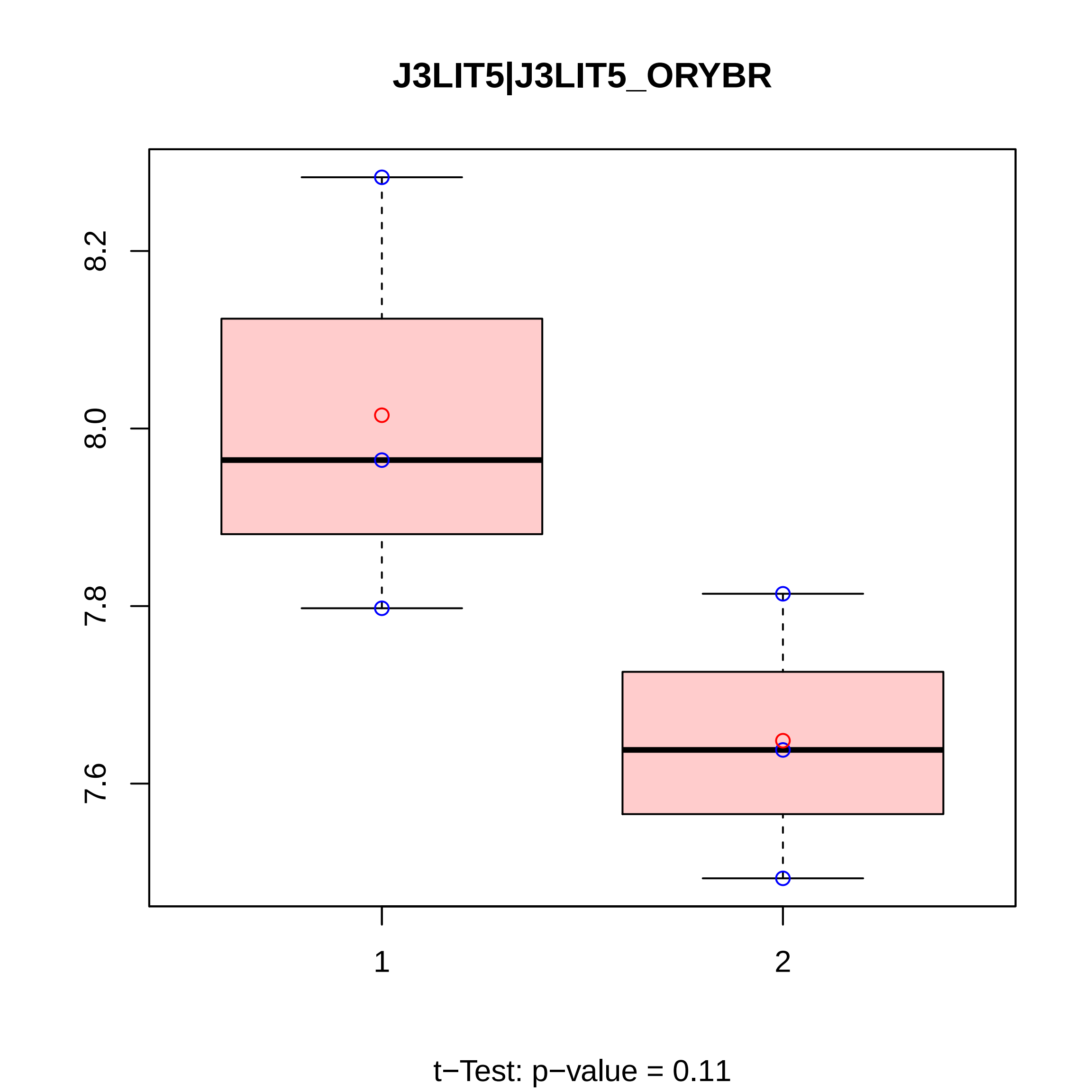
J3LIT5|J3LIT5_ORYBR
8.2
8.0
7.8
7.6
1
2
t−Test: p−value = 0.11

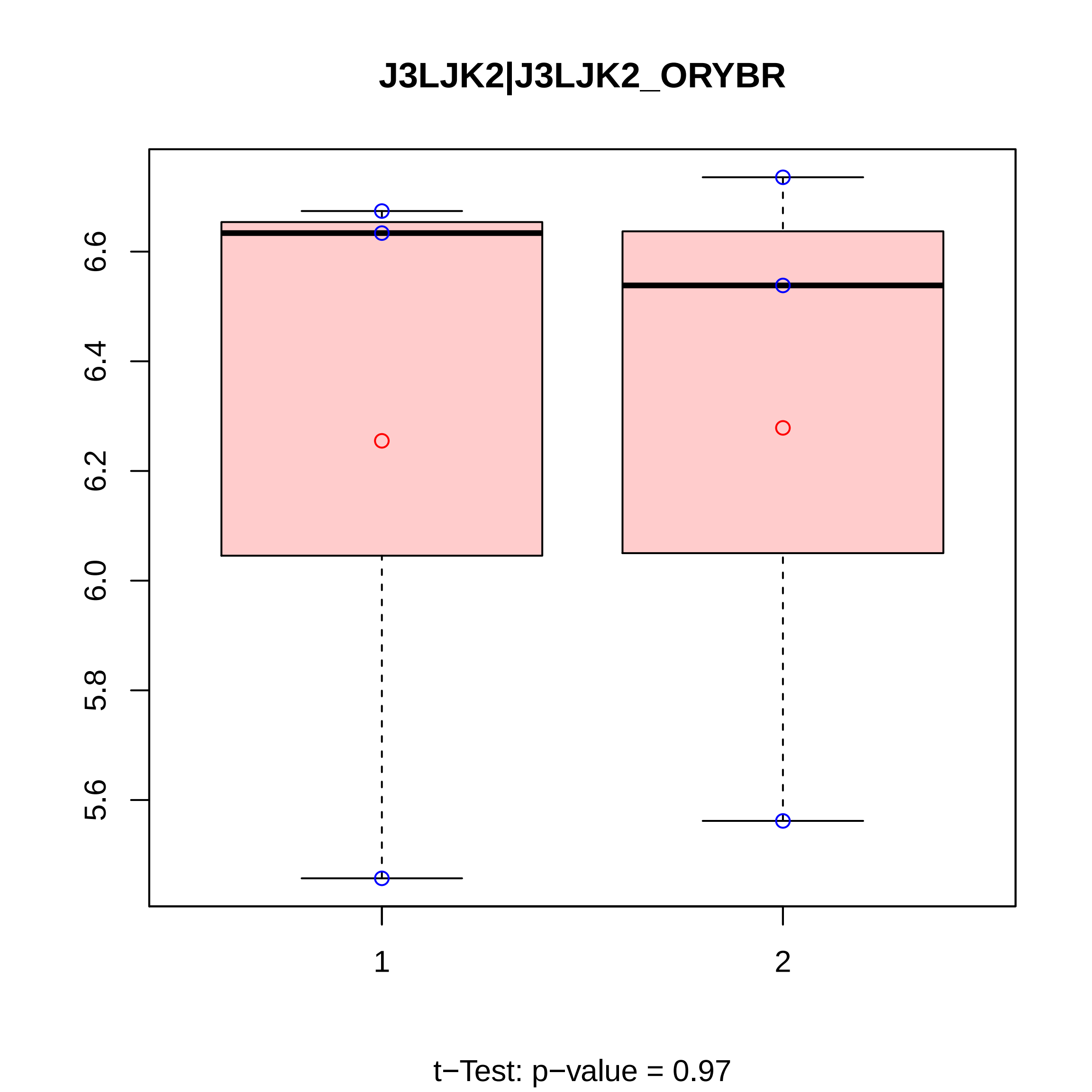
J3LJK2|J3LJK2_ORYBR
6.6
6.4
6.2
6.0
5.8
5.6
1
2
t−Test: p−value = 0.97

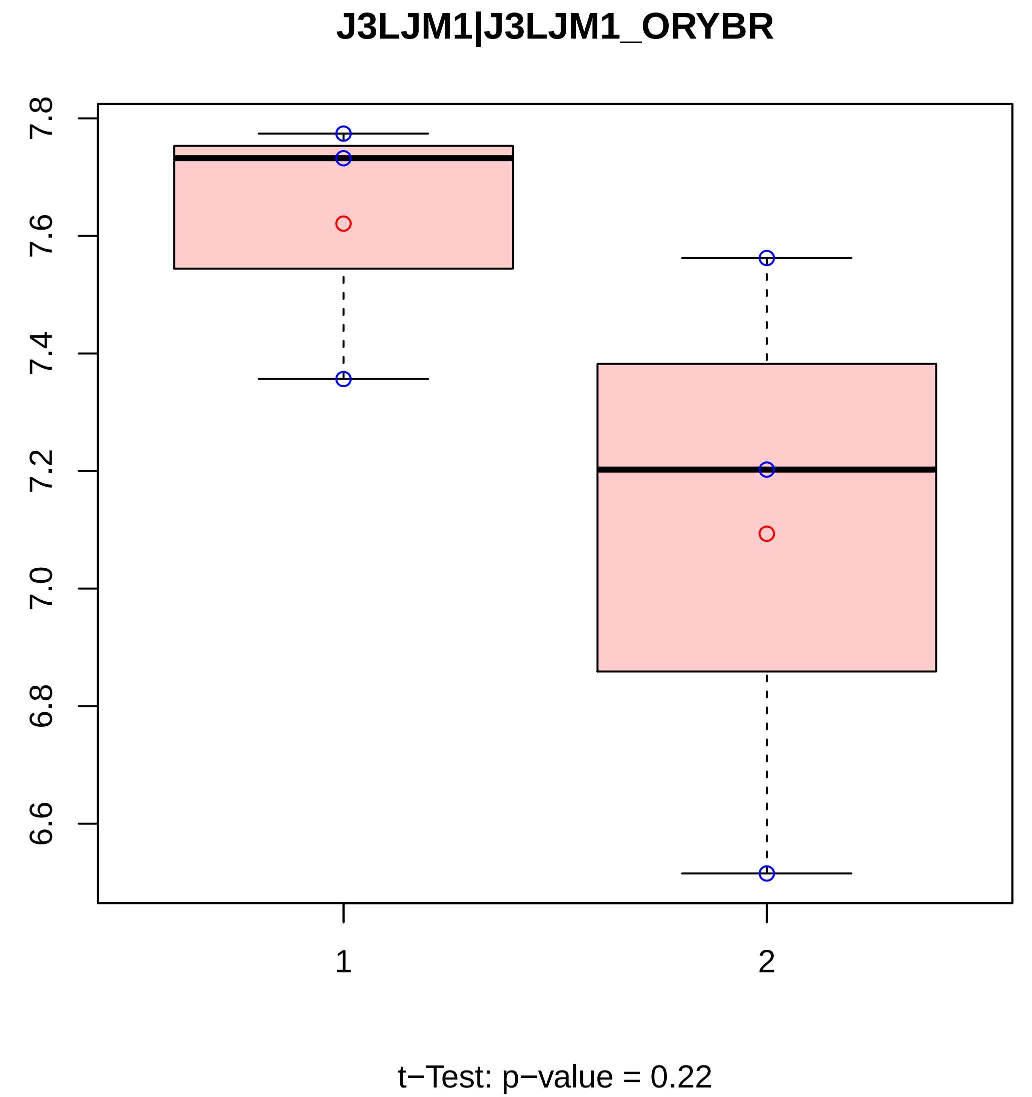
J3LJM1|J3LJM1_ORYBR
7.8
7.6
7.4
7.2
7.0
6.8
6.6
1
2
t−Test: p−value = 0.22

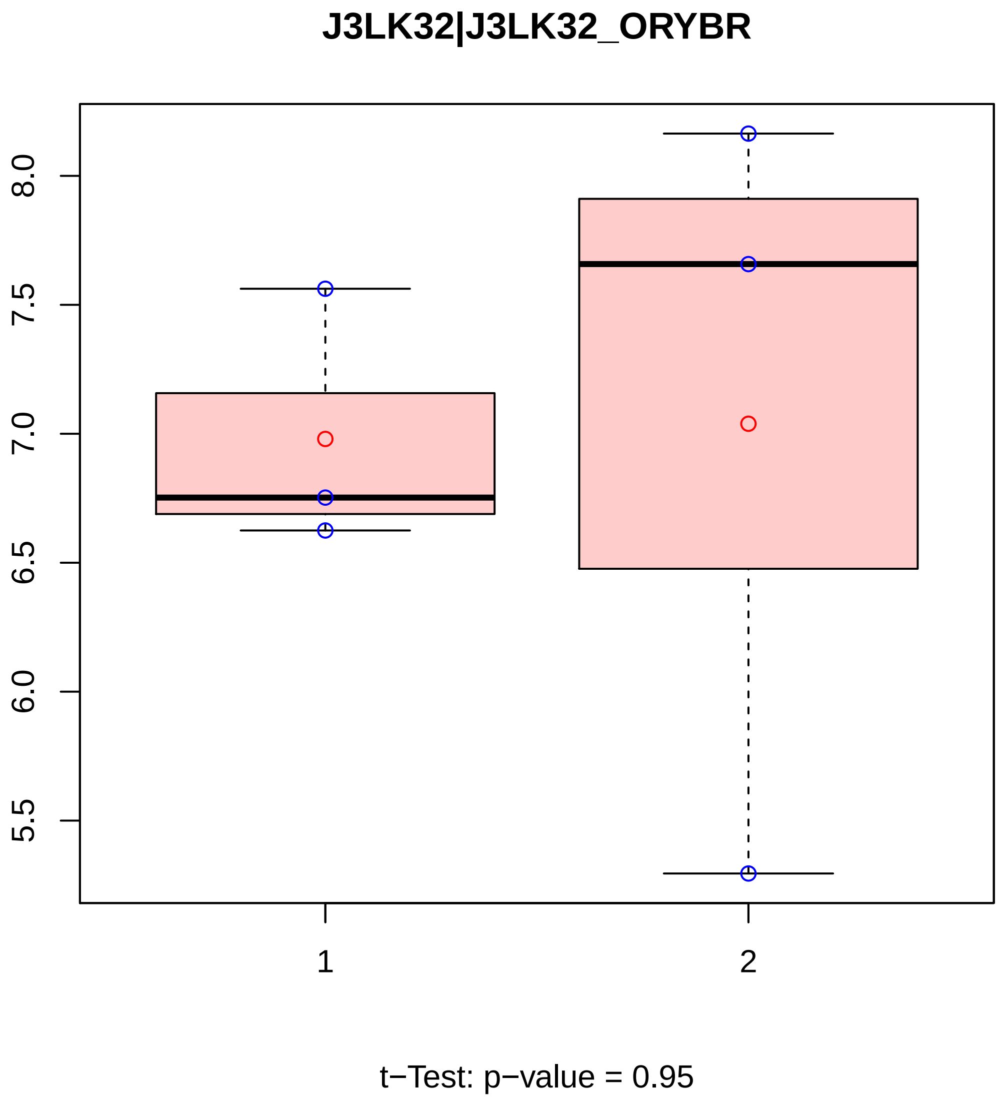
J3LK32|J3LK32_ORYBR
8.0
7.5
7.0
6.5
6.0
5.5
1
2
t−Test: p−value = 0.95

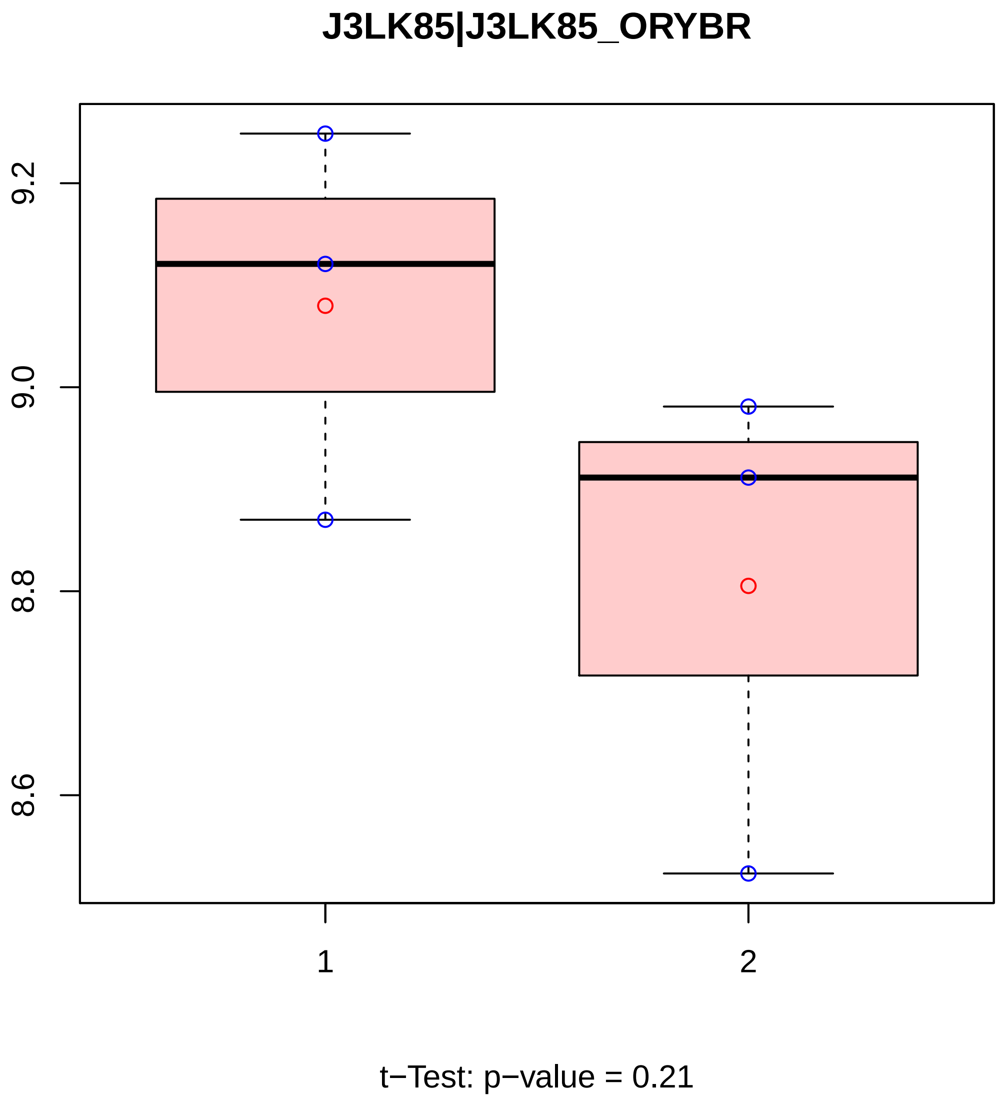
J3LK85|J3LK85_ORYBR
9.2
9.0
8.8
8.6
1
2
t−Test: p−value = 0.21

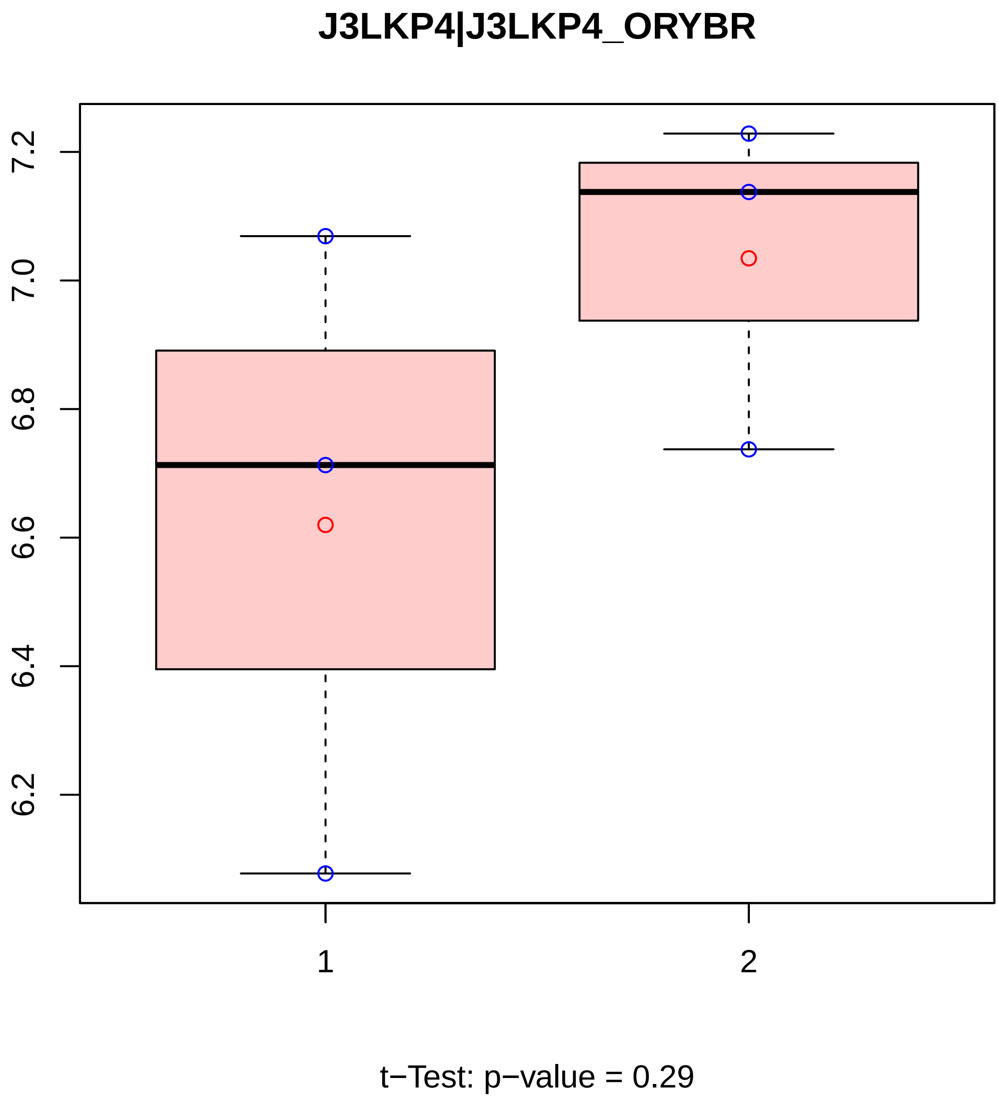
J3LKP4|J3LKP4_ORYBR
7.2
7.0
6.8
6.6
6.4
6.2
1
2
t−Test: p−value = 0.29

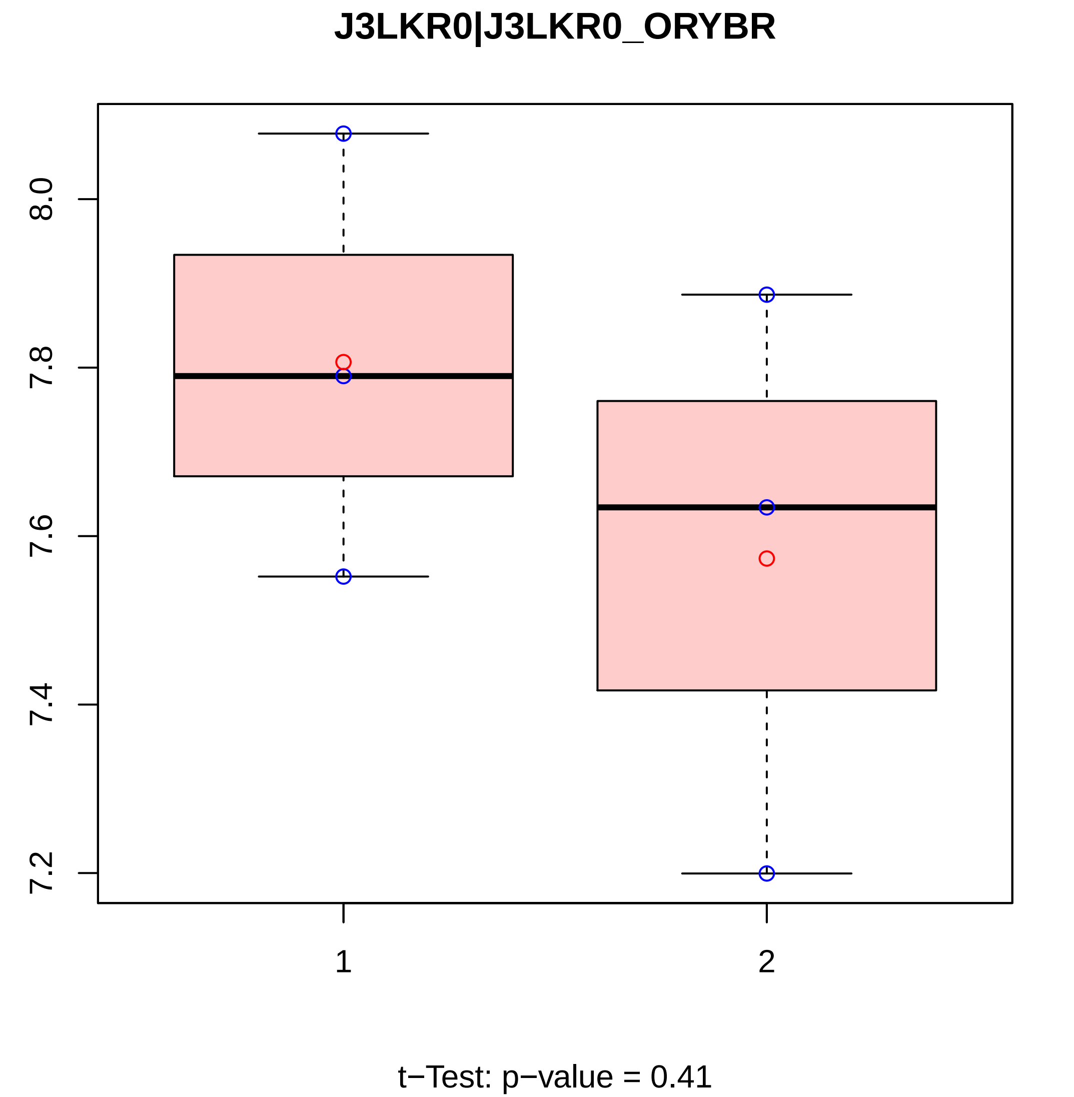

J3LKR0|J3LKR0_ORYBR
8.0
7.8
7.6
7.4
7.2
1
2
t−Test: p−value = 0.41

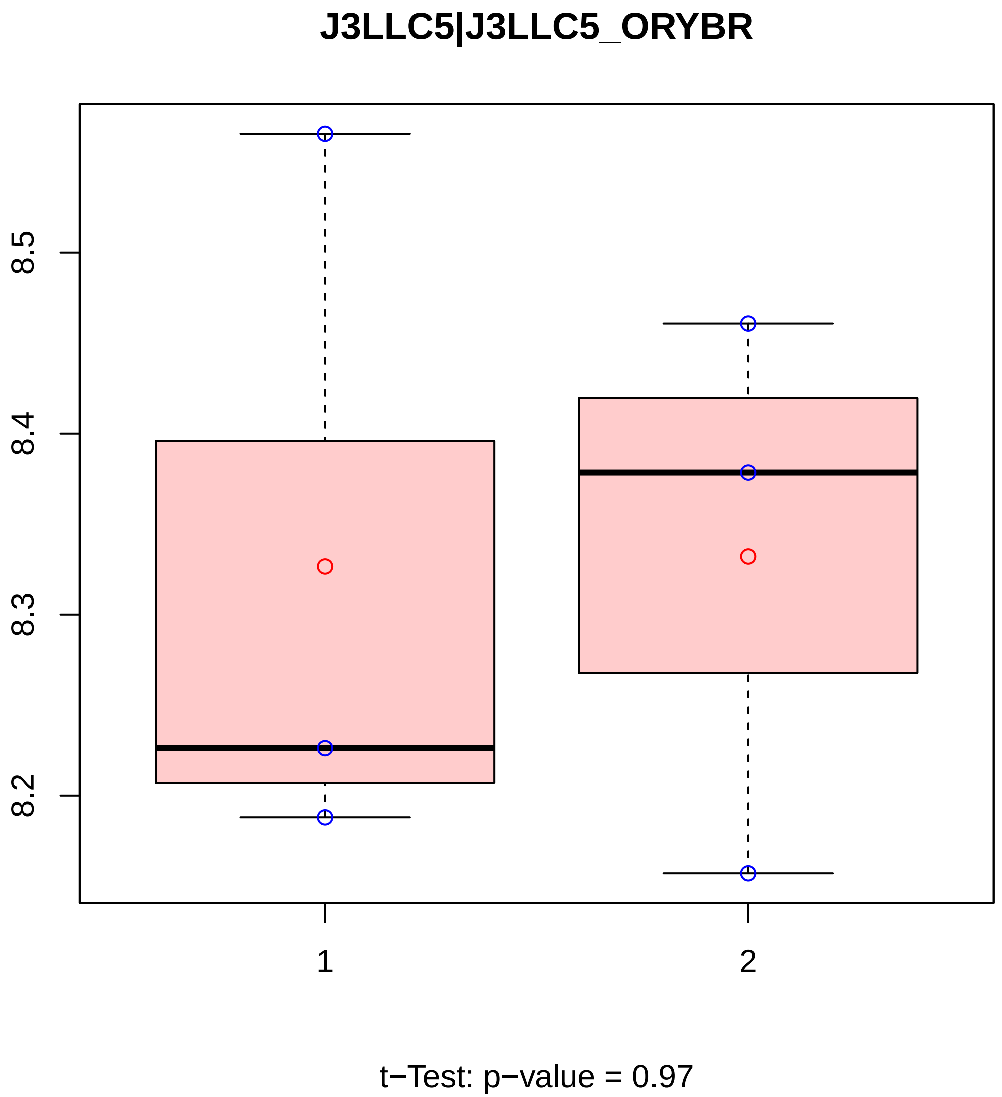
J3LLC5|J3LLC5_ORYBR
8.2
8.3
8.4
8.5
1
2
t−Test: p−value = 0.97

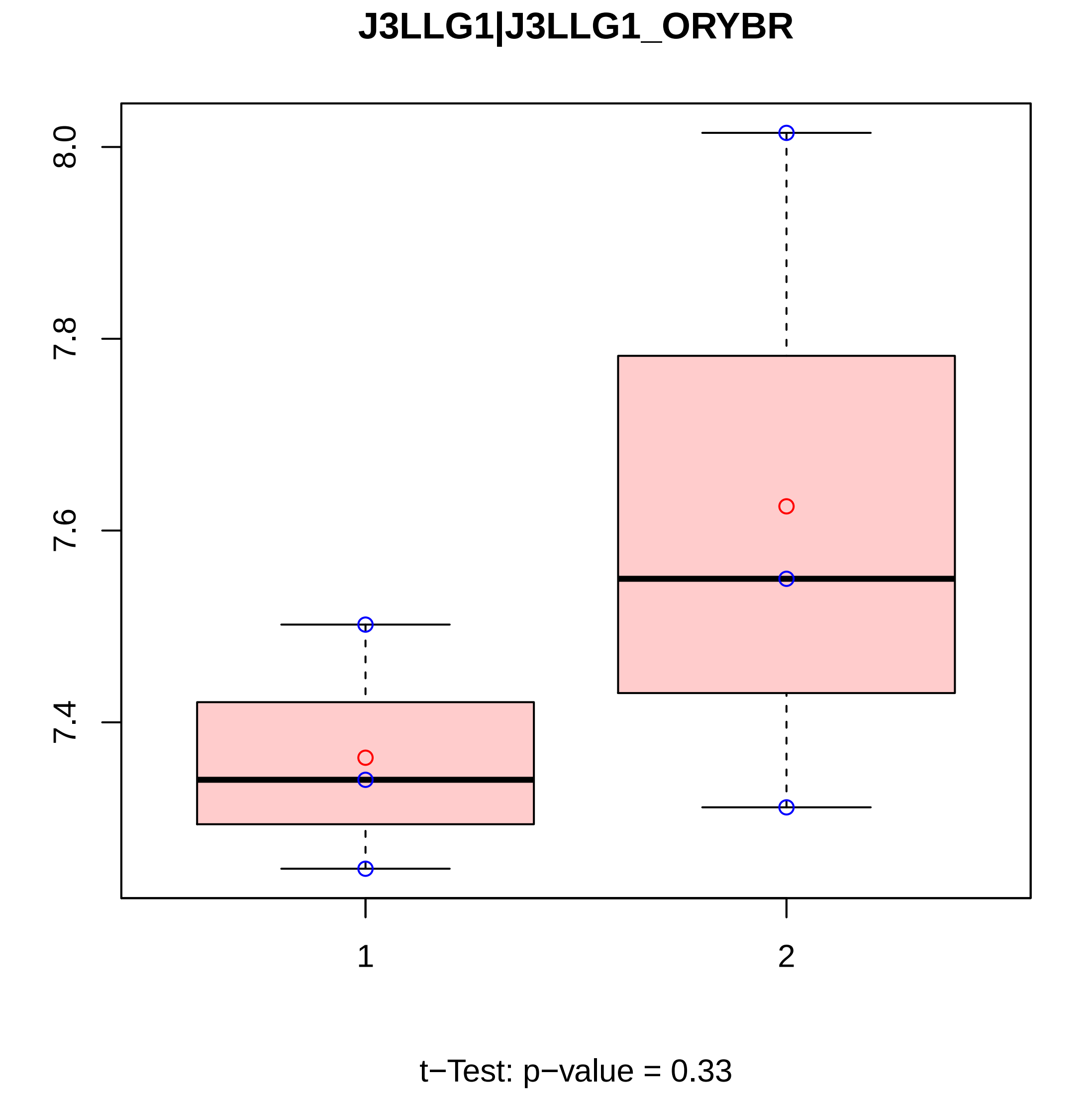
J3LLG1|J3LLG1_ORYBR
8.0
7.8
7.6
7.4
1
2
t−Test: p−value = 0.33

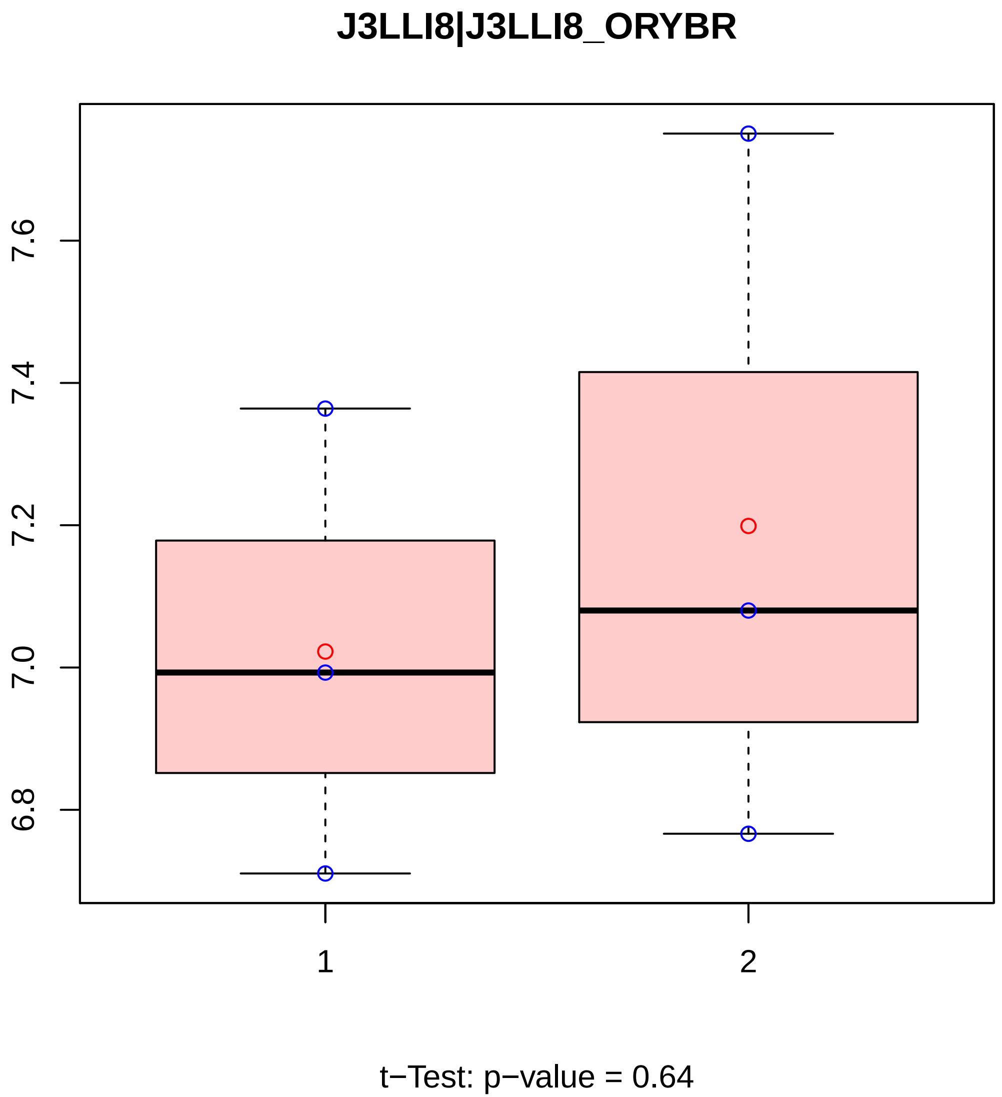
J3LLI8|J3LLI8_ORYBR
7.6
7.4
7.2
7.0
6.8
1
2
t−Test: p−value = 0.64

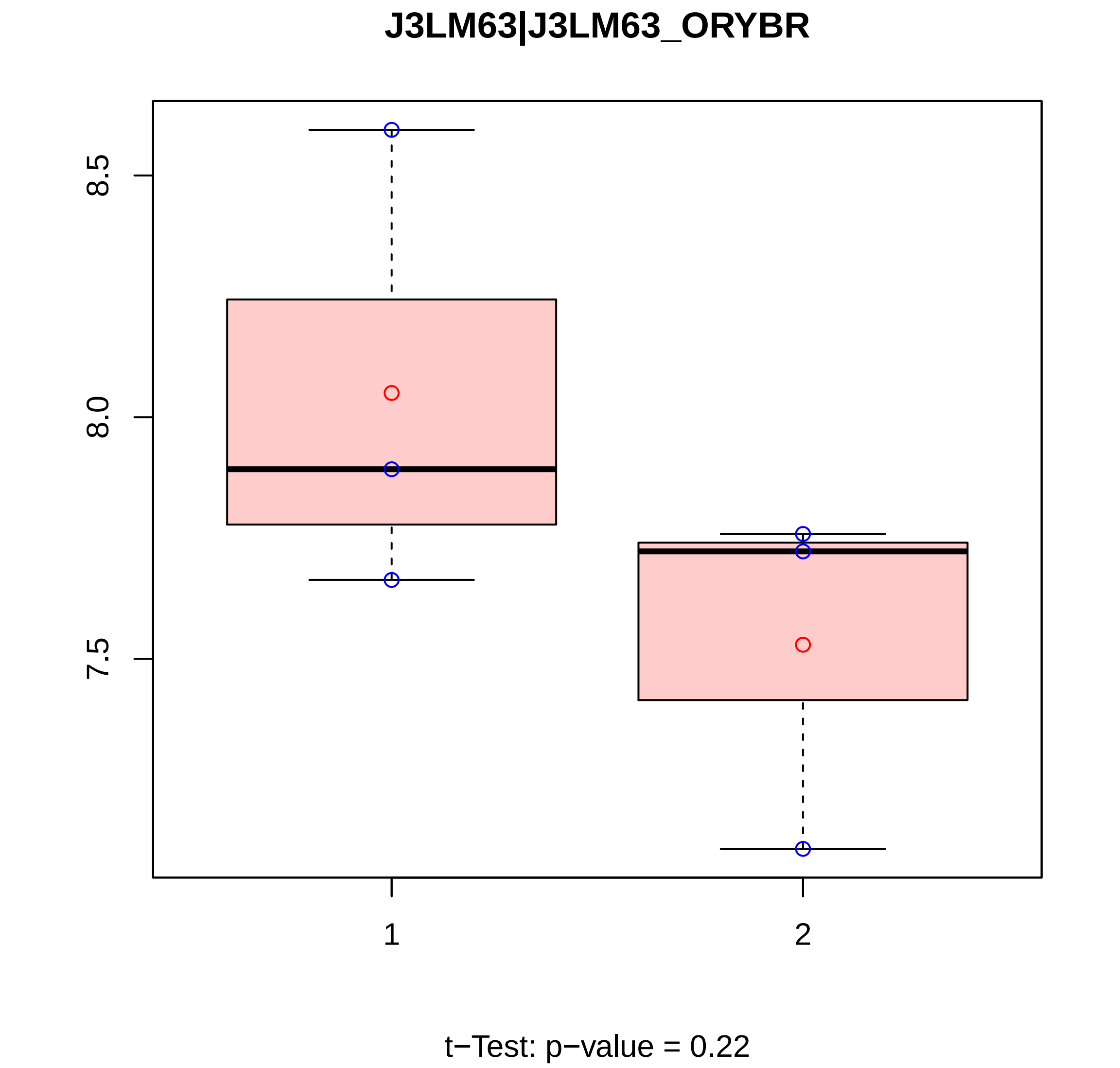

J3LM63|J3LM63_ORYBR
8.5
8.0
7.5
1
2
t−Test: p−value = 0.22

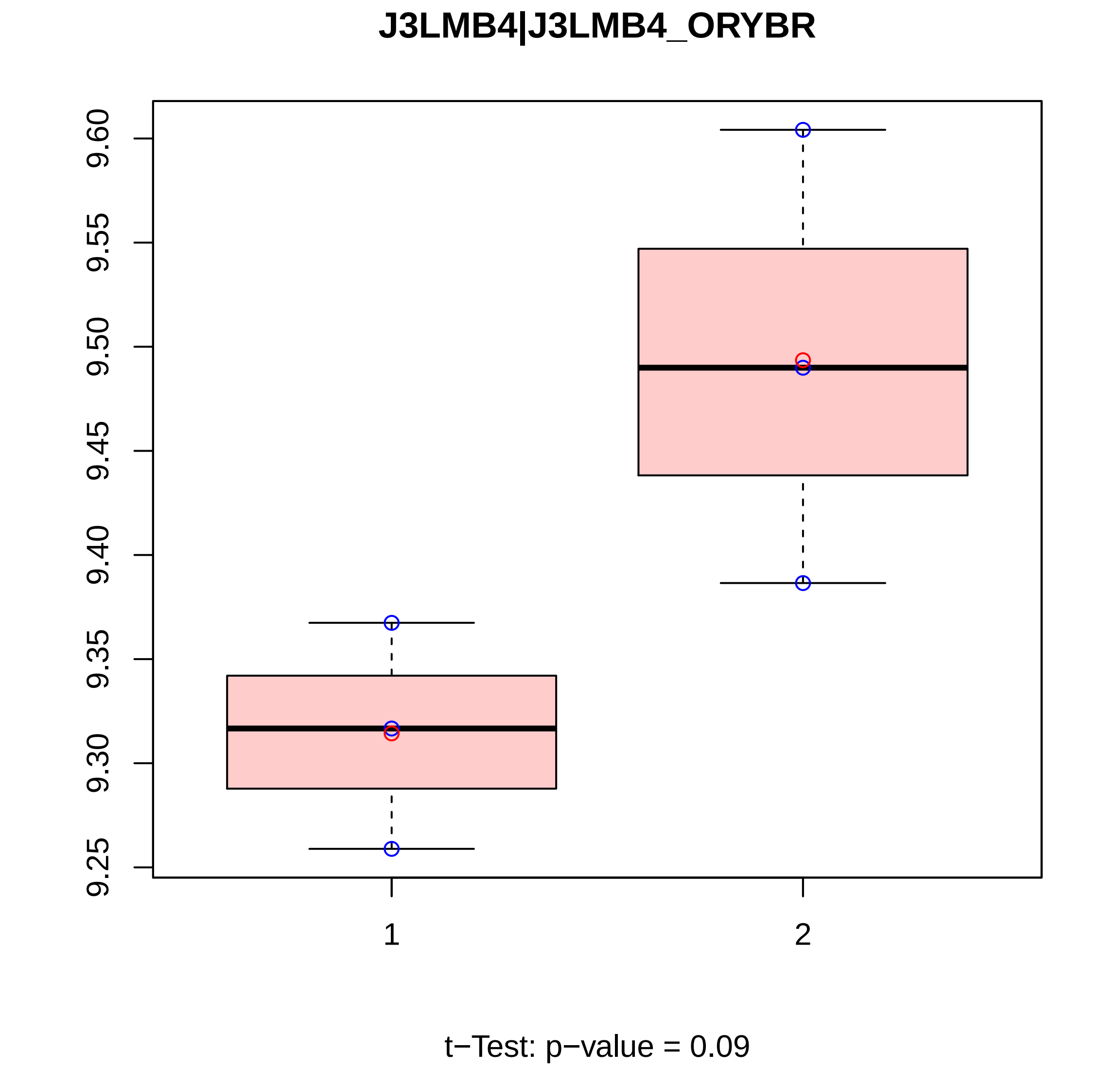

J3LMB4|J3LMB4_ORYBR
9.25
9.30
9.35
9.40
9.45
9.50
9.55
9.60
1
2
t−Test: p−value = 0.09

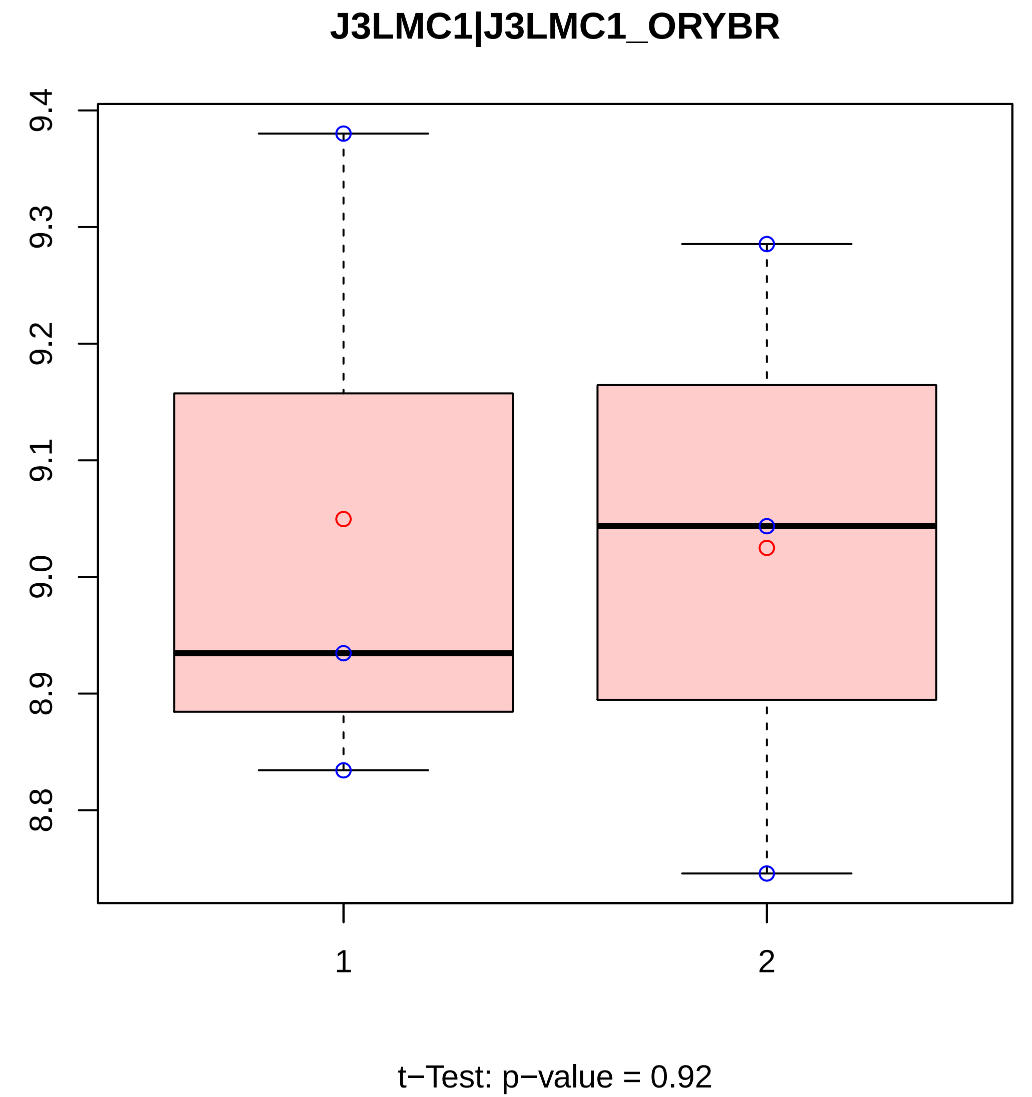
J3LMC1|J3LMC1_ORYBR
9.4
9.3
9.2
9.1
9.0
8.9
8.8
1
2
t−Test: p−value = 0.92

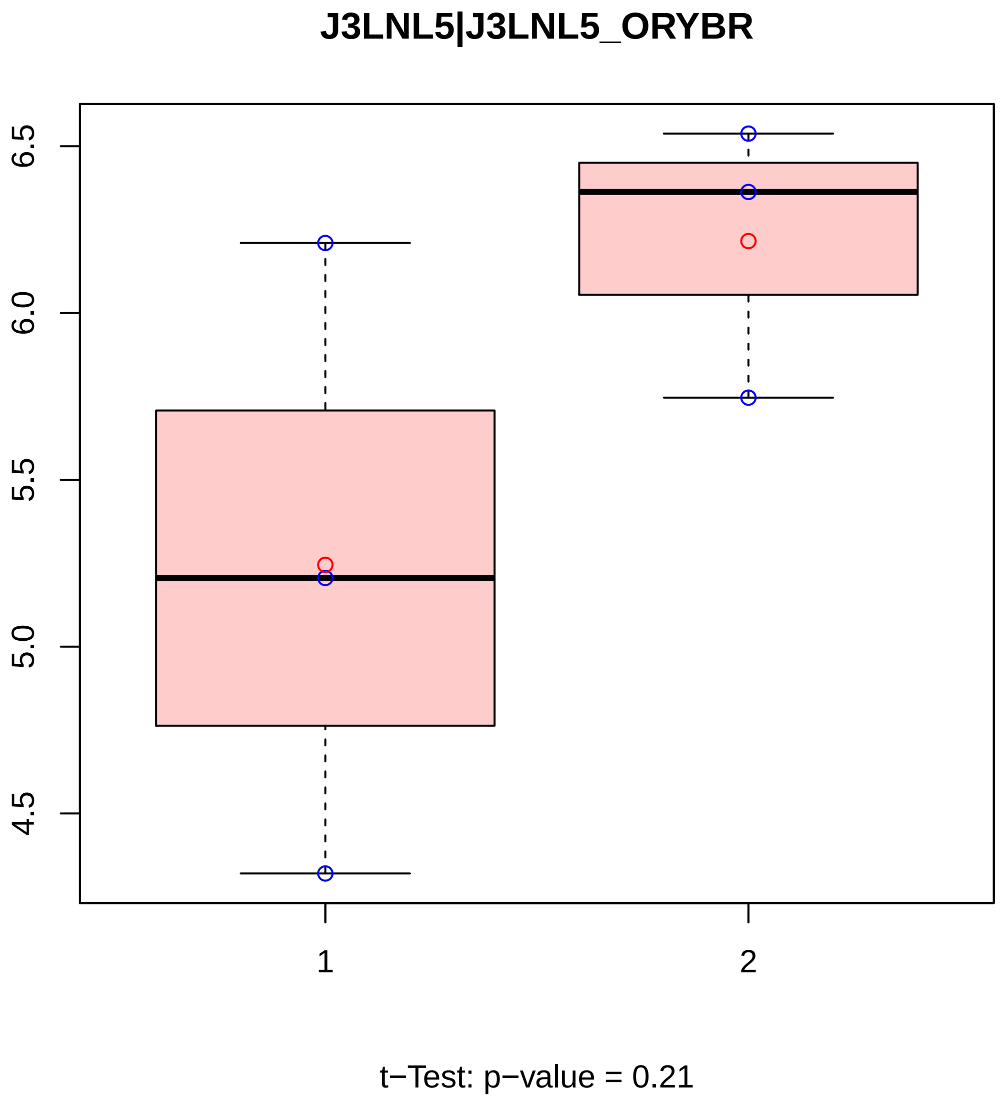

J3LNL5|J3LNL5_ORYBR
6.5
6.0
5.5
5.0
4.5
1
2
t−Test: p−value = 0.21

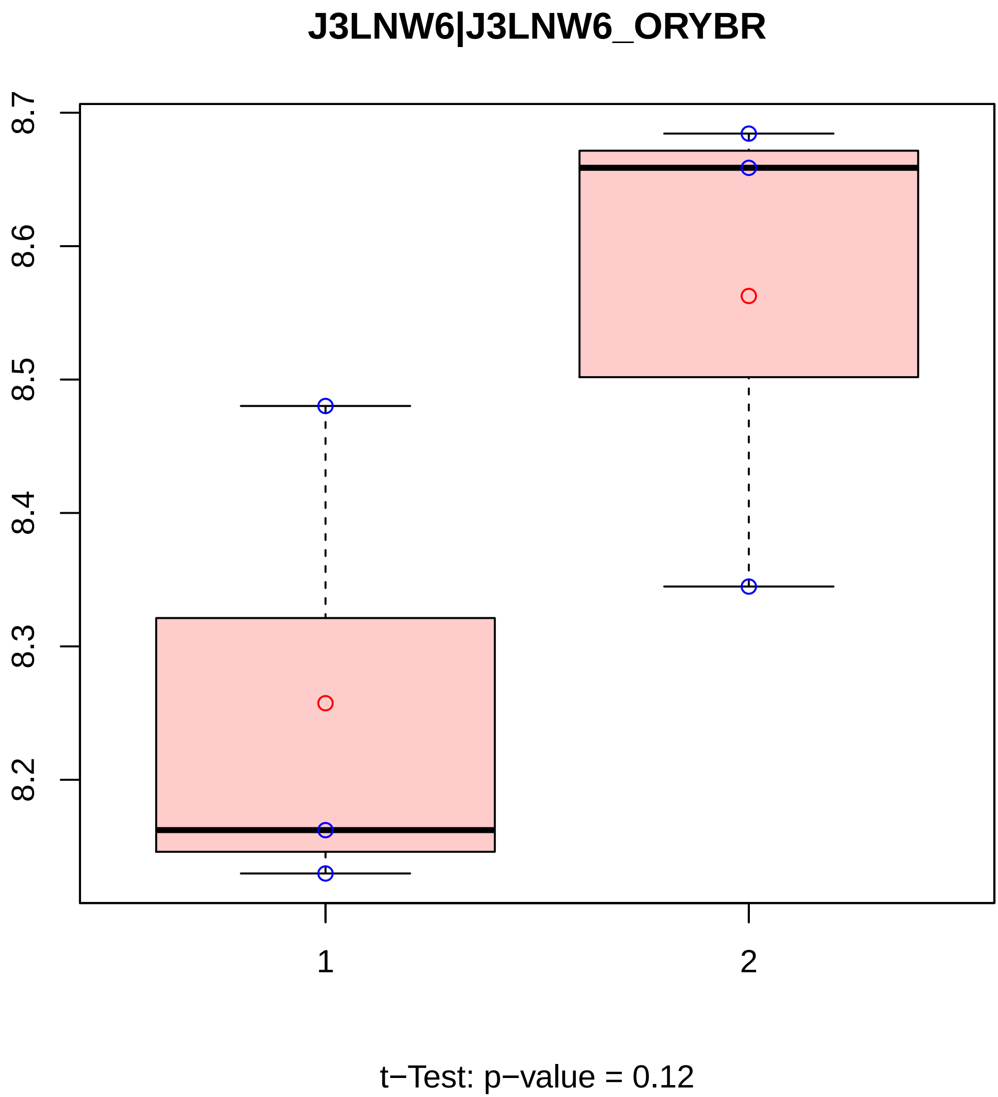
J3LNW6|J3LNW6_ORYBR
8.7
8.6
8.5
8.4
8.3
8.2
1
2
t−Test: p−value = 0.12

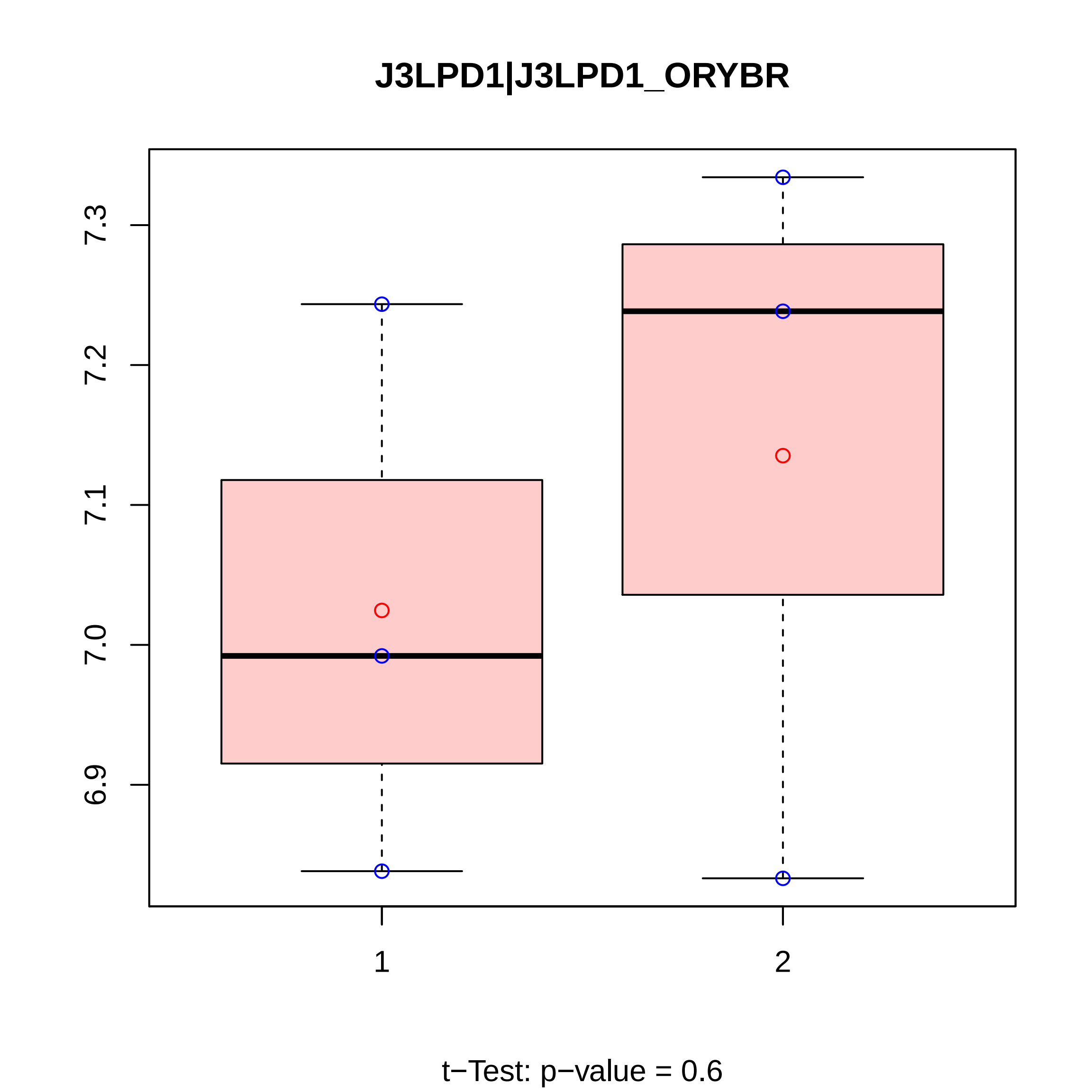
J3LPD1|J3LPD1_ORYBR
7.3
7.2
7.1
7.0
6.9
1
2
t−Test: p−value = 0.6

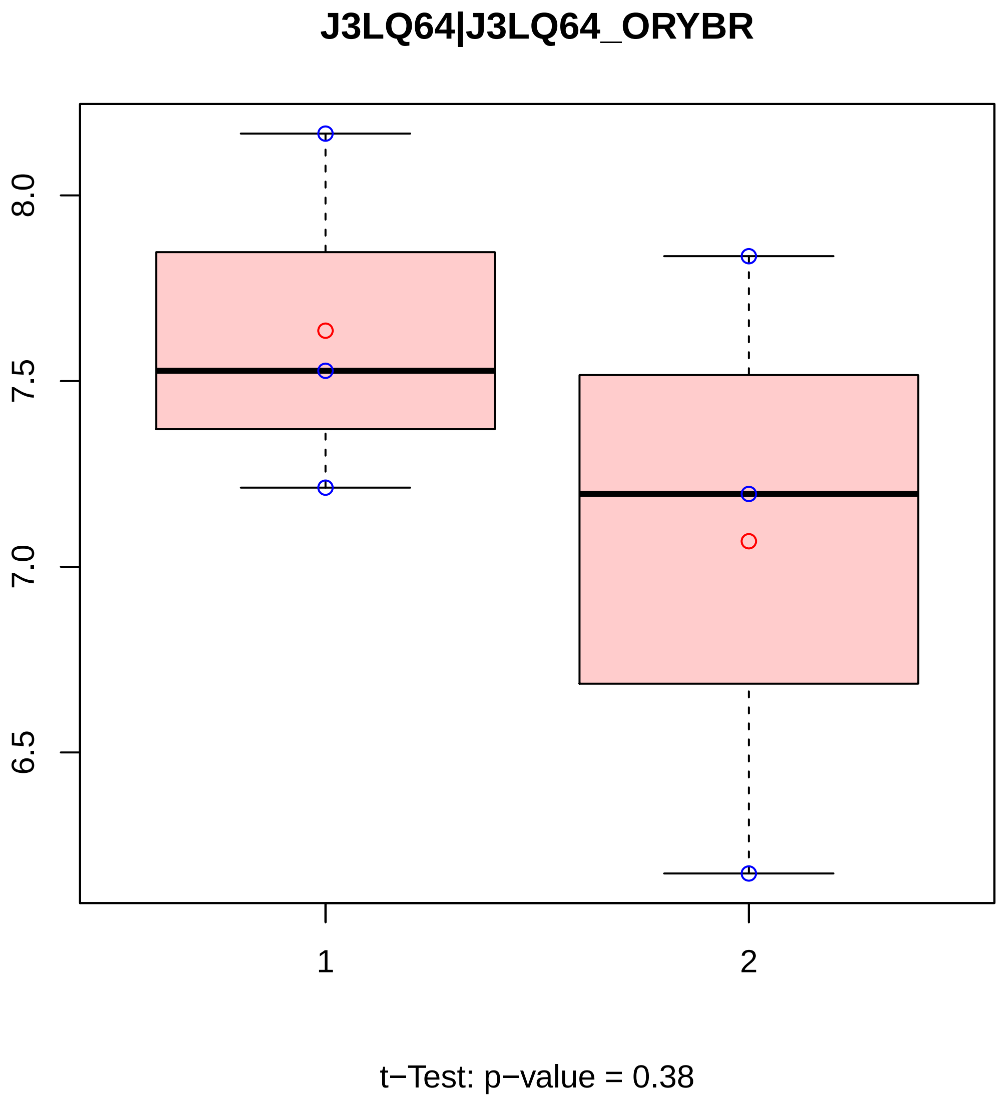

J3LQ64|J3LQ64_ORYBR
8.0
7.5
7.0
6.5
1
2
t−Test: p−value = 0.38

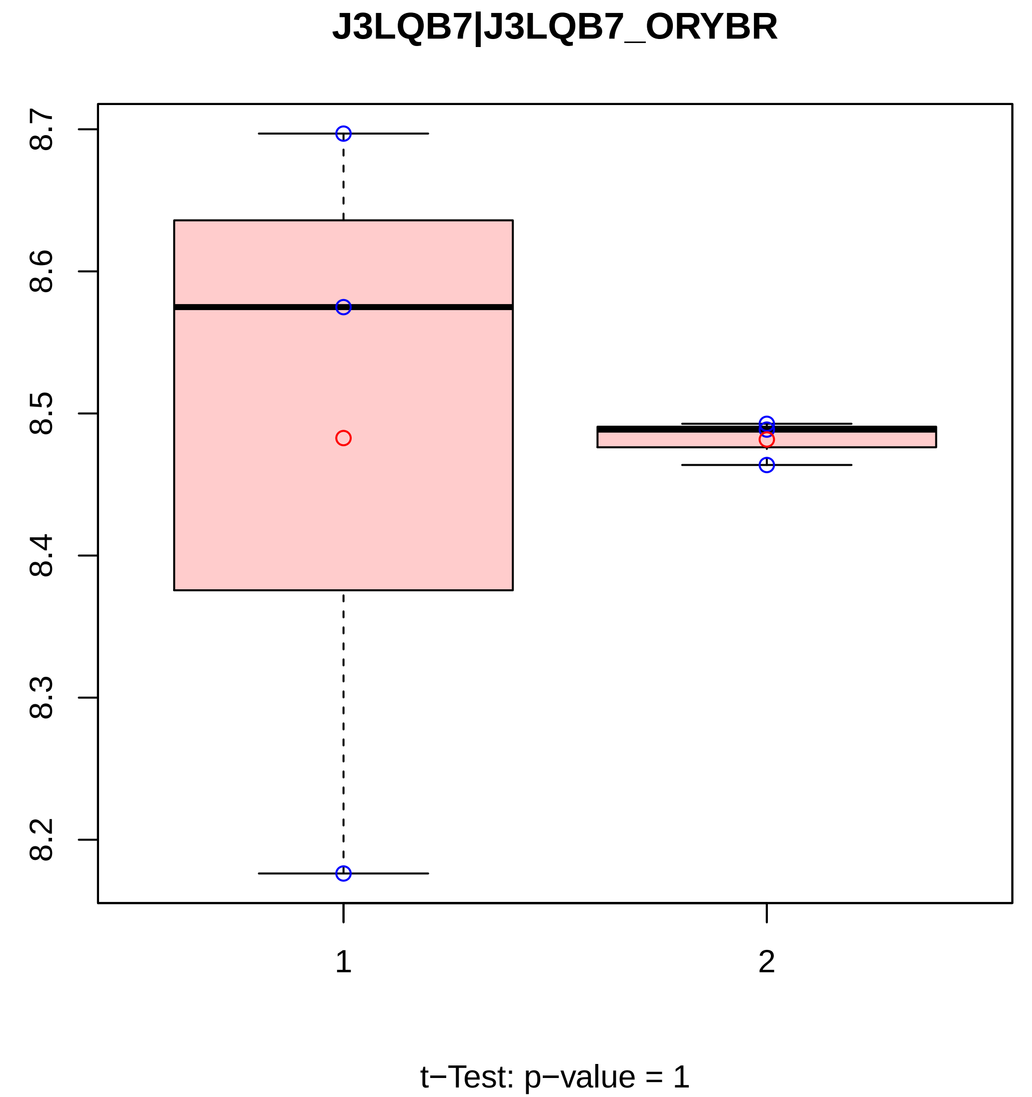
J3LQB7|J3LQB7_ORYBR
8.2
8.3
8.4
8.5
8.6
8.7
1
2
t−Test: p−value = 1

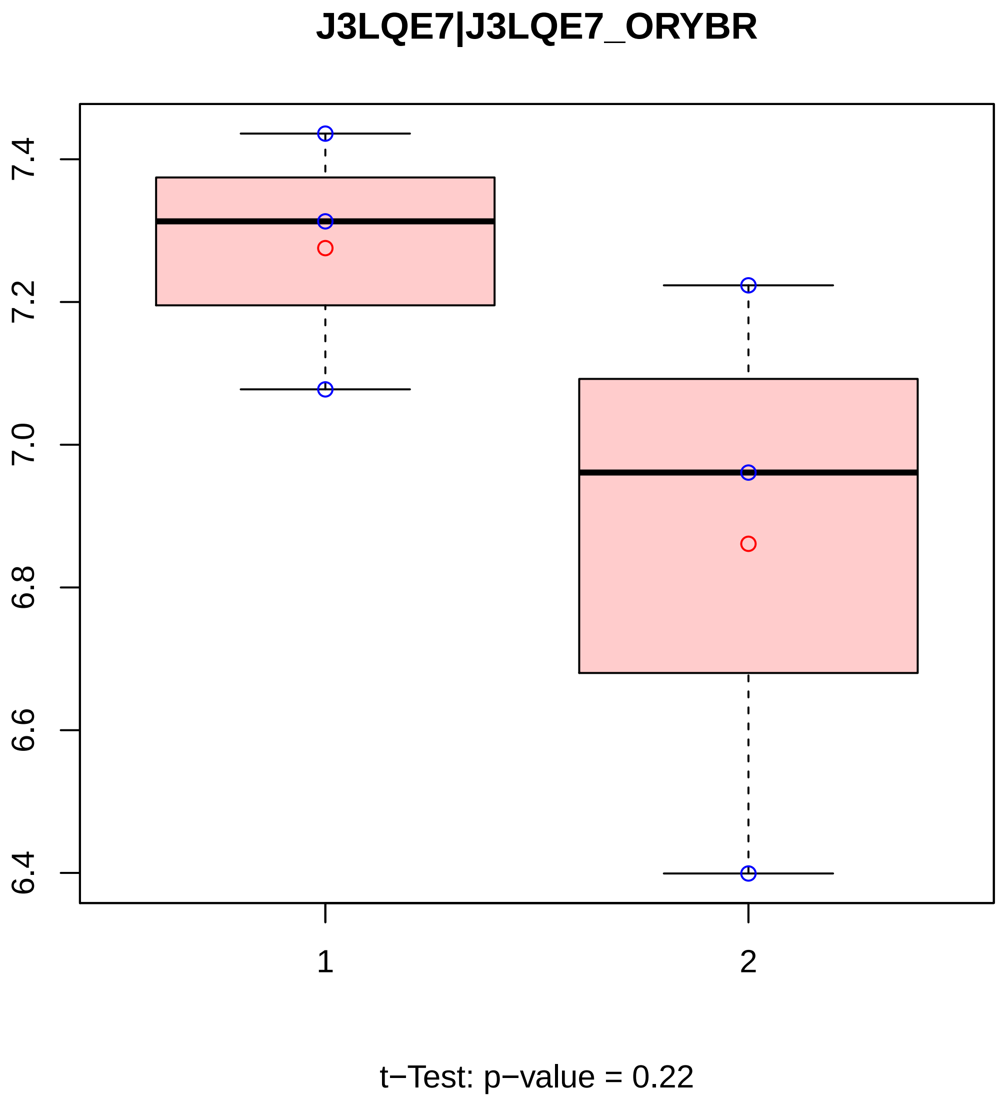

J3LQE7|J3LQE7_ORYBR
7.4
7.2
7.0
6.8
6.6
6.4
1
2
t−Test: p−value = 0.22

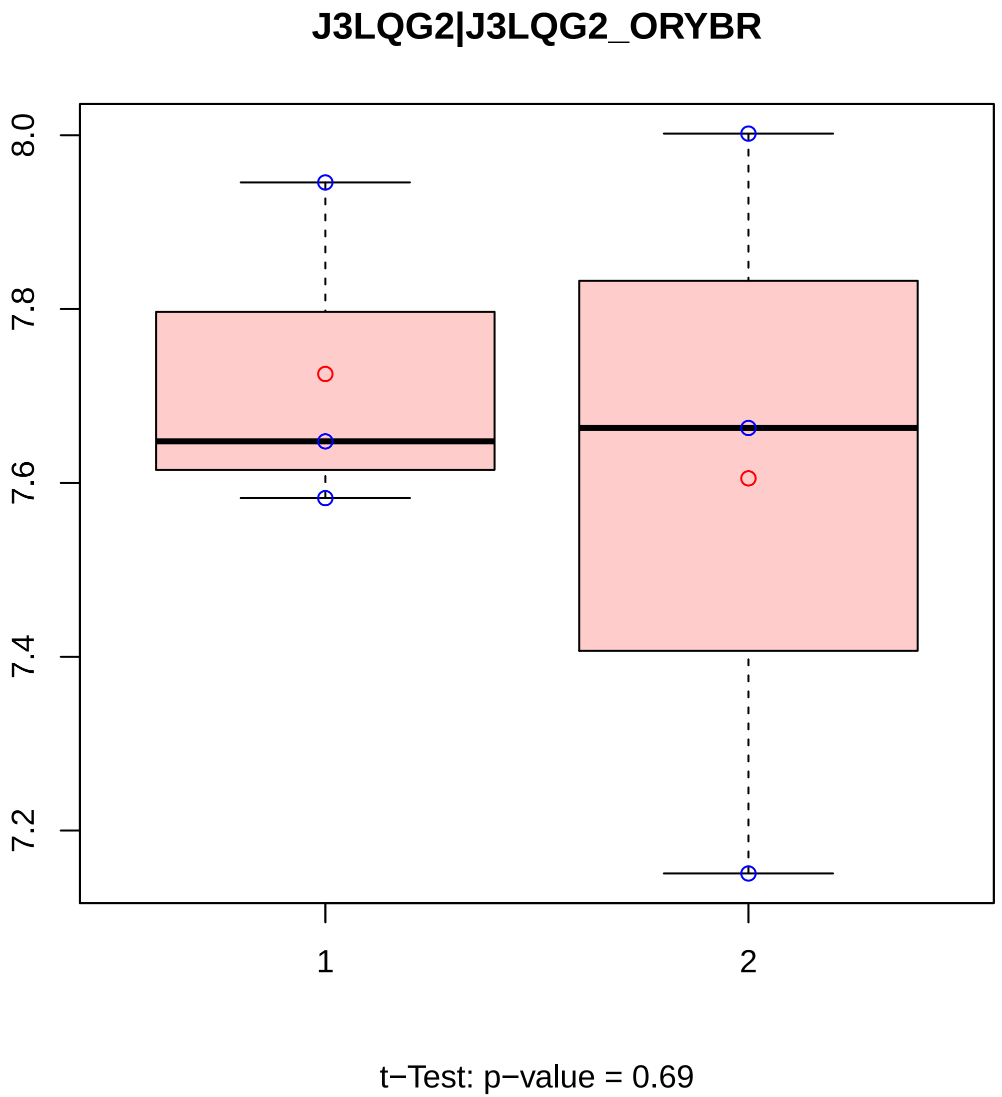

J3LQG2|J3LQG2_ORYBR
8.0
7.8
7.6
7.4
7.2
1
2
t−Test: p−value = 0.69

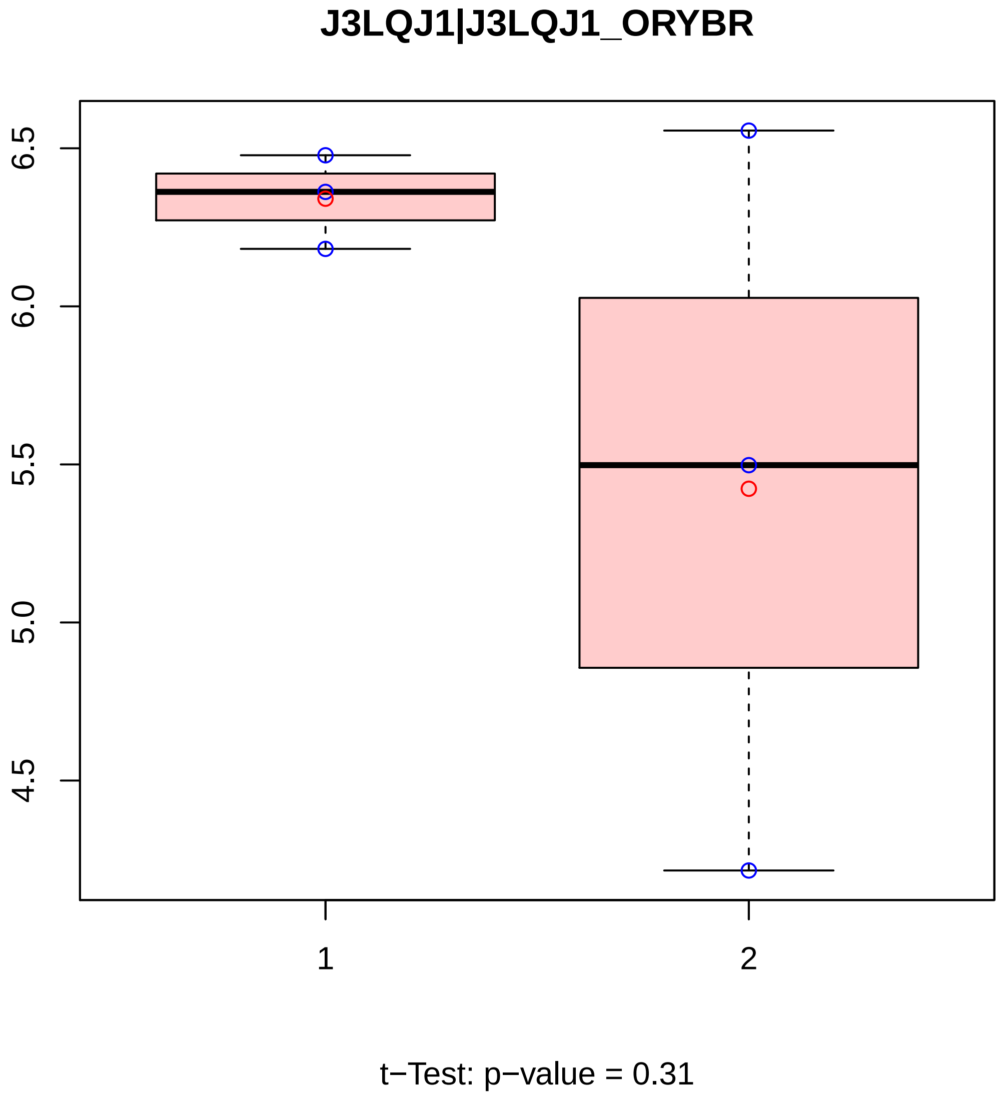

J3LQJ1|J3LQJ1_ORYBR
6.5
6.0
5.5
5.0
4.5
1
2
t−Test: p−value = 0.31

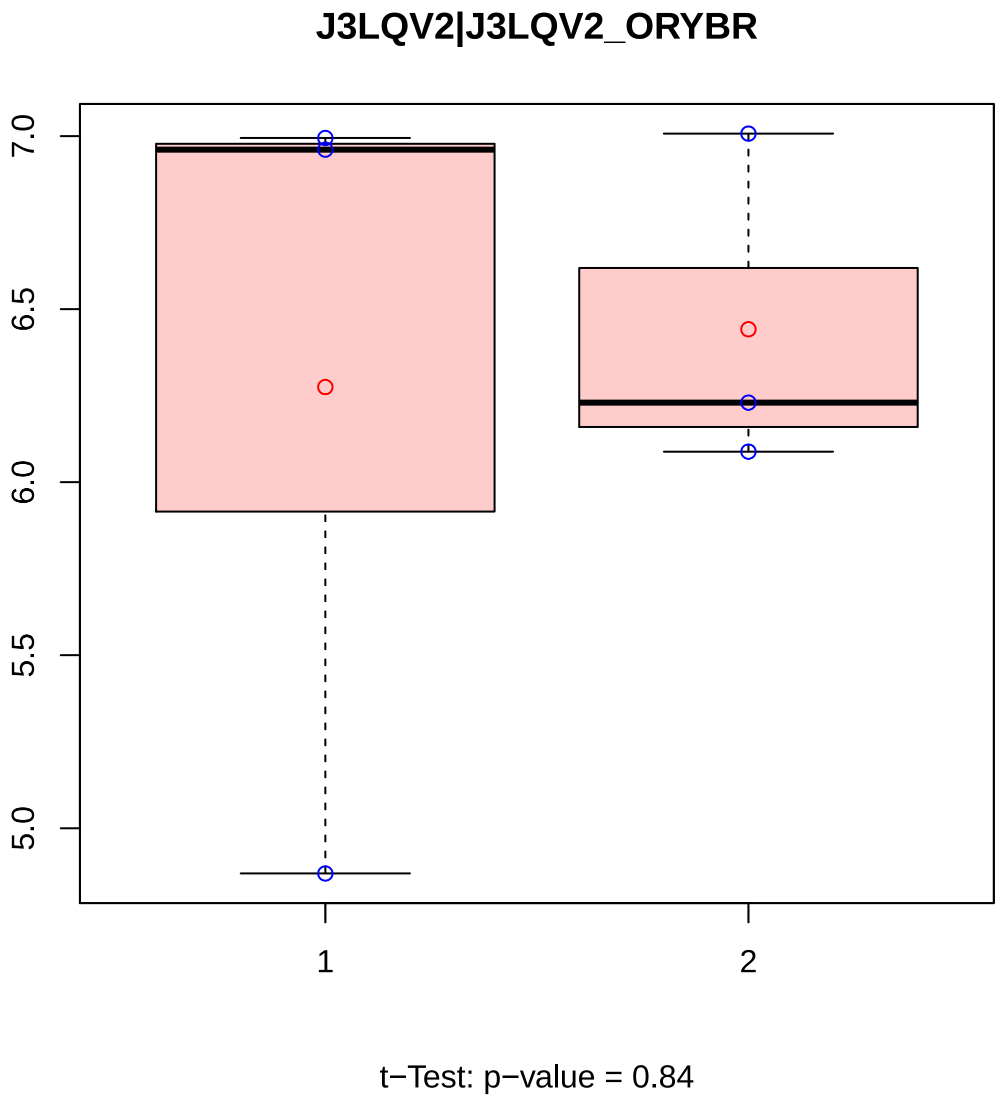
J3LQV2|J3LQV2_ORYBR
7.0
6.5
6.0
5.5
5.0
1
2
t−Test: p−value = 0.84

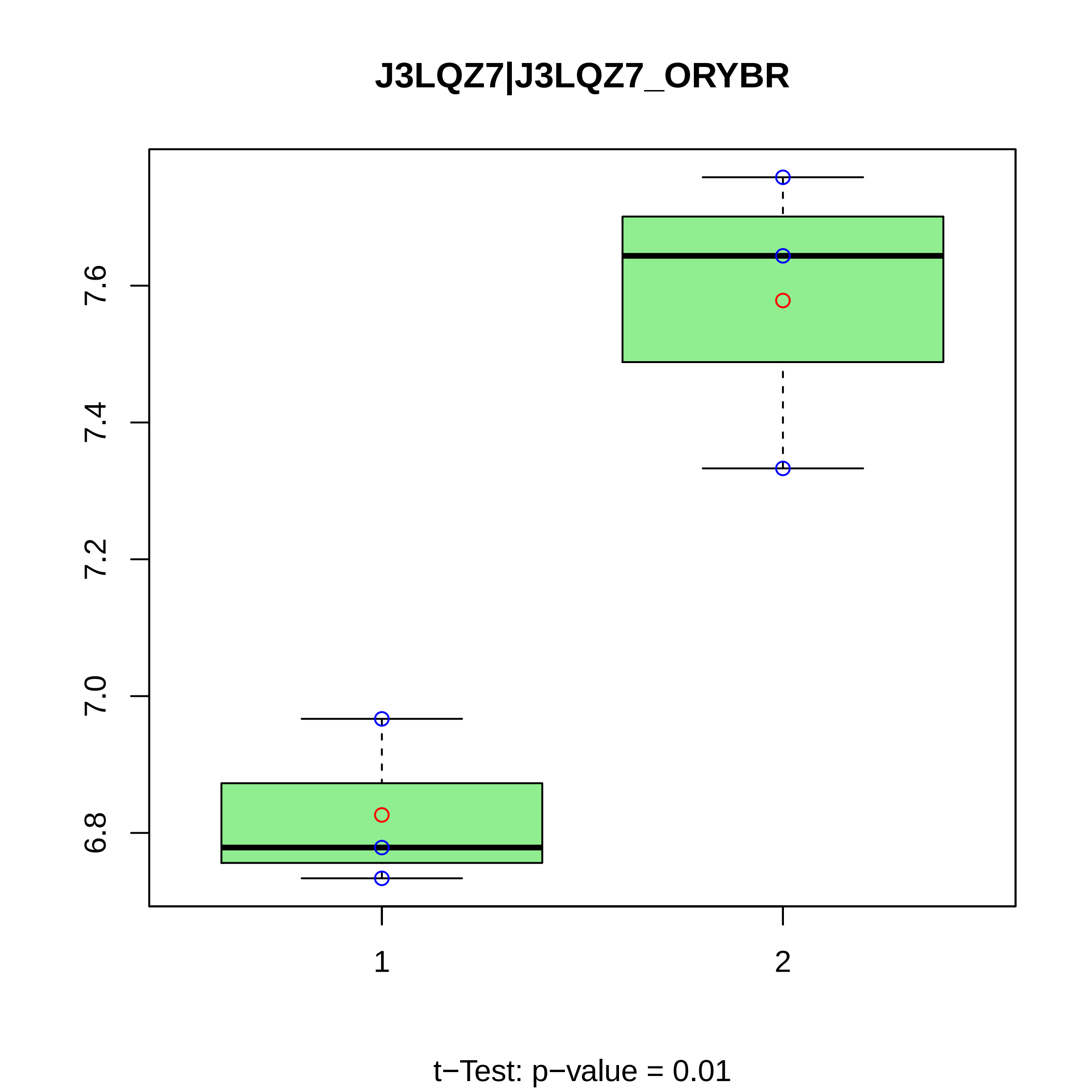
J3LQZ7|J3LQZ7_ORYBR
7.6
7.4
7.2
7.0
6.8
1
2
t−Test: p−value = 0.01

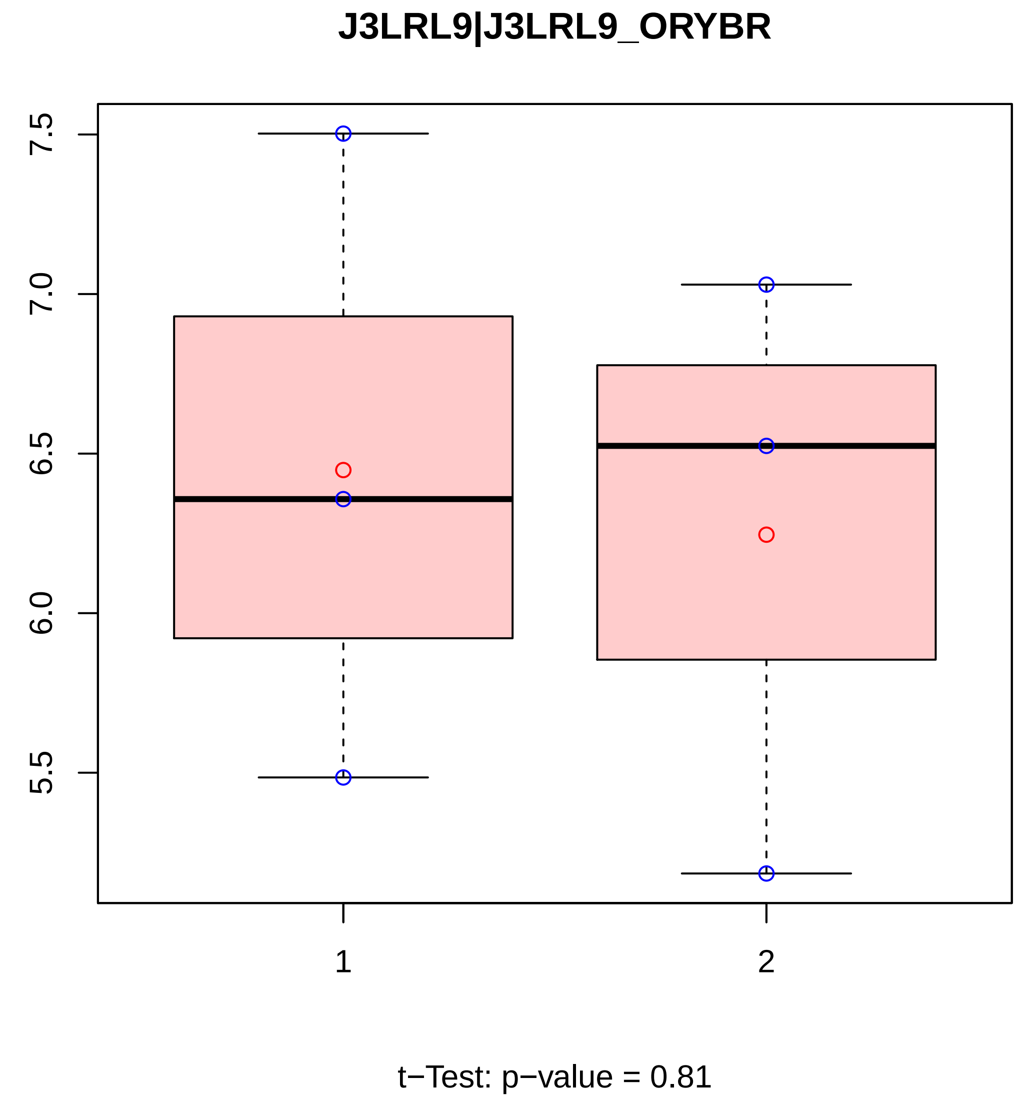
J3LRL9|J3LRL9_ORYBR
7.5
7.0
6.5
6.0
5.5
1
2
t−Test: p−value = 0.81

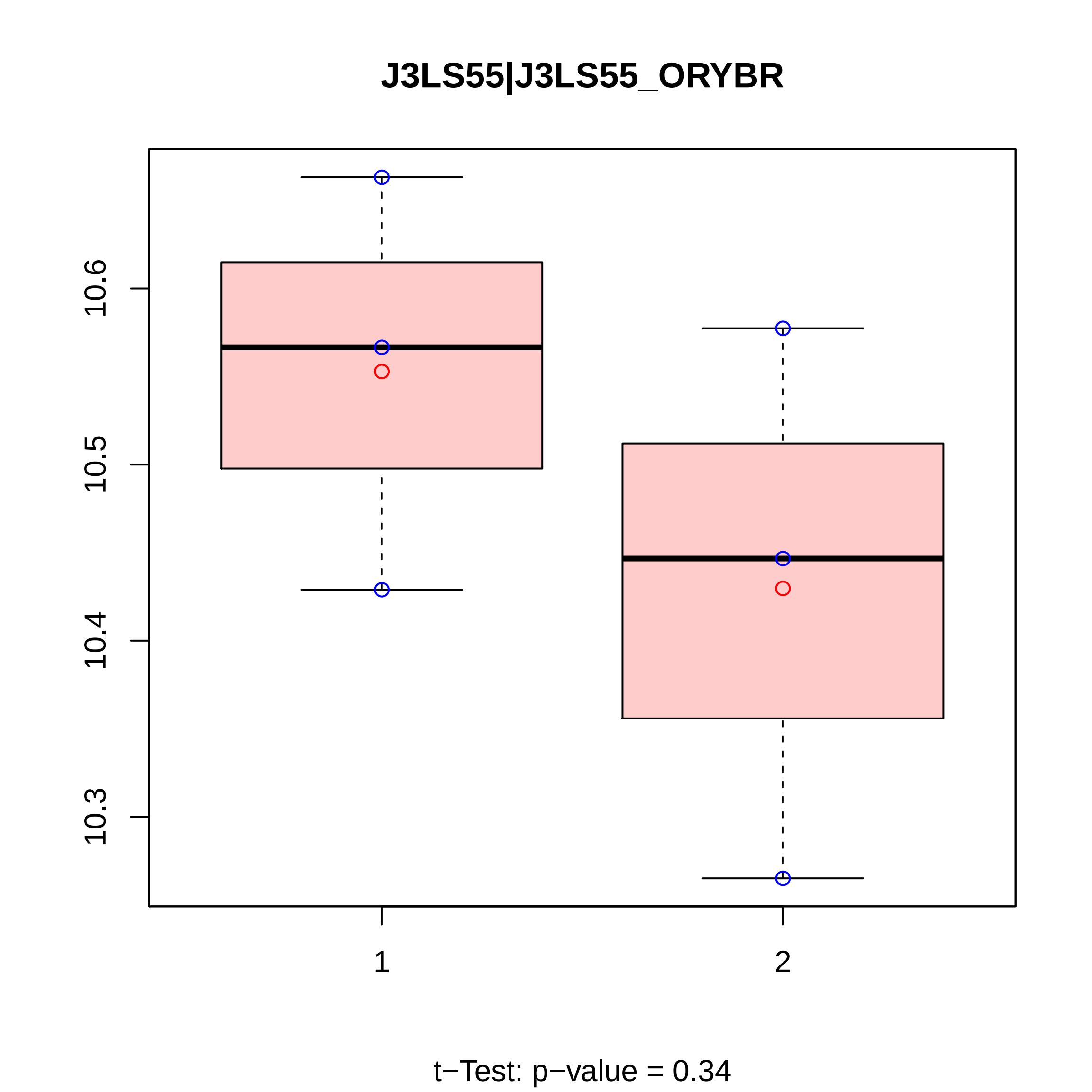
J3LS55|J3LS55_ORYBR
10.3
10.4
10.5
10.6
1
2
t−Test: p−value = 0.34

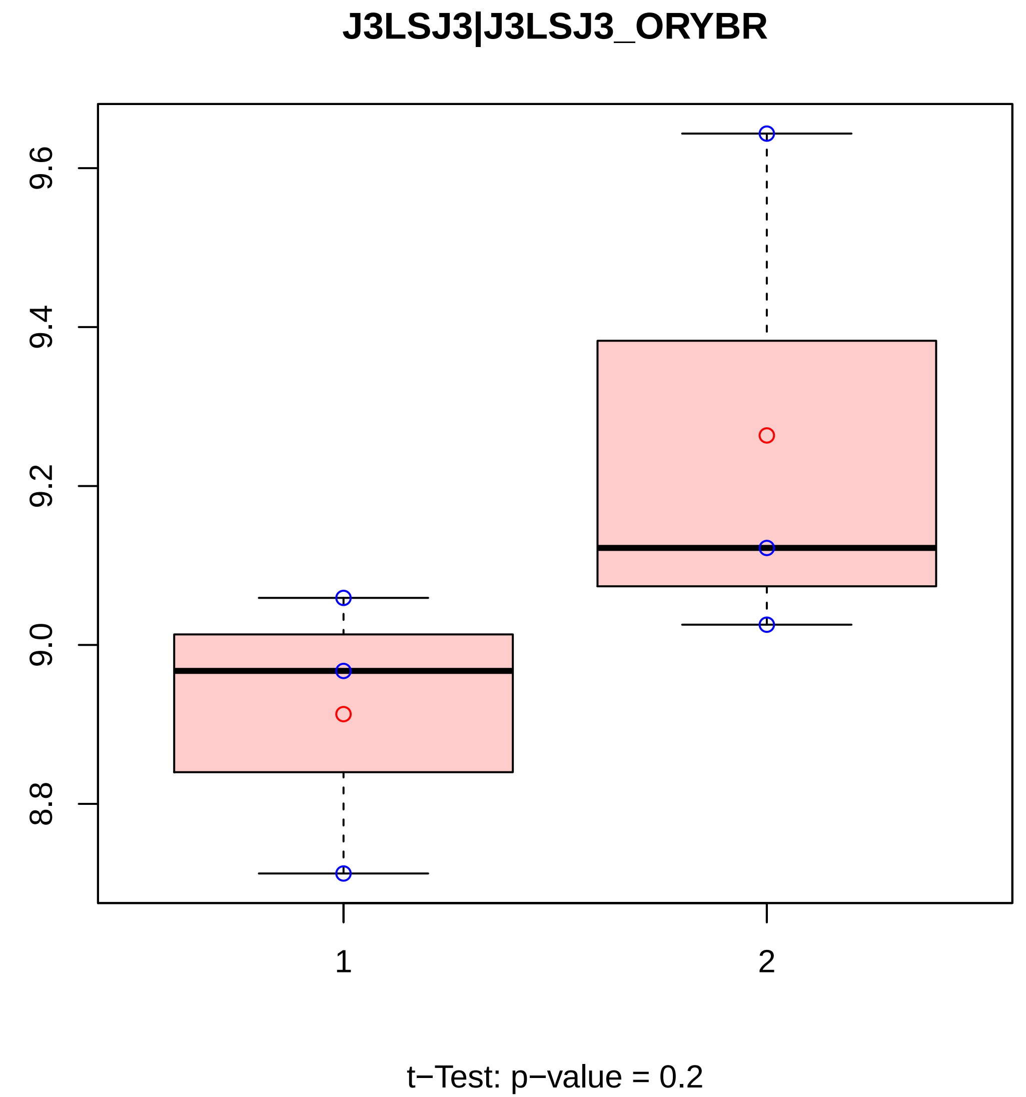
J3LSJ3|J3LSJ3_ORYBR
9.6
9.4
9.2
9.0
8.8
1
2
t−Test: p−value = 0.2

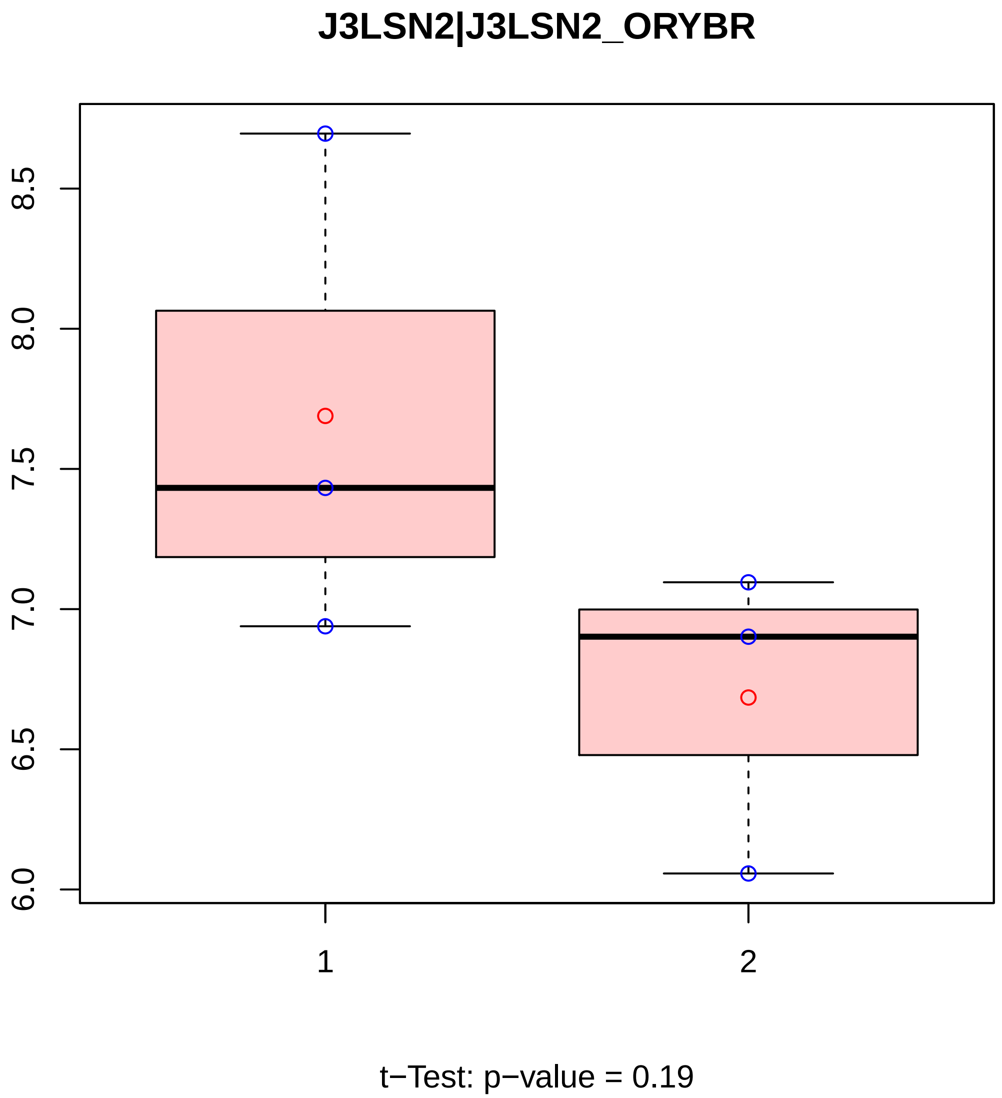
J3LSN2|J3LSN2_ORYBR
8.5
8.0
7.5
7.0
6.5
6.0
1
2
t−Test: p−value = 0.19

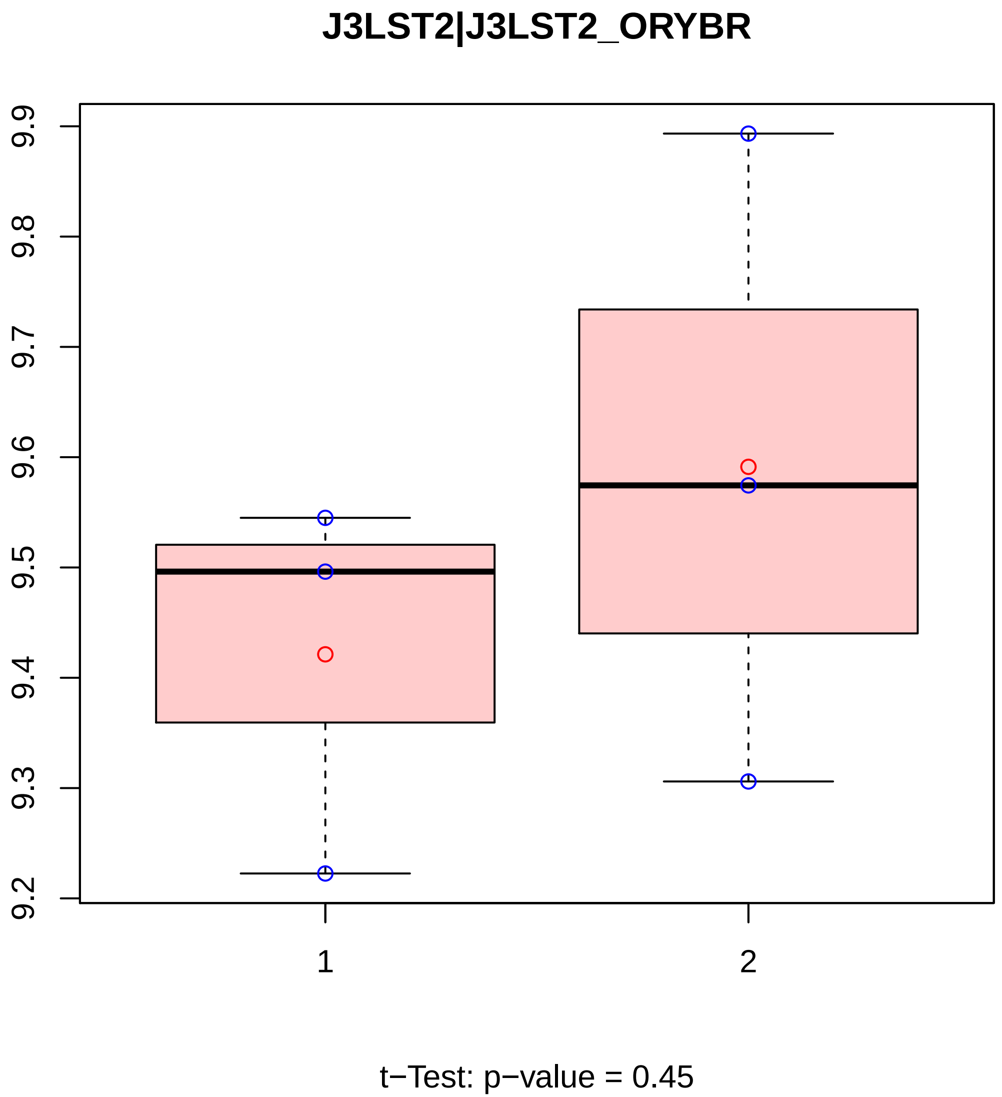
J3LST2|J3LST2_ORYBR
9.2
9.3
9.4
9.5
9.6
9.7
9.8
9.9
1
2
t−Test: p−value = 0.45

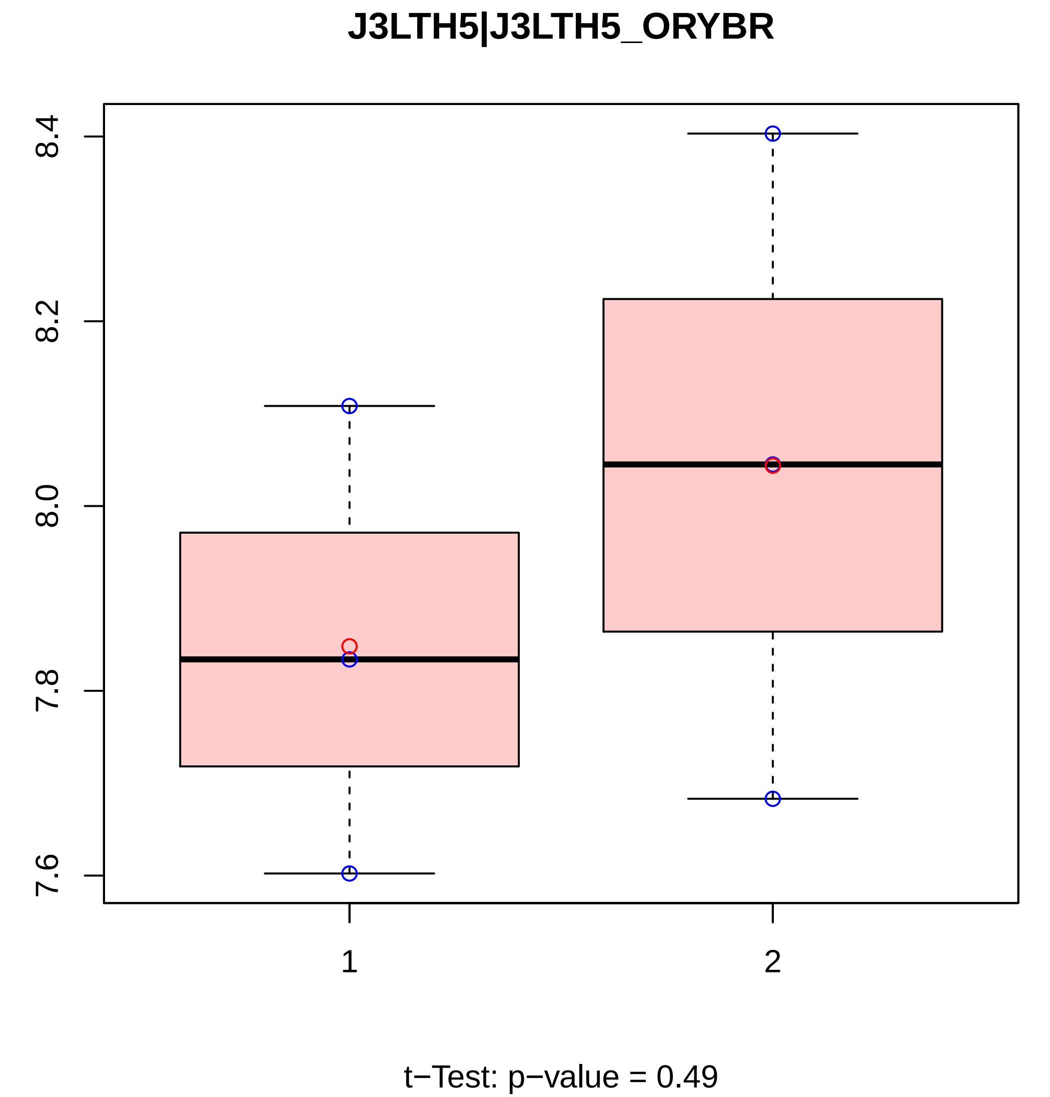
J3LTH5|J3LTH5_ORYBR
8.4
8.2
8.0
7.8
7.6
1
2
t−Test: p−value = 0.49

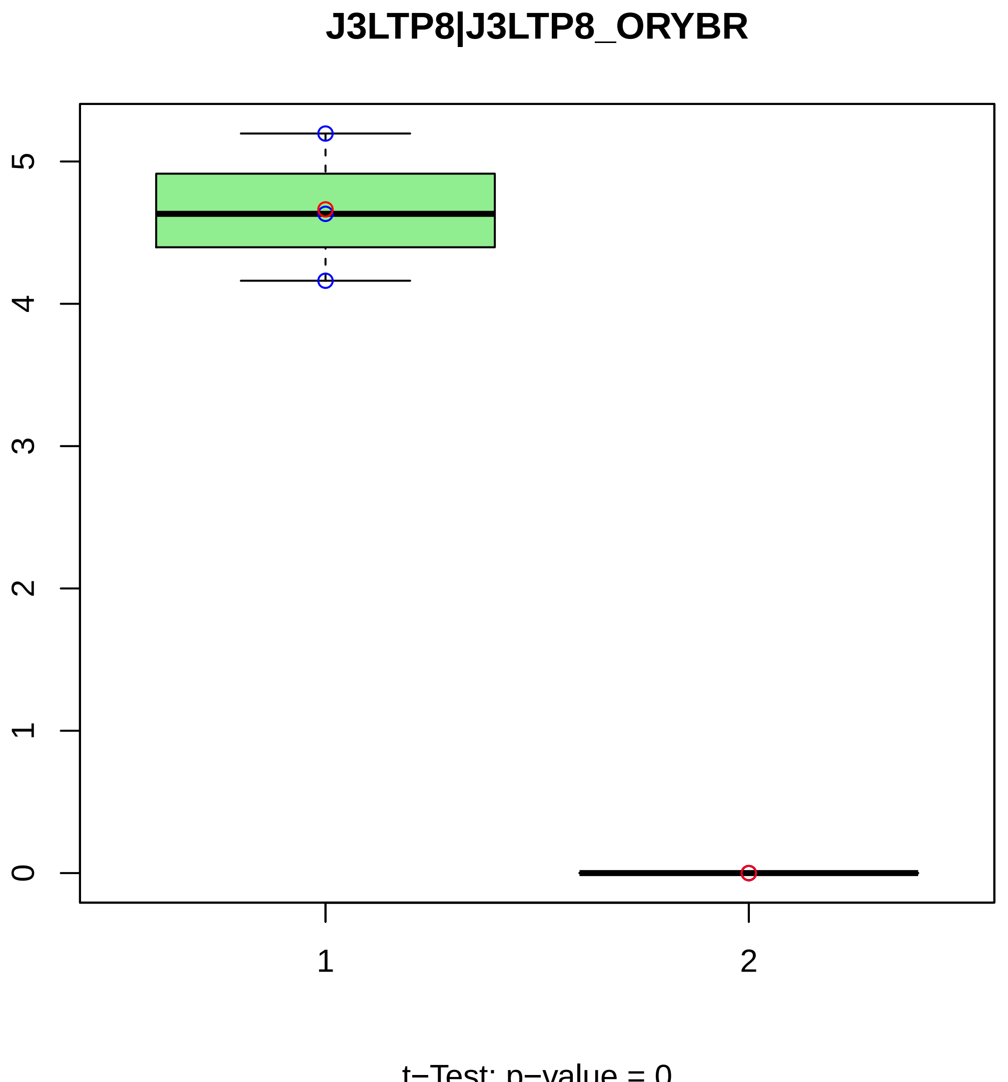

J3LTP8|J3LTP8_ORYBR
0
1
2
3
4
5
1
2
t−Test: p−value = 0

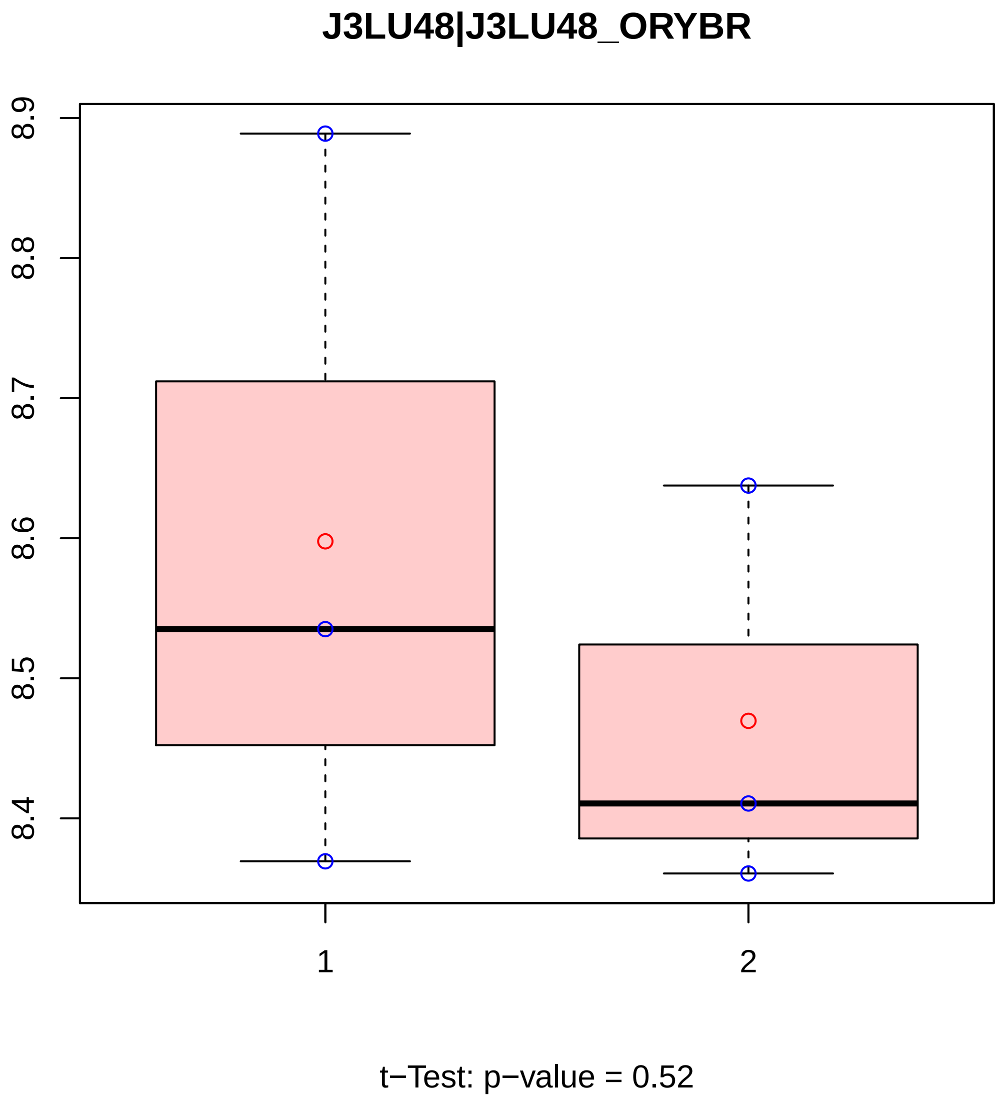
J3LU48|J3LU48_ORYBR
8.4
8.5
8.6
8.7
8.8
8.9
1
2
t−Test: p−value = 0.52

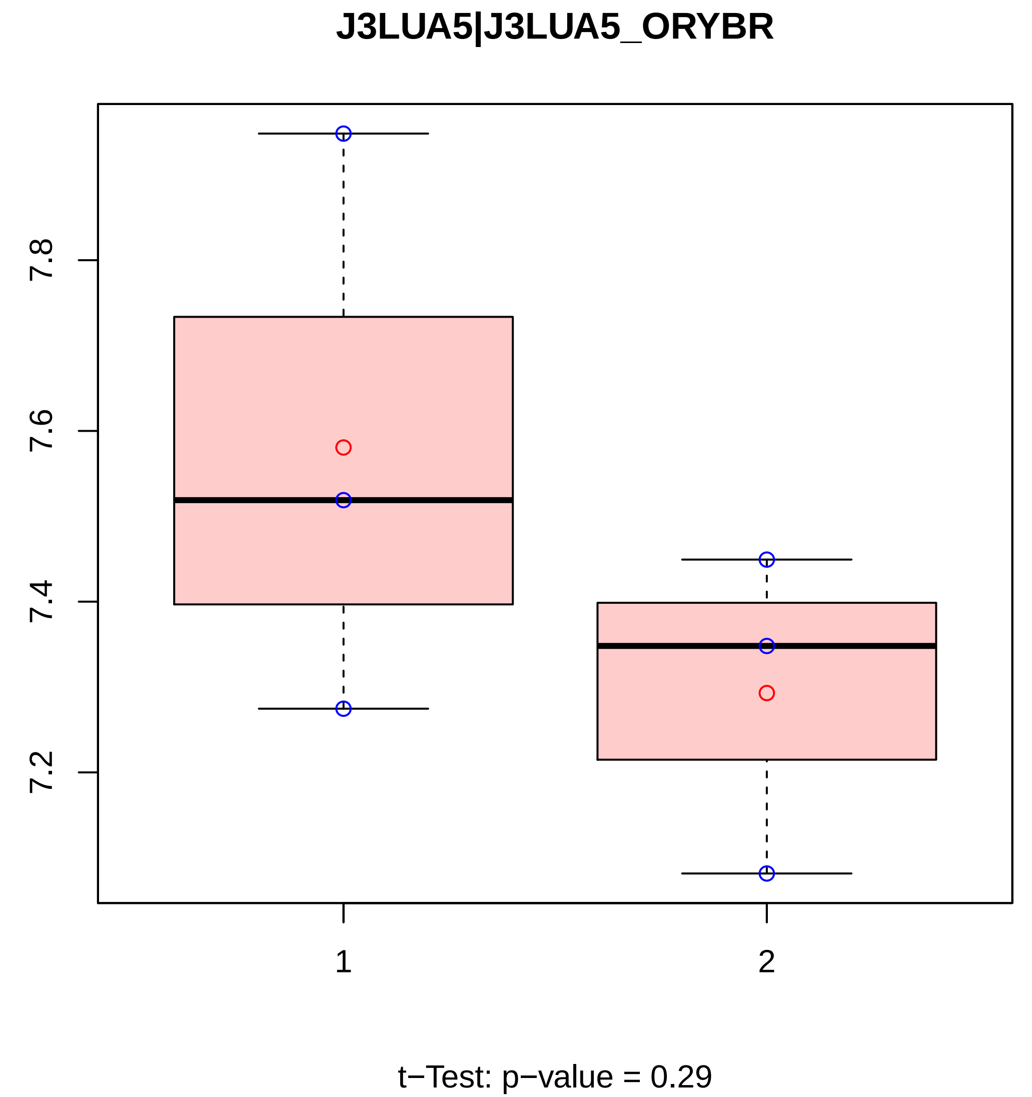

J3LUA5|J3LUA5_ORYBR
7.2
7.4
7.6
7.8
1
2
t−Test: p−value = 0.29

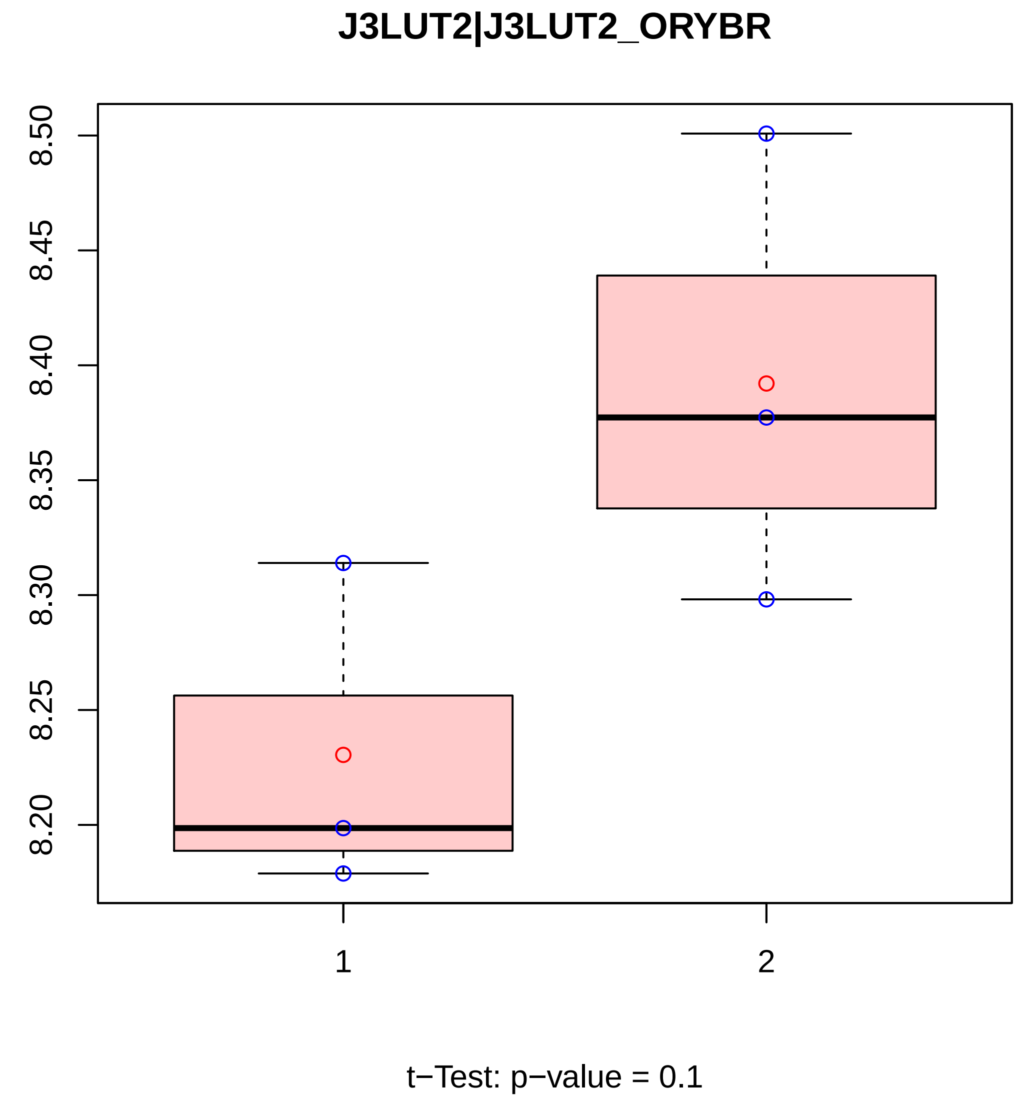
J3LUT2|J3LUT2_ORYBR
8.20
8.25
8.30
8.35
8.40
8.45
8.50
1
2
t−Test: p−value = 0.1

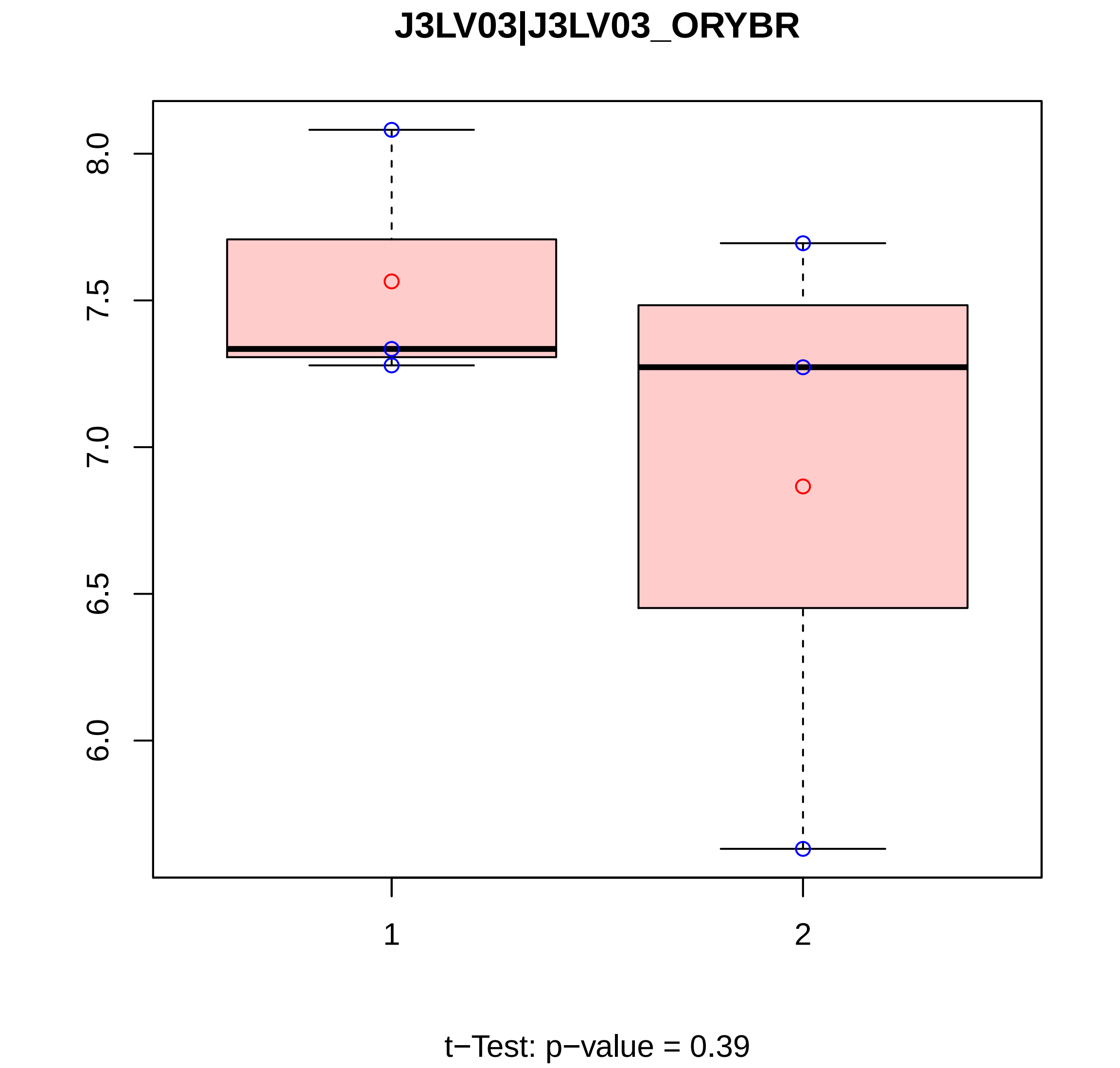
J3LV03|J3LV03_ORYBR
8.0
7.5
7.0
6.5
6.0
1
2
t−Test: p−value = 0.39

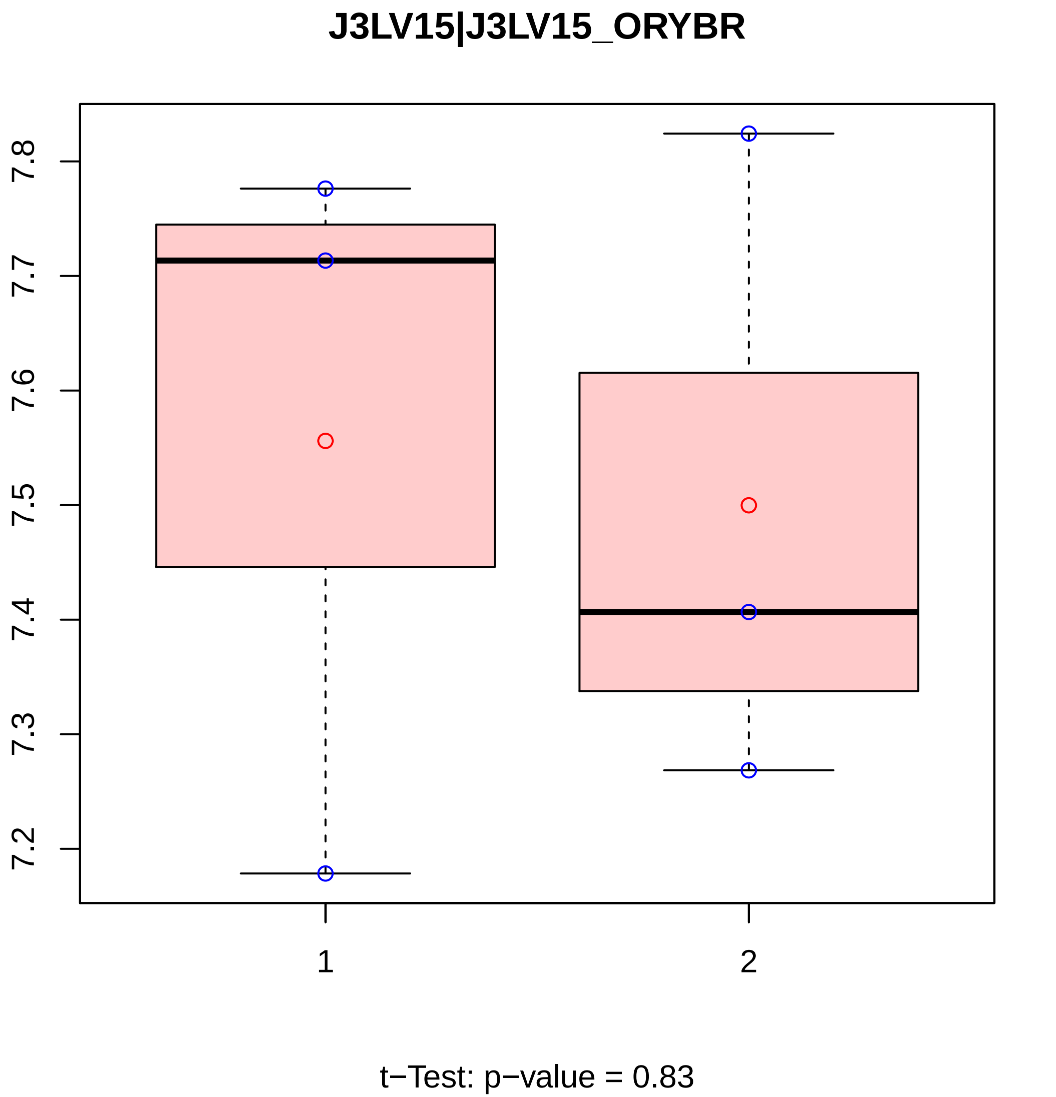

J3LV15|J3LV15_ORYBR
7.2
7.3
7.4
7.5
7.6
7.7
7.8
1
2
t−Test: p−value = 0.83

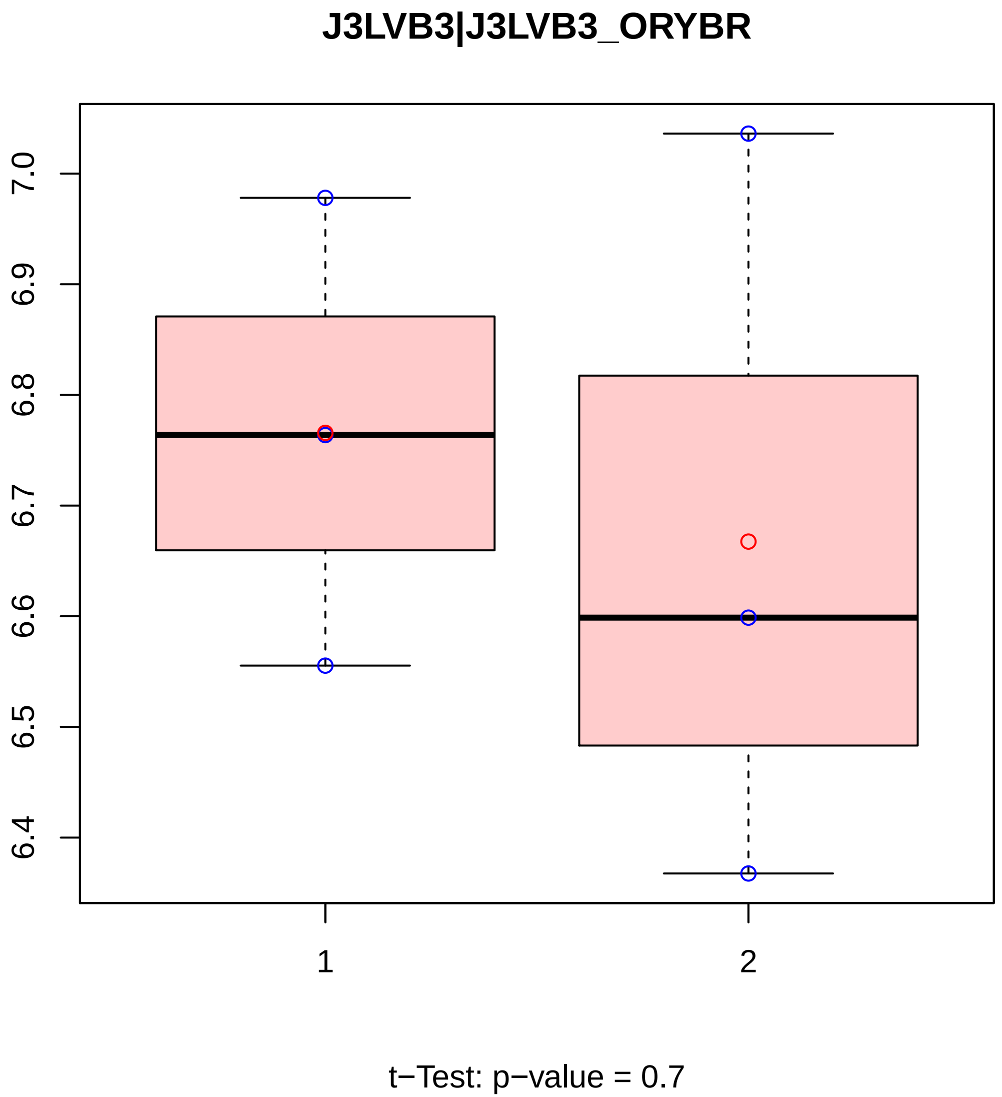
J3LVB3|J3LVB3_ORYBR
7.0
6.9
6.8
6.7
6.6
6.5
6.4
1
2
t−Test: p−value = 0.7

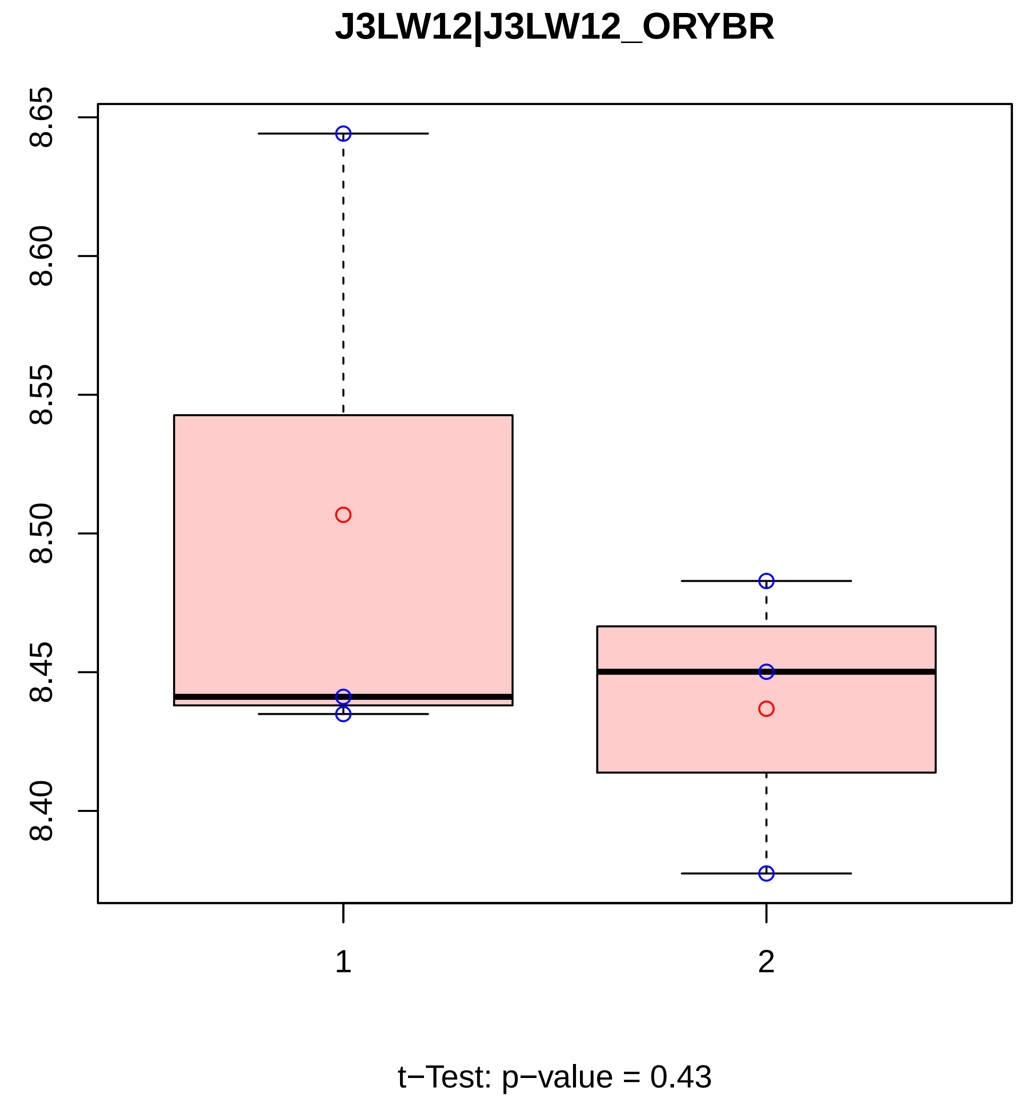
J3LW12|J3LW12_ORYBR
8.40
8.45
8.50
8.55
8.60
8.65
1
2
t−Test: p−value = 0.43

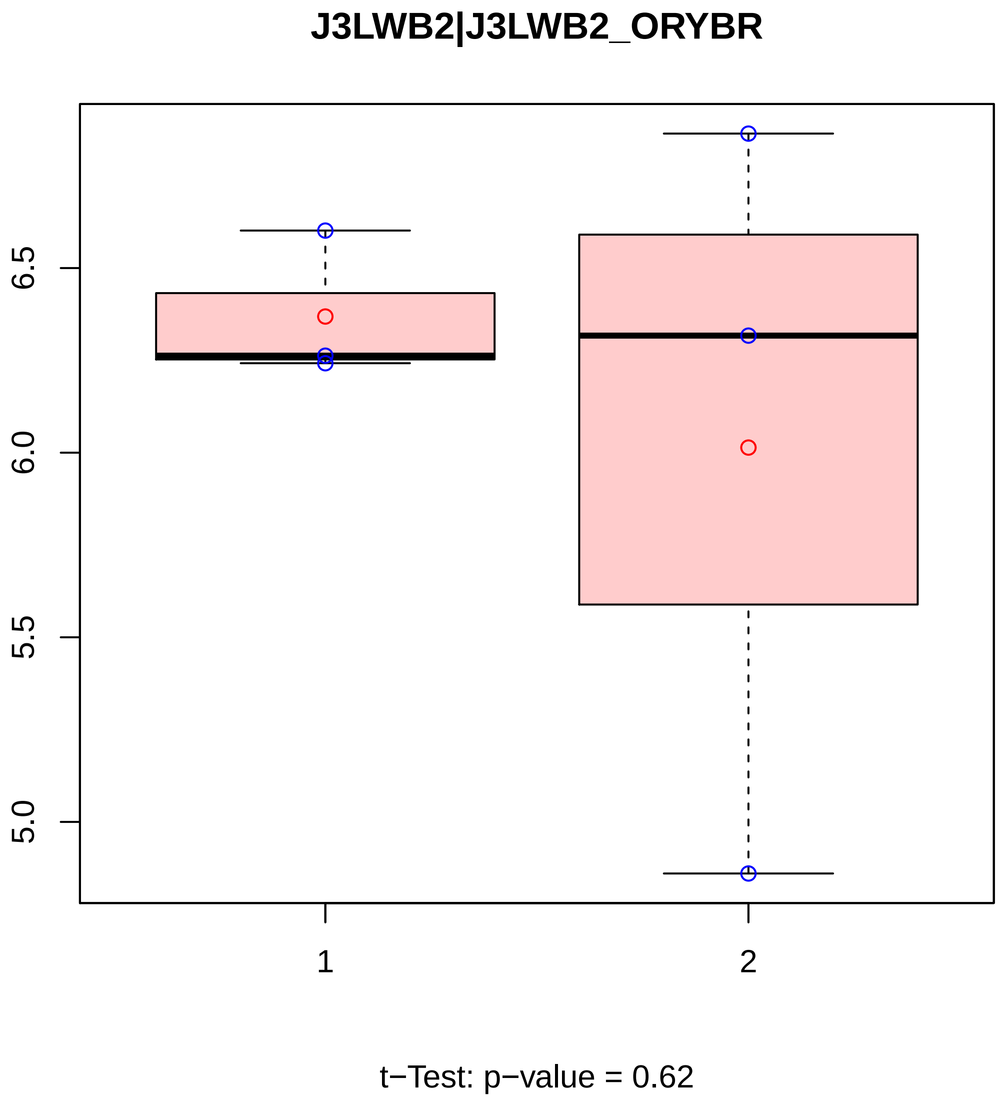
J3LWB2|J3LWB2_ORYBR
6.5
6.0
5.5
5.0
1
2
t−Test: p−value = 0.62

J3LWQ2|J3LWQ2_ORYBR

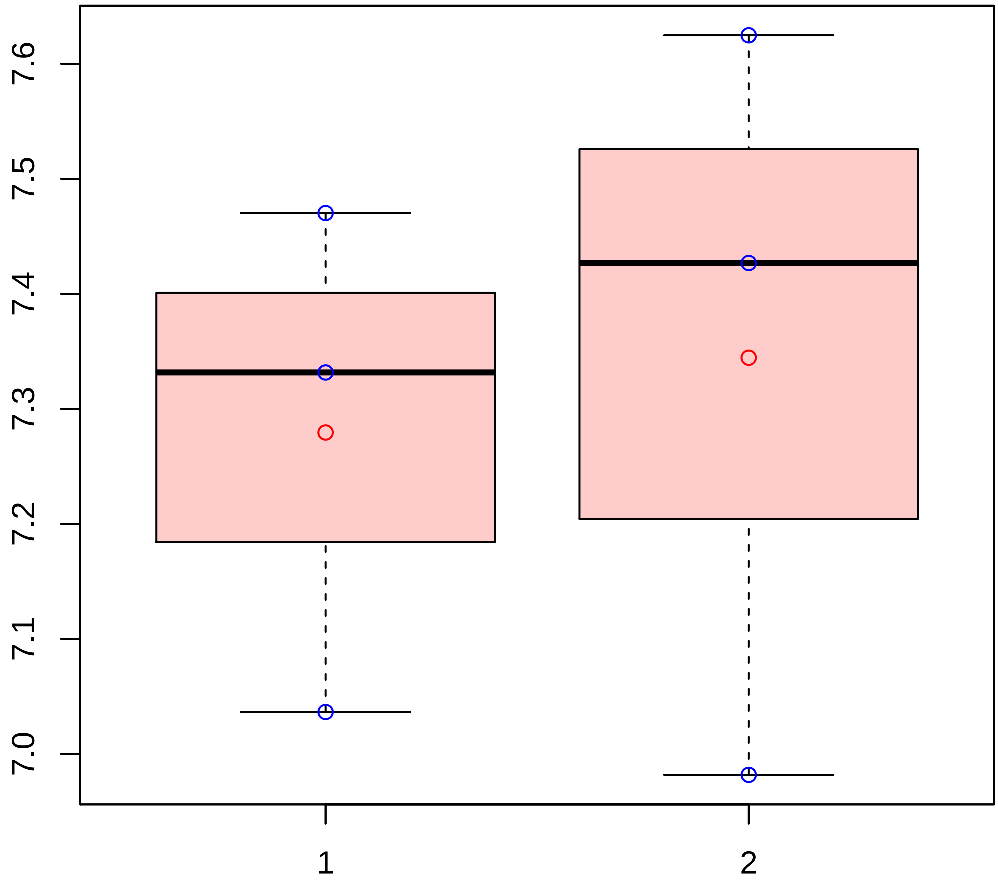

t−Test: p−value = 0.79

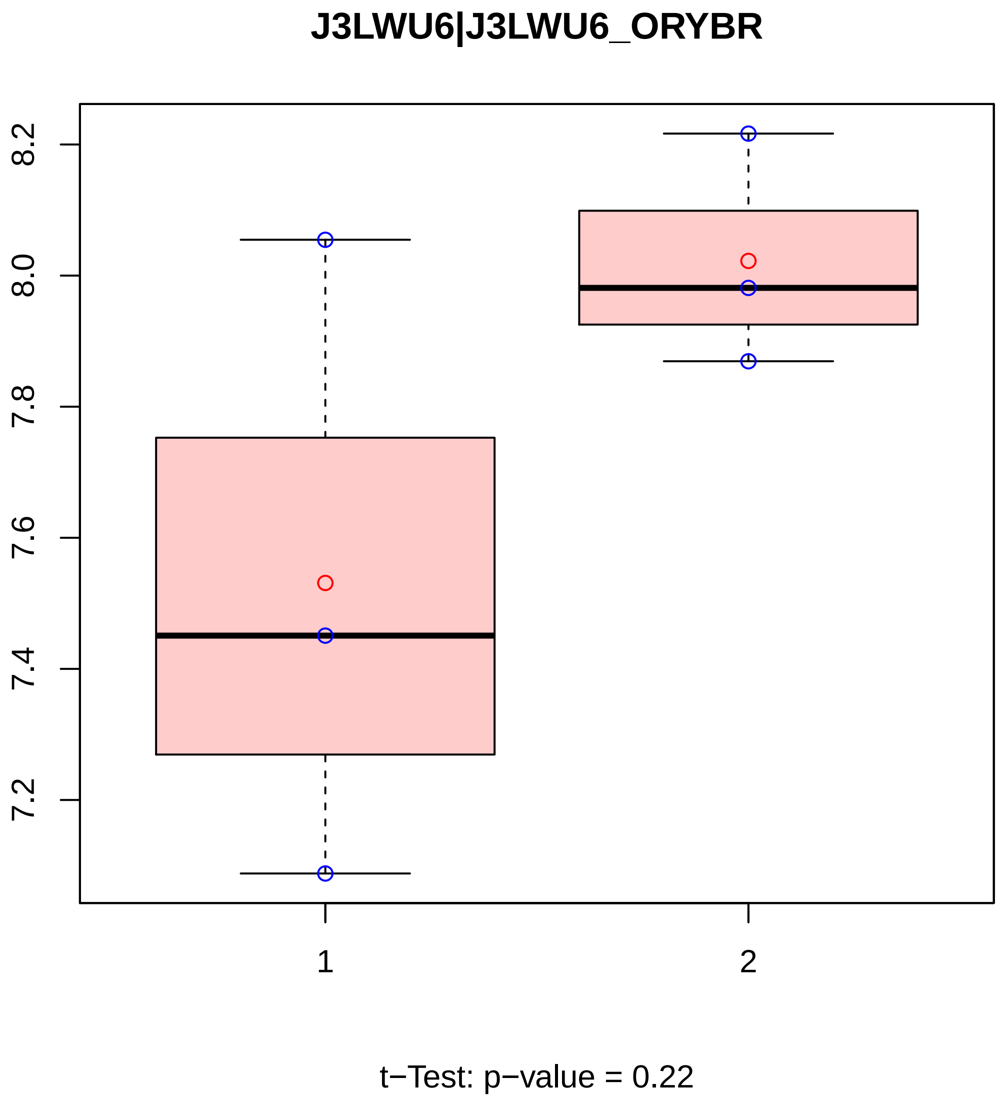
J3LWU6|J3LWU6_ORYBR
8.2
8.0
7.8
7.6
7.4
7.2
1
2
t−Test: p−value = 0.22

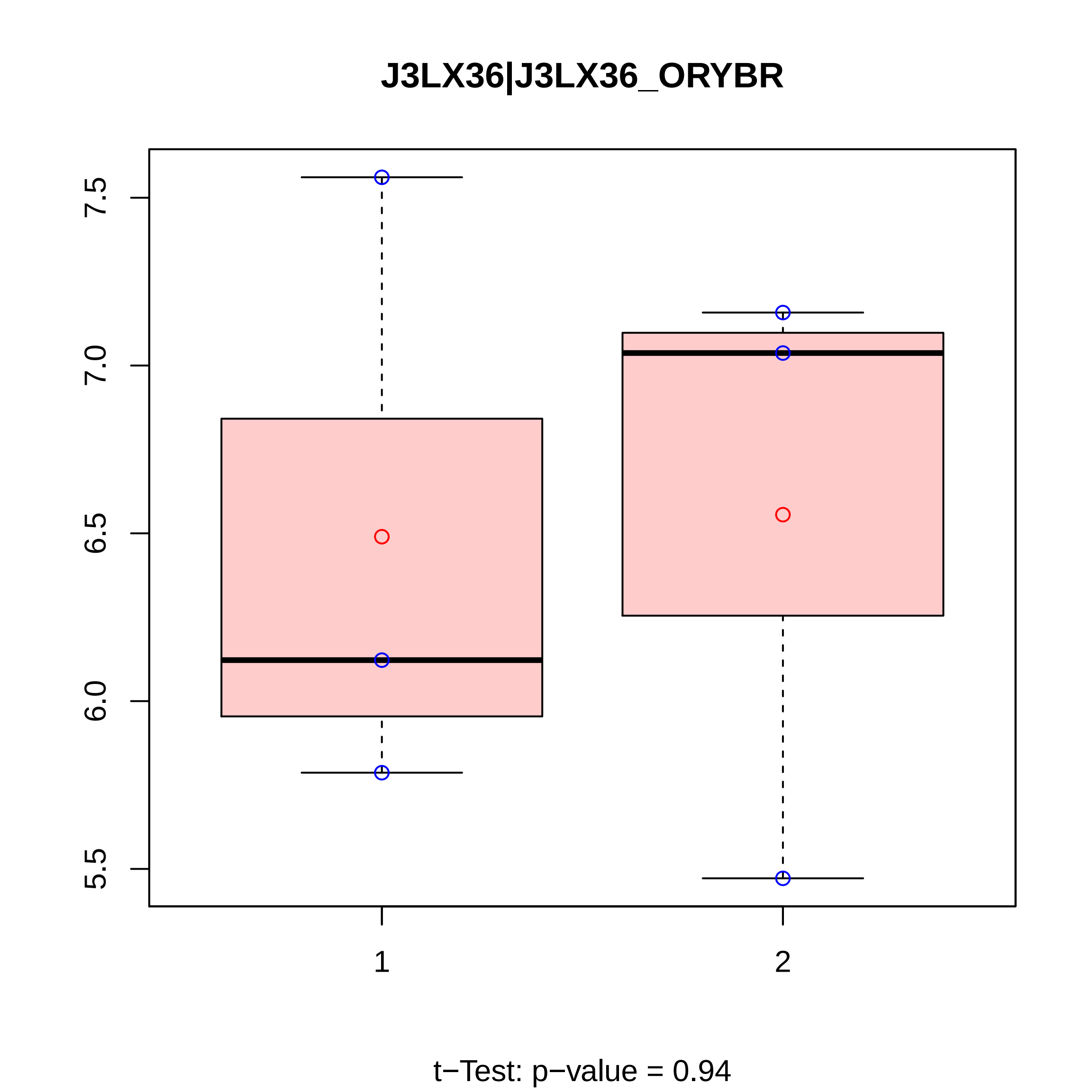
J3LX36|J3LX36_ORYBR
7.5
7.0
6.5
6.0
5.5
1
2
t−Test: p−value = 0.94

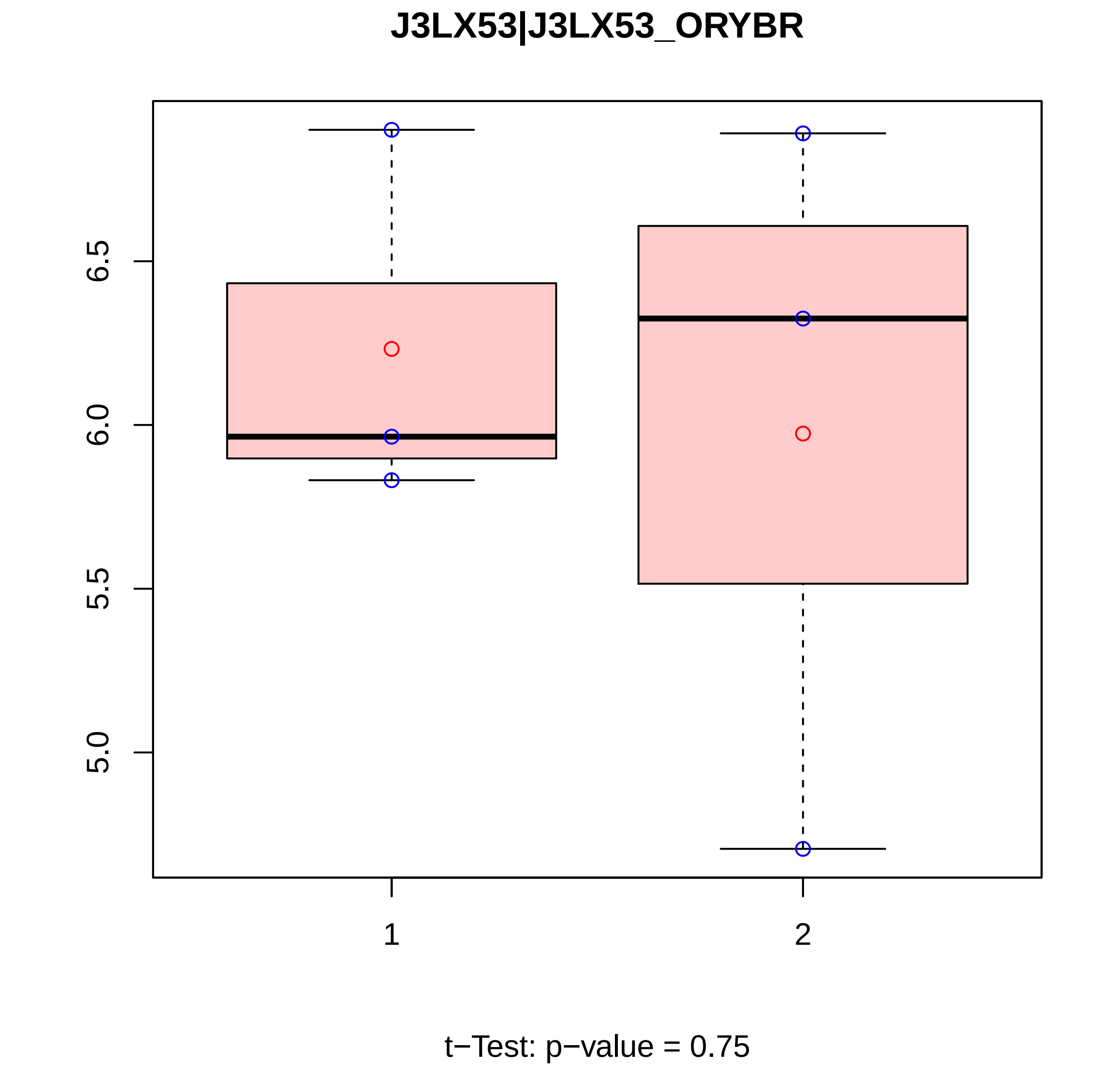
J3LX53|J3LX53_ORYBR
6.5
6.0
5.5
5.0
1
2
t−Test: p−value = 0.75

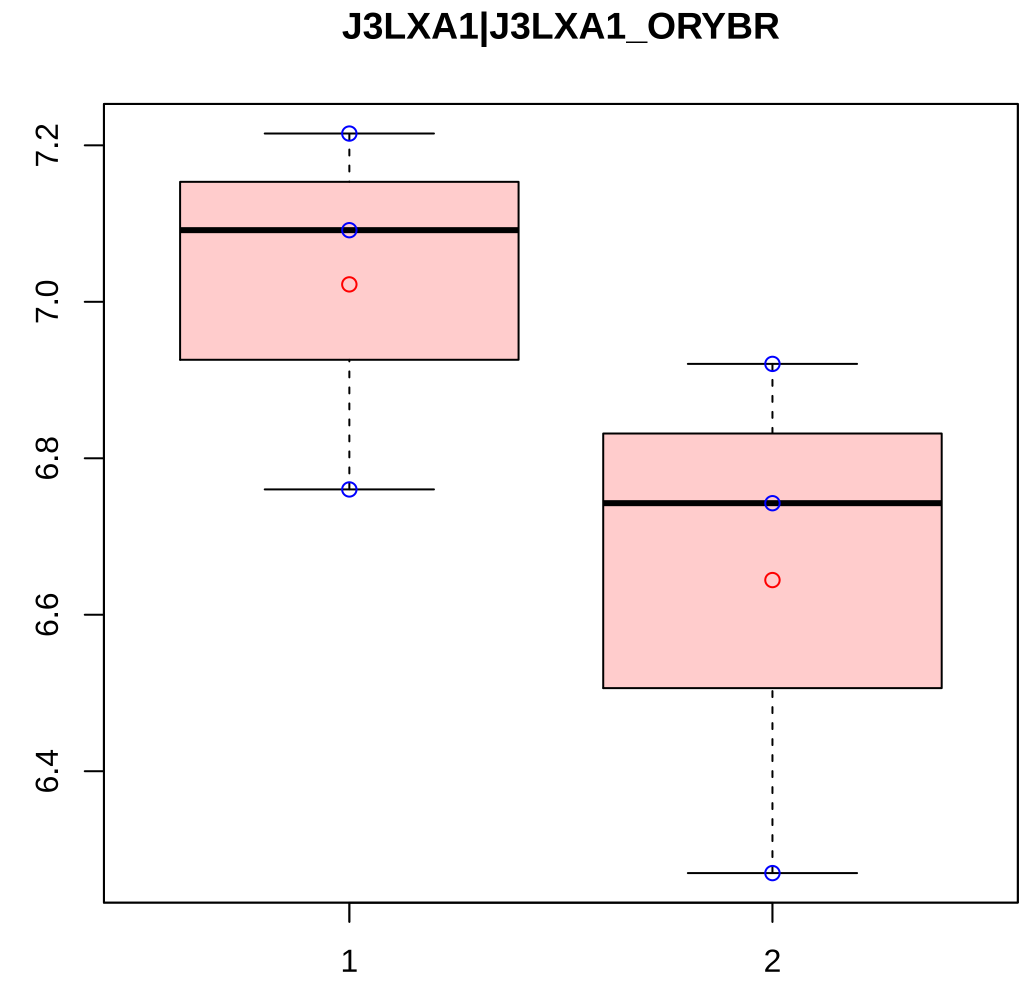

t−Test: p−value = 0.19

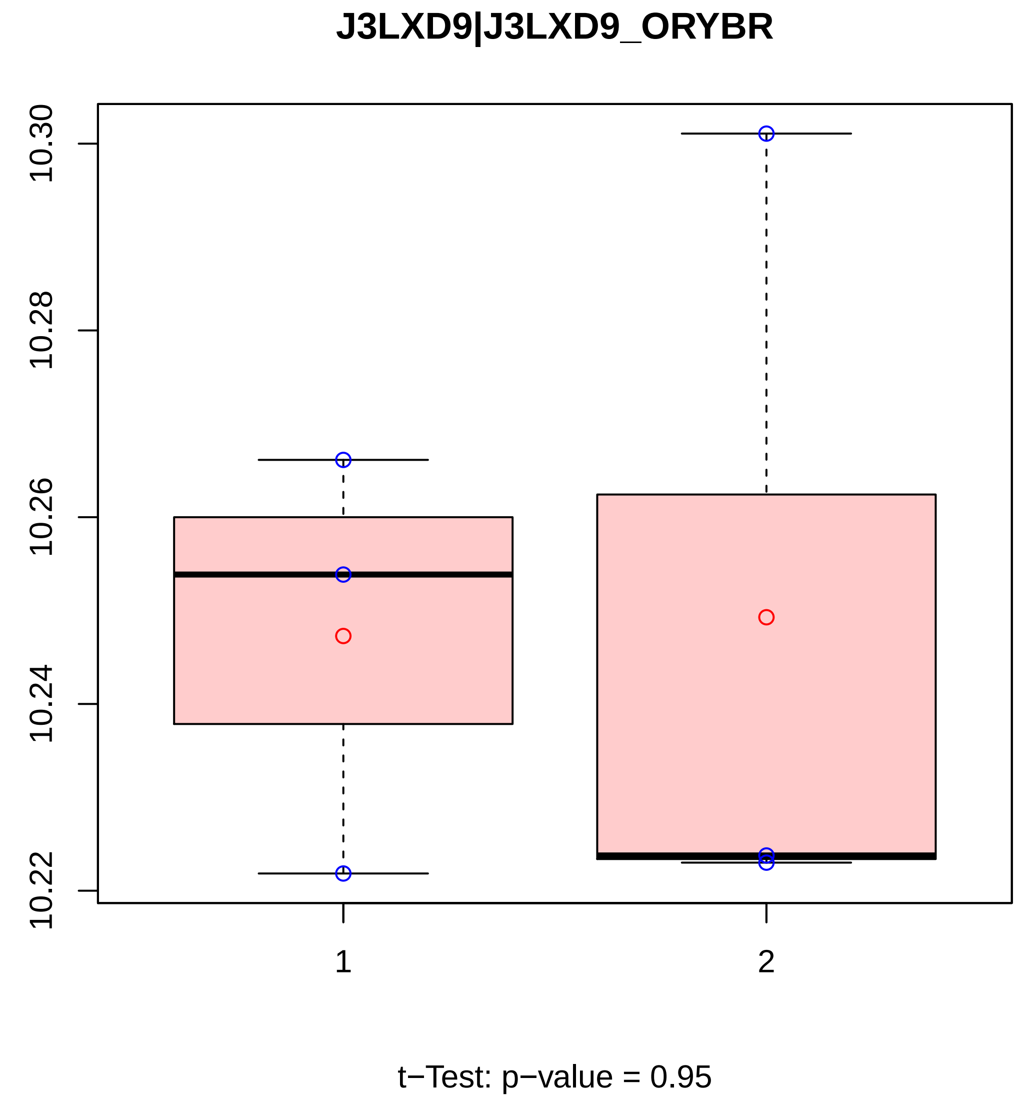
J3LXD9|J3LXD9_ORYBR
10.22
10.24
10.26
10.28
10.30
1
2
t−Test: p−value = 0.95

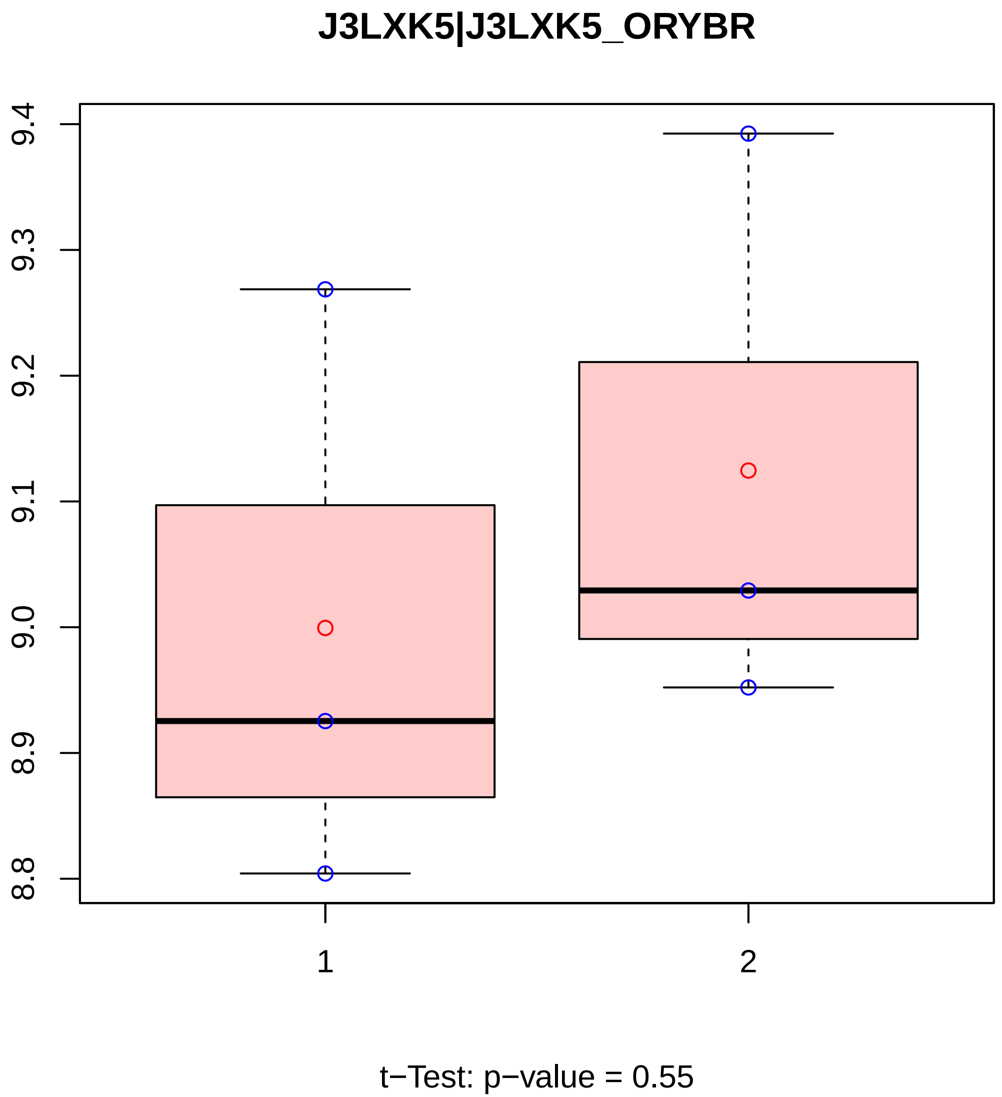
J3LXK5|J3LXK5_ORYBR
9.4
9.3
9.2
9.1
9.0
8.9
8.8
1
2
t−Test: p−value = 0.55

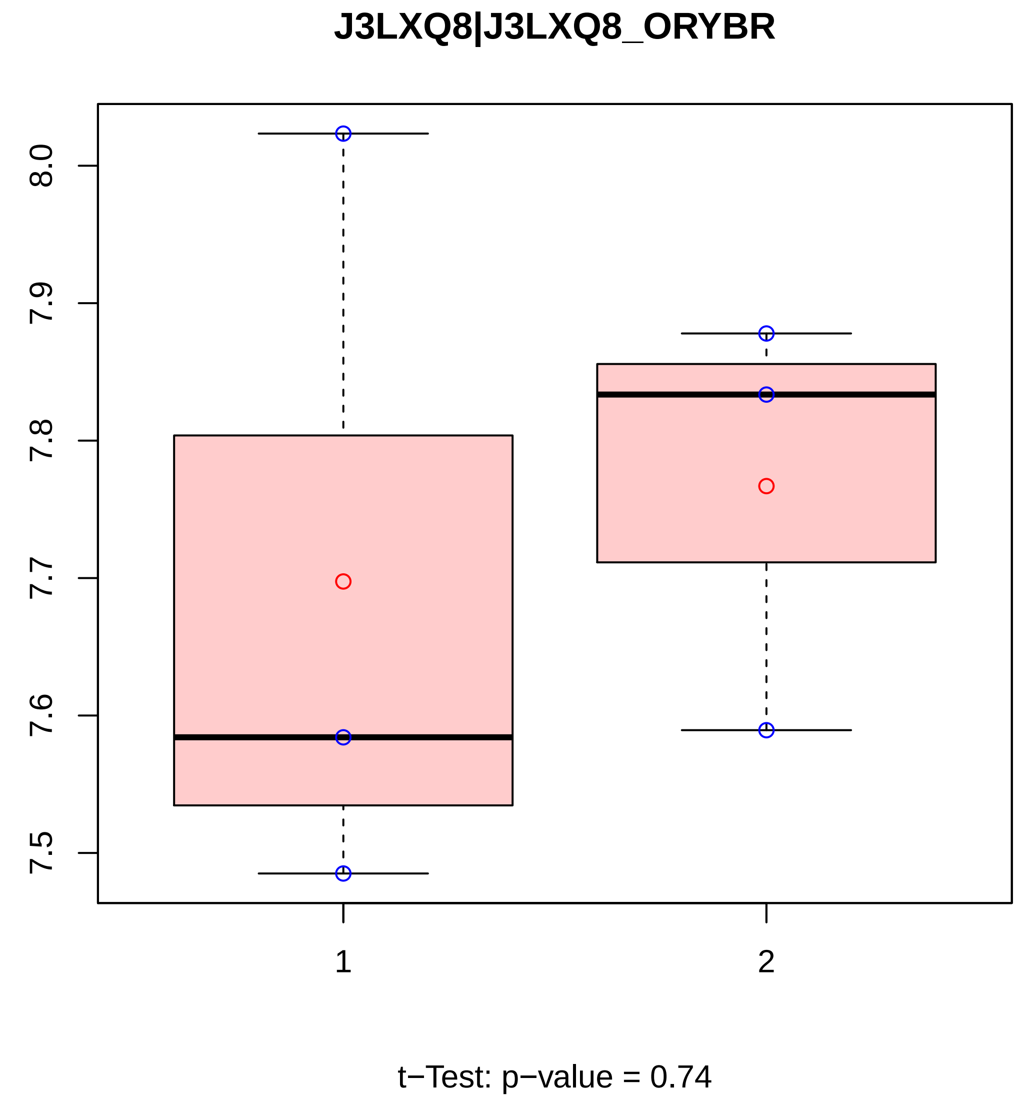
J3LXQ8|J3LXQ8_ORYBR
8.0
7.9
7.8
7.7
7.6
7.5
1
2
t−Test: p−value = 0.74

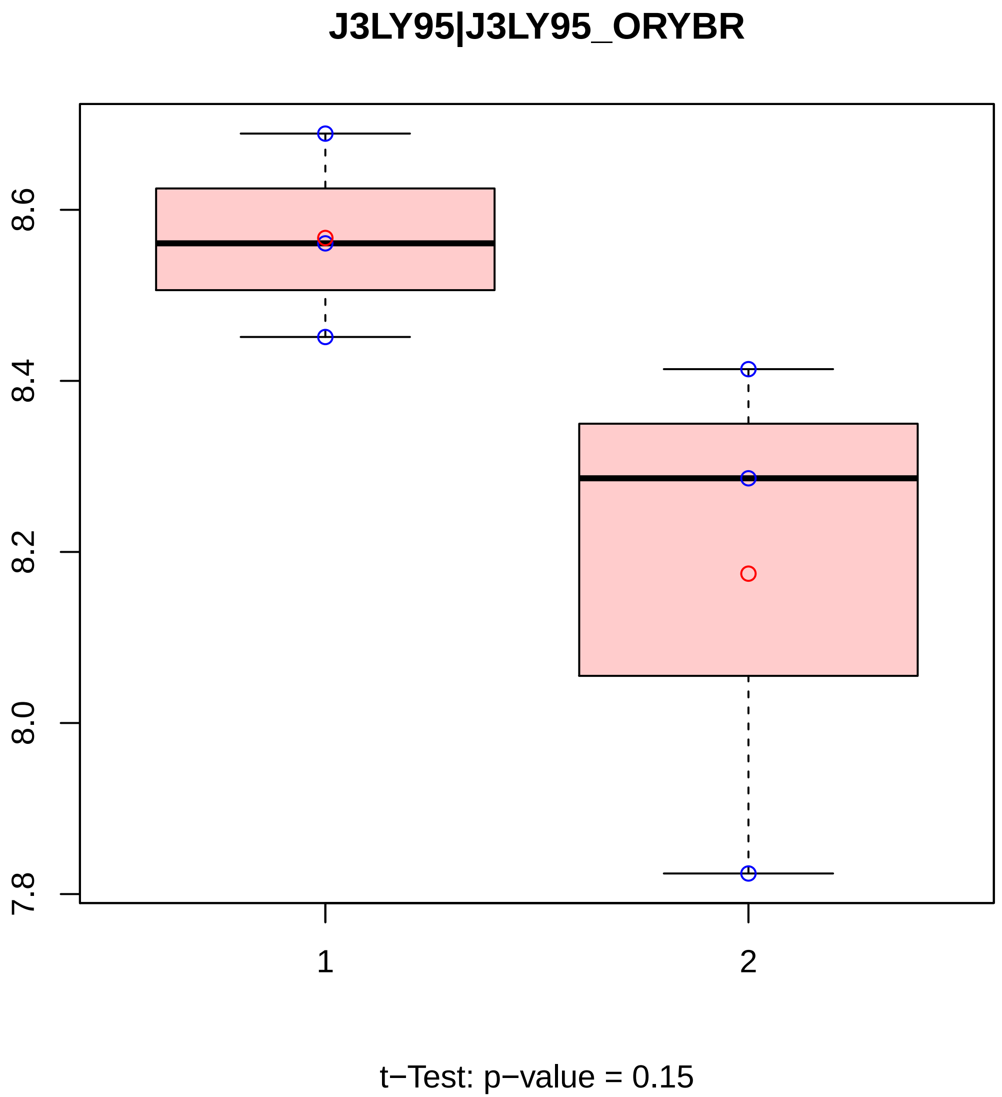
J3LY95|J3LY95_ORYBR
8.6
8.4
8.2
8.0
7.8
1
2
t−Test: p−value = 0.15

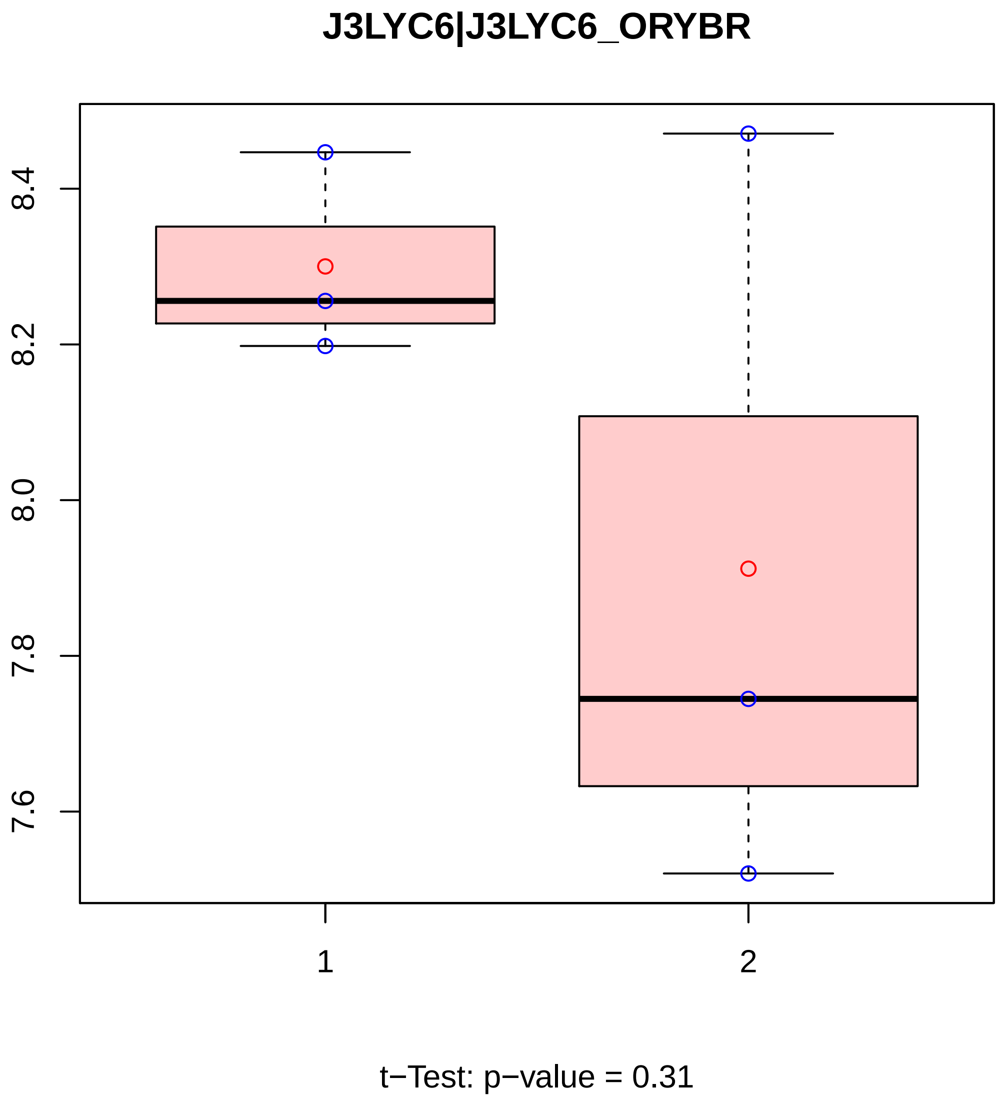
J3LYC6|J3LYC6_ORYBR
8.4
8.2
8.0
7.8
7.6
1
2
t−Test: p−value = 0.31

J3LYY4|J3LYY4_ORYBR

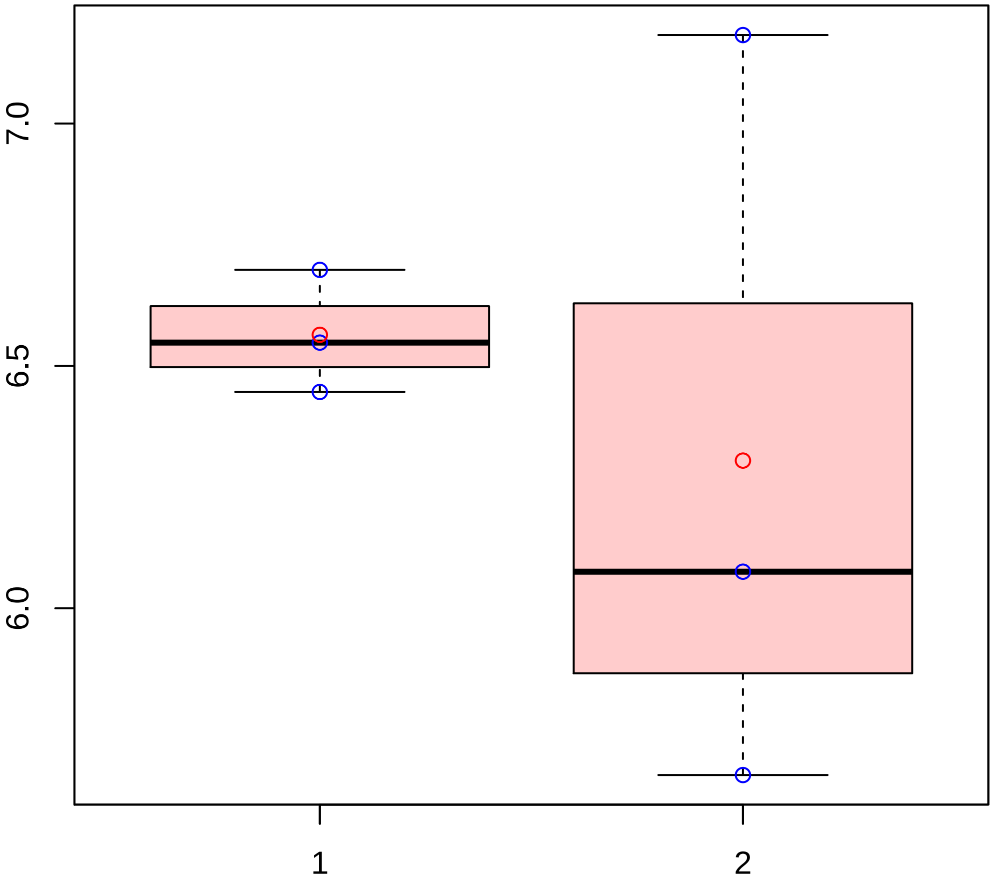

t−Test: p−value = 0.63

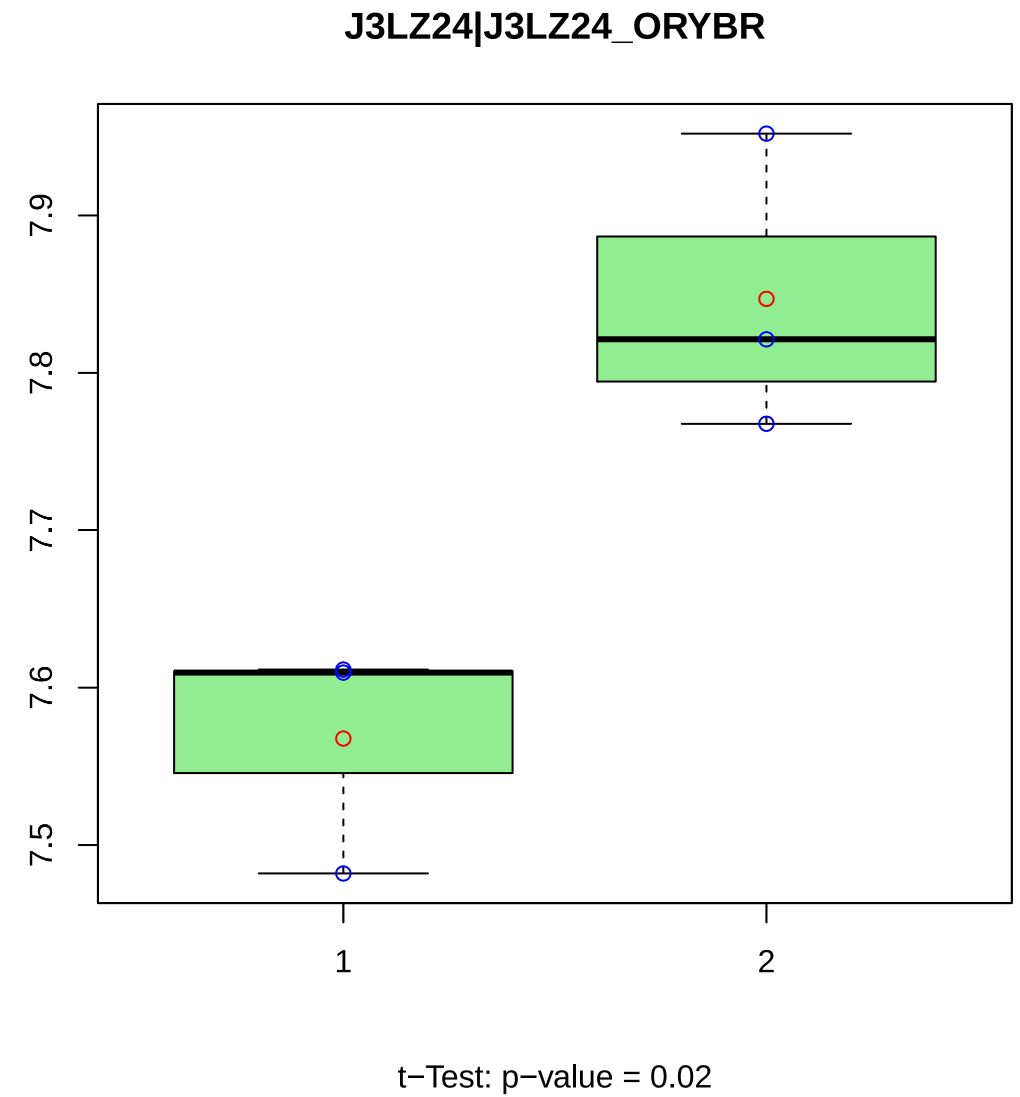

J3LZ24|J3LZ24_ORYBR
7.5
7.6
7.7
7.8
7.9
1
2
t−Test: p−value = 0.02

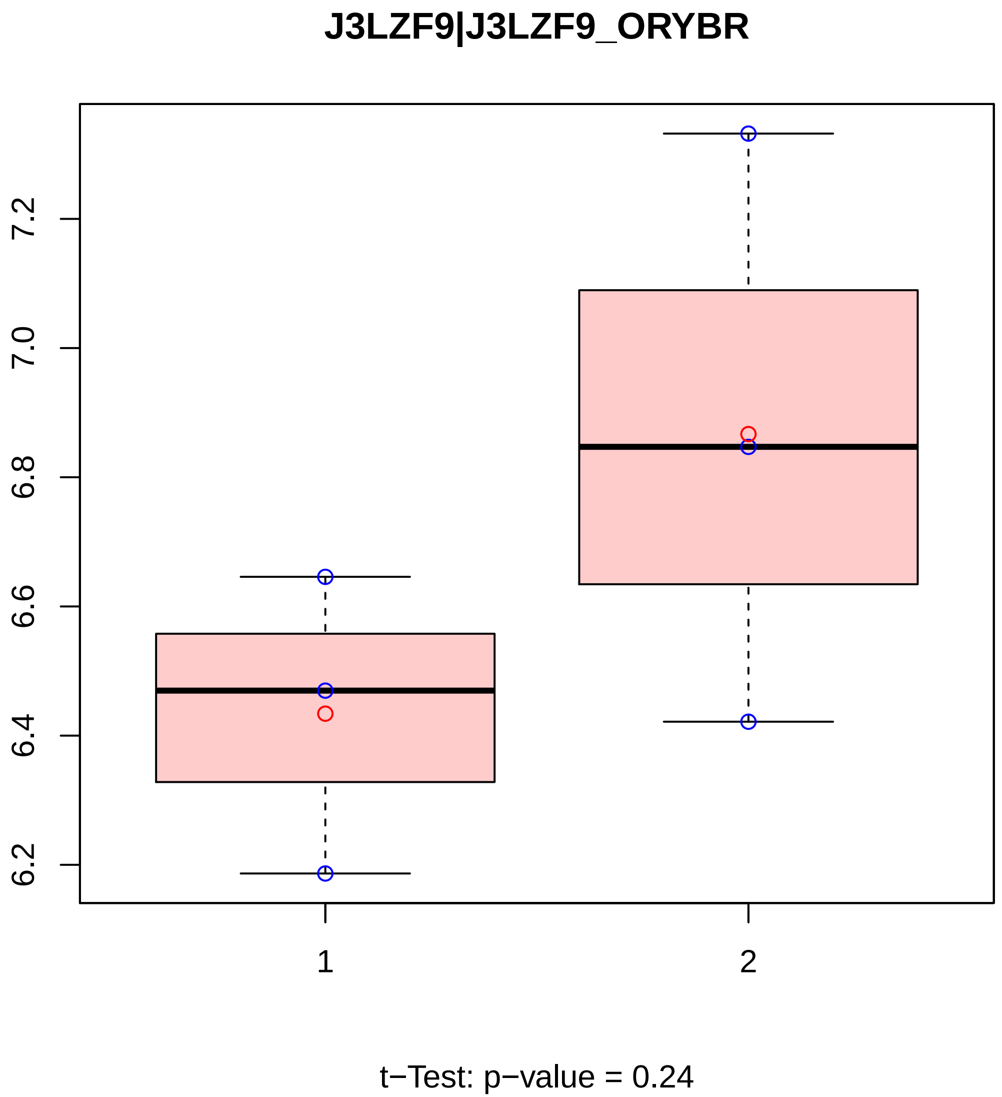
J3LZF9|J3LZF9_ORYBR
7.2
7.0
6.8
6.6
6.4
6.2
1
2
t−Test: p−value = 0.24

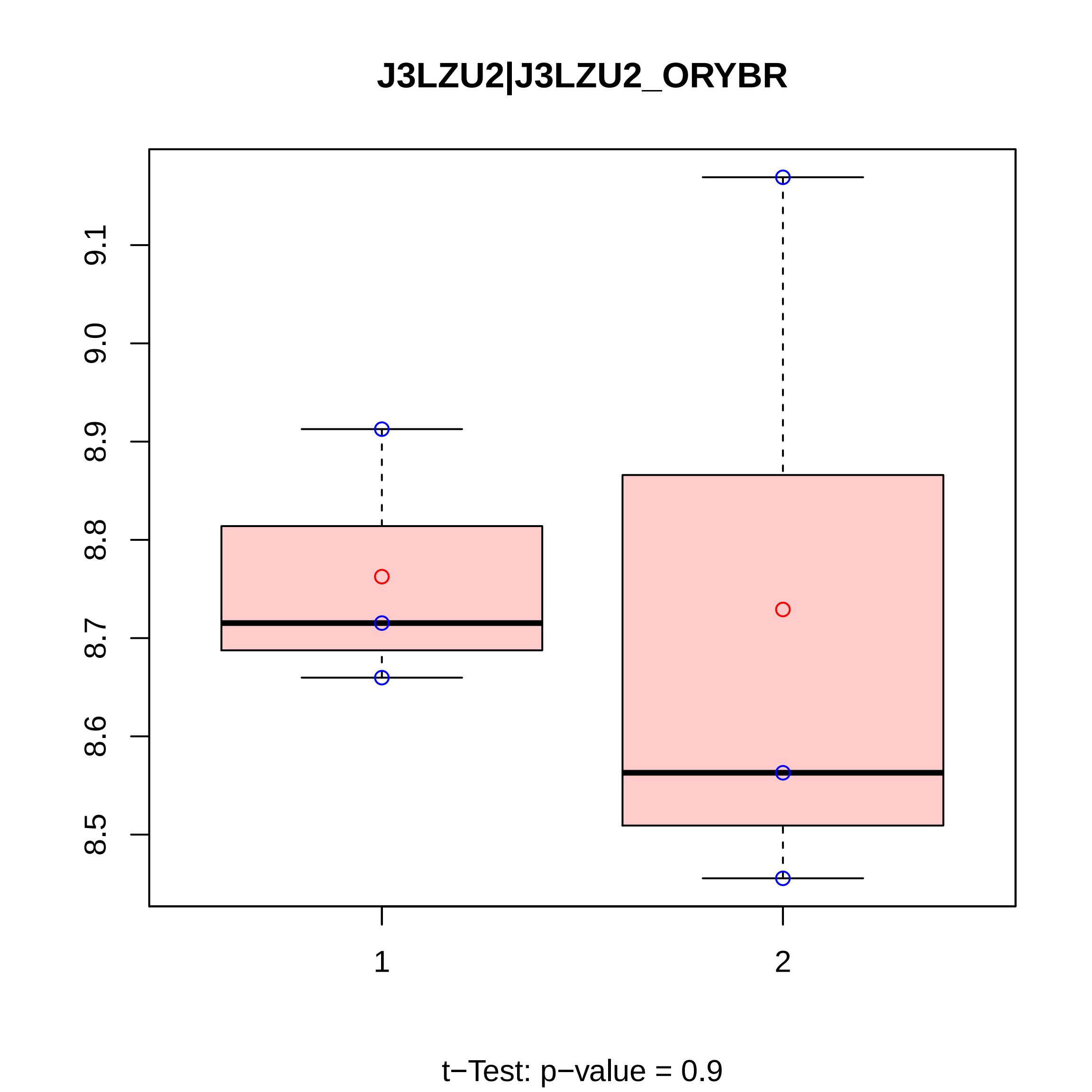
J3LZU2|J3LZU2_ORYBR
9.1
9.0
8.9
8.8
8.7
8.6
8.5
1
2
t−Test: p−value = 0.9

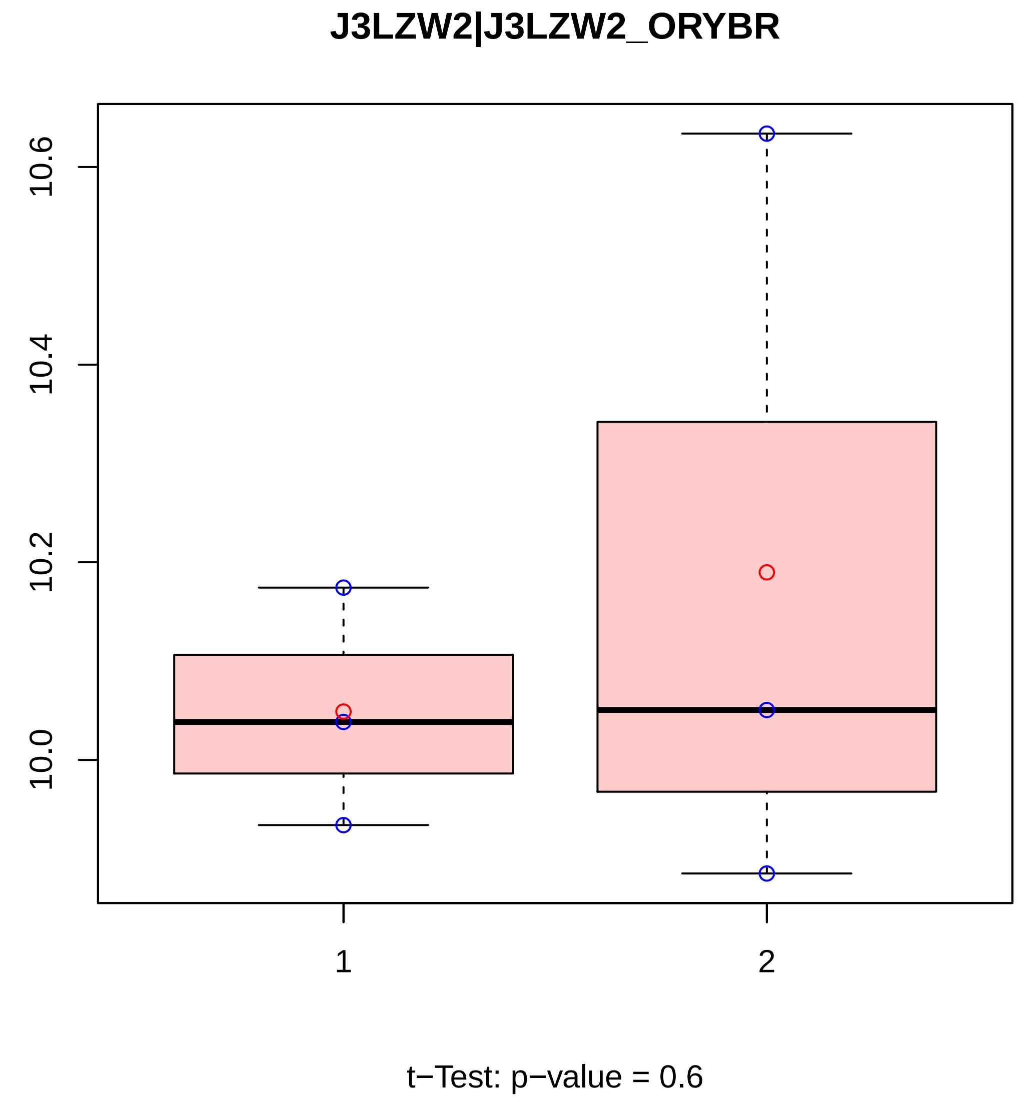
J3LZW2|J3LZW2_ORYBR
10.0
10.2
10.4
10.6
1
2
t−Test: p−value = 0.6

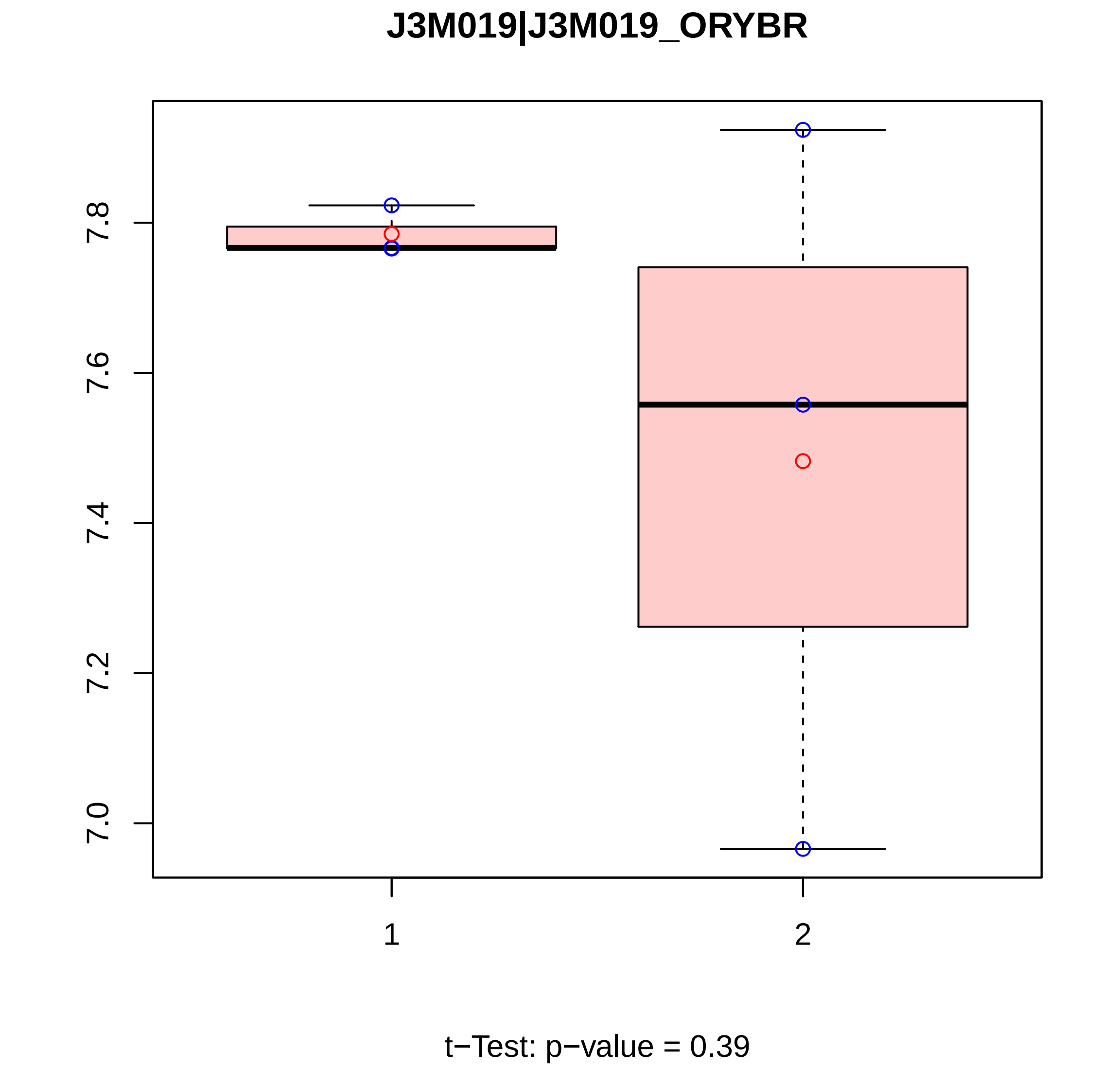
J3M019|J3M019_ORYBR
7.0
7.2
7.4
7.6
7.8
1
2
t−Test: p−value = 0.39

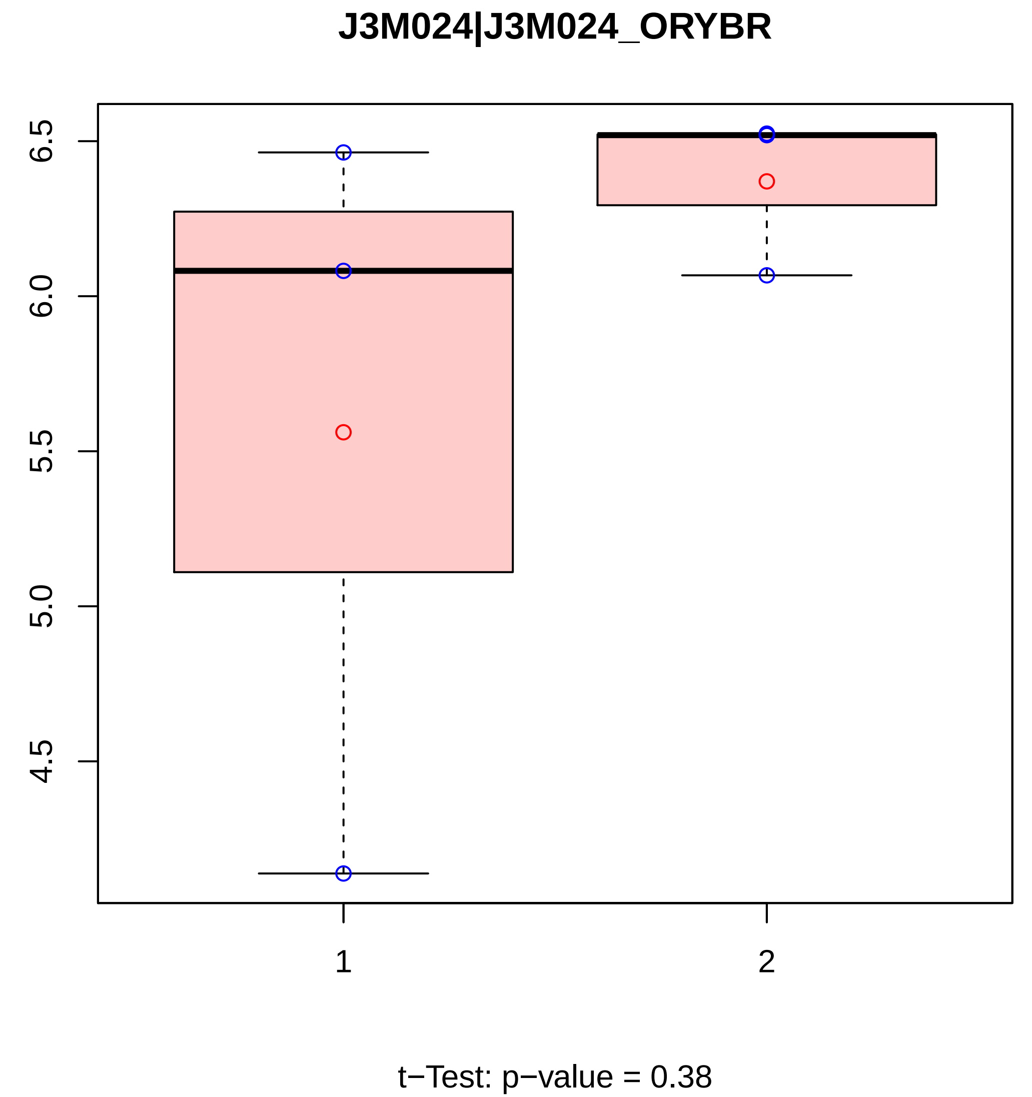
J3M024|J3M024_ORYBR
6.5
6.0
5.5
5.0
4.5
1
2
t−Test: p−value = 0.38

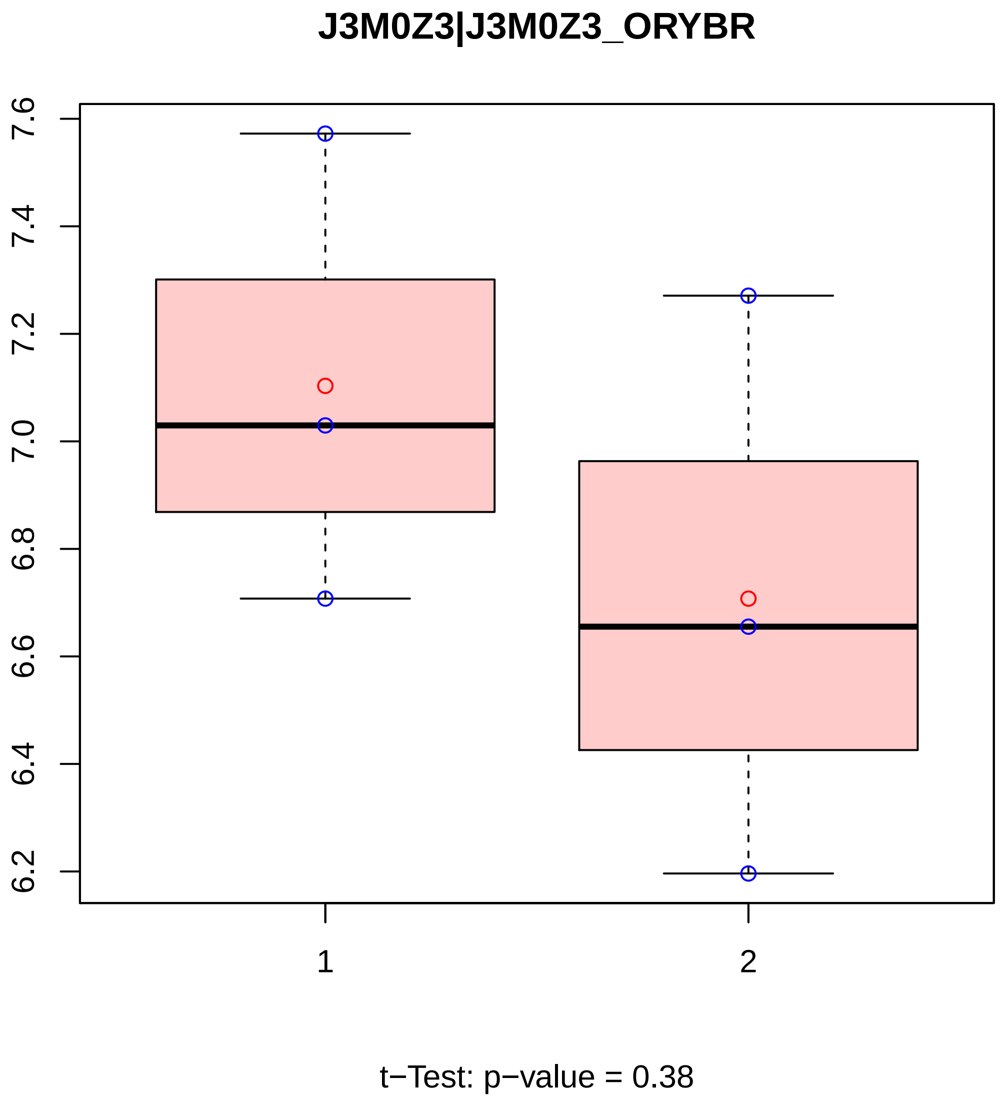
J3M0Z3|J3M0Z3_ORYBR
7.6
7.4
7.2
7.0
6.8
6.6
6.4
6.2
1
2
t−Test: p−value = 0.38

J3M103|J3M103_ORYBR

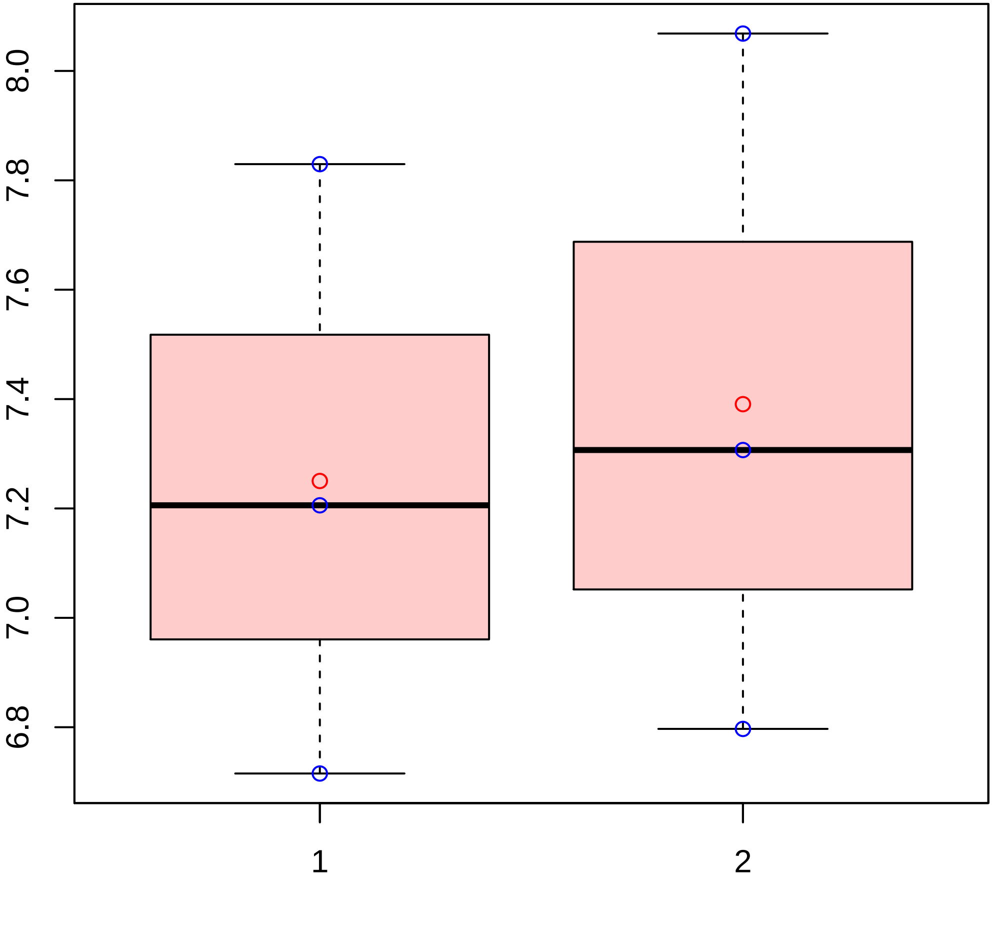

t−Test: p−value = 0.79

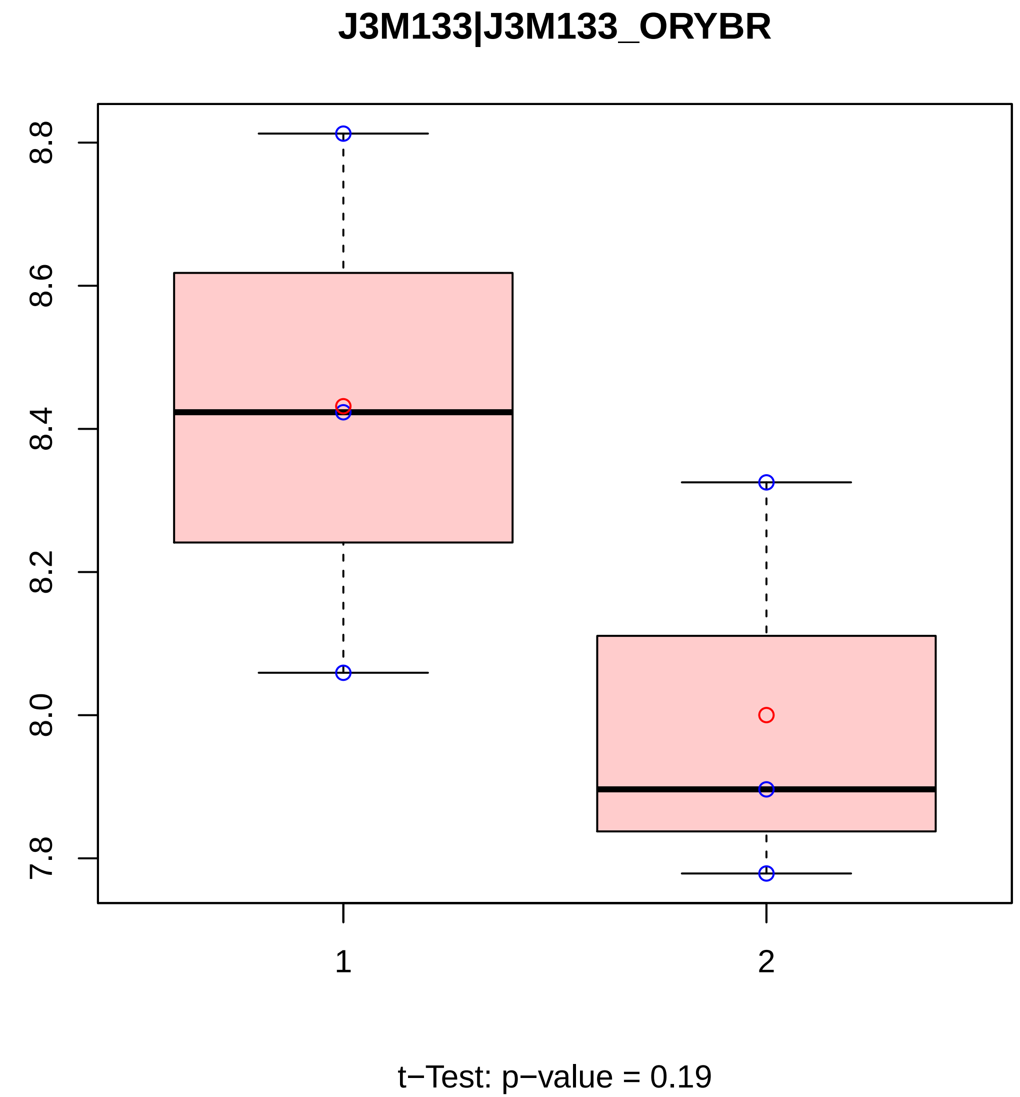
J3M133|J3M133_ORYBR
8.8
8.6
8.4
8.2
8.0
7.8
1
2
t−Test: p−value = 0.19

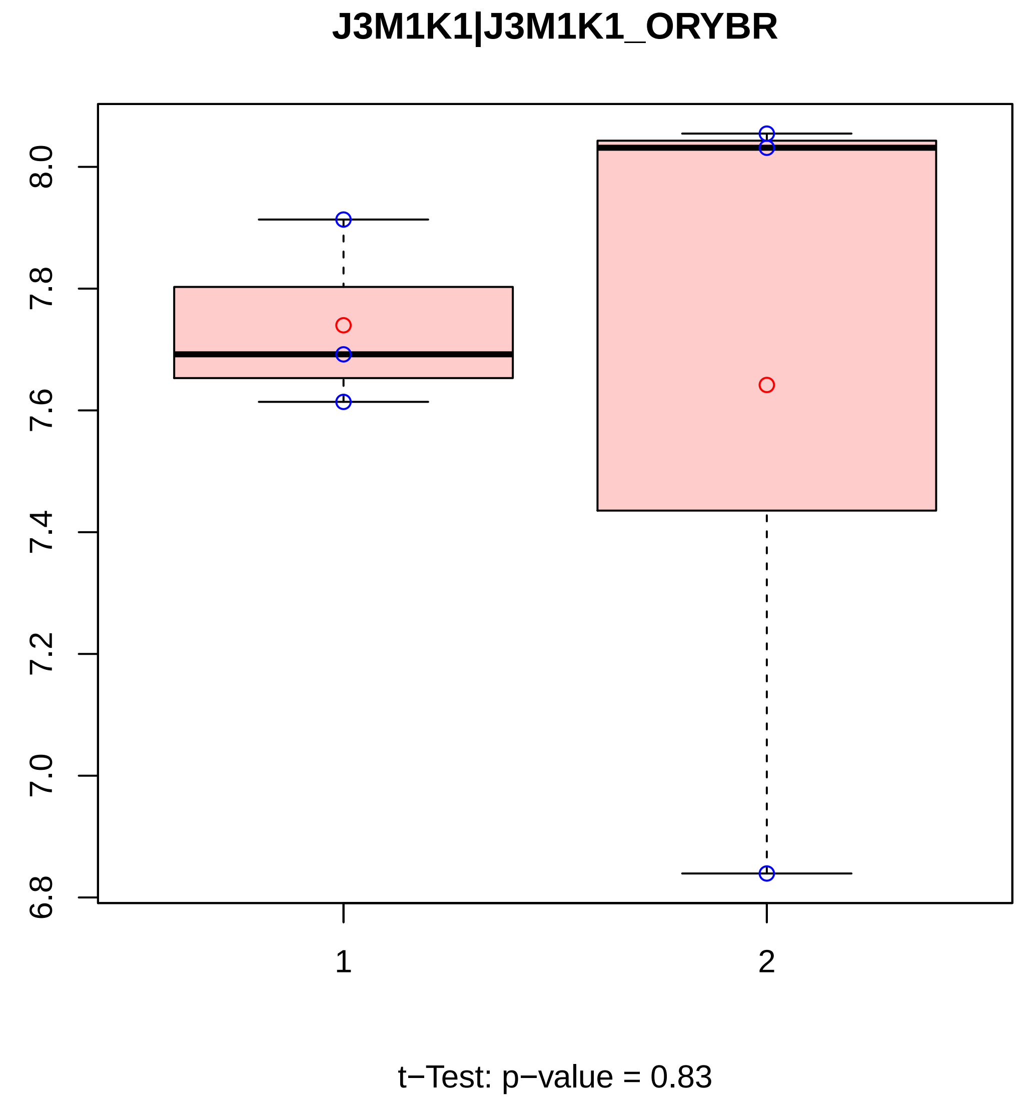

J3M1K1|J3M1K1_ORYBR
8.0
7.8
7.6
7.4
7.2
7.0
6.8
1
2
t−Test: p−value = 0.83

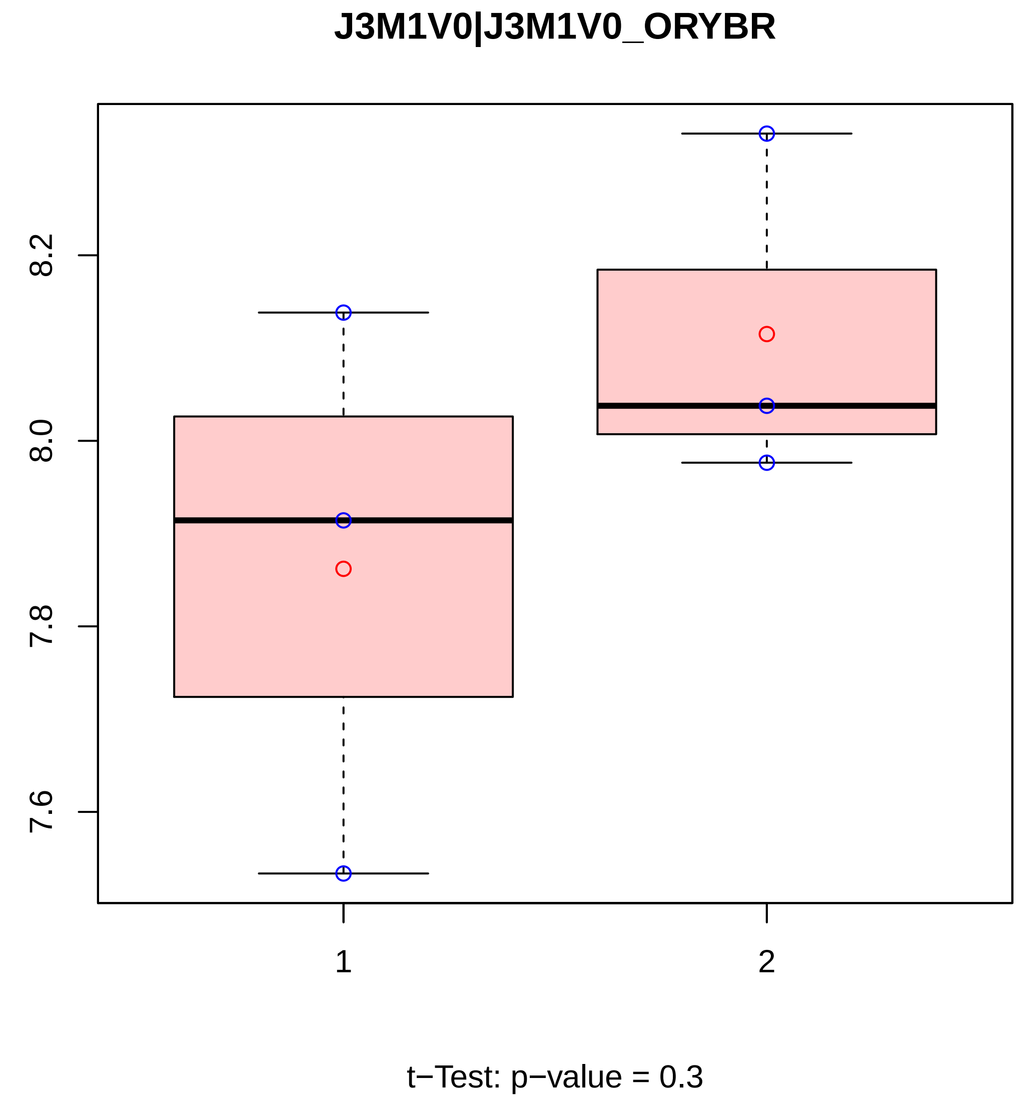

J3M1V0|J3M1V0_ORYBR
7.6
7.8
8.0
8.2
1
2
t−Test: p−value = 0.3

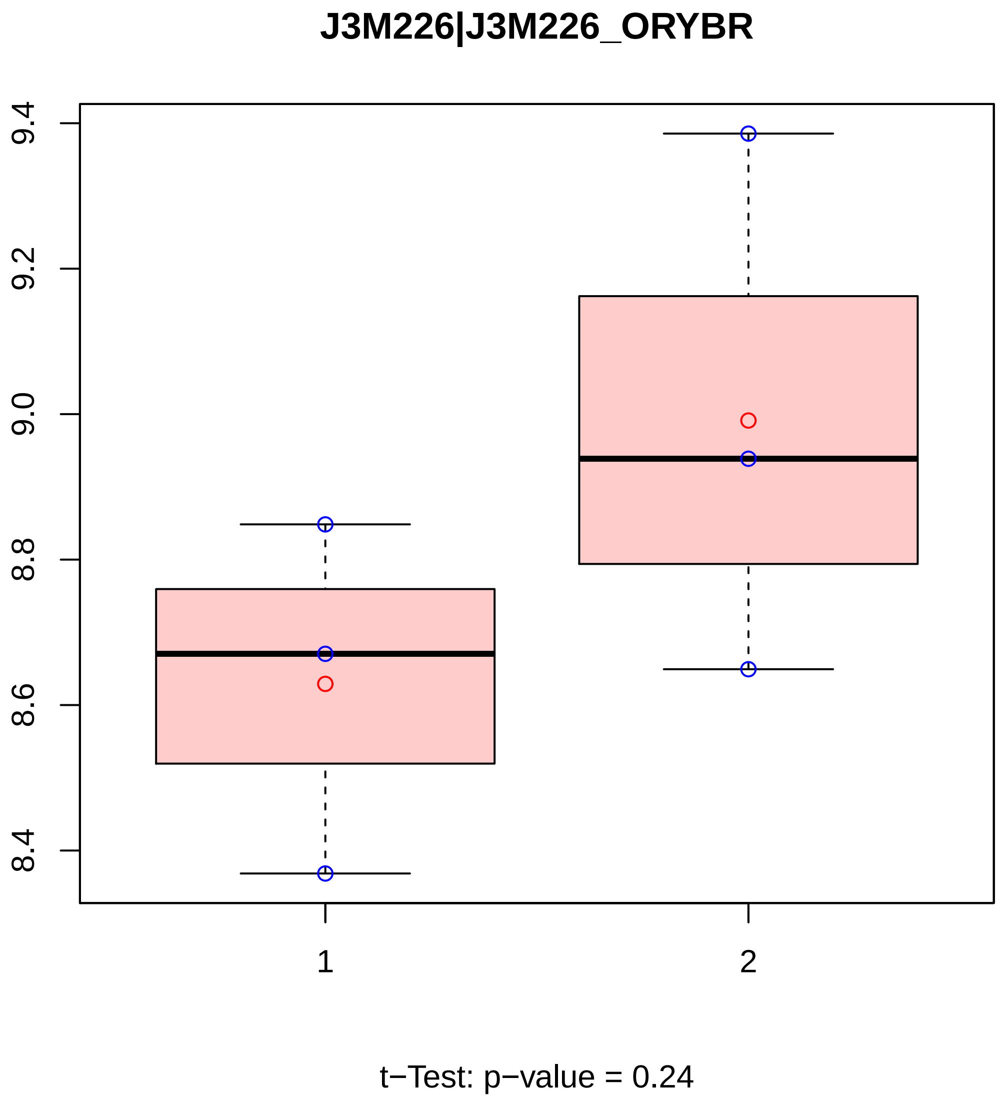

J3M226|J3M226_ORYBR
9.4
9.2
9.0
8.8
8.6
8.4
1
2
t−Test: p−value = 0.24

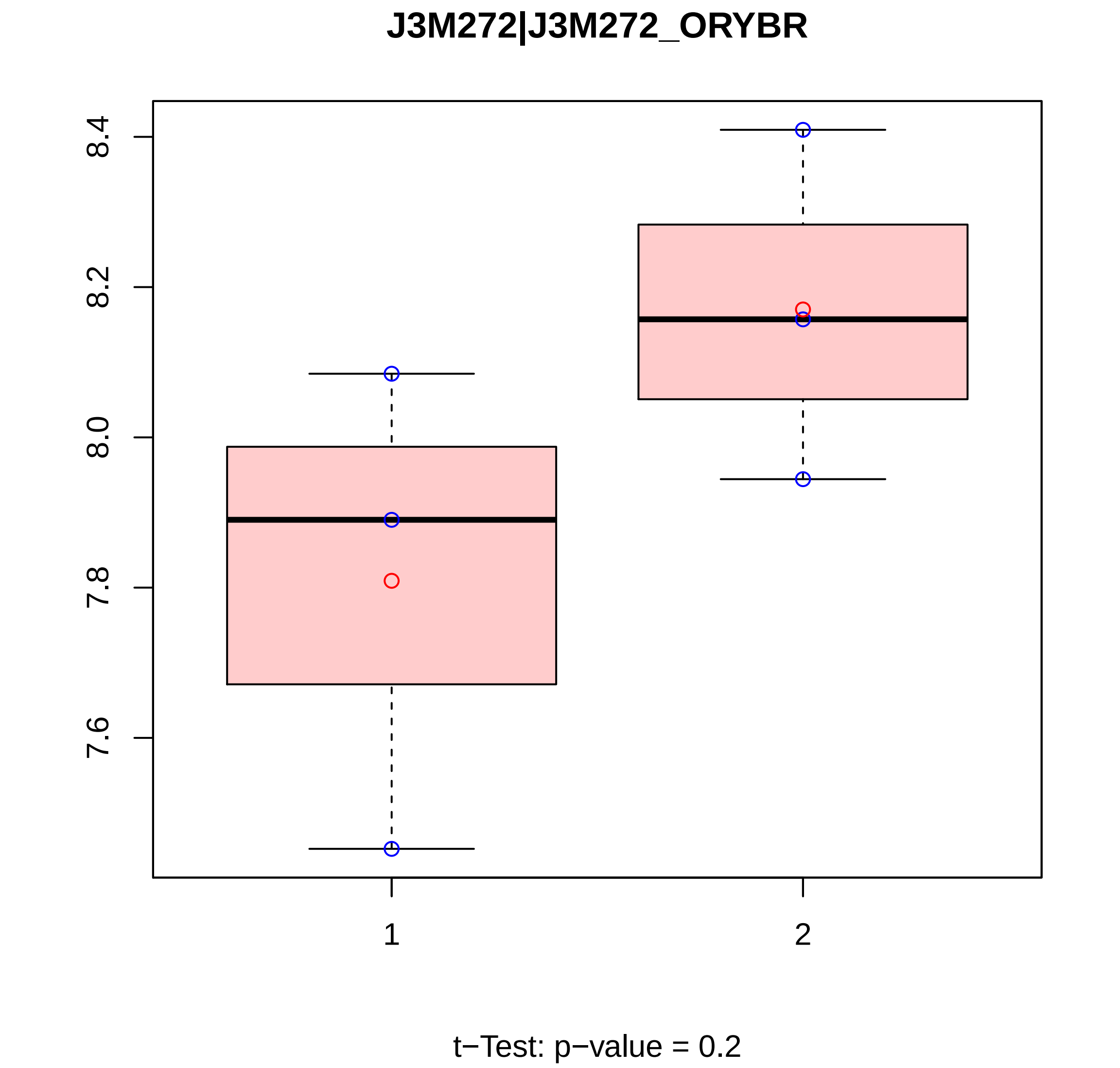
J3M272|J3M272_ORYBR
8.4
8.2
8.0
7.8
7.6
1
2
t−Test: p−value = 0.2

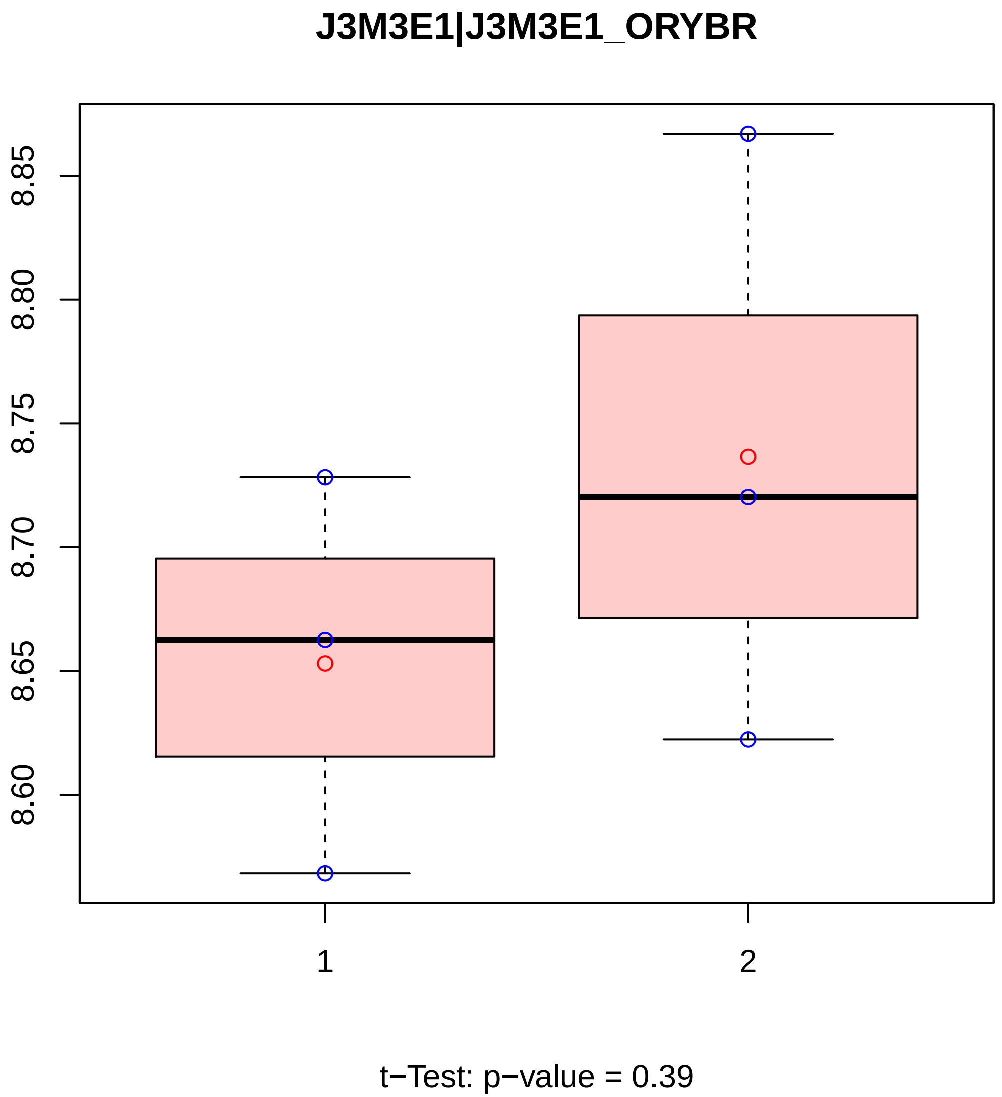
J3M3E1|J3M3E1_ORYBR
8.60
8.65
8.70
8.75
8.80
8.85
1
2
t−Test: p−value = 0.39

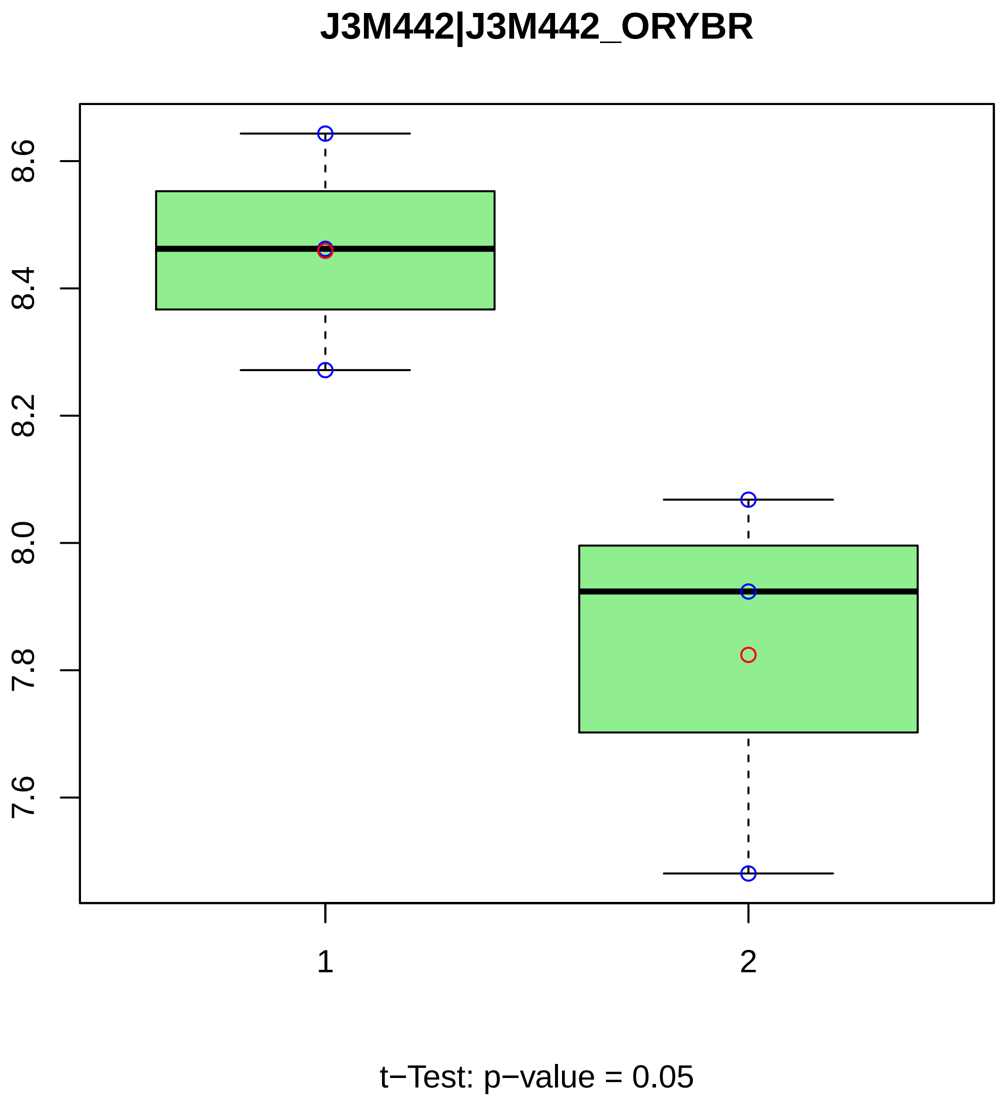
J3M442|J3M442_ORYBR
8.6
8.4
8.2
8.0
7.8
7.6
1
2
t−Test: p−value = 0.05

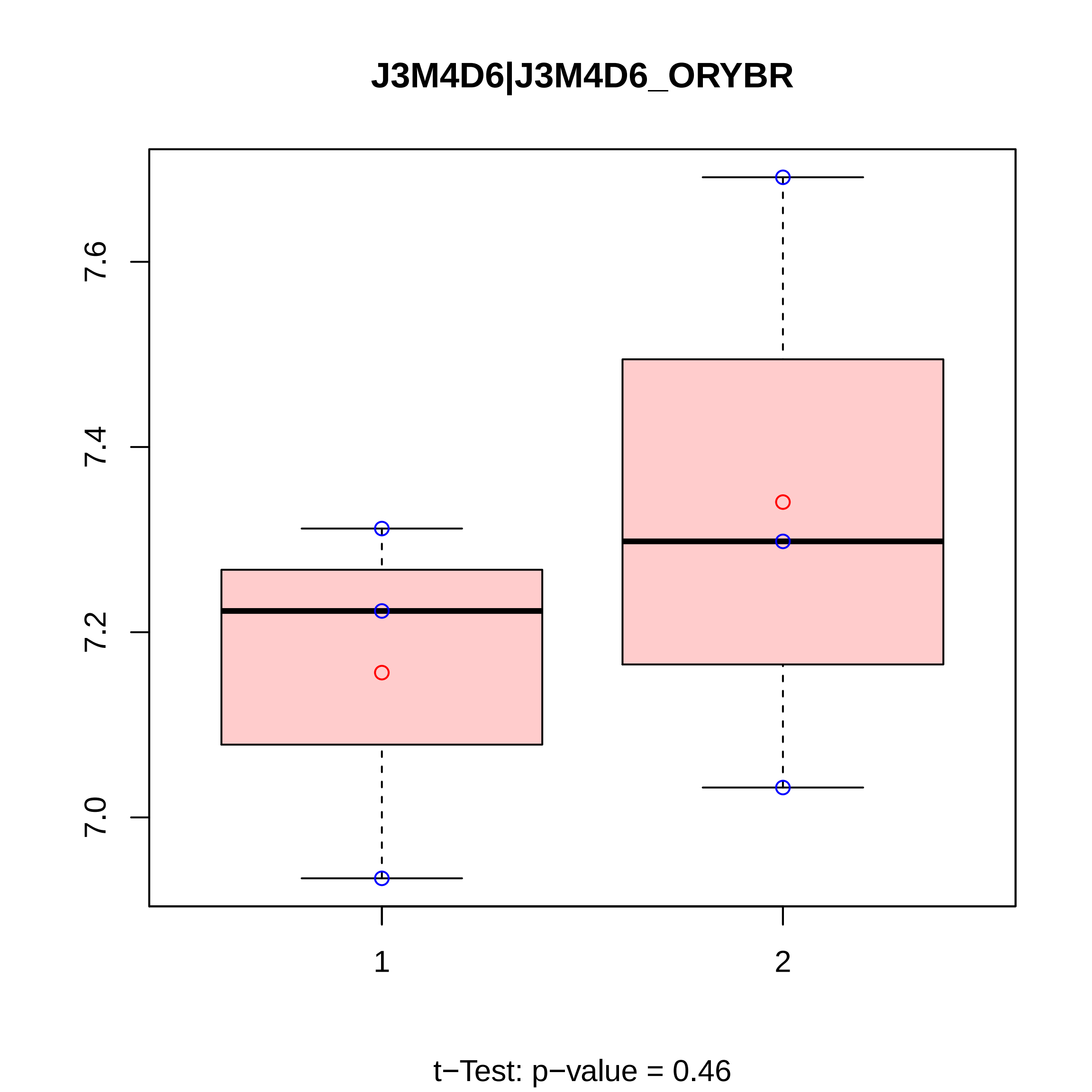
J3M4D6|J3M4D6_ORYBR
7.6
7.4
7.2
7.0
1
2
t−Test: p−value = 0.46

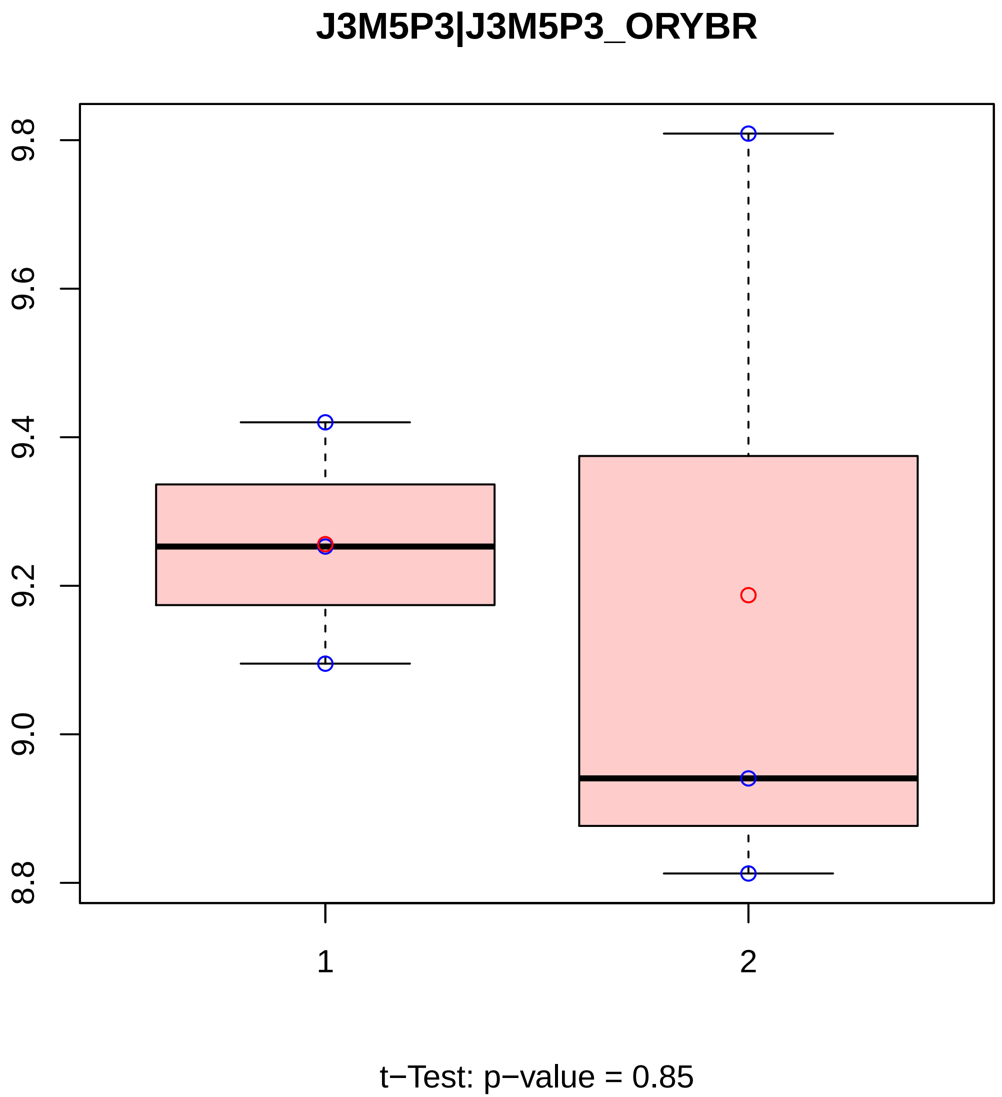
J3M5P3|J3M5P3_ORYBR
9.8
9.6
9.4
9.2
9.0
8.8
1
2
t−Test: p−value = 0.85

J3M5V2|J3M5V2_ORYBR

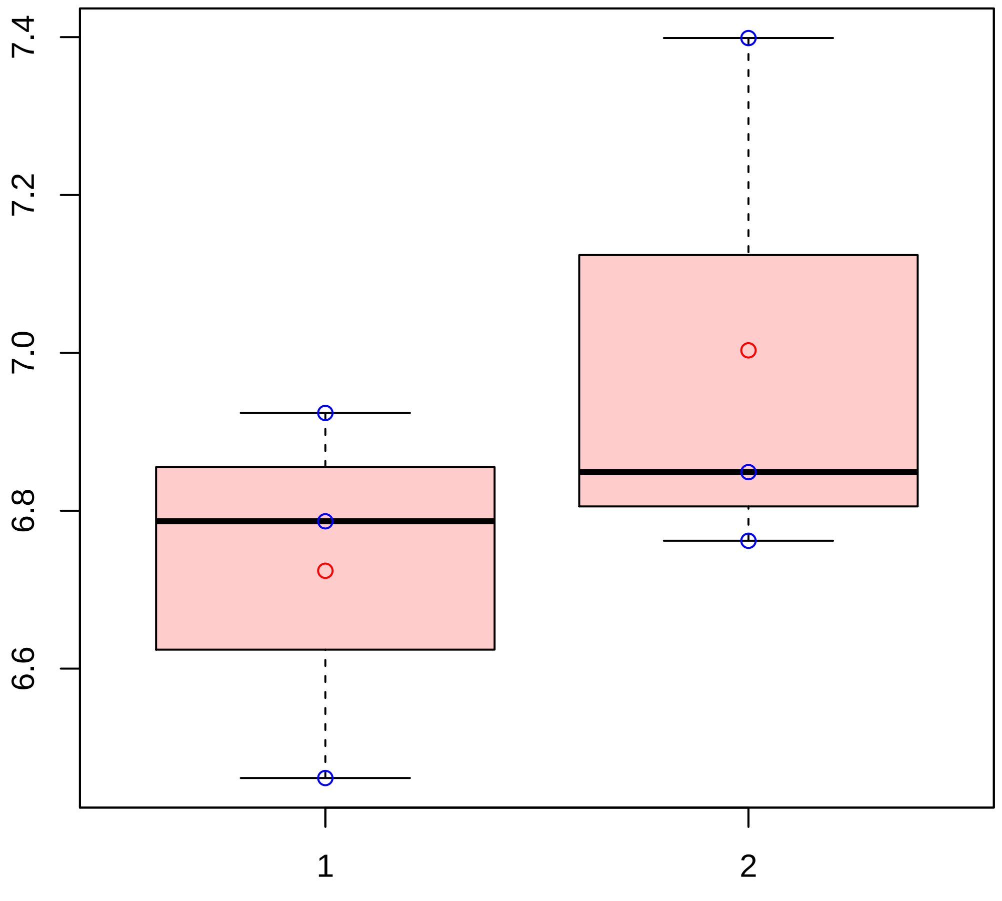

t−Test: p−value = 0.32

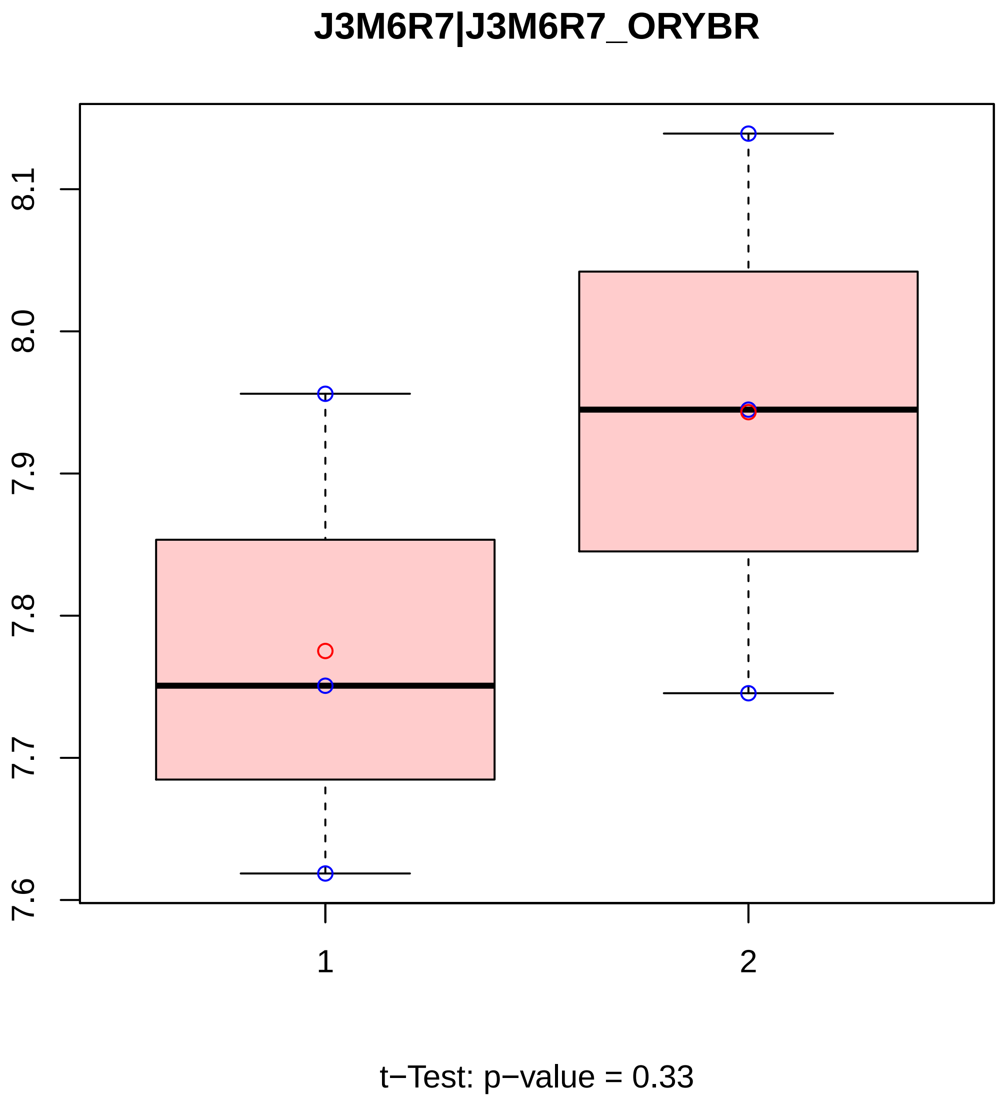

J3M6R7|J3M6R7_ORYBR
8.1
8.0
7.9
7.8
7.7
7.6
1
2
t−Test: p−value = 0.33

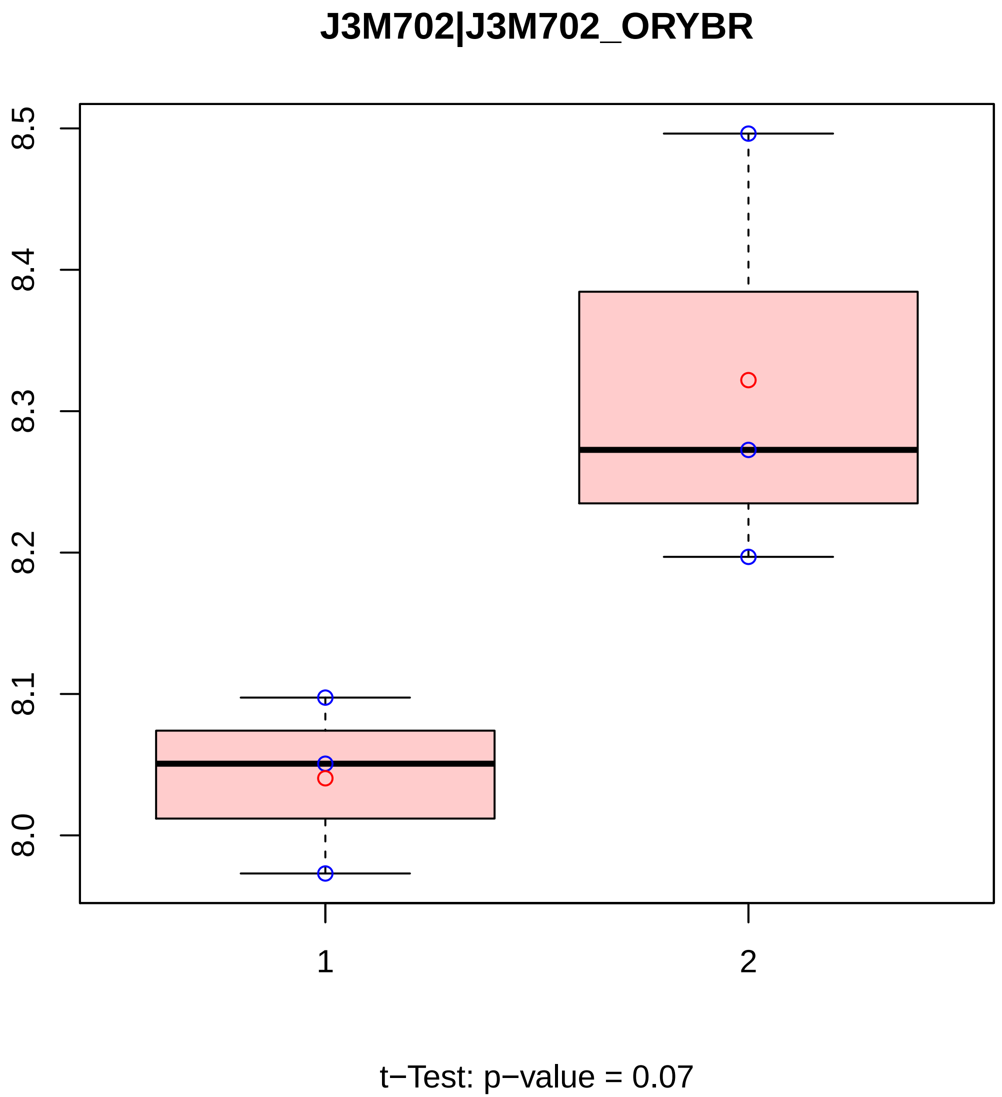
J3M702|J3M702_ORYBR
8.0
8.1
8.2
8.3
8.4
8.5
1
2
t−Test: p−value = 0.07

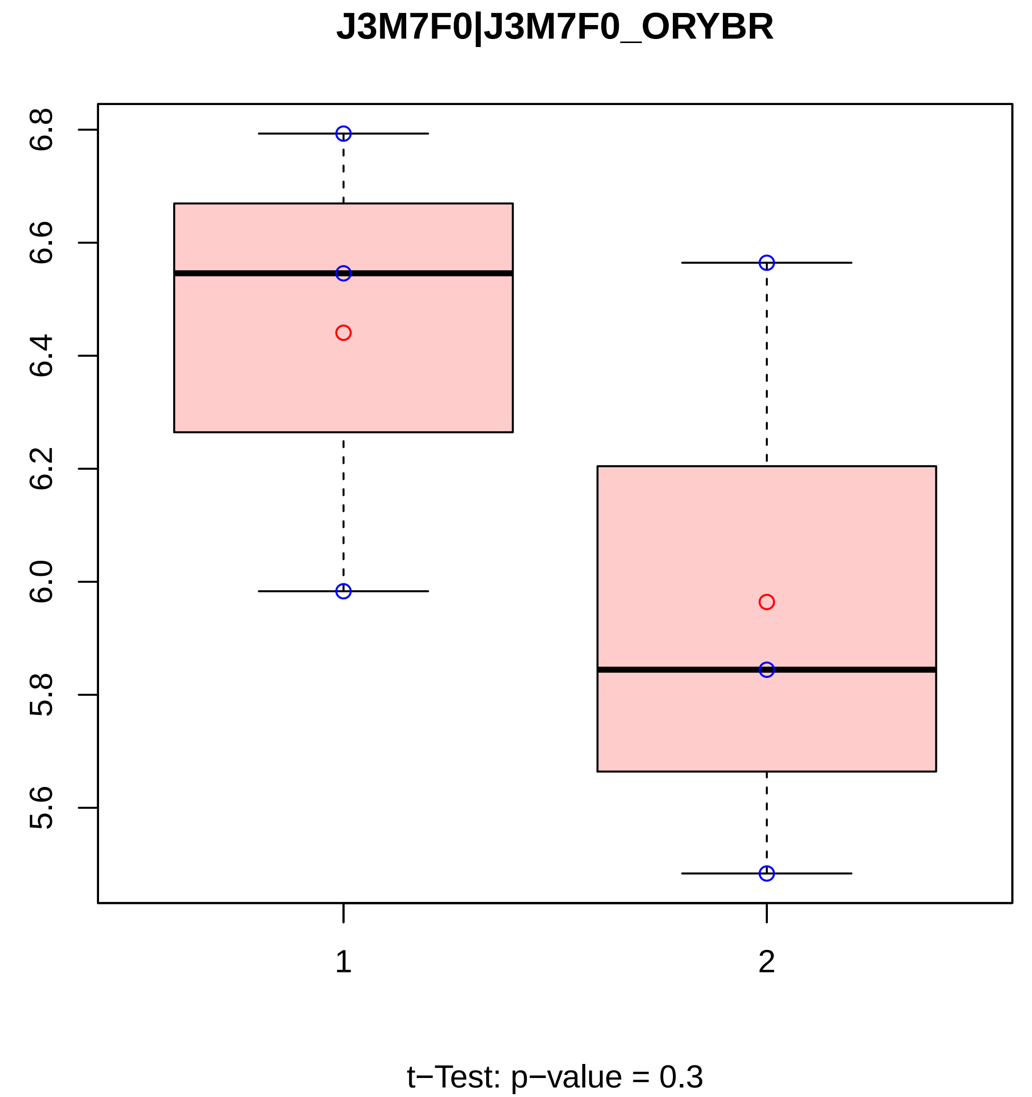
J3M7F0|J3M7F0_ORYBR
6.8
6.6
6.4
6.2
6.0
5.8
5.6
1
2
t−Test: p−value = 0.3

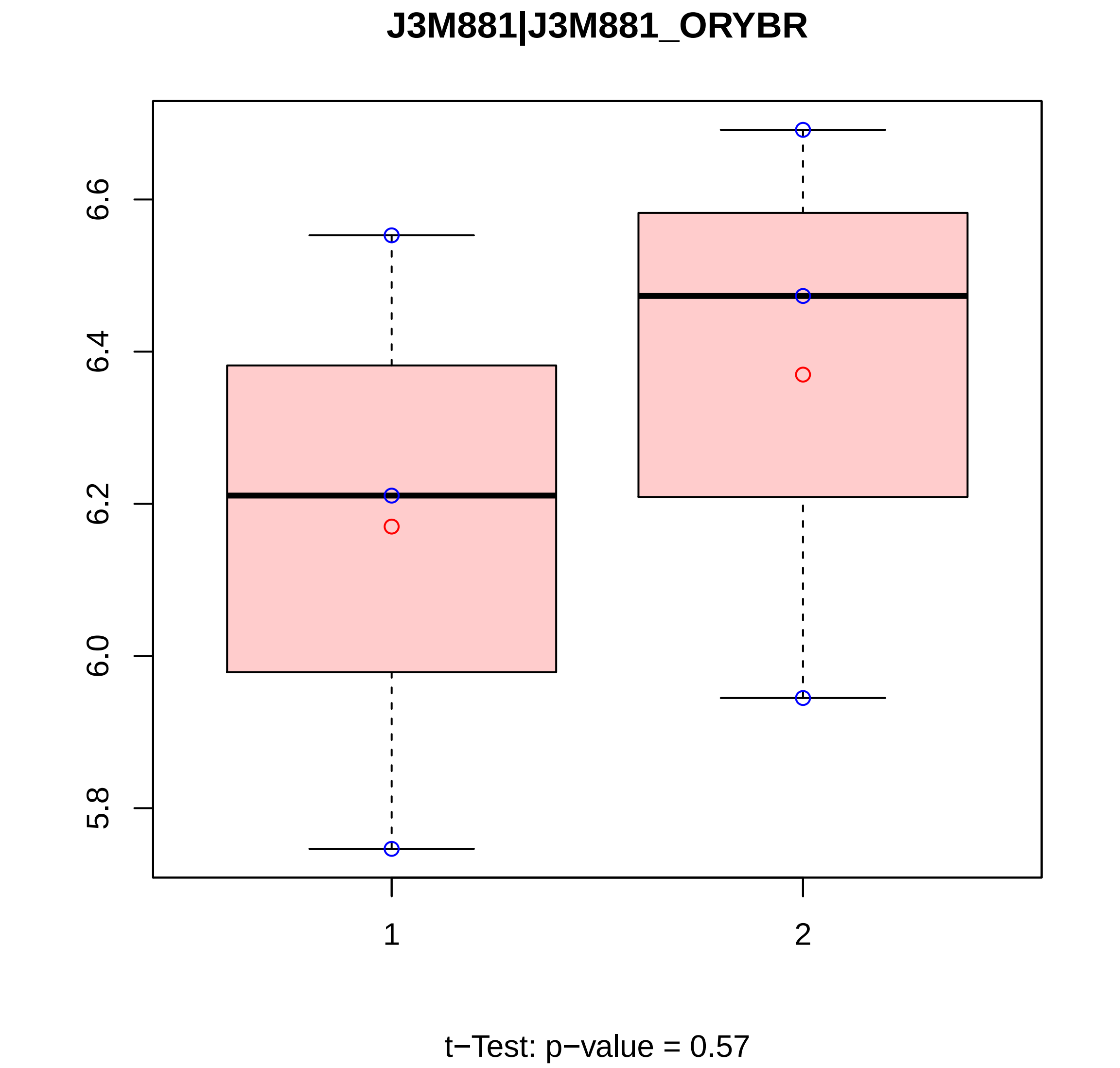

J3M881|J3M881_ORYBR
6.6
6.4
6.2
6.0
5.8
1
2
t−Test: p−value = 0.57

J3M8D4|J3M8D4_ORYBR

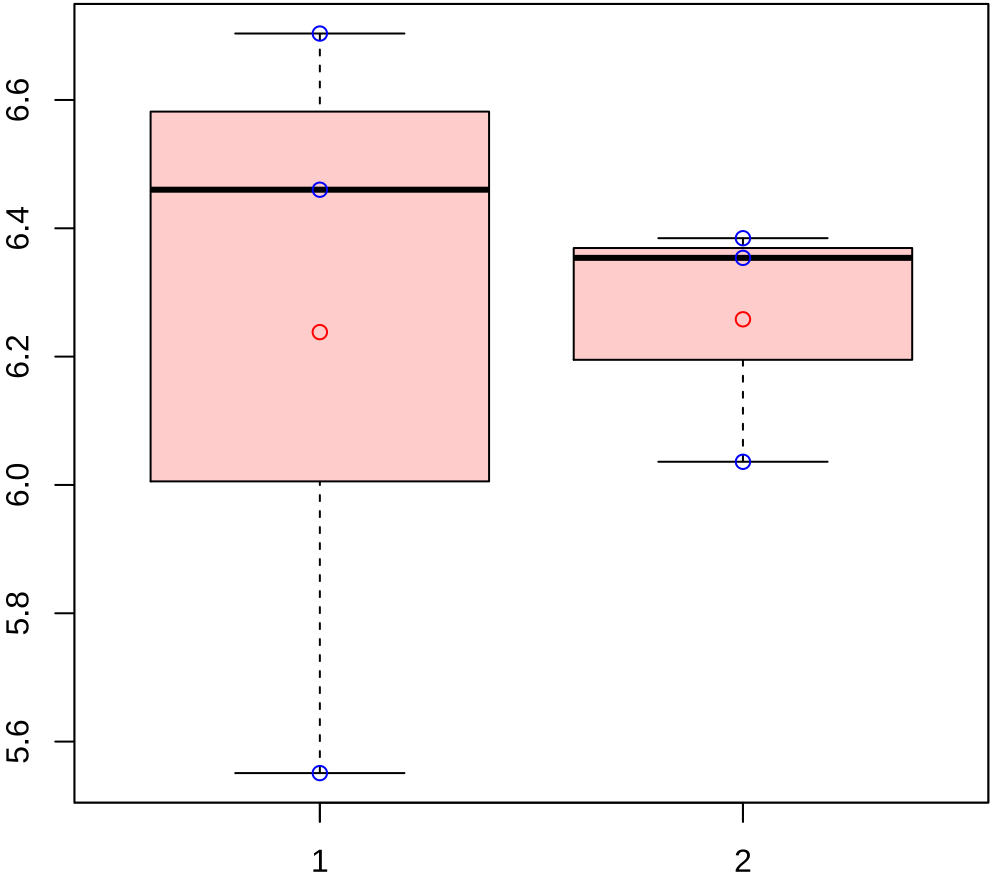

t−Test: p−value = 0.96

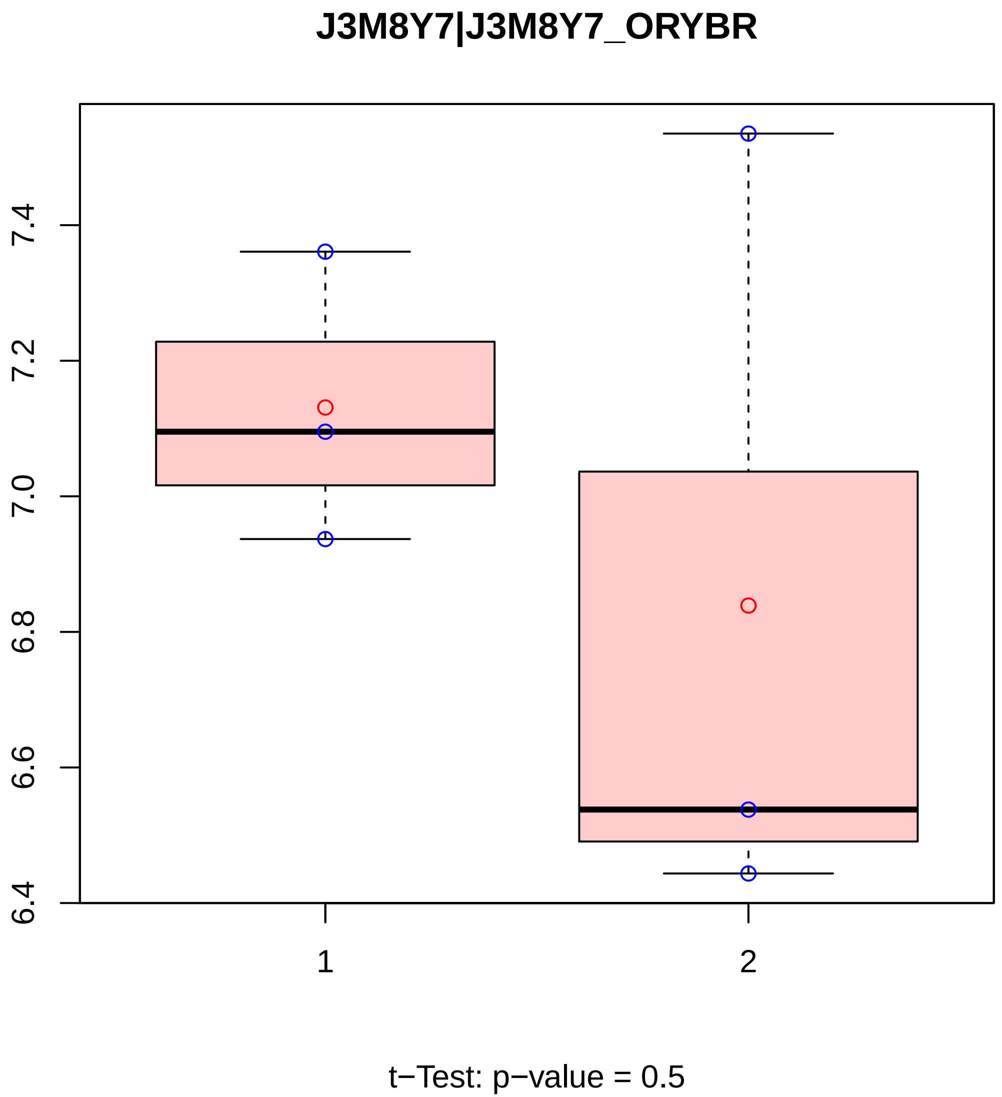
J3M8Y7|J3M8Y7_ORYBR
7.4
7.2
7.0
6.8
6.6
6.4
1
2
t−Test: p−value = 0.5

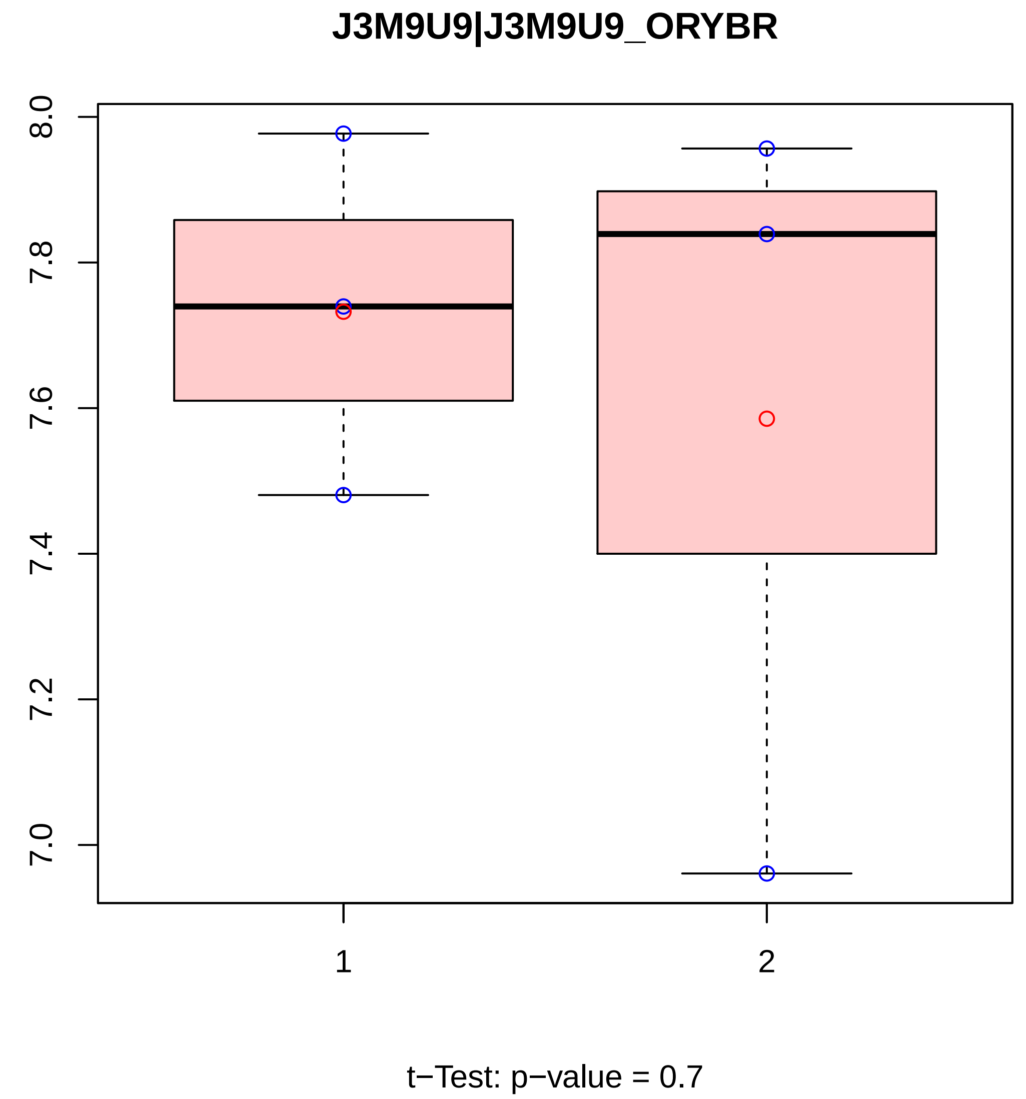
J3M9U9|J3M9U9_ORYBR
8.0
7.8
7.6
7.4
7.2
7.0
1
2
t−Test: p−value = 0.7

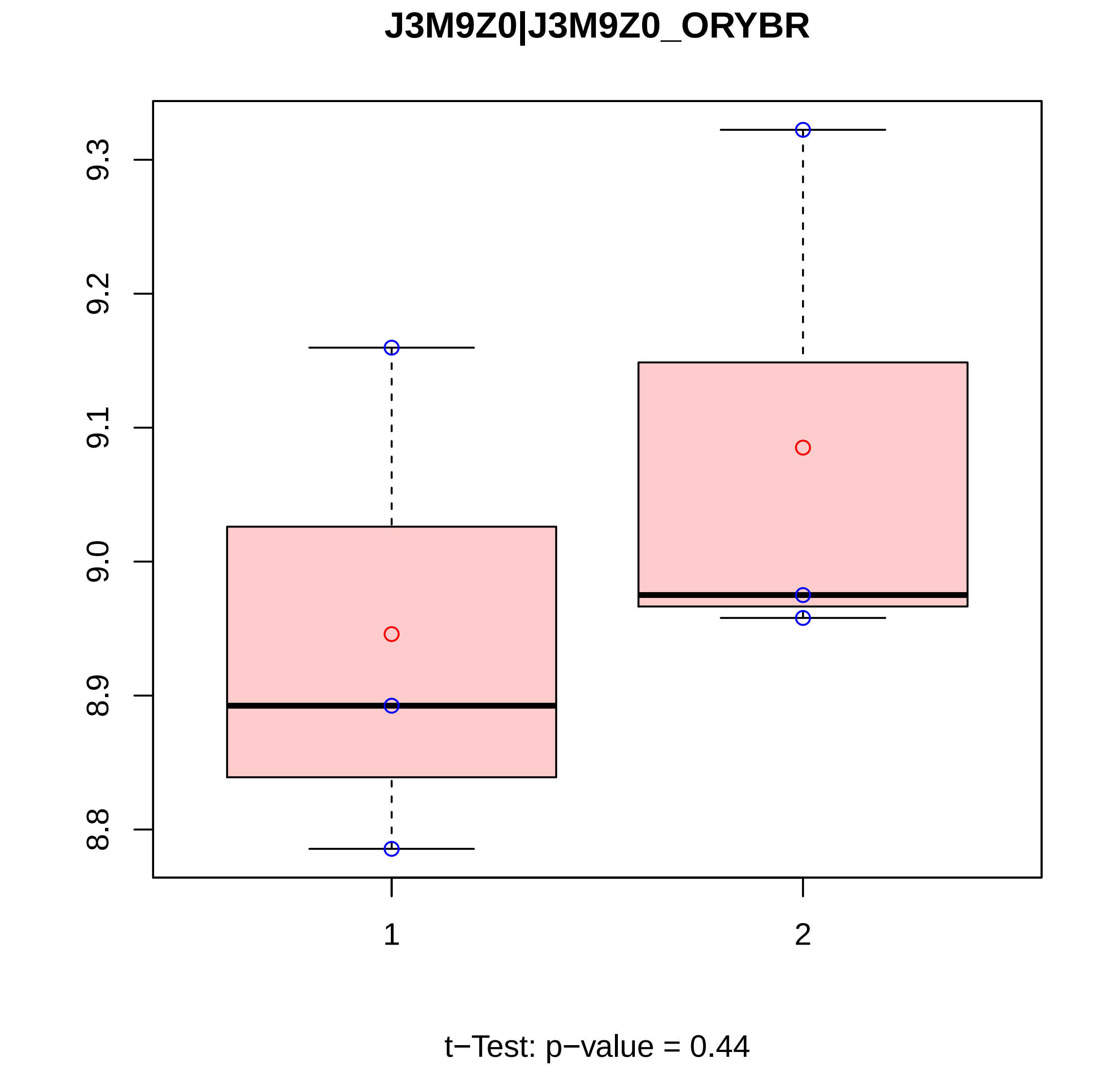
J3M9Z0|J3M9Z0_ORYBR
9.3
9.2
9.1
9.0
8.9
8.8
1
2
t−Test: p−value = 0.44

J3M9Z5|J3M9Z5_ORYBR

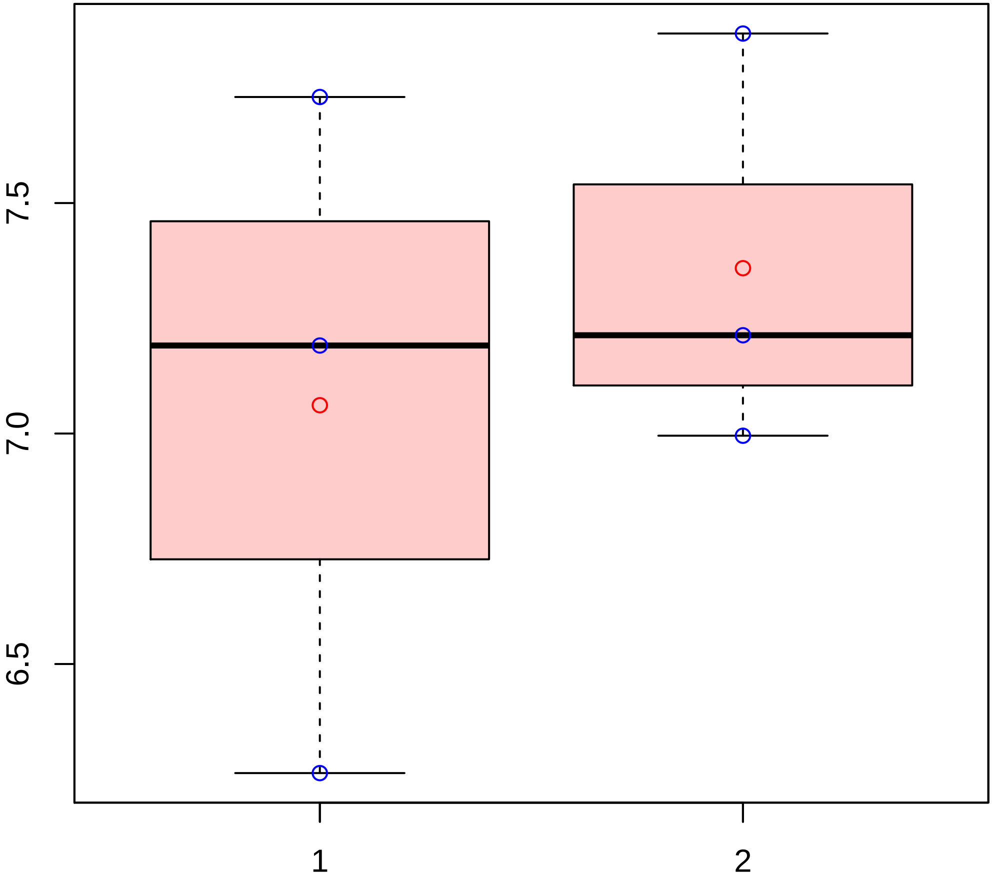

t−Test: p−value = 0.59

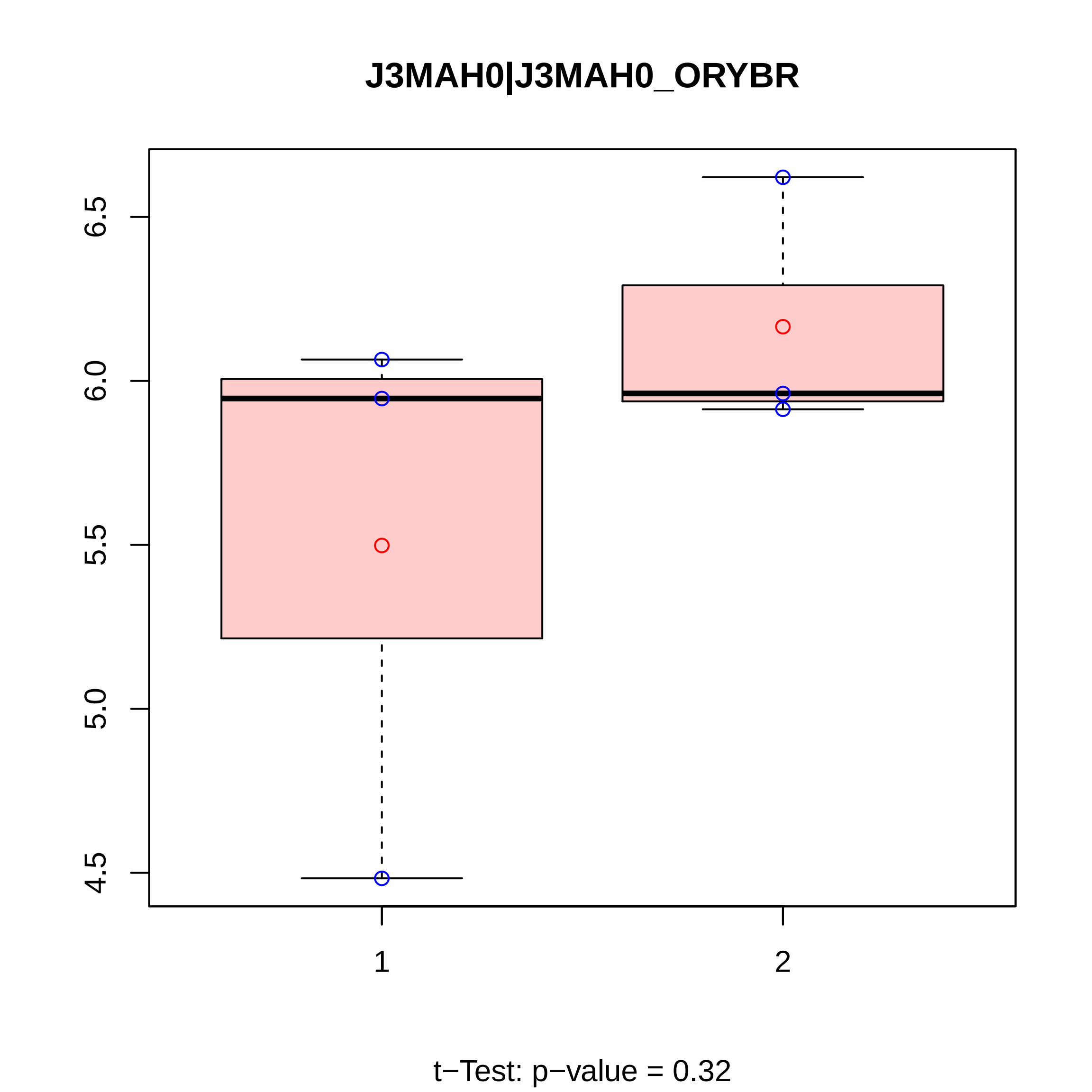
J3MAH0|J3MAH0_ORYBR
6.5
6.0
5.5
5.0
4.5
1
2
t−Test: p−value = 0.32

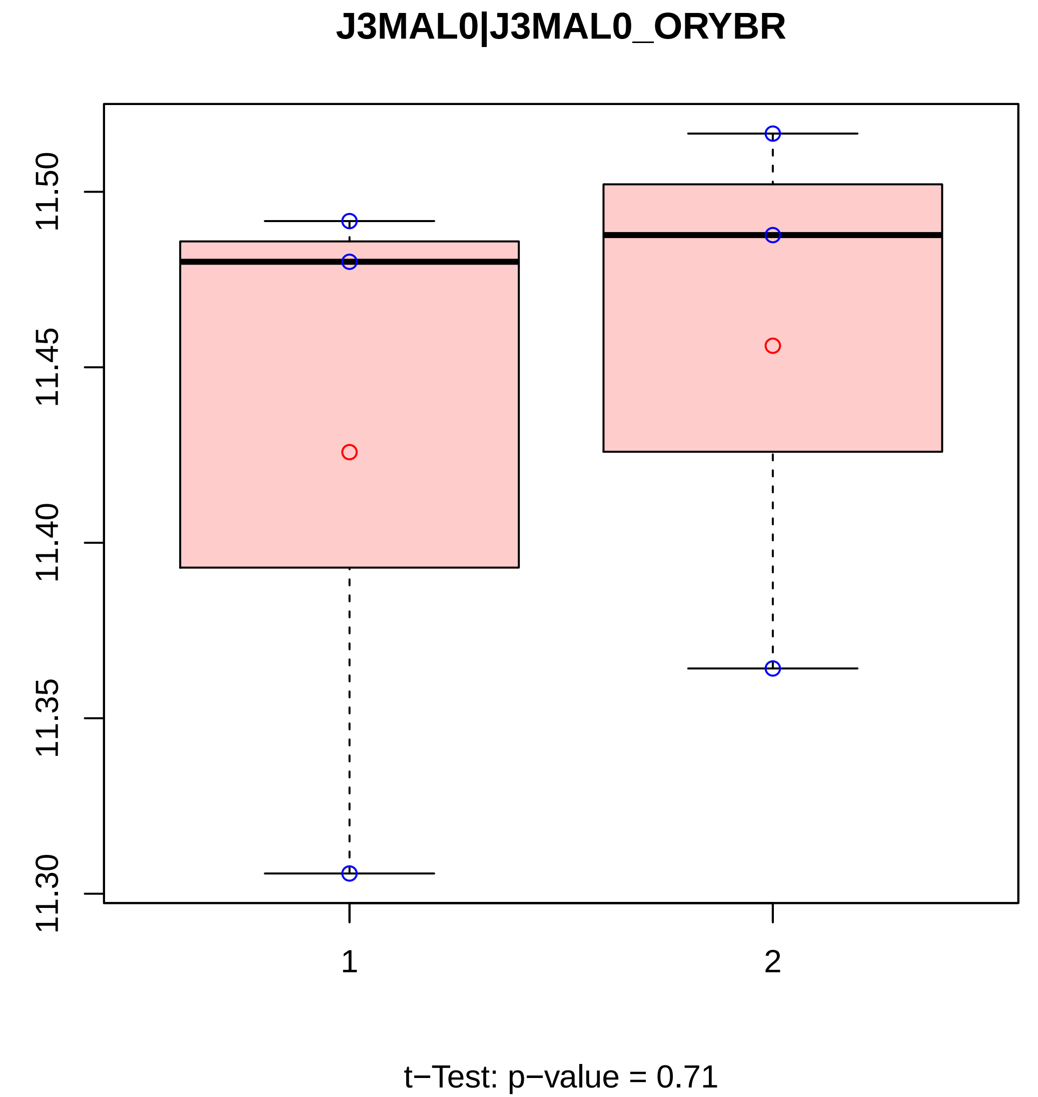
J3MAL0|J3MAL0_ORYBR
11.30
11.35
11.40
11.45
11.50
1
2
t−Test: p−value = 0.71

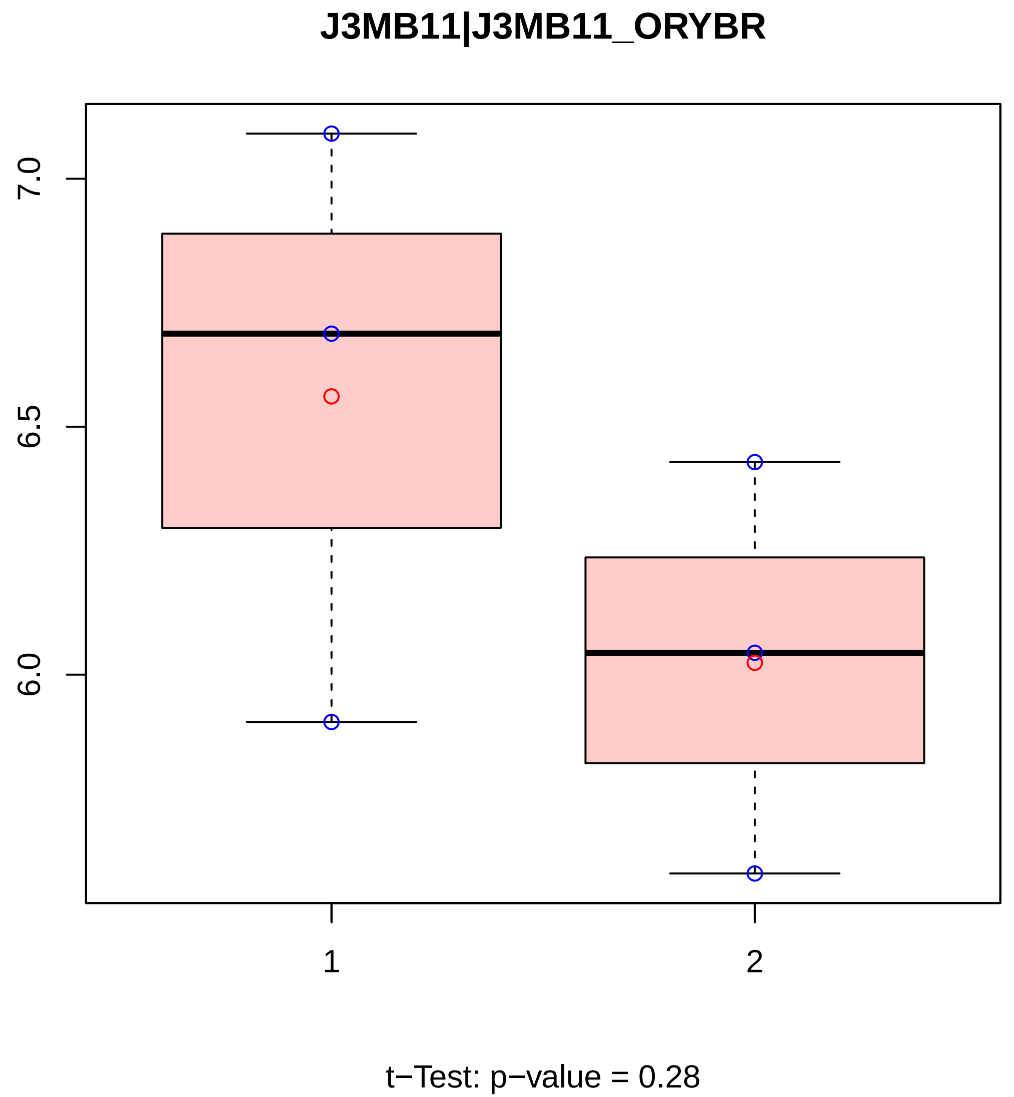
J3MB11|J3MB11_ORYBR
7.0
6.5
6.0
1
2
t−Test: p−value = 0.28

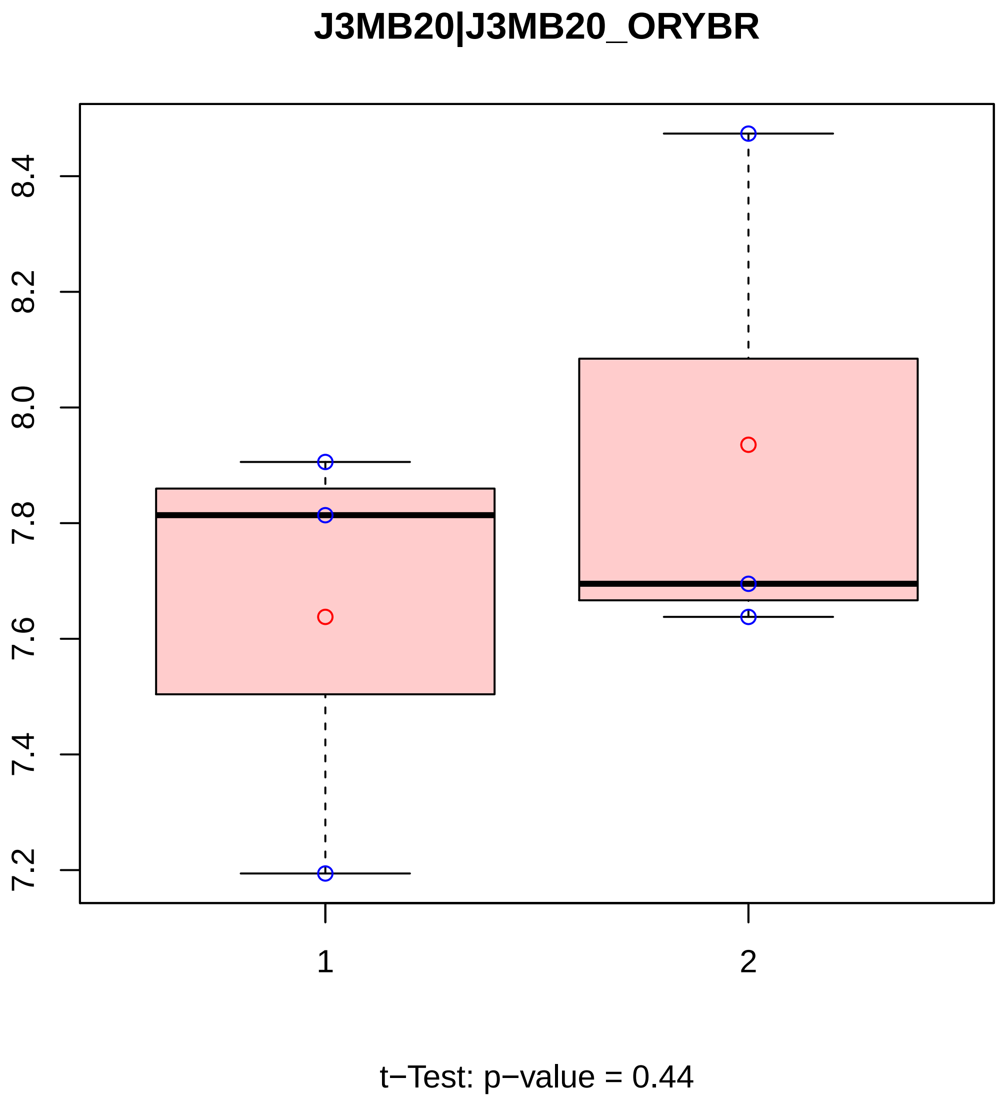
J3MB20|J3MB20_ORYBR
7.2
7.4
7.6
7.8
8.0
8.2
8.4
1
2
t−Test: p−value = 0.44

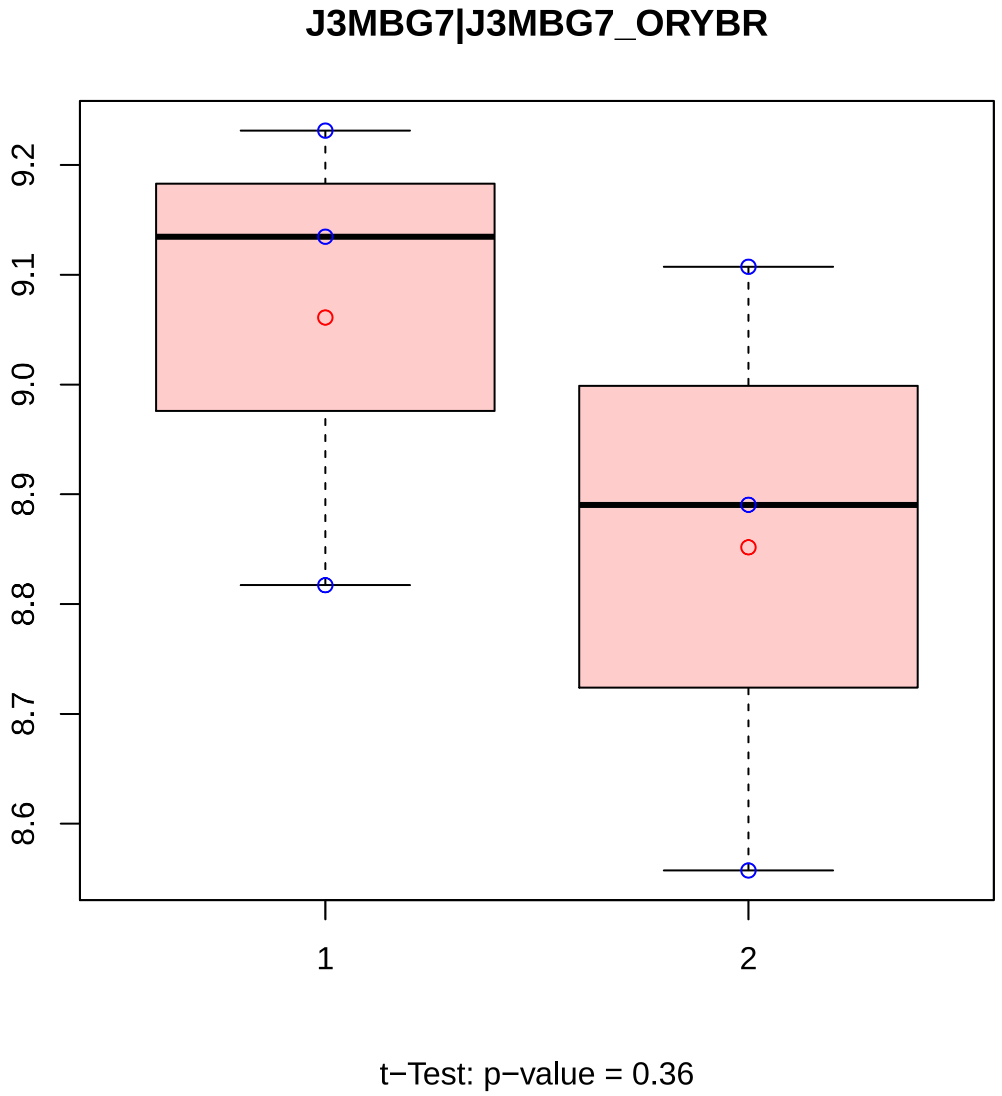

J3MBG7|J3MBG7_ORYBR
9.2
9.1
9.0
8.9
8.8
8.7
8.6
1
2
t−Test: p−value = 0.36

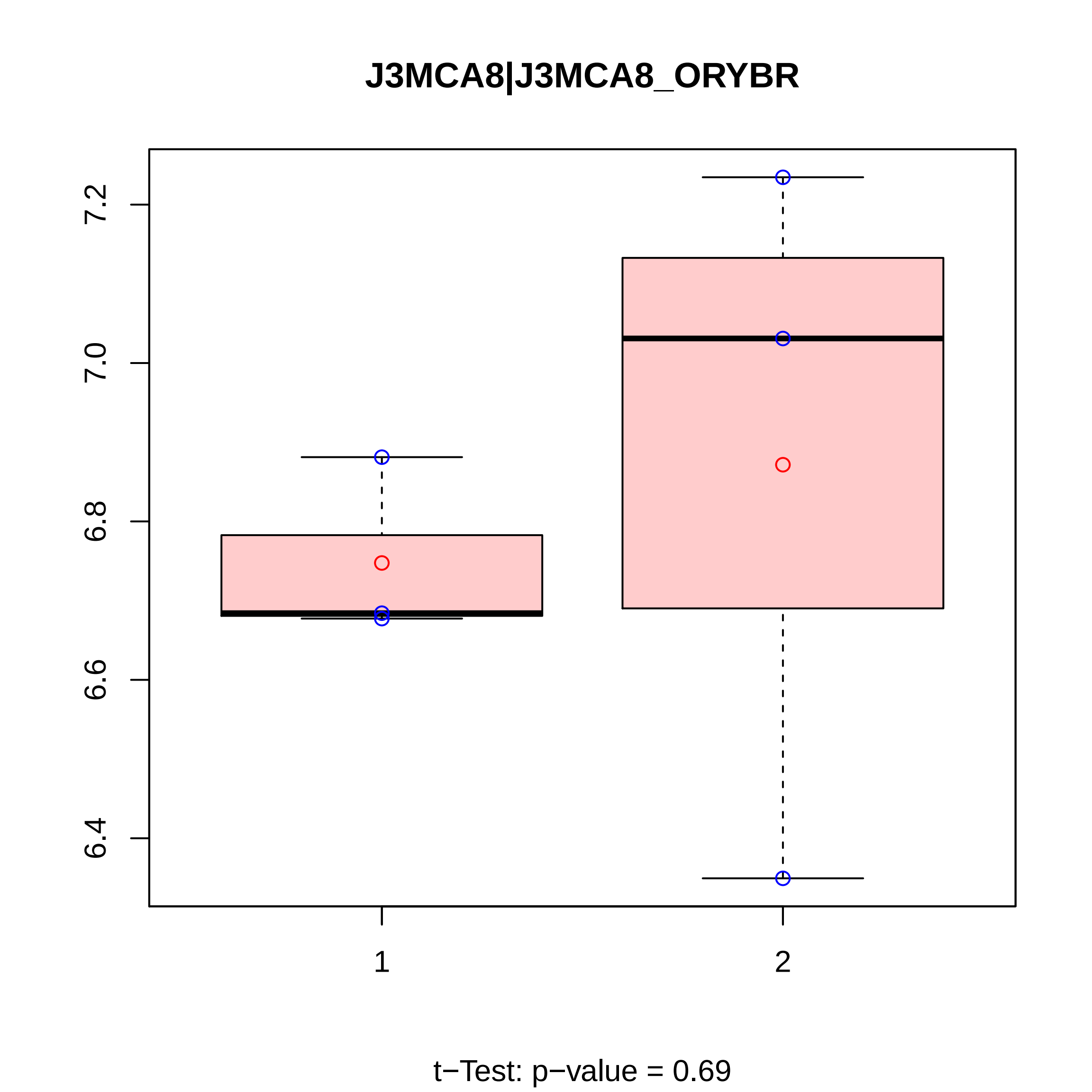
J3MCA8|J3MCA8_ORYBR
7.2
7.0
6.8
6.6
6.4
1
2
t−Test: p−value = 0.69

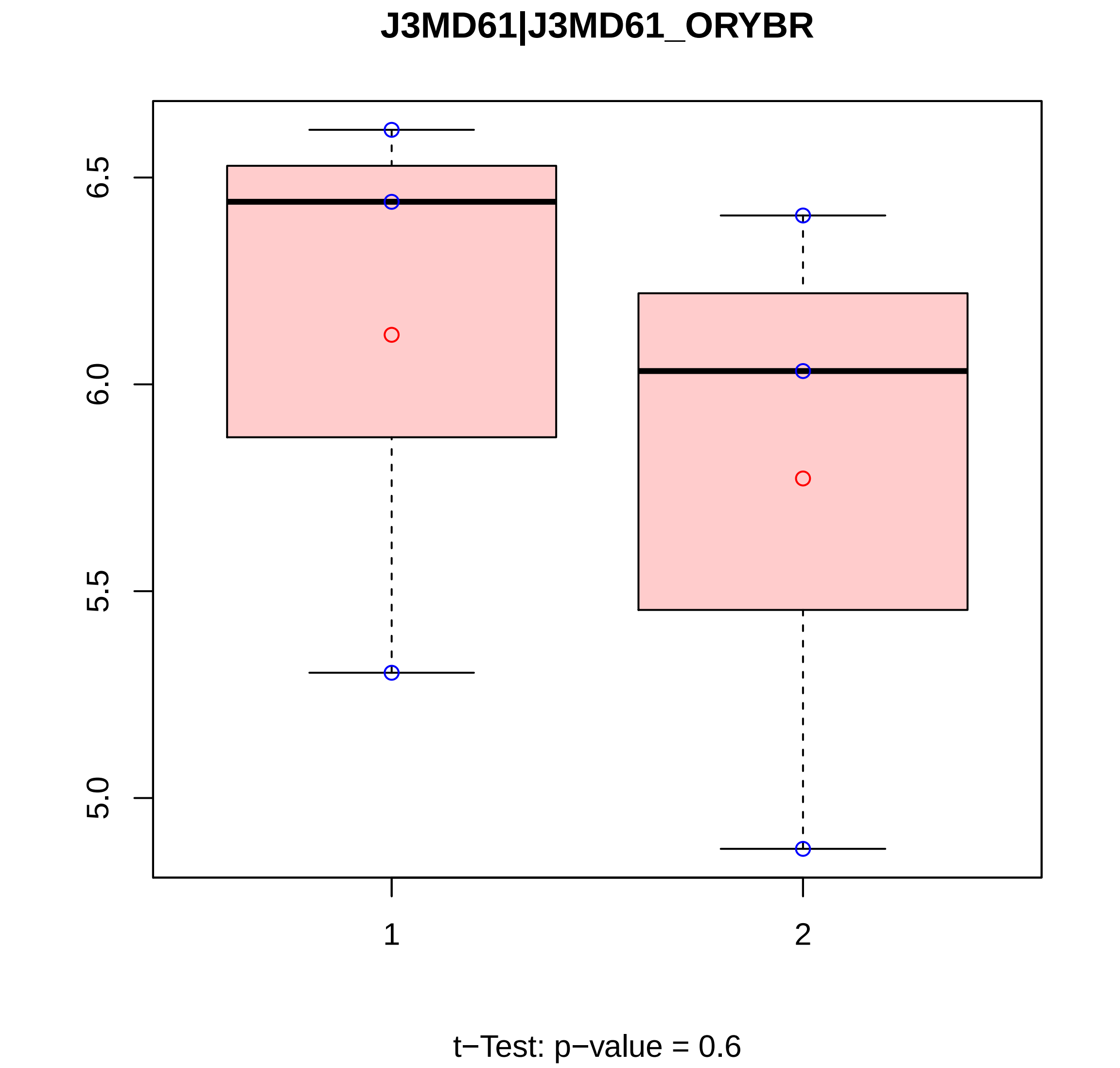

J3MD61|J3MD61_ORYBR
6.5
6.0
5.5
5.0
1
2
t−Test: p−value = 0.6

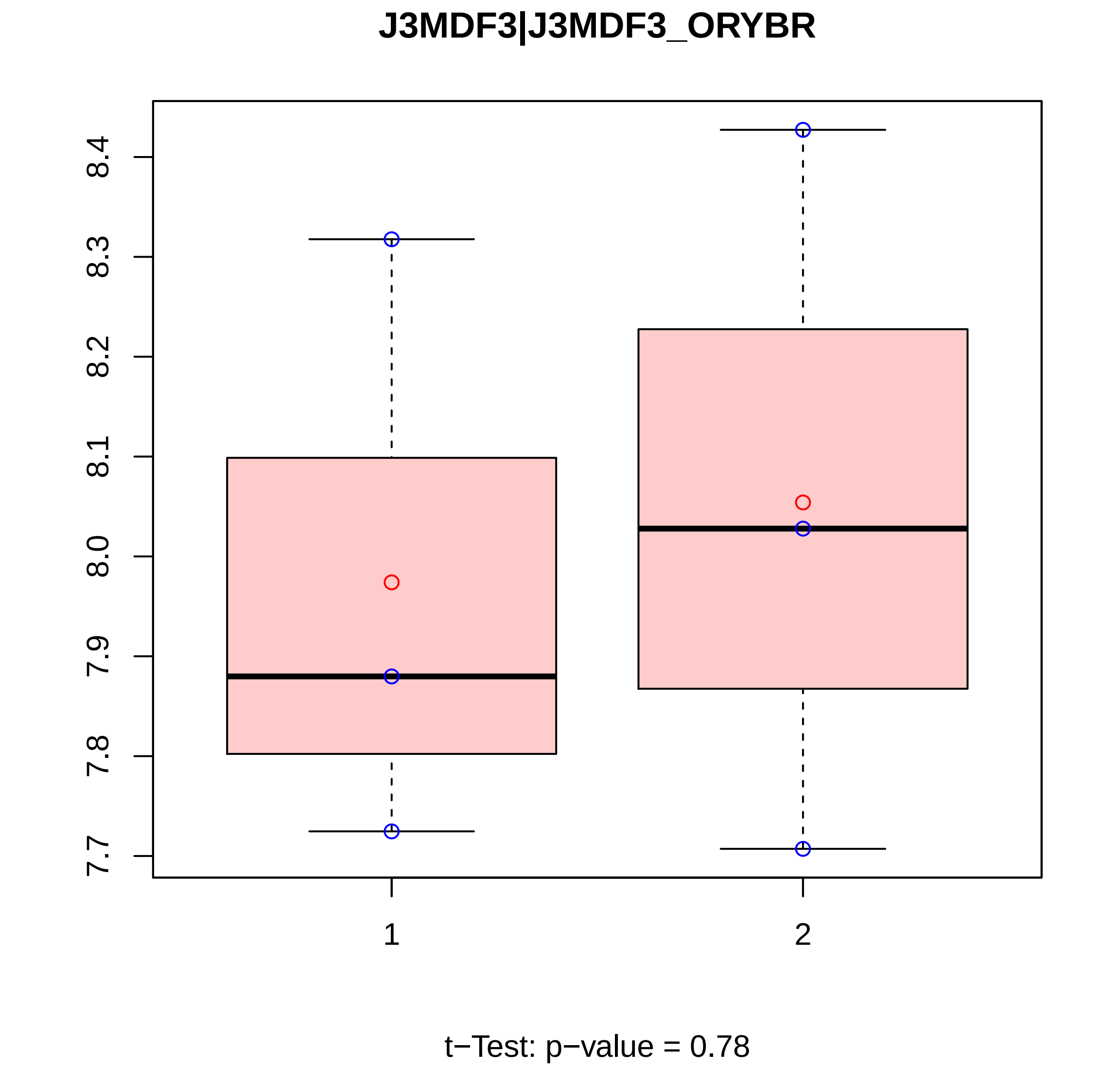

J3MDF3|J3MDF3_ORYBR
8.4
8.3
8.2
8.1
8.0
7.9
7.8
7.7
1
2
t−Test: p−value = 0.78

J3MDN2|J3MDN2_ORYBR

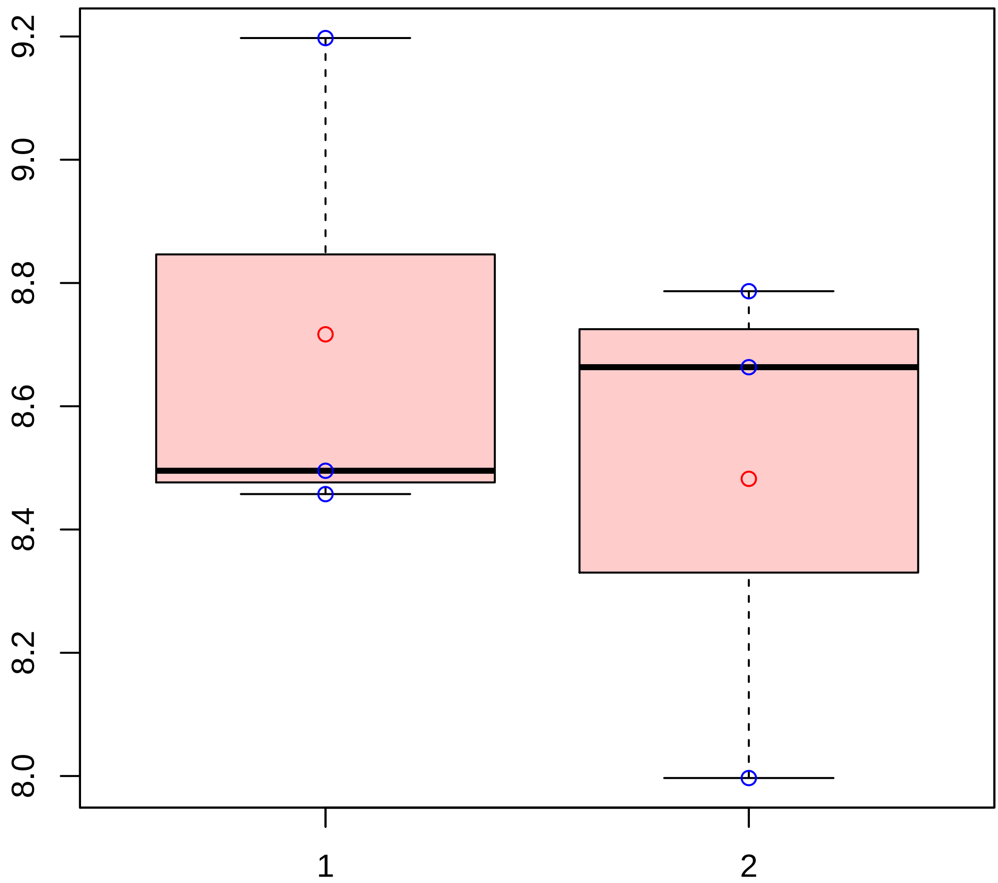

t−Test: p−value = 0.53

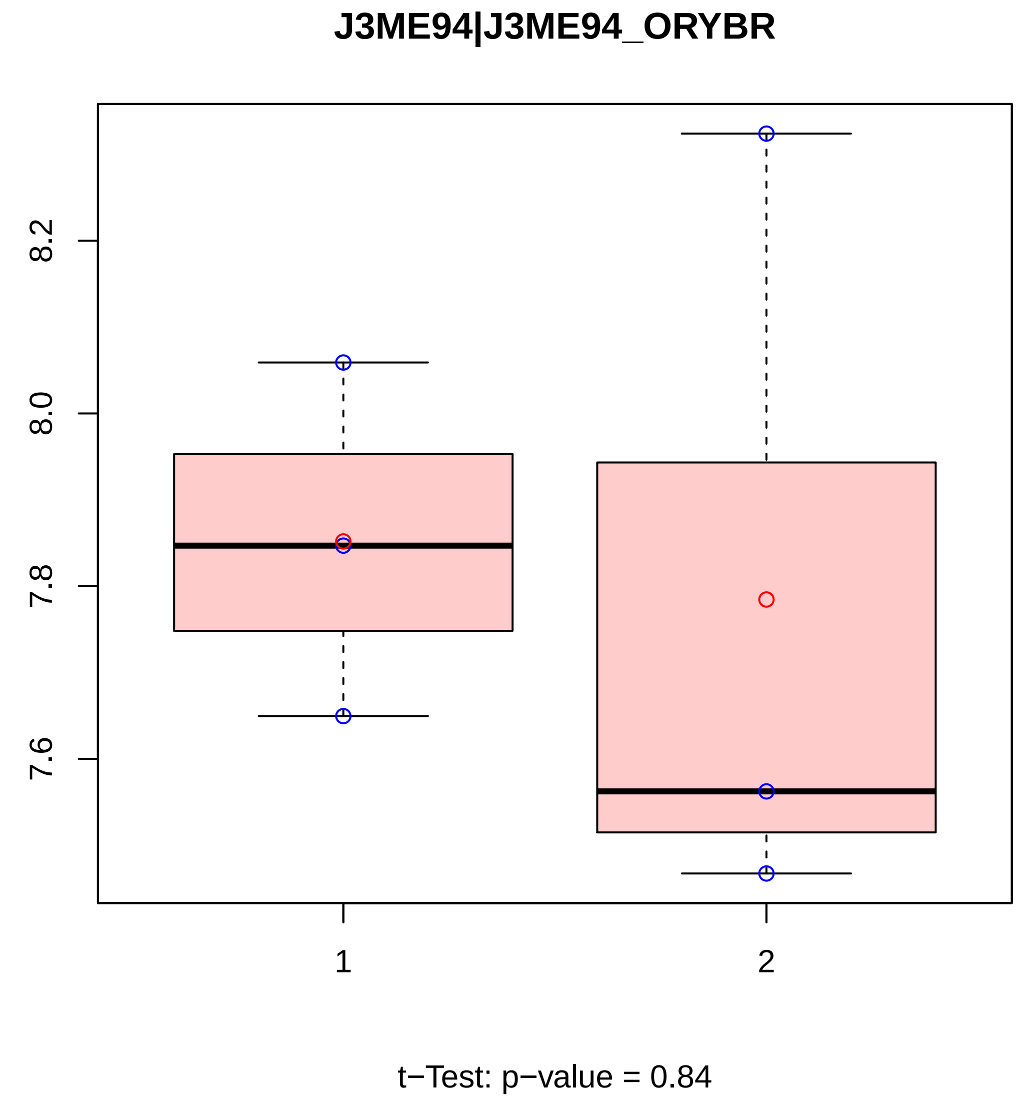
J3ME94|J3ME94_ORYBR
8.2
8.0
7.8
7.6
1
2
t−Test: p−value = 0.84

J3MEP9|J3MEP9_ORYBR

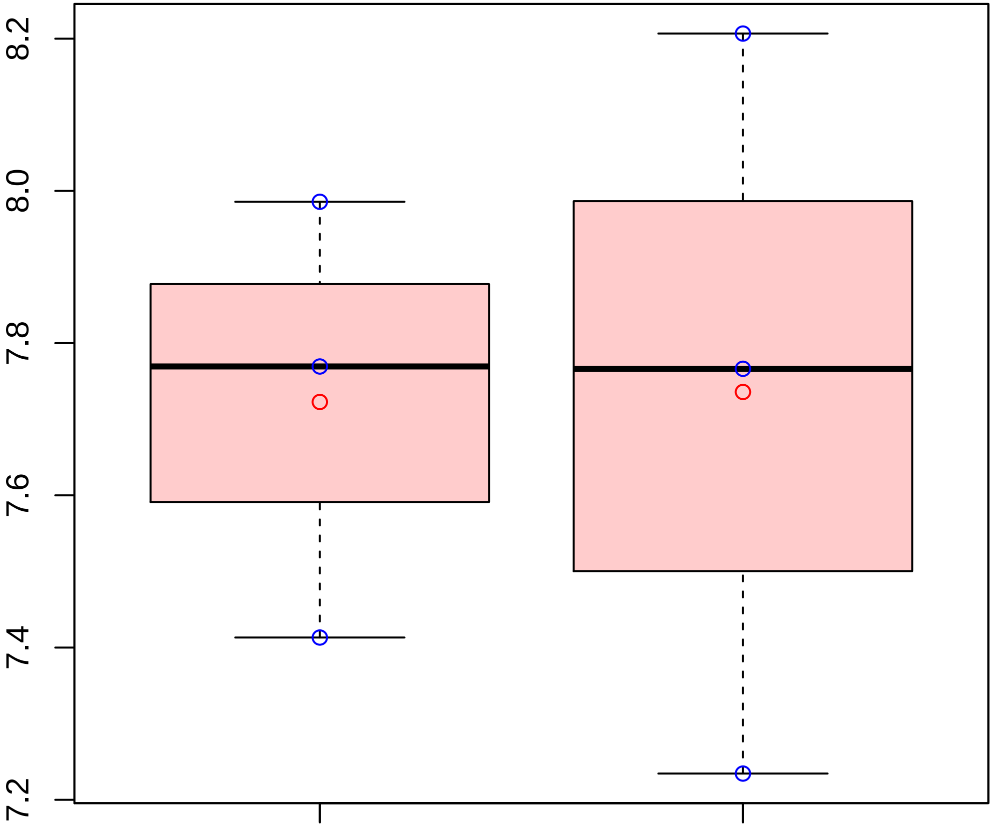

t−Test: p−value = 0.97

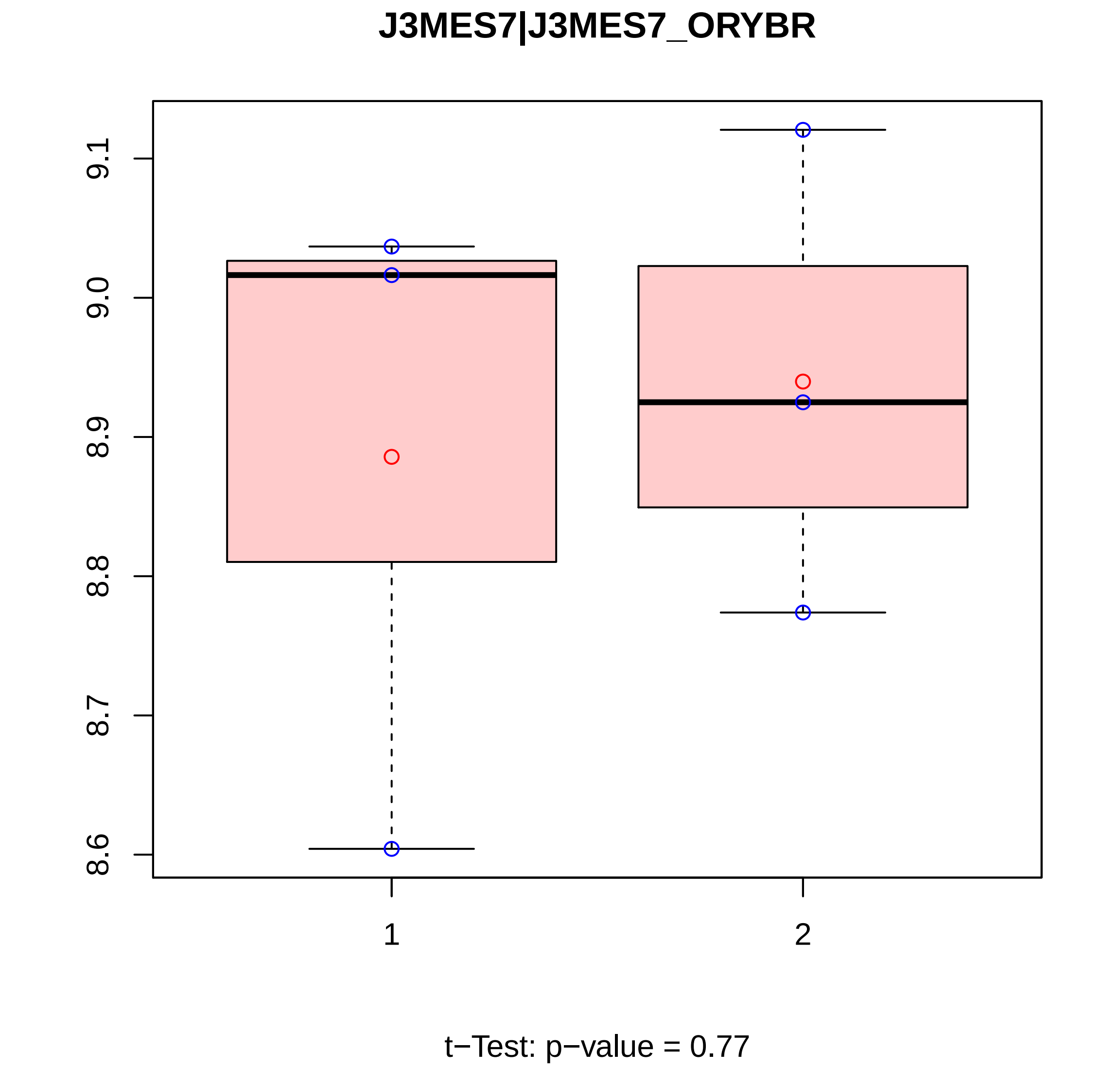
J3MES7|J3MES7_ORYBR
9.1
9.0
8.9
8.8
8.7
8.6
1
2
t−Test: p−value = 0.77

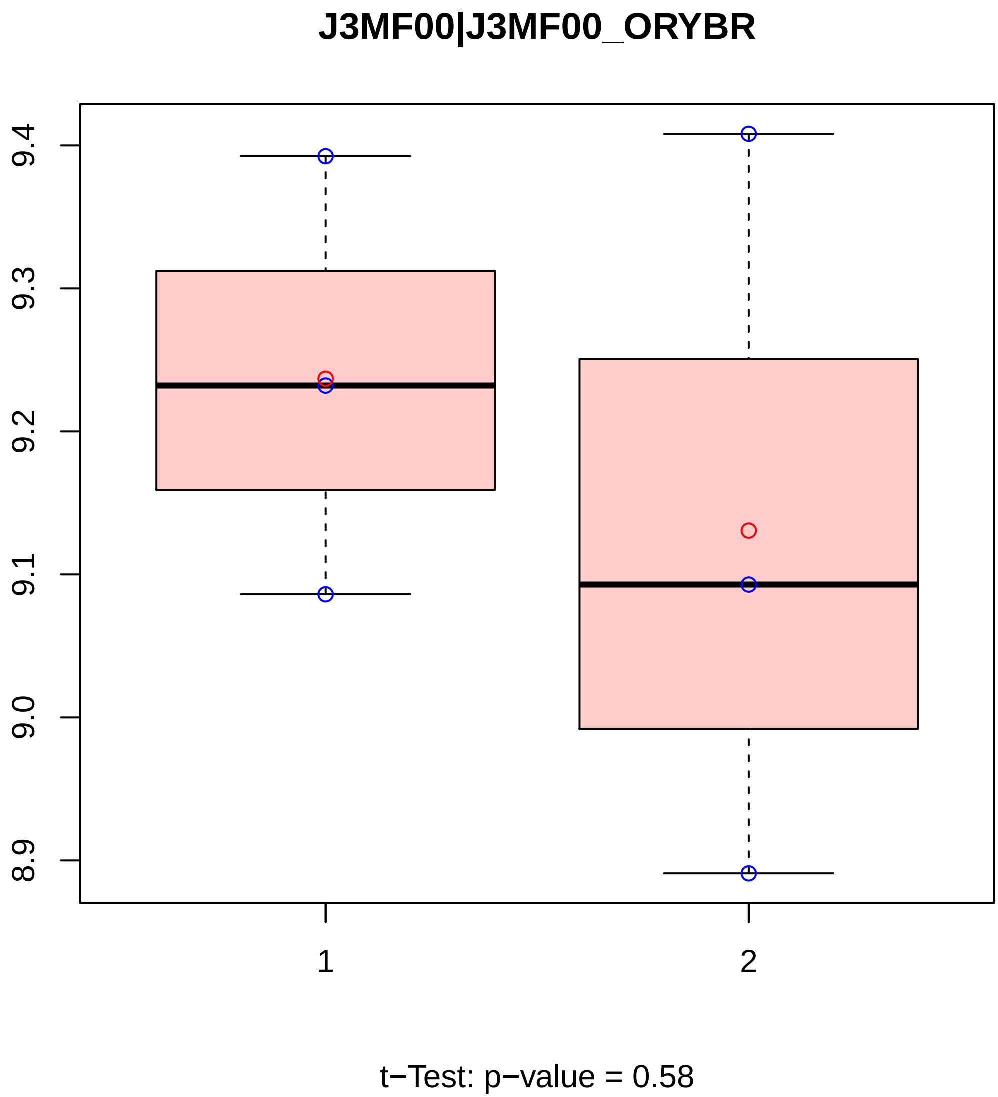
J3MF00|J3MF00_ORYBR
9.4
9.3
9.2
9.1
9.0
8.9
1
2
t−Test: p−value = 0.58

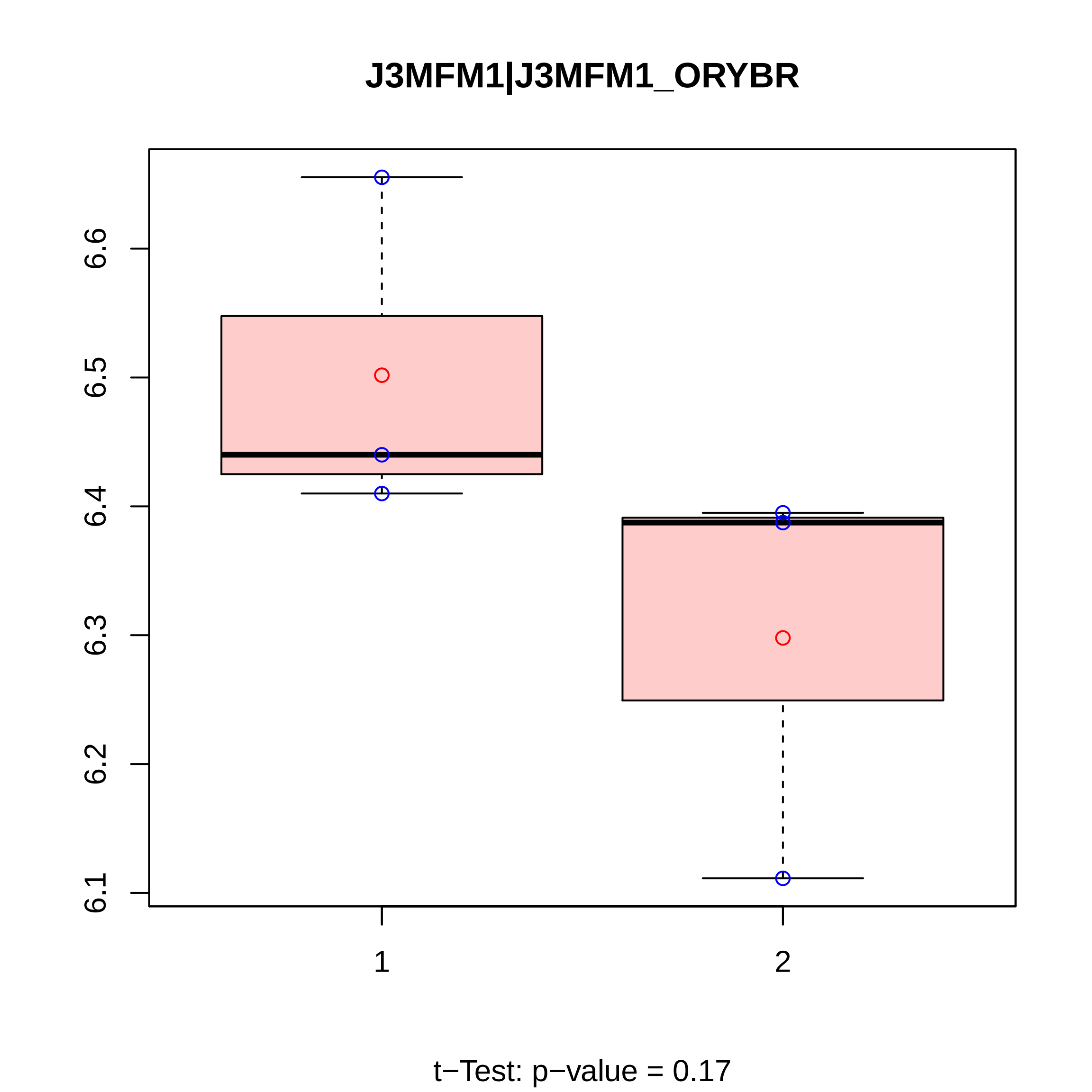
J3MFM1|J3MFM1_ORYBR
6.1
6.2
6.3
6.4
6.5
6.6
1
2
t−Test: p−value = 0.17

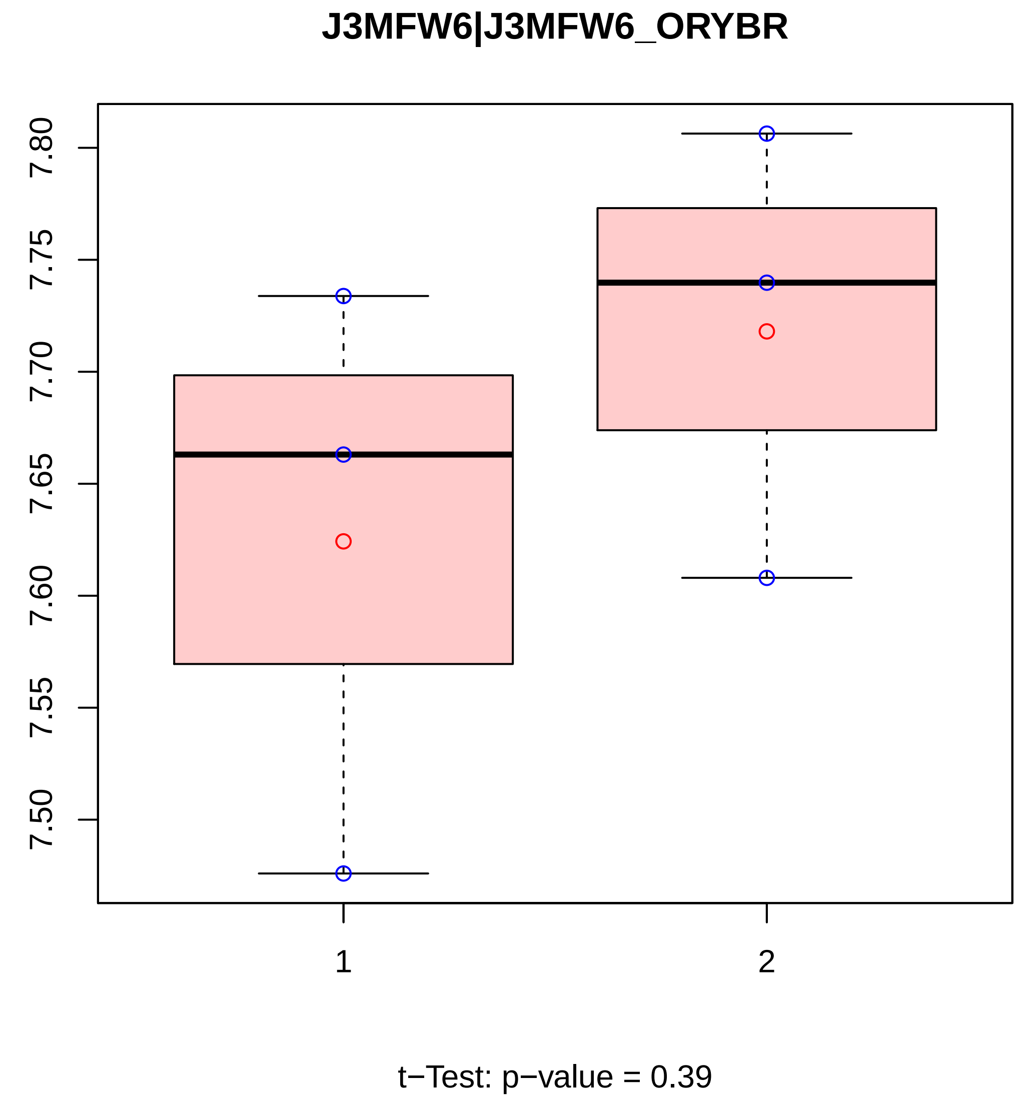
J3MFW6|J3MFW6_ORYBR
7.50
7.55
7.60
7.65
7.70
7.75
7.80
1
2
t−Test: p−value = 0.39

J3MGF6|J3MGF6_ORYBR

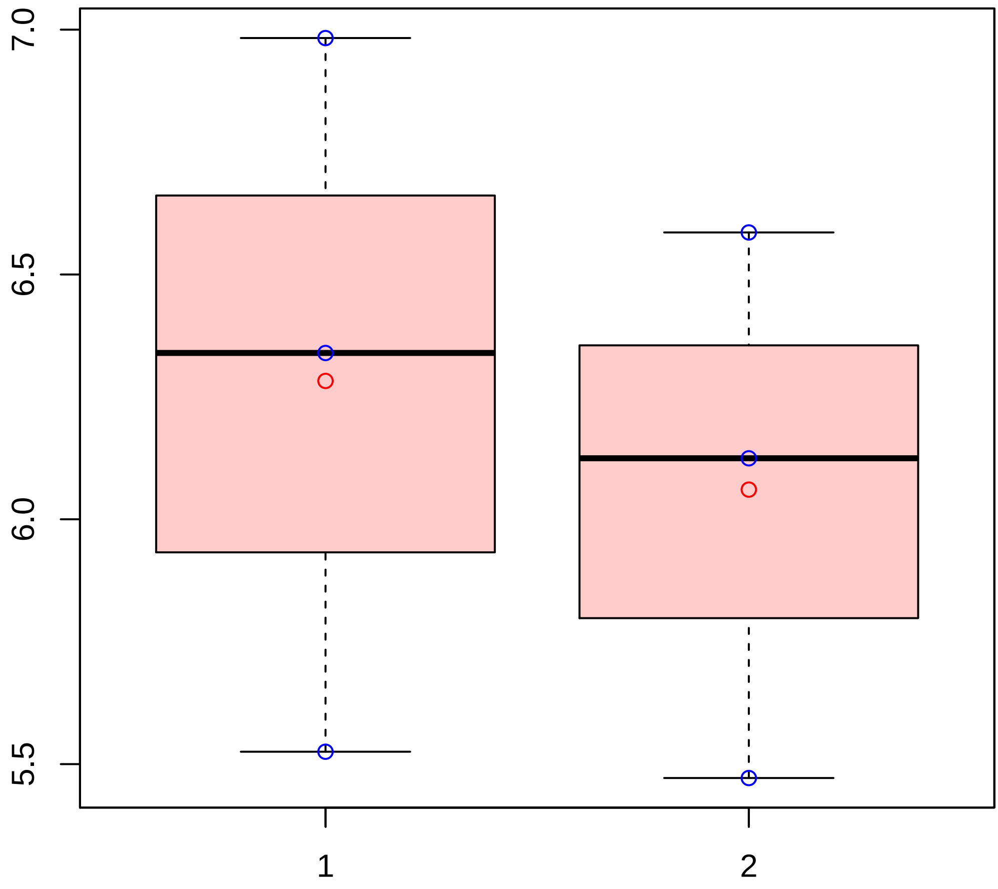

t−Test: p−value = 0.7

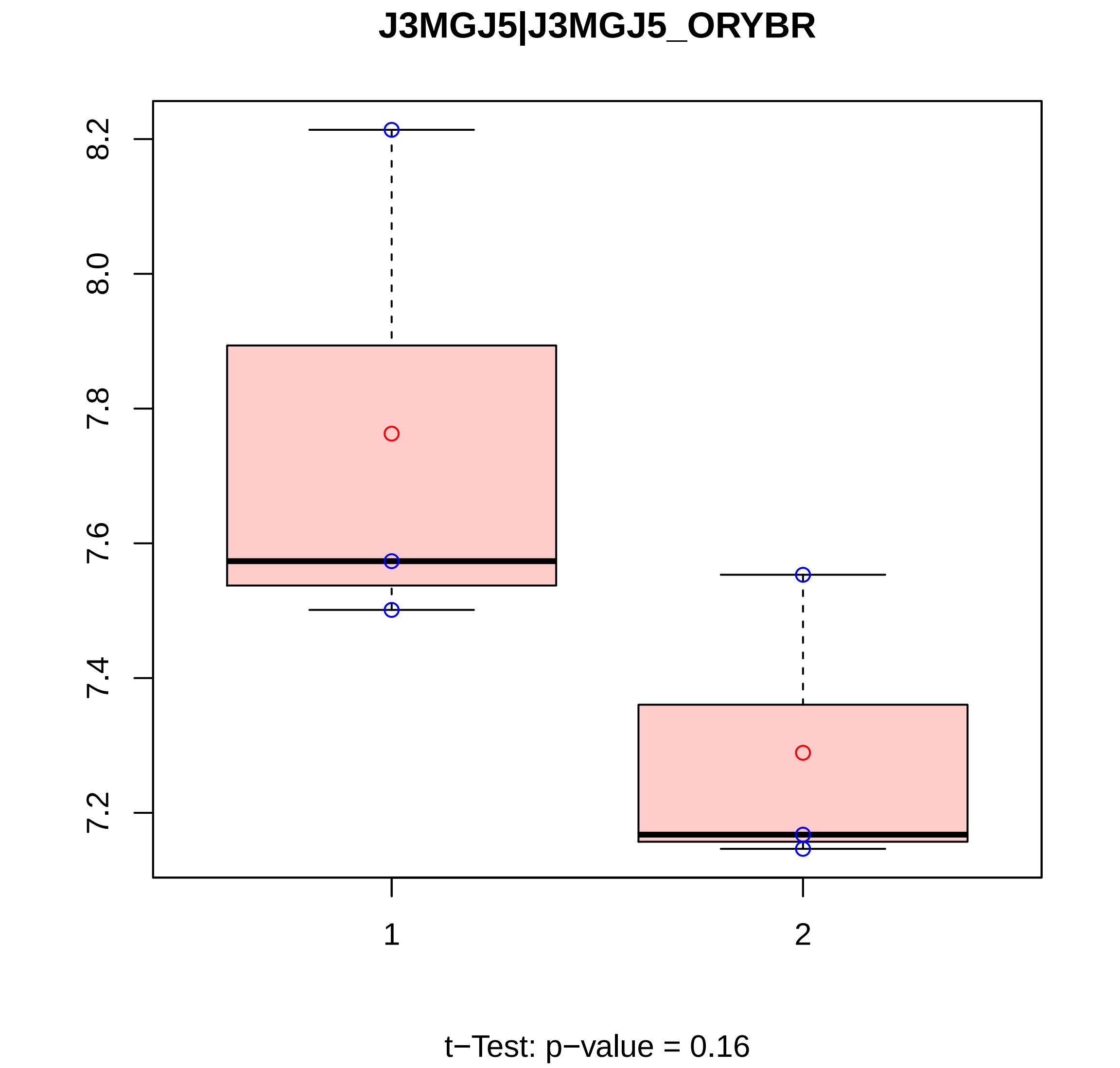
J3MGJ5|J3MGJ5_ORYBR
8.2
8.0
7.8
7.6
7.4
7.2
1
2
t−Test: p−value = 0.16

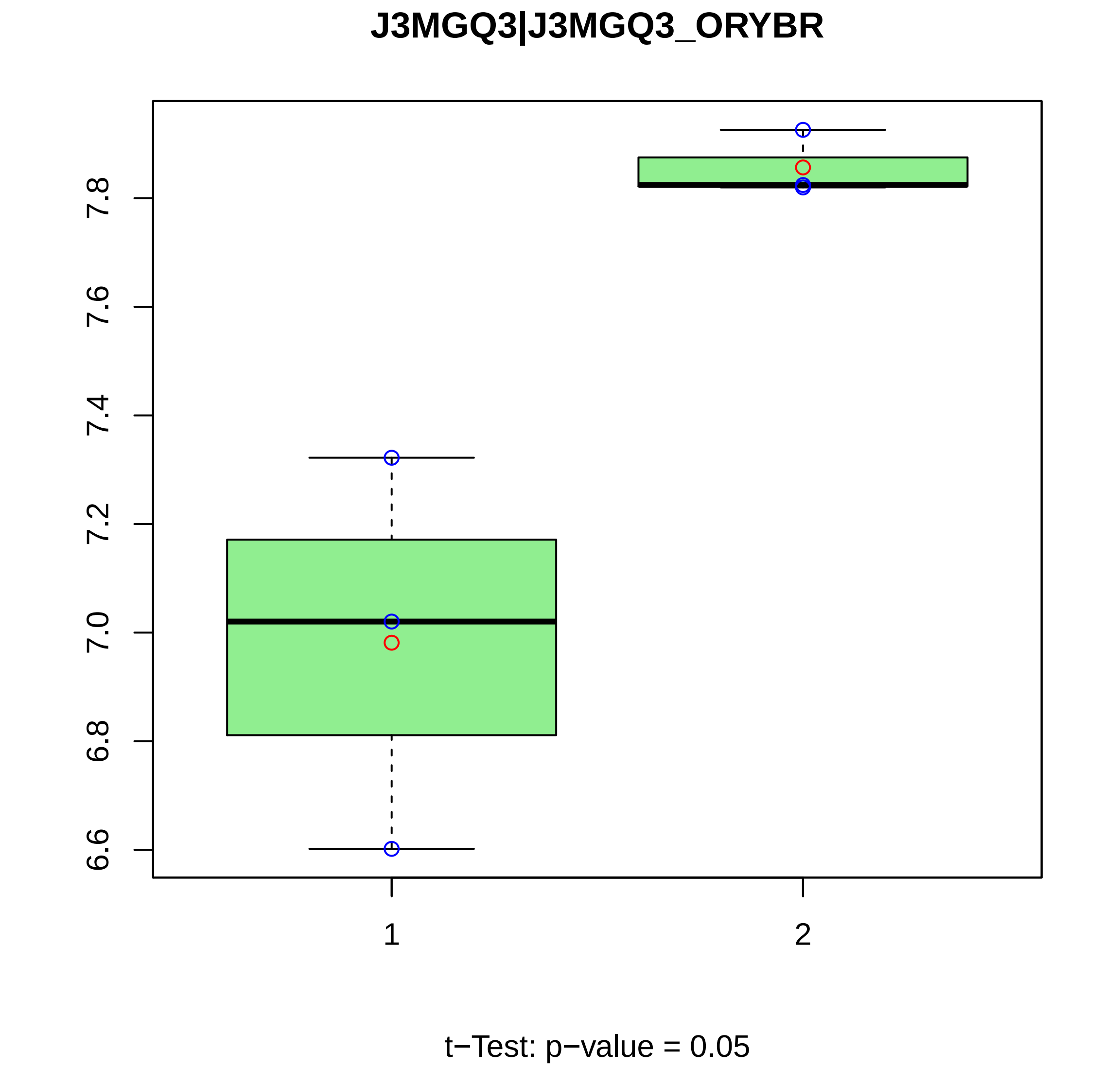
J3MGQ3|J3MGQ3_ORYBR
7.8
7.6
7.4
7.2
7.0
6.8
6.6
1
2
t−Test: p−value = 0.05

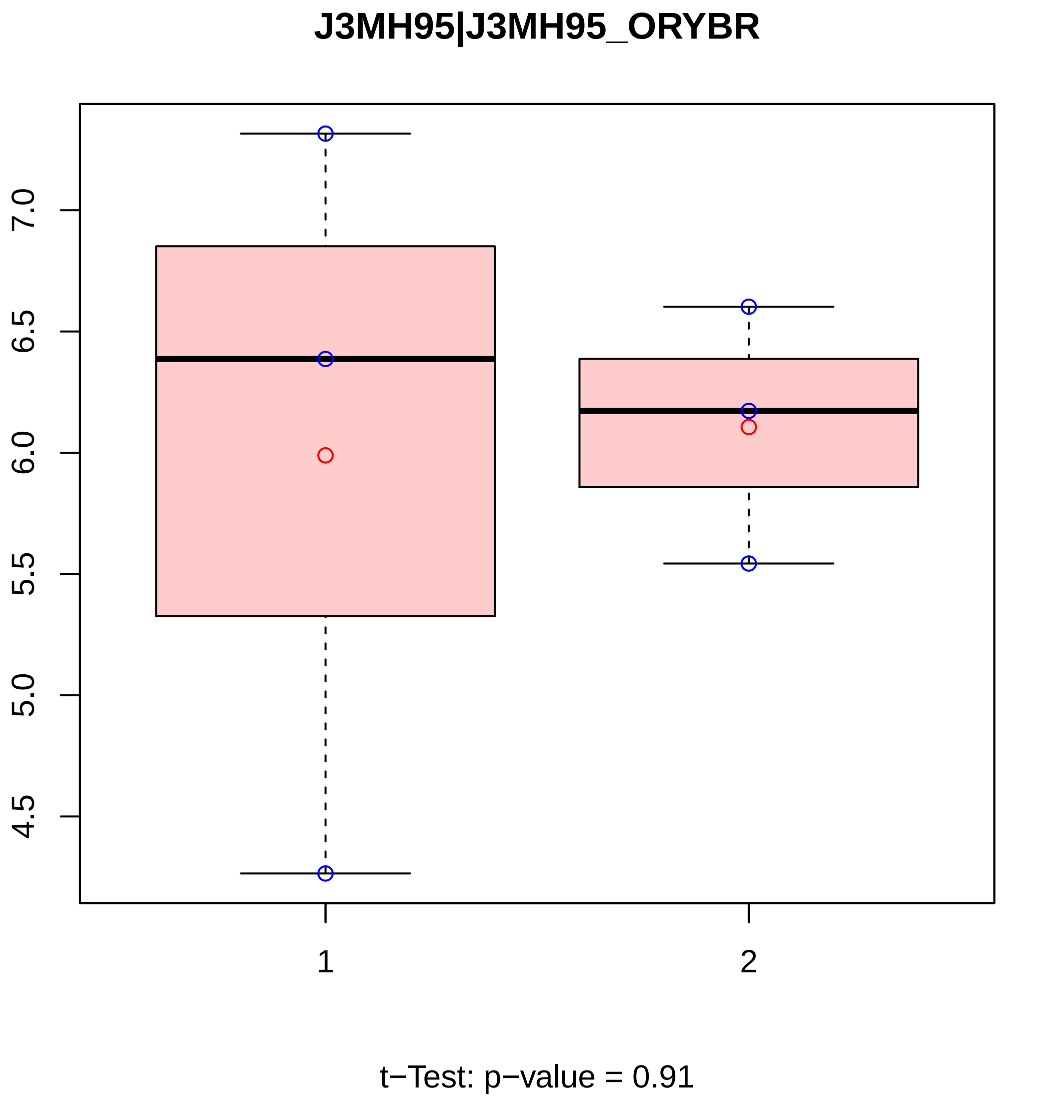

J3MH95|J3MH95_ORYBR
4.5
5.0
5.5
6.0
6.5
7.0
1
2
t−Test: p−value = 0.91

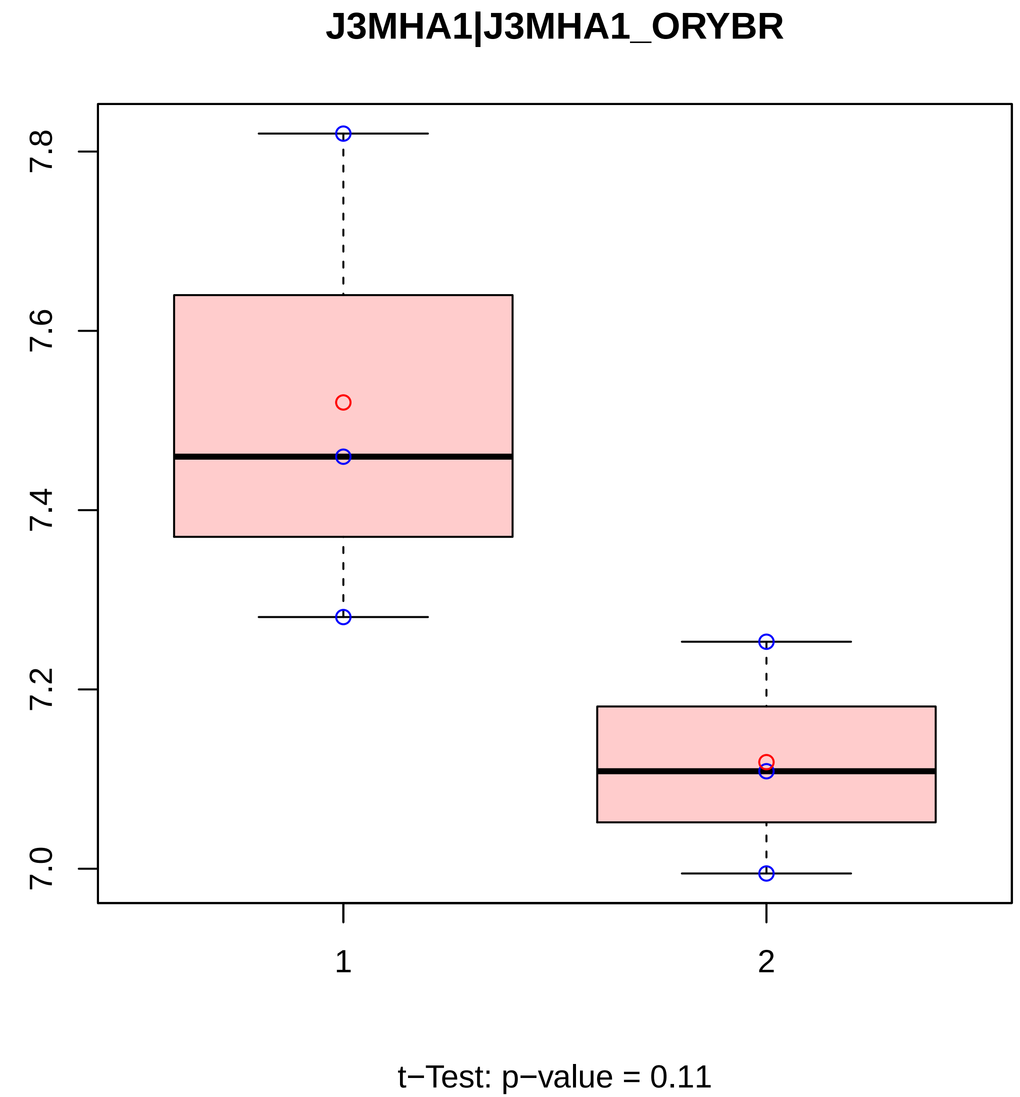
J3MHA1|J3MHA1_ORYBR
7.0
7.2
7.4
7.6
7.8
1
2
t−Test: p−value = 0.11

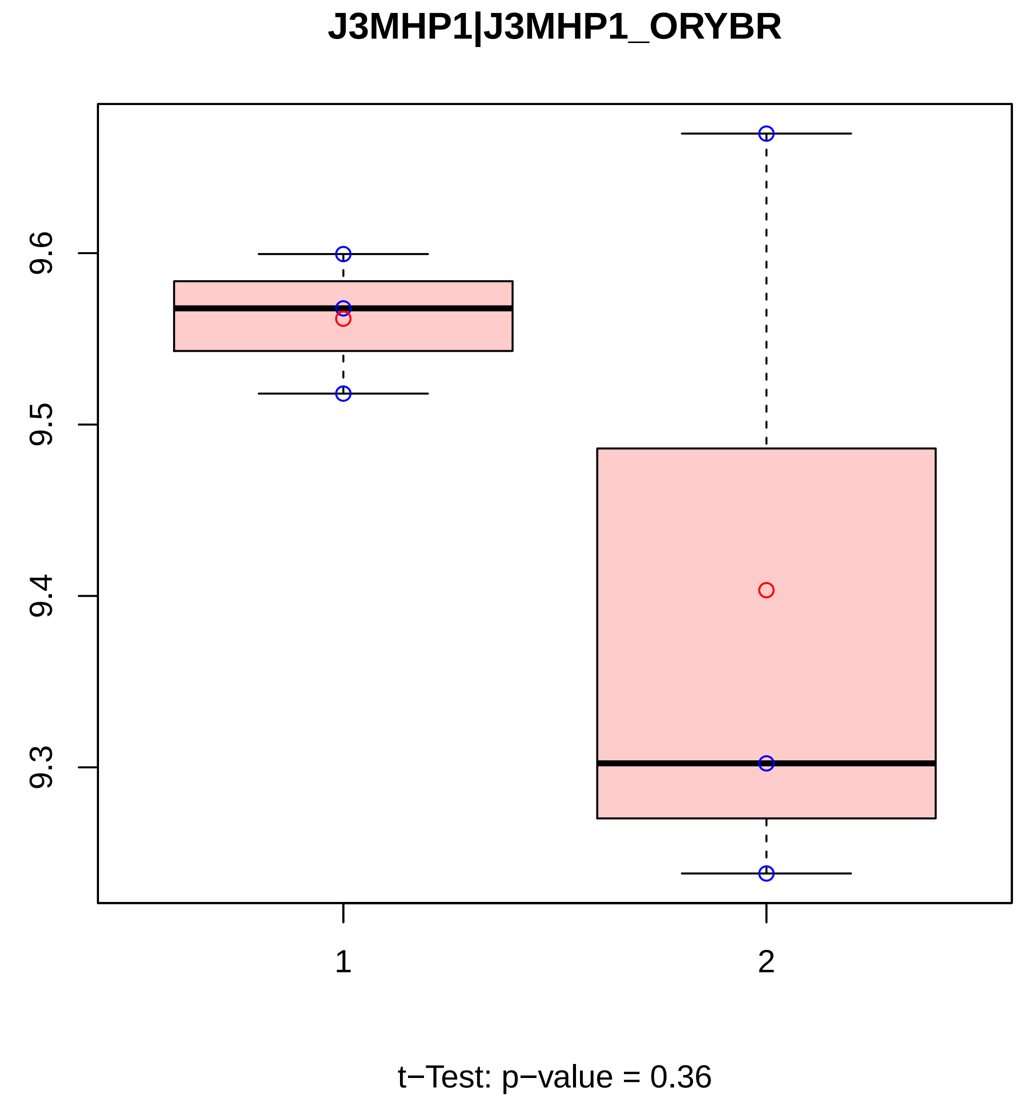
J3MHP1|J3MHP1_ORYBR
9.3
9.4
9.5
9.6
1
2
t−Test: p−value = 0.36

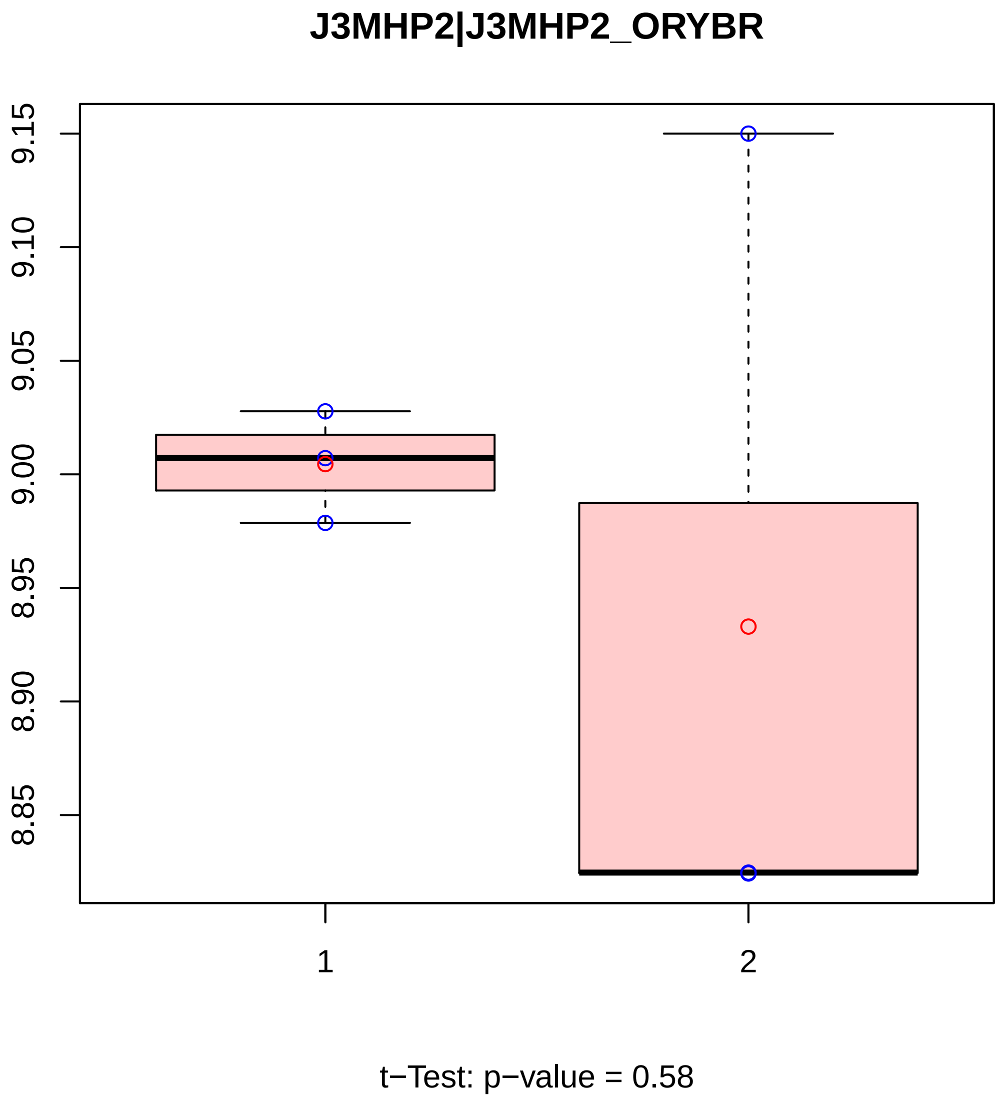
J3MHP2|J3MHP2_ORYBR
9.15
9.10
9.05
9.00
8.95
8.90
8.85
1
2
t−Test: p−value = 0.58

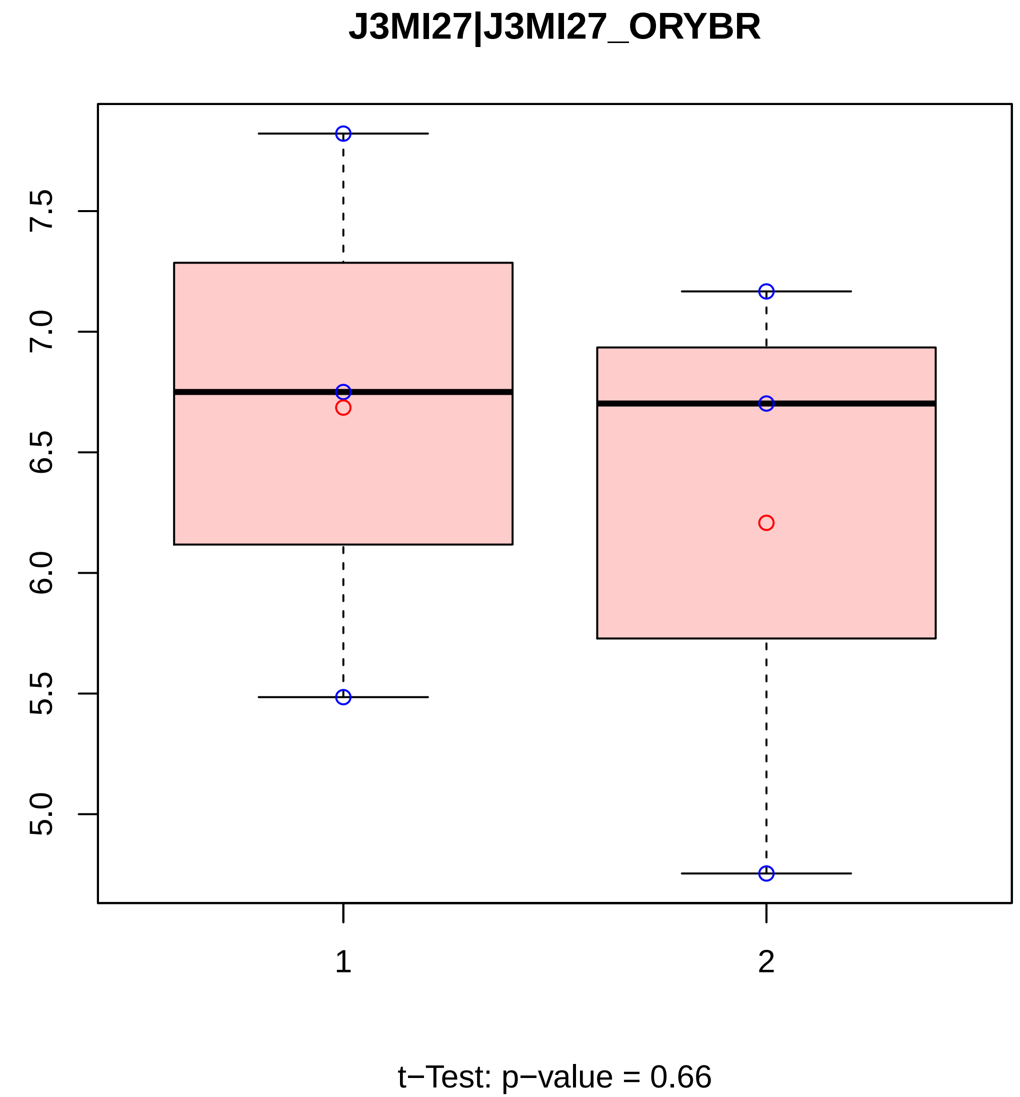
J3MI27|J3MI27_ORYBR
7.5
7.0
6.5
6.0
5.5
5.0
1
2
t−Test: p−value = 0.66

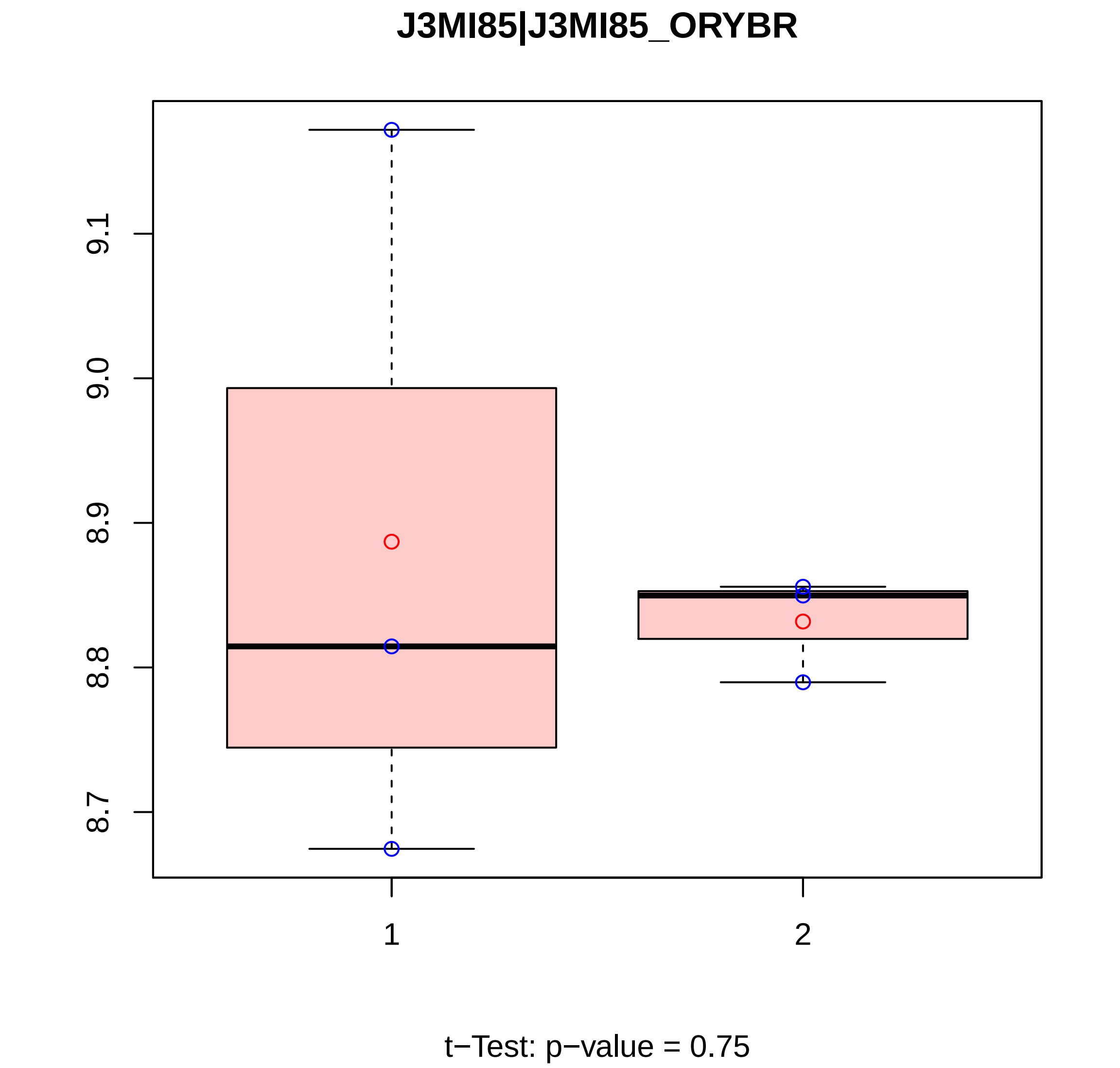

J3MI85|J3MI85_ORYBR
9.1
9.0
8.9
8.8
8.7
1
2
t−Test: p−value = 0.75

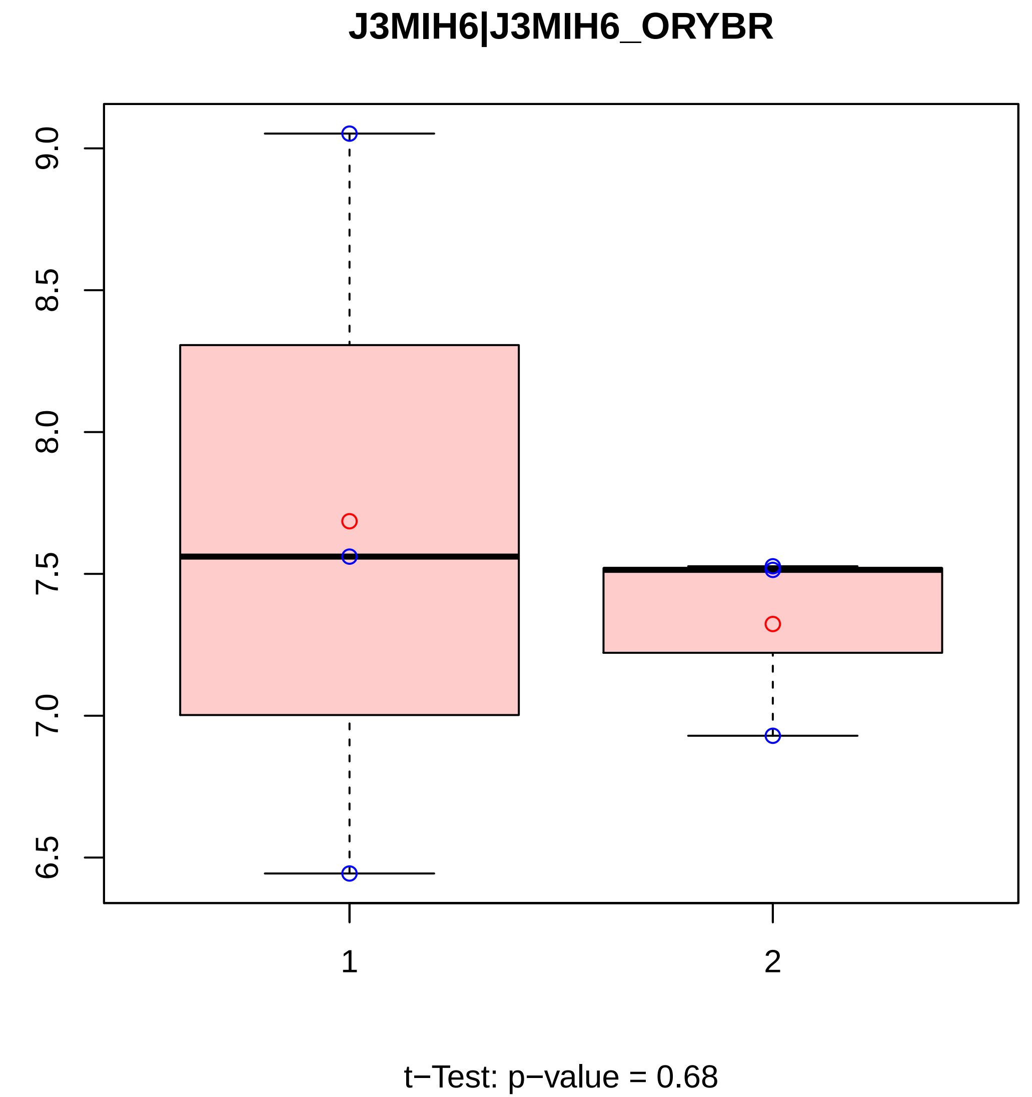

J3MIH6|J3MIH6_ORYBR
9.0
8.5
8.0
7.5
7.0
6.5
1
2
t−Test: p−value = 0.68

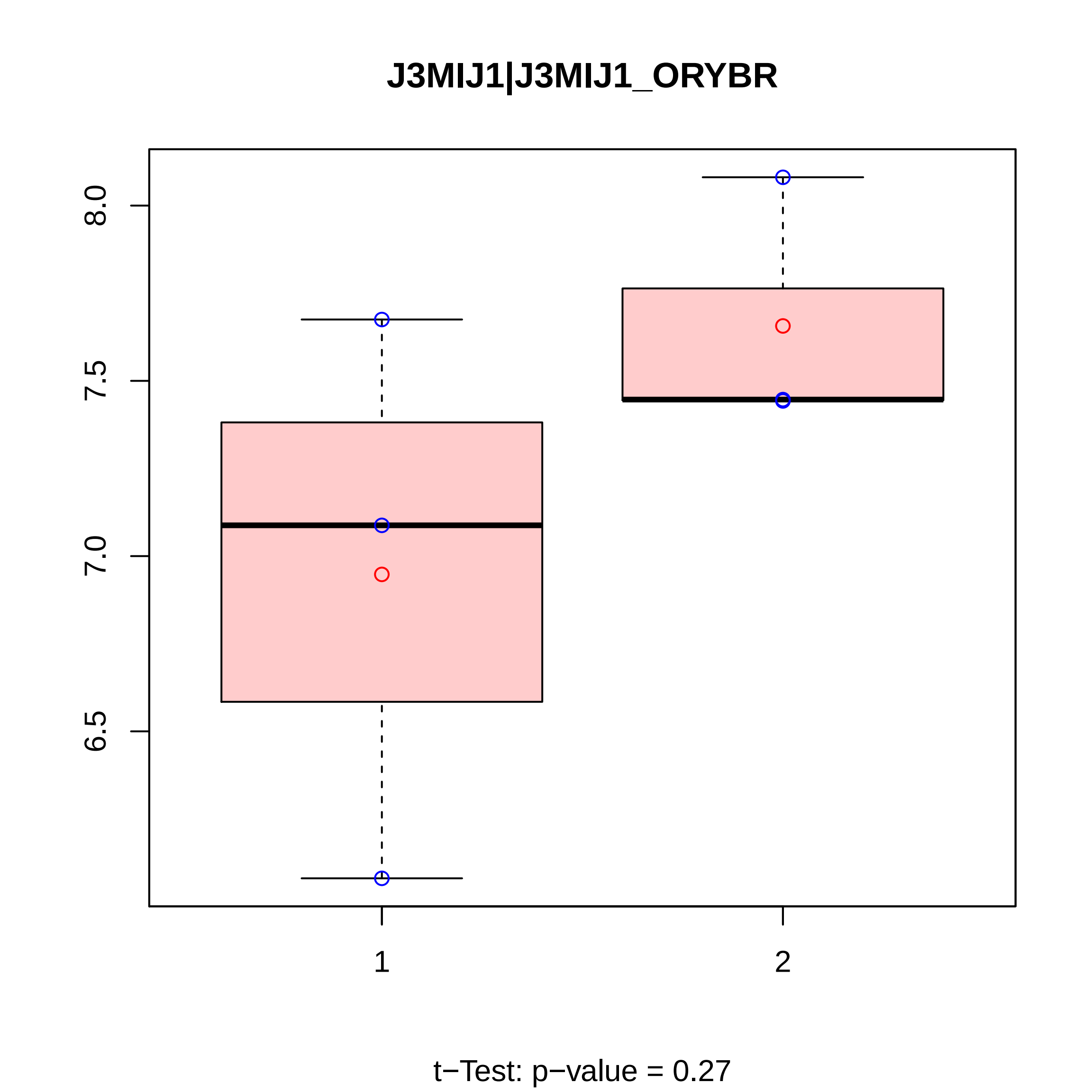
J3MIJ1|J3MIJ1_ORYBR
8.0
7.5
7.0
6.5
1
2
t−Test: p−value = 0.27

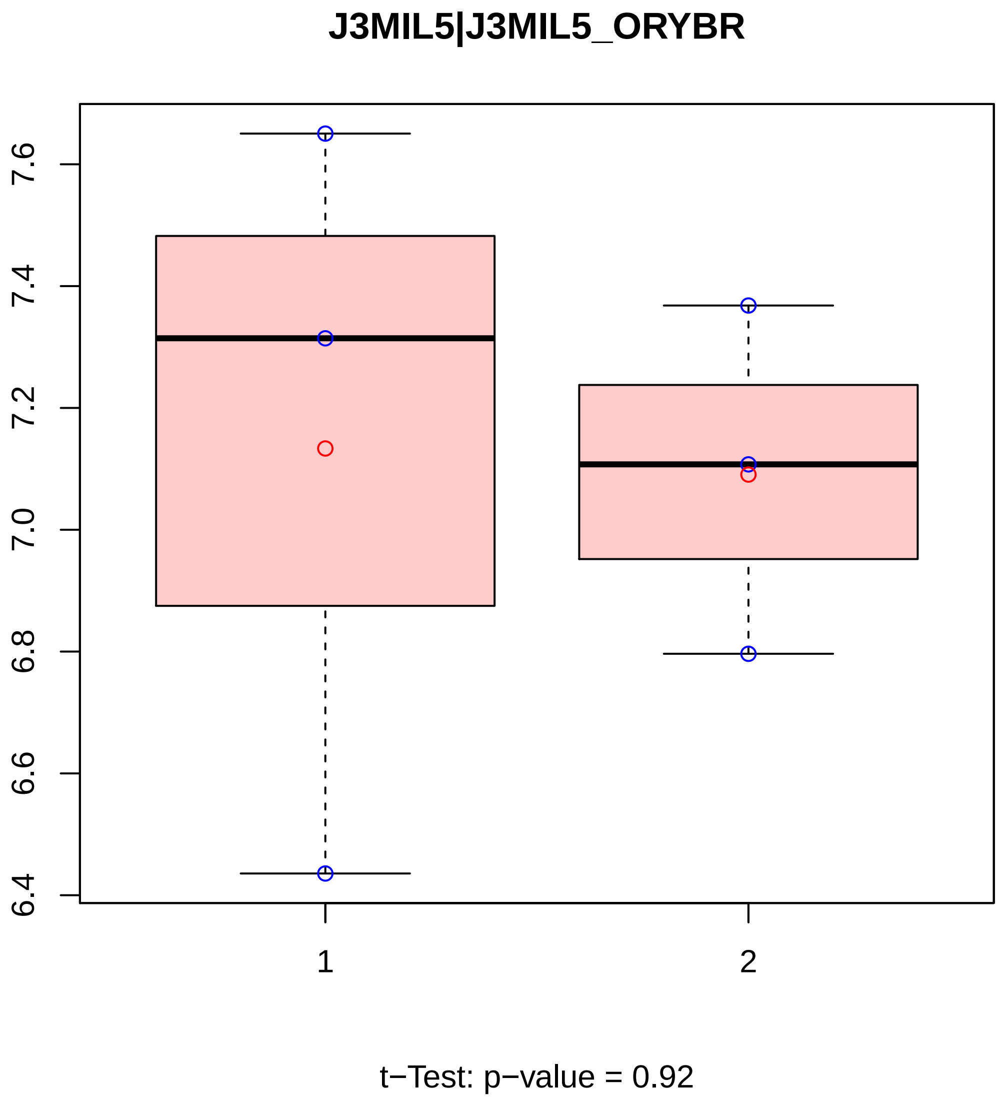
J3MIL5|J3MIL5_ORYBR
7.6
7.4
7.2
7.0
6.8
6.6
6.4
1
2
t−Test: p−value = 0.92

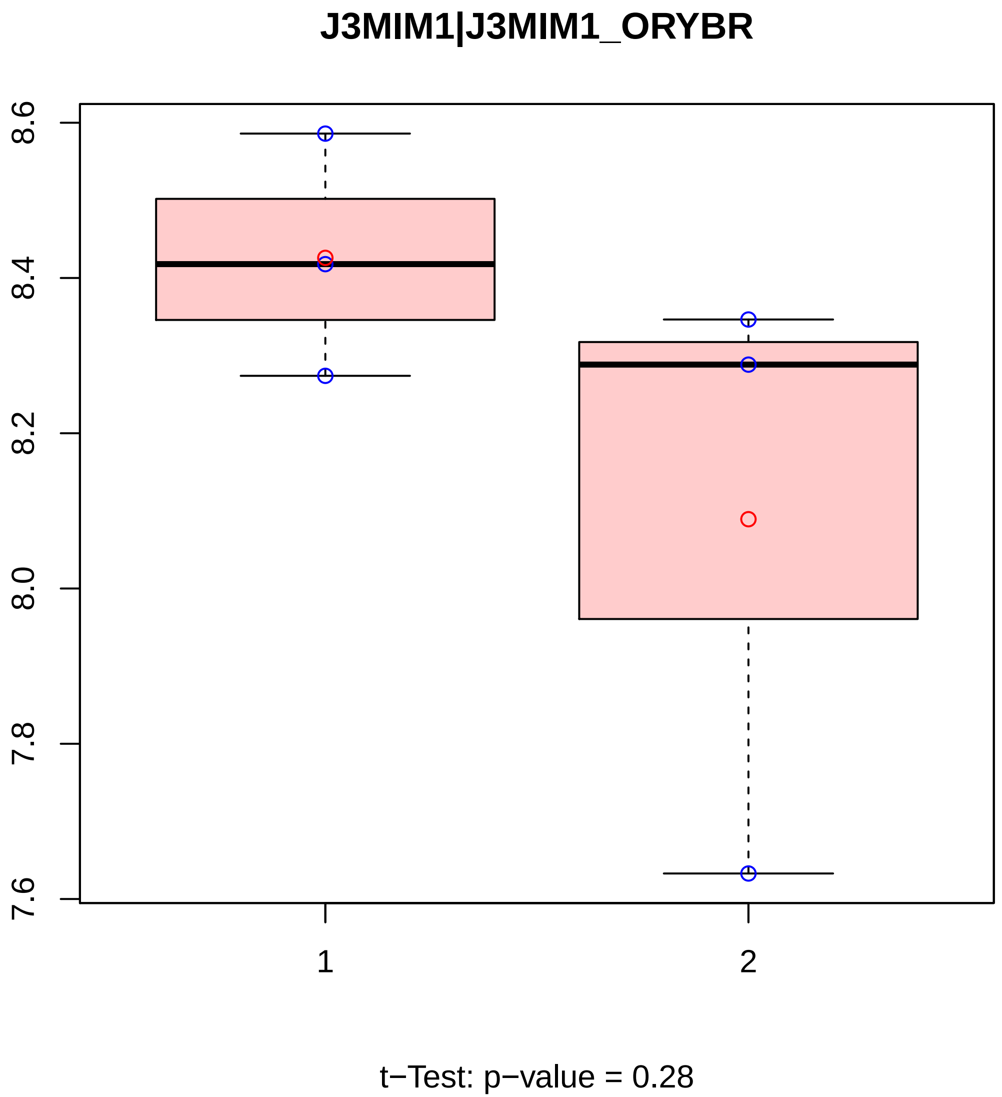
J3MIM1|J3MIM1_ORYBR
8.6
8.4
8.2
8.0
7.8
7.6
1
2
t−Test: p−value = 0.28

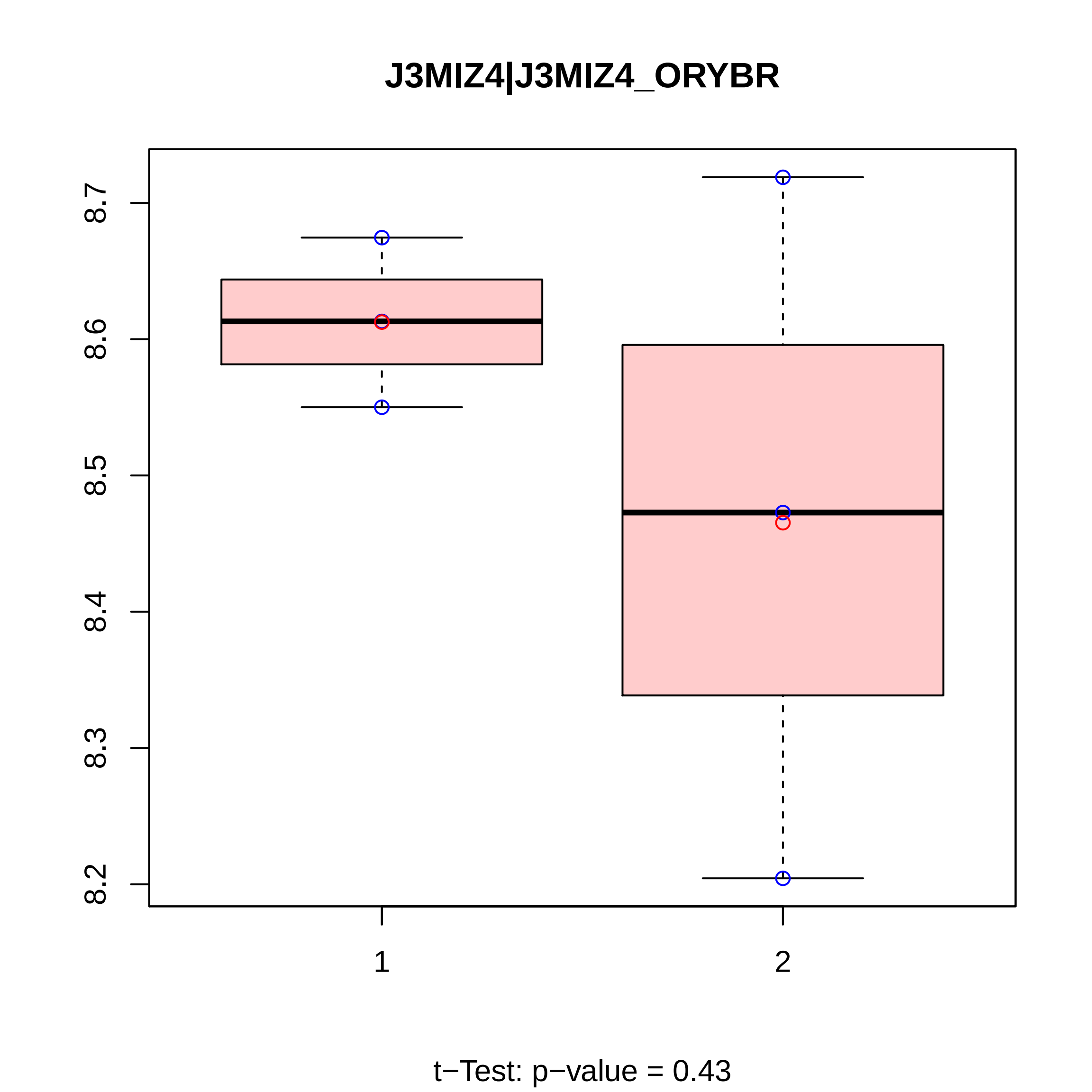
J3MIZ4|J3MIZ4_ORYBR
8.2
8.3
8.4
8.5
8.6
8.7
1
2
t−Test: p−value = 0.43

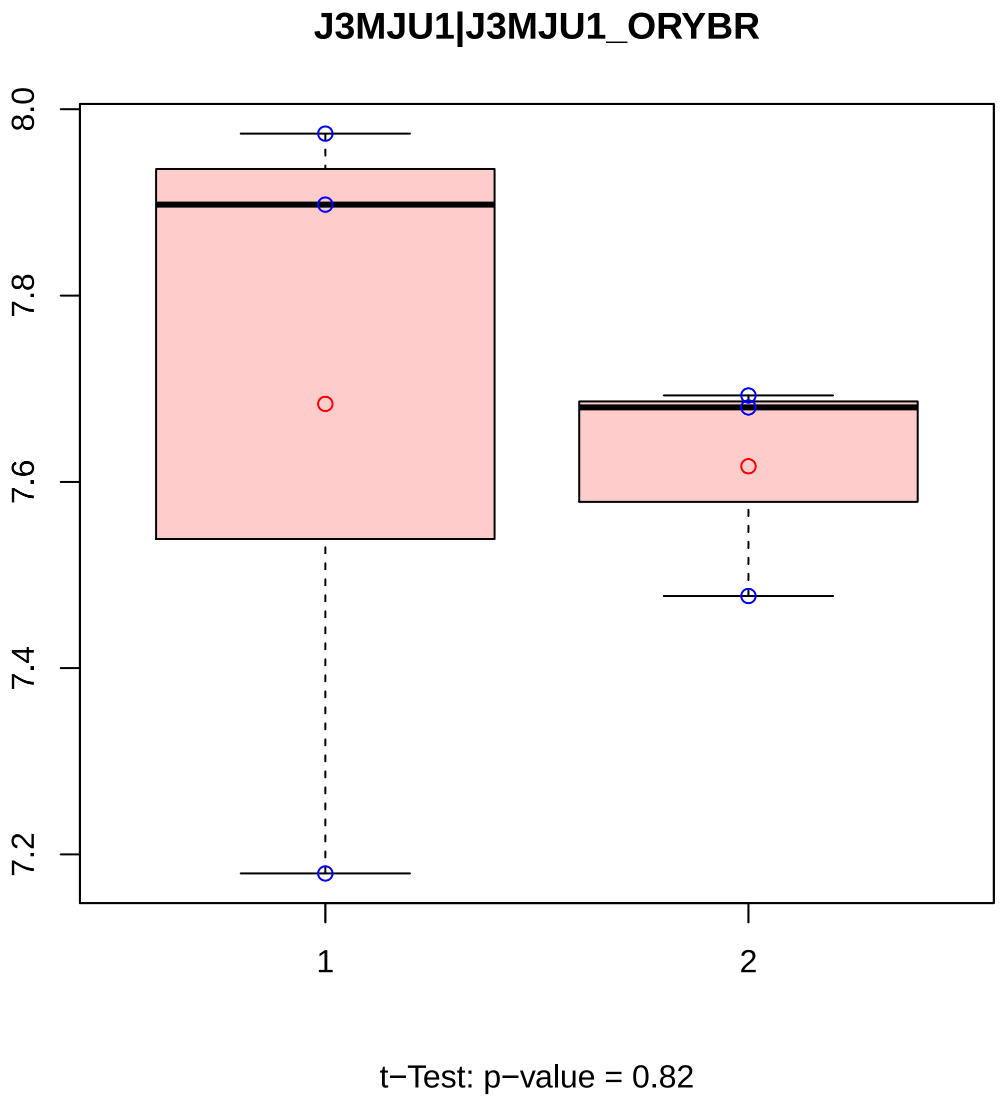
J3MJU1|J3MJU1_ORYBR
8.0
7.8
7.6
7.4
7.2
1
2
t−Test: p−value = 0.82

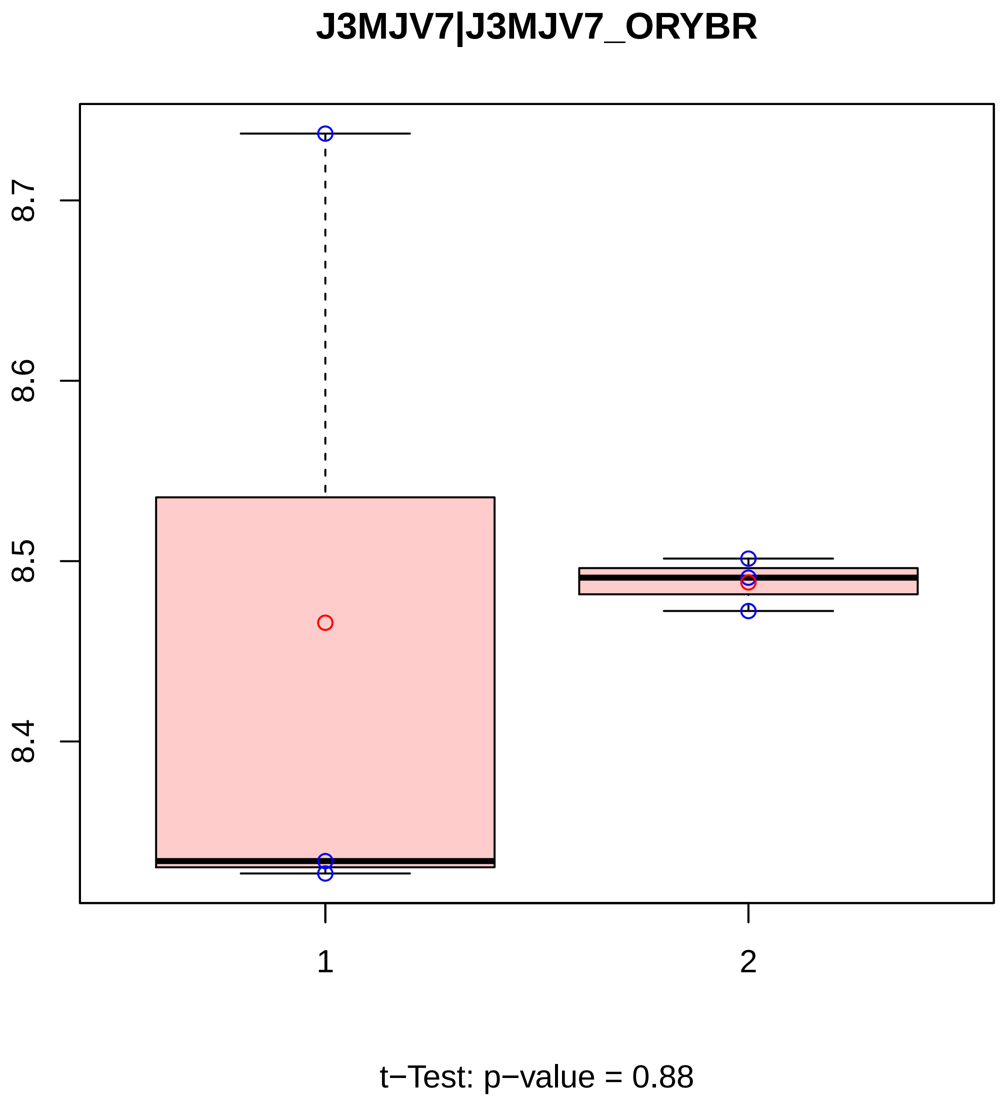
J3MJV7|J3MJV7_ORYBR
8.4
8.5
8.6
8.7
1
2
t−Test: p−value = 0.88

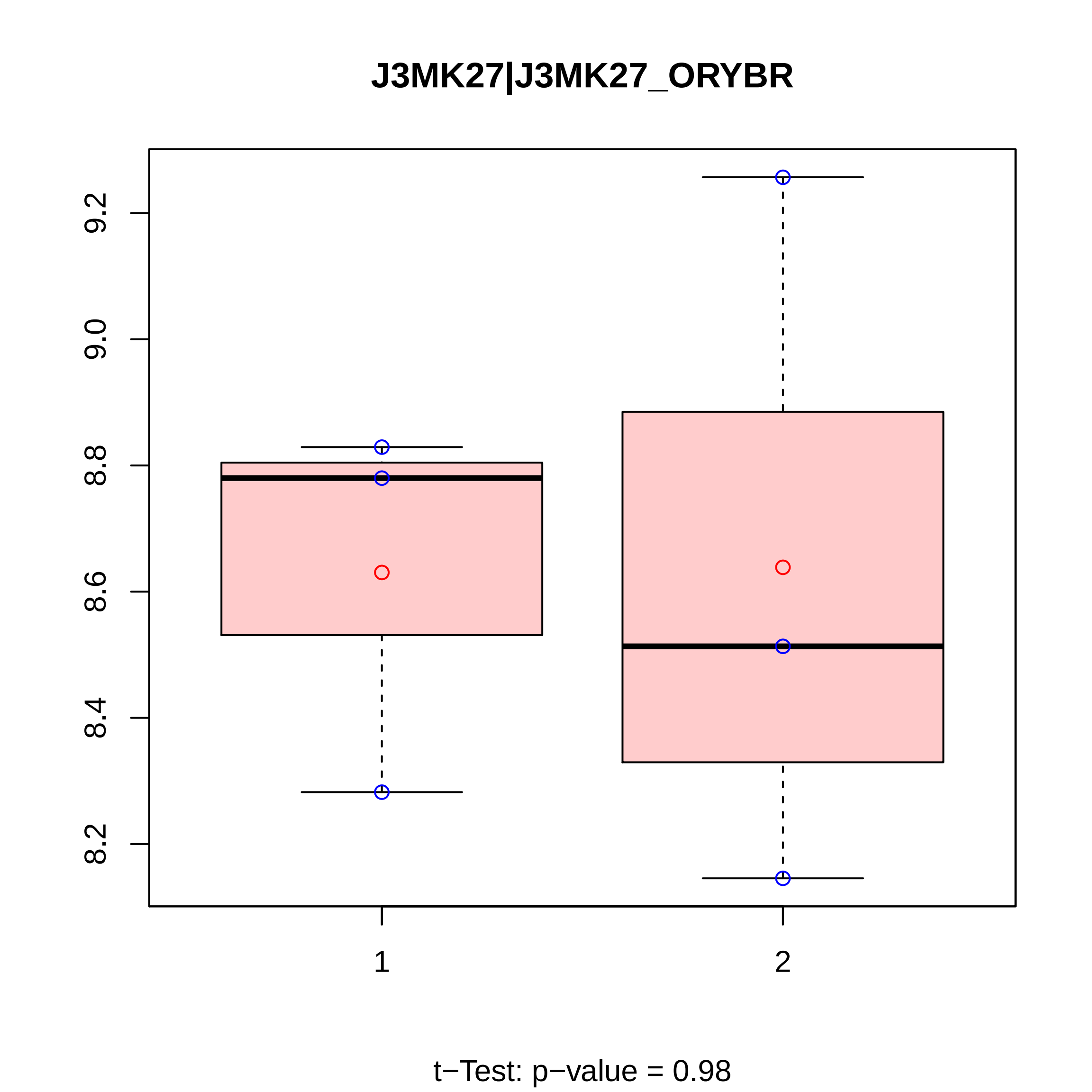
J3MK27|J3MK27_ORYBR
9.2
9.0
8.8
8.6
8.4
8.2
1
2
t−Test: p−value = 0.98

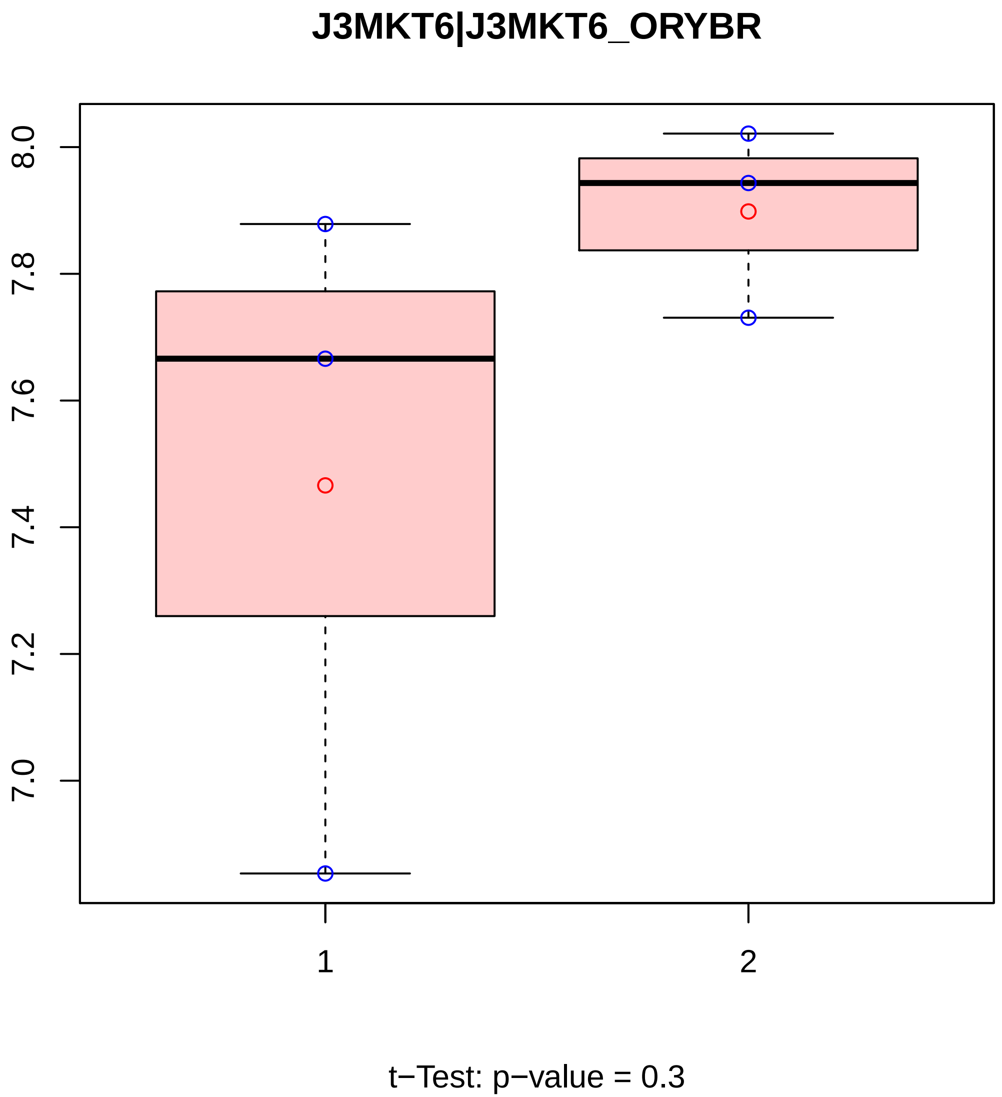
J3MKT6|J3MKT6_ORYBR
8.0
7.8
7.6
7.4
7.2
7.0
1
2
t−Test: p−value = 0.3

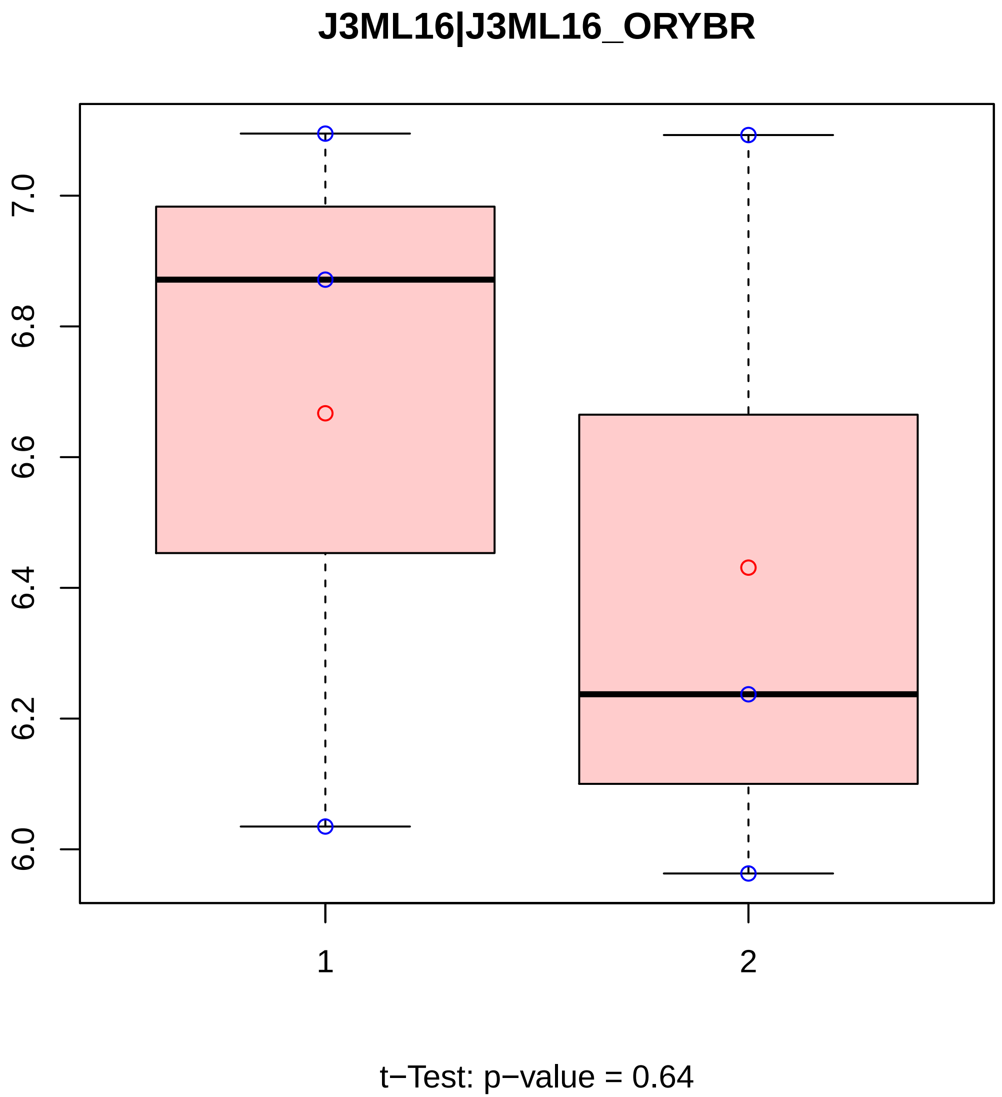

J3ML16|J3ML16_ORYBR
7.0
6.8
6.6
6.4
6.2
6.0
1
2
t−Test: p−value = 0.64

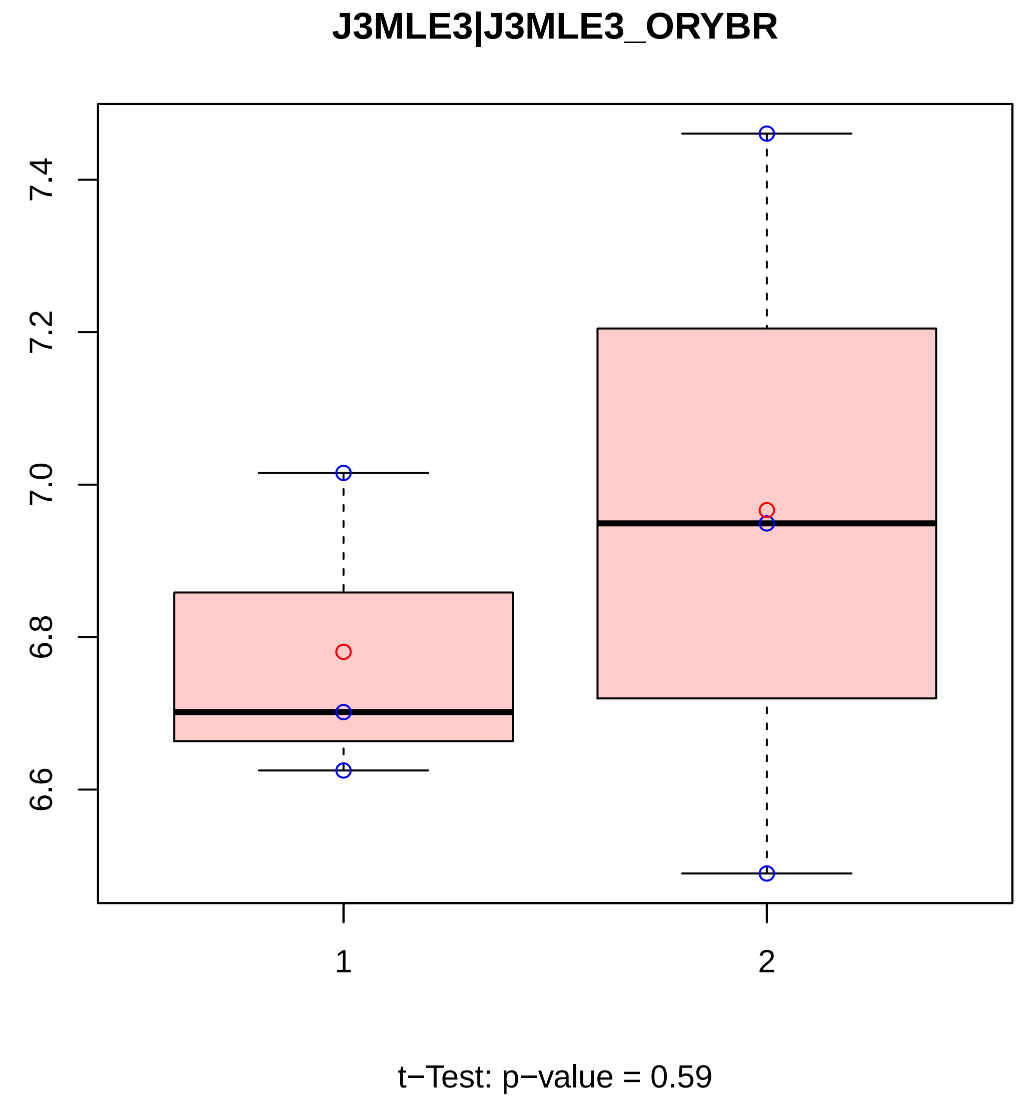

J3MLE3|J3MLE3_ORYBR
7.4
7.2
7.0
6.8
6.6
1
2
t−Test: p−value = 0.59

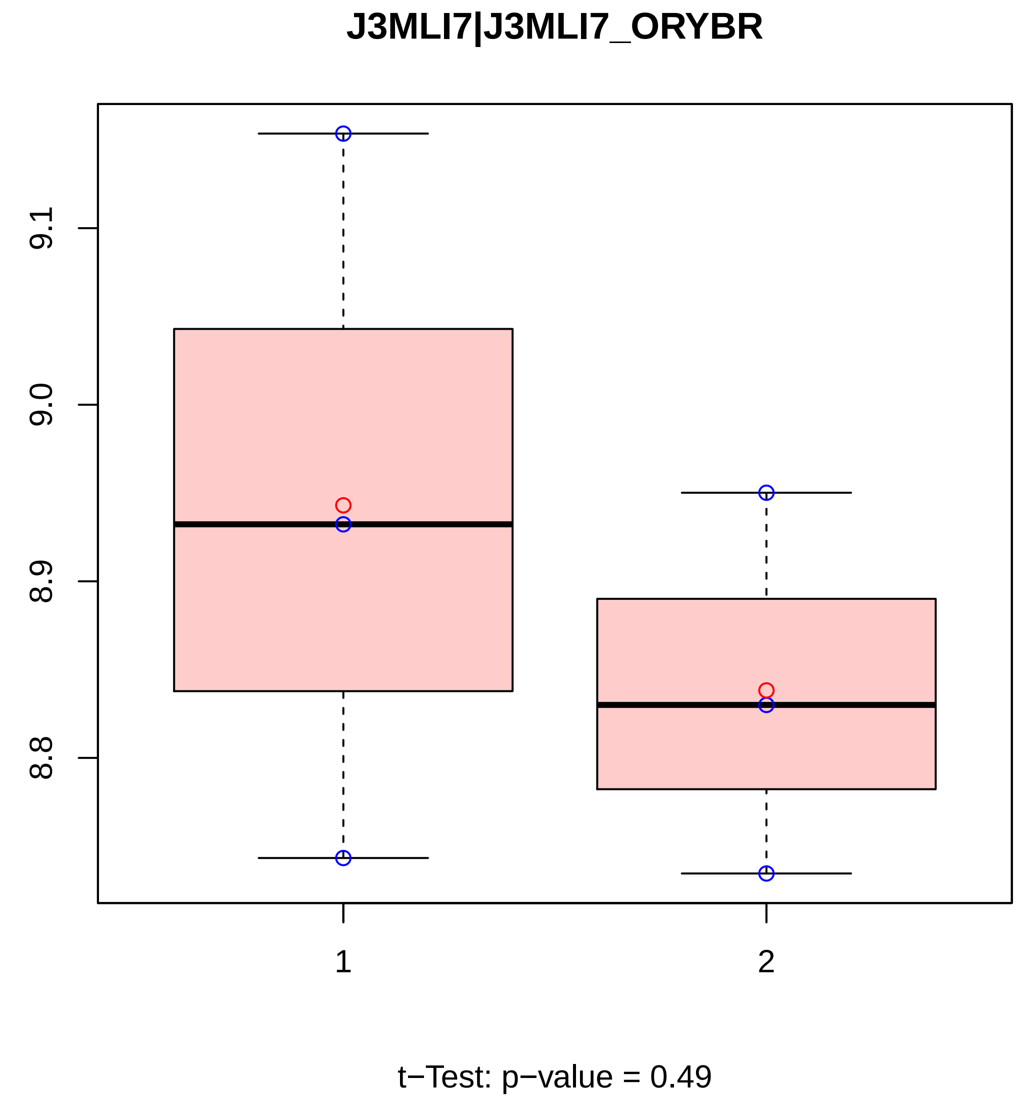
J3MLI7|J3MLI7_ORYBR
9.1
9.0
8.9
8.8
1
2
t−Test: p−value = 0.49

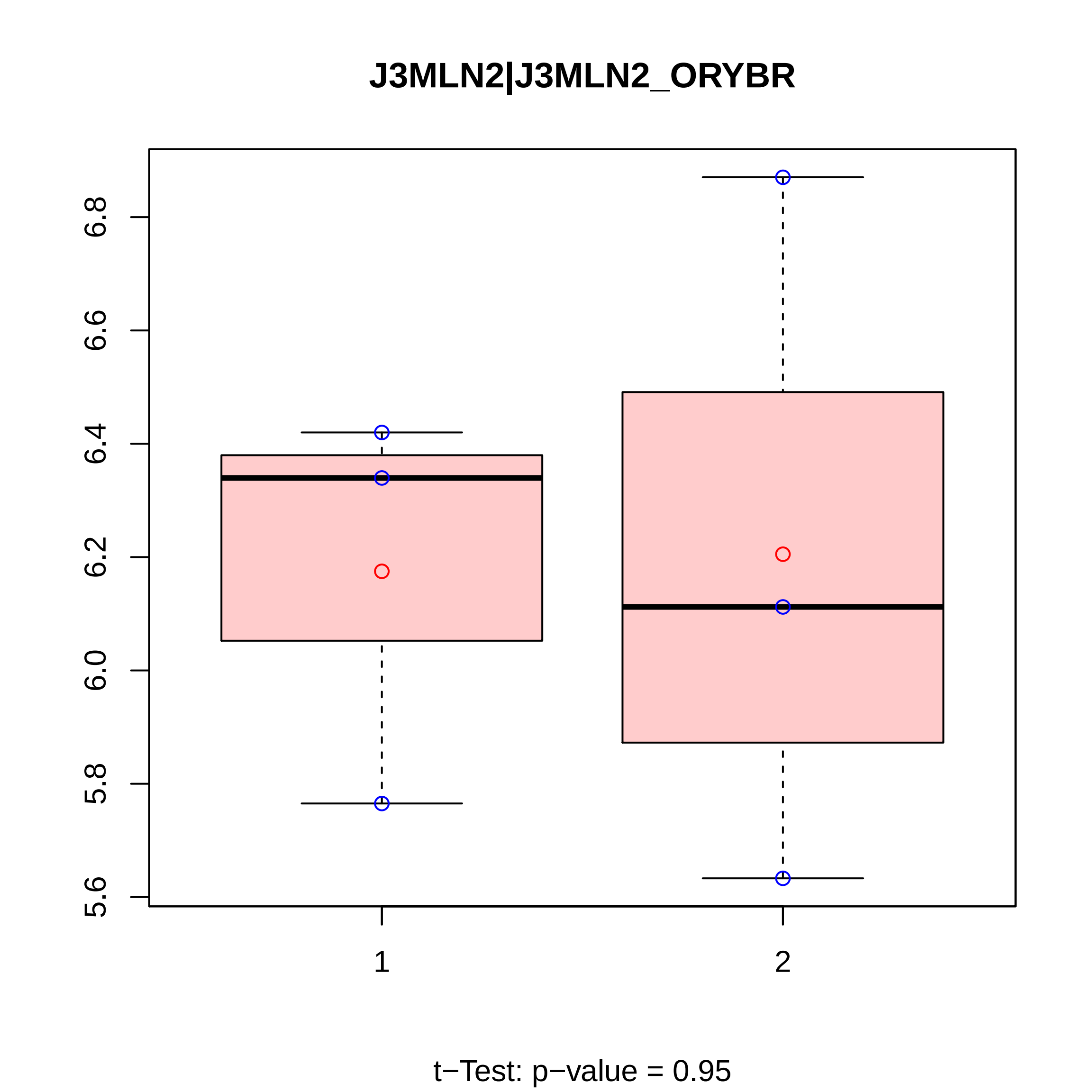
J3MLN2|J3MLN2_ORYBR
6.8
6.6
6.4
6.2
6.0
5.8
5.6
1
2
t−Test: p−value = 0.95

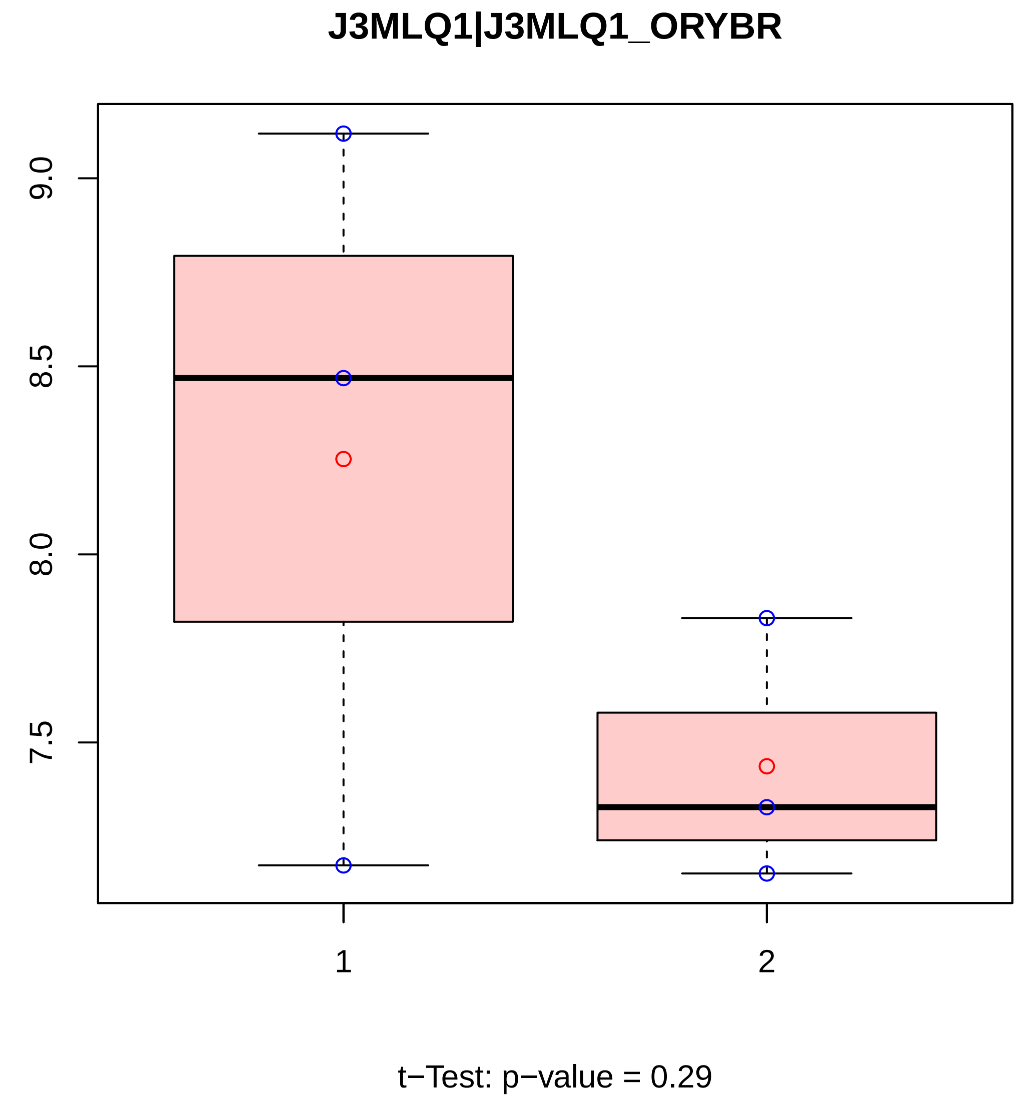

J3MLQ1|J3MLQ1_ORYBR
9.0
8.5
8.0
7.5
1
2
t−Test: p−value = 0.29

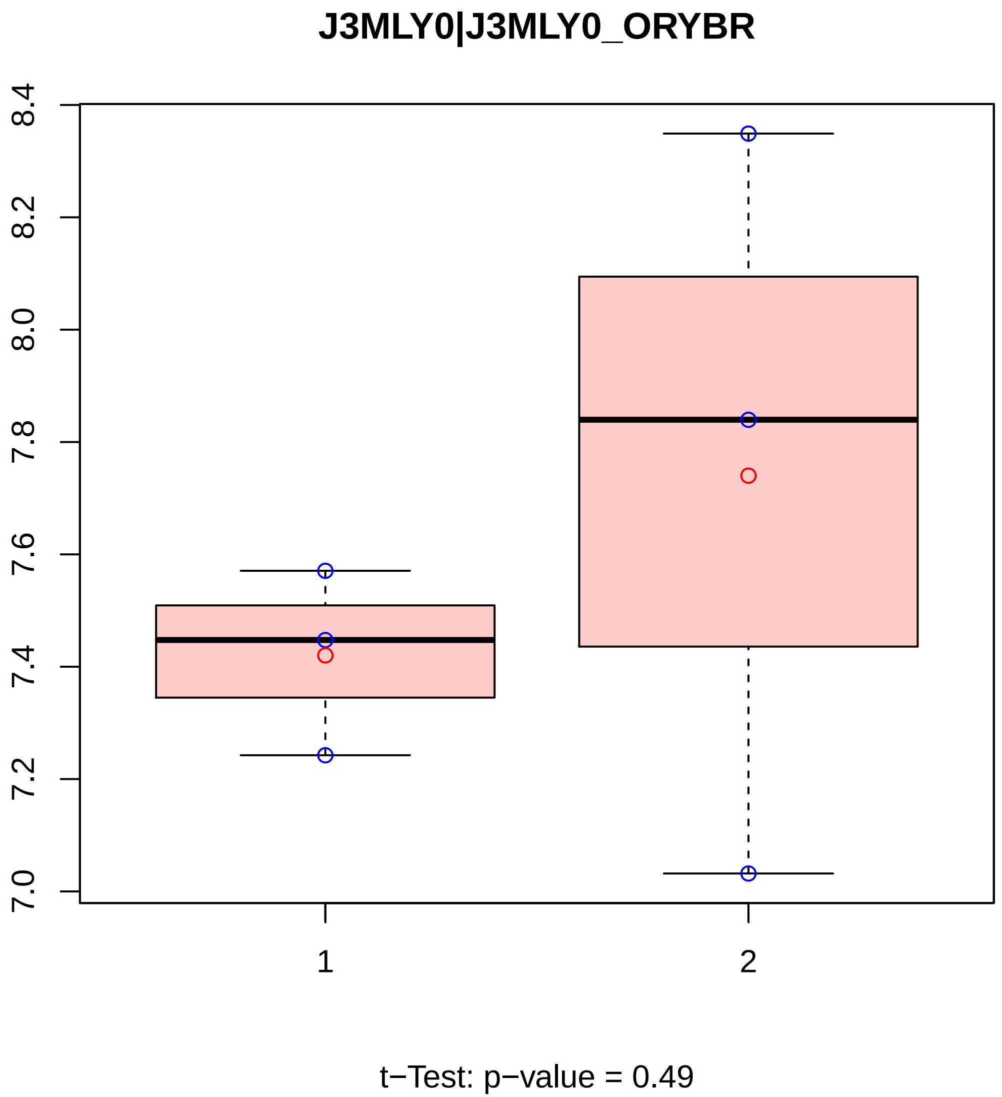
J3MLY0|J3MLY0_ORYBR
8.4
8.2
8.0
7.8
7.6
7.4
7.2
7.0
1
2
t−Test: p−value = 0.49

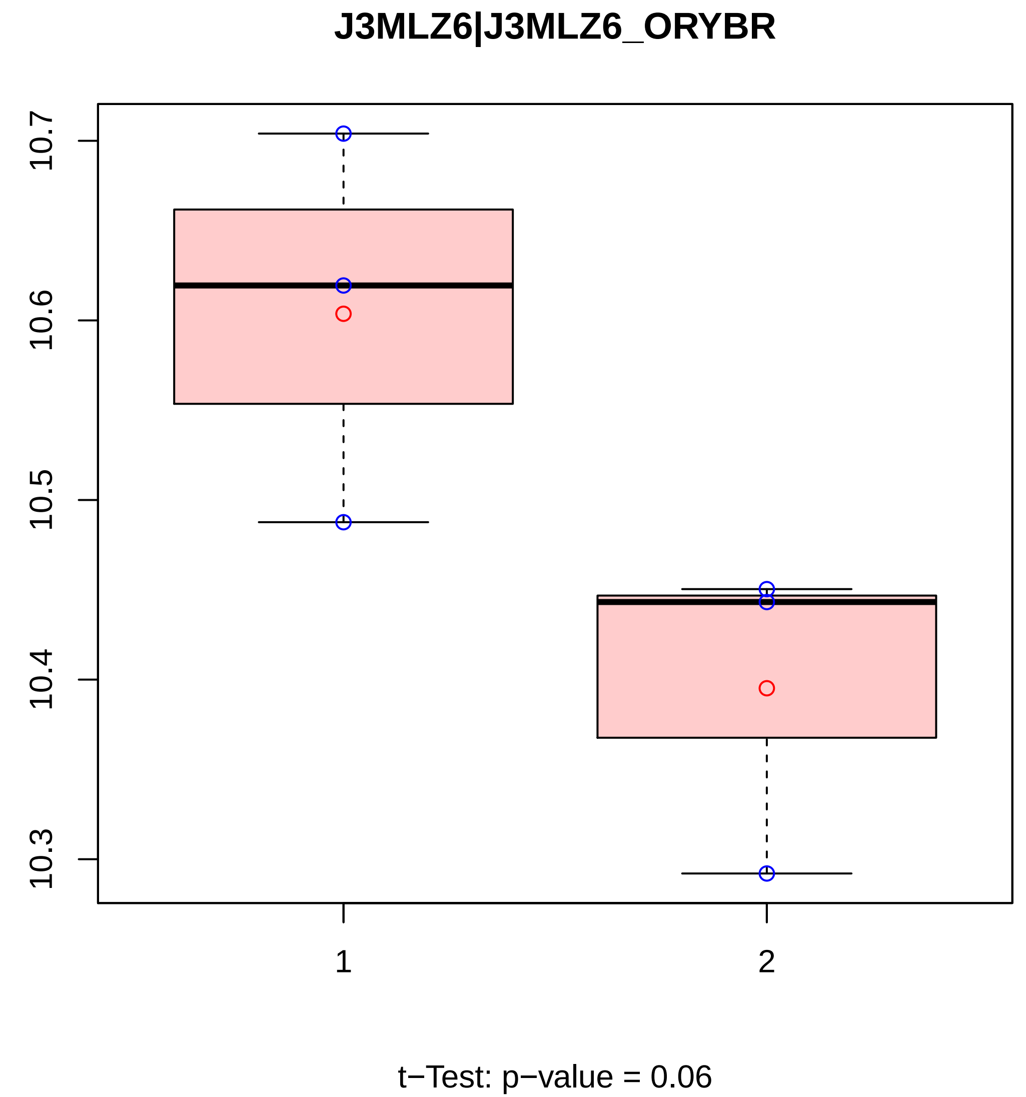
J3MLZ6|J3MLZ6_ORYBR
10.3
10.4
10.5
10.6
10.7
1
2
t−Test: p−value = 0.06

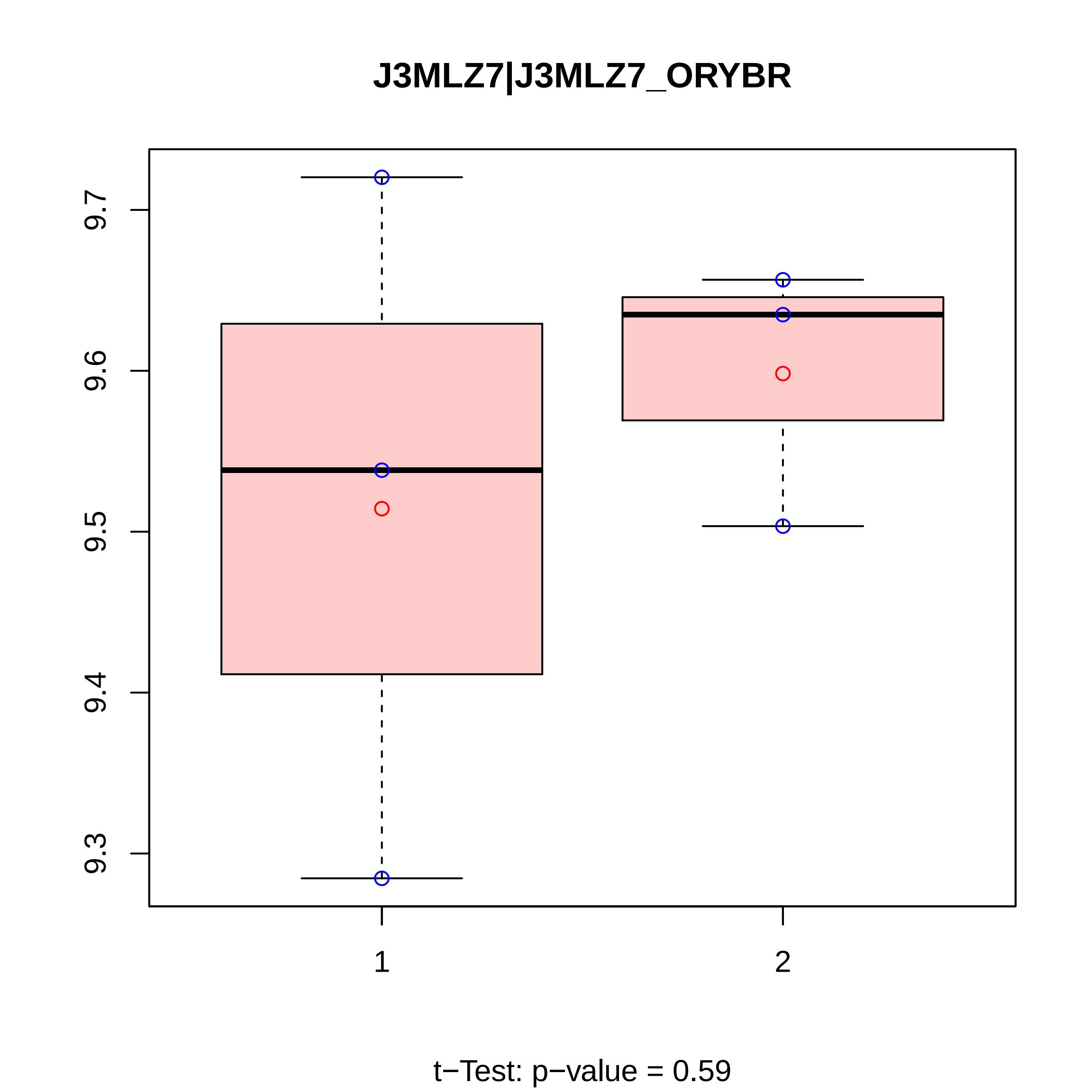

J3MLZ7|J3MLZ7_ORYBR
9.3
9.4
9.5
9.6
9.7
1
2
t−Test: p−value = 0.59

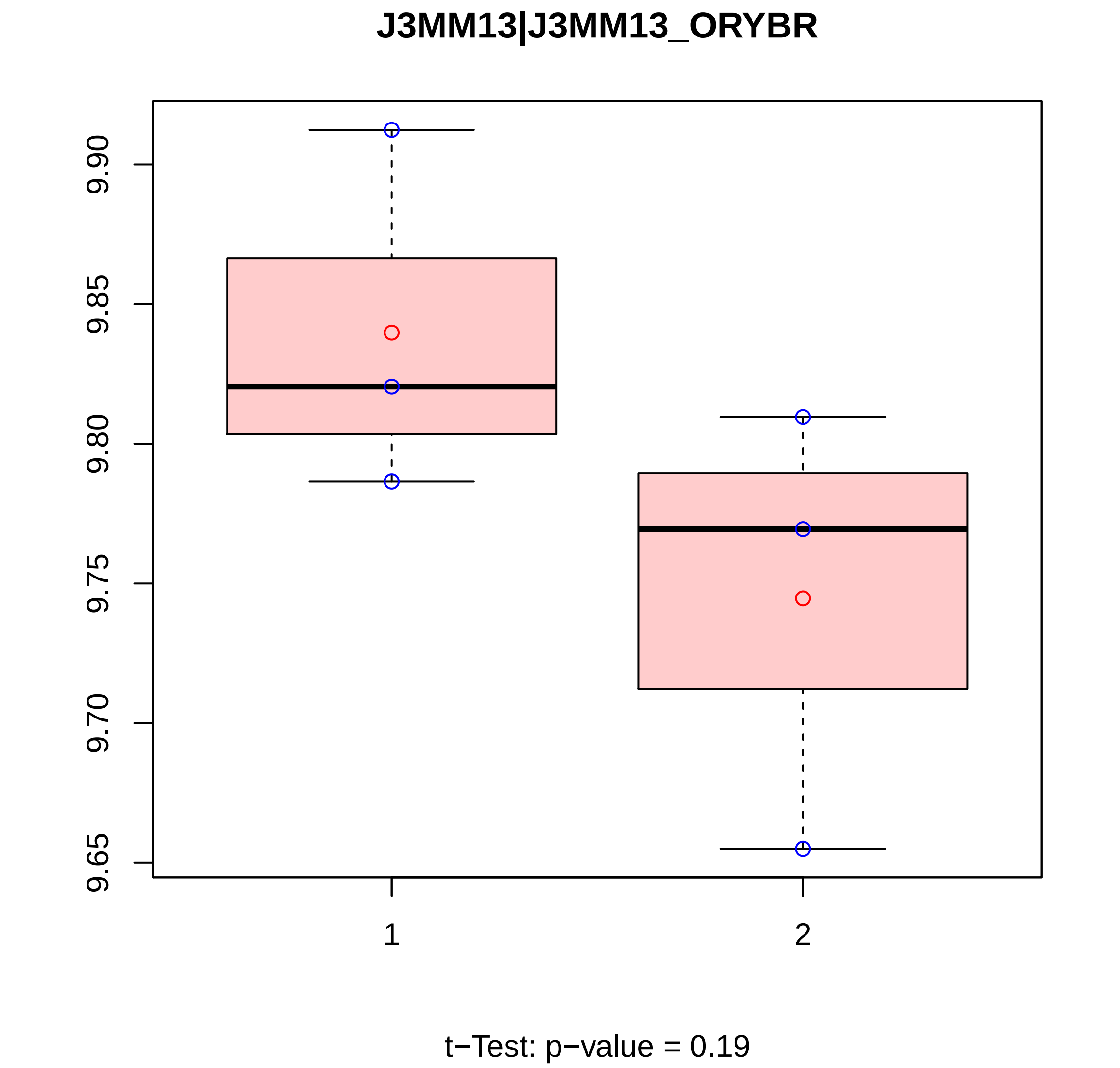

J3MM13|J3MM13_ORYBR
9.65
9.70
9.75
9.80
9.85
9.90
1
2
t−Test: p−value = 0.19

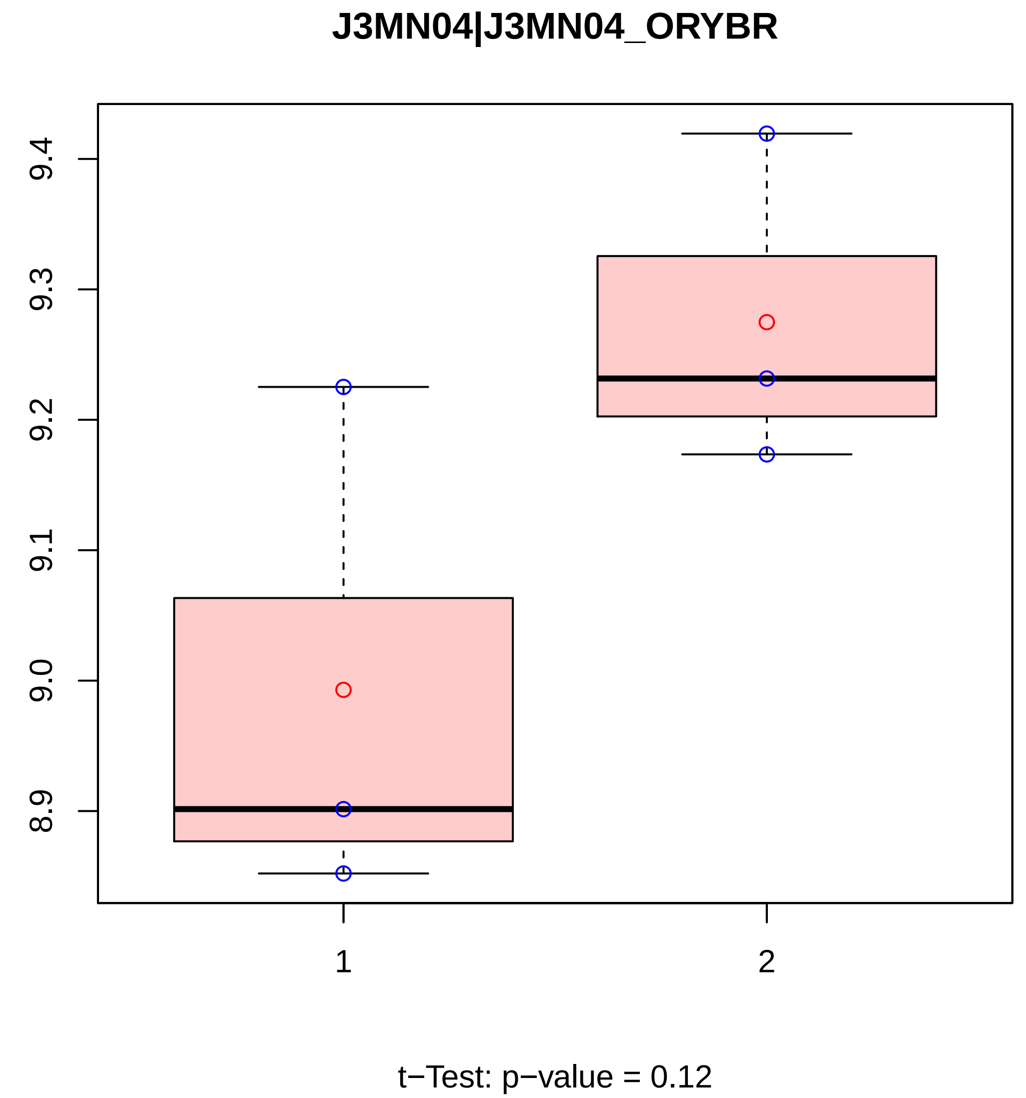
J3MN04|J3MN04_ORYBR
9.4
9.3
9.2
9.1
9.0
8.9
1
2
t−Test: p−value = 0.12

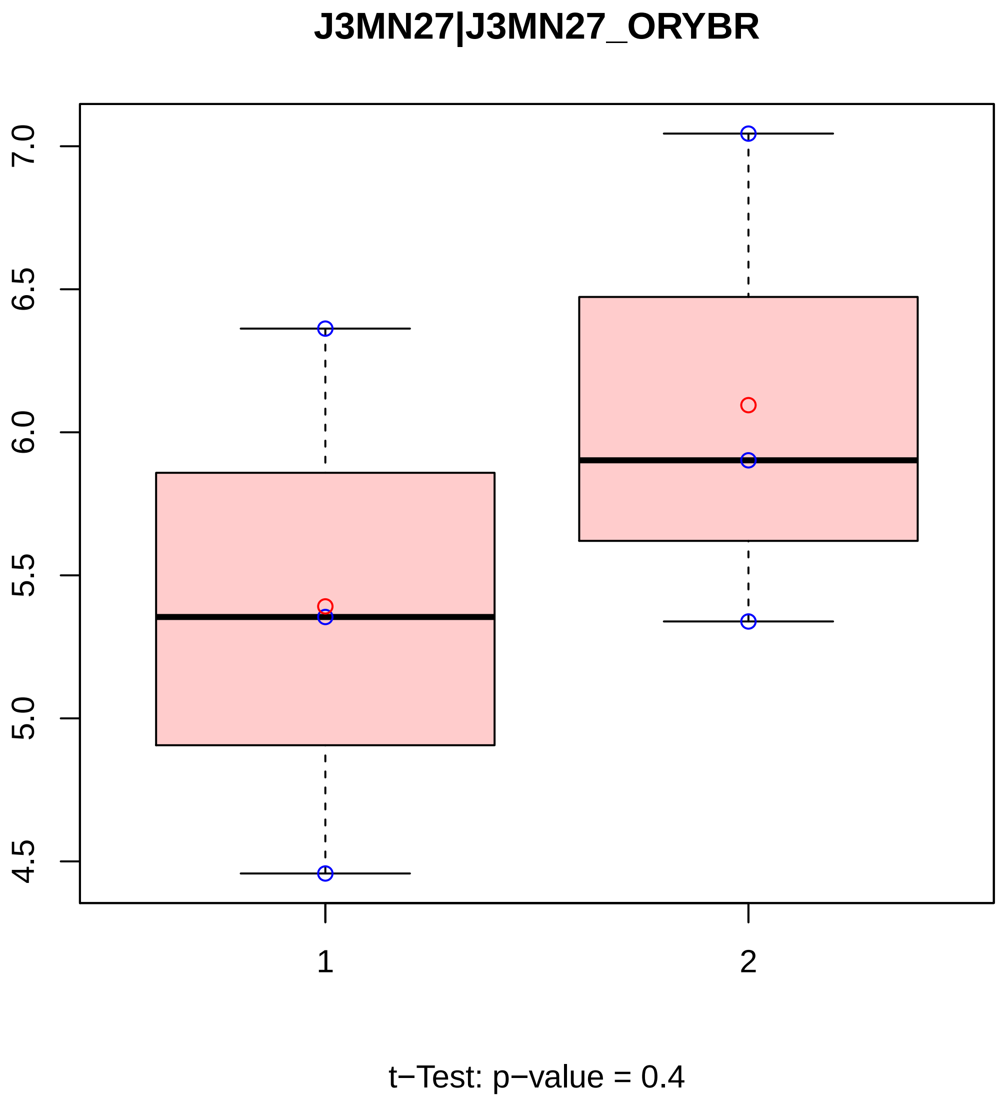
J3MN27|J3MN27_ORYBR
7.0
6.5
6.0
5.5
5.0
4.5
1
2
t−Test: p−value = 0.4

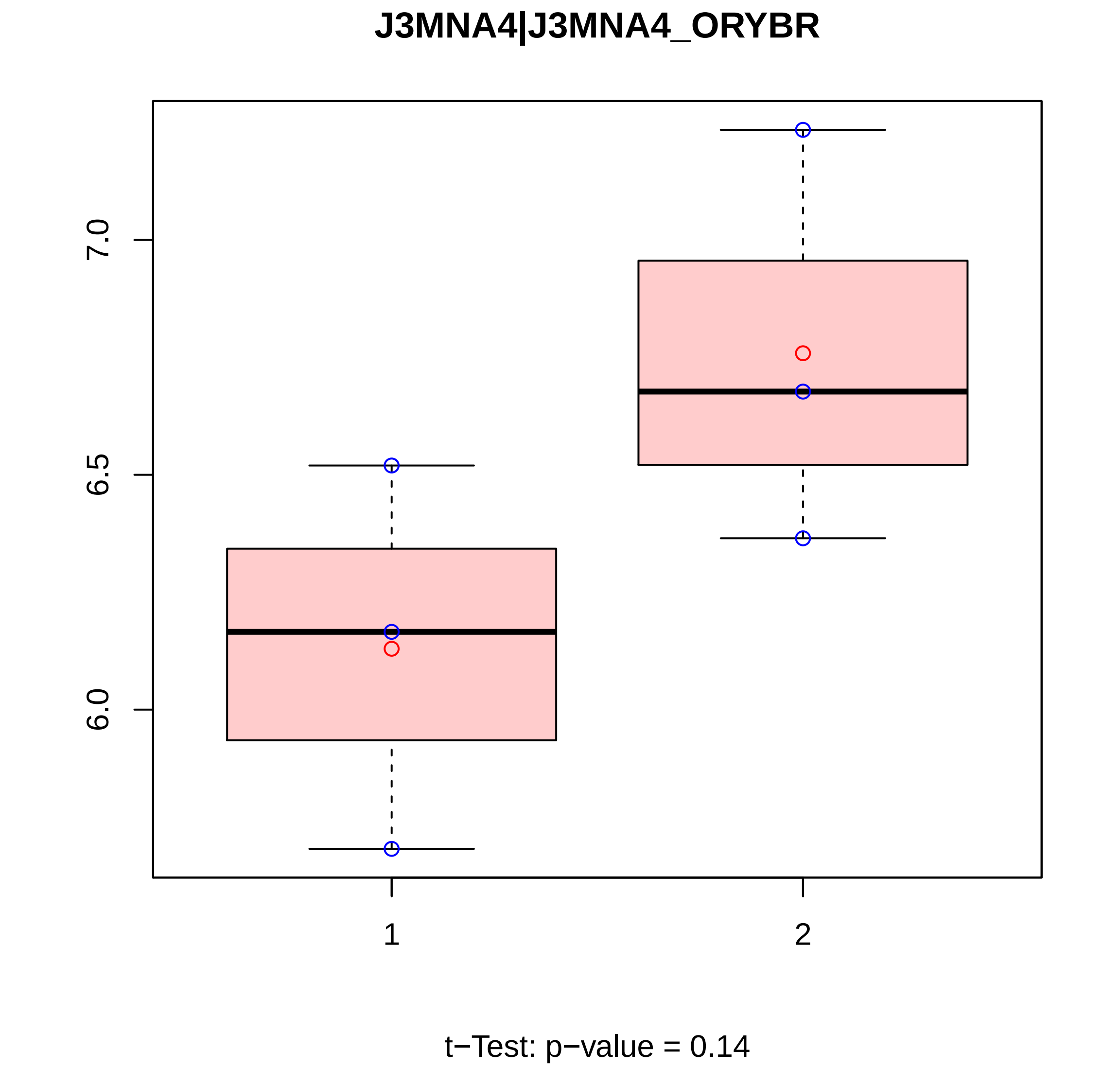

J3MNA4|J3MNA4_ORYBR
7.0
6.5
6.0
1
2
t−Test: p−value = 0.14

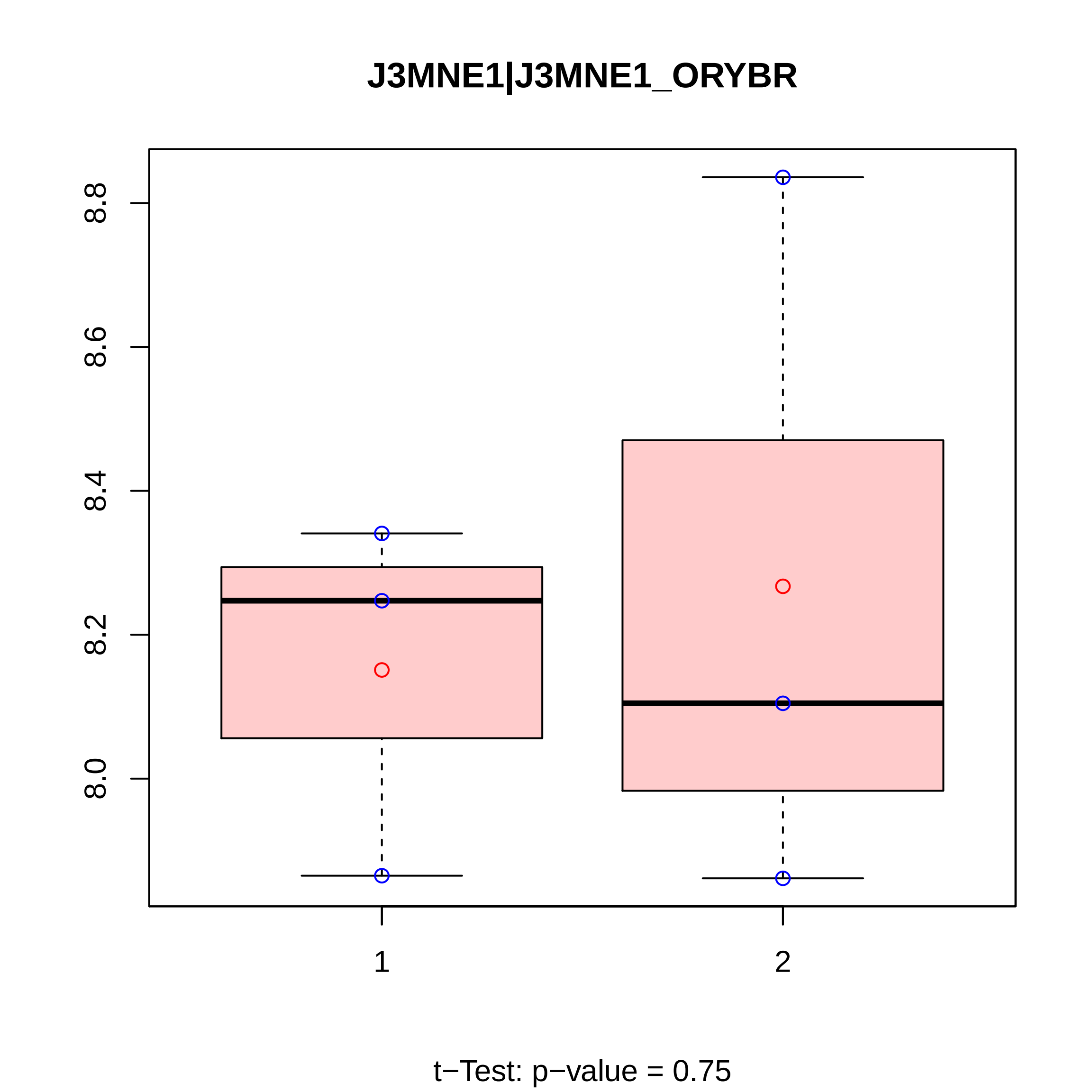
J3MNE1|J3MNE1_ORYBR
8.0
8.2
8.4
8.6
8.8
1
2
t−Test: p−value = 0.75

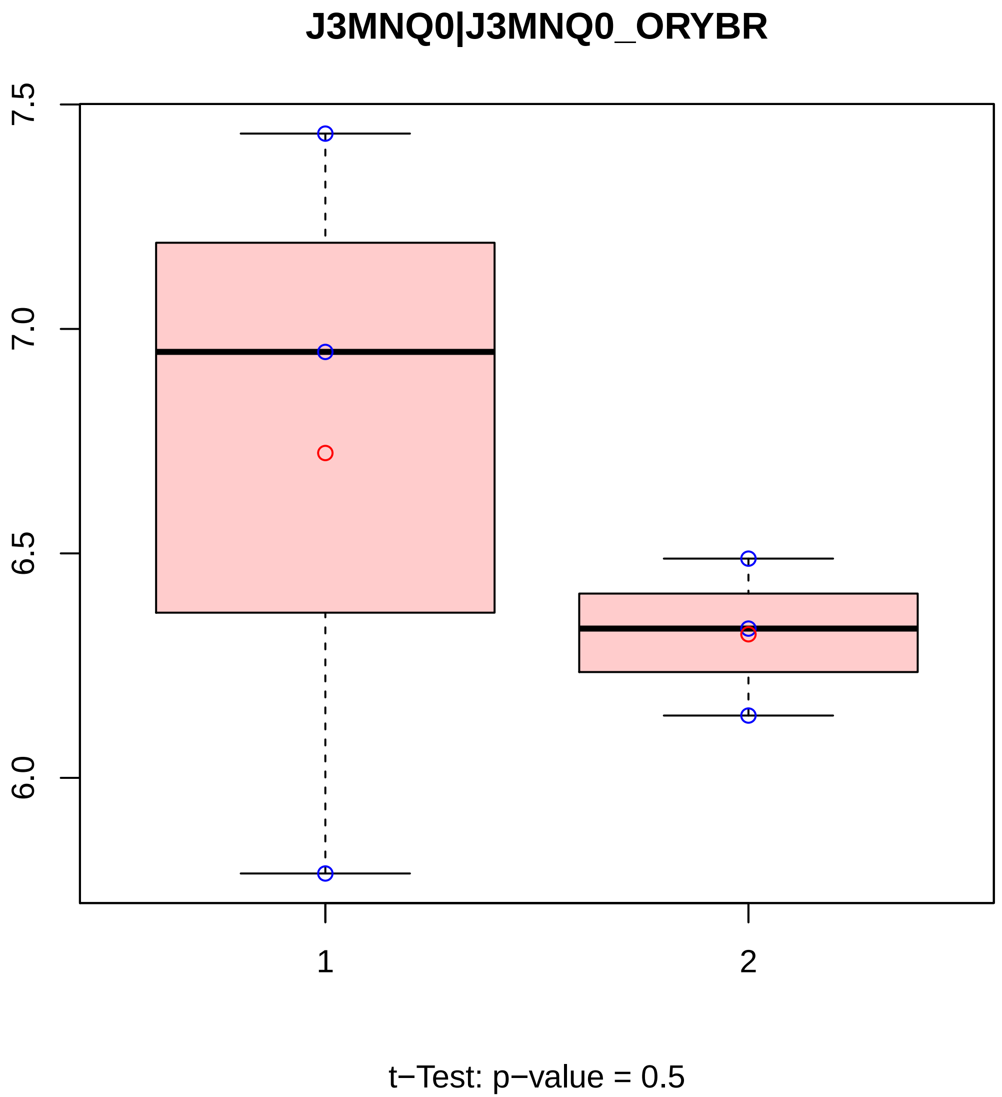
J3MNQ0|J3MNQ0_ORYBR
7.5
7.0
6.5
6.0
1
2
t−Test: p−value = 0.5

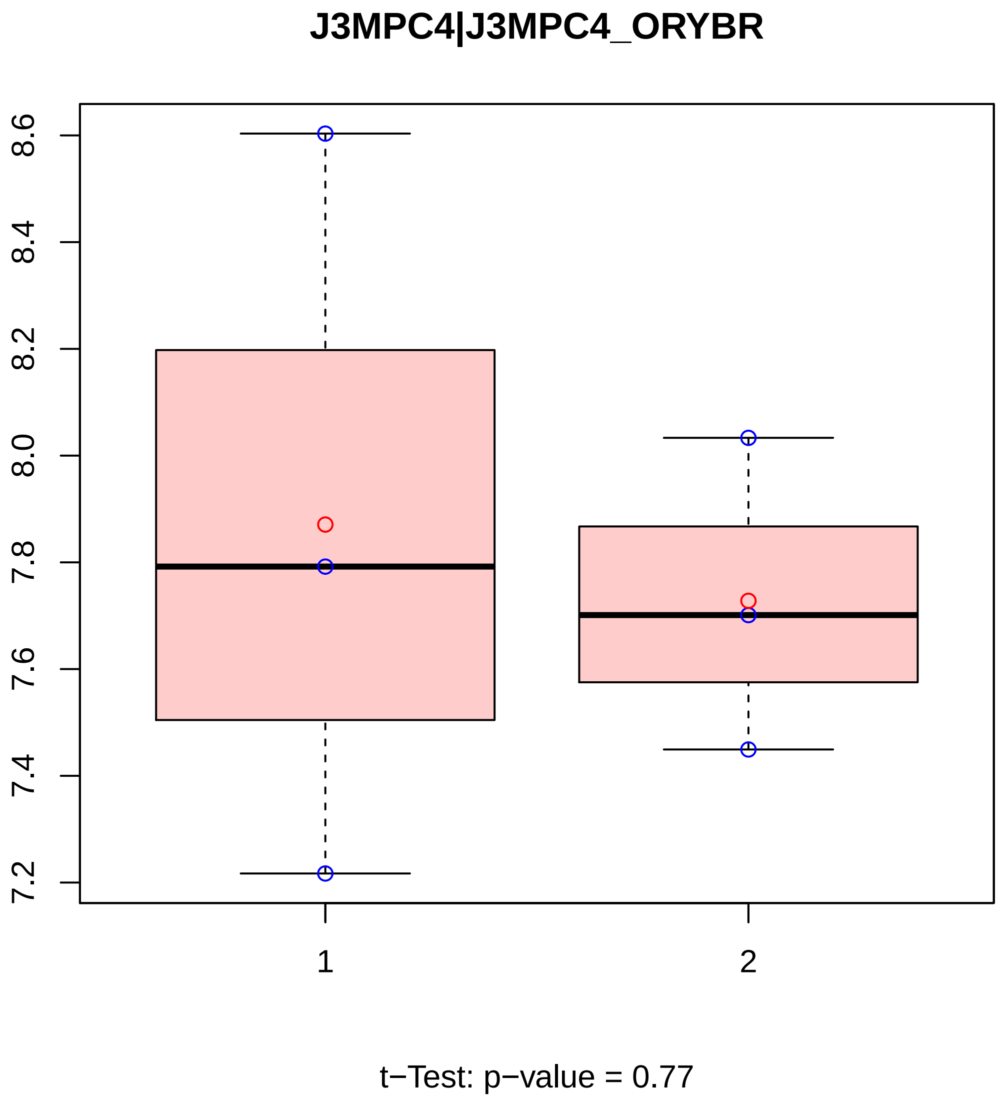
J3MPC4|J3MPC4_ORYBR
8.6
8.4
8.2
8.0
7.8
7.6
7.4
7.2
1
2
t−Test: p−value = 0.77

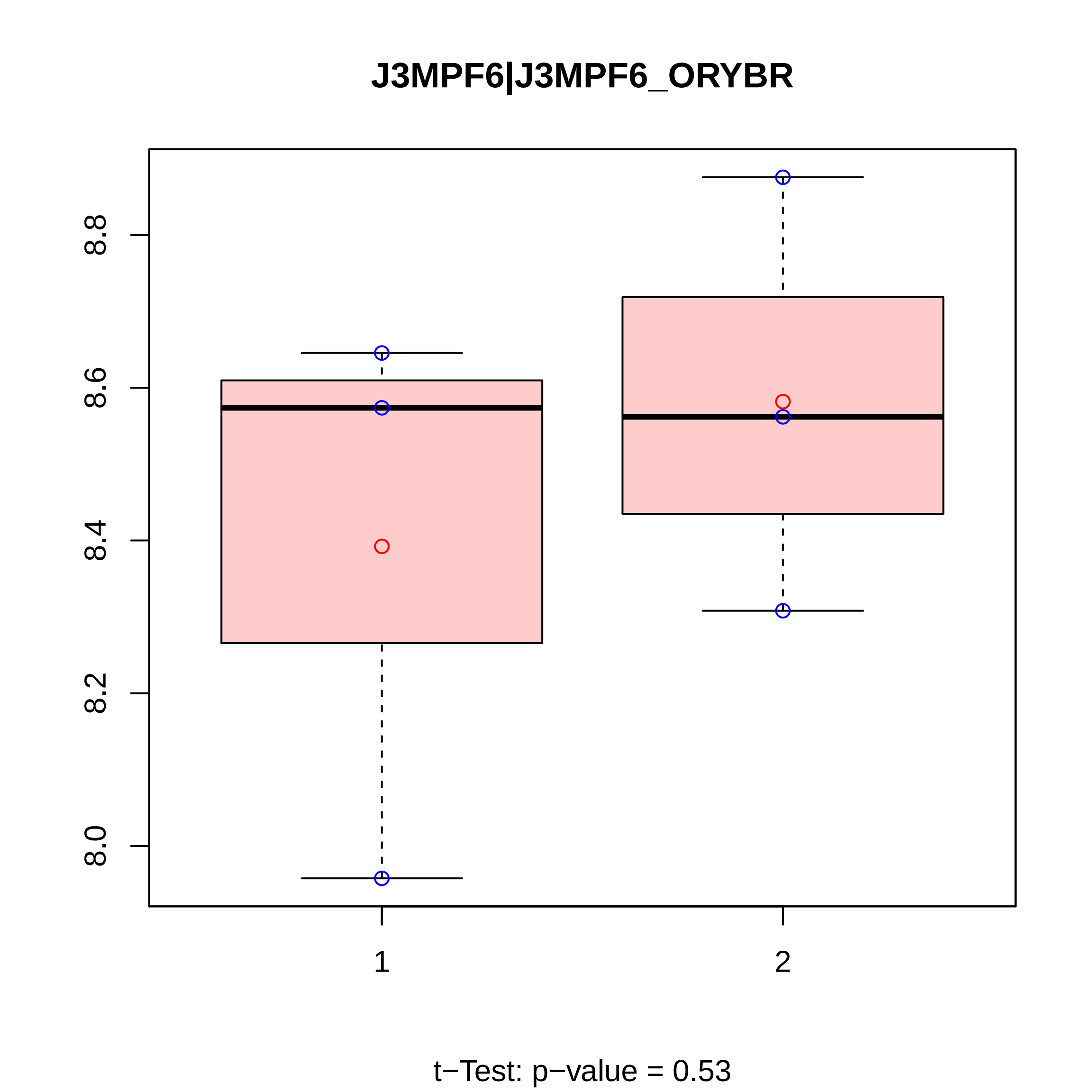
J3MPF6|J3MPF6_ORYBR
8.0
8.2
8.4
8.6
8.8
1
2
t−Test: p−value = 0.53

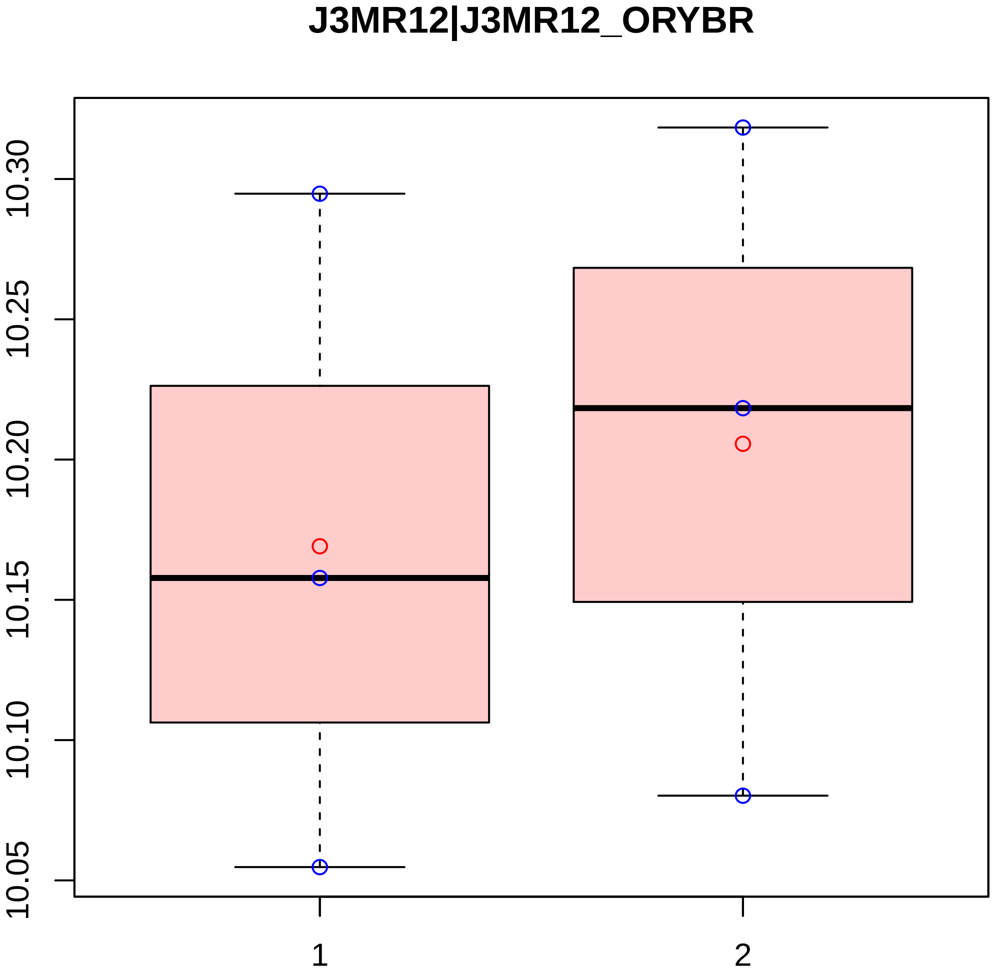

t−Test: p−value = 0.73

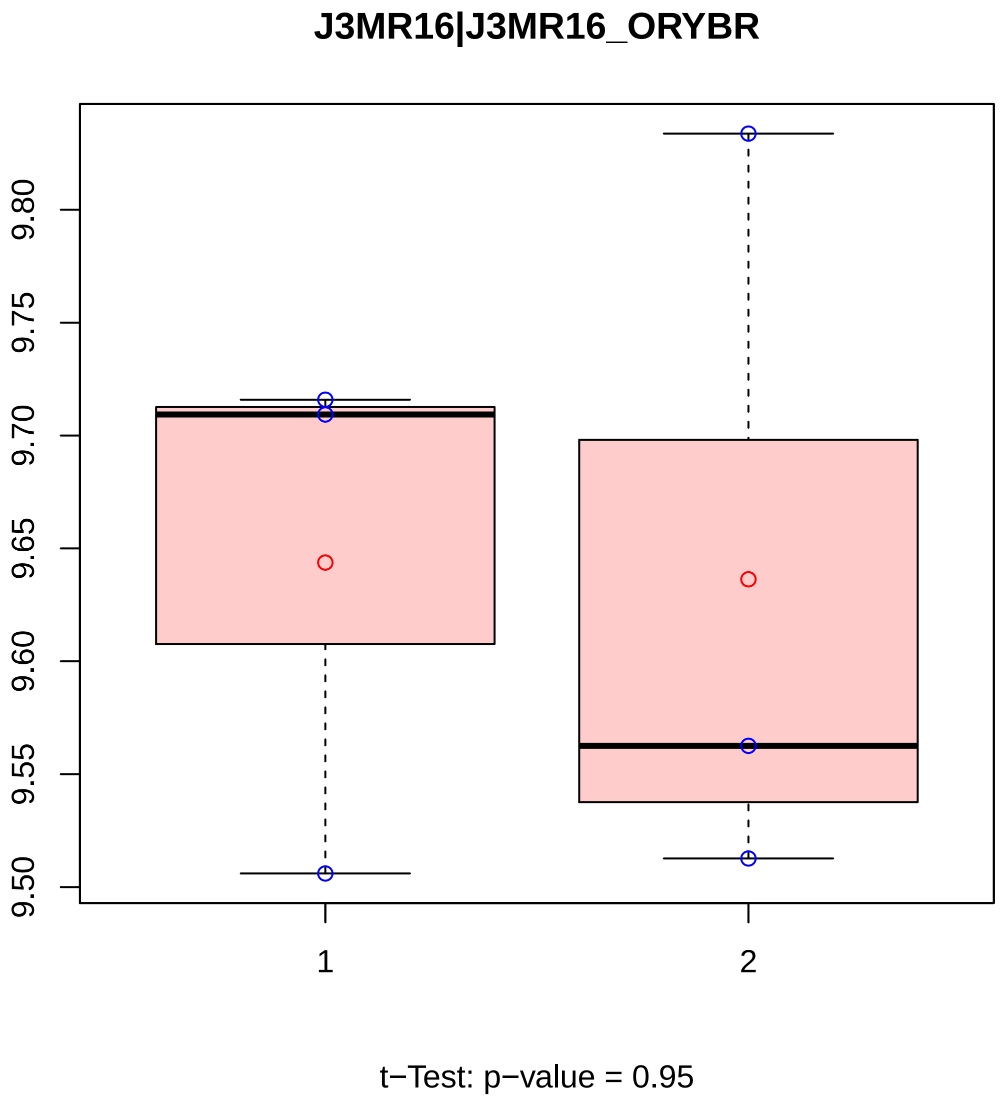
J3MR16|J3MR16_ORYBR
9.50
9.55
9.60
9.65
9.70
9.75
9.80
1
2
t−Test: p−value = 0.95

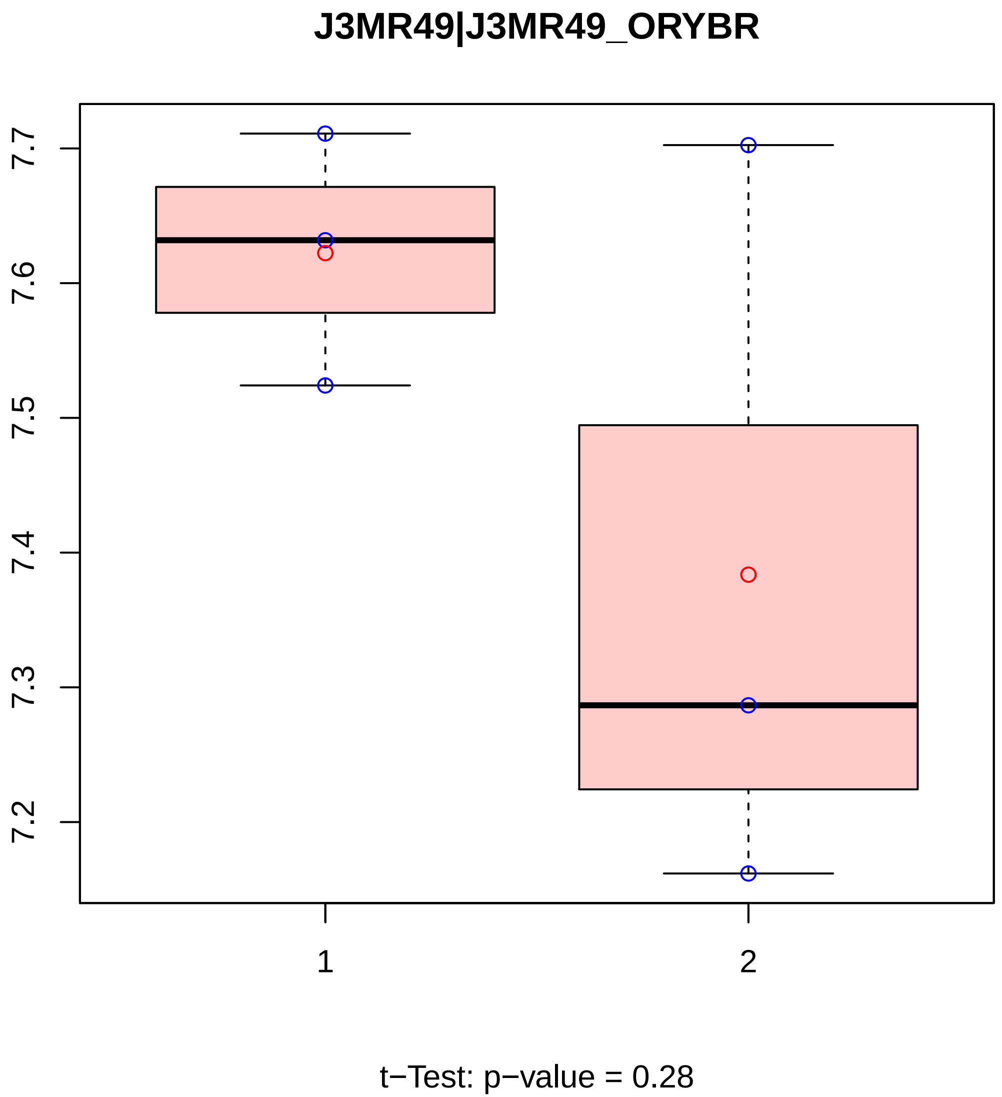

J3MR49|J3MR49_ORYBR
7.2
7.3
7.4
7.5
7.6
7.7
1
2
t−Test: p−value = 0.28

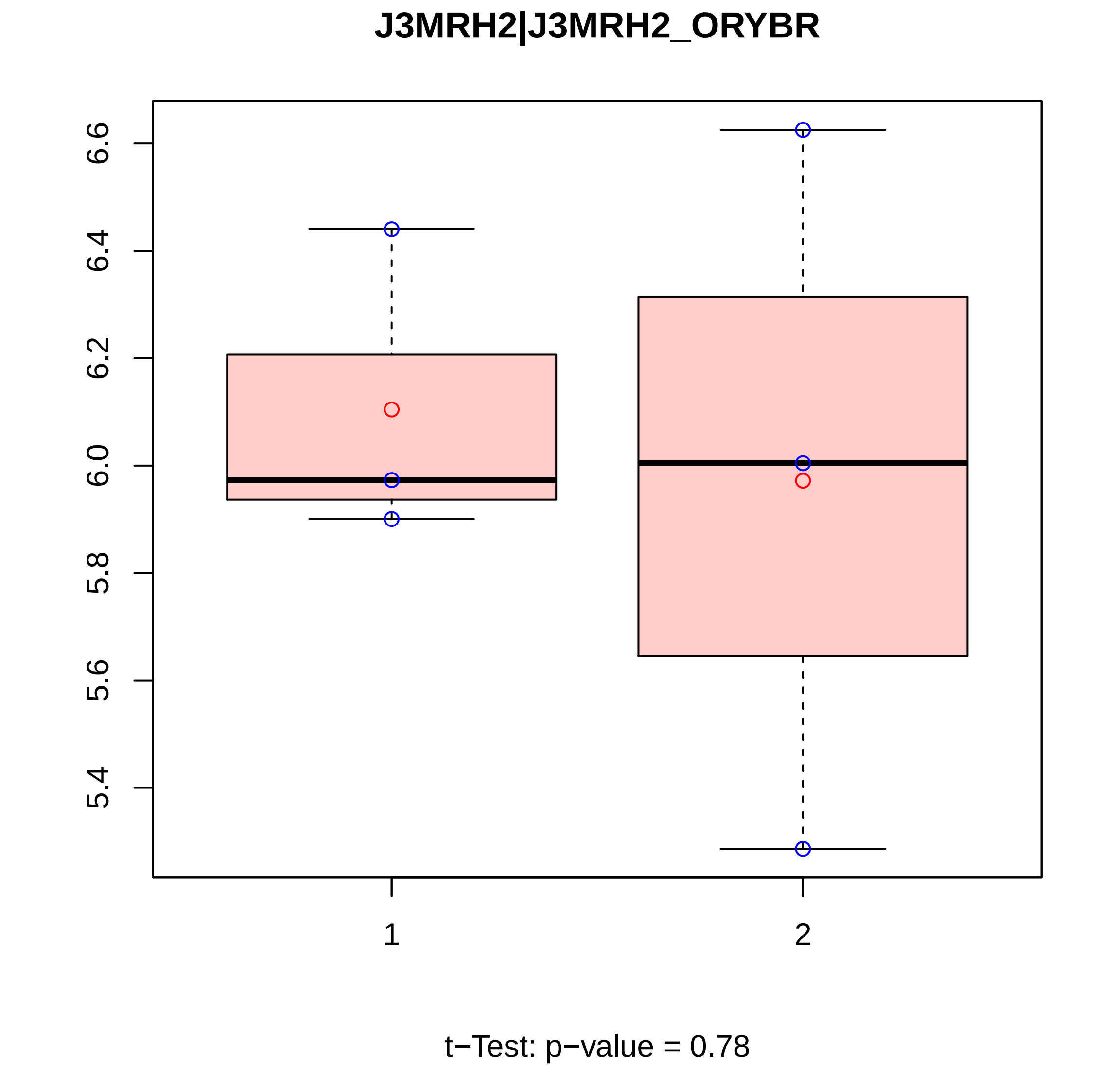
J3MRH2|J3MRH2_ORYBR
6.6
6.4
6.2
6.0
5.8
5.6
5.4
1
2
t−Test: p−value = 0.78

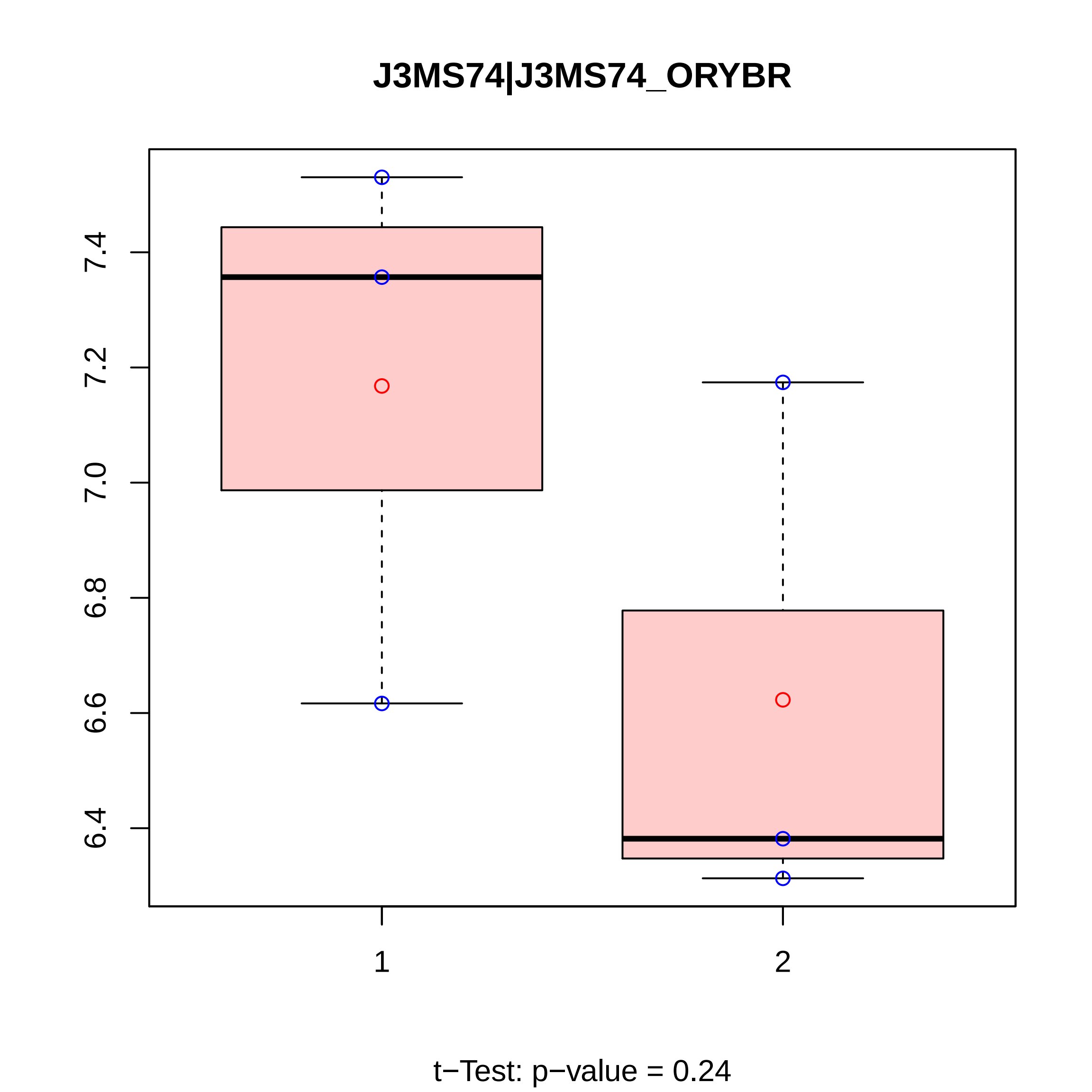
J3MS74|J3MS74_ORYBR
7.4
7.2
7.0
6.8
6.6
6.4
1
2
t−Test: p−value = 0.24

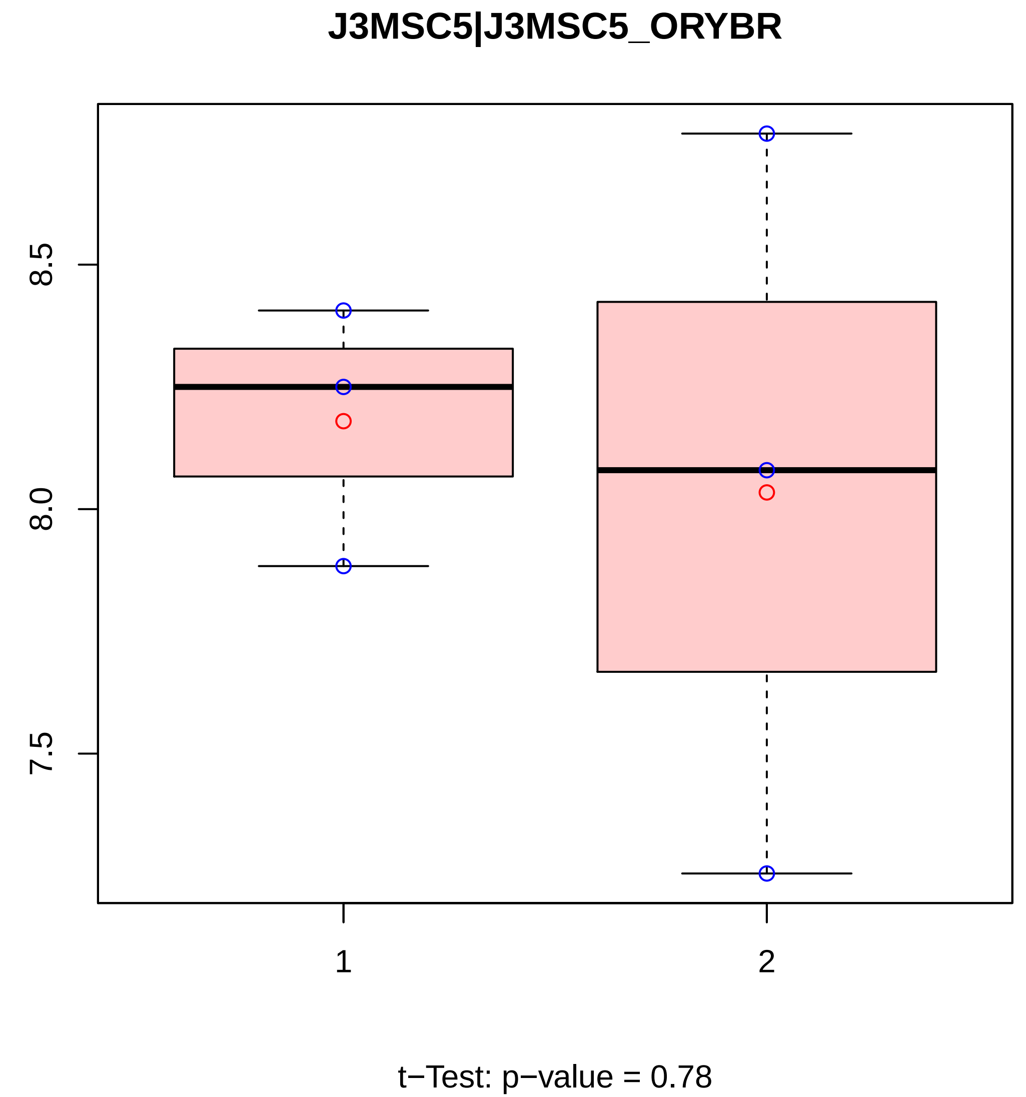
J3MSC5|J3MSC5_ORYBR
8.5
8.0
7.5
1
2
t−Test: p−value = 0.78

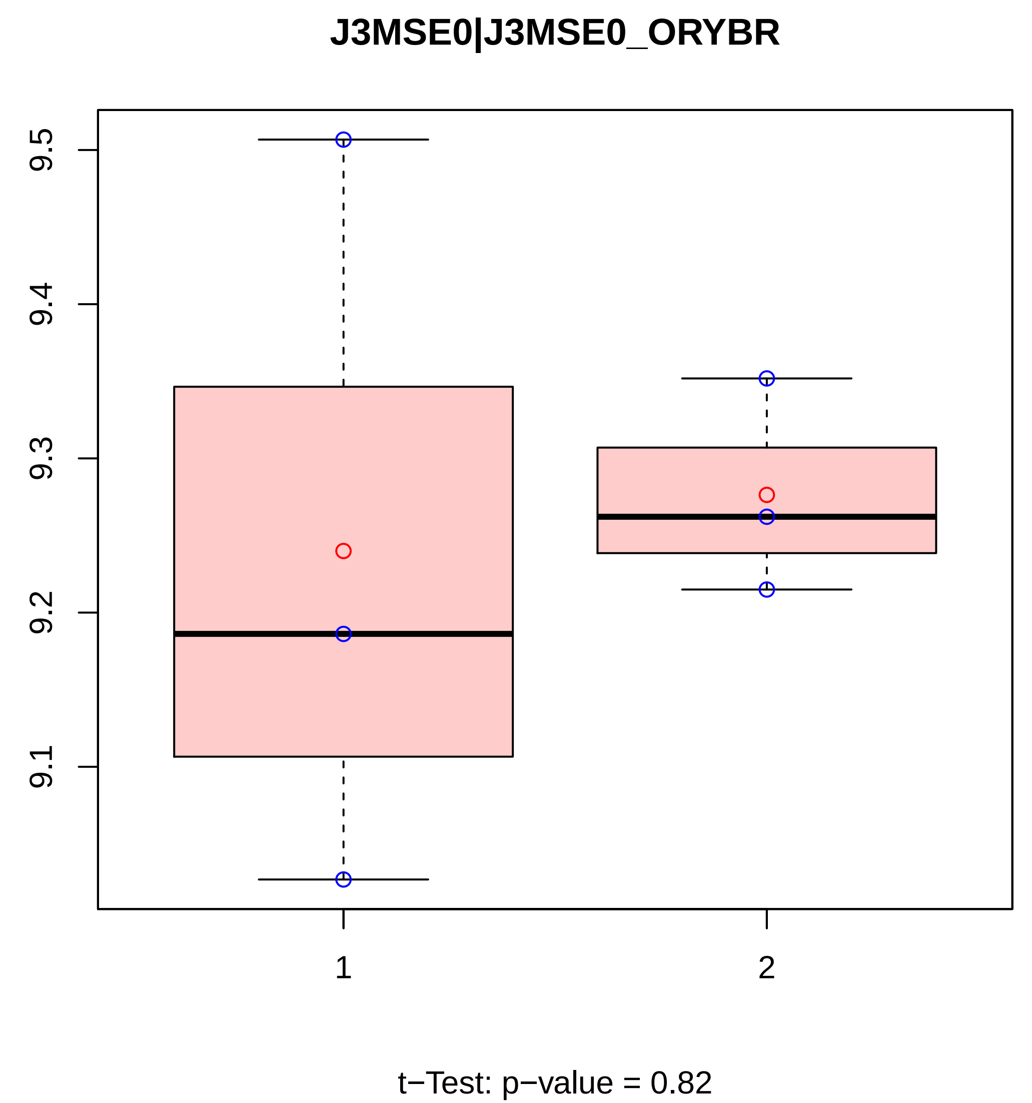
J3MSE0|J3MSE0_ORYBR
9.1
9.2
9.3
9.4
9.5
1
2
t−Test: p−value = 0.82

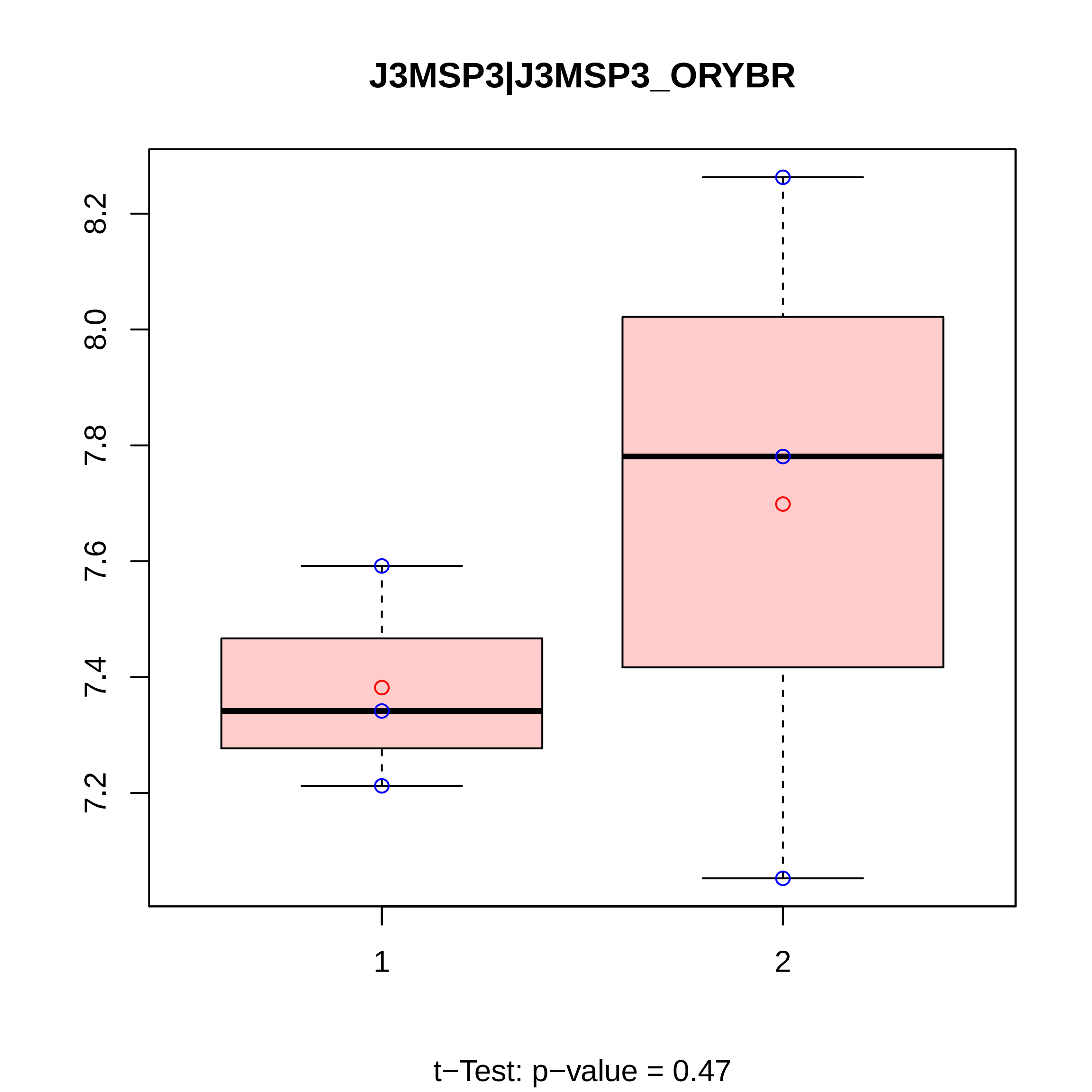
J3MSP3|J3MSP3_ORYBR
8.2
8.0
7.8
7.6
7.4
7.2
1
2
t−Test: p−value = 0.47

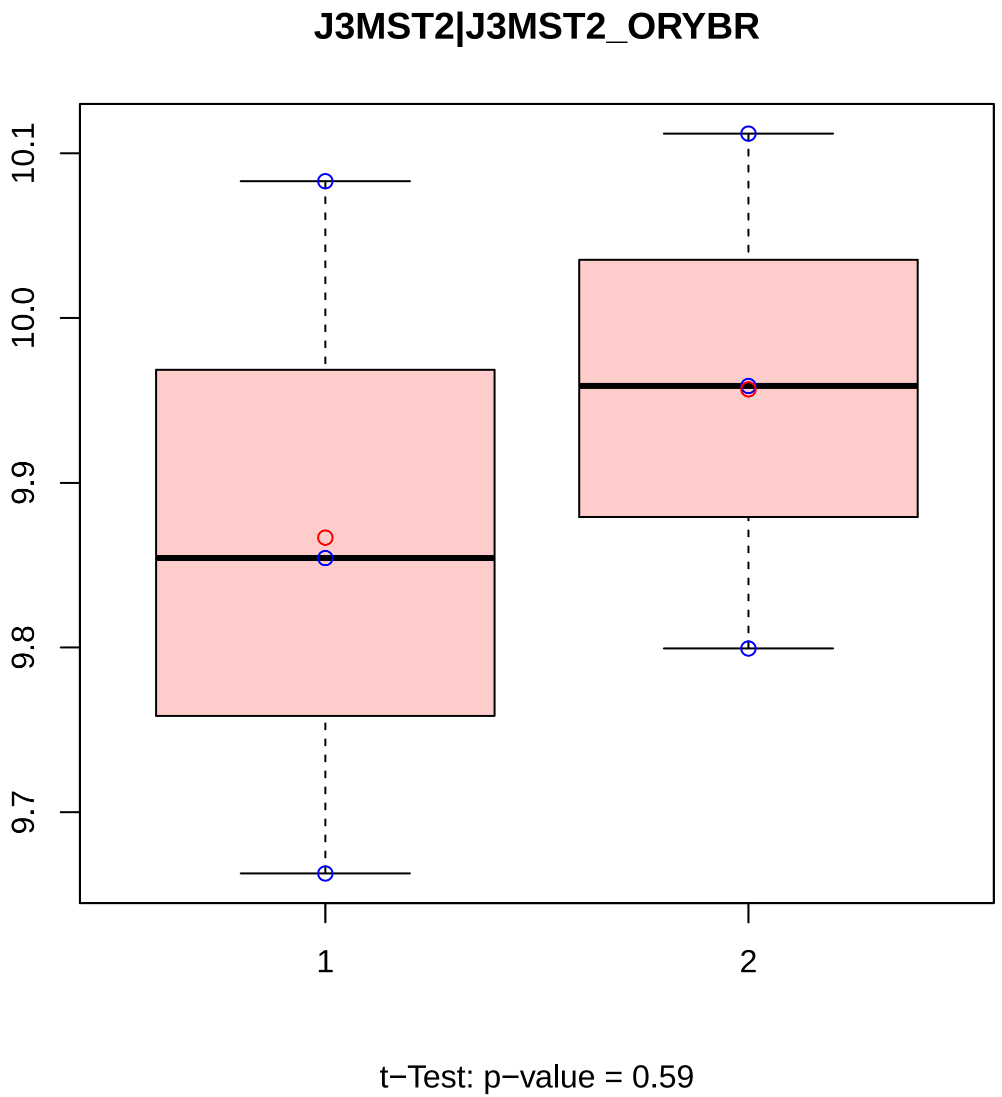
J3MST2|J3MST2_ORYBR
10.1
10.0
9.9
9.8
9.7
1
2
t−Test: p−value = 0.59

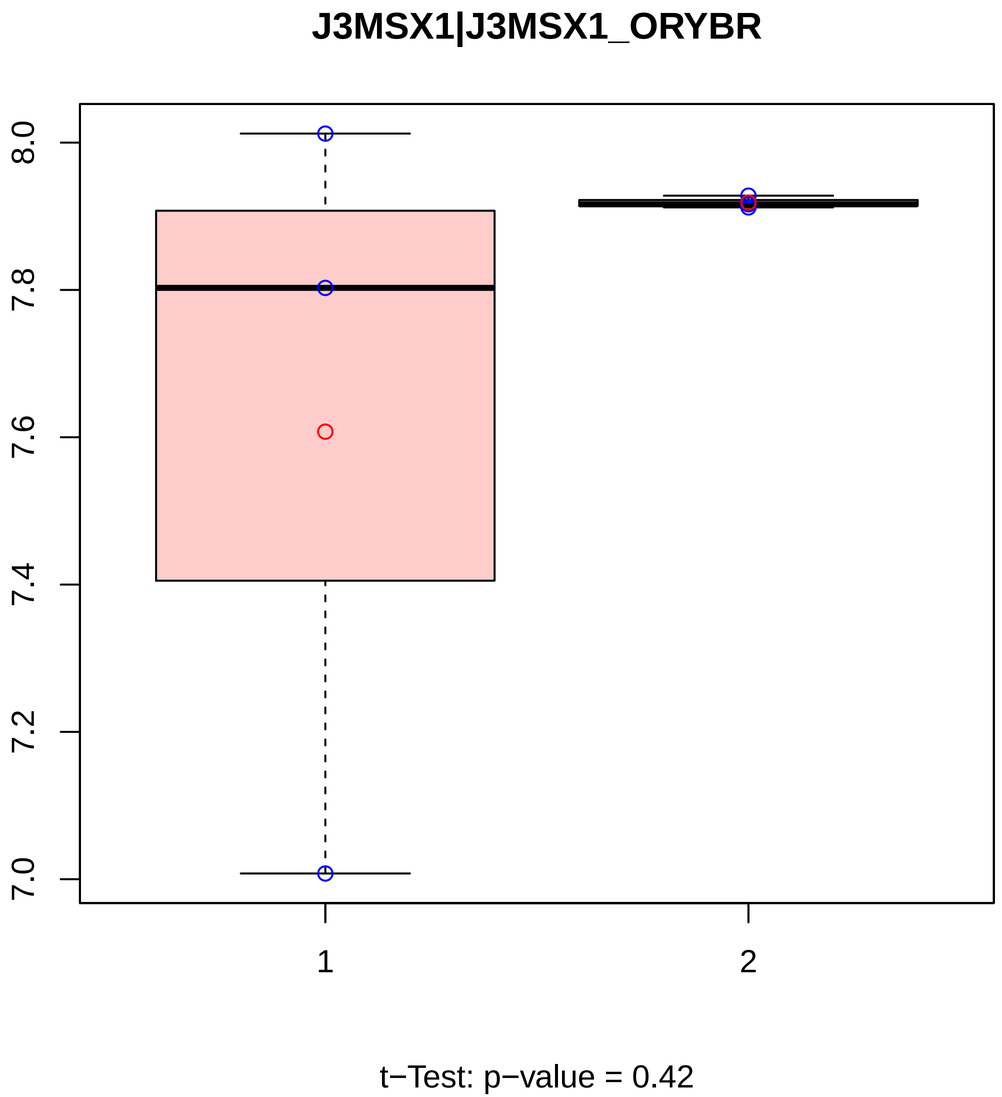
J3MSX1|J3MSX1_ORYBR
8.0
7.8
7.6
7.4
7.2
7.0
1
2
t−Test: p−value = 0.42

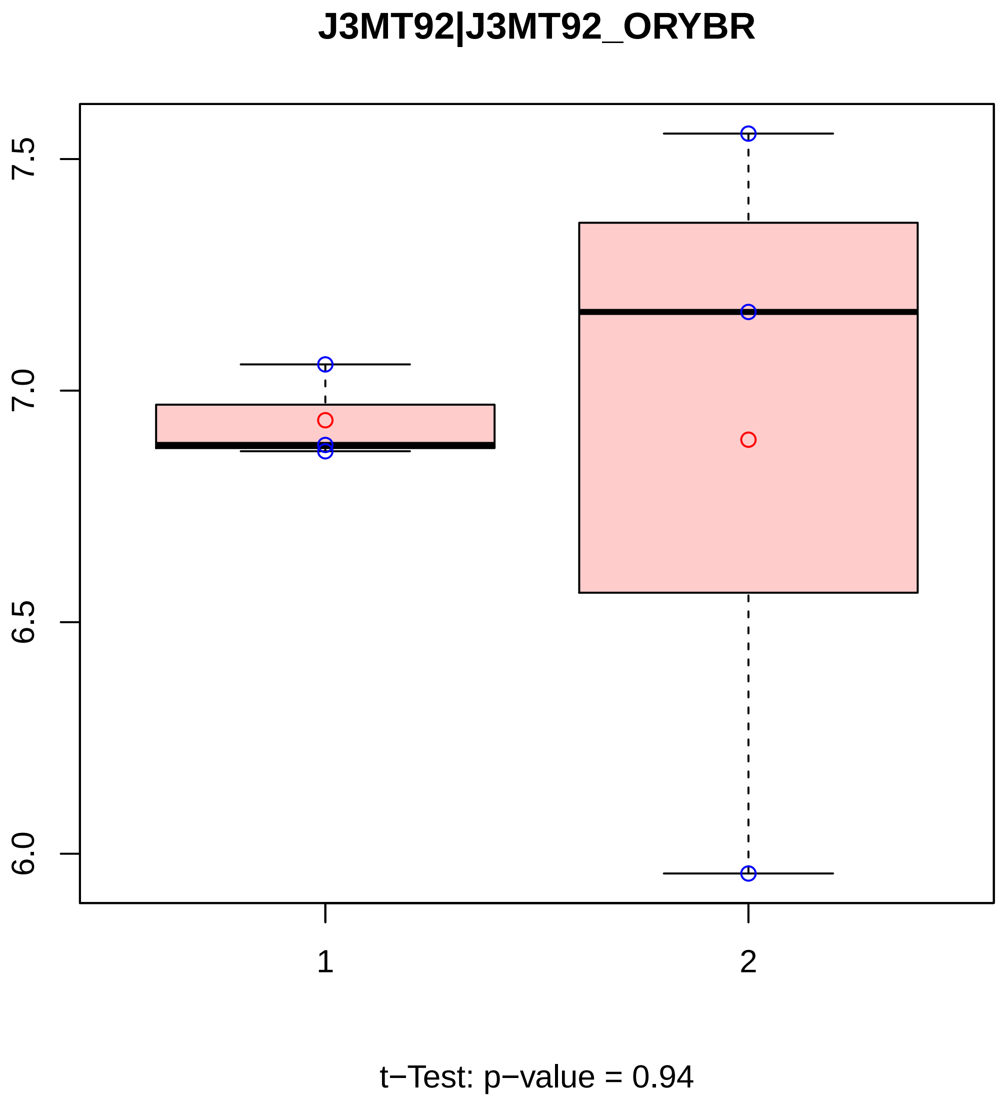
J3MT92|J3MT92_ORYBR
7.5
7.0
6.5
6.0
1
2
t−Test: p−value = 0.94

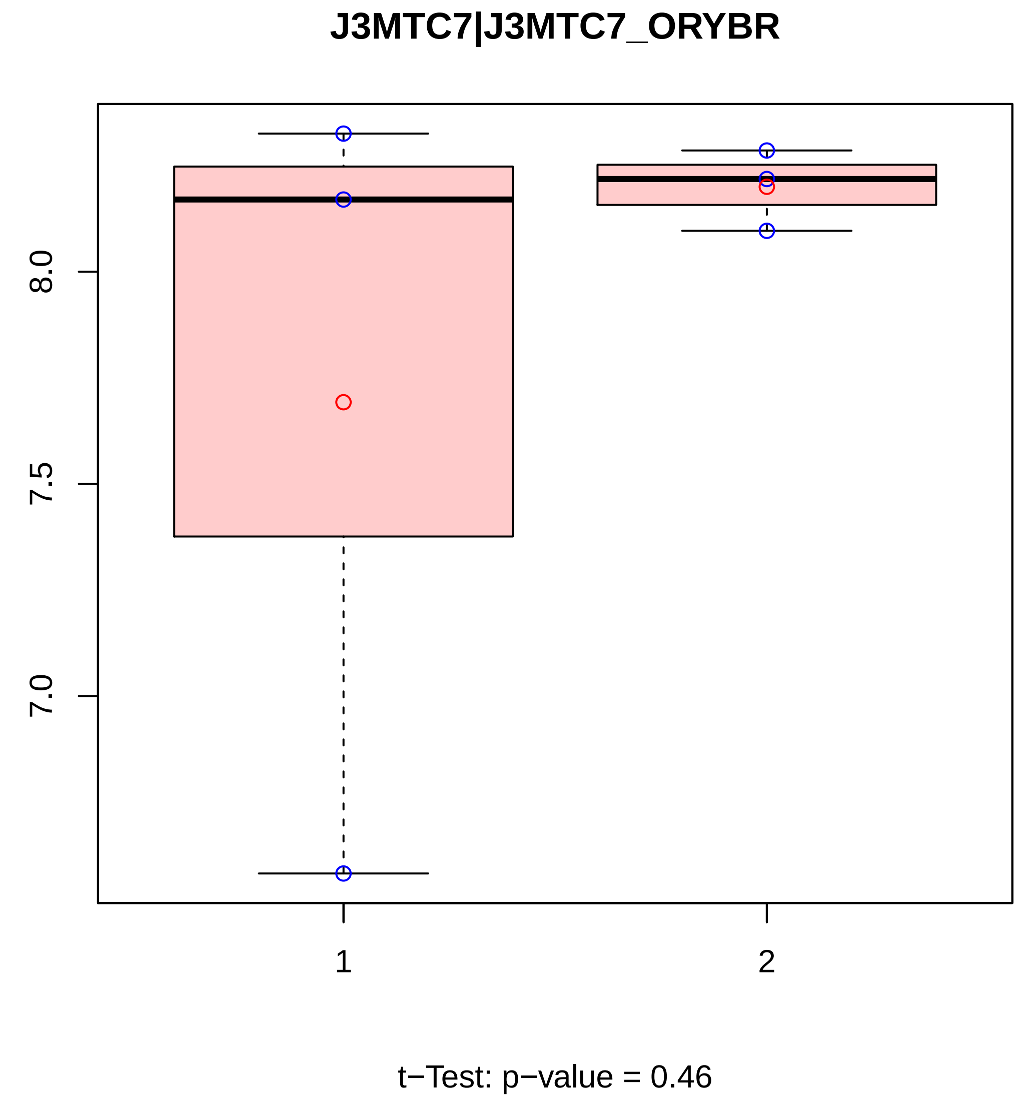
J3MTC7|J3MTC7_ORYBR
8.0
7.5
7.0
1
2
t−Test: p−value = 0.46

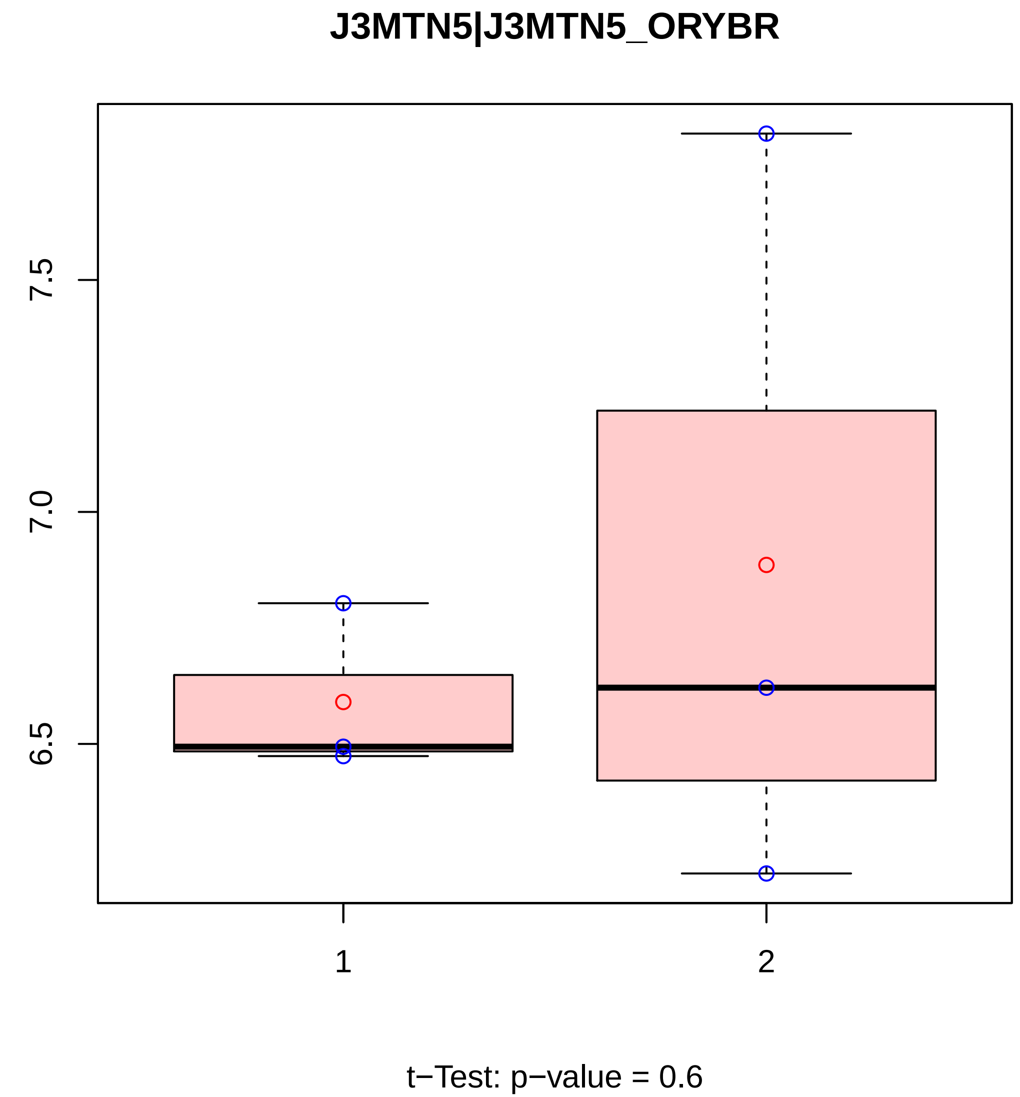
J3MTN5|J3MTN5_ORYBR
7.5
7.0
6.5
1
2
t−Test: p−value = 0.6

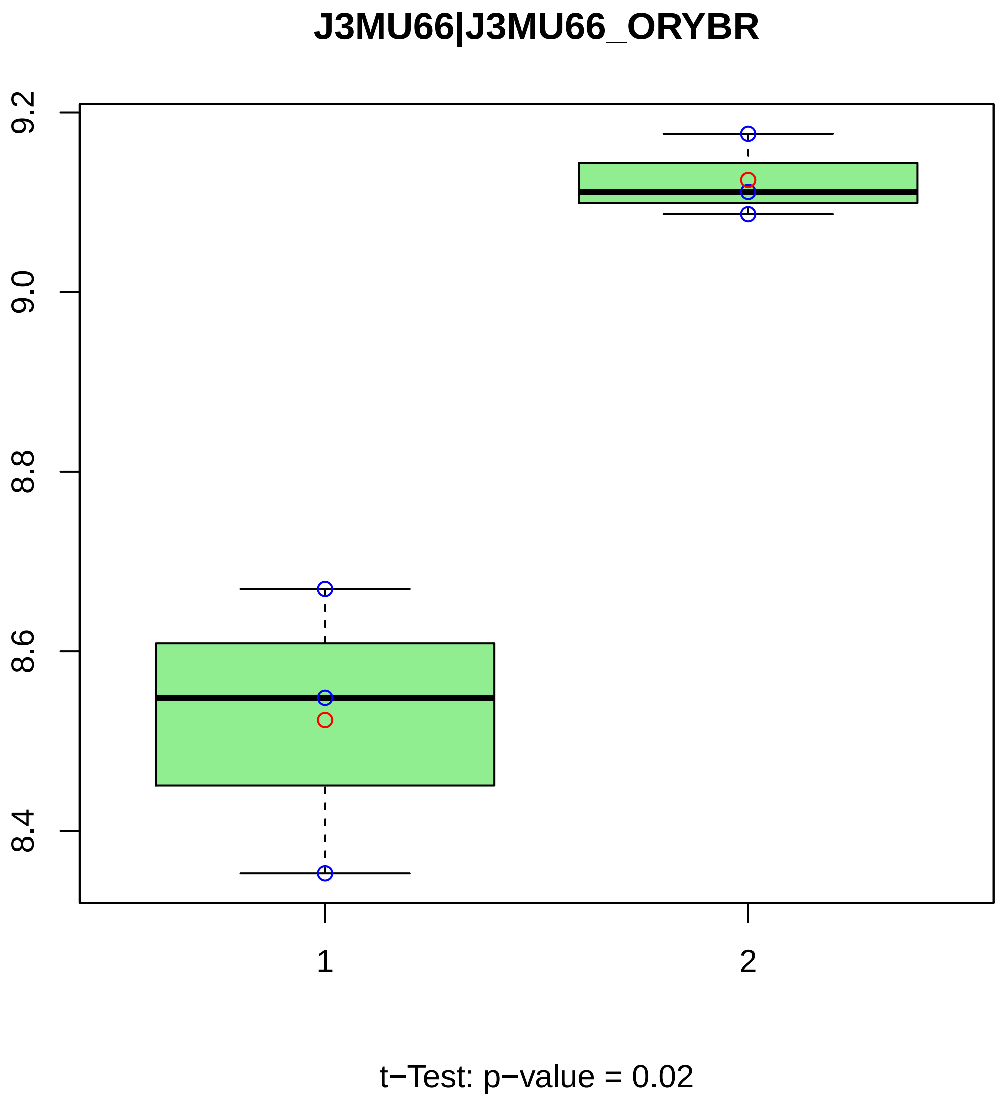
J3MU66|J3MU66_ORYBR
9.2
9.0
8.8
8.6
8.4
1
2
t−Test: p−value = 0.02

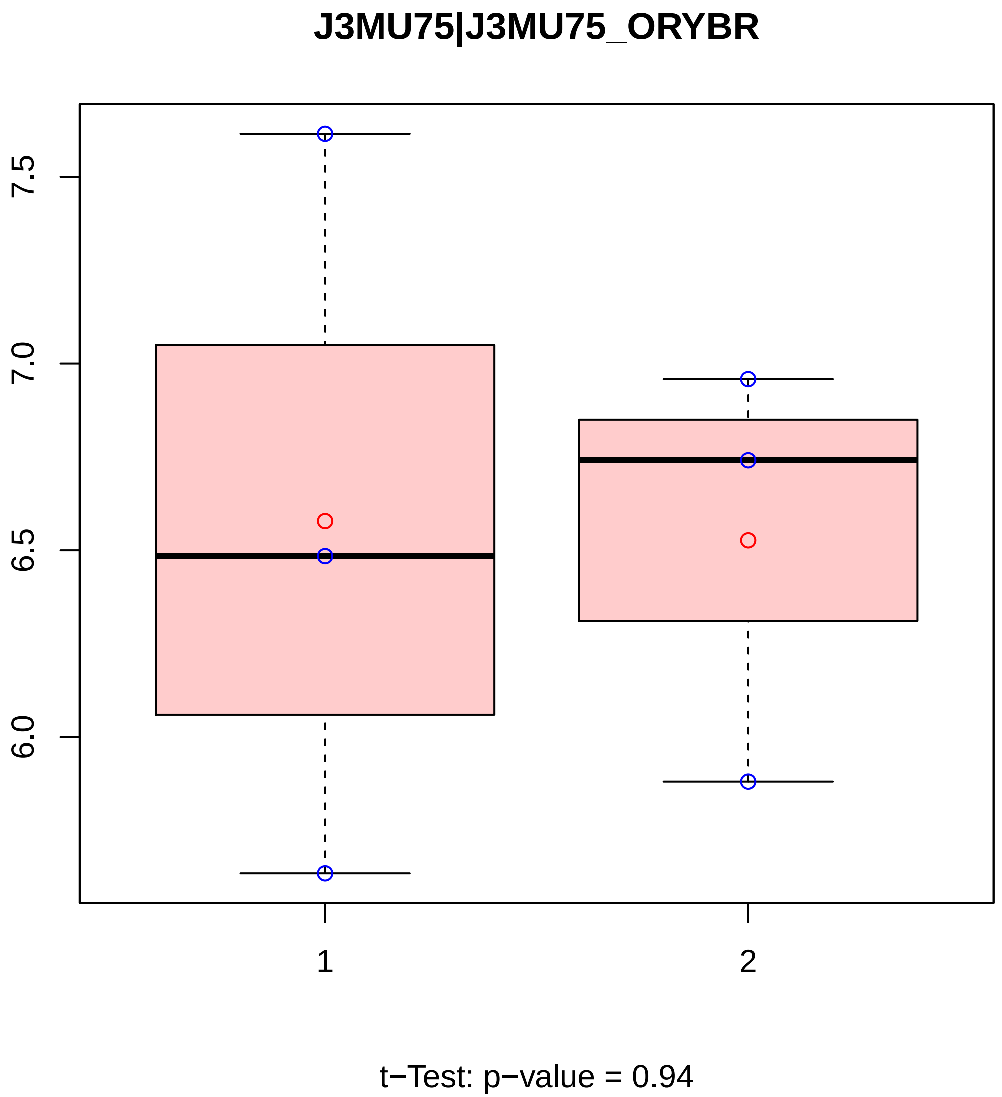
J3MU75|J3MU75_ORYBR
7.5
7.0
6.5
6.0
1
2
t−Test: p−value = 0.94

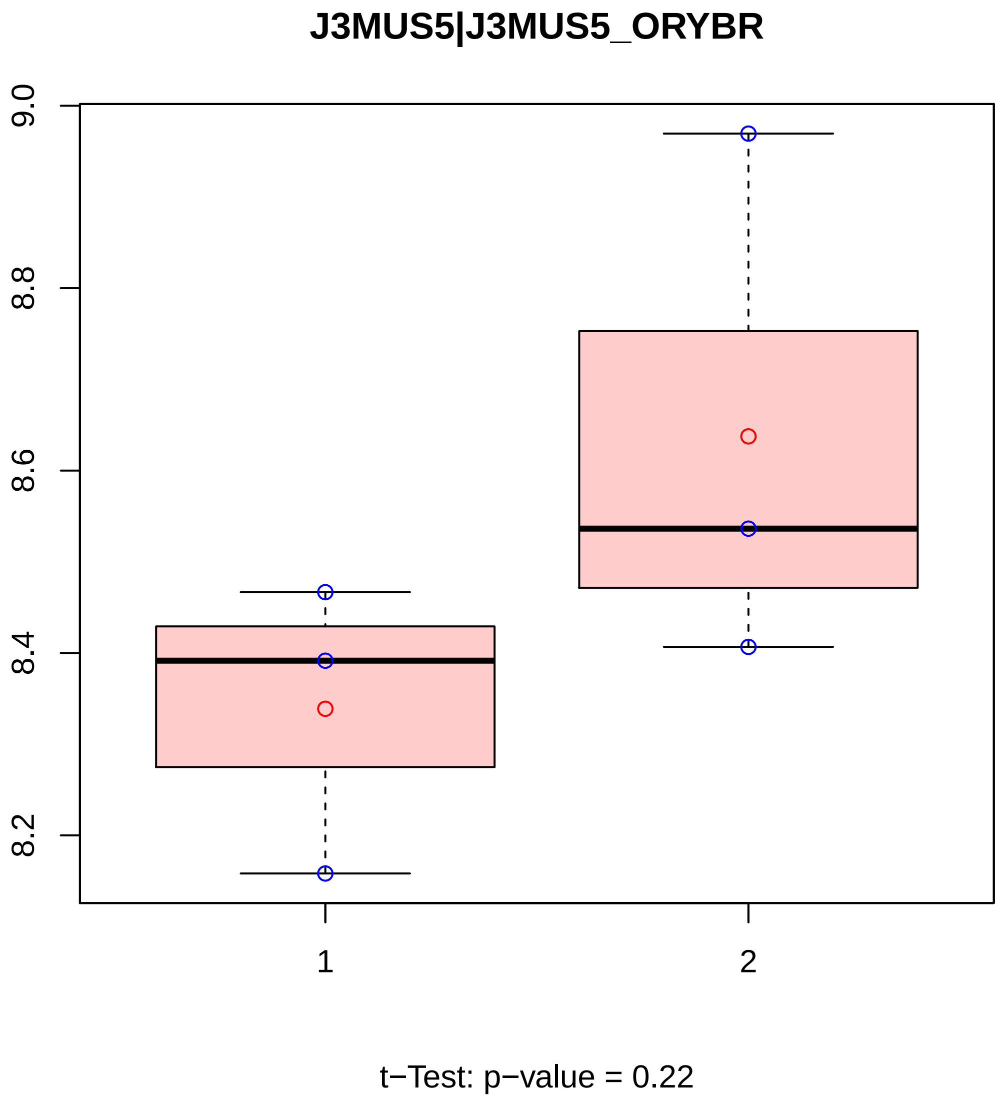
J3MUS5|J3MUS5_ORYBR
9.0
8.8
8.6
8.4
8.2
1
2
t−Test: p−value = 0.22

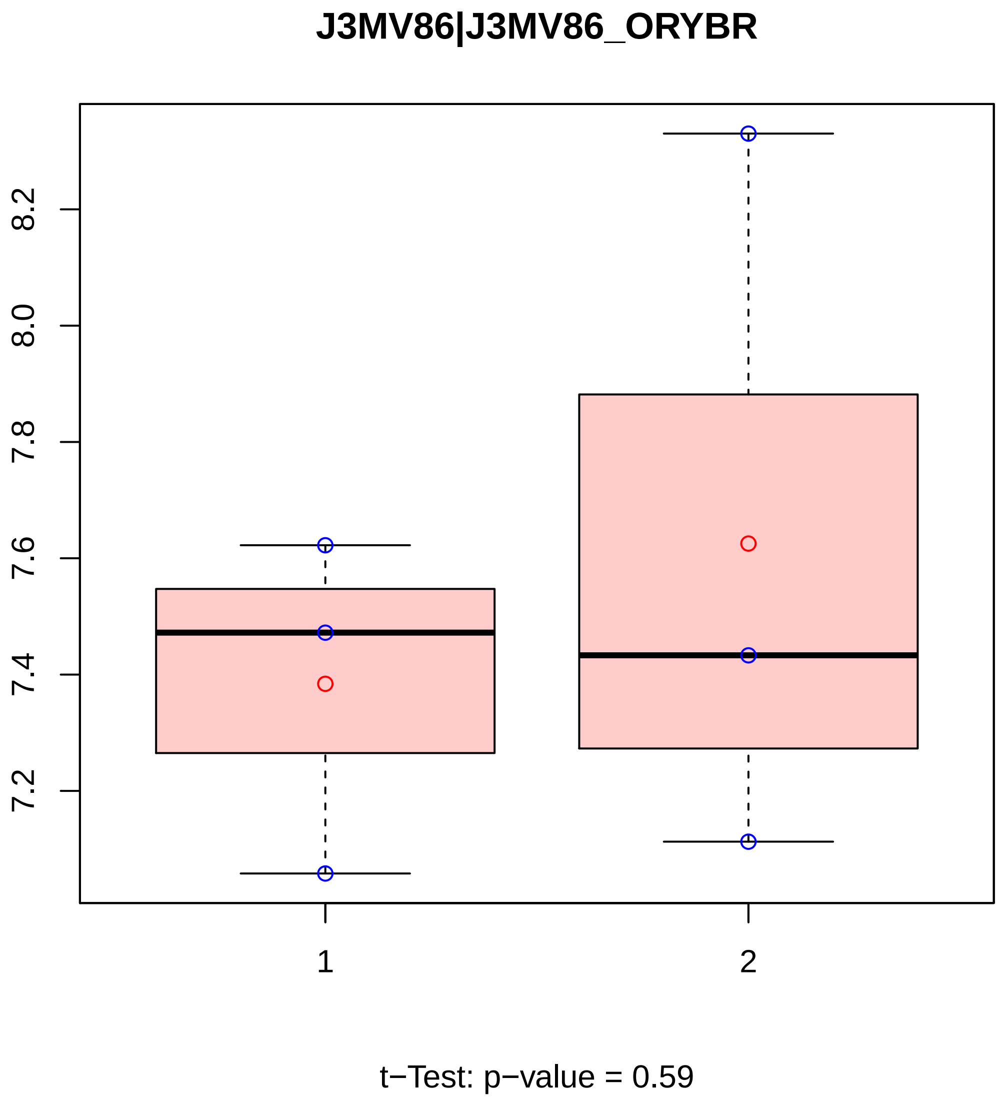
J3MV86|J3MV86_ORYBR
7.2
7.4
7.6
7.8
8.0
8.2
1
2
t−Test: p−value = 0.59

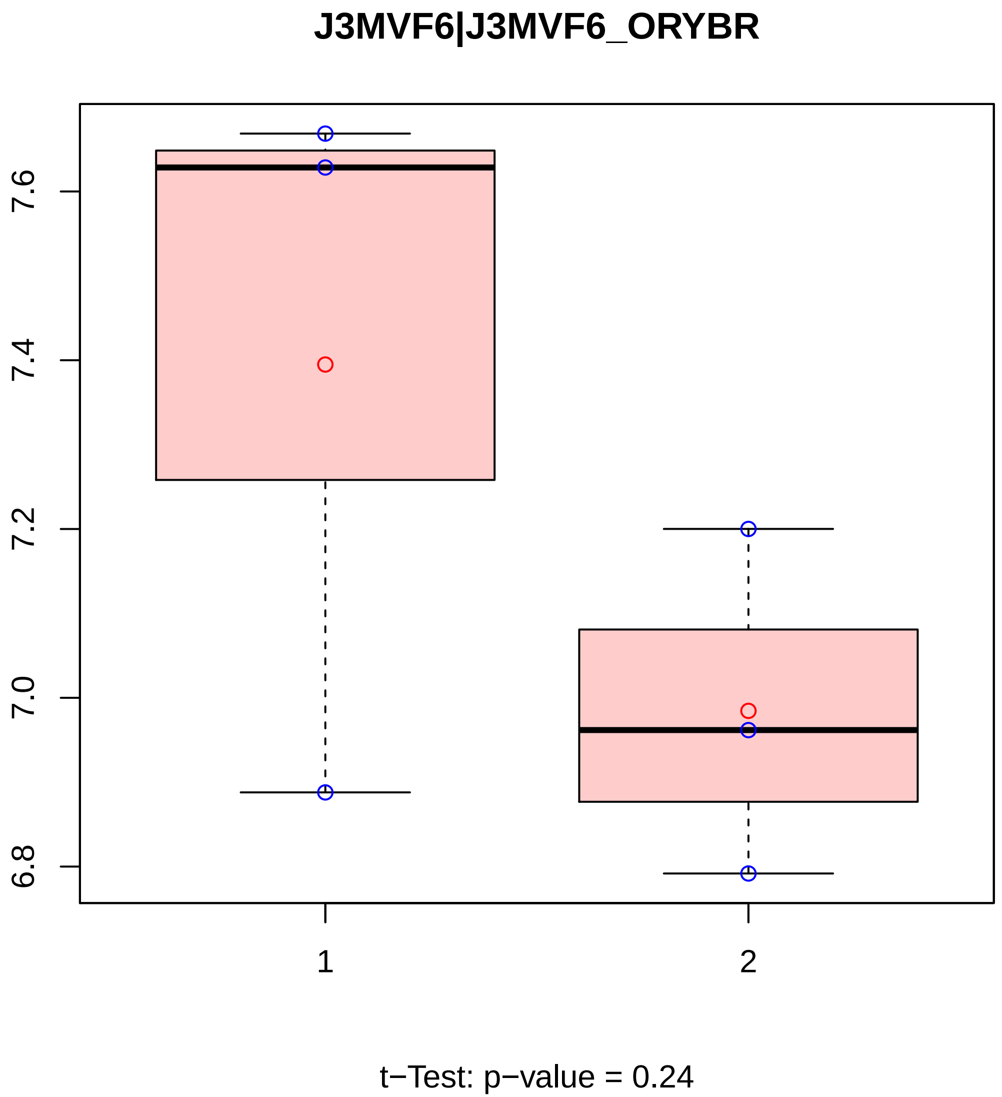
J3MVF6|J3MVF6_ORYBR
7.6
7.4
7.2
7.0
6.8
1
2
t−Test: p−value = 0.24

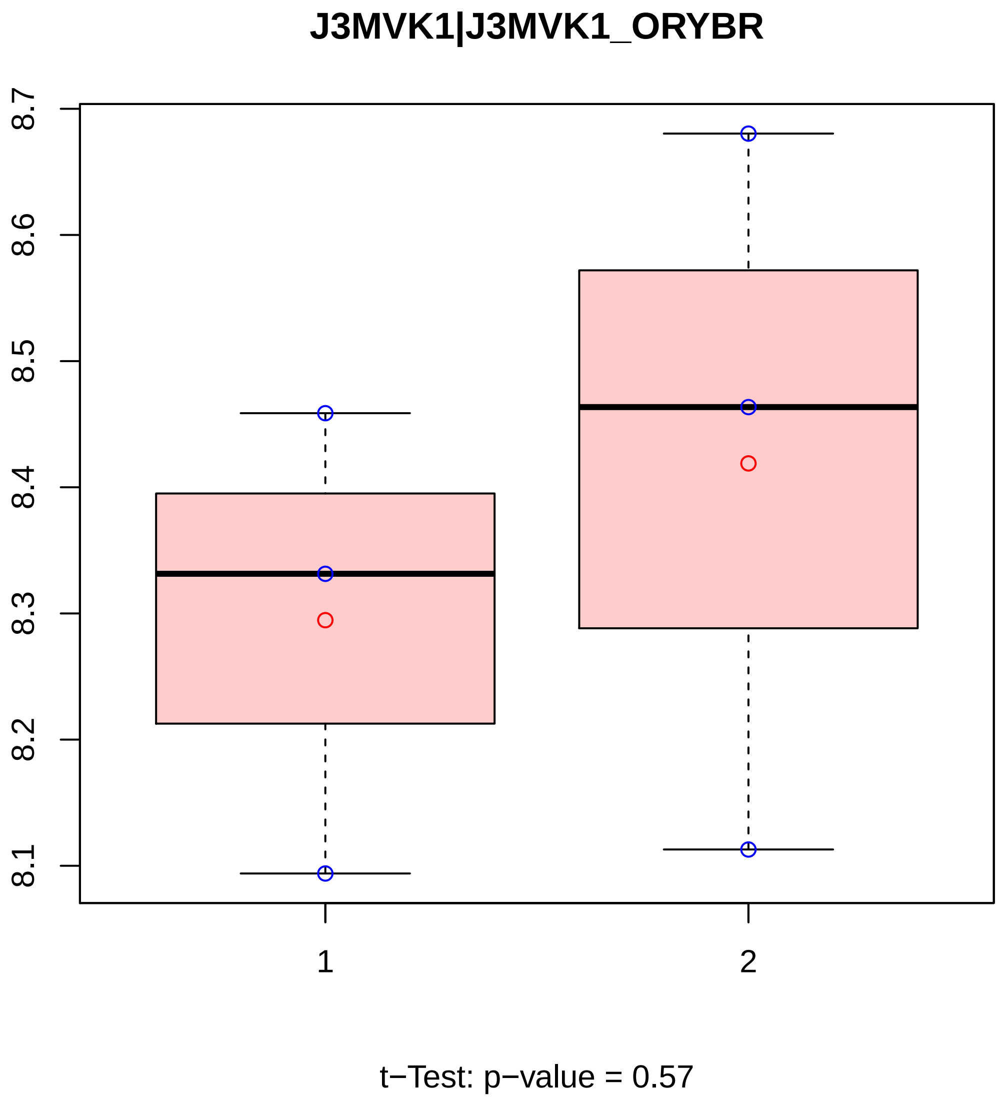
J3MVK1|J3MVK1_ORYBR
8.1
8.2
8.3
8.4
8.5
8.6
8.7
1
2
t−Test: p−value = 0.57

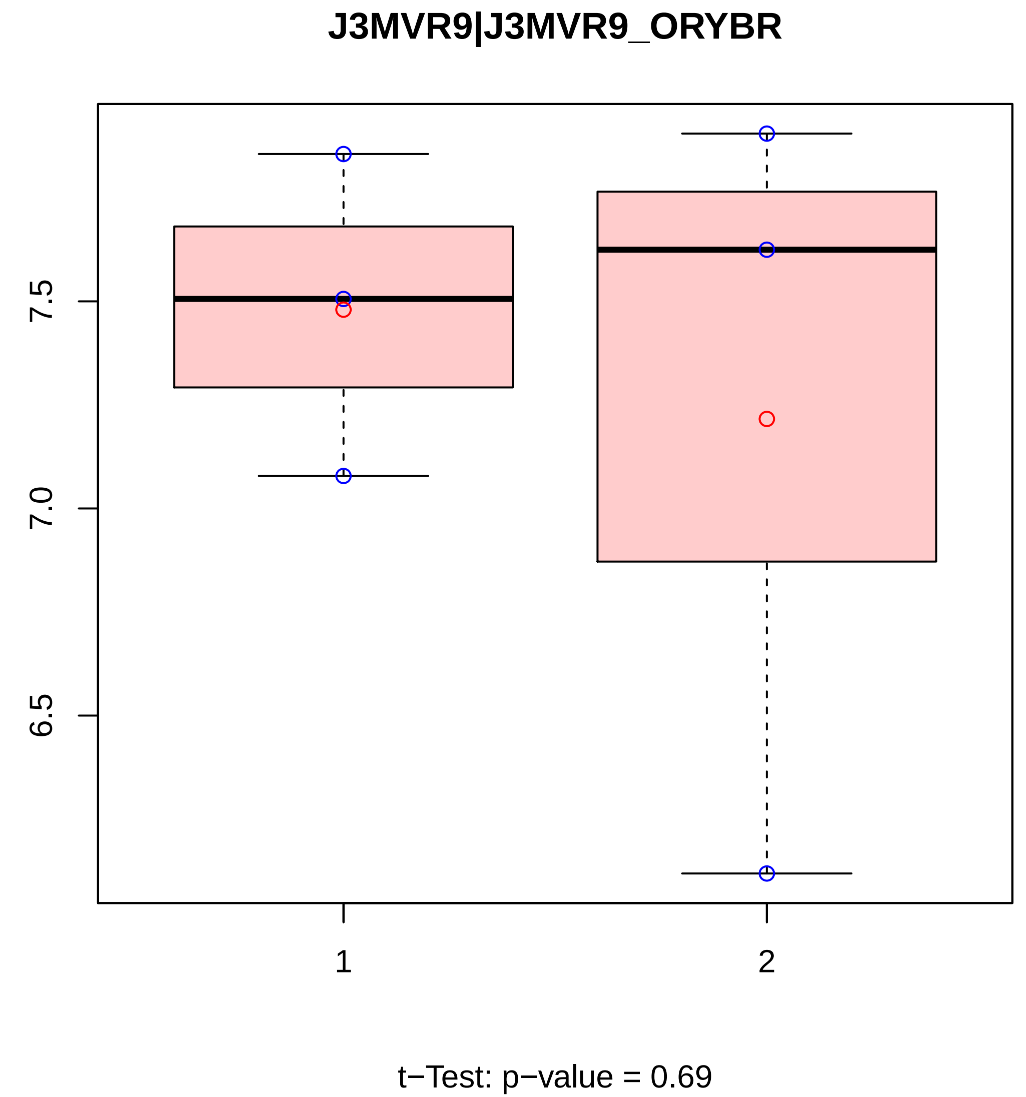

J3MVR9|J3MVR9_ORYBR
7.5
7.0
6.5
1
2
t−Test: p−value = 0.69

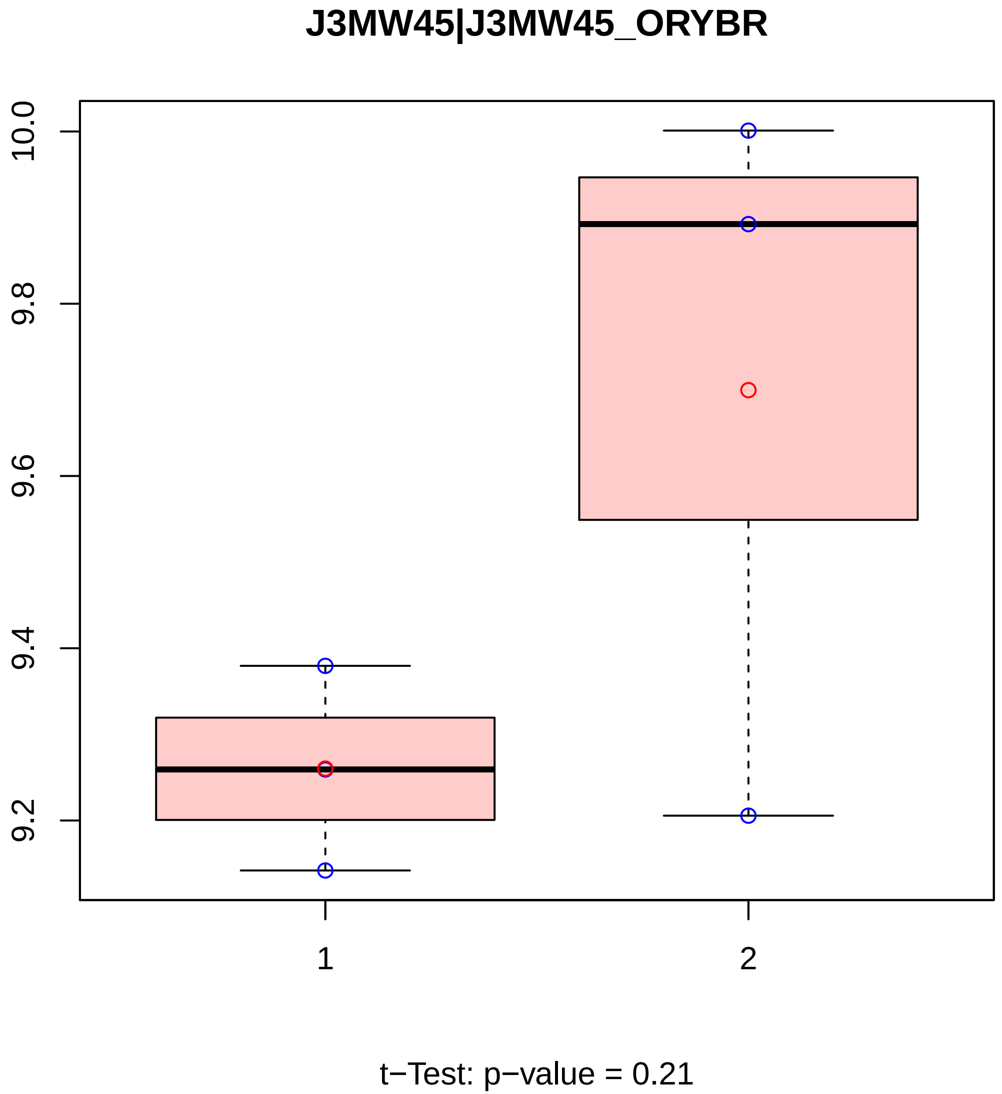

J3MW45|J3MW45_ORYBR
10.0
9.8
9.6
9.4
9.2
1
2
t−Test: p−value = 0.21

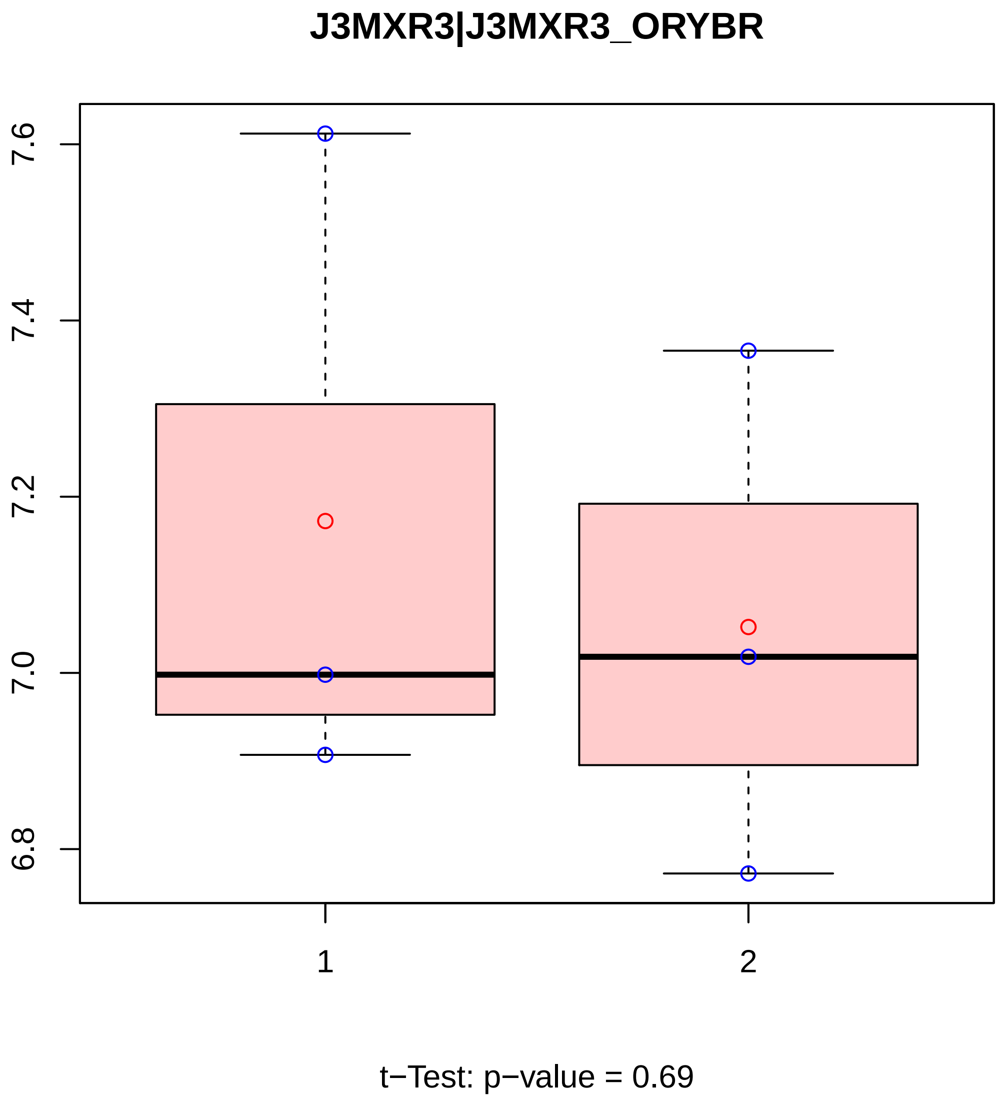
J3MXR3|J3MXR3_ORYBR
7.6
7.4
7.2
7.0
6.8
1
2
t−Test: p−value = 0.69

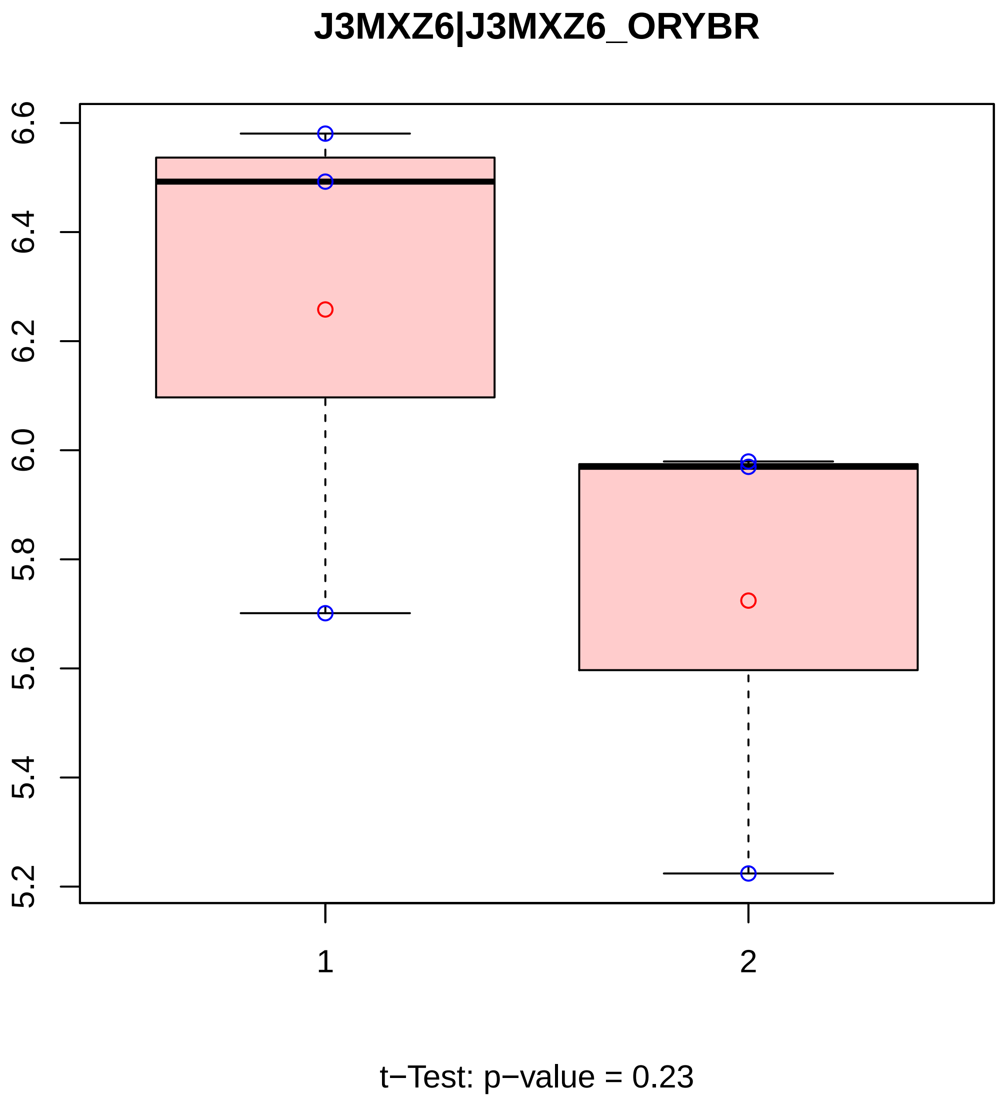

J3MXZ6|J3MXZ6_ORYBR
6.6
6.4
6.2
6.0
5.8
5.6
5.4
5.2
1
2
t−Test: p−value = 0.23

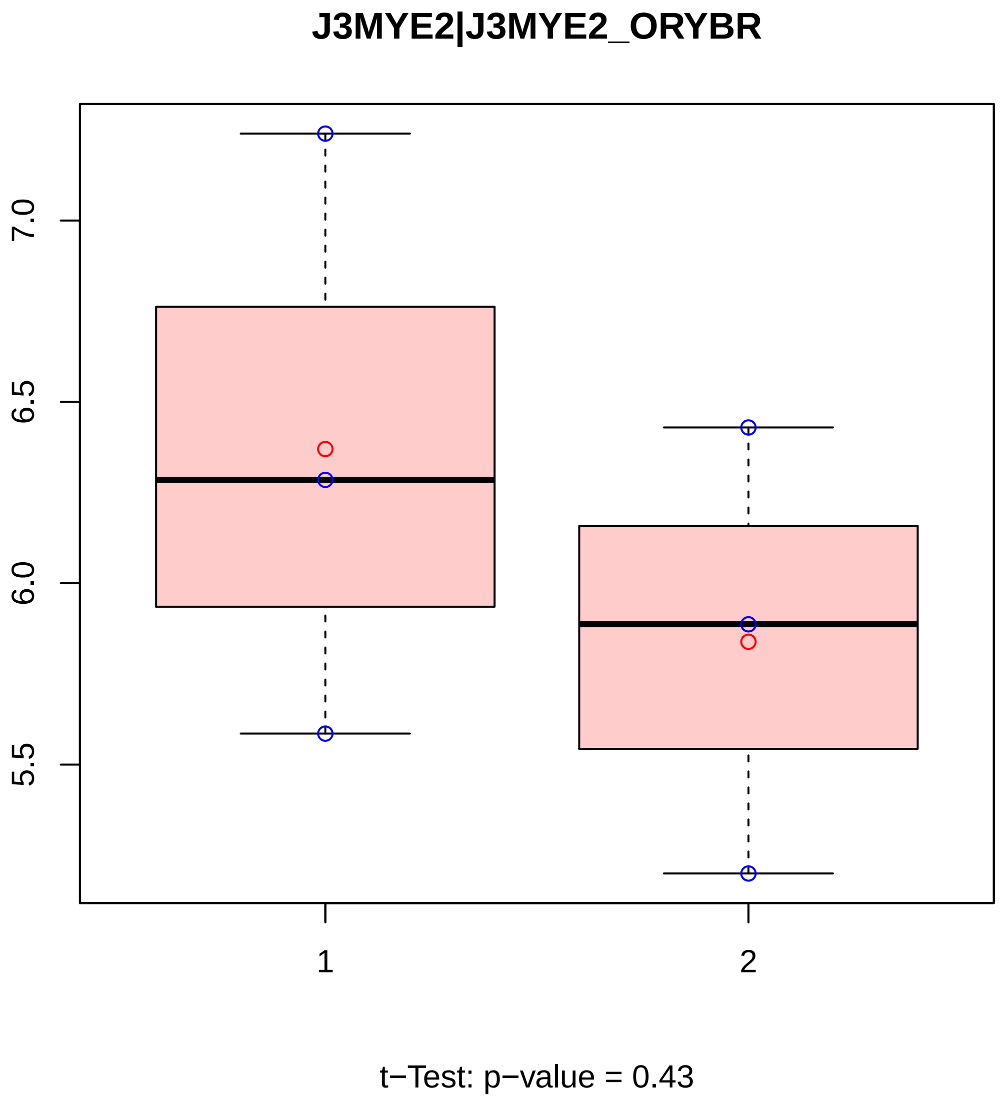
J3MYE2|J3MYE2_ORYBR
7.0
6.5
6.0
5.5
1
2
t−Test: p−value = 0.43

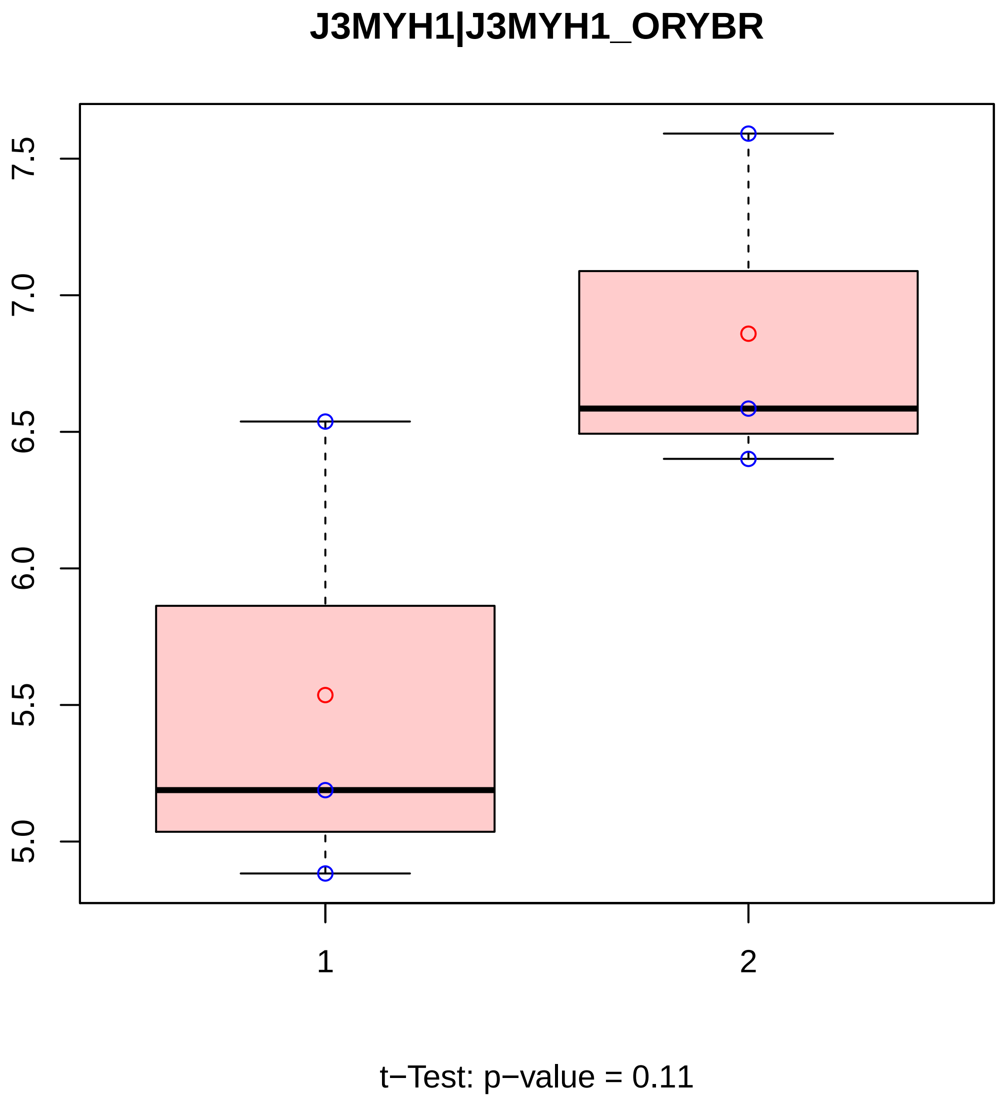

J3MYH1|J3MYH1_ORYBR
5.0
5.5
6.0
6.5
7.0
7.5
1
2
t−Test: p−value = 0.11

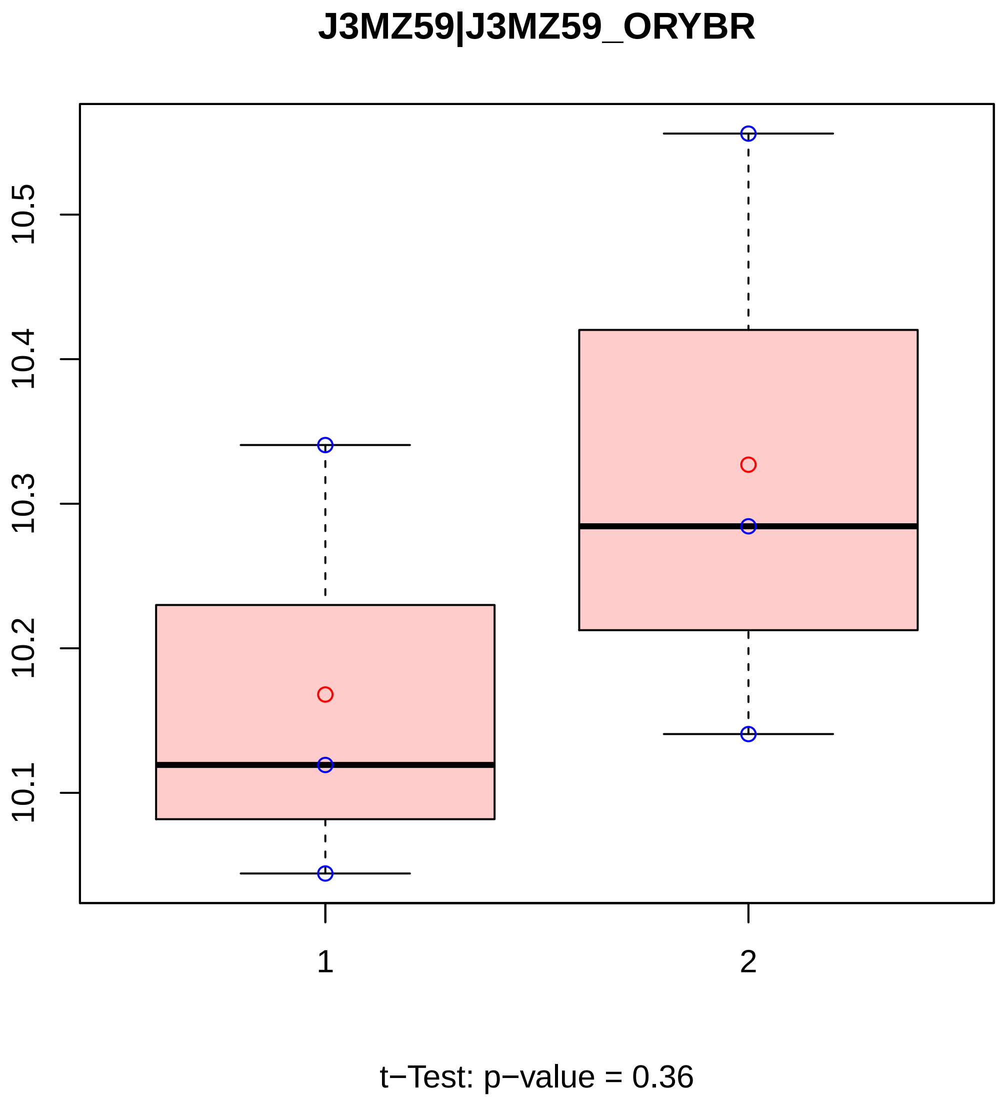
J3MZ59|J3MZ59_ORYBR
10.1
10.2
10.3
10.4
10.5
1
2
t−Test: p−value = 0.36

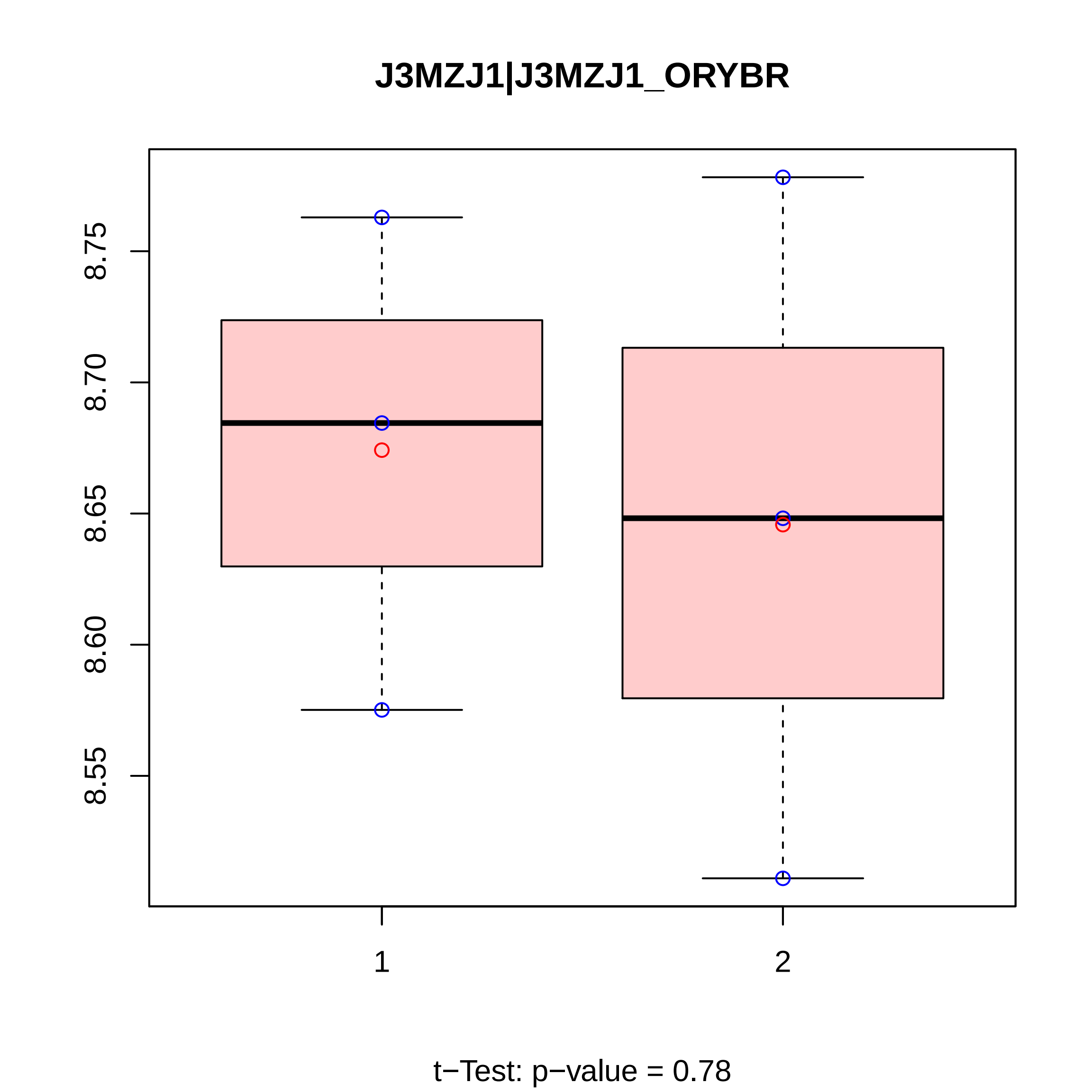
J3MZJ1|J3MZJ1_ORYBR
8.75
8.70
8.65
8.60
8.55
1
2
t−Test: p−value = 0.78

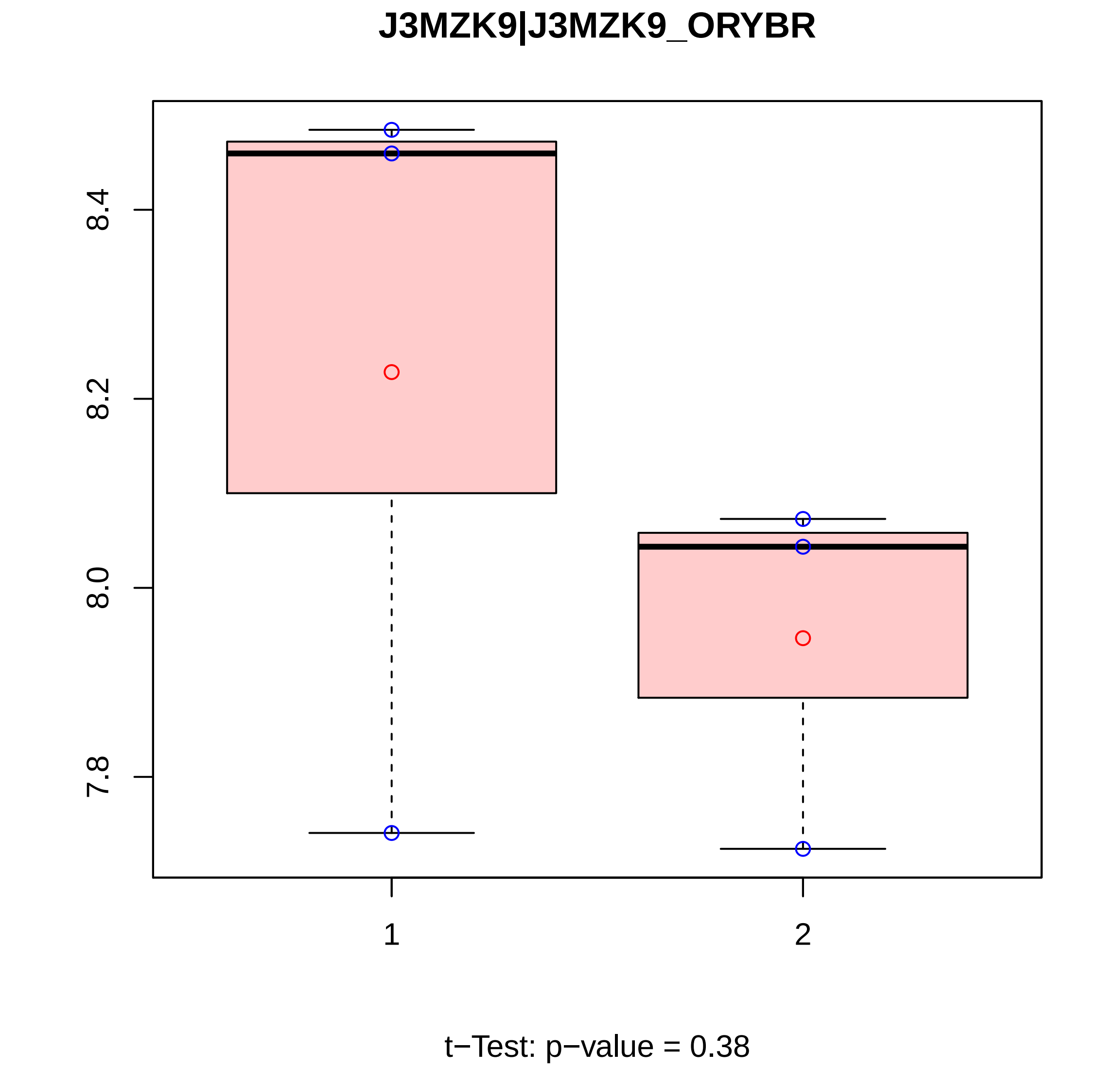

J3MZK9|J3MZK9_ORYBR
8.4
8.2
8.0
7.8
1
2
t−Test: p−value = 0.38

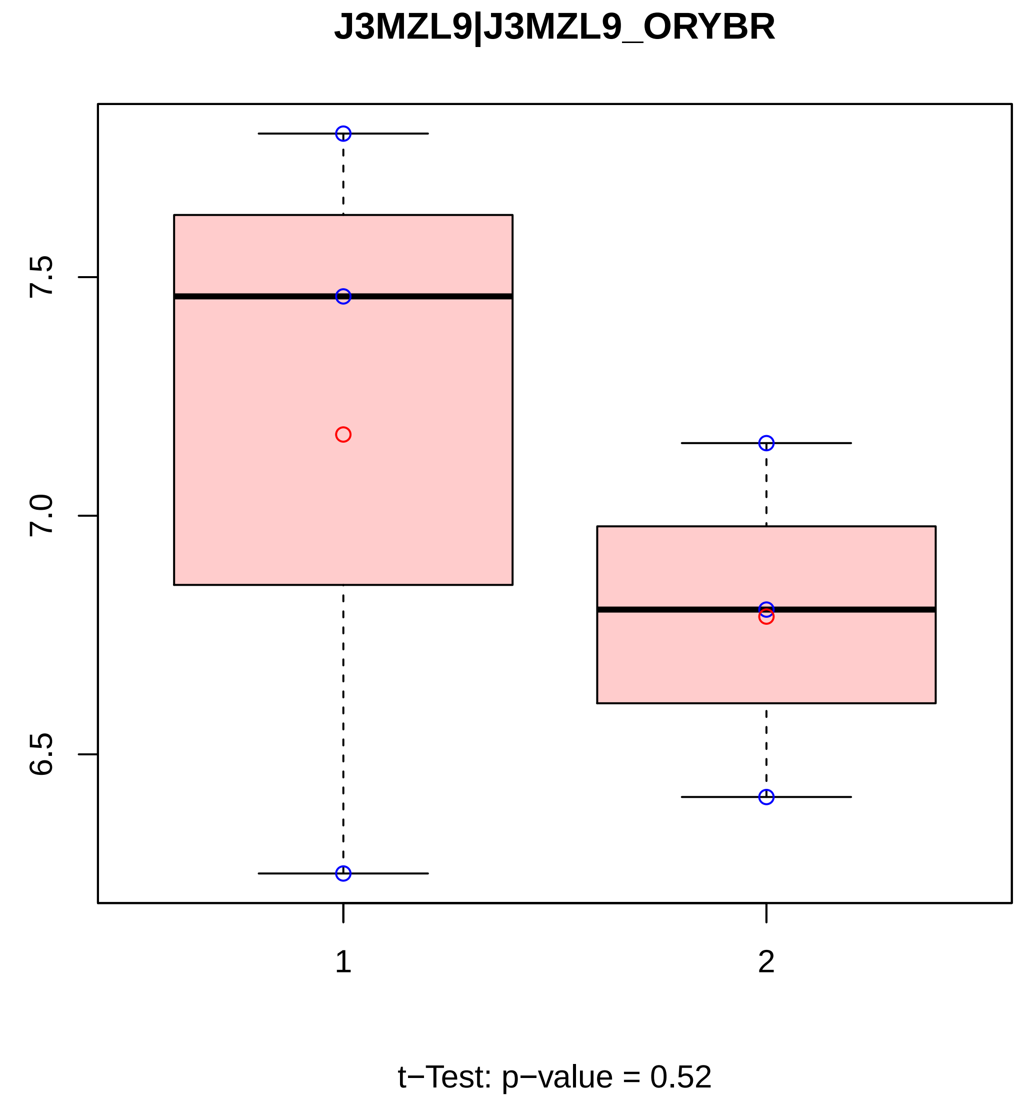
J3MZL9|J3MZL9_ORYBR
7.5
7.0
6.5
1
2
t−Test: p−value = 0.52

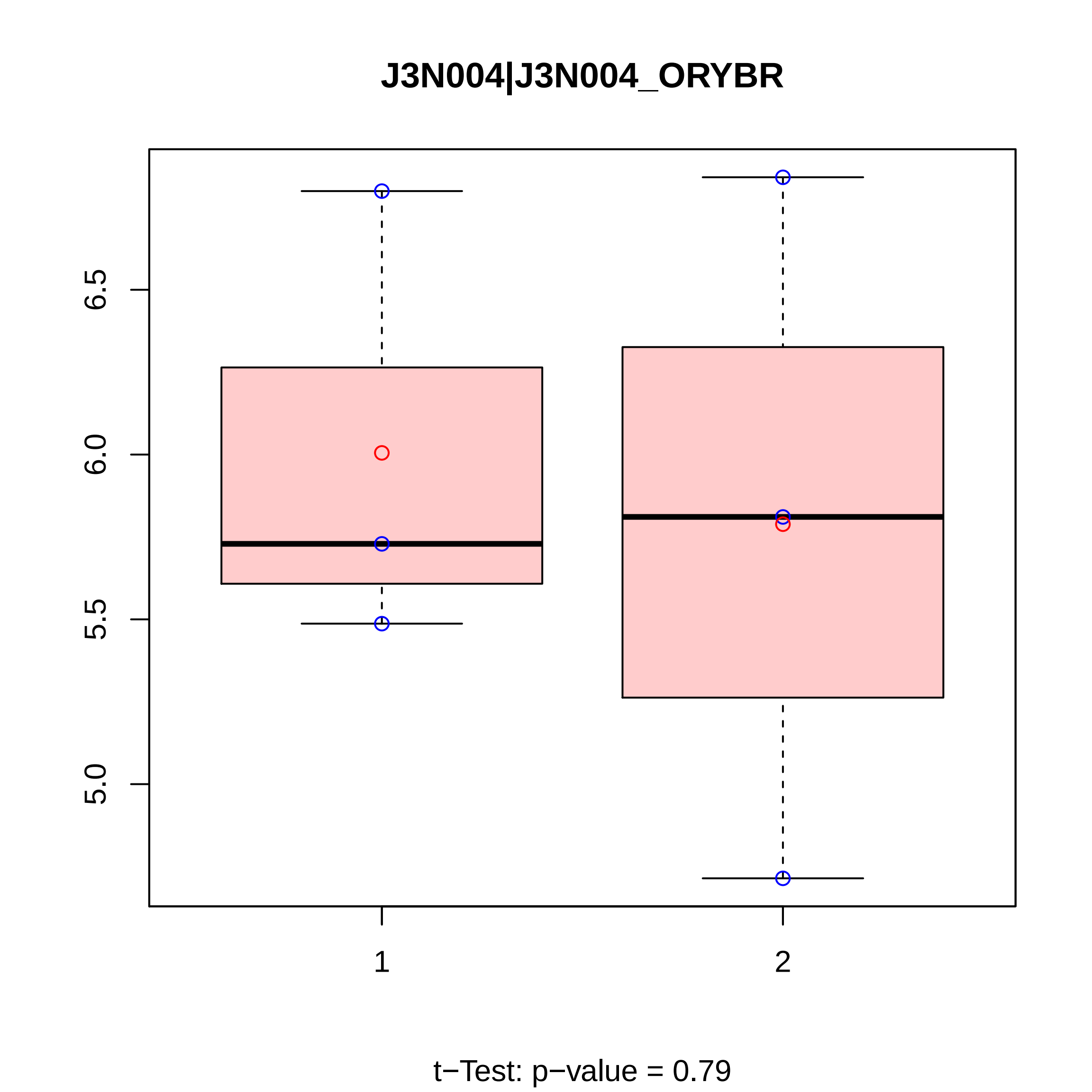
J3N004|J3N004_ORYBR
6.5
6.0
5.5
5.0
1
2
t−Test: p−value = 0.79

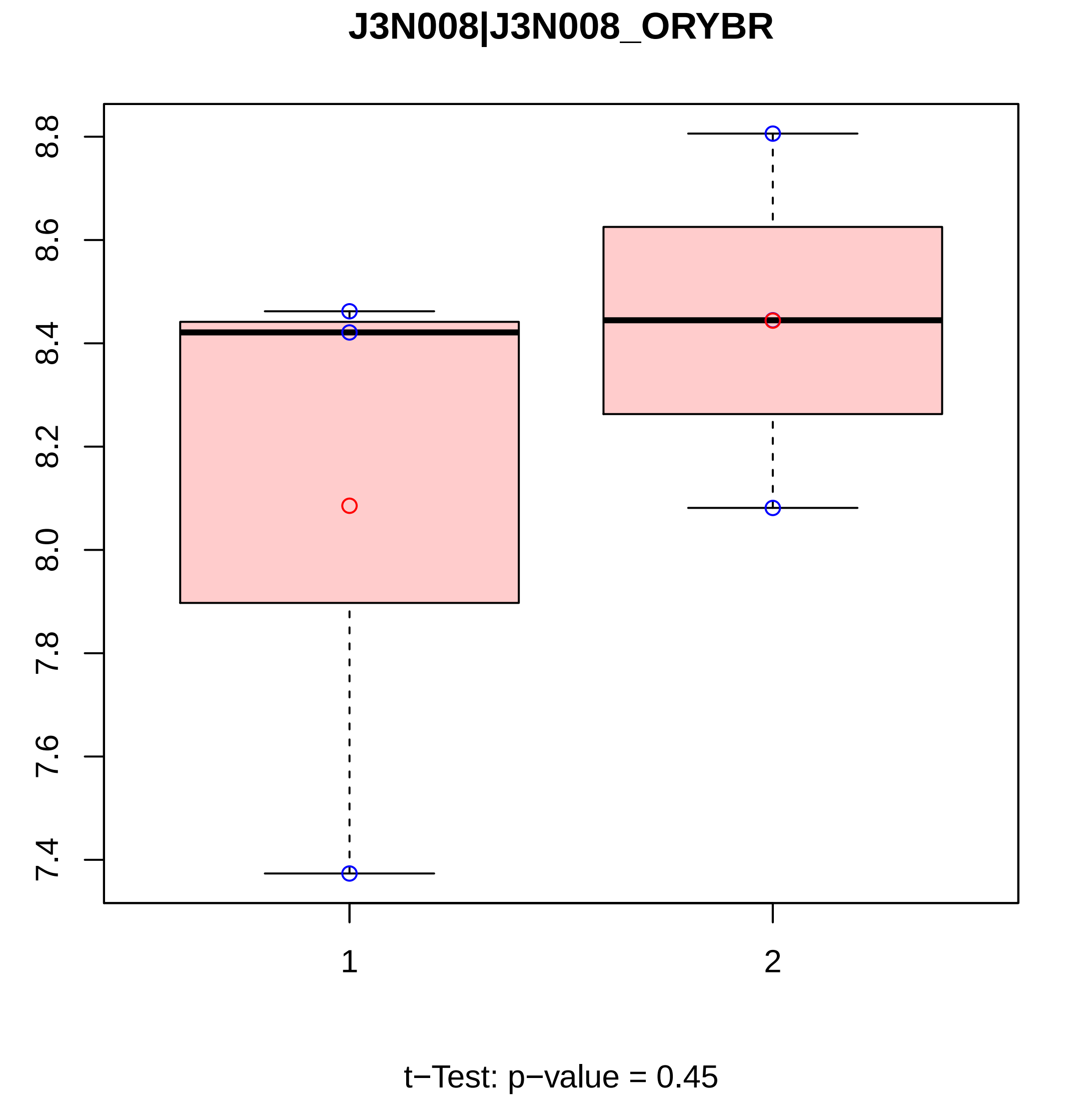
J3N008|J3N008_ORYBR
8.8
8.6
8.4
8.2
8.0
7.8
7.6
7.4
1
2
t−Test: p−value = 0.45

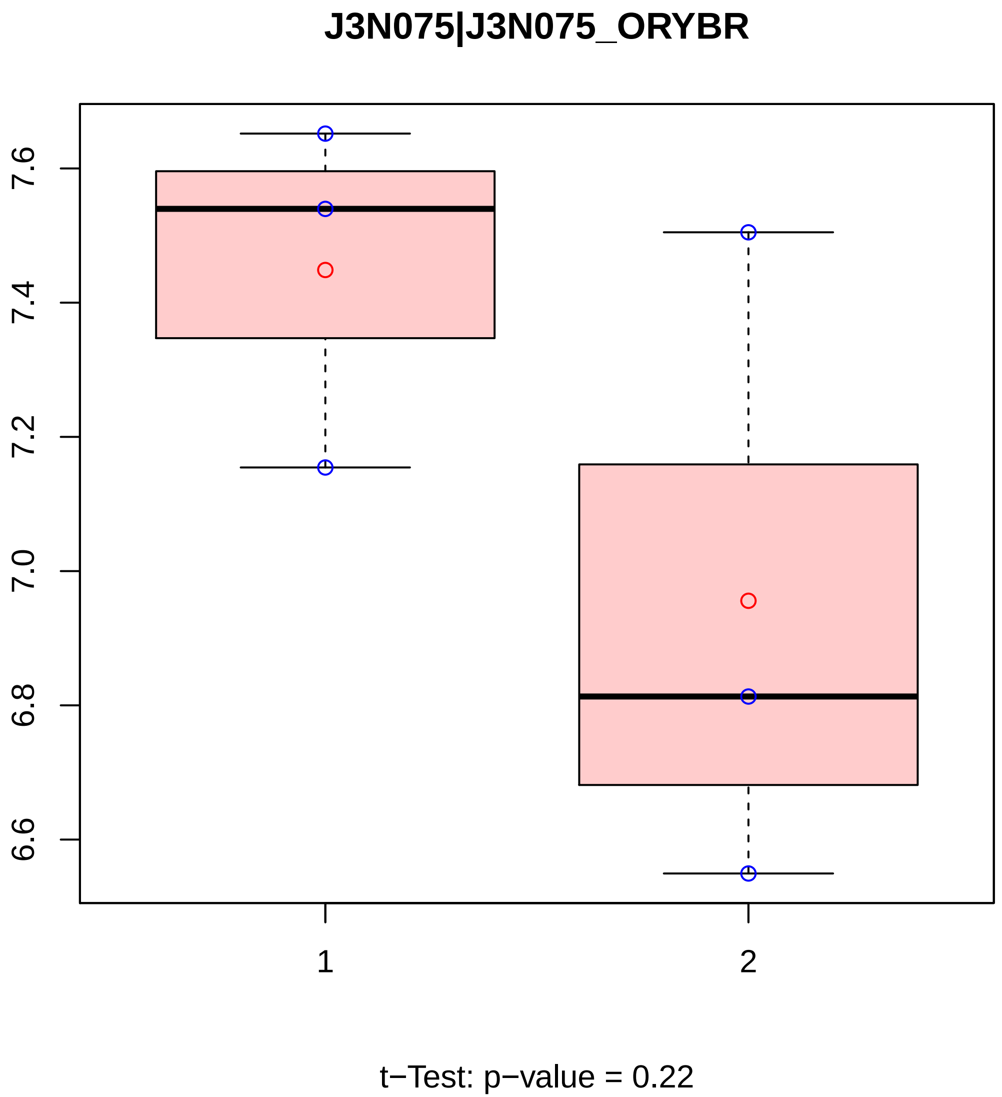
J3N075|J3N075_ORYBR
7.6
7.4
7.2
7.0
6.8
6.6
1
2
t−Test: p−value = 0.22

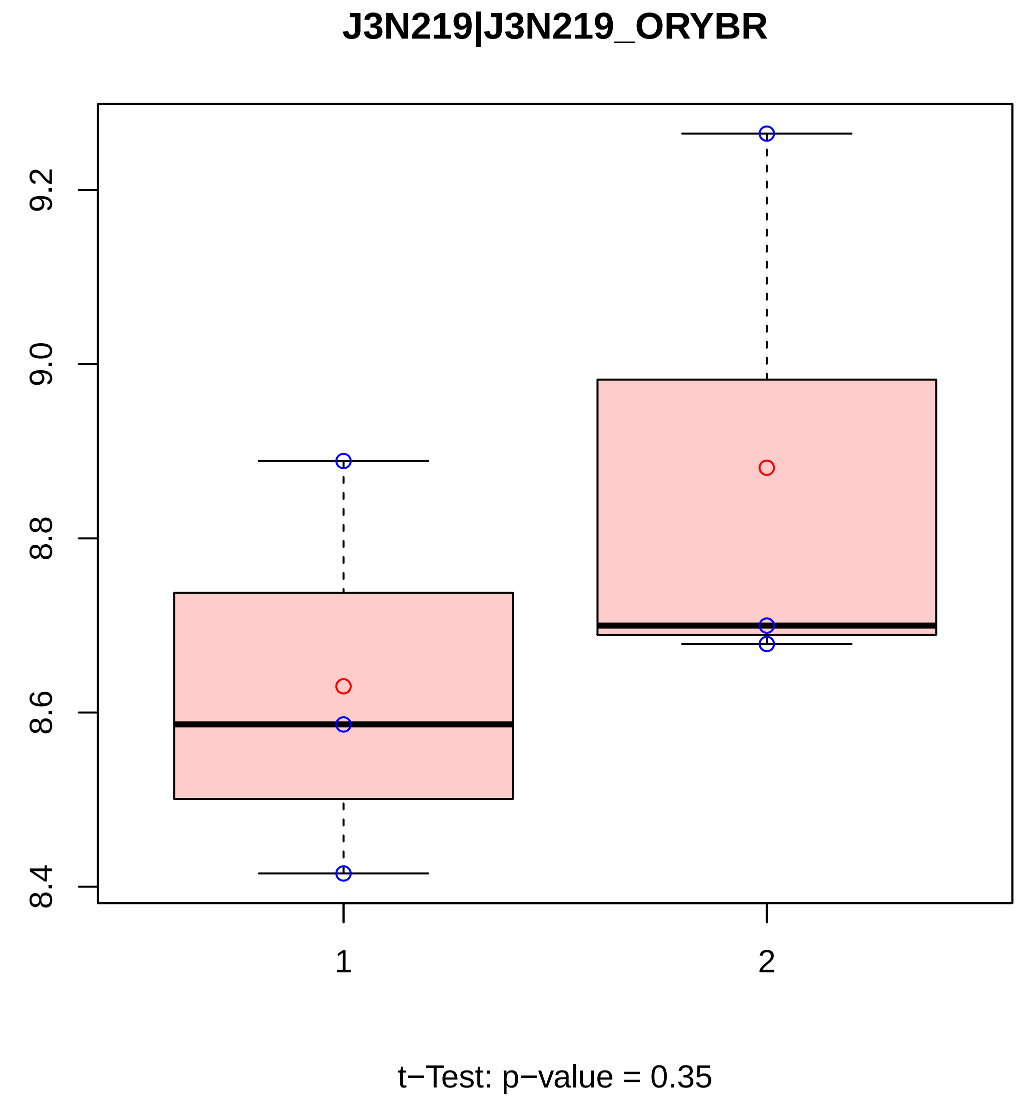
J3N219|J3N219_ORYBR
9.2
9.0
8.8
8.6
8.4
1
2
t−Test: p−value = 0.35

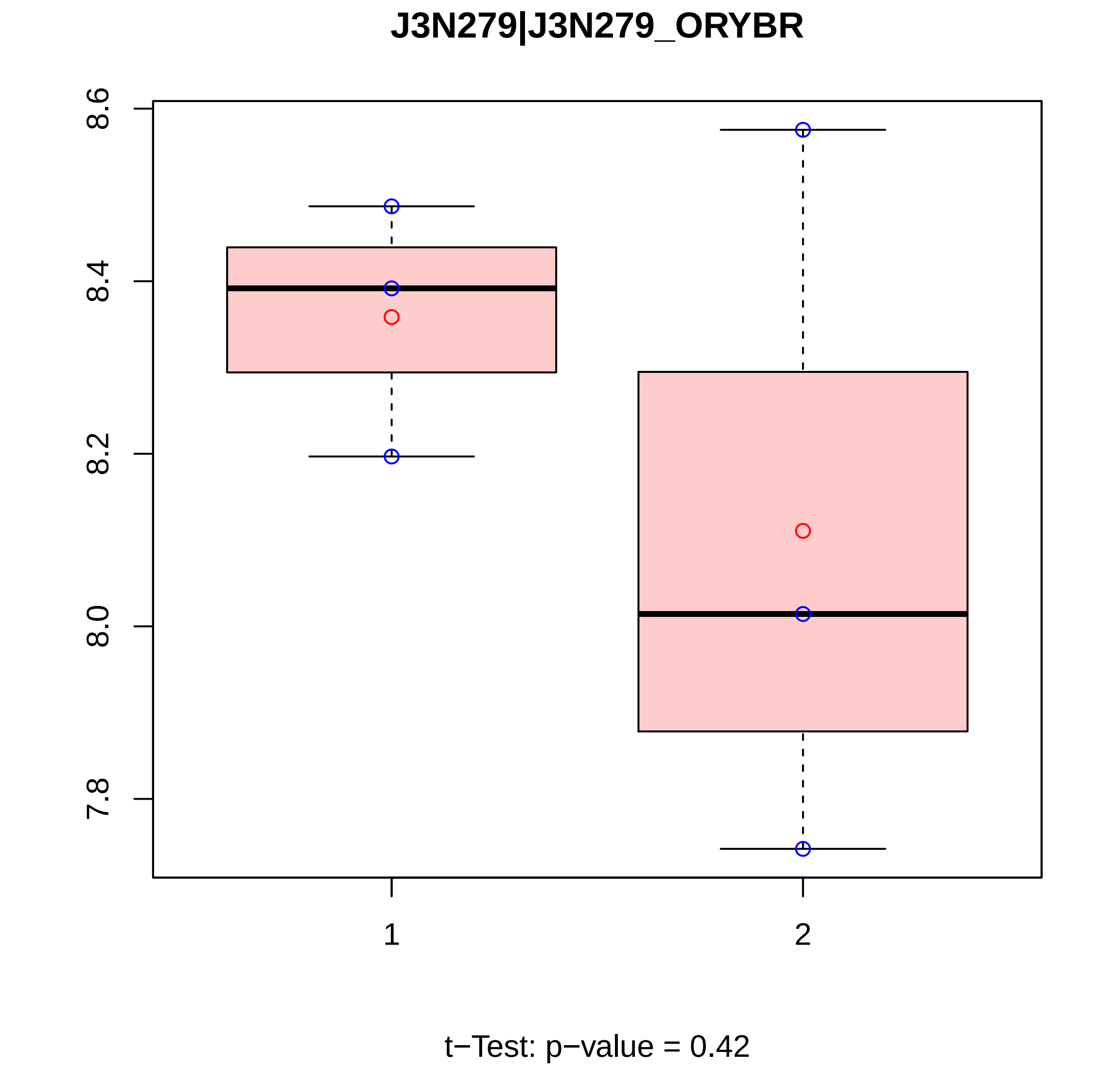
J3N279|J3N279_ORYBR
8.6
8.4
8.2
8.0
7.8
1
2
t−Test: p−value = 0.42

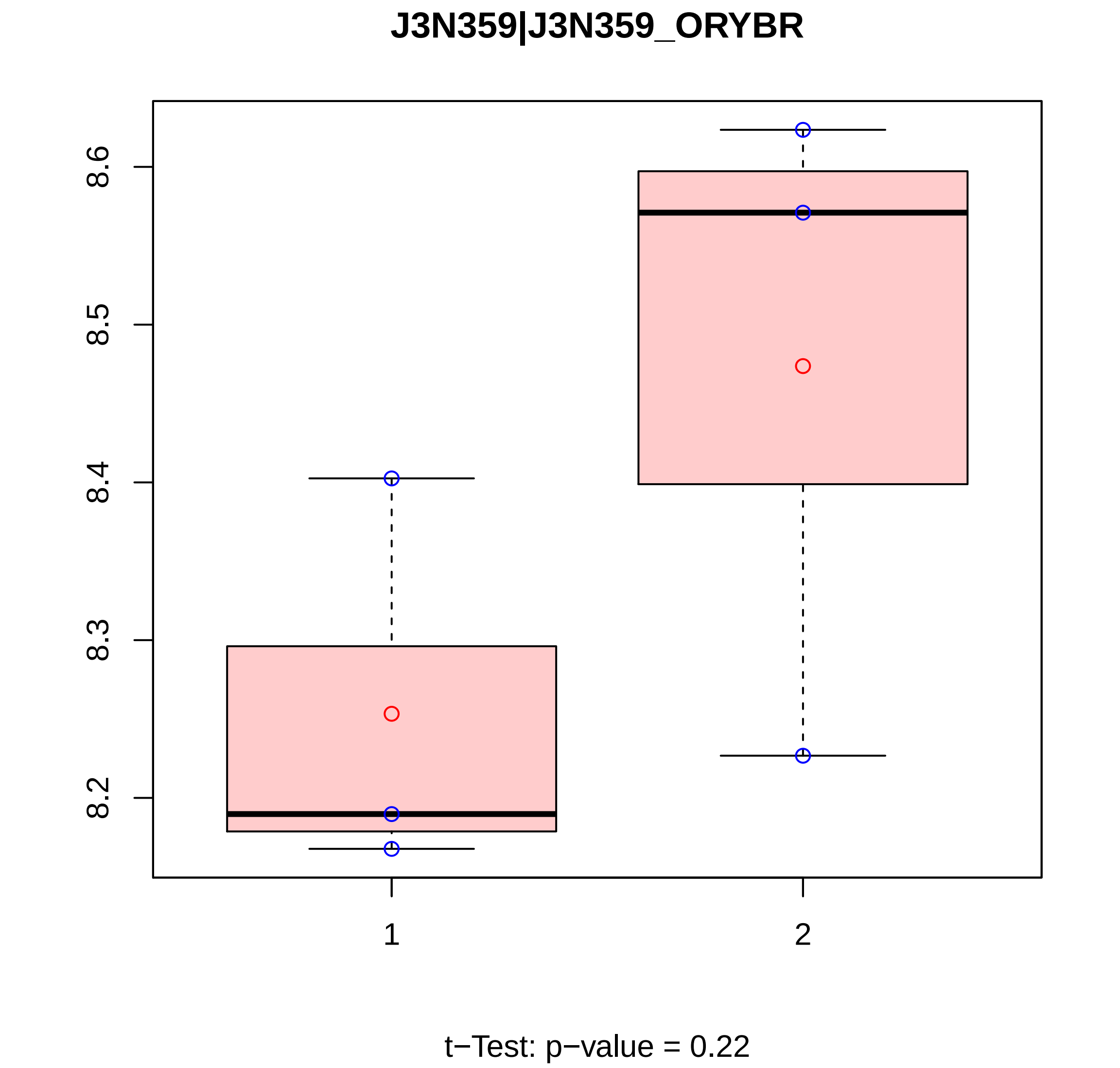
J3N359|J3N359_ORYBR
8.2
8.3
8.4
8.5
8.6
1
2
t−Test: p−value = 0.22

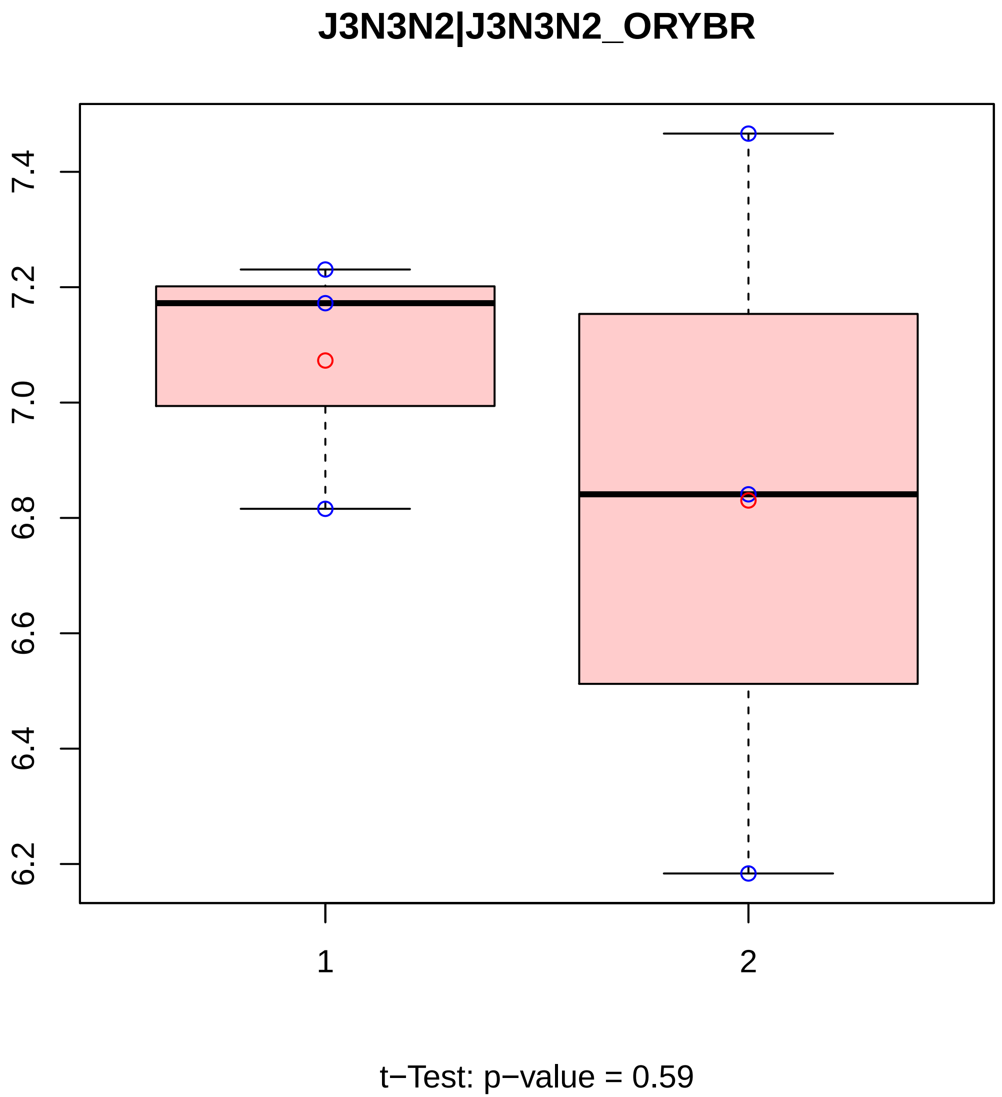
J3N3N2|J3N3N2_ORYBR
7.4
7.2
7.0
6.8
6.6
6.4
6.2
1
2
t−Test: p−value = 0.59

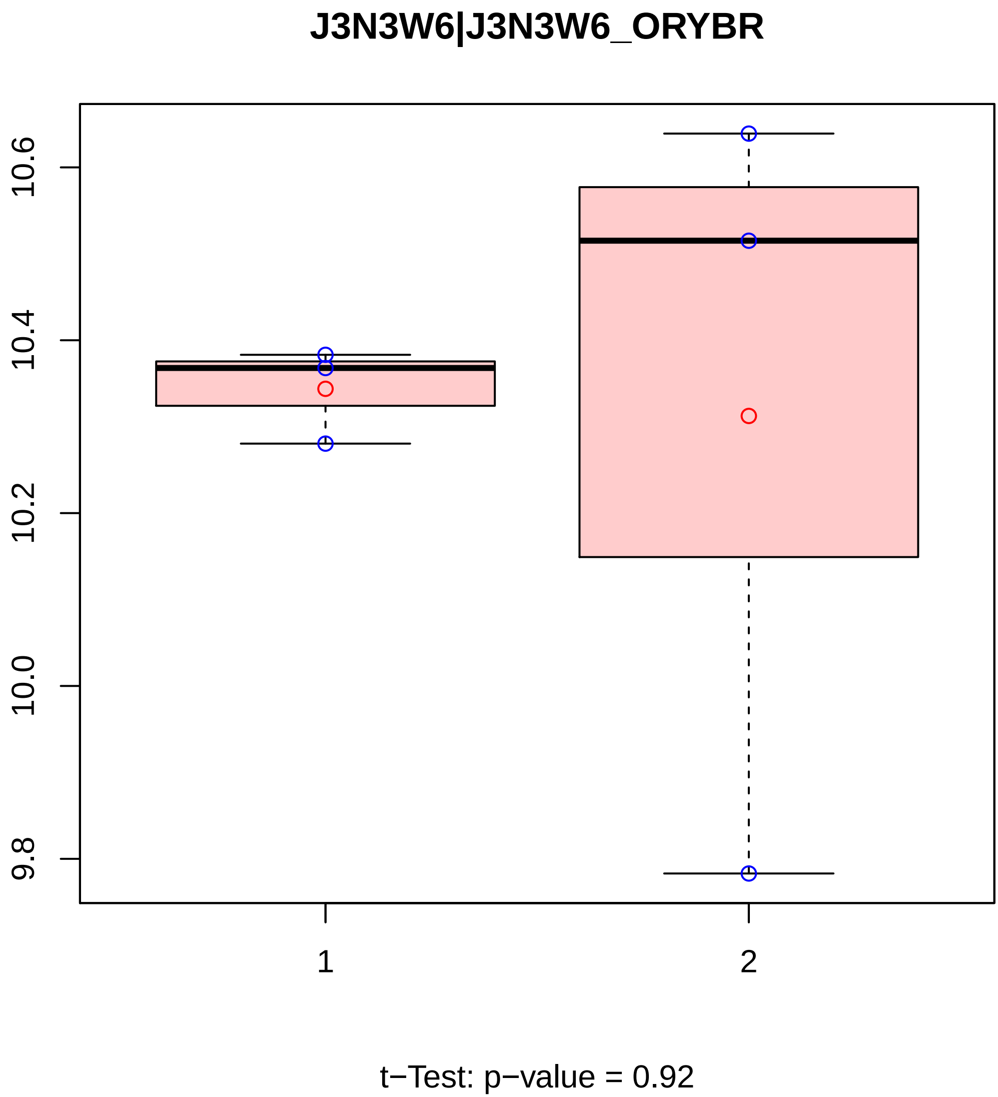
J3N3W6|J3N3W6_ORYBR
10.6
10.4
10.2
10.0
9.8
1
2
t−Test: p−value = 0.92

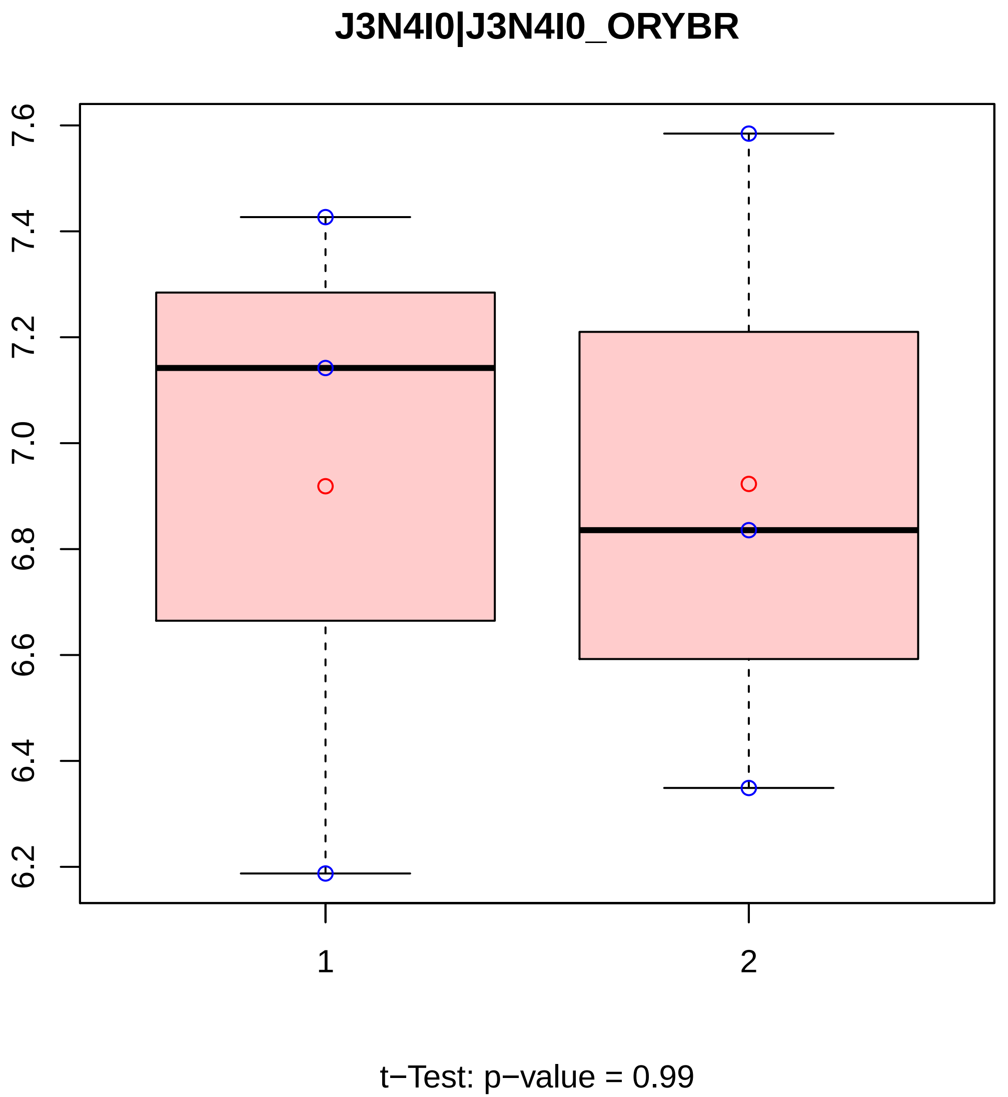
J3N4I0|J3N4I0_ORYBR
7.6
7.4
7.2
7.0
6.8
6.6
6.4
6.2
1
2
t−Test: p−value = 0.99

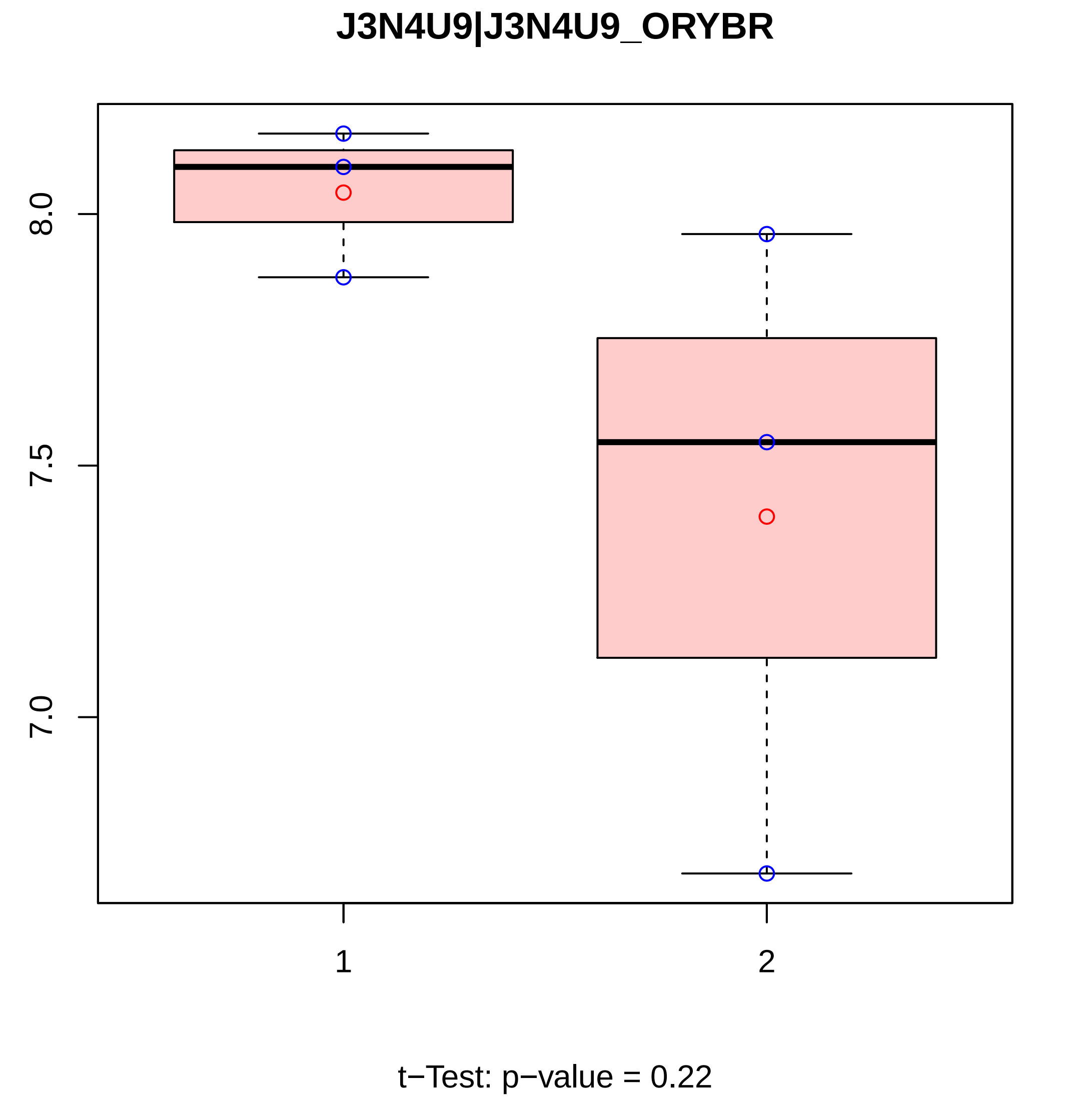
J3N4U9|J3N4U9_ORYBR
8.0
7.5
7.0
1
2
t−Test: p−value = 0.22

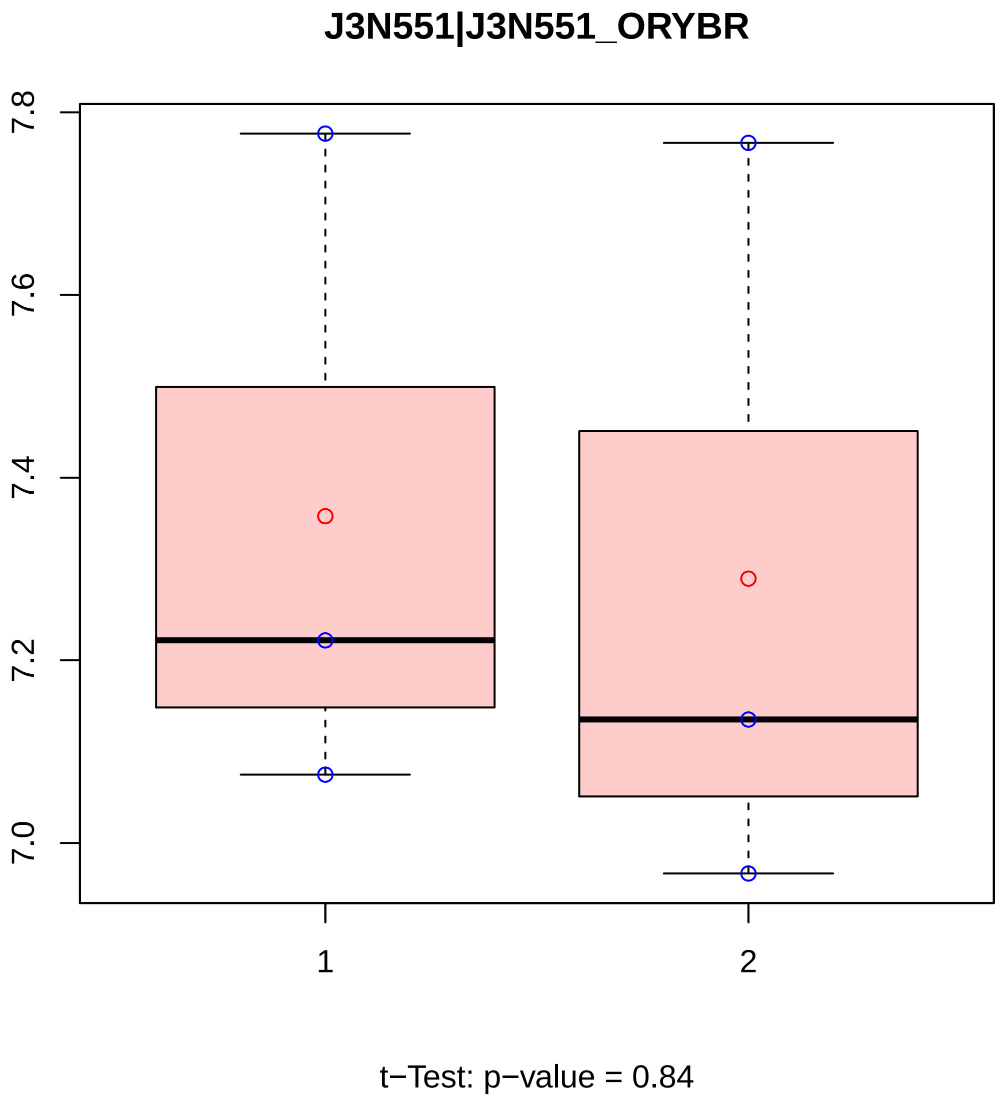
J3N551|J3N551_ORYBR
7.0
7.2
7.4
7.6
7.8
1
2
t−Test: p−value = 0.84

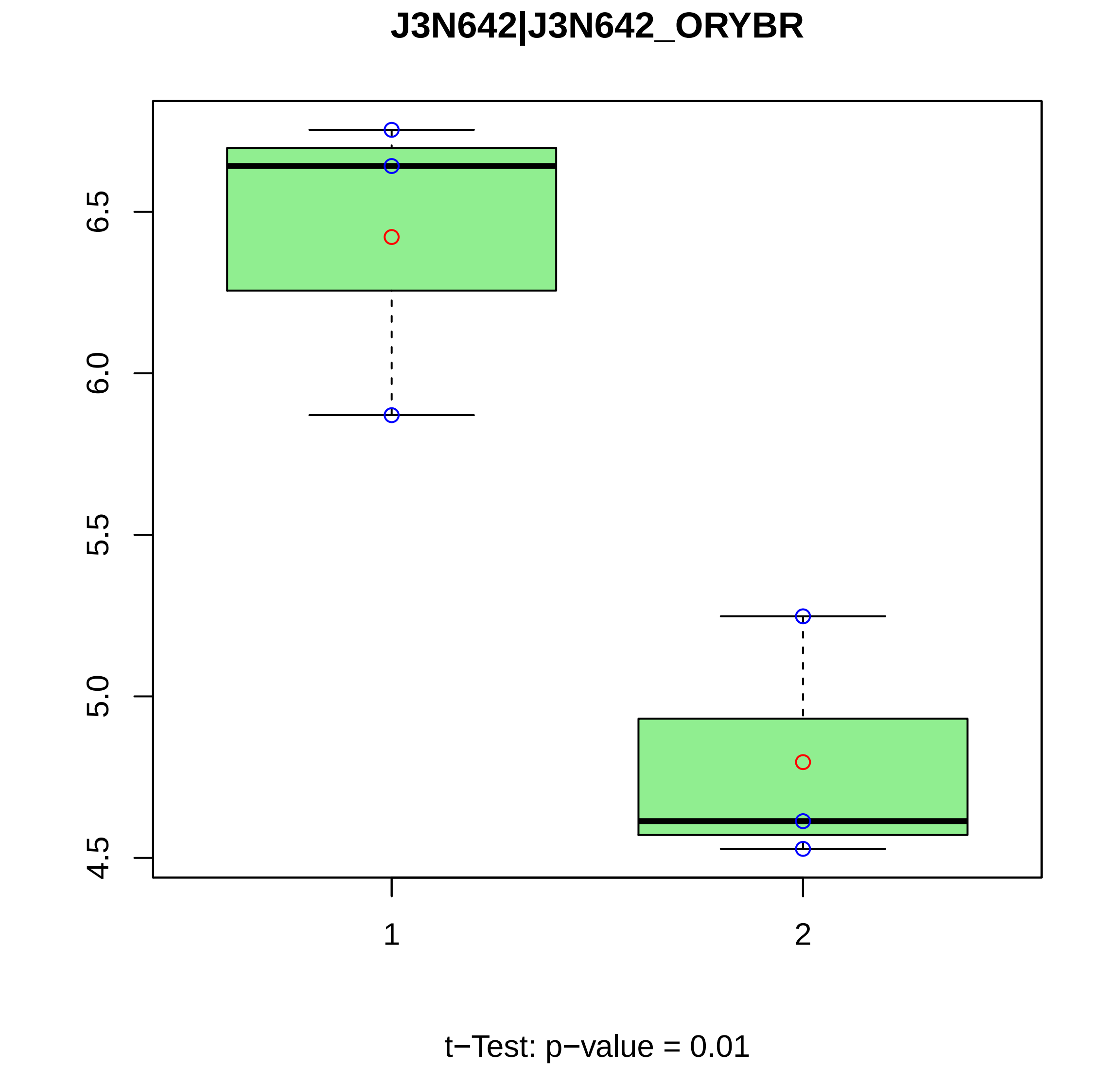

J3N642|J3N642_ORYBR
6.5
6.0
5.5
5.0
4.5
1
2
t−Test: p−value = 0.01

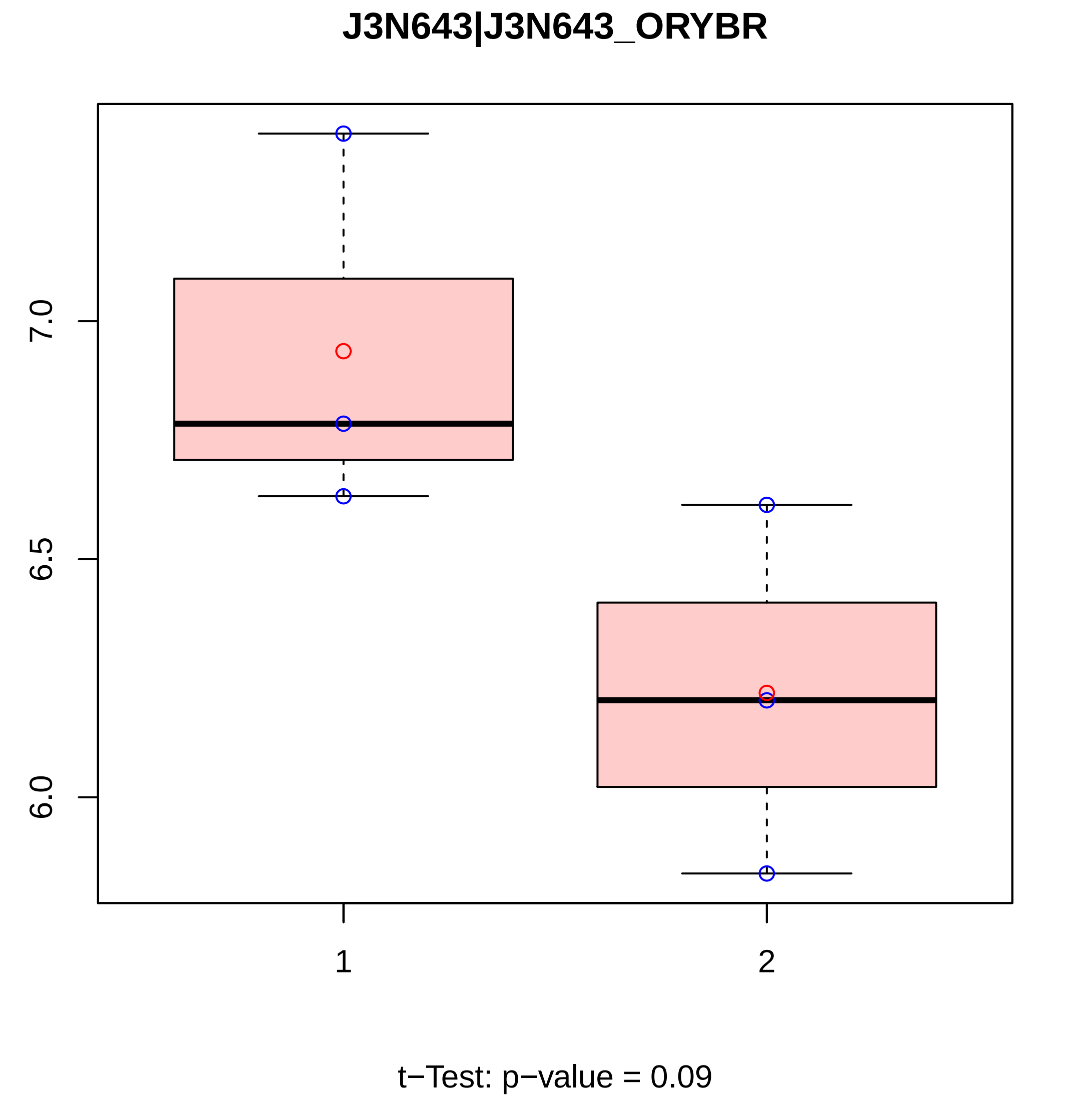
J3N643|J3N643_ORYBR
7.0
6.5
6.0
1
2
t−Test: p−value = 0.09

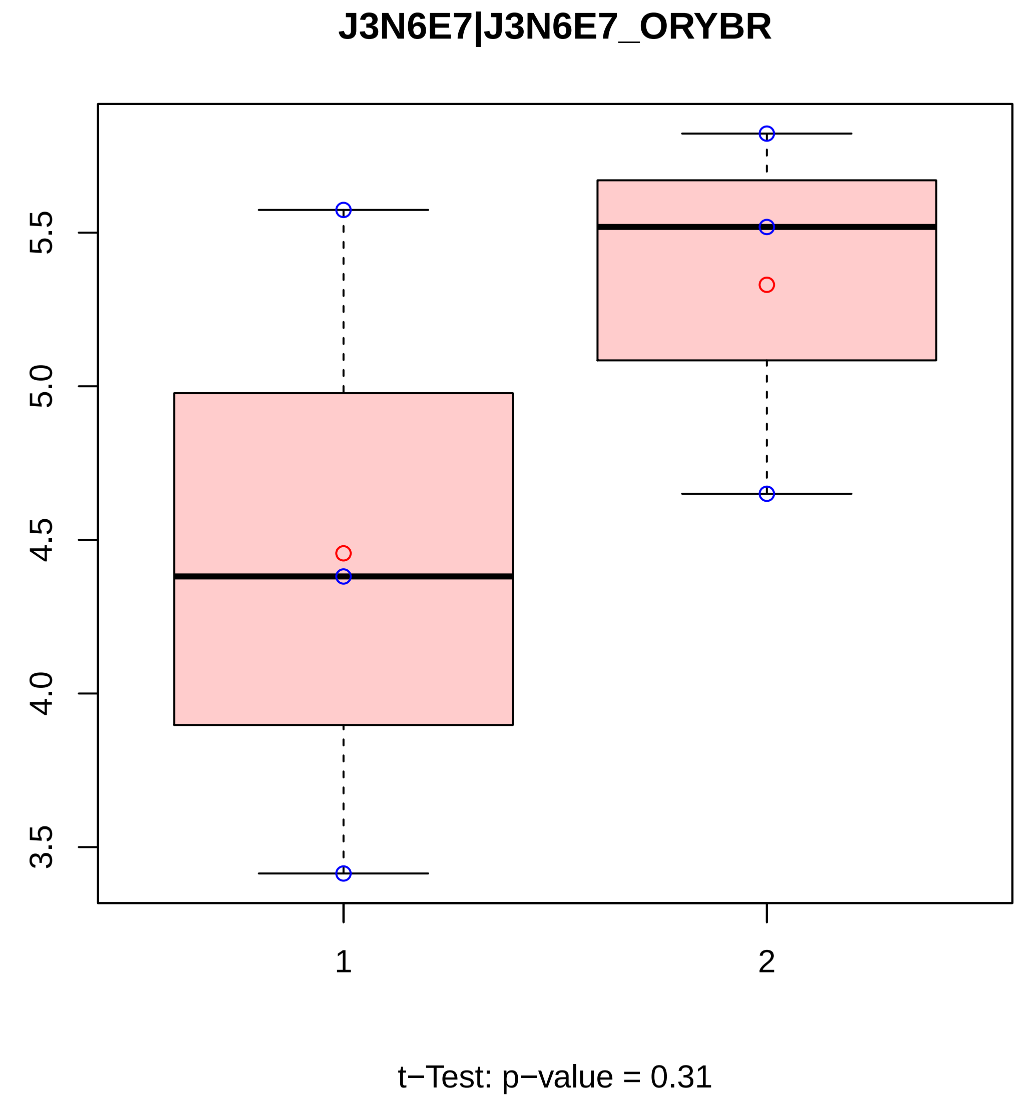

J3N6E7|J3N6E7_ORYBR
3.5
4.0
4.5
5.0
5.5
1
2
t−Test: p−value = 0.31

J3N6N4|J3N6N4_ORYBR

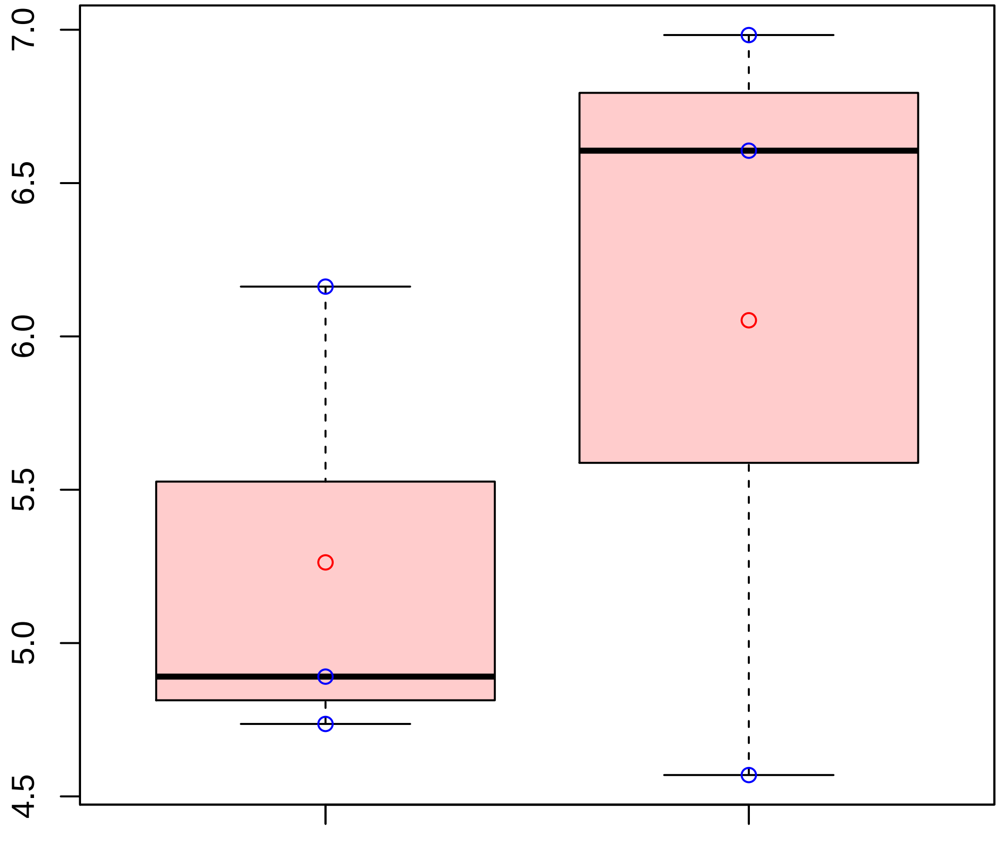

t−Test: p−value = 0.43

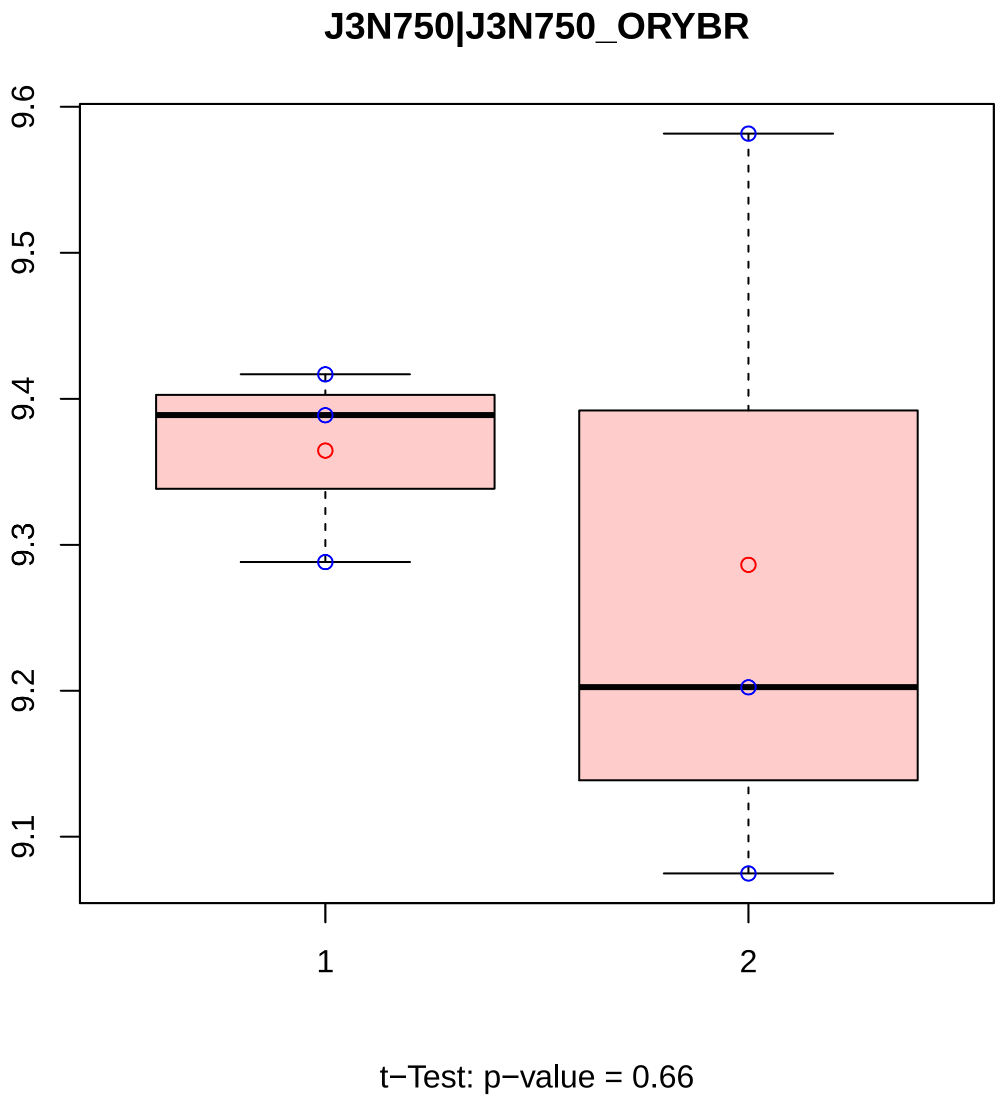
J3N750|J3N750_ORYBR
9.1
9.2
9.3
9.4
9.5
9.6
1
2
t−Test: p−value = 0.66

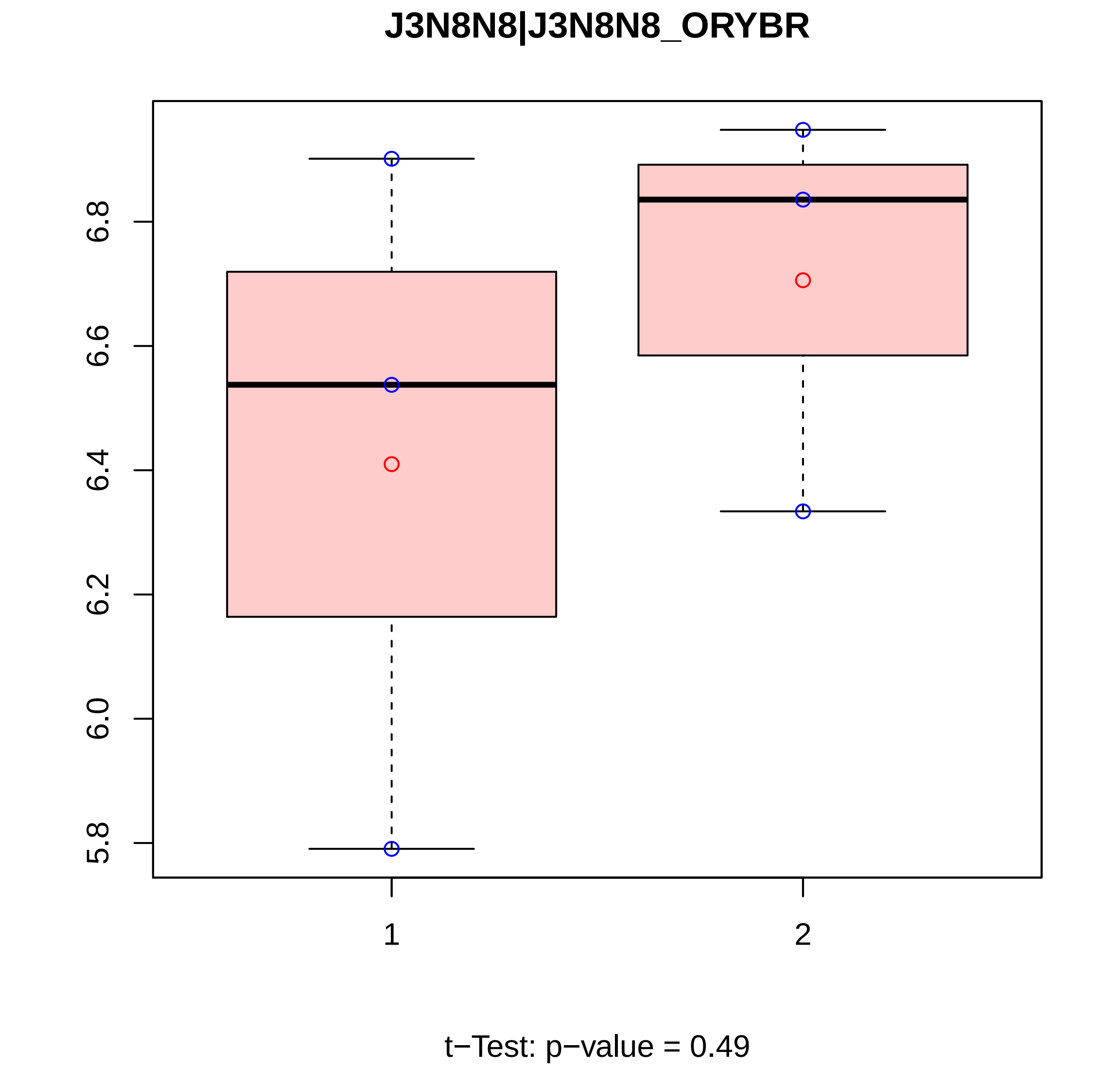

J3N8N8|J3N8N8_ORYBR
6.8
6.6
6.4
6.2
6.0
5.8
1
2
t−Test: p−value = 0.49

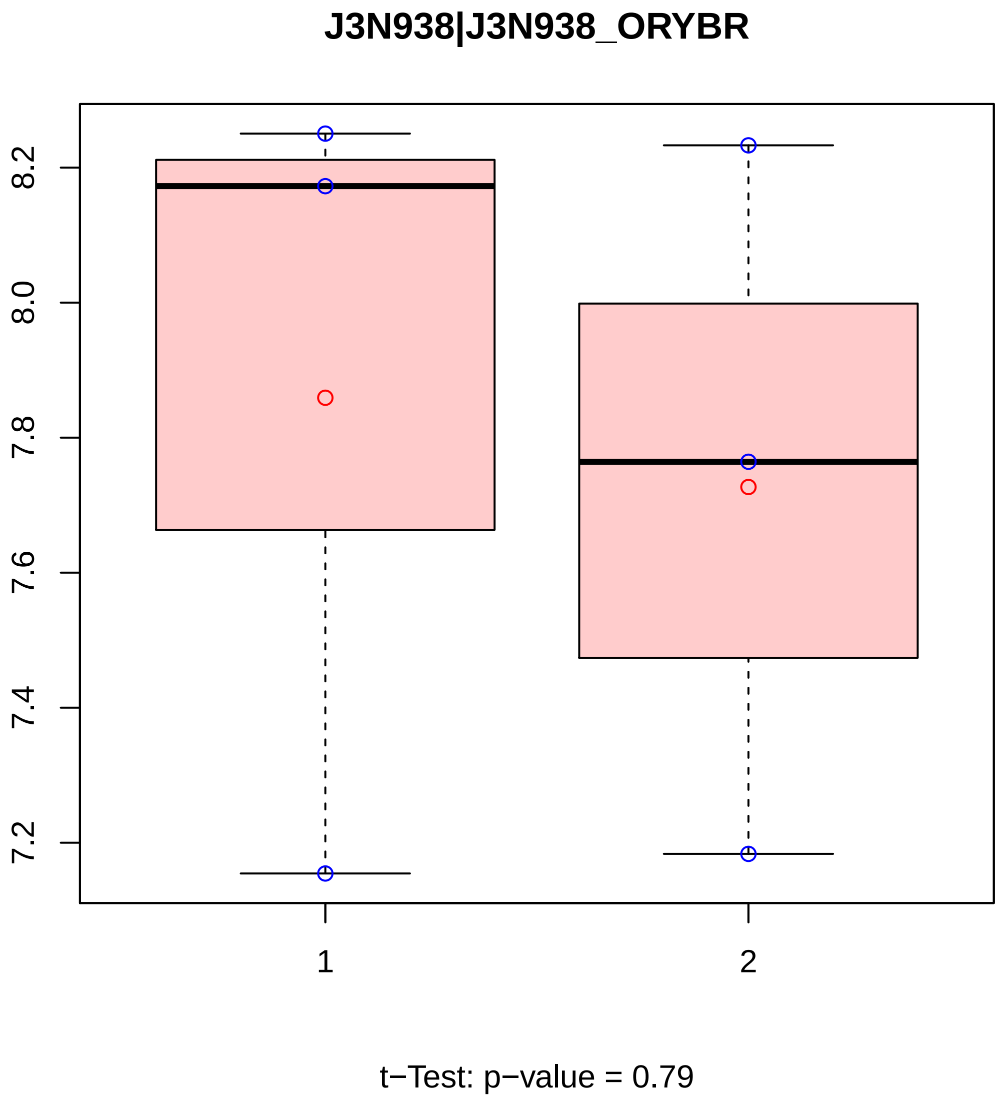
J3N938|J3N938_ORYBR
8.2
8.0
7.8
7.6
7.4
7.2
1
2
t−Test: p−value = 0.79

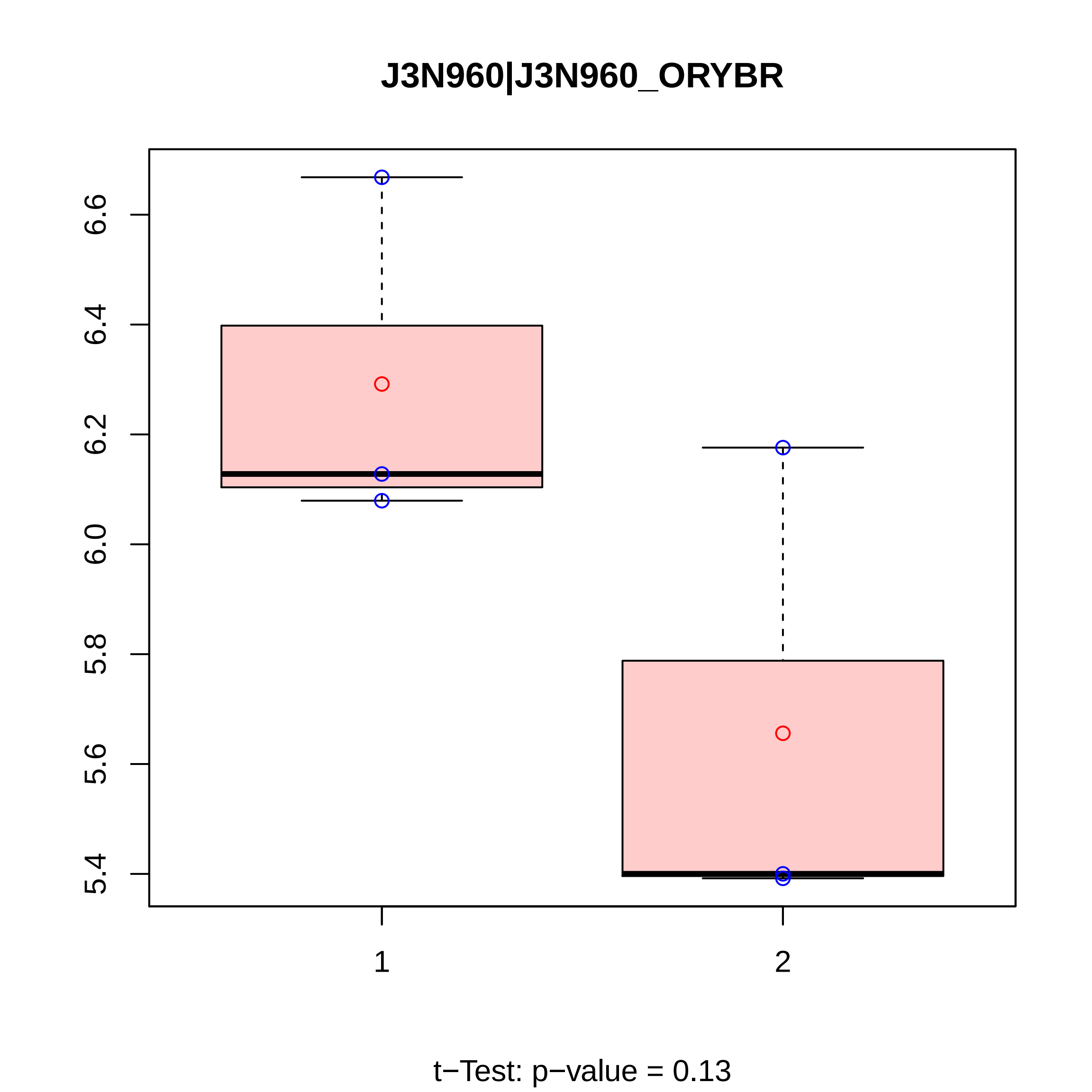

J3N960|J3N960_ORYBR
6.6
6.4
6.2
6.0
5.8
5.6
5.4
1
2
t−Test: p−value = 0.13

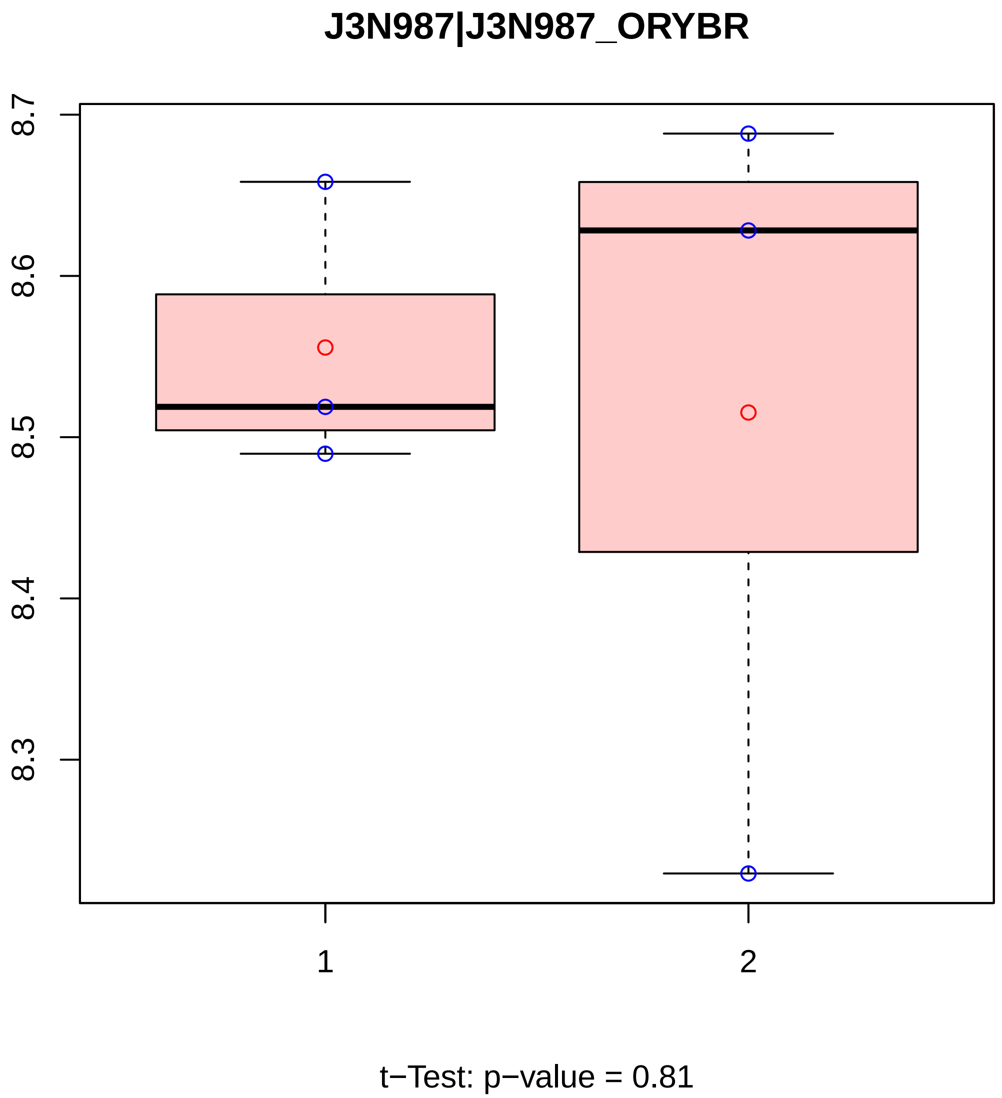
J3N987|J3N987_ORYBR
8.3
8.4
8.5
8.6
8.7
1
2
t−Test: p−value = 0.81

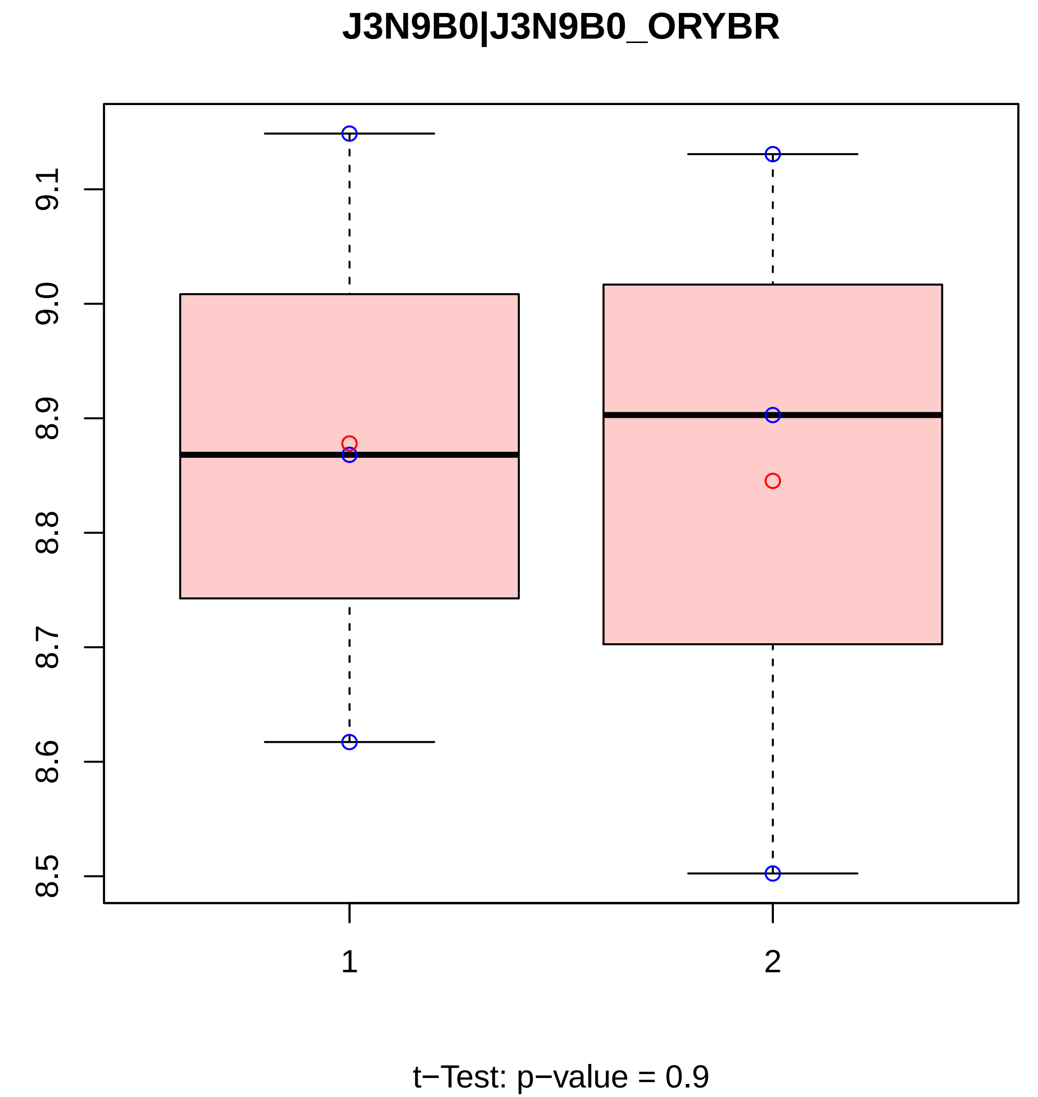
J3N9B0|J3N9B0_ORYBR
9.1
9.0
8.9
8.8
8.7
8.6
8.5
1
2
t−Test: p−value = 0.9

J3NA21|J3NA21_ORYBR

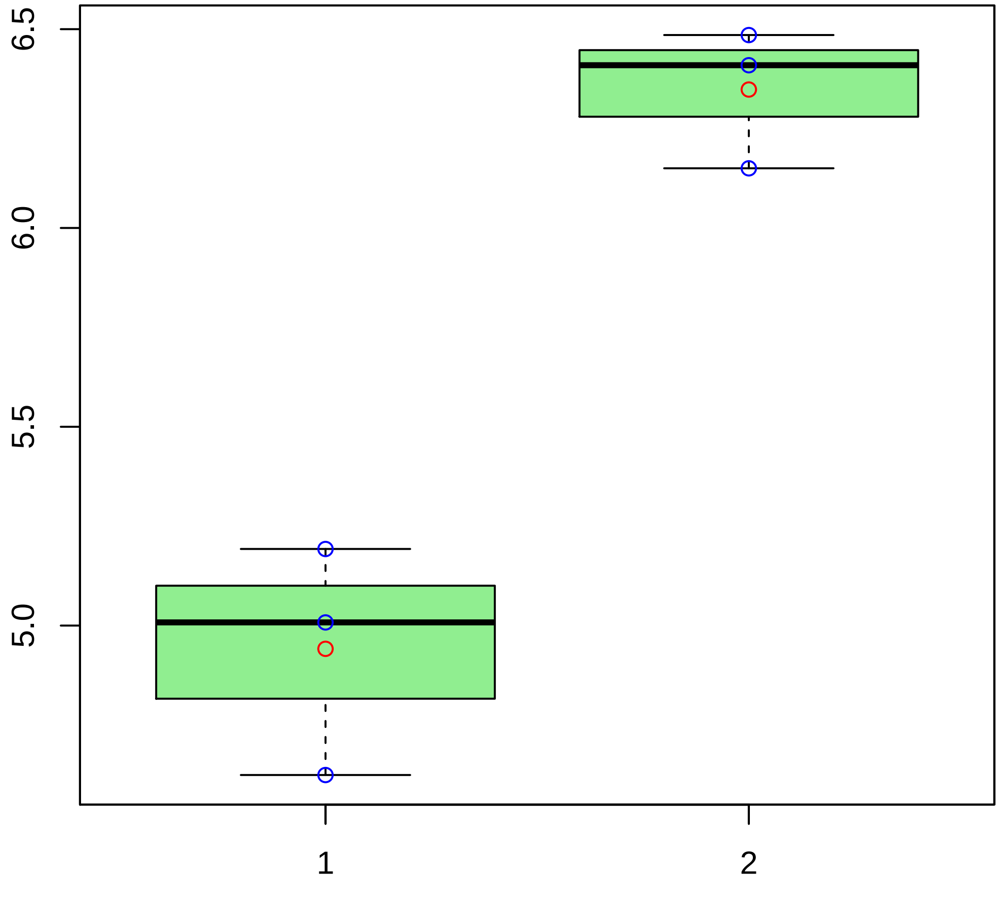

t−Test: p−value = 0

J3NA27|J3NA27_ORYBR

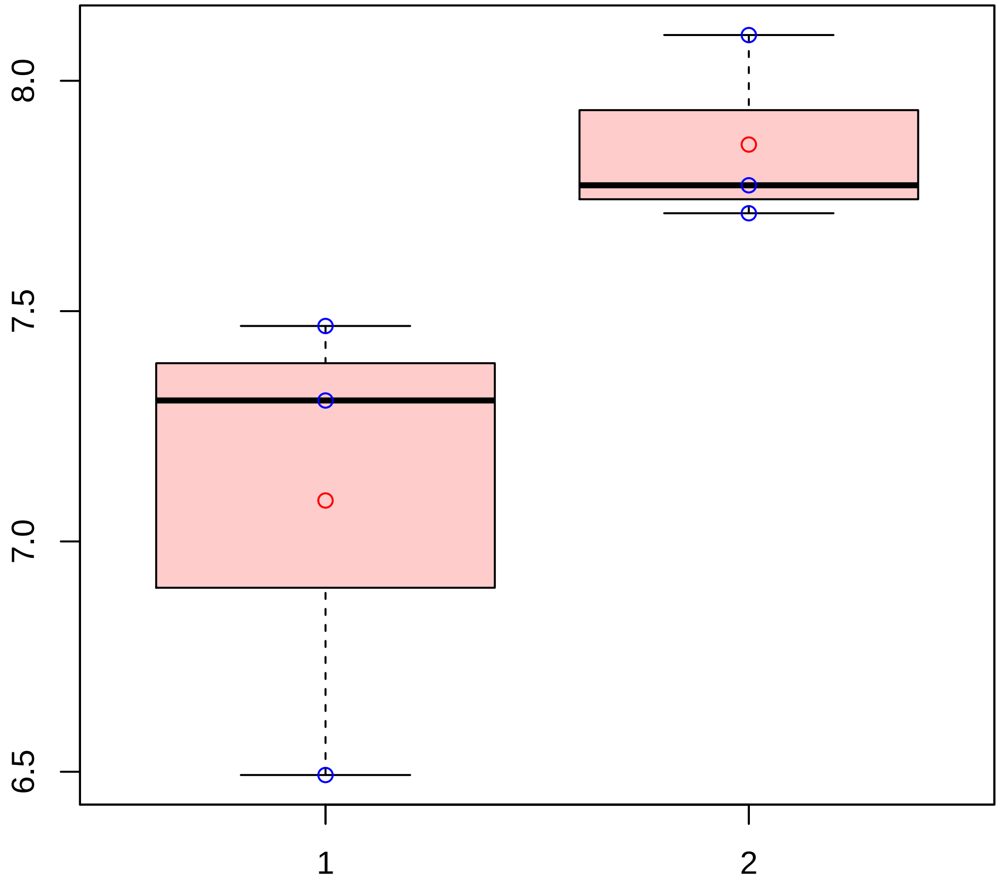

t−Test: p−value = 0.11

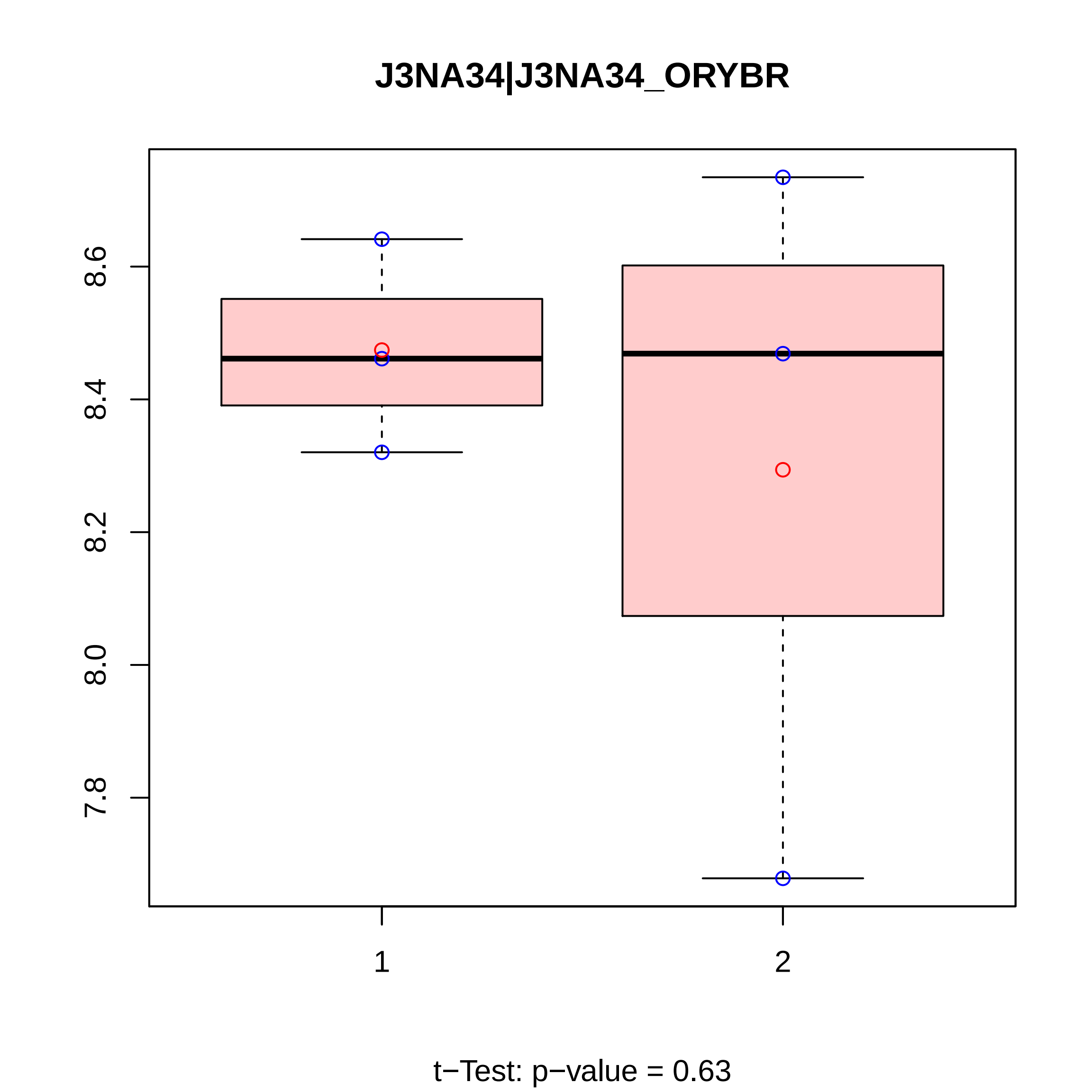
J3NA34|J3NA34_ORYBR
8.6
8.4
8.2
8.0
7.8
1
2
t−Test: p−value = 0.63

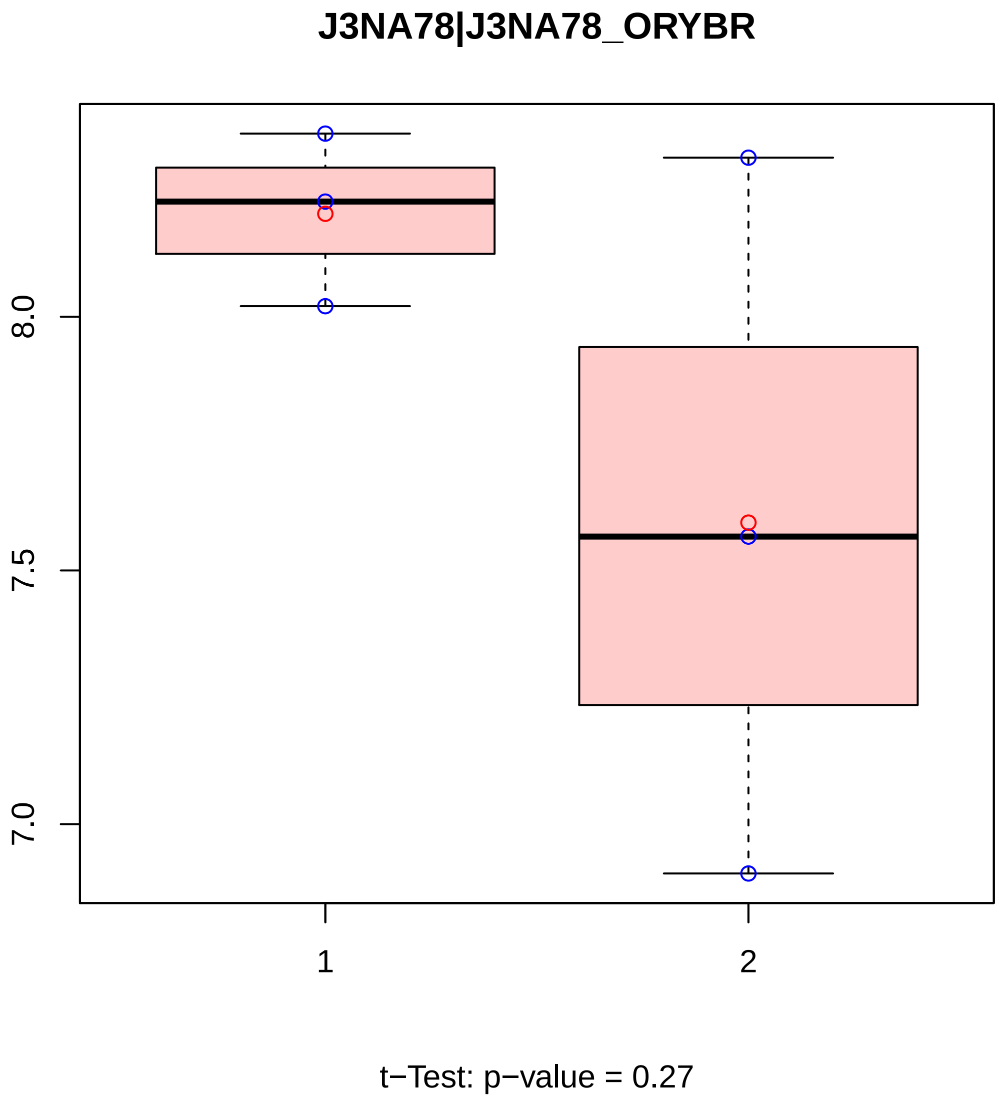
J3NA78|J3NA78_ORYBR
8.0
7.5
7.0
1
2
t−Test: p−value = 0.27

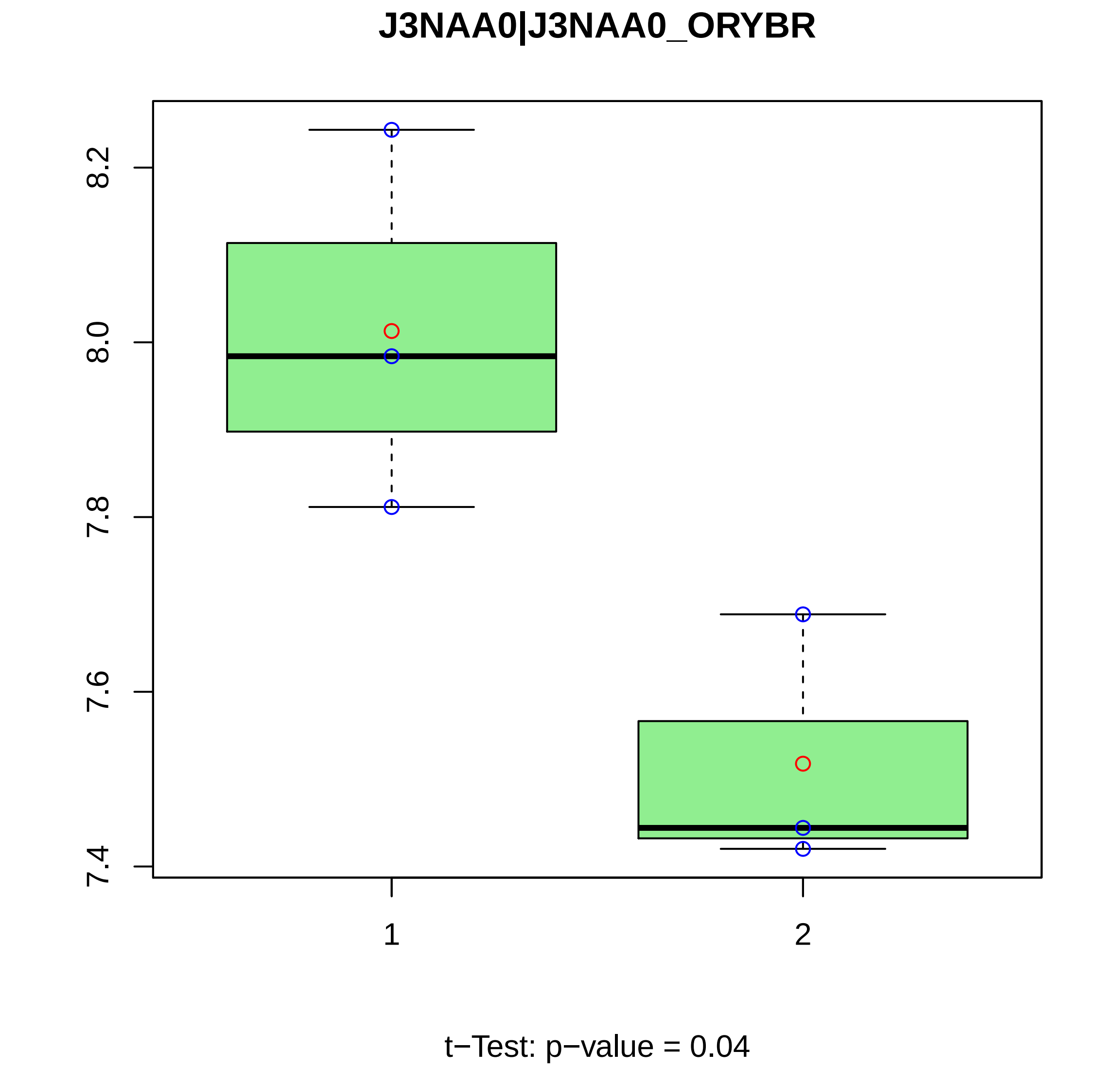
J3NAA0|J3NAA0_ORYBR
8.2
8.0
7.8
7.6
7.4
1
2
t−Test: p−value = 0.04

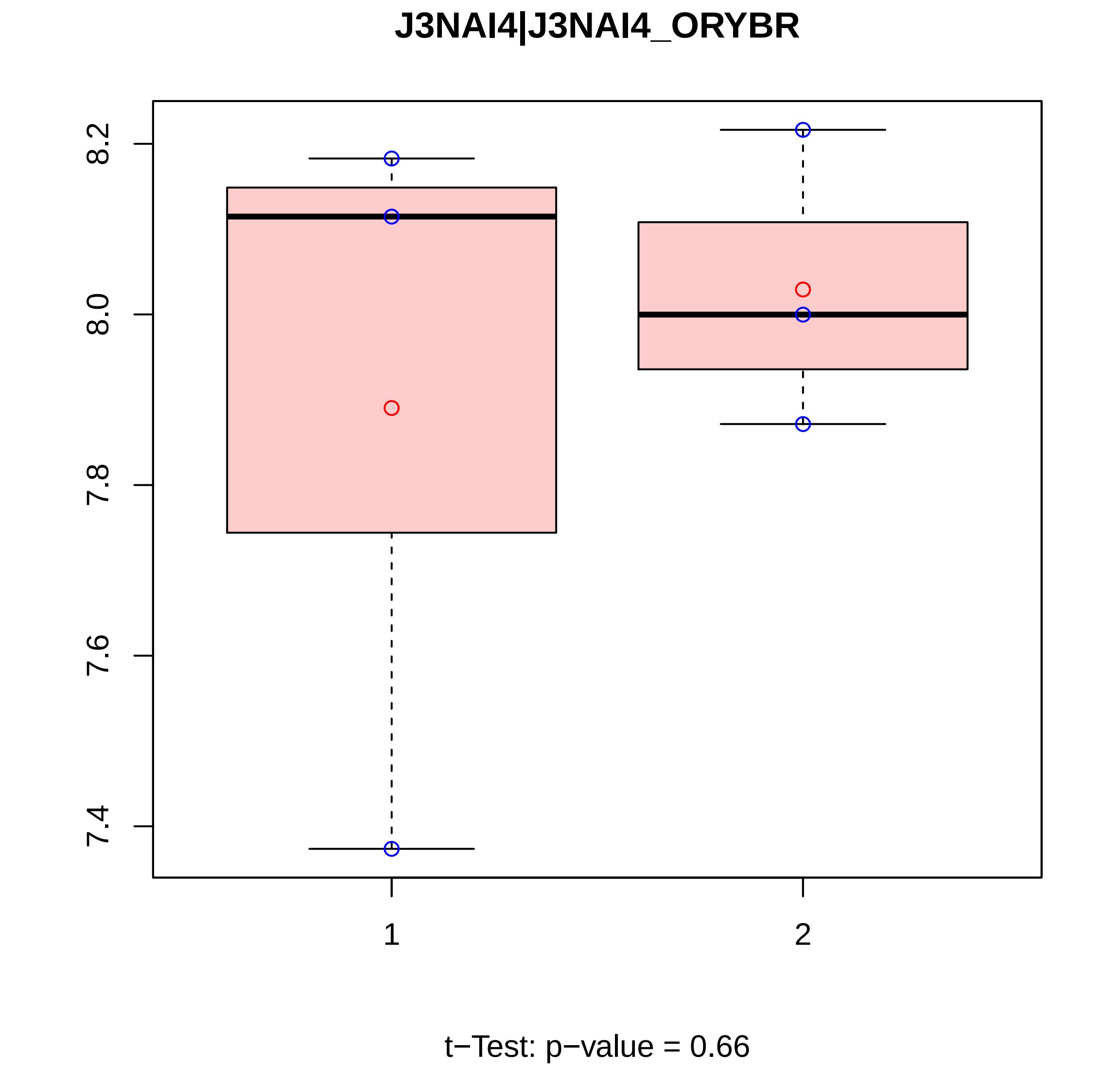
J3NAI4|J3NAI4_ORYBR
8.2
8.0
7.8
7.6
7.4
1
2
t−Test: p−value = 0.66

J3NAI9|J3NAI9_ORYBR

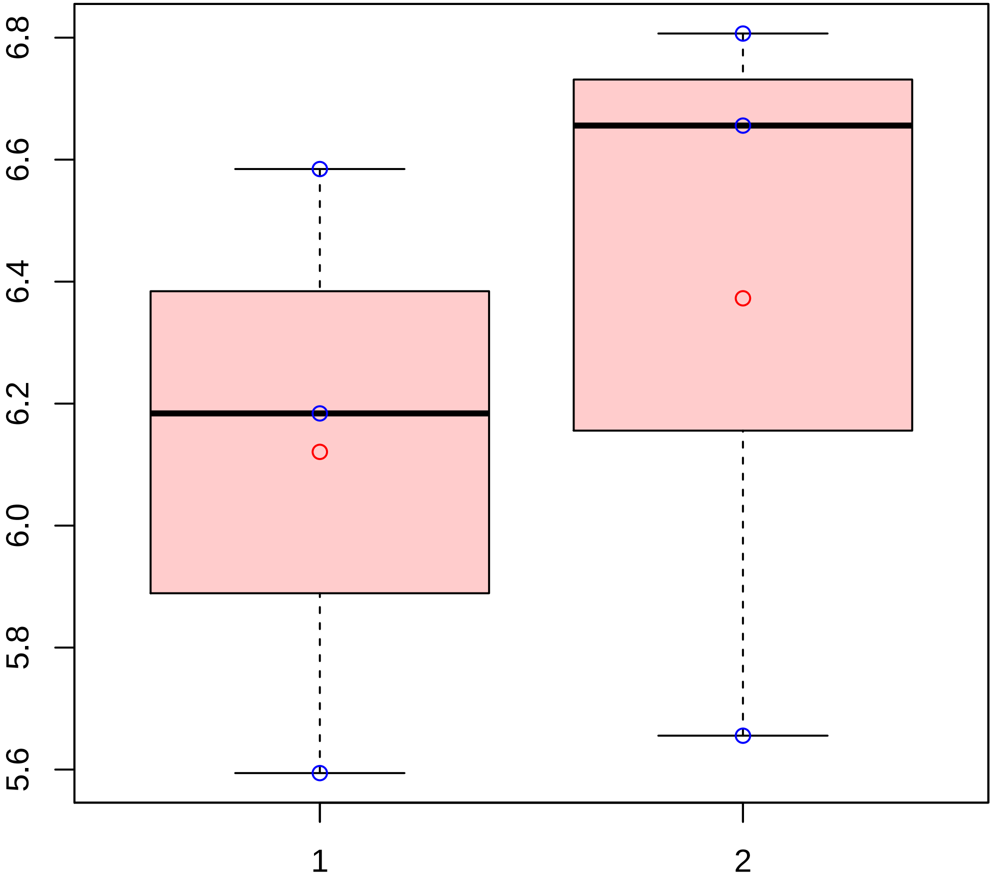

t−Test: p−value = 0.62

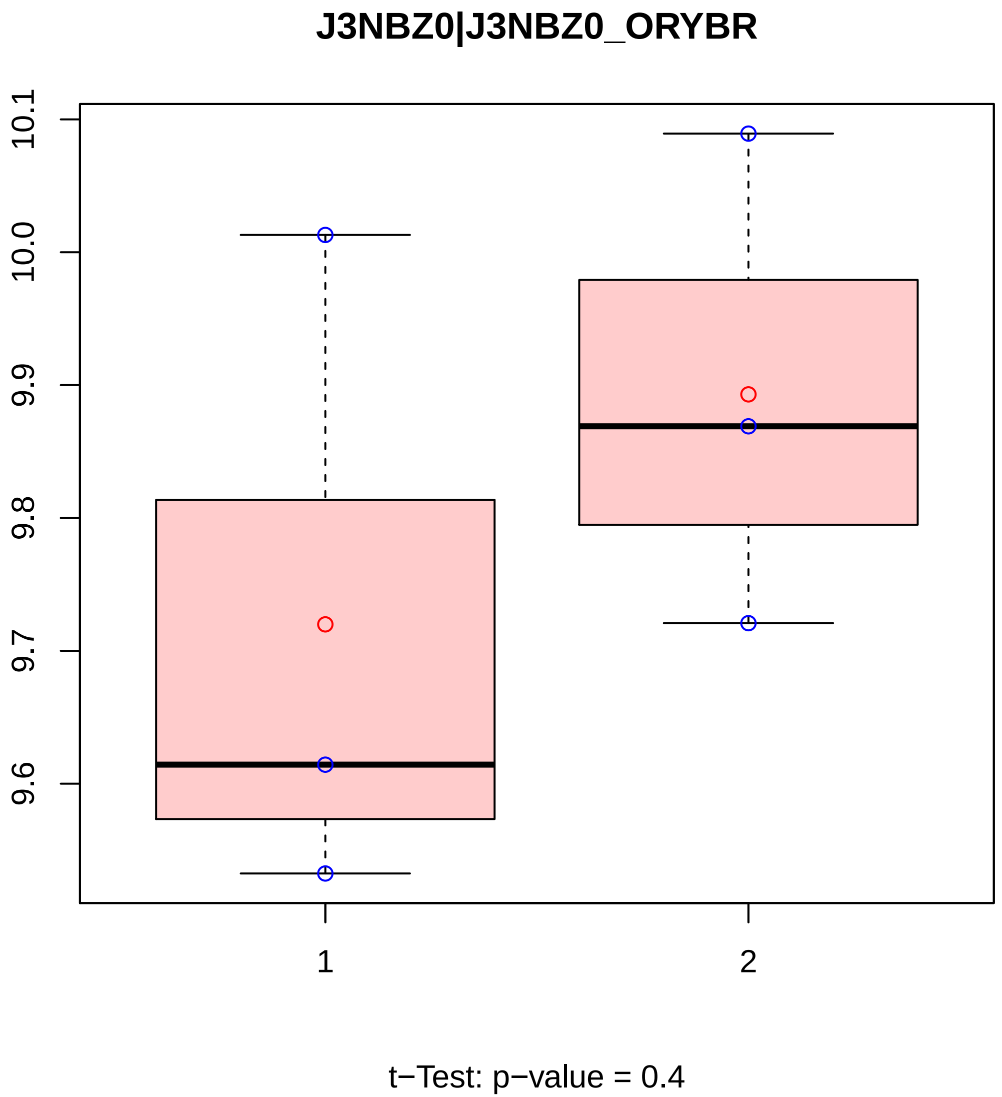

J3NBZ0|J3NBZ0_ORYBR
10.1
10.0
9.9
9.8
9.7
9.6
1
2
t−Test: p−value = 0.4

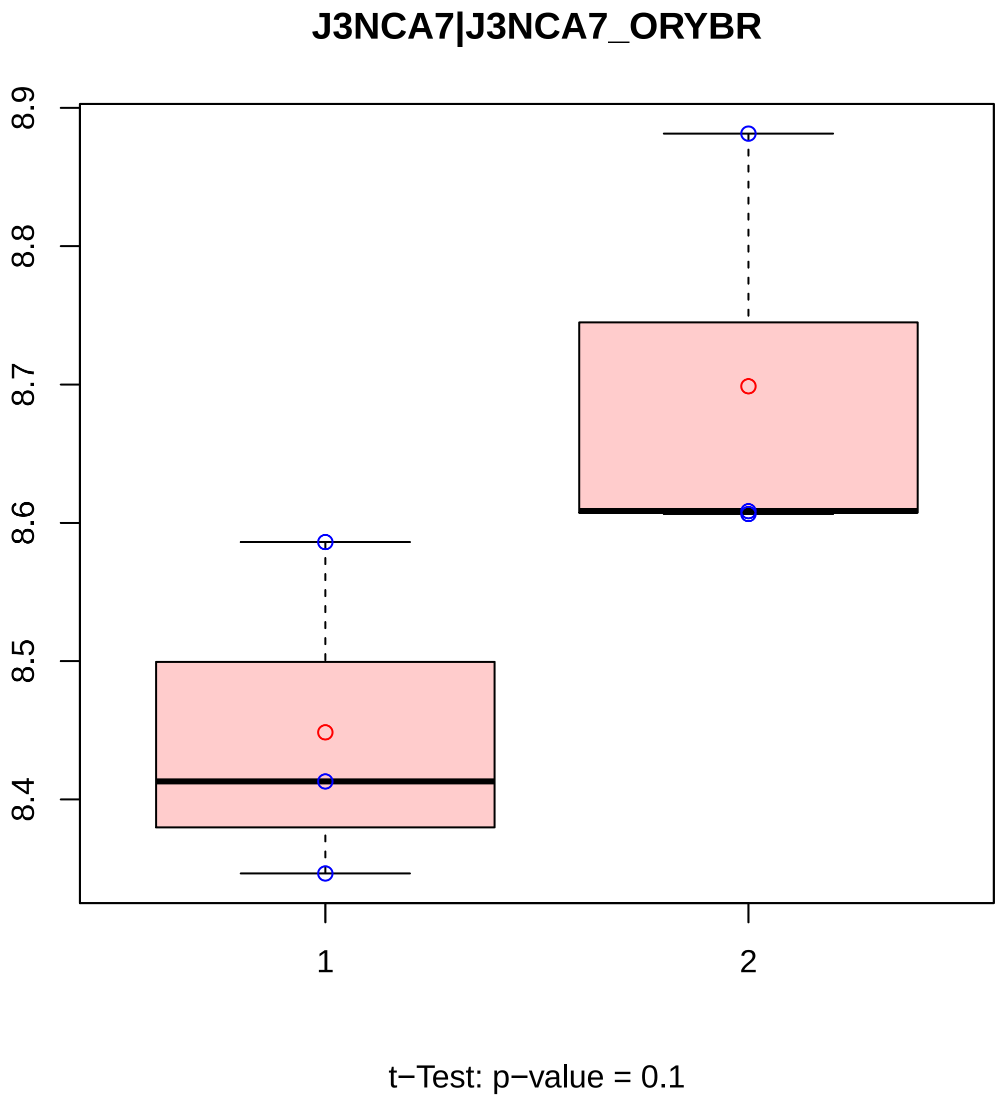
J3NCA7|J3NCA7_ORYBR
8.4
8.5
8.6
8.7
8.8
8.9
1
2
t−Test: p−value = 0.1

J3NCC7|J3NCC7_ORYBR

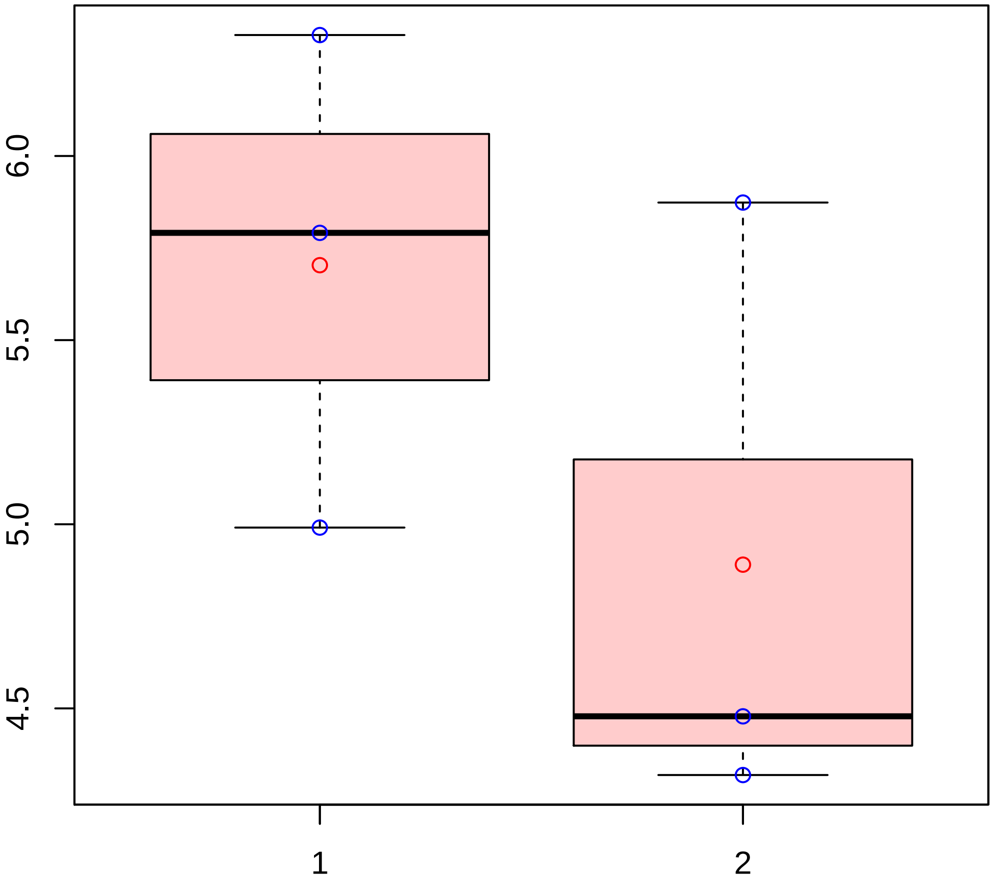

t−Test: p−value = 0.27

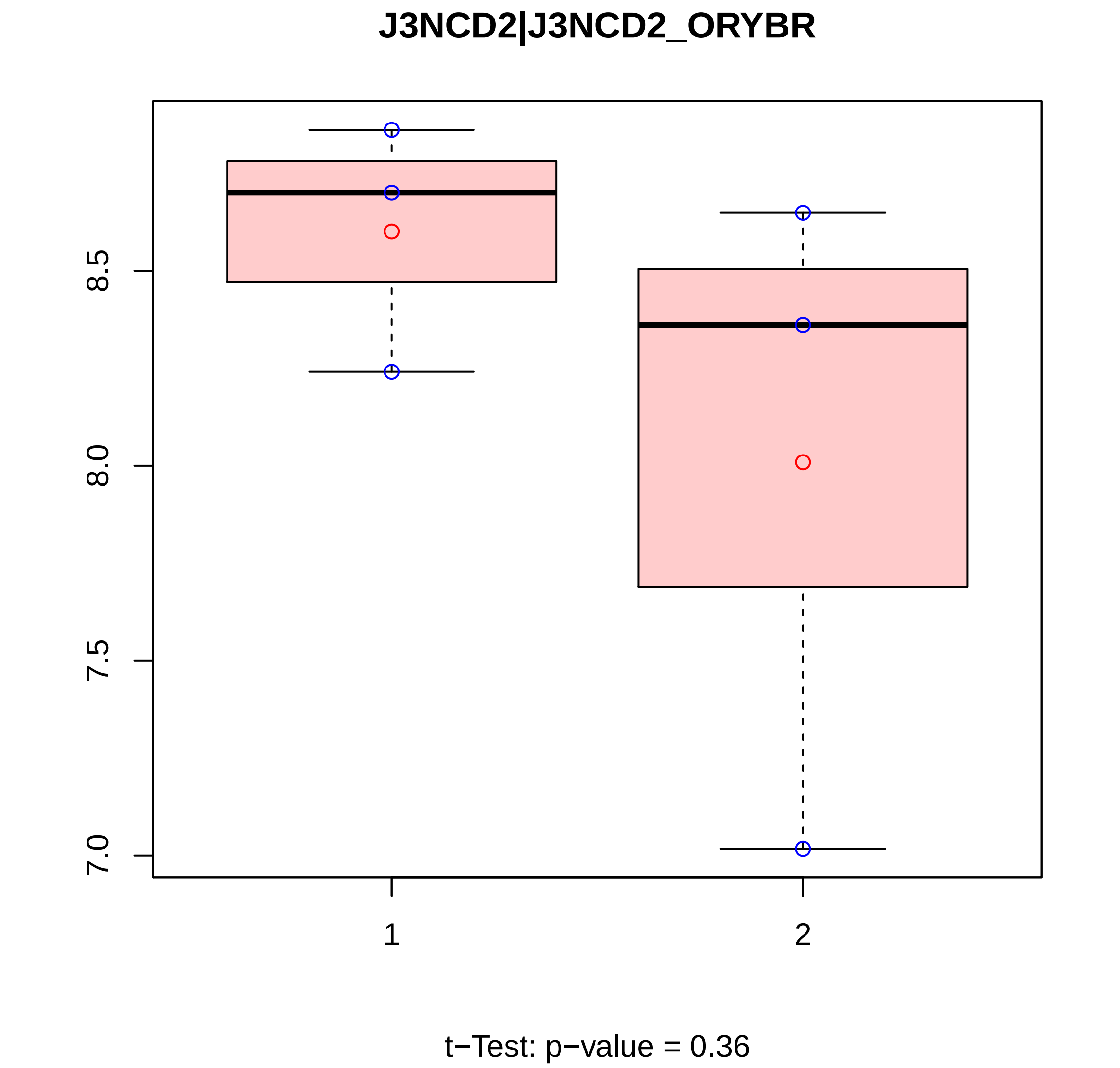
J3NCD2|J3NCD2_ORYBR
8.5
8.0
7.5
7.0
1
2
t−Test: p−value = 0.36

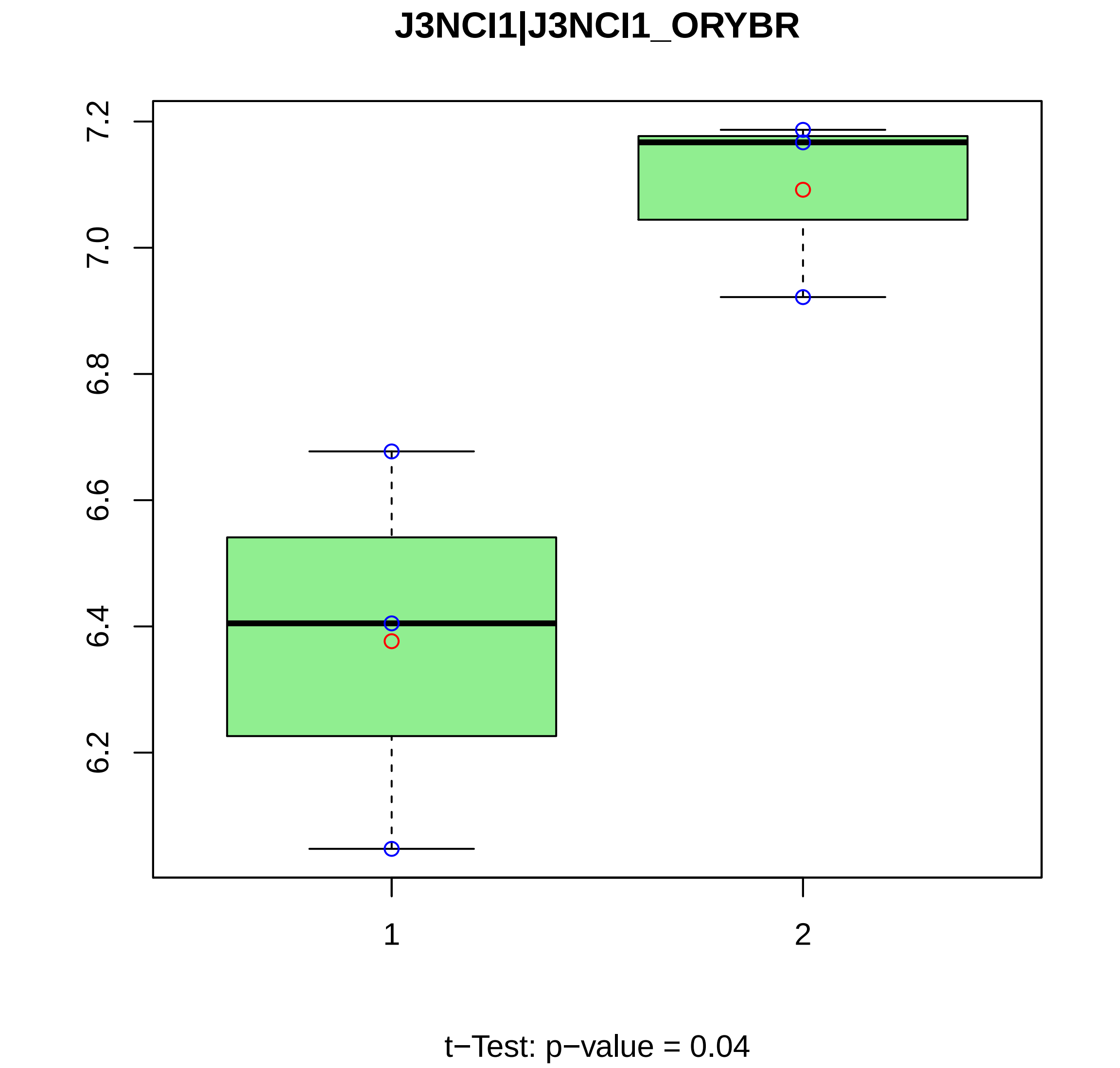

J3NCI1|J3NCI1_ORYBR
7.2
7.0
6.8
6.6
6.4
6.2
1
2
t−Test: p−value = 0.04

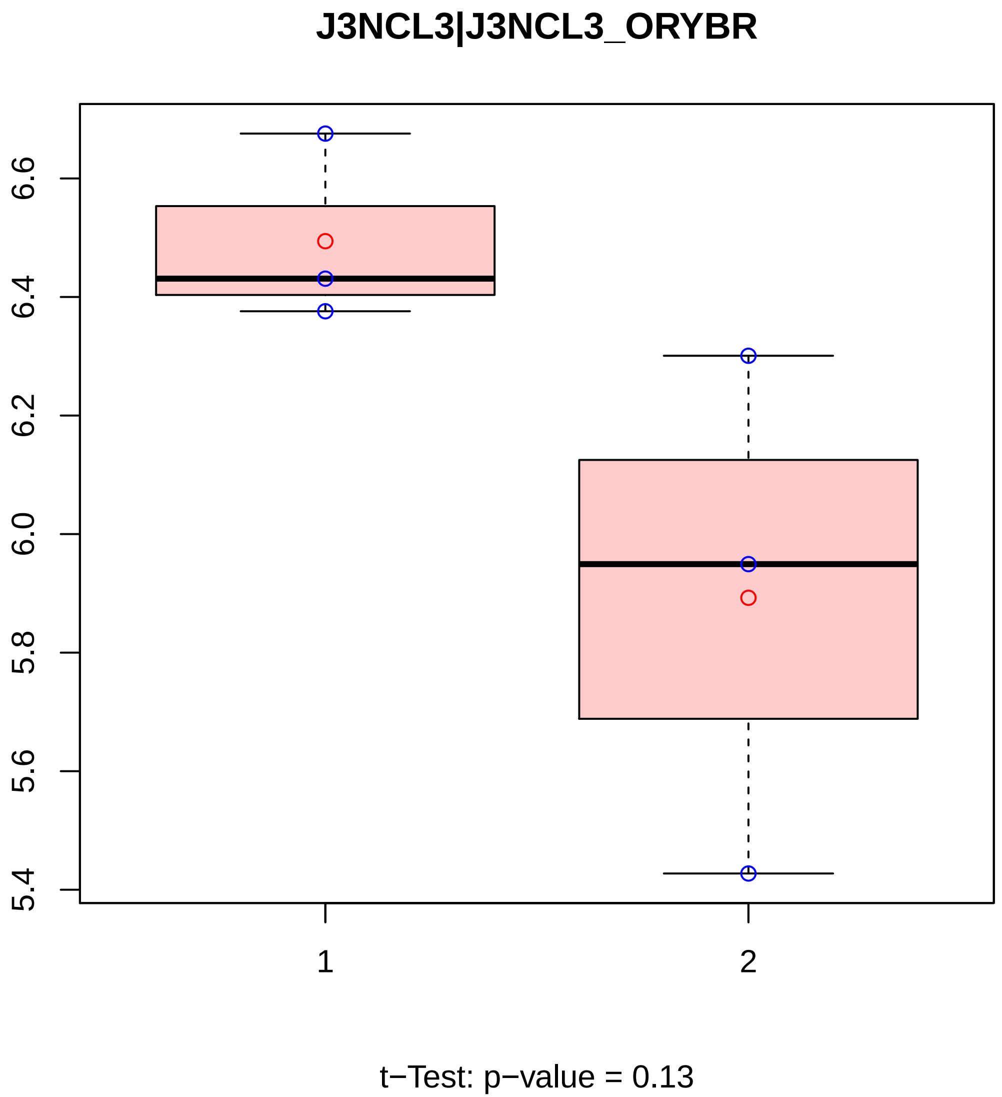
J3NCL3|J3NCL3_ORYBR
6.6
6.4
6.2
6.0
5.8
5.6
5.4
1
2
t−Test: p−value = 0.13

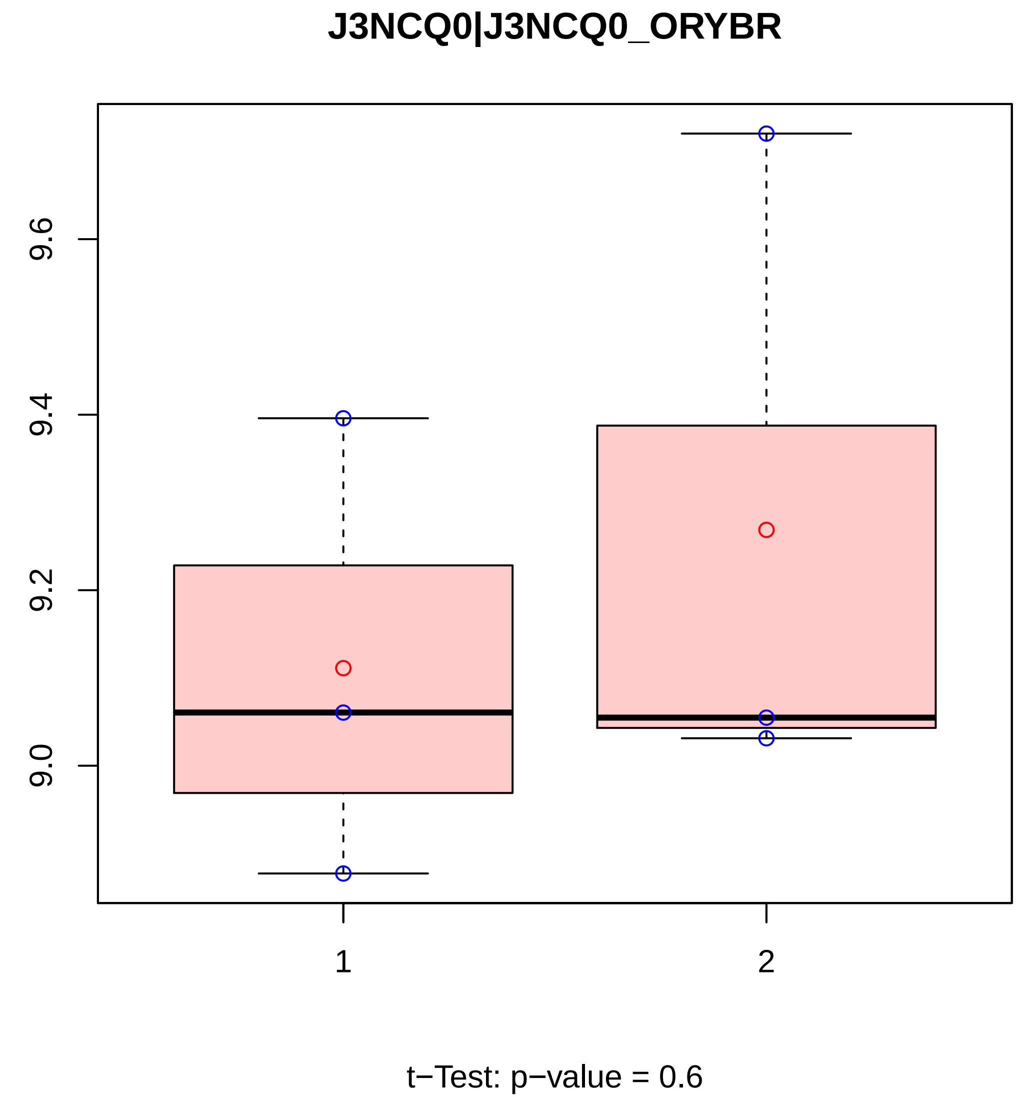
J3NCQ0|J3NCQ0_ORYBR
9.0
9.2
9.4
9.6
1
2
t−Test: p−value = 0.6

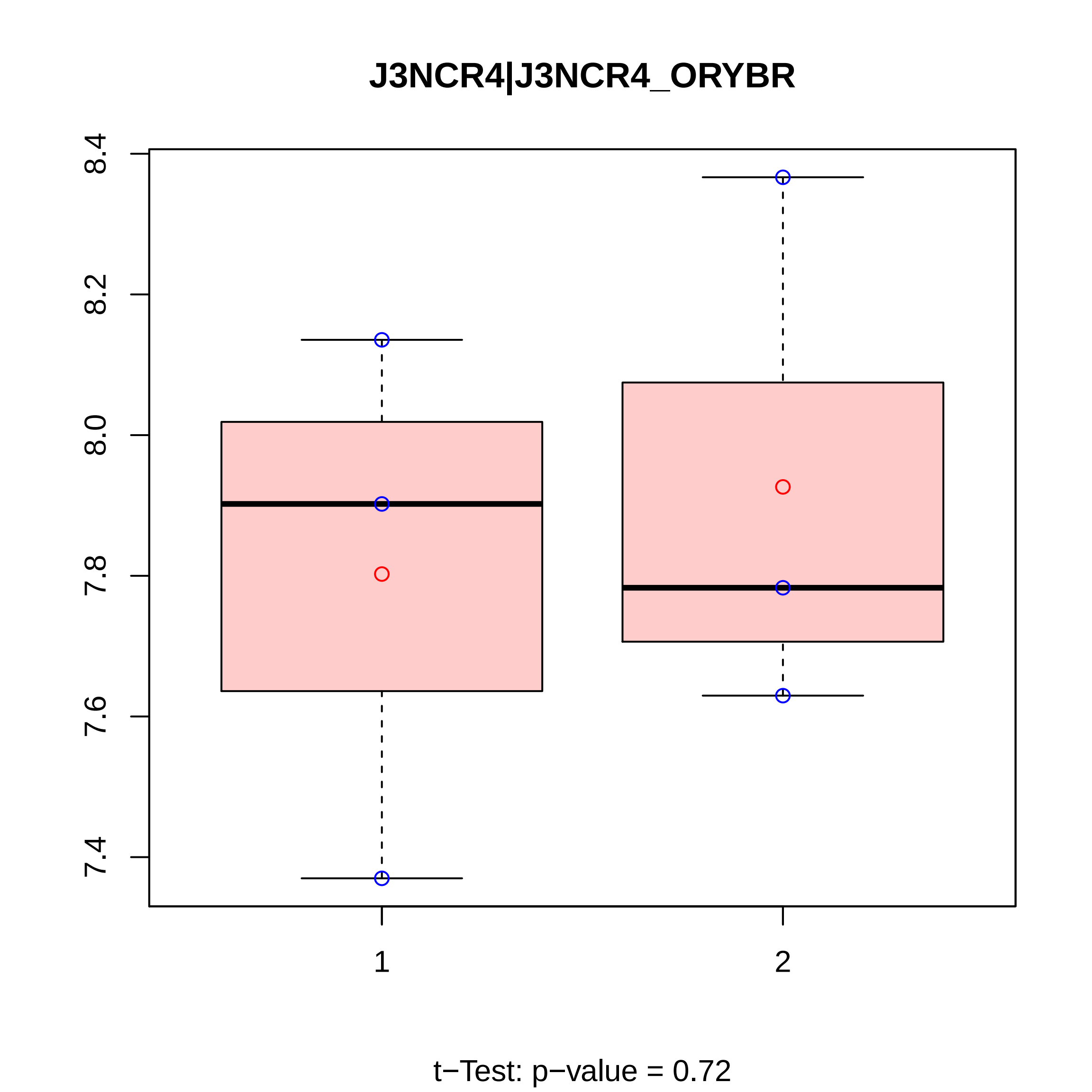

J3NCR4|J3NCR4_ORYBR
8.4
8.2
8.0
7.8
7.6
7.4
1
2
t−Test: p−value = 0.72

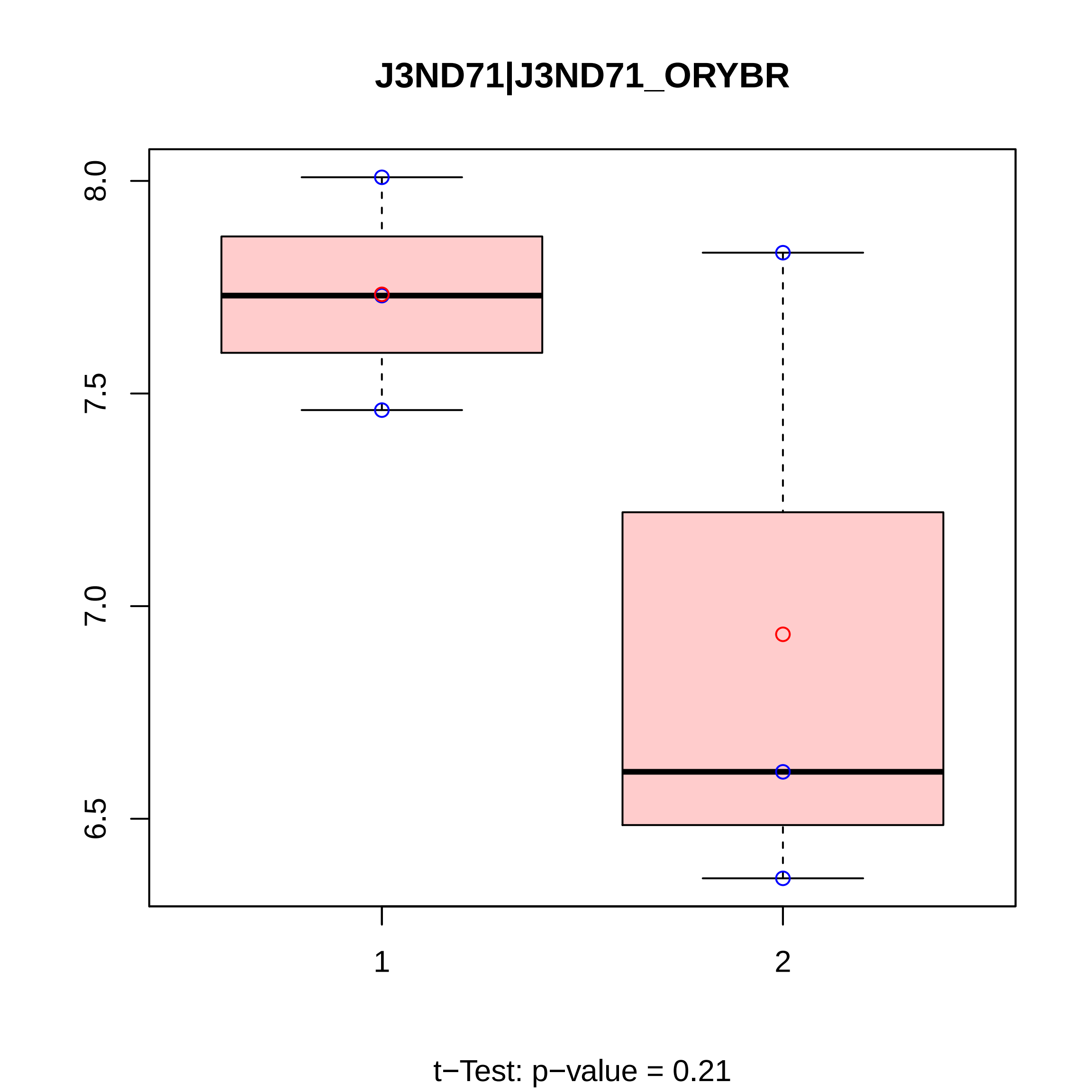
J3ND71|J3ND71_ORYBR
8.0
7.5
7.0
6.5
1
2
t−Test: p−value = 0.21

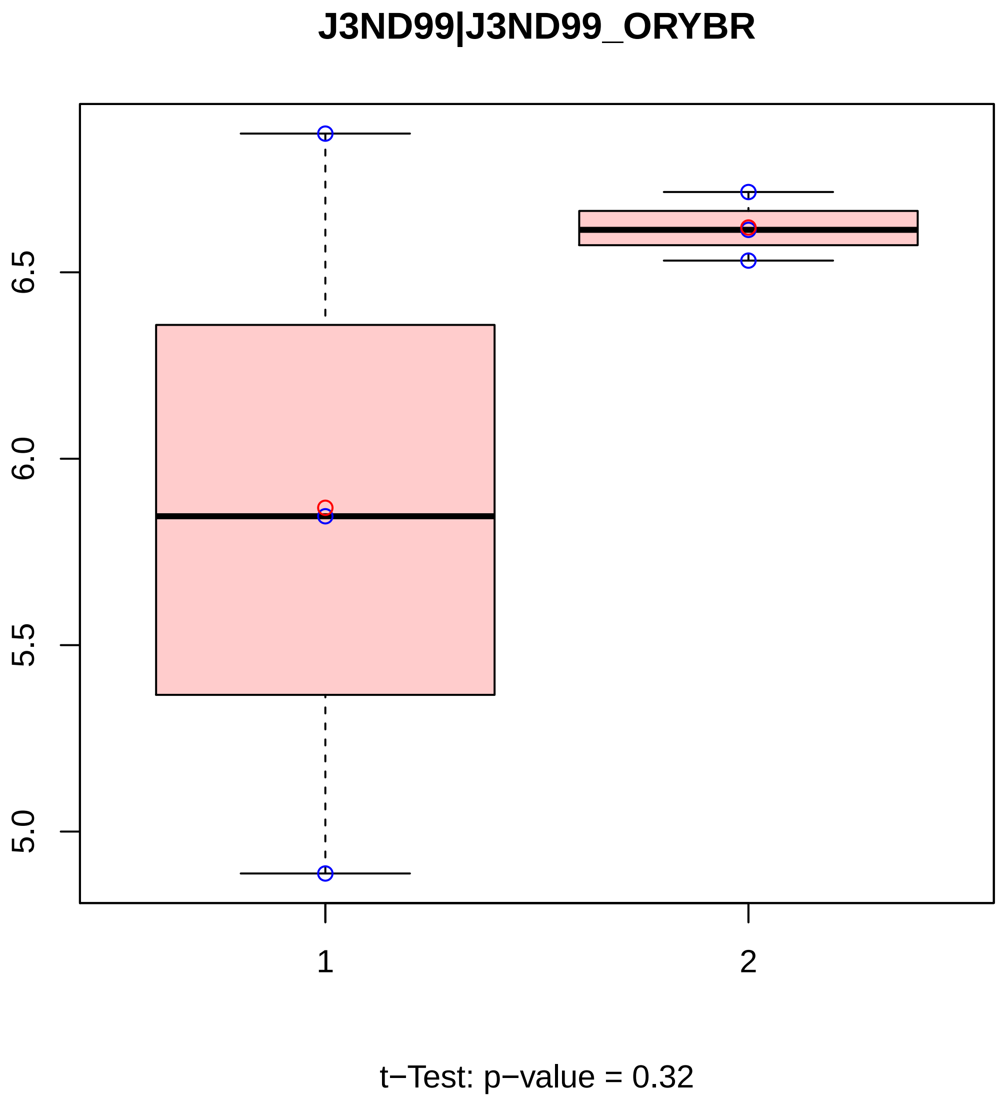

J3ND99|J3ND99_ORYBR
5.0
5.5
6.0
6.5
1
2
t−Test: p−value = 0.32

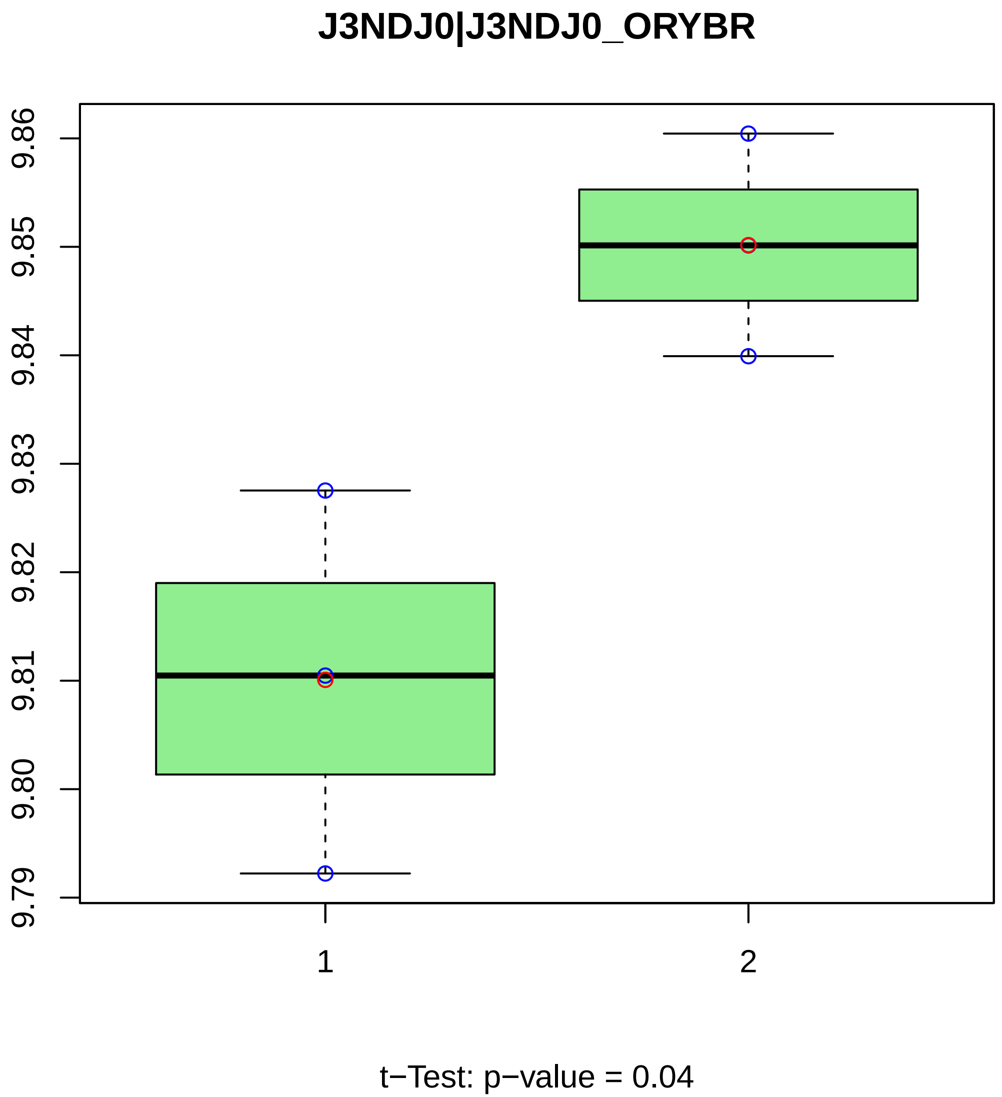
J3NDJ0|J3NDJ0_ORYBR
9.79
9.80
9.81
9.82
9.83
9.84
9.85
9.86
1
2
t−Test: p−value = 0.04

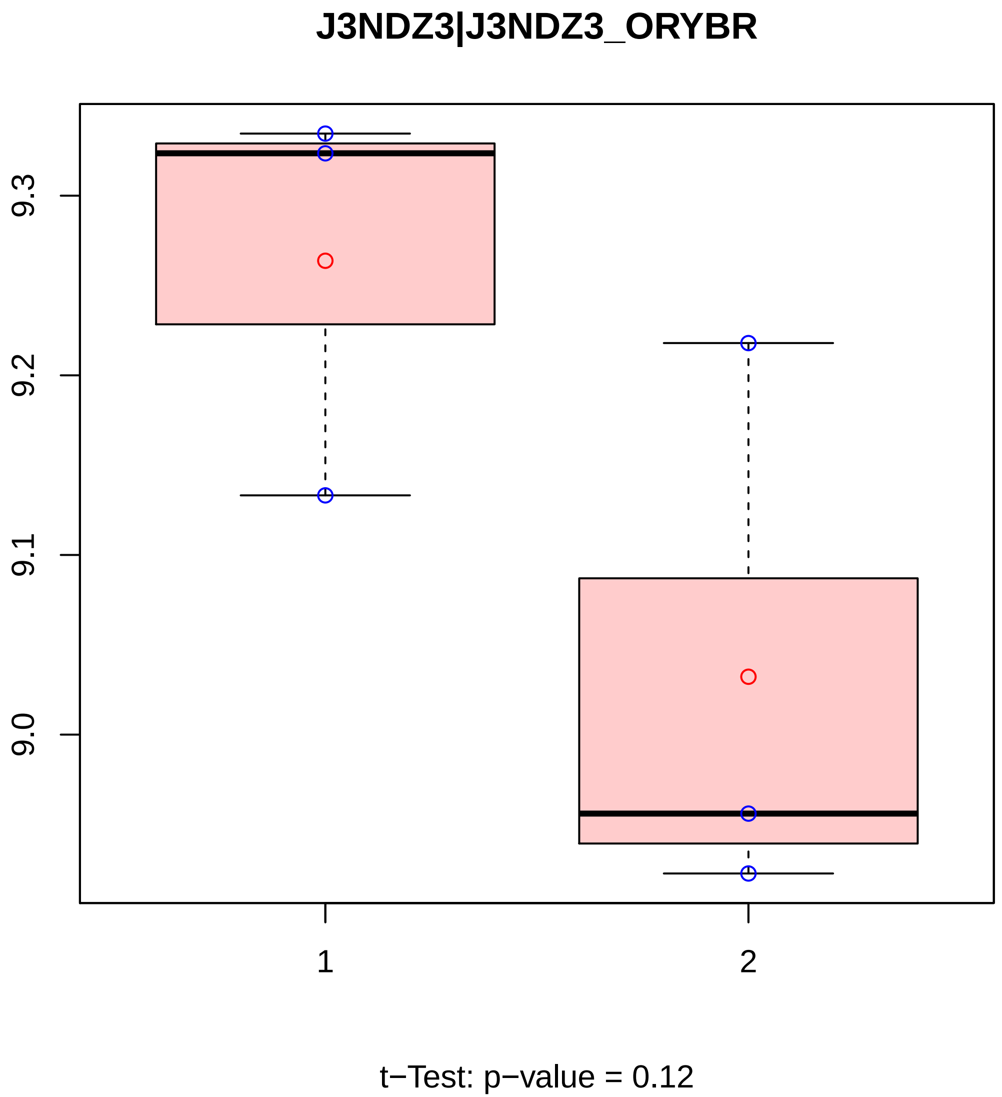
J3NDZ3|J3NDZ3_ORYBR
9.3
9.2
9.1
9.0
1
2
t−Test: p−value = 0.12

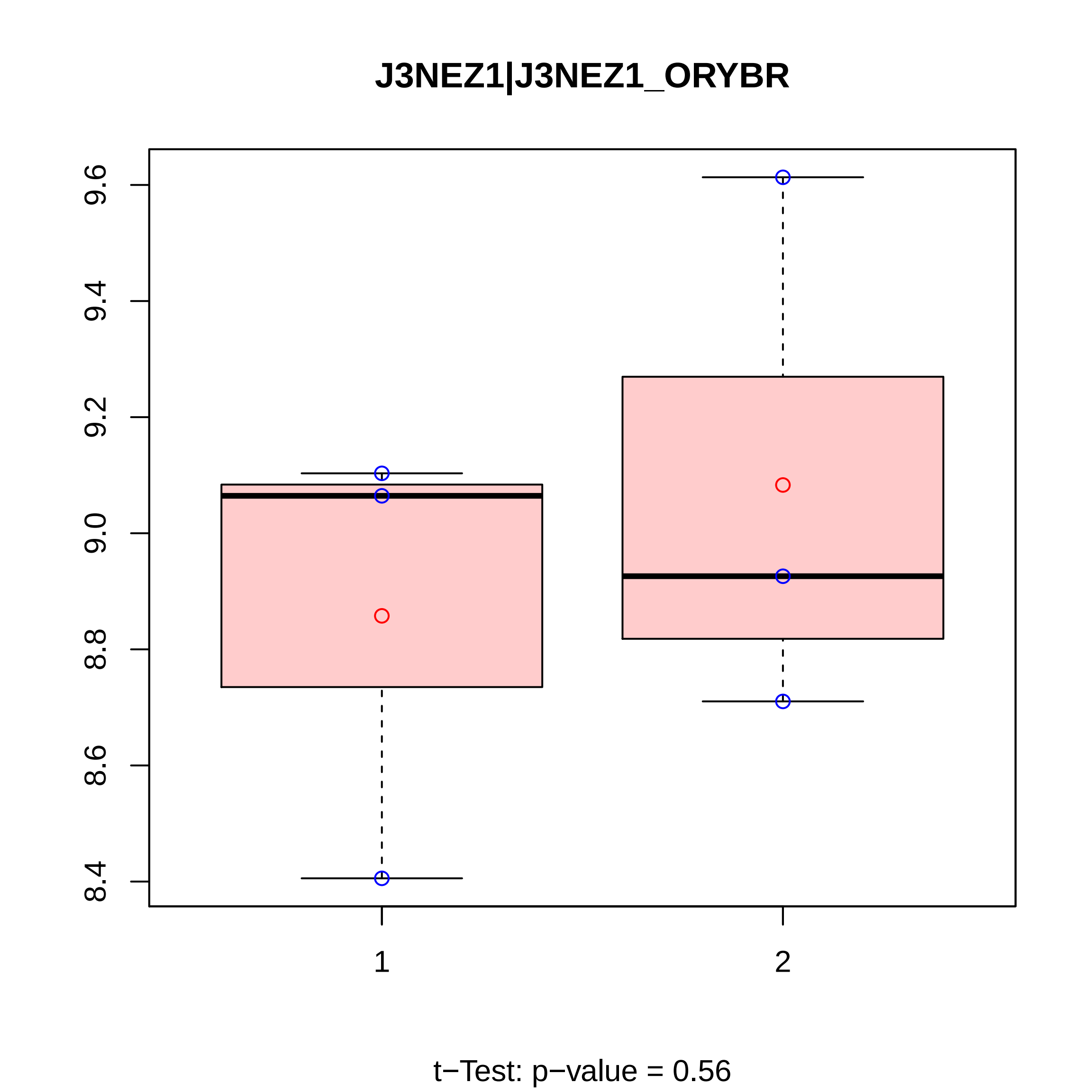
J3NEZ1|J3NEZ1_ORYBR
9.6
9.4
9.2
9.0
8.8
8.6
8.4
1
2
t−Test: p−value = 0.56

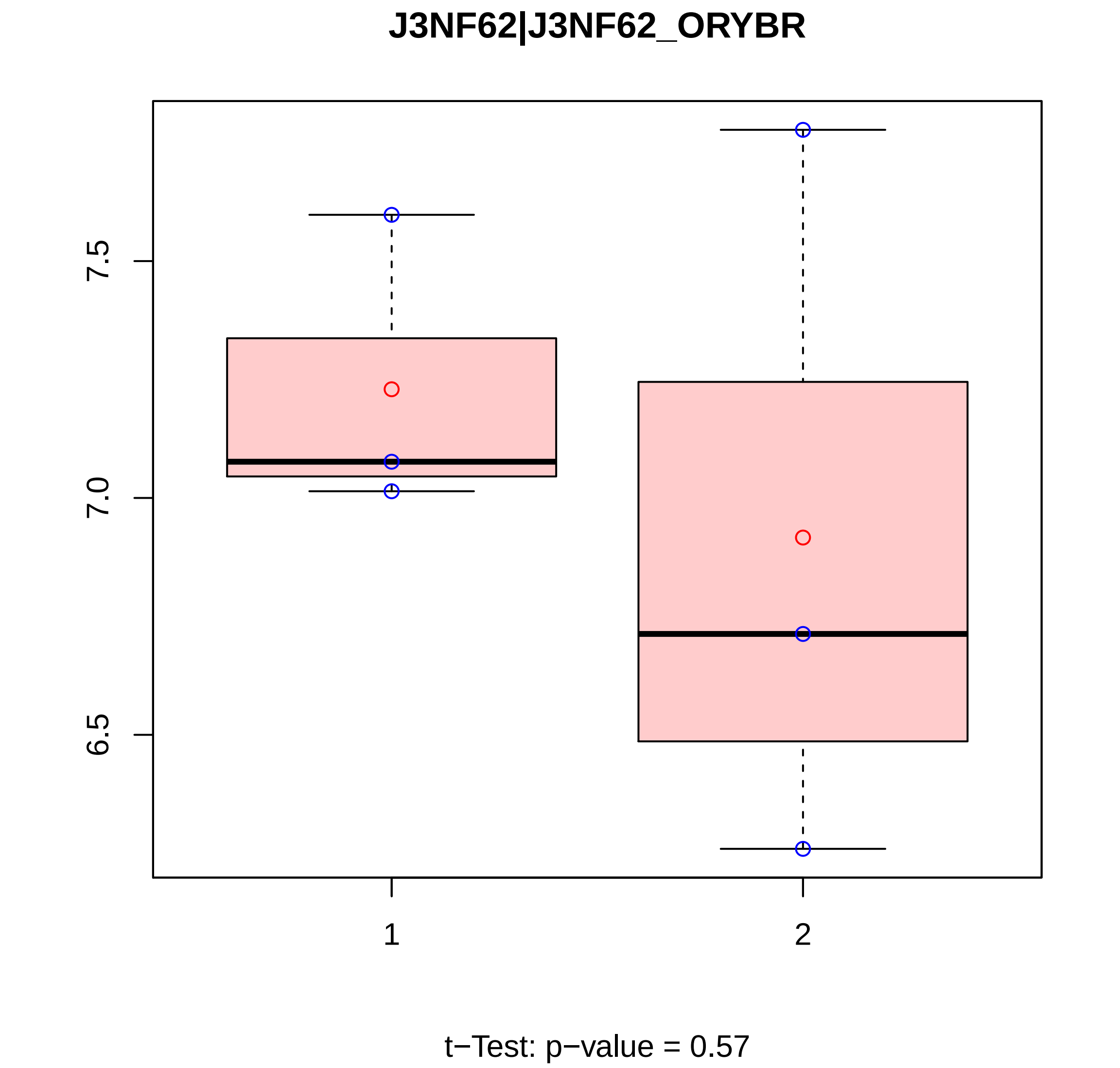
J3NF62|J3NF62_ORYBR
7.5
7.0
6.5
1
2
t−Test: p−value = 0.57

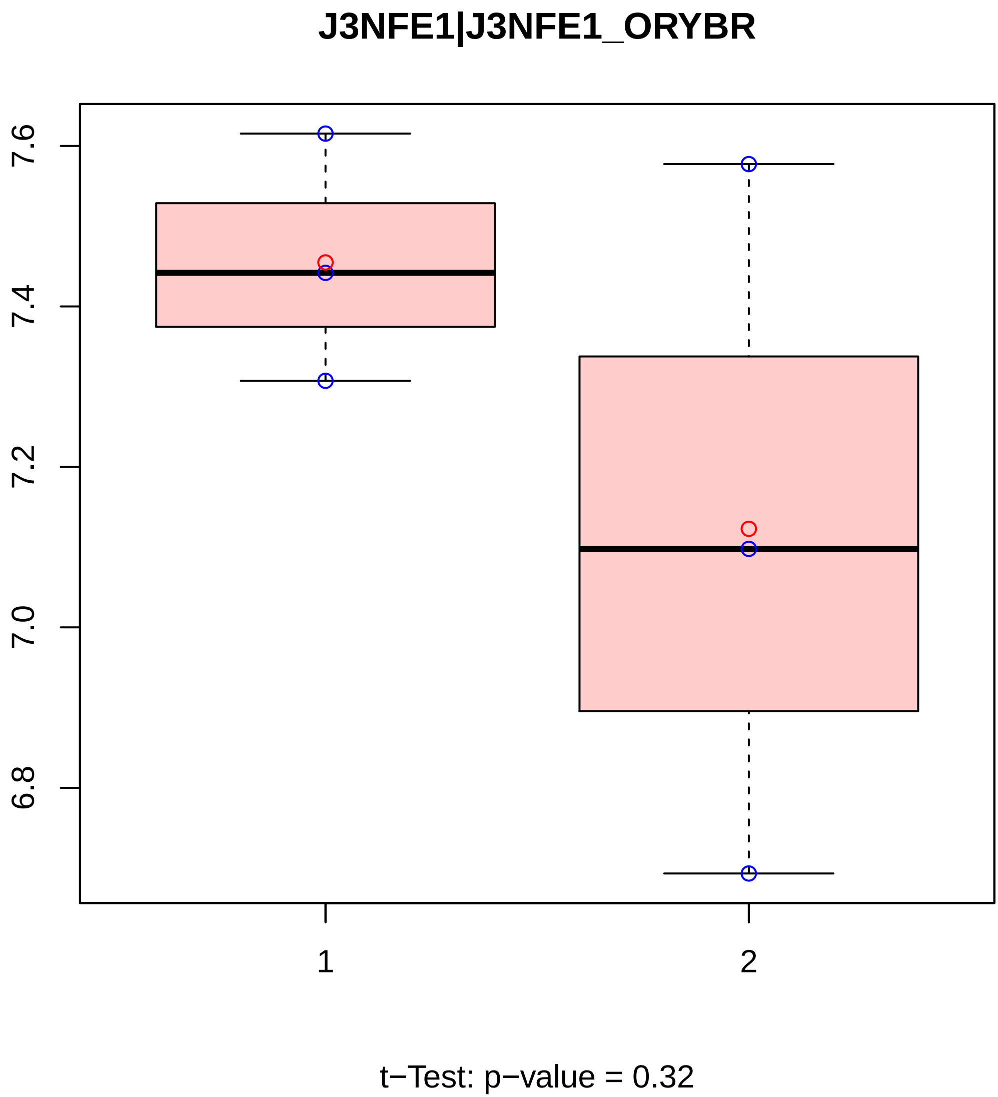
J3NFE1|J3NFE1_ORYBR
7.6
7.4
7.2
7.0
6.8
1
2
t−Test: p−value = 0.32

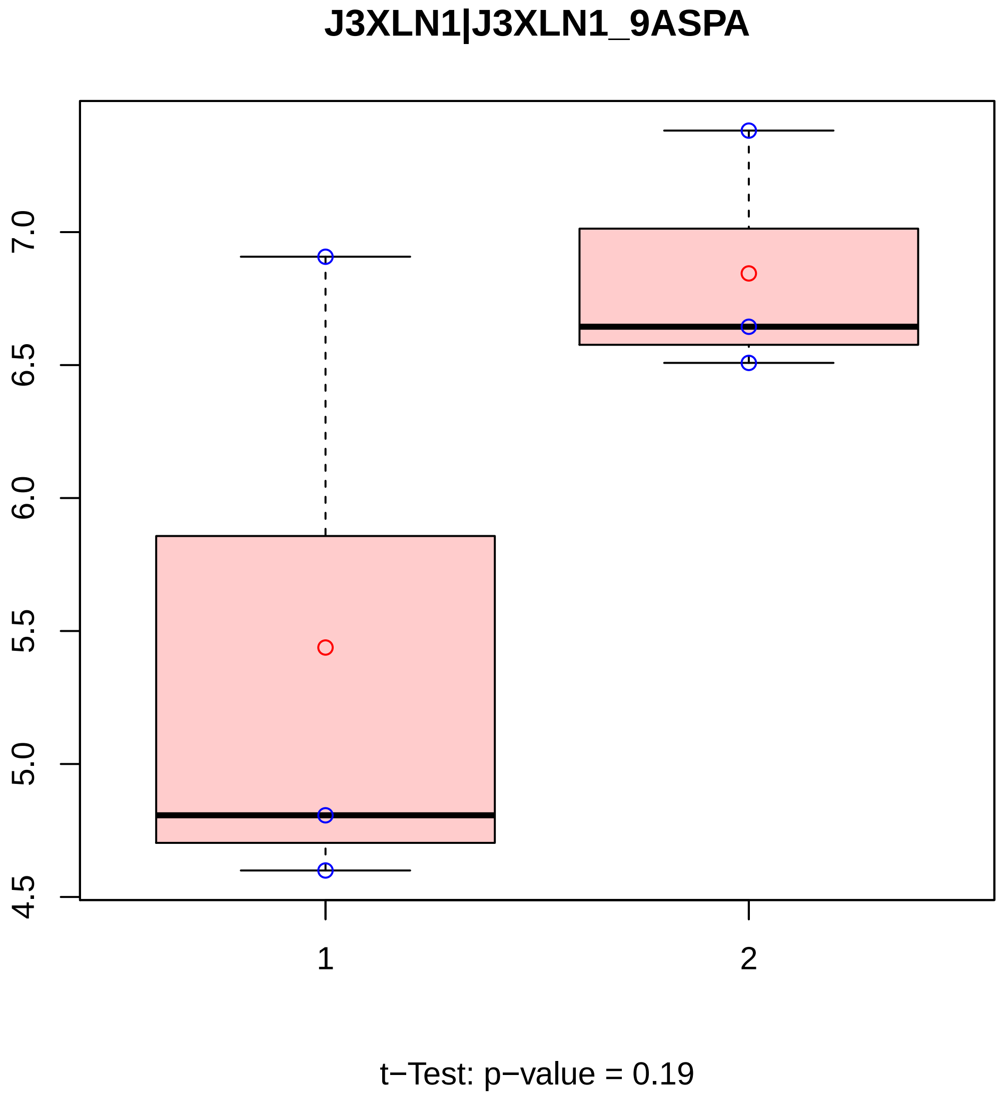
J3XLN1|J3XLN1_9ASPA
7.0
6.5
6.0
5.5
5.0
4.5
1
2
t−Test: p−value = 0.19

J7F224|J7F224_ORYSI

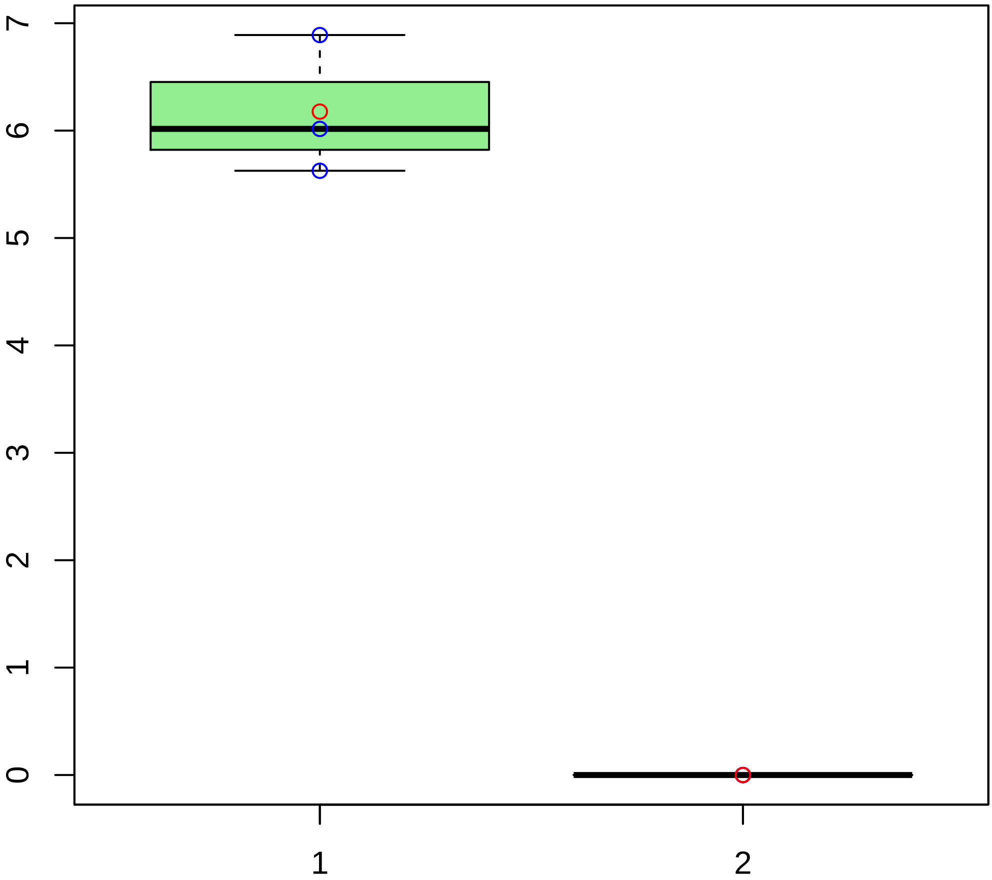

t−Test: p−value = 0

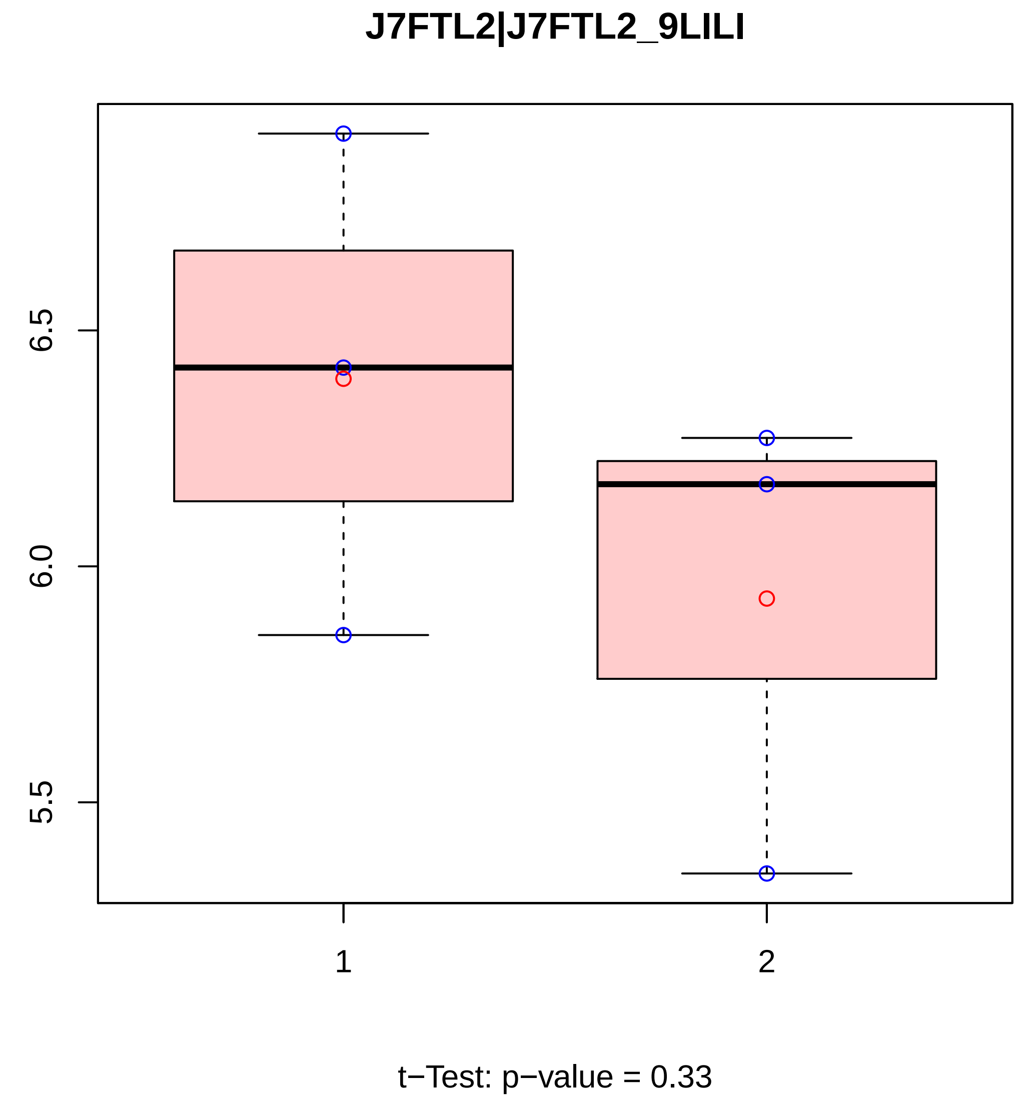
J7FTL2|J7FTL2_9LILI
6.5
6.0
5.5
1
2
t−Test: p−value = 0.33

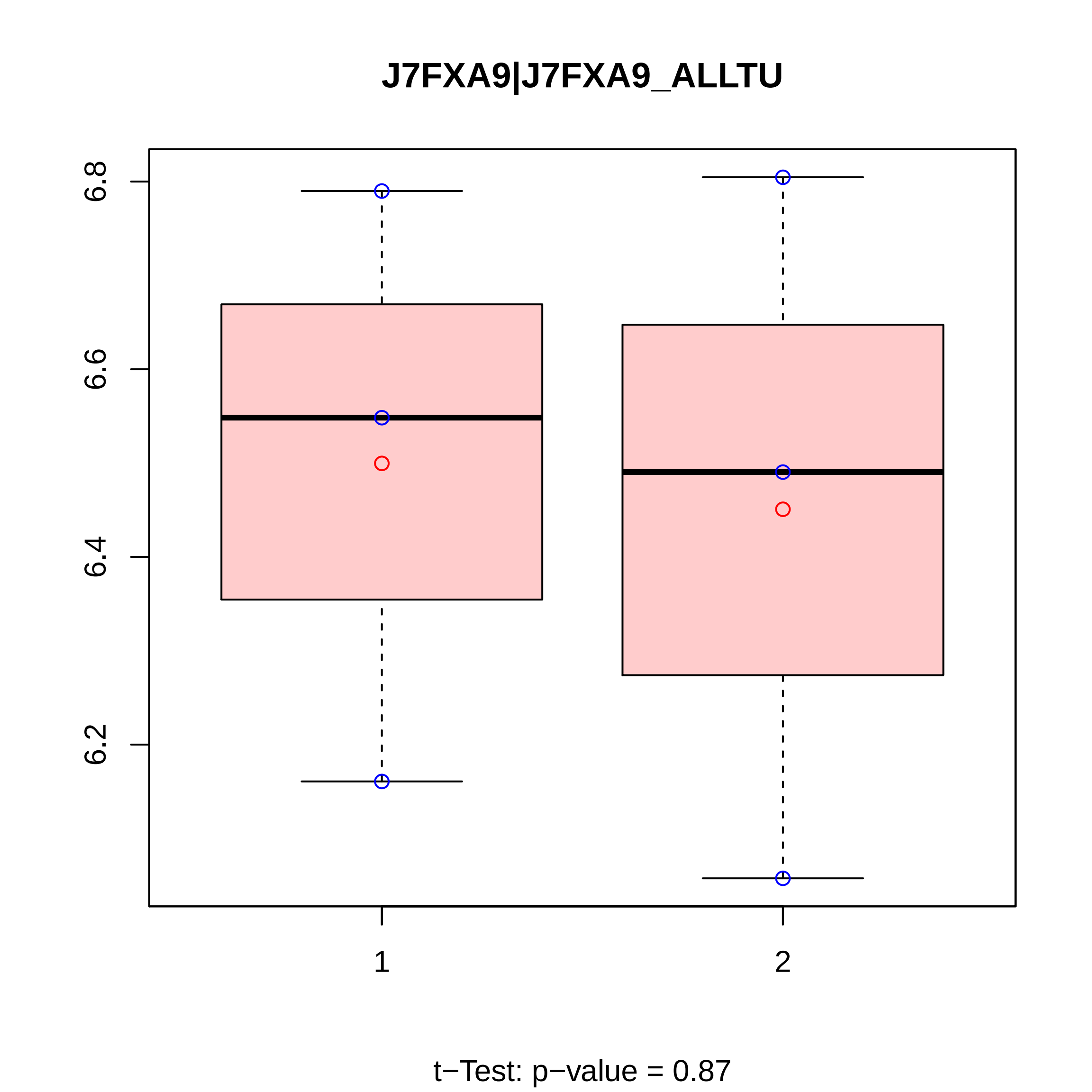
J7FXA9|J7FXA9_ALLTU
6.8
6.6
6.4
6.2
1
2
t−Test: p−value = 0.87

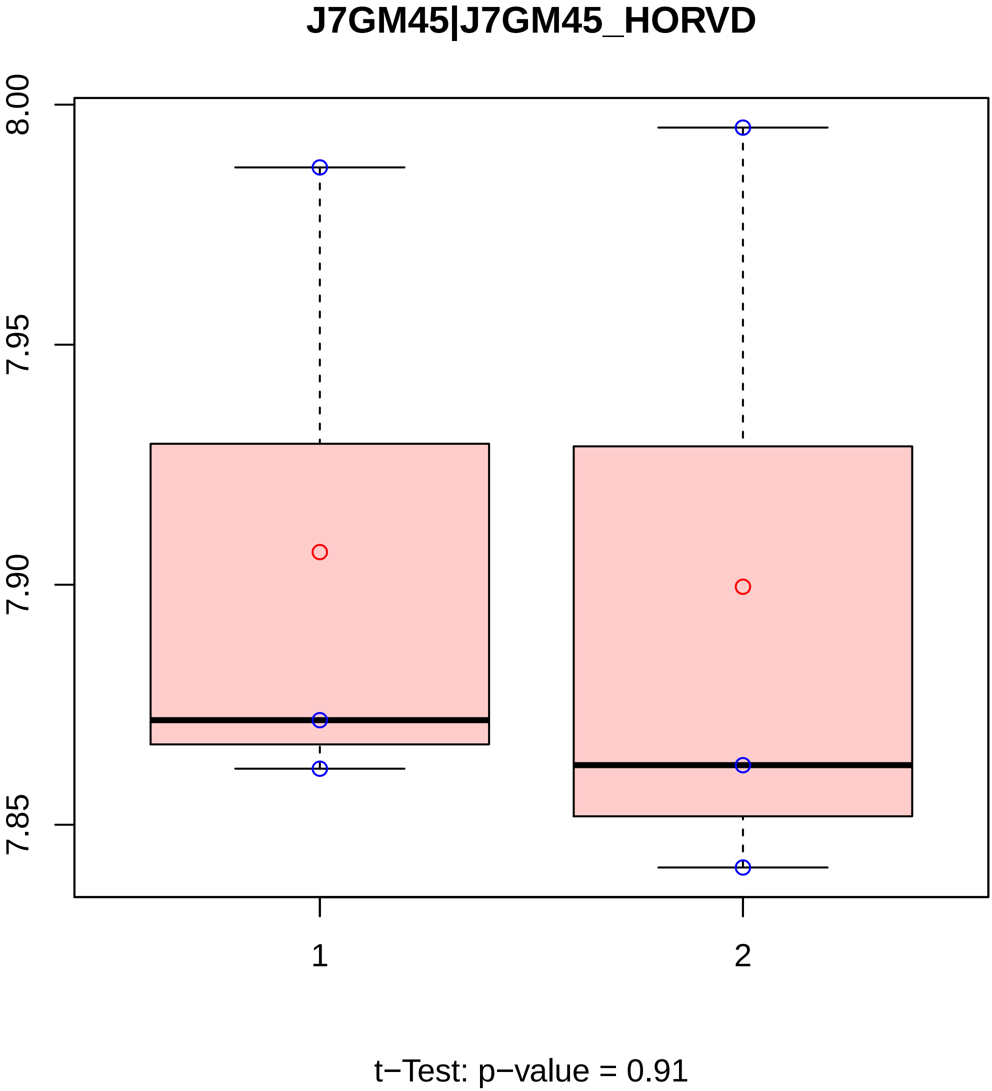
J7GM45|J7GM45_HORVD
8.00
7.95
7.90
7.85
1
2
t−Test: p−value = 0.91

J7I008|J7I008_9POAL

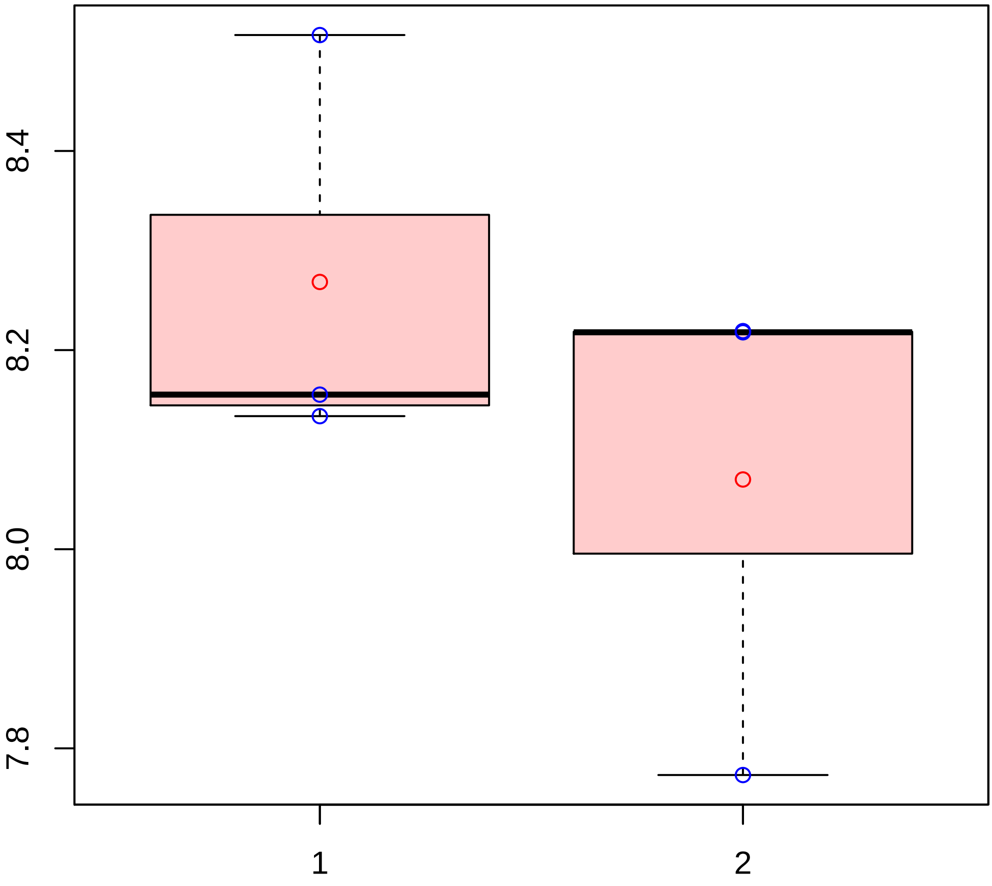

t−Test: p−value = 0.36

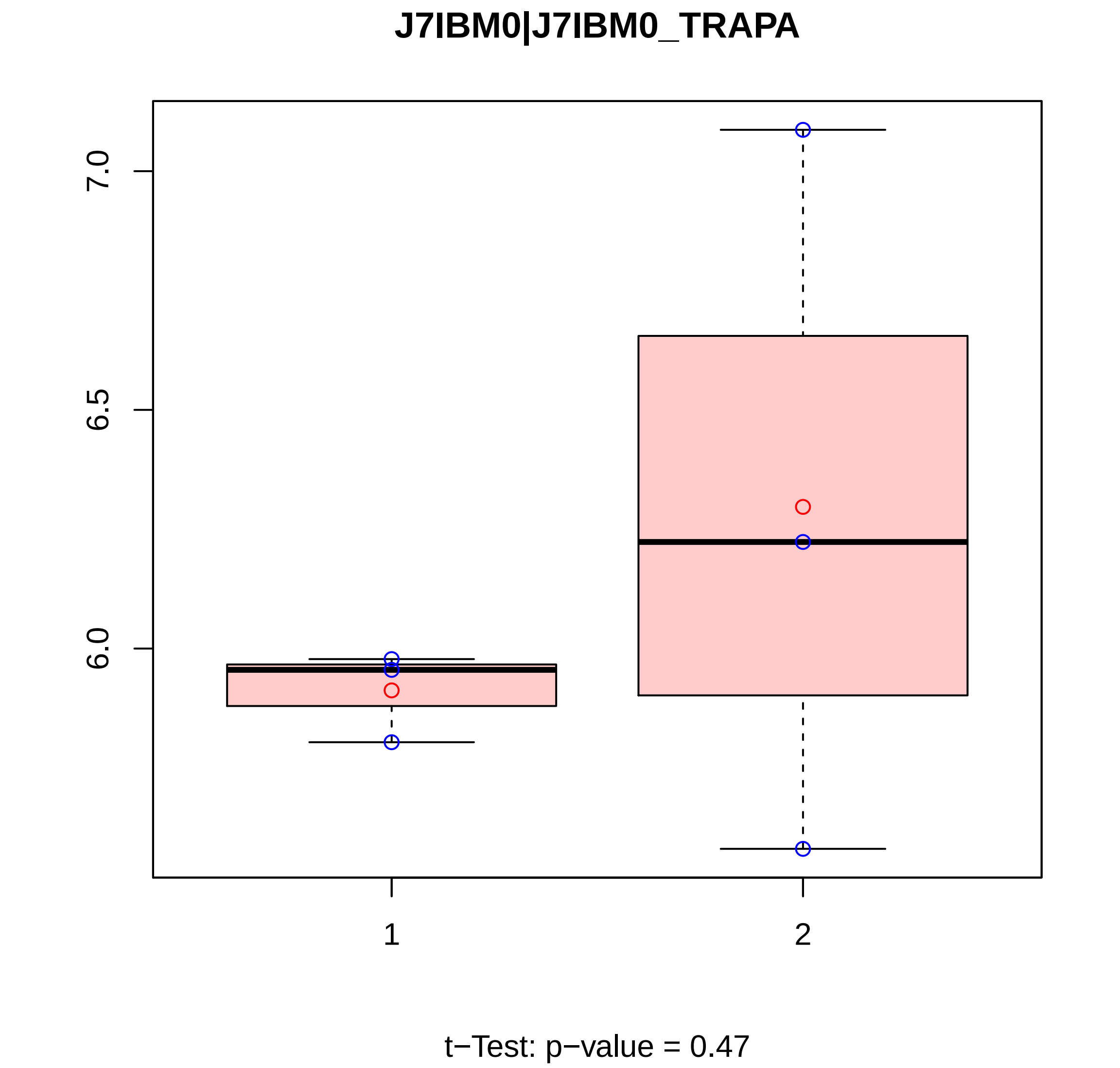

J7IBM0|J7IBM0_TRAPA
7.0
6.5
6.0
1
2
t−Test: p−value = 0.47

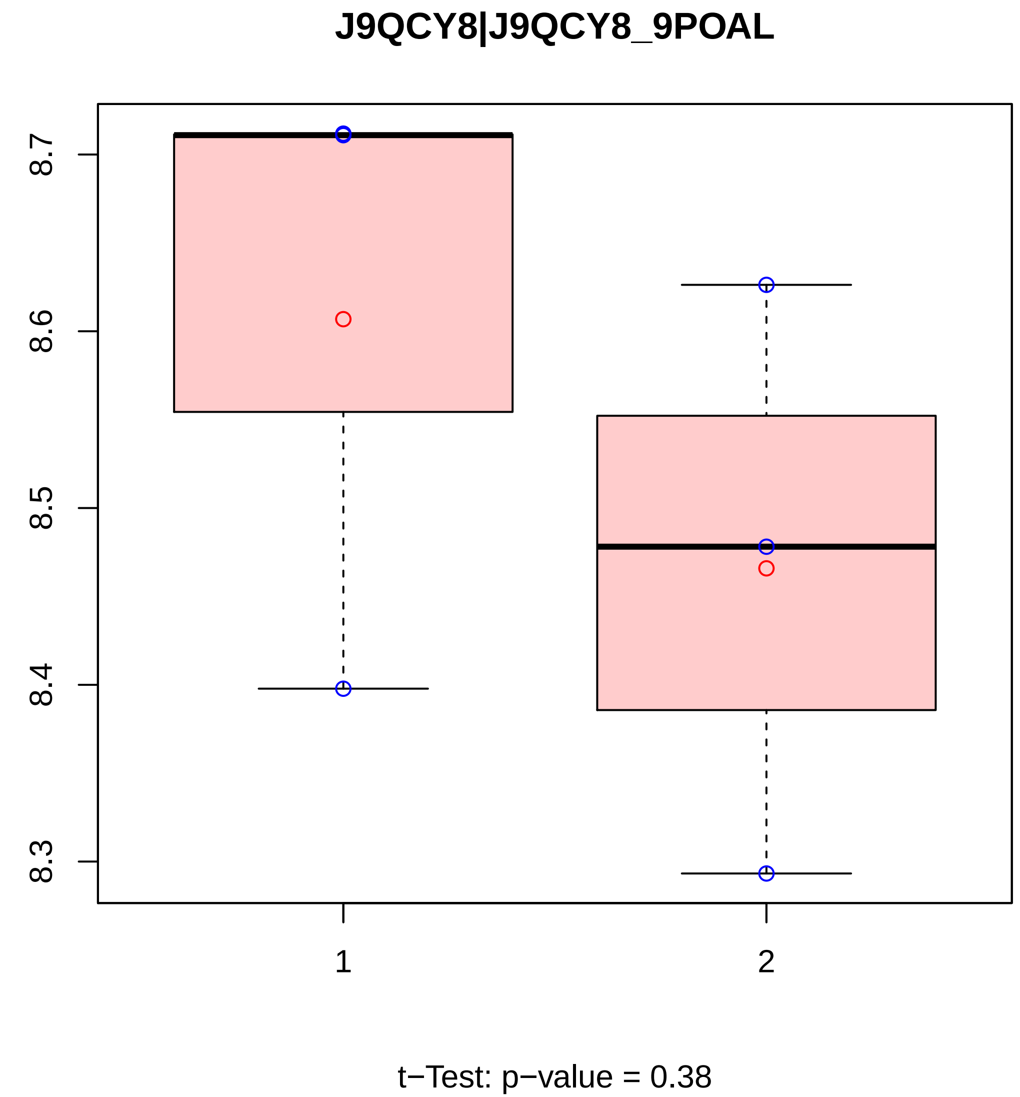

J9QCY8|J9QCY8_9POAL
8.7
8.6
8.5
8.4
8.3
1
2
t−Test: p−value = 0.38

J9QDZ6|J9QDZ6_9POAL

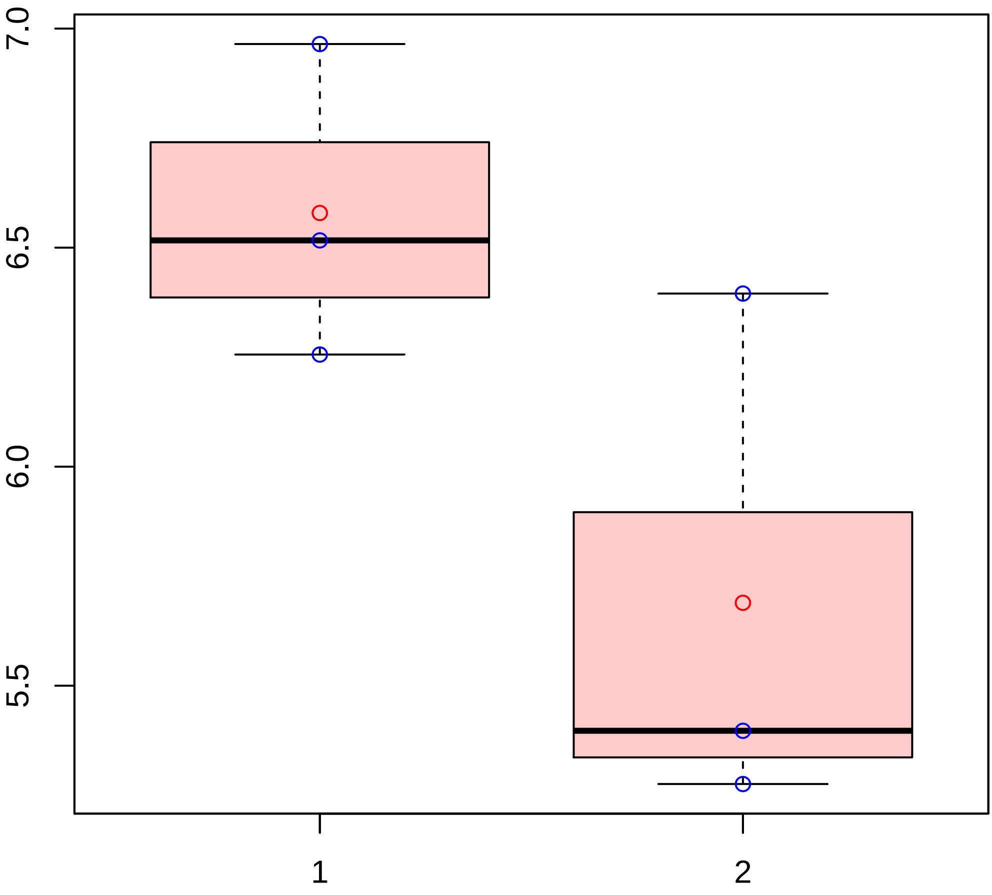

t−Test: p−value = 0.11

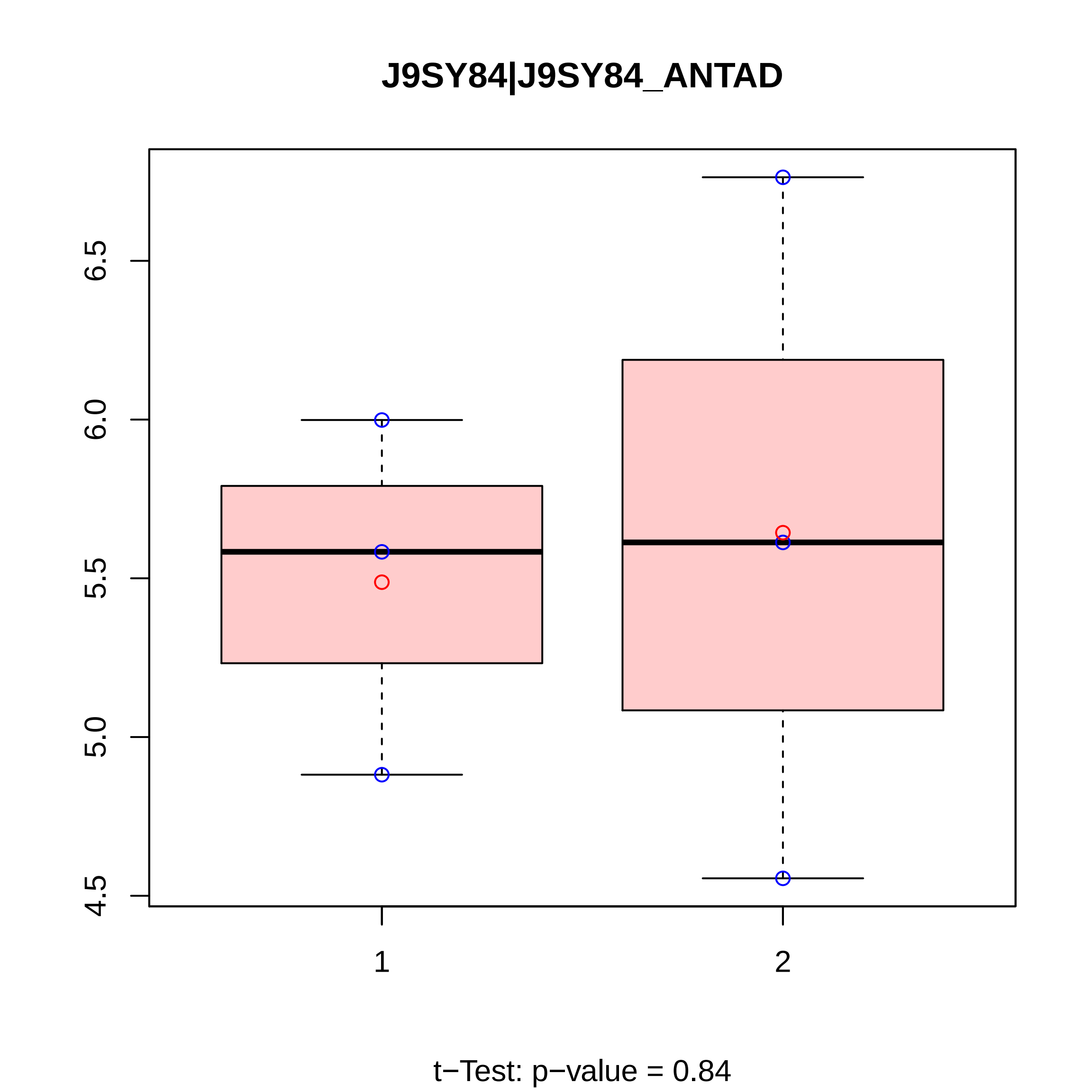
J9SY84|J9SY84_ANTAD
4.5
5.0
5.5
6.0
6.5
1
2
t−Test: p−value = 0.84

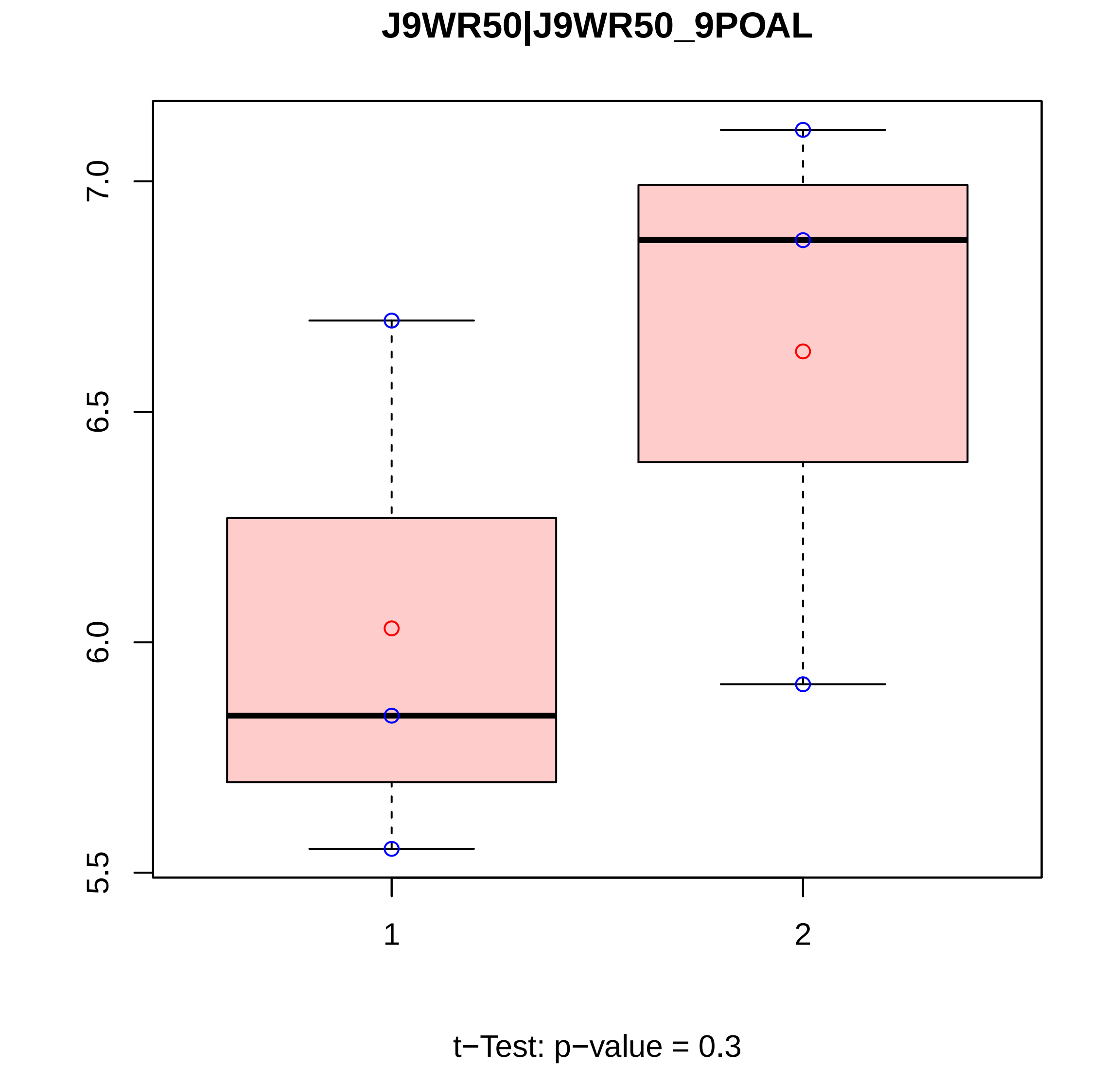
J9WR50|J9WR50_9POAL
7.0
6.5
6.0
5.5
1
2
t−Test: p−value = 0.3

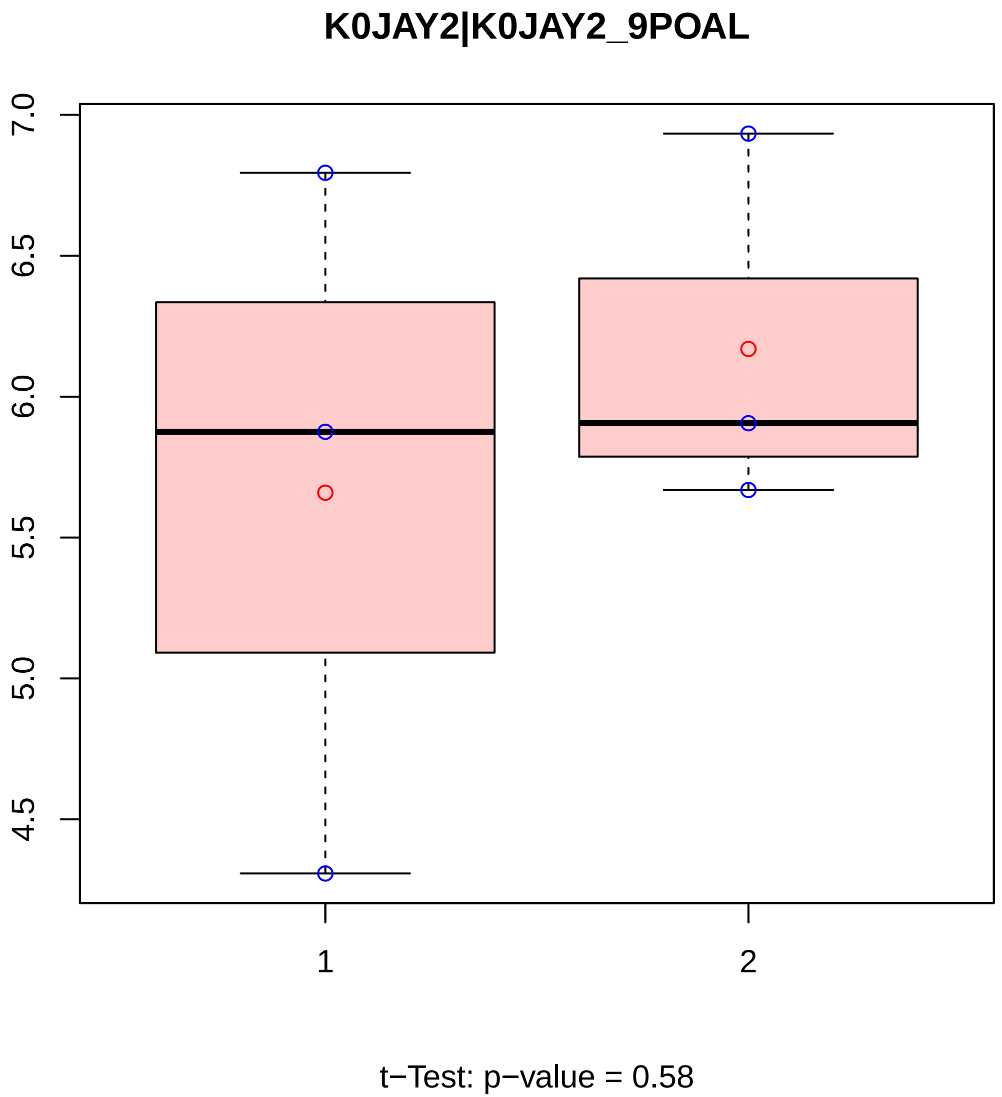
K0JAY2|K0JAY2_9POAL
7.0
6.5
6.0
5.5
5.0
4.5
1
2
t−Test: p−value = 0.58

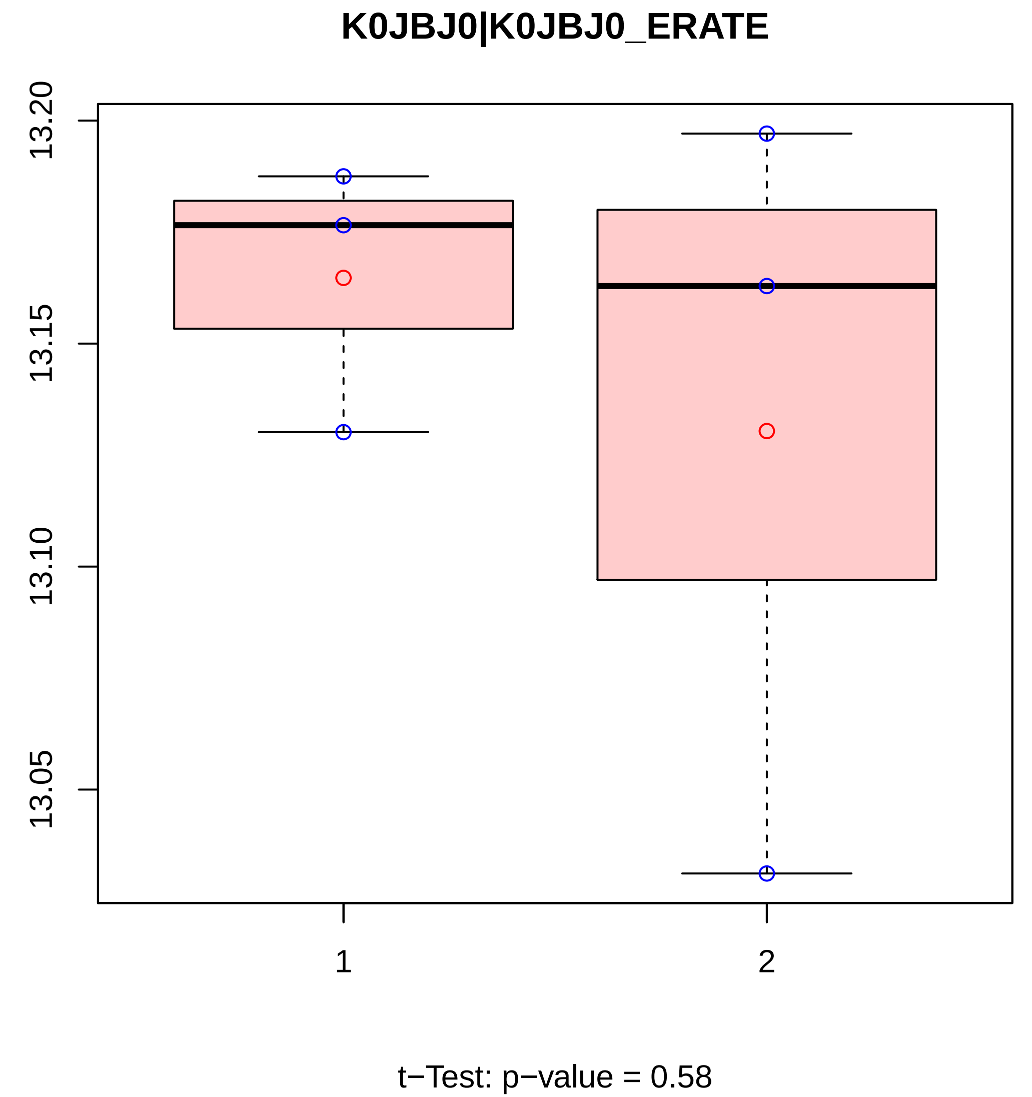
K0JBJ0|K0JBJ0_ERATE
13.20
13.15
13.10
13.05
1
2
t−Test: p−value = 0.58

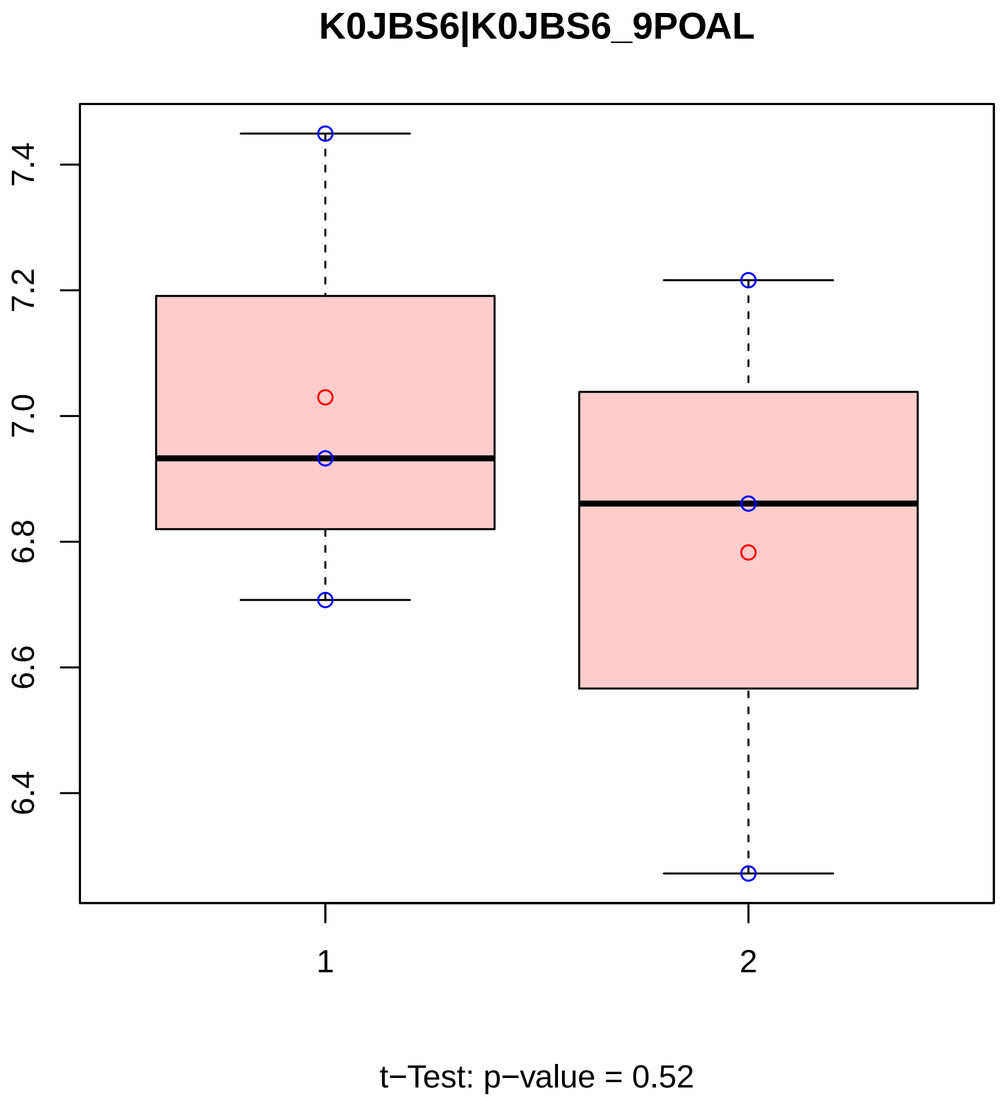
K0JBS6|K0JBS6_9POAL
7.4
7.2
7.0
6.8
6.6
6.4
1
2
t−Test: p−value = 0.52

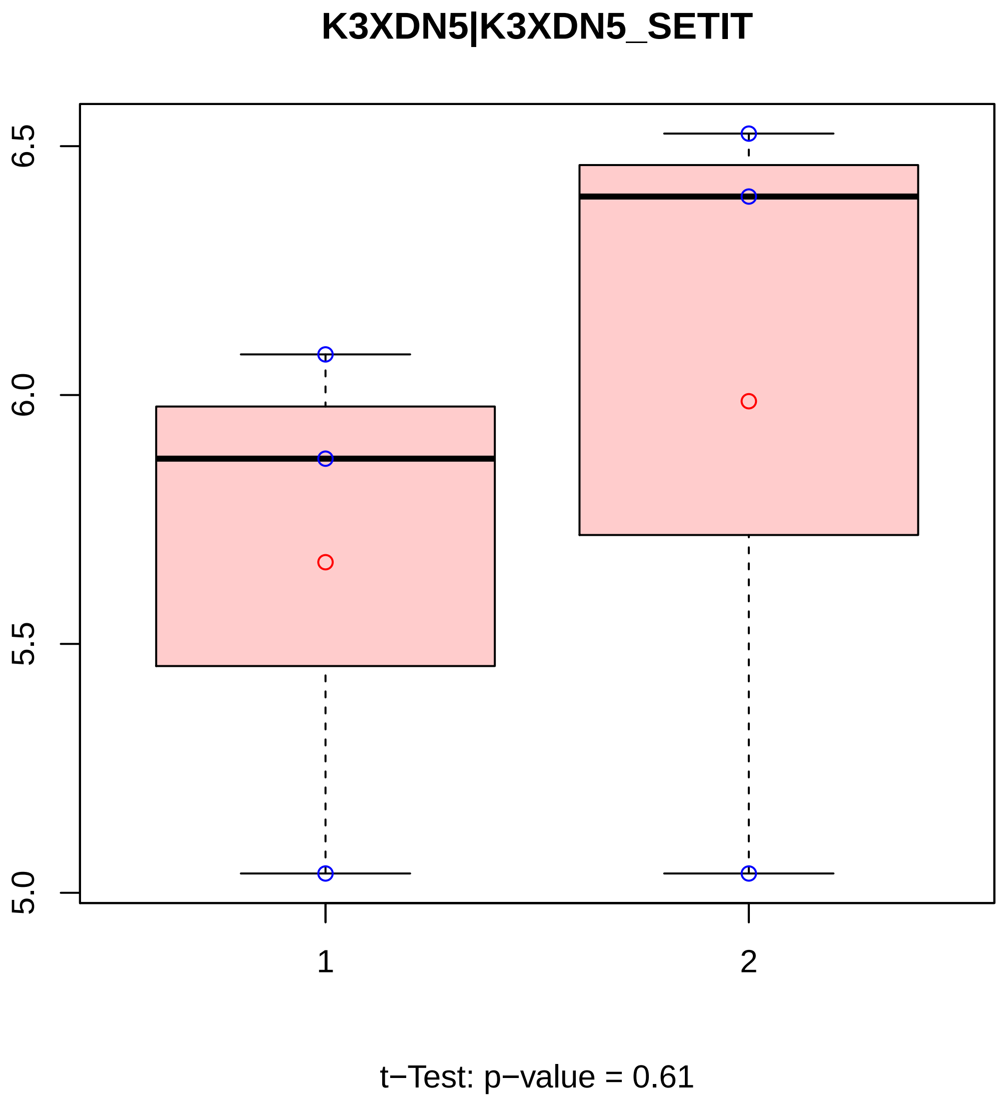
K3XDN5|K3XDN5_SETIT
6.5
6.0
5.5
5.0
1
2
t−Test: p−value = 0.61

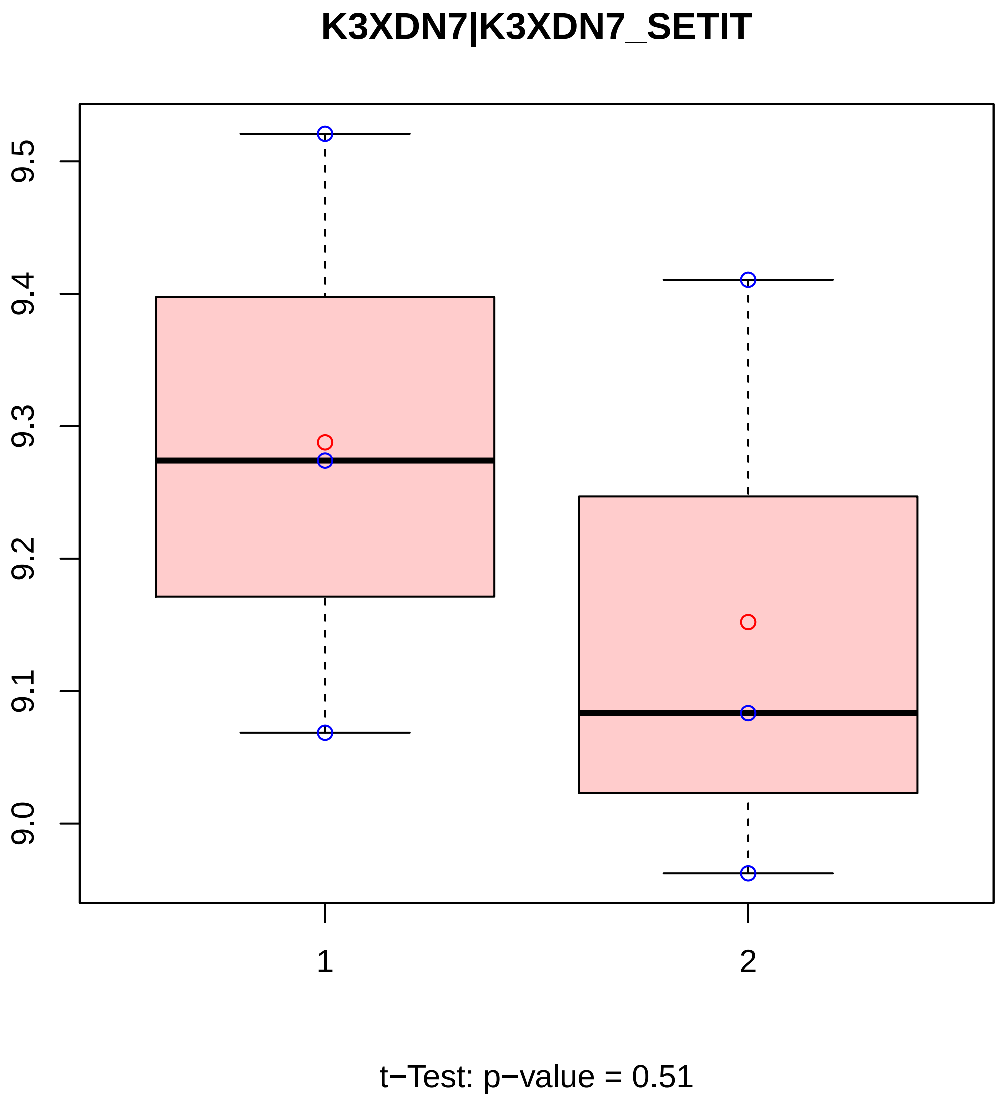

K3XDN7|K3XDN7_SETIT
9.0
9.1
9.2
9.3
9.4
9.5
1
2
t−Test: p−value = 0.51

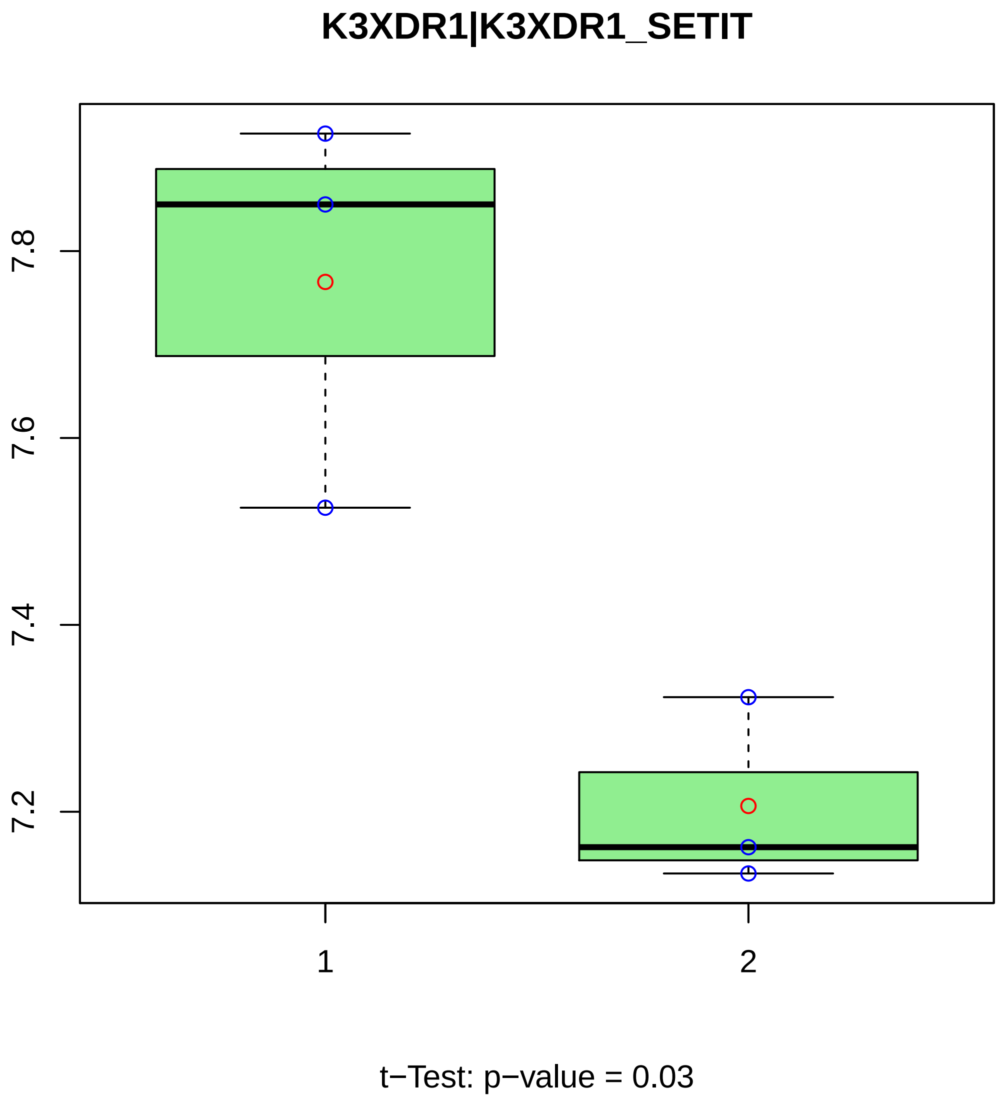
K3XDR1|K3XDR1_SETIT
7.2
7.4
7.6
7.8
1
2
t−Test: p−value = 0.03

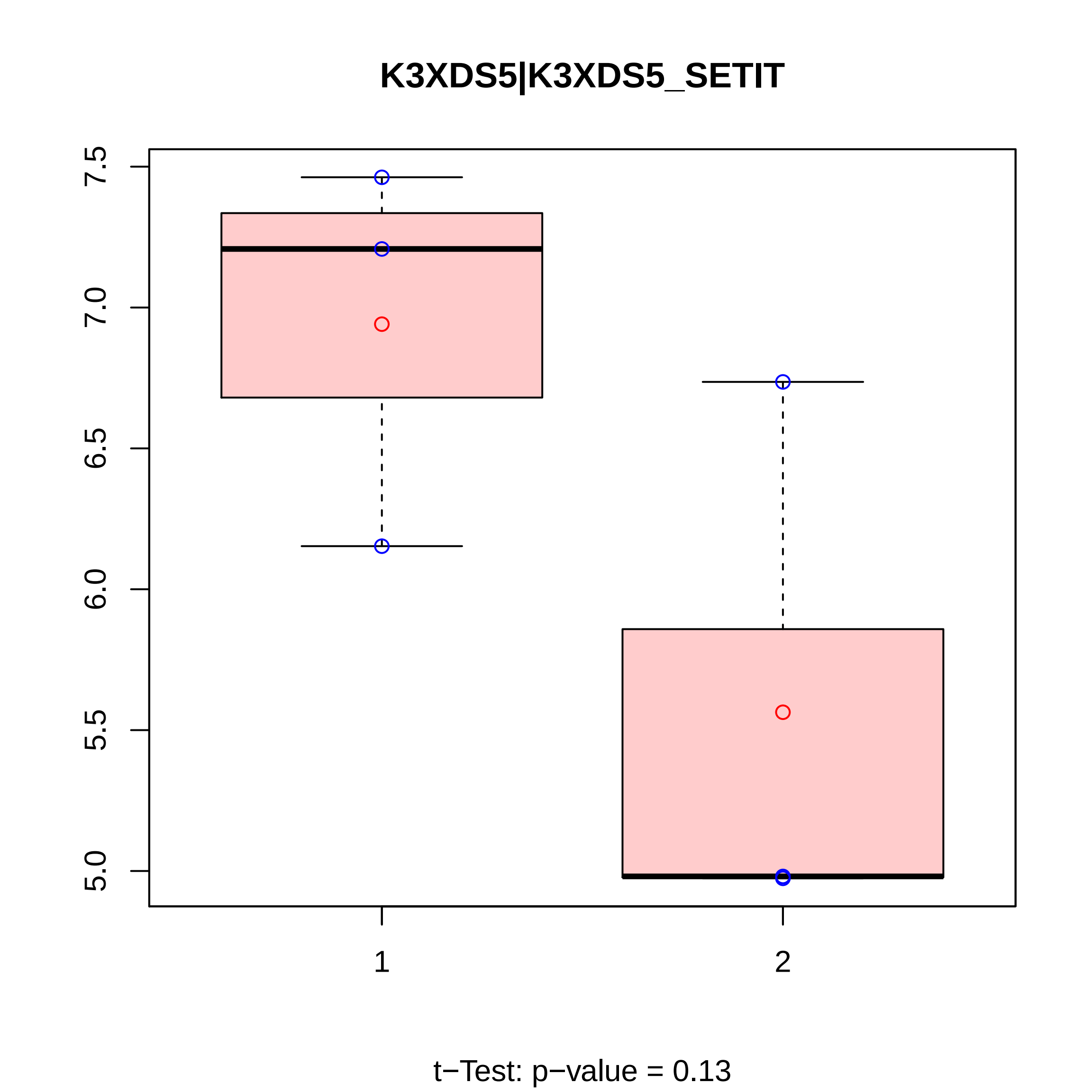
K3XDS5|K3XDS5_SETIT
7.5
7.0
6.5
6.0
5.5
5.0
1
2
t−Test: p−value = 0.13

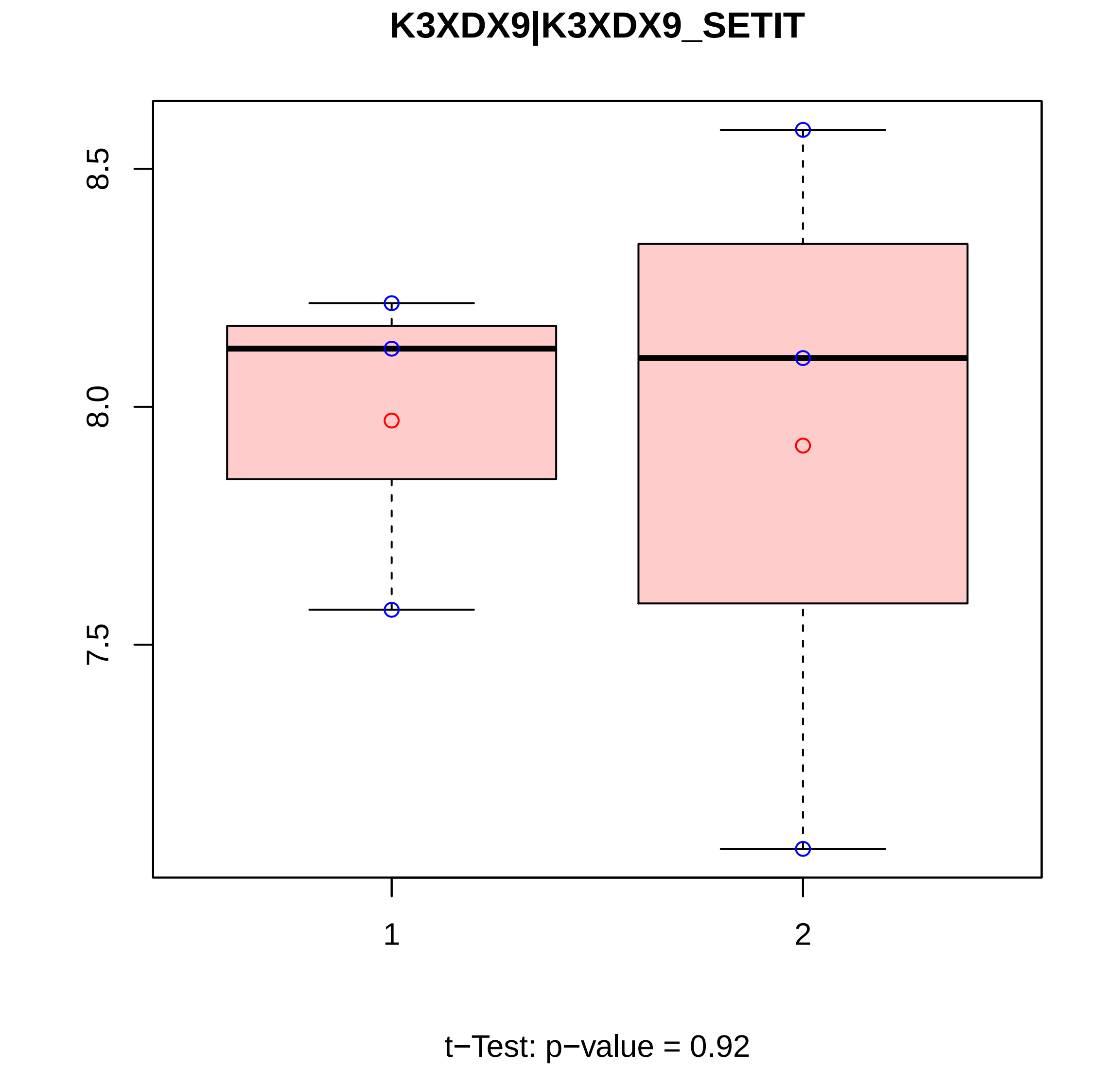
K3XDX9|K3XDX9_SETIT
8.5
8.0
7.5
1
2
t−Test: p−value = 0.92

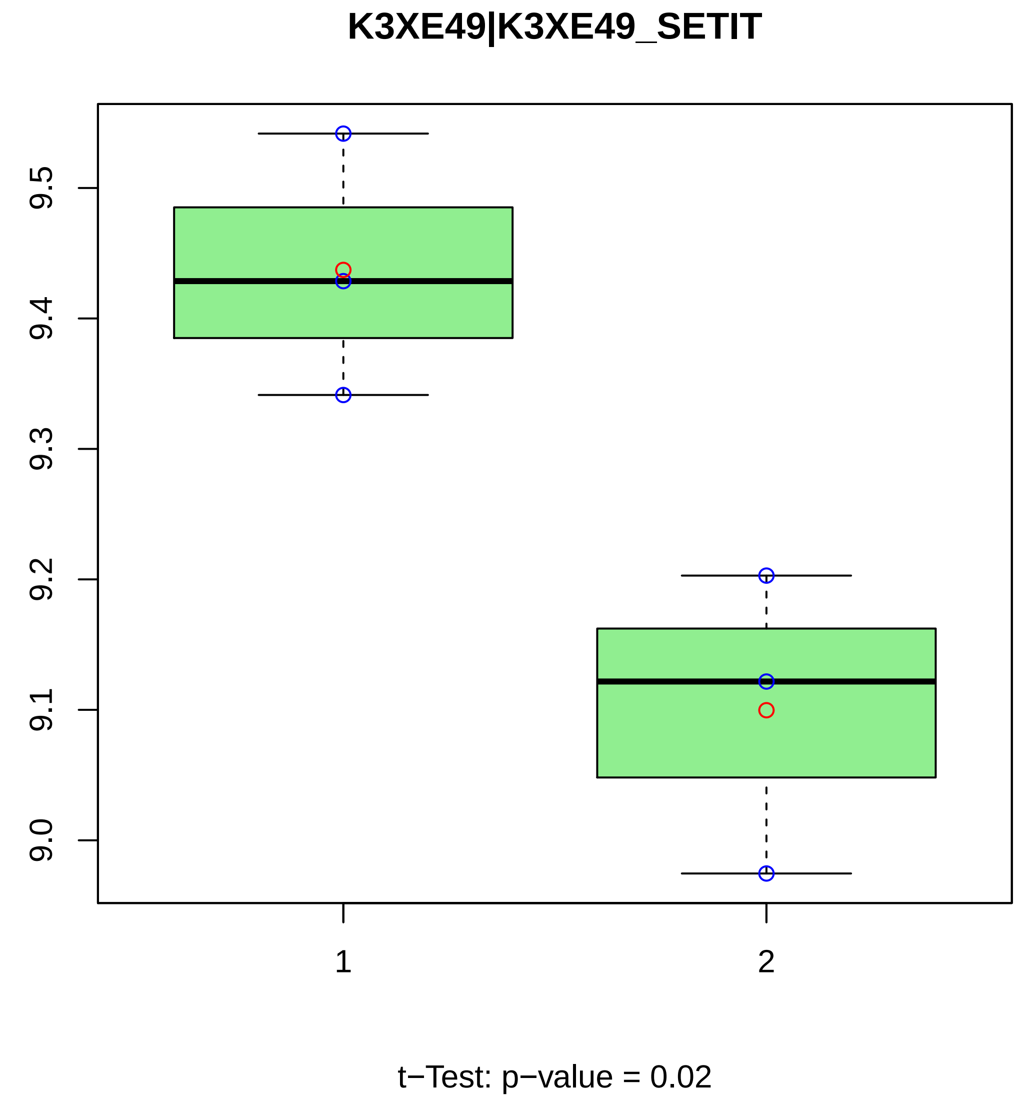
K3XE49|K3XE49_SETIT
9.0
9.1
9.2
9.3
9.4
9.5
1
2
t−Test: p−value = 0.02

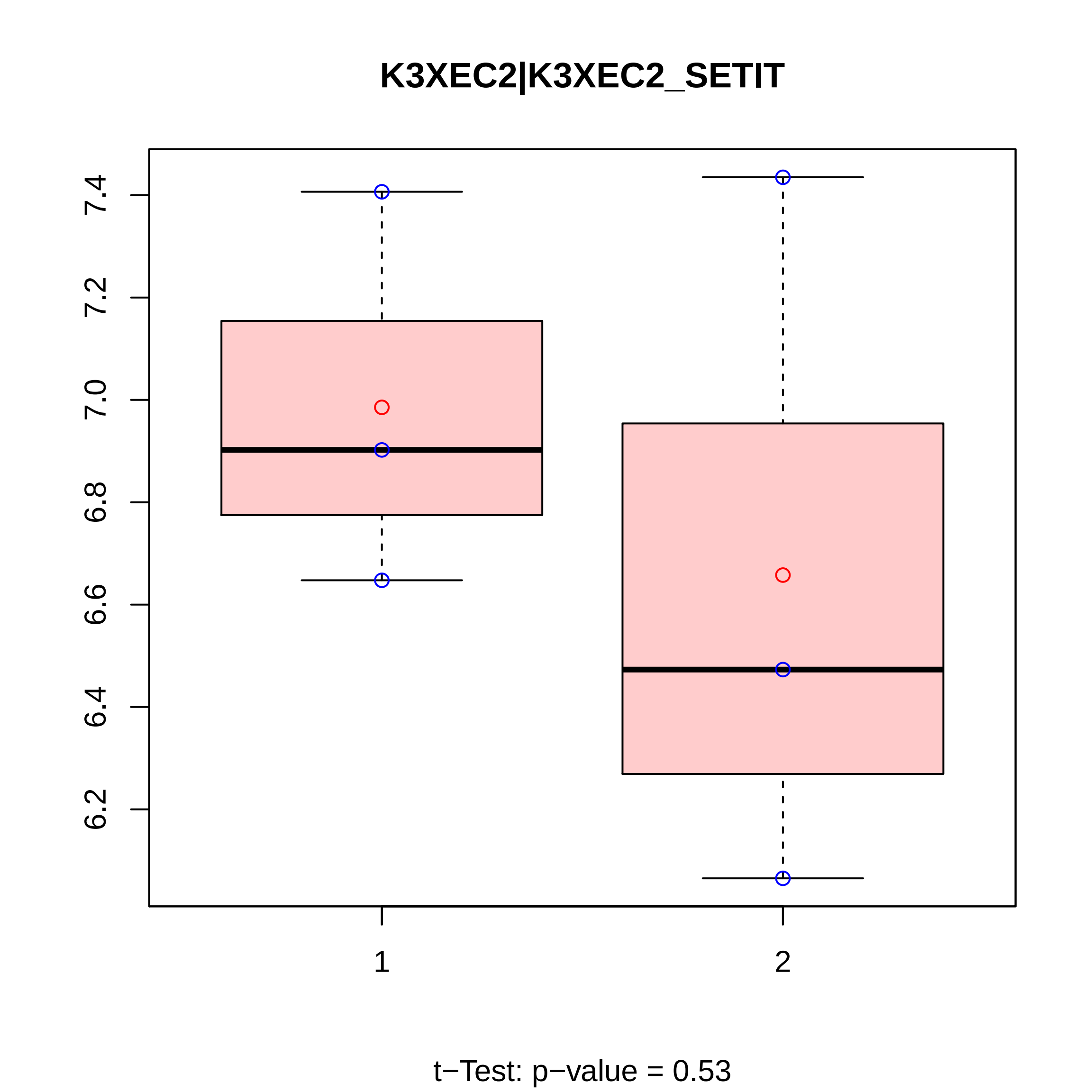

K3XEC2|K3XEC2_SETIT
7.4
7.2
7.0
6.8
6.6
6.4
6.2
1
2
t−Test: p−value = 0.53

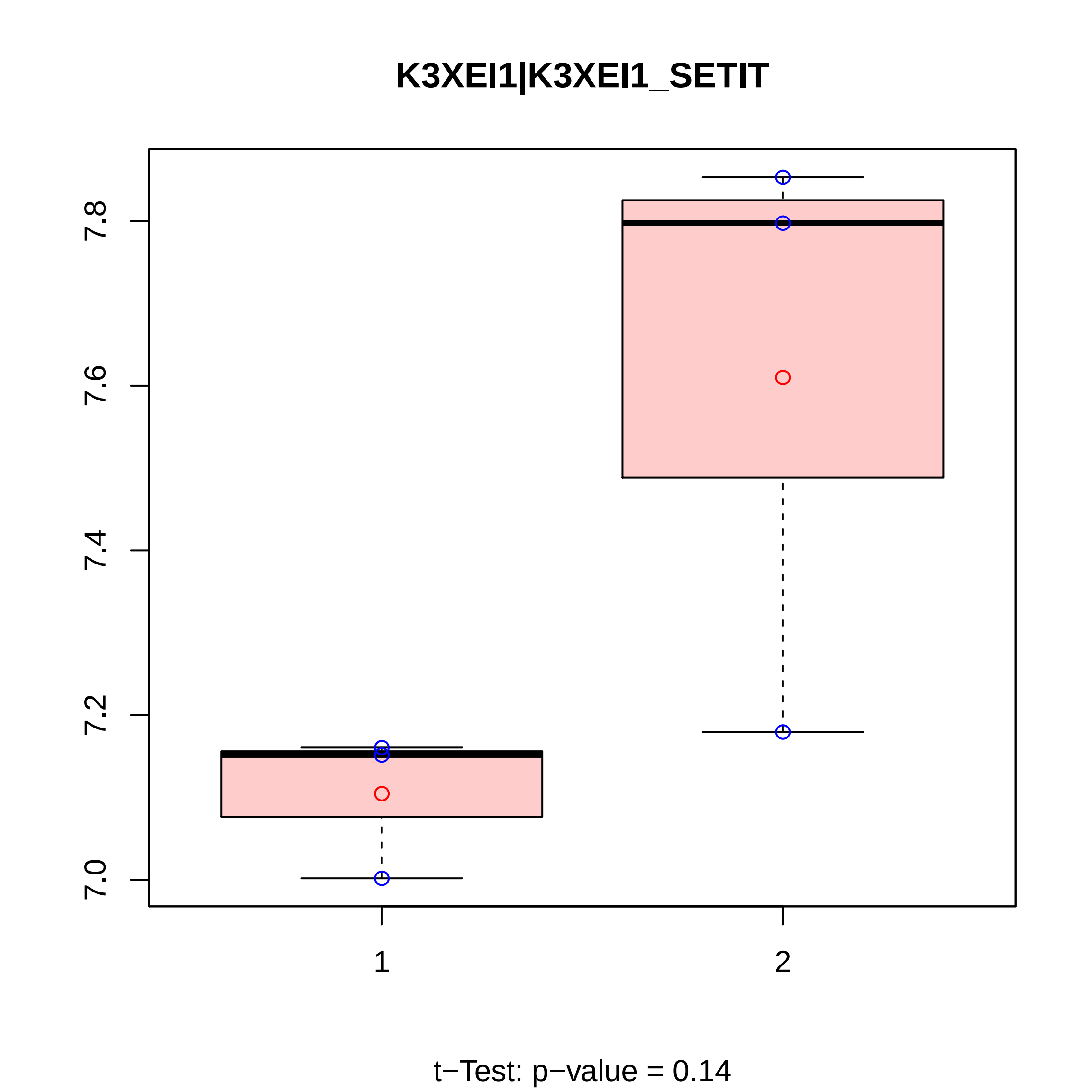
K3XEI1|K3XEI1_SETIT
7.0
7.2
7.4
7.6
7.8
1
2
t−Test: p−value = 0.14

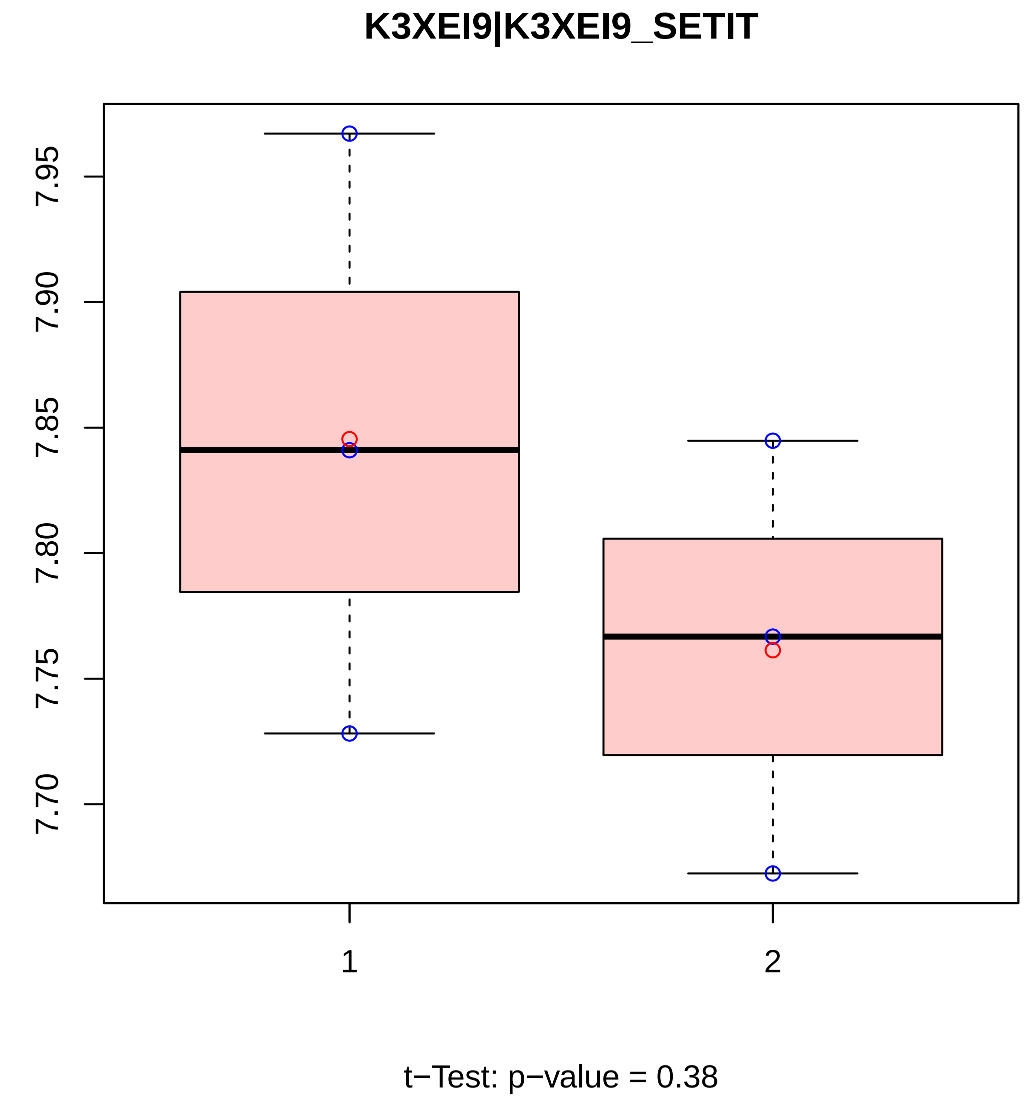
K3XEI9|K3XEI9_SETIT
7.70
7.75
7.80
7.85
7.90
7.95
1
2
t−Test: p−value = 0.38

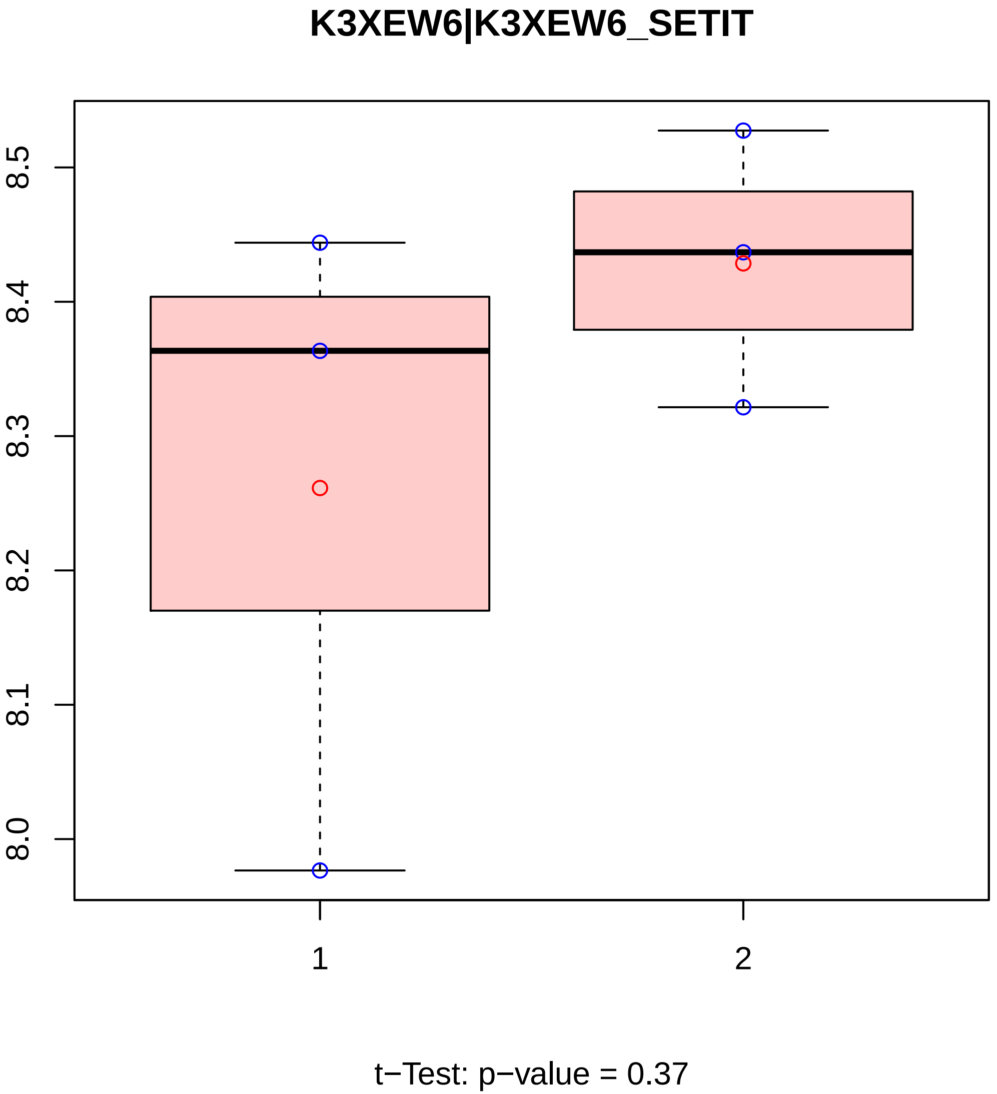
K3XEW6|K3XEW6_SETIT
8.0
8.1
8.2
8.3
8.4
8.5
1
2
t−Test: p−value = 0.37

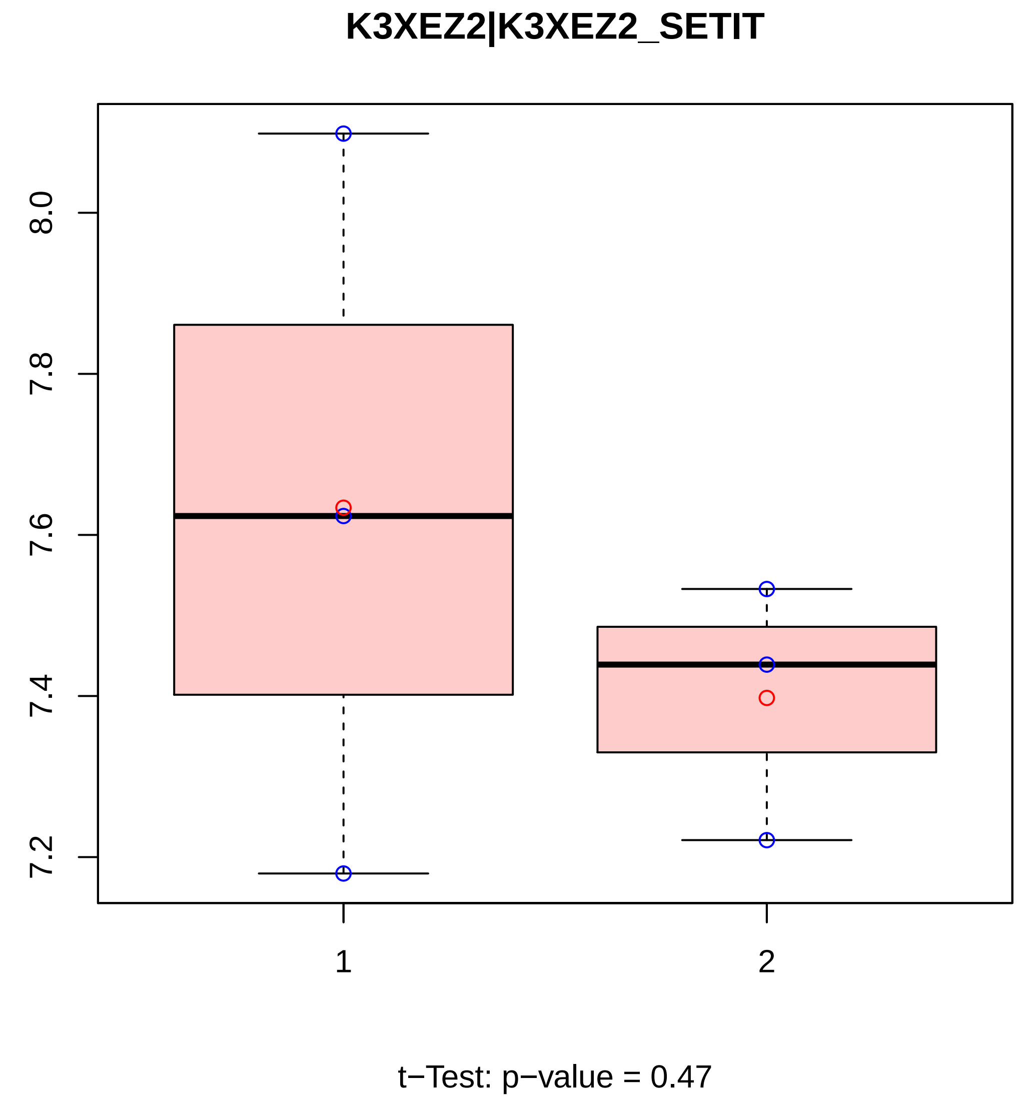
K3XEZ2|K3XEZ2_SETIT
8.0
7.8
7.6
7.4
7.2
1
2
t−Test: p−value = 0.47

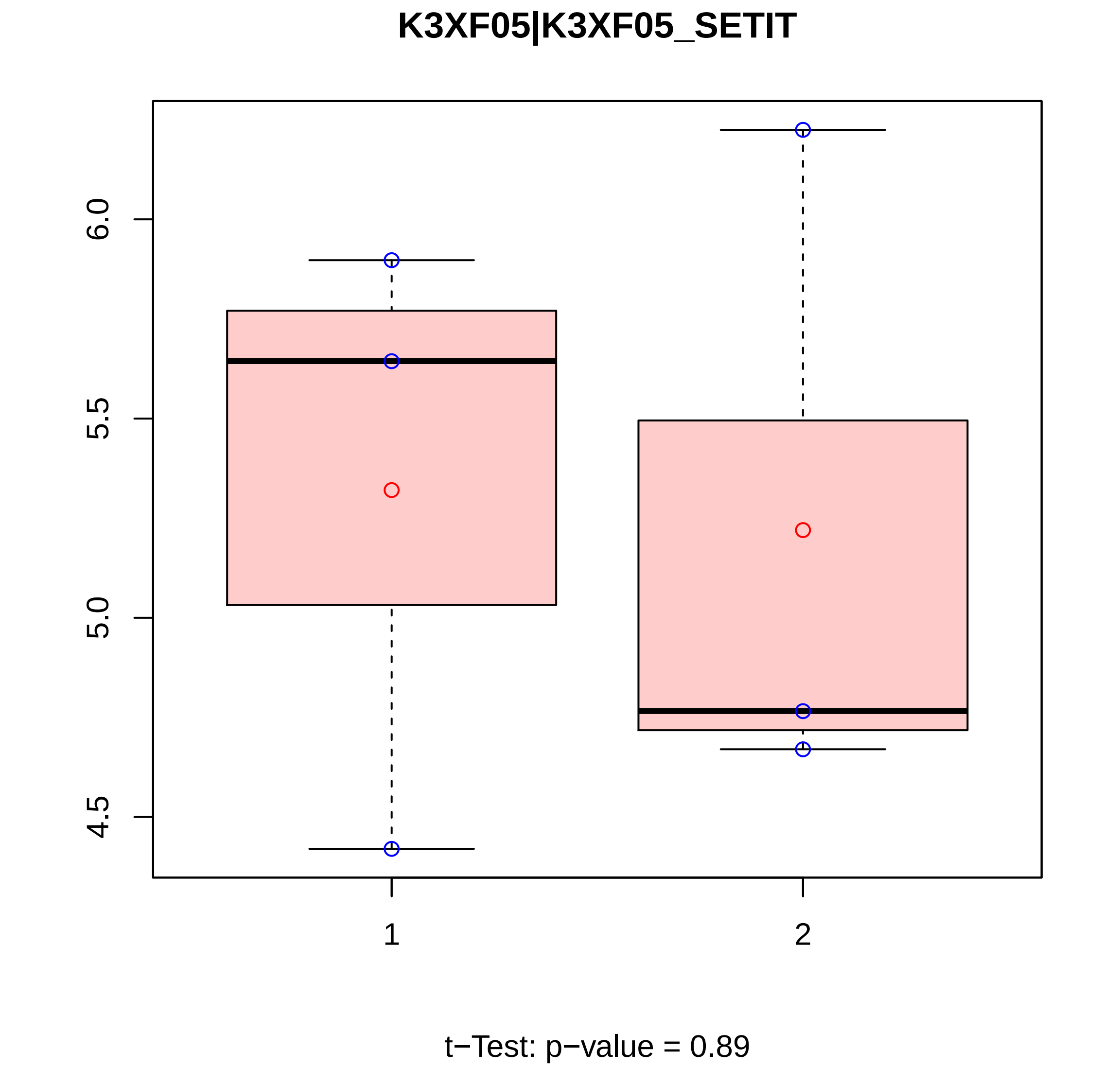
K3XF05|K3XF05_SETIT
6.0
5.5
5.0
4.5
1
2
t−Test: p−value = 0.89

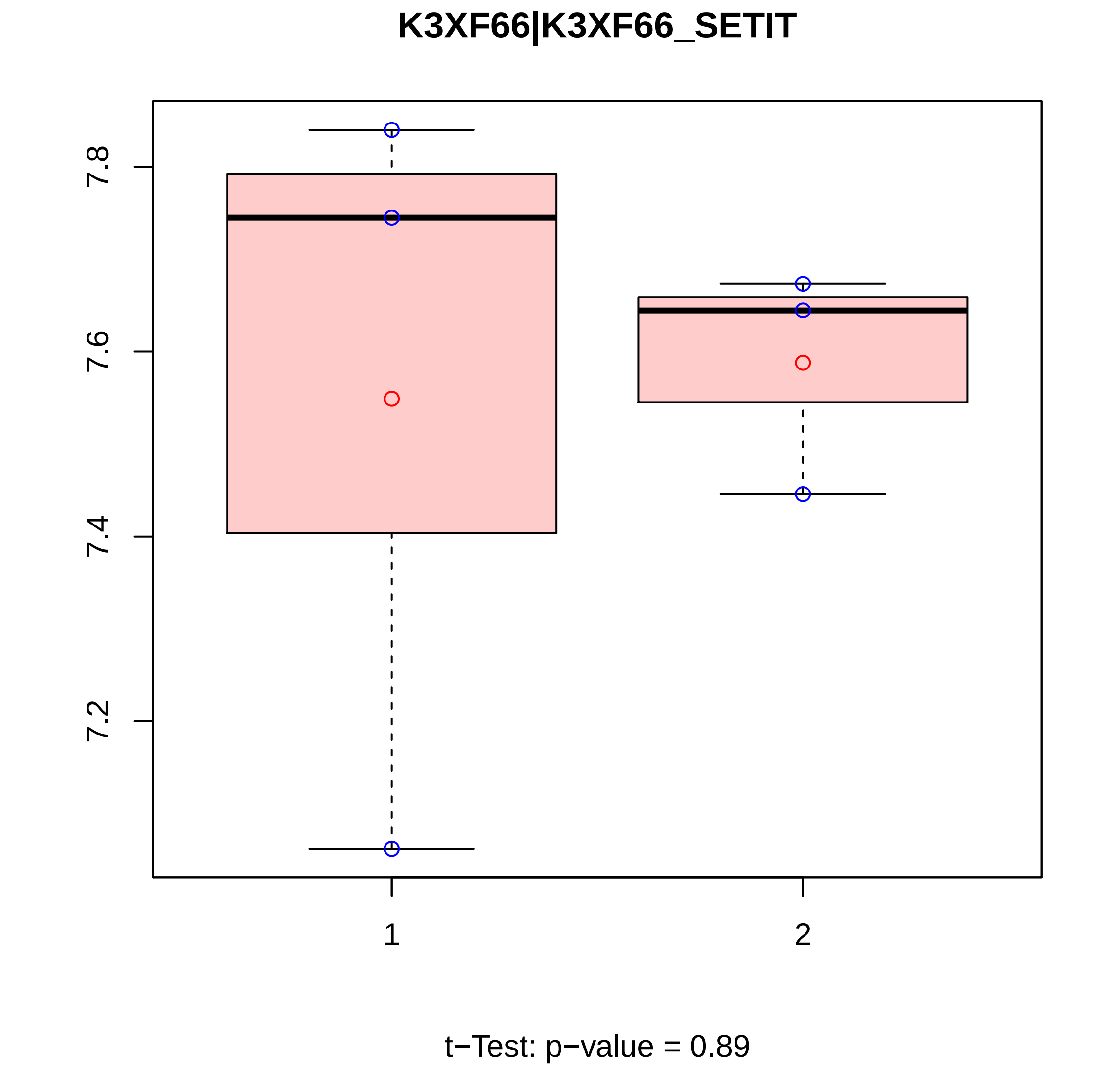
K3XF66|K3XF66_SETIT
7.8
7.6
7.4
7.2
1
2
t−Test: p−value = 0.89

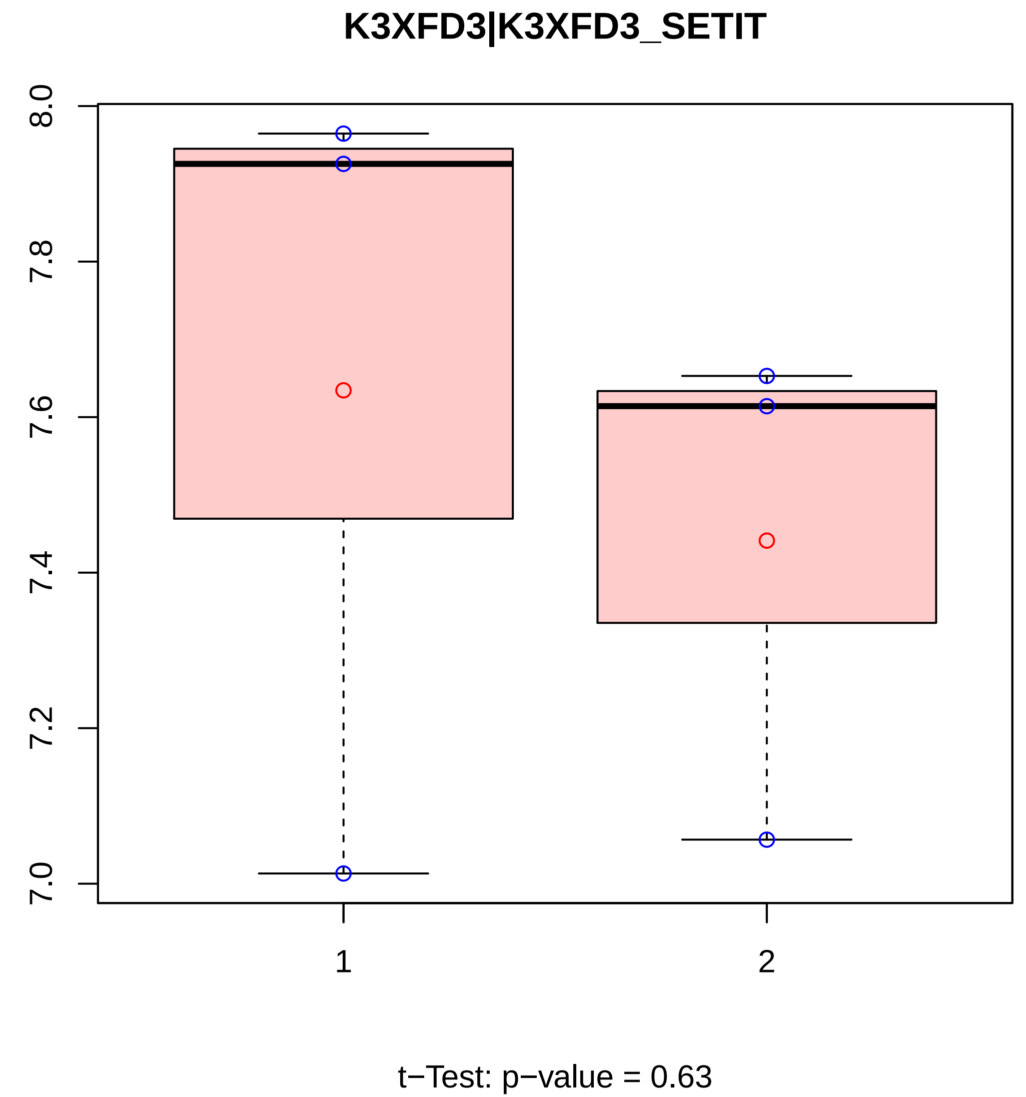
K3XFD3|K3XFD3_SETIT
8.0
7.8
7.6
7.4
7.2
7.0
1
2
t−Test: p−value = 0.63

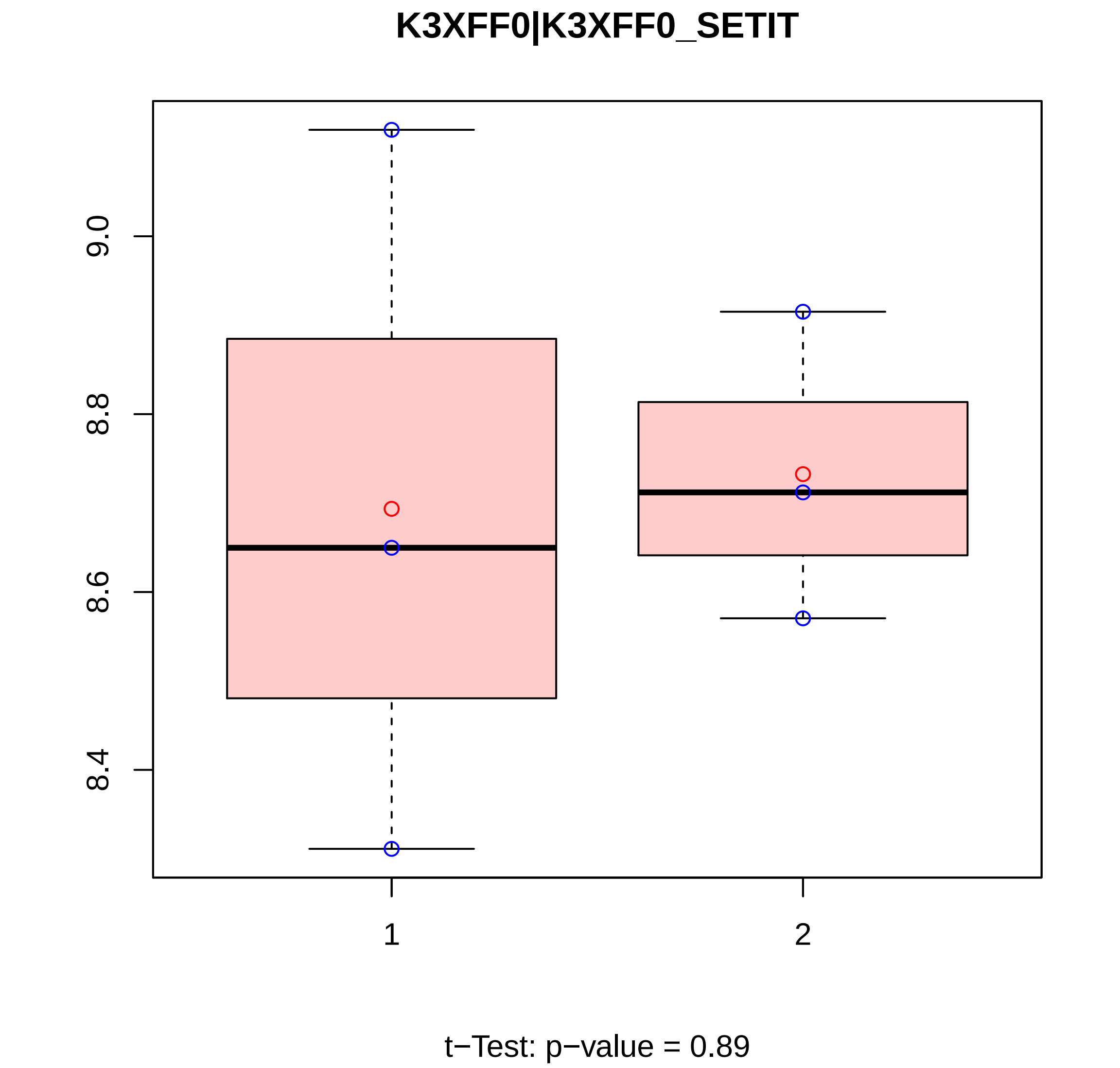
K3XFF0|K3XFF0_SETIT
9.0
8.8
8.6
8.4
1
2
t−Test: p−value = 0.89

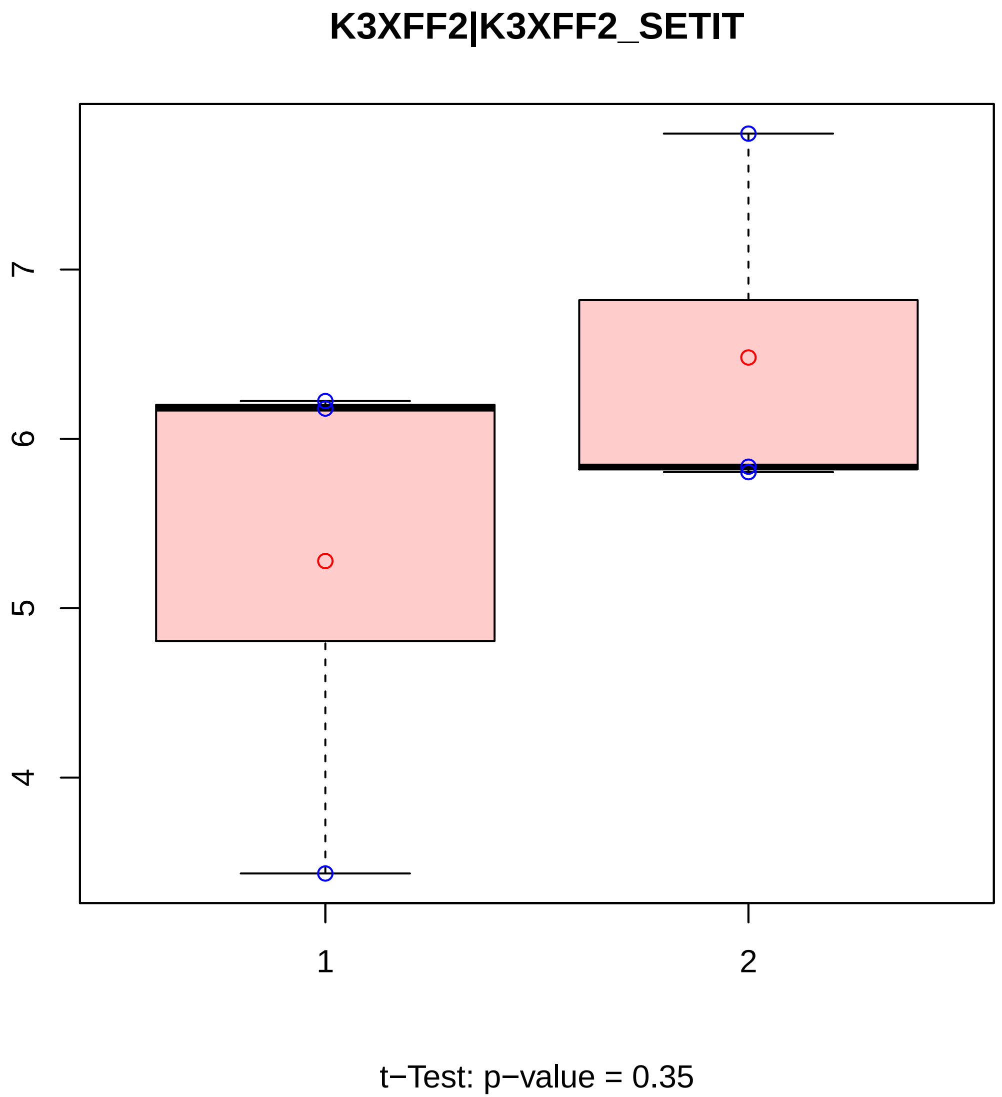
K3XFF2|K3XFF2_SETIT
4
5
6
7
1
2
t−Test: p−value = 0.35

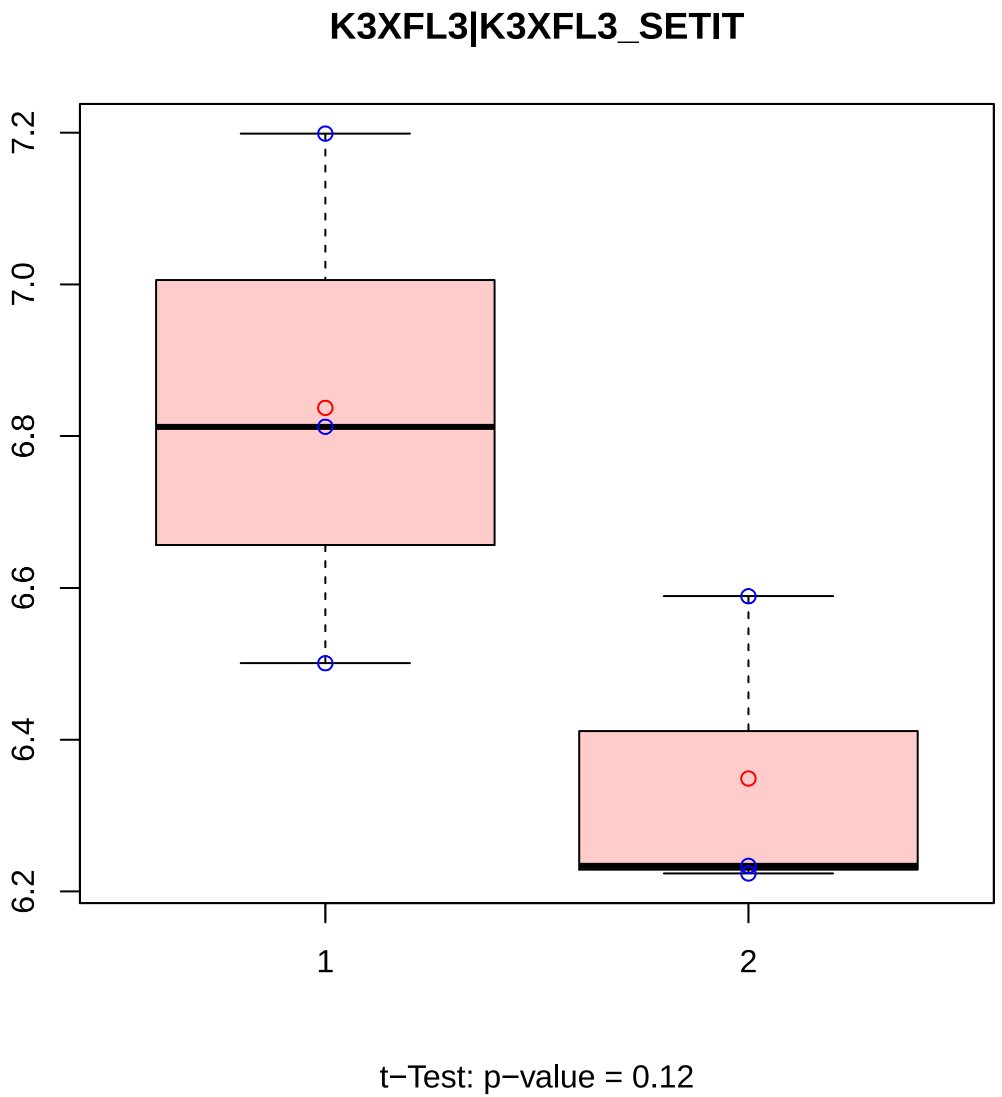
K3XFL3|K3XFL3_SETIT
7.2
7.0
6.8
6.6
6.4
6.2
1
2
t−Test: p−value = 0.12

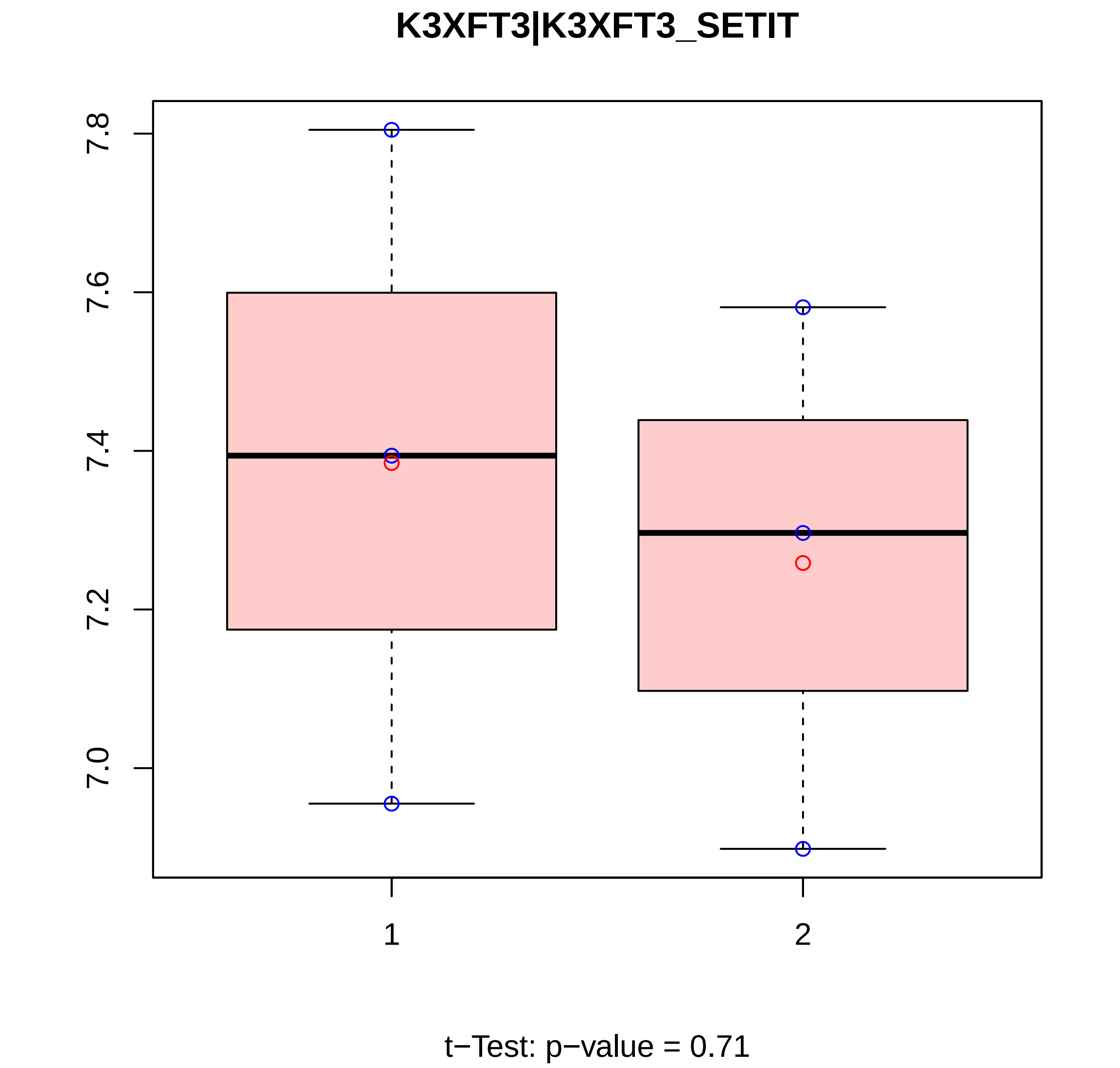
K3XFT3|K3XFT3_SETIT
7.8
7.6
7.4
7.2
7.0
1
2
t−Test: p−value = 0.71

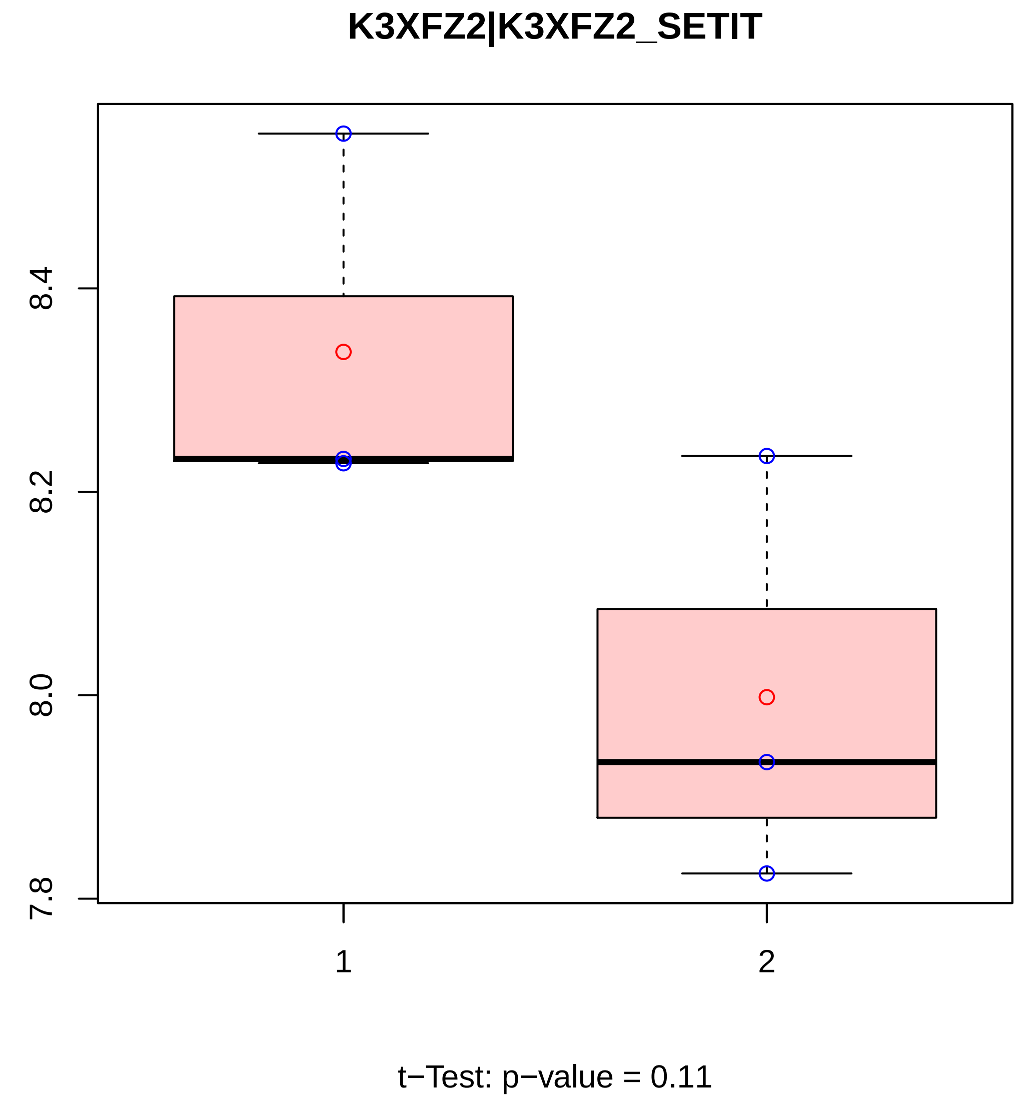
K3XFZ2|K3XFZ2_SETIT
8.4
8.2
8.0
7.8
1
2
t−Test: p−value = 0.11

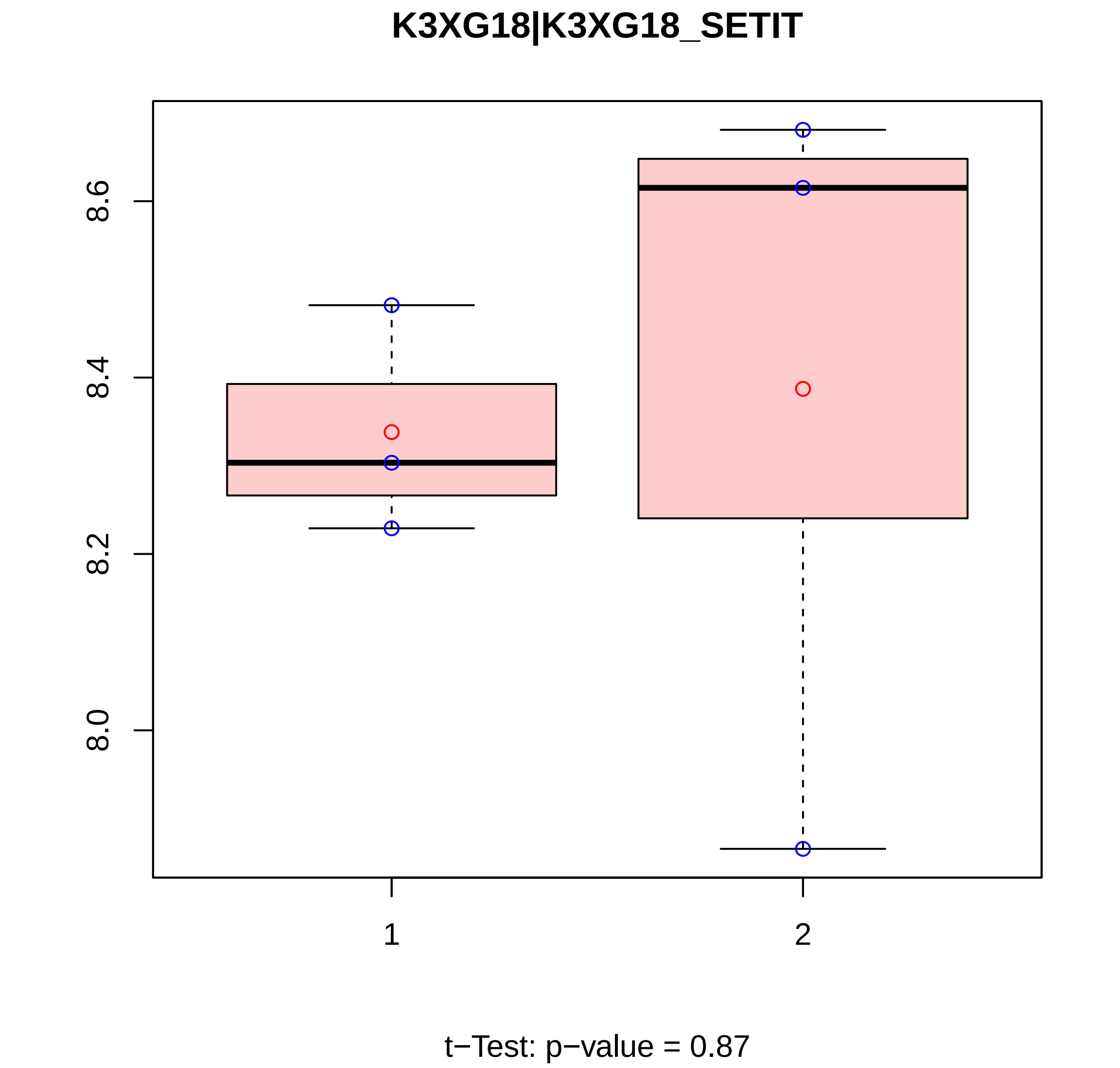
K3XG18|K3XG18_SETIT
8.0
8.2
8.4
8.6
1
2
t−Test: p−value = 0.87

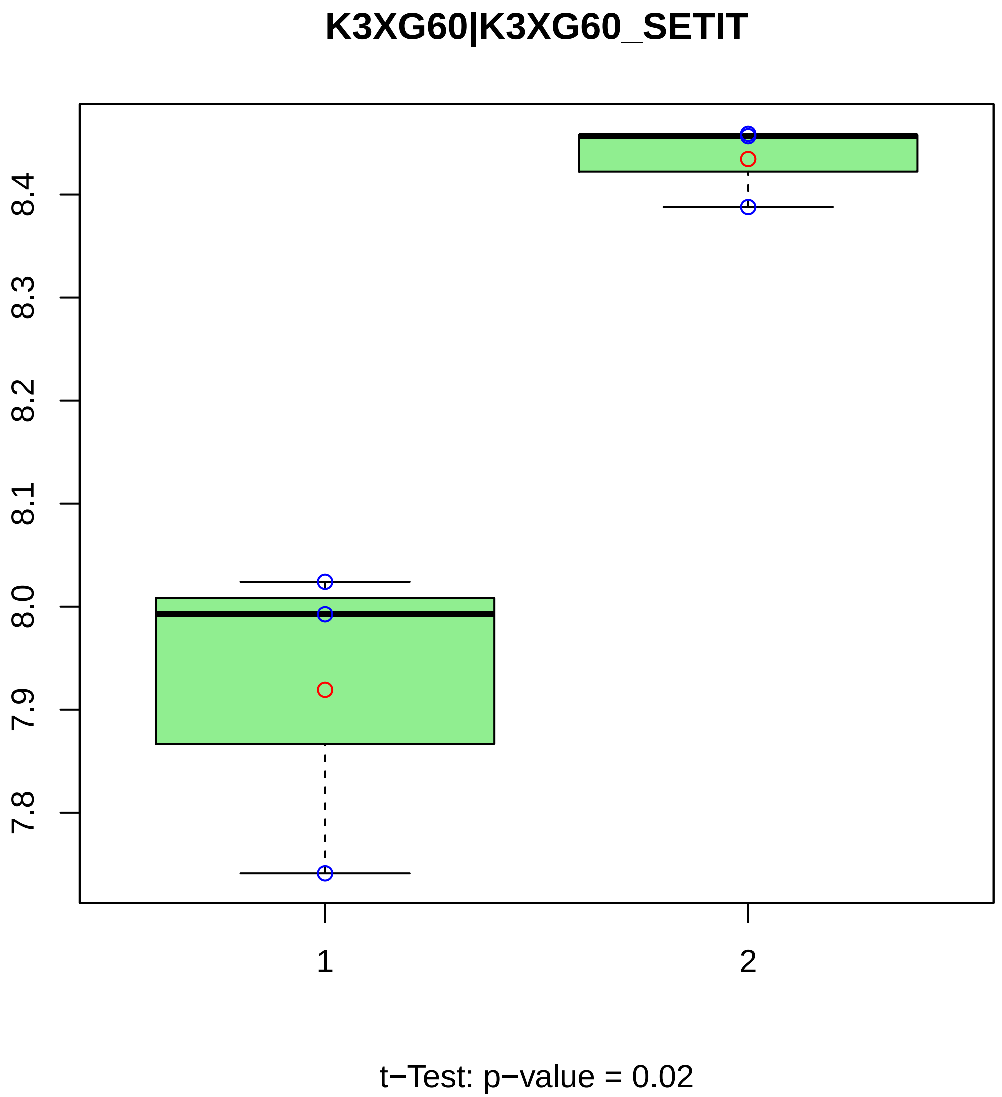
K3XG60|K3XG60_SETIT
8.4
8.3
8.2
8.1
8.0
7.9
7.8
1
2
t−Test: p−value = 0.02

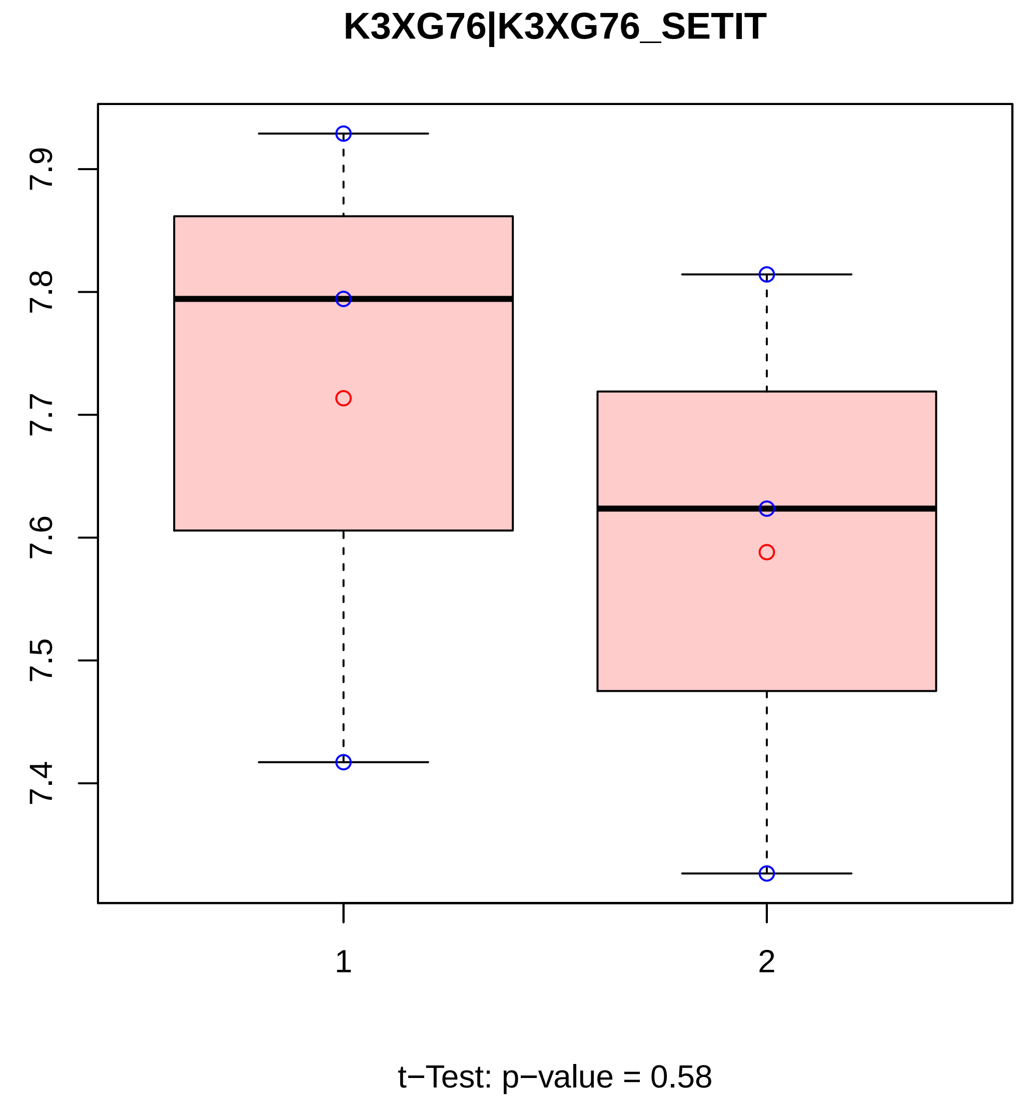
K3XG76|K3XG76_SETIT
7.9
7.8
7.7
7.6
7.5
7.4
1
2
t−Test: p−value = 0.58

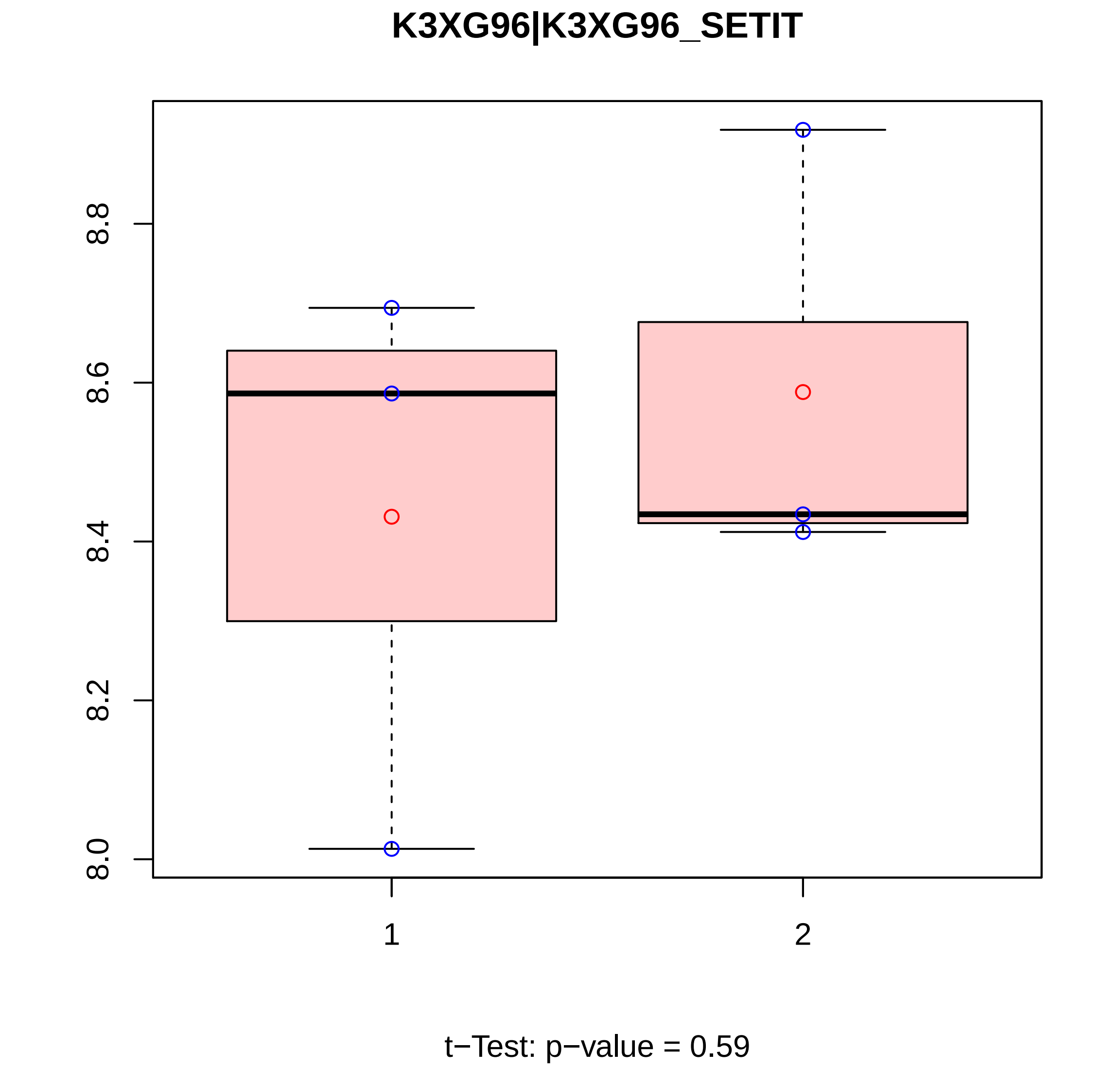
K3XG96|K3XG96_SETIT
8.0
8.2
8.4
8.6
8.8
1
2
t−Test: p−value = 0.59

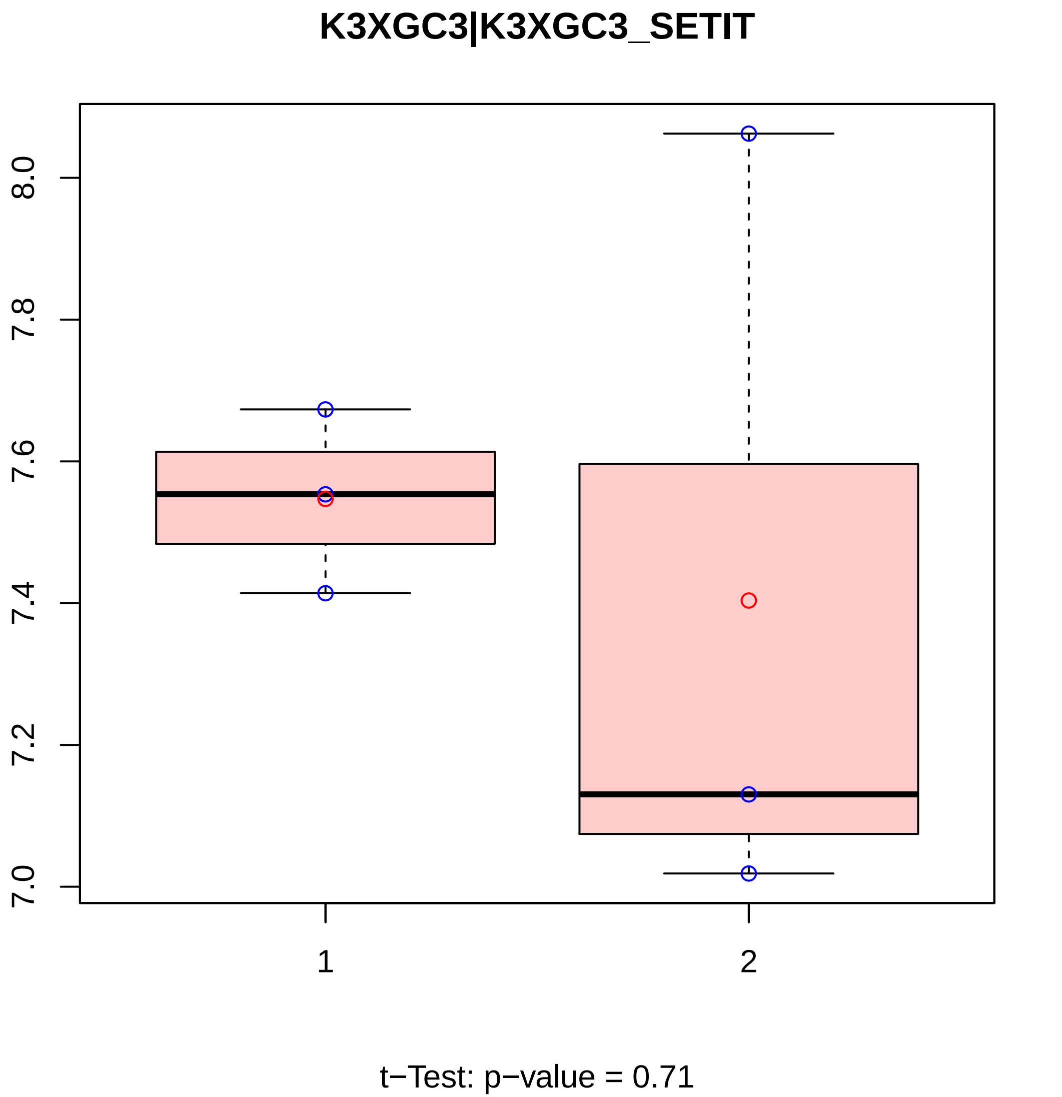
K3XGC3|K3XGC3_SETIT
8.0
7.8
7.6
7.4
7.2
7.0
1
2
t−Test: p−value = 0.71

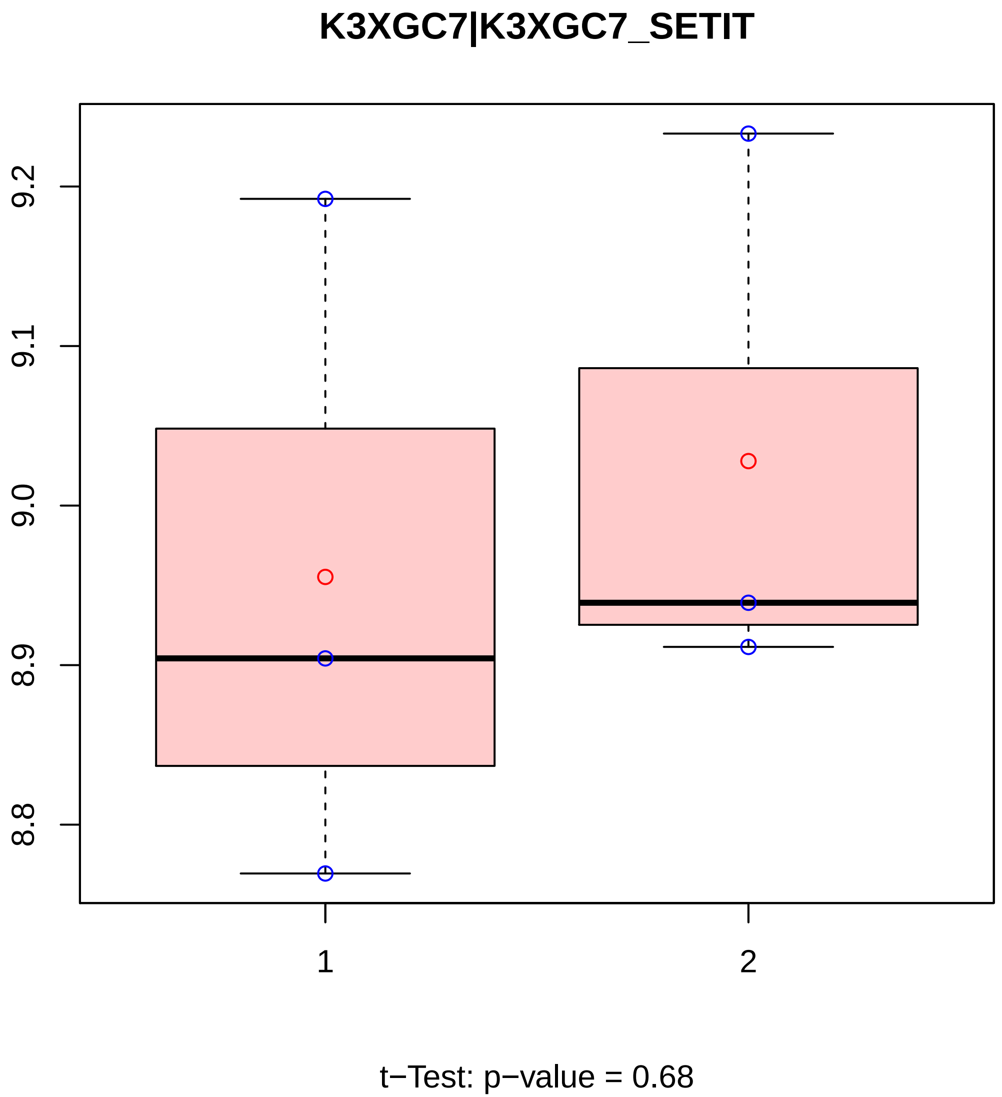
K3XGC7|K3XGC7_SETIT
9.2
9.1
9.0
8.9
8.8
1
2
t−Test: p−value = 0.68

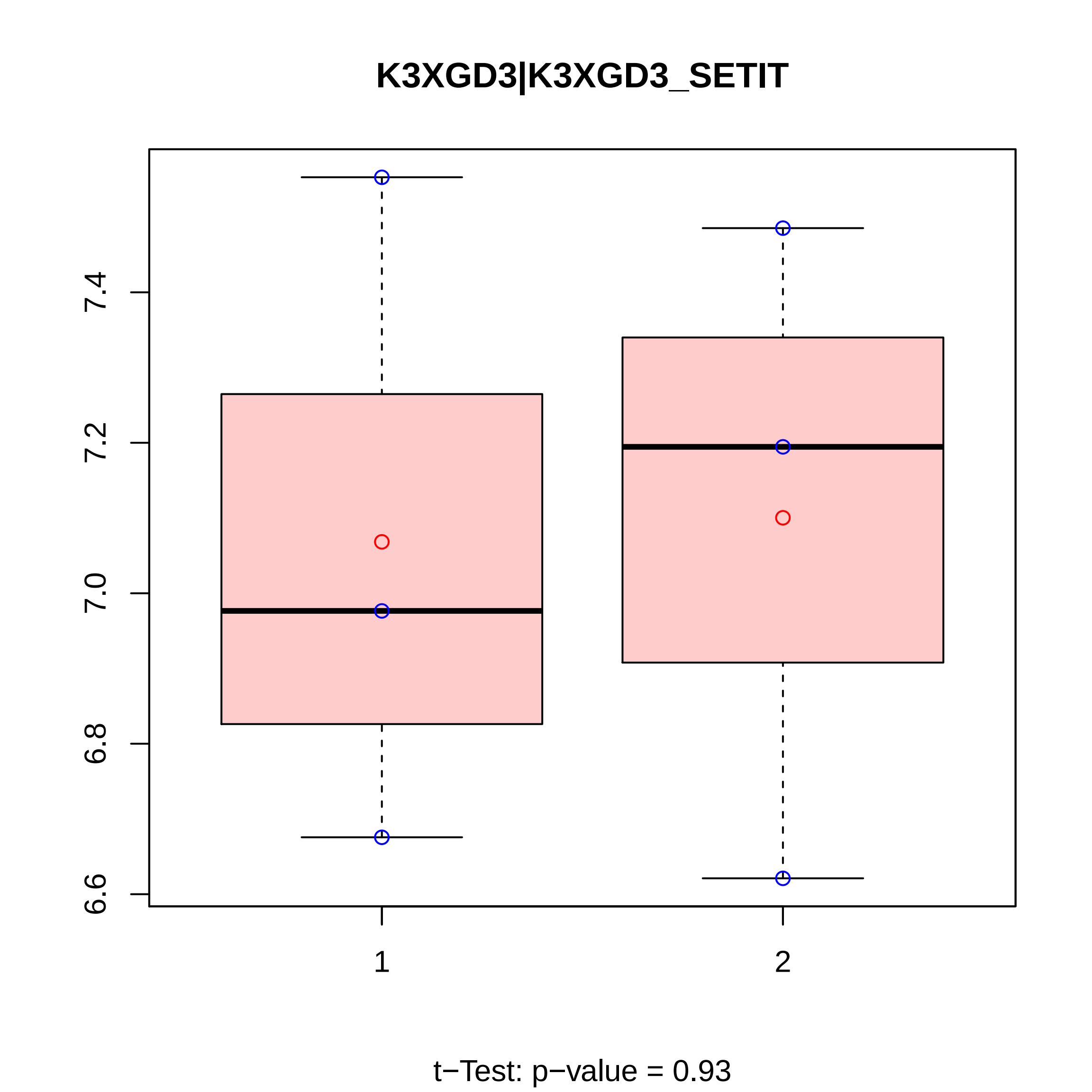

K3XGD3|K3XGD3_SETIT
7.4
7.2
7.0
6.8
6.6
1
2
t−Test: p−value = 0.93

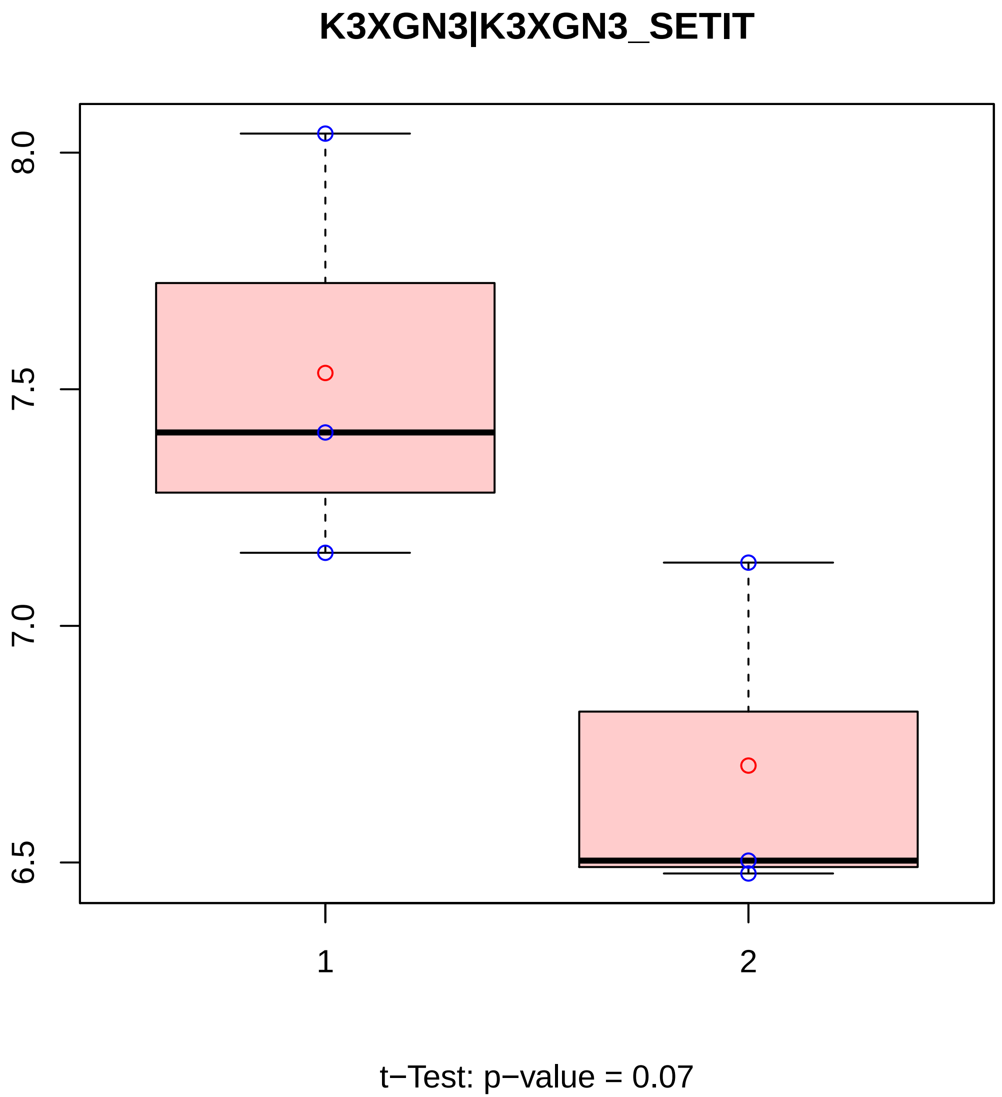
K3XGN3|K3XGN3_SETIT
8.0
7.5
7.0
6.5
1
2
t−Test: p−value = 0.07

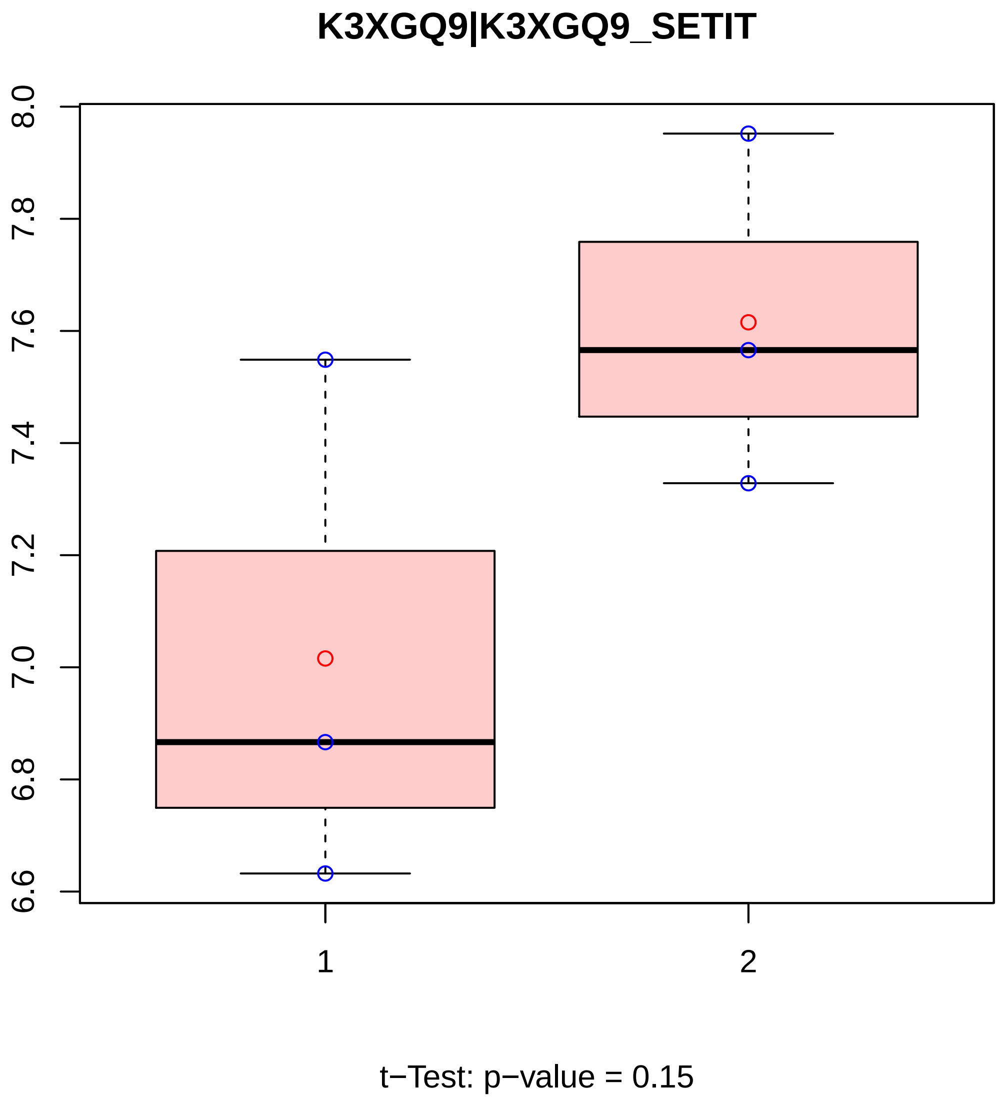
K3XGQ9|K3XGQ9_SETIT
8.0
7.8
7.6
7.4
7.2
7.0
6.8
6.6
1
2
t−Test: p−value = 0.15

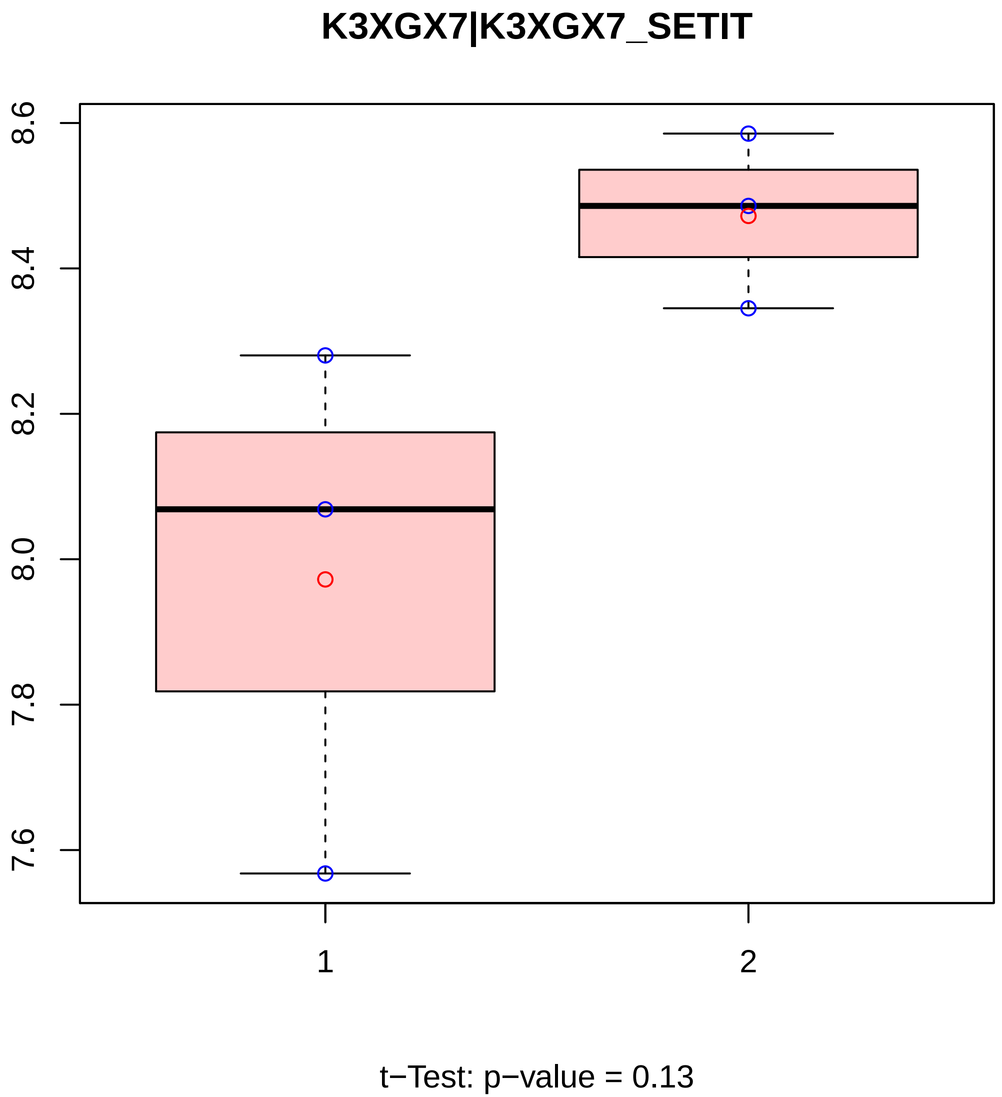
K3XGX7|K3XGX7_SETIT
8.6
8.4
8.2
8.0
7.8
7.6
1
2
t−Test: p−value = 0.13

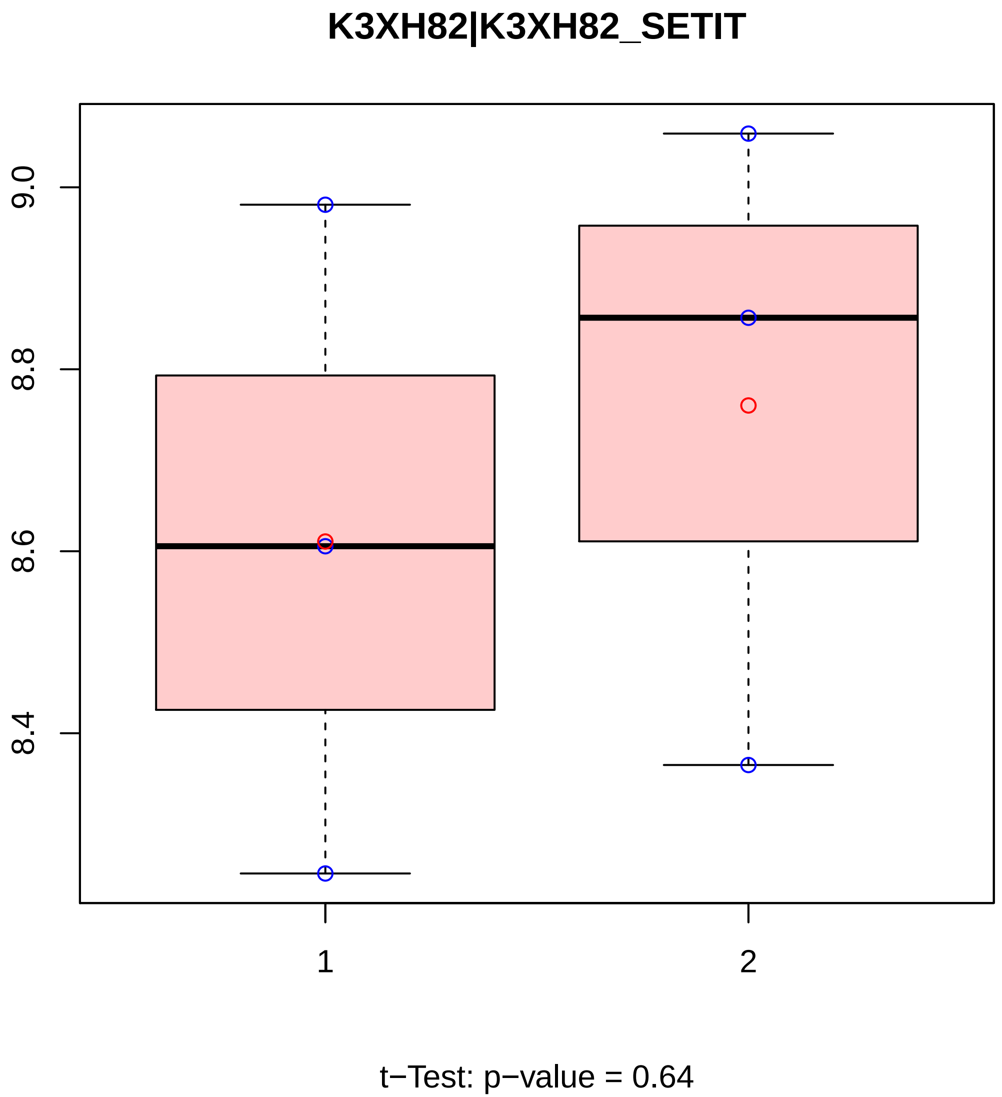
K3XH82|K3XH82_SETIT
9.0
8.8
8.6
8.4
1
2
t−Test: p−value = 0.64

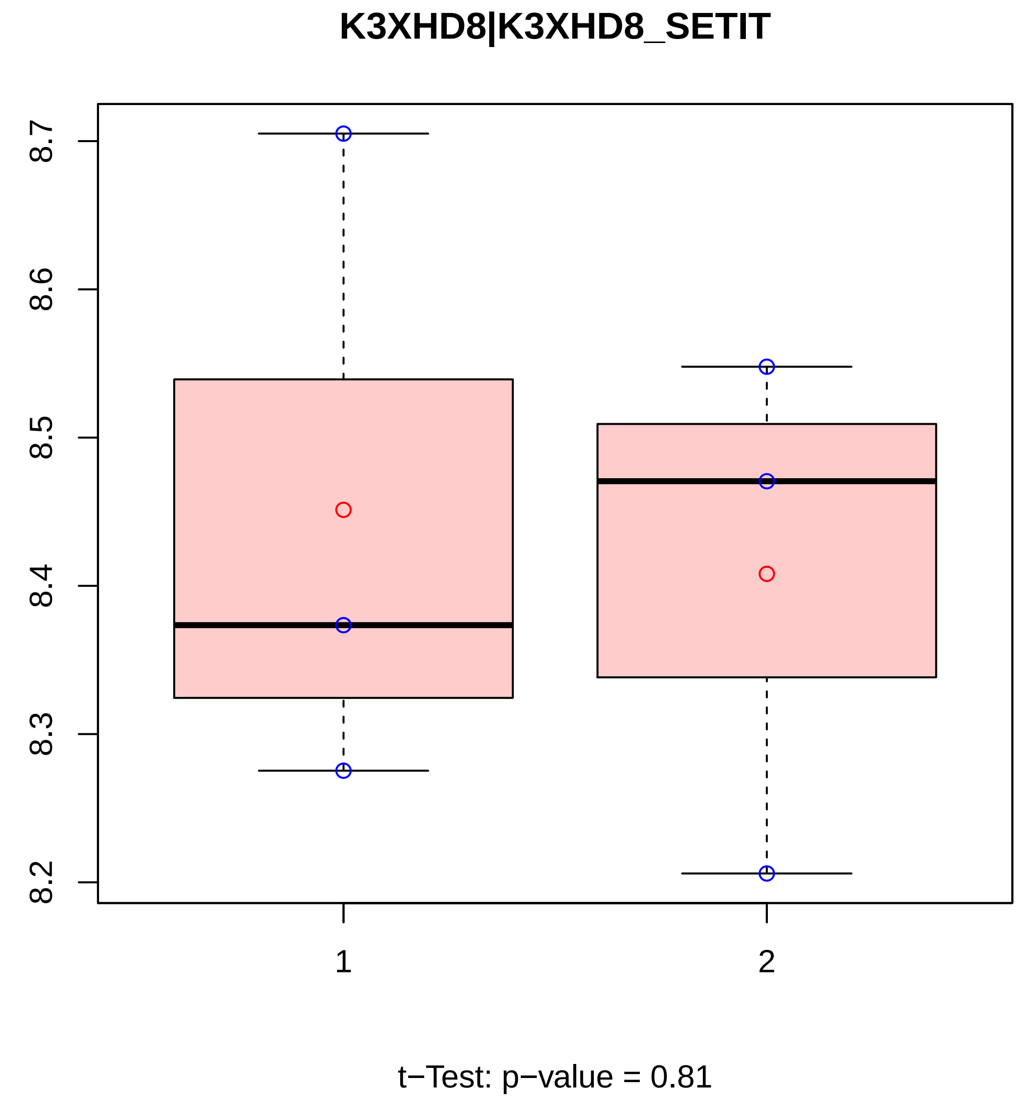
K3XHD8|K3XHD8_SETIT
8.2
8.3
8.4
8.5
8.6
8.7
1
2
t−Test: p−value = 0.81

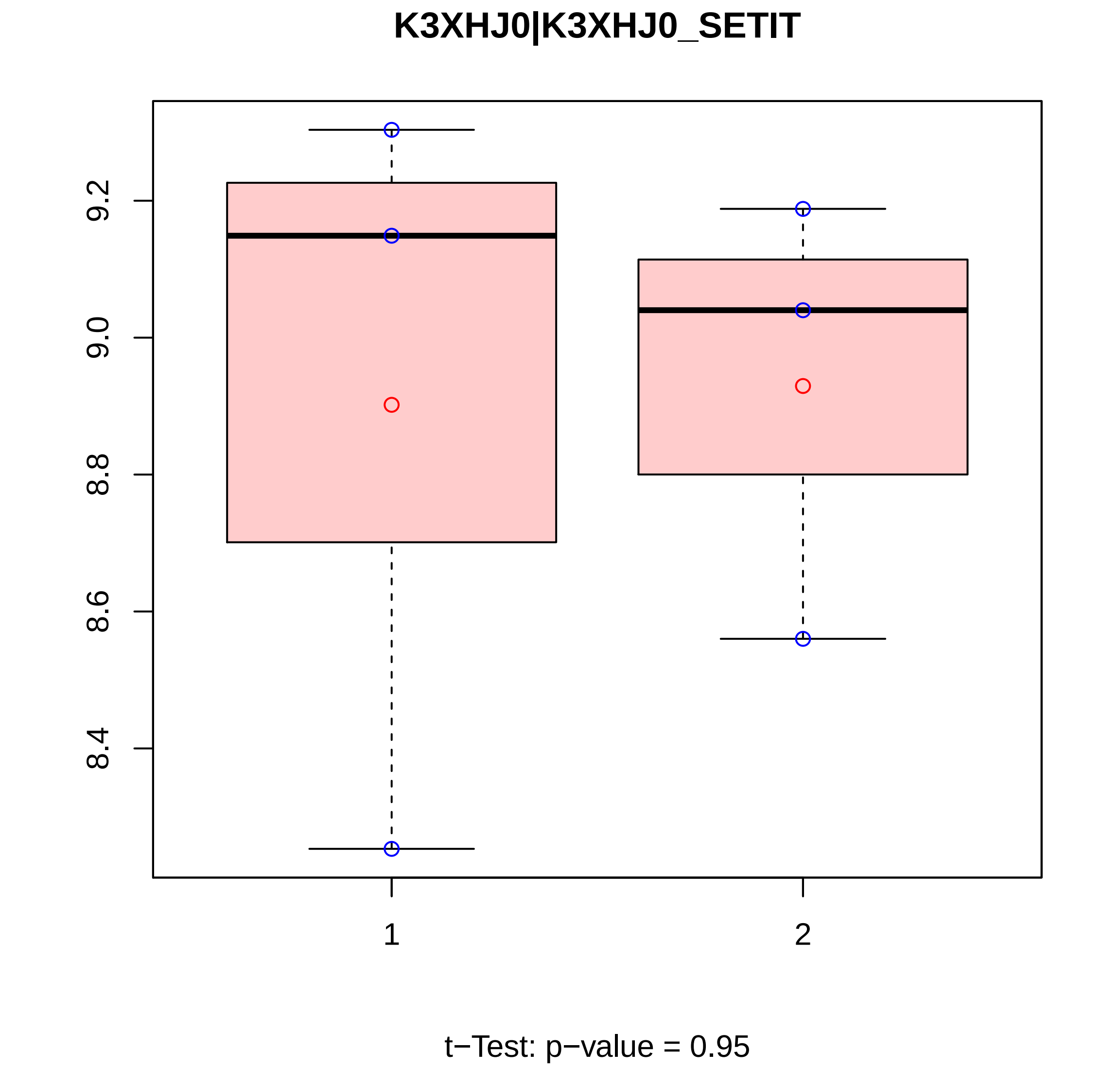
K3XHJ0|K3XHJ0_SETIT
9.2
9.0
8.8
8.6
8.4
1
2
t−Test: p−value = 0.95

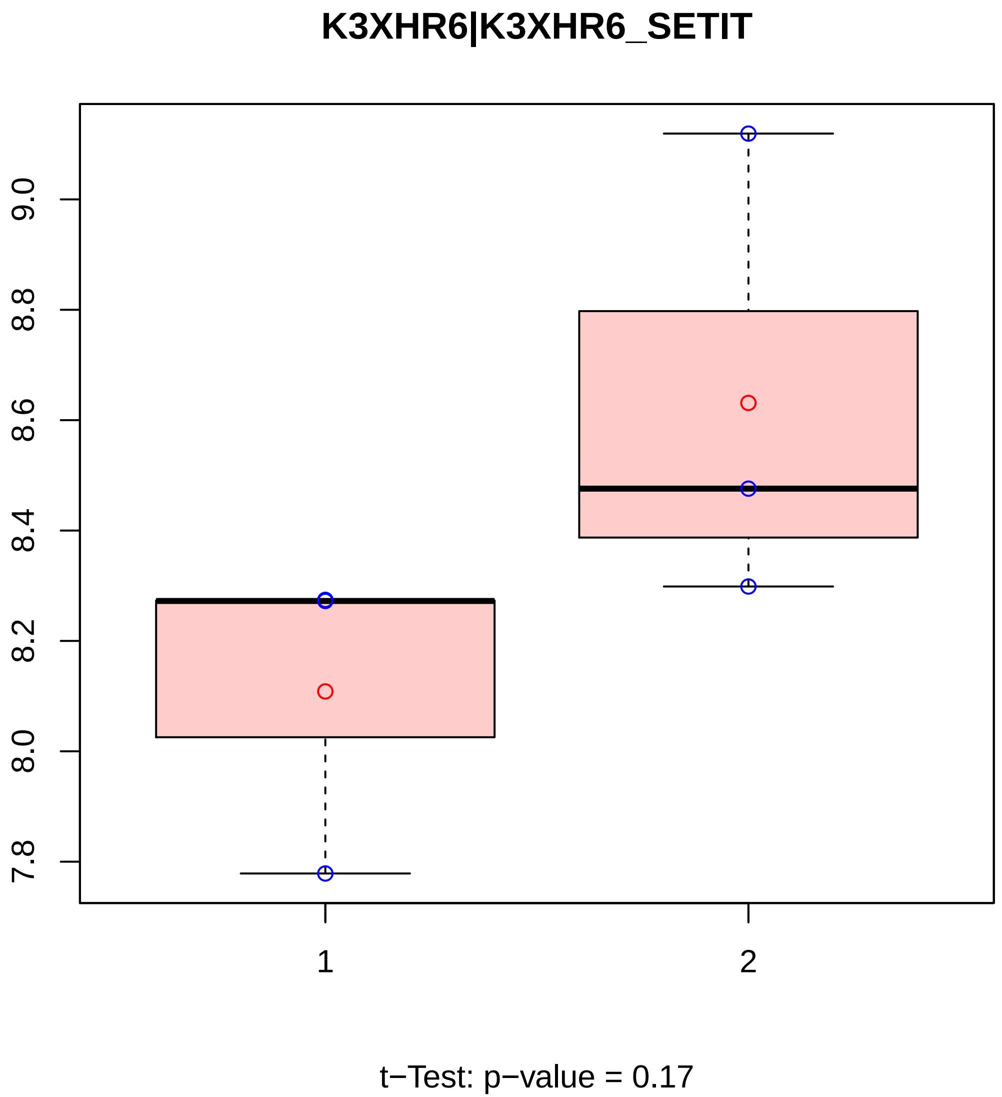

K3XHR6|K3XHR6_SETIT
9.0
8.8
8.6
8.4
8.2
8.0
7.8
1
2
t−Test: p−value = 0.17

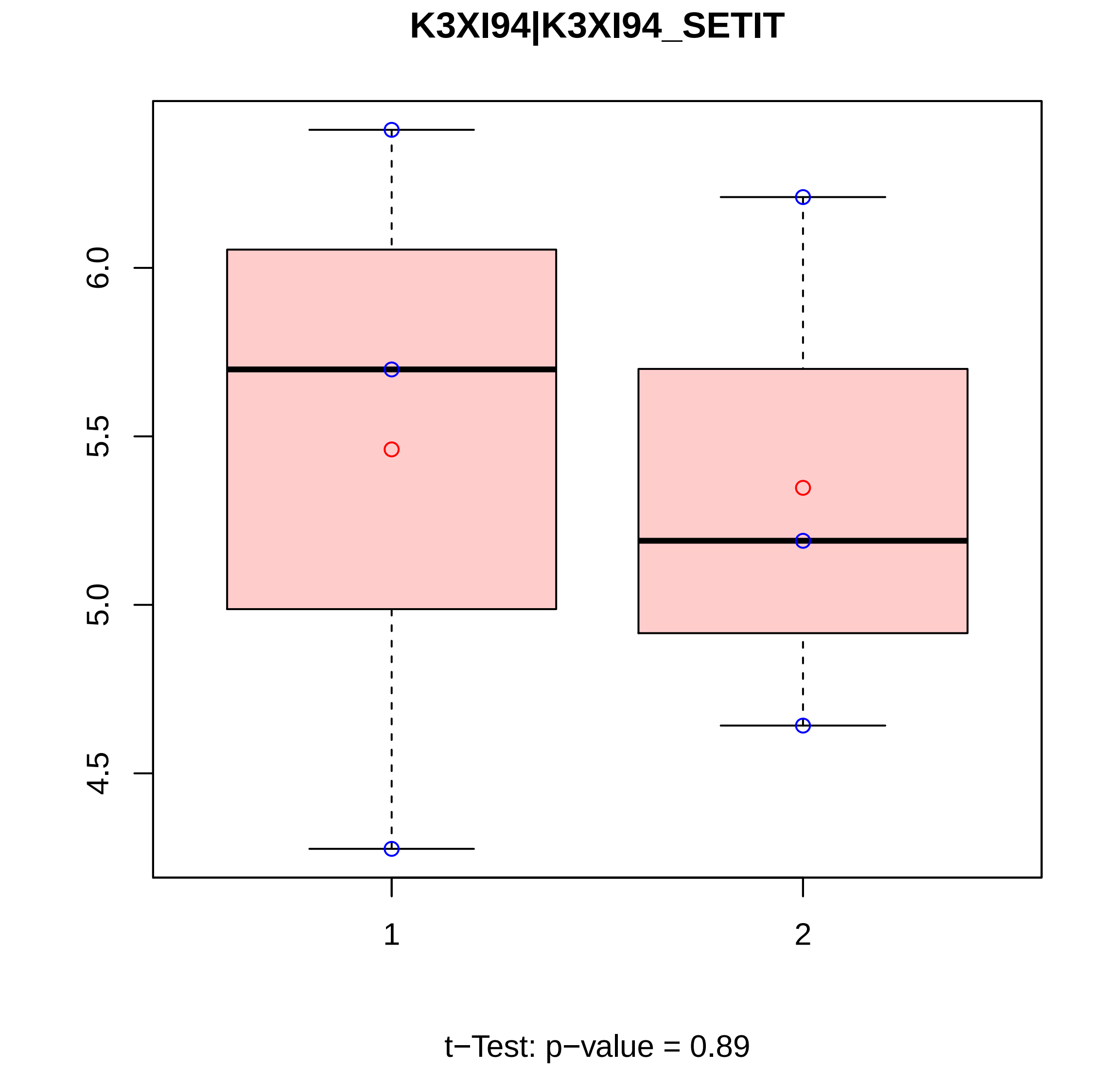
K3XI94|K3XI94_SETIT
6.0
5.5
5.0
4.5
1
2
t−Test: p−value = 0.89

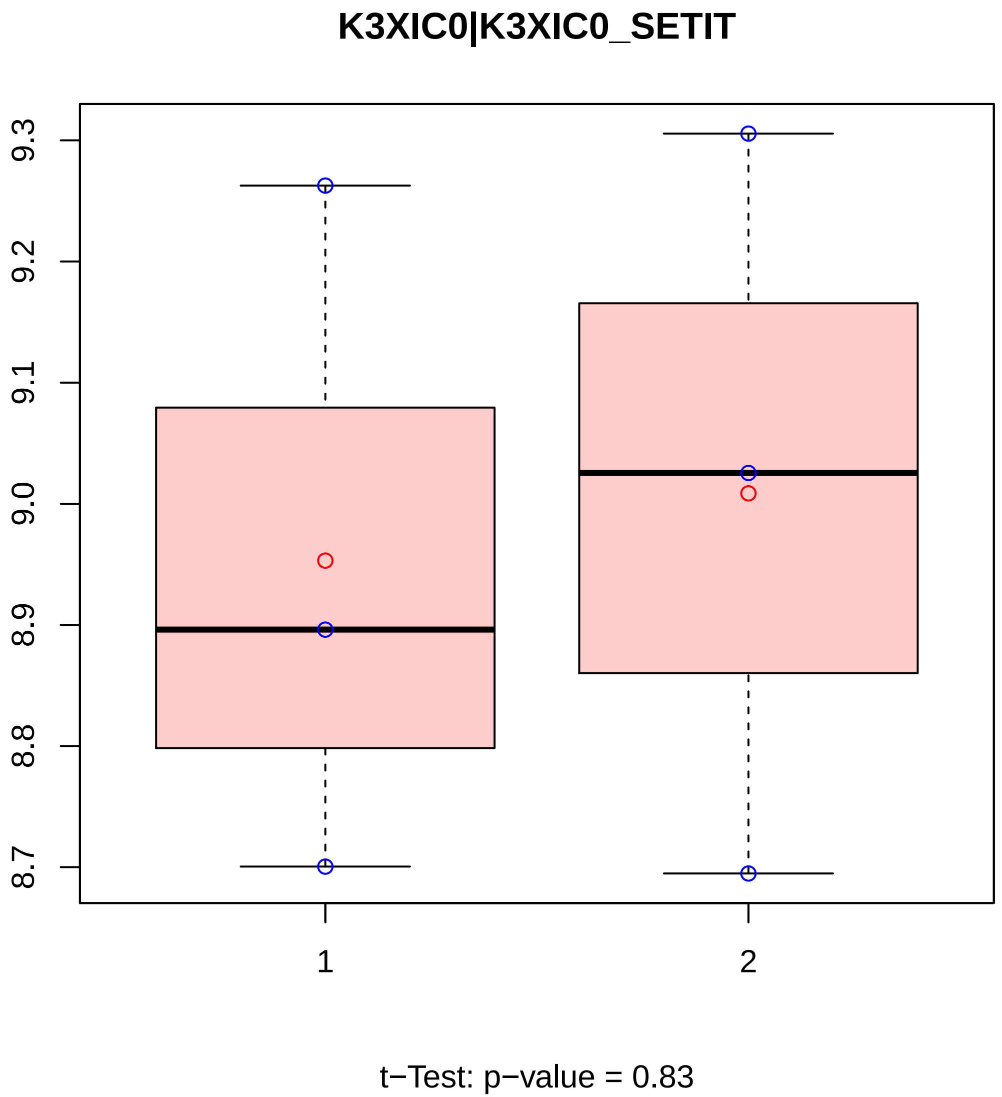
K3XIC0|K3XIC0_SETIT
9.3
9.2
9.1
9.0
8.9
8.8
8.7
1
2
t−Test: p−value = 0.83

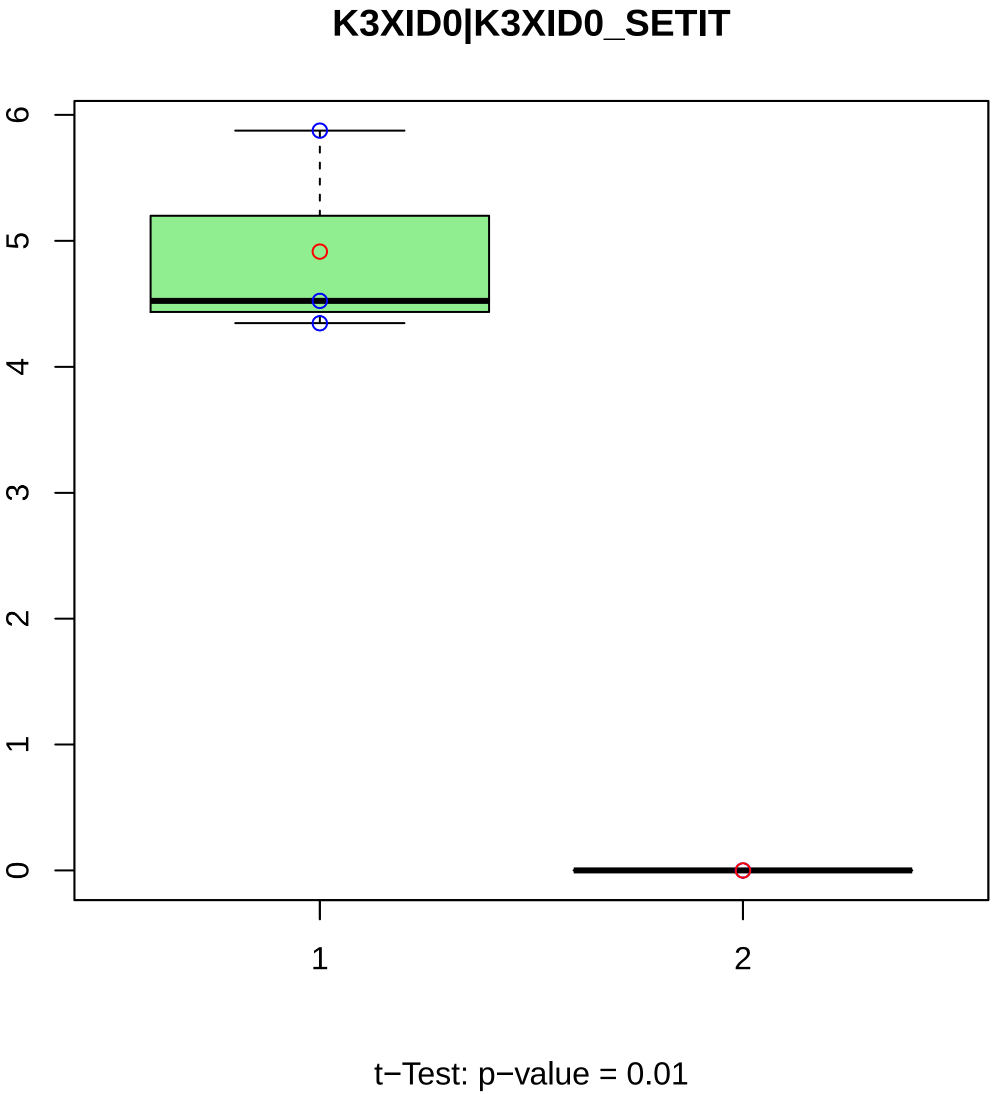
K3XID0|K3XID0_SETIT
0
1
2
3
4
5
6
1
2
t−Test: p−value = 0.01

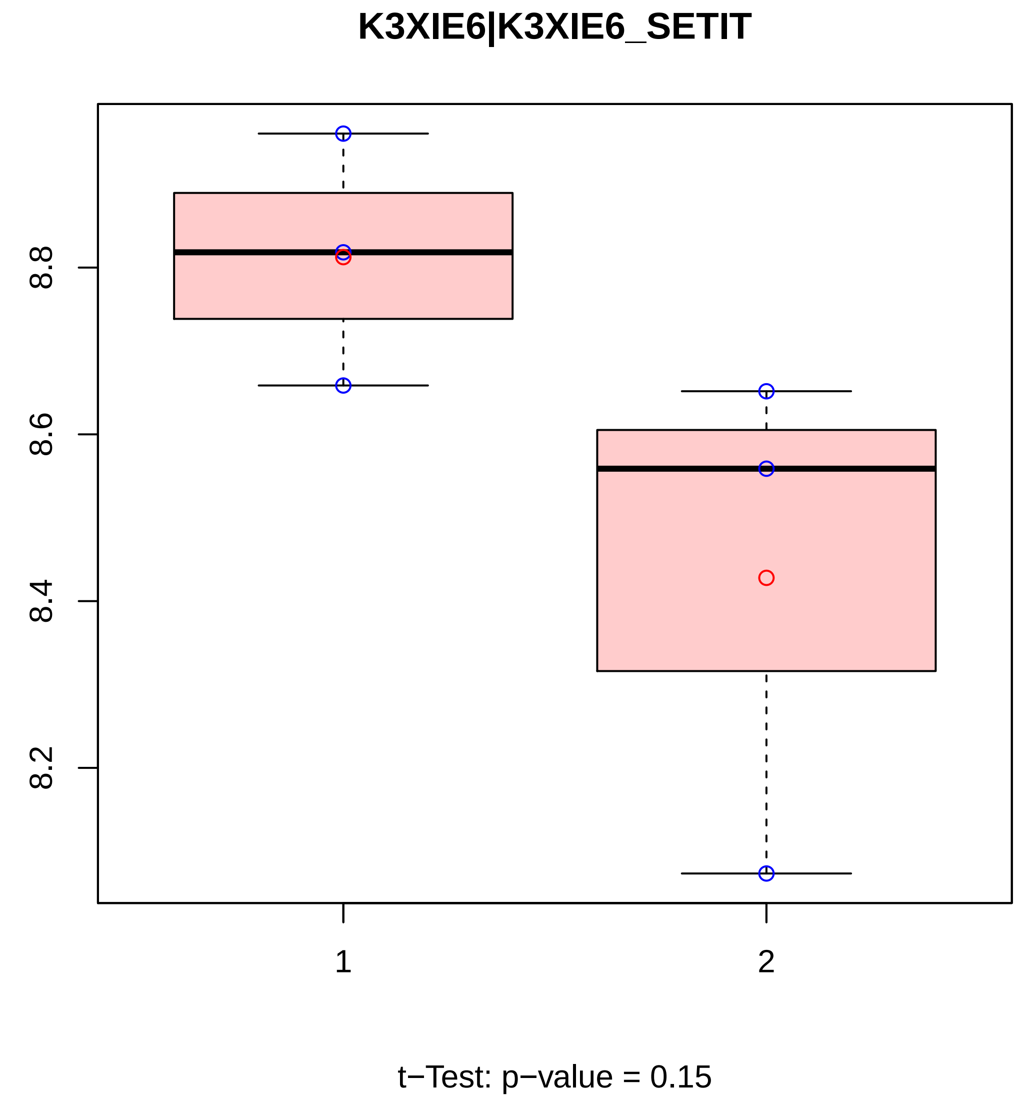
K3XIE6|K3XIE6_SETIT
8.2
8.4
8.6
8.8
1
2
t−Test: p−value = 0.15

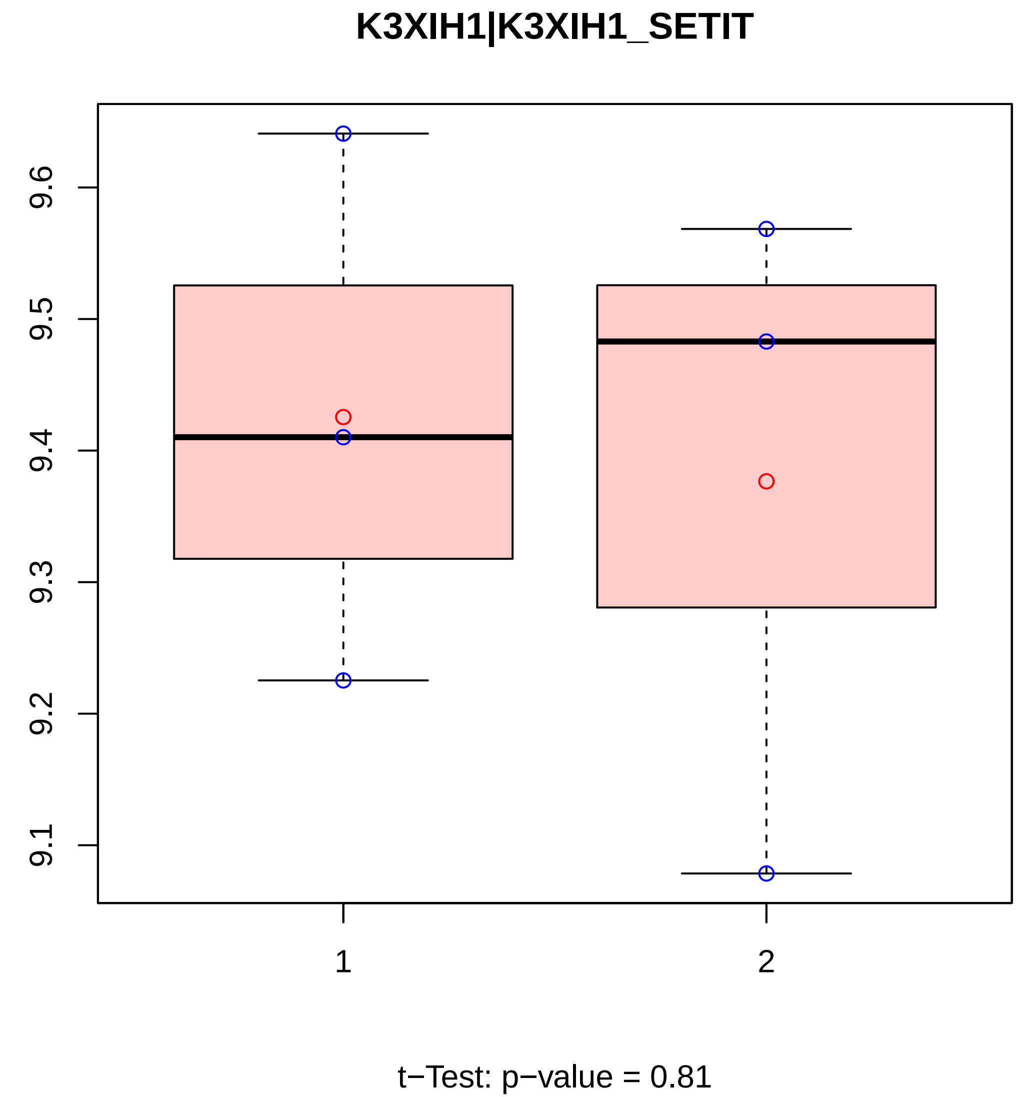

K3XIH1|K3XIH1_SETIT
9.1
9.2
9.3
9.4
9.5
9.6
1
2
t−Test: p−value = 0.81

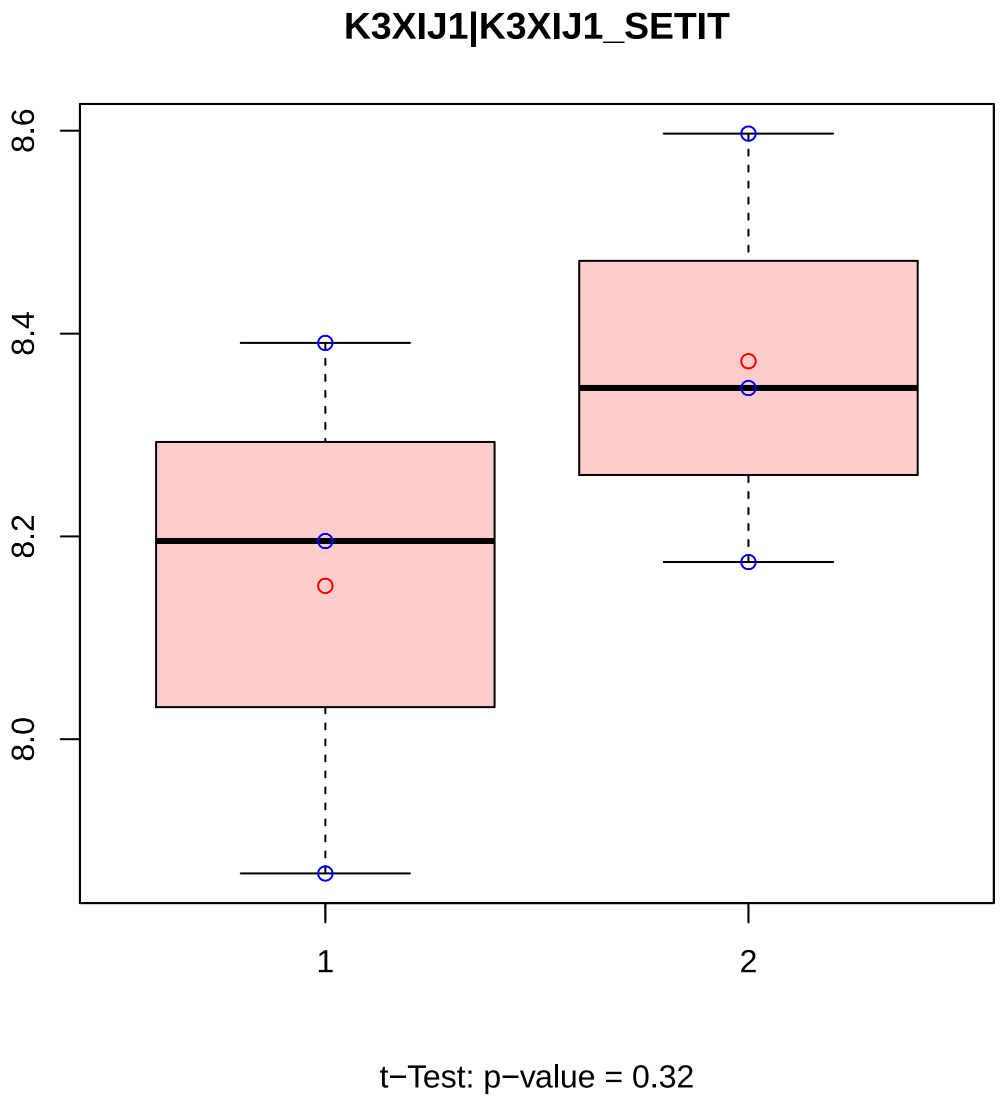
K3XIJ1|K3XIJ1_SETIT
8.0
8.2
8.4
8.6
1
2
t−Test: p−value = 0.32

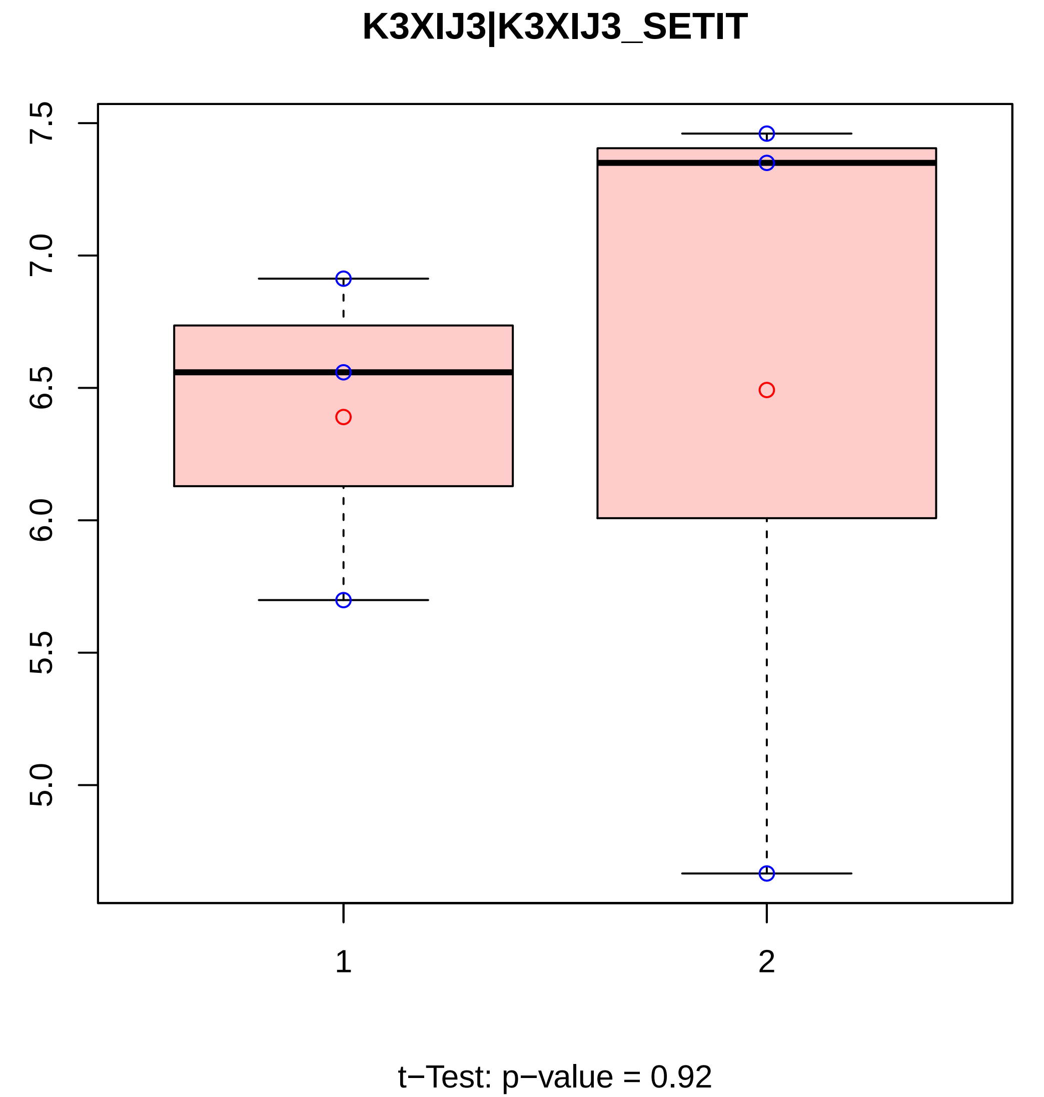
K3XIJ3|K3XIJ3_SETIT
7.5
7.0
6.5
6.0
5.5
5.0
1
2
t−Test: p−value = 0.92

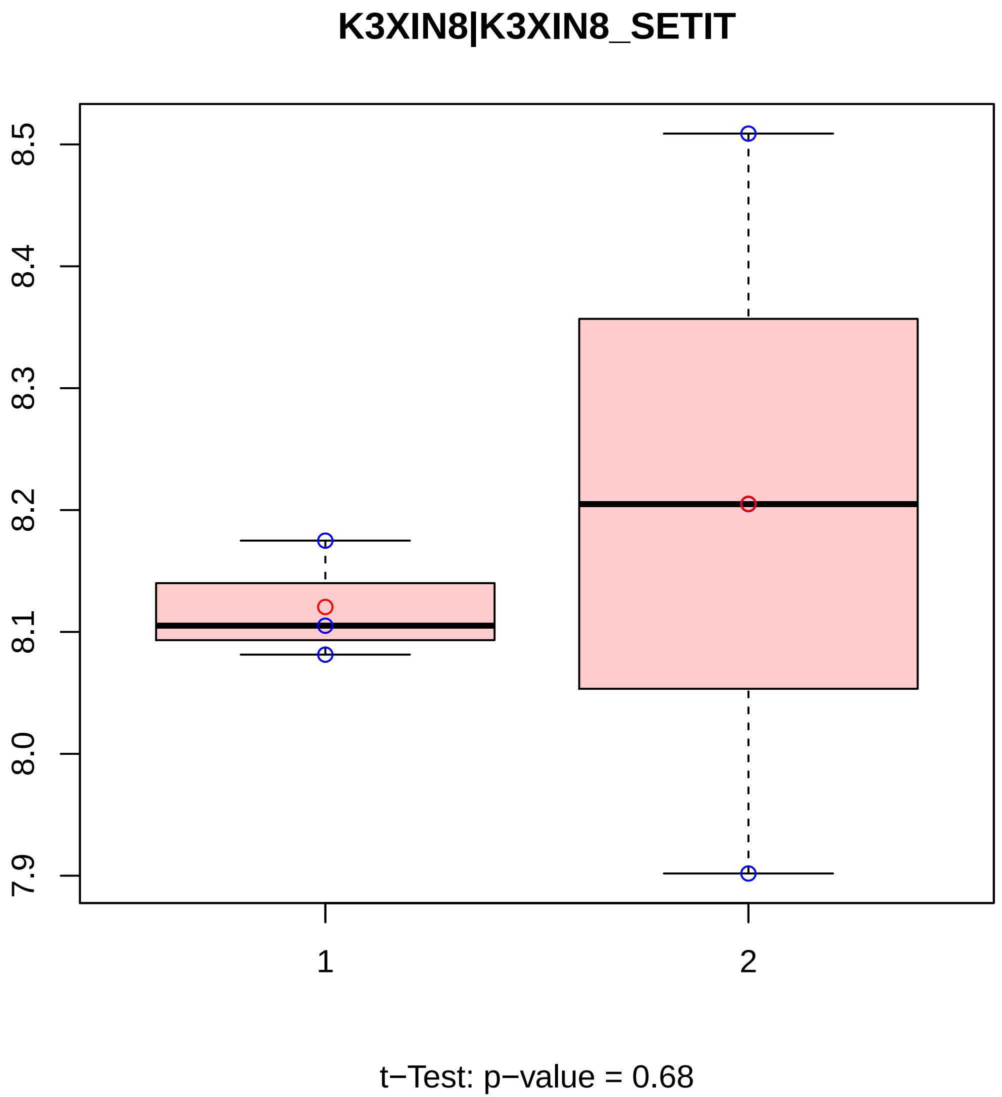
K3XIN8|K3XIN8_SETIT
8.5
8.4
8.3
8.2
8.1
8.0
7.9
1
2
t−Test: p−value = 0.68

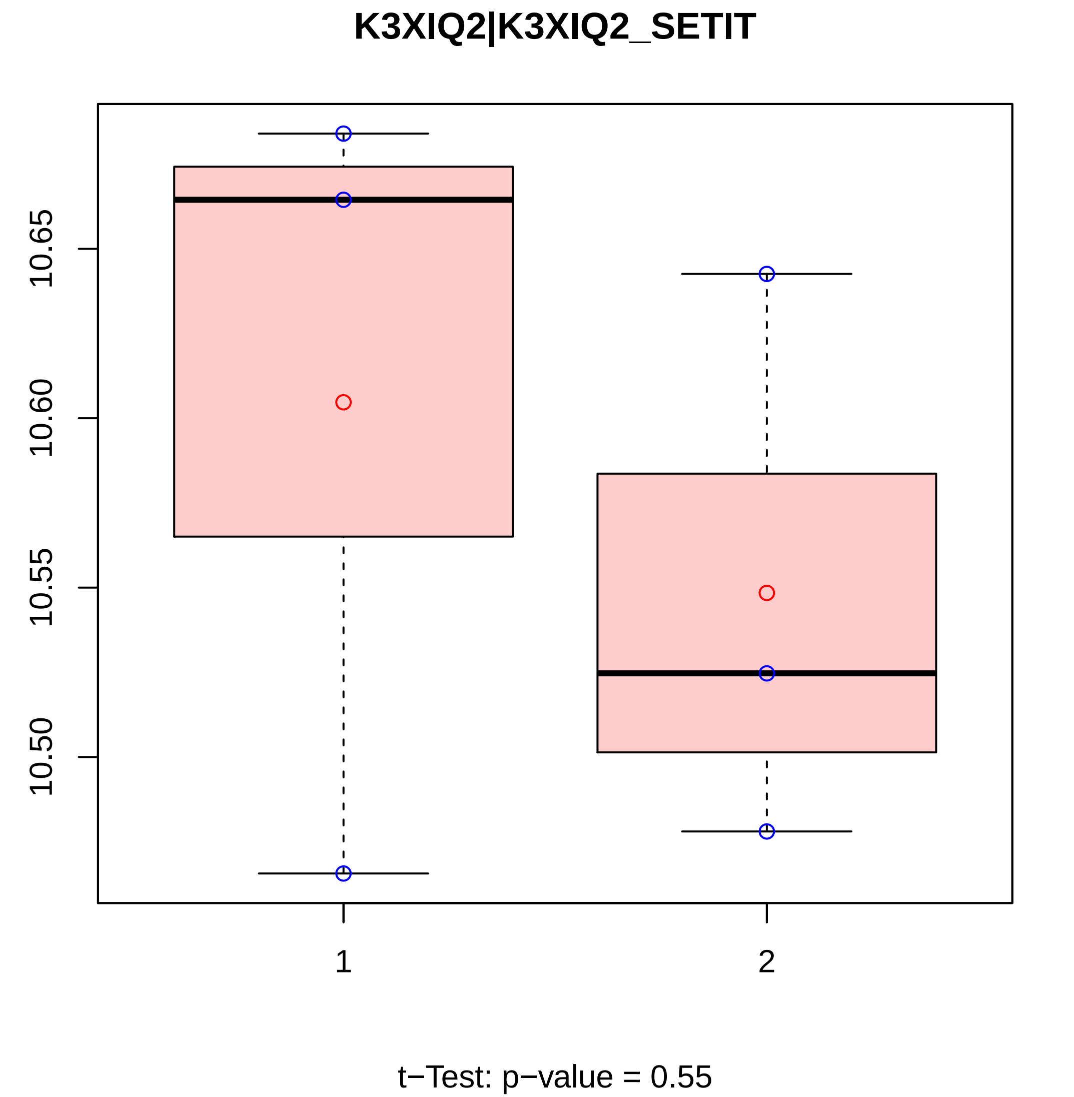
K3XIQ2|K3XIQ2_SETIT
10.65
10.60
10.55
10.50
1
2
t−Test: p−value = 0.55

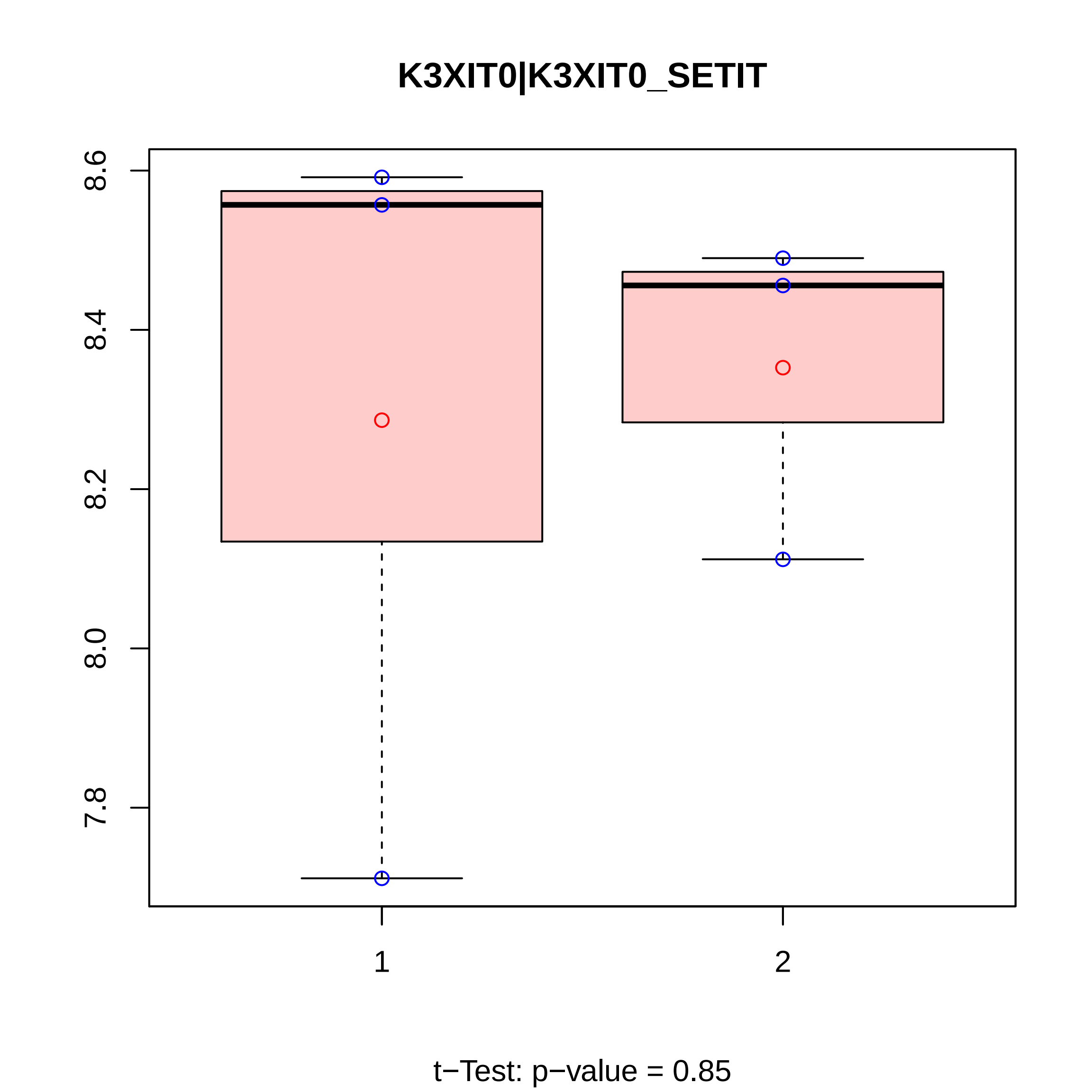
K3XIT0|K3XIT0_SETIT
8.6
8.4
8.2
8.0
7.8
1
2
t−Test: p−value = 0.85

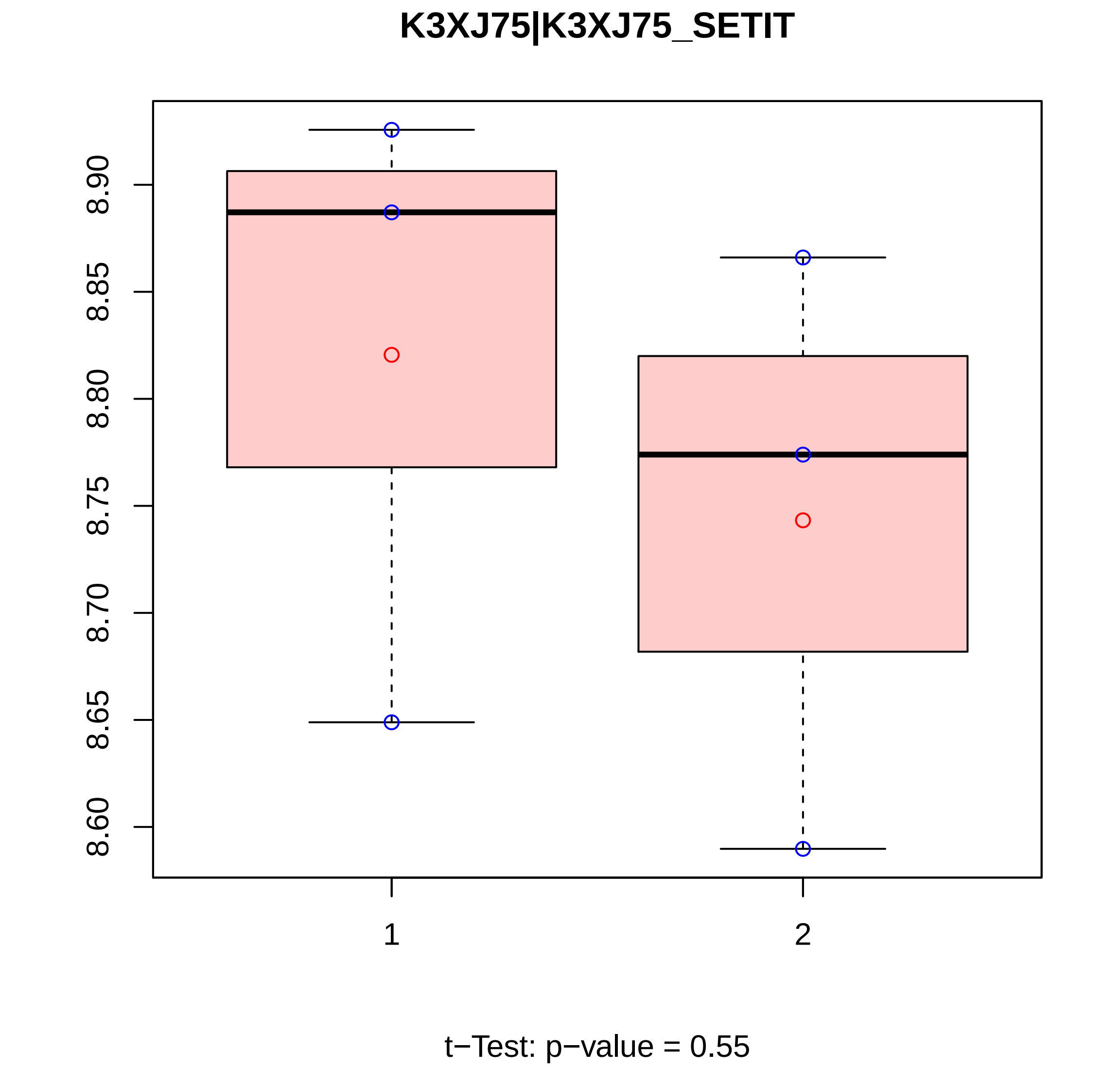
K3XJ75|K3XJ75_SETIT
8.60
8.65
8.70
8.75
8.80
8.85
8.90
1
2
t−Test: p−value = 0.55

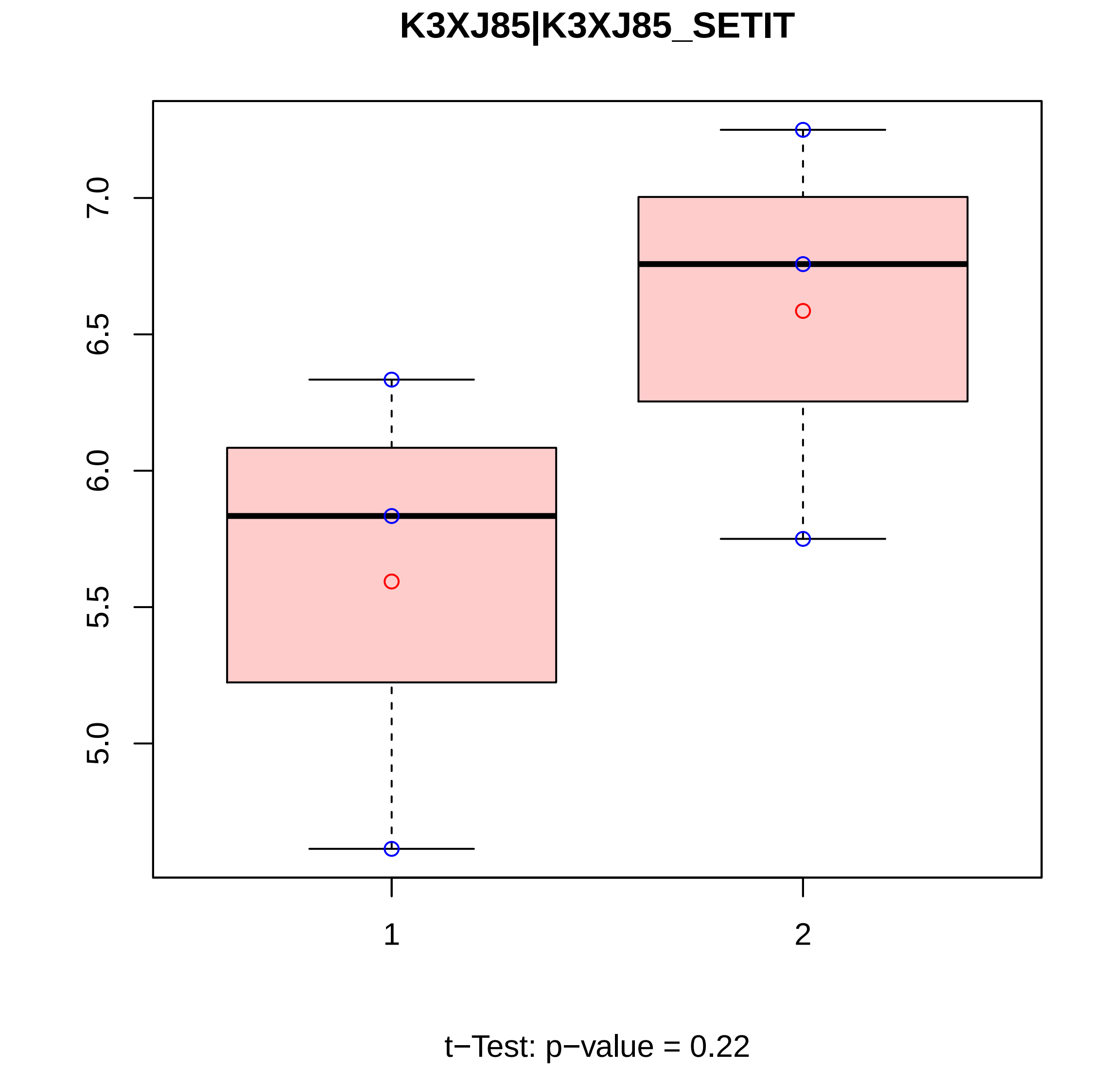
K3XJ85|K3XJ85_SETIT
7.0
6.5
6.0
5.5
5.0
1
2
t−Test: p−value = 0.22

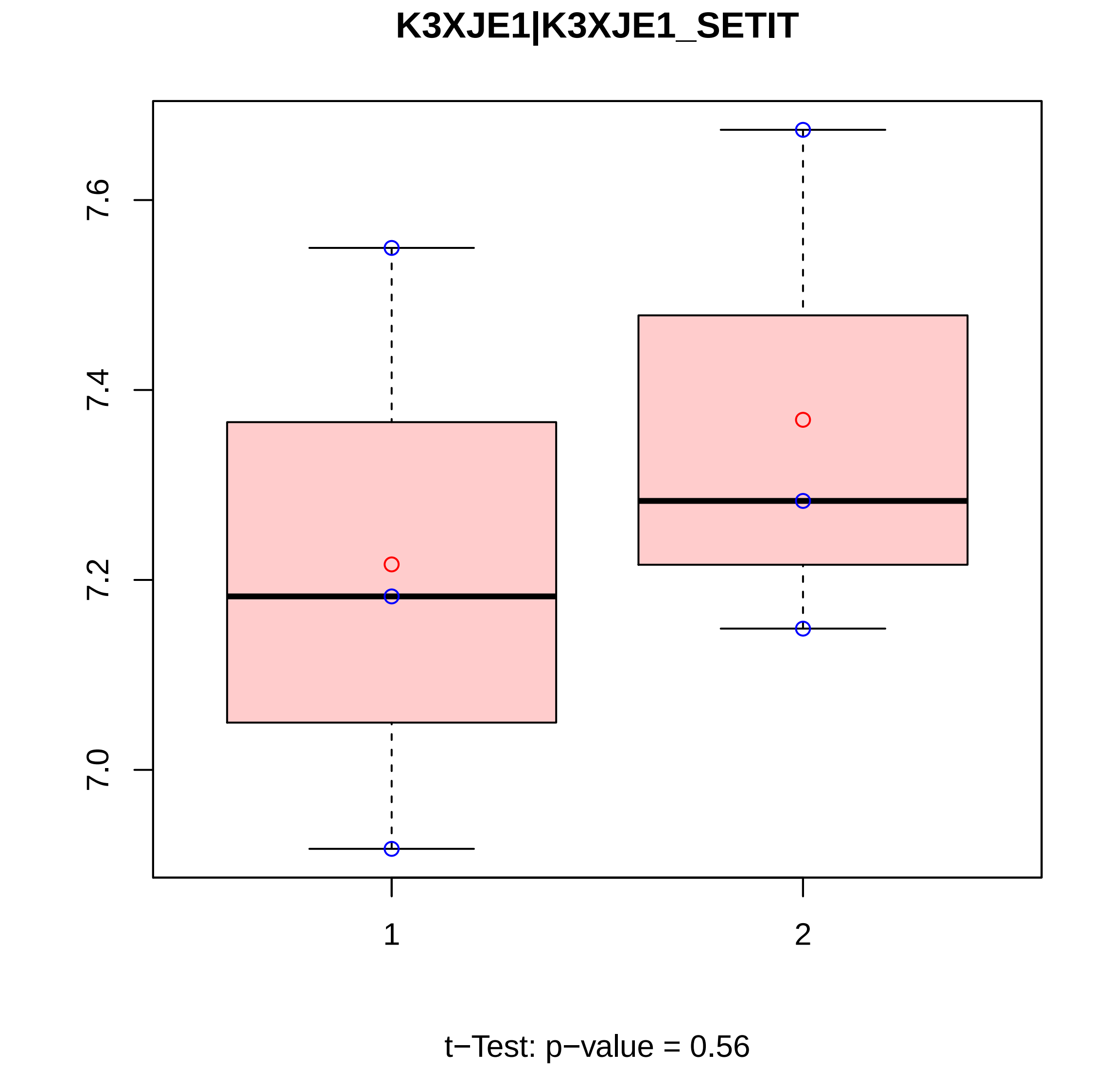

K3XJE1|K3XJE1_SETIT
7.0
7.2
7.4
7.6
1
2
t−Test: p−value = 0.56

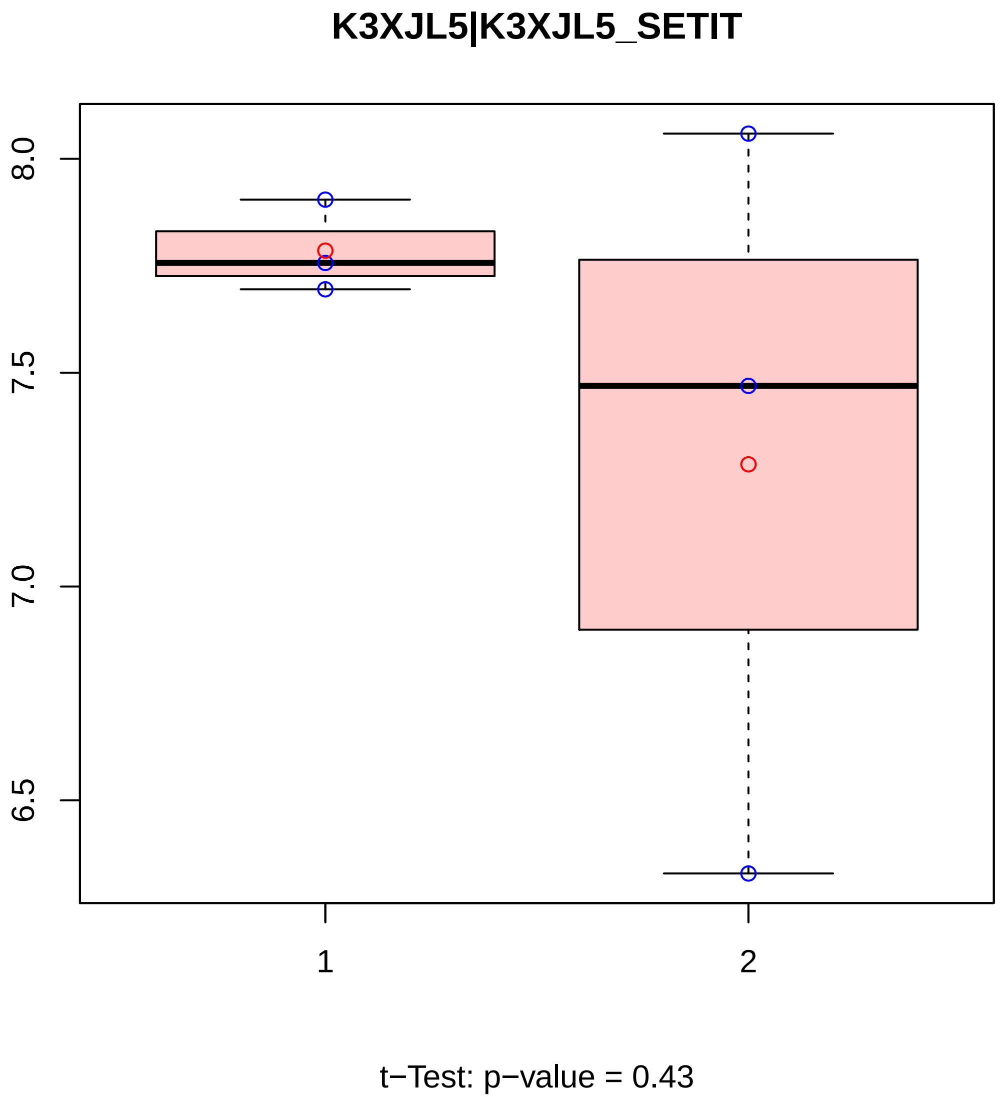
K3XJL5|K3XJL5_SETIT
8.0
7.5
7.0
6.5
1
2
t−Test: p−value = 0.43

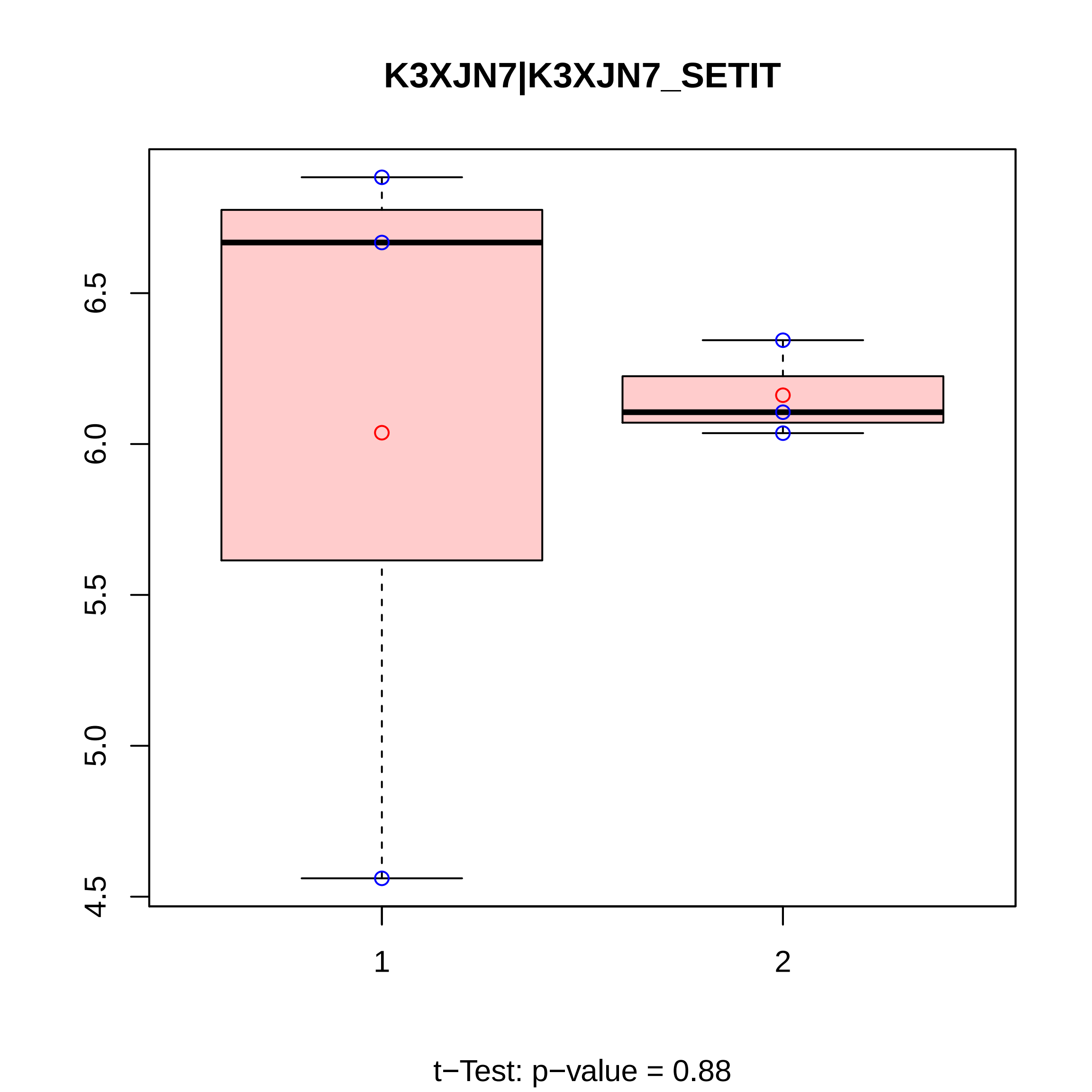
K3XJN7|K3XJN7_SETIT
6.5
6.0
5.5
5.0
4.5
1
2
t−Test: p−value = 0.88

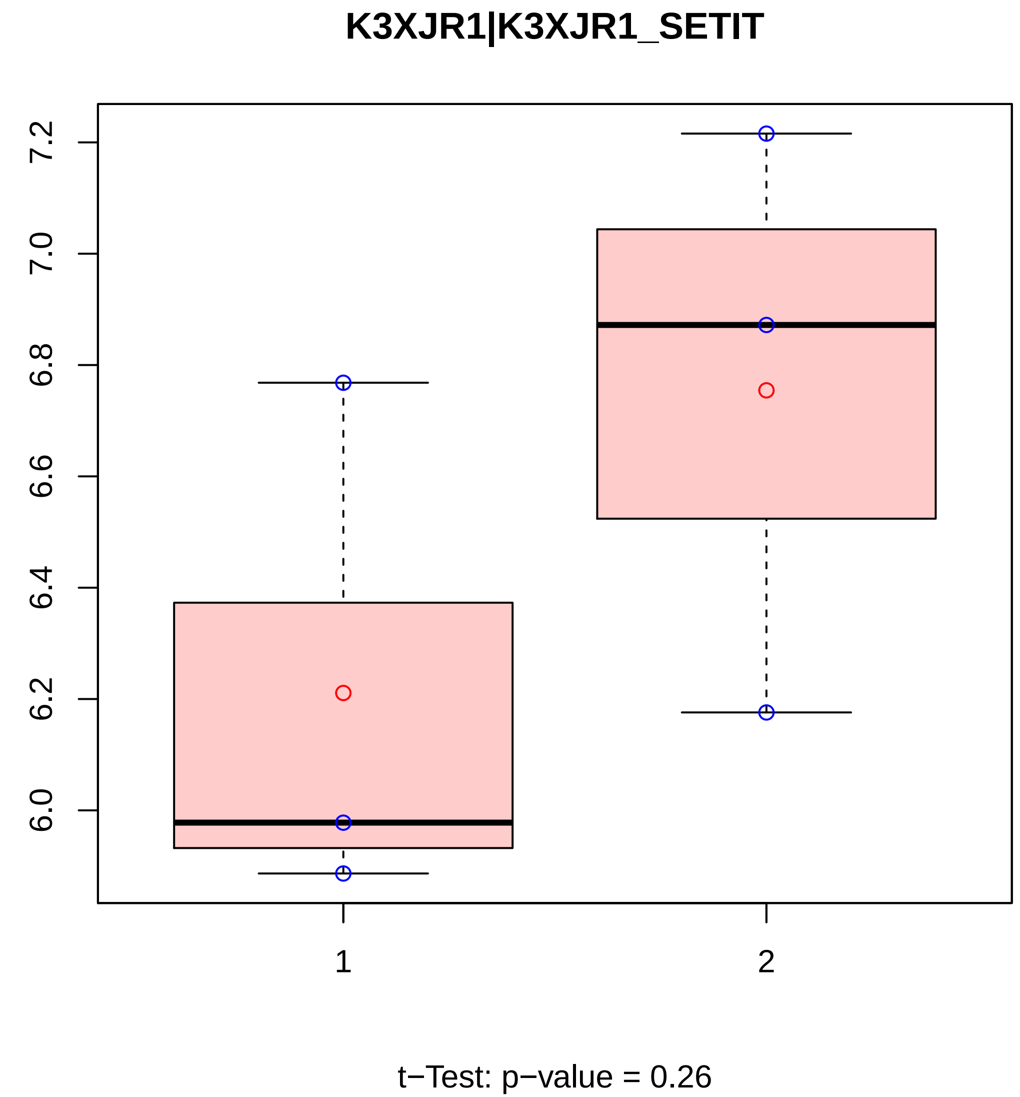
K3XJR1|K3XJR1_SETIT
7.2
7.0
6.8
6.6
6.4
6.2
6.0
1
2
t−Test: p−value = 0.26

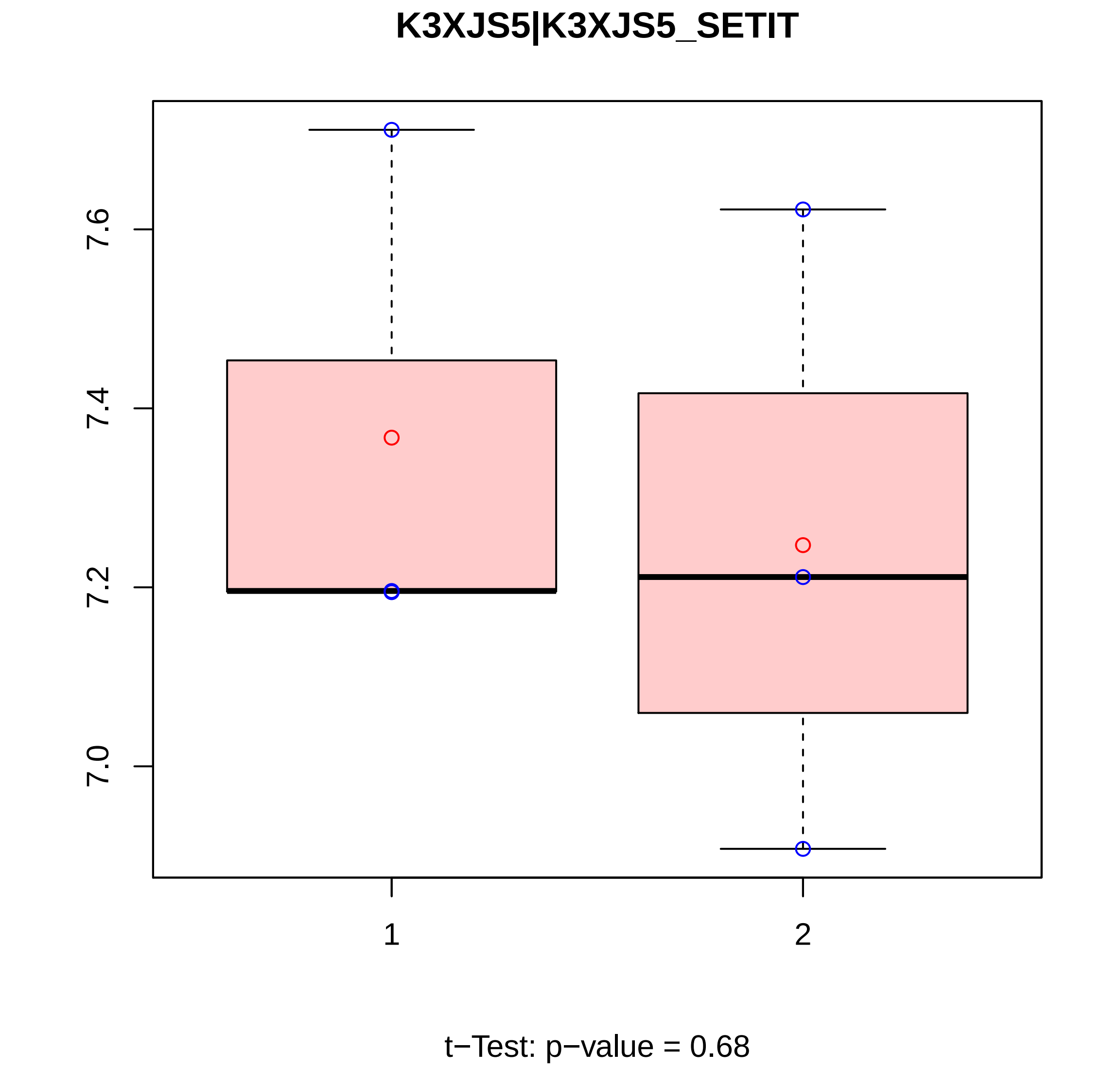
K3XJS5|K3XJS5_SETIT
7.0
7.2
7.4
7.6
1
2
t−Test: p−value = 0.68

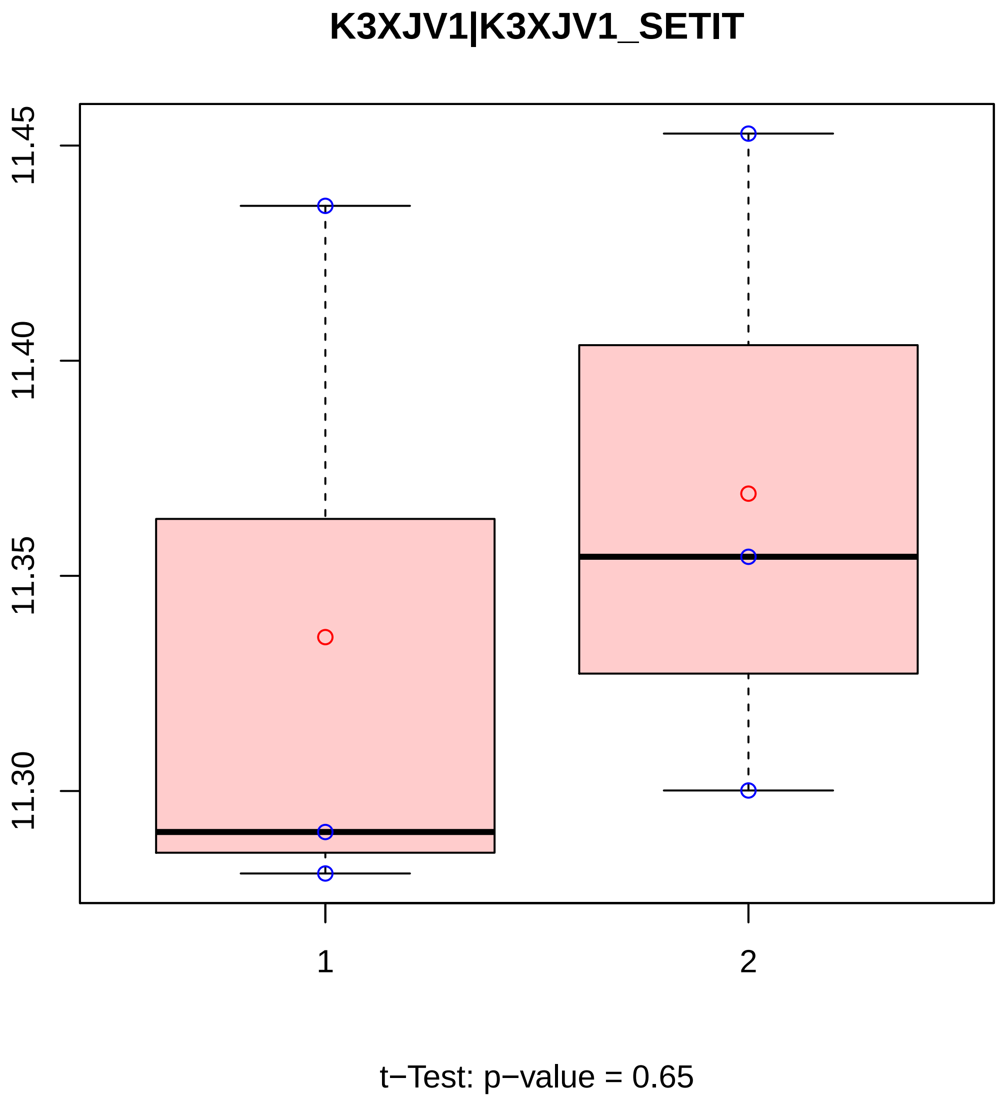
K3XJV1|K3XJV1_SETIT
11.45
11.40
11.35
11.30
1
2
t−Test: p−value = 0.65

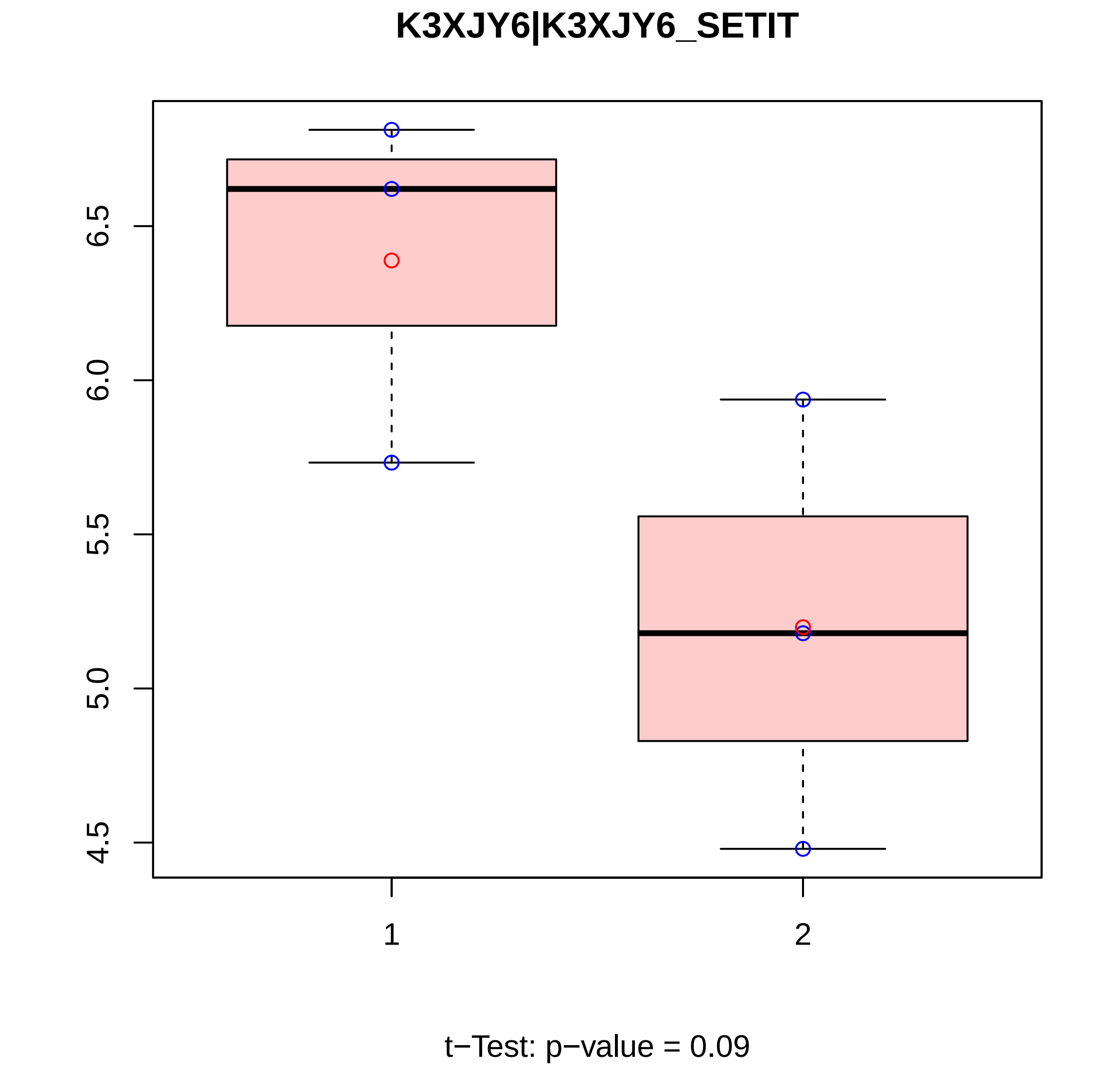
K3XJY6|K3XJY6_SETIT
6.5
6.0
5.5
5.0
4.5
1
2
t−Test: p−value = 0.09

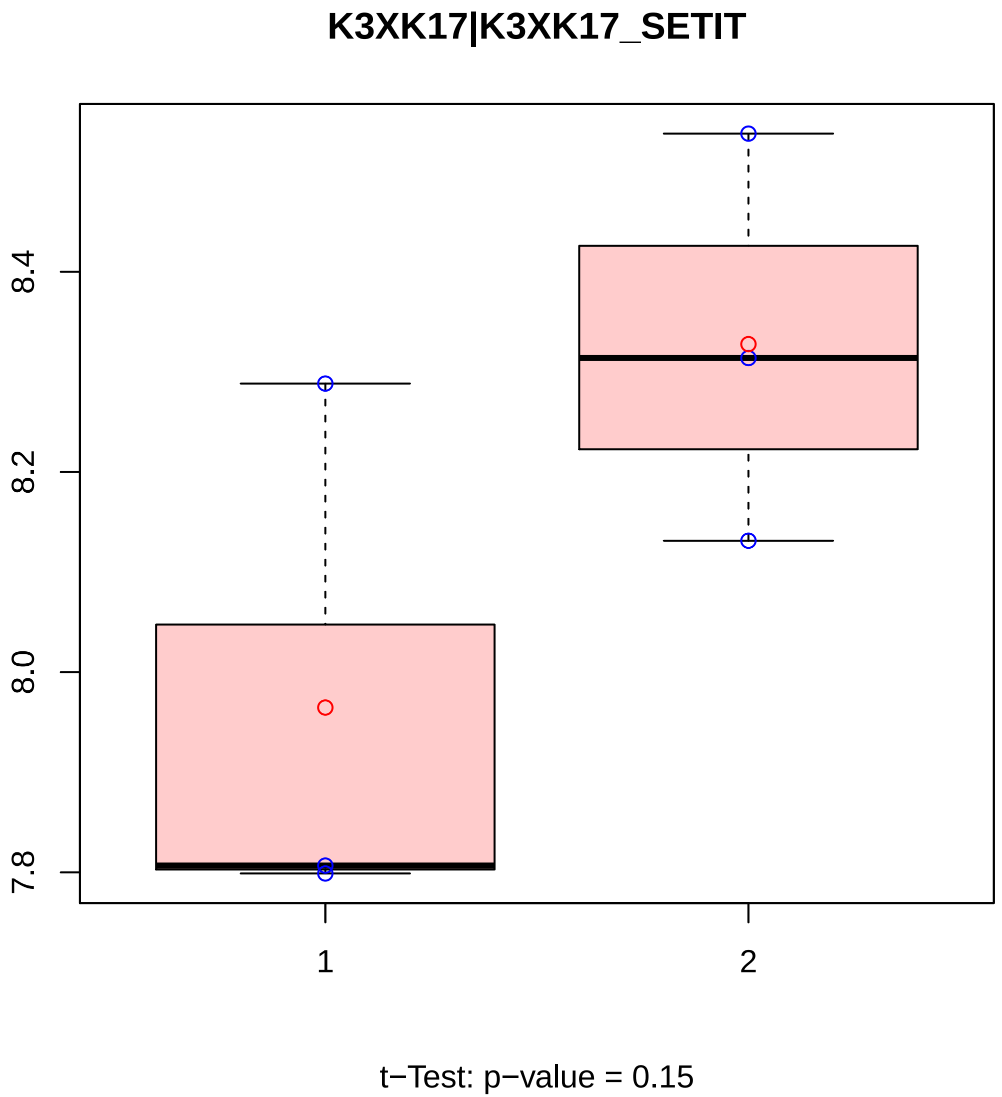
K3XK17|K3XK17_SETIT
8.4
8.2
8.0
7.8
1
2
t−Test: p−value = 0.15

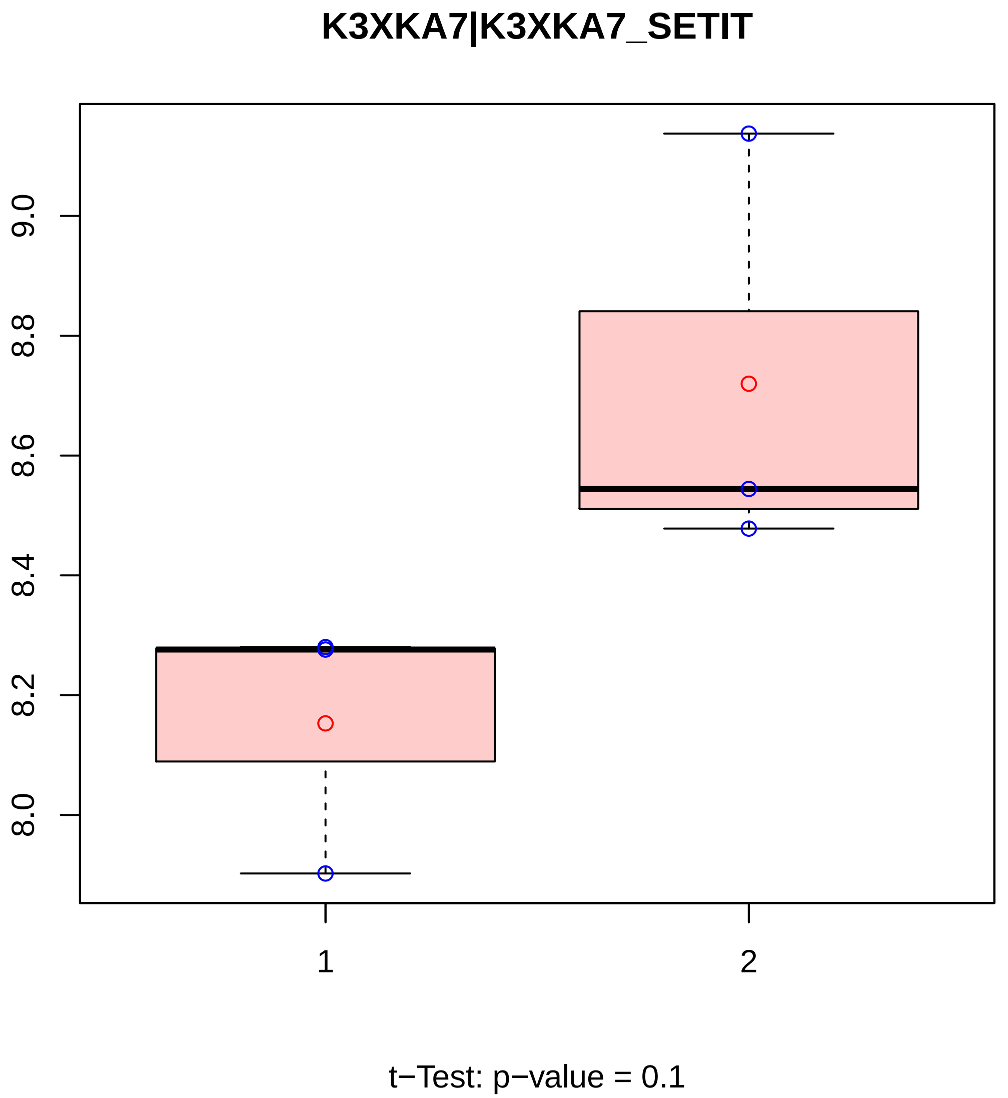
K3XKA7|K3XKA7_SETIT
9.0
8.8
8.6
8.4
8.2
8.0
1
2
t−Test: p−value = 0.1

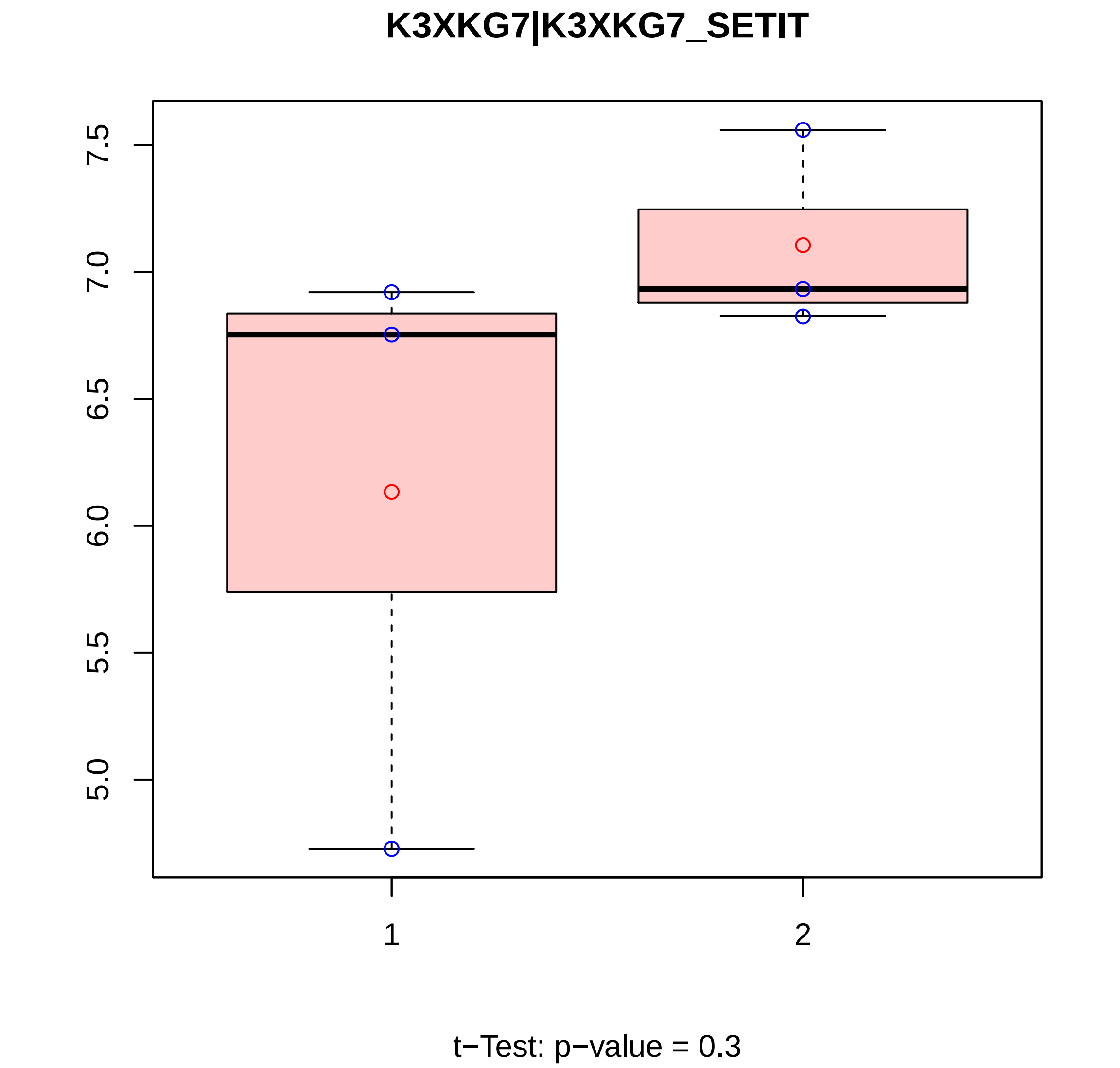
K3XKG7|K3XKG7_SETIT
7.5
7.0
6.5
6.0
5.5
5.0
1
2
t−Test: p−value = 0.3

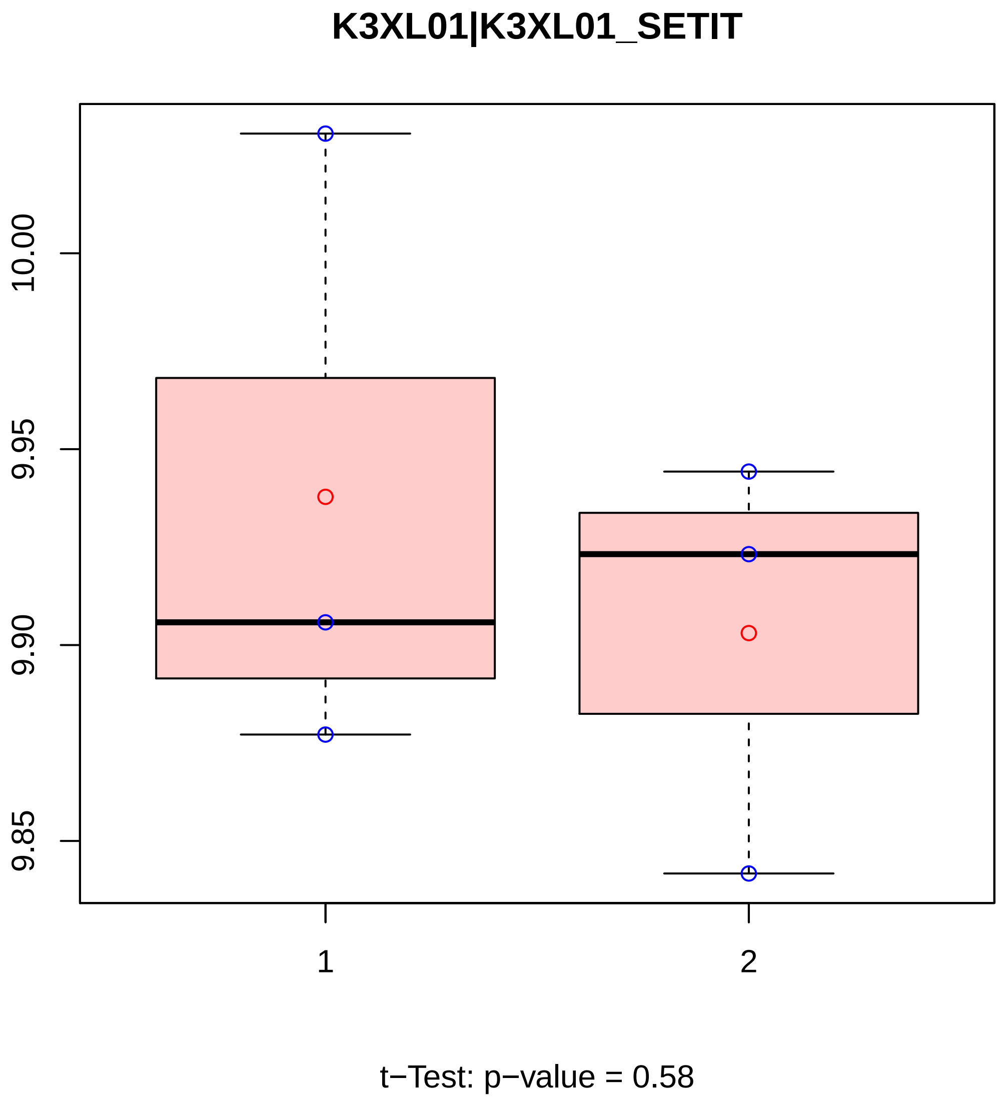

K3XL01|K3XL01_SETIT
10.00
9.95
9.90
9.85
1
2
t−Test: p−value = 0.58

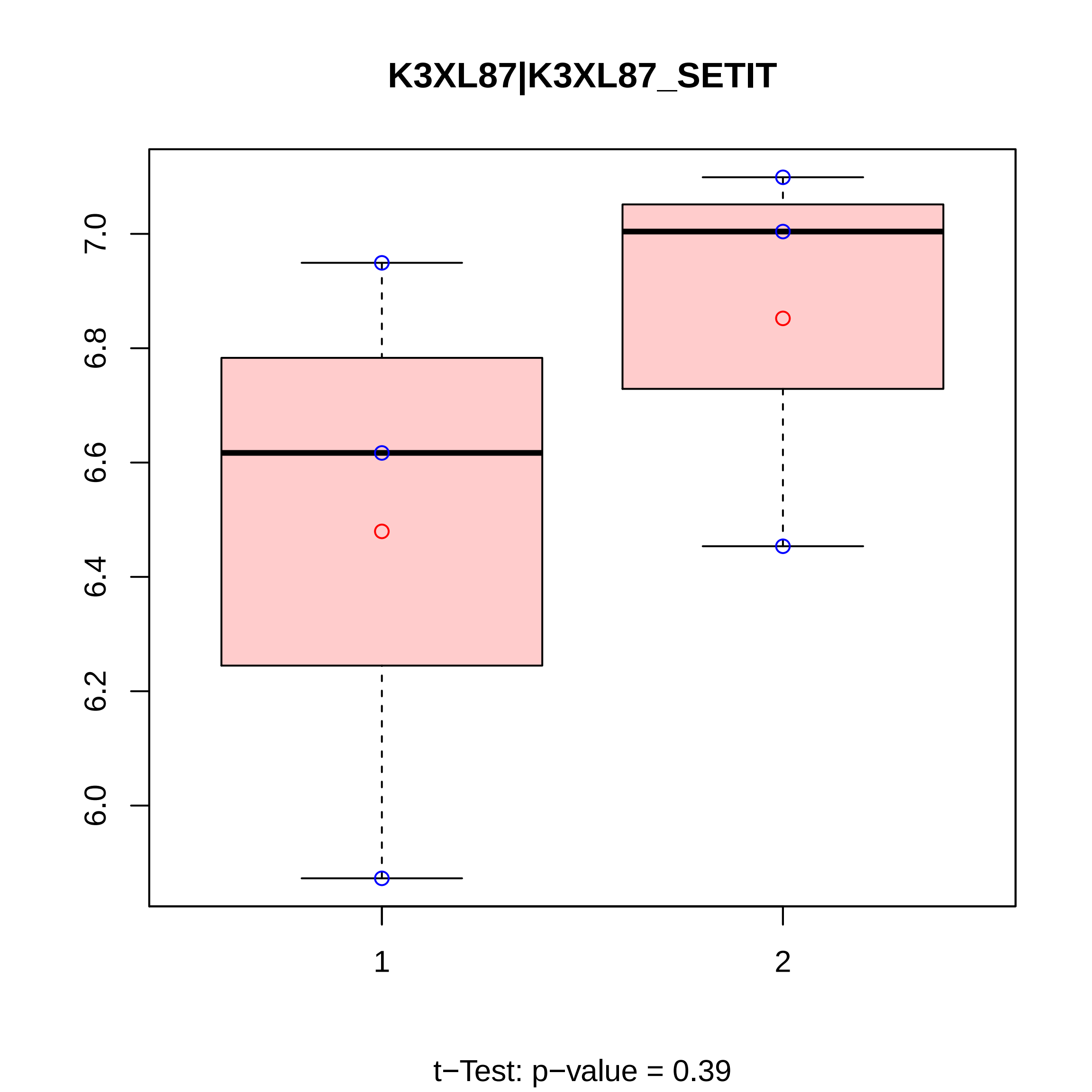
K3XL87|K3XL87_SETIT
7.0
6.8
6.6
6.4
6.2
6.0
1
2
t−Test: p−value = 0.39

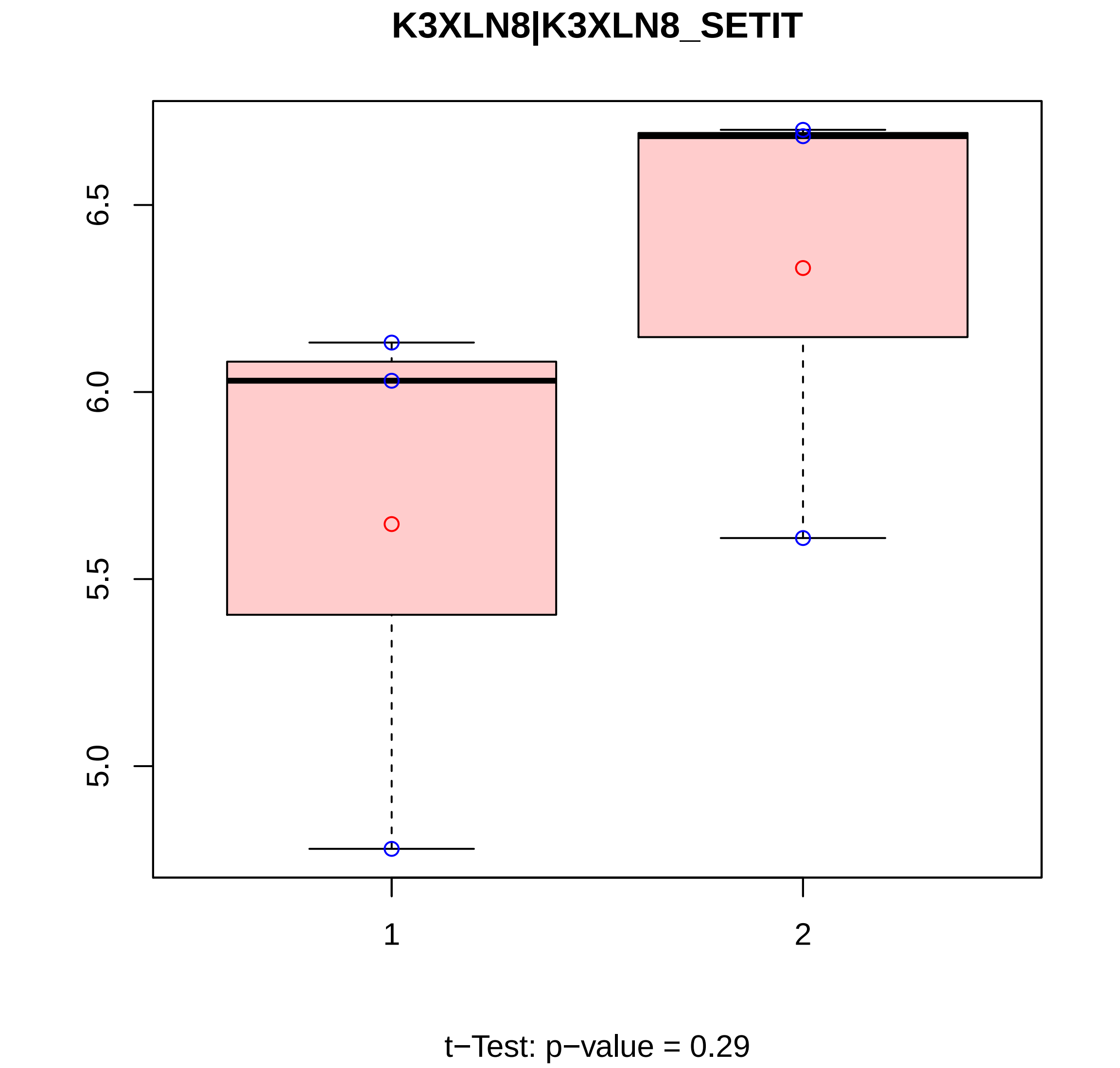
K3XLN8|K3XLN8_SETIT
5.0
5.5
6.0
6.5
1
2
t−Test: p−value = 0.29

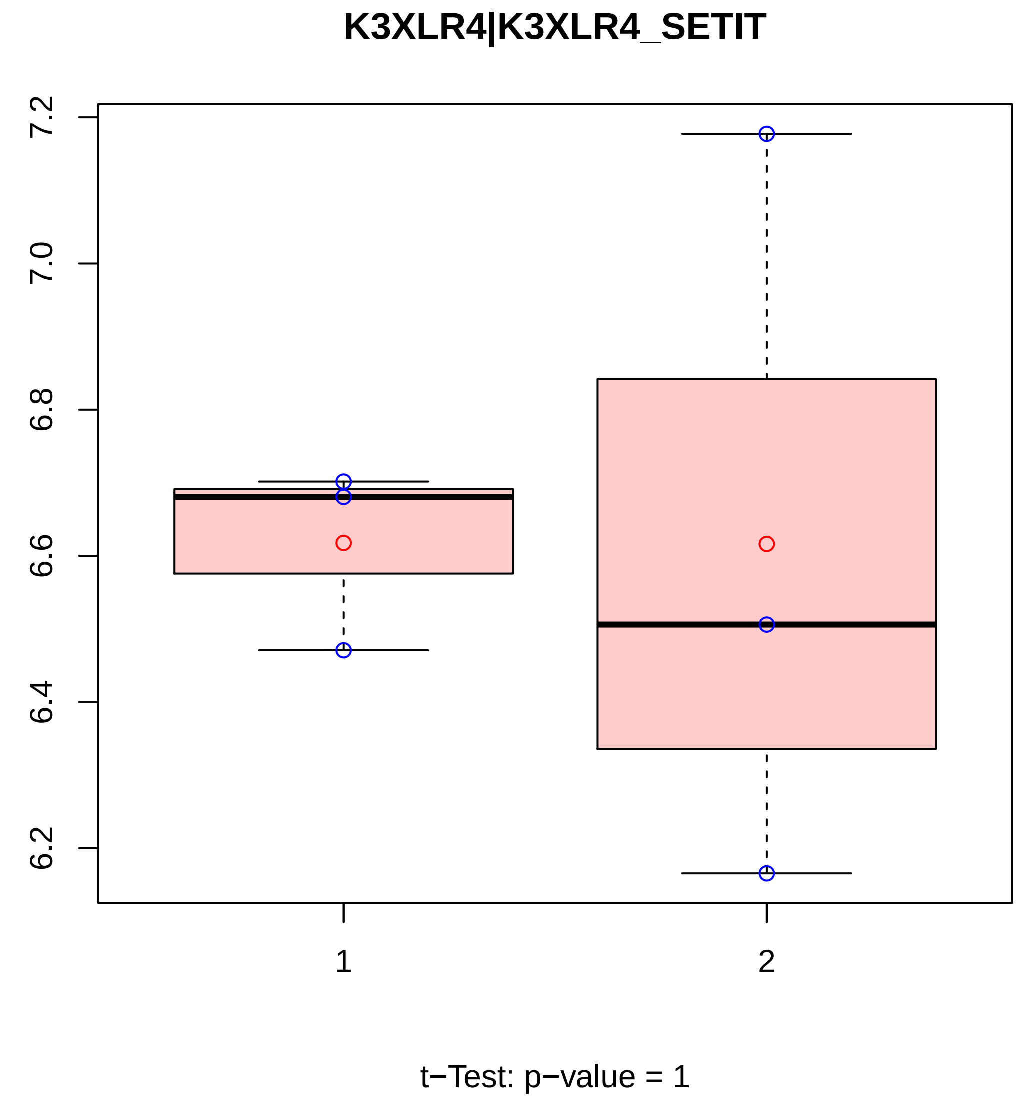
K3XLR4|K3XLR4_SETIT
7.2
7.0
6.8
6.6
6.4
6.2
1
2
t−Test: p−value = 1

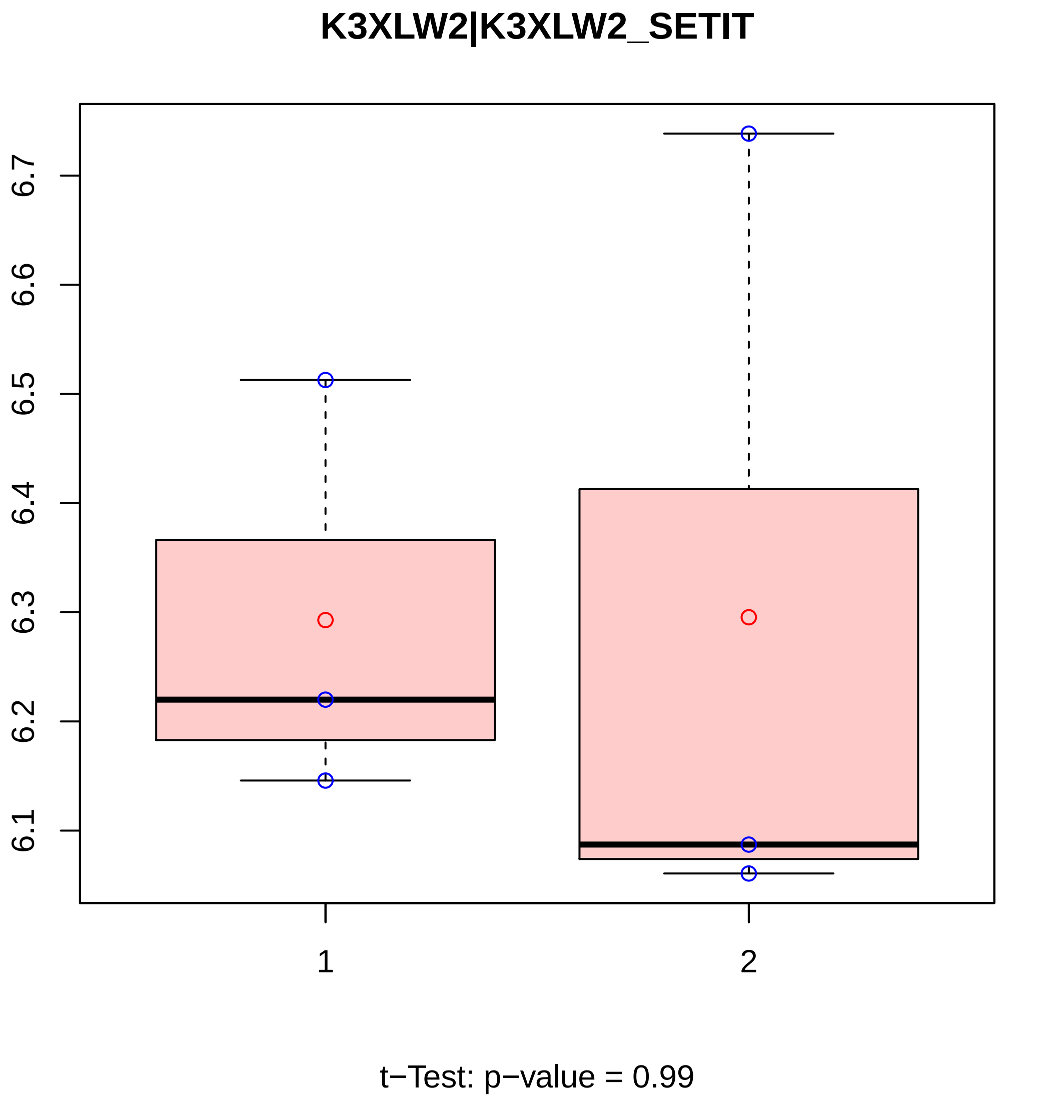
K3XLW2|K3XLW2_SETIT
6.1
6.2
6.3
6.4
6.5
6.6
6.7
1
2
t−Test: p−value = 0.99

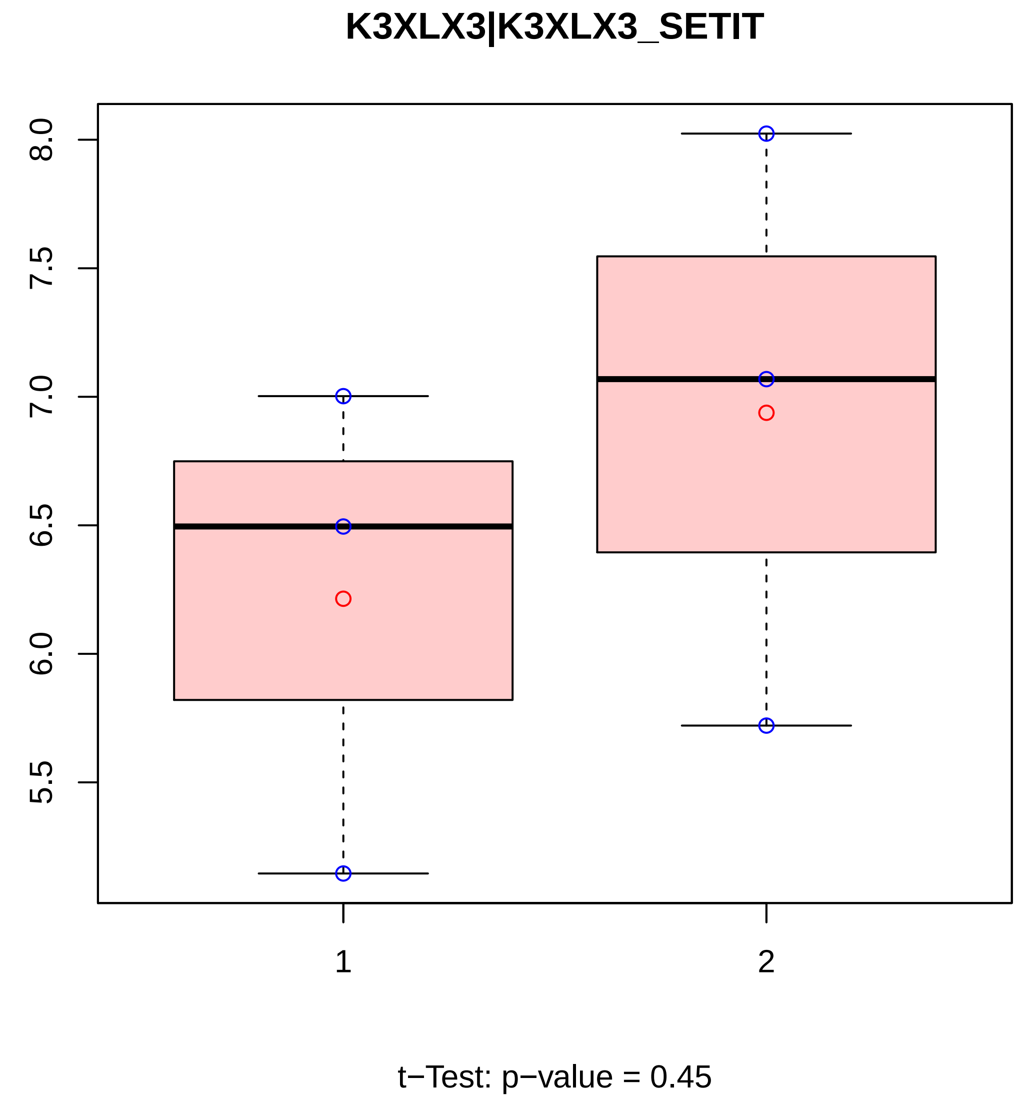

K3XLX3|K3XLX3_SETIT
8.0
7.5
7.0
6.5
6.0
5.5
1
2
t−Test: p−value = 0.45

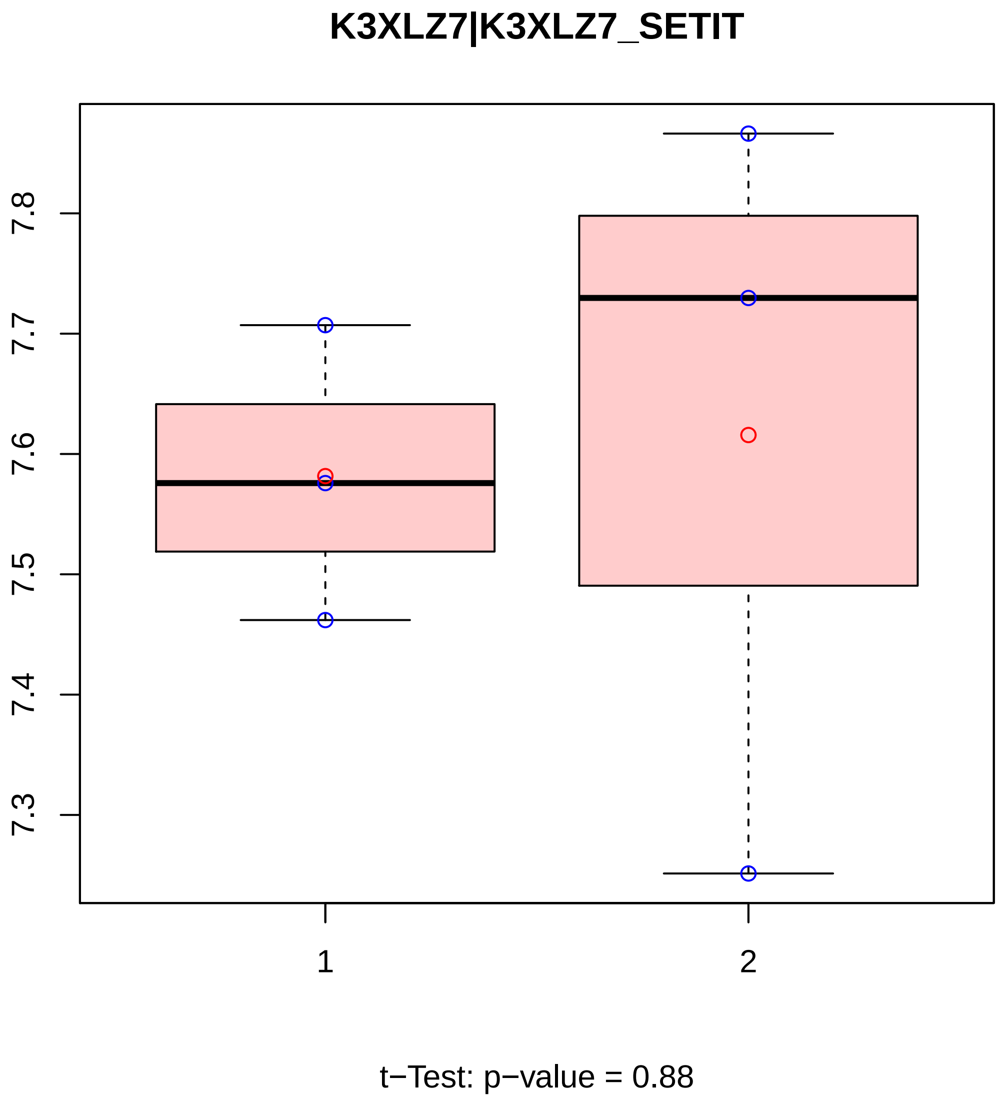
K3XLZ7|K3XLZ7_SETIT
7.3
7.4
7.5
7.6
7.7
7.8
1
2
t−Test: p−value = 0.88

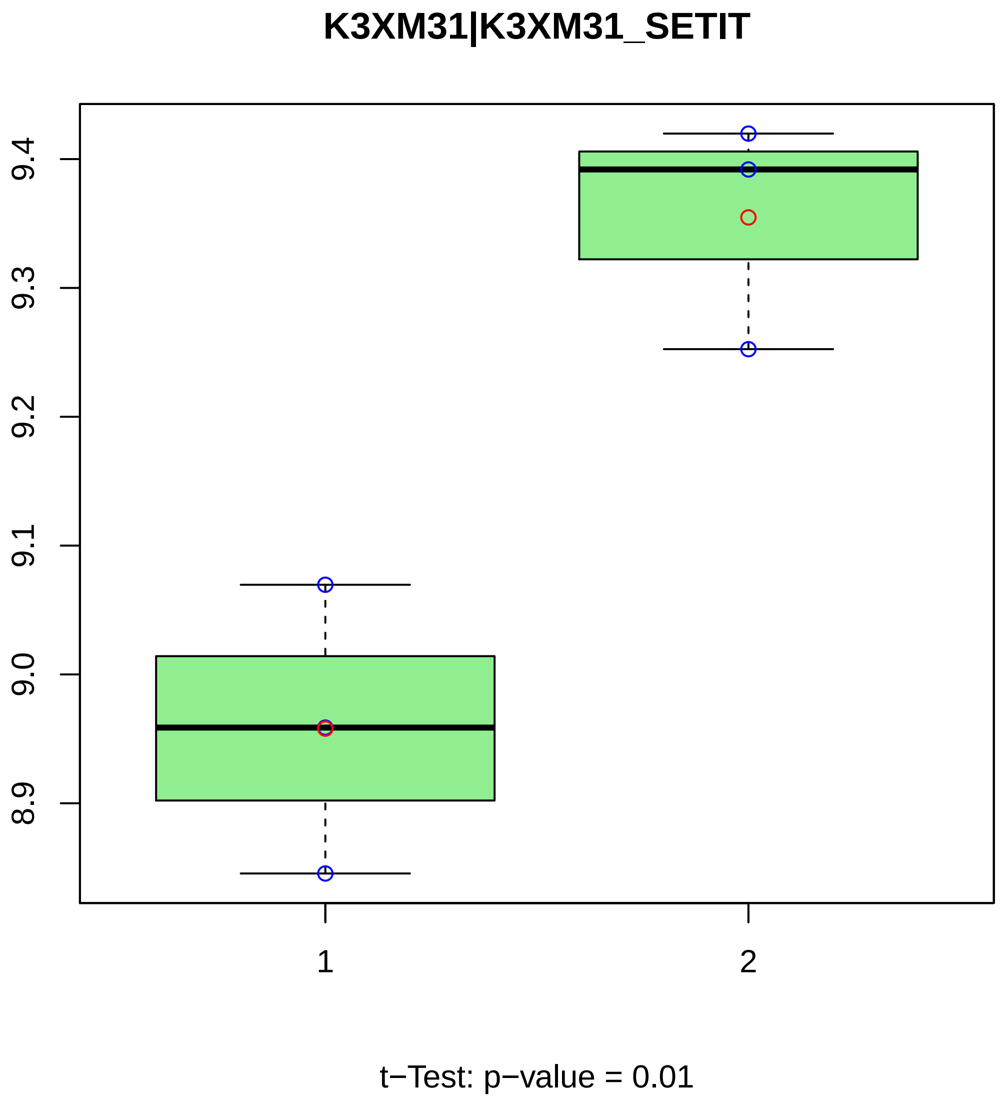
K3XM31|K3XM31_SETIT
9.4
9.3
9.2
9.1
9.0
8.9
1
2
t−Test: p−value = 0.01

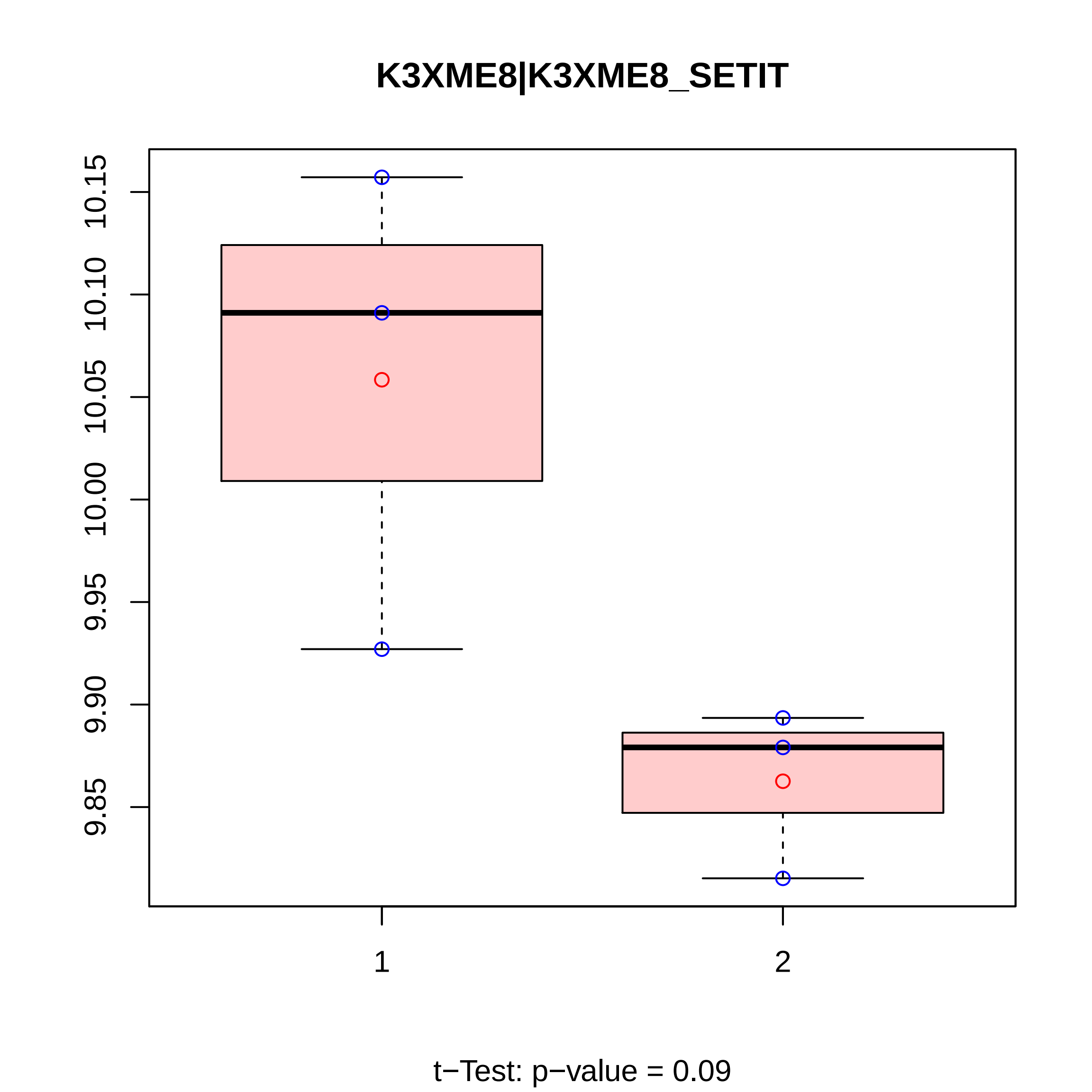
K3XME8|K3XME8_SETIT
10.15
10.10
10.05
10.00
9.95
9.90
9.85
1
2
t−Test: p−value = 0.09

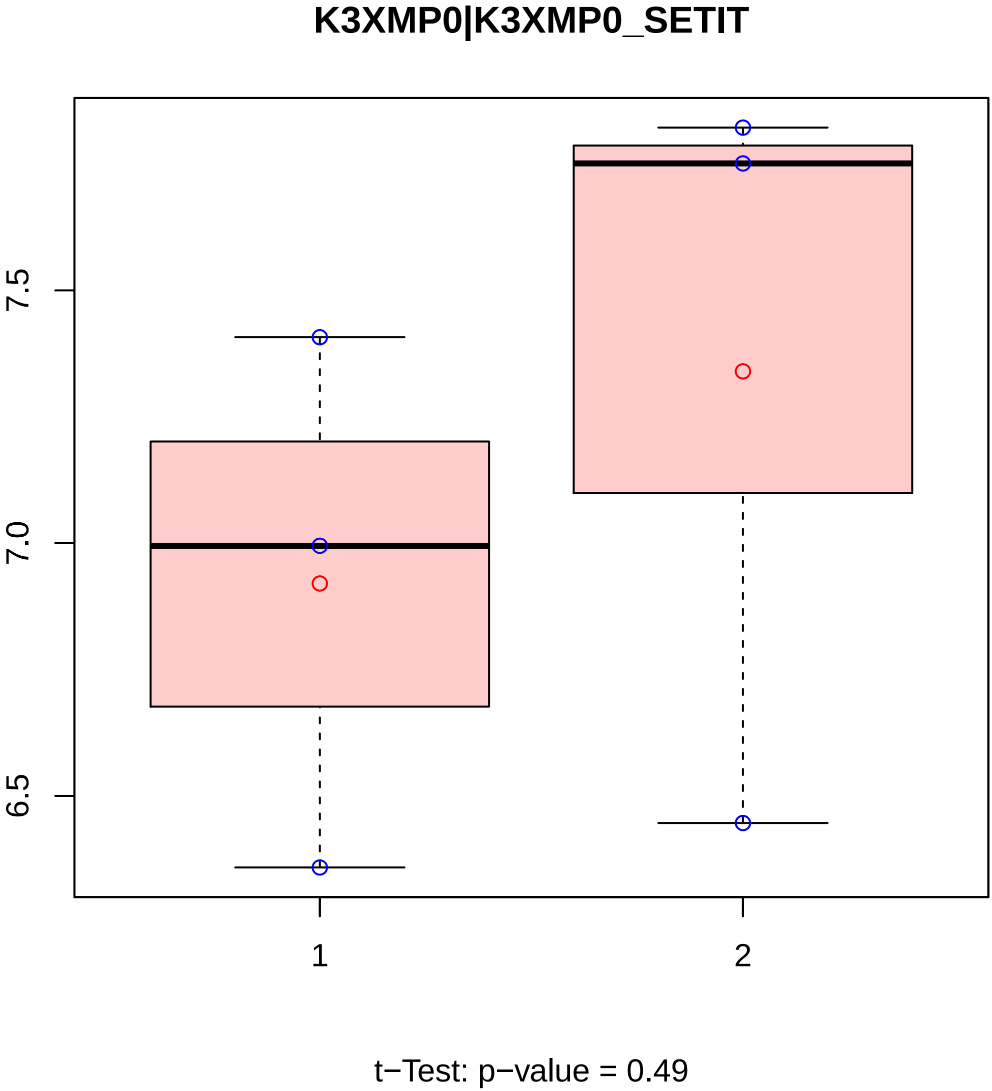
K3XMP0|K3XMP0_SETIT
7.5
7.0
6.5
1
2
t−Test: p−value = 0.49

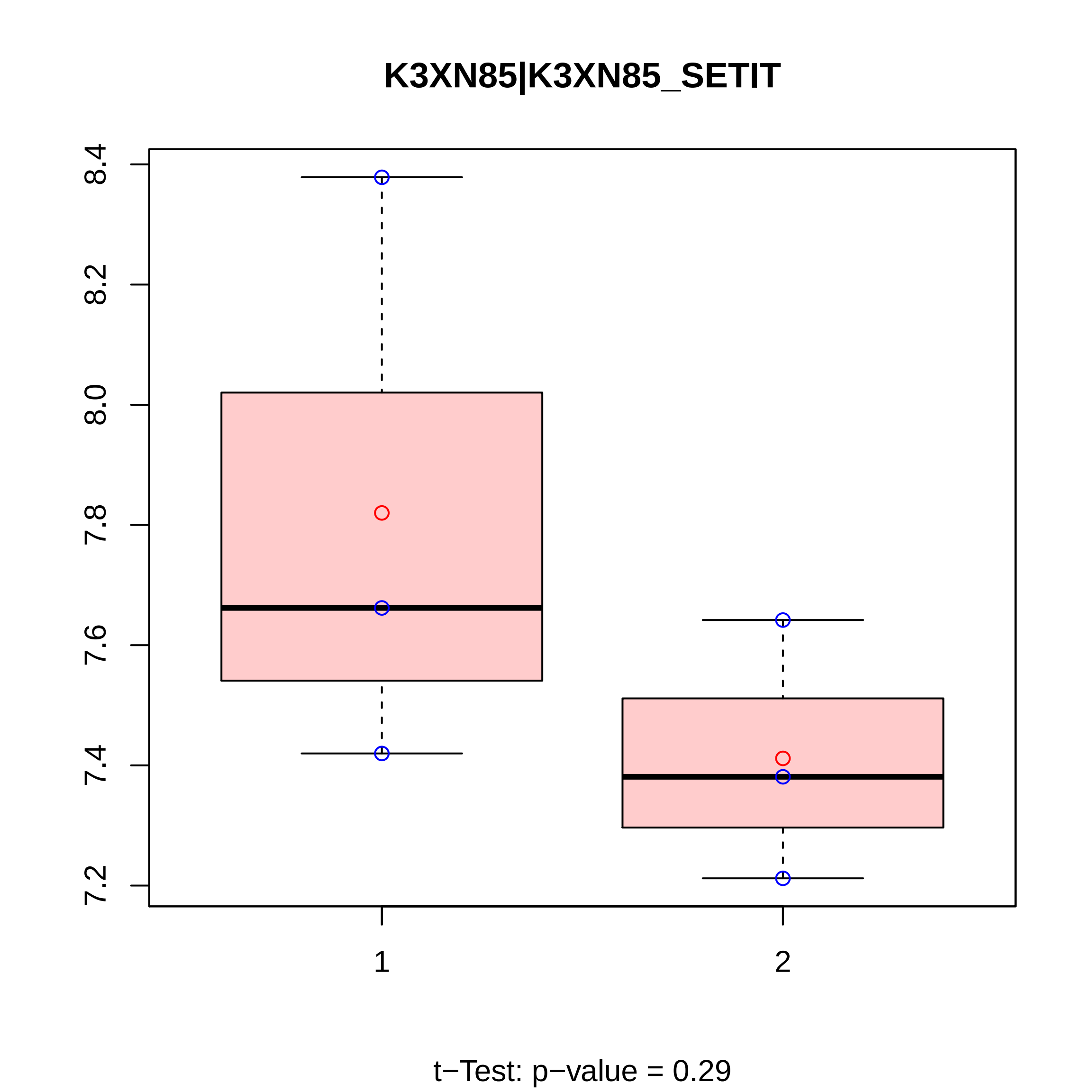

K3XN85|K3XN85_SETIT
8.4
8.2
8.0
7.8
7.6
7.4
7.2
1
2
t−Test: p−value = 0.29

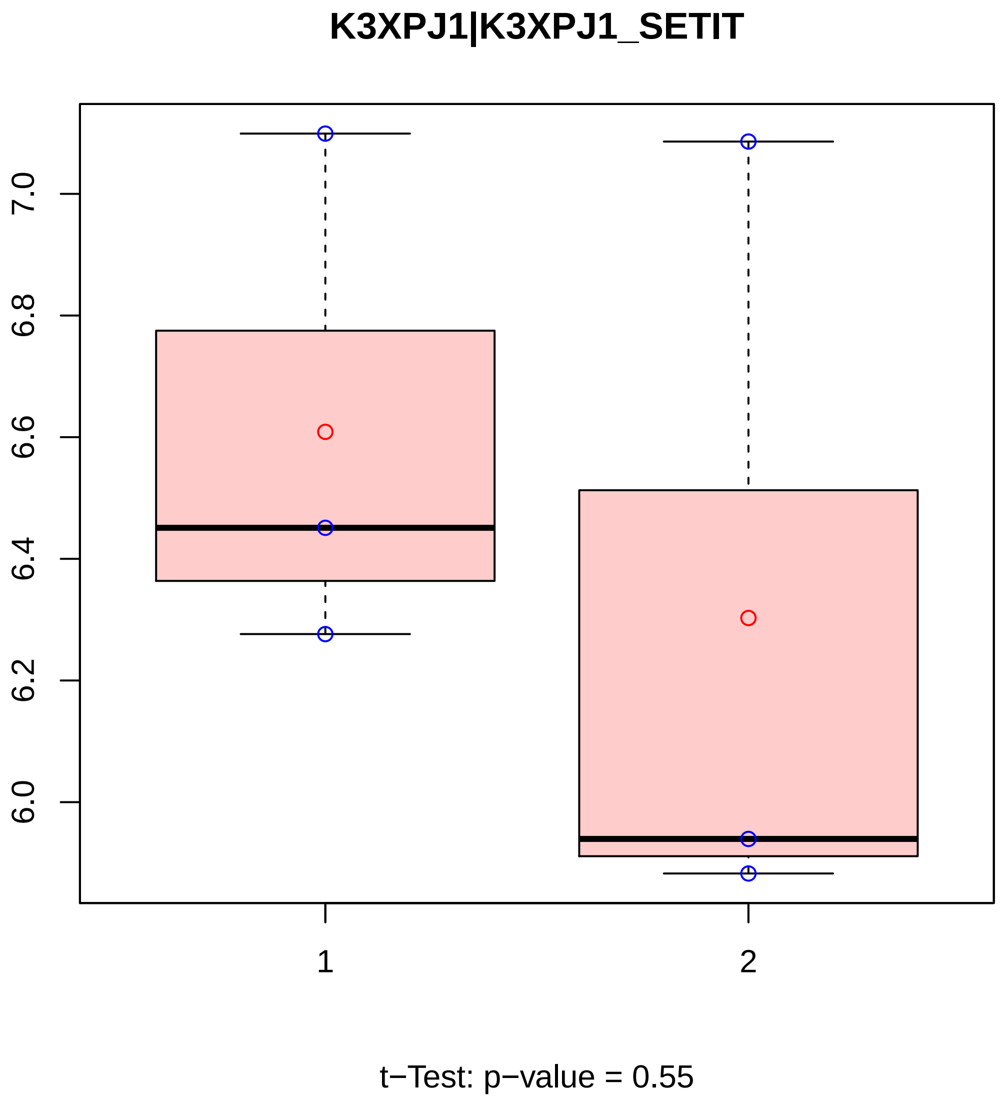
K3XPJ1|K3XPJ1_SETIT
7.0
6.8
6.6
6.4
6.2
6.0
1
2
t−Test: p−value = 0.55

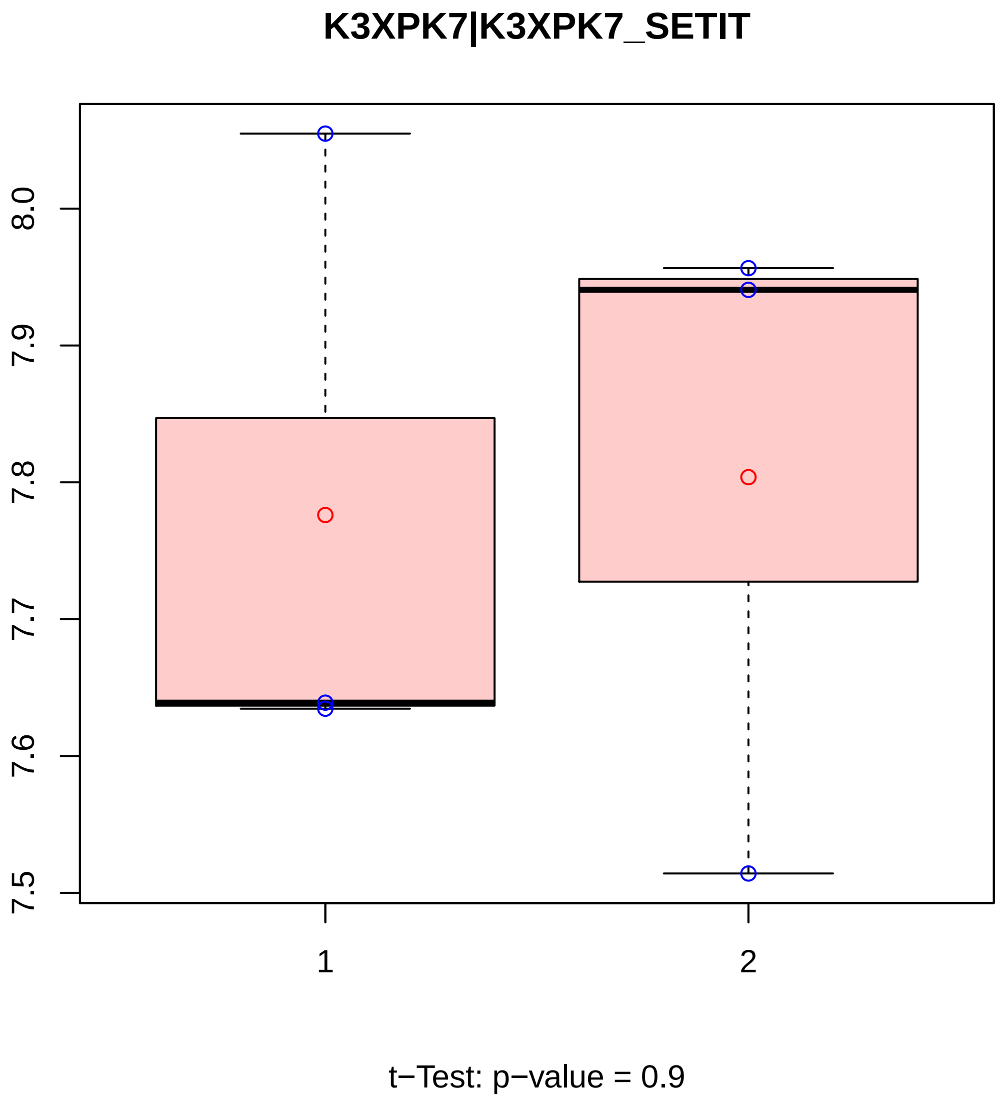
K3XPK7|K3XPK7_SETIT
8.0
7.9
7.8
7.7
7.6
7.5
1
2
t−Test: p−value = 0.9

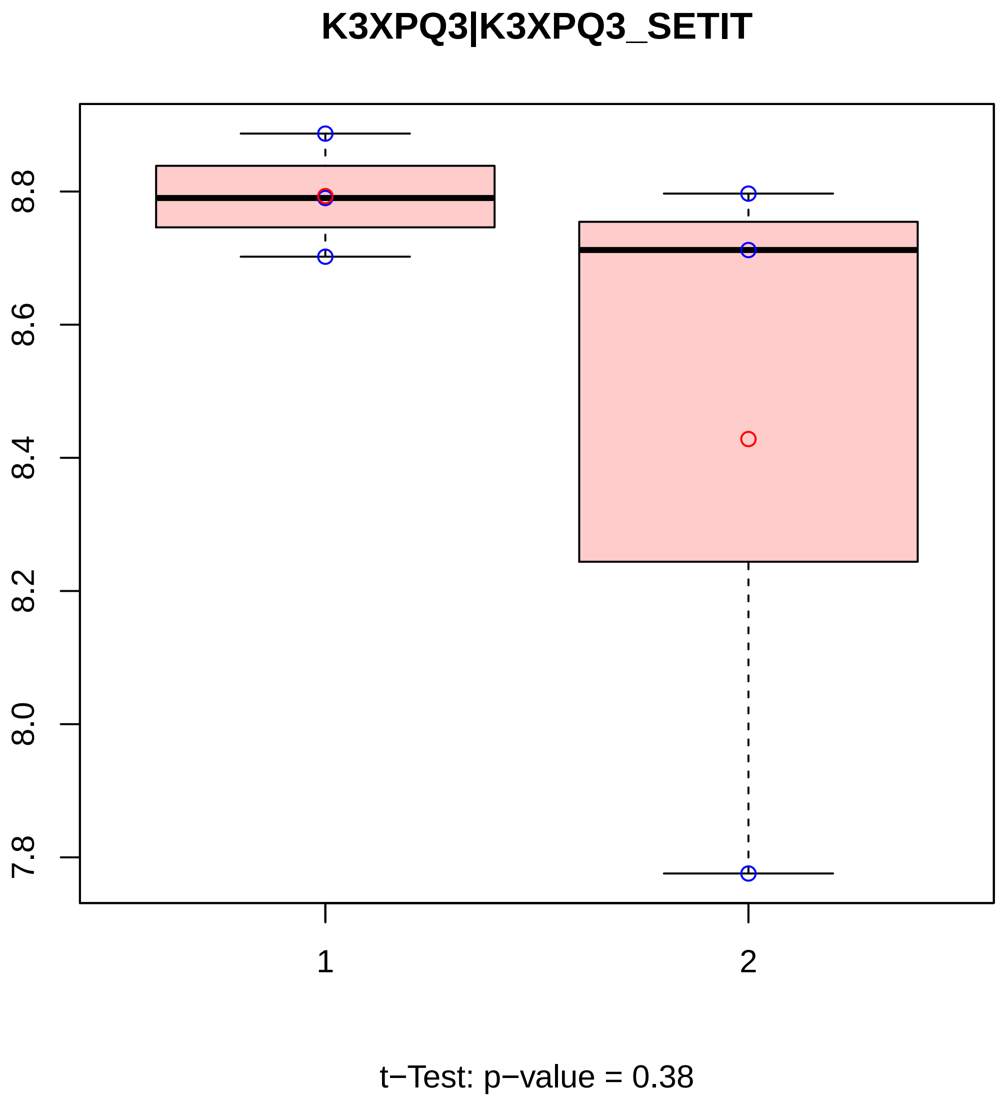

K3XPQ3|K3XPQ3_SETIT
8.8
8.6
8.4
8.2
8.0
7.8
1
2
t−Test: p−value = 0.38

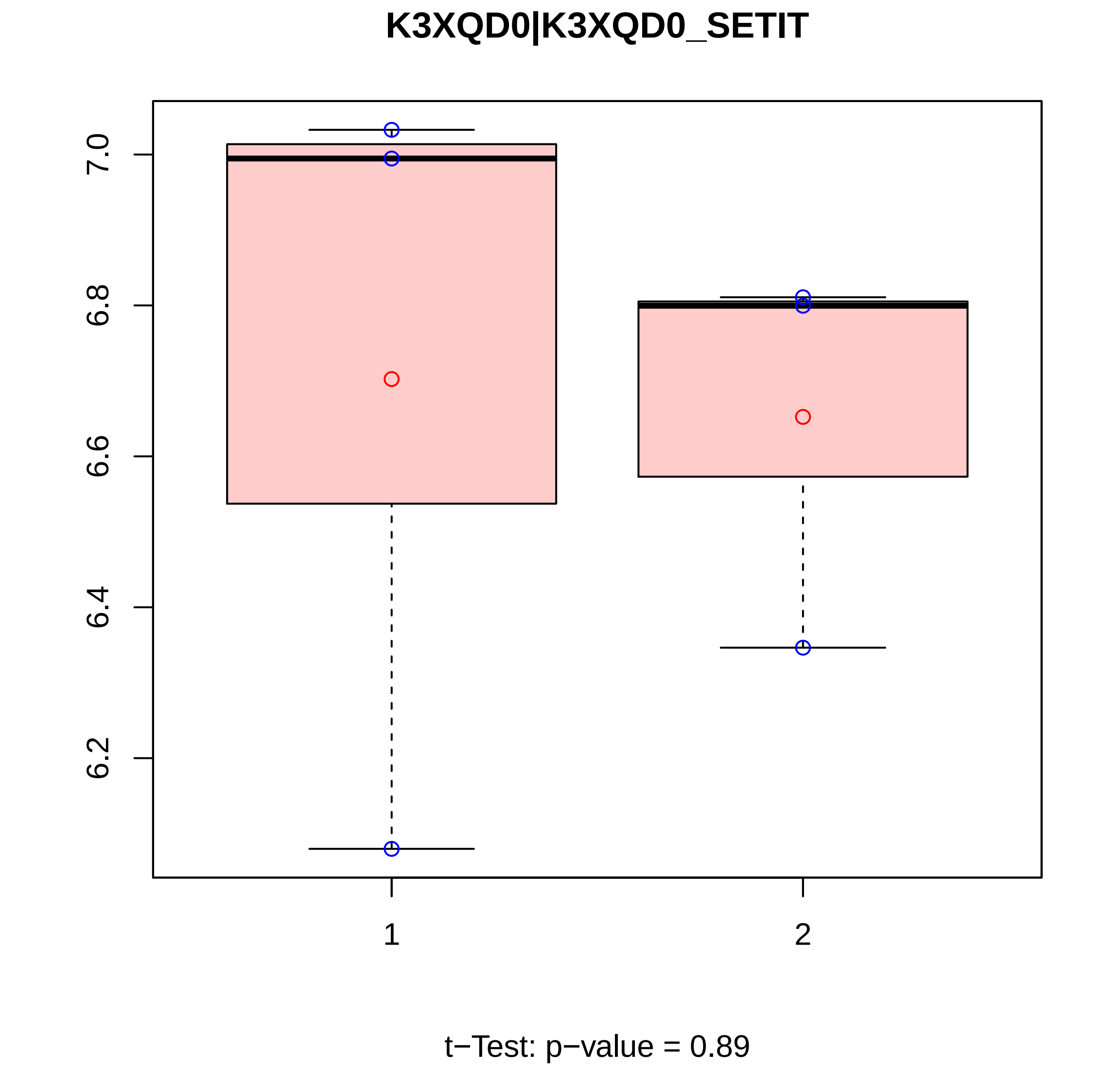
K3XQD0|K3XQD0_SETIT
7.0
6.8
6.6
6.4
6.2
1
2
t−Test: p−value = 0.89

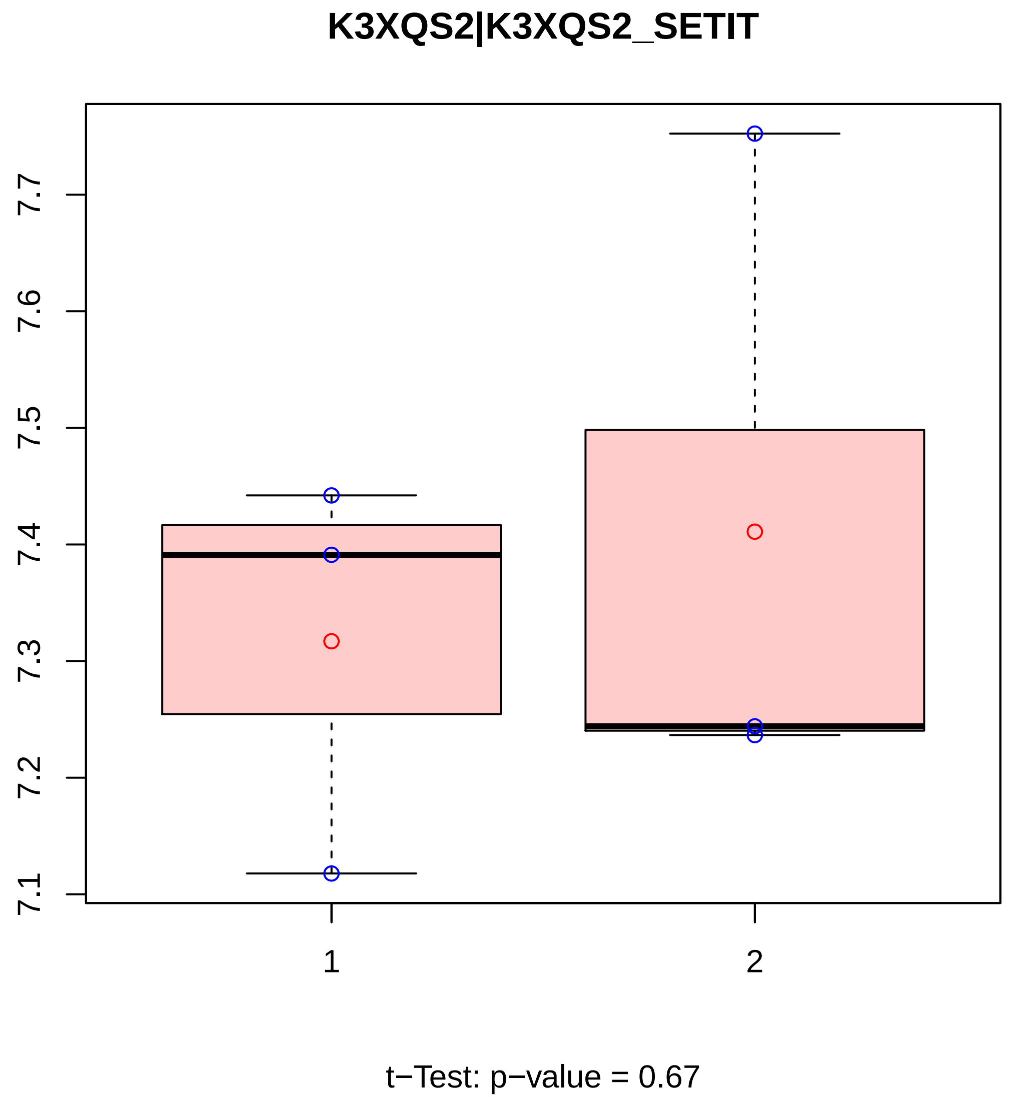

K3XQS2|K3XQS2_SETIT
7.1
7.2
7.3
7.4
7.5
7.6
7.7
1
2
t−Test: p−value = 0.67

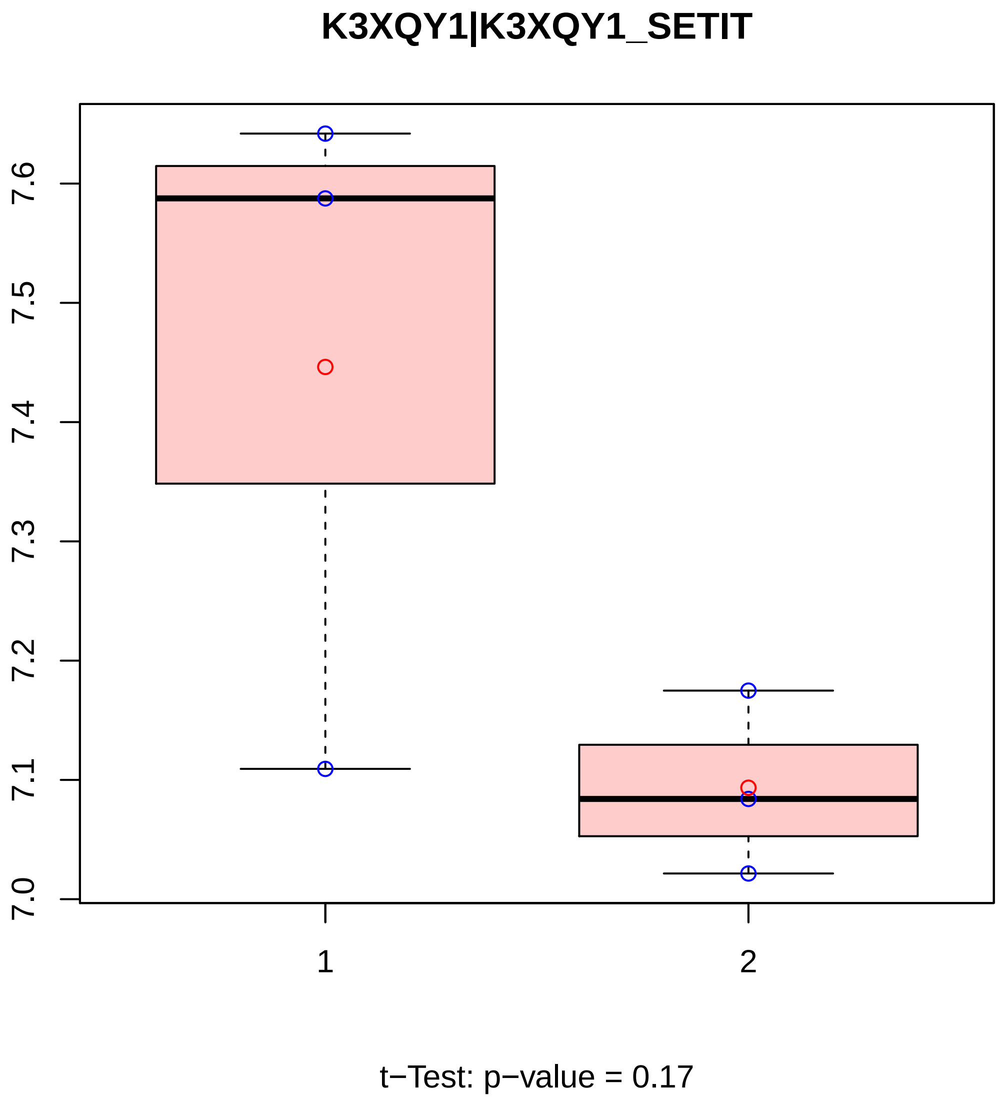
K3XQY1|K3XQY1_SETIT
7.0
7.1
7.2
7.3
7.4
7.5
7.6
1
2
t−Test: p−value = 0.17

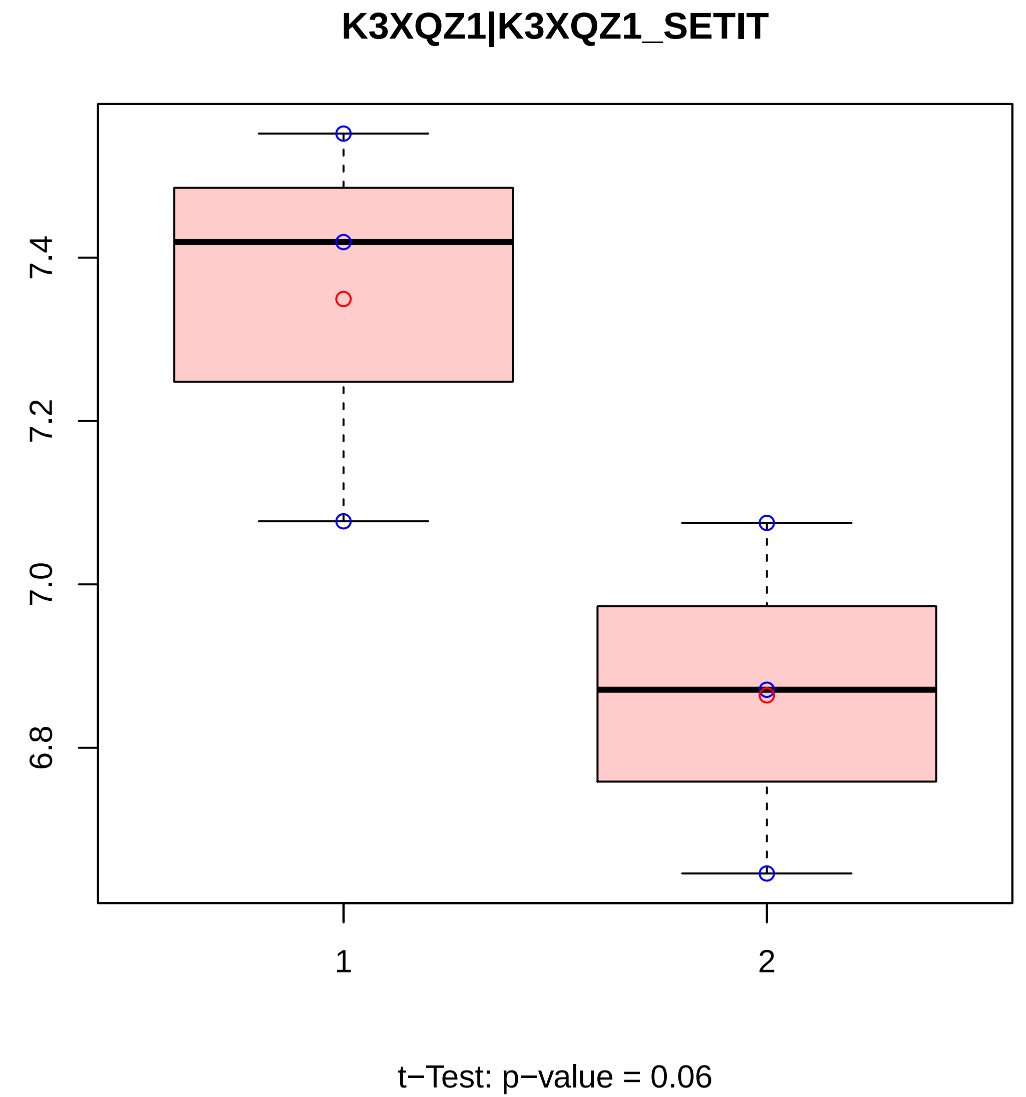

K3XQZ1|K3XQZ1_SETIT
7.4
7.2
7.0
6.8
1
2
t−Test: p−value = 0.06

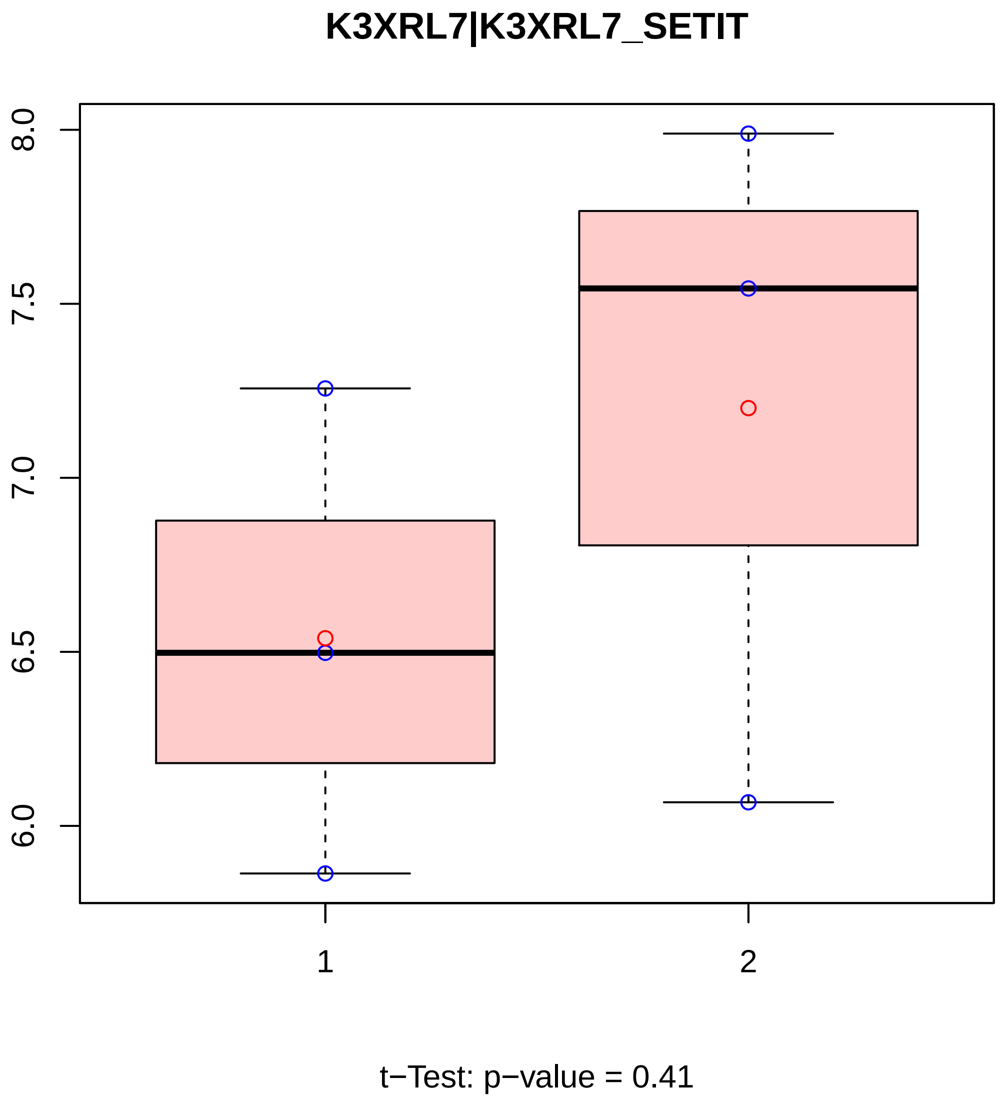
K3XRL7|K3XRL7_SETIT
8.0
7.5
7.0
6.5
6.0
1
2
t−Test: p−value = 0.41

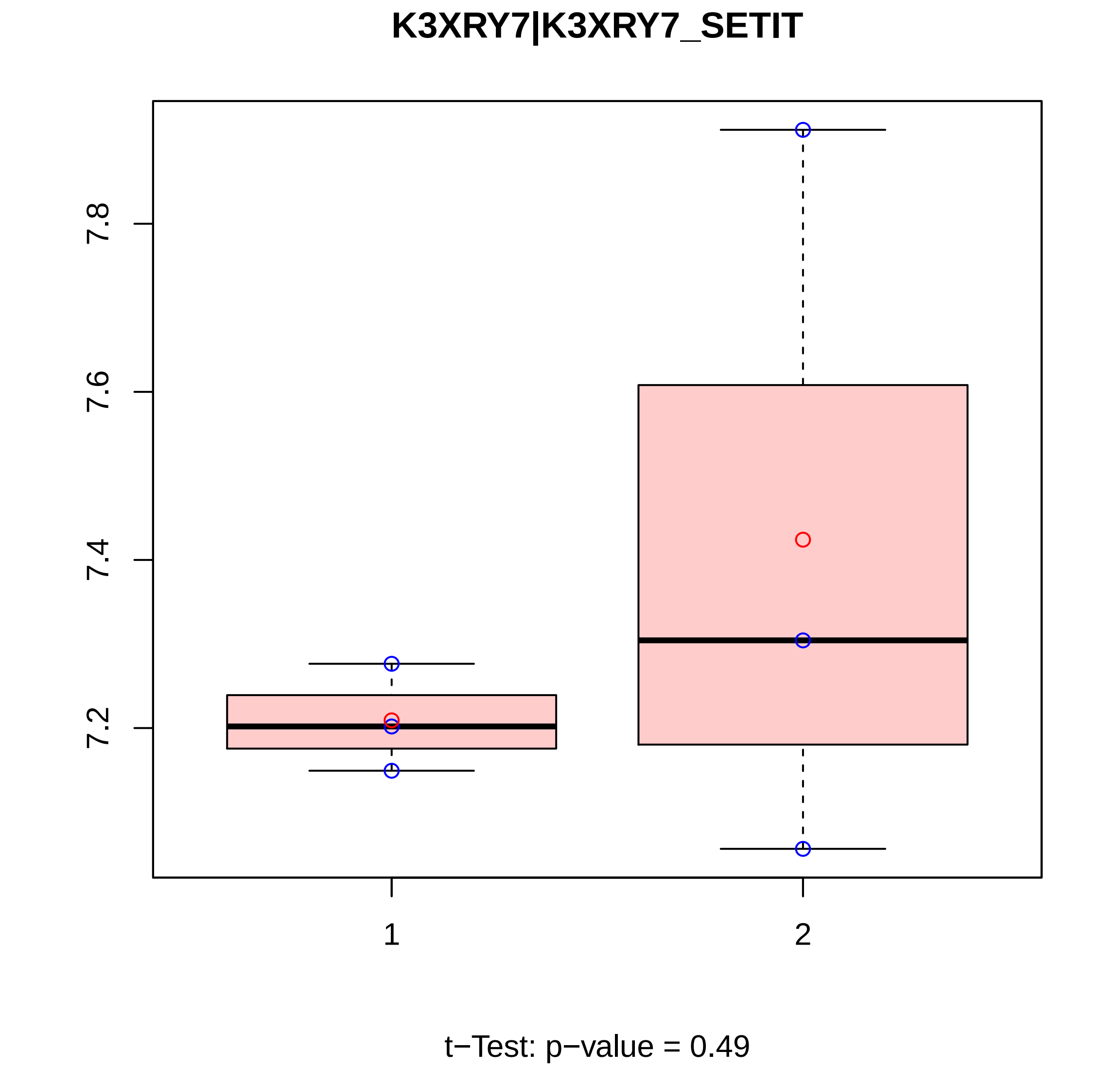
K3XRY7|K3XRY7_SETIT
7.2
7.4
7.6
7.8
1
2
t−Test: p−value = 0.49

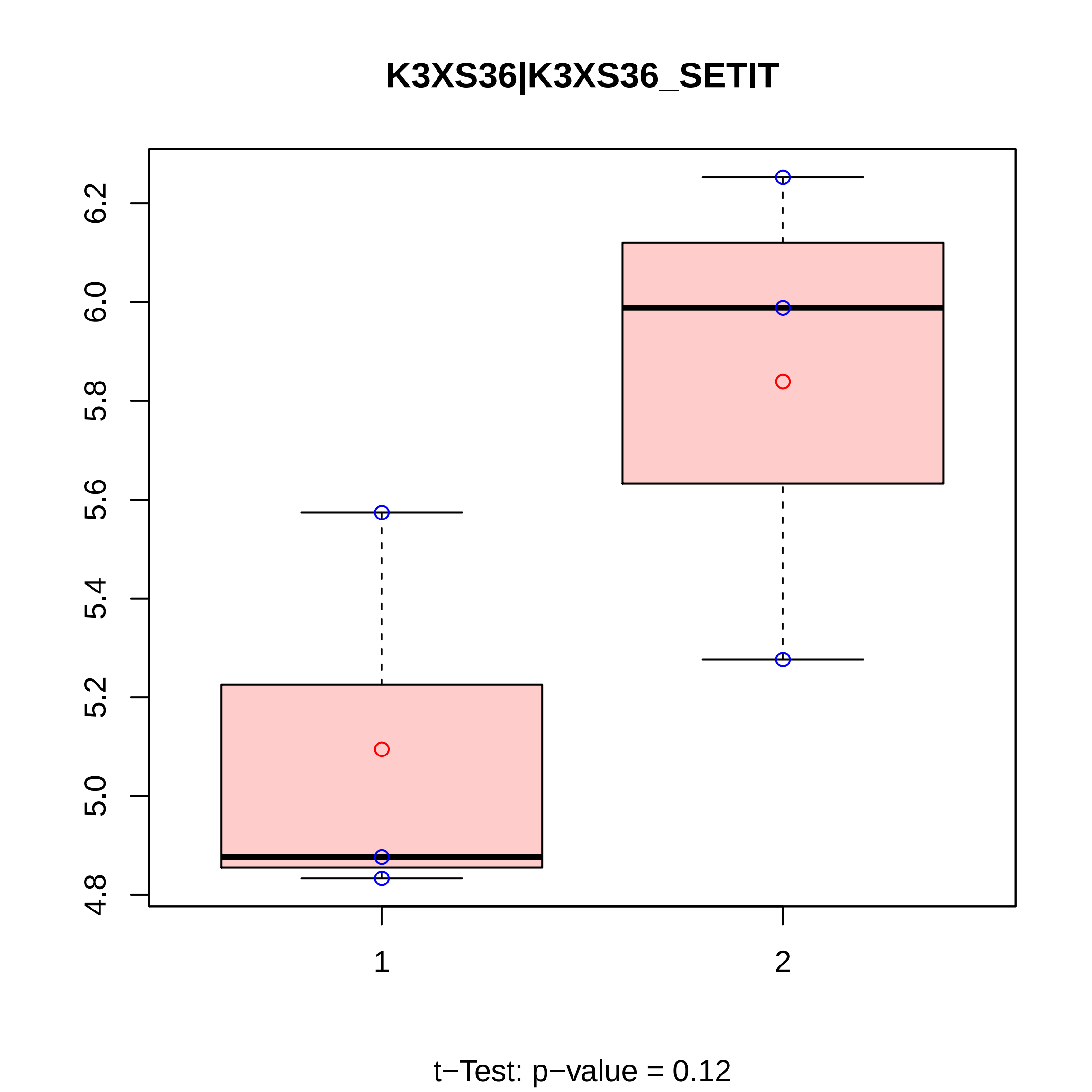
K3XS36|K3XS36_SETIT
6.2
6.0
5.8
5.6
5.4
5.2
5.0
4.8
1
2
t−Test: p−value = 0.12

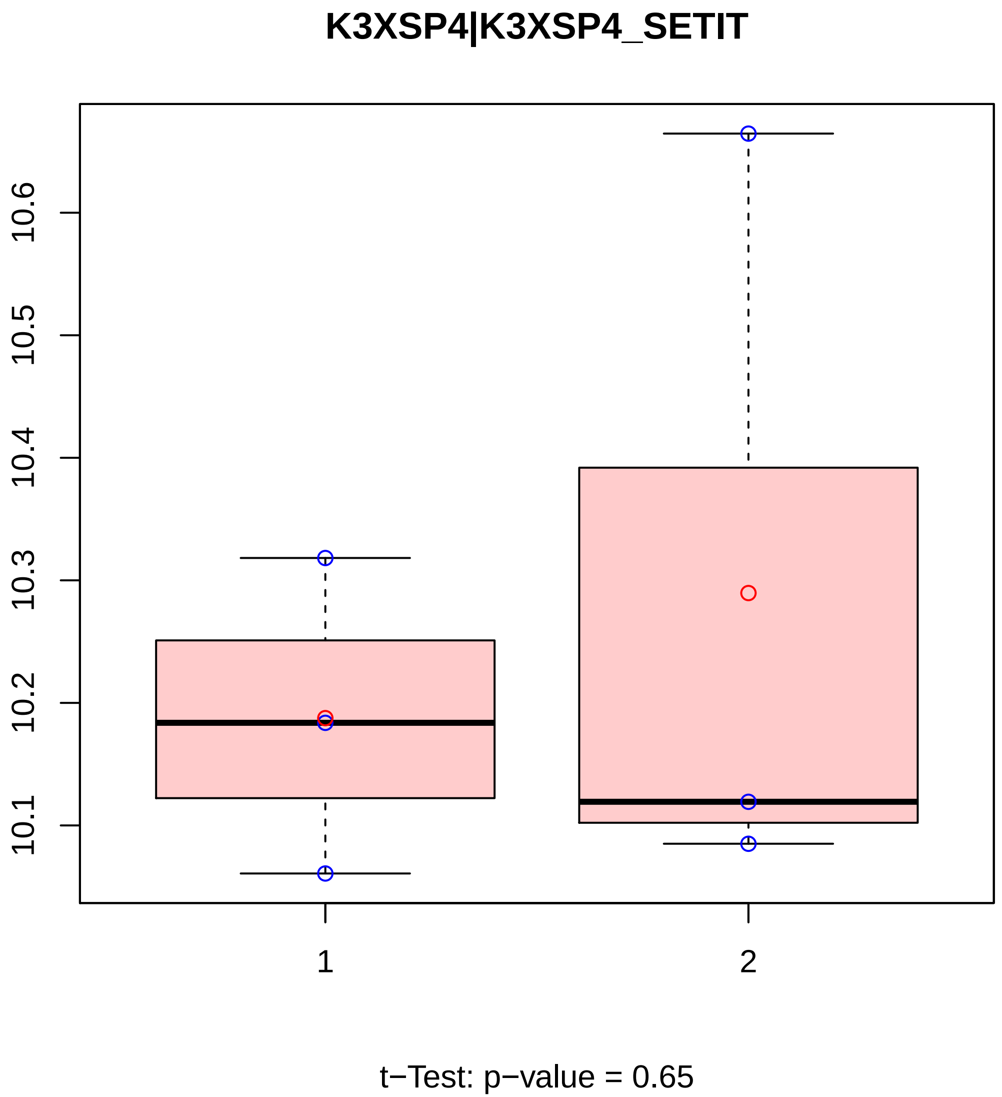
K3XSP4|K3XSP4_SETIT
10.1
10.2
10.3
10.4
10.5
10.6
1
2
t−Test: p−value = 0.65

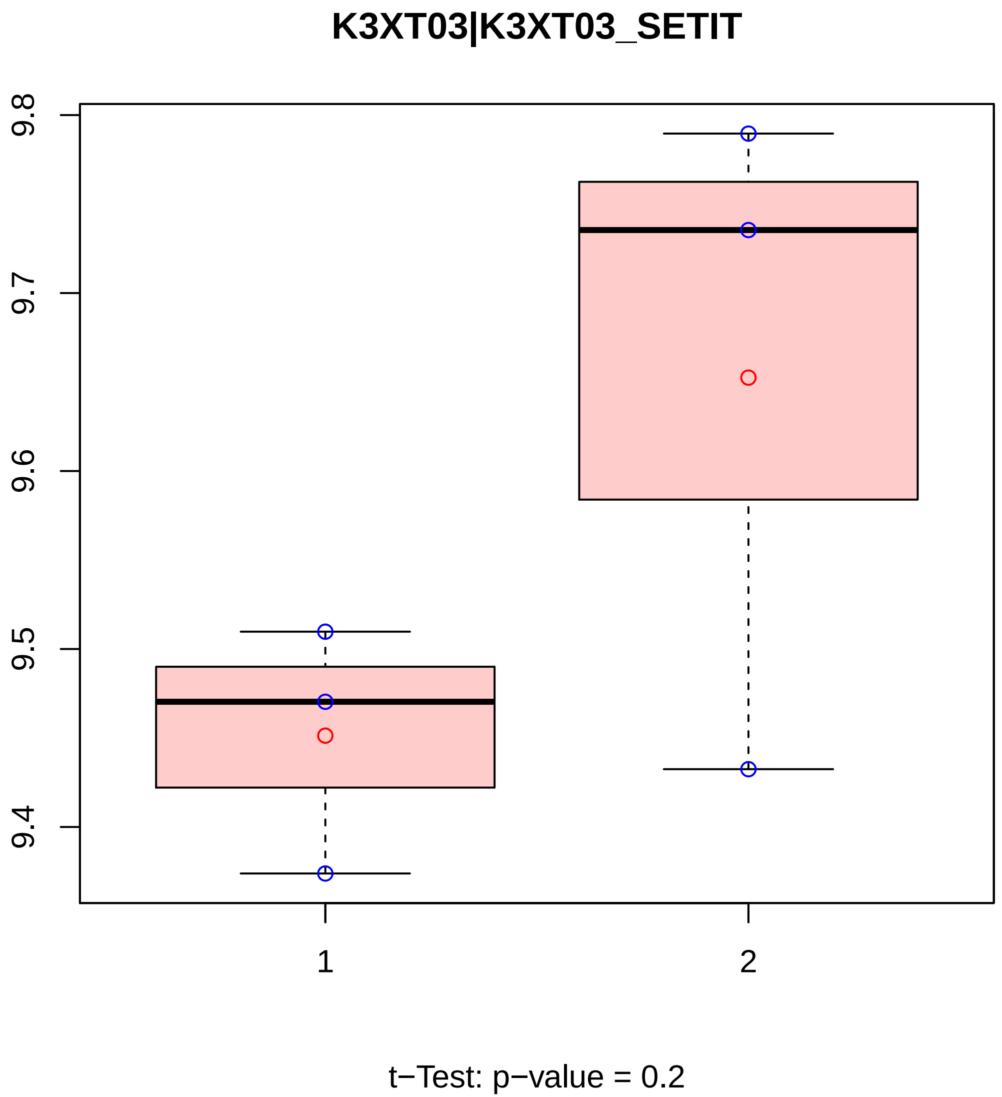
K3XT03|K3XT03_SETIT
9.4
9.5
9.6
9.7
9.8
1
2
t−Test: p−value = 0.2

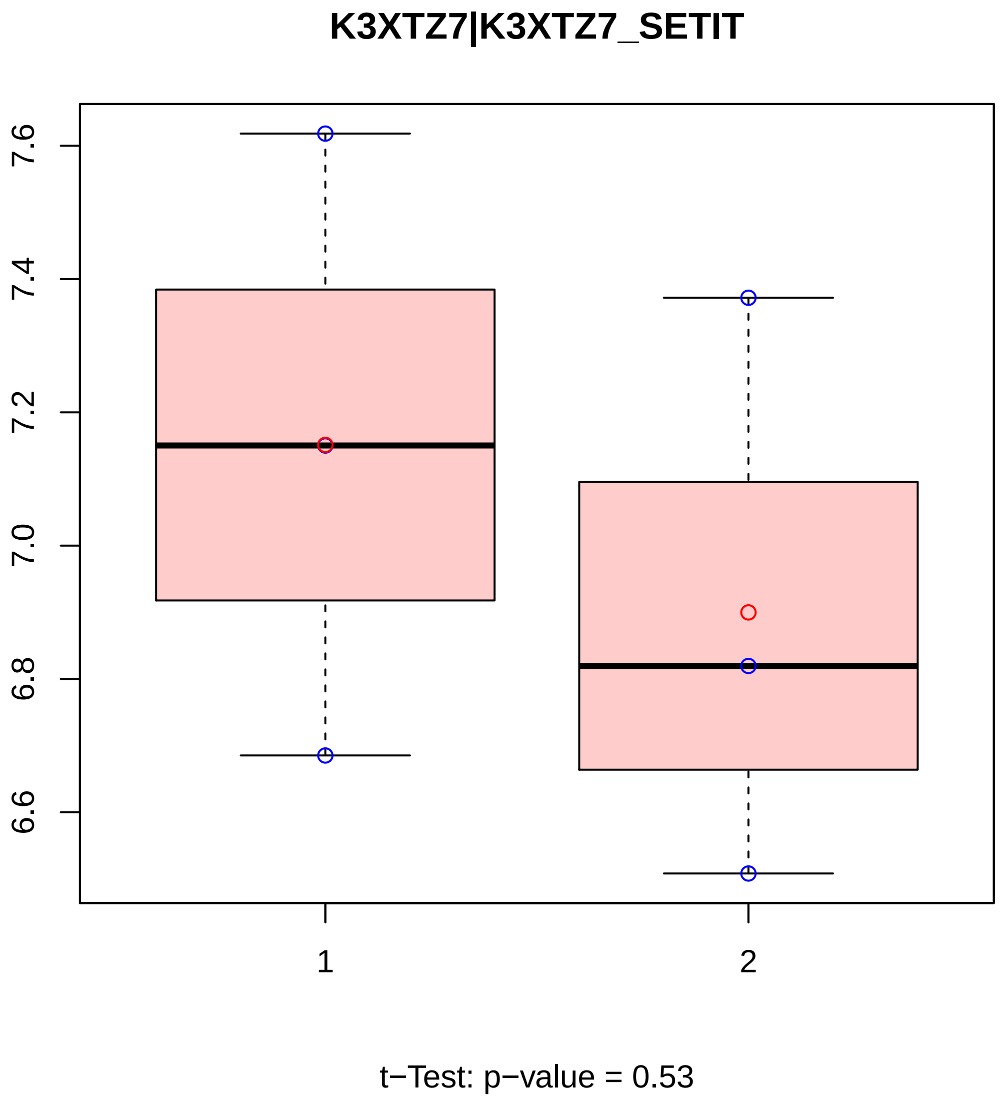
K3XTZ7|K3XTZ7_SETIT
7.6
7.4
7.2
7.0
6.8
6.6
1
2
t−Test: p−value = 0.53

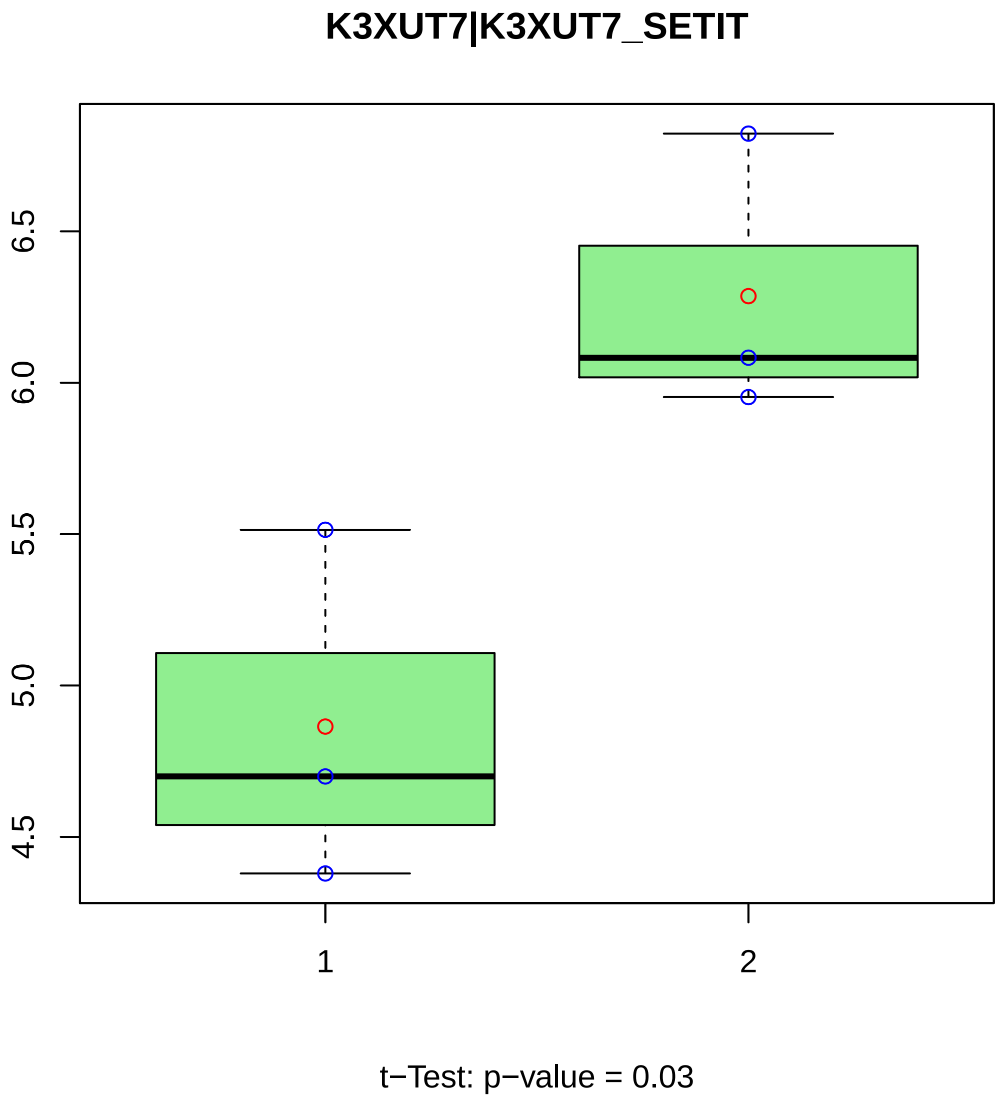
K3XUT7|K3XUT7_SETIT
6.5
6.0
5.5
5.0
4.5
1
2
t−Test: p−value = 0.03

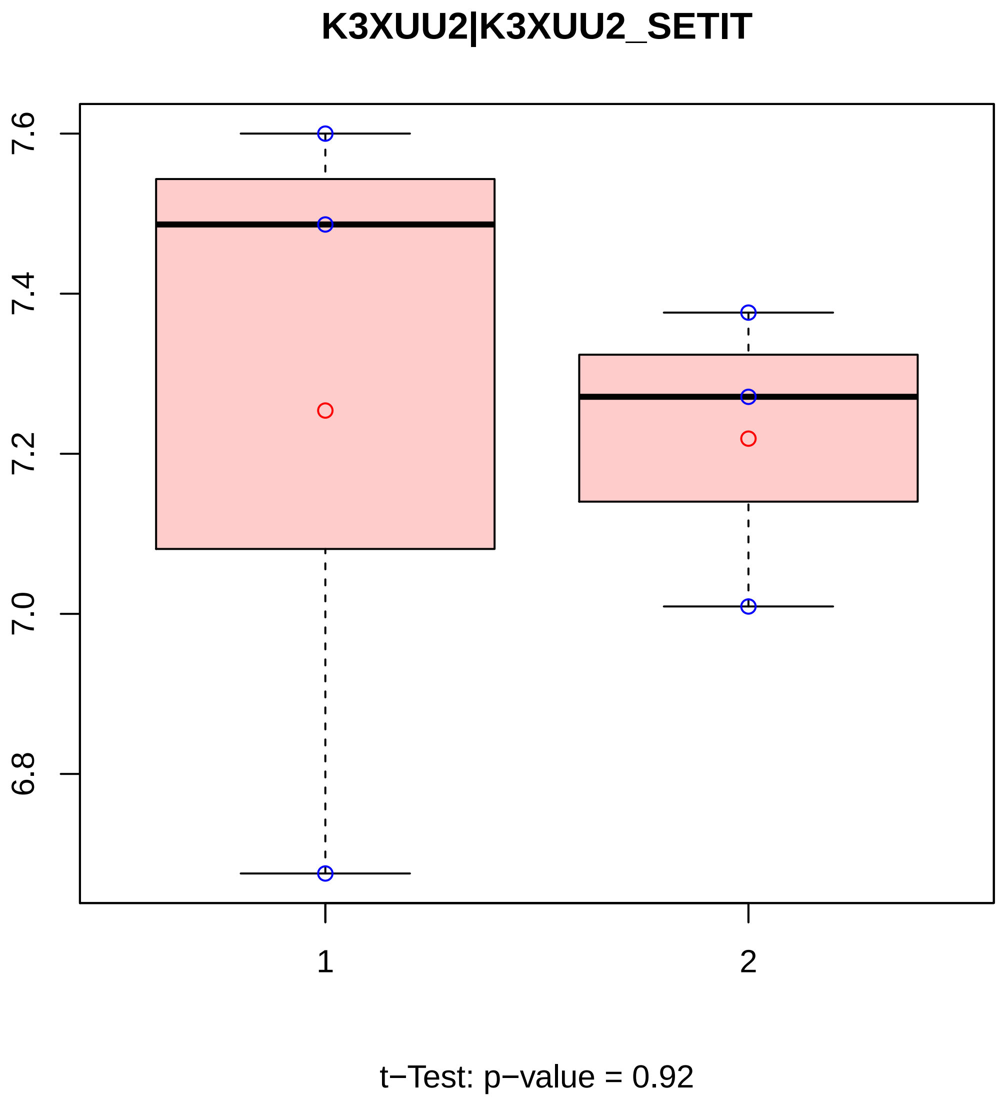
K3XUU2|K3XUU2_SETIT
7.6
7.4
7.2
7.0
6.8
1
2
t−Test: p−value = 0.92

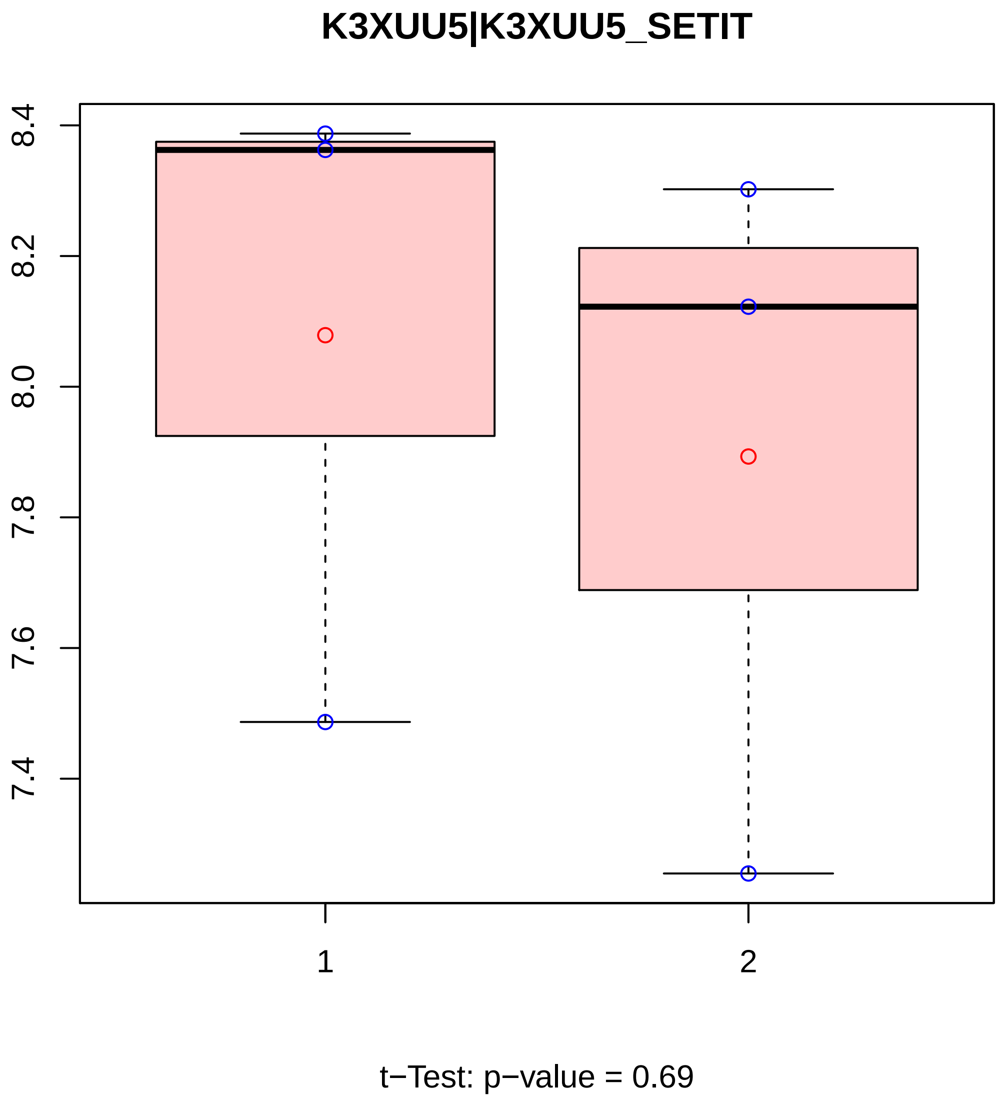
K3XUU5|K3XUU5_SETIT
8.4
8.2
8.0
7.8
7.6
7.4
1
2
t−Test: p−value = 0.69

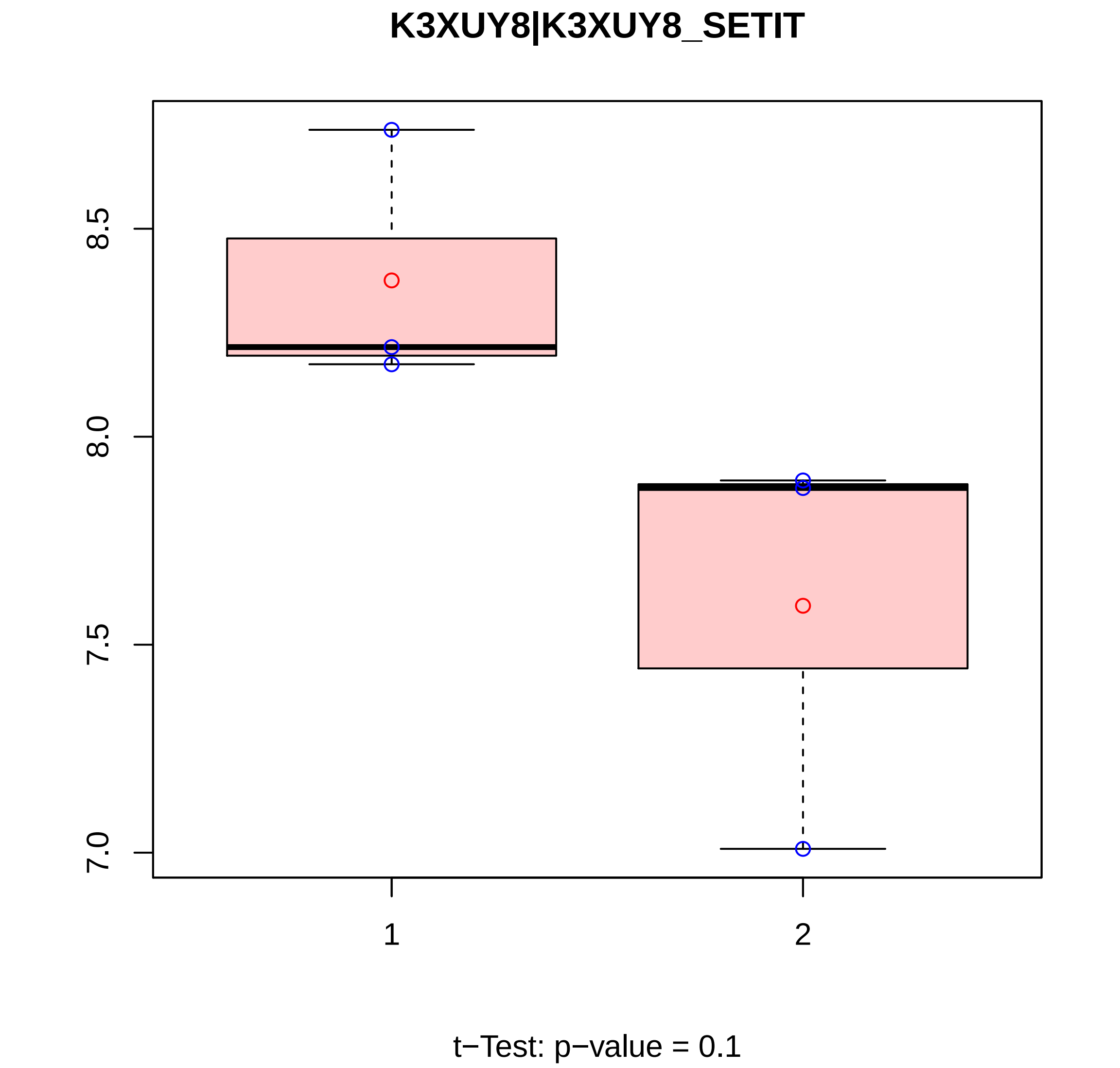
K3XUY8|K3XUY8_SETIT
8.5
8.0
7.5
7.0
1
2
t−Test: p−value = 0.1

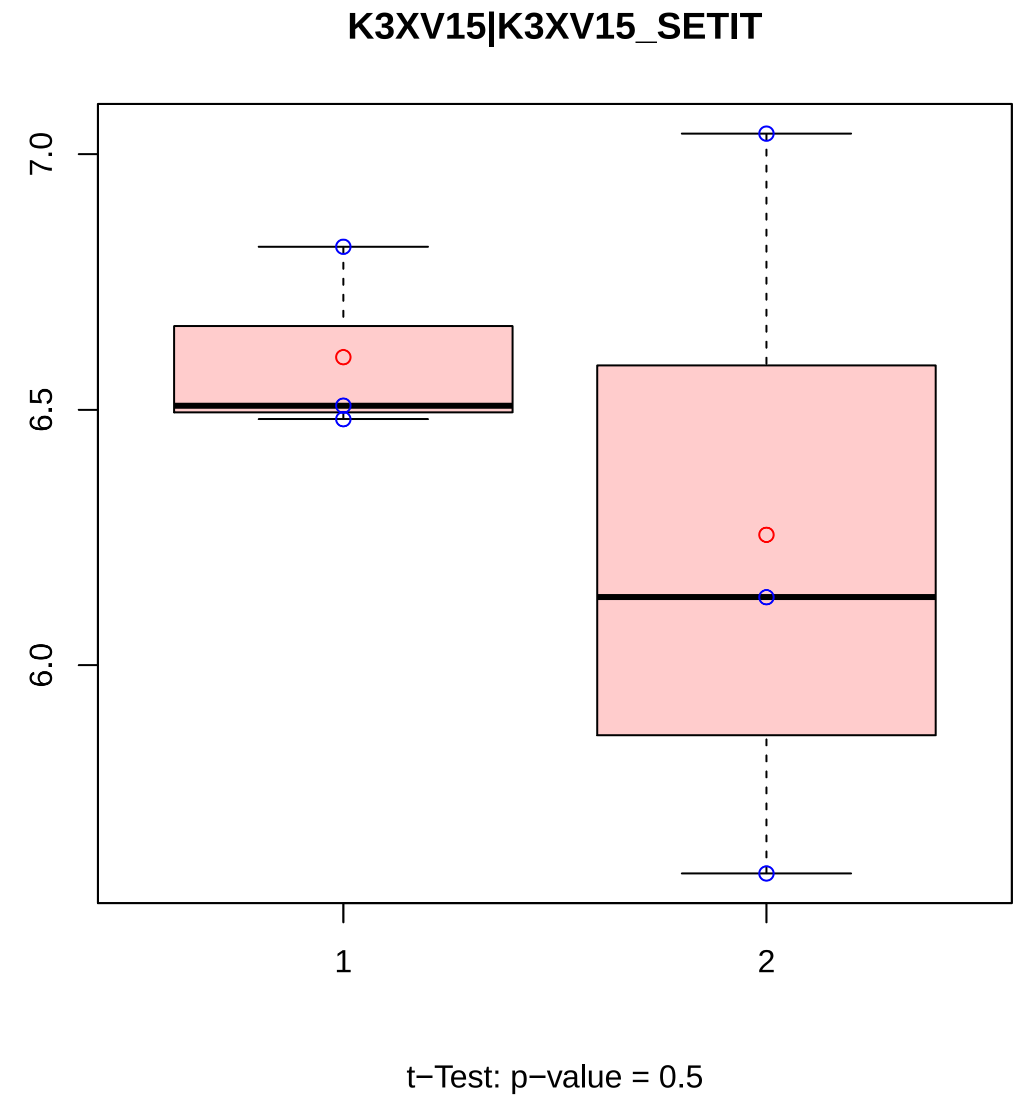
K3XV15|K3XV15_SETIT
7.0
6.5
6.0
1
2
t−Test: p−value = 0.5

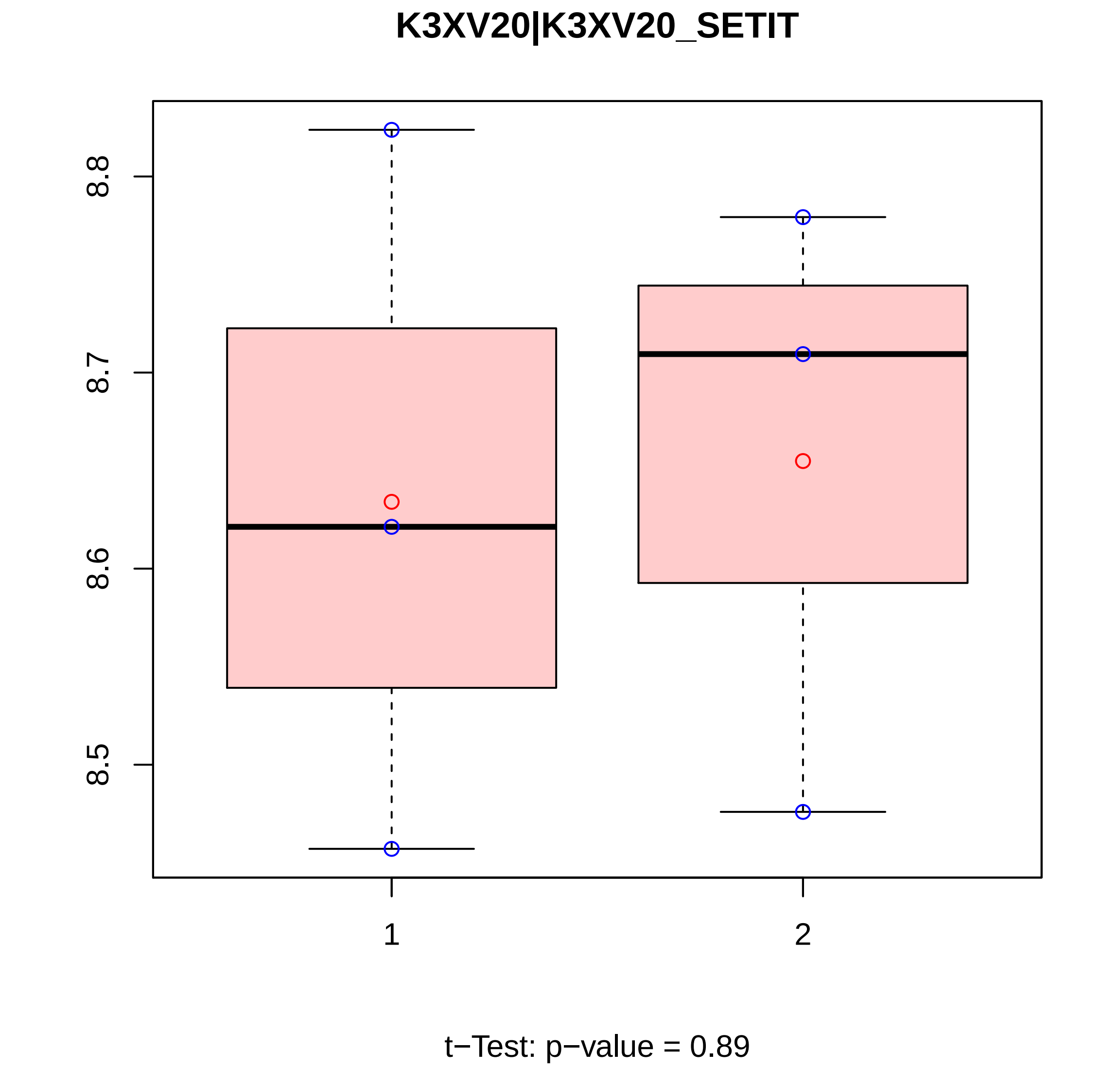
K3XV20|K3XV20_SETIT
8.5
8.6
8.7
8.8
1
2
t−Test: p−value = 0.89

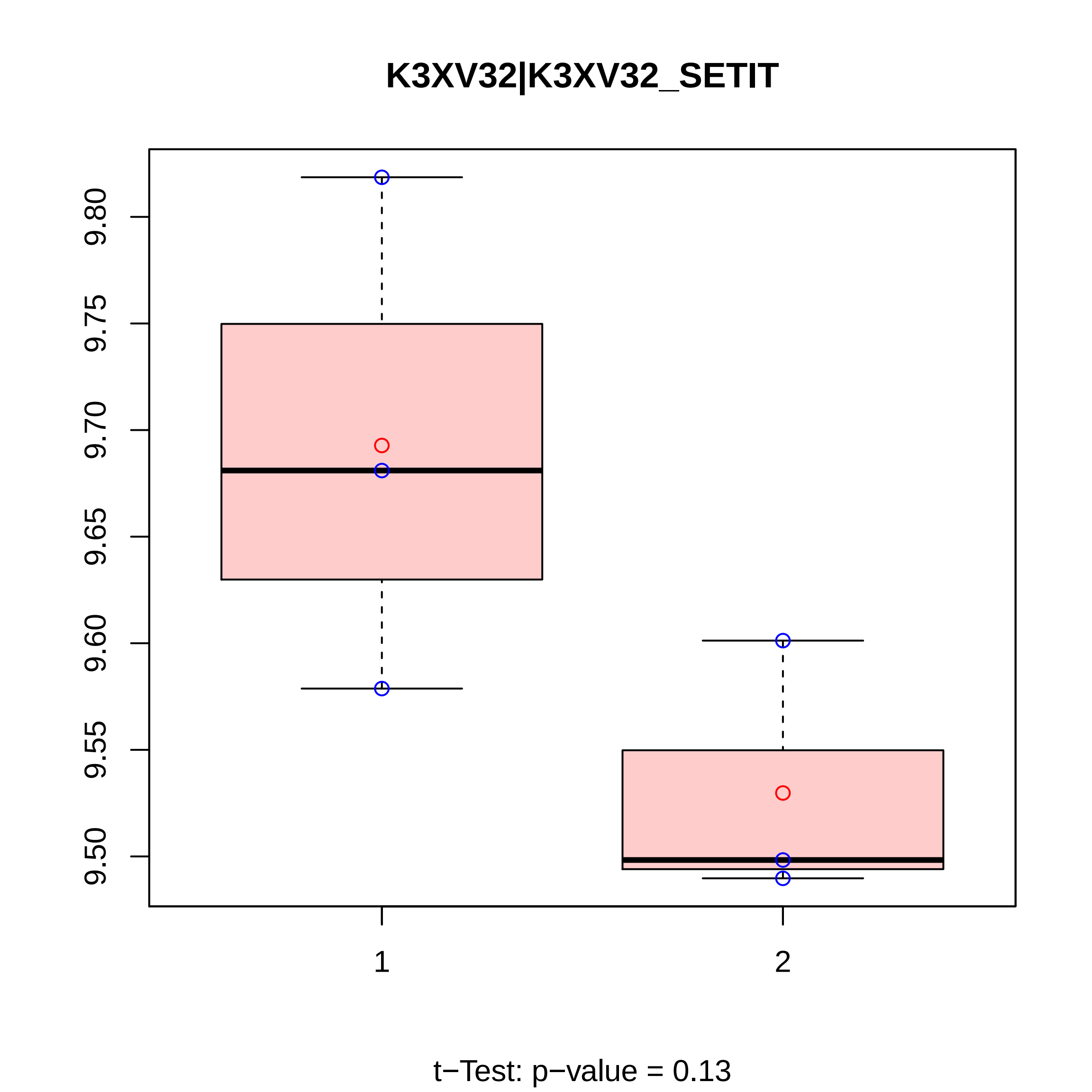
K3XV32|K3XV32_SETIT
9.80
9.75
9.70
9.65
9.60
9.55
9.50
1
2
t−Test: p−value = 0.13

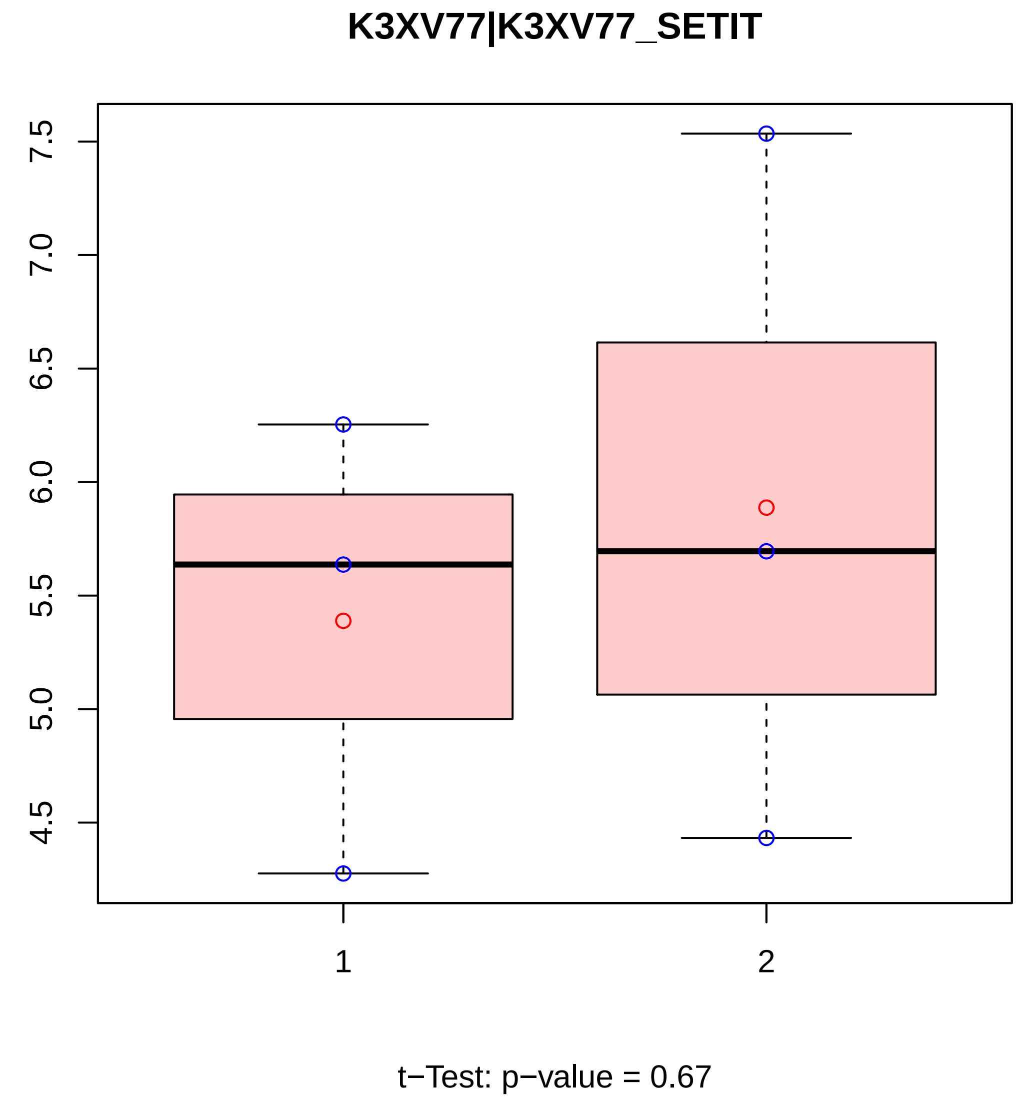
K3XV77|K3XV77_SETIT
7.5
7.0
6.5
6.0
5.5
5.0
4.5
1
2
t−Test: p−value = 0.67

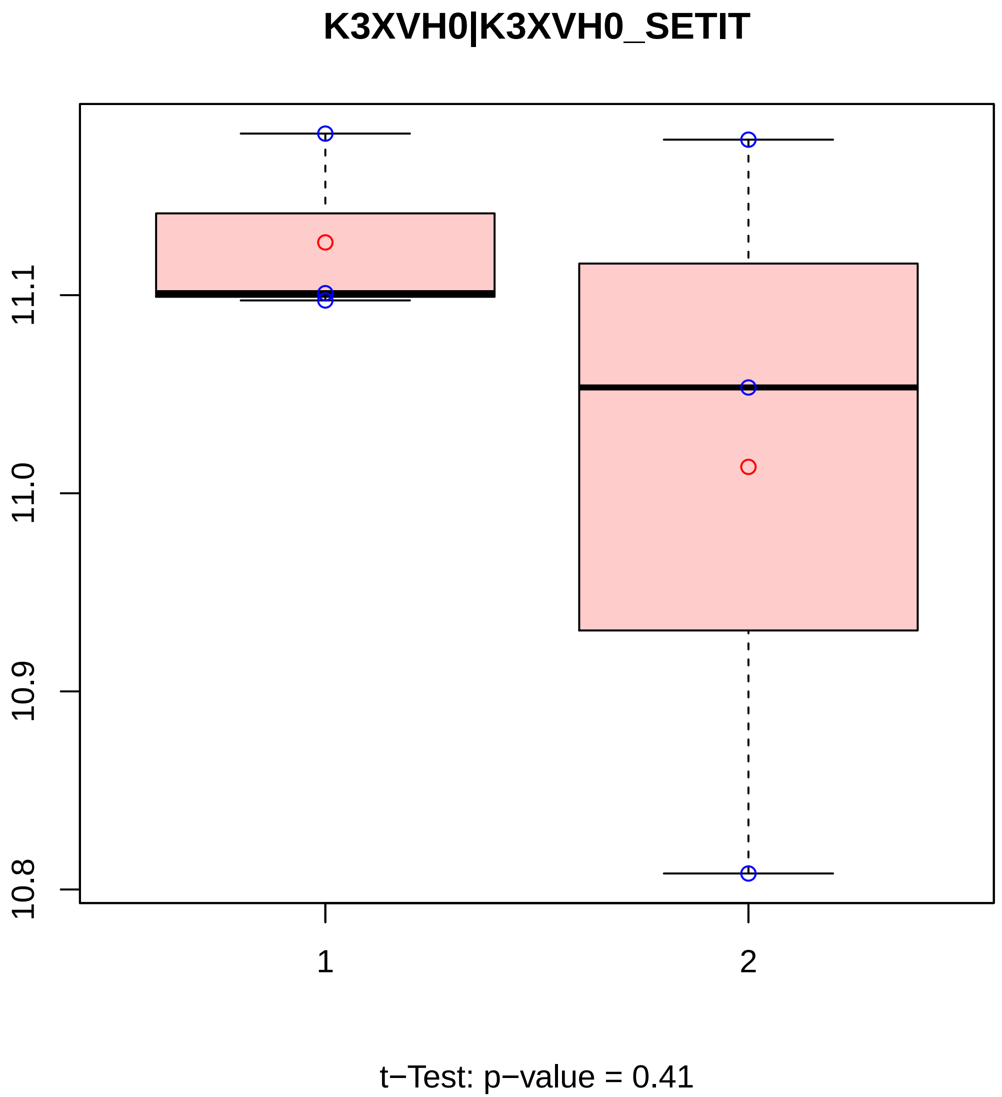
K3XVH0|K3XVH0_SETIT
11.1
11.0
10.9
10.8
1
2
t−Test: p−value = 0.41

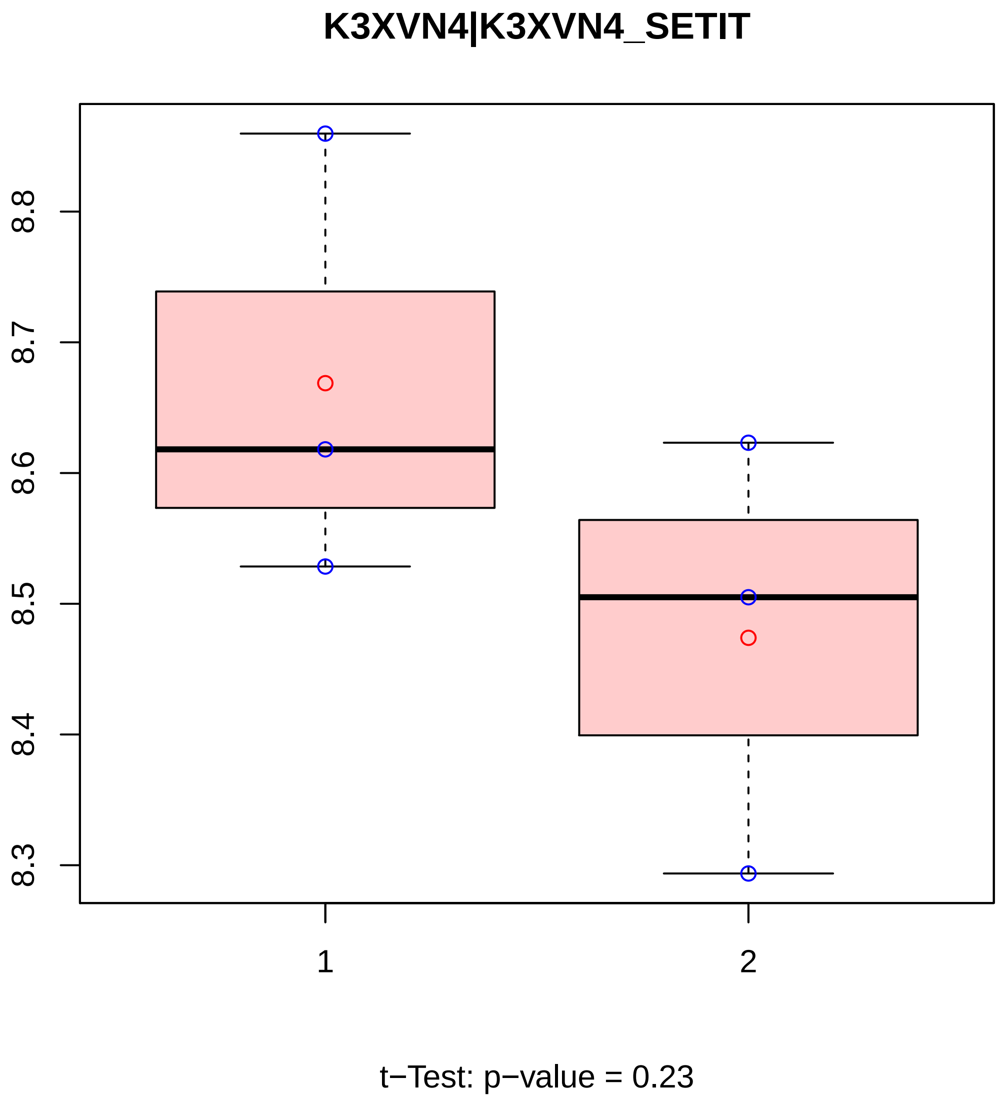
K3XVN4|K3XVN4_SETIT
8.3
8.4
8.5
8.6
8.7
8.8
1
2
t−Test: p−value = 0.23

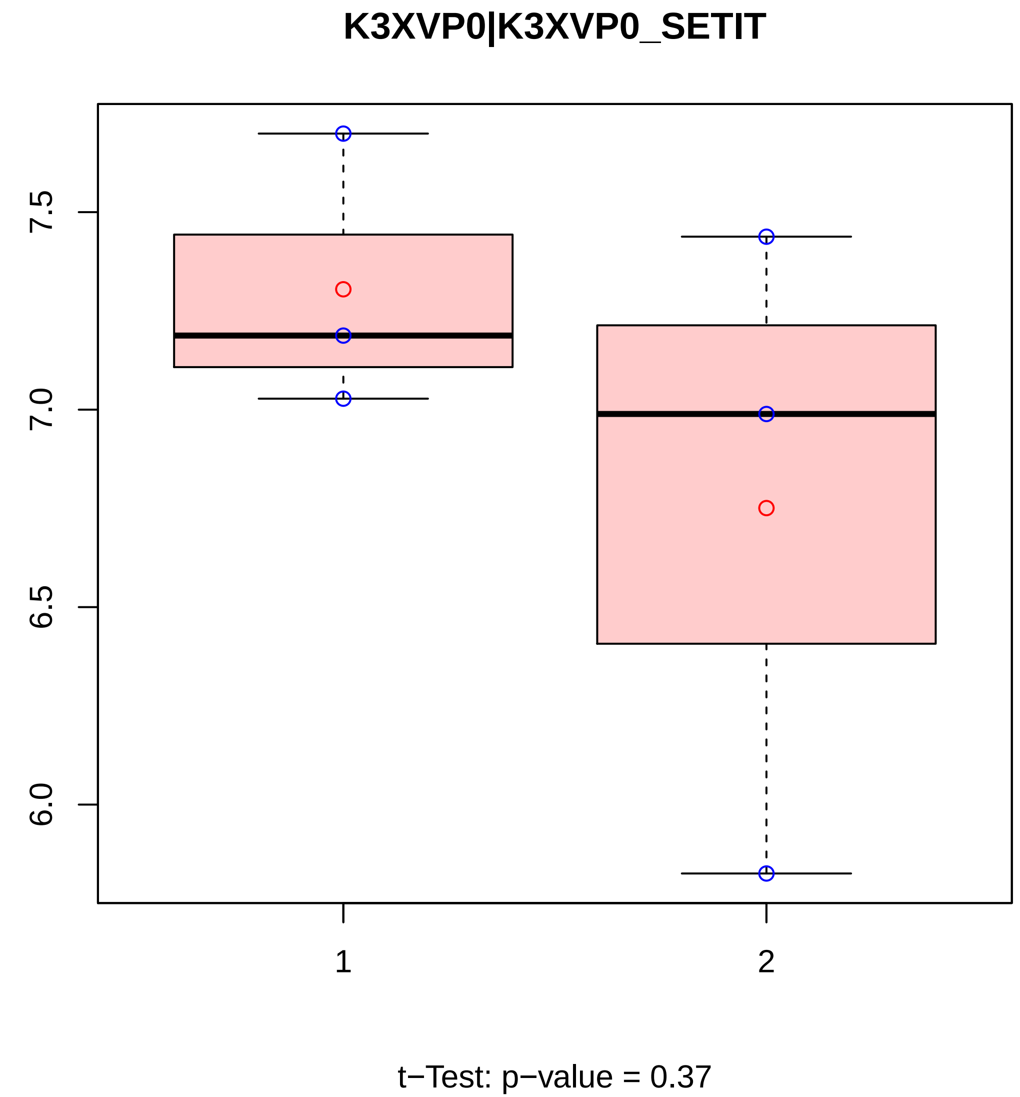

K3XVP0|K3XVP0_SETIT
7.5
7.0
6.5
6.0
1
2
t−Test: p−value = 0.37

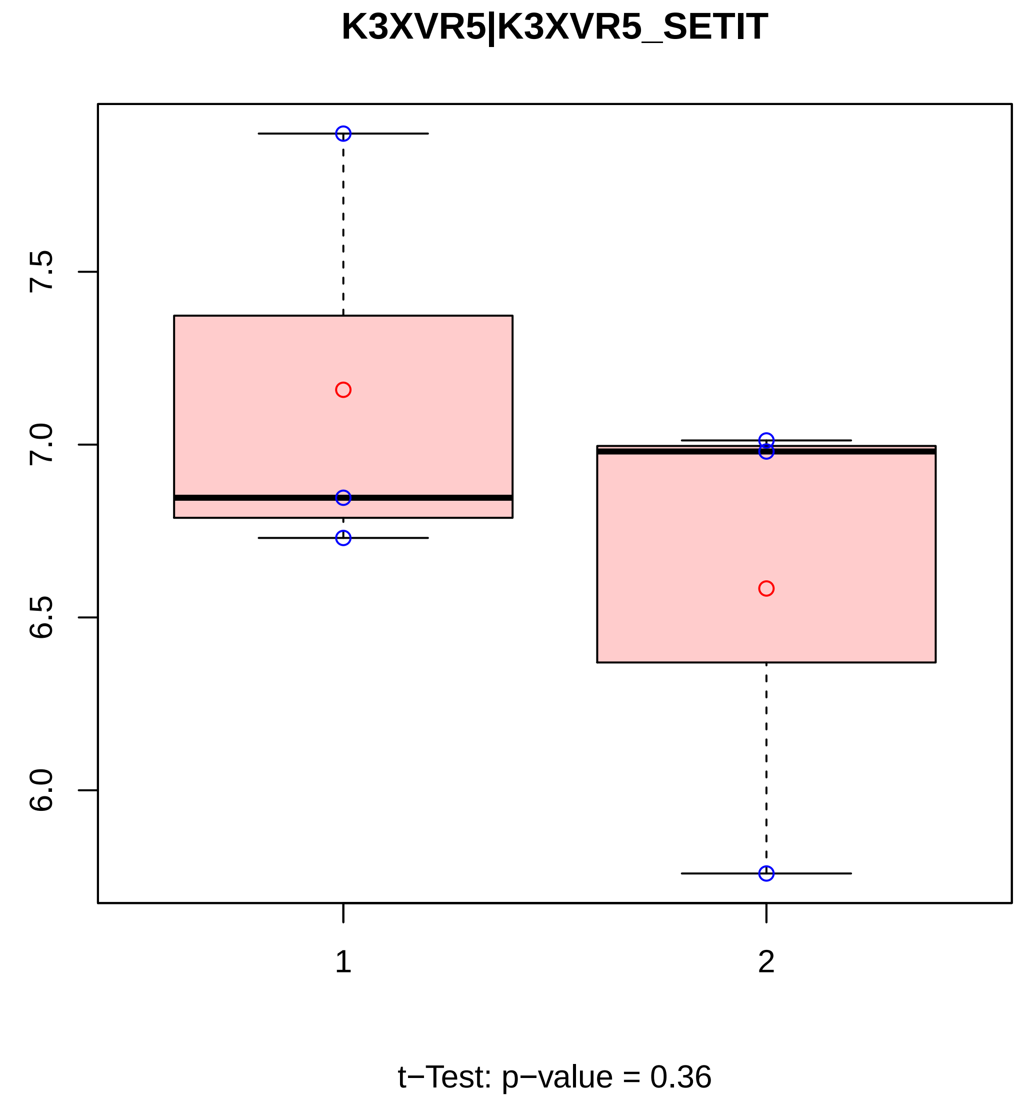
K3XVR5|K3XVR5_SETIT
7.5
7.0
6.5
6.0
1
2
t−Test: p−value = 0.36

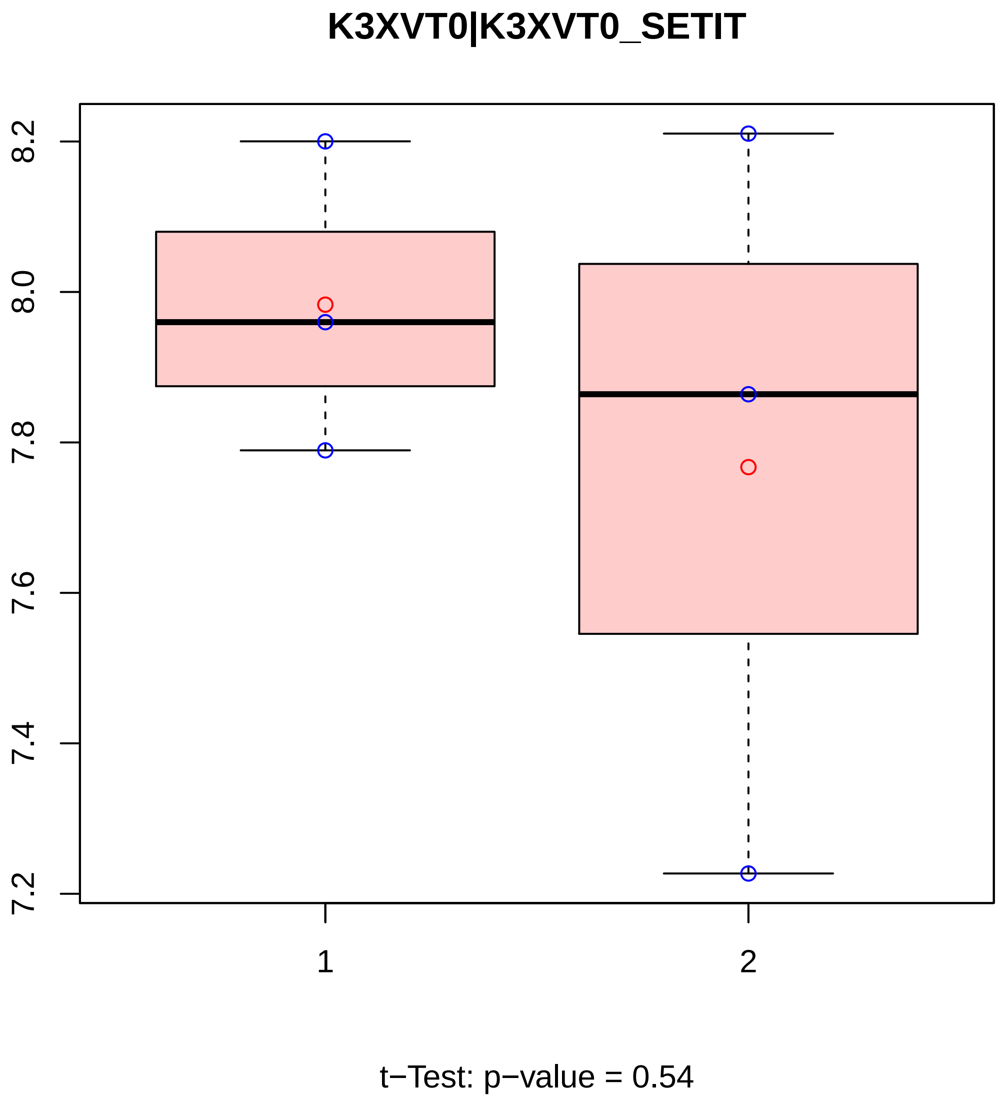
K3XVT0|K3XVT0_SETIT
8.2
8.0
7.8
7.6
7.4
7.2
1
2
t−Test: p−value = 0.54

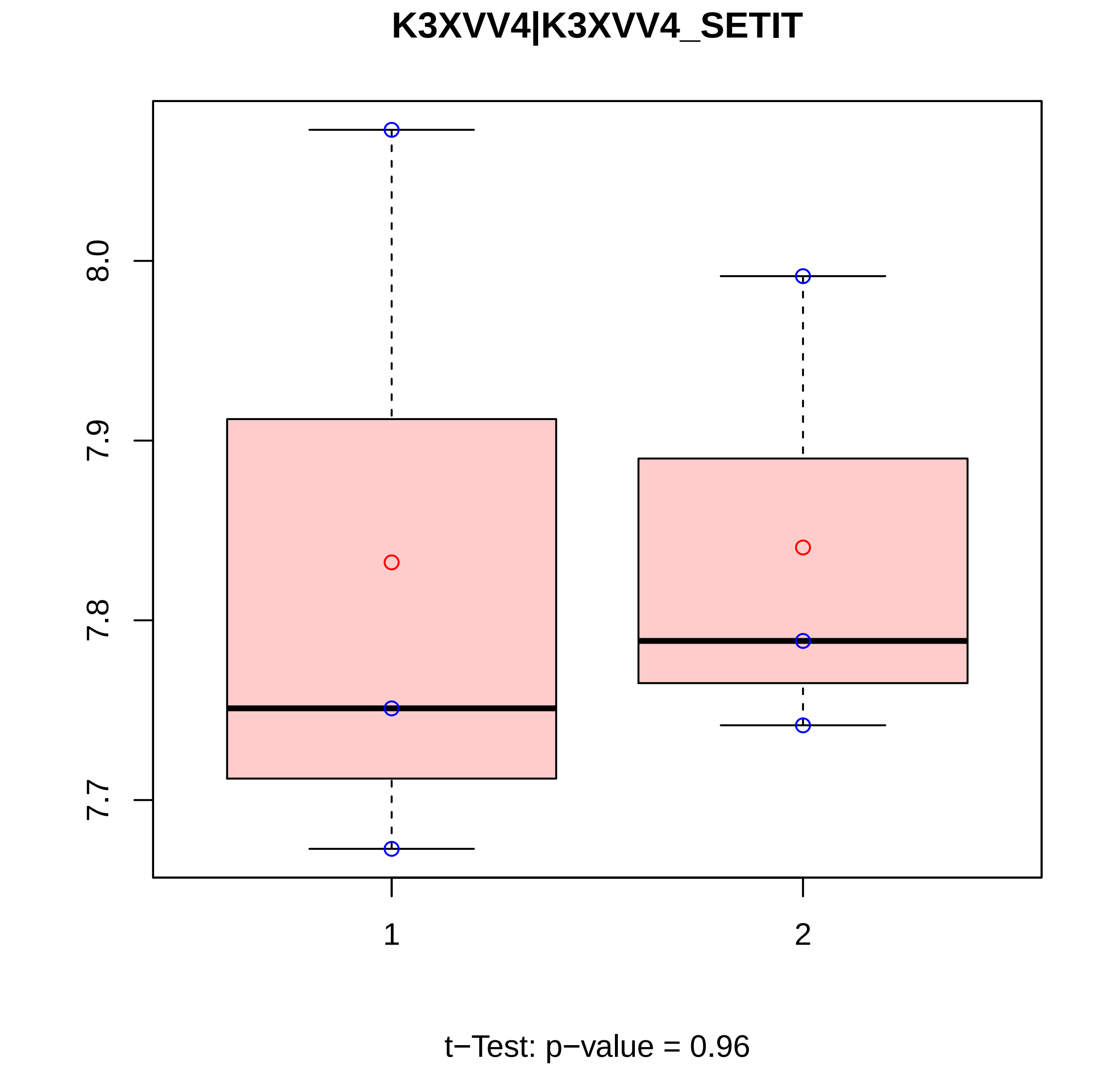
K3XVV4|K3XVV4_SETIT
8.0
7.9
7.8
7.7
1
2
t−Test: p−value = 0.96

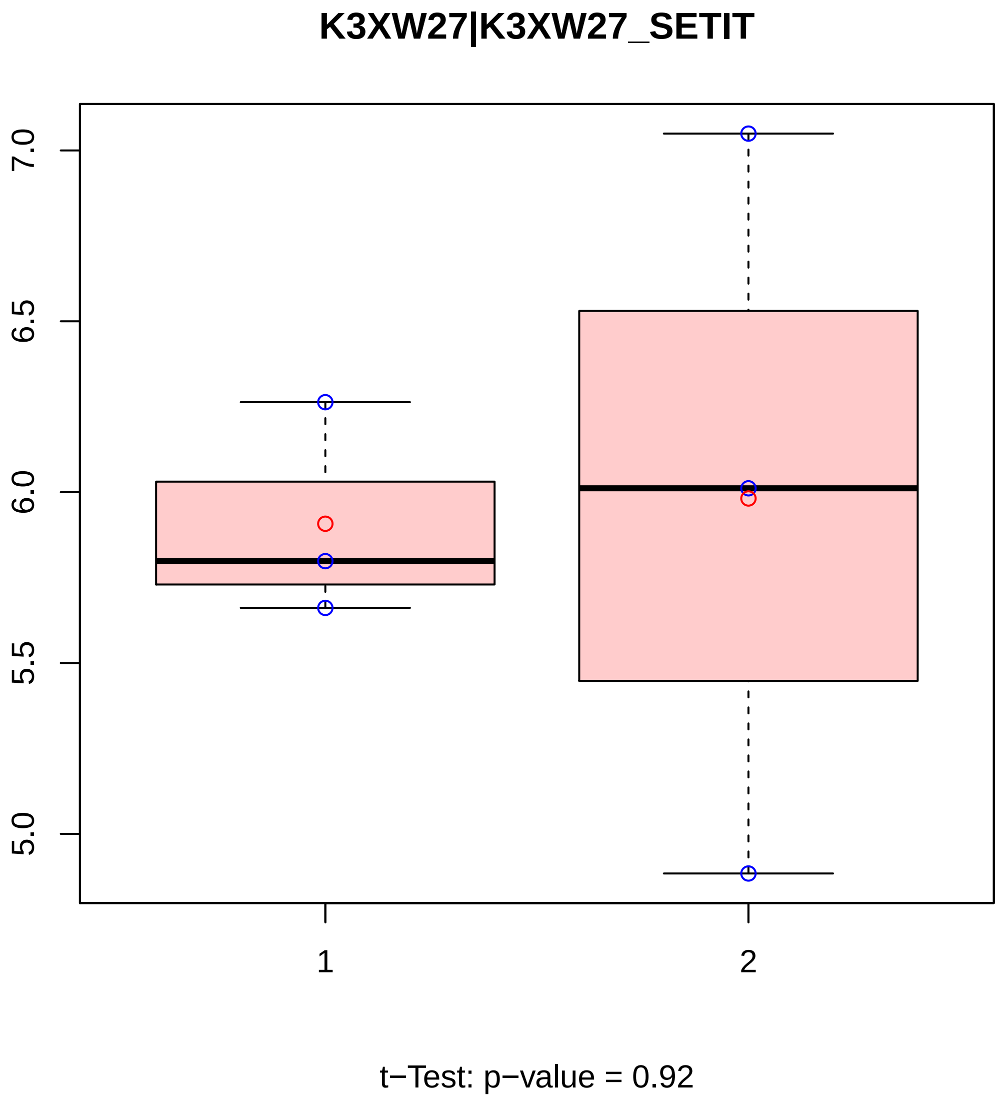
K3XW27|K3XW27_SETIT
7.0
6.5
6.0
5.5
5.0
1
2
t−Test: p−value = 0.92

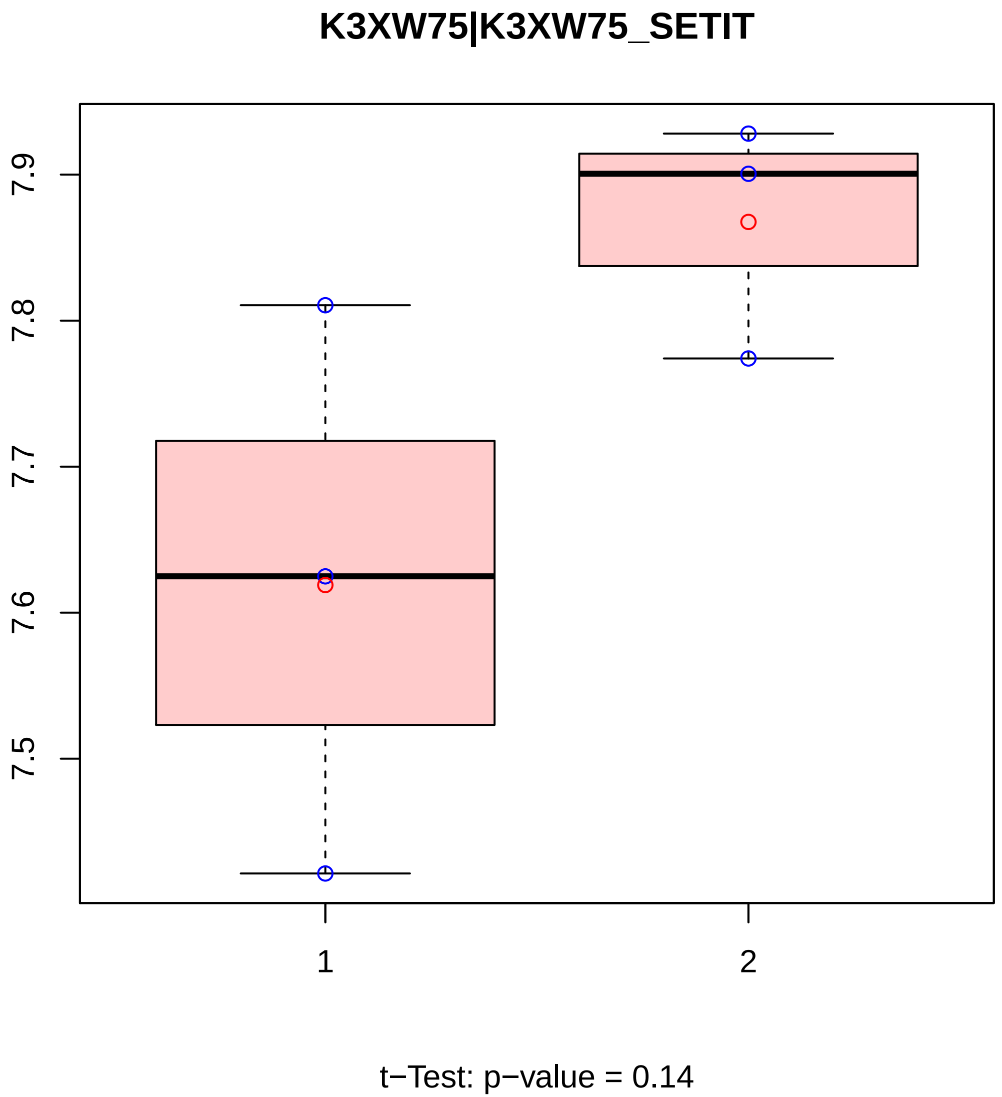

K3XW75|K3XW75_SETIT
7.5
7.6
7.7
7.8
7.9
1
2
t−Test: p−value = 0.14

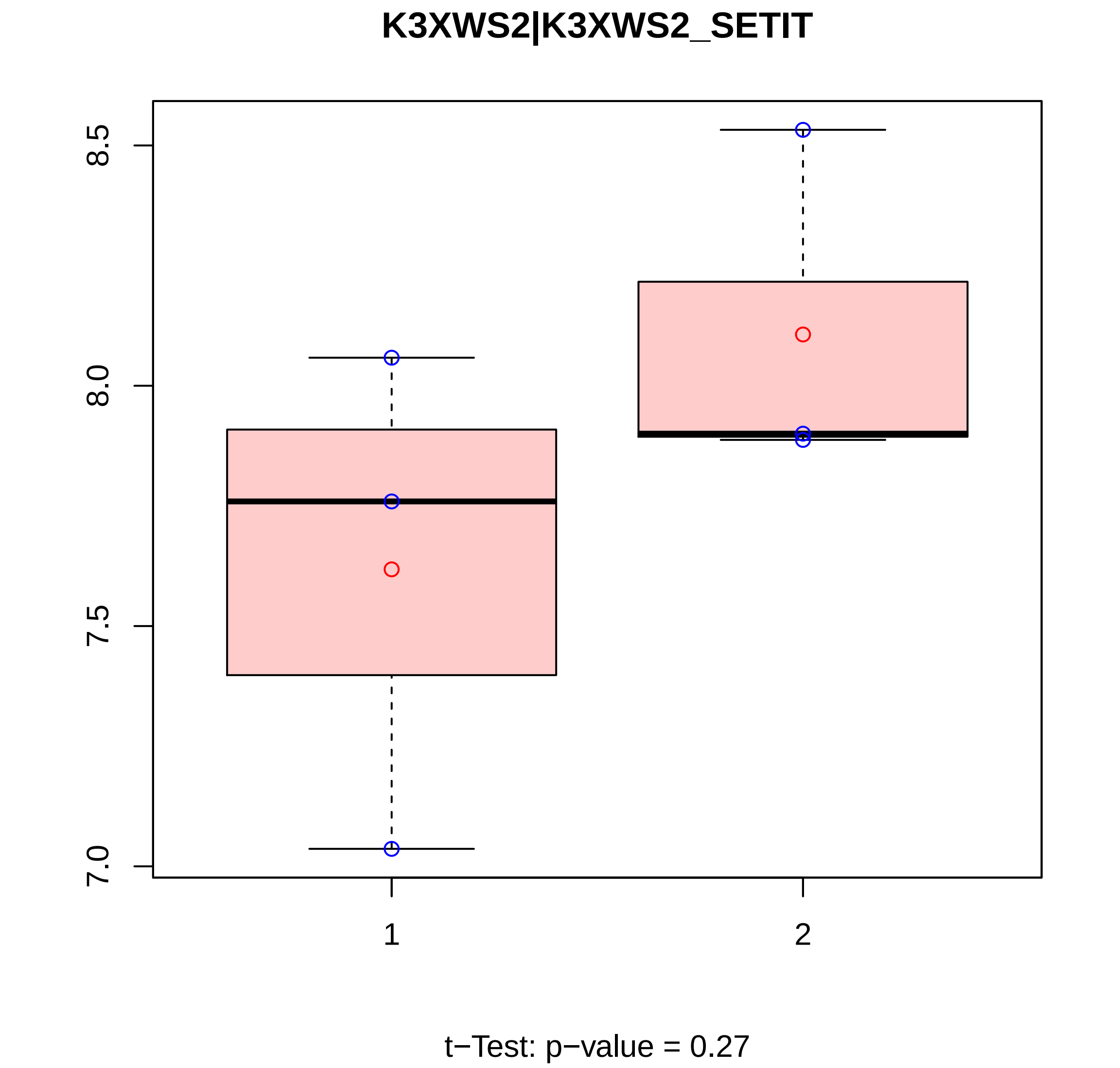
K3XWS2|K3XWS2_SETIT
8.5
8.0
7.5
7.0
1
2
t−Test: p−value = 0.27

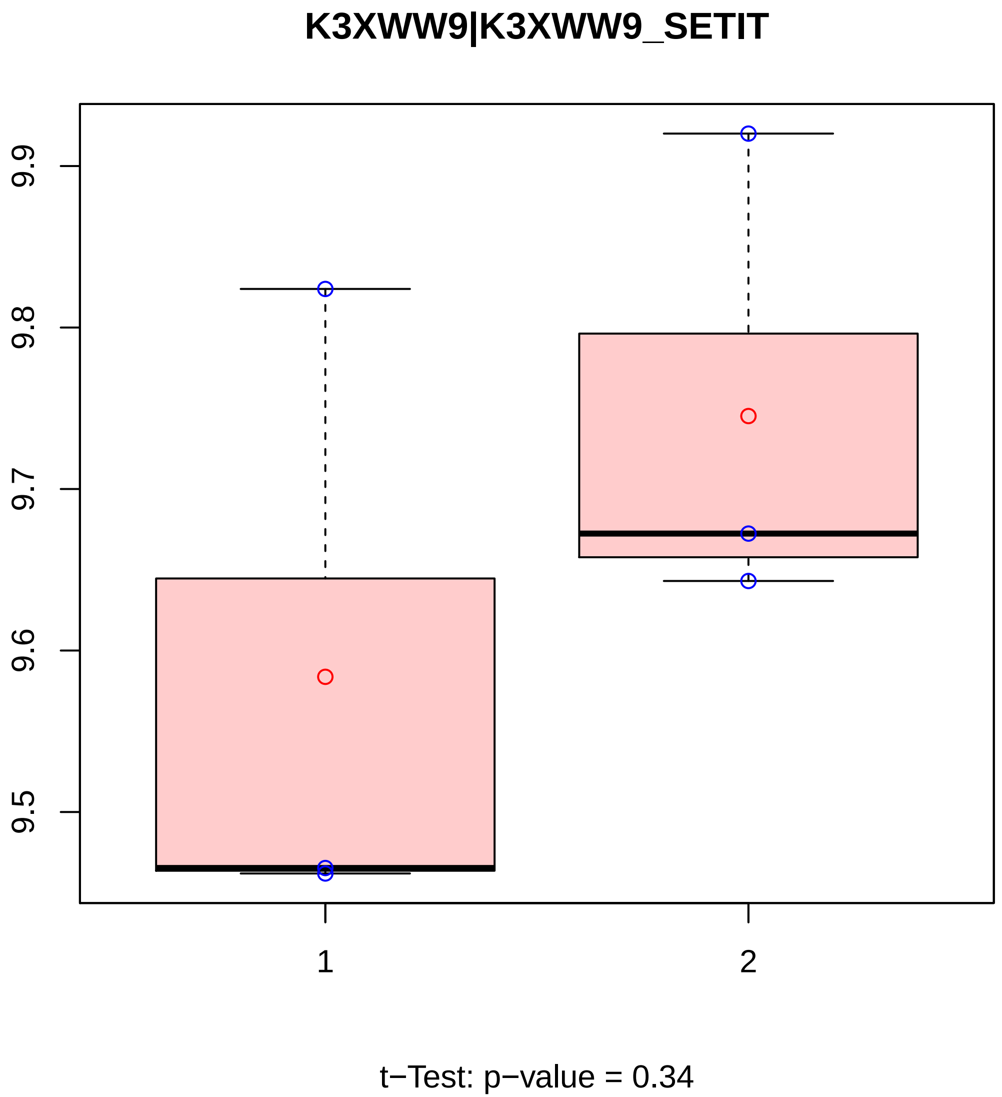
K3XWW9|K3XWW9_SETIT
9.5
9.6
9.7
9.8
9.9
1
2
t−Test: p−value = 0.34

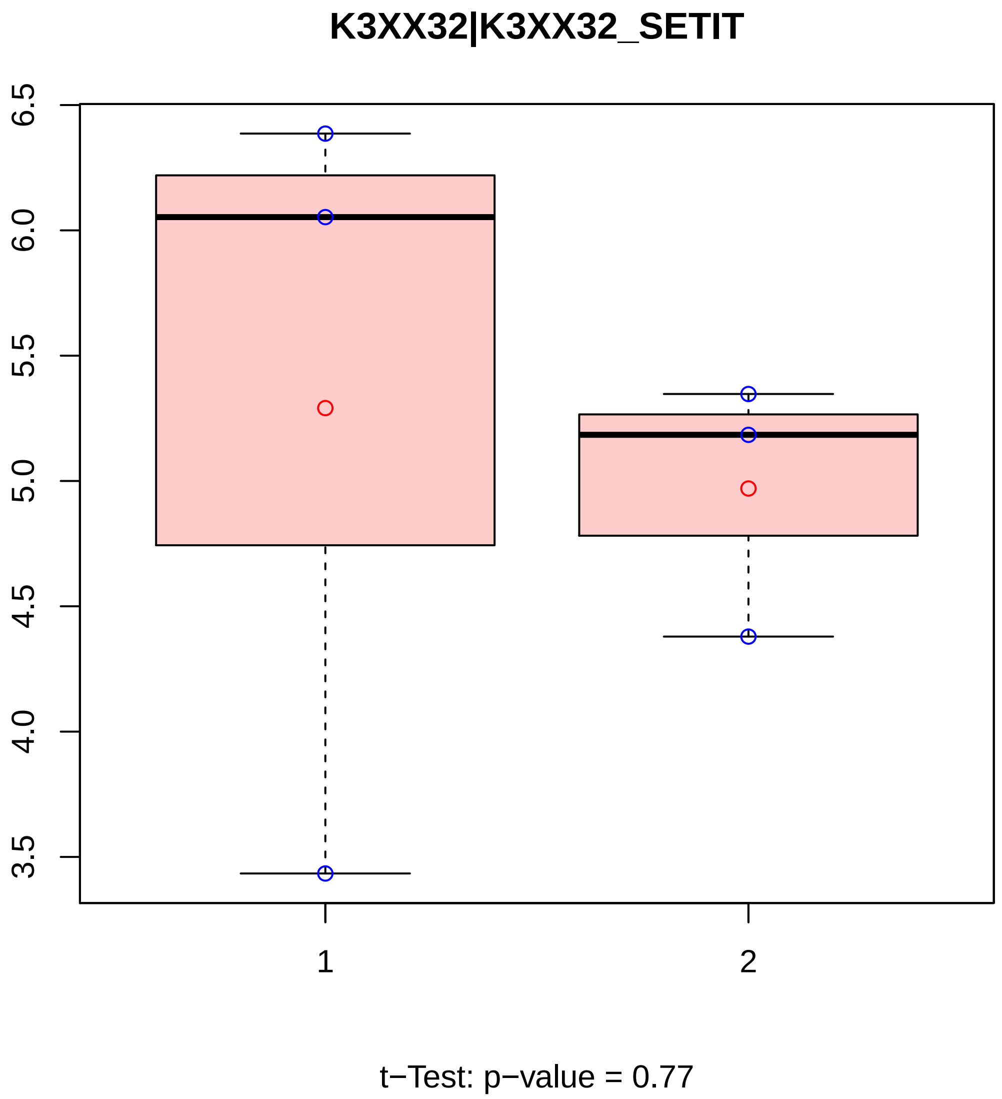
K3XX32|K3XX32_SETIT
6.5
6.0
5.5
5.0
4.5
4.0
3.5
1
2
t−Test: p−value = 0.77

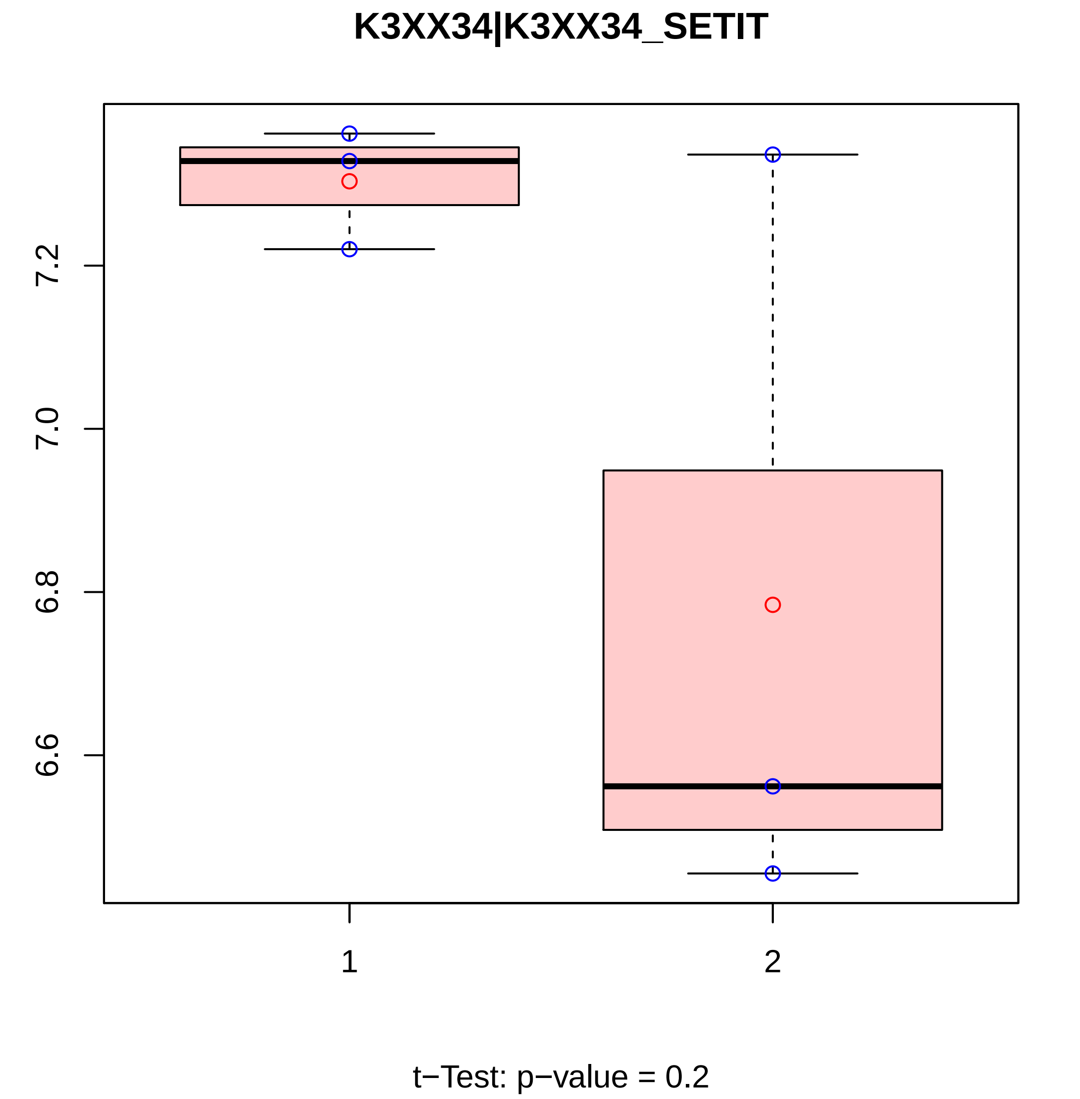
K3XX34|K3XX34_SETIT
7.2
7.0
6.8
6.6
1
2
t−Test: p−value = 0.2

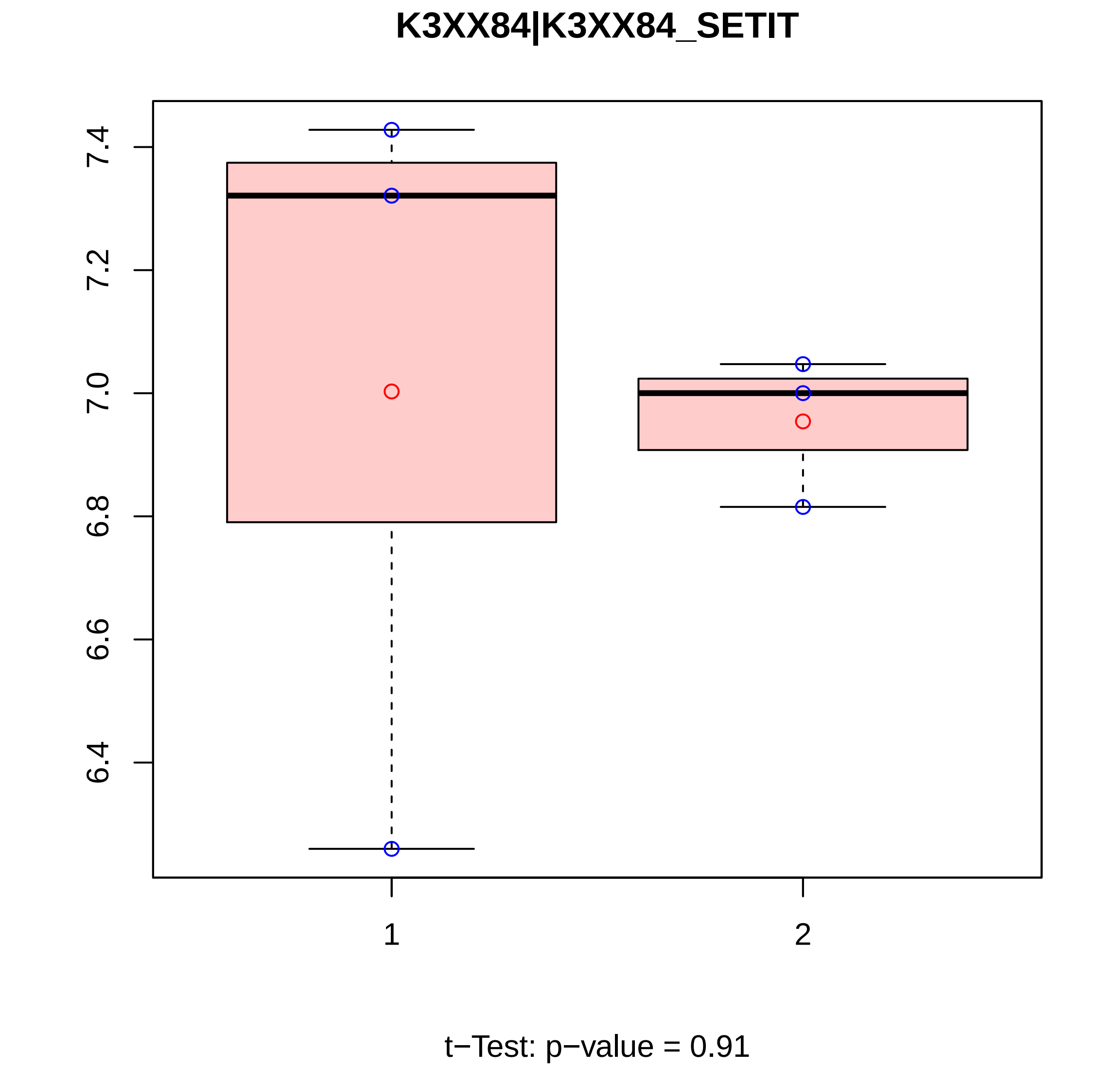
K3XX84|K3XX84_SETIT
7.4
7.2
7.0
6.8
6.6
6.4
1
2
t−Test: p−value = 0.91

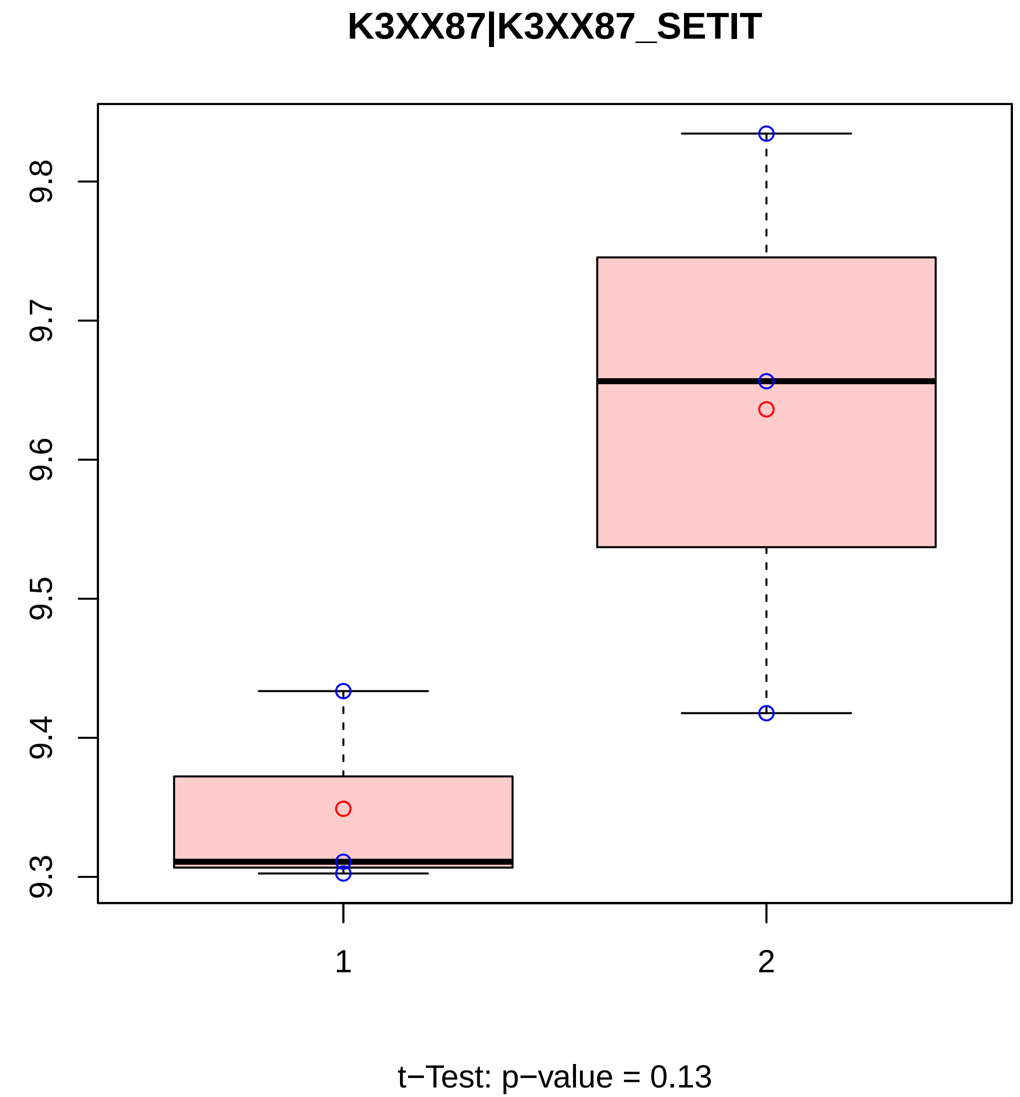
K3XX87|K3XX87_SETIT
9.3
9.4
9.5
9.6
9.7
9.8
1
2
t−Test: p−value = 0.13

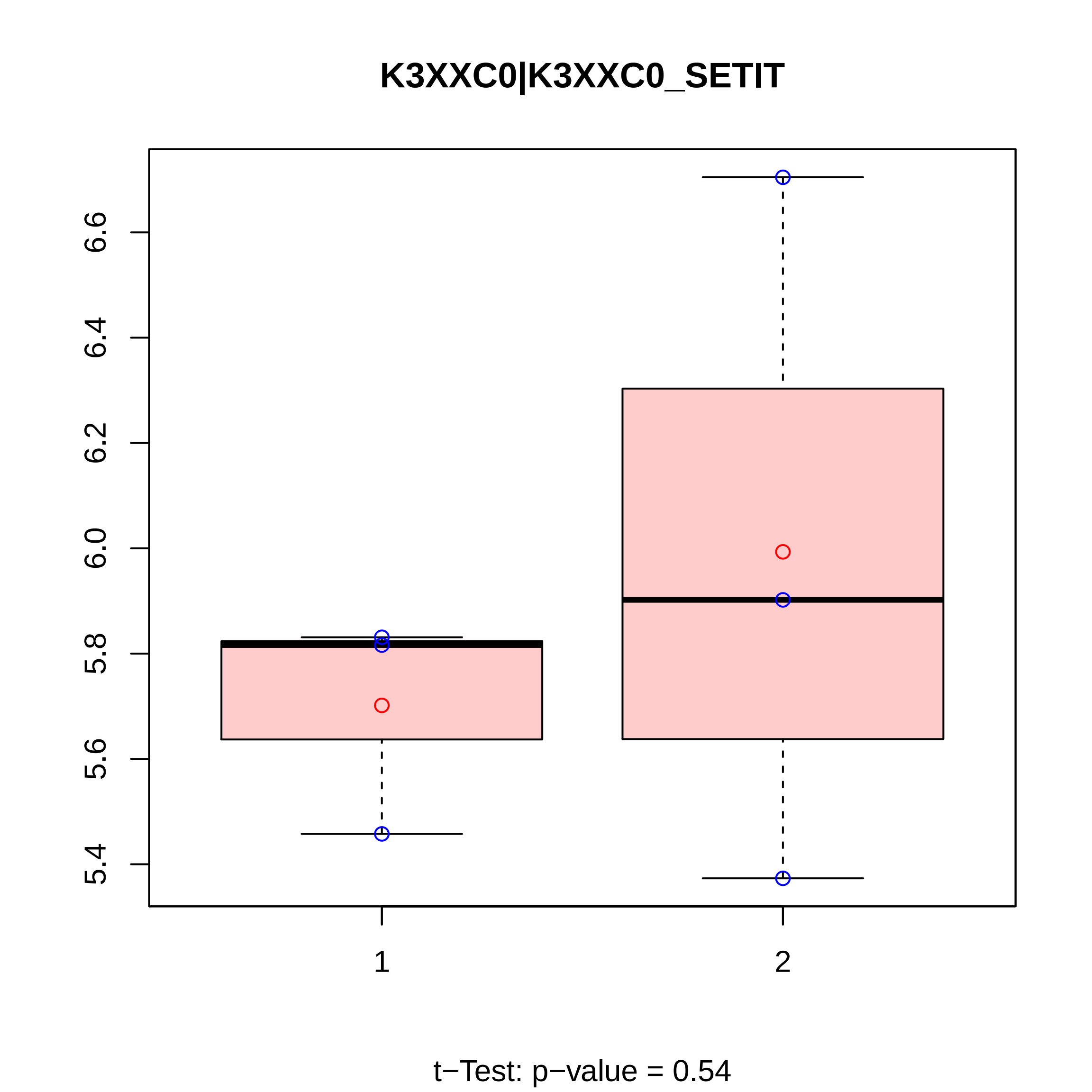

K3XXC0|K3XXC0_SETIT
6.6
6.4
6.2
6.0
5.8
5.6
5.4
1
2
t−Test: p−value = 0.54

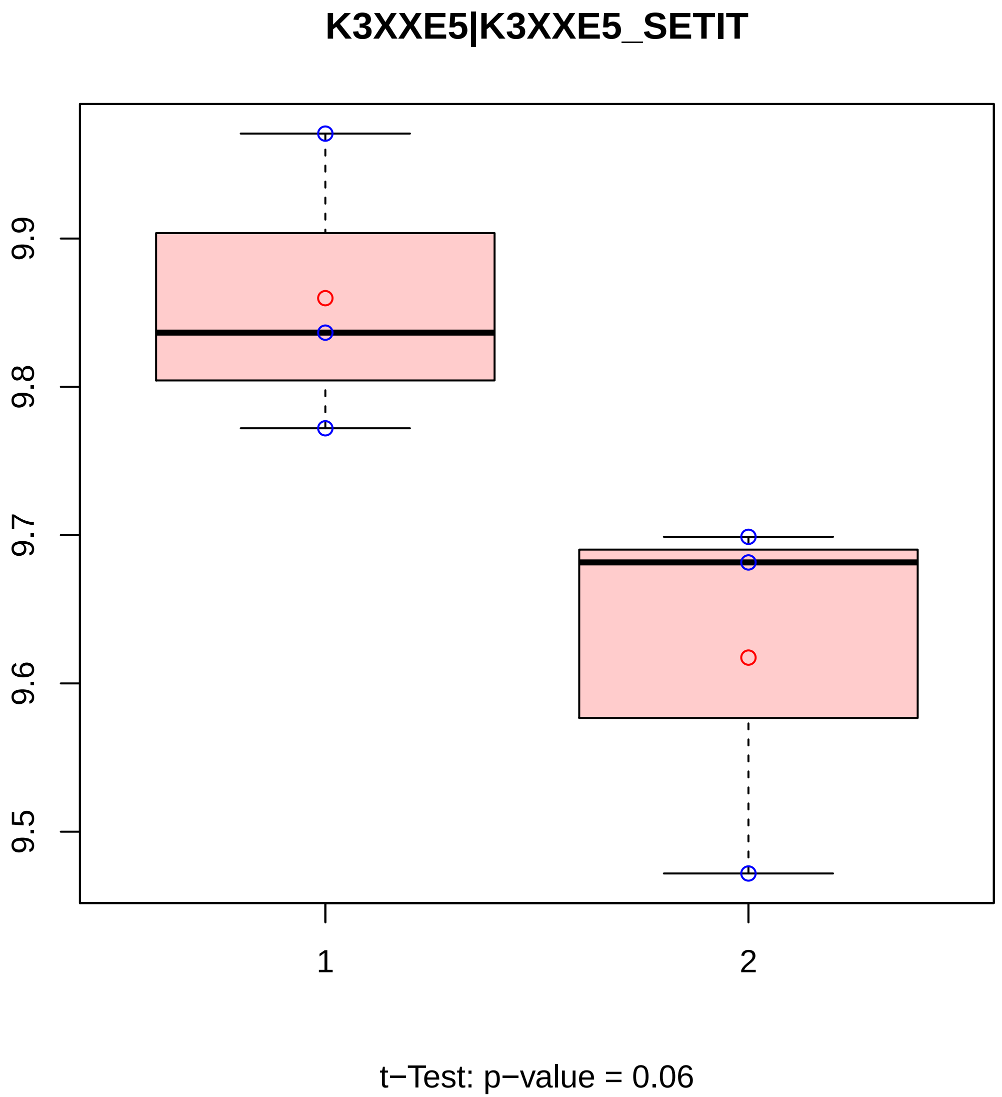
K3XXE5|K3XXE5_SETIT
9.5
9.6
9.7
9.8
9.9
1
2
t−Test: p−value = 0.06

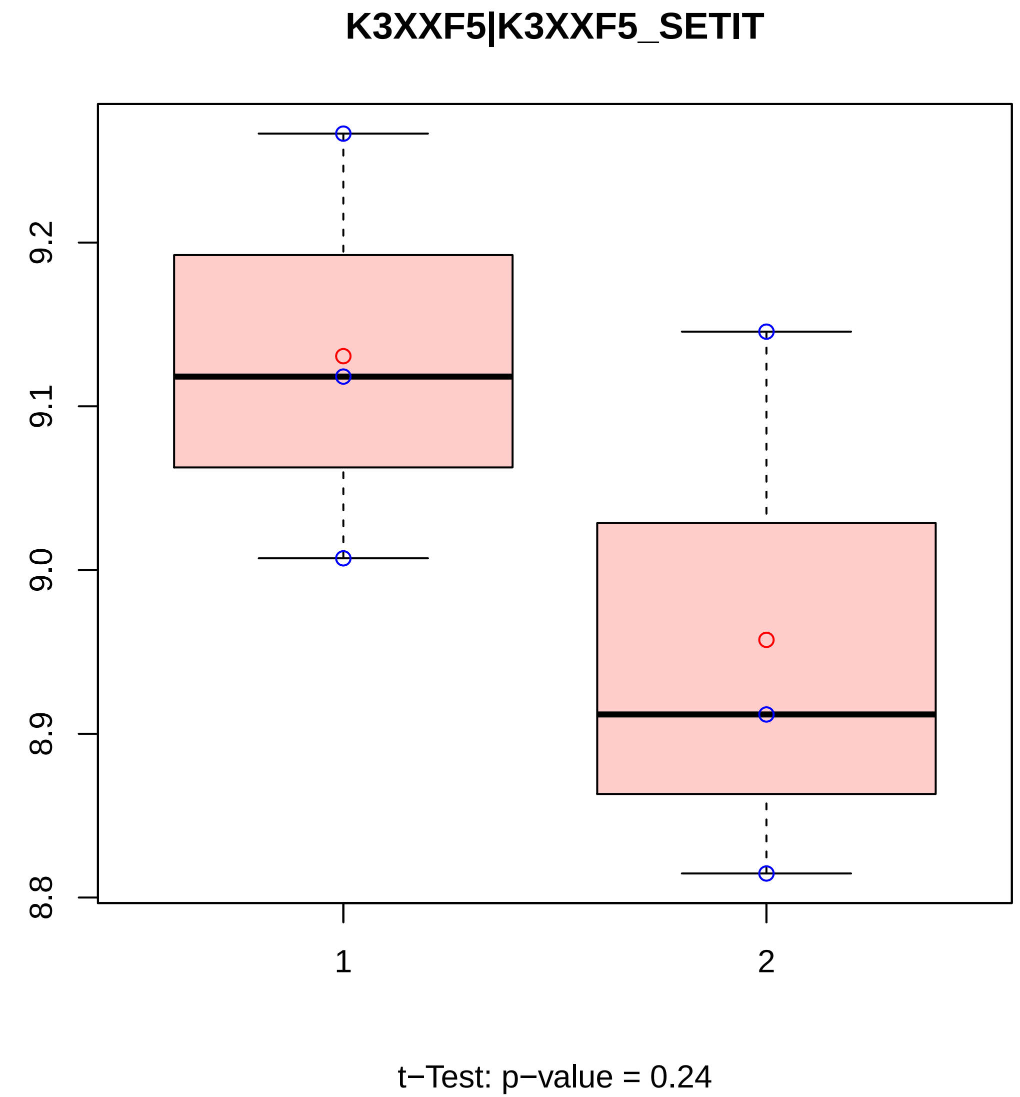
K3XXF5|K3XXF5_SETIT
9.2
9.1
9.0
8.9
8.8
1
2
t−Test: p−value = 0.24

K3XXN9|K3XXN9_SETIT

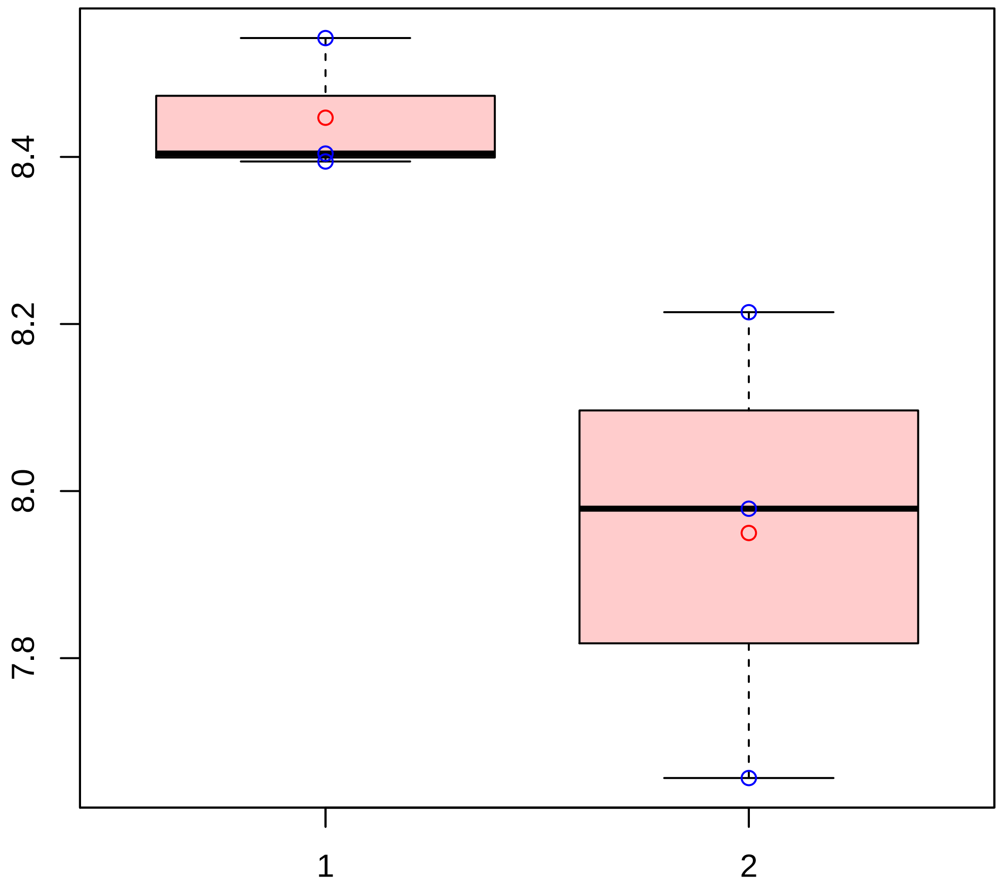

t−Test: p−value = 0.08

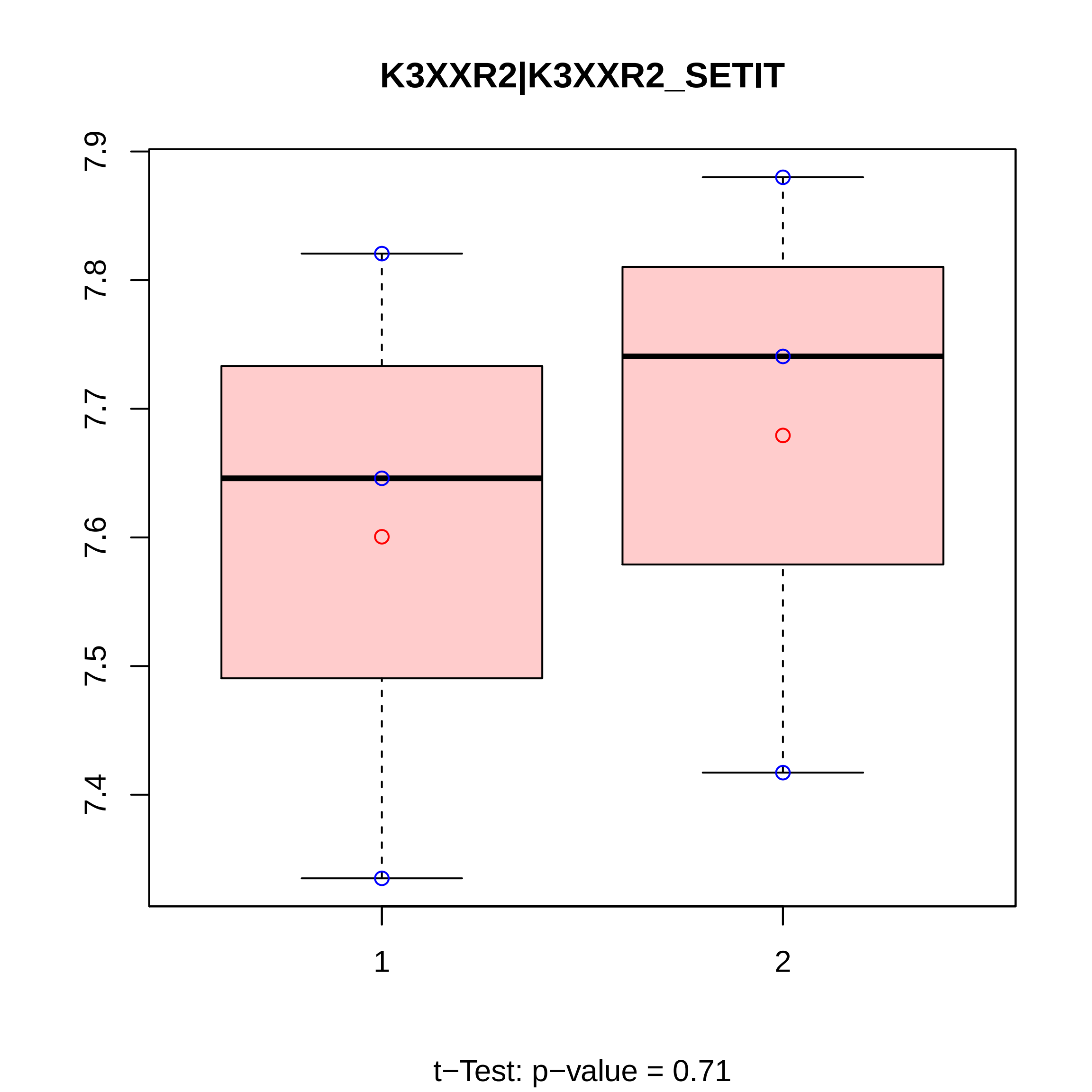
K3XXR2|K3XXR2_SETIT
7.9
7.8
7.7
7.6
7.5
7.4
1
2
t−Test: p−value = 0.71

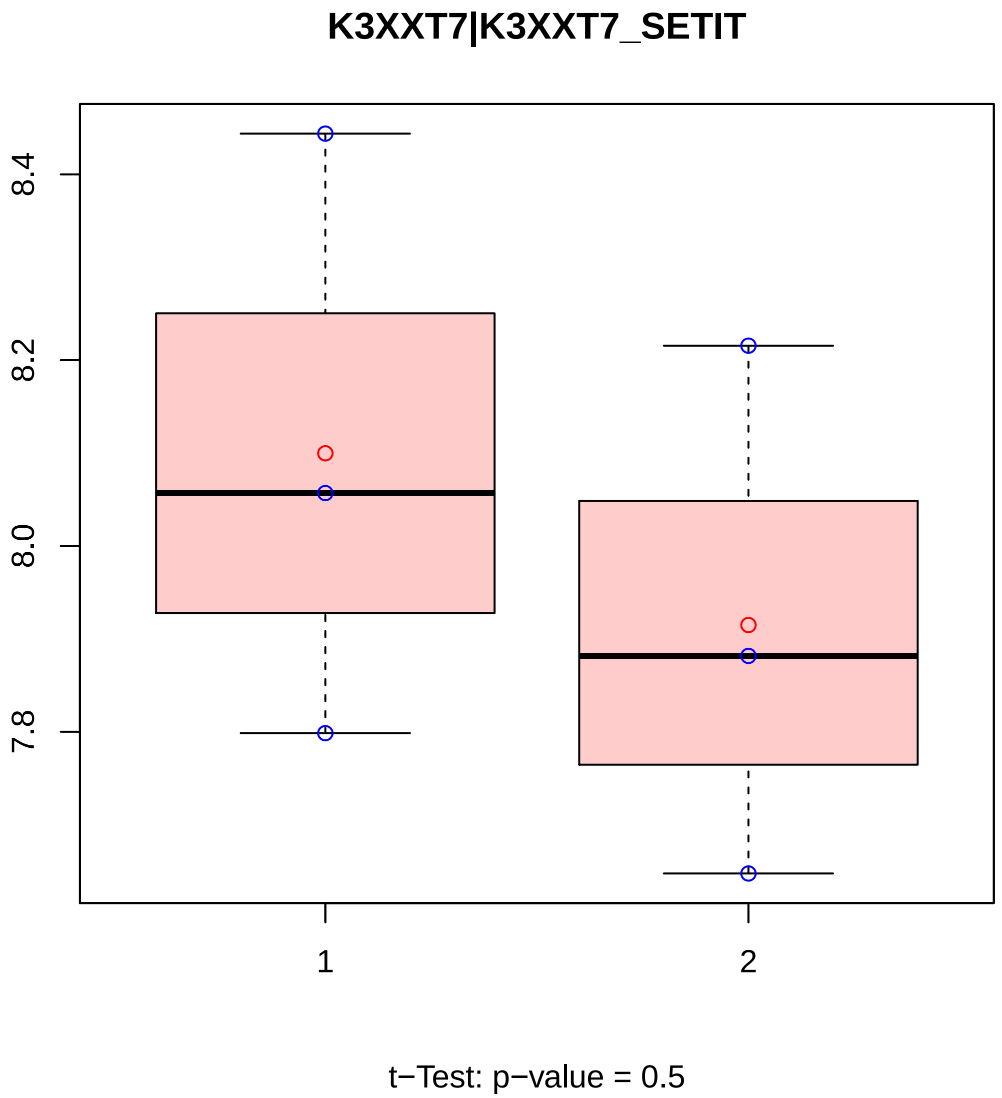
K3XXT7|K3XXT7_SETIT
8.4
8.2
8.0
7.8
1
2
t−Test: p−value = 0.5

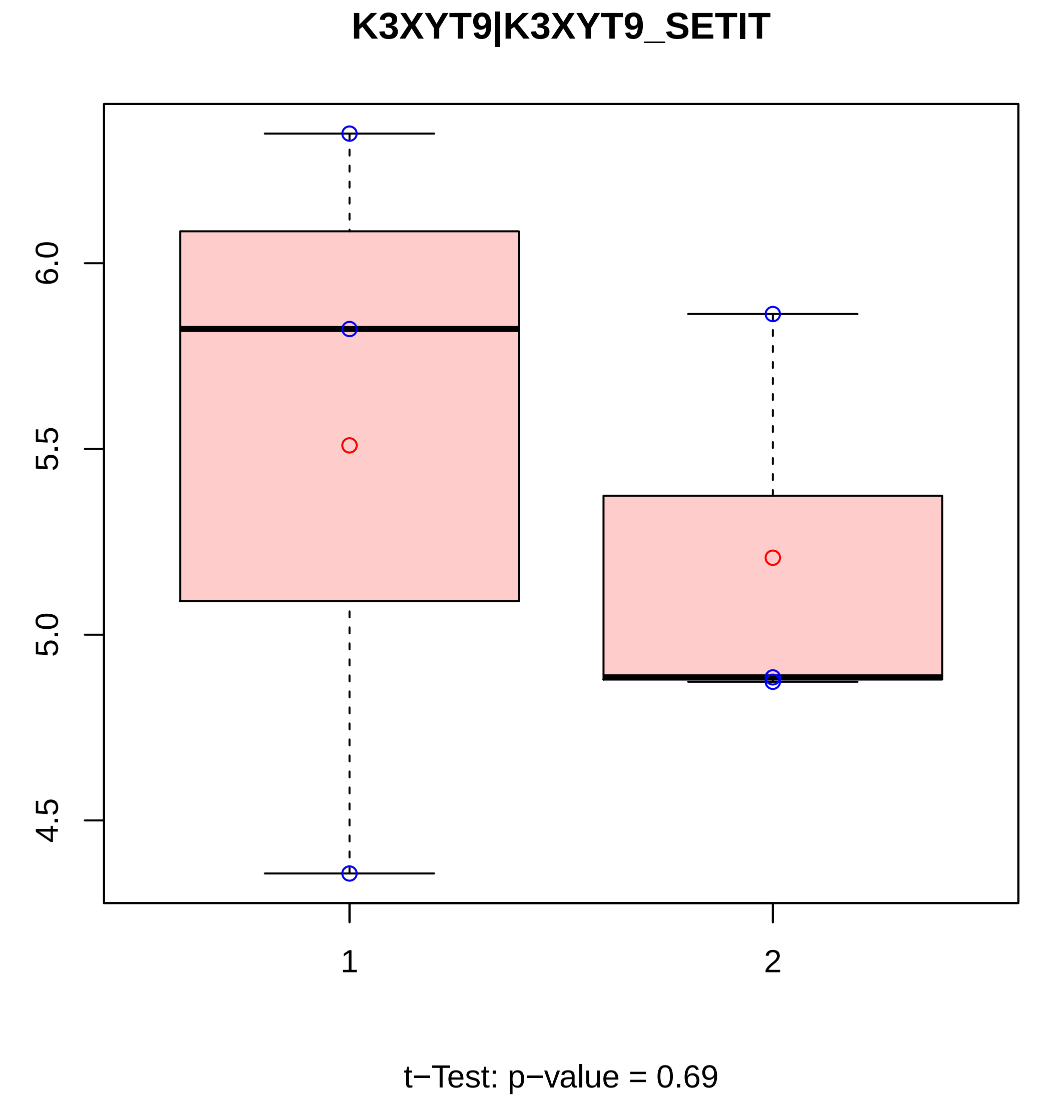
K3XYT9|K3XYT9_SETIT
6.0
5.5
5.0
4.5
1
2
t−Test: p−value = 0.69

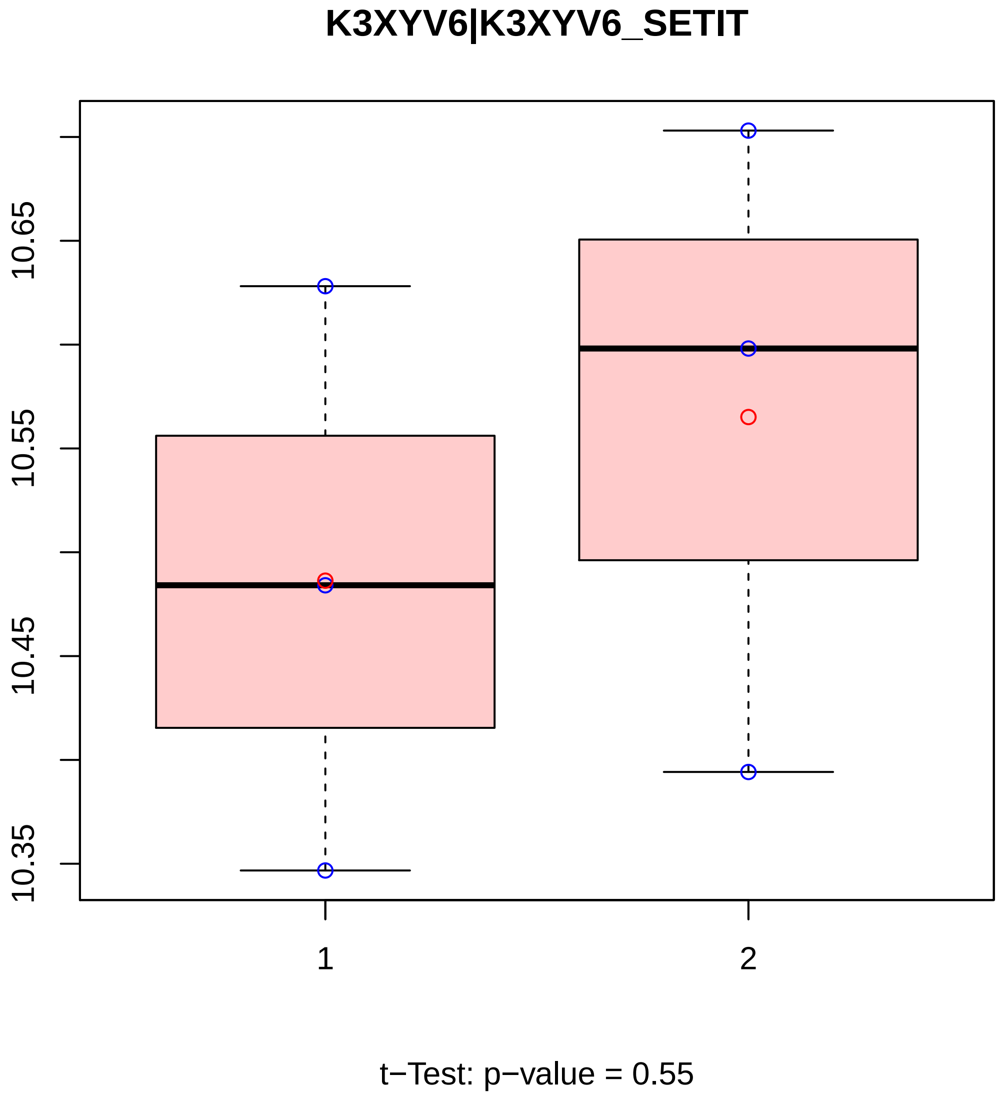
K3XYV6|K3XYV6_SETIT
10.35
10.45
10.55
10.65
1
2
t−Test: p−value = 0.55

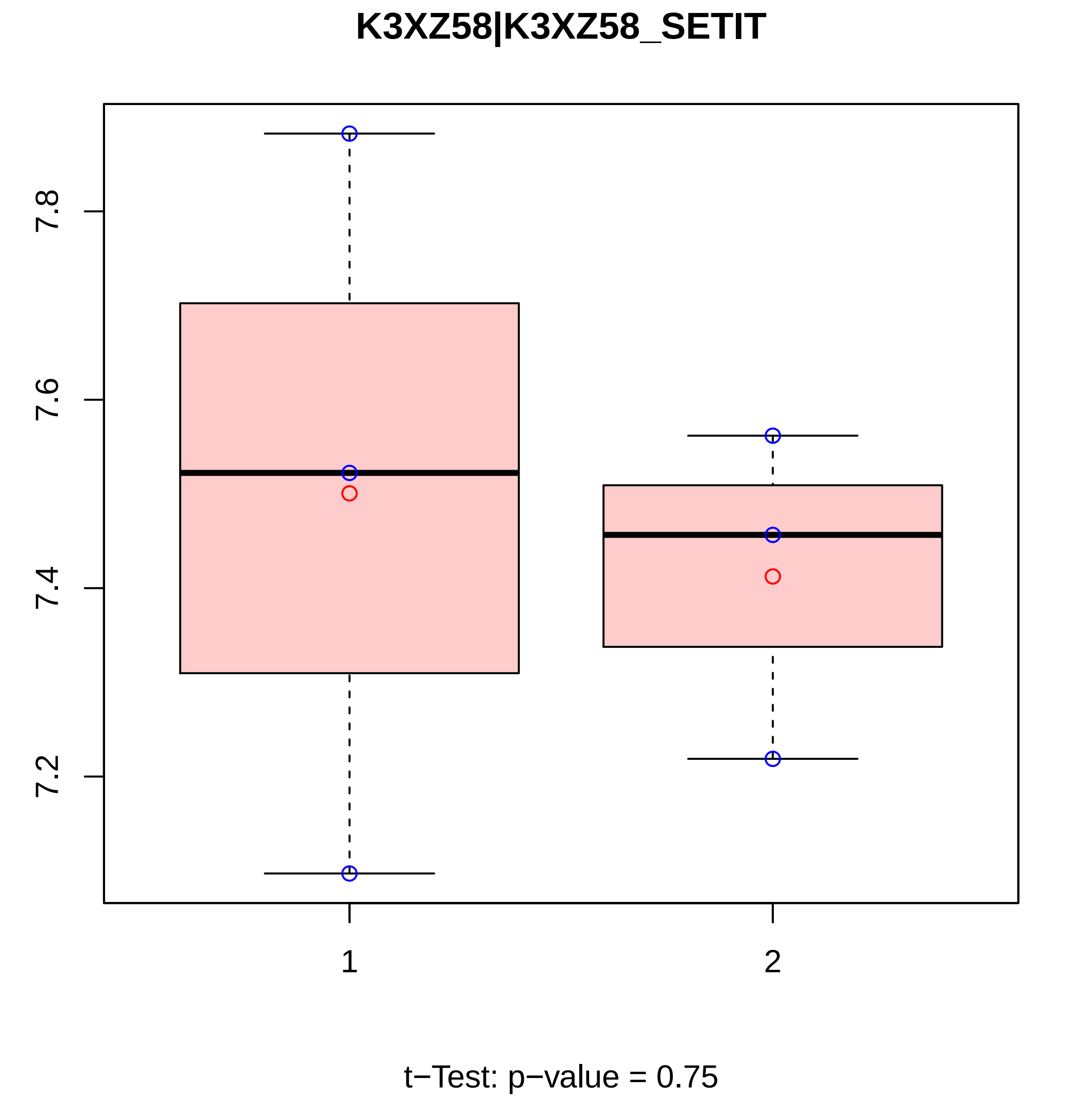
K3XZ58|K3XZ58_SETIT
7.8
7.6
7.4
7.2
1
2
t−Test: p−value = 0.75

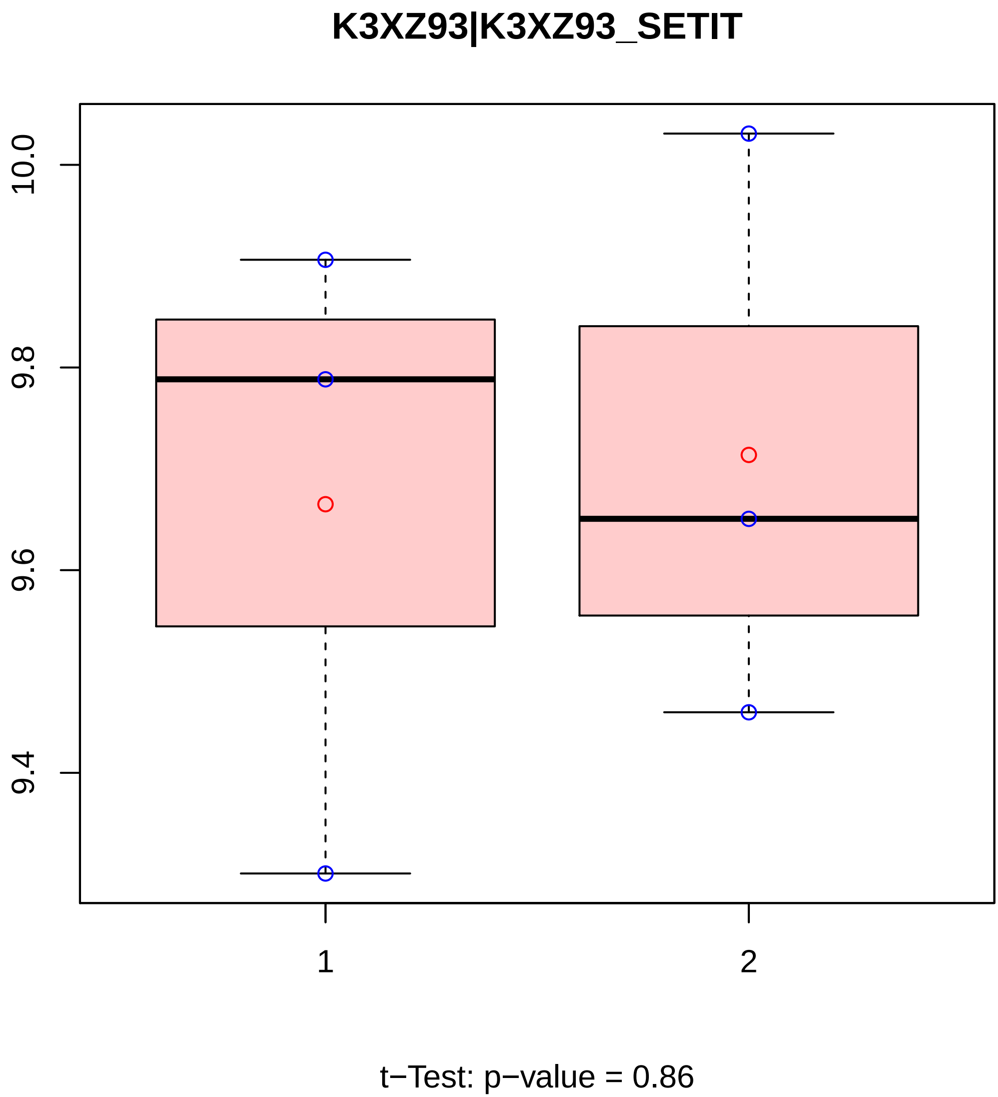
K3XZ93|K3XZ93_SETIT
10.0
9.8
9.6
9.4
1
2
t−Test: p−value = 0.86

K3Y157|K3Y157_SETIT

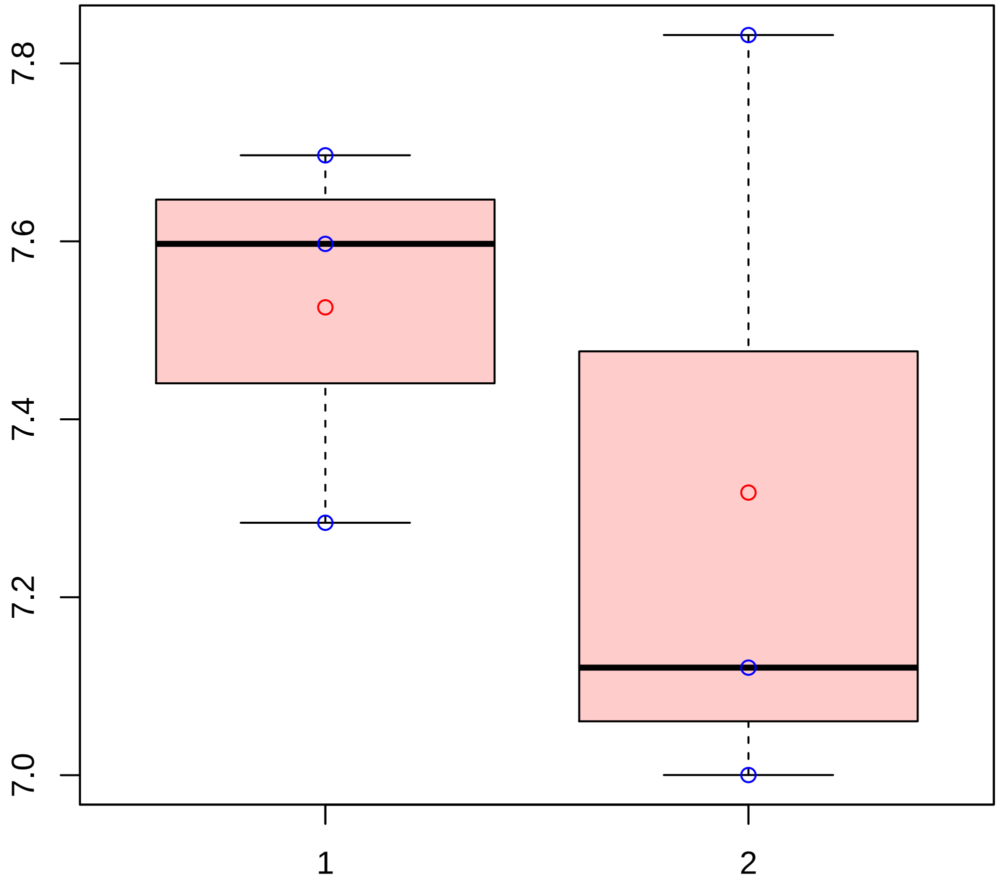

t−Test: p−value = 0.52

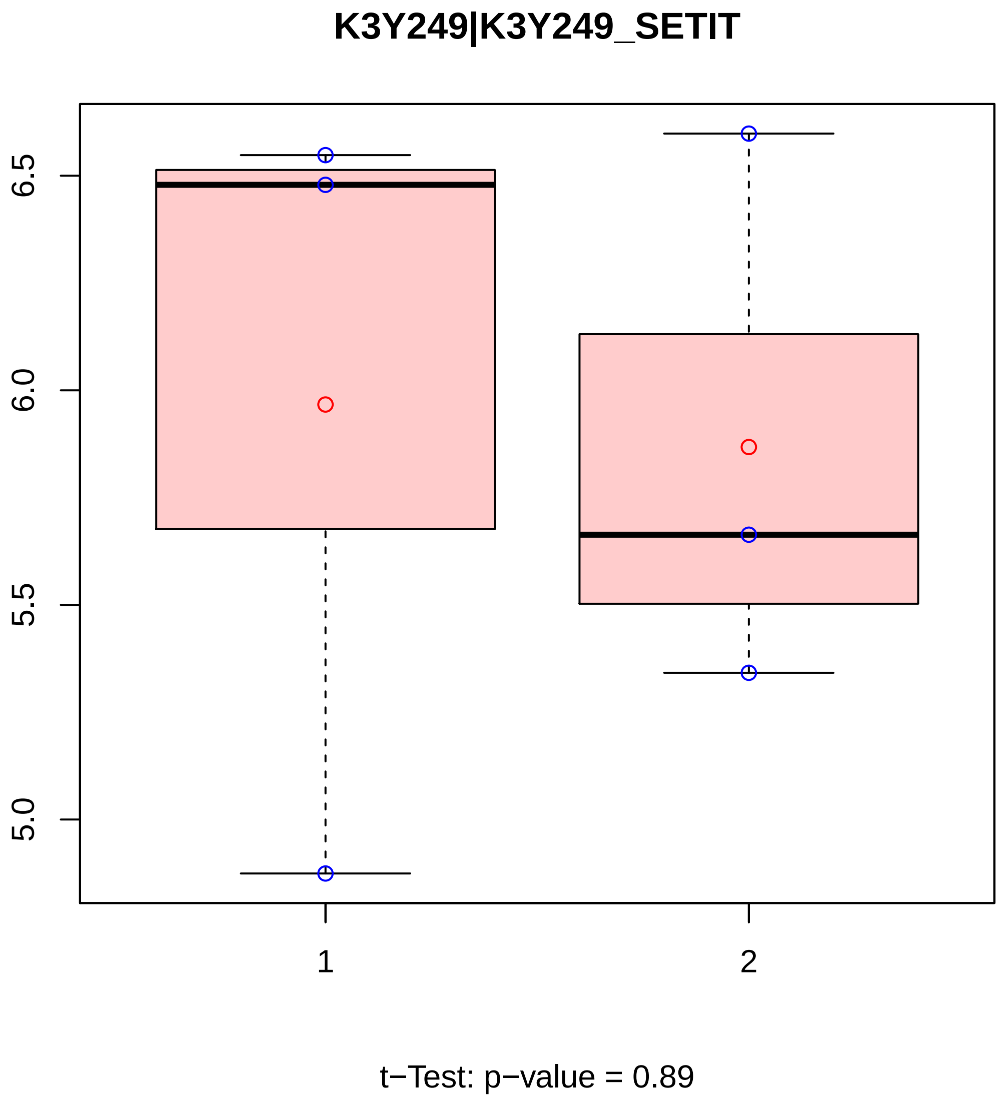

K3Y249|K3Y249_SETIT
6.5
6.0
5.5
5.0
1
2
t−Test: p−value = 0.89

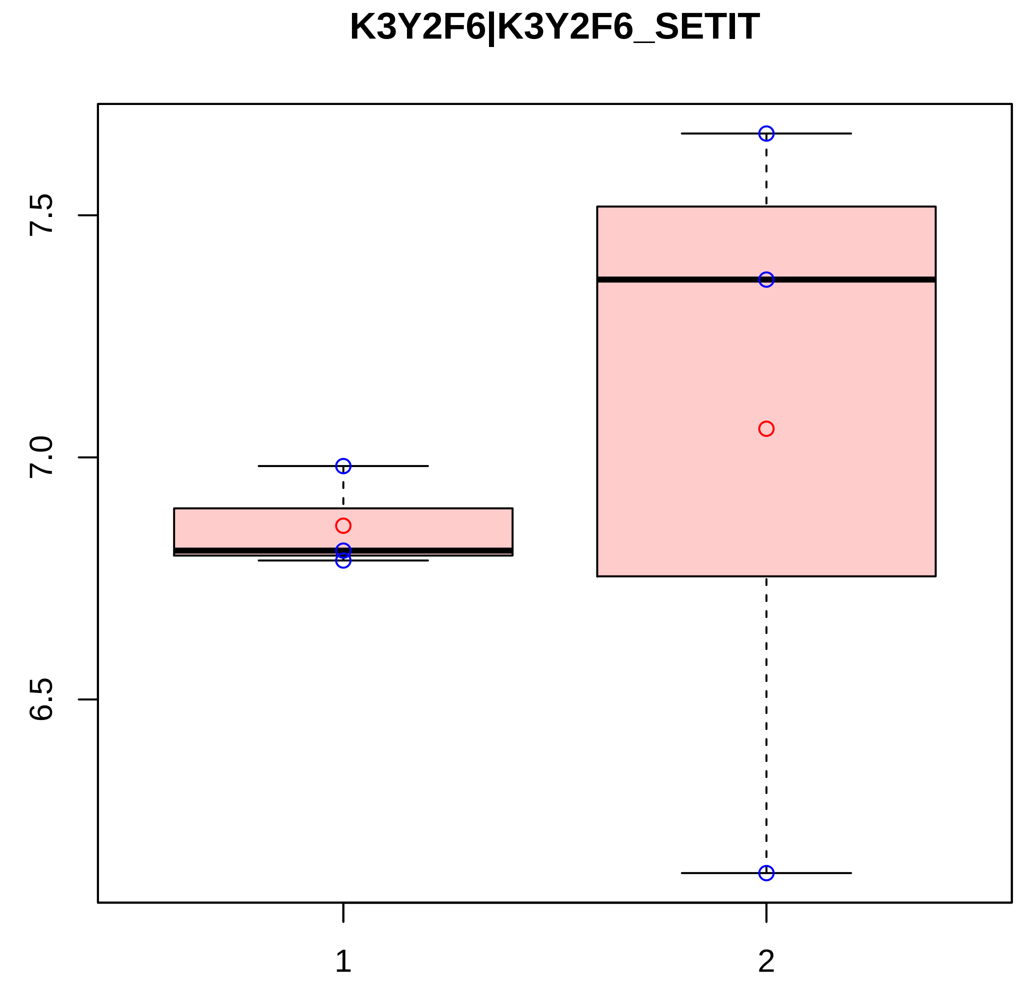

t−Test: p−value = 0.71

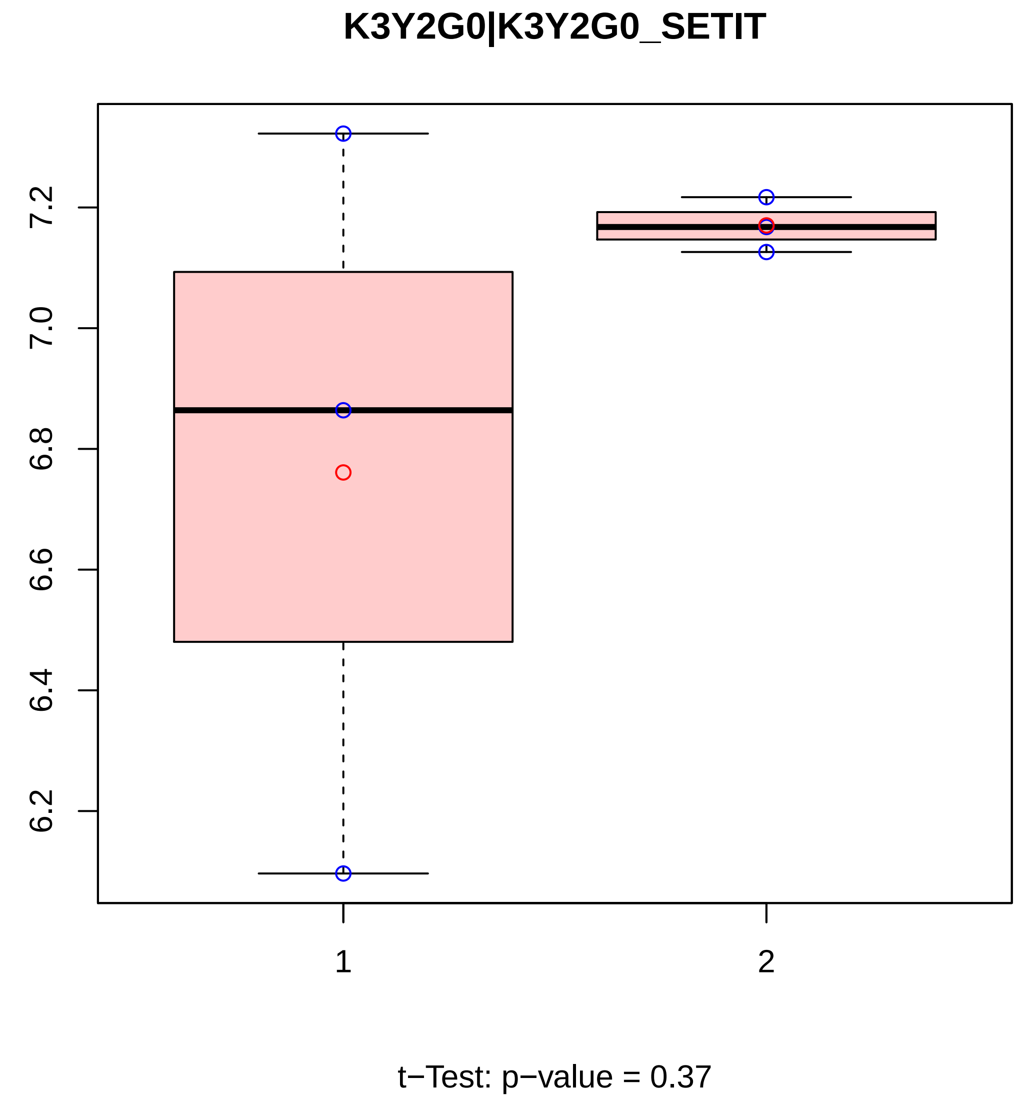
K3Y2G0|K3Y2G0_SETIT
7.2
7.0
6.8
6.6
6.4
6.2
1
2
t−Test: p−value = 0.37

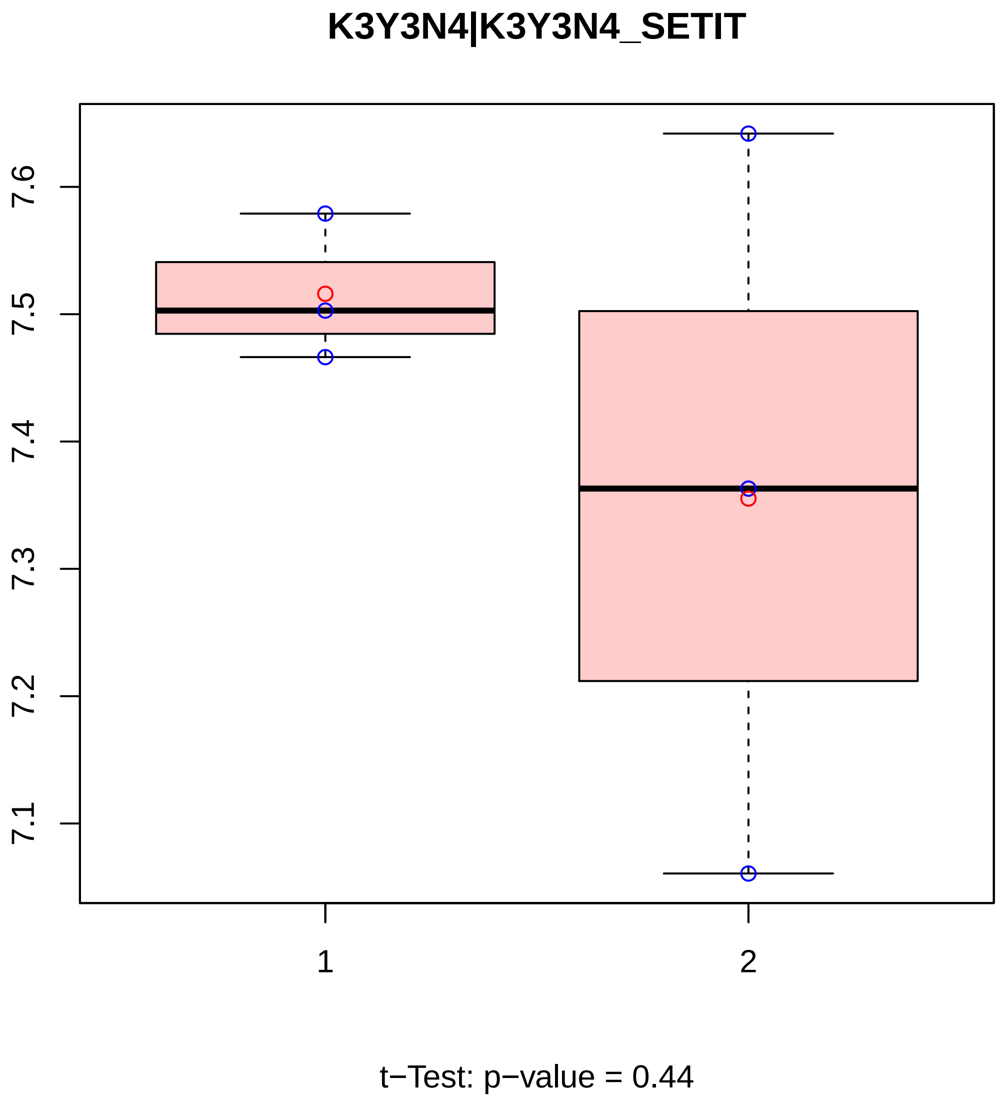

K3Y3N4|K3Y3N4_SETIT
7.1
7.2
7.3
7.4
7.5
7.6
1
2
t−Test: p−value = 0.44

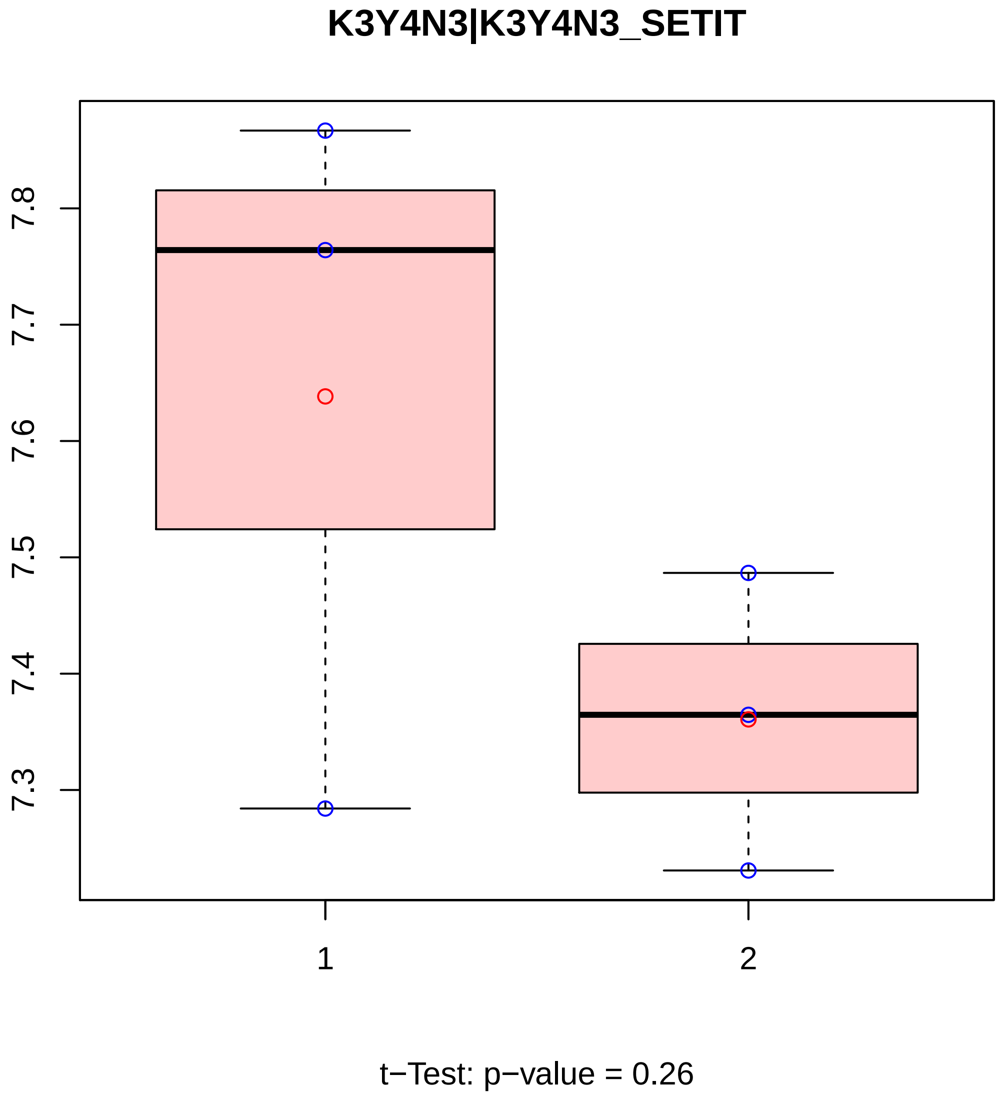

K3Y4N3|K3Y4N3_SETIT
7.3
7.4
7.5
7.6
7.7
7.8
1
2
t−Test: p−value = 0.26

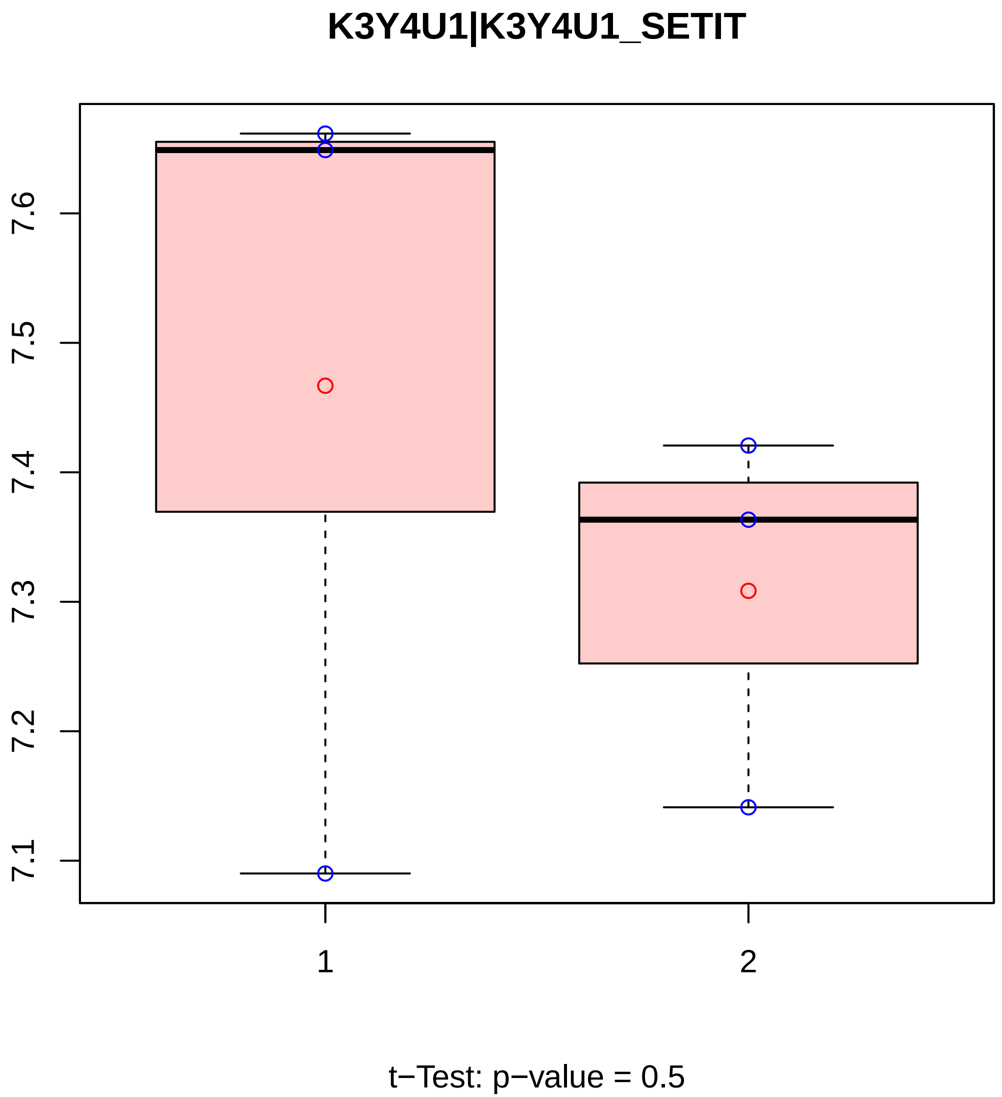
K3Y4U1|K3Y4U1_SETIT
7.1
7.2
7.3
7.4
7.5
7.6
1
2
t−Test: p−value = 0.5

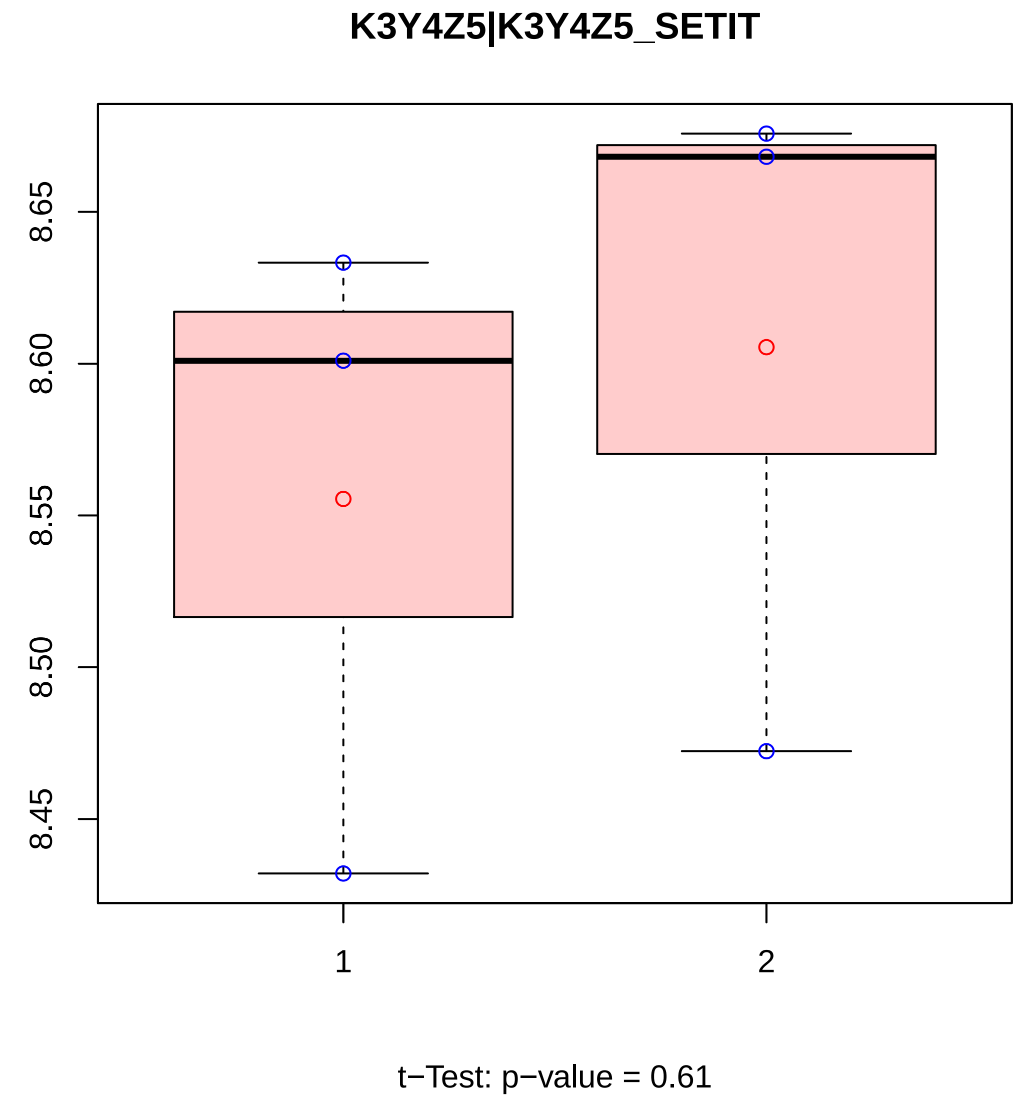
K3Y4Z5|K3Y4Z5_SETIT
8.45
8.50
8.55
8.60
8.65
1
2
t−Test: p−value = 0.61

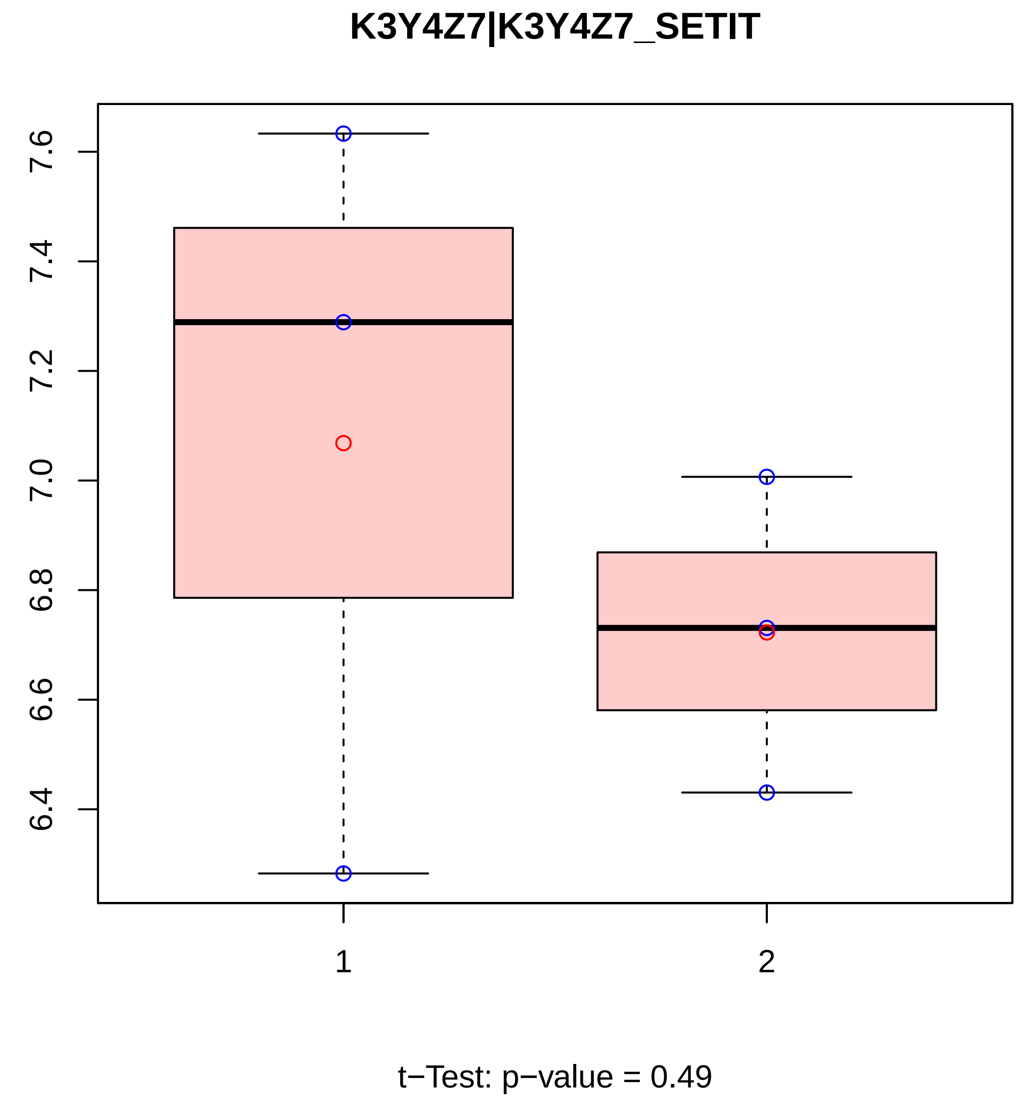
K3Y4Z7|K3Y4Z7_SETIT
7.6
7.4
7.2
7.0
6.8
6.6
6.4
1
2
t−Test: p−value = 0.49

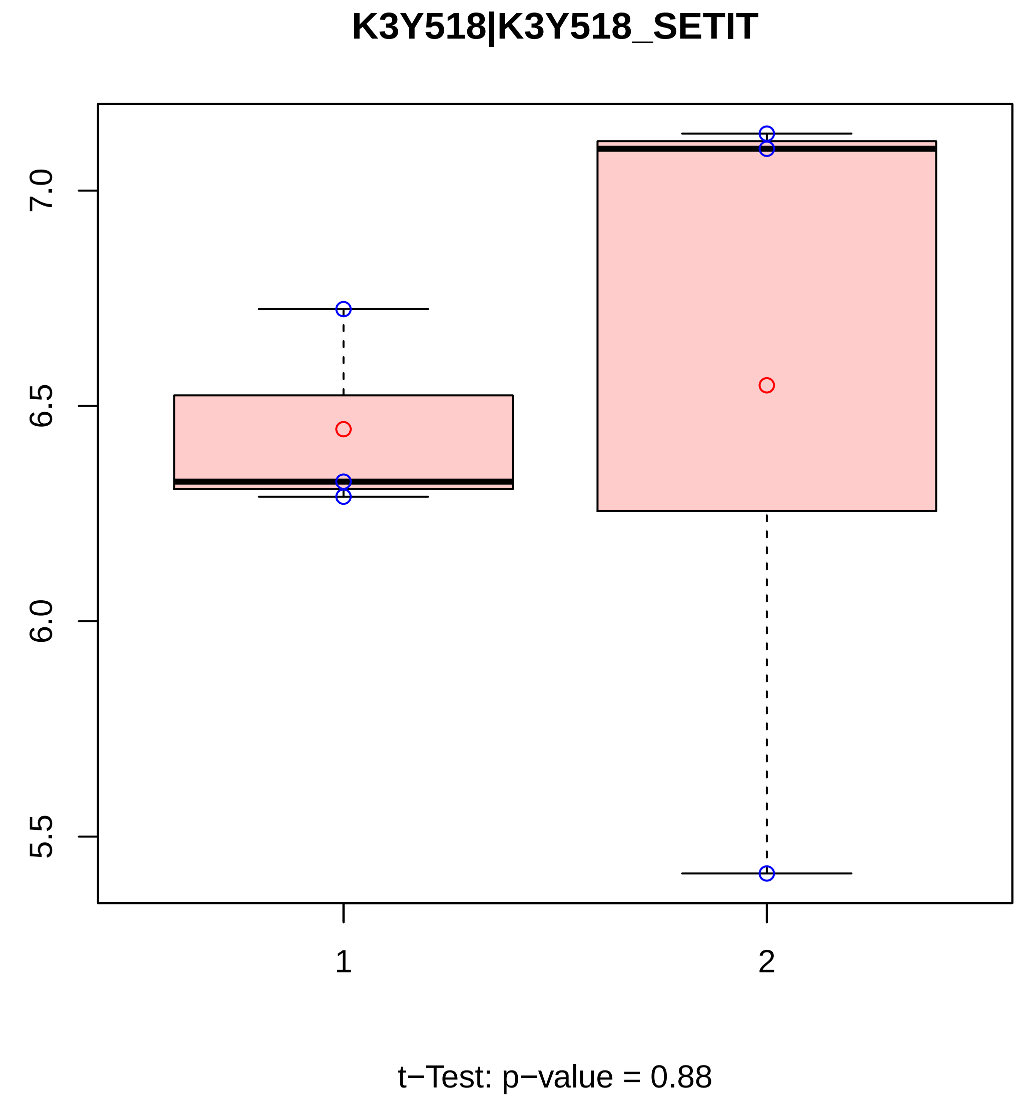

K3Y518|K3Y518_SETIT
7.0
6.5
6.0
5.5
1
2
t−Test: p−value = 0.88

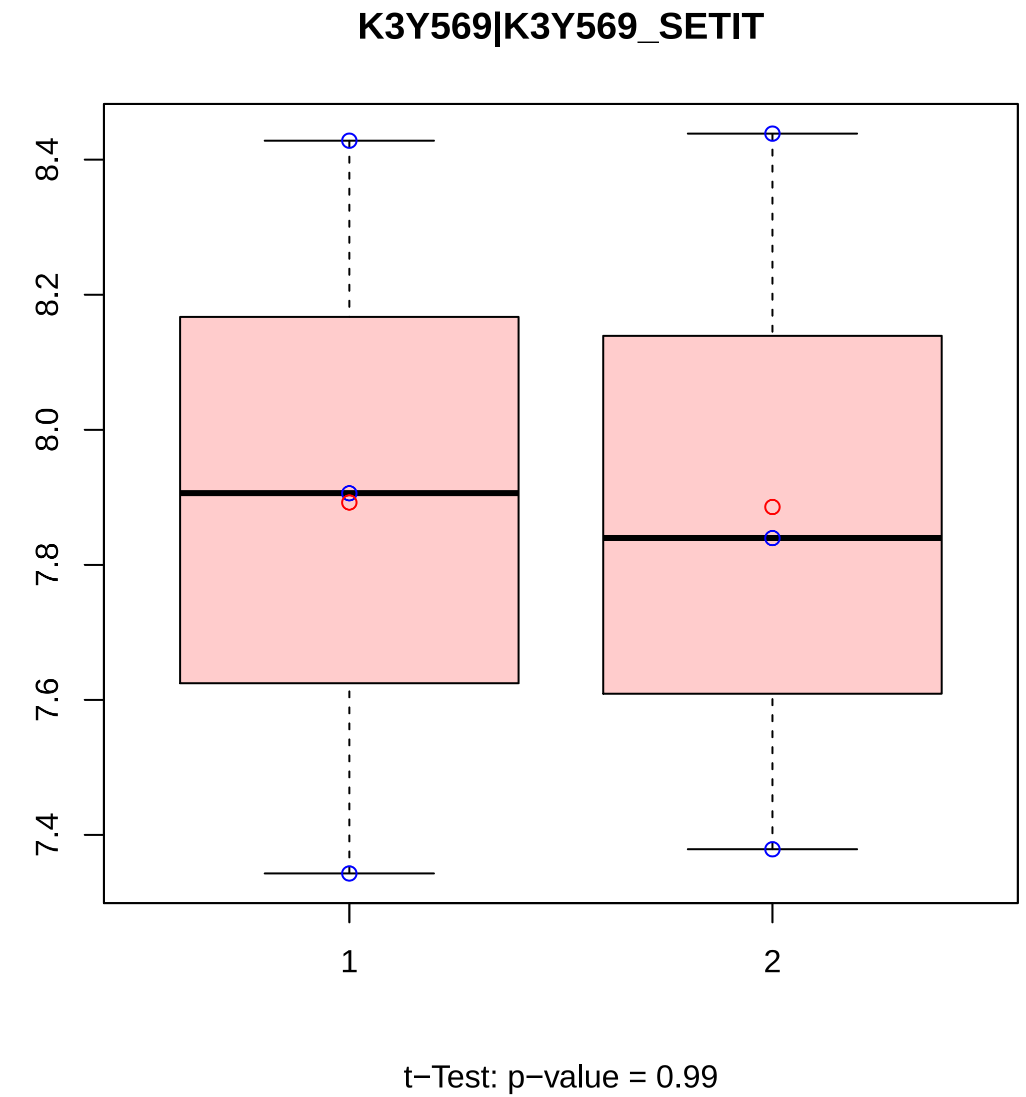
K3Y569|K3Y569_SETIT
8.4
8.2
8.0
7.8
7.6
7.4
1
2
t−Test: p−value = 0.99

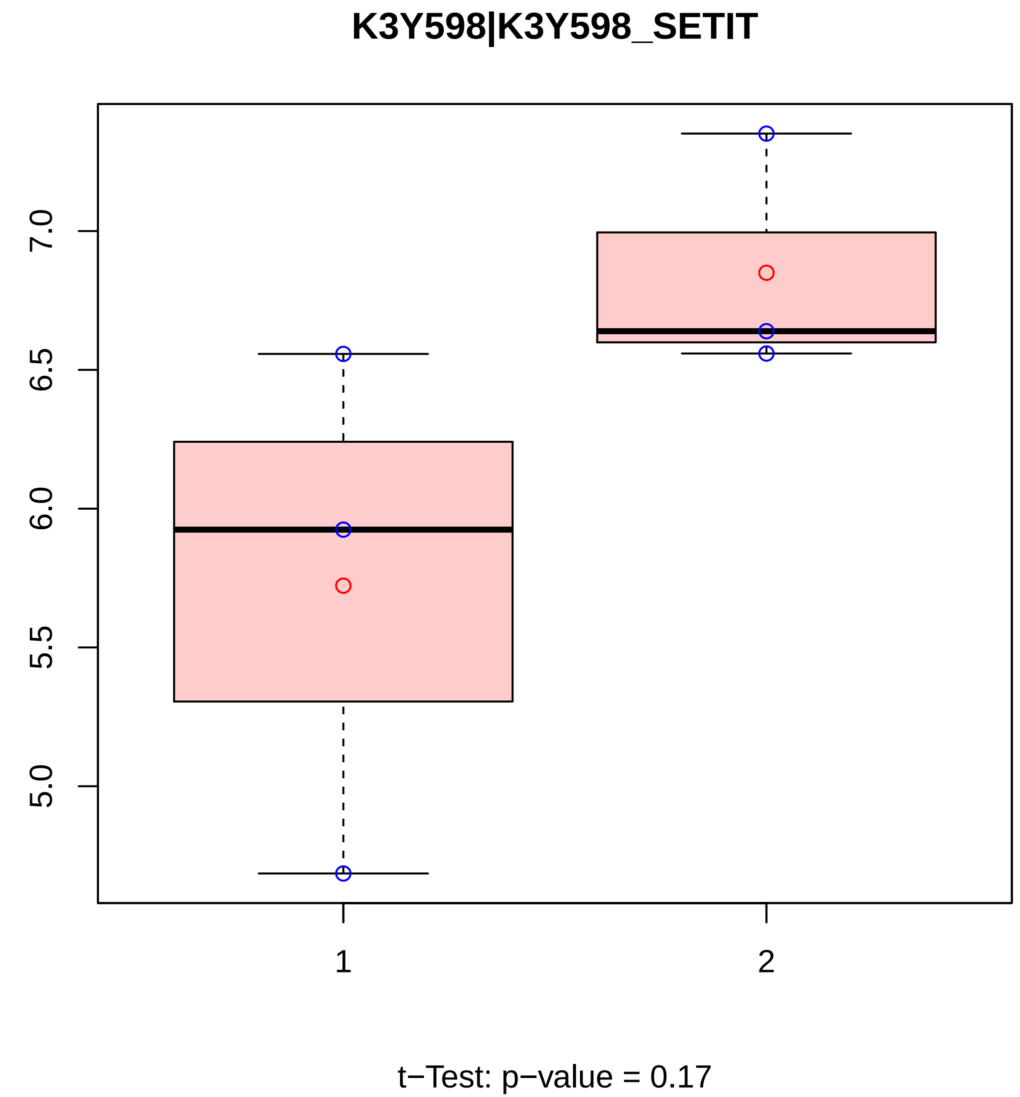
K3Y598|K3Y598_SETIT
7.0
6.5
6.0
5.5
5.0
1
2
t−Test: p−value = 0.17

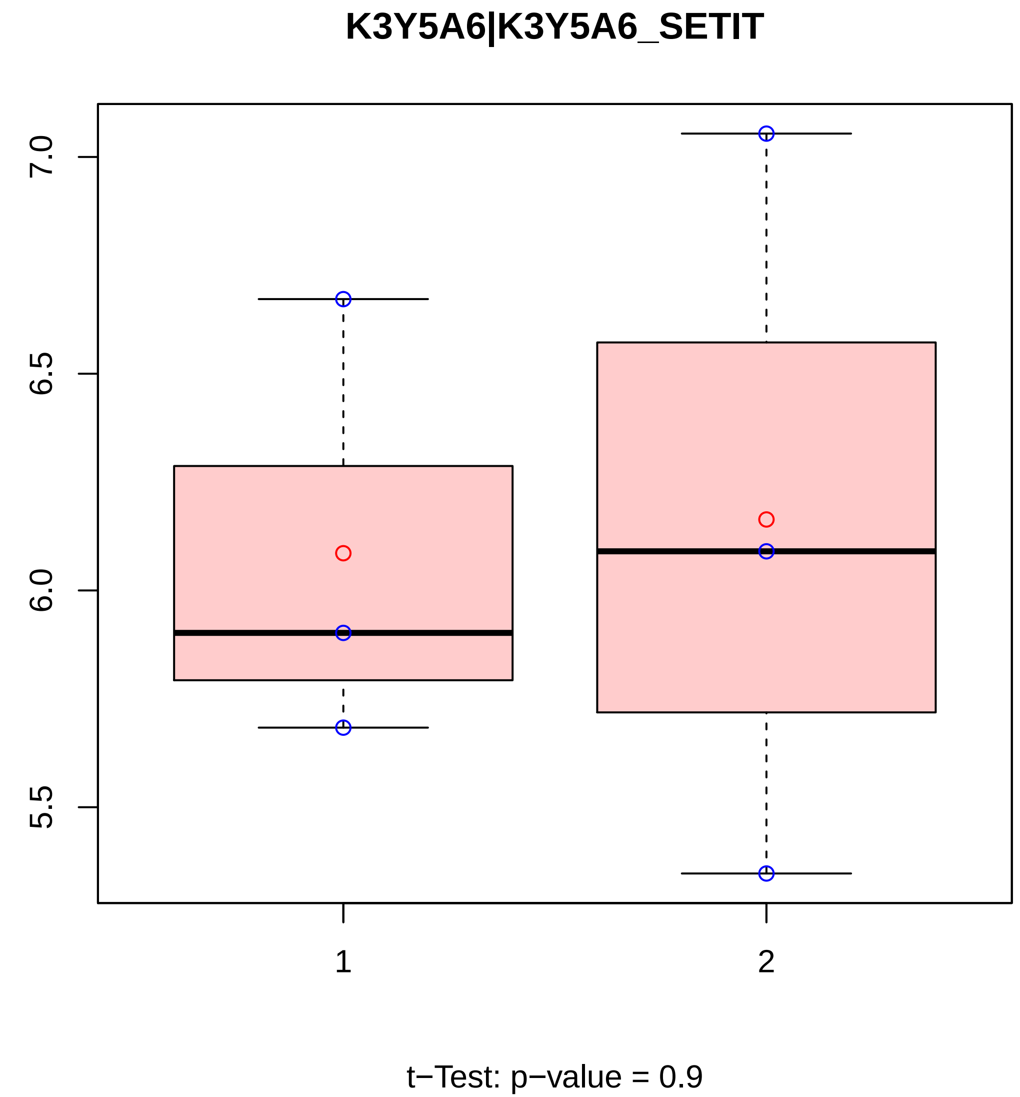
K3Y5A6|K3Y5A6_SETIT
7.0
6.5
6.0
5.5
1
2
t−Test: p−value = 0.9

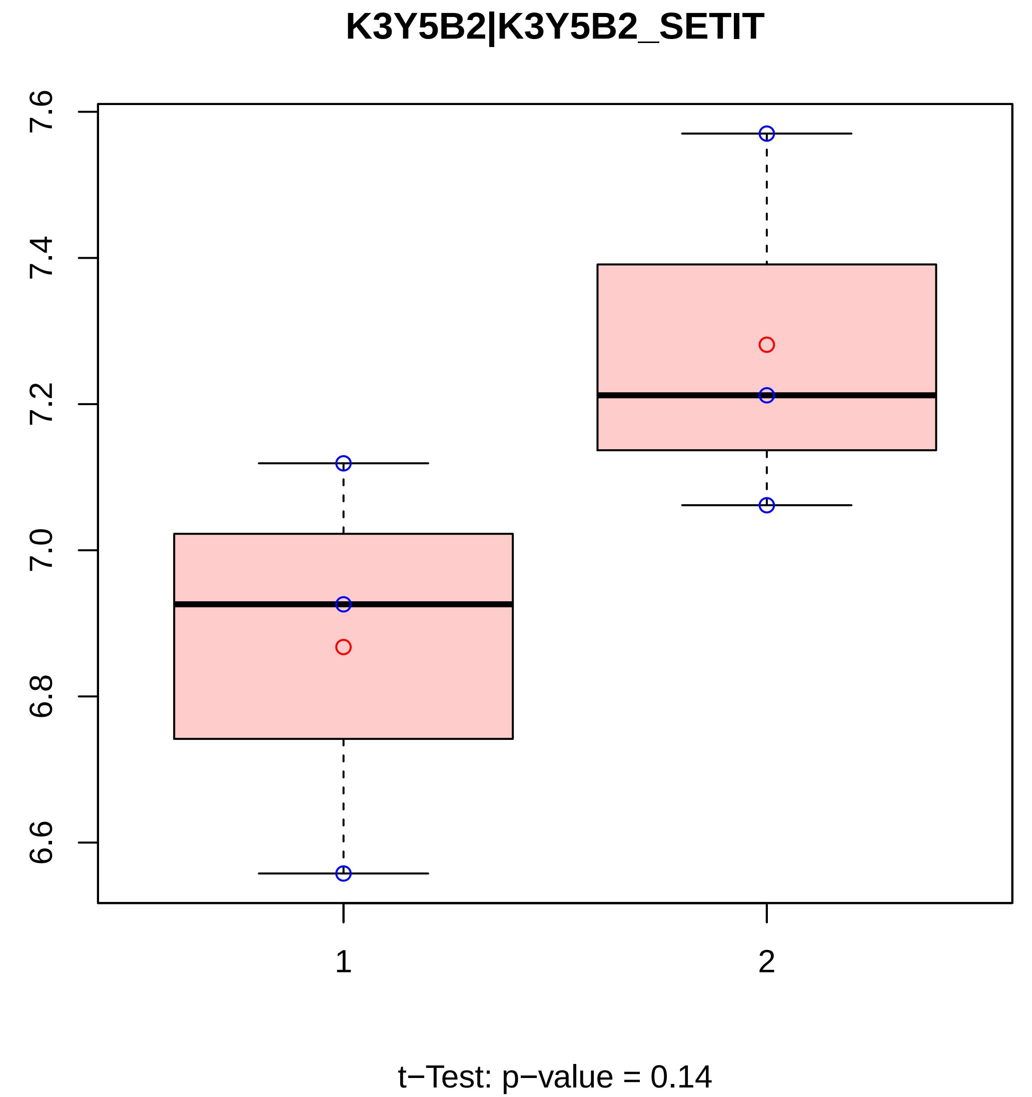
K3Y5B2|K3Y5B2_SETIT
7.6
7.4
7.2
7.0
6.8
6.6
1
2
t−Test: p−value = 0.14

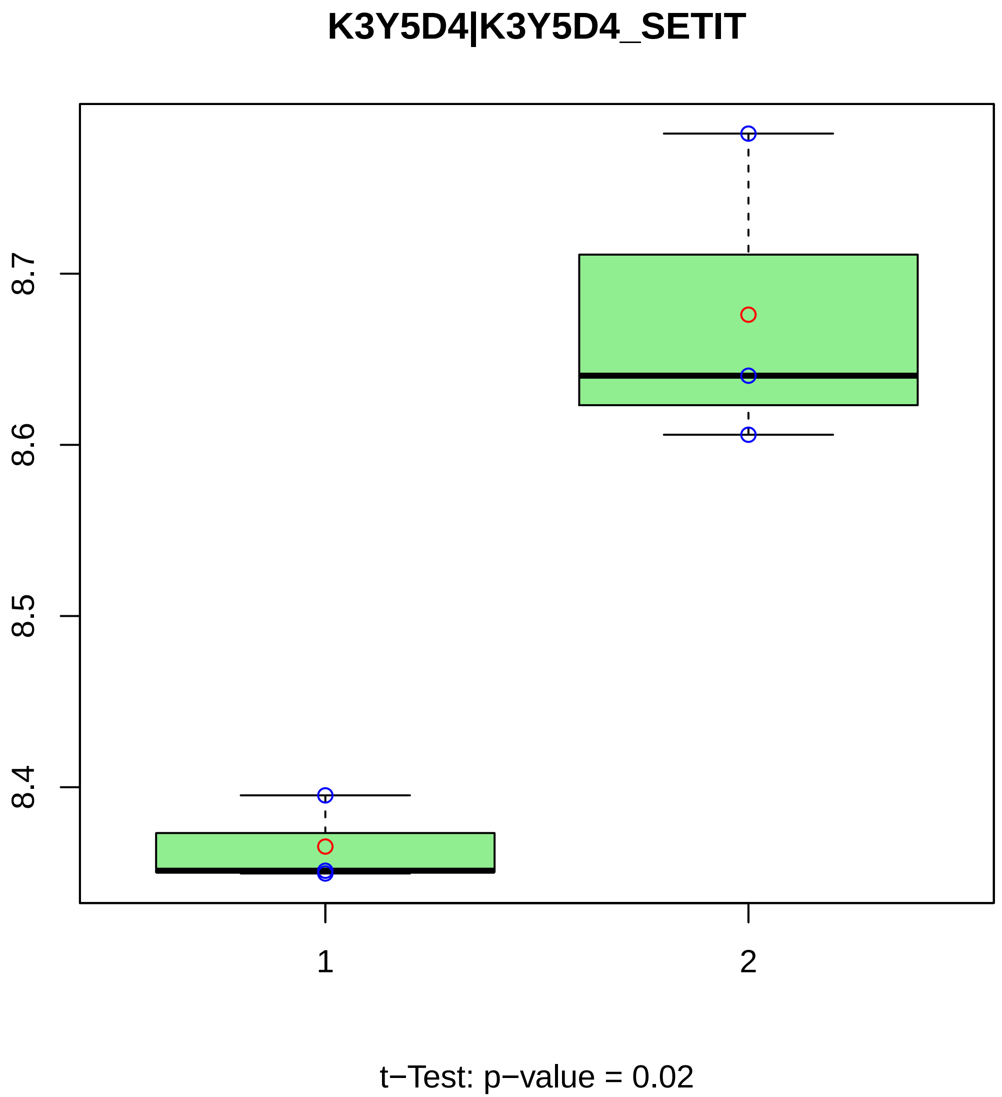
K3Y5D4|K3Y5D4_SETIT
8.4
8.5
8.6
8.7
1
2
t−Test: p−value = 0.02

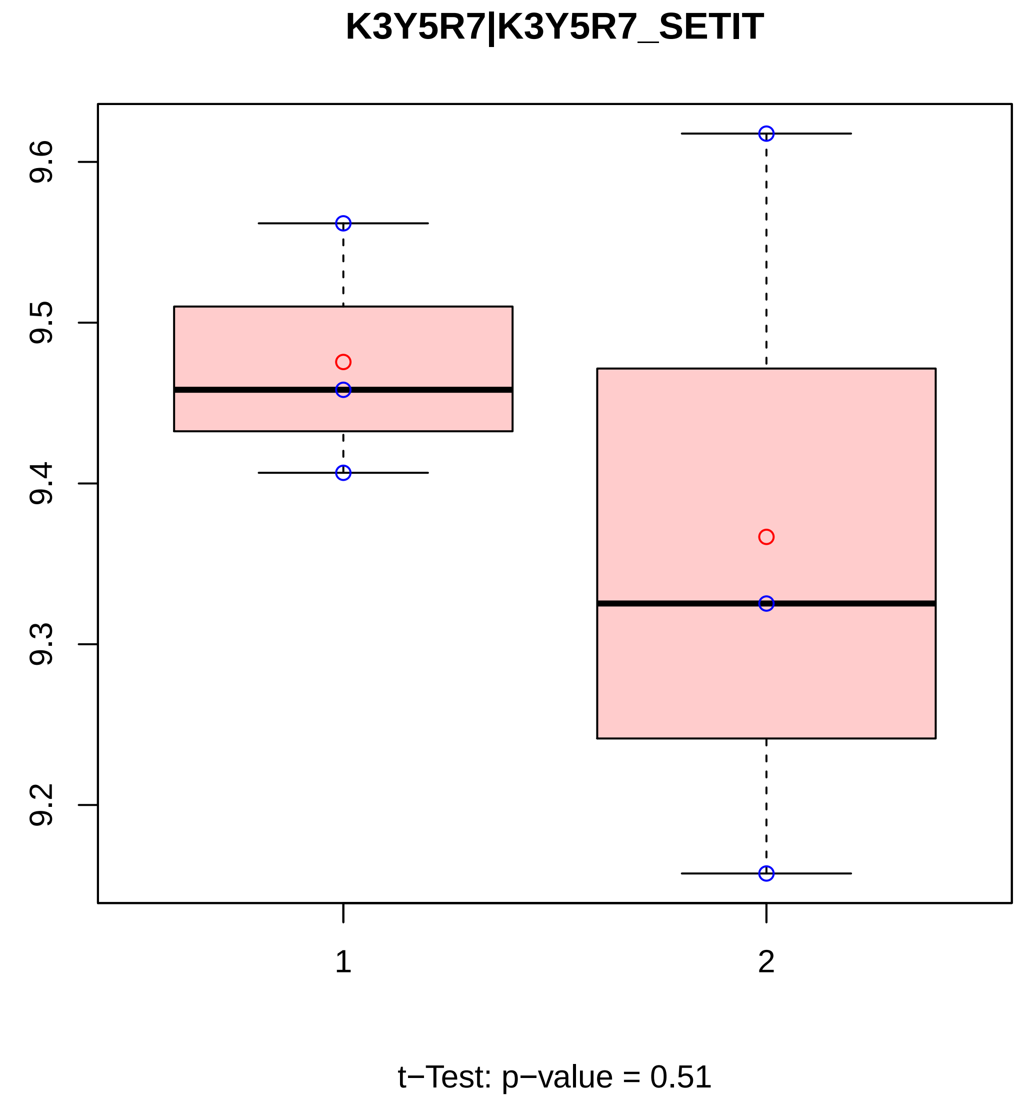

K3Y5R7|K3Y5R7_SETIT
9.2
9.3
9.4
9.5
9.6
1
2
t−Test: p−value = 0.51

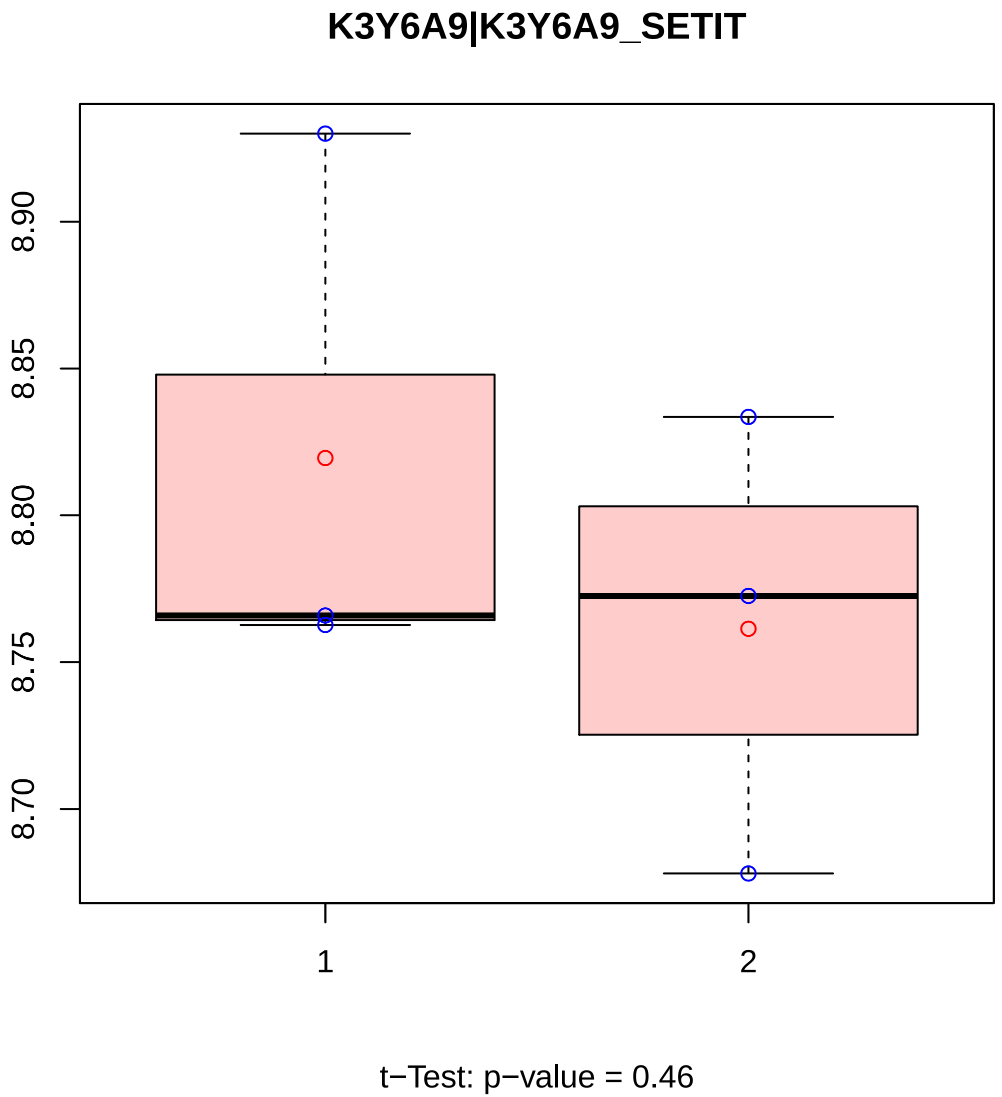
K3Y6A9|K3Y6A9_SETIT
8.70
8.75
8.80
8.85
8.90
1
2
t−Test: p−value = 0.46

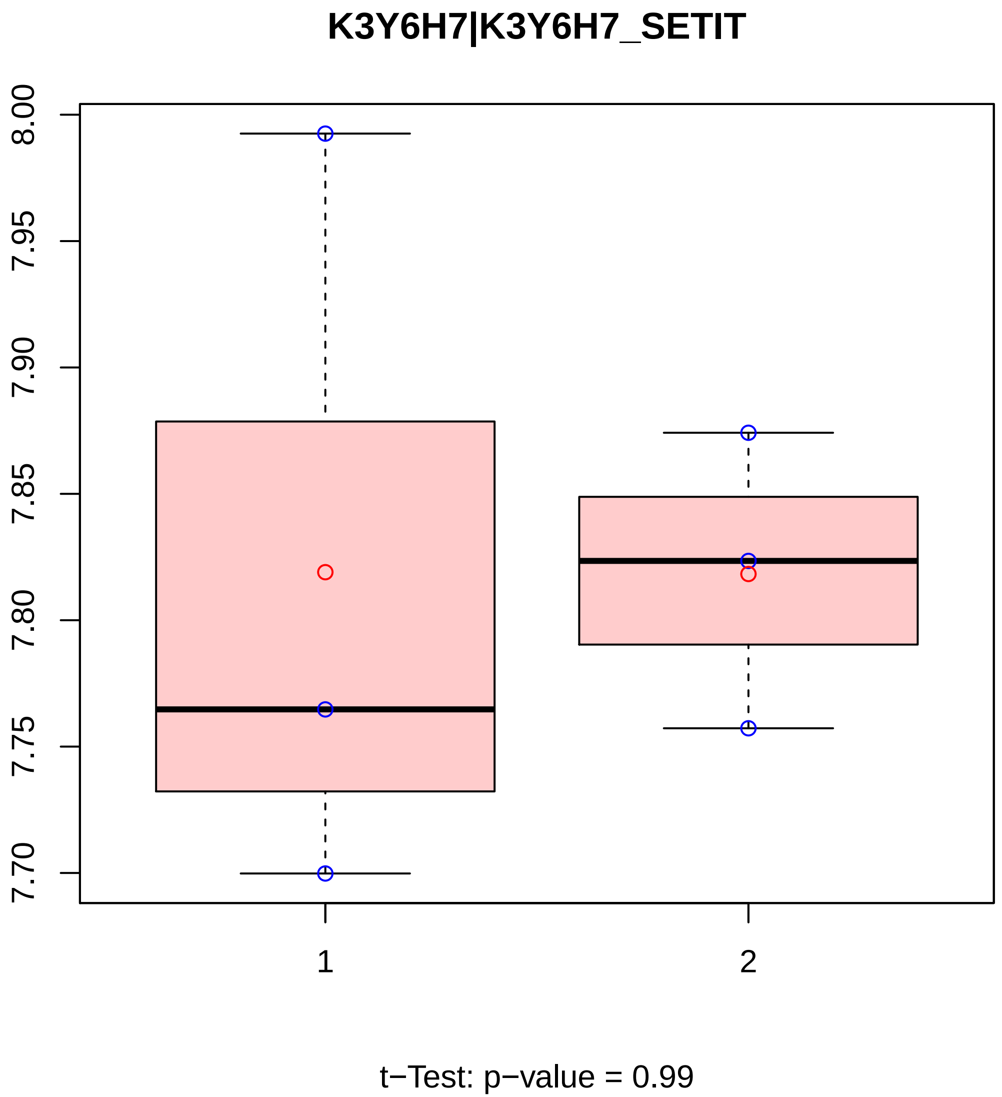
K3Y6H7|K3Y6H7_SETIT
7.70
7.75
7.80
7.85
7.90
7.95
8.00
1
2
t−Test: p−value = 0.99

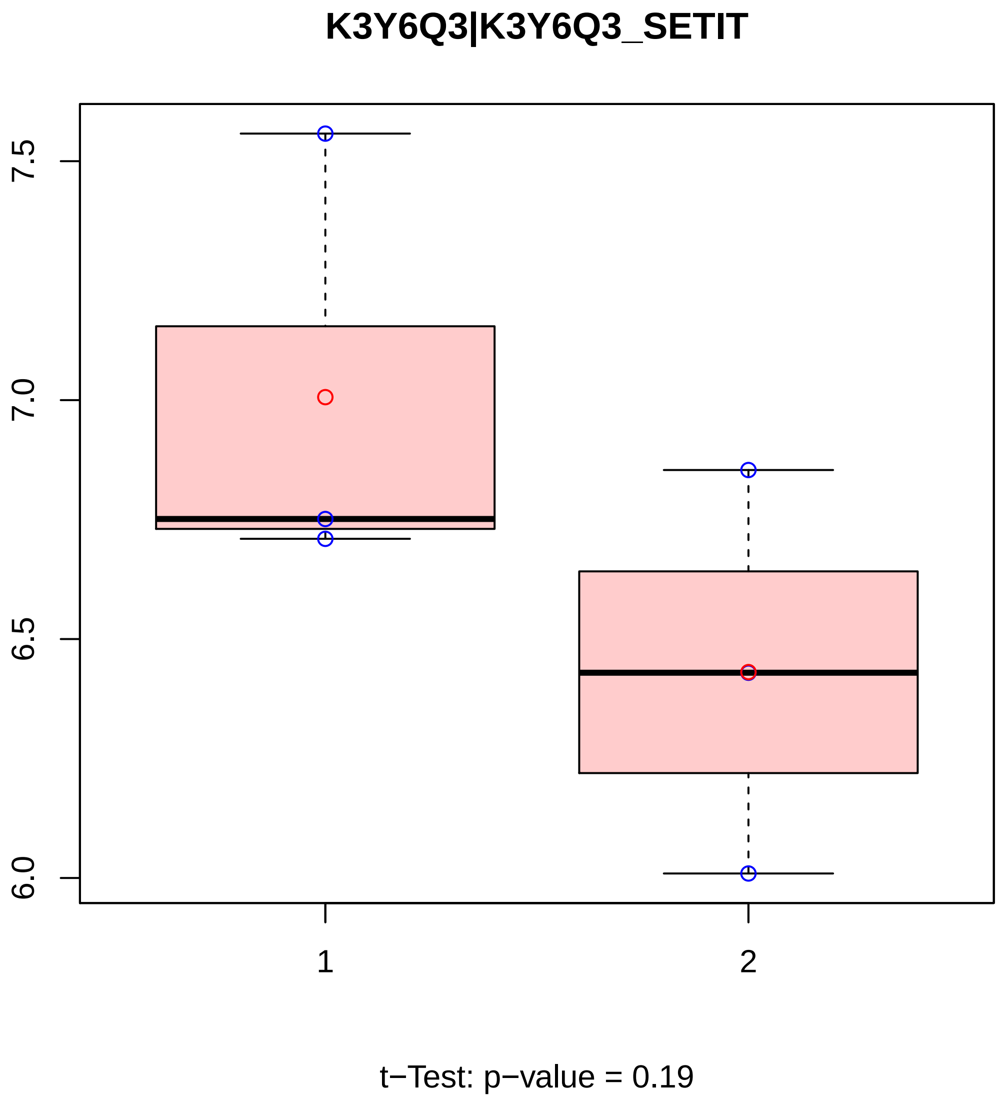
K3Y6Q3|K3Y6Q3_SETIT
7.5
7.0
6.5
6.0
1
2
t−Test: p−value = 0.19

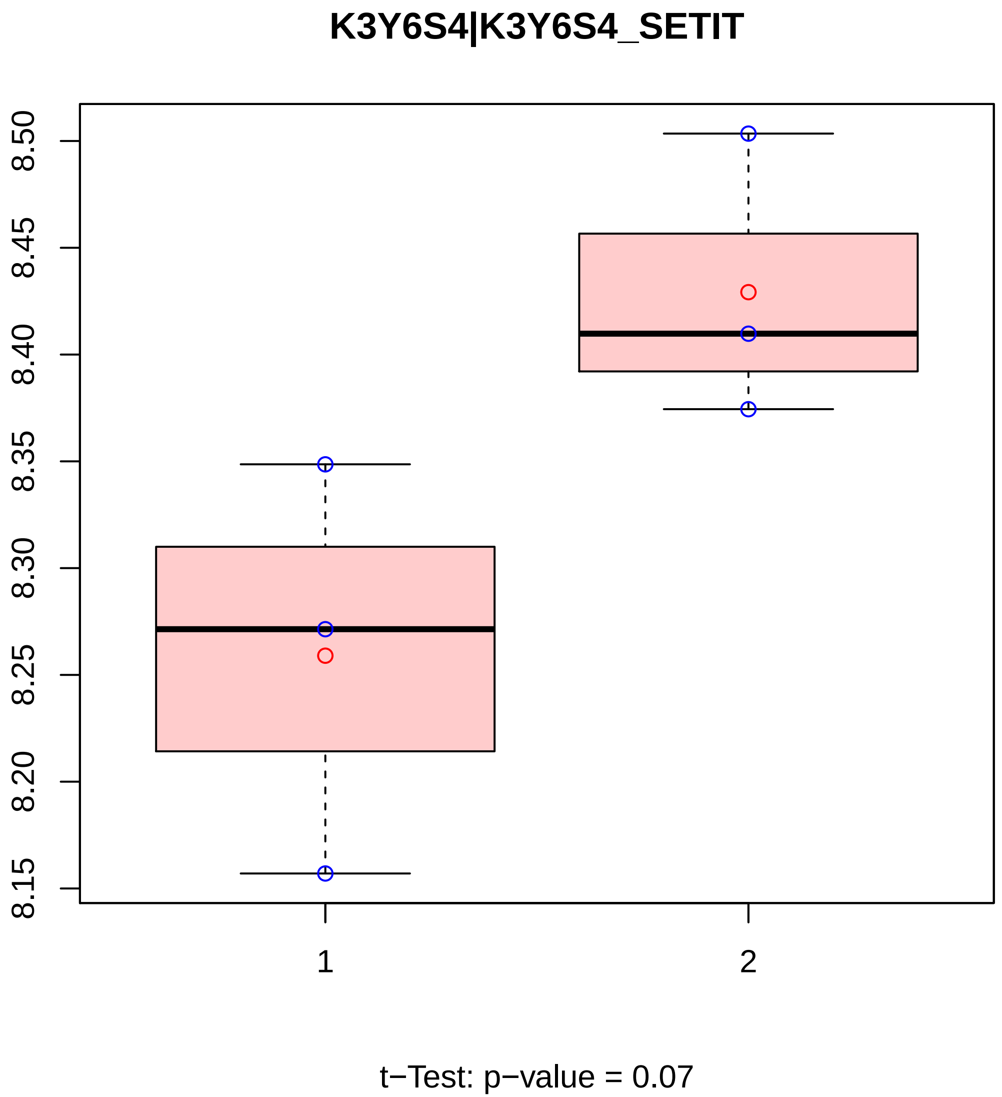

K3Y6S4|K3Y6S4_SETIT
8.15
8.20
8.25
8.30
8.35
8.40
8.45
8.50
1
2
t−Test: p−value = 0.07

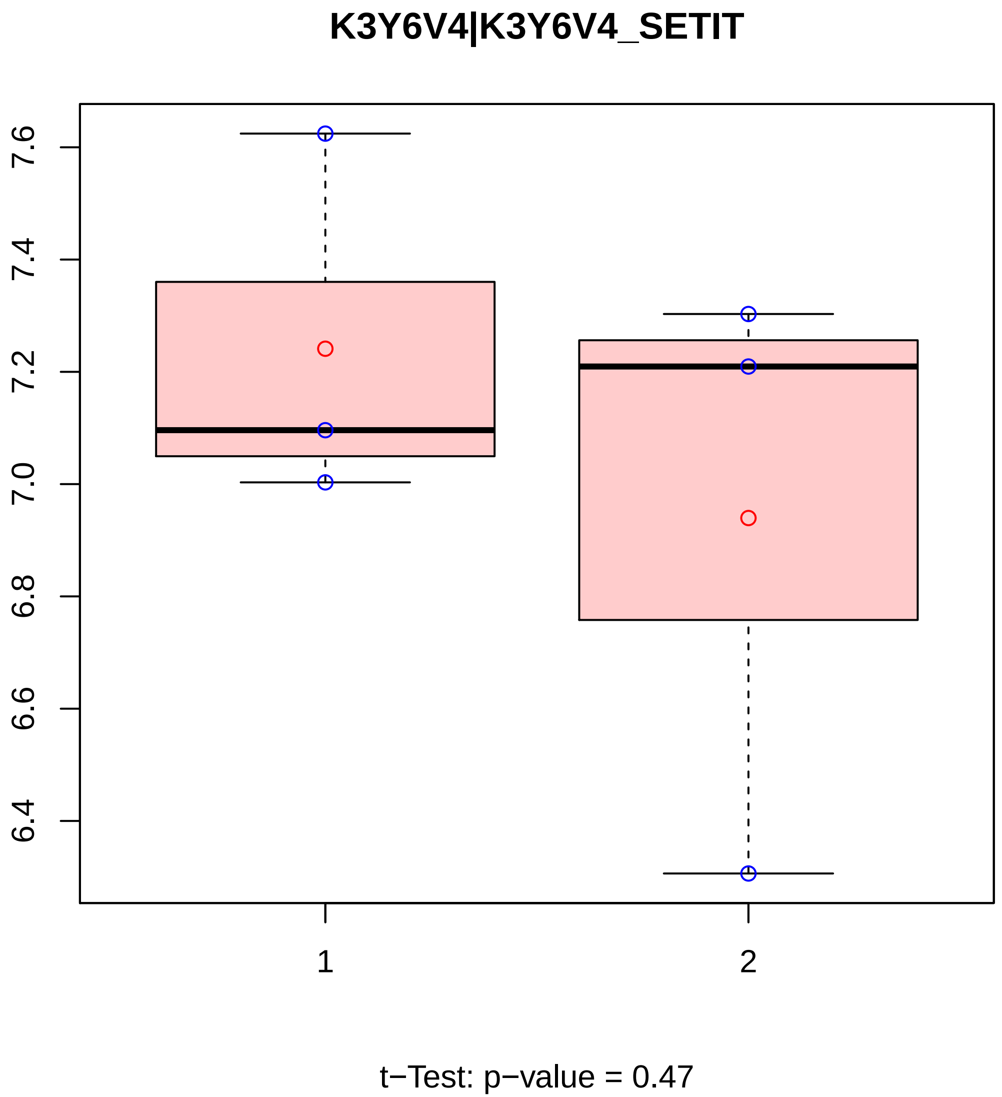
K3Y6V4|K3Y6V4_SETIT
7.6
7.4
7.2
7.0
6.8
6.6
6.4
1
2
t−Test: p−value = 0.47

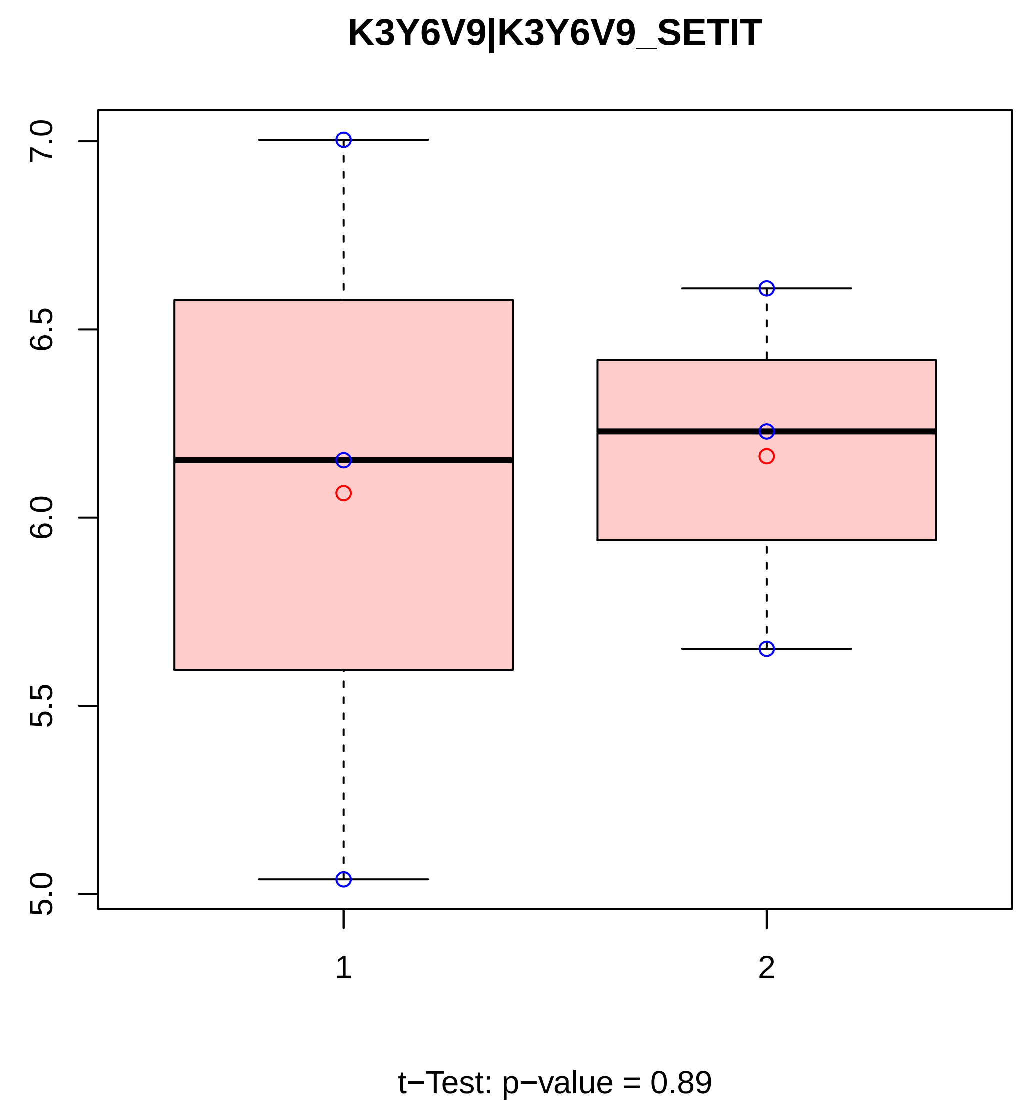
K3Y6V9|K3Y6V9_SETIT
7.0
6.5
6.0
5.5
5.0
1
2
t−Test: p−value = 0.89

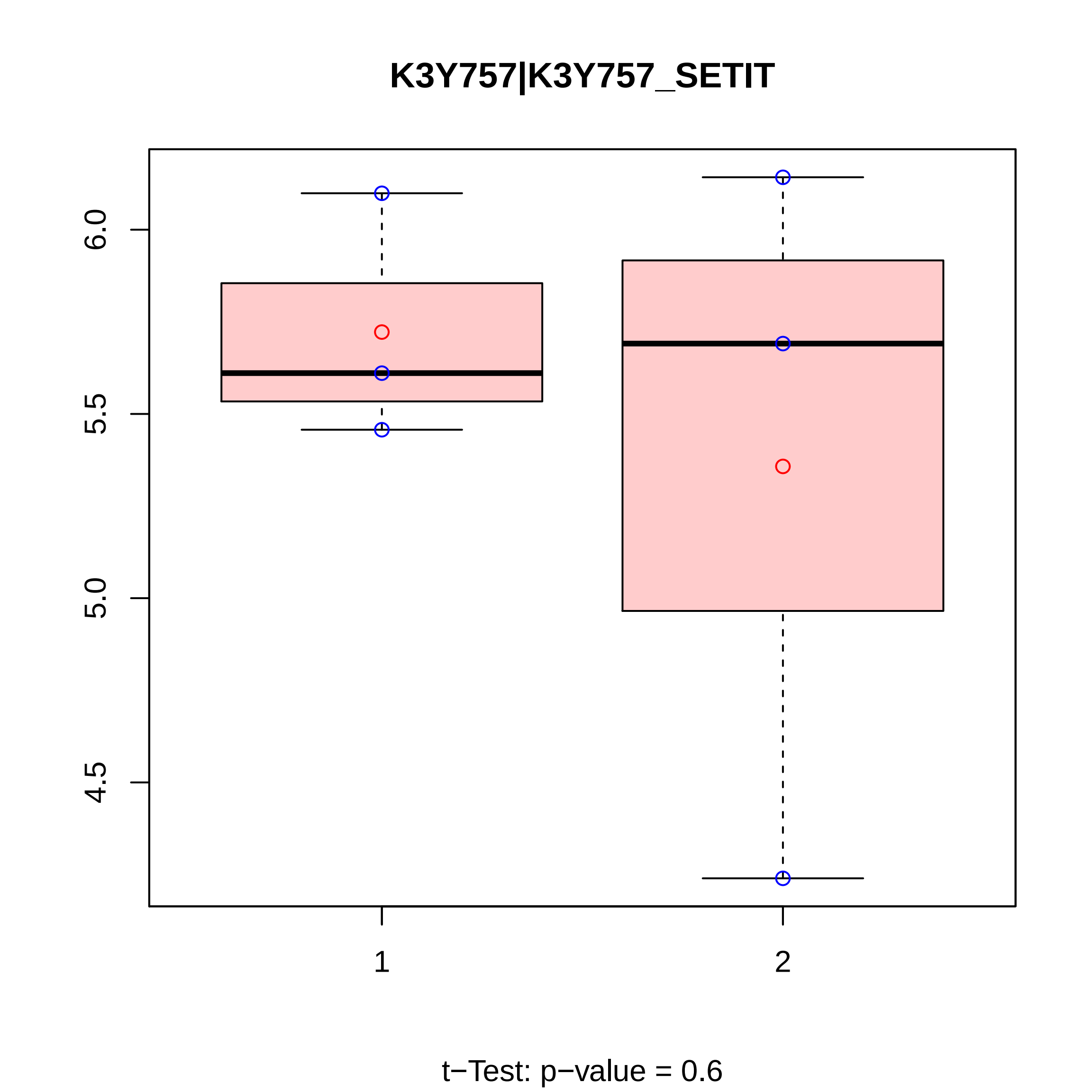
K3Y757|K3Y757_SETIT
6.0
5.5
5.0
4.5
1
2
t−Test: p−value = 0.6

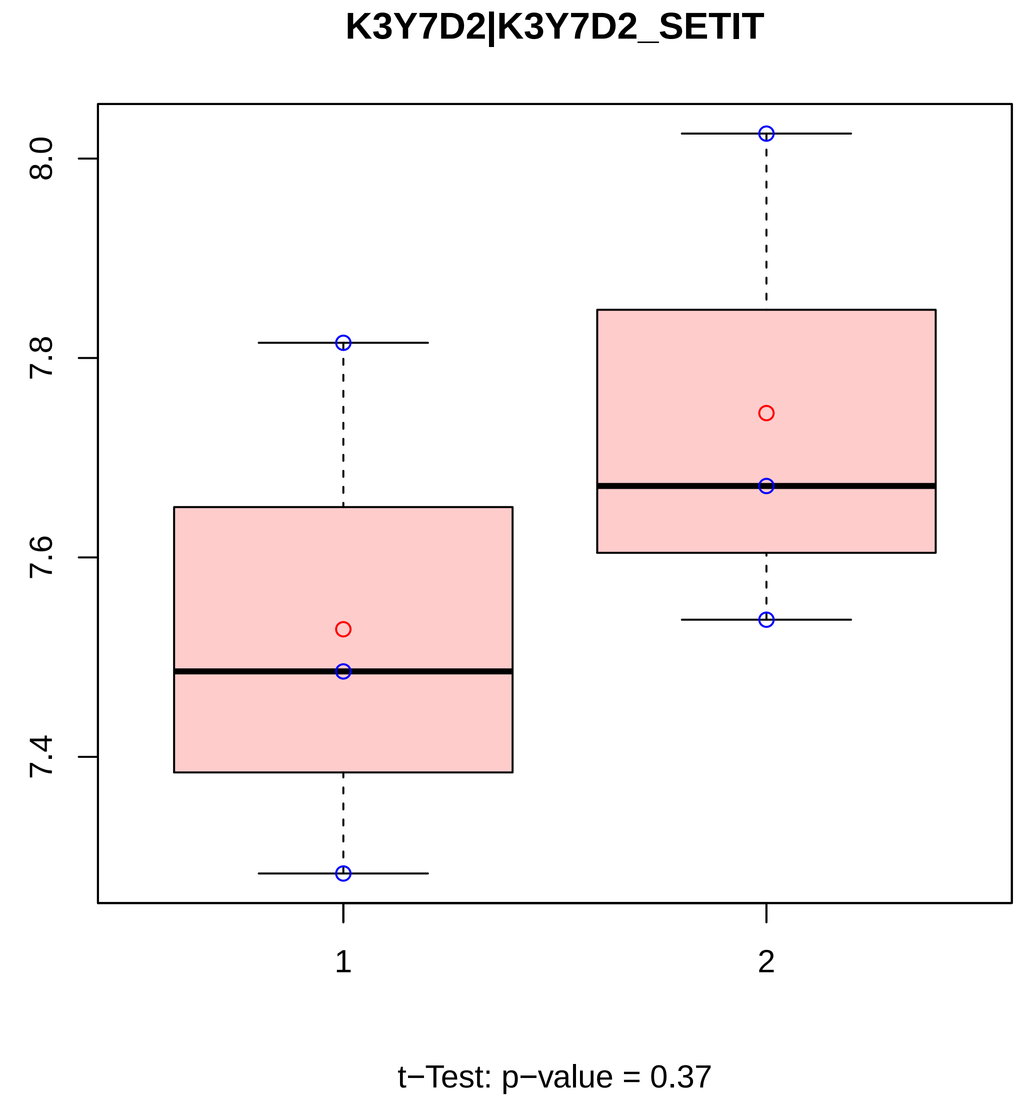
K3Y7D2|K3Y7D2_SETIT
8.0
7.8
7.6
7.4
1
2
t−Test: p−value = 0.37

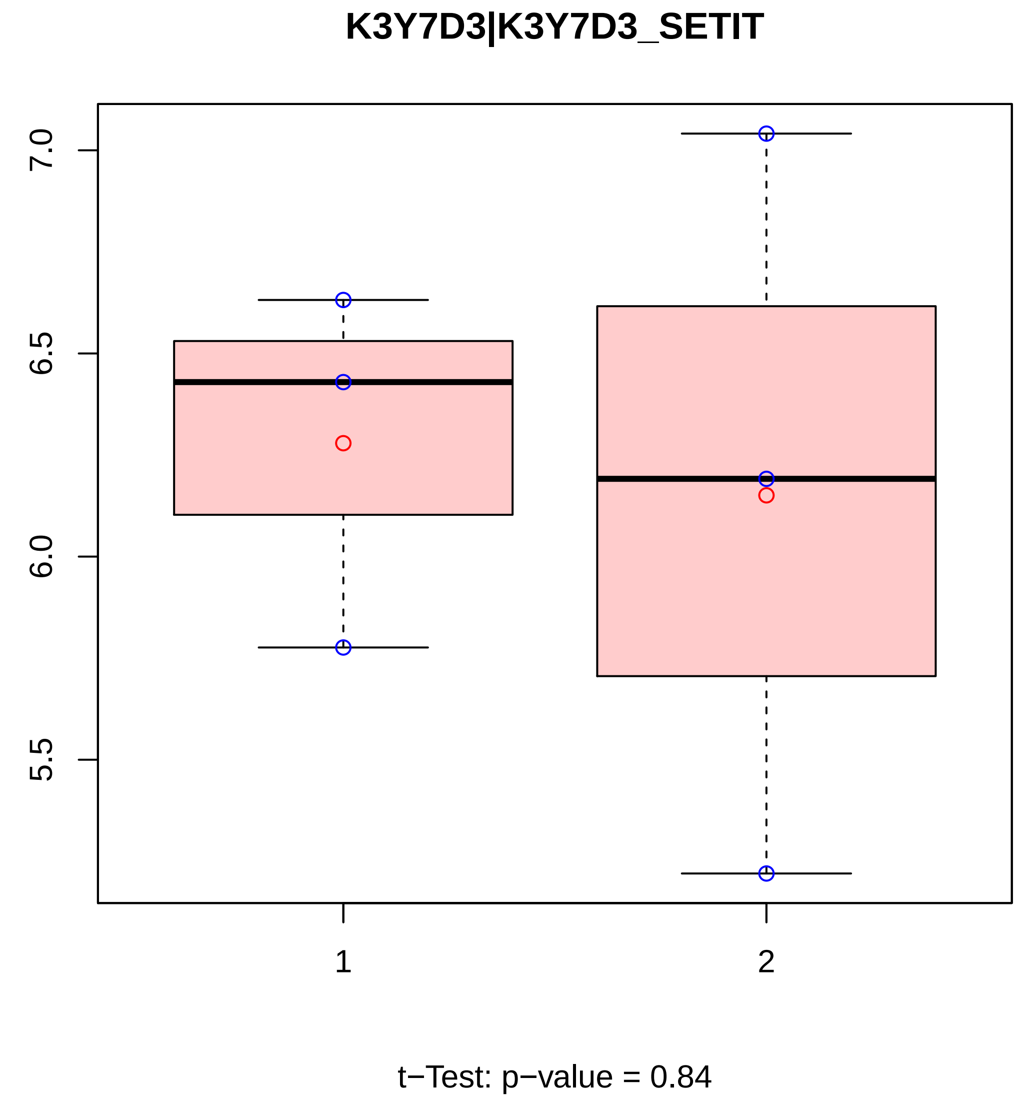
K3Y7D3|K3Y7D3_SETIT
7.0
6.5
6.0
5.5
1
2
t−Test: p−value = 0.84

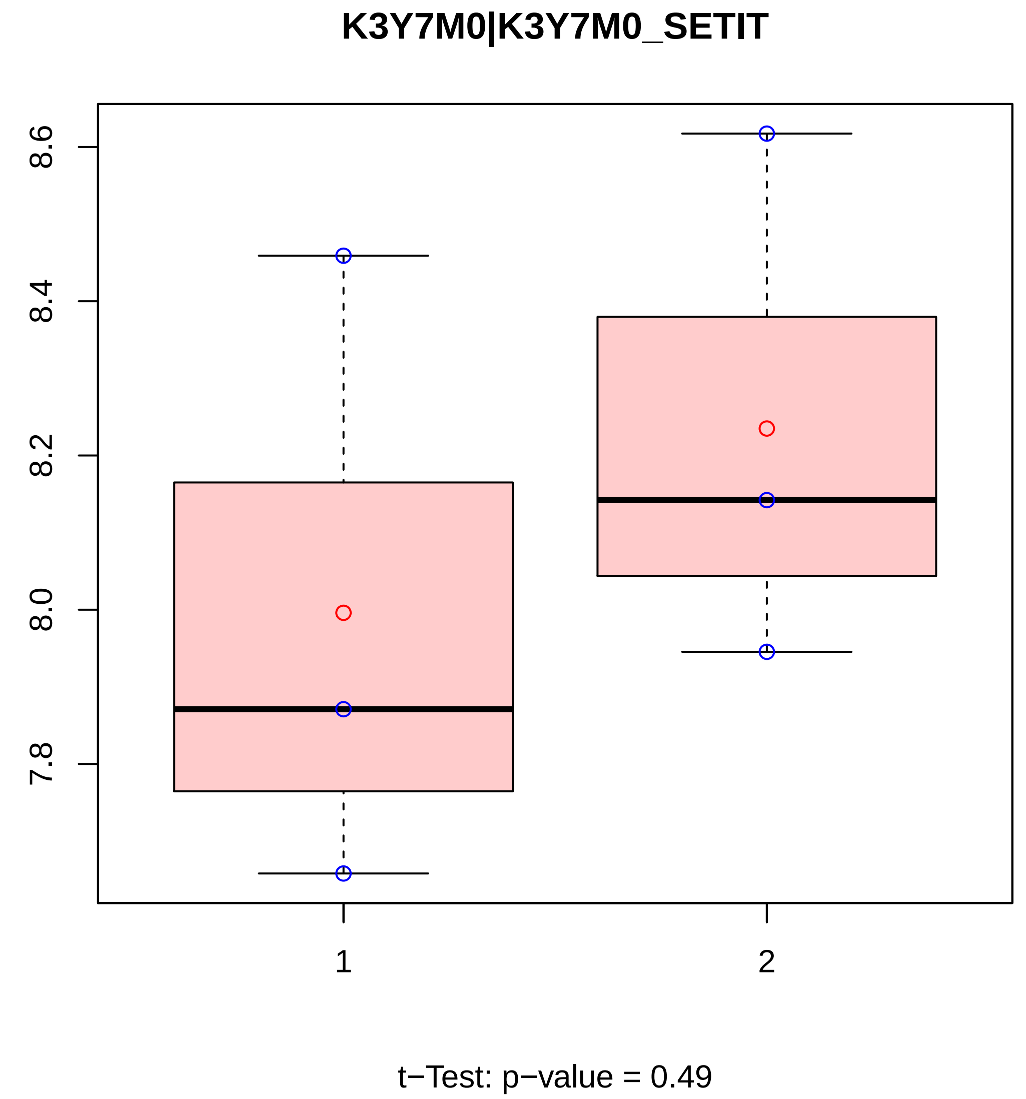
K3Y7M0|K3Y7M0_SETIT
8.6
8.4
8.2
8.0
7.8
1
2
t−Test: p−value = 0.49

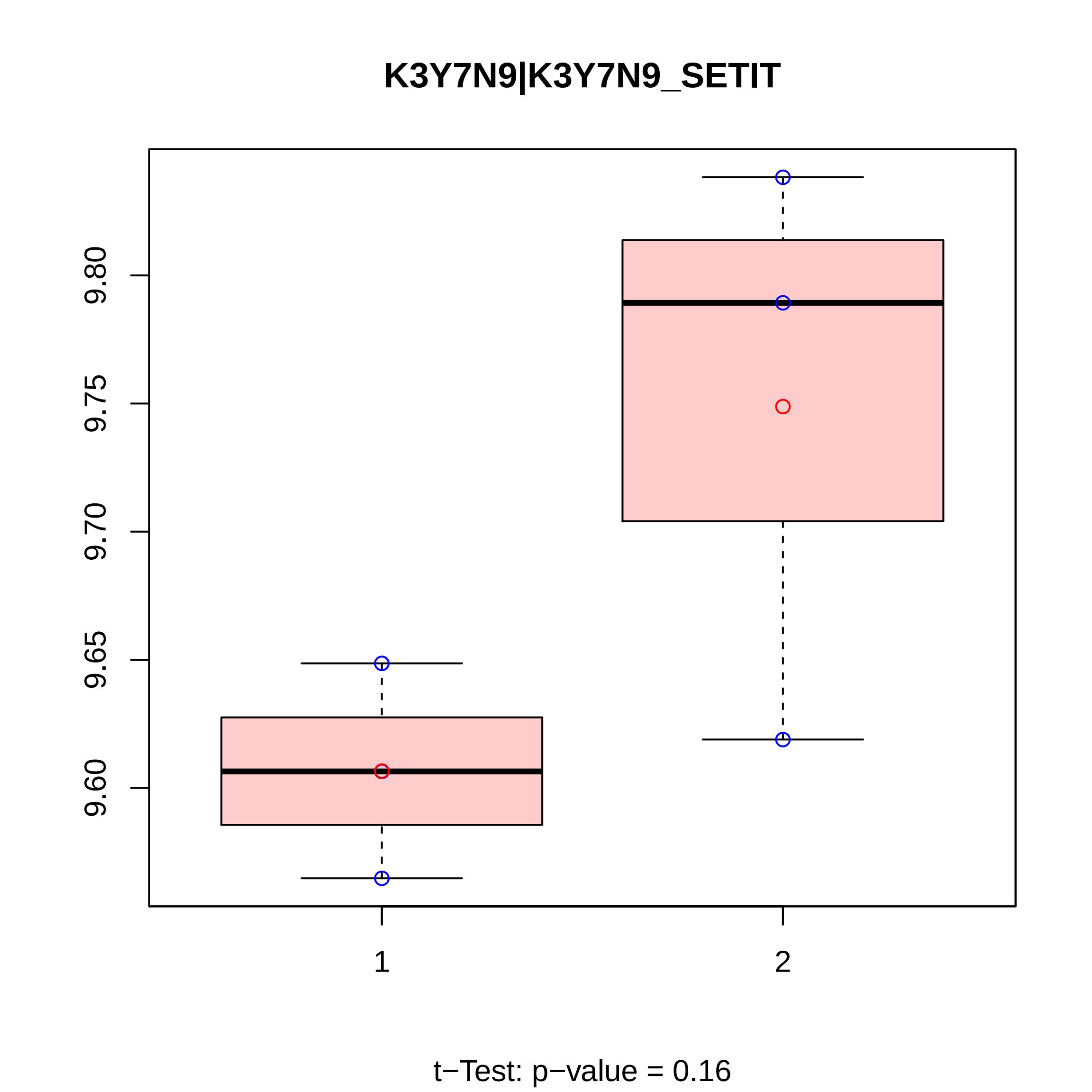
K3Y7N9|K3Y7N9_SETIT
9.60
9.65
9.70
9.75
9.80
1
2
t−Test: p−value = 0.16

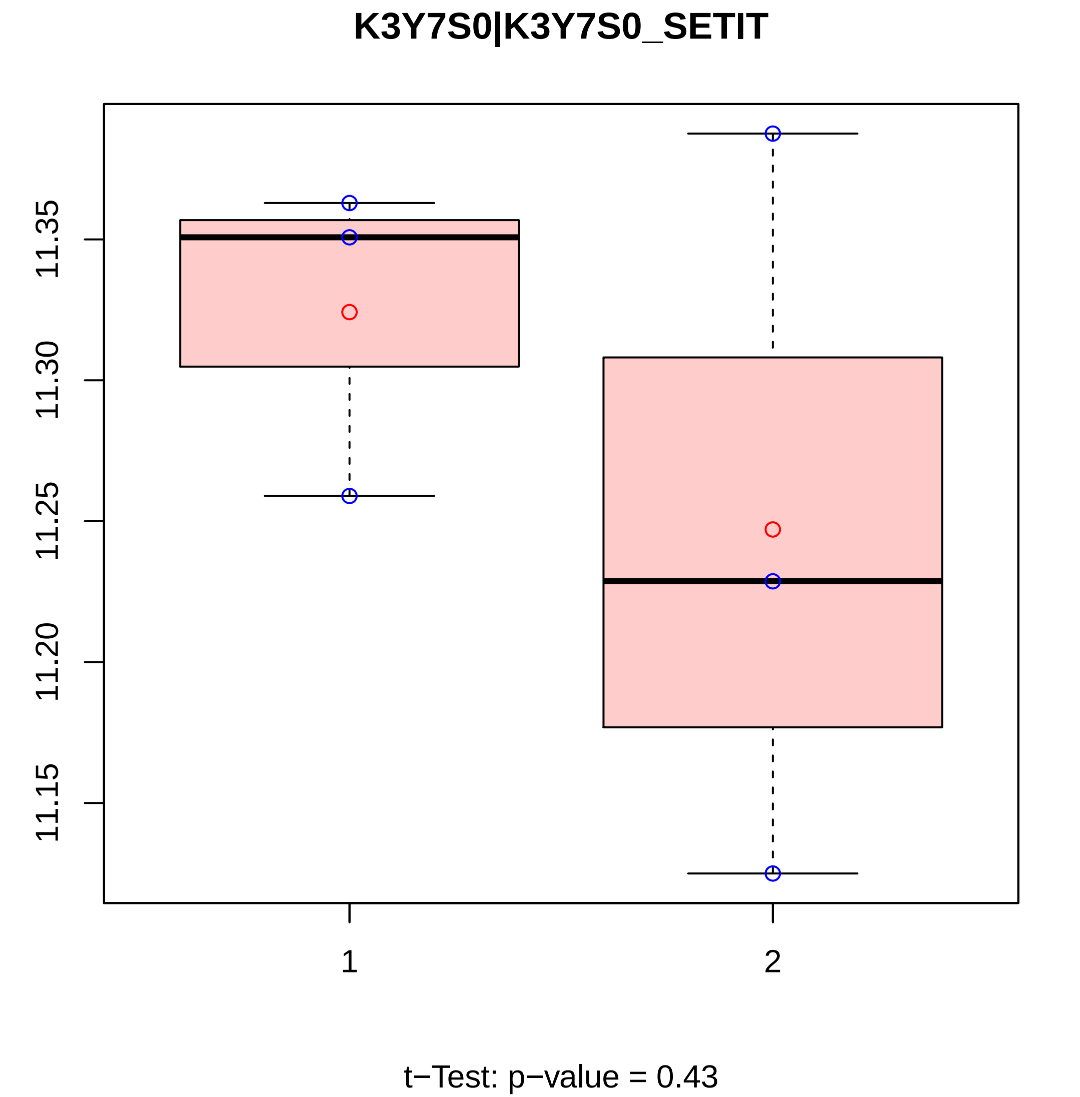

K3Y7S0|K3Y7S0_SETIT
11.15
11.20
11.25
11.30
11.35
1
2
t−Test: p−value = 0.43

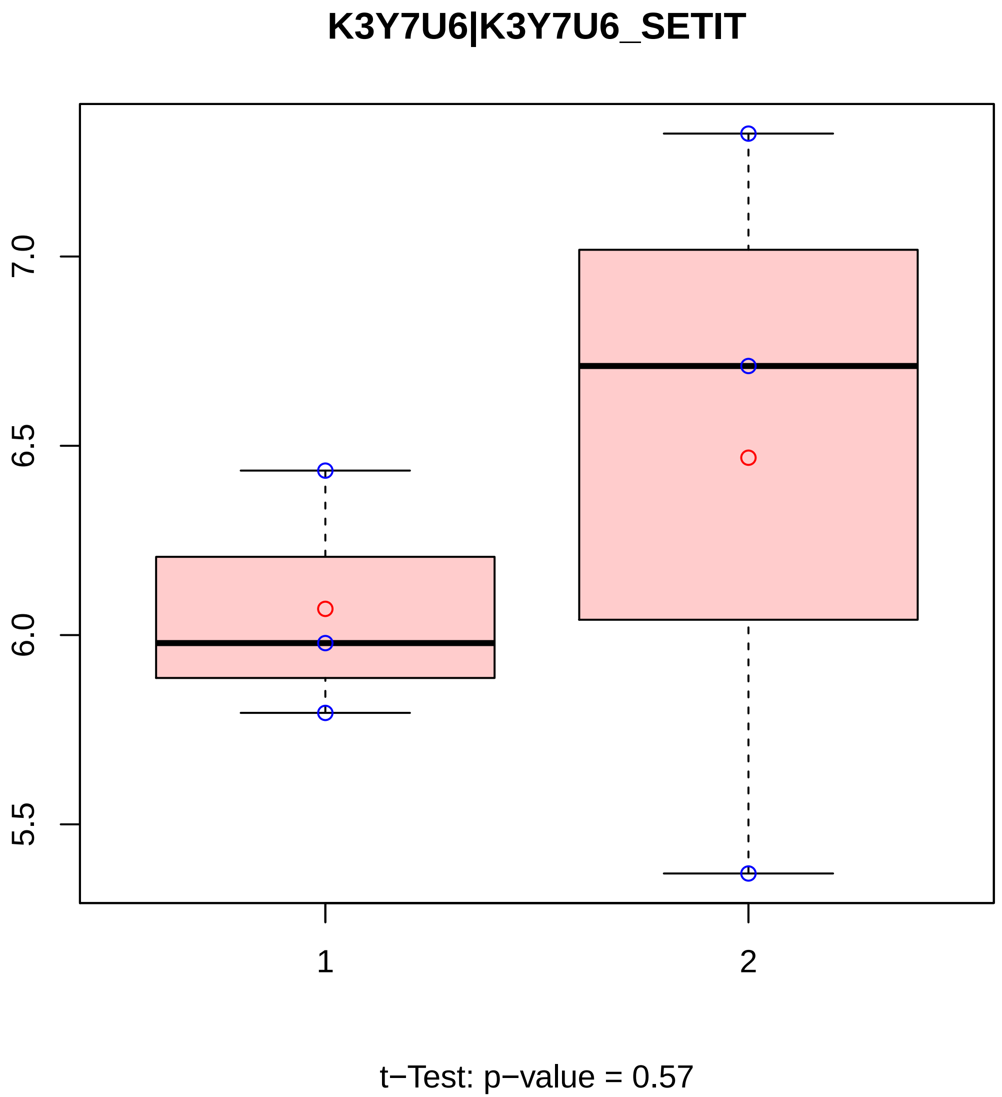
K3Y7U6|K3Y7U6_SETIT
7.0
6.5
6.0
5.5
1
2
t−Test: p−value = 0.57

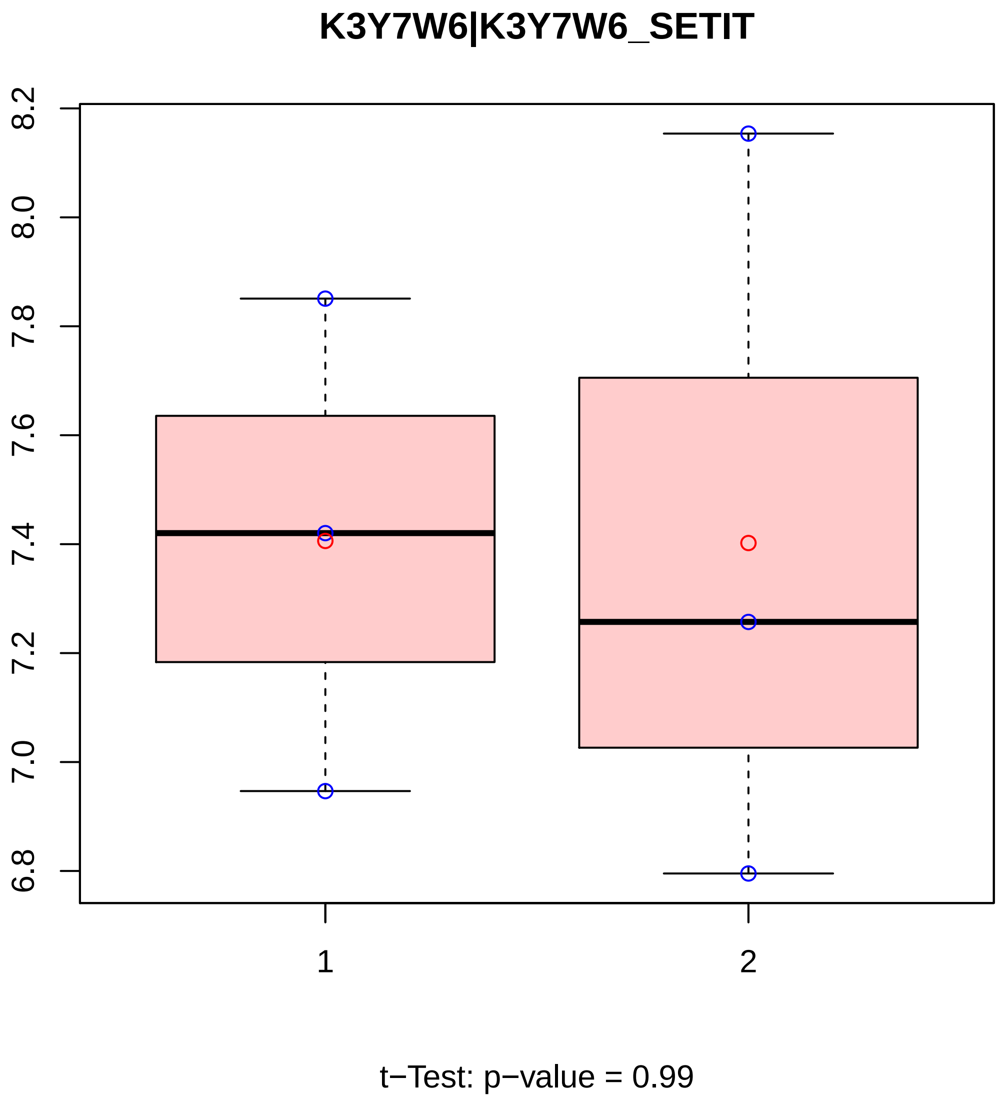

K3Y7W6|K3Y7W6_SETIT
8.2
8.0
7.8
7.6
7.4
7.2
7.0
6.8
1
2
t−Test: p−value = 0.99

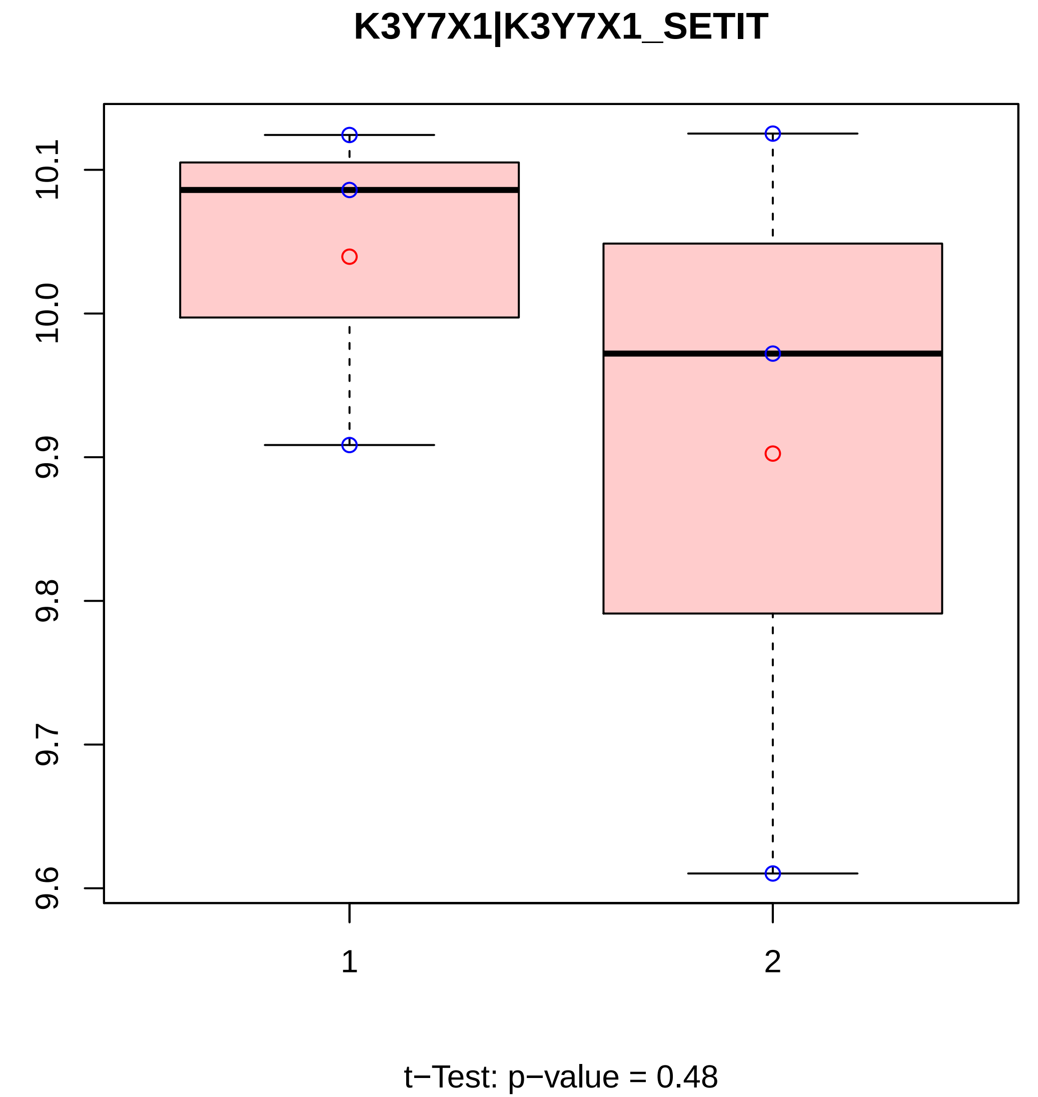
K3Y7X1|K3Y7X1_SETIT
10.1
10.0
9.9
9.8
9.7
9.6
1
2
t−Test: p−value = 0.48

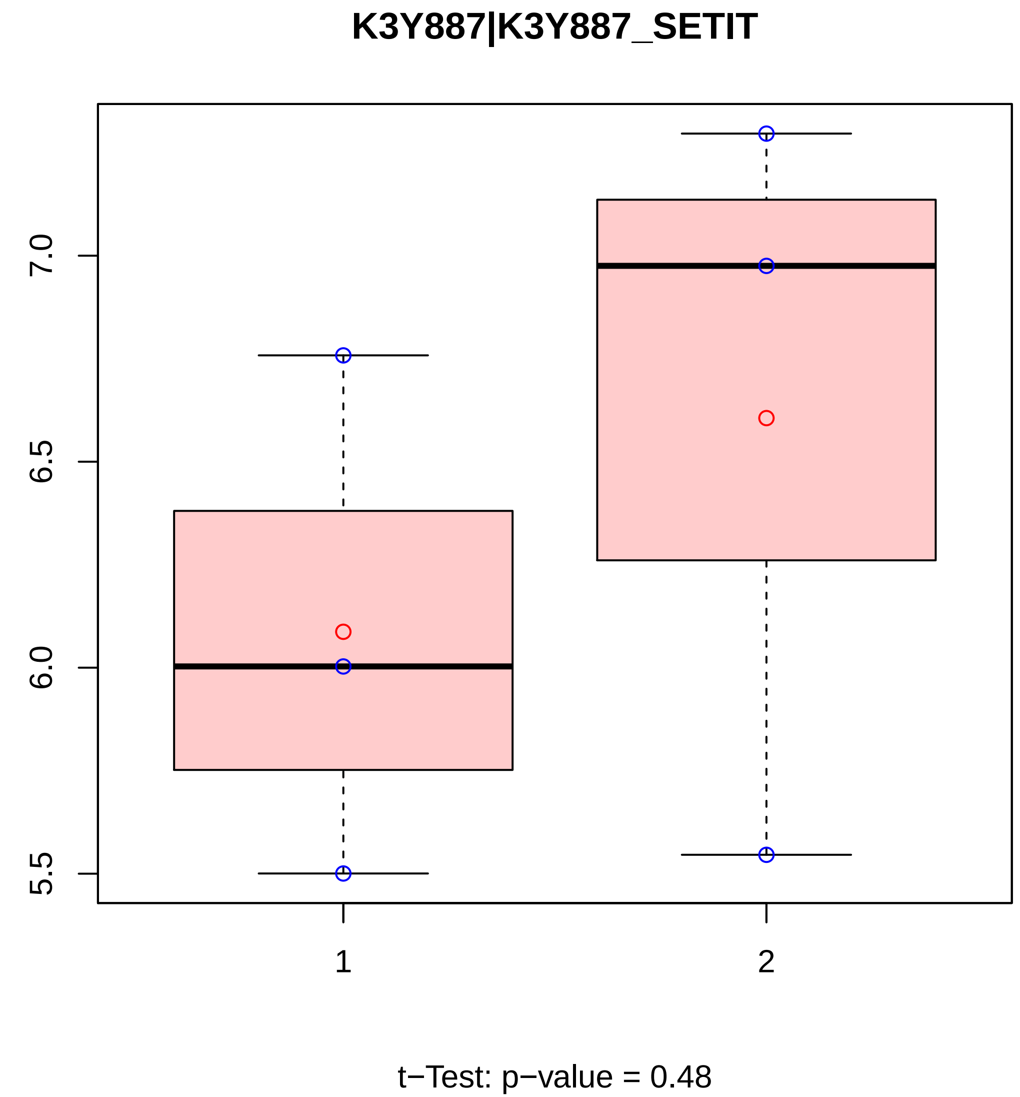

K3Y887|K3Y887_SETIT
7.0
6.5
6.0
5.5
1
2
t−Test: p−value = 0.48

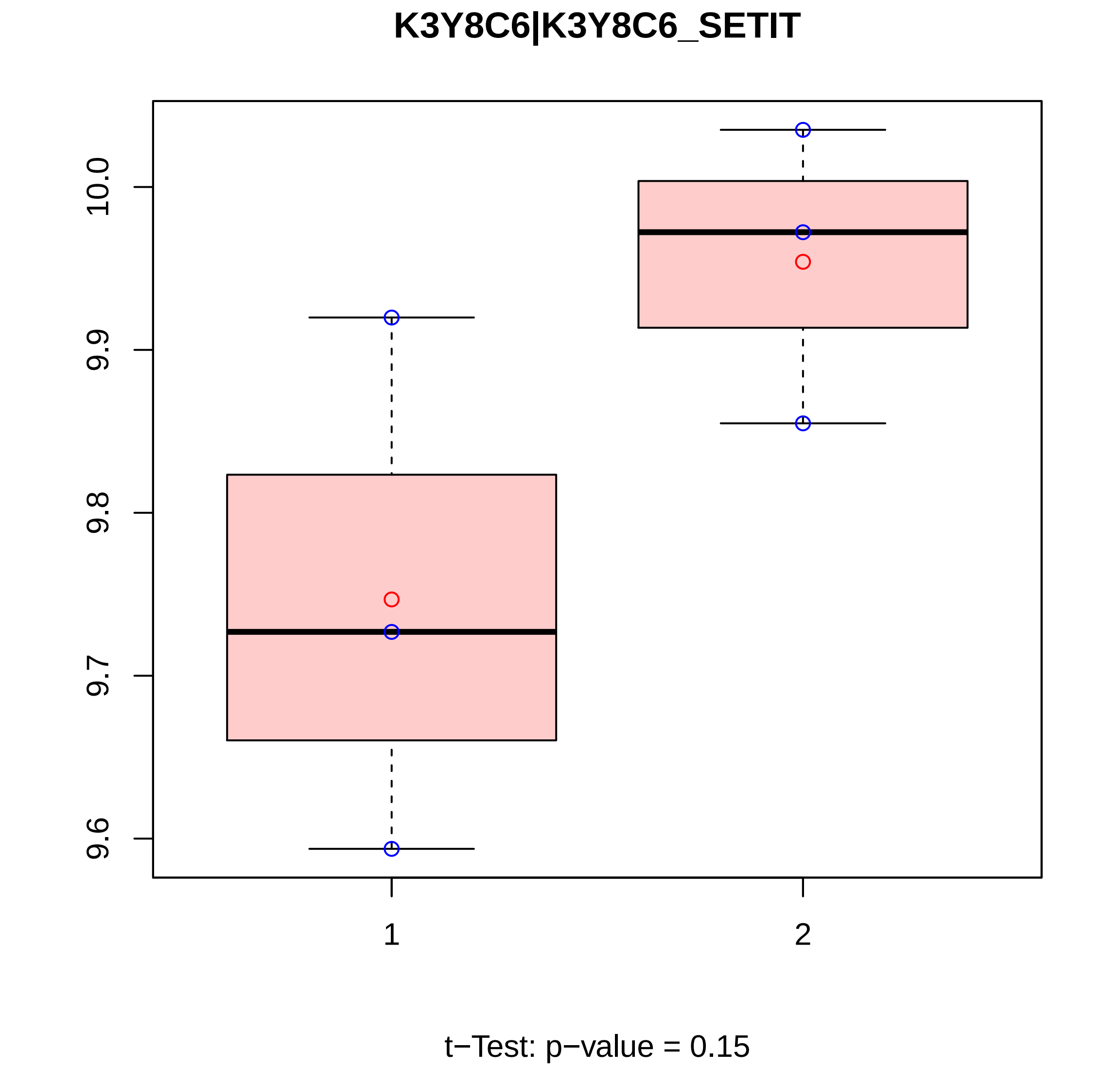
K3Y8C6|K3Y8C6_SETIT
10.0
9.9
9.8
9.7
9.6
1
2
t−Test: p−value = 0.15

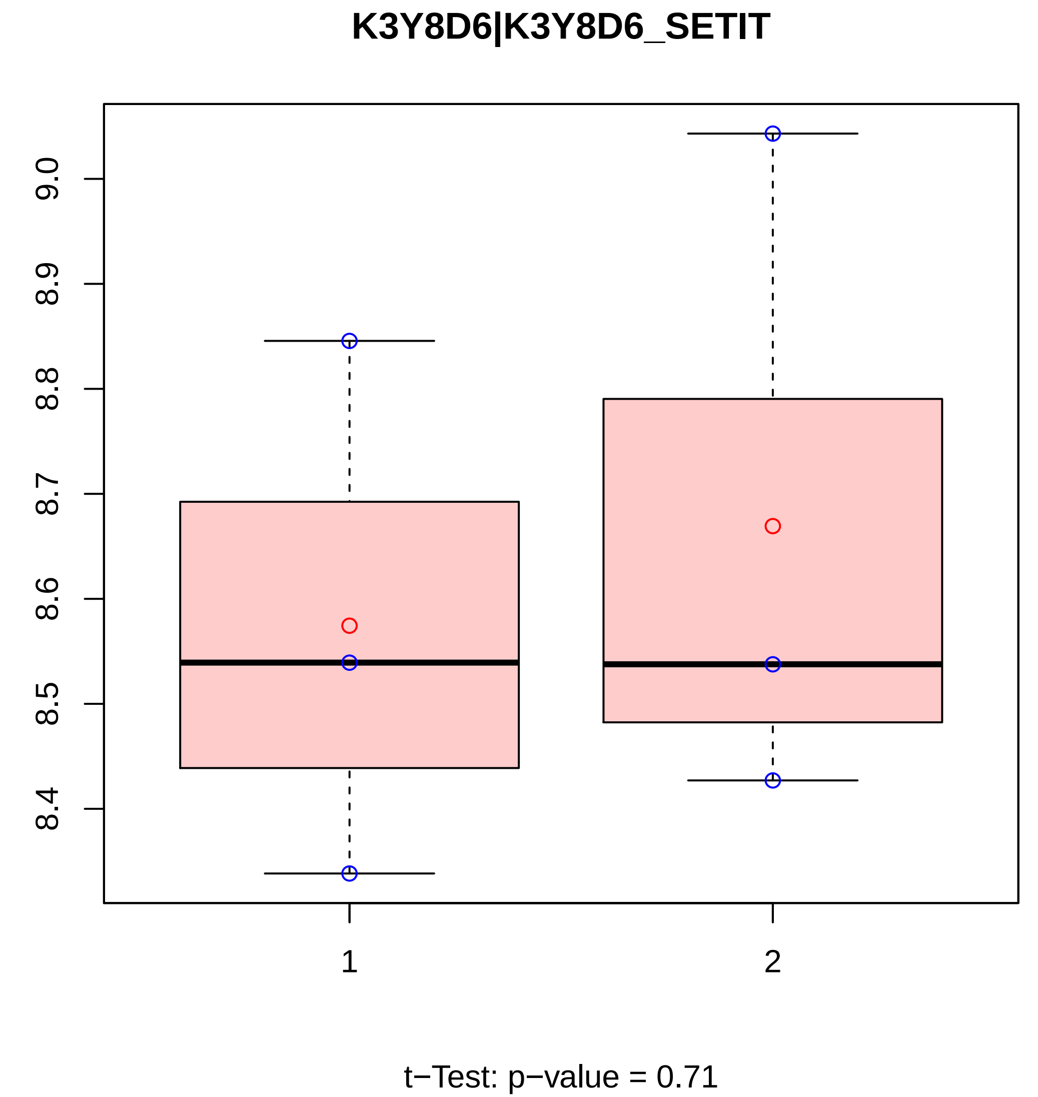
K3Y8D6|K3Y8D6_SETIT
9.0
8.9
8.8
8.7
8.6
8.5
8.4
1
2
t−Test: p−value = 0.71

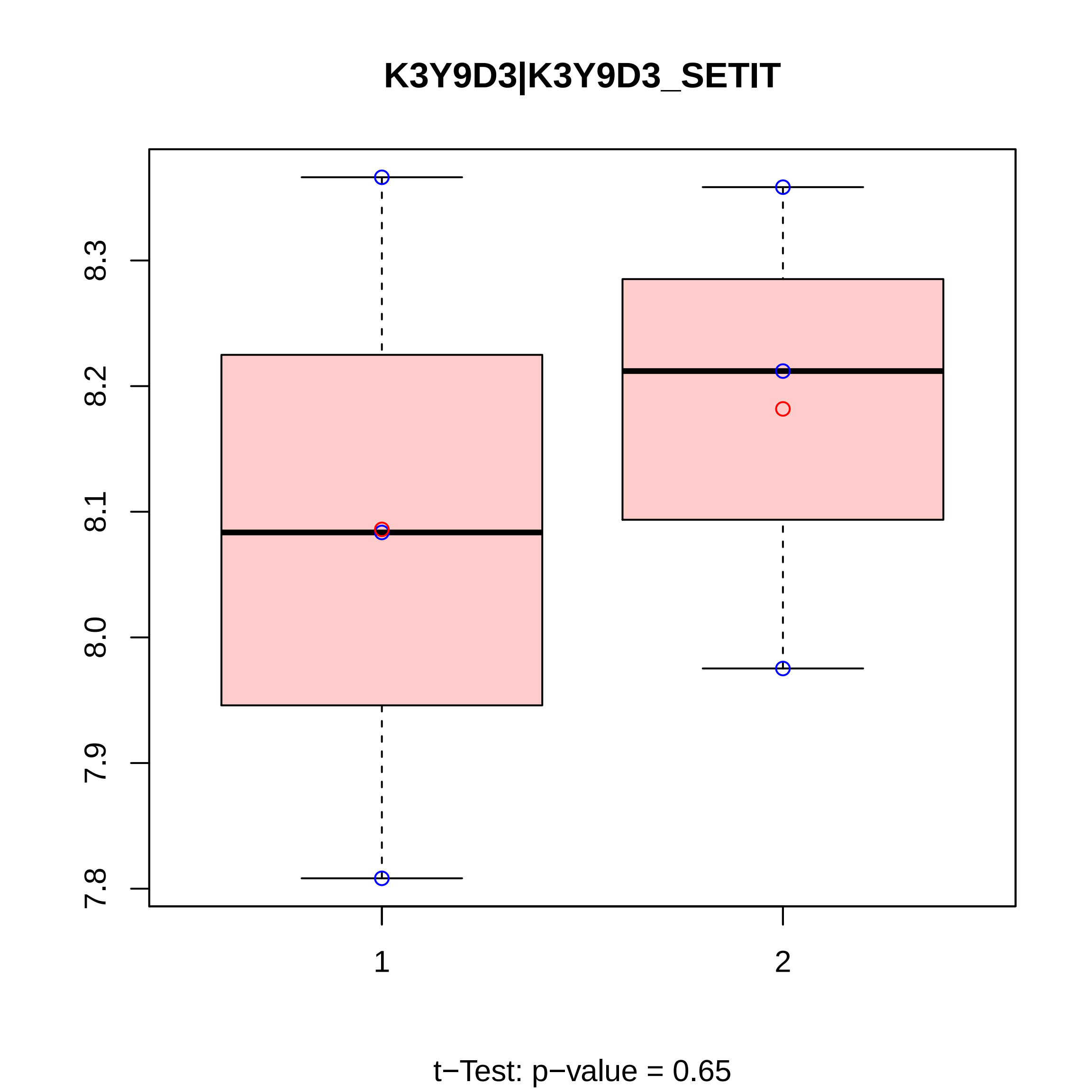
K3Y9D3|K3Y9D3_SETIT
8.3
8.2
8.1
8.0
7.9
7.8
1
2
t−Test: p−value = 0.65

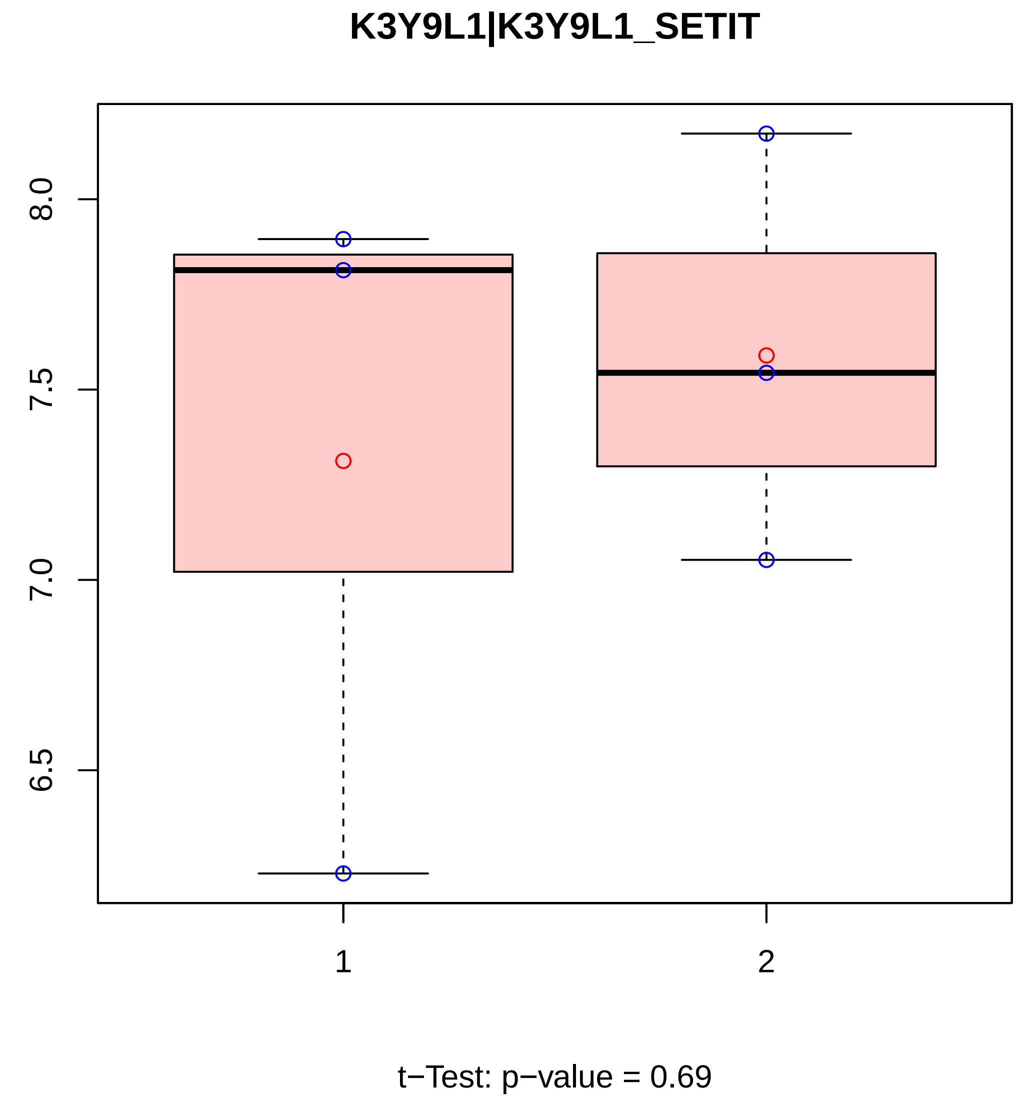
K3Y9L1|K3Y9L1_SETIT
8.0
7.5
7.0
6.5
1
2
t−Test: p−value = 0.69

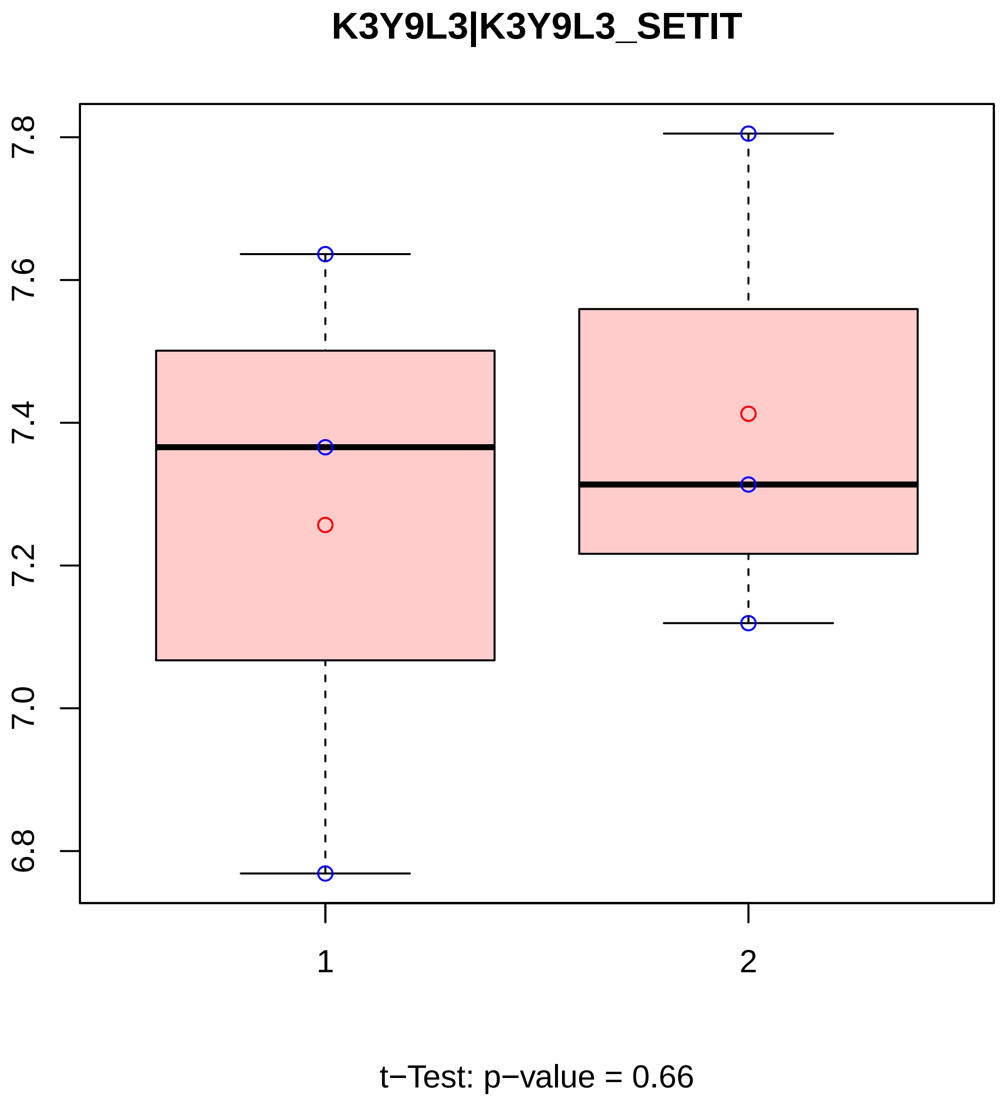
K3Y9L3|K3Y9L3_SETIT
7.8
7.6
7.4
7.2
7.0
6.8
1
2
t−Test: p−value = 0.66

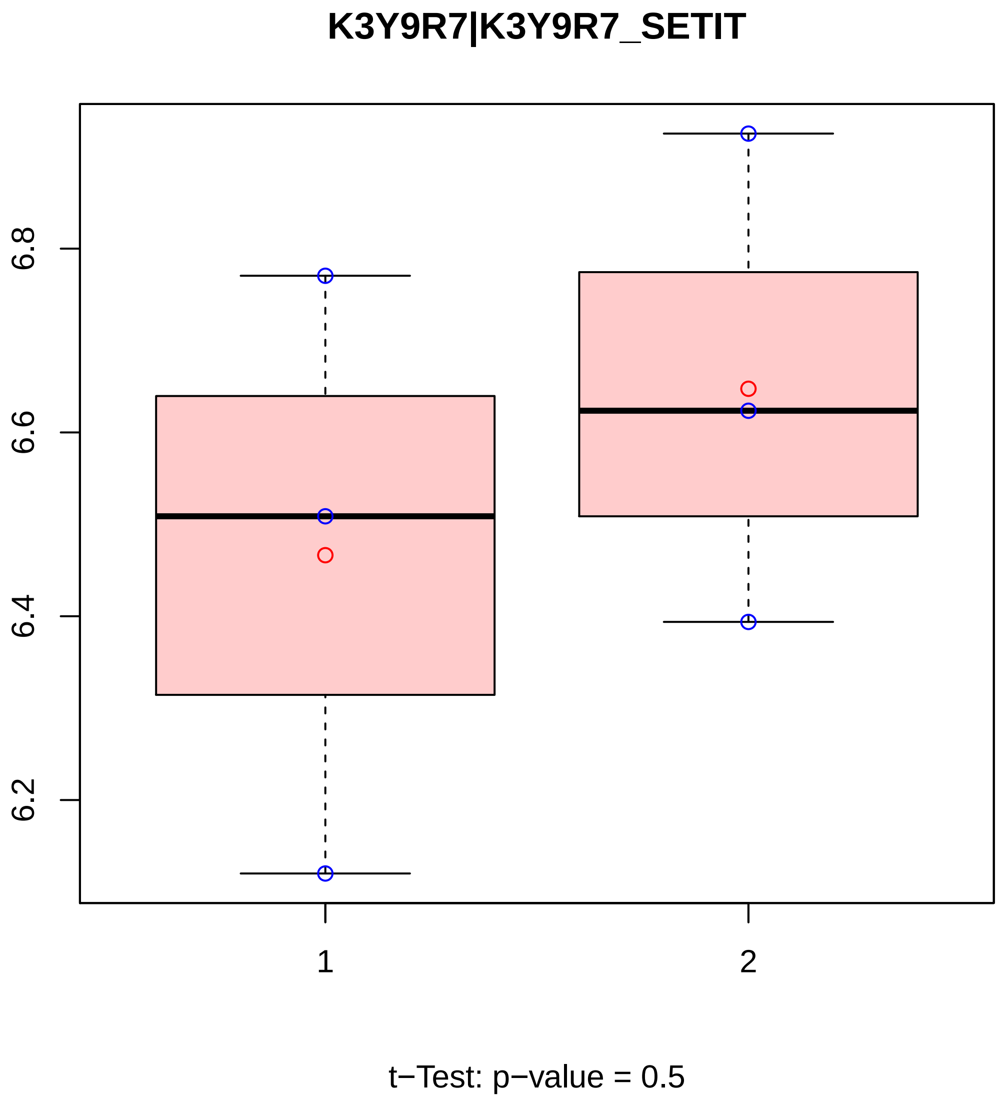
K3Y9R7|K3Y9R7_SETIT
6.2
6.4
6.6
6.8
1
2
t−Test: p−value = 0.5

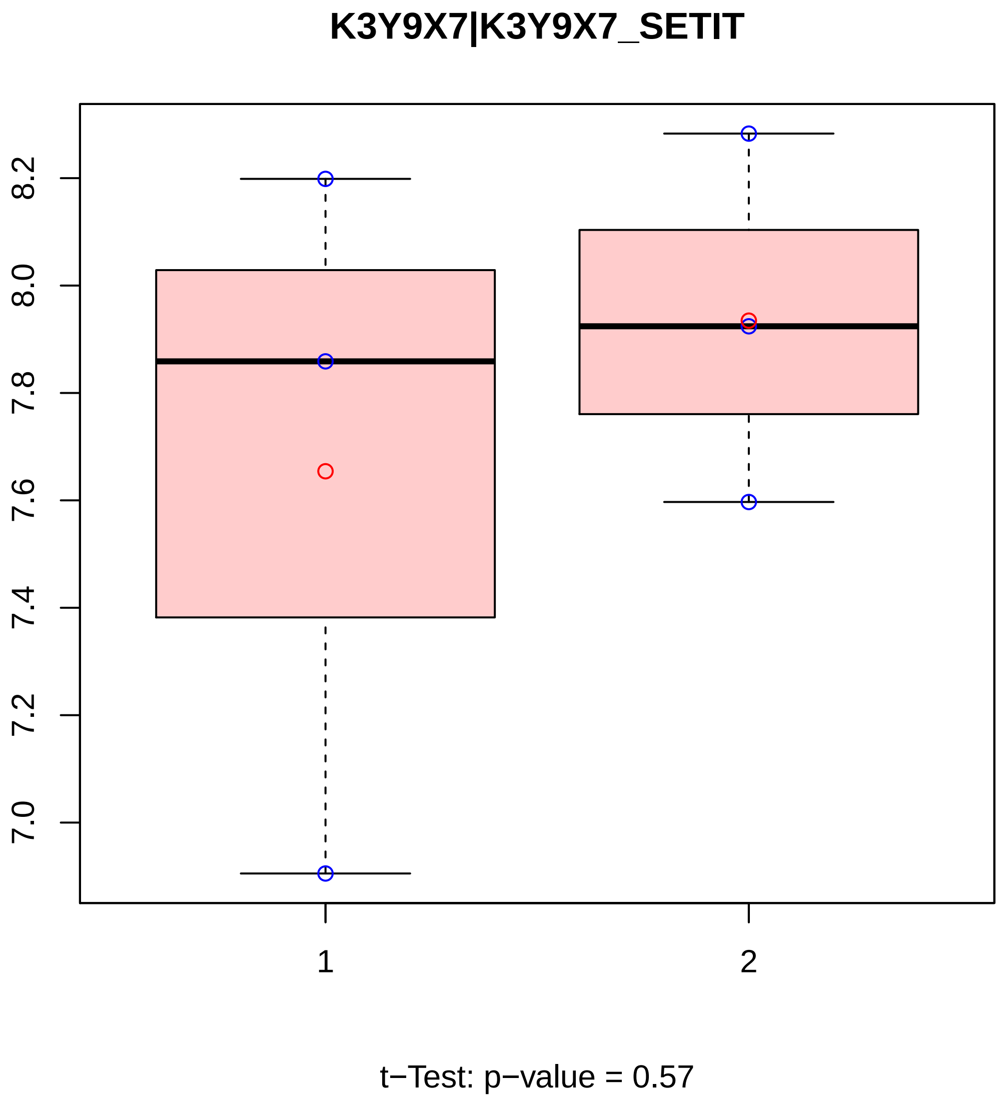
K3Y9X7|K3Y9X7_SETIT
8.2
8.0
7.8
7.6
7.4
7.2
7.0
1
2
t−Test: p−value = 0.57

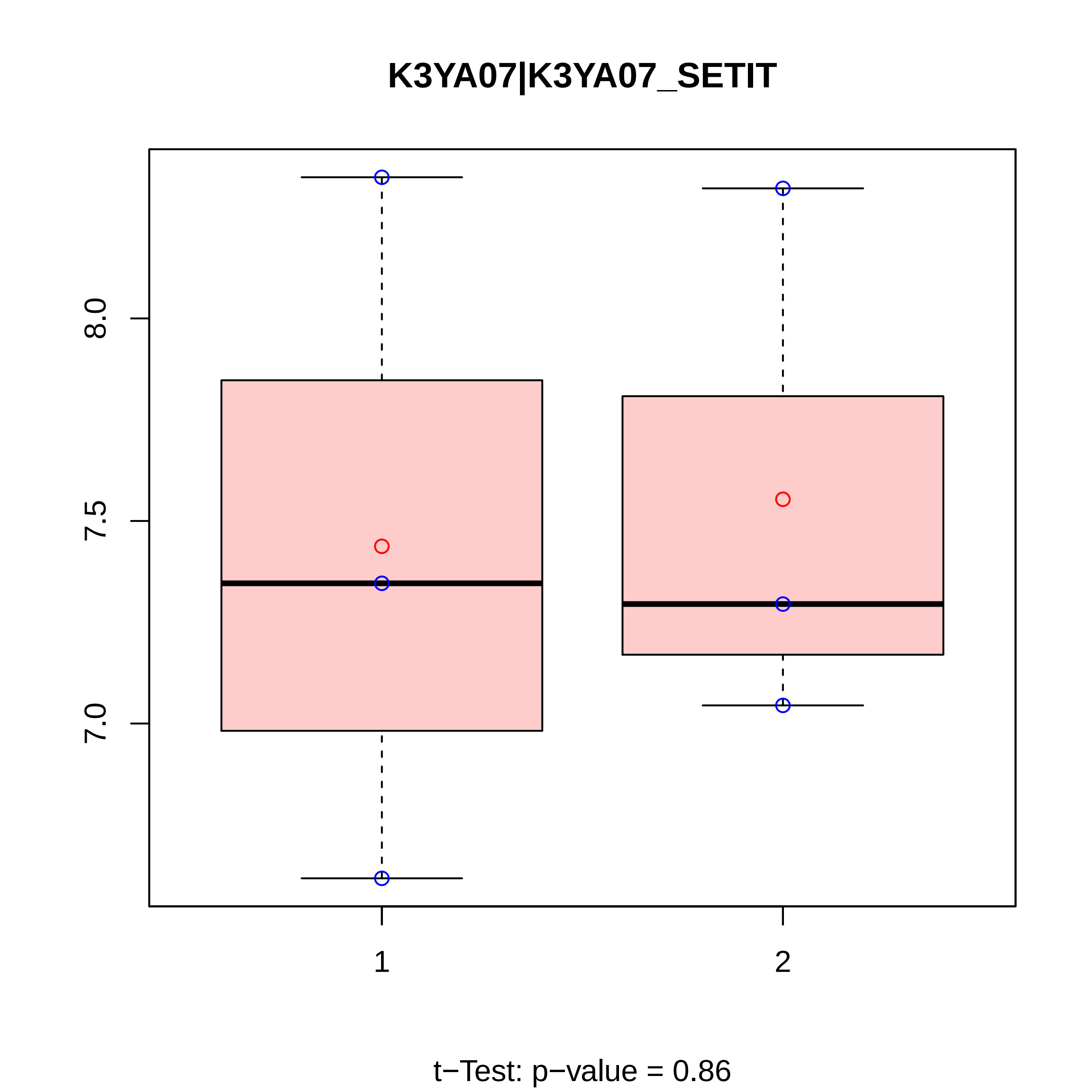
K3YA07|K3YA07_SETIT
8.0
7.5
7.0
1
2
t−Test: p−value = 0.86

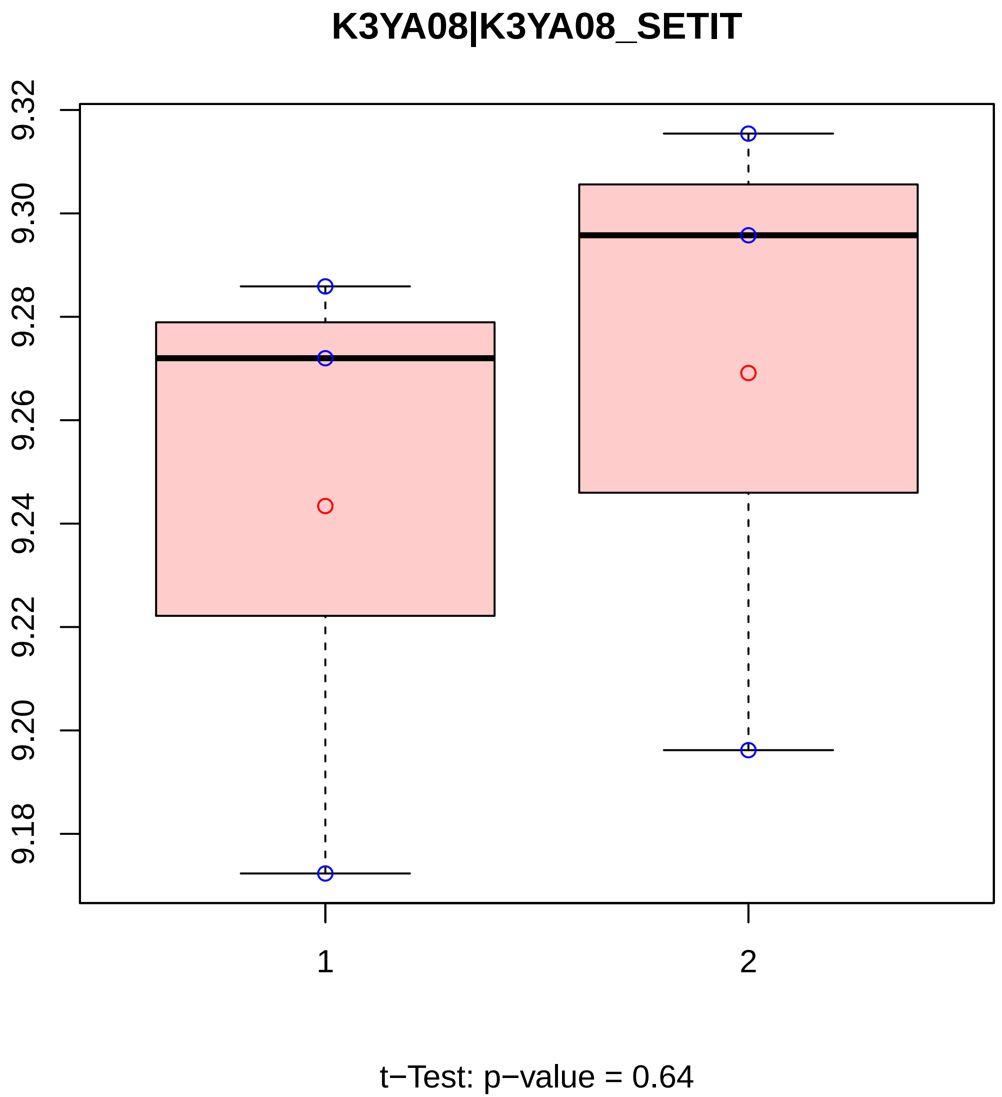
K3YA08|K3YA08_SETIT
9.18
9.20
9.22
9.24
9.26
9.28
9.30
9.32
1
2
t−Test: p−value = 0.64

K3YA18|K3YA18_SETIT

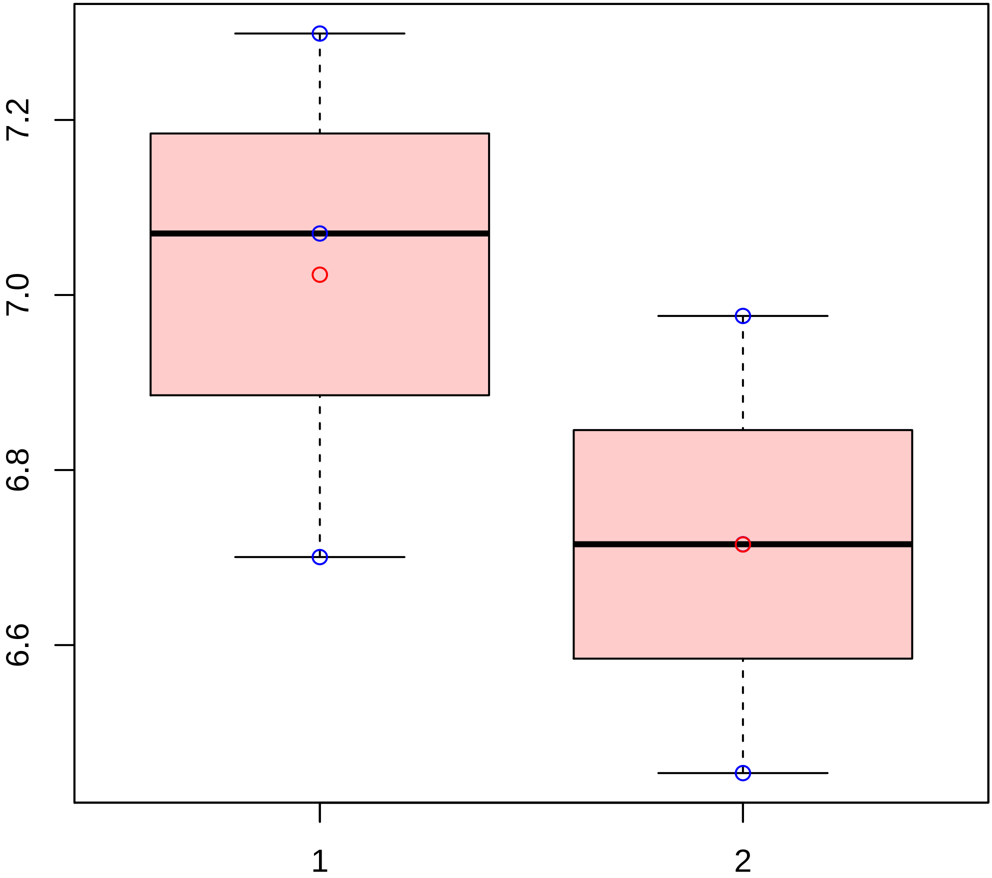

t−Test: p−value = 0.25

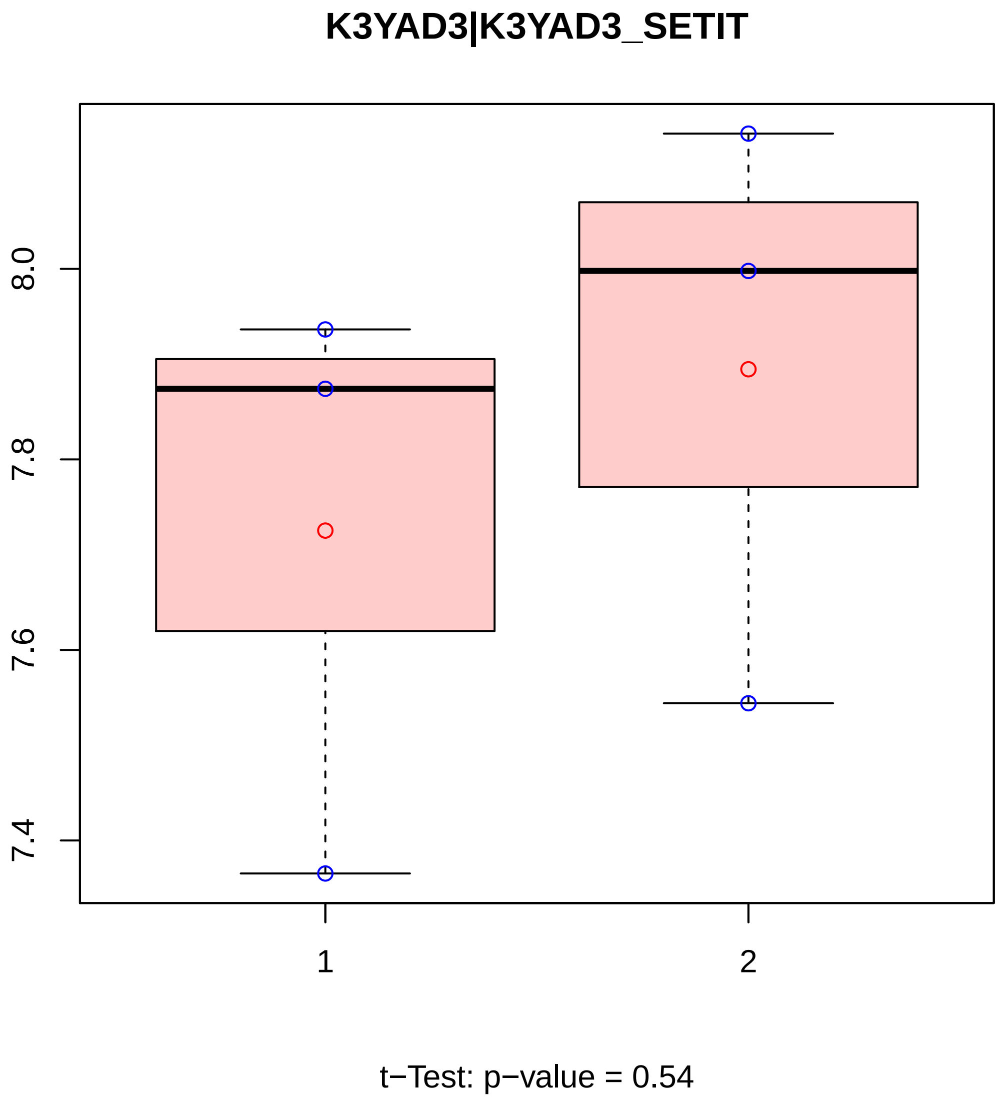
K3YAD3|K3YAD3_SETIT
8.0
7.8
7.6
7.4
1
2
t−Test: p−value = 0.54

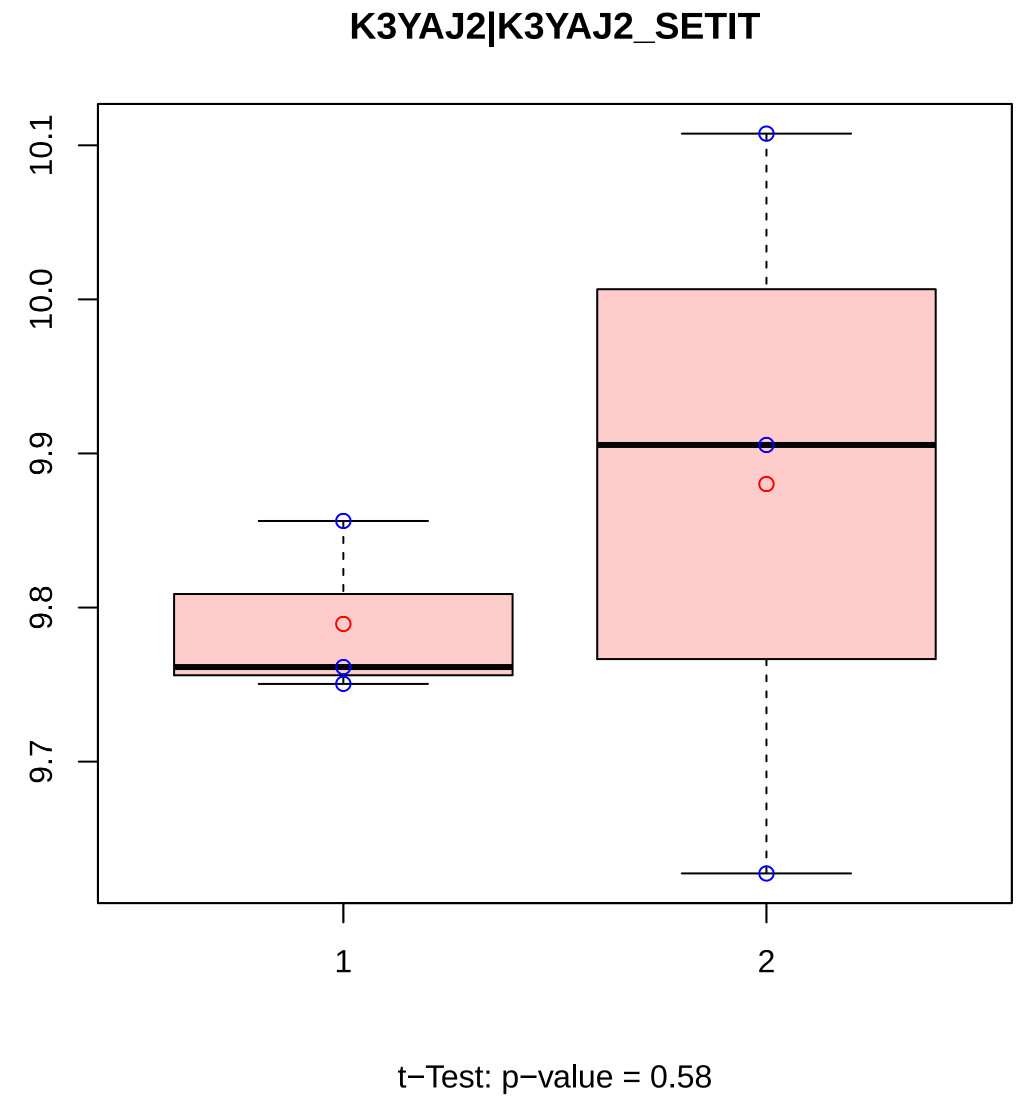
K3YAJ2|K3YAJ2_SETIT
10.1
10.0
9.9
9.8
9.7
1
2
t−Test: p−value = 0.58

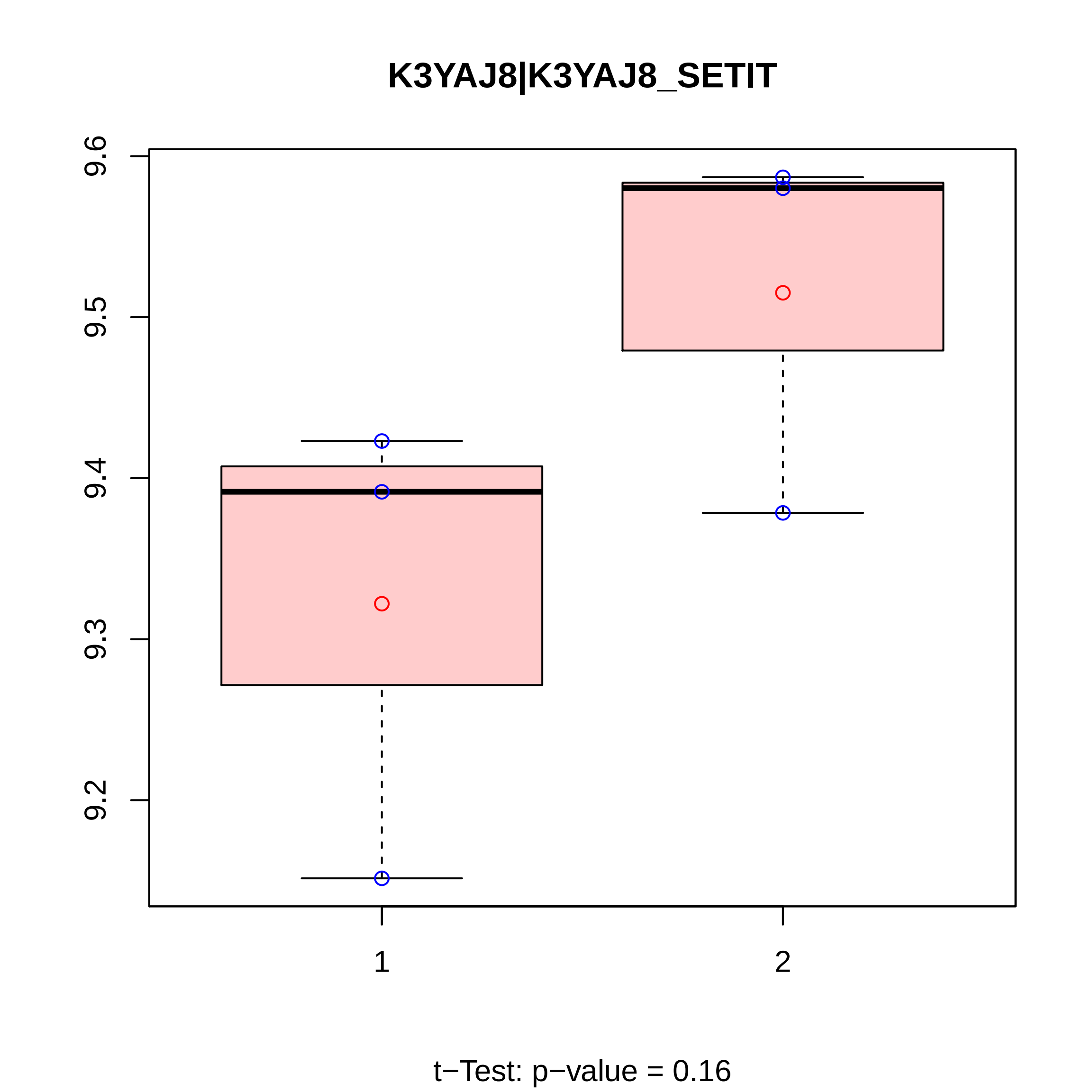
K3YAJ8|K3YAJ8_SETIT
9.2
9.3
9.4
9.5
9.6
1
2
t−Test: p−value = 0.16

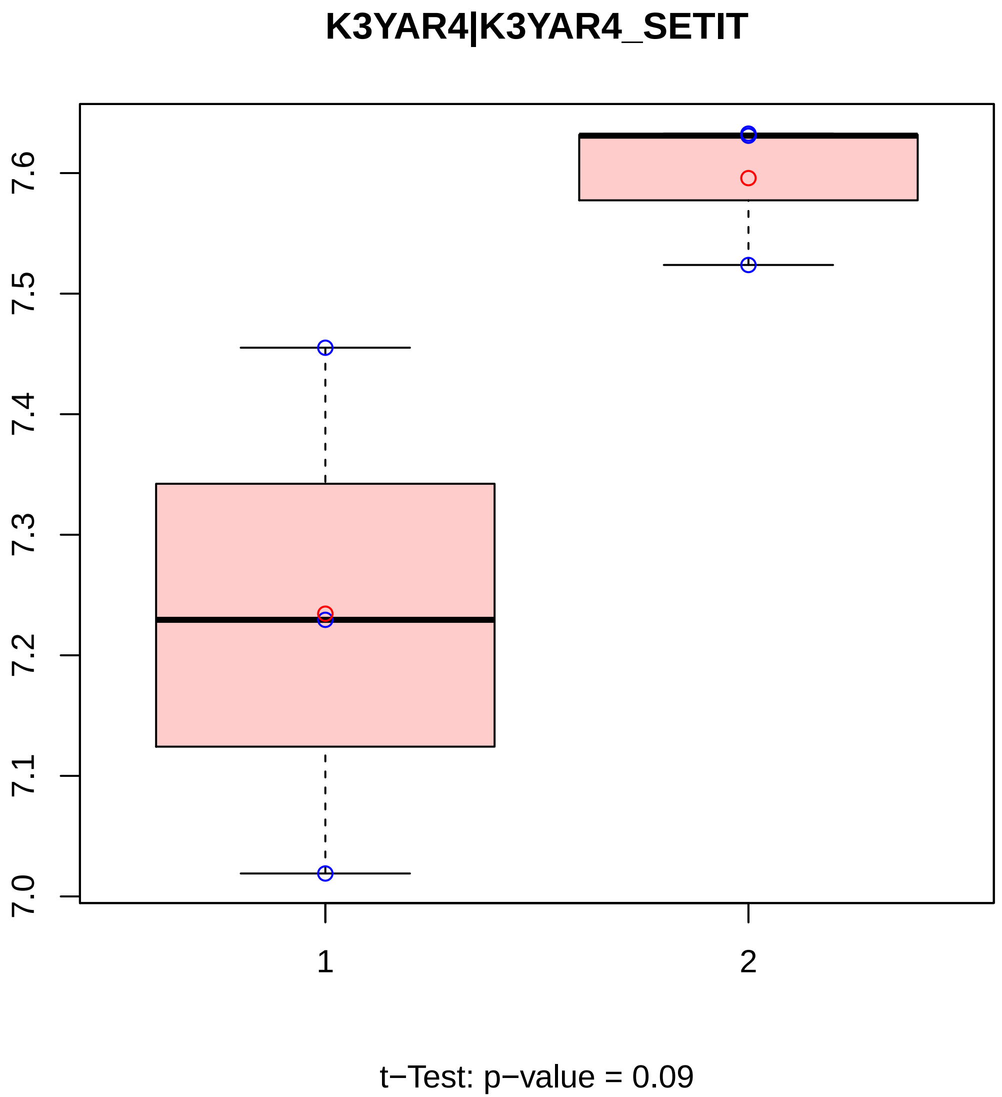
K3YAR4|K3YAR4_SETIT
7.0
7.1
7.2
7.3
7.4
7.5
7.6
1
2
t−Test: p−value = 0.09

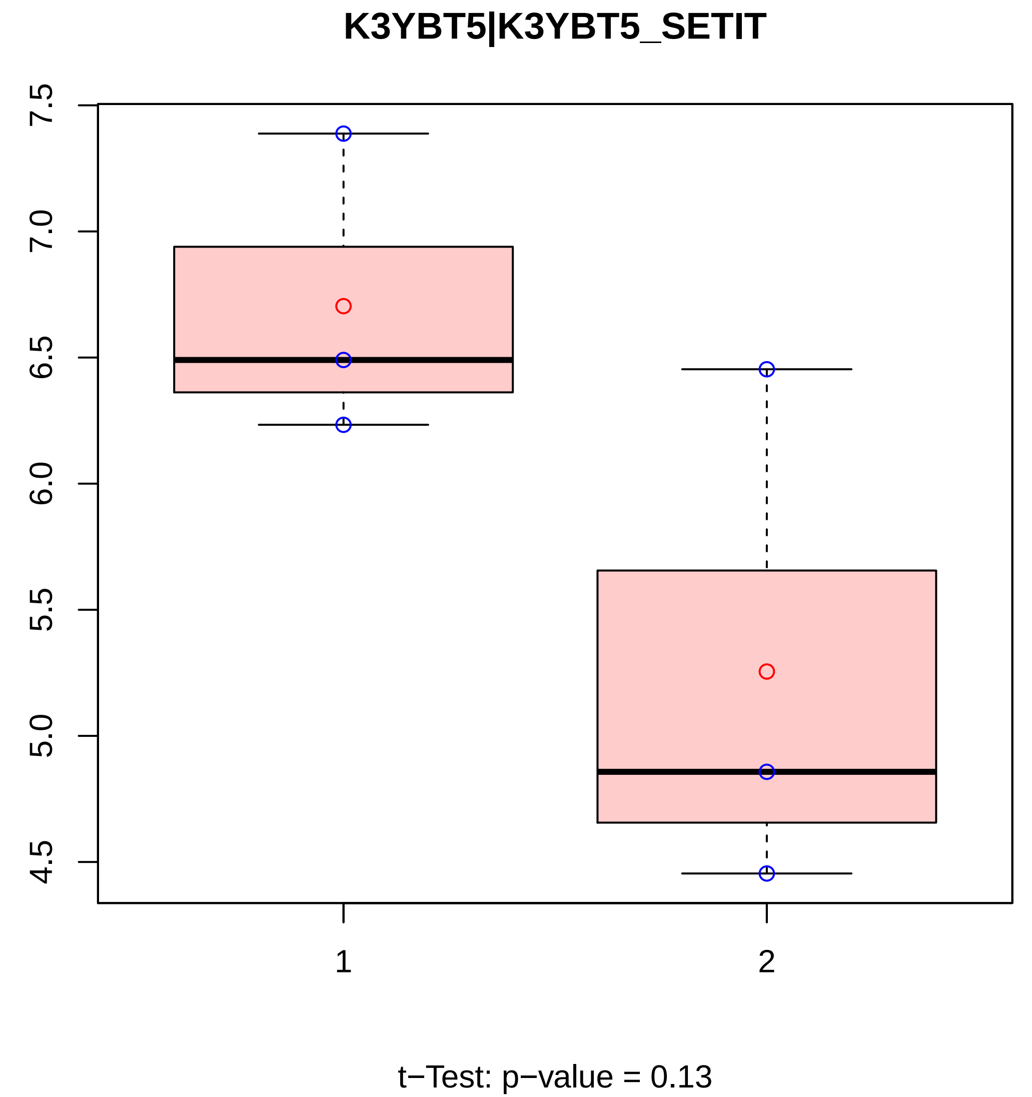
K3YBT5|K3YBT5_SETIT
7.5
7.0
6.5
6.0
5.5
5.0
4.5
1
2
t−Test: p−value = 0.13

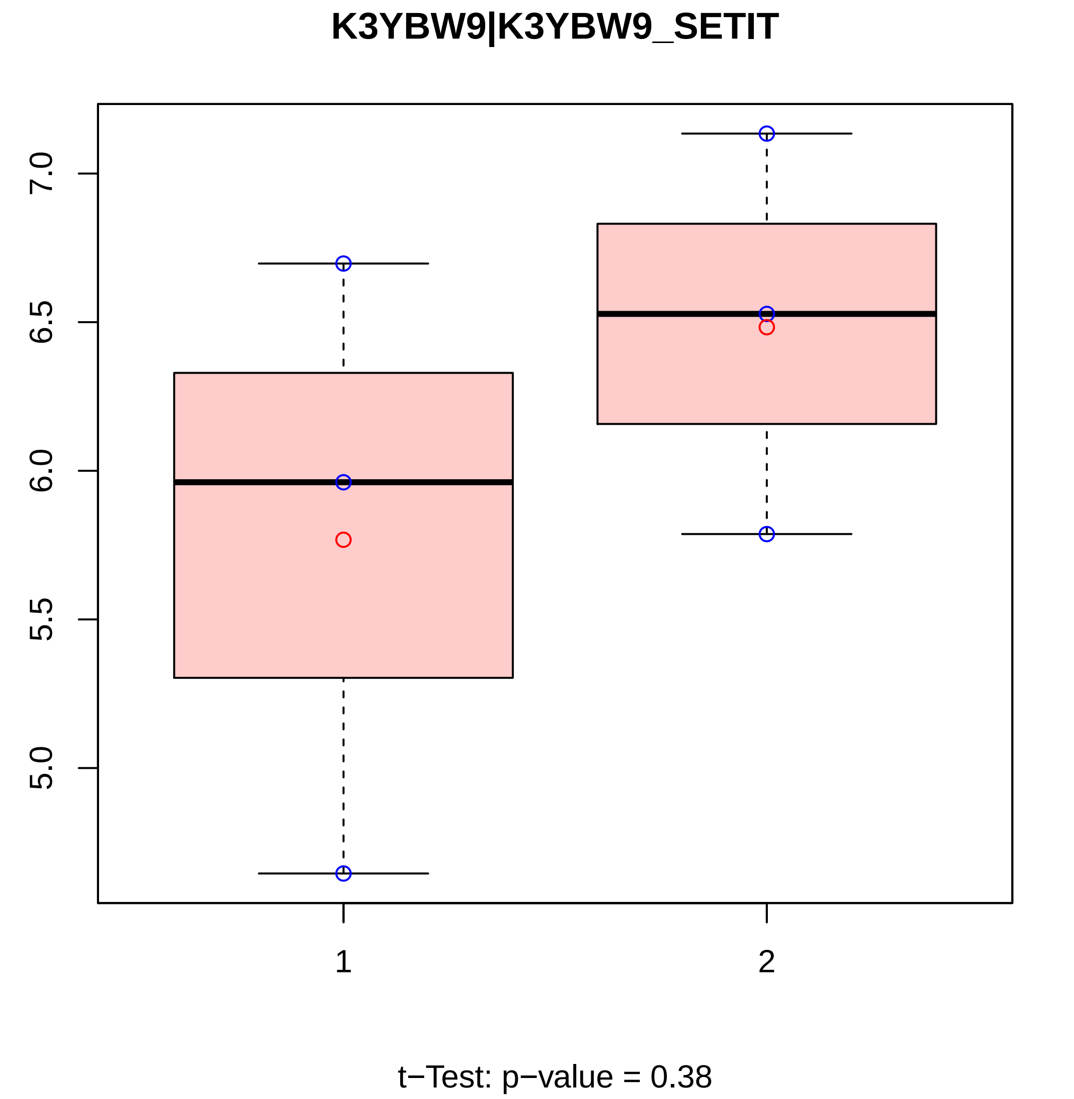
K3YBW9|K3YBW9_SETIT
7.0
6.5
6.0
5.5
5.0
1
2
t−Test: p−value = 0.38

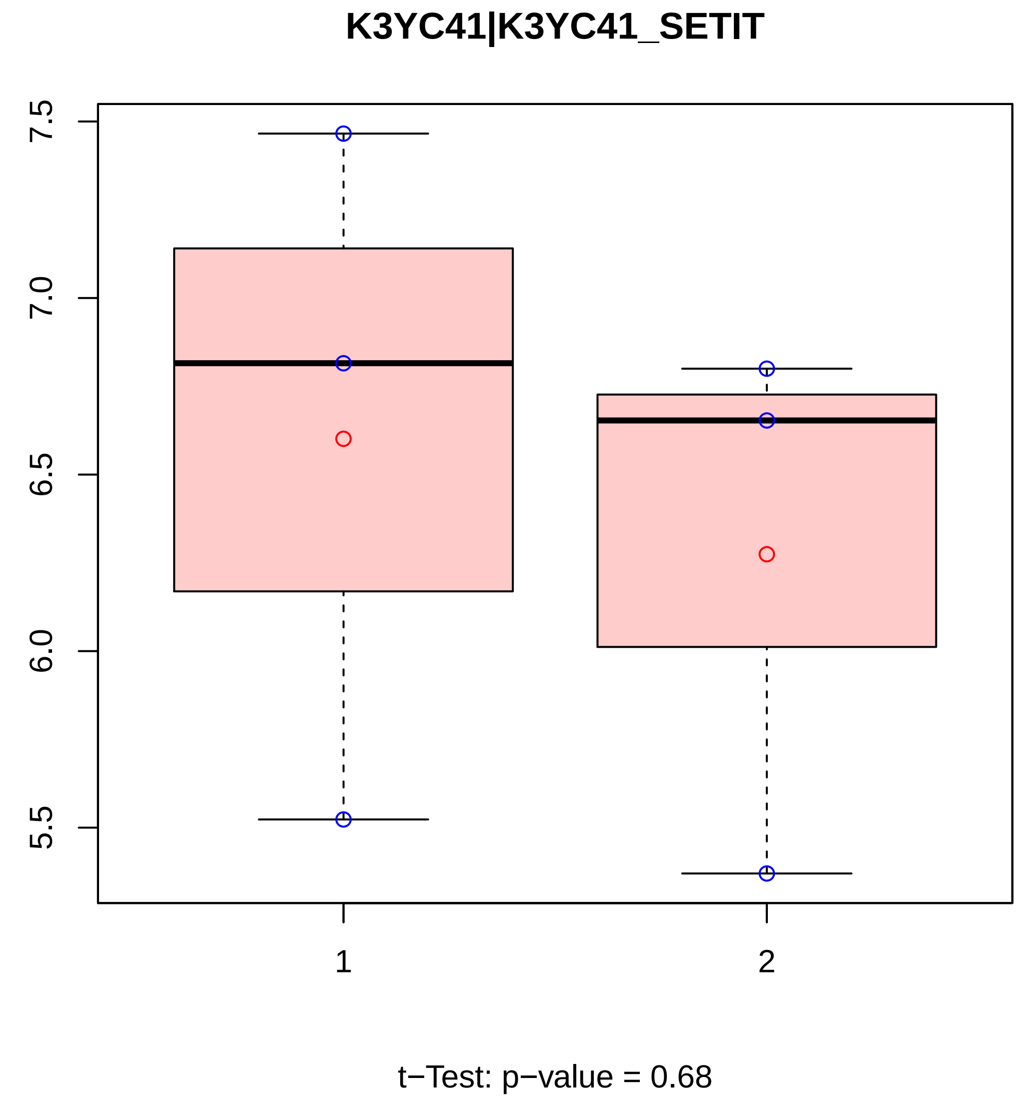
K3YC41|K3YC41_SETIT
7.5
7.0
6.5
6.0
5.5
1
2
t−Test: p−value = 0.68

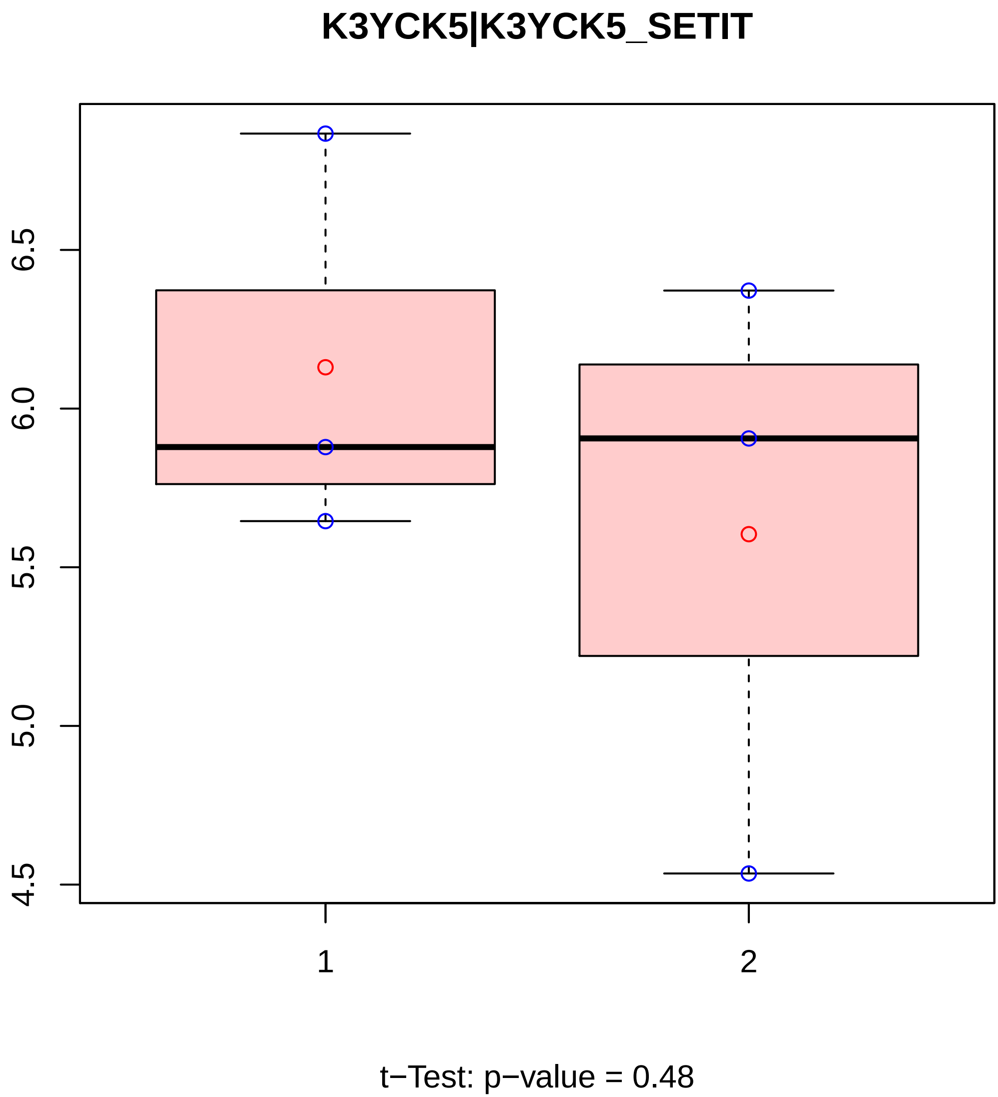
K3YCK5|K3YCK5_SETIT
4.5
5.0
5.5
6.0
6.5
1
2
t−Test: p−value = 0.48

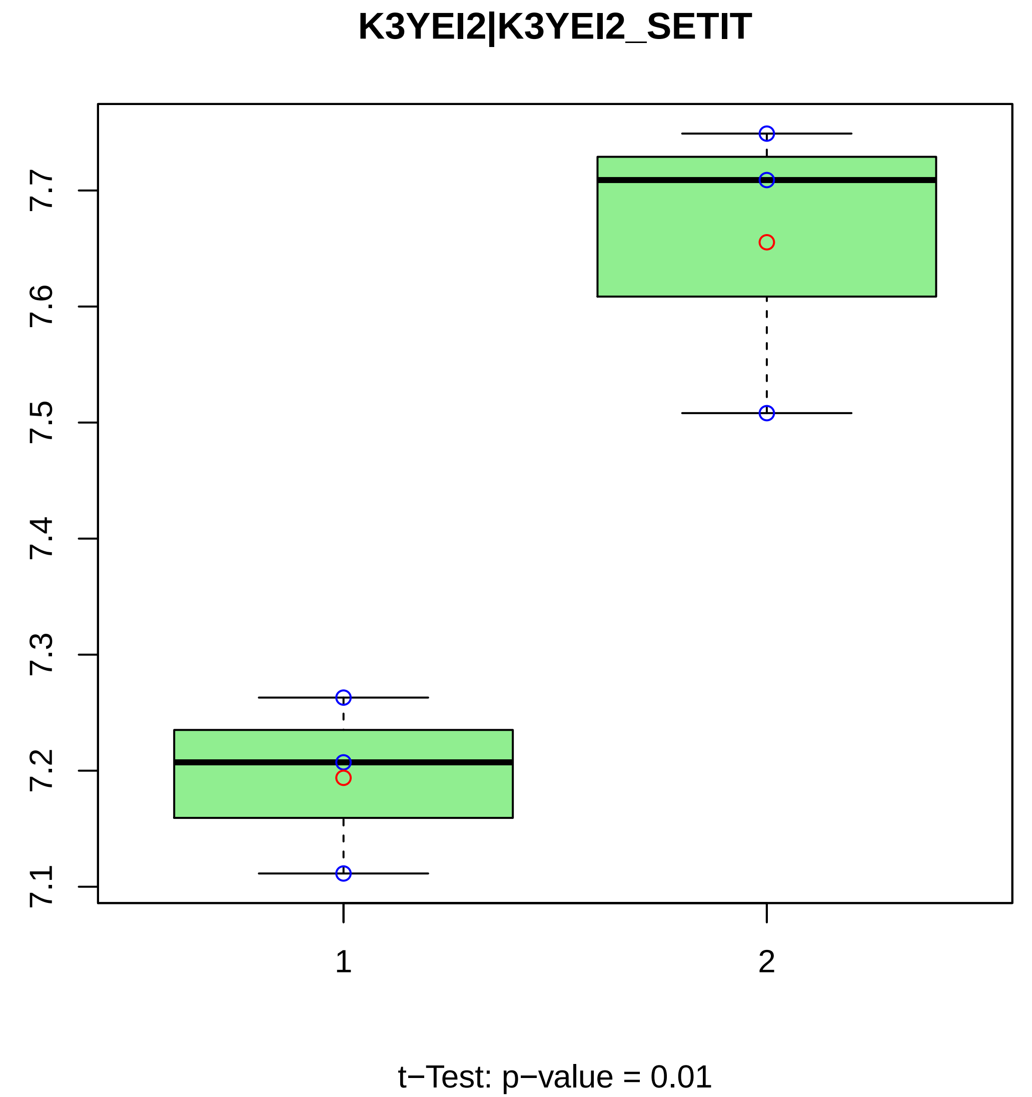
K3YEI2|K3YEI2_SETIT
7.1
7.2
7.3
7.4
7.5
7.6
7.7
1
2
t−Test: p−value = 0.01

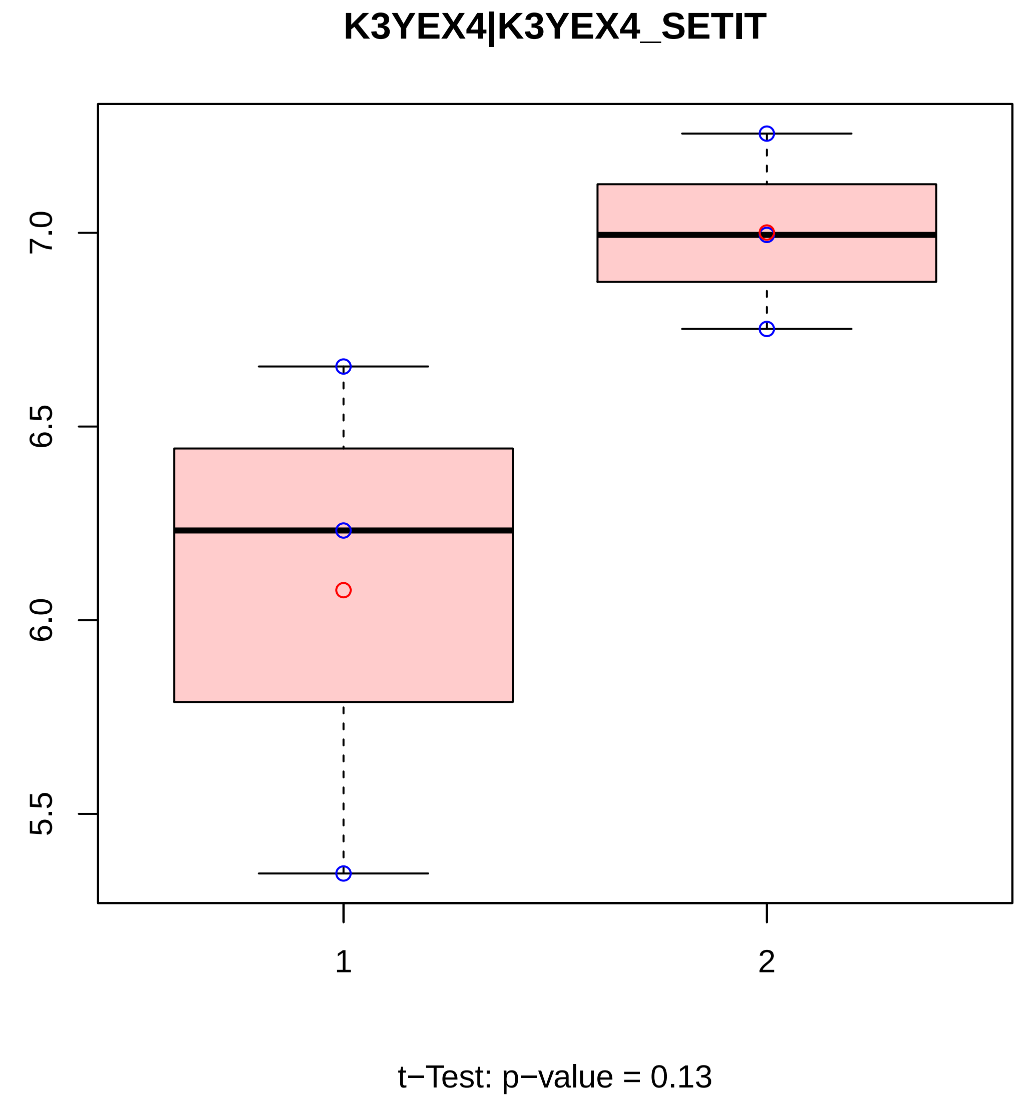

K3YEX4|K3YEX4_SETIT
7.0
6.5
6.0
5.5
1
2
t−Test: p−value = 0.13

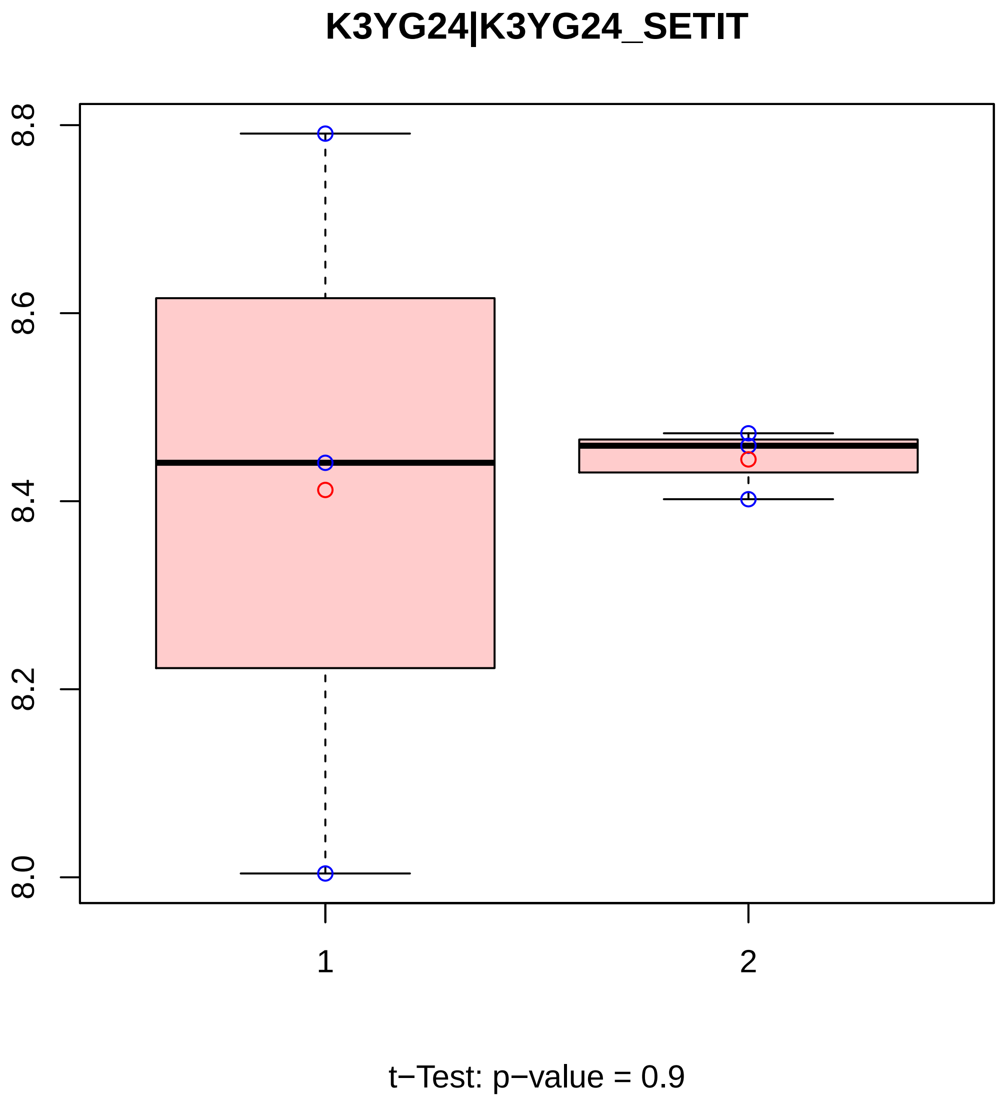
K3YG24|K3YG24_SETIT
8.8
8.6
8.4
8.2
8.0
1
2
t−Test: p−value = 0.9

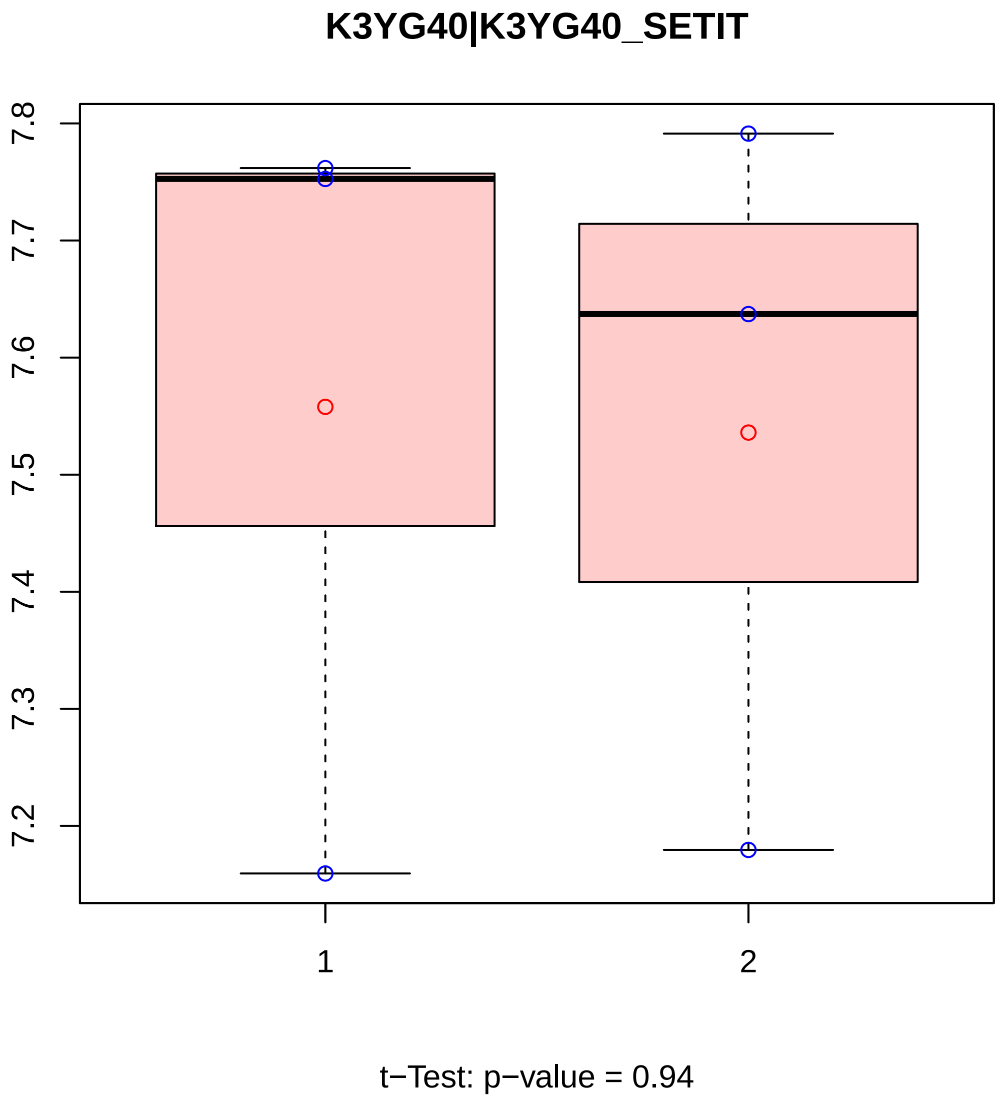
K3YG40|K3YG40_SETIT
7.2
7.3
7.4
7.5
7.6
7.7
7.8
1
2
t−Test: p−value = 0.94

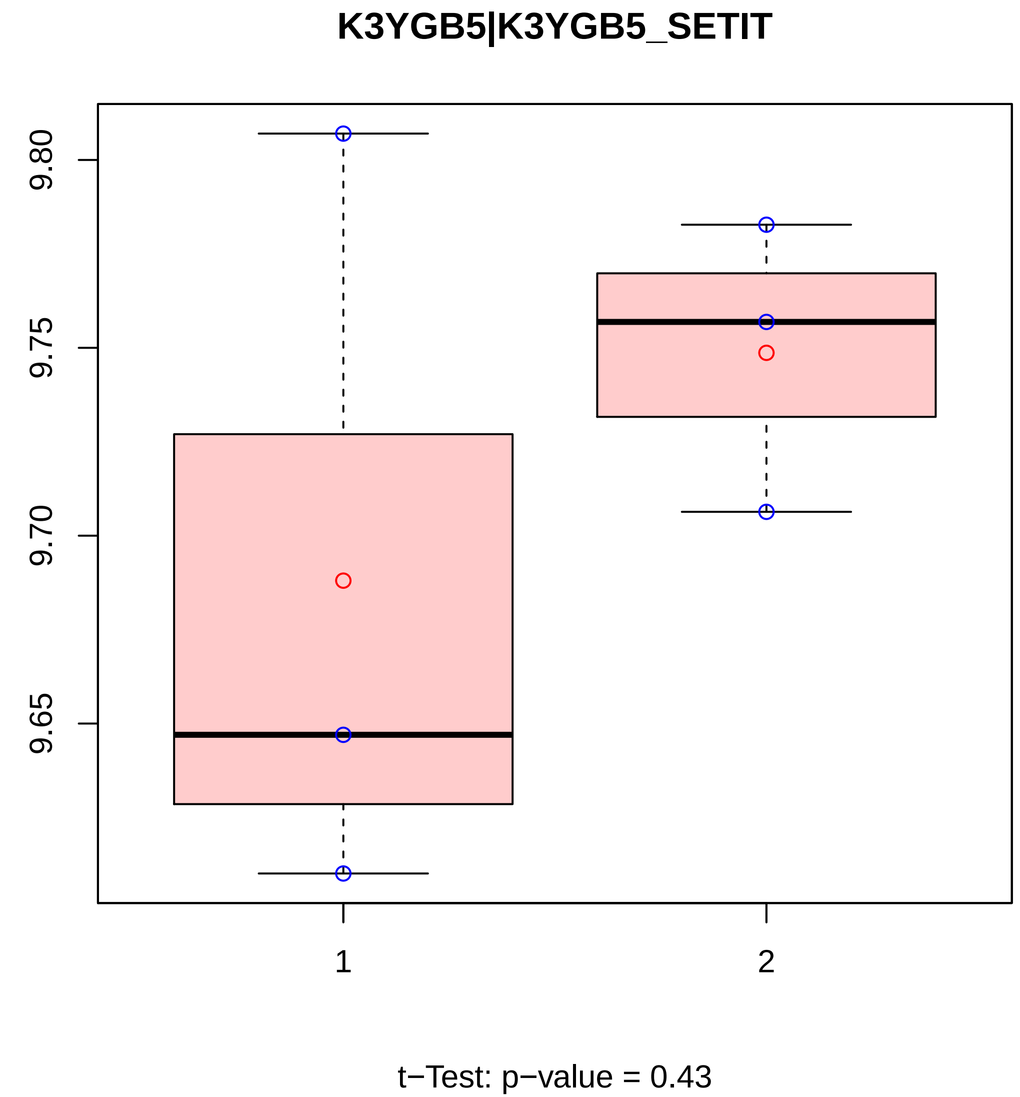
K3YGB5|K3YGB5_SETIT
9.80
9.75
9.70
9.65
1
2
t−Test: p−value = 0.43

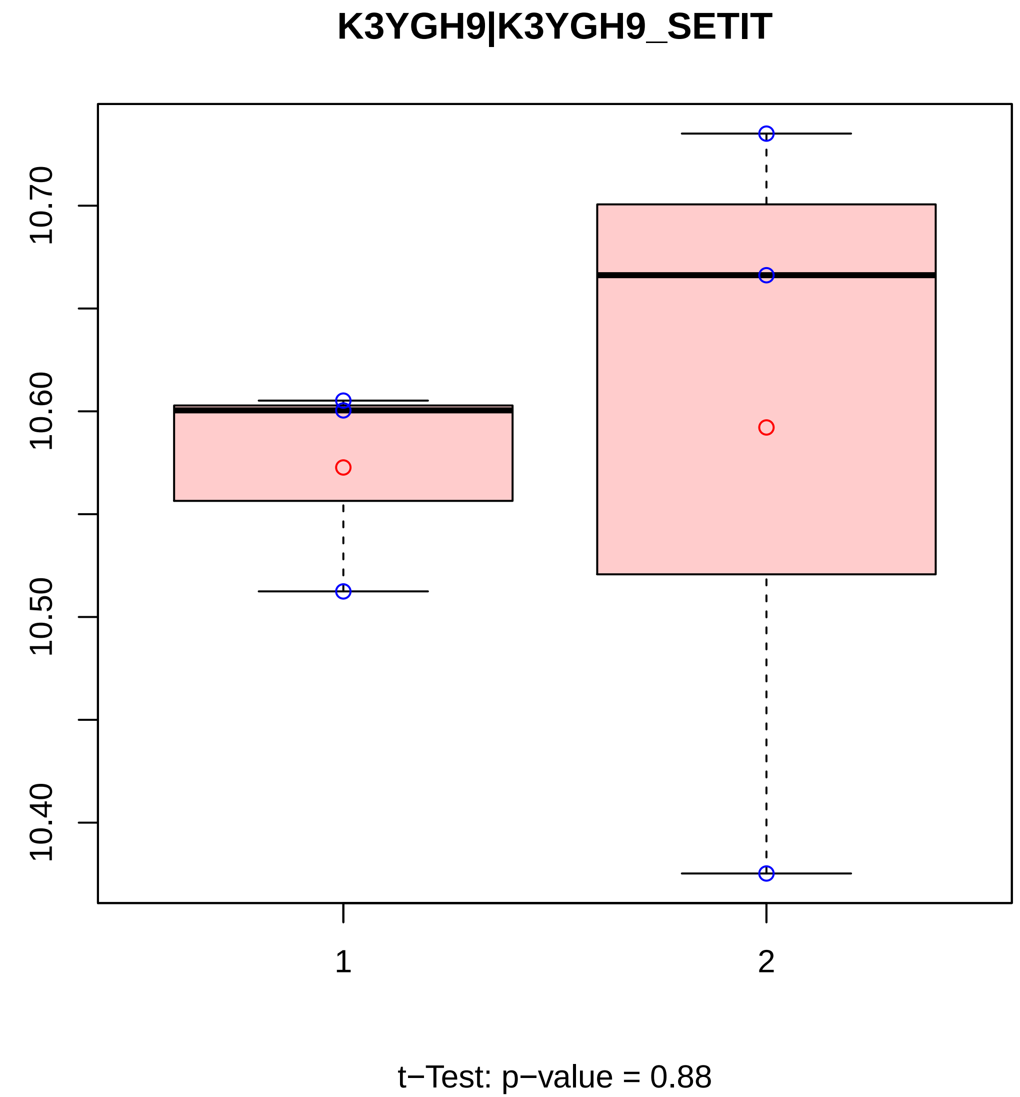
K3YGH9|K3YGH9_SETIT
10.40
10.50
10.60
10.70
1
2
t−Test: p−value = 0.88

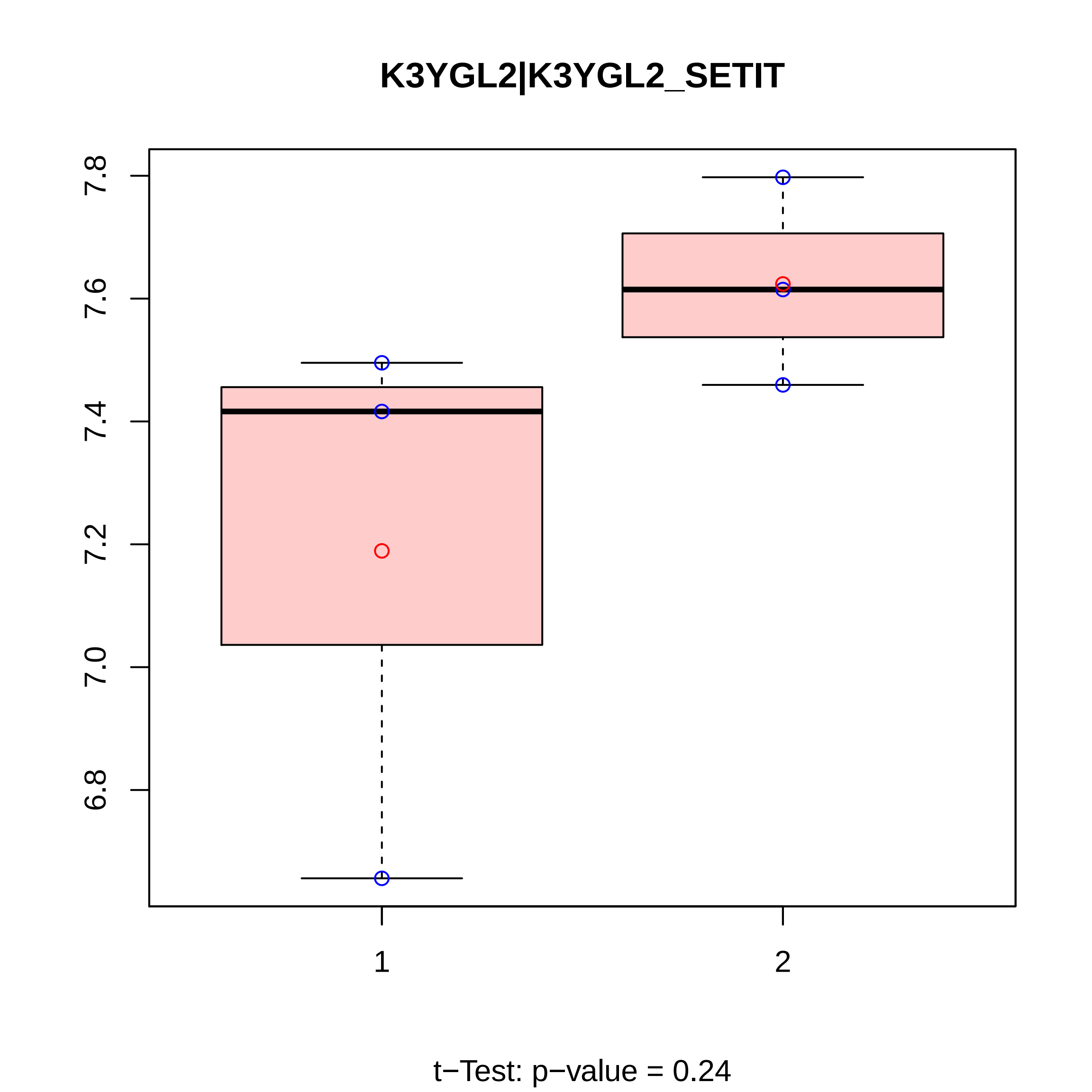
K3YGL2|K3YGL2_SETIT
7.8
7.6
7.4
7.2
7.0
6.8
1
2
t−Test: p−value = 0.24

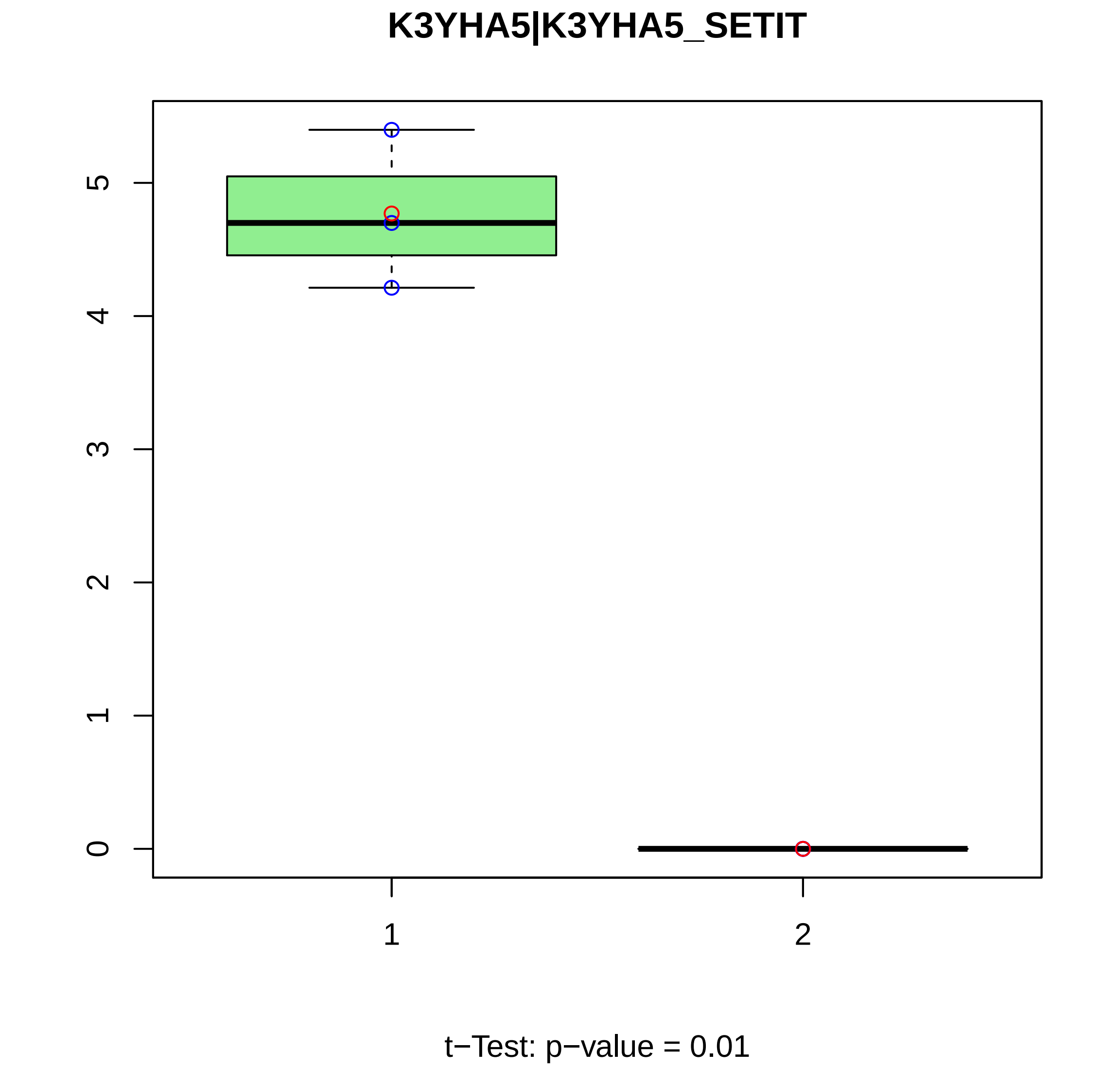
K3YHA5|K3YHA5_SETIT
0
1
2
3
4
5
1
2
t−Test: p−value = 0.01

K3YHF4|K3YHF4_SETIT

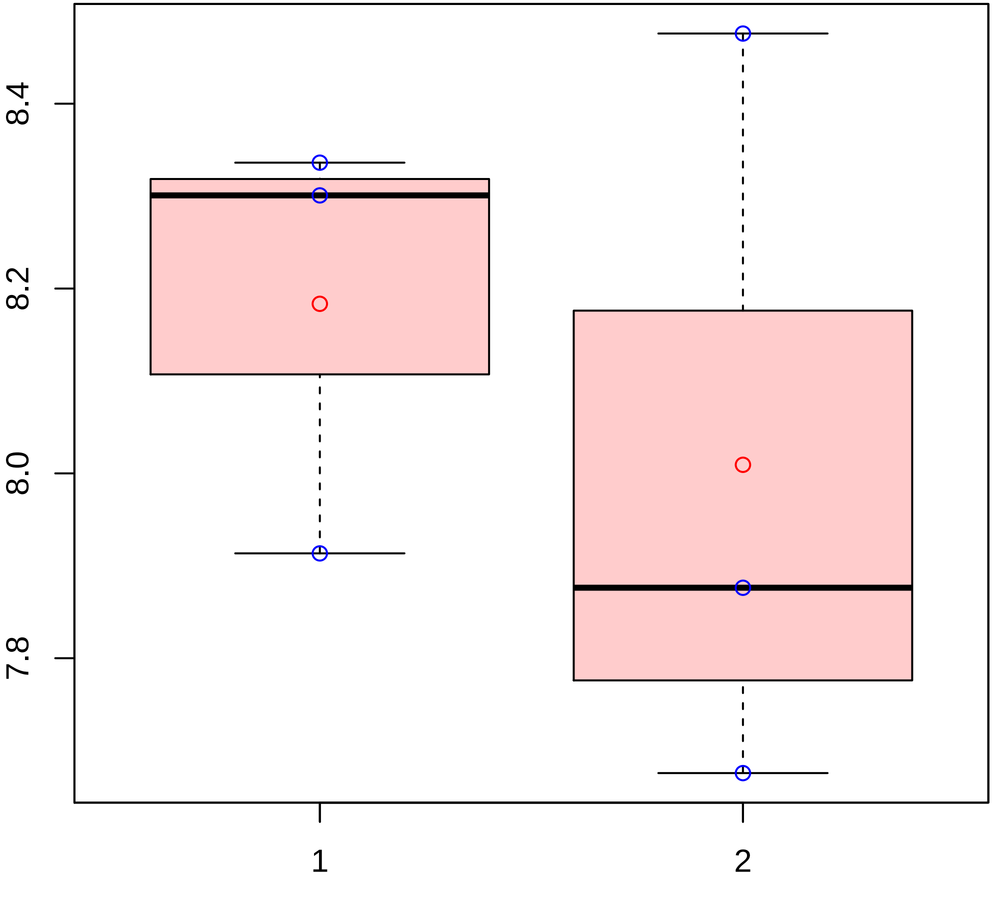

t−Test: p−value = 0.57

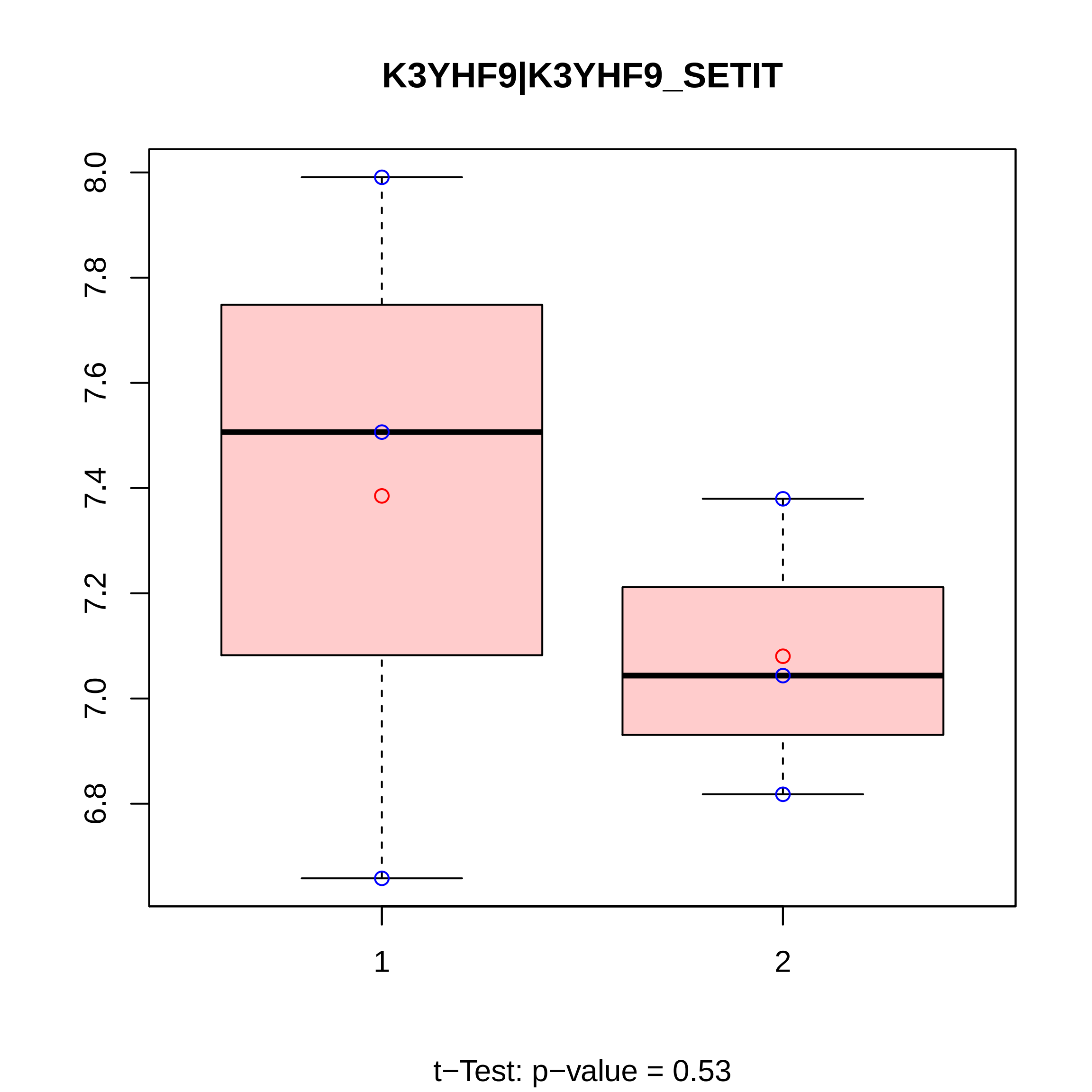
K3YHF9|K3YHF9_SETIT
8.0
7.8
7.6
7.4
7.2
7.0
6.8
1
2
t−Test: p−value = 0.53

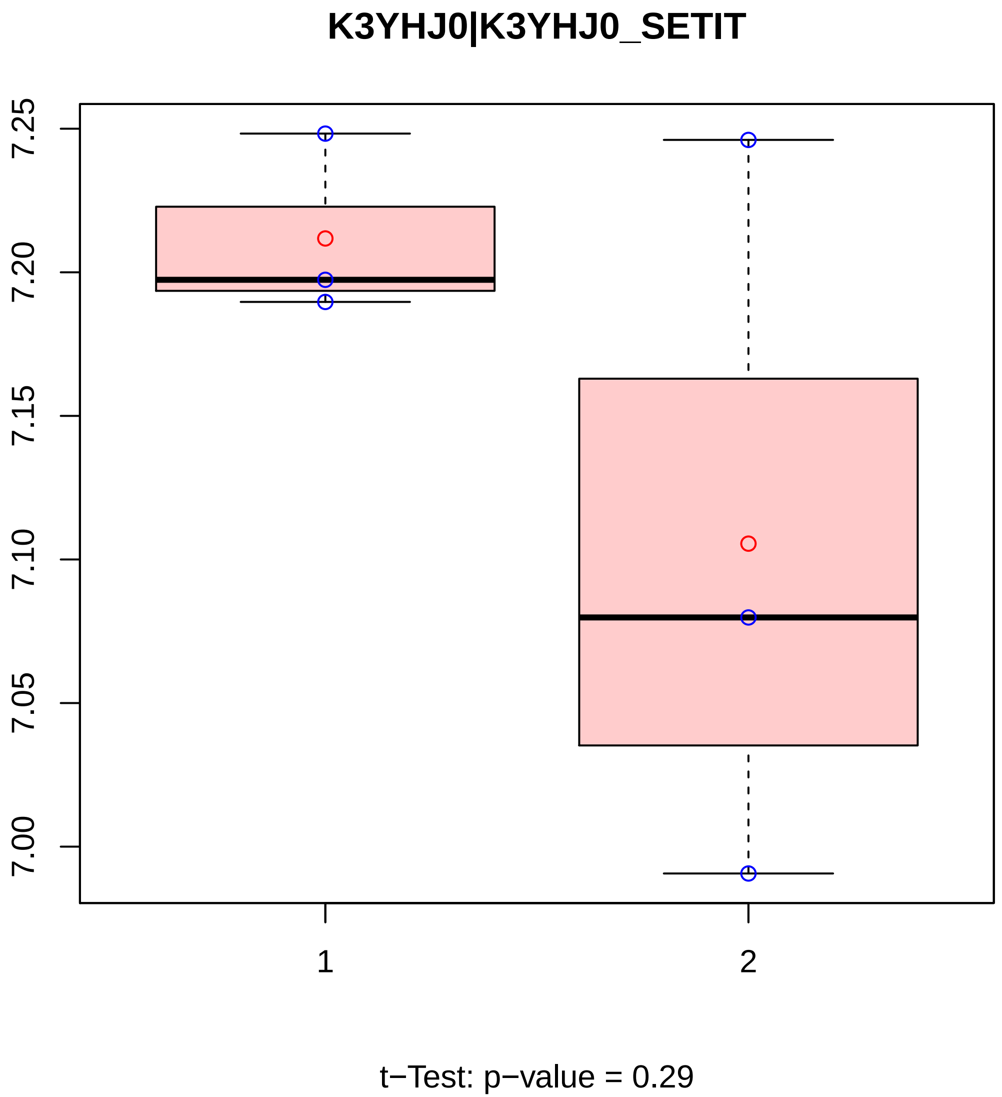
K3YHJ0|K3YHJ0_SETIT
7.25
7.20
7.15
7.10
7.05
7.00
1
2
t−Test: p−value = 0.29

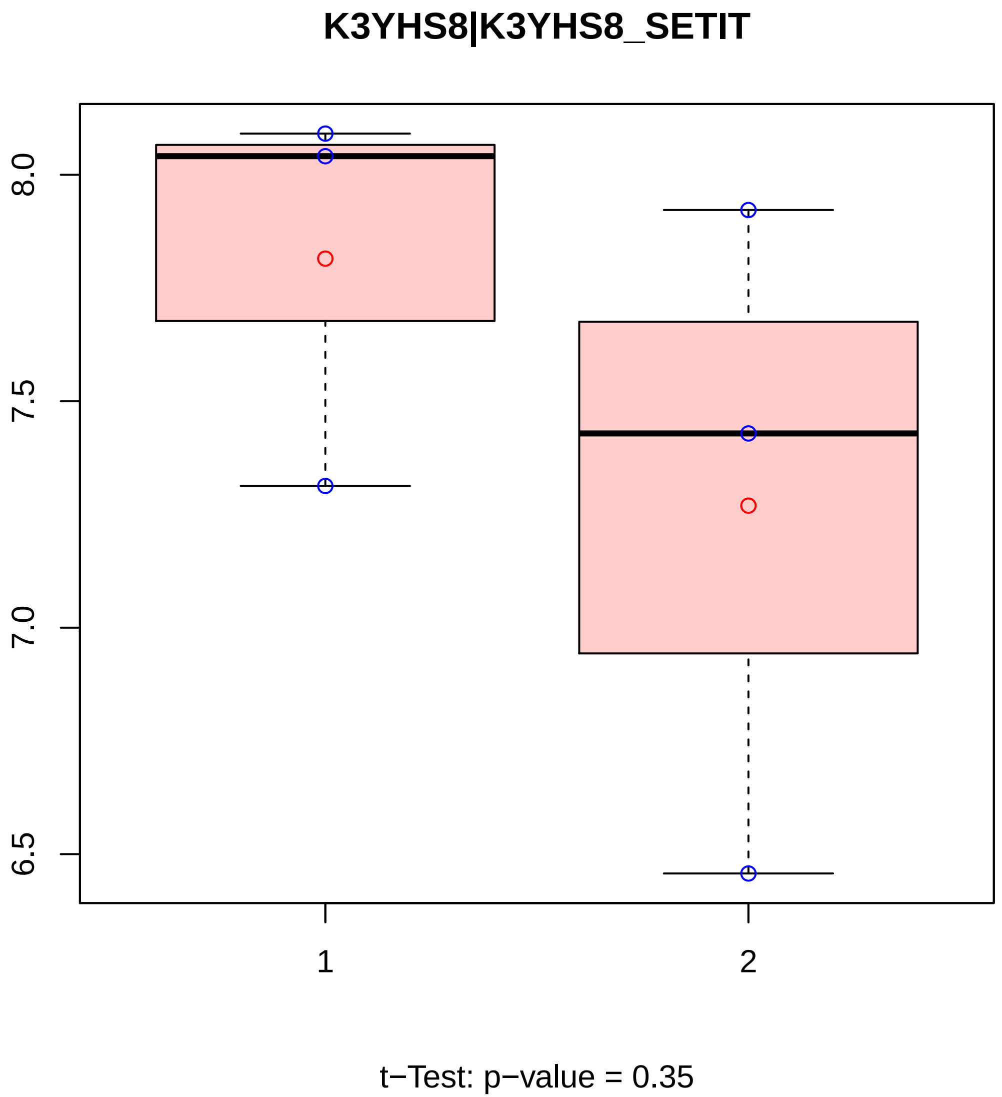
K3YHS8|K3YHS8_SETIT
8.0
7.5
7.0
6.5
1
2
t−Test: p−value = 0.35

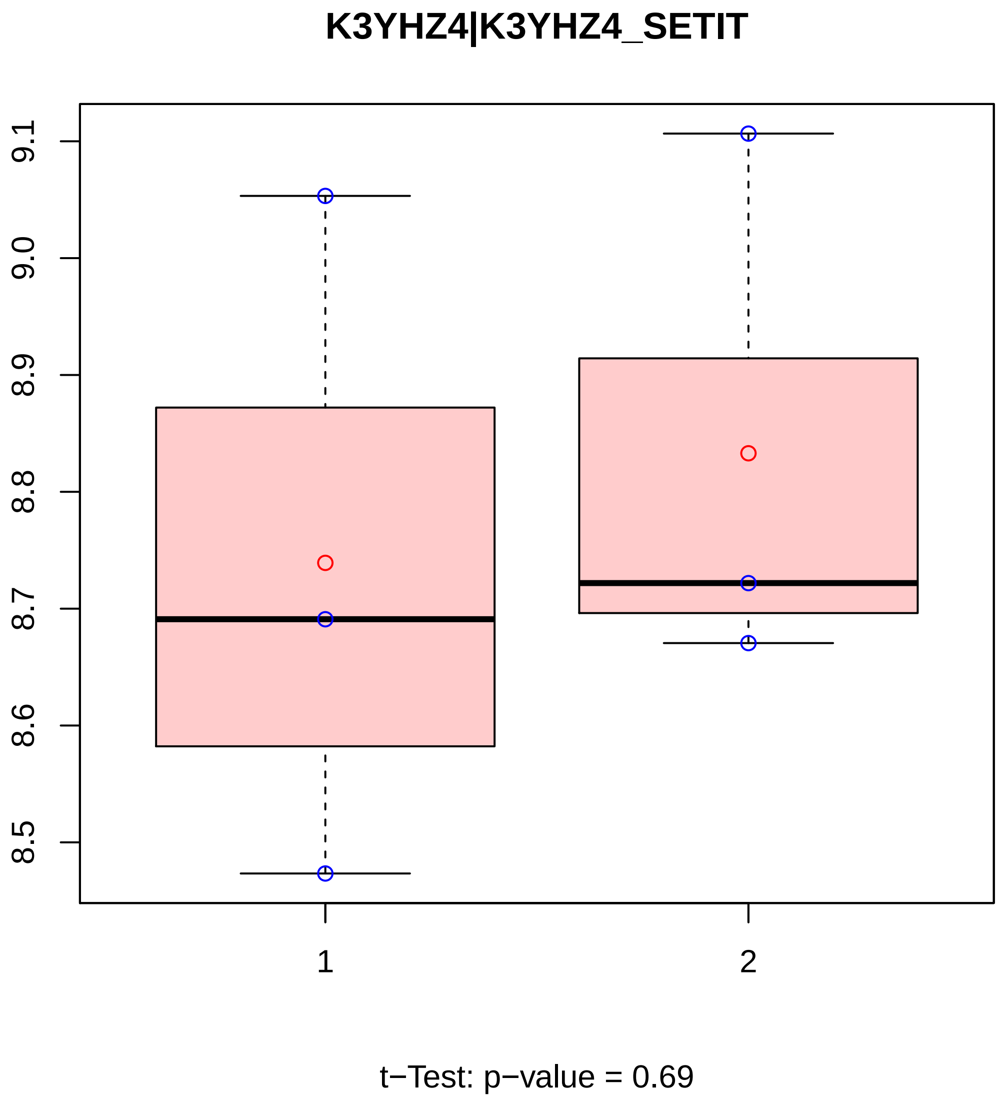

K3YHZ4|K3YHZ4_SETIT
9.1
9.0
8.9
8.8
8.7
8.6
8.5
1
2
t−Test: p−value = 0.69

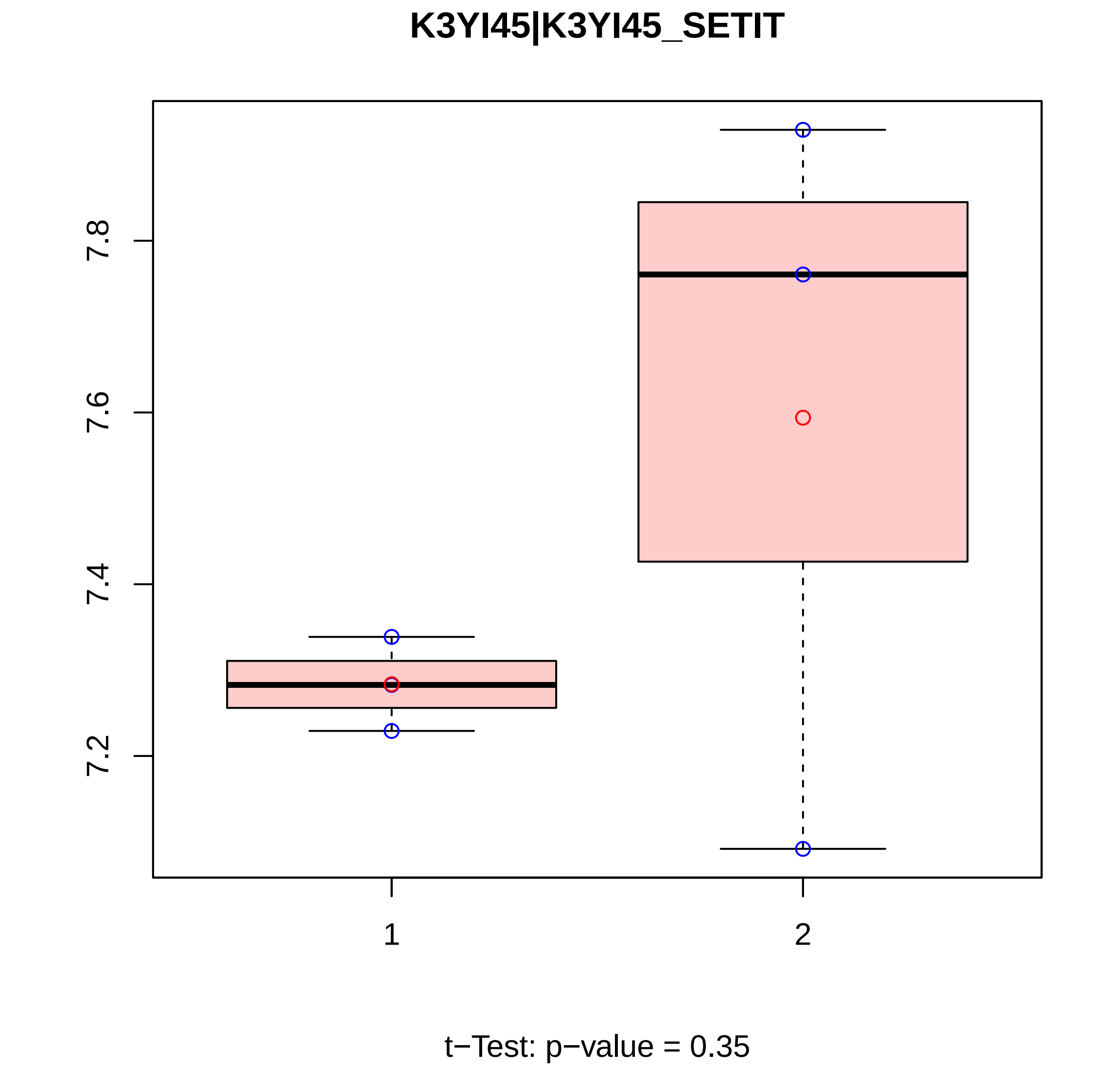
K3YI45|K3YI45_SETIT
7.2
7.4
7.6
7.8
1
2
t−Test: p−value = 0.35

K3YI82|K3YI82_SETIT

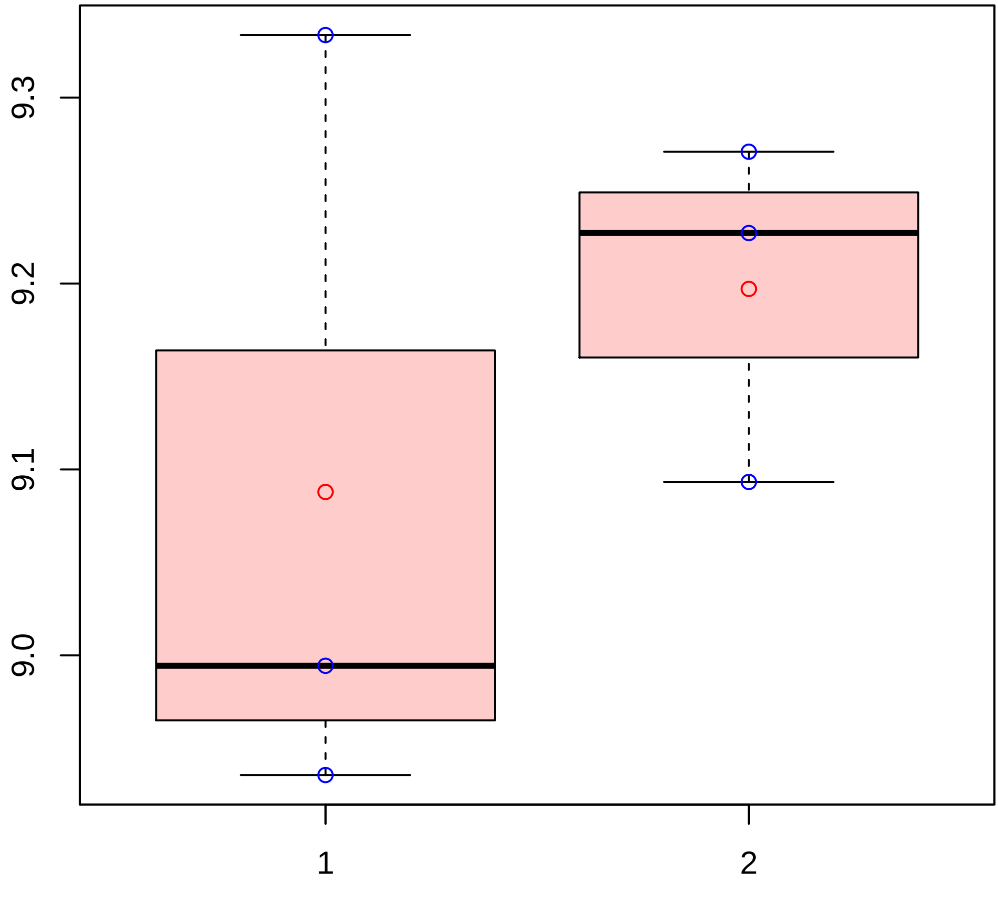

t−Test: p−value = 0.48

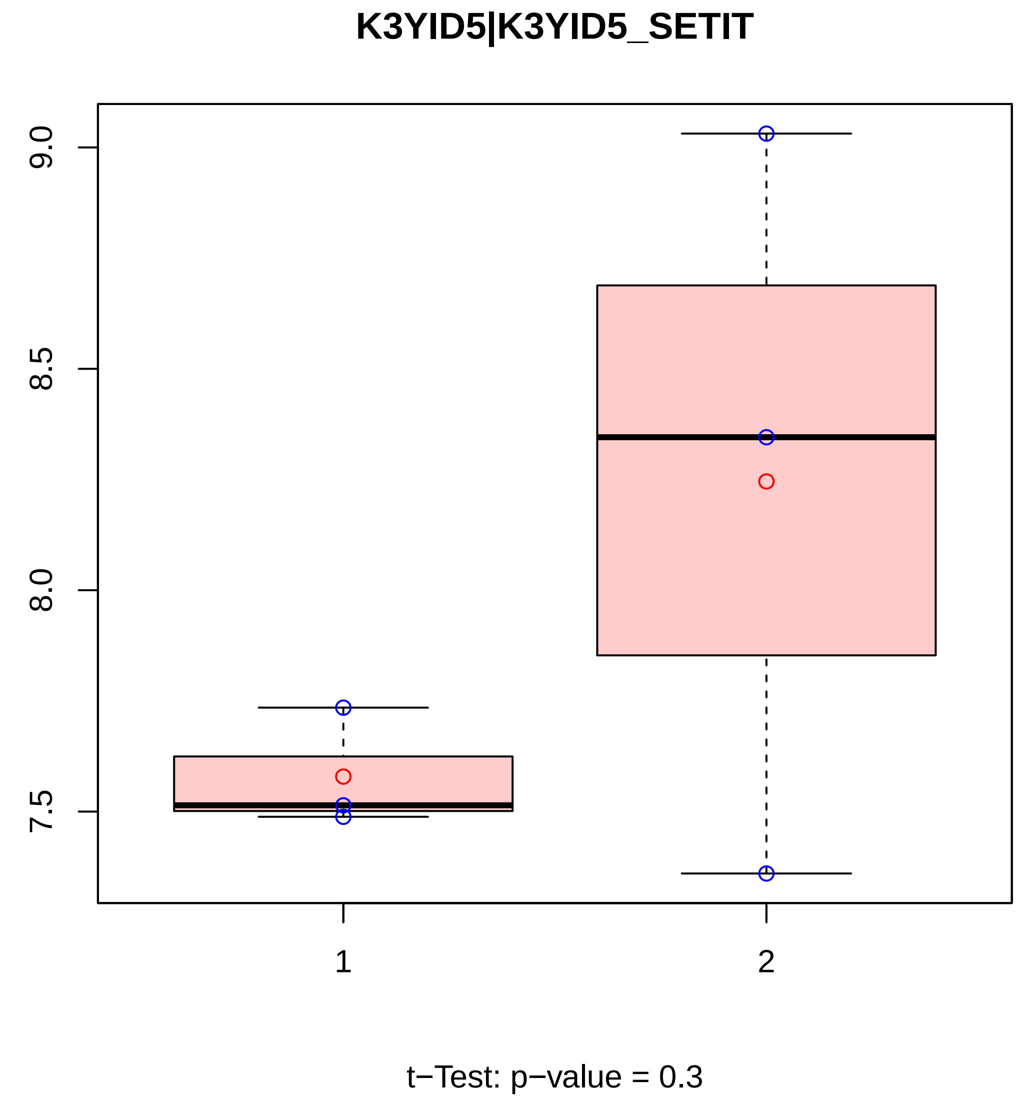

K3YID5|K3YID5_SETIT
9.0
8.5
8.0
7.5
1
2
t−Test: p−value = 0.3

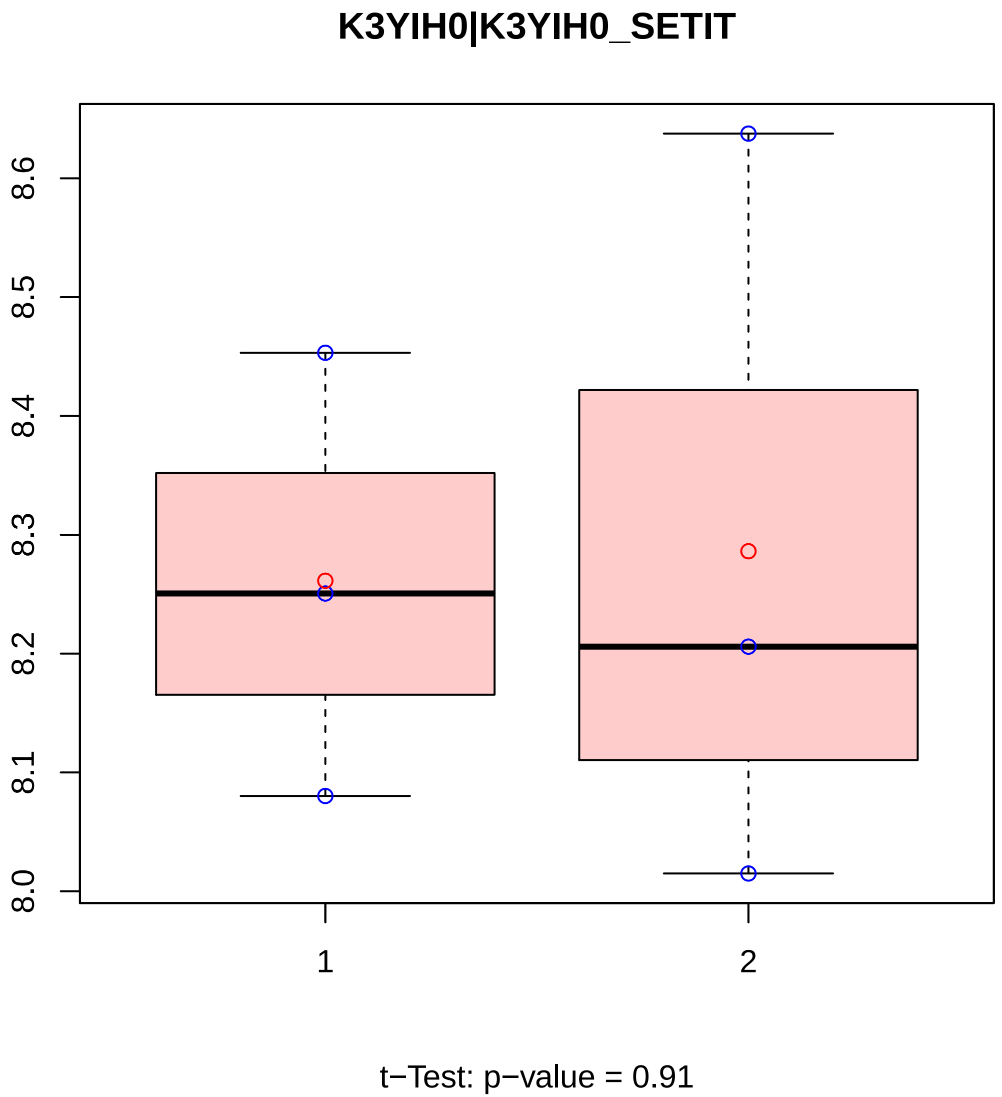

K3YIH0|K3YIH0_SETIT
8.0
8.1
8.2
8.3
8.4
8.5
8.6
1
2
t−Test: p−value = 0.91

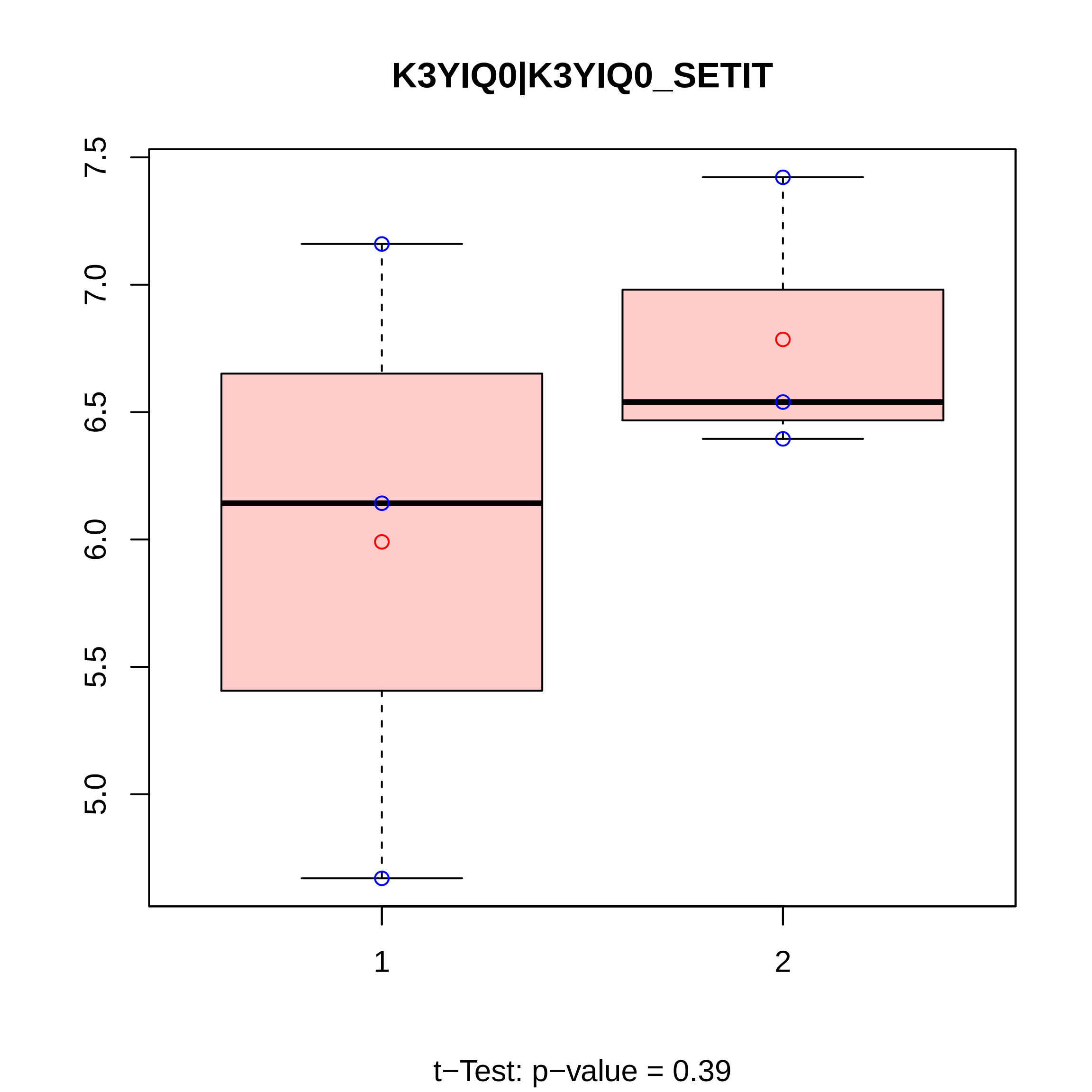
K3YIQ0|K3YIQ0_SETIT
7.5
7.0
6.5
6.0
5.5
5.0
1
2
t−Test: p−value = 0.39

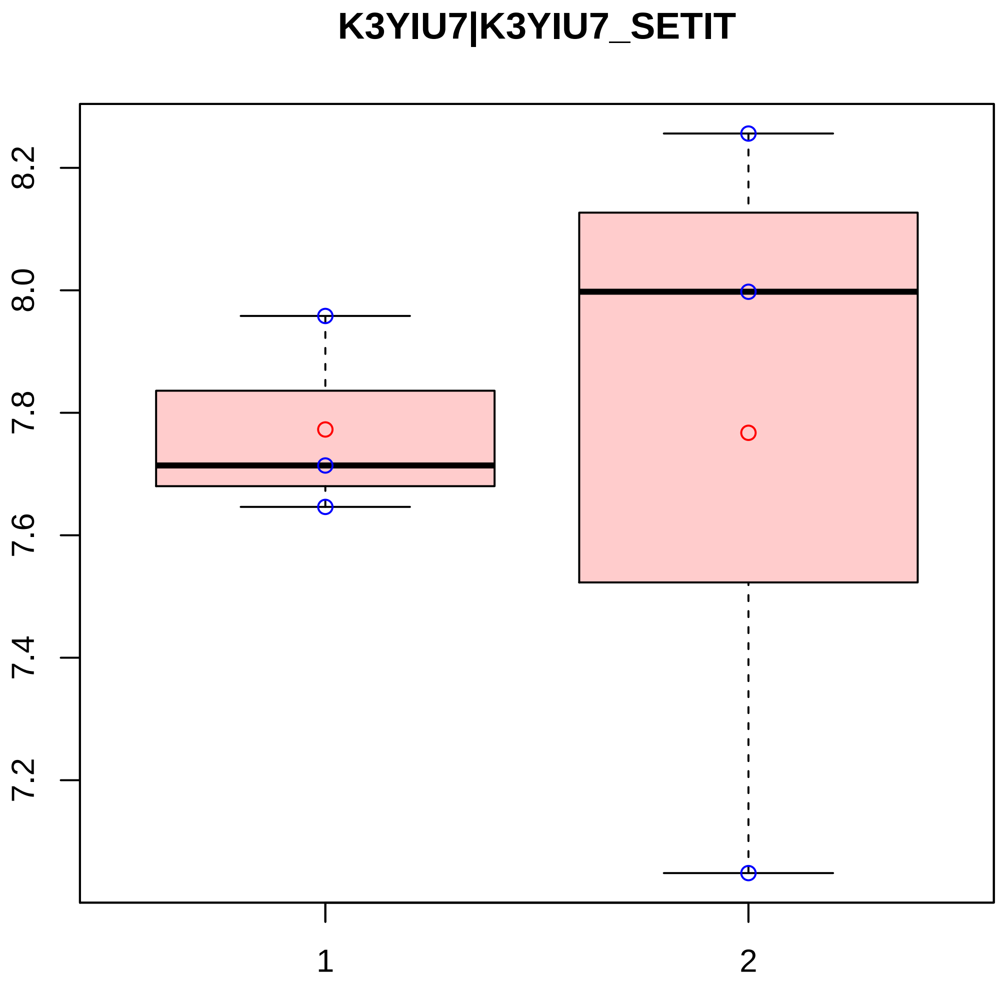

t−Test: p−value = 0.99

K3YIW7|K3YIW7_SETIT

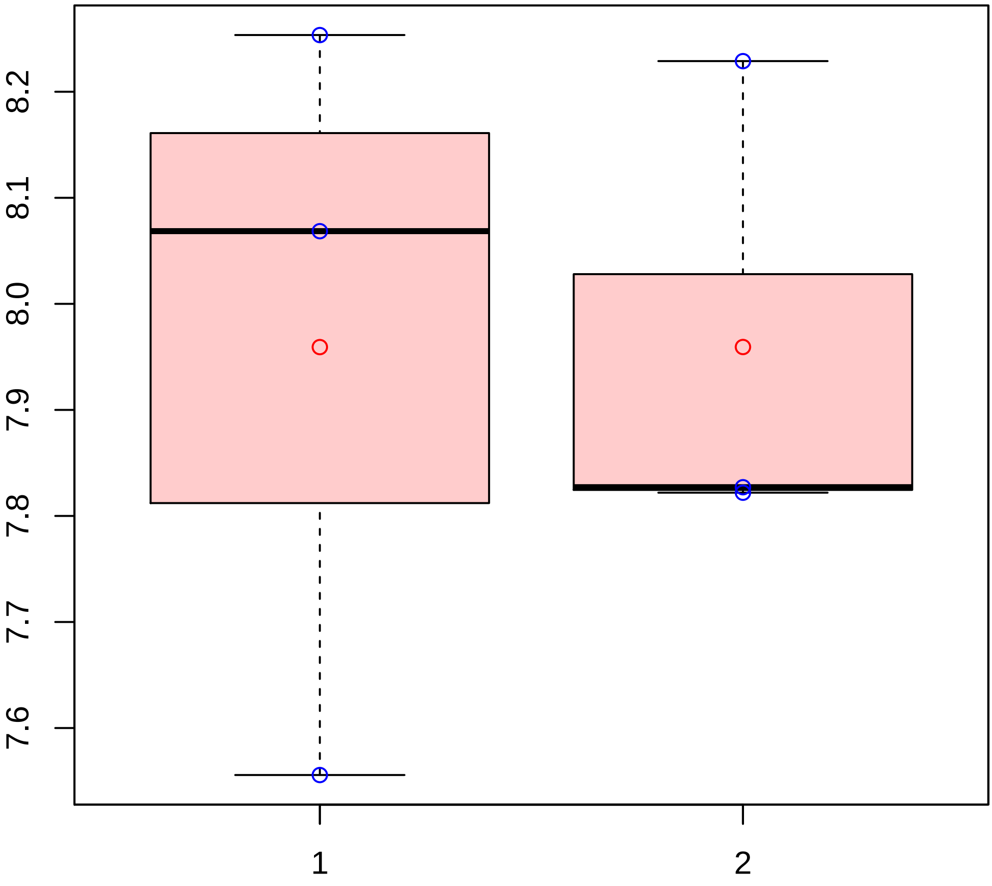

t−Test: p−value = 1

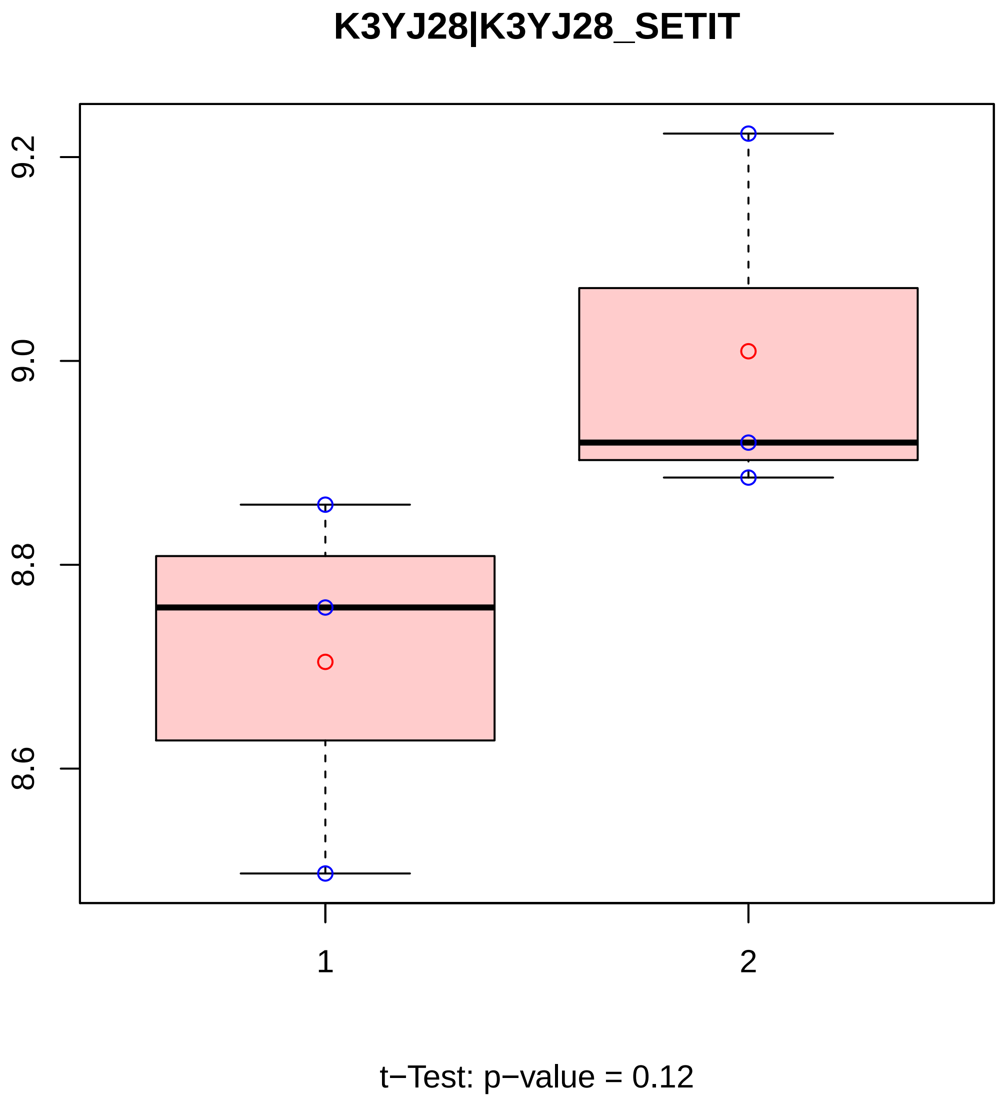
K3YJ28|K3YJ28_SETIT
9.2
9.0
8.8
8.6
1
2
t−Test: p−value = 0.12

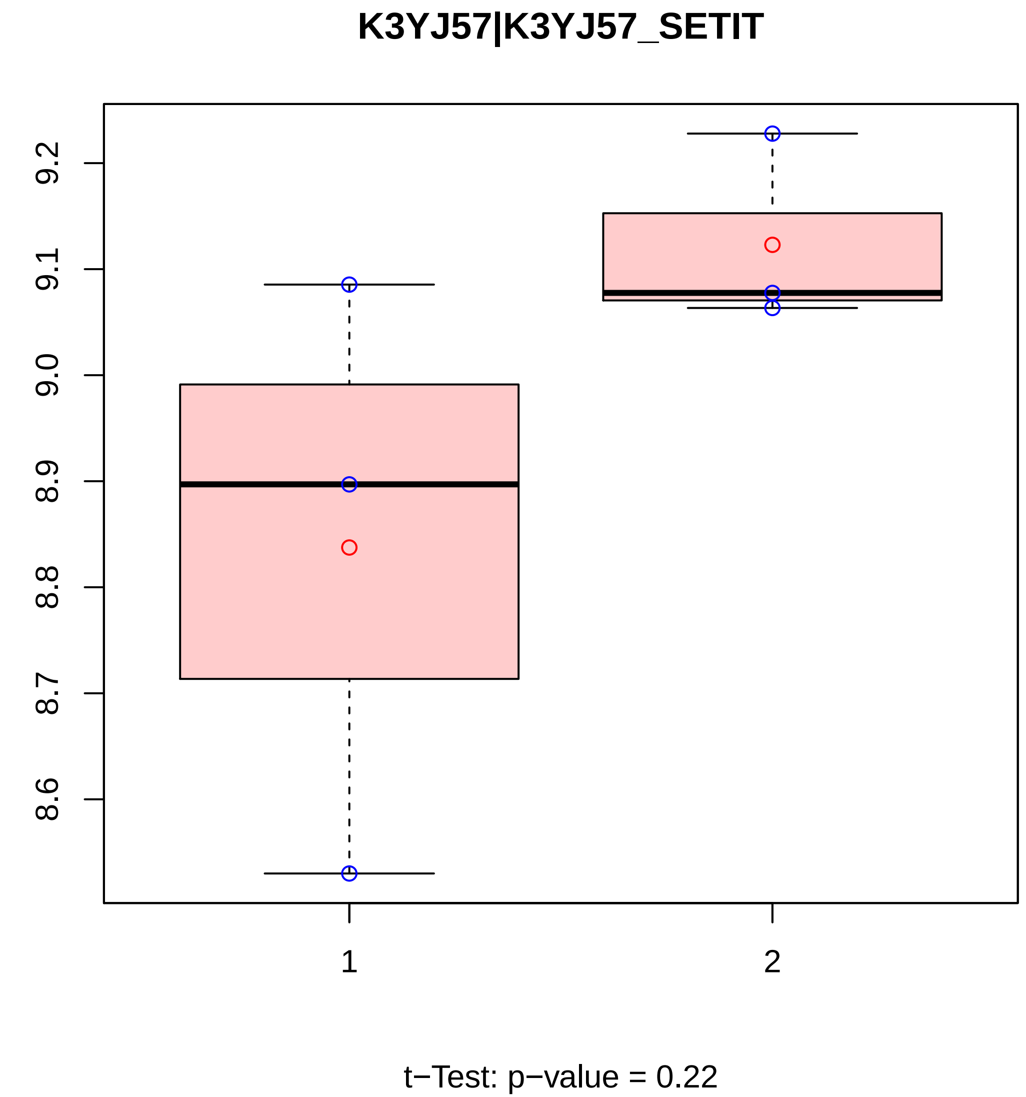
K3YJ57|K3YJ57_SETIT
9.2
9.1
9.0
8.9
8.8
8.7
8.6
1
2
t−Test: p−value = 0.22

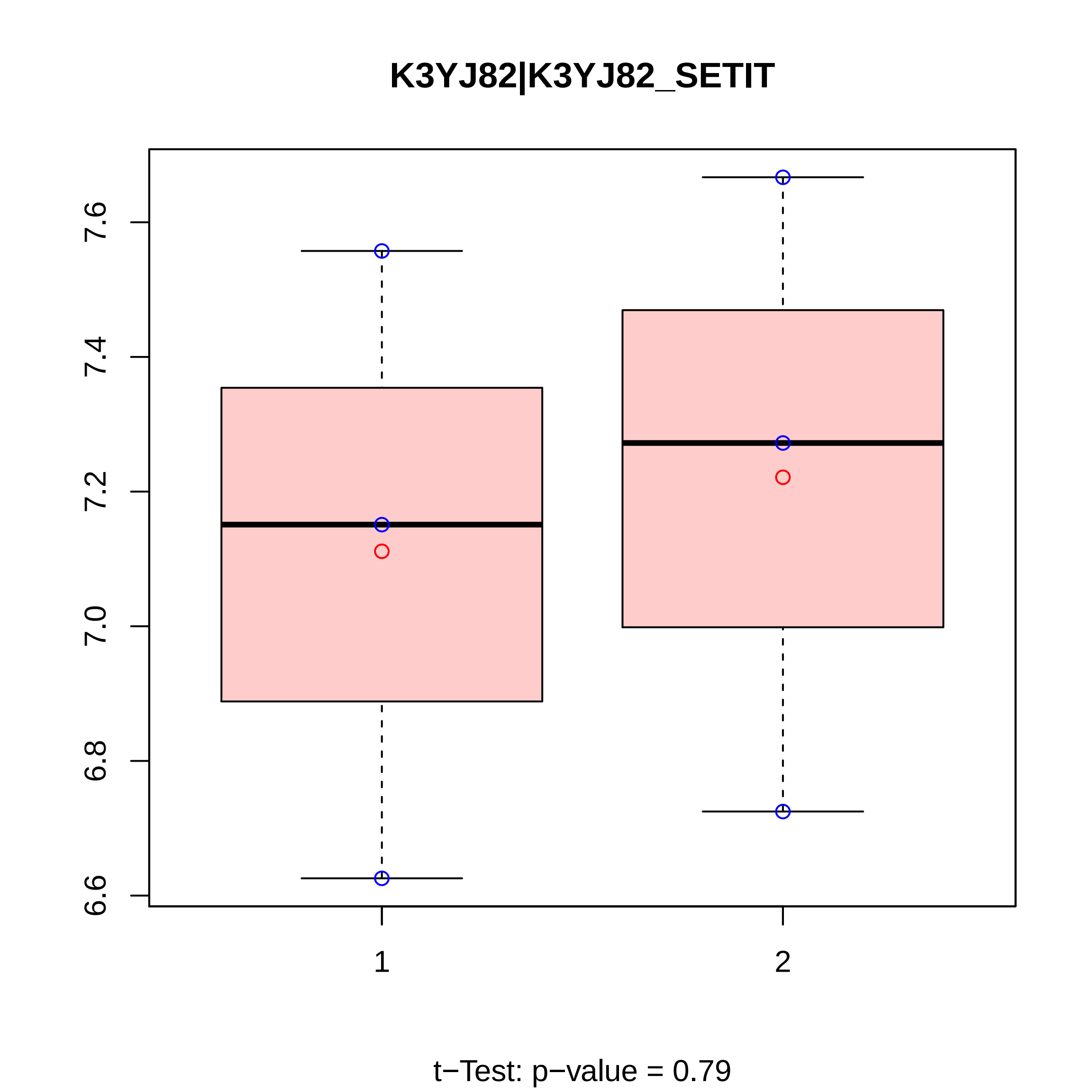
K3YJ82|K3YJ82_SETIT
7.6
7.4
7.2
7.0
6.8
6.6
1
2
t−Test: p−value = 0.79

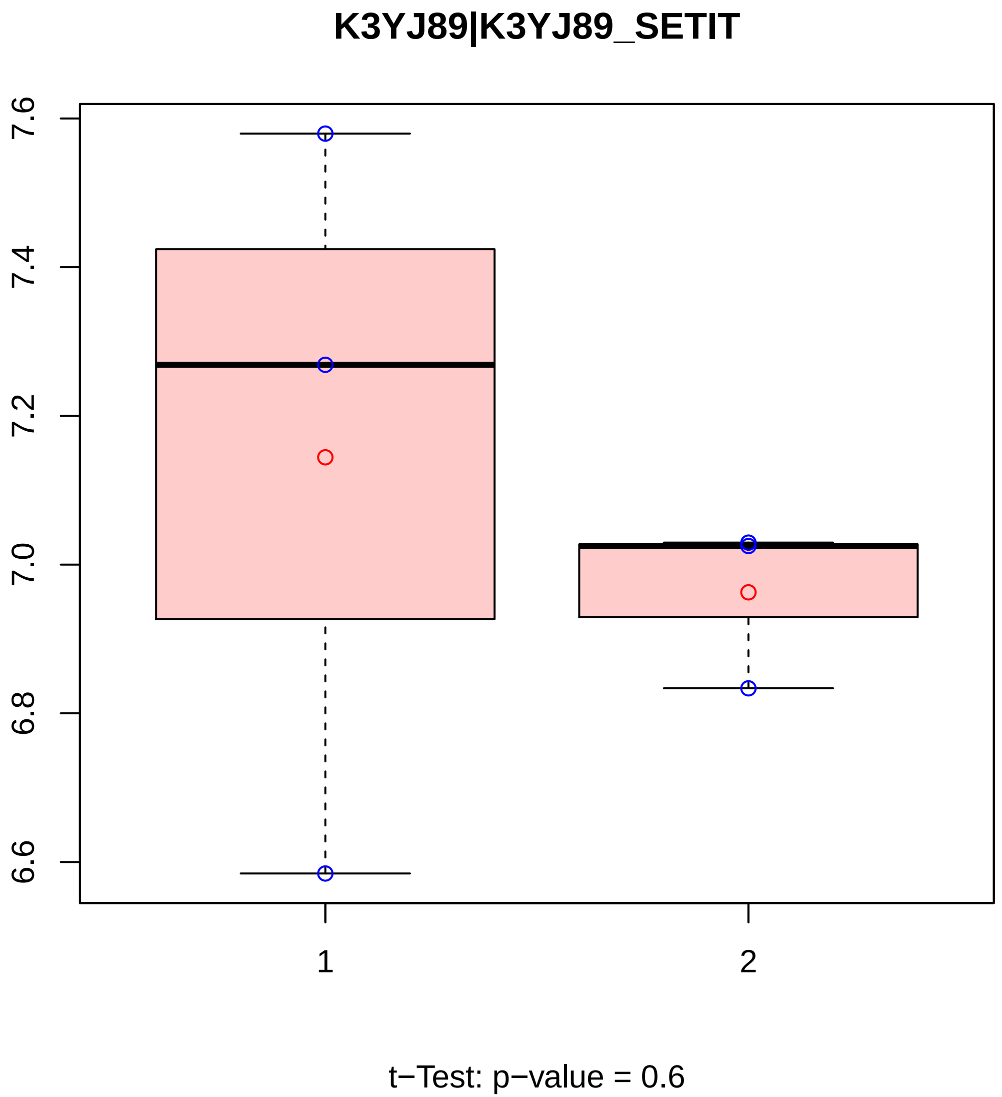
K3YJ89|K3YJ89_SETIT
7.6
7.4
7.2
7.0
6.8
6.6
1
2
t−Test: p−value = 0.6

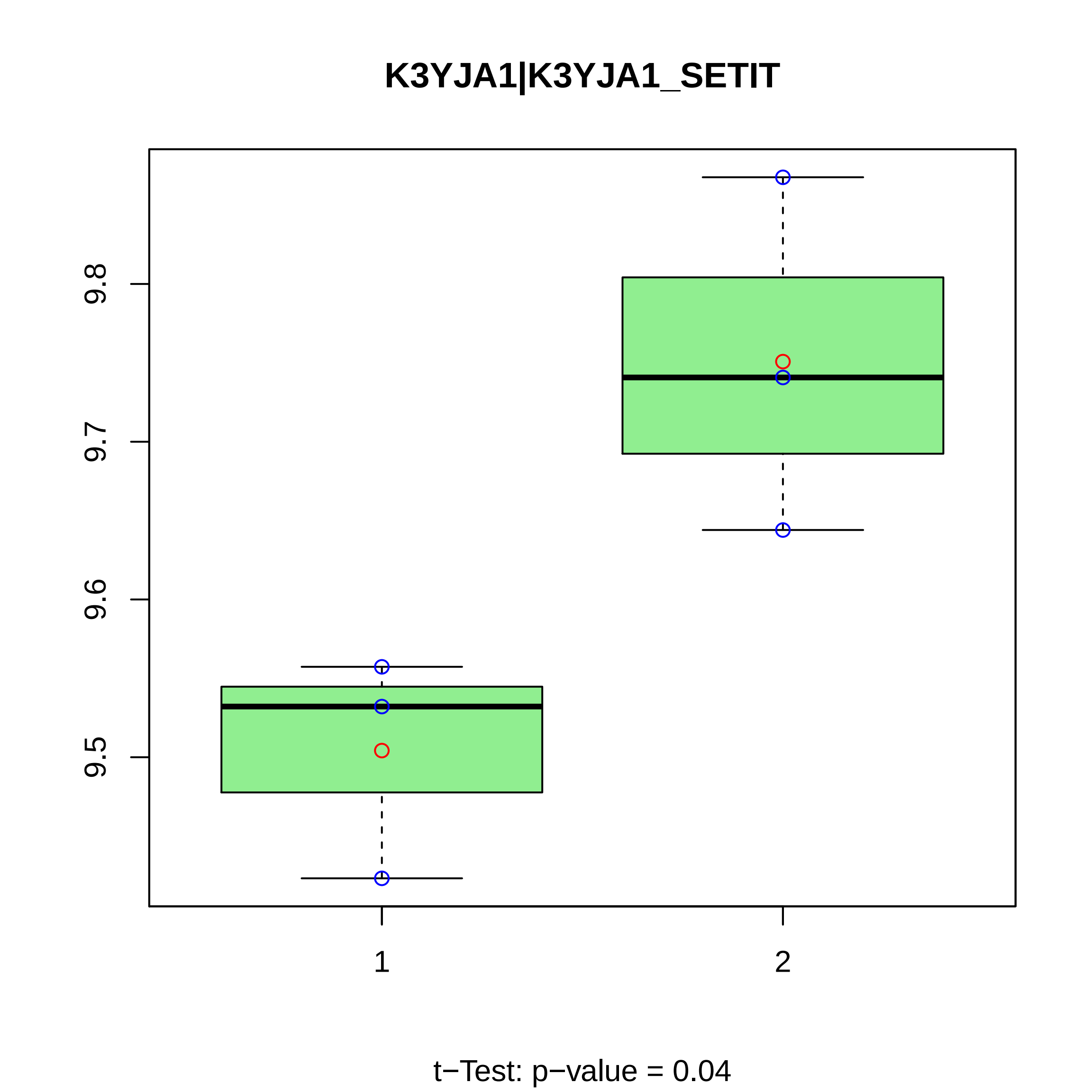
K3YJA1|K3YJA1_SETIT
9.5
9.6
9.7
9.8
1
2
t−Test: p−value = 0.04

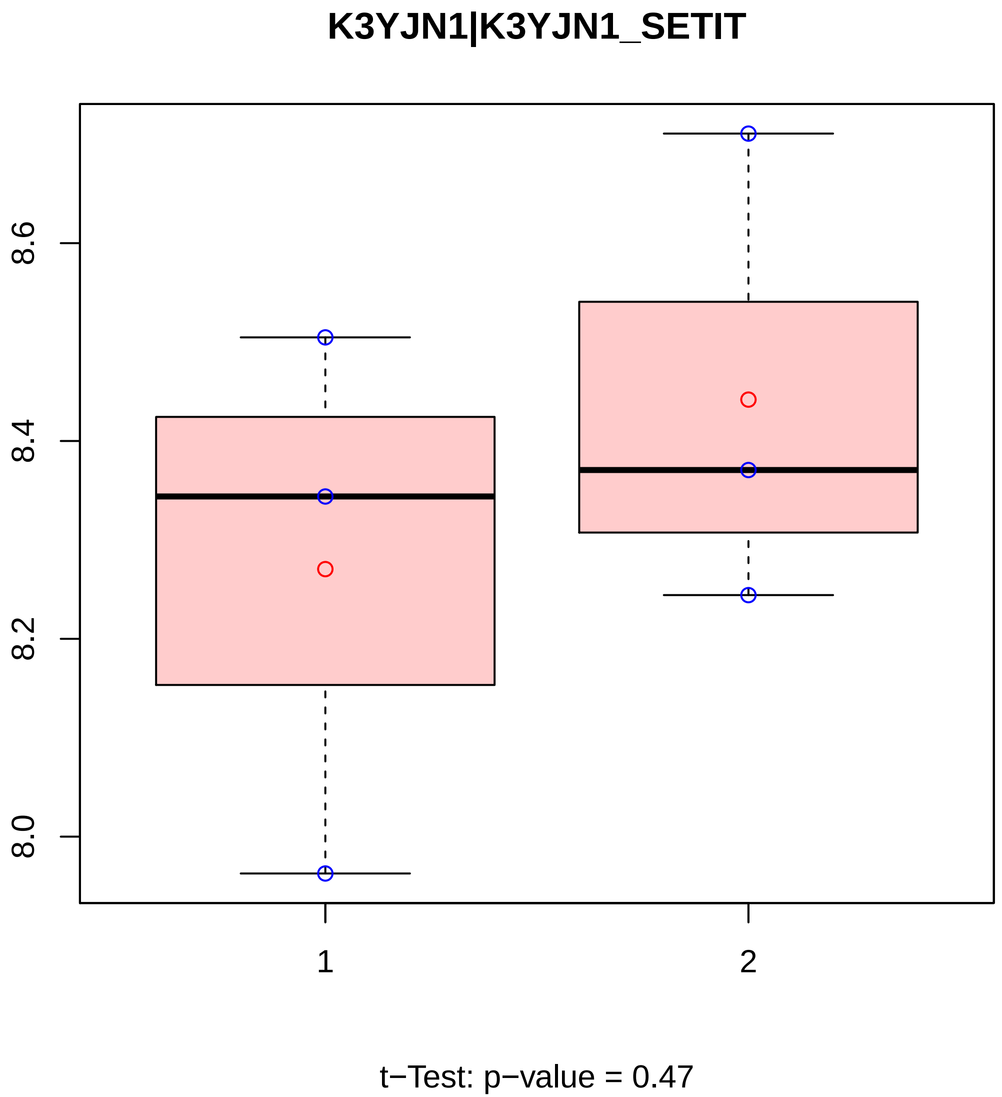
K3YJN1|K3YJN1_SETIT
8.0
8.2
8.4
8.6
1
2
t−Test: p−value = 0.47

K3YJP2|K3YJP2_SETIT

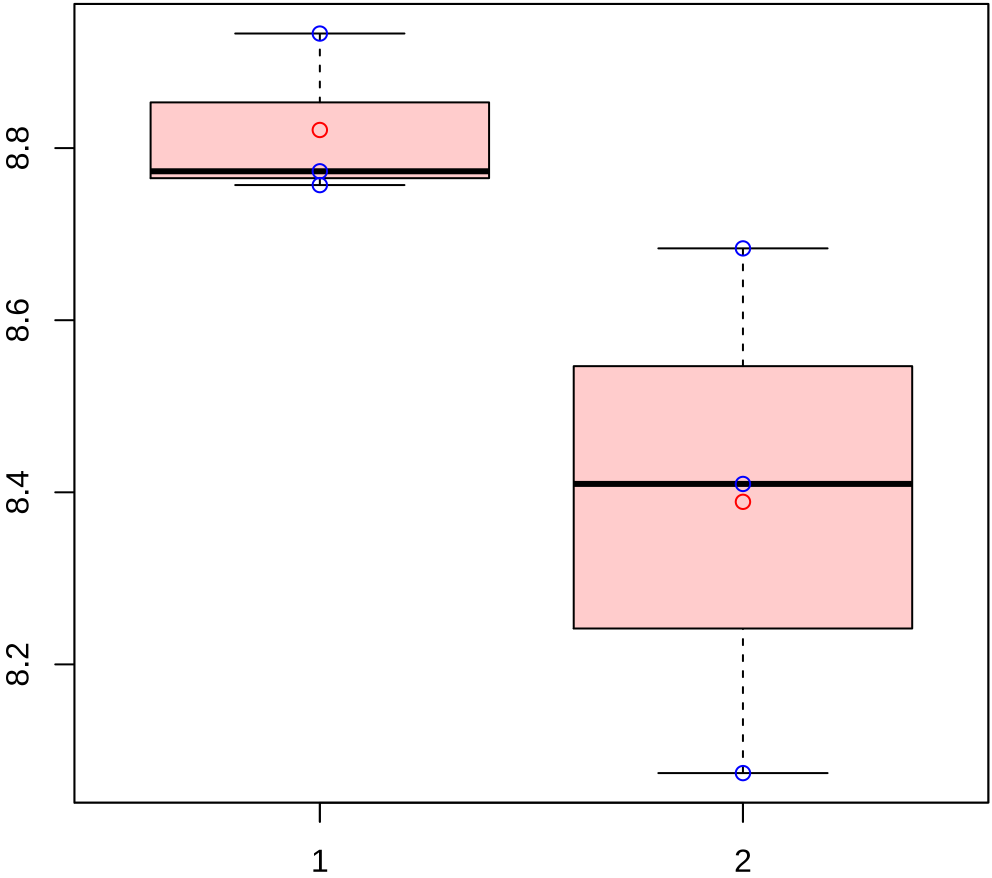

t−Test: p−value = 0.12

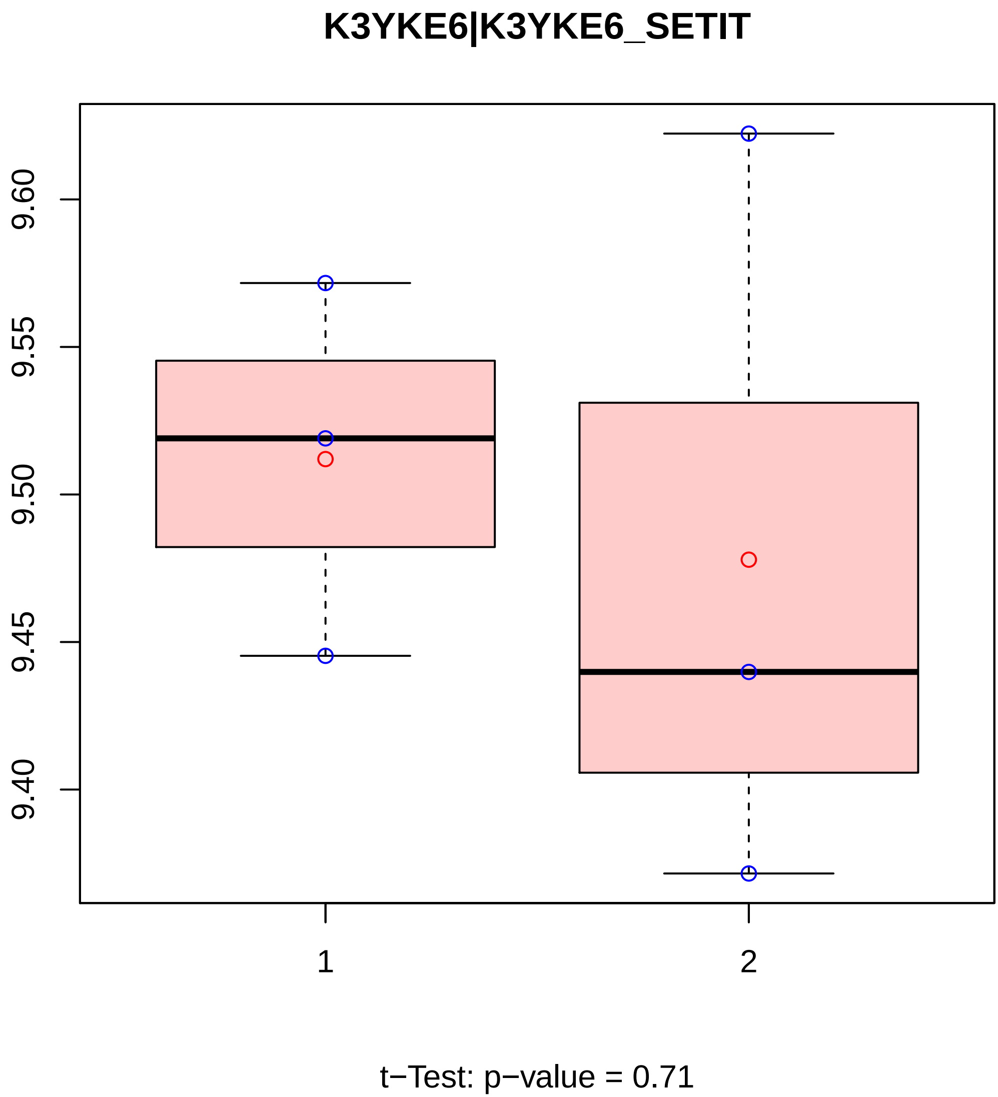
K3YKE6|K3YKE6_SETIT
9.40
9.45
9.50
9.55
9.60
1
2
t−Test: p−value = 0.71

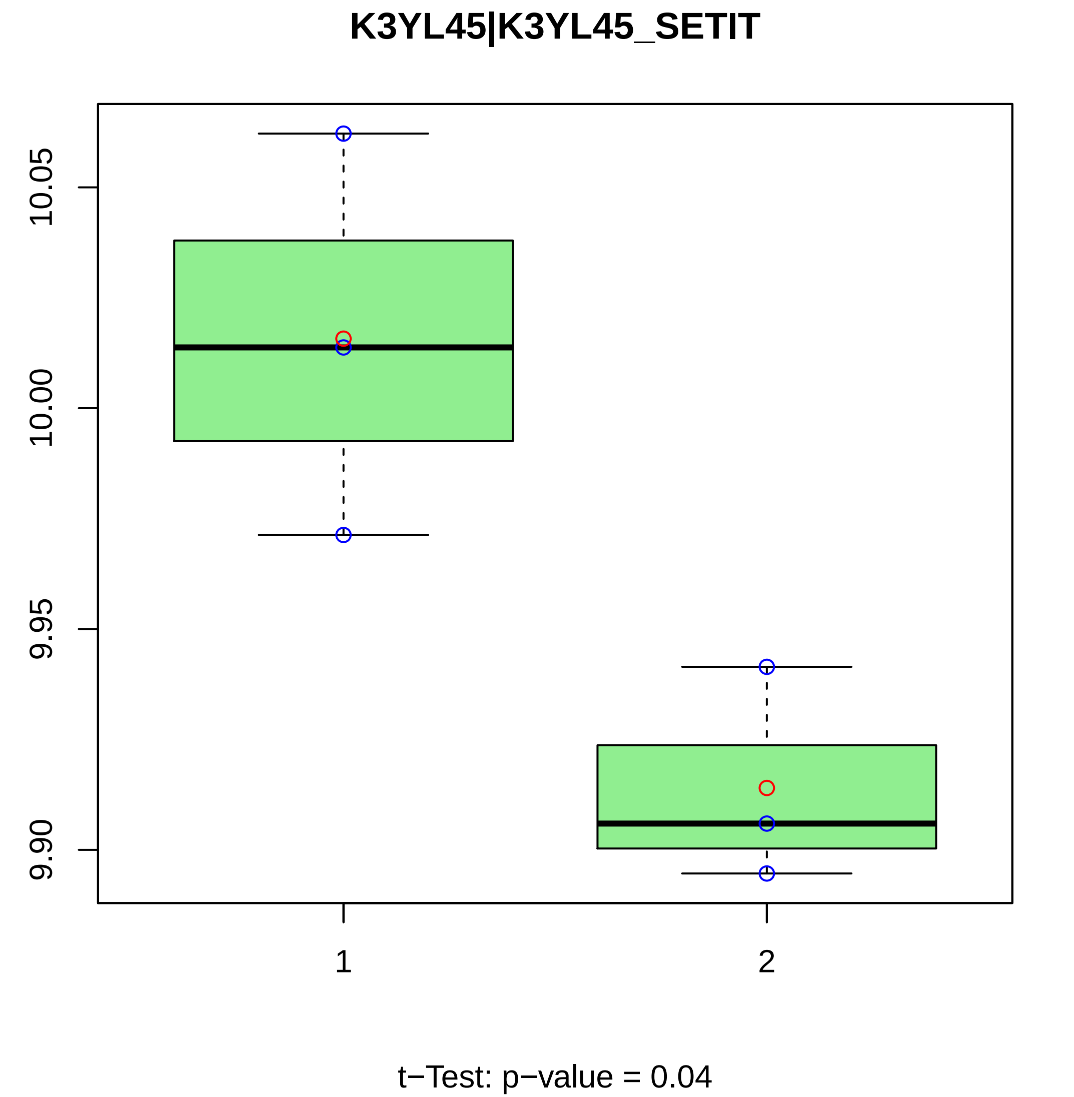
K3YL45|K3YL45_SETIT
10.05
10.00
9.95
9.90
1
2
t−Test: p−value = 0.04

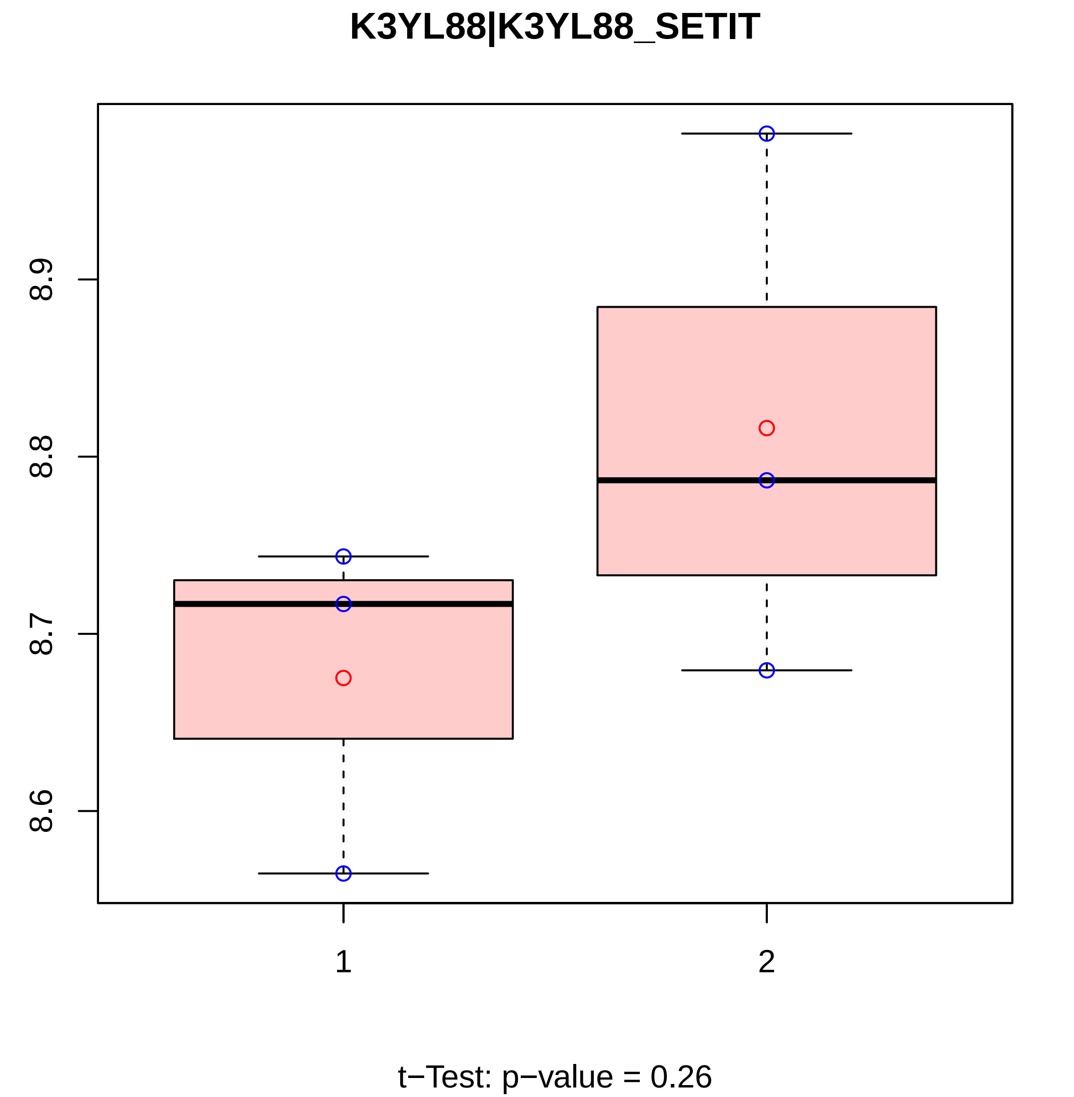
K3YL88|K3YL88_SETIT
8.6
8.7
8.8
8.9
1
2
t−Test: p−value = 0.26

K3YLC2|K3YLC2_SETIT

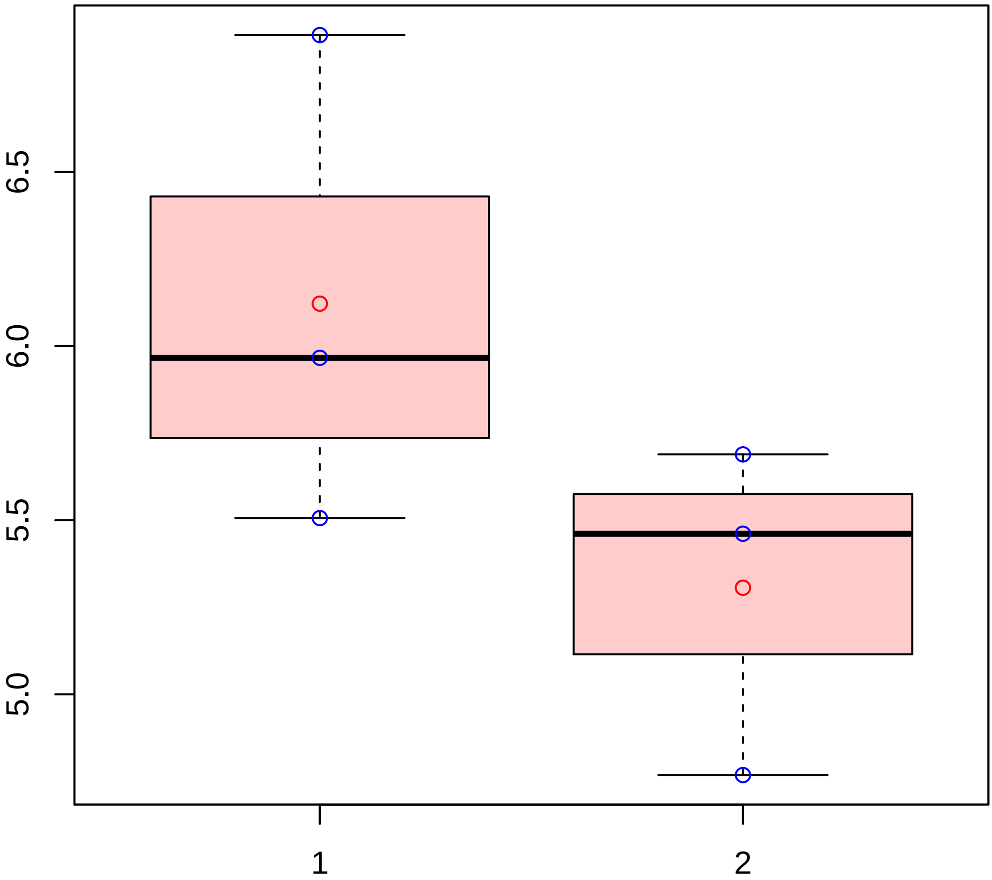

t−Test: p−value = 0.18

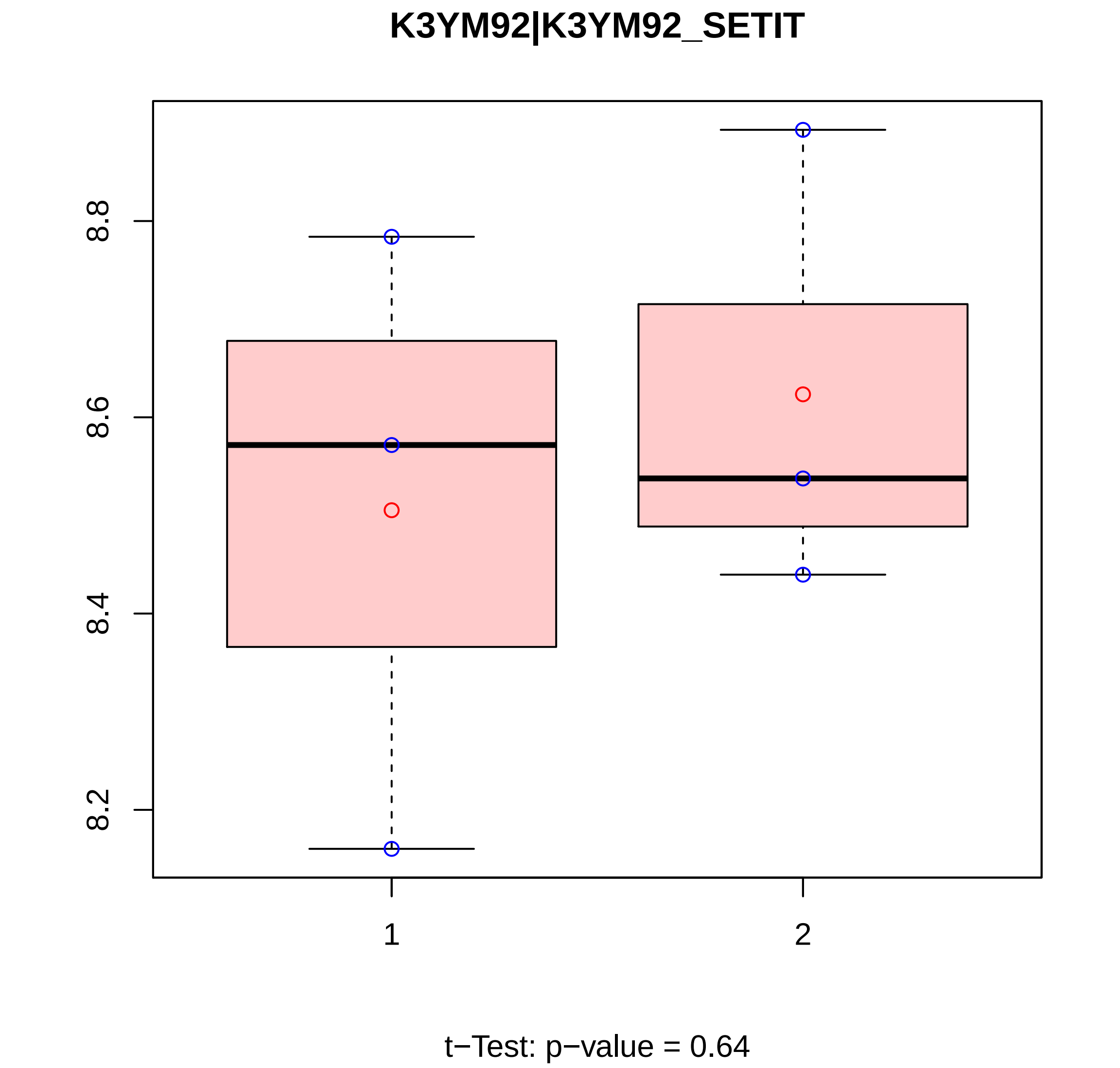
K3YM92|K3YM92_SETIT
8.2
8.4
8.6
8.8
1
2
t−Test: p−value = 0.64

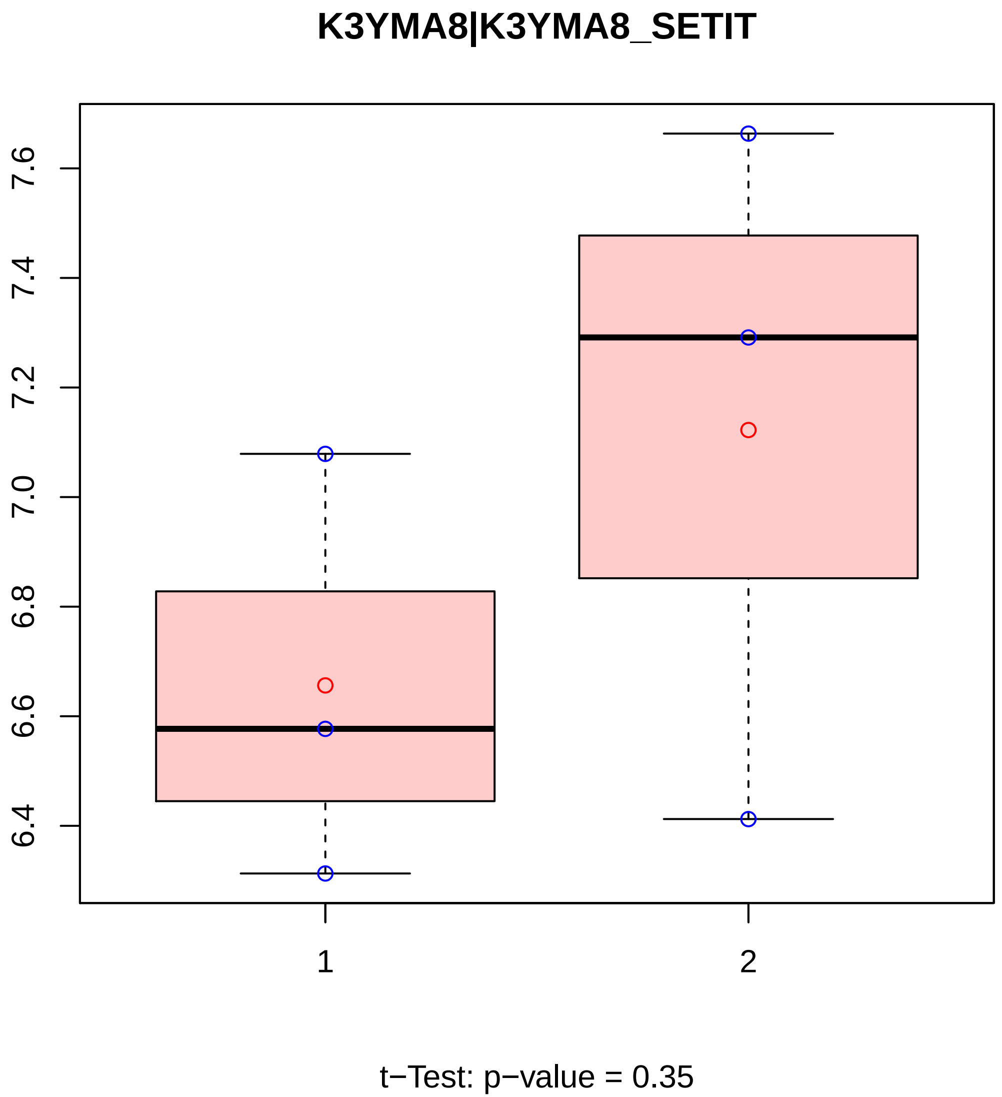
K3YMA8|K3YMA8_SETIT
7.6
7.4
7.2
7.0
6.8
6.6
6.4
1
2
t−Test: p−value = 0.35

K3YMI4|K3YMI4_SETIT

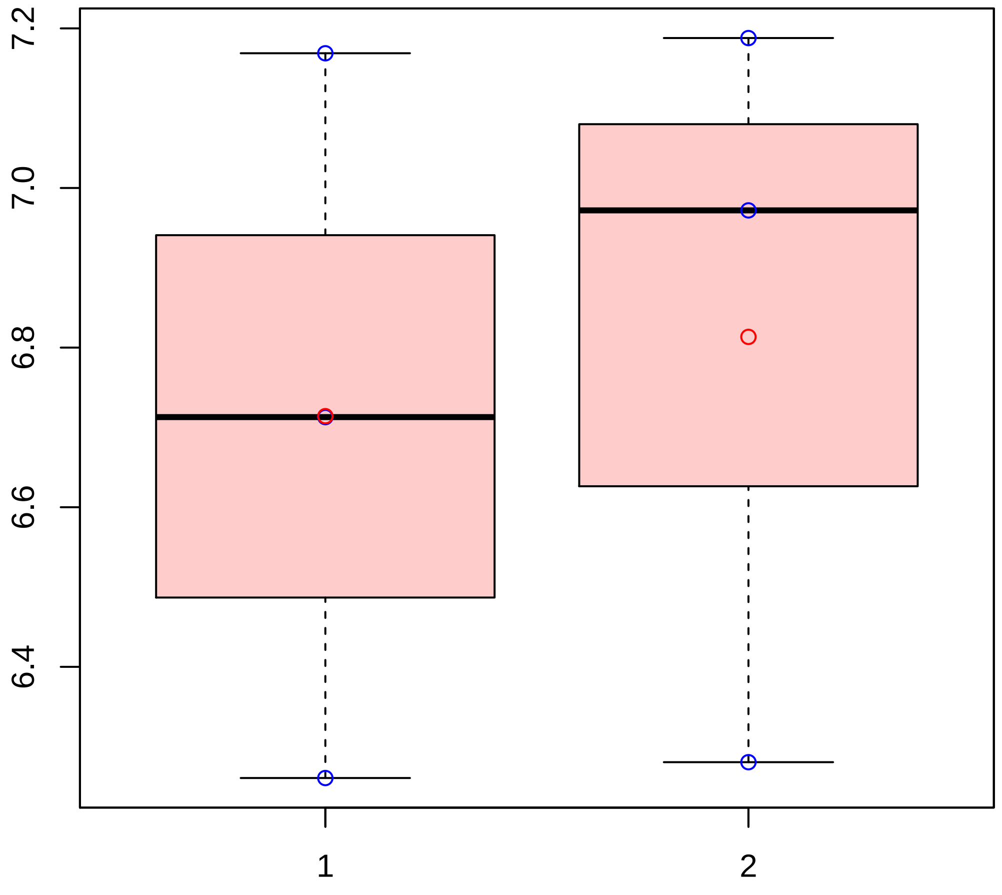

t−Test: p−value = 0.81

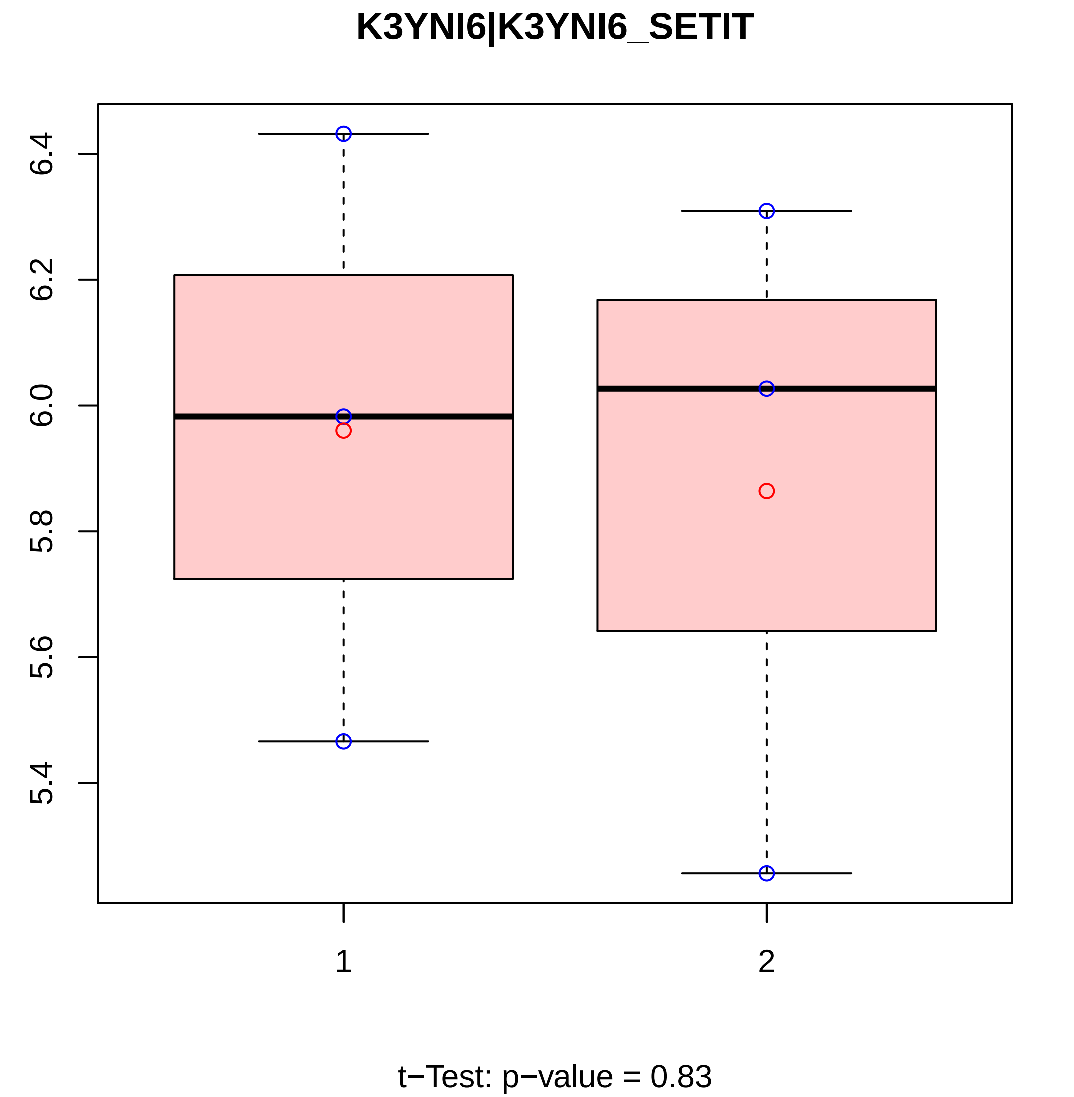

K3YNI6|K3YNI6_SETIT
6.4
6.2
6.0
5.8
5.6
5.4
1
2
t−Test: p−value = 0.83

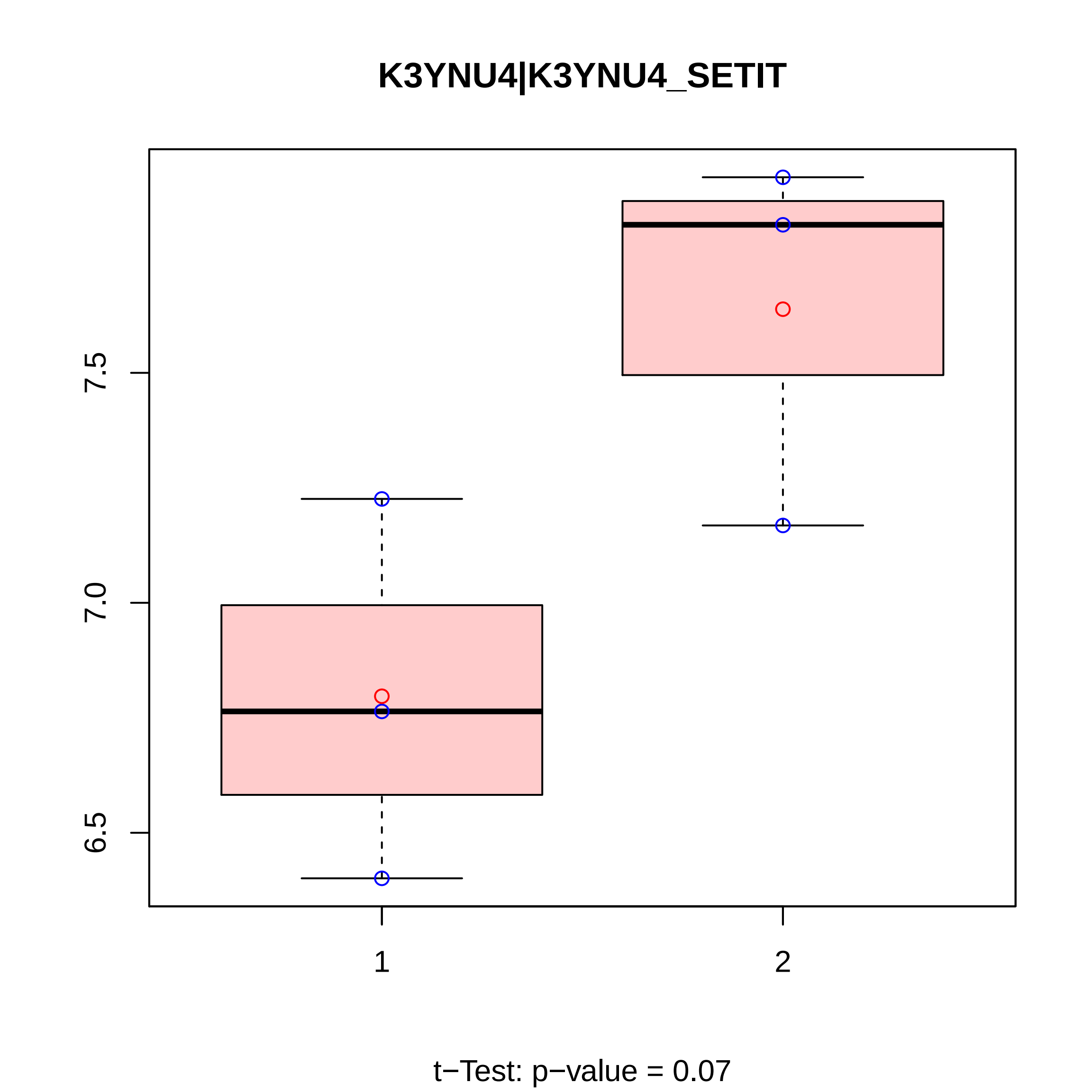

K3YNU4|K3YNU4_SETIT
6.5
7.0
7.5
1
2
t−Test: p−value = 0.07

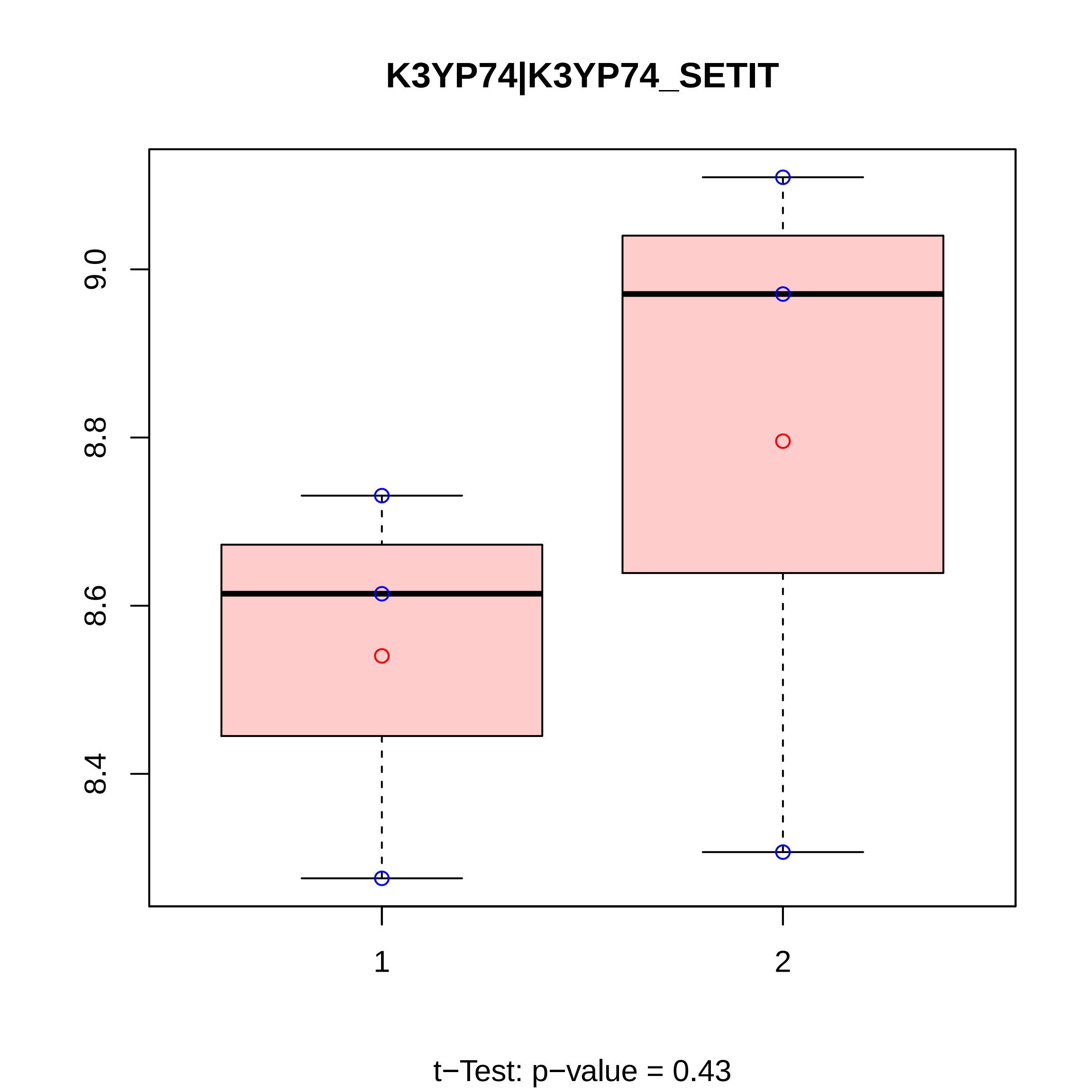

K3YP74|K3YP74_SETIT
9.0
8.8
8.6
8.4
1
2
t−Test: p−value = 0.43

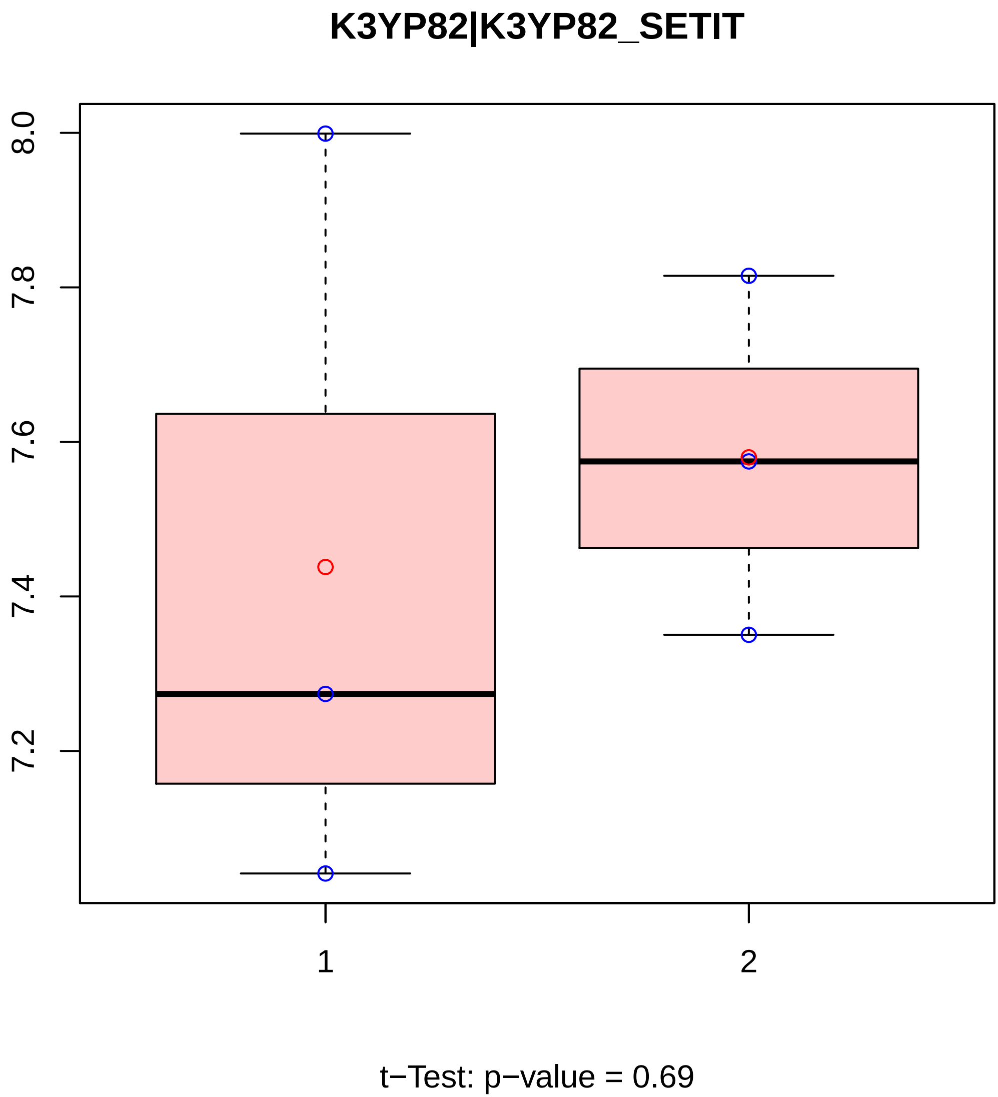
K3YP82|K3YP82_SETIT
8.0
7.8
7.6
7.4
7.2
1
2
t−Test: p−value = 0.69

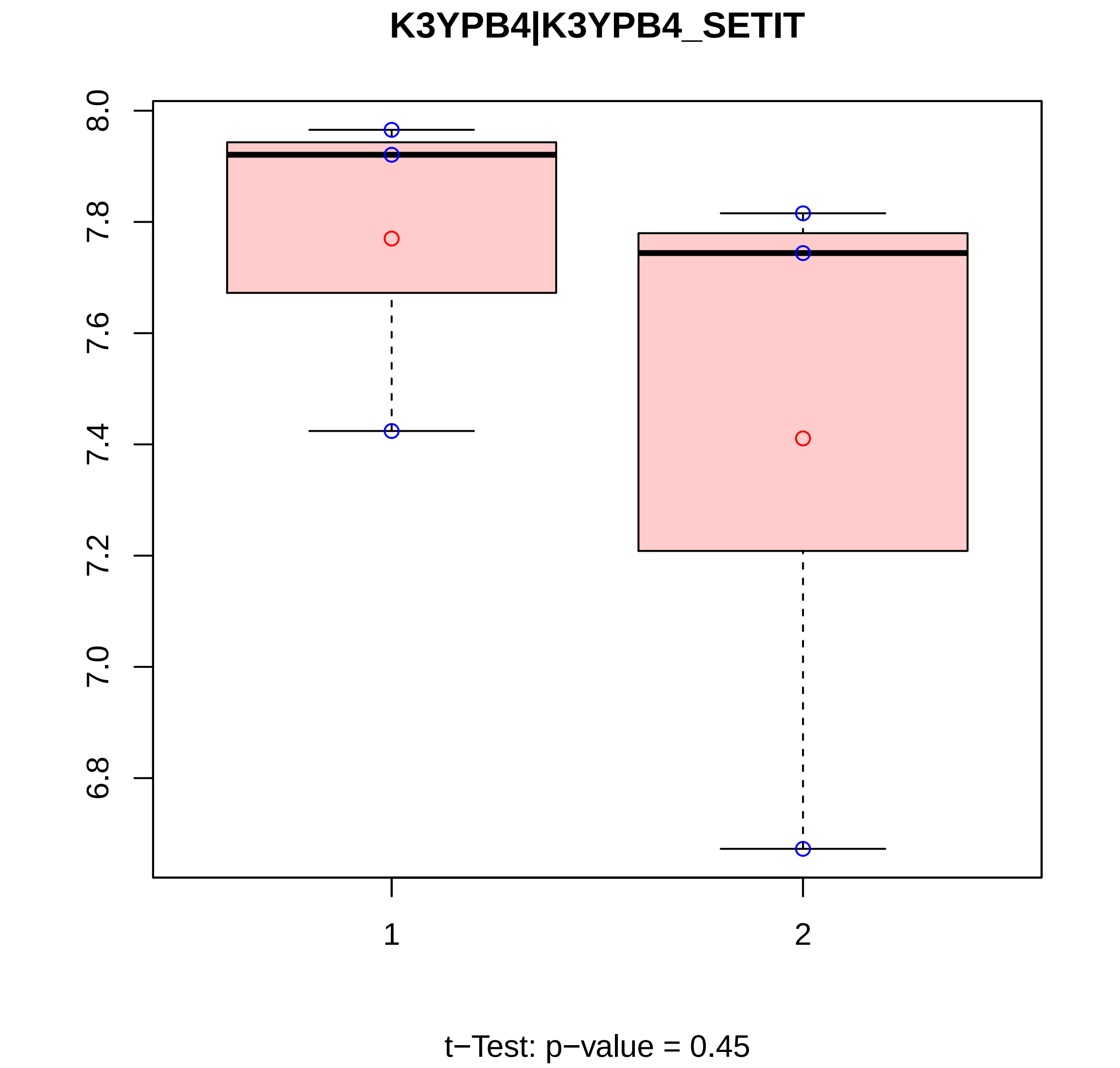

K3YPB4|K3YPB4_SETIT
8.0
7.8
7.6
7.4
7.2
7.0
6.8
1
2
t−Test: p−value = 0.45

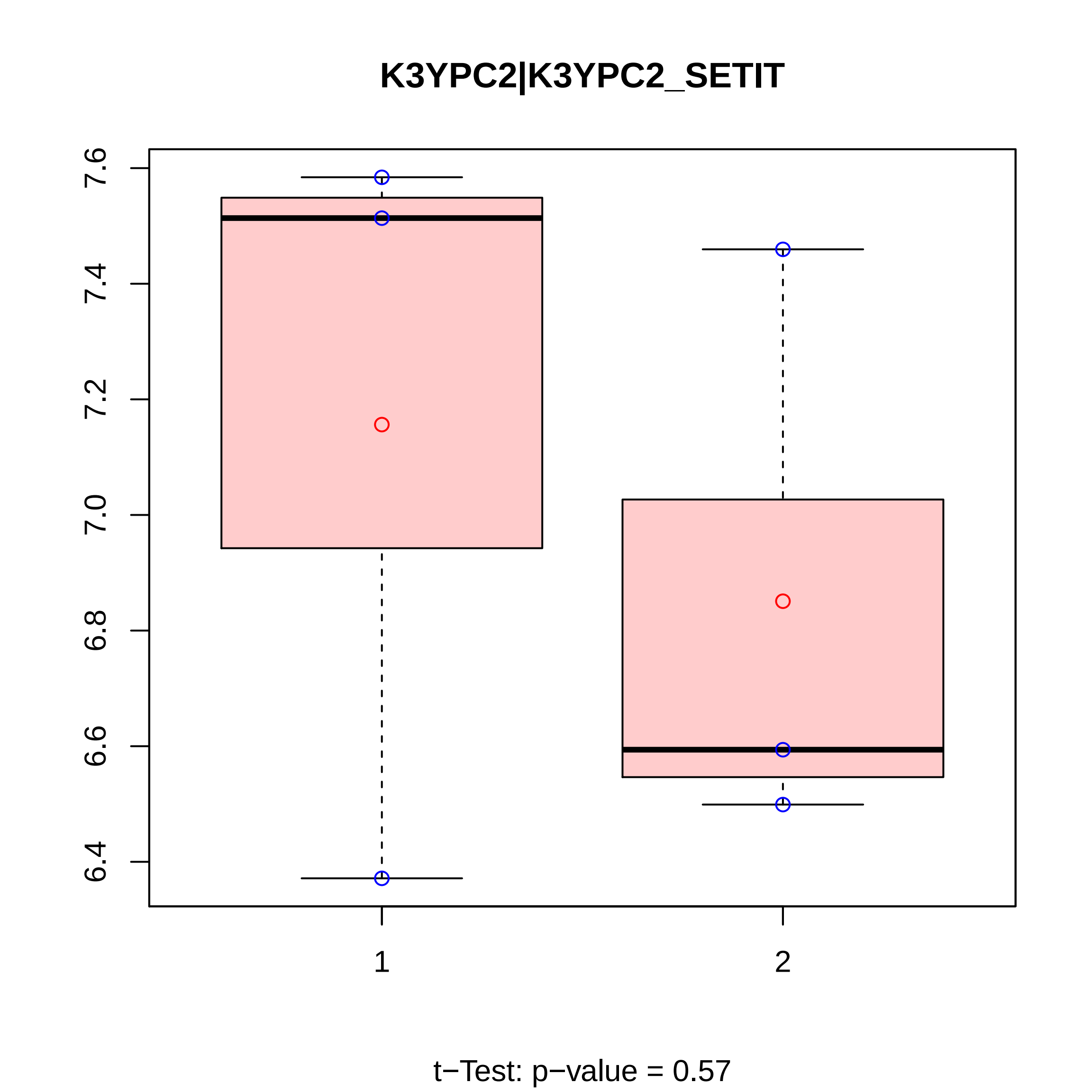

K3YPC2|K3YPC2_SETIT
7.6
7.4
7.2
7.0
6.8
6.6
6.4
1
2
t−Test: p−value = 0.57

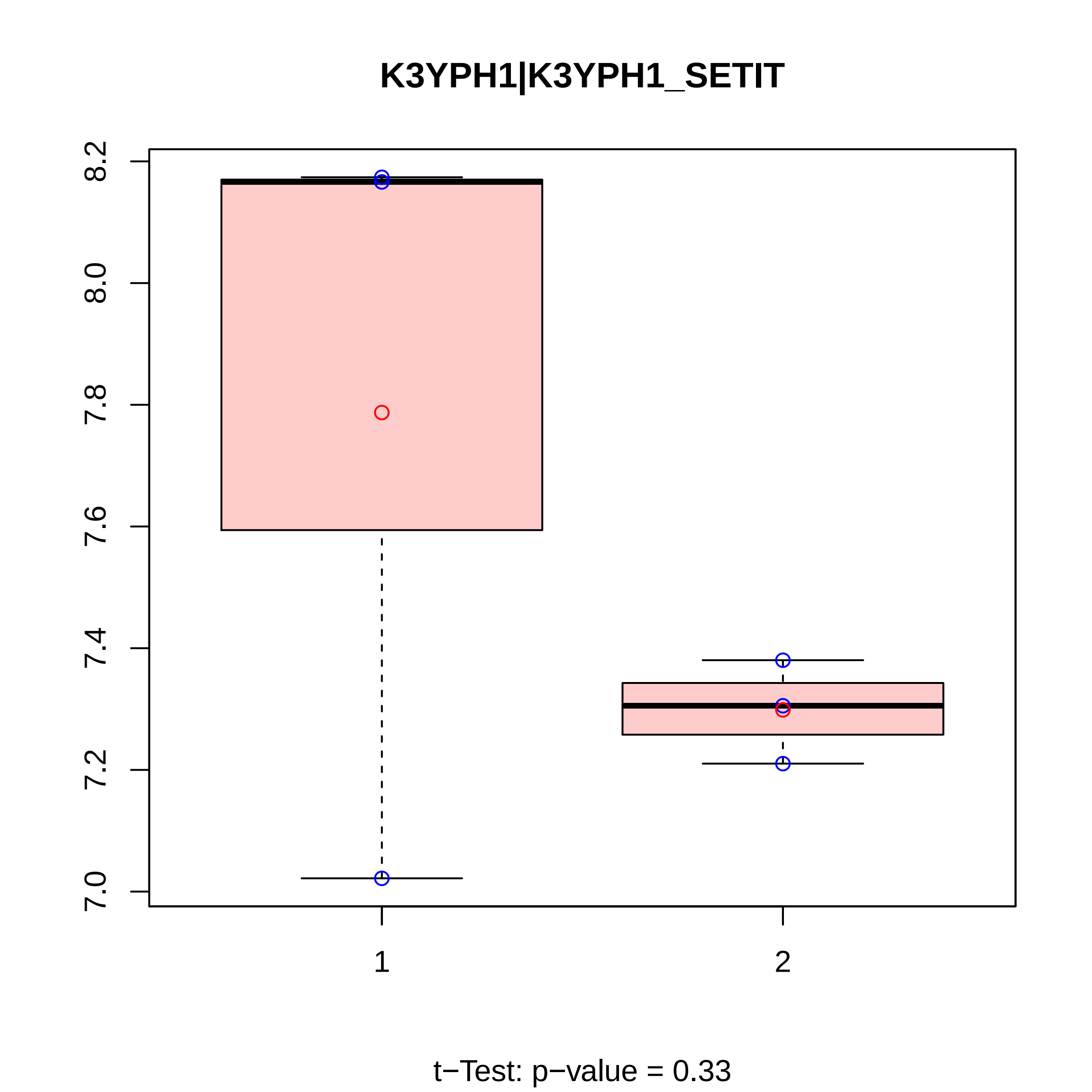

K3YPH1|K3YPH1_SETIT
8.2
8.0
7.8
7.6
7.4
7.2
7.0
1
2
t−Test: p−value = 0.33

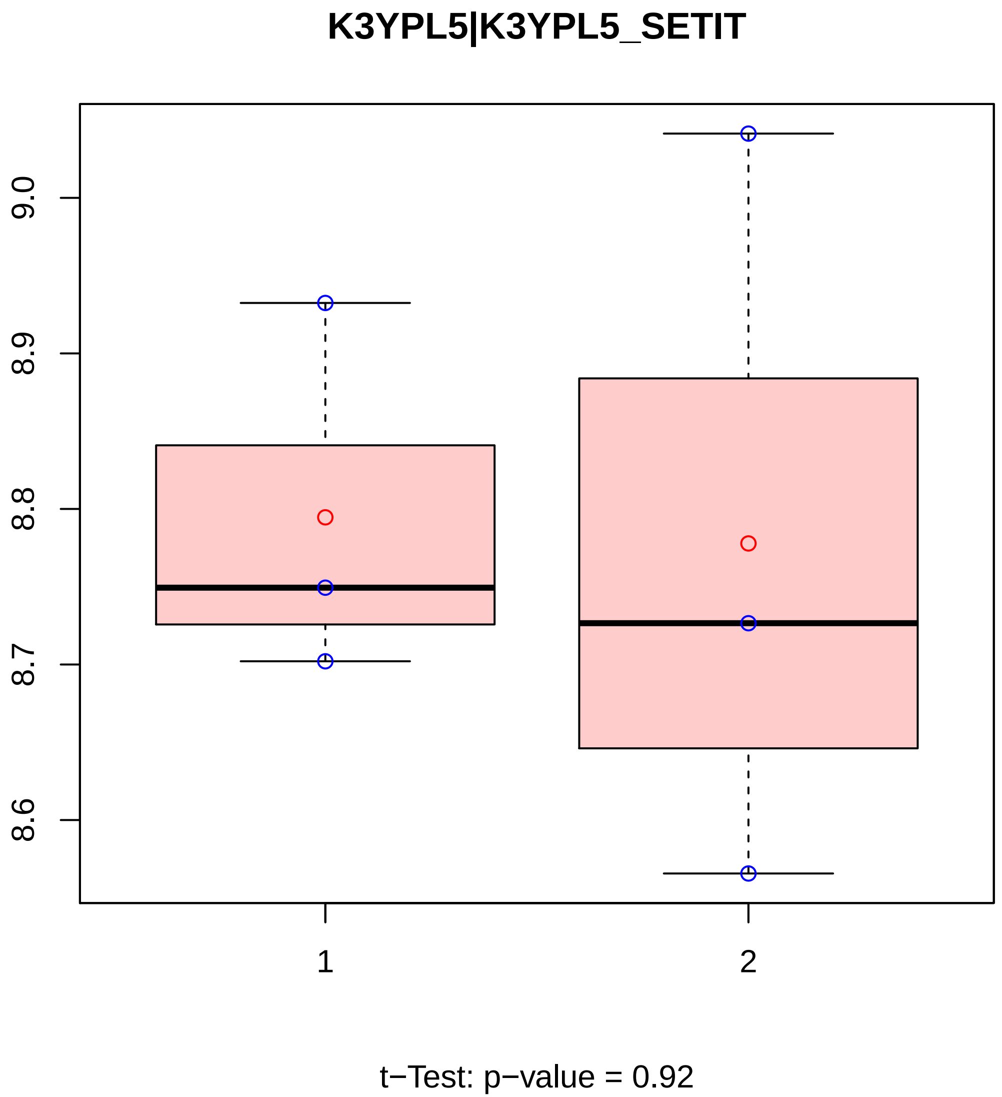
K3YPL5|K3YPL5_SETIT
9.0
8.9
8.8
8.7
8.6
1
2
t−Test: p−value = 0.92

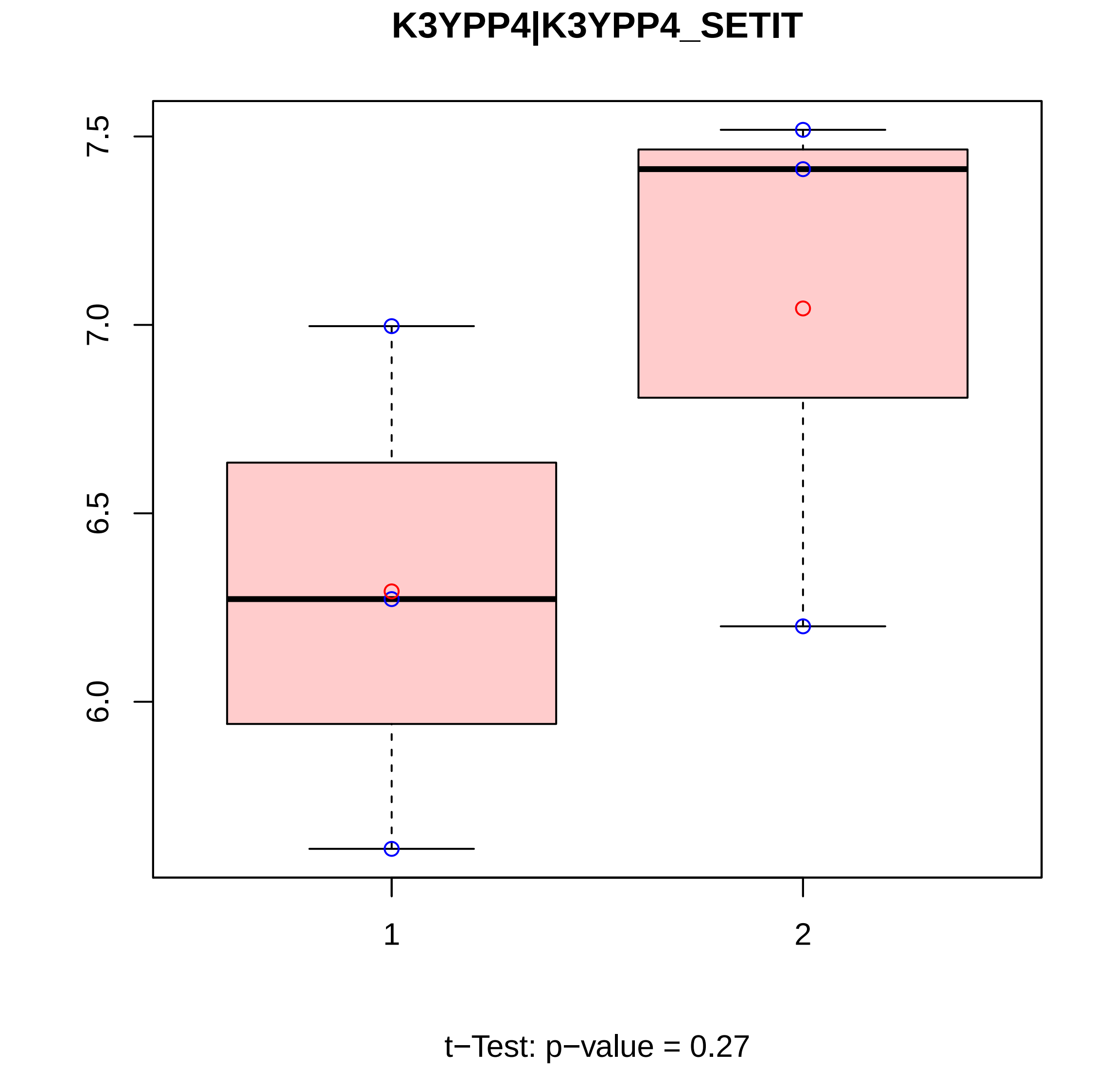

K3YPP4|K3YPP4_SETIT
7.5
7.0
6.5
6.0
1
2
t−Test: p−value = 0.27

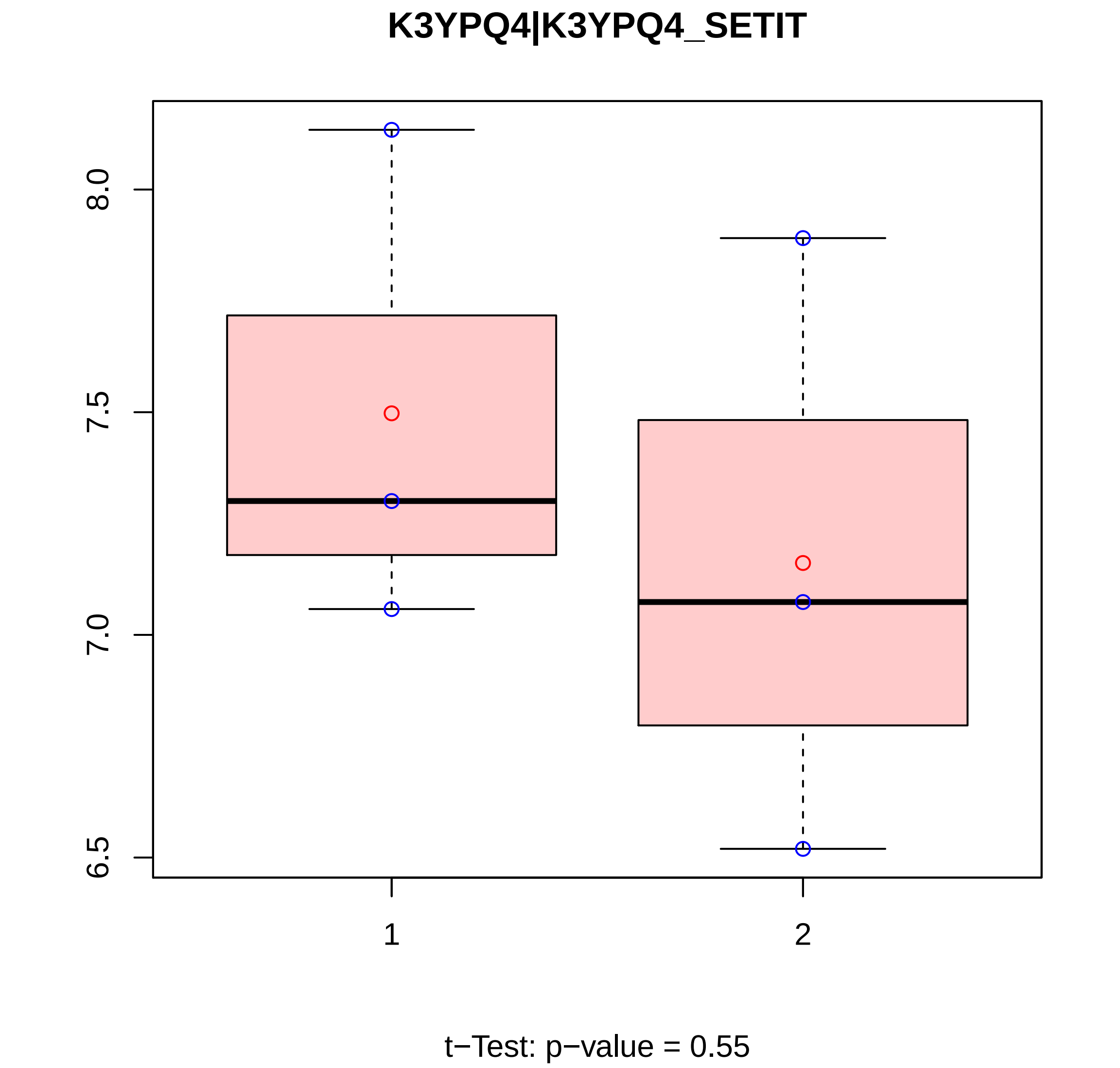
K3YPQ4|K3YPQ4_SETIT
8.0
7.5
7.0
6.5
1
2
t−Test: p−value = 0.55

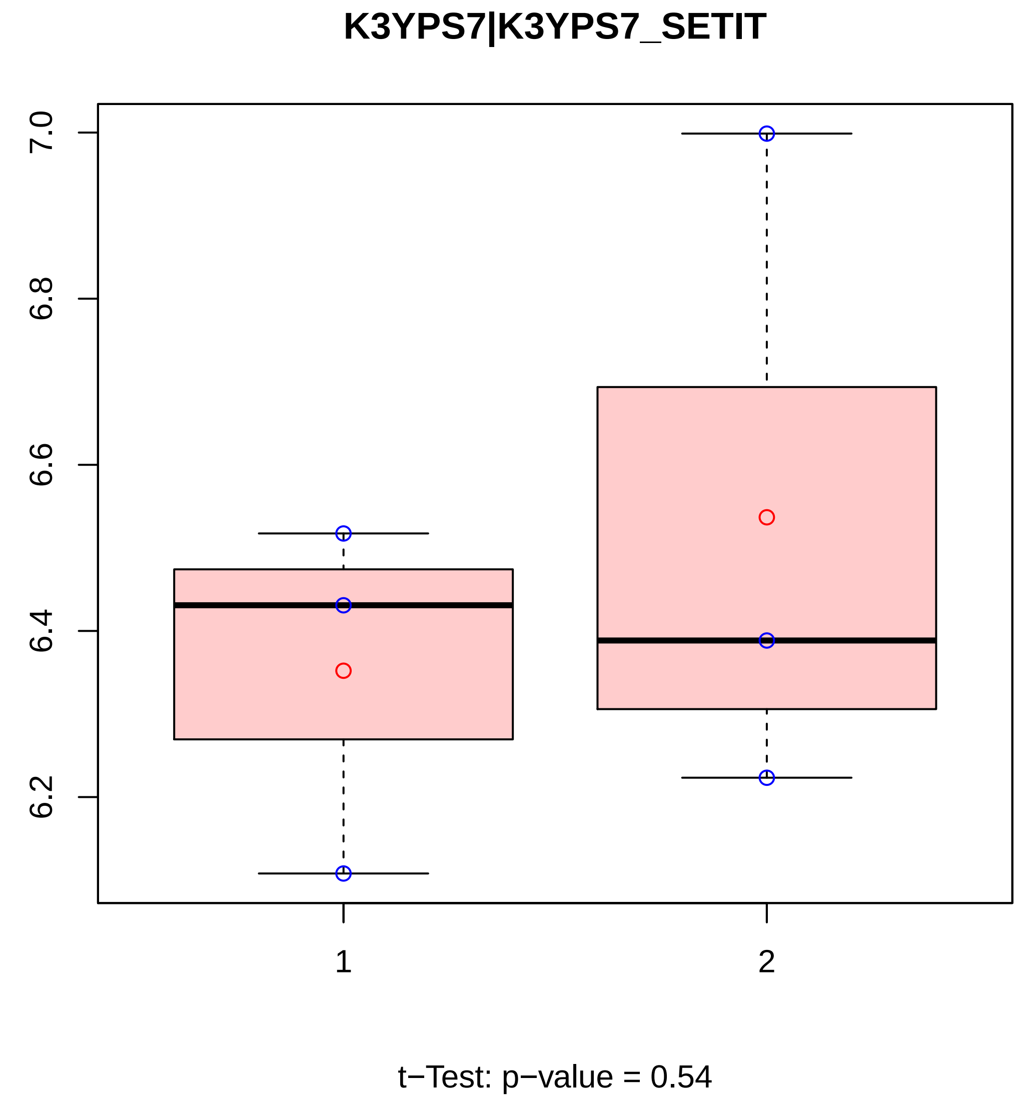
K3YPS7|K3YPS7_SETIT
7.0
6.8
6.6
6.4
6.2
1
2
t−Test: p−value = 0.54

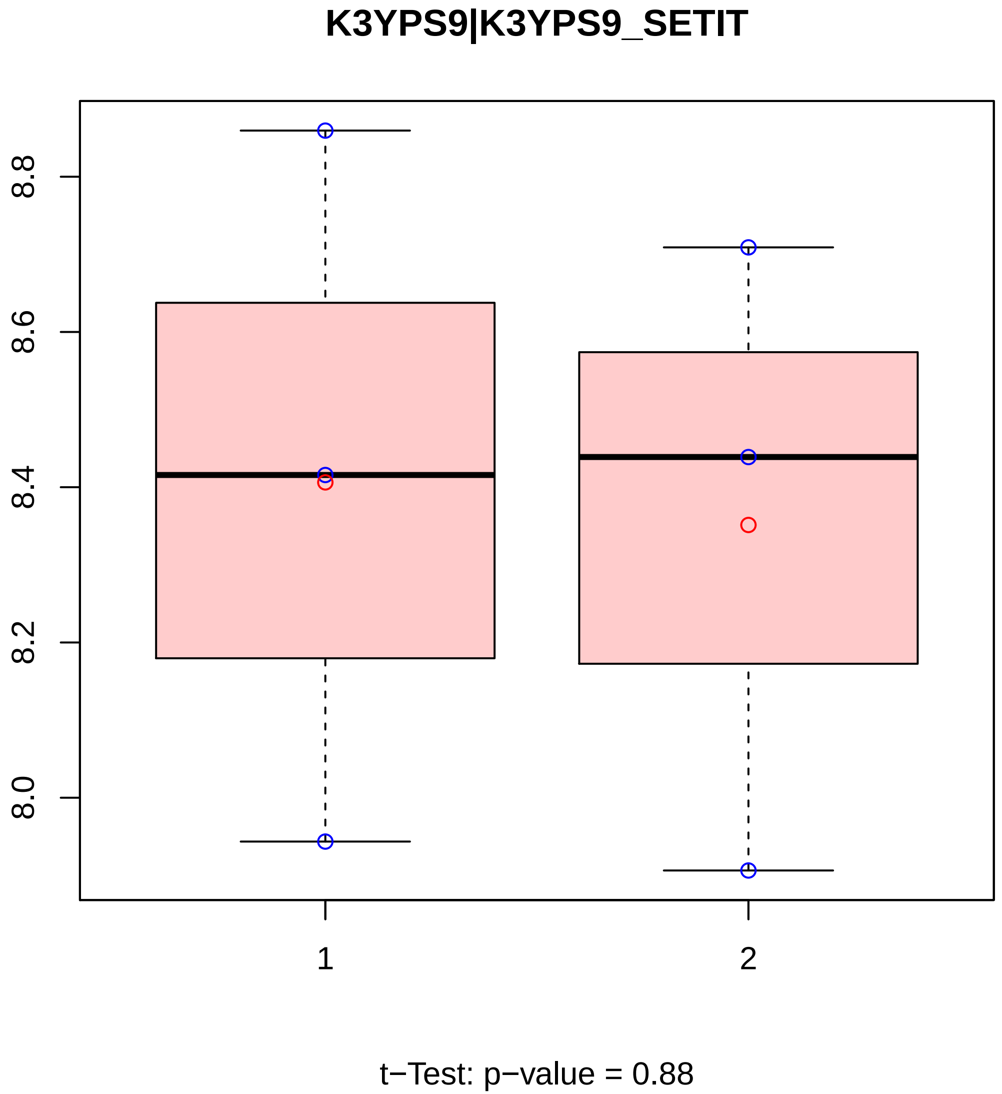
K3YPS9|K3YPS9_SETIT
8.0
8.2
8.4
8.6
8.8
1
2
t−Test: p−value = 0.88

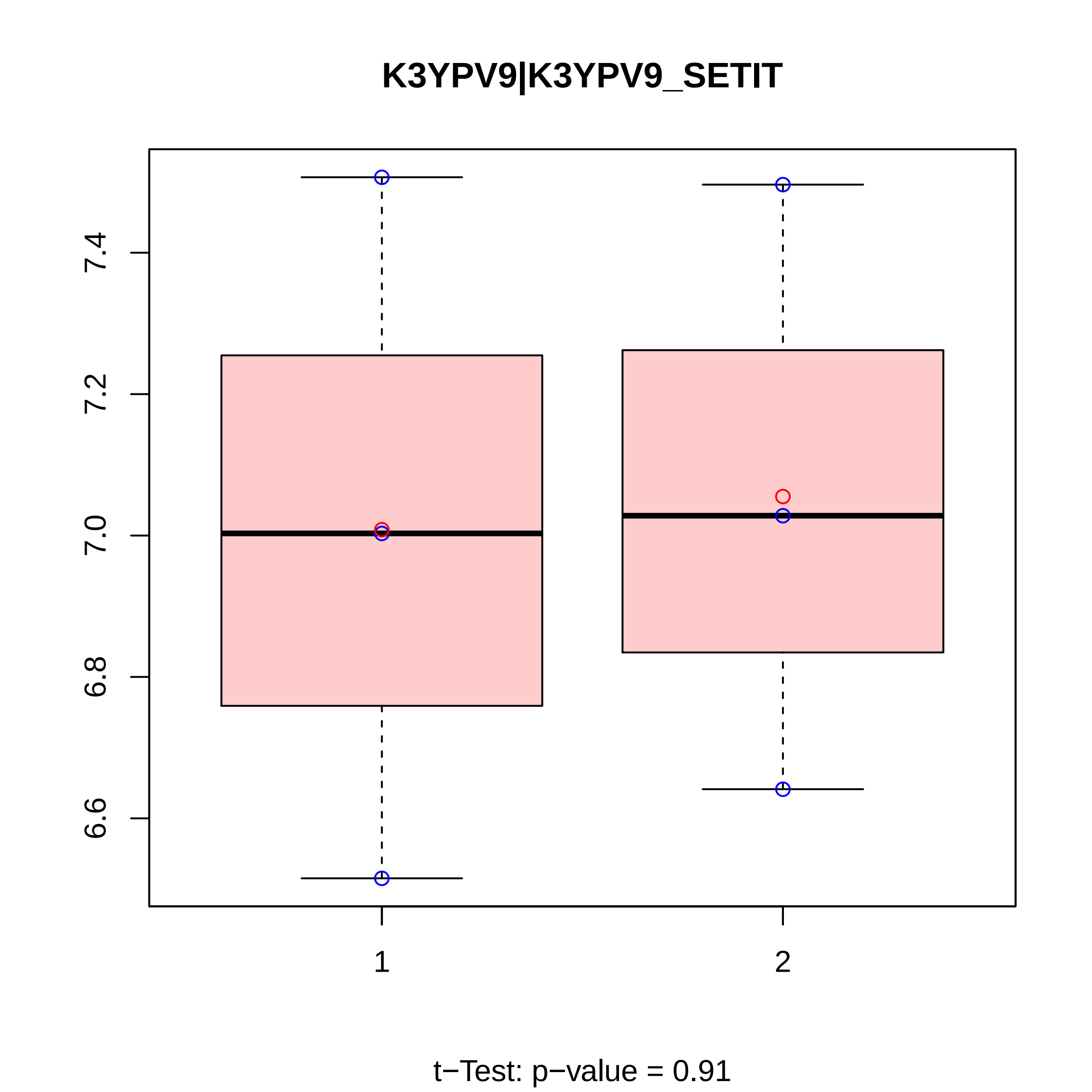
K3YPV9|K3YPV9_SETIT
7.4
7.2
7.0
6.8
6.6
1
2
t−Test: p−value = 0.91

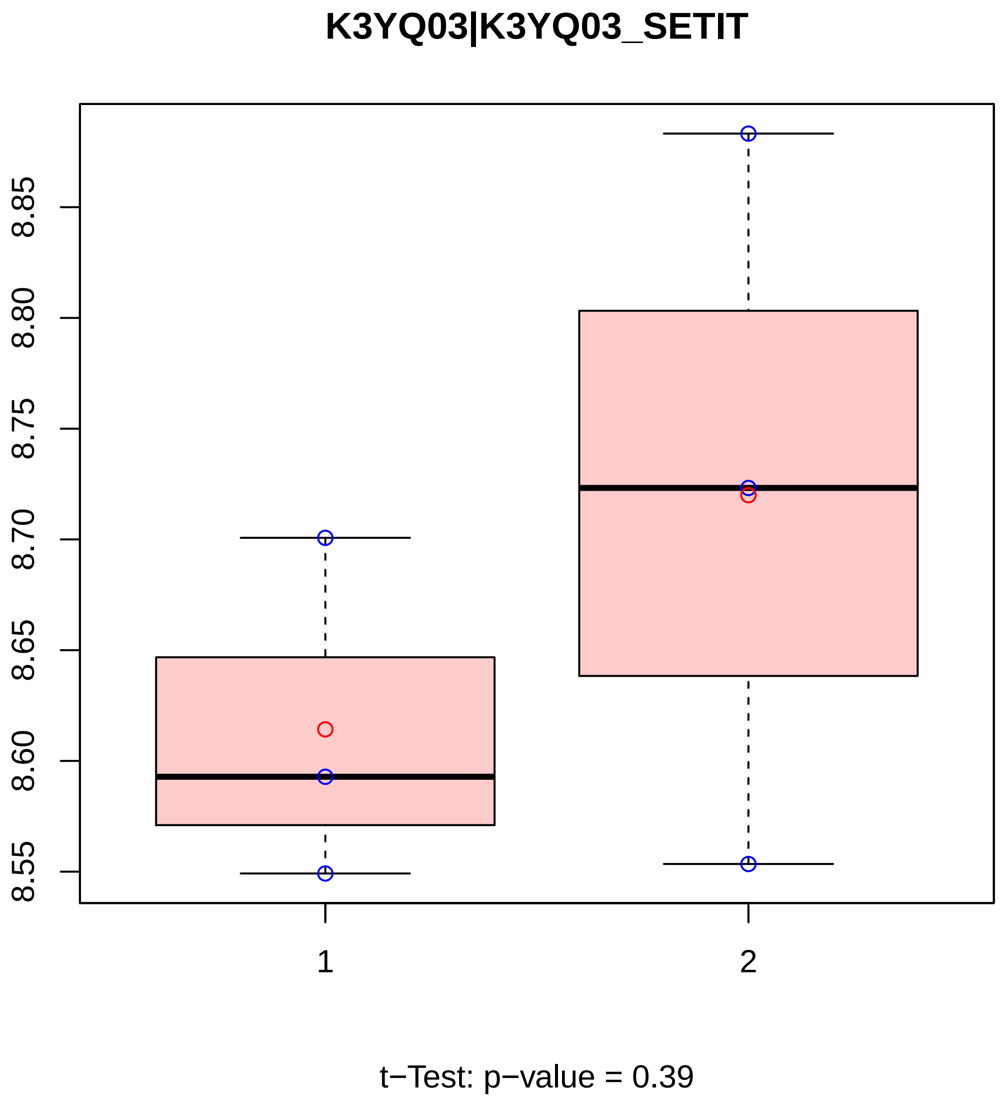

K3YQ03|K3YQ03_SETIT
8.55
8.60
8.65
8.70
8.75
8.80
8.85
1
2
t−Test: p−value = 0.39

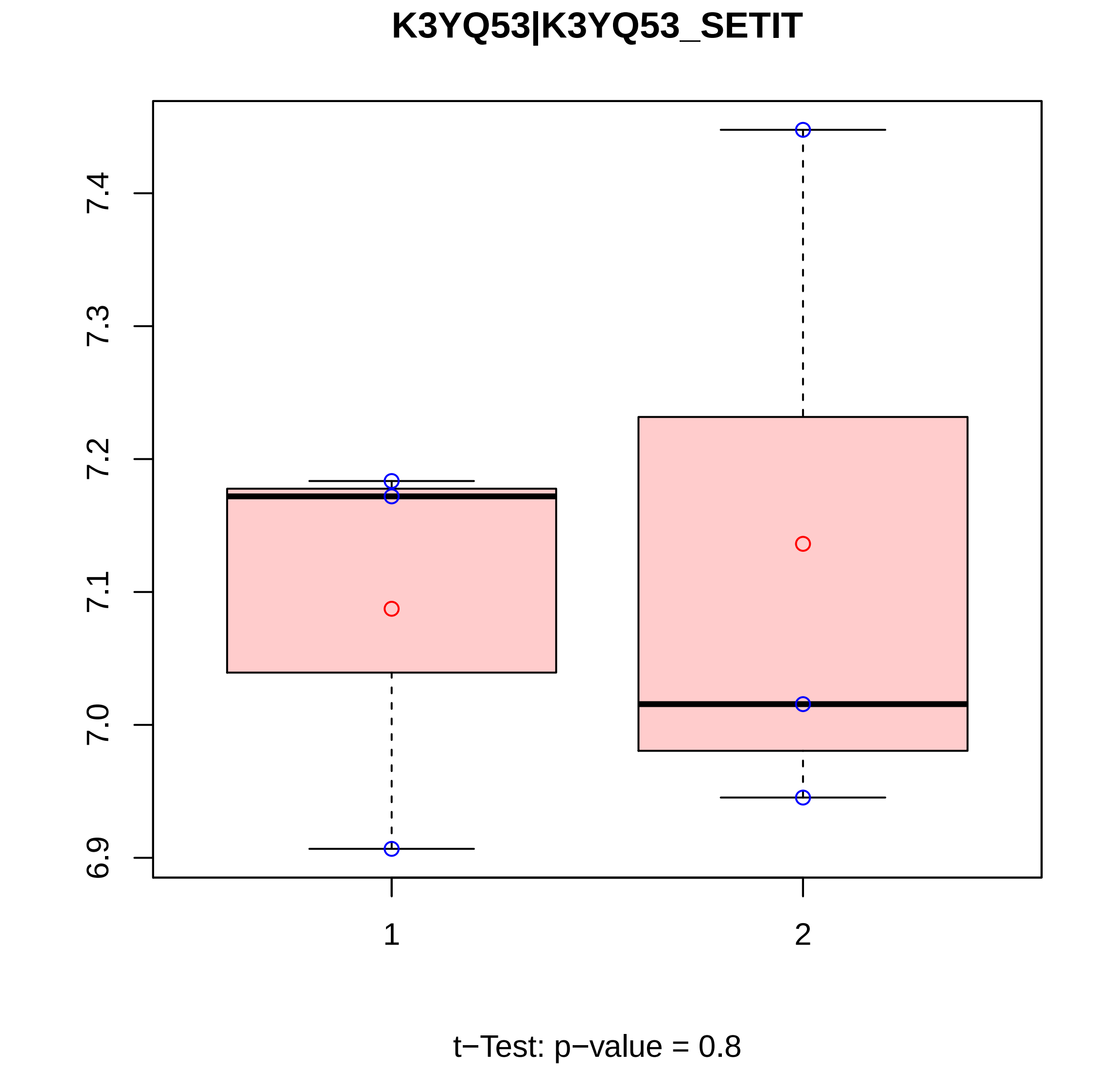

K3YQ53|K3YQ53_SETIT
7.4
7.3
7.2
7.1
7.0
6.9
1
2
t−Test: p−value = 0.8

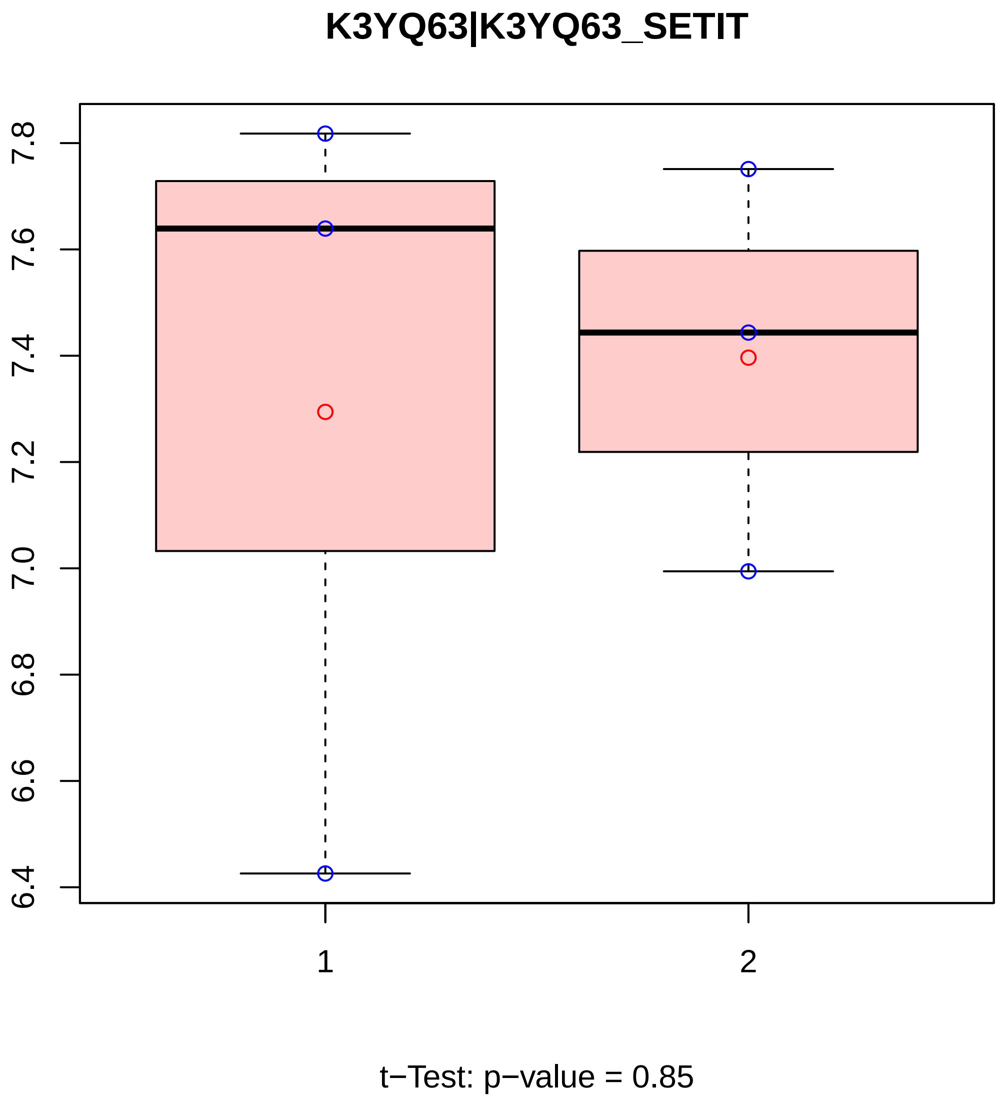
K3YQ63|K3YQ63_SETIT
7.8
7.6
7.4
7.2
7.0
6.8
6.6
6.4
1
2
t−Test: p−value = 0.85

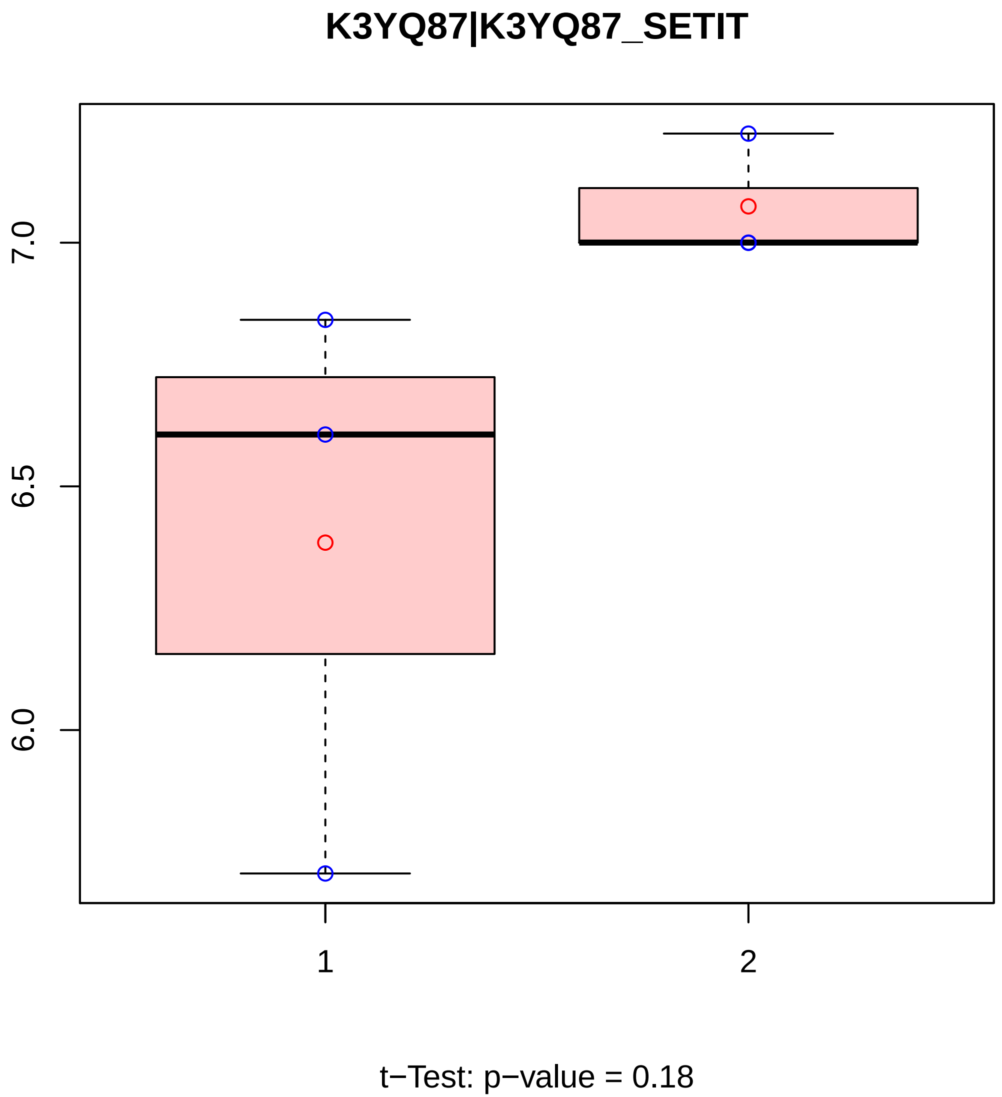
K3YQ87|K3YQ87_SETIT
7.0
6.5
6.0
1
2
t−Test: p−value = 0.18

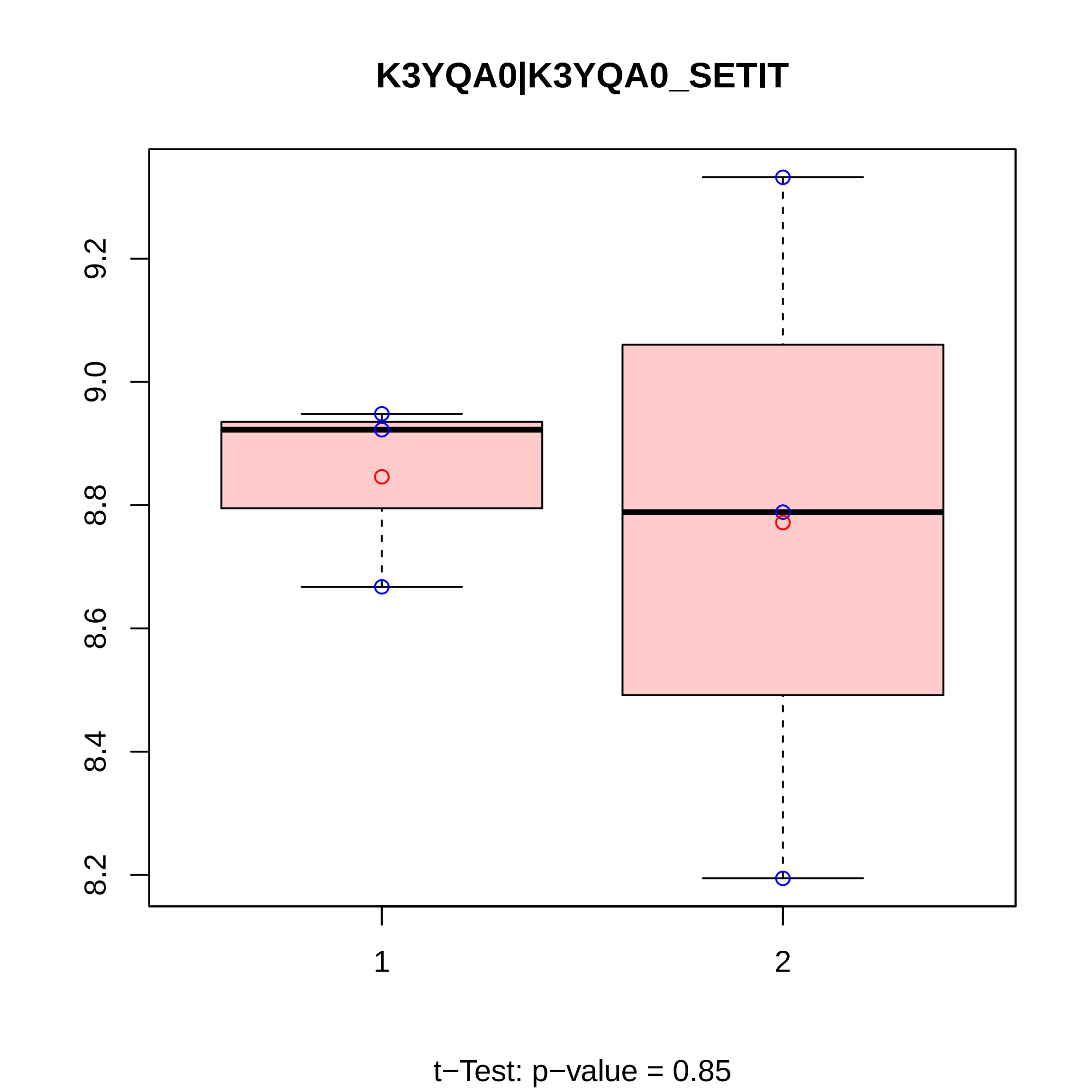
K3YQA0|K3YQA0_SETIT
9.2
9.0
8.8
8.6
8.4
8.2
1
2
t−Test: p−value = 0.85

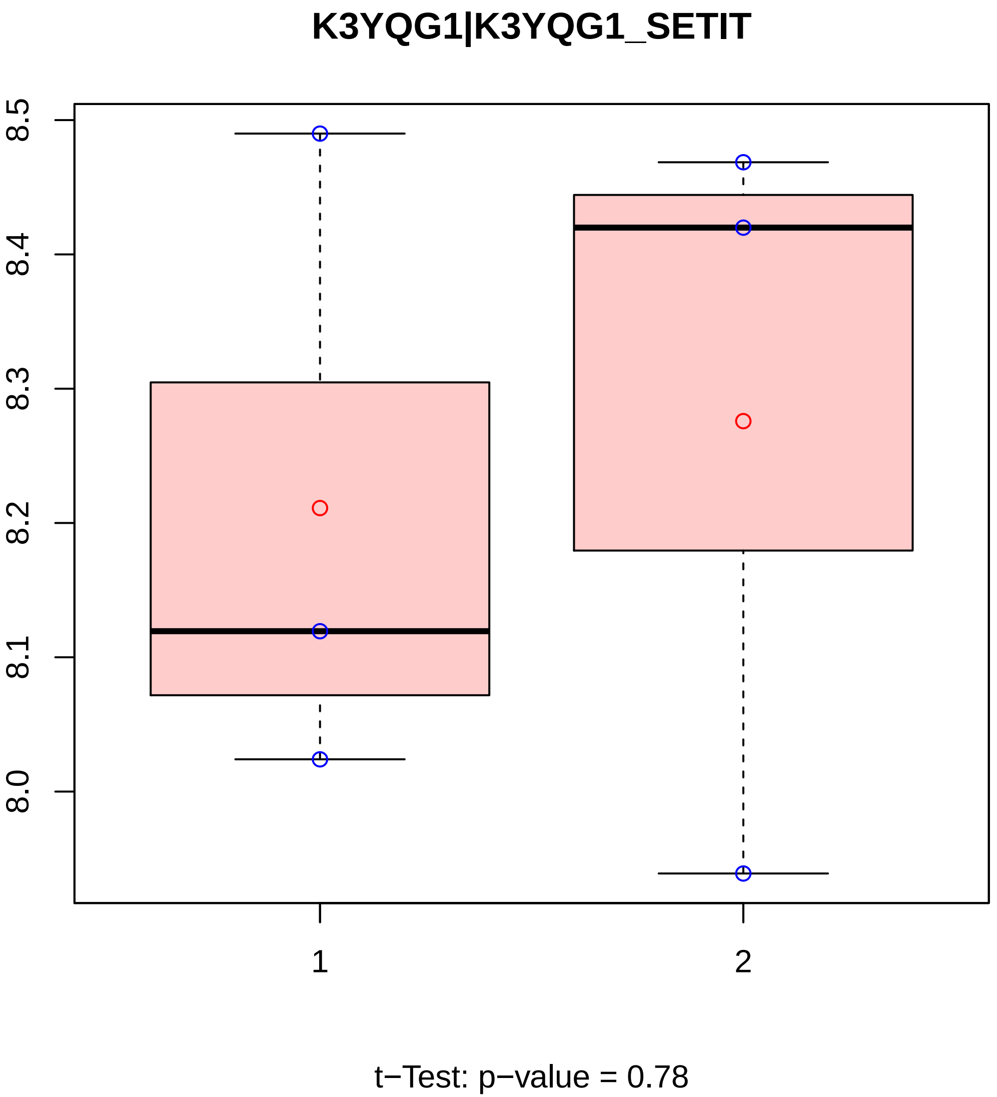

K3YQG1|K3YQG1_SETIT
8.5
8.4
8.3
8.2
8.1
8.0
1
2
t−Test: p−value = 0.78

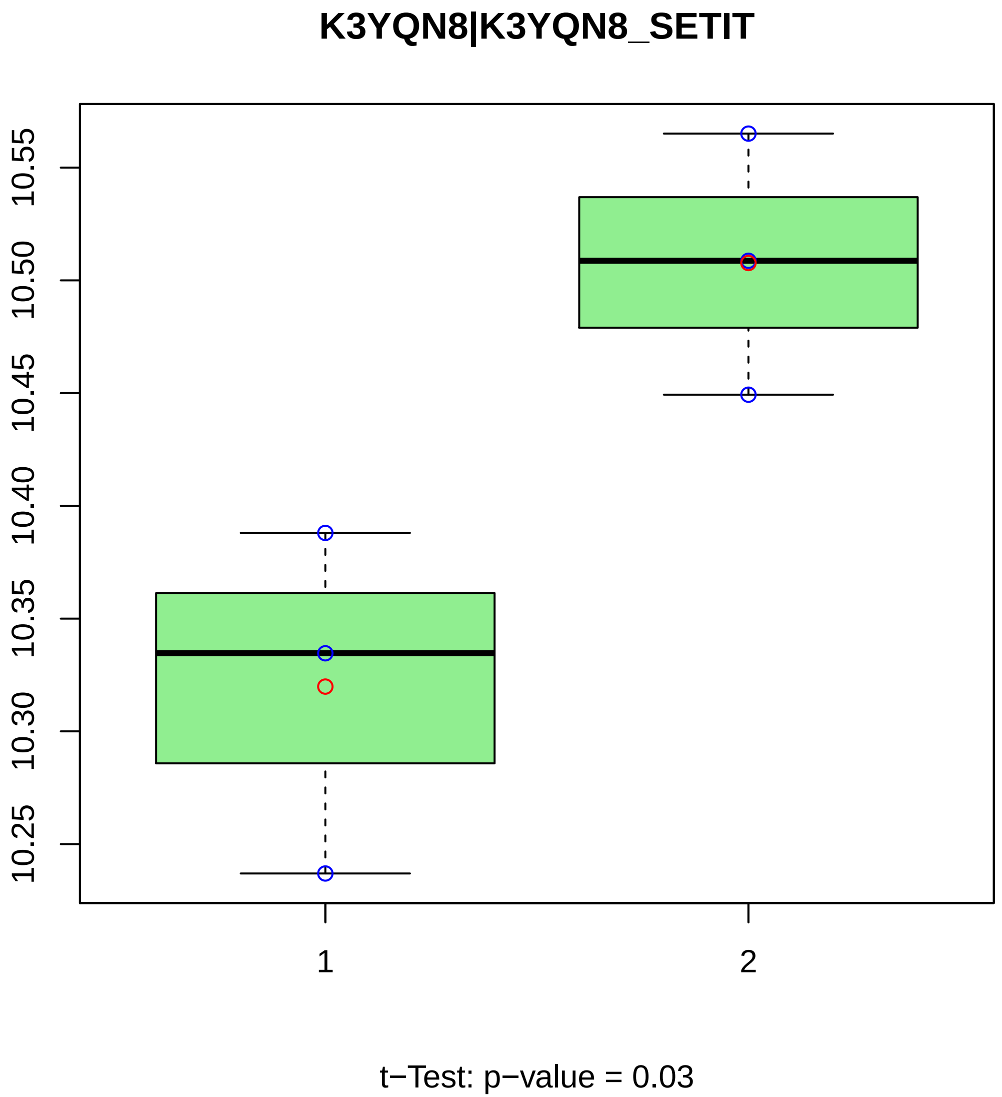
K3YQN8|K3YQN8_SETIT
10.25
10.30
10.35
10.40
10.45
10.50
10.55
1
2
t−Test: p−value = 0.03

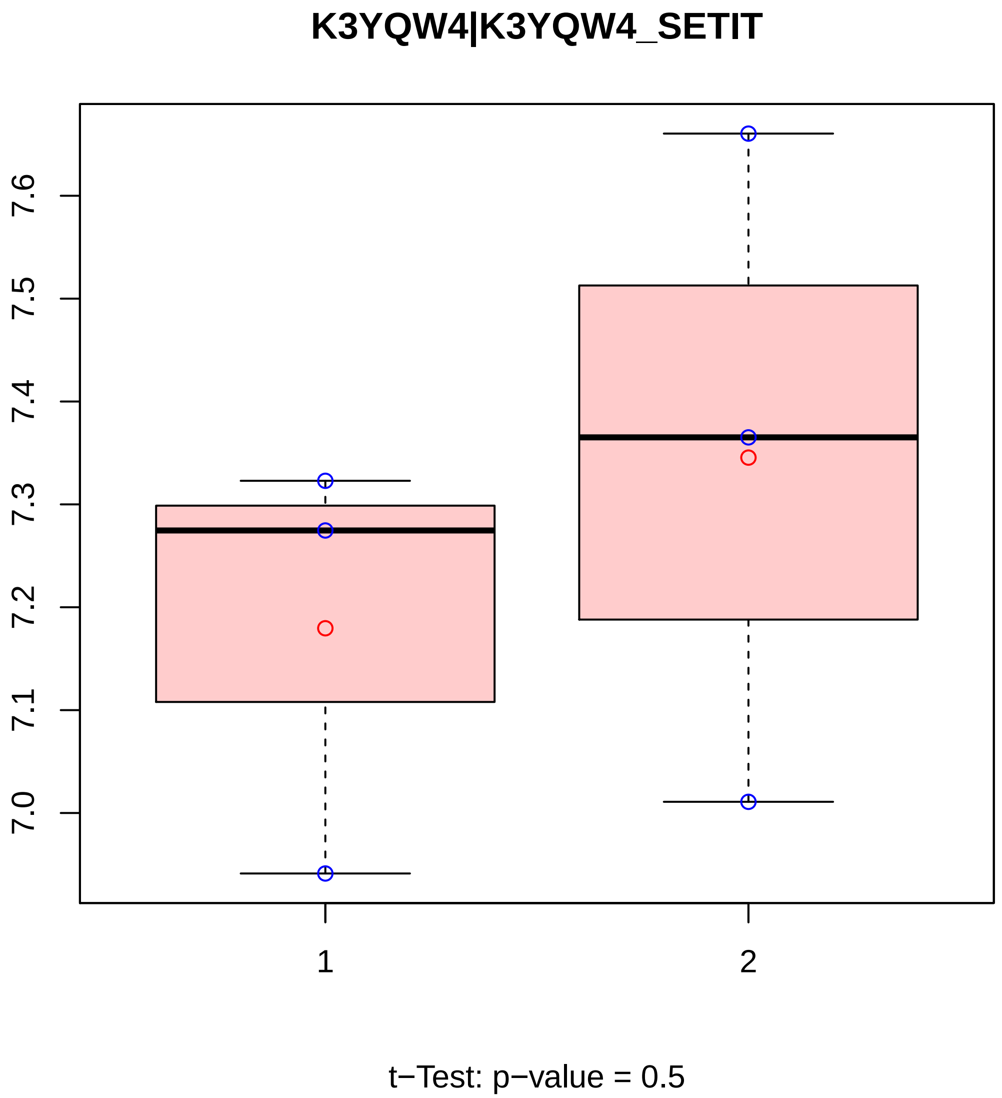
K3YQW4|K3YQW4_SETIT
7.0
7.1
7.2
7.3
7.4
7.5
7.6
1
2
t−Test: p−value = 0.5

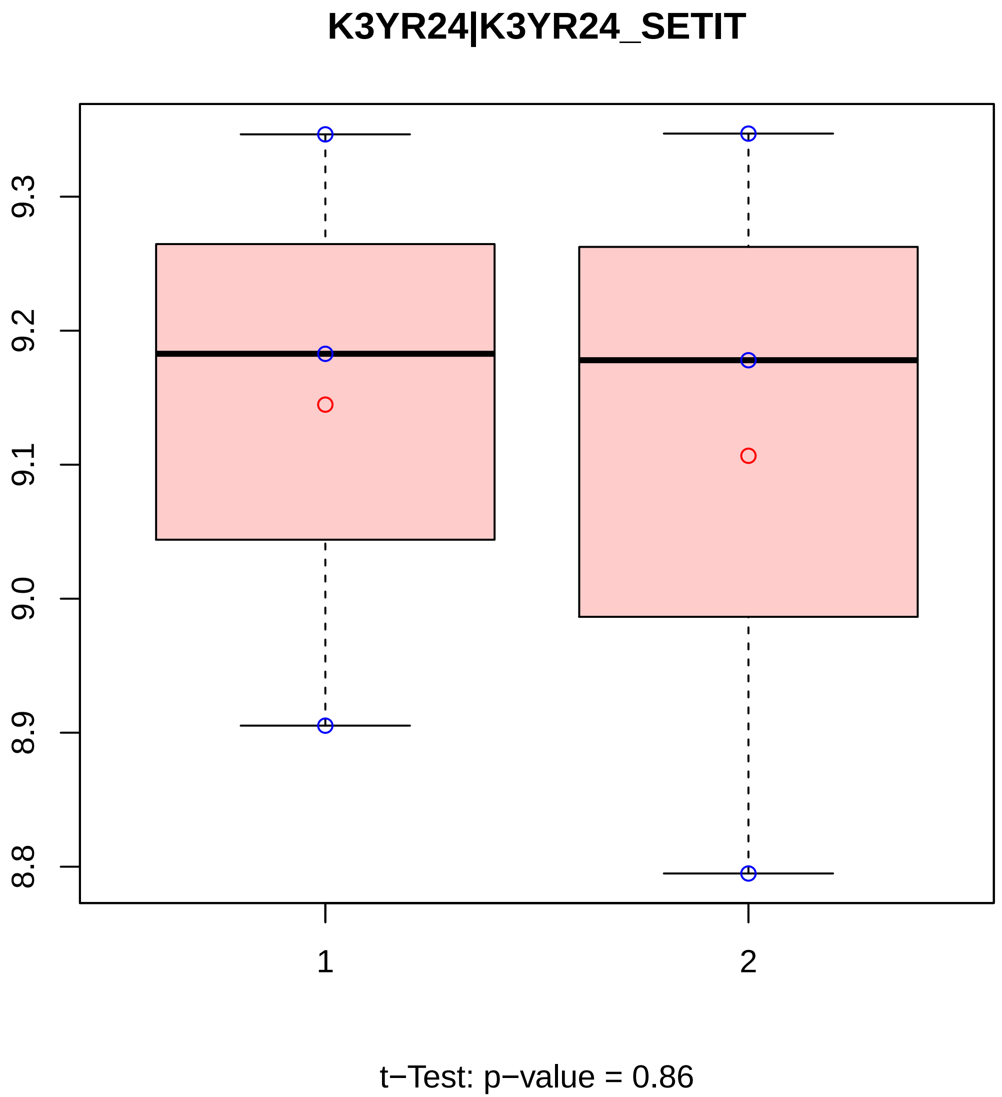

K3YR24|K3YR24_SETIT
9.3
9.2
9.1
9.0
8.9
8.8
1
2
t−Test: p−value = 0.86

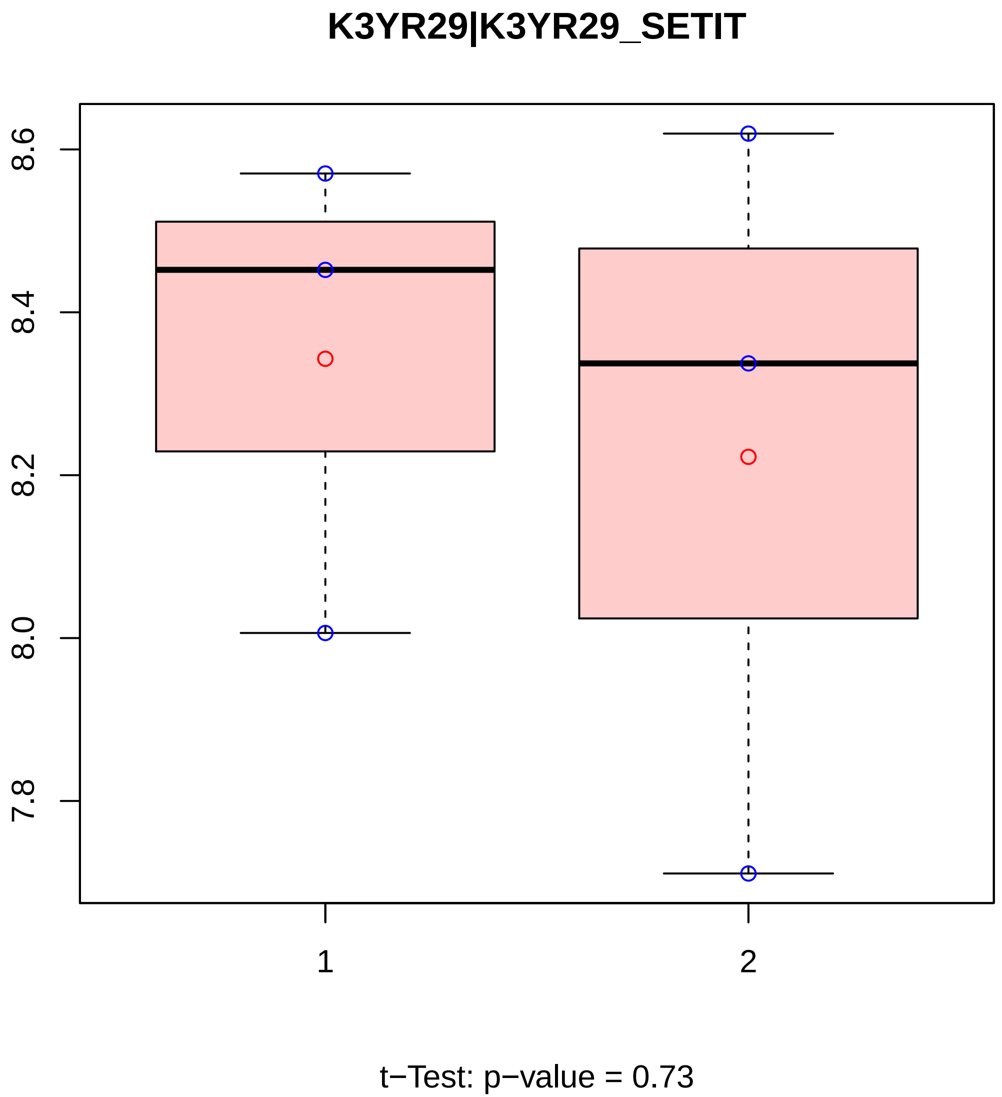

K3YR29|K3YR29_SETIT
8.6
8.4
8.2
8.0
7.8
1
2
t−Test: p−value = 0.73

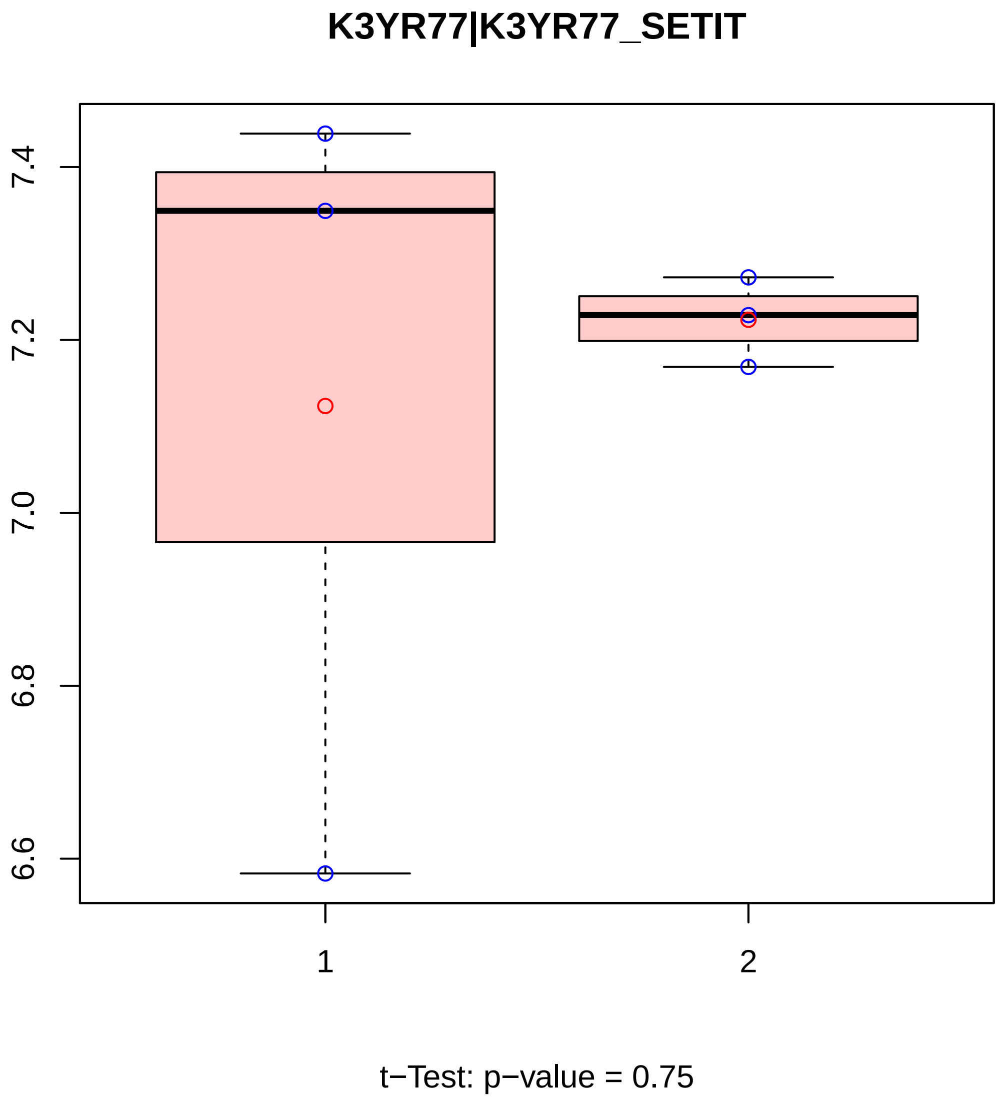

K3YR77|K3YR77_SETIT
7.4
7.2
7.0
6.8
6.6
1
2
t−Test: p−value = 0.75

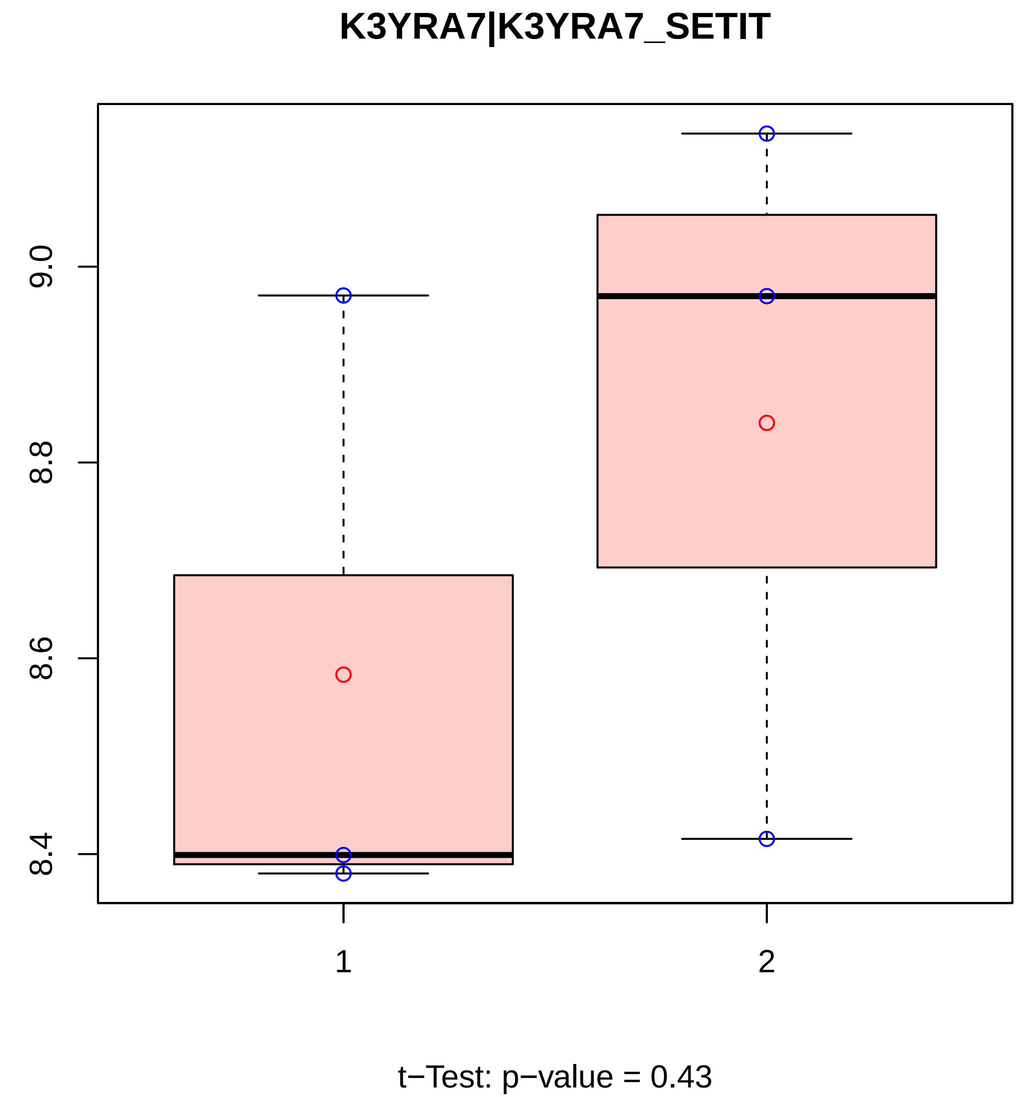
K3YRA7|K3YRA7_SETIT
9.0
8.8
8.6
8.4
1
2
t−Test: p−value = 0.43

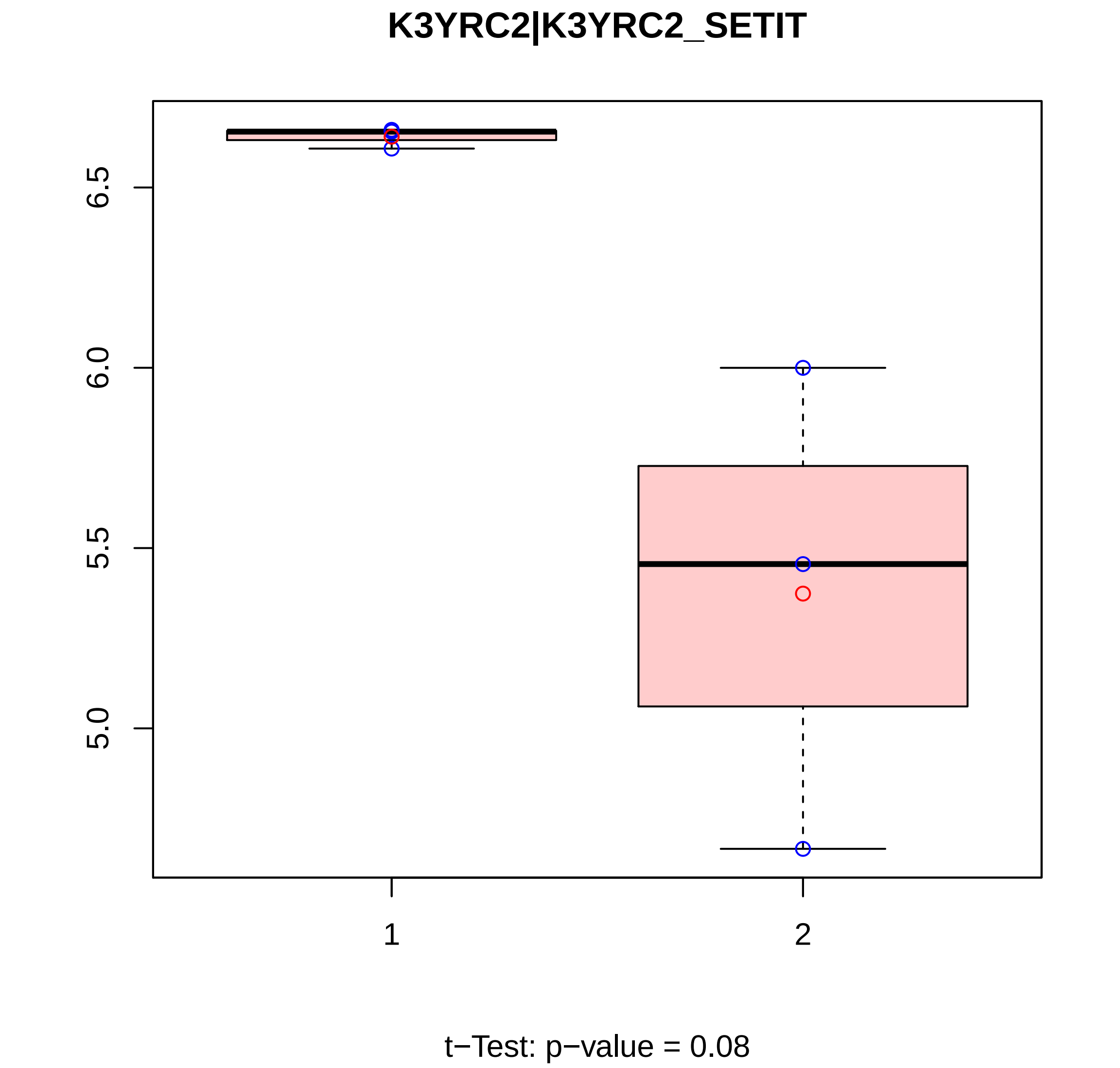

K3YRC2|K3YRC2_SETIT
6.5
6.0
5.5
5.0
1
2
t−Test: p−value = 0.08

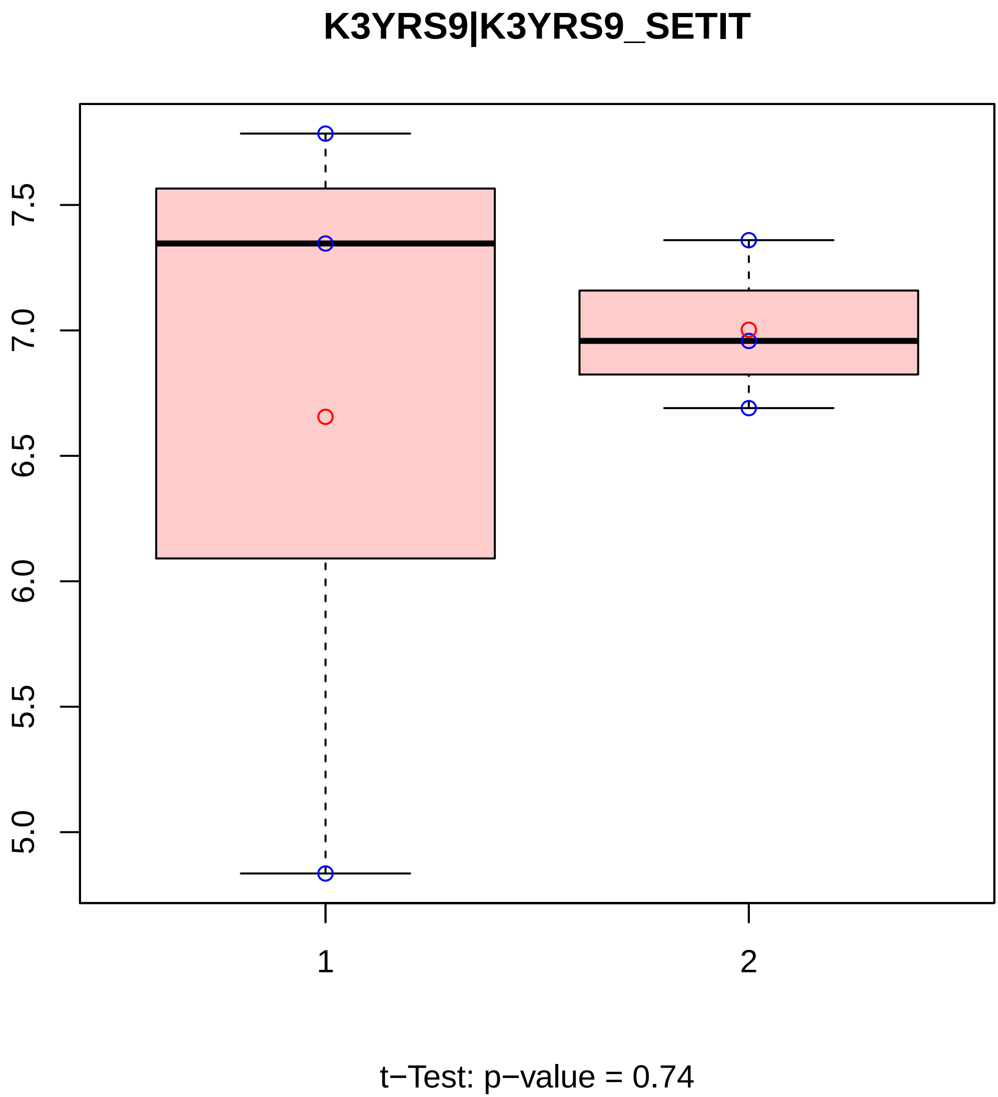
K3YRS9|K3YRS9_SETIT
7.5
7.0
6.5
6.0
5.5
5.0
1
2
t−Test: p−value = 0.74

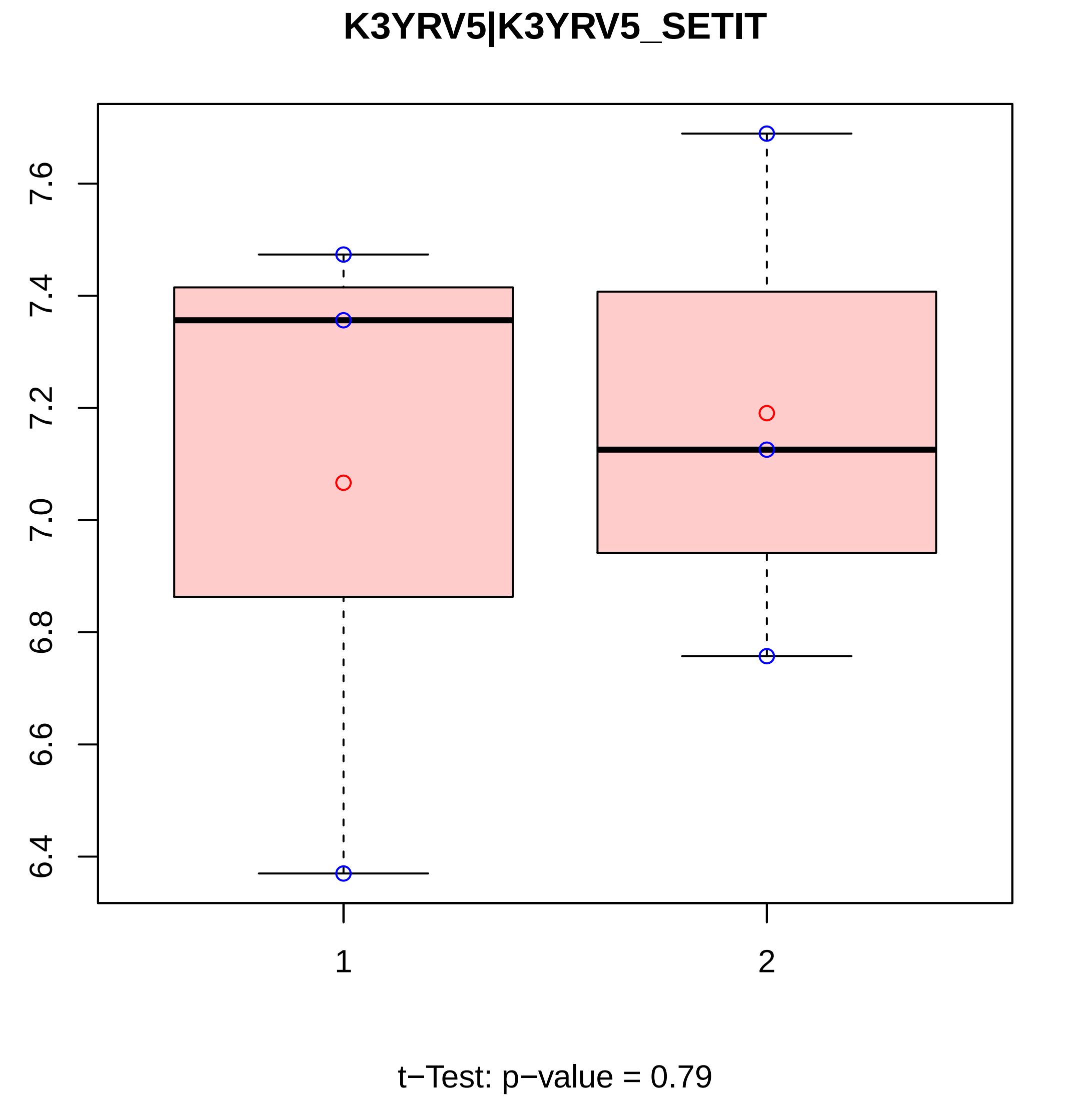

K3YRV5|K3YRV5_SETIT
7.6
7.4
7.2
7.0
6.8
6.6
6.4
1
2
t−Test: p−value = 0.79

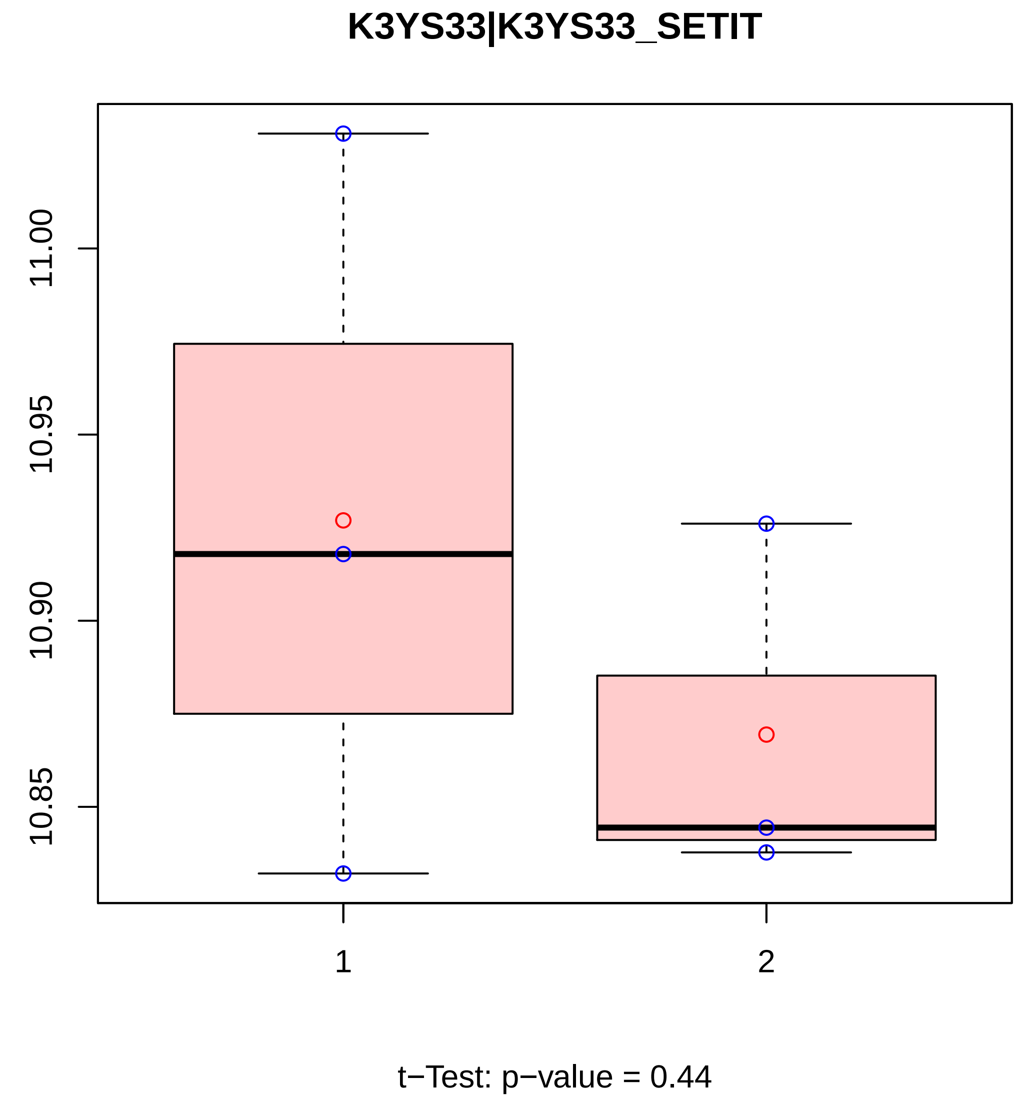
K3YS33|K3YS33_SETIT
11.00
10.95
10.90
10.85
1
2
t−Test: p−value = 0.44

K3YS38|K3YS38_SETIT

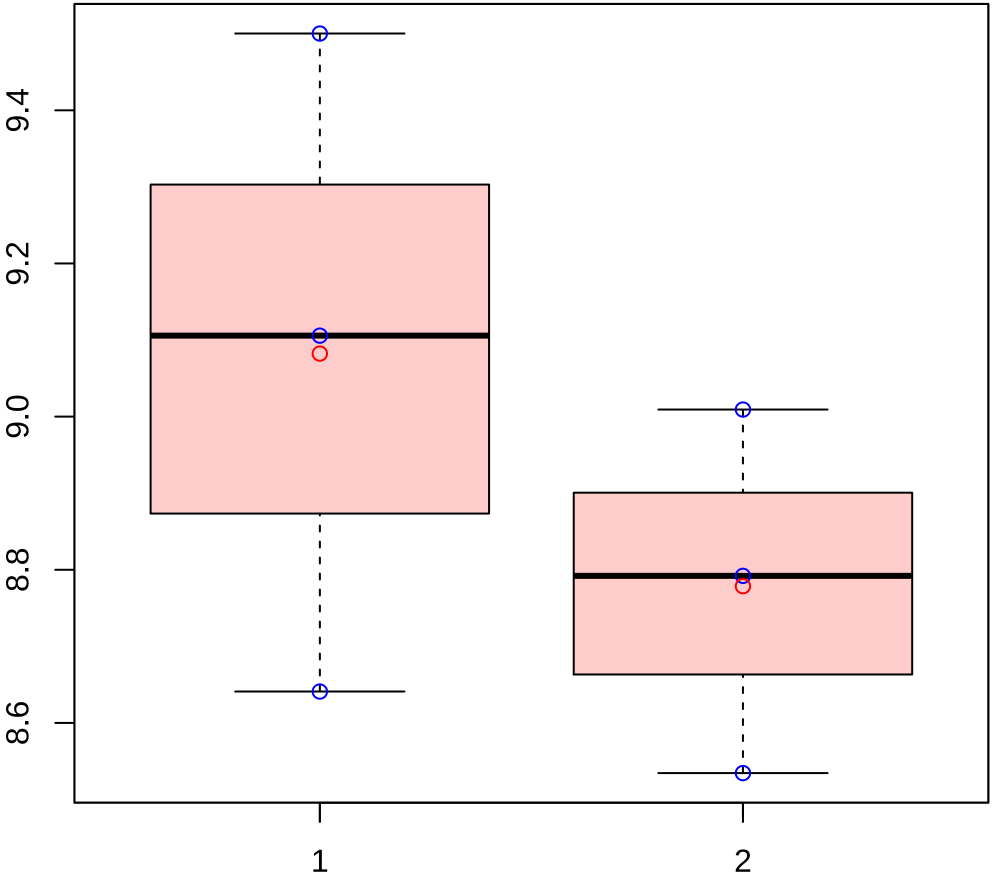

t−Test: p−value = 0.36

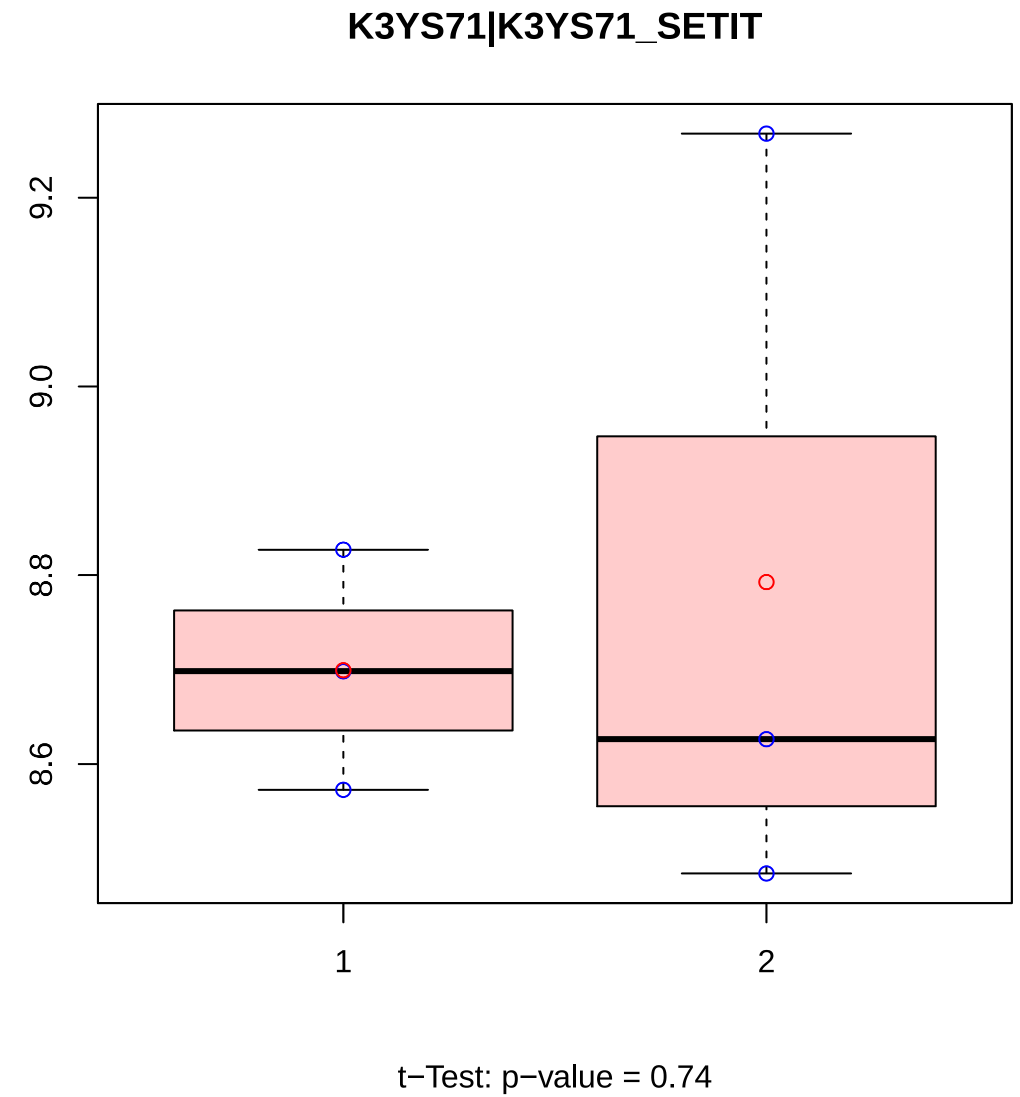
K3YS71|K3YS71_SETIT
9.2
9.0
8.8
8.6
1
2
t−Test: p−value = 0.74

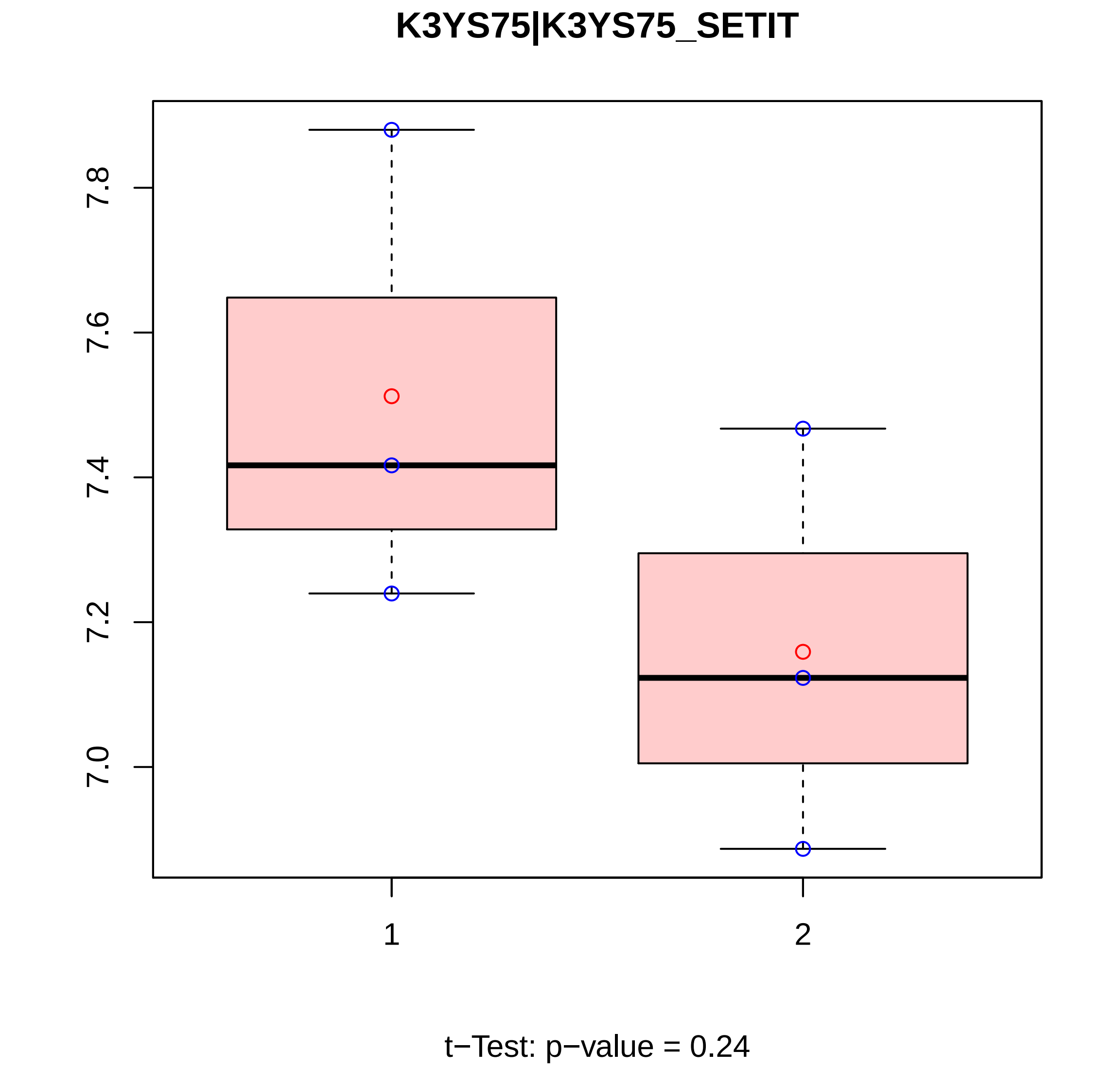
K3YS75|K3YS75_SETIT
7.0
7.2
7.4
7.6
7.8
1
2
t−Test: p−value = 0.24

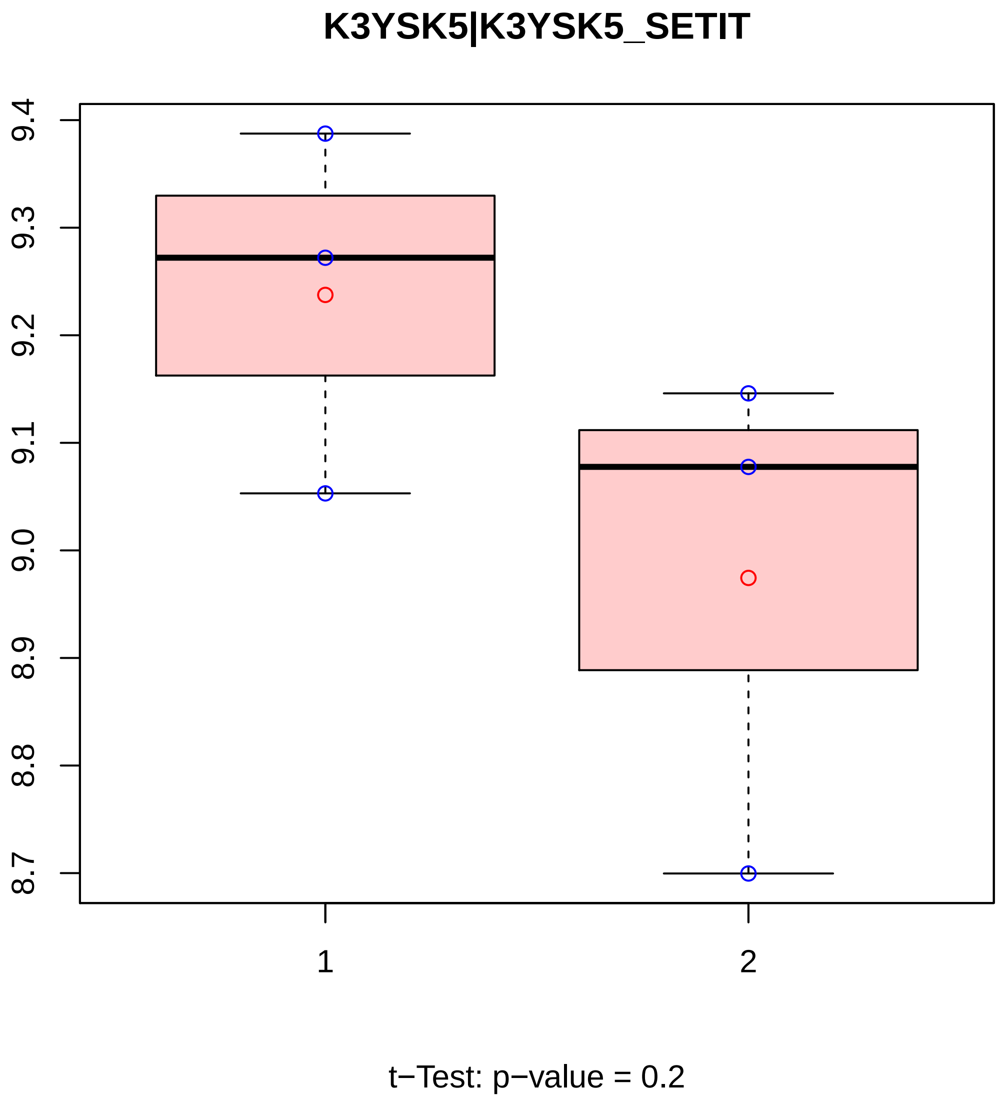
K3YSK5|K3YSK5_SETIT
9.4
9.3
9.2
9.1
9.0
8.9
8.8
8.7
1
2
t−Test: p−value = 0.2

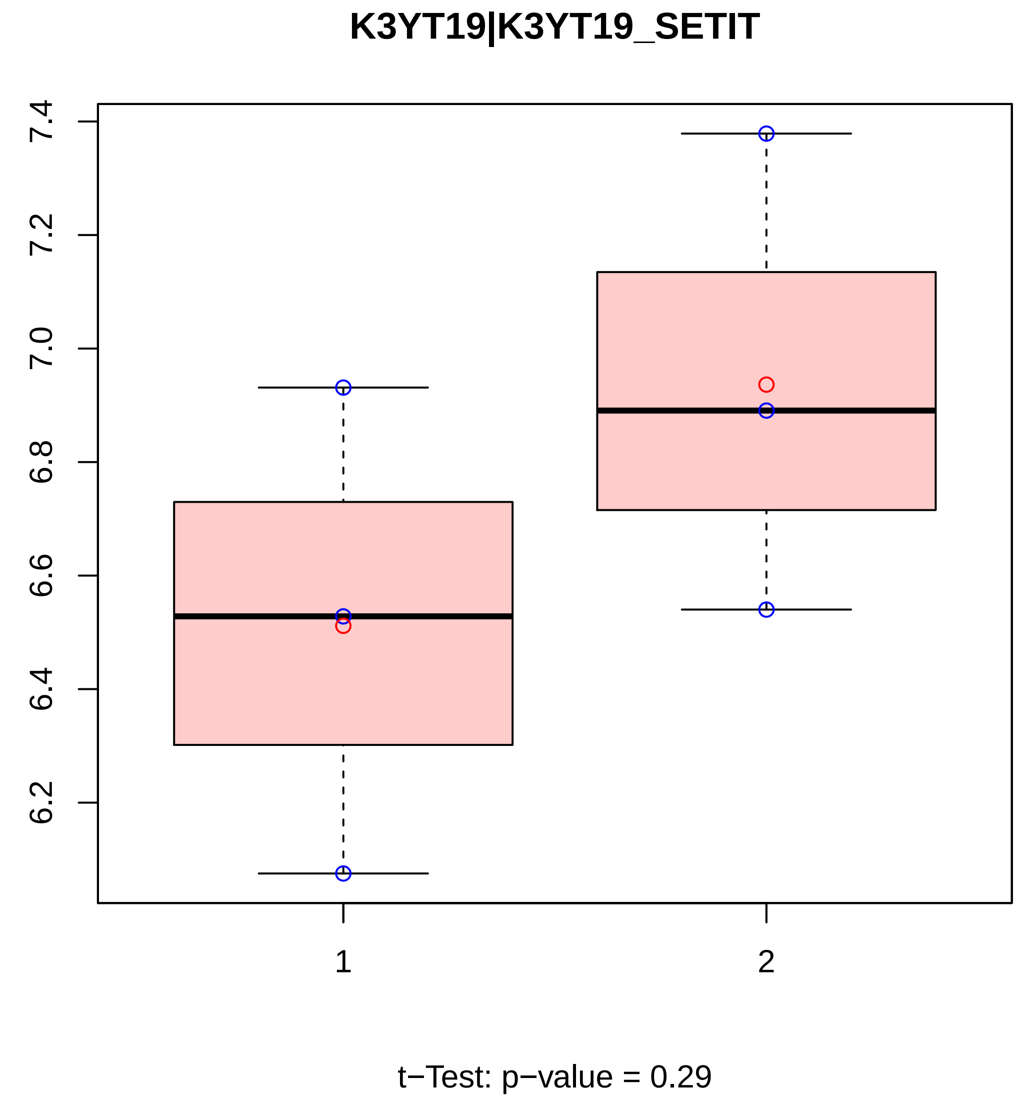
K3YT19|K3YT19_SETIT
7.4
7.2
7.0
6.8
6.6
6.4
6.2
1
2
t−Test: p−value = 0.29

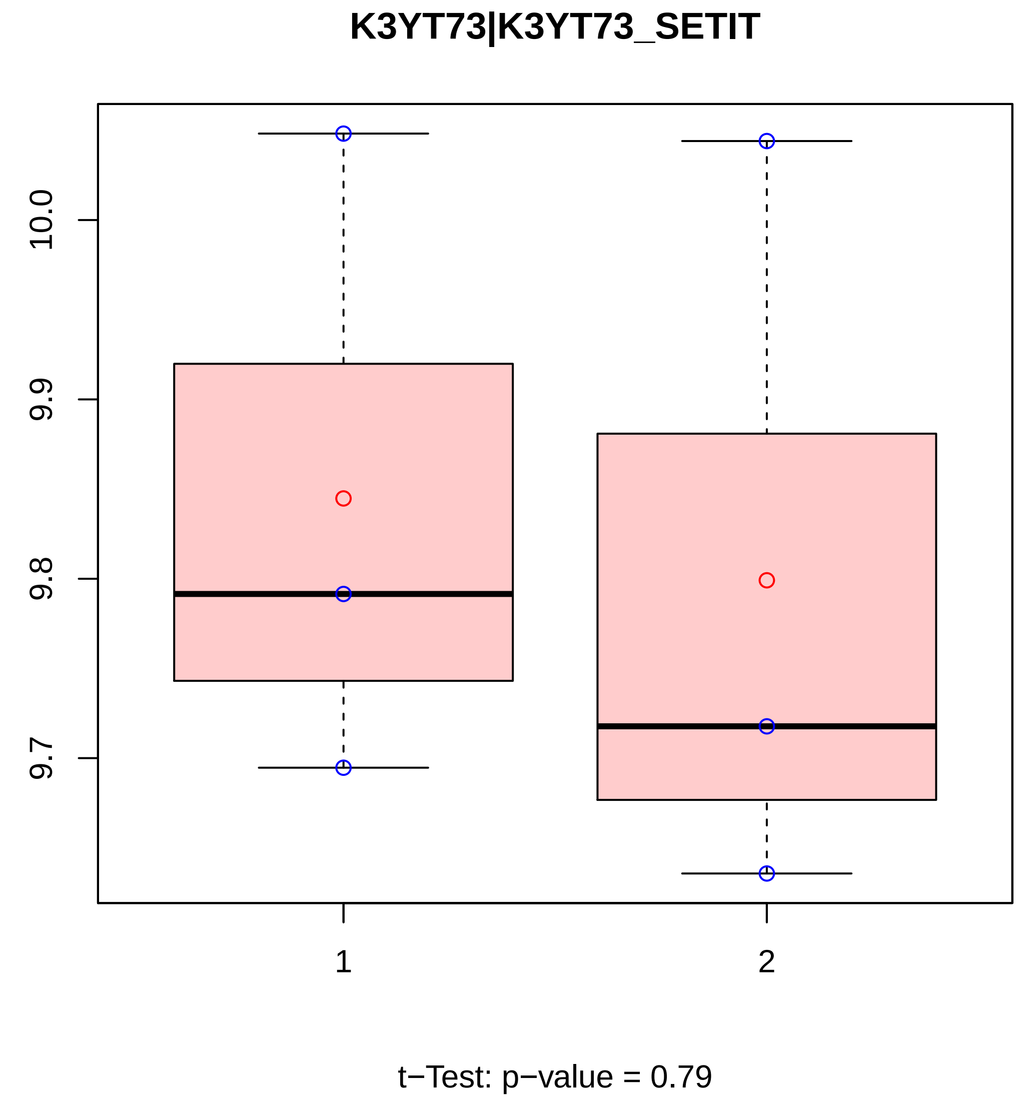
K3YT73|K3YT73_SETIT
10.0
9.9
9.8
9.7
1
2
t−Test: p−value = 0.79

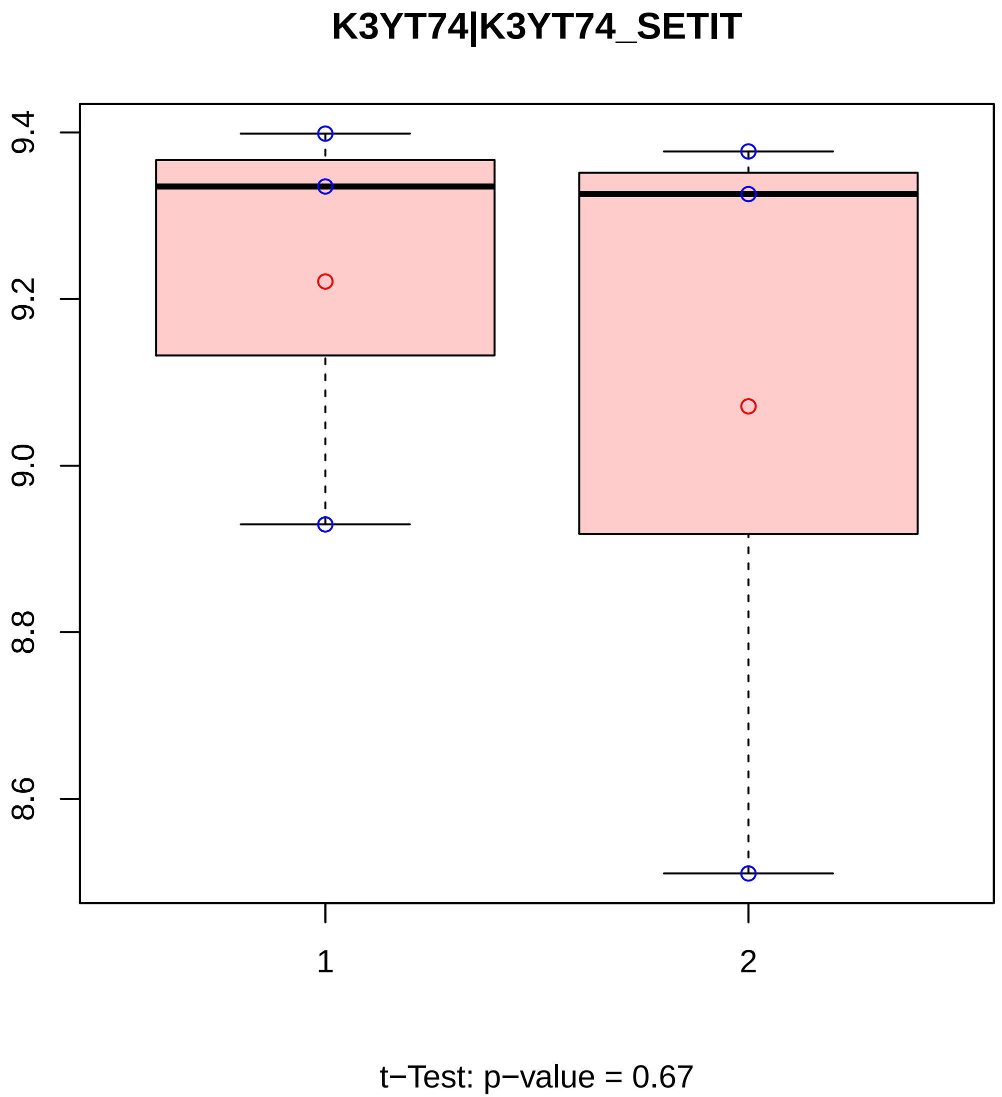
K3YT74|K3YT74_SETIT
9.4
9.2
9.0
8.8
8.6
1
2
t−Test: p−value = 0.67

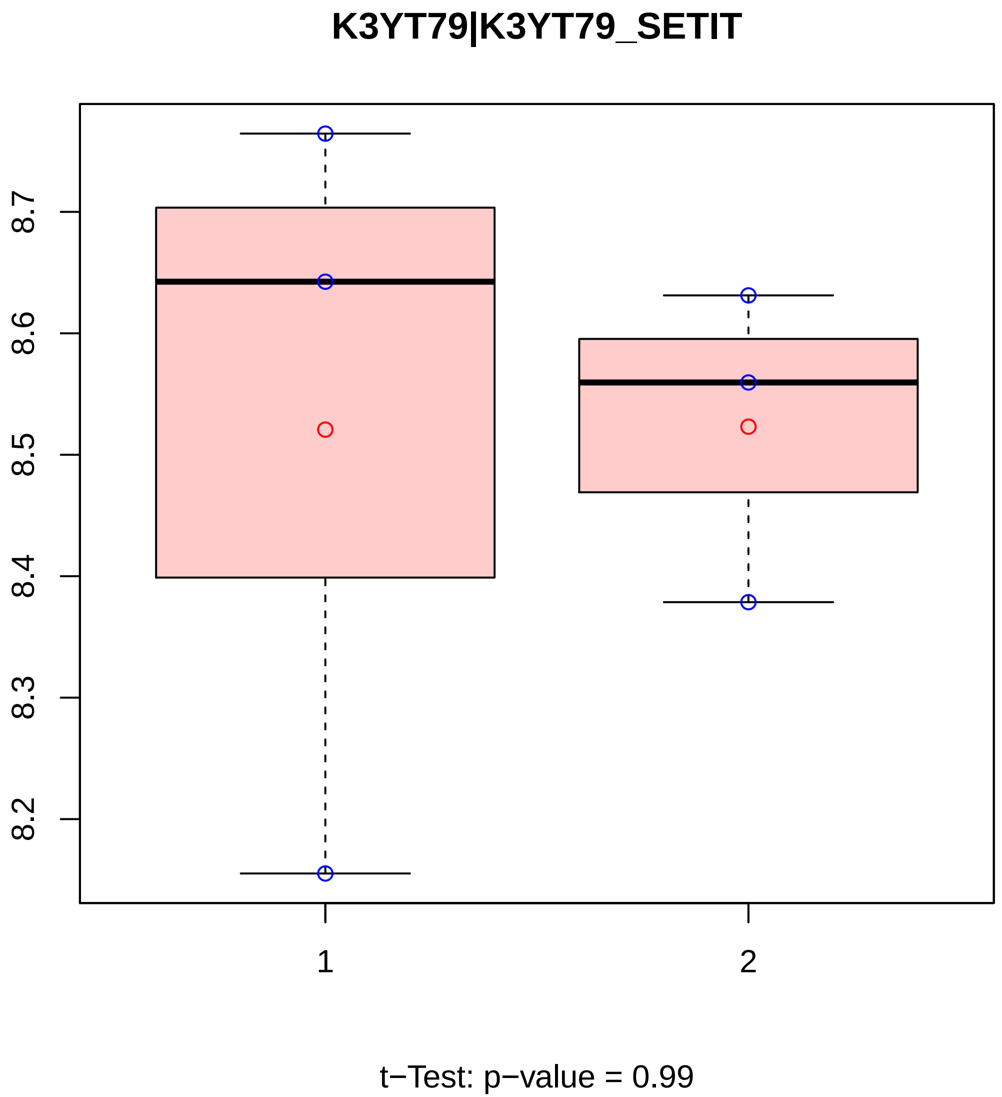

K3YT79|K3YT79_SETIT
8.2
8.3
8.4
8.5
8.6
8.7
1
2
t−Test: p−value = 0.99

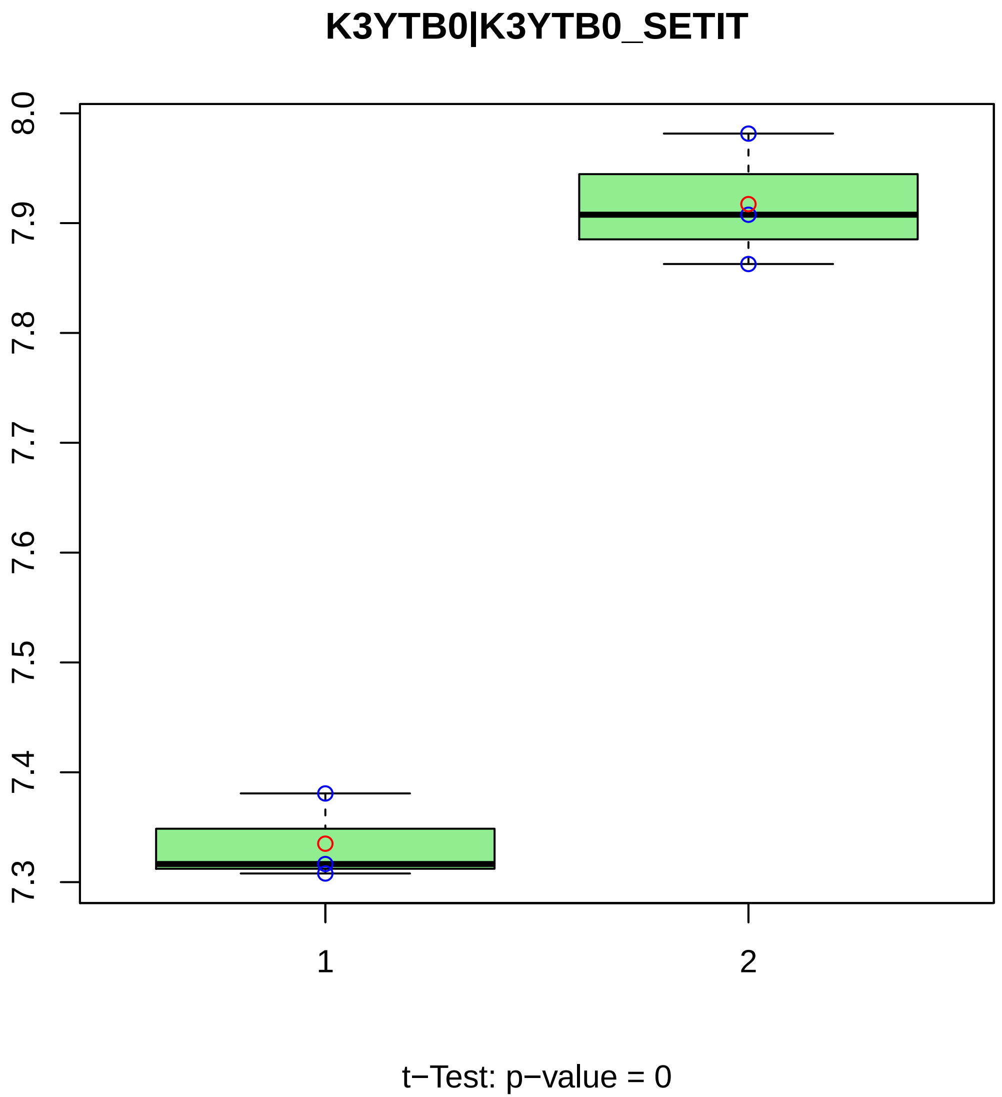
K3YTB0|K3YTB0_SETIT
8.0
7.9
7.8
7.7
7.6
7.5
7.4
7.3
1
2
t−Test: p−value = 0

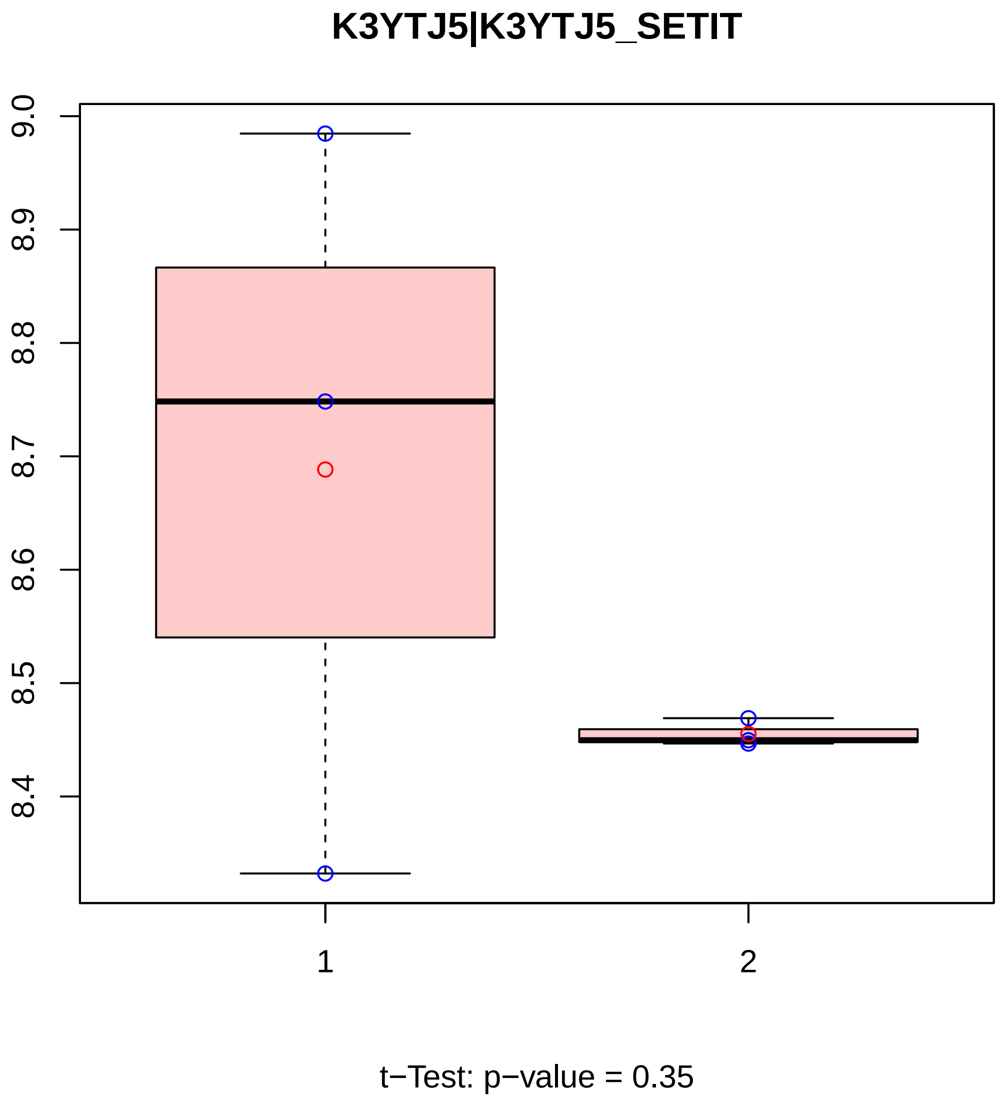
K3YTJ5|K3YTJ5_SETIT
9.0
8.9
8.8
8.7
8.6
8.5
8.4
1
2
t−Test: p−value = 0.35

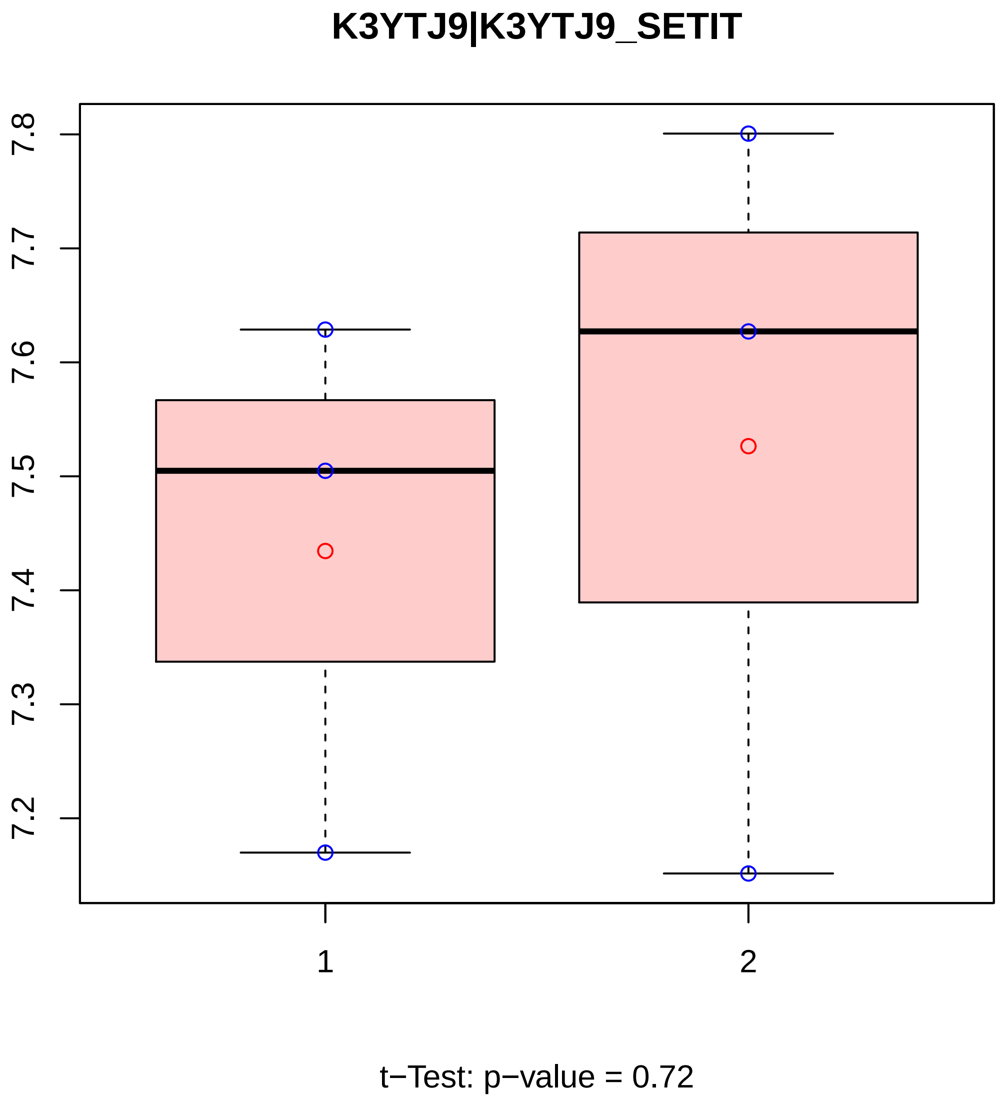
K3YTJ9|K3YTJ9_SETIT
7.2
7.3
7.4
7.5
7.6
7.7
7.8
1
2
t−Test: p−value = 0.72

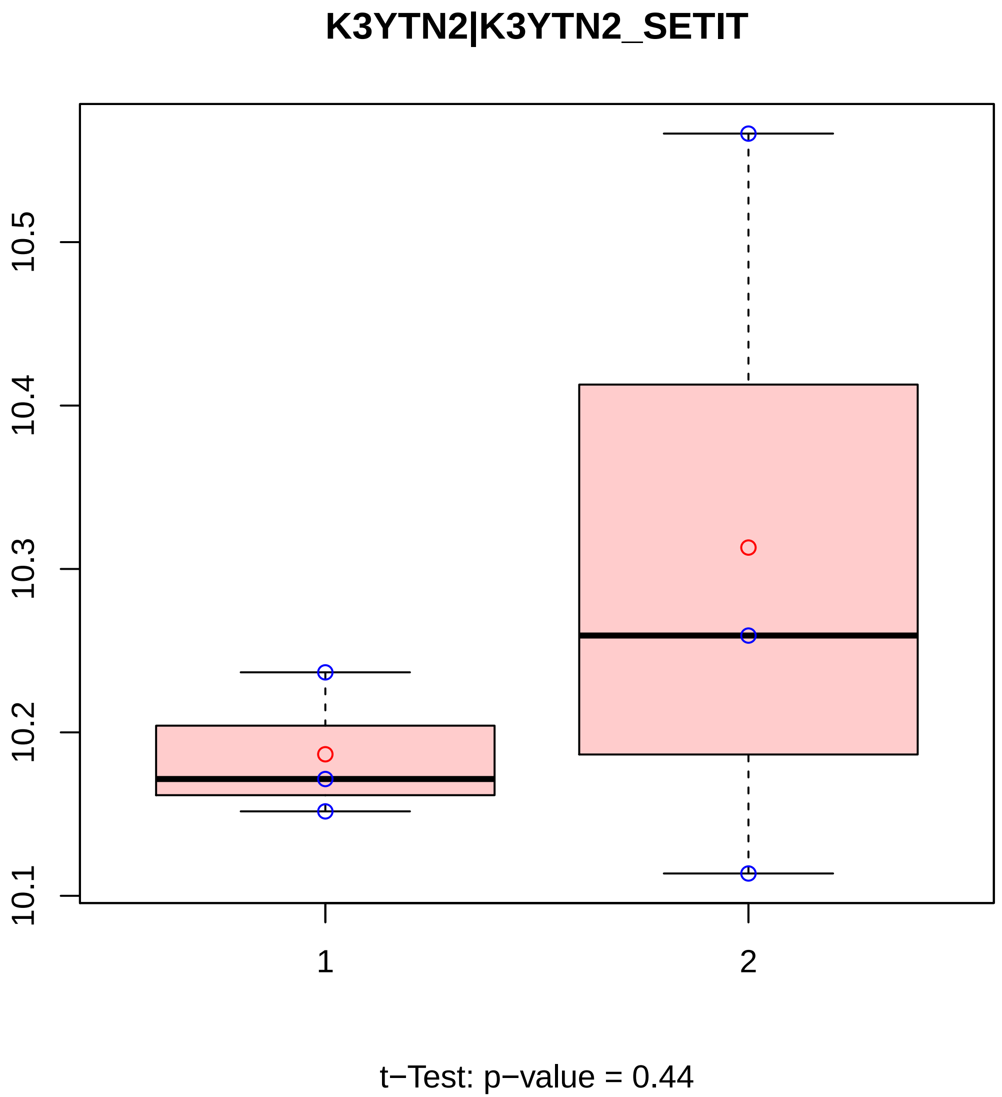
K3YTN2|K3YTN2_SETIT
10.1
10.2
10.3
10.4
10.5
1
2
t−Test: p−value = 0.44

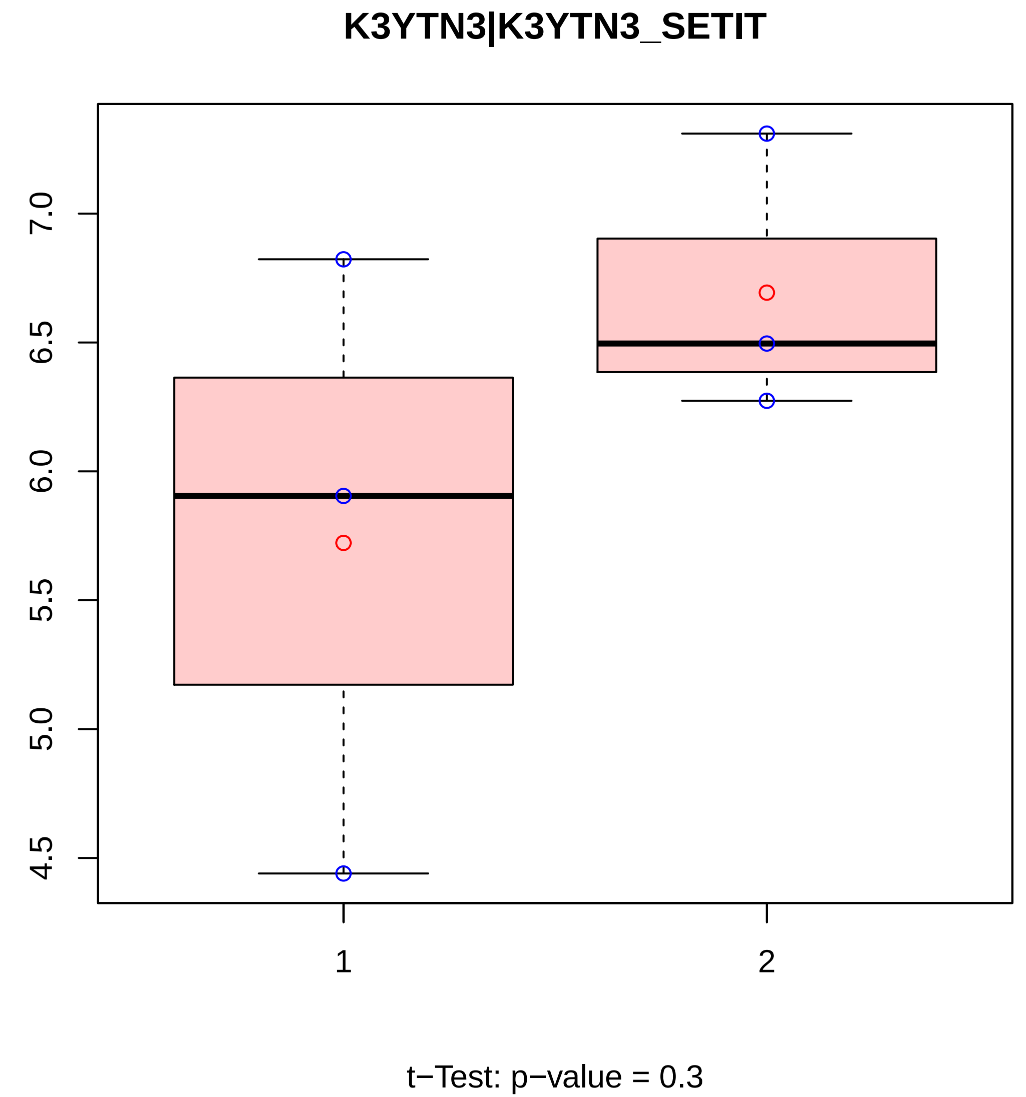
K3YTN3|K3YTN3_SETIT
7.0
6.5
6.0
5.5
5.0
4.5
1
2
t−Test: p−value = 0.3

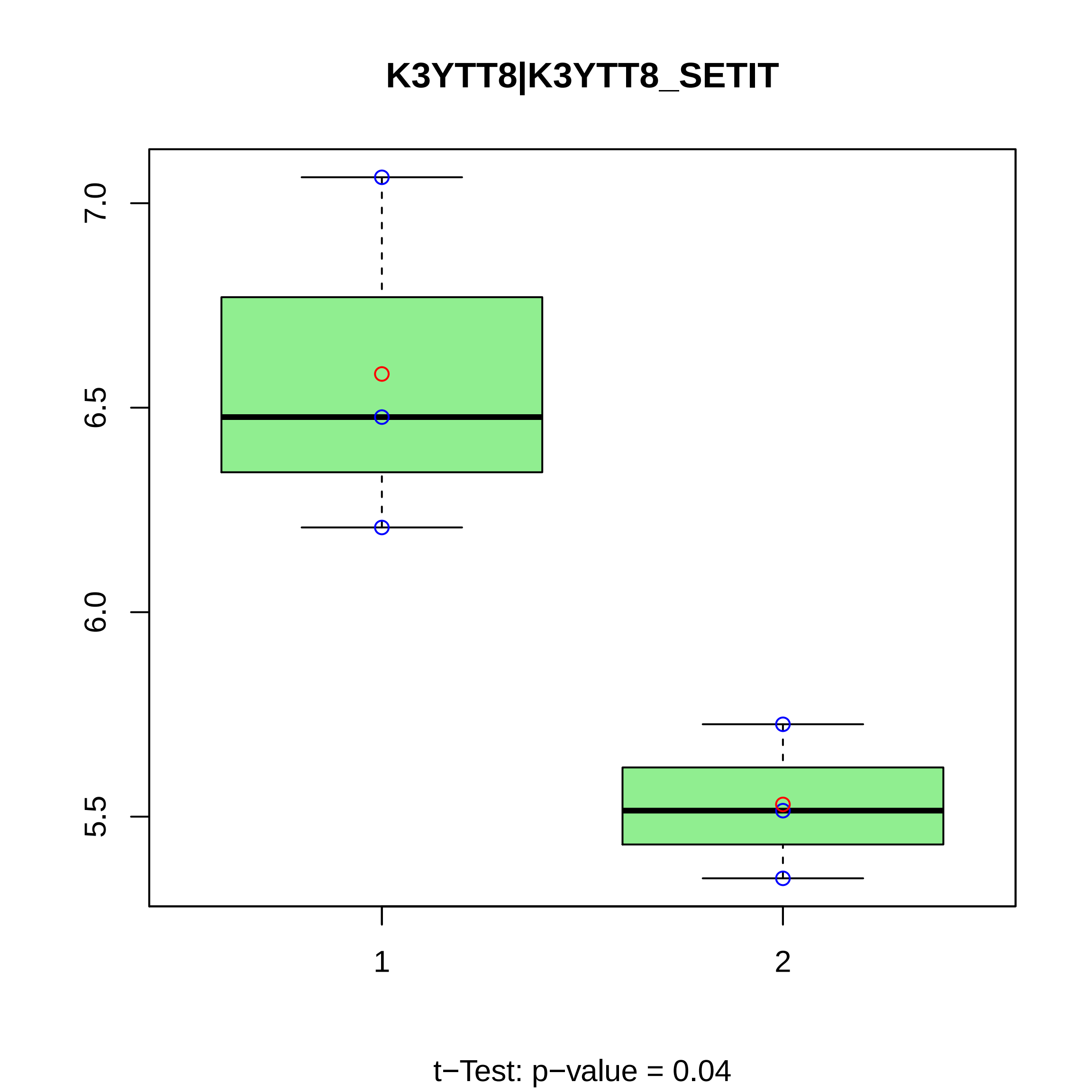
K3YTT8|K3YTT8_SETIT
7.0
6.5
6.0
5.5
1
2
t−Test: p−value = 0.04

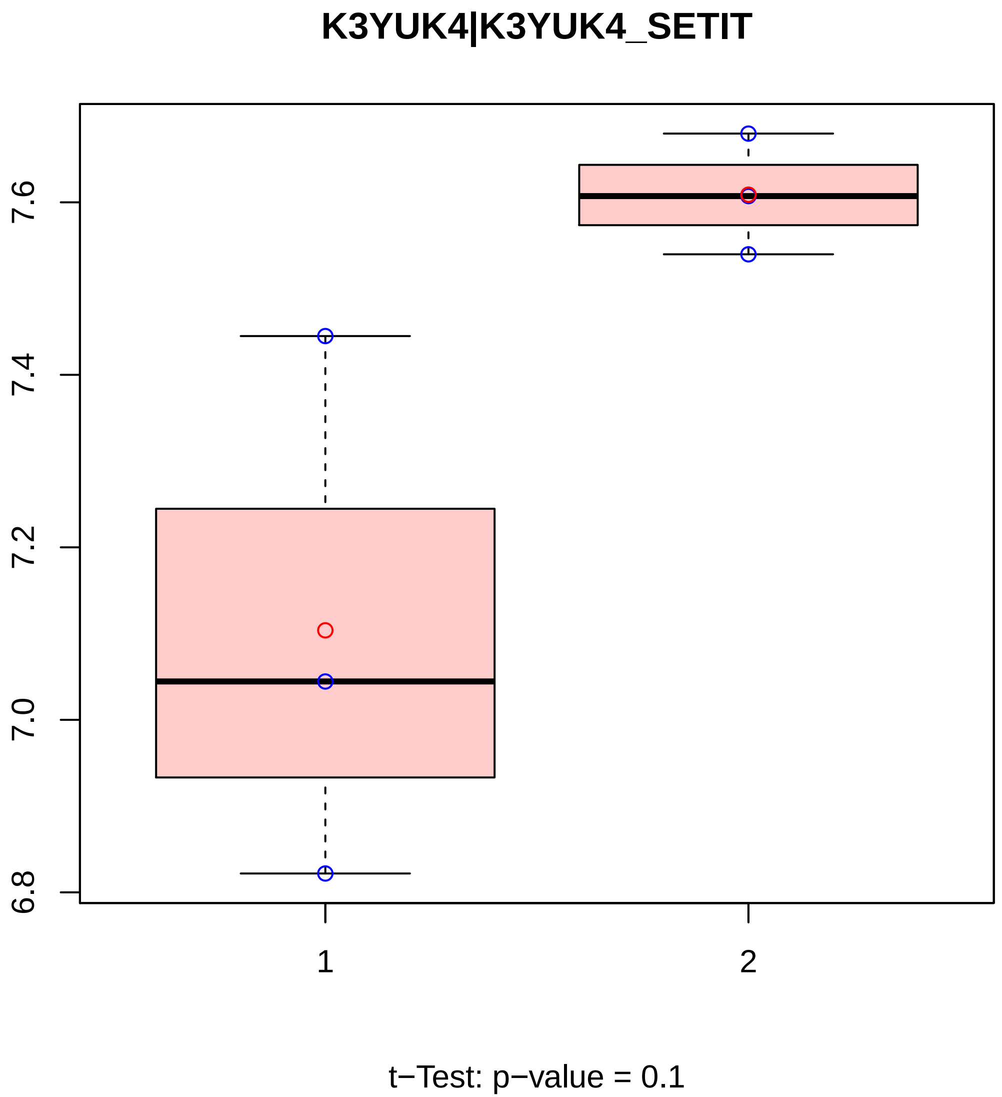
K3YUK4|K3YUK4_SETIT
7.6
7.4
7.2
7.0
6.8
1
2
t−Test: p−value = 0.1

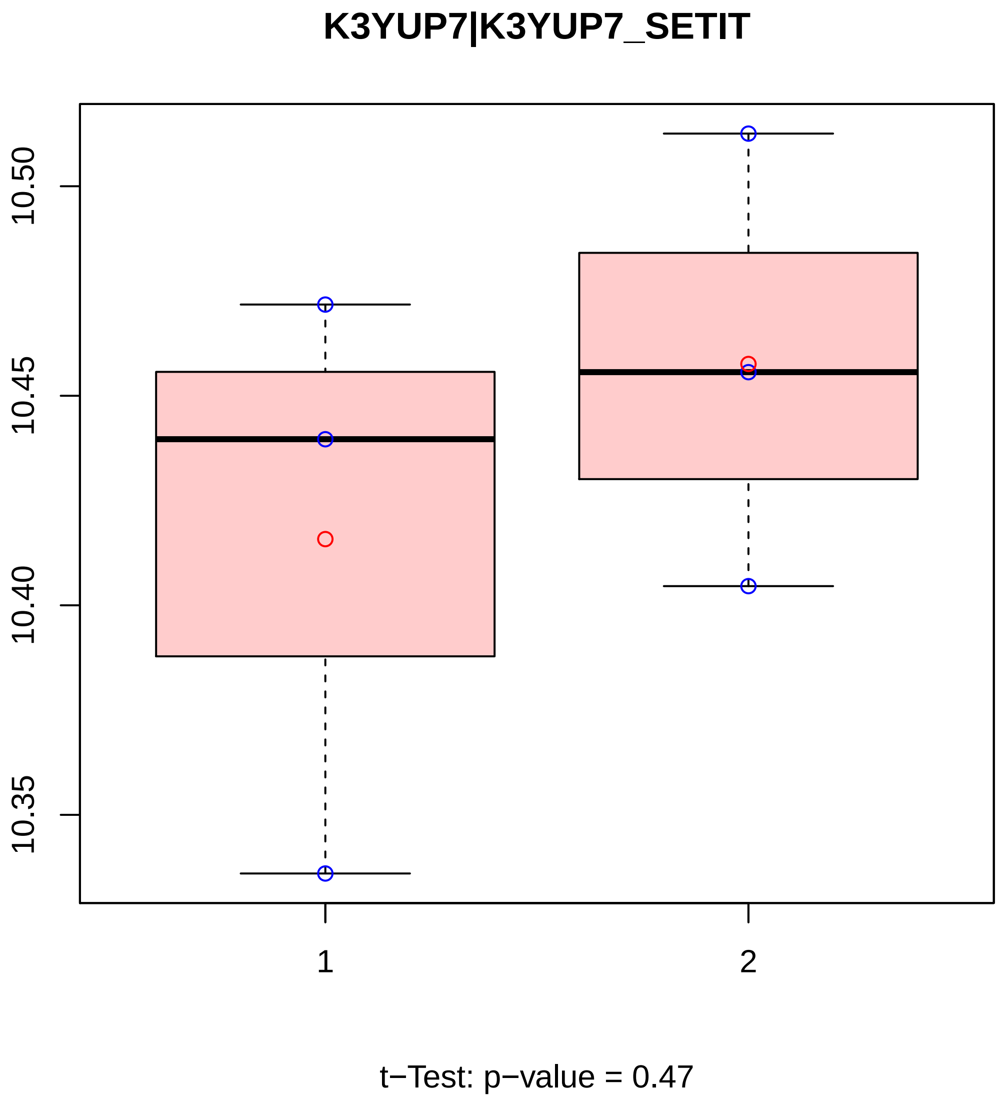
K3YUP7|K3YUP7_SETIT
10.35
10.40
10.45
10.50
1
2
t−Test: p−value = 0.47

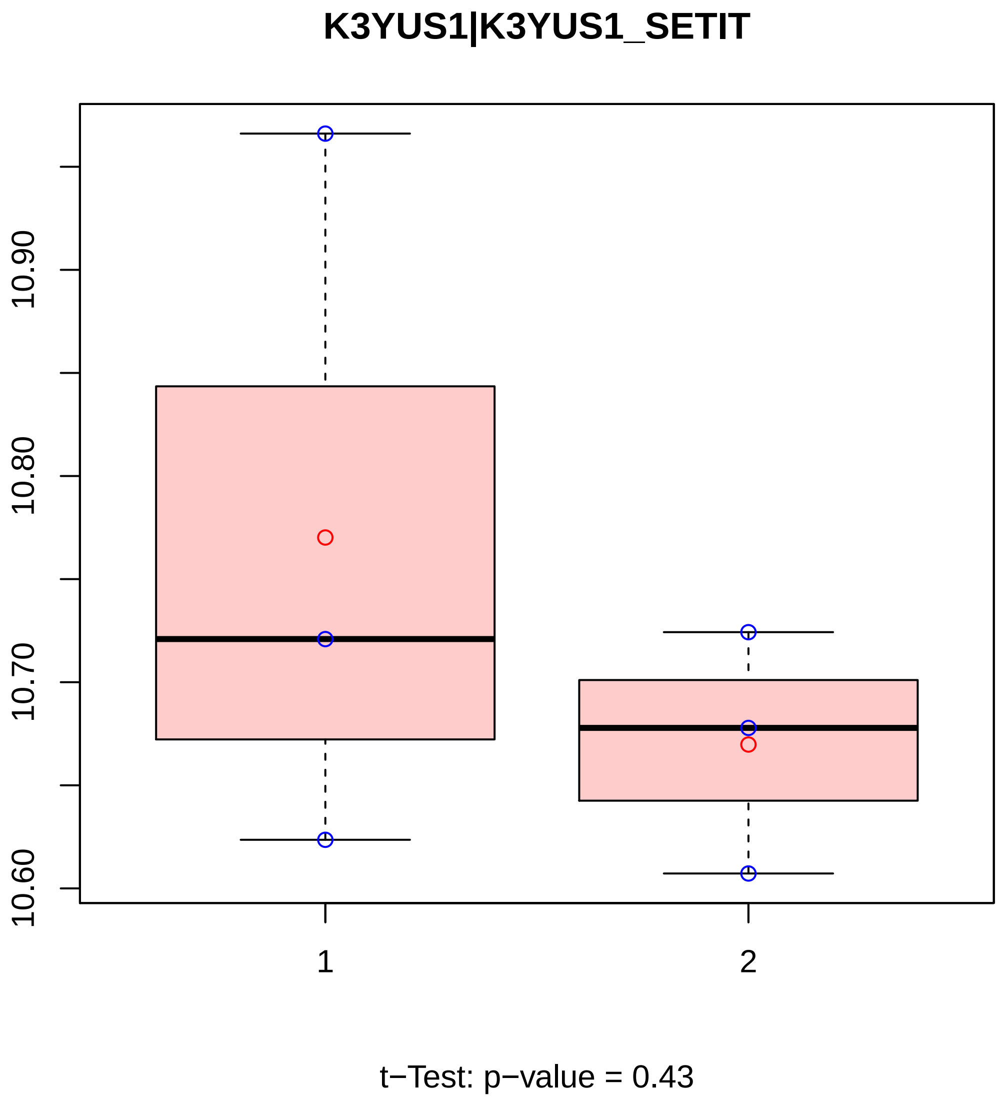
K3YUS1|K3YUS1_SETIT
10.60
10.70
10.80
10.90
1
2
t−Test: p−value = 0.43

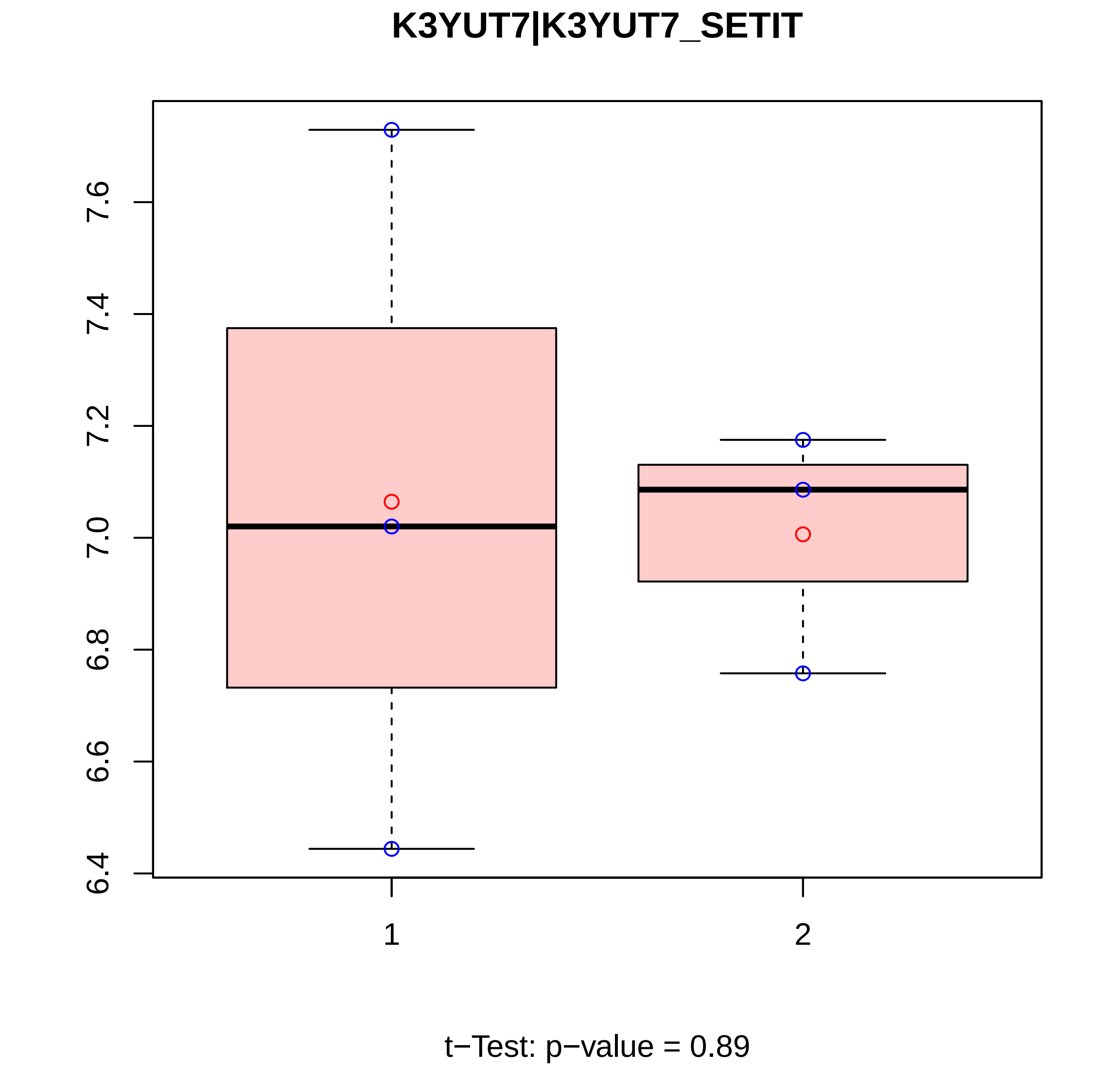

K3YUT7|K3YUT7_SETIT
7.6
7.4
7.2
7.0
6.8
6.6
6.4
1
2
t−Test: p−value = 0.89

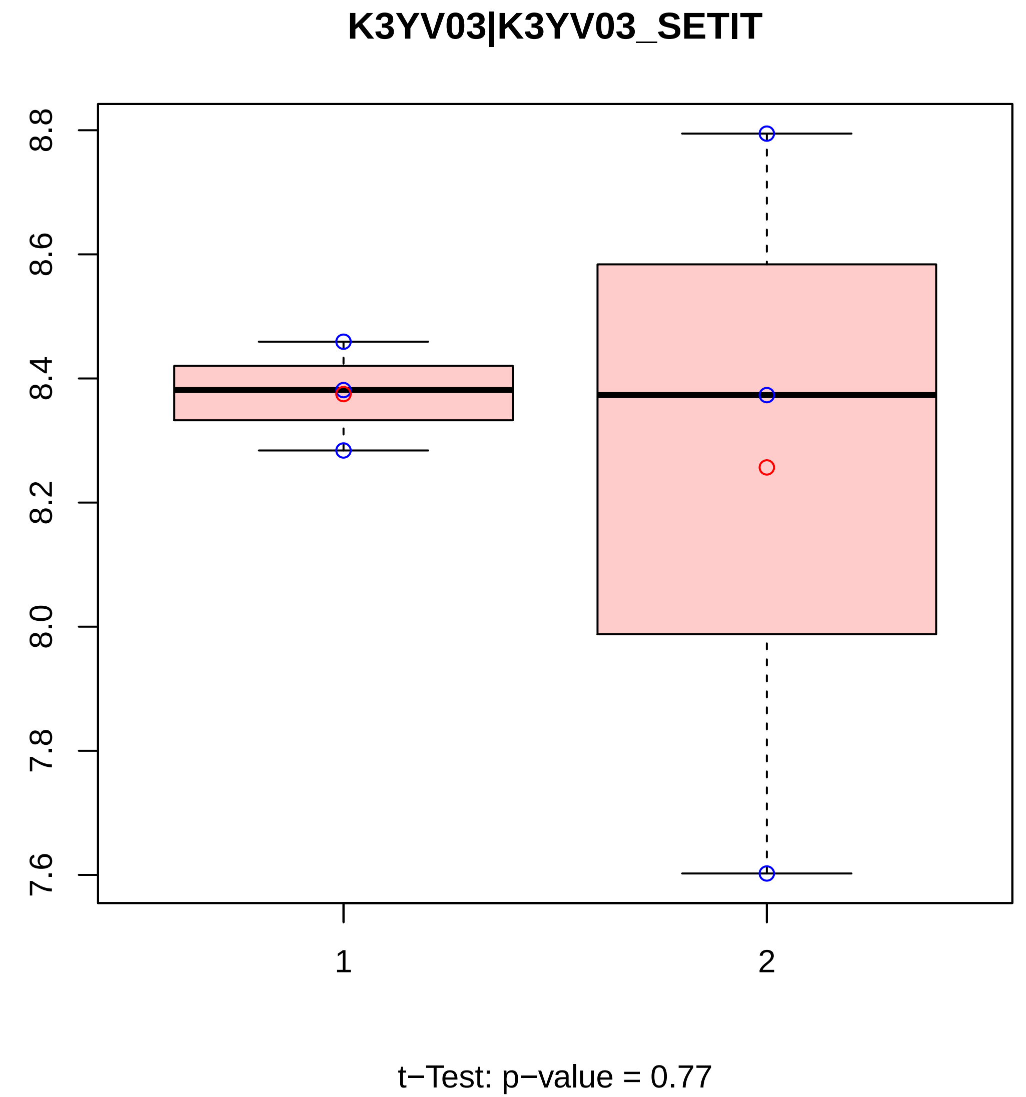
K3YV03|K3YV03_SETIT
8.8
8.6
8.4
8.2
8.0
7.8
7.6
1
2
t−Test: p−value = 0.77

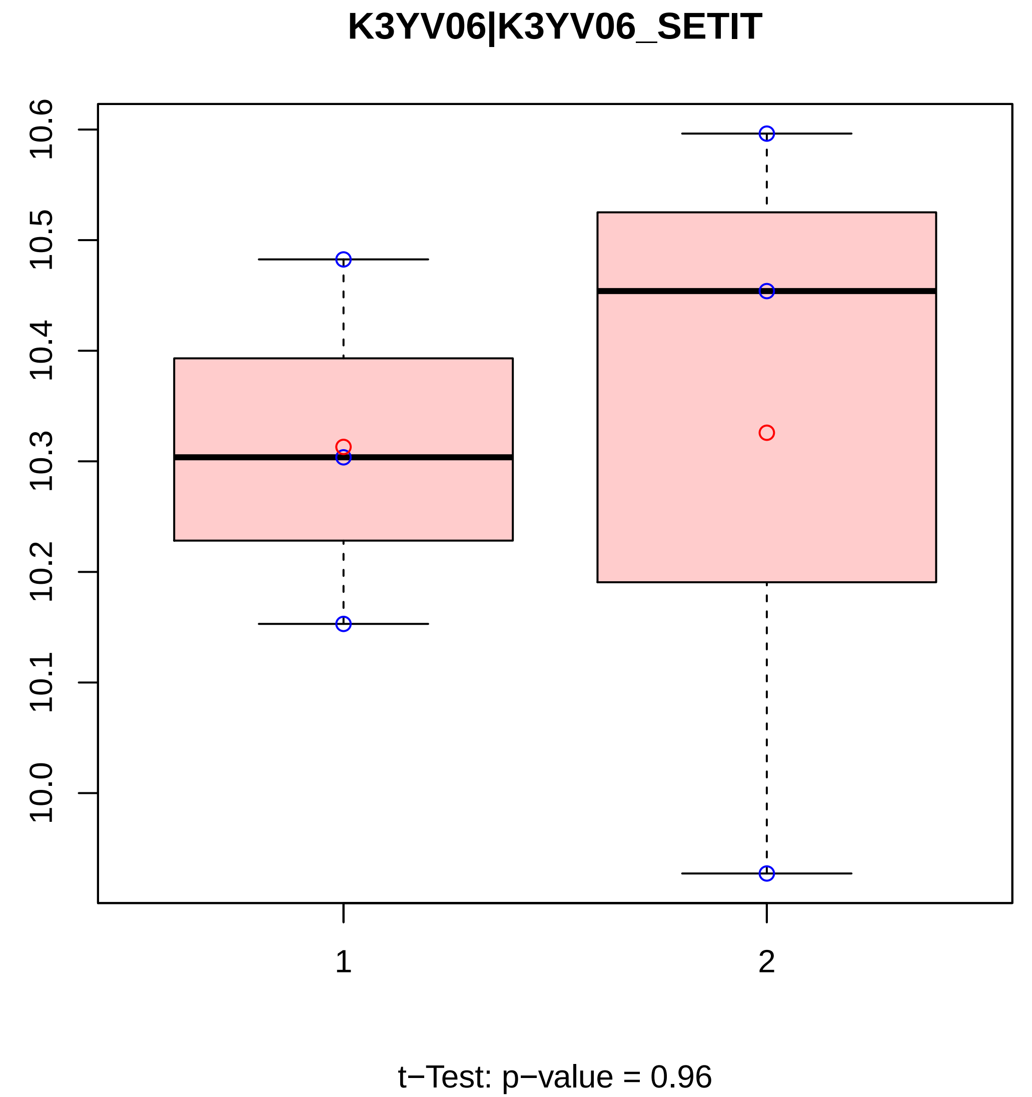
K3YV06|K3YV06_SETIT
10.6
10.5
10.4
10.3
10.2
10.1
10.0
1
2
t−Test: p−value = 0.96

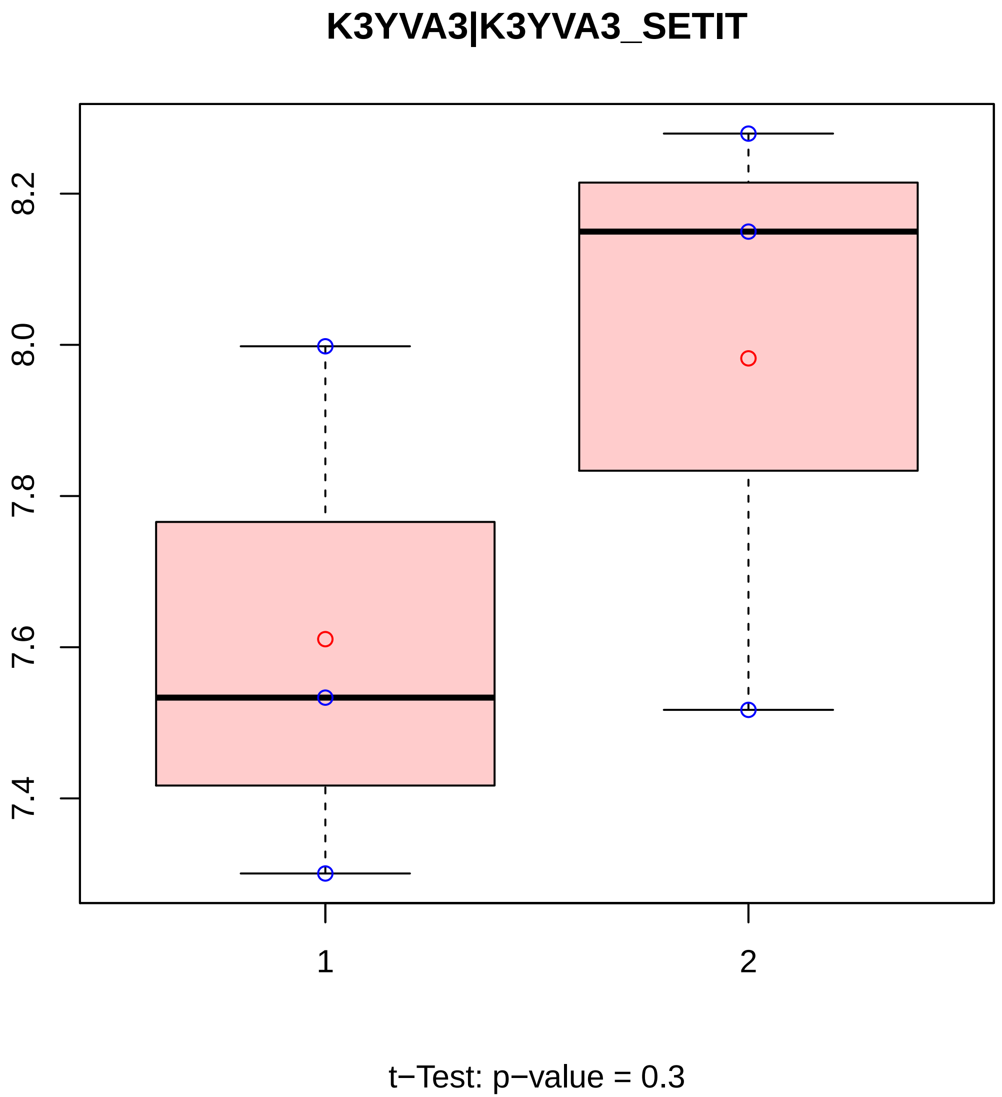
K3YVA3|K3YVA3_SETIT
8.2
8.0
7.8
7.6
7.4
1
2
t−Test: p−value = 0.3

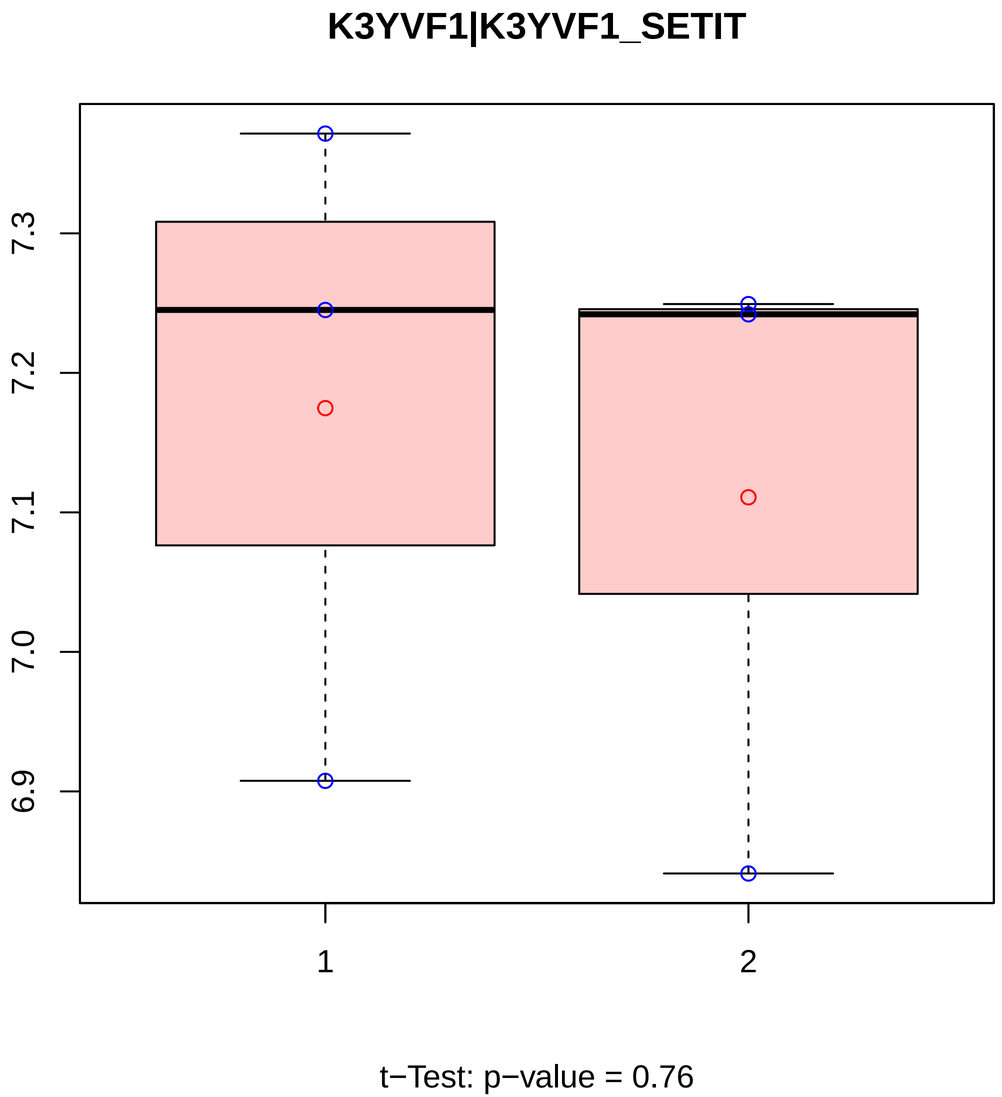
K3YVF1|K3YVF1_SETIT
7.3
7.2
7.1
7.0
6.9
1
2
t−Test: p−value = 0.76

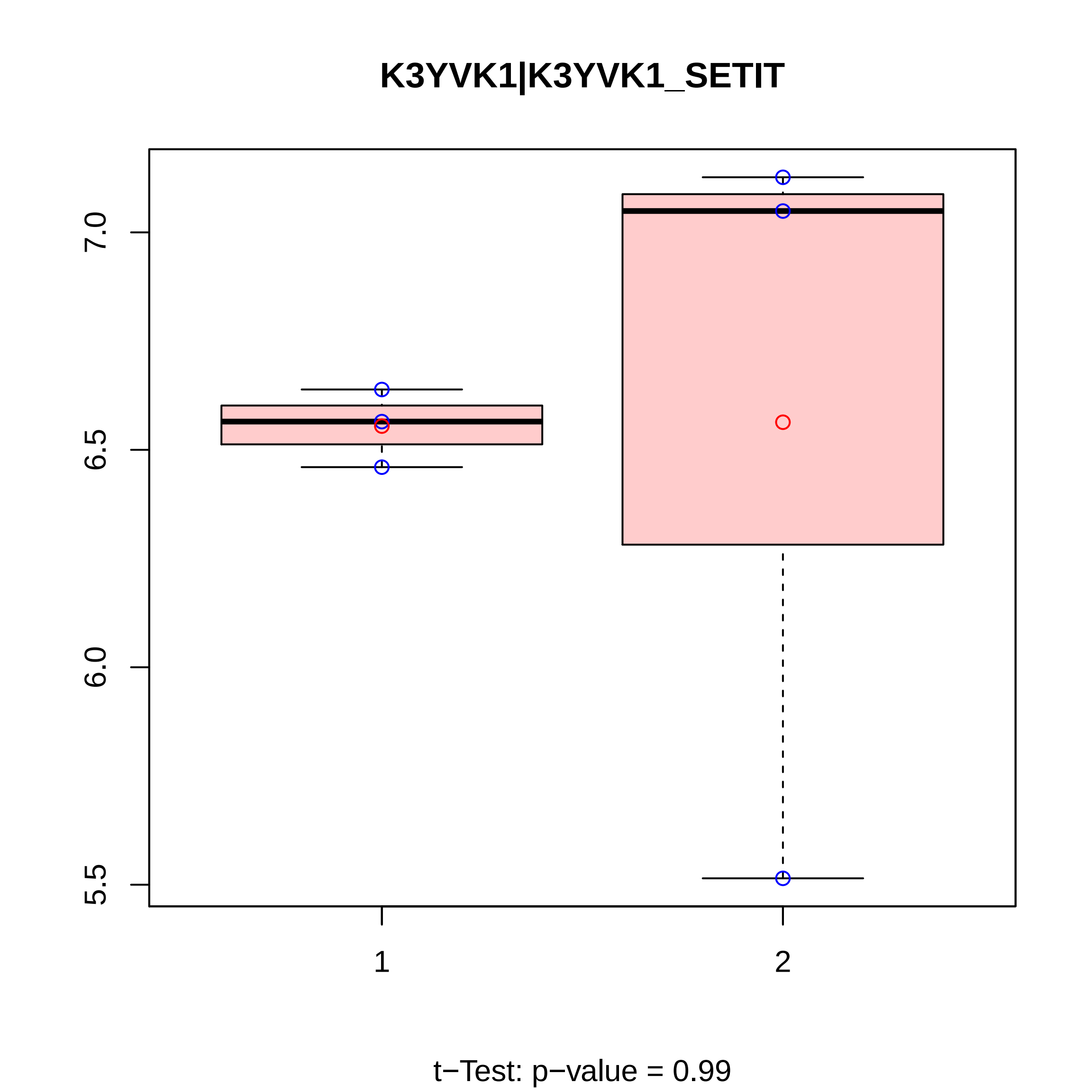
K3YVK1|K3YVK1_SETIT
7.0
6.5
6.0
5.5
1
2
t−Test: p−value = 0.99

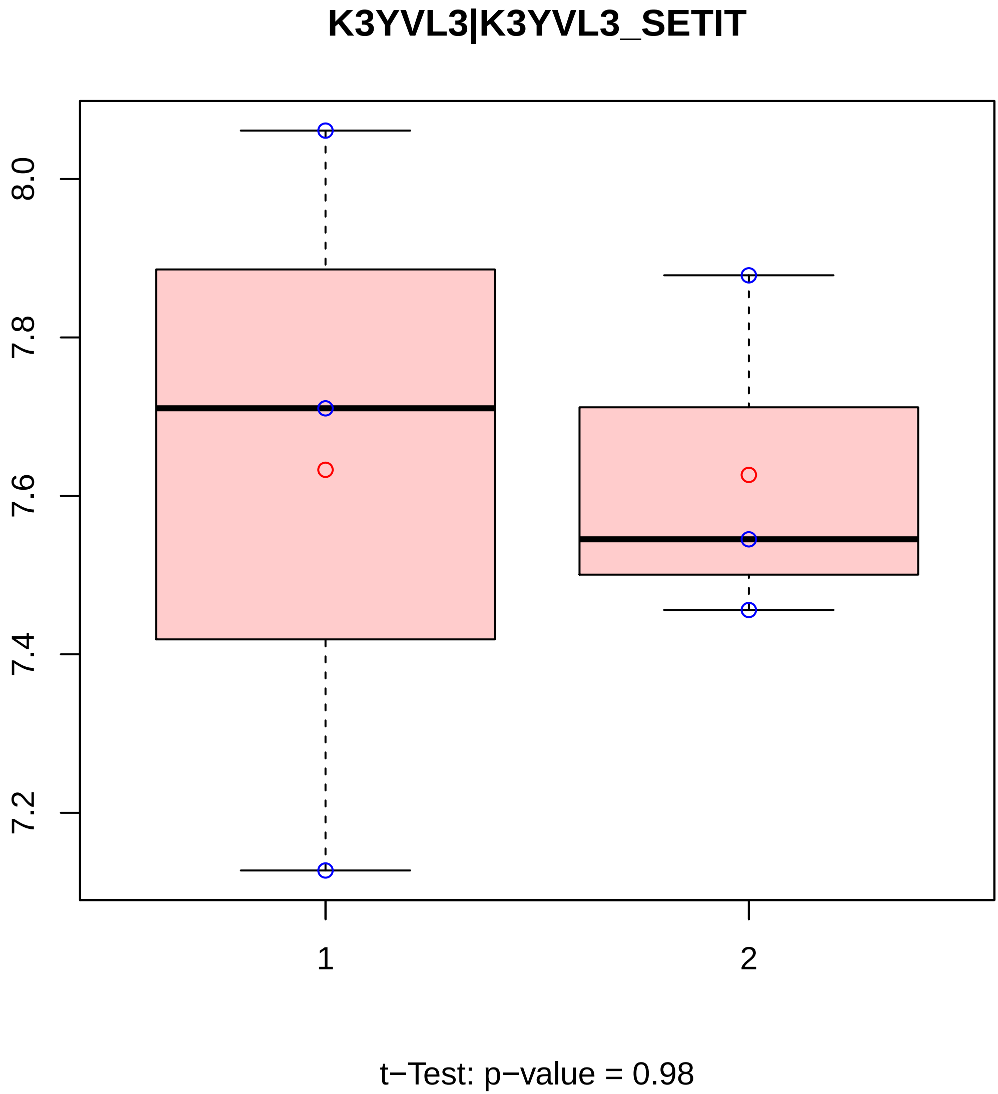
K3YVL3|K3YVL3_SETIT
8.0
7.8
7.6
7.4
7.2
1
2
t−Test: p−value = 0.98

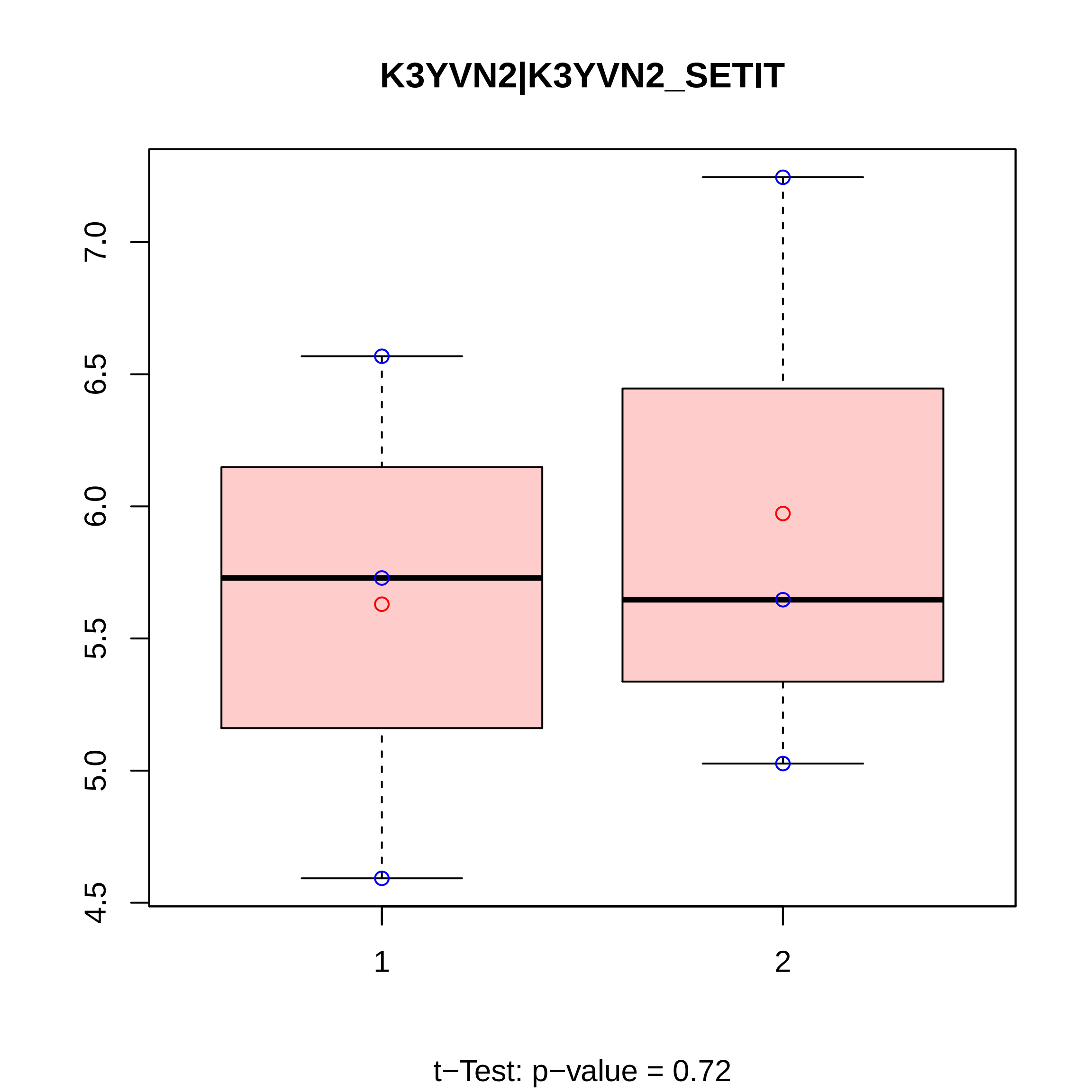
K3YVN2|K3YVN2_SETIT
7.0
6.5
6.0
5.5
5.0
4.5
1
2
t−Test: p−value = 0.72

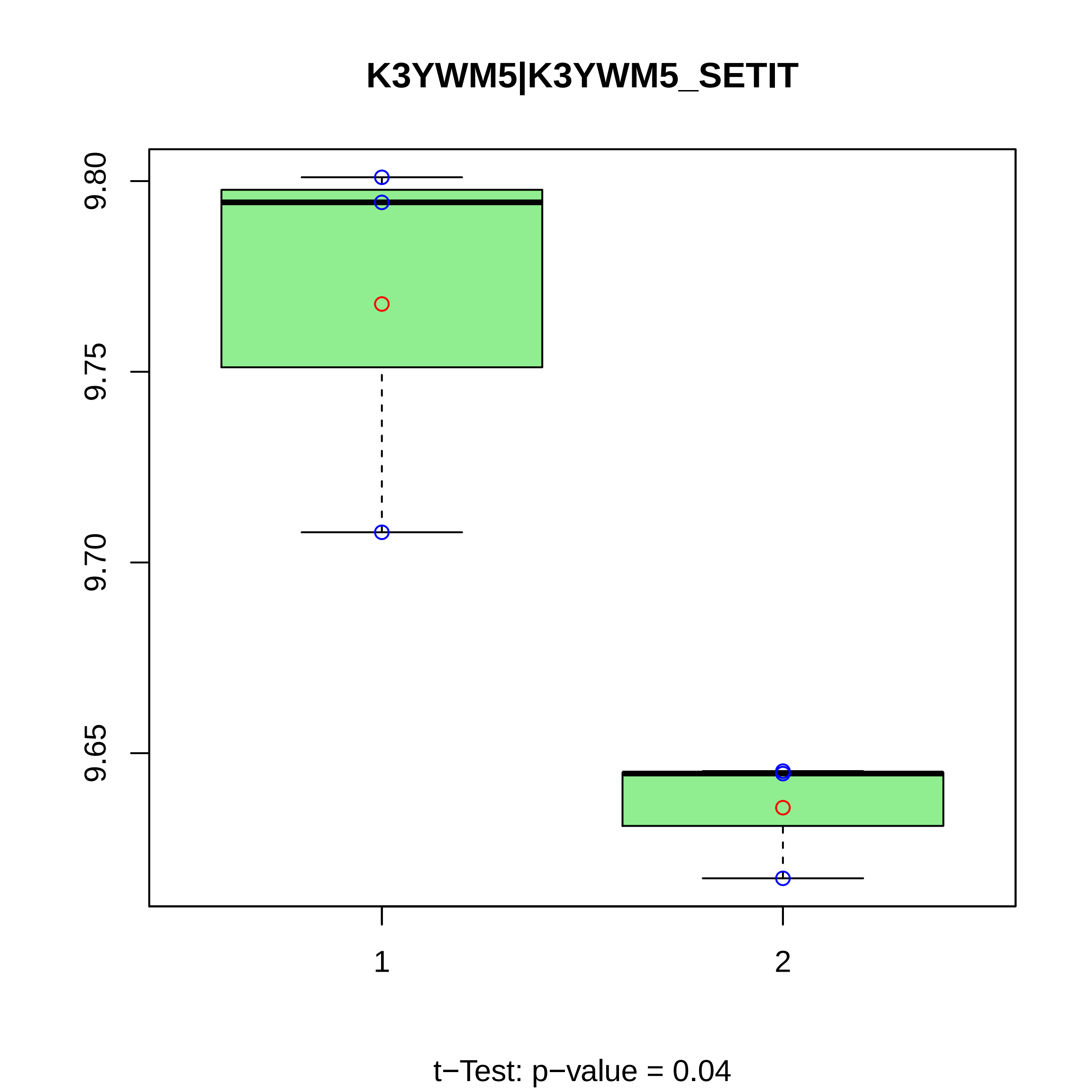
K3YWM5|K3YWM5_SETIT
9.80
9.75
9.70
9.65
1
2
t−Test: p−value = 0.04

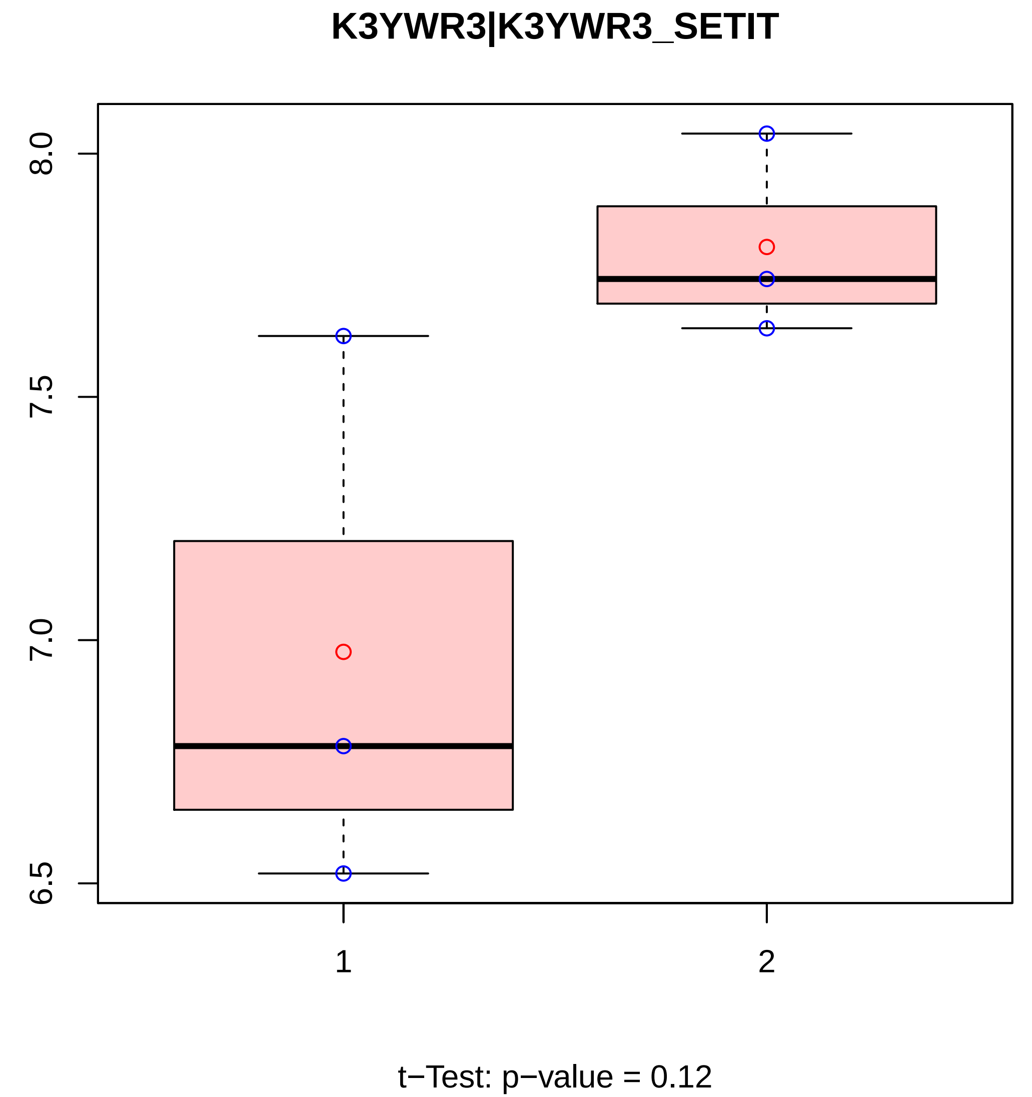
K3YWR3|K3YWR3_SETIT
8.0
7.5
7.0
6.5
1
2
t−Test: p−value = 0.12

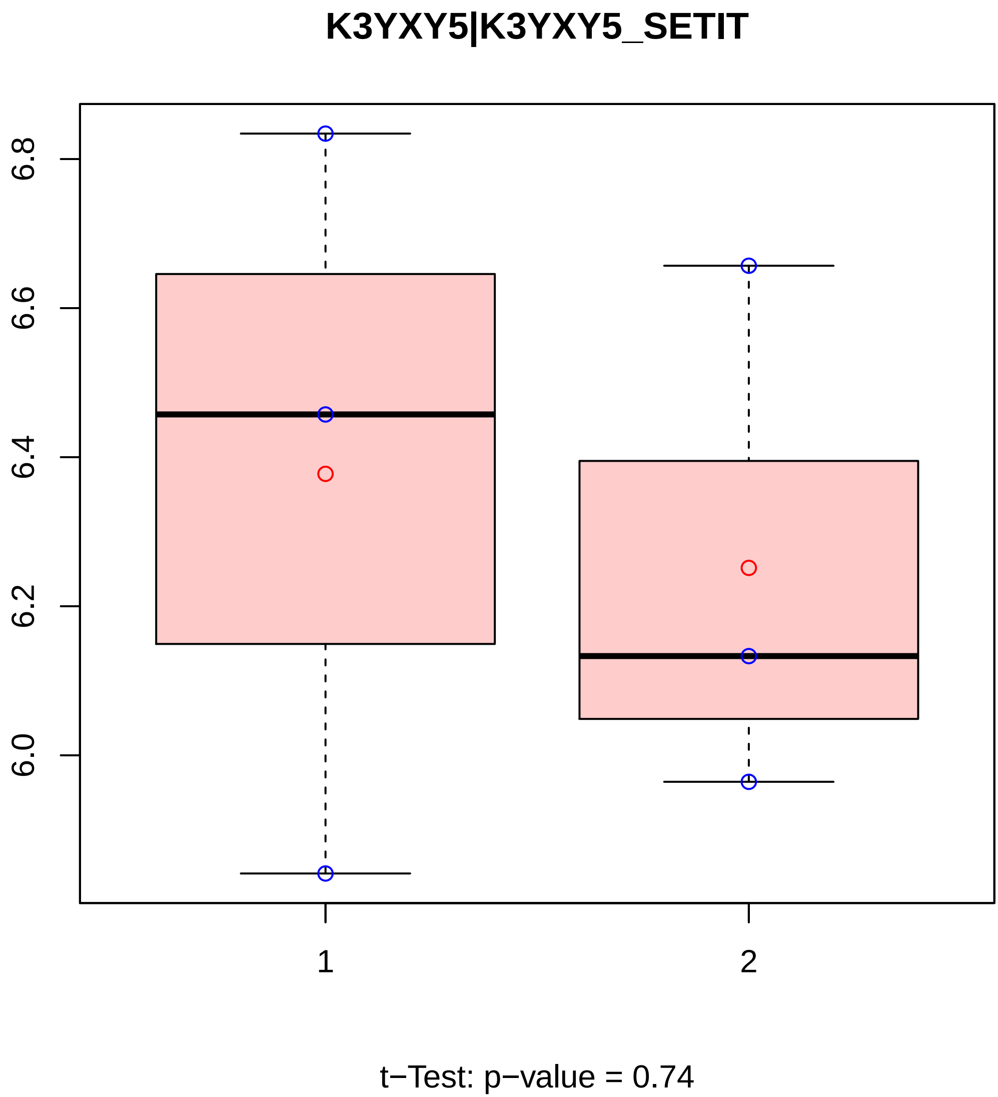
K3YXY5|K3YXY5_SETIT
6.0
6.2
6.4
6.6
6.8
1
2
t−Test: p−value = 0.74

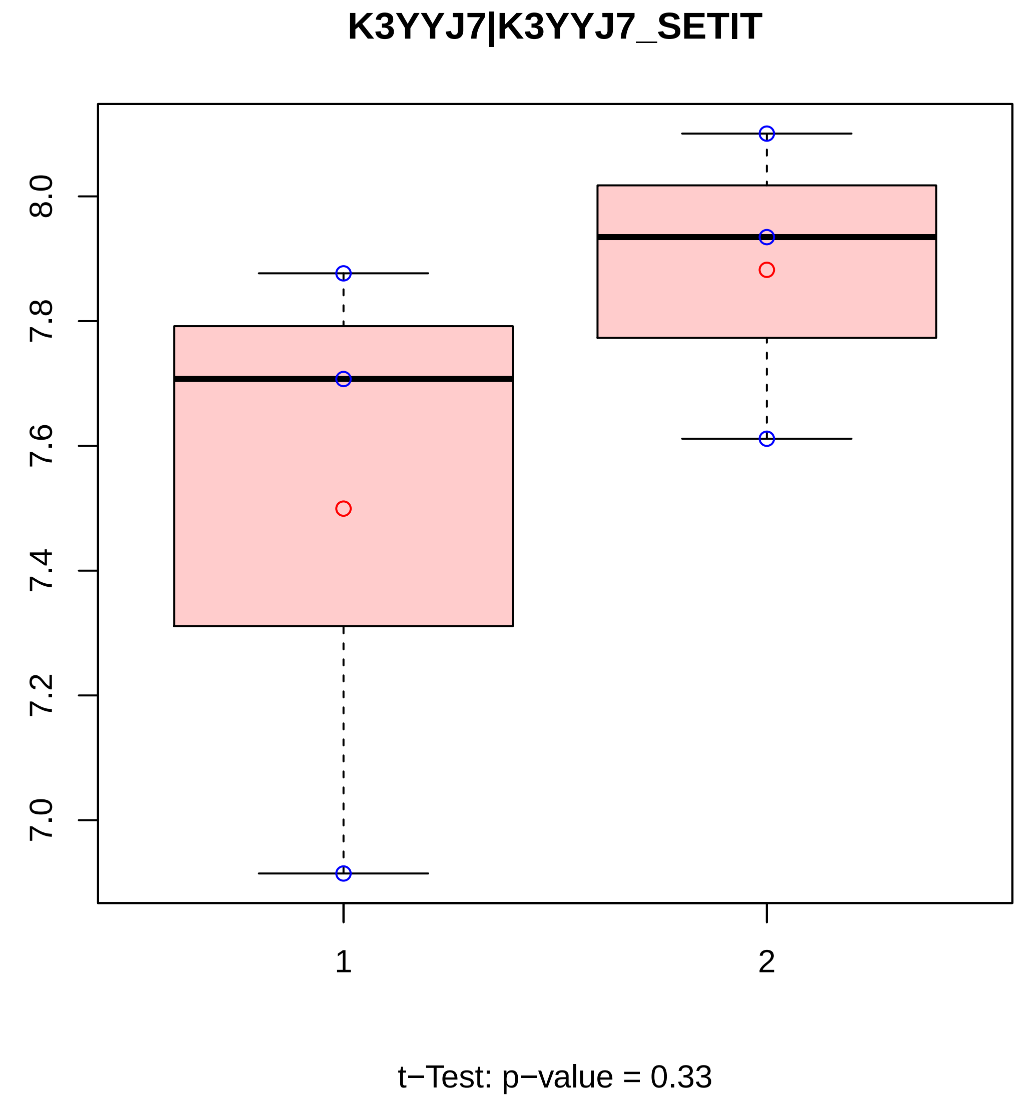

K3YYJ7|K3YYJ7_SETIT
7.0
7.2
7.4
7.6
7.8
8.0
1
2
t−Test: p−value = 0.33

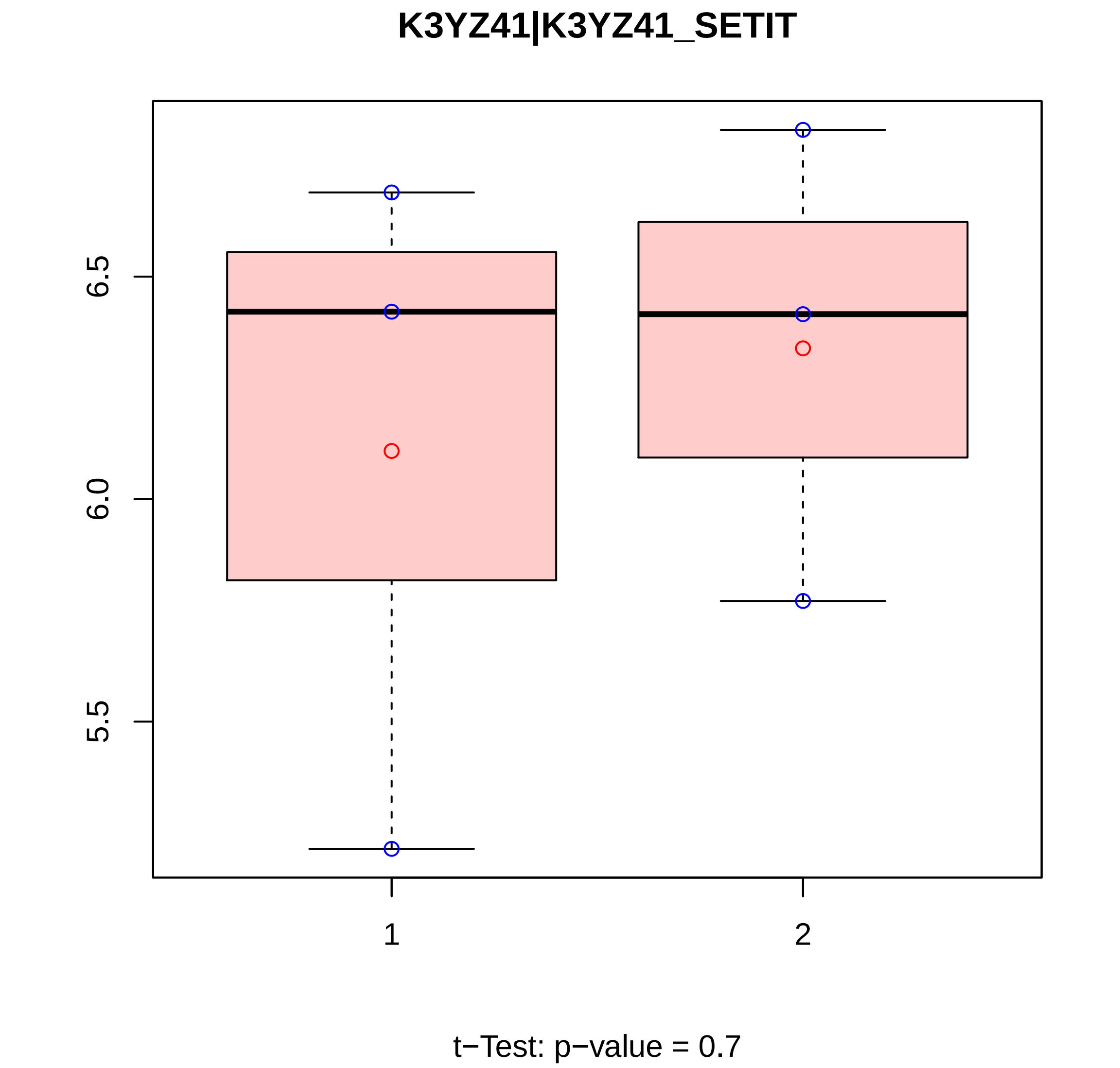
K3YZ41|K3YZ41_SETIT
6.5
6.0
5.5
1
2
t−Test: p−value = 0.7

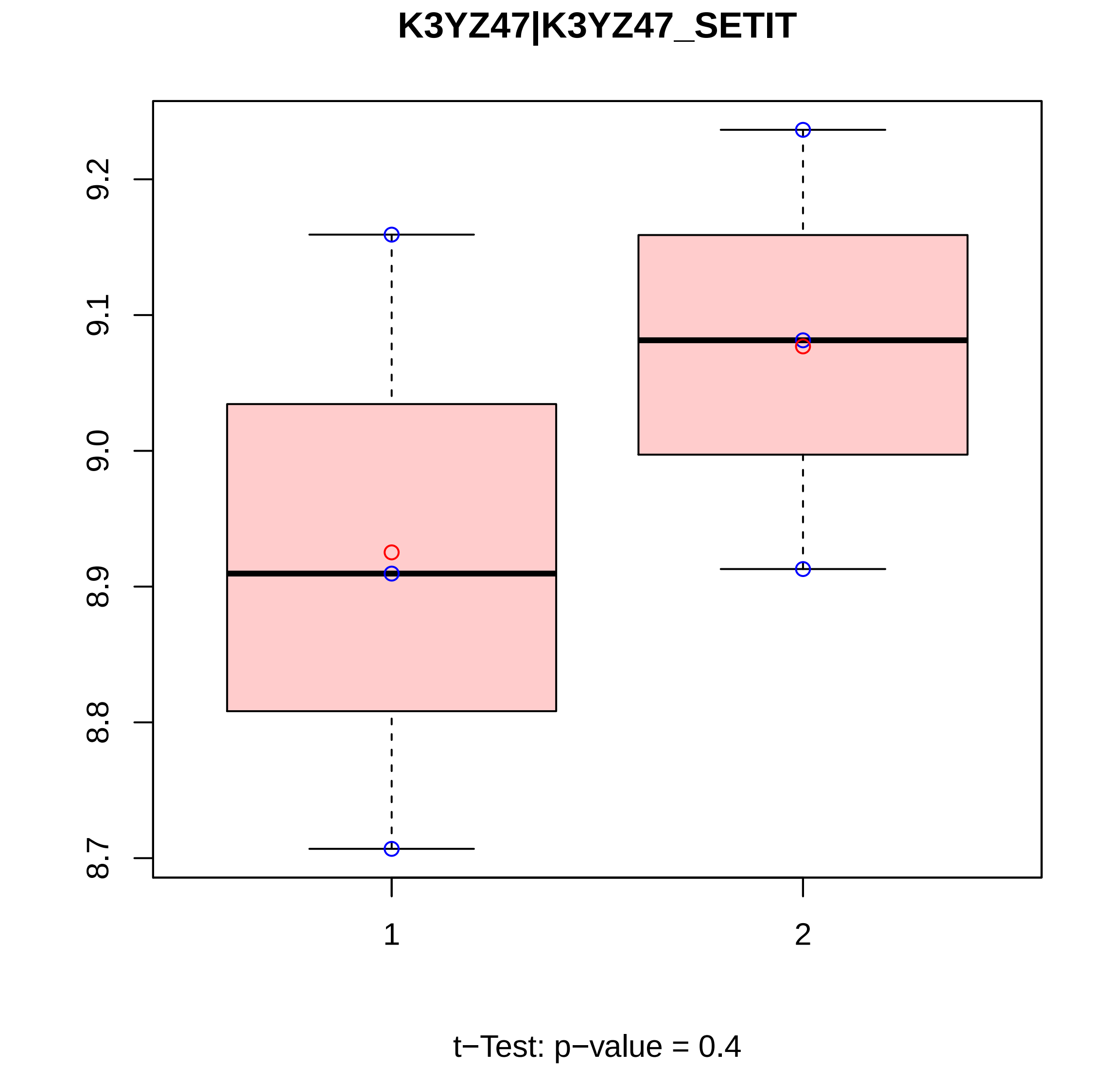
K3YZ47|K3YZ47_SETIT
9.2
9.1
9.0
8.9
8.8
8.7
1
2
t−Test: p−value = 0.4

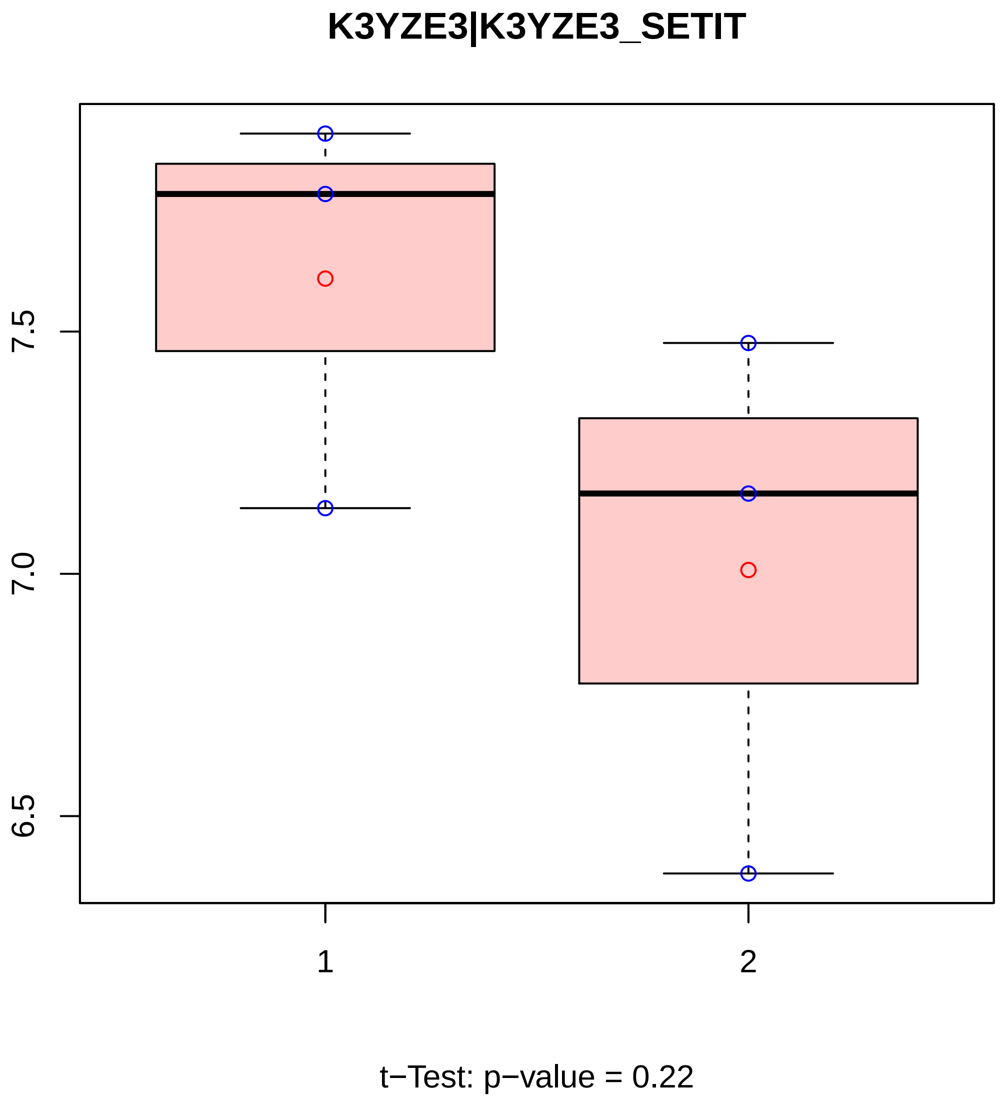
K3YZE3|K3YZE3_SETIT
7.5
7.0
6.5
1
2
t−Test: p−value = 0.22

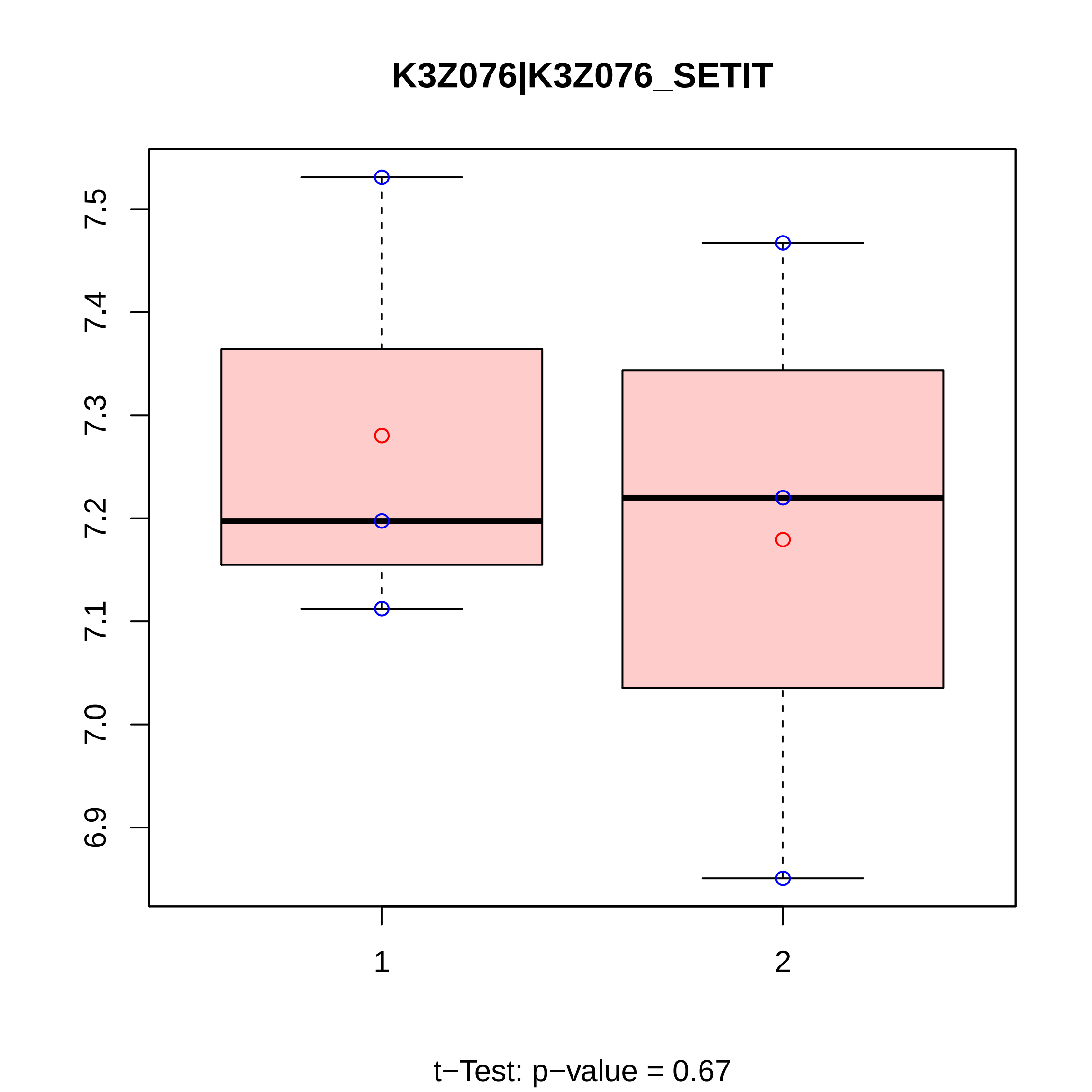
K3Z076|K3Z076_SETIT
7.5
7.4
7.3
7.2
7.1
7.0
6.9
1
2
t−Test: p−value = 0.67

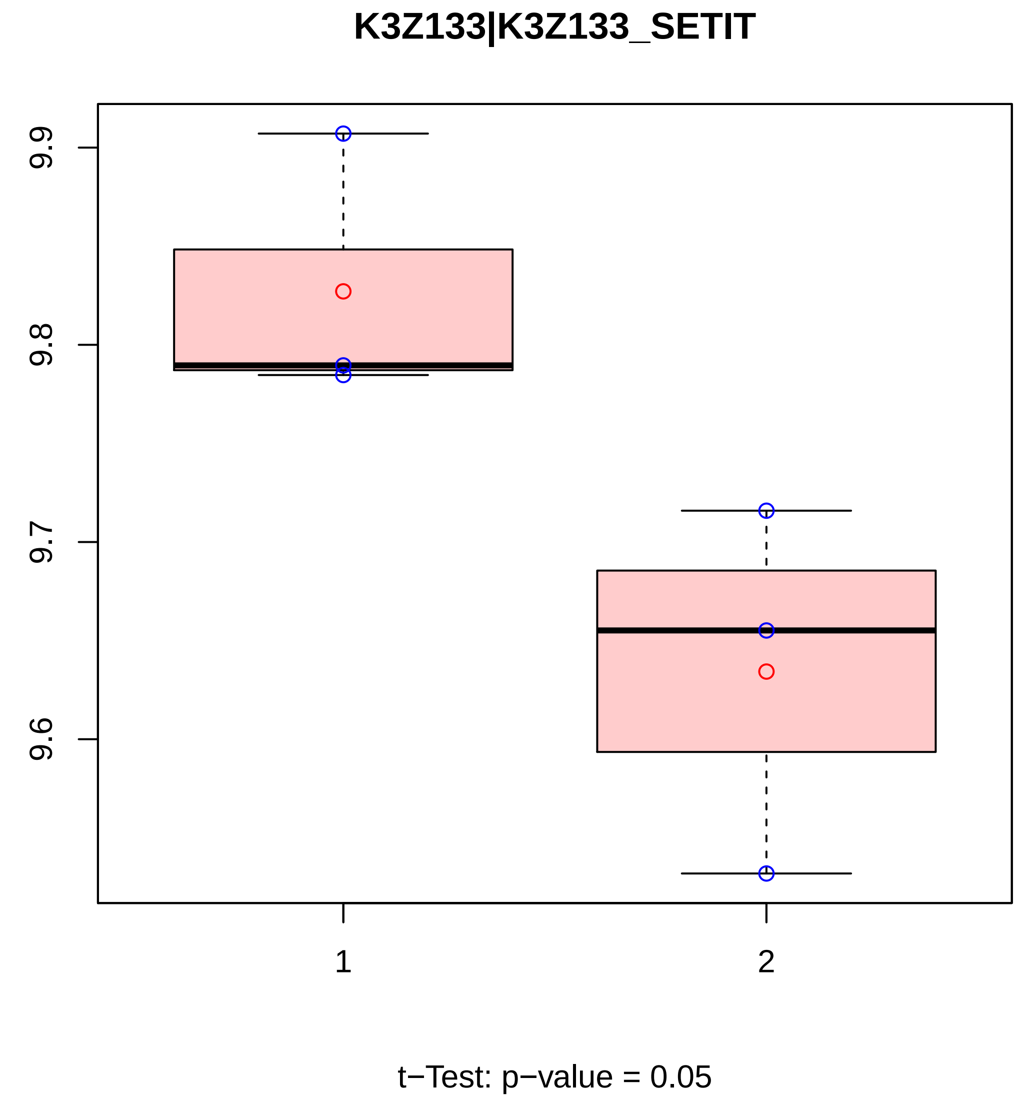
K3Z133|K3Z133_SETIT
9.9
9.8
9.7
9.6
1
2
t−Test: p−value = 0.05

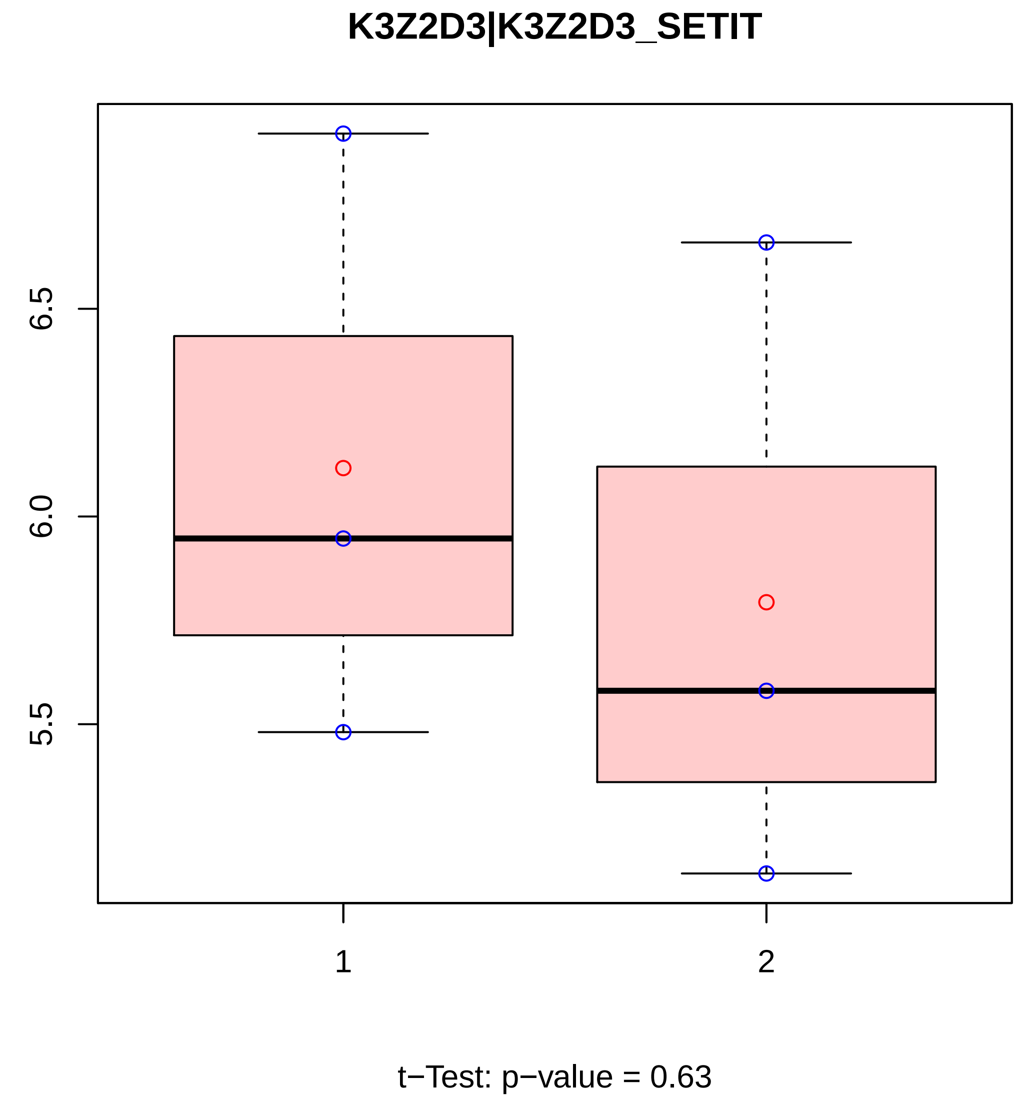
K3Z2D3|K3Z2D3_SETIT
6.5
6.0
5.5
1
2
t−Test: p−value = 0.63

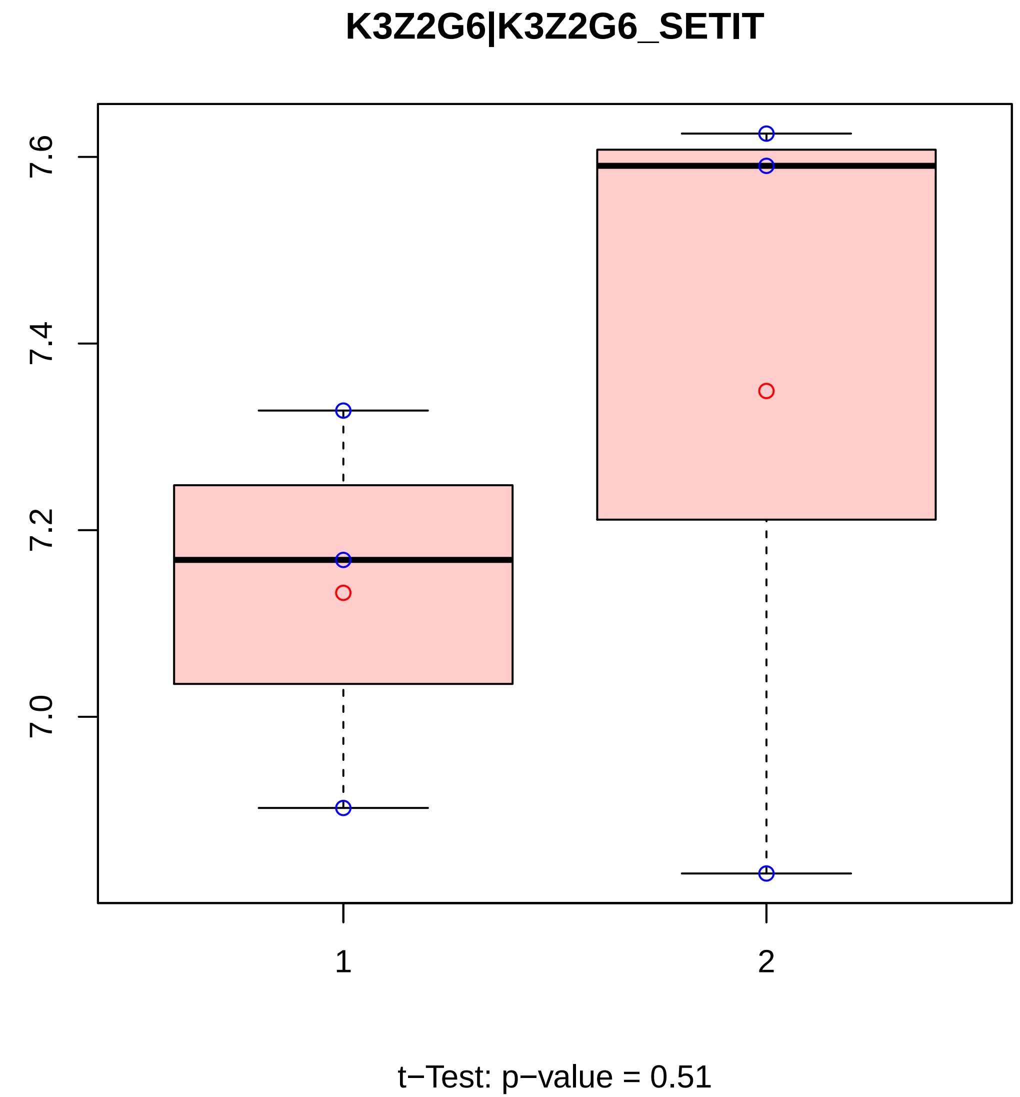
K3Z2G6|K3Z2G6_SETIT
7.6
7.4
7.2
7.0
1
2
t−Test: p−value = 0.51

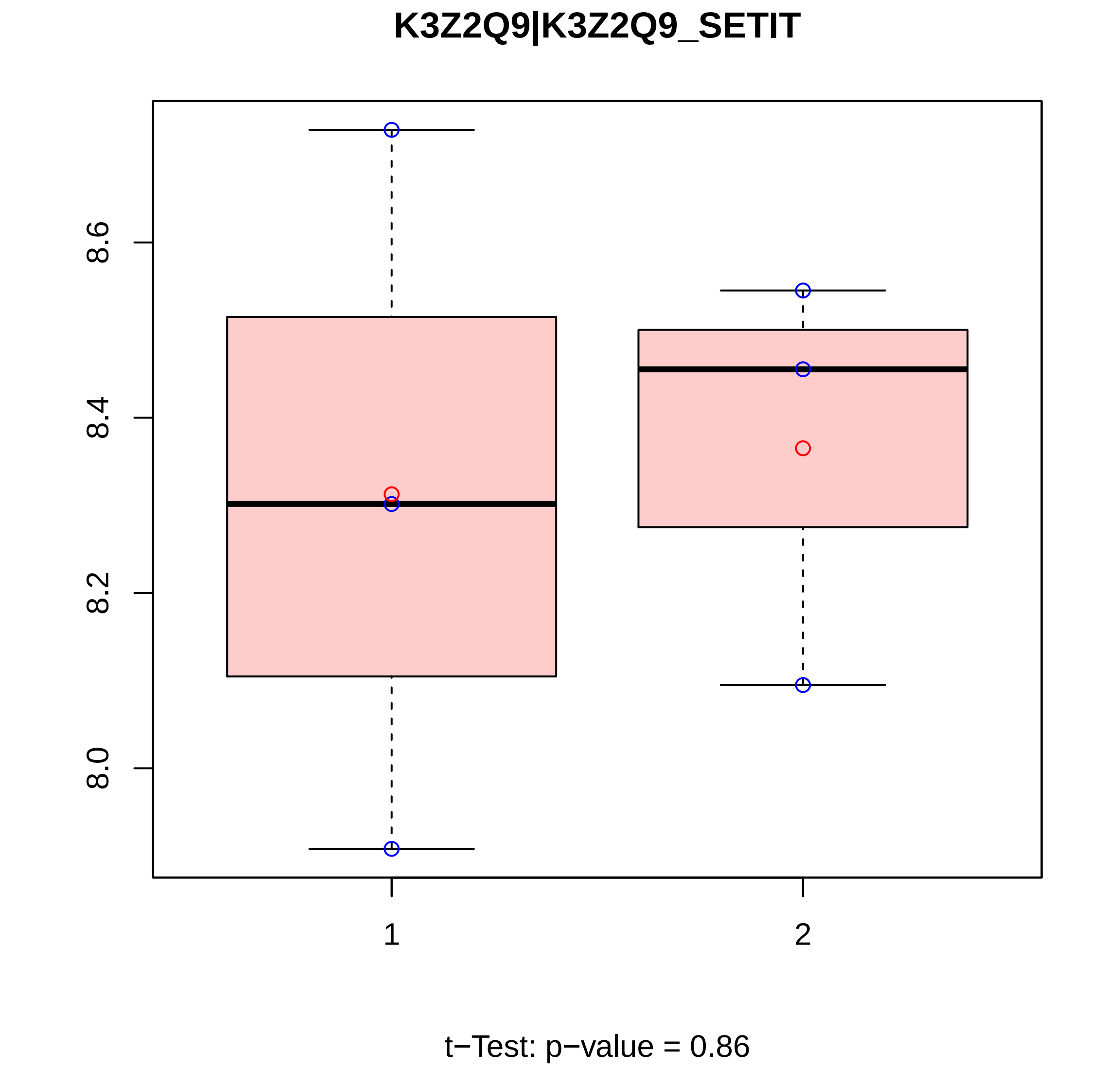

K3Z2Q9|K3Z2Q9_SETIT
8.0
8.2
8.4
8.6
1
2
t−Test: p−value = 0.86

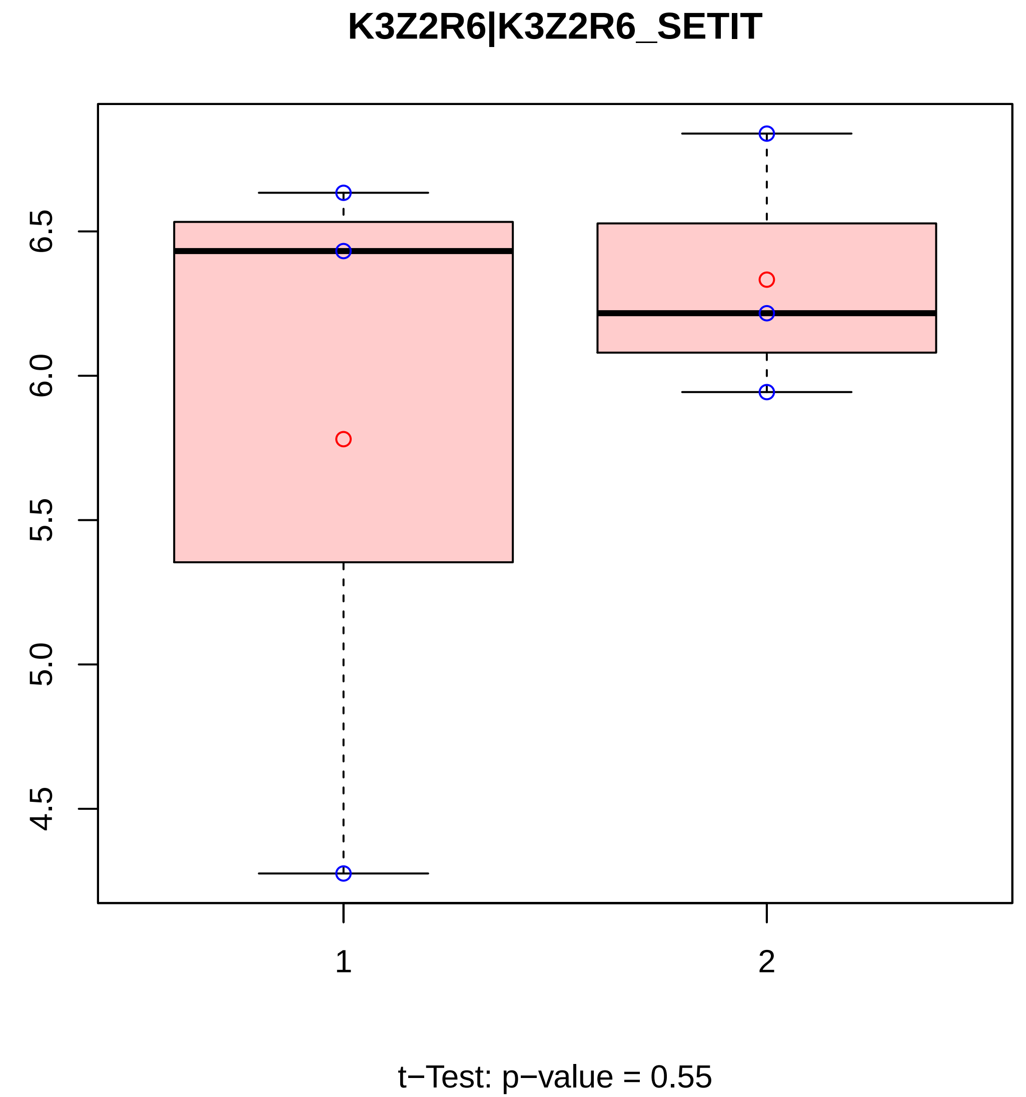
K3Z2R6|K3Z2R6_SETIT
4.5
5.0
5.5
6.0
6.5
1
2
t−Test: p−value = 0.55

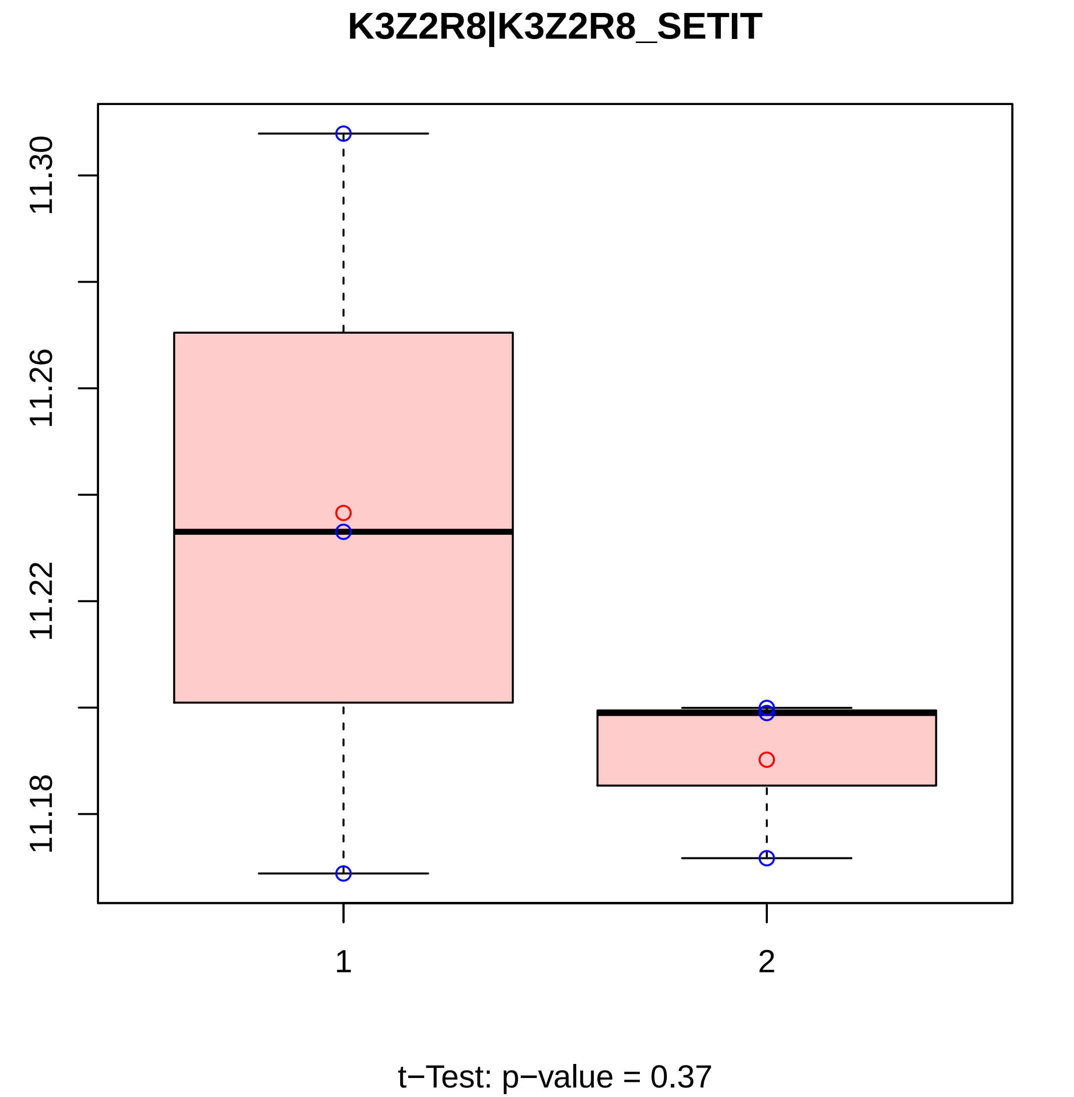
K3Z2R8|K3Z2R8_SETIT
11.18
11.22
11.26
11.30
1
2
t−Test: p−value = 0.37

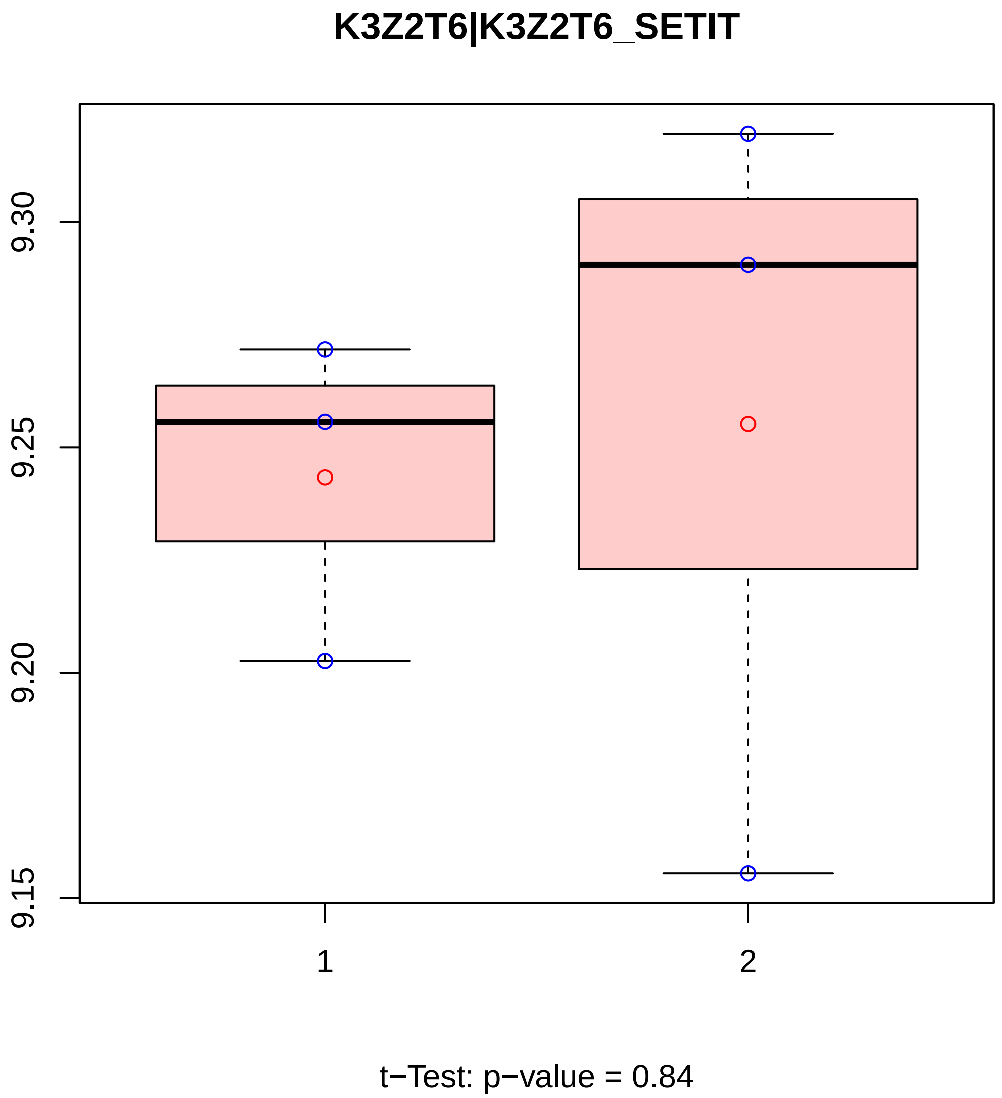

K3Z2T6|K3Z2T6_SETIT
9.15
9.20
9.25
9.30
1
2
t−Test: p−value = 0.84

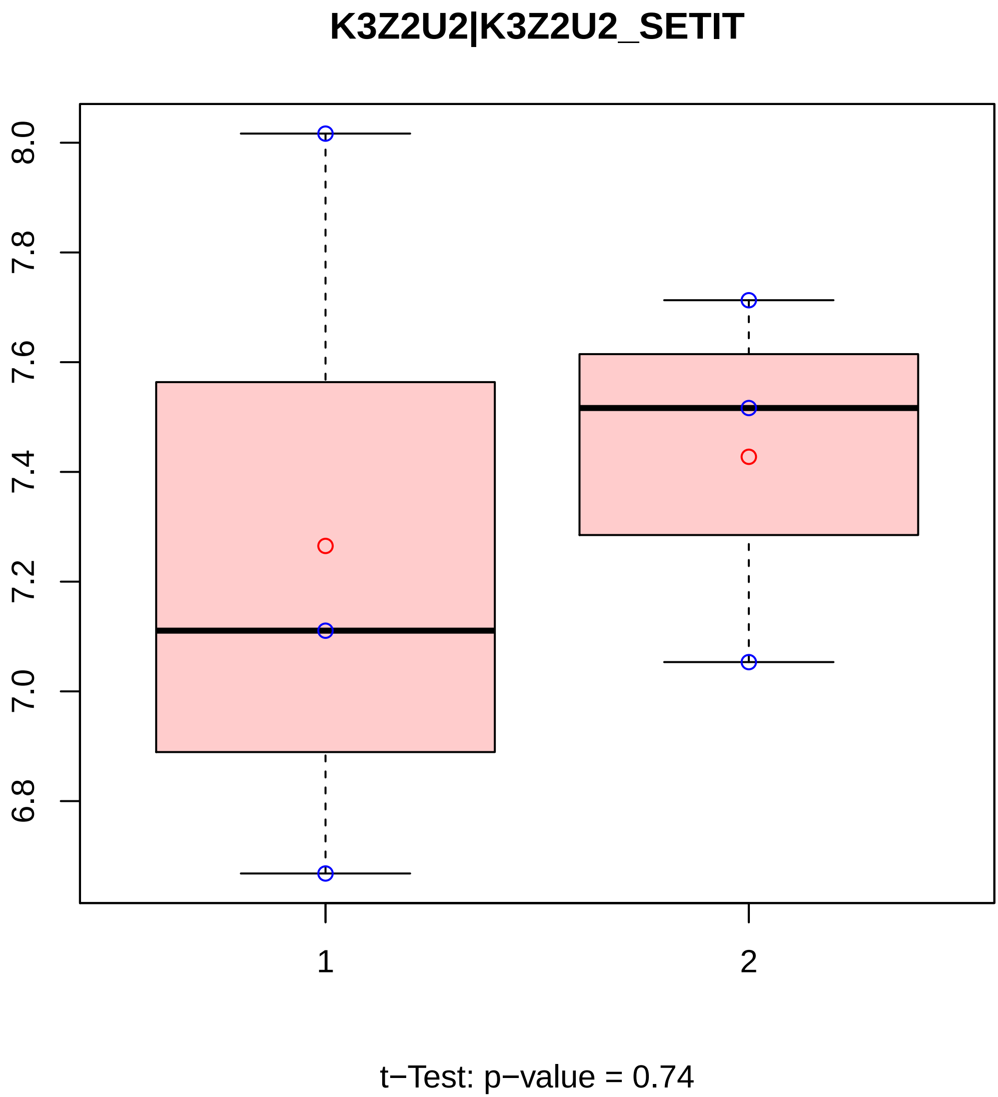

K3Z2U2|K3Z2U2_SETIT
8.0
7.8
7.6
7.4
7.2
7.0
6.8
1
2
t−Test: p−value = 0.74

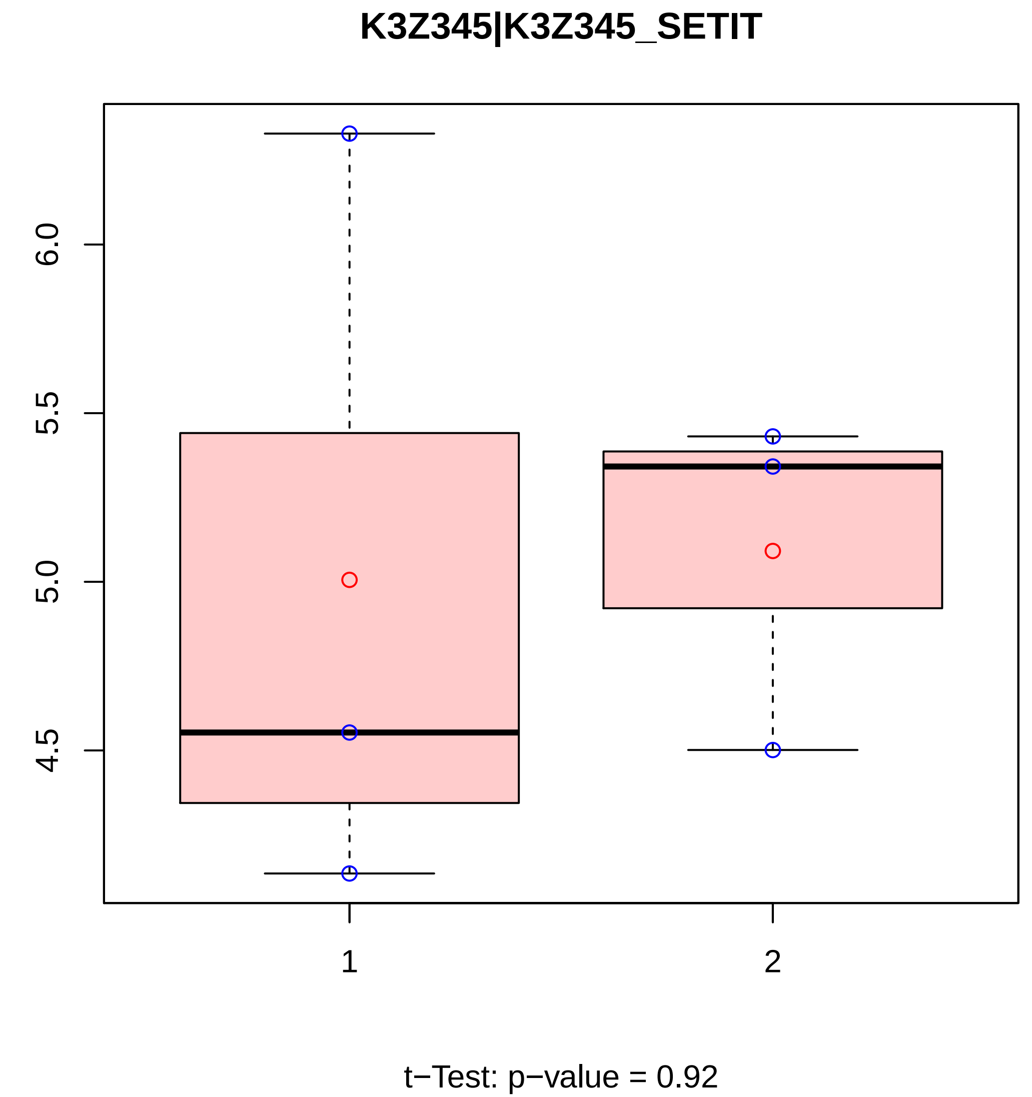
K3Z345|K3Z345_SETIT
6.0
5.5
5.0
4.5
1
2
t−Test: p−value = 0.92

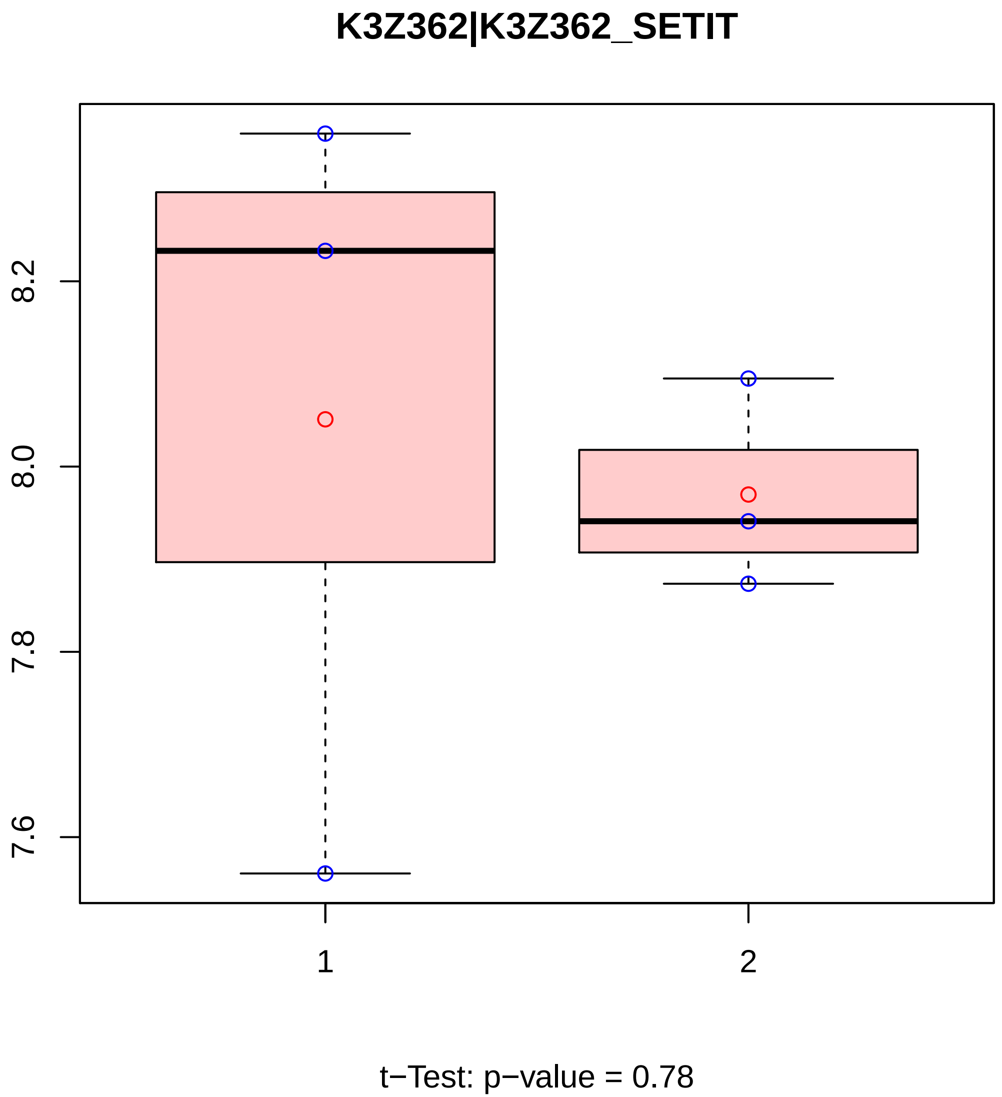
K3Z362|K3Z362_SETIT
8.2
8.0
7.8
7.6
1
2
t−Test: p−value = 0.78

K3Z3A2|K3Z3A2_SETIT

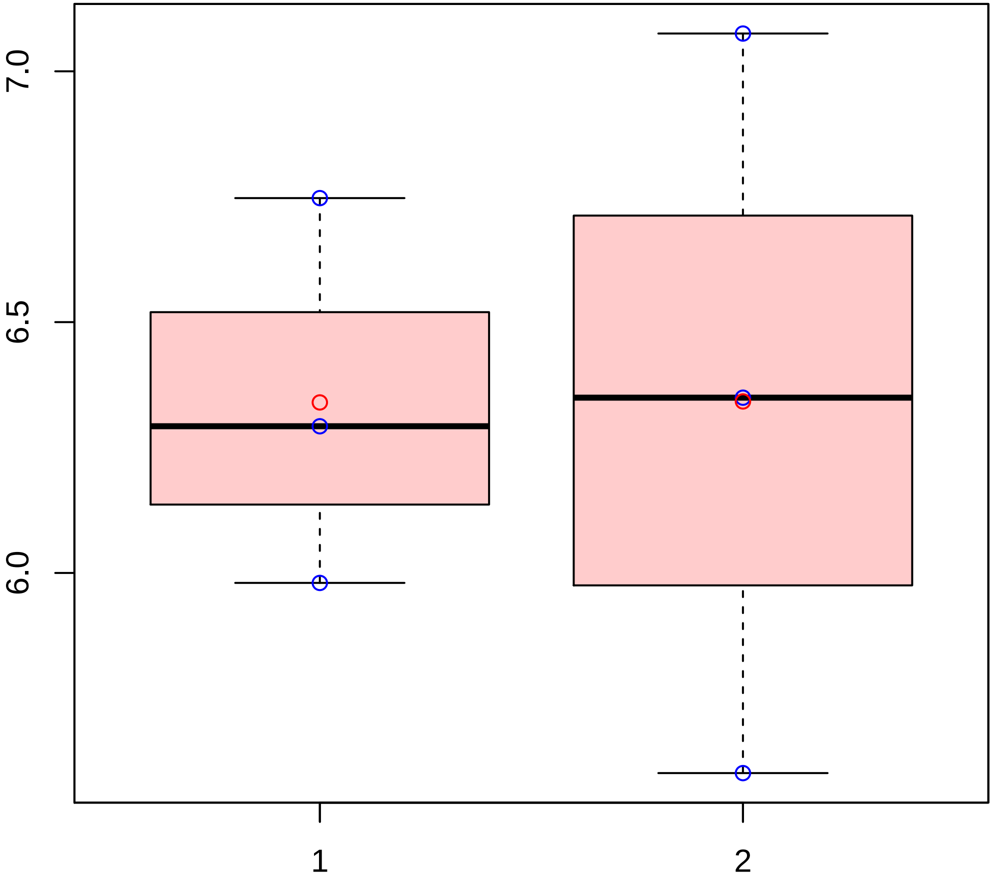

t−Test: p−value = 1

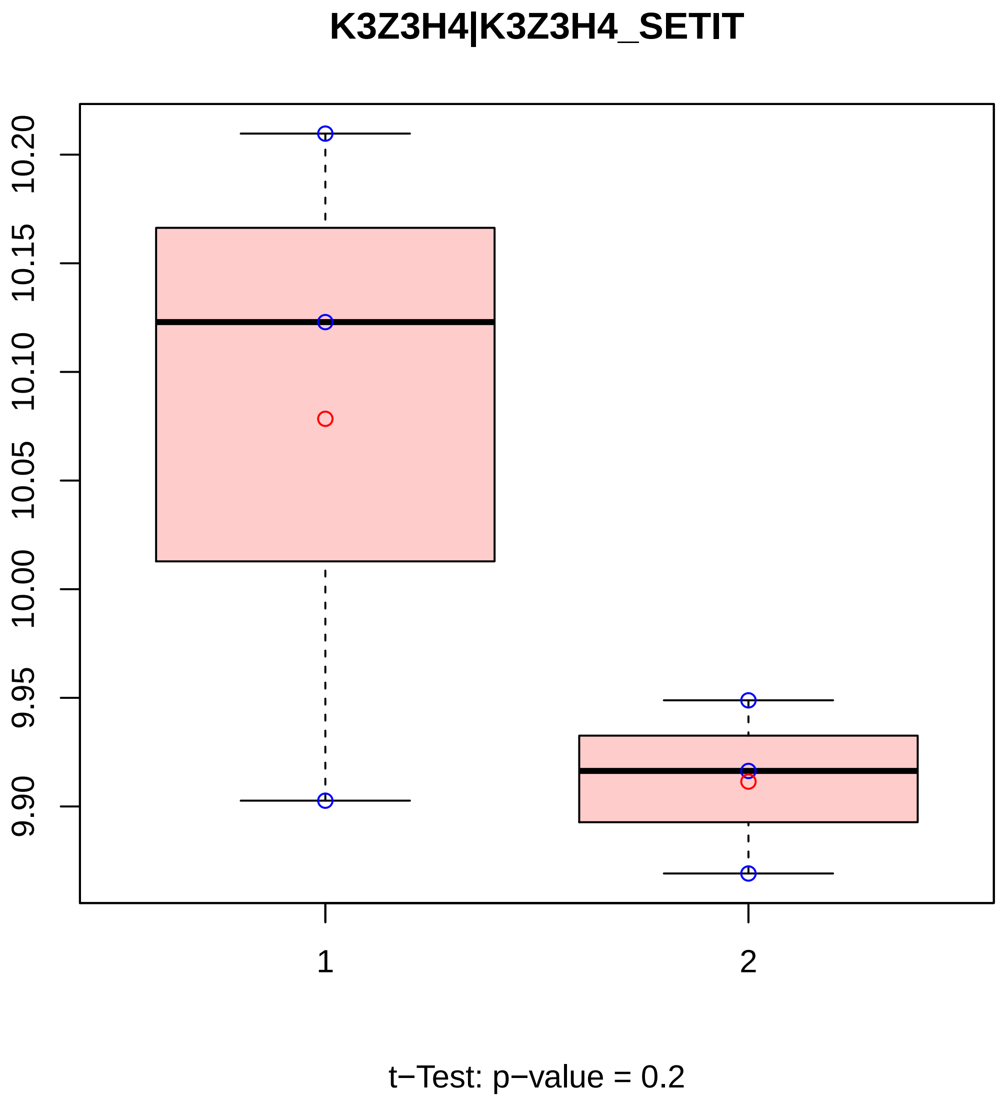
K3Z3H4|K3Z3H4_SETIT
10.20
10.15
10.10
10.05
10.00
9.95
9.90
1
2
t−Test: p−value = 0.2

K3Z3I6|K3Z3I6_SETIT

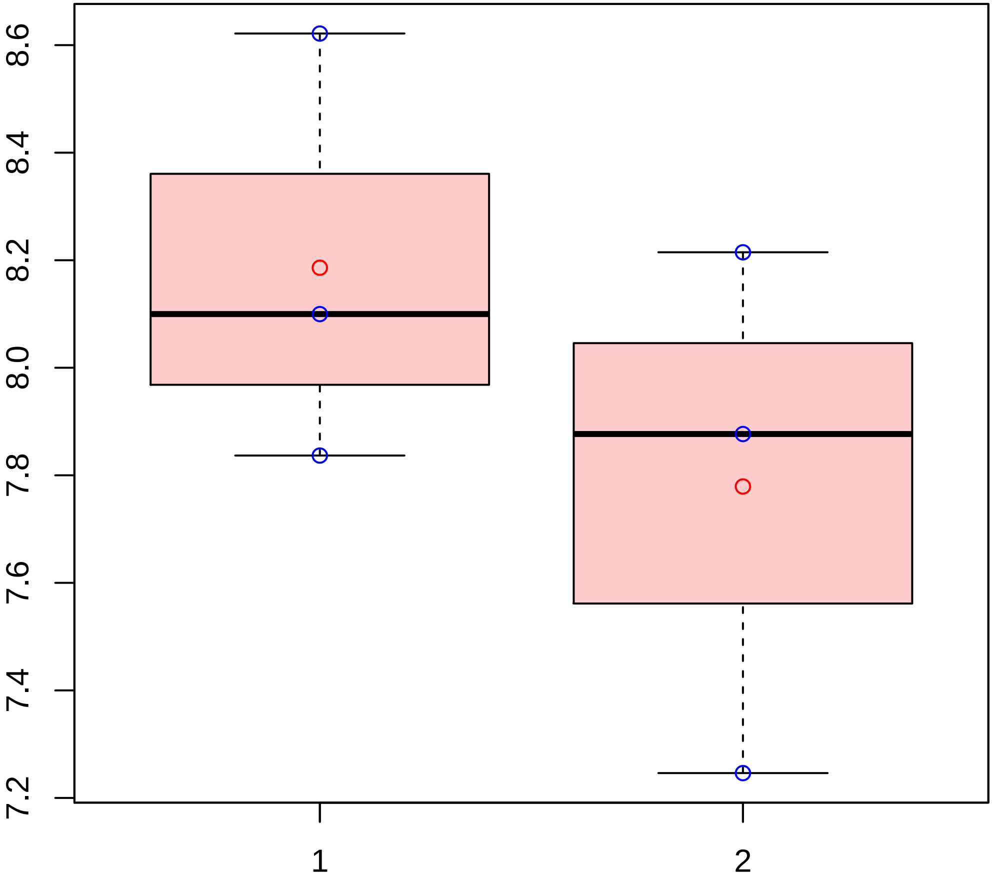

t−Test: p−value = 0.33

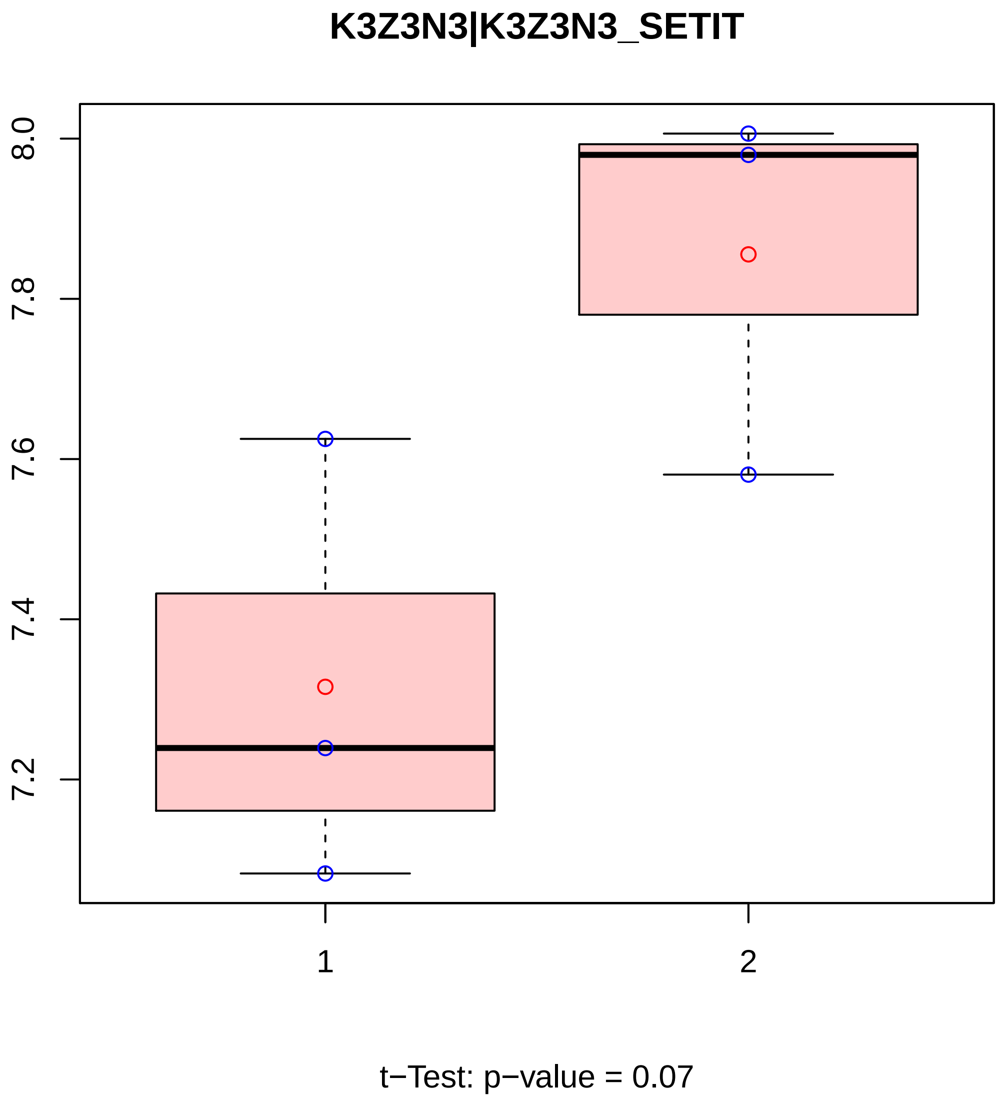
K3Z3N3|K3Z3N3_SETIT
8.0
7.8
7.6
7.4
7.2
1
2
t−Test: p−value = 0.07

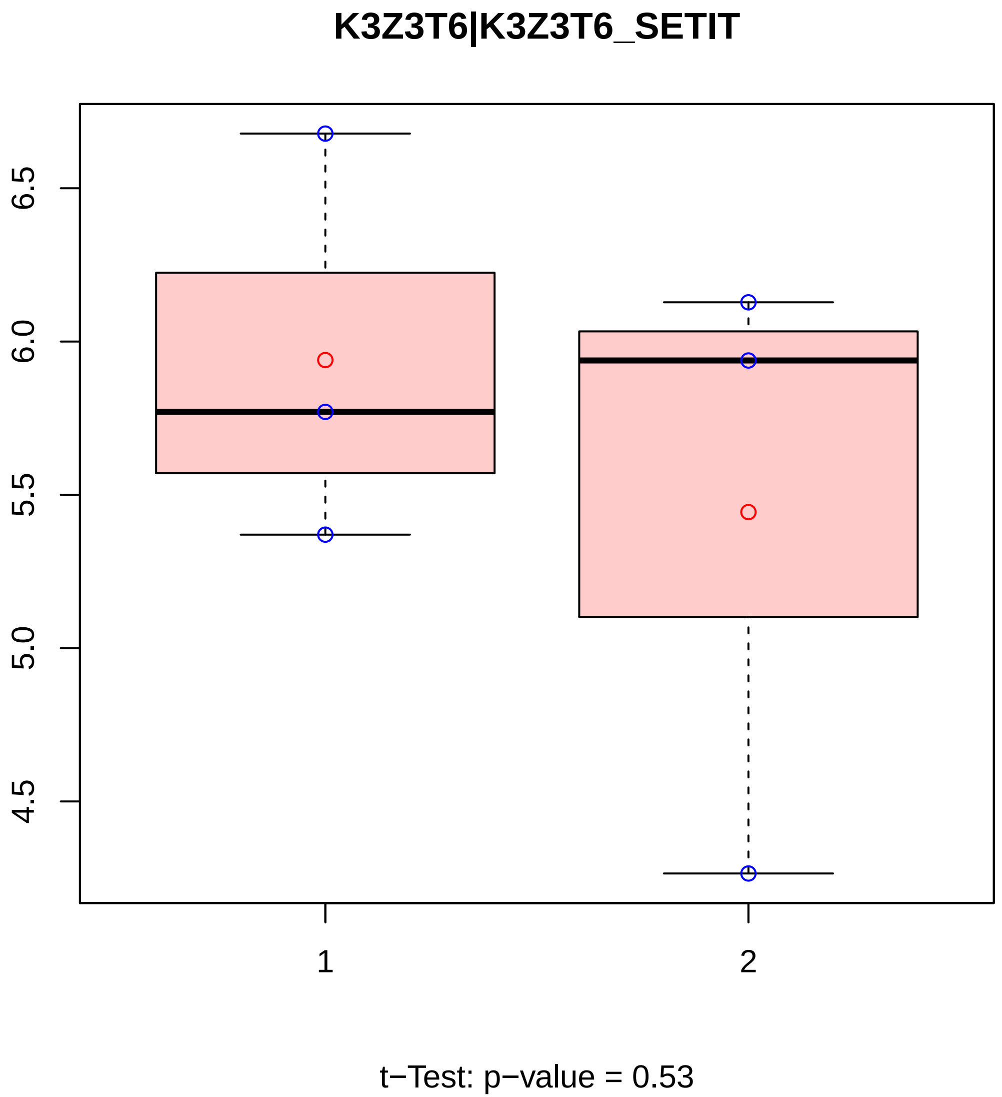
K3Z3T6|K3Z3T6_SETIT
6.5
6.0
5.5
5.0
4.5
1
2
t−Test: p−value = 0.53

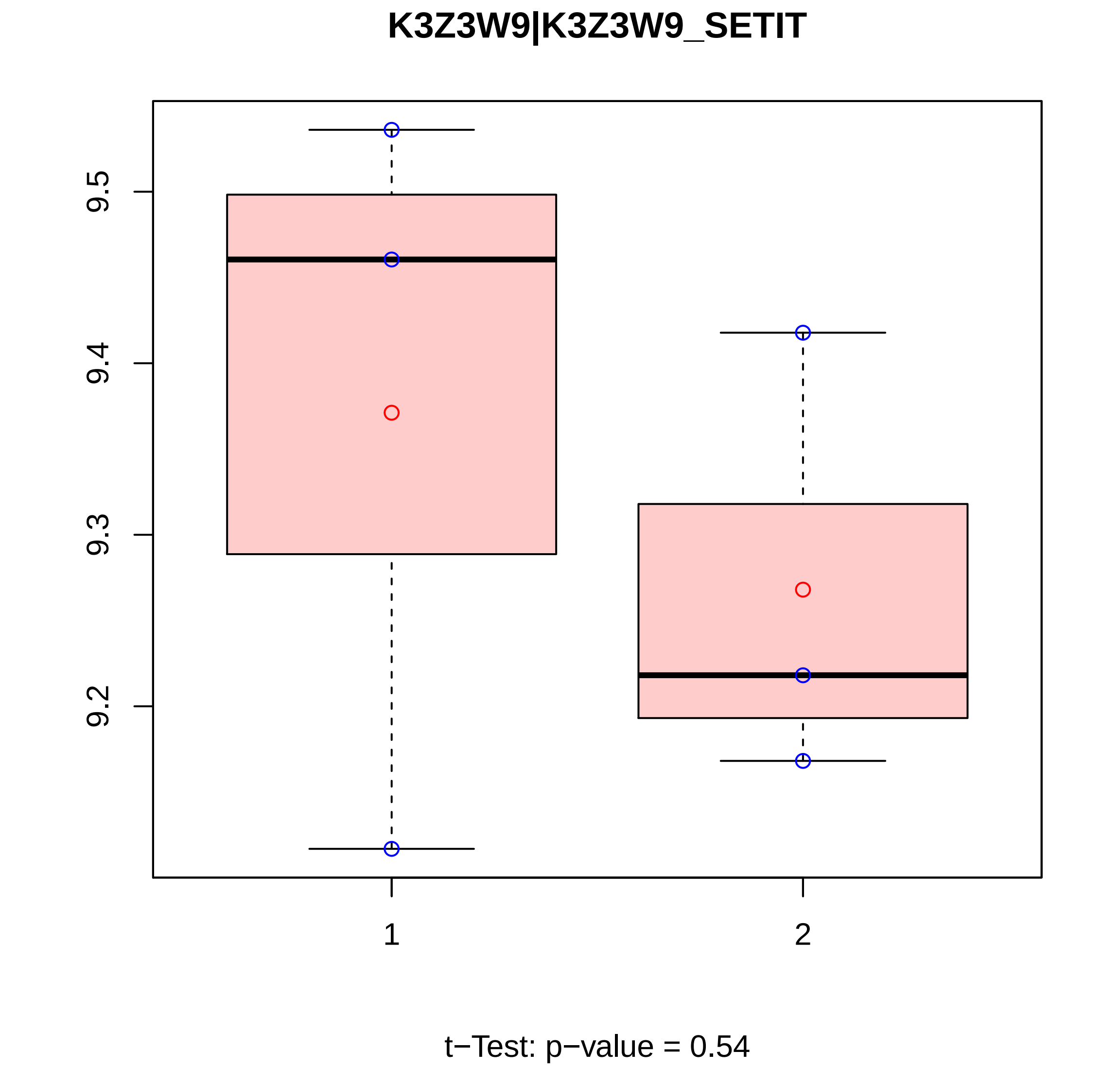
K3Z3W9|K3Z3W9_SETIT
9.2
9.3
9.4
9.5
1
2
t−Test: p−value = 0.54

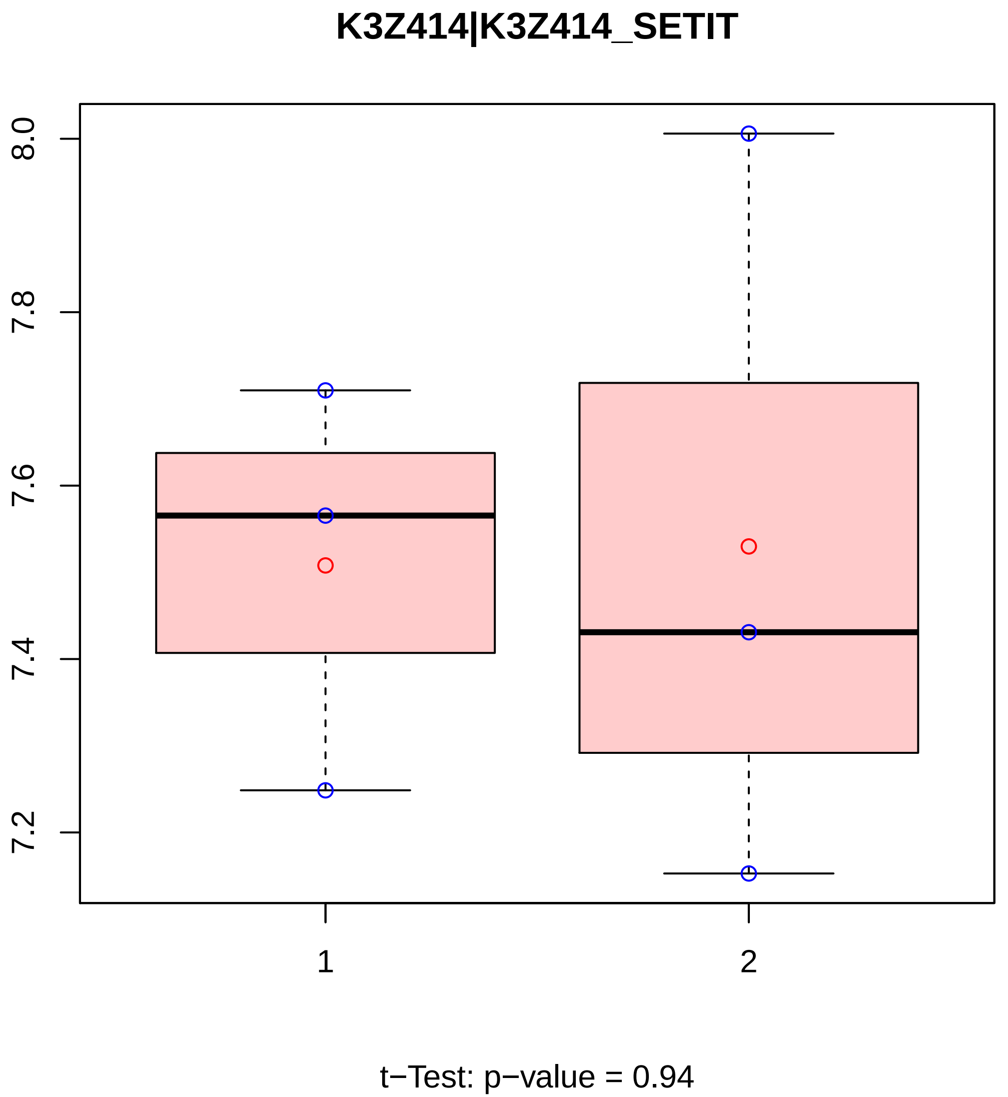

K3Z414|K3Z414_SETIT
8.0
7.8
7.6
7.4
7.2
1
2
t−Test: p−value = 0.94

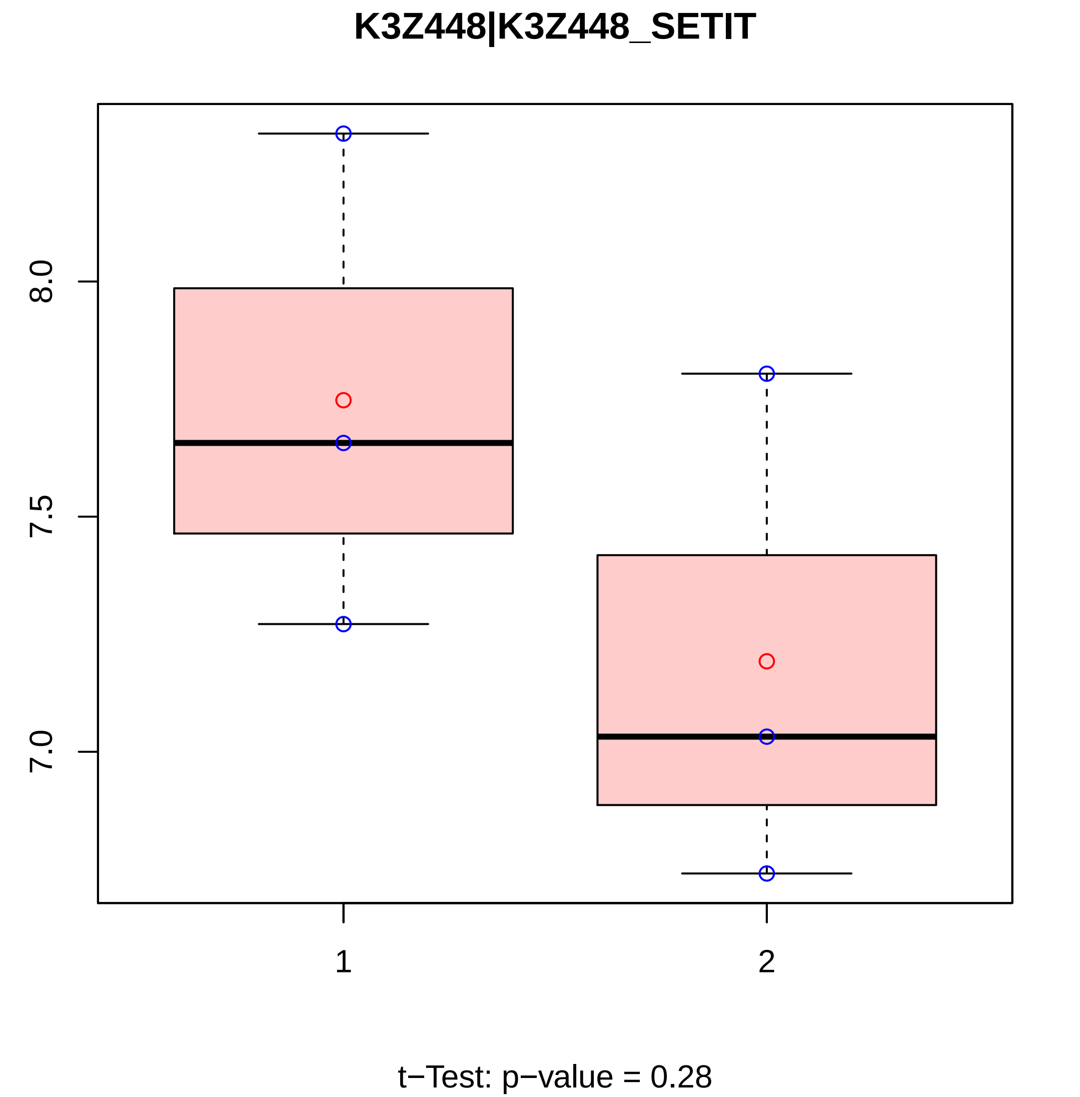
K3Z448|K3Z448_SETIT
8.0
7.5
7.0
1
2
t−Test: p−value = 0.28

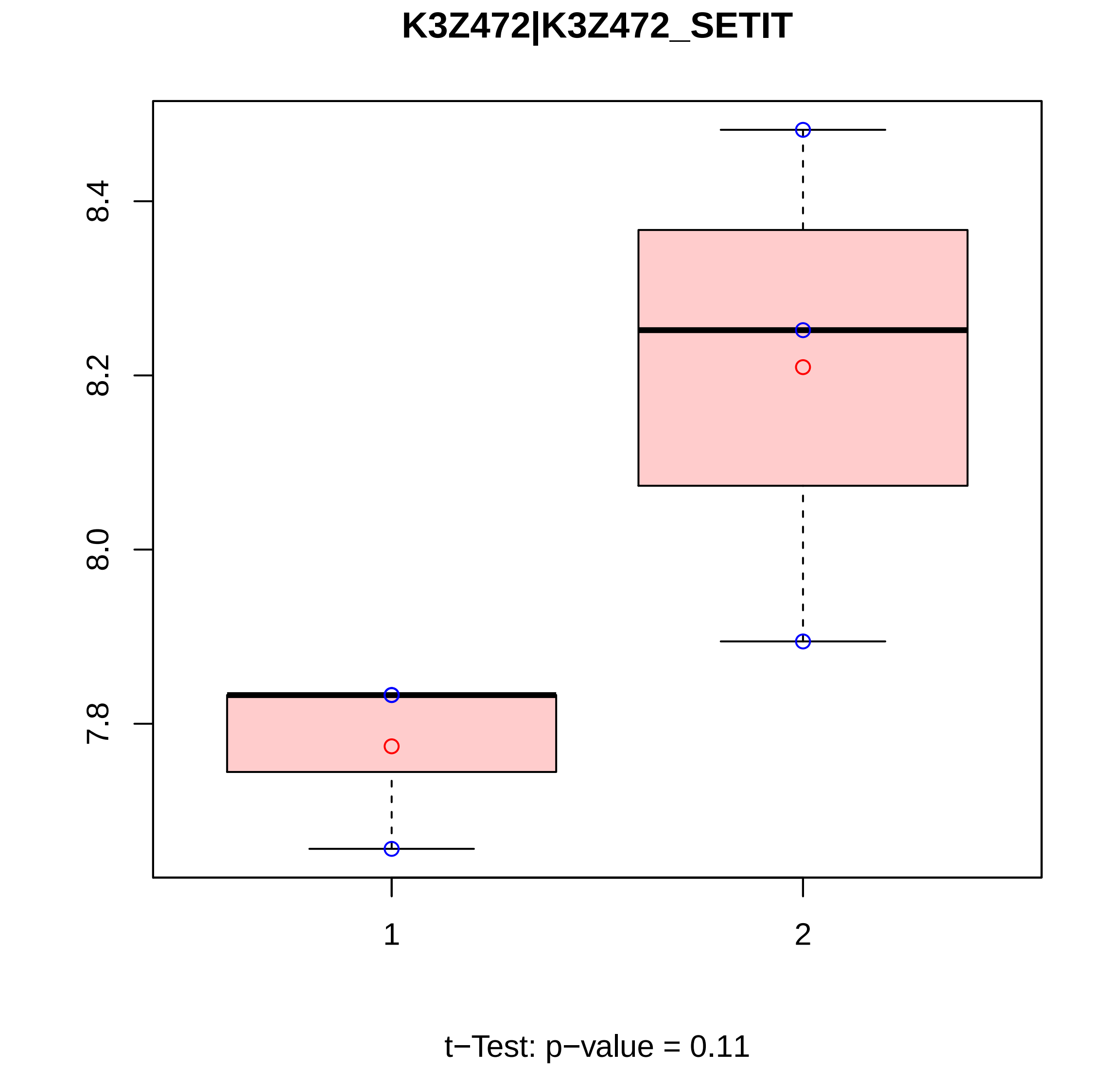
K3Z472|K3Z472_SETIT
8.4
8.2
8.0
7.8
1
2
t−Test: p−value = 0.11

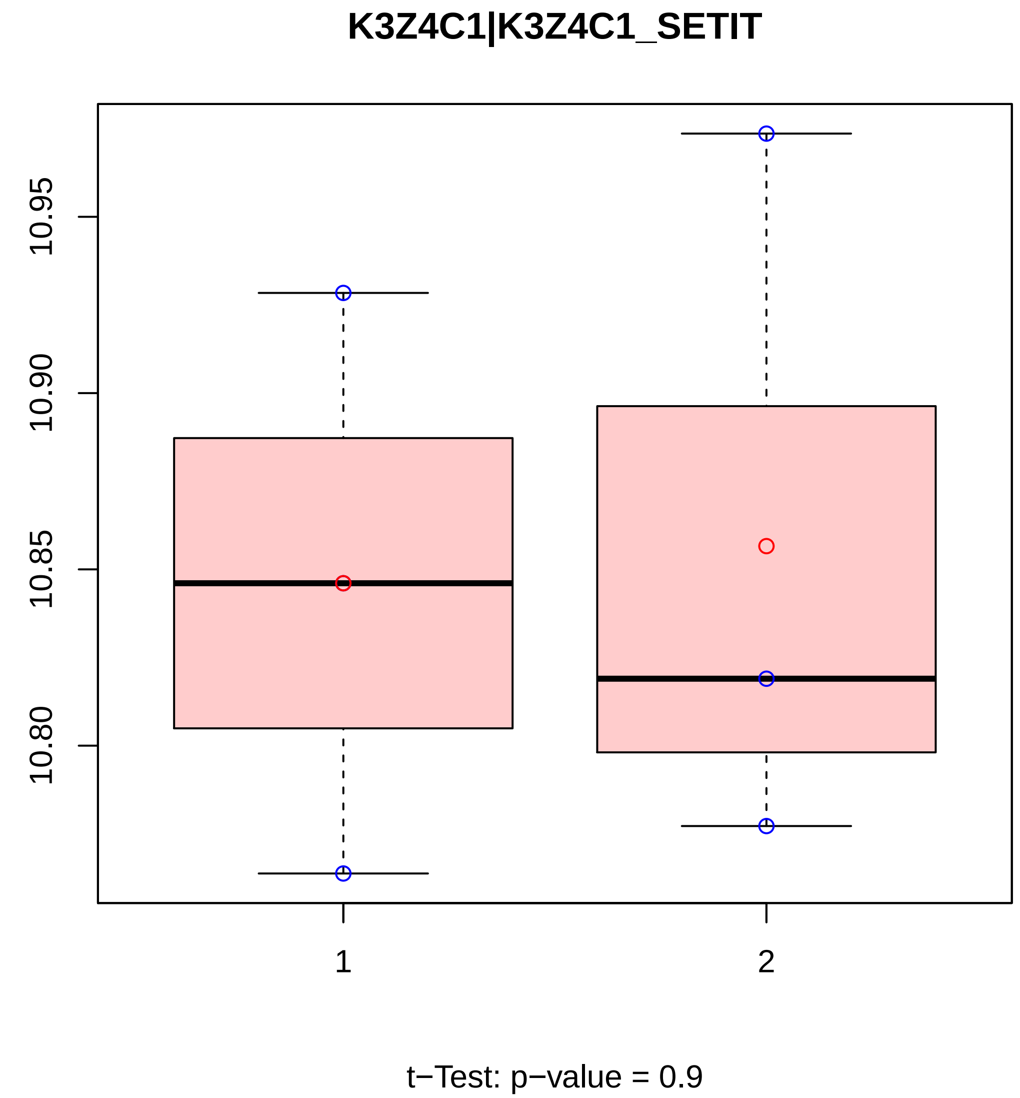

K3Z4C1|K3Z4C1_SETIT
10.80
10.85
10.90
10.95
1
2
t−Test: p−value = 0.9

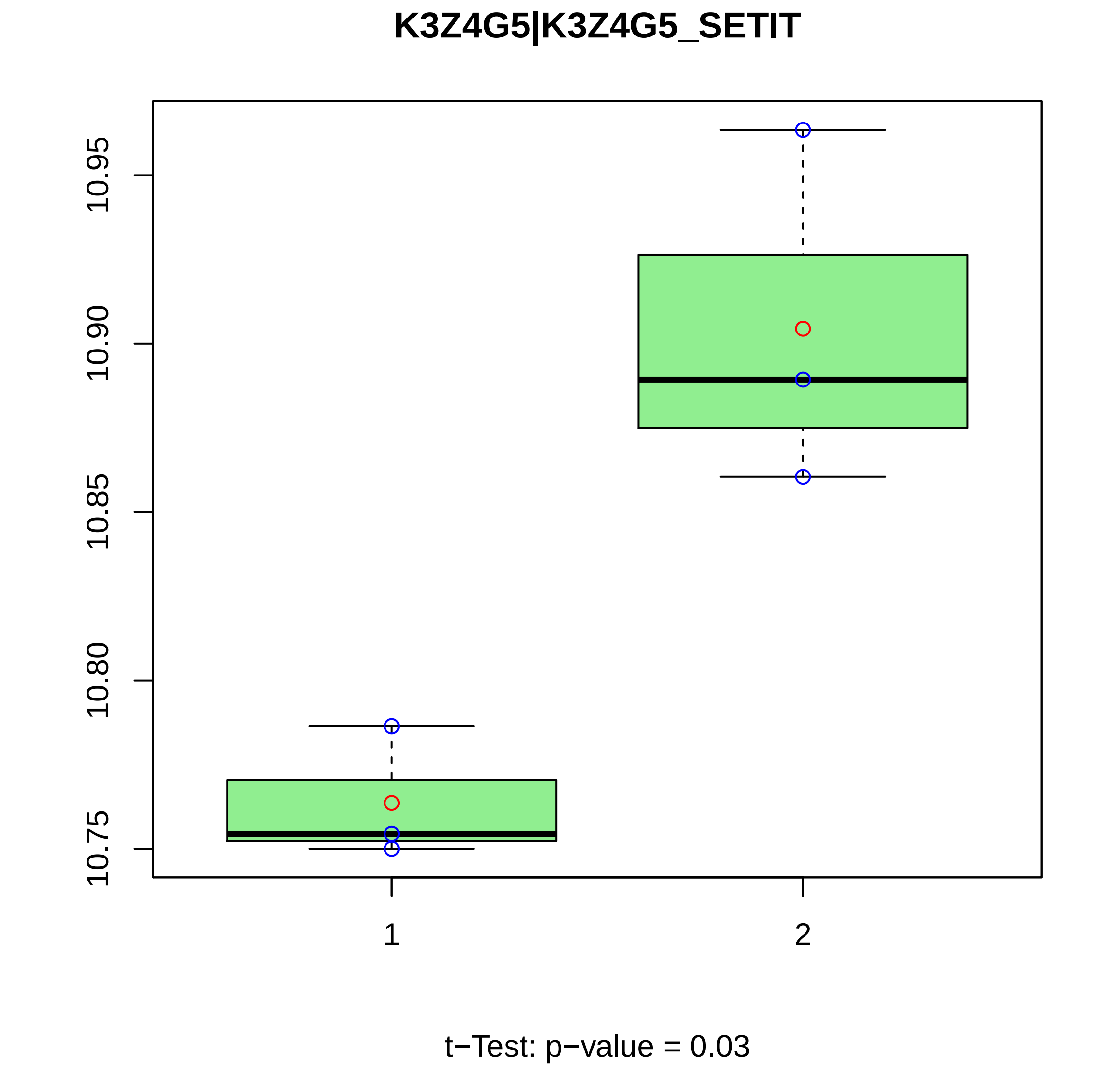
K3Z4G5|K3Z4G5_SETIT
10.75
10.80
10.85
10.90
10.95
1
2
t−Test: p−value = 0.03

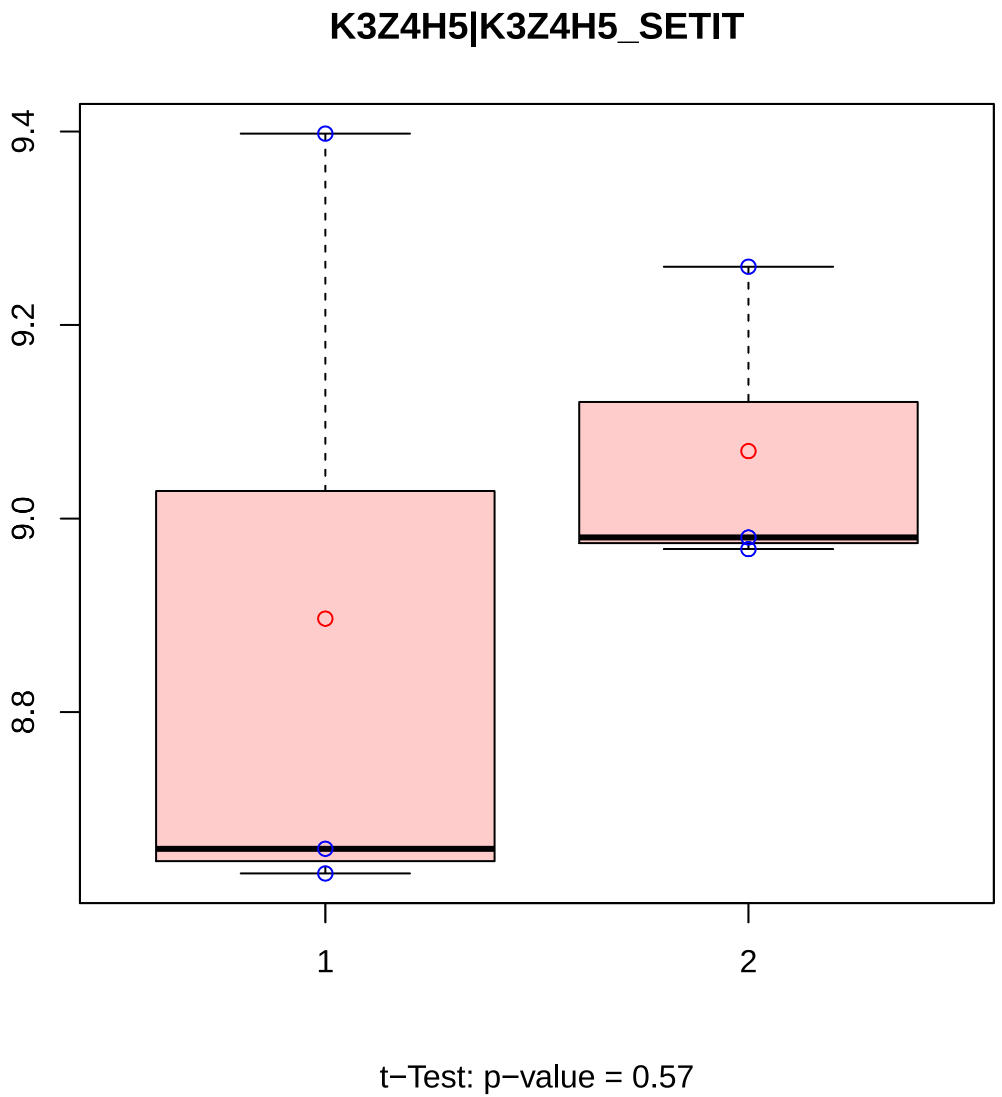

K3Z4H5|K3Z4H5_SETIT
9.4
9.2
9.0
8.8
1
2
t−Test: p−value = 0.57

K3Z4J6|K3Z4J6_SETIT

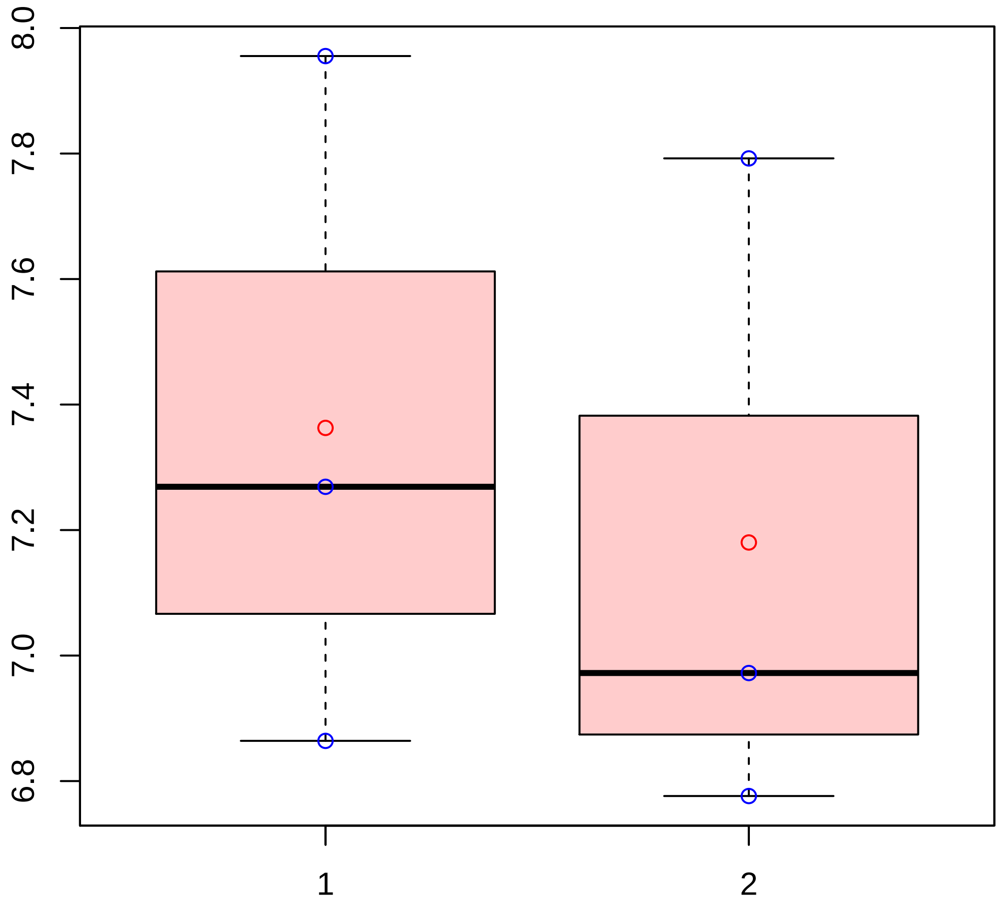

t−Test: p−value = 0.7

K3Z4X2|K3Z4X2_SETIT

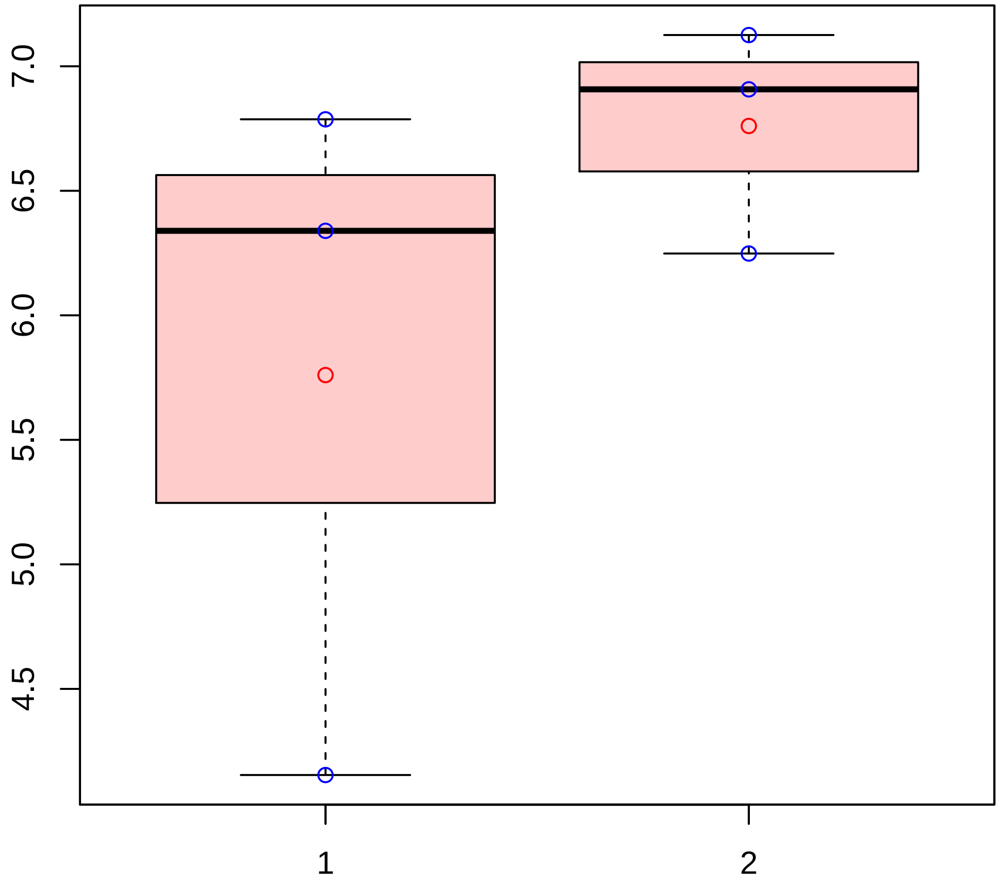

t−Test: p−value = 0.34

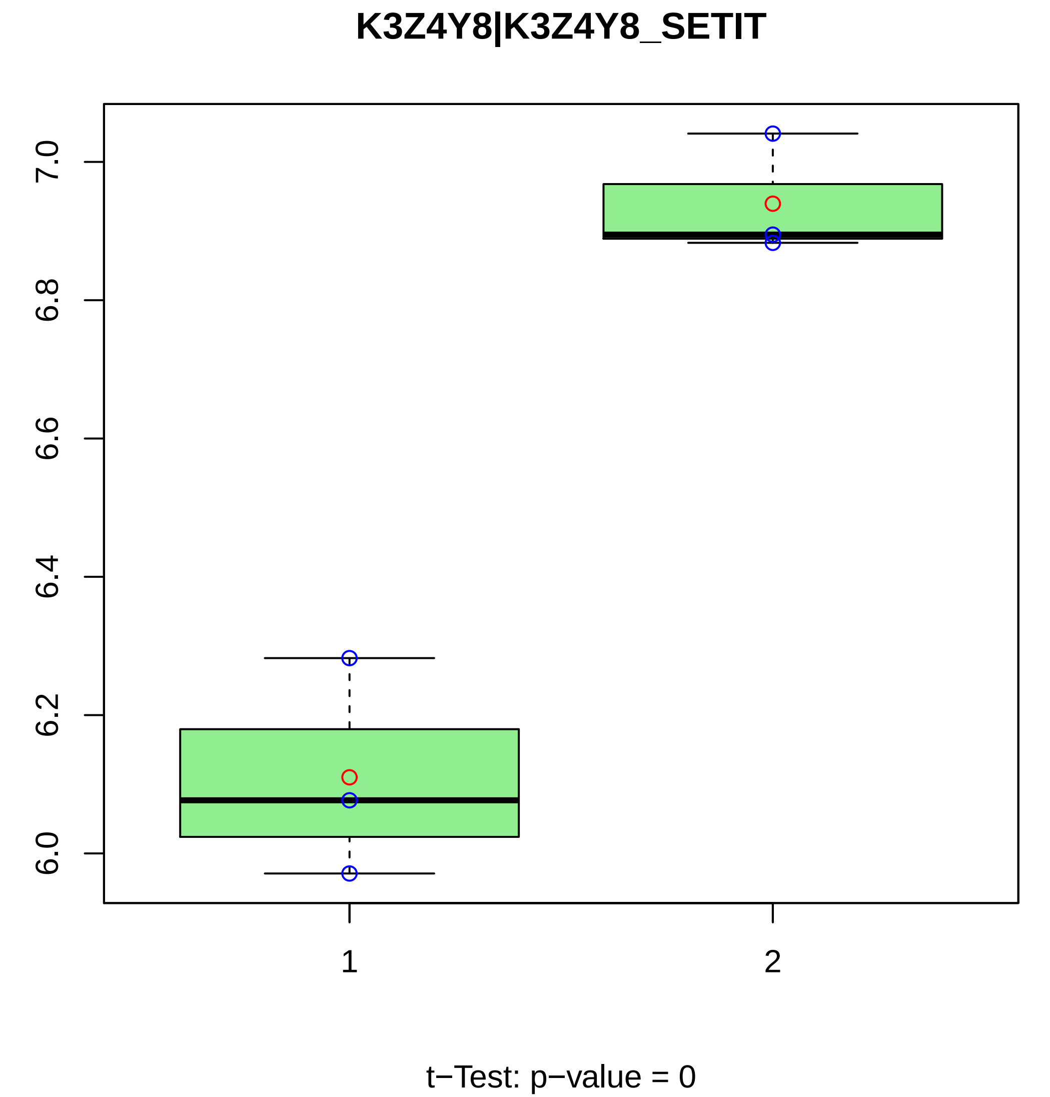
K3Z4Y8|K3Z4Y8_SETIT
7.0
6.8
6.6
6.4
6.2
6.0
1
2
t−Test: p−value = 0

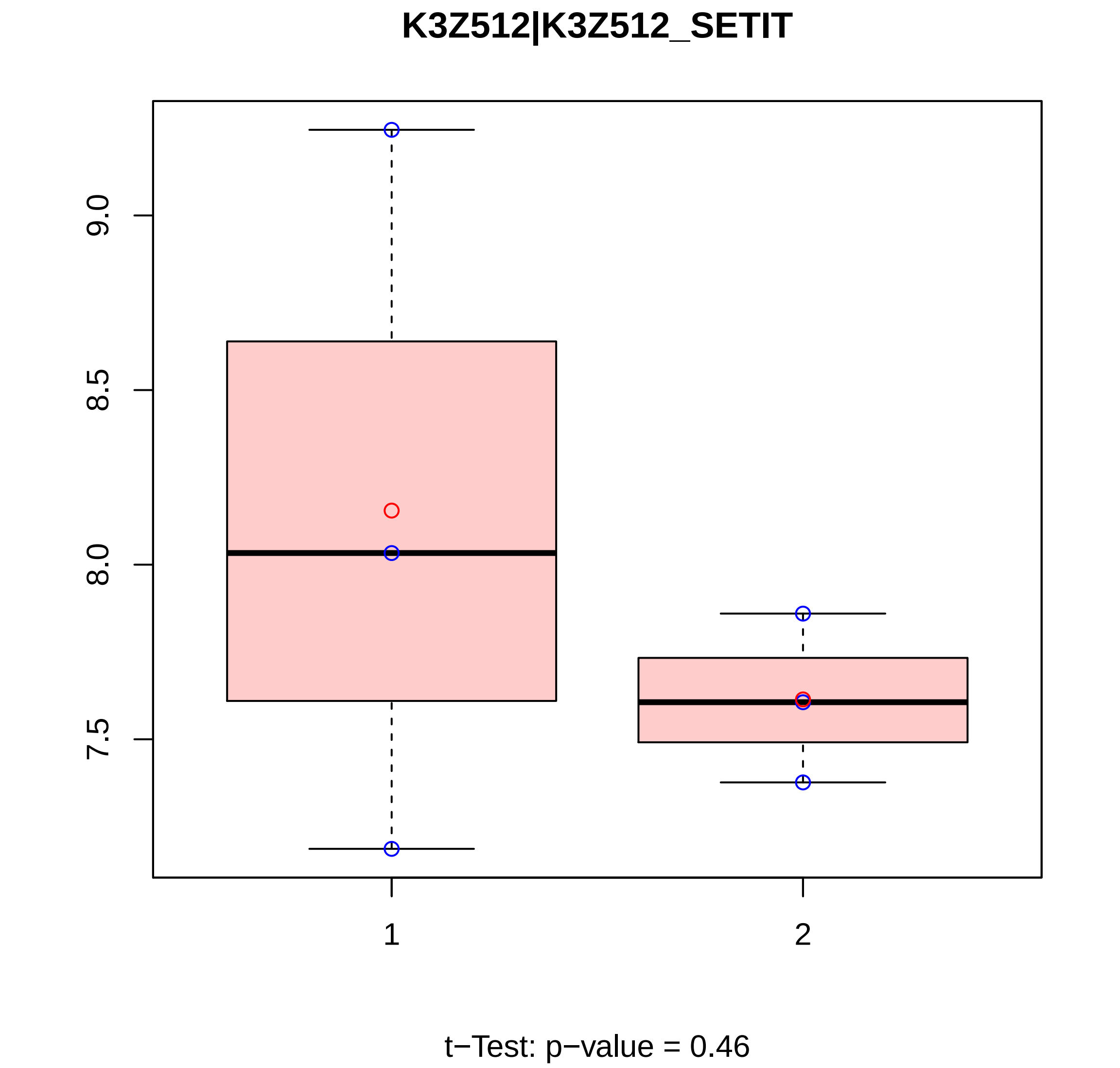
K3Z512|K3Z512_SETIT
9.0
8.5
8.0
7.5
1
2
t−Test: p−value = 0.46

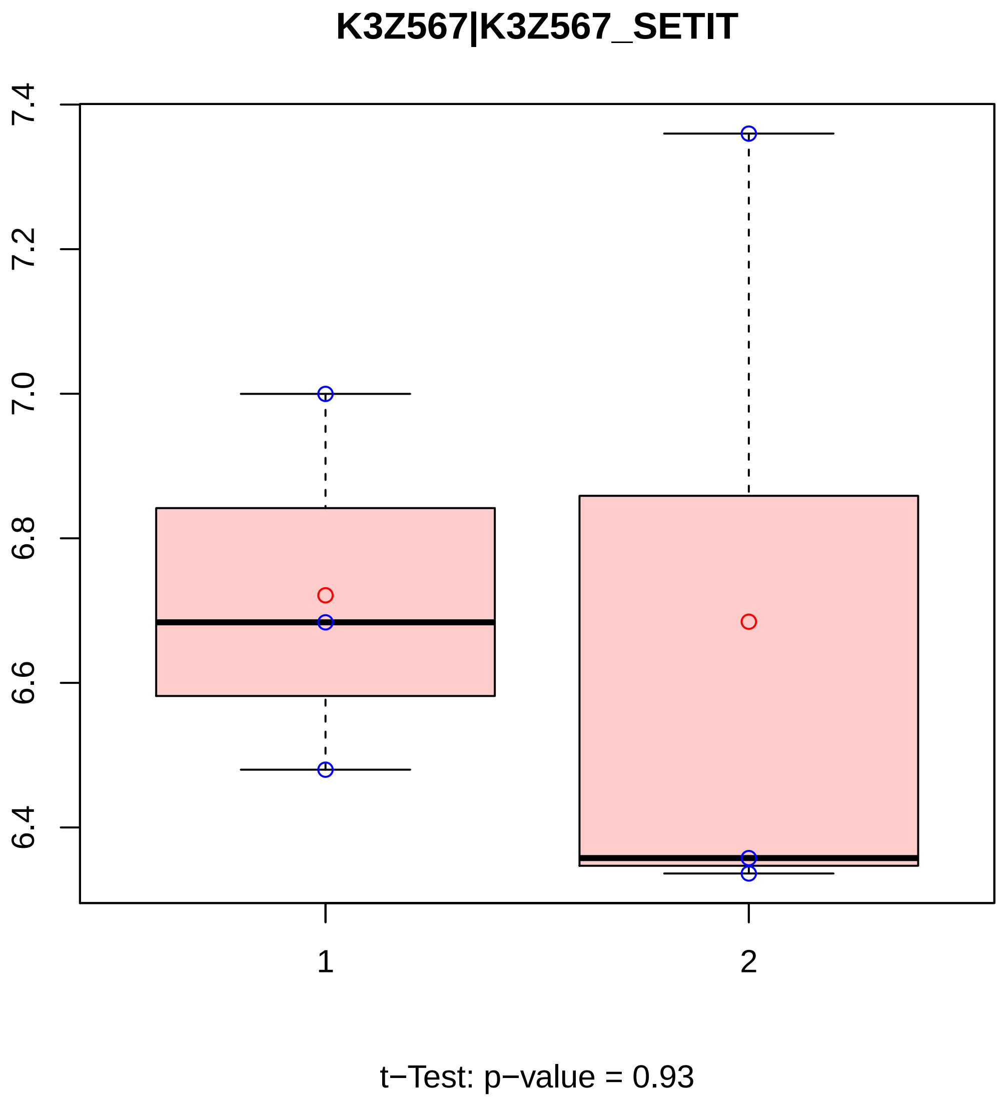
K3Z567|K3Z567_SETIT
7.4
7.2
7.0
6.8
6.6
6.4
1
2
t−Test: p−value = 0.93

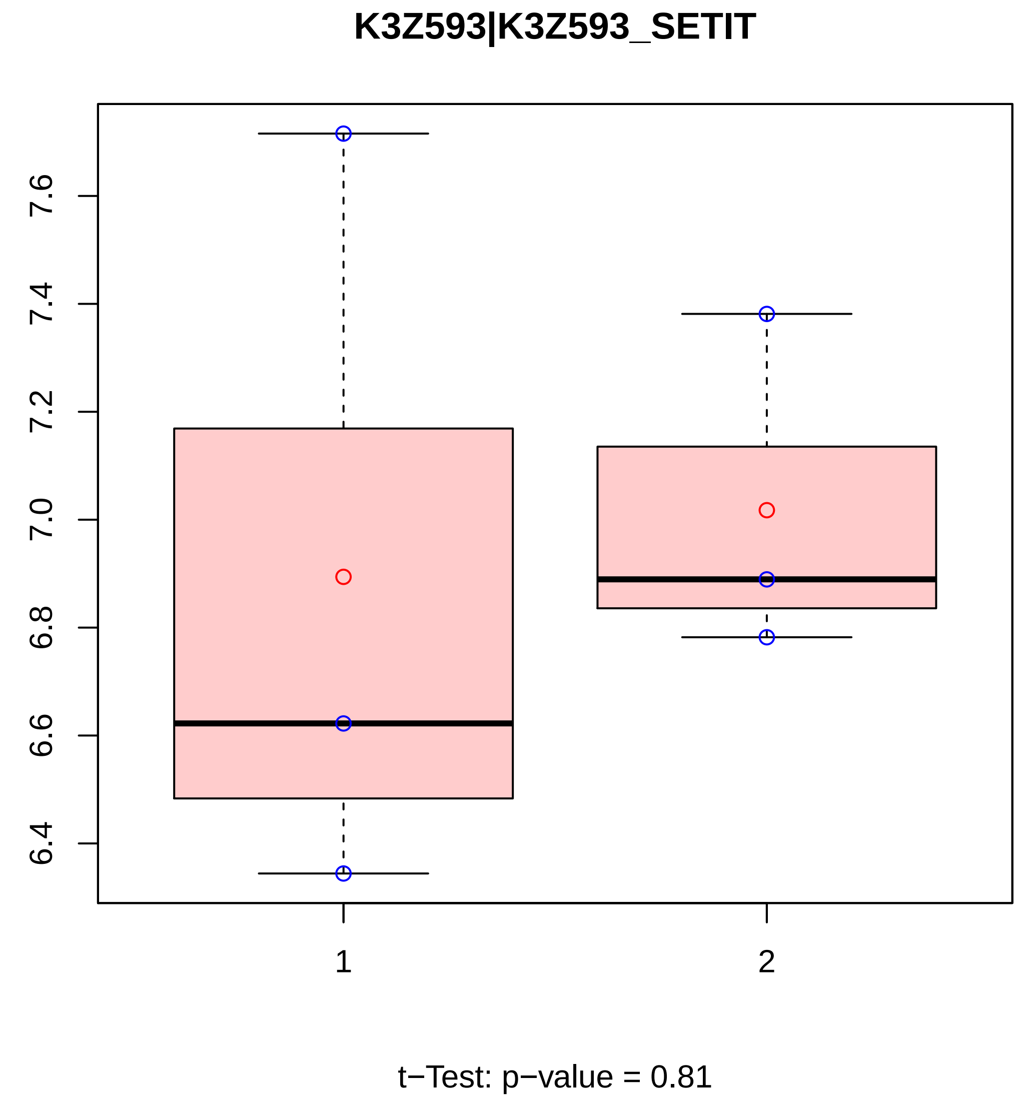
K3Z593|K3Z593_SETIT
7.6
7.4
7.2
7.0
6.8
6.6
6.4
1
2
t−Test: p−value = 0.81

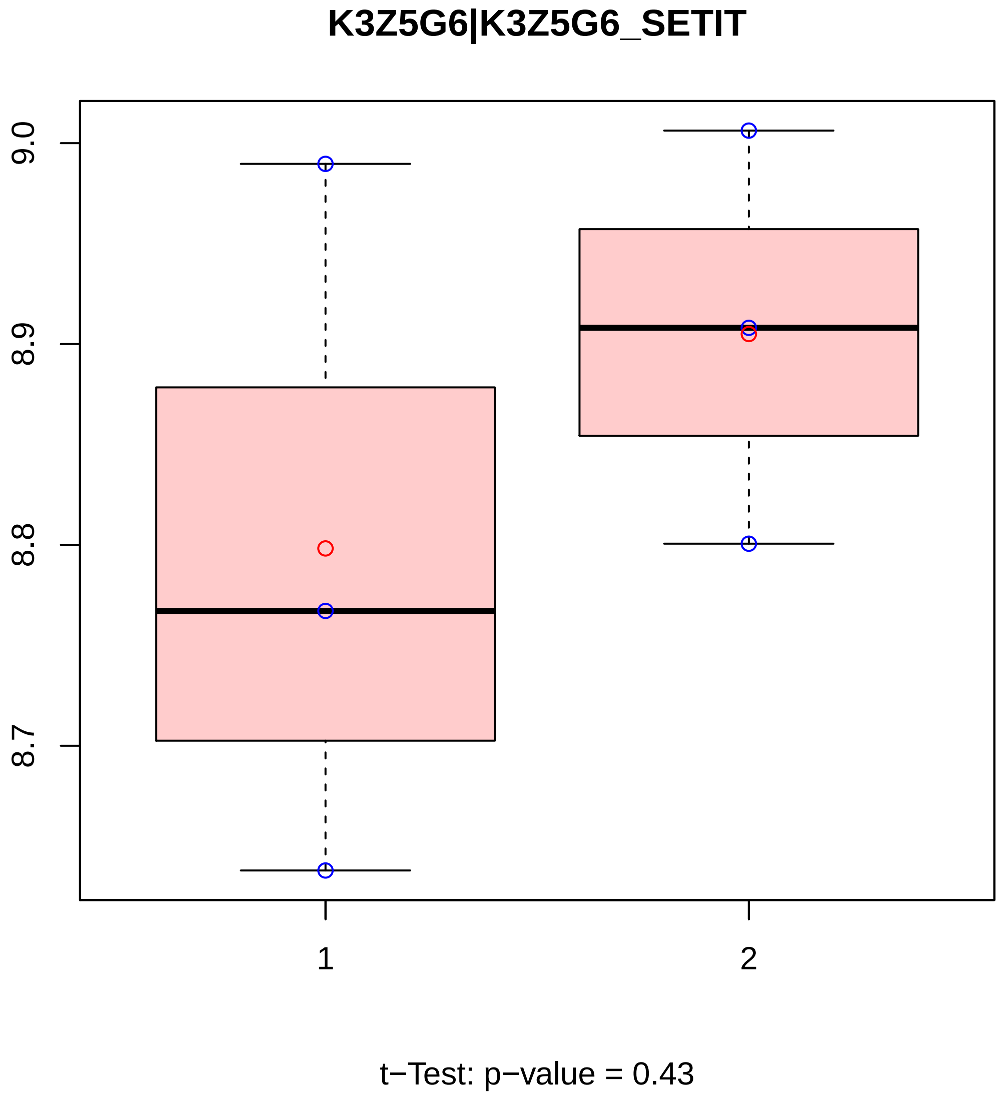
K3Z5G6|K3Z5G6_SETIT
9.0
8.9
8.8
8.7
1
2
t−Test: p−value = 0.43

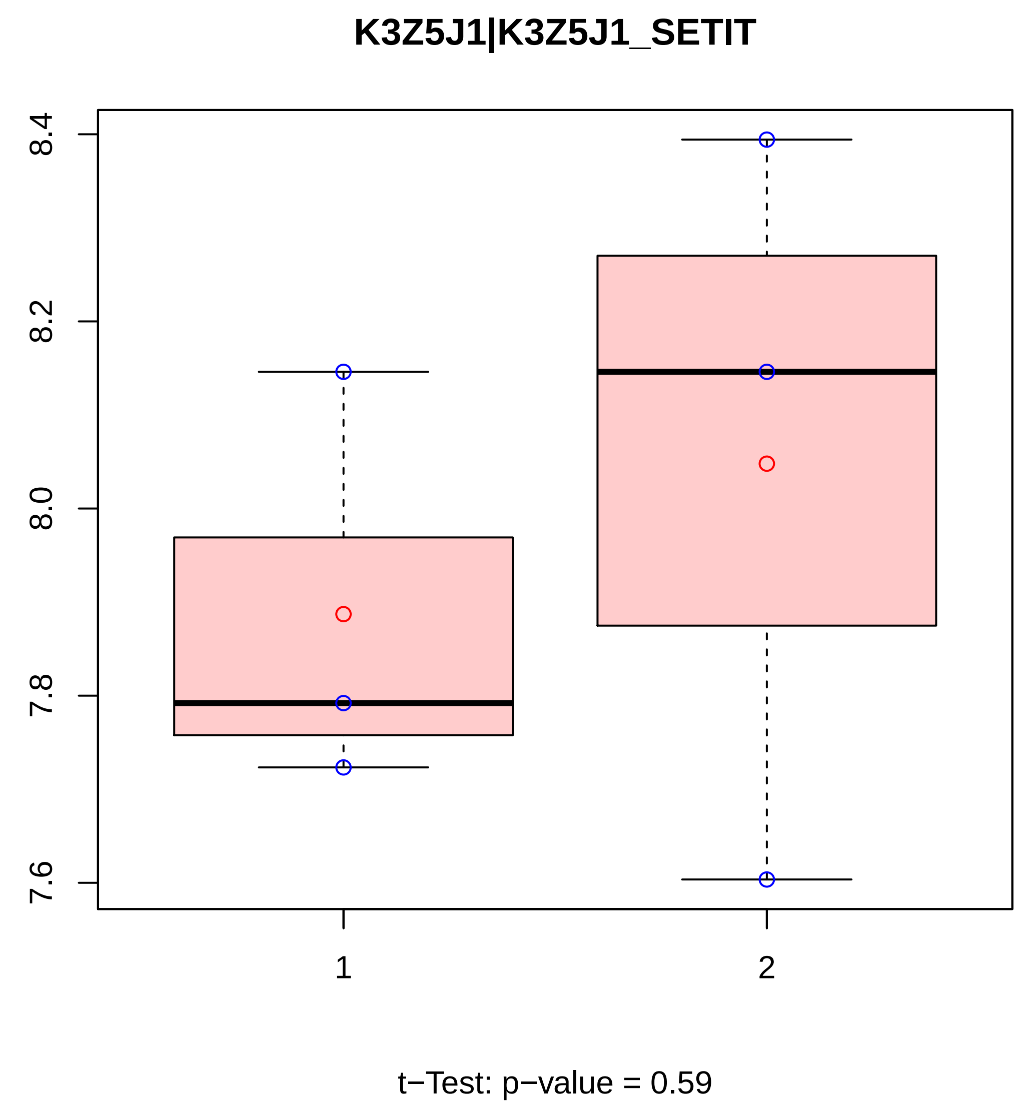
K3Z5J1|K3Z5J1_SETIT
8.4
8.2
8.0
7.8
7.6
1
2
t−Test: p−value = 0.59

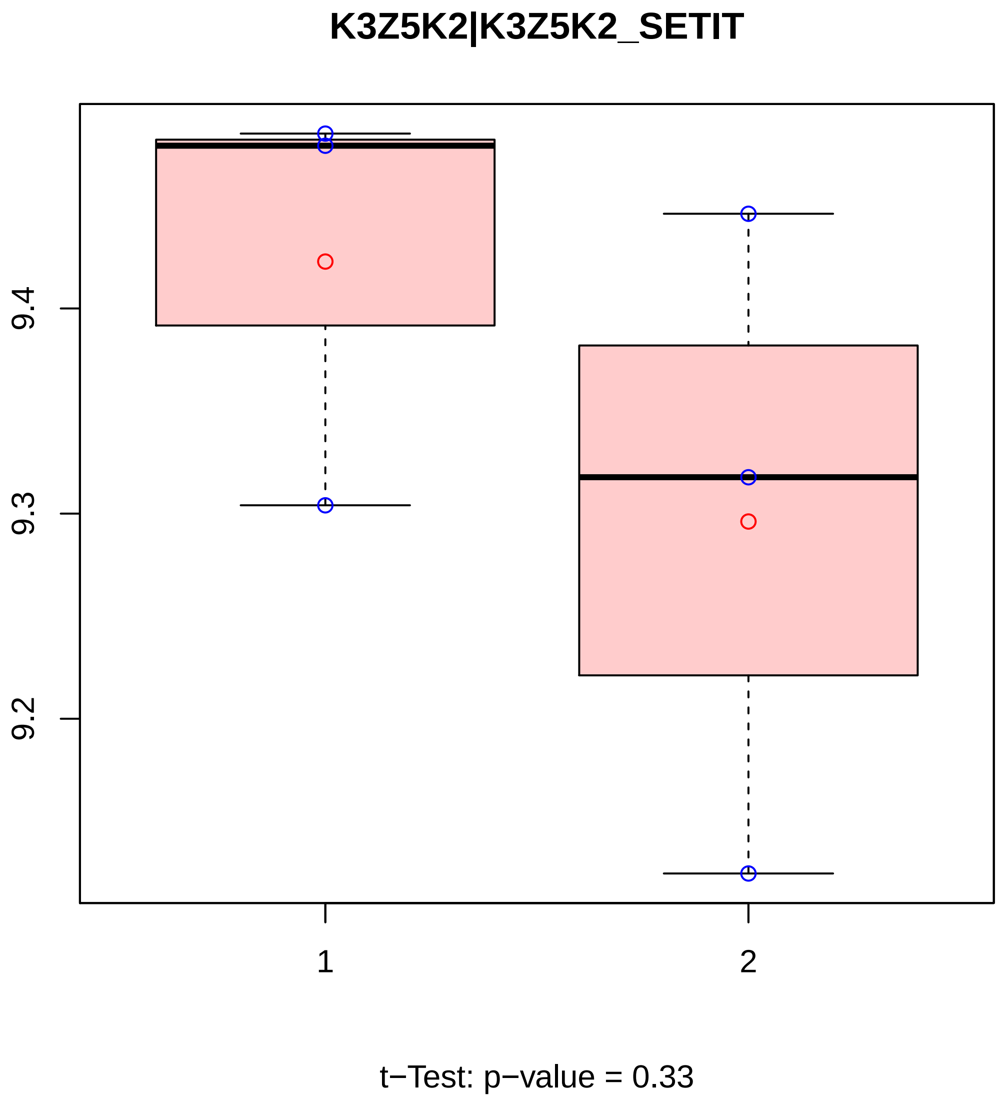

K3Z5K2|K3Z5K2_SETIT
9.2
9.3
9.4
1
2
t−Test: p−value = 0.33

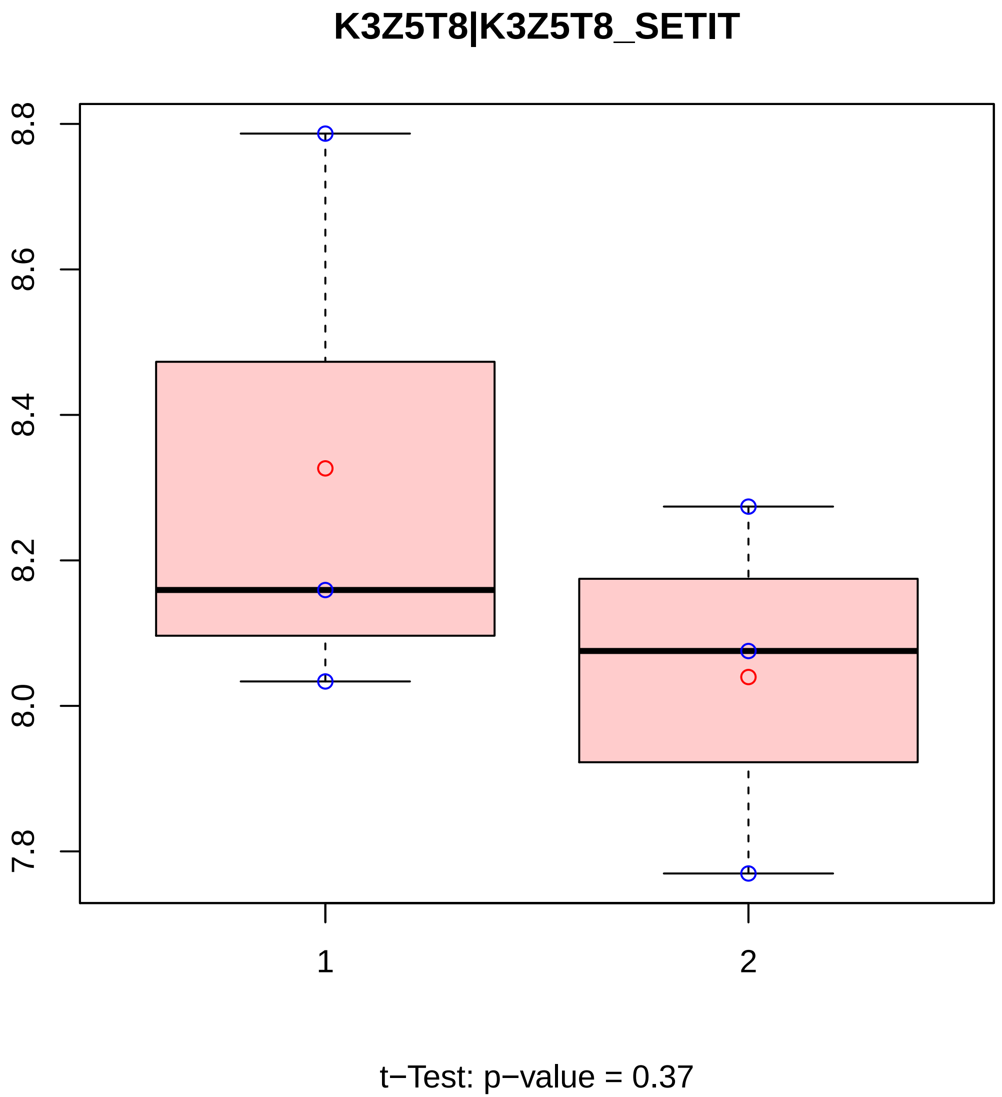
K3Z5T8|K3Z5T8_SETIT
8.8
8.6
8.4
8.2
8.0
7.8
1
2
t−Test: p−value = 0.37

K3Z5U9|K3Z5U9_SETIT

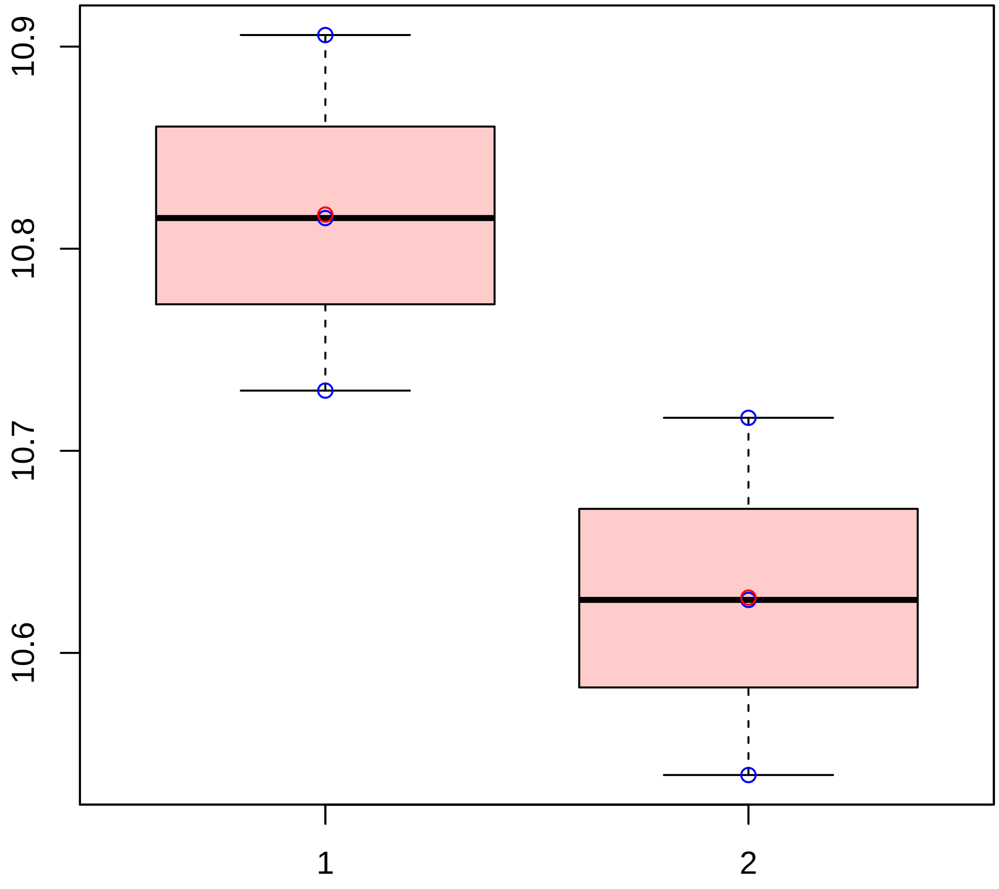

t−Test: p−value = 0.06

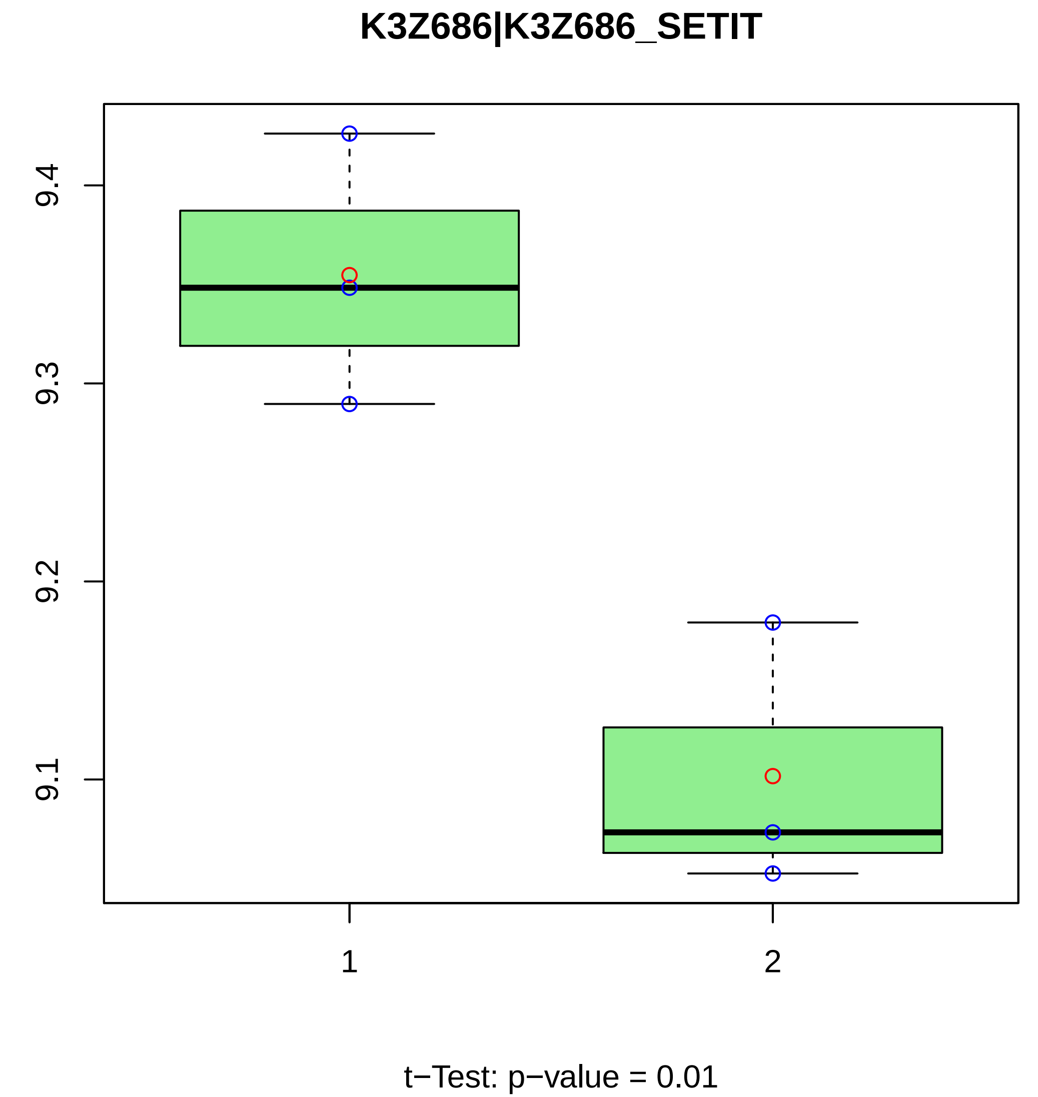
K3Z686|K3Z686_SETIT
9.1
9.2
9.3
9.4
1
2
t−Test: p−value = 0.01

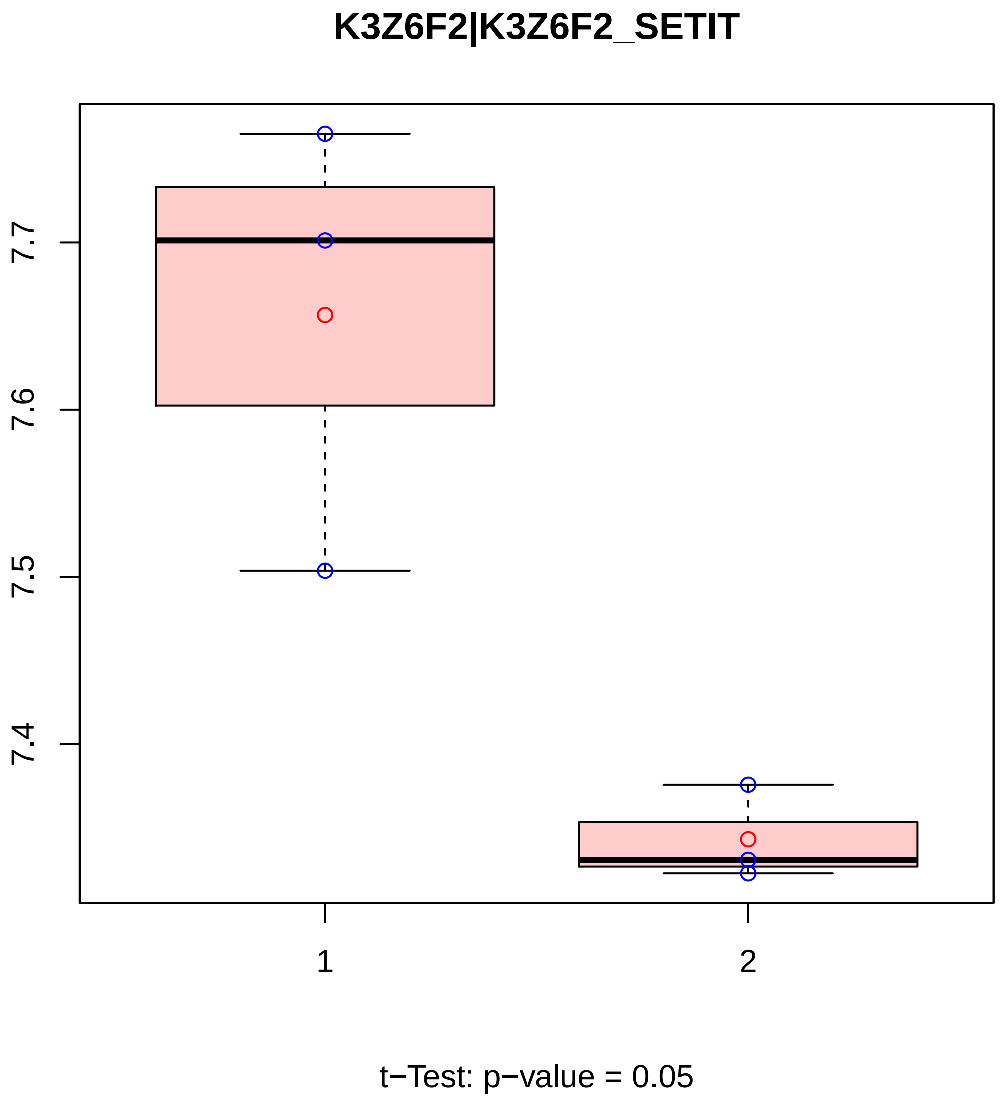
K3Z6F2|K3Z6F2_SETIT
7.4
7.5
7.6
7.7
1
2
t−Test: p−value = 0.05

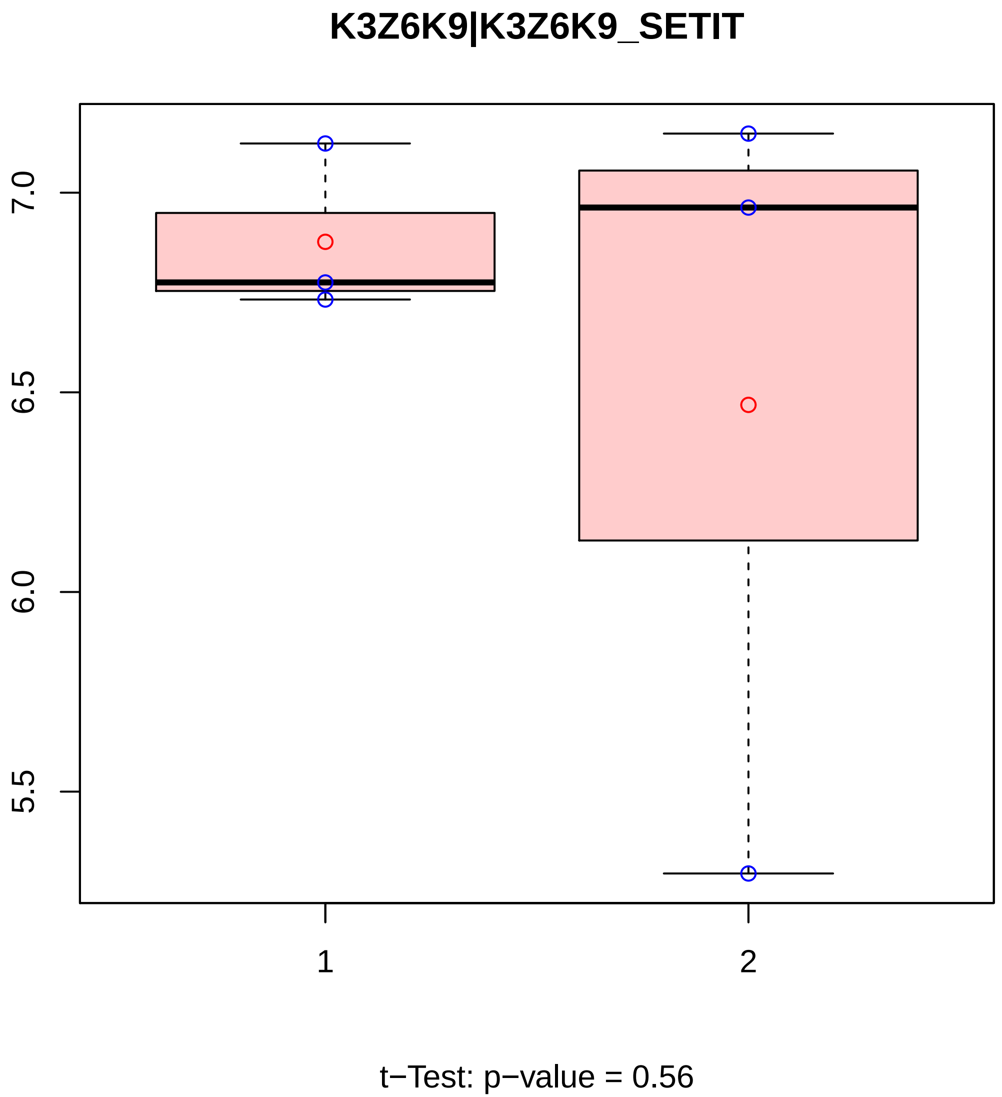
K3Z6K9|K3Z6K9_SETIT
7.0
6.5
6.0
5.5
1
2
t−Test: p−value = 0.56

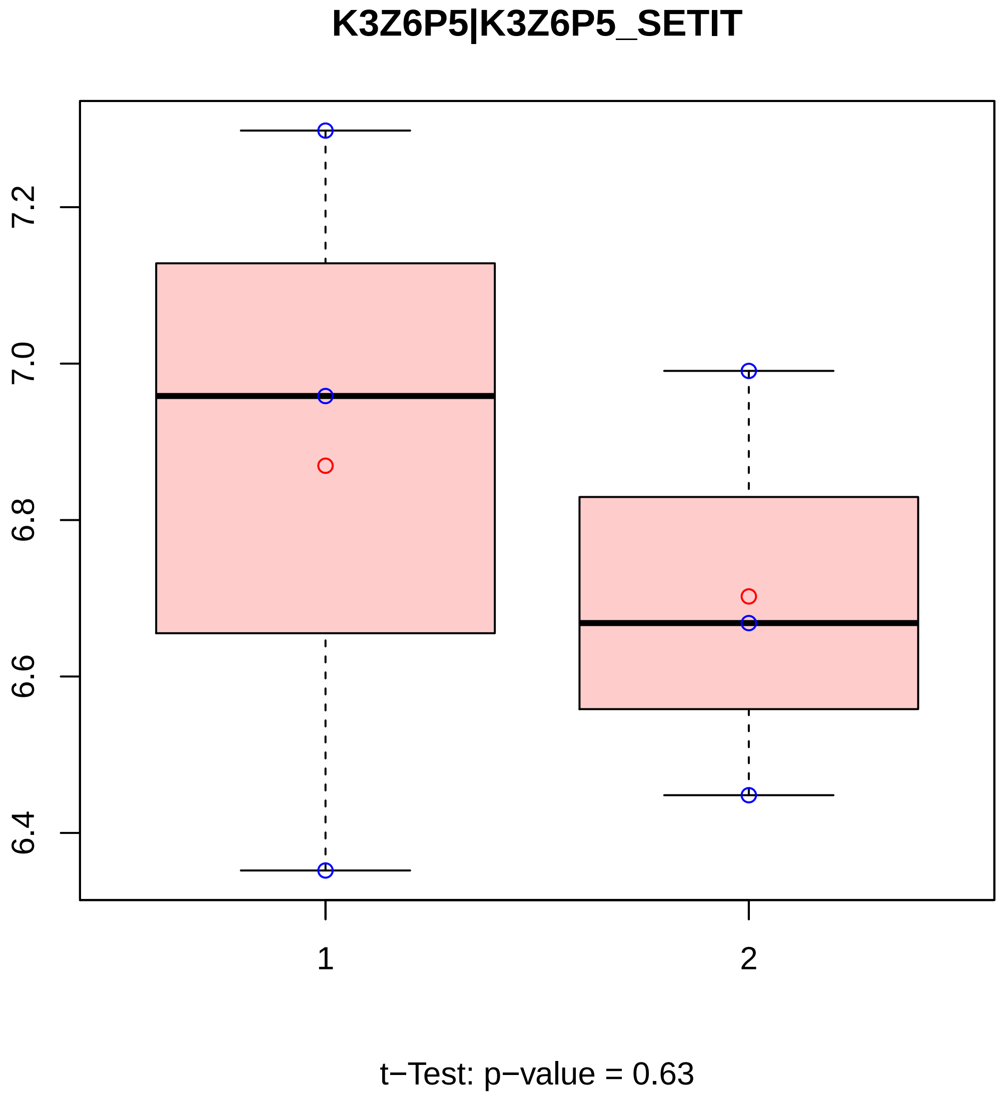

K3Z6P5|K3Z6P5_SETIT
7.2
7.0
6.8
6.6
6.4
1
2
t−Test: p−value = 0.63

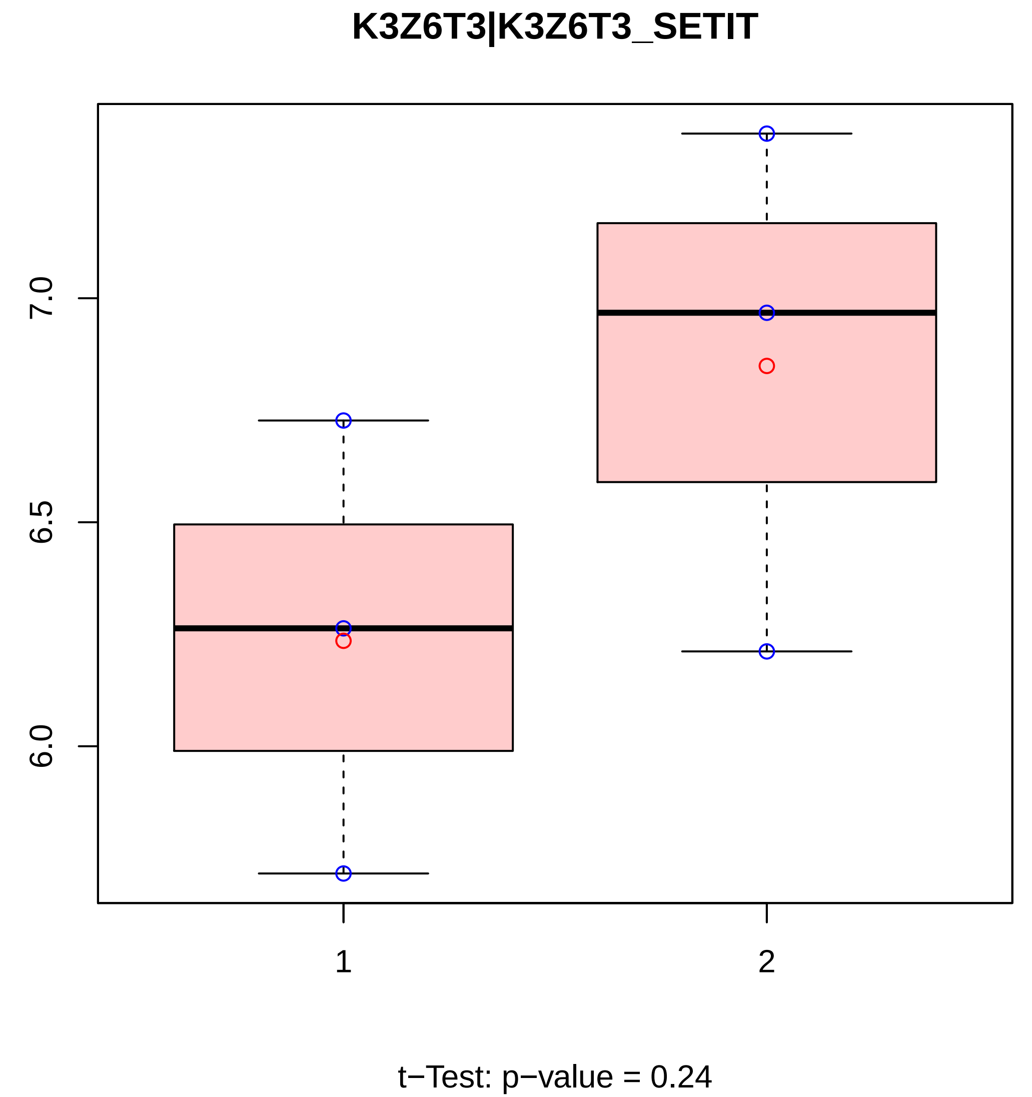
K3Z6T3|K3Z6T3_SETIT
7.0
6.5
6.0
1
2
t−Test: p−value = 0.24

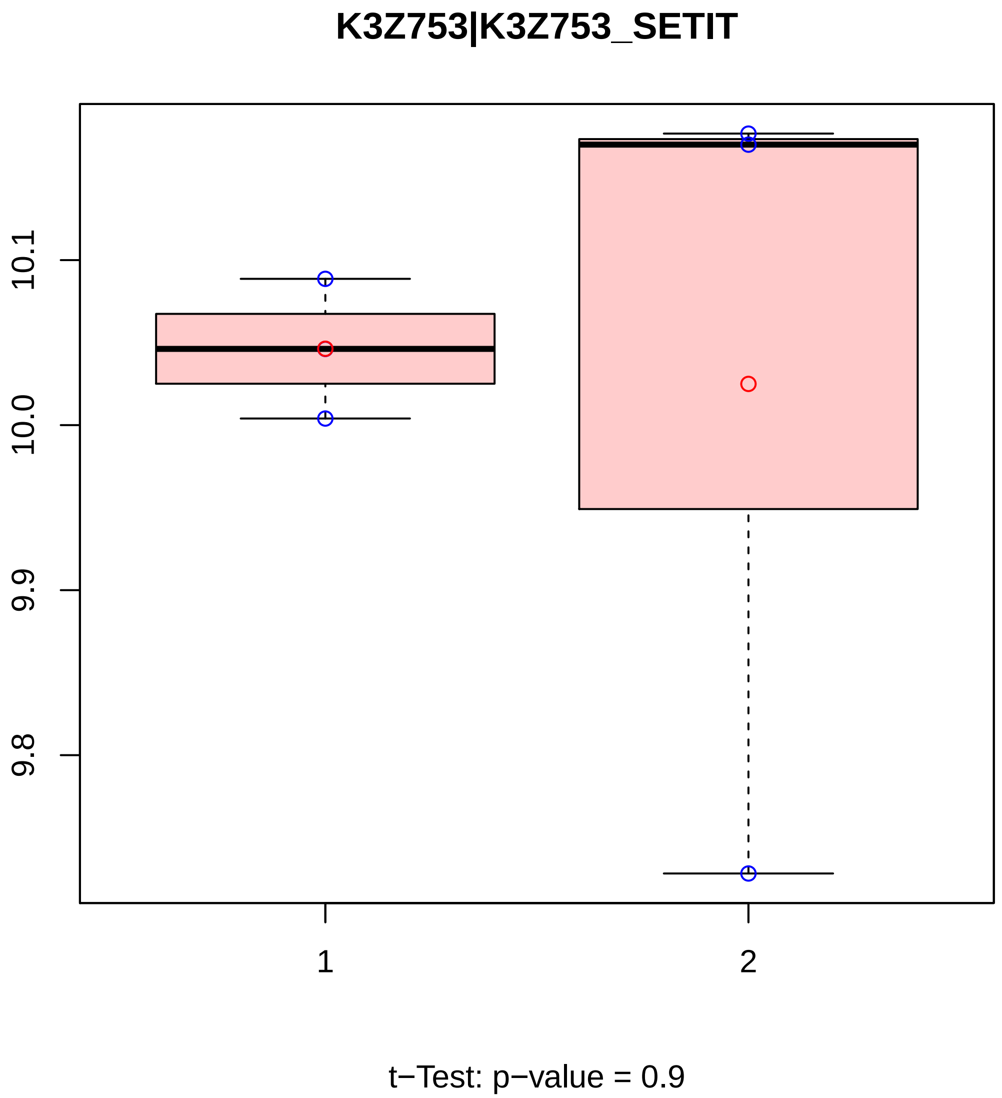

K3Z753|K3Z753_SETIT
10.1
10.0
9.9
9.8
1
2
t−Test: p−value = 0.9

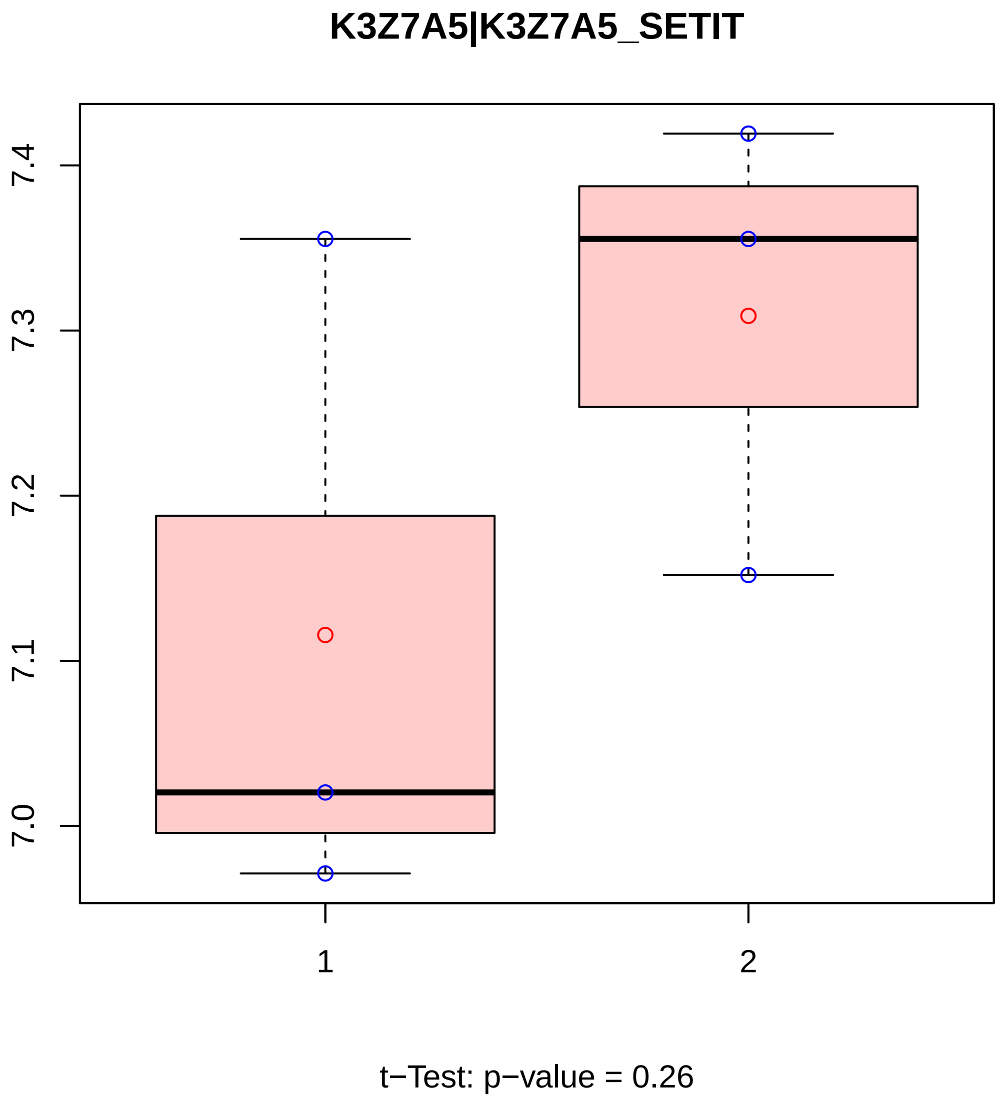
K3Z7A5|K3Z7A5_SETIT
7.0
7.1
7.2
7.3
7.4
1
2
t−Test: p−value = 0.26

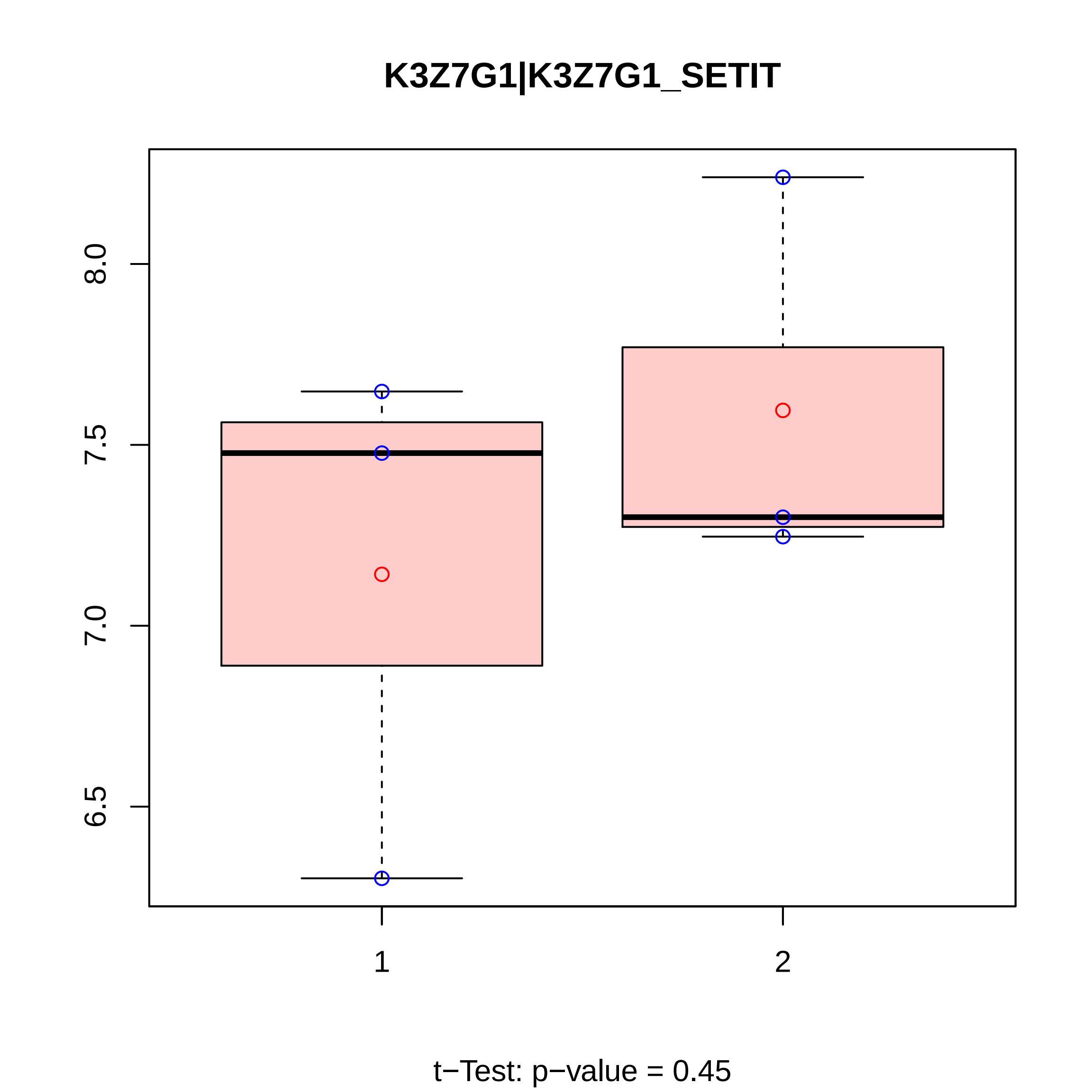
K3Z7G1|K3Z7G1_SETIT
8.0
7.5
7.0
6.5
1
2
t−Test: p−value = 0.45

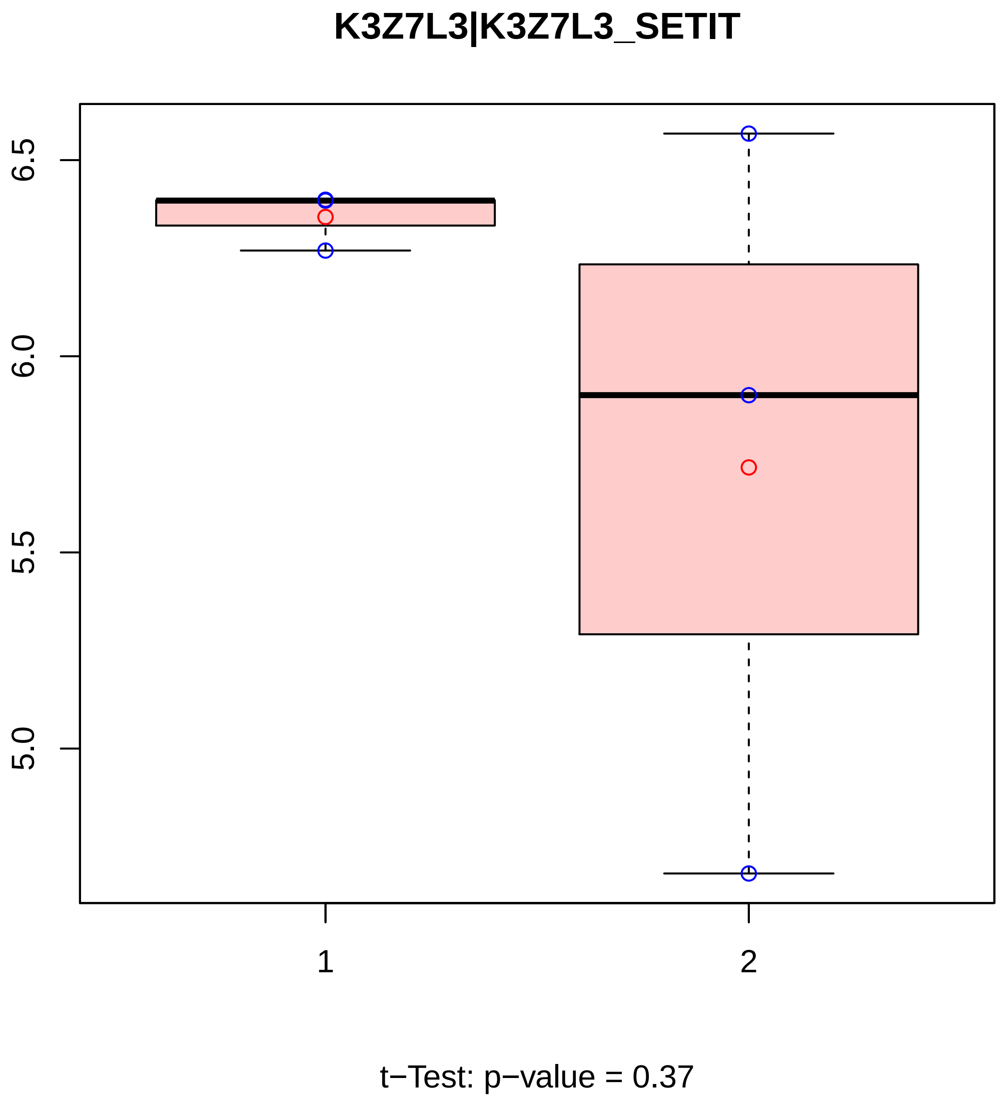
K3Z7L3|K3Z7L3_SETIT
6.5
6.0
5.5
5.0
1
2
t−Test: p−value = 0.37

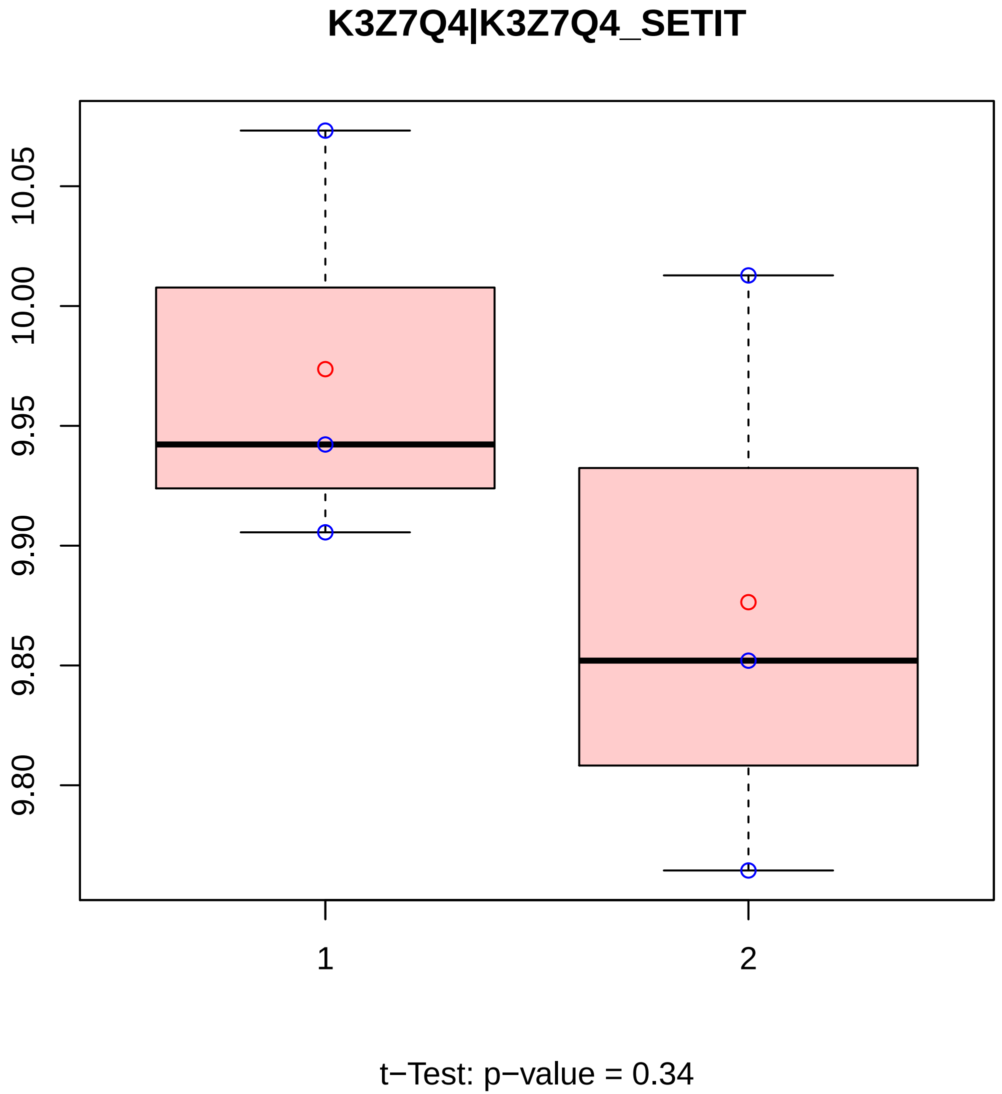
K3Z7Q4|K3Z7Q4_SETIT
10.05
10.00
9.95
9.90
9.85
9.80
1
2
t−Test: p−value = 0.34

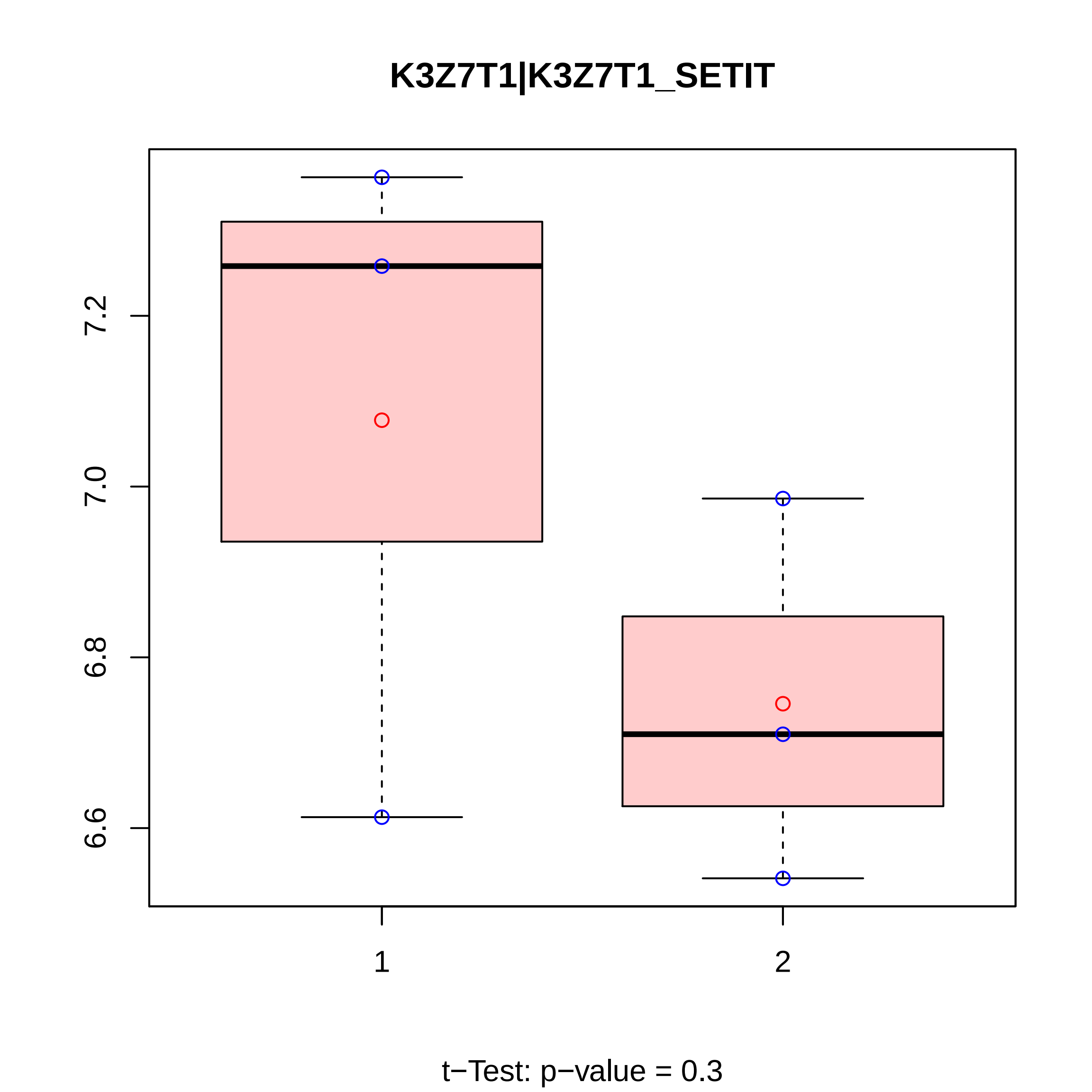
K3Z7T1|K3Z7T1_SETIT
7.2
7.0
6.8
6.6
1
2
t−Test: p−value = 0.3

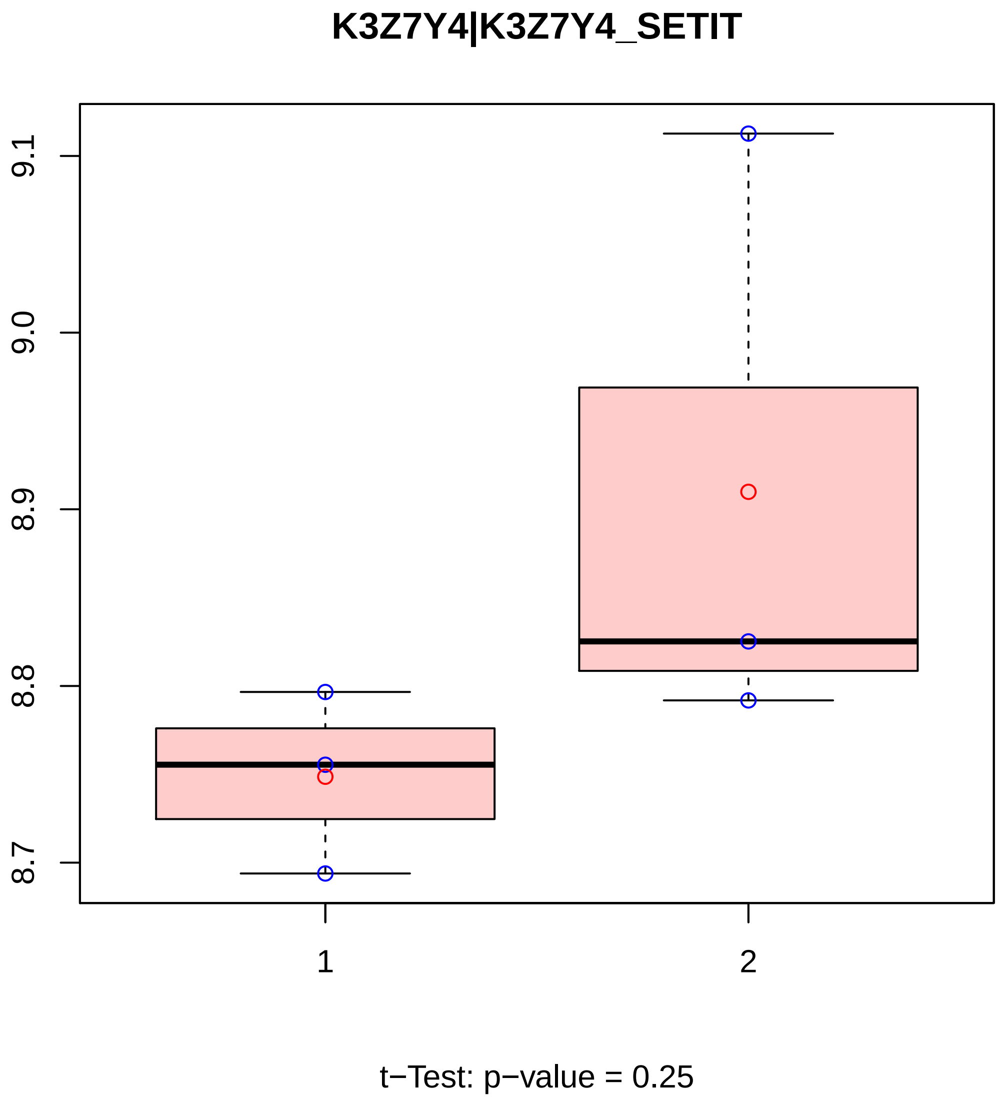

K3Z7Y4|K3Z7Y4_SETIT
9.1
9.0
8.9
8.8
8.7
1
2
t−Test: p−value = 0.25

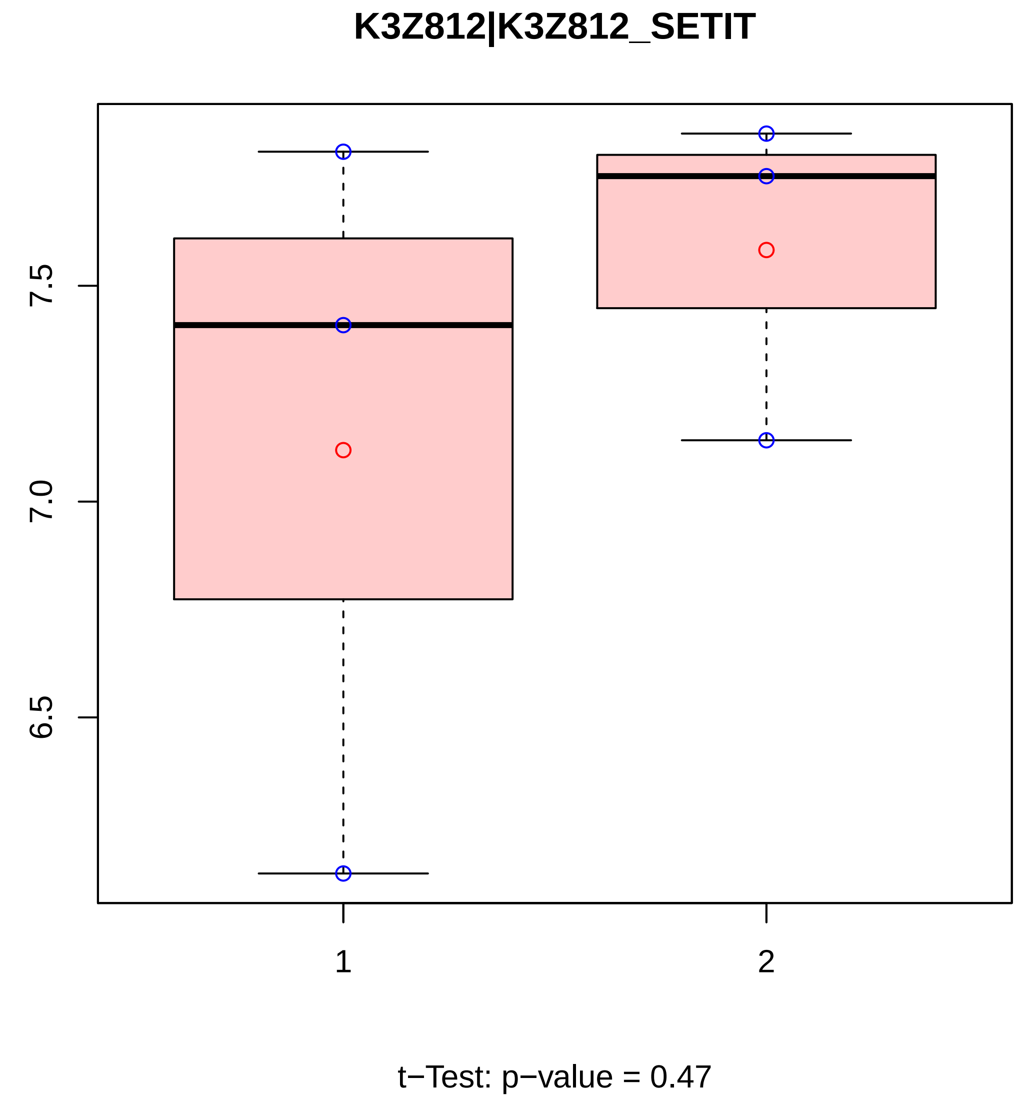
K3Z812|K3Z812_SETIT
7.5
7.0
6.5
1
2
t−Test: p−value = 0.47

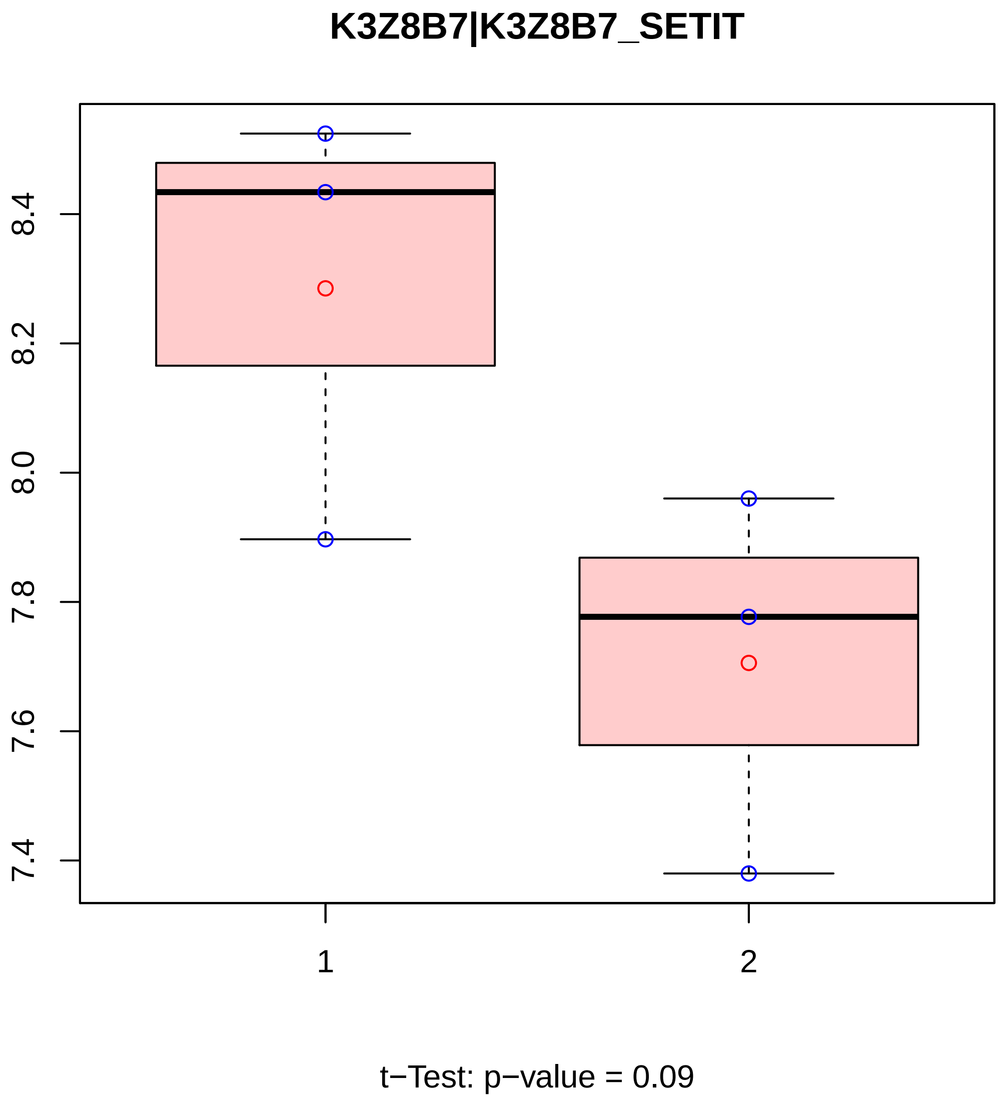
K3Z8B7|K3Z8B7_SETIT
8.4
8.2
8.0
7.8
7.6
7.4
1
2
t−Test: p−value = 0.09

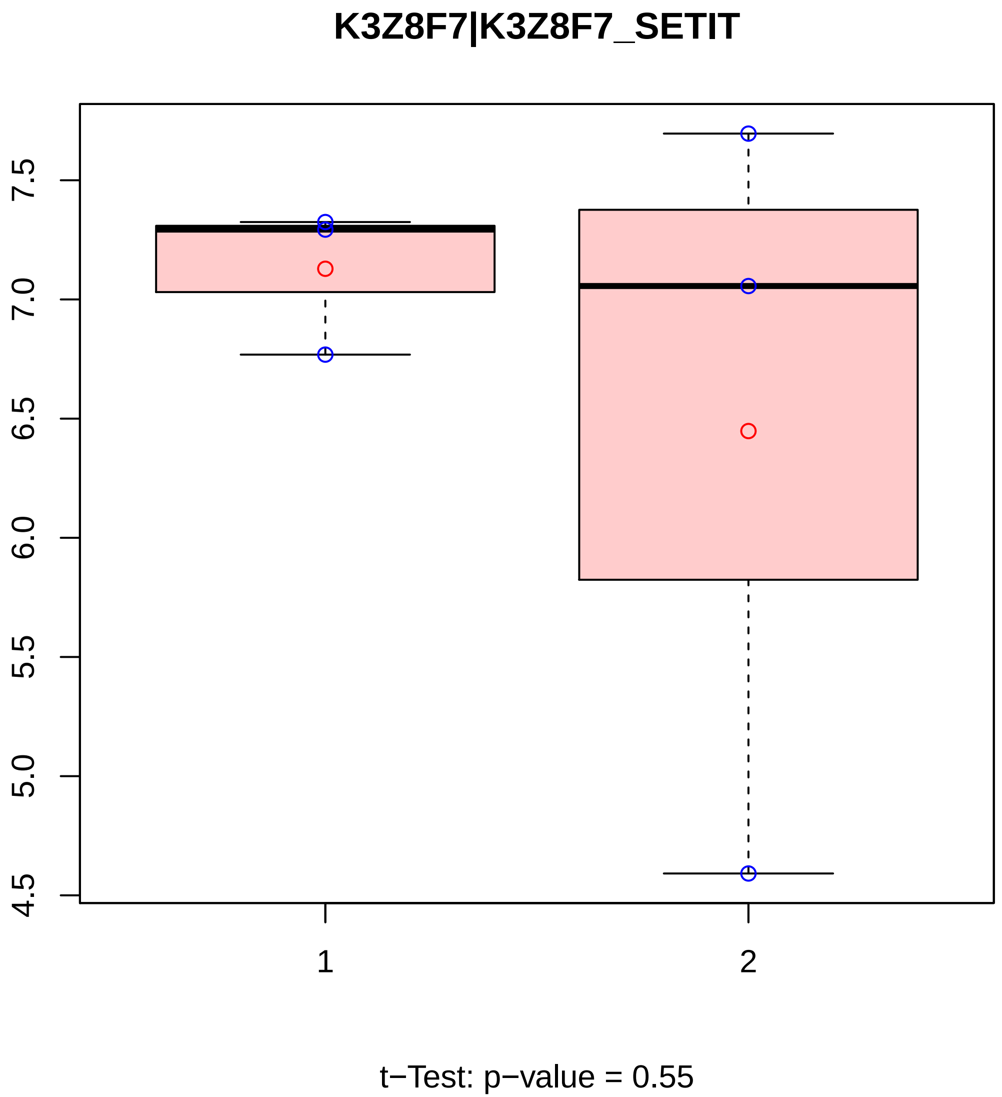
K3Z8F7|K3Z8F7_SETIT
7.5
7.0
6.5
6.0
5.5
5.0
4.5
1
2
t−Test: p−value = 0.55

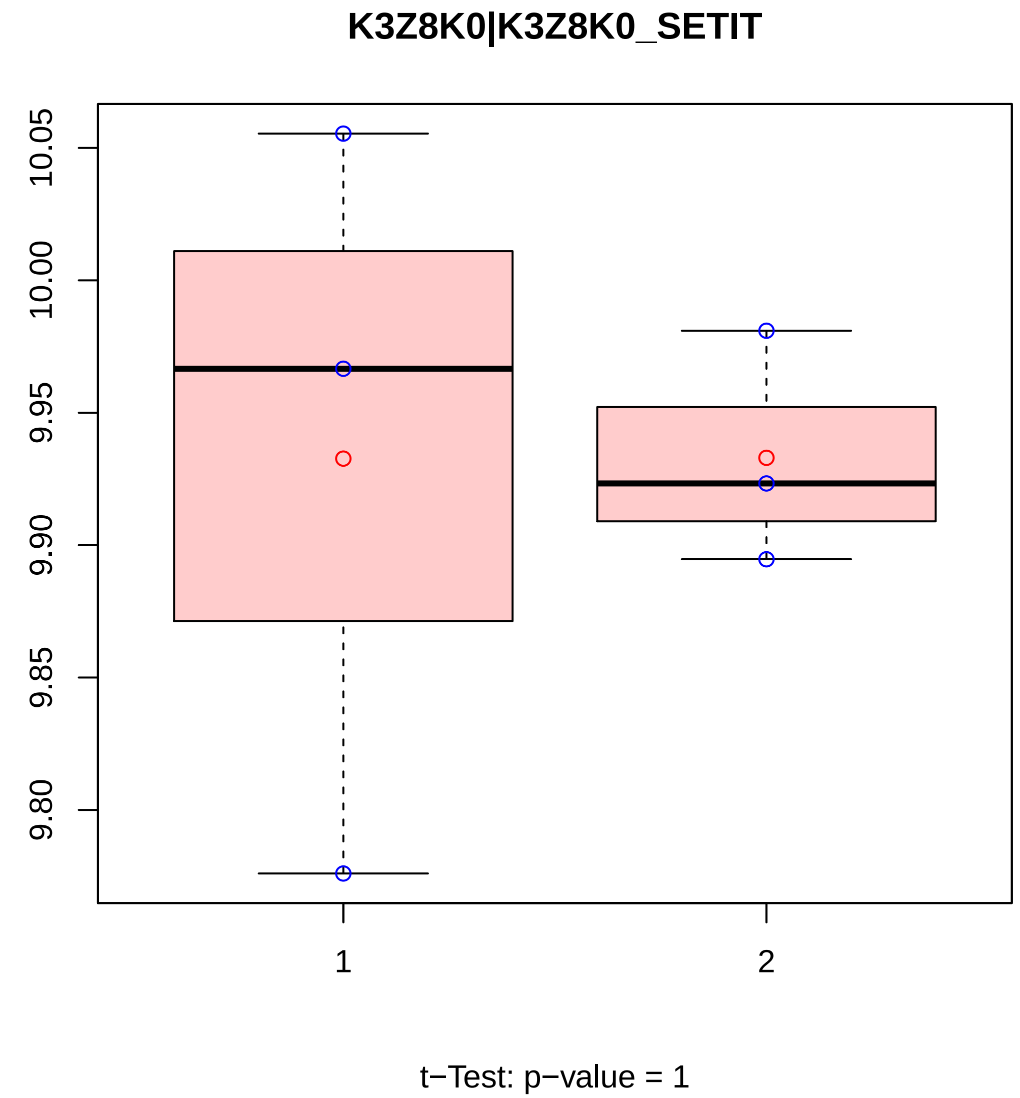
K3Z8K0|K3Z8K0_SETIT
10.05
10.00
9.95
9.90
9.85
9.80
1
2
t−Test: p−value = 1

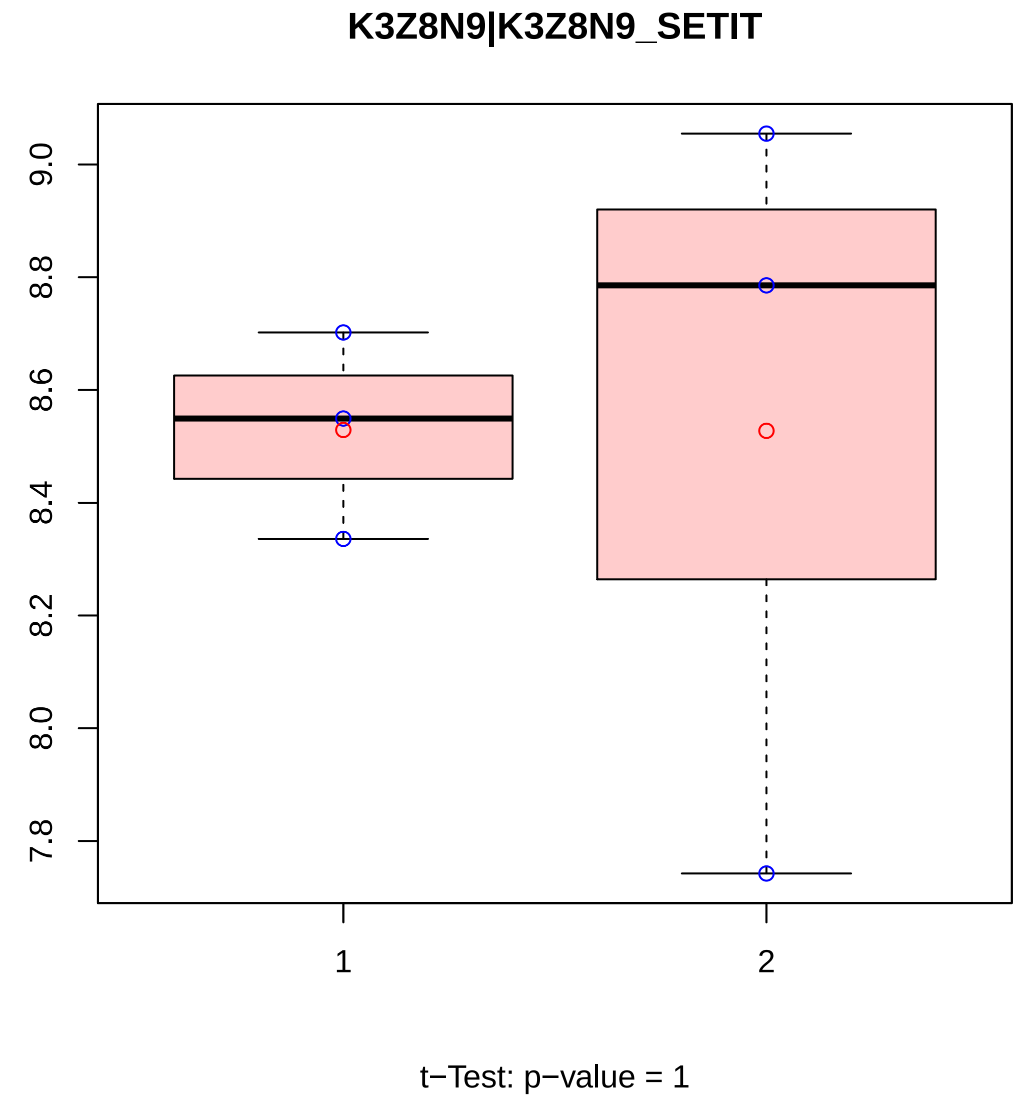
K3Z8N9|K3Z8N9_SETIT
9.0
8.8
8.6
8.4
8.2
8.0
7.8
1
2
t−Test: p−value = 1

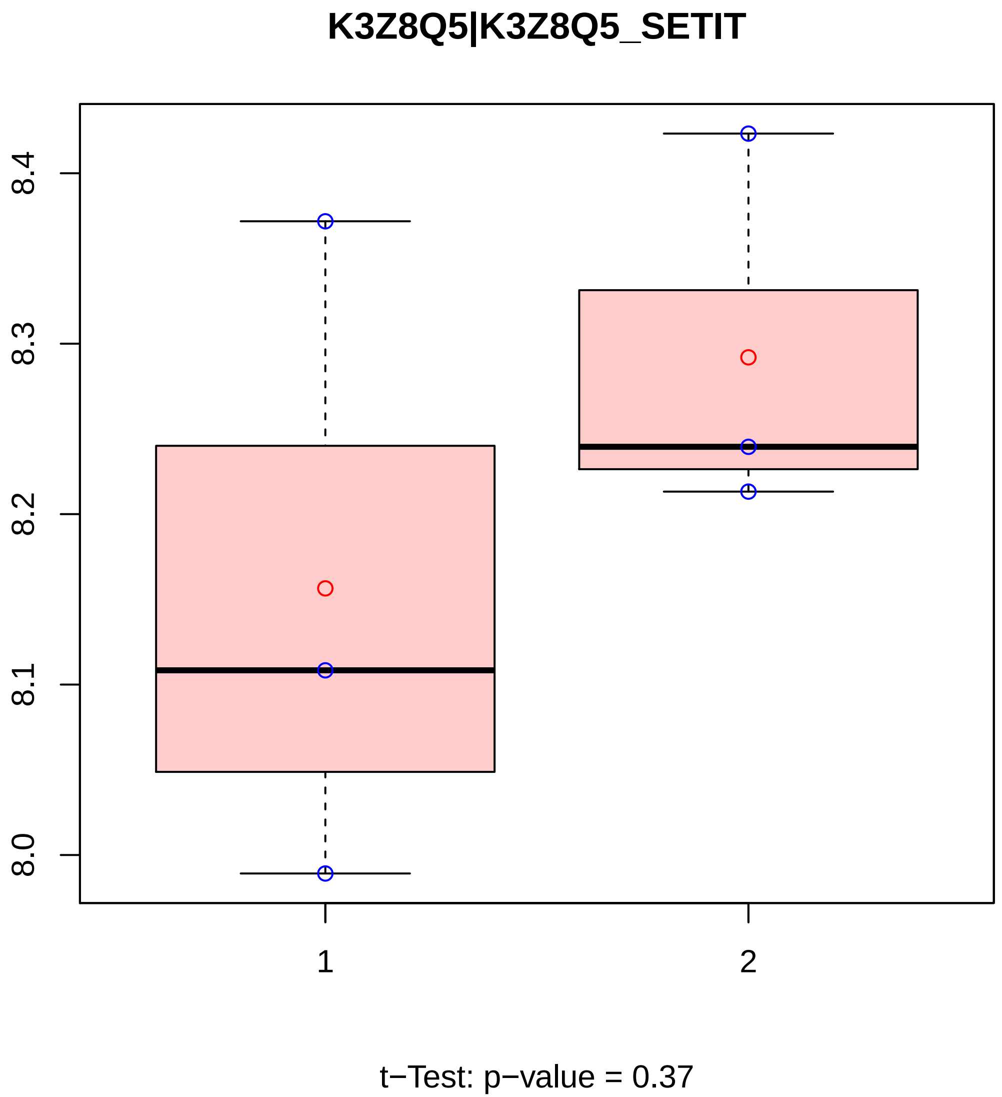
K3Z8Q5|K3Z8Q5_SETIT
8.0
8.1
8.2
8.3
8.4
1
2
t−Test: p−value = 0.37

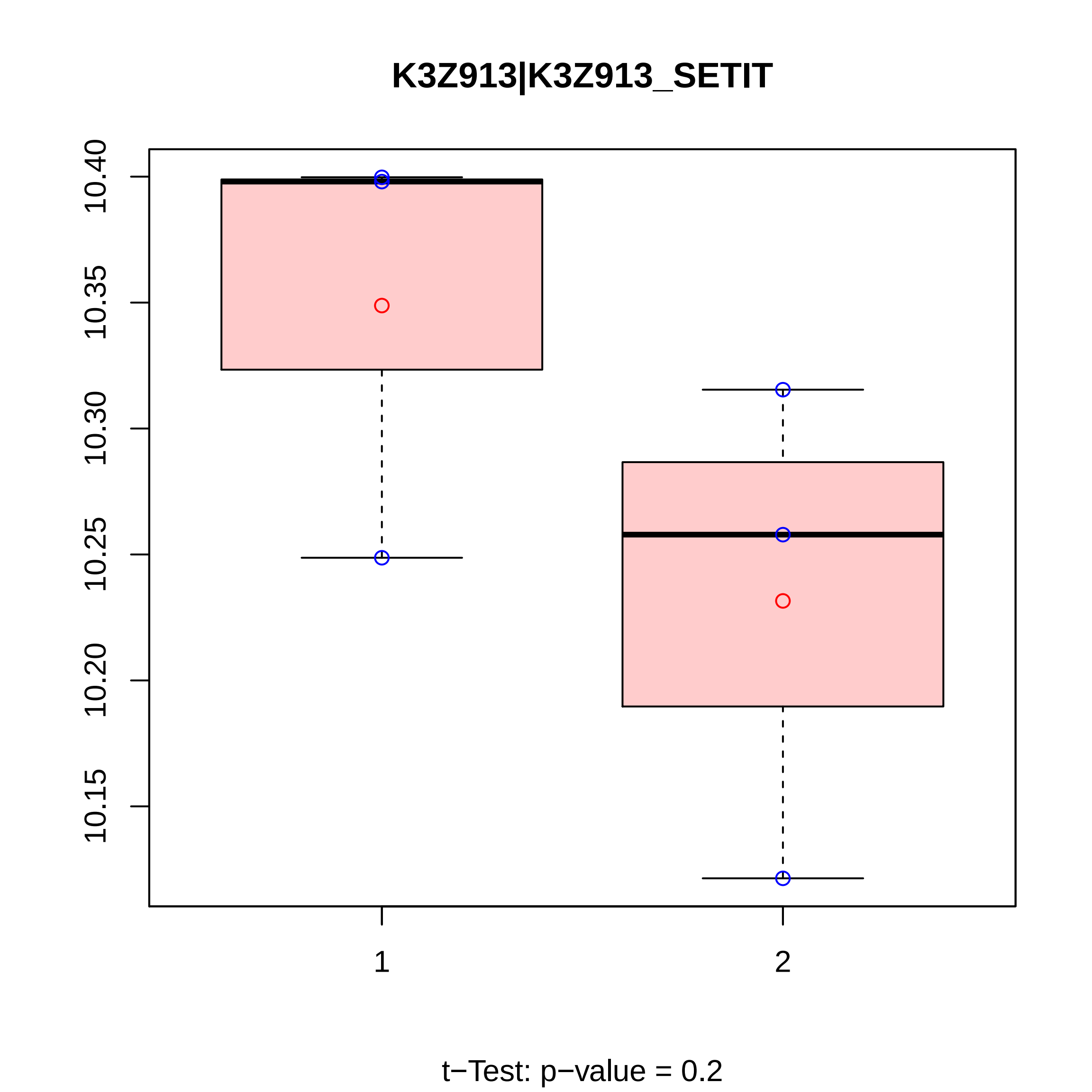
K3Z913|K3Z913_SETIT
10.40
10.35
10.30
10.25
10.20
10.15
1
2
t−Test: p−value = 0.2

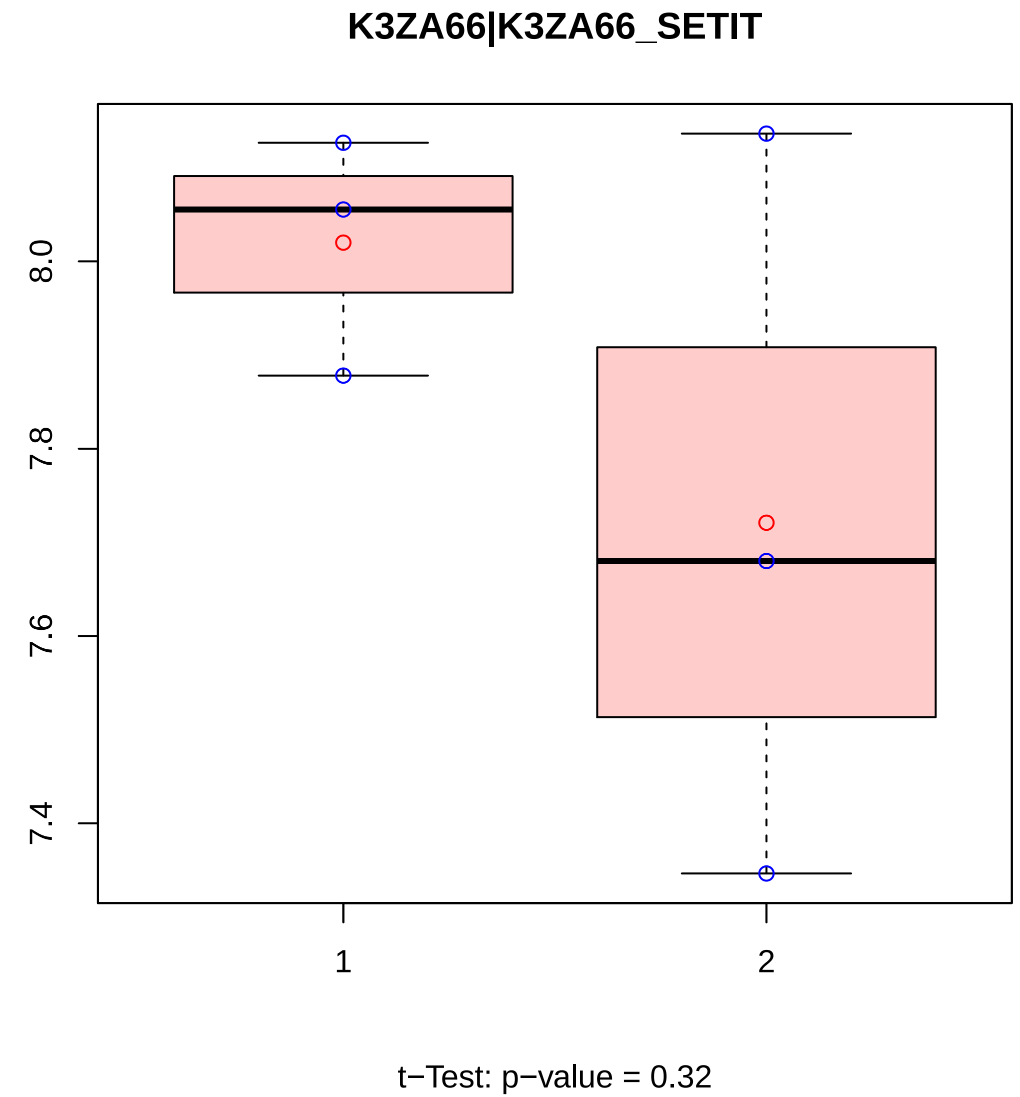
K3ZA66|K3ZA66_SETIT
8.0
7.8
7.6
7.4
1
2
t−Test: p−value = 0.32

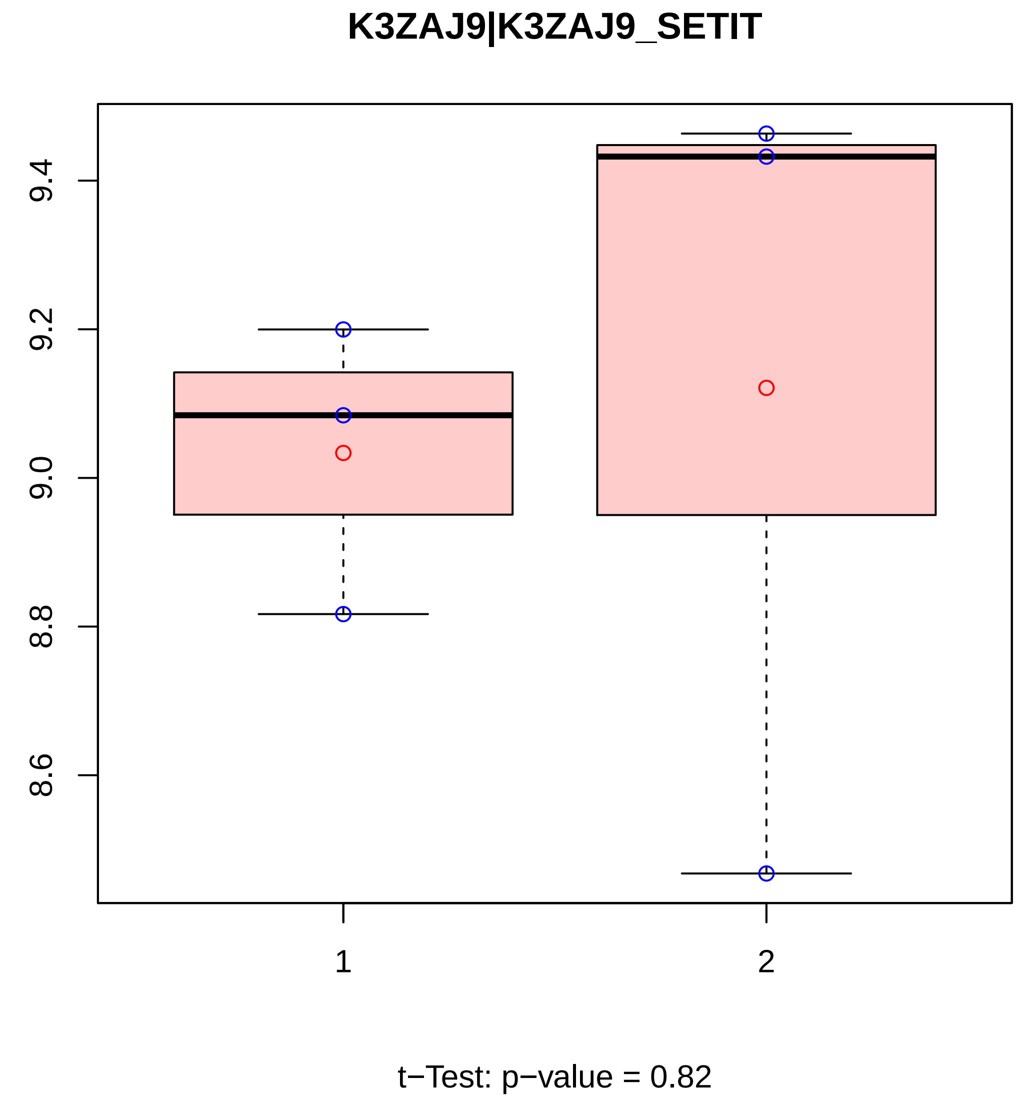
K3ZAJ9|K3ZAJ9_SETIT
9.4
9.2
9.0
8.8
8.6
1
2
t−Test: p−value = 0.82

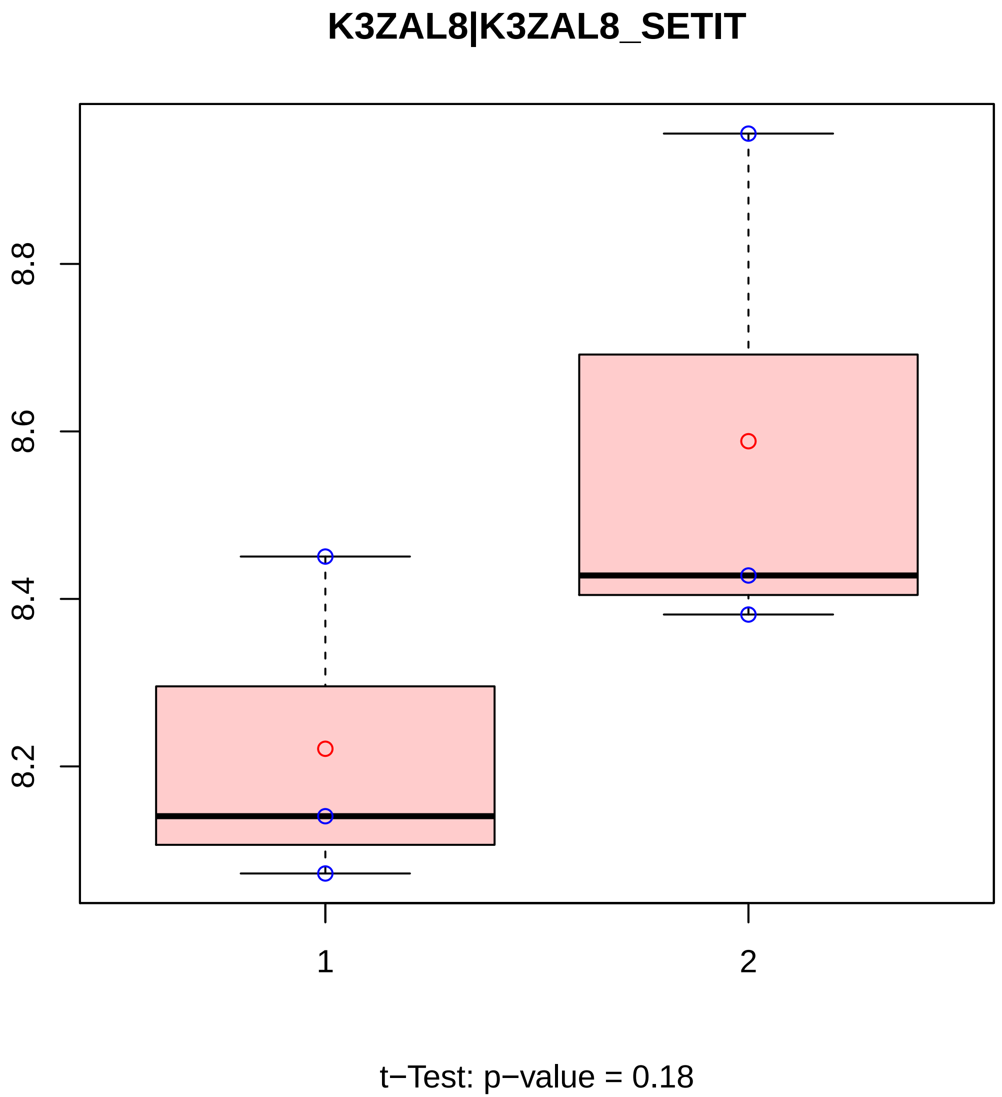
K3ZAL8|K3ZAL8_SETIT
8.2
8.4
8.6
8.8
1
2
t−Test: p−value = 0.18

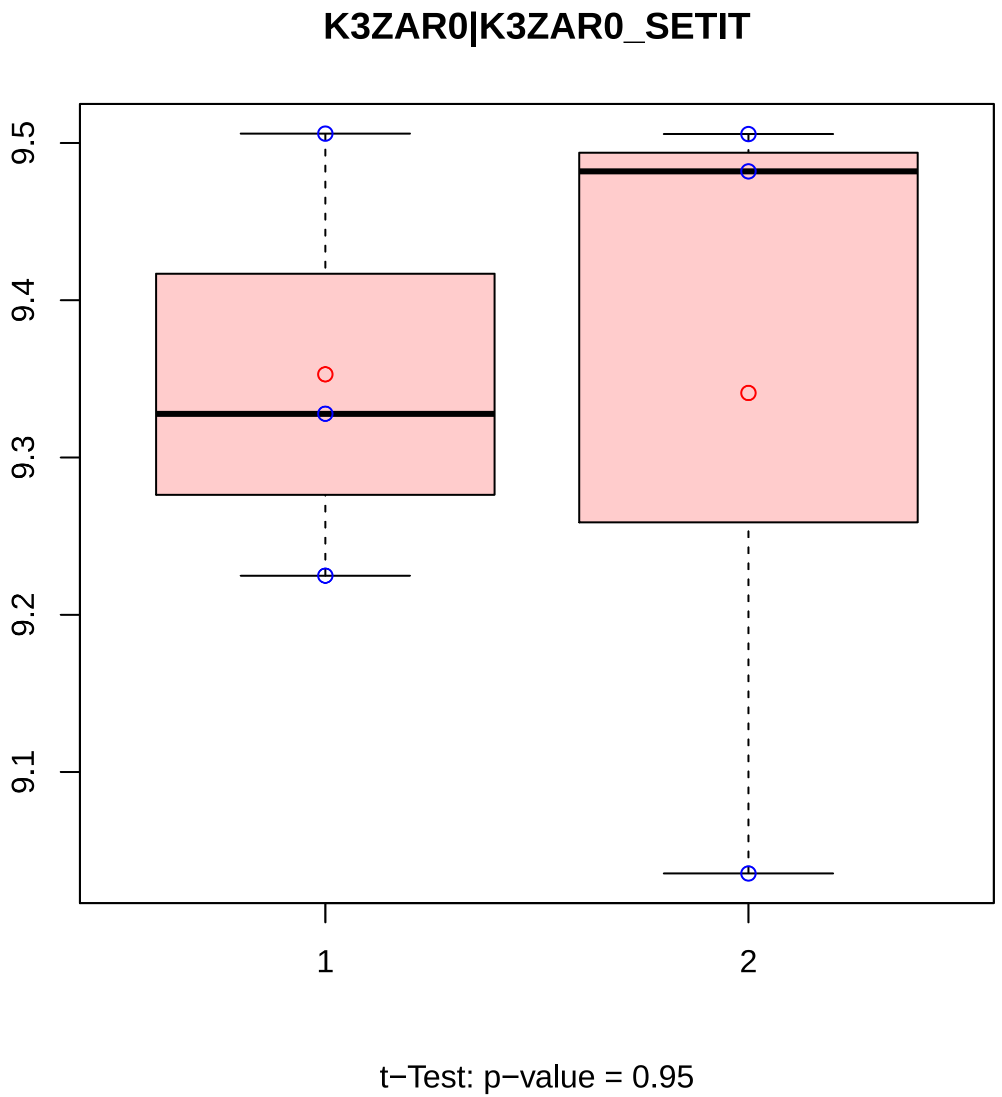
K3ZAR0|K3ZAR0_SETIT
9.1
9.2
9.3
9.4
9.5
1
2
t−Test: p−value = 0.95

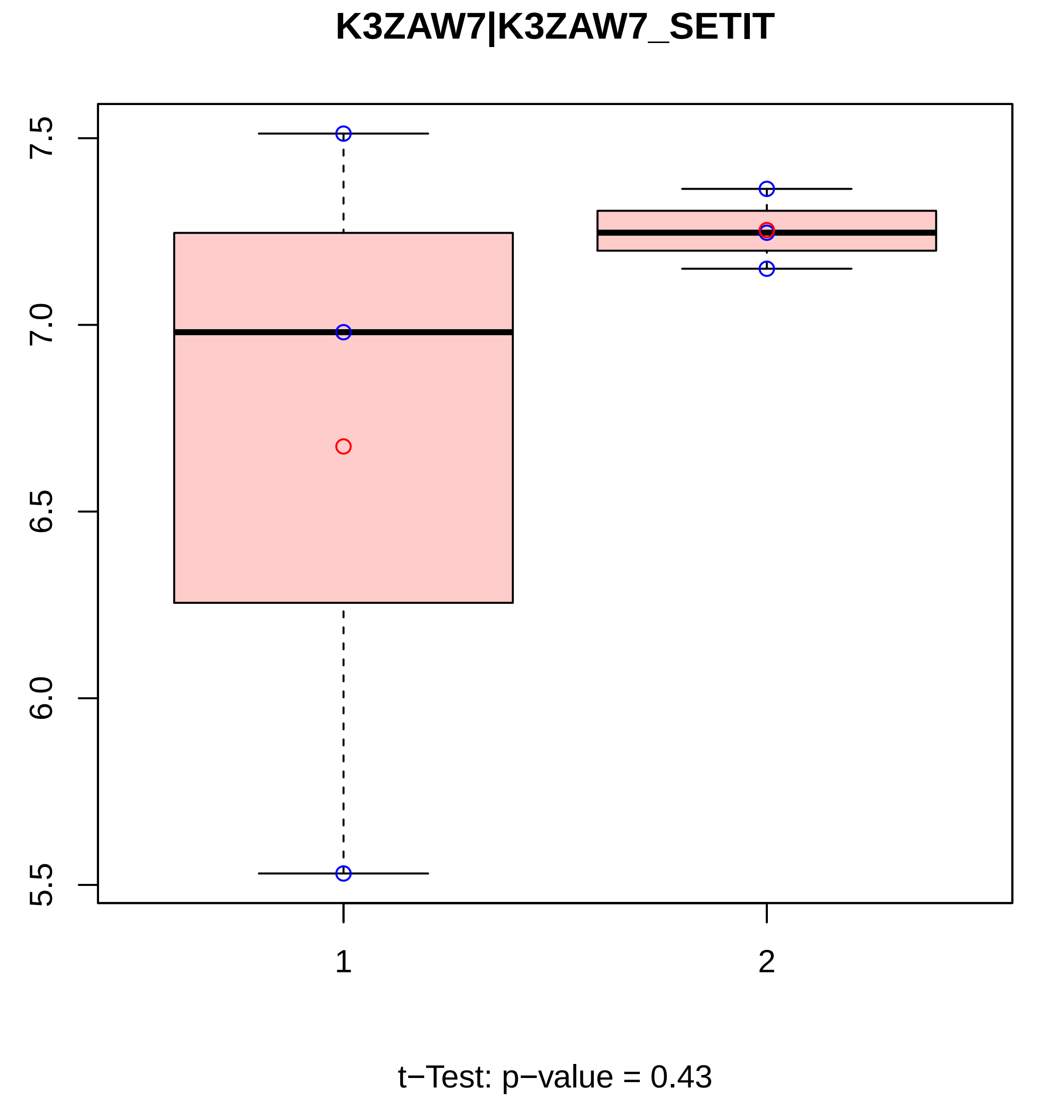

K3ZAW7|K3ZAW7_SETIT
7.5
7.0
6.5
6.0
5.5
1
2
t−Test: p−value = 0.43

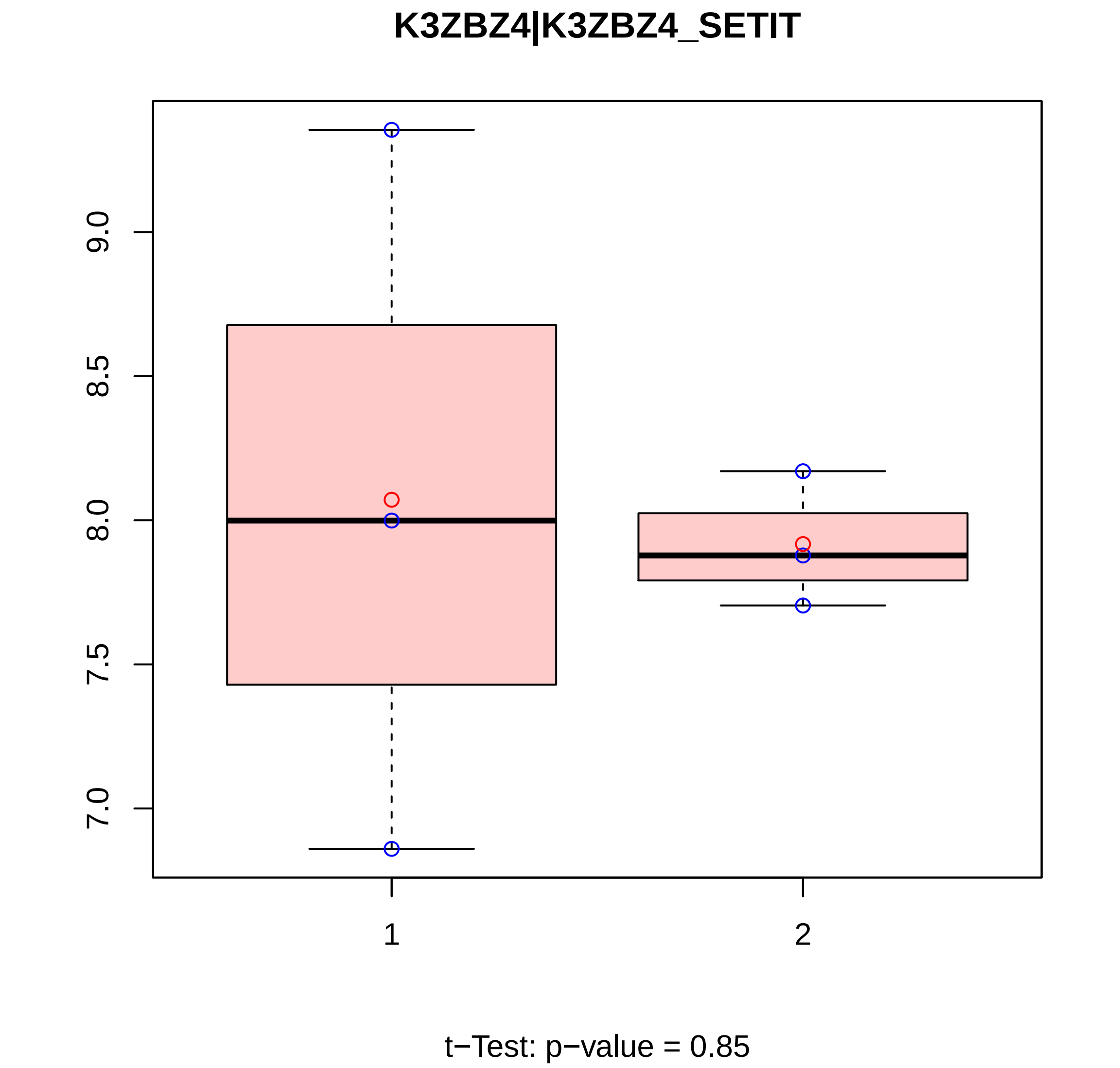
K3ZBZ4|K3ZBZ4_SETIT
9.0
8.5
8.0
7.5
7.0
1
2
t−Test: p−value = 0.85

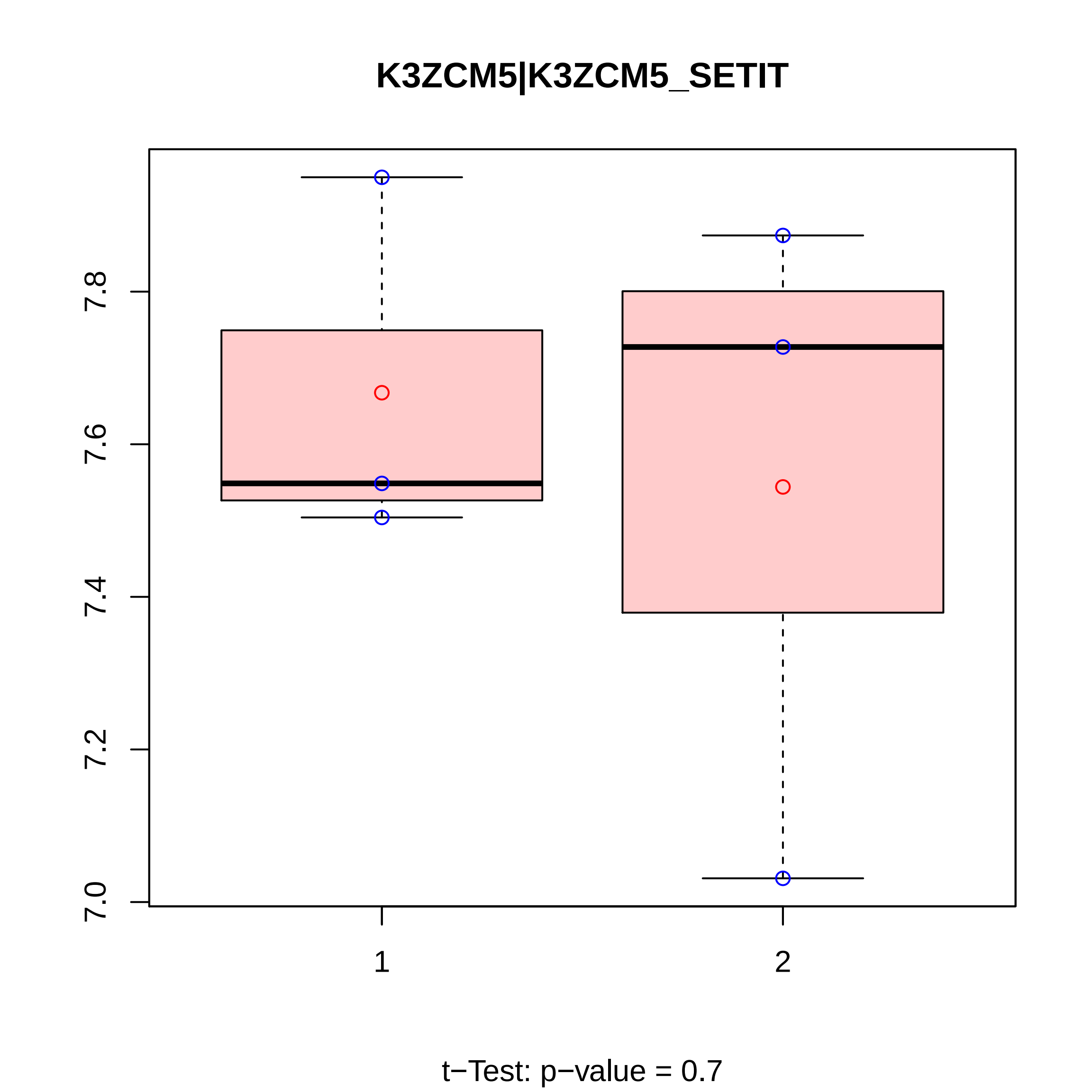

K3ZCM5|K3ZCM5_SETIT
7.0
7.2
7.4
7.6
7.8
1
2
t−Test: p−value = 0.7

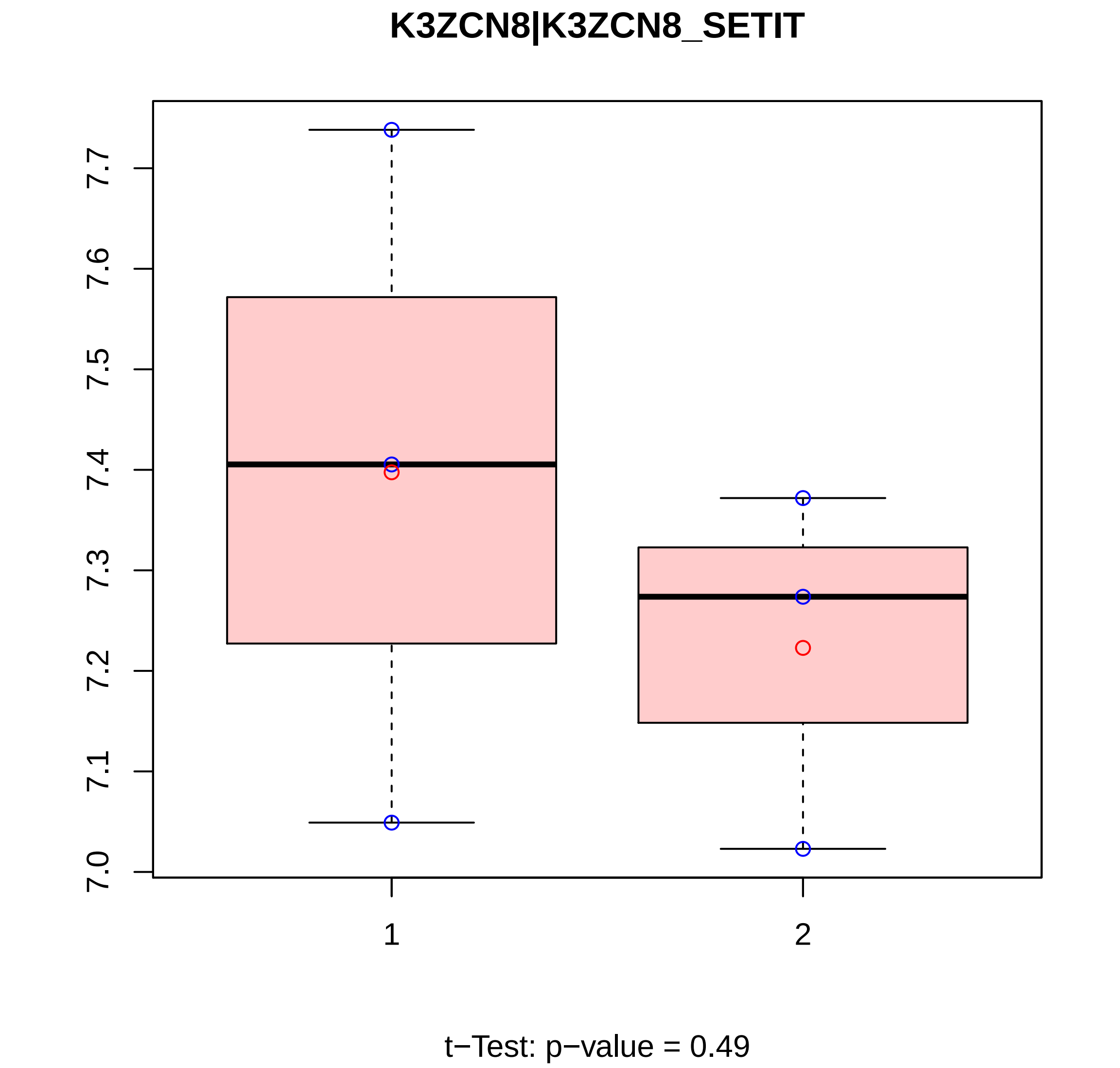

K3ZCN8|K3ZCN8_SETIT
7.0
7.1
7.2
7.3
7.4
7.5
7.6
7.7
1
2
t−Test: p−value = 0.49

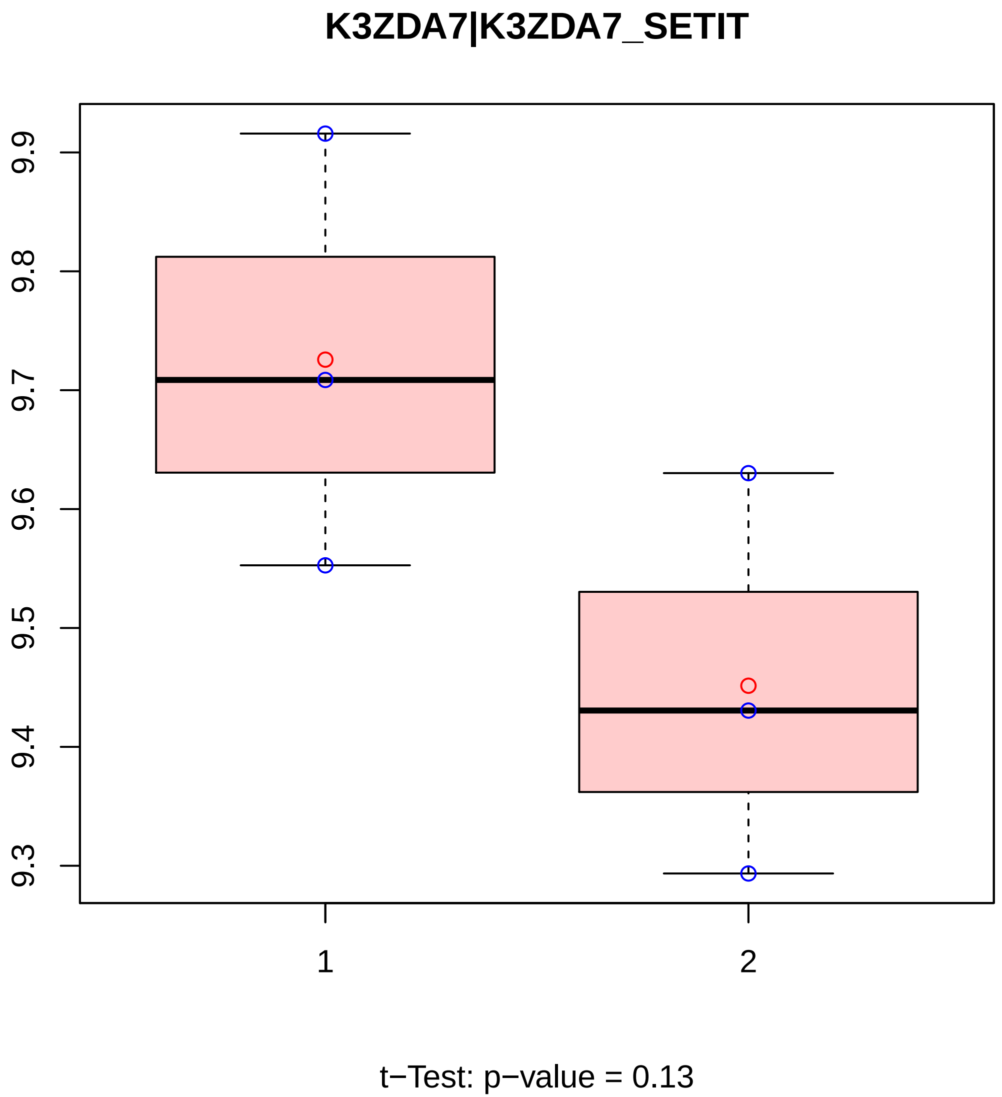

K3ZDA7|K3ZDA7_SETIT
9.3
9.4
9.5
9.6
9.7
9.8
9.9
1
2
t−Test: p−value = 0.13

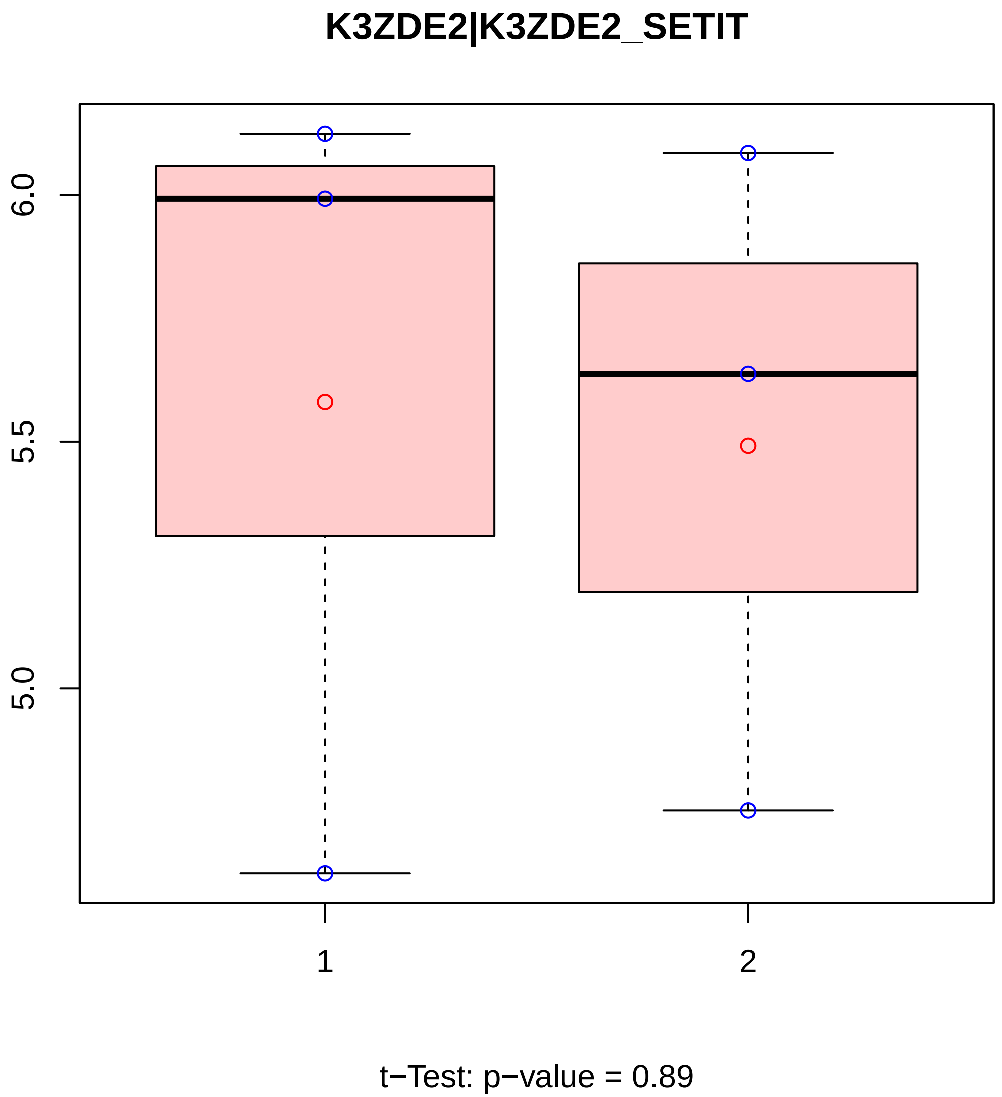
K3ZDE2|K3ZDE2_SETIT
6.0
5.5
5.0
1
2
t−Test: p−value = 0.89

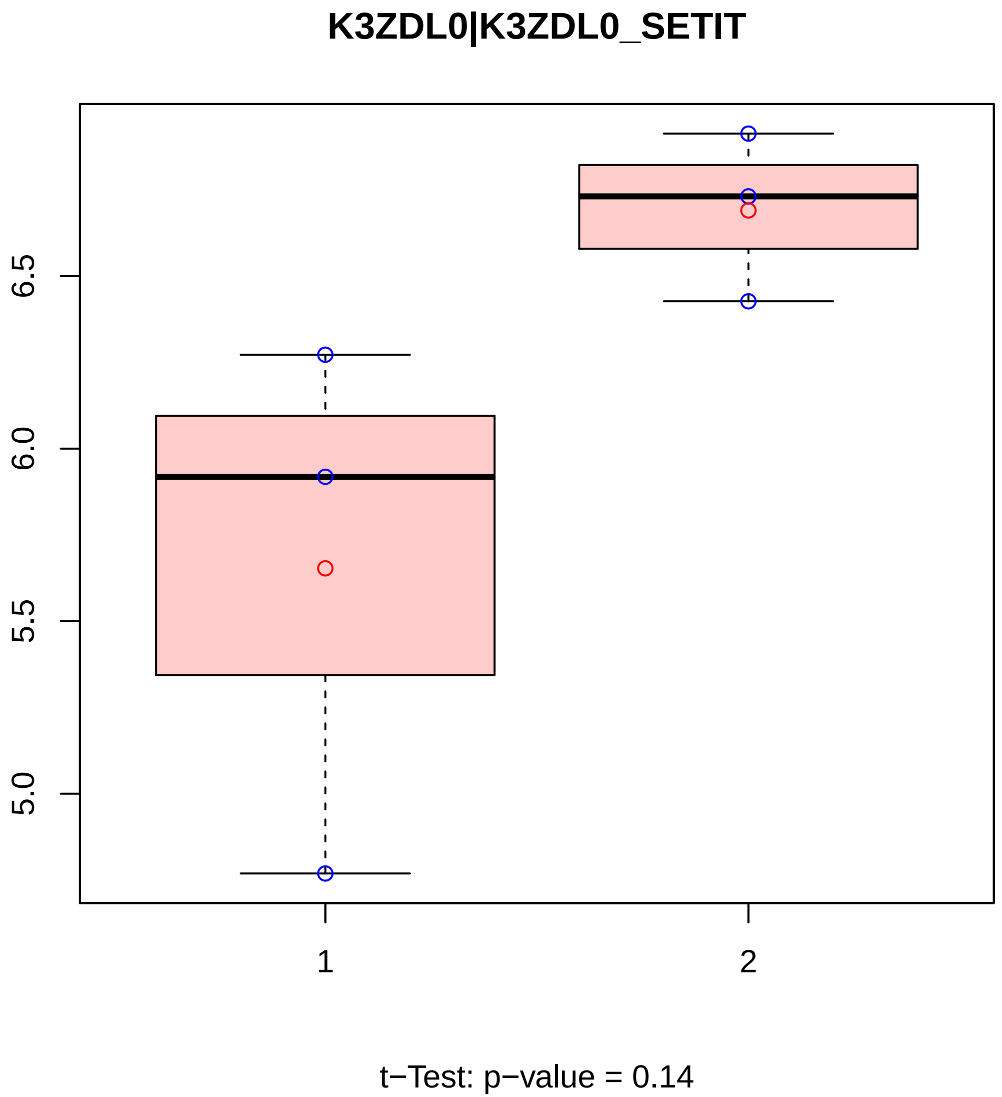

K3ZDL0|K3ZDL0_SETIT
5.0
5.5
6.0
6.5
1
2
t−Test: p−value = 0.14

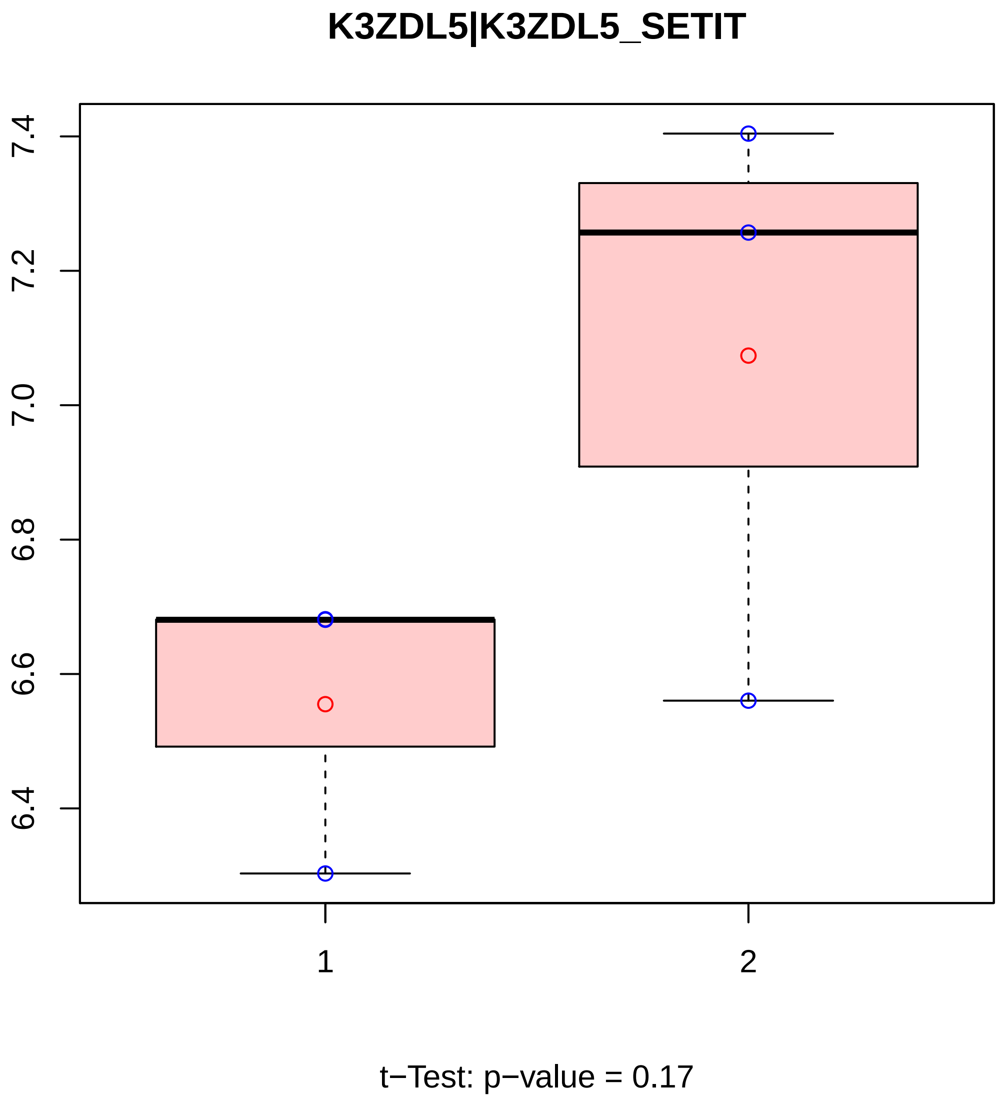
K3ZDL5|K3ZDL5_SETIT
7.4
7.2
7.0
6.8
6.6
6.4
1
2
t−Test: p−value = 0.17

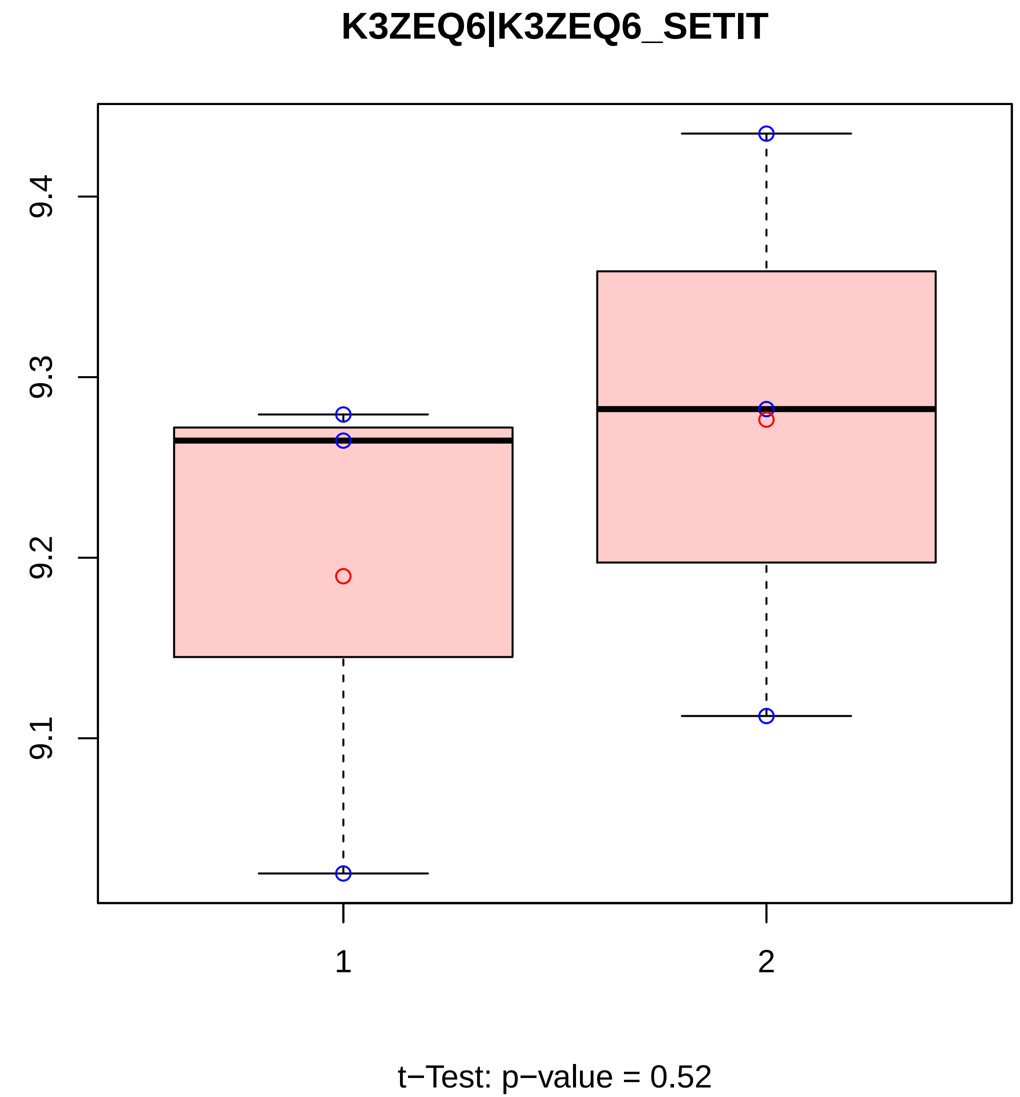
K3ZEQ6|K3ZEQ6_SETIT
9.1
9.2
9.3
9.4
1
2
t−Test: p−value = 0.52

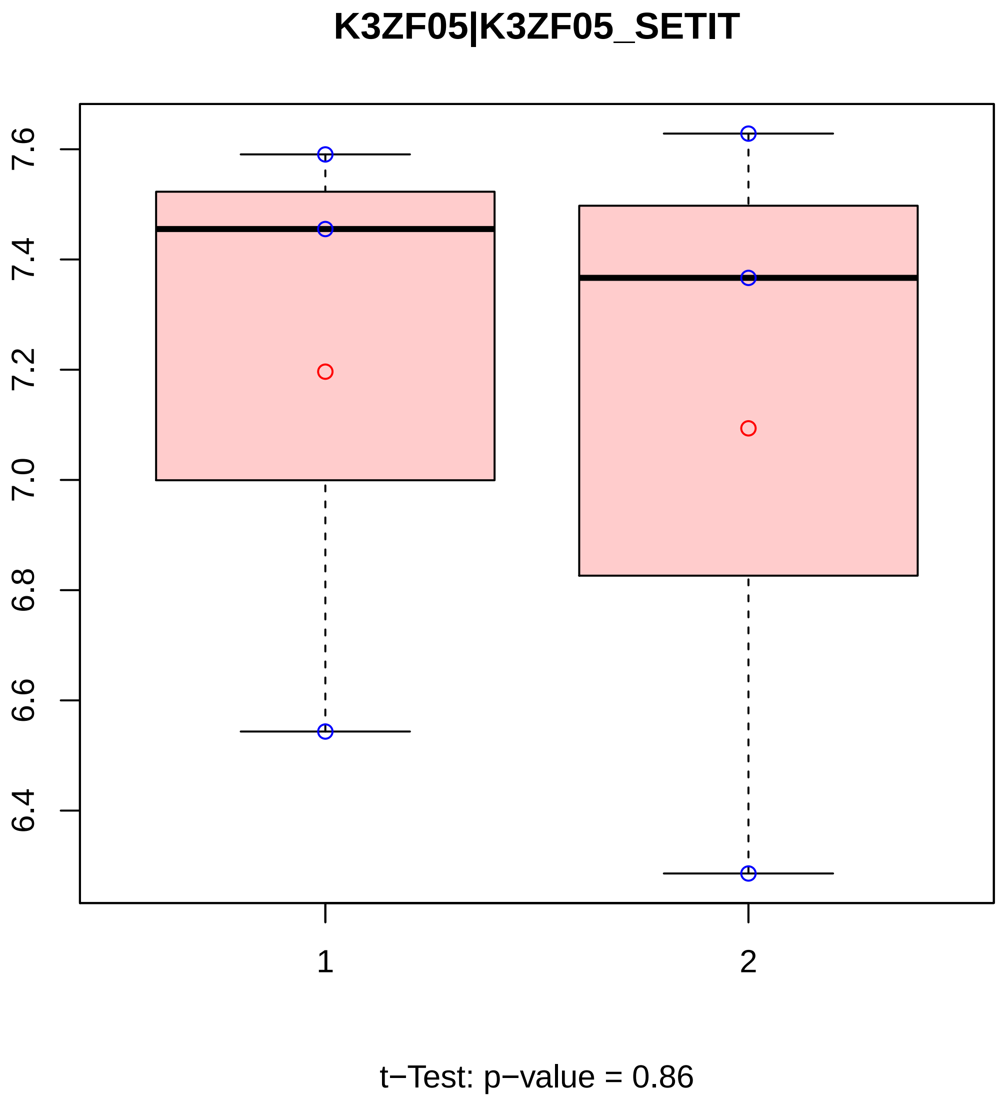
K3ZF05|K3ZF05_SETIT
7.6
7.4
7.2
7.0
6.8
6.6
6.4
1
2
t−Test: p−value = 0.86

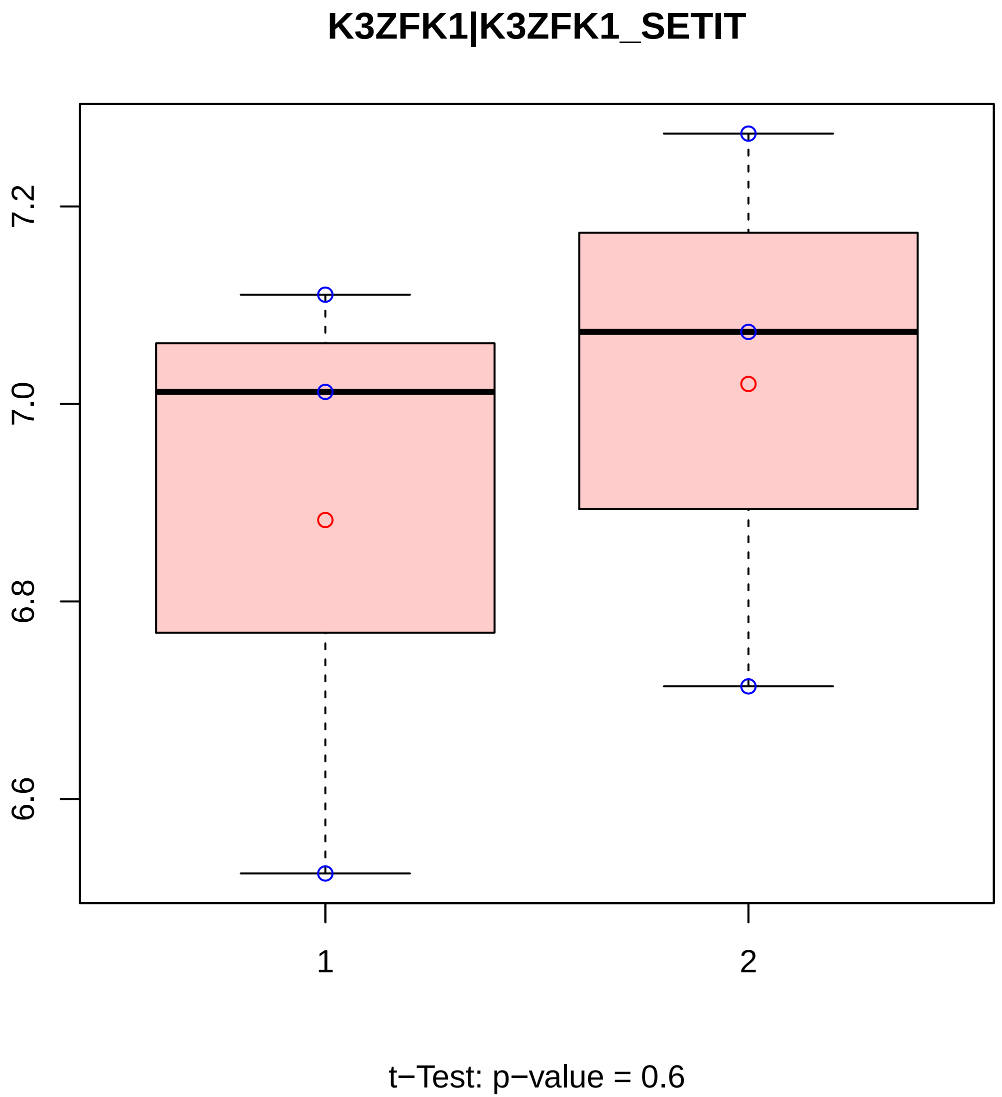
K3ZFK1|K3ZFK1_SETIT
7.2
7.0
6.8
6.6
1
2
t−Test: p−value = 0.6

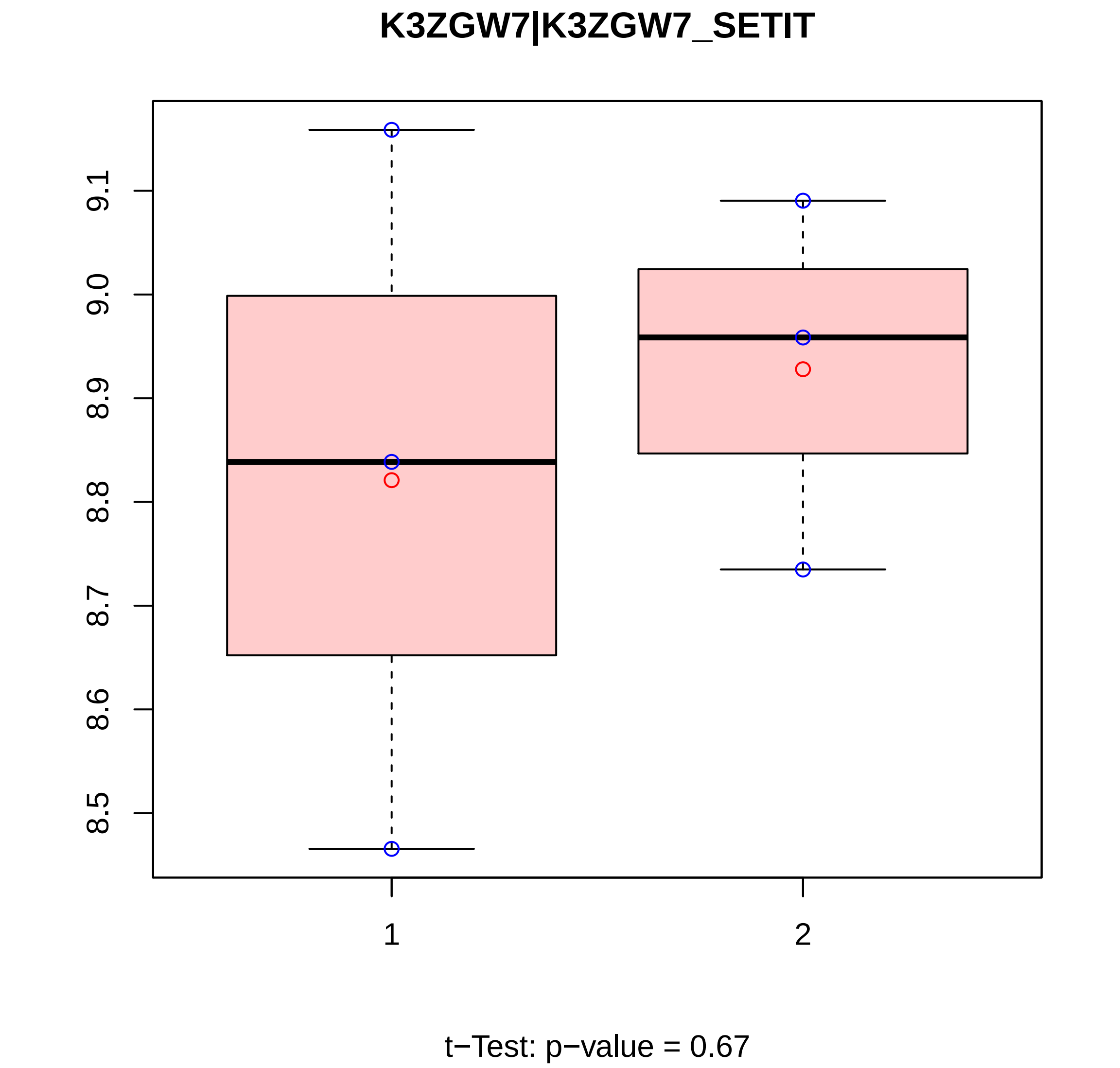

K3ZGW7|K3ZGW7_SETIT
9.1
9.0
8.9
8.8
8.7
8.6
8.5
1
2
t−Test: p−value = 0.67

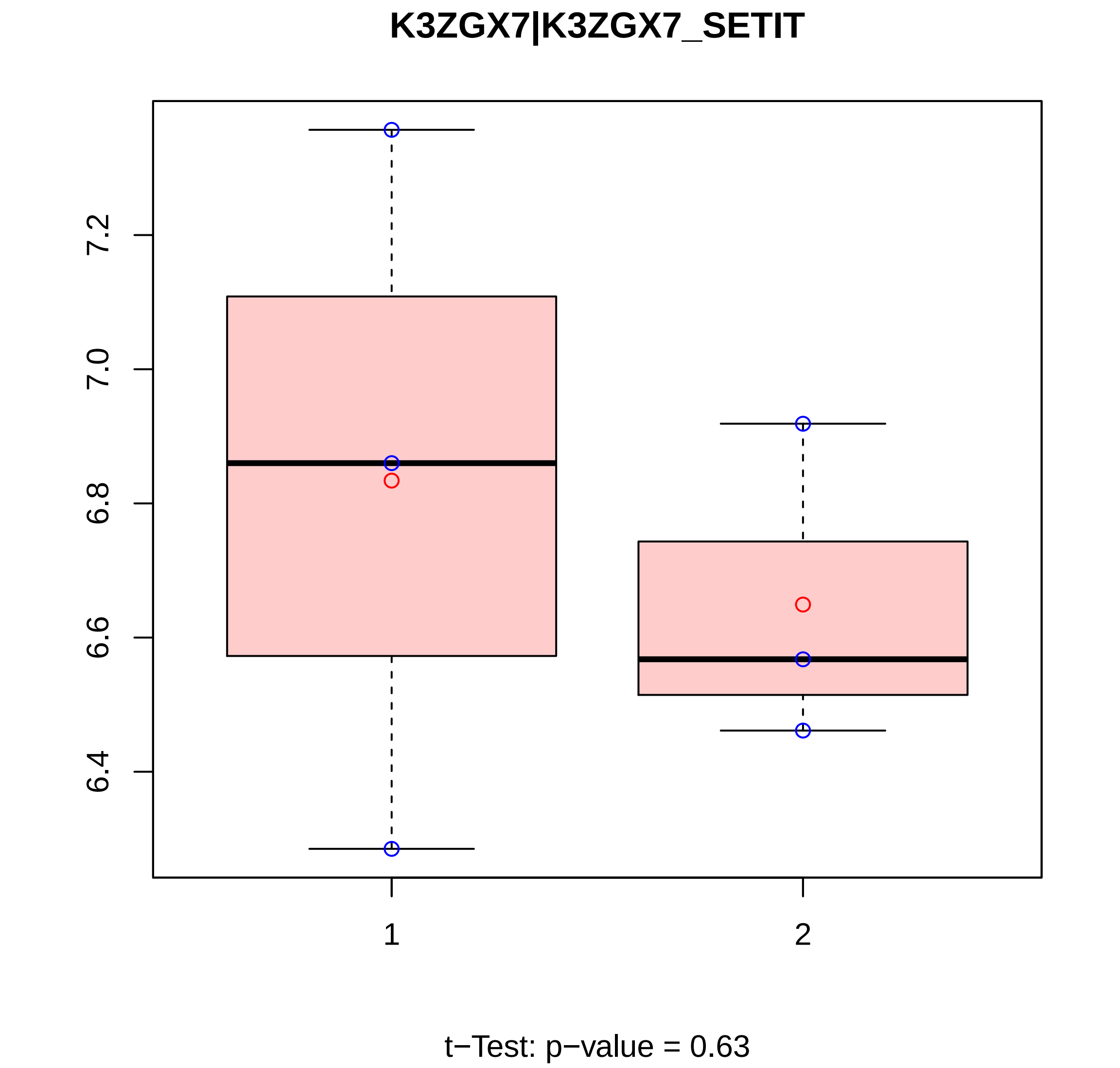
K3ZGX7|K3ZGX7_SETIT
6.4
6.6
6.8
7.0
7.2
1
2
t−Test: p−value = 0.63

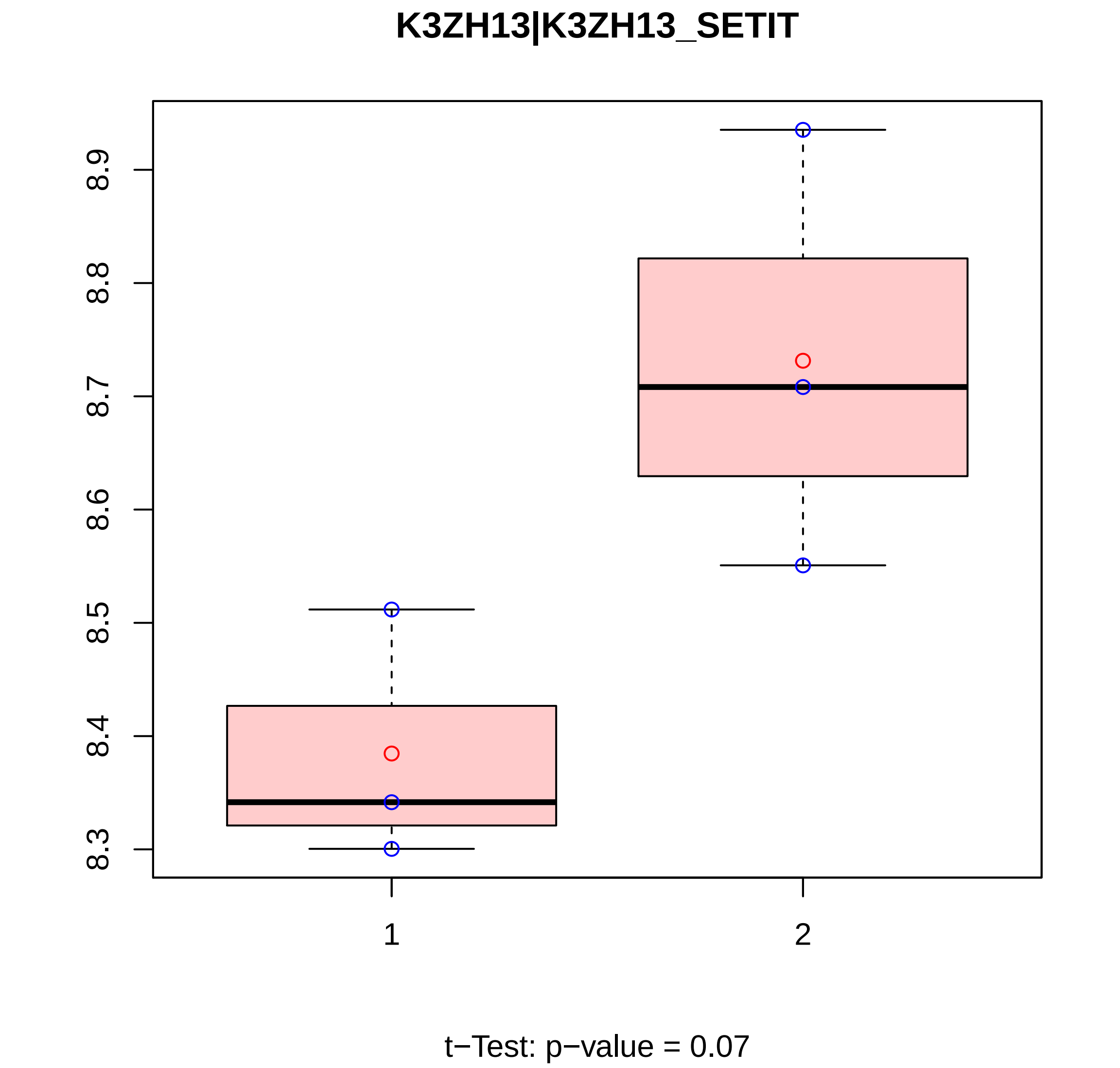
K3ZH13|K3ZH13_SETIT
8.3
8.4
8.5
8.6
8.7
8.8
8.9
1
2
t−Test: p−value = 0.07

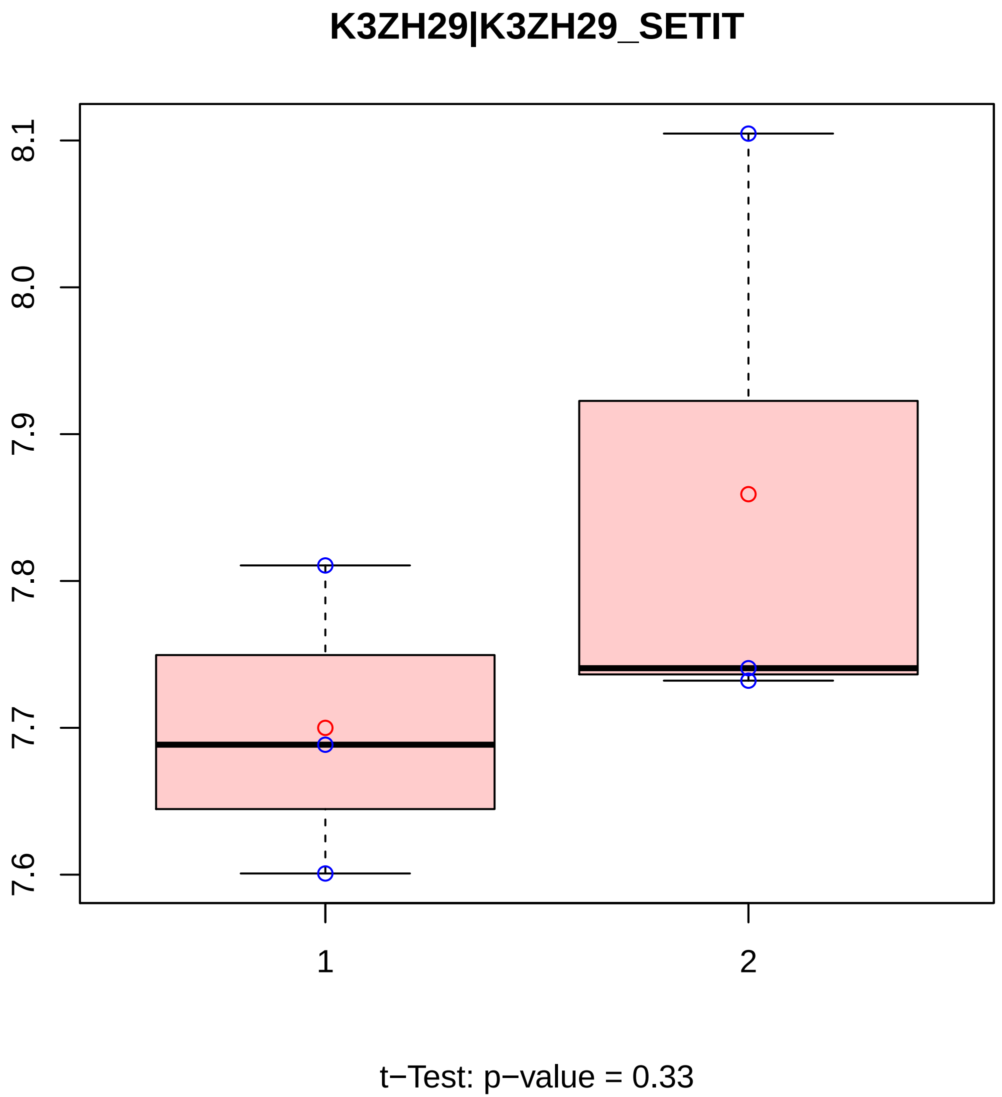

K3ZH29|K3ZH29_SETIT
8.1
8.0
7.9
7.8
7.7
7.6
1
2
t−Test: p−value = 0.33

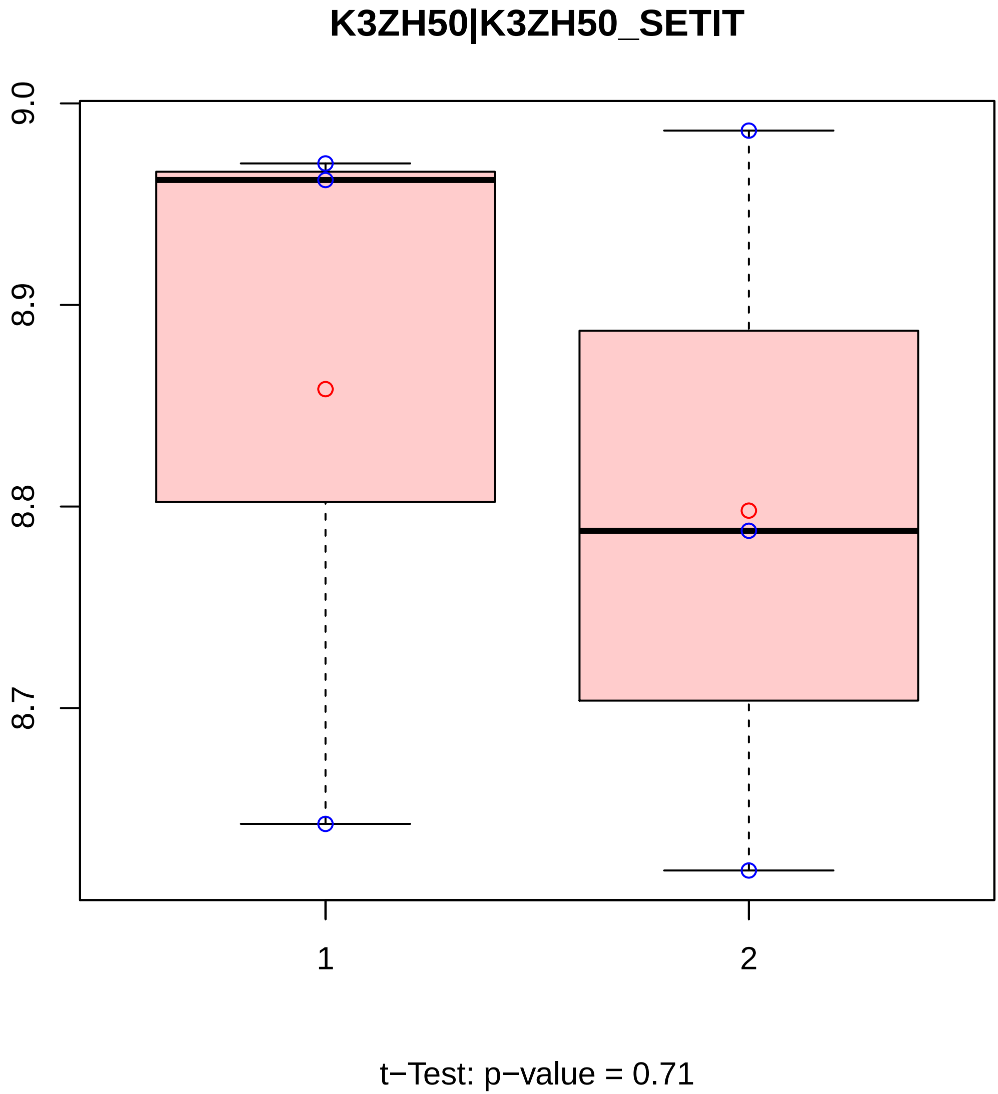
K3ZH50|K3ZH50_SETIT
9.0
8.9
8.8
8.7
1
2
t−Test: p−value = 0.71

K3ZHI1|K3ZHI1_SETIT

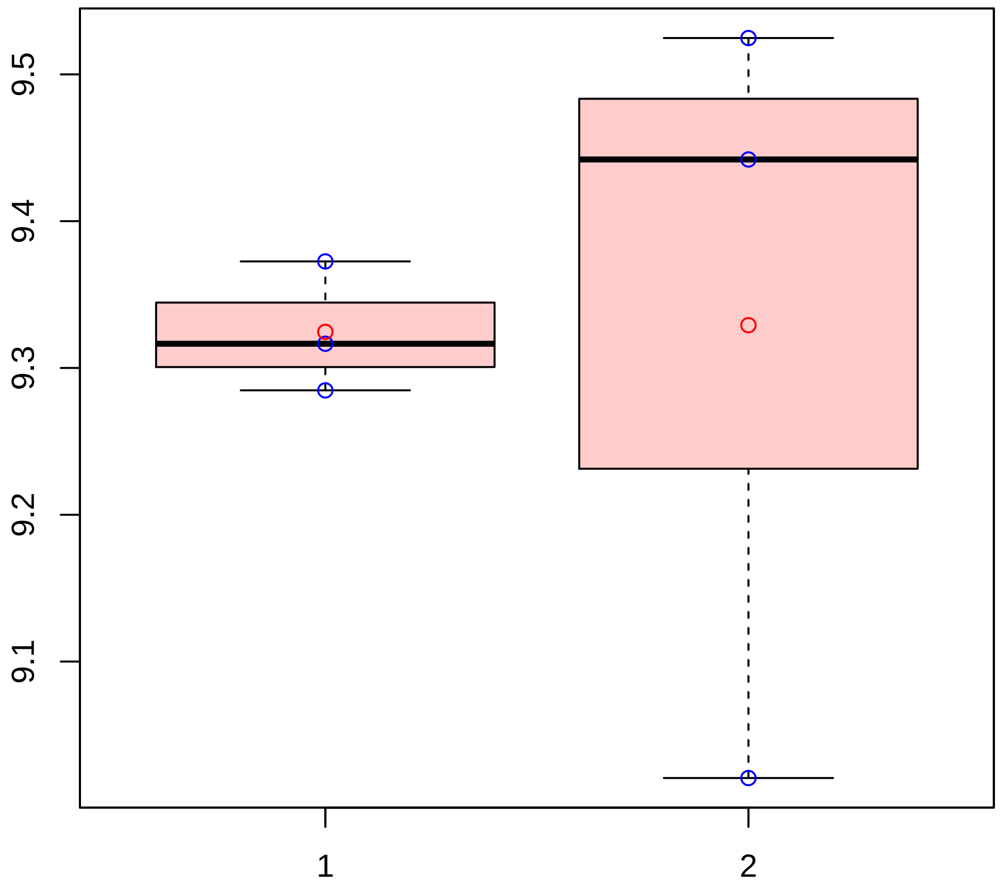

t−Test: p−value = 0.98

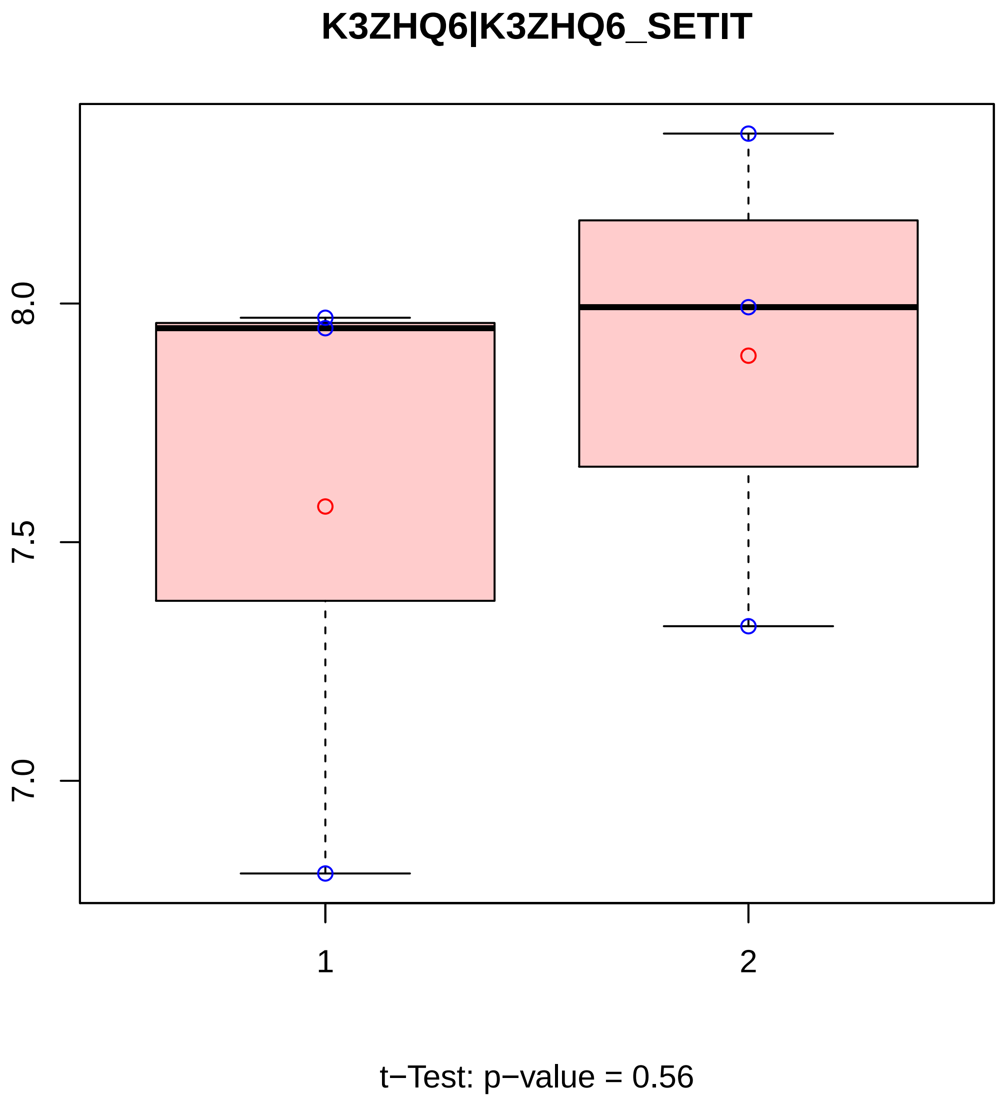
K3ZHQ6|K3ZHQ6_SETIT
8.0
7.5
7.0
1
2
t−Test: p−value = 0.56

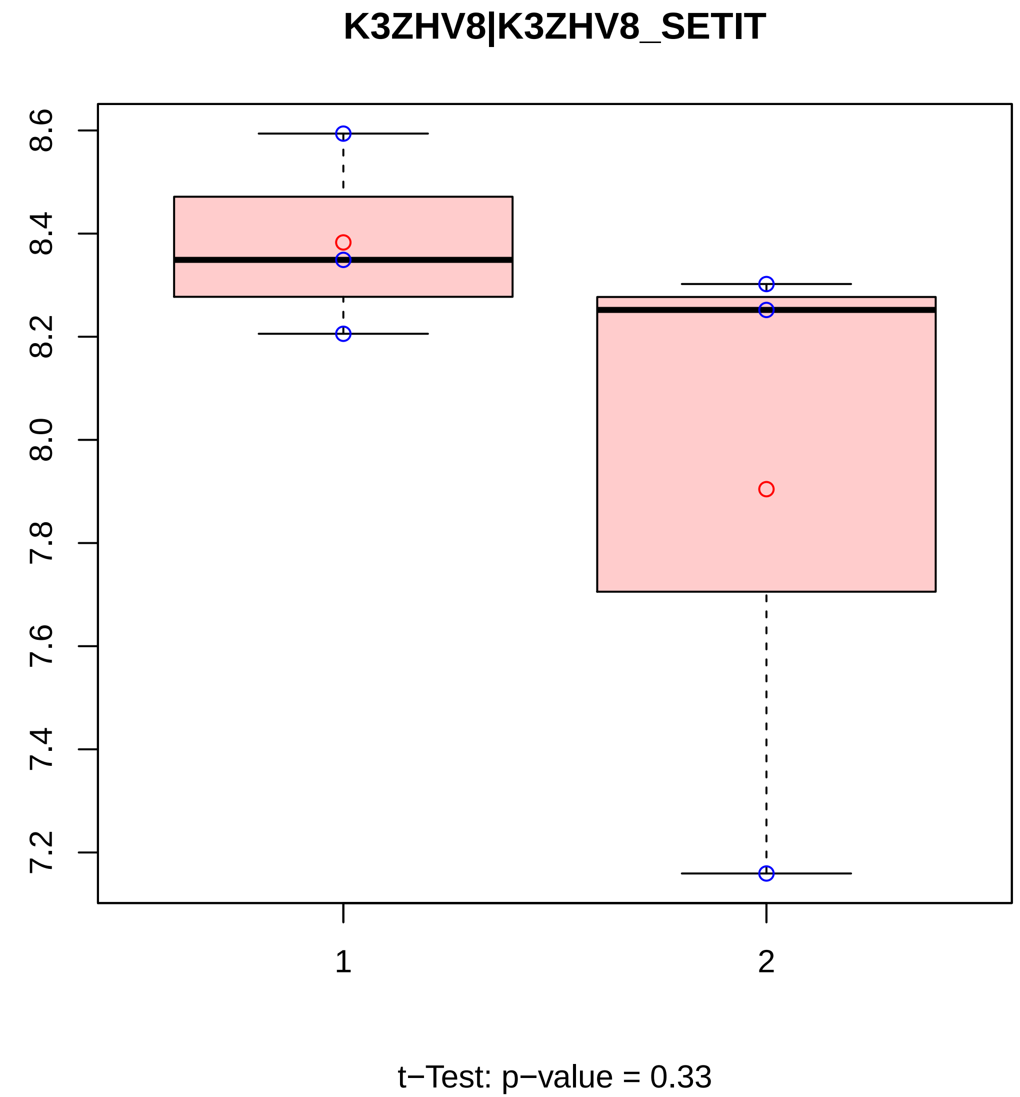
K3ZHV8|K3ZHV8_SETIT
8.6
8.4
8.2
8.0
7.8
7.6
7.4
7.2
1
2
t−Test: p−value = 0.33

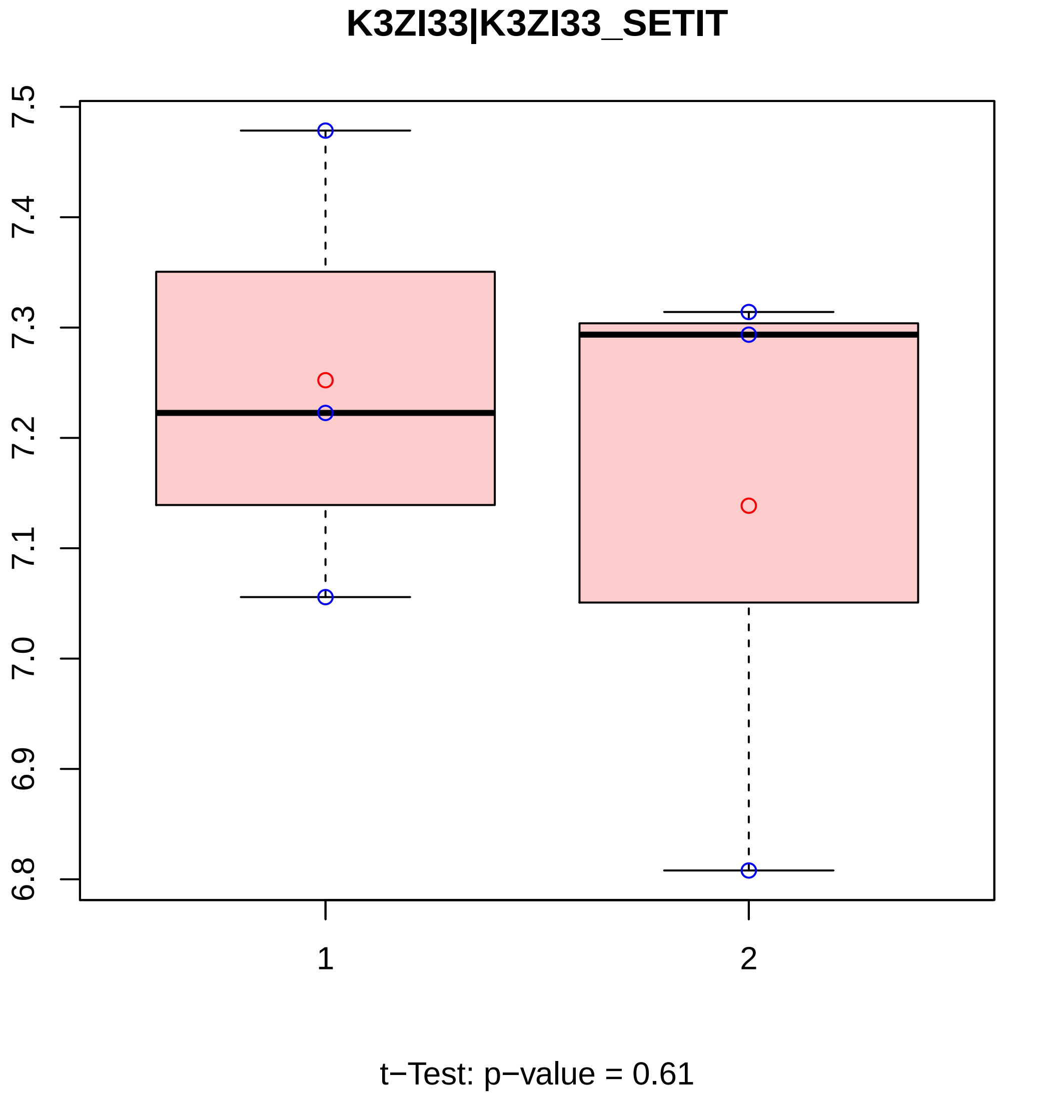
K3ZI33|K3ZI33_SETIT
7.5
7.4
7.3
7.2
7.1
7.0
6.9
6.8
1
2
t−Test: p−value = 0.61

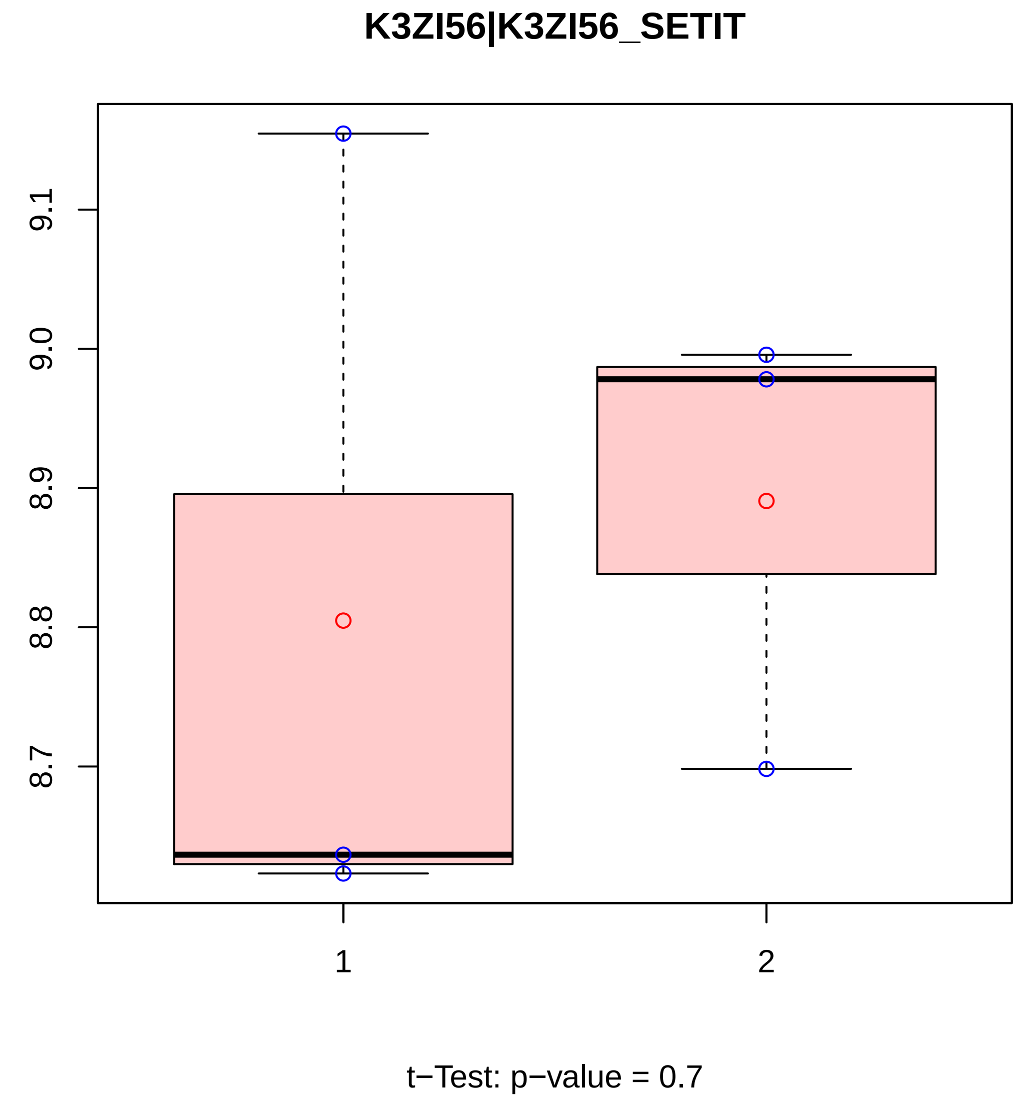
K3ZI56|K3ZI56_SETIT
9.1
9.0
8.9
8.8
8.7
1
2
t−Test: p−value = 0.7

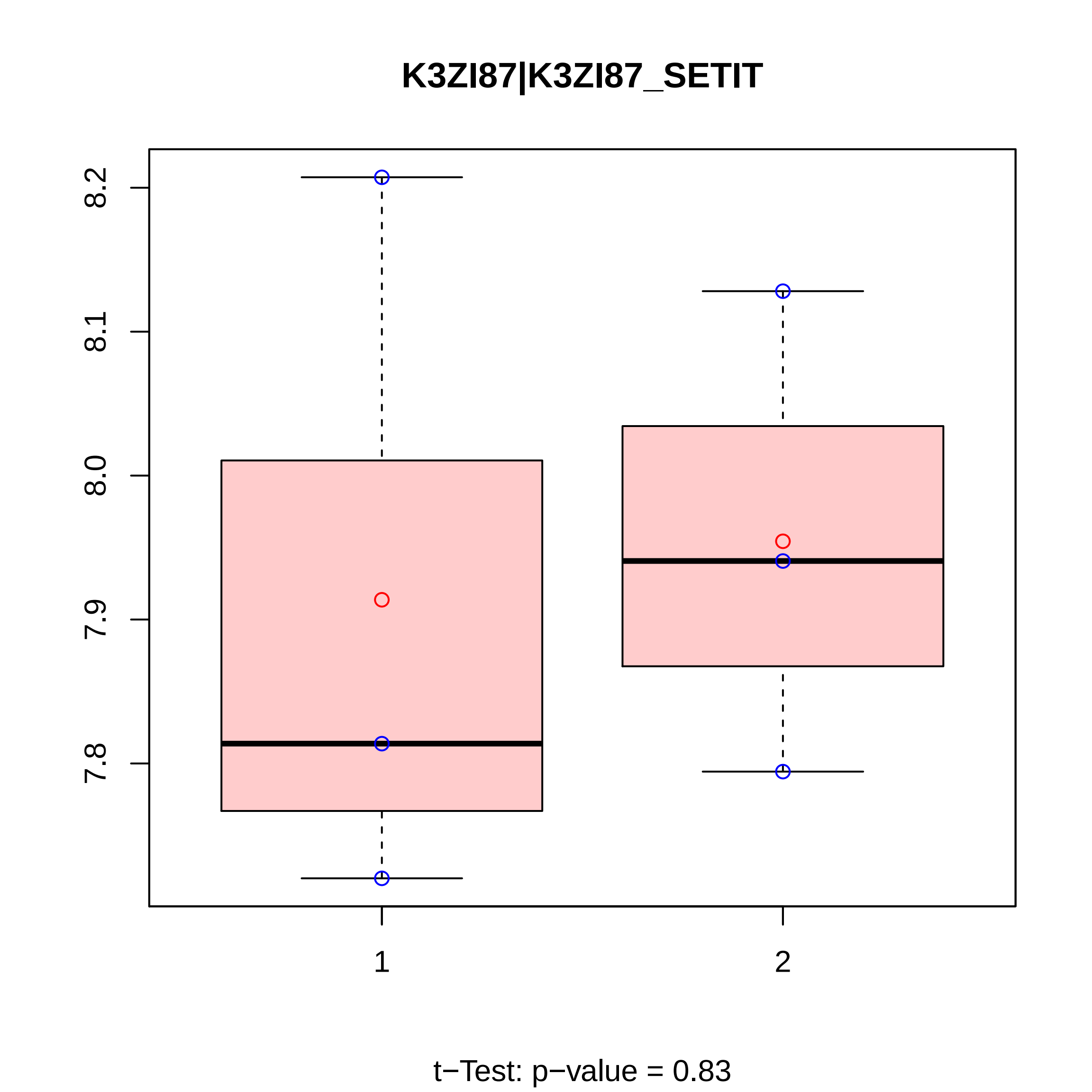
K3ZI87|K3ZI87_SETIT
8.2
8.1
8.0
7.9
7.8
1
2
t−Test: p−value = 0.83

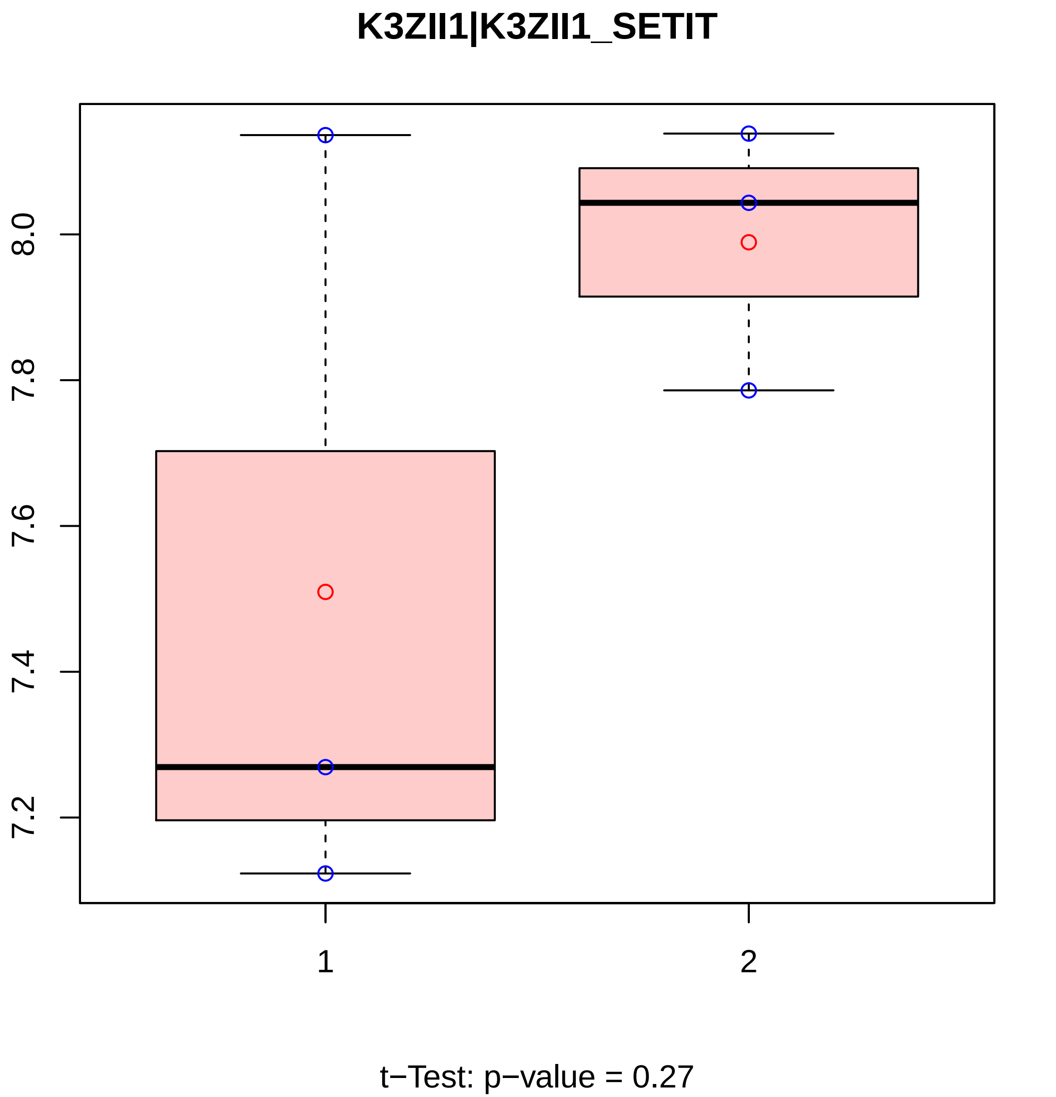
K3ZII1|K3ZII1_SETIT
8.0
7.8
7.6
7.4
7.2
1
2
t−Test: p−value = 0.27

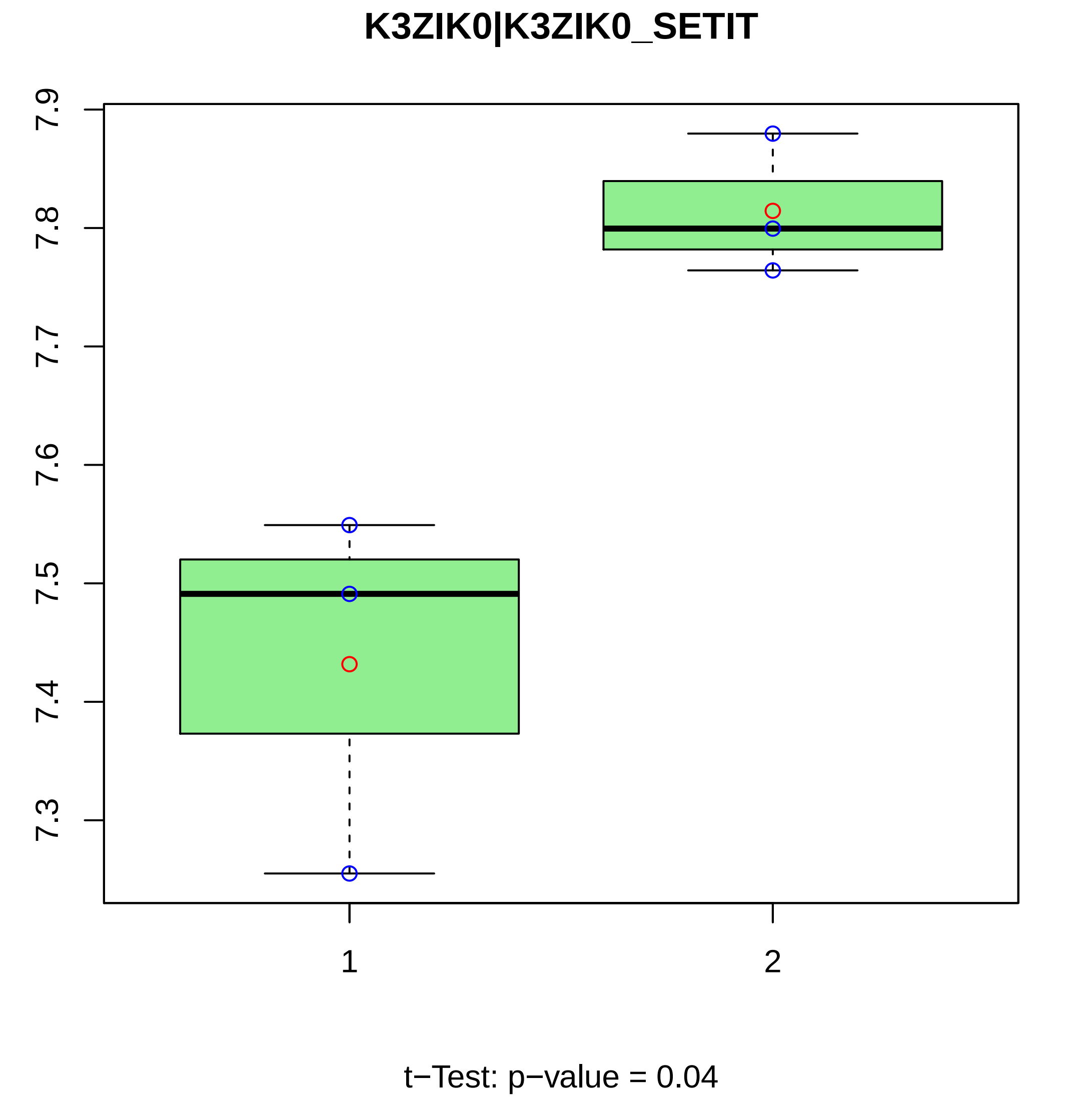
K3ZIK0|K3ZIK0_SETIT
7.9
7.8
7.7
7.6
7.5
7.4
7.3
1
2
t−Test: p−value = 0.04

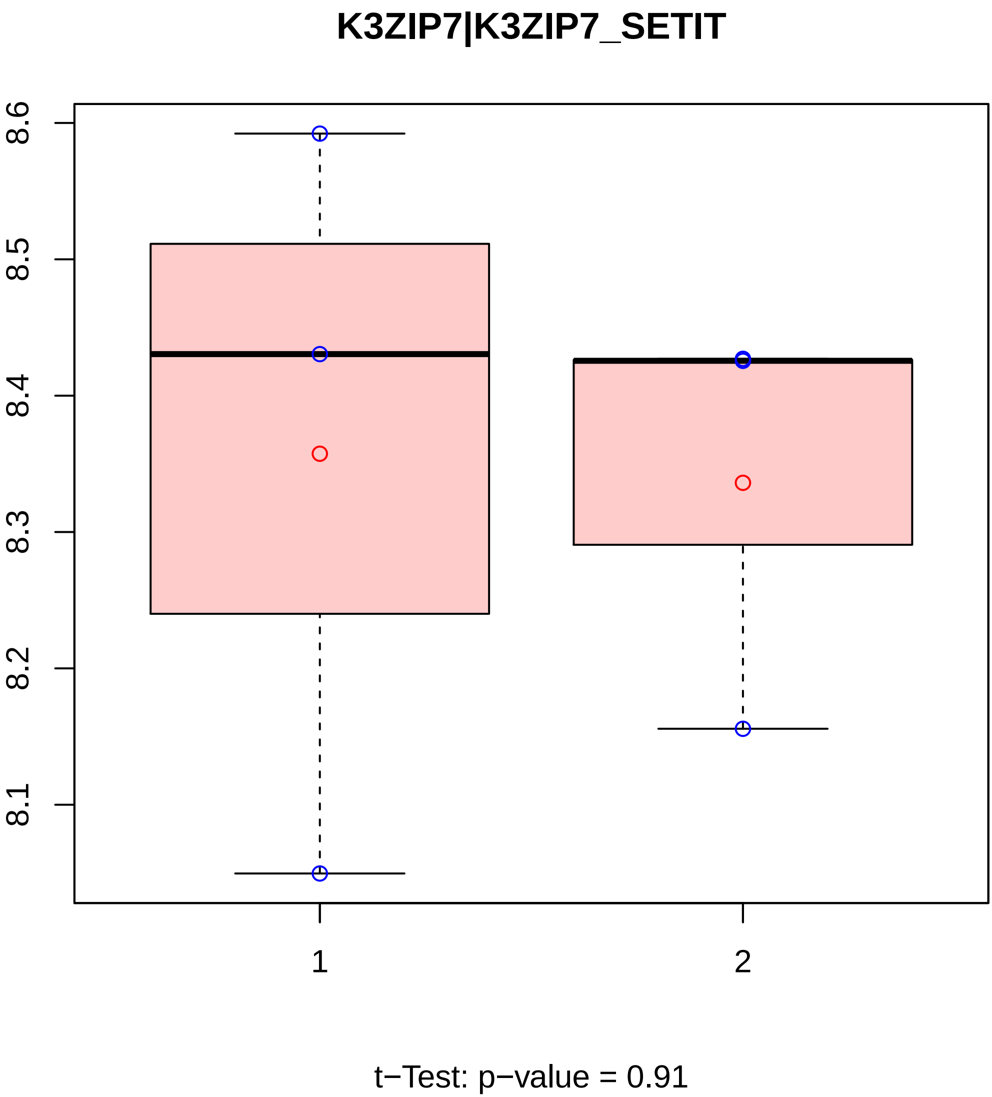
K3ZIP7|K3ZIP7_SETIT
8.6
8.5
8.4
8.3
8.2
8.1
1
2
t−Test: p−value = 0.91

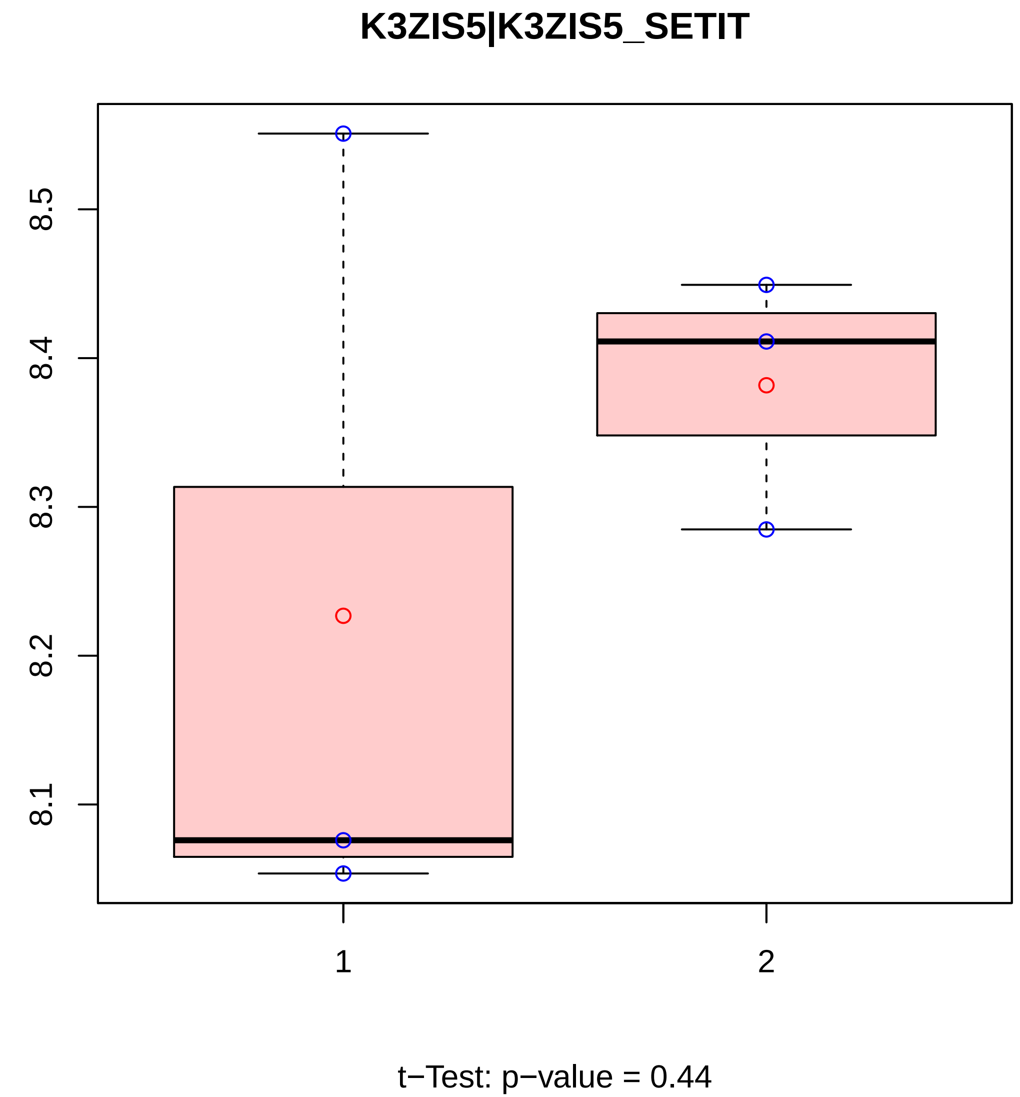
K3ZIS5|K3ZIS5_SETIT
8.1
8.2
8.3
8.4
8.5
1
2
t−Test: p−value = 0.44

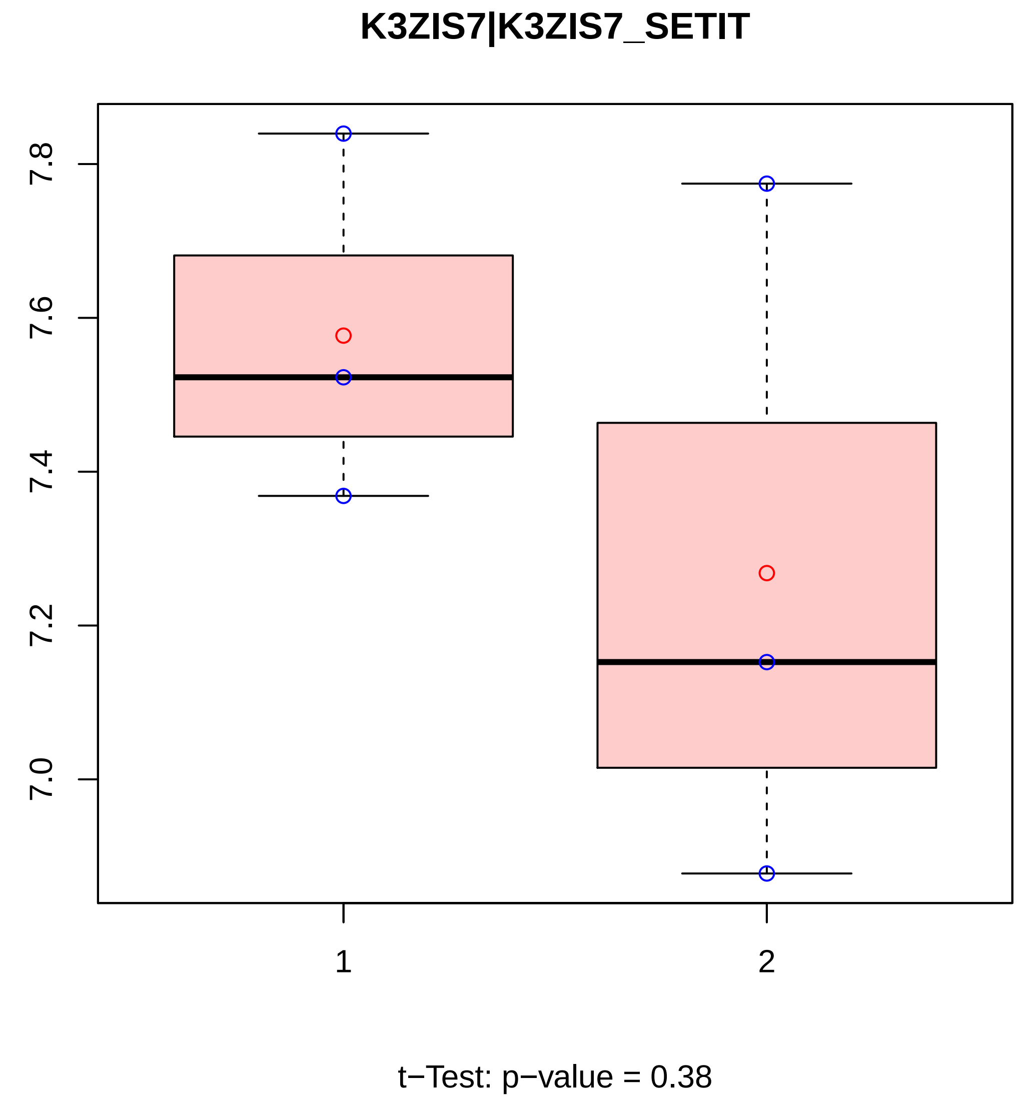
K3ZIS7|K3ZIS7_SETIT
7.0
7.2
7.4
7.6
7.8
1
2
t−Test: p−value = 0.38

K3ZJ12|K3ZJ12_SETIT

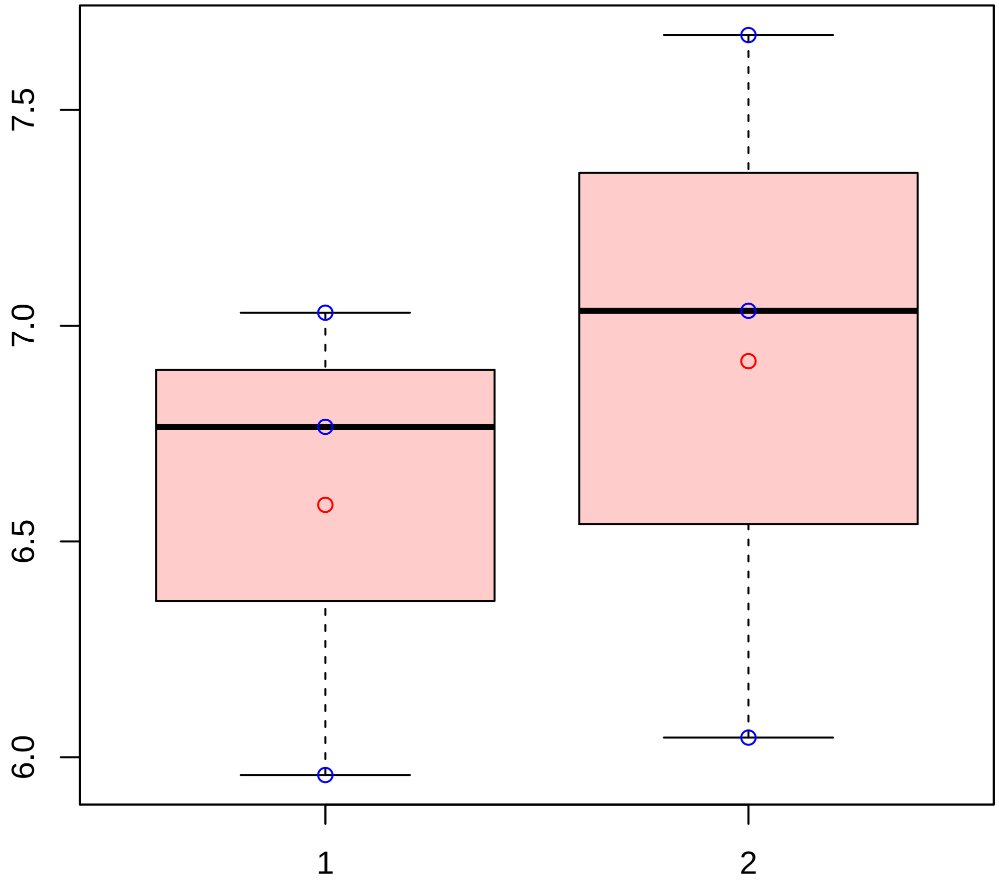

t−Test: p−value = 0.6

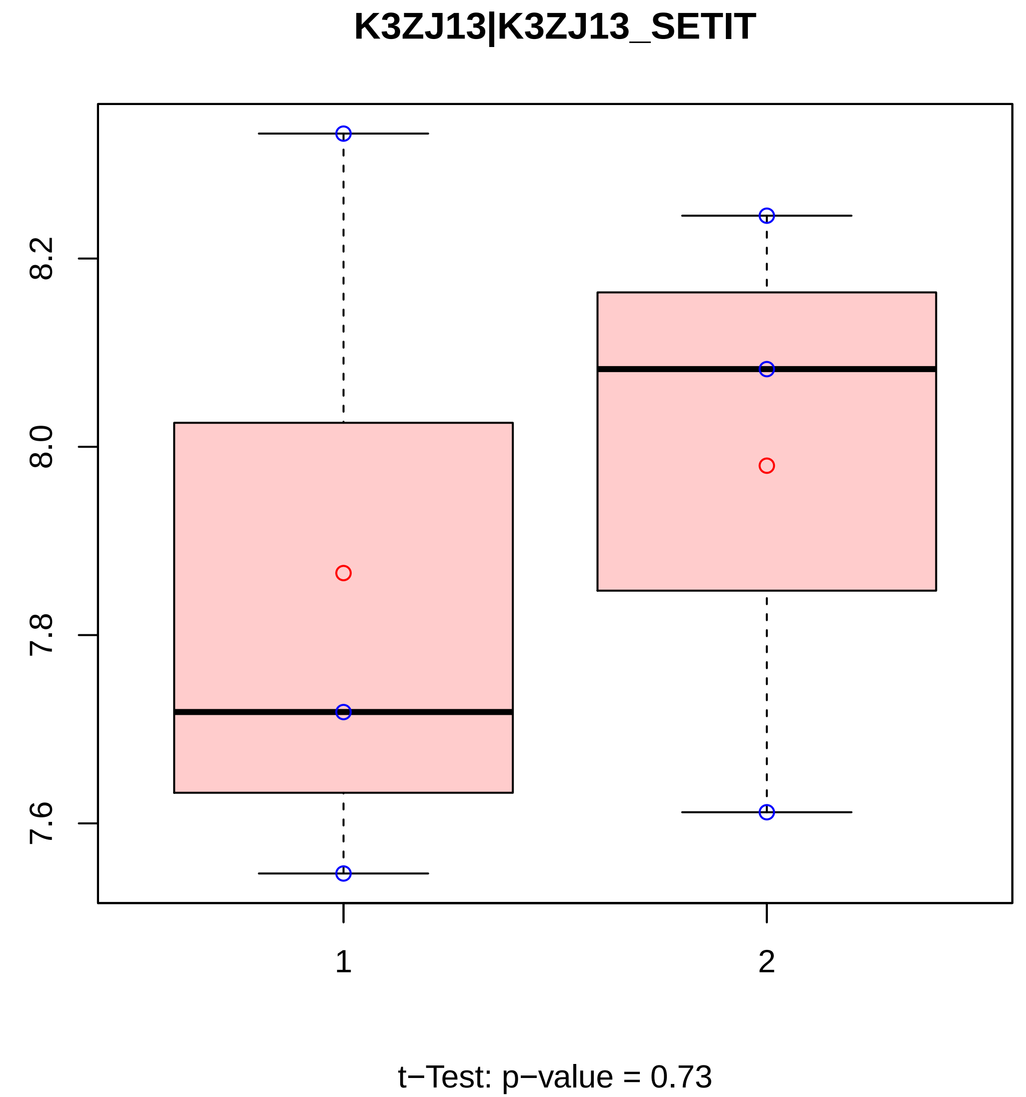
K3ZJ13|K3ZJ13_SETIT
8.2
8.0
7.8
7.6
1
2
t−Test: p−value = 0.73

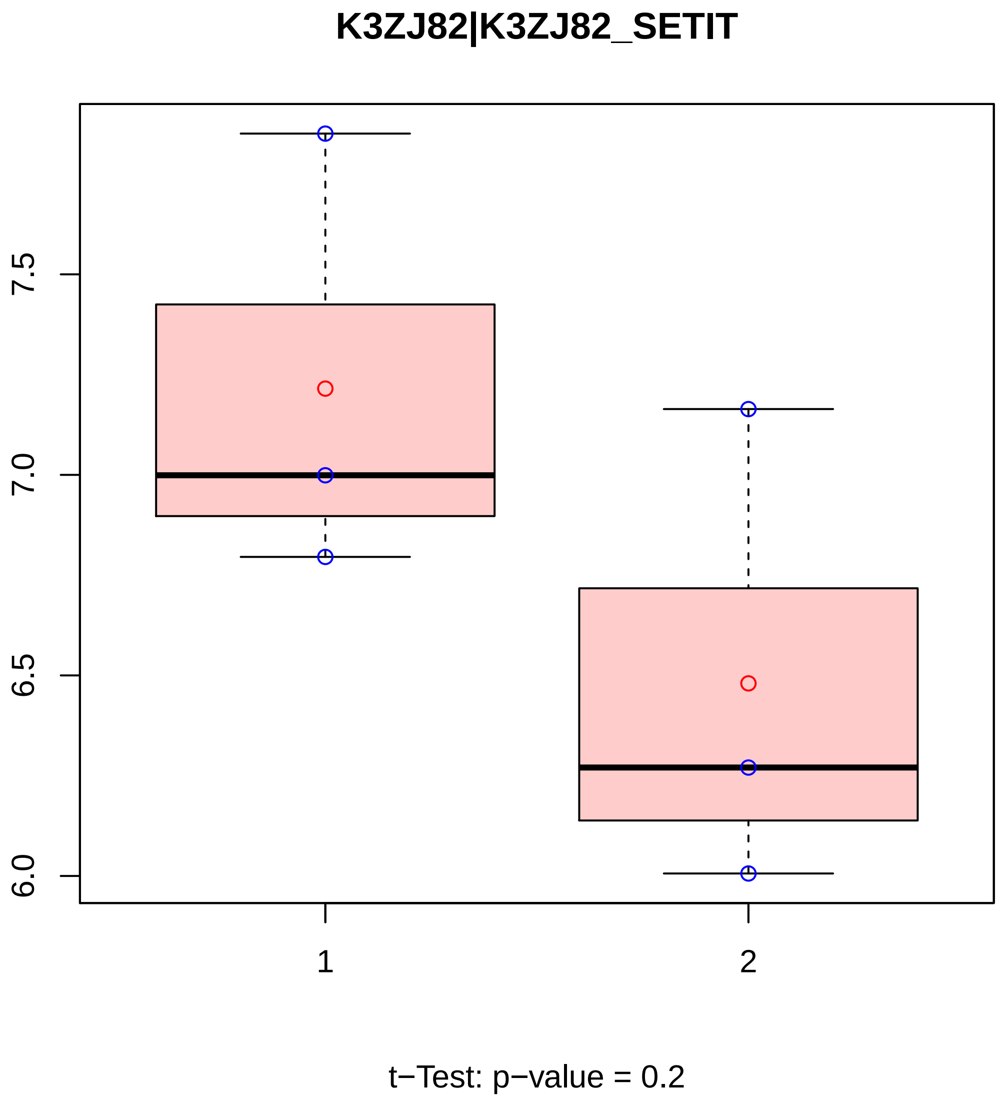

K3ZJ82|K3ZJ82_SETIT
7.5
7.0
6.5
6.0
1
2
t−Test: p−value = 0.2

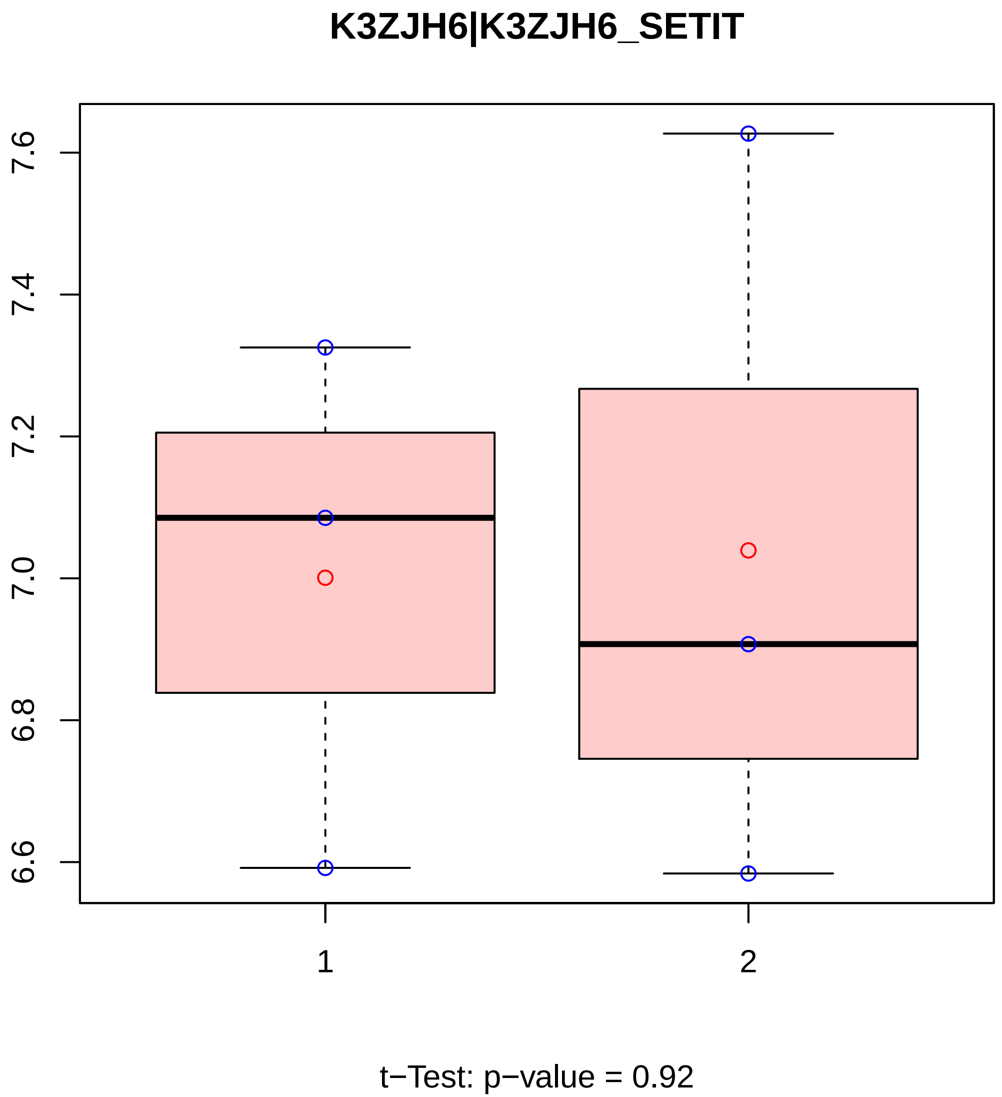
K3ZJH6|K3ZJH6_SETIT
7.6
7.4
7.2
7.0
6.8
6.6
1
2
t−Test: p−value = 0.92

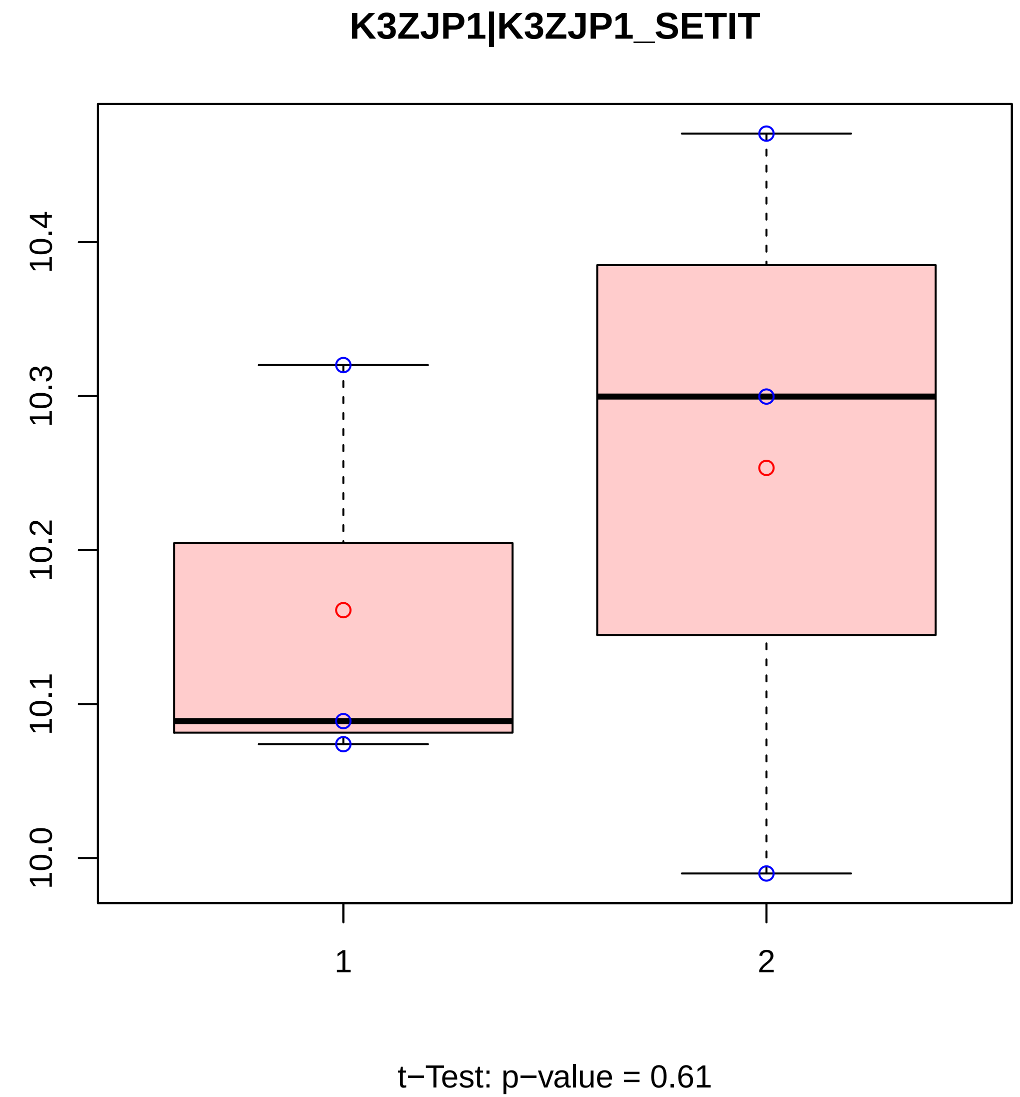
K3ZJP1|K3ZJP1_SETIT
10.0
10.1
10.2
10.3
10.4
1
2
t−Test: p−value = 0.61

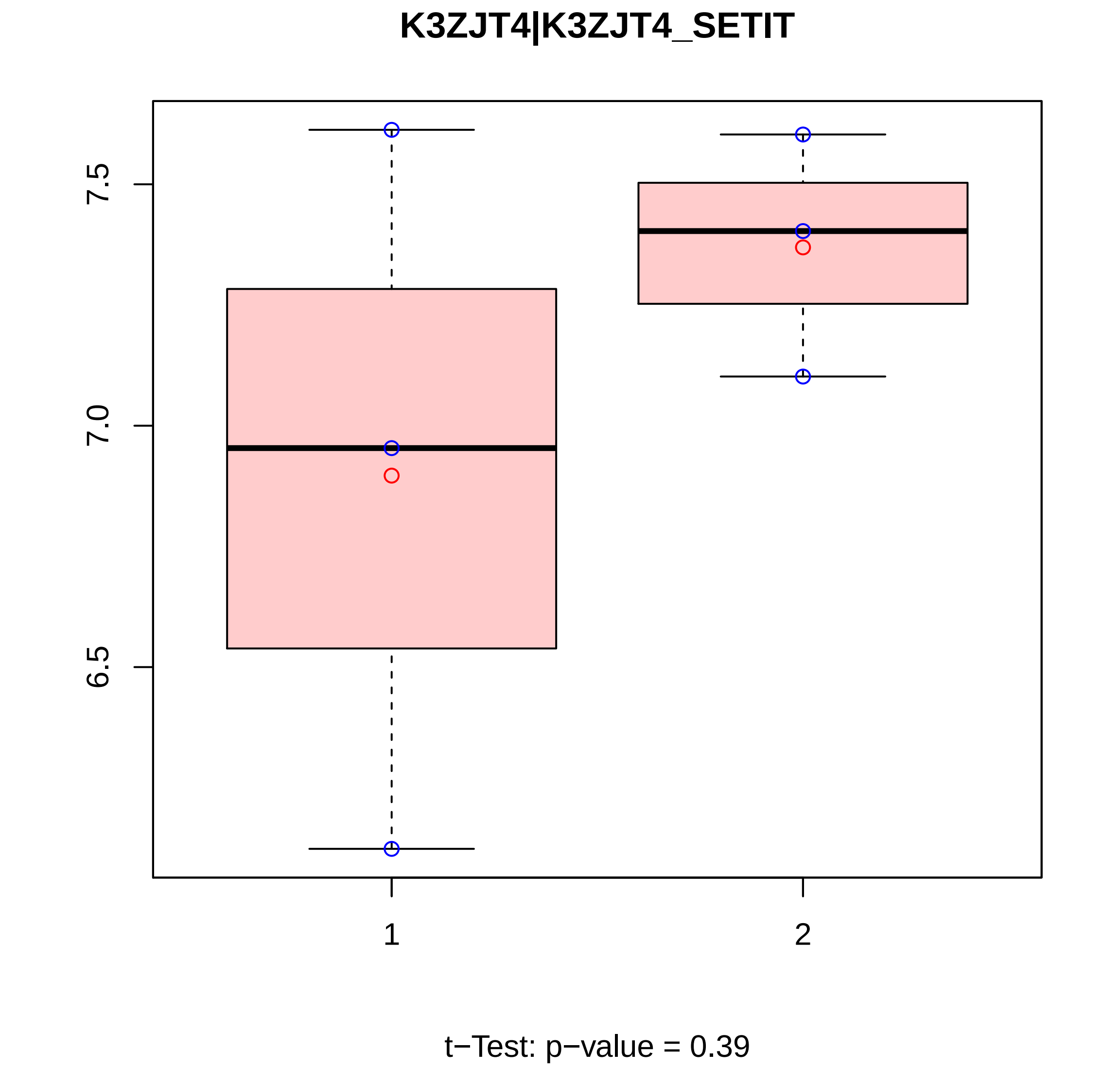
K3ZJT4|K3ZJT4_SETIT
7.5
7.0
6.5
1
2
t−Test: p−value = 0.39

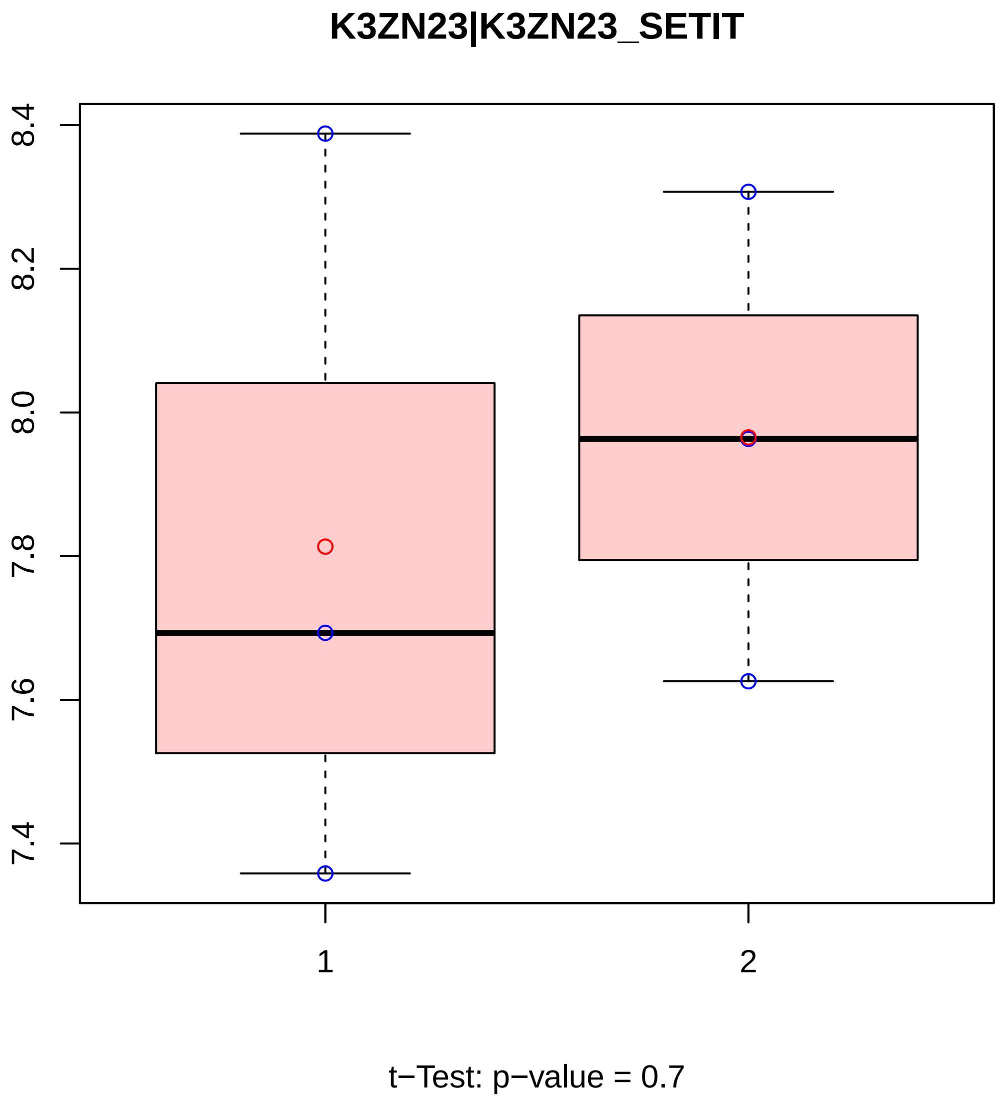
K3ZN23|K3ZN23_SETIT
8.4
8.2
8.0
7.8
7.6
7.4
1
2
t−Test: p−value = 0.7

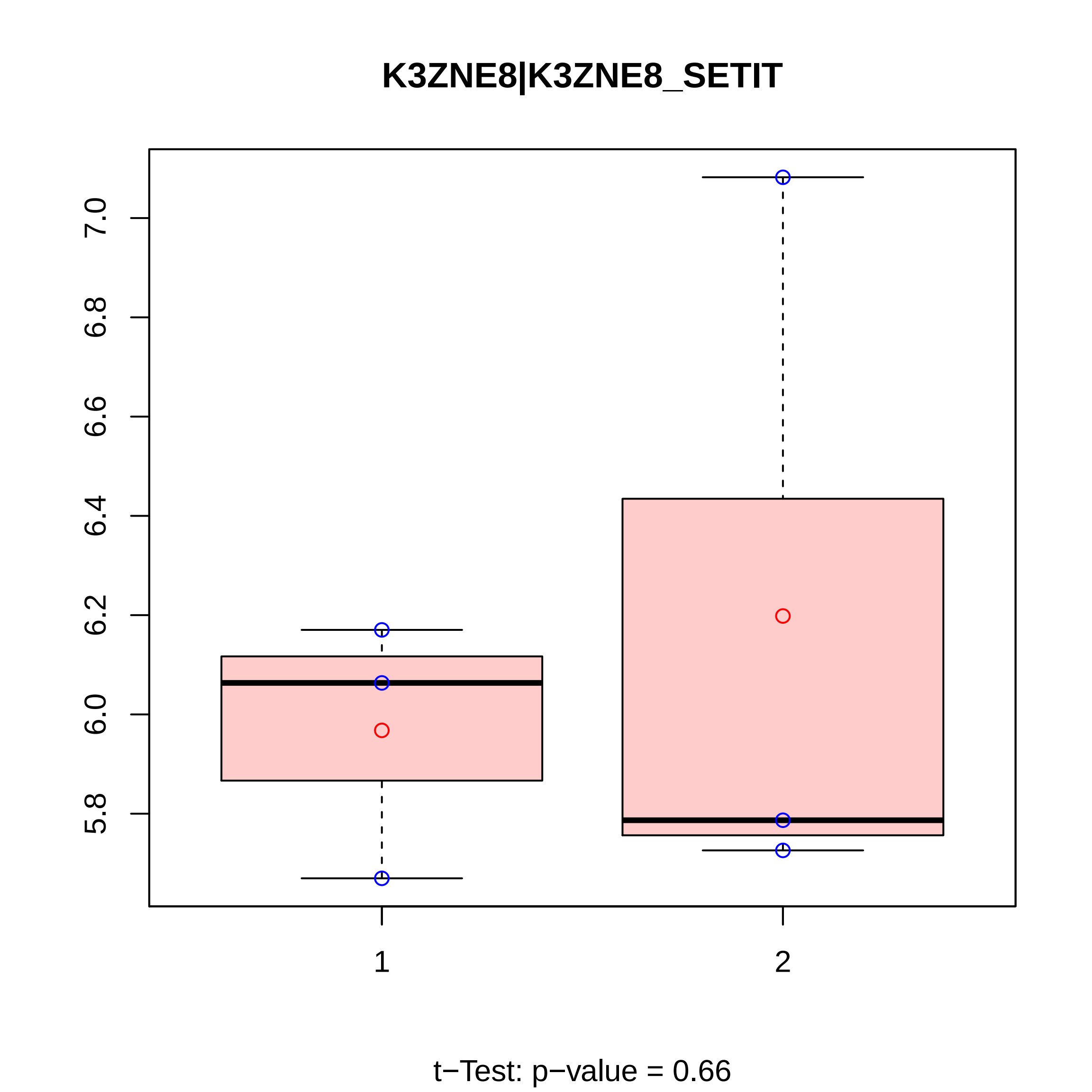
K3ZNE8|K3ZNE8_SETIT
7.0
6.8
6.6
6.4
6.2
6.0
5.8
1
2
t−Test: p−value = 0.66

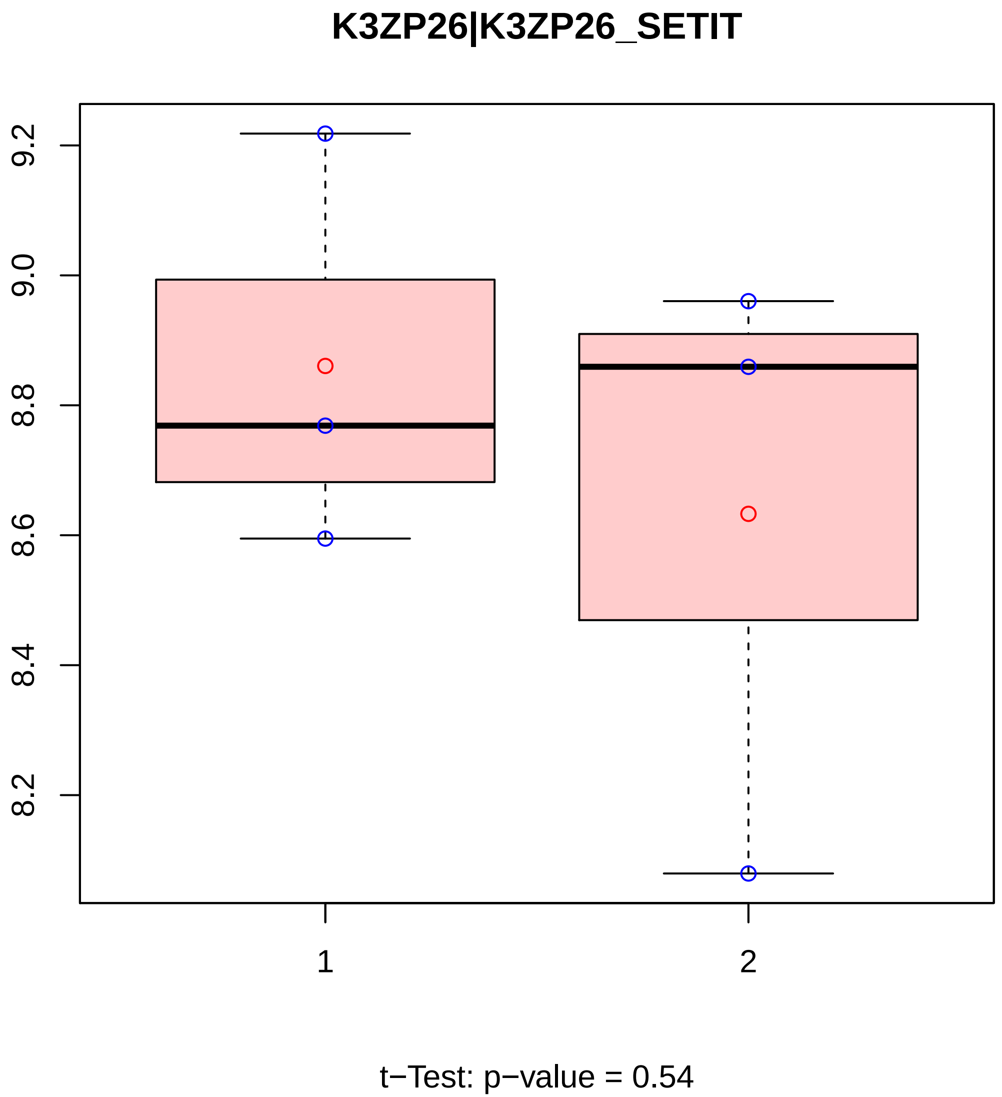
K3ZP26|K3ZP26_SETIT
9.2
9.0
8.8
8.6
8.4
8.2
1
2
t−Test: p−value = 0.54

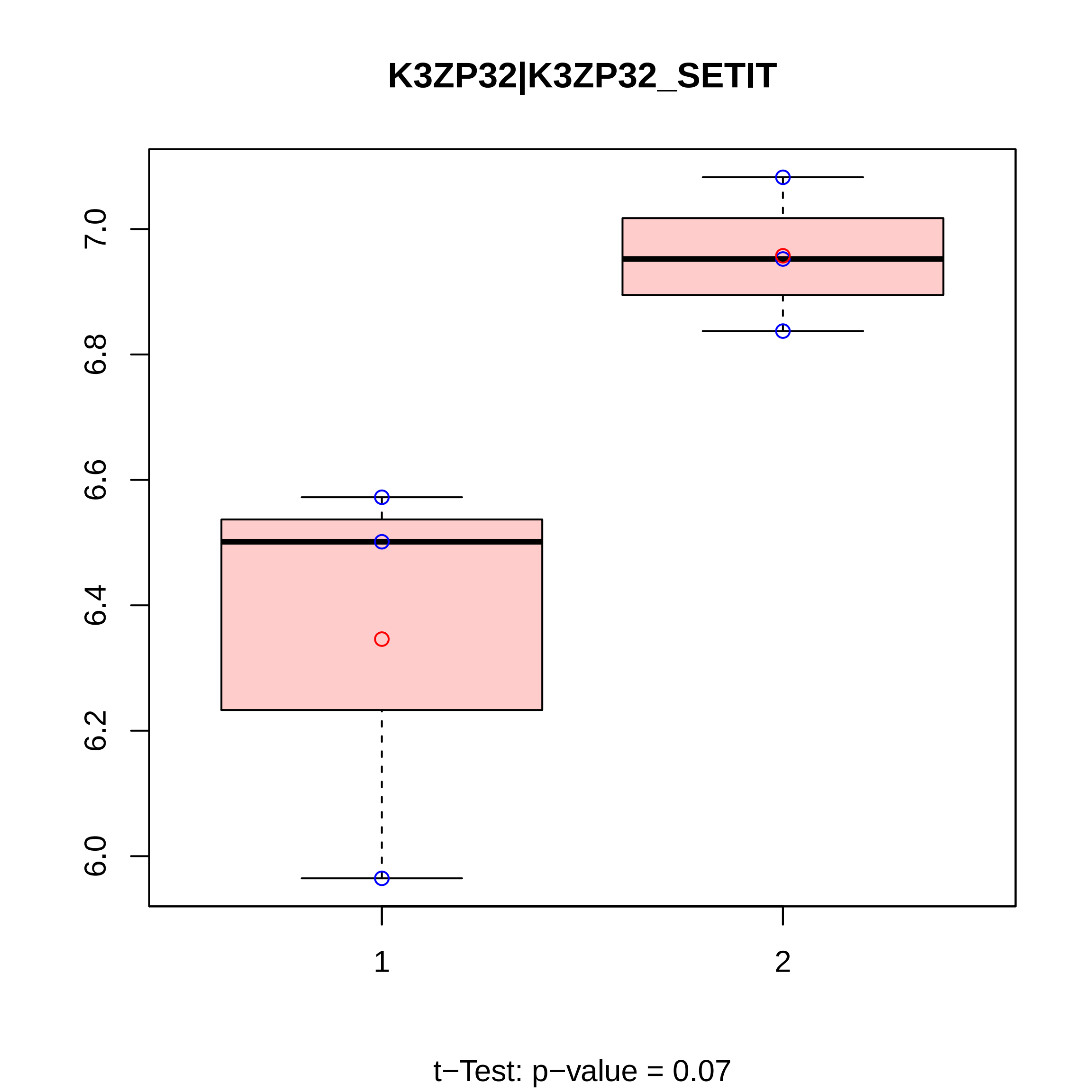
K3ZP32|K3ZP32_SETIT
7.0
6.8
6.6
6.4
6.2
6.0
1
2
t−Test: p−value = 0.07

K3ZPW5|K3ZPW5_SETIT

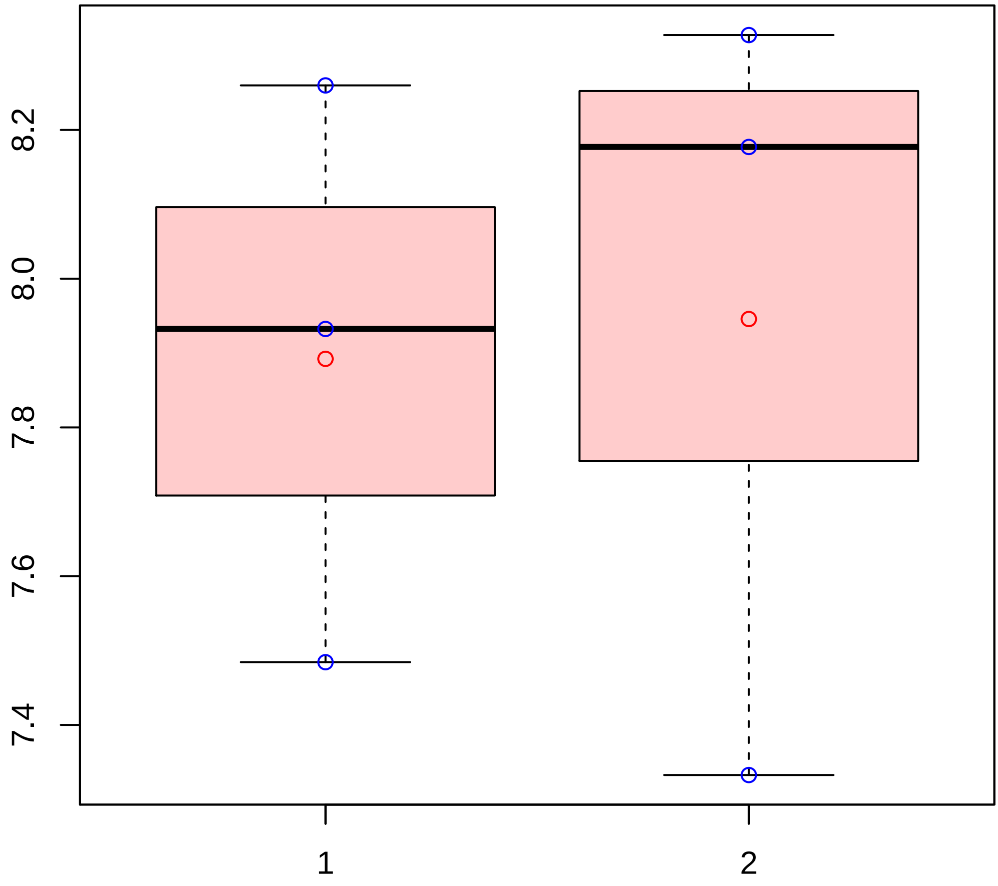

t−Test: p−value = 0.9

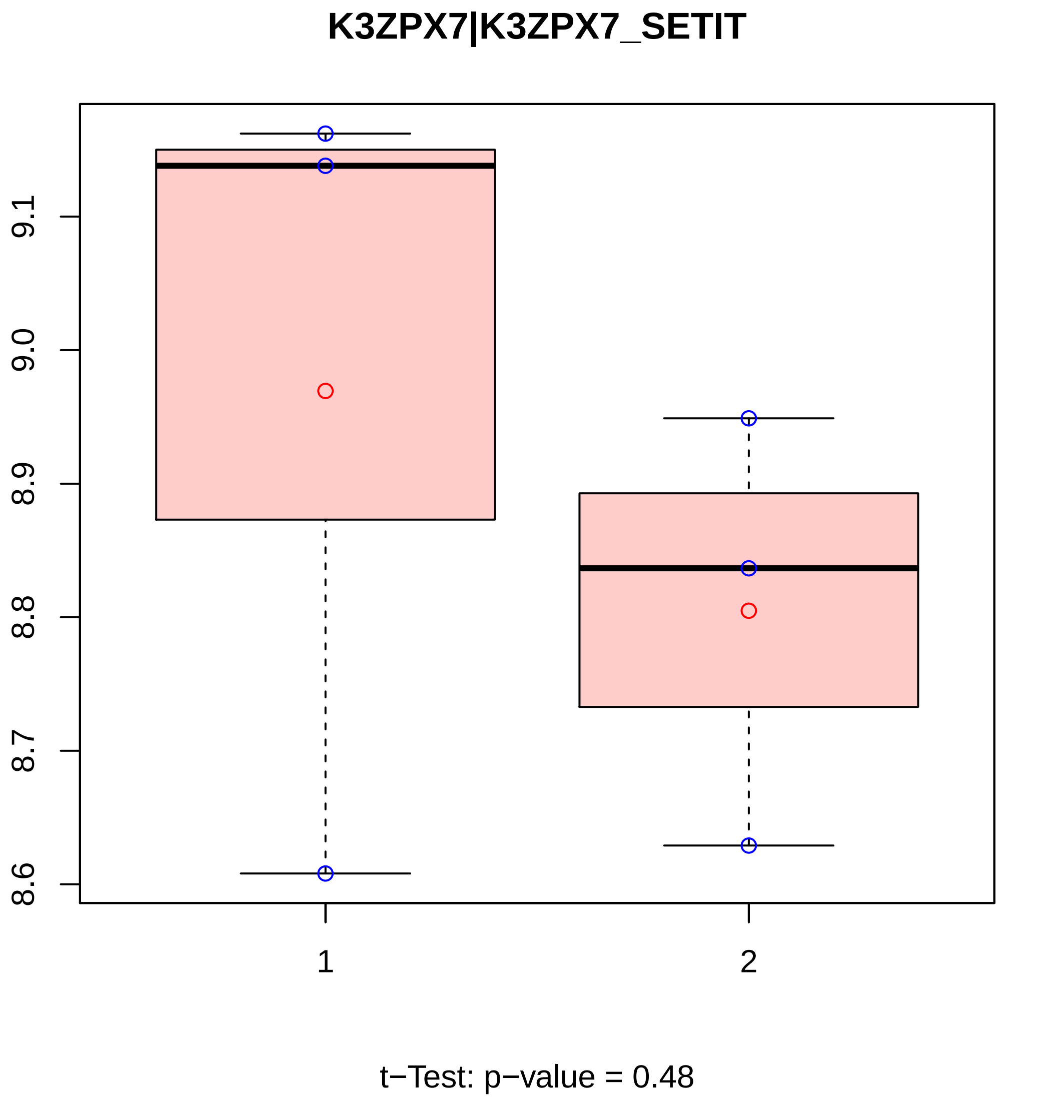
K3ZPX7|K3ZPX7_SETIT
9.1
9.0
8.9
8.8
8.7
8.6
1
2
t−Test: p−value = 0.48

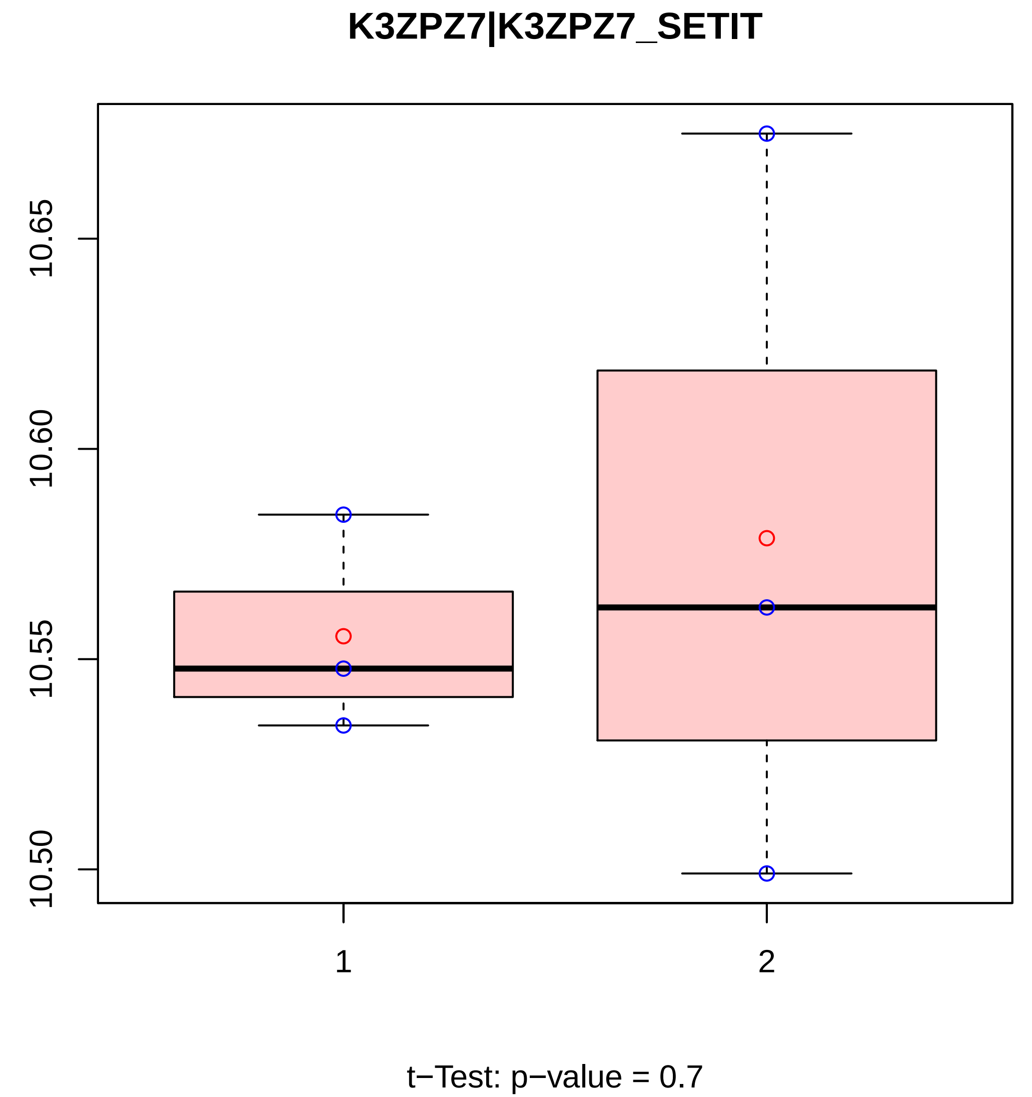
K3ZPZ7|K3ZPZ7_SETIT
10.50
10.55
10.60
10.65
1
2
t−Test: p−value = 0.7

K3ZQ57|K3ZQ57_SETIT

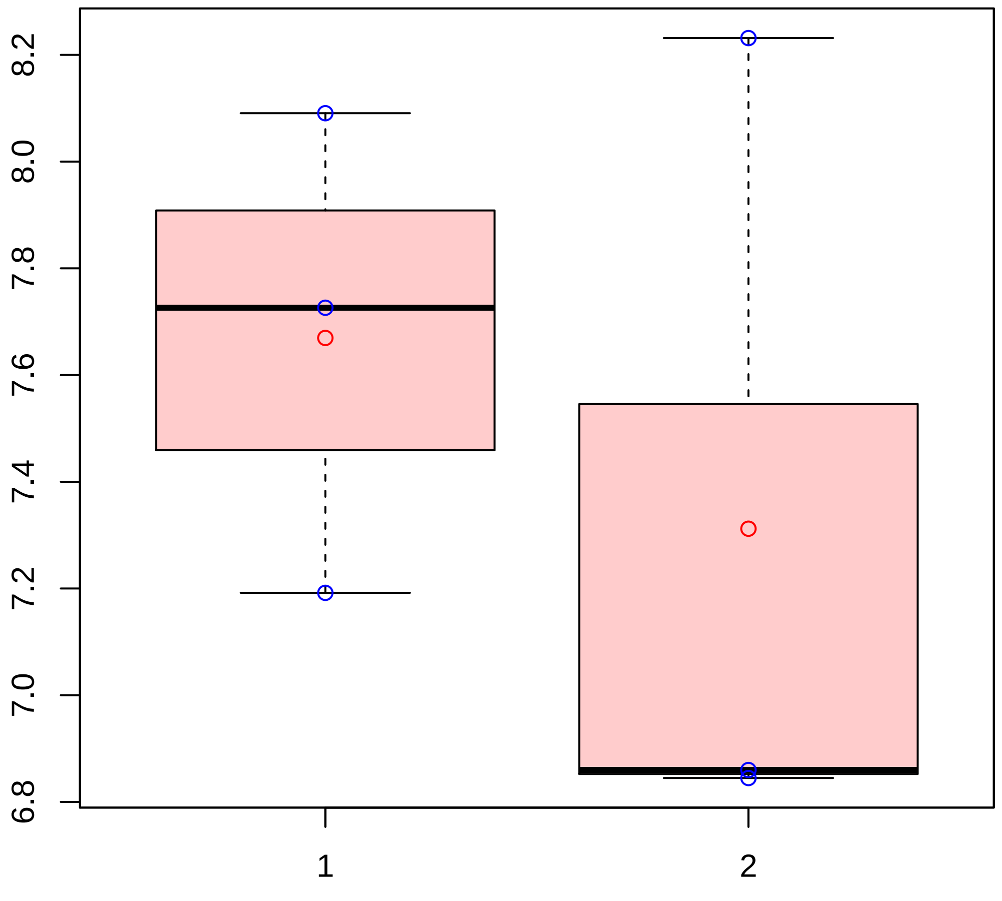

t−Test: p−value = 0.54

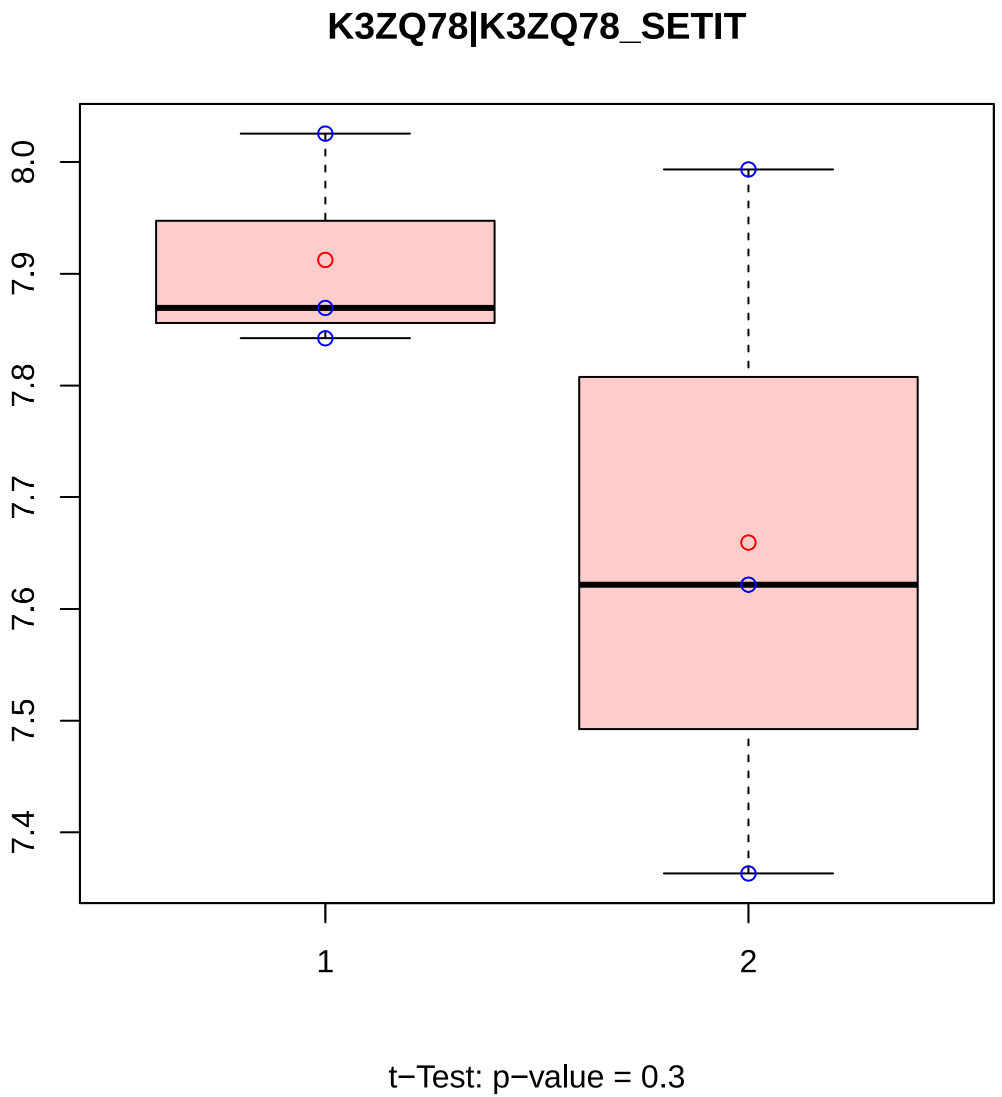
K3ZQ78|K3ZQ78_SETIT
8.0
7.9
7.8
7.7
7.6
7.5
7.4
1
2
t−Test: p−value = 0.3

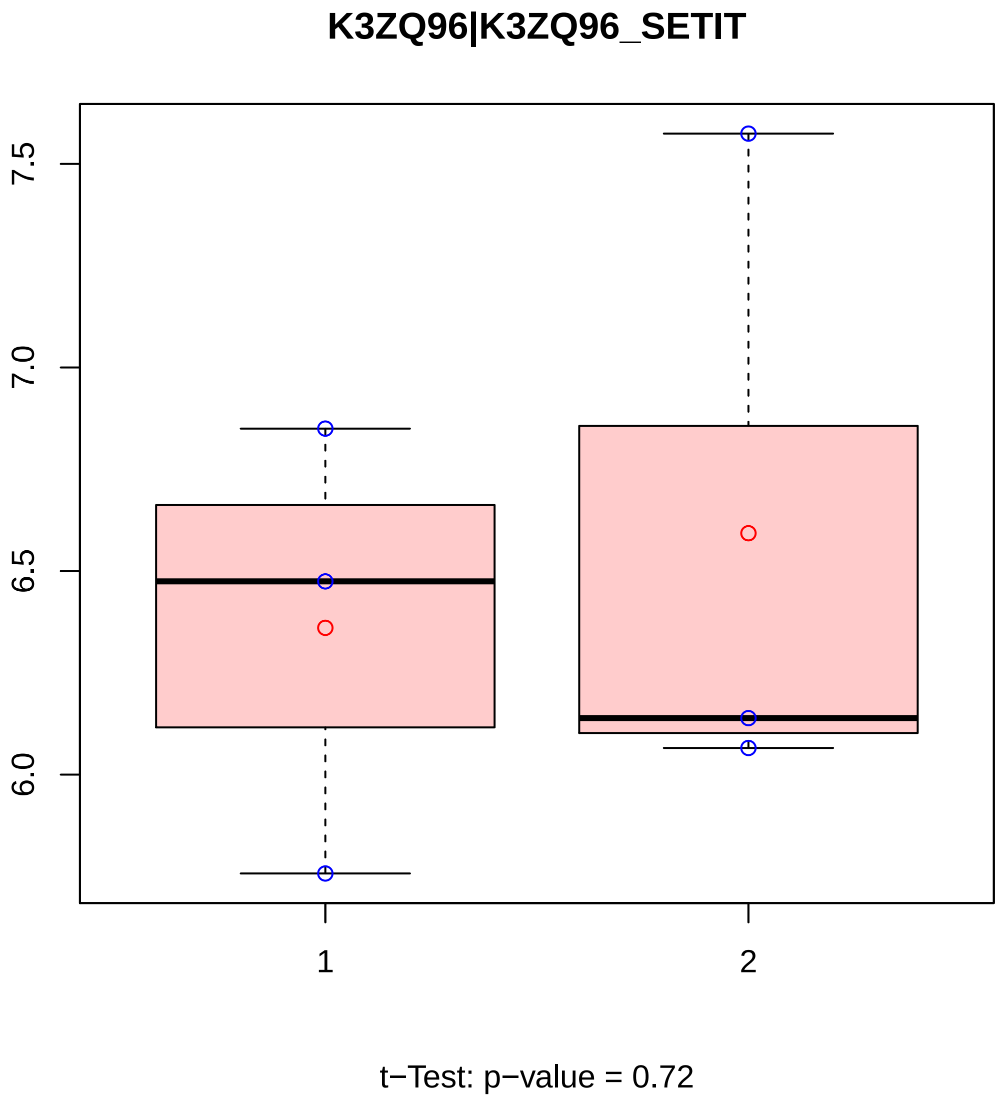

K3ZQ96|K3ZQ96_SETIT
7.5
7.0
6.5
6.0
1
2
t−Test: p−value = 0.72

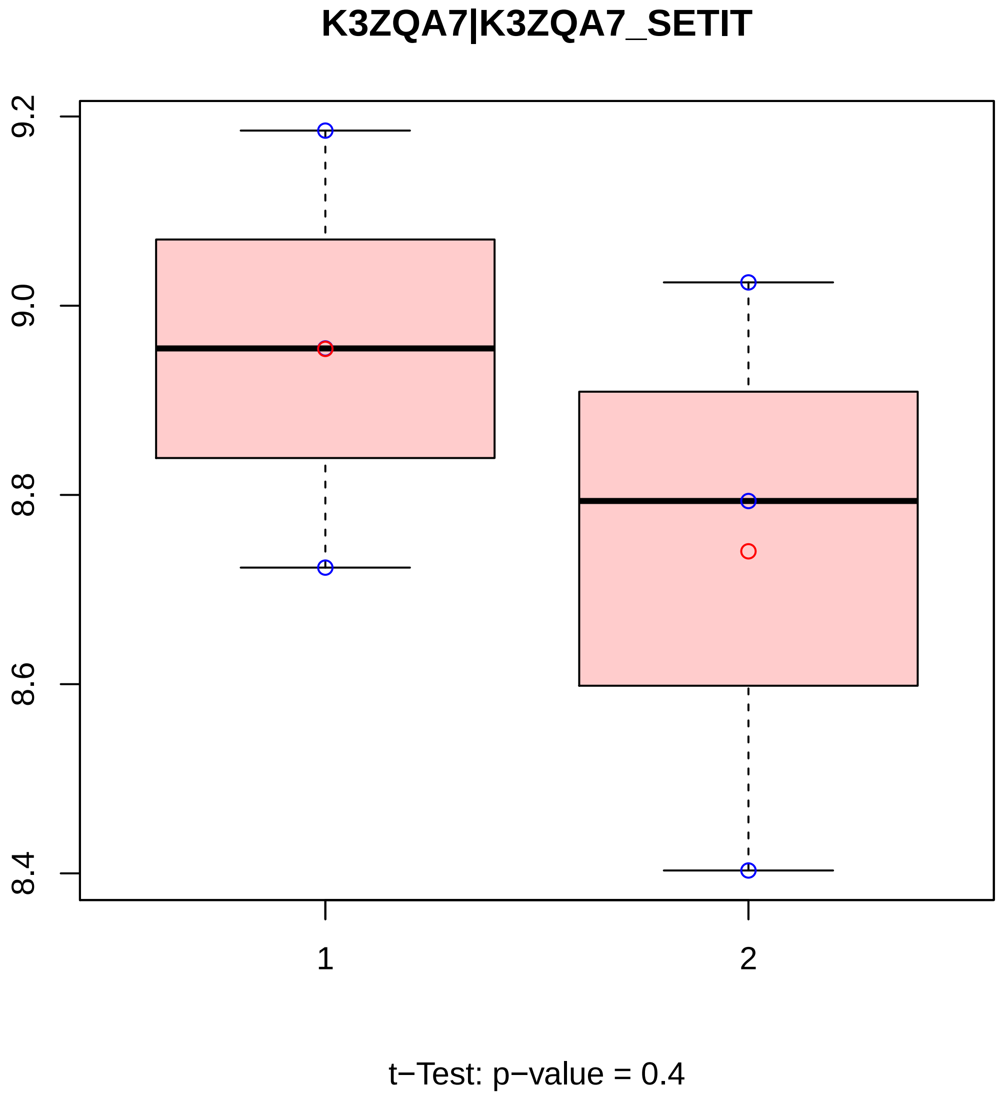
K3ZQA7|K3ZQA7_SETIT
9.2
9.0
8.8
8.6
8.4
1
2
t−Test: p−value = 0.4

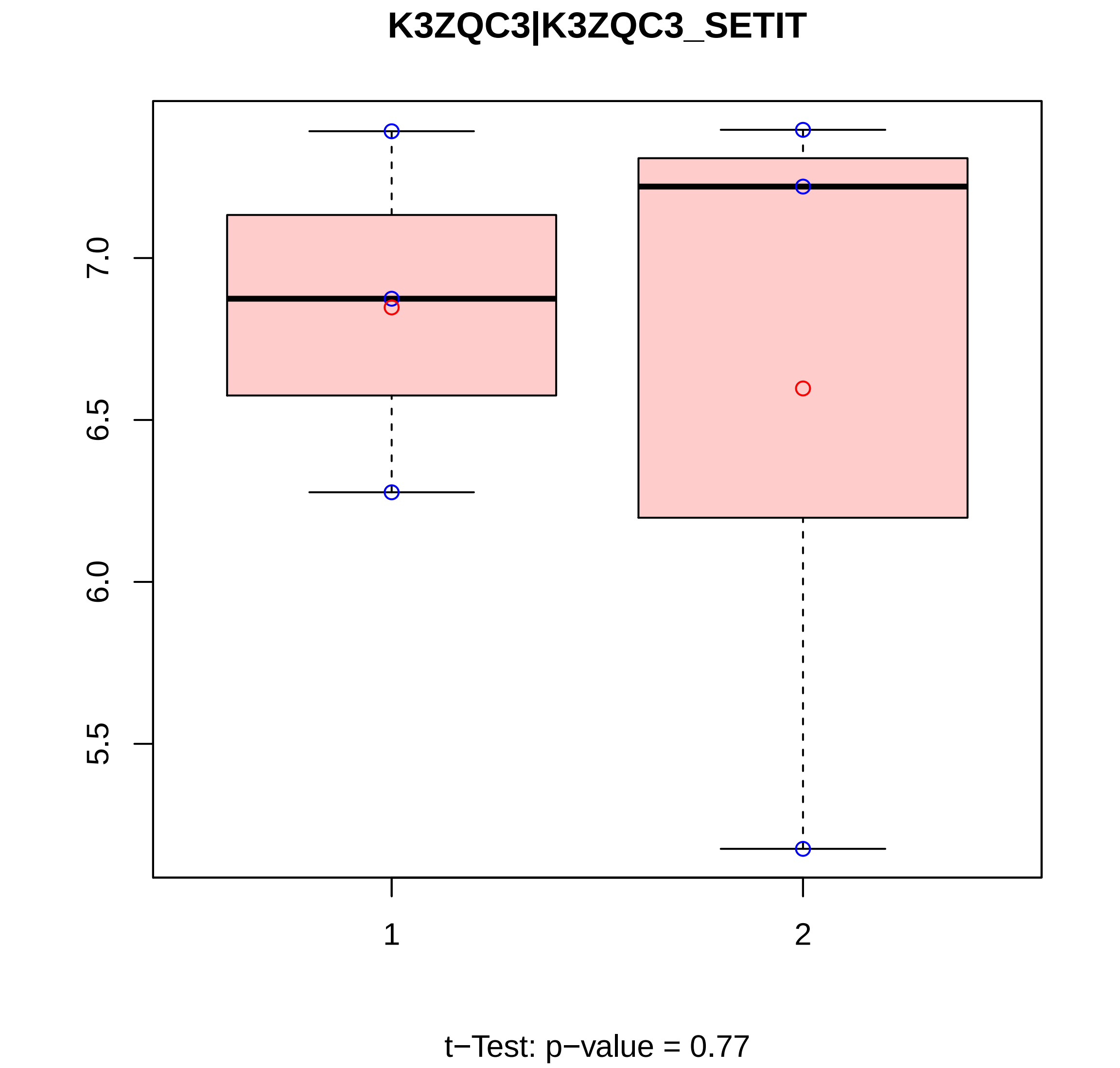
K3ZQC3|K3ZQC3_SETIT
7.0
6.5
6.0
5.5
1
2
t−Test: p−value = 0.77

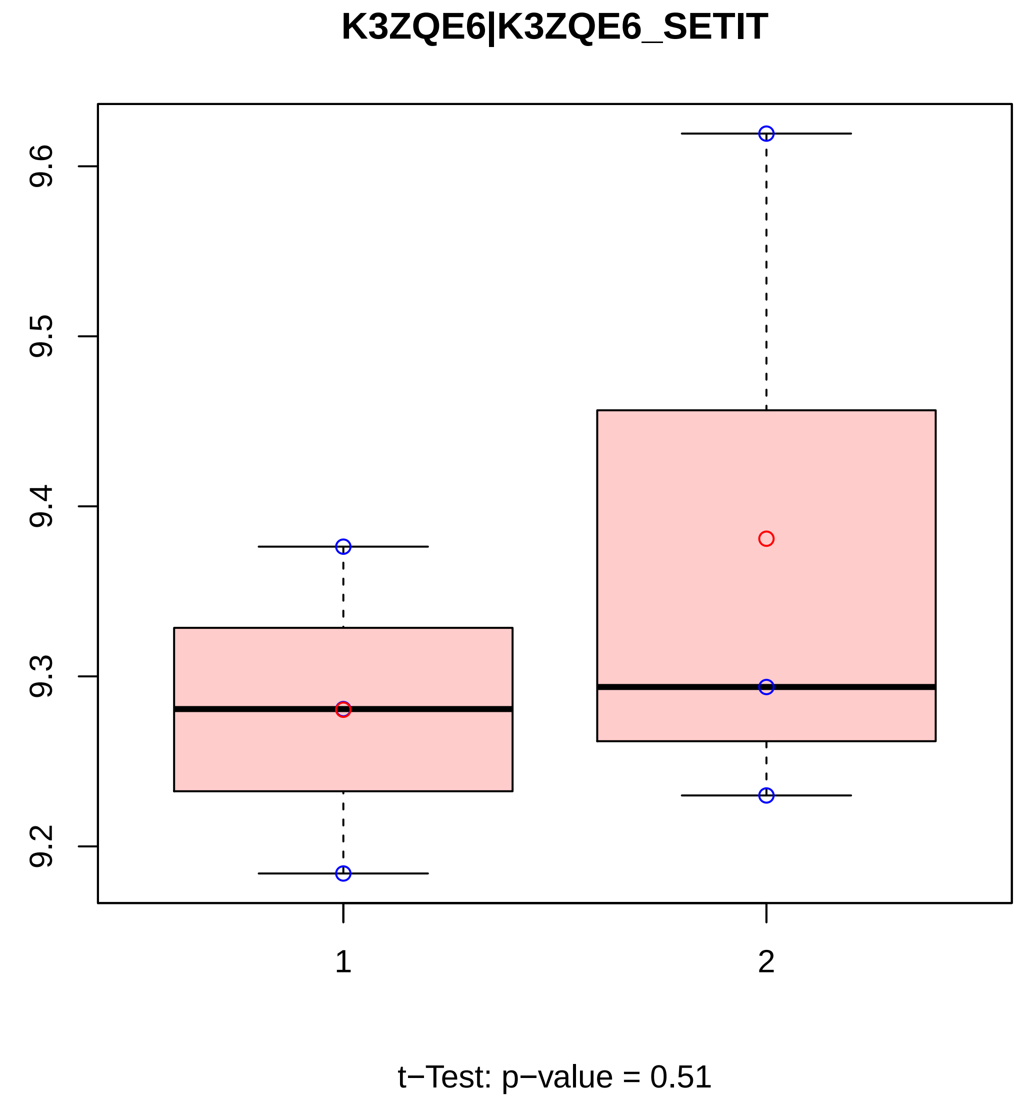
K3ZQE6|K3ZQE6_SETIT
9.2
9.3
9.4
9.5
9.6
1
2
t−Test: p−value = 0.51

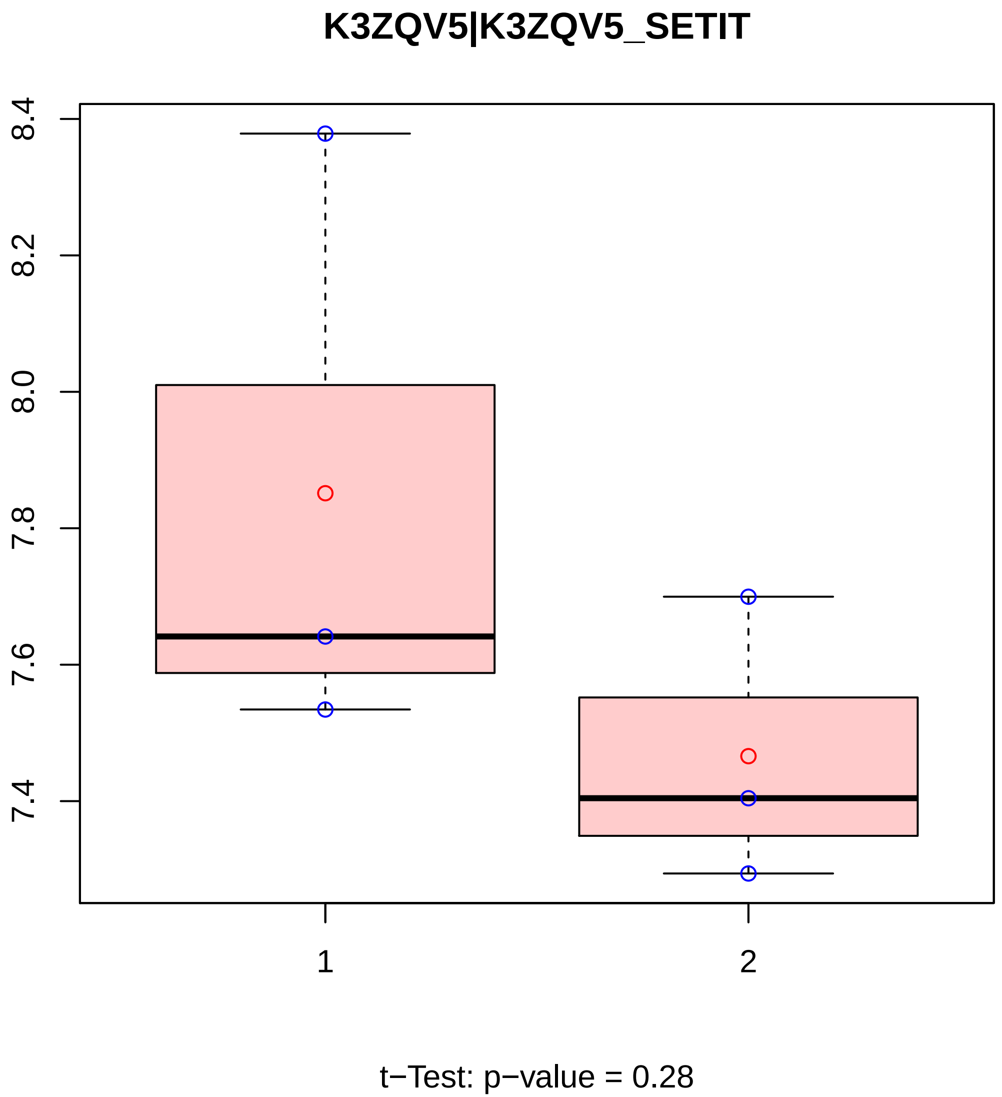
K3ZQV5|K3ZQV5_SETIT
8.4
8.2
8.0
7.8
7.6
7.4
1
2
t−Test: p−value = 0.28

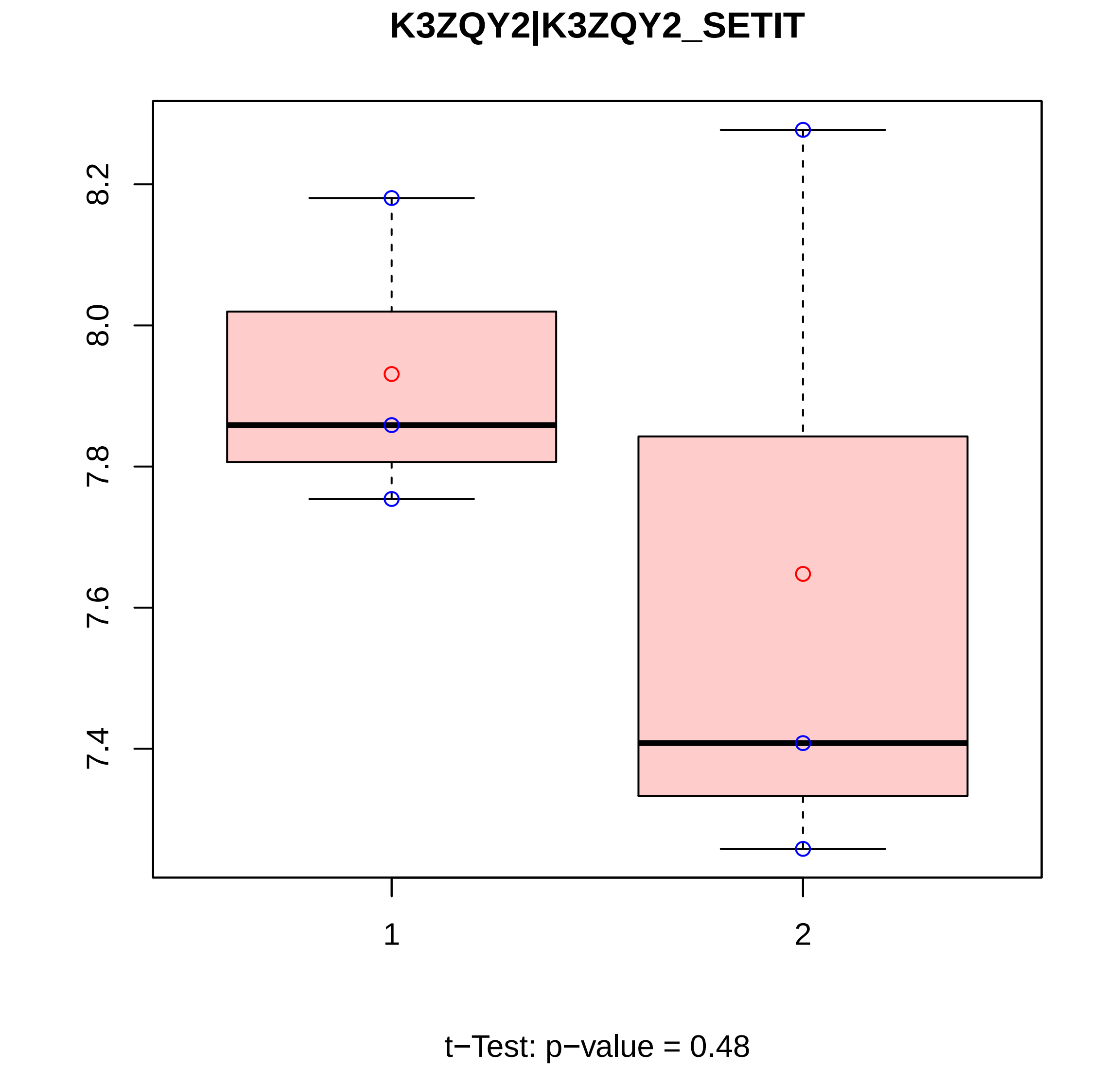
K3ZQY2|K3ZQY2_SETIT
8.2
8.0
7.8
7.6
7.4
1
2
t−Test: p−value = 0.48

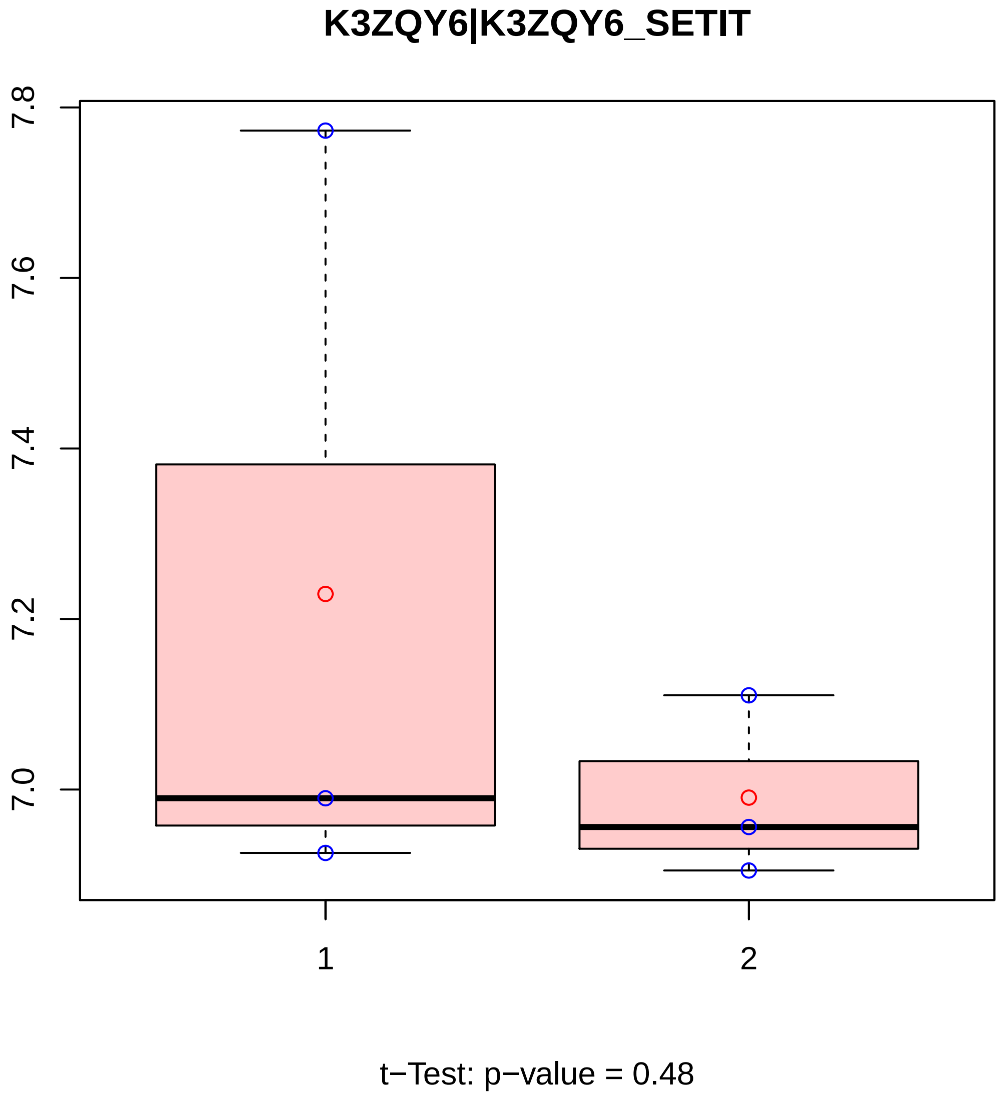

K3ZQY6|K3ZQY6_SETIT
7.8
7.6
7.4
7.2
7.0
1
2
t−Test: p−value = 0.48

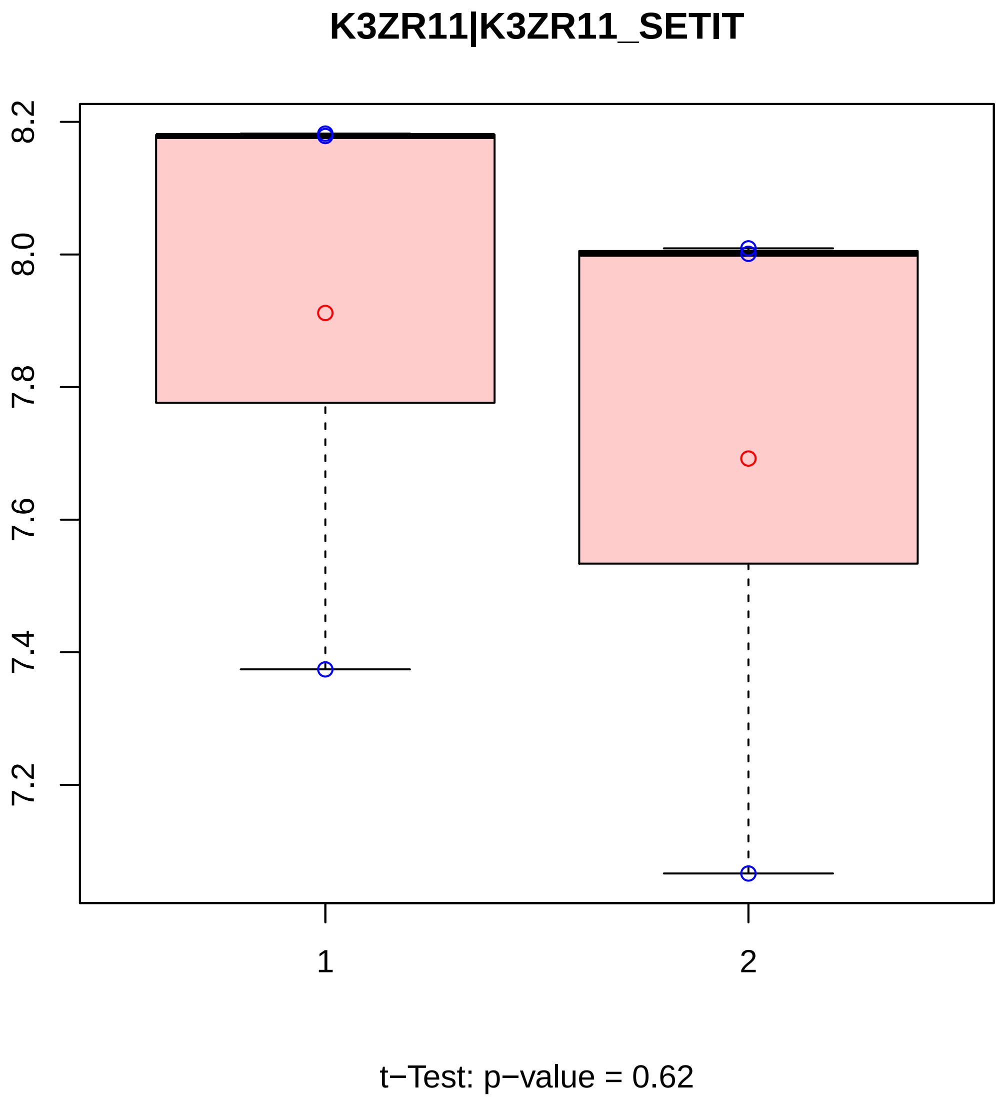
K3ZR11|K3ZR11_SETIT
8.2
8.0
7.8
7.6
7.4
7.2
1
2
t−Test: p−value = 0.62

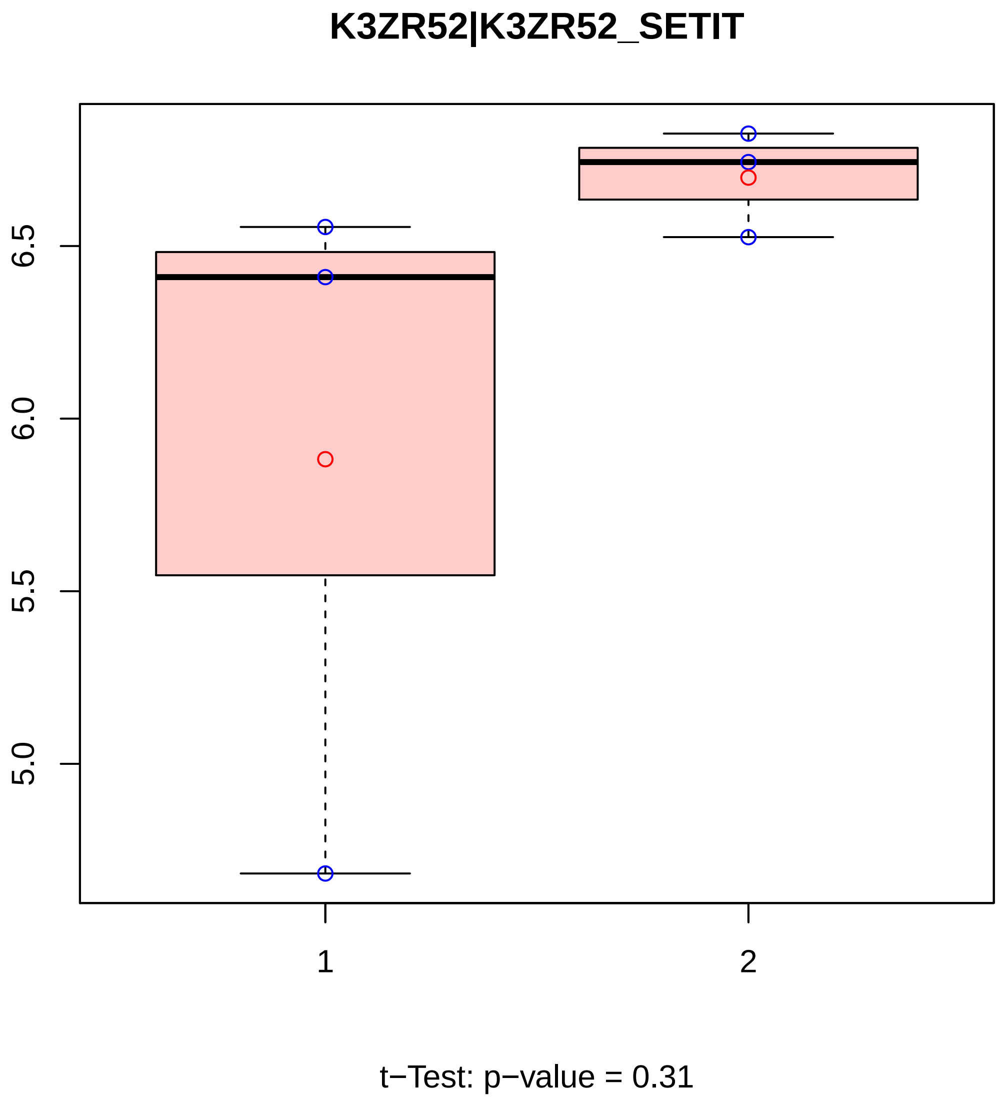

K3ZR52|K3ZR52_SETIT
6.5
6.0
5.5
5.0
1
2
t−Test: p−value = 0.31

K3ZR74|K3ZR74_SETIT

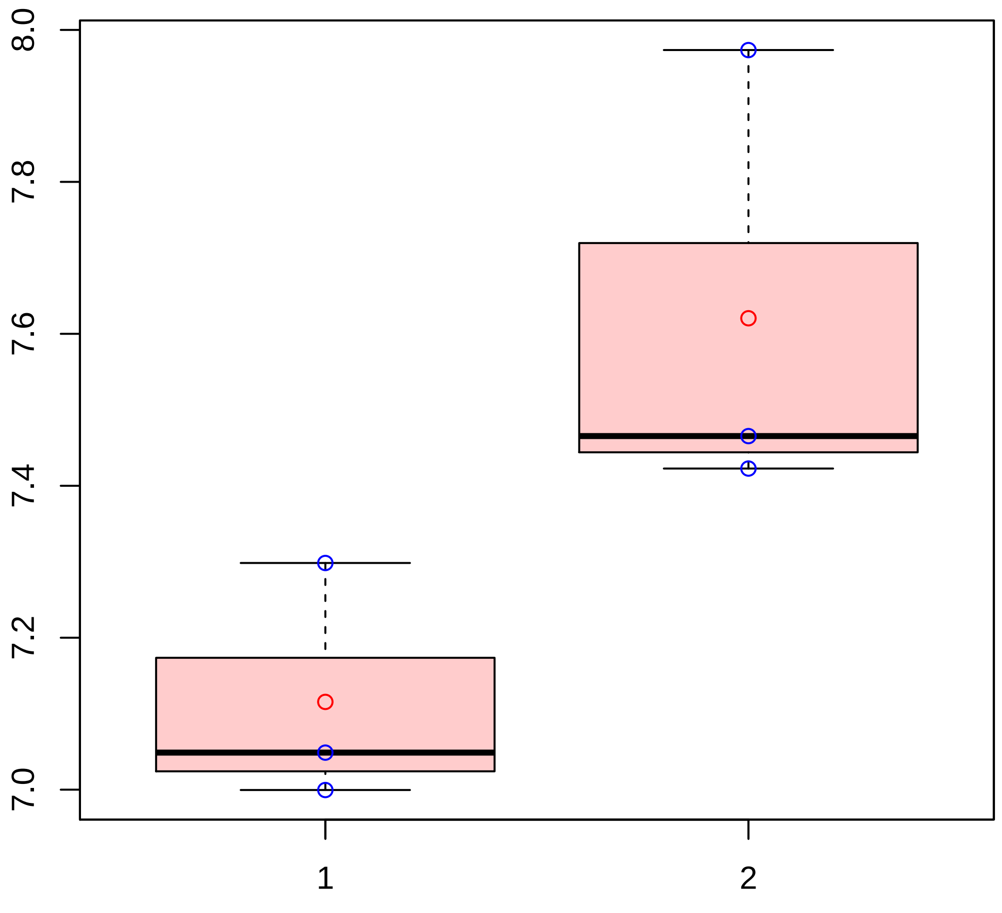

t−Test: p−value = 0.08

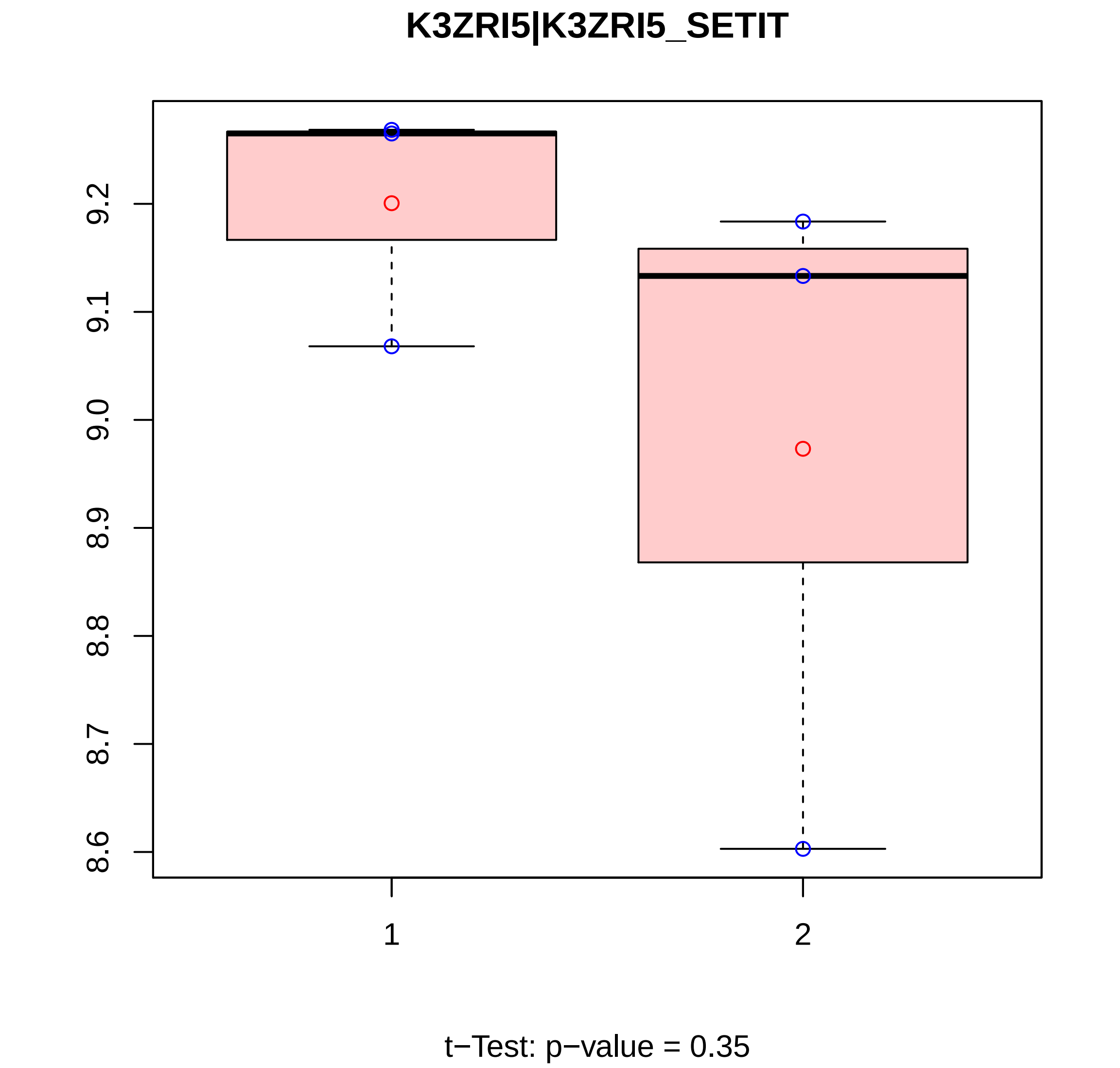

K3ZRI5|K3ZRI5_SETIT
9.2
9.1
9.0
8.9
8.8
8.7
8.6
1
2
t−Test: p−value = 0.35

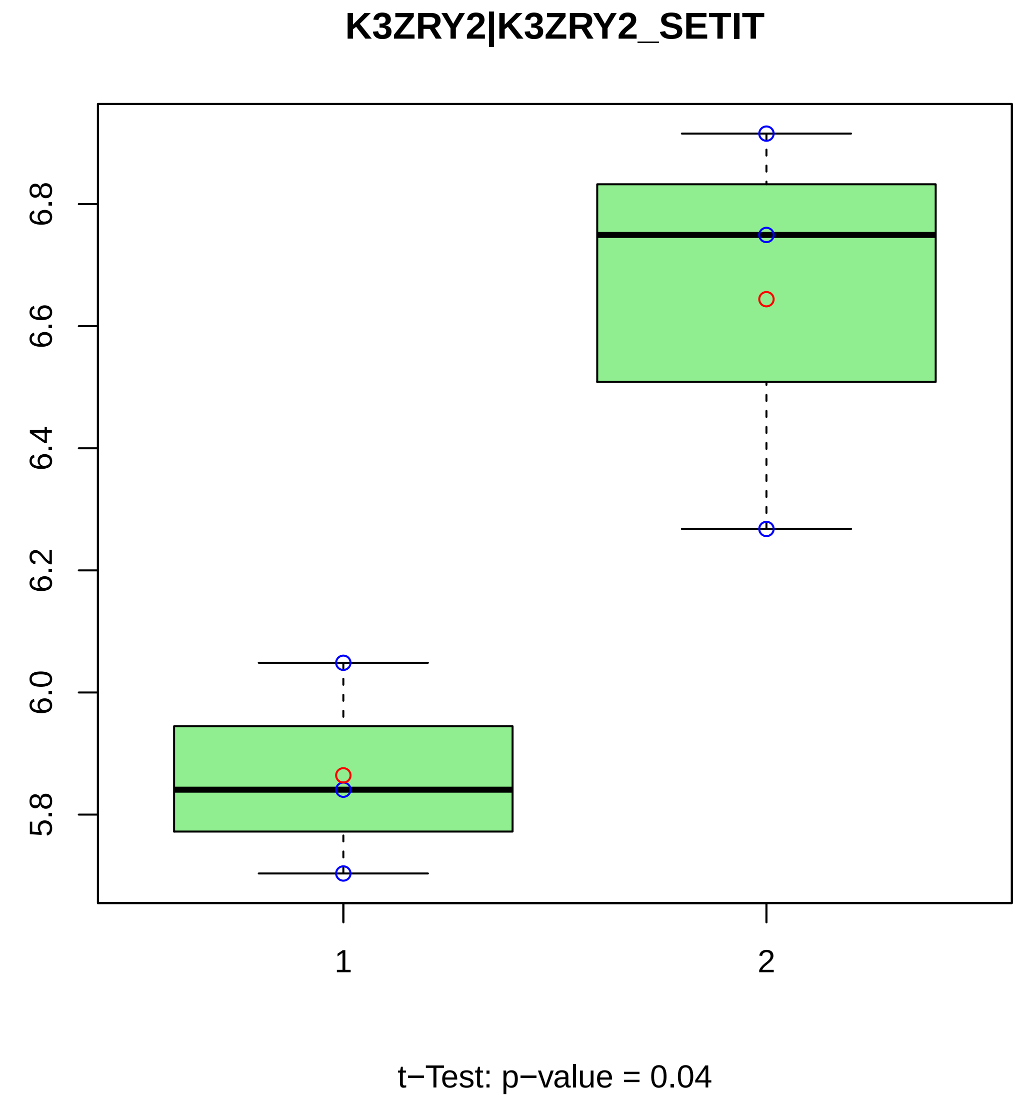
K3ZRY2|K3ZRY2_SETIT
6.8
6.6
6.4
6.2
6.0
5.8
1
2
t−Test: p−value = 0.04

K3ZSG5|K3ZSG5_SETIT

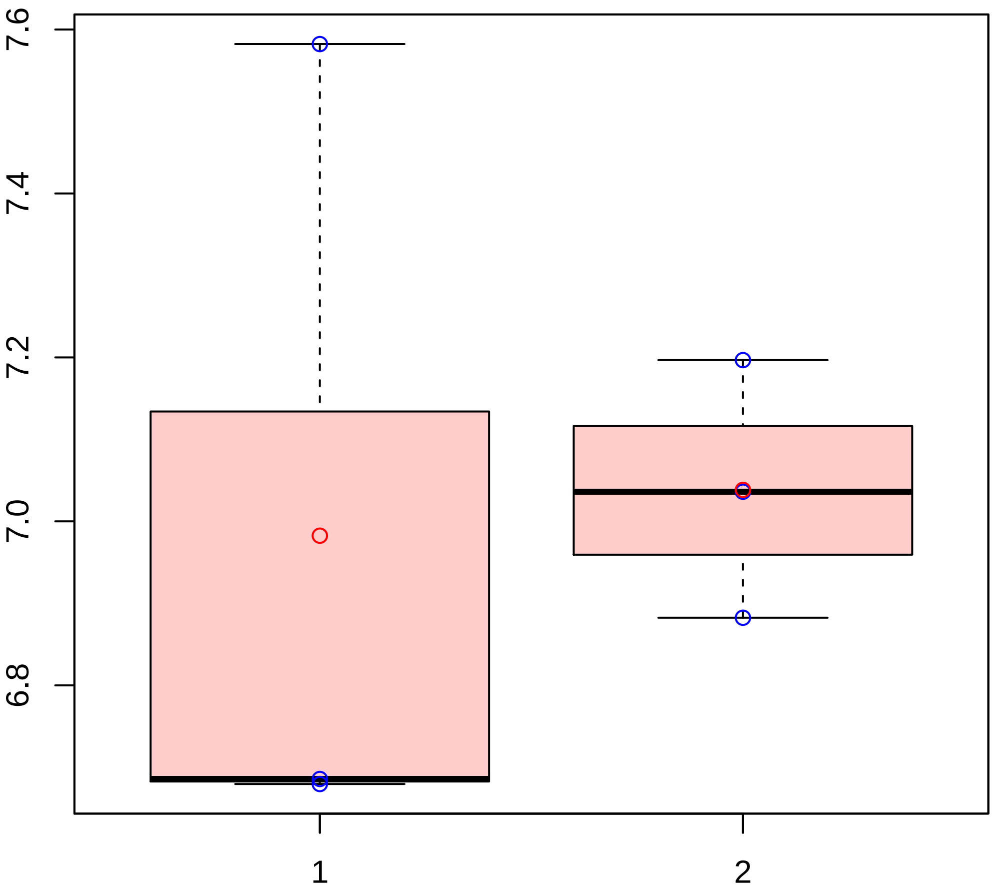

t−Test: p−value = 0.87

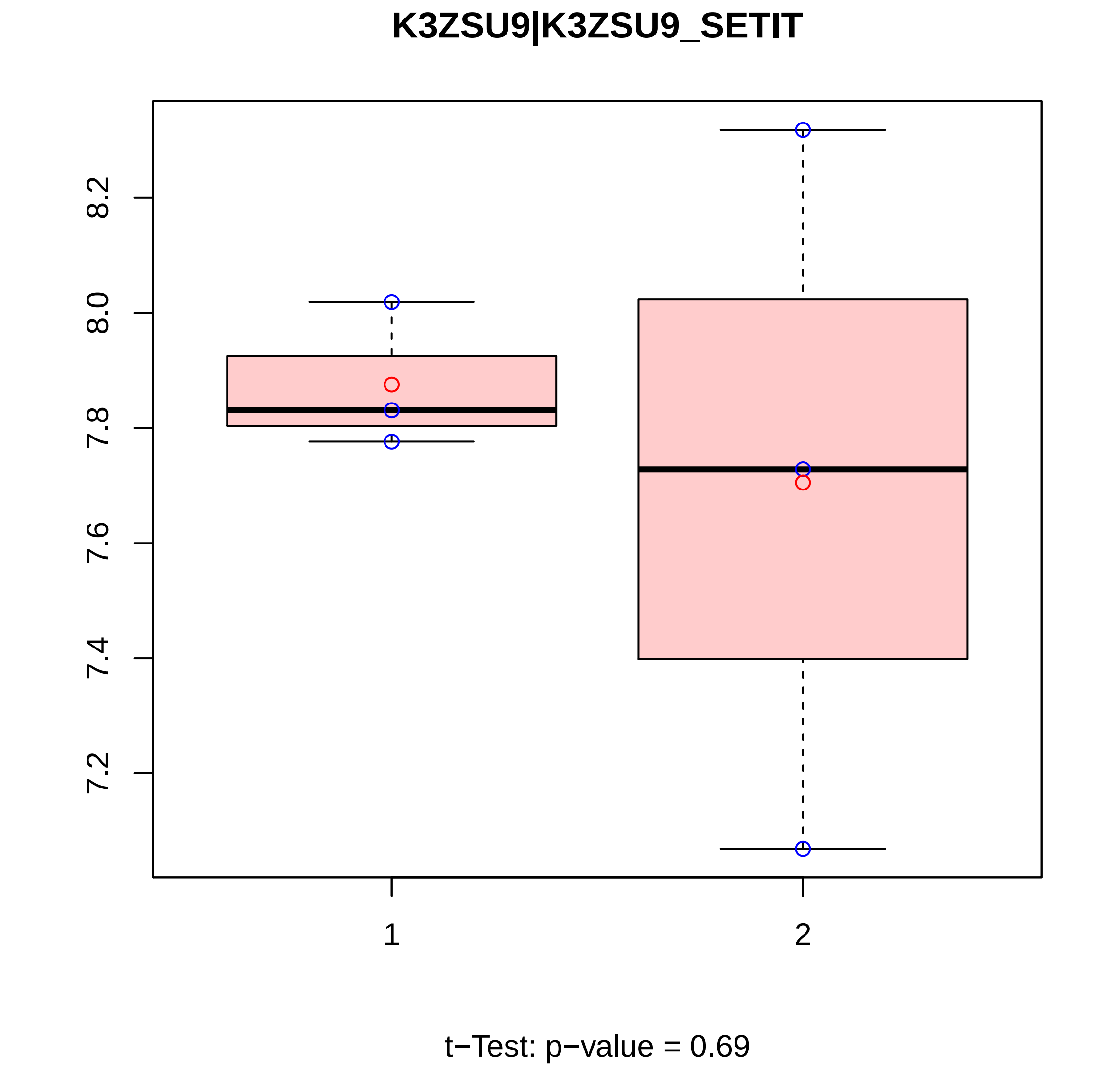
K3ZSU9|K3ZSU9_SETIT
8.2
8.0
7.8
7.6
7.4
7.2
1
2
t−Test: p−value = 0.69

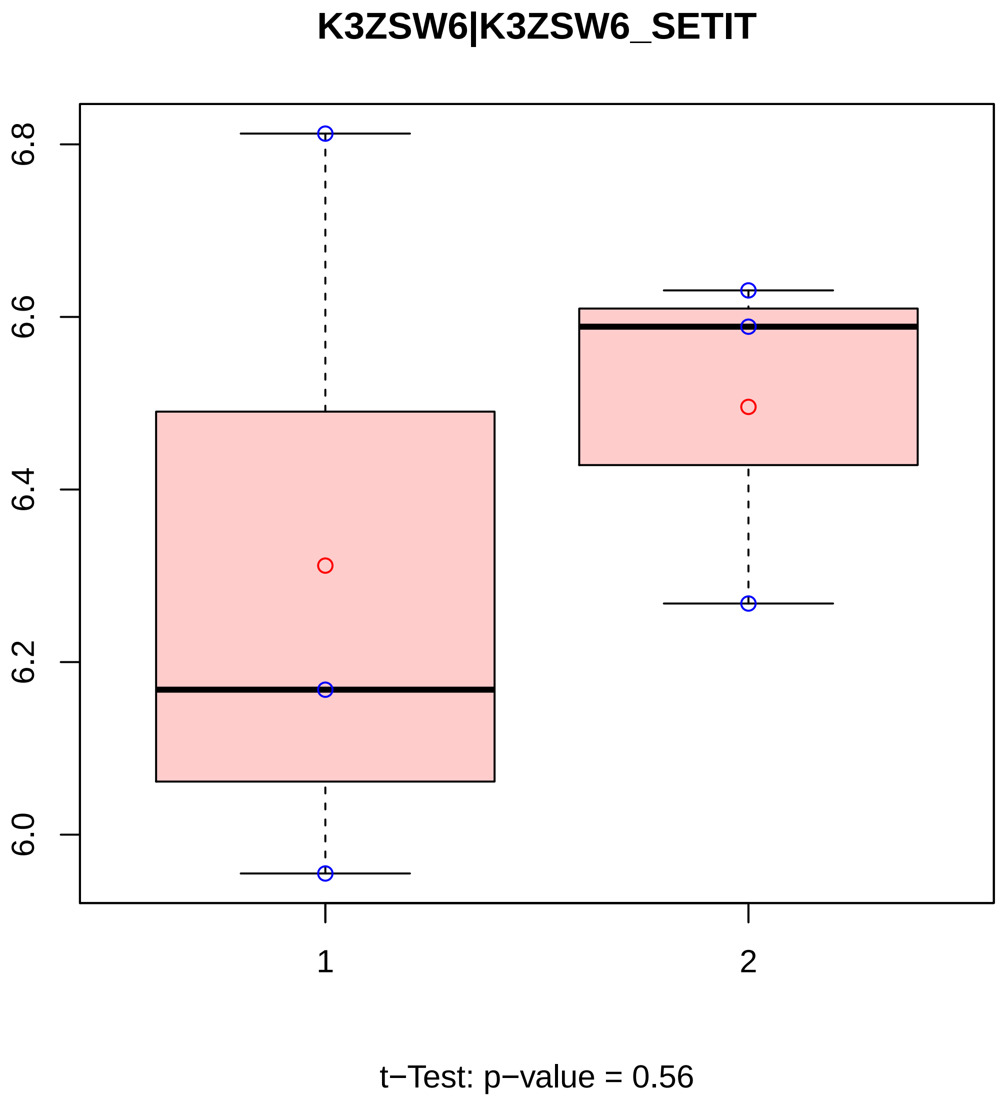
K3ZSW6|K3ZSW6_SETIT
6.0
6.2
6.4
6.6
6.8
1
2
t−Test: p−value = 0.56

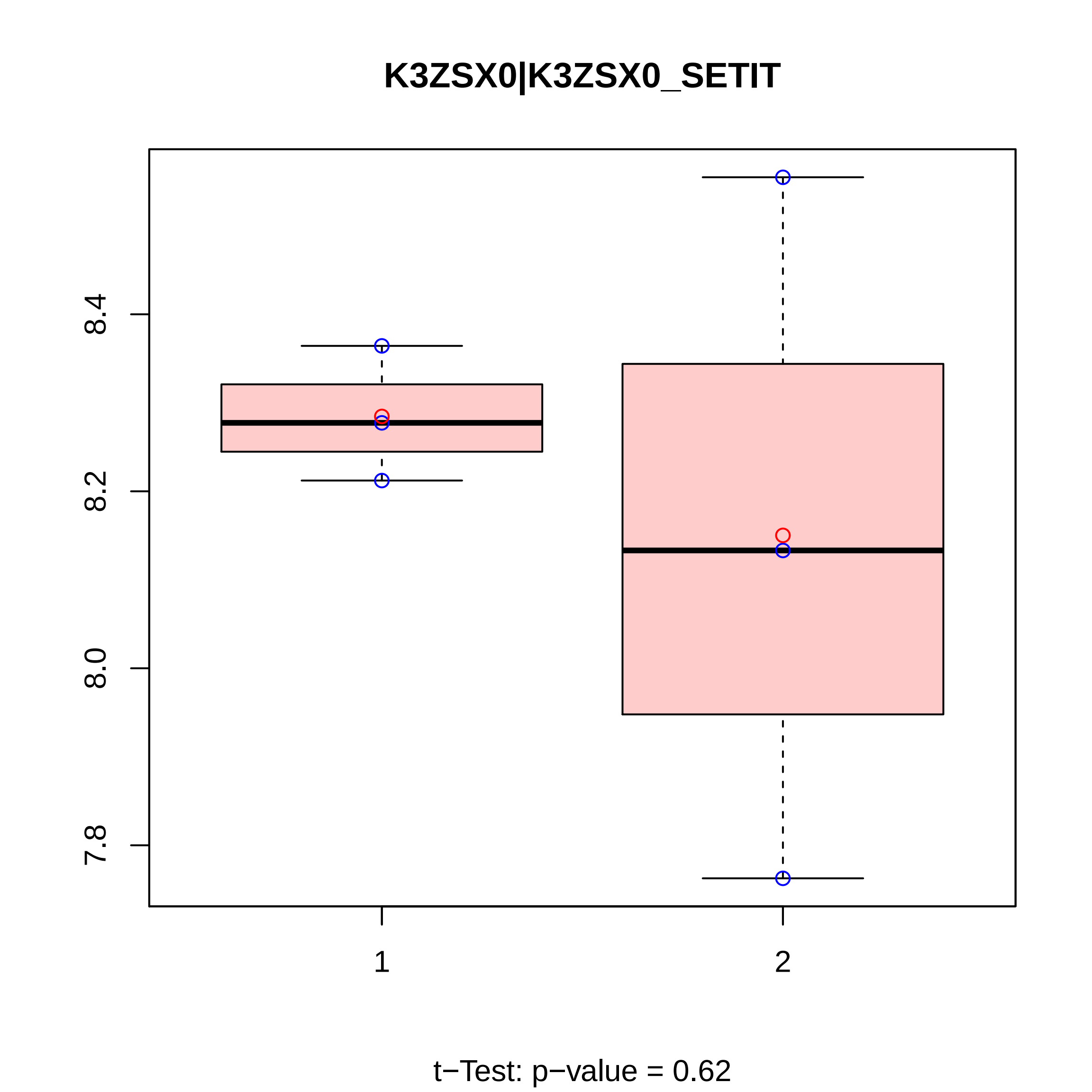

K3ZSX0|K3ZSX0_SETIT
8.4
8.2
8.0
7.8
1
2
t−Test: p−value = 0.62

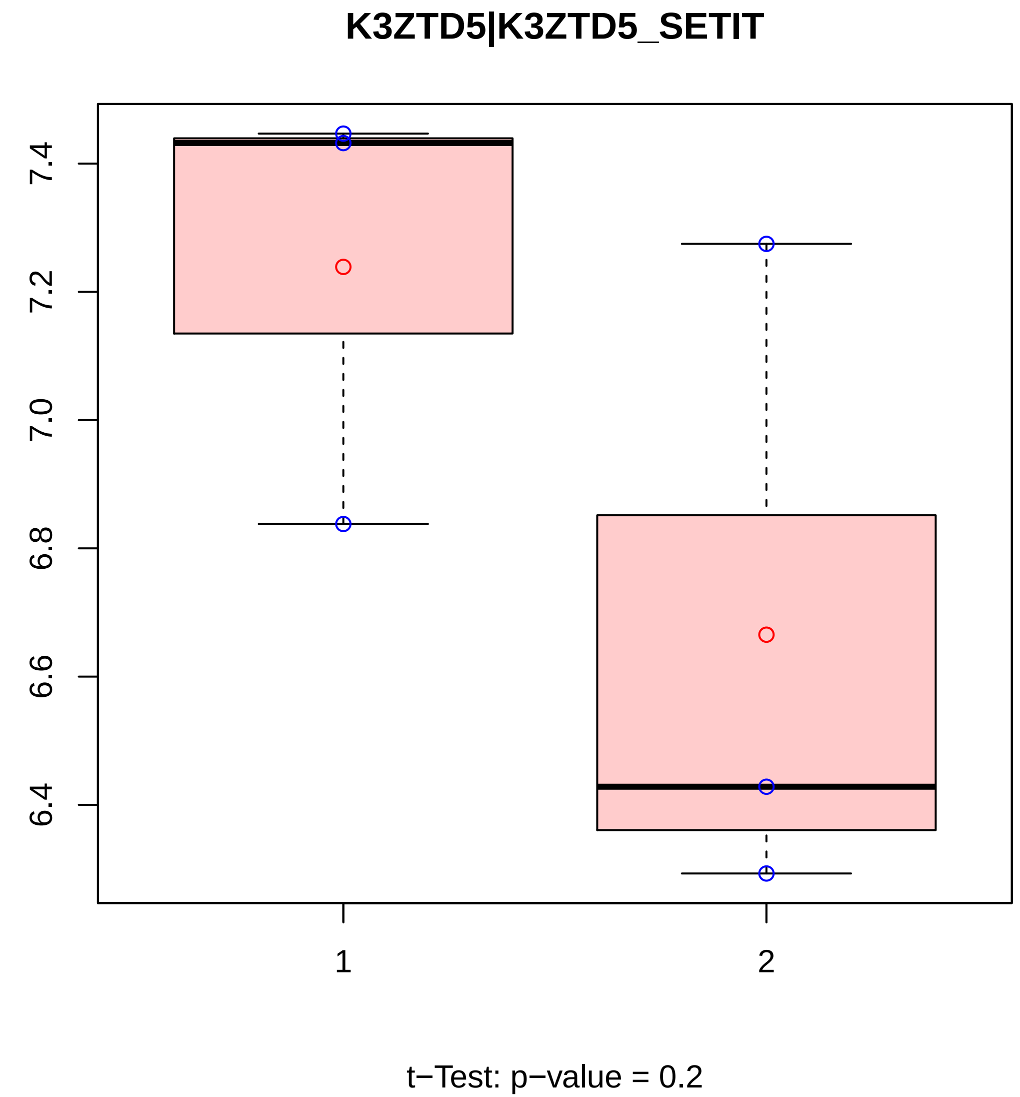

K3ZTD5|K3ZTD5_SETIT
7.4
7.2
7.0
6.8
6.6
6.4
1
2
t−Test: p−value = 0.2

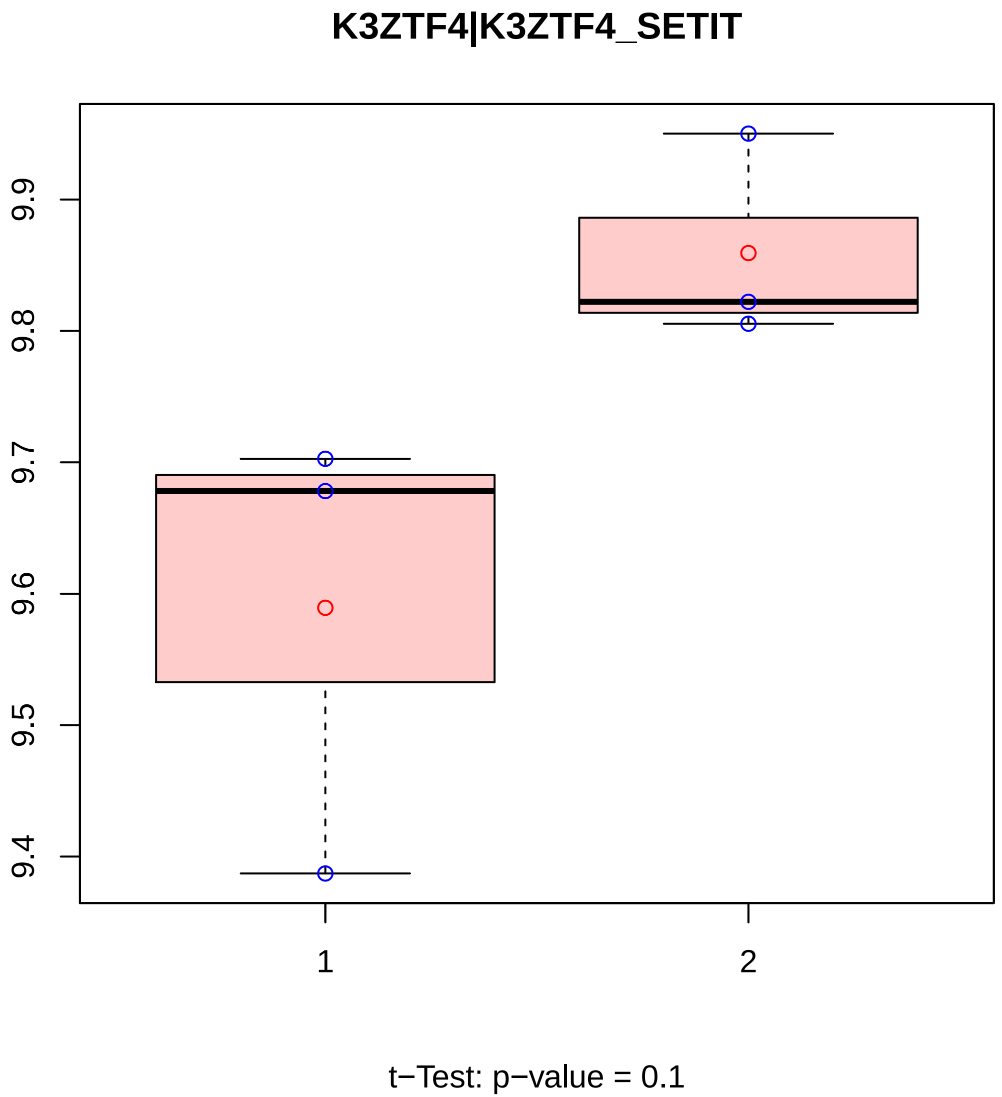
K3ZTF4|K3ZTF4_SETIT
9.4
9.5
9.6
9.7
9.8
9.9
1
2
t−Test: p−value = 0.1

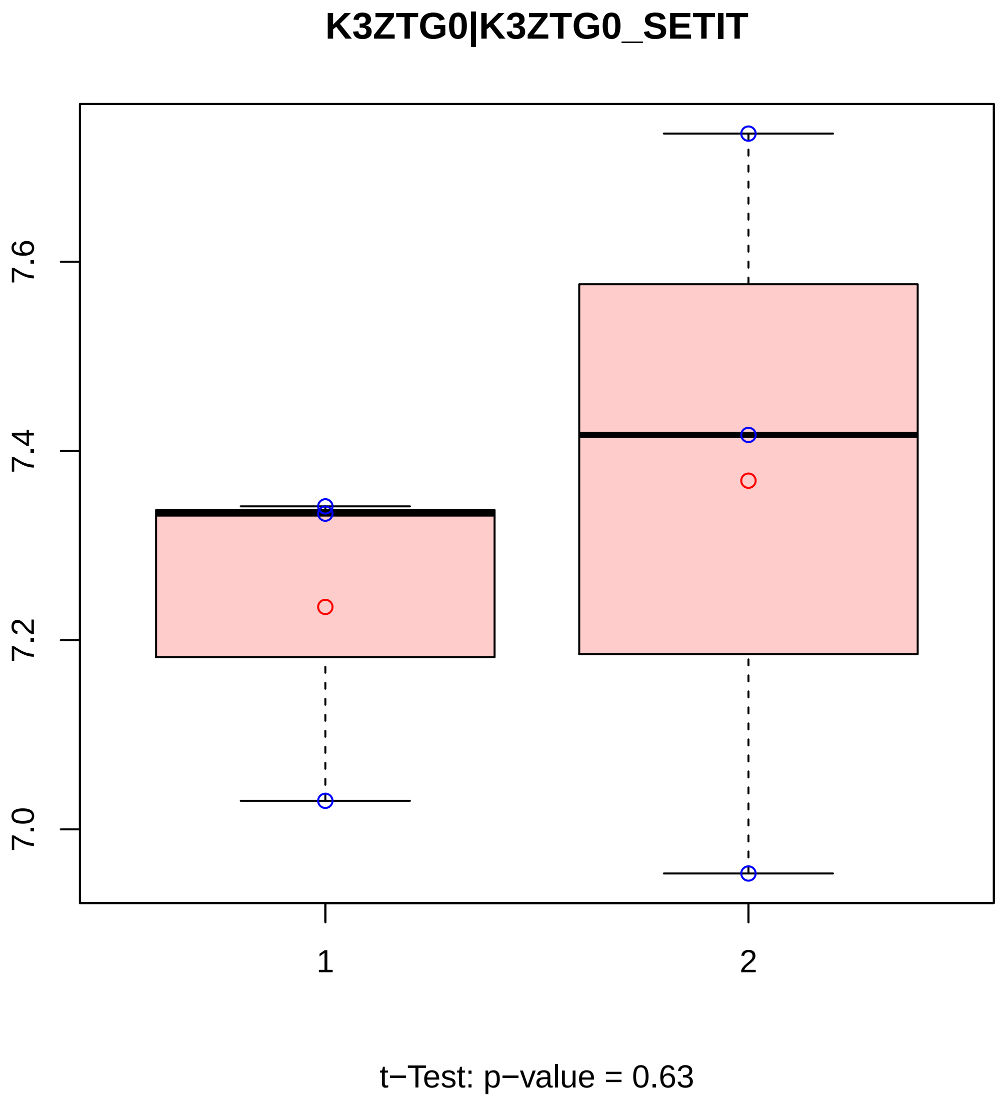
K3ZTG0|K3ZTG0_SETIT
7.0
7.2
7.4
7.6
1
2
t−Test: p−value = 0.63

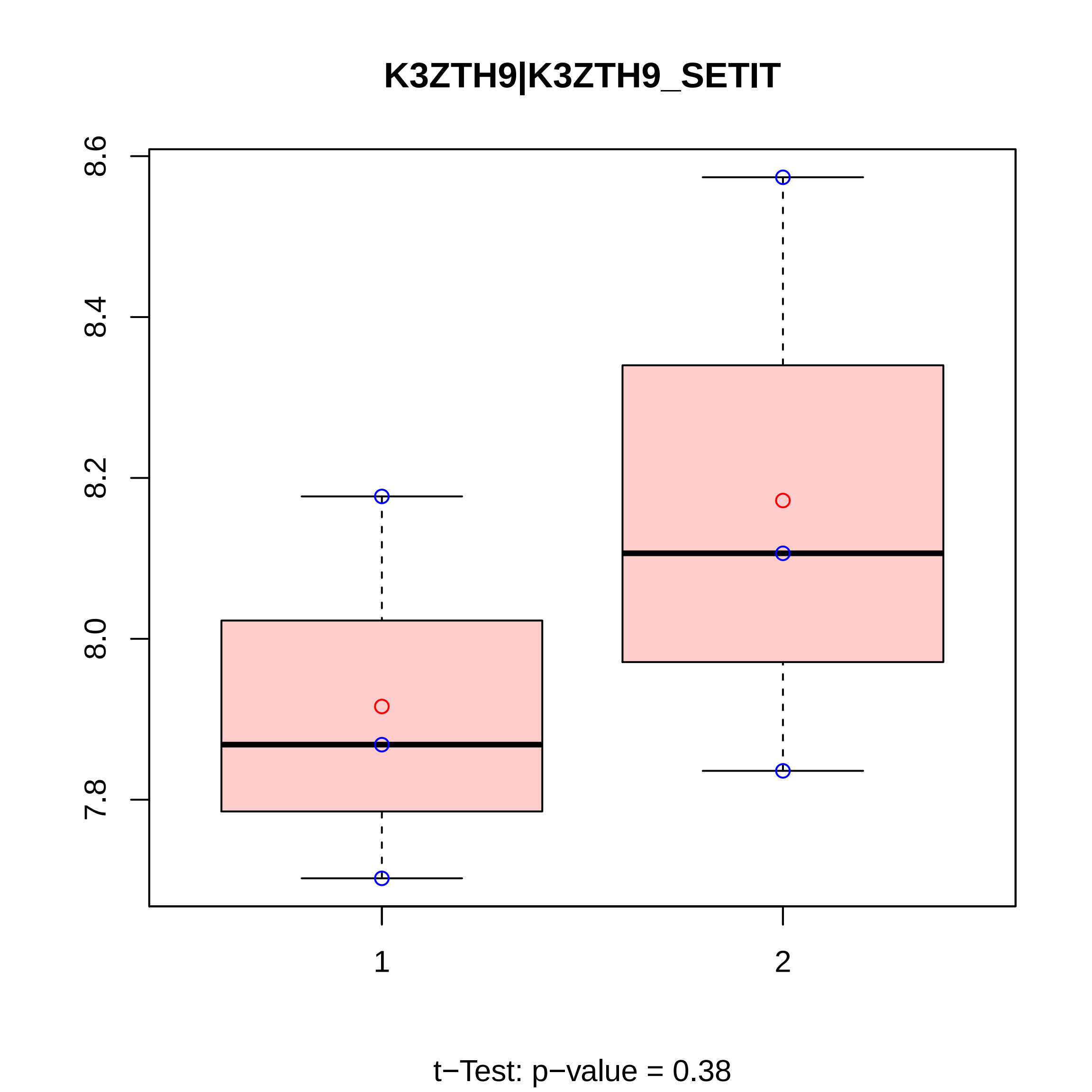
K3ZTH9|K3ZTH9_SETIT
8.6
8.4
8.2
8.0
7.8
1
2
t−Test: p−value = 0.38

K3ZTJ3|K3ZTJ3_SETIT

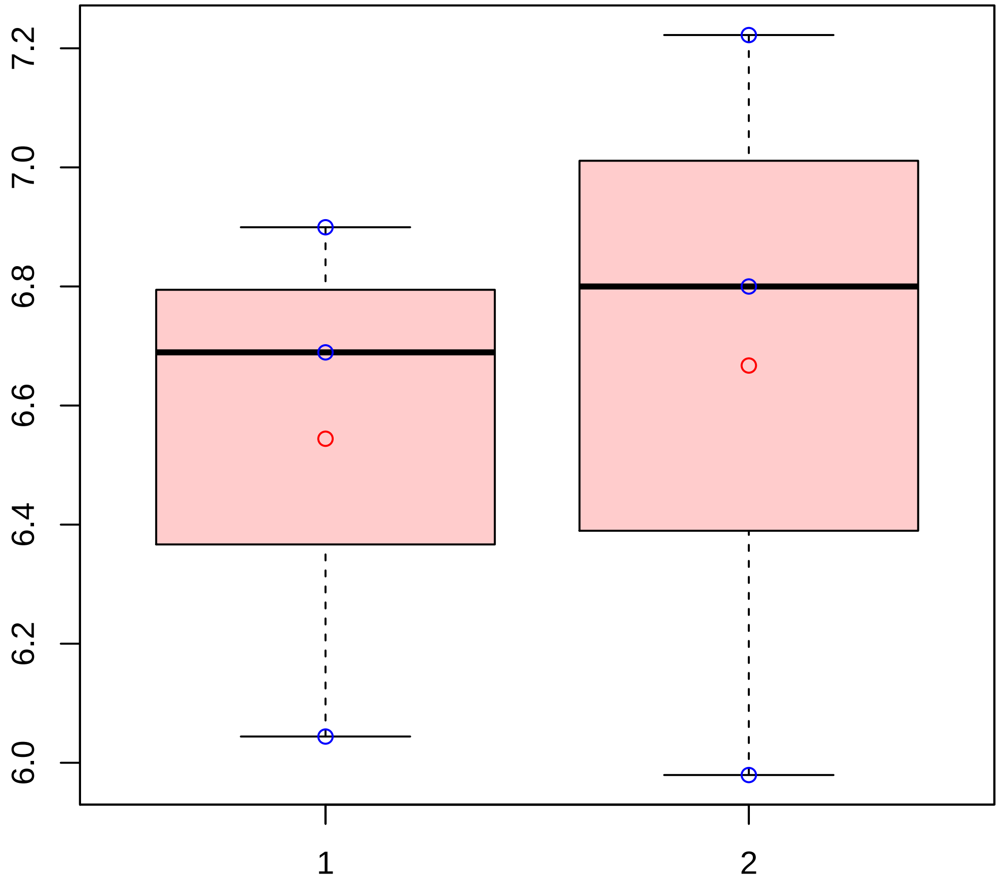

t−Test: p−value = 0.8

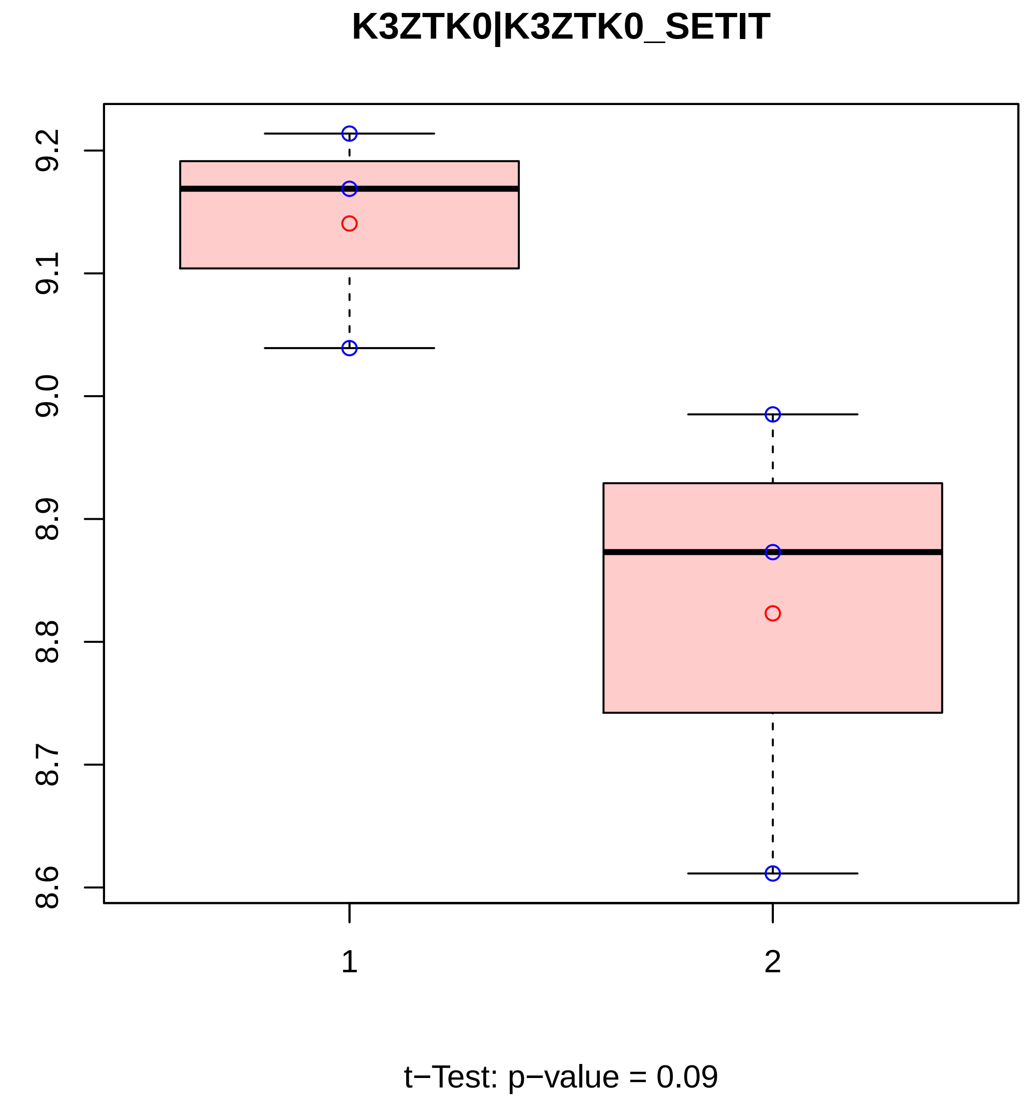
K3ZTK0|K3ZTK0_SETIT
9.2
9.1
9.0
8.9
8.8
8.7
8.6
1
2
t−Test: p−value = 0.09

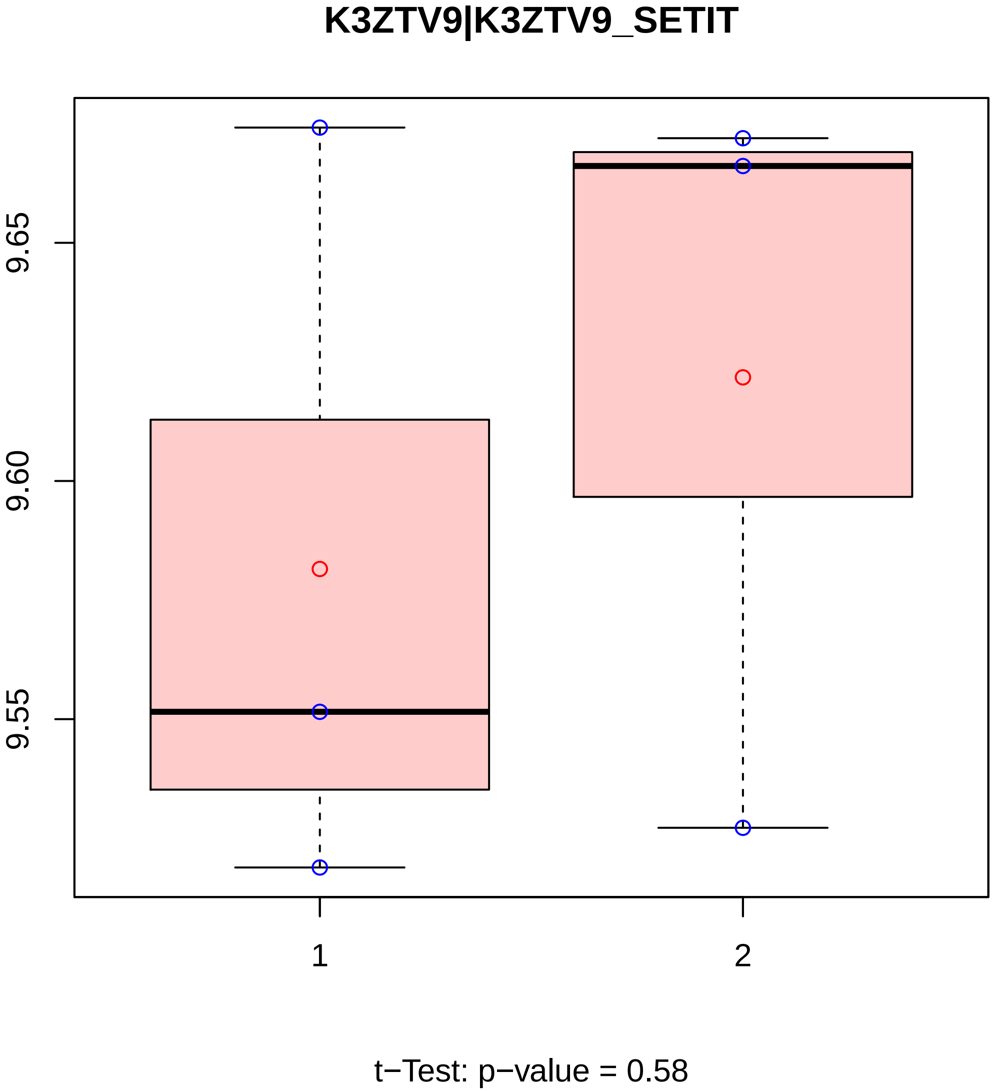

K3ZTV9|K3ZTV9_SETIT
9.55
9.60
9.65
1
2
t−Test: p−value = 0.58

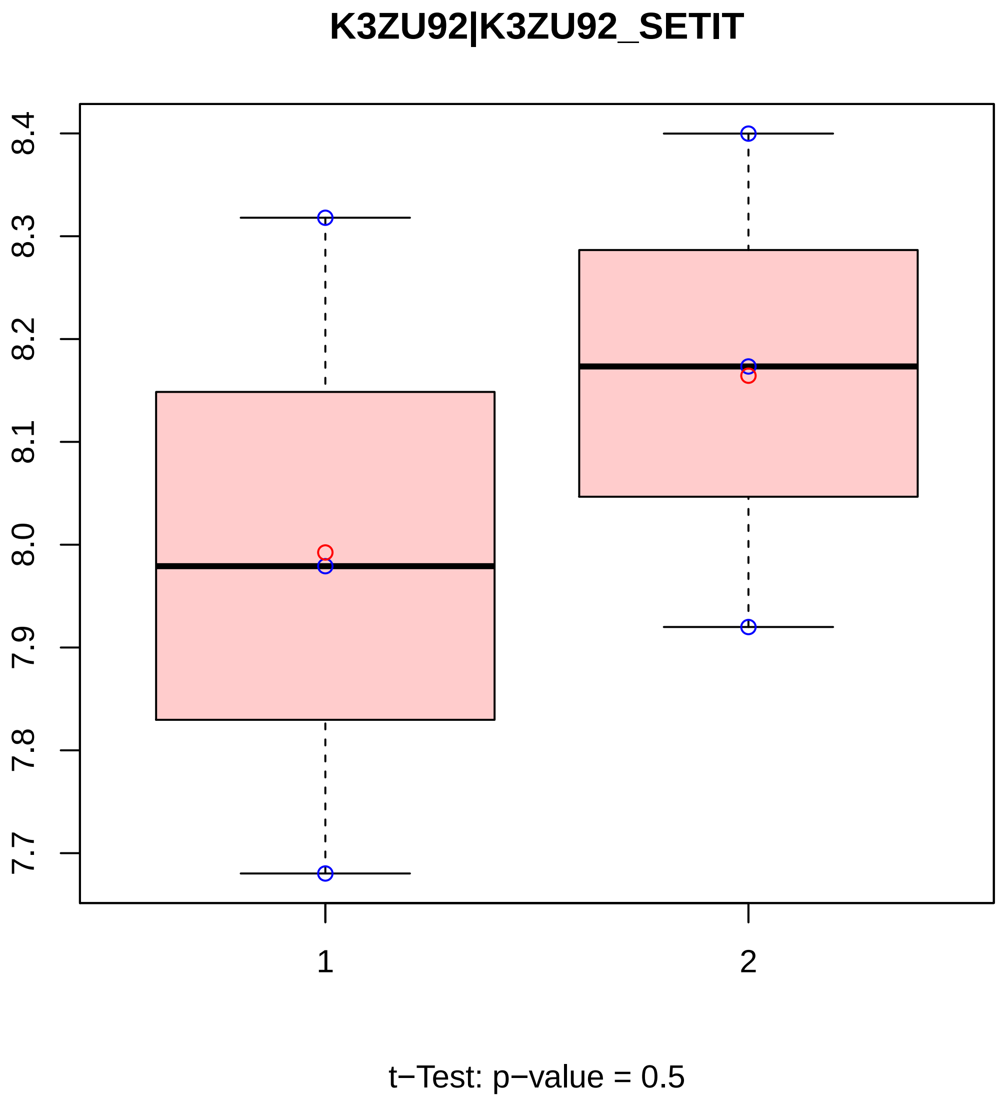
K3ZU92|K3ZU92_SETIT
8.4
8.3
8.2
8.1
8.0
7.9
7.8
7.7
1
2
t−Test: p−value = 0.5

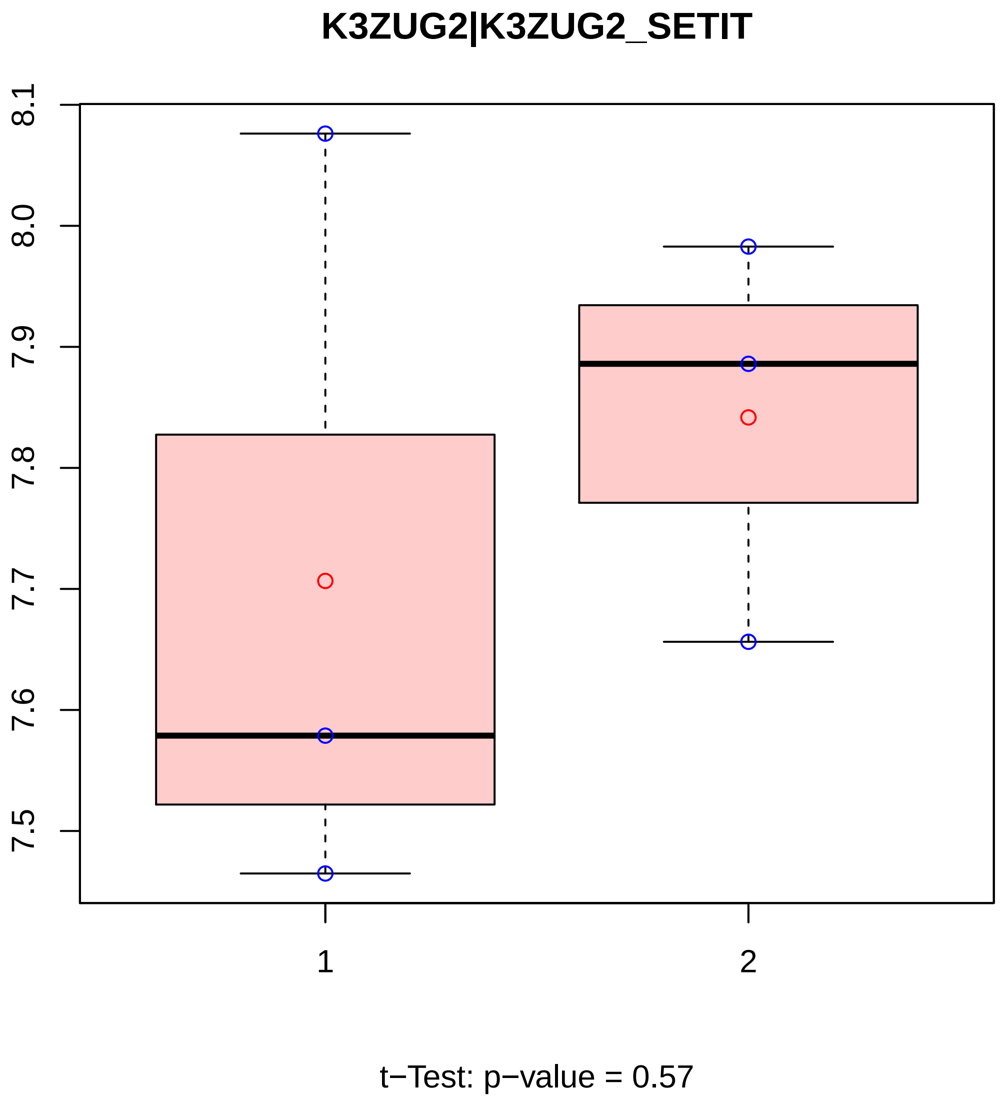
K3ZUG2|K3ZUG2_SETIT
8.1
8.0
7.9
7.8
7.7
7.6
7.5
1
2
t−Test: p−value = 0.57

K3ZUL5|K3ZUL5_SETIT

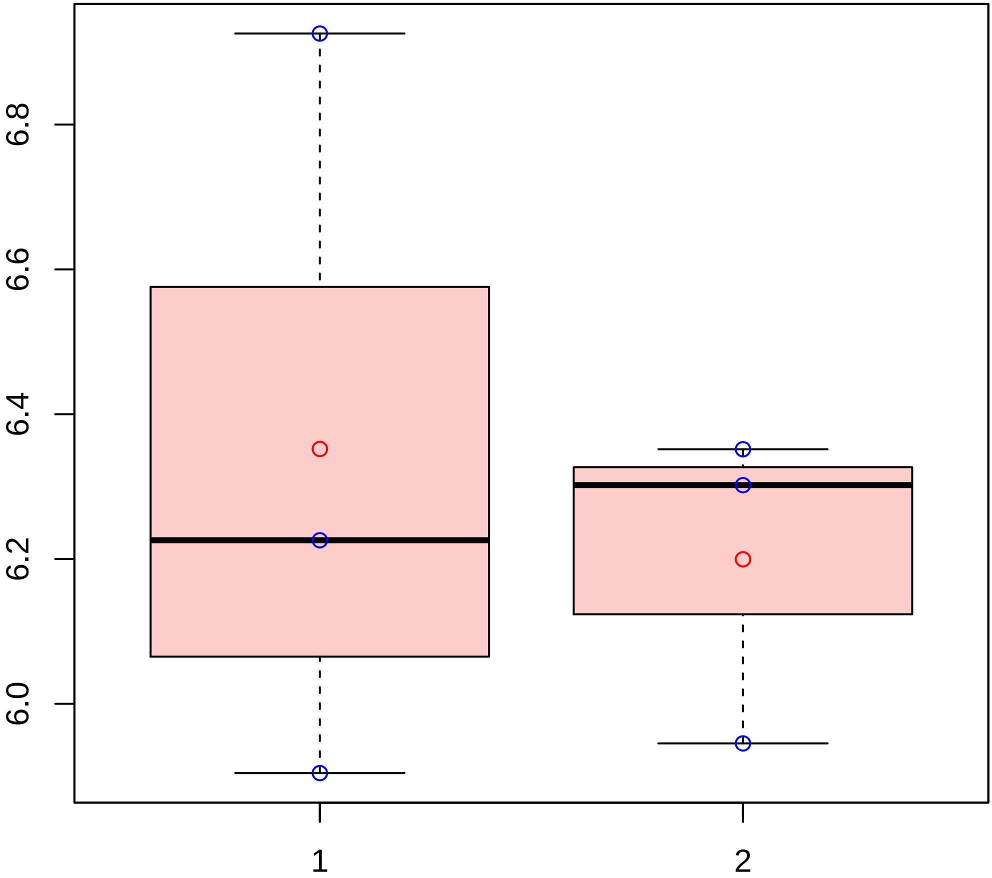

t−Test: p−value = 0.68

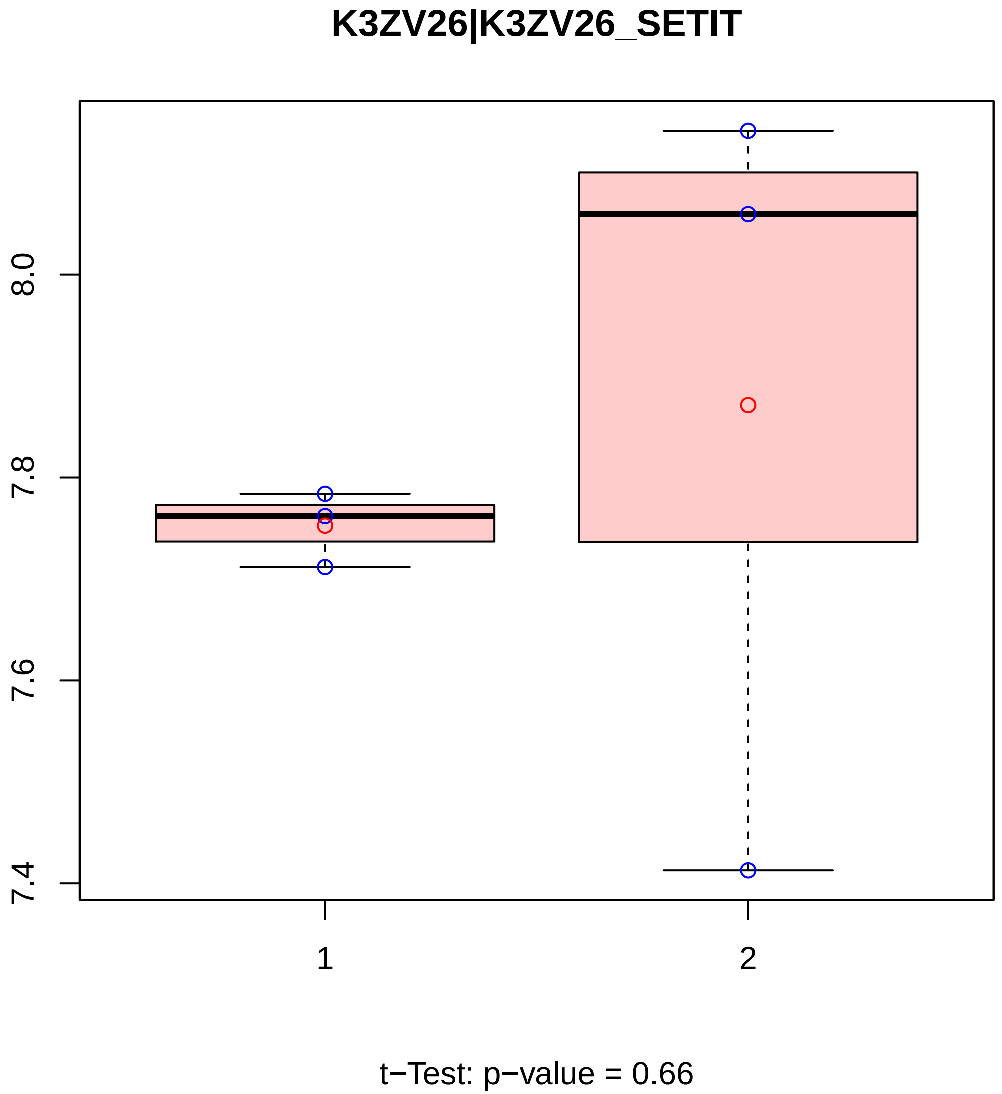

K3ZV26|K3ZV26_SETIT
8.0
7.8
7.6
7.4
1
2
t−Test: p−value = 0.66

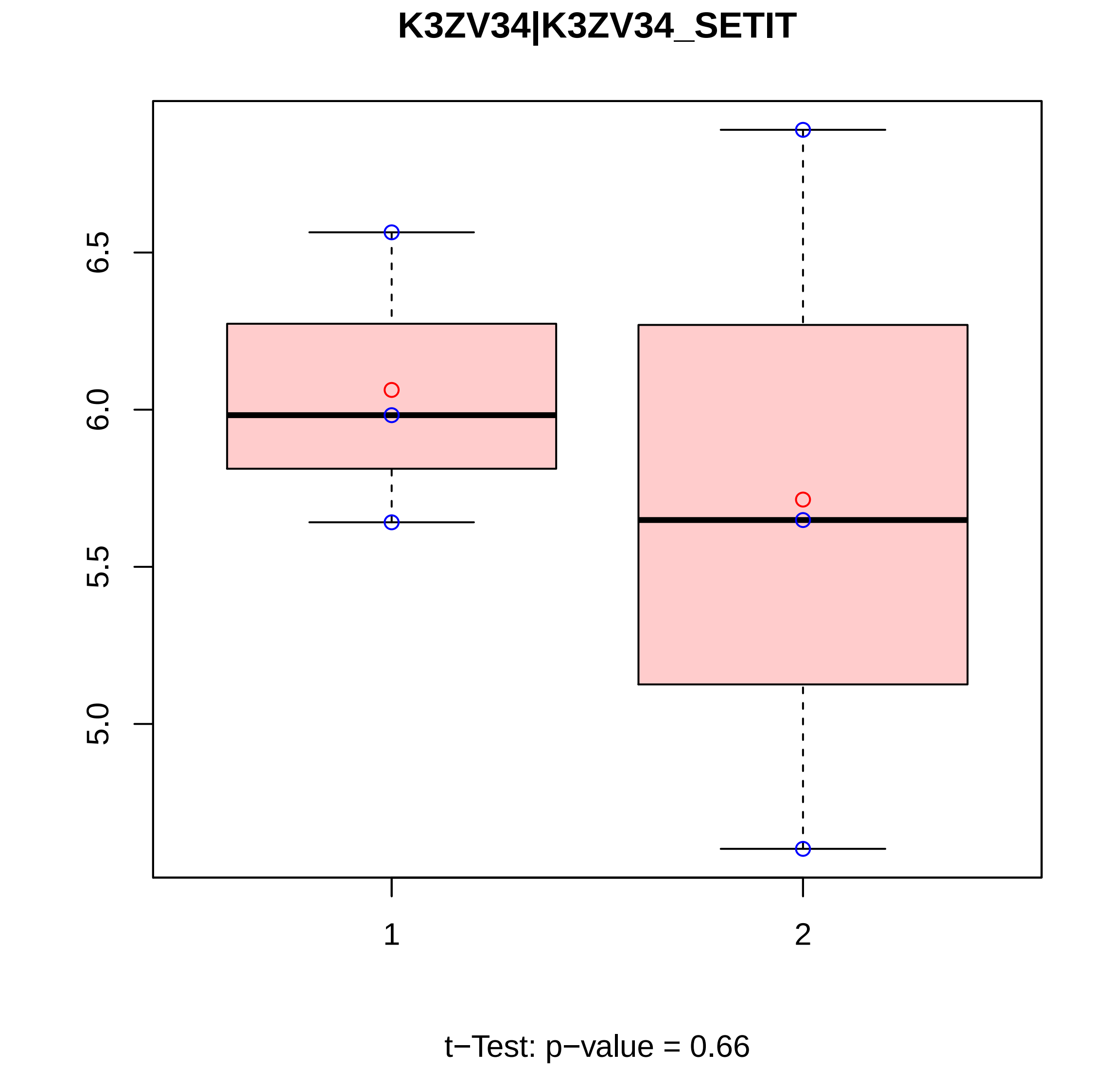
K3ZV34|K3ZV34_SETIT
6.5
6.0
5.5
5.0
1
2
t−Test: p−value = 0.66

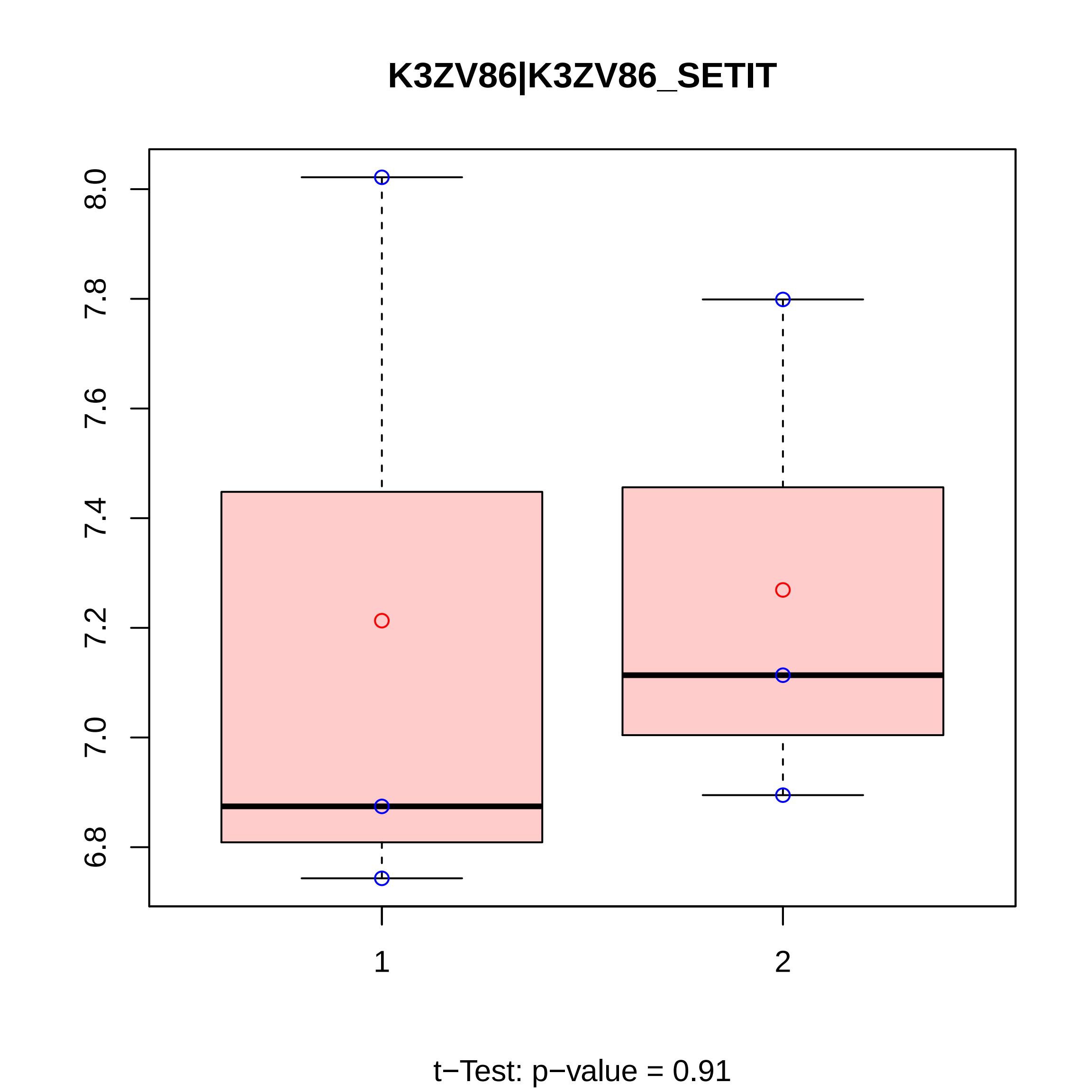
K3ZV86|K3ZV86_SETIT
8.0
7.8
7.6
7.4
7.2
7.0
6.8
1
2
t−Test: p−value = 0.91

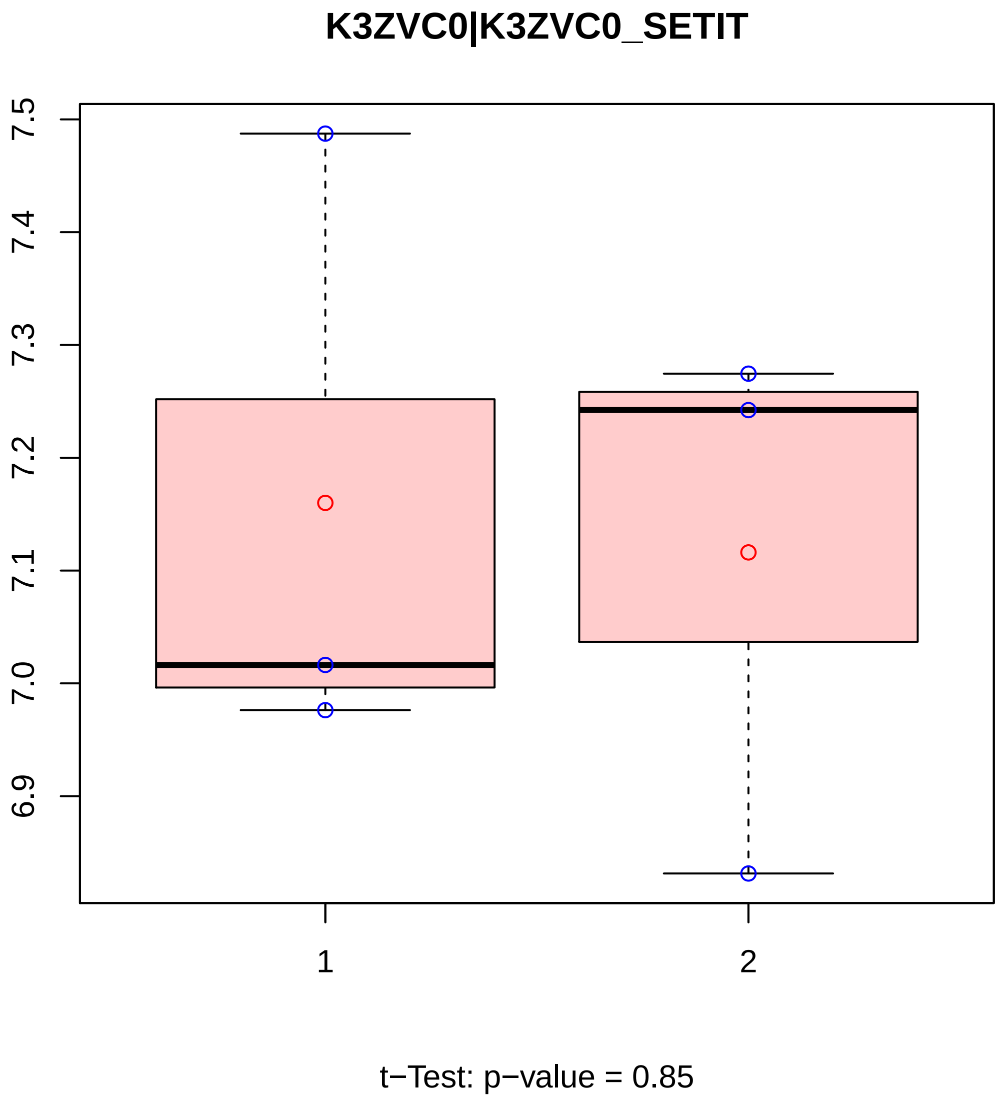
K3ZVC0|K3ZVC0_SETIT
7.5
7.4
7.3
7.2
7.1
7.0
6.9
1
2
t−Test: p−value = 0.85

K3ZVC8|K3ZVC8_SETIT

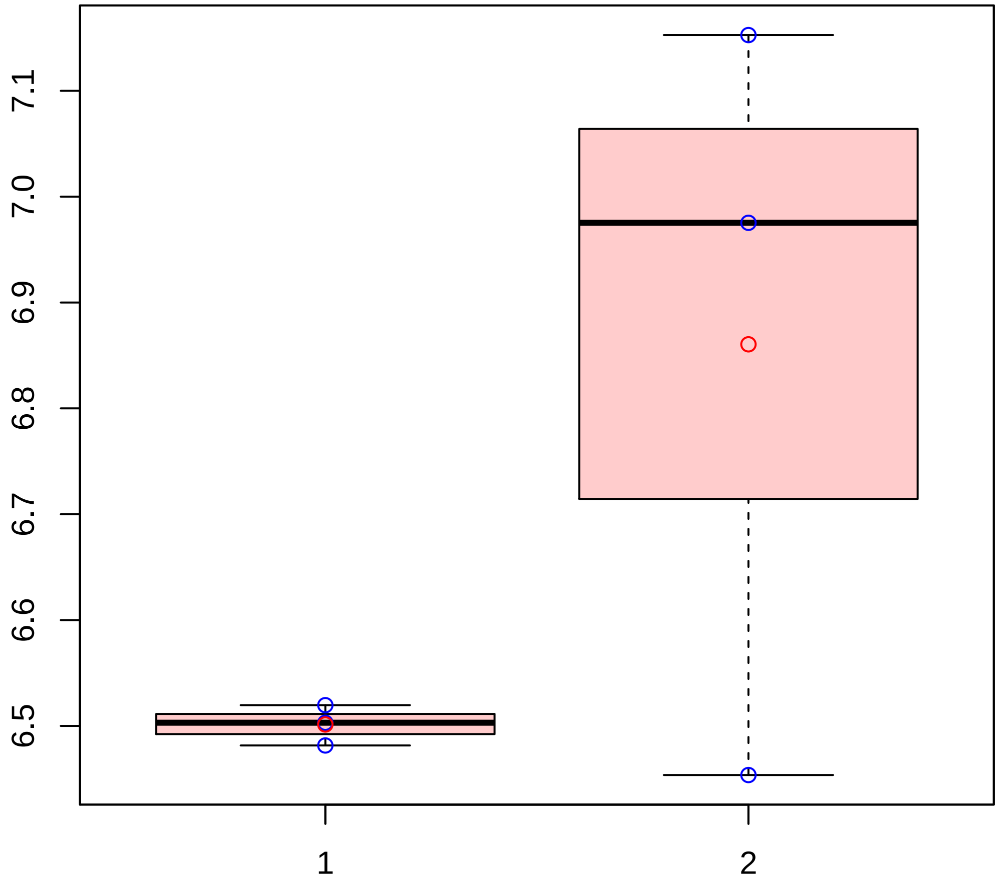

t−Test: p−value = 0.23

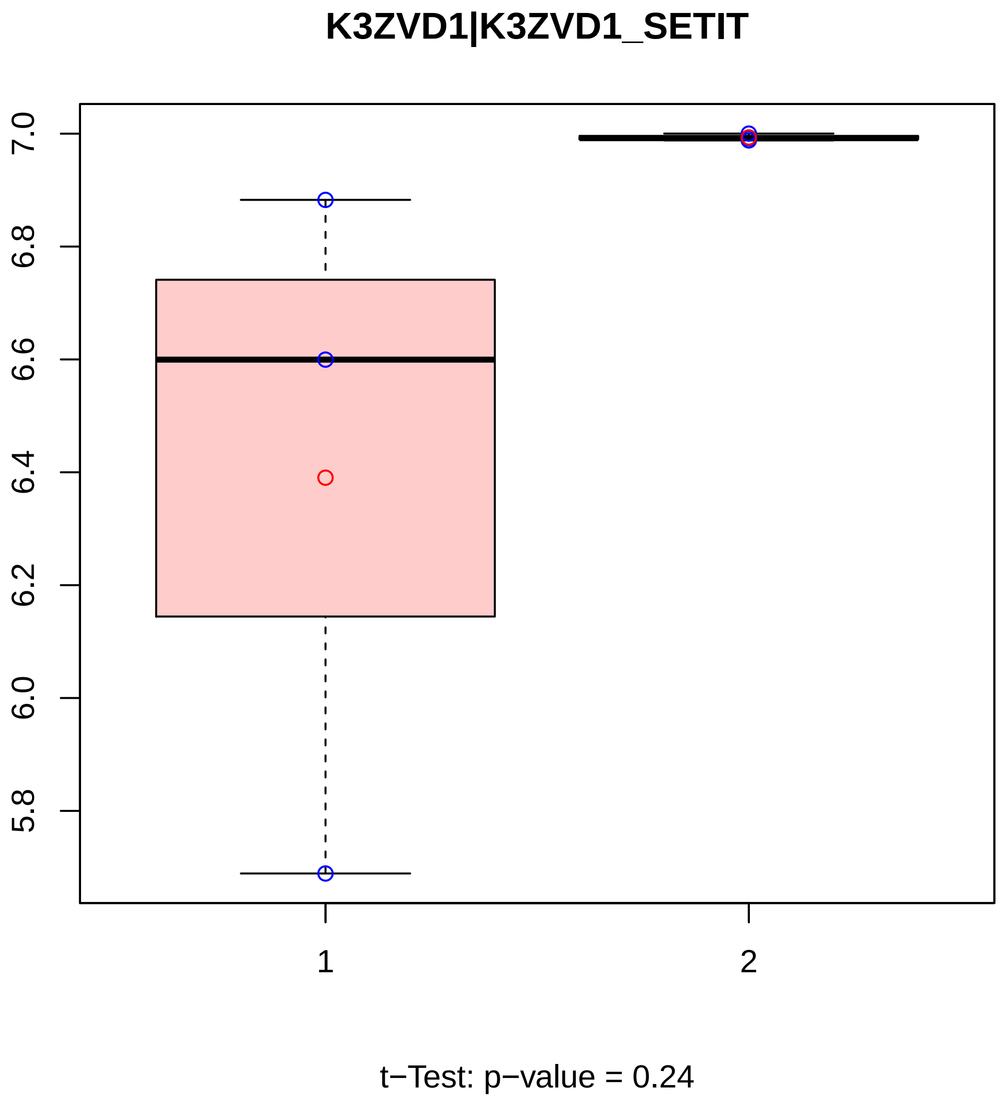
K3ZVD1|K3ZVD1_SETIT
7.0
6.8
6.6
6.4
6.2
6.0
5.8
1
2
t−Test: p−value = 0.24

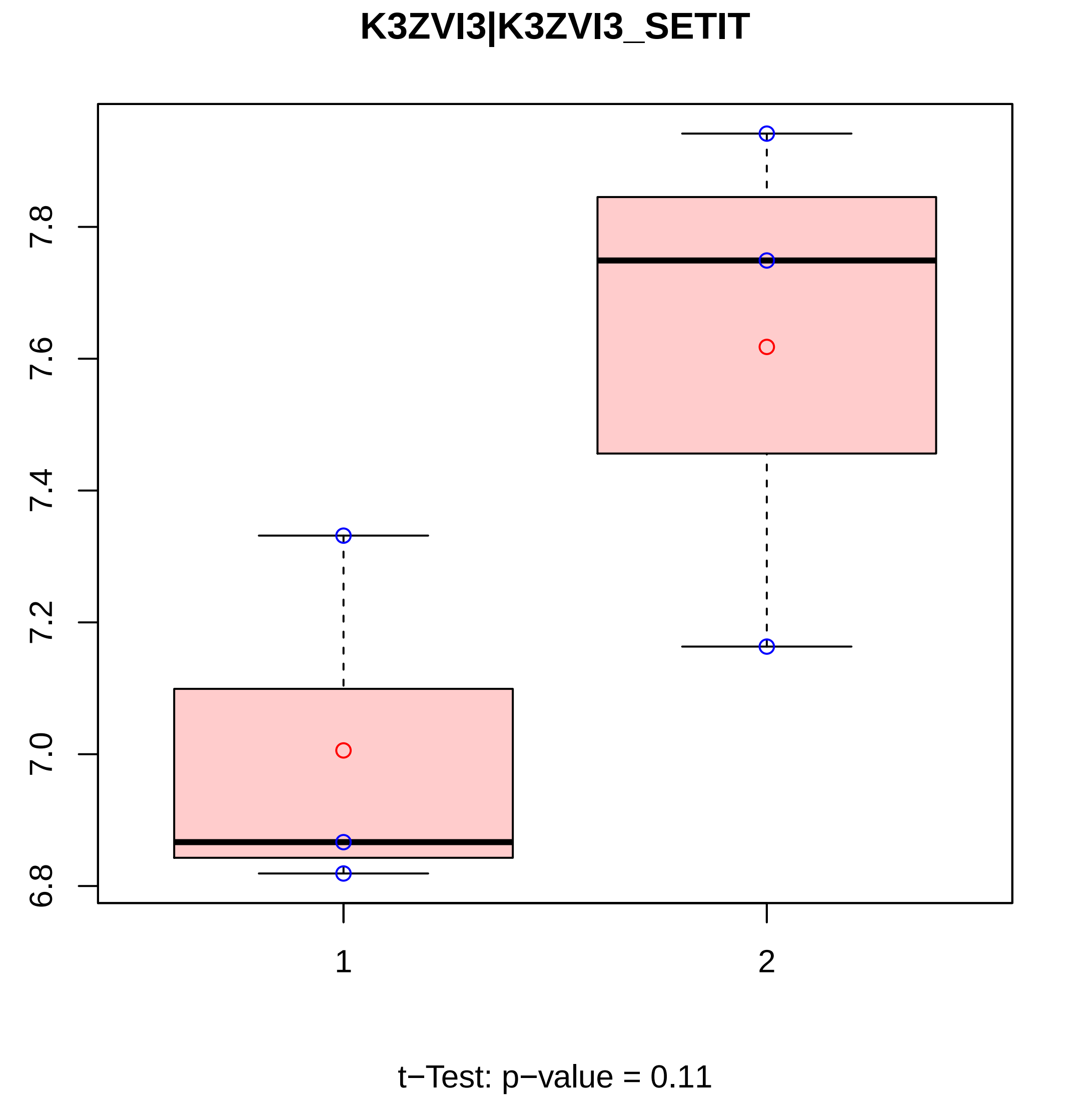
K3ZVI3|K3ZVI3_SETIT
7.8
7.6
7.4
7.2
7.0
6.8
1
2
t−Test: p−value = 0.11

K3ZVK5|K3ZVK5_SETIT

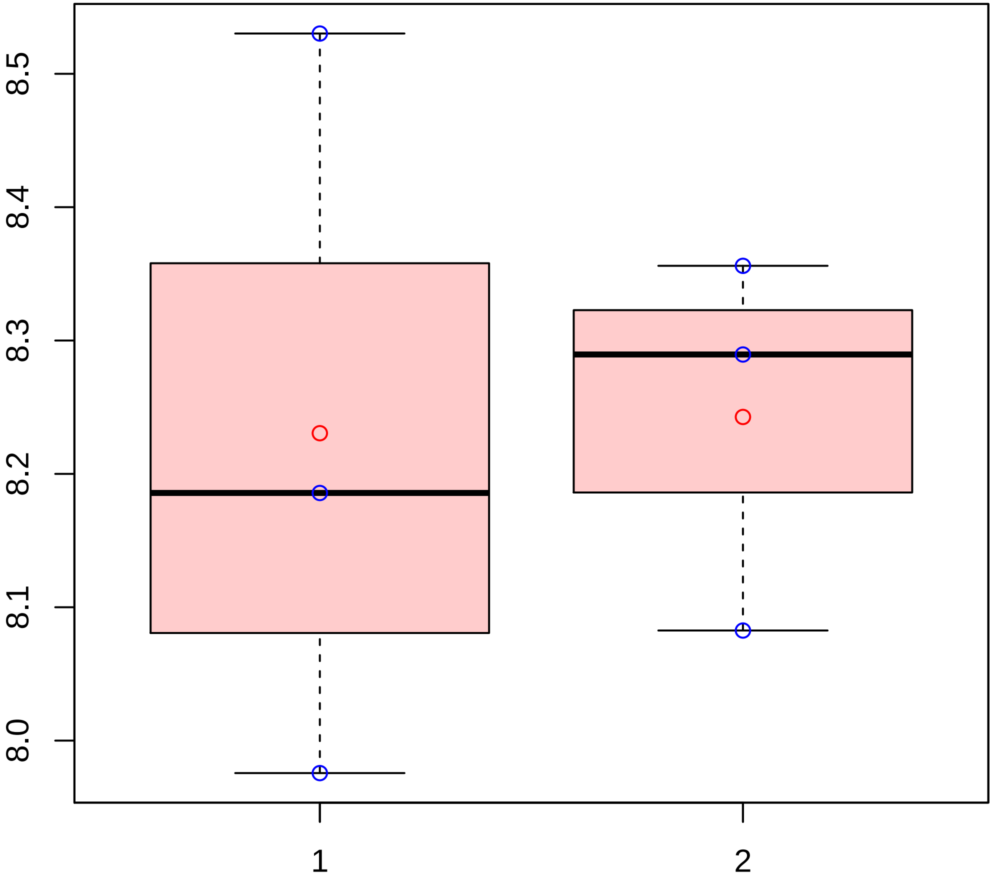

t−Test: p−value = 0.95

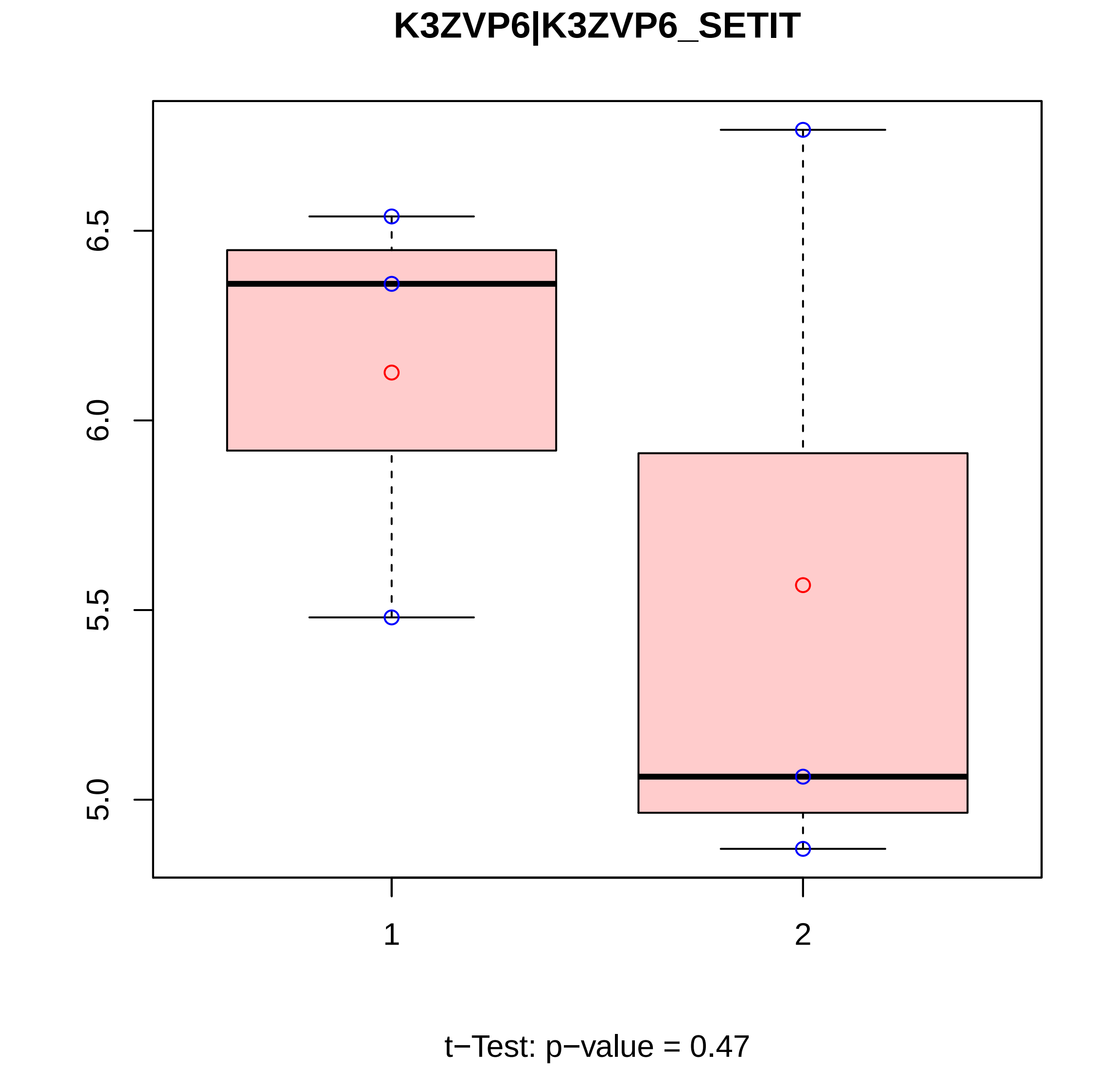
K3ZVP6|K3ZVP6_SETIT
6.5
6.0
5.5
5.0
1
2
t−Test: p−value = 0.47

K3ZVS1|K3ZVS1_SETIT

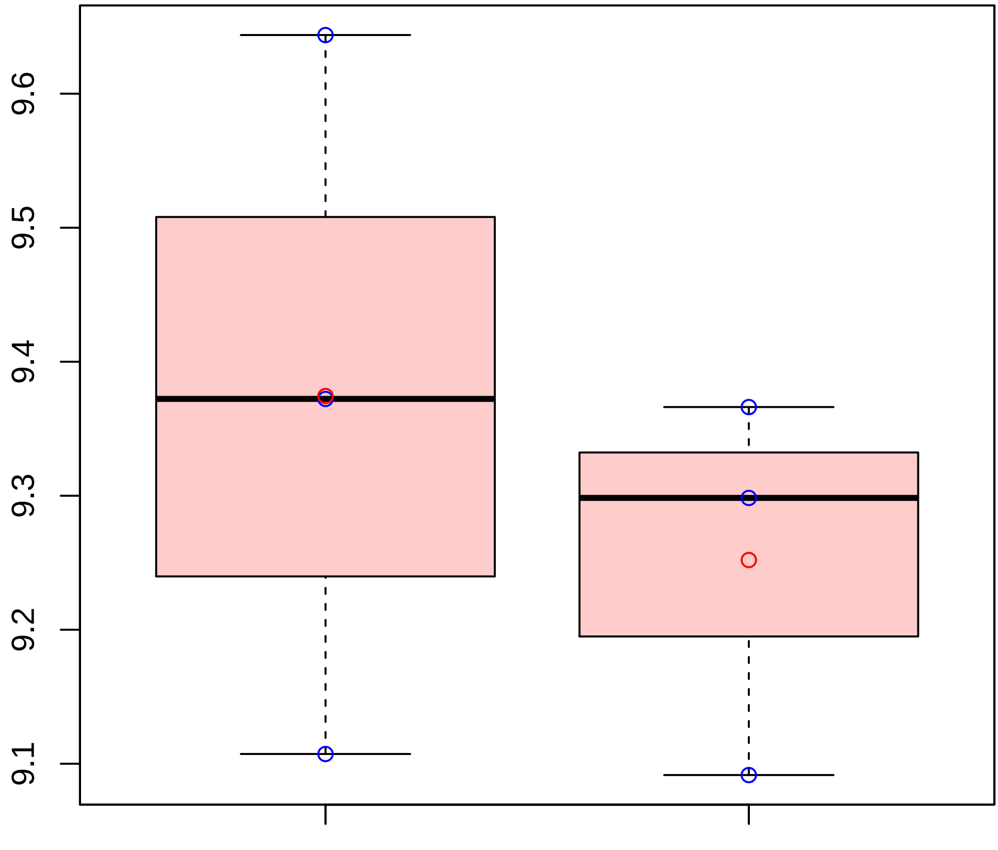

t−Test: p−value = 0.53

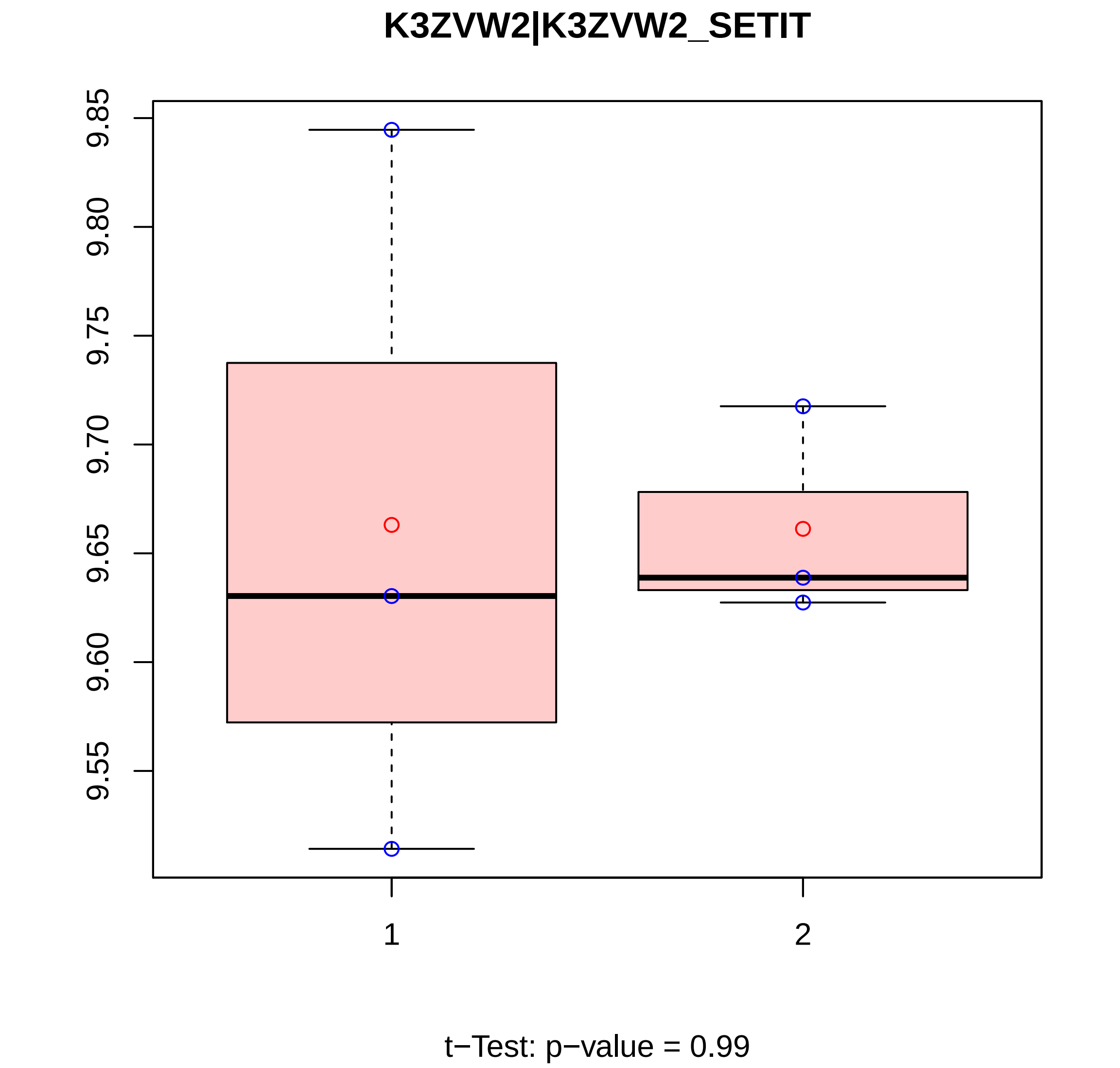
K3ZVW2|K3ZVW2_SETIT
9.55
9.60
9.65
9.70
9.75
9.80
9.85
1
2
t−Test: p−value = 0.99

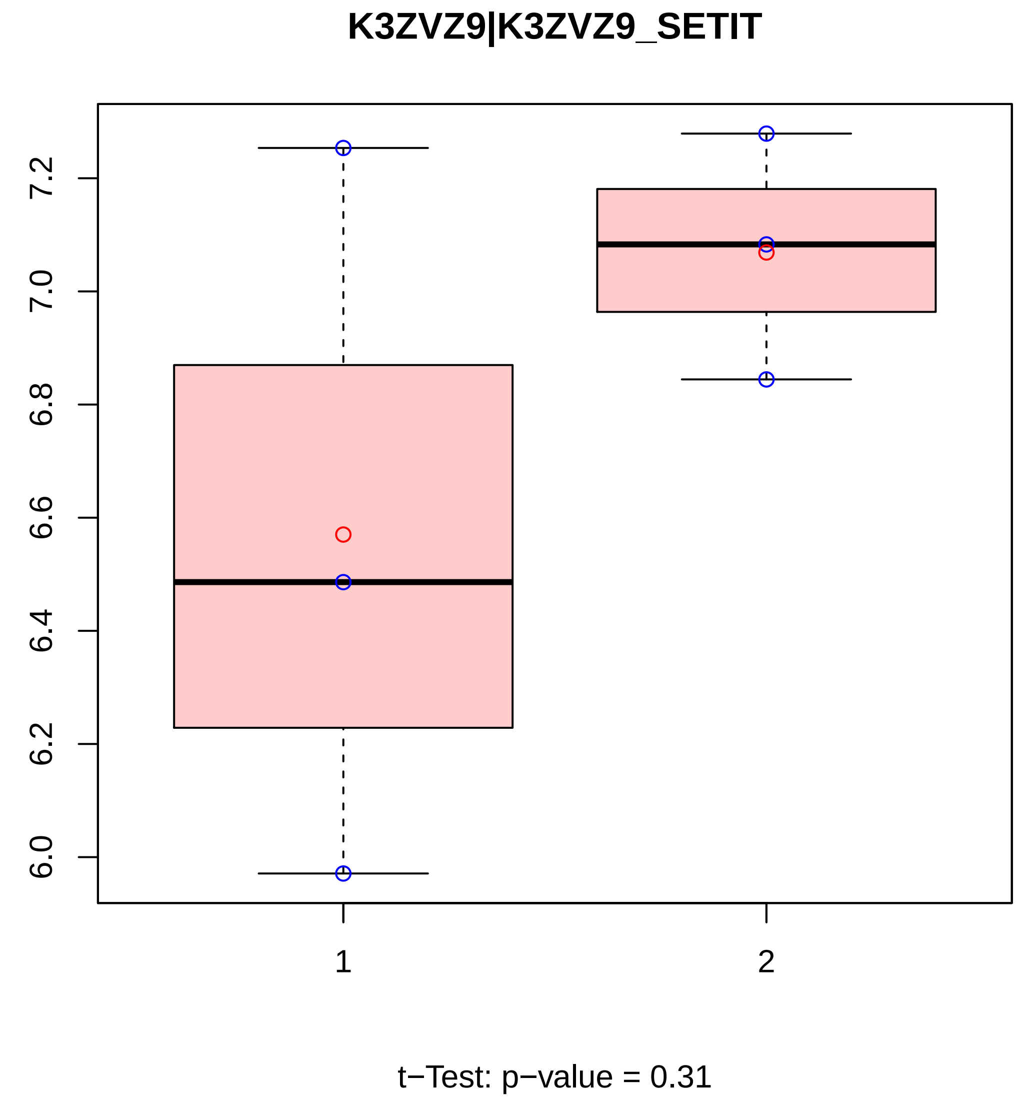

K3ZVZ9|K3ZVZ9_SETIT
7.2
7.0
6.8
6.6
6.4
6.2
6.0
1
2
t−Test: p−value = 0.31

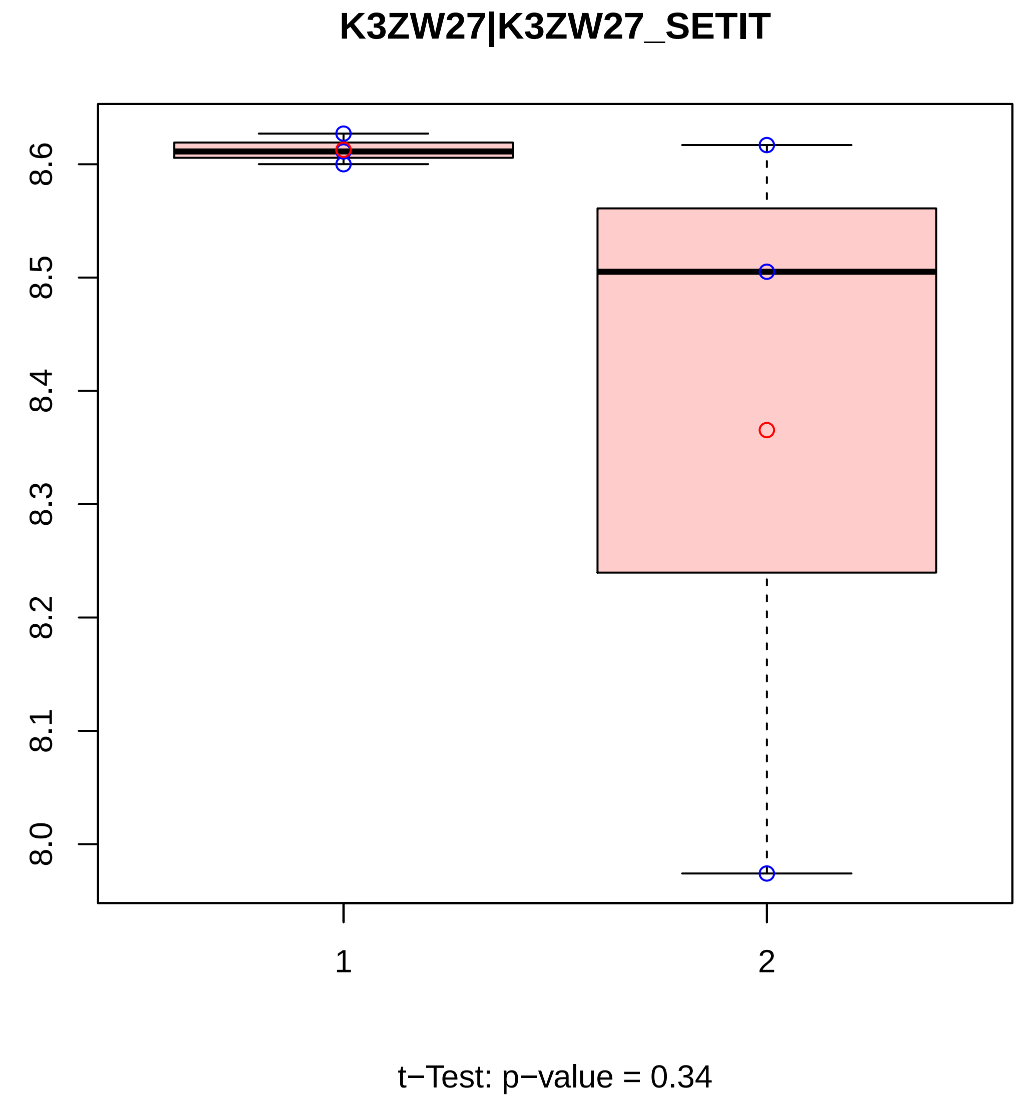
K3ZW27|K3ZW27_SETIT
8.0
8.1
8.2
8.3
8.4
8.5
8.6
1
2
t−Test: p−value = 0.34

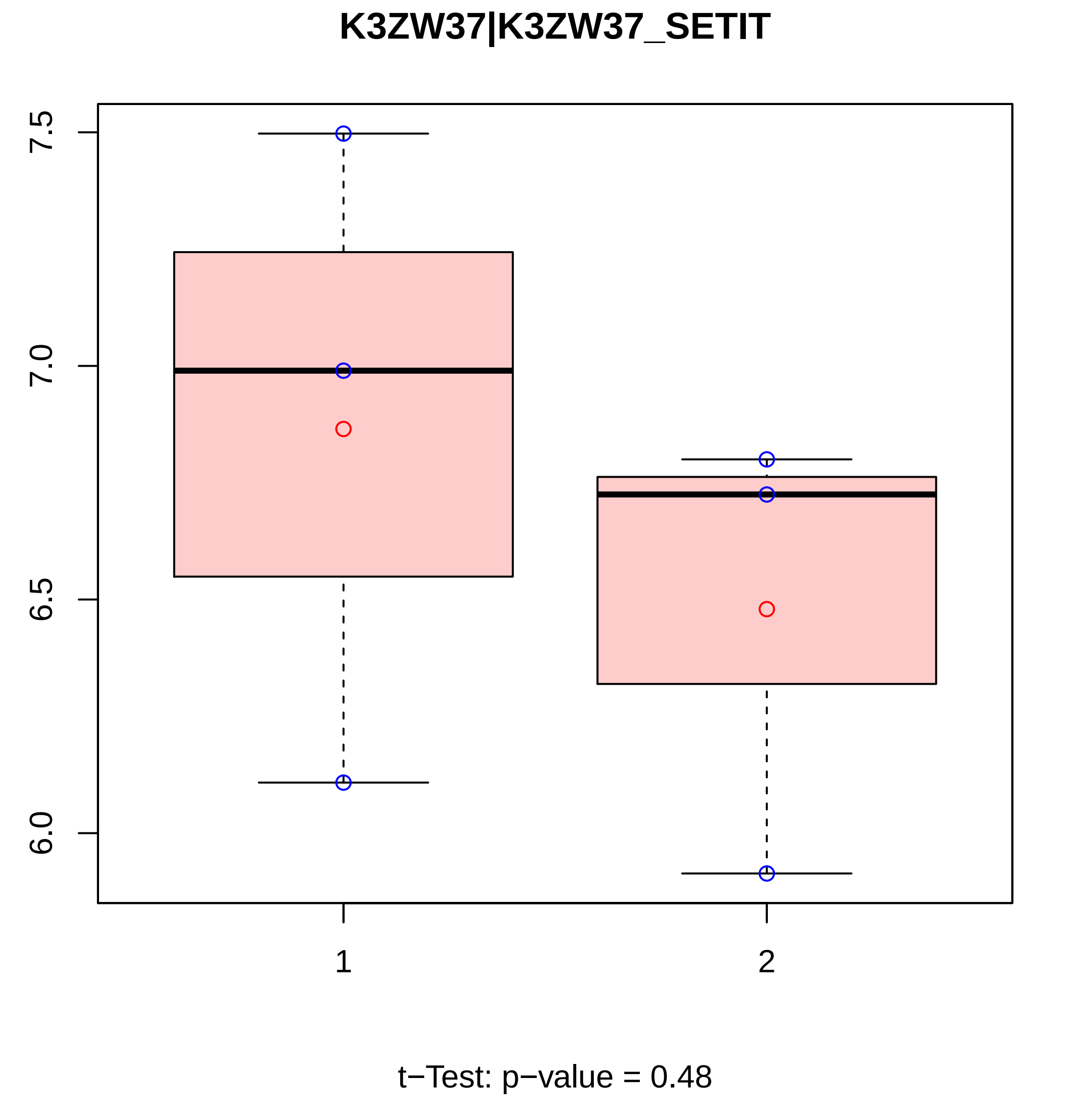
K3ZW37|K3ZW37_SETIT
7.5
7.0
6.5
6.0
1
2
t−Test: p−value = 0.48

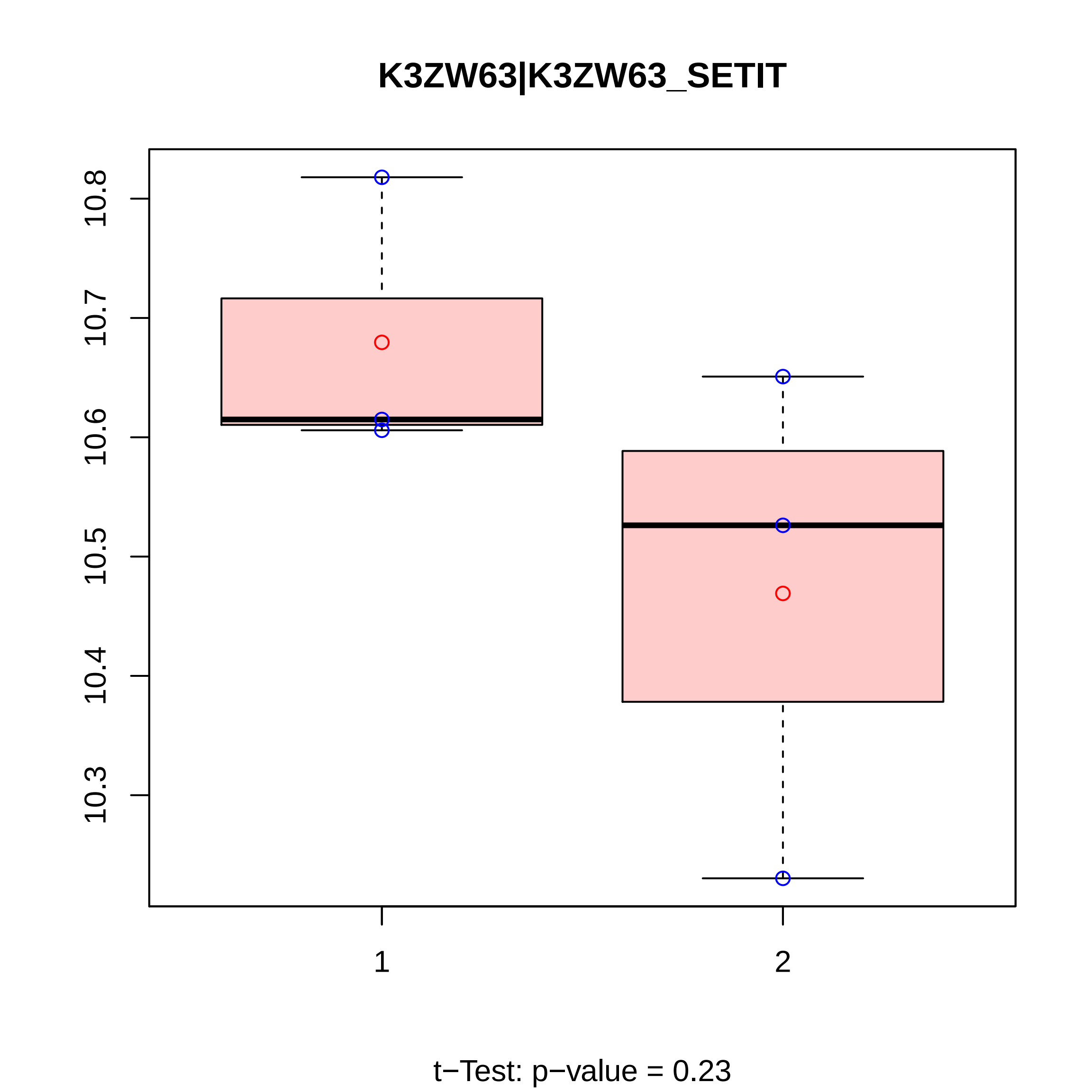
K3ZW63|K3ZW63_SETIT
10.3
10.4
10.5
10.6
10.7
10.8
1
2
t−Test: p−value = 0.23

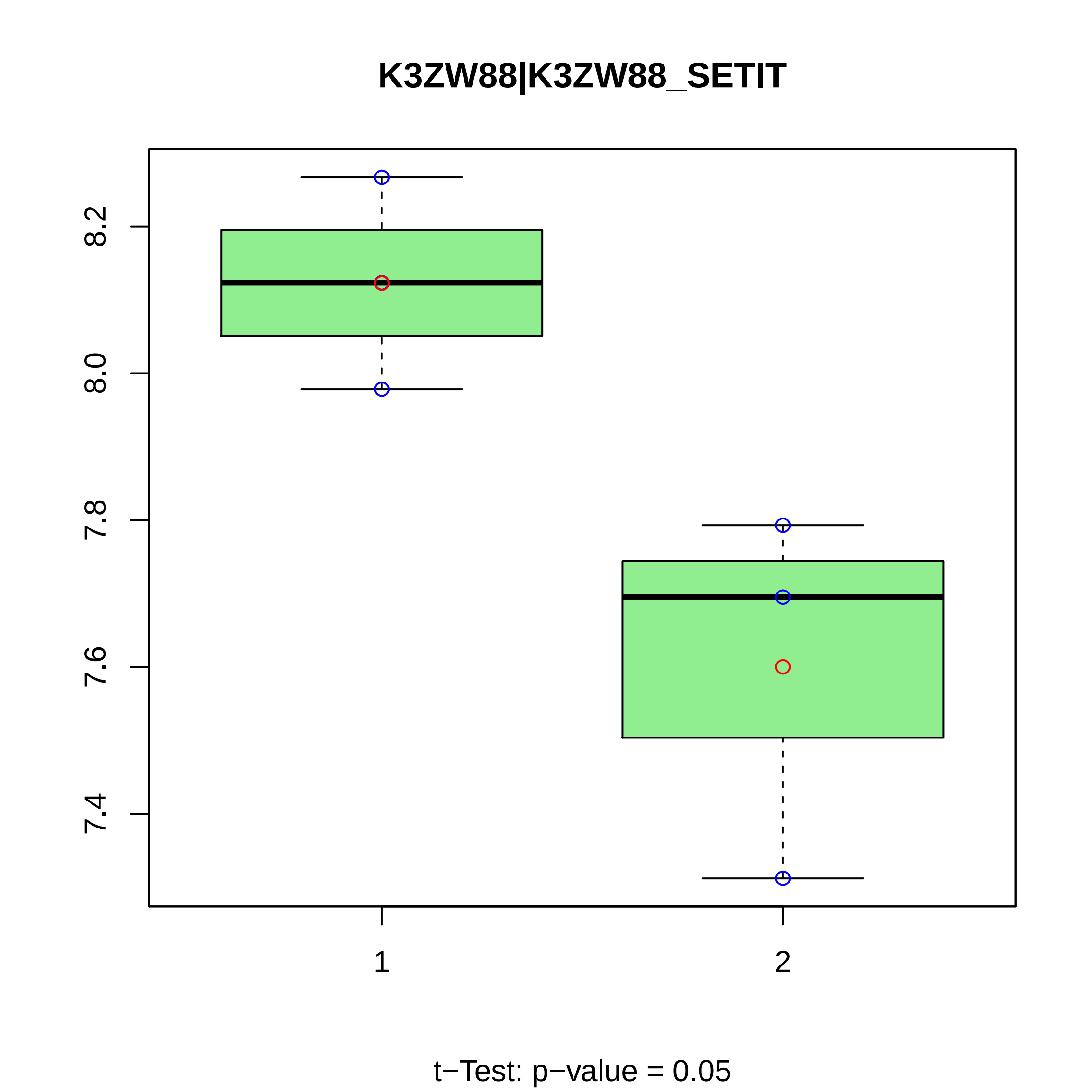
K3ZW88|K3ZW88_SETIT
8.2
8.0
7.8
7.6
7.4
1
2
t−Test: p−value = 0.05

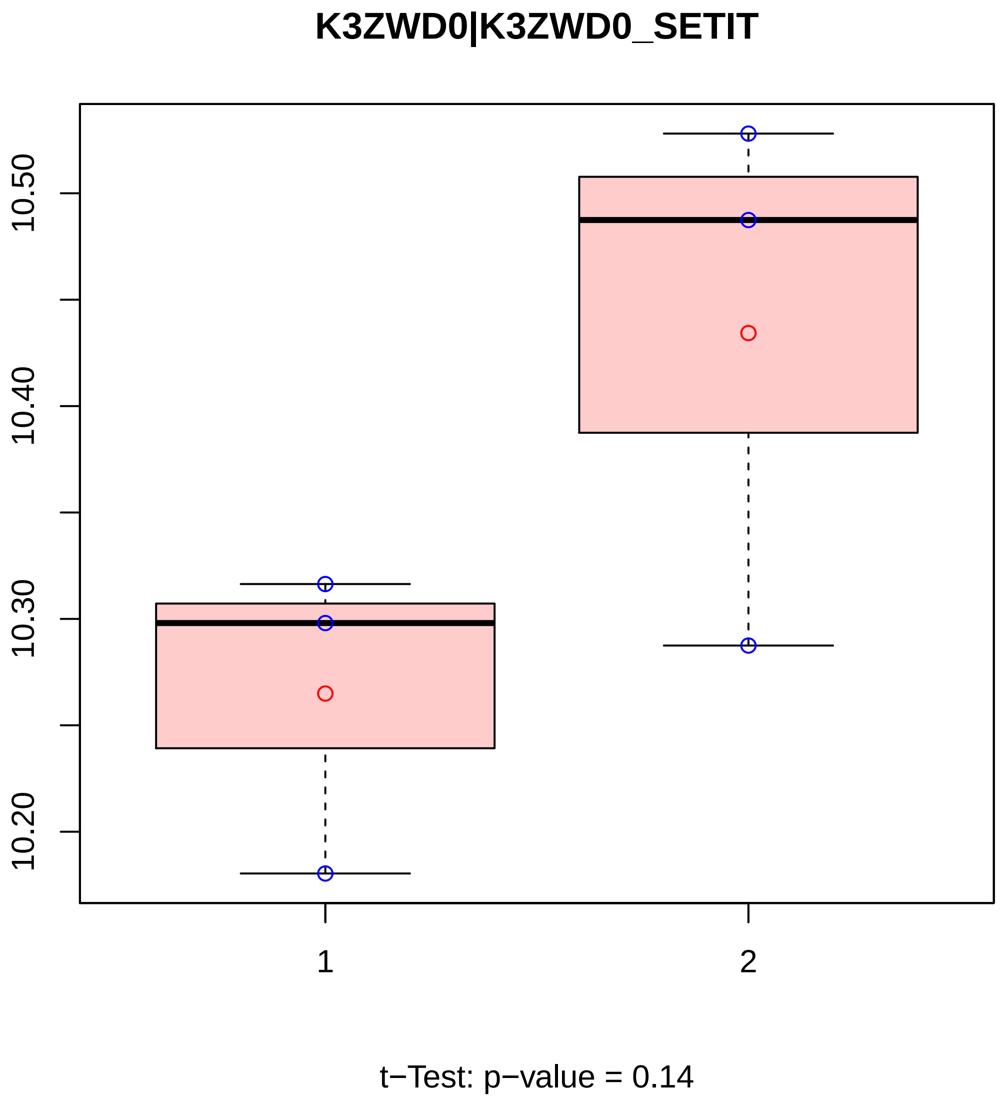

K3ZWD0|K3ZWD0_SETIT
10.20
10.30
10.40
10.50
1
2
t−Test: p−value = 0.14

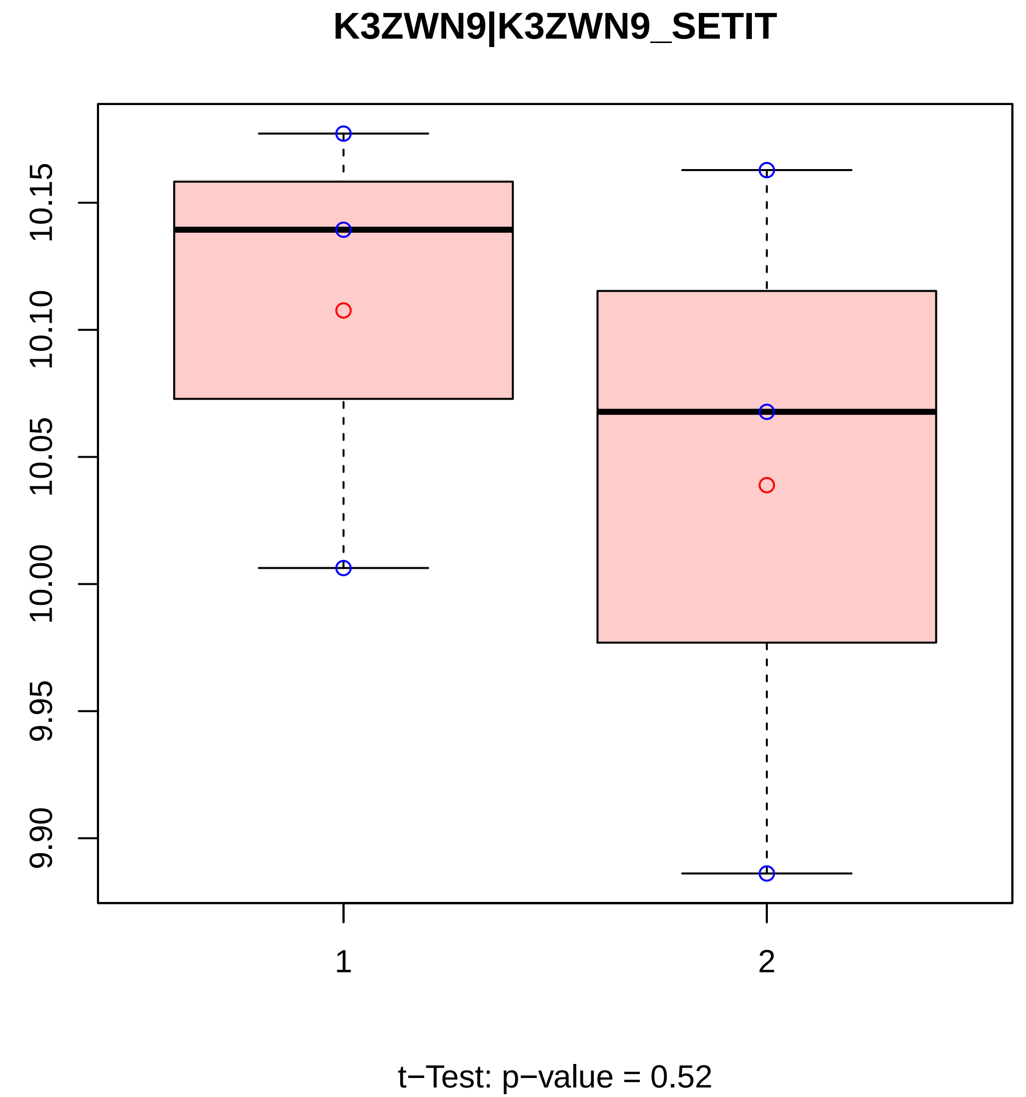
K3ZWN9|K3ZWN9_SETIT
10.15
10.10
10.05
10.00
9.95
9.90
1
2
t−Test: p−value = 0.52

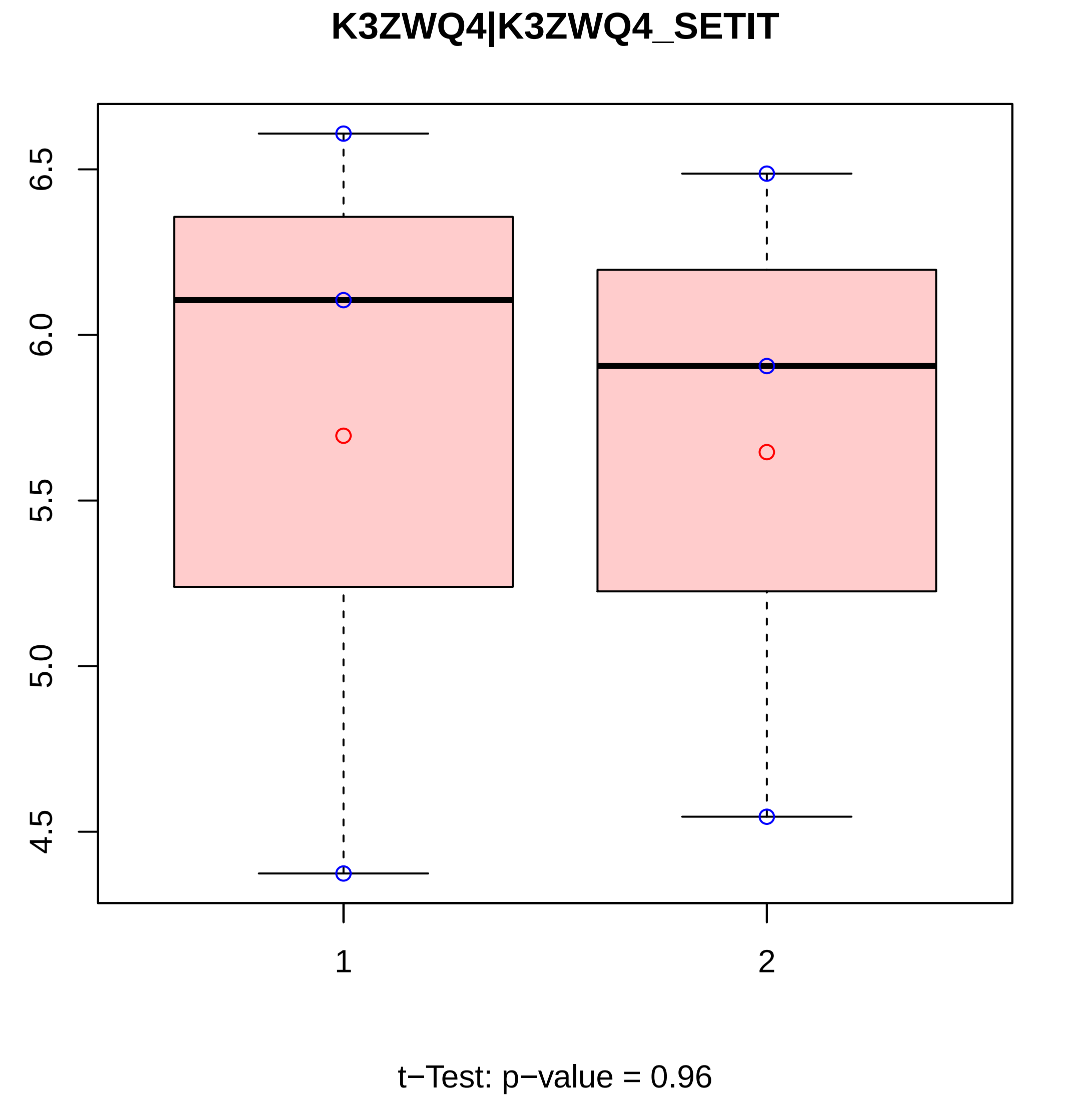
K3ZWQ4|K3ZWQ4_SETIT
6.5
6.0
5.5
5.0
4.5
1
2
t−Test: p−value = 0.96

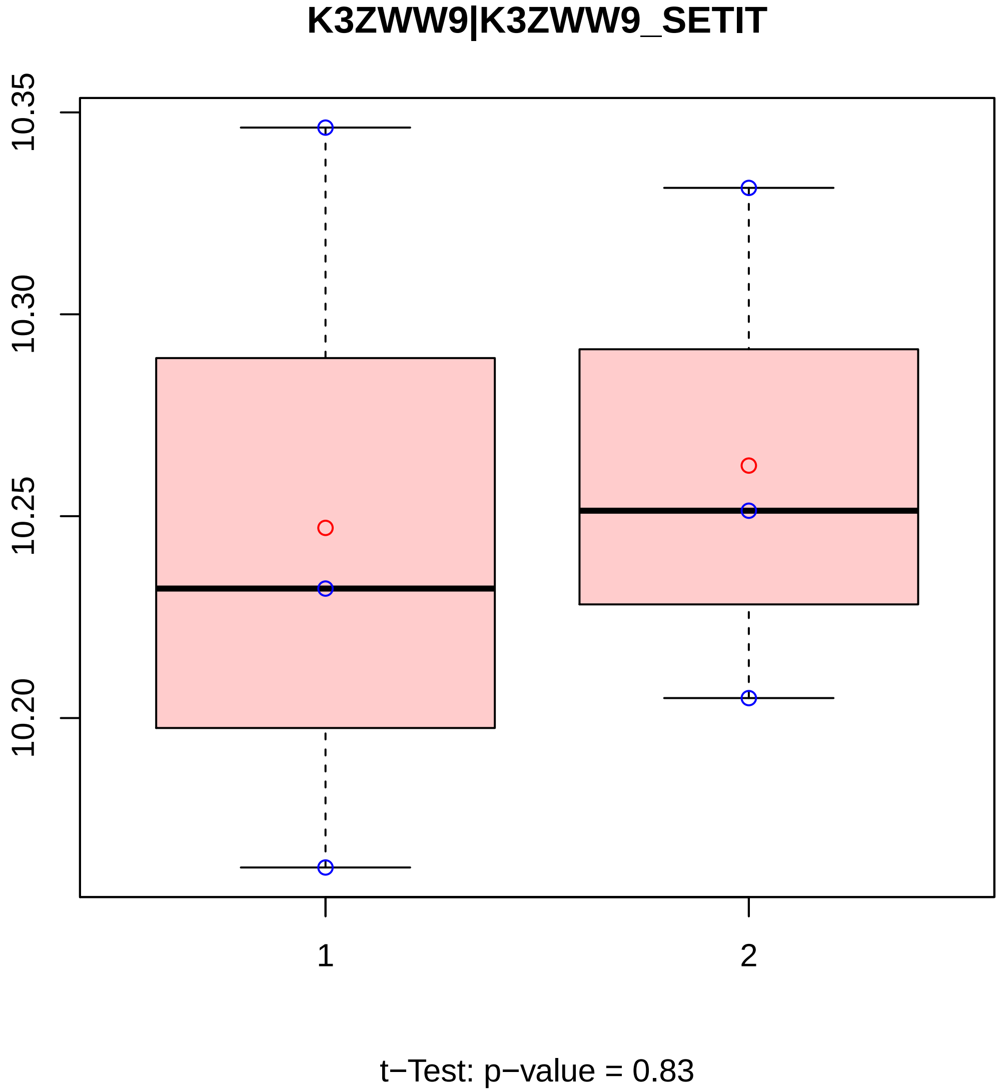

K3ZWW9|K3ZWW9_SETIT
10.35
10.30
10.25
10.20
1
2
t−Test: p−value = 0.83

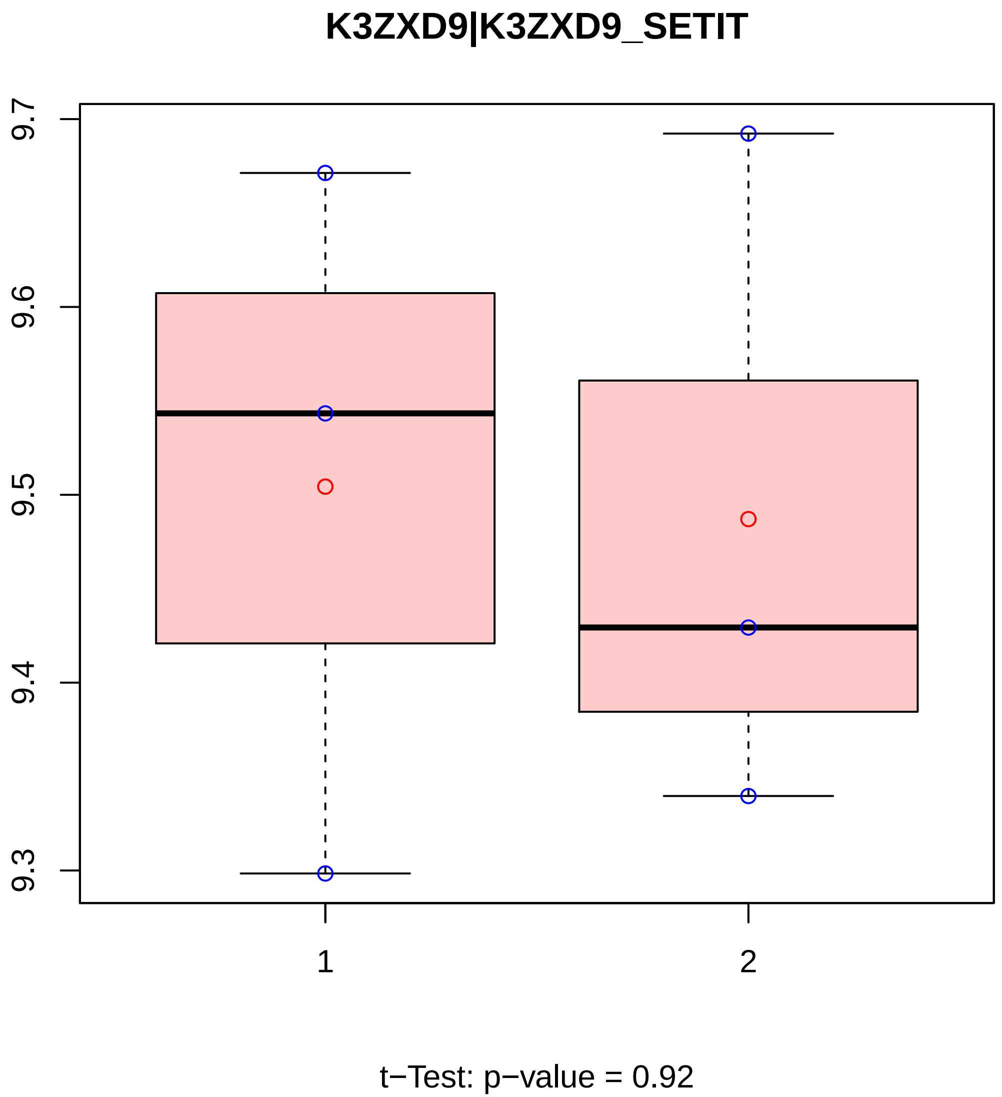
K3ZXD9|K3ZXD9_SETIT
9.7
9.6
9.5
9.4
9.3
1
2
t−Test: p−value = 0.92

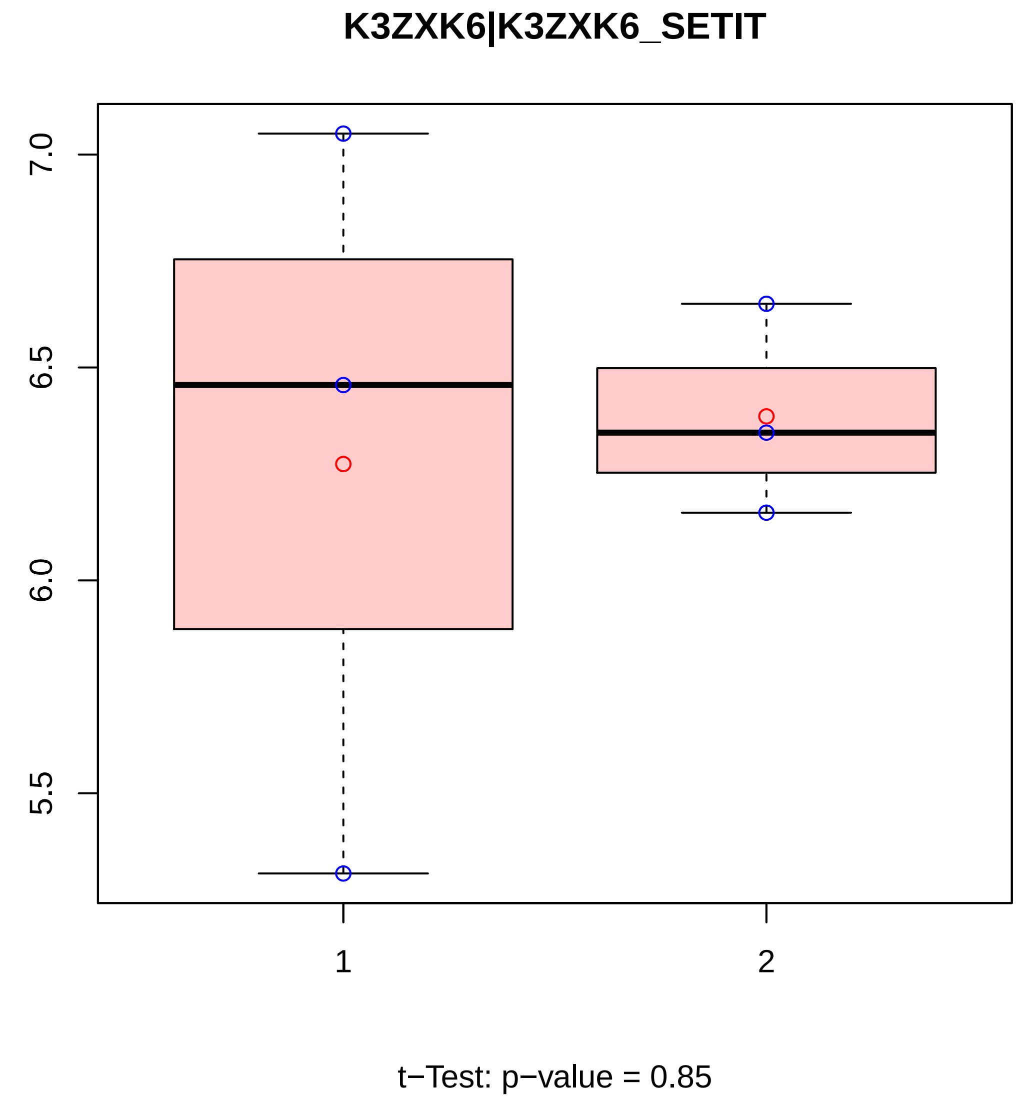

K3ZXK6|K3ZXK6_SETIT
7.0
6.5
6.0
5.5
1
2
t−Test: p−value = 0.85

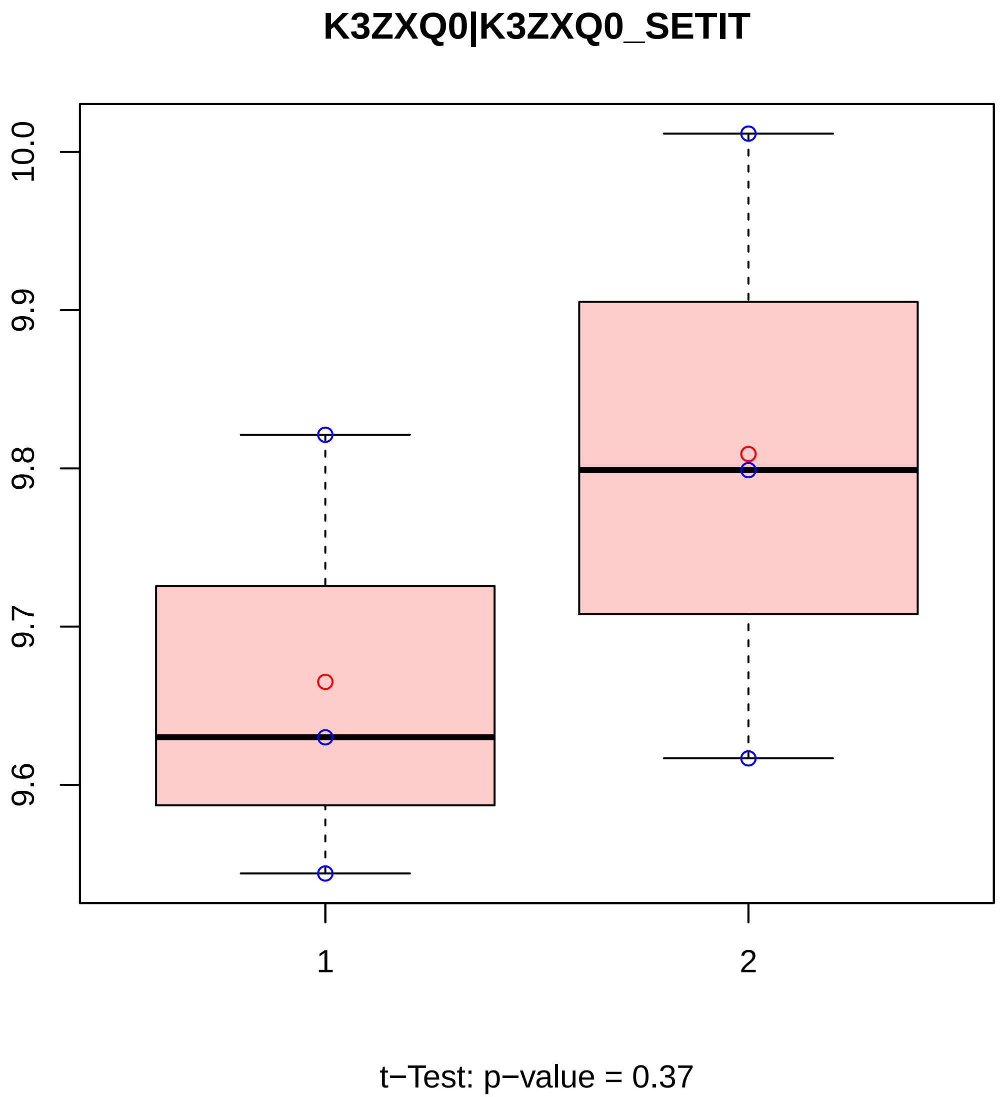
K3ZXQ0|K3ZXQ0_SETIT
10.0
9.9
9.8
9.7
9.6
1
2
t−Test: p−value = 0.37

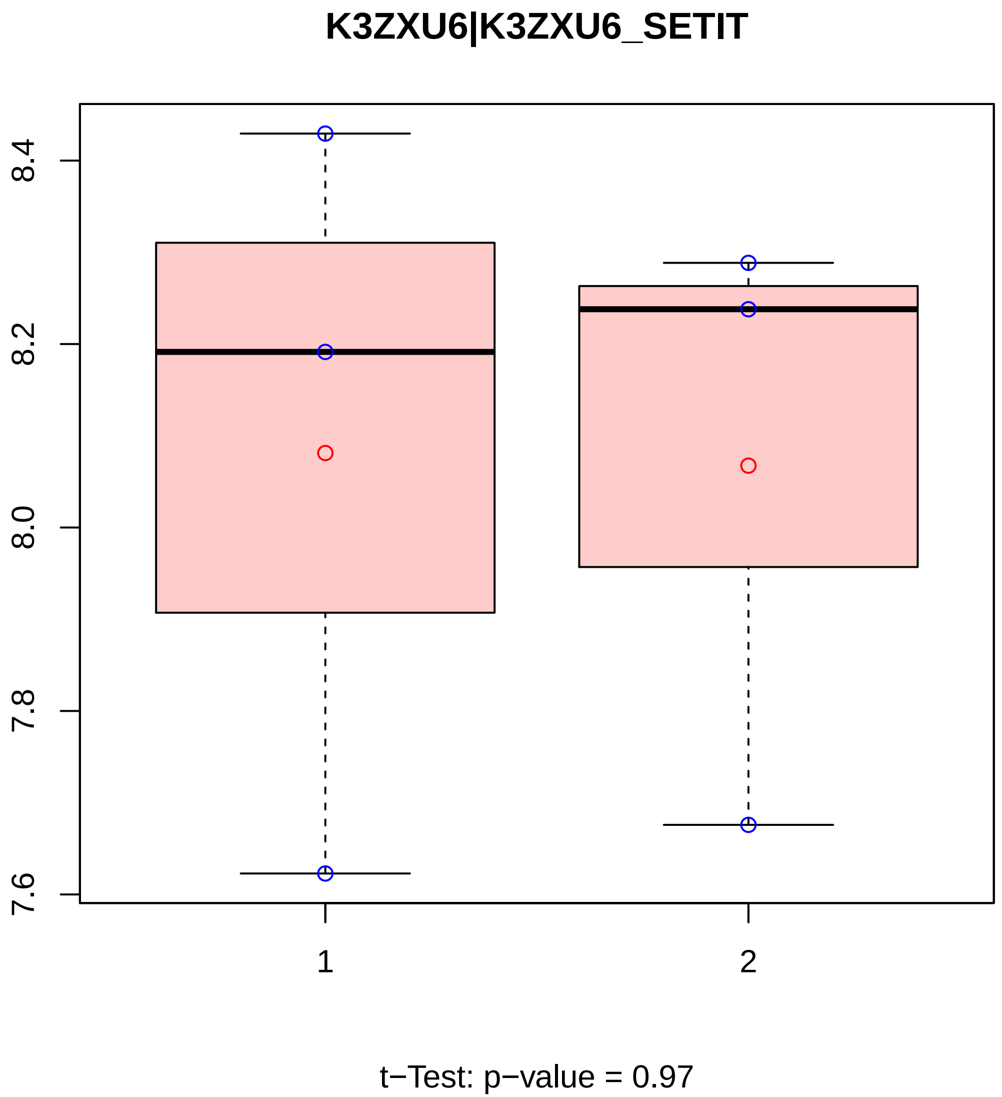

K3ZXU6|K3ZXU6_SETIT
8.4
8.2
8.0
7.8
7.6
1
2
t−Test: p−value = 0.97

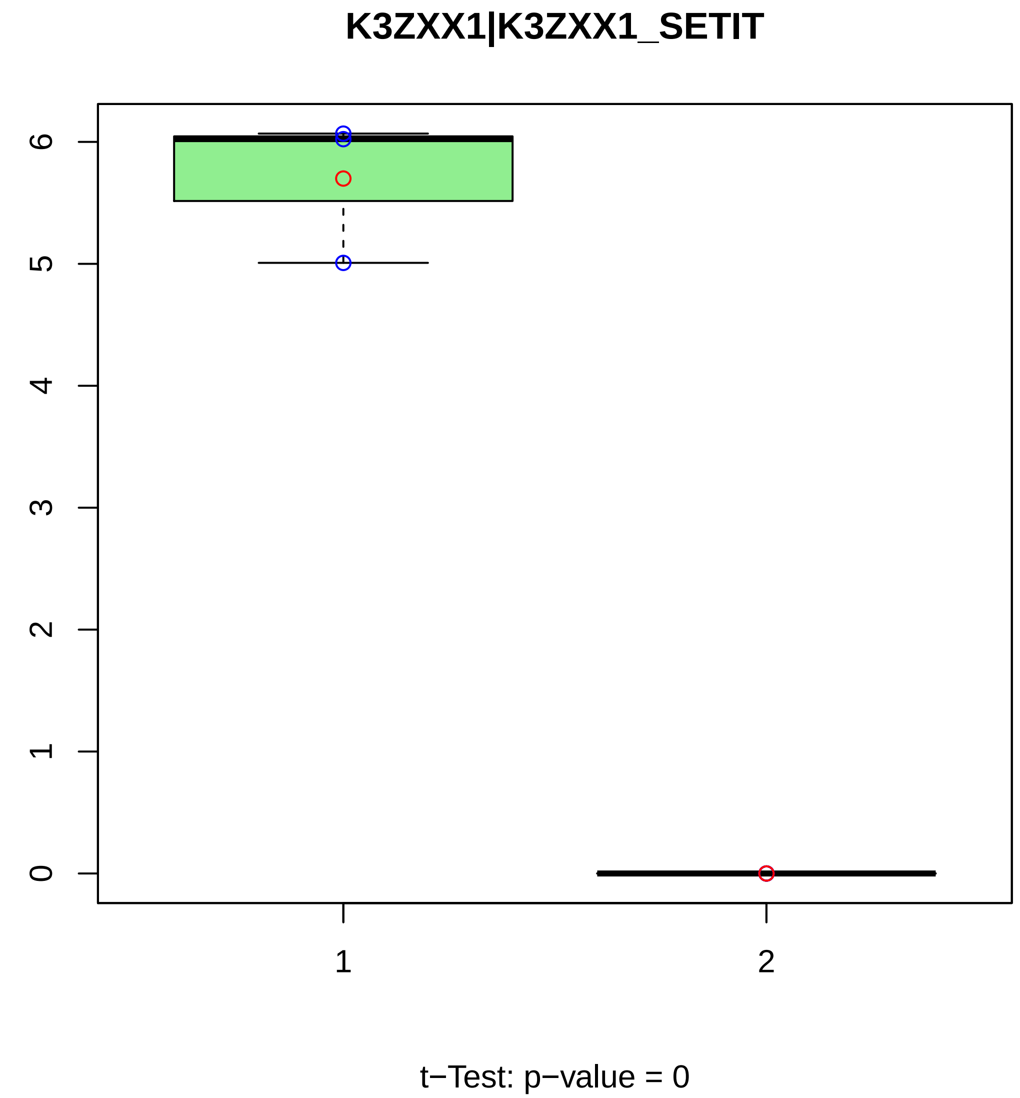

K3ZXX1|K3ZXX1_SETIT
0
1
2
3
4
5
6
1
2
t−Test: p−value = 0

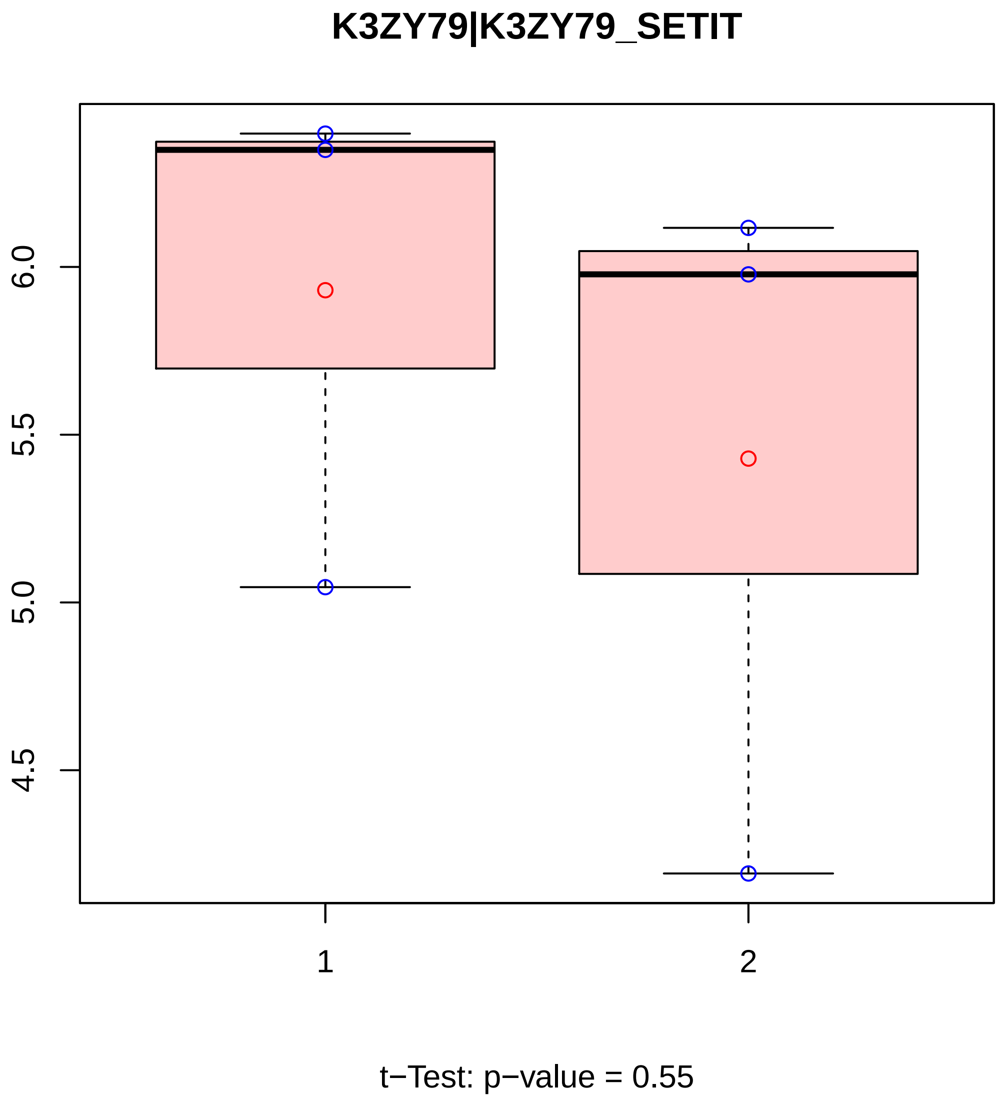
K3ZY79|K3ZY79_SETIT
6.0
5.5
5.0
4.5
1
2
t−Test: p−value = 0.55

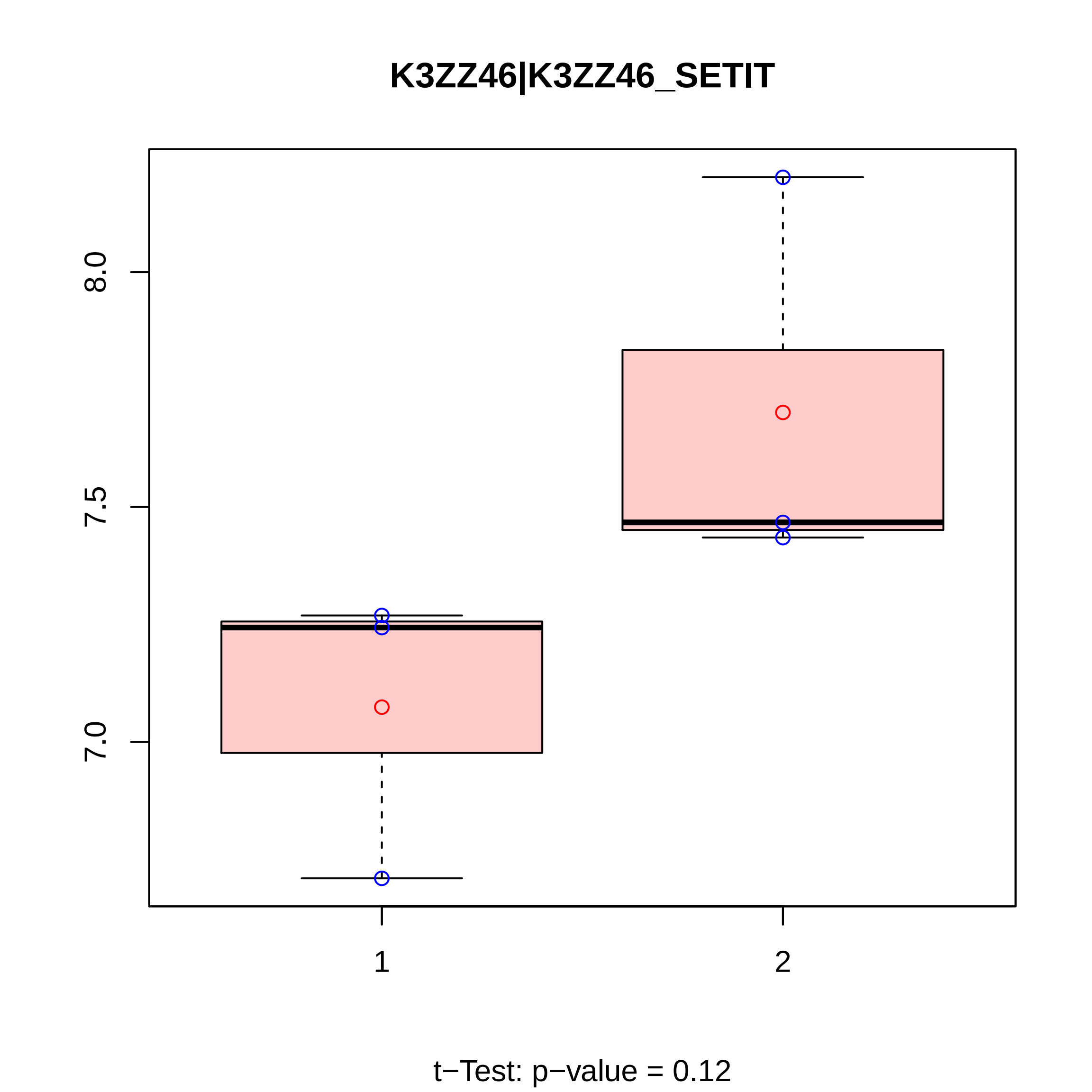

K3ZZ46|K3ZZ46_SETIT
8.0
7.5
7.0
1
2
t−Test: p−value = 0.12

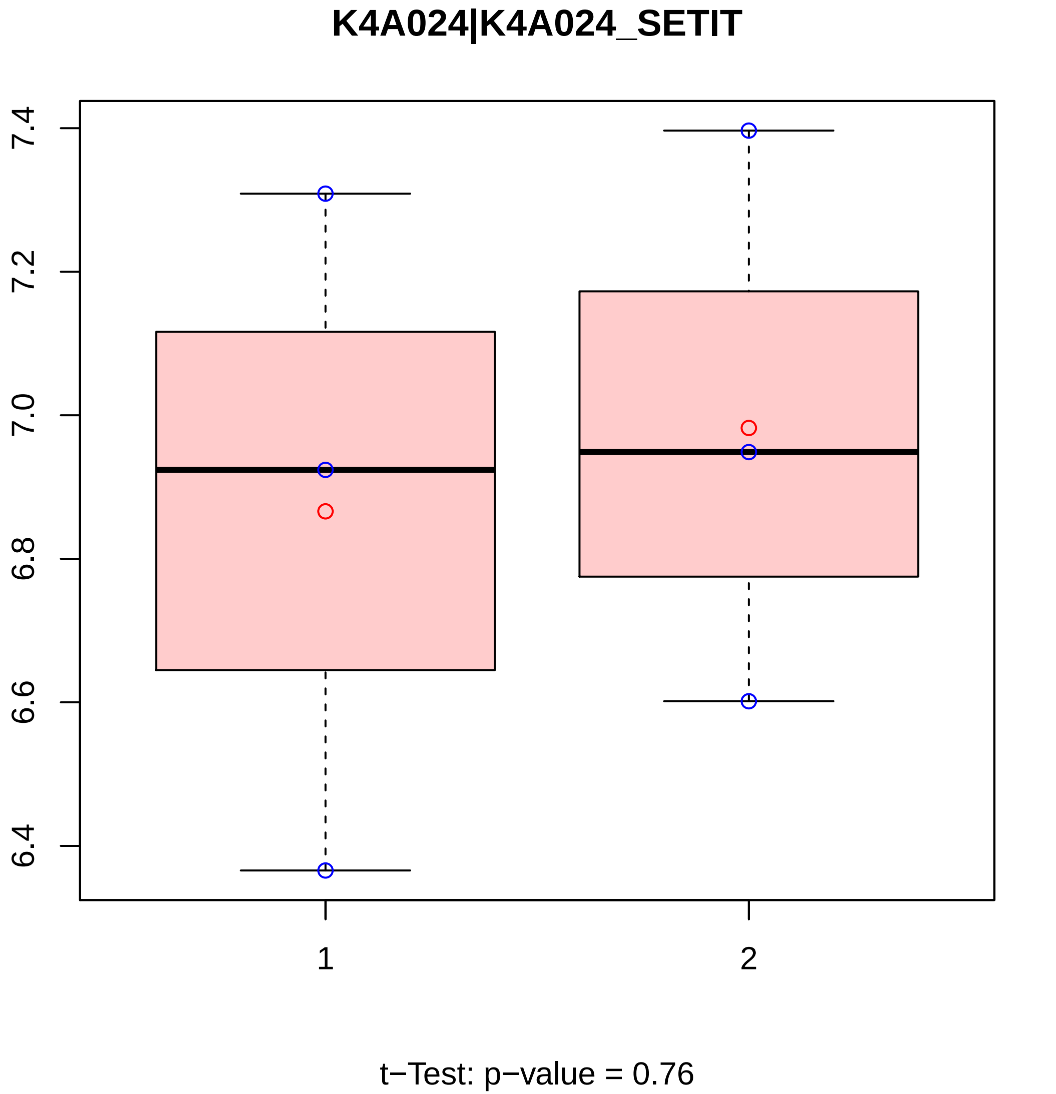
K4A024|K4A024_SETIT
7.4
7.2
7.0
6.8
6.6
6.4
1
2
t−Test: p−value = 0.76

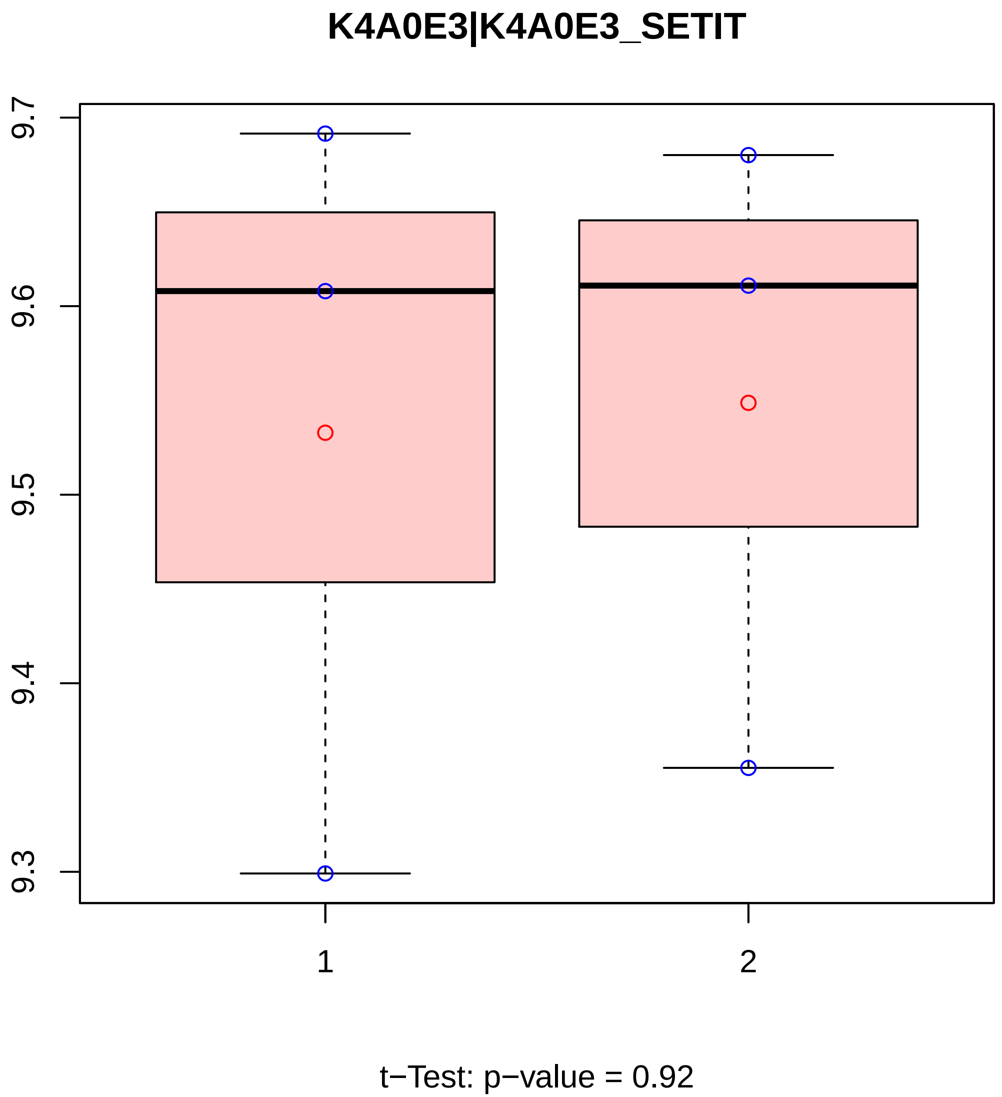
K4A0E3|K4A0E3_SETIT
9.7
9.6
9.5
9.4
9.3
1
2
t−Test: p−value = 0.92

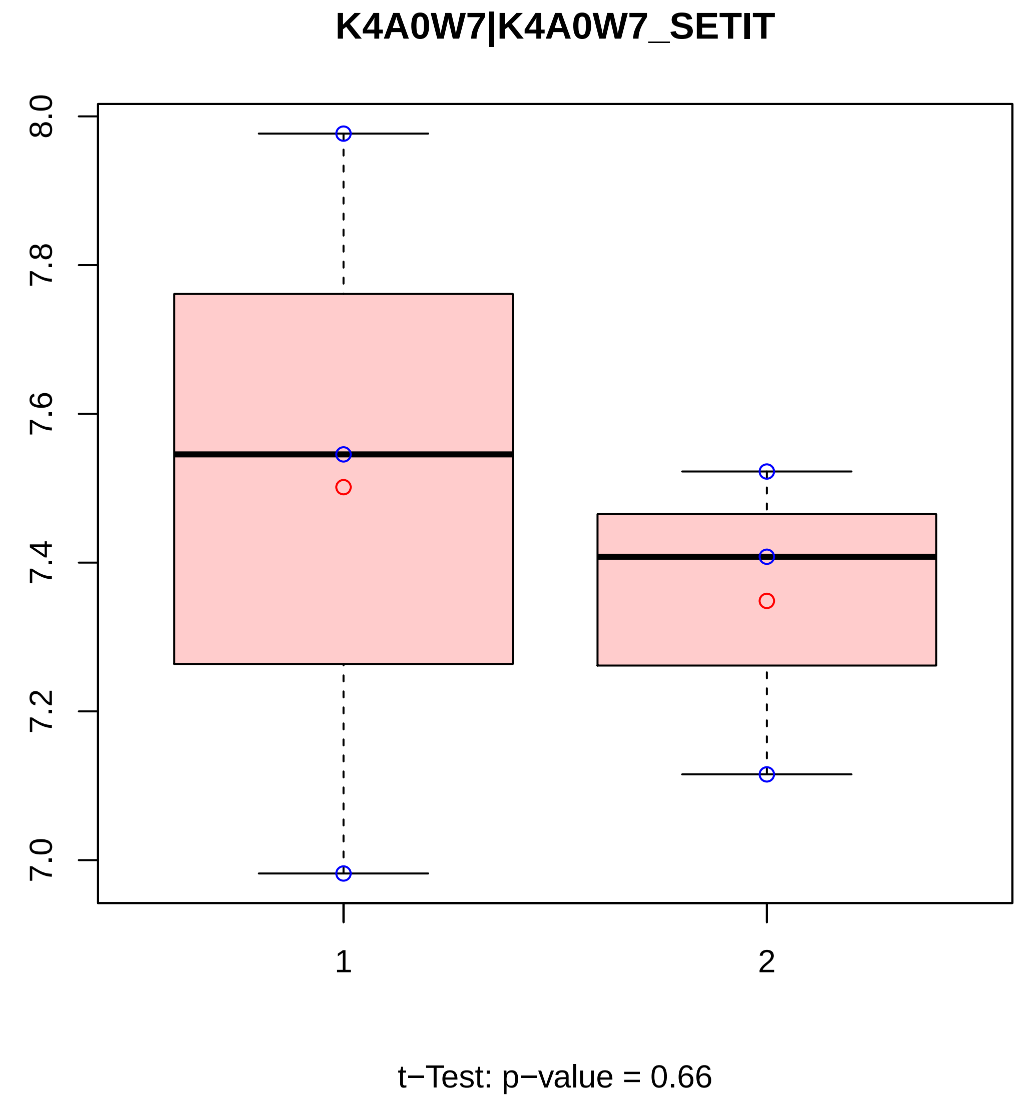
K4A0W7|K4A0W7_SETIT
8.0
7.8
7.6
7.4
7.2
7.0
1
2
t−Test: p−value = 0.66

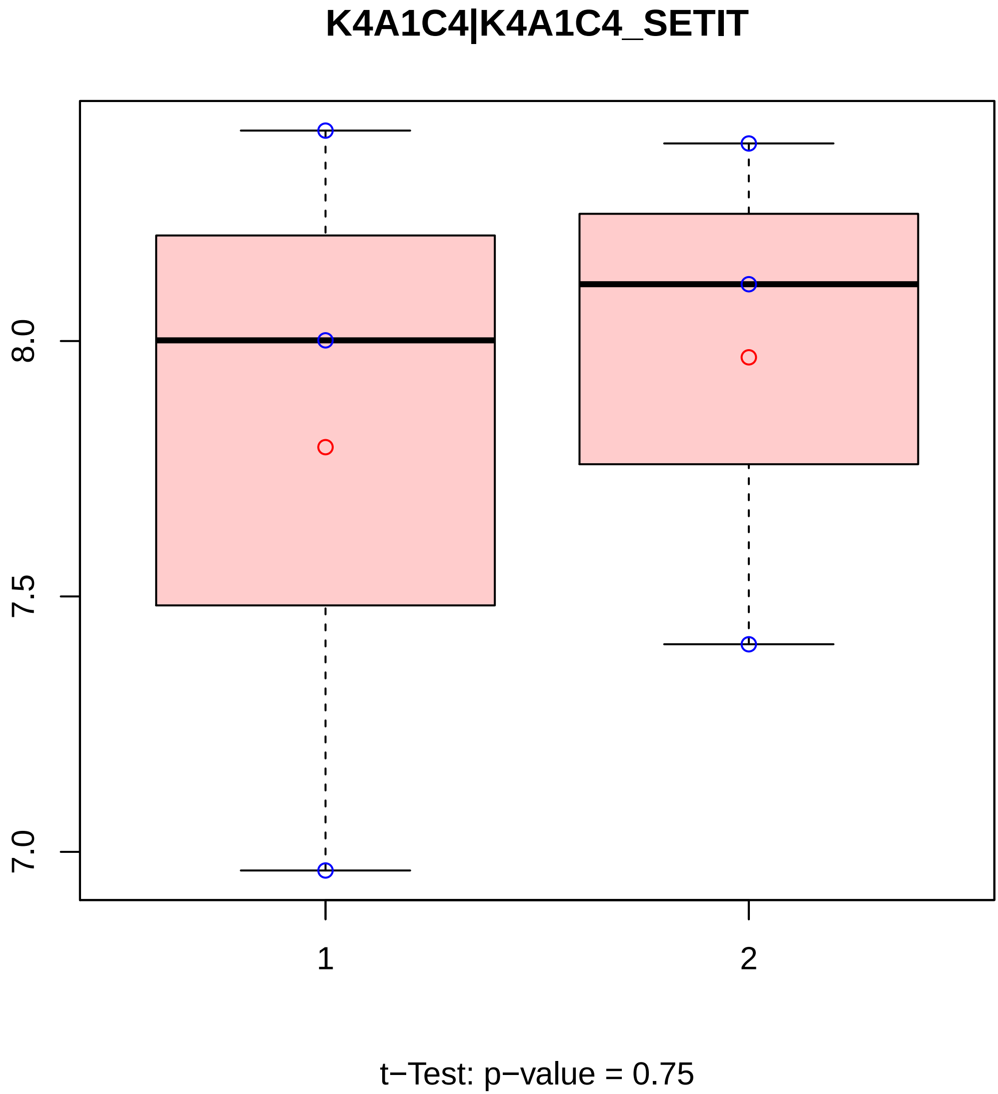
K4A1C4|K4A1C4_SETIT
8.0
7.5
7.0
1
2
t−Test: p−value = 0.75

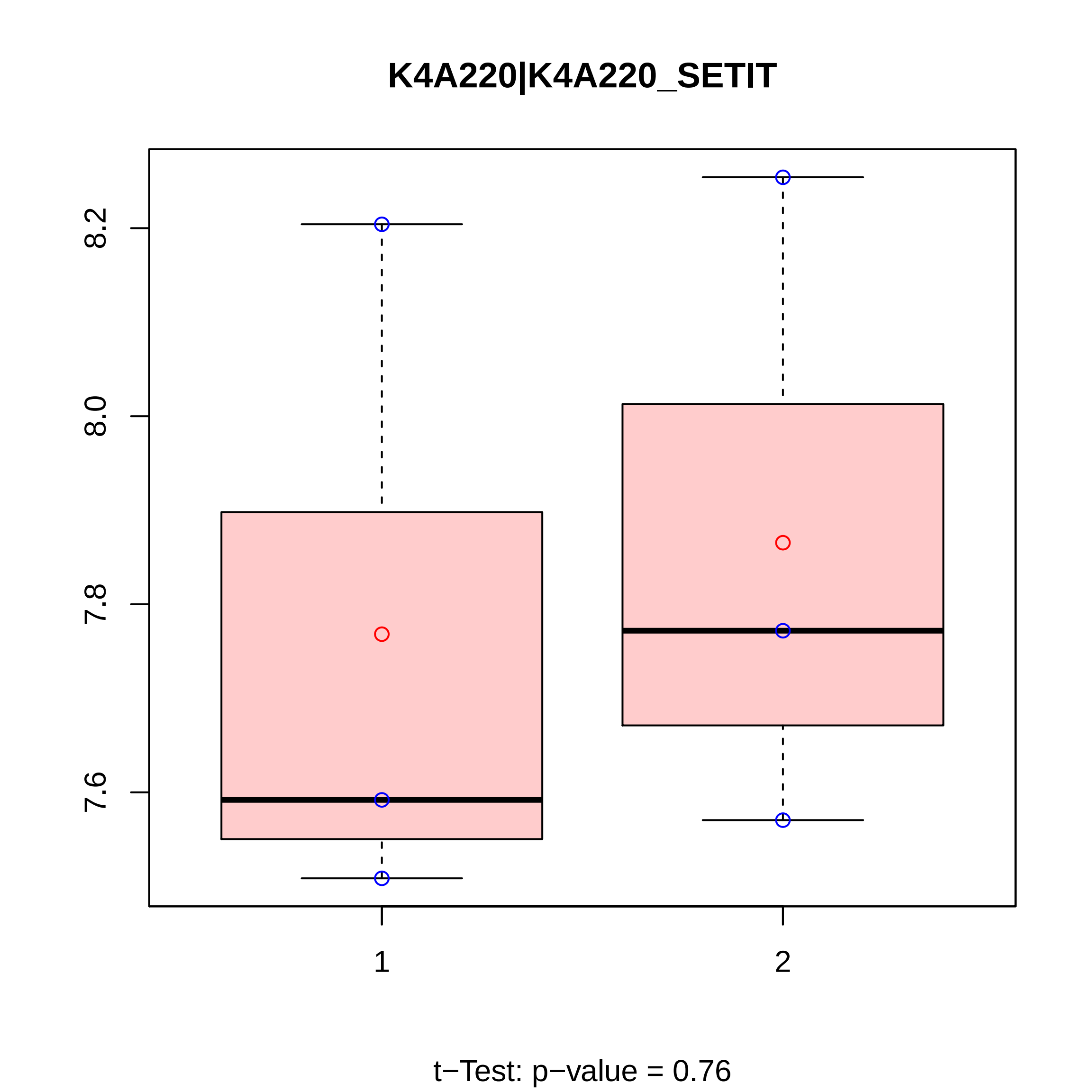

K4A220|K4A220_SETIT
8.2
8.0
7.8
7.6
1
2
t−Test: p−value = 0.76

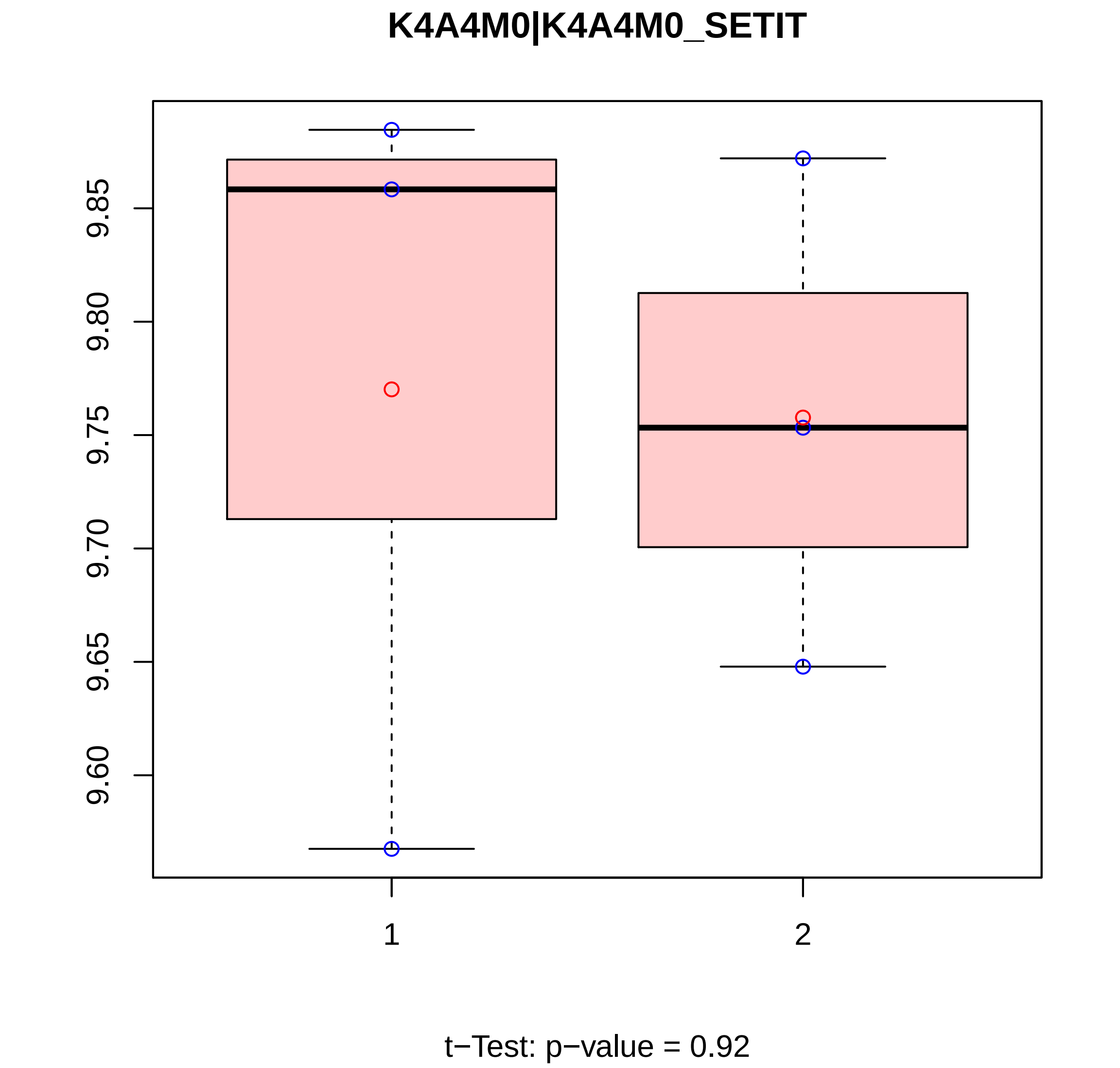
K4A4M0|K4A4M0_SETIT
9.60
9.65
9.70
9.75
9.80
9.85
1
2
t−Test: p−value = 0.92

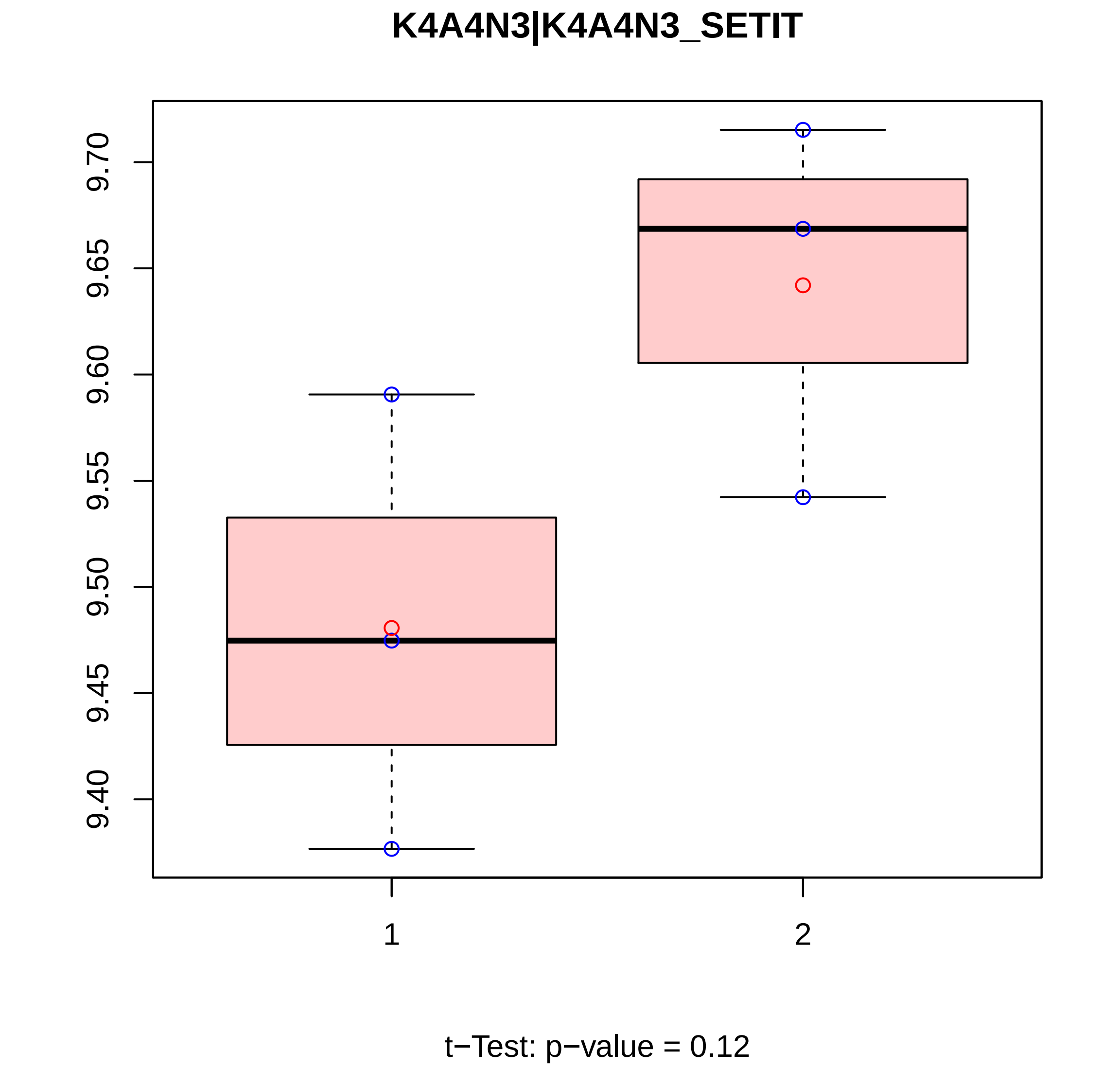
K4A4N3|K4A4N3_SETIT
9.40
9.45
9.50
9.55
9.60
9.65
9.70
1
2
t−Test: p−value = 0.12

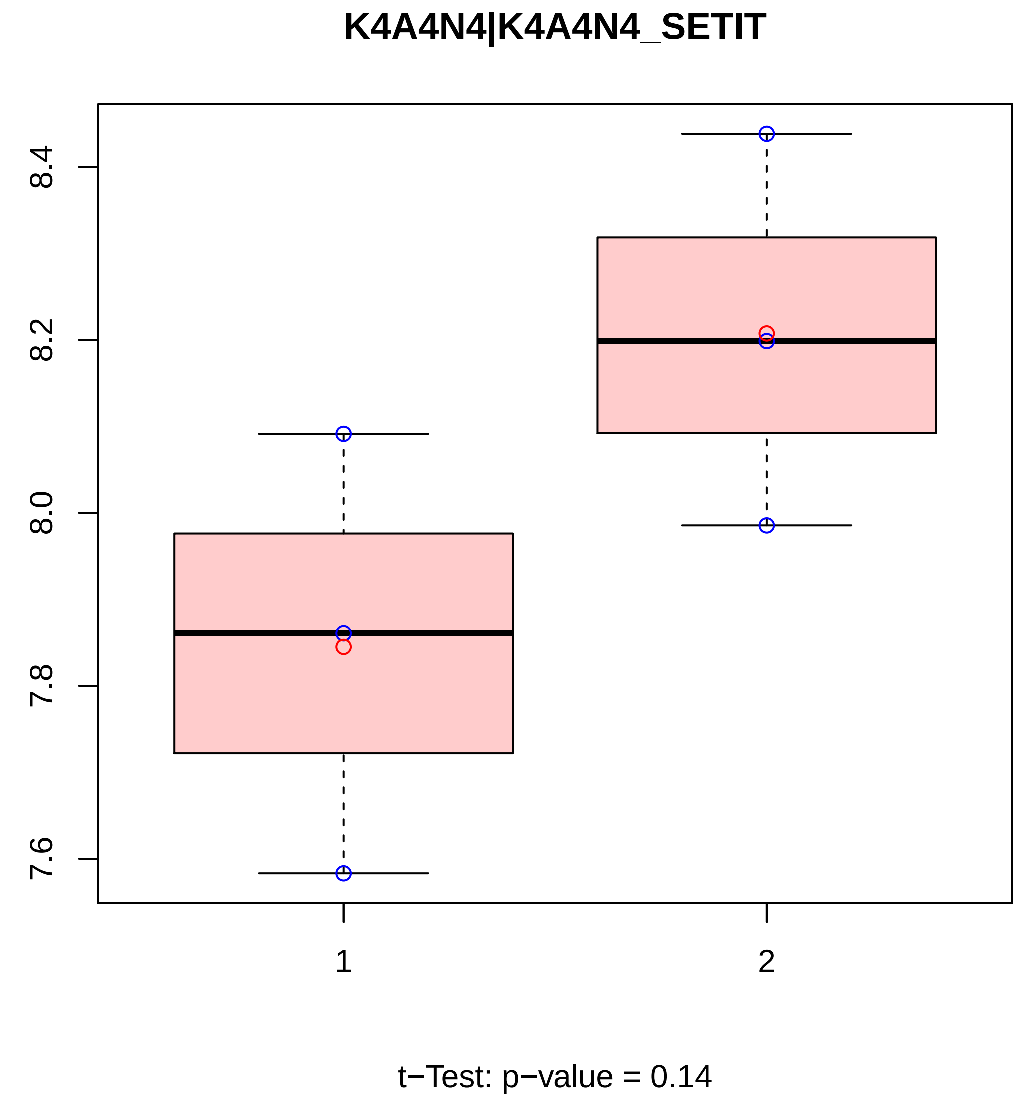
K4A4N4|K4A4N4_SETIT
8.4
8.2
8.0
7.8
7.6
1
2
t−Test: p−value = 0.14

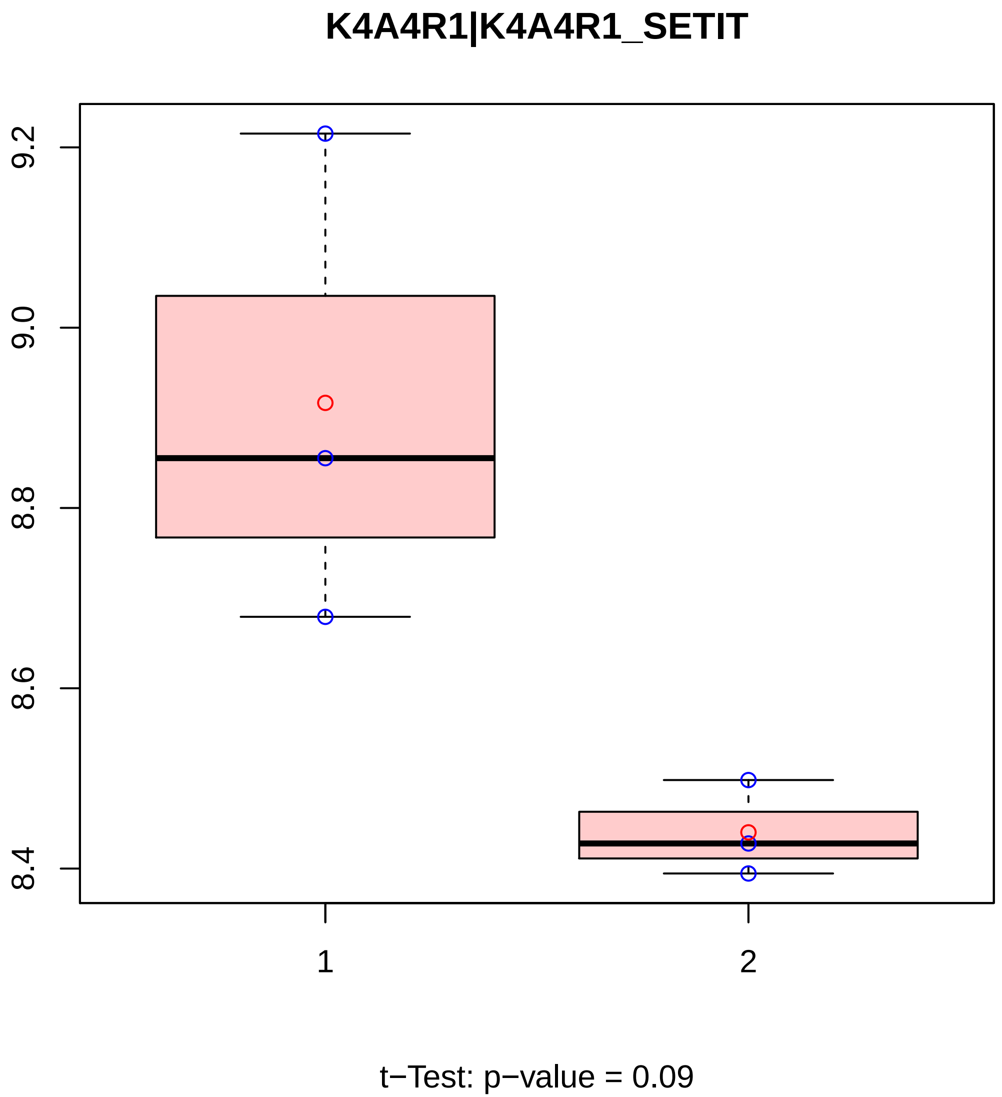
K4A4R1|K4A4R1_SETIT
9.2
9.0
8.8
8.6
8.4
1
2
t−Test: p−value = 0.09

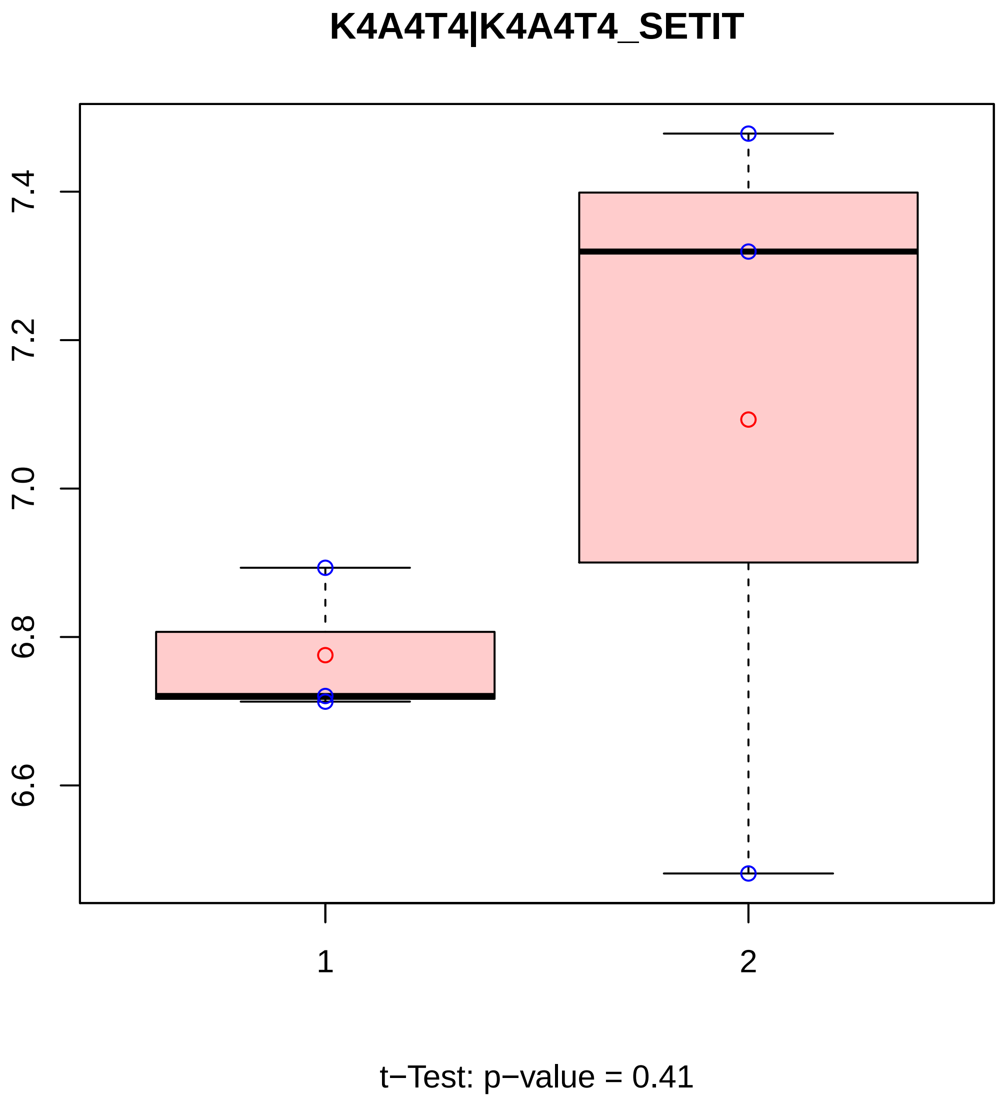
K4A4T4|K4A4T4_SETIT
7.4
7.2
7.0
6.8
6.6
1
2
t−Test: p−value = 0.41

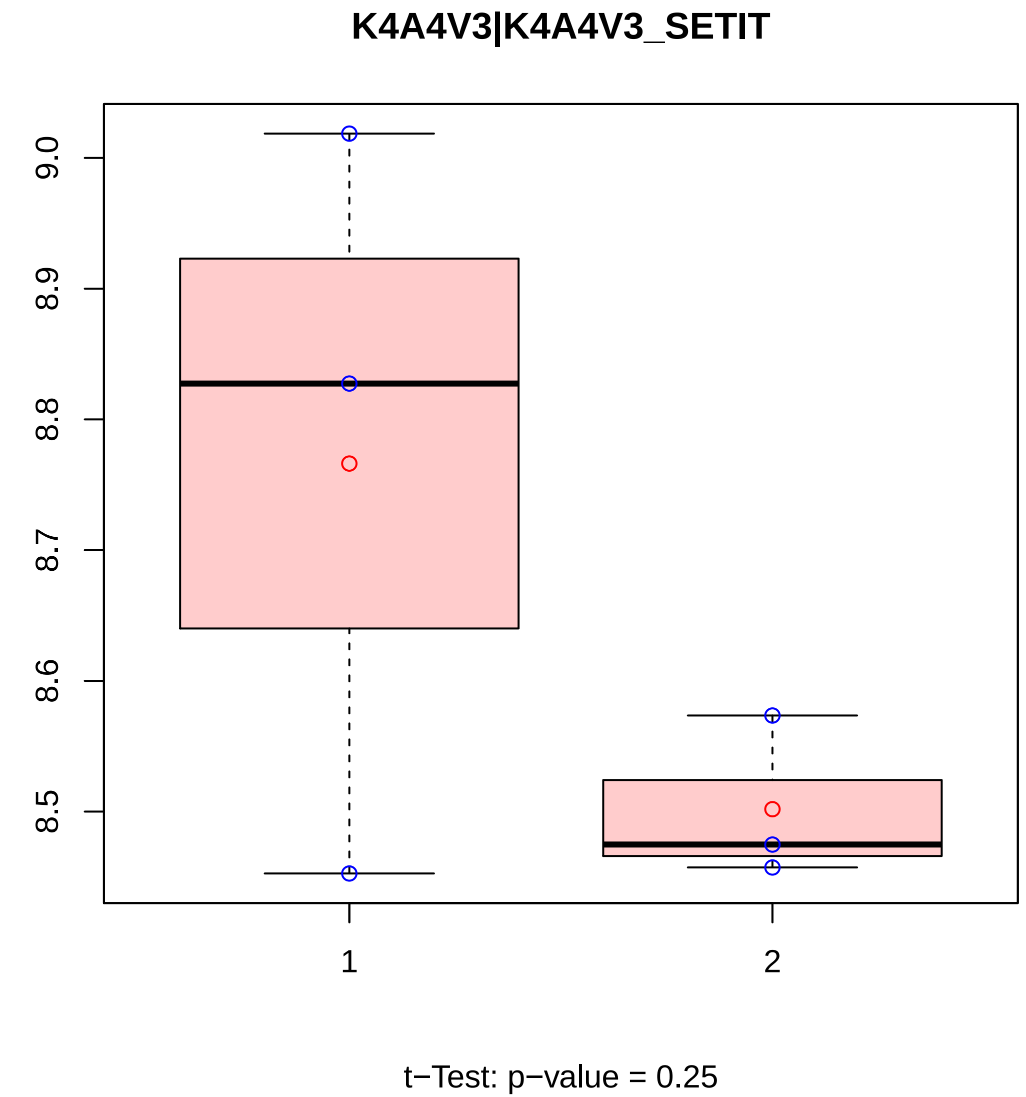

K4A4V3|K4A4V3_SETIT
9.0
8.9
8.8
8.7
8.6
8.5
1
2
t−Test: p−value = 0.25

K4A4Y6|K4A4Y6_SETIT

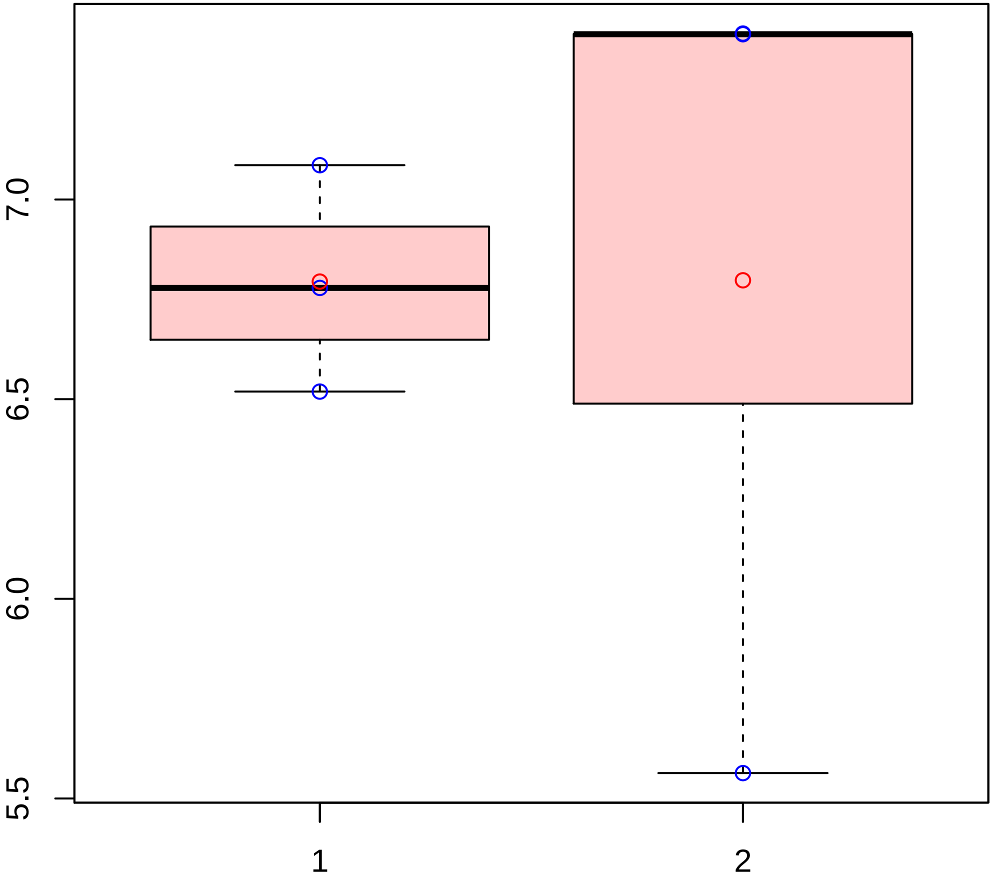

t−Test: p−value = 1

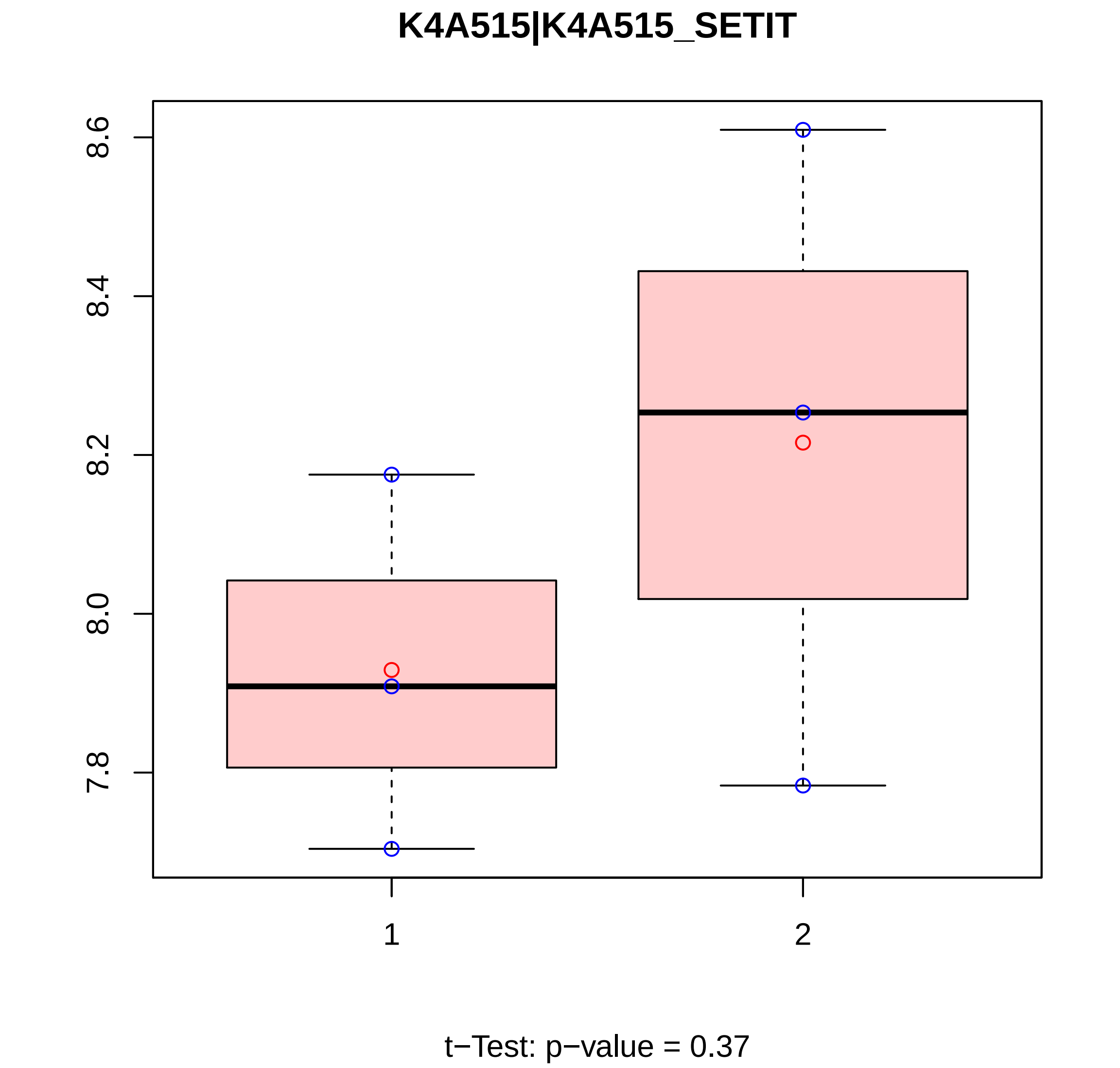
K4A515|K4A515_SETIT
8.6
8.4
8.2
8.0
7.8
1
2
t−Test: p−value = 0.37

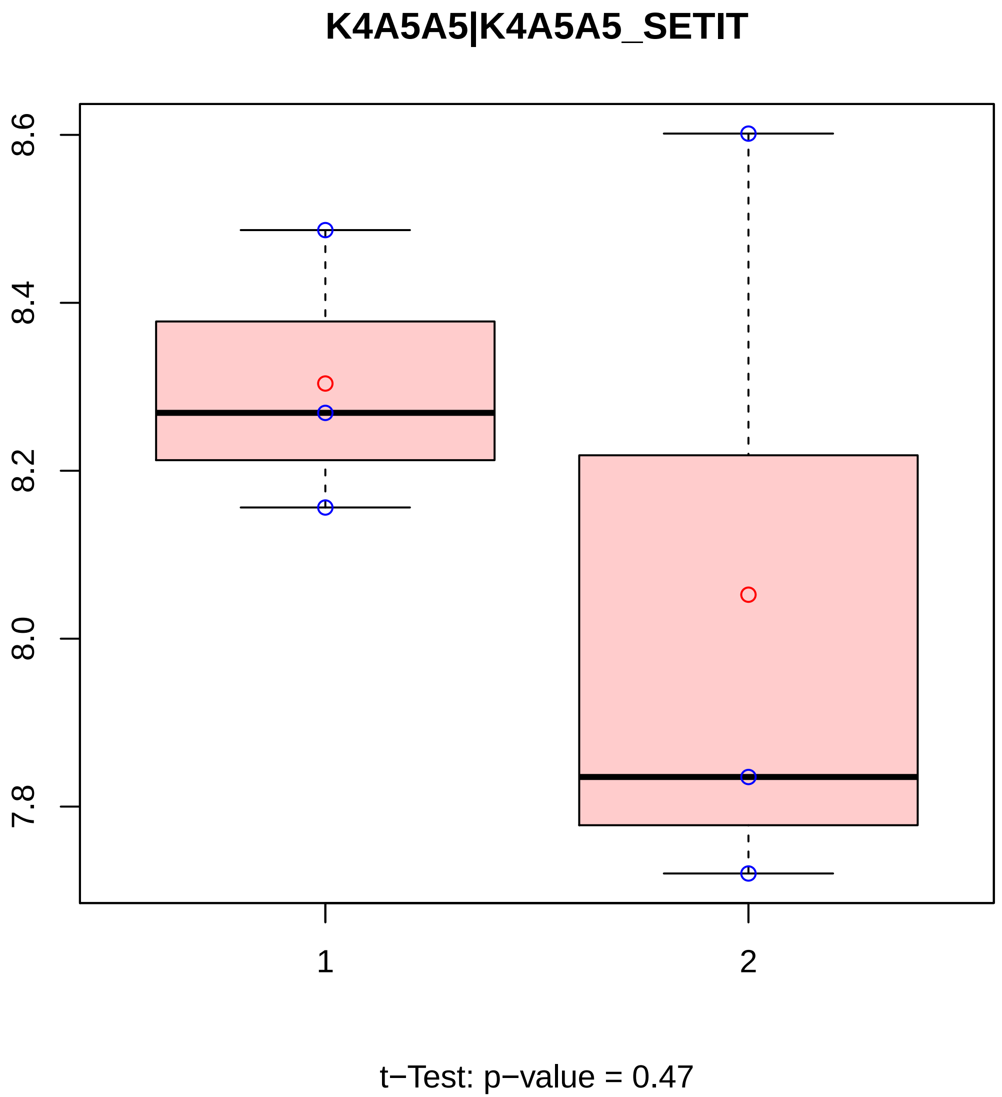

K4A5A5|K4A5A5_SETIT
8.6
8.4
8.2
8.0
7.8
1
2
t−Test: p−value = 0.47

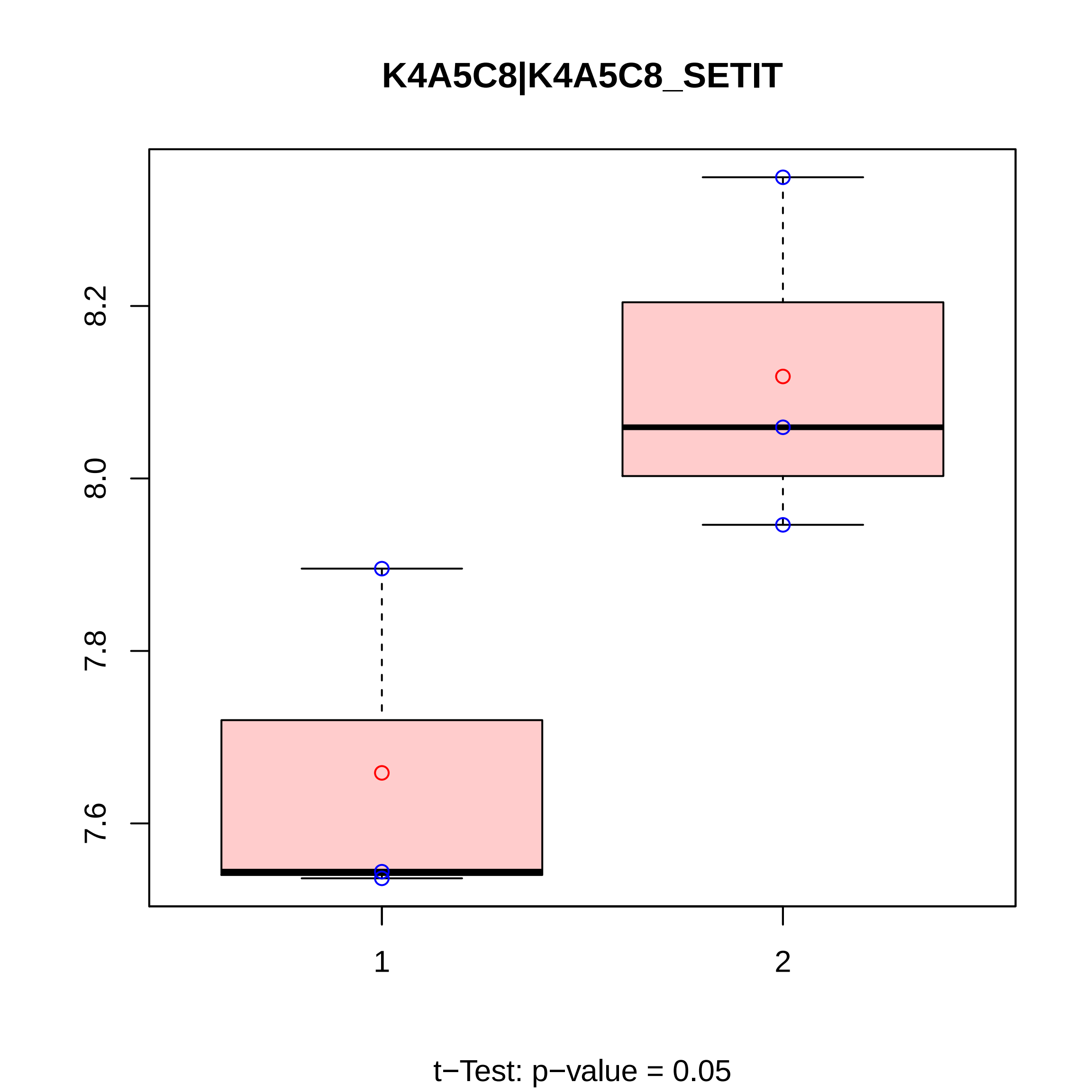

K4A5C8|K4A5C8_SETIT
7.6
7.8
8.0
8.2
1
2
t−Test: p−value = 0.05

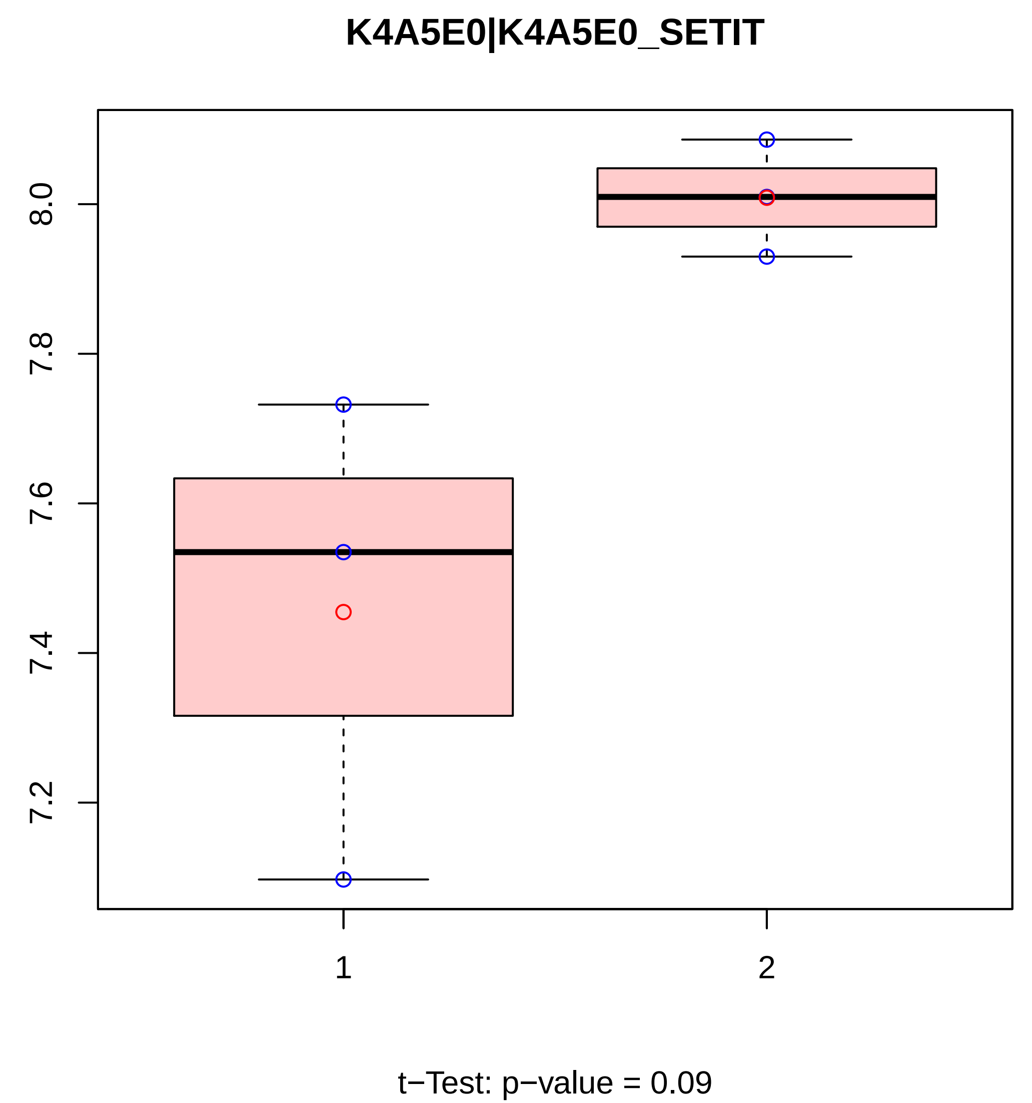
K4A5E0|K4A5E0_SETIT
7.2
7.4
7.6
7.8
8.0
1
2
t−Test: p−value = 0.09

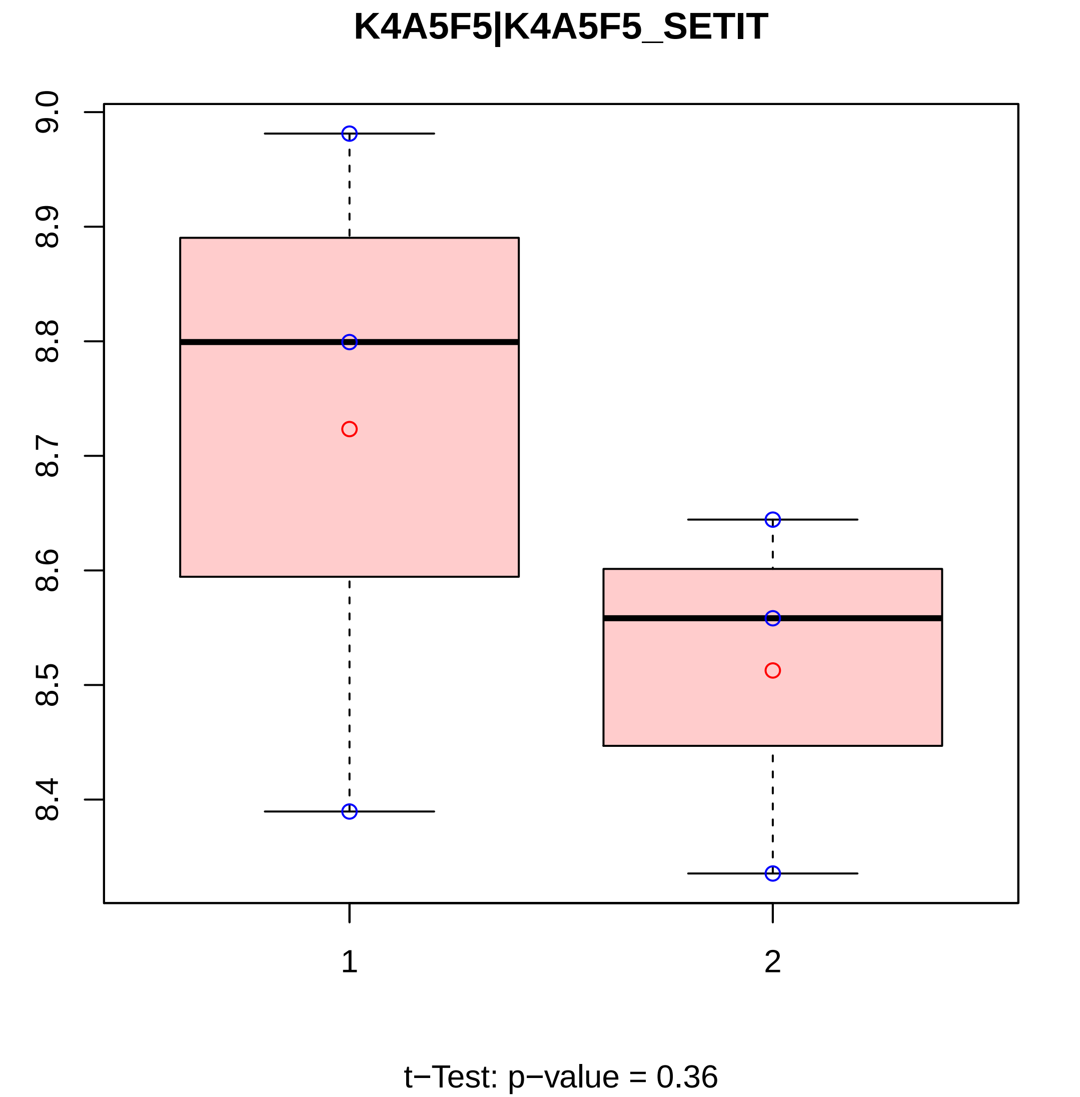

K4A5F5|K4A5F5_SETIT
9.0
8.9
8.8
8.7
8.6
8.5
8.4
1
2
t−Test: p−value = 0.36

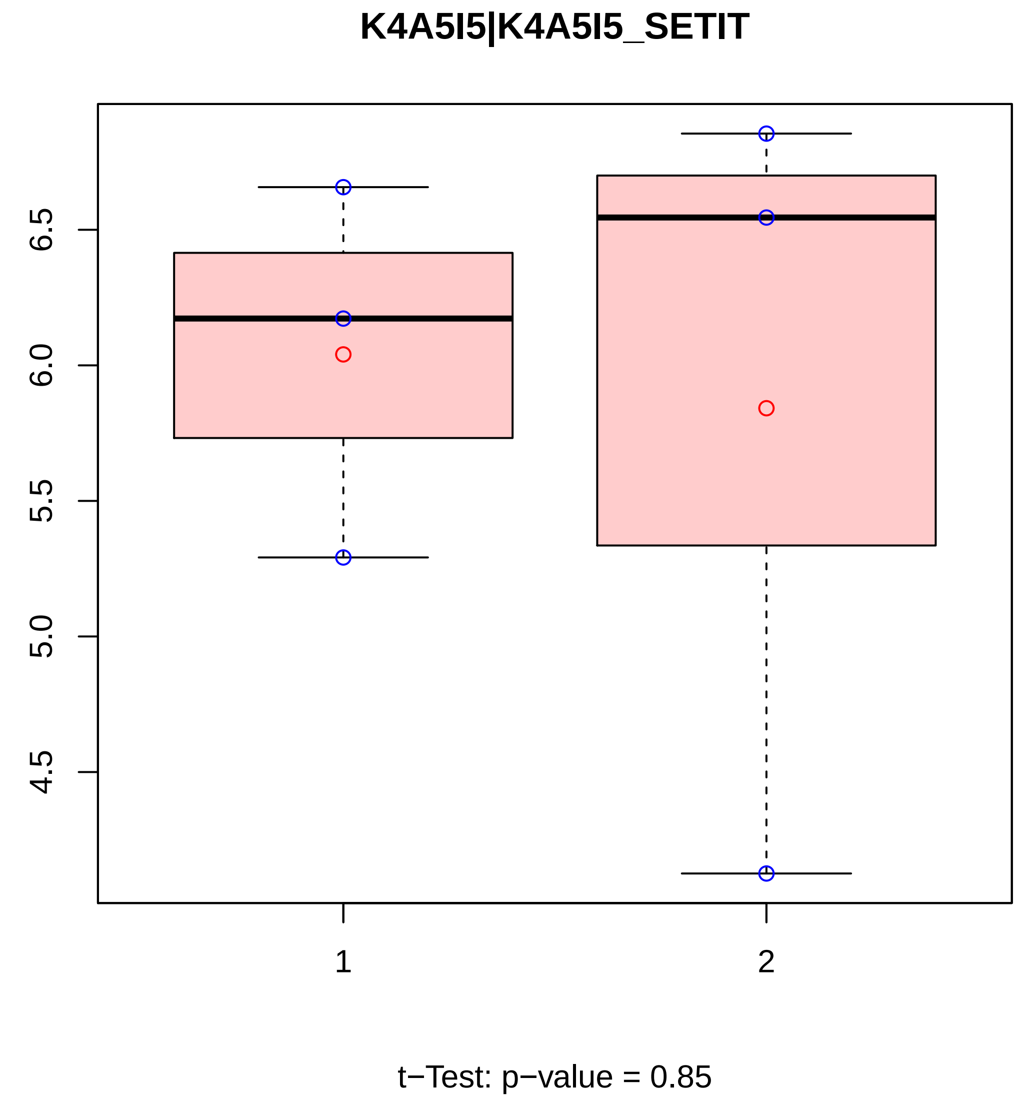
K4A5I5|K4A5I5_SETIT
6.5
6.0
5.5
5.0
4.5
1
2
t−Test: p−value = 0.85

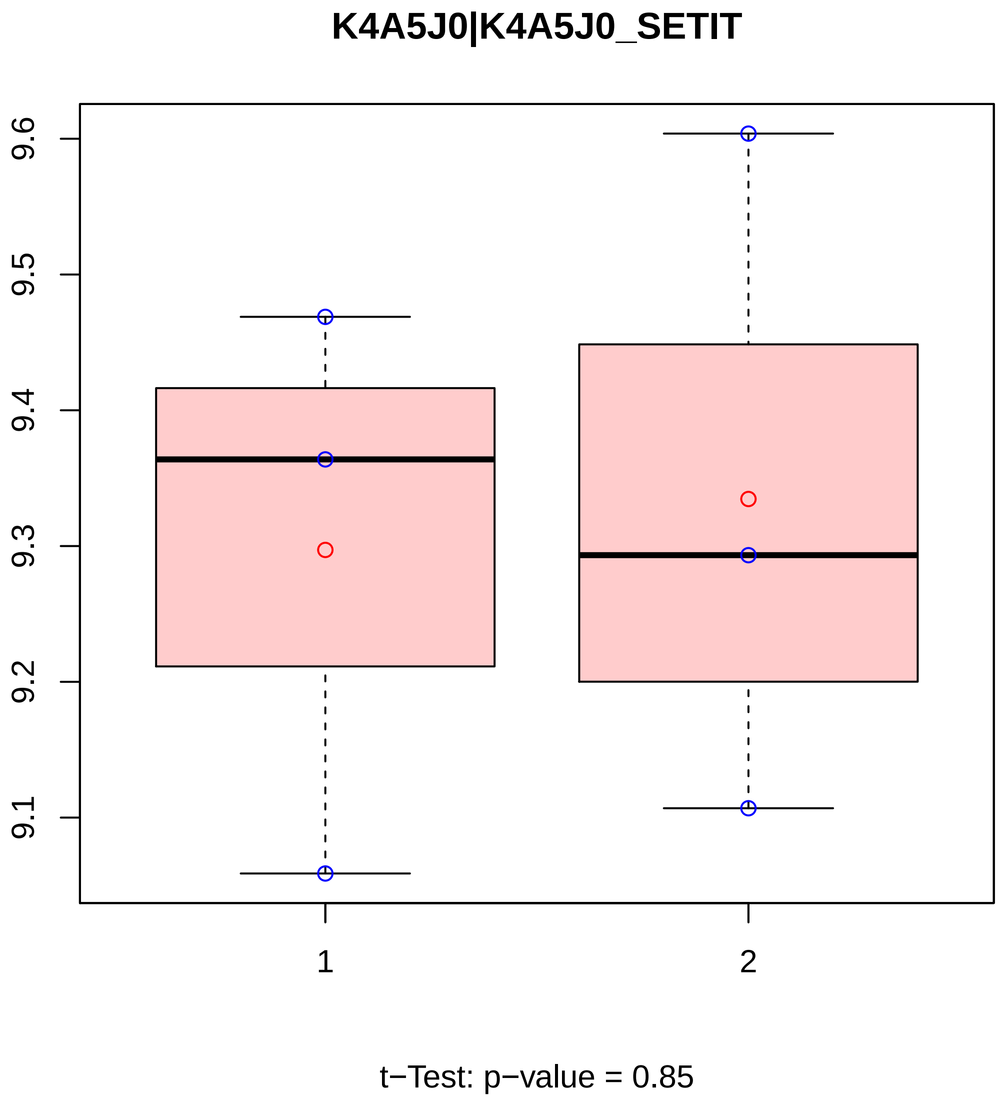
K4A5J0|K4A5J0_SETIT
9.1
9.2
9.3
9.4
9.5
9.6
1
2
t−Test: p−value = 0.85

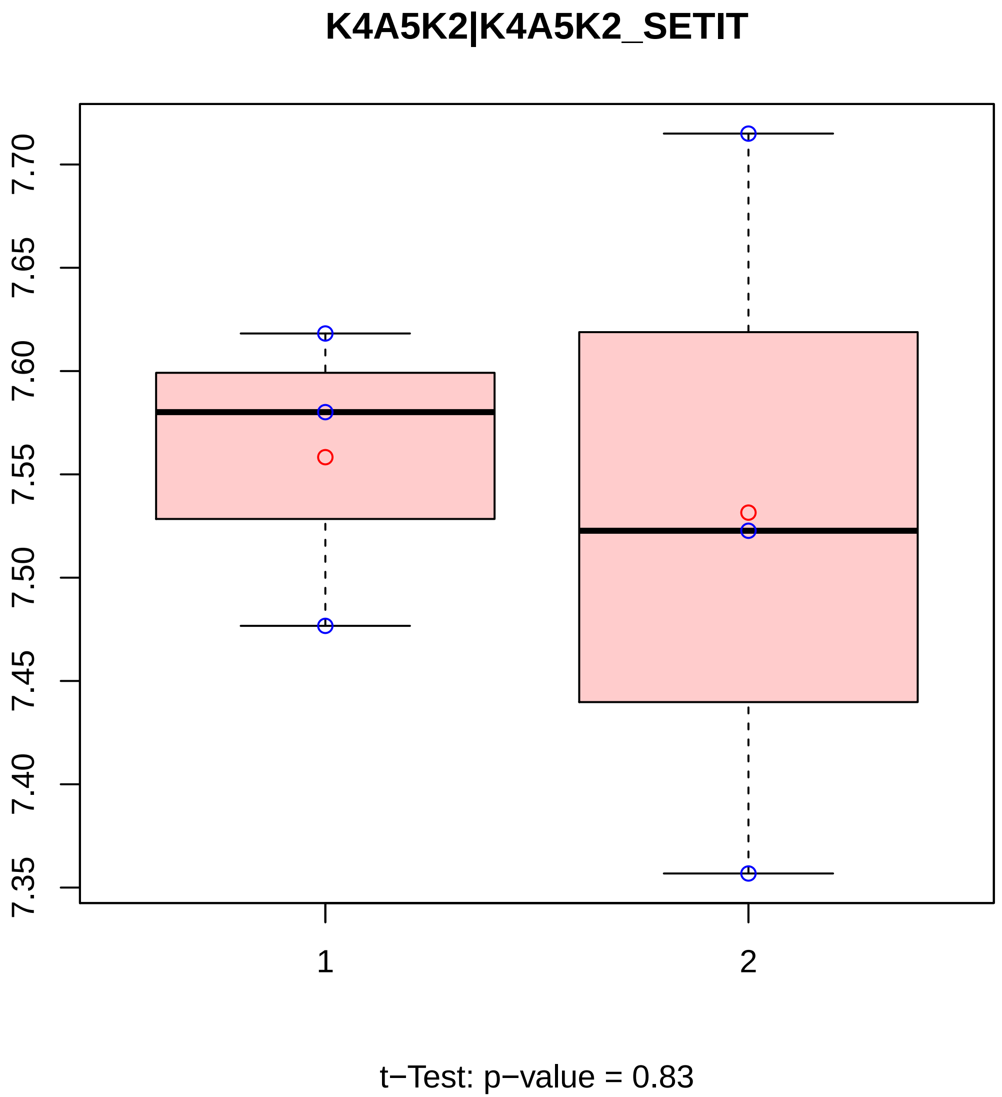

K4A5K2|K4A5K2_SETIT
7.35
7.40
7.45
7.50
7.55
7.60
7.65
7.70
1
2
t−Test: p−value = 0.83

K4A5K9|K4A5K9_SETIT

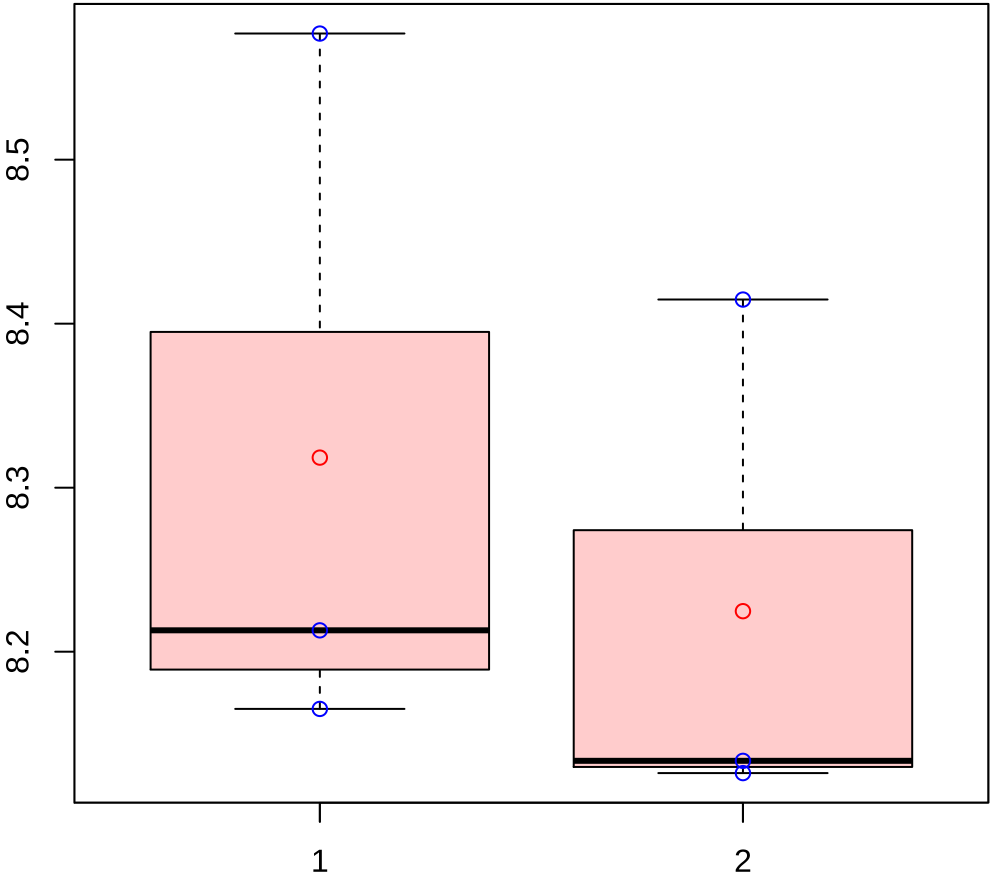

t−Test: p−value = 0.59

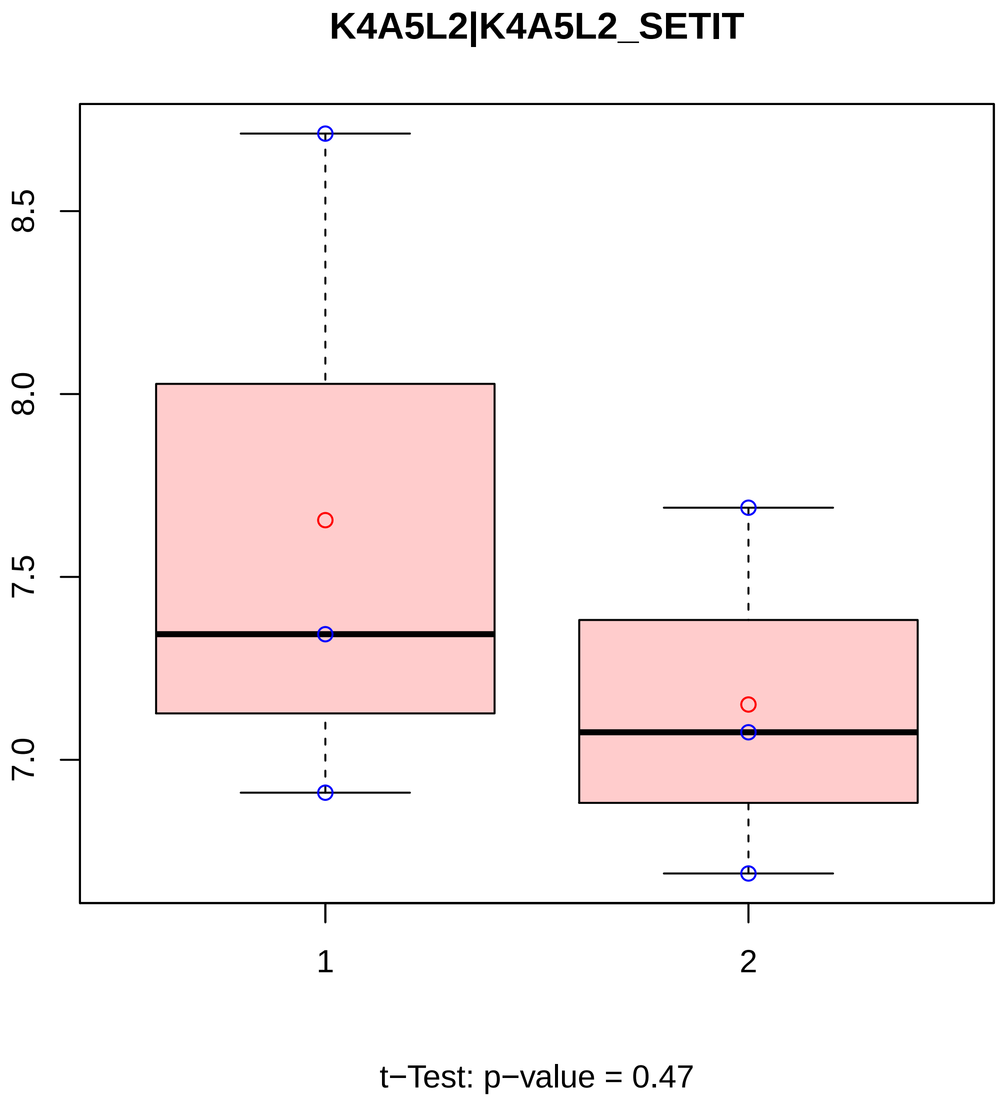

K4A5L2|K4A5L2_SETIT
8.5
8.0
7.5
7.0
1
2
t−Test: p−value = 0.47

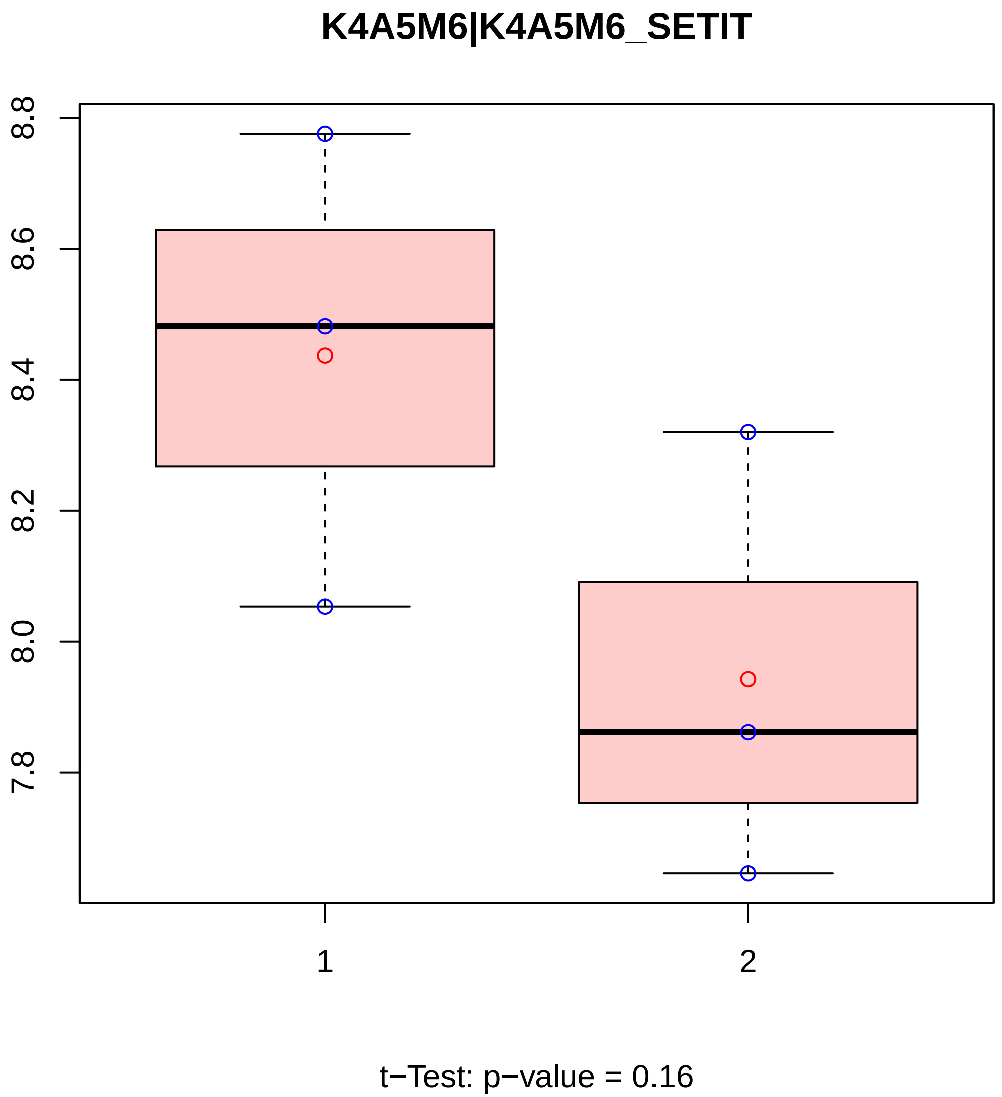
K4A5M6|K4A5M6_SETIT
8.8
8.6
8.4
8.2
8.0
7.8
1
2
t−Test: p−value = 0.16

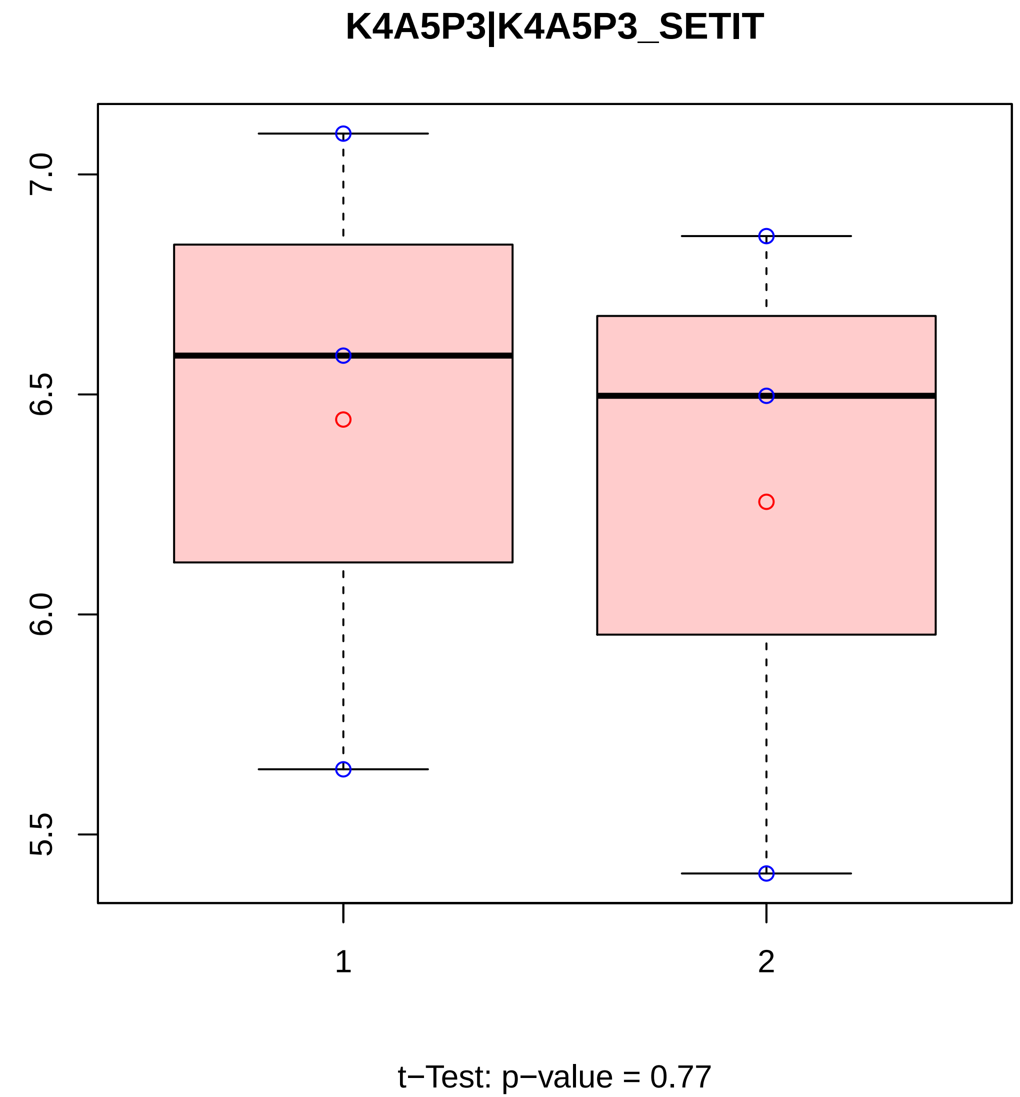
K4A5P3|K4A5P3_SETIT
7.0
6.5
6.0
5.5
1
2
t−Test: p−value = 0.77

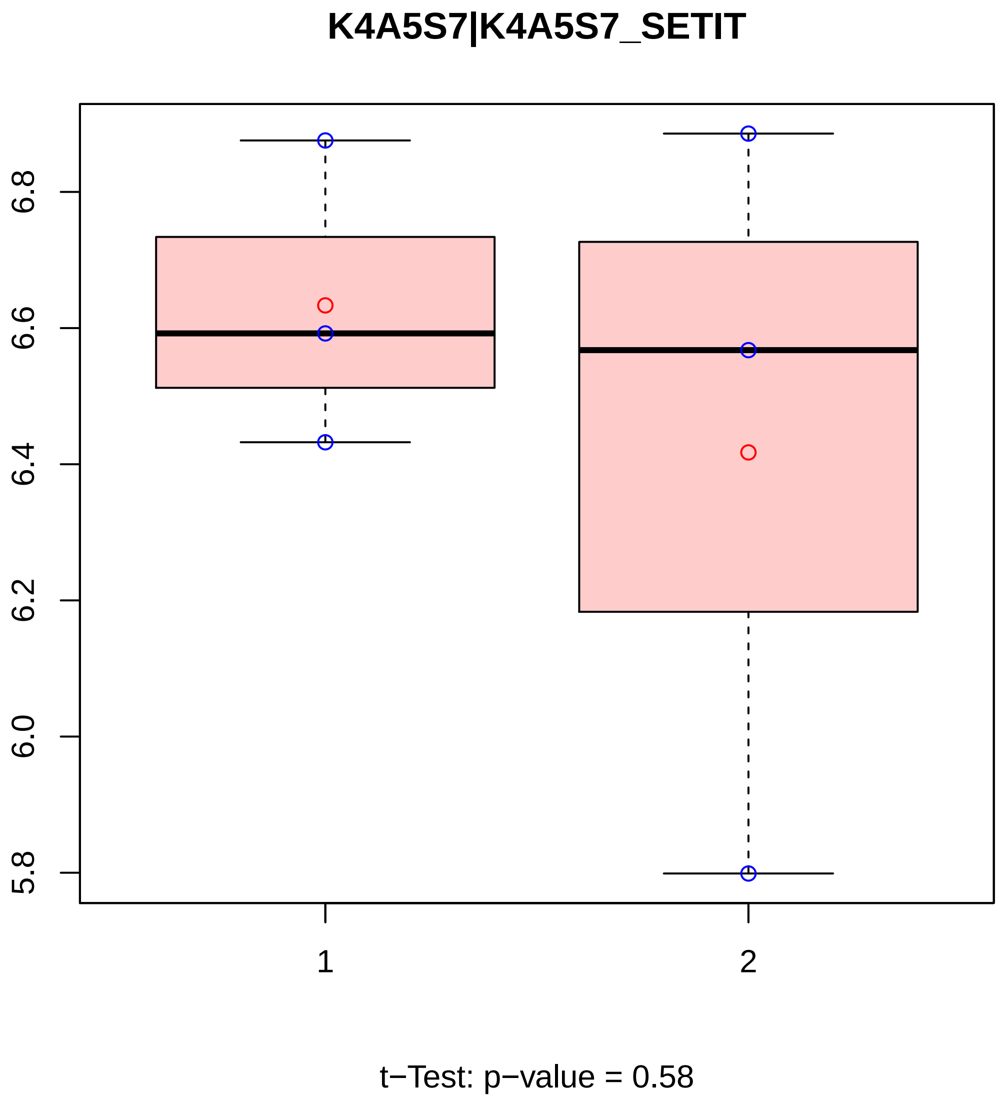
K4A5S7|K4A5S7_SETIT
6.8
6.6
6.4
6.2
6.0
5.8
1
2
t−Test: p−value = 0.58

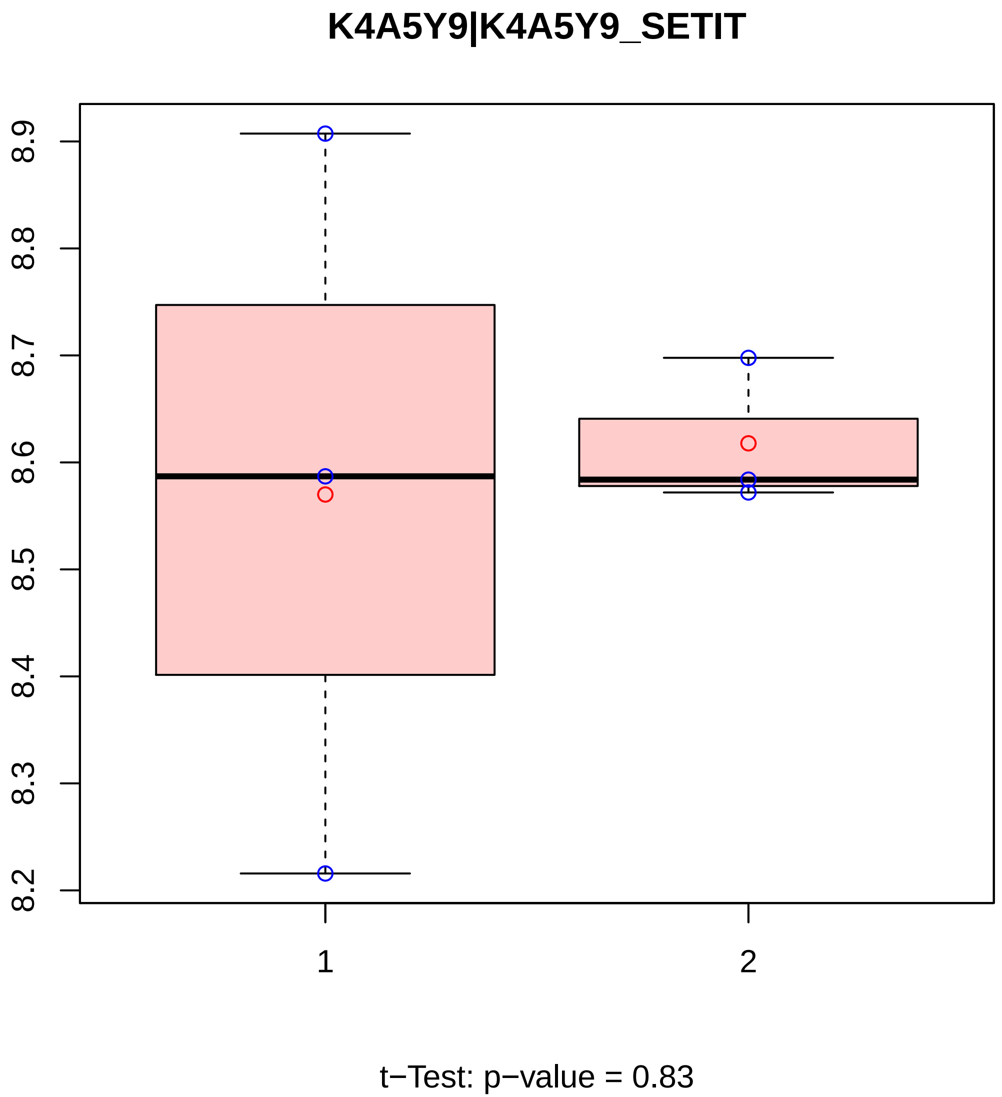

K4A5Y9|K4A5Y9_SETIT
8.2
8.3
8.4
8.5
8.6
8.7
8.8
8.9
1
2
t−Test: p−value = 0.83

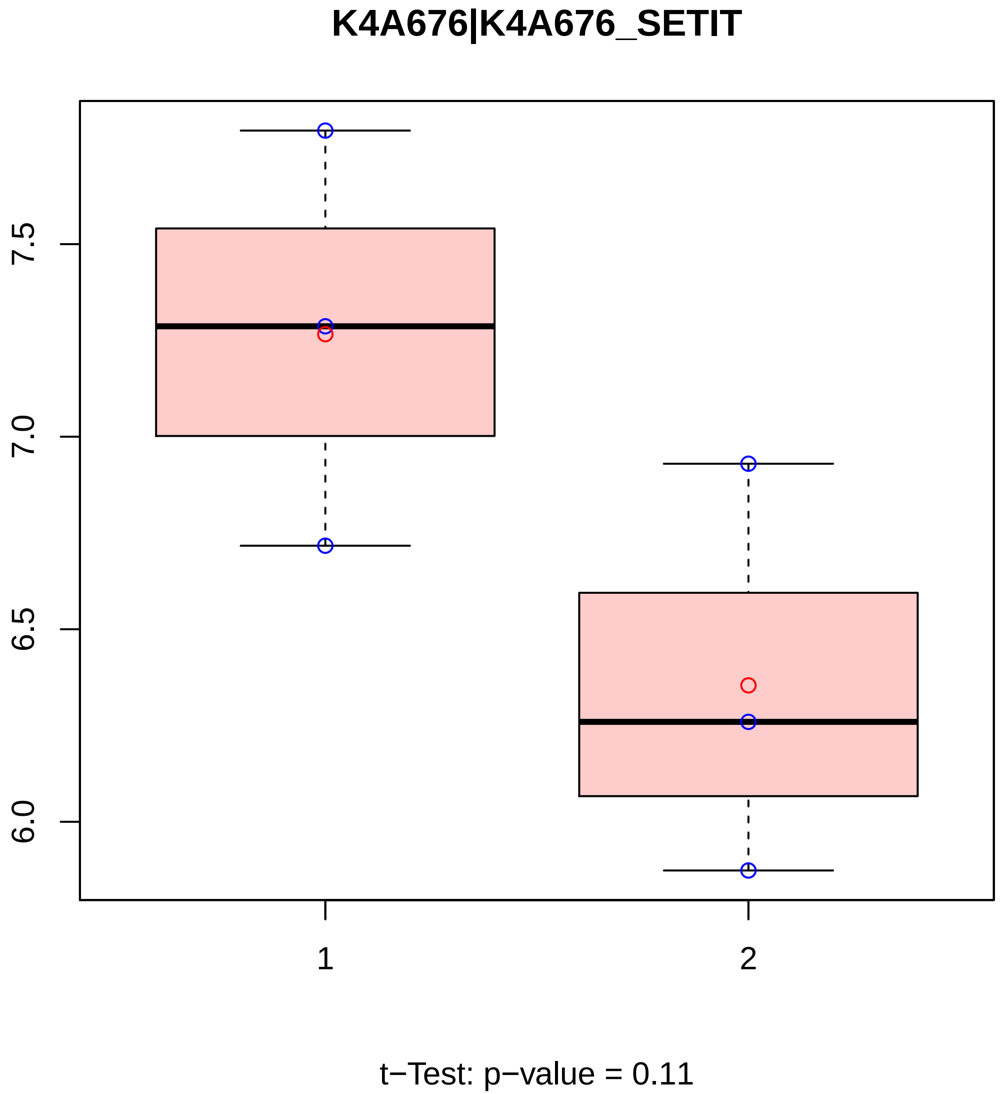
K4A676|K4A676_SETIT
7.5
7.0
6.5
6.0
1
2
t−Test: p−value = 0.11

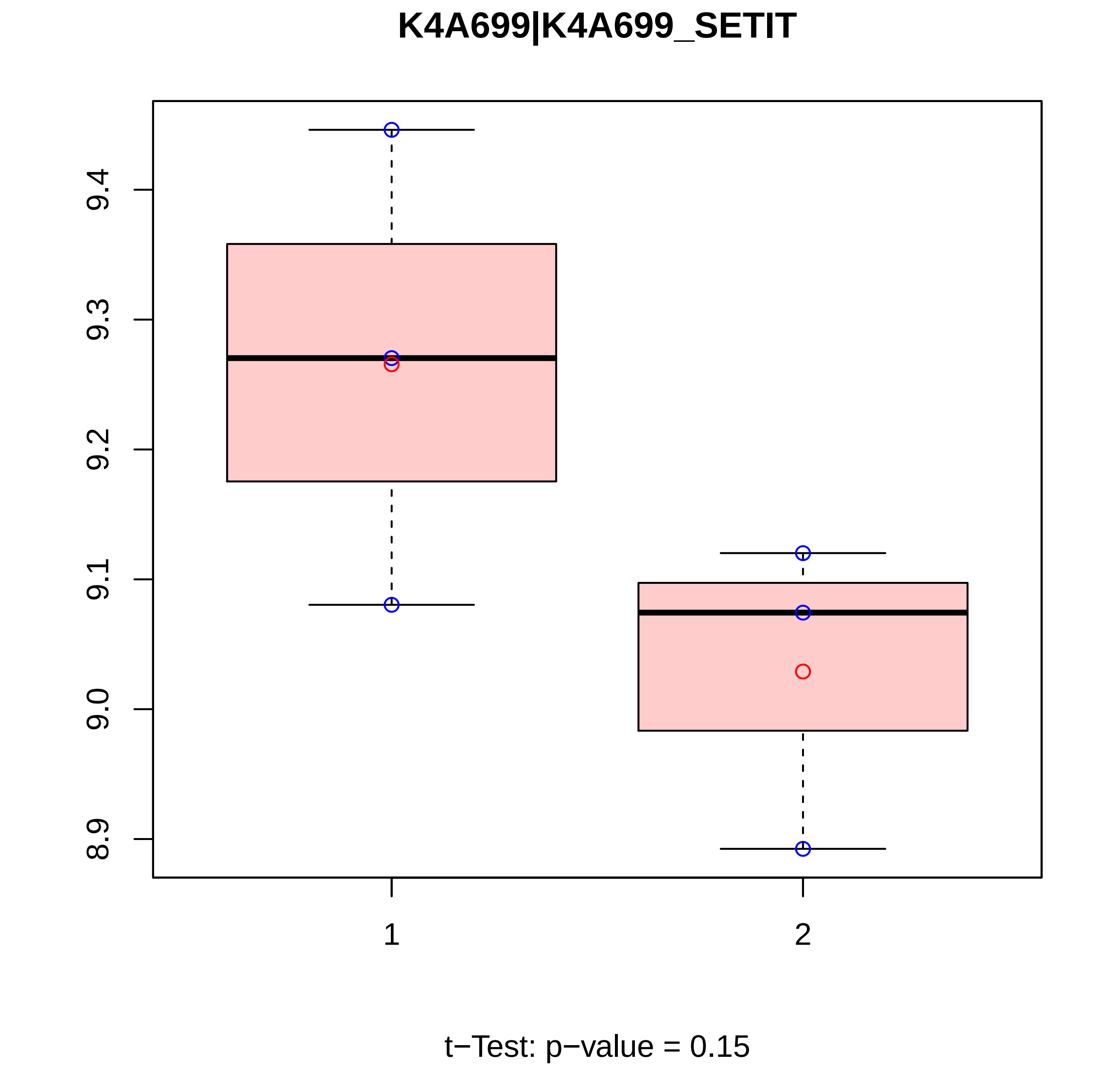
K4A699|K4A699_SETIT
9.4
9.3
9.2
9.1
9.0
8.9
1
2
t−Test: p−value = 0.15

K4A6A7|K4A6A7_SETIT

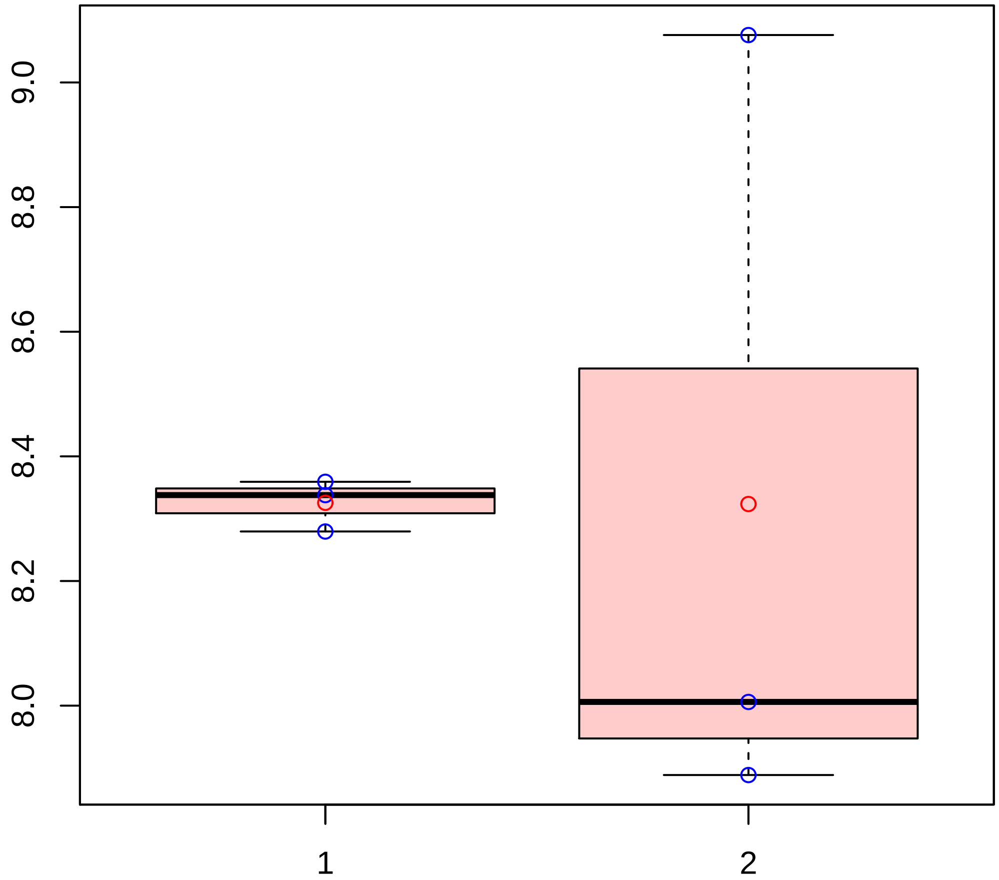

t−Test: p−value = 1

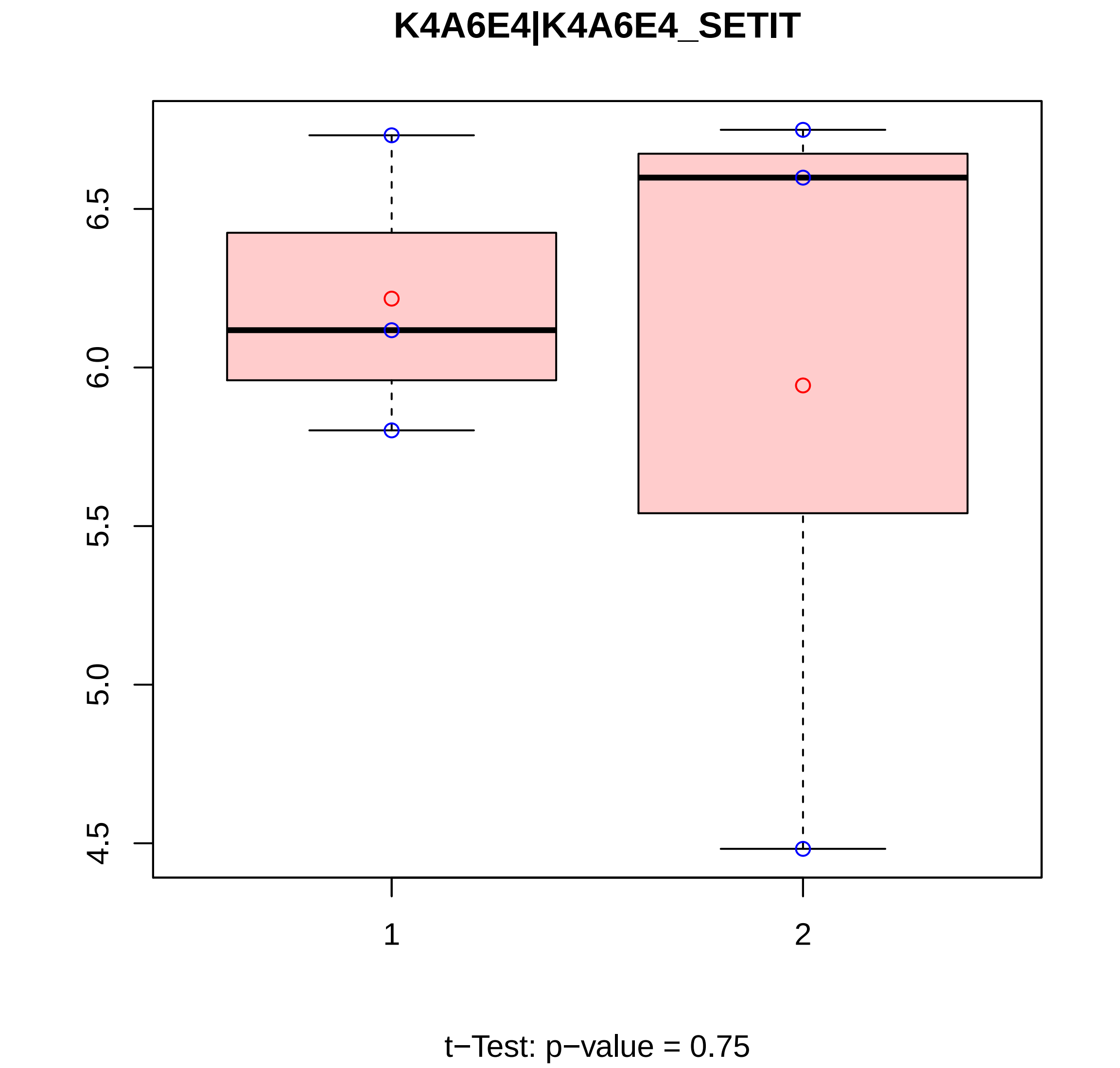
K4A6E4|K4A6E4_SETIT
6.5
6.0
5.5
5.0
4.5
1
2
t−Test: p−value = 0.75

K4A6N2|K4A6N2_SETIT

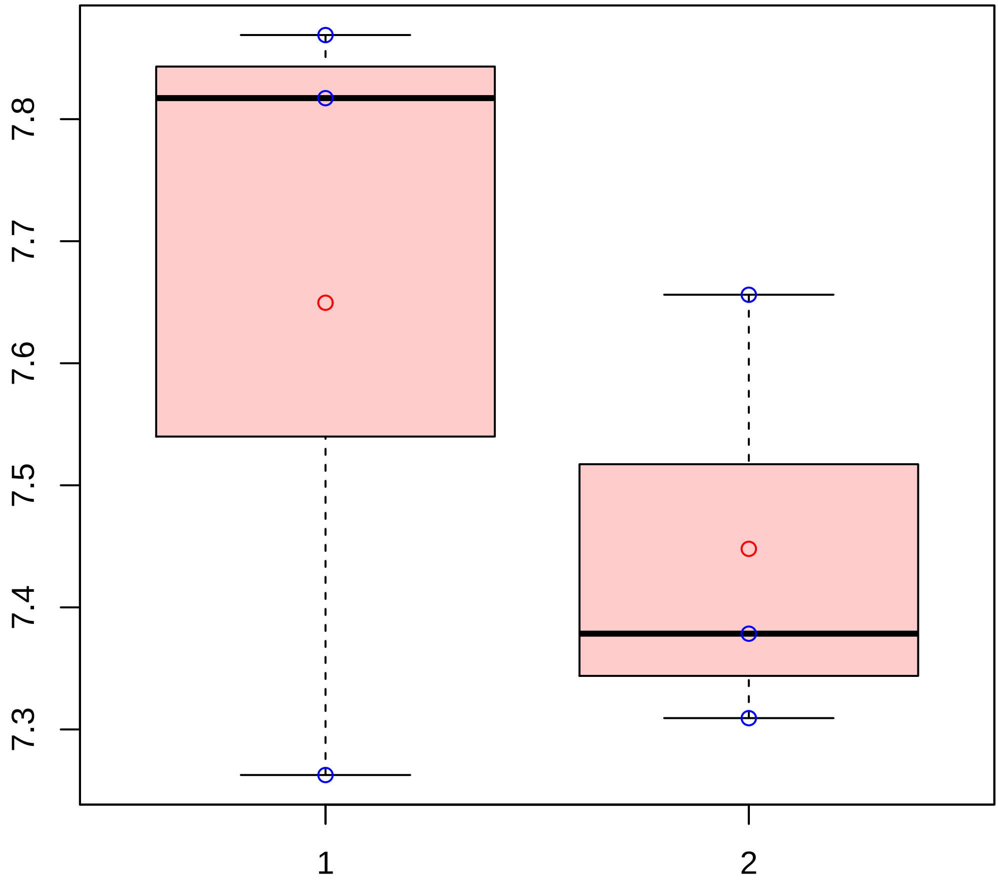

t−Test: p−value = 0.43

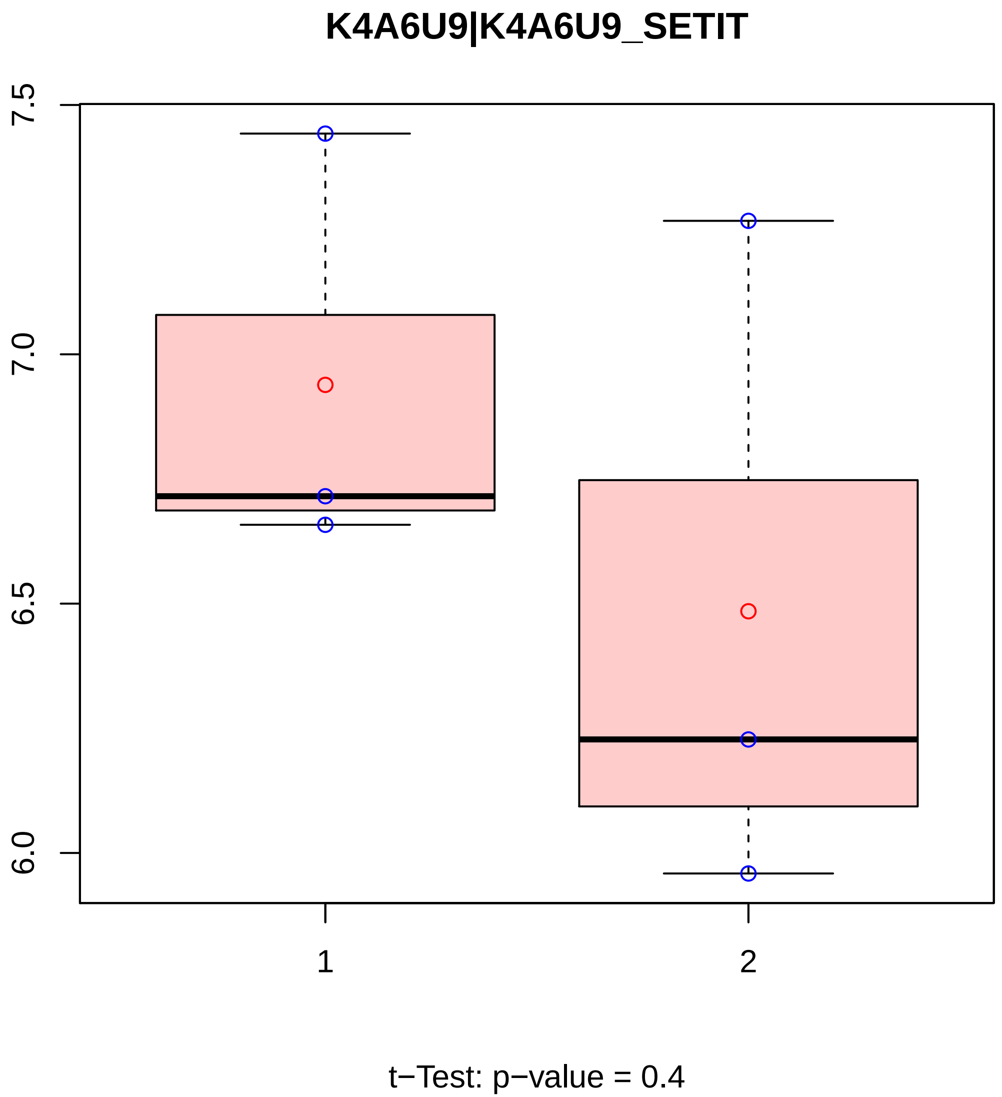

K4A6U9|K4A6U9_SETIT
7.5
7.0
6.5
6.0
1
2
t−Test: p−value = 0.4

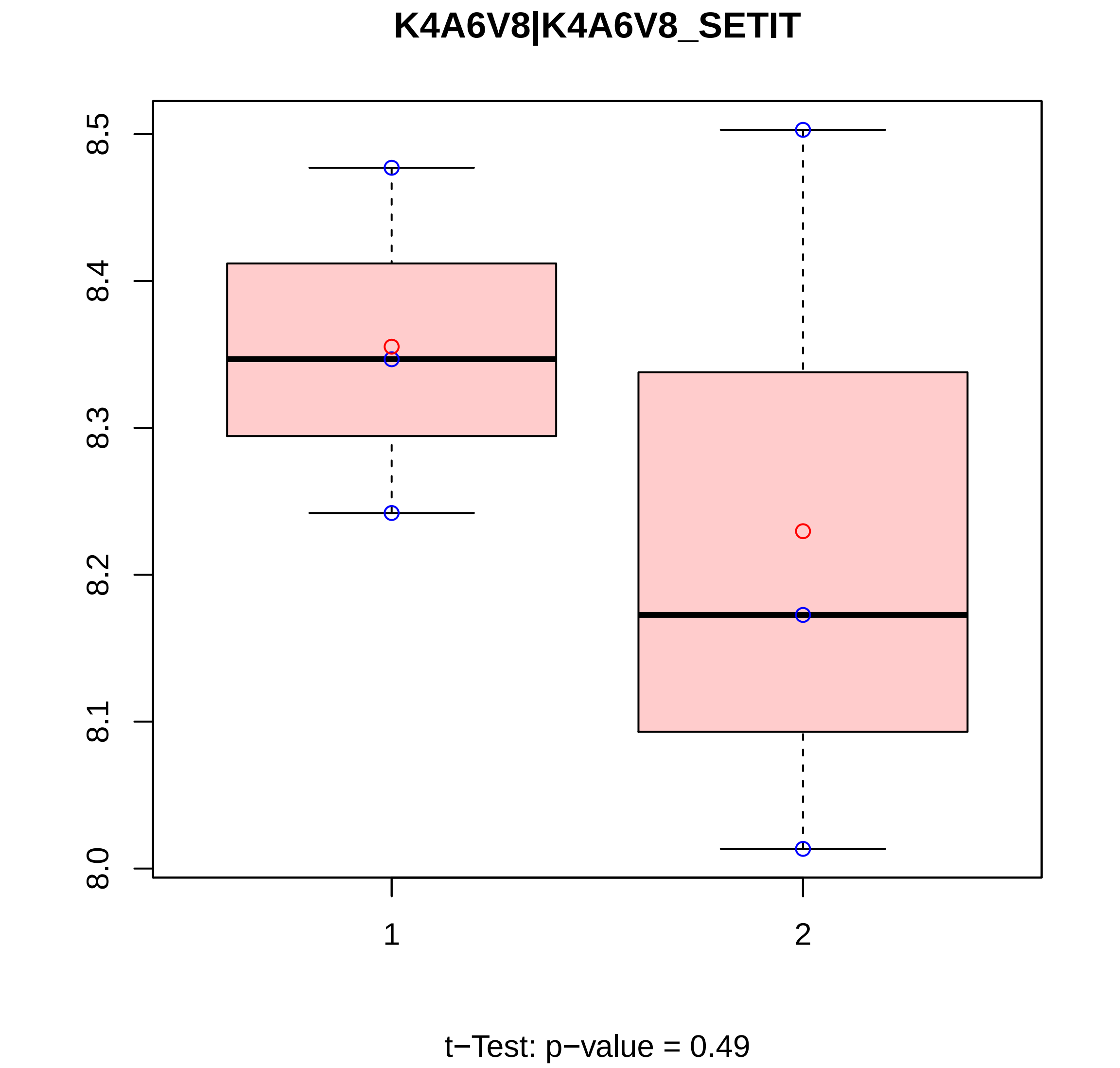
K4A6V8|K4A6V8_SETIT
8.0
8.1
8.2
8.3
8.4
8.5
1
2
t−Test: p−value = 0.49

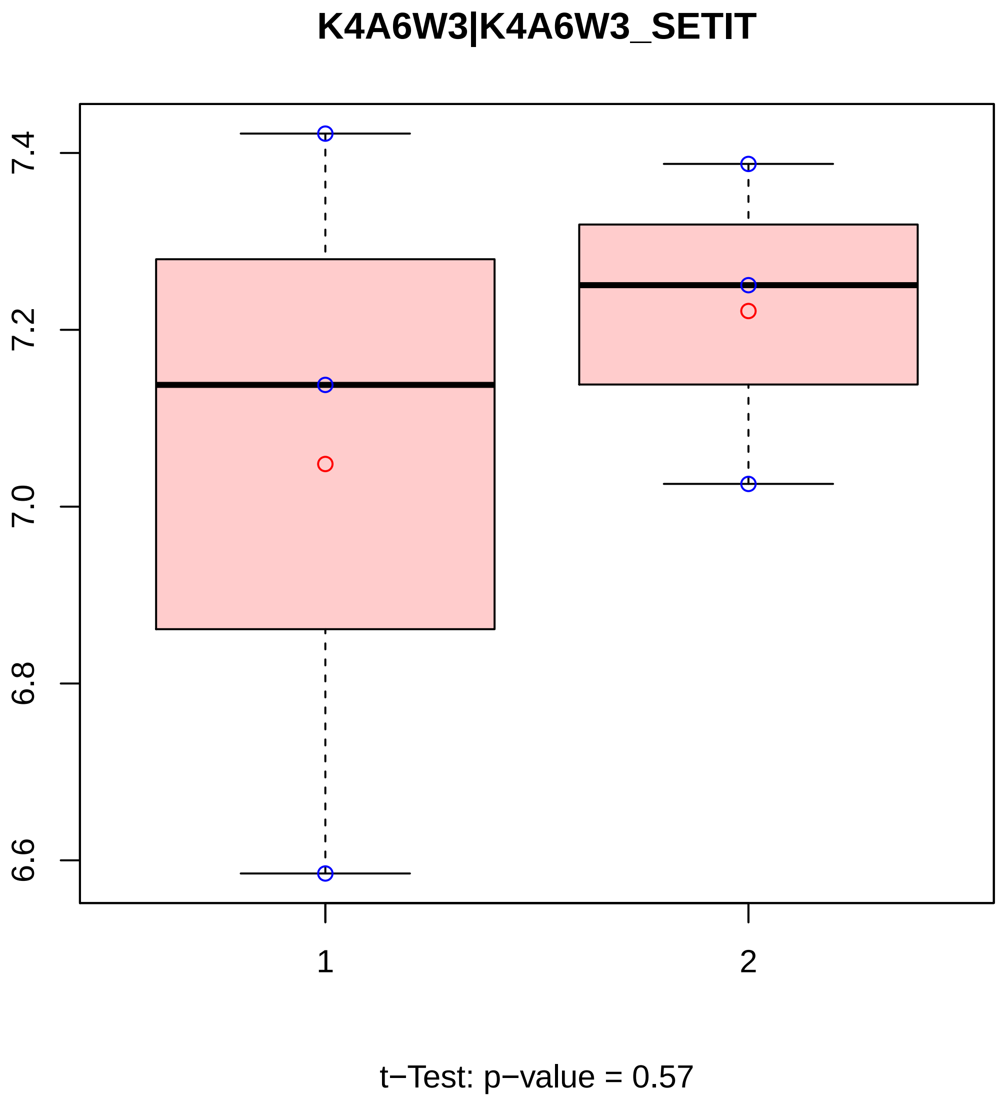

K4A6W3|K4A6W3_SETIT
7.4
7.2
7.0
6.8
6.6
1
2
t−Test: p−value = 0.57

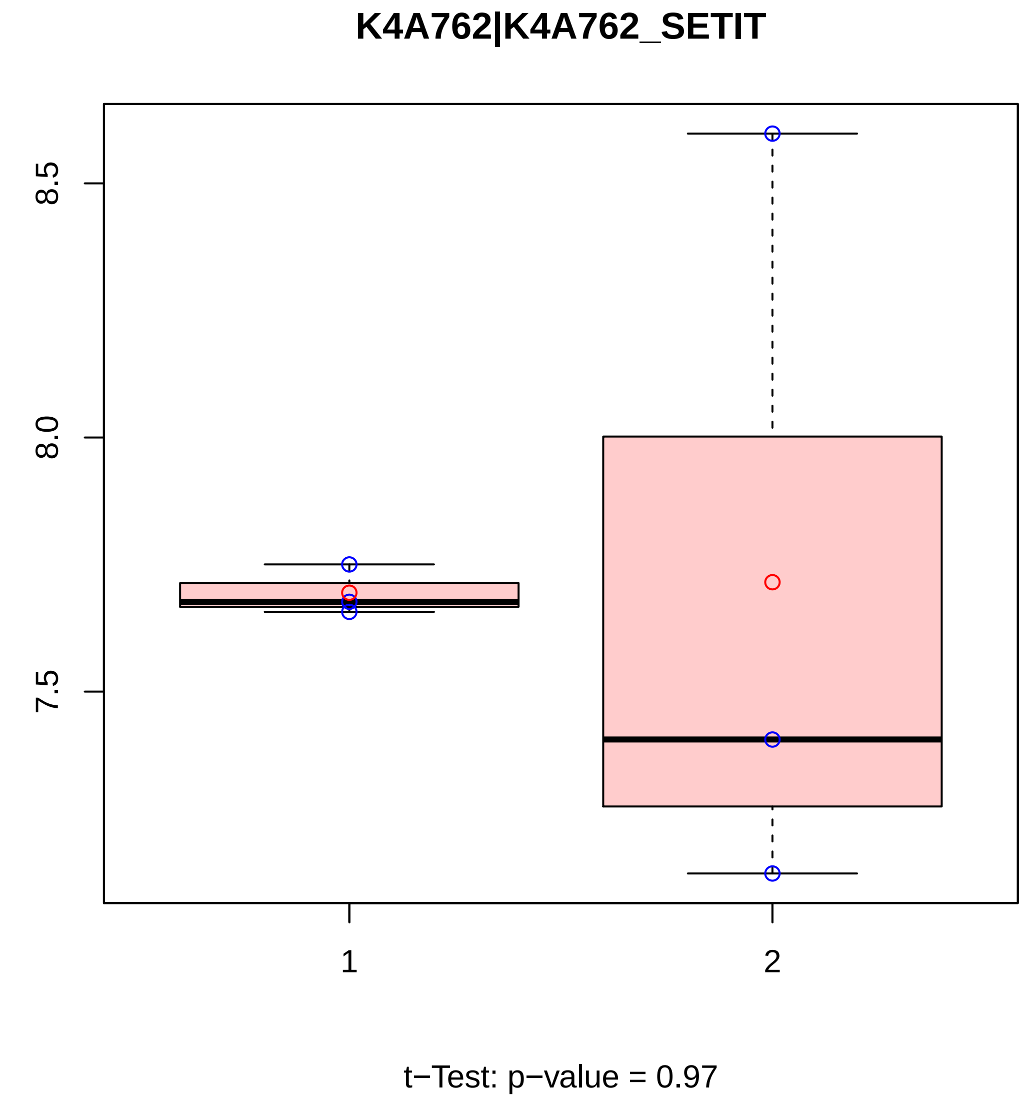
K4A762|K4A762_SETIT
8.5
8.0
7.5
1
2
t−Test: p−value = 0.97

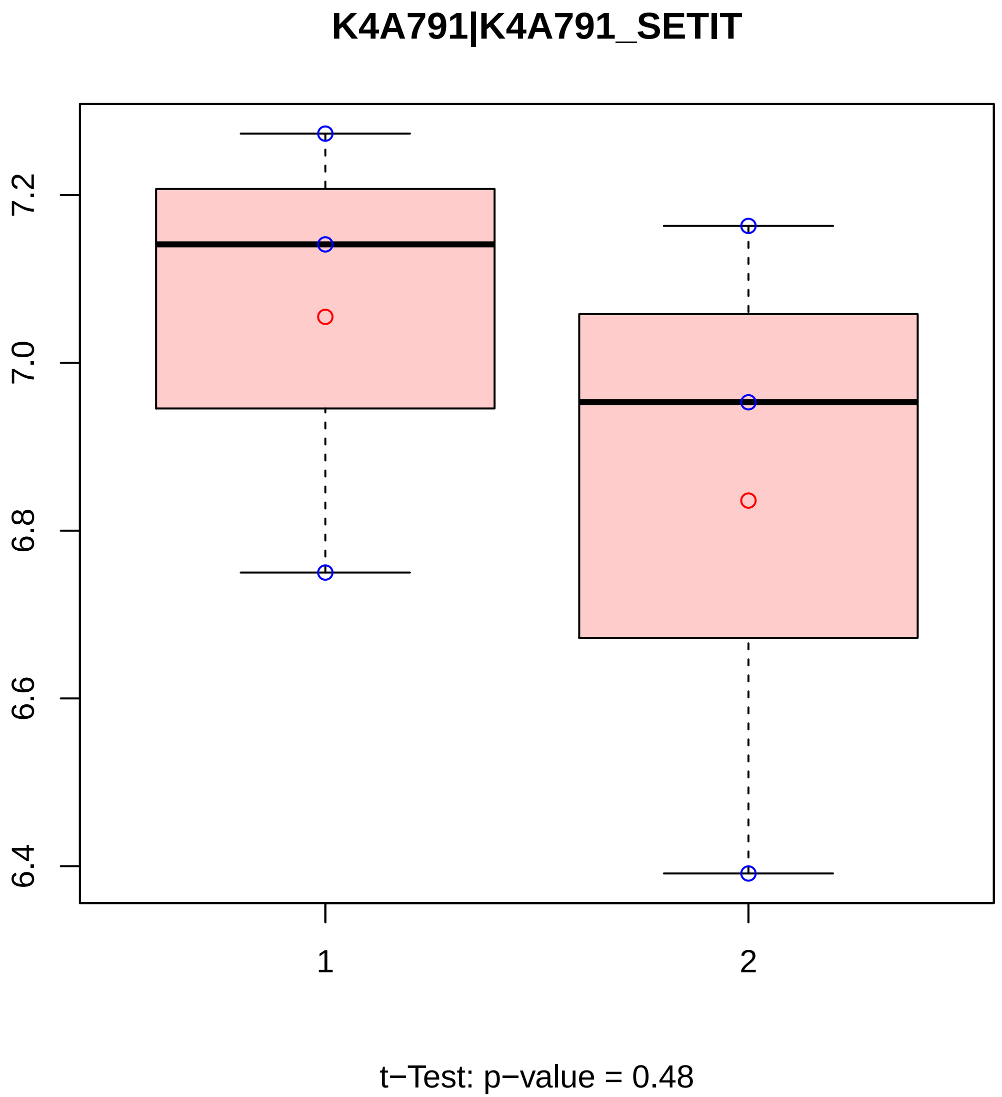

K4A791|K4A791_SETIT
7.2
7.0
6.8
6.6
6.4
1
2
t−Test: p−value = 0.48

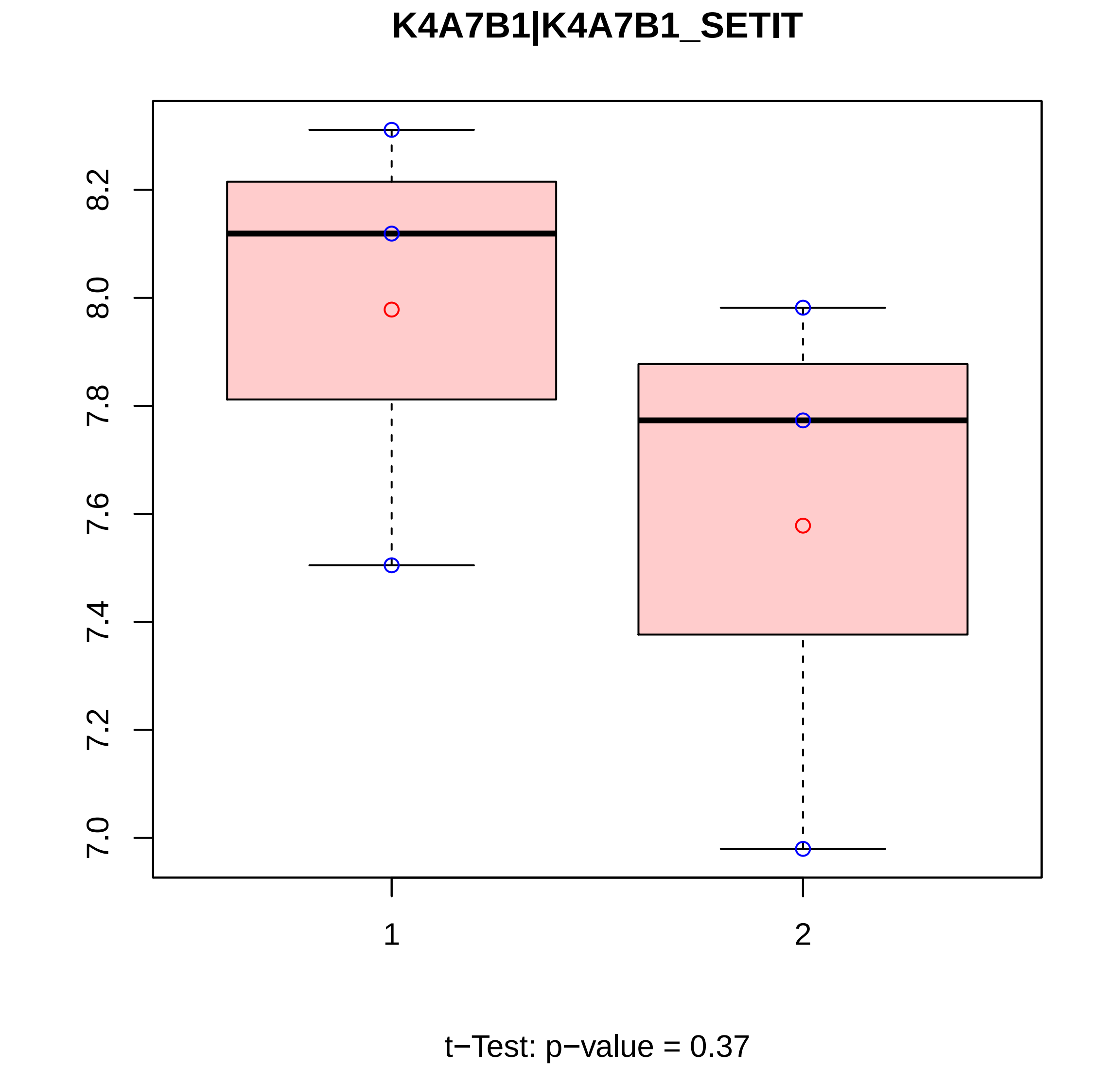
K4A7B1|K4A7B1_SETIT
8.2
8.0
7.8
7.6
7.4
7.2
7.0
1
2
t−Test: p−value = 0.37

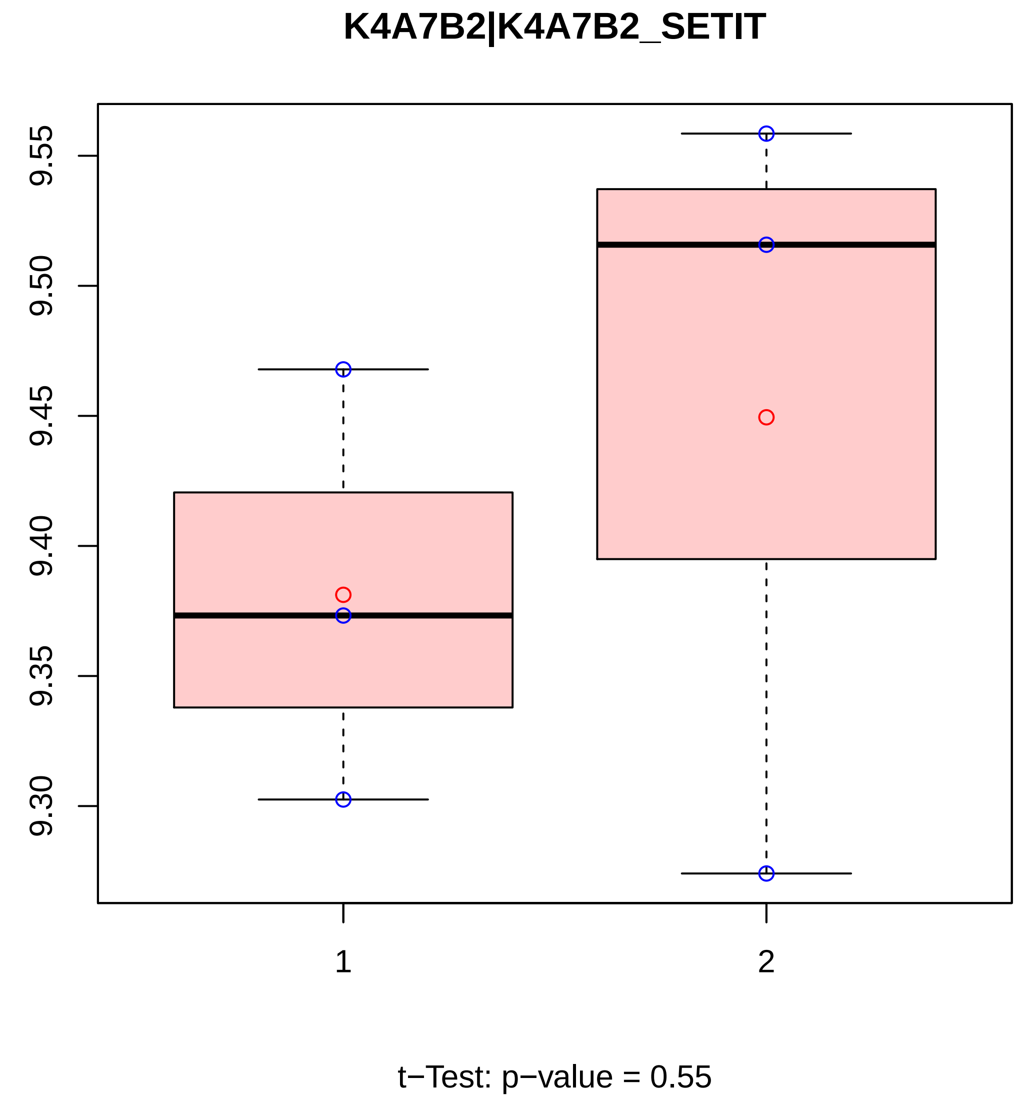
K4A7B2|K4A7B2_SETIT
9.30
9.35
9.40
9.45
9.50
9.55
1
2
t−Test: p−value = 0.55

K4A7I4|K4A7I4_SETIT

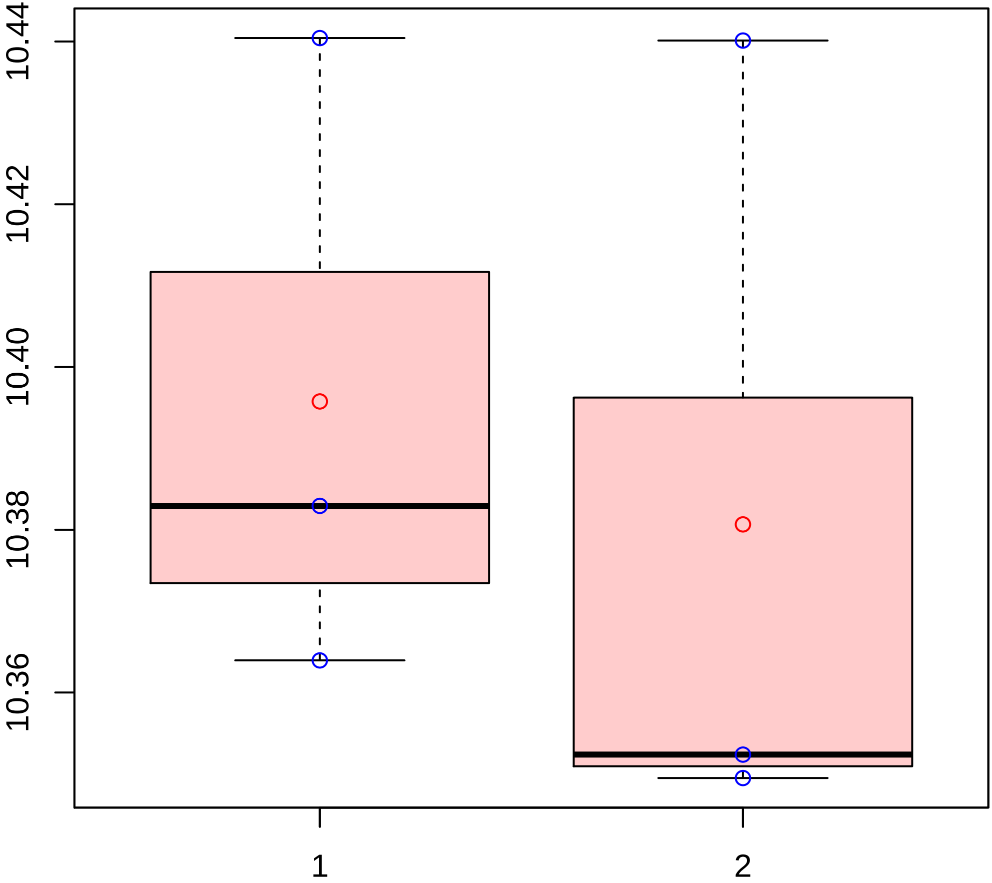

t−Test: p−value = 0.71

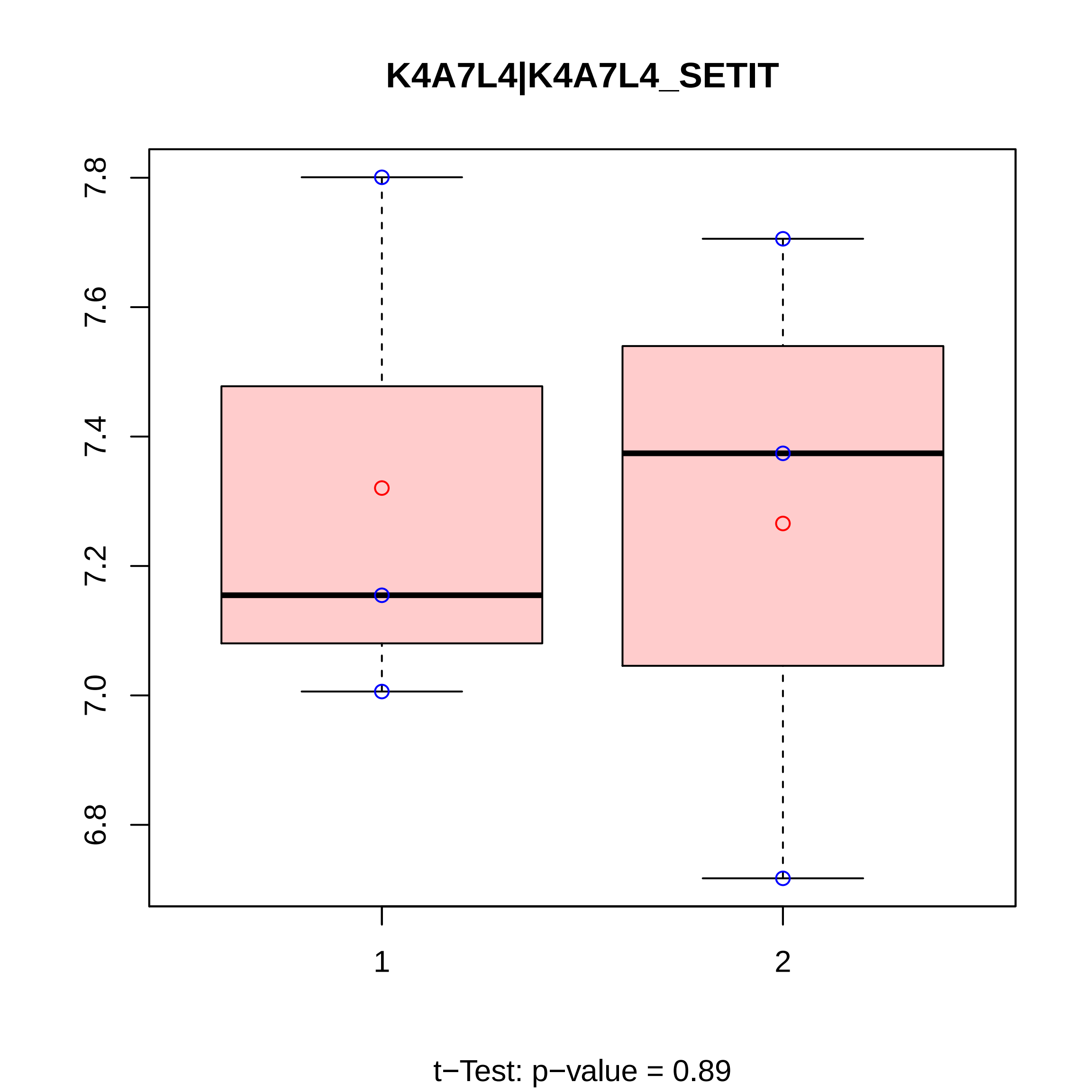

K4A7L4|K4A7L4_SETIT
7.8
7.6
7.4
7.2
7.0
6.8
1
2
t−Test: p−value = 0.89

K4A7W7|K4A7W7_SETIT

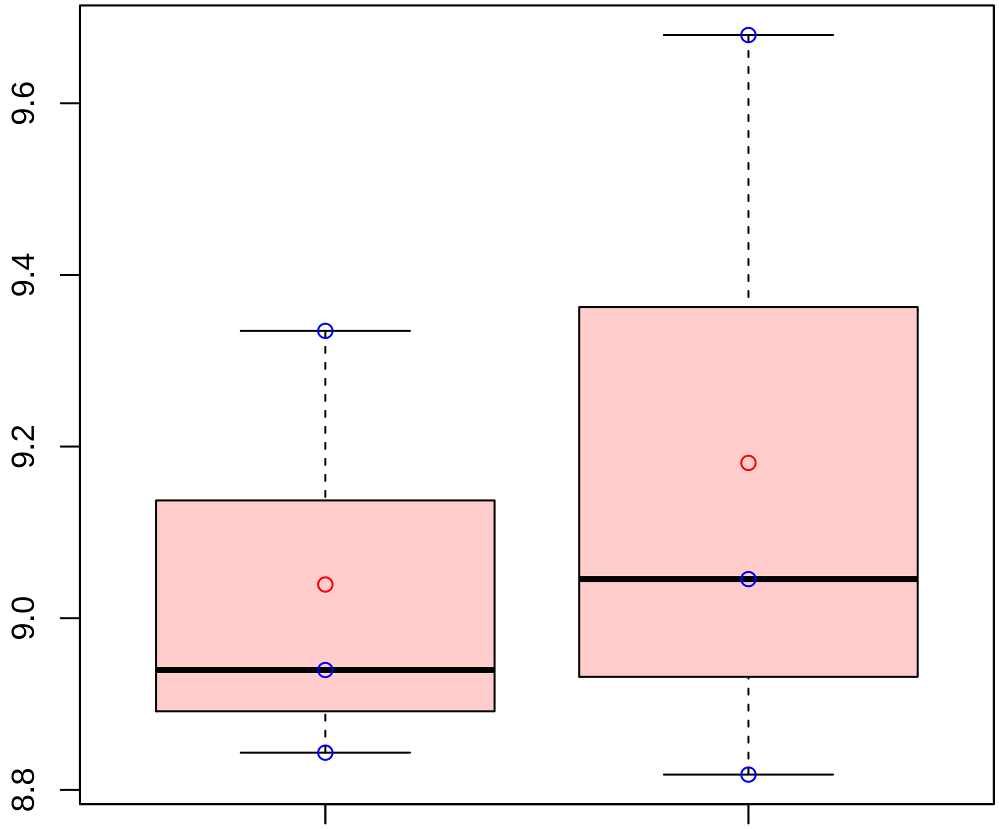

t−Test: p−value = 0.67

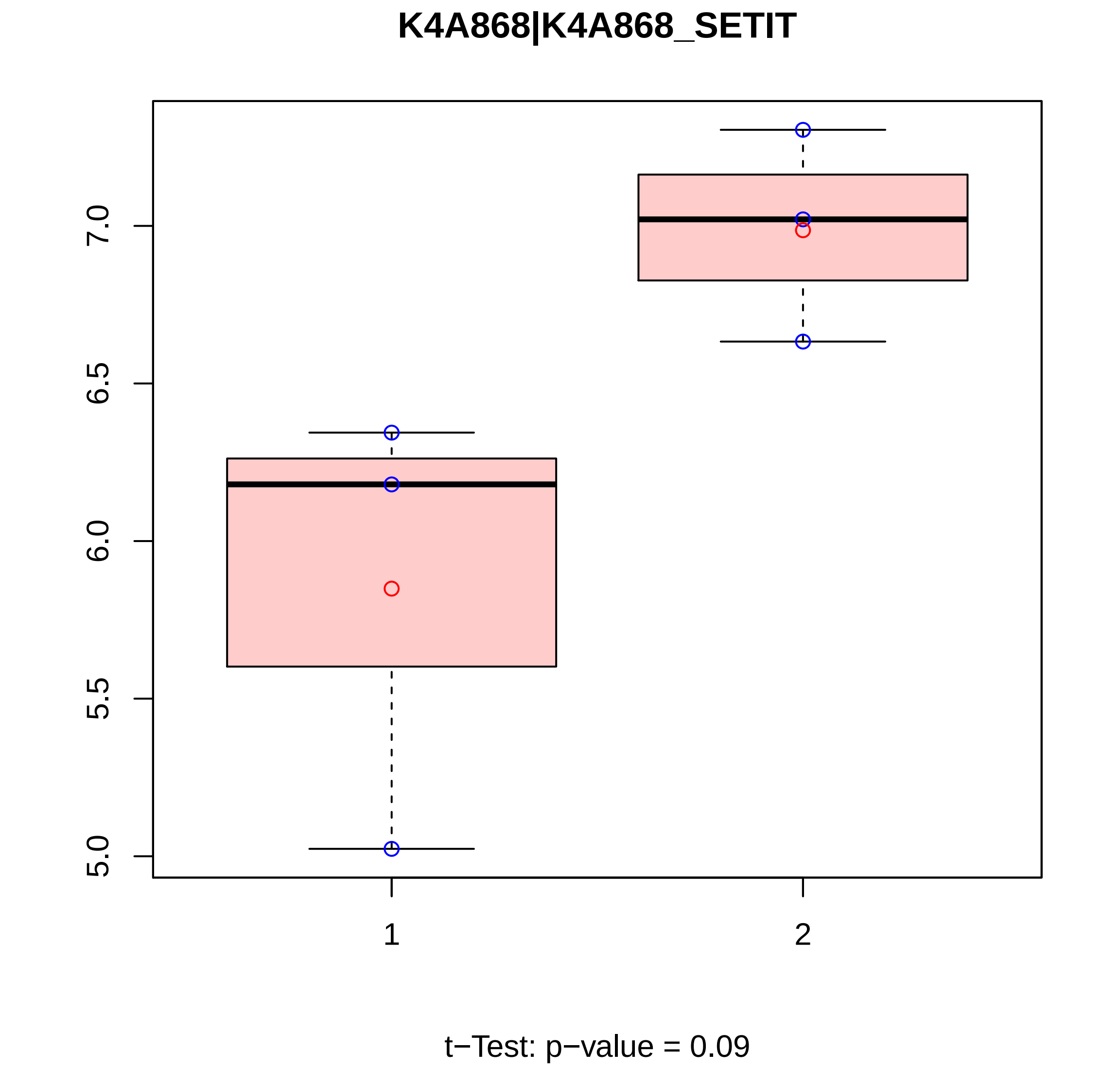
K4A868|K4A868_SETIT
7.0
6.5
6.0
5.5
5.0
1
2
t−Test: p−value = 0.09

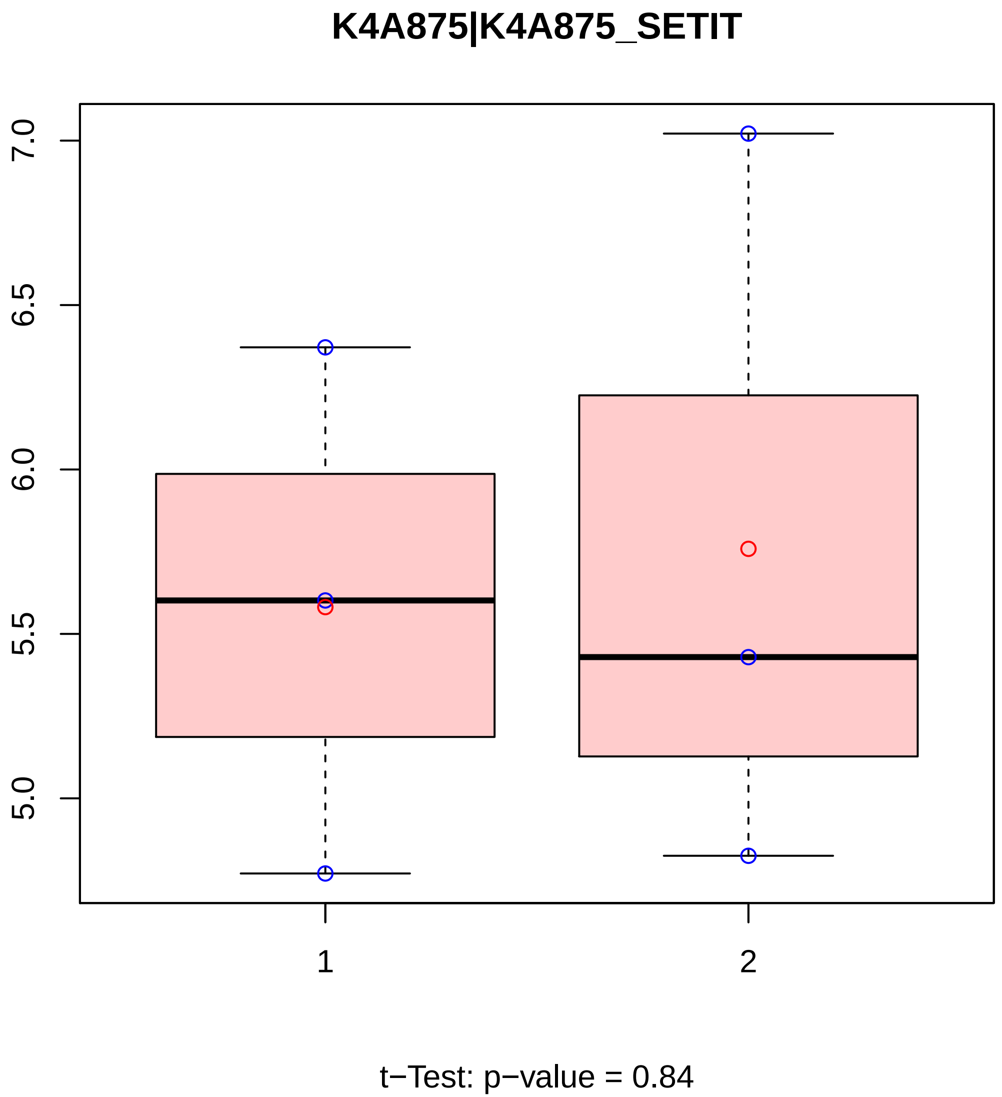
K4A875|K4A875_SETIT
7.0
6.5
6.0
5.5
5.0
1
2
t−Test: p−value = 0.84

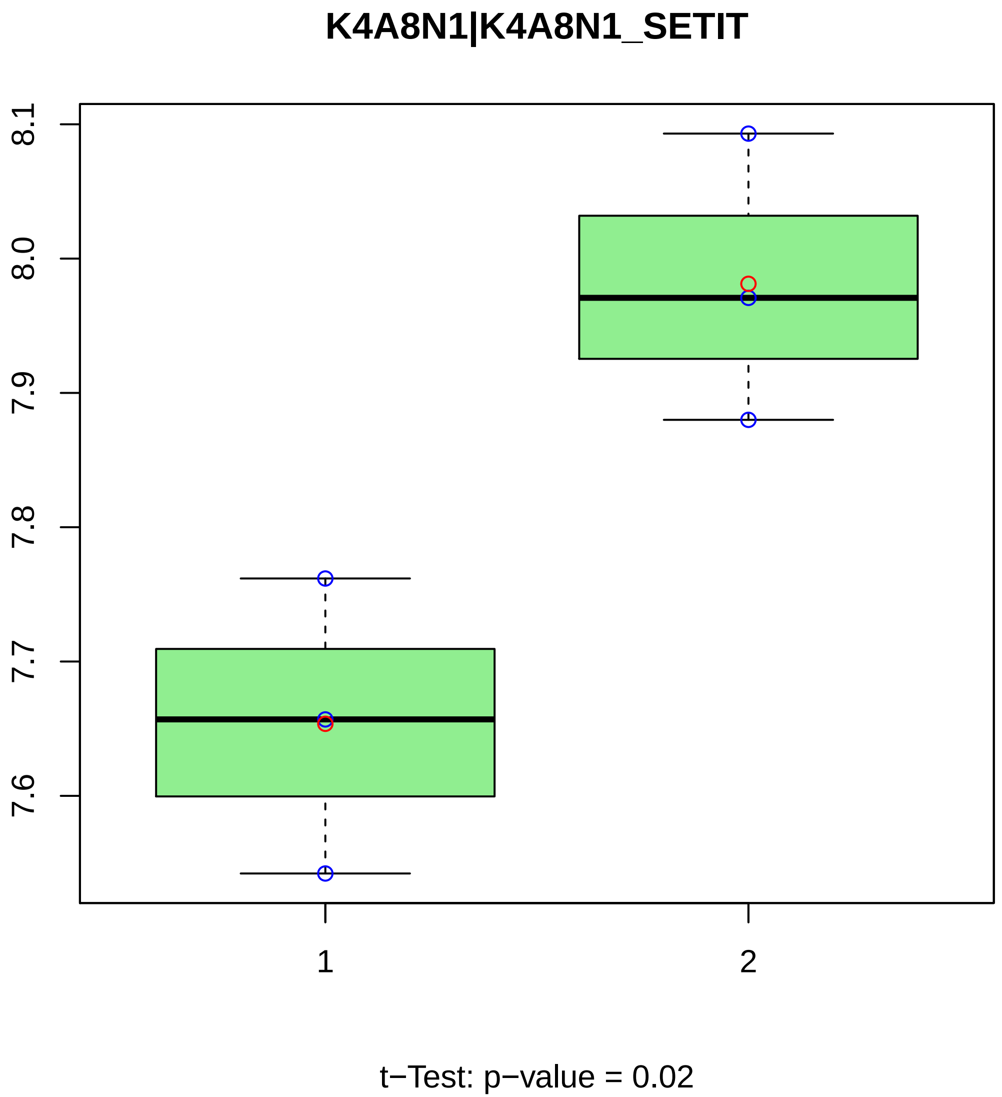
K4A8N1|K4A8N1_SETIT
8.1
8.0
7.9
7.8
7.7
7.6
1
2
t−Test: p−value = 0.02

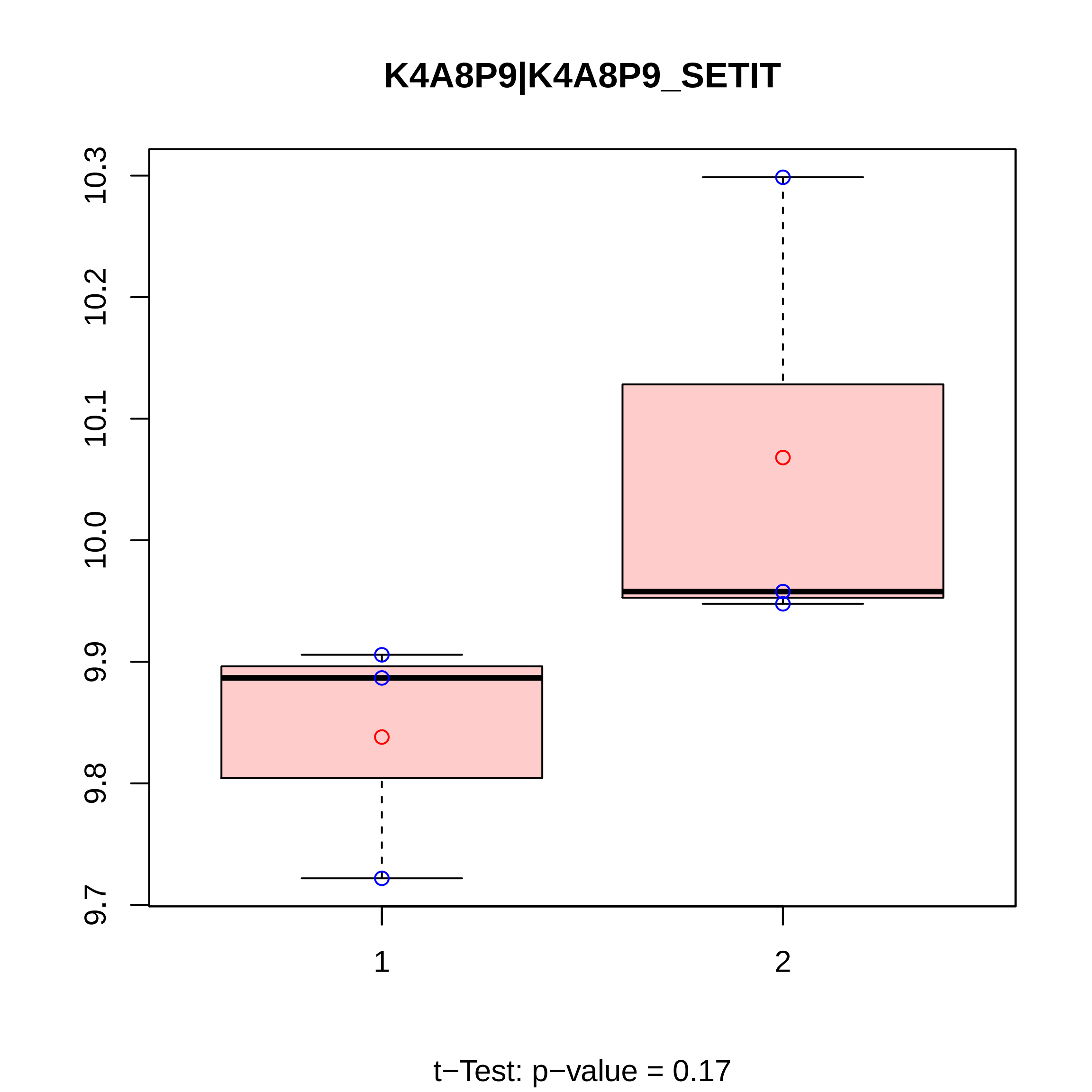

K4A8P9|K4A8P9_SETIT
10.3
10.2
10.1
10.0
9.9
9.8
9.7
1
2
t−Test: p−value = 0.17

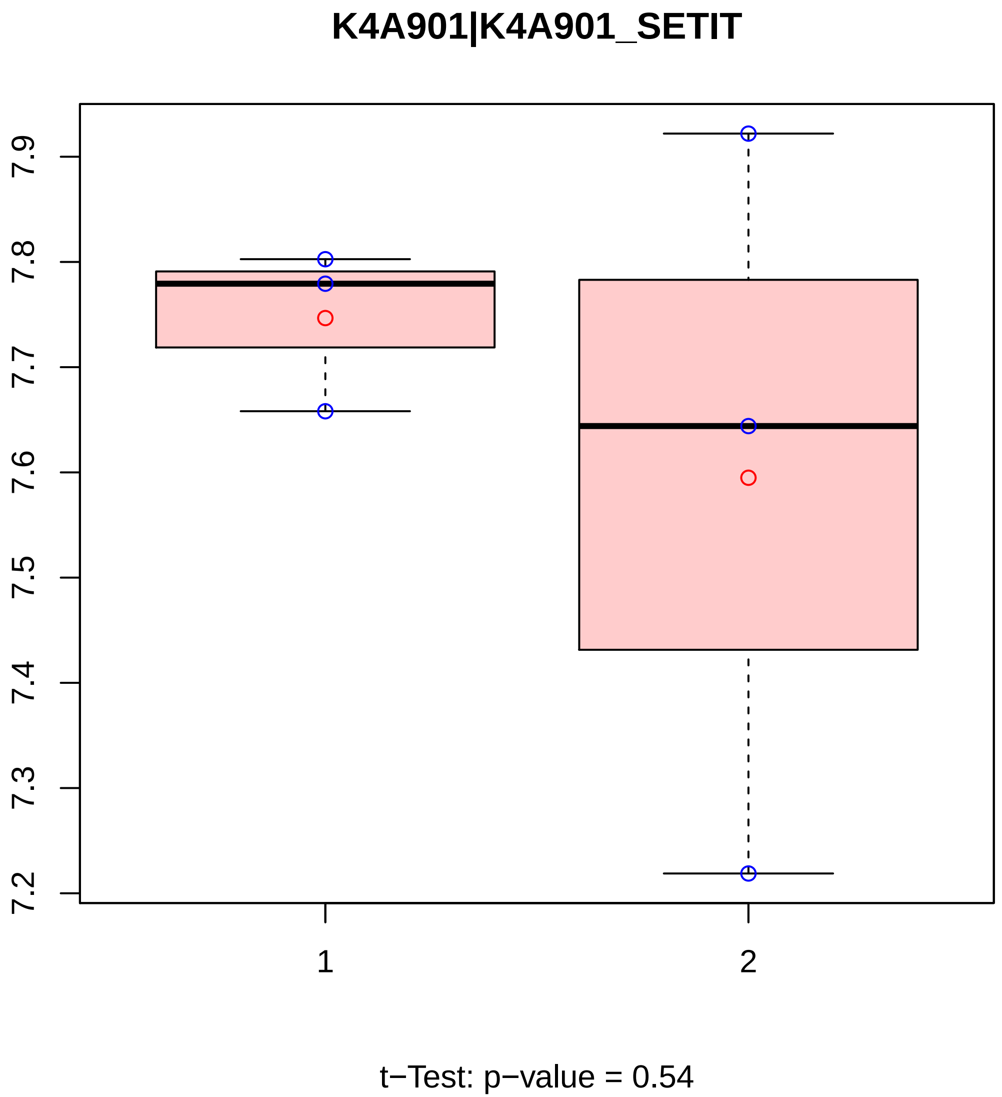
K4A901|K4A901_SETIT
7.9
7.8
7.7
7.6
7.5
7.4
7.3
7.2
1
2
t−Test: p−value = 0.54

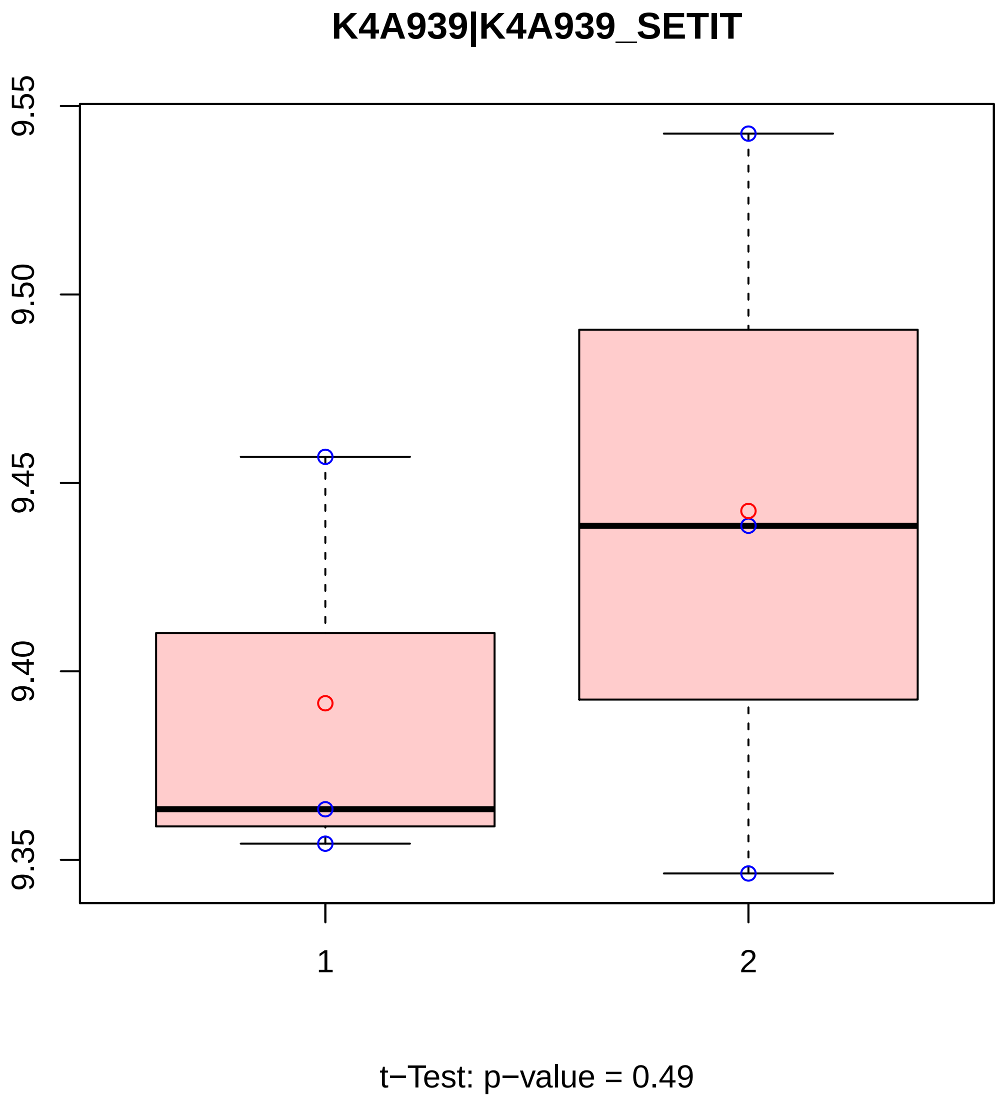

K4A939|K4A939_SETIT
9.55
9.50
9.45
9.40
9.35
1
2
t−Test: p−value = 0.49

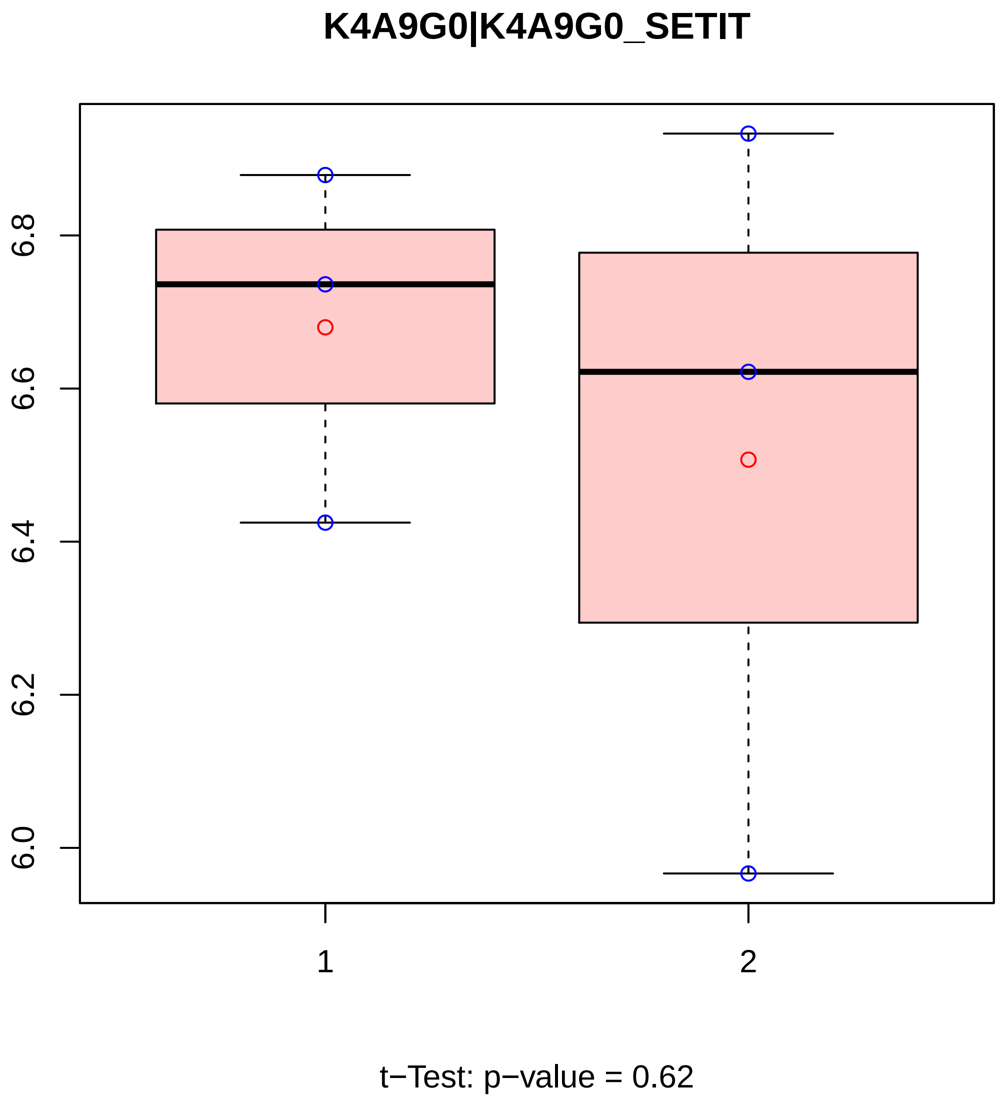
K4A9G0|K4A9G0_SETIT
6.0
6.2
6.4
6.6
6.8
1
2
t−Test: p−value = 0.62

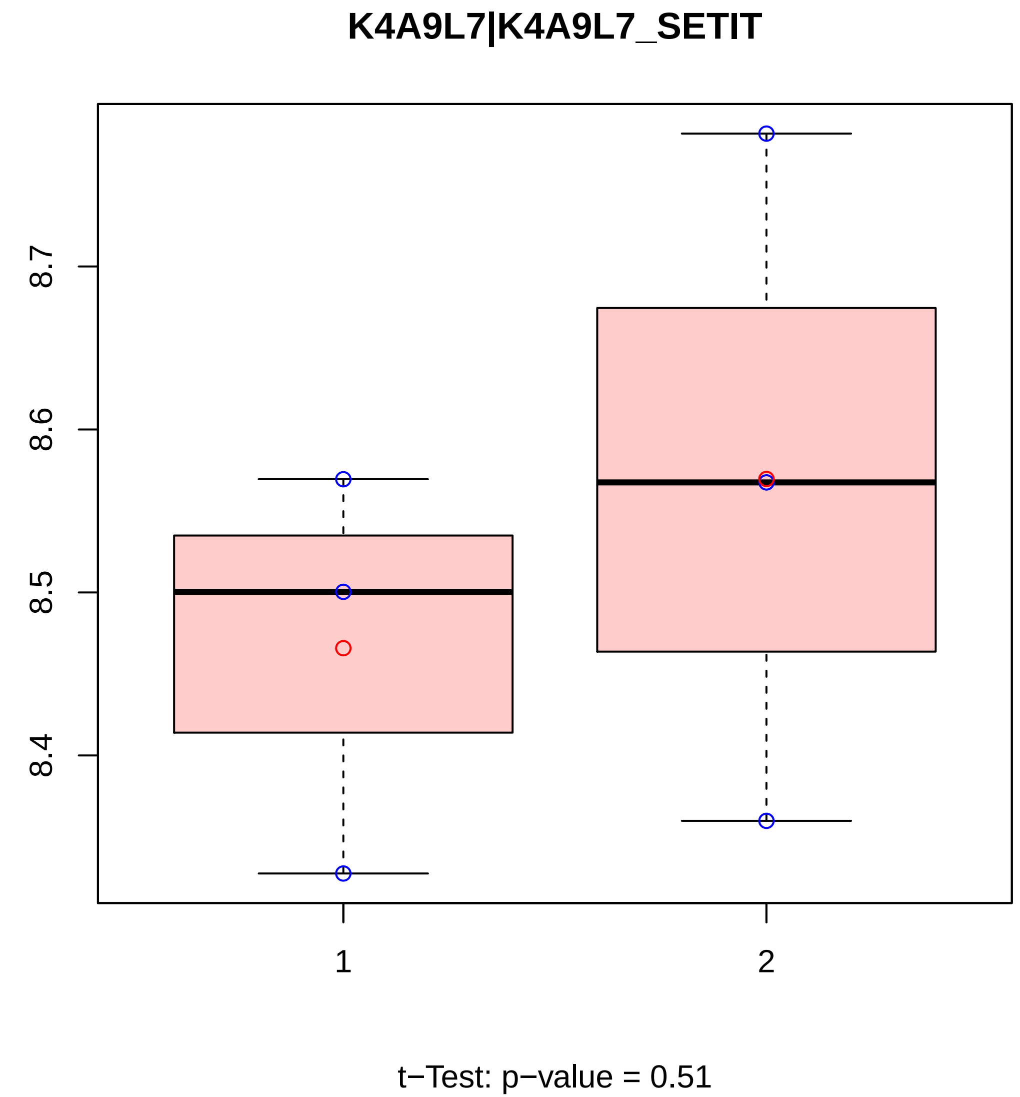
K4A9L7|K4A9L7_SETIT
8.4
8.5
8.6
8.7
1
2
t−Test: p−value = 0.51

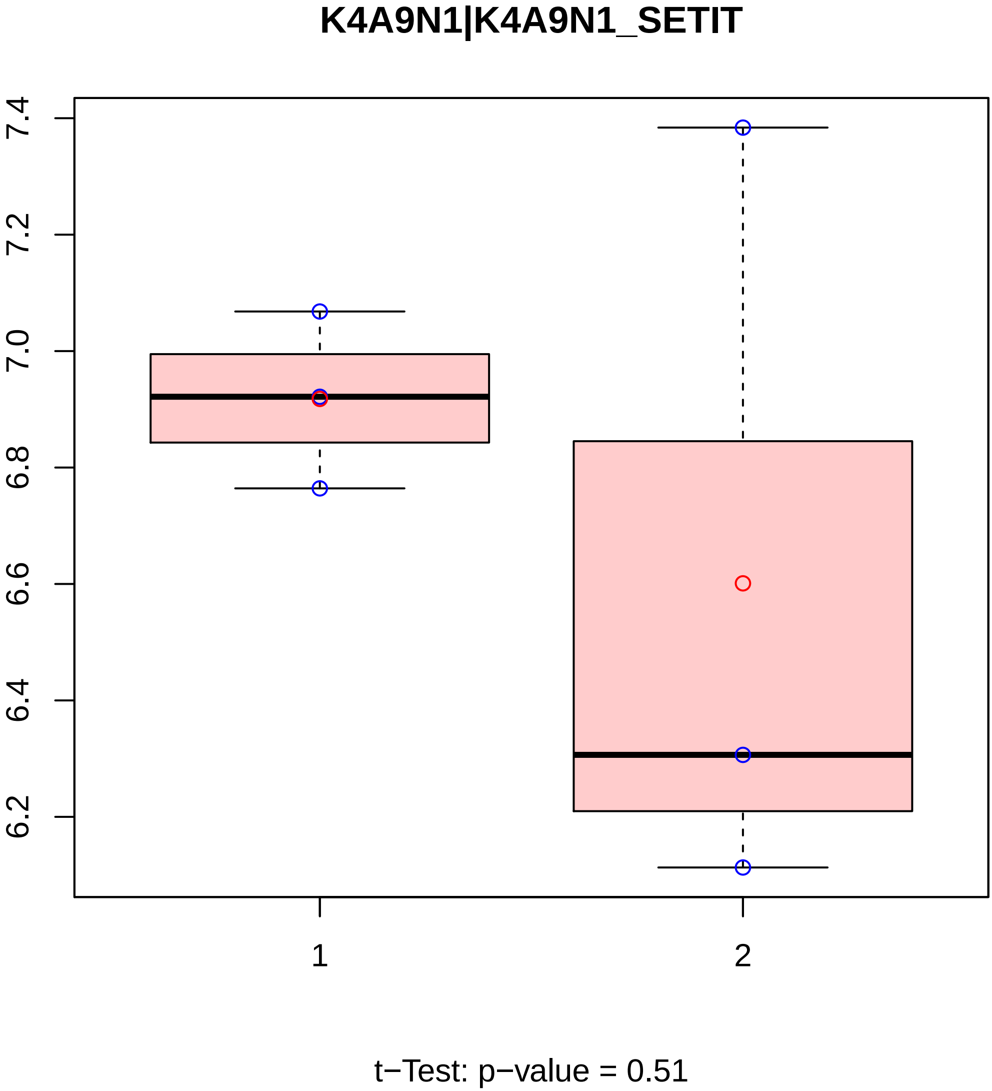
K4A9N1|K4A9N1_SETIT
7.4
7.2
7.0
6.8
6.6
6.4
6.2
1
2
t−Test: p−value = 0.51

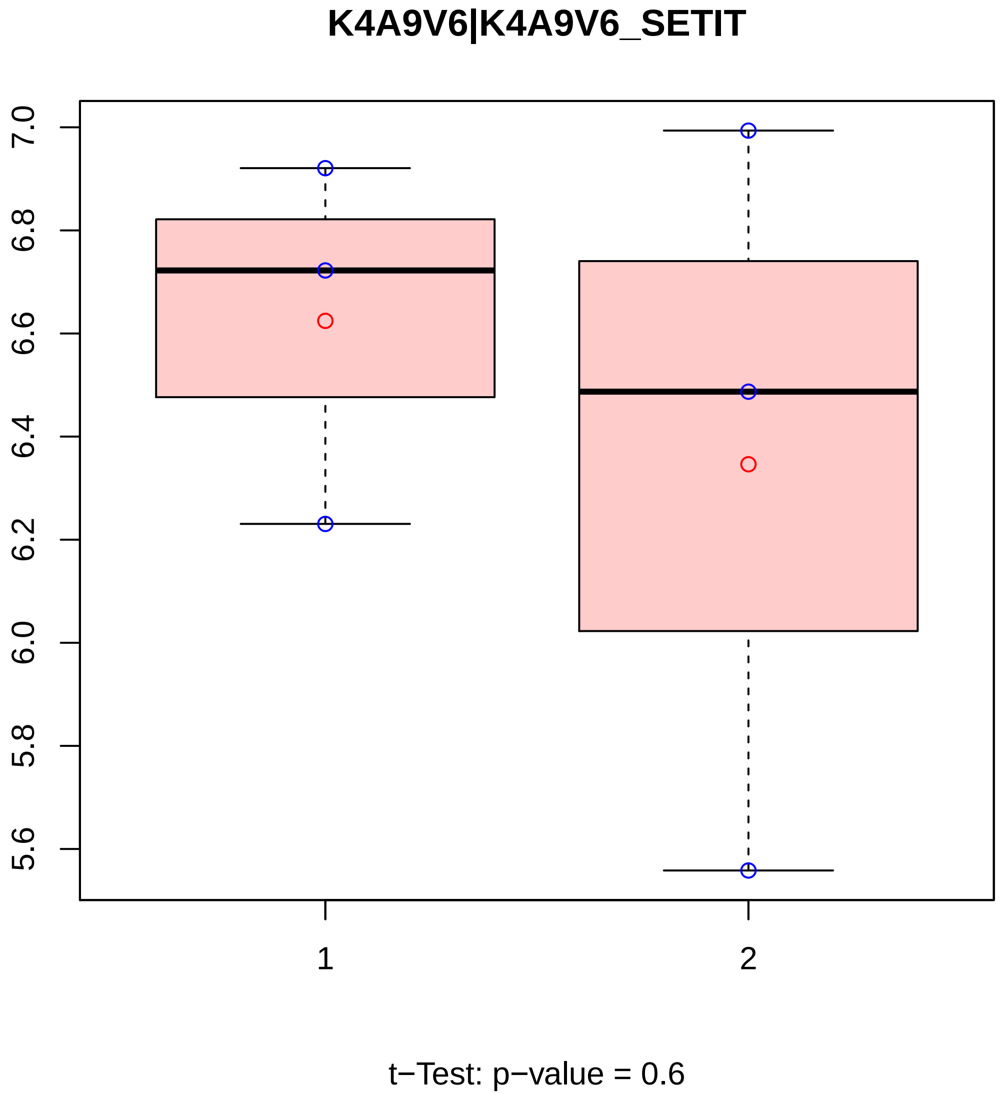
K4A9V6|K4A9V6_SETIT
7.0
6.8
6.6
6.4
6.2
6.0
5.8
5.6
1
2
t−Test: p−value = 0.6

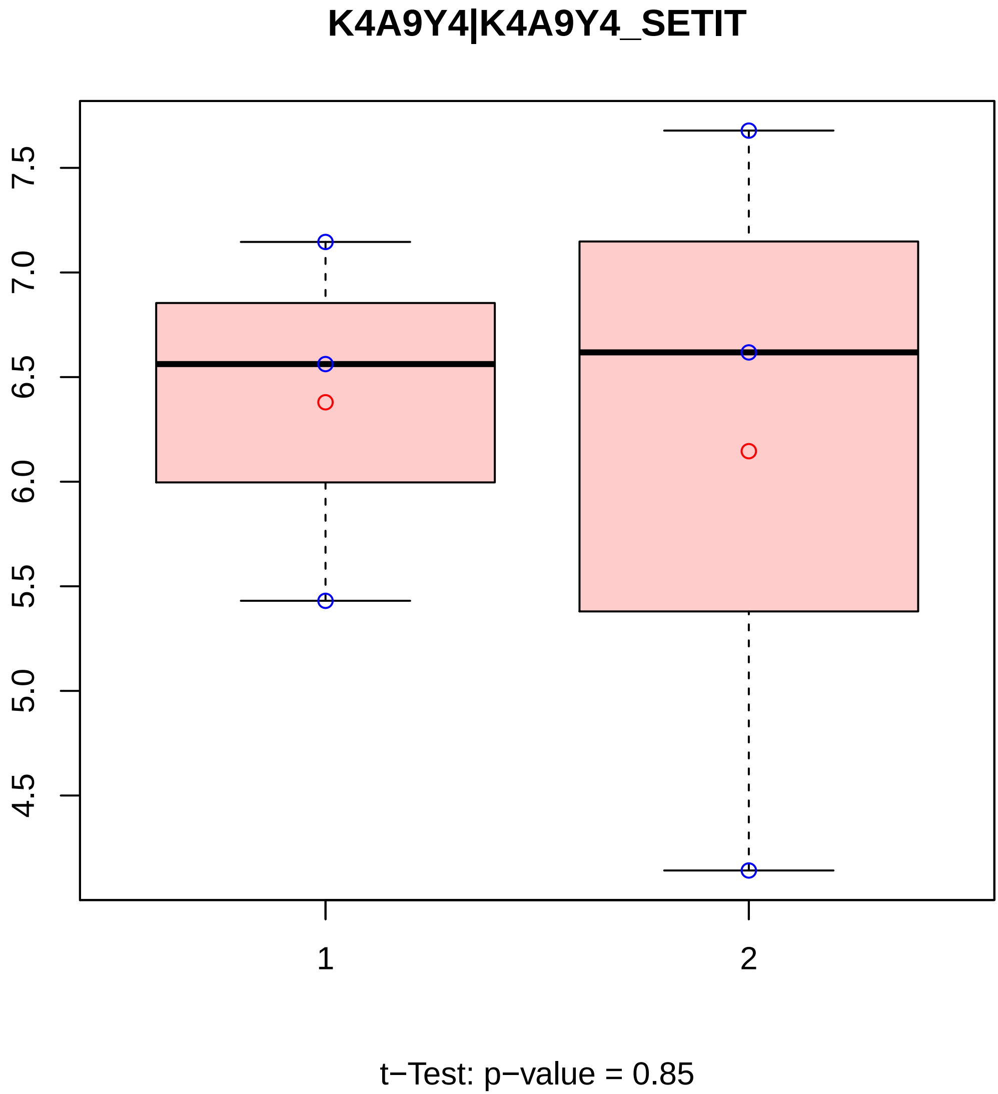

K4A9Y4|K4A9Y4_SETIT
7.5
7.0
6.5
6.0
5.5
5.0
4.5
1
2
t−Test: p−value = 0.85

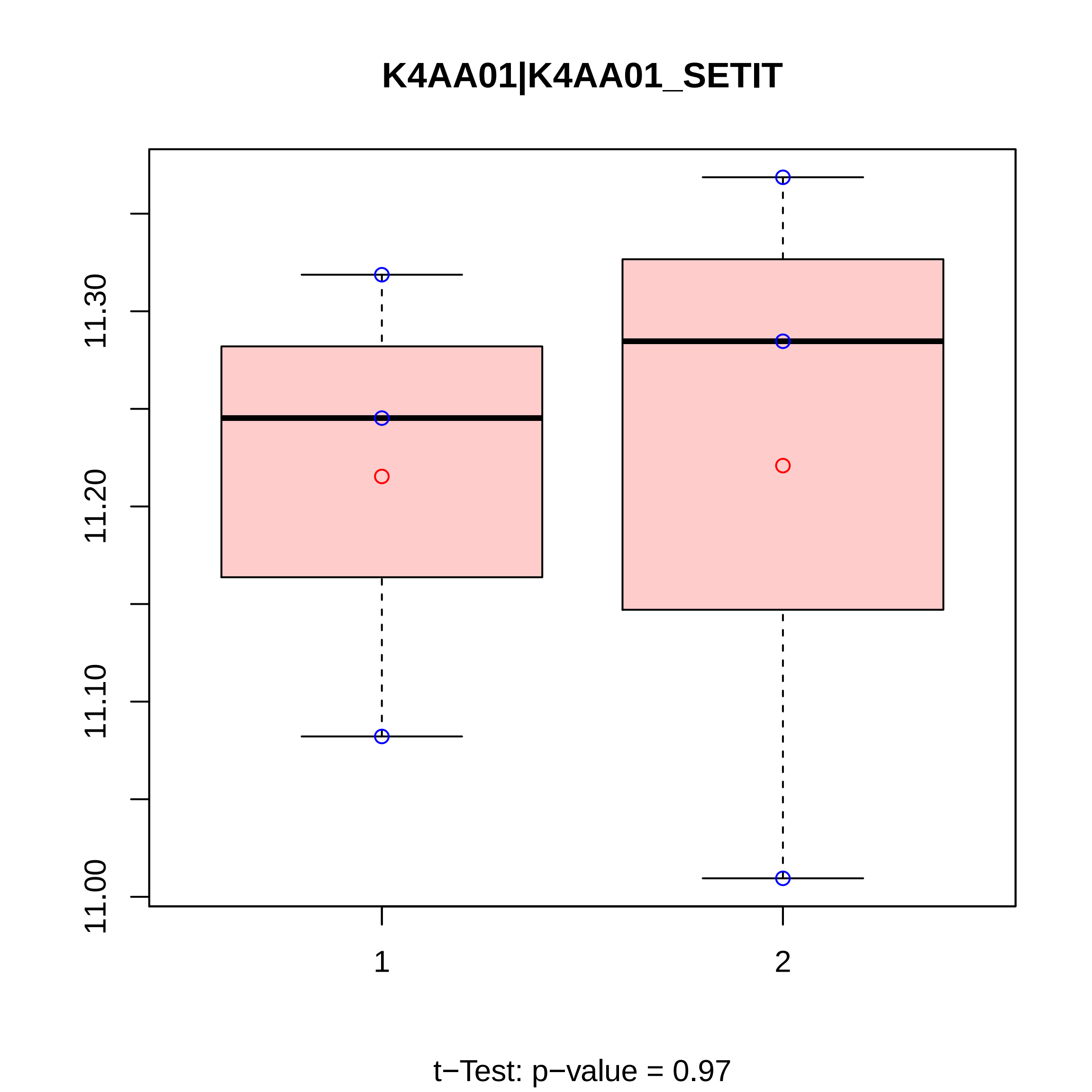

K4AA01|K4AA01_SETIT
11.00
11.10
11.20
11.30
1
2
t−Test: p−value = 0.97

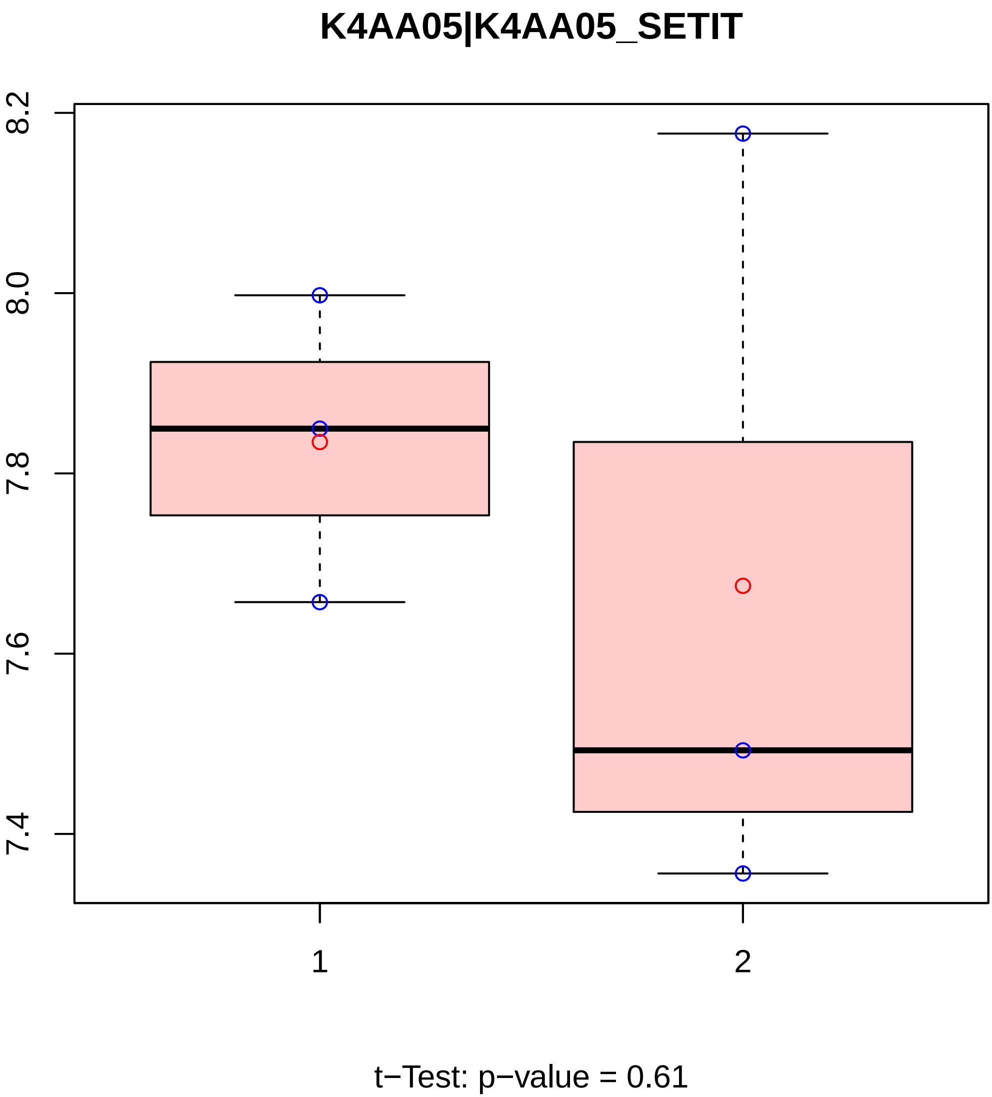
K4AA05|K4AA05_SETIT
8.2
8.0
7.8
7.6
7.4
1
2
t−Test: p−value = 0.61

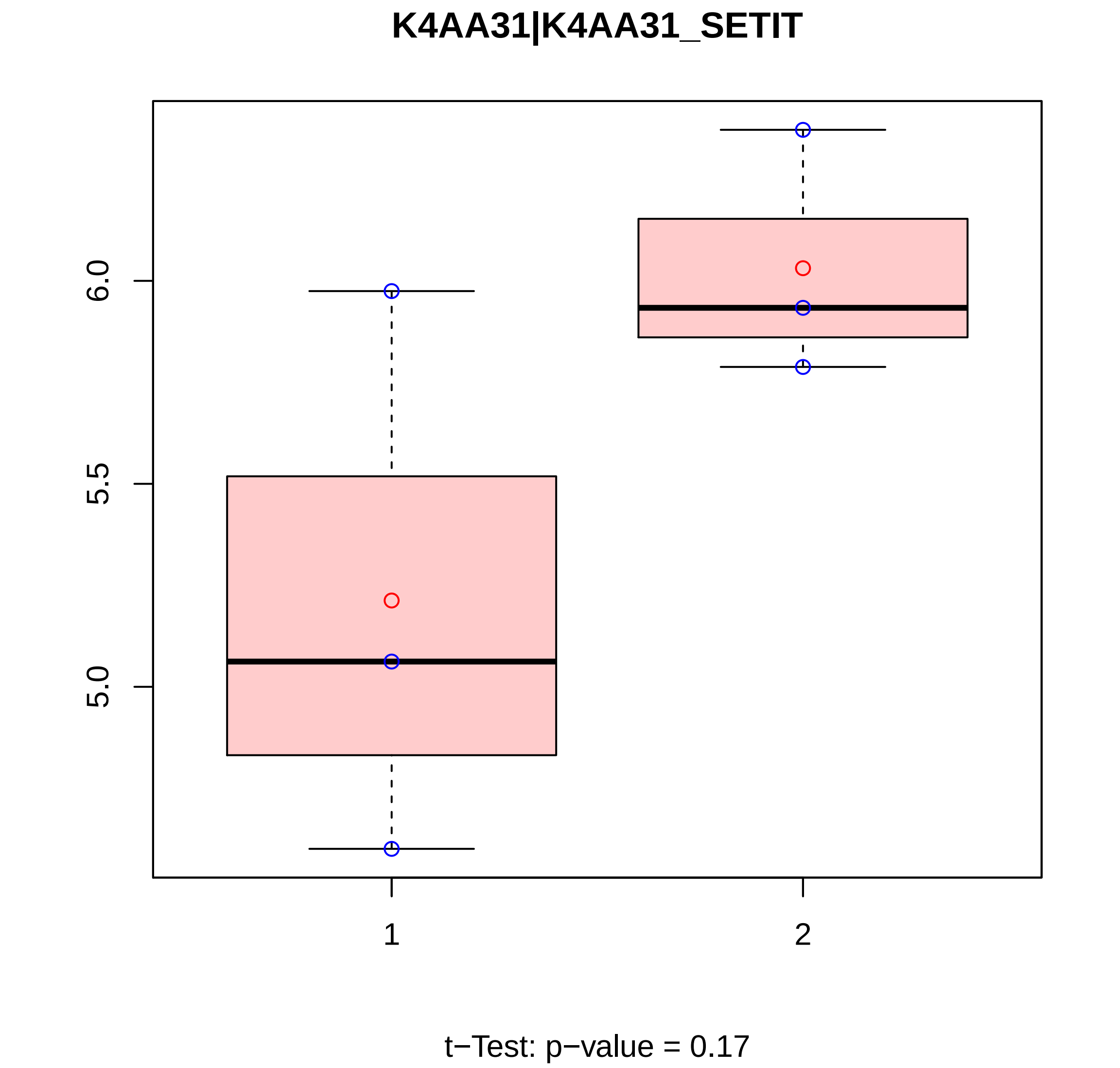
K4AA31|K4AA31_SETIT
6.0
5.5
5.0
1
2
t−Test: p−value = 0.17

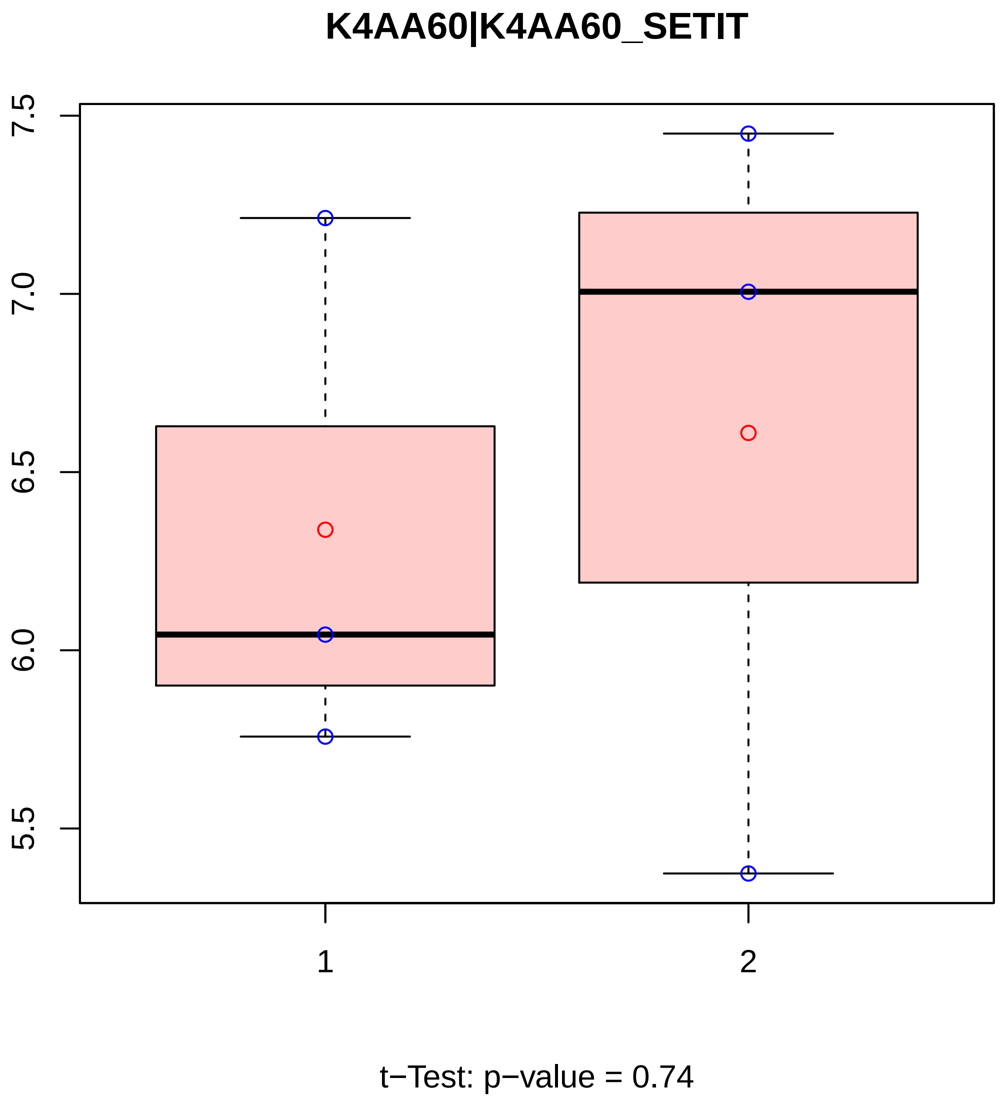

K4AA60|K4AA60_SETIT
7.5
7.0
6.5
6.0
5.5
1
2
t−Test: p−value = 0.74

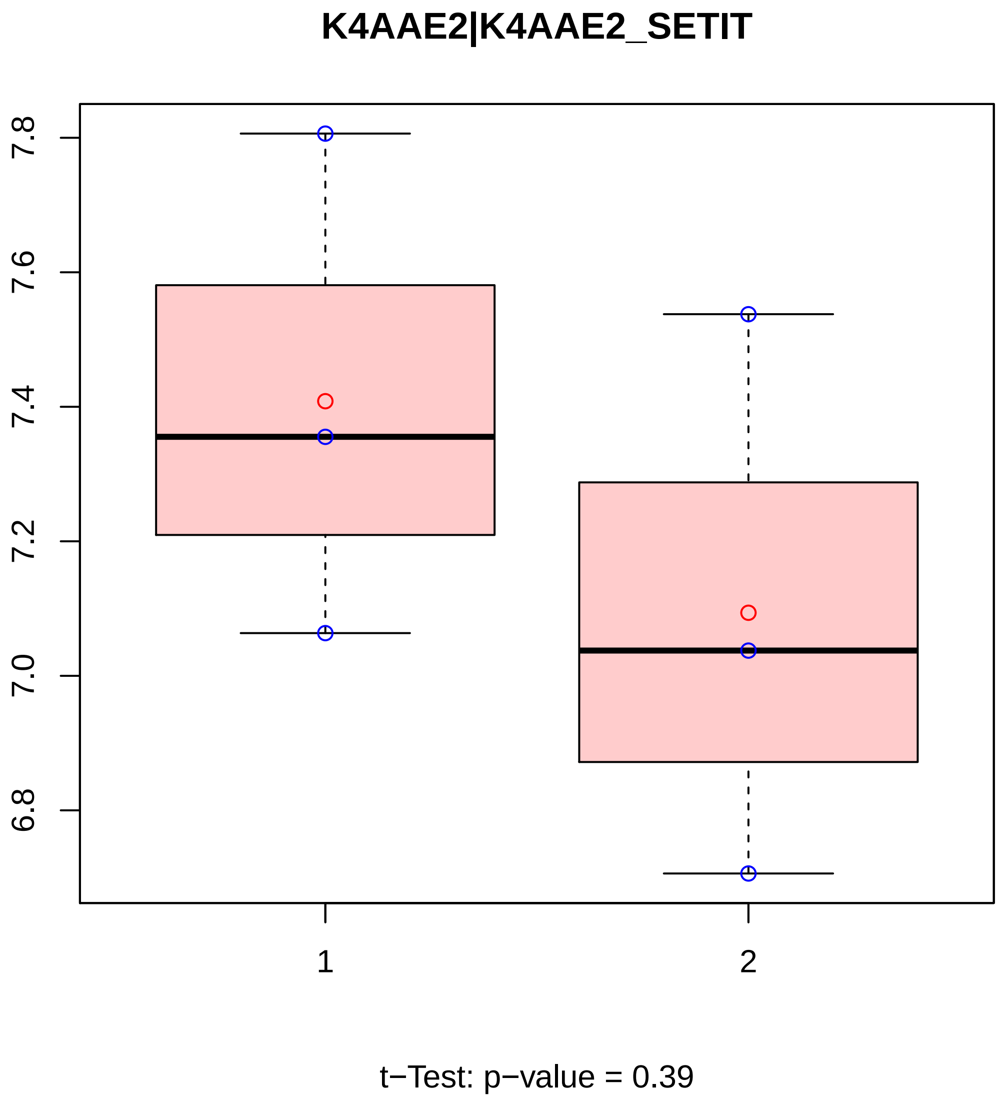
K4AAE2|K4AAE2_SETIT
7.8
7.6
7.4
7.2
7.0
6.8
1
2
t−Test: p−value = 0.39

K4AAG2|K4AAG2_SETIT

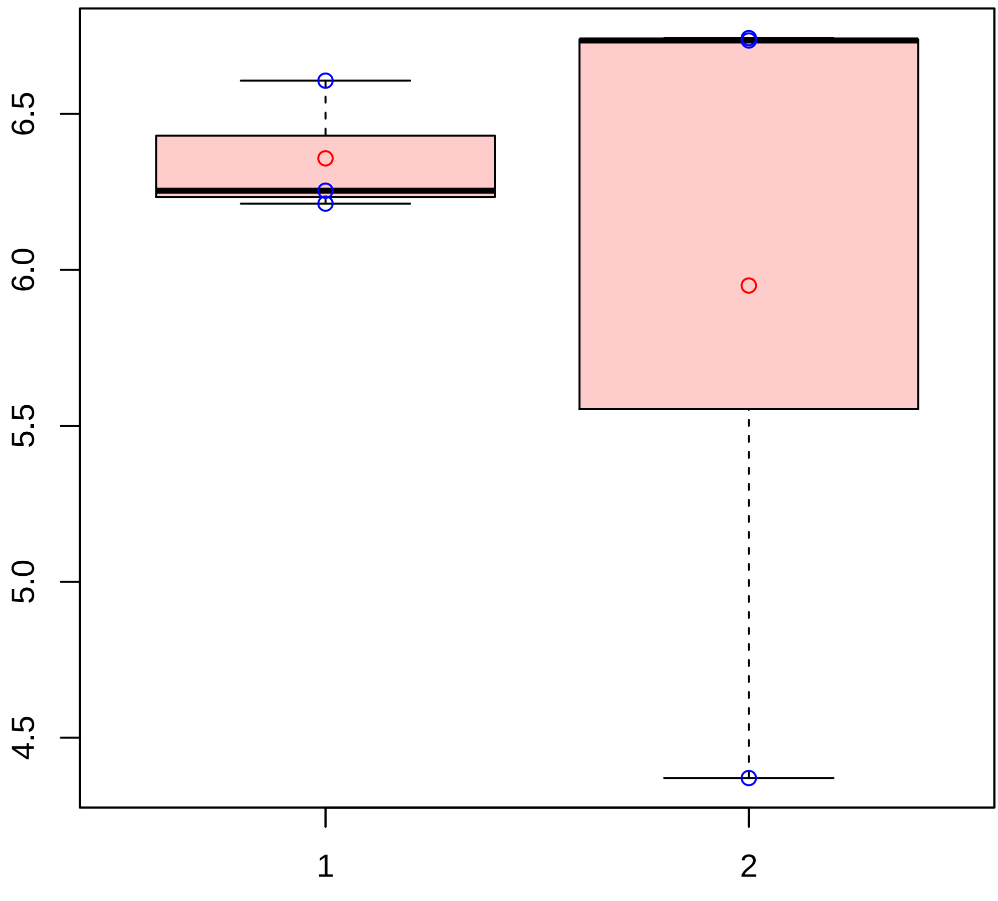

t−Test: p−value = 0.66

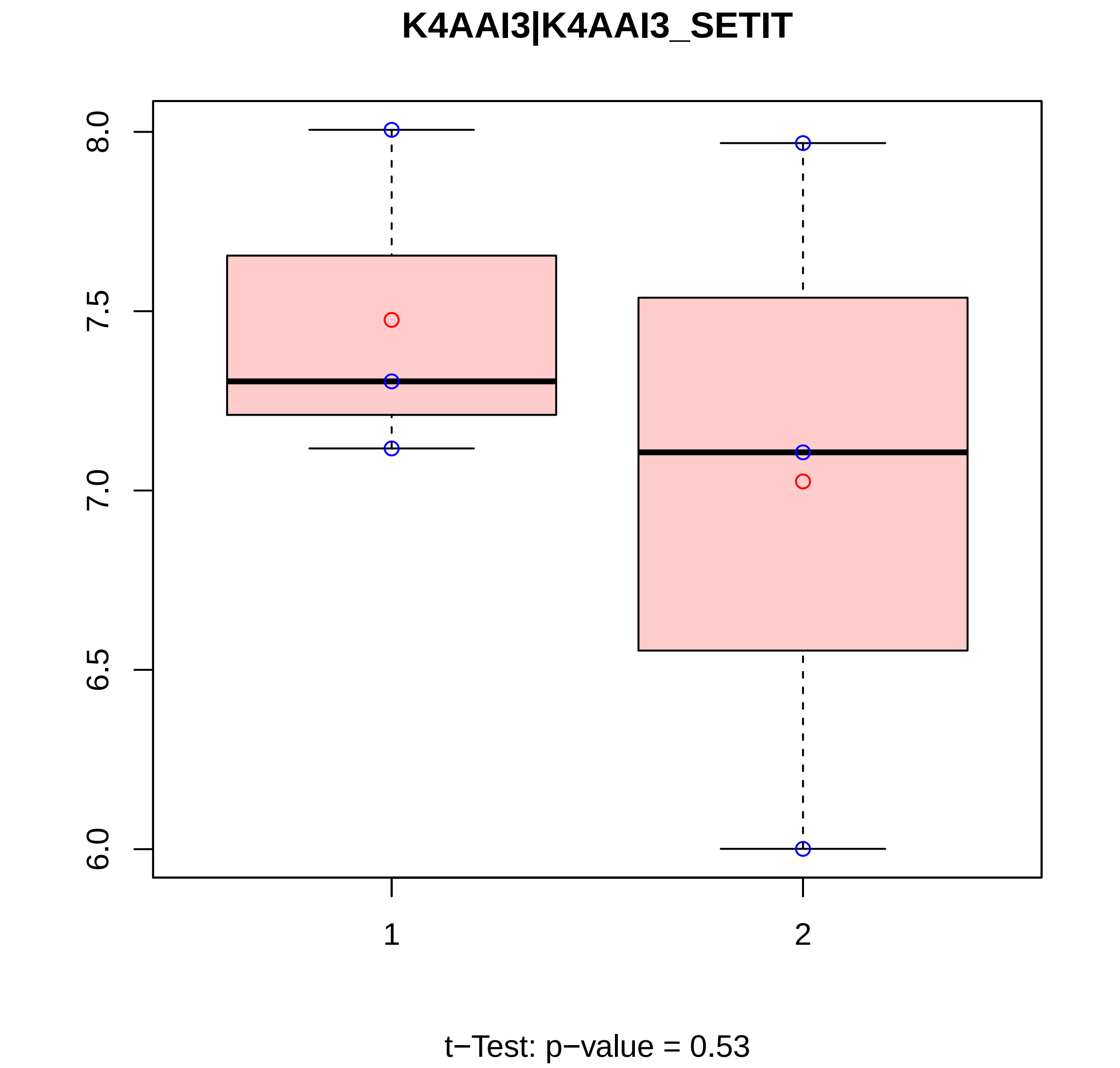
K4AAI3|K4AAI3_SETIT
8.0
7.5
7.0
6.5
6.0
1
2
t−Test: p−value = 0.53

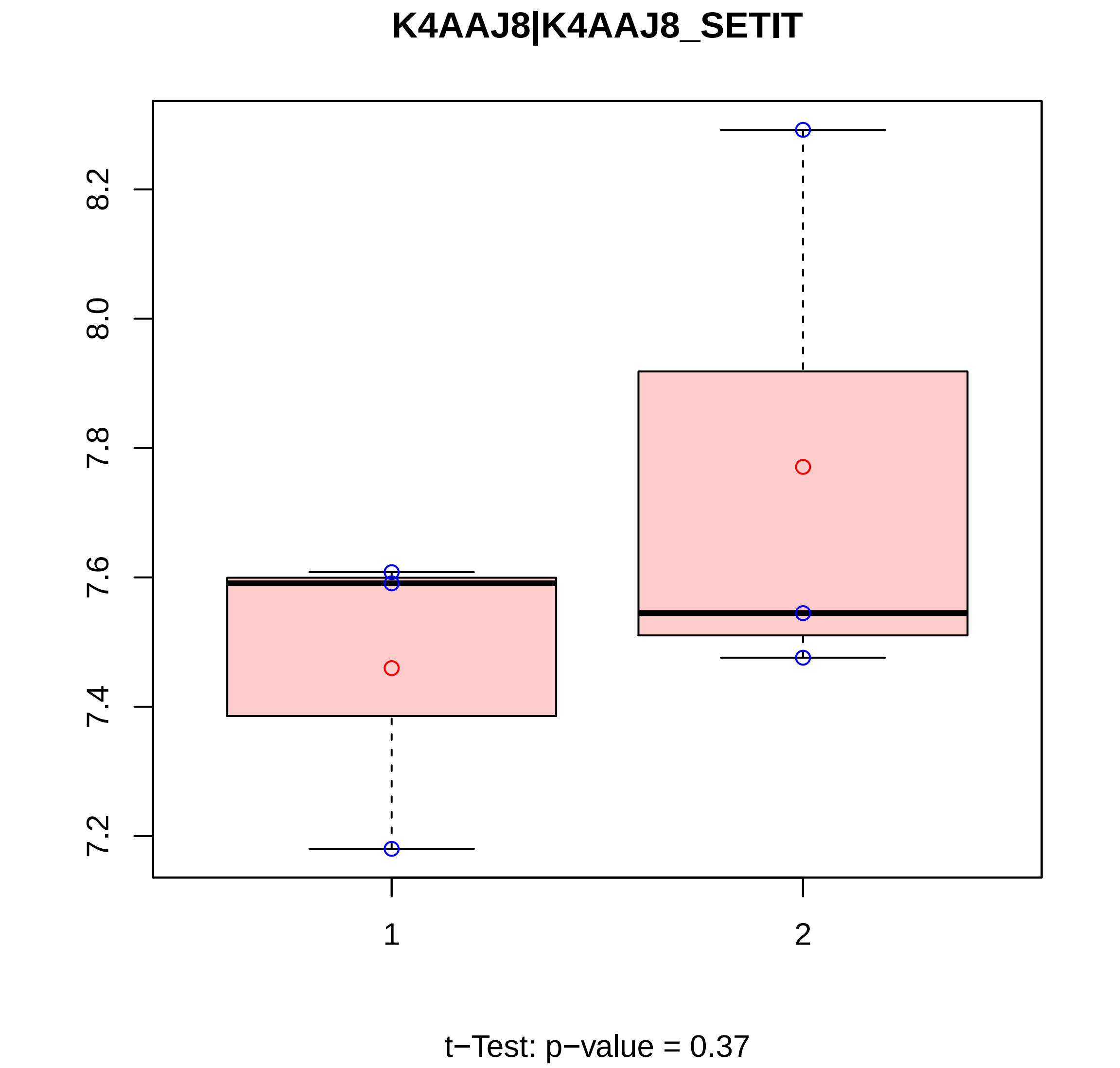

K4AAJ8|K4AAJ8_SETIT
7.2
7.4
7.6
7.8
8.0
8.2
1
2
t−Test: p−value = 0.37

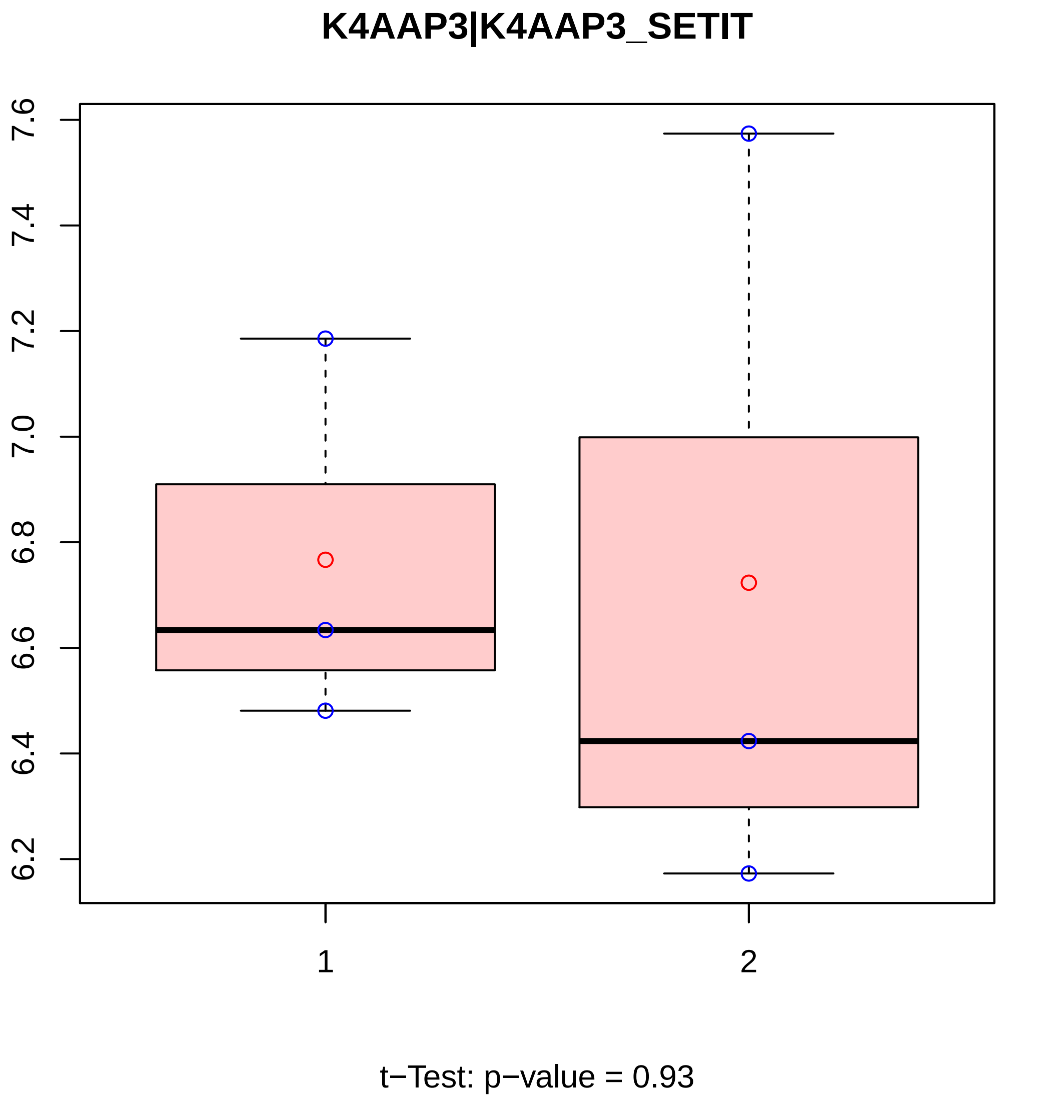
K4AAP3|K4AAP3_SETIT
7.6
7.4
7.2
7.0
6.8
6.6
6.4
6.2
1
2
t−Test: p−value = 0.93

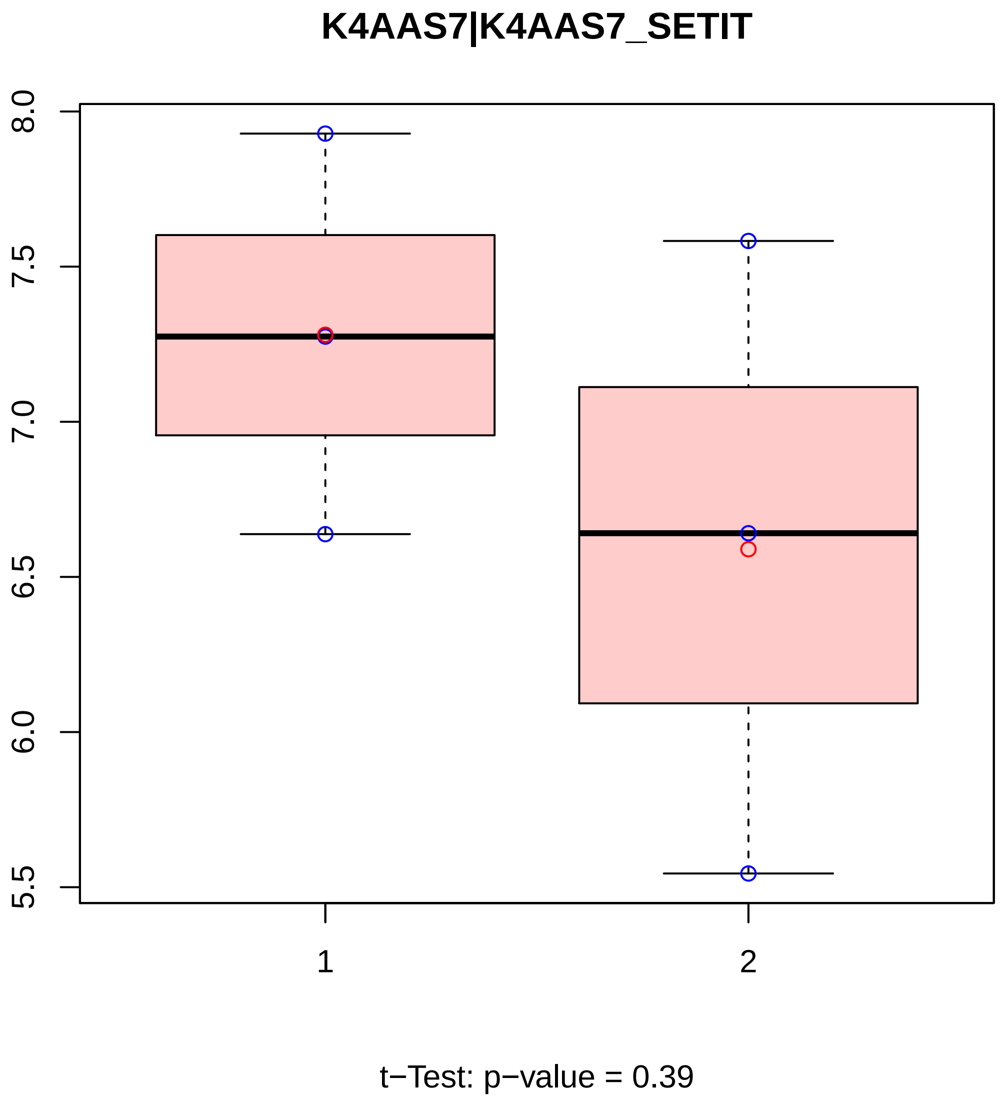
K4AAS7|K4AAS7_SETIT
8.0
7.5
7.0
6.5
6.0
5.5
1
2
t−Test: p−value = 0.39

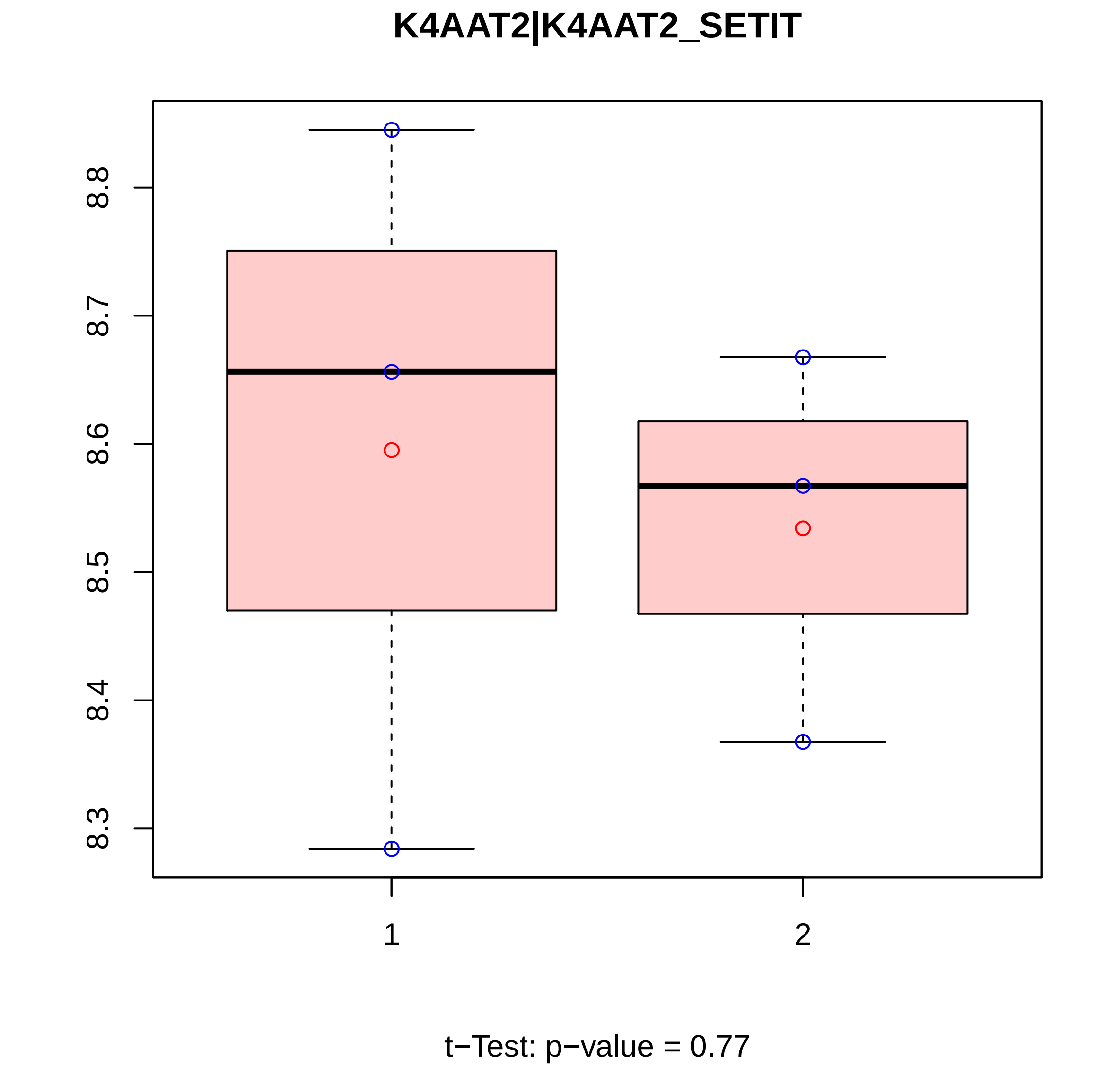
K4AAT2|K4AAT2_SETIT
8.3
8.4
8.5
8.6
8.7
8.8
1
2
t−Test: p−value = 0.77

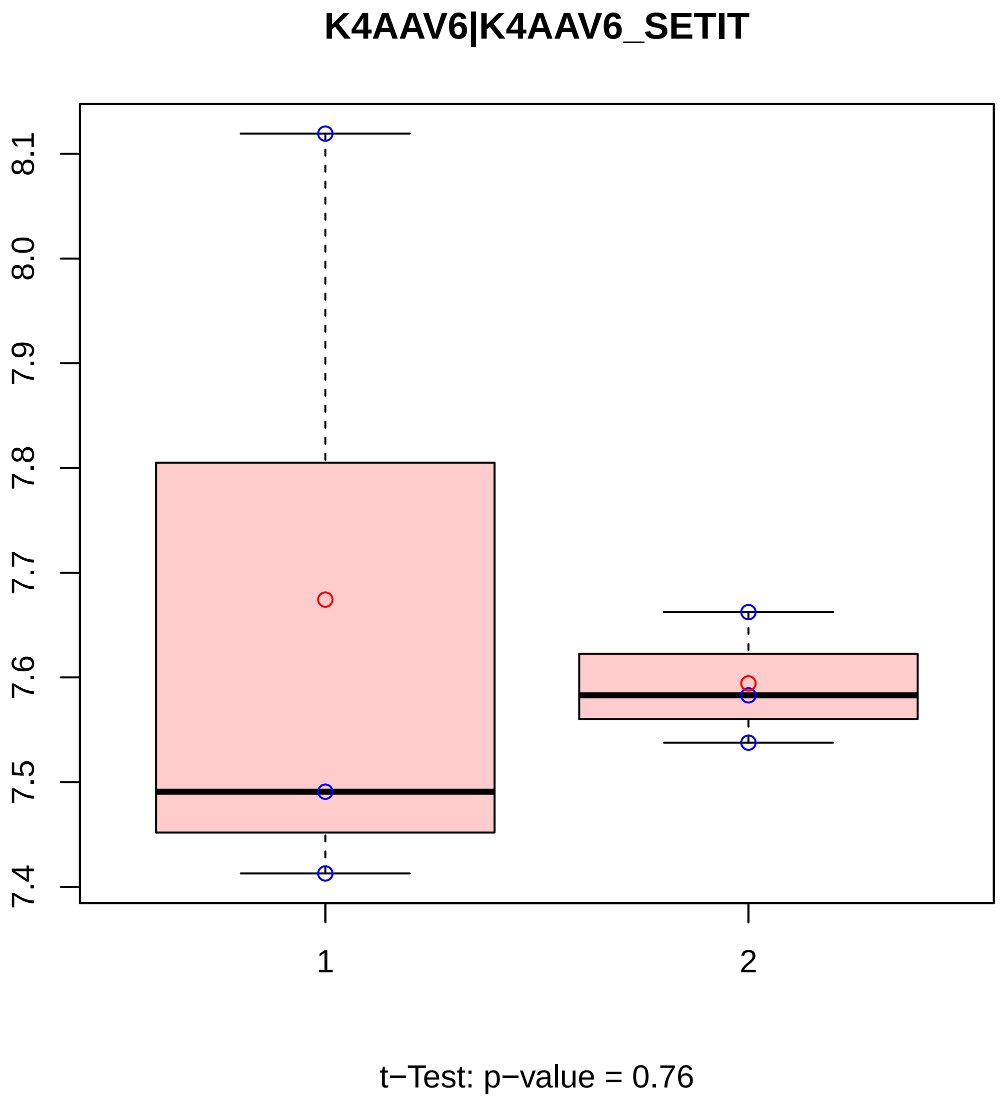
K4AAV6|K4AAV6_SETIT
8.1
8.0
7.9
7.8
7.7
7.6
7.5
7.4
1
2
t−Test: p−value = 0.76

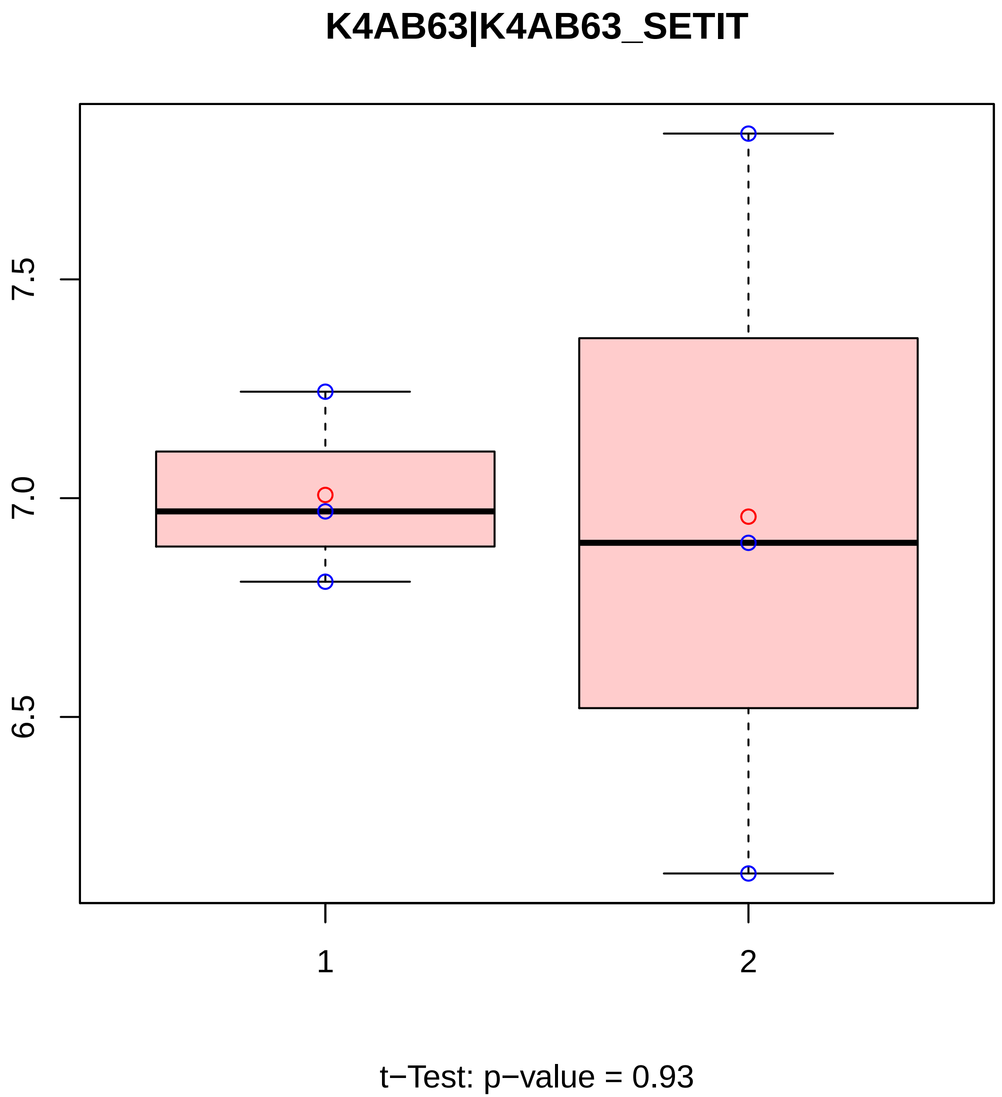
K4AB63|K4AB63_SETIT
7.5
7.0
6.5
1
2
t−Test: p−value = 0.93

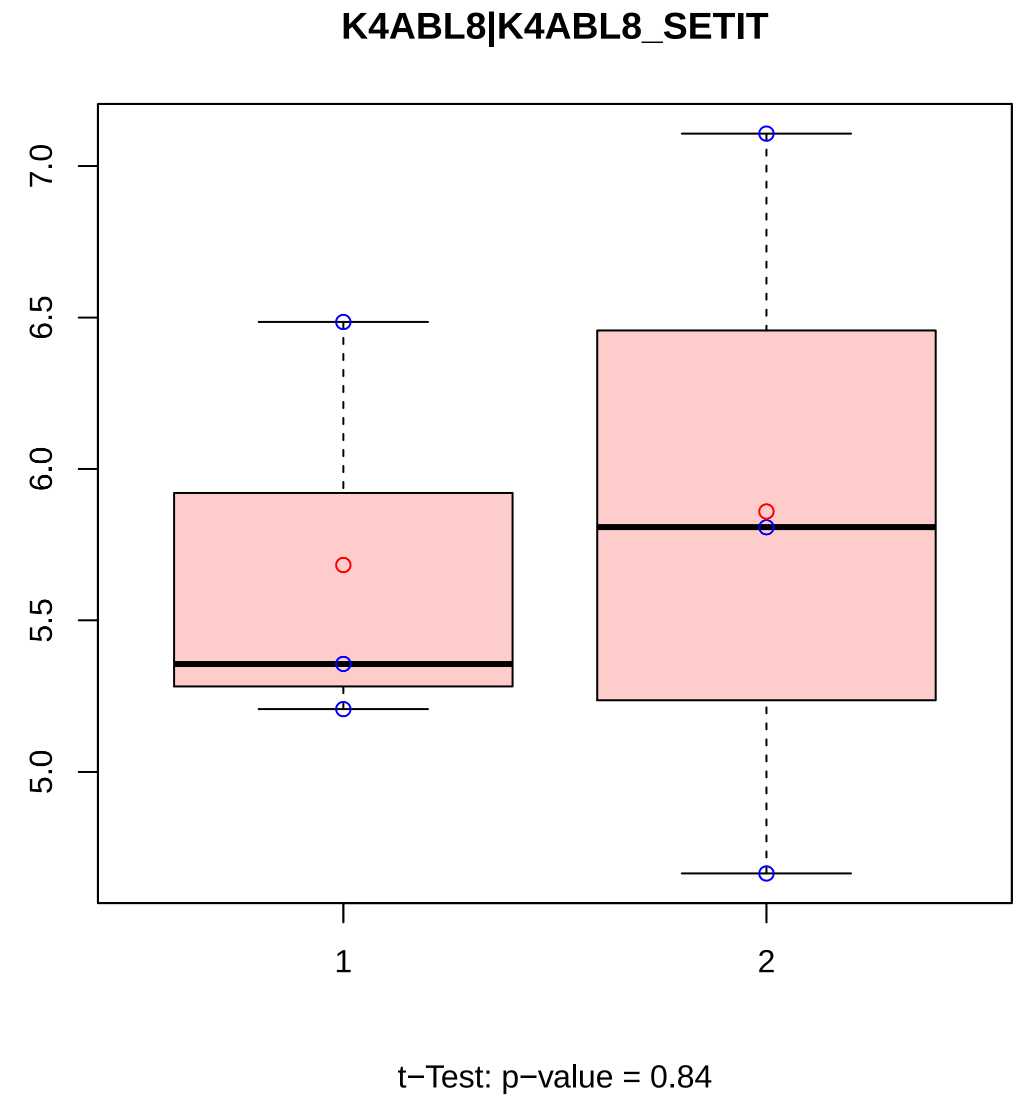

K4ABL8|K4ABL8_SETIT
7.0
6.5
6.0
5.5
5.0
1
2
t−Test: p−value = 0.84

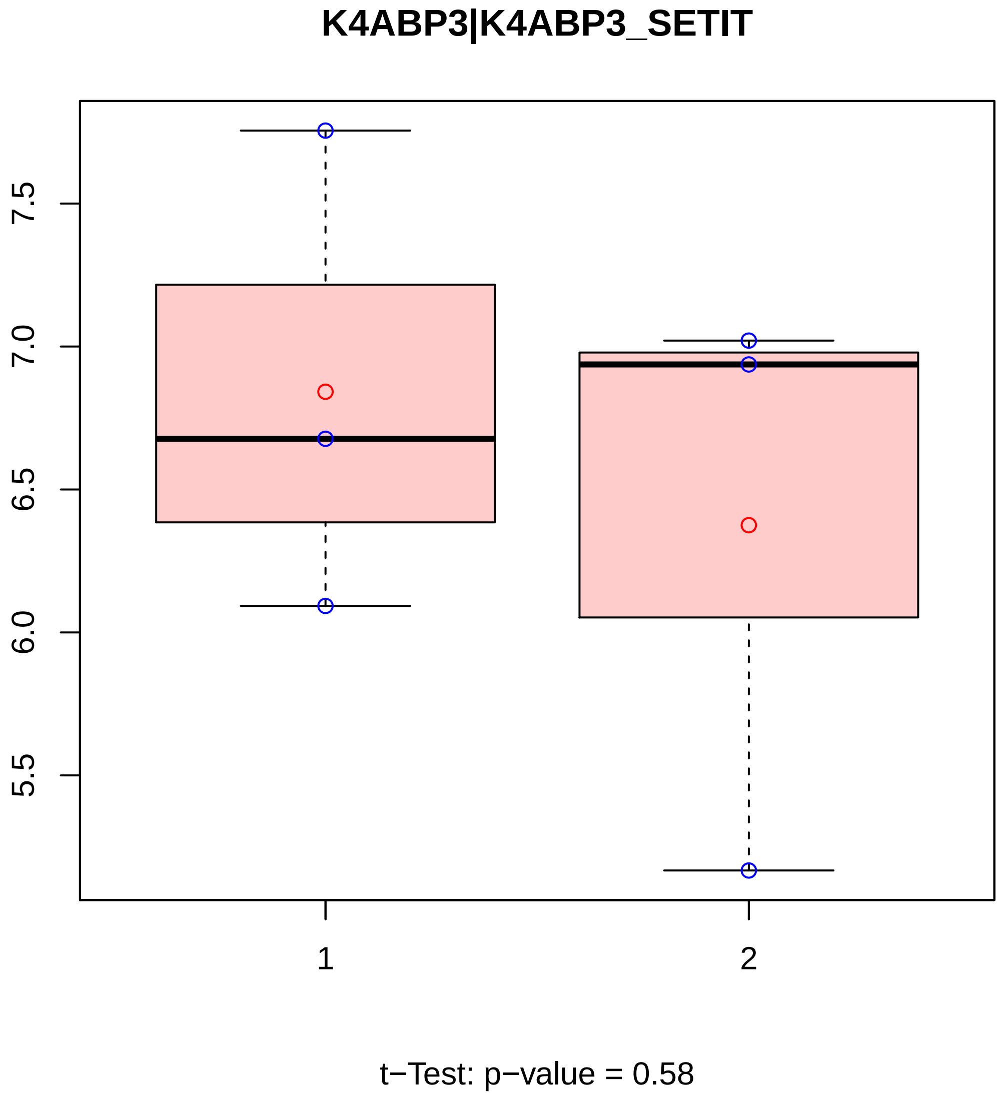
K4ABP3|K4ABP3_SETIT
7.5
7.0
6.5
6.0
5.5
1
2
t−Test: p−value = 0.58

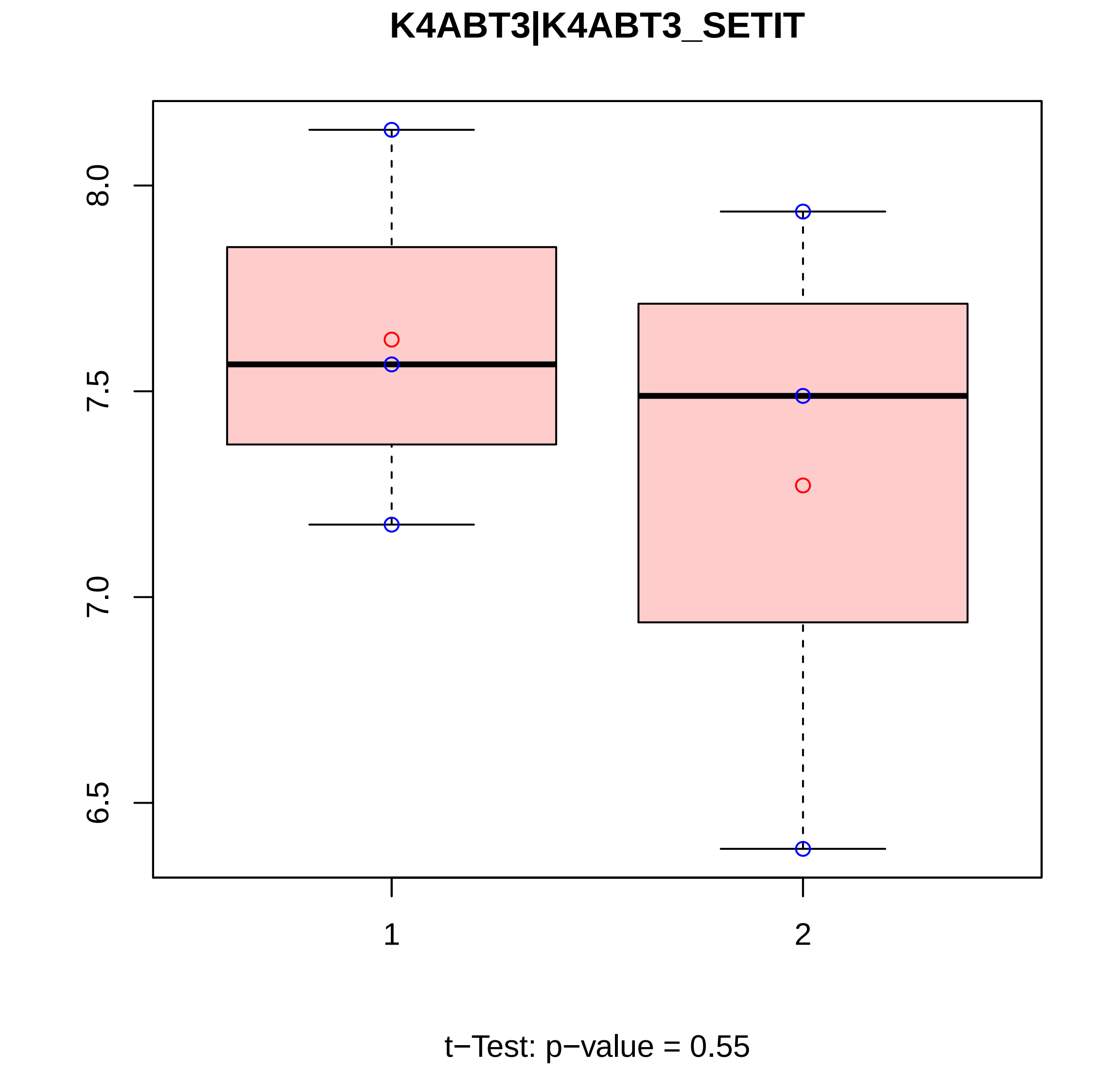
K4ABT3|K4ABT3_SETIT
8.0
7.5
7.0
6.5
1
2
t−Test: p−value = 0.55

K4ABV8|K4ABV8_SETIT

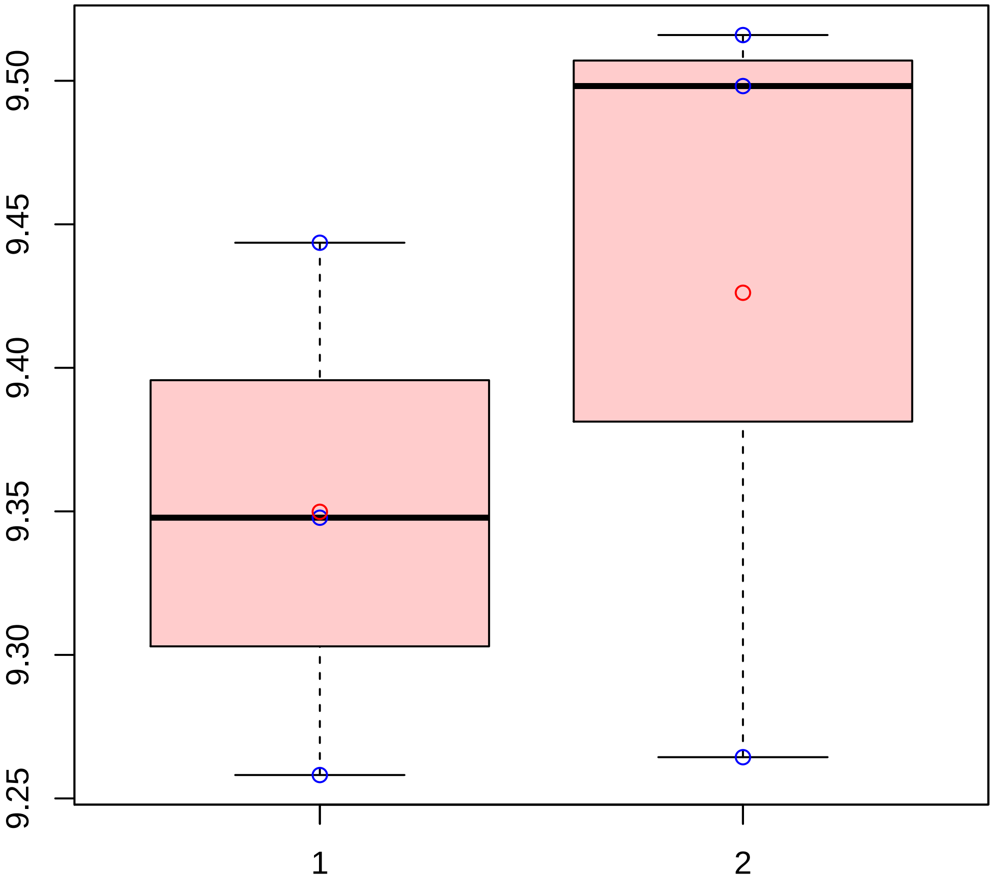

t−Test: p−value = 0.48

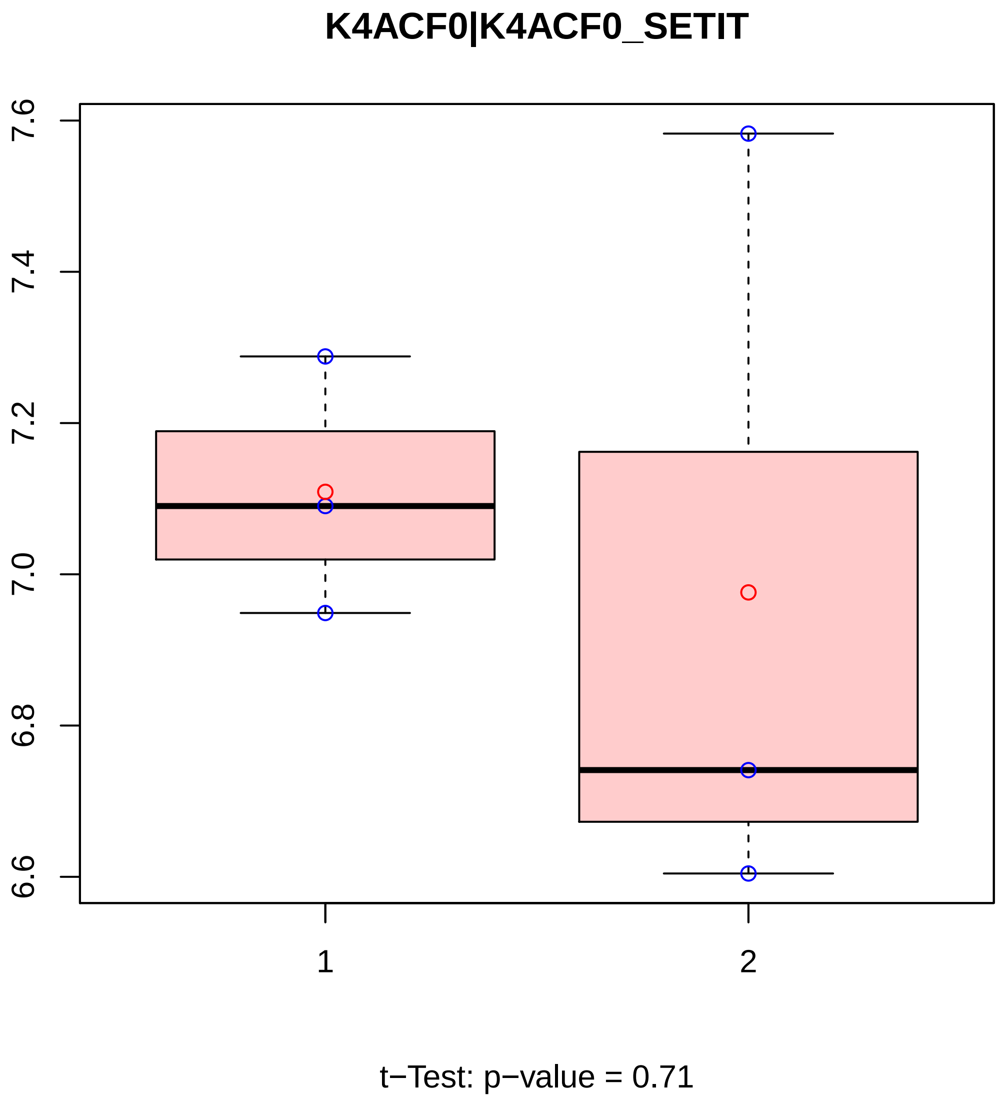
K4ACF0|K4ACF0_SETIT
7.6
7.4
7.2
7.0
6.8
6.6
1
2
t−Test: p−value = 0.71

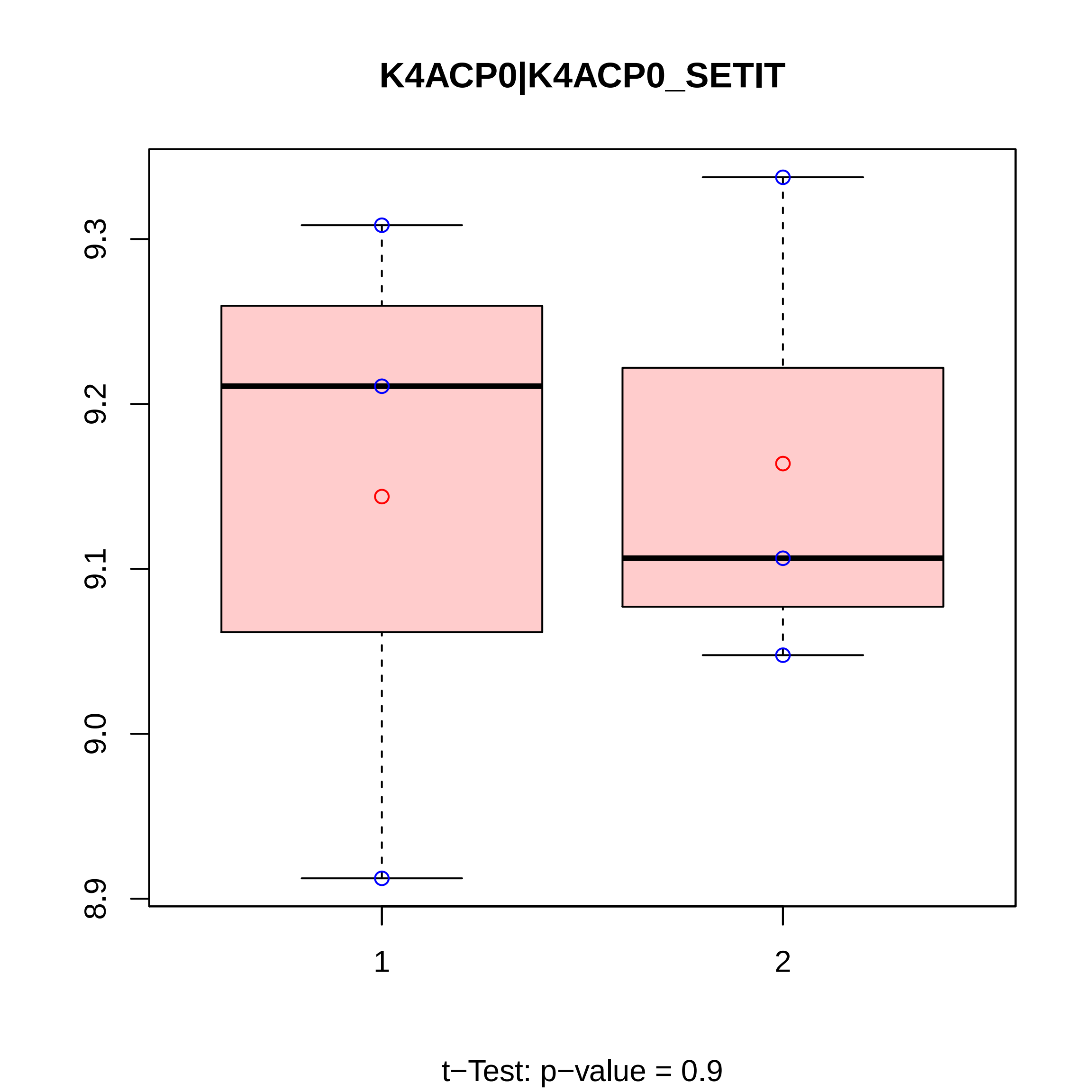
K4ACP0|K4ACP0_SETIT
9.3
9.2
9.1
9.0
8.9
1
2
t−Test: p−value = 0.9

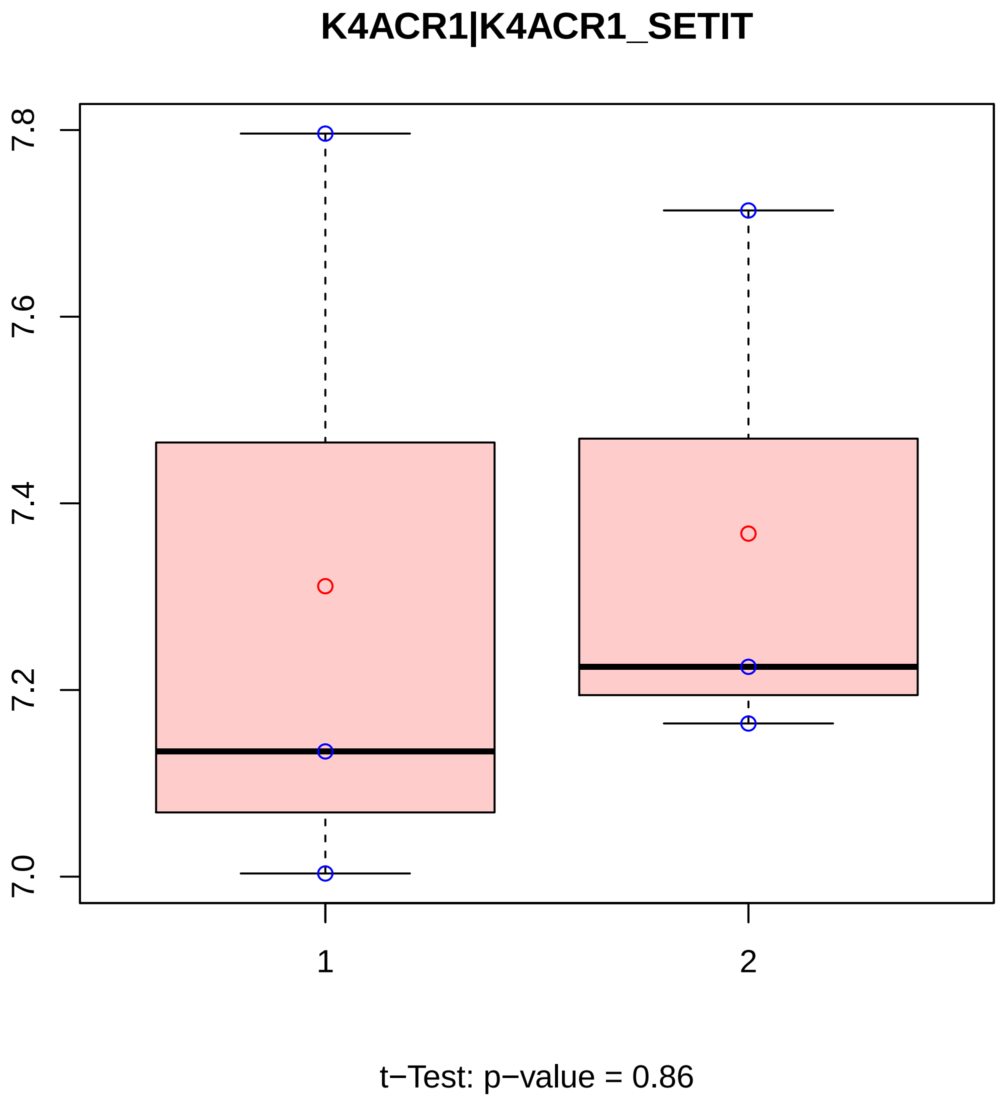
K4ACR1|K4ACR1_SETIT
7.8
7.6
7.4
7.2
7.0
1
2
t−Test: p−value = 0.86

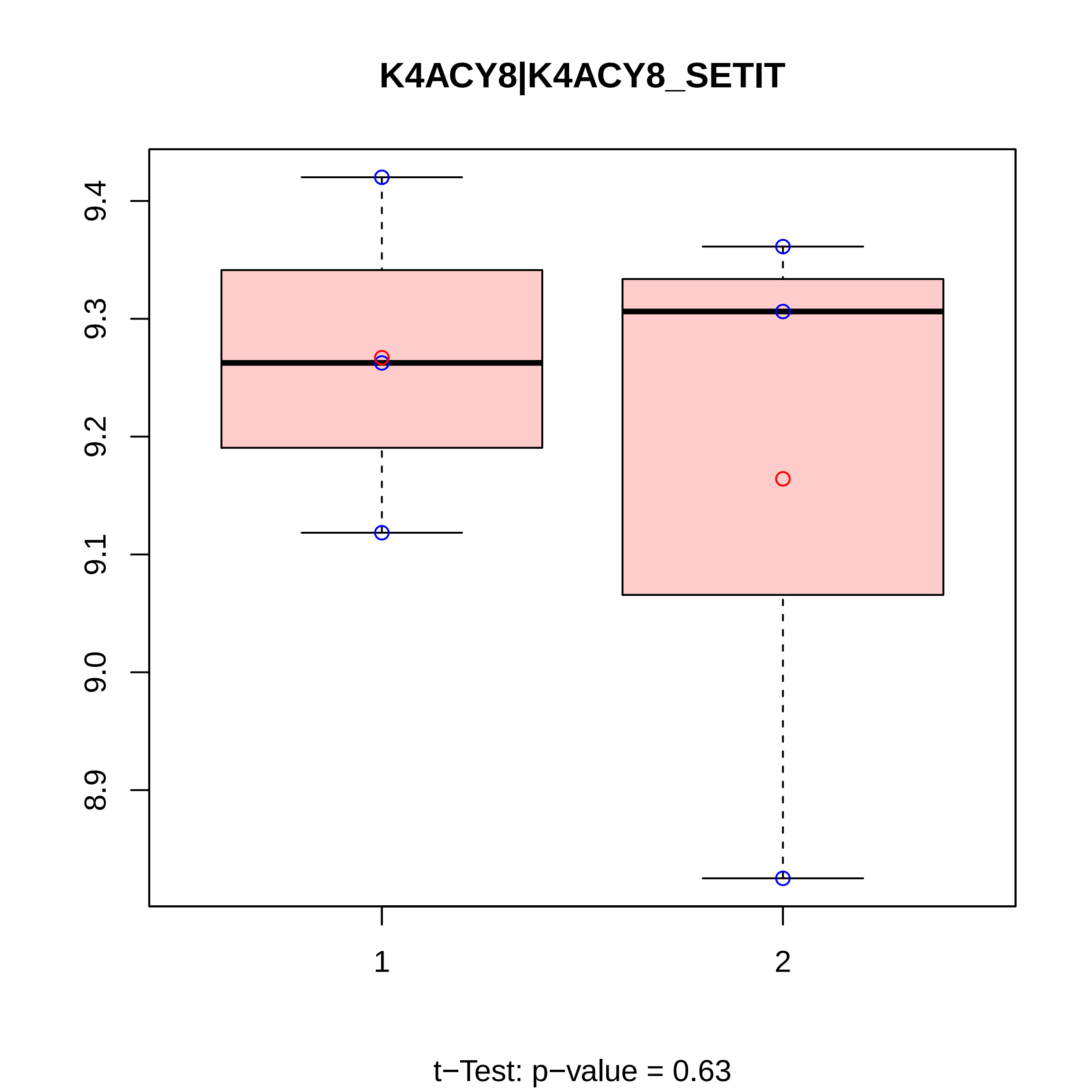

K4ACY8|K4ACY8_SETIT
9.4
9.3
9.2
9.1
9.0
8.9
1
2
t−Test: p−value = 0.63

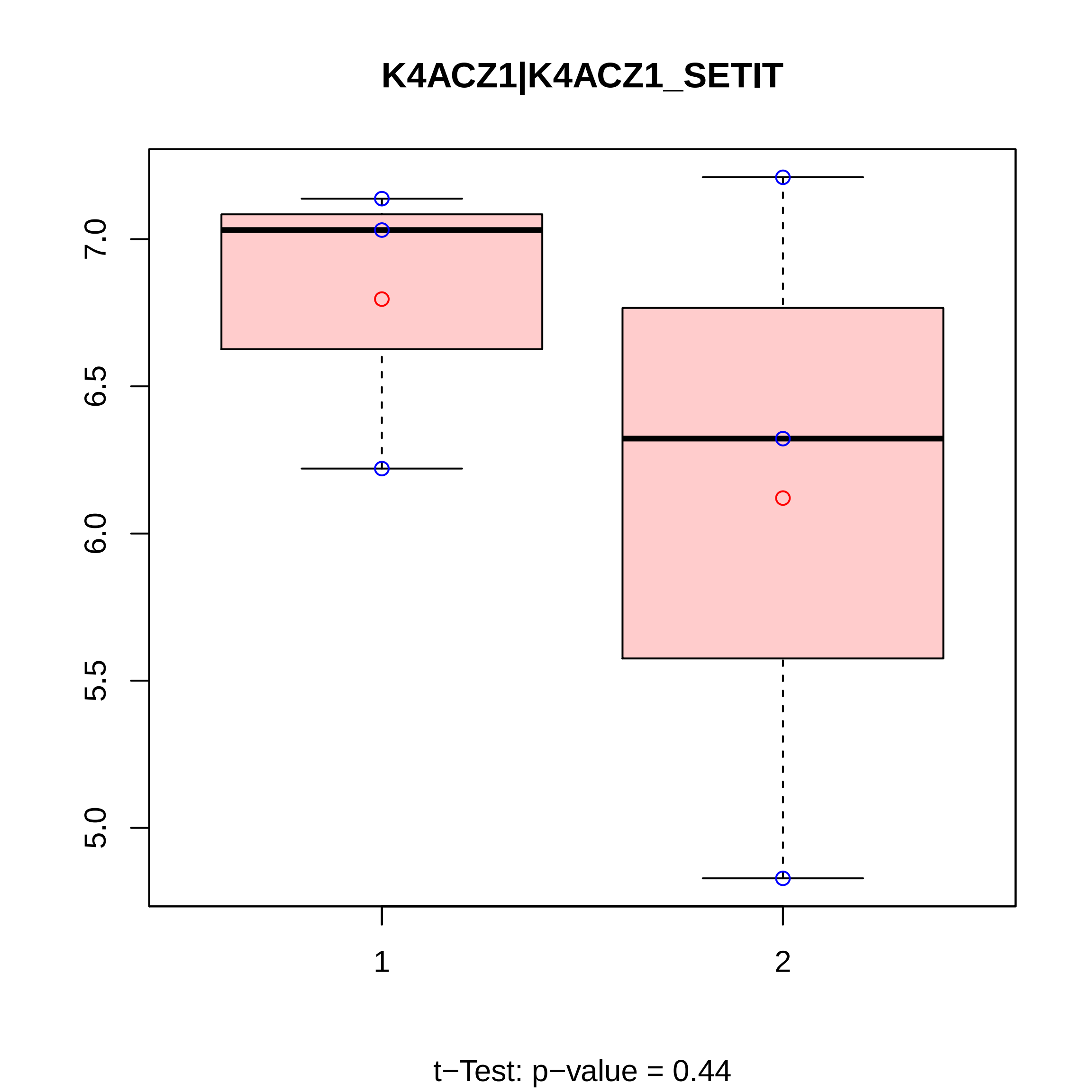
K4ACZ1|K4ACZ1_SETIT
7.0
6.5
6.0
5.5
5.0
1
2
t−Test: p−value = 0.44

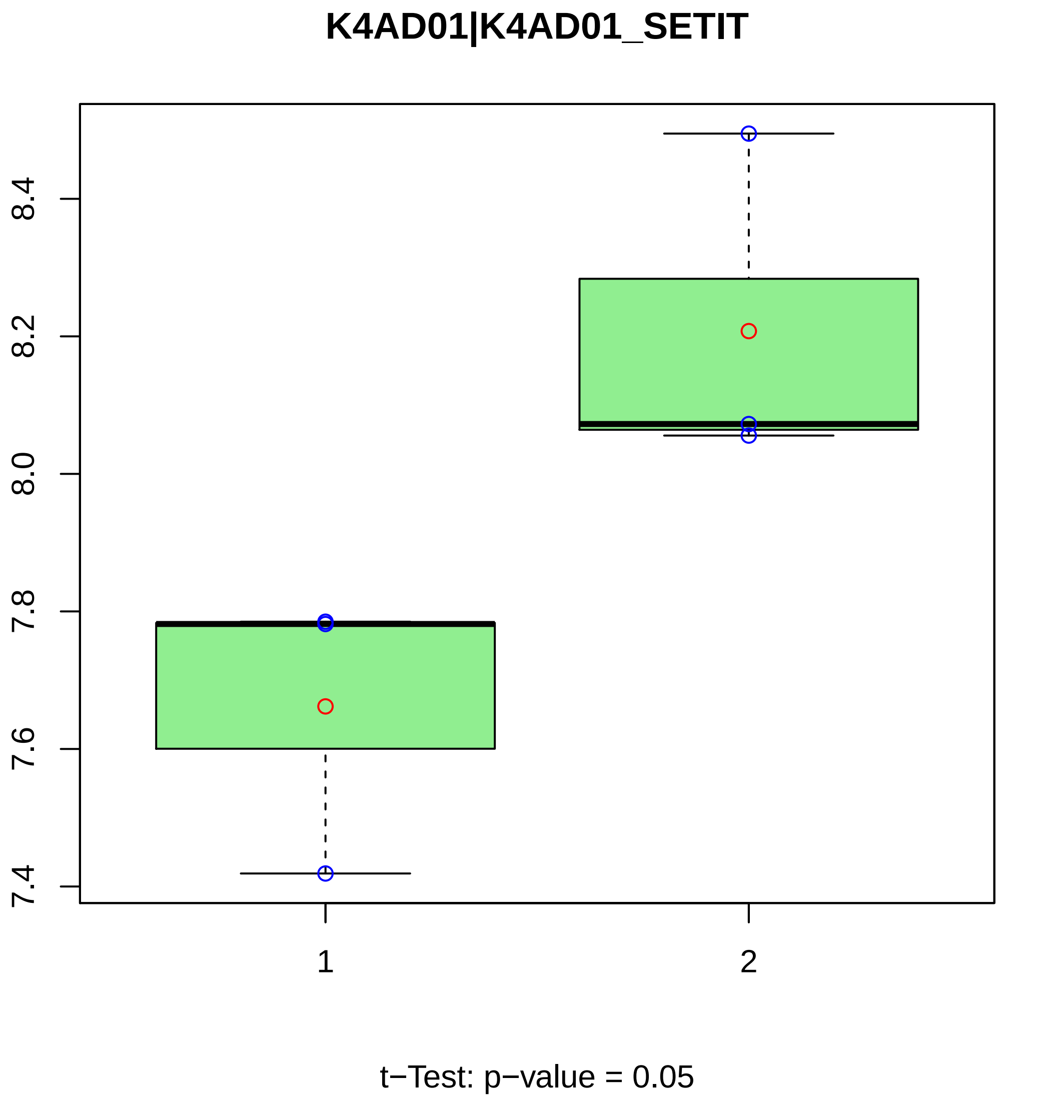
K4AD01|K4AD01_SETIT
8.4
8.2
8.0
7.8
7.6
7.4
1
2
t−Test: p−value = 0.05

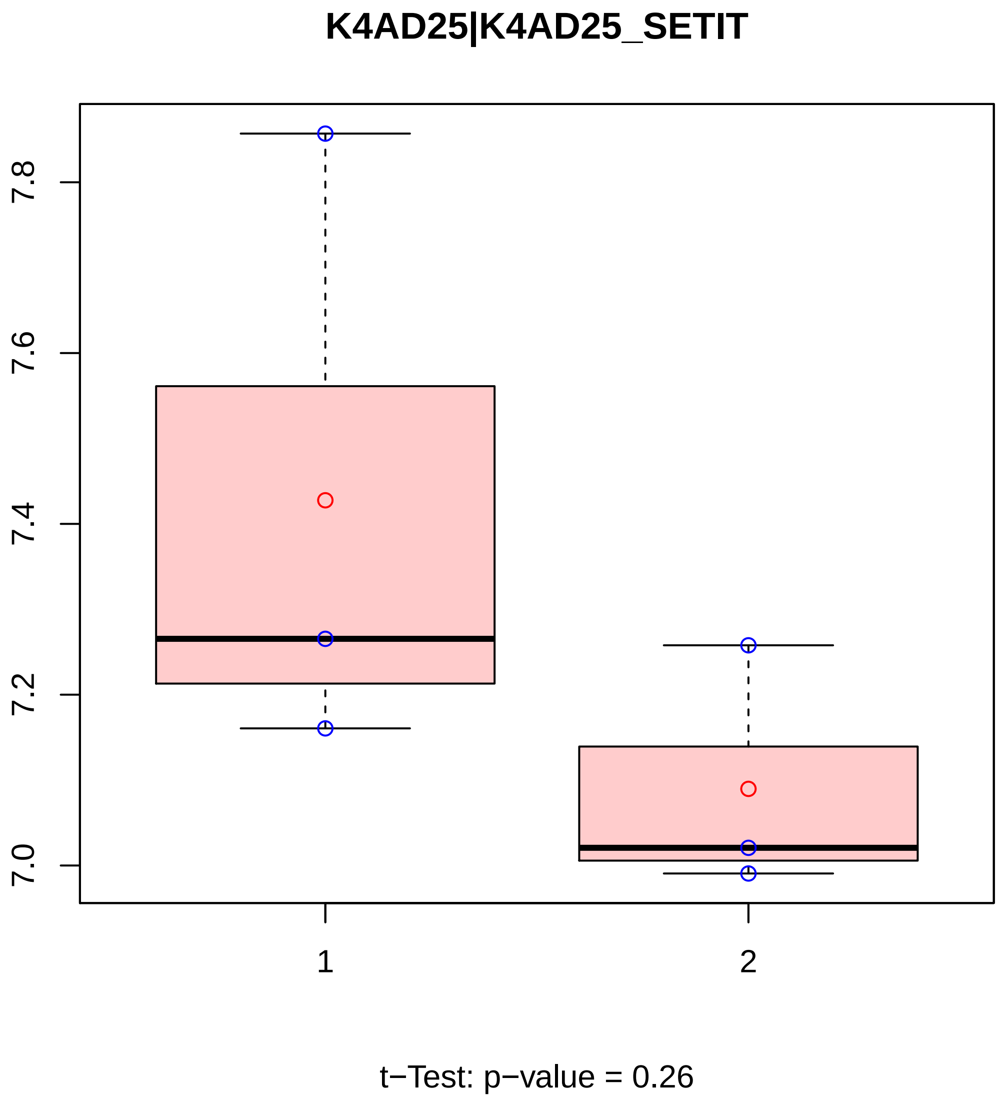
K4AD25|K4AD25_SETIT
7.0
7.2
7.4
7.6
7.8
1
2
t−Test: p−value = 0.26

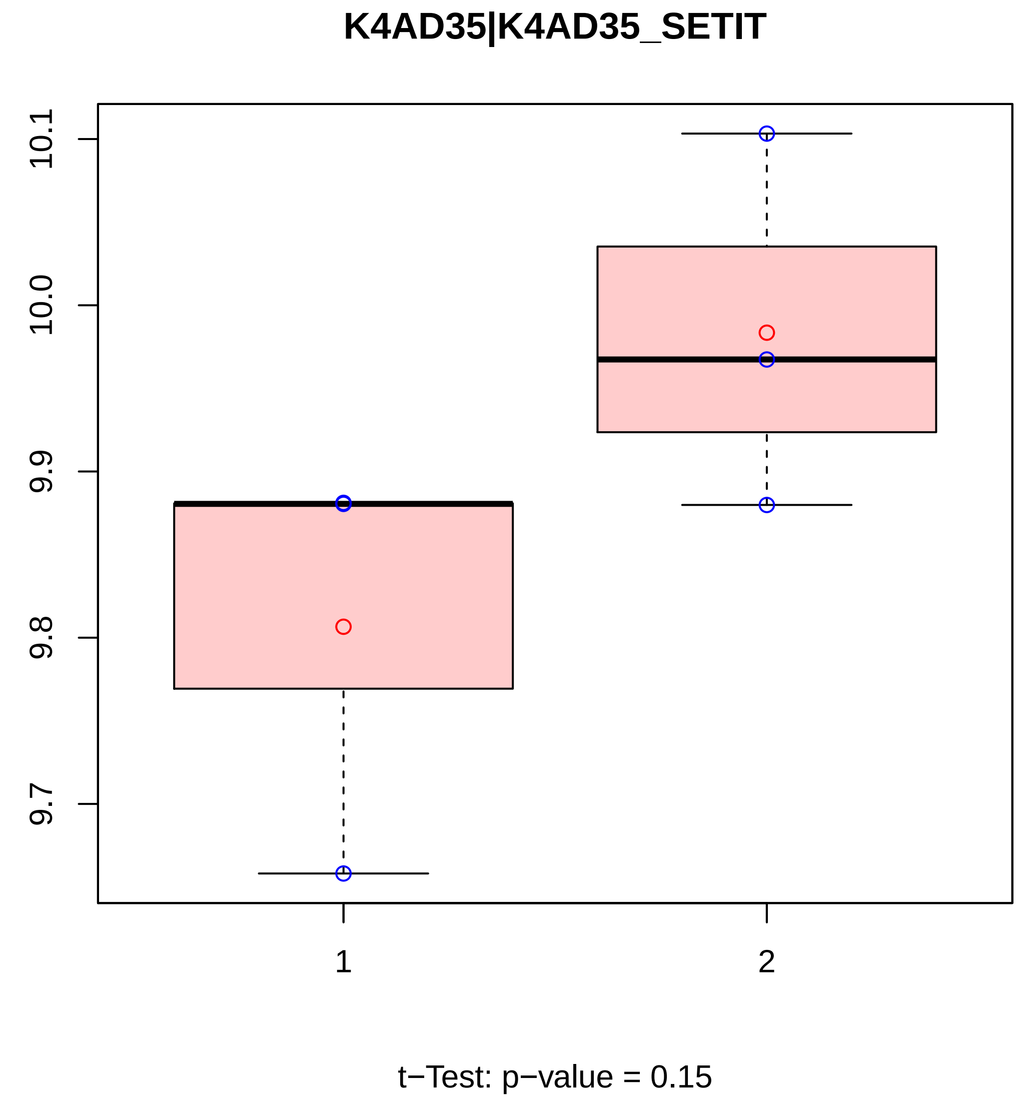
K4AD35|K4AD35_SETIT
10.1
10.0
9.9
9.8
9.7
1
2
t−Test: p−value = 0.15

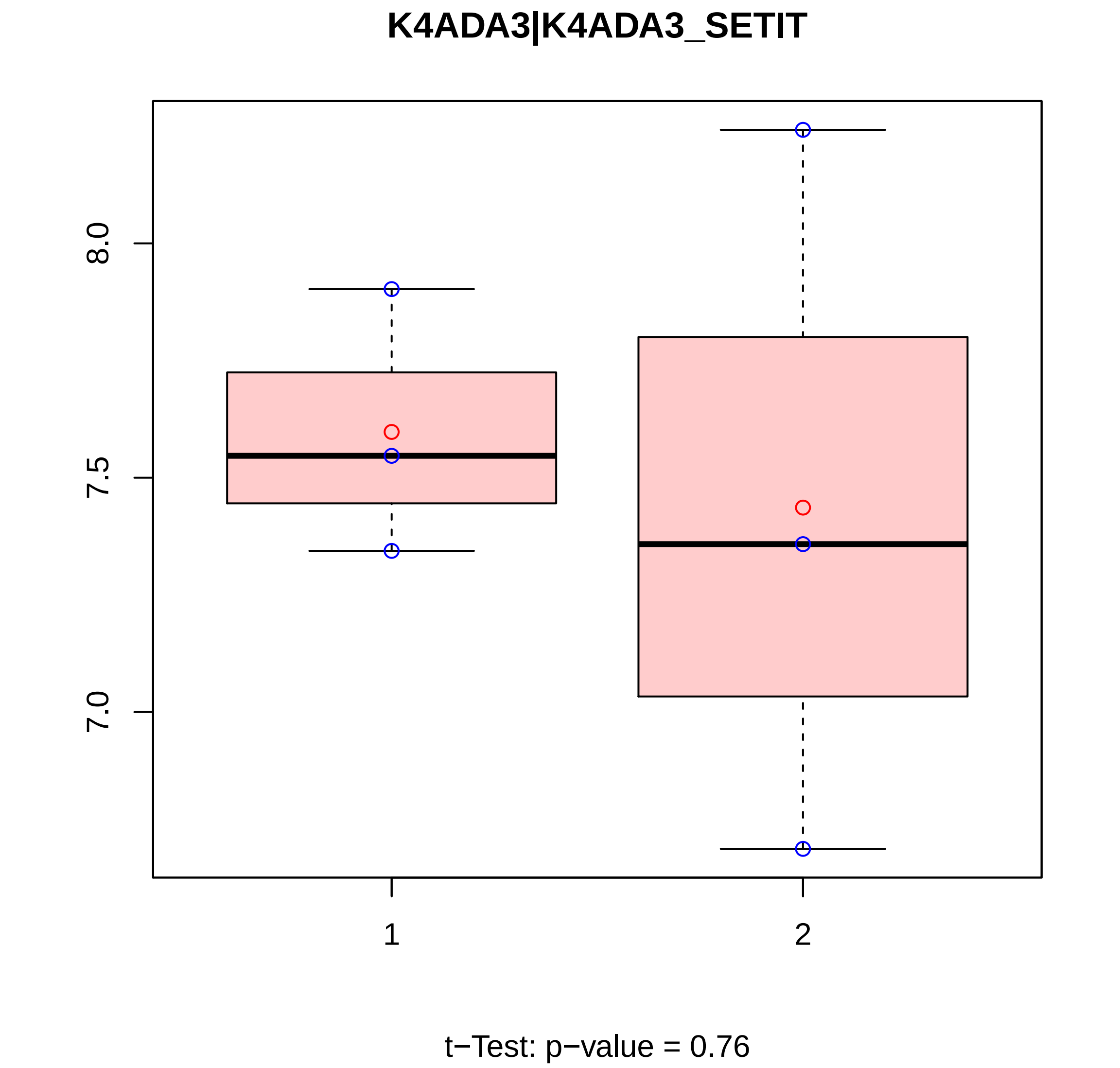
K4ADA3|K4ADA3_SETIT
8.0
7.5
7.0
1
2
t−Test: p−value = 0.76

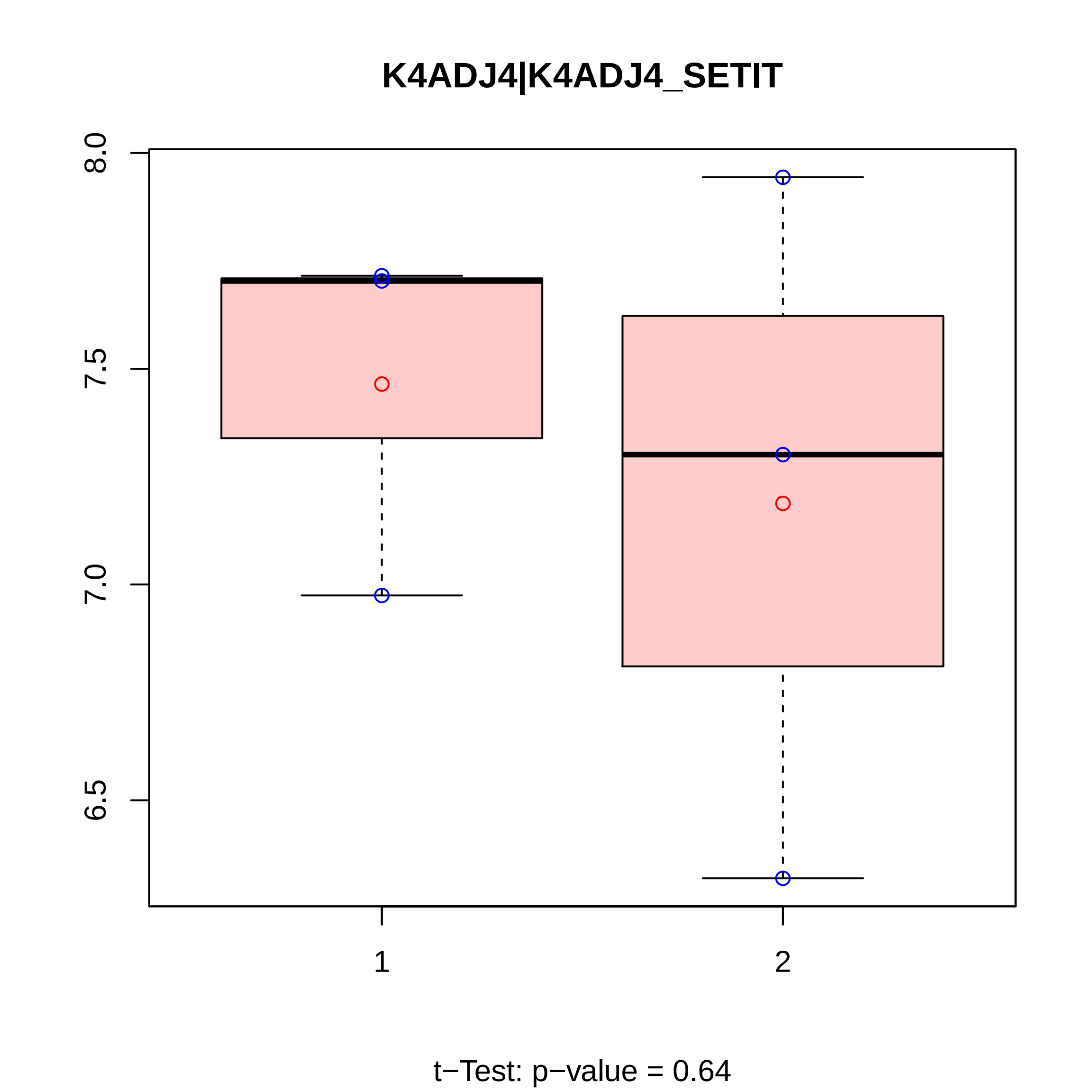
K4ADJ4|K4ADJ4_SETIT
8.0
7.5
7.0
6.5
1
2
t−Test: p−value = 0.64

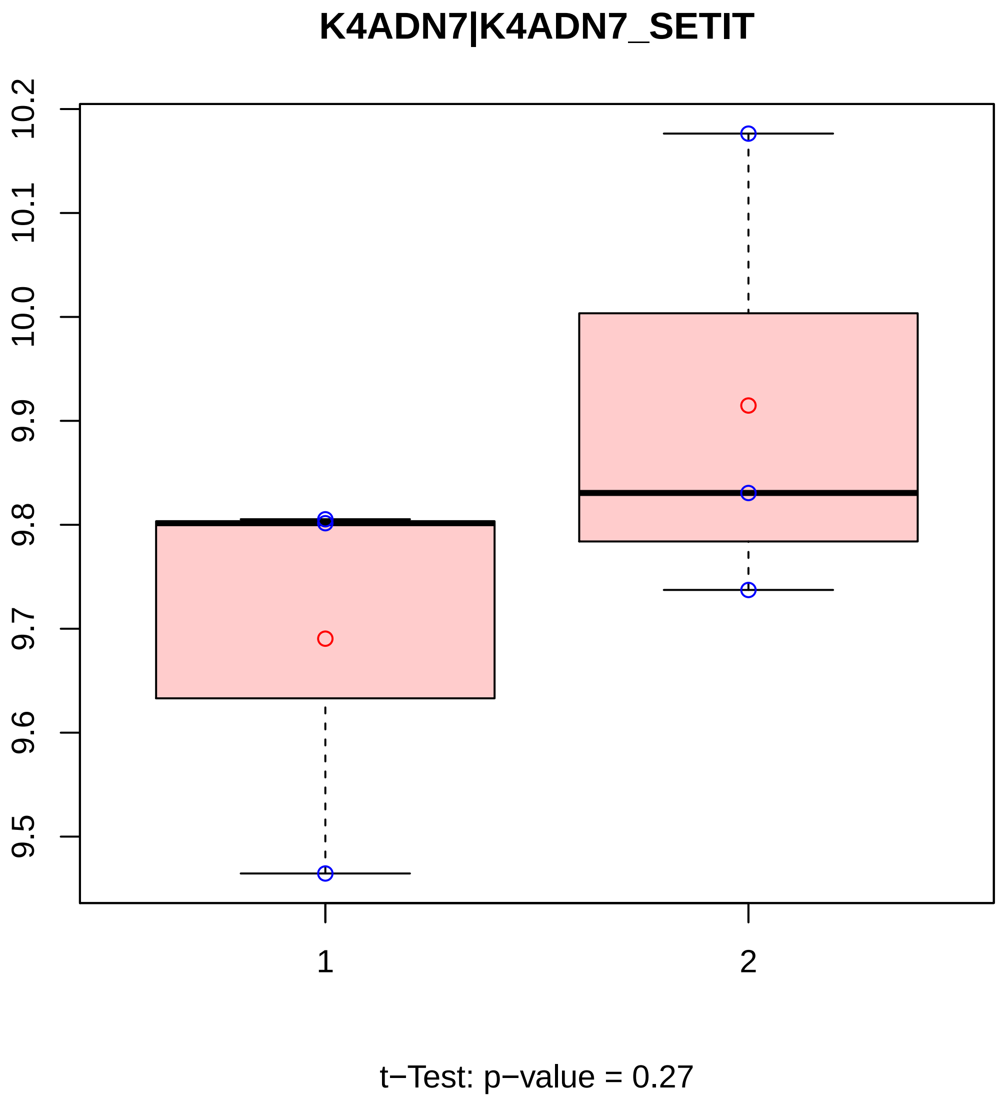
K4ADN7|K4ADN7_SETIT
10.2
10.1
10.0
9.9
9.8
9.7
9.6
9.5
1
2
t−Test: p−value = 0.27

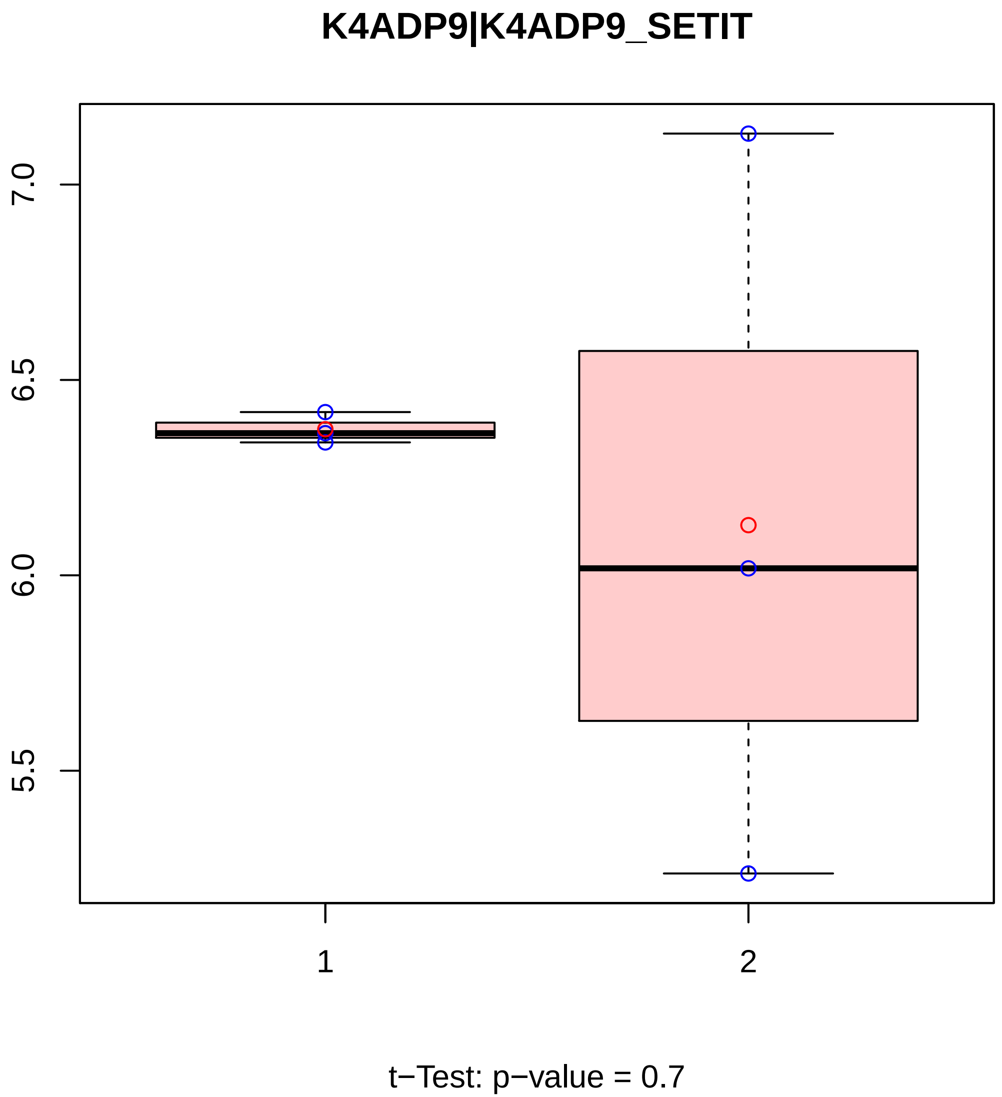
K4ADP9|K4ADP9_SETIT
7.0
6.5
6.0
5.5
1
2
t−Test: p−value = 0.7

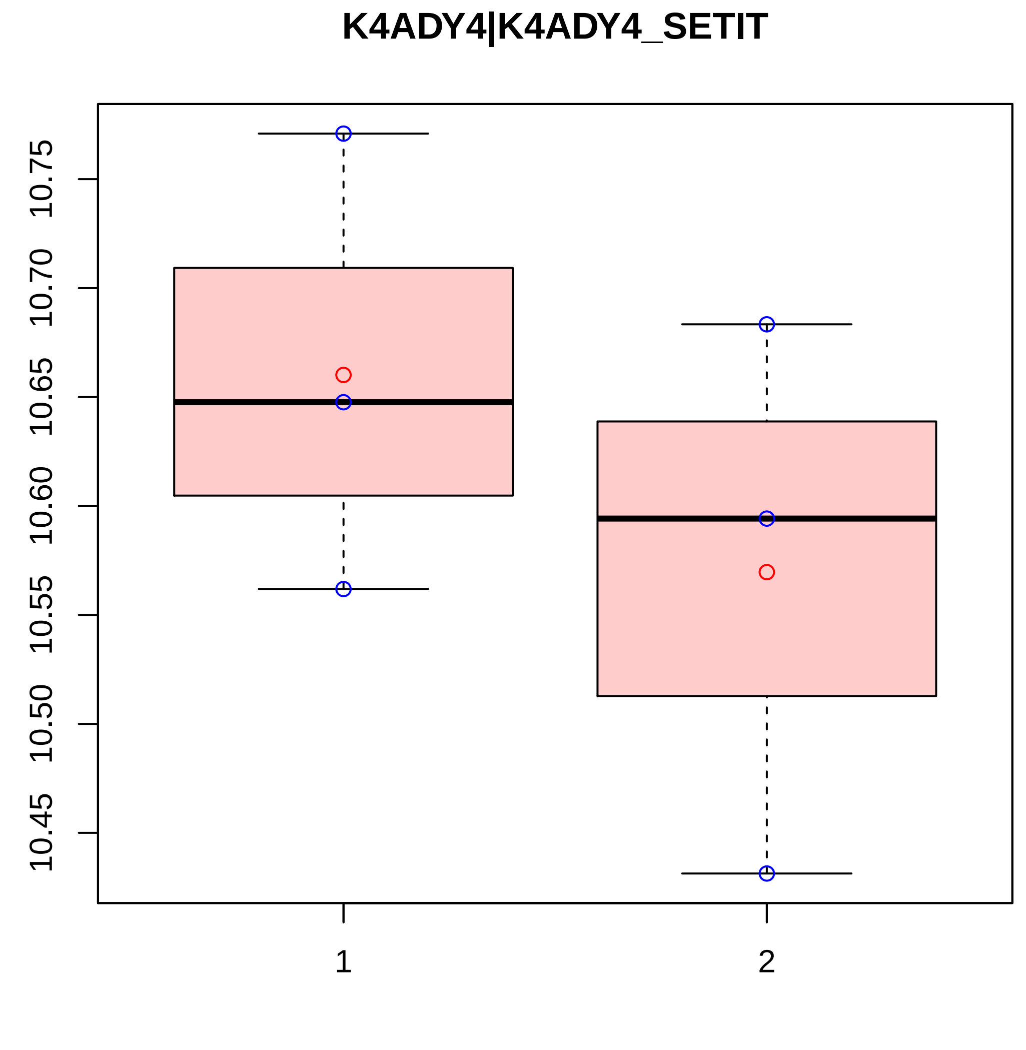
K4ADY4|K4ADY4_SETIT
10.45
10.50
10.55
10.60
10.65
10.70
10.75
1
2
t−Test: p−value = 0.4

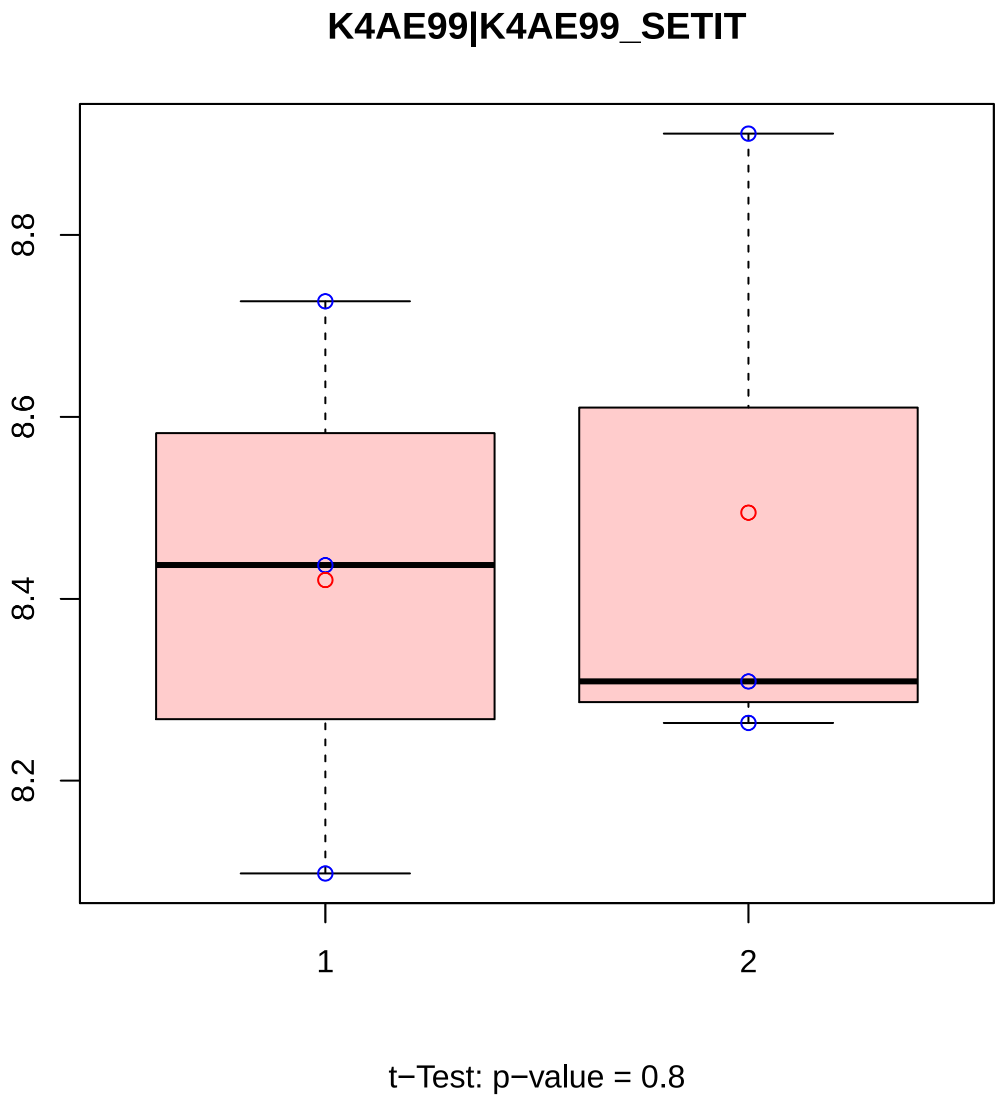

K4AE99|K4AE99_SETIT
8.2
8.4
8.6
8.8
1
2
t−Test: p−value = 0.8

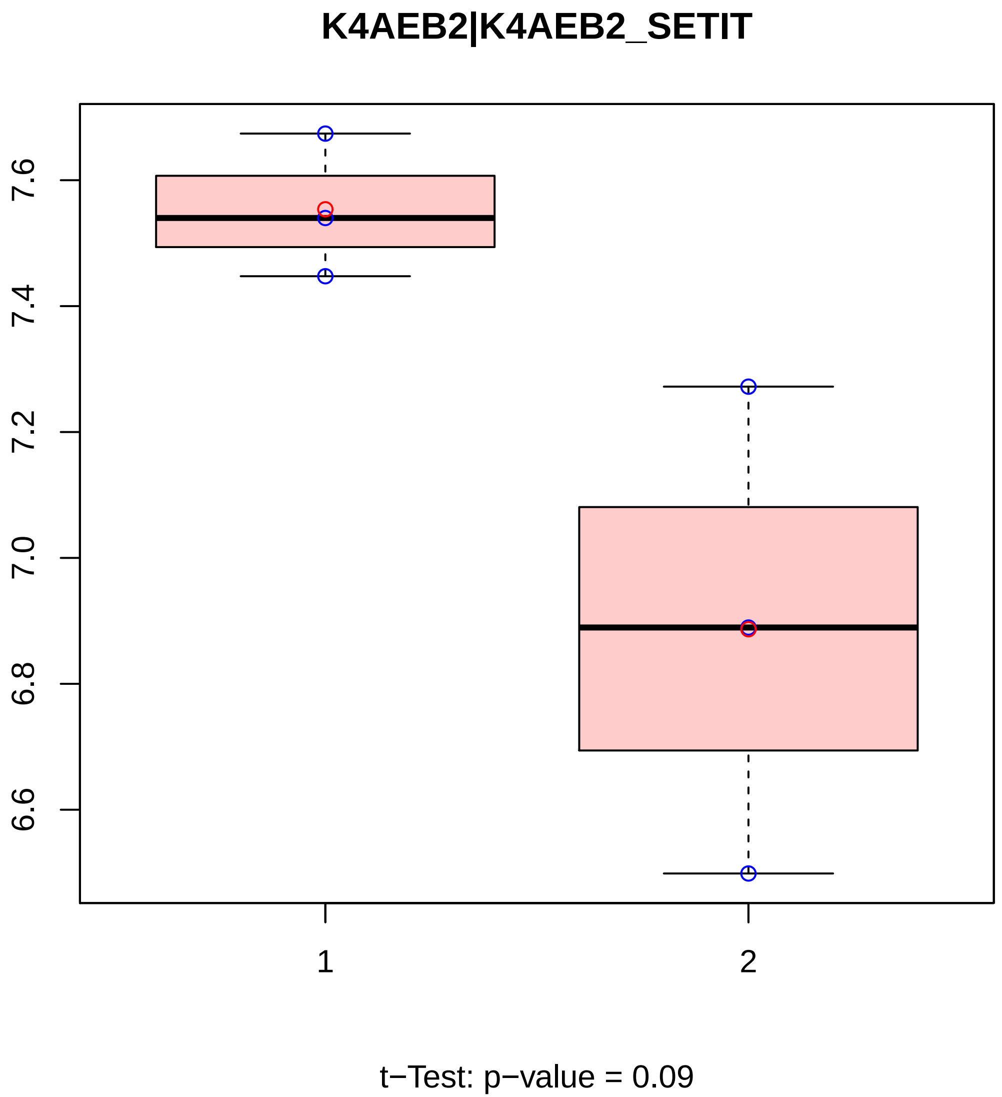
K4AEB2|K4AEB2_SETIT
7.6
7.4
7.2
7.0
6.8
6.6
1
2
t−Test: p−value = 0.09

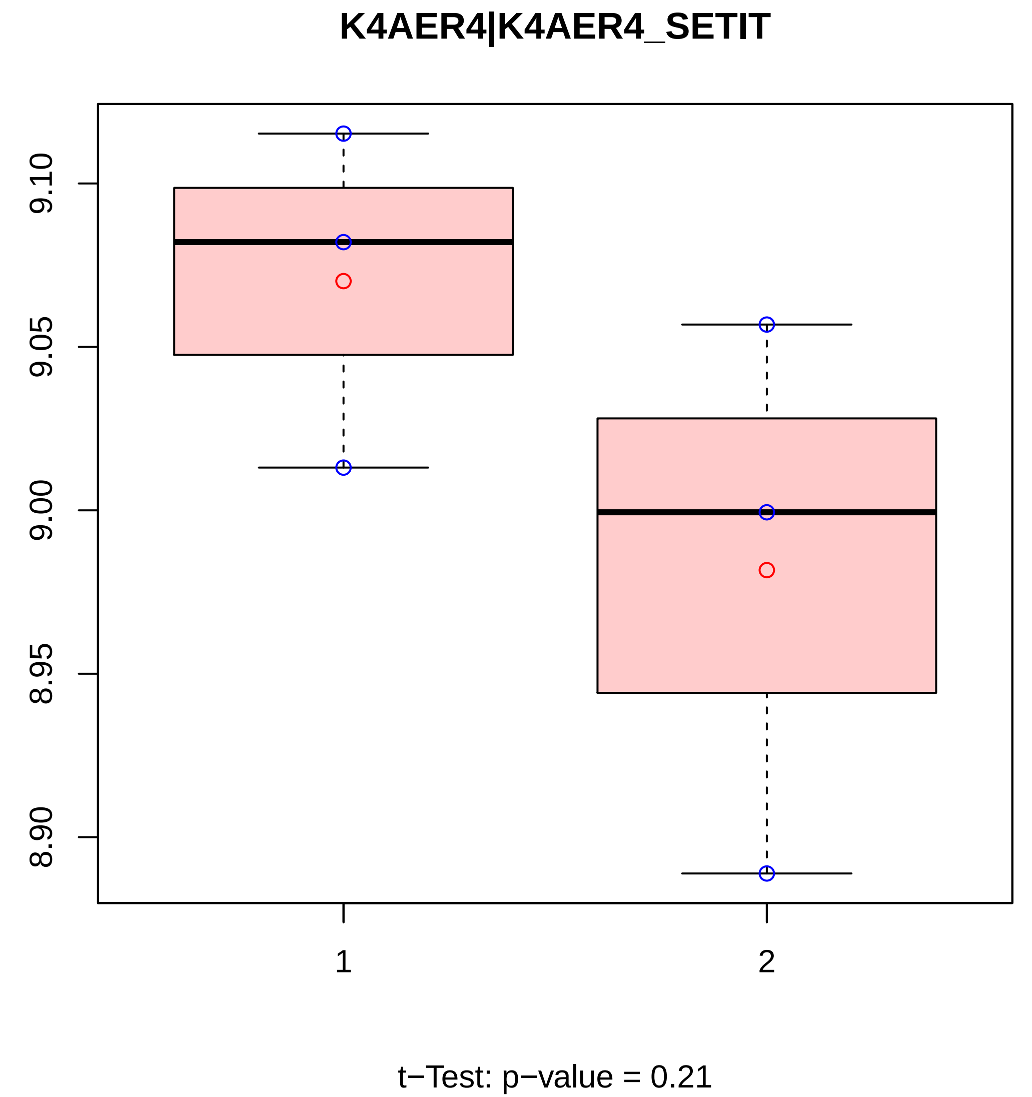
K4AER4|K4AER4_SETIT
9.10
9.05
9.00
8.95
8.90
1
2
t−Test: p−value = 0.21

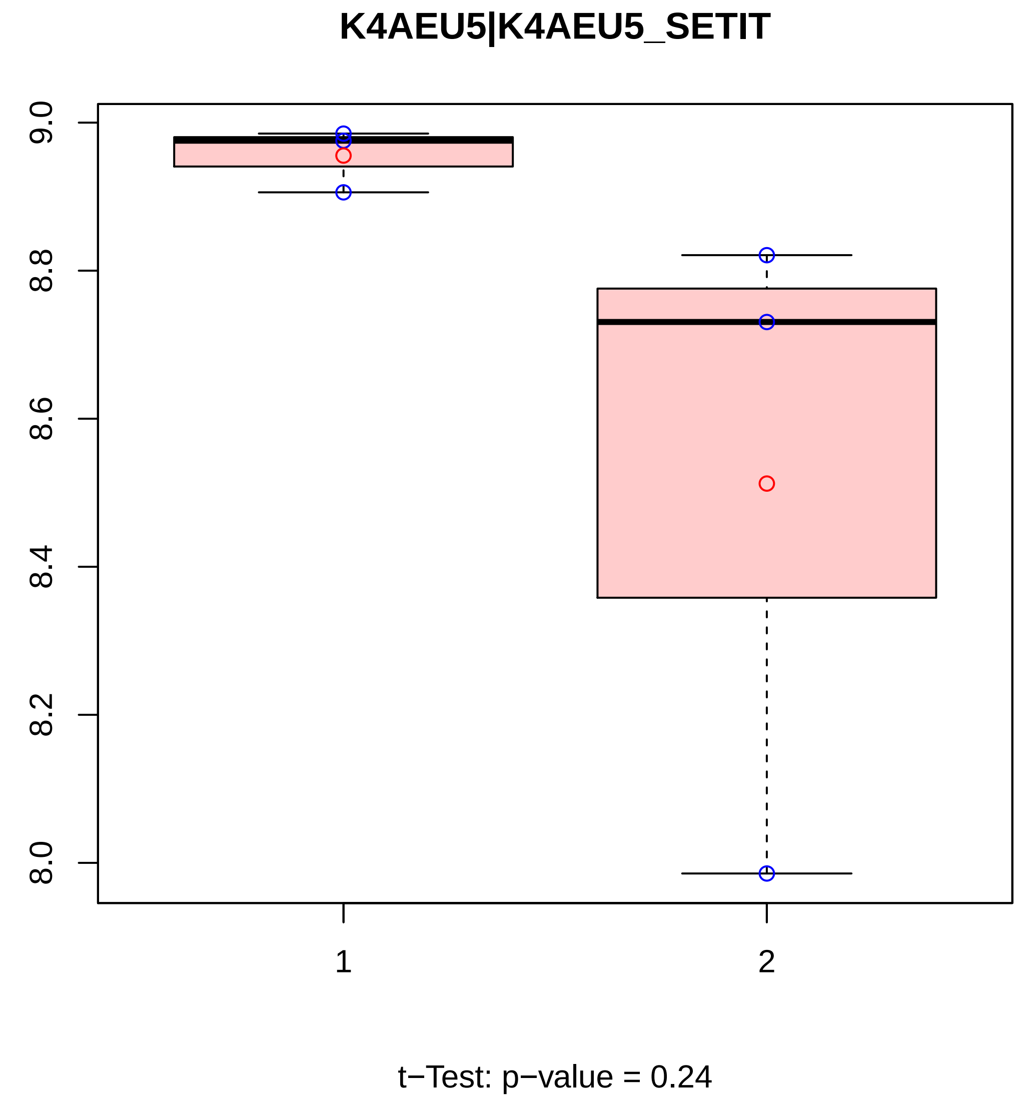
K4AEU5|K4AEU5_SETIT
9.0
8.8
8.6
8.4
8.2
8.0
1
2
t−Test: p−value = 0.24

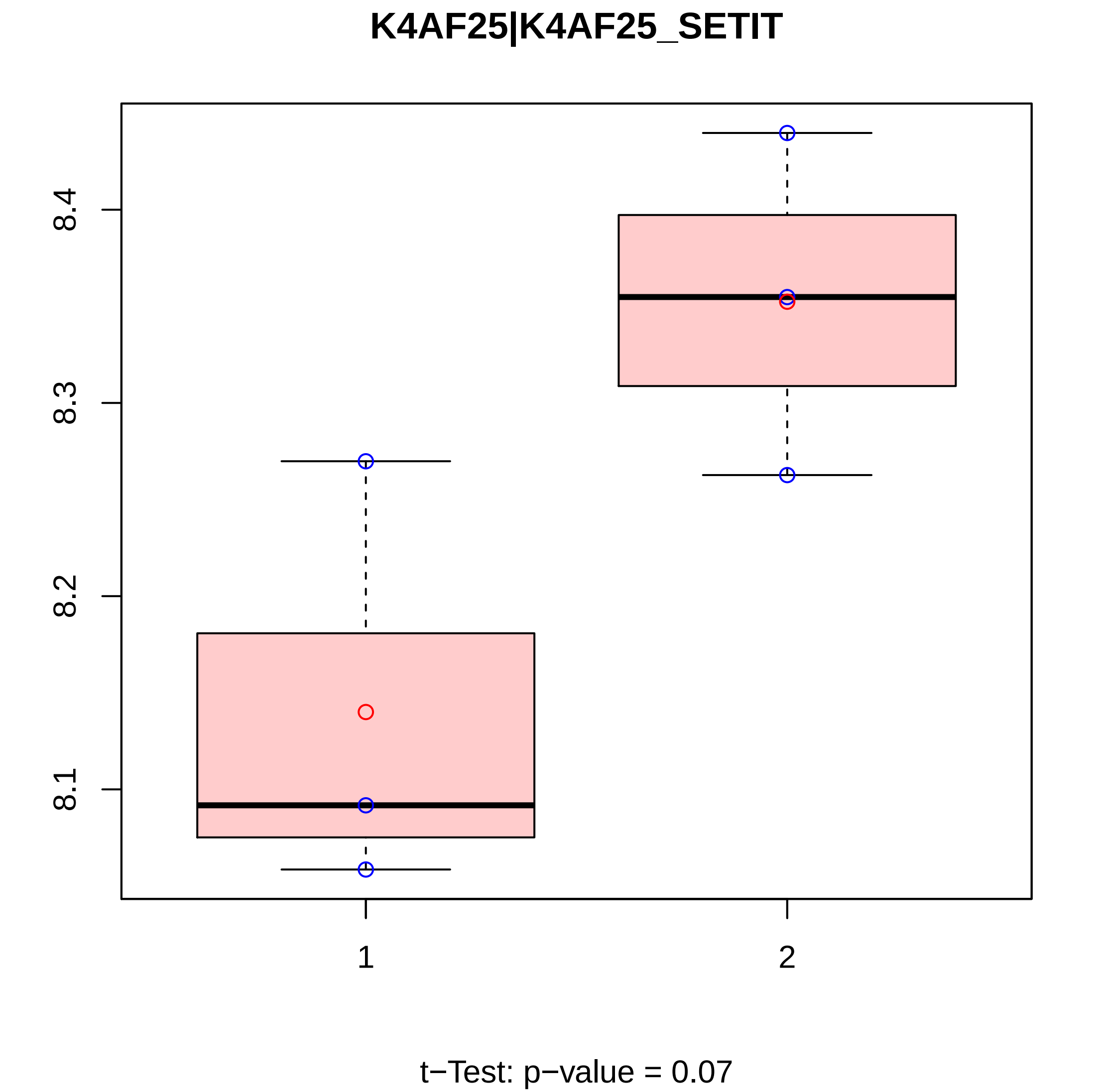
K4AF25|K4AF25_SETIT
8.1
8.2
8.3
8.4
1
2
t−Test: p−value = 0.07

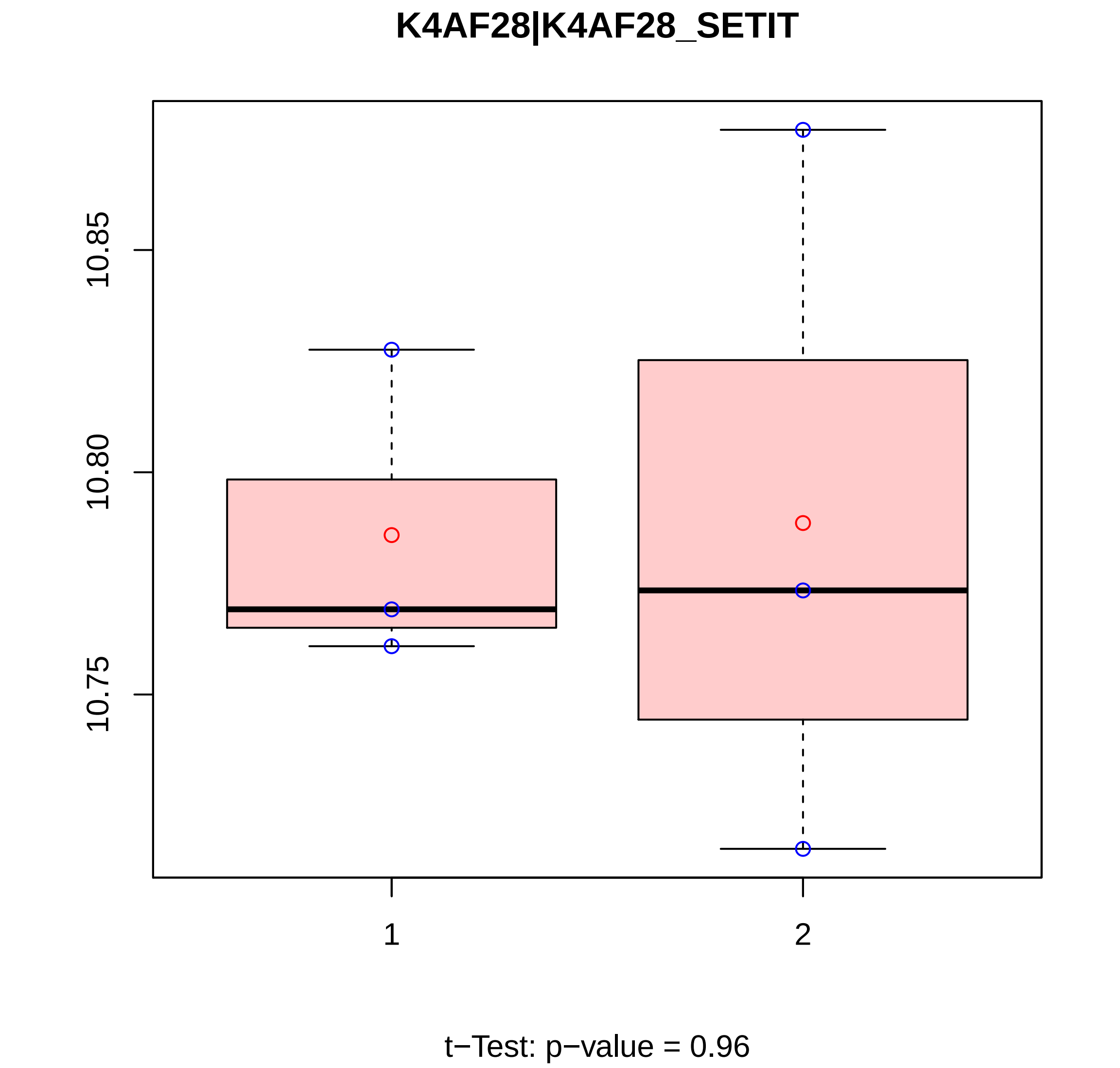
K4AF28|K4AF28_SETIT
10.75
10.80
10.85
1
2
t−Test: p−value = 0.96

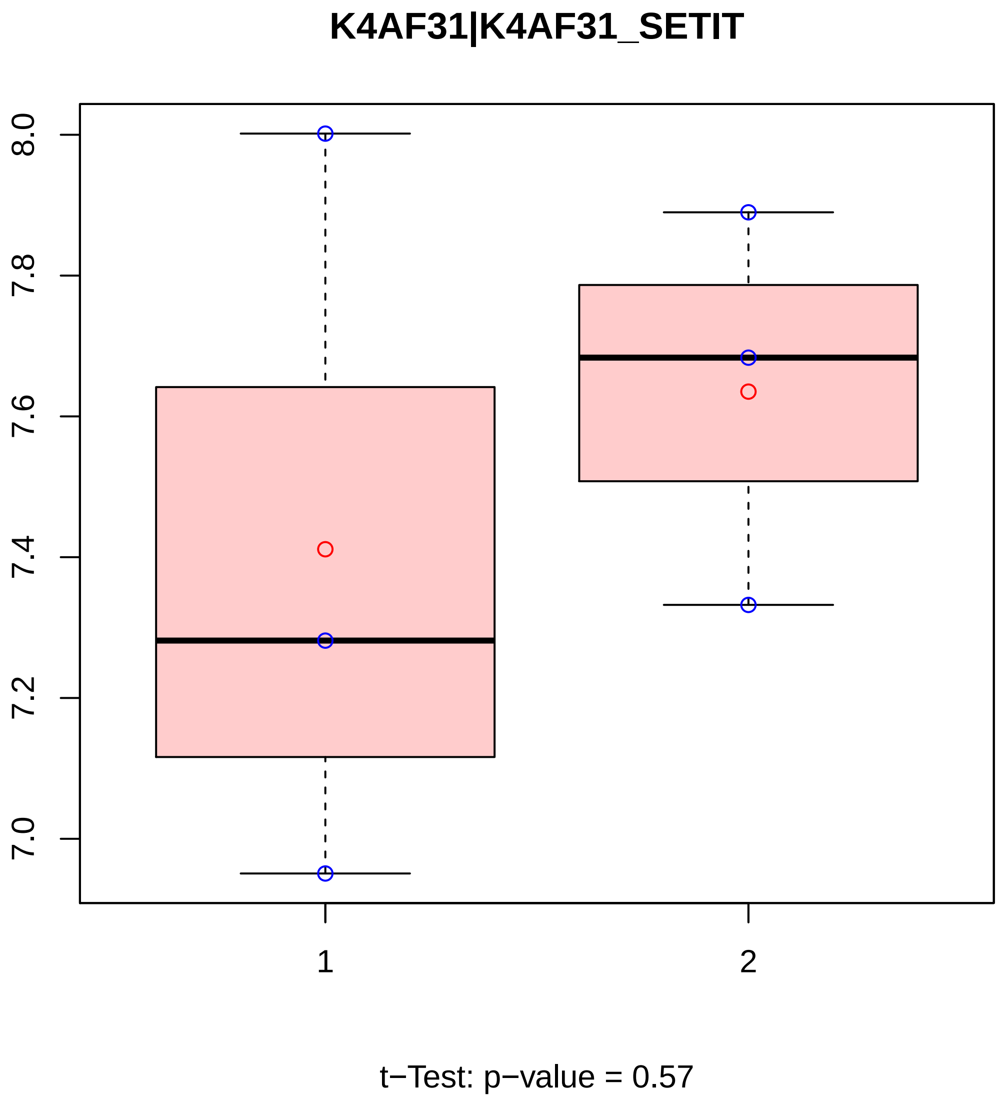
K4AF31|K4AF31_SETIT
8.0
7.8
7.6
7.4
7.2
7.0
1
2
t−Test: p−value = 0.57

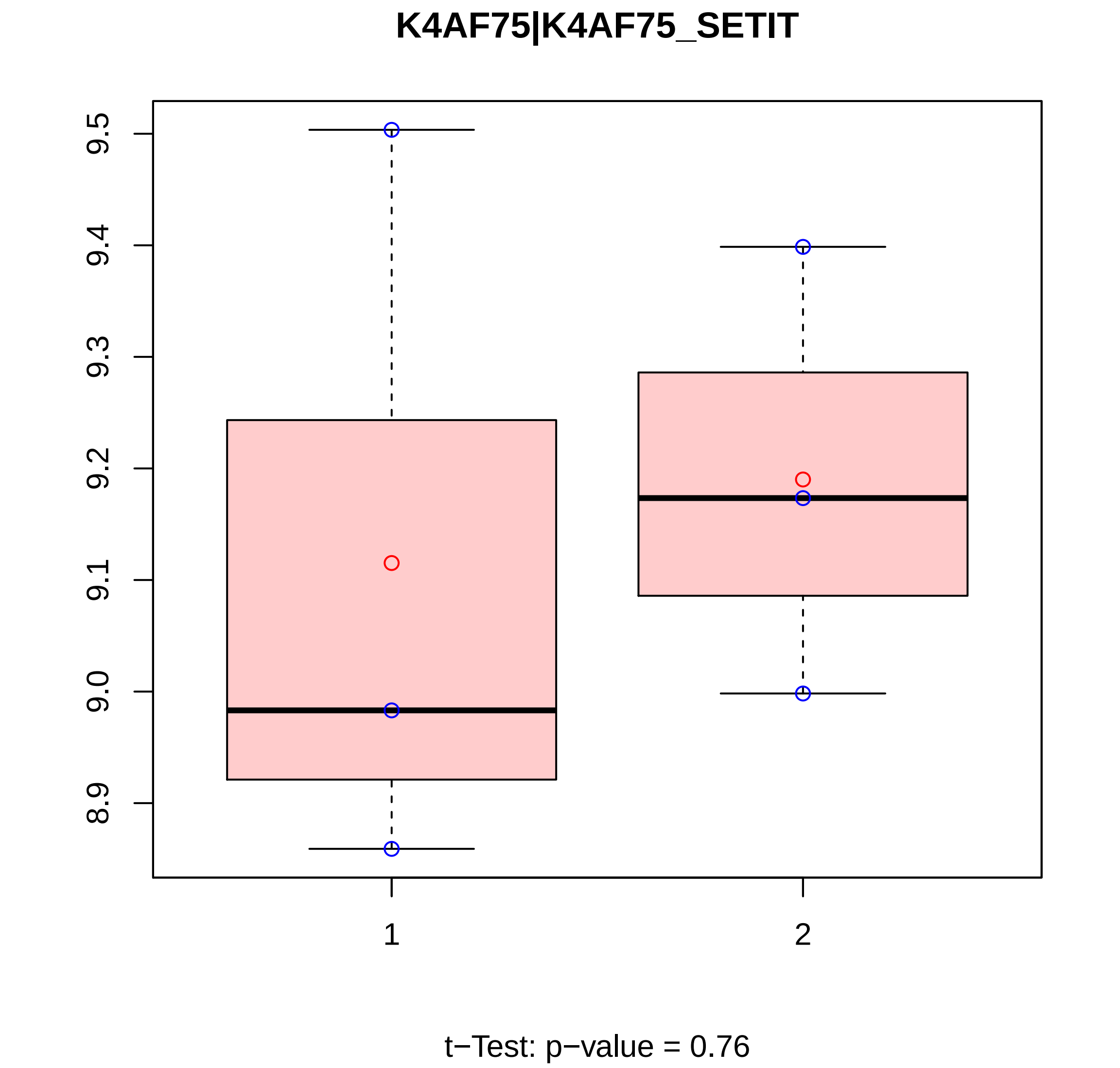
K4AF75|K4AF75_SETIT
9.5
9.4
9.3
9.2
9.1
9.0
8.9
1
2
t−Test: p−value = 0.76

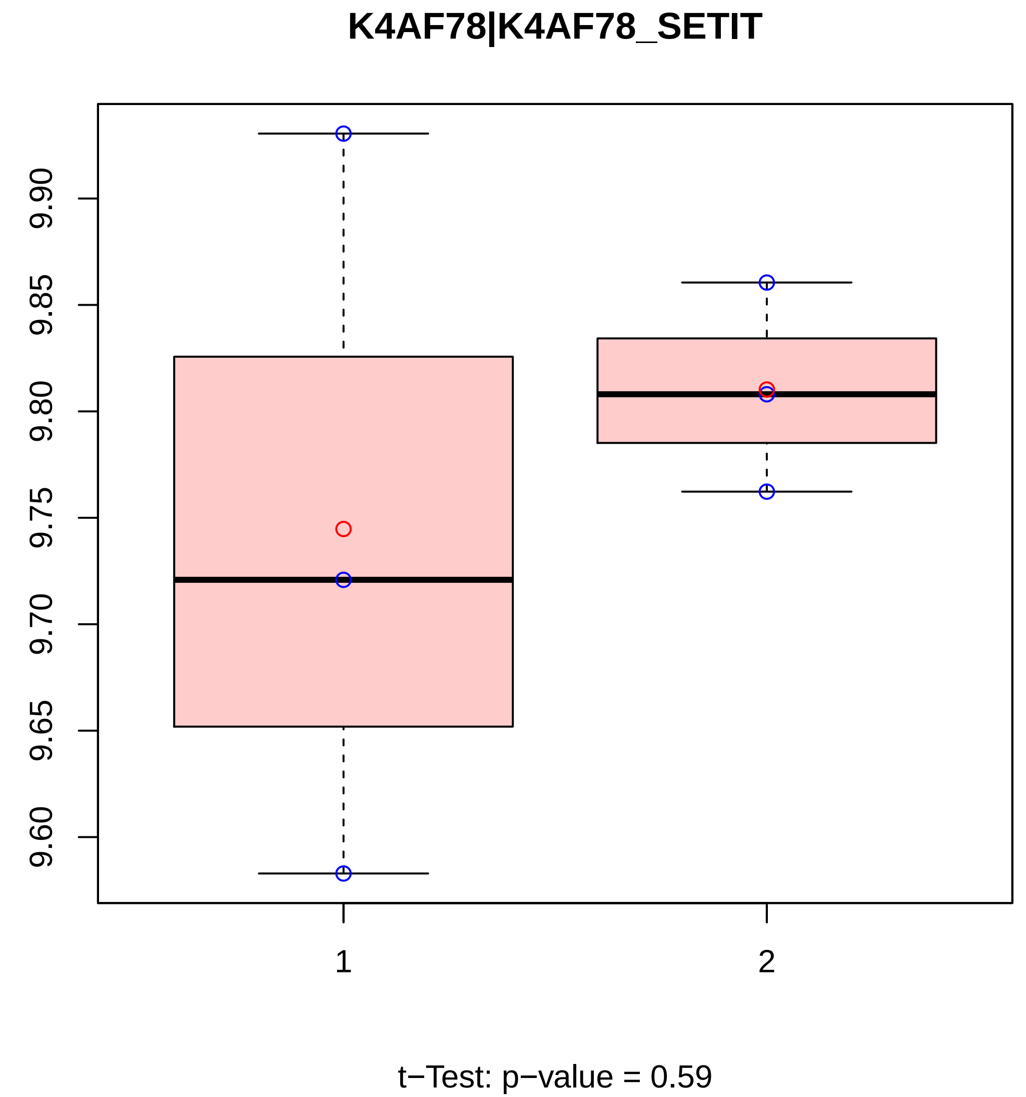
K4AF78|K4AF78_SETIT
9.60
9.65
9.70
9.75
9.80
9.85
9.90
1
2
t−Test: p−value = 0.59

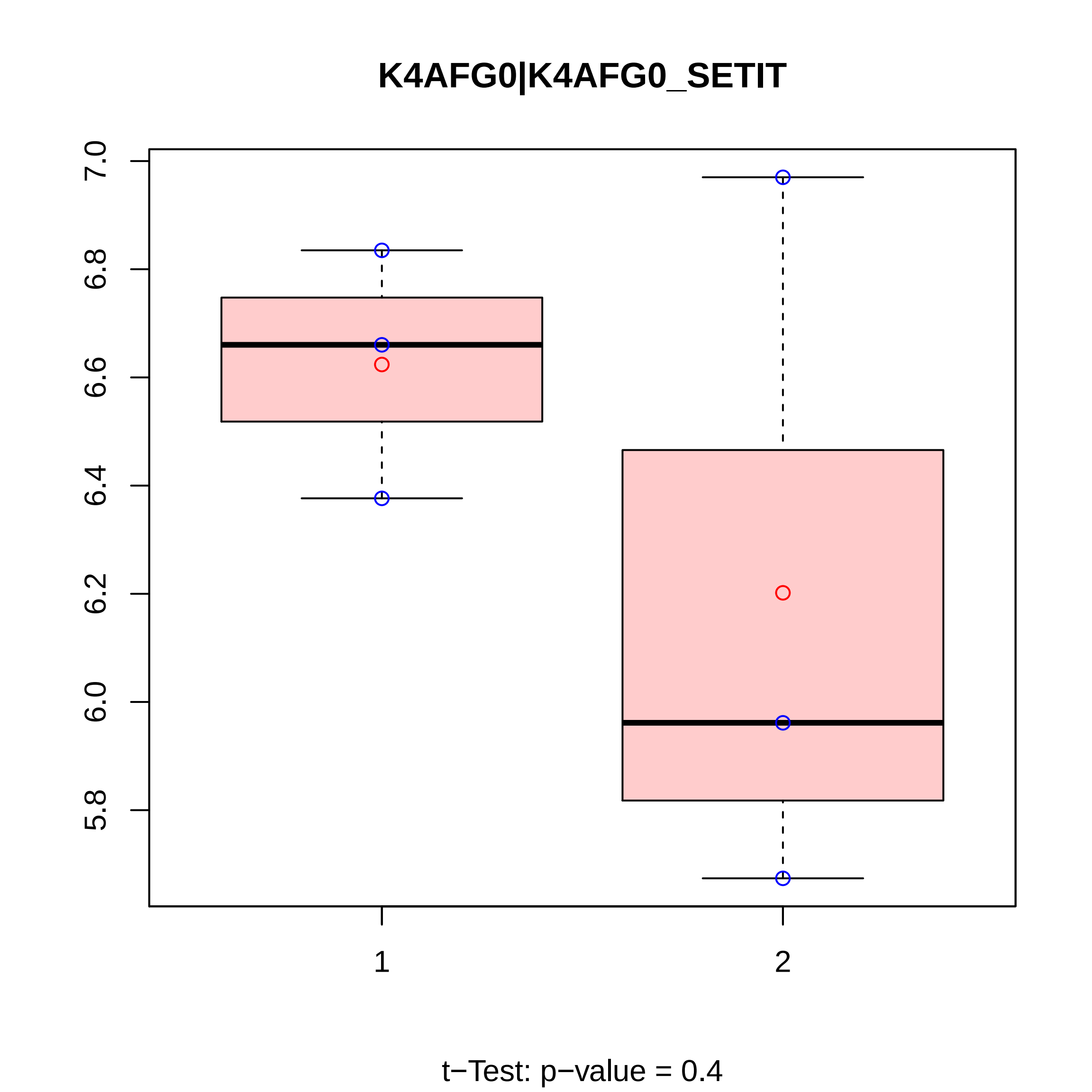

K4AFG0|K4AFG0_SETIT
7.0
6.8
6.6
6.4
6.2
6.0
5.8
1
2
t−Test: p−value = 0.4

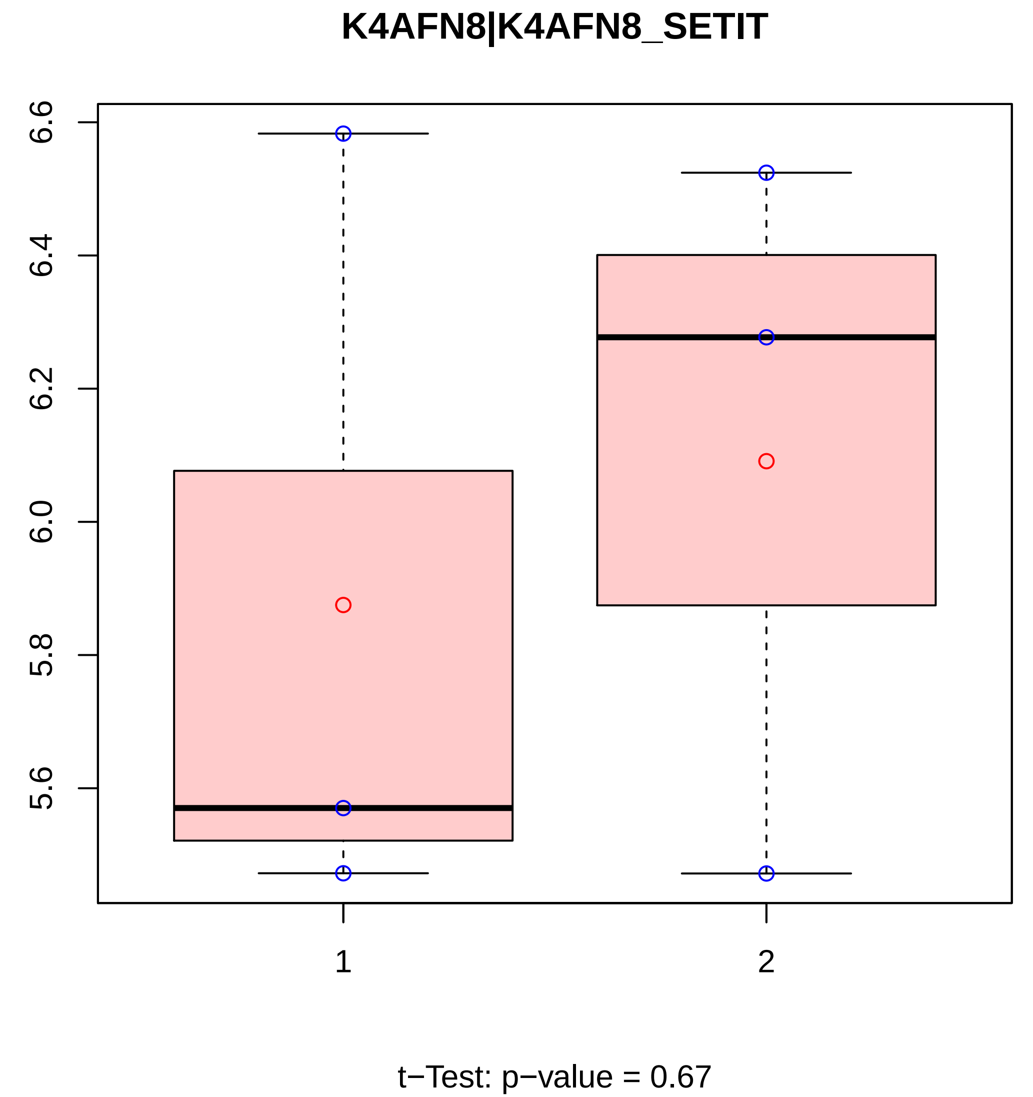
K4AFN8|K4AFN8_SETIT
6.6
6.4
6.2
6.0
5.8
5.6
1
2
t−Test: p−value = 0.67

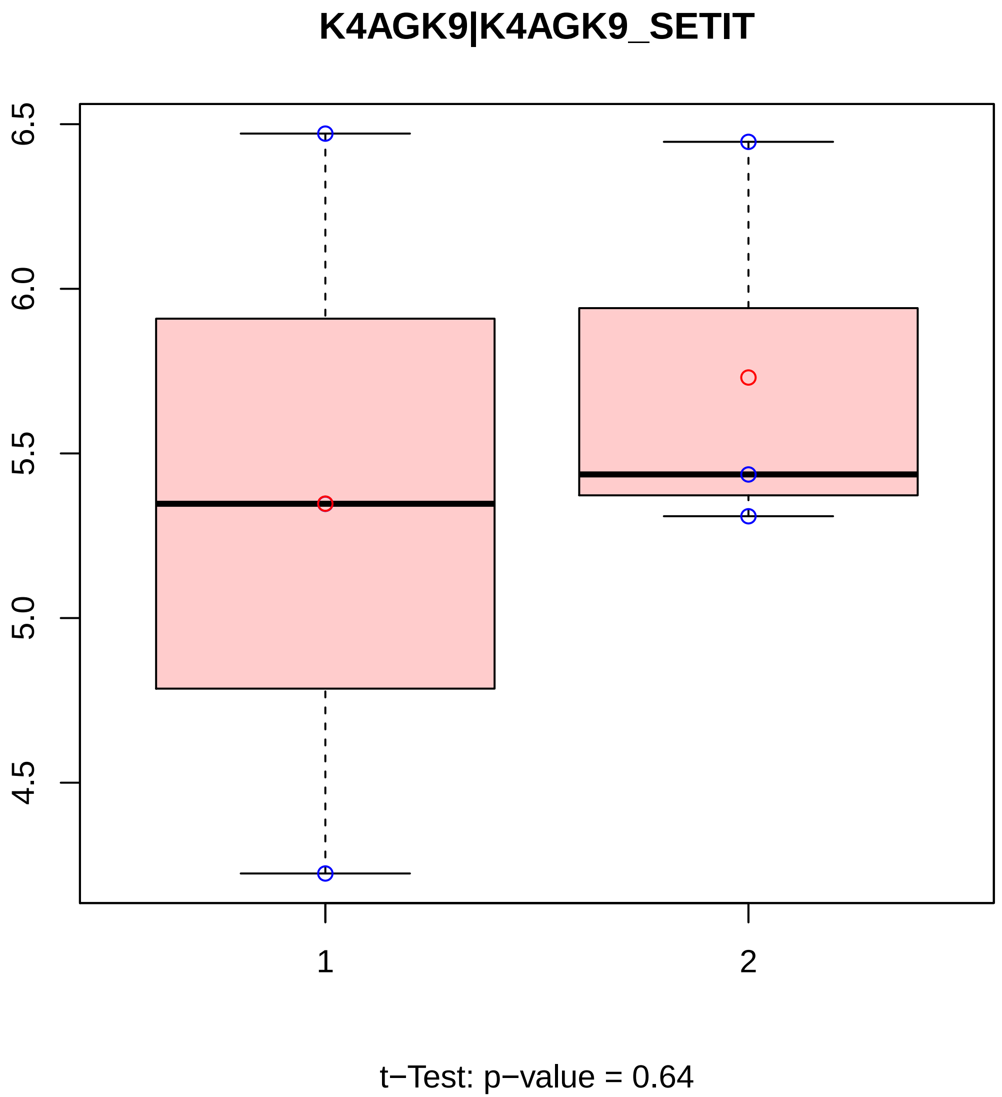

K4AGK9|K4AGK9_SETIT
6.5
6.0
5.5
5.0
4.5
1
2
t−Test: p−value = 0.64

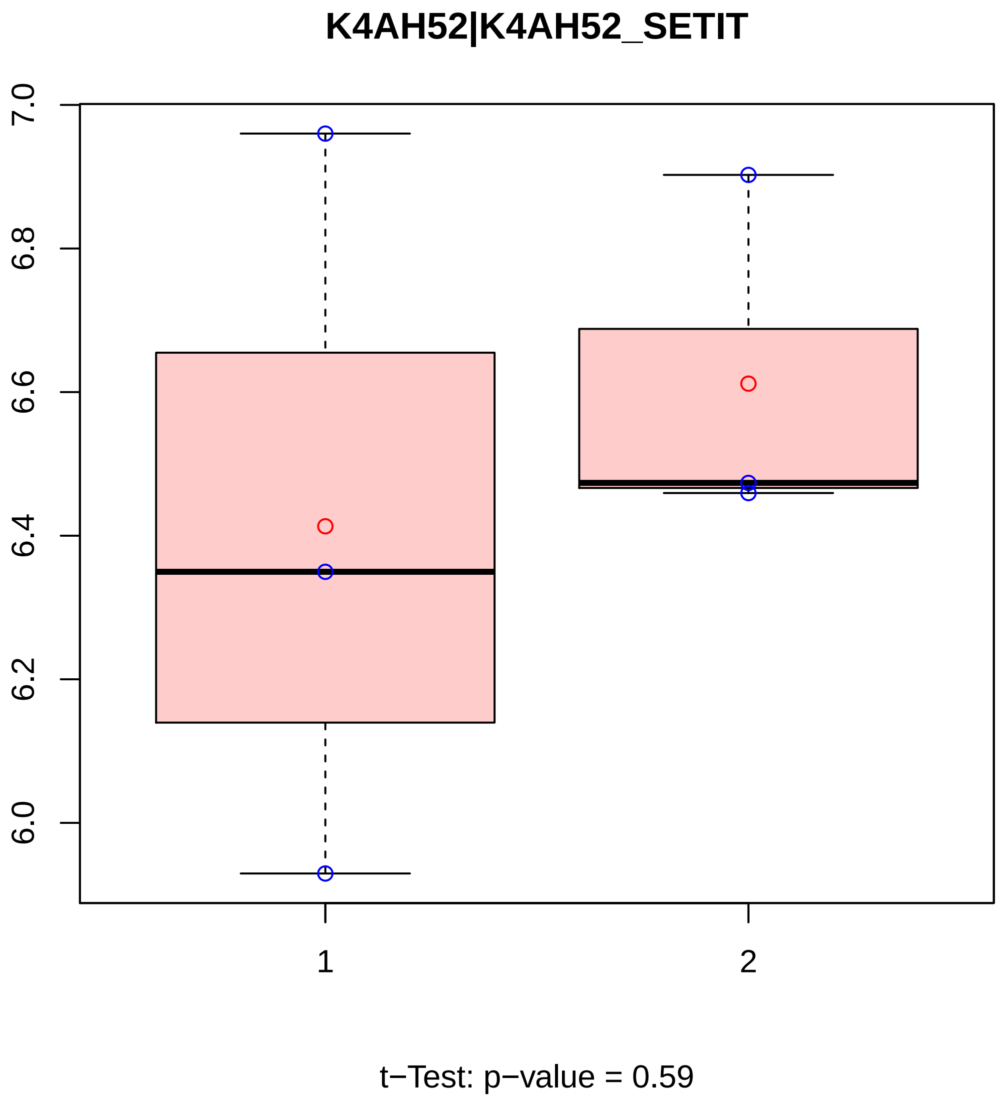
K4AH52|K4AH52_SETIT
7.0
6.8
6.6
6.4
6.2
6.0
1
2
t−Test: p−value = 0.59

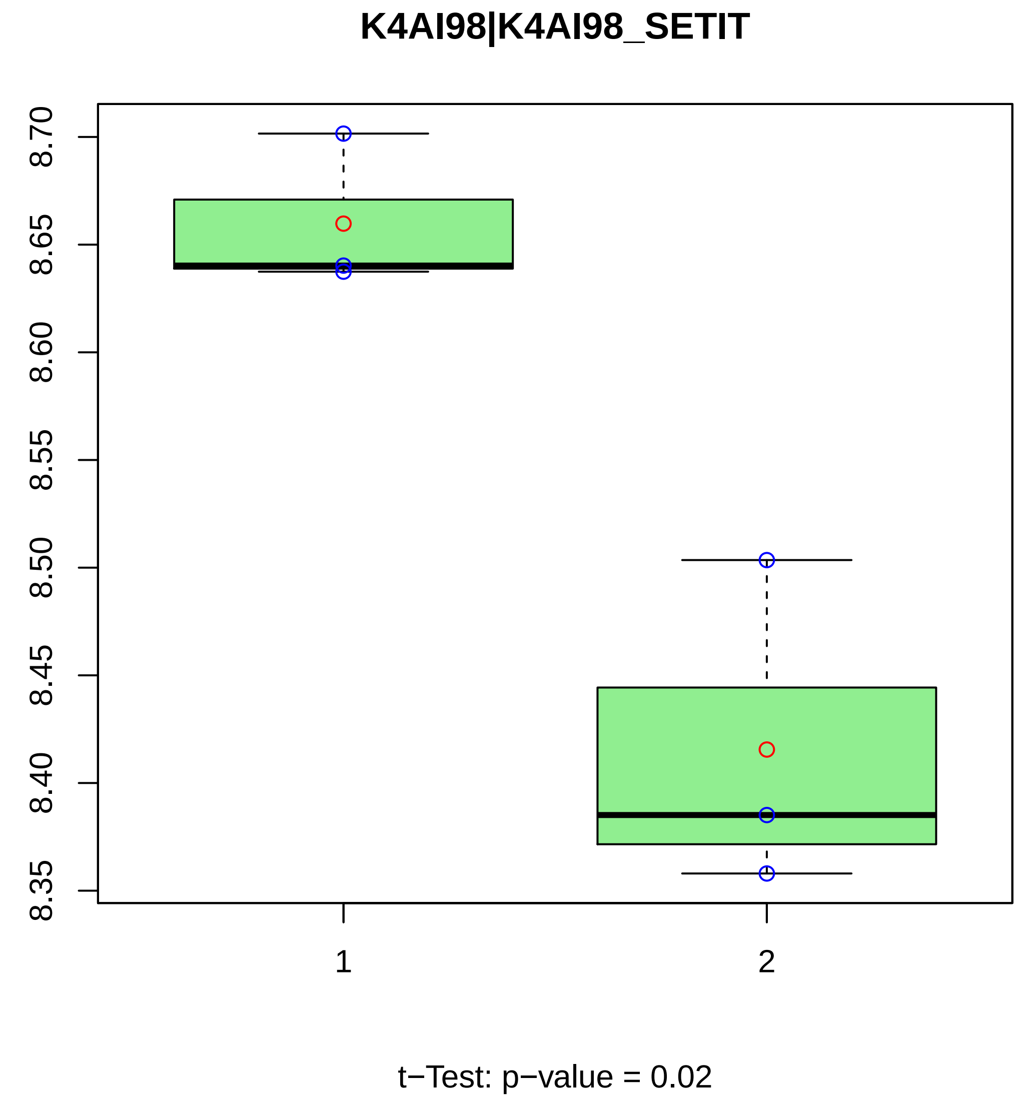
K4AI98|K4AI98_SETIT
8.35
8.40
8.45
8.50
8.55
8.60
8.65
8.70
1
2
t−Test: p−value = 0.02

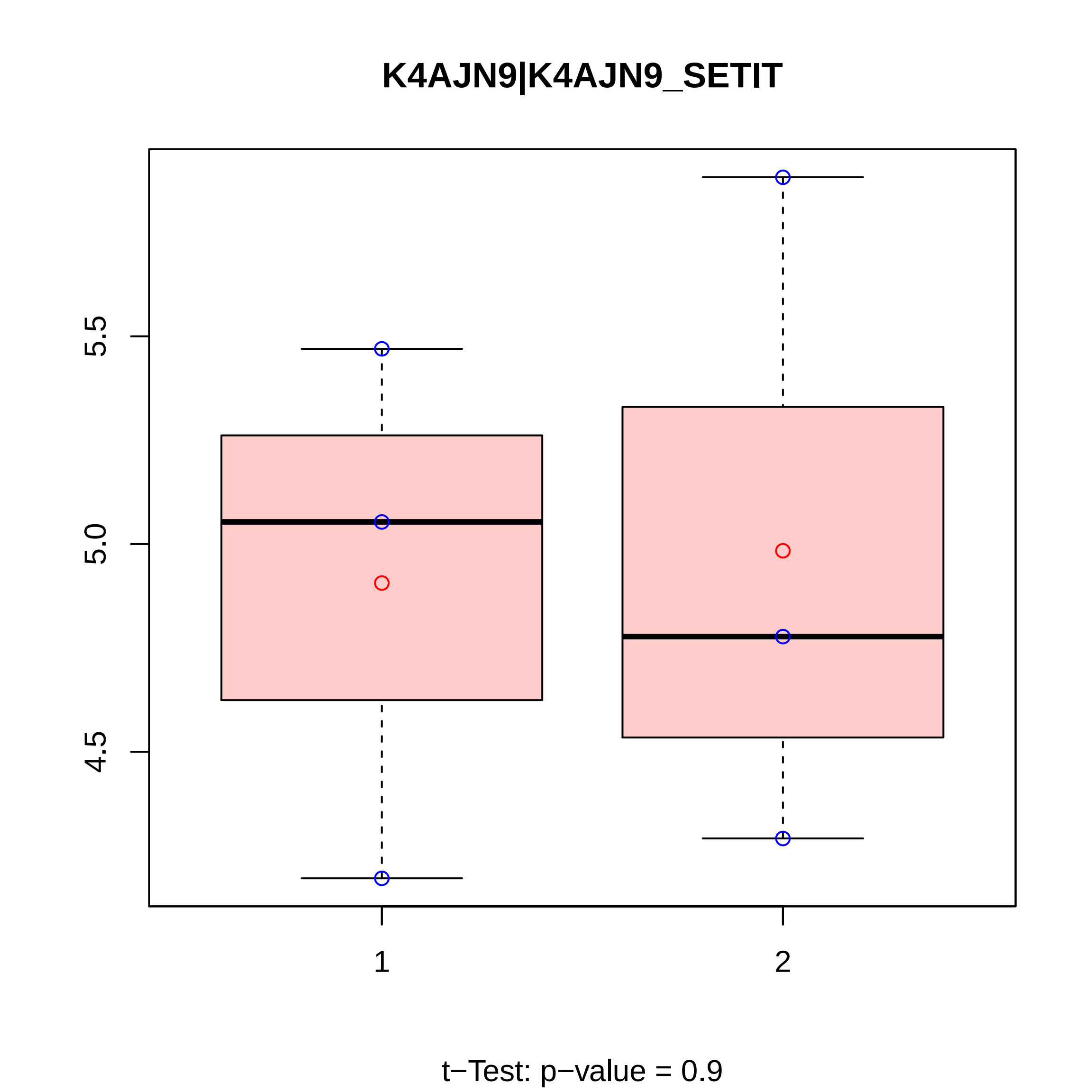

K4AJN9|K4AJN9_SETIT
5.5
5.0
4.5
1
2
t−Test: p−value = 0.9

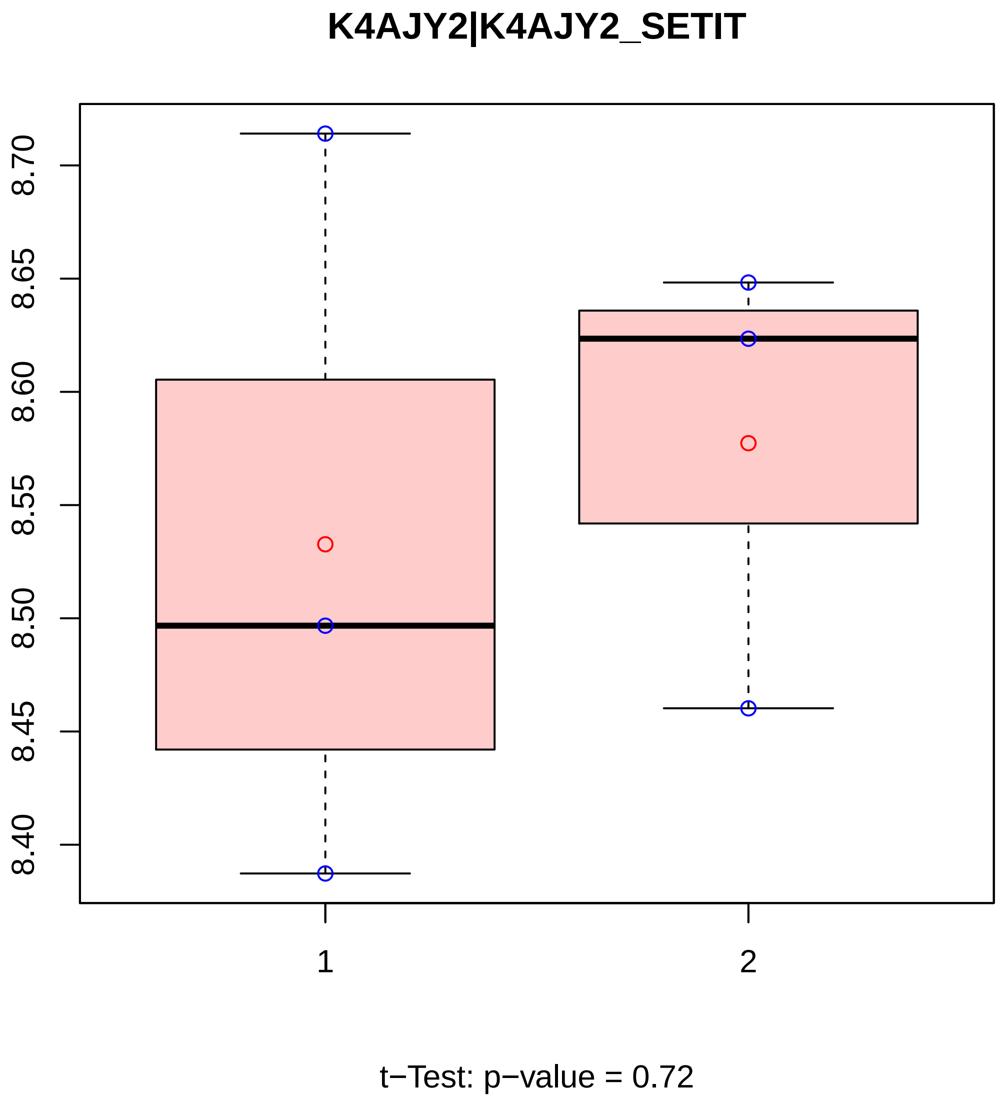

K4AJY2|K4AJY2_SETIT
8.40
8.45
8.50
8.55
8.60
8.65
8.70
1
2
t−Test: p−value = 0.72

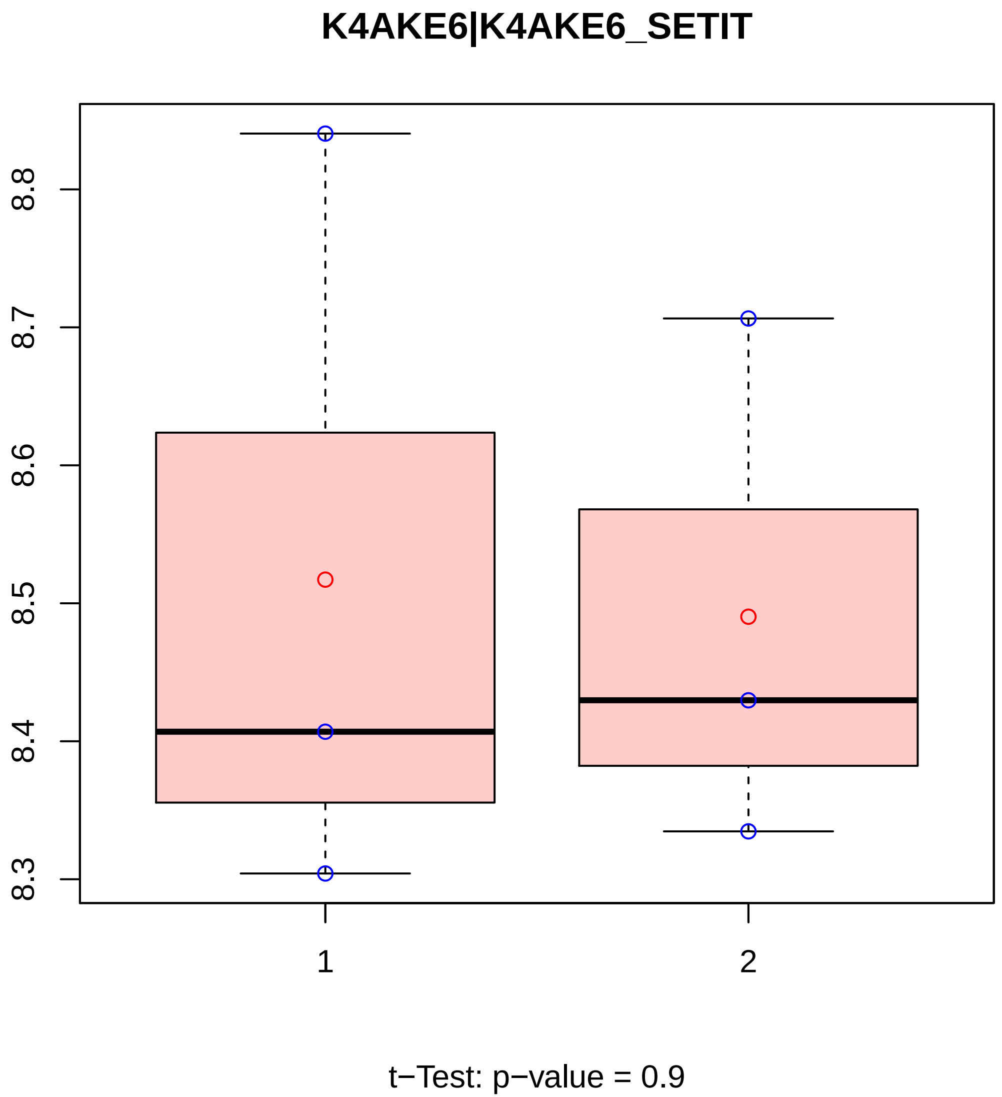

K4AKE6|K4AKE6_SETIT
8.3
8.4
8.5
8.6
8.7
8.8
1
2
t−Test: p−value = 0.9

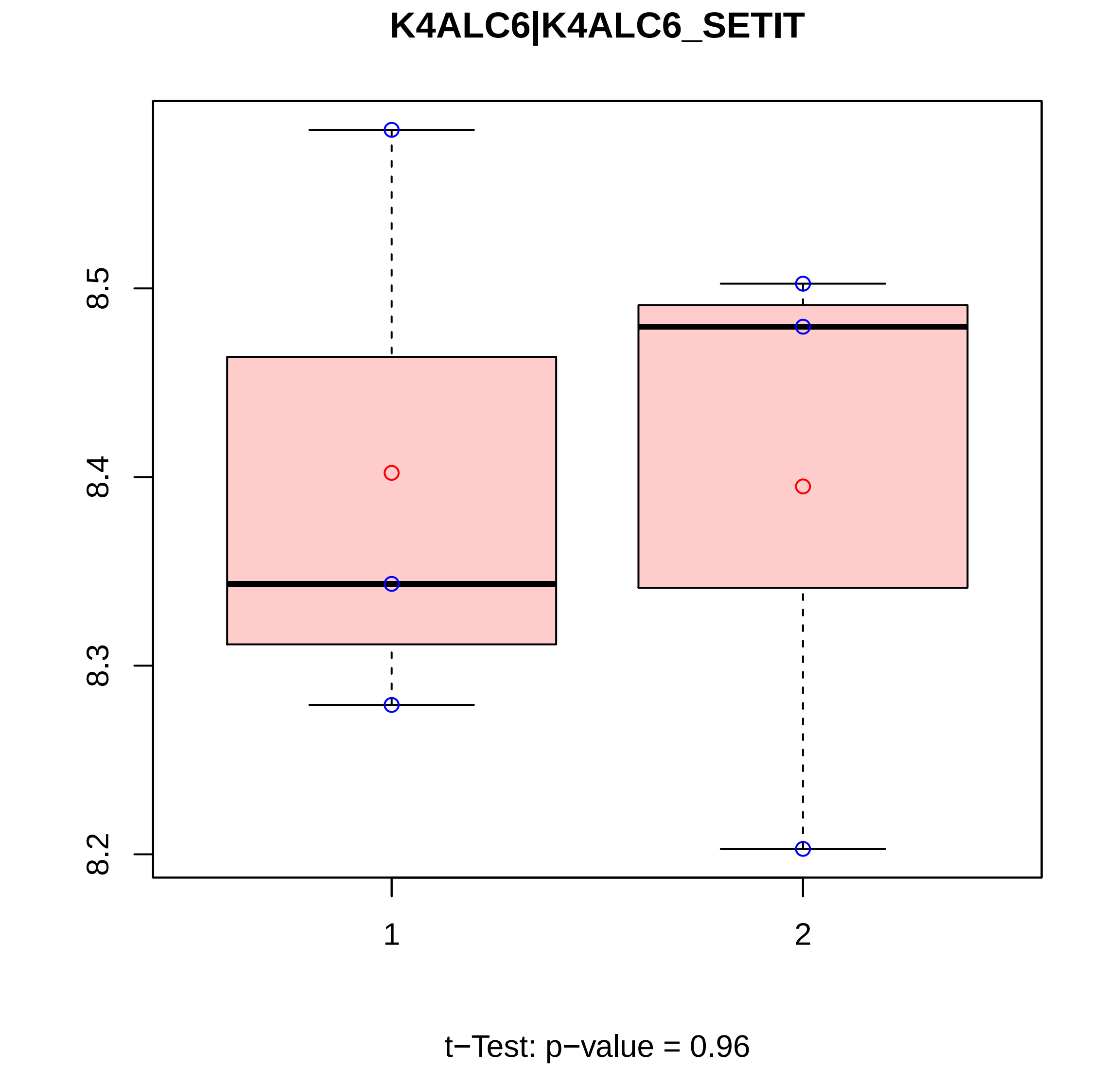
K4ALC6|K4ALC6_SETIT
8.2
8.3
8.4
8.5
1
2
t−Test: p−value = 0.96

K4ALQ0|K4ALQ0_SETIT

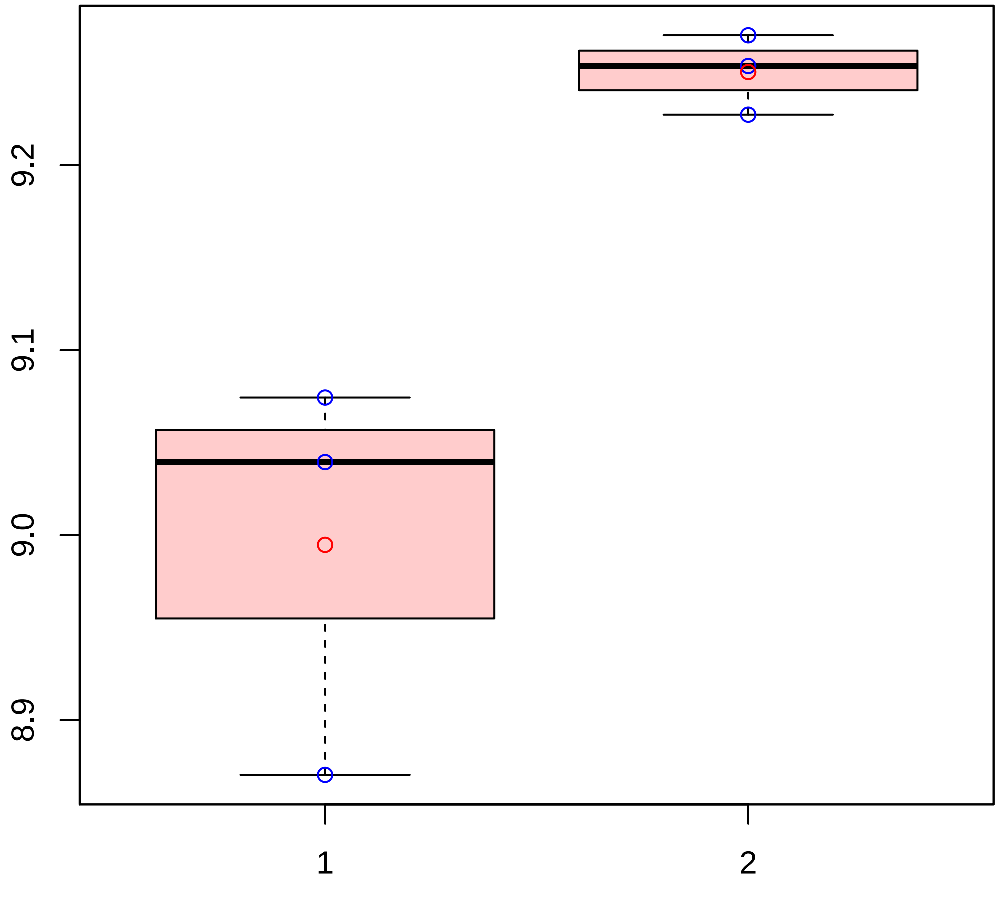

t−Test: p−value = 0.05

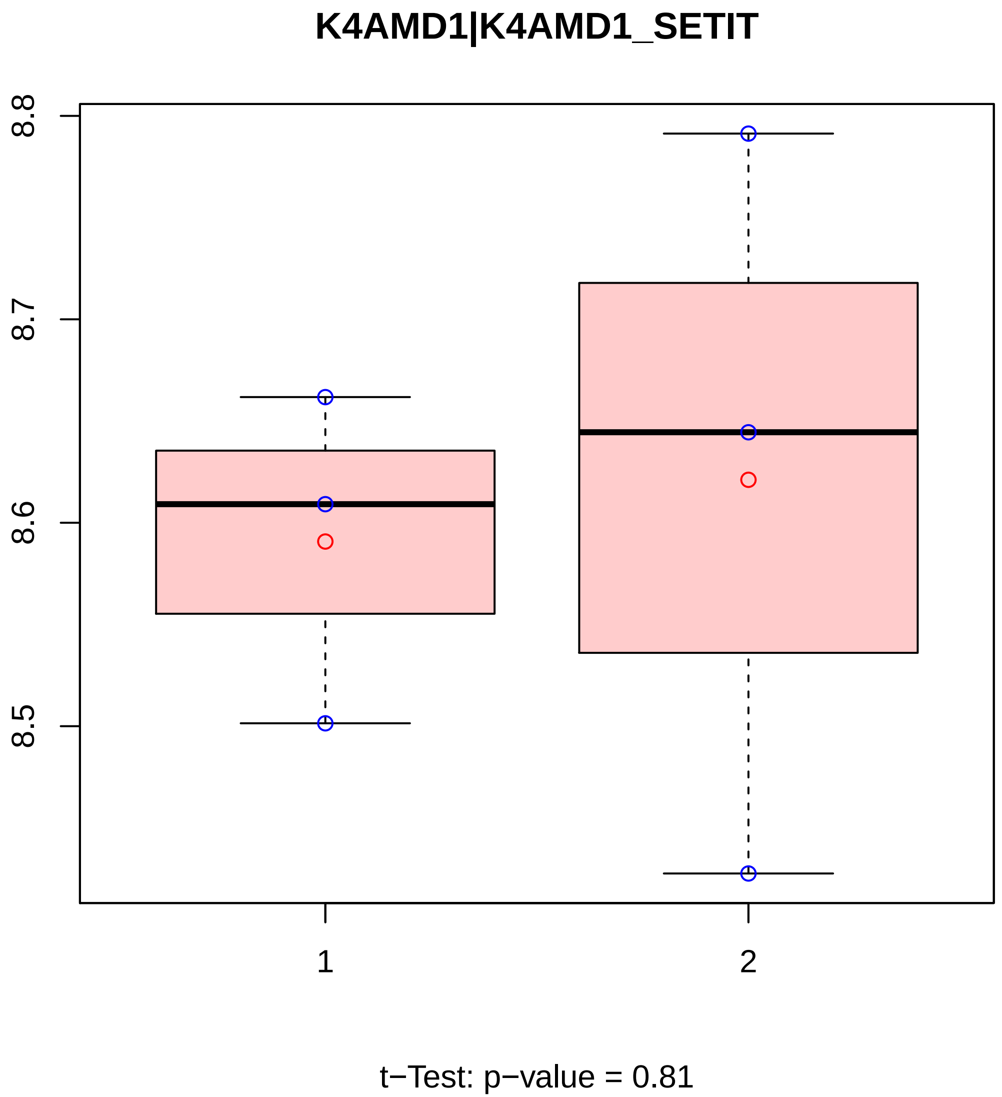

K4AMD1|K4AMD1_SETIT
8.8
8.7
8.6
8.5
1
2
t−Test: p−value = 0.81

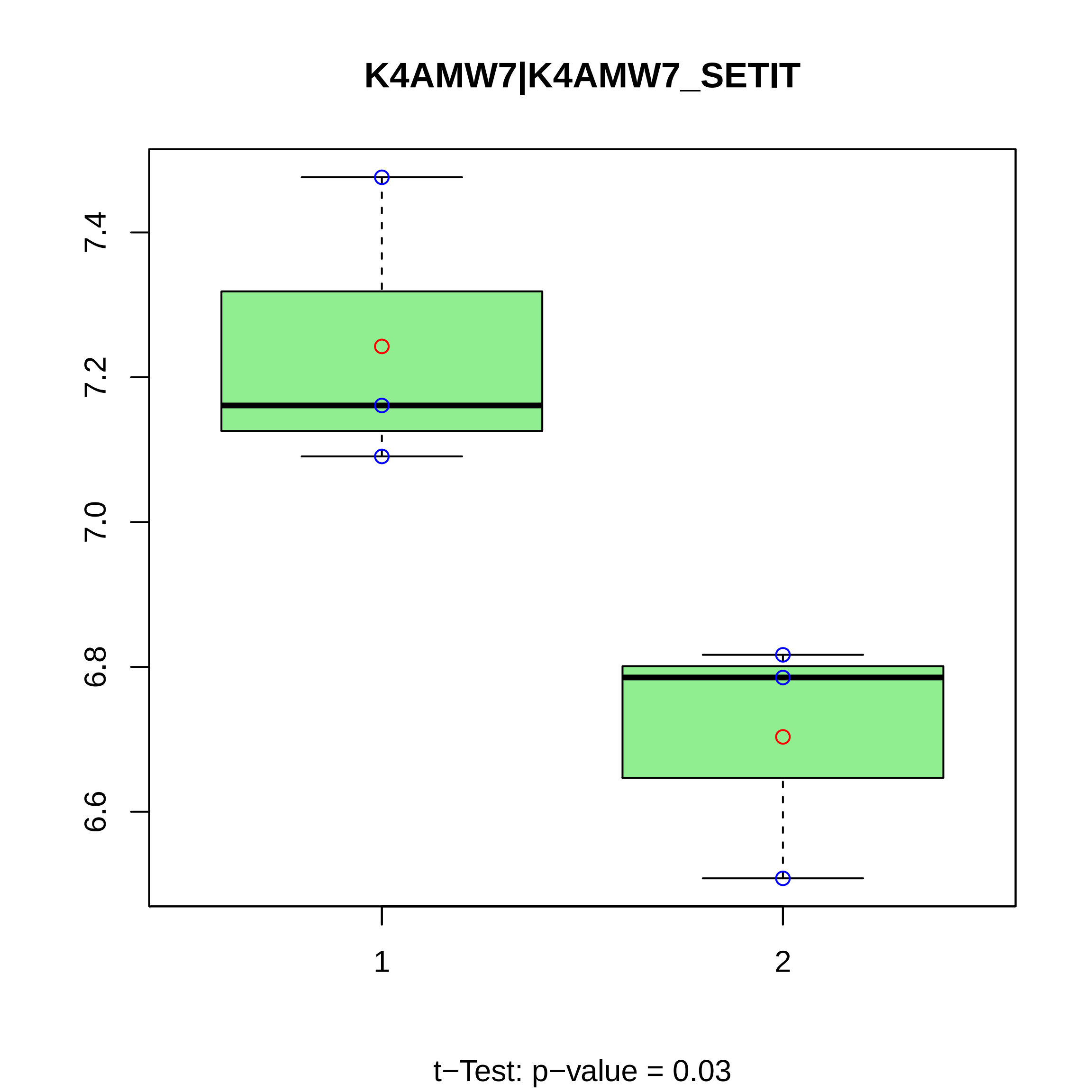
K4AMW7|K4AMW7_SETIT
7.4
7.2
7.0
6.8
6.6
1
2
t−Test: p−value = 0.03

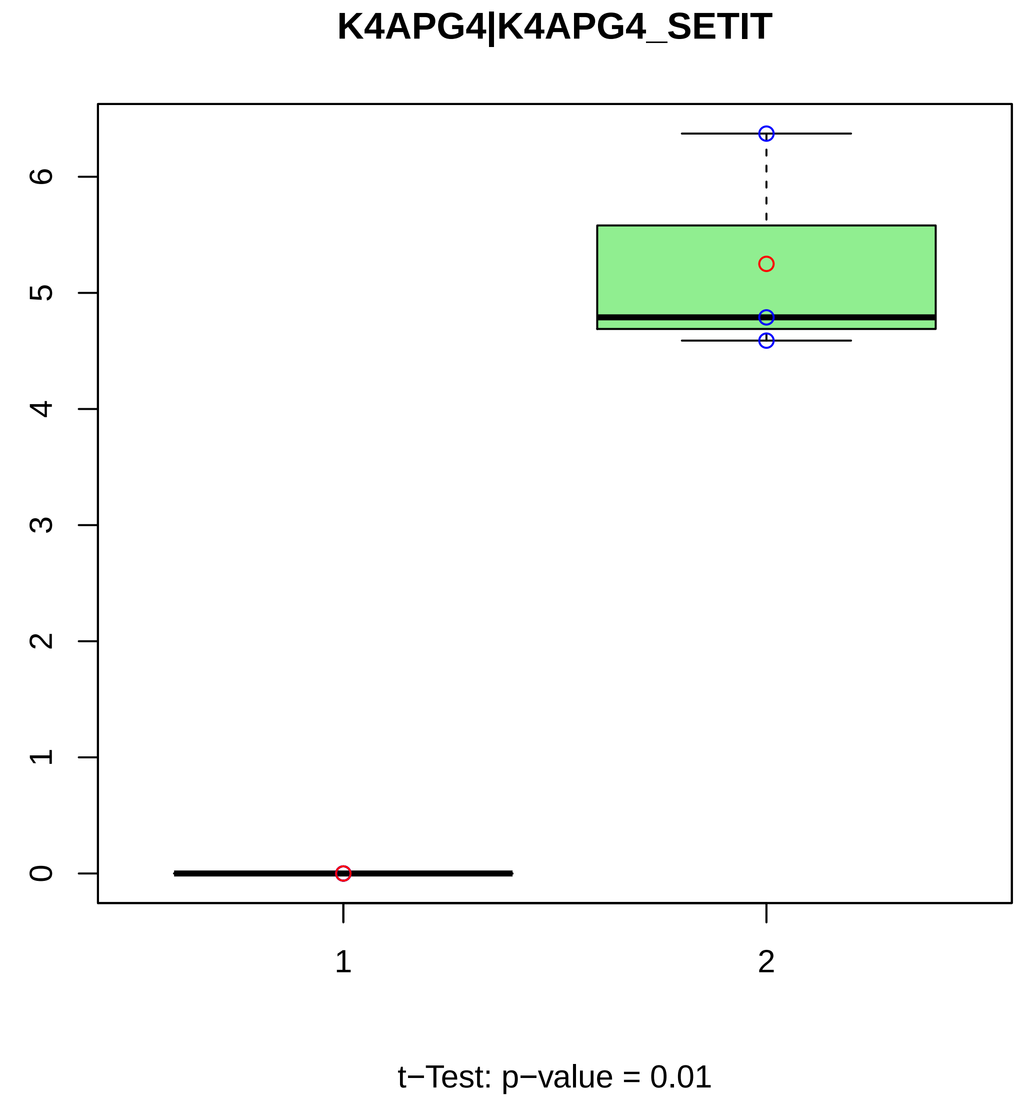
K4APG4|K4APG4_SETIT
0
1
2
3
4
5
6
1
2
t−Test: p−value = 0.01

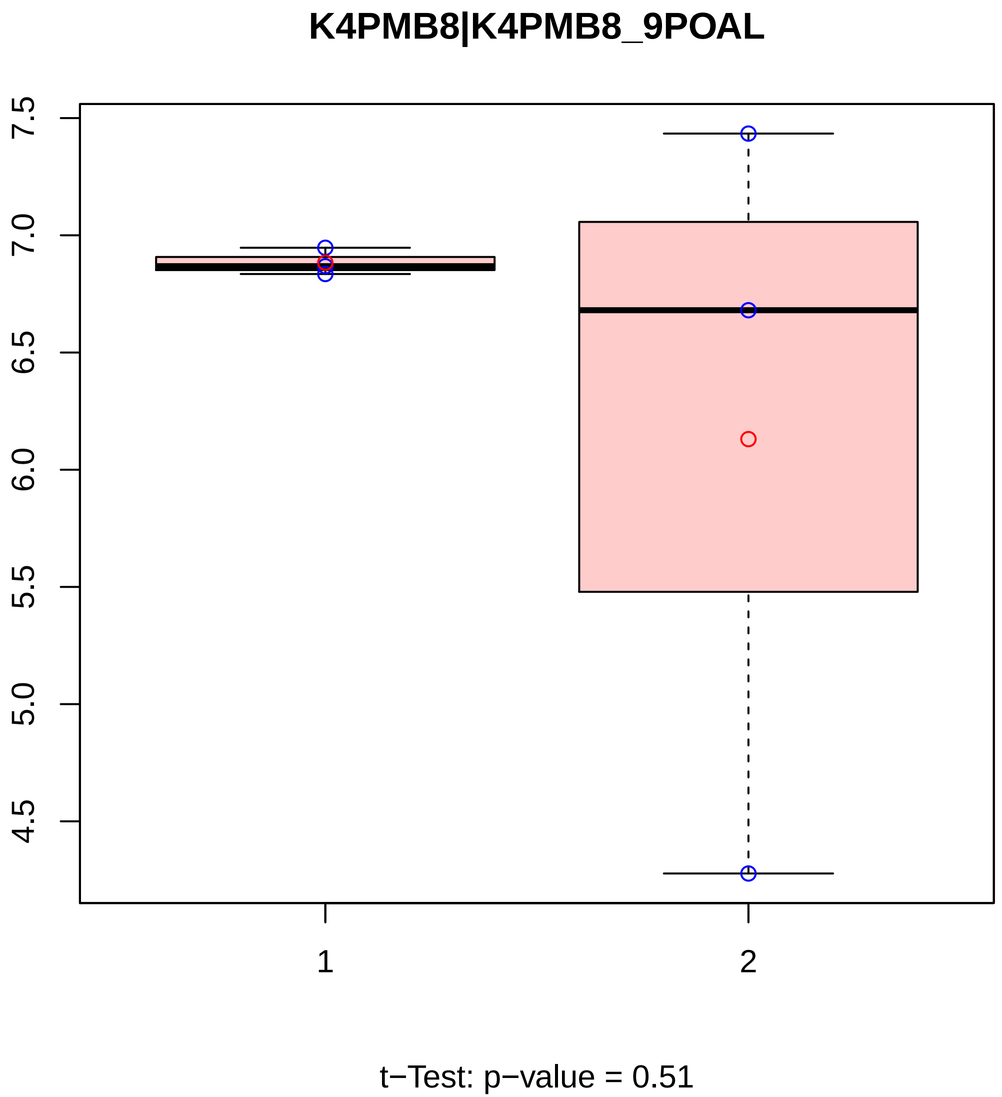
K4PMB8|K4PMB8_9POAL
7.5
7.0
6.5
6.0
5.5
5.0
4.5
1
2
t−Test: p−value = 0.51

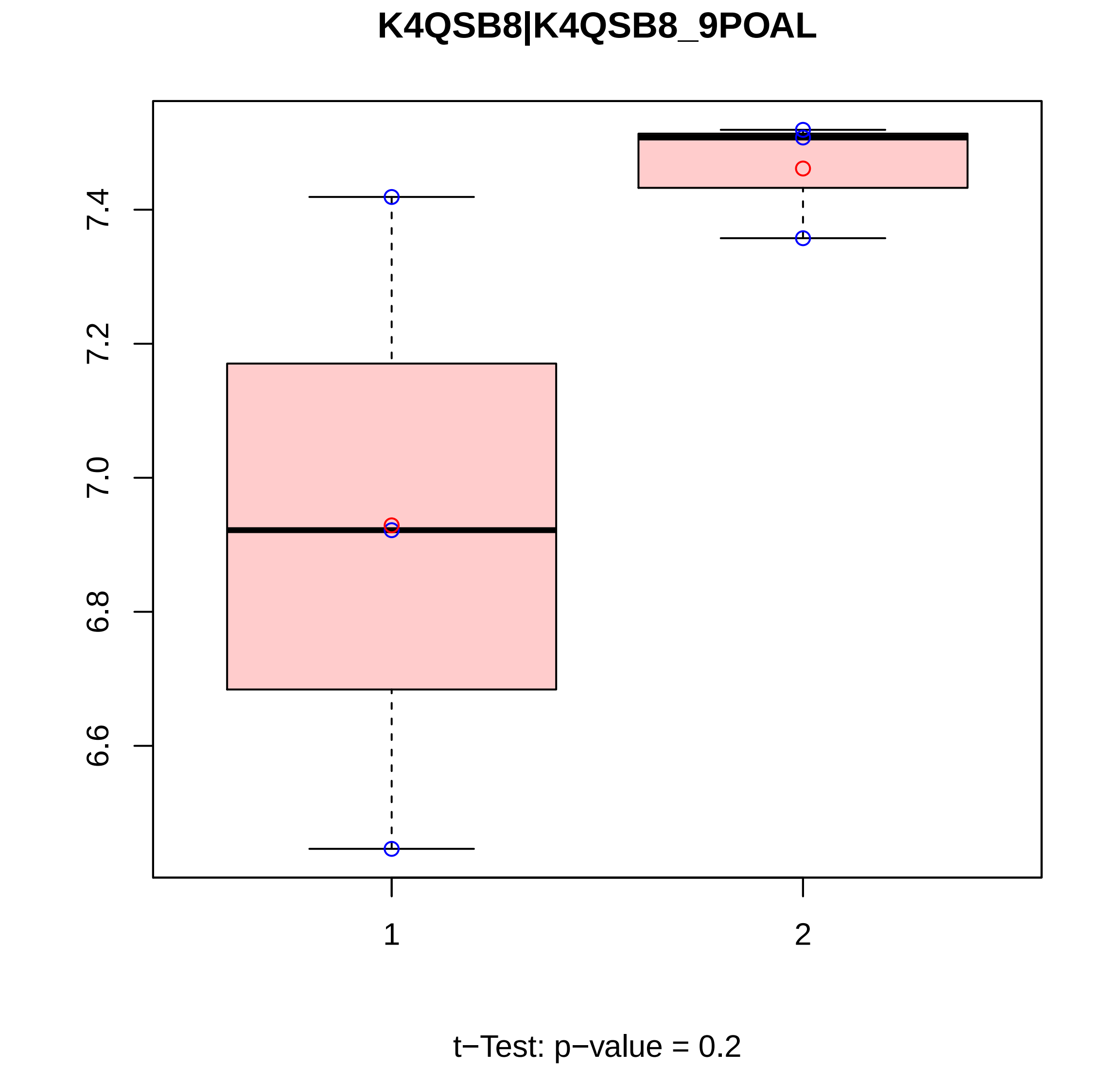
K4QSB8|K4QSB8_9POAL
7.4
7.2
7.0
6.8
6.6
1
2
t−Test: p−value = 0.2

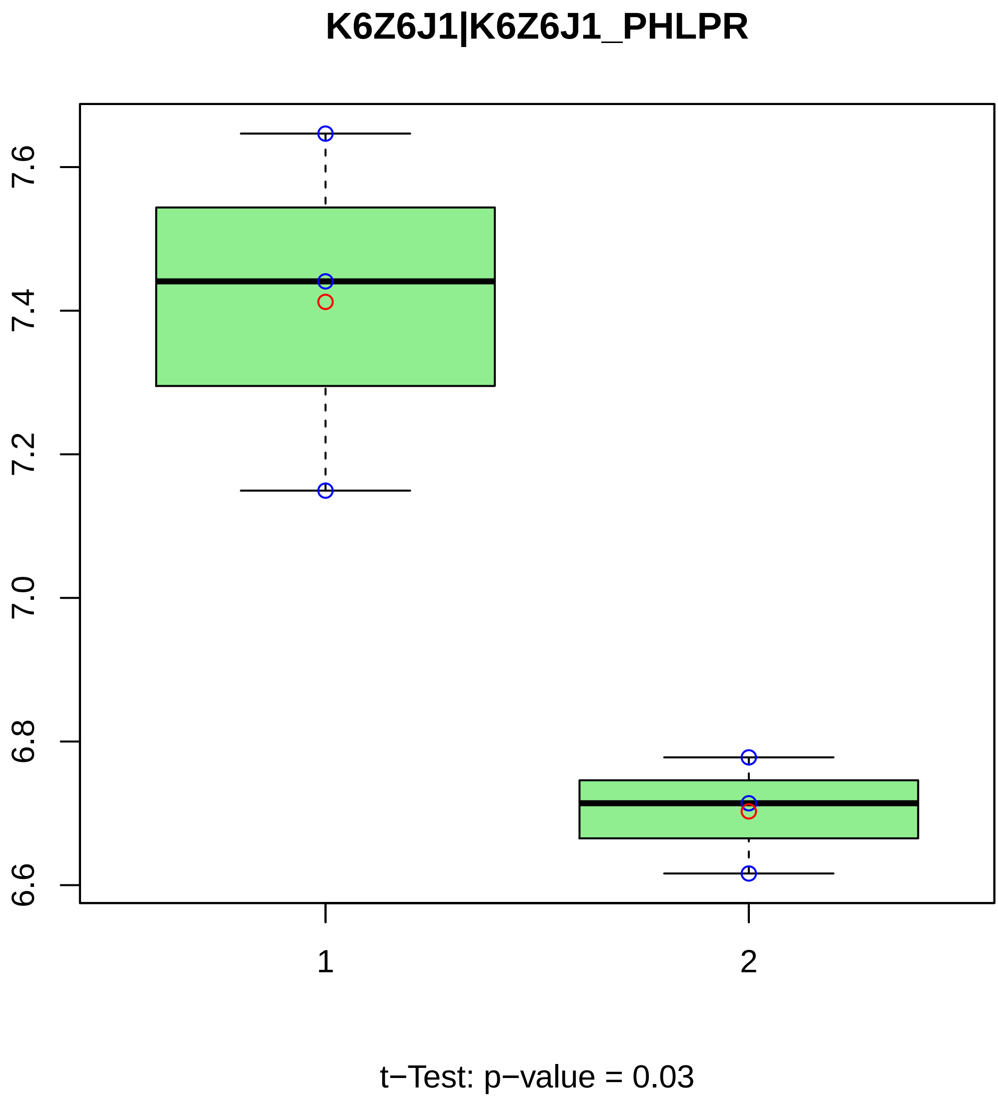

K6Z6J1|K6Z6J1_PHLPR
7.6
7.4
7.2
7.0
6.8
6.6
1
2
t−Test: p−value = 0.03

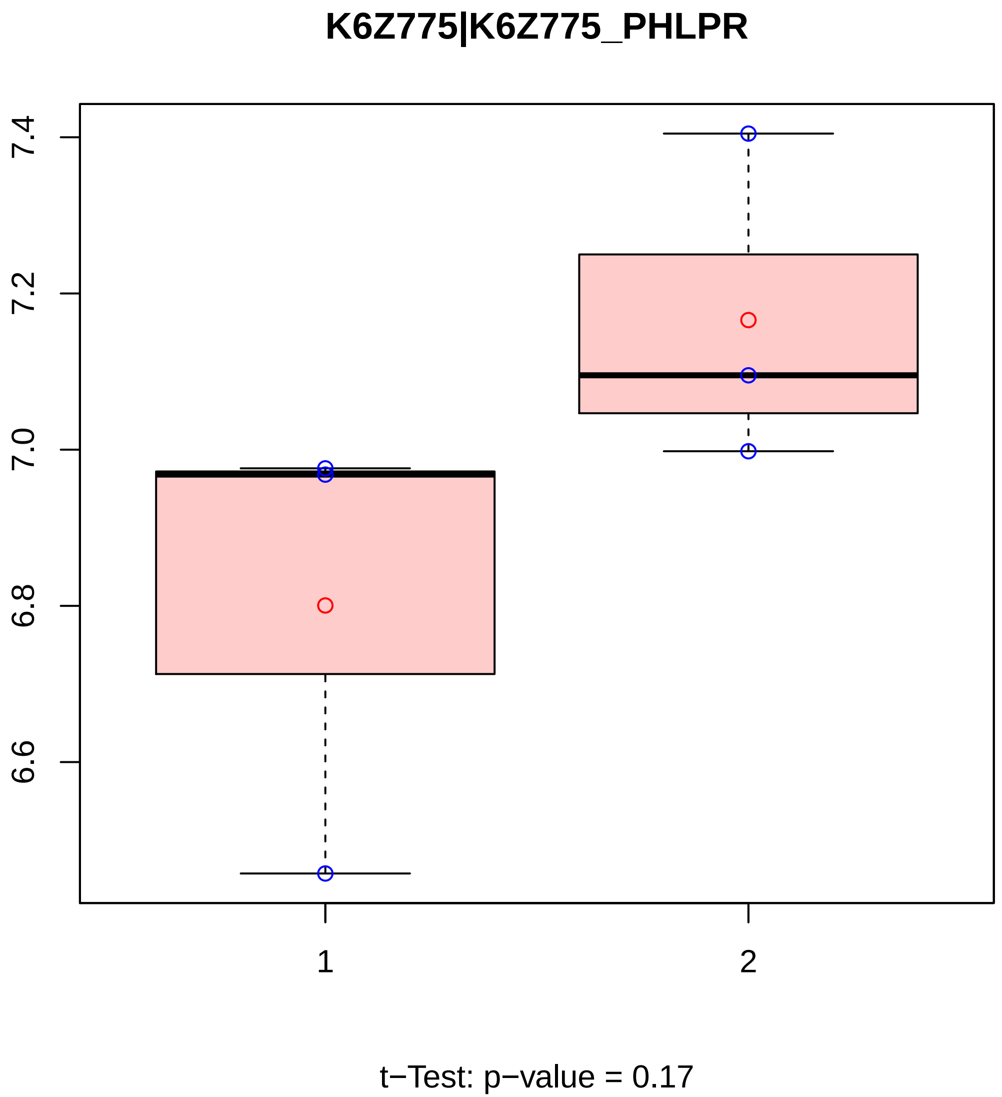
K6Z775|K6Z775_PHLPR
7.4
7.2
7.0
6.8
6.6
1
2
t−Test: p−value = 0.17

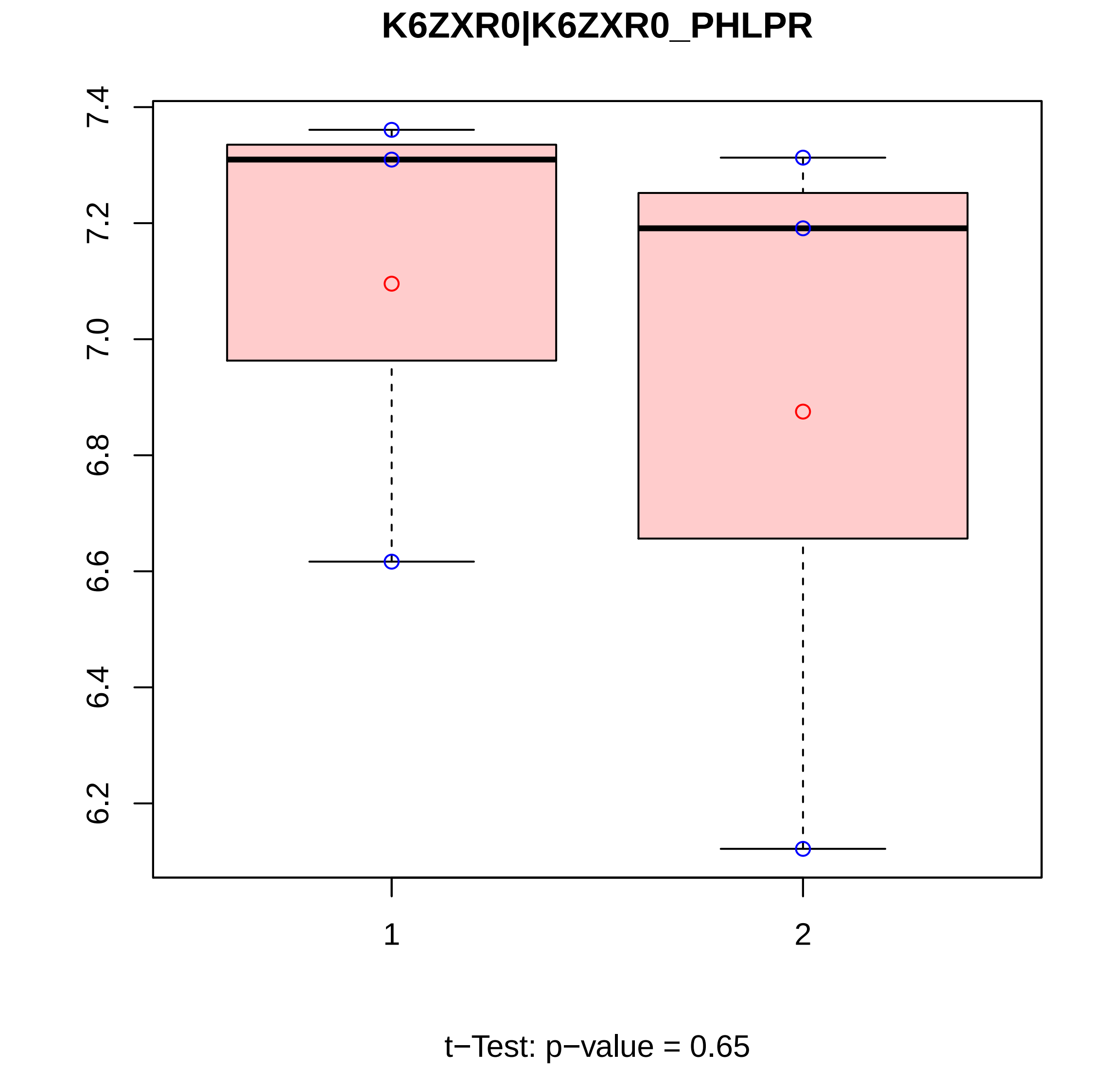
K6ZXR0|K6ZXR0_PHLPR
7.4
7.2
7.0
6.8
6.6
6.4
6.2
1
2
t−Test: p−value = 0.65

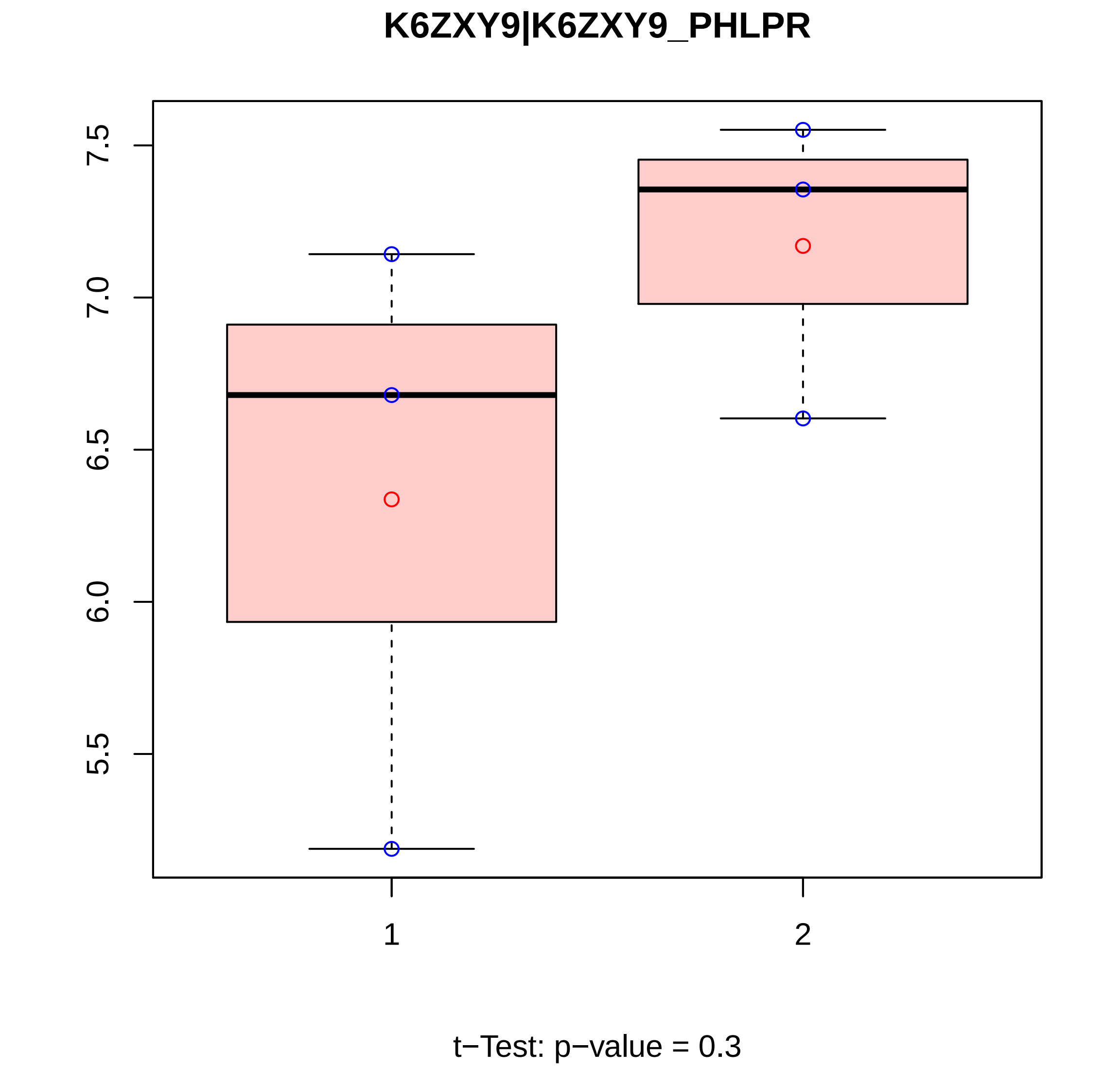
K6ZXY9|K6ZXY9_PHLPR
7.5
7.0
6.5
6.0
5.5
1
2
t−Test: p−value = 0.3

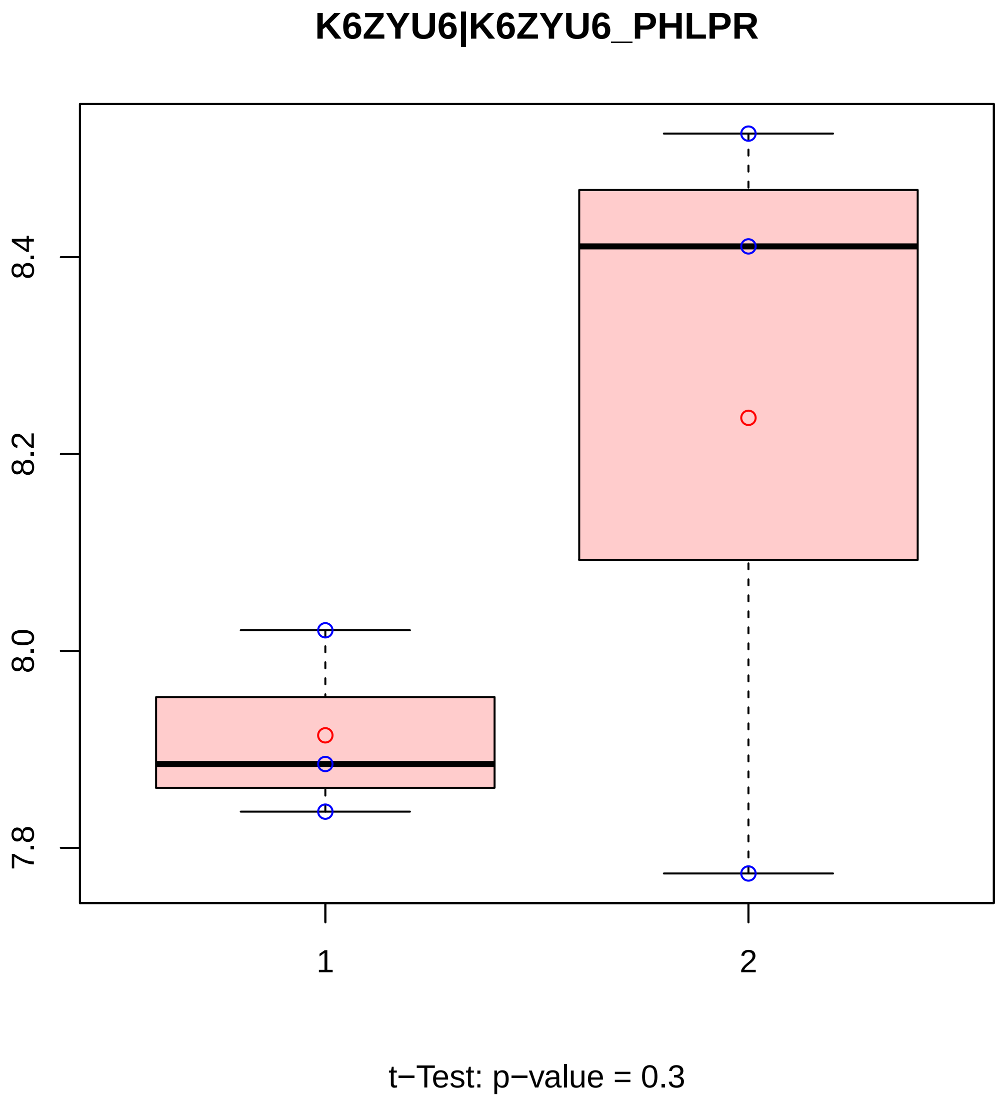
K6ZYU6|K6ZYU6_PHLPR
8.4
8.2
8.0
7.8
1
2
t−Test: p−value = 0.3

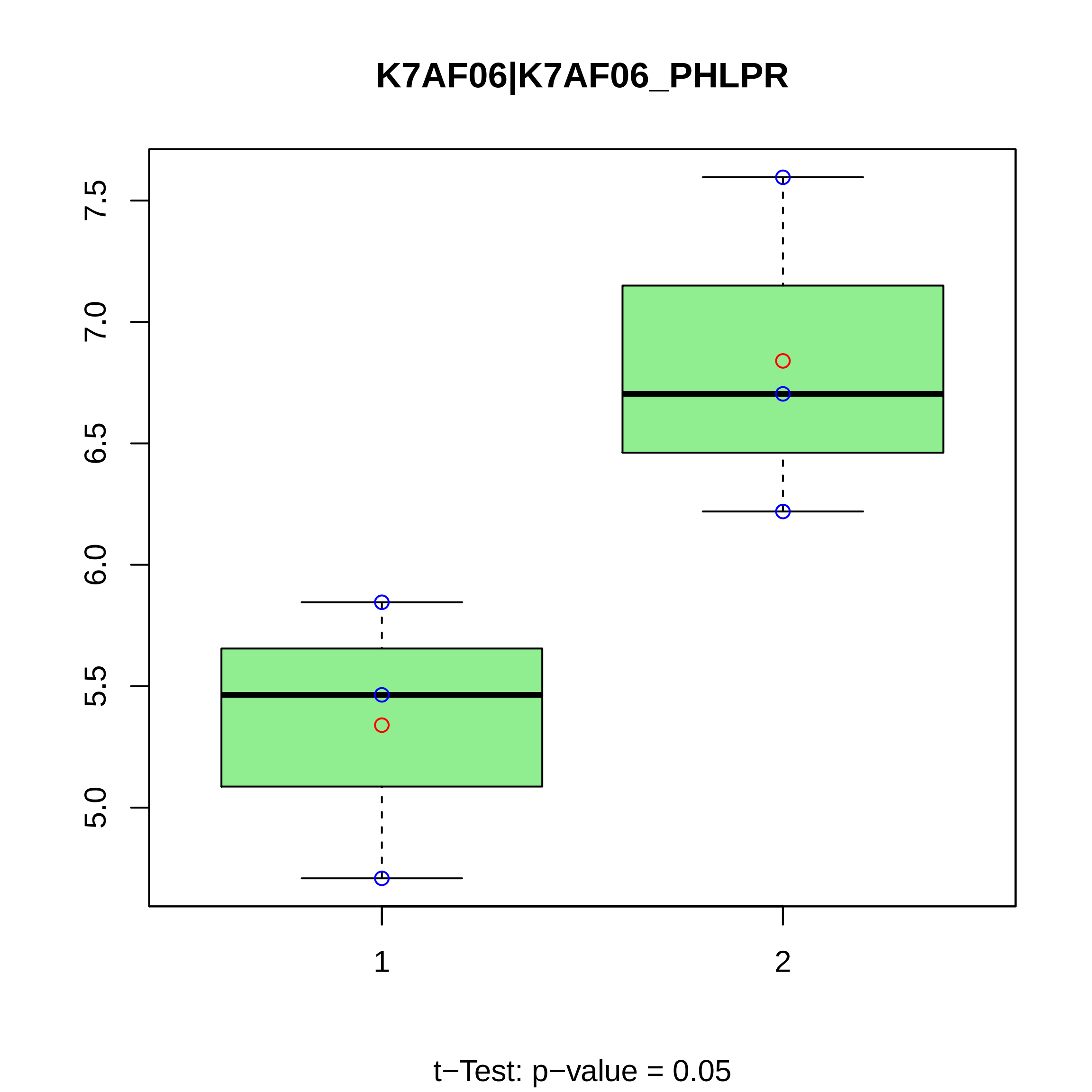
K7AF06|K7AF06_PHLPR
7.5
7.0
6.5
6.0
5.5
5.0
1
2
t−Test: p−value = 0.05

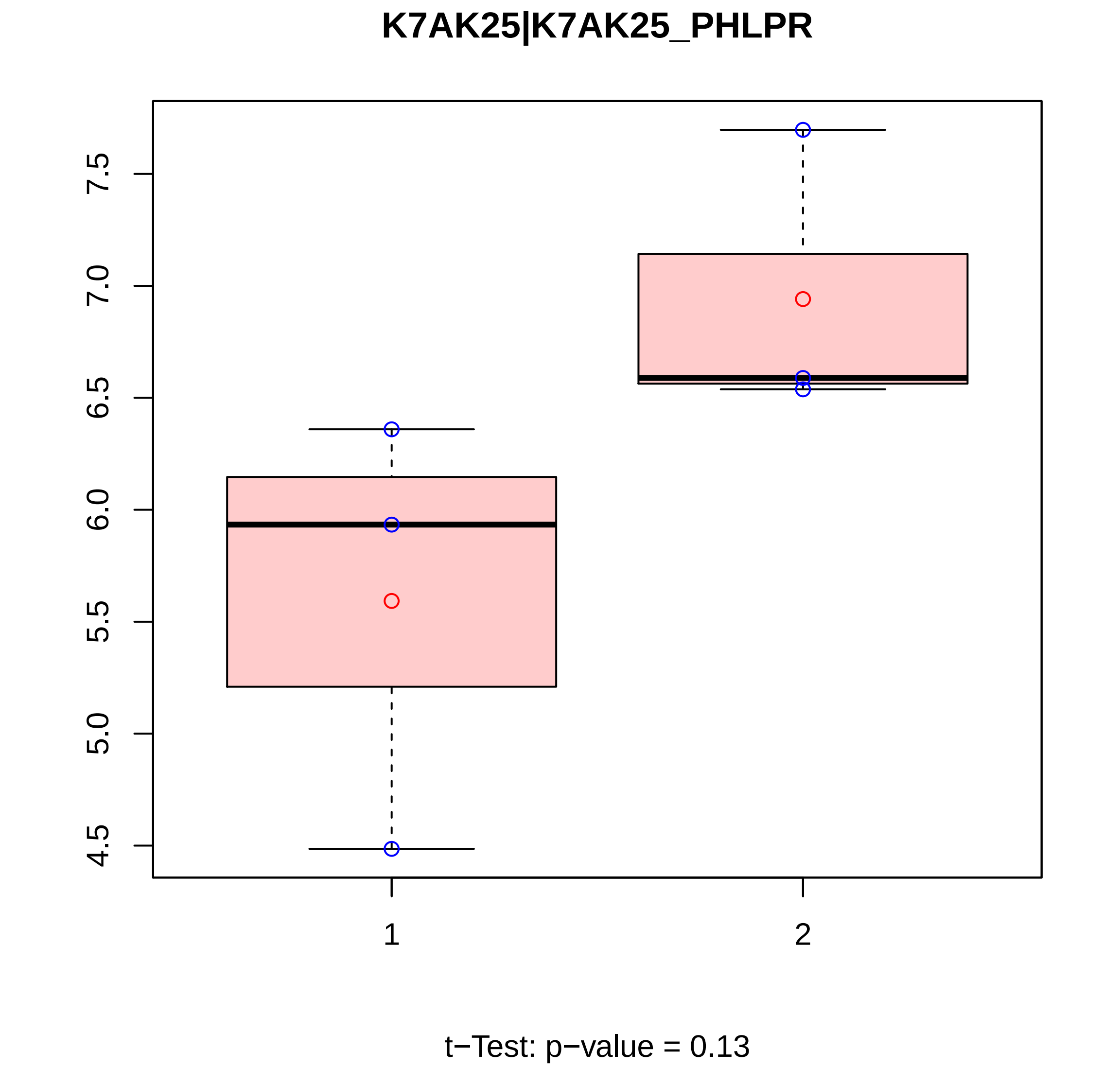

K7AK25|K7AK25_PHLPR
4.5
5.0
5.5
6.0
6.5
7.0
7.5
1
2
t−Test: p−value = 0.13

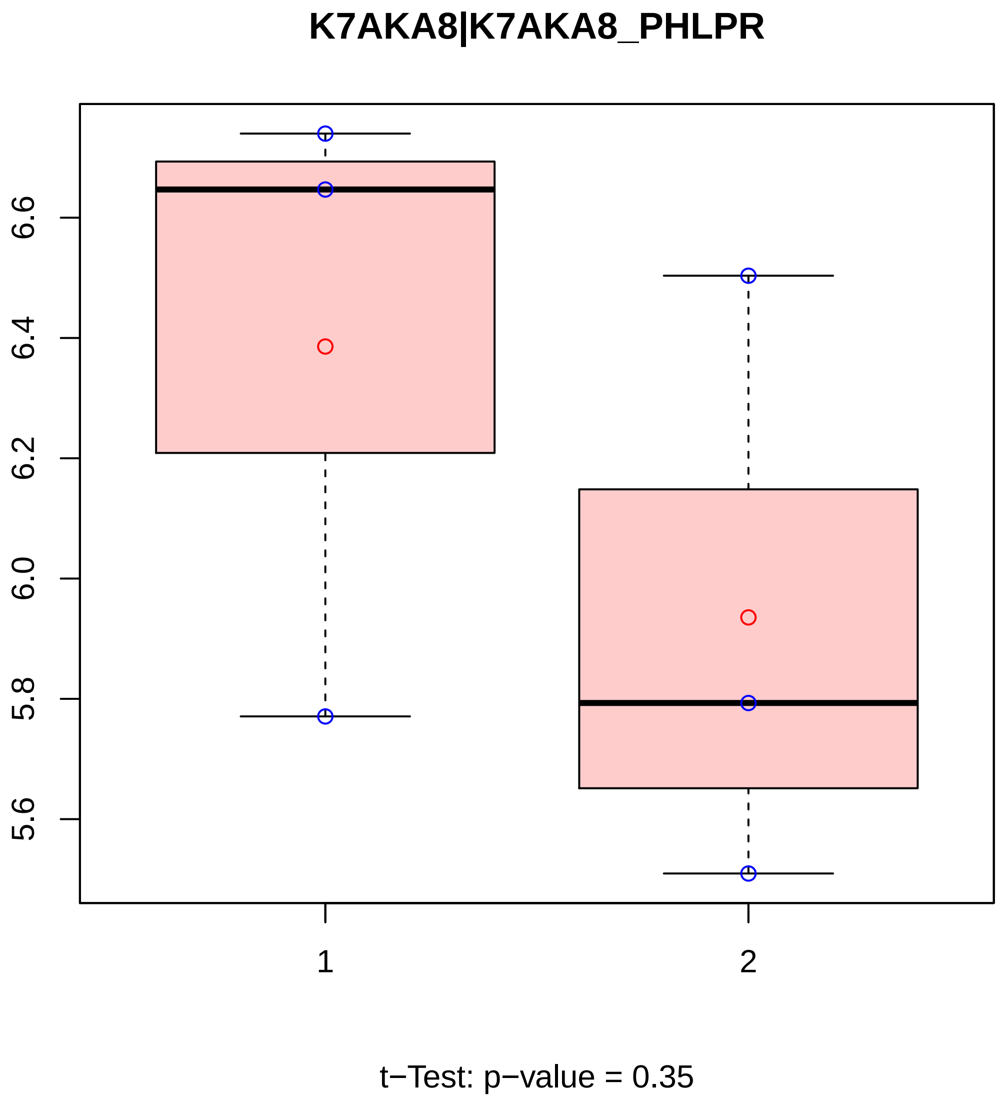

K7AKA8|K7AKA8_PHLPR
6.6
6.4
6.2
6.0
5.8
5.6
1
2
t−Test: p−value = 0.35

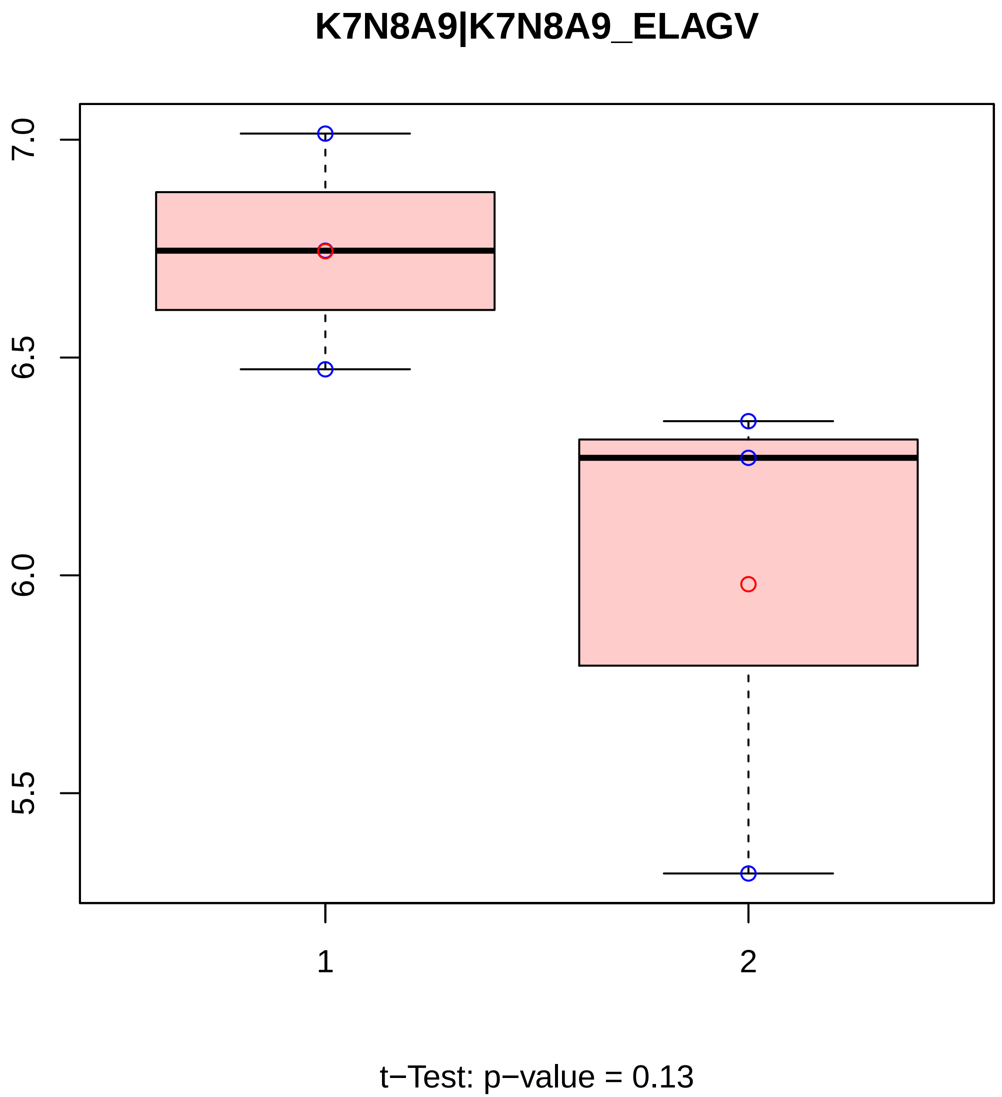
K7N8A9|K7N8A9_ELAGV
7.0
6.5
6.0
5.5
1
2
t−Test: p−value = 0.13

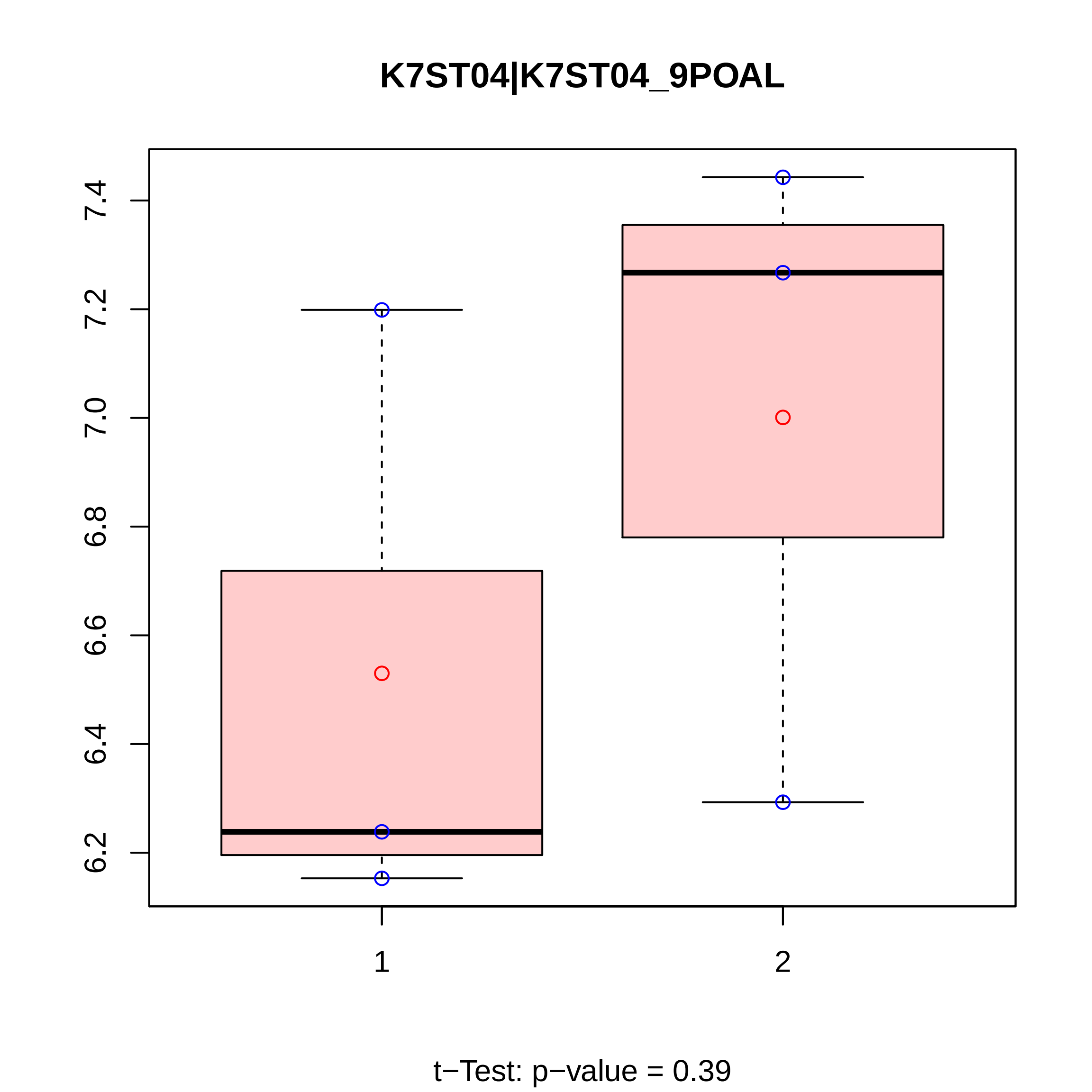
K7ST04|K7ST04_9POAL
7.4
7.2
7.0
6.8
6.6
6.4
6.2
1
2
t−Test: p−value = 0.39

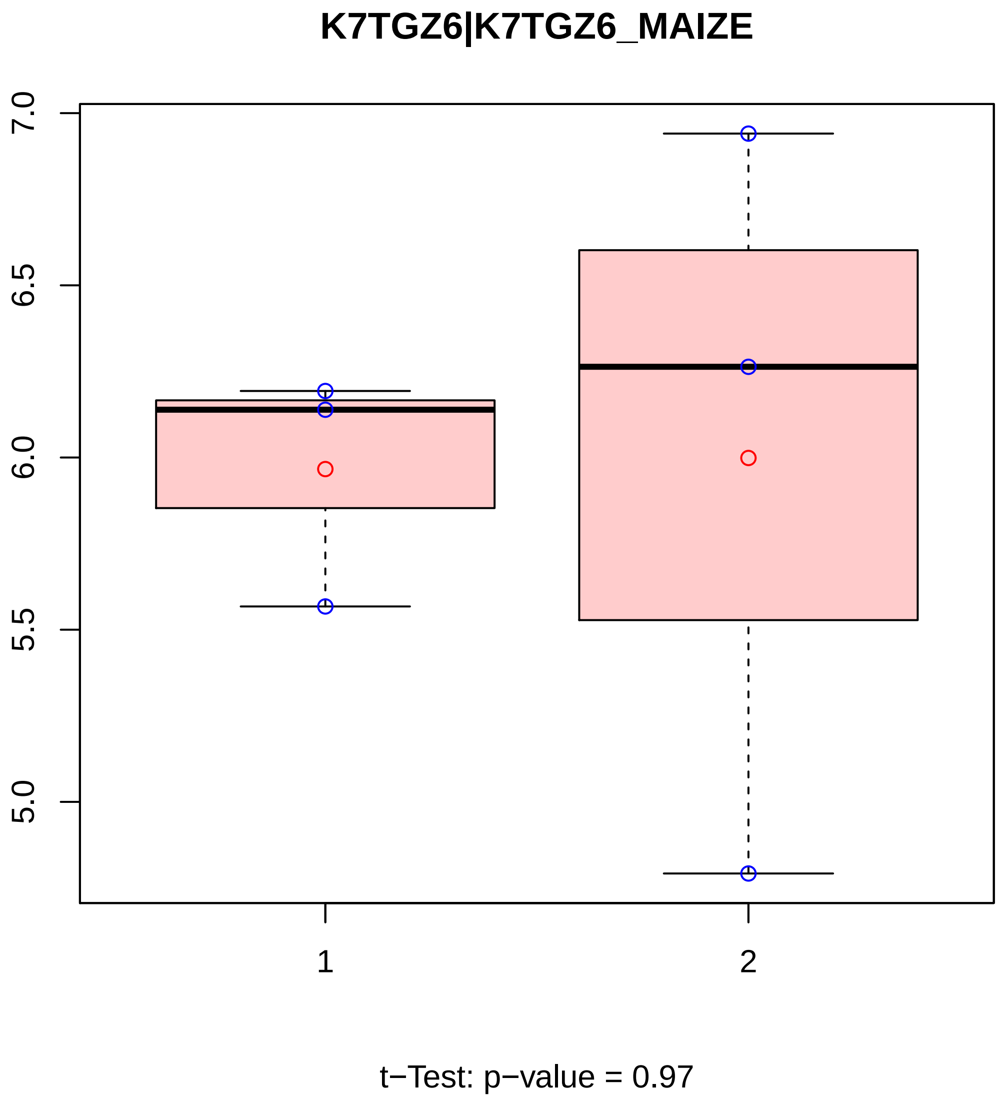

K7TGZ6|K7TGZ6_MAIZE
7.0
6.5
6.0
5.5
5.0
1
2
t−Test: p−value = 0.97

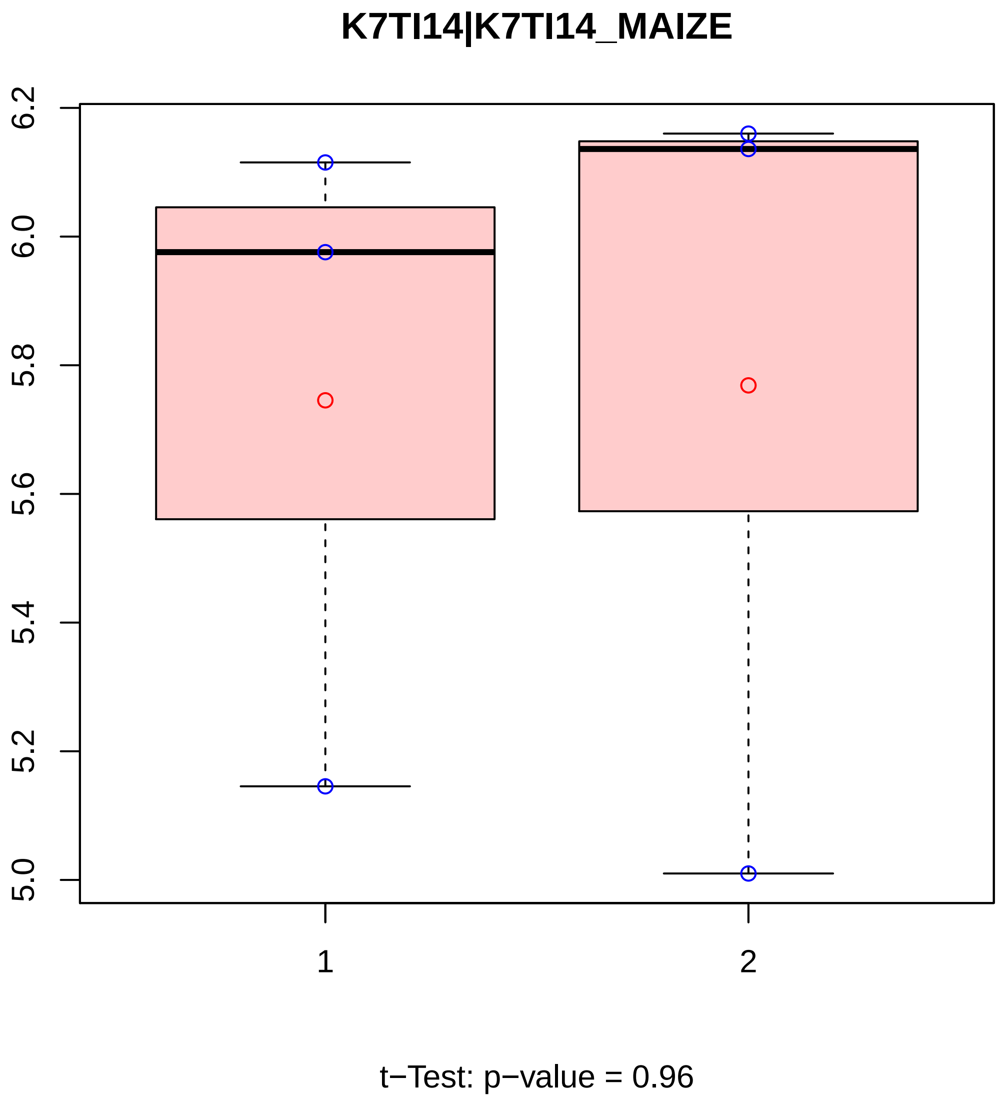
K7TI14|K7TI14_MAIZE
6.2
6.0
5.8
5.6
5.4
5.2
5.0
1
2
t−Test: p−value = 0.96

K7TIB4|K7TIB4_MAIZE

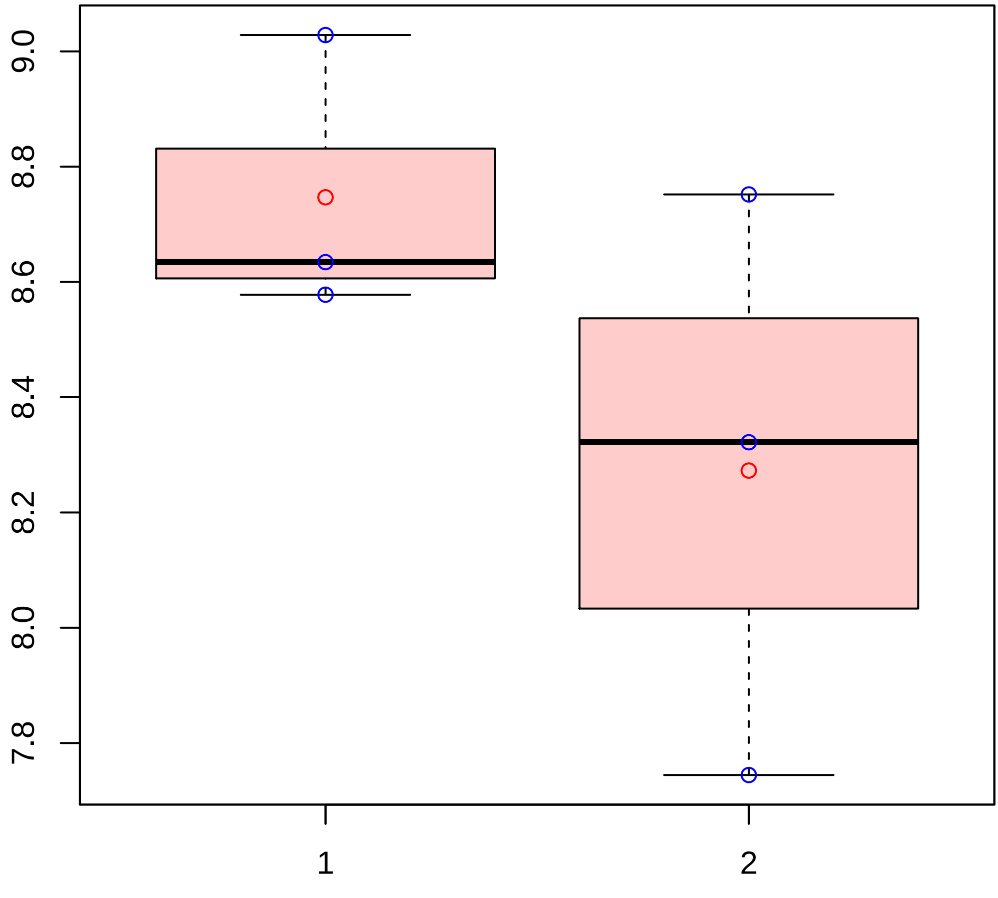

t−Test: p−value = 0.24

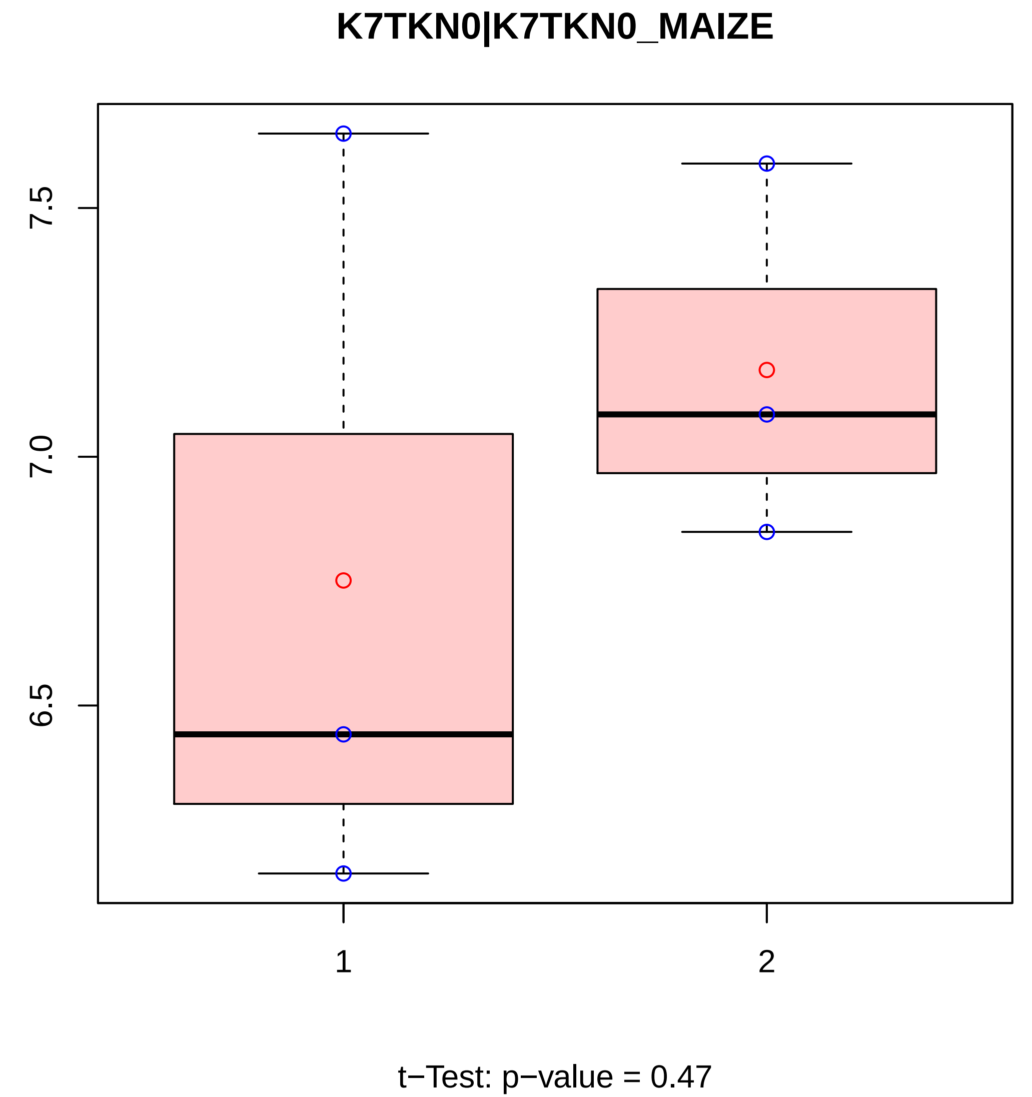
K7TKN0|K7TKN0_MAIZE
7.5
7.0
6.5
1
2
t−Test: p−value = 0.47

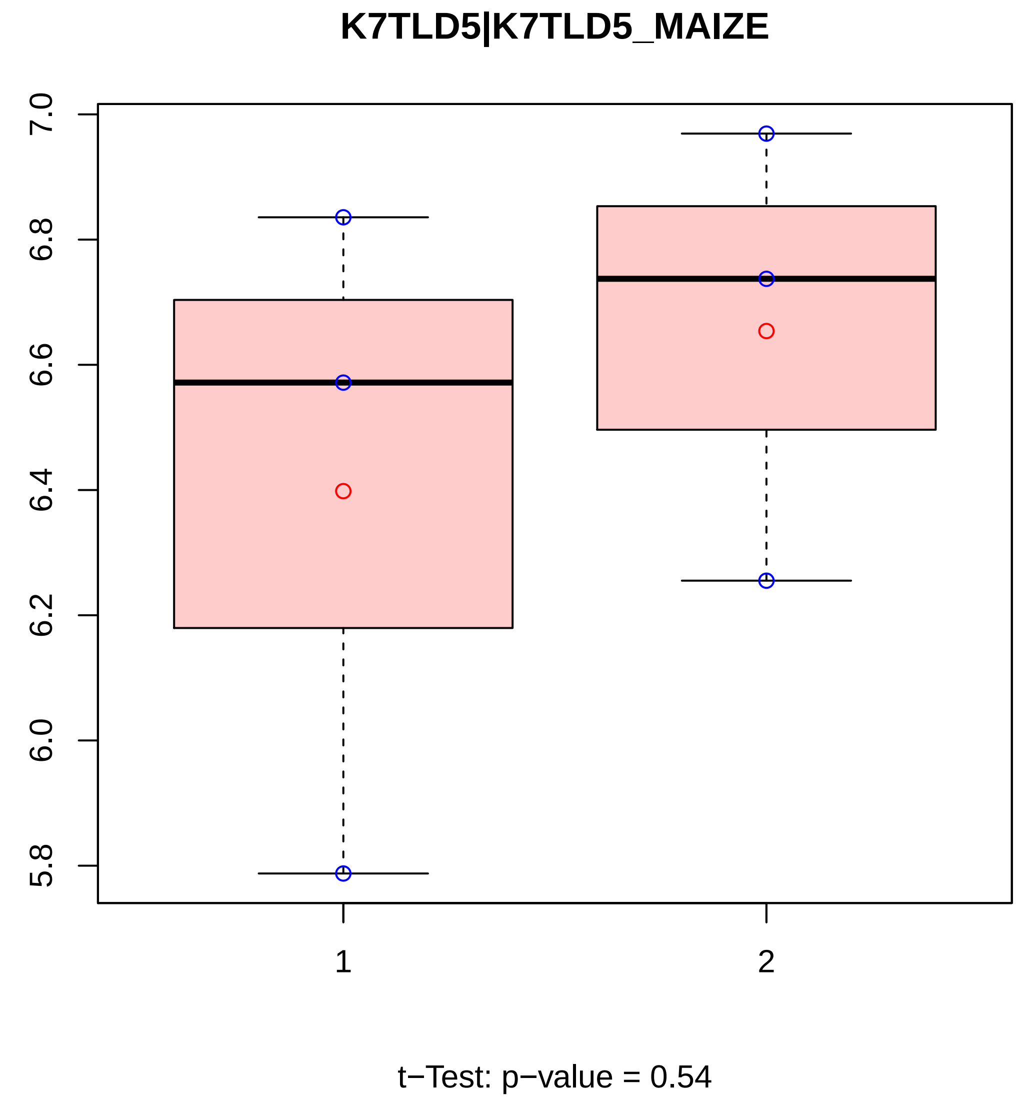
K7TLD5|K7TLD5_MAIZE
7.0
6.8
6.6
6.4
6.2
6.0
5.8
1
2
t−Test: p−value = 0.54

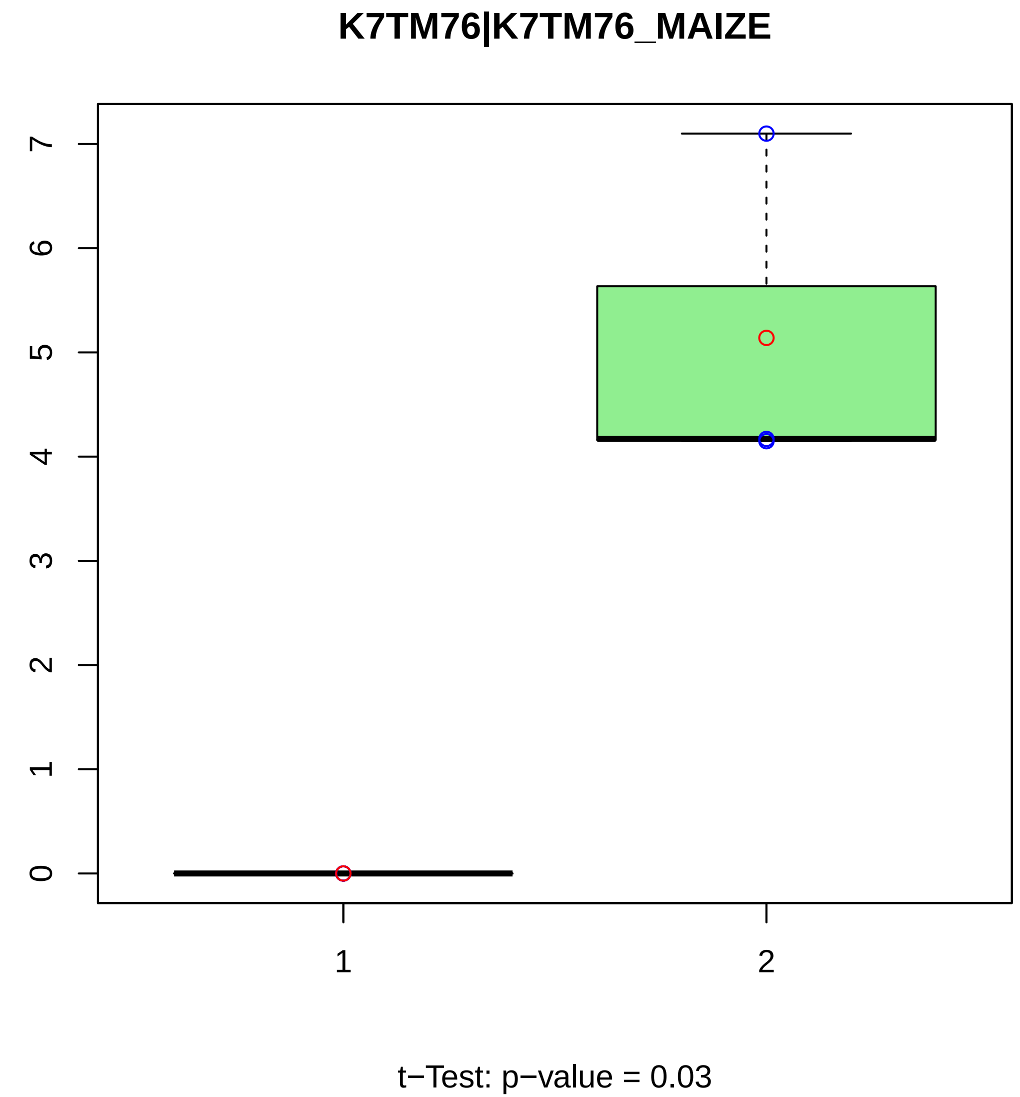

K7TM76|K7TM76_MAIZE
0
1
2
3
4
5
6
7
1
2
t−Test: p−value = 0.03

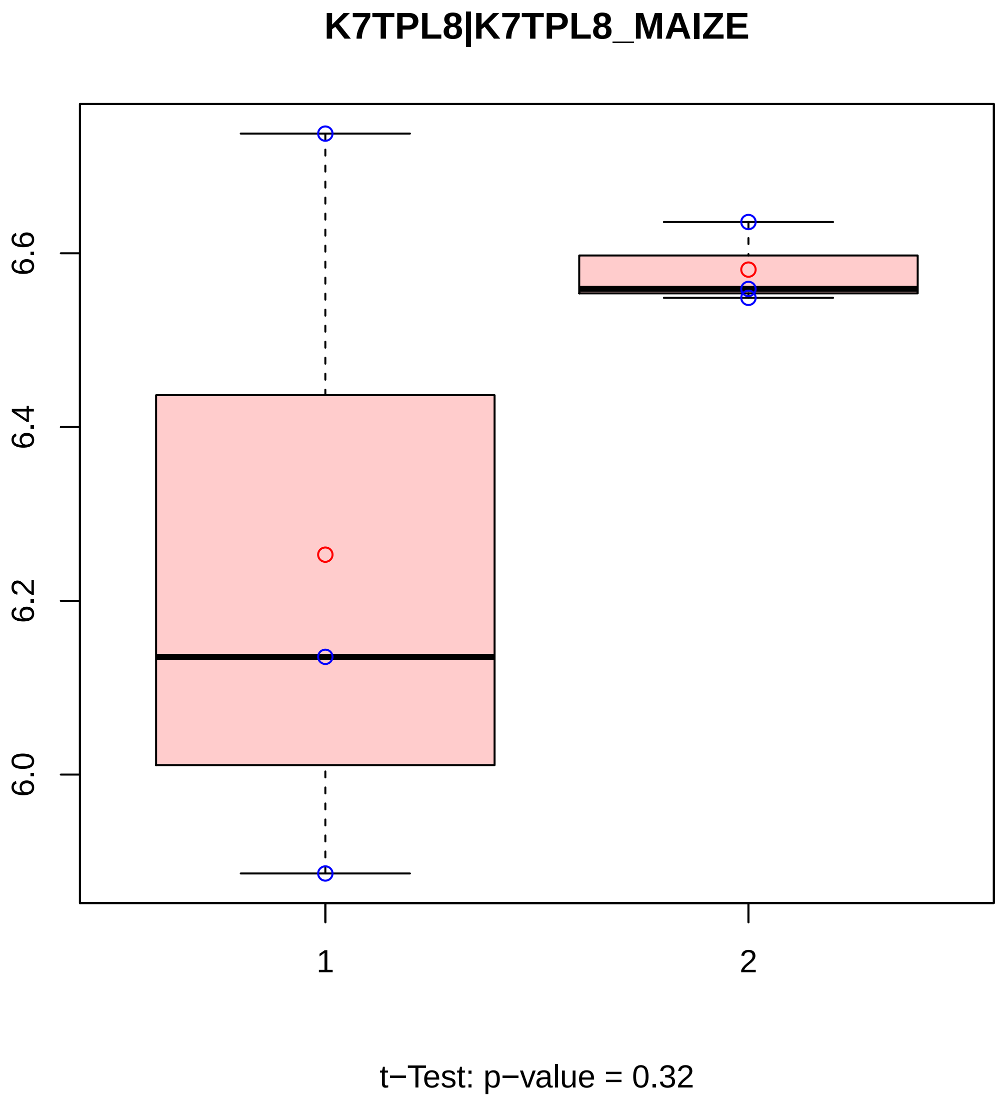
K7TPL8|K7TPL8_MAIZE
6.0
6.2
6.4
6.6
1
2
t−Test: p−value = 0.32

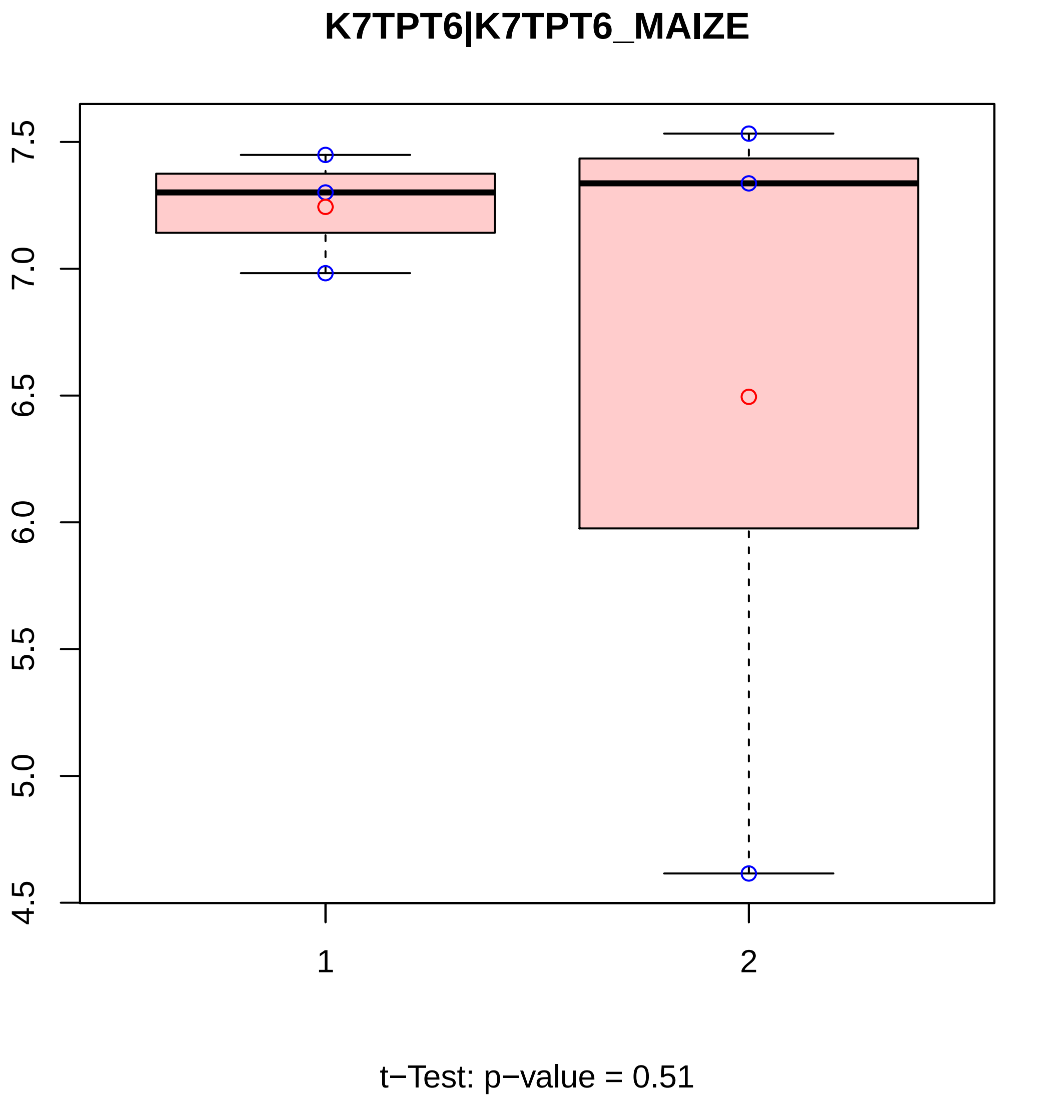
K7TPT6|K7TPT6_MAIZE
7.5
7.0
6.5
6.0
5.5
5.0
4.5
1
2
t−Test: p−value = 0.51

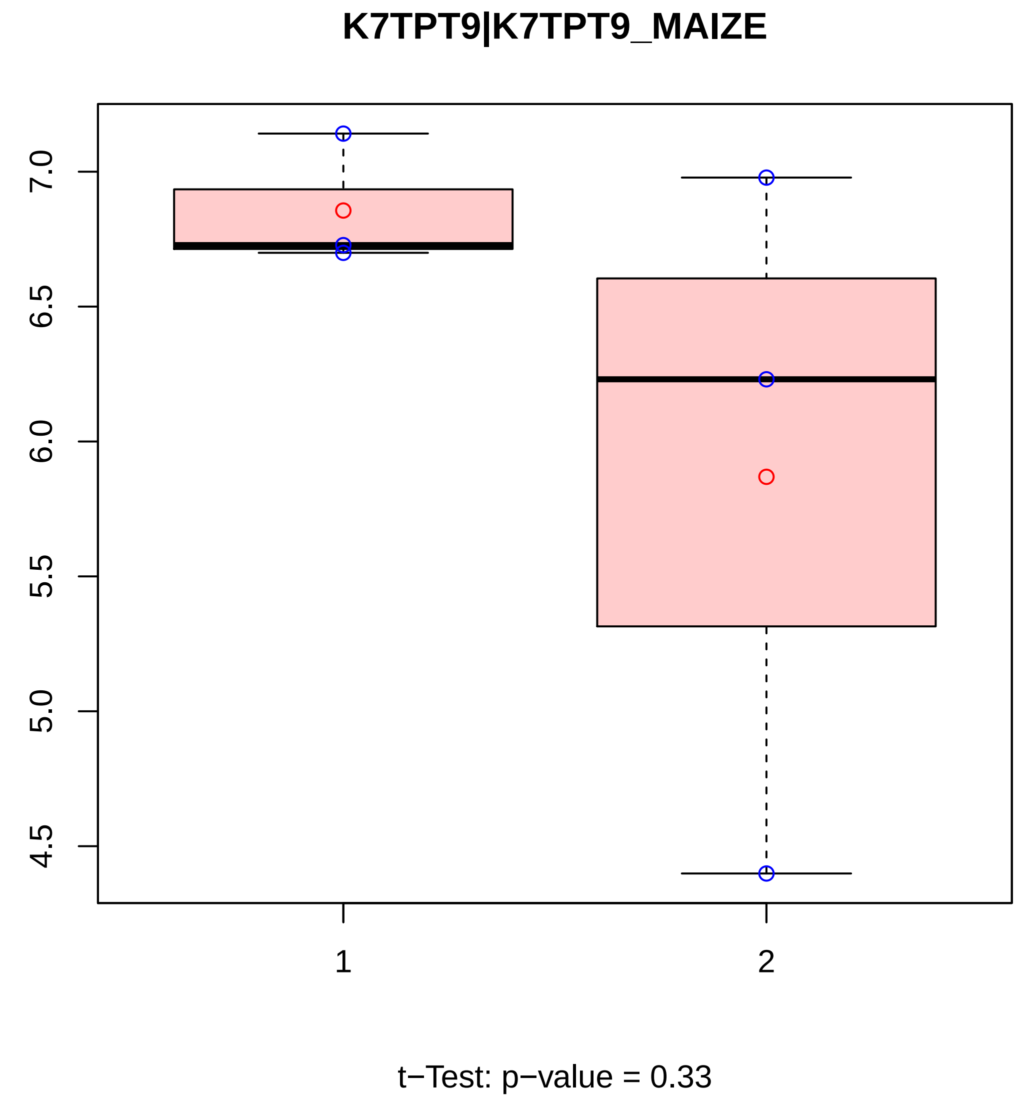
K7TPT9|K7TPT9_MAIZE
7.0
6.5
6.0
5.5
5.0
4.5
1
2
t−Test: p−value = 0.33

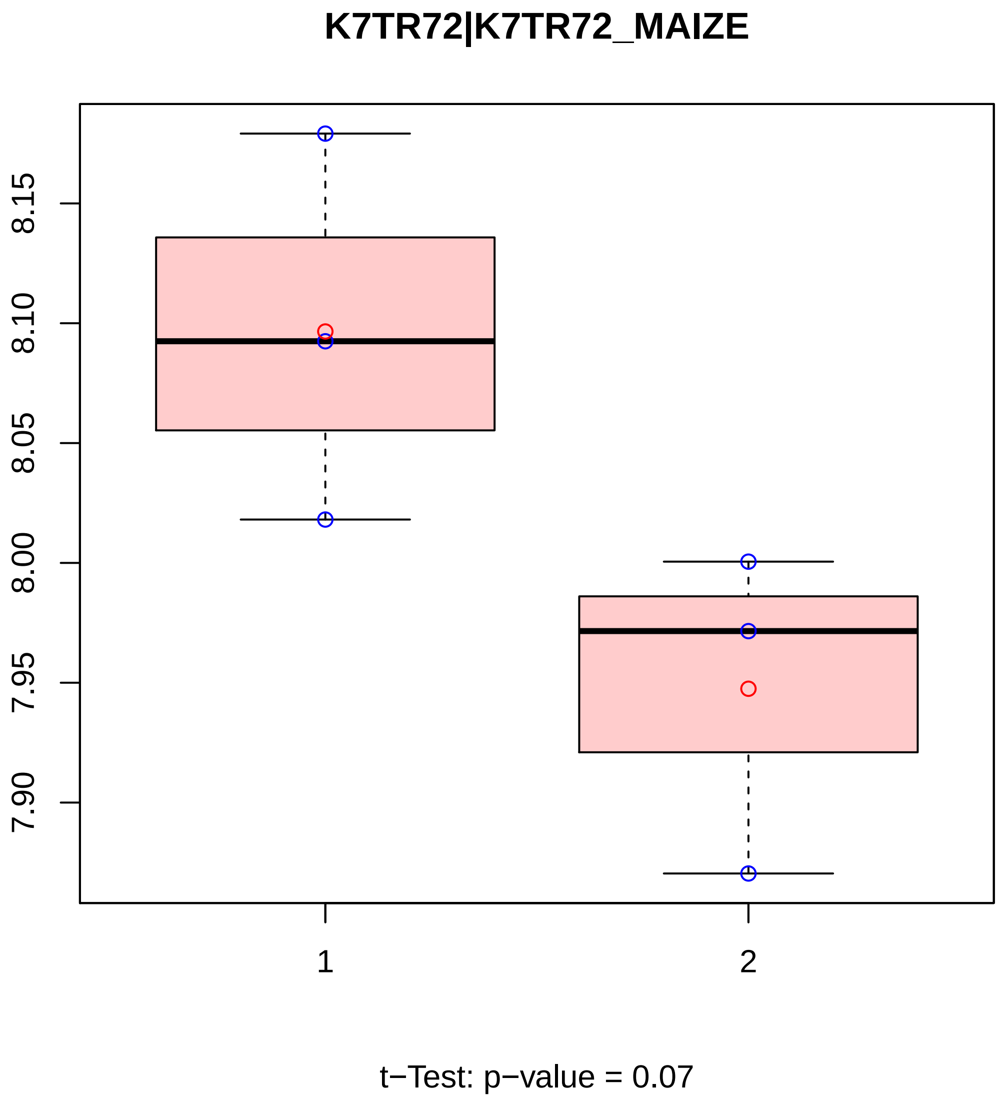

K7TR72|K7TR72_MAIZE
8.15
8.10
8.05
8.00
7.95
7.90
1
2
t−Test: p−value = 0.07

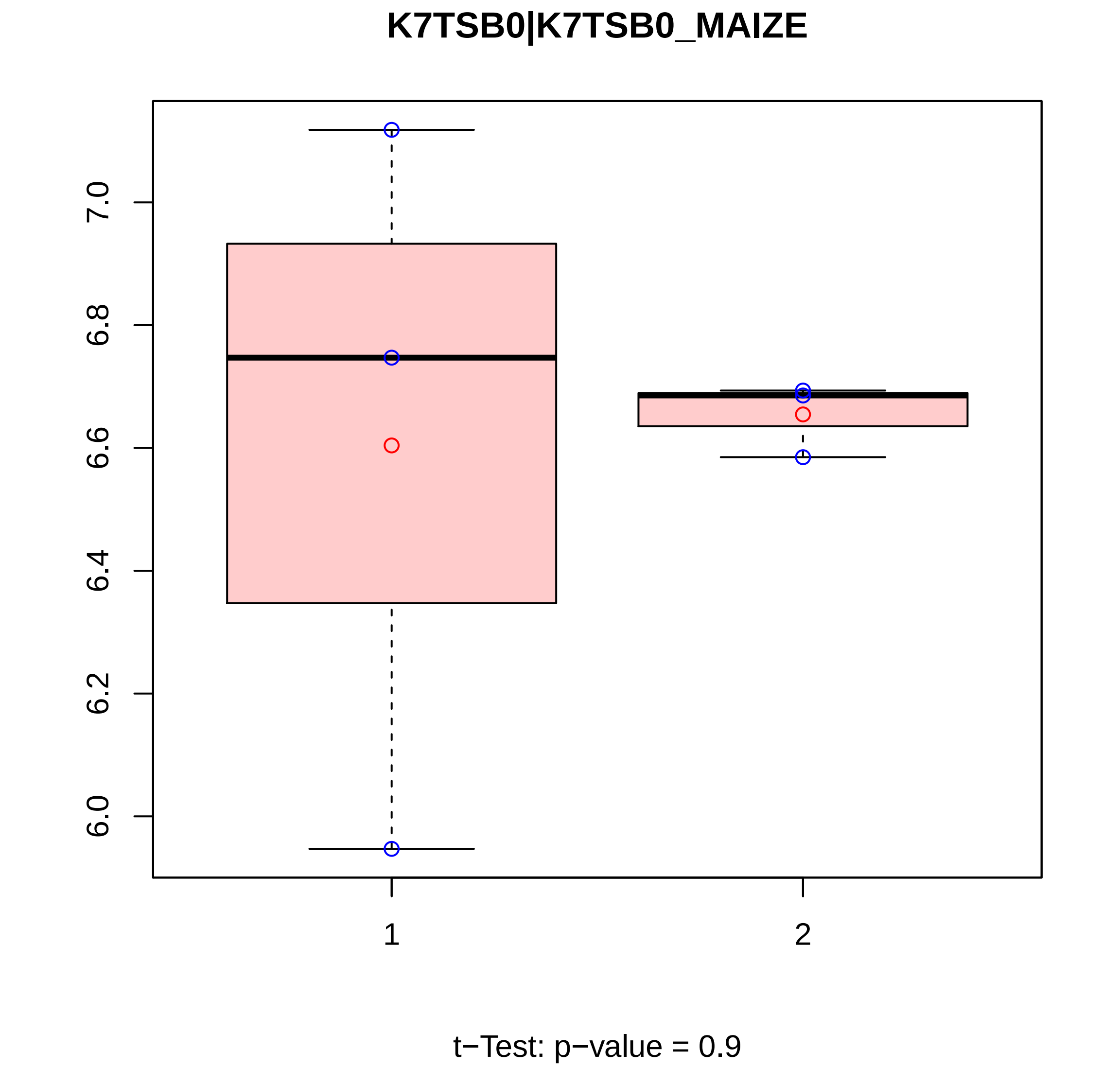

K7TSB0|K7TSB0_MAIZE
7.0
6.8
6.6
6.4
6.2
6.0
1
2
t−Test: p−value = 0.9

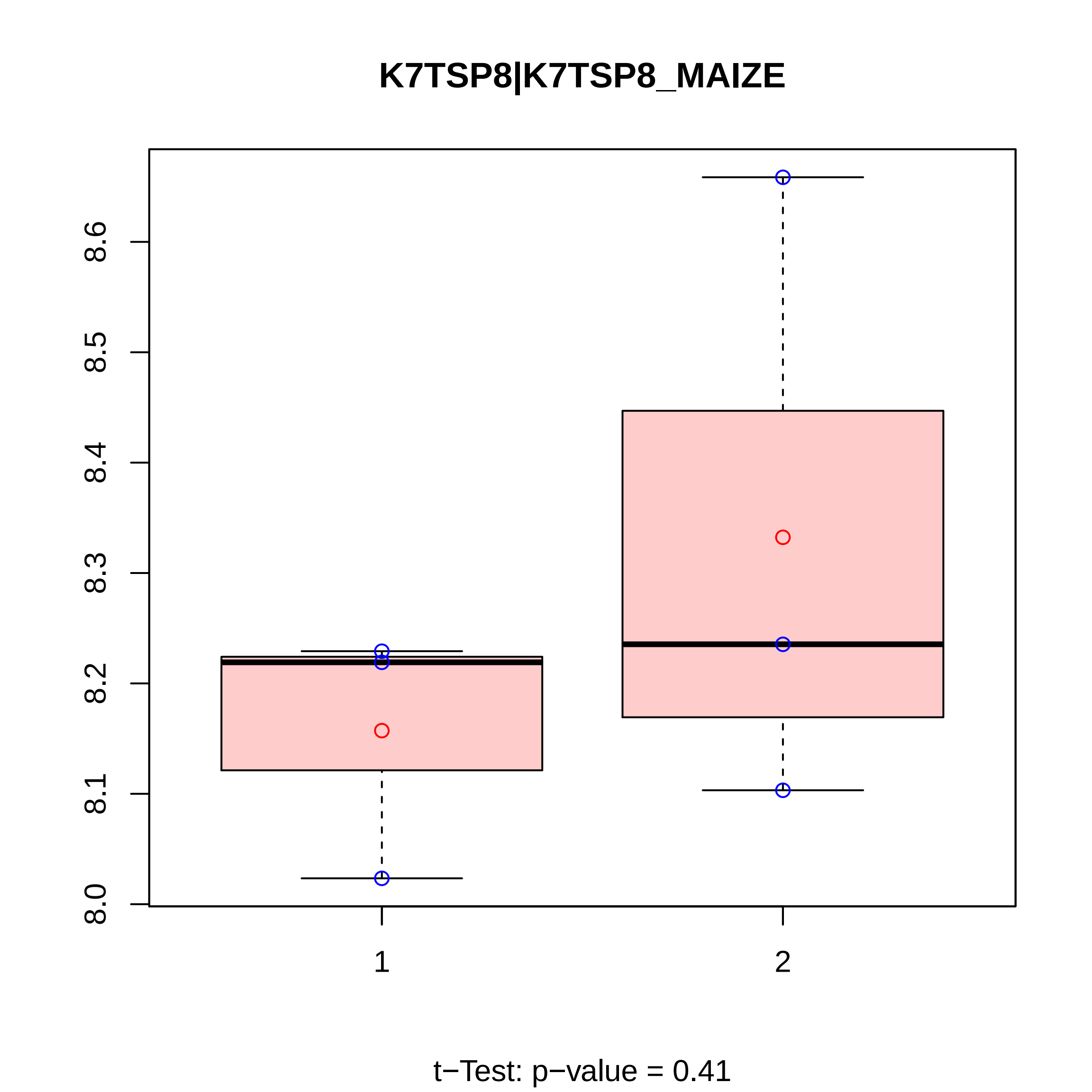

K7TSP8|K7TSP8_MAIZE
8.0
8.1
8.2
8.3
8.4
8.5
8.6
1
2
t−Test: p−value = 0.41

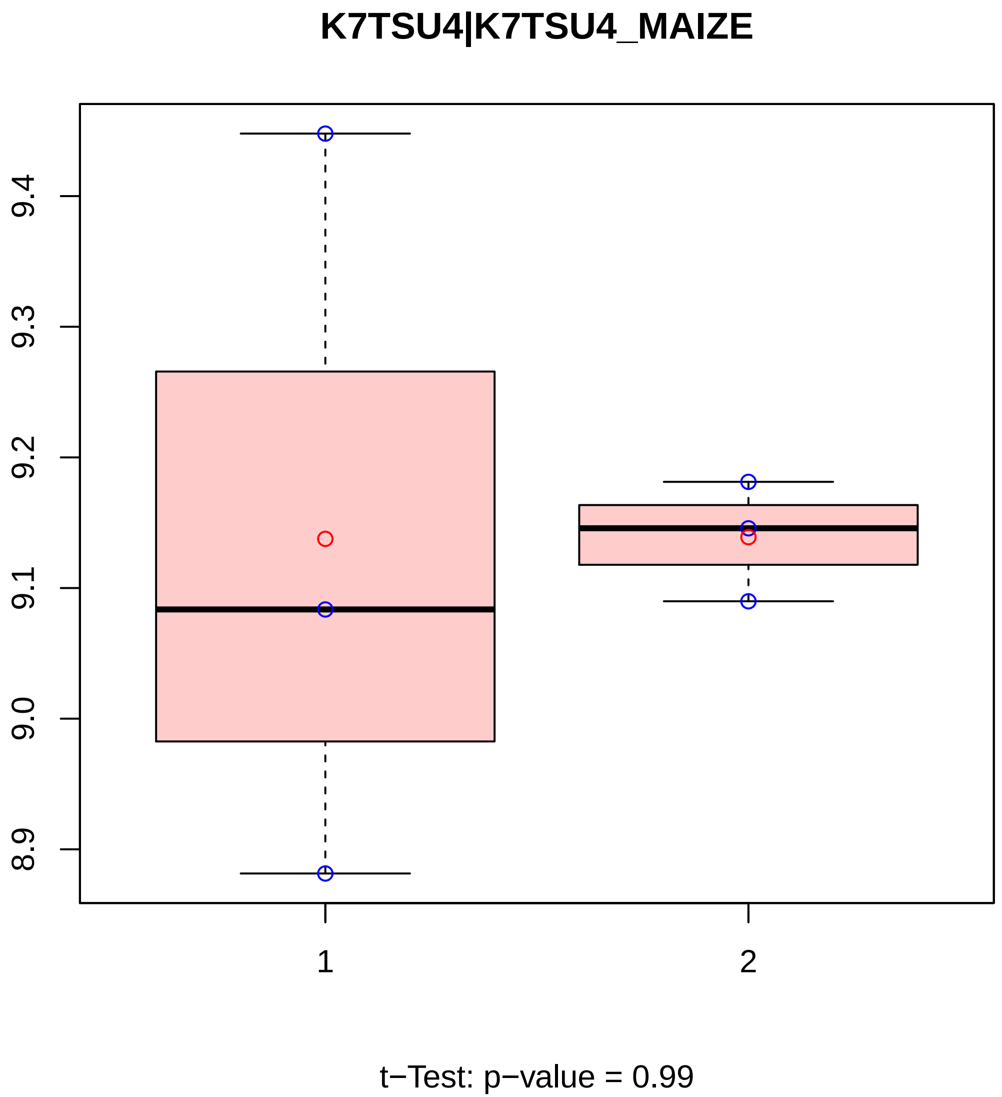
K7TSU4|K7TSU4_MAIZE
9.4
9.3
9.2
9.1
9.0
8.9
1
2
t−Test: p−value = 0.99

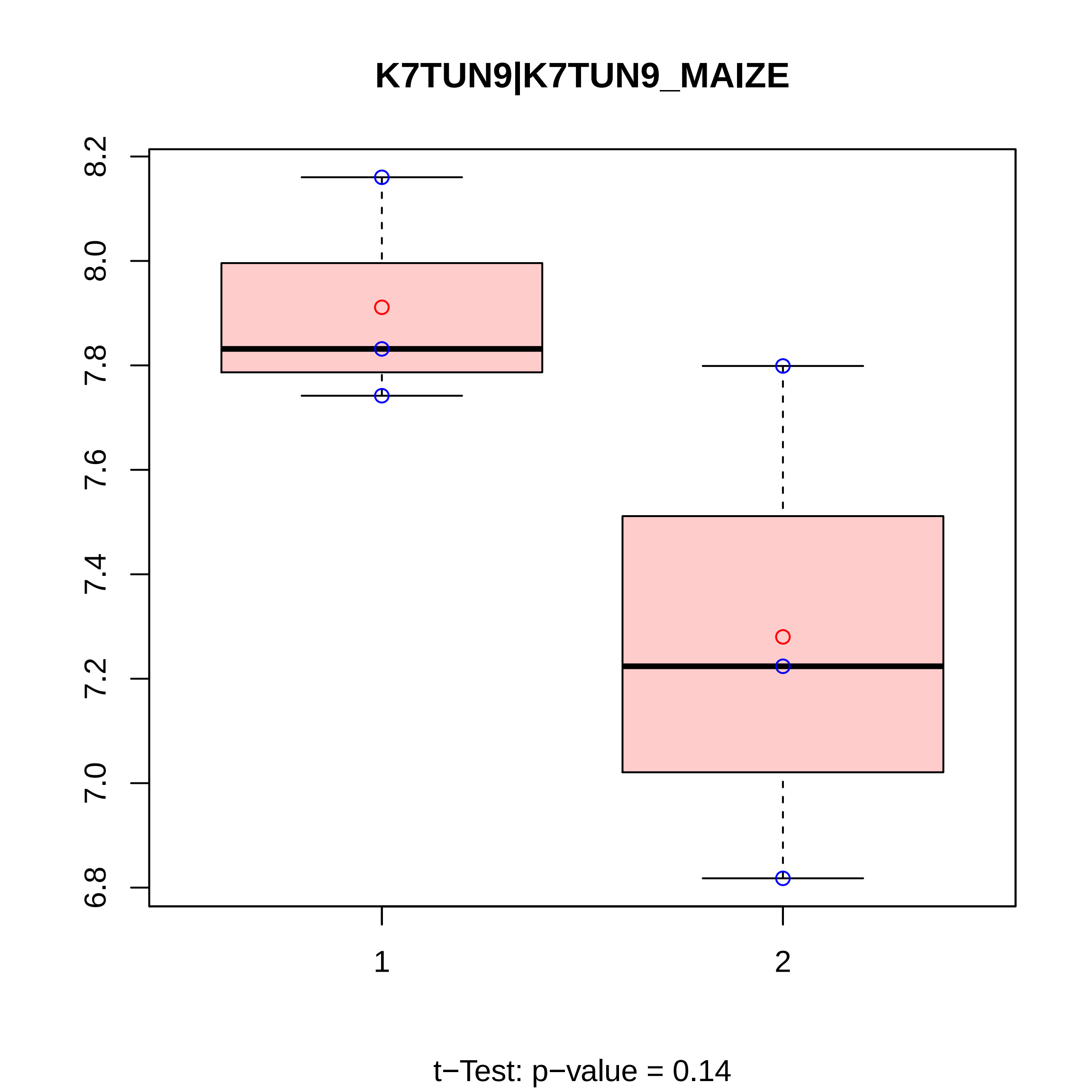
K7TUN9|K7TUN9_MAIZE
8.2
8.0
7.8
7.6
7.4
7.2
7.0
6.8
1
2
t−Test: p−value = 0.14

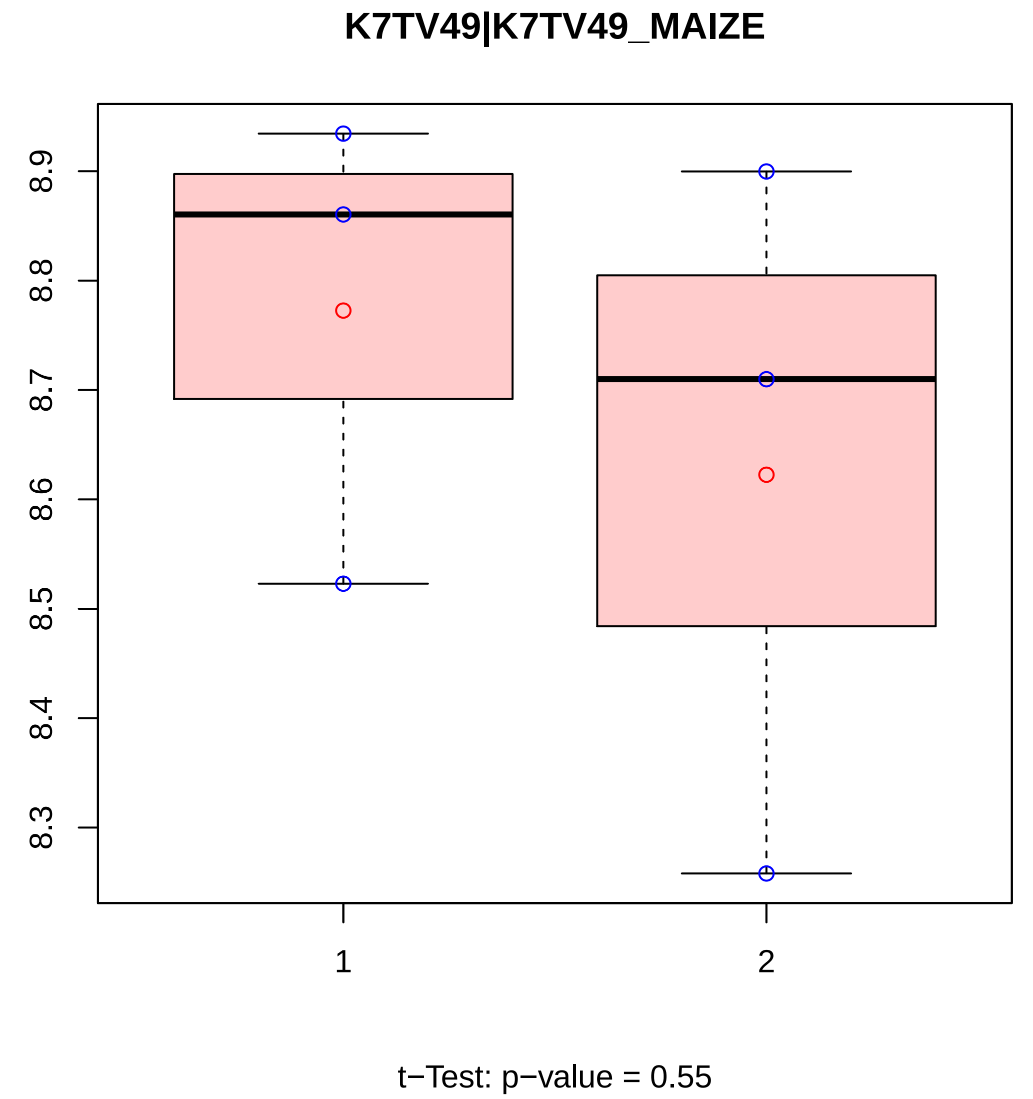
K7TV49|K7TV49_MAIZE
8.3
8.4
8.5
8.6
8.7
8.8
8.9
1
2
t−Test: p−value = 0.55

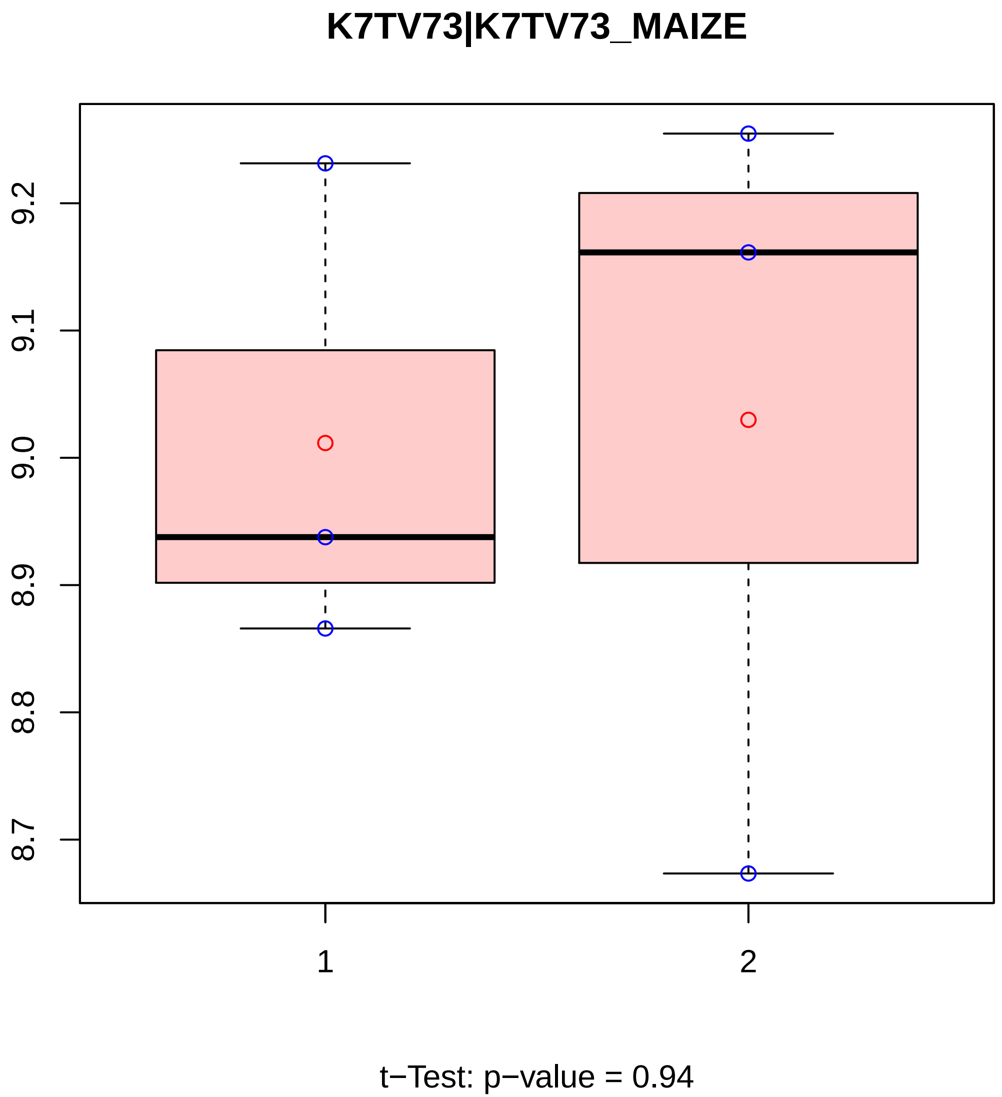
K7TV73|K7TV73_MAIZE
9.2
9.1
9.0
8.9
8.8
8.7
1
2
t−Test: p−value = 0.94

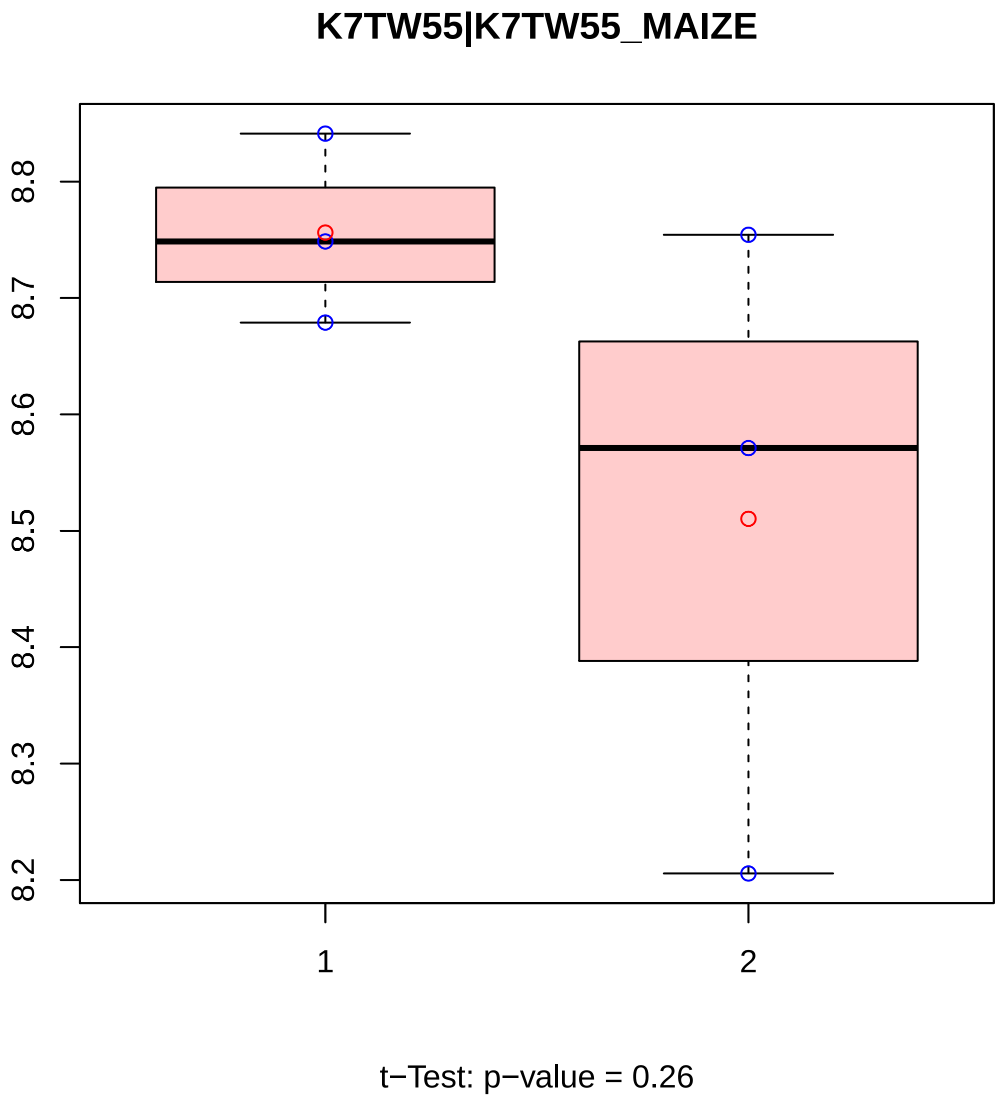
K7TW55|K7TW55_MAIZE
8.2
8.3
8.4
8.5
8.6
8.7
8.8
1
2
t−Test: p−value = 0.26

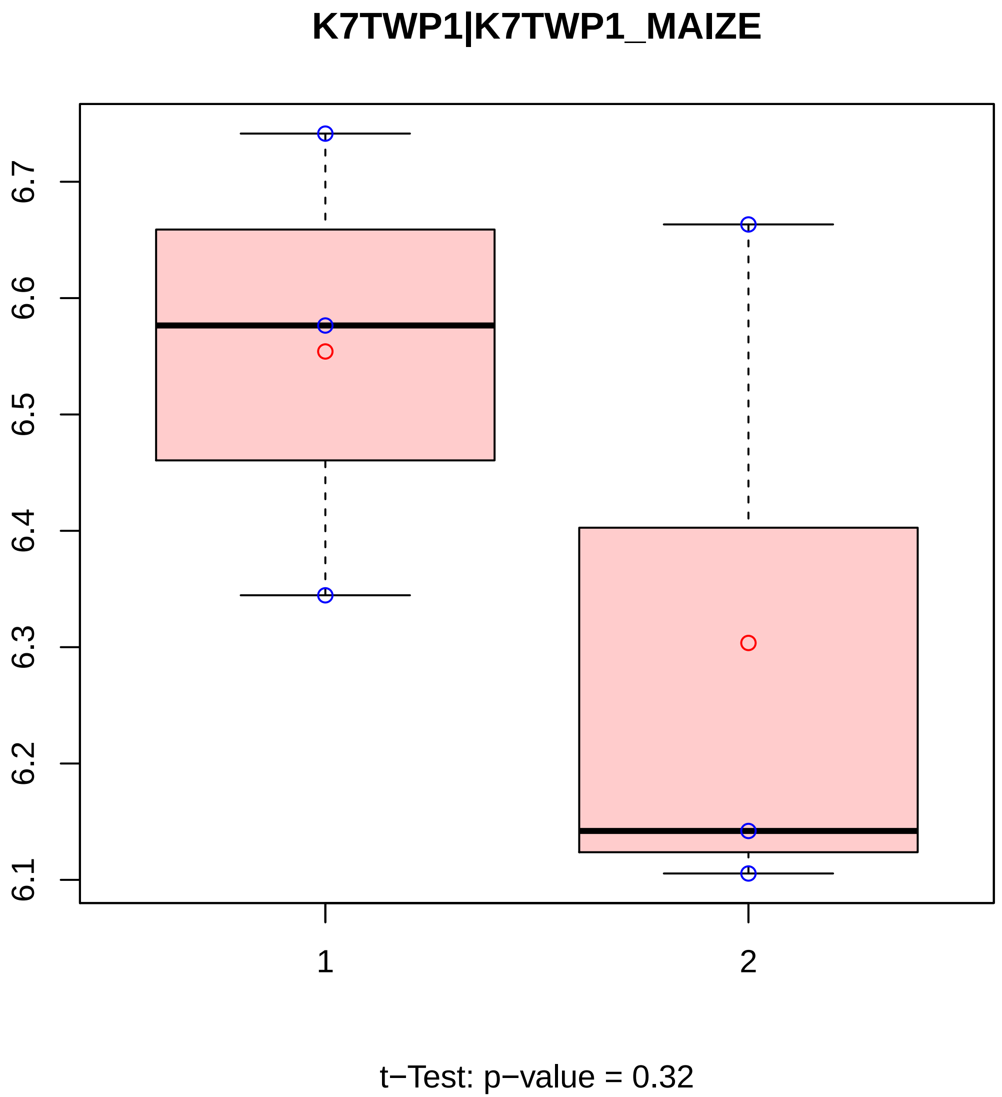
K7TWP1|K7TWP1_MAIZE
6.1
6.2
6.3
6.4
6.5
6.6
6.7
1
2
t−Test: p−value = 0.32

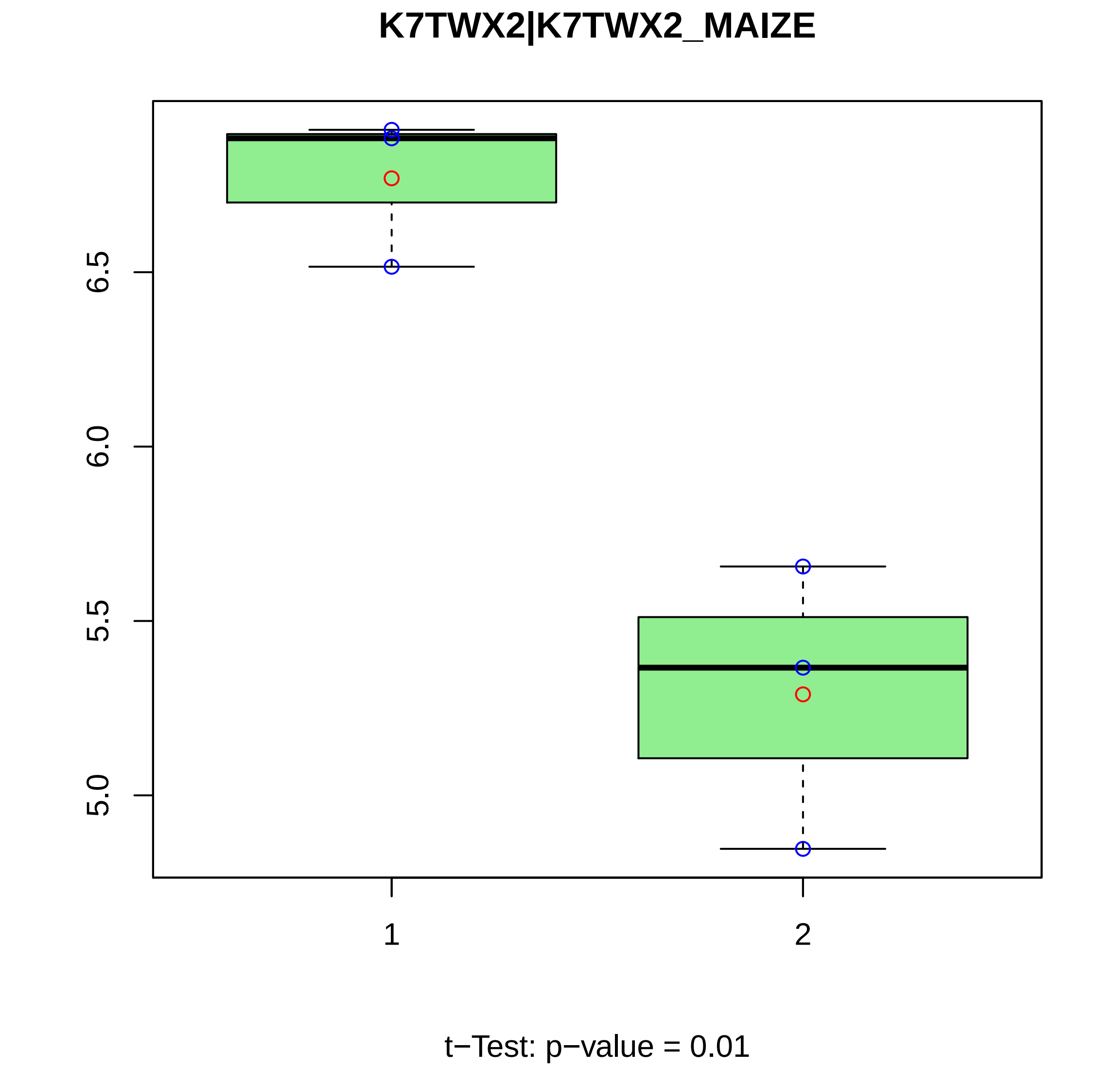
K7TWX2|K7TWX2_MAIZE
6.5
6.0
5.5
5.0
1
2
t−Test: p−value = 0.01

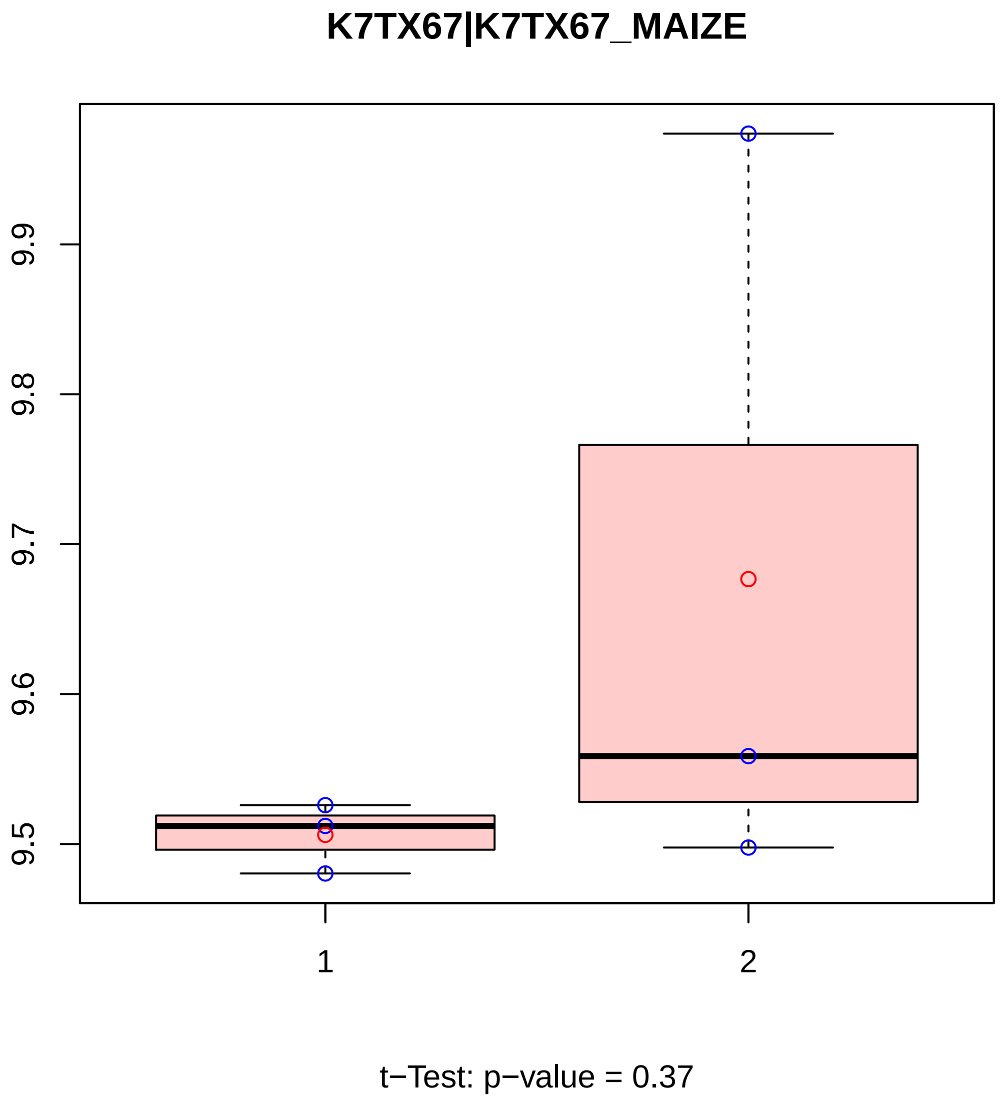

K7TX67|K7TX67_MAIZE
9.5
9.6
9.7
9.8
9.9
1
2
t−Test: p−value = 0.37

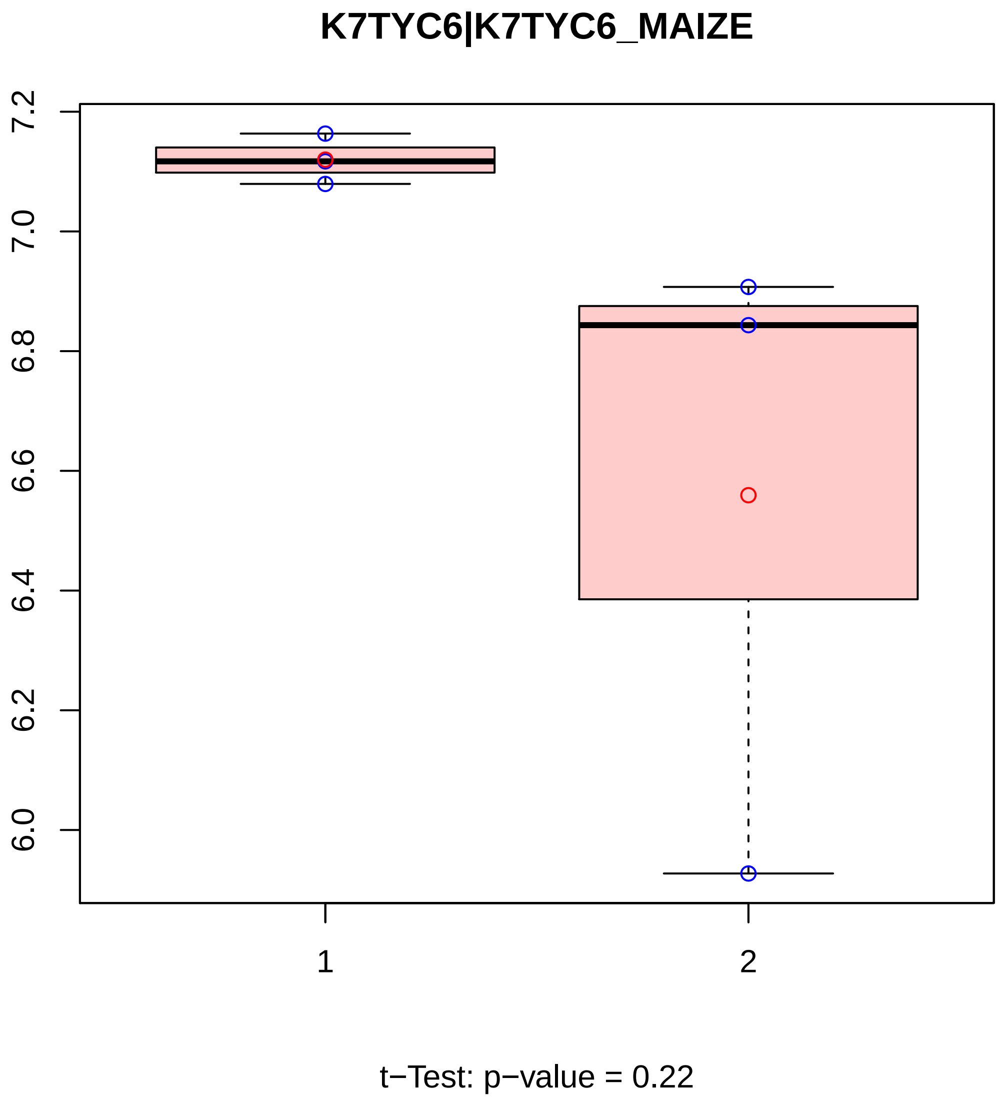
K7TYC6|K7TYC6_MAIZE
7.2
7.0
6.8
6.6
6.4
6.2
6.0
1
2
t−Test: p−value = 0.22

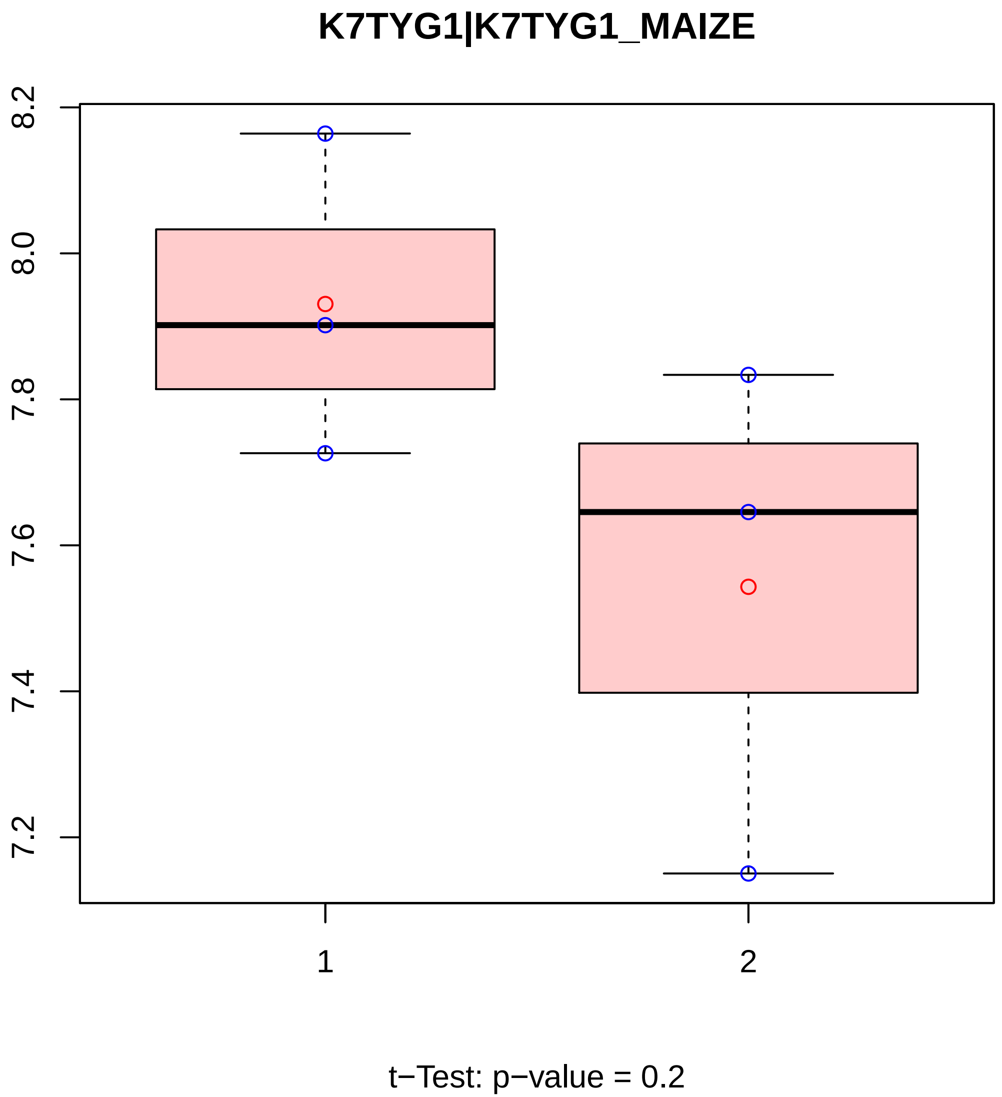
K7TYG1|K7TYG1_MAIZE
8.2
8.0
7.8
7.6
7.4
7.2
1
2
t−Test: p−value = 0.2

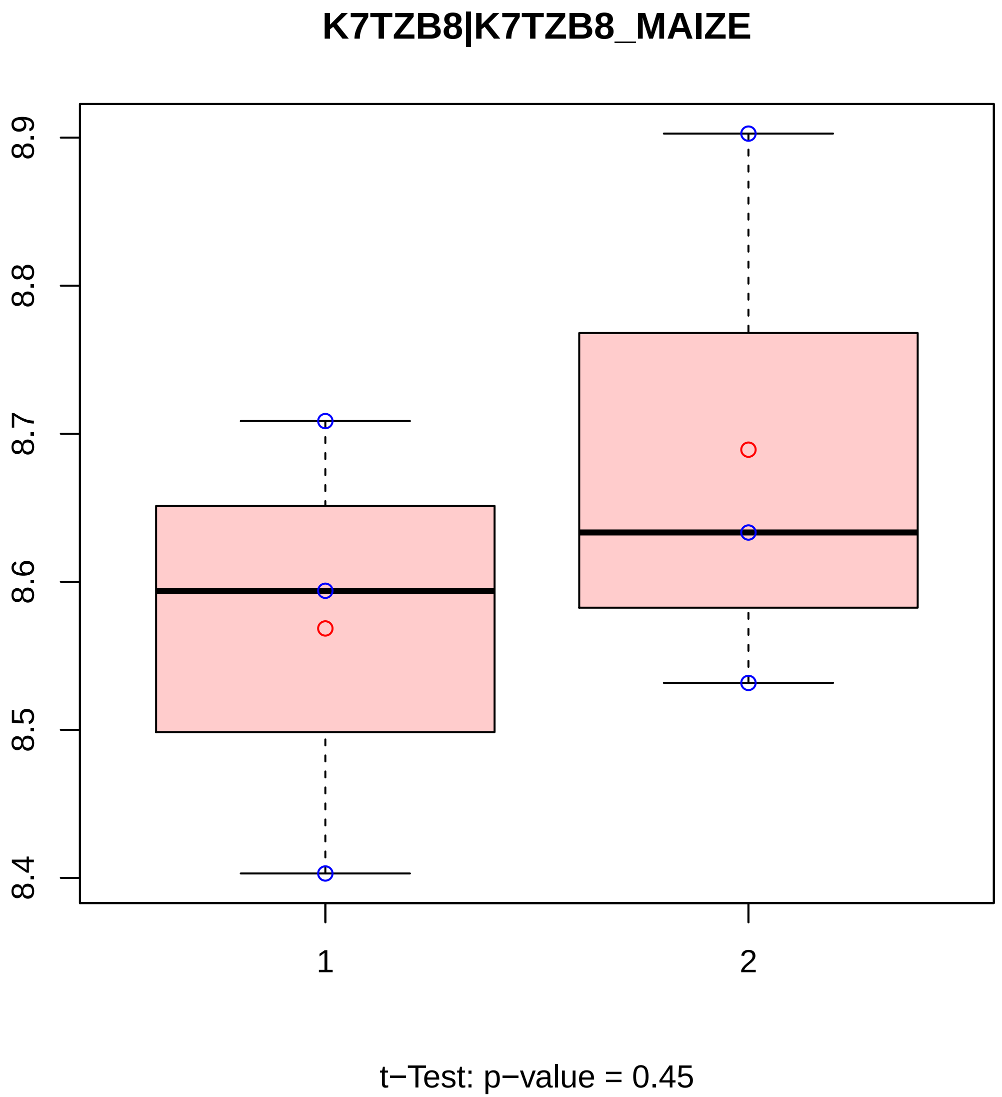
K7TZB8|K7TZB8_MAIZE
8.4
8.5
8.6
8.7
8.8
8.9
1
2
t−Test: p−value = 0.45

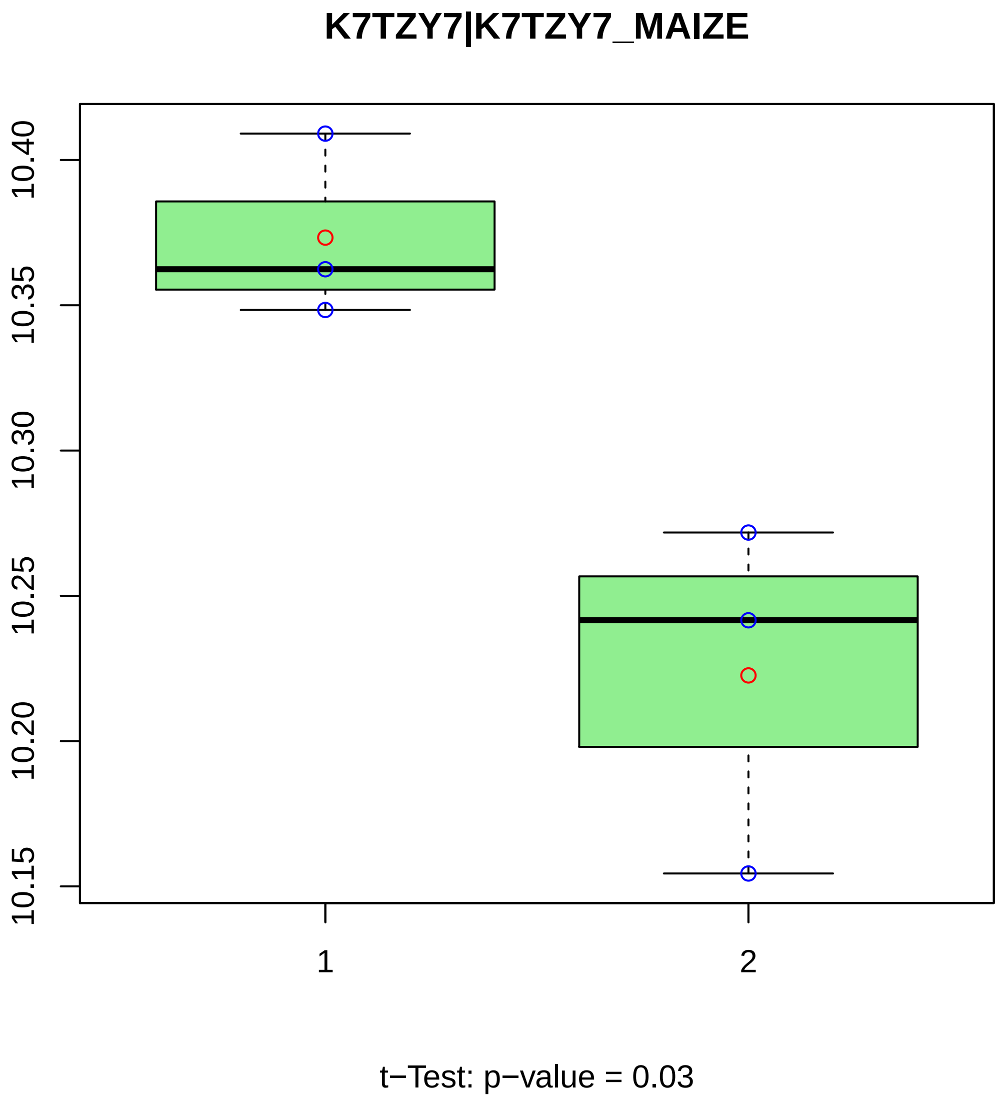

K7TZY7|K7TZY7_MAIZE
10.40
10.35
10.30
10.25
10.20
10.15
1
2
t−Test: p−value = 0.03

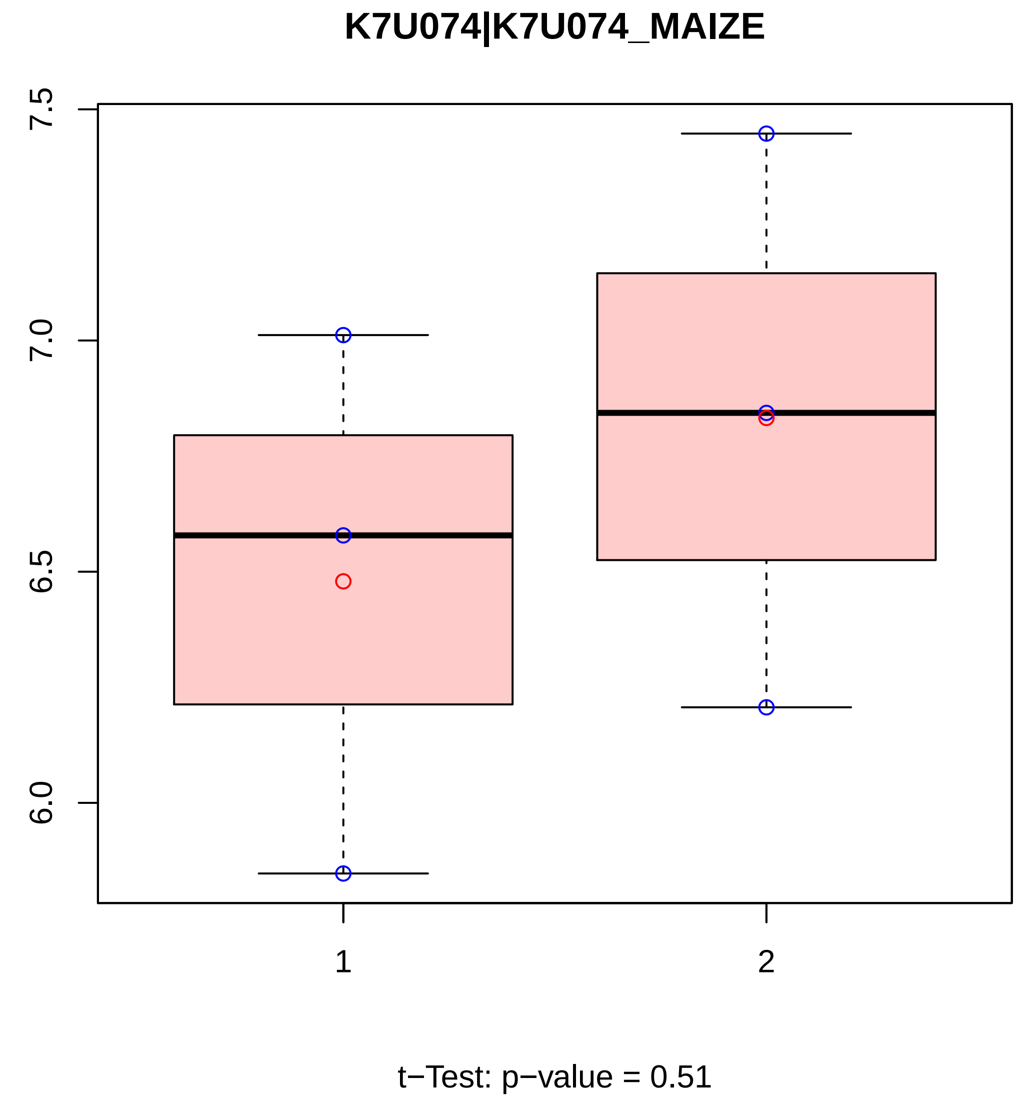

K7U074|K7U074_MAIZE
7.5
7.0
6.5
6.0
1
2
t−Test: p−value = 0.51

K7U135|K7U135_MAIZE

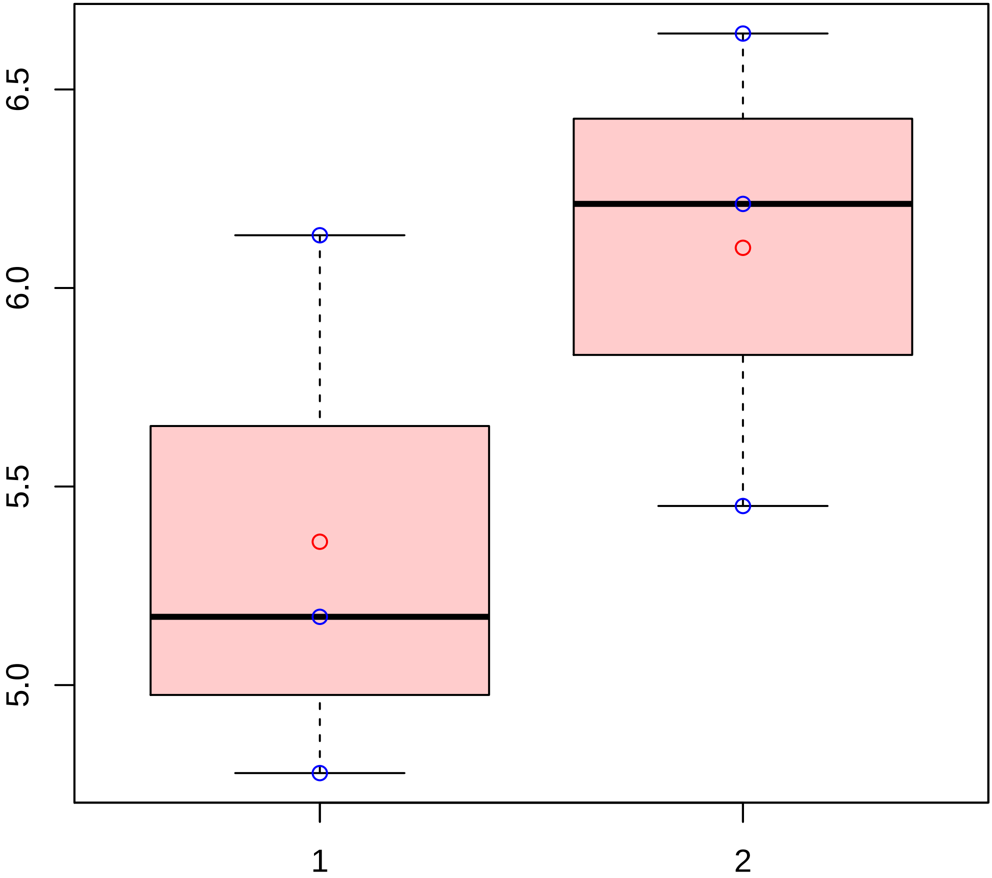

t−Test: p−value = 0.24

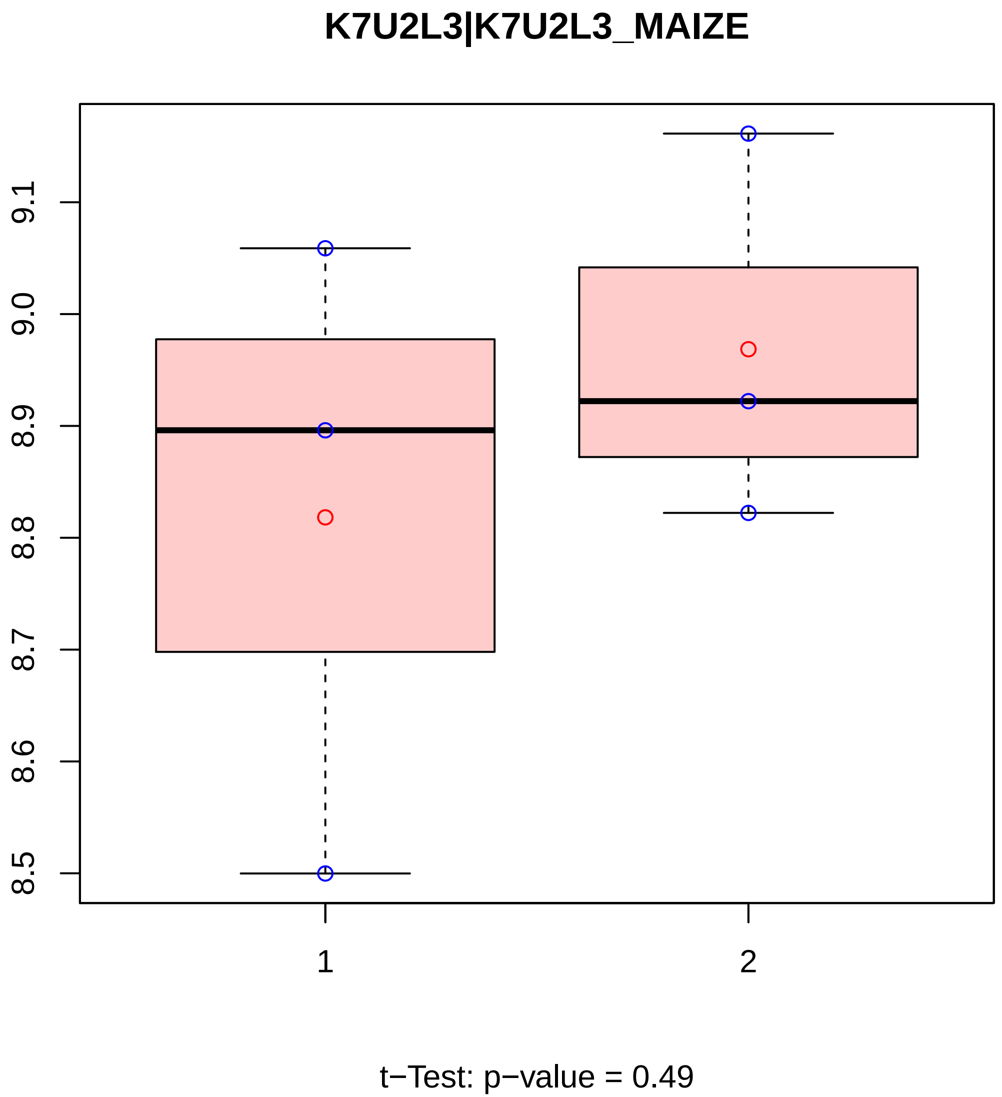
K7U2L3|K7U2L3_MAIZE
9.1
9.0
8.9
8.8
8.7
8.6
8.5
1
2
t−Test: p−value = 0.49

K7U3B1|K7U3B1_MAIZE

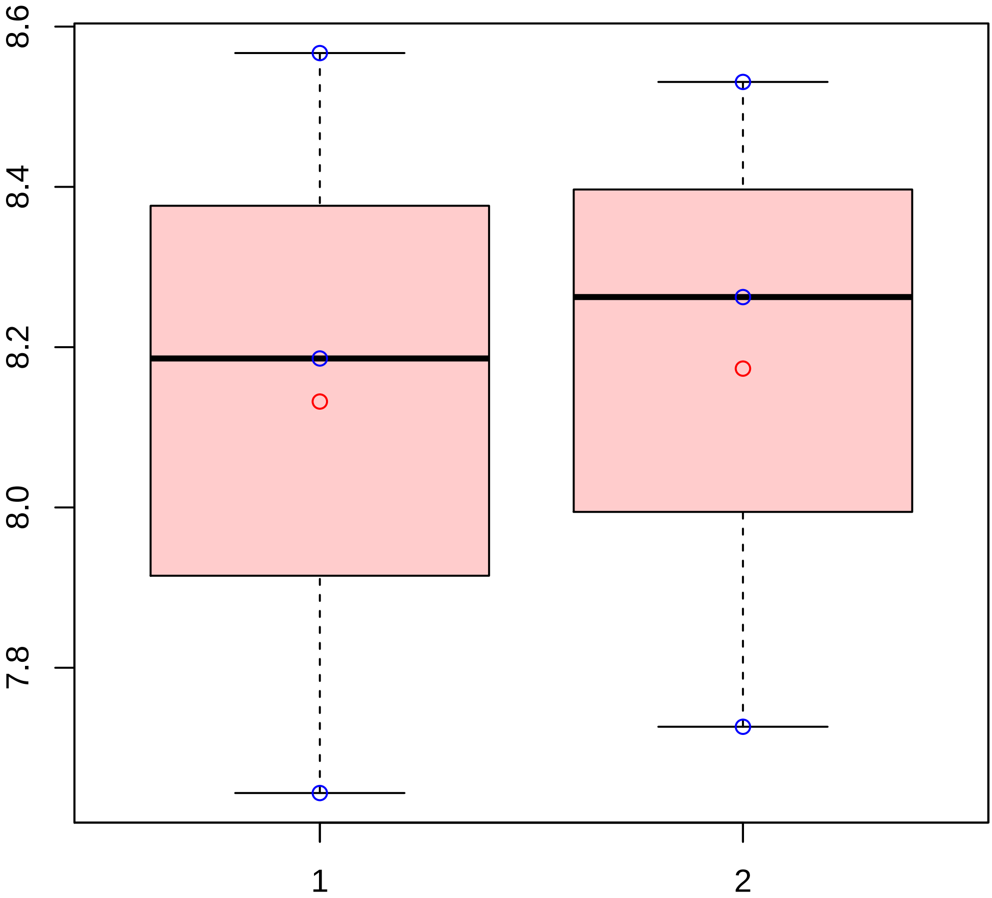

t−Test: p−value = 0.91

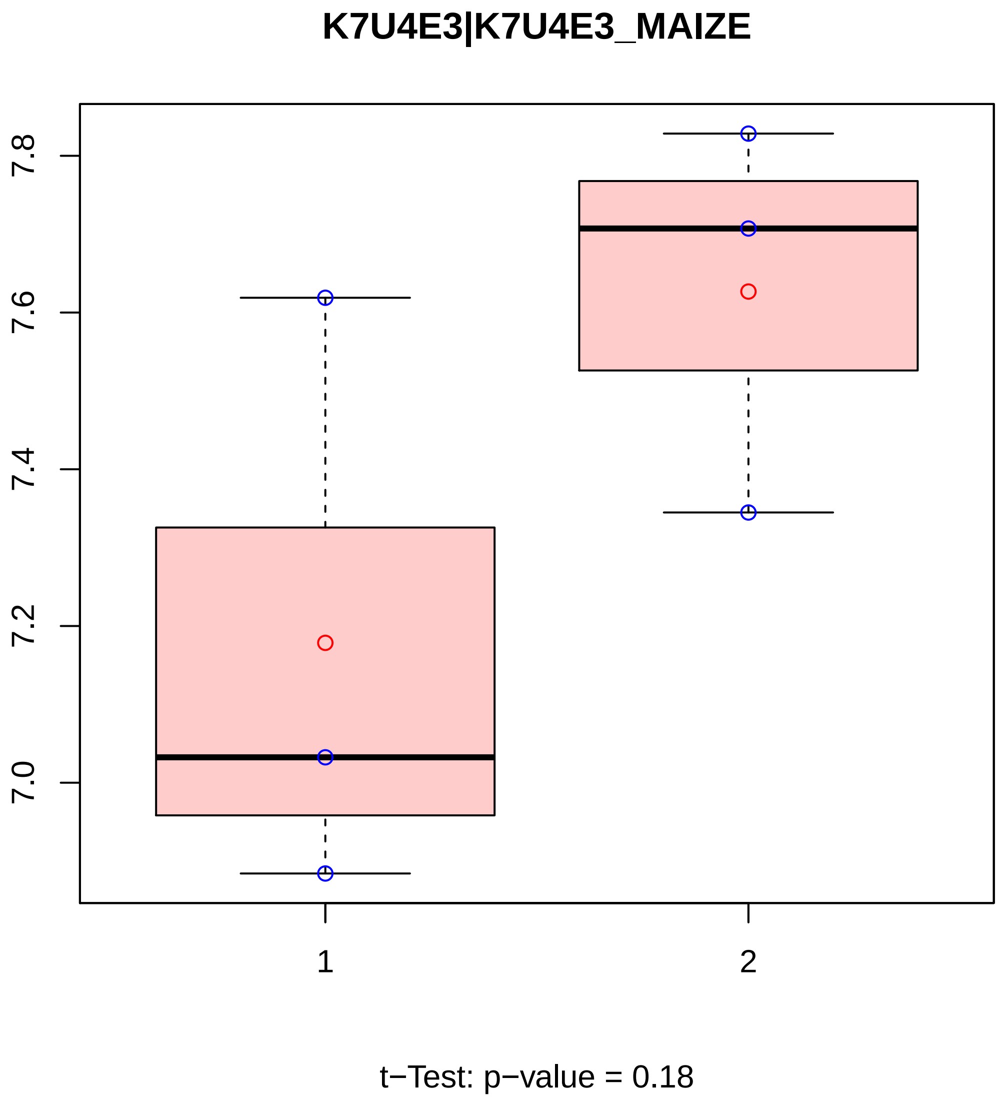
K7U4E3|K7U4E3_MAIZE
7.0
7.2
7.4
7.6
7.8
1
2
t−Test: p−value = 0.18

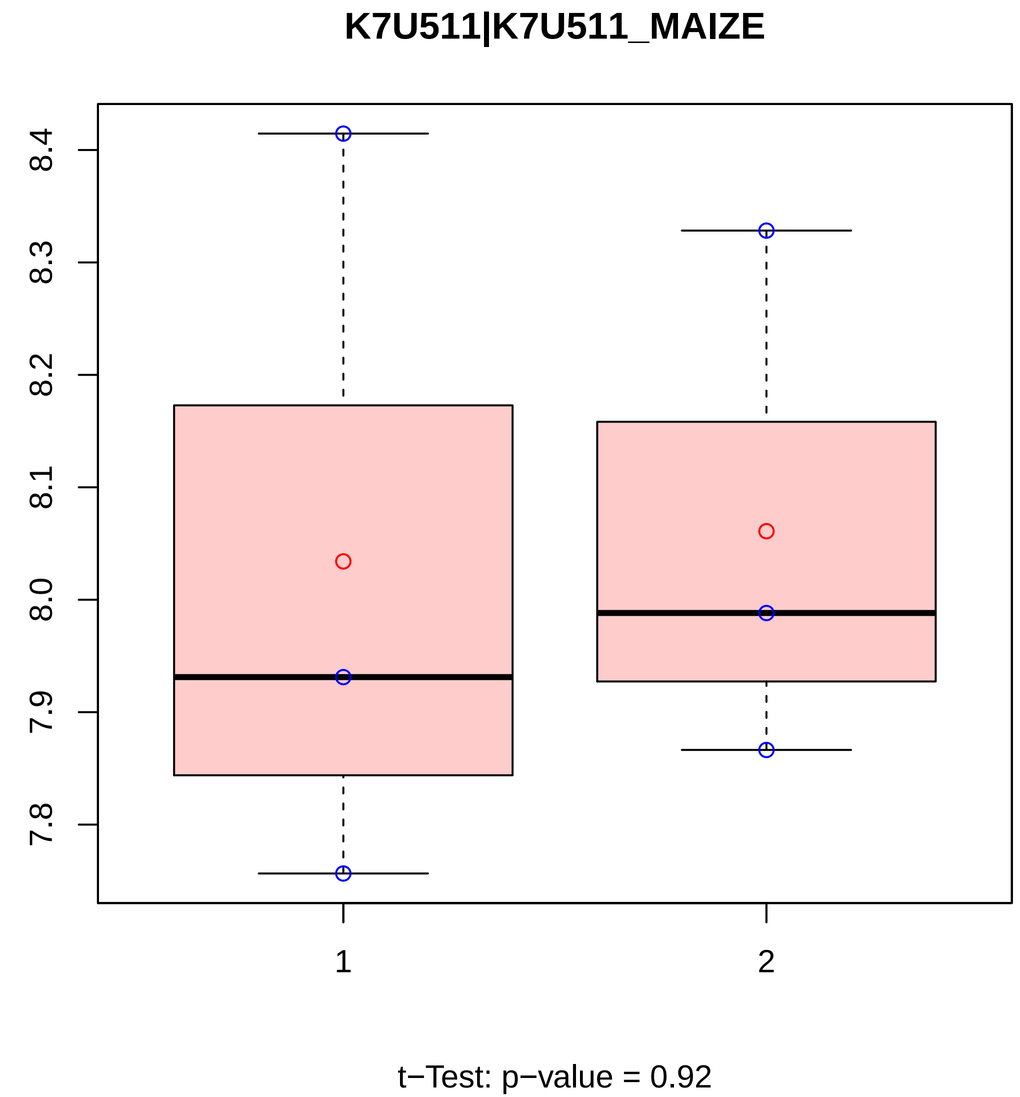
K7U511|K7U511_MAIZE
8.4
8.3
8.2
8.1
8.0
7.9
7.8
1
2
t−Test: p−value = 0.92

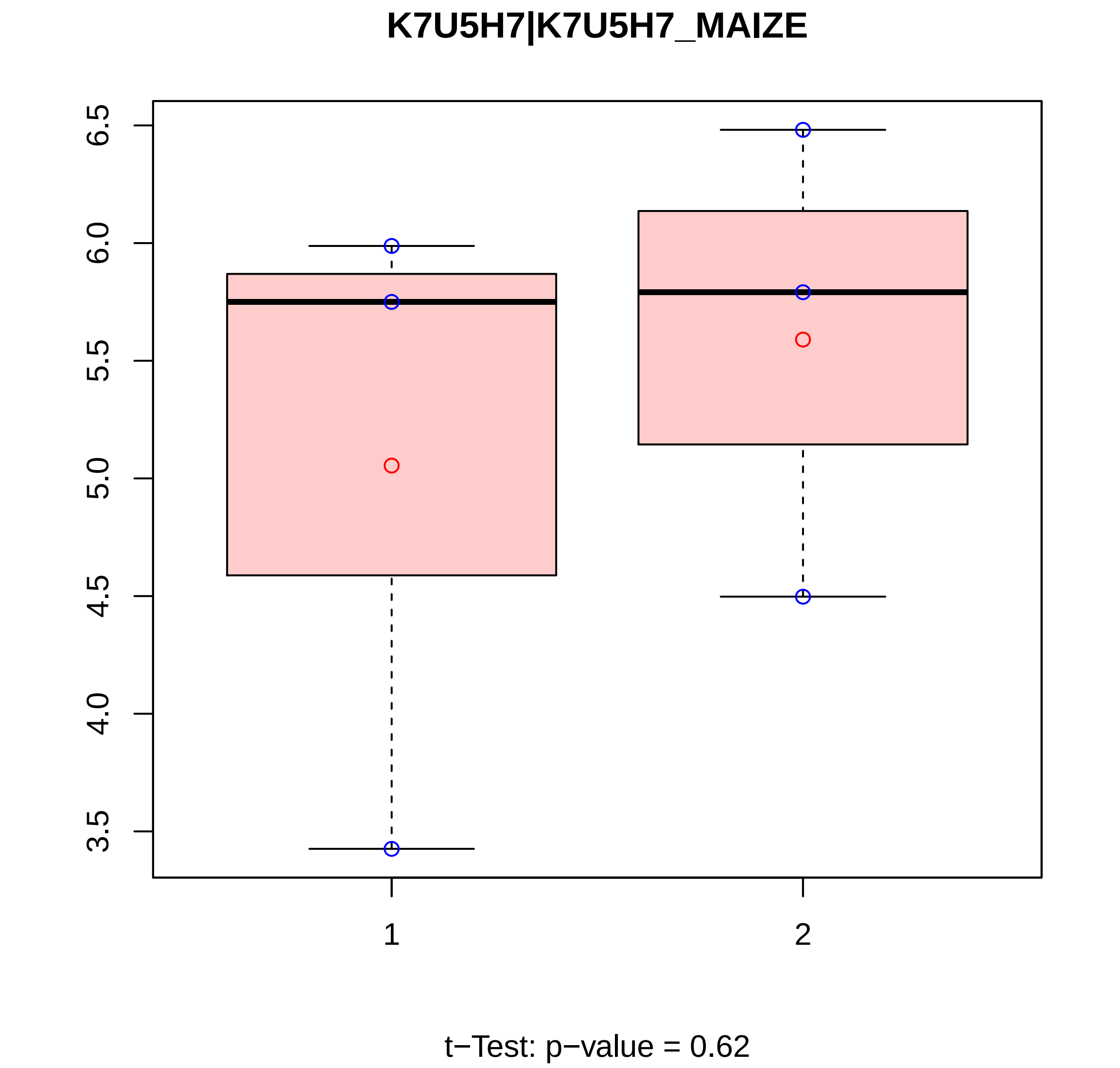
K7U5H7|K7U5H7_MAIZE
6.5
6.0
5.5
5.0
4.5
4.0
3.5
1
2
t−Test: p−value = 0.62

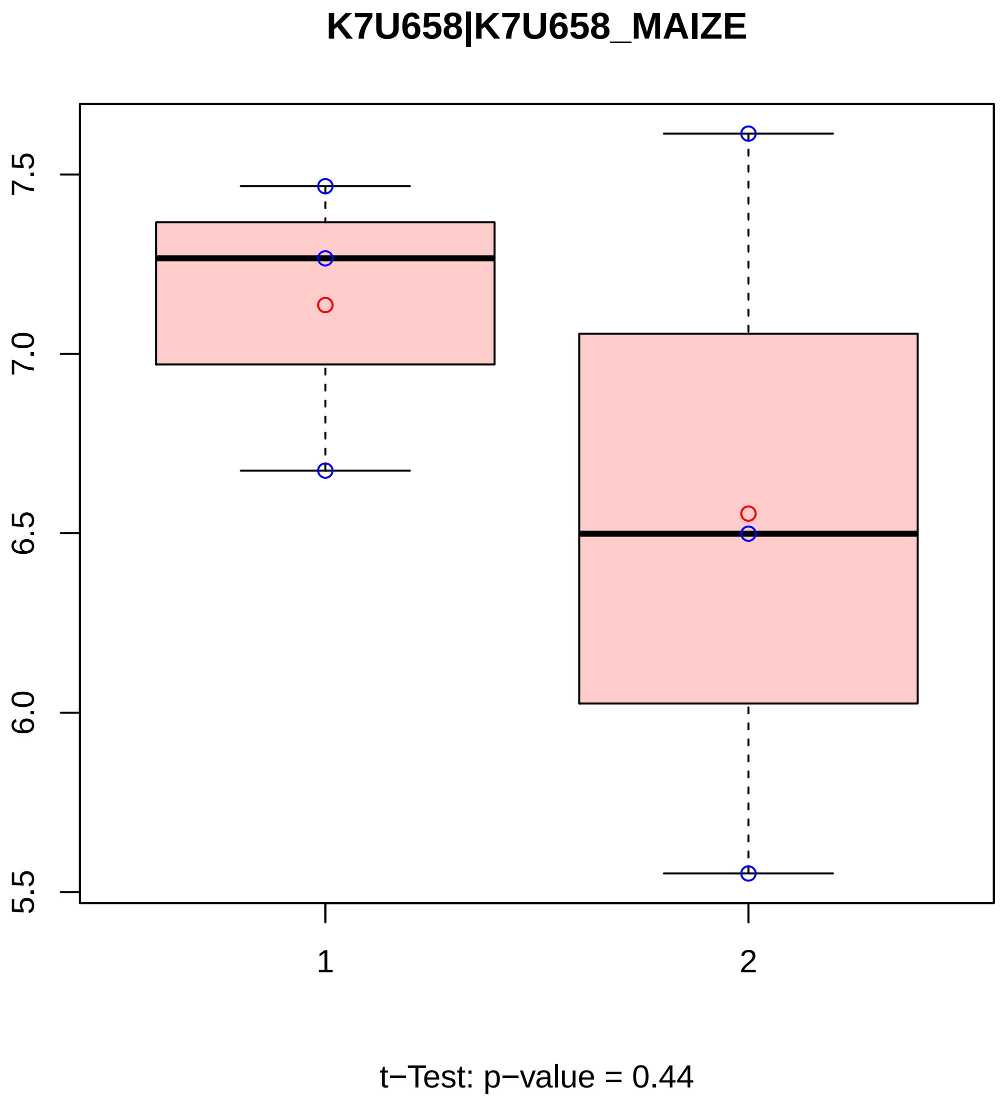
K7U658|K7U658_MAIZE
7.5
7.0
6.5
6.0
5.5
1
2
t−Test: p−value = 0.44

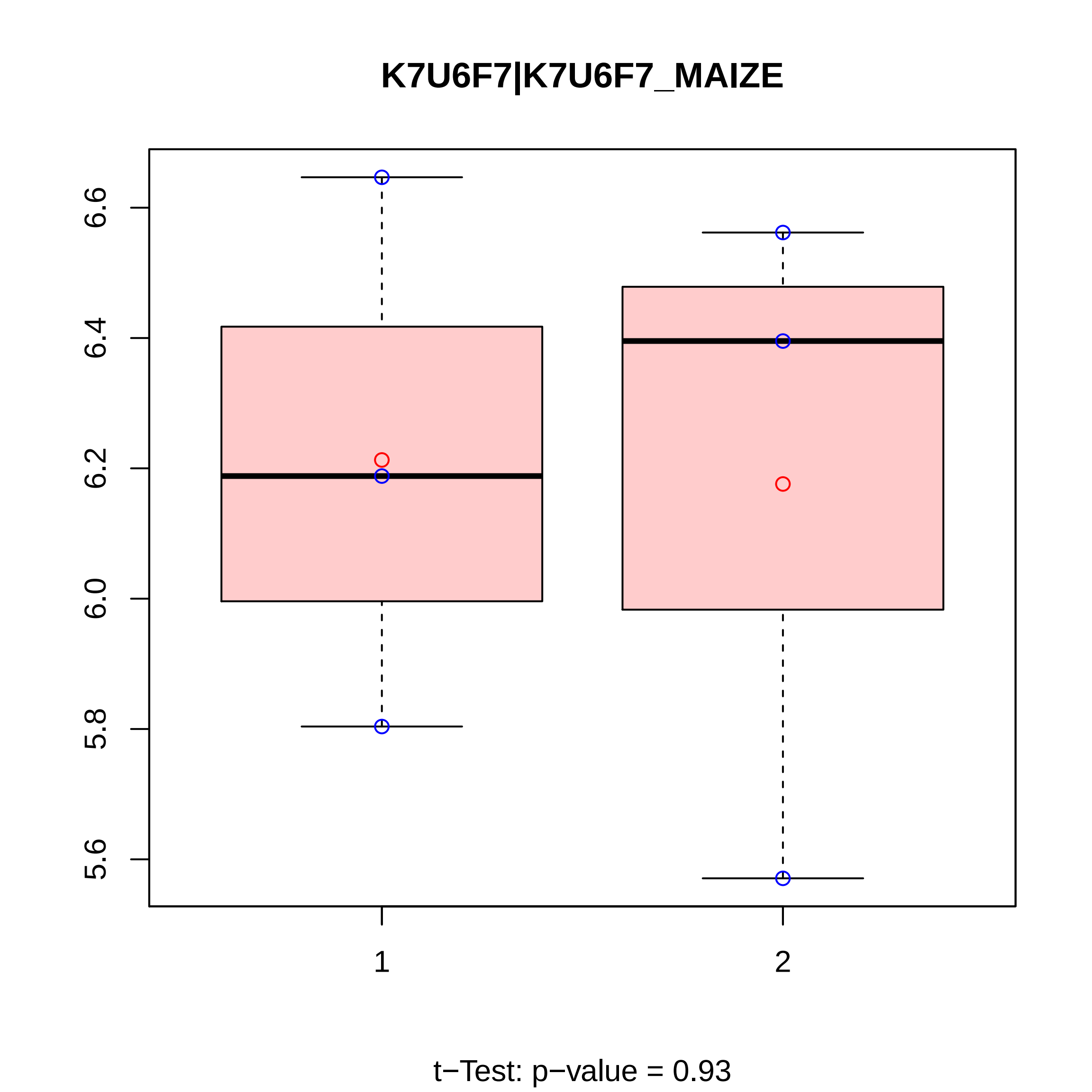
K7U6F7|K7U6F7_MAIZE
6.6
6.4
6.2
6.0
5.8
5.6
1
2
t−Test: p−value = 0.93

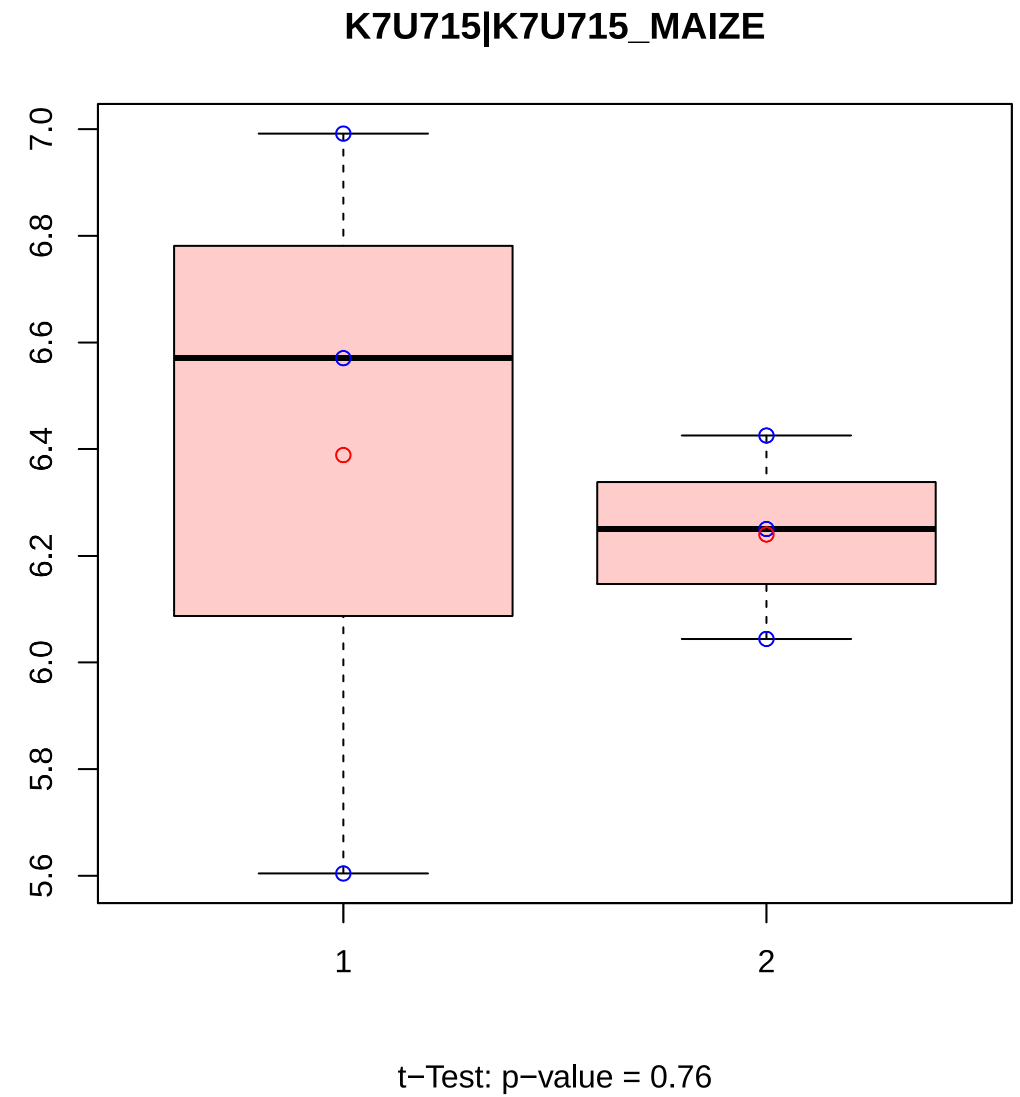

K7U715|K7U715_MAIZE
7.0
6.8
6.6
6.4
6.2
6.0
5.8
5.6
1
2
t−Test: p−value = 0.76

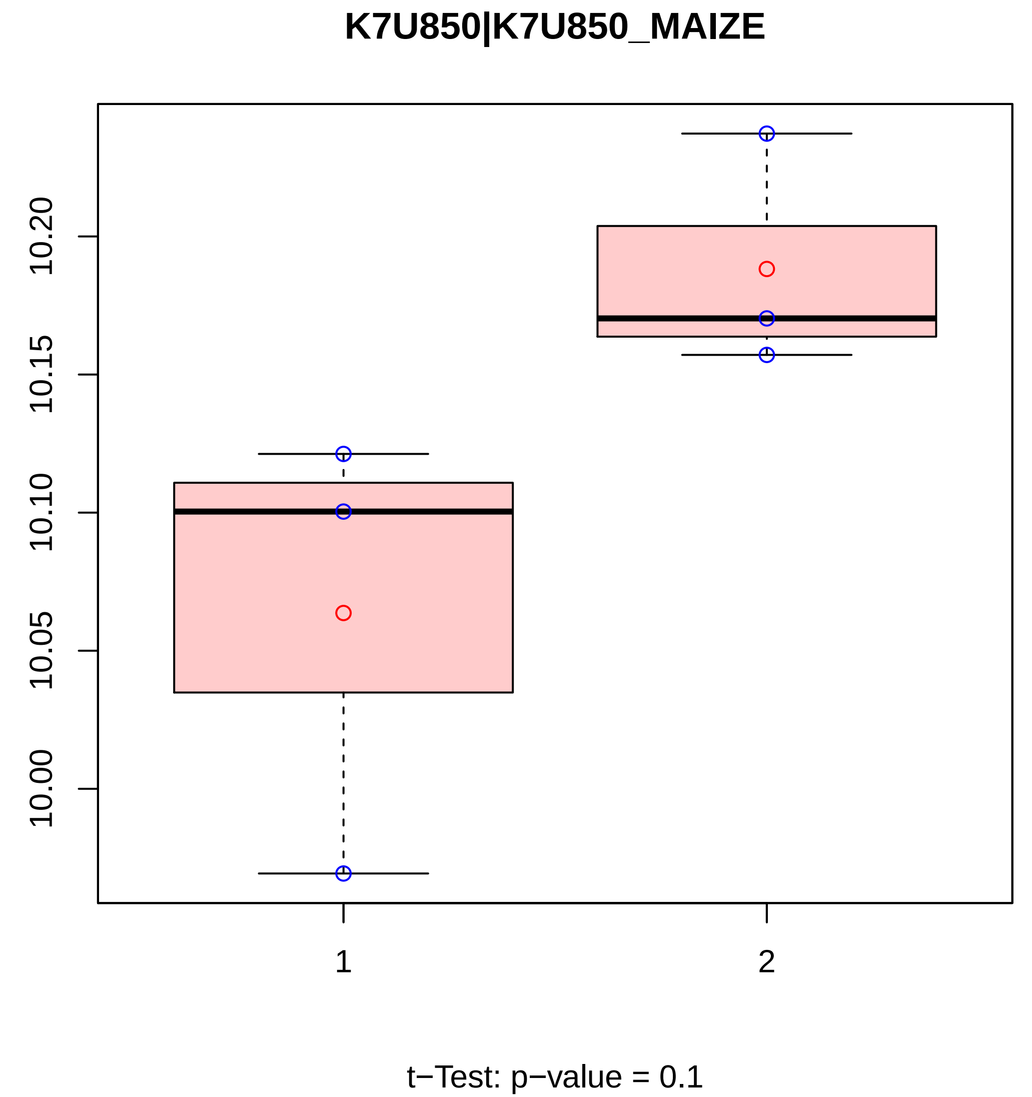
K7U850|K7U850_MAIZE
10.00
10.05
10.10
10.15
10.20
1
2
t−Test: p−value = 0.1

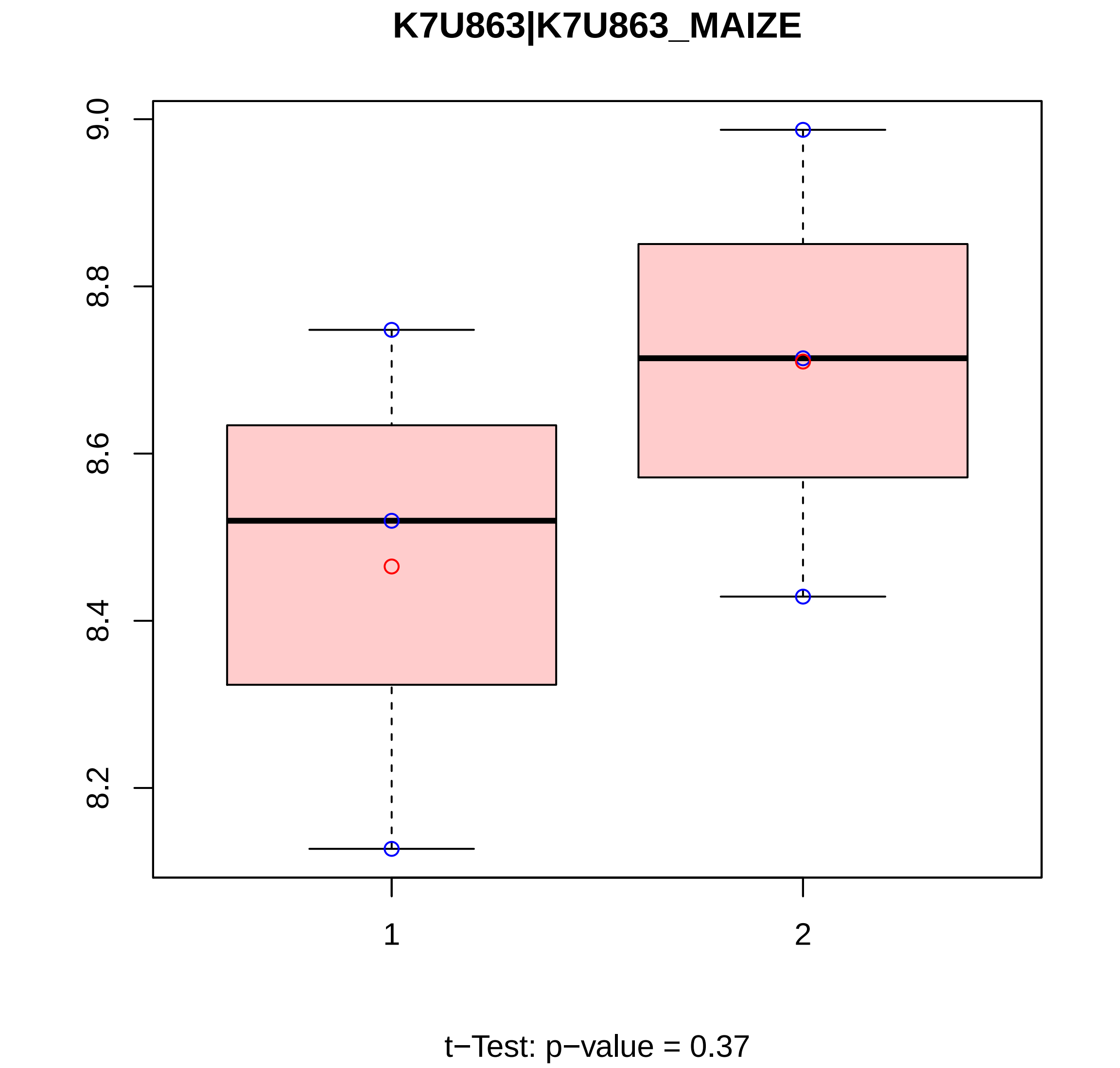

K7U863|K7U863_MAIZE
9.0
8.8
8.6
8.4
8.2
1
2
t−Test: p−value = 0.37

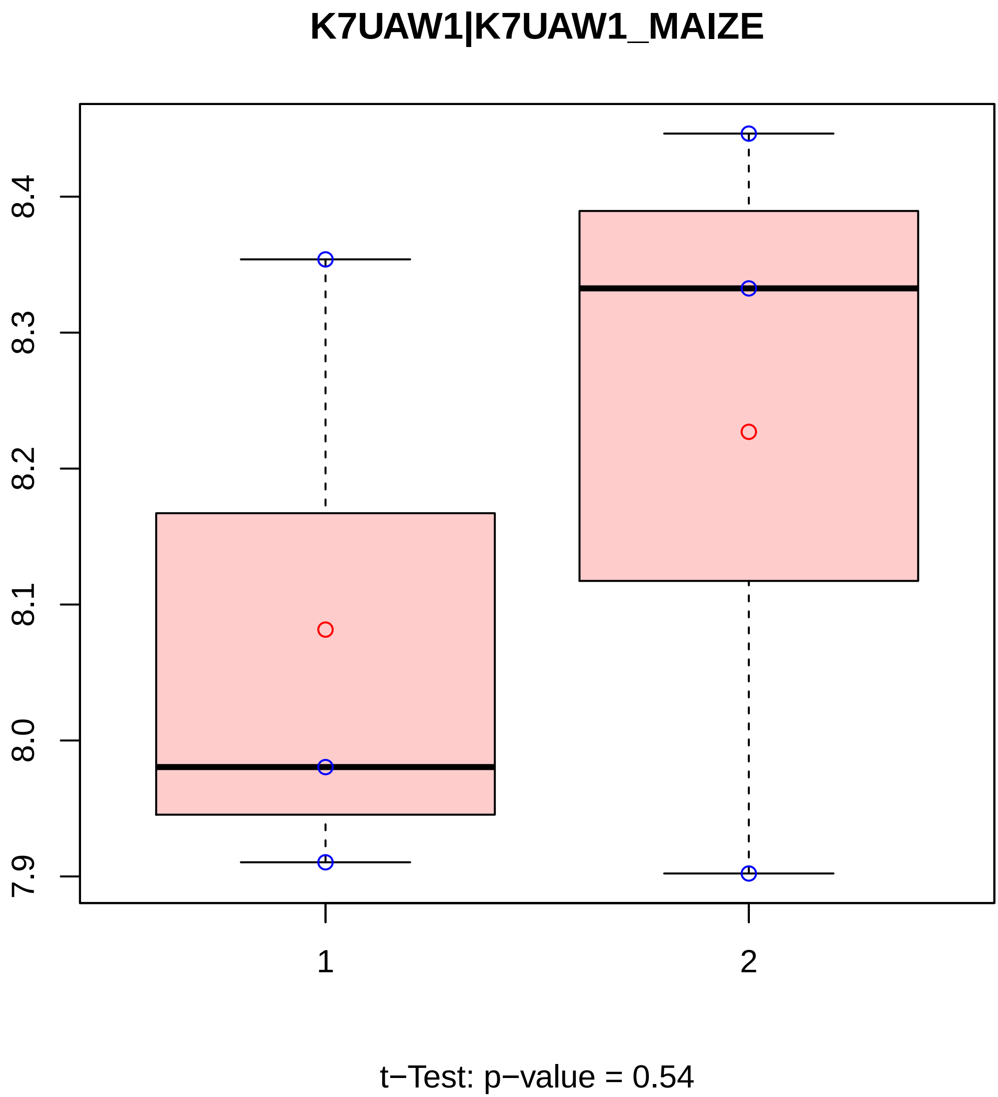
K7UAW1|K7UAW1_MAIZE
8.4
8.3
8.2
8.1
8.0
7.9
1
2
t−Test: p−value = 0.54

K7UB00|K7UB00_MAIZE

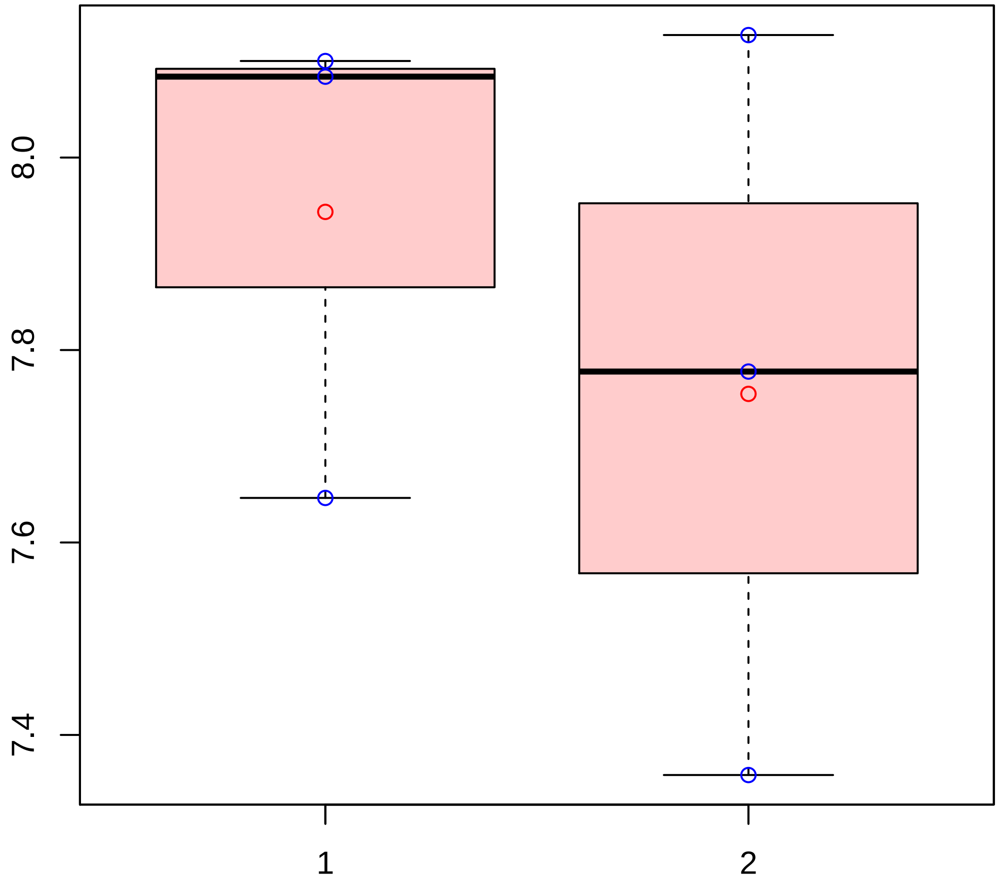

t−Test: p−value = 0.52

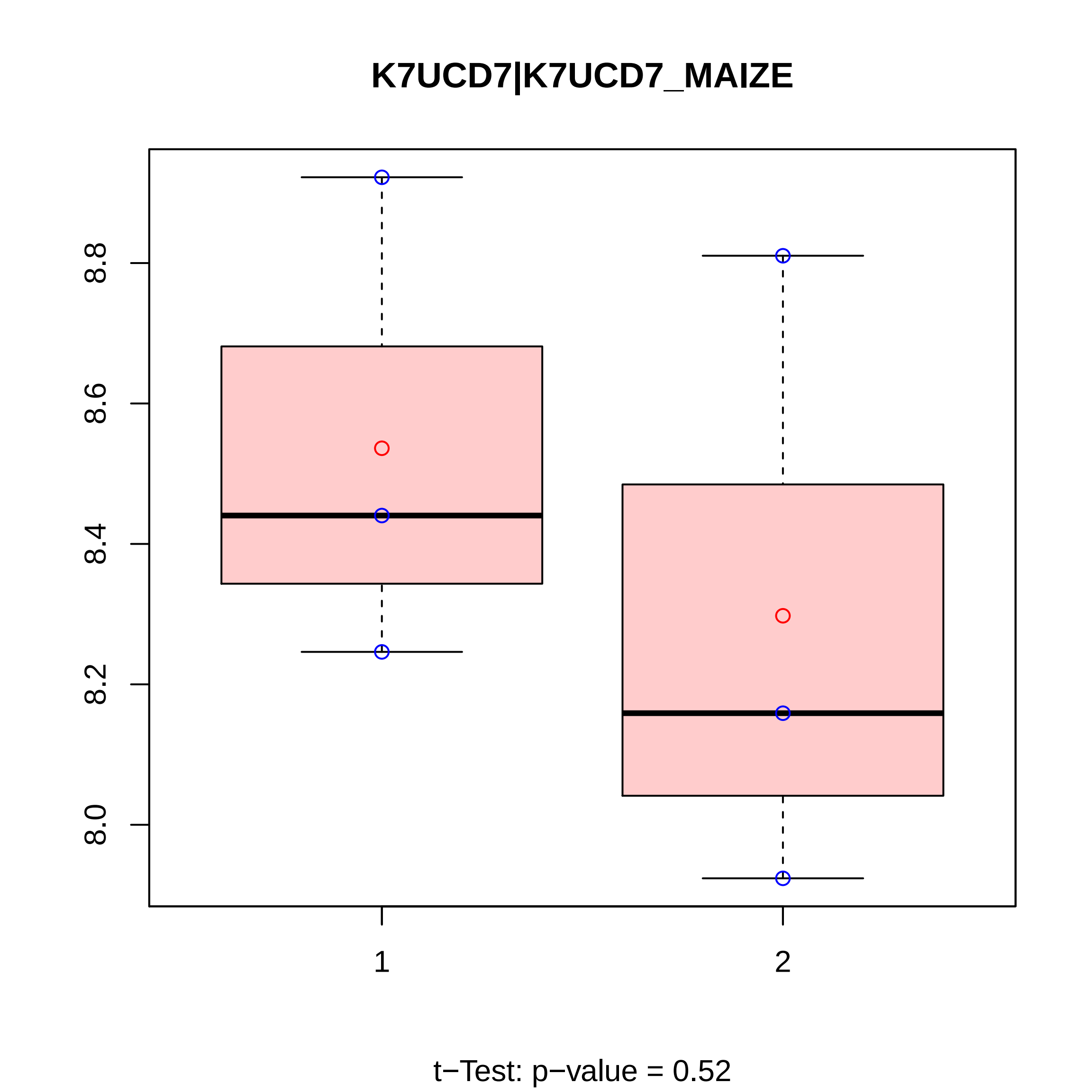
K7UCD7|K7UCD7_MAIZE
8.0
8.2
8.4
8.6
8.8
1
2
t−Test: p−value = 0.52

K7UCV5|K7UCV5_MAIZE

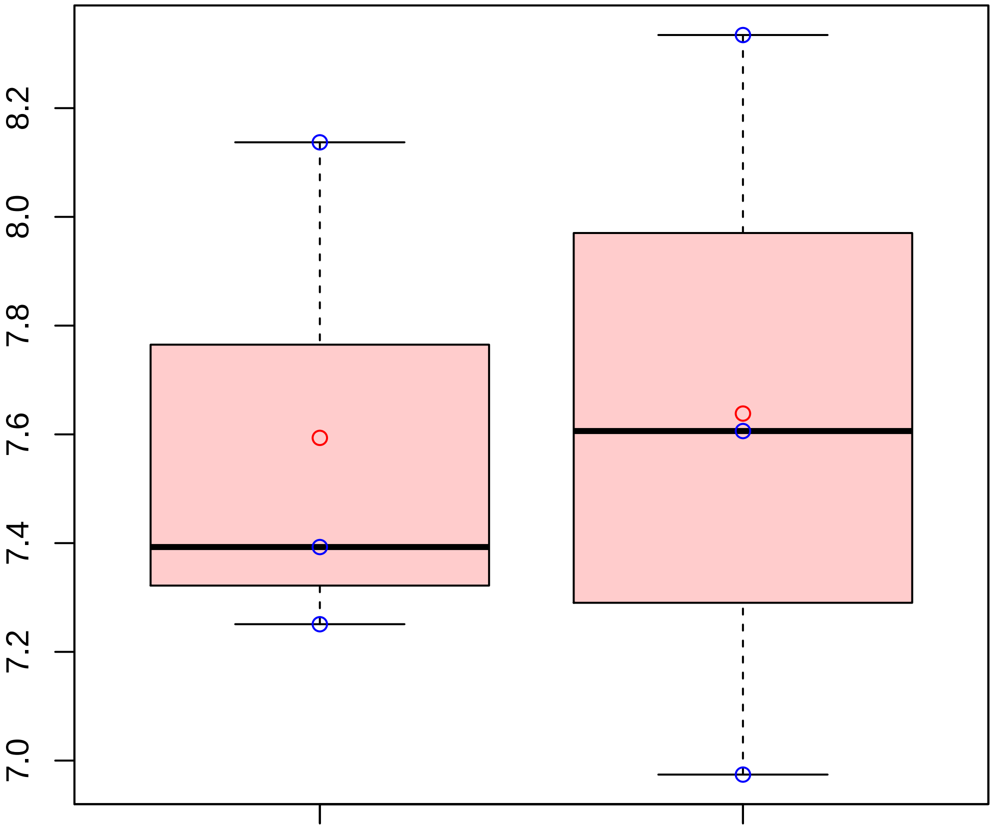

t−Test: p−value = 0.93

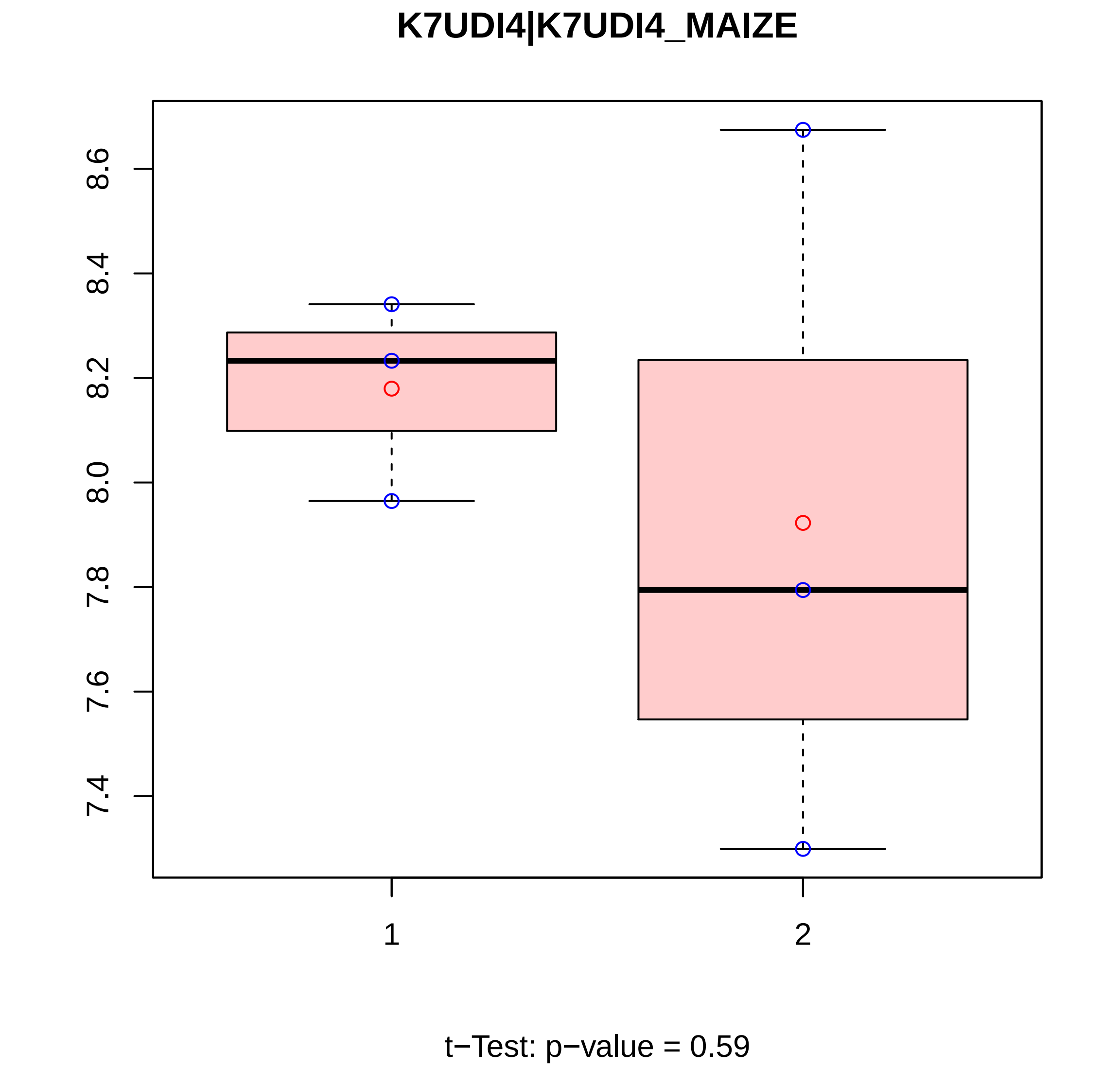
K7UDI4|K7UDI4_MAIZE
8.6
8.4
8.2
8.0
7.8
7.6
7.4
1
2
t−Test: p−value = 0.59

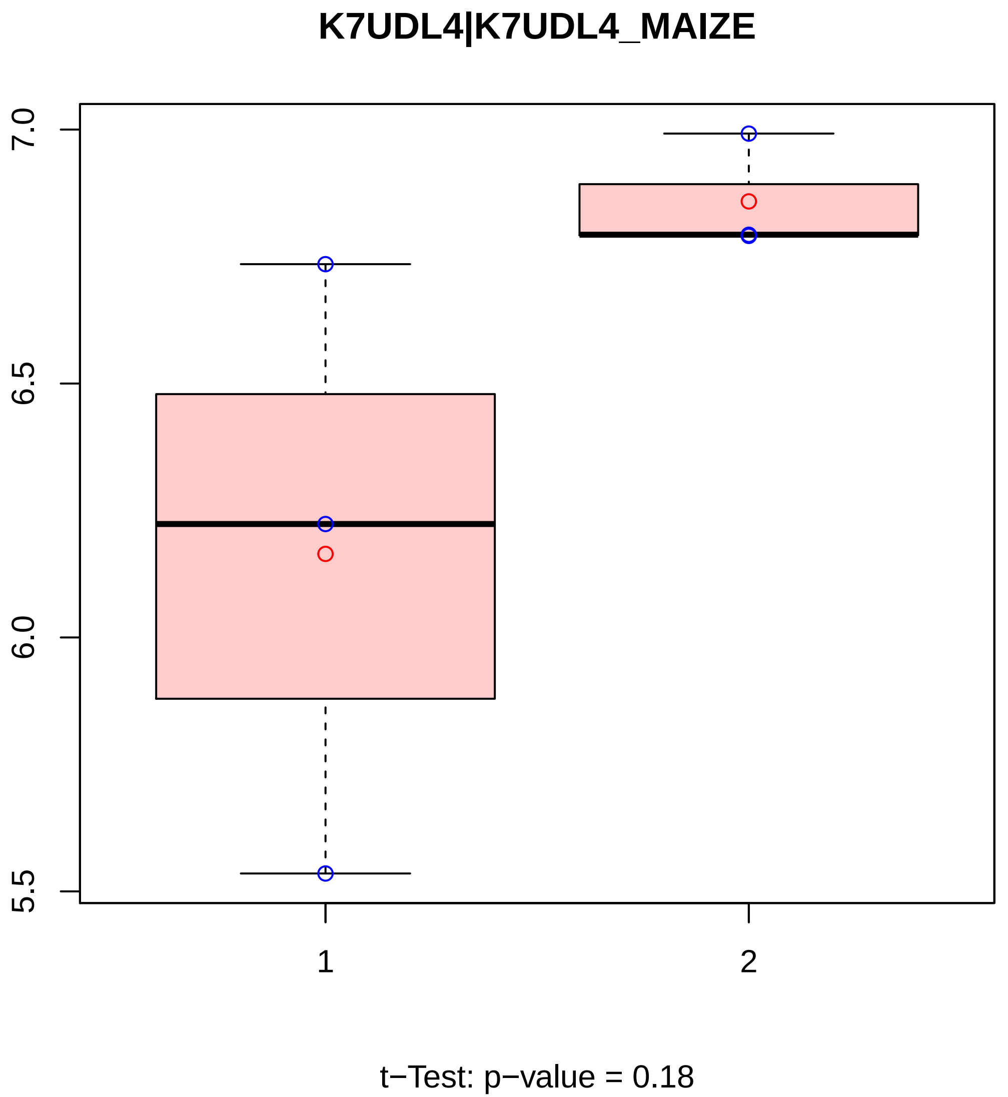
K7UDL4|K7UDL4_MAIZE
7.0
6.5
6.0
5.5
1
2
t−Test: p−value = 0.18

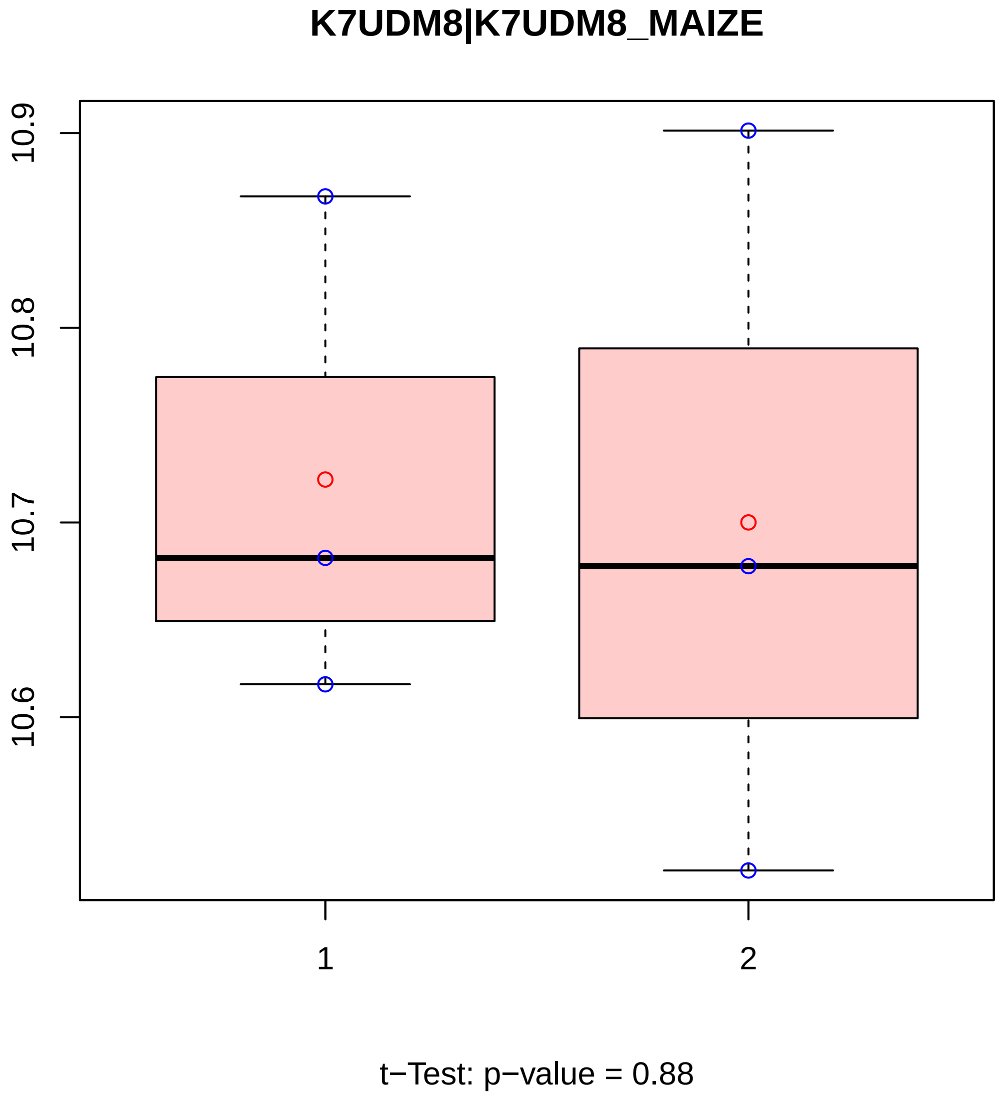
K7UDM8|K7UDM8_MAIZE
10.9
10.8
10.7
10.6
1
2
t−Test: p−value = 0.88

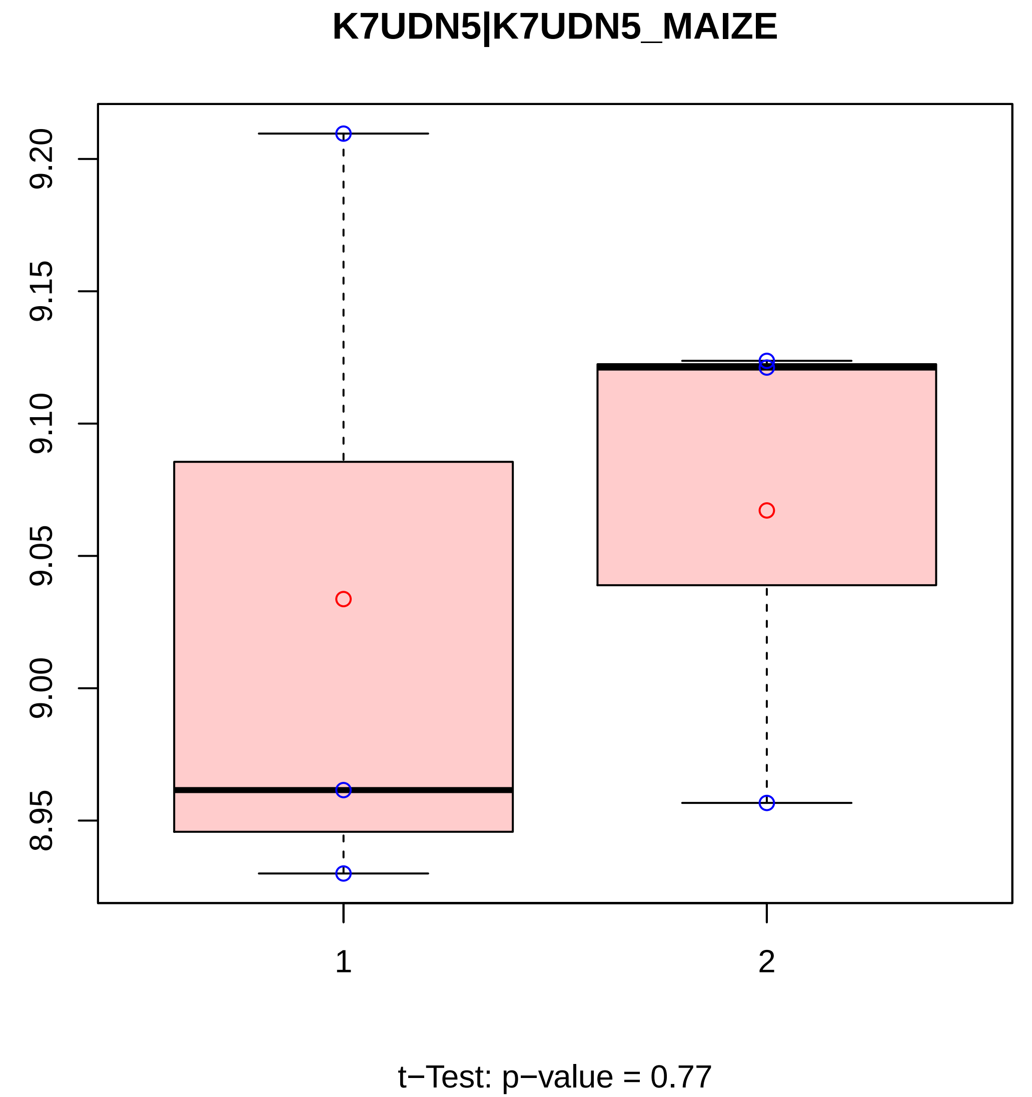
K7UDN5|K7UDN5_MAIZE
9.20
9.15
9.10
9.05
9.00
8.95
1
2
t−Test: p−value = 0.77

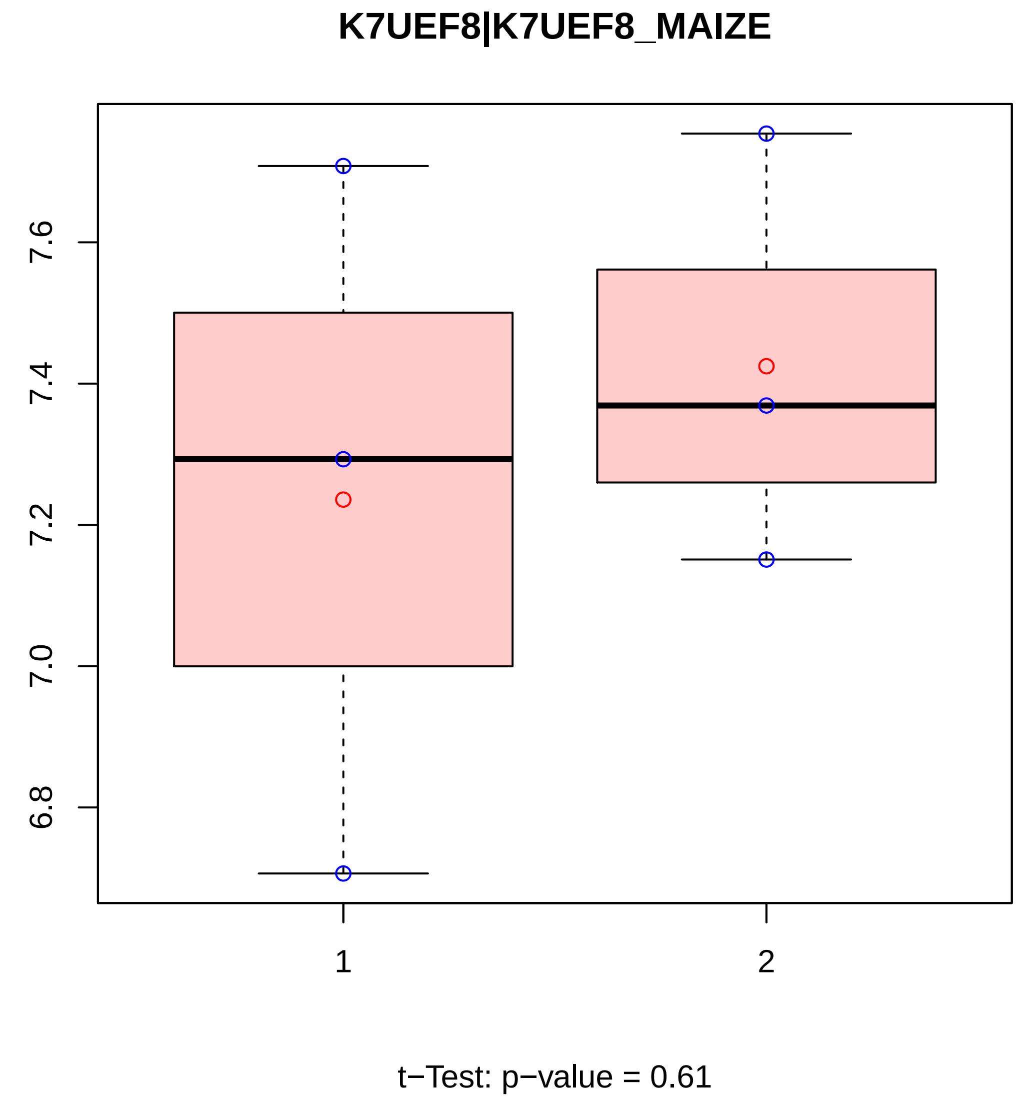
K7UEF8|K7UEF8_MAIZE
6.8
7.0
7.2
7.4
7.6
1
2
t−Test: p−value = 0.61

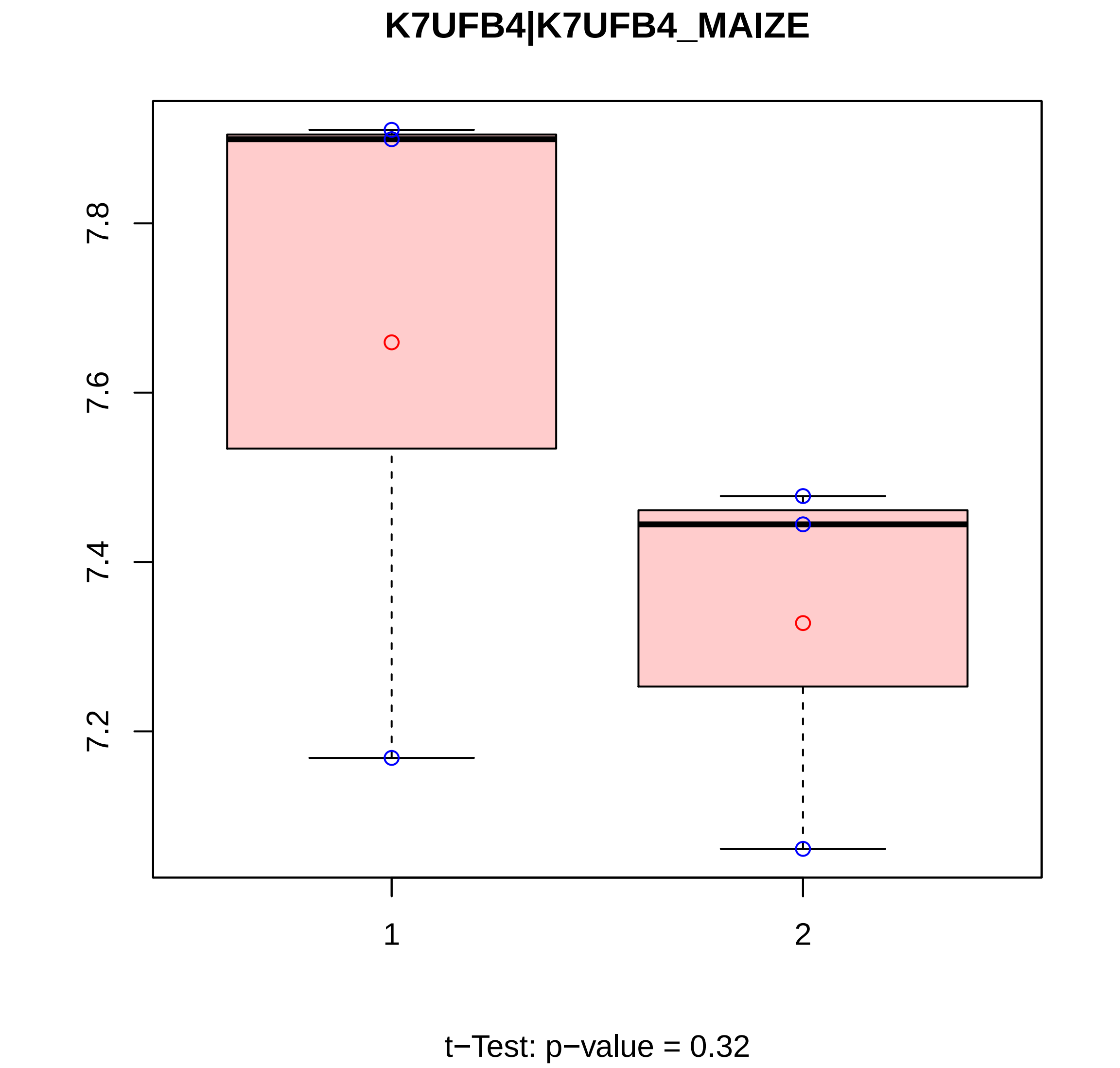
K7UFB4|K7UFB4_MAIZE
7.2
7.4
7.6
7.8
1
2
t−Test: p−value = 0.32

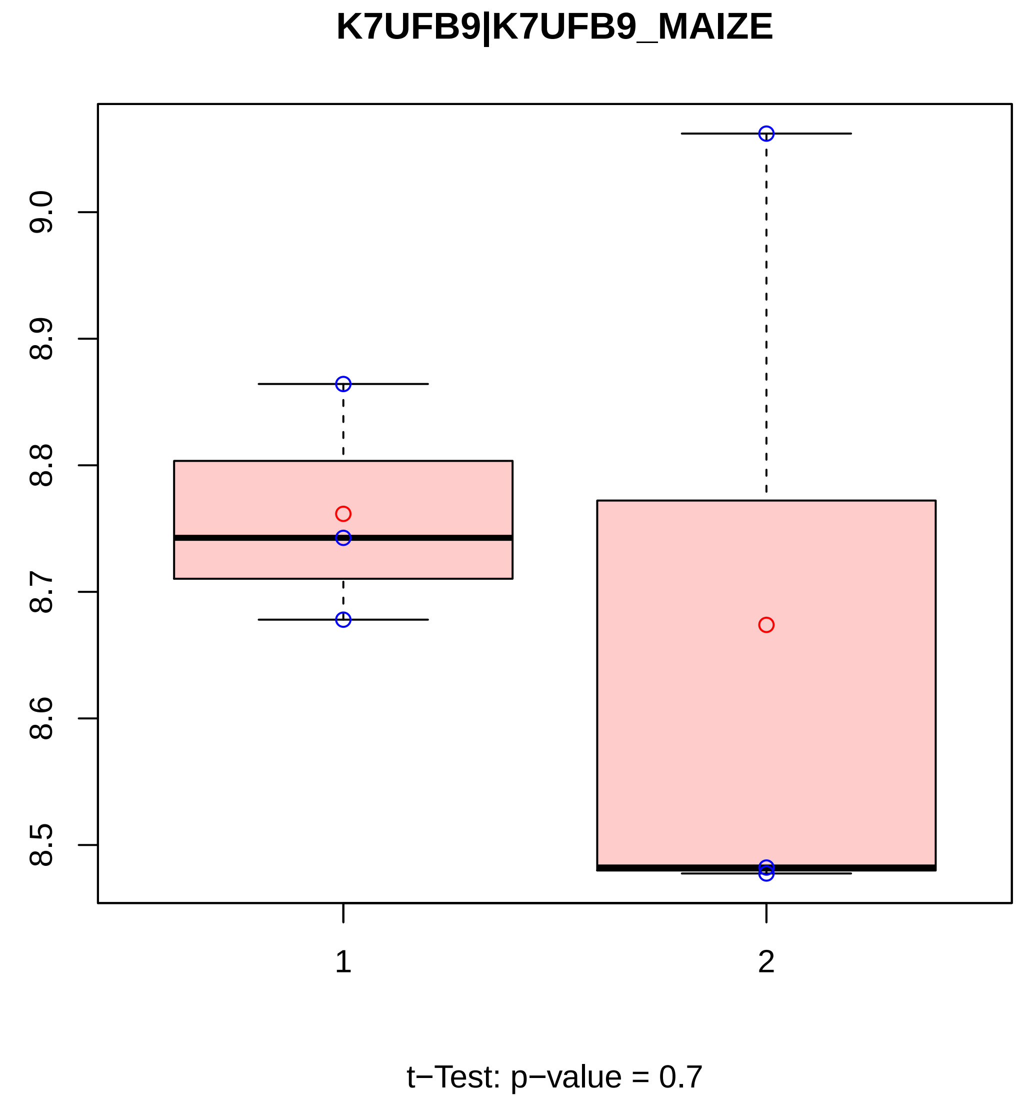

K7UFB9|K7UFB9_MAIZE
9.0
8.9
8.8
8.7
8.6
8.5
1
2
t−Test: p−value = 0.7

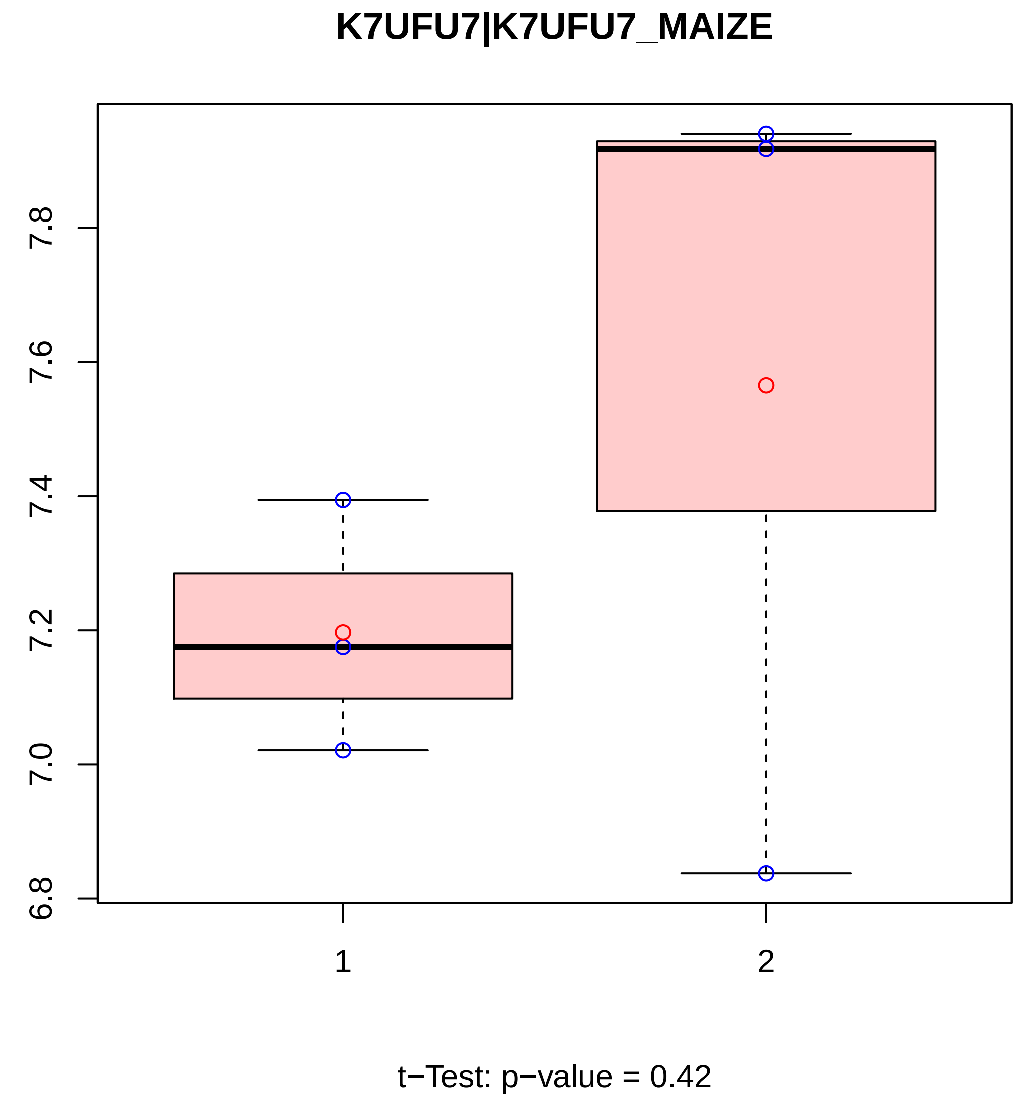
K7UFU7|K7UFU7_MAIZE
7.8
7.6
7.4
7.2
7.0
6.8
1
2
t−Test: p−value = 0.42

K7UFW0|K7UFW0_MAIZE

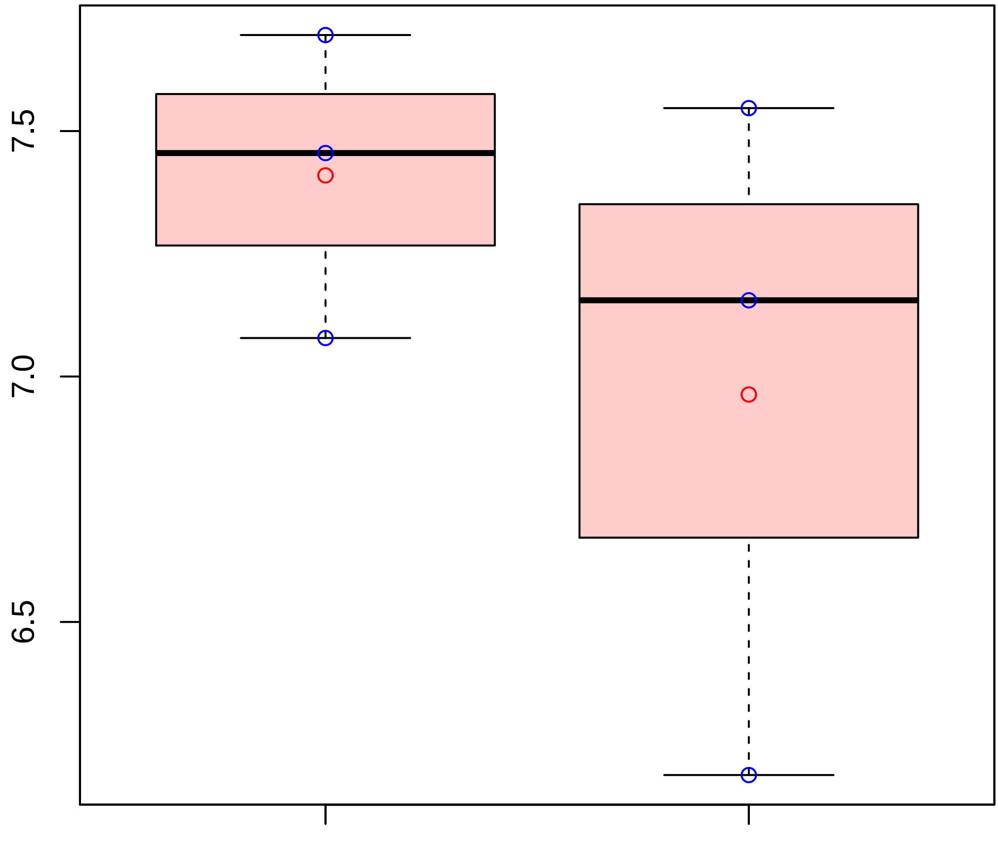

t−Test: p−value = 0.39

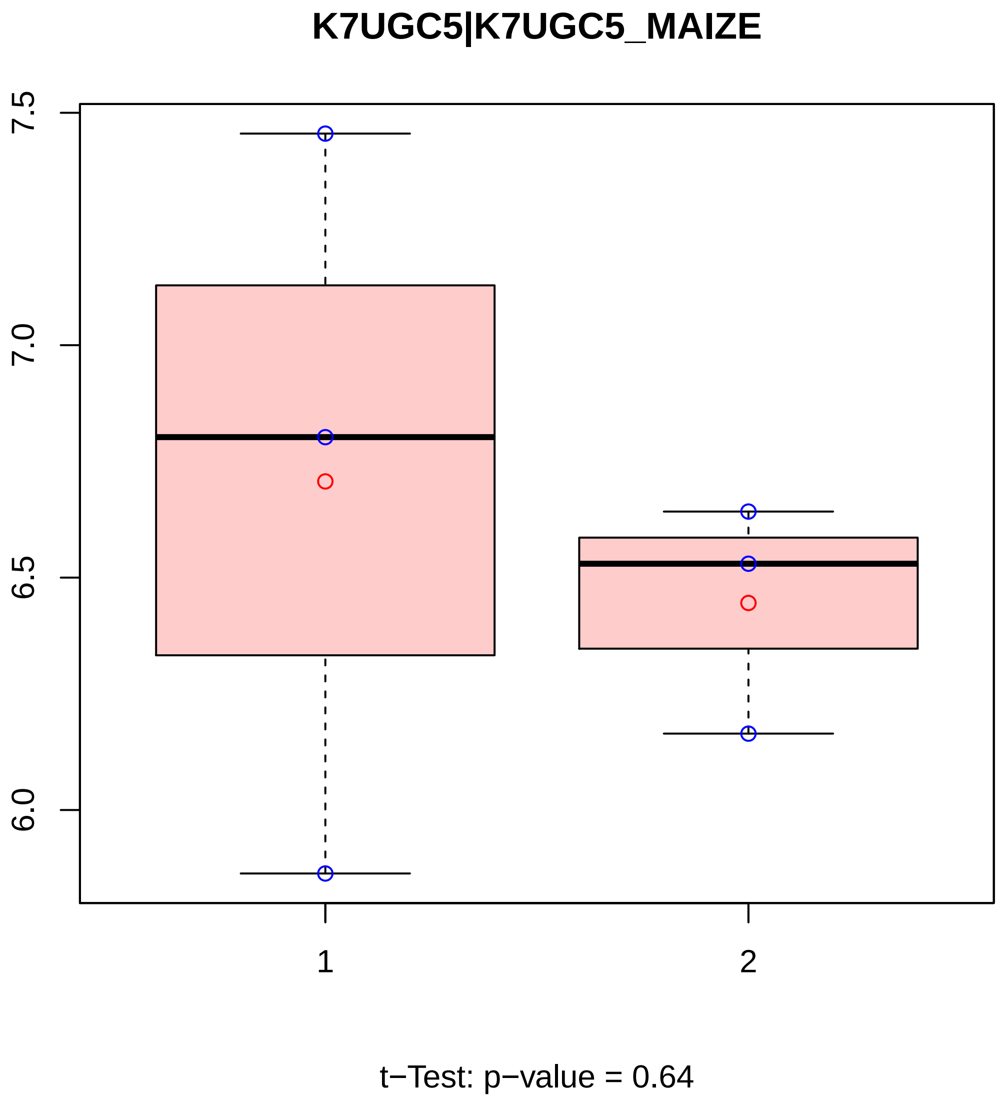

K7UGC5|K7UGC5_MAIZE
7.5
7.0
6.5
6.0
1
2
t−Test: p−value = 0.64

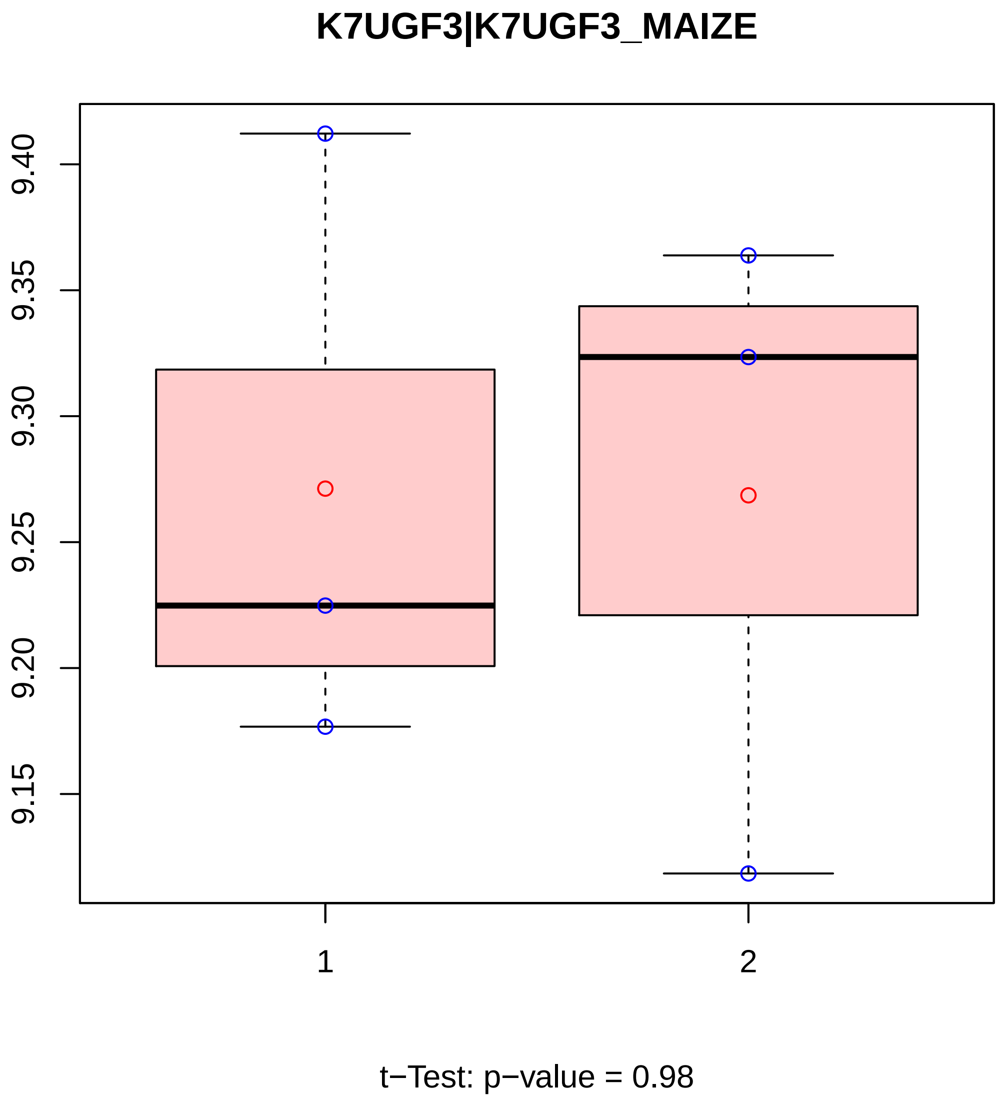
K7UGF3|K7UGF3_MAIZE
9.40
9.35
9.30
9.25
9.20
9.15
1
2
t−Test: p−value = 0.98

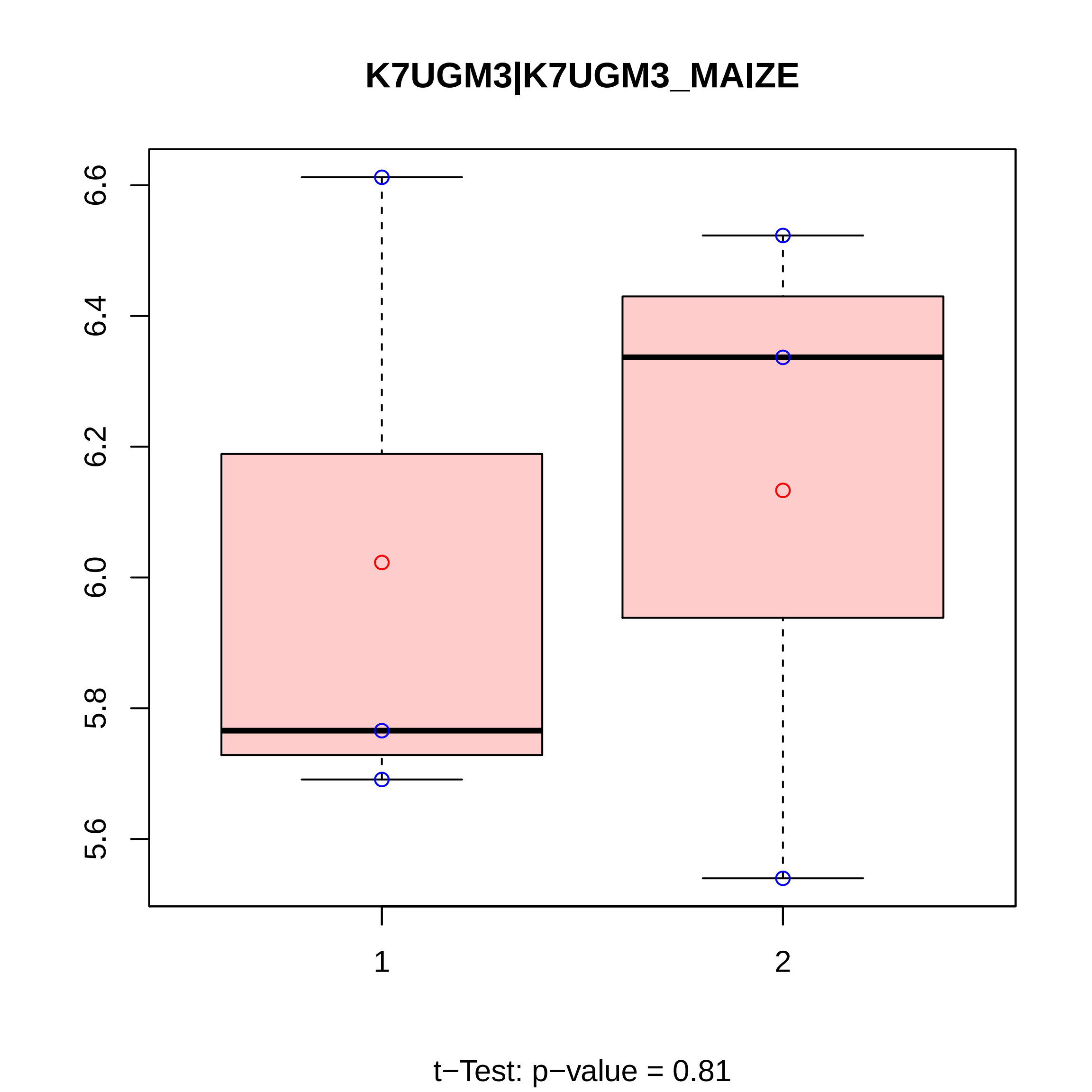
K7UGM3|K7UGM3_MAIZE
6.6
6.4
6.2
6.0
5.8
5.6
1
2
t−Test: p−value = 0.81

K7UHV6|K7UHV6_MAIZE

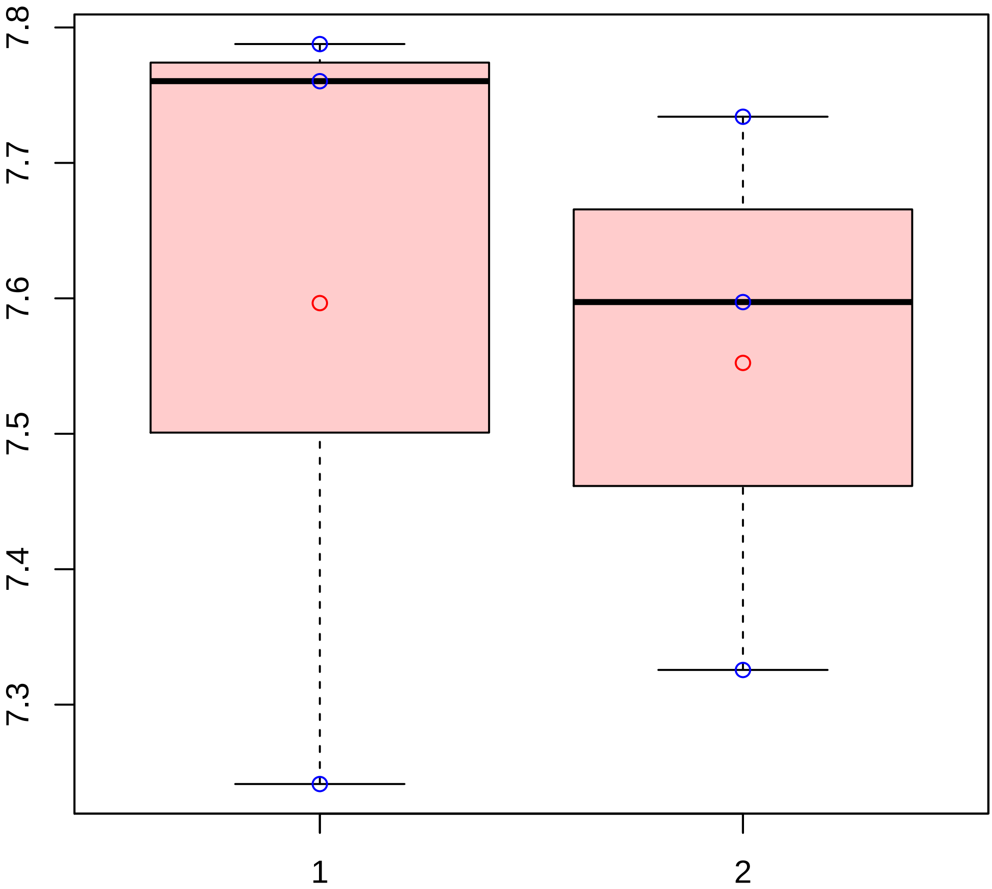

t−Test: p−value = 0.85

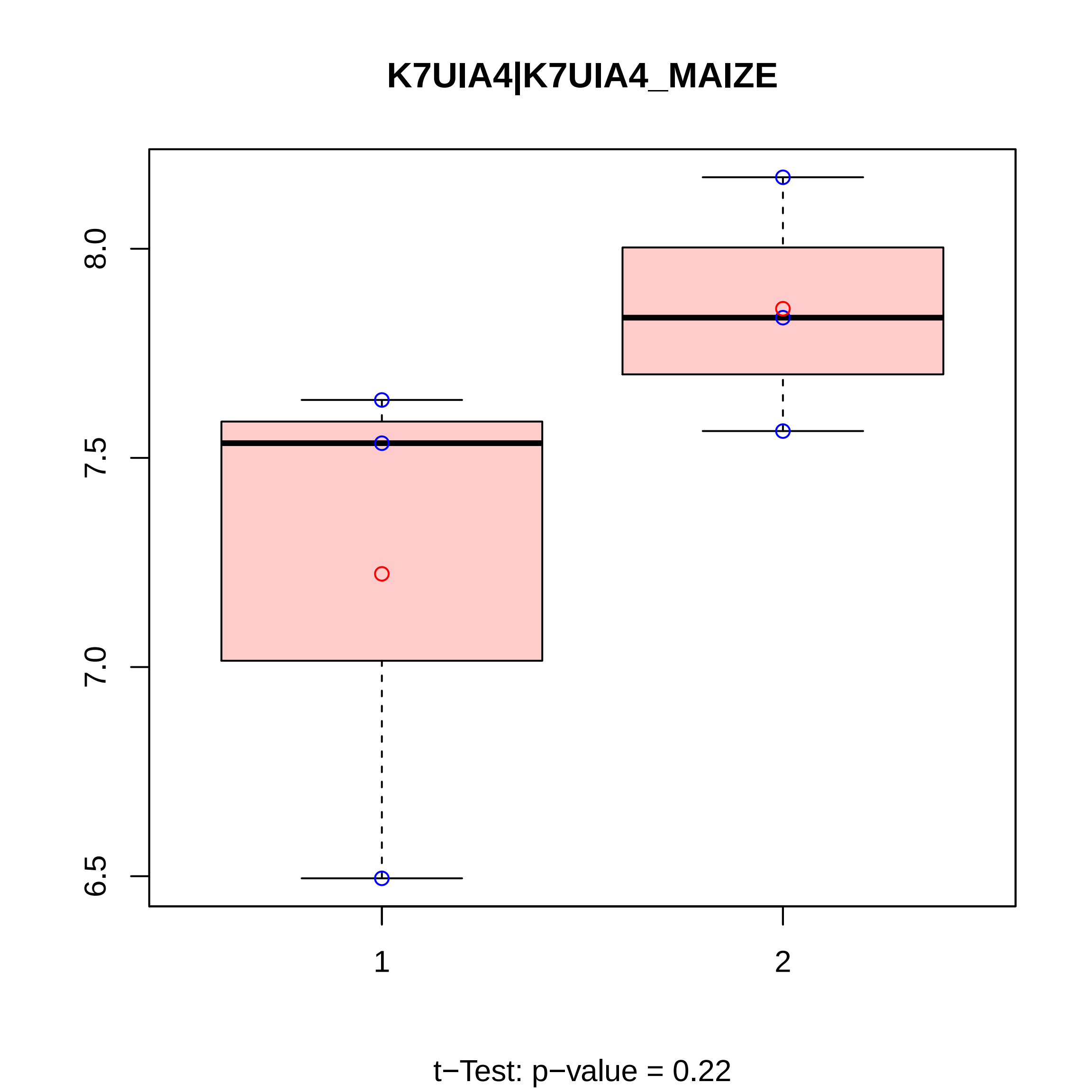
K7UIA4|K7UIA4_MAIZE
8.0
7.5
7.0
6.5
1
2
t−Test: p−value = 0.22

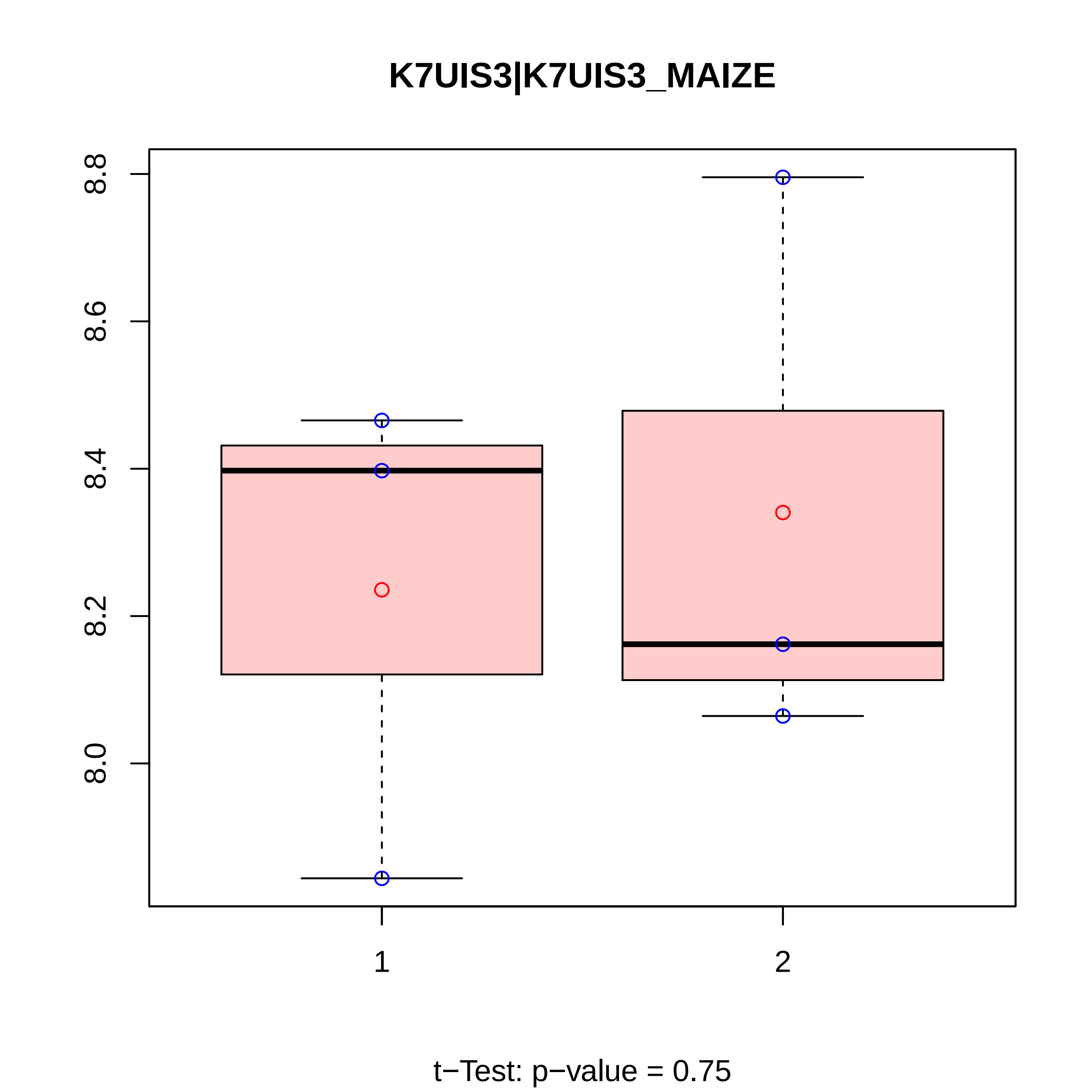
K7UIS3|K7UIS3_MAIZE
8.0
8.2
8.4
8.6
8.8
1
2
t−Test: p−value = 0.75

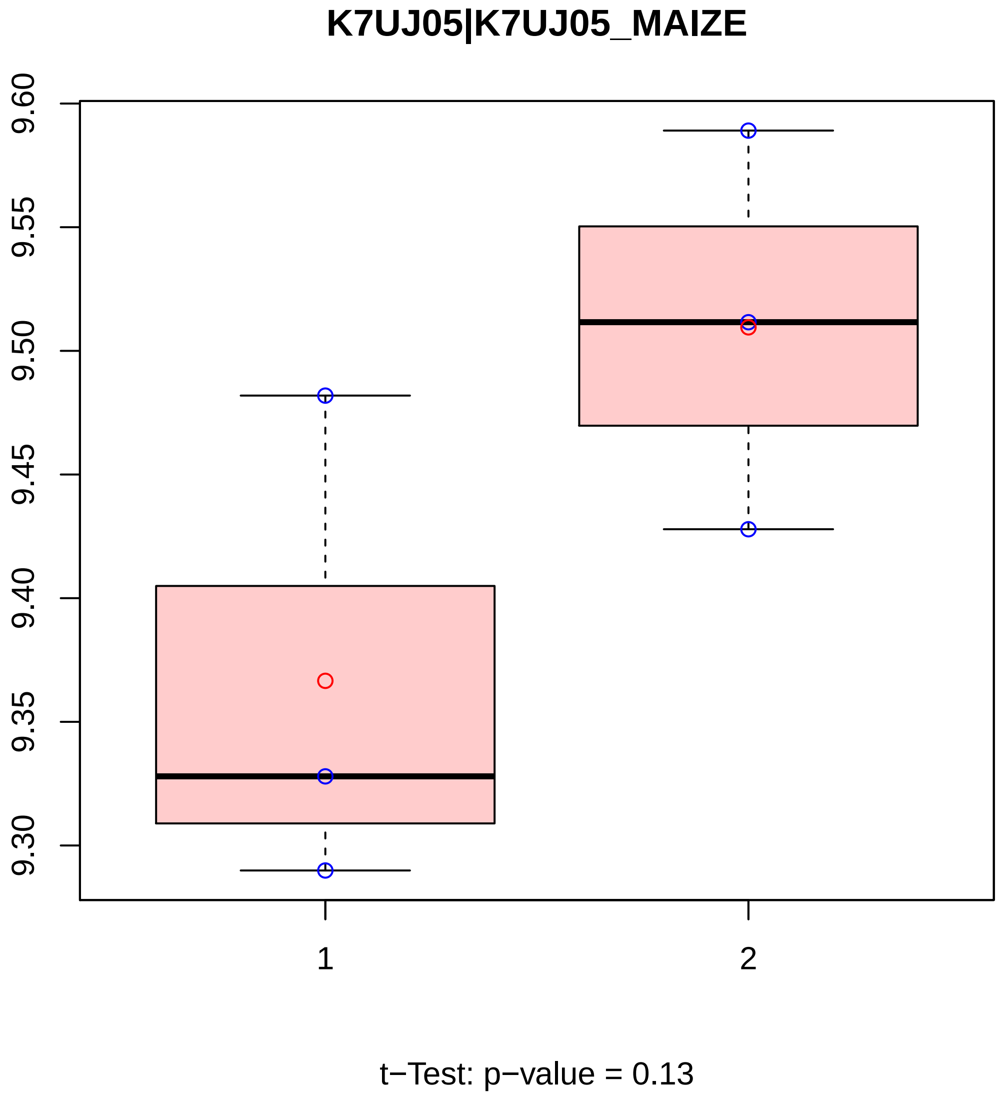
K7UJ05|K7UJ05_MAIZE
9.60
9.55
9.50
9.45
9.40
9.35
9.30
1
2
t−Test: p−value = 0.13

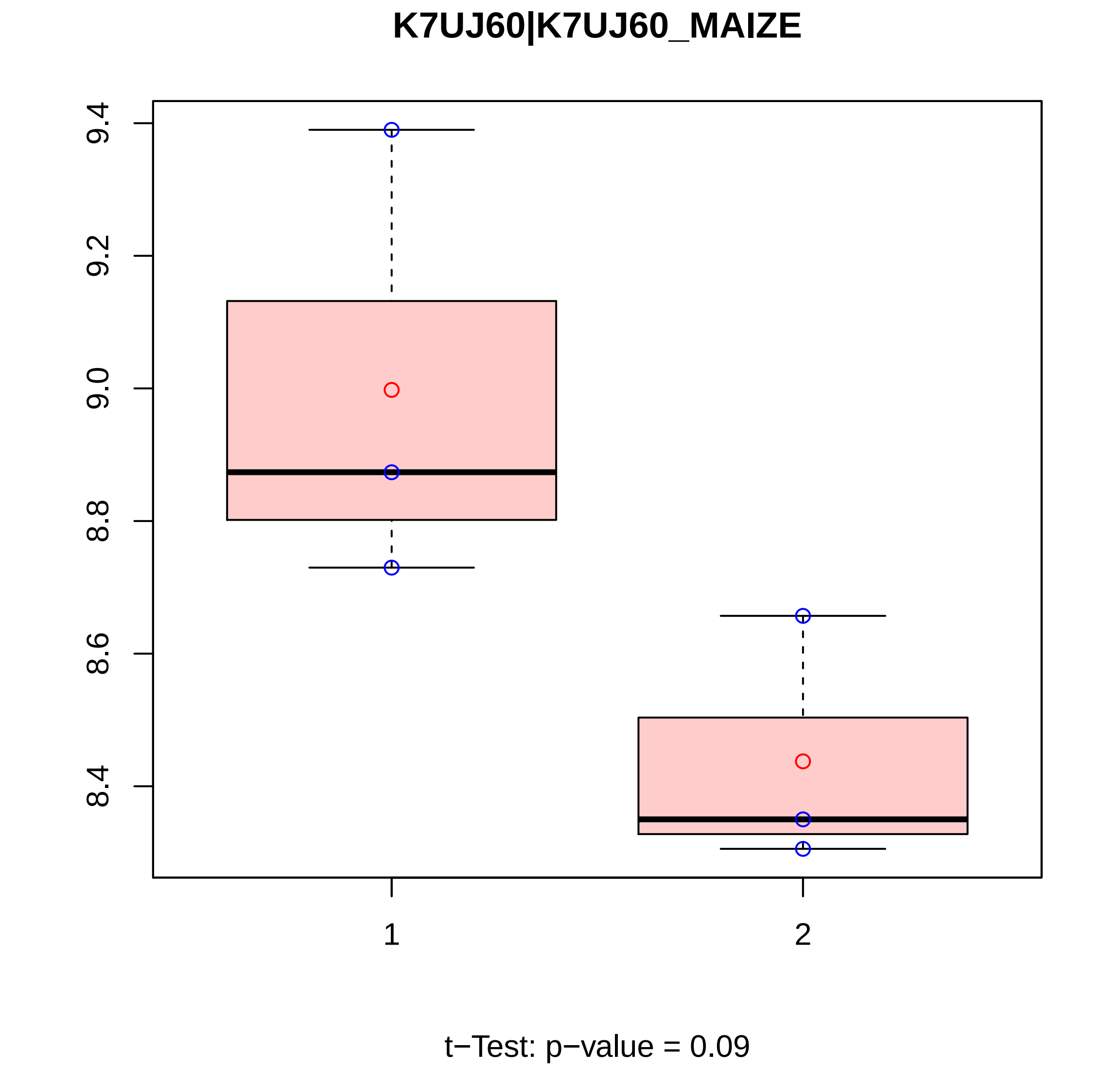

K7UJ60|K7UJ60_MAIZE
9.4
9.2
9.0
8.8
8.6
8.4
1
2
t−Test: p−value = 0.09

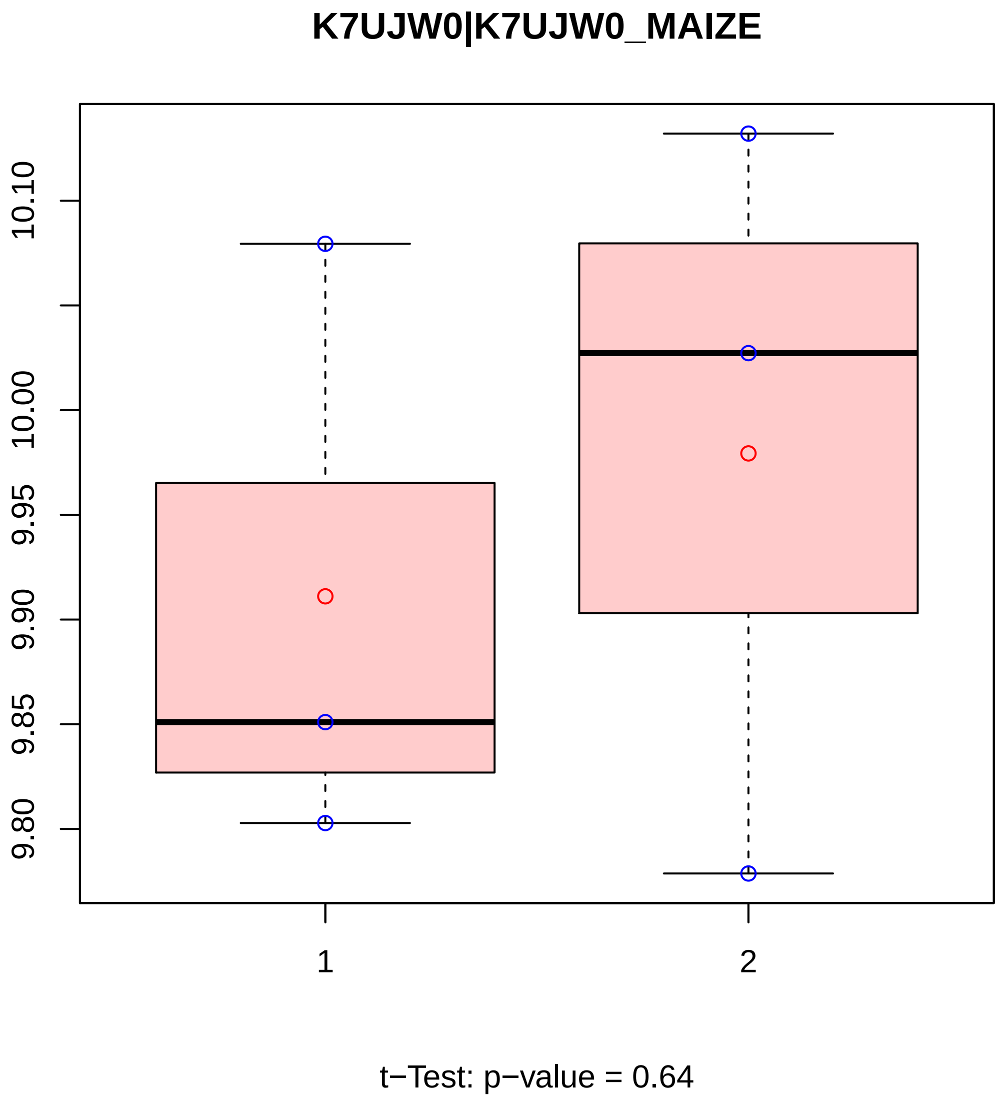
K7UJW0|K7UJW0_MAIZE
10.10
10.00
9.95
9.90
9.85
9.80
1
2
t−Test: p−value = 0.64

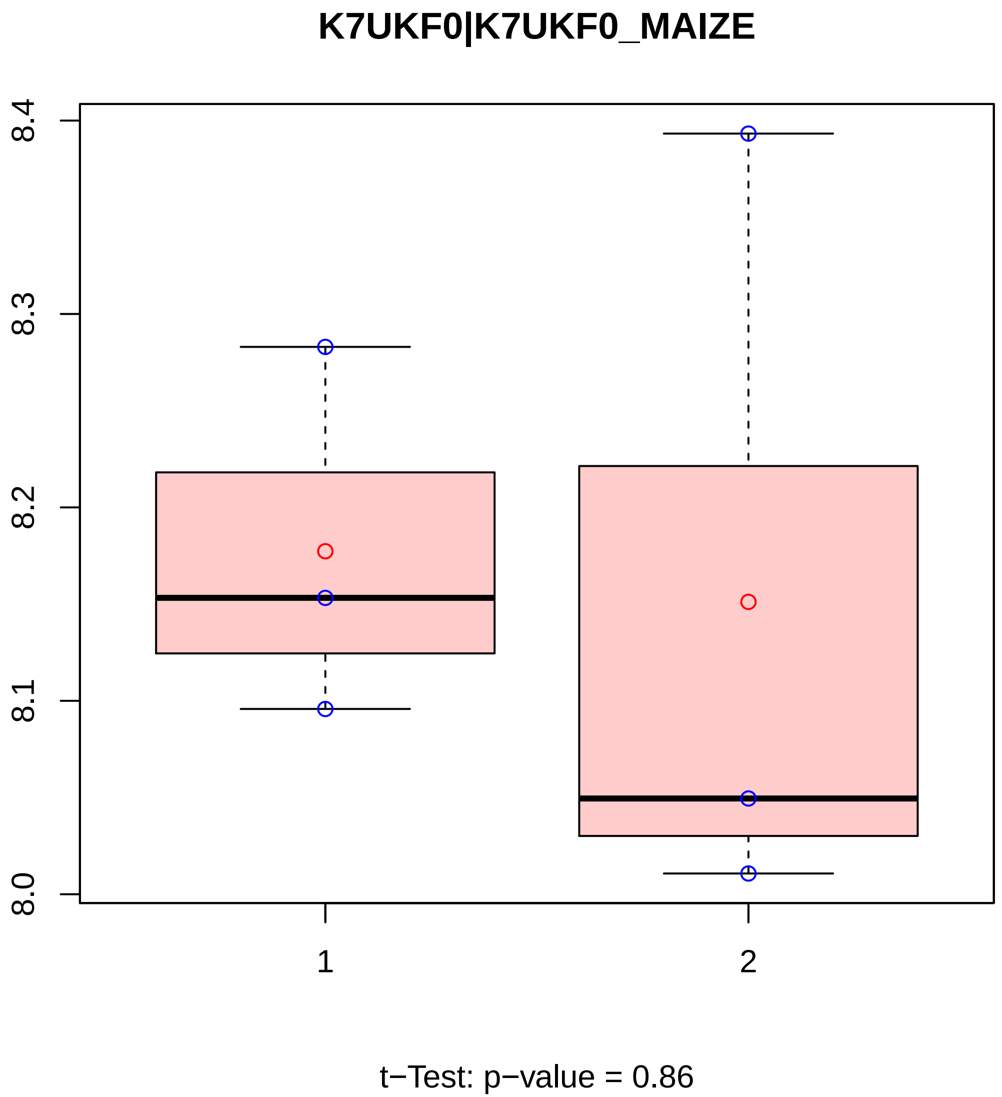
K7UKF0|K7UKF0_MAIZE
8.0
8.1
8.2
8.3
8.4
1
2
t−Test: p−value = 0.86

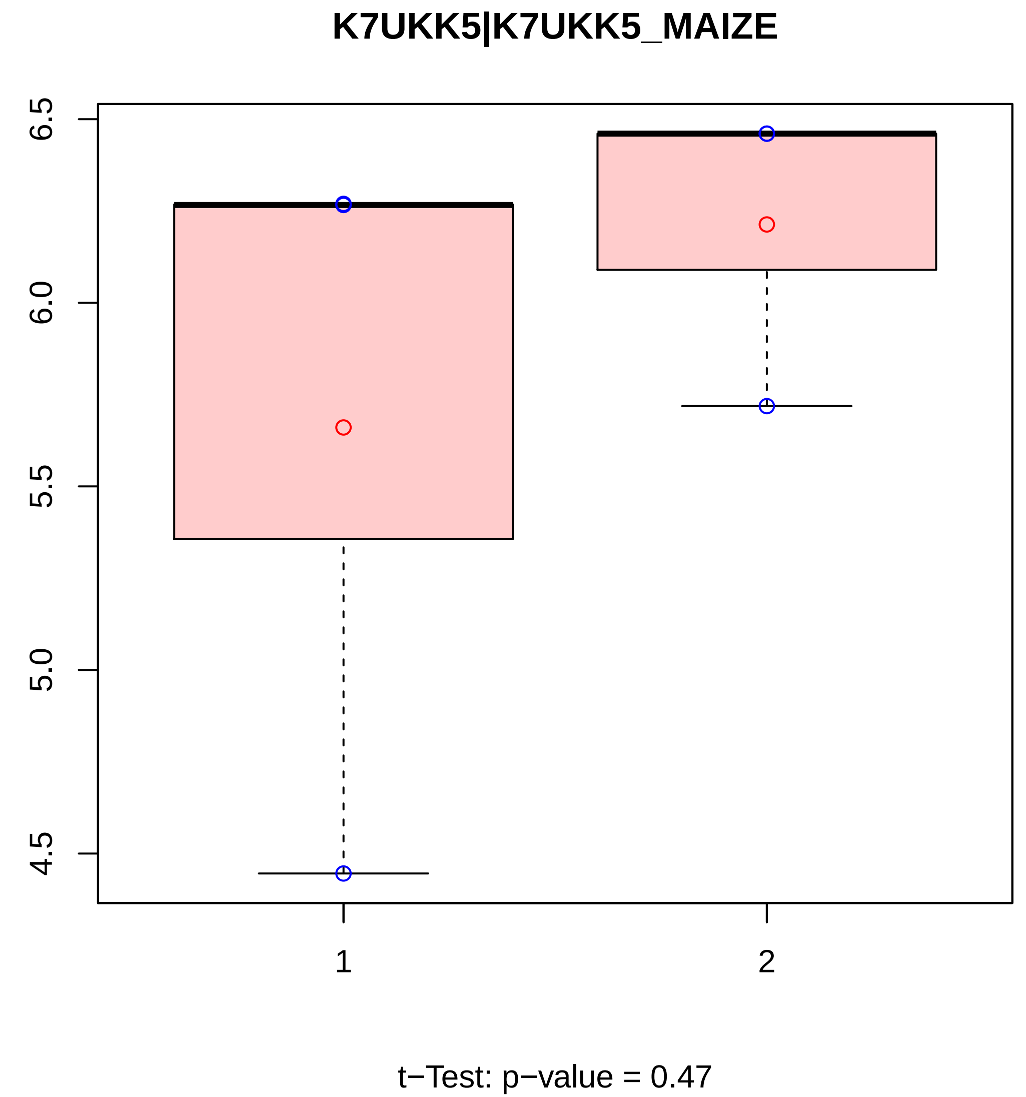

K7UKK5|K7UKK5_MAIZE
6.5
6.0
5.5
5.0
4.5
1
2
t−Test: p−value = 0.47

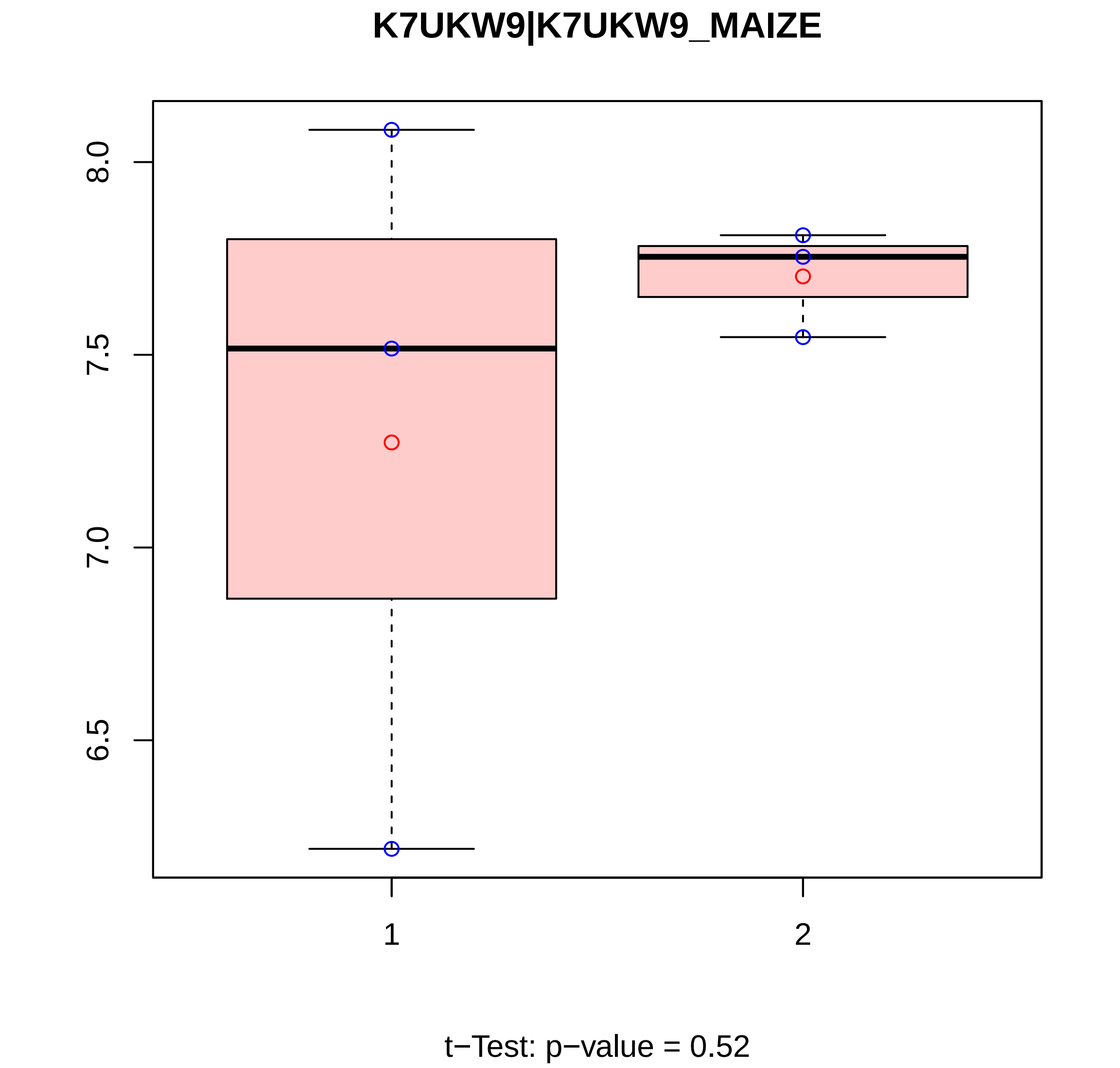
K7UKW9|K7UKW9_MAIZE
8.0
7.5
7.0
6.5
1
2
t−Test: p−value = 0.52

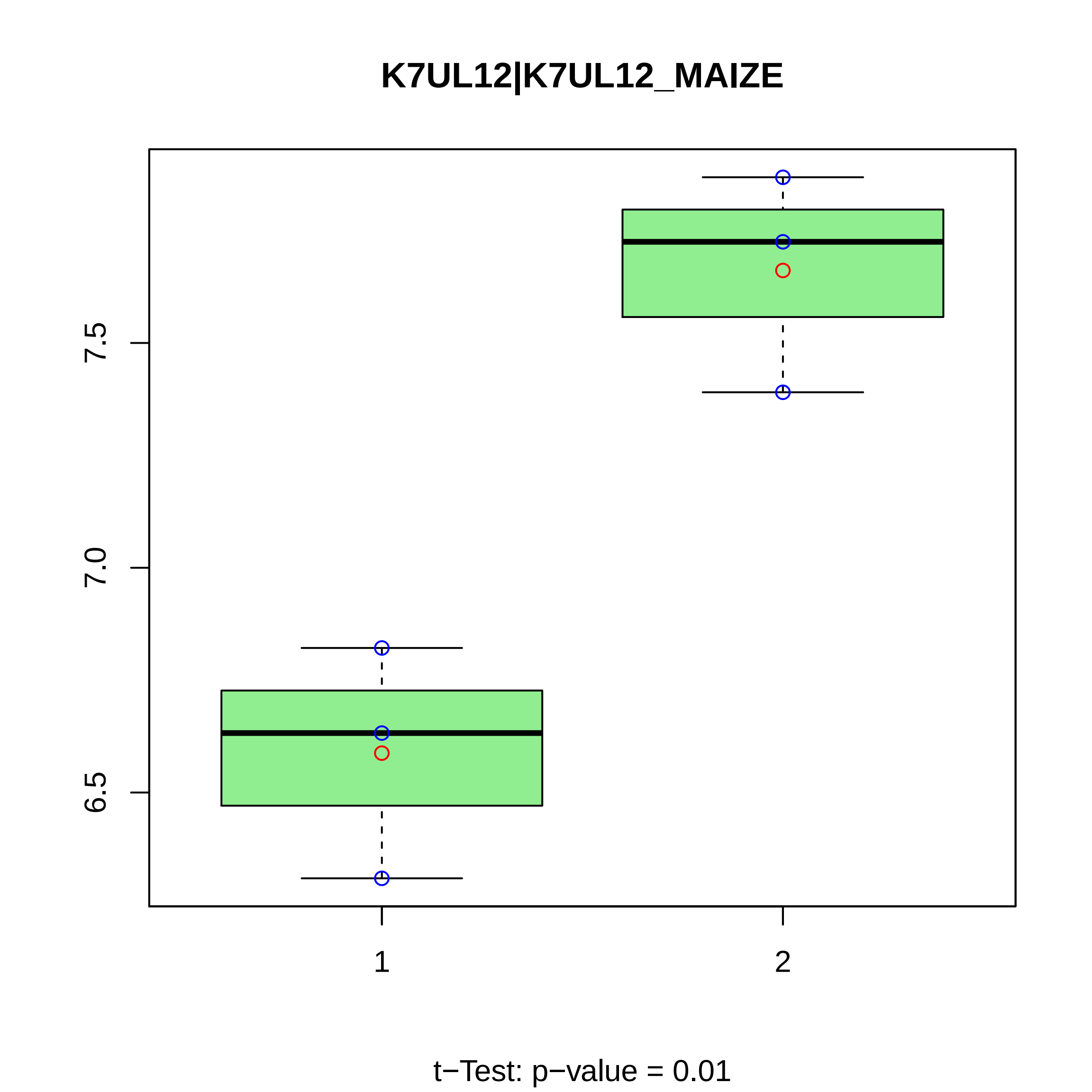

K7UL12|K7UL12_MAIZE
7.5
7.0
6.5
1
2
t−Test: p−value = 0.01

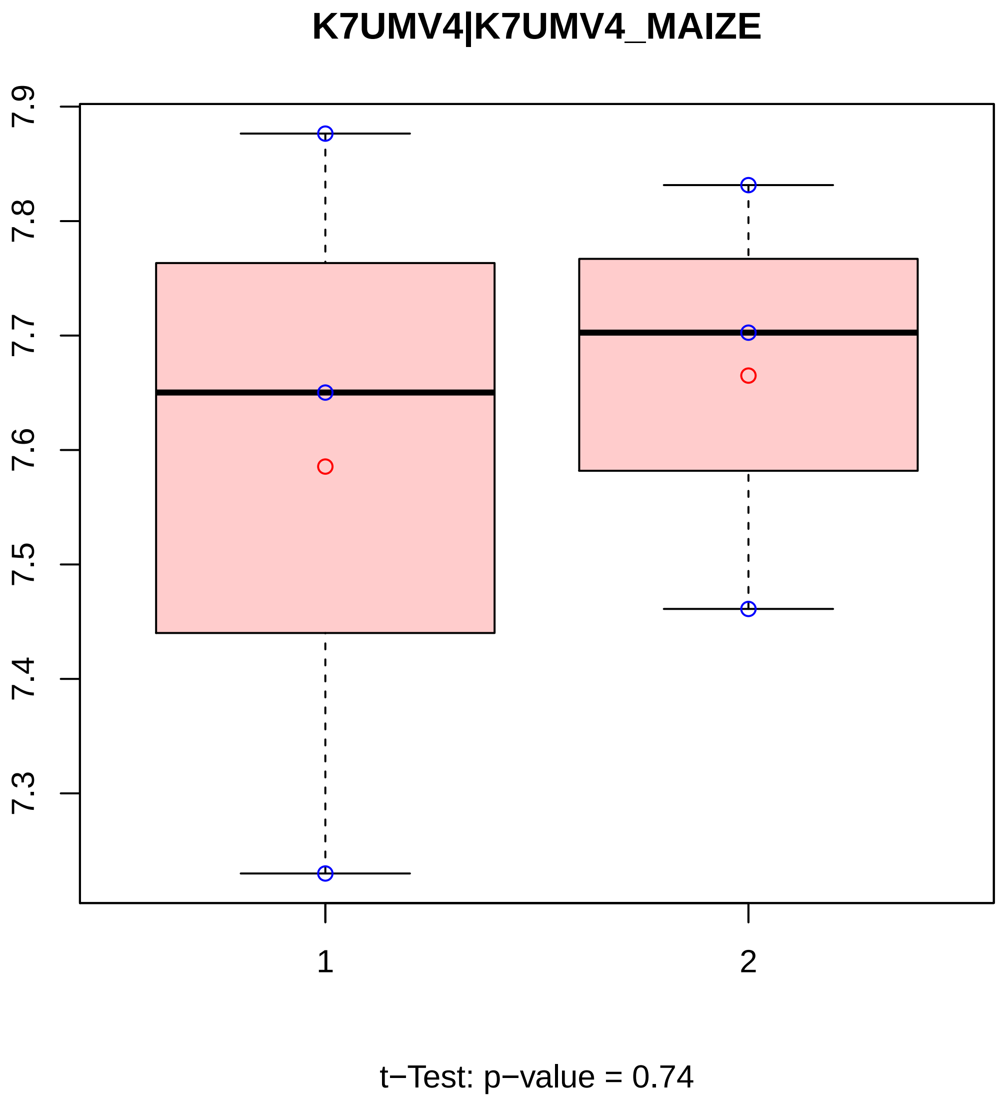
K7UMV4|K7UMV4_MAIZE
7.9
7.8
7.7
7.6
7.5
7.4
7.3
1
2
t−Test: p−value = 0.74

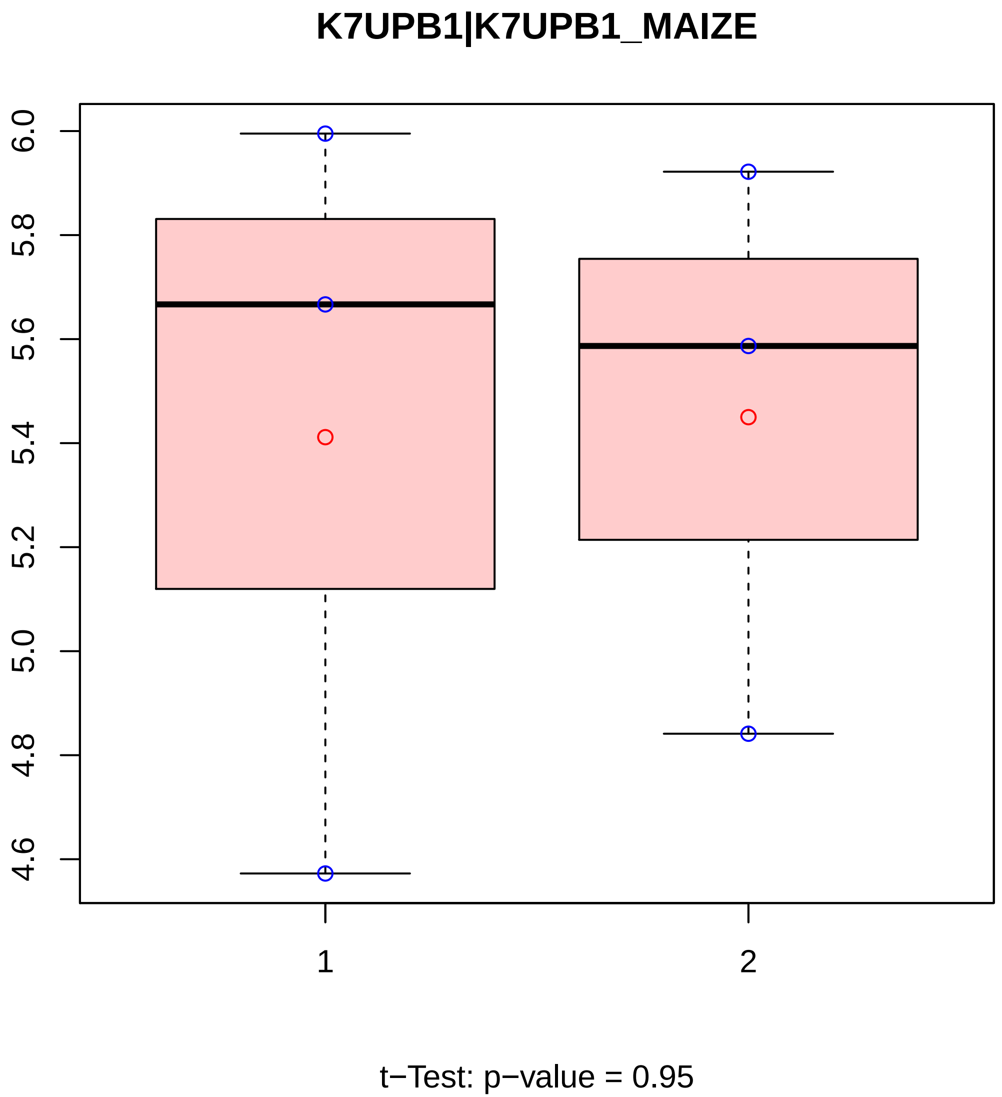

K7UPB1|K7UPB1_MAIZE
6.0
5.8
5.6
5.4
5.2
5.0
4.8
4.6
1
2
t−Test: p−value = 0.95

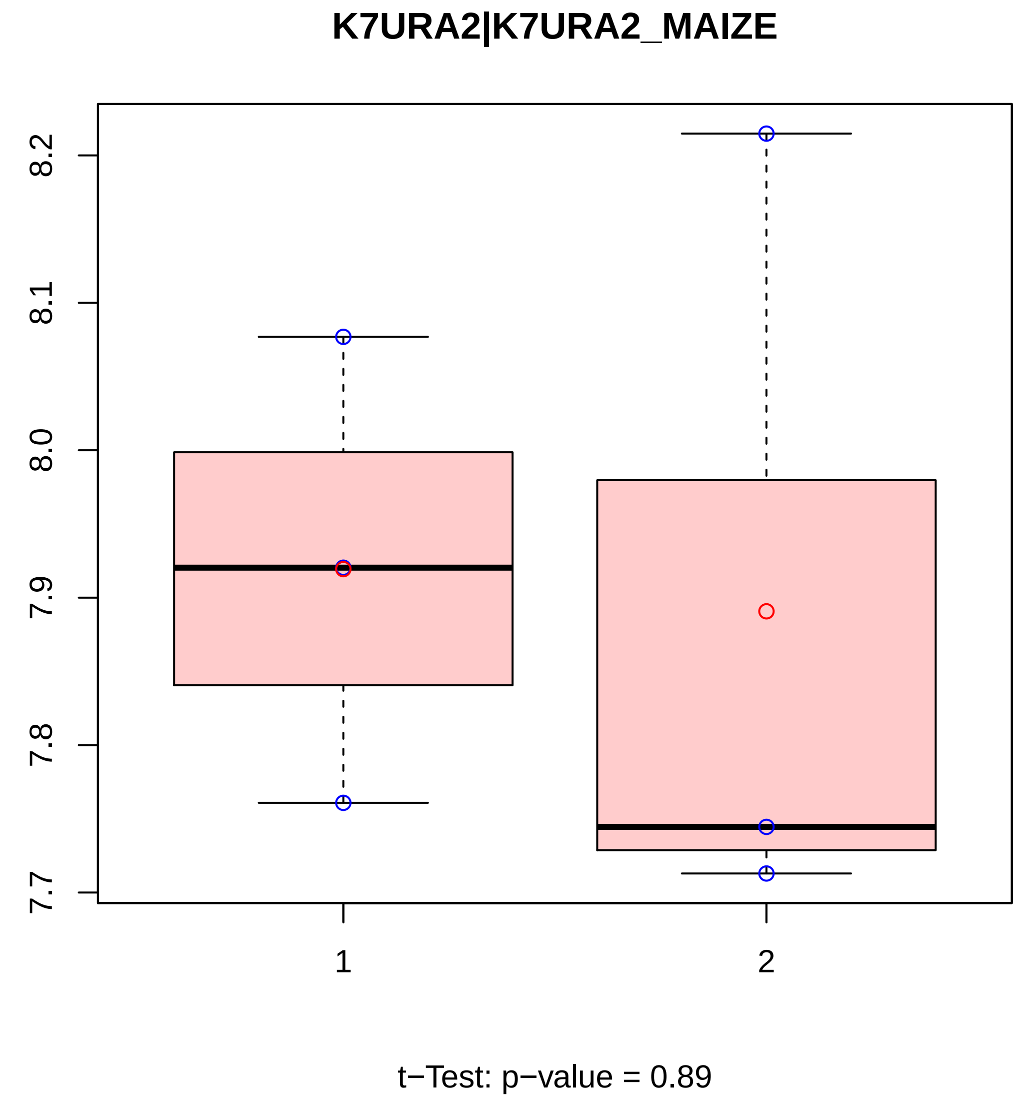
K7URA2|K7URA2_MAIZE
8.2
8.1
8.0
7.9
7.8
7.7
1
2
t−Test: p−value = 0.89

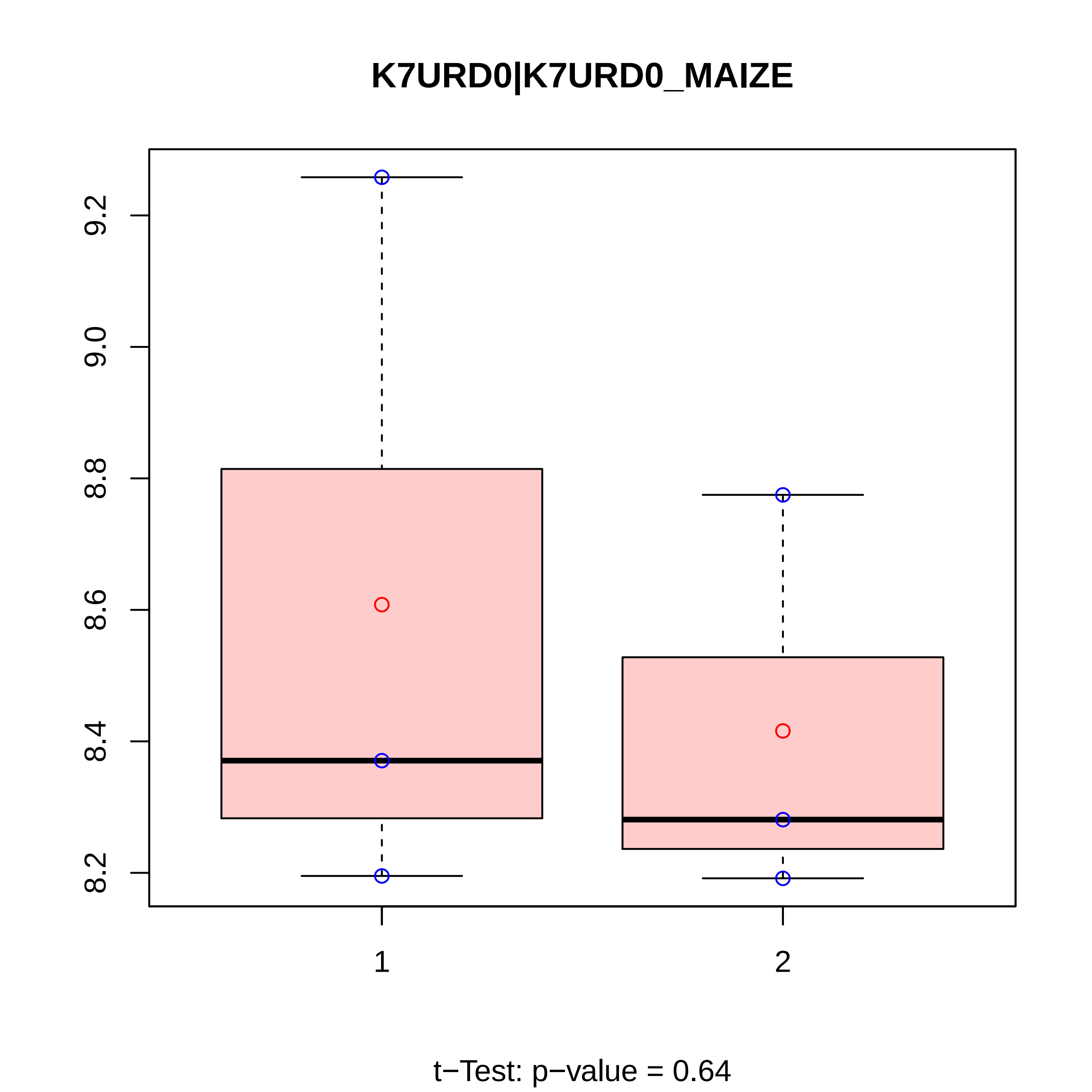
K7URD0|K7URD0_MAIZE
9.2
9.0
8.8
8.6
8.4
8.2
1
2
t−Test: p−value = 0.64

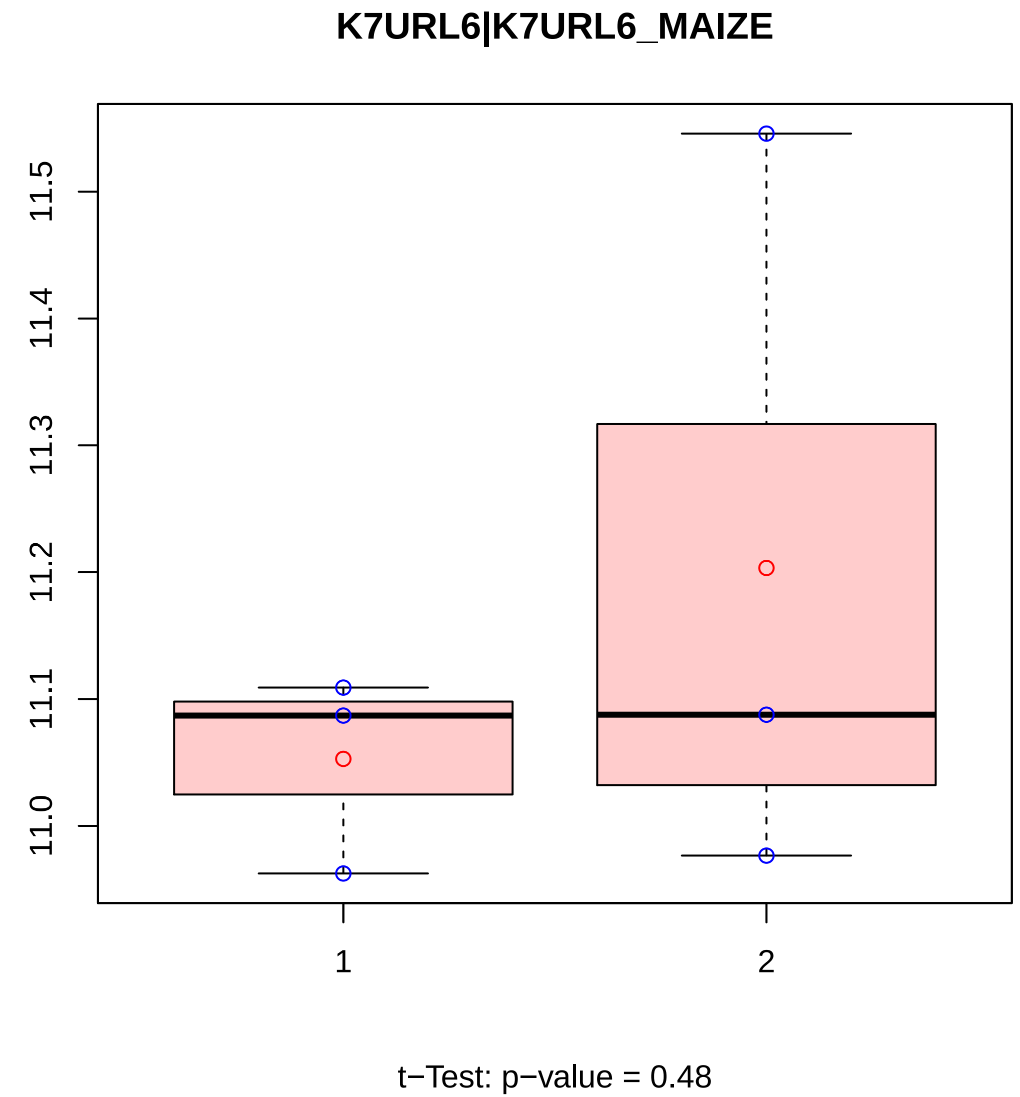
K7URL6|K7URL6_MAIZE
11.0
11.1
11.2
11.3
11.4
11.5
1
2
t−Test: p−value = 0.48

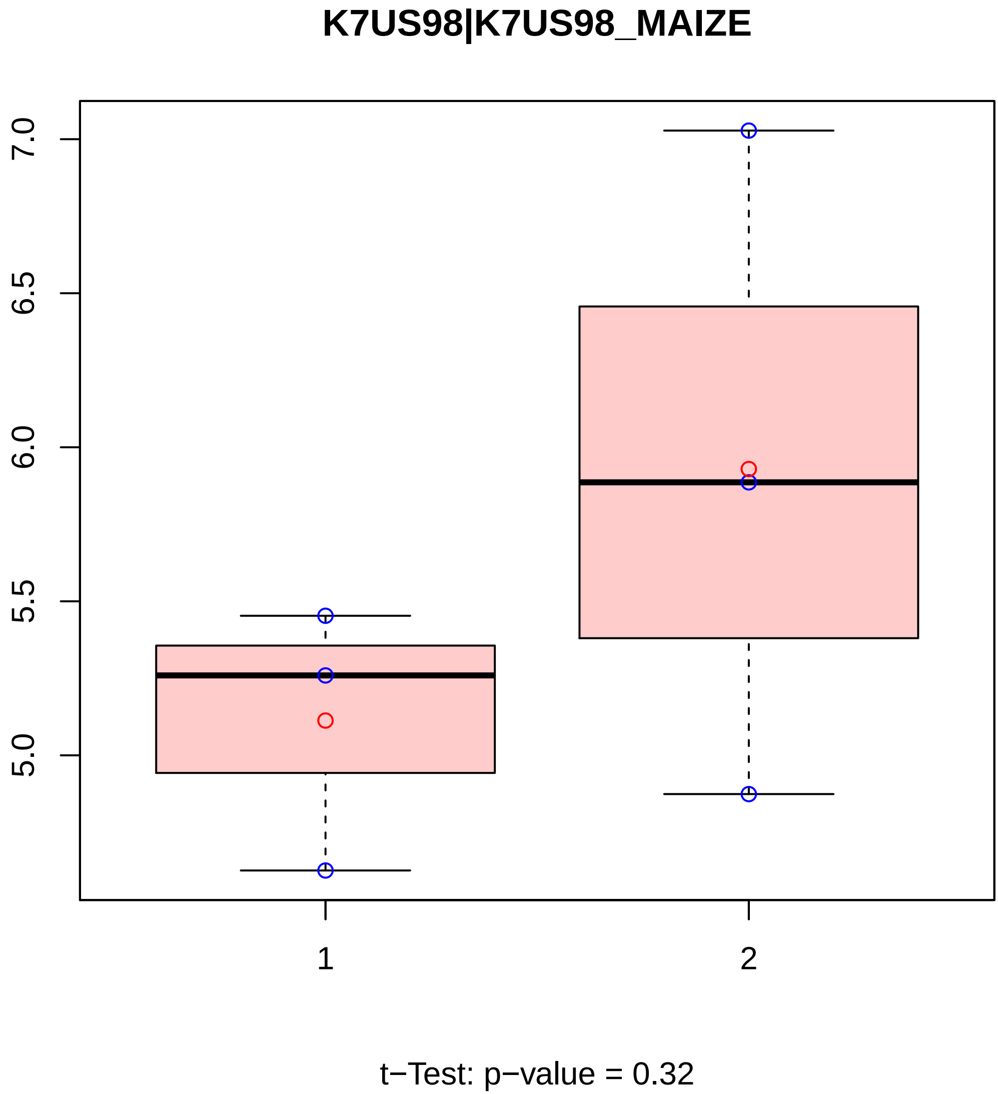
K7US98|K7US98_MAIZE
7.0
6.5
6.0
5.5
5.0
1
2
t−Test: p−value = 0.32

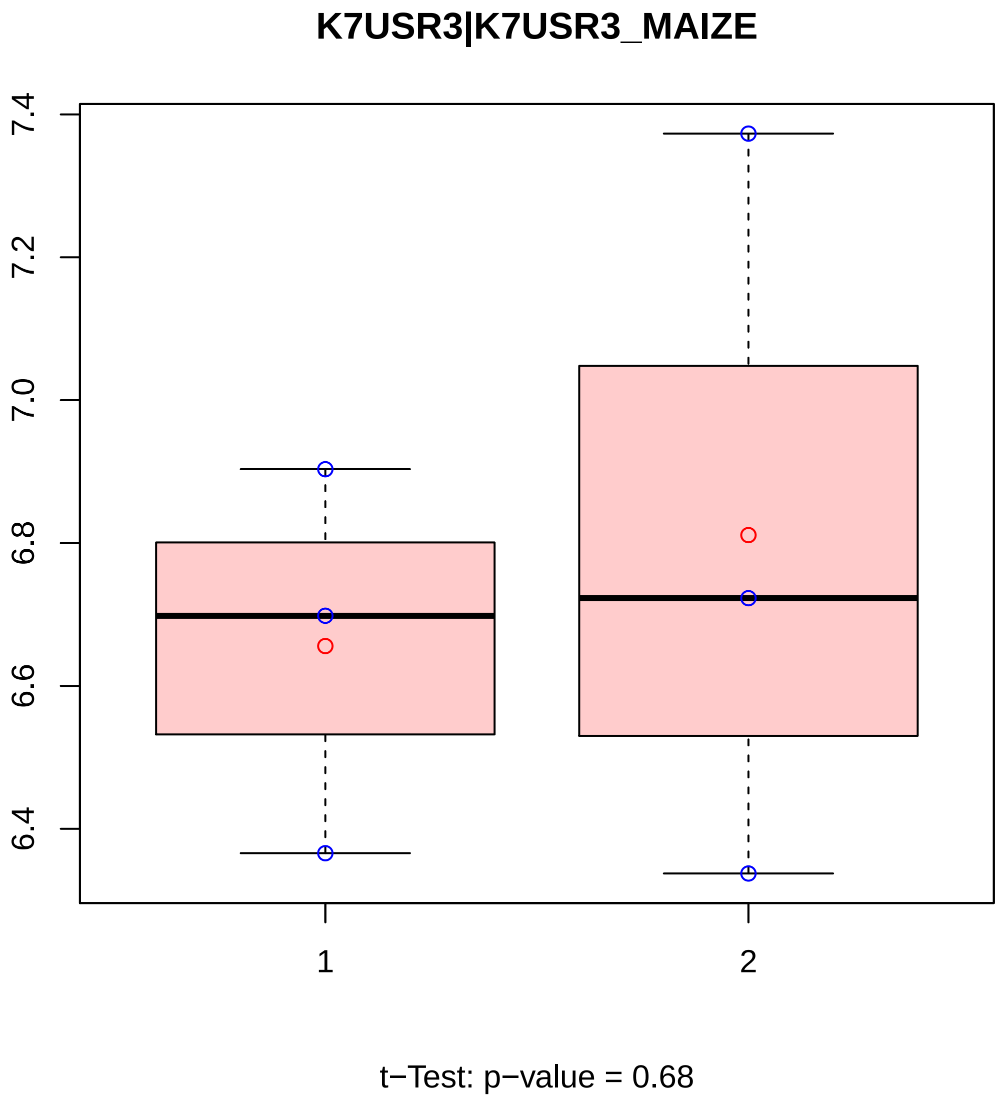
K7USR3|K7USR3_MAIZE
7.4
7.2
7.0
6.8
6.6
6.4
1
2
t−Test: p−value = 0.68

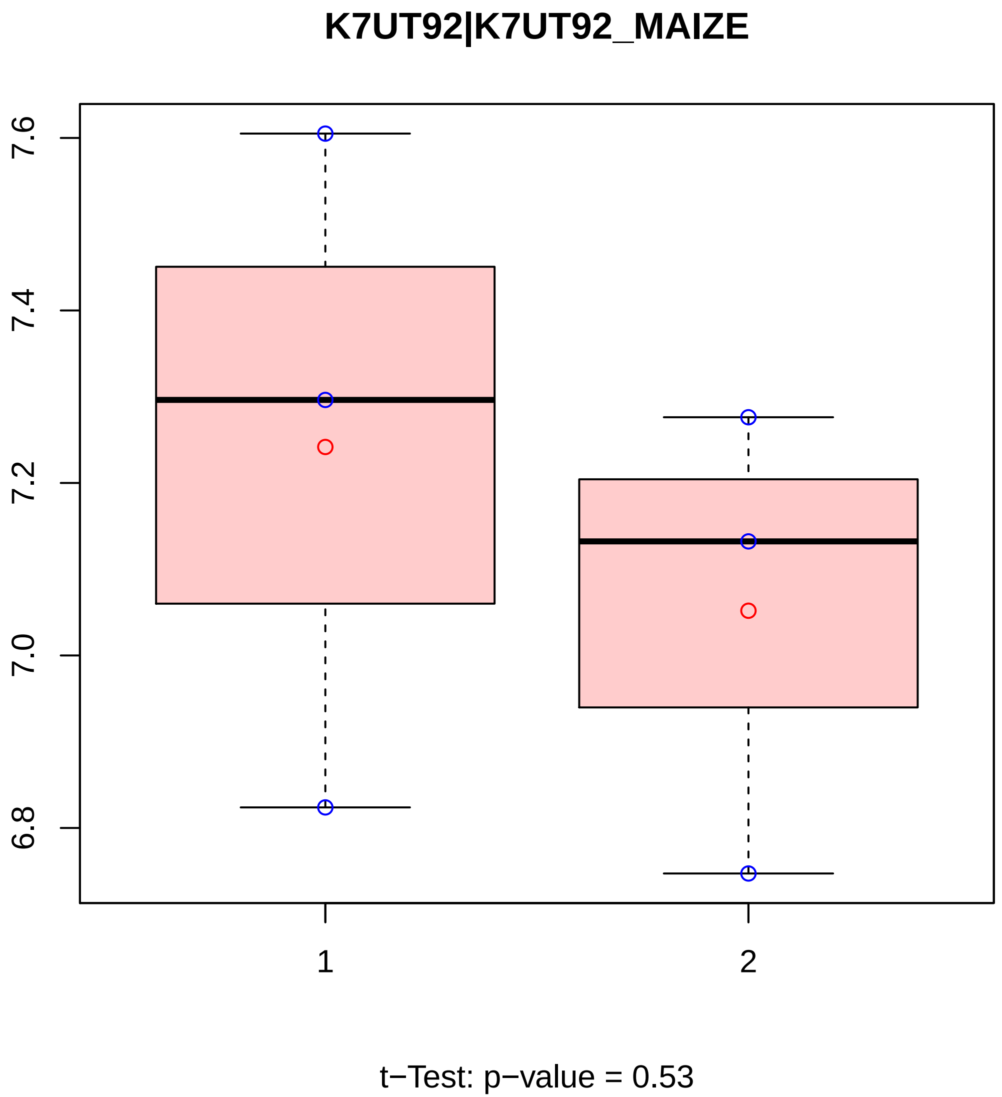
K7UT92|K7UT92_MAIZE
7.6
7.4
7.2
7.0
6.8
1
2
t−Test: p−value = 0.53

K7UTZ2|K7UTZ2_MAIZE

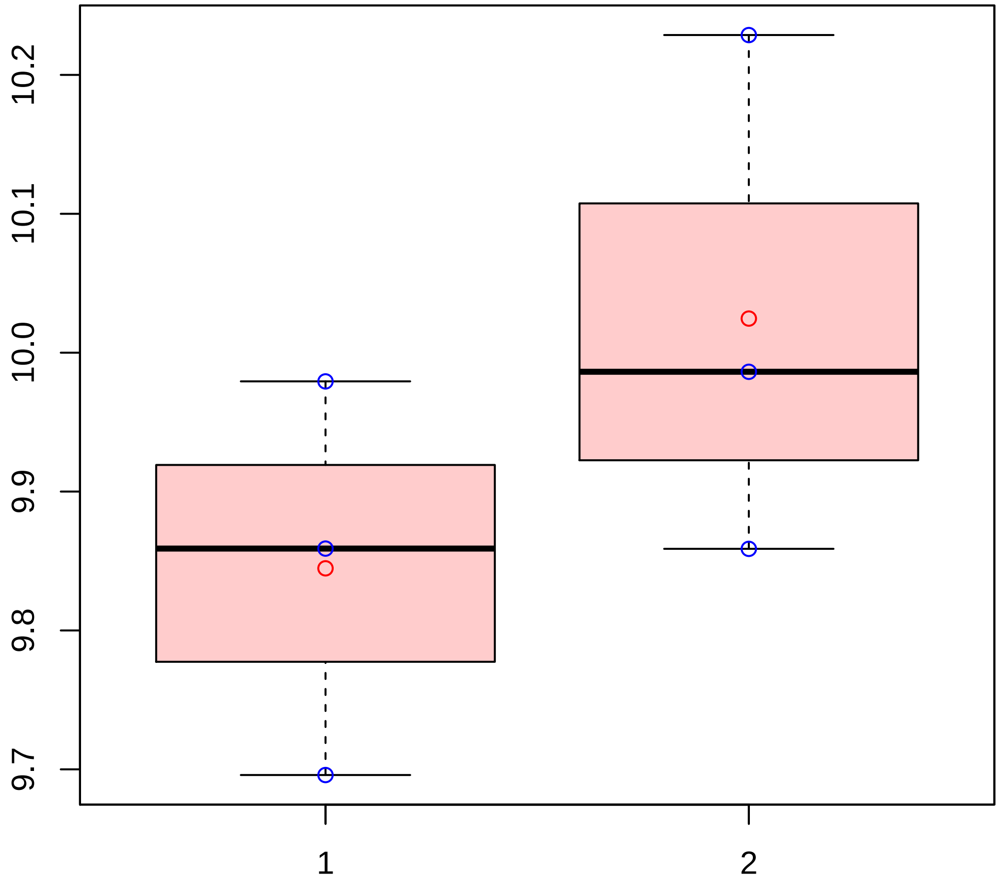

t−Test: p−value = 0.26

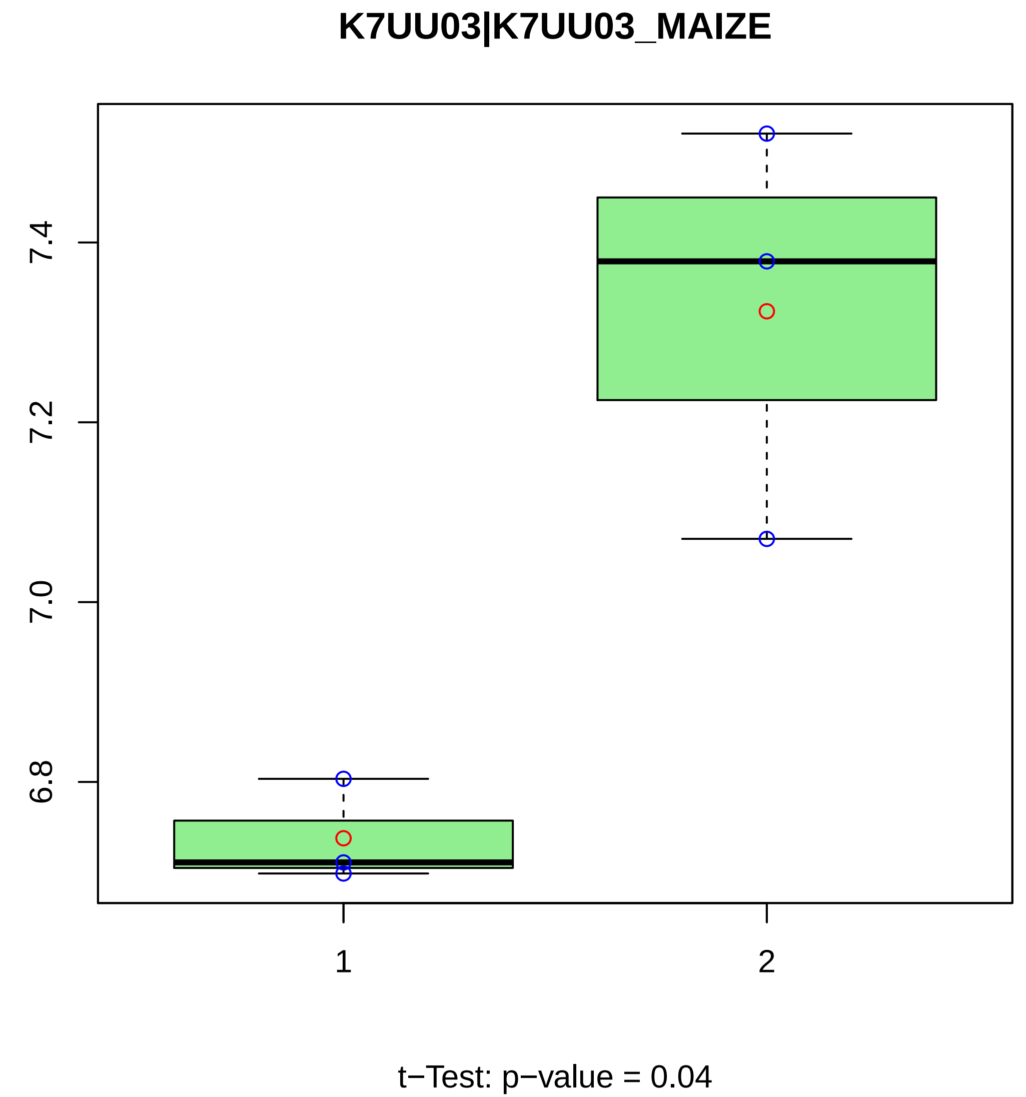
K7UU03|K7UU03_MAIZE
7.4
7.2
7.0
6.8
1
2
t−Test: p−value = 0.04

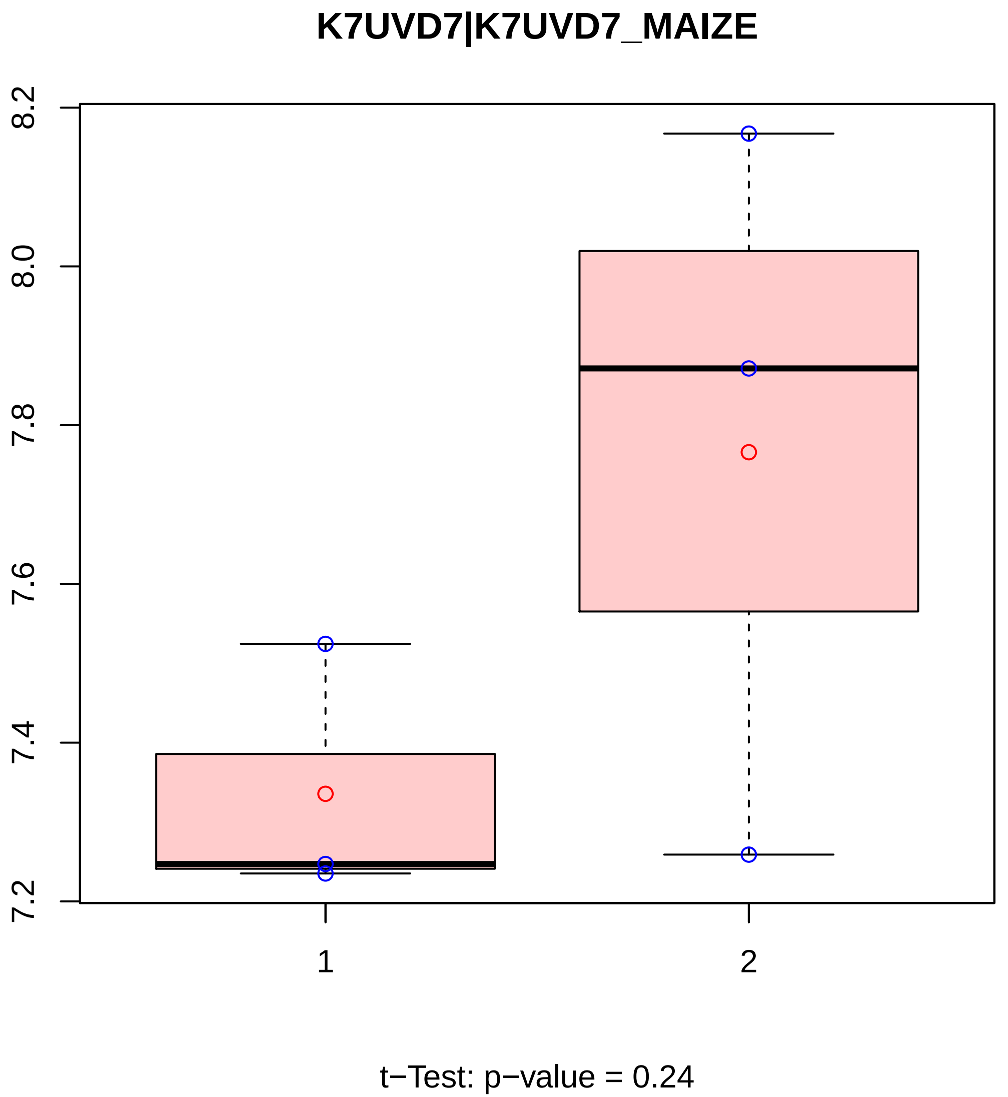
K7UVD7|K7UVD7_MAIZE
8.2
8.0
7.8
7.6
7.4
7.2
1
2
t−Test: p−value = 0.24

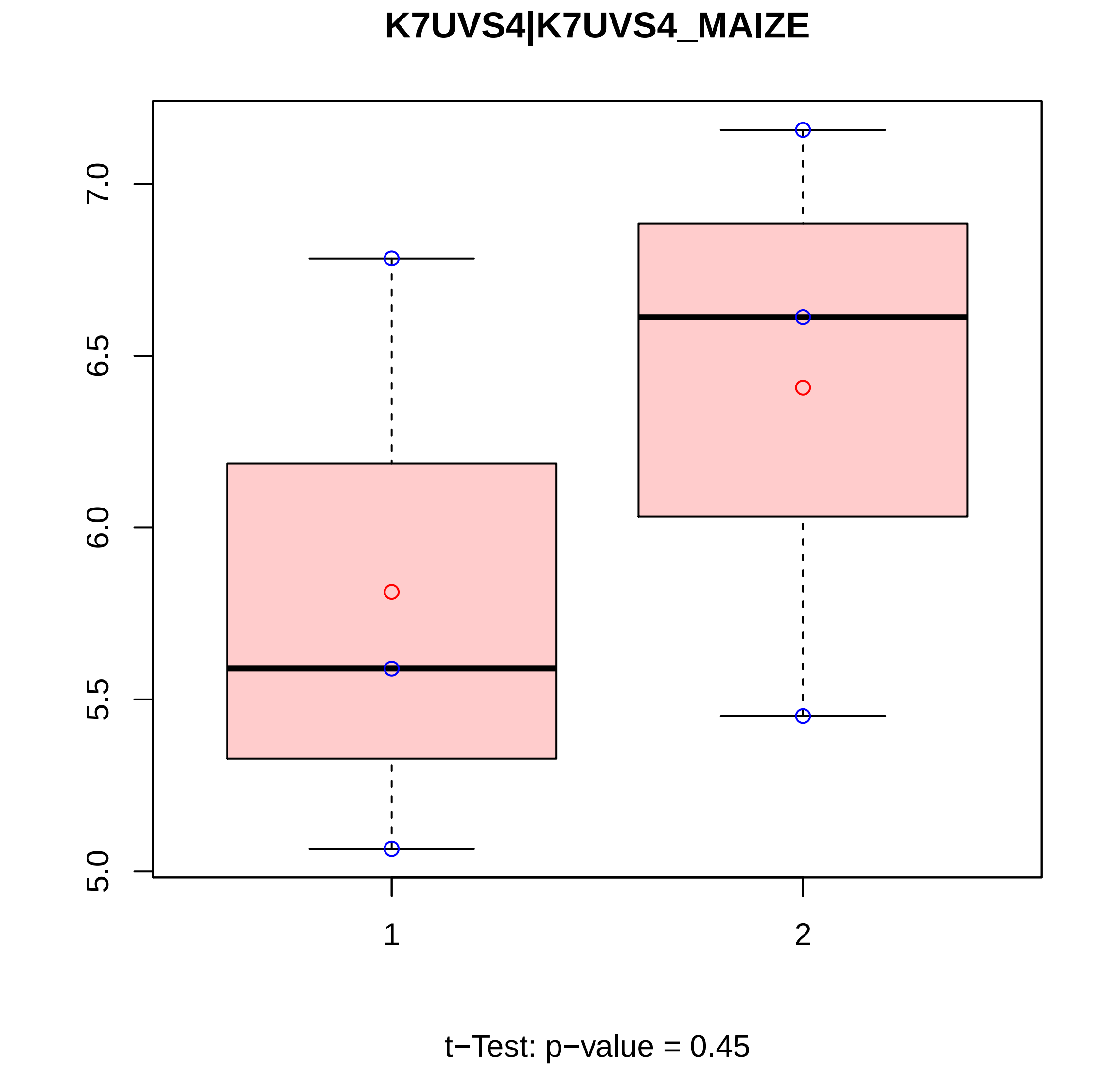
K7UVS4|K7UVS4_MAIZE
7.0
6.5
6.0
5.5
5.0
1
2
t−Test: p−value = 0.45

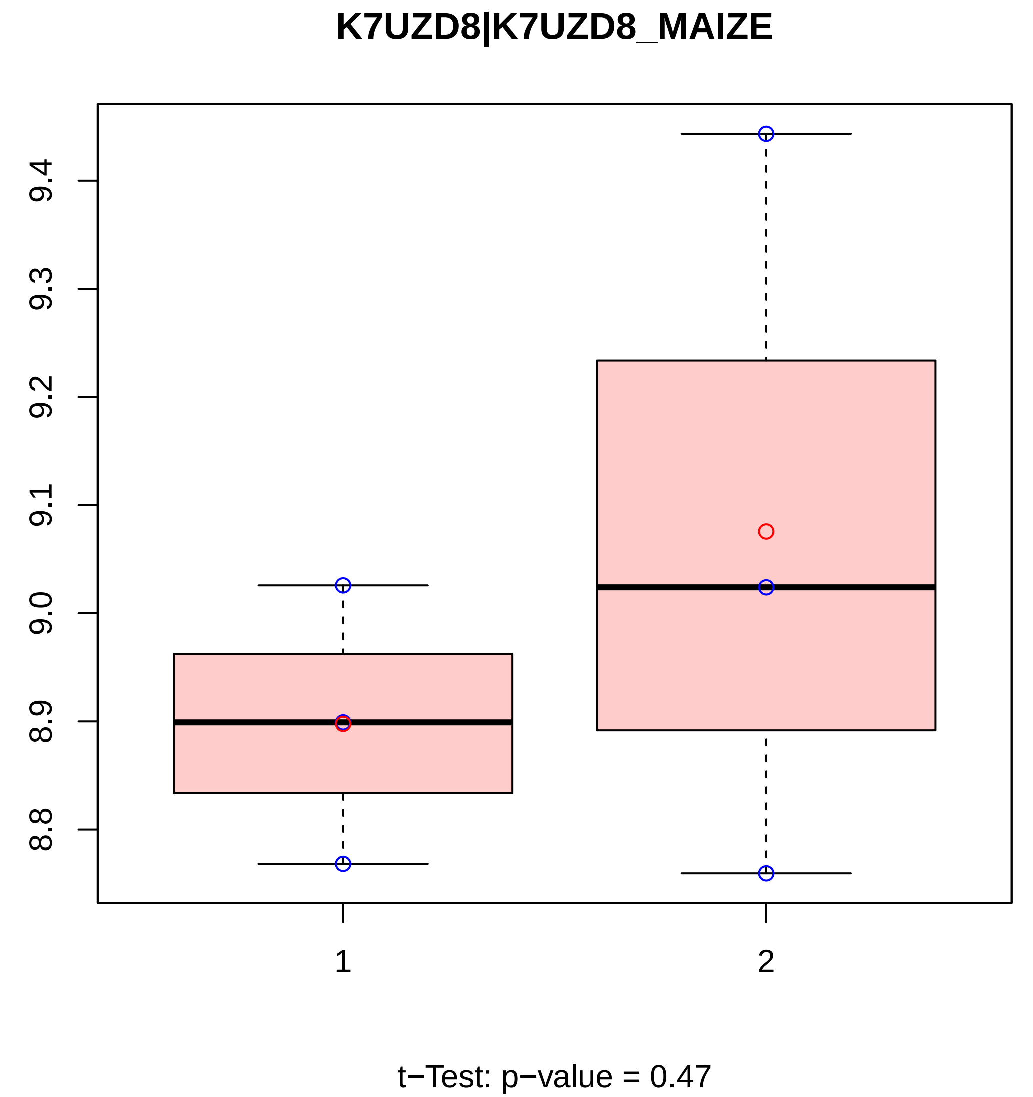
K7UZD8|K7UZD8_MAIZE
9.4
9.3
9.2
9.1
9.0
8.9
8.8
1
2
t−Test: p−value = 0.47

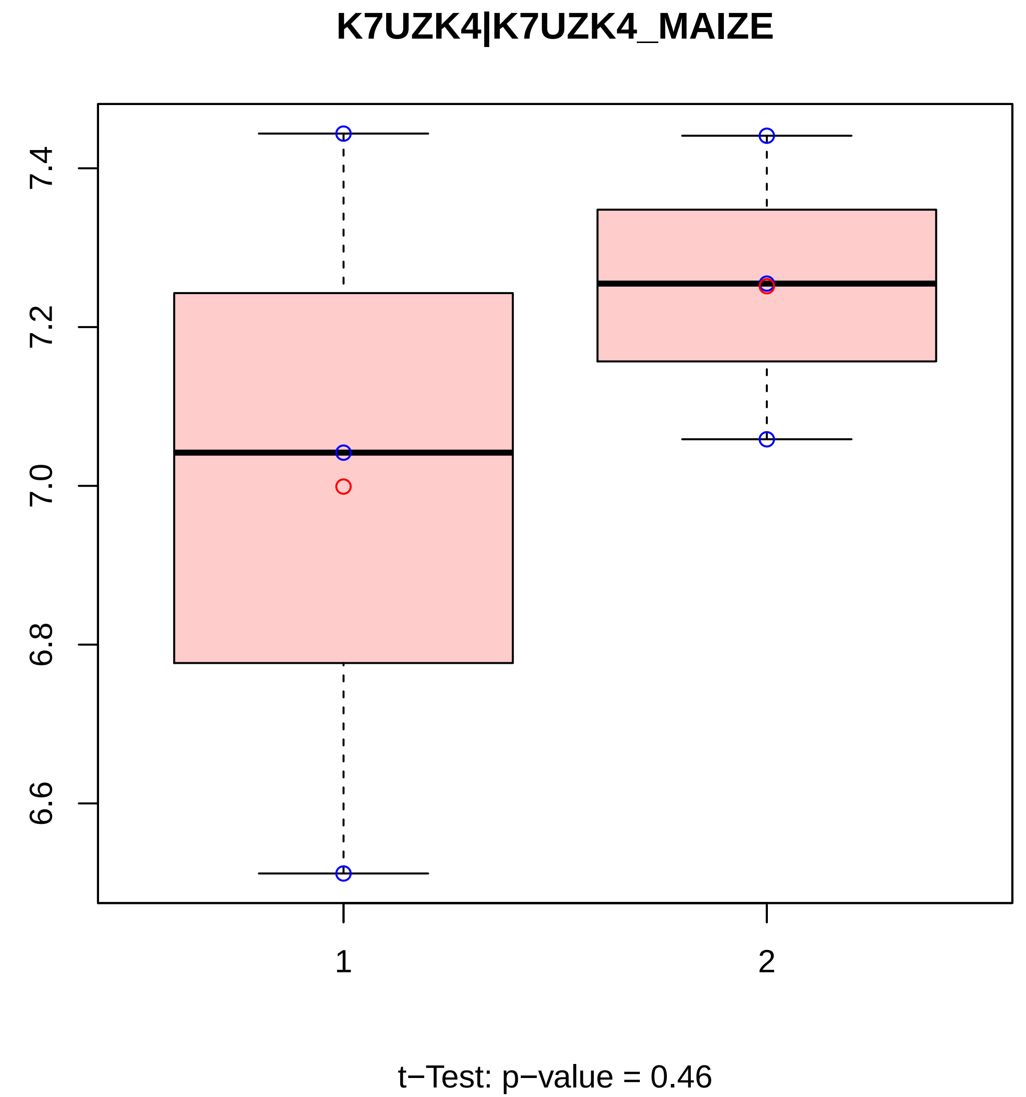
K7UZK4|K7UZK4_MAIZE
7.4
7.2
7.0
6.8
6.6
1
2
t−Test: p−value = 0.46

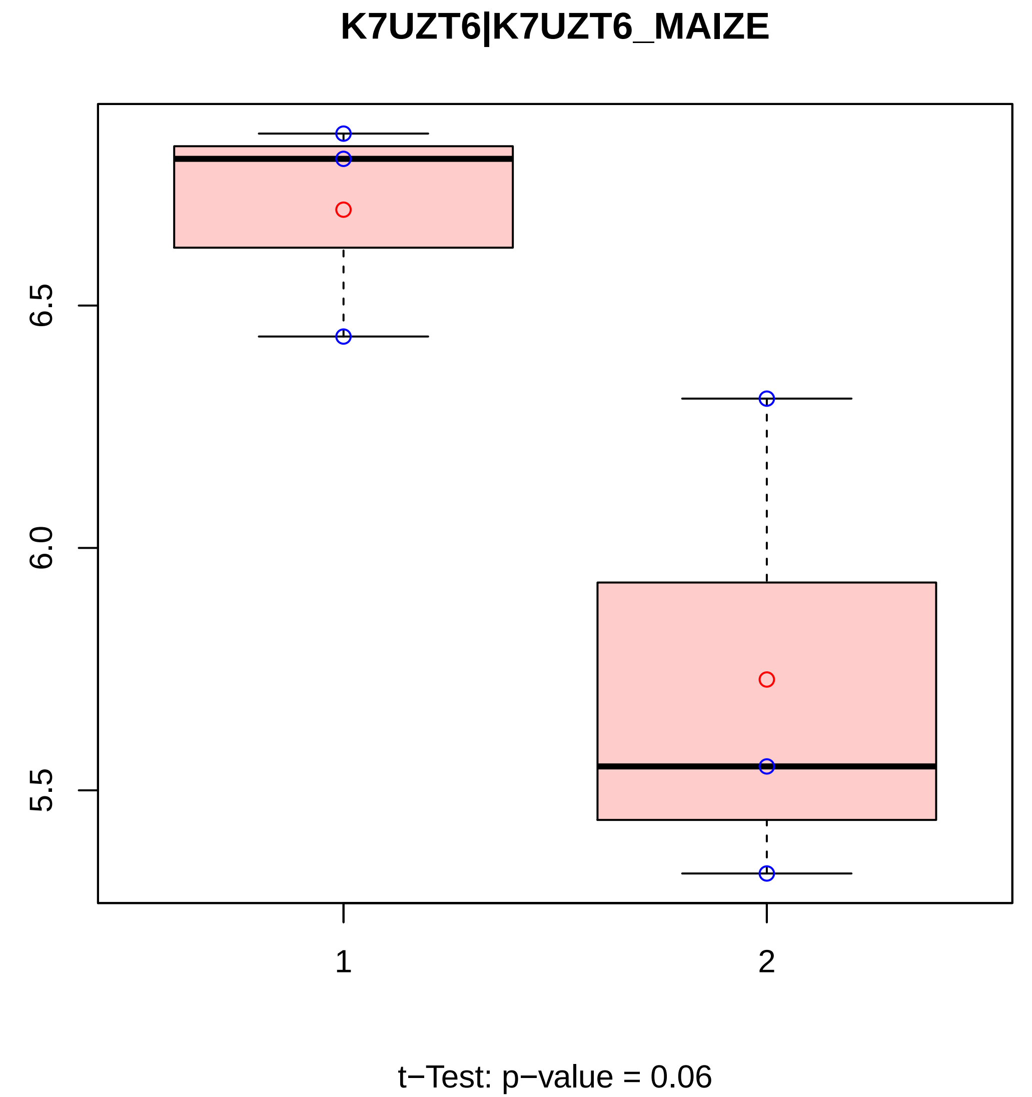

K7UZT6|K7UZT6_MAIZE
6.5
6.0
5.5
1
2
t−Test: p−value = 0.06

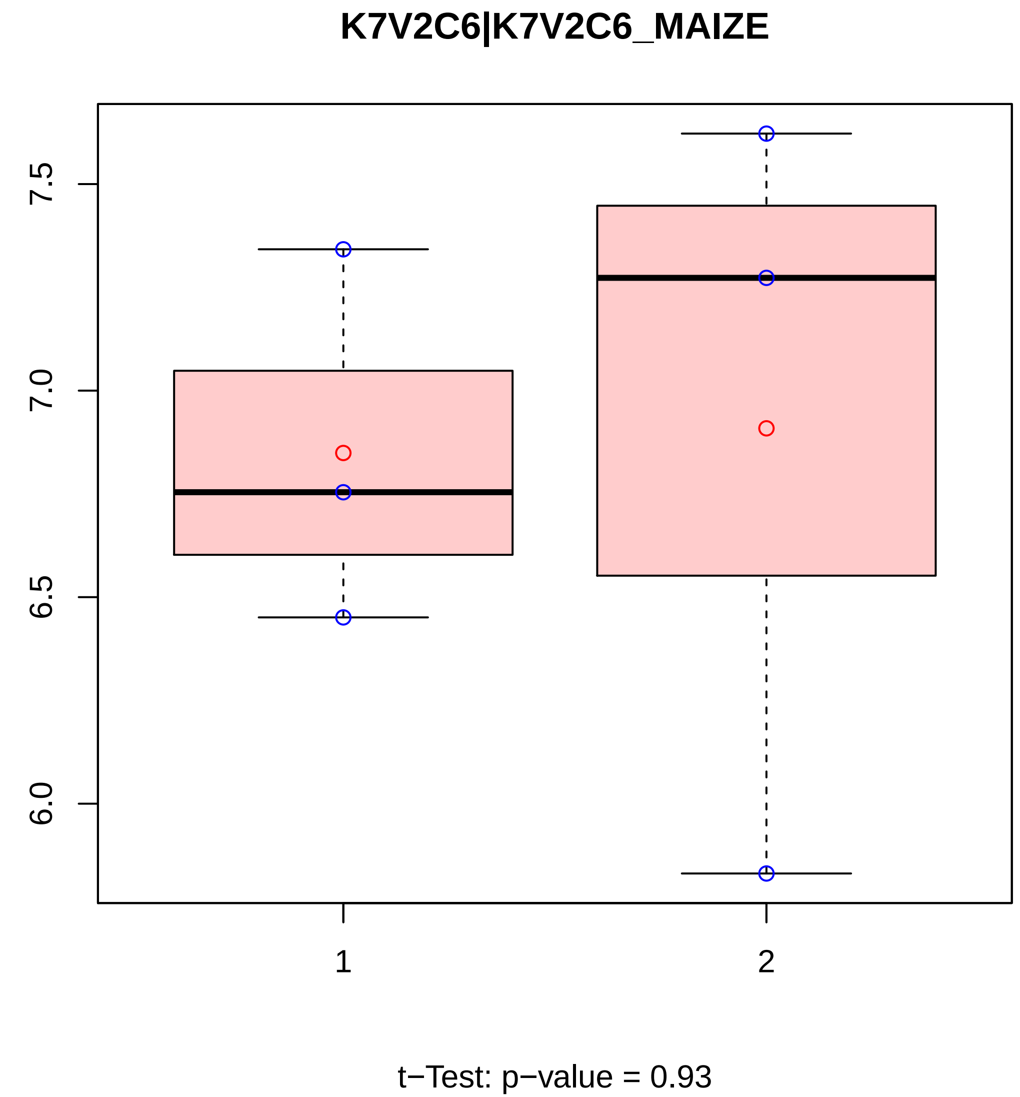
K7V2C6|K7V2C6_MAIZE
7.5
7.0
6.5
6.0
1
2
t−Test: p−value = 0.93

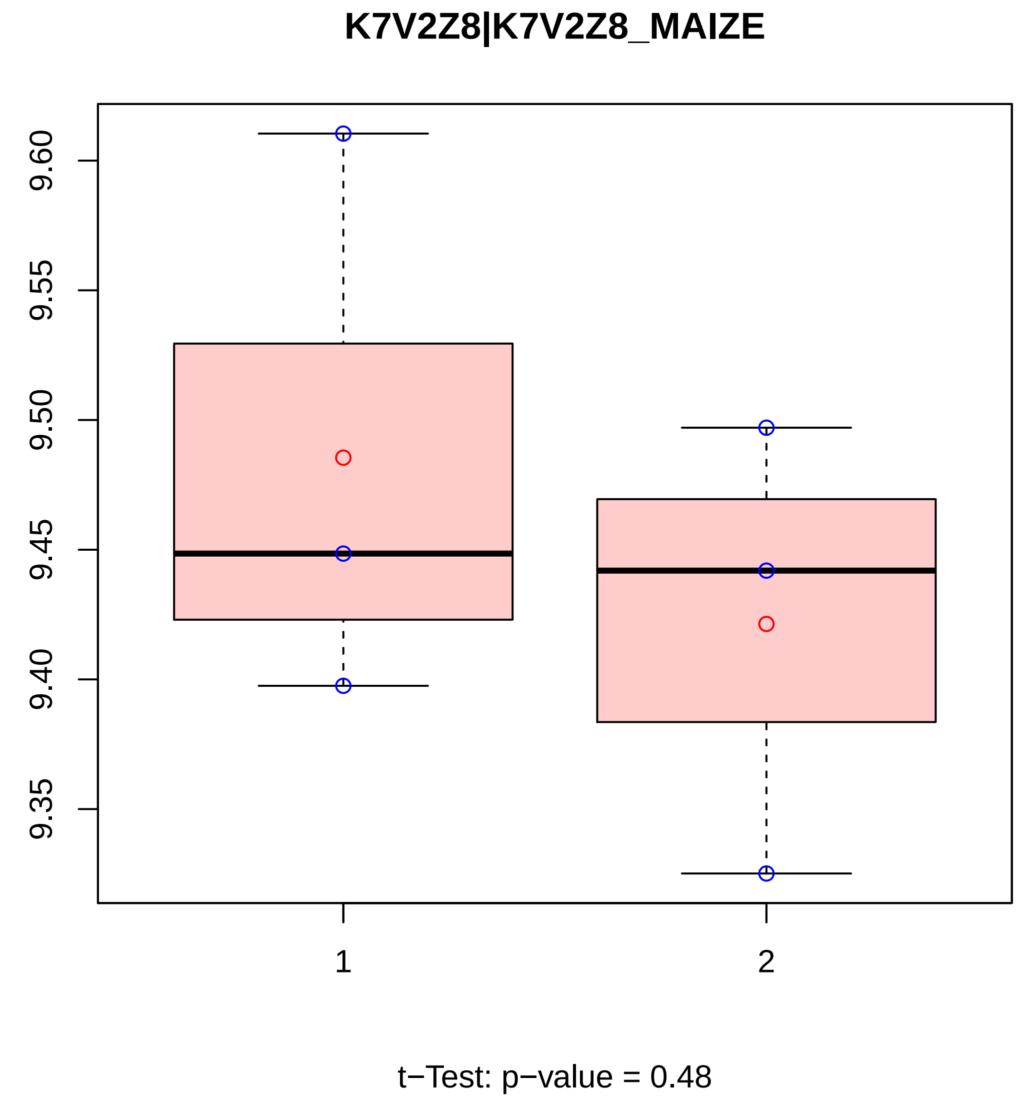
K7V2Z8|K7V2Z8_MAIZE
9.60
9.55
9.50
9.45
9.40
9.35
1
2
t−Test: p−value = 0.48

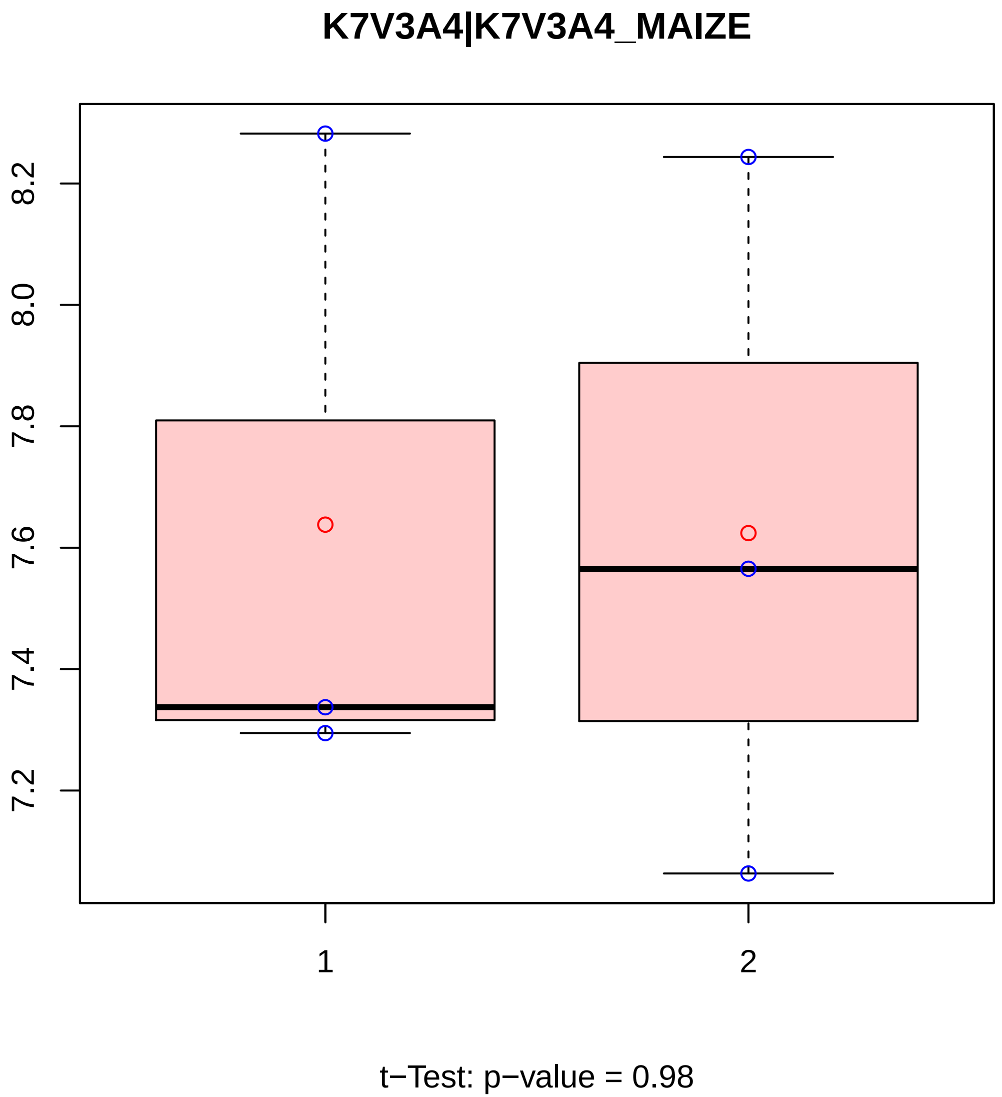
K7V3A4|K7V3A4_MAIZE
8.2
8.0
7.8
7.6
7.4
7.2
1
2
t−Test: p−value = 0.98

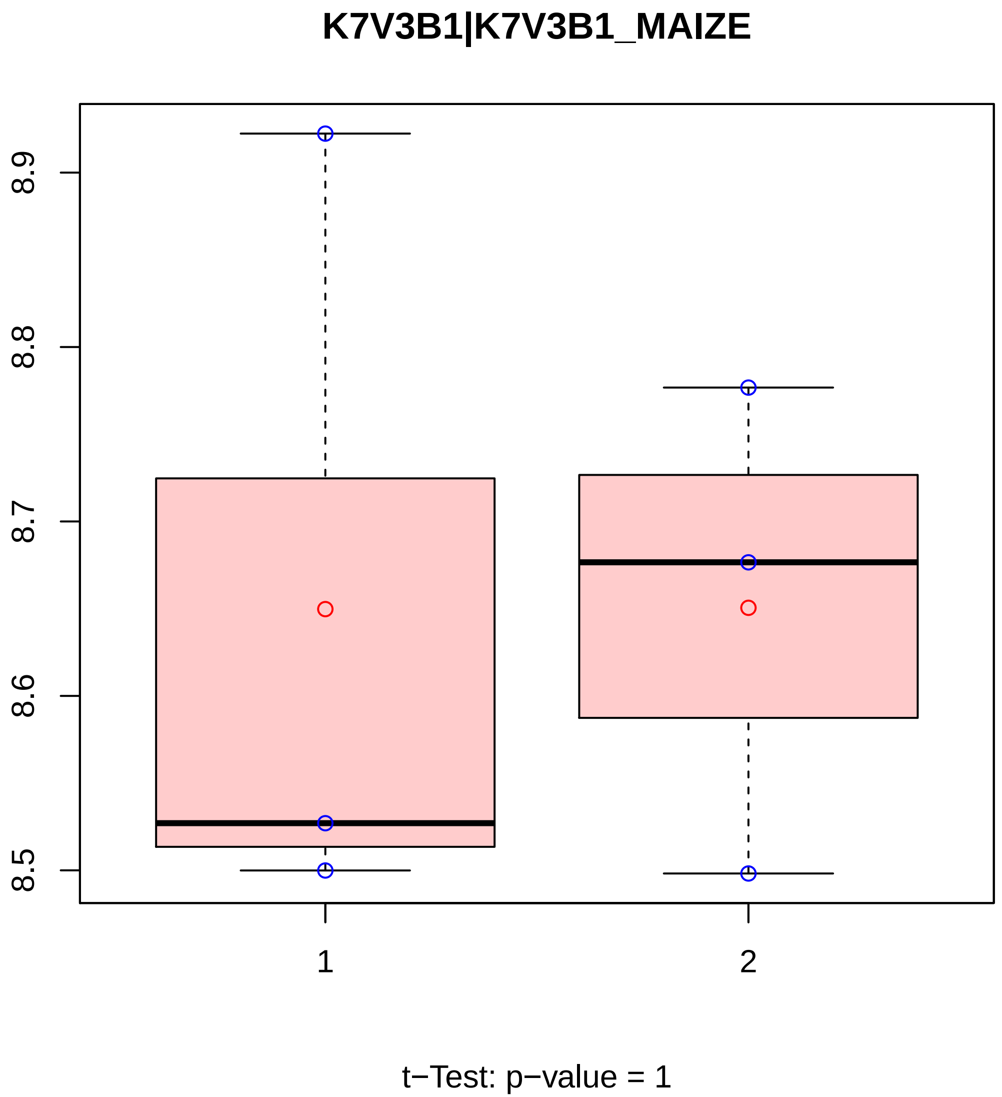

K7V3B1|K7V3B1_MAIZE
8.5
8.6
8.7
8.8
8.9
1
2
t−Test: p−value = 1

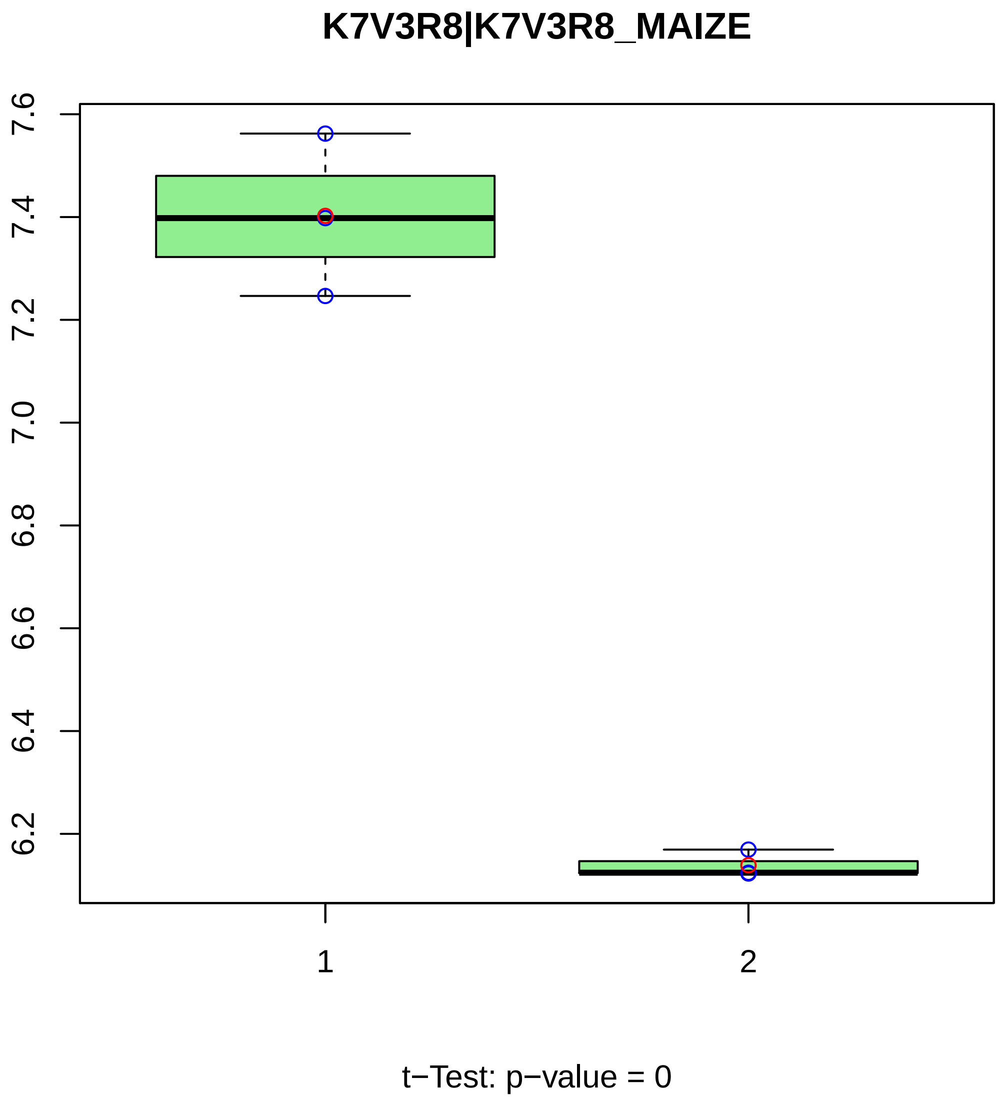
K7V3R8|K7V3R8_MAIZE
7.6
7.4
7.2
7.0
6.8
6.6
6.4
6.2
1
2
t−Test: p−value = 0

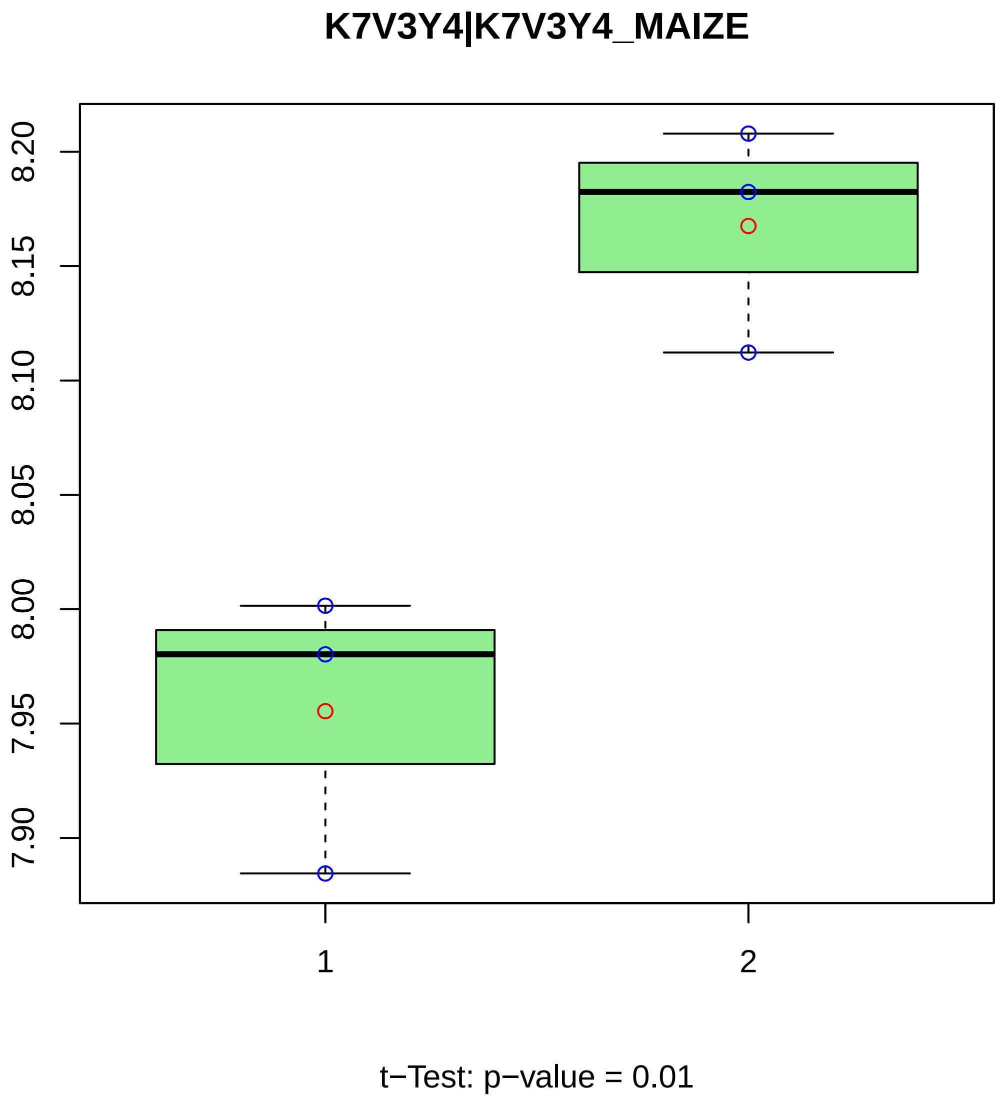
K7V3Y4|K7V3Y4_MAIZE
8.20
8.15
8.10
8.05
8.00
7.95
7.90
1
2
t−Test: p−value = 0.01

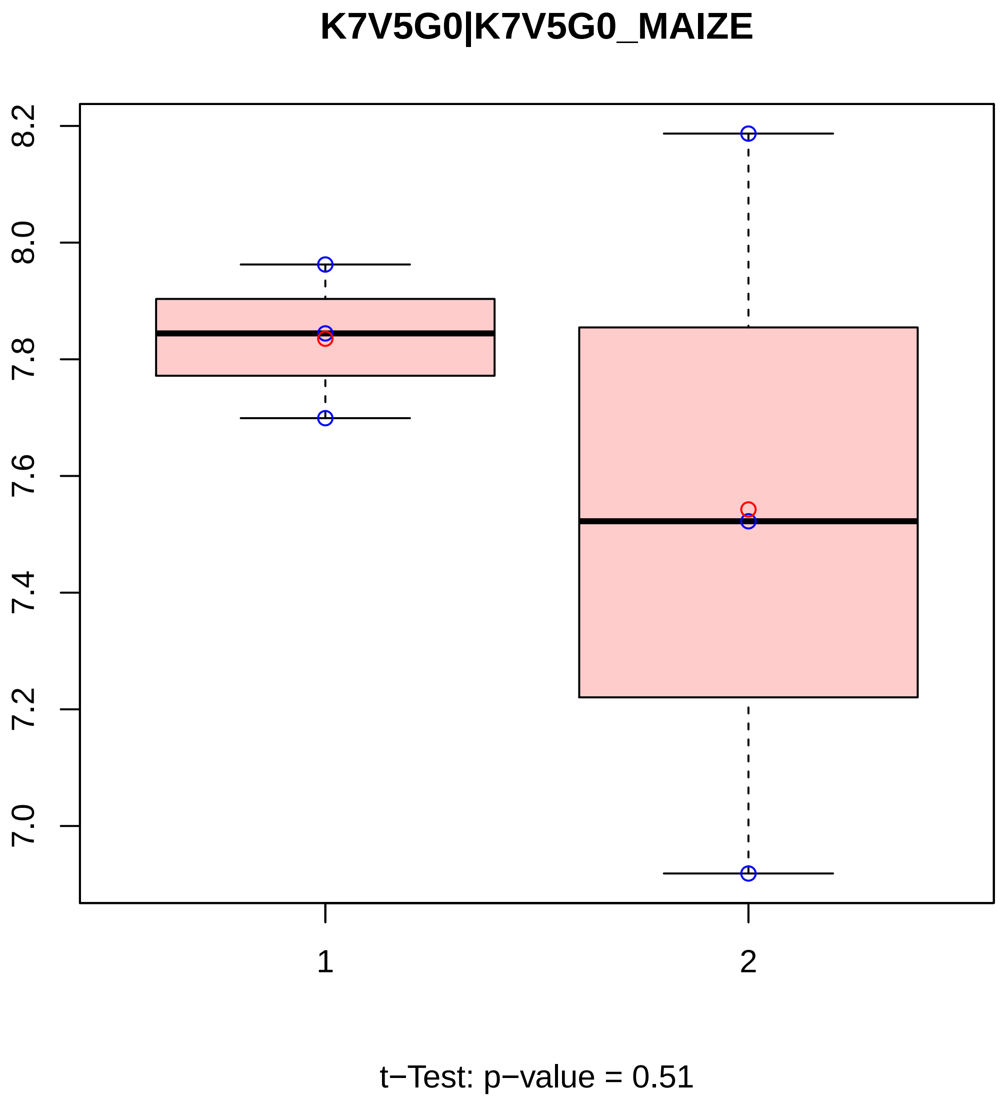
K7V5G0|K7V5G0_MAIZE
8.2
8.0
7.8
7.6
7.4
7.2
7.0
1
2
t−Test: p−value = 0.51

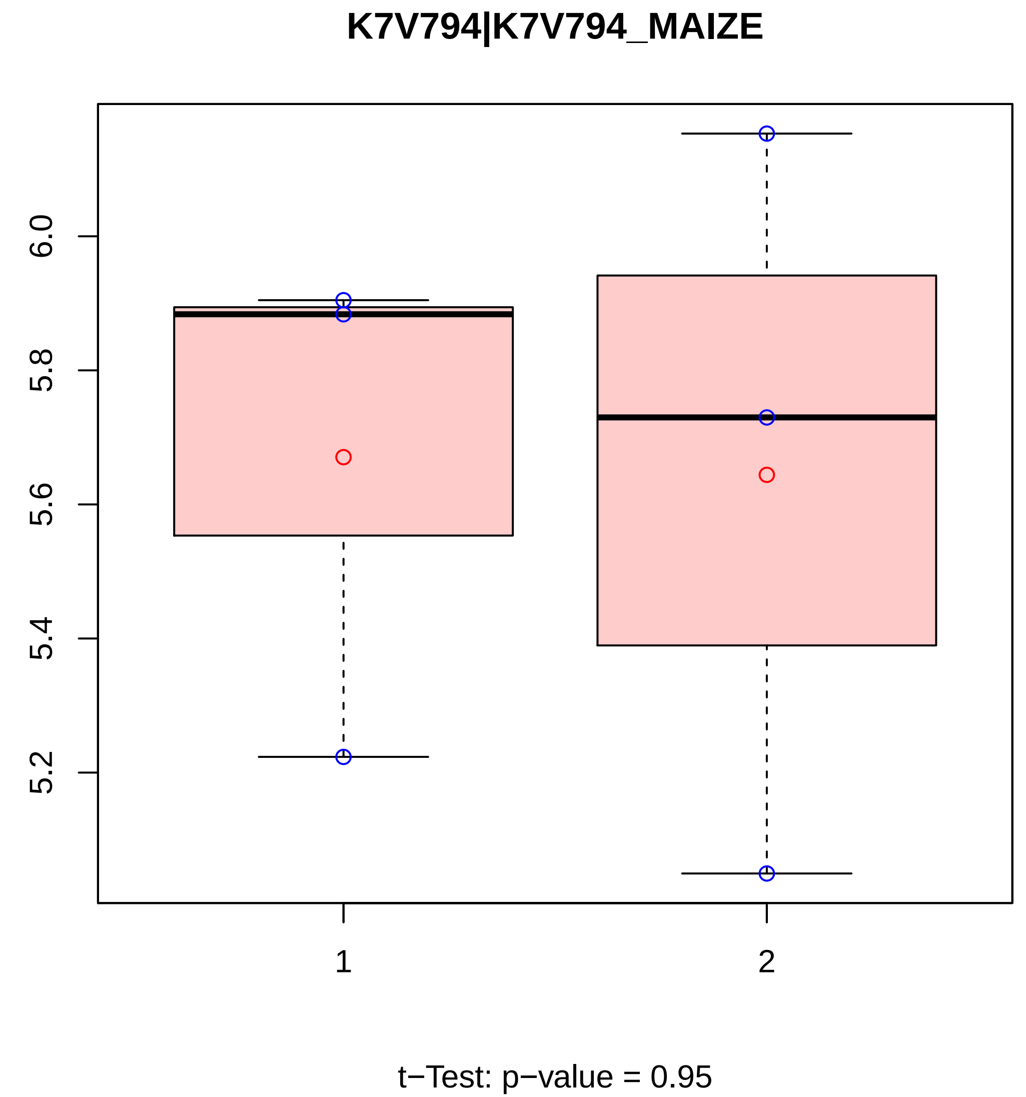
K7V794|K7V794_MAIZE
6.0
5.8
5.6
5.4
5.2
1
2
t−Test: p−value = 0.95

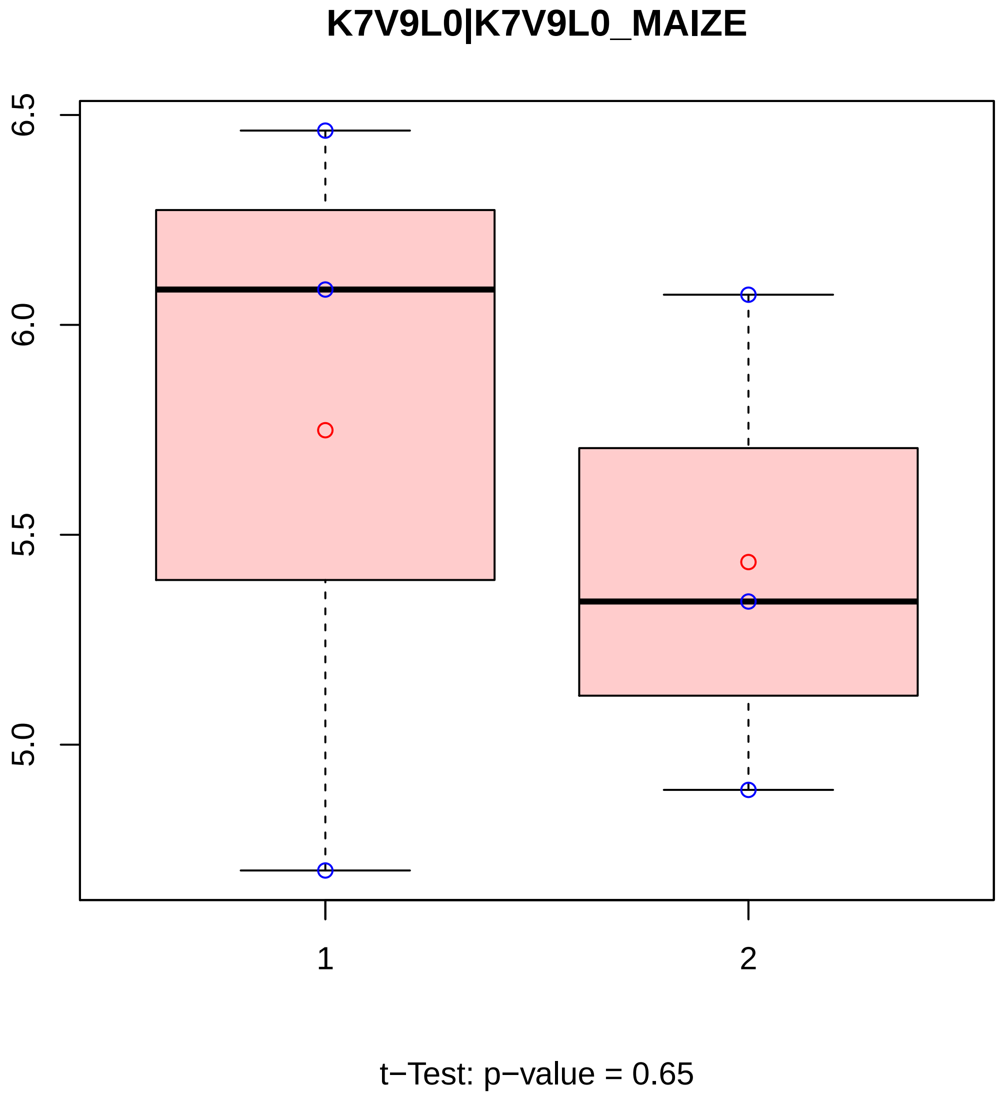
K7V9L0|K7V9L0_MAIZE
6.5
6.0
5.5
5.0
1
2
t−Test: p−value = 0.65

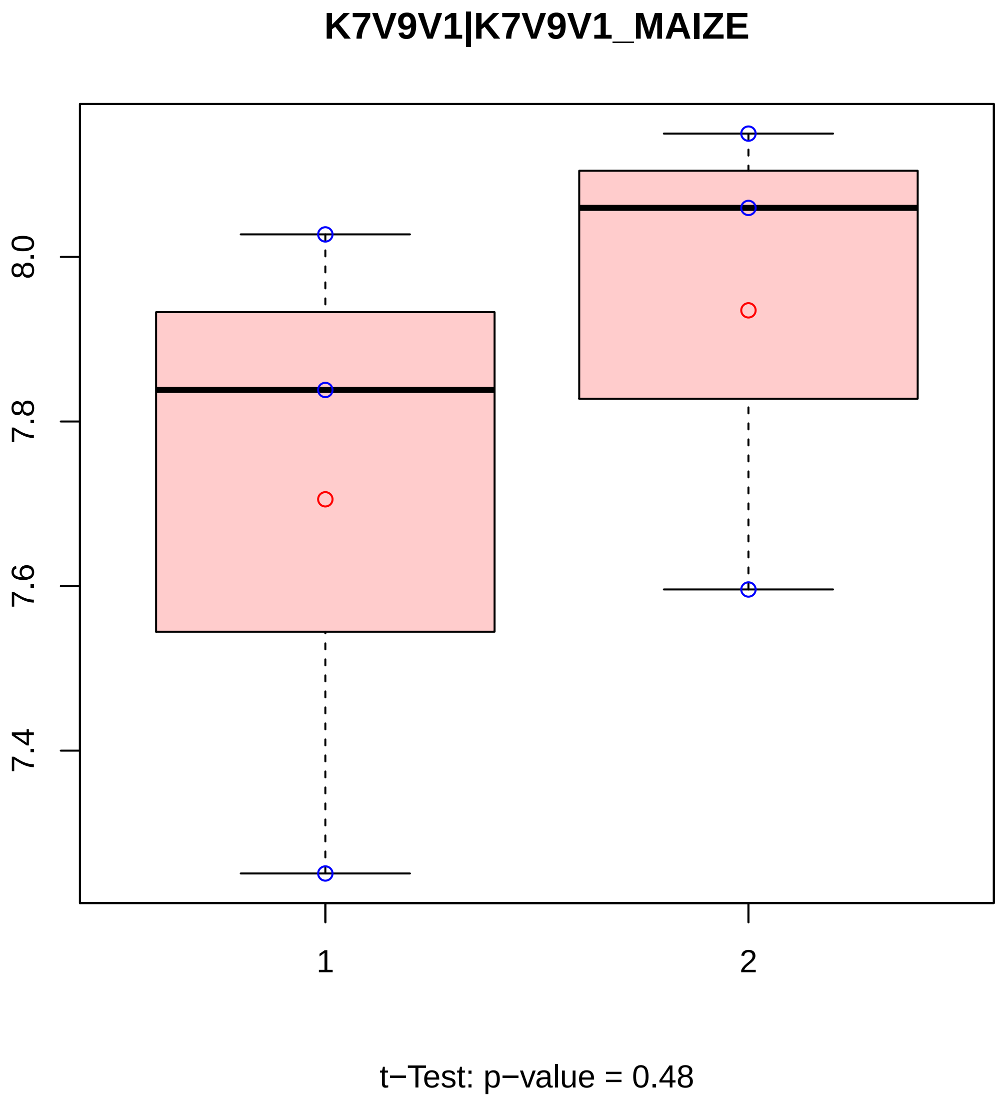
K7V9V1|K7V9V1_MAIZE
8.0
7.8
7.6
7.4
1
2
t−Test: p−value = 0.48

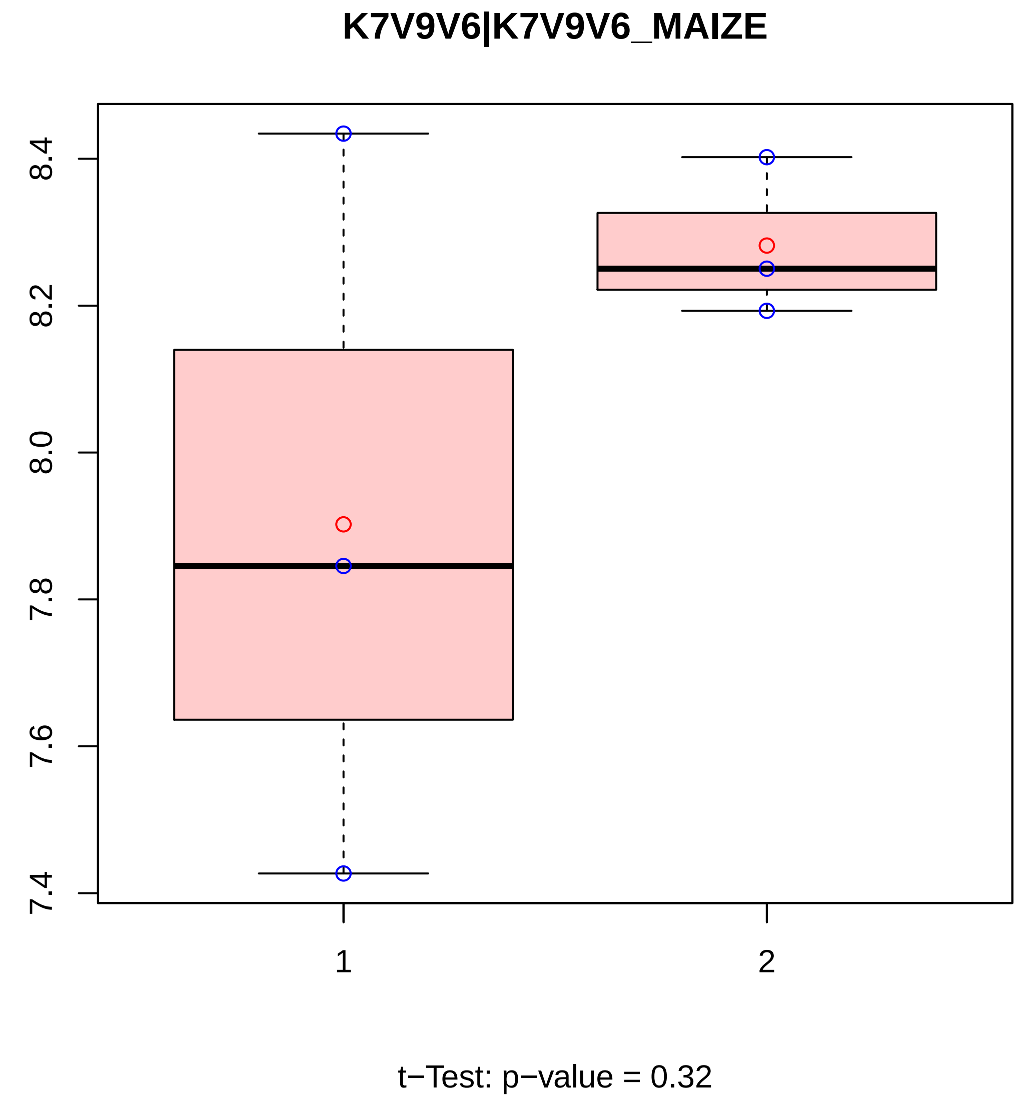
K7V9V6|K7V9V6_MAIZE
8.4
8.2
8.0
7.8
7.6
7.4
1
2
t−Test: p−value = 0.32

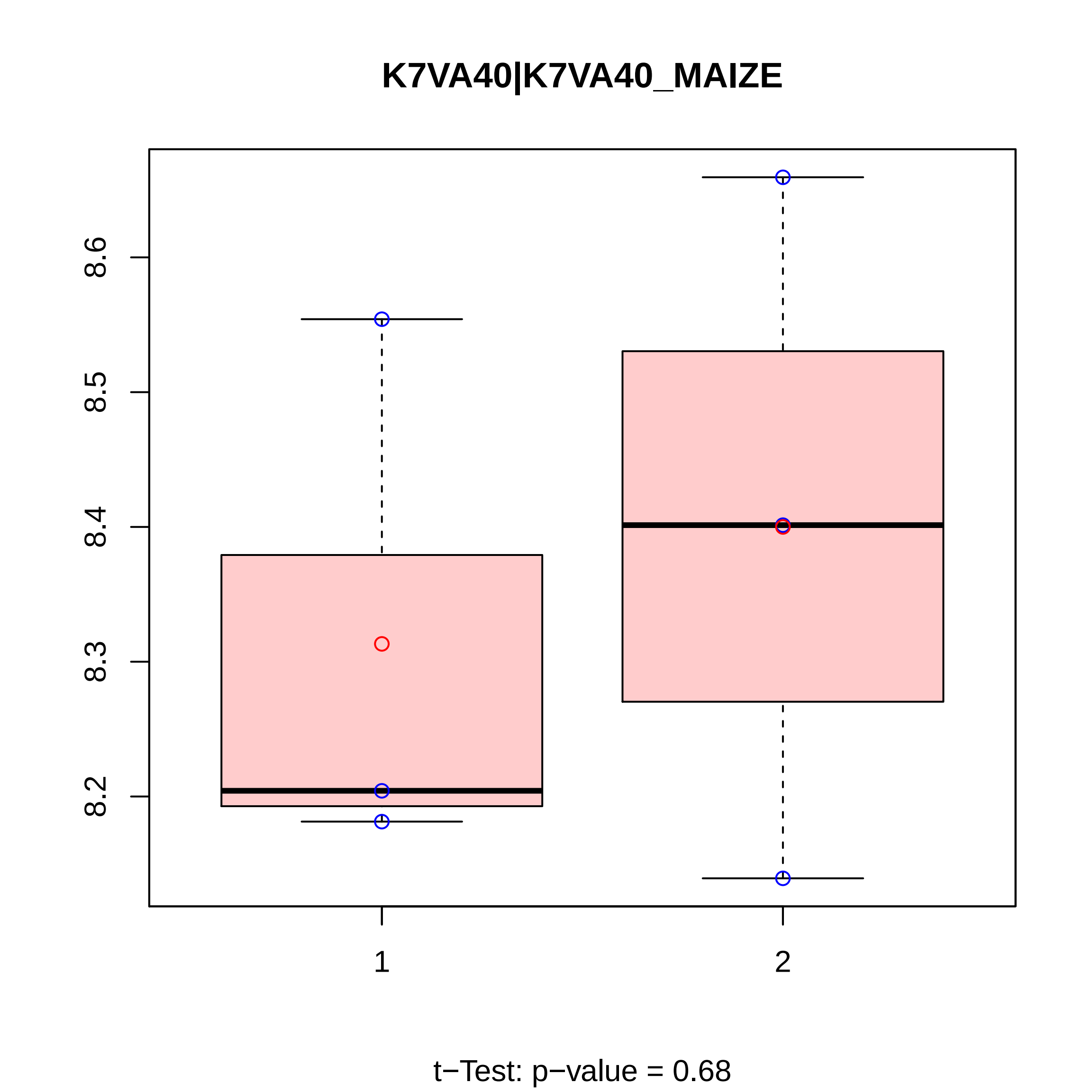
K7VA40|K7VA40_MAIZE
8.2
8.3
8.4
8.5
8.6
1
2
t−Test: p−value = 0.68

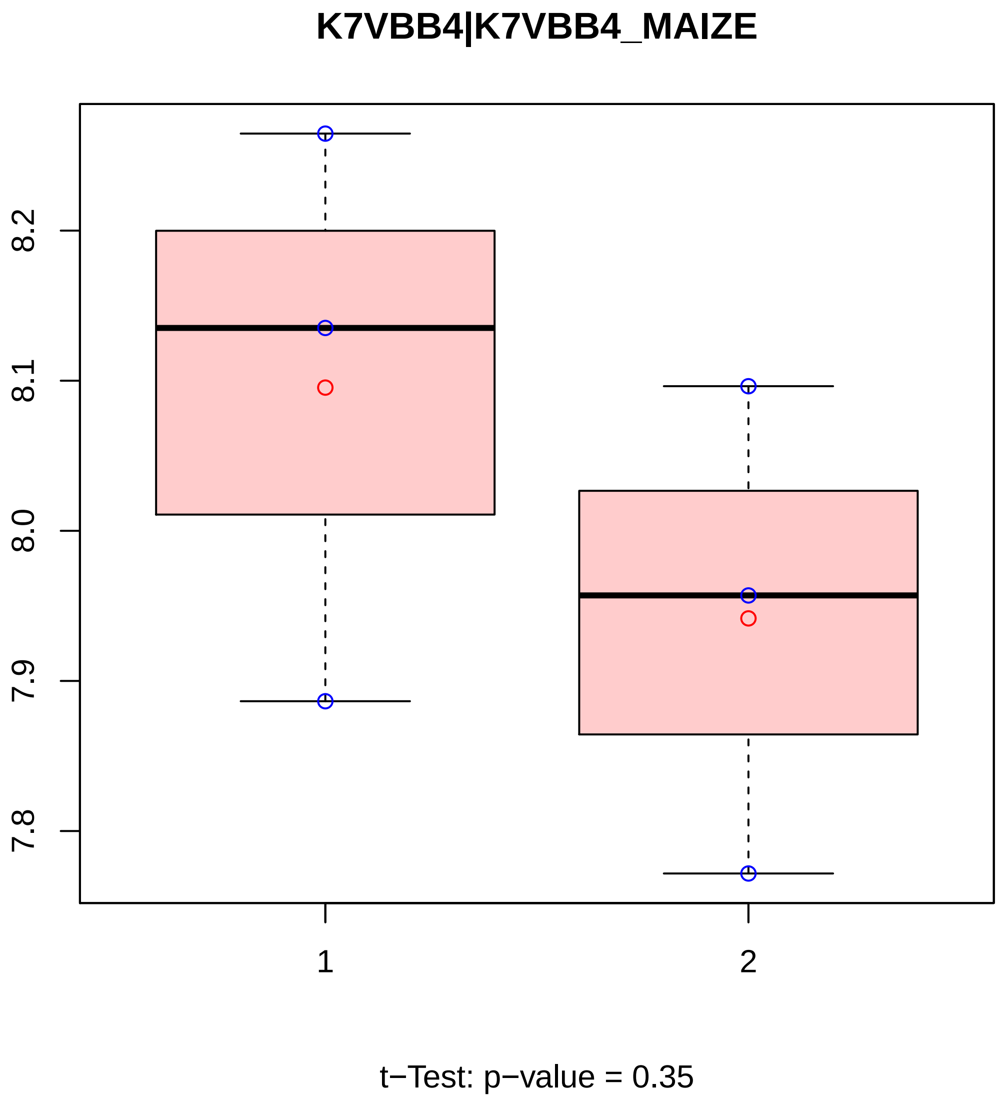

K7VBB4|K7VBB4_MAIZE
8.2
8.1
8.0
7.9
7.8
1
2
t−Test: p−value = 0.35

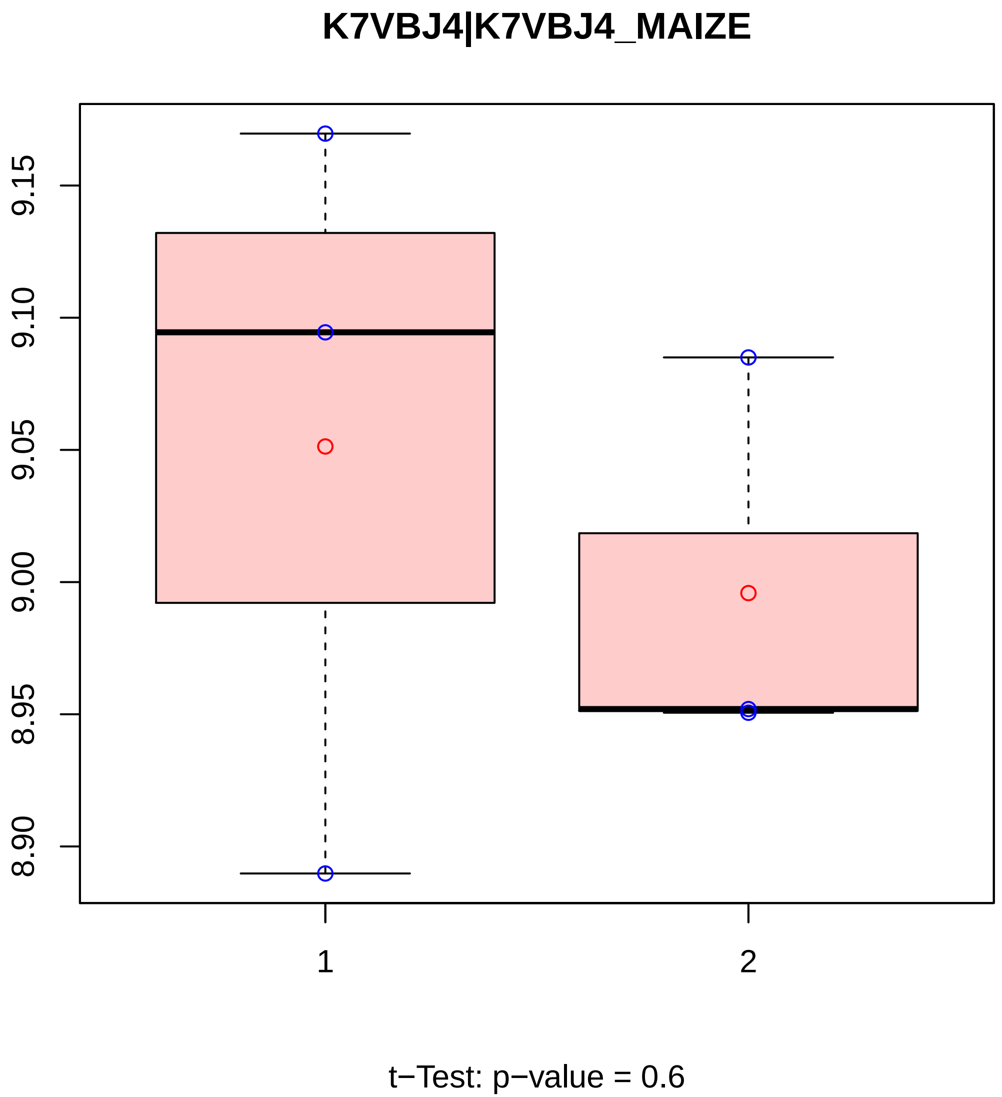
K7VBJ4|K7VBJ4_MAIZE
9.15
9.10
9.05
9.00
8.95
8.90
1
2
t−Test: p−value = 0.6

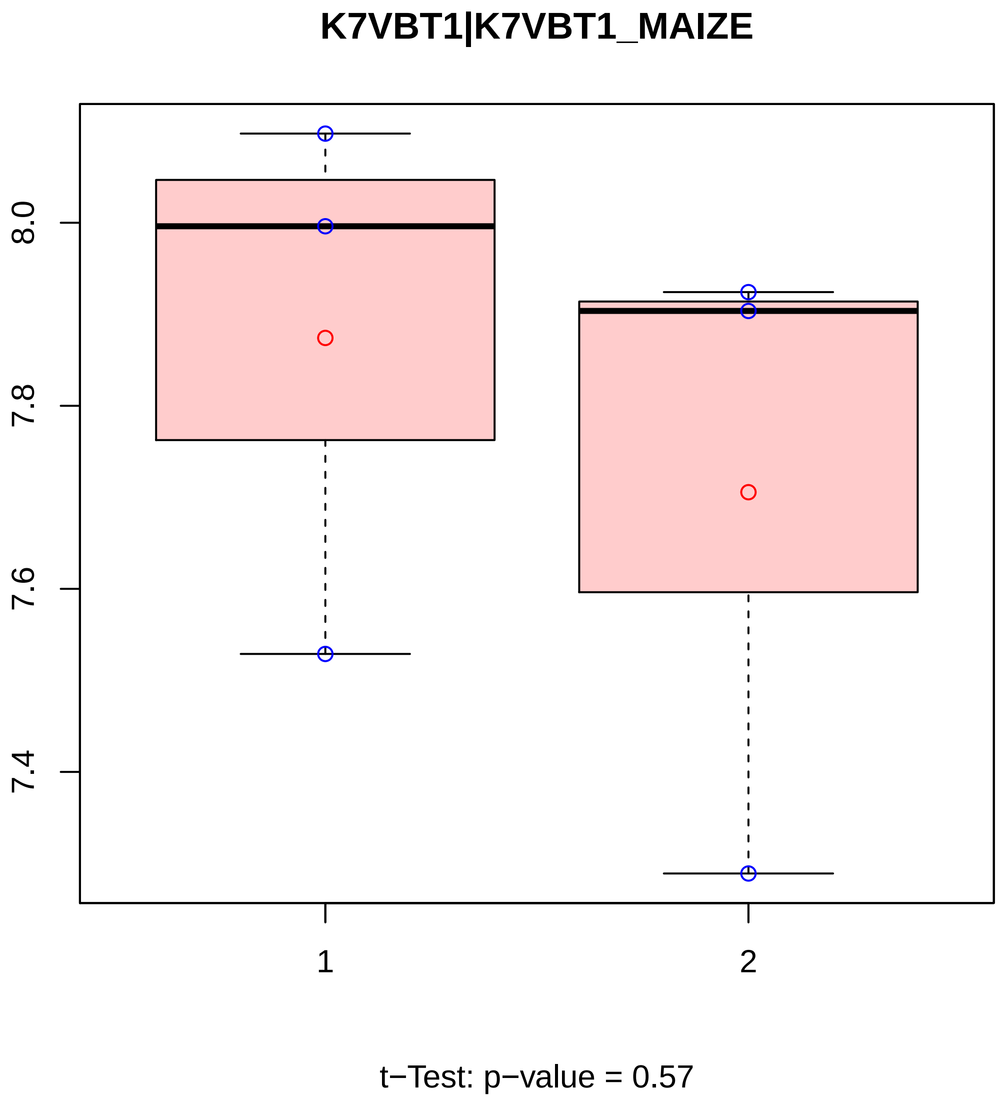
K7VBT1|K7VBT1_MAIZE
8.0
7.8
7.6
7.4
1
2
t−Test: p−value = 0.57

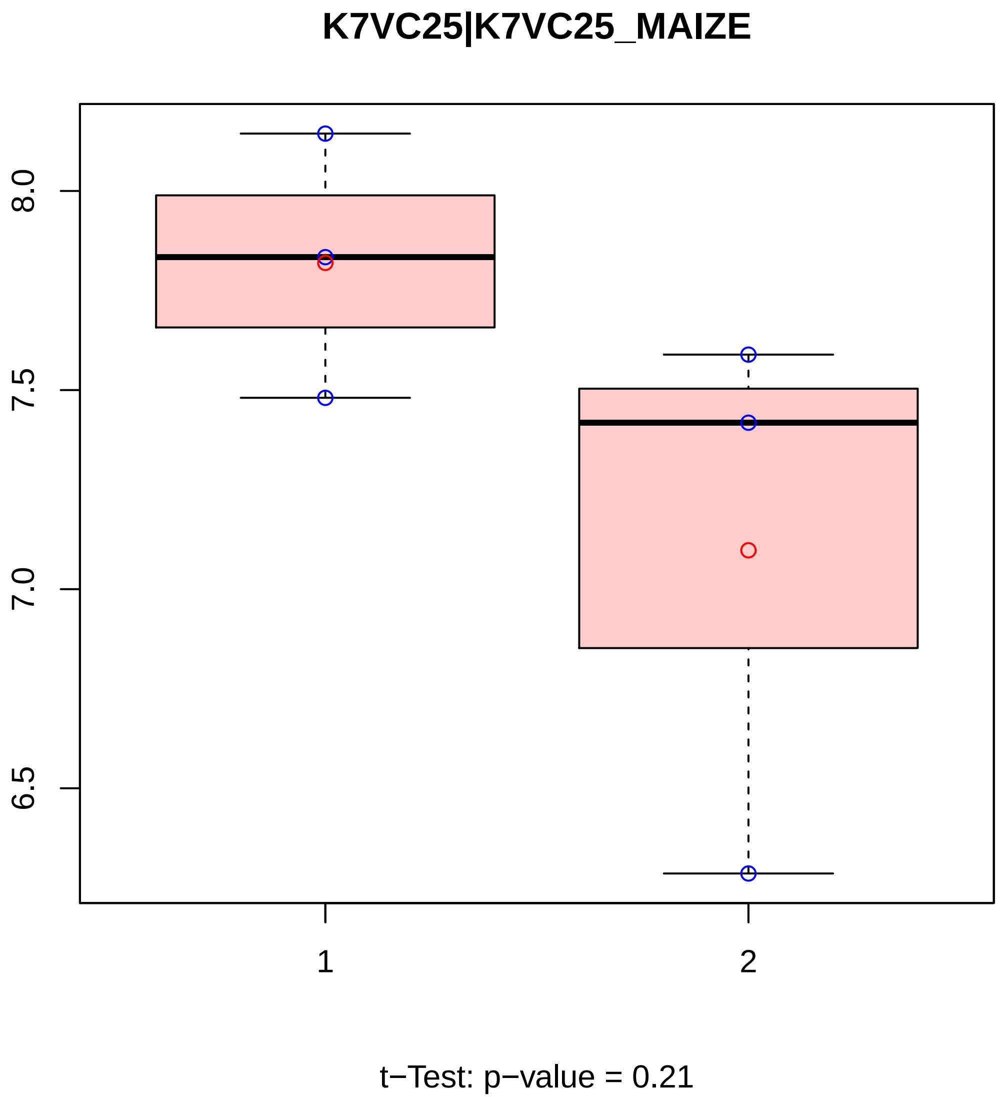
K7VC25|K7VC25_MAIZE
8.0
7.5
7.0
6.5
1
2
t−Test: p−value = 0.21

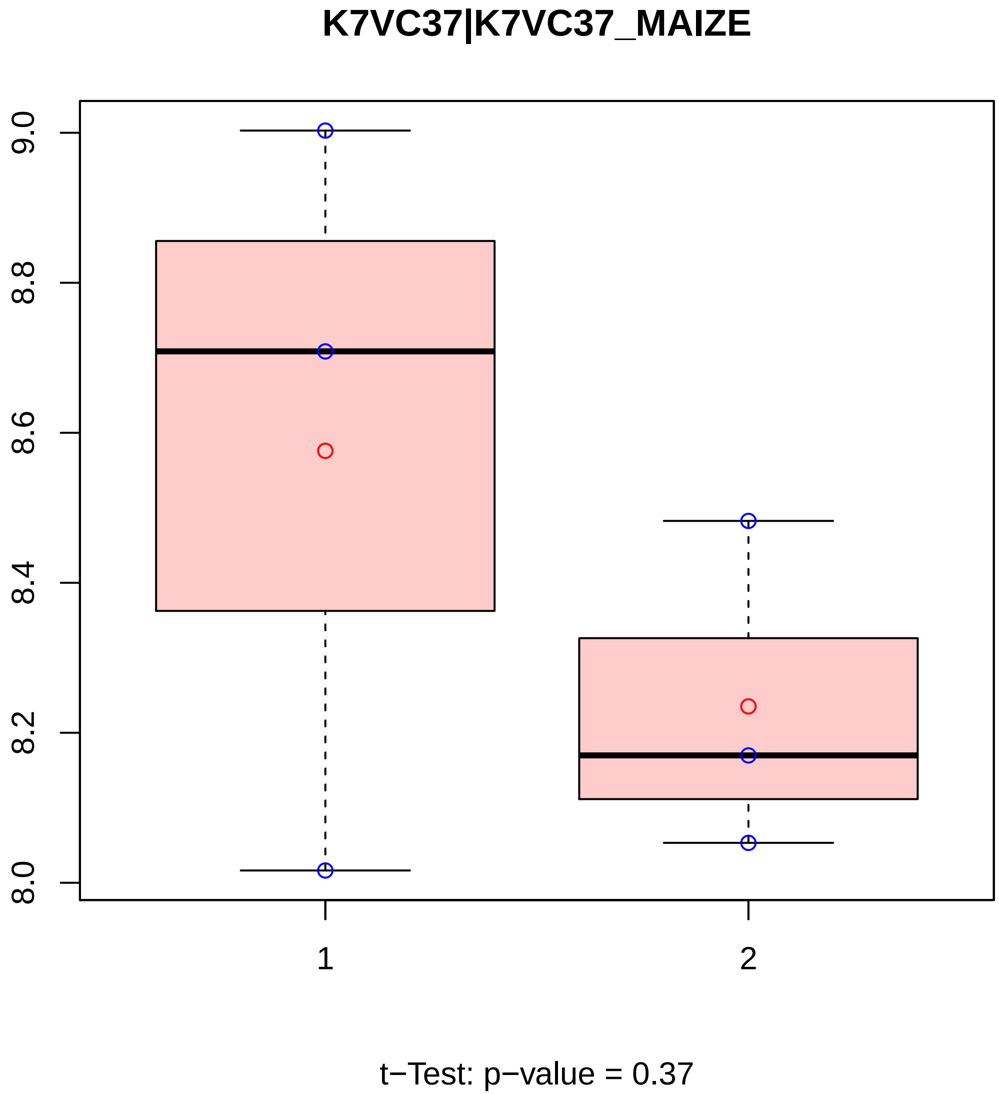
K7VC37|K7VC37_MAIZE
9.0
8.8
8.6
8.4
8.2
8.0
1
2
t−Test: p−value = 0.37

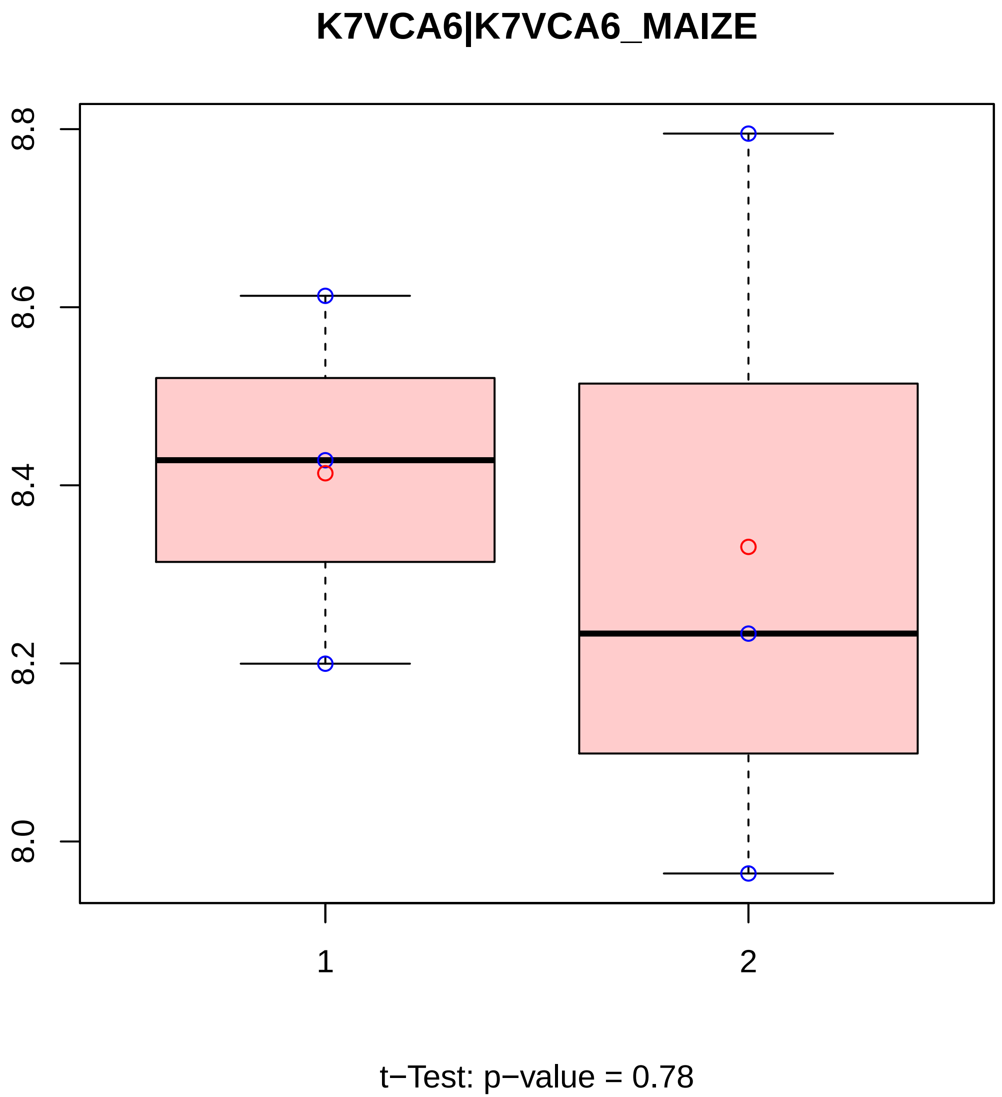
K7VCA6|K7VCA6_MAIZE
8.0
8.2
8.4
8.6
8.8
1
2
t−Test: p−value = 0.78

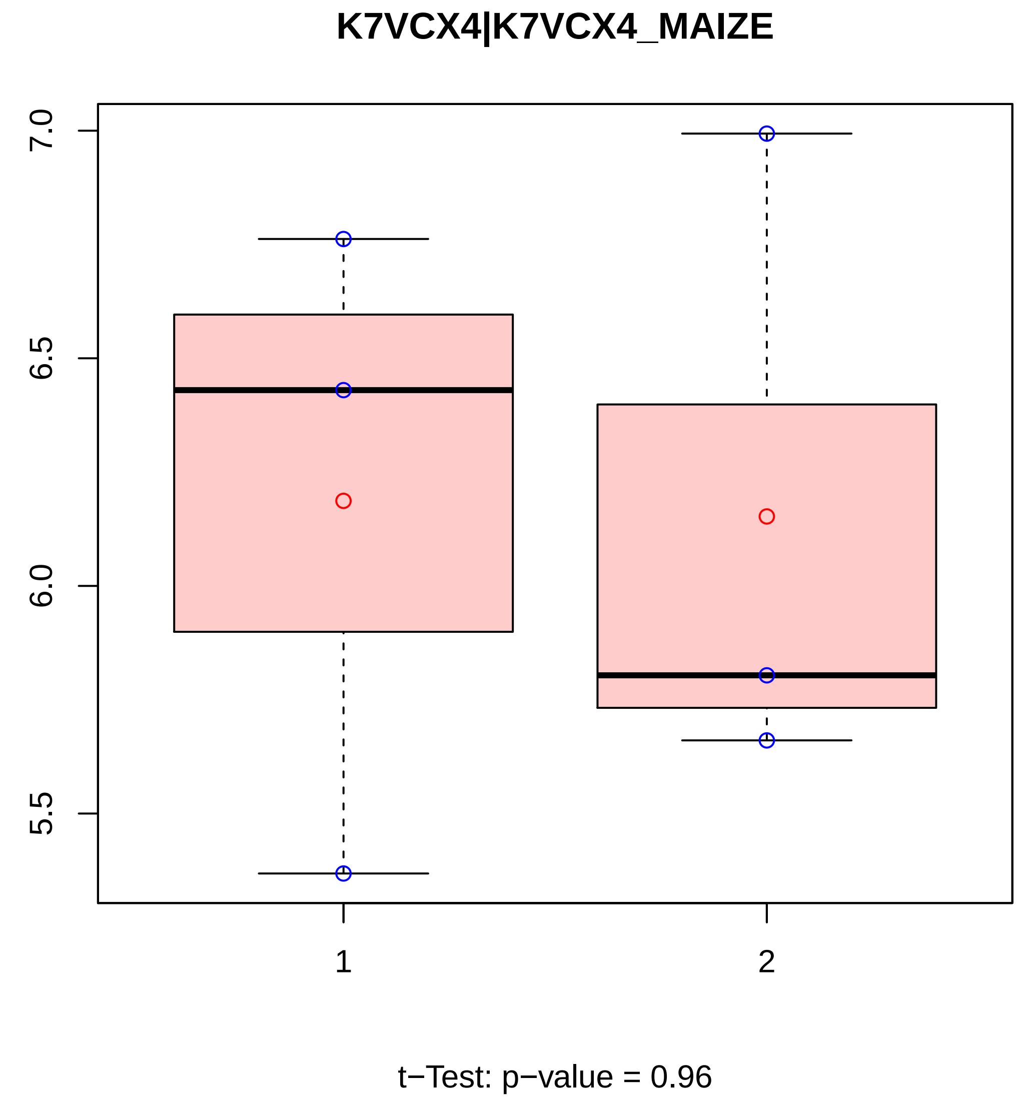
K7VCX4|K7VCX4_MAIZE
7.0
6.5
6.0
5.5
1
2
t−Test: p−value = 0.96

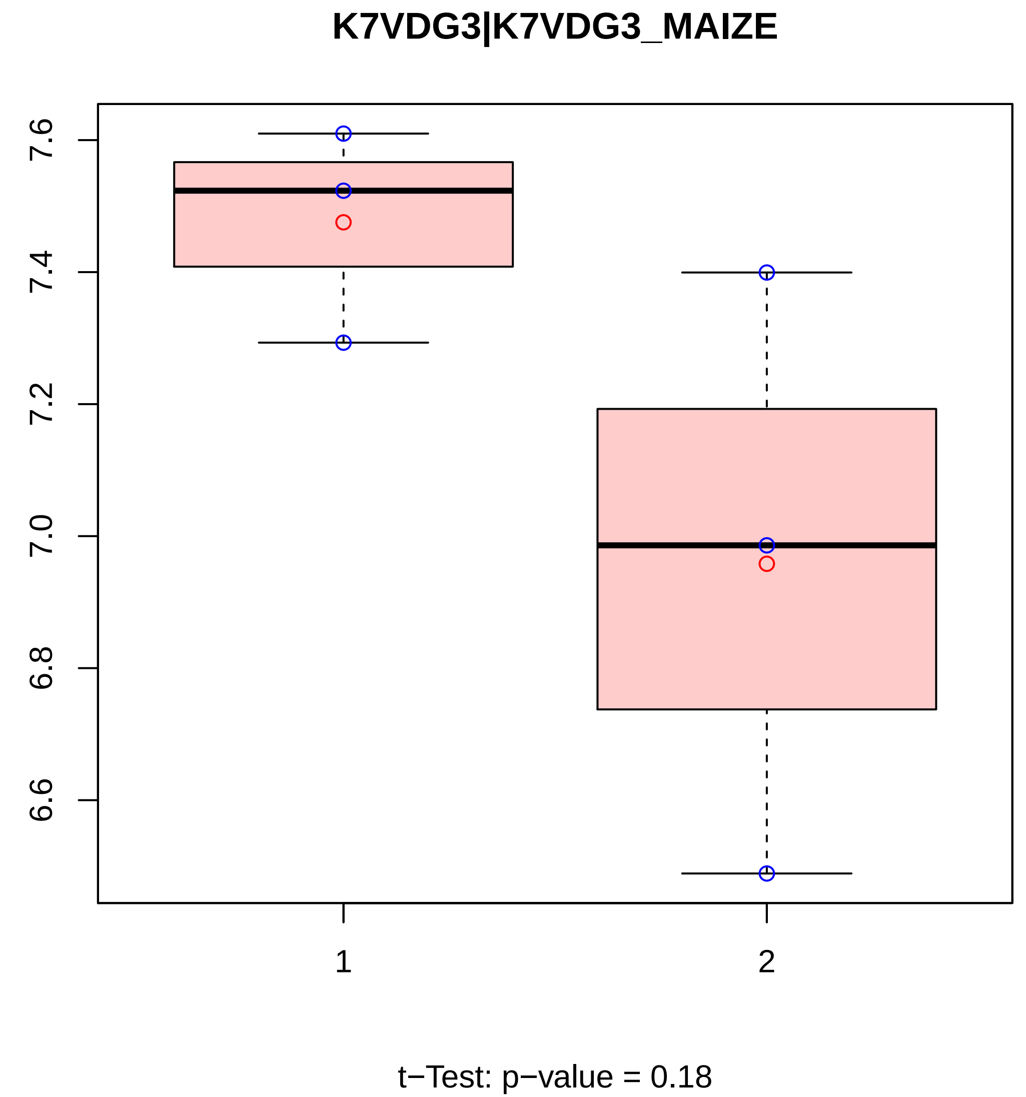
K7VDG3|K7VDG3_MAIZE
7.6
7.4
7.2
7.0
6.8
6.6
1
2
t−Test: p−value = 0.18

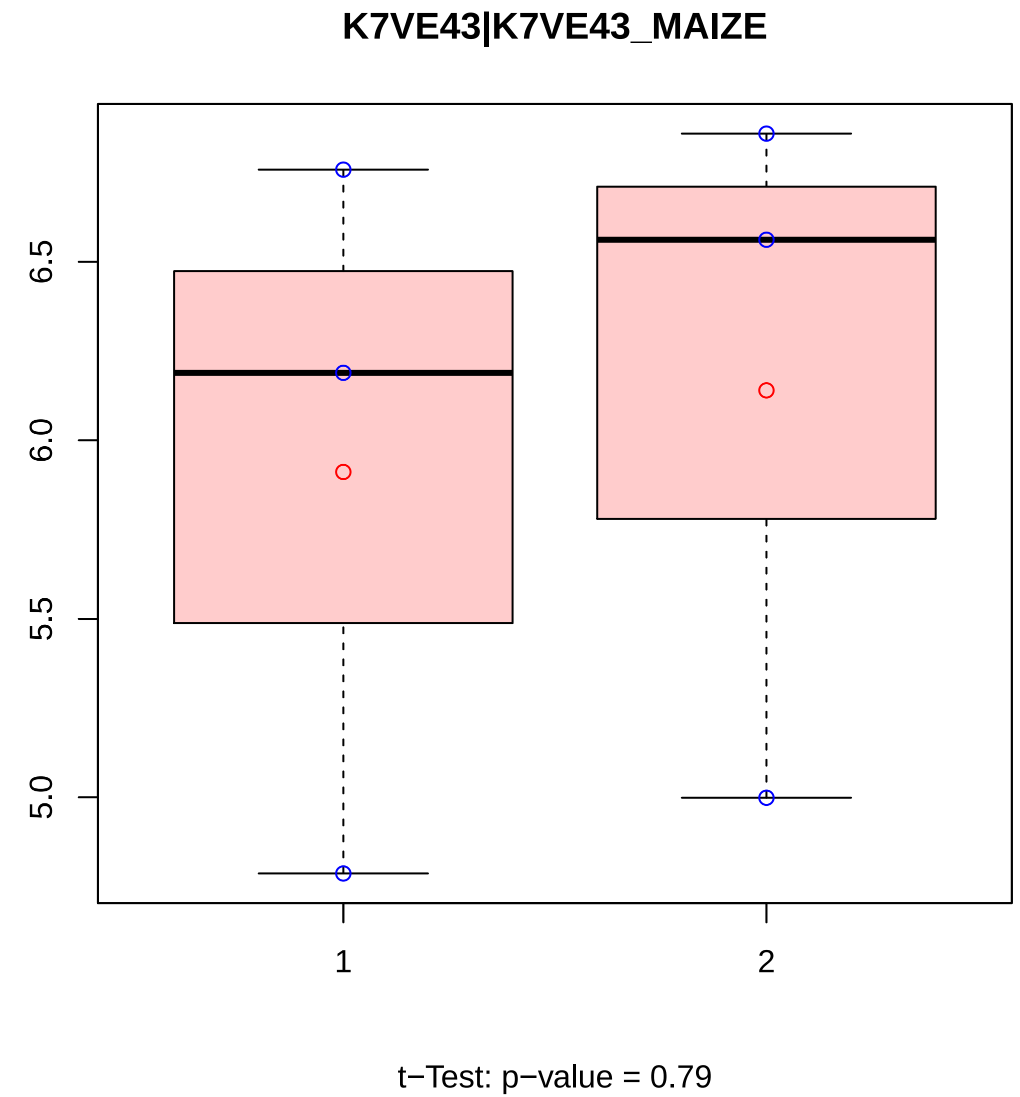

K7VE43|K7VE43_MAIZE
5.0
5.5
6.0
6.5
1
2
t−Test: p−value = 0.79

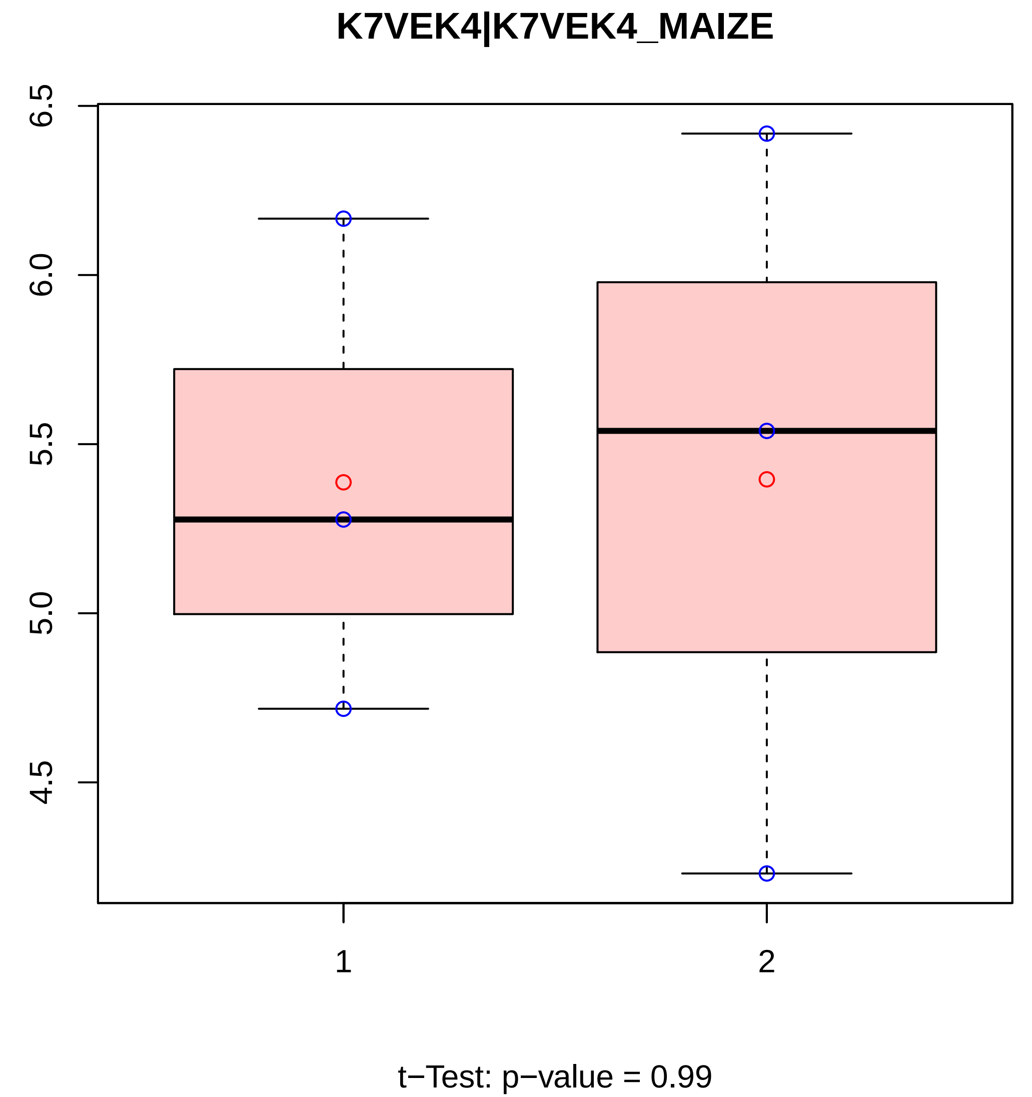
K7VEK4|K7VEK4_MAIZE
6.5
6.0
5.5
5.0
4.5
1
2
t−Test: p−value = 0.99

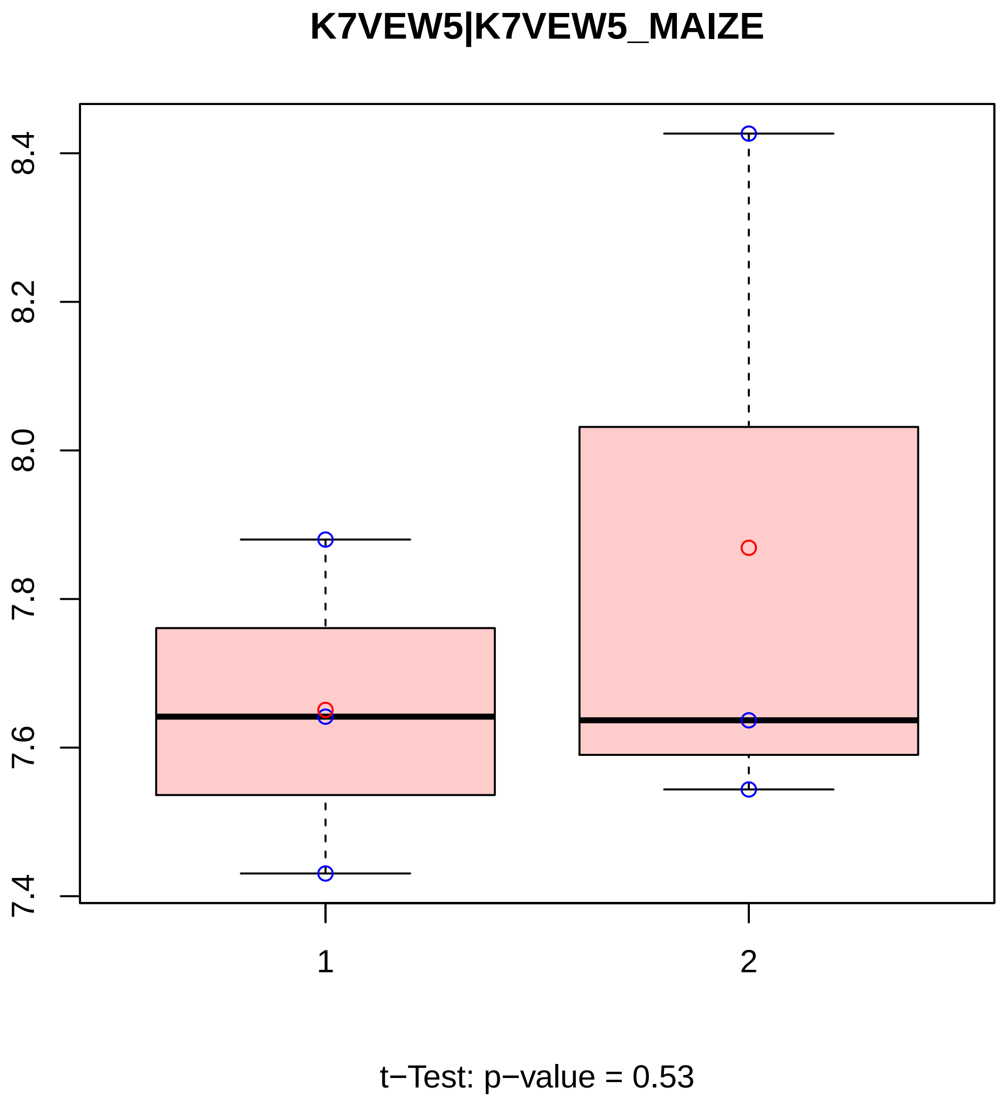
K7VEW5|K7VEW5_MAIZE
8.4
8.2
8.0
7.8
7.6
7.4
1
2
t−Test: p−value = 0.53

K7VG66|K7VG66_MAIZE

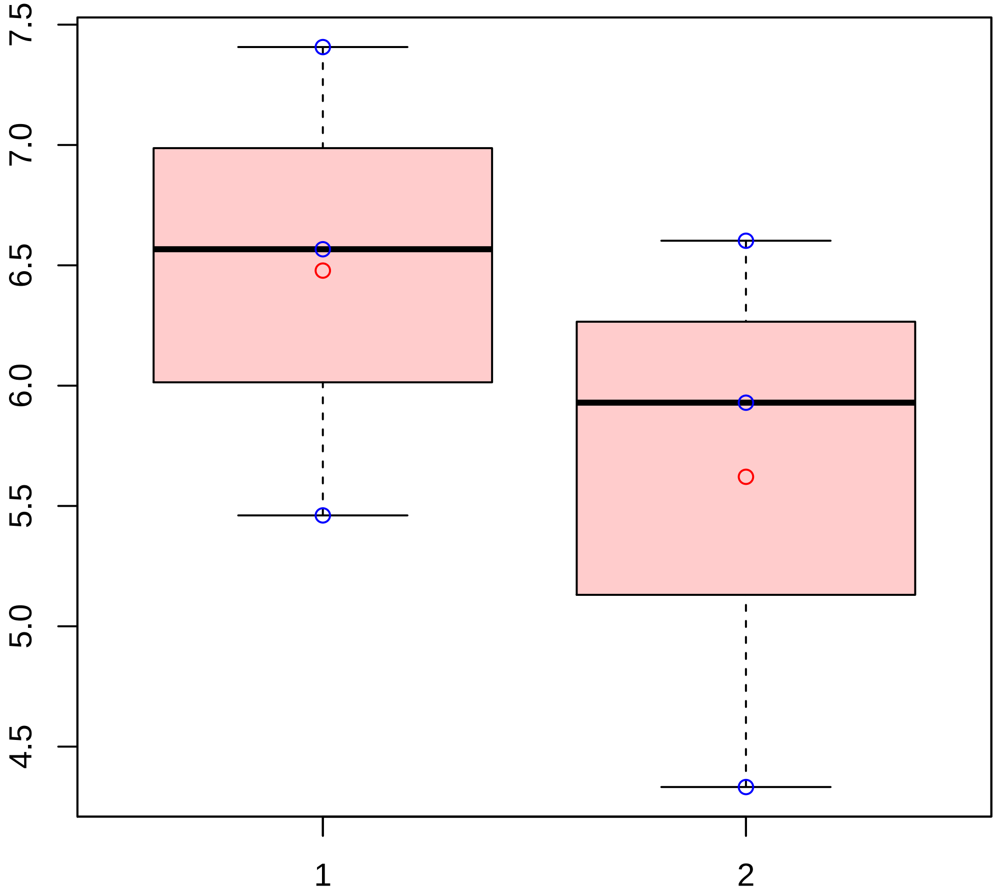

t−Test: p−value = 0.39

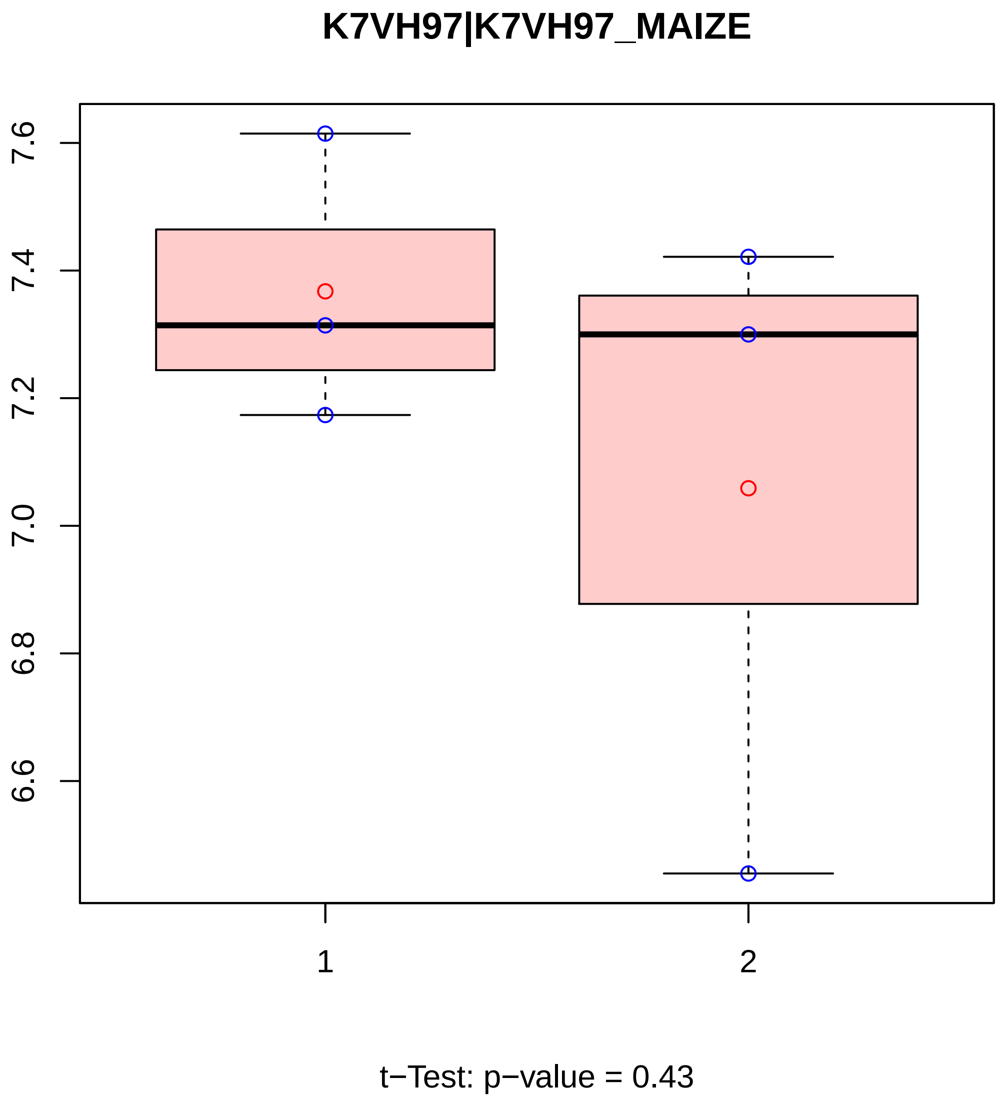
K7VH97|K7VH97_MAIZE
7.6
7.4
7.2
7.0
6.8
6.6
1
2
t−Test: p−value = 0.43

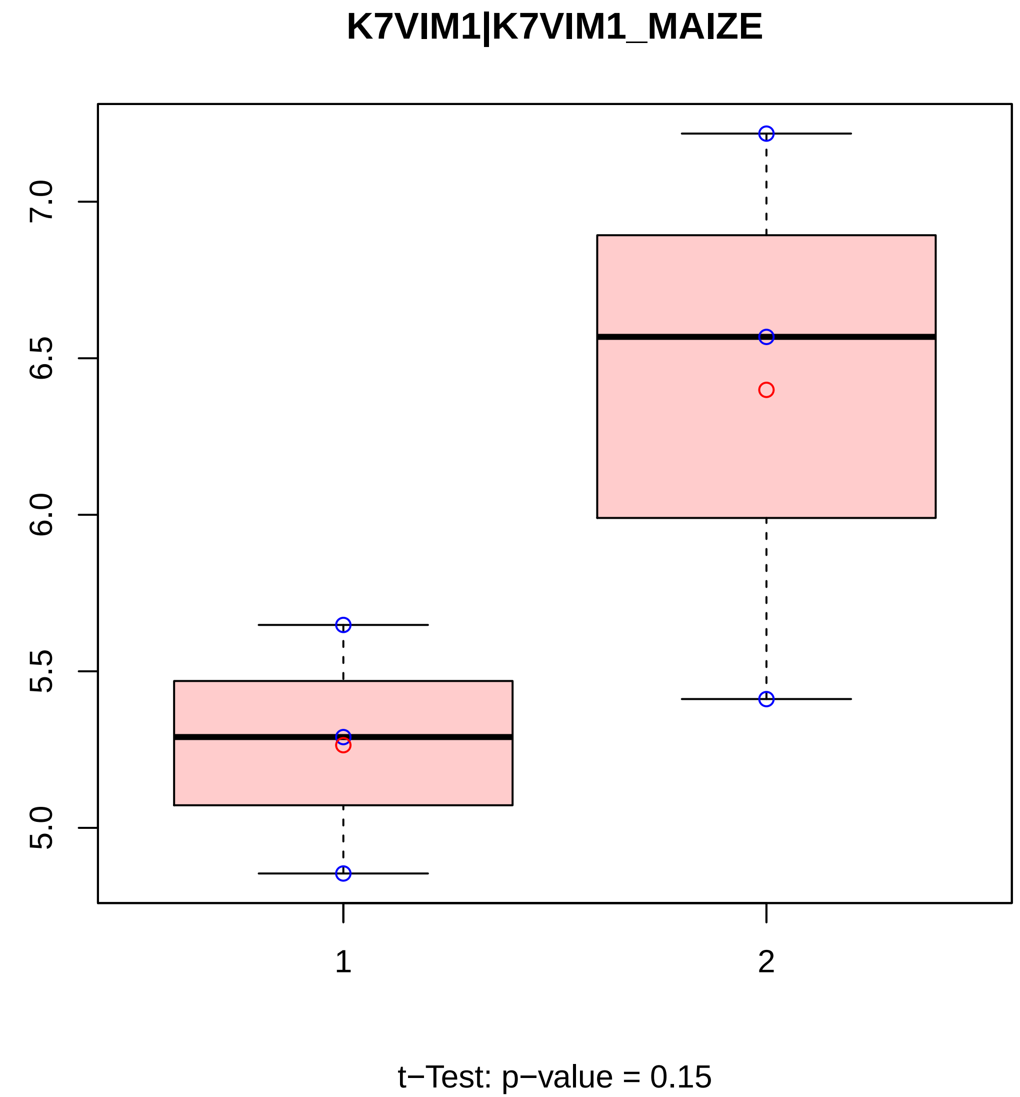

K7VIM1|K7VIM1_MAIZE
7.0
6.5
6.0
5.5
5.0
1
2
t−Test: p−value = 0.15

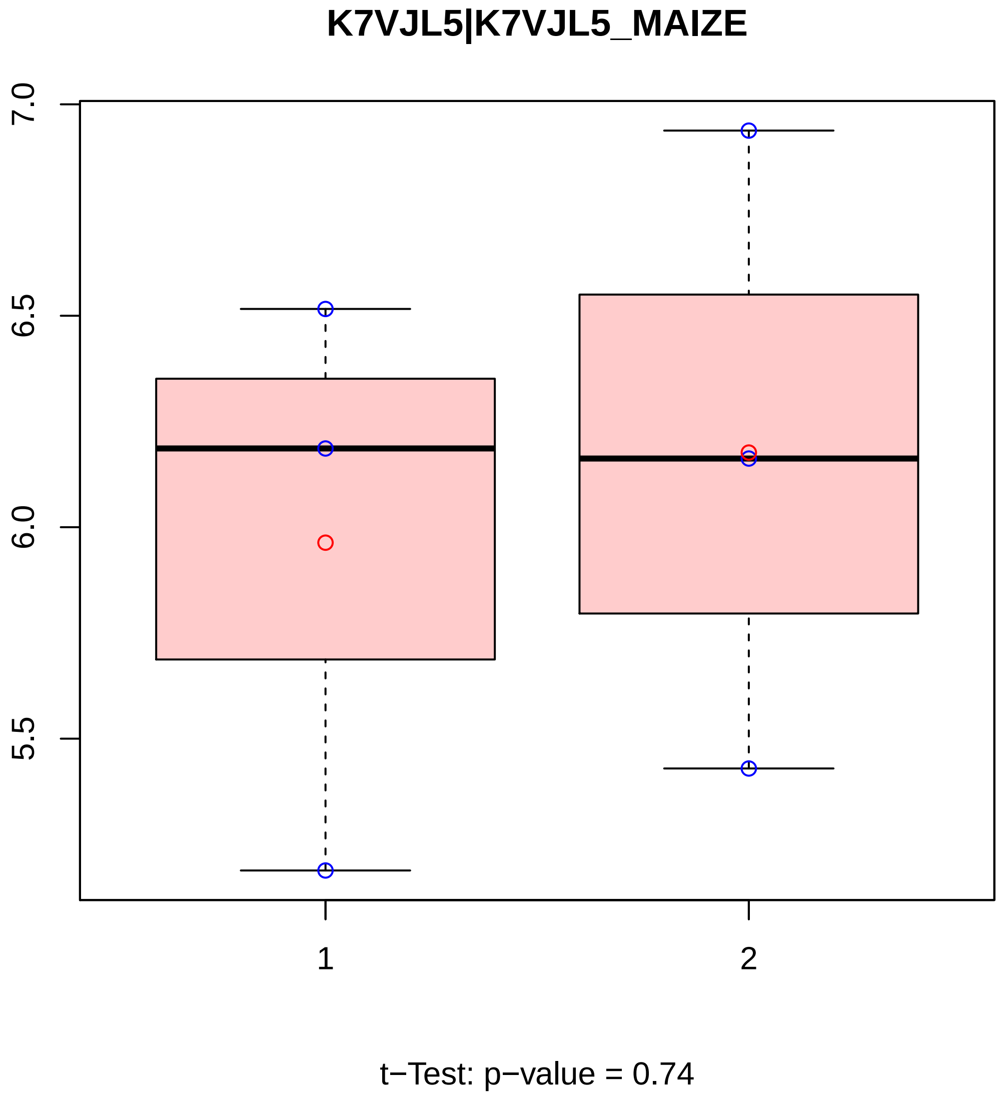
K7VJL5|K7VJL5_MAIZE
7.0
6.5
6.0
5.5
1
2
t−Test: p−value = 0.74

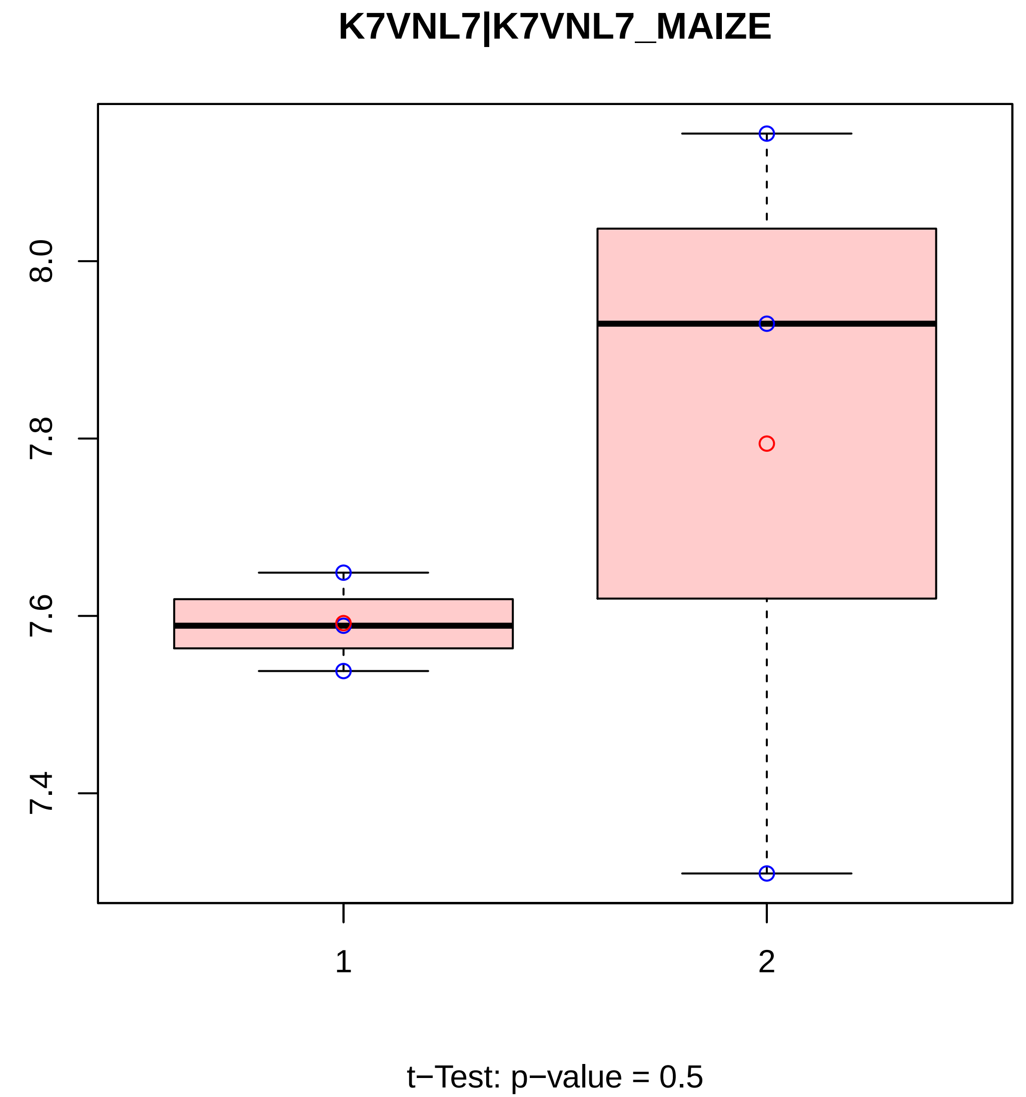
K7VNL7|K7VNL7_MAIZE
8.0
7.8
7.6
7.4
1
2
t−Test: p−value = 0.5

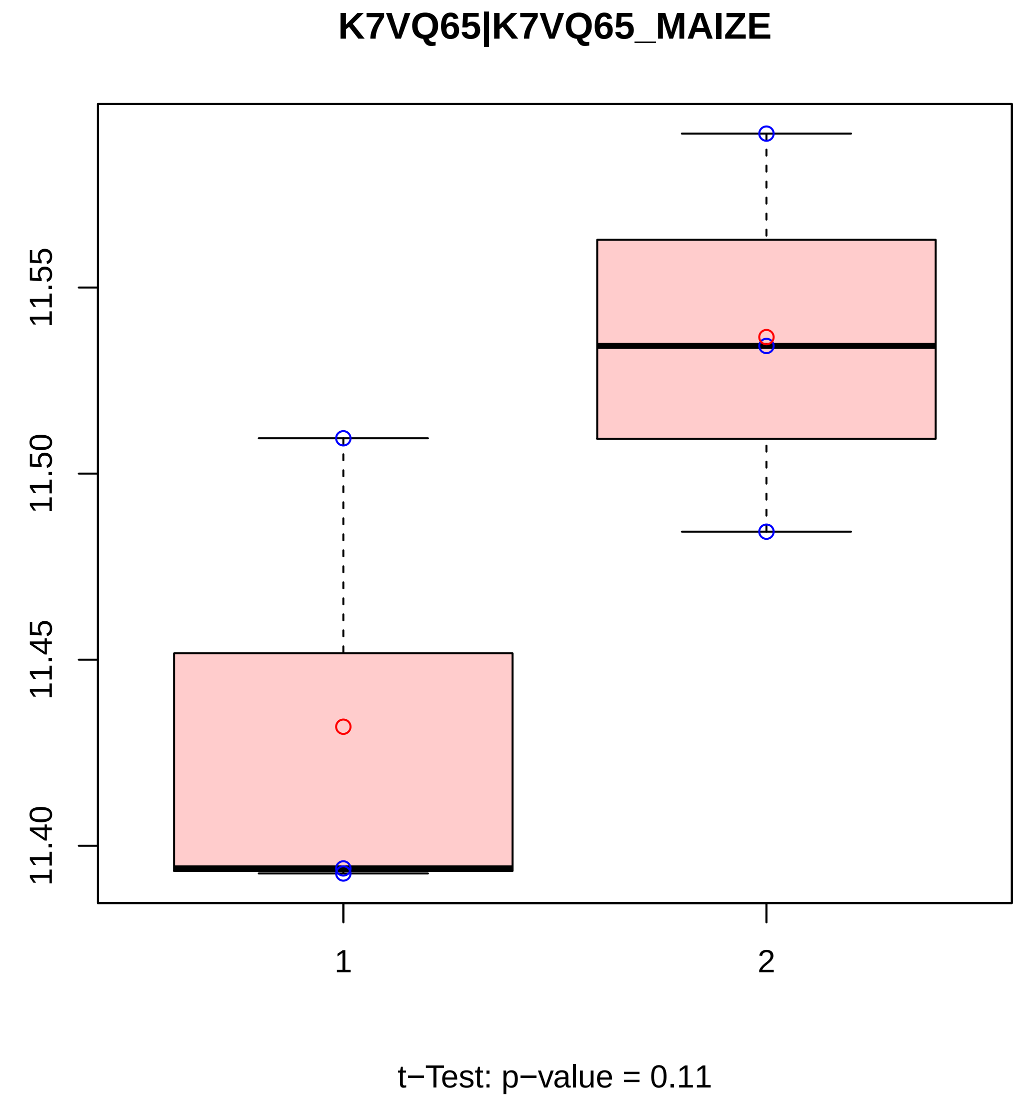
K7VQ65|K7VQ65_MAIZE
11.40
11.45
11.50
11.55
1
2
t−Test: p−value = 0.11

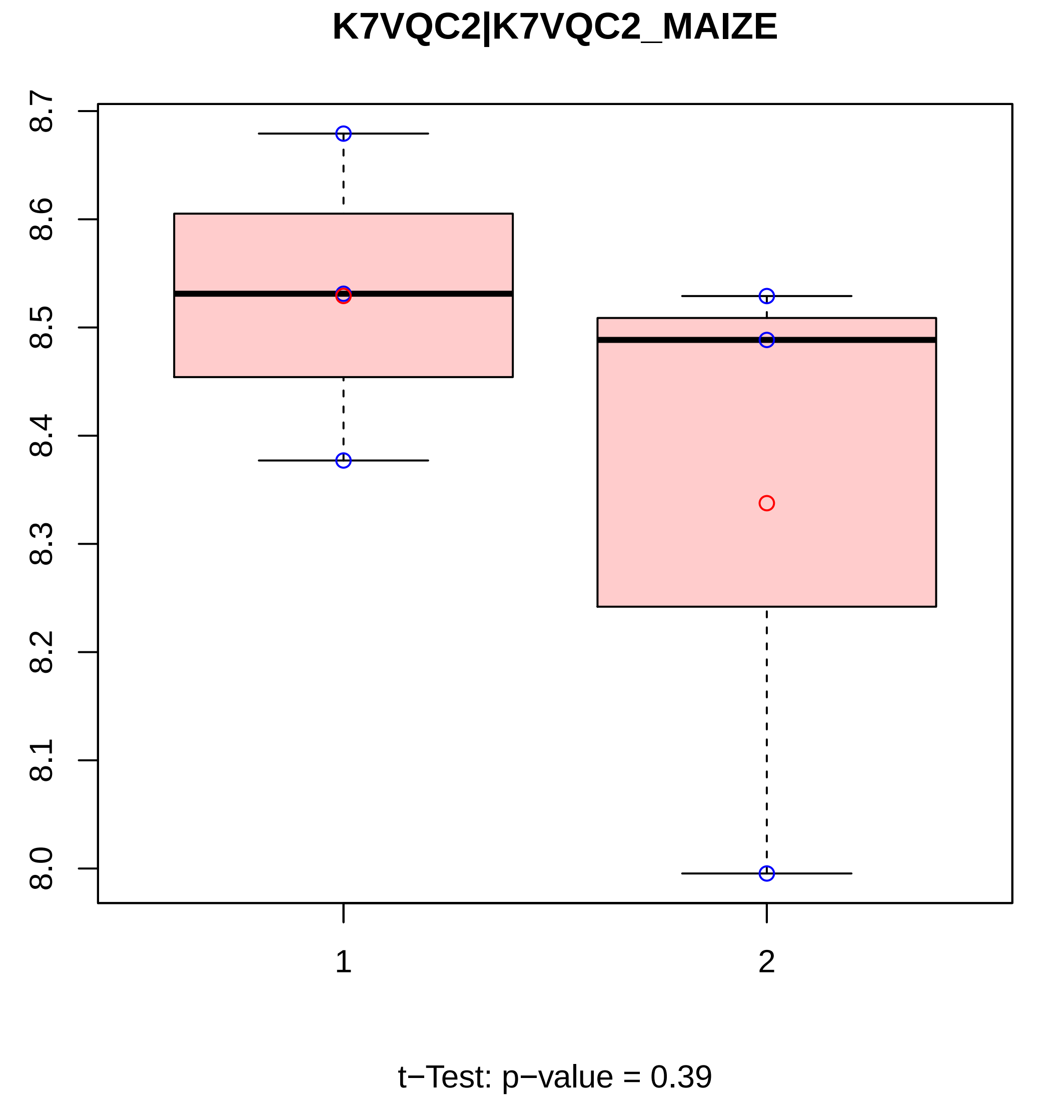

K7VQC2|K7VQC2_MAIZE
8.0
8.1
8.2
8.3
8.4
8.5
8.6
8.7
1
2
t−Test: p−value = 0.39

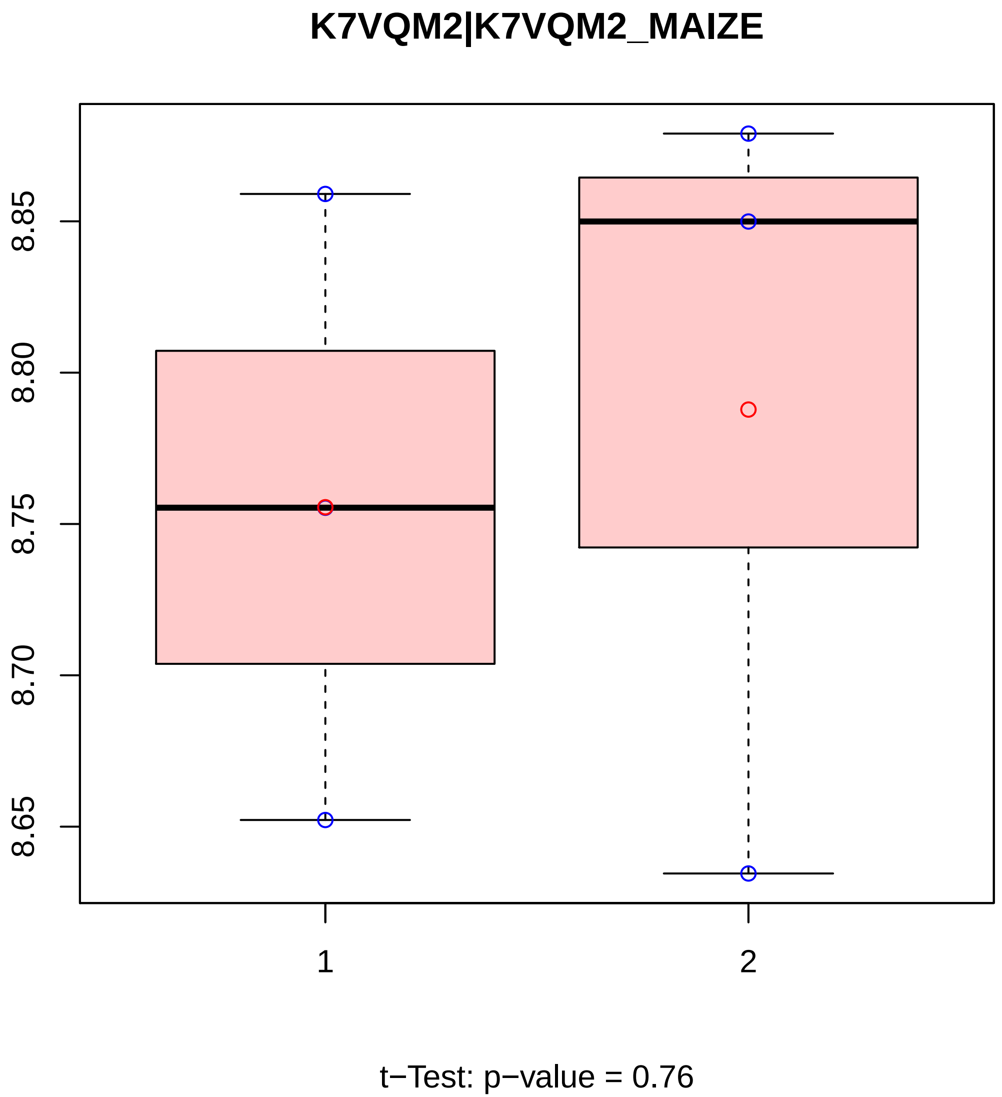
K7VQM2|K7VQM2_MAIZE
8.65
8.70
8.75
8.80
8.85
1
2
t−Test: p−value = 0.76

K7VQP2|K7VQP2_MAIZE

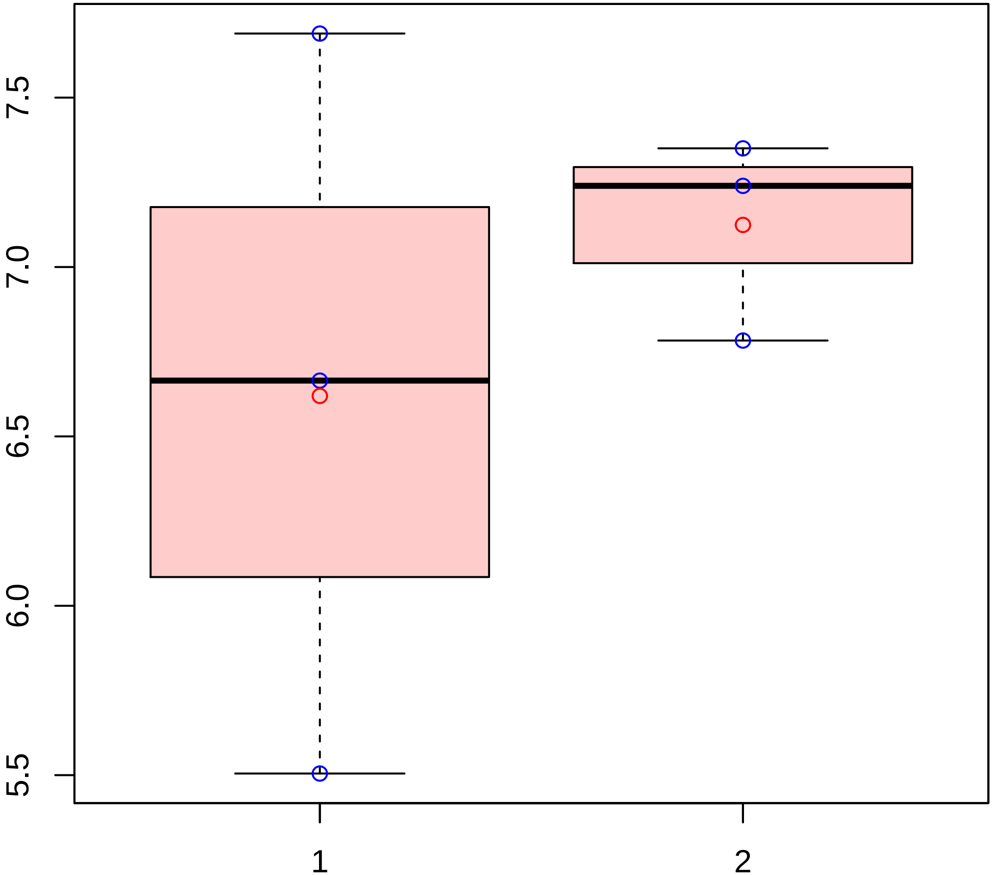

t−Test: p−value = 0.51

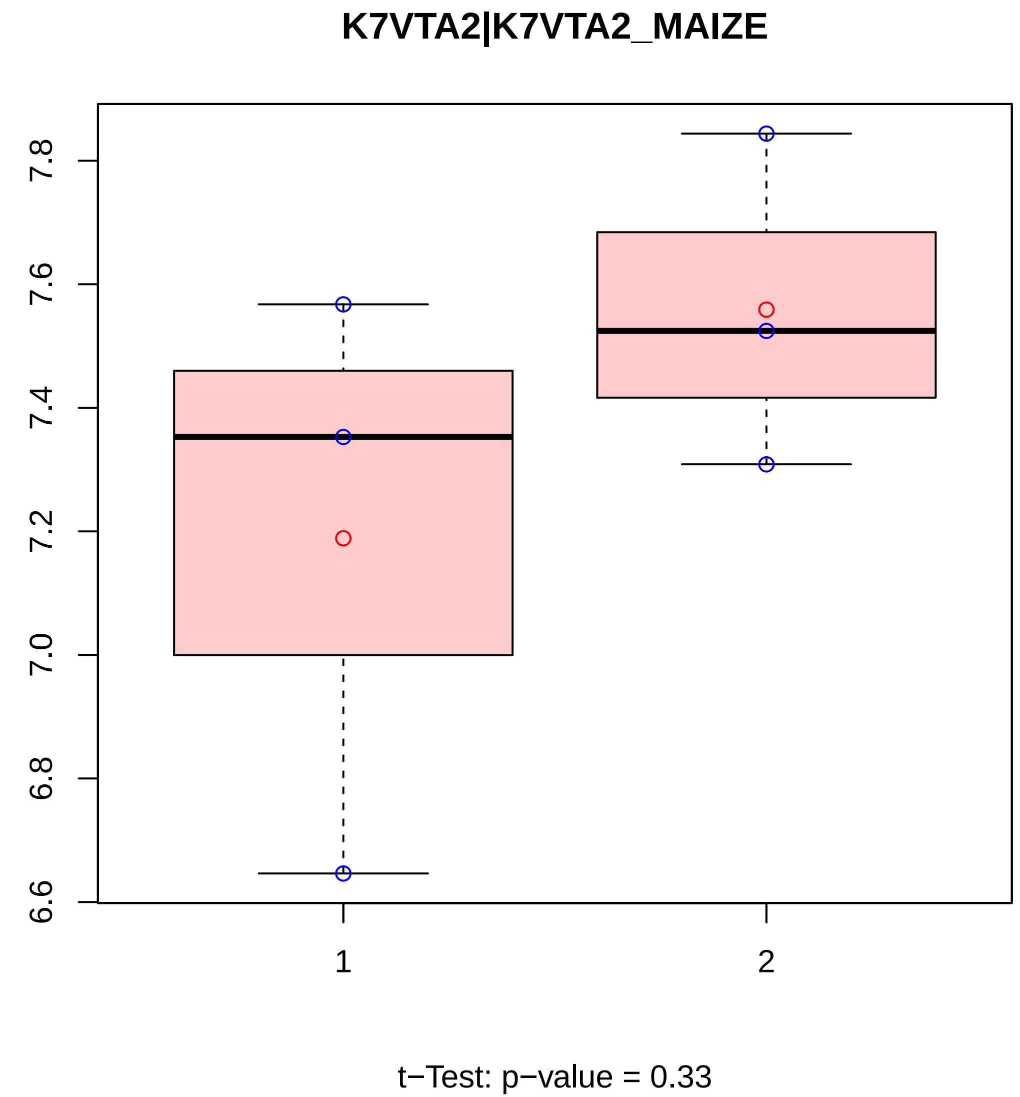
K7VTA2|K7VTA2_MAIZE
7.8
7.6
7.4
7.2
7.0
6.8
6.6
1
2
t−Test: p−value = 0.33

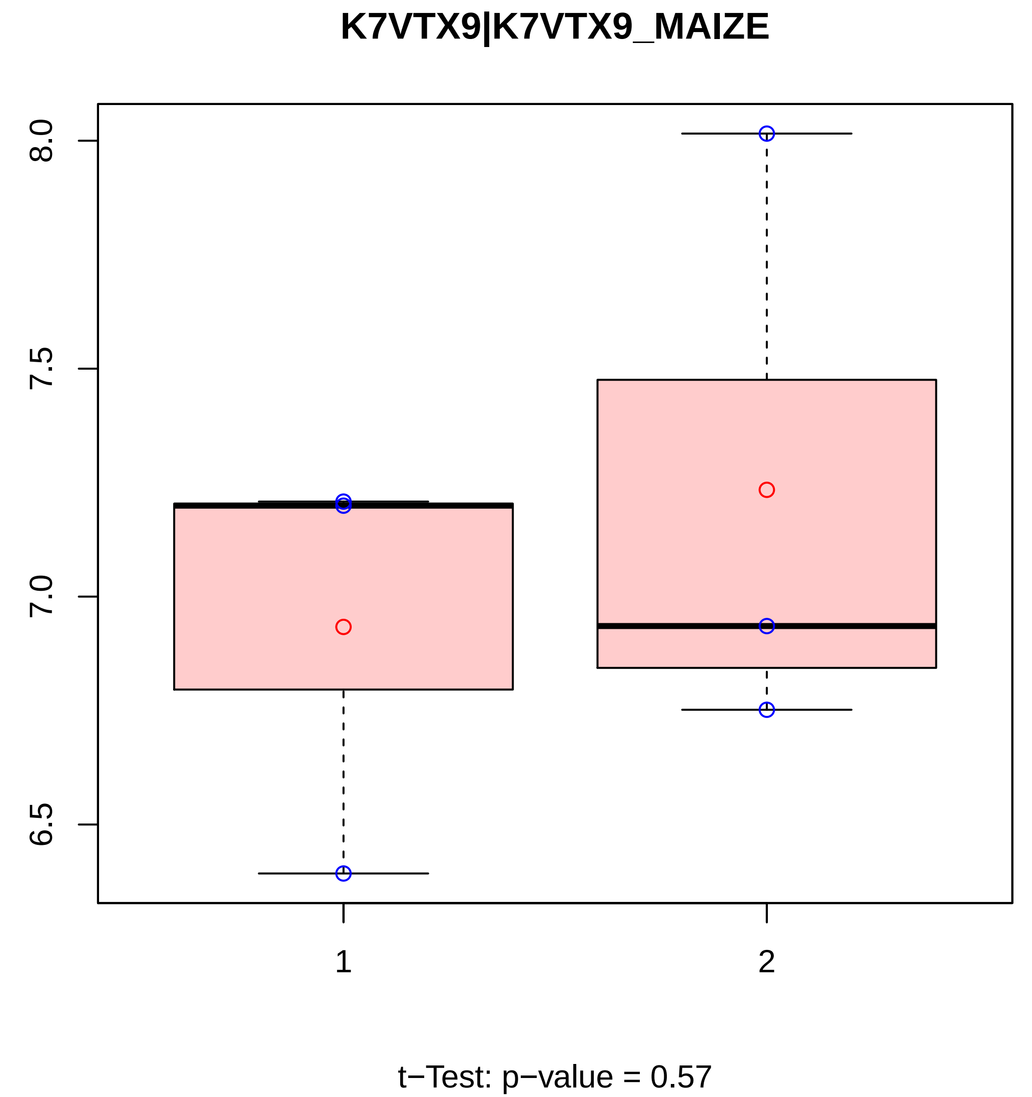
K7VTX9|K7VTX9_MAIZE
8.0
7.5
7.0
6.5
1
2
t−Test: p−value = 0.57

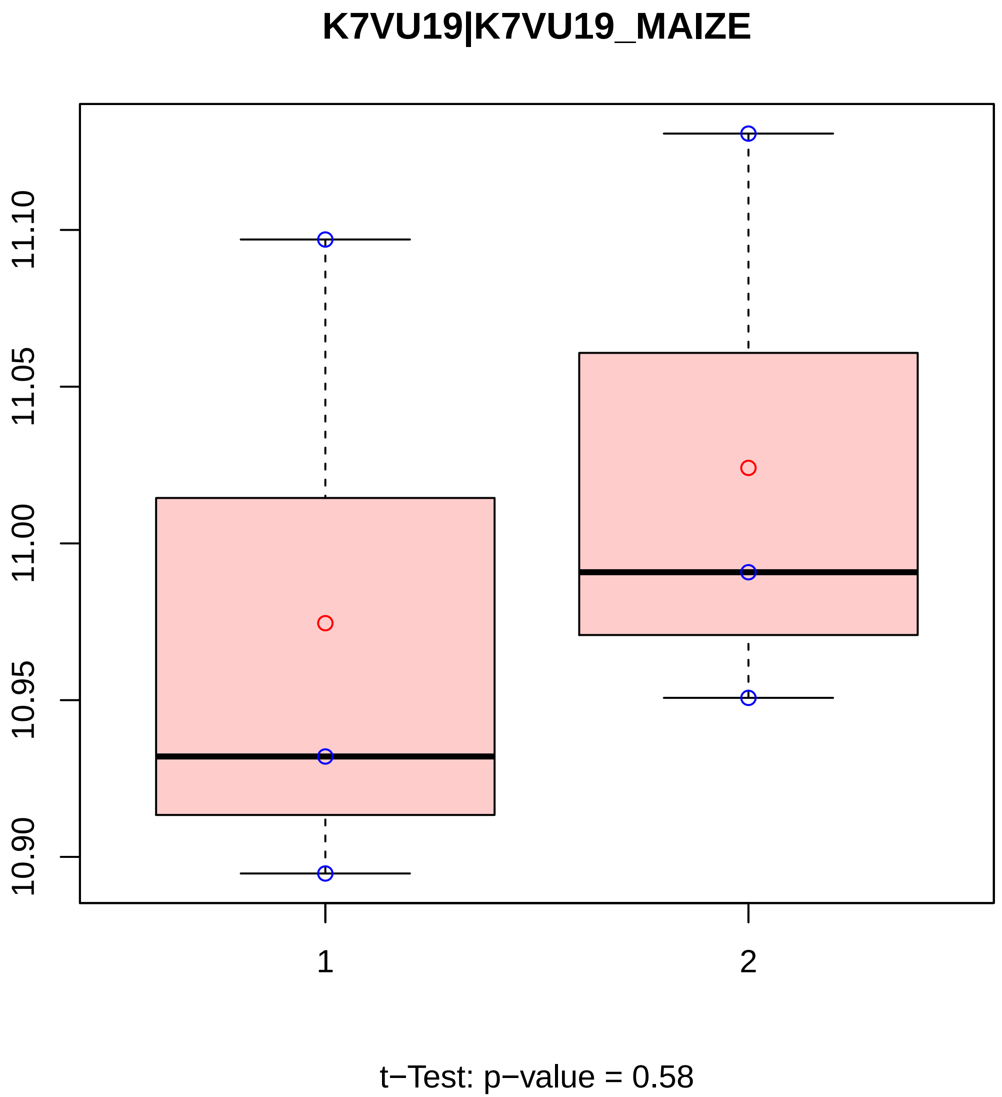
K7VU19|K7VU19_MAIZE
11.10
11.05
11.00
10.95
10.90
1
2
t−Test: p−value = 0.58

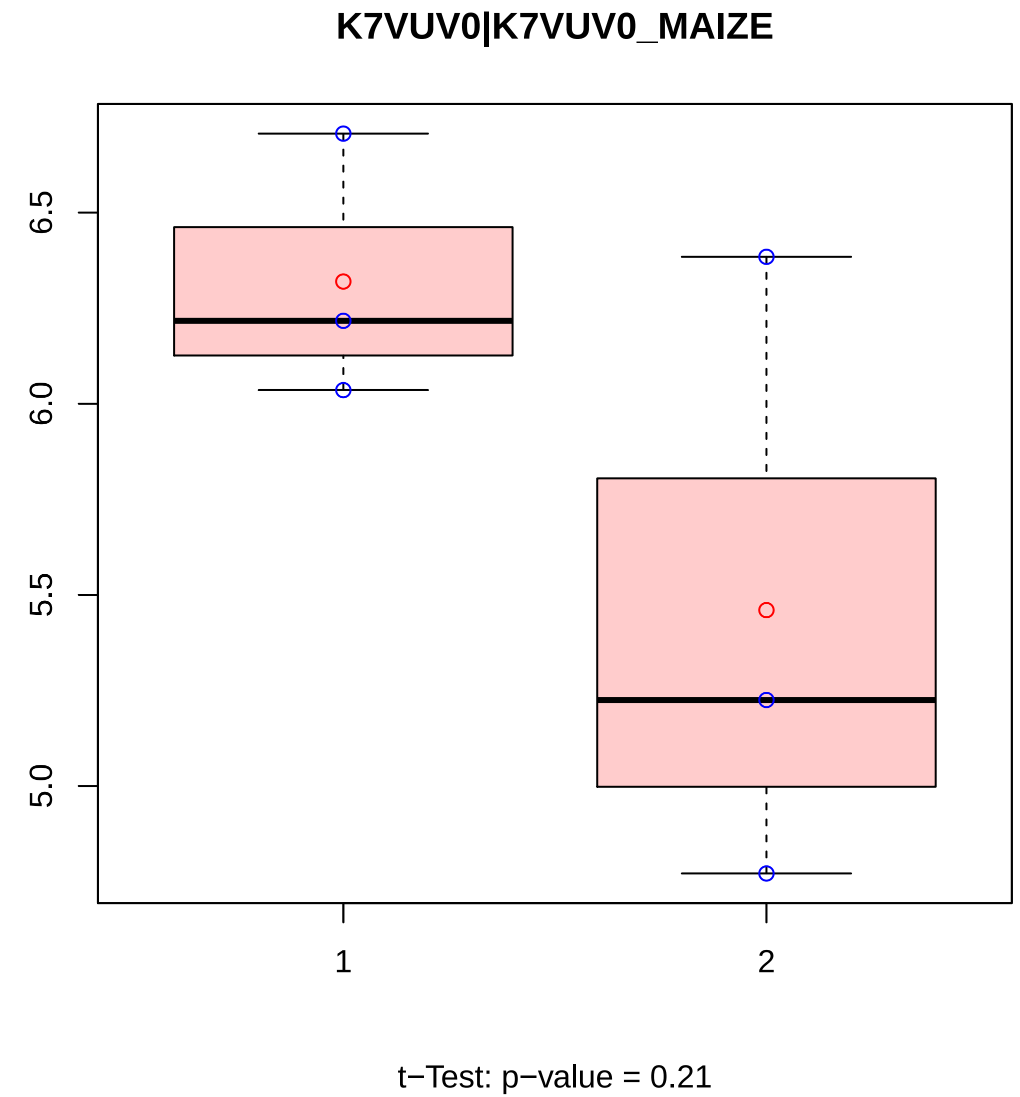
K7VUV0|K7VUV0_MAIZE
6.5
6.0
5.5
5.0
1
2
t−Test: p−value = 0.21

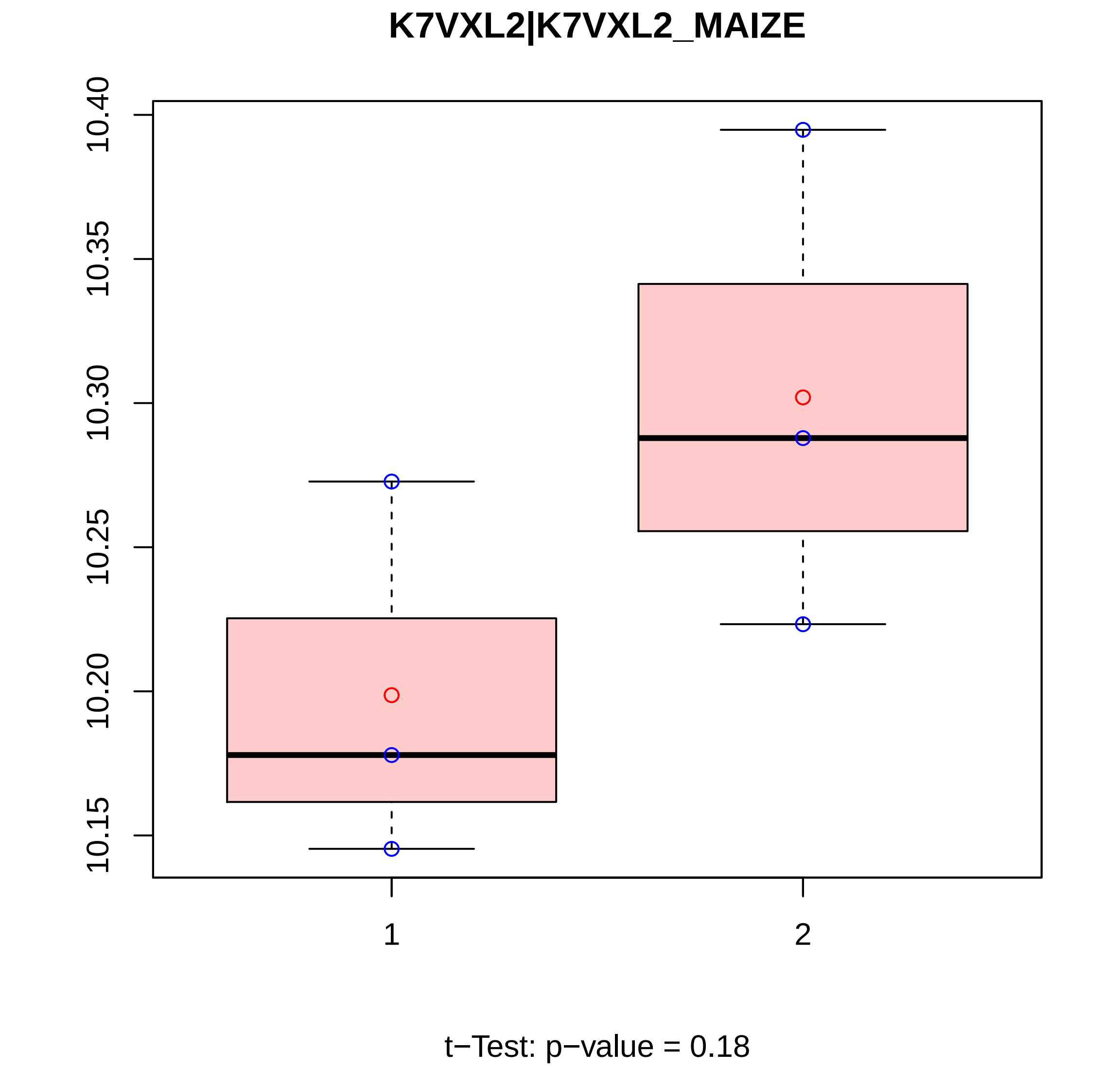
K7VXL2|K7VXL2_MAIZE
10.40
10.35
10.30
10.25
10.20
10.15
1
2
t−Test: p−value = 0.18

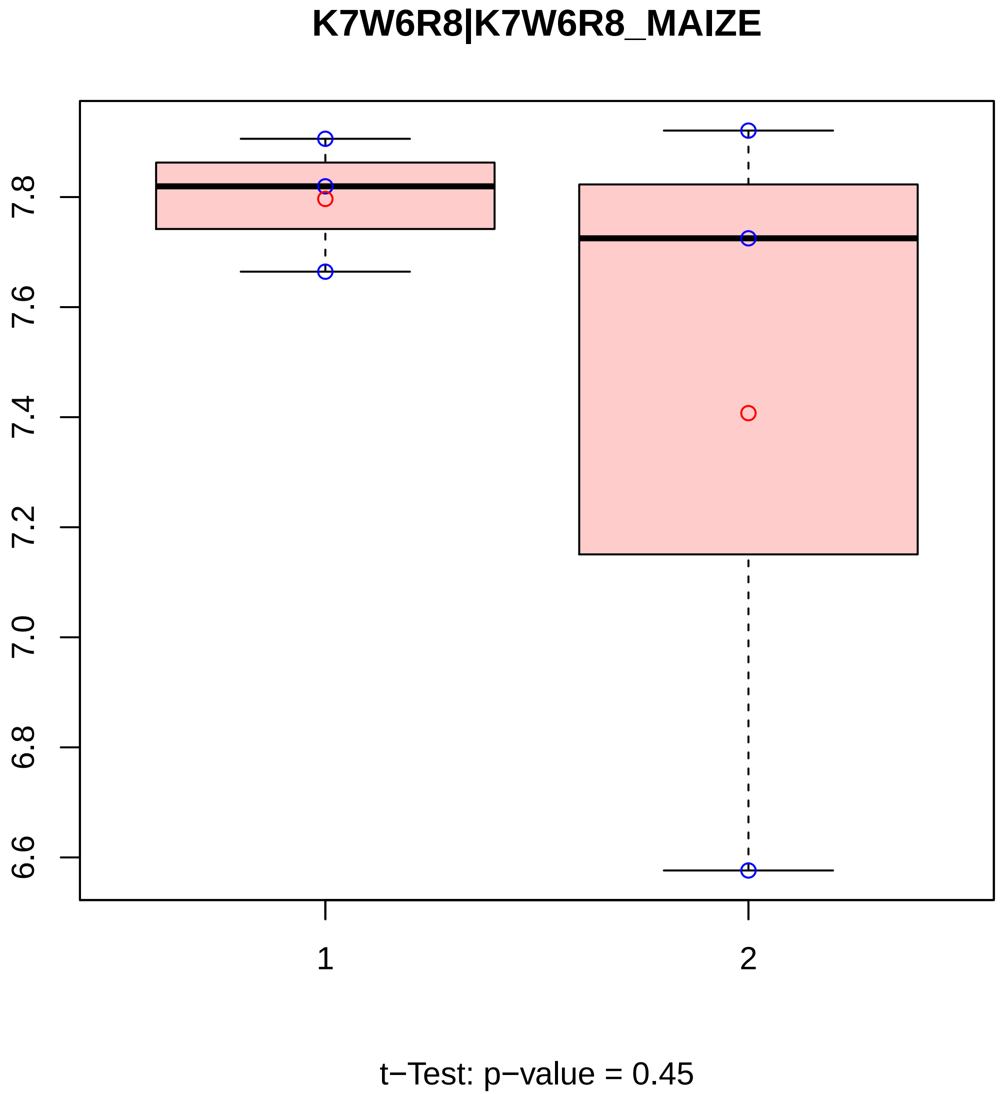
K7W6R8|K7W6R8_MAIZE
7.8
7.6
7.4
7.2
7.0
6.8
6.6
1
2
t−Test: p−value = 0.45

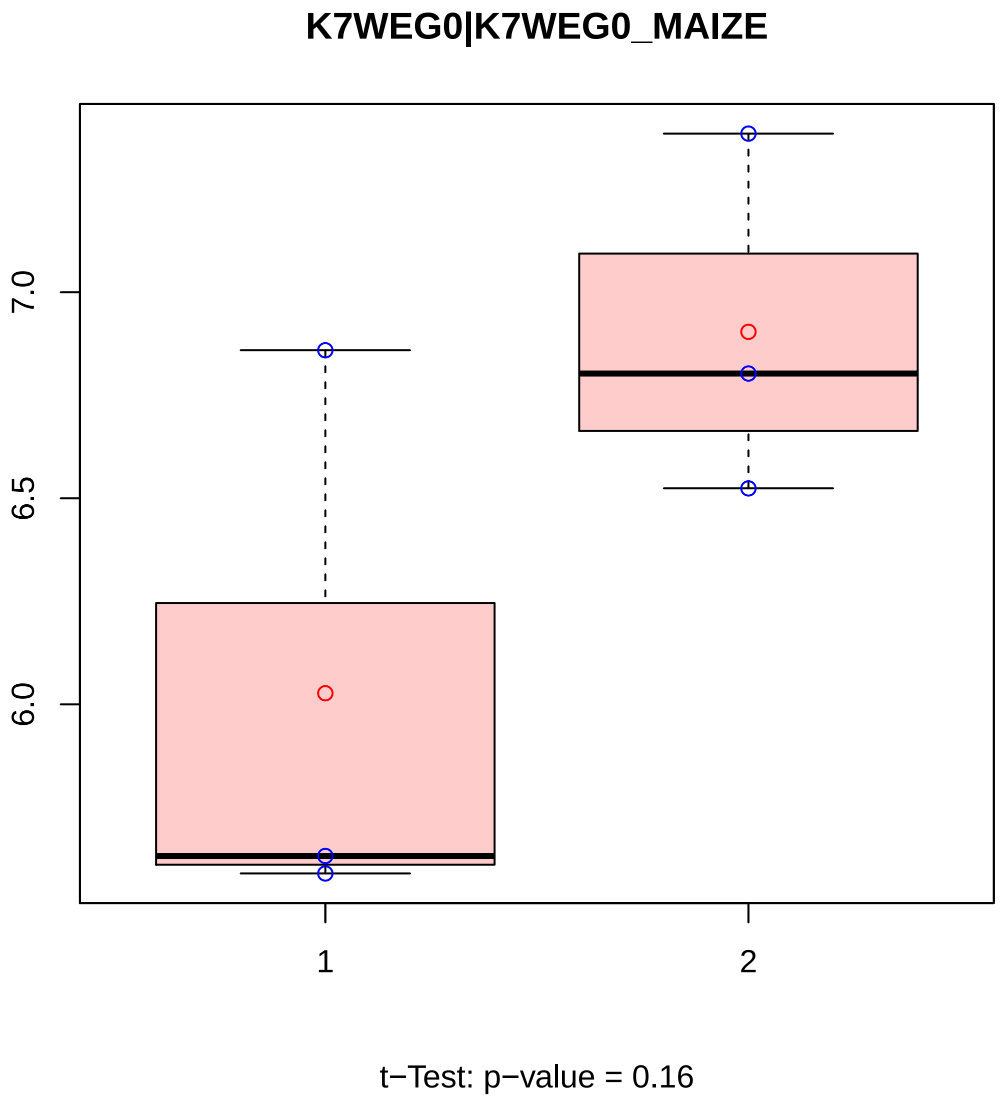
K7WEG0|K7WEG0_MAIZE
7.0
6.5
6.0
1
2
t−Test: p−value = 0.16

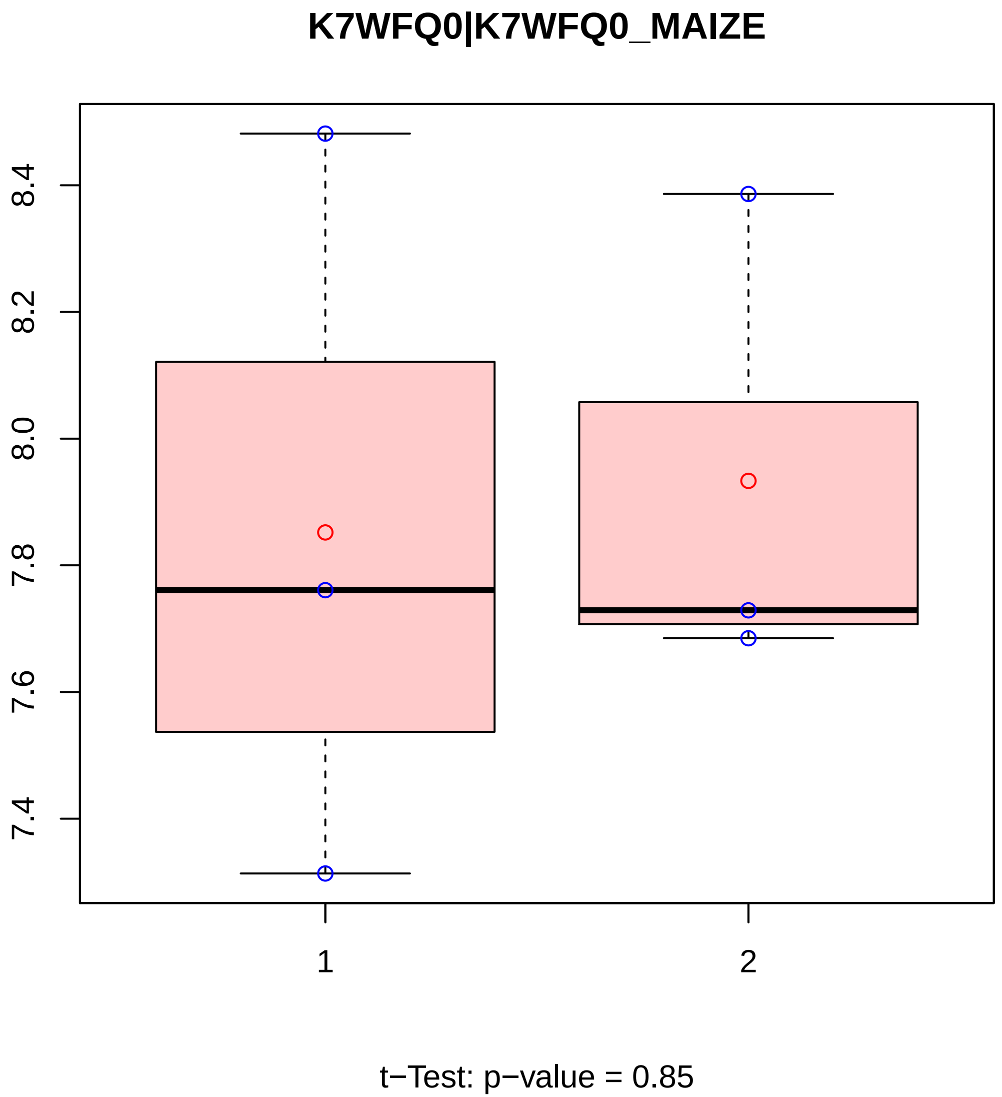
K7WFQ0|K7WFQ0_MAIZE
8.4
8.2
8.0
7.8
7.6
7.4
1
2
t−Test: p−value = 0.85

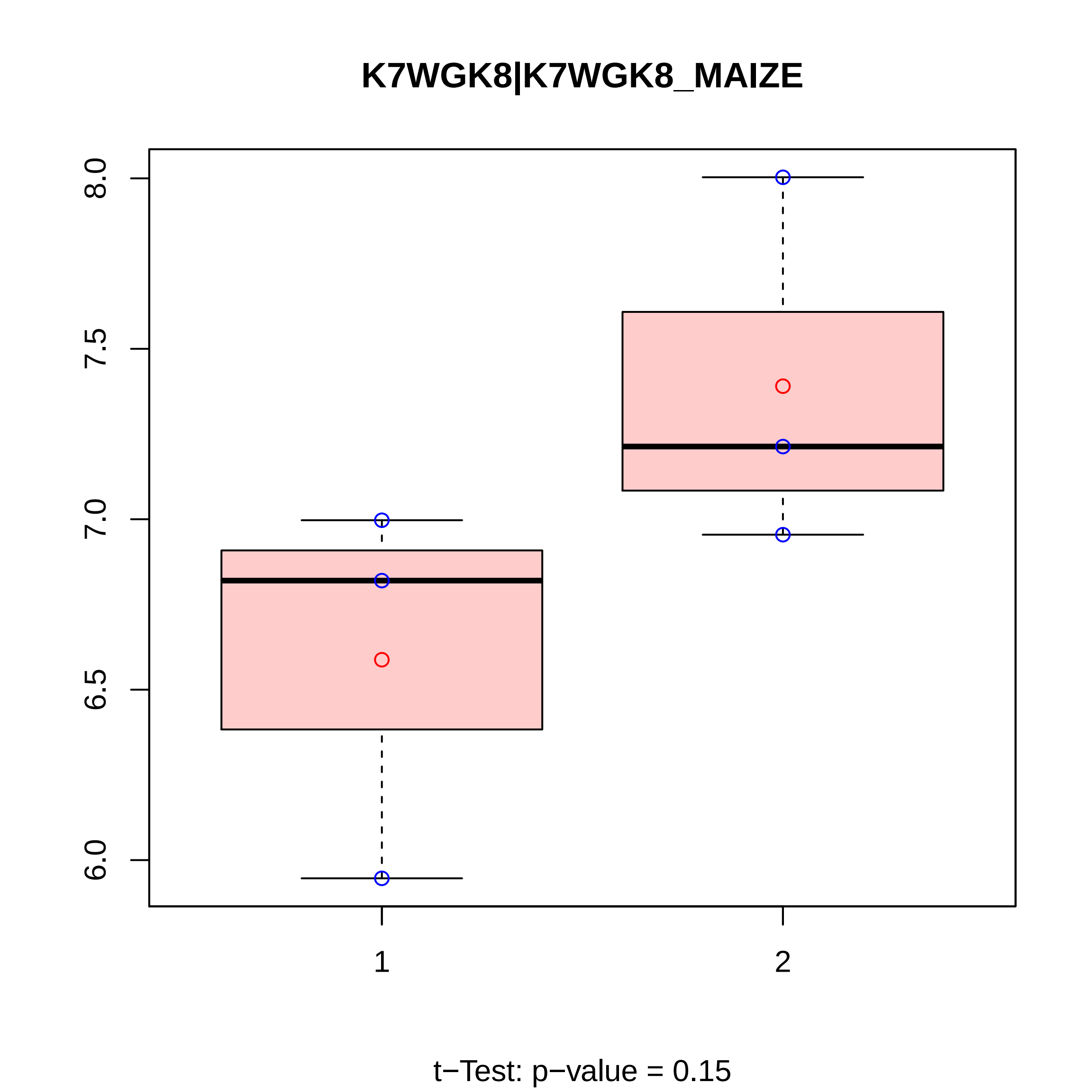
K7WGK8|K7WGK8_MAIZE
8.0
7.5
7.0
6.5
6.0
1
2
t−Test: p−value = 0.15

K7WHB6|K7WHB6_MAIZE

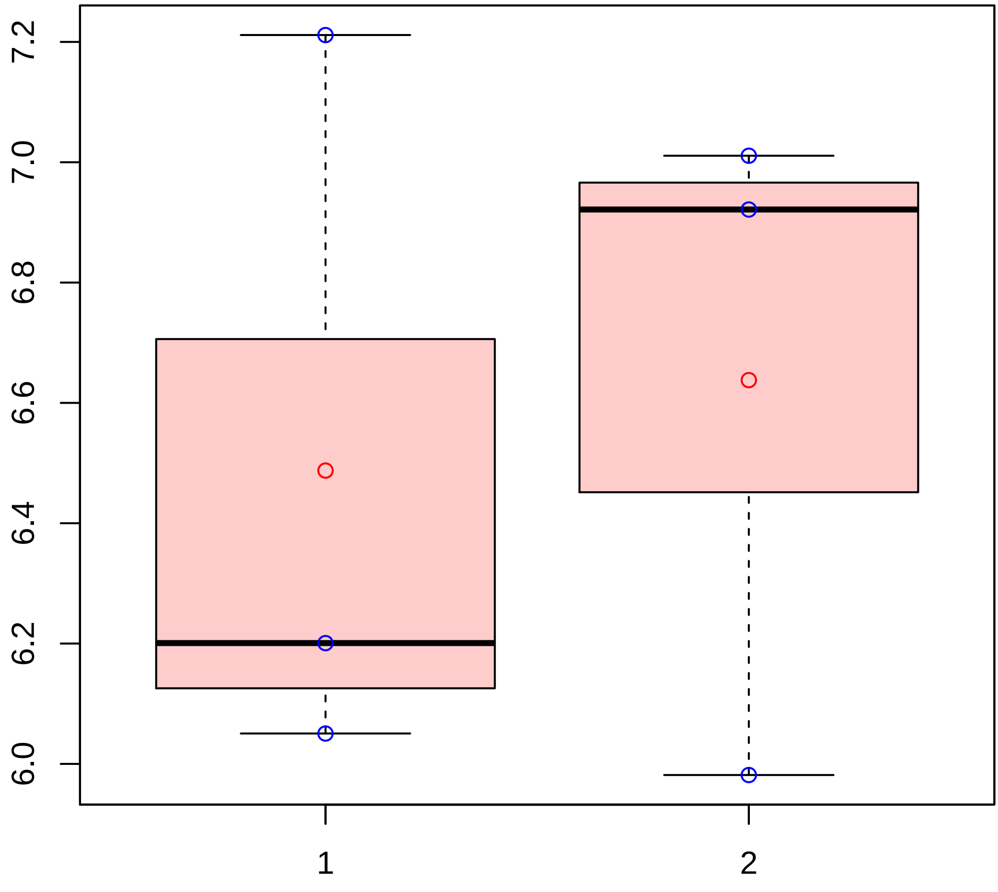

t−Test: p−value = 0.77

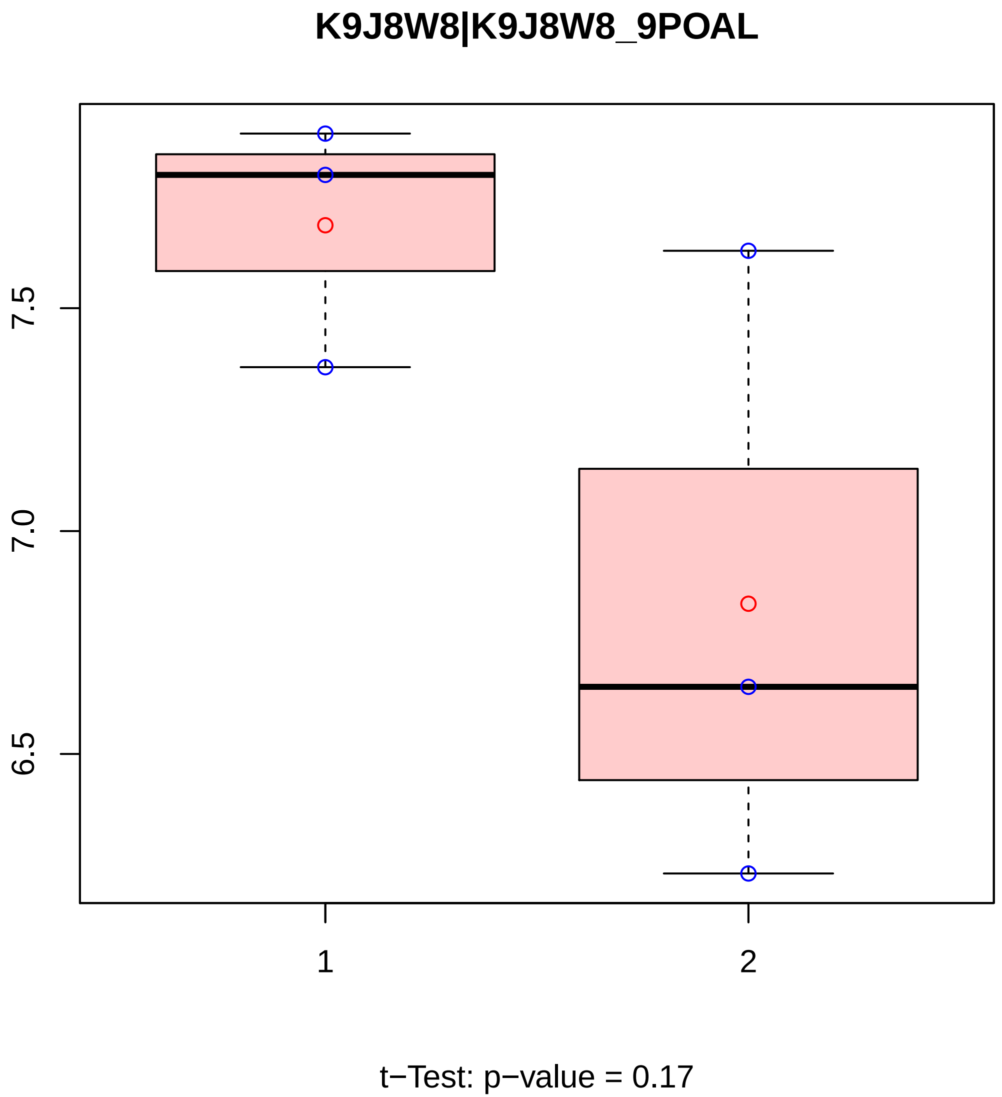
K9J8W8|K9J8W8_9POAL
7.5
7.0
6.5
1
2
t−Test: p−value = 0.17

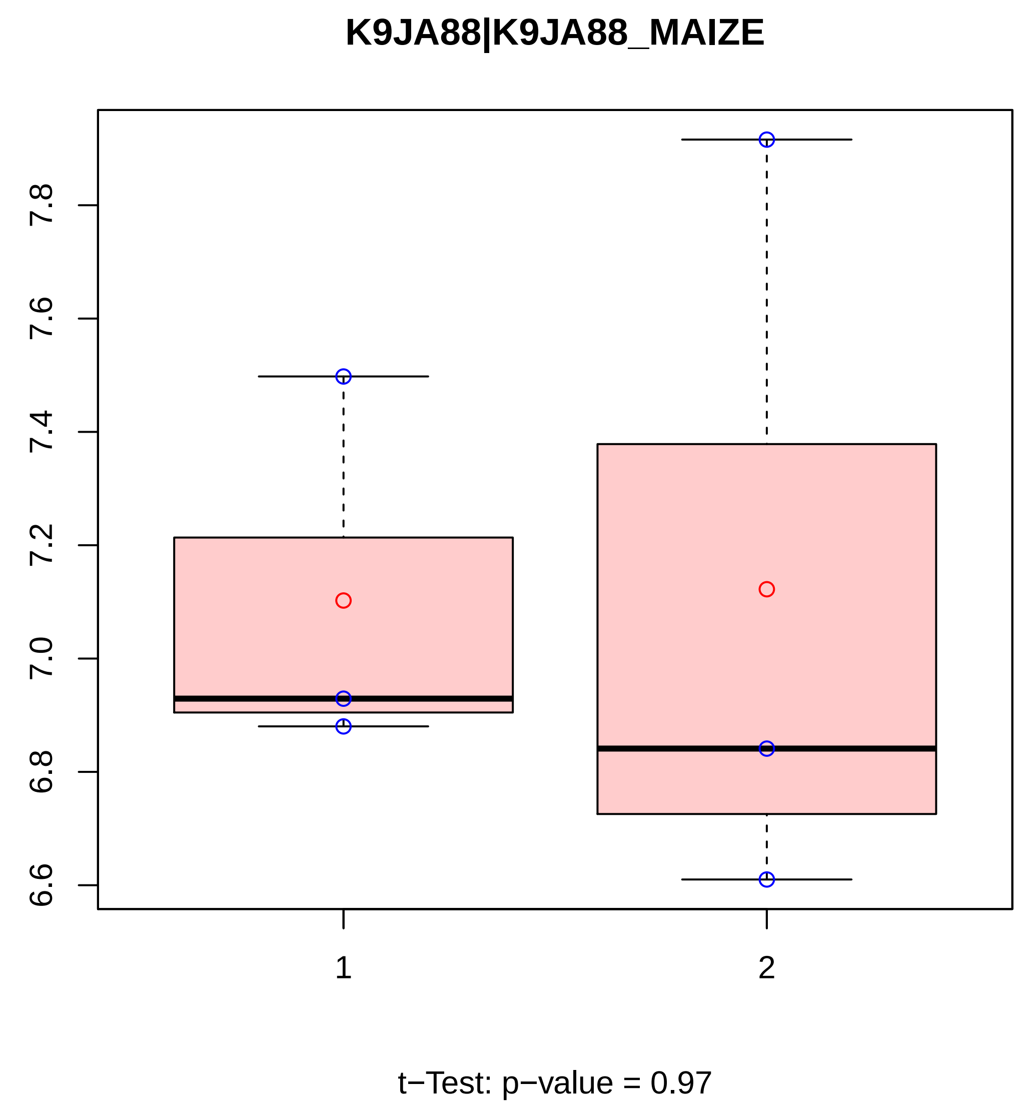
K9JA88|K9JA88_MAIZE
7.8
7.6
7.4
7.2
7.0
6.8
6.6
1
2
t−Test: p−value = 0.97

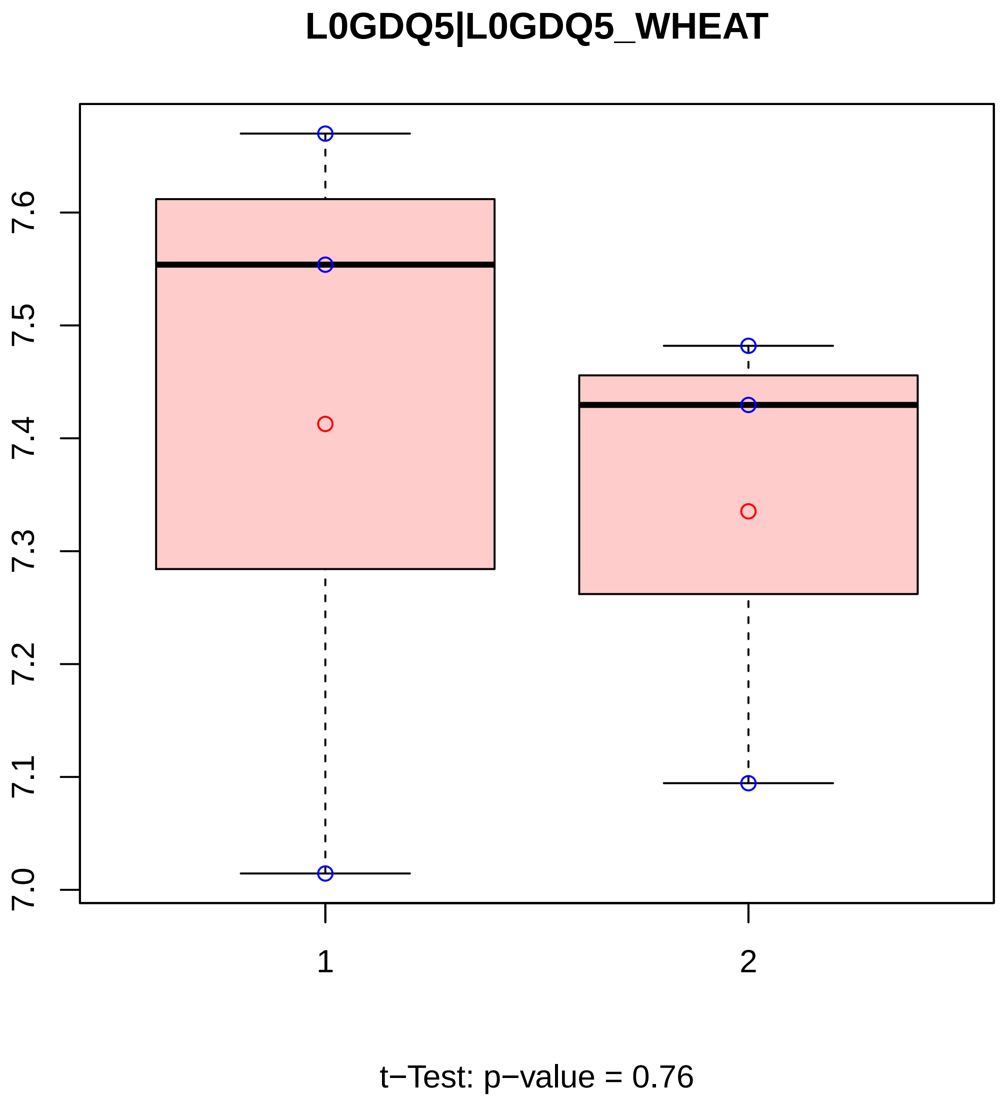
L0GDQ5|L0GDQ5_WHEAT
7.0
7.1
7.2
7.3
7.4
7.5
7.6
1
2
t−Test: p−value = 0.76

L7S1T0|L7S1T0_MUSAC

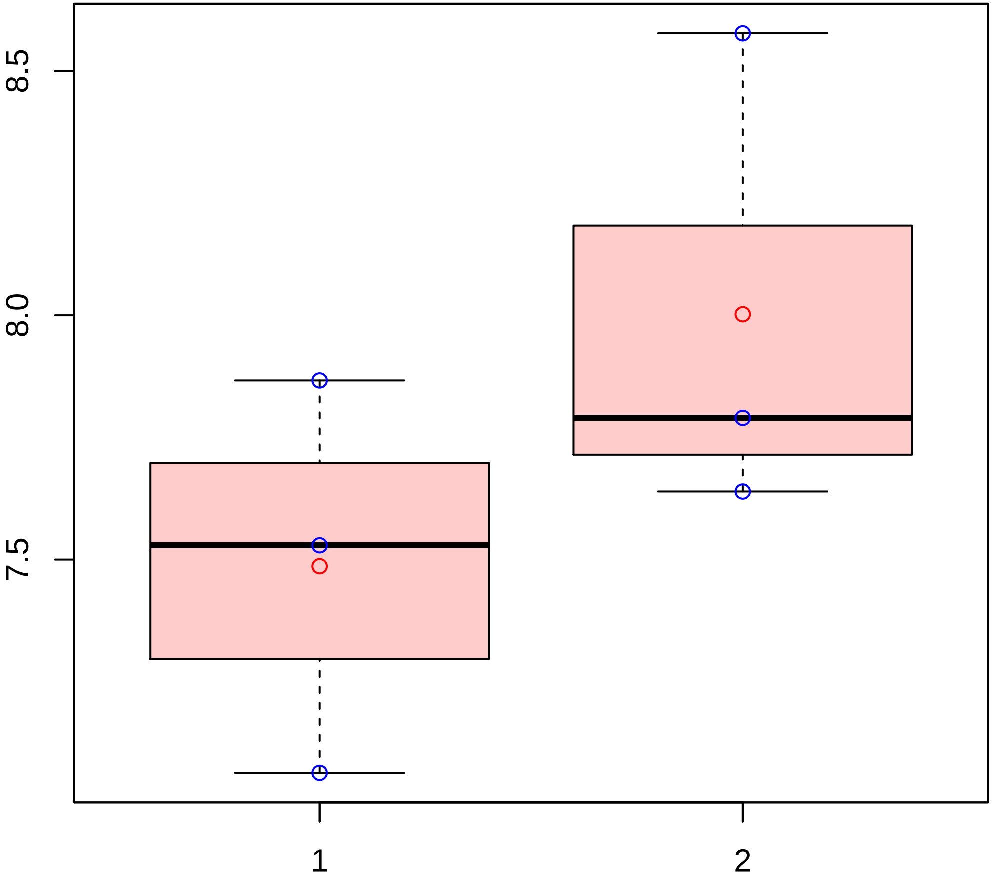

t−Test: p−value = 0.24

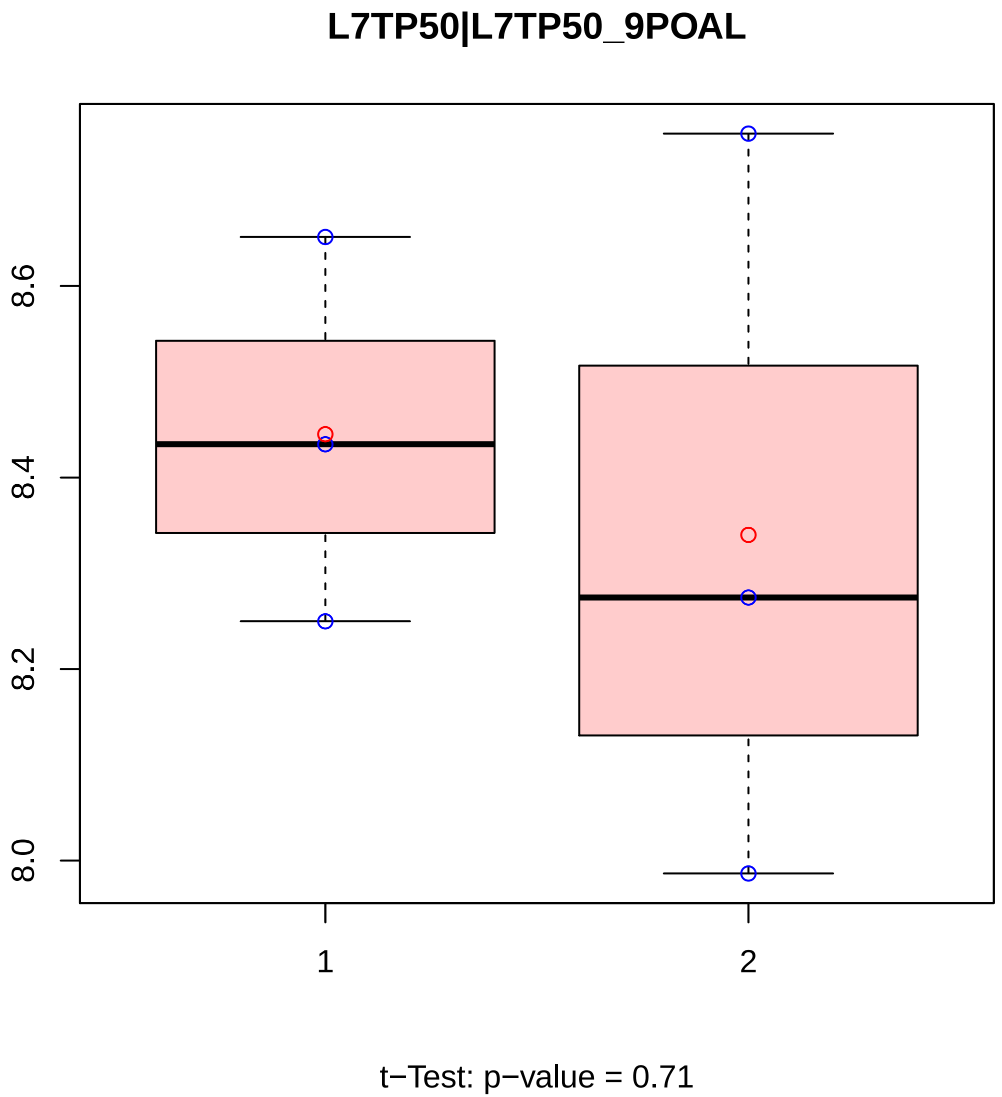

L7TP50|L7TP50_9POAL
8.0
8.2
8.4
8.6
1
2
t−Test: p−value = 0.71

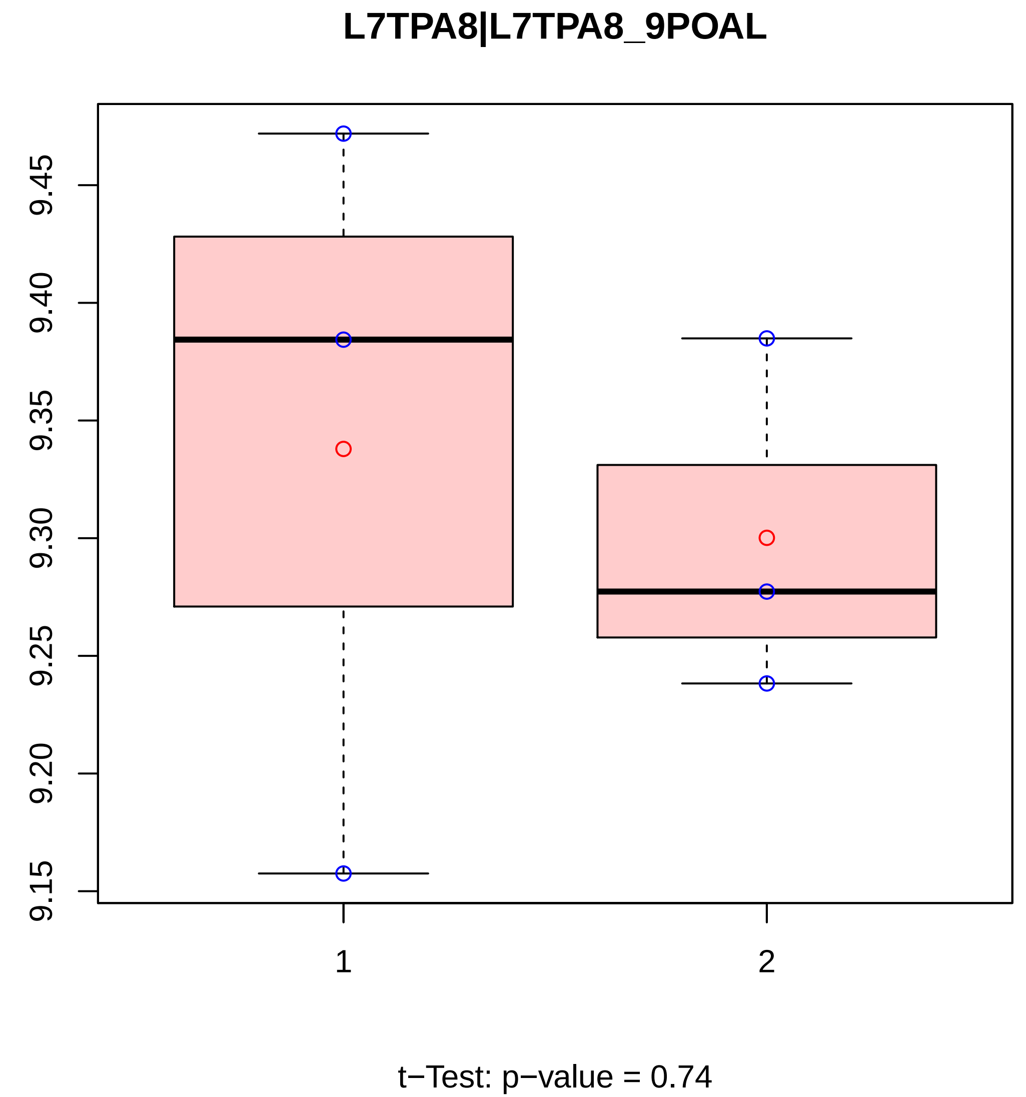
L7TPA8|L7TPA8_9POAL
9.15
9.20
9.25
9.30
9.35
9.40
9.45
1
2
t−Test: p−value = 0.74

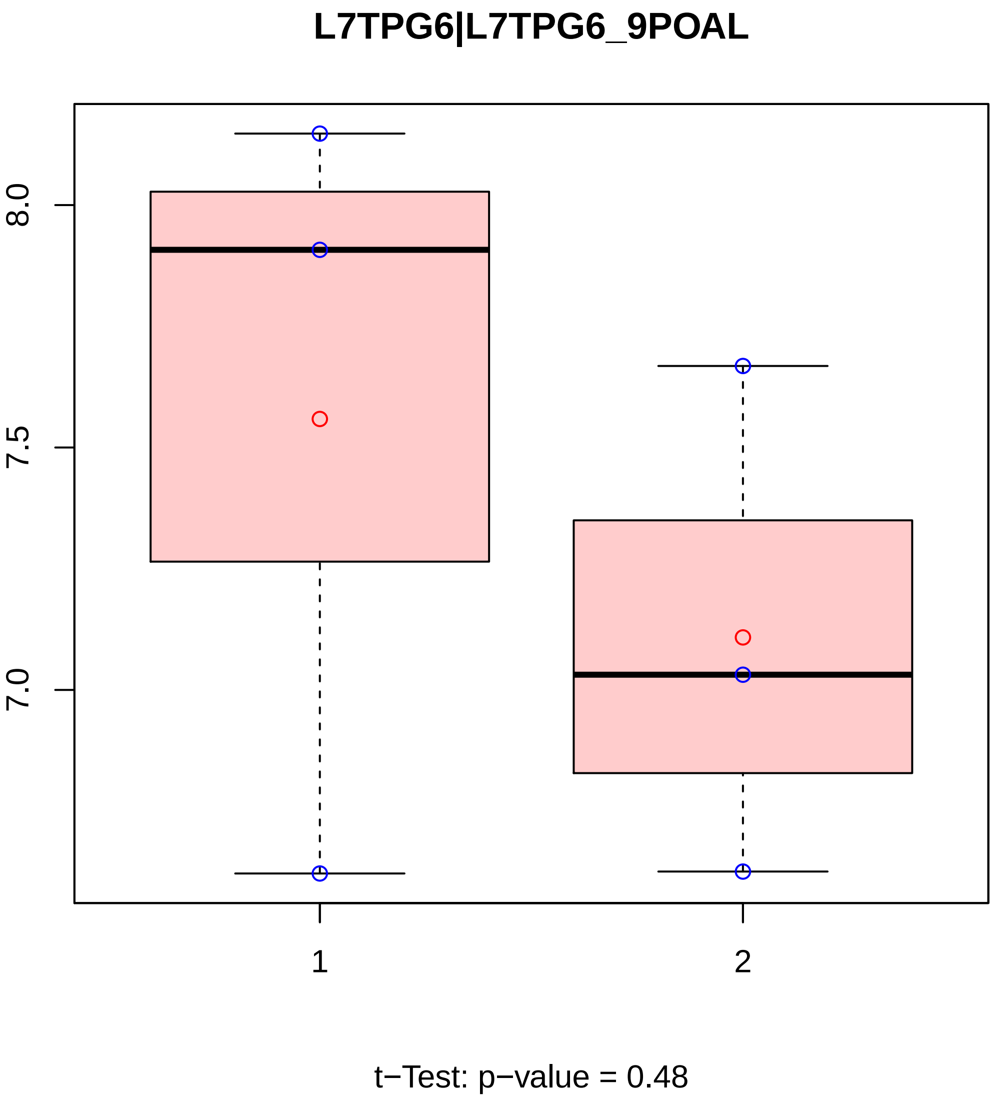
L7TPG6|L7TPG6_9POAL
8.0
7.5
7.0
1
2
t−Test: p−value = 0.48

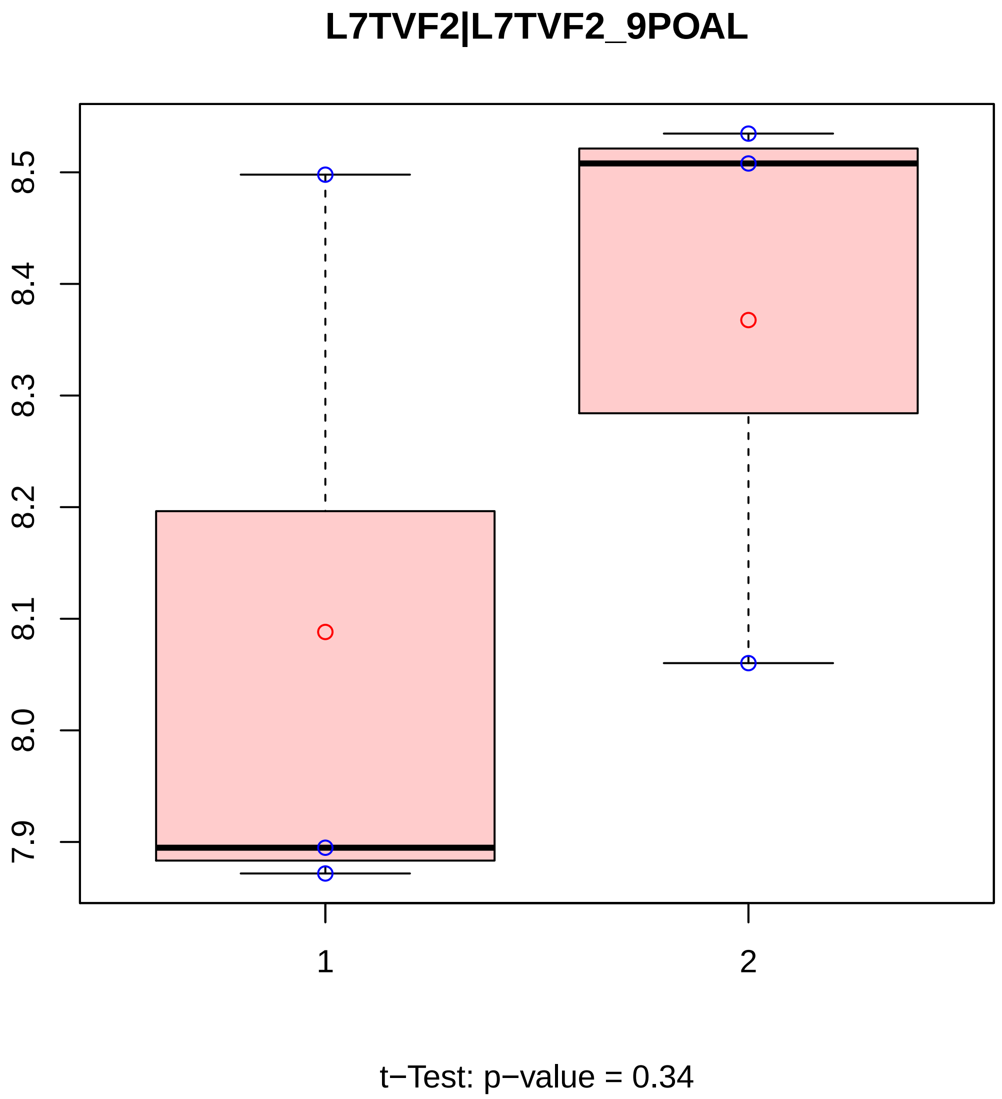
L7TVF2|L7TVF2_9POAL
8.5
8.4
8.3
8.2
8.1
8.0
7.9
1
2
t−Test: p−value = 0.34

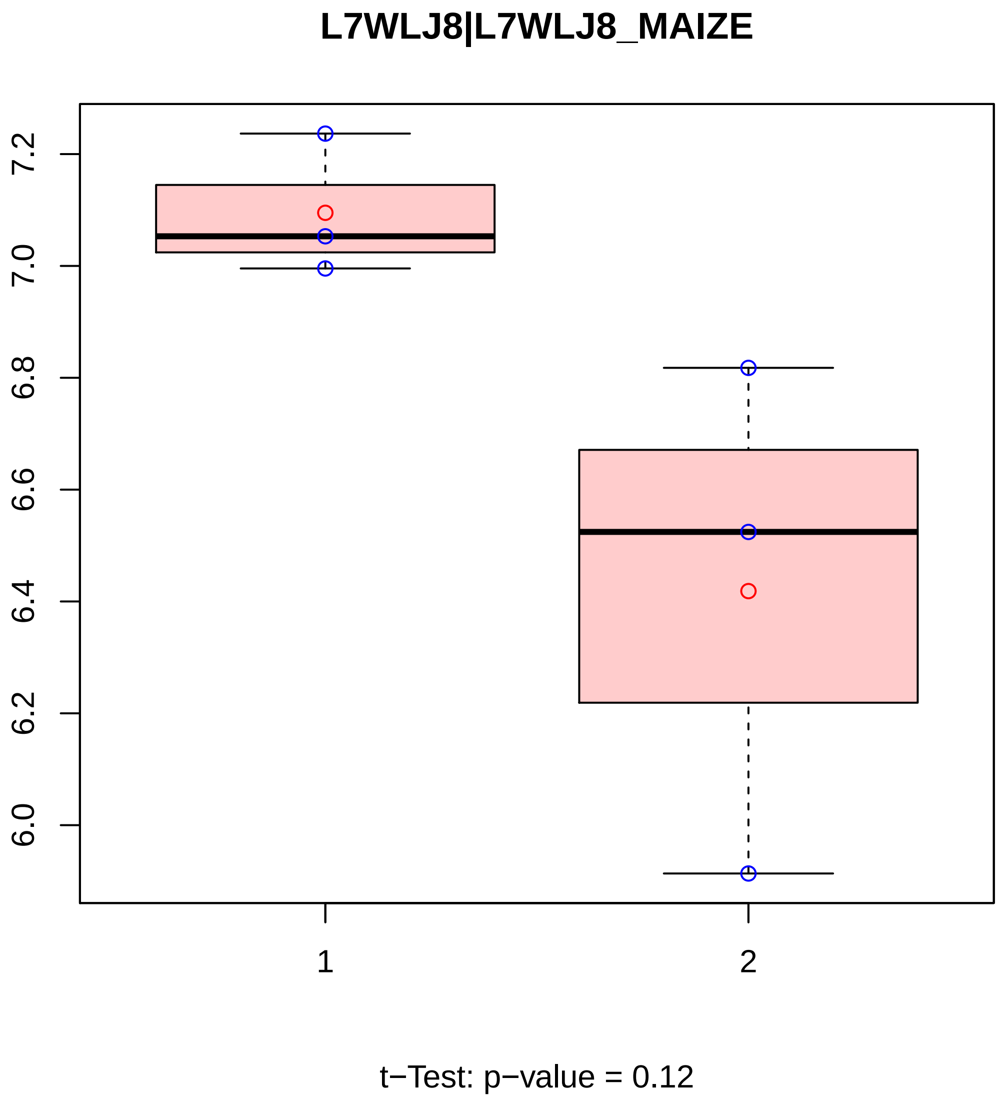
L7WLJ8|L7WLJ8_MAIZE
7.2
7.0
6.8
6.6
6.4
6.2
6.0
1
2
t−Test: p−value = 0.12

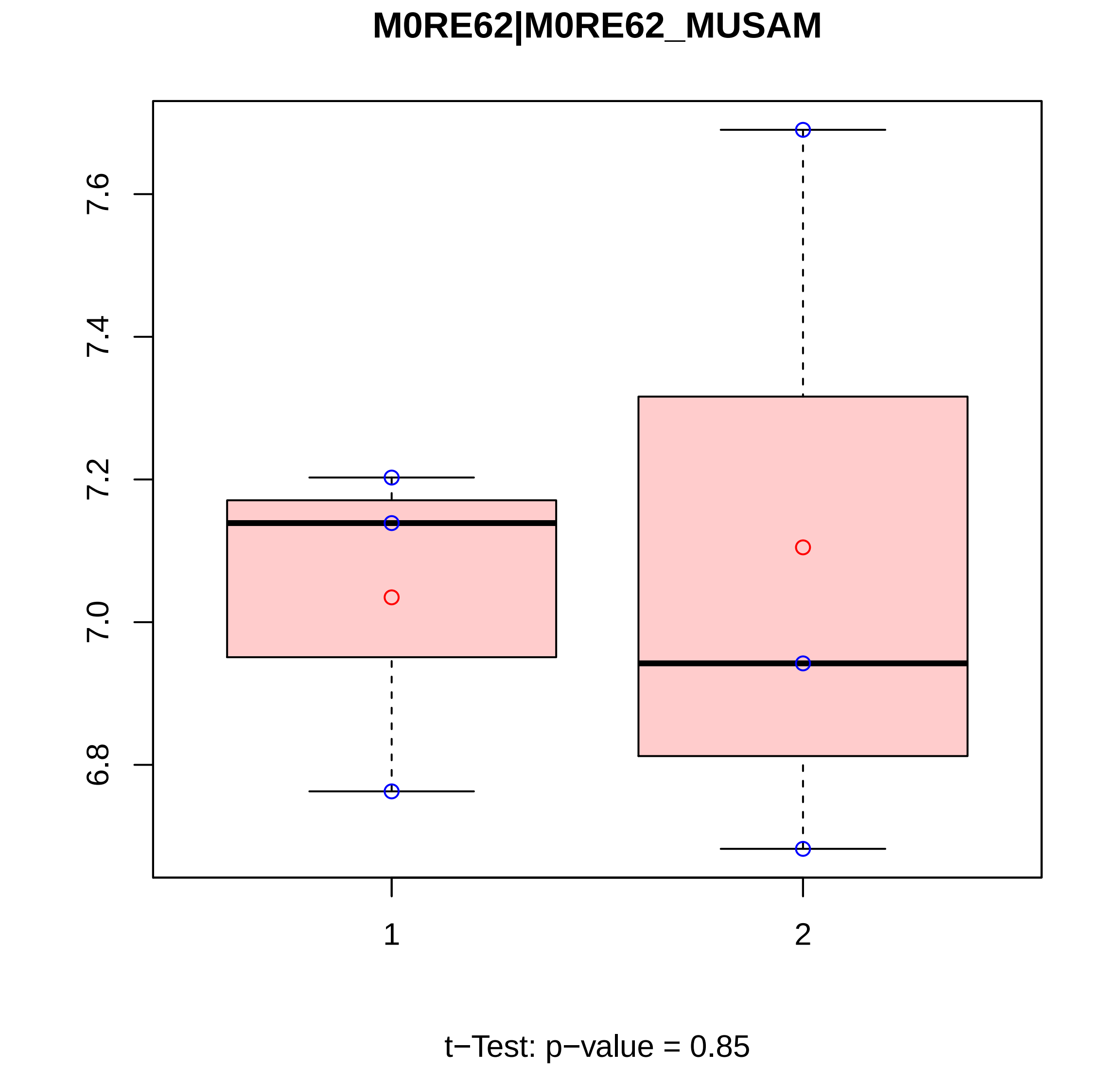
M0RE62|M0RE62_MUSAM
7.6
7.4
7.2
7.0
6.8
1
2
t−Test: p−value = 0.85

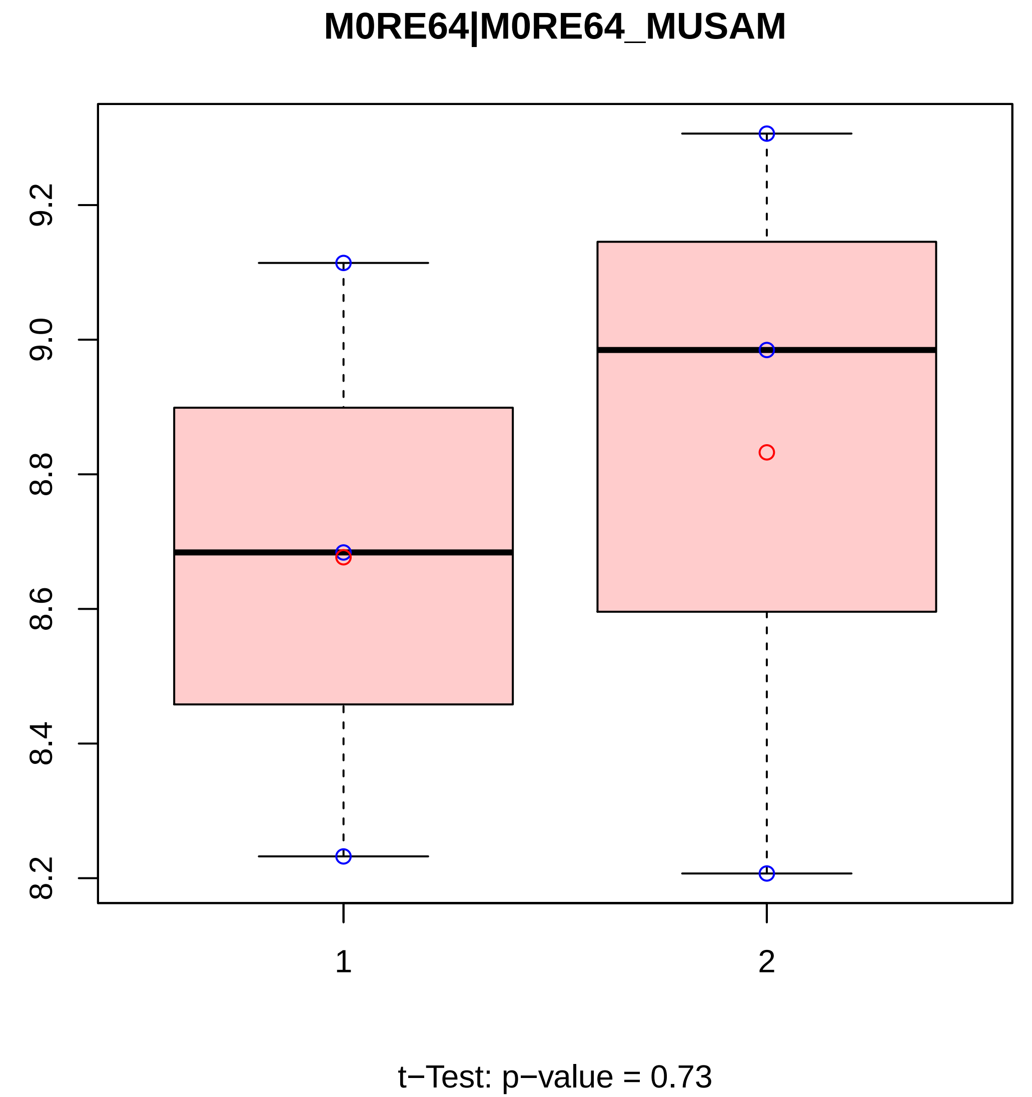
M0RE64|M0RE64_MUSAM
9.2
9.0
8.8
8.6
8.4
8.2
1
2
t−Test: p−value = 0.73

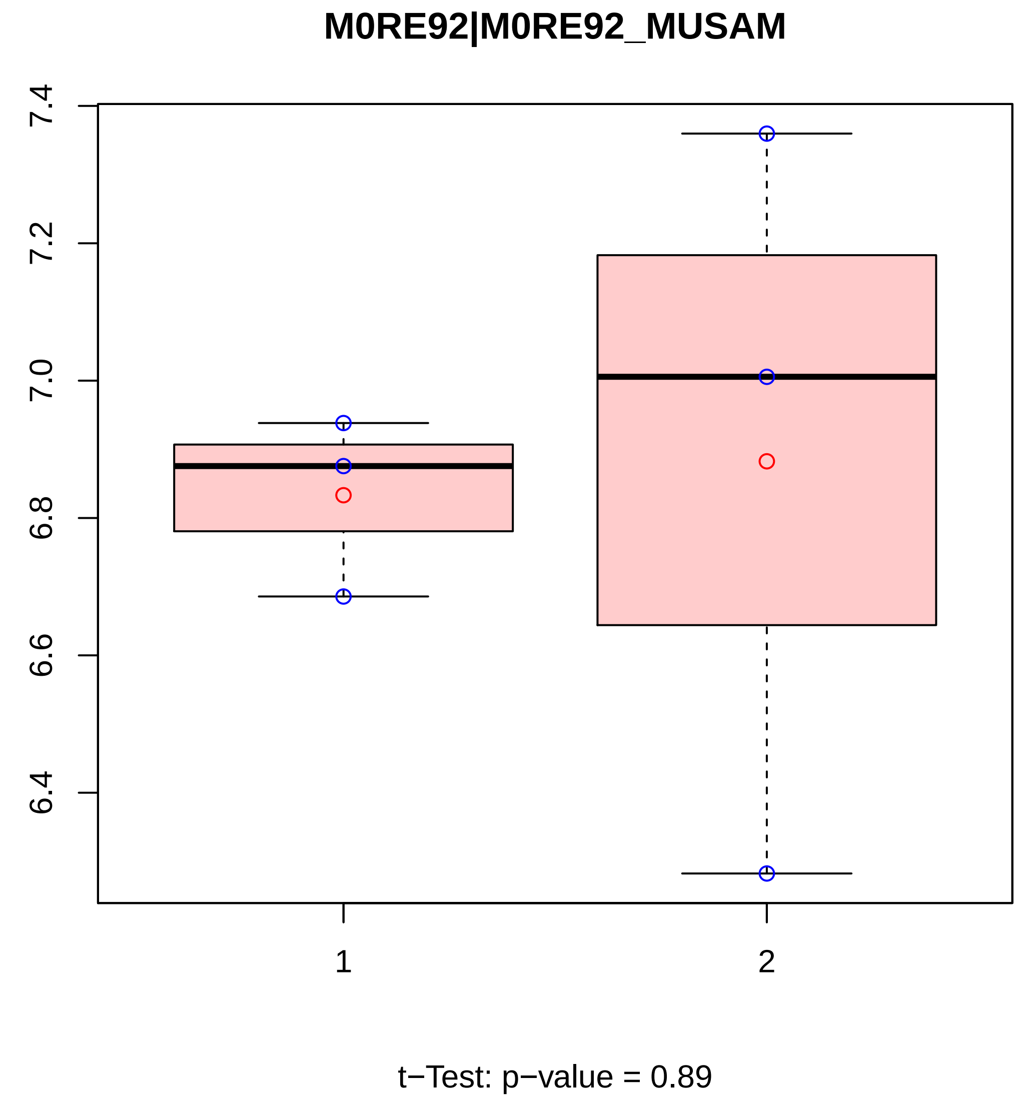
M0RE92|M0RE92_MUSAM
7.4
7.2
7.0
6.8
6.6
6.4
1
2
t−Test: p−value = 0.89

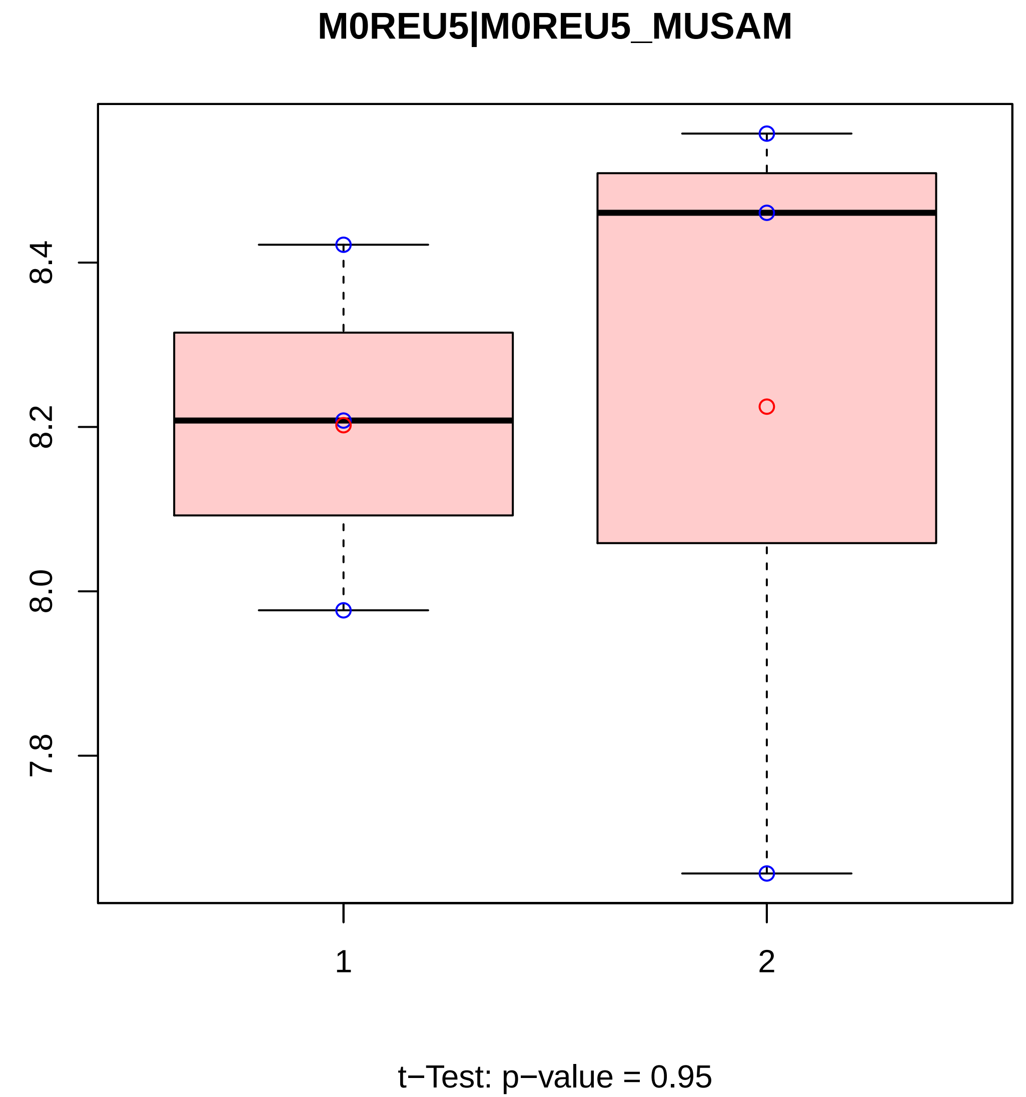
M0REU5|M0REU5_MUSAM
8.4
8.2
8.0
7.8
1
2
t−Test: p−value = 0.95

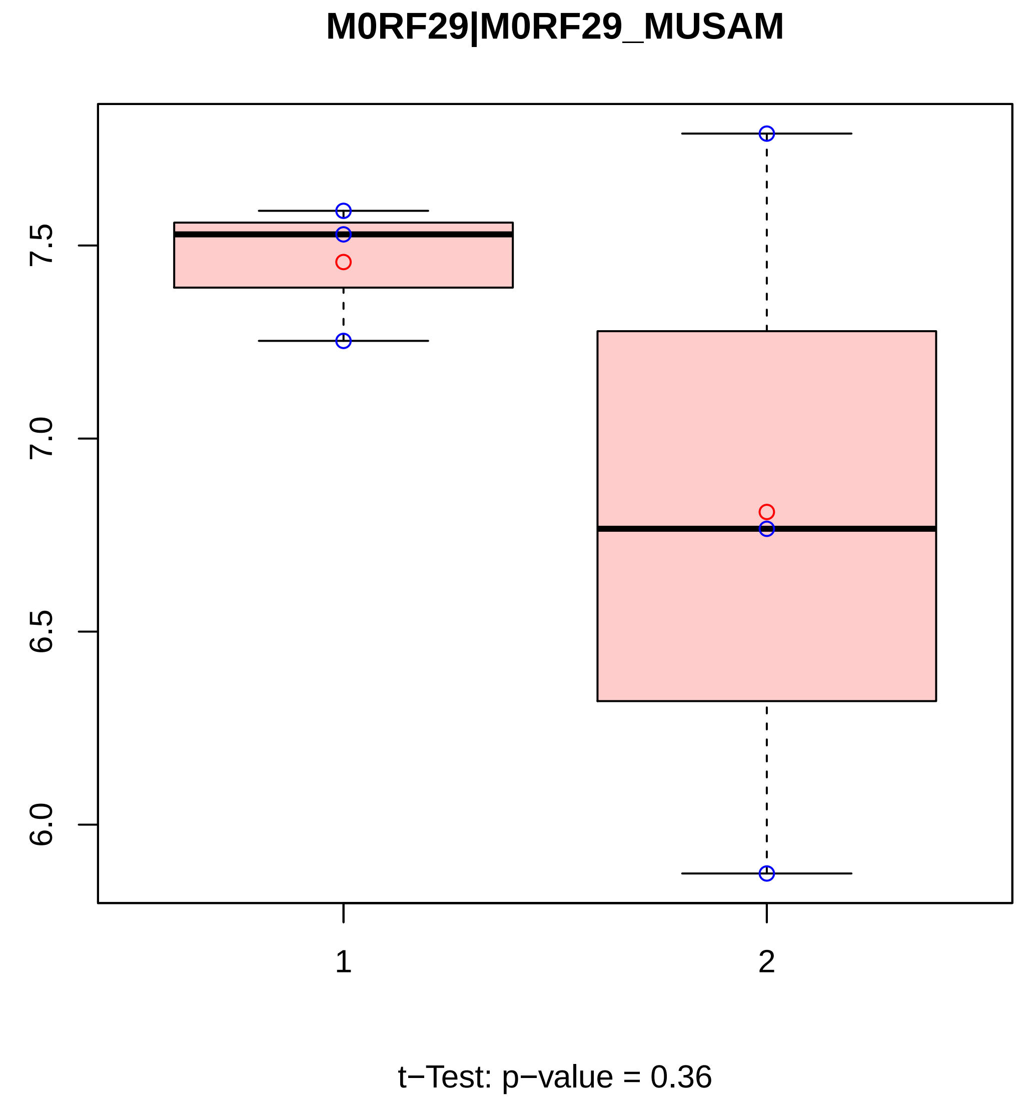
M0RF29|M0RF29_MUSAM
7.5
7.0
6.5
6.0
1
2
t−Test: p−value = 0.36

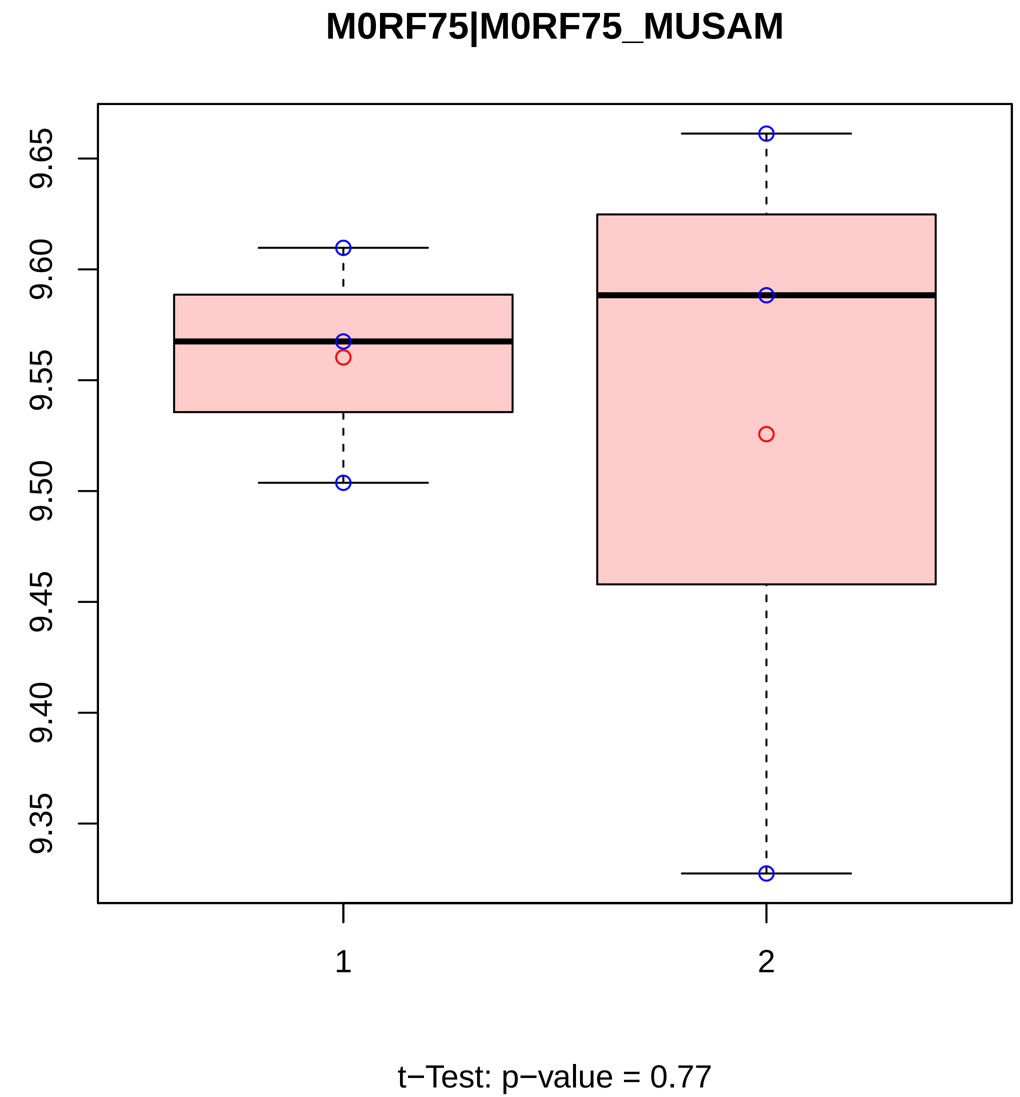
M0RF75|M0RF75_MUSAM
9.35
9.40
9.45
9.50
9.55
9.60
9.65
1
2
t−Test: p−value = 0.77

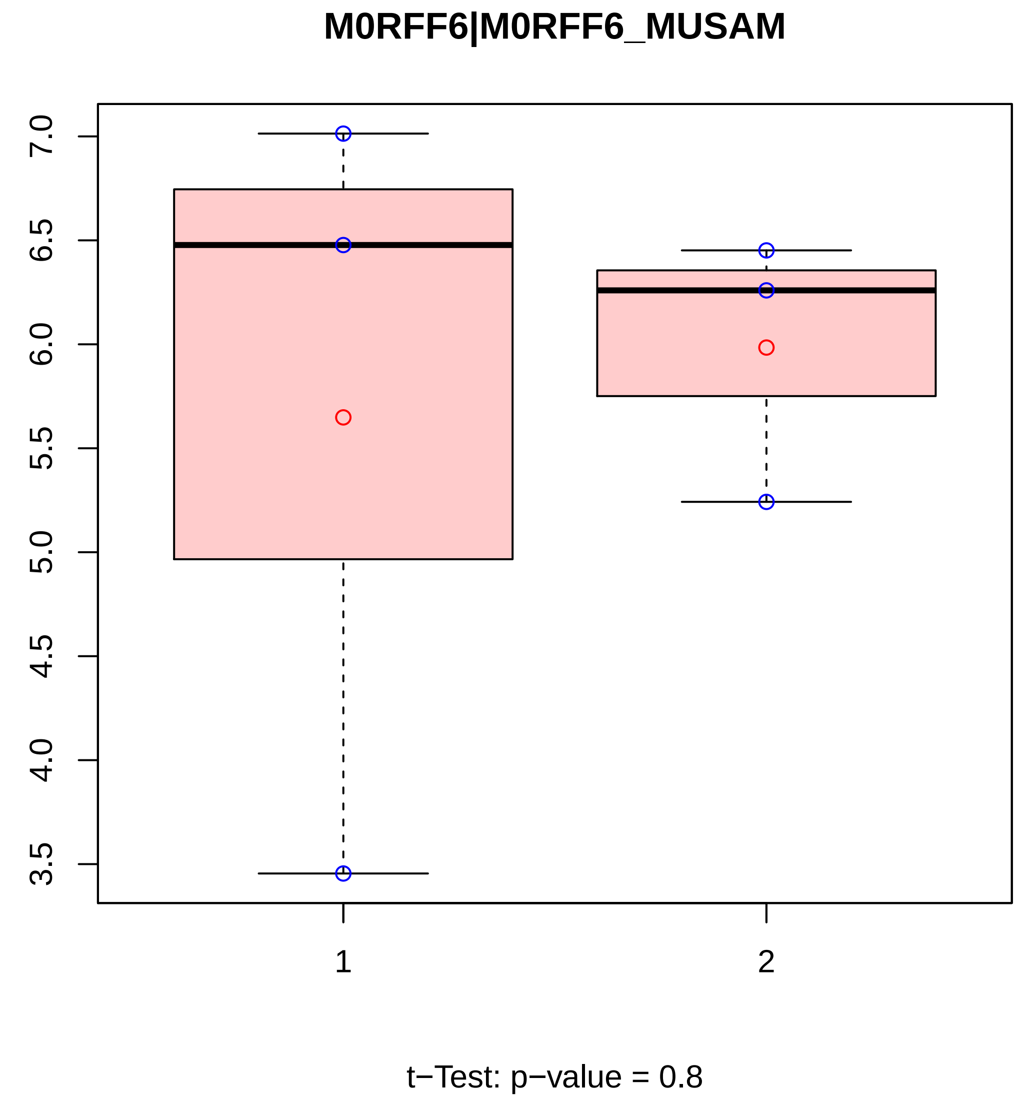

M0RFF6|M0RFF6_MUSAM
7.0
6.5
6.0
5.5
5.0
4.5
4.0
3.5
1
2
t−Test: p−value = 0.8

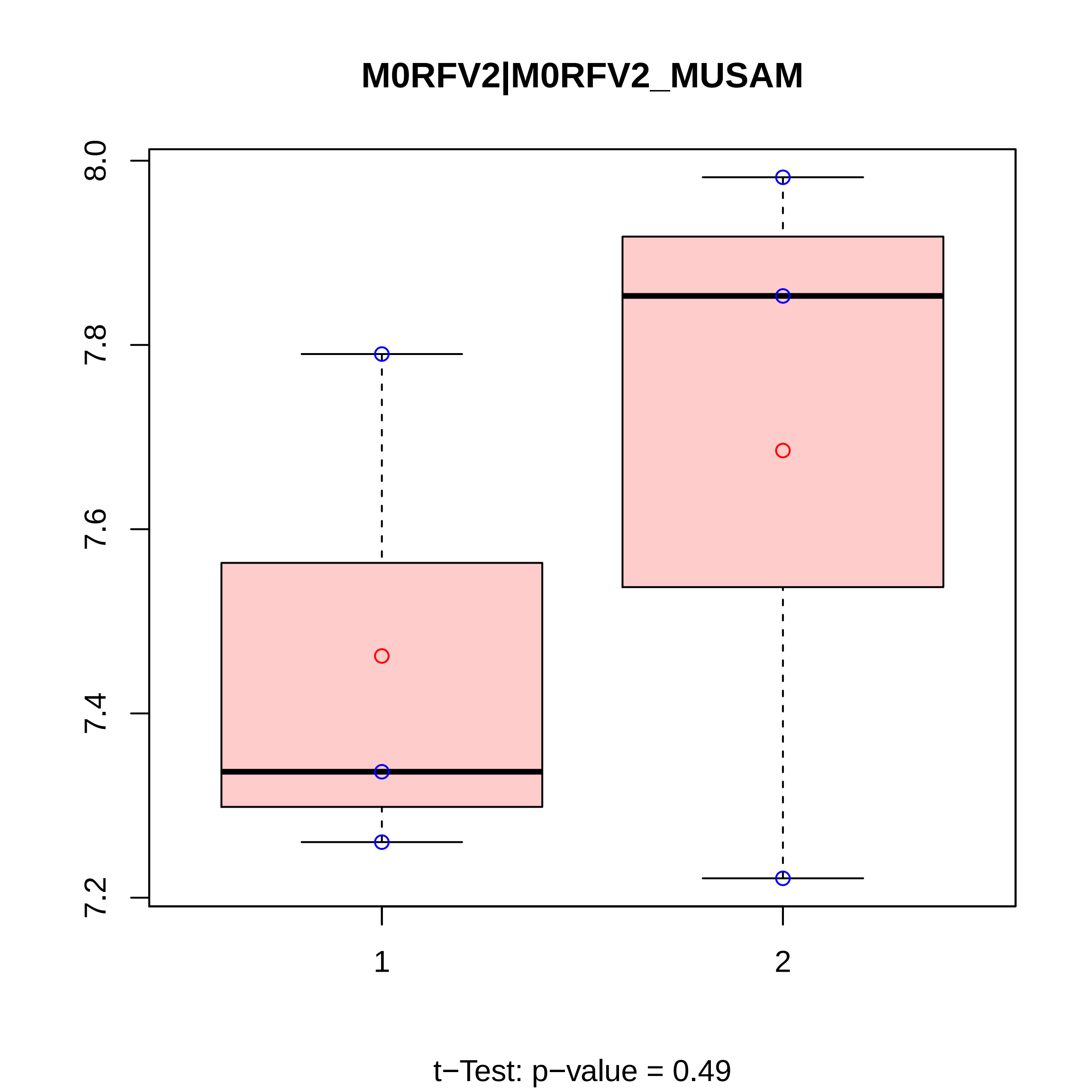
M0RFV2|M0RFV2_MUSAM
8.0
7.8
7.6
7.4
7.2
1
2
t−Test: p−value = 0.49

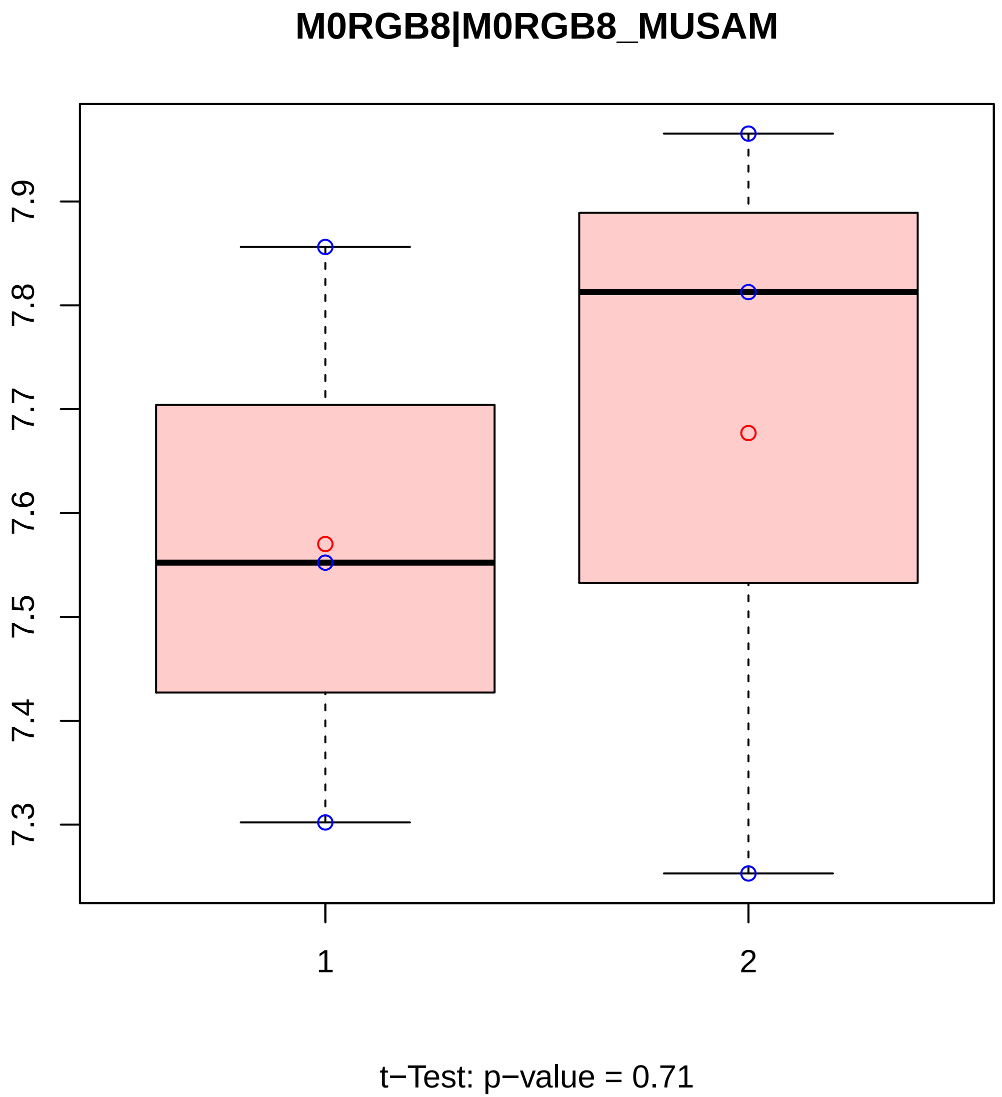
M0RGB8|M0RGB8_MUSAM
7.3
7.4
7.5
7.6
7.7
7.8
7.9
1
2
t−Test: p−value = 0.71

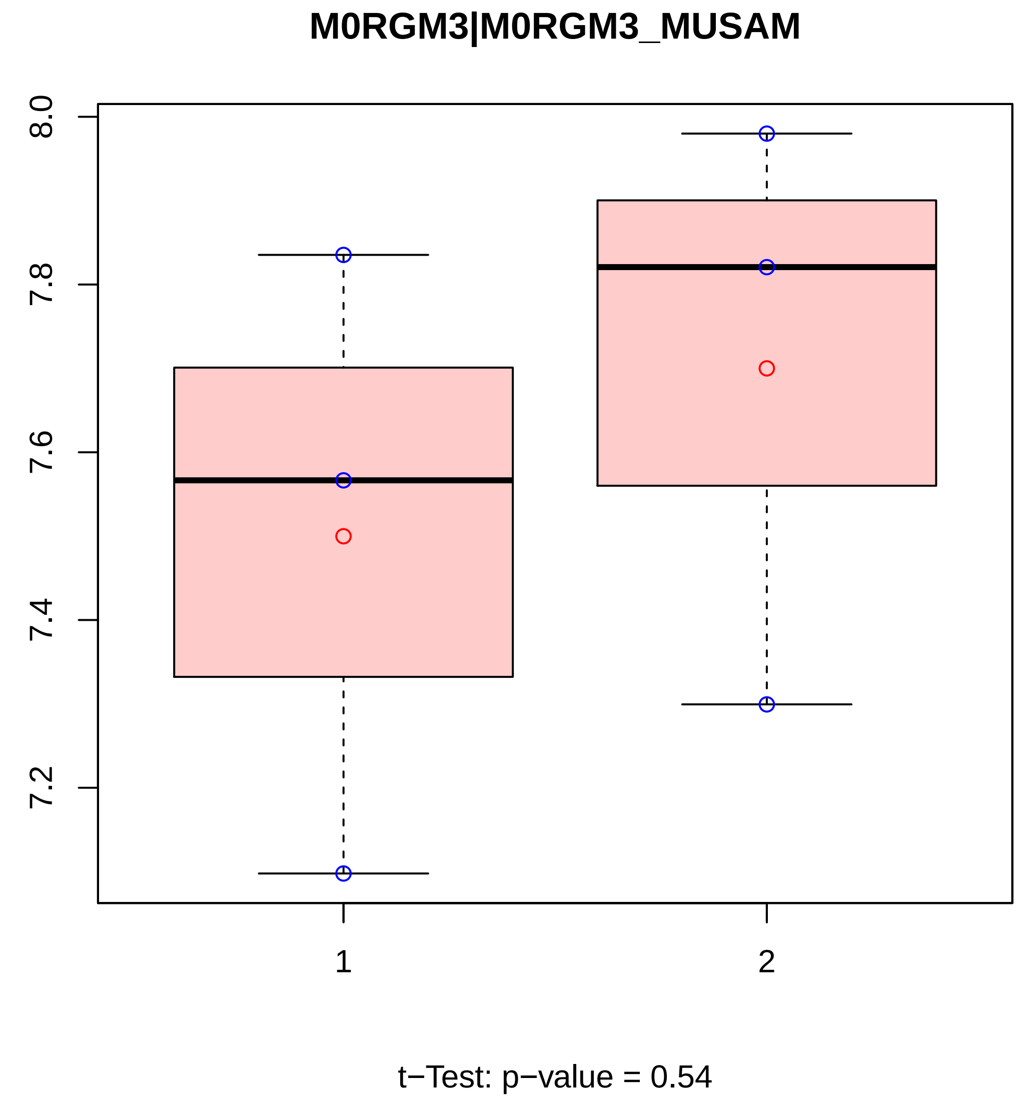
M0RGM3|M0RGM3_MUSAM
8.0
7.8
7.6
7.4
7.2
1
2
t−Test: p−value = 0.54

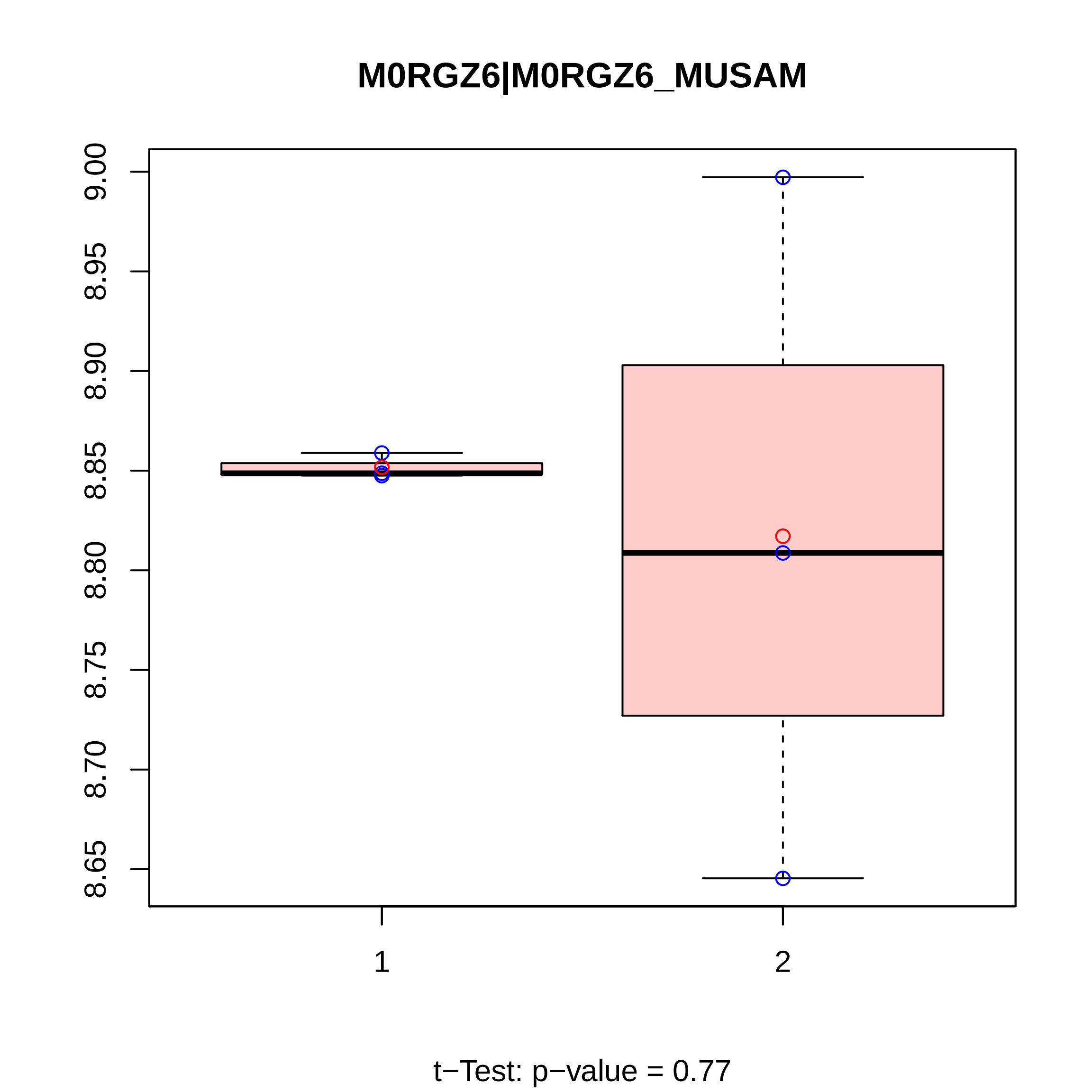
M0RGZ6|M0RGZ6_MUSAM
9.00
8.95
8.90
8.85
8.80
8.75
8.70
8.65
1
2
t−Test: p−value = 0.77

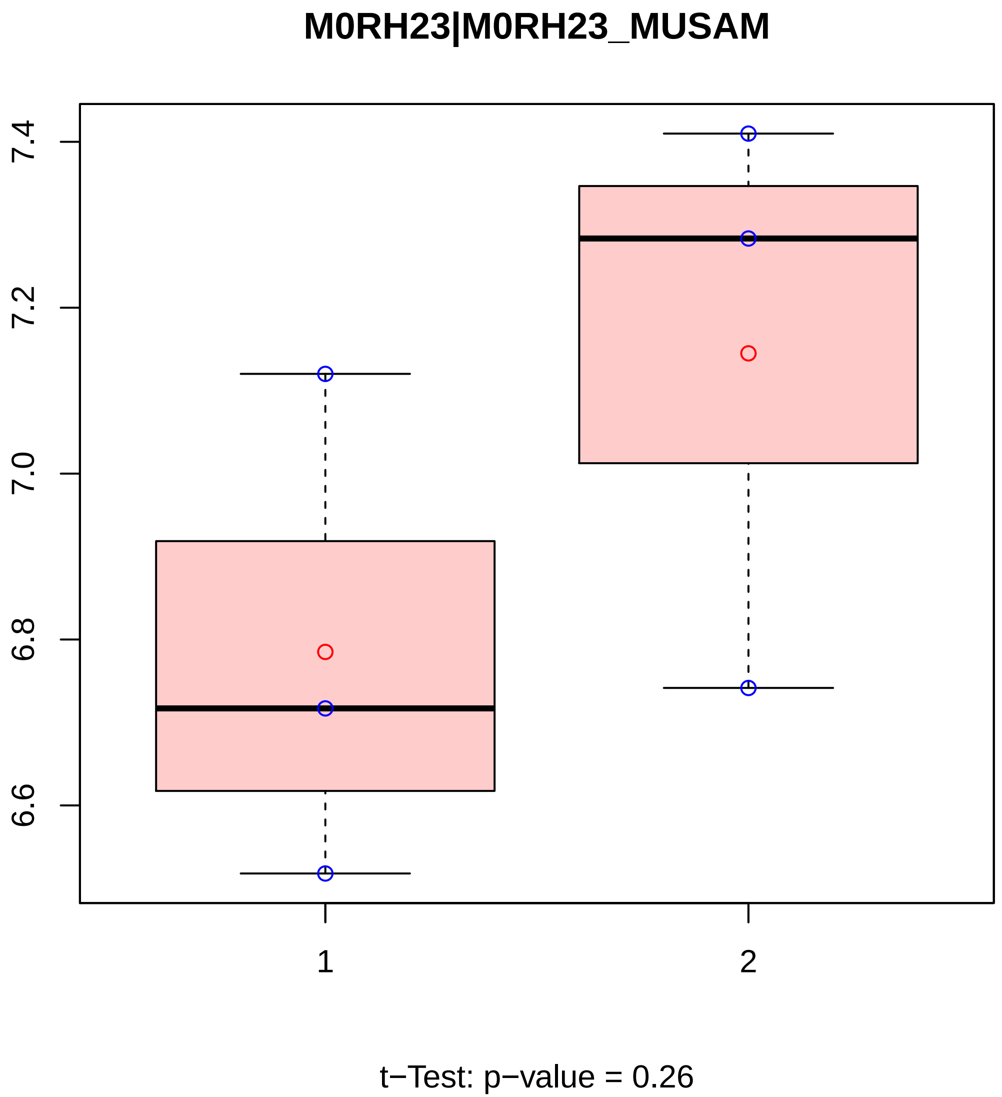

M0RH23|M0RH23_MUSAM
7.4
7.2
7.0
6.8
6.6
1
2
t−Test: p−value = 0.26

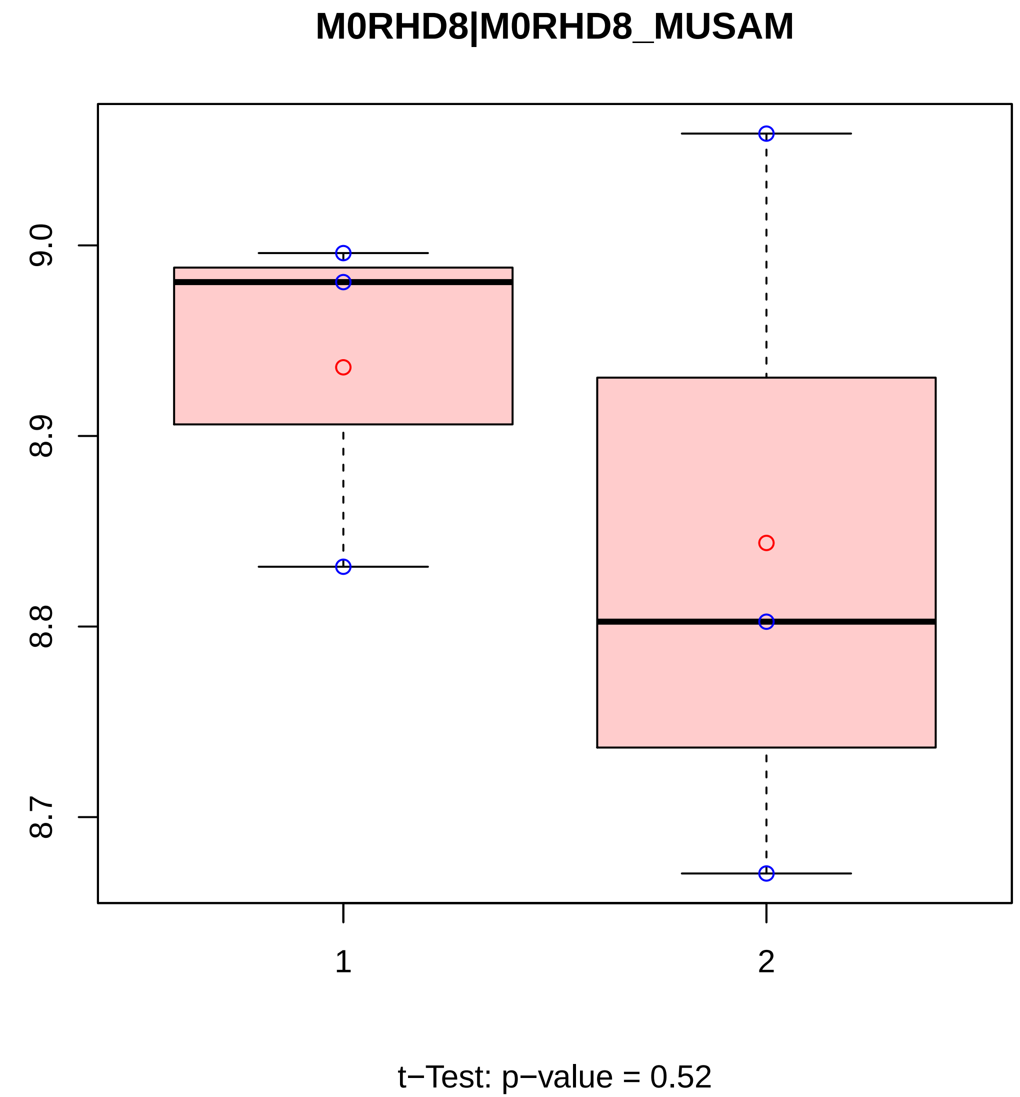
M0RHD8|M0RHD8_MUSAM
9.0
8.9
8.8
8.7
1
2
t−Test: p−value = 0.52

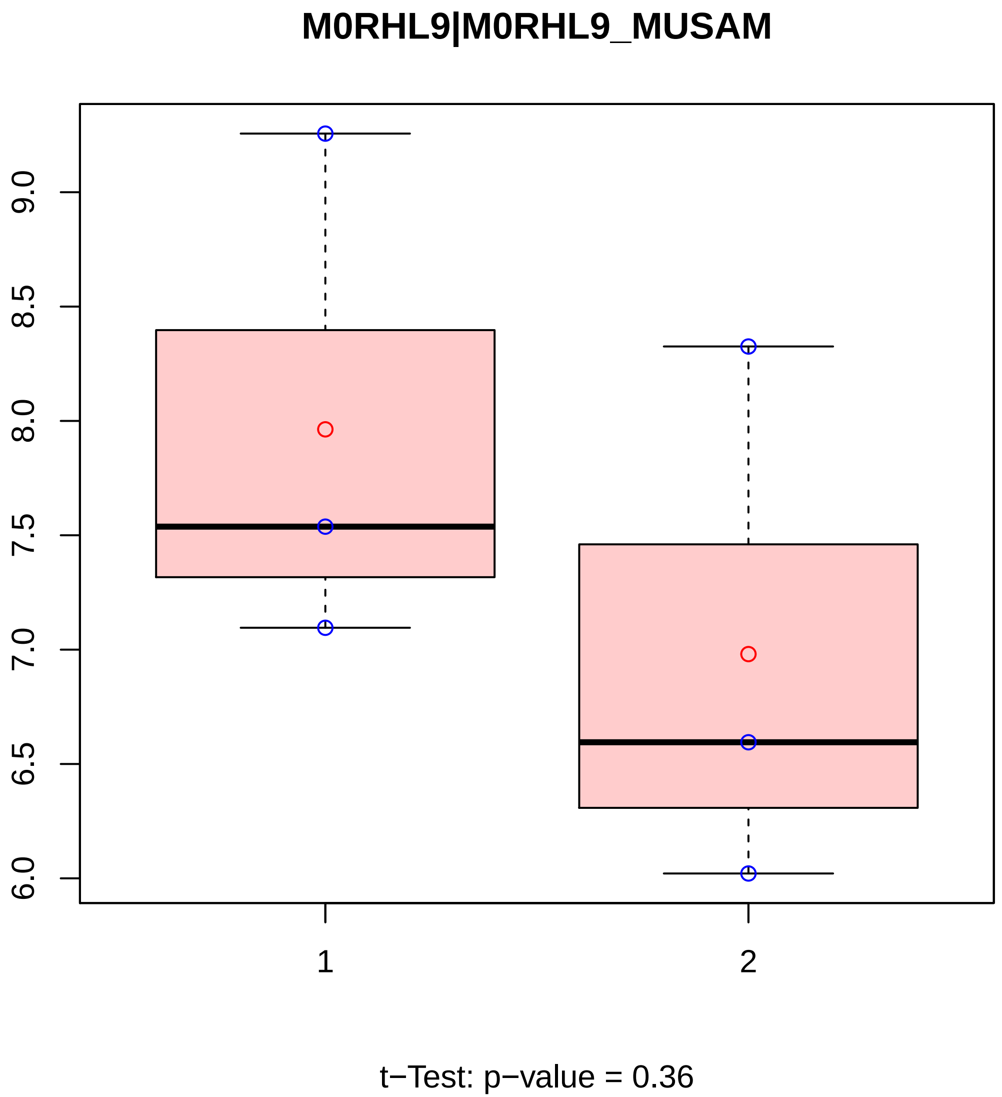
M0RHL9|M0RHL9_MUSAM
9.0
8.5
8.0
7.5
7.0
6.5
6.0
1
2
t−Test: p−value = 0.36

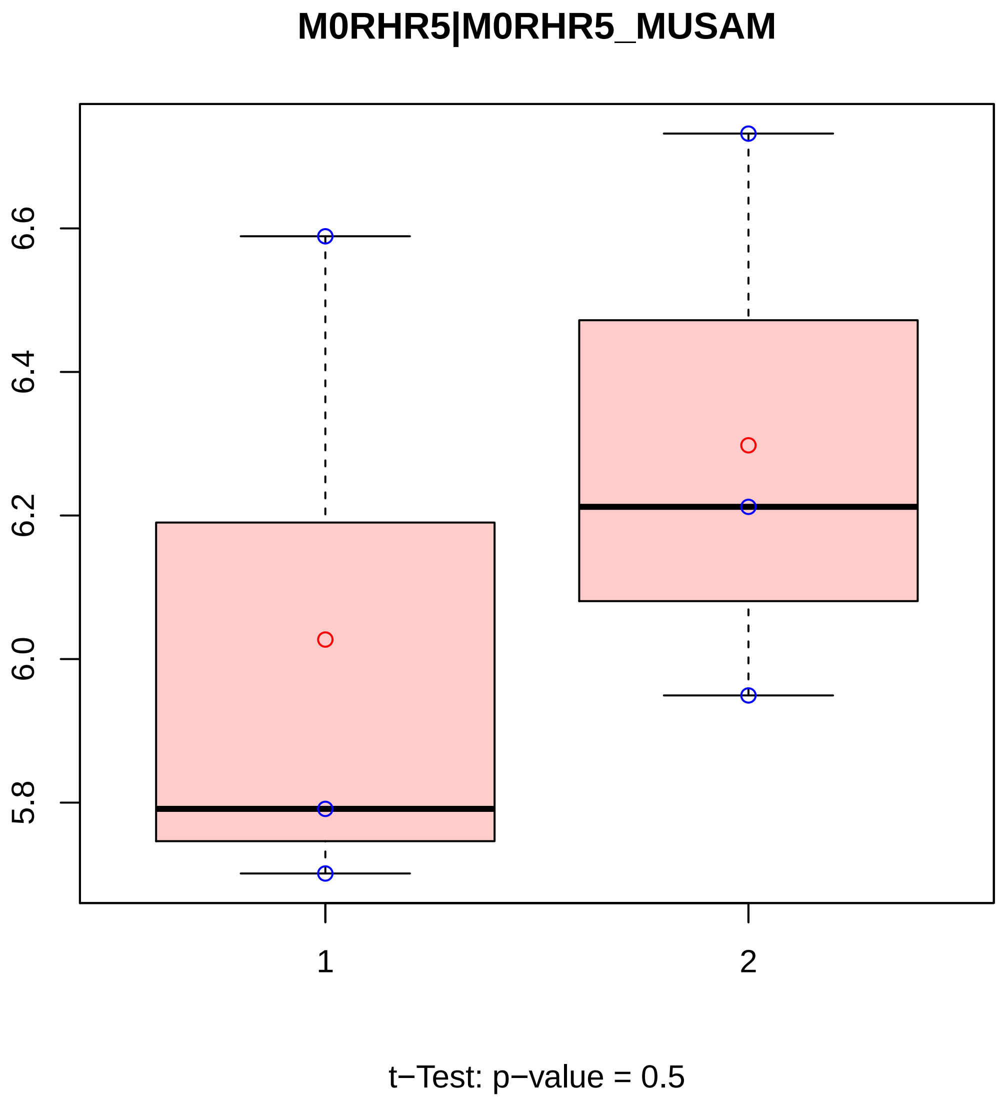

M0RHR5|M0RHR5_MUSAM
6.6
6.4
6.2
6.0
5.8
1
2
t−Test: p−value = 0.5

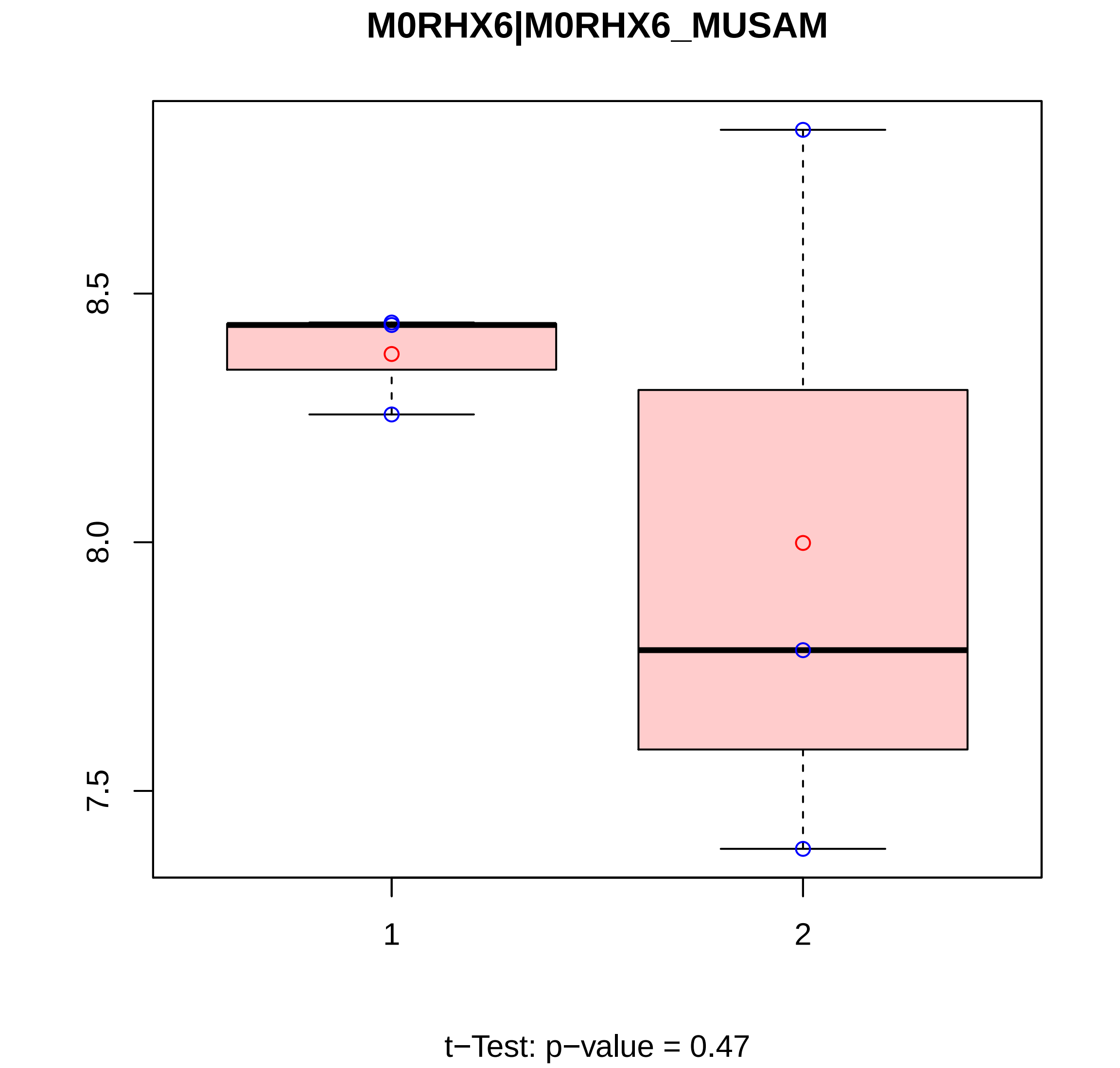
M0RHX6|M0RHX6_MUSAM
8.5
8.0
7.5
1
2
t−Test: p−value = 0.47

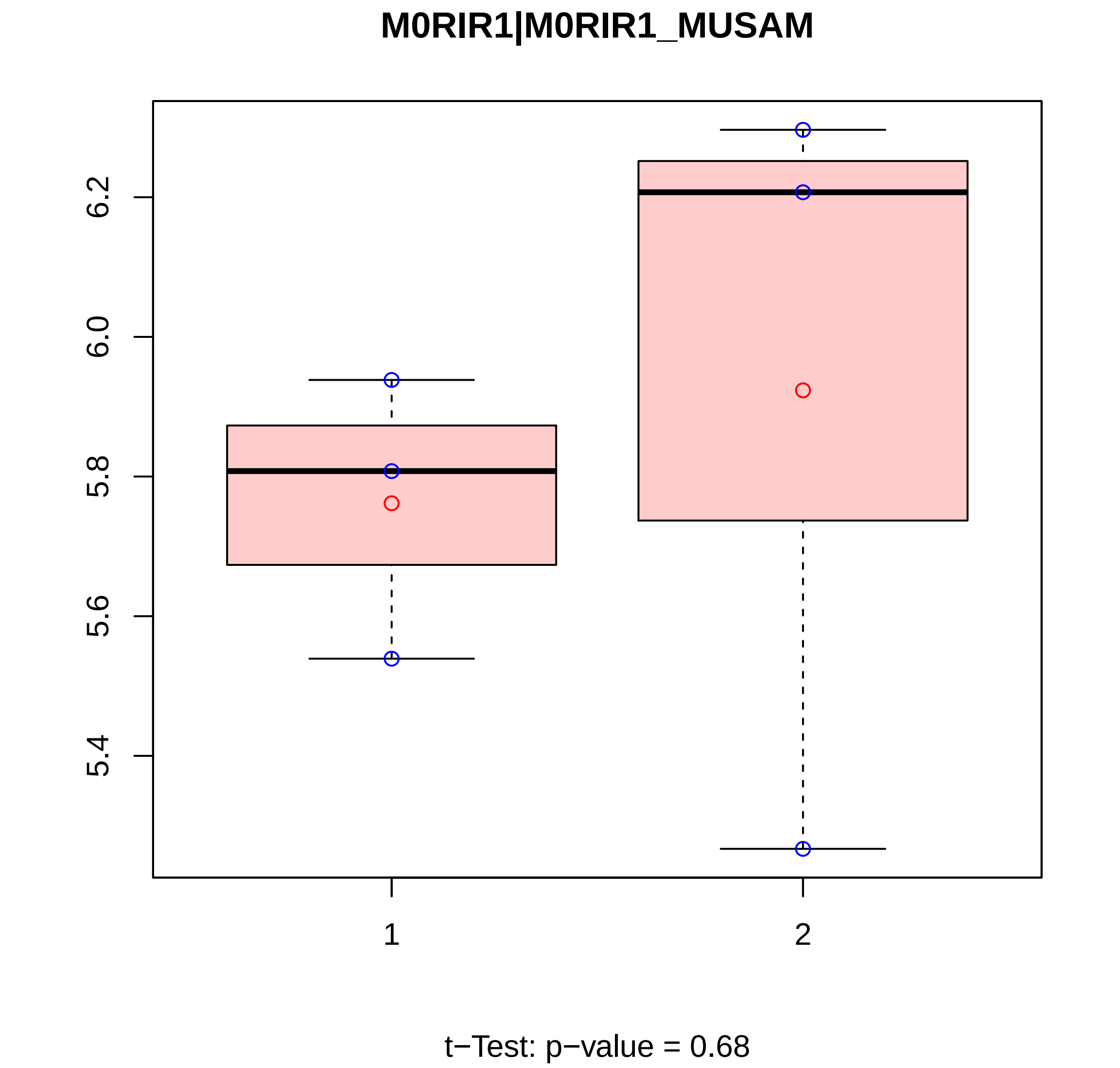
M0RIR1|M0RIR1_MUSAM
6.2
6.0
5.8
5.6
5.4
1
2
t−Test: p−value = 0.68

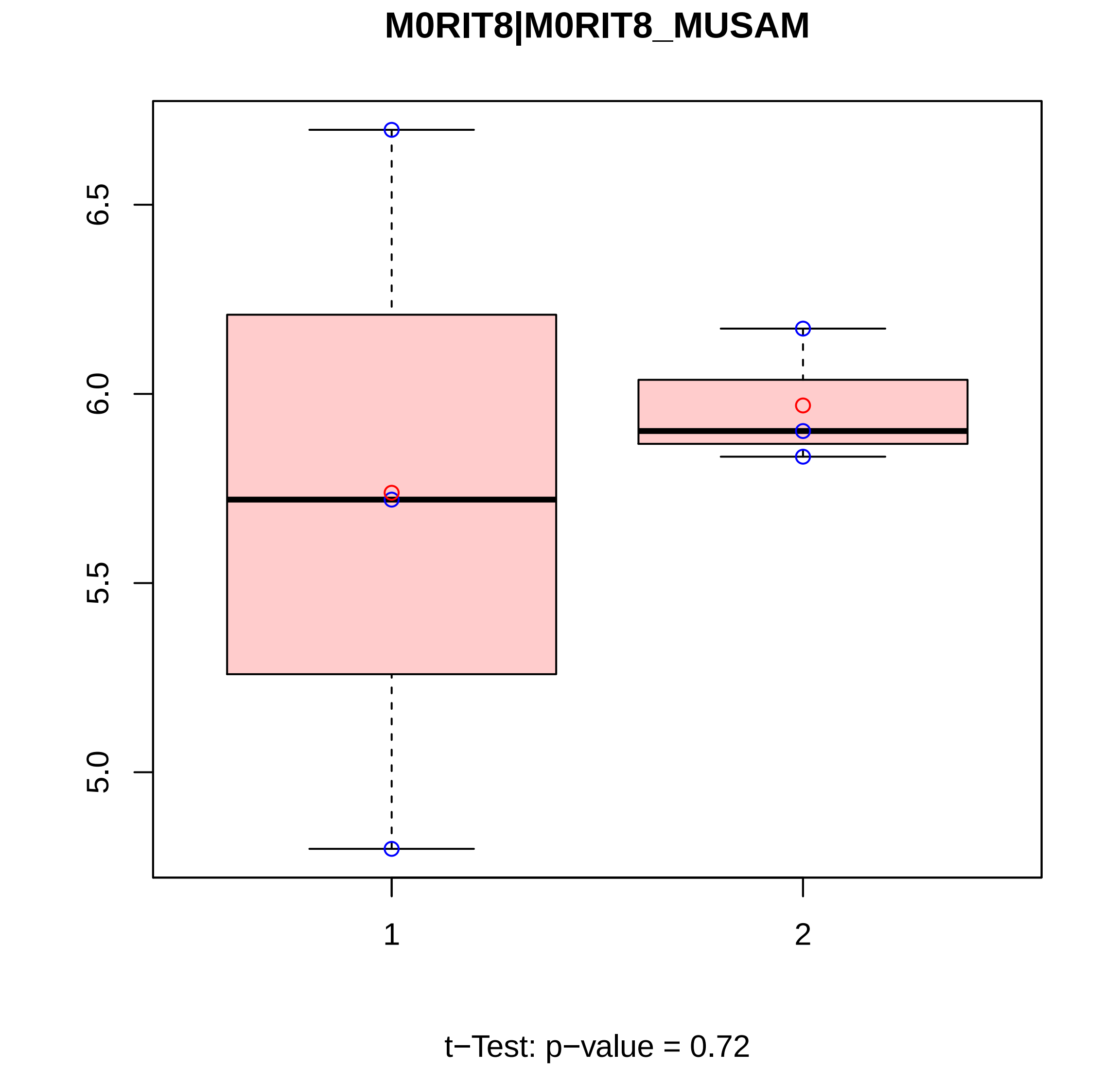
M0RIT8|M0RIT8_MUSAM
6.5
6.0
5.5
5.0
1
2
t−Test: p−value = 0.72

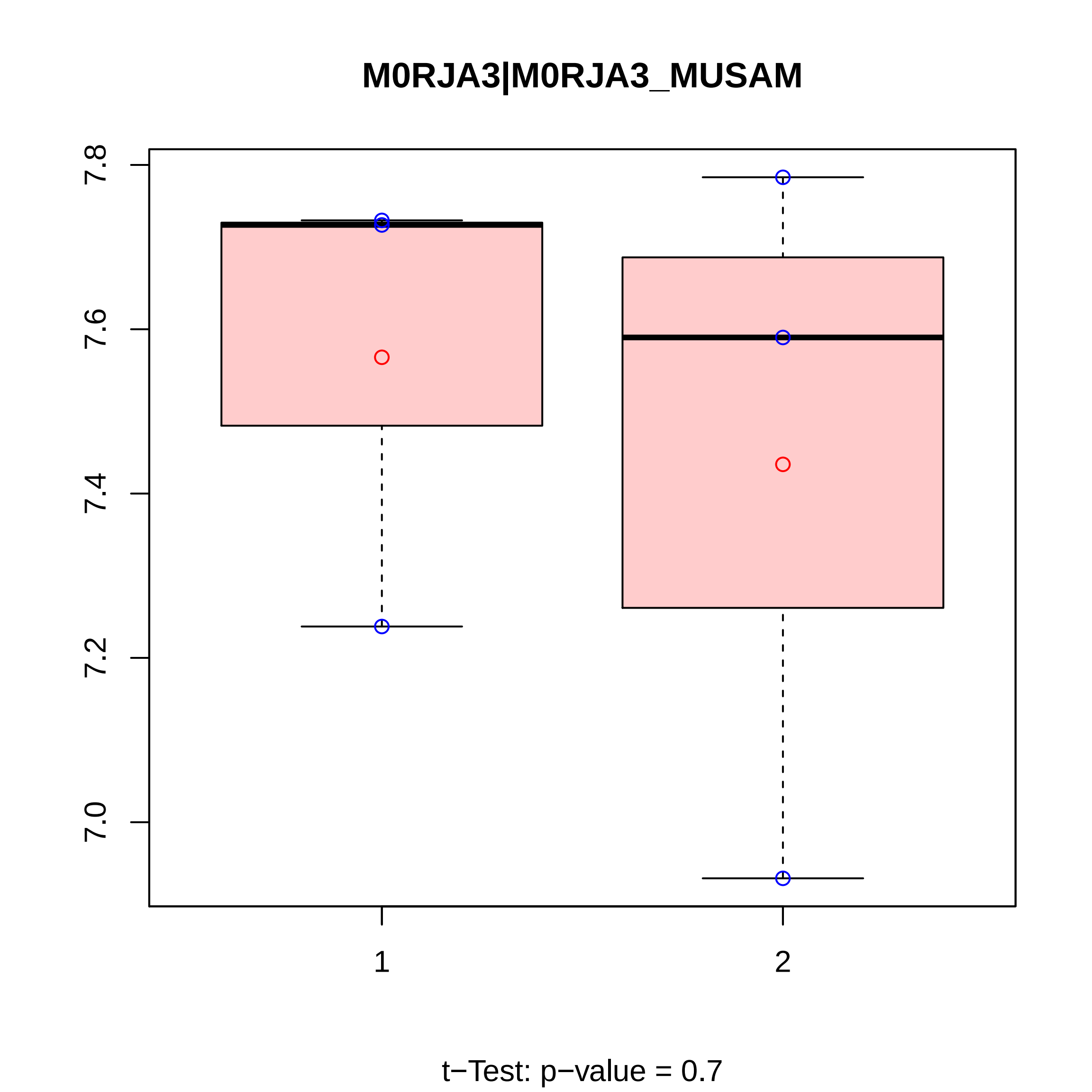

M0RJA3|M0RJA3_MUSAM
7.8
7.6
7.4
7.2
7.0
1
2
t−Test: p−value = 0.7

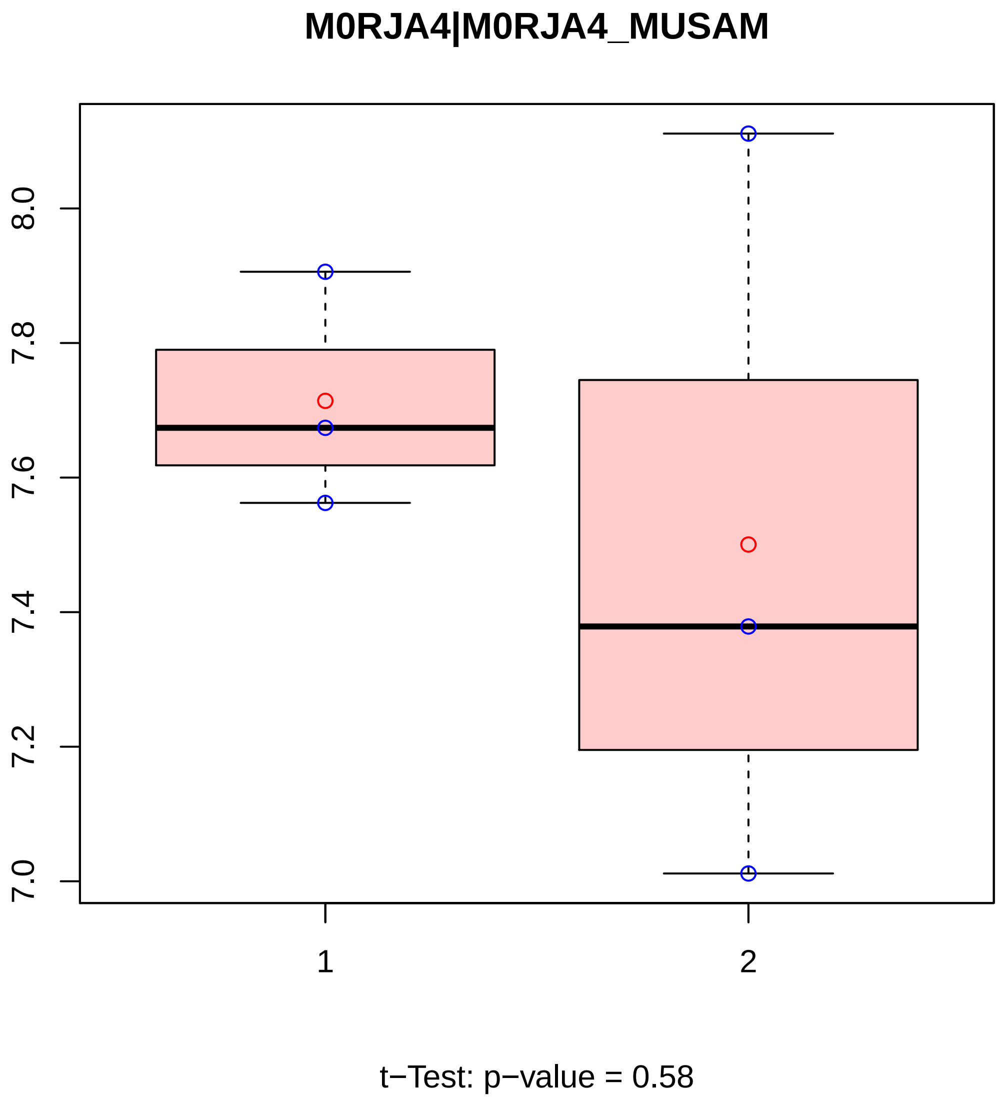
M0RJA4|M0RJA4_MUSAM
8.0
7.8
7.6
7.4
7.2
7.0
1
2
t−Test: p−value = 0.58

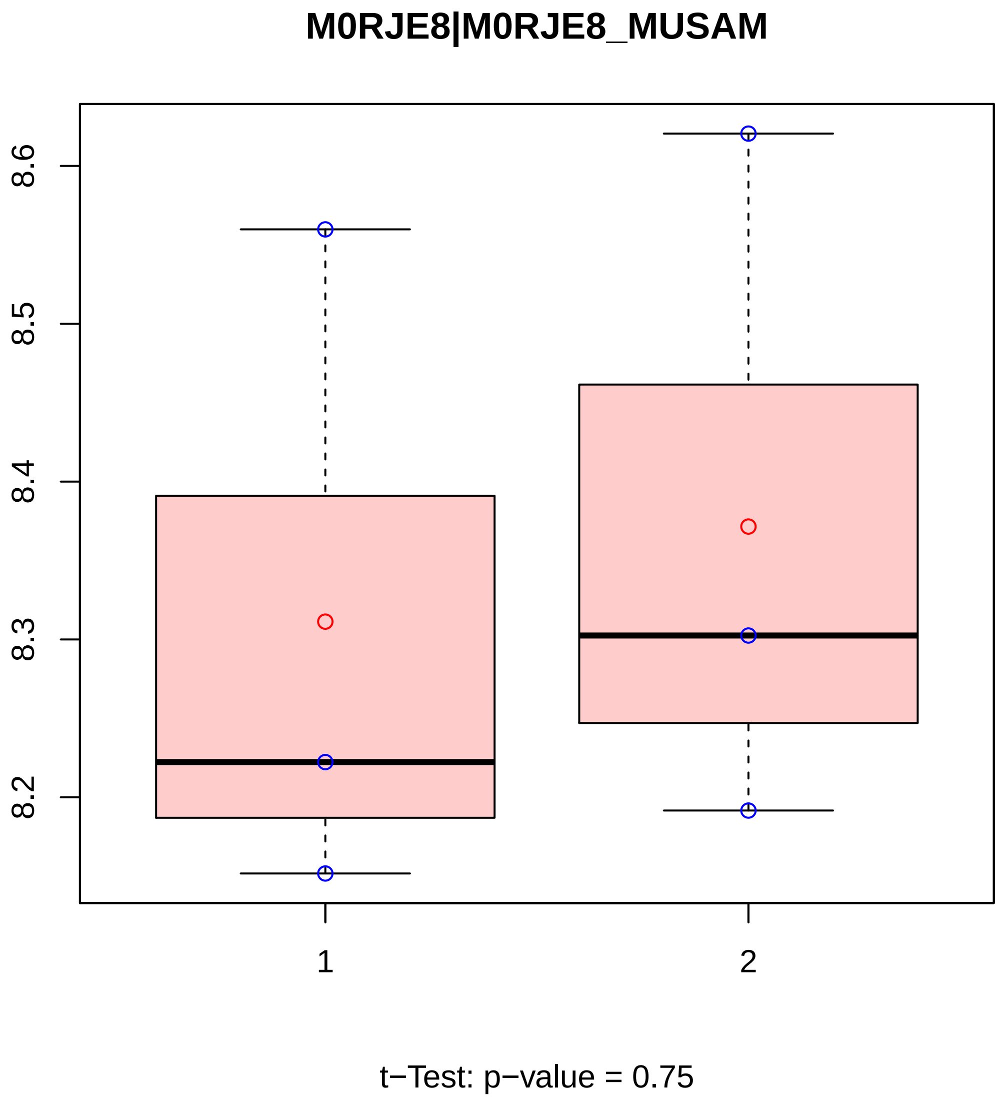
M0RJE8|M0RJE8_MUSAM
8.2
8.3
8.4
8.5
8.6
1
2
t−Test: p−value = 0.75

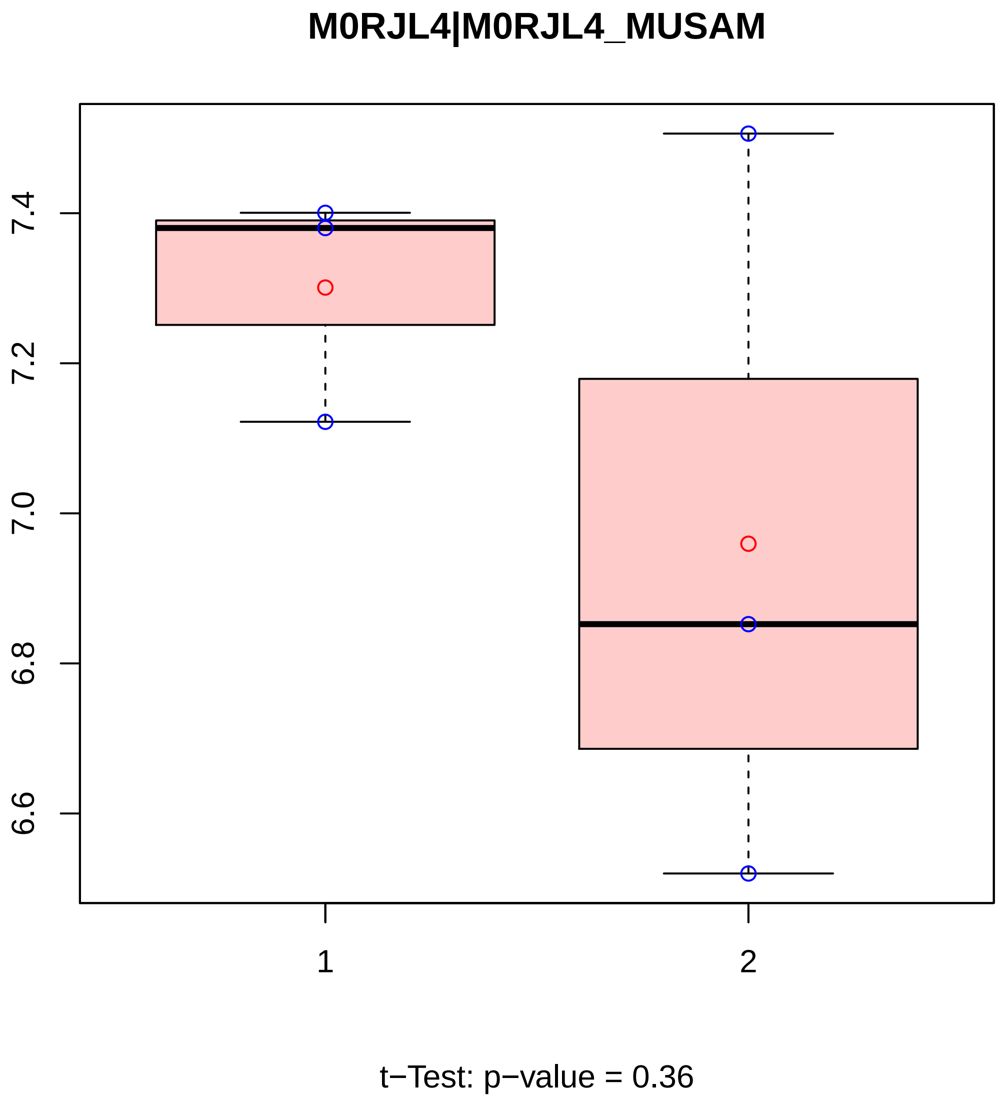
M0RJL4|M0RJL4_MUSAM
7.4
7.2
7.0
6.8
6.6
1
2
t−Test: p−value = 0.36

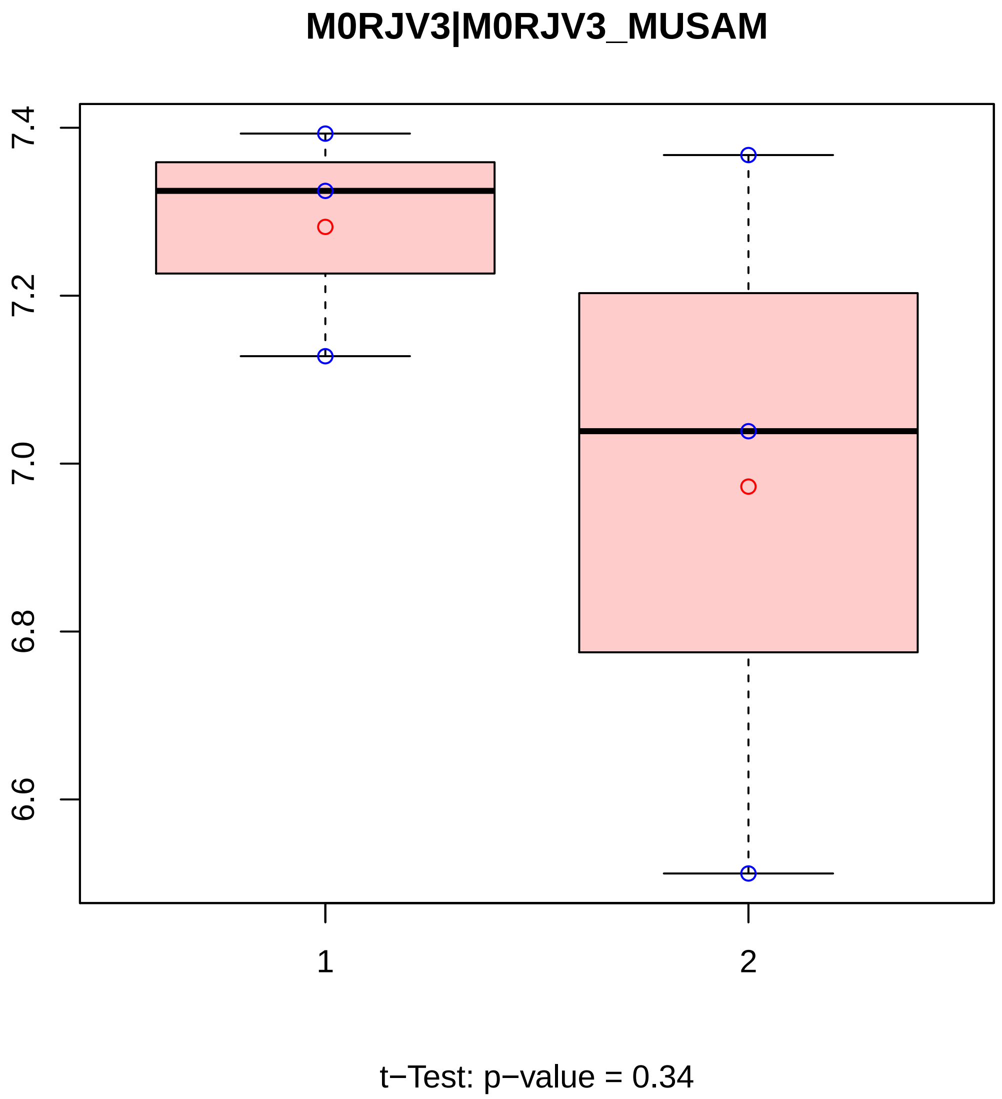
M0RJV3|M0RJV3_MUSAM
7.4
7.2
7.0
6.8
6.6
1
2
t−Test: p−value = 0.34

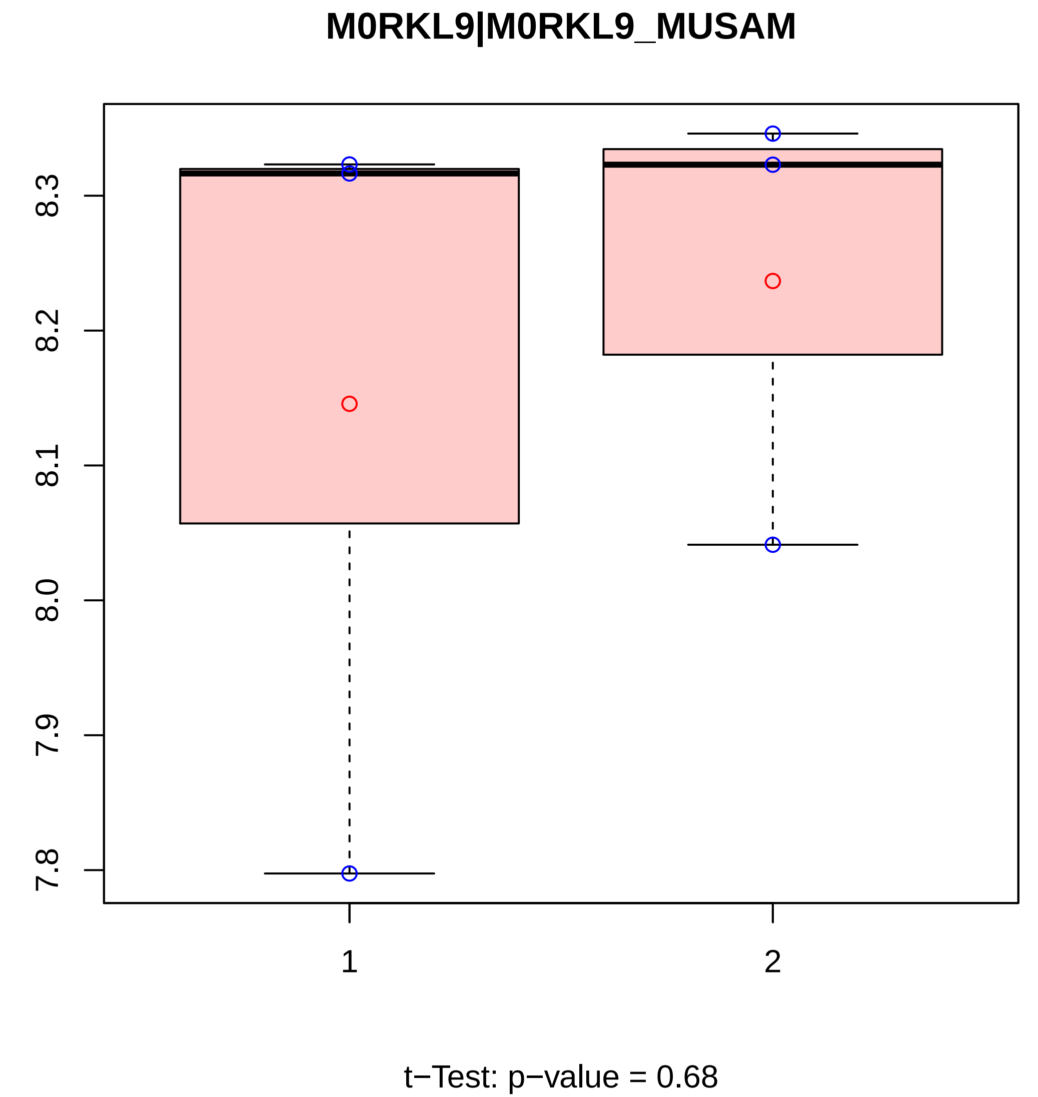
M0RKL9|M0RKL9_MUSAM
8.3
8.2
8.1
8.0
7.9
7.8
1
2
t−Test: p−value = 0.68

M0RKX7|M0RKX7_MUSAM

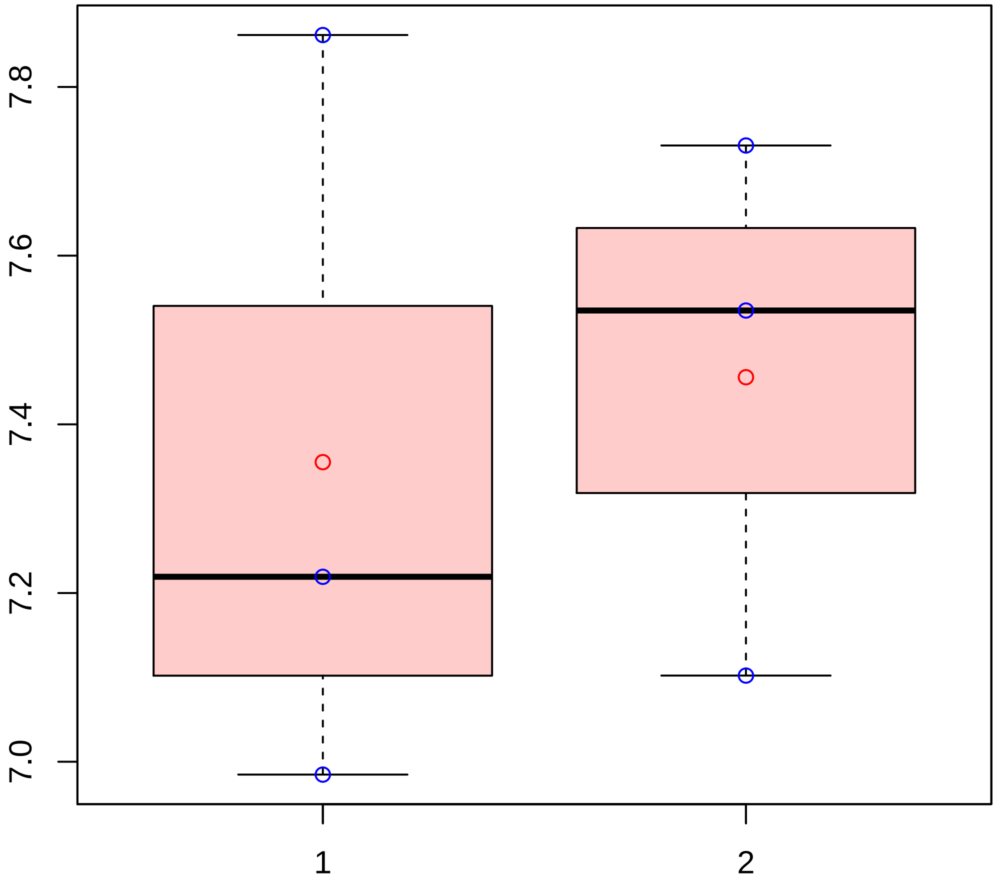

t−Test: p−value = 0.77

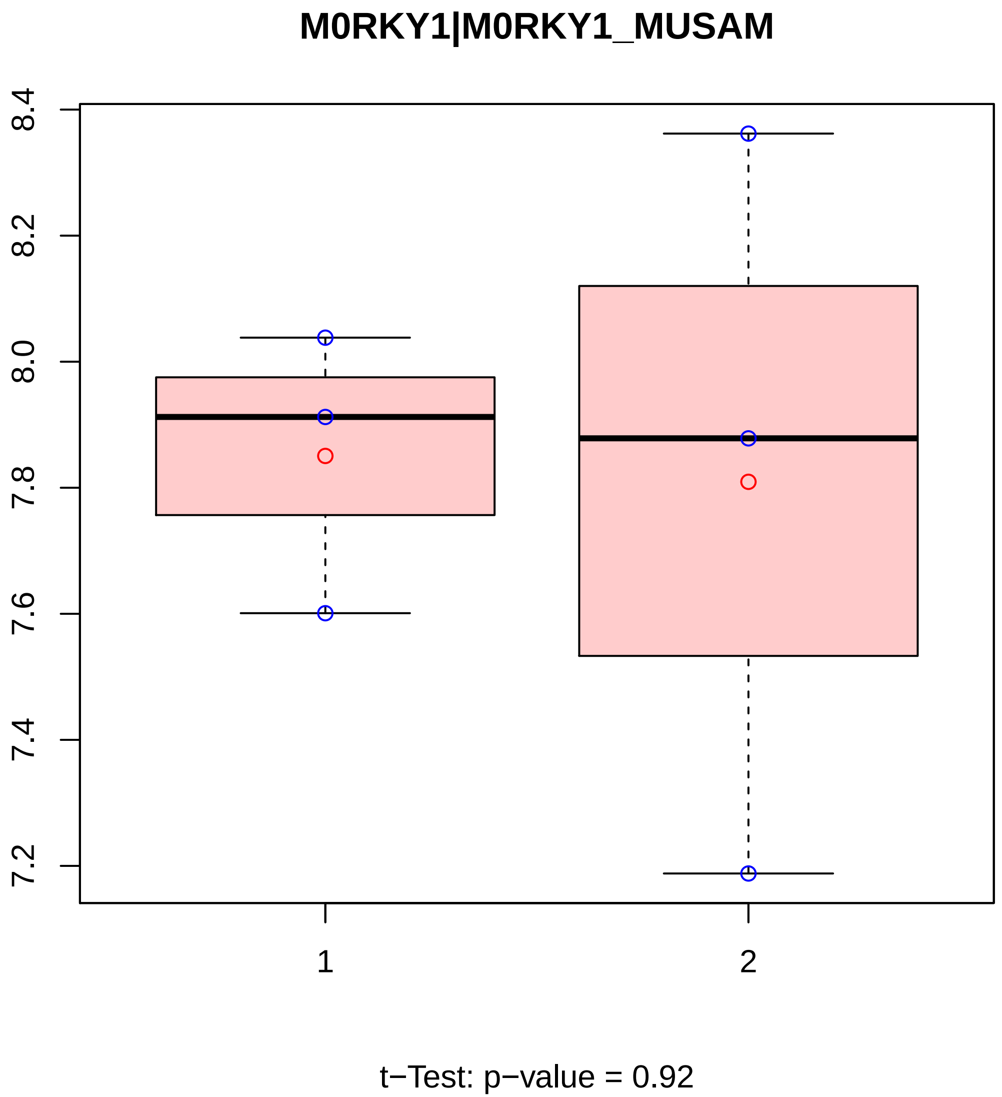
M0RKY1|M0RKY1_MUSAM
8.4
8.2
8.0
7.8
7.6
7.4
7.2
1
2
t−Test: p−value = 0.92

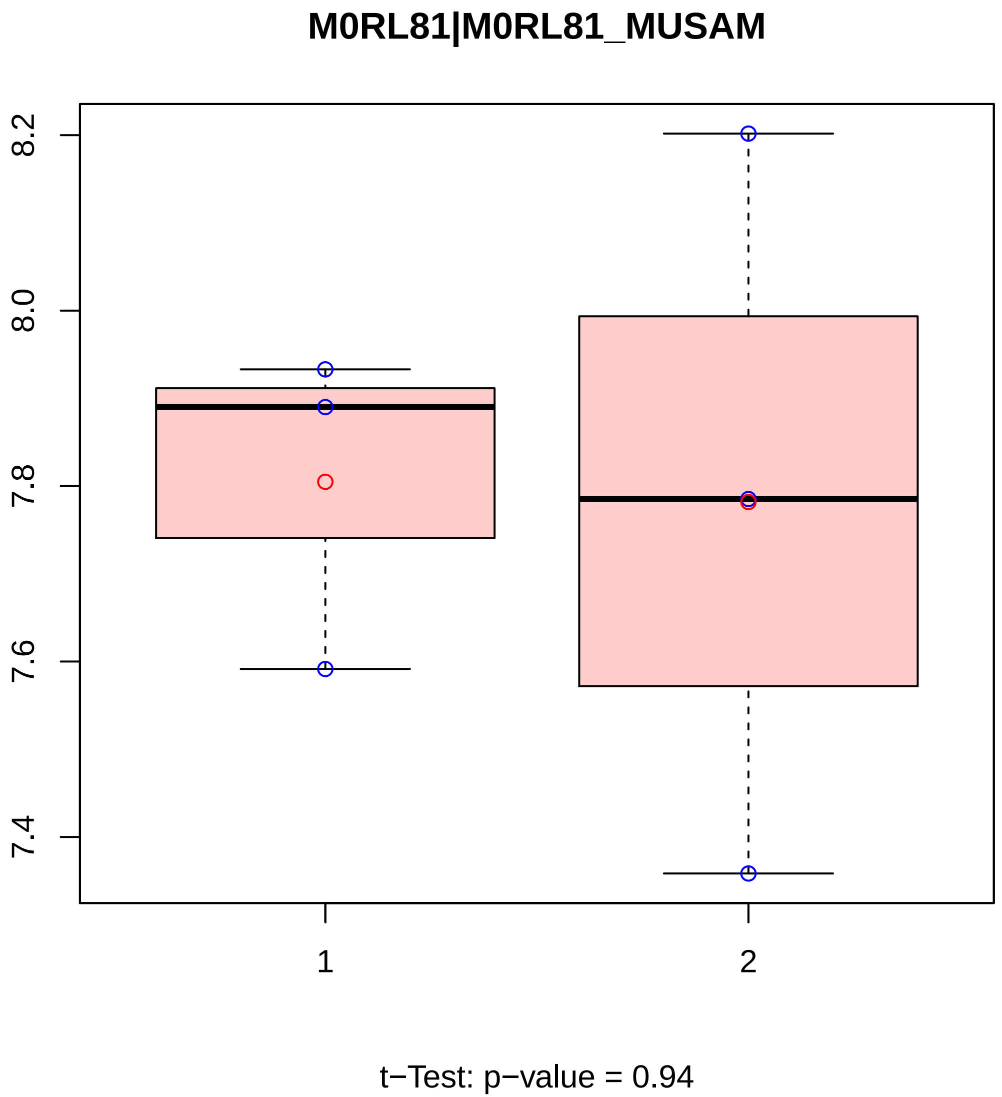
M0RL81|M0RL81_MUSAM
8.2
8.0
7.8
7.6
7.4
1
2
t−Test: p−value = 0.94

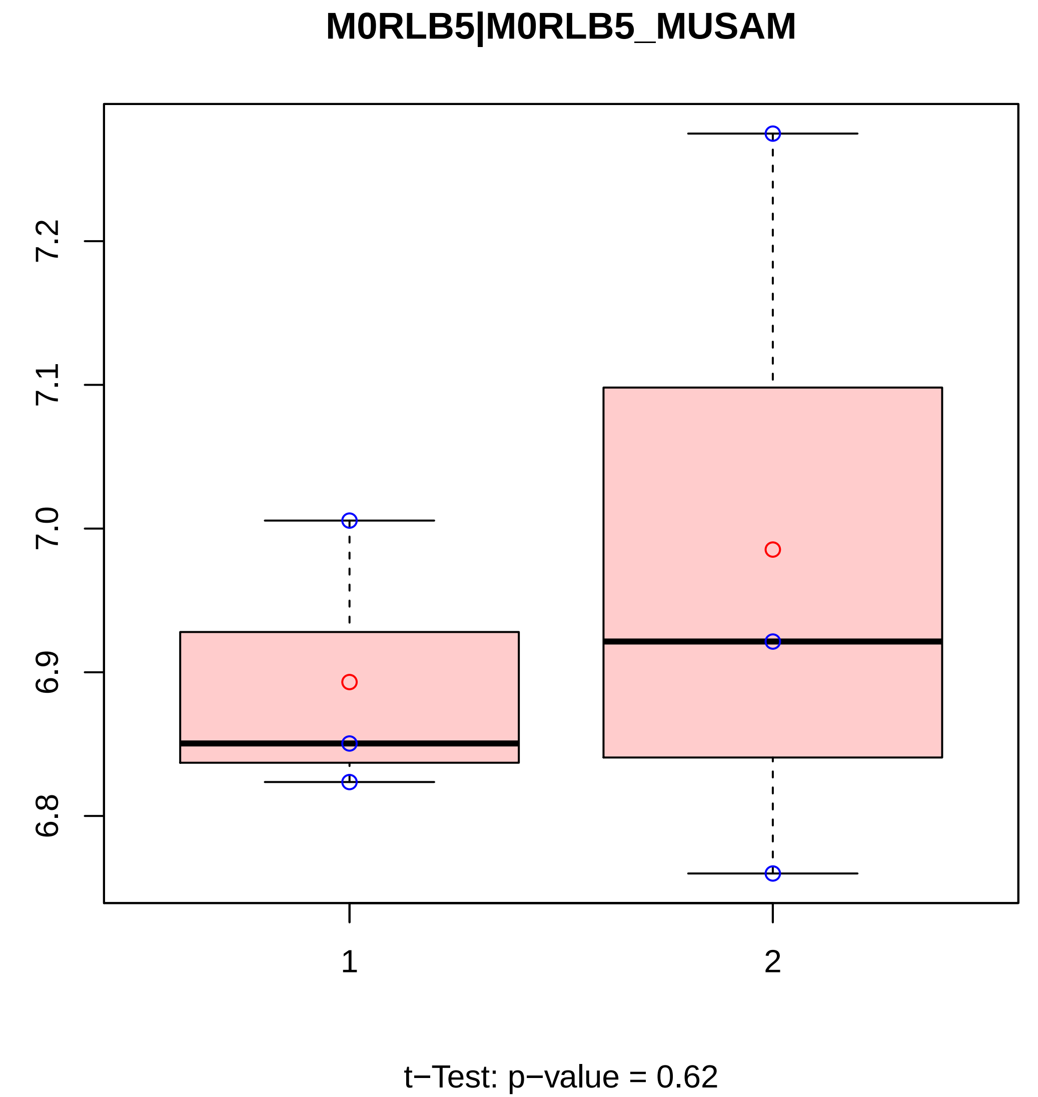

M0RLB5|M0RLB5_MUSAM
7.2
7.1
7.0
6.9
6.8
1
2
t−Test: p−value = 0.62

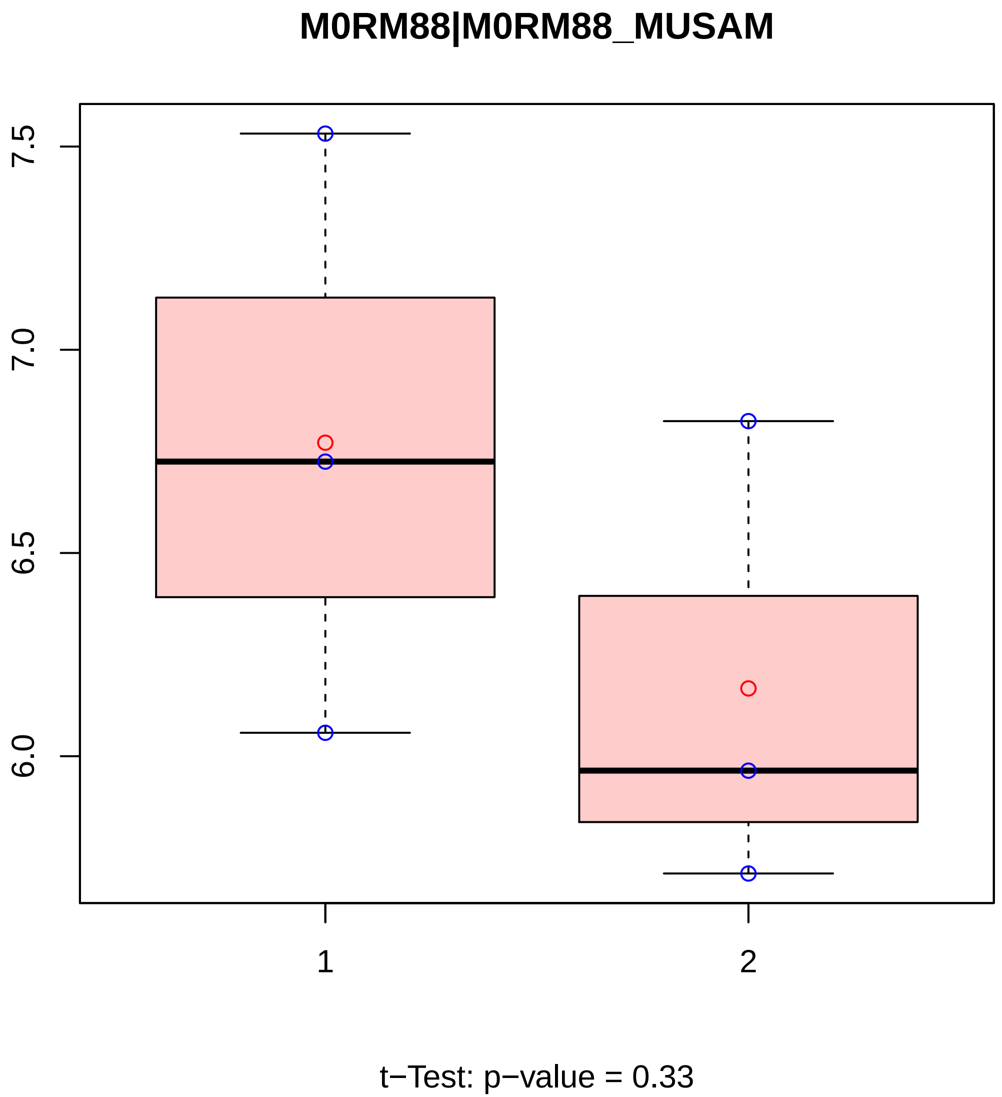
M0RM88|M0RM88_MUSAM
7.5
7.0
6.5
6.0
1
2
t−Test: p−value = 0.33

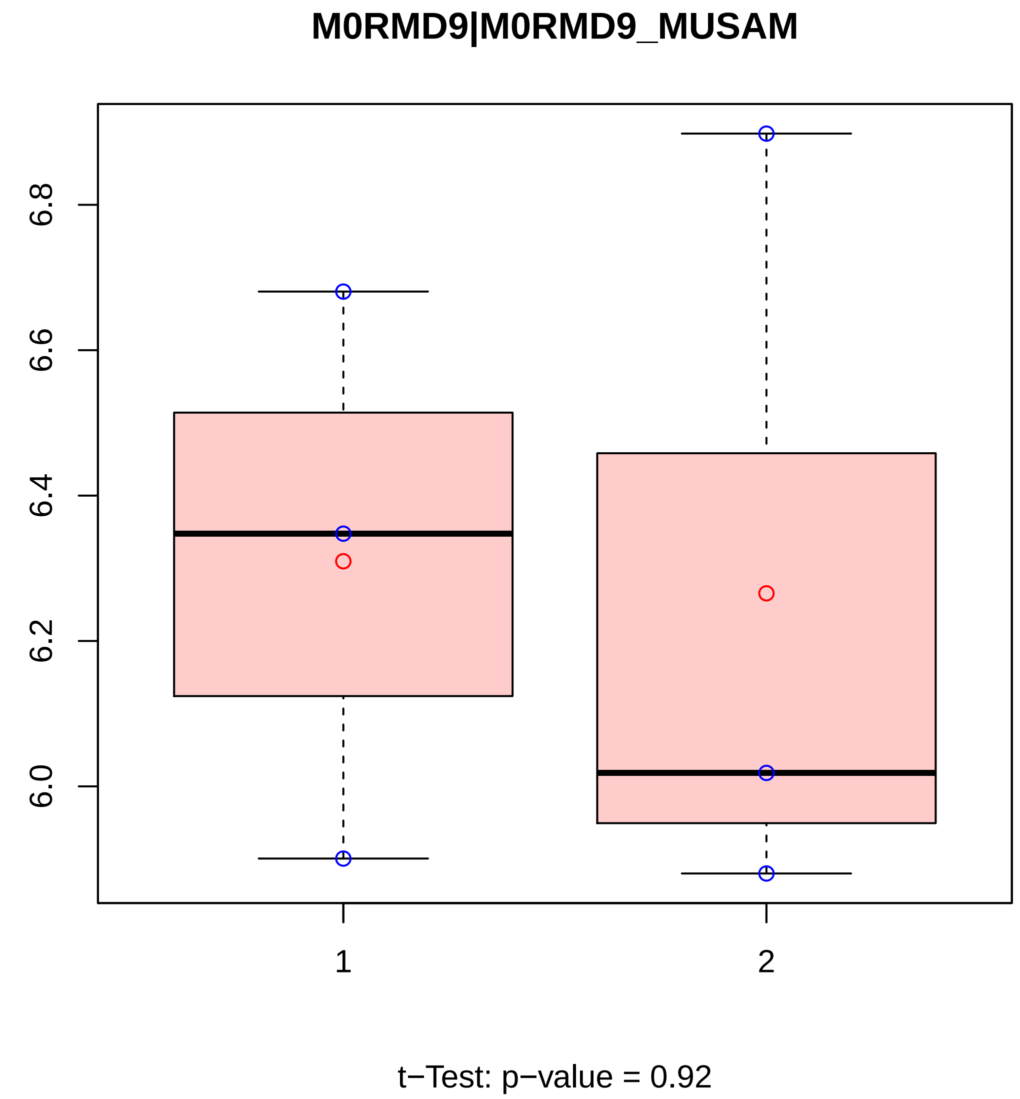
M0RMD9|M0RMD9_MUSAM
6.0
6.2
6.4
6.6
6.8
1
2
t−Test: p−value = 0.92

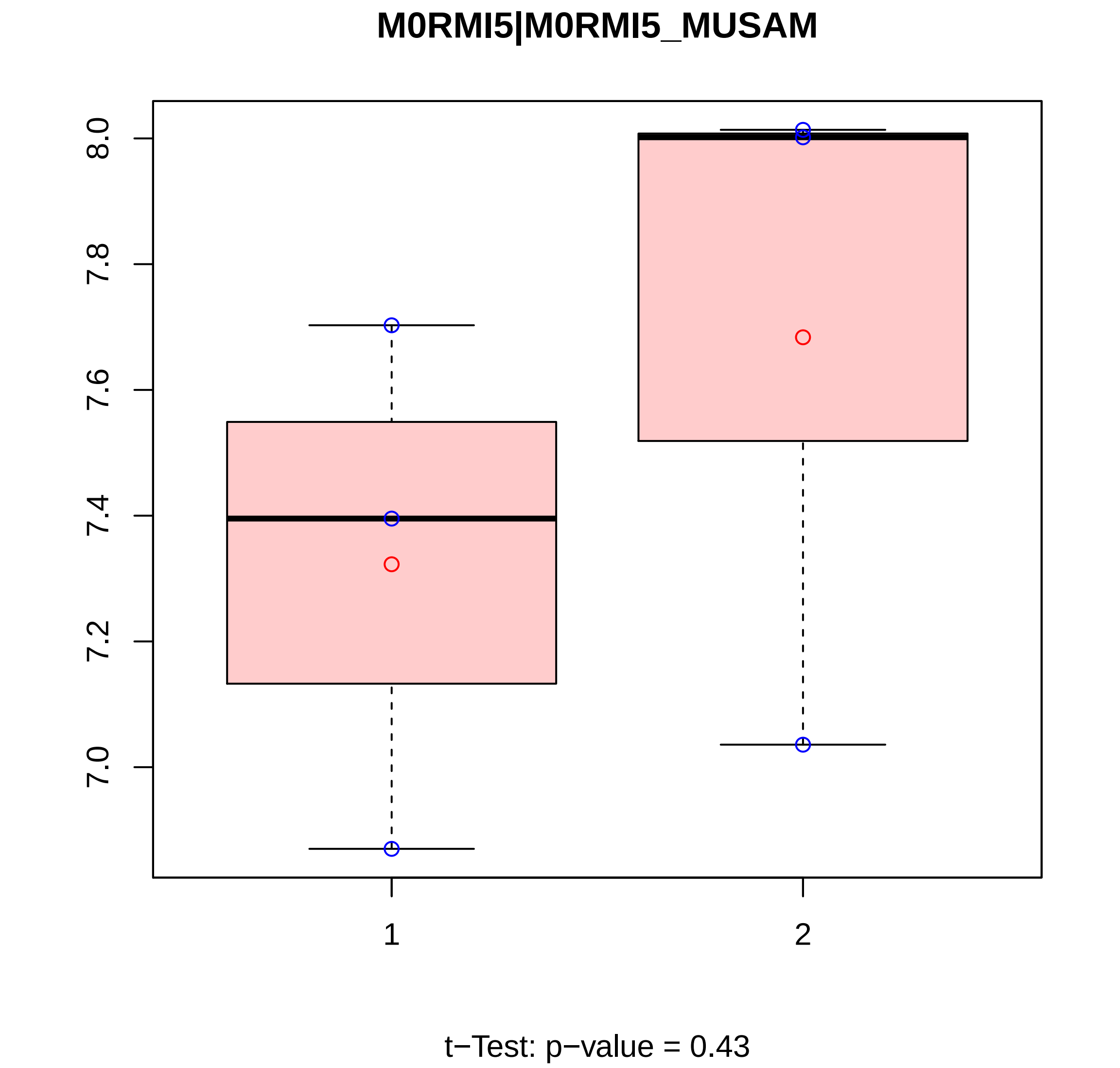
M0RMI5|M0RMI5_MUSAM
8.0
7.8
7.6
7.4
7.2
7.0
1
2
t−Test: p−value = 0.43

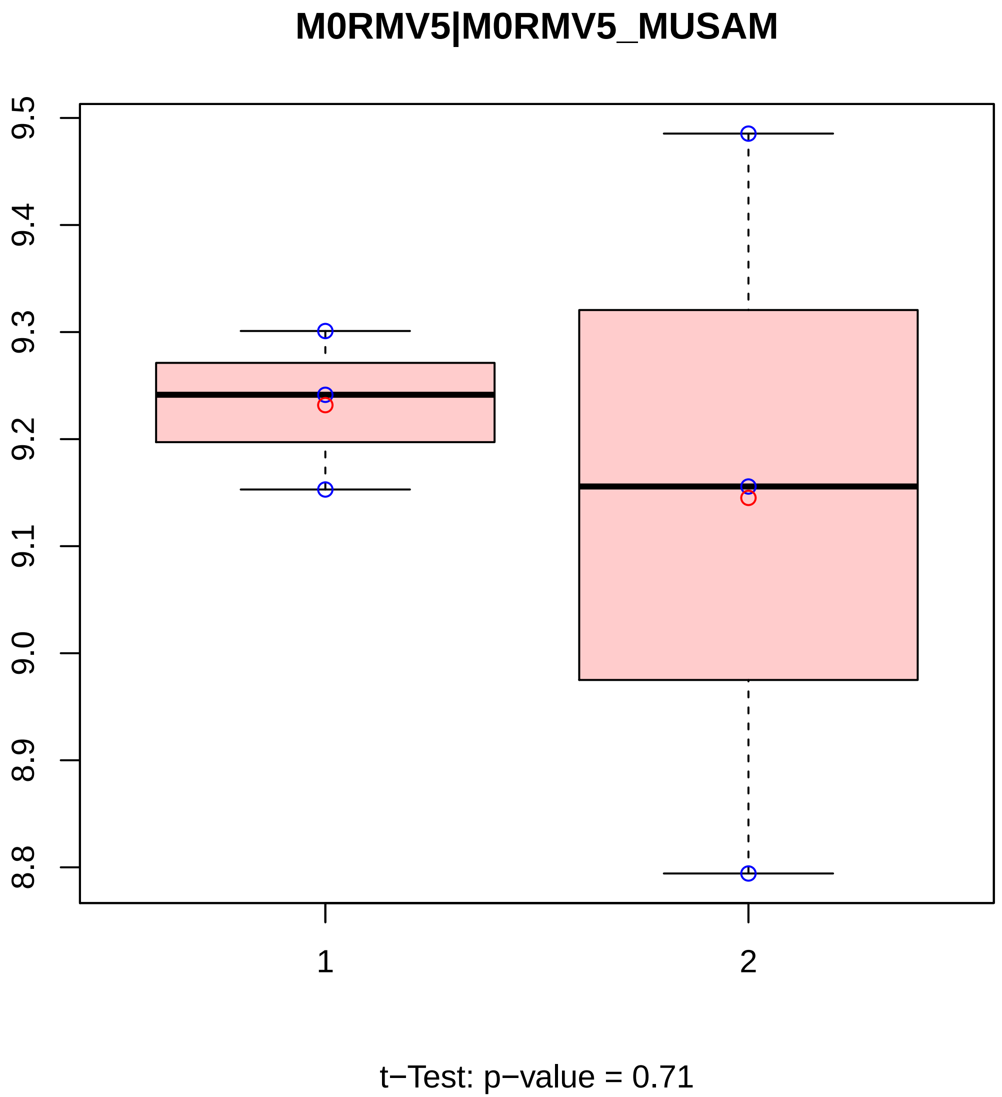
M0RMV5|M0RMV5_MUSAM
9.5
9.4
9.3
9.2
9.1
9.0
8.9
8.8
1
2
t−Test: p−value = 0.71

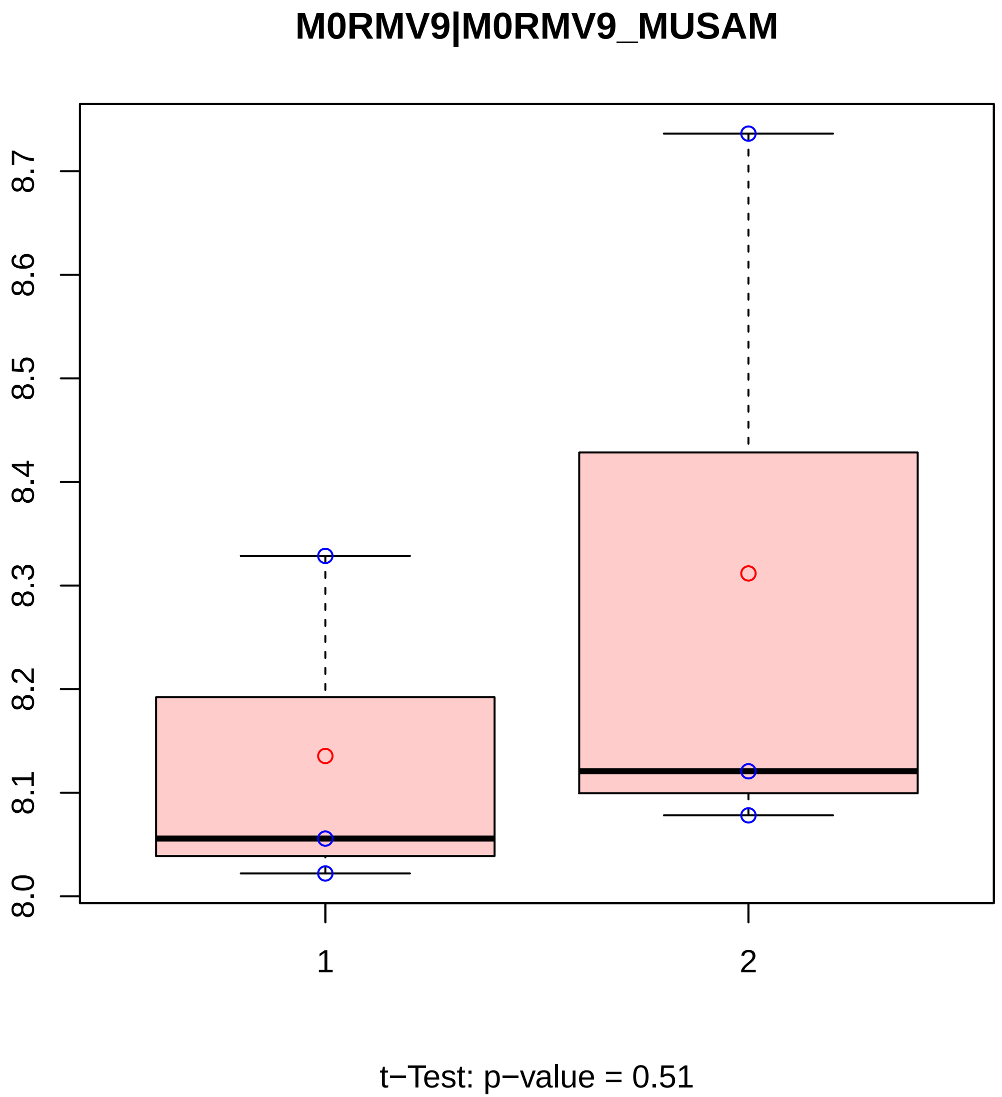
M0RMV9|M0RMV9_MUSAM
8.0
8.1
8.2
8.3
8.4
8.5
8.6
8.7
1
2
t−Test: p−value = 0.51

M0RN55|M0RN55_MUSAM

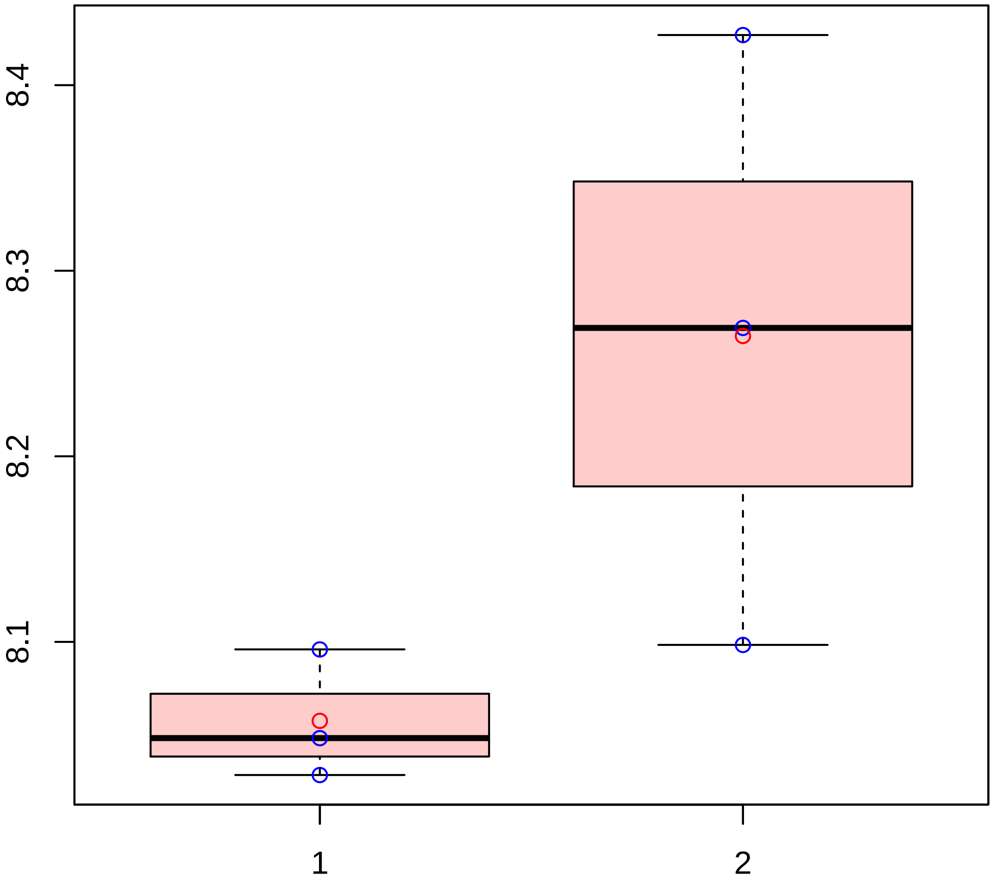

t−Test: p−value = 0.16

M0RNE8|M0RNE8_MUSAM

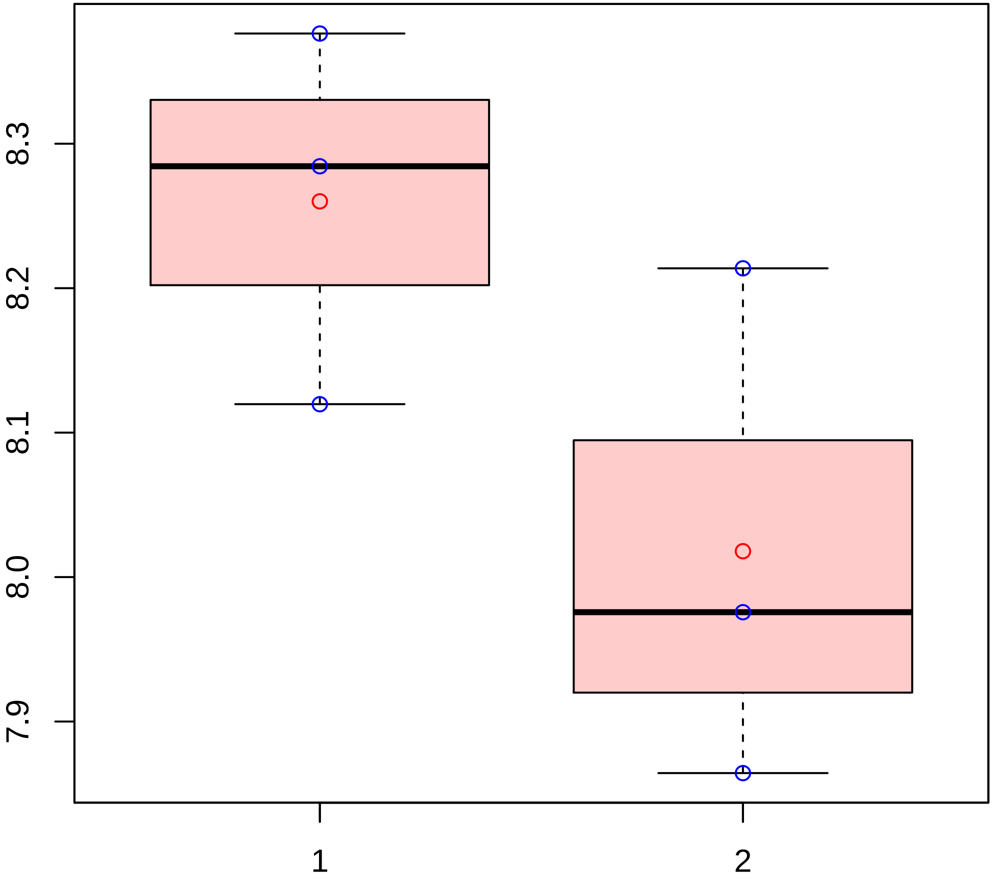

t−Test: p−value = 0.14

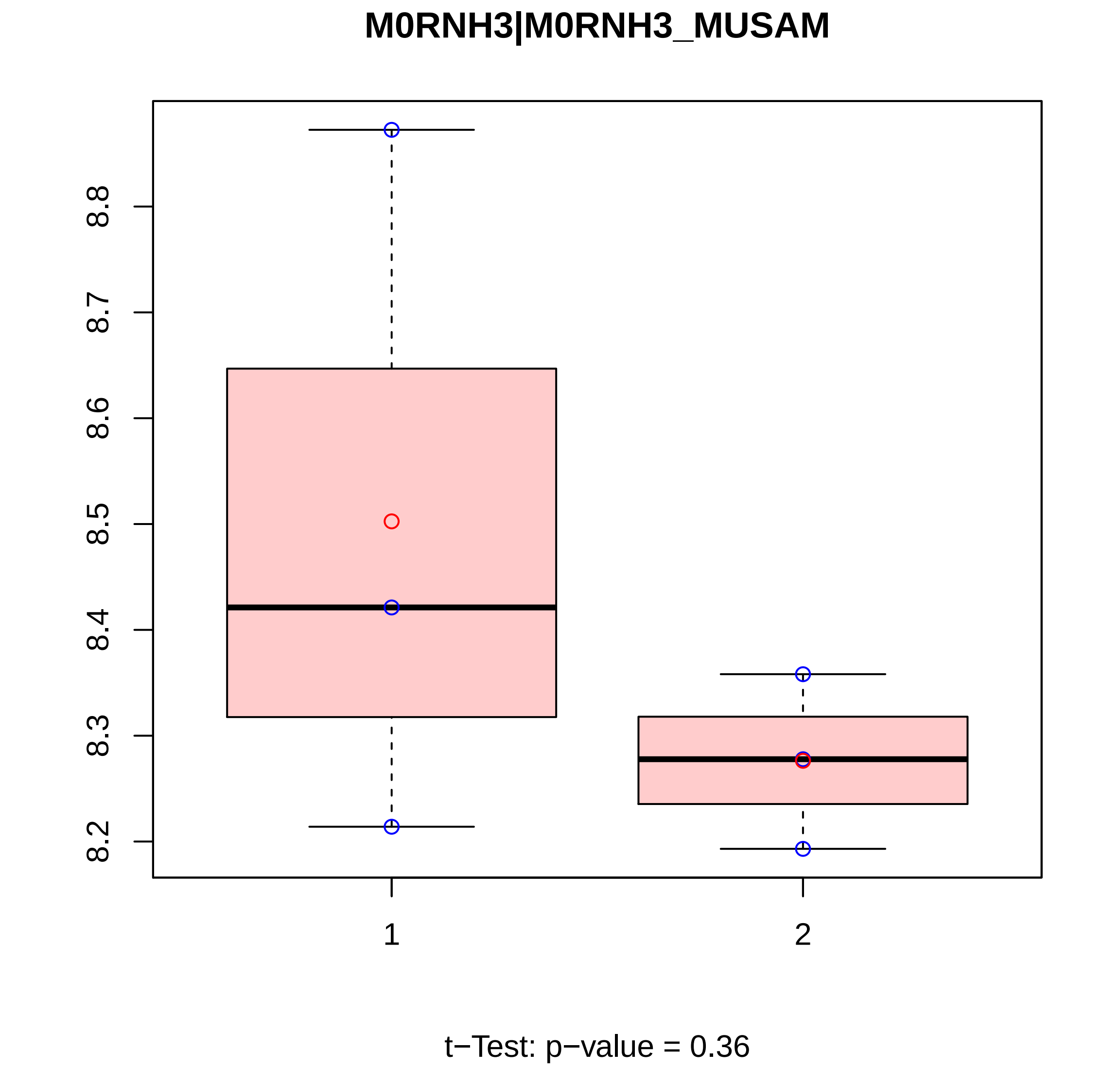
M0RNH3|M0RNH3_MUSAM
8.2
8.3
8.4
8.5
8.6
8.7
8.8
1
2
t−Test: p−value = 0.36

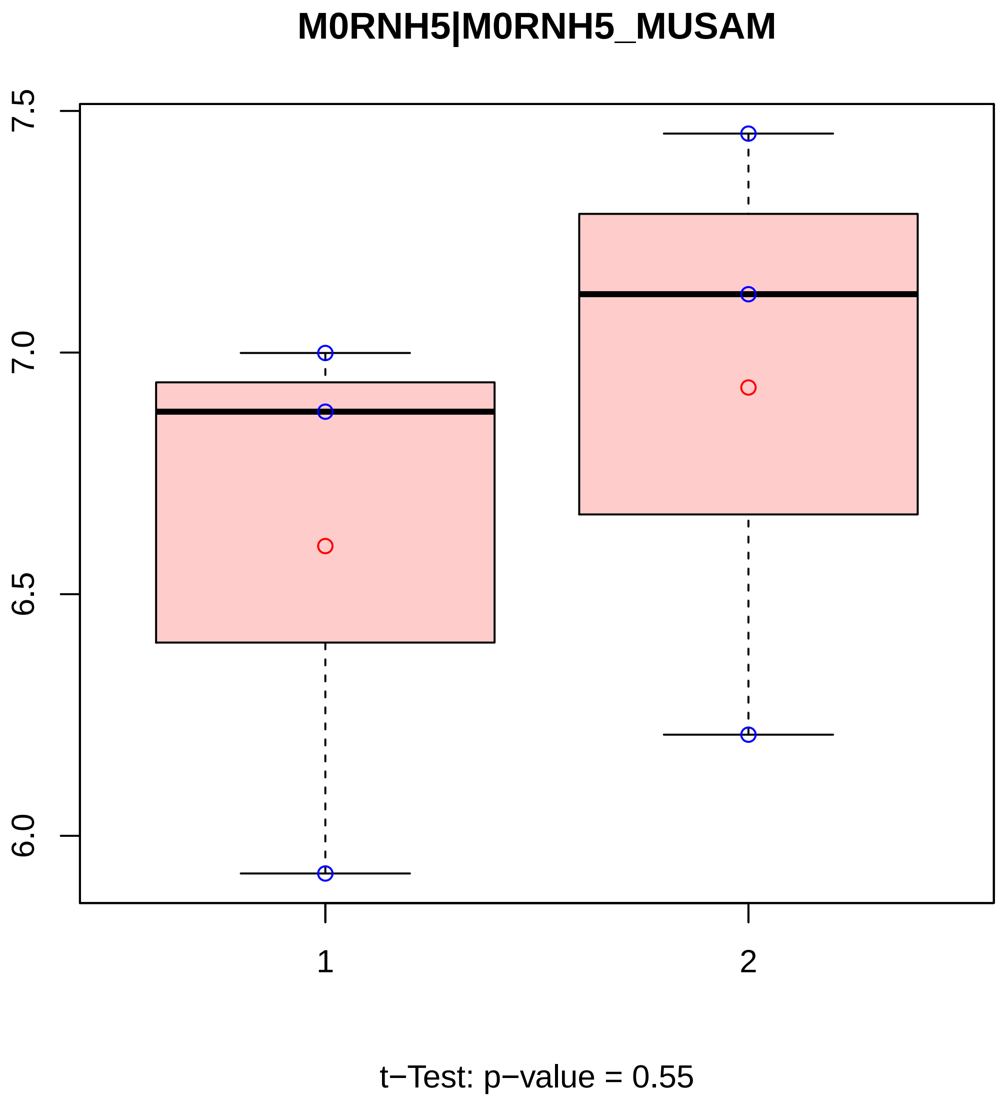
M0RNH5|M0RNH5_MUSAM
7.5
7.0
6.5
6.0
1
2
t−Test: p−value = 0.55

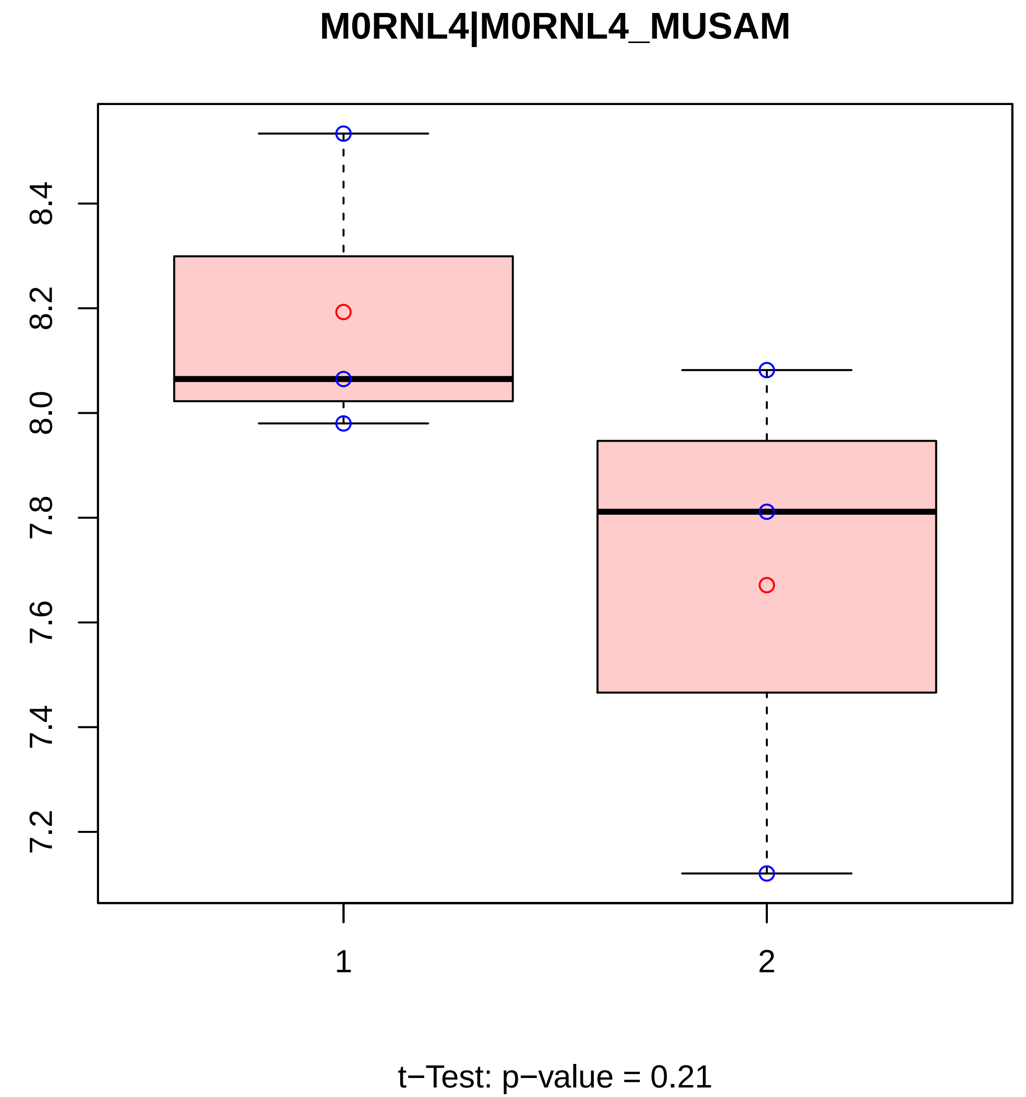
M0RNL4|M0RNL4_MUSAM
8.4
8.2
8.0
7.8
7.6
7.4
7.2
1
2
t−Test: p−value = 0.21

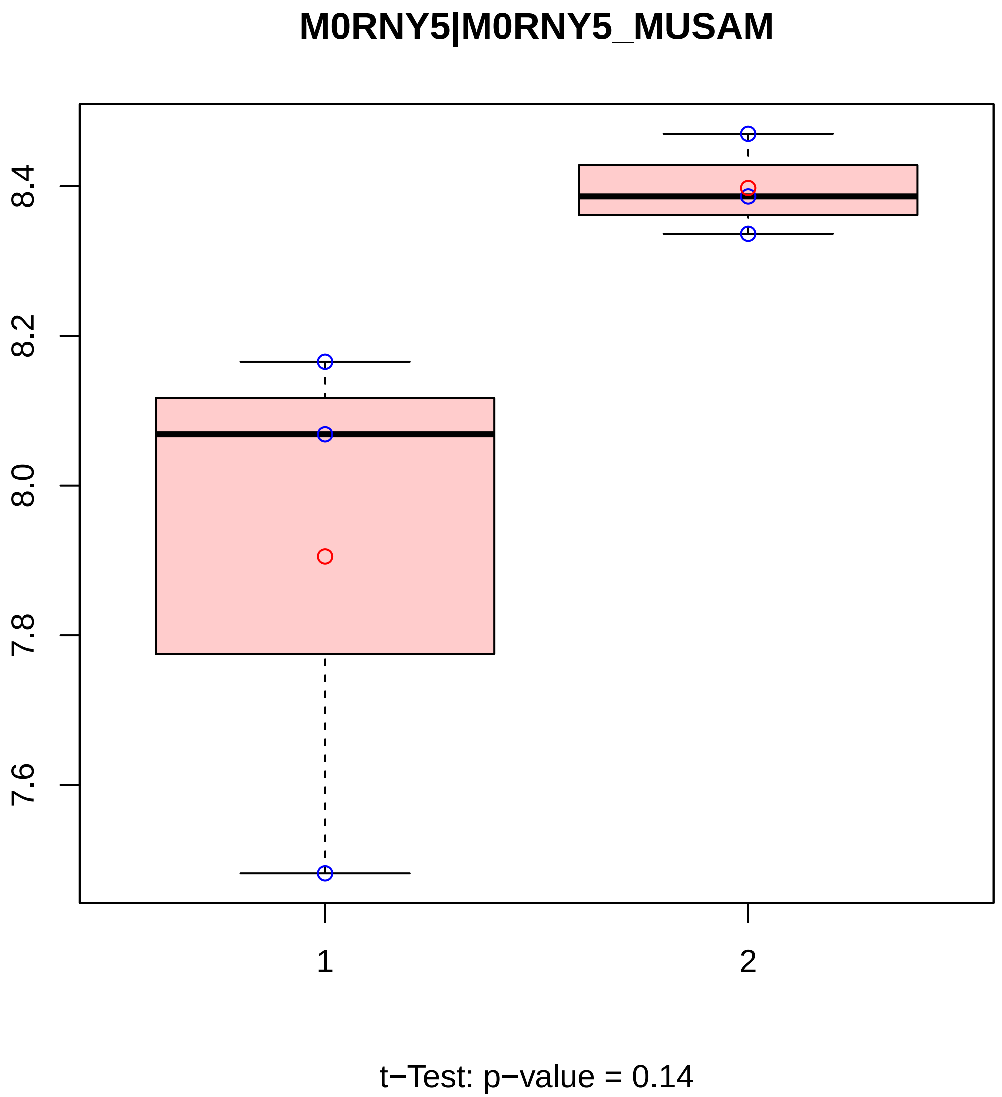

M0RNY5|M0RNY5_MUSAM
8.4
8.2
8.0
7.8
7.6
1
2
t−Test: p−value = 0.14

M0RPG0|M0RPG0_MUSAM

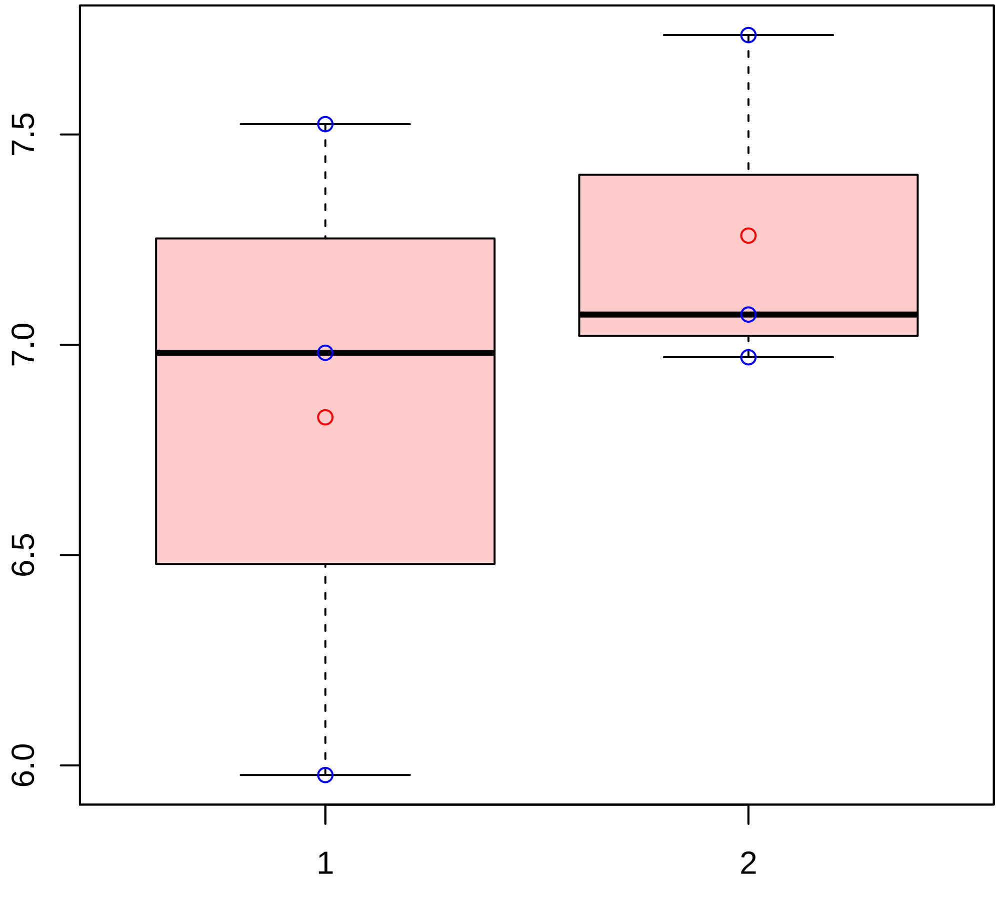

t−Test: p−value = 0.46

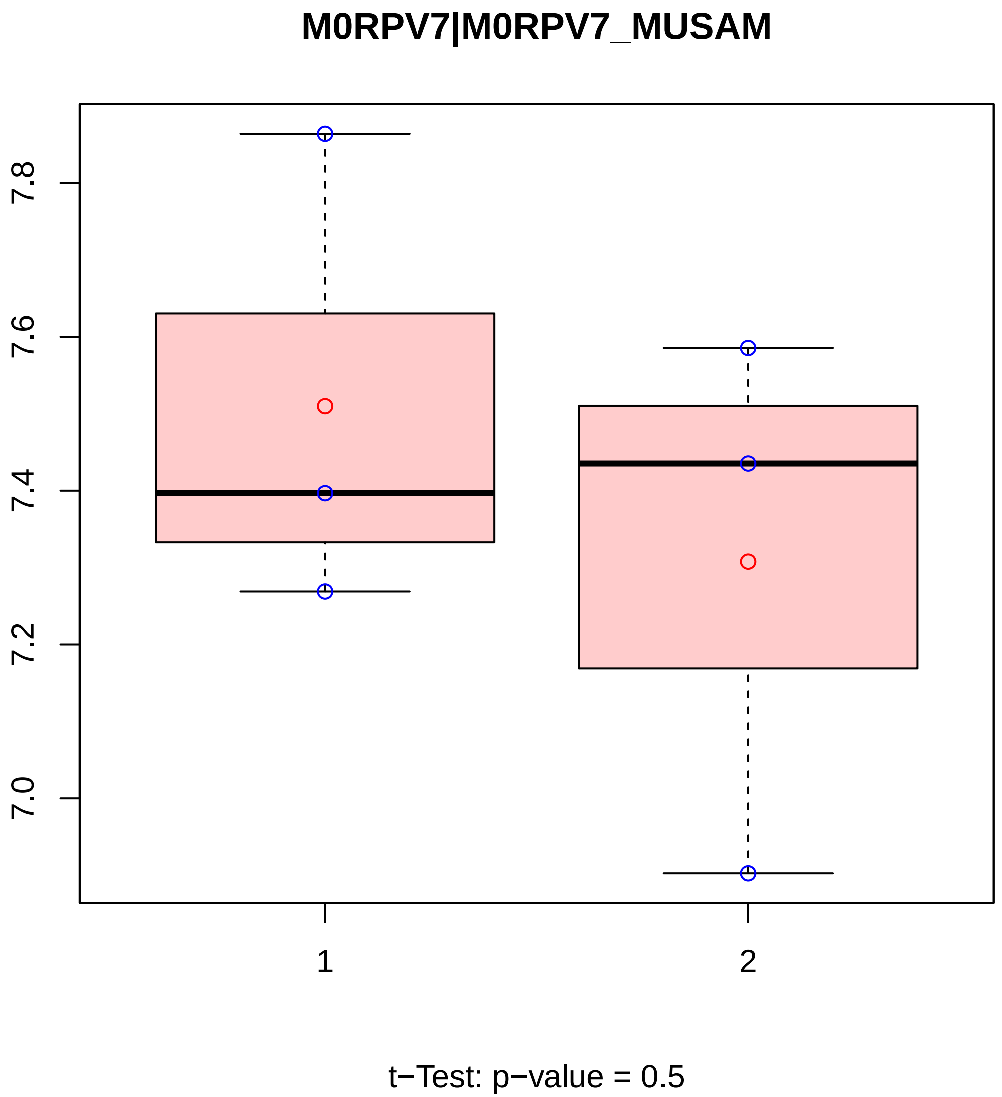
M0RPV7|M0RPV7_MUSAM
7.0
7.2
7.4
7.6
7.8
1
2
t−Test: p−value = 0.5

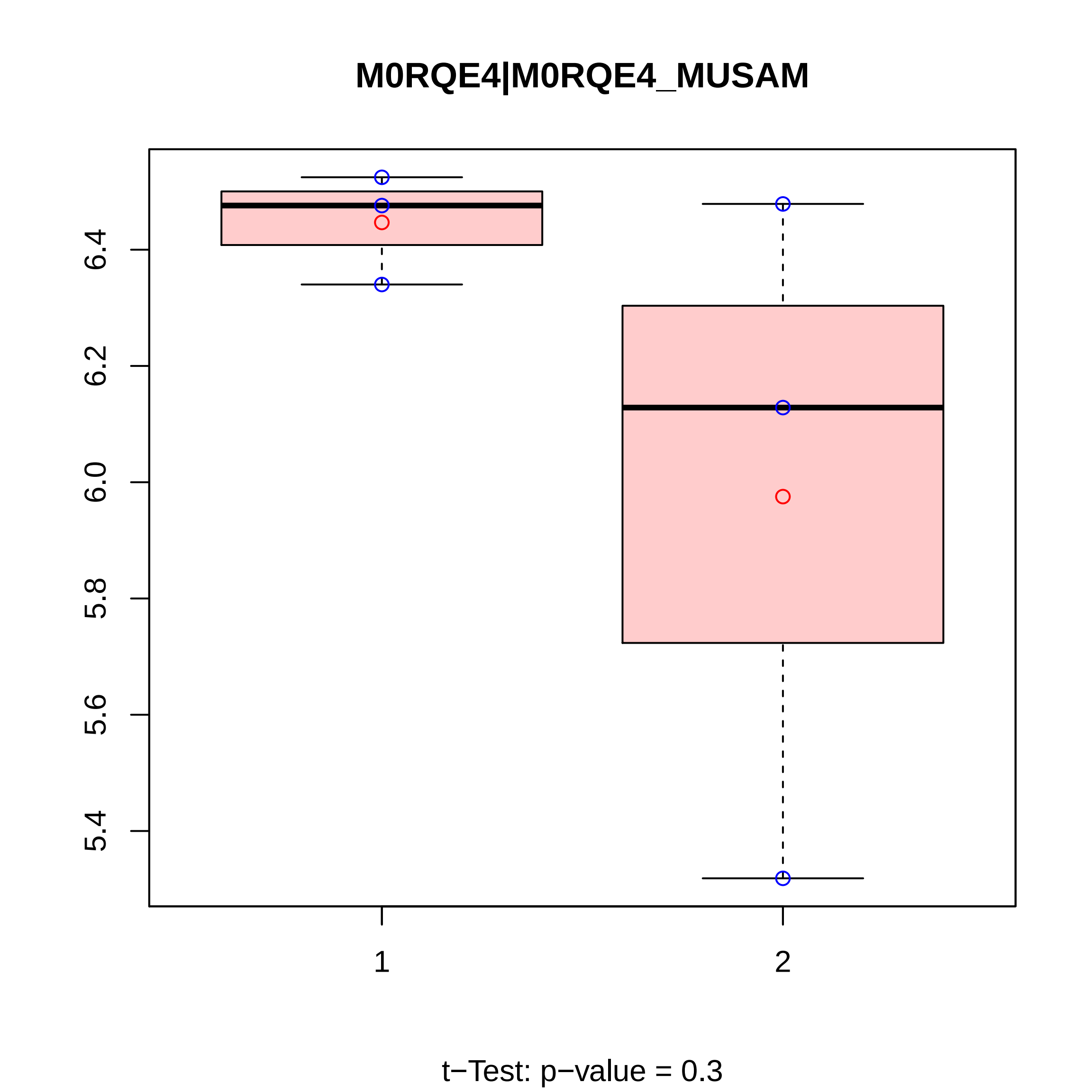
M0RQE4|M0RQE4_MUSAM
6.4
6.2
6.0
5.8
5.6
5.4
1
2
t−Test: p−value = 0.3

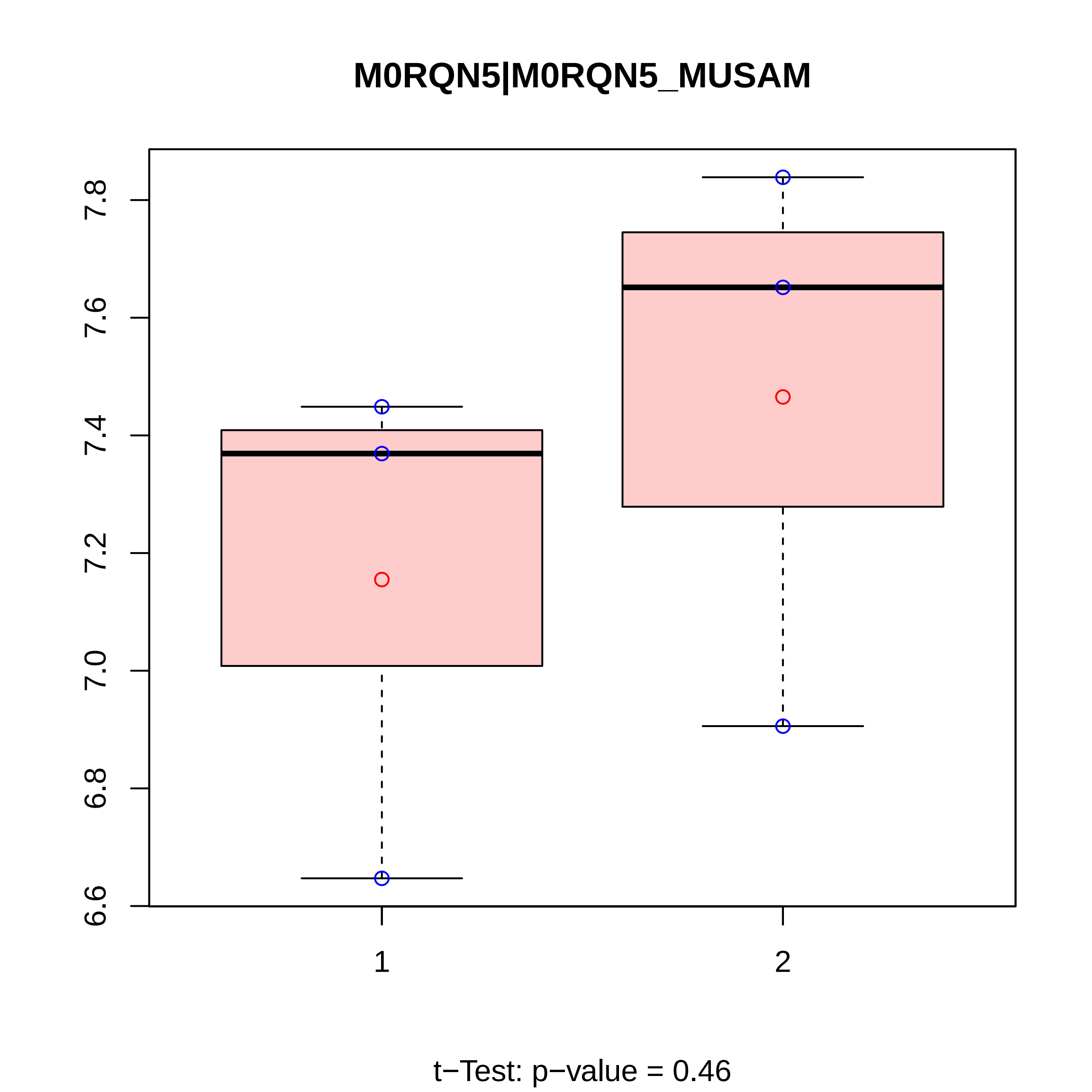
M0RQN5|M0RQN5_MUSAM
7.8
7.6
7.4
7.2
7.0
6.8
6.6
1
2
t−Test: p−value = 0.46

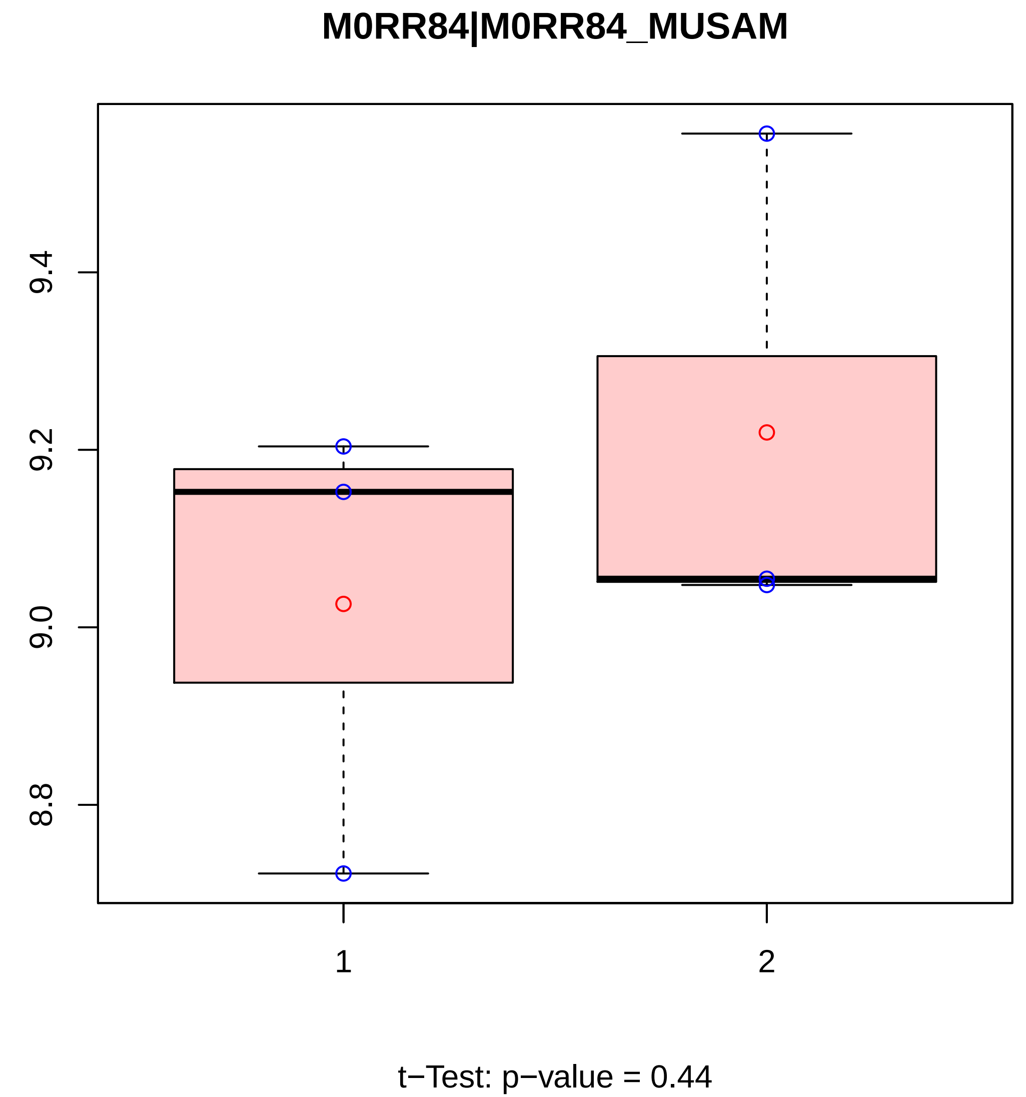

M0RR84|M0RR84_MUSAM
9.4
9.2
9.0
8.8
1
2
t−Test: p−value = 0.44

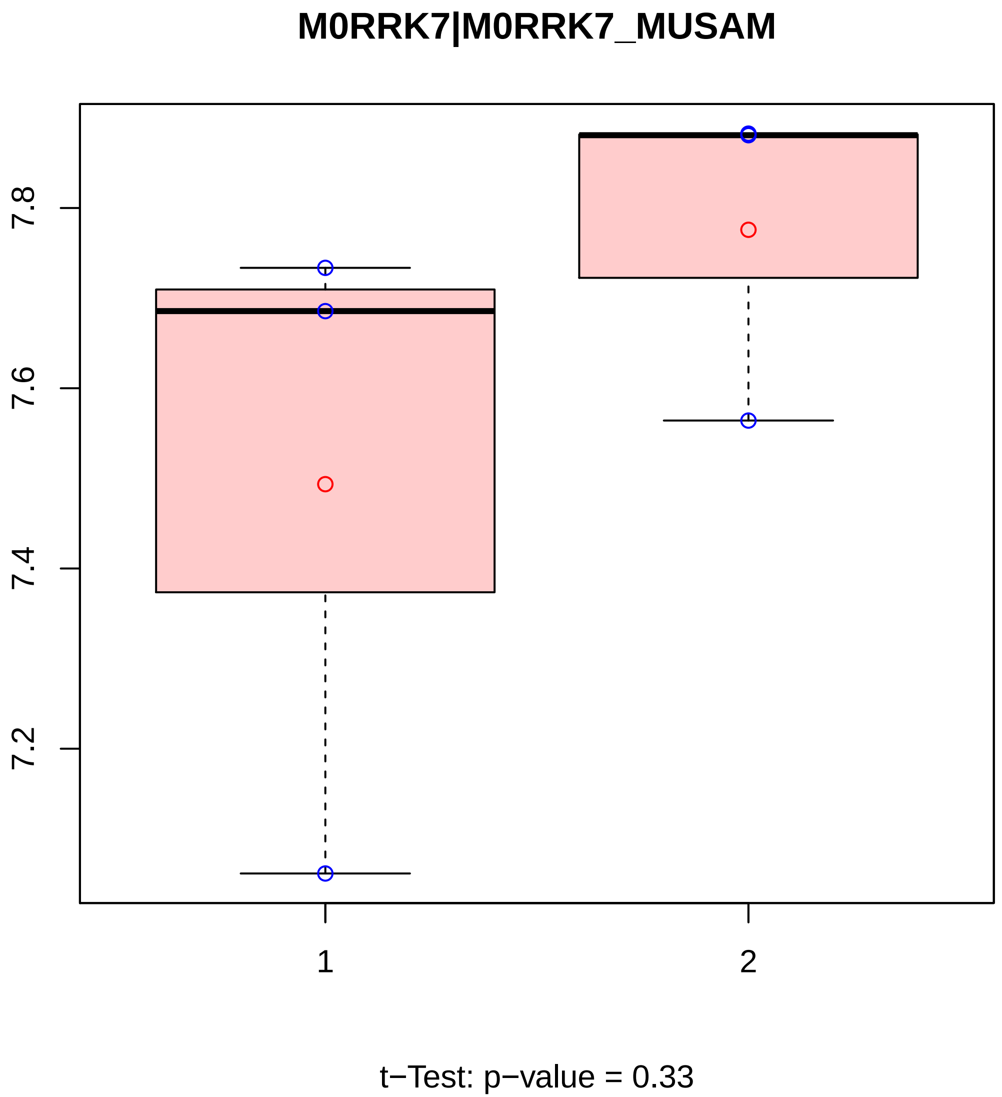
M0RRK7|M0RRK7_MUSAM
7.2
7.4
7.6
7.8
1
2
t−Test: p−value = 0.33

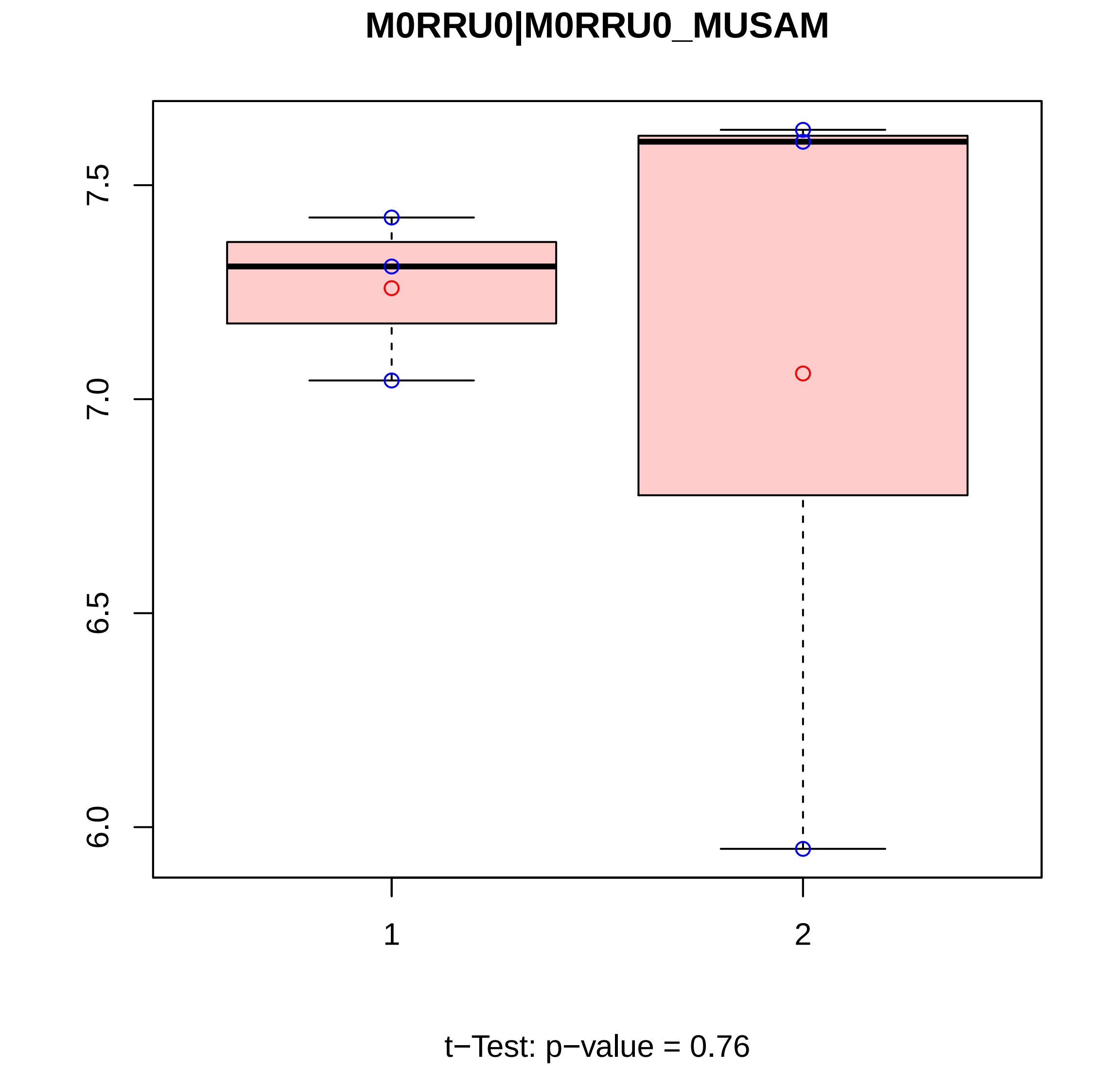
M0RRU0|M0RRU0_MUSAM
7.5
7.0
6.5
6.0
1
2
t−Test: p−value = 0.76

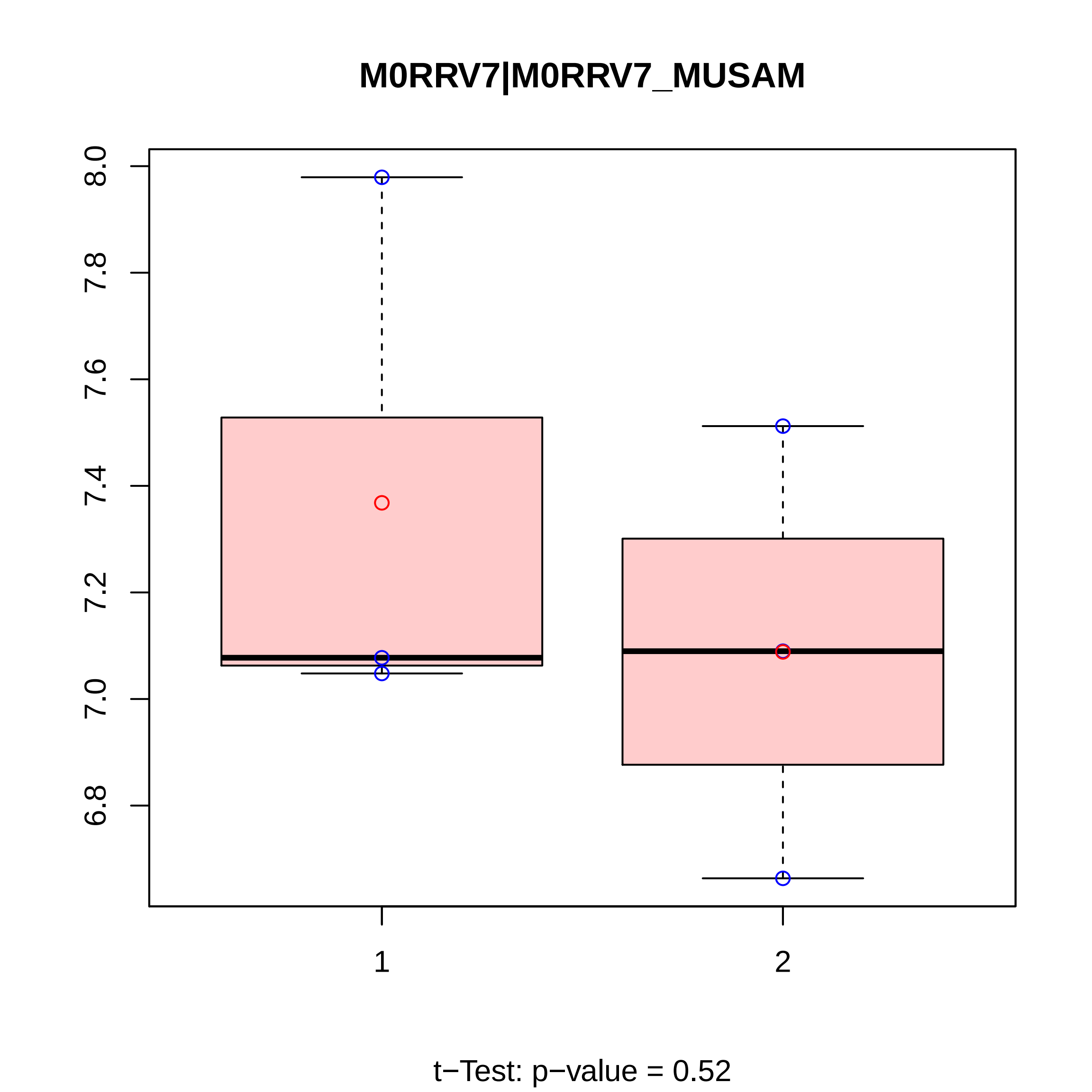

M0RRV7|M0RRV7_MUSAM
8.0
7.8
7.6
7.4
7.2
7.0
6.8
1
2
t−Test: p−value = 0.52

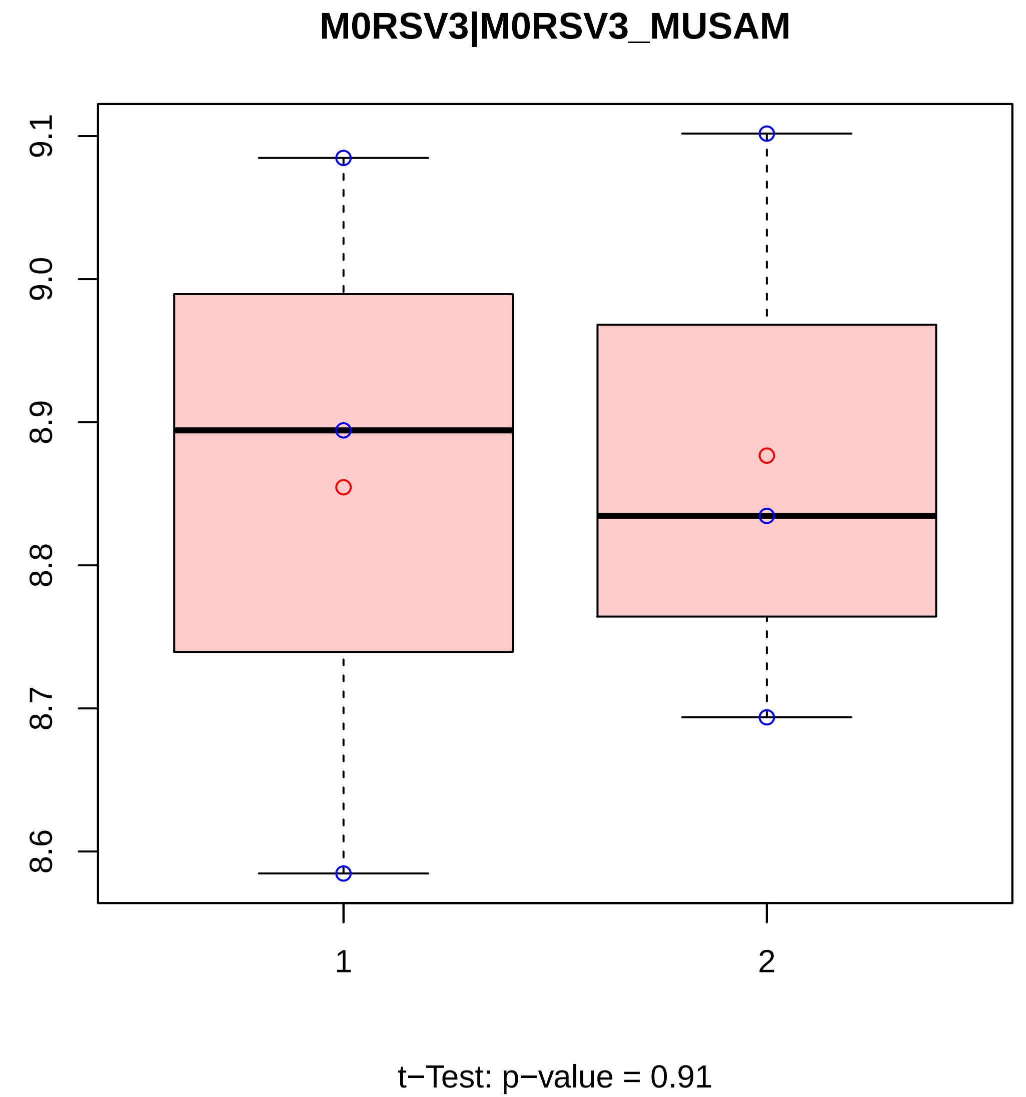
M0RSV3|M0RSV3_MUSAM
9.1
9.0
8.9
8.8
8.7
8.6
1
2
t−Test: p−value = 0.91

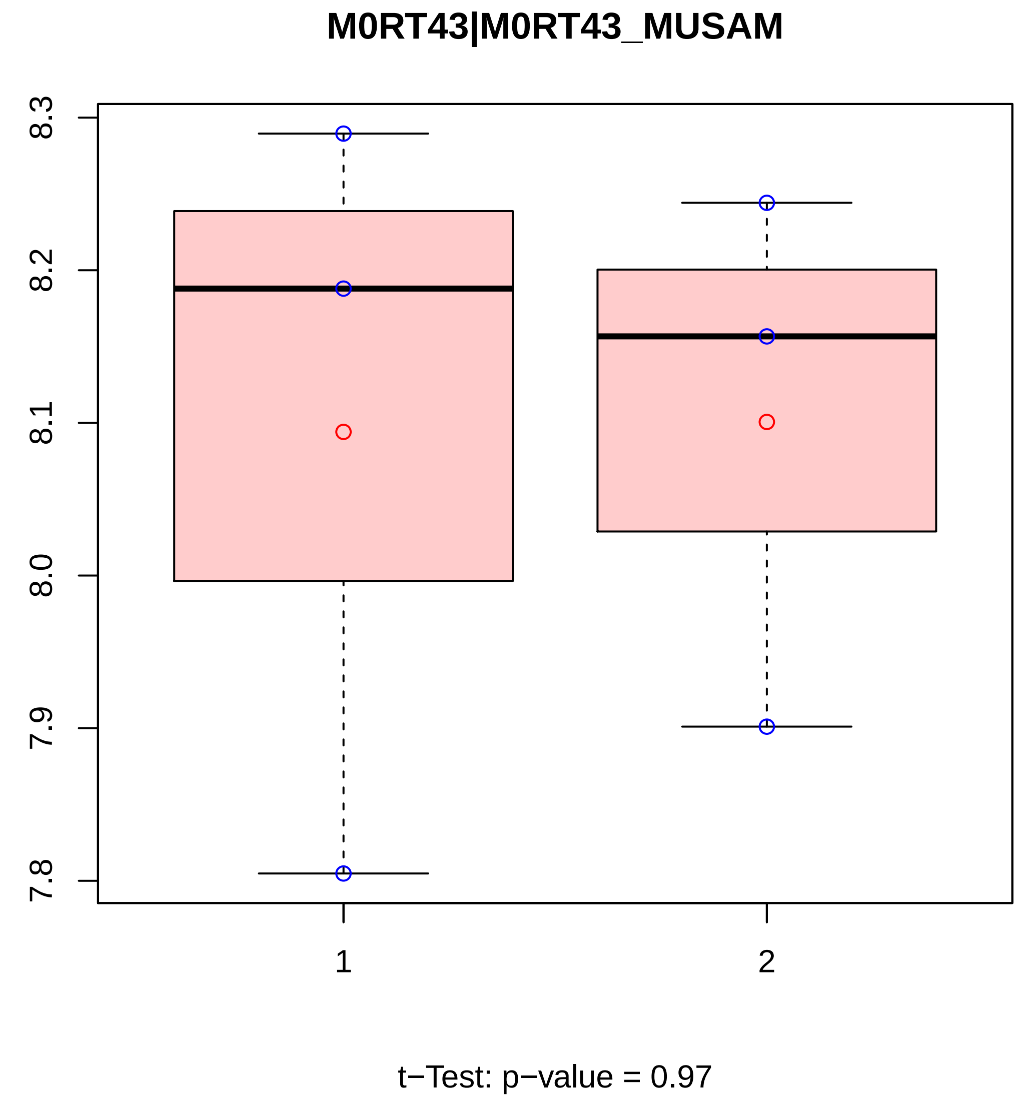
M0RT43|M0RT43_MUSAM
8.3
8.2
8.1
8.0
7.9
7.8
1
2
t−Test: p−value = 0.97

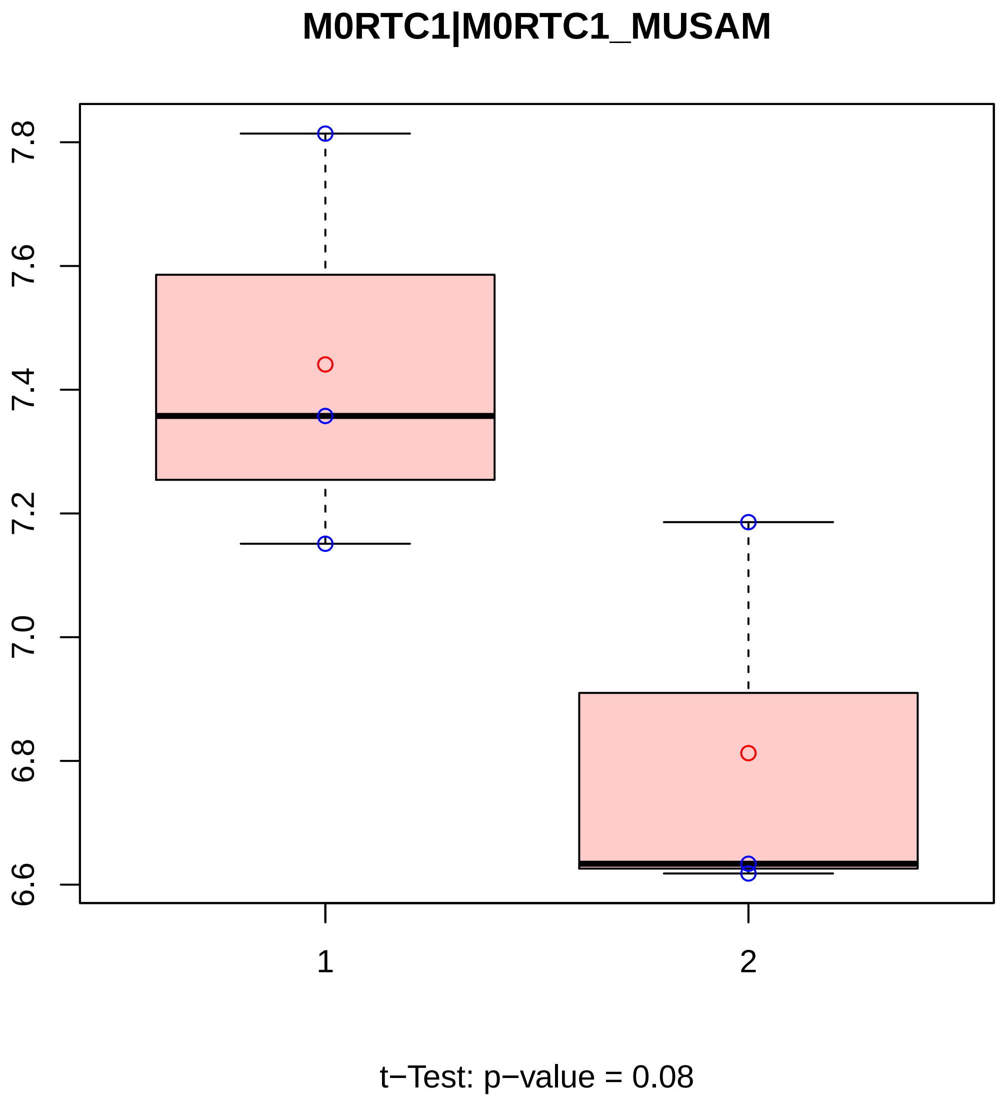
M0RTC1|M0RTC1_MUSAM
7.8
7.6
7.4
7.2
7.0
6.8
6.6
1
2
t−Test: p−value = 0.08

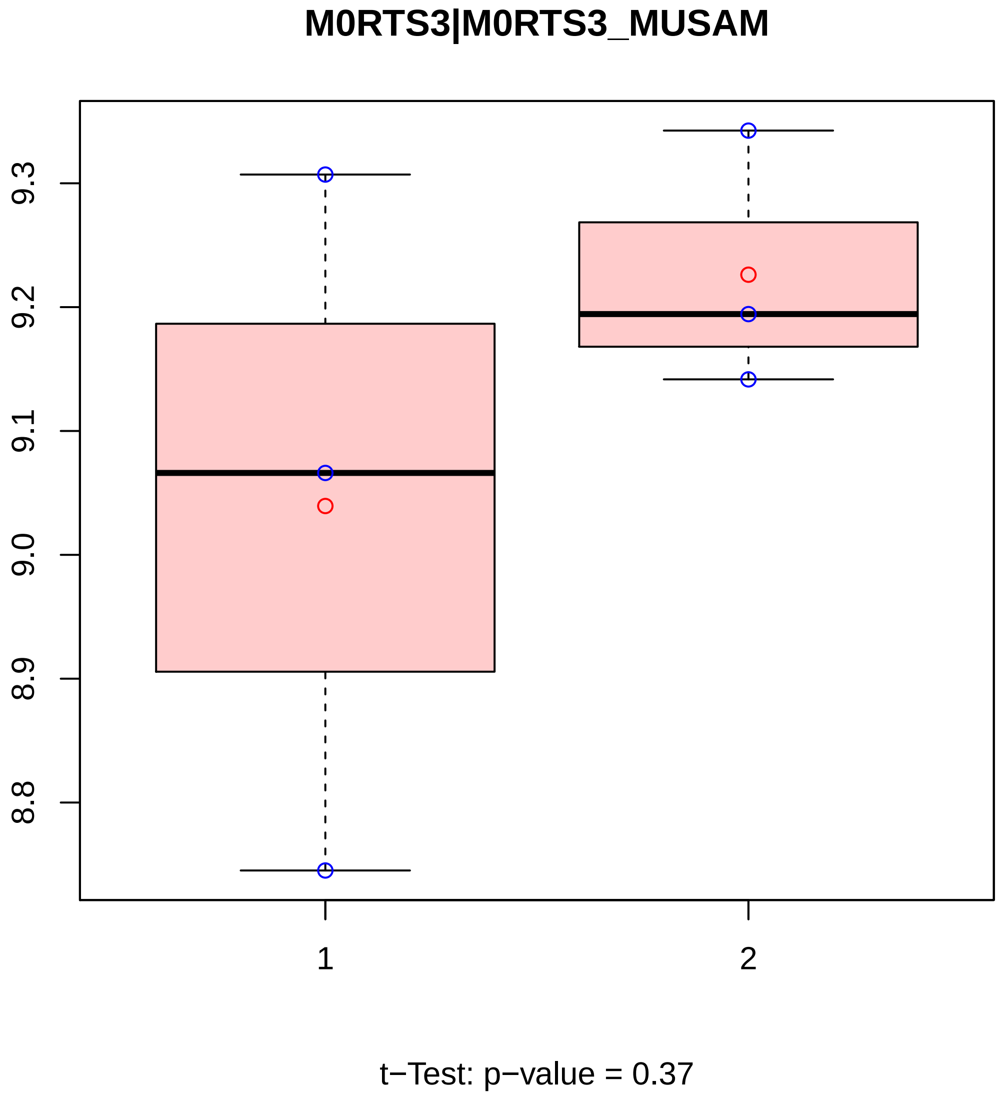

M0RTS3|M0RTS3_MUSAM
9.3
9.2
9.1
9.0
8.9
8.8
1
2
t−Test: p−value = 0.37

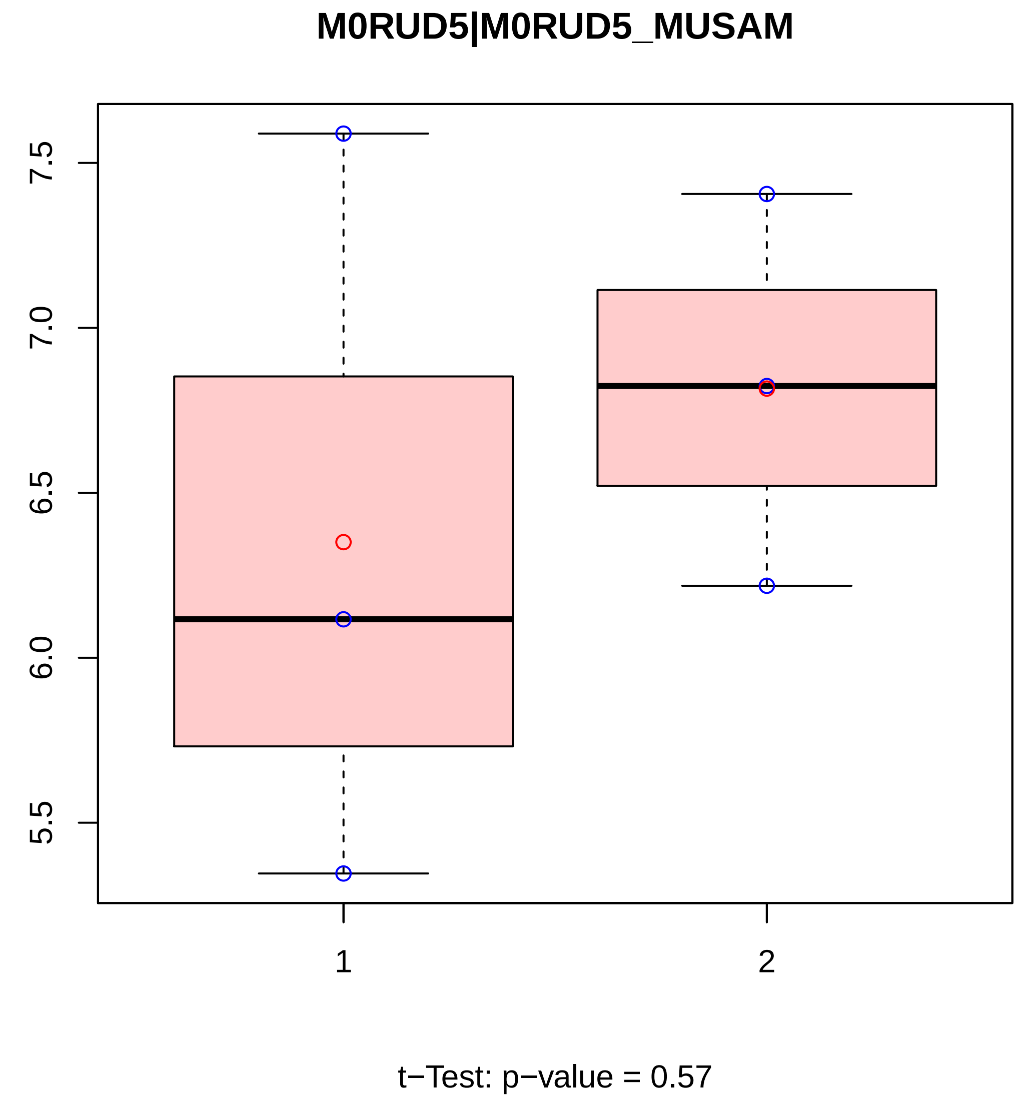
M0RUD5|M0RUD5_MUSAM
7.5
7.0
6.5
6.0
5.5
1
2
t−Test: p−value = 0.57

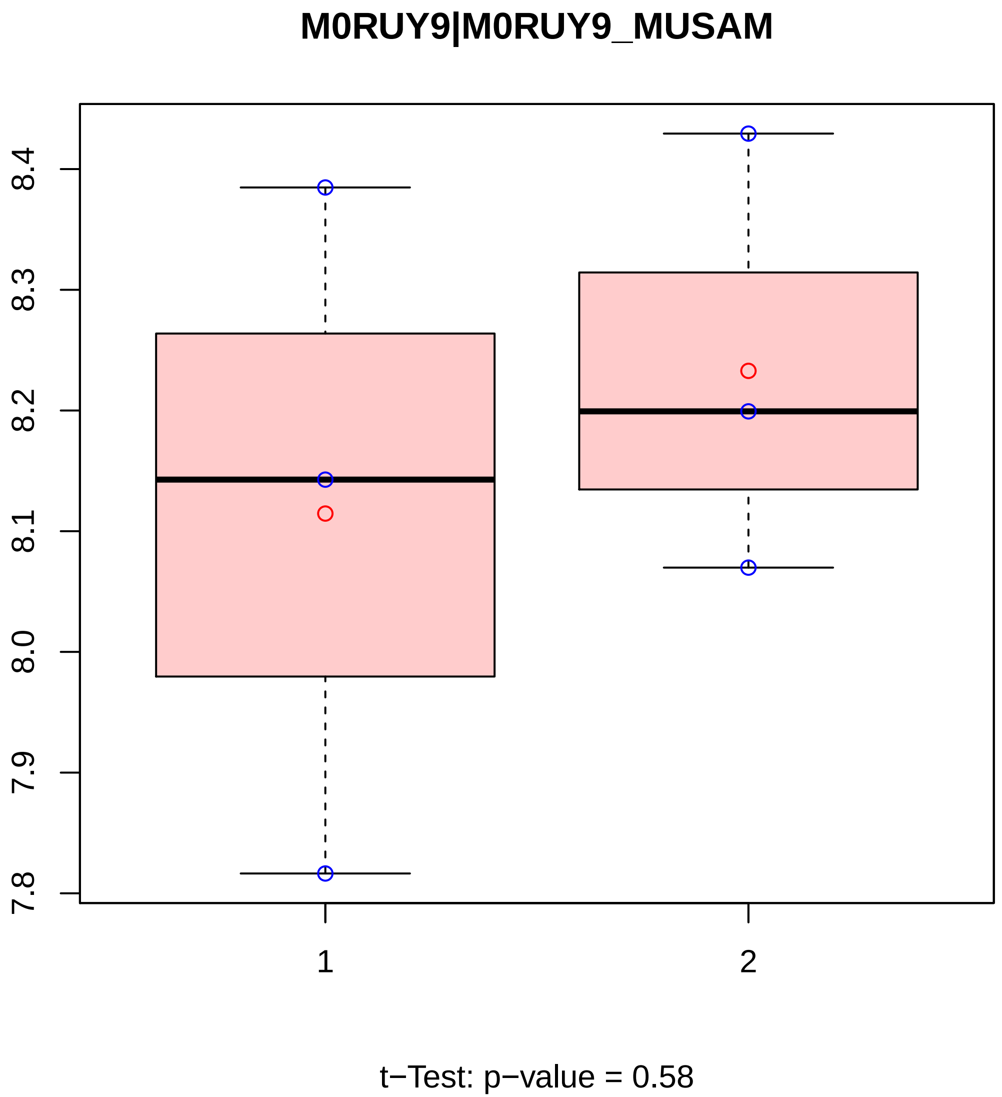
M0RUY9|M0RUY9_MUSAM
8.4
8.3
8.2
8.1
8.0
7.9
7.8
1
2
t−Test: p−value = 0.58

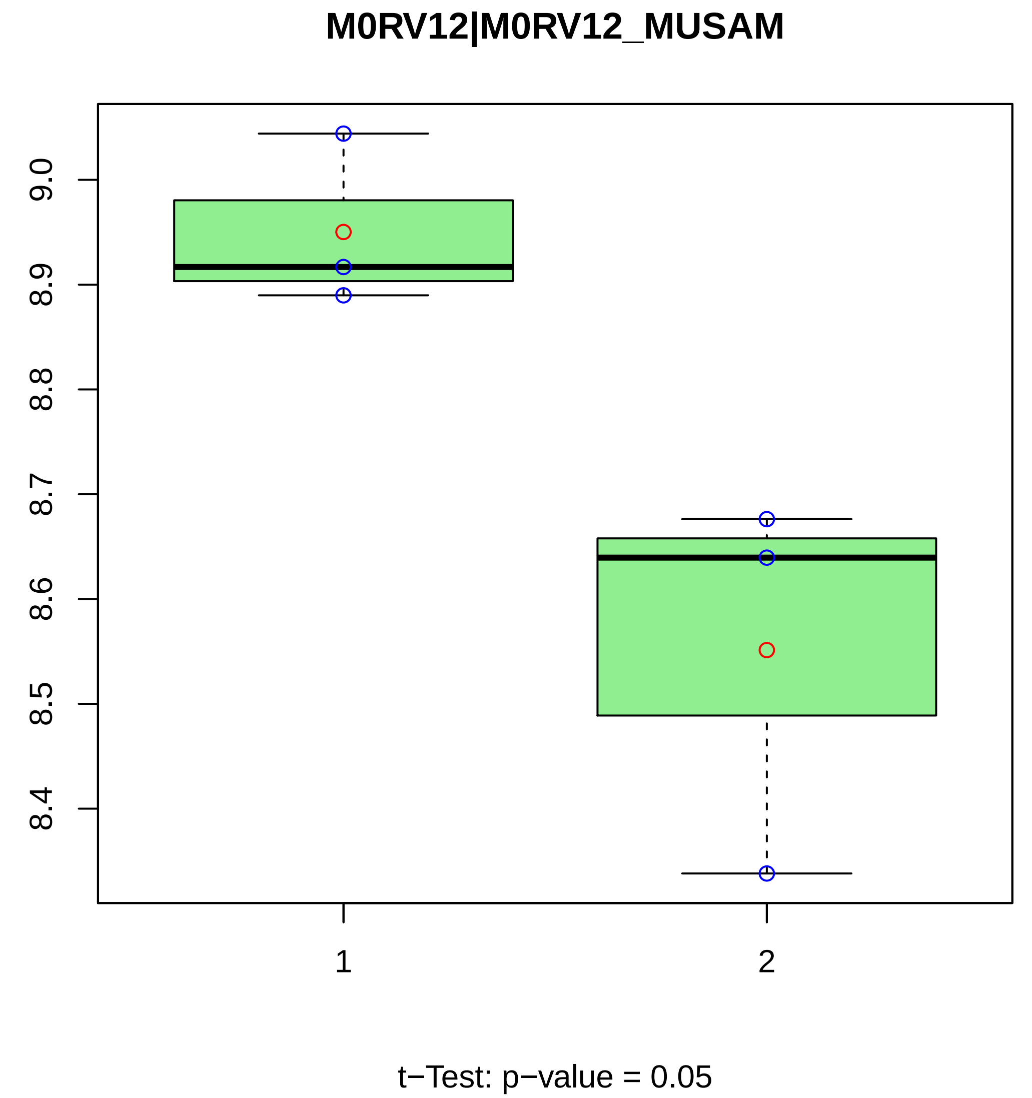
M0RV12|M0RV12_MUSAM
9.0
8.9
8.8
8.7
8.6
8.5
8.4
1
2
t−Test: p−value = 0.05

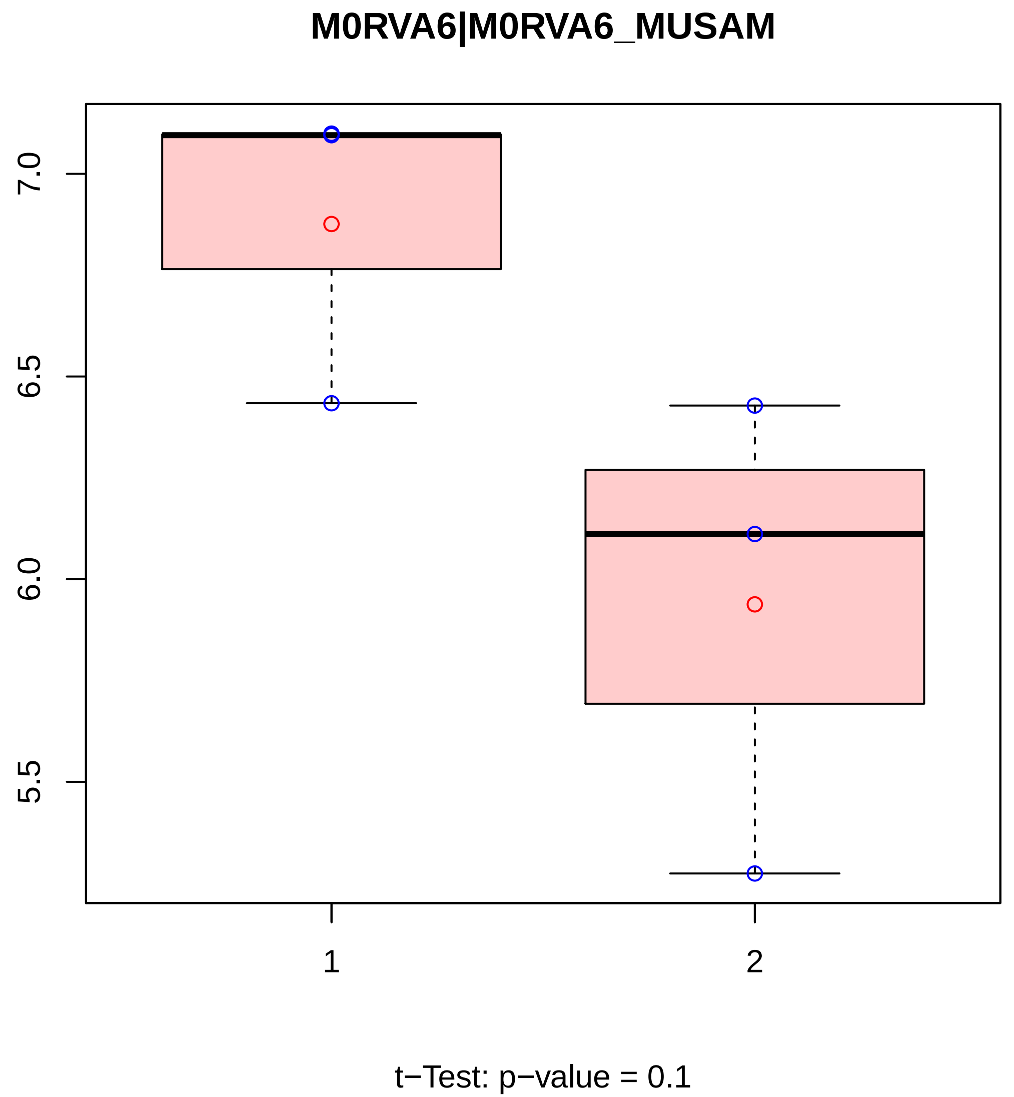

M0RVA6|M0RVA6_MUSAM
7.0
6.5
6.0
5.5
1
2
t−Test: p−value = 0.1

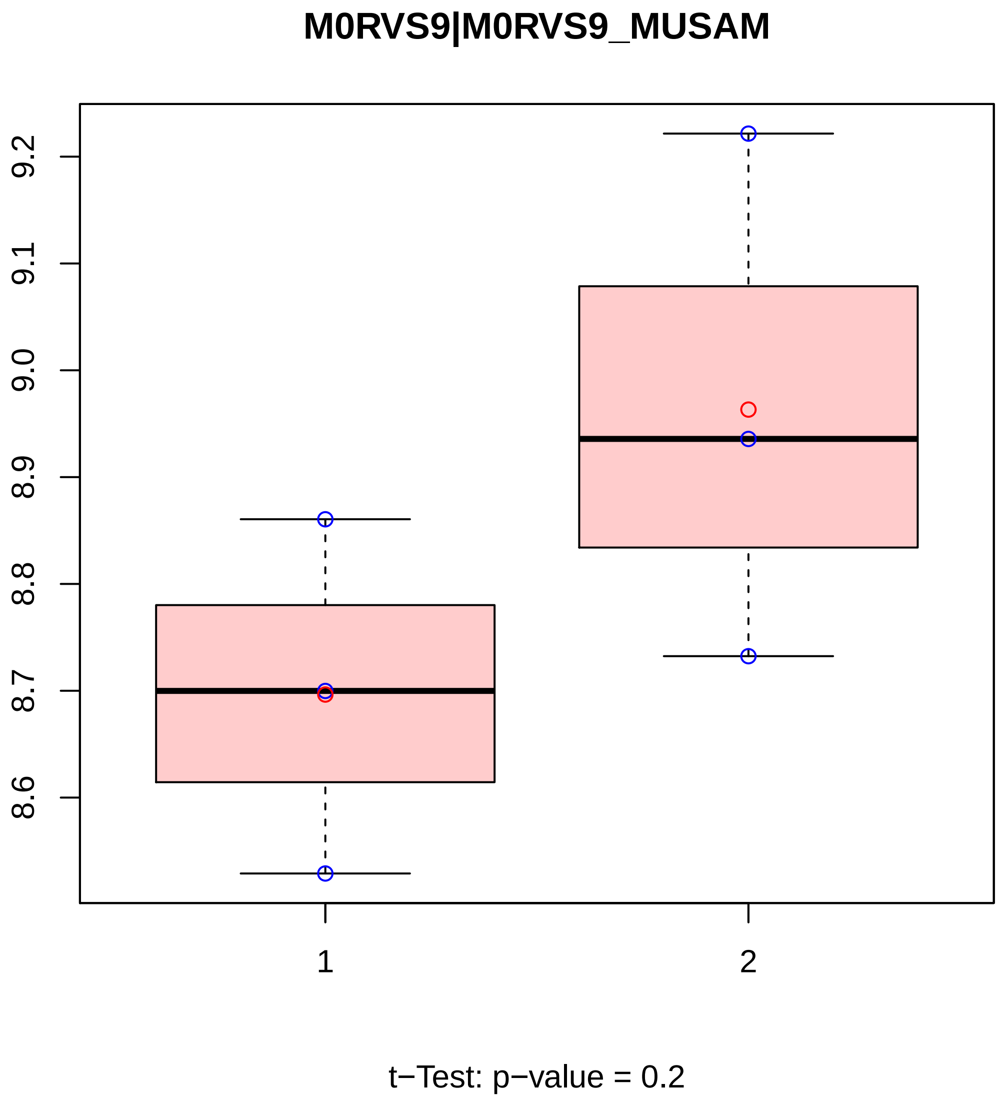
M0RVS9|M0RVS9_MUSAM
9.2
9.1
9.0
8.9
8.8
8.7
8.6
1
2
t−Test: p−value = 0.2

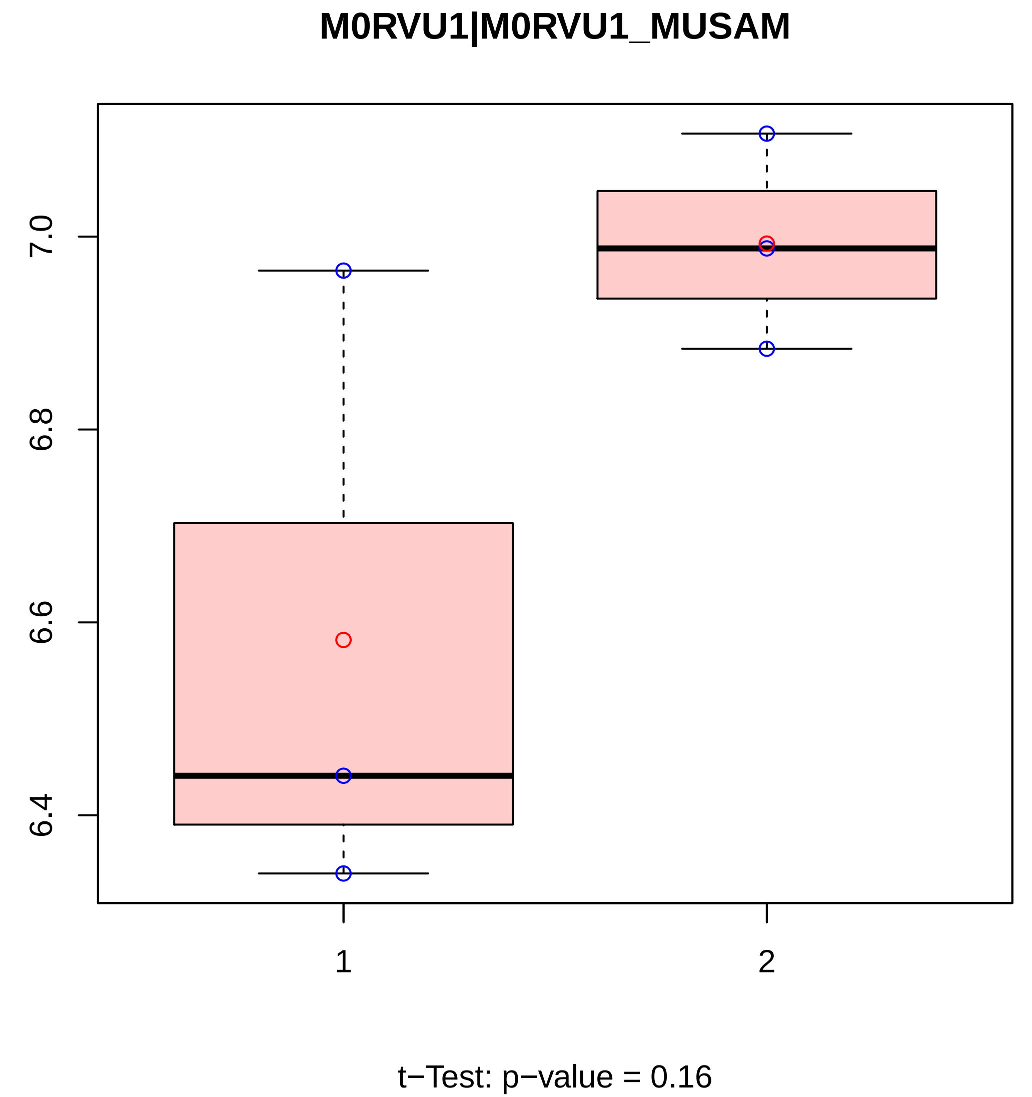
M0RVU1|M0RVU1_MUSAM
7.0
6.8
6.6
6.4
1
2
t−Test: p−value = 0.16

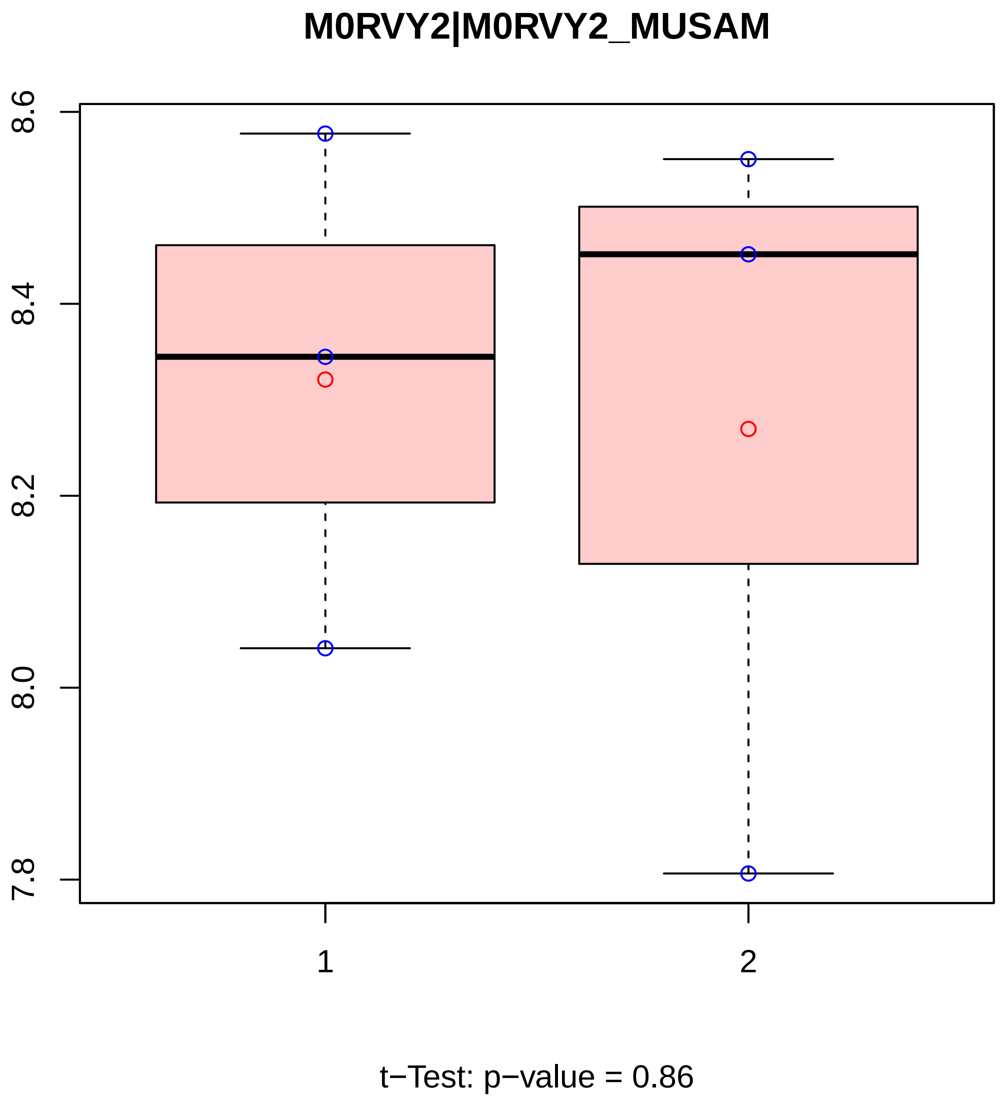
M0RVY2|M0RVY2_MUSAM
8.6
8.4
8.2
8.0
7.8
1
2
t−Test: p−value = 0.86

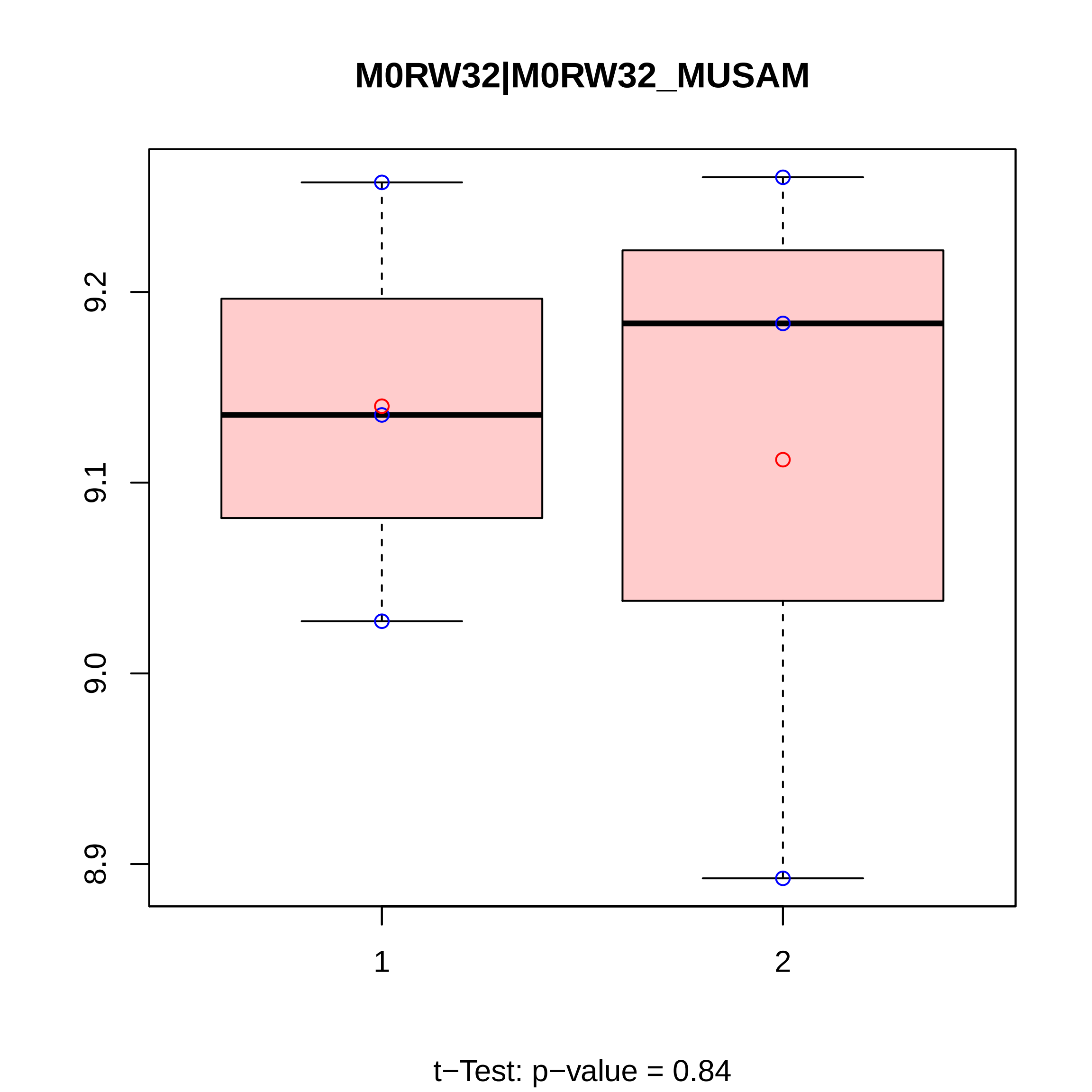
M0RW32|M0RW32_MUSAM
9.2
9.1
9.0
8.9
1
2
t−Test: p−value = 0.84

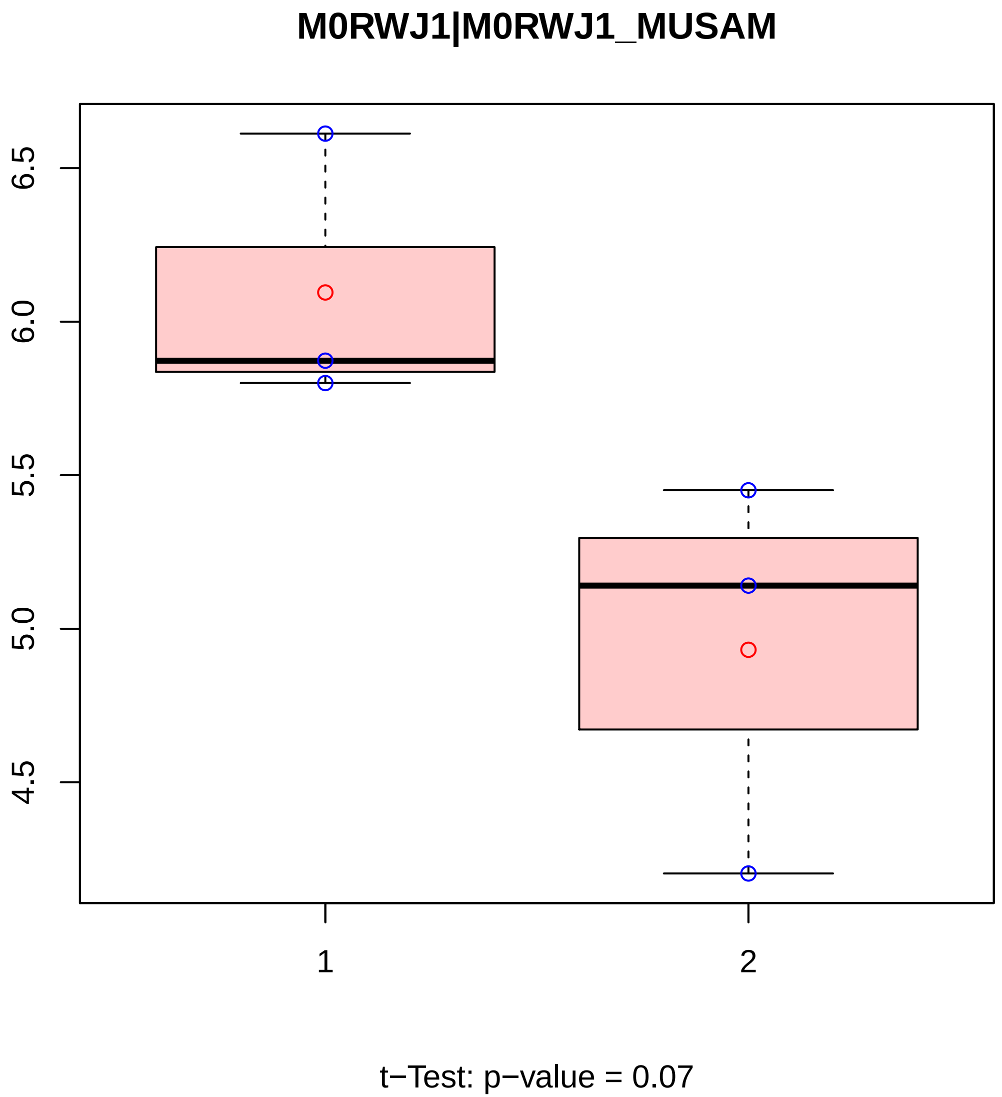

M0RWJ1|M0RWJ1_MUSAM
6.5
6.0
5.5
5.0
4.5
1
2
t−Test: p−value = 0.07

M0RWT6|M0RWT6_MUSAM

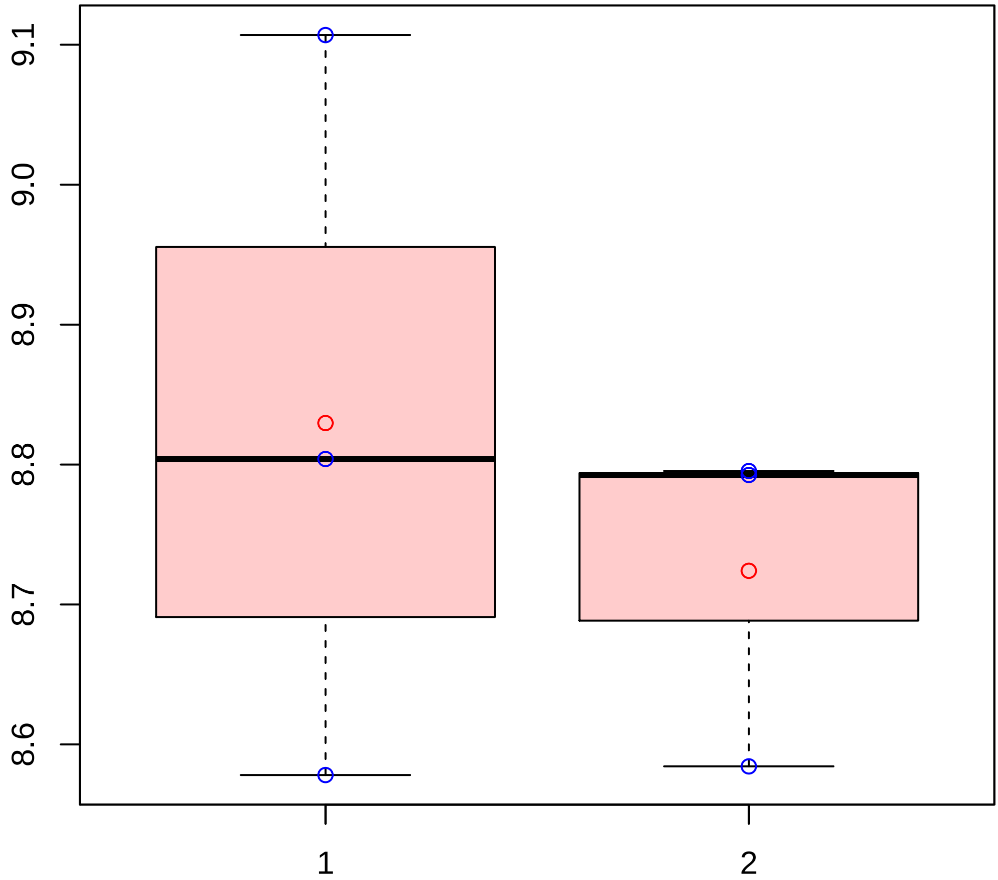

t−Test: p−value = 0.58

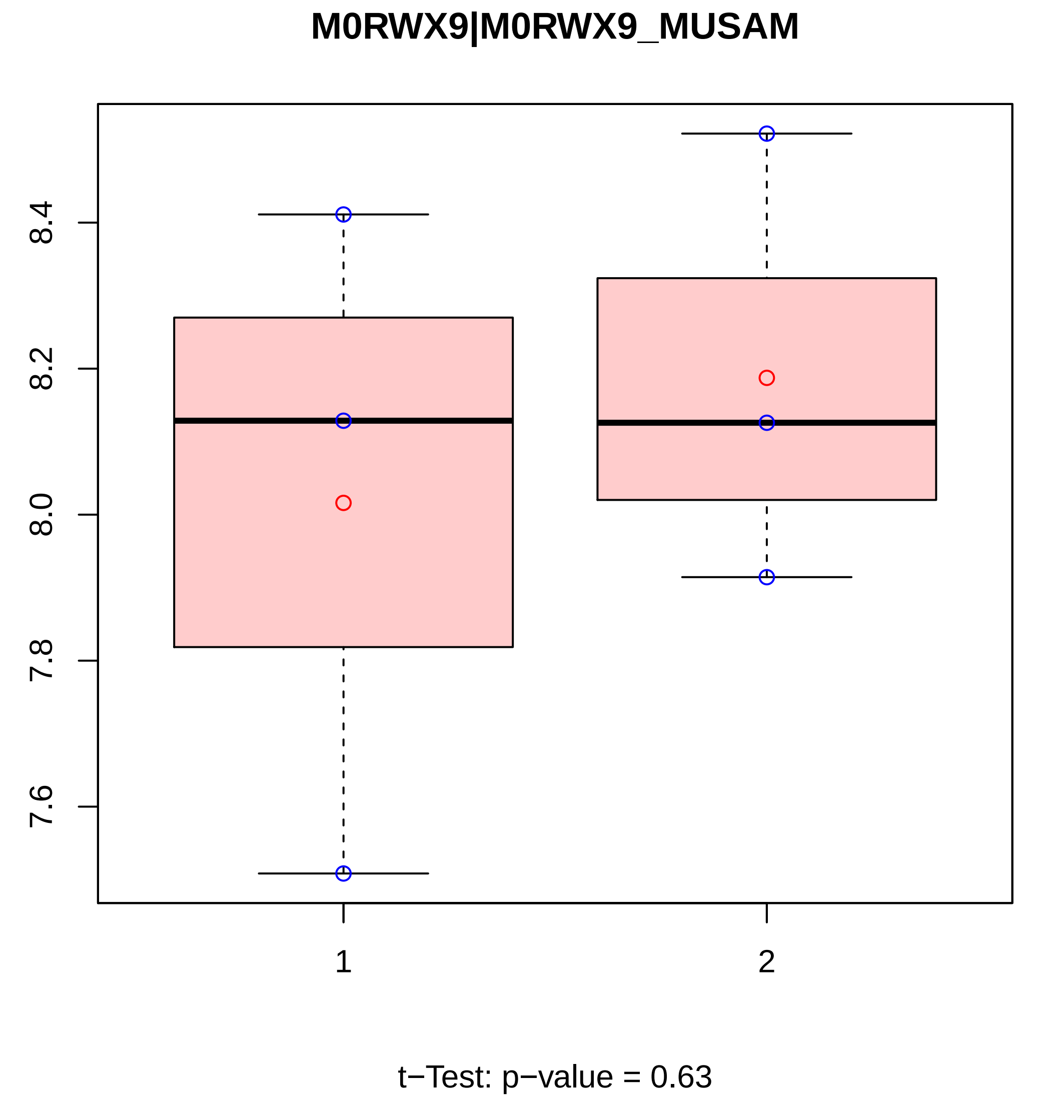
M0RWX9|M0RWX9_MUSAM
8.4
8.2
8.0
7.8
7.6
1
2
t−Test: p−value = 0.63

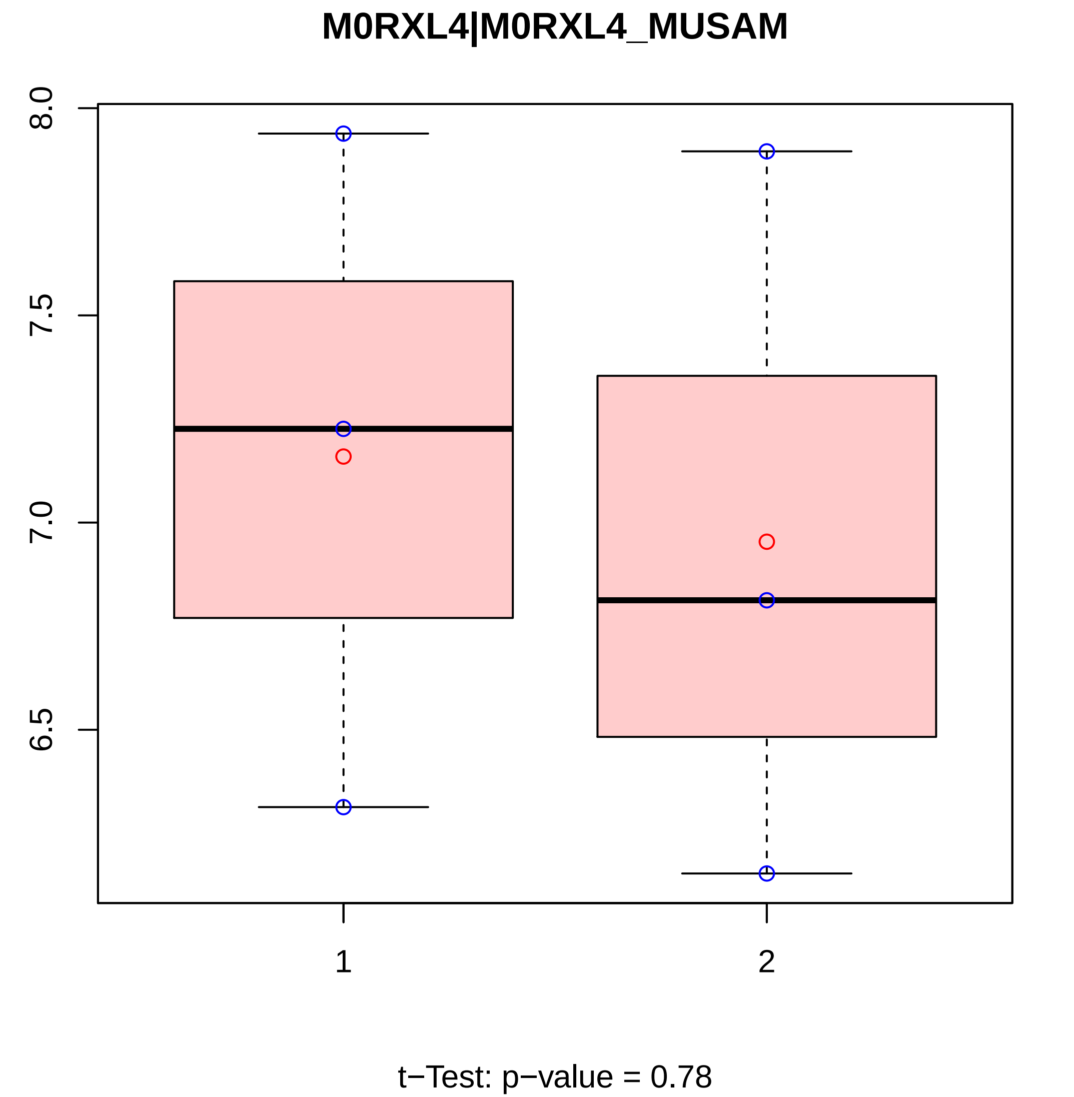
M0RXL4|M0RXL4_MUSAM
8.0
7.5
7.0
6.5
1
2
t−Test: p−value = 0.78

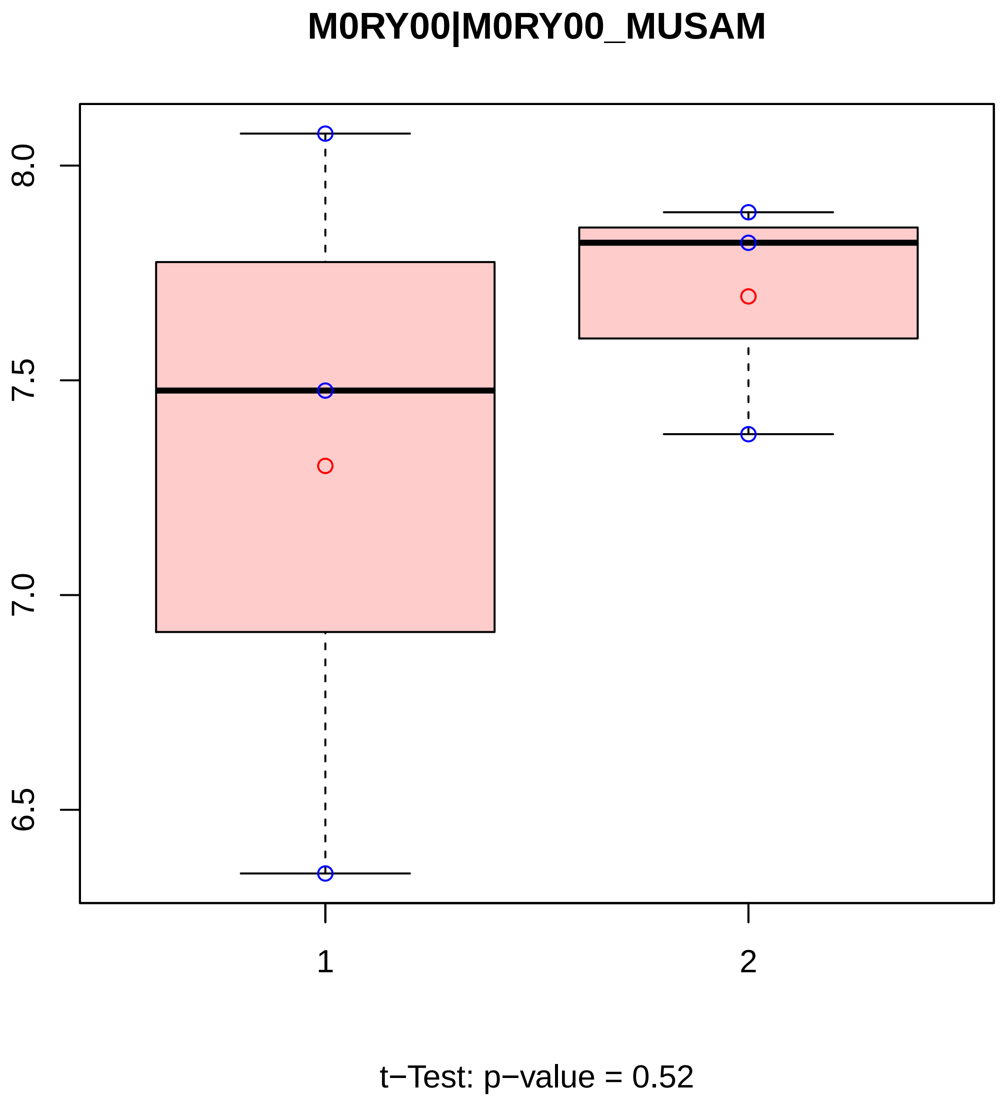
M0RY00|M0RY00_MUSAM
8.0
7.5
7.0
6.5
1
2
t−Test: p−value = 0.52

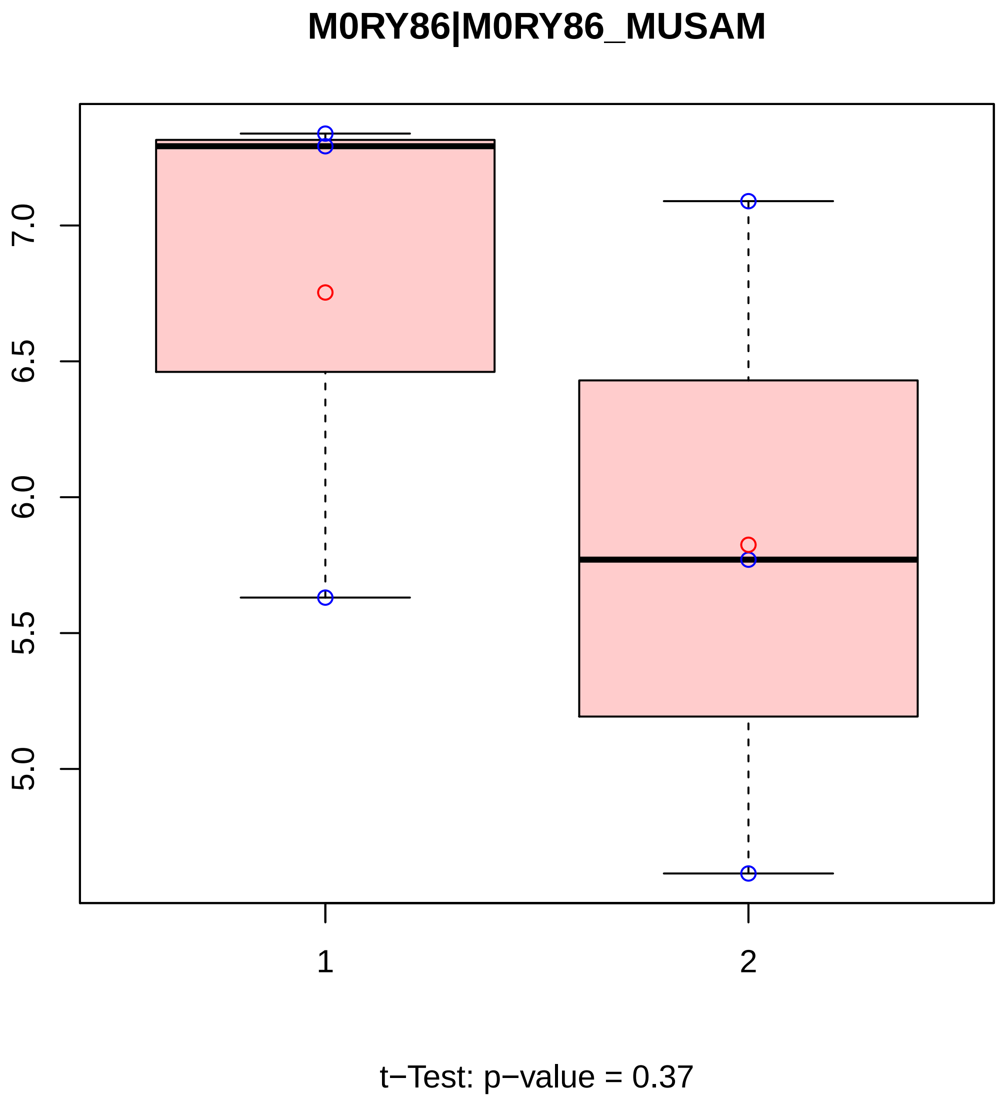
M0RY86|M0RY86_MUSAM
7.0
6.5
6.0
5.5
5.0
1
2
t−Test: p−value = 0.37

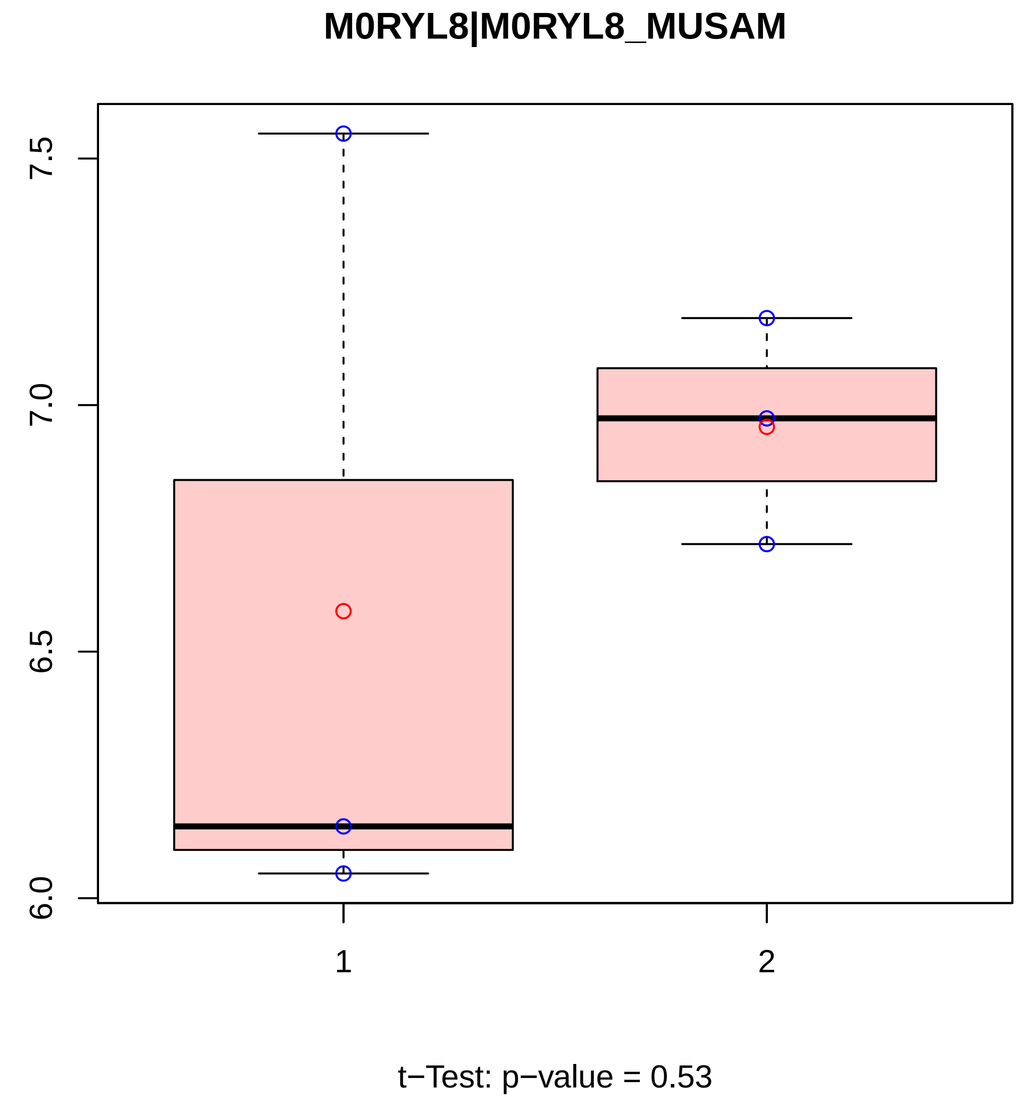
M0RYL8|M0RYL8_MUSAM
6.0
6.5
7.0
7.5
1
2
t−Test: p−value = 0.53

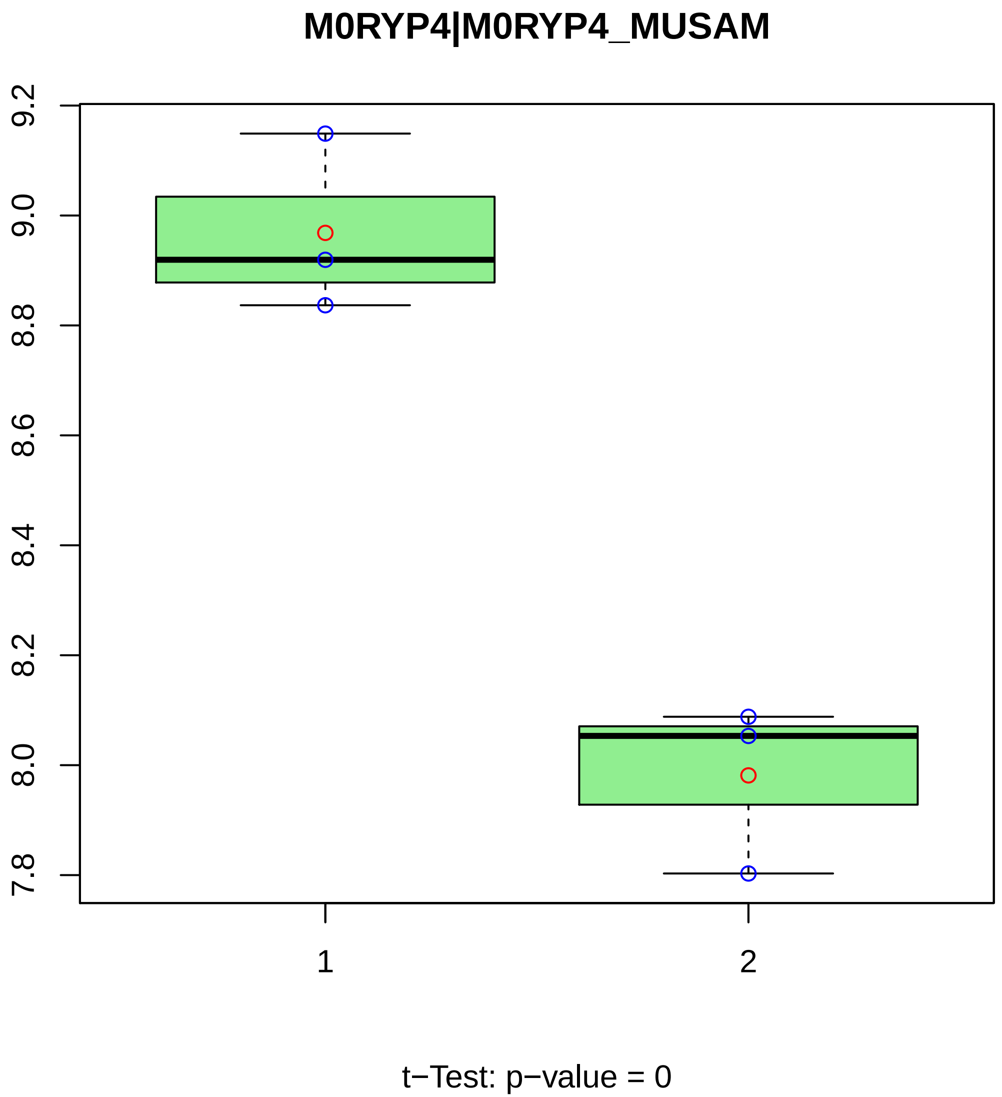
M0RYP4|M0RYP4_MUSAM
9.2
9.0
8.8
8.6
8.4
8.2
8.0
7.8
1
2
t−Test: p−value = 0

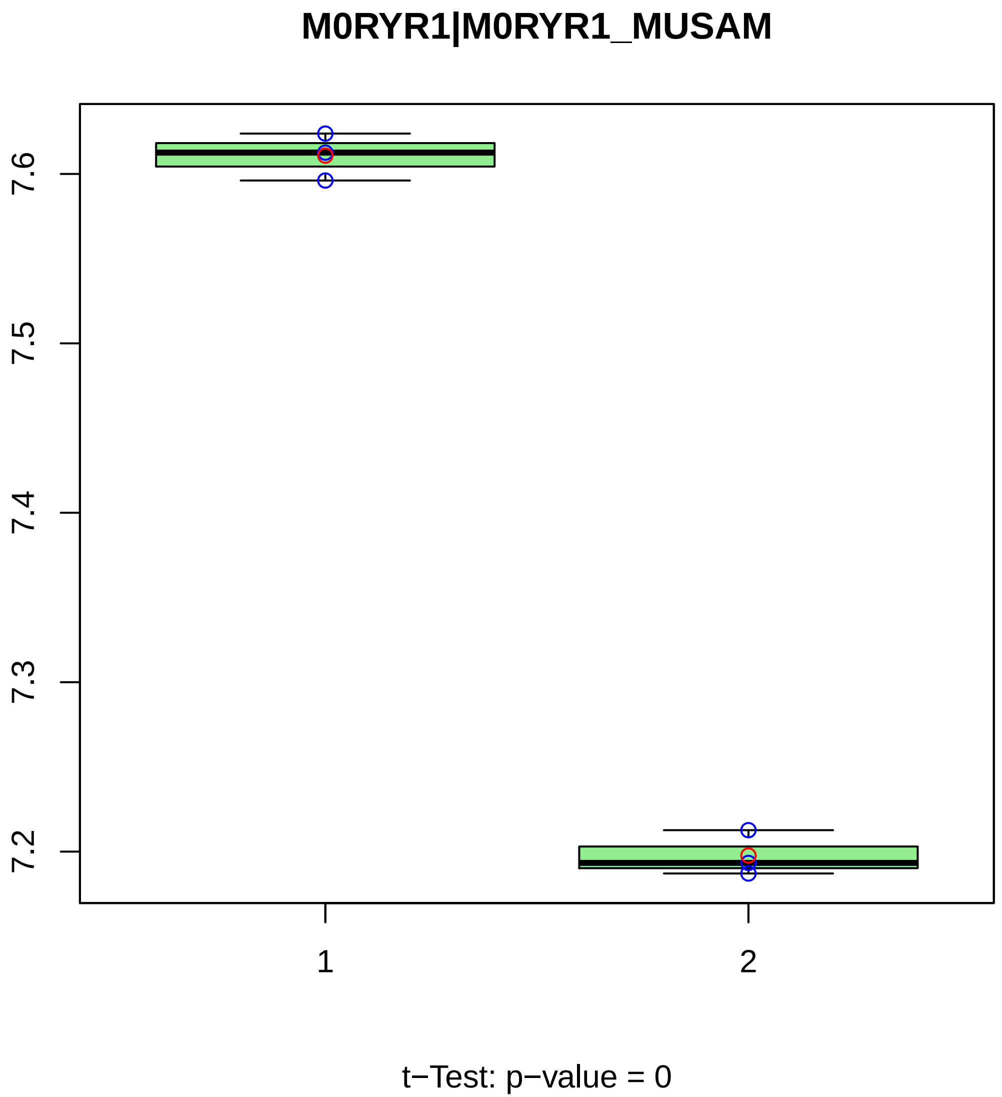
M0RYR1|M0RYR1_MUSAM
7.6
7.5
7.4
7.3
7.2
1
2
t−Test: p−value = 0

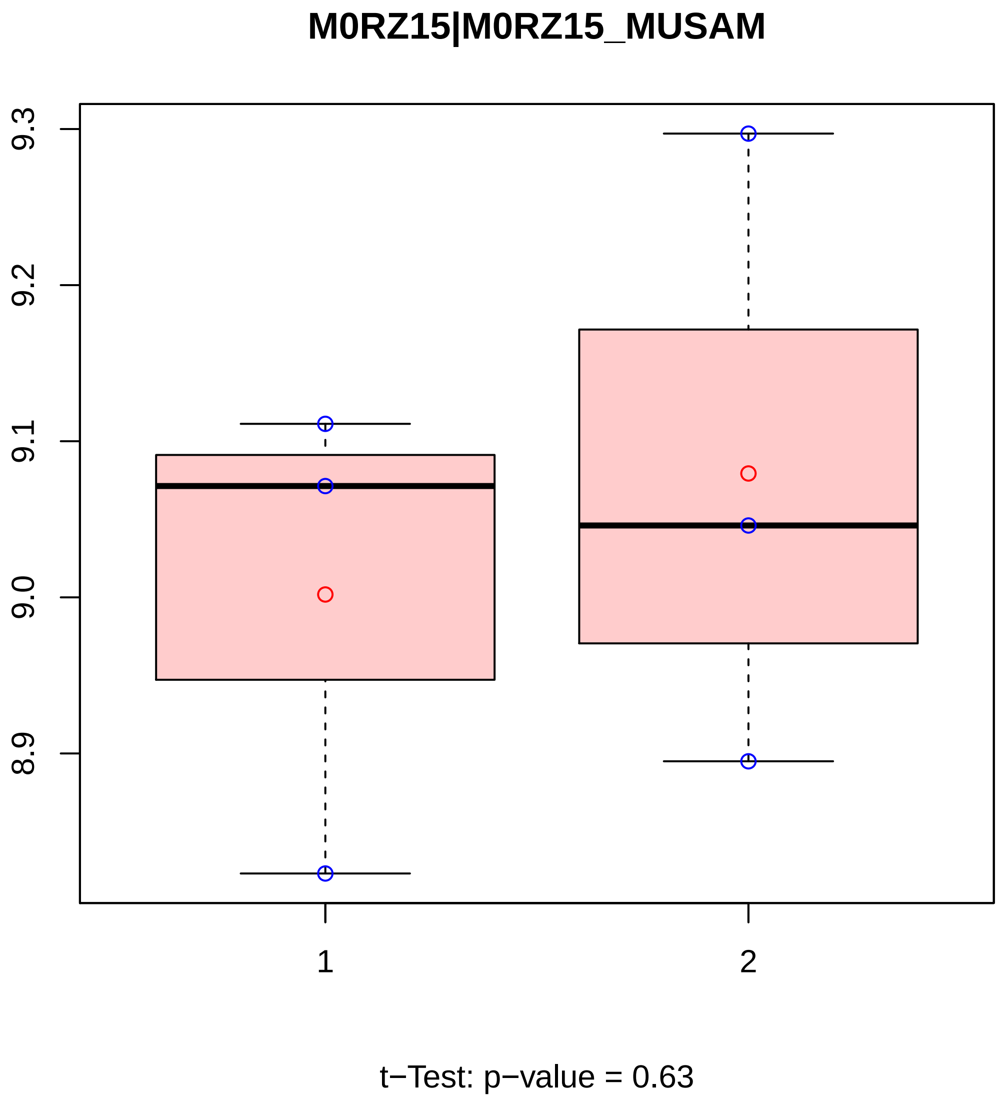
M0RZ15|M0RZ15_MUSAM
9.3
9.2
9.1
9.0
8.9
1
2
t−Test: p−value = 0.63

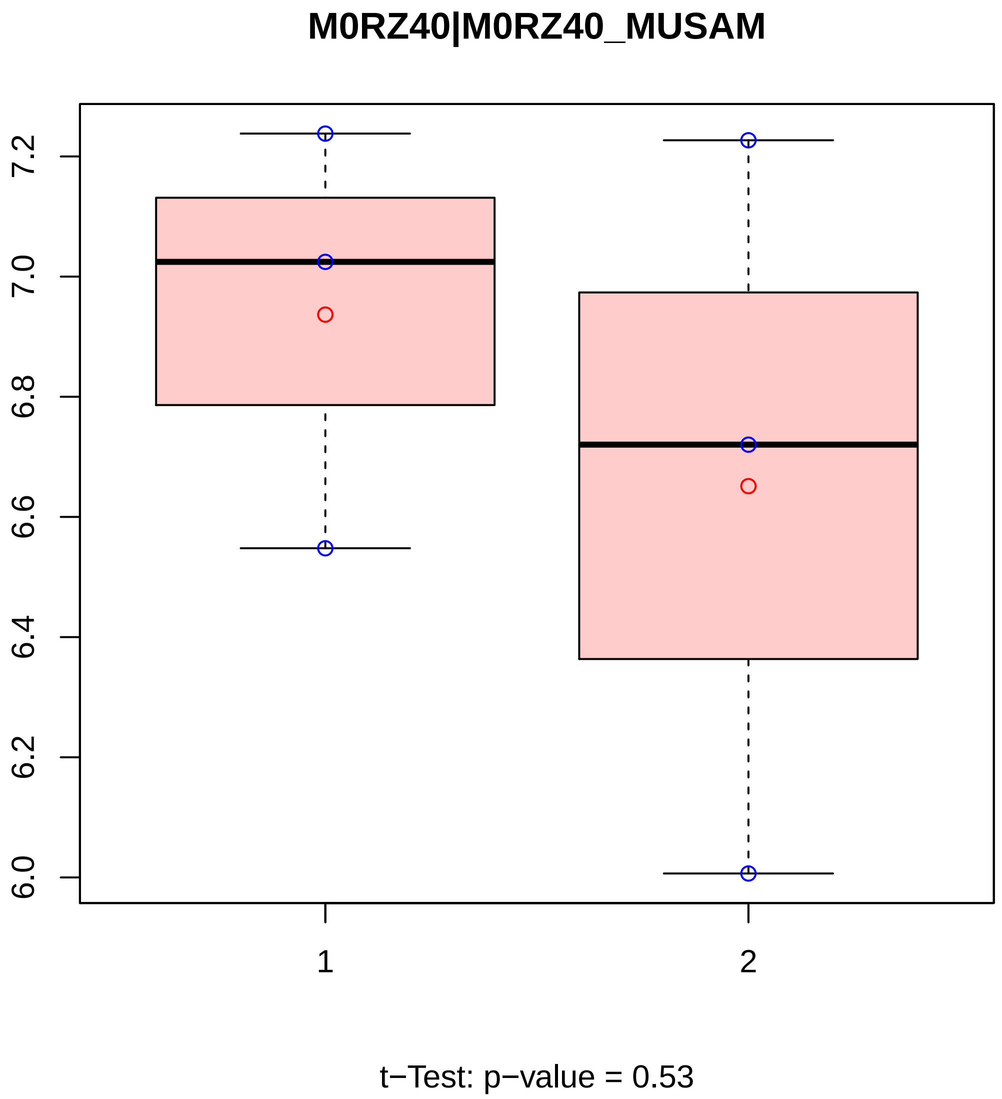
M0RZ40|M0RZ40_MUSAM
7.2
7.0
6.8
6.6
6.4
6.2
6.0
1
2
t−Test: p−value = 0.53

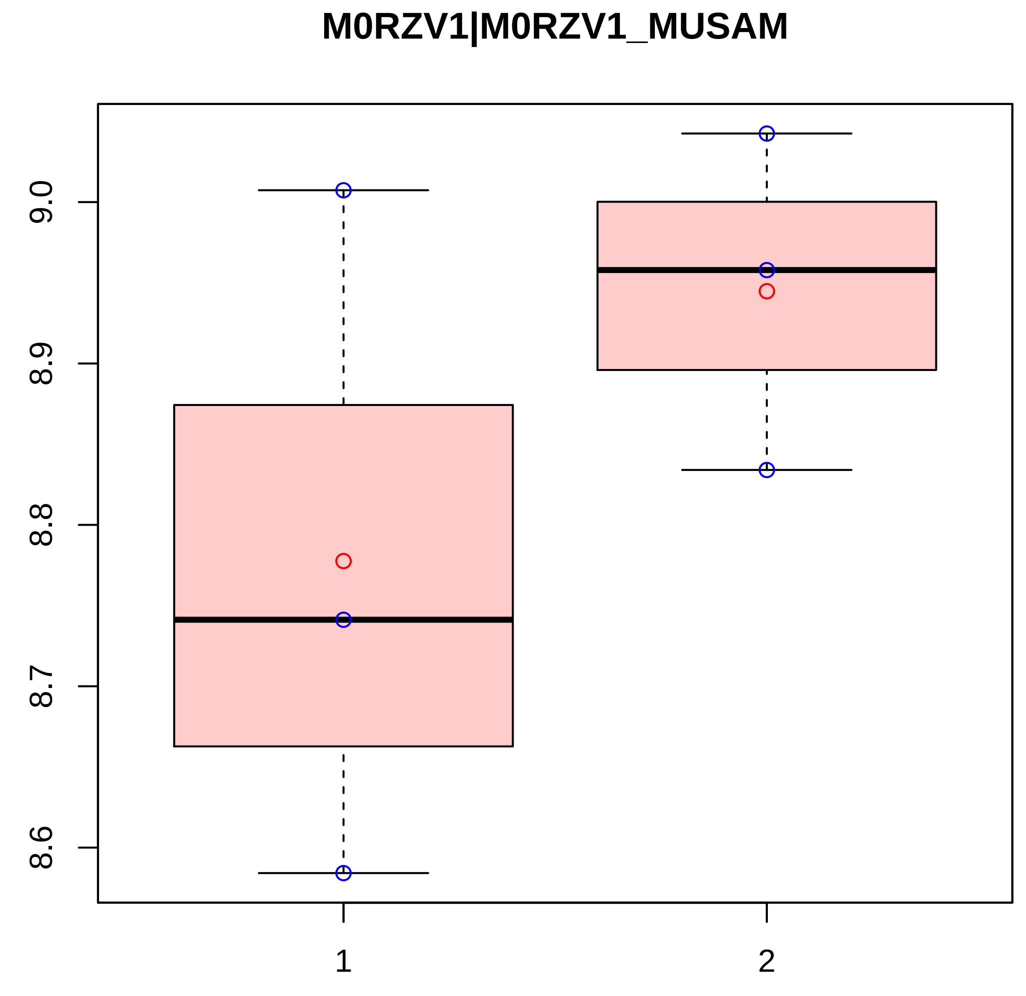

t−Test: p−value = 0.31

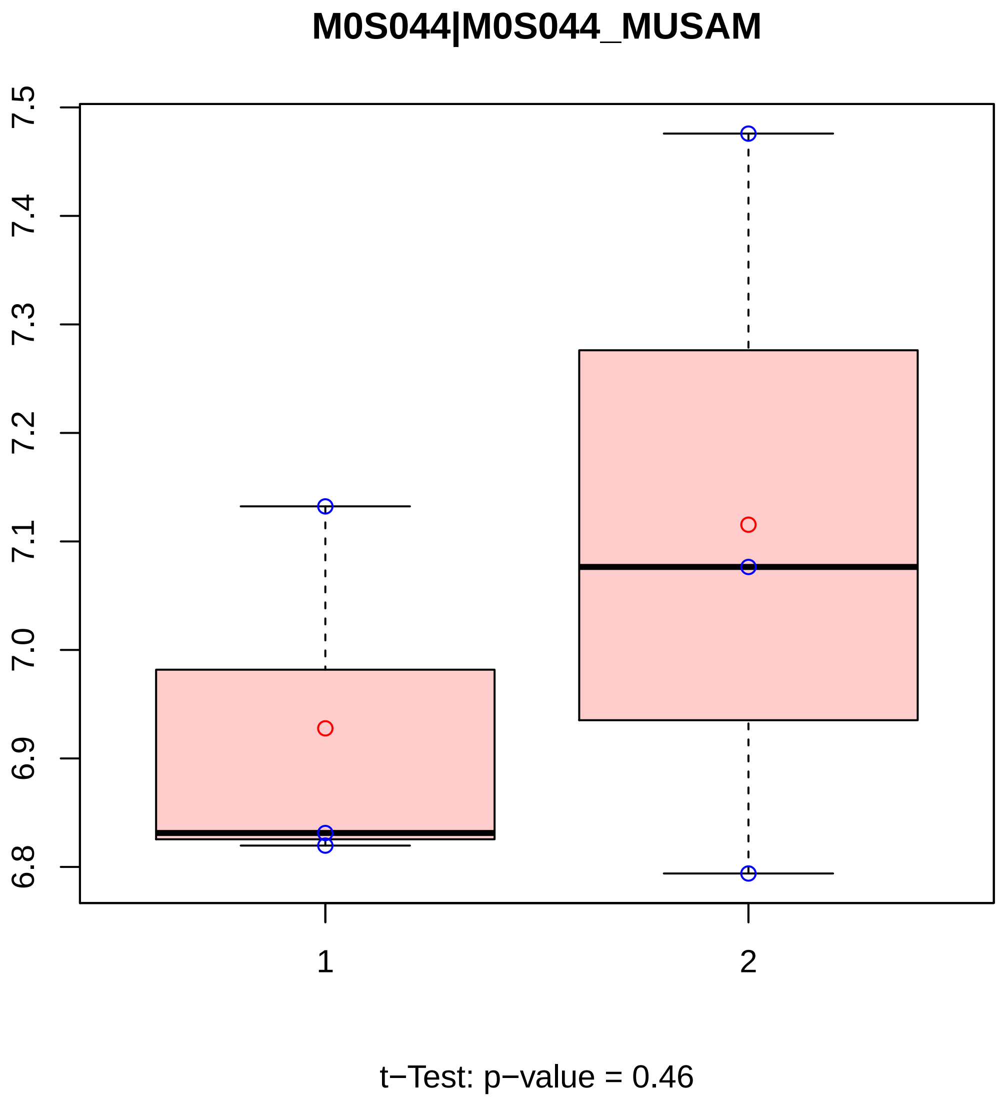
M0S044|M0S044_MUSAM
7.5
7.4
7.3
7.2
7.1
7.0
6.9
6.8
1
2
t−Test: p−value = 0.46

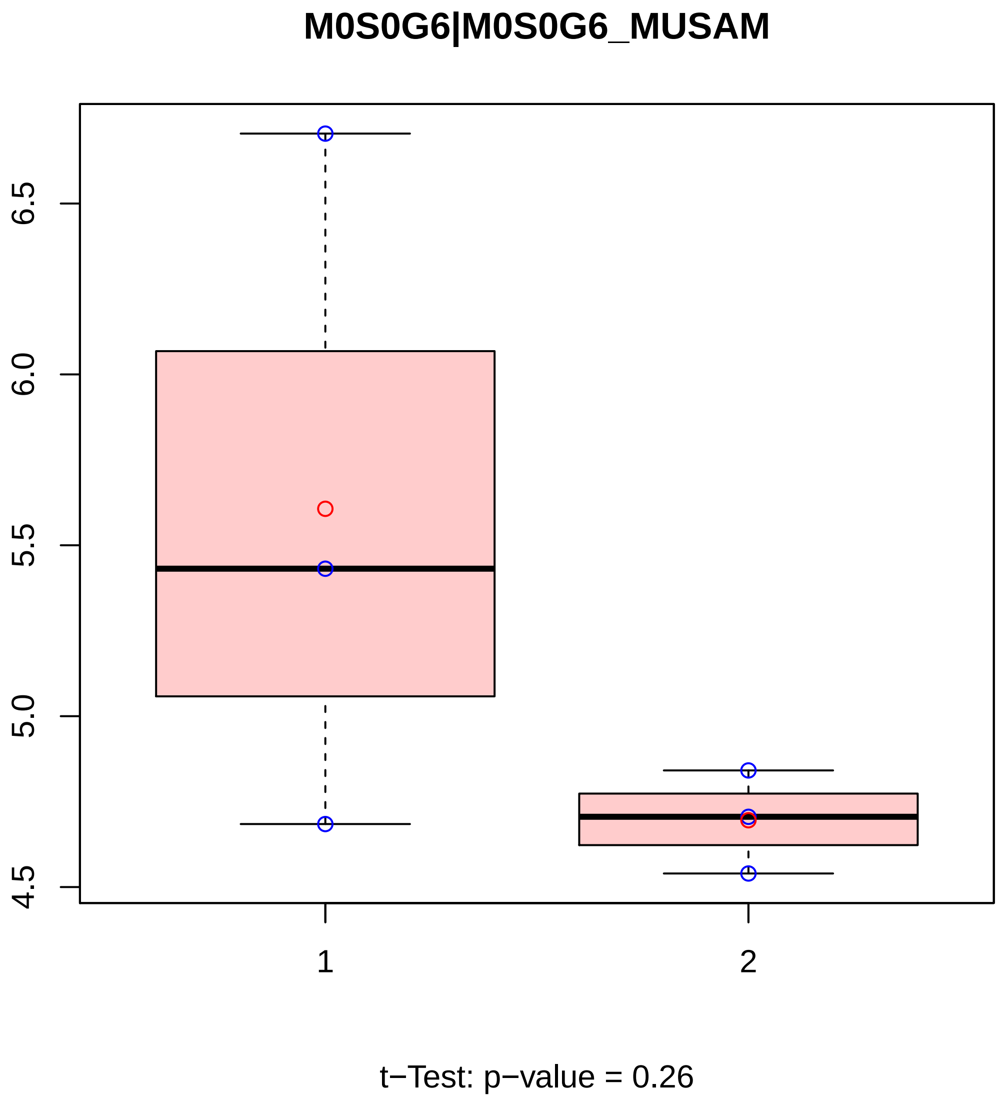
M0S0G6|M0S0G6_MUSAM
6.5
6.0
5.5
5.0
4.5
1
2
t−Test: p−value = 0.26

M0S0N5|M0S0N5_MUSAM

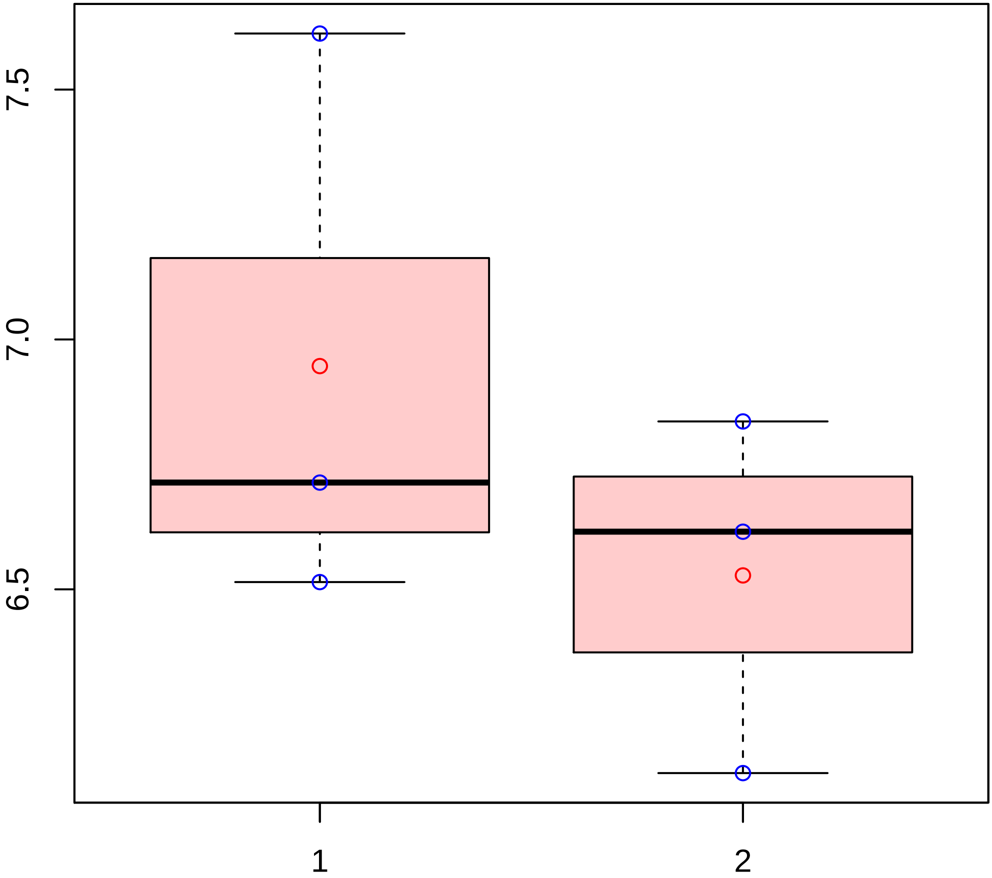

t−Test: p−value = 0.36

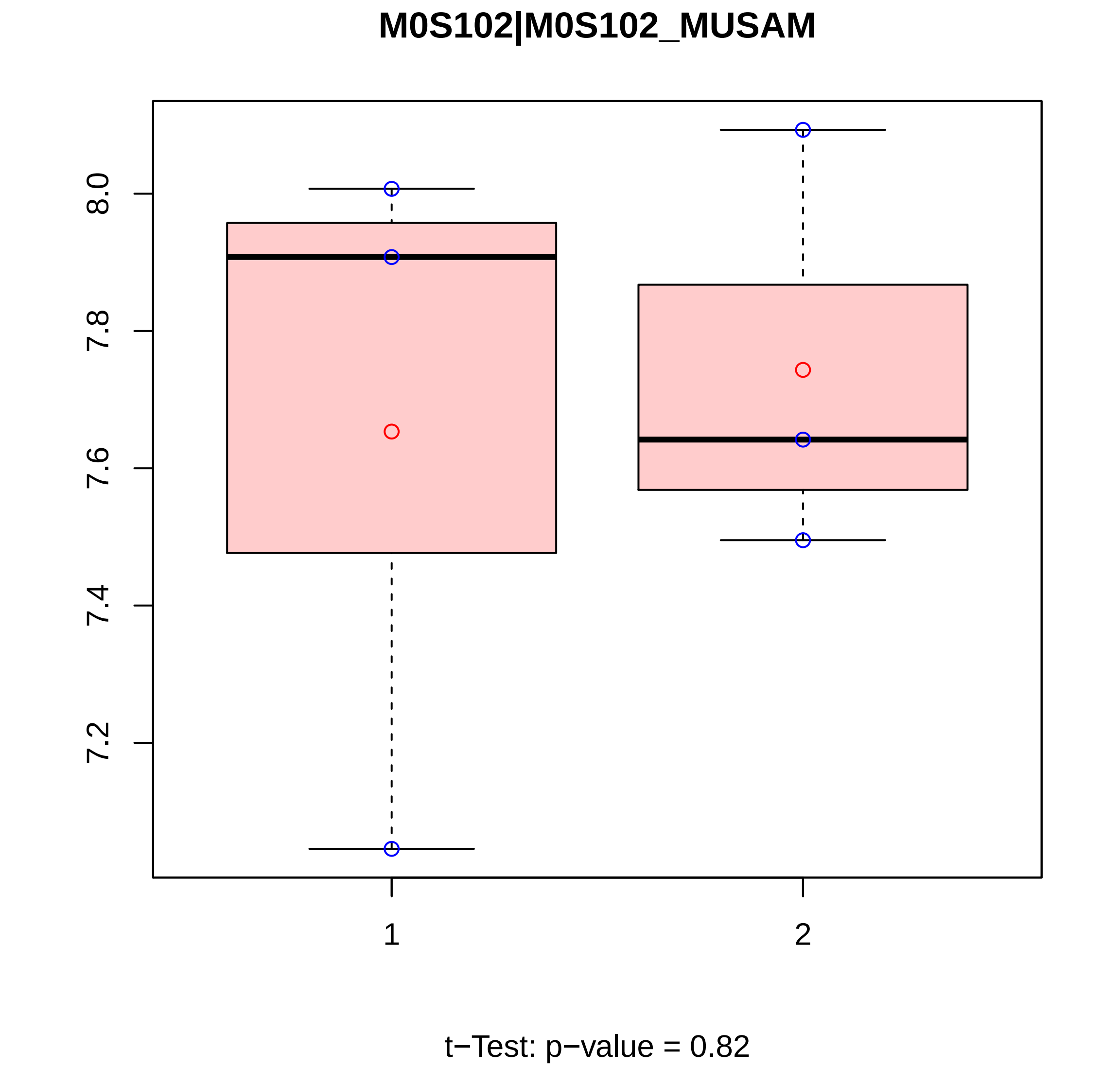
M0S102|M0S102_MUSAM
8.0
7.8
7.6
7.4
7.2
1
2
t−Test: p−value = 0.82

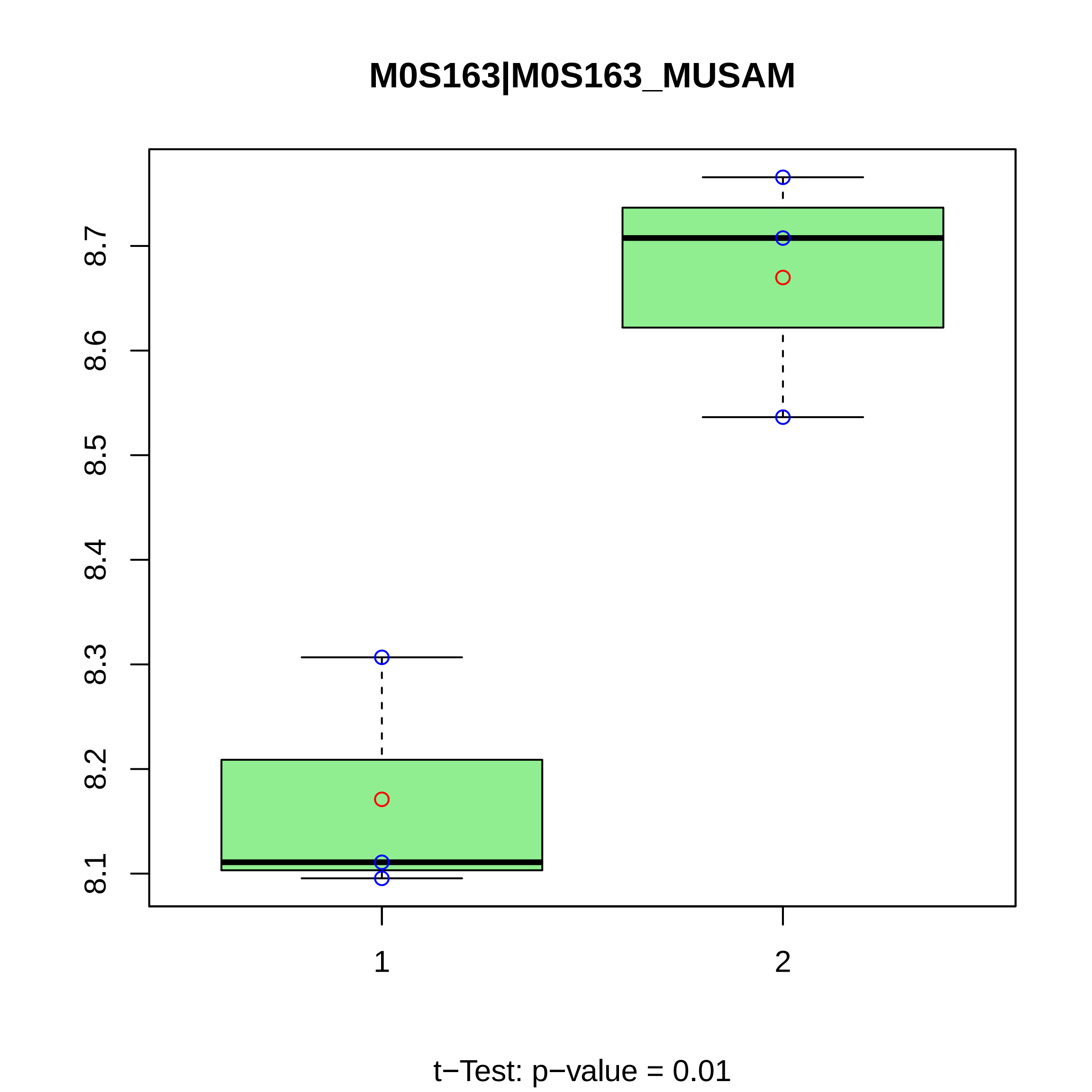

M0S163|M0S163_MUSAM
8.1
8.2
8.3
8.4
8.5
8.6
8.7
1
2
t−Test: p−value = 0.01

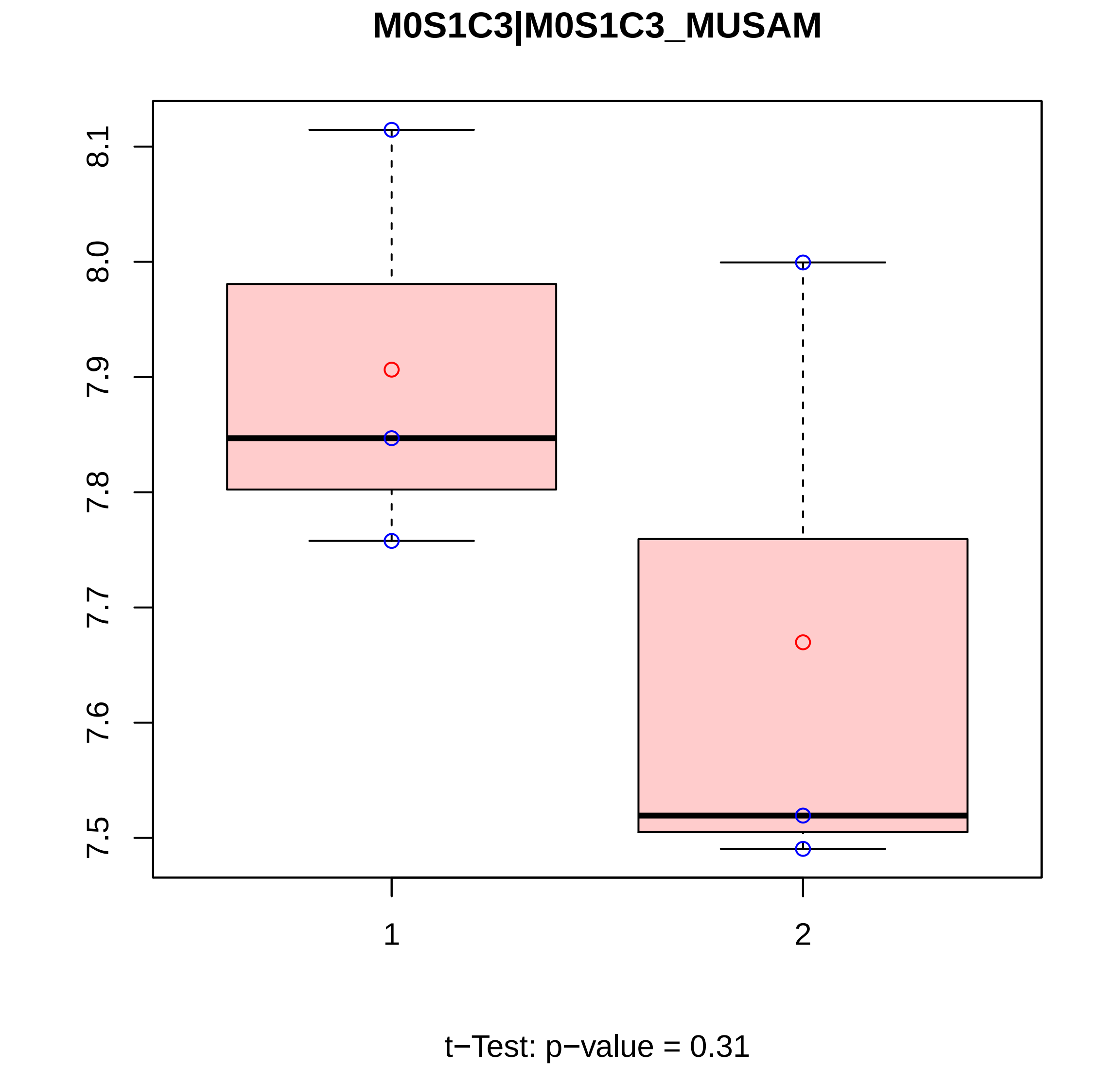
M0S1C3|M0S1C3_MUSAM
8.1
8.0
7.9
7.8
7.7
7.6
7.5
1
2
t−Test: p−value = 0.31

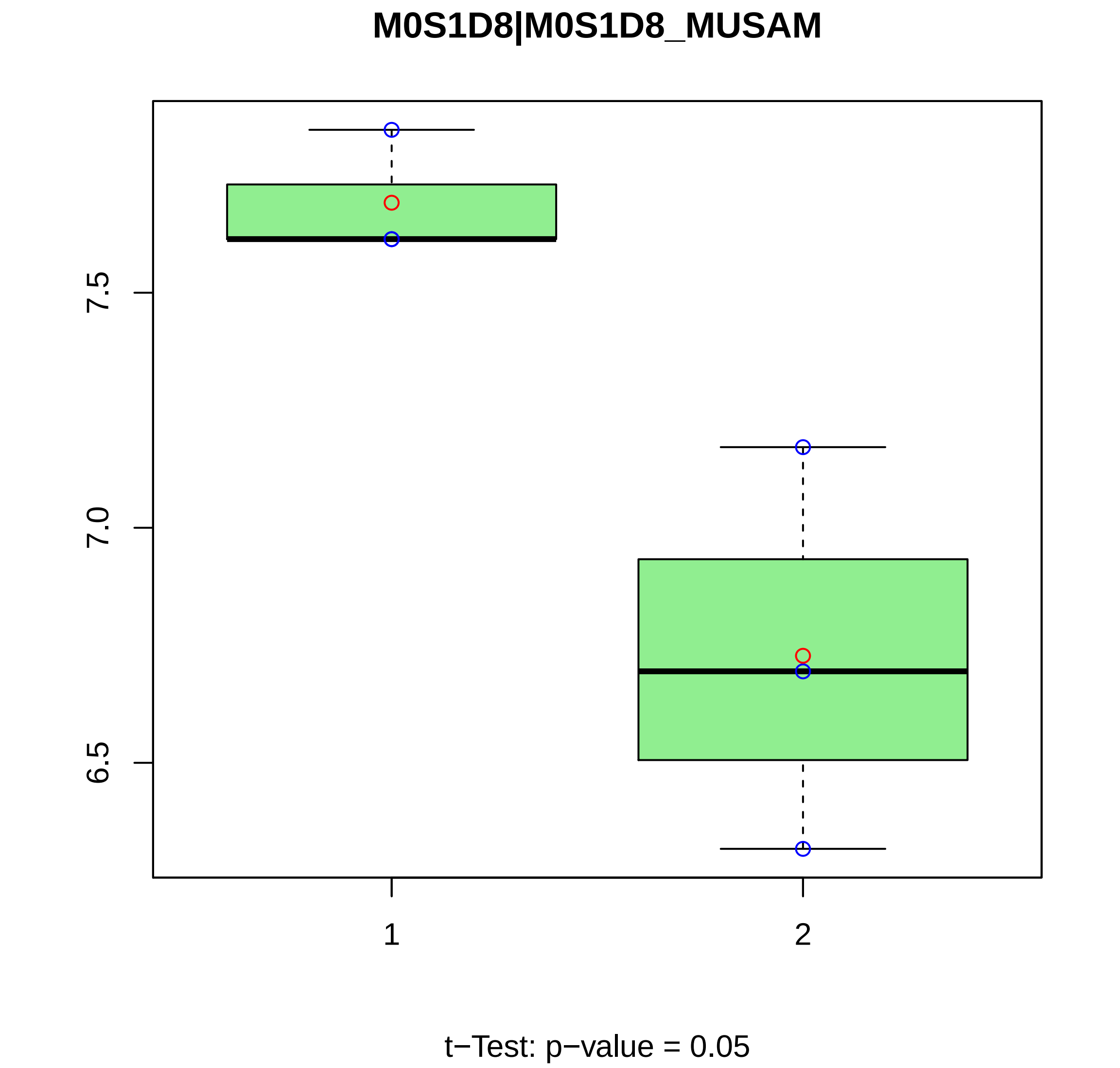
M0S1D8|M0S1D8_MUSAM
7.5
7.0
6.5
1
2
t−Test: p−value = 0.05

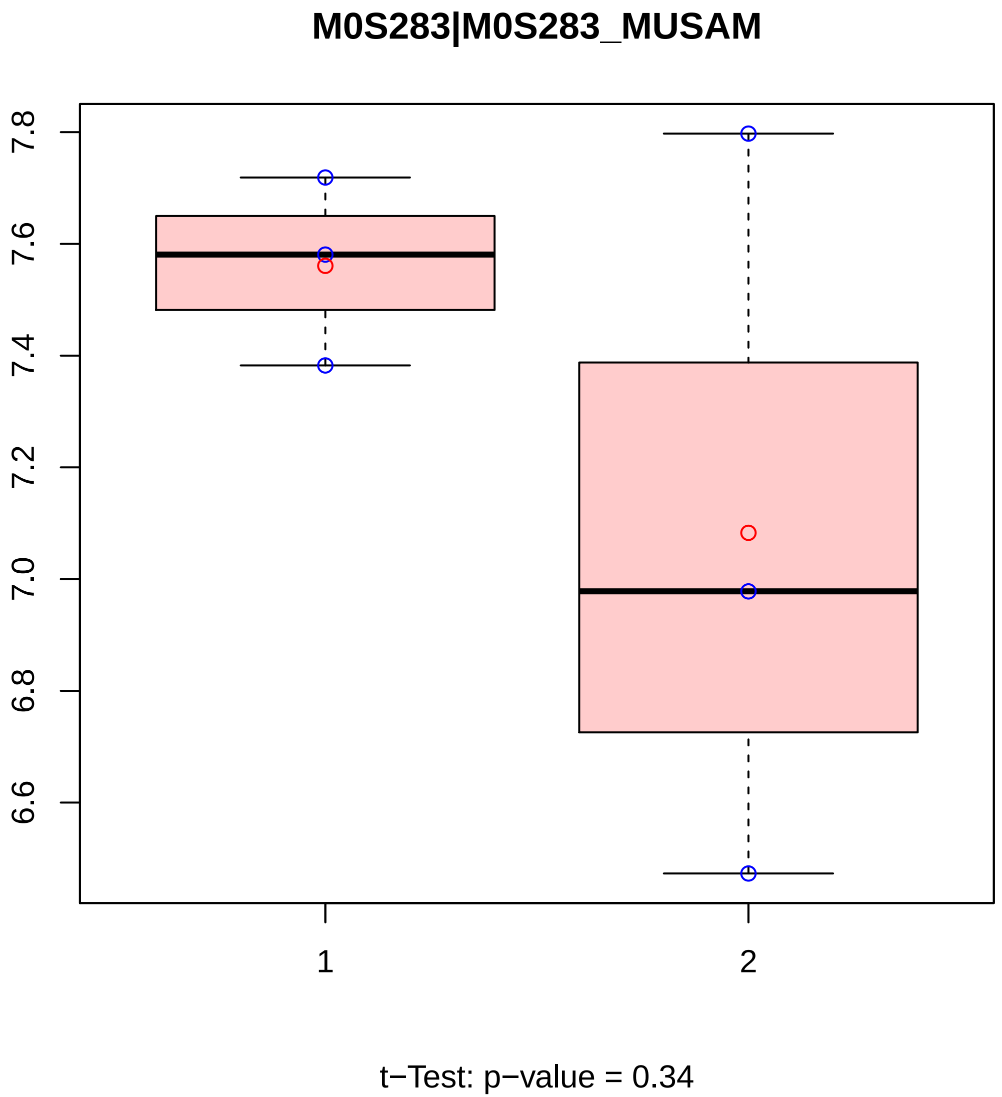
M0S283|M0S283_MUSAM
7.8
7.6
7.4
7.2
7.0
6.8
6.6
1
2
t−Test: p−value = 0.34

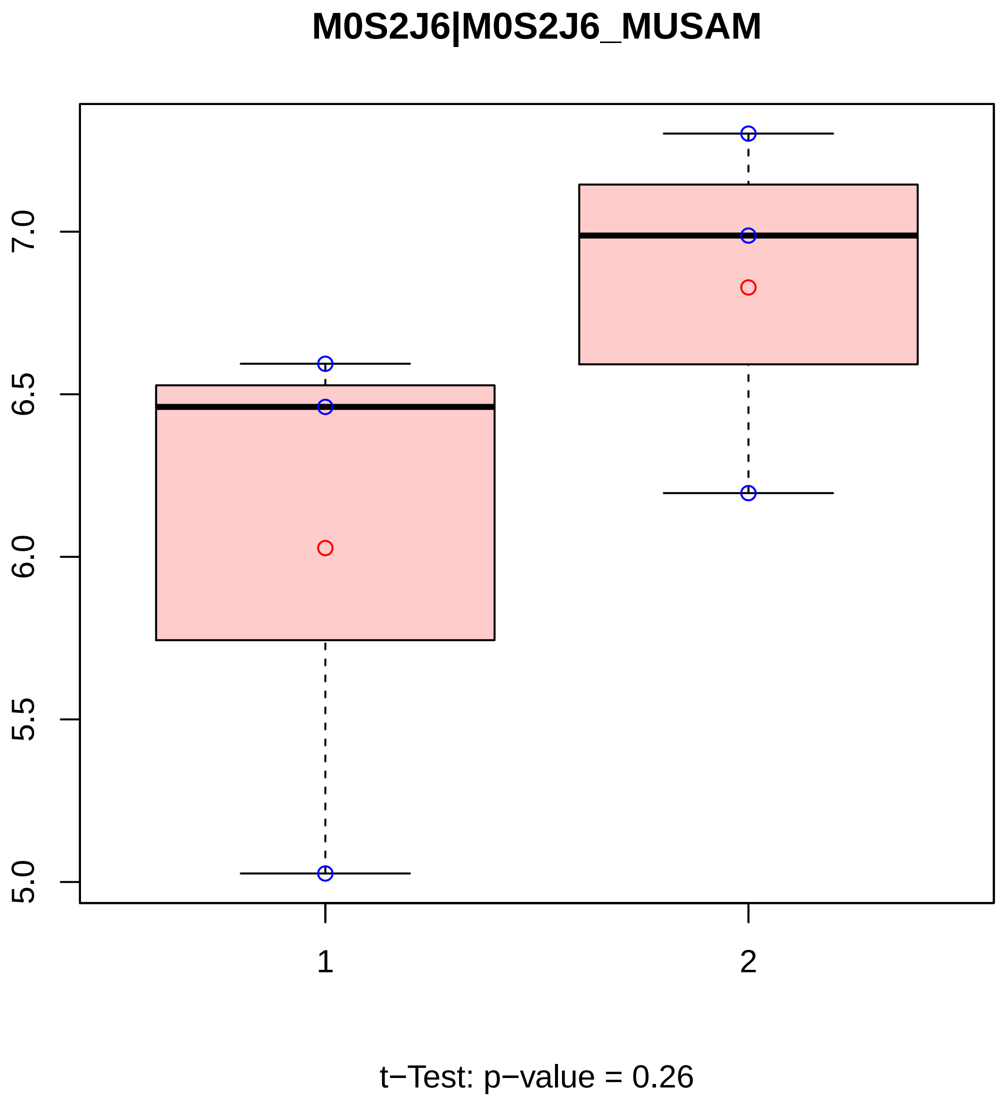
M0S2J6|M0S2J6_MUSAM
7.0
6.5
6.0
5.5
5.0
1
2
t−Test: p−value = 0.26

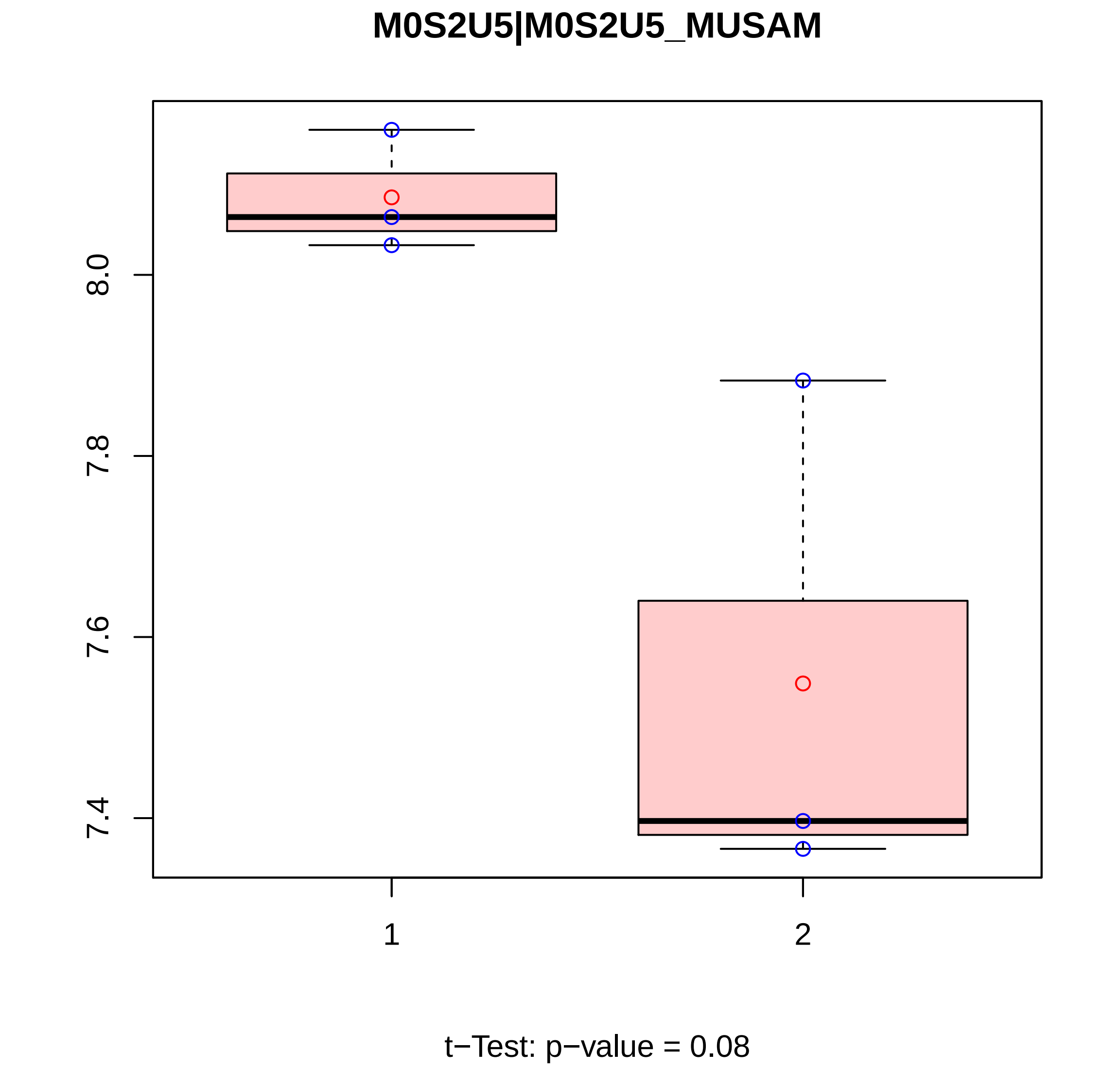
M0S2U5|M0S2U5_MUSAM
8.0
7.8
7.6
7.4
1
2
t−Test: p−value = 0.08

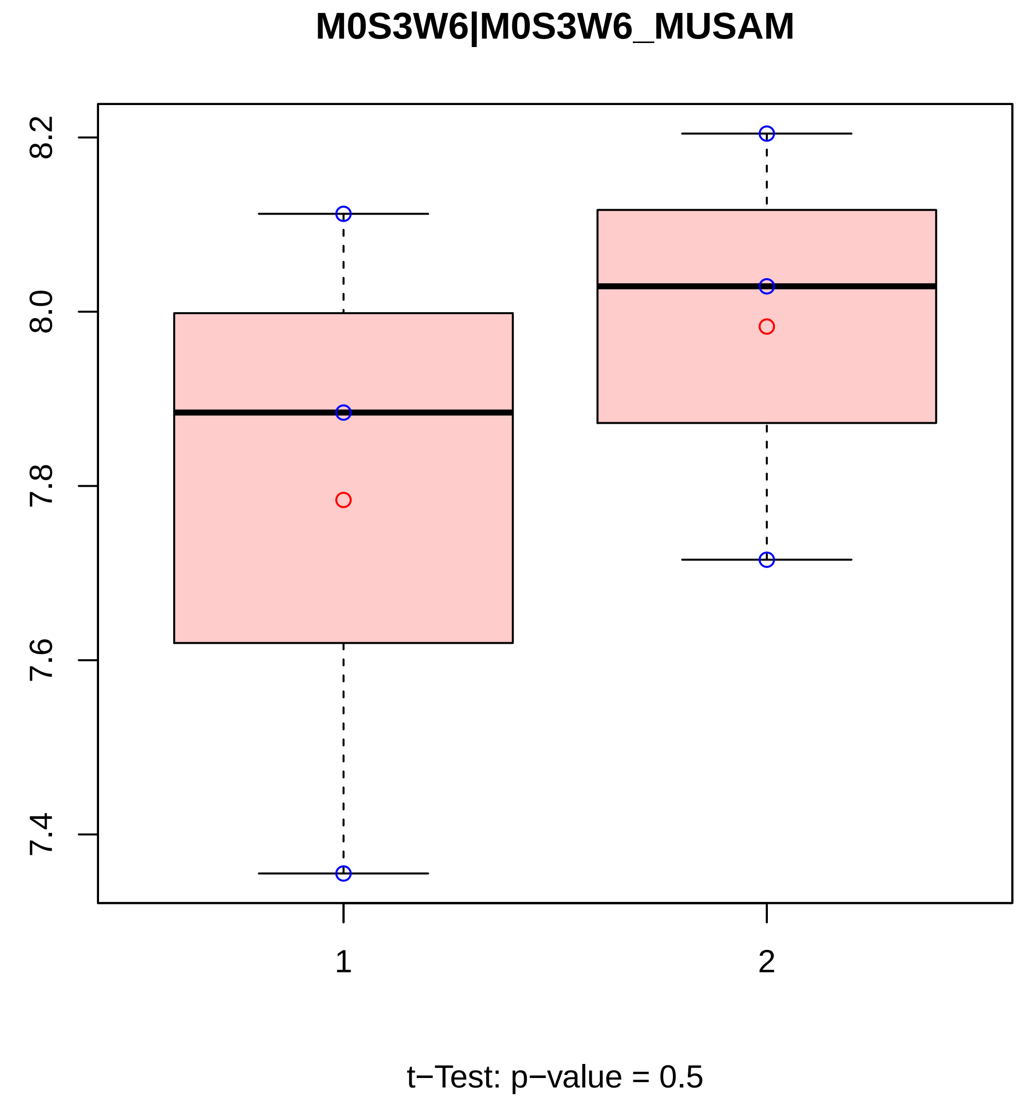
M0S3W6|M0S3W6_MUSAM
8.2
8.0
7.8
7.6
7.4
1
2
t−Test: p−value = 0.5

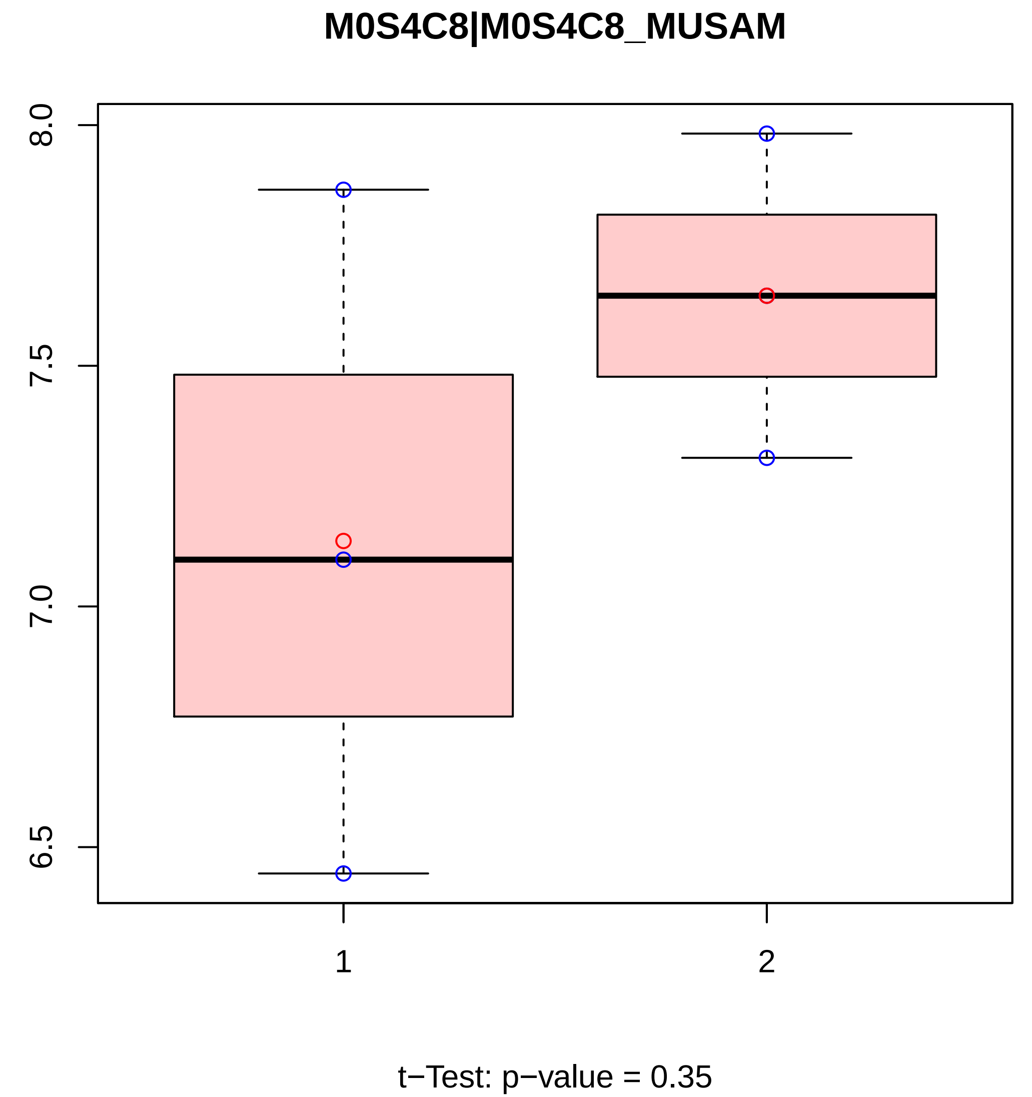
M0S4C8|M0S4C8_MUSAM
8.0
7.5
7.0
6.5
1
2
t−Test: p−value = 0.35

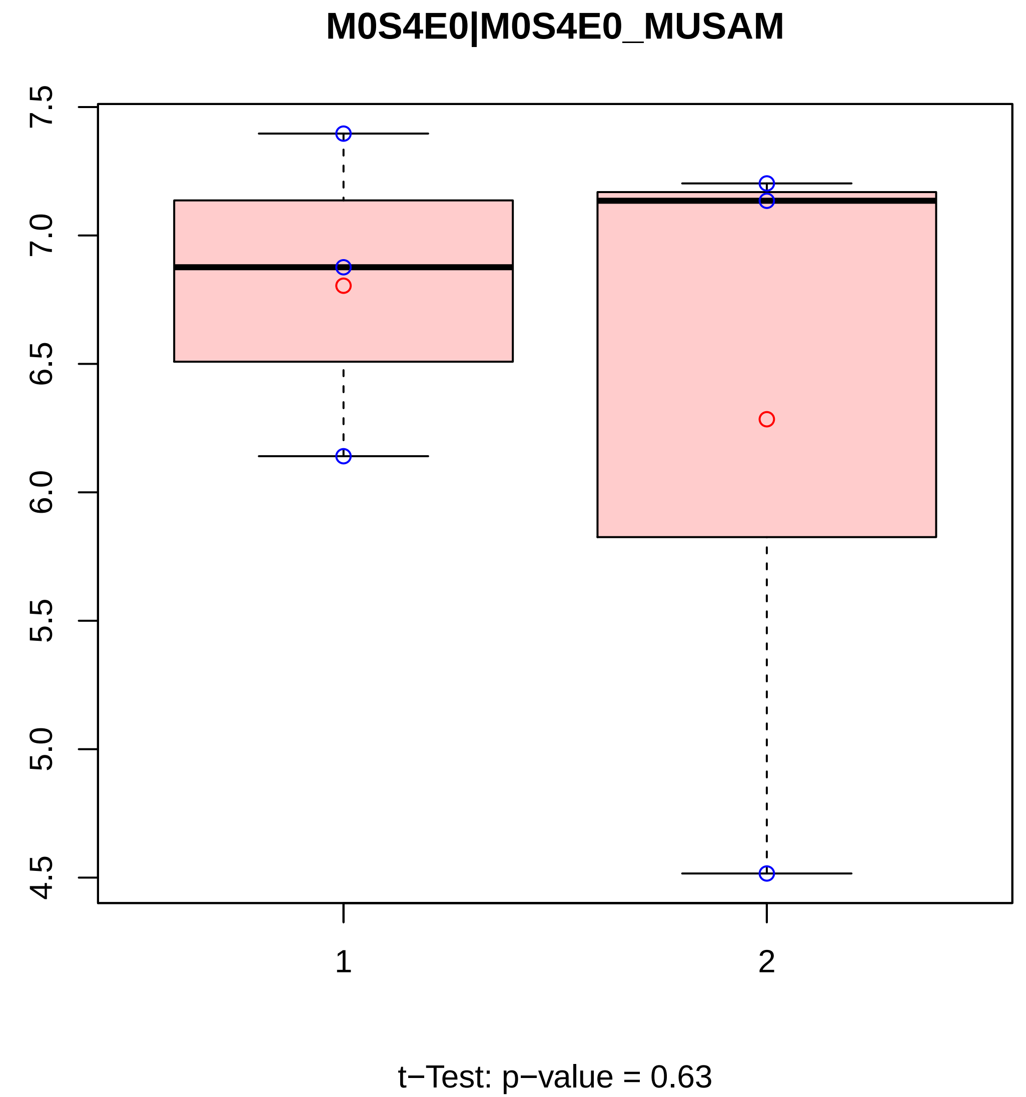

M0S4E0|M0S4E0_MUSAM
7.5
7.0
6.5
6.0
5.5
5.0
4.5
1
2
t−Test: p−value = 0.63

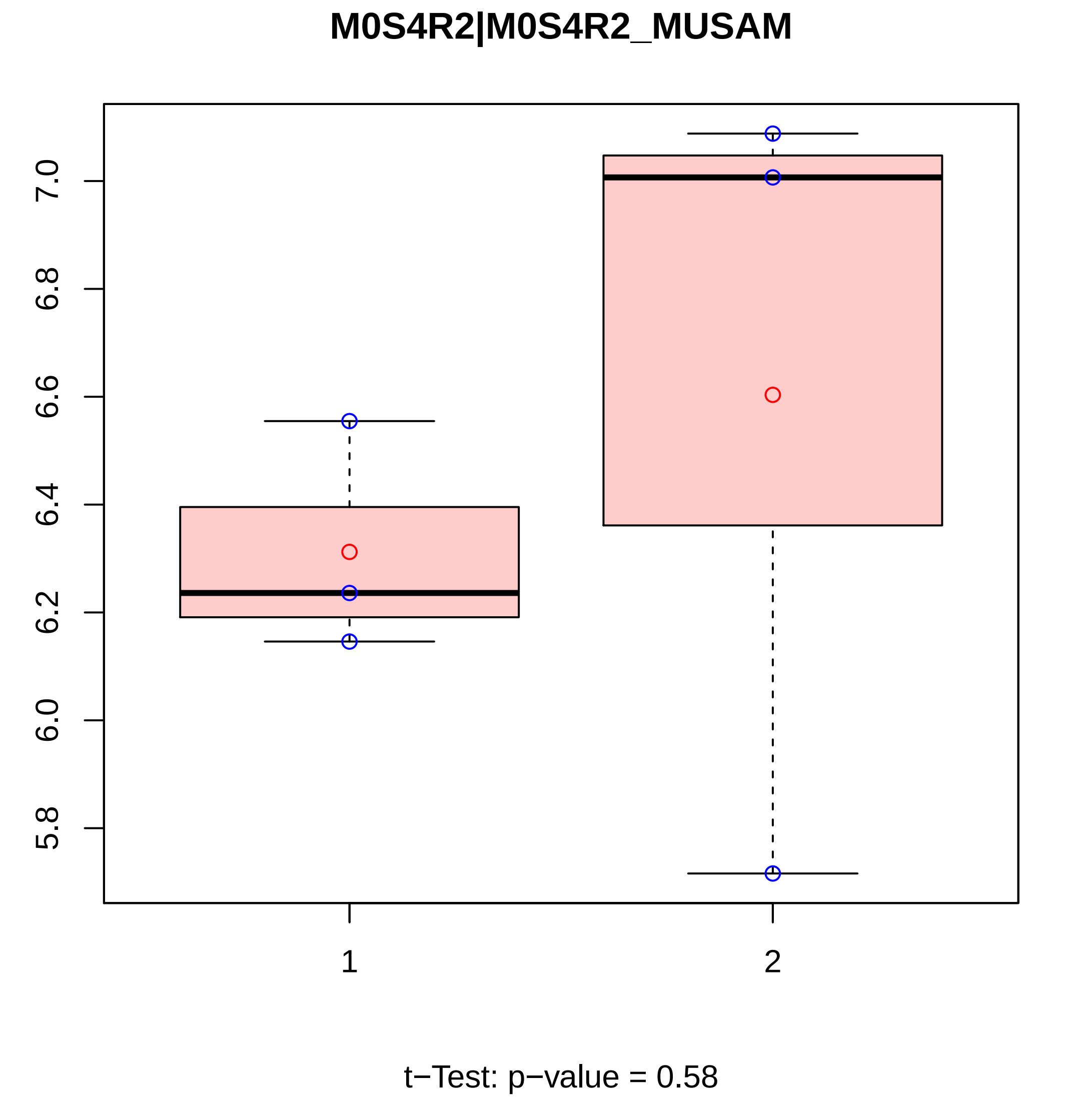

M0S4R2|M0S4R2_MUSAM
7.0
6.8
6.6
6.4
6.2
6.0
5.8
1
2
t−Test: p−value = 0.58

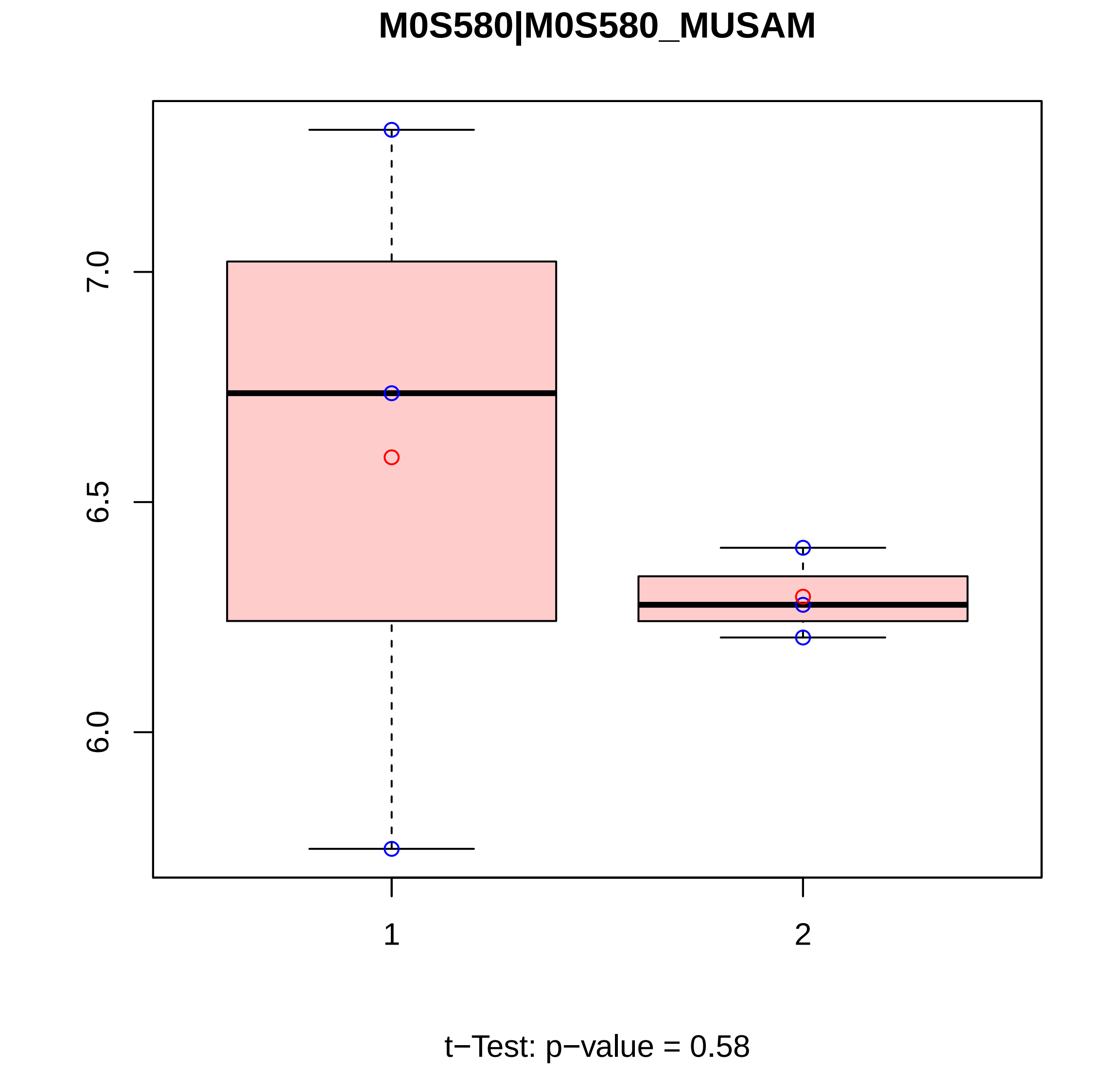
M0S580|M0S580_MUSAM
7.0
6.5
6.0
1
2
t−Test: p−value = 0.58

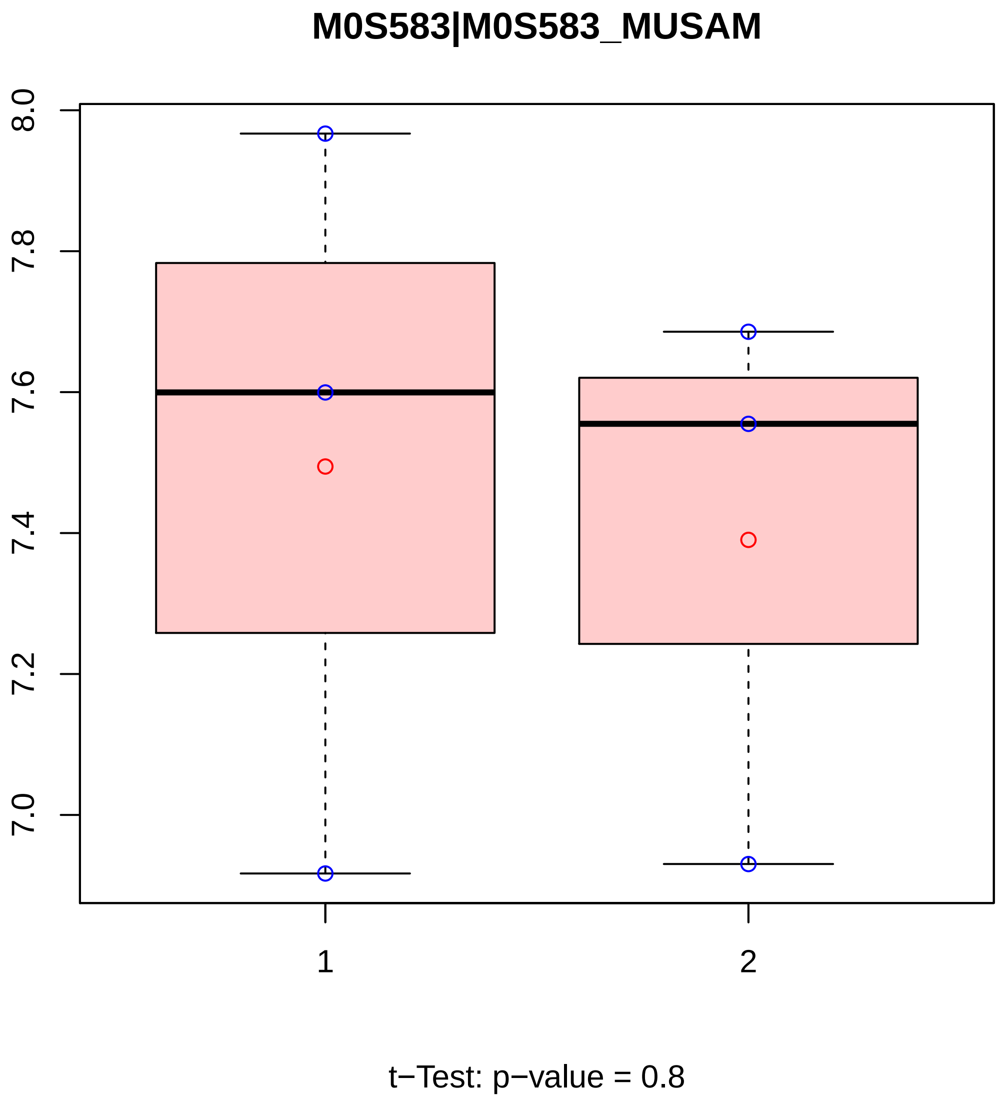
M0S583|M0S583_MUSAM
8.0
7.8
7.6
7.4
7.2
7.0
1
2
t−Test: p−value = 0.8

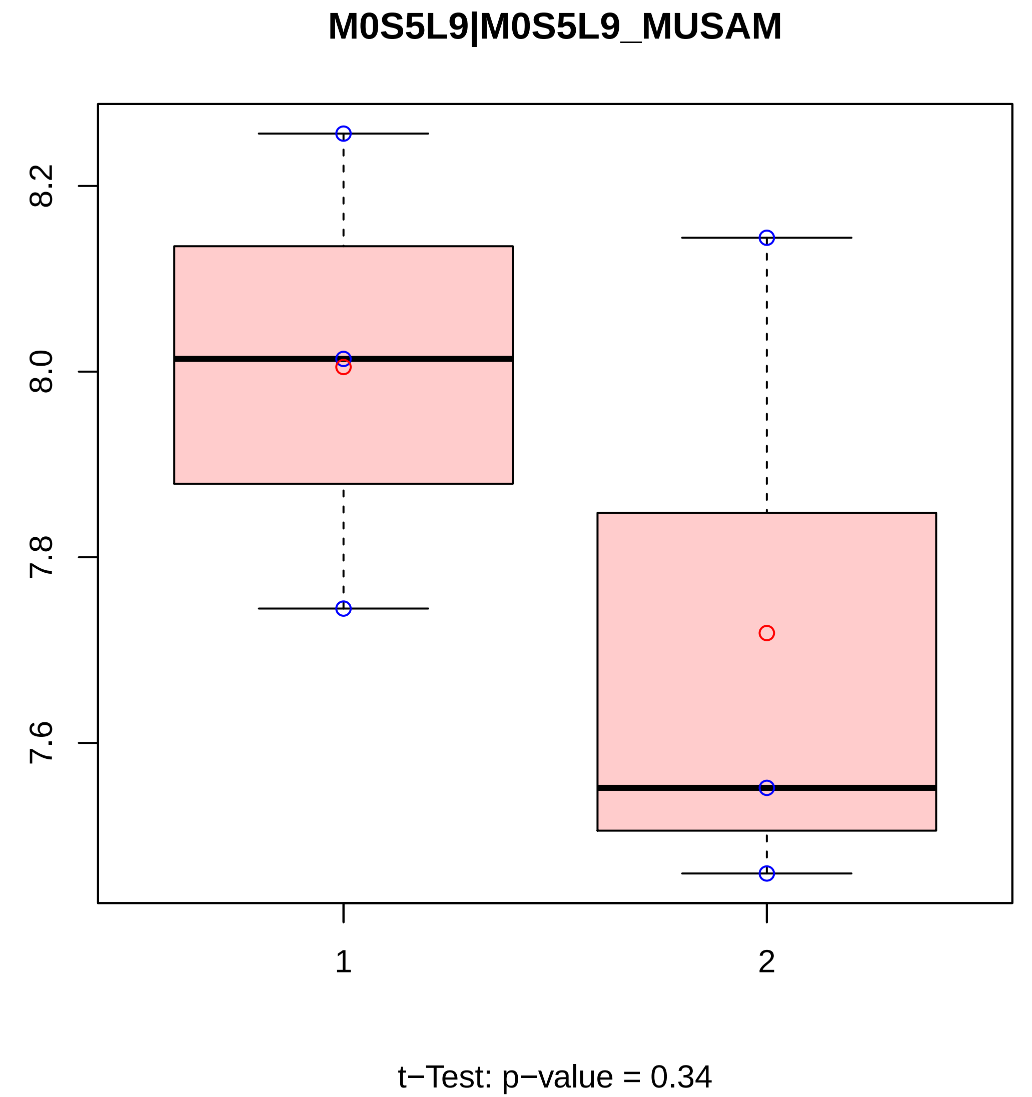
M0S5L9|M0S5L9_MUSAM
8.2
8.0
7.8
7.6
1
2
t−Test: p−value = 0.34

M0S5S1|M0S5S1_MUSAM

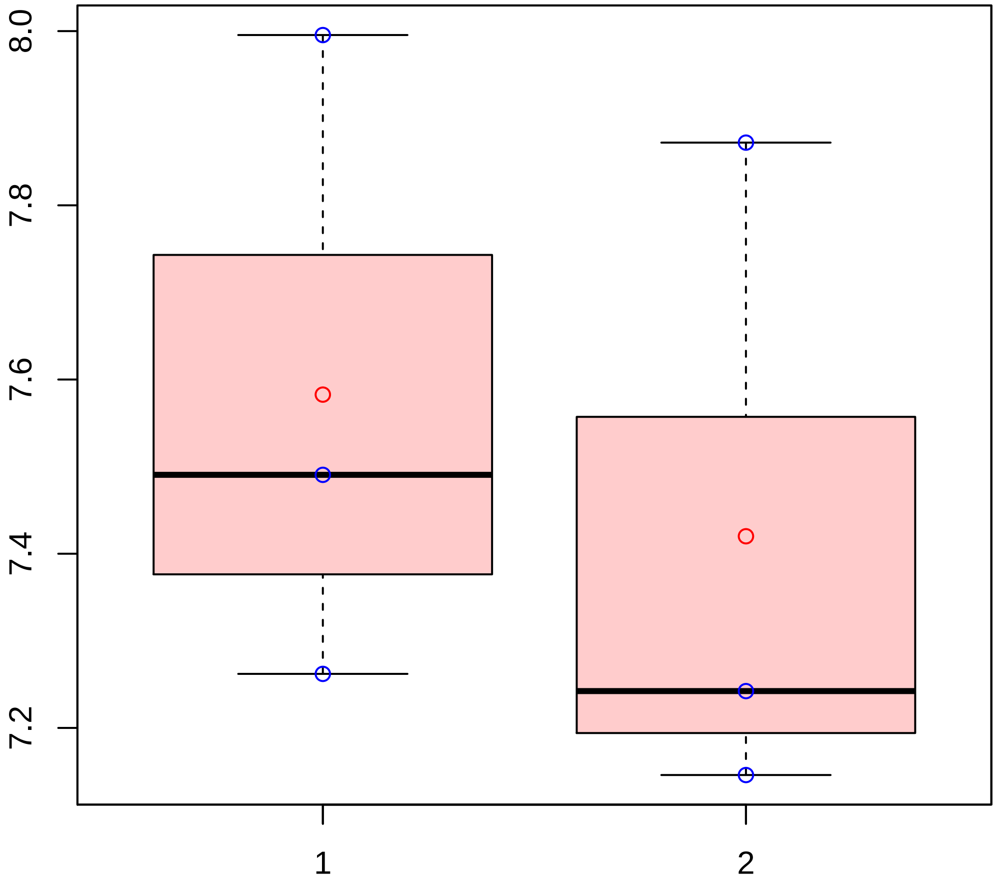

t−Test: p−value = 0.63

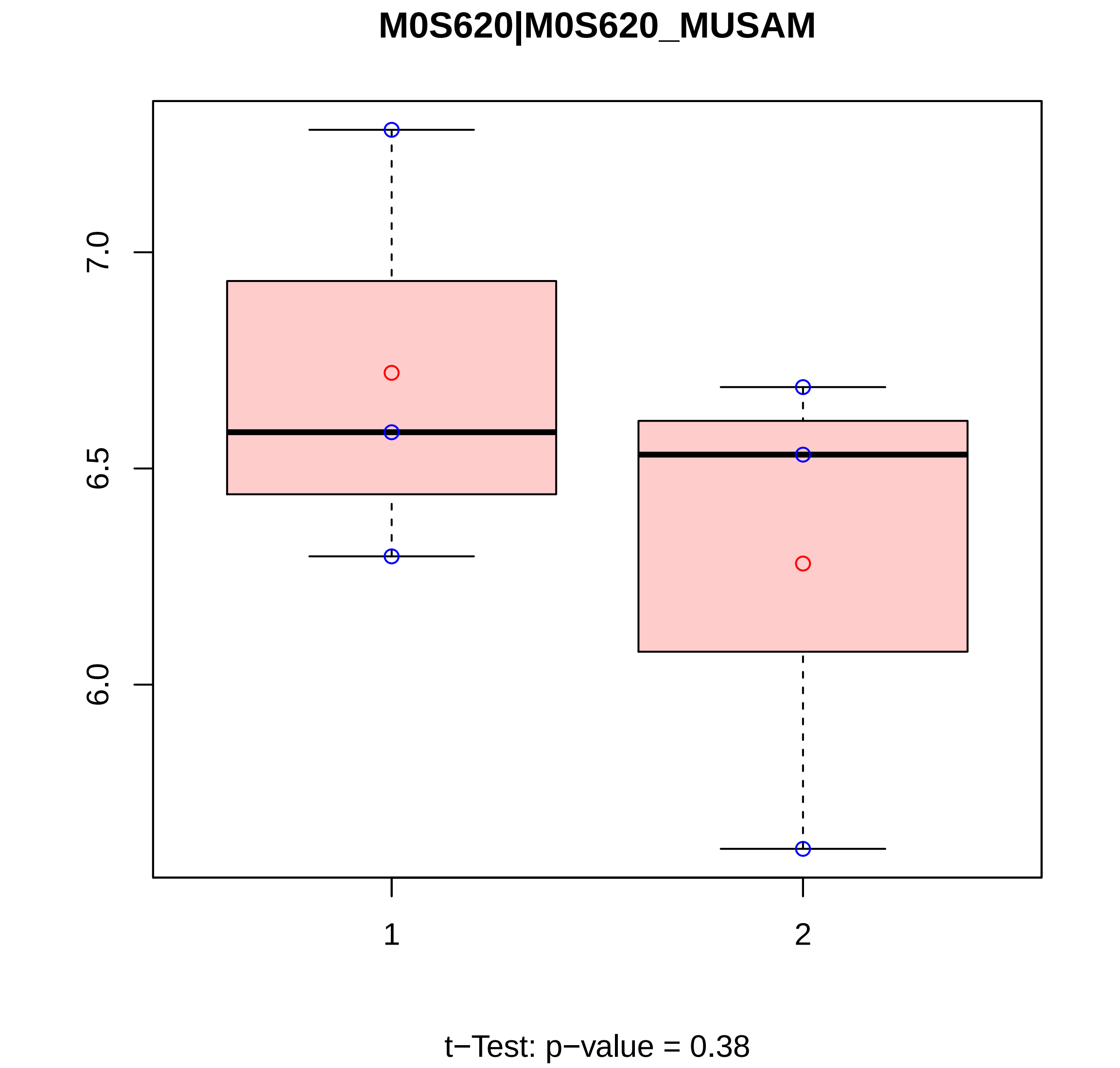
M0S620|M0S620_MUSAM
7.0
6.5
6.0
1
2
t−Test: p−value = 0.38

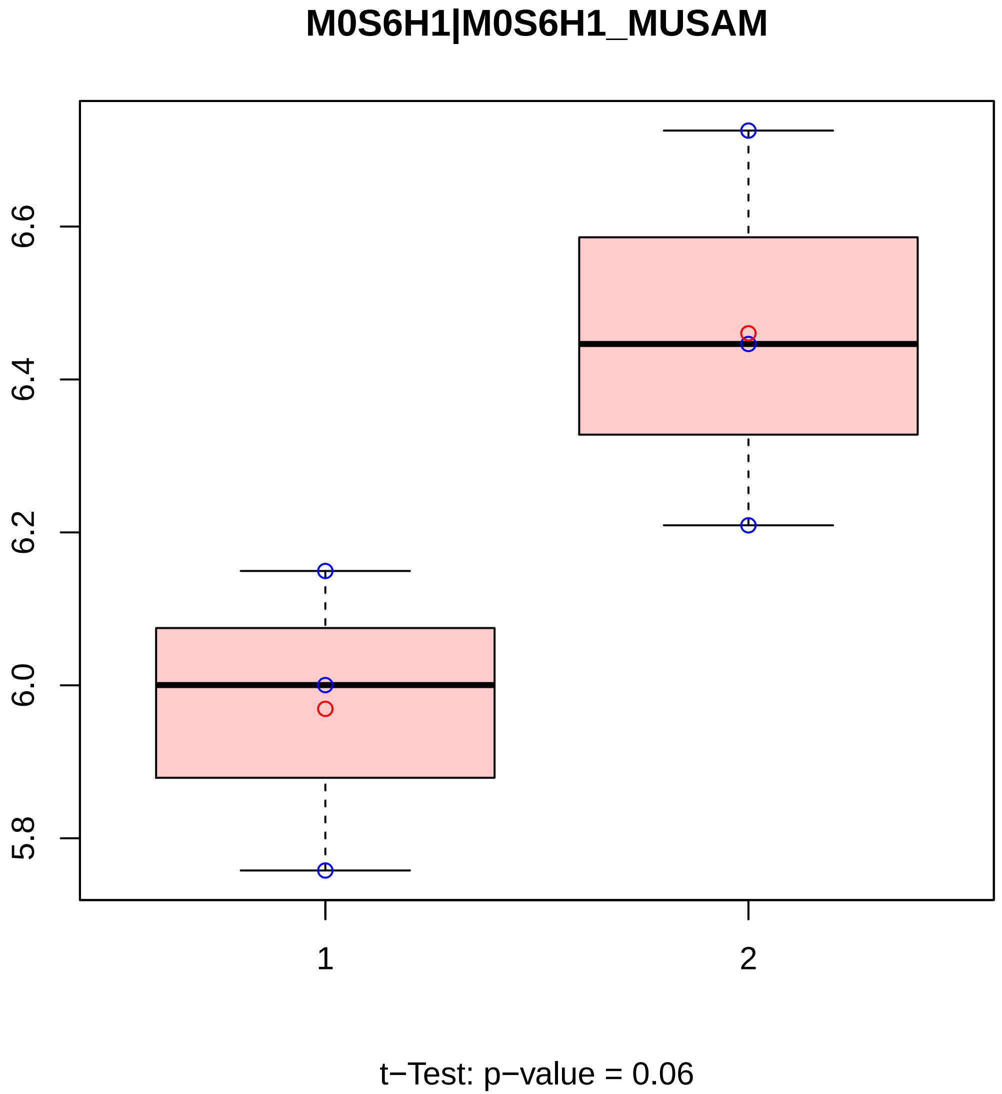
M0S6H1|M0S6H1_MUSAM
6.6
6.4
6.2
6.0
5.8
1
2
t−Test: p−value = 0.06

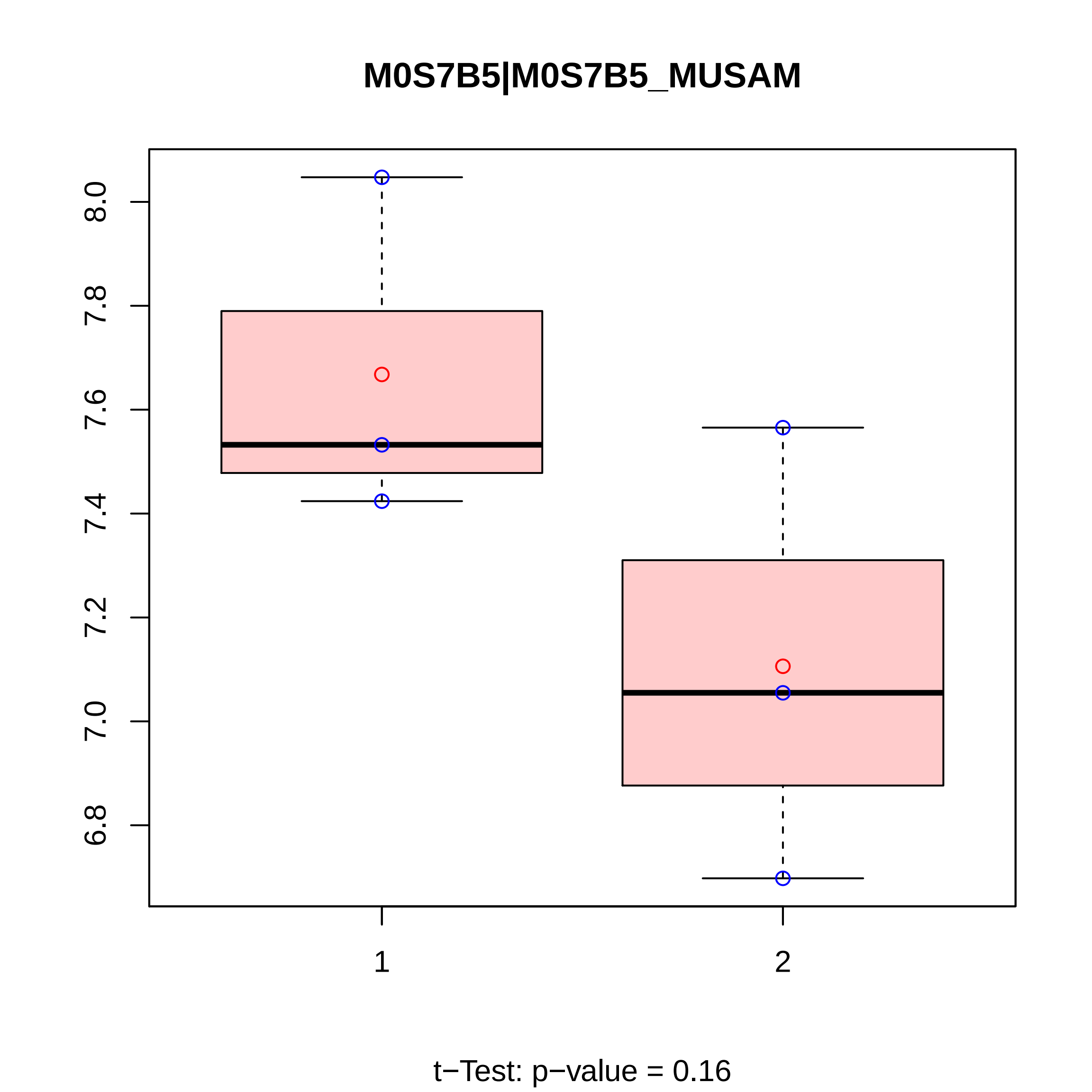
M0S7B5|M0S7B5_MUSAM
8.0
7.8
7.6
7.4
7.2
7.0
6.8
1
2
t−Test: p−value = 0.16

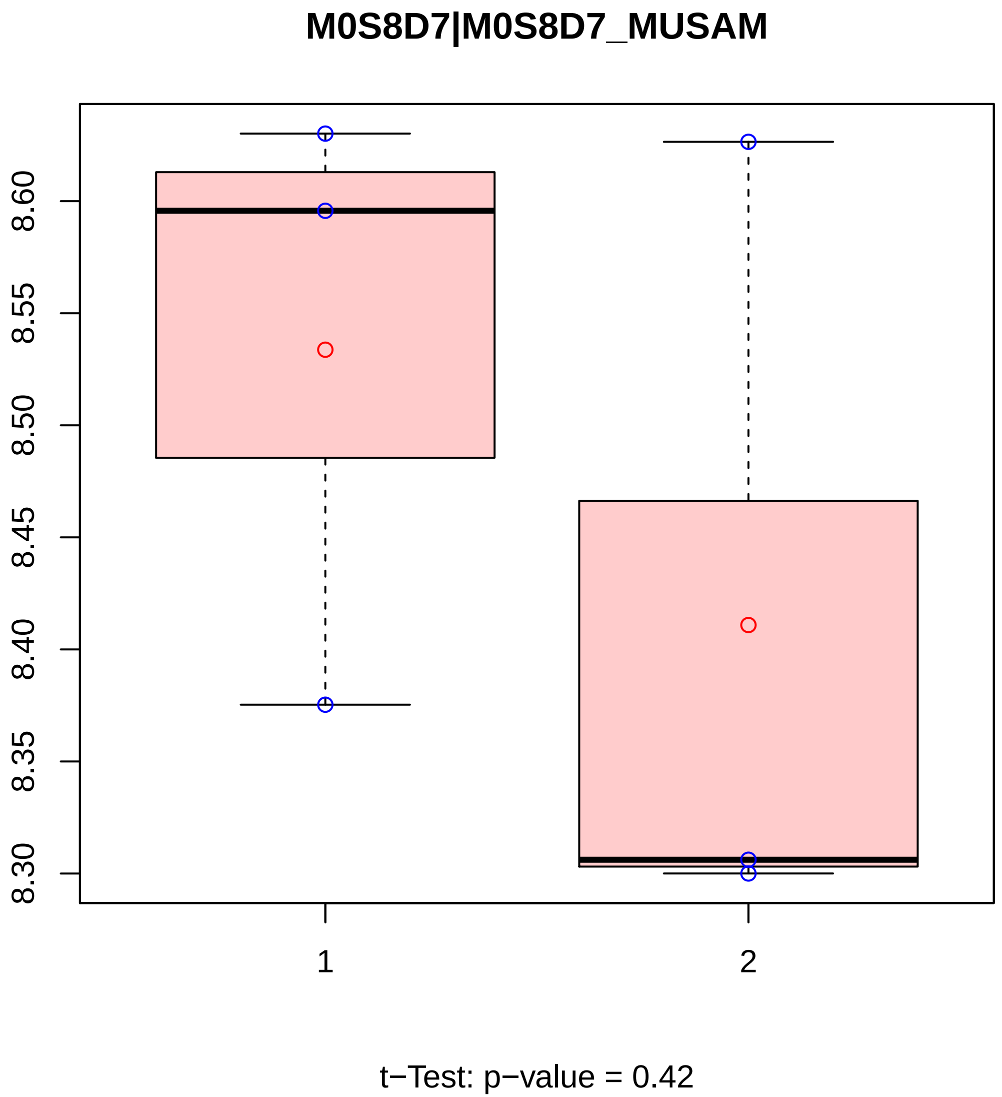
M0S8D7|M0S8D7_MUSAM
8.30
8.35
8.40
8.45
8.50
8.55
8.60
1
2
t−Test: p−value = 0.42

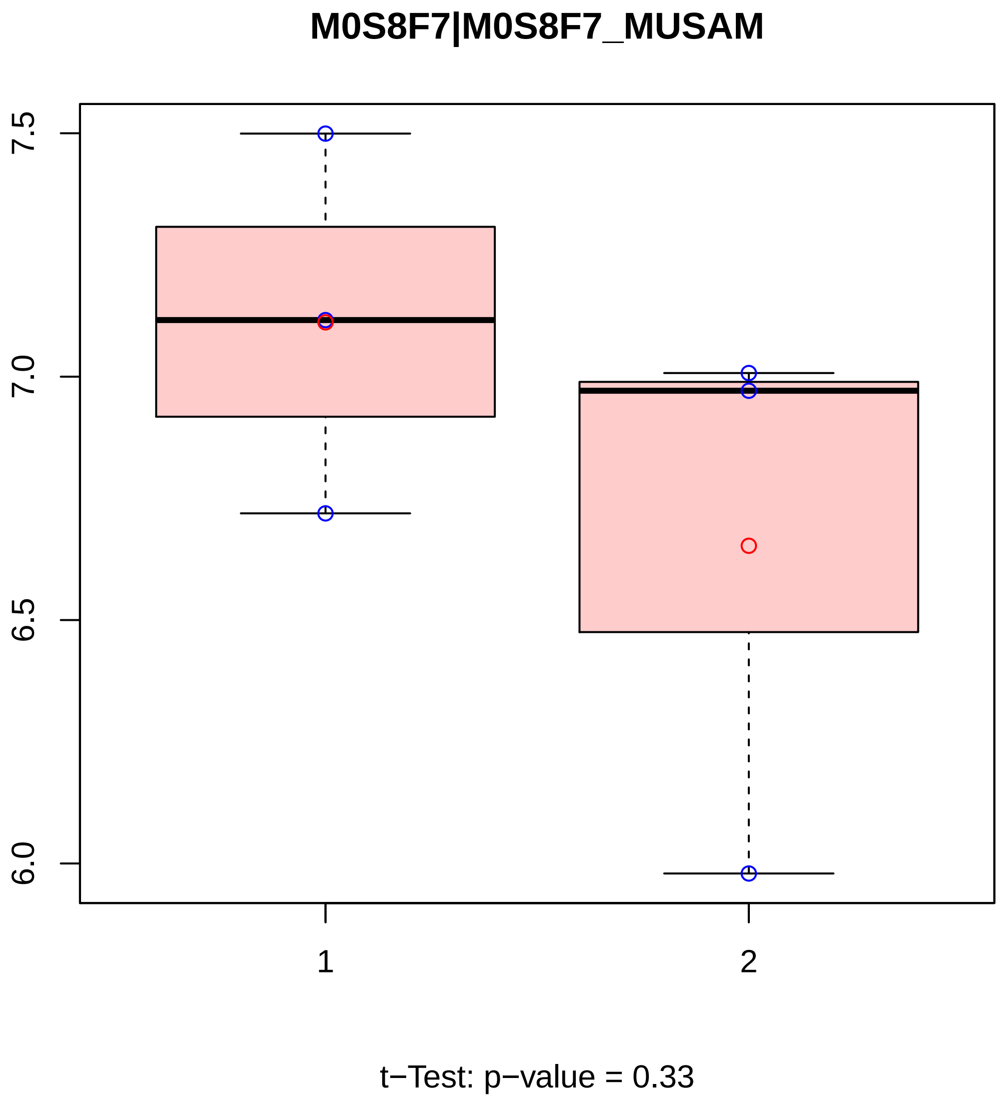
M0S8F7|M0S8F7_MUSAM
7.5
7.0
6.5
6.0
1
2
t−Test: p−value = 0.33

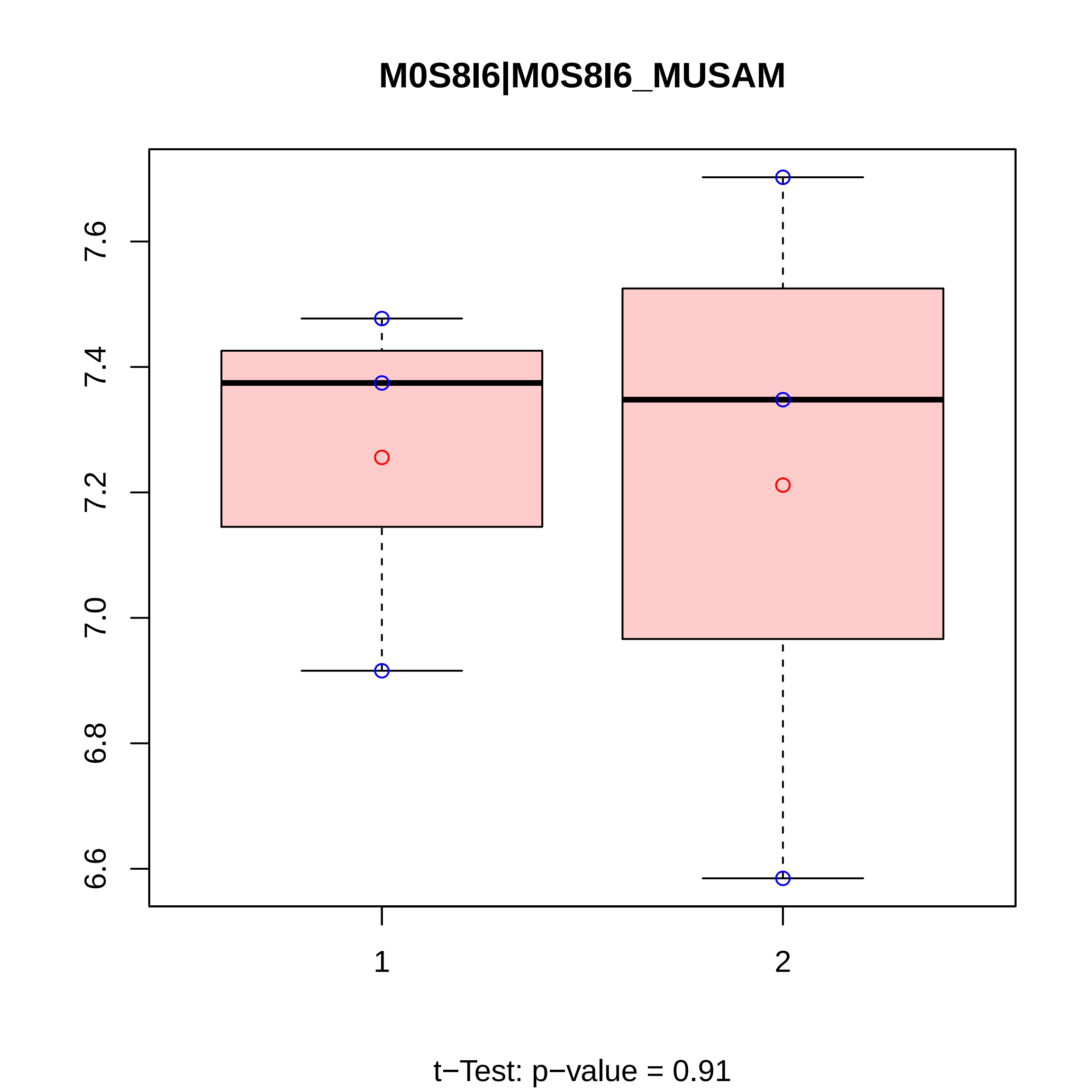
M0S8I6|M0S8I6_MUSAM
7.6
7.4
7.2
7.0
6.8
6.6
1
2
t−Test: p−value = 0.91

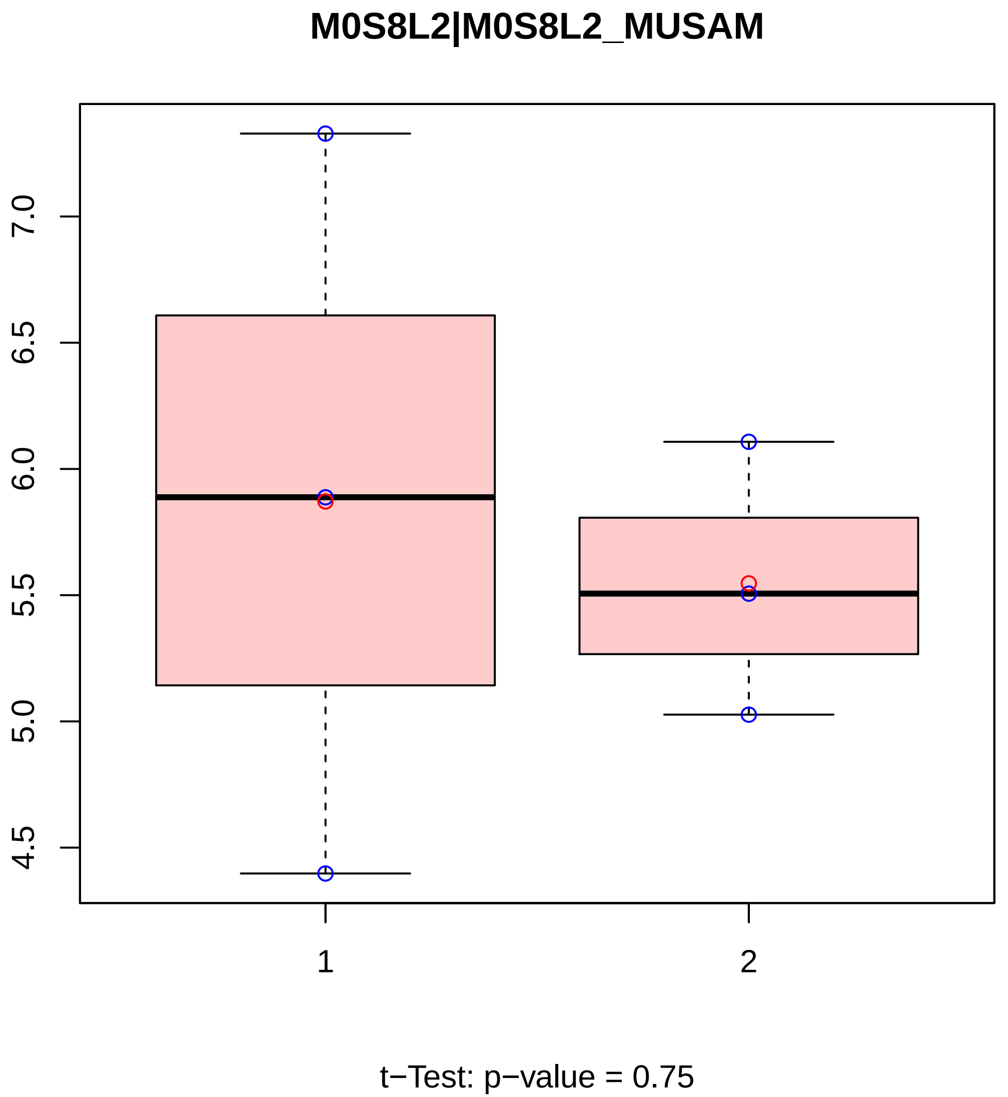

M0S8L2|M0S8L2_MUSAM
4.5
5.0
5.5
6.0
6.5
7.0
1
2
t−Test: p−value = 0.75

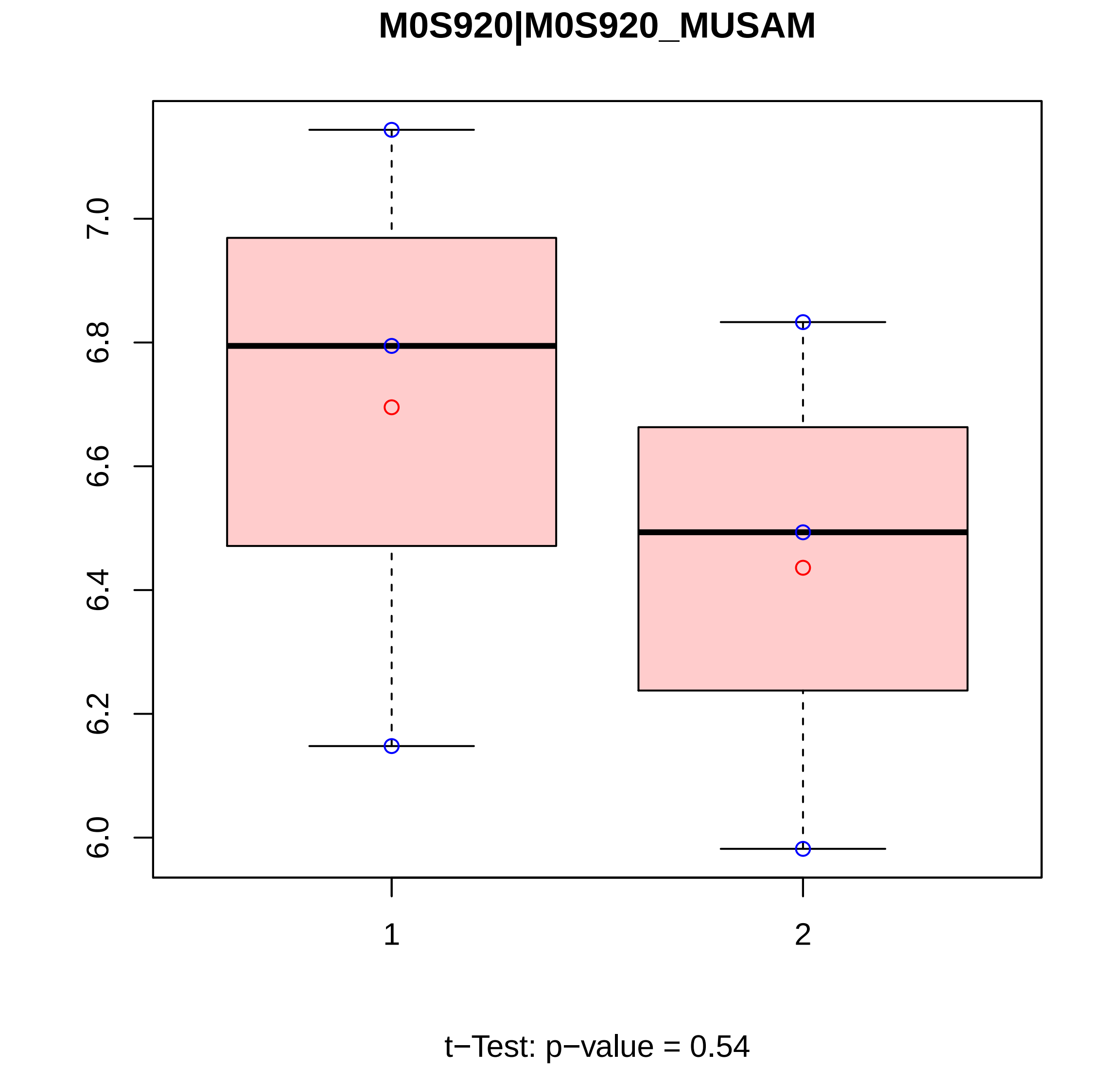
M0S920|M0S920_MUSAM
7.0
6.8
6.6
6.4
6.2
6.0
1
2
t−Test: p−value = 0.54

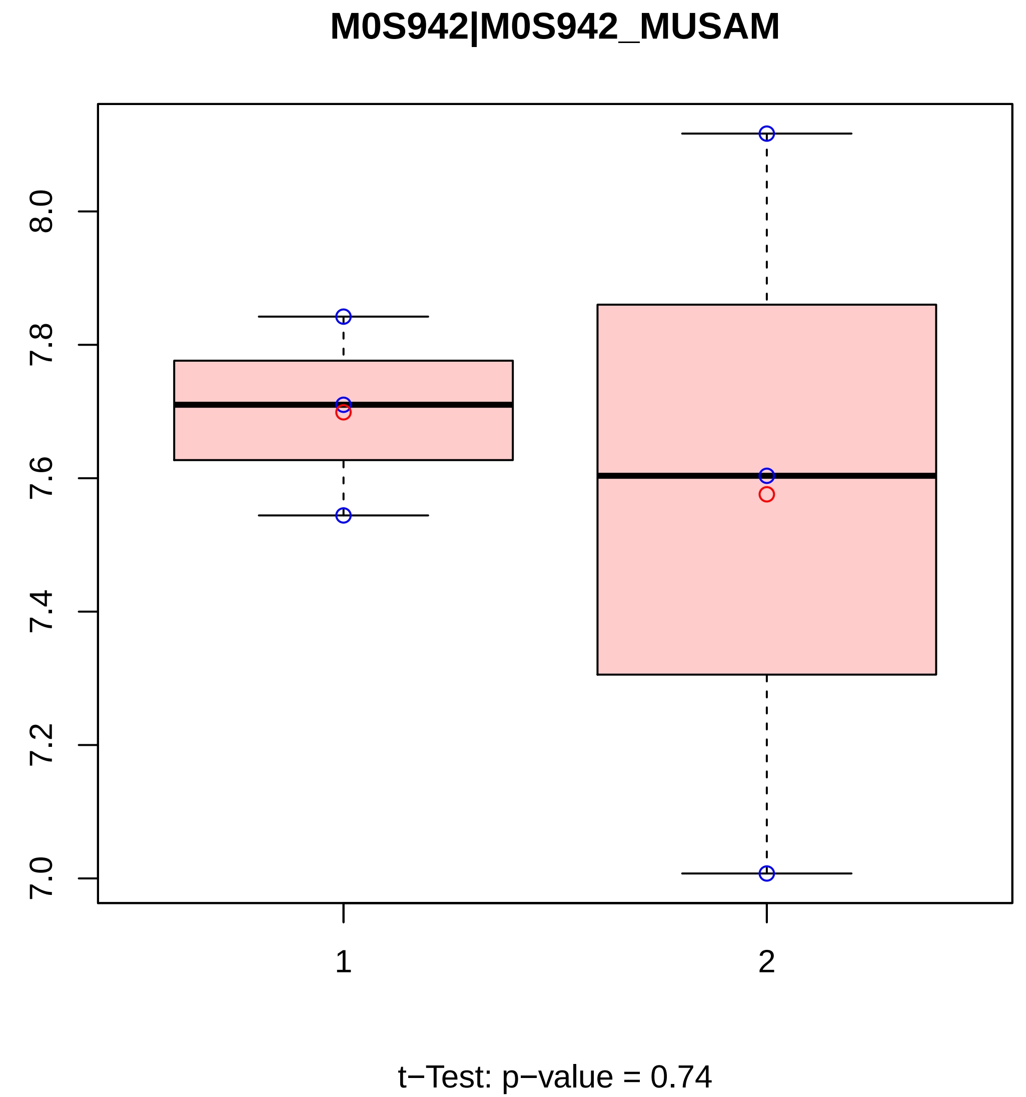
M0S942|M0S942_MUSAM
8.0
7.8
7.6
7.4
7.2
7.0
1
2
t−Test: p−value = 0.74

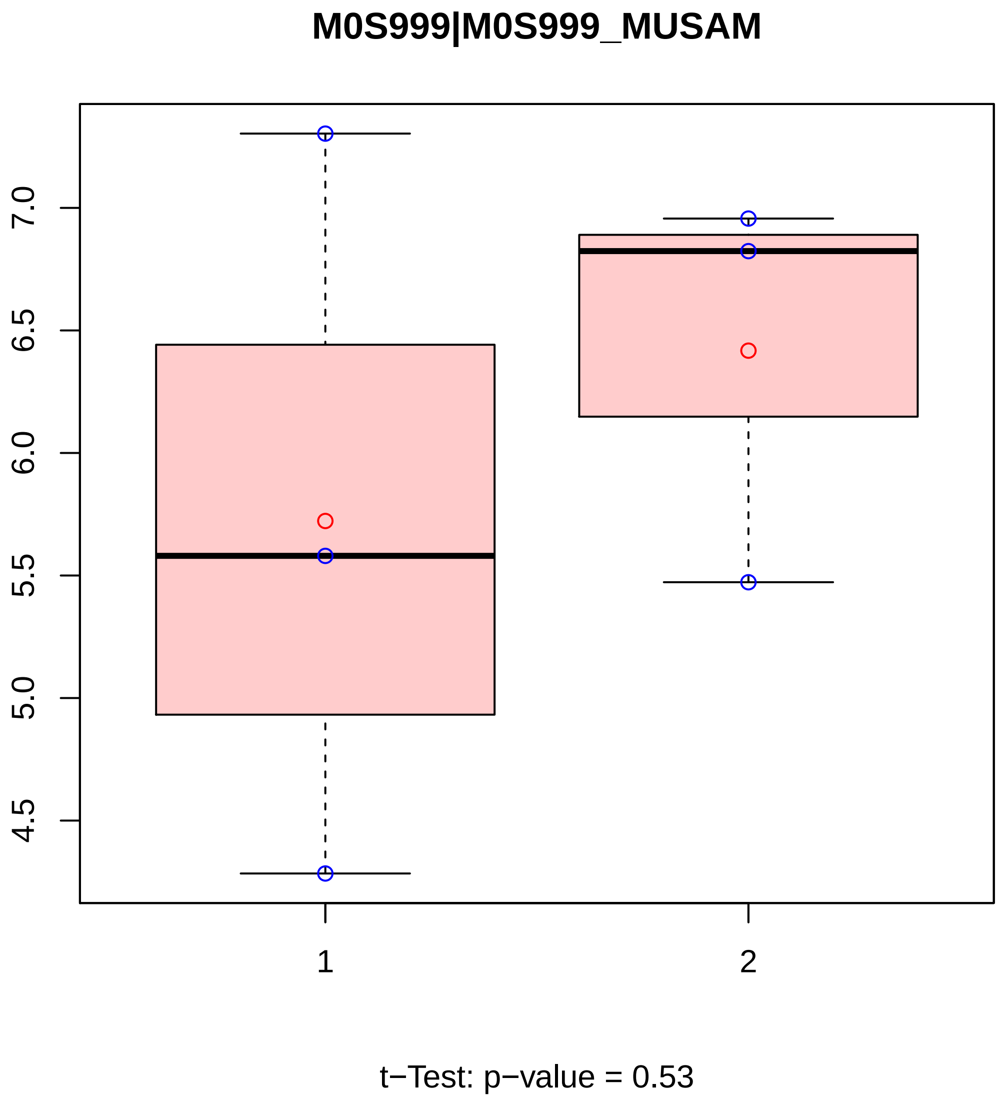
M0S999|M0S999_MUSAM
7.0
6.5
6.0
5.5
5.0
4.5
1
2
t−Test: p−value = 0.53

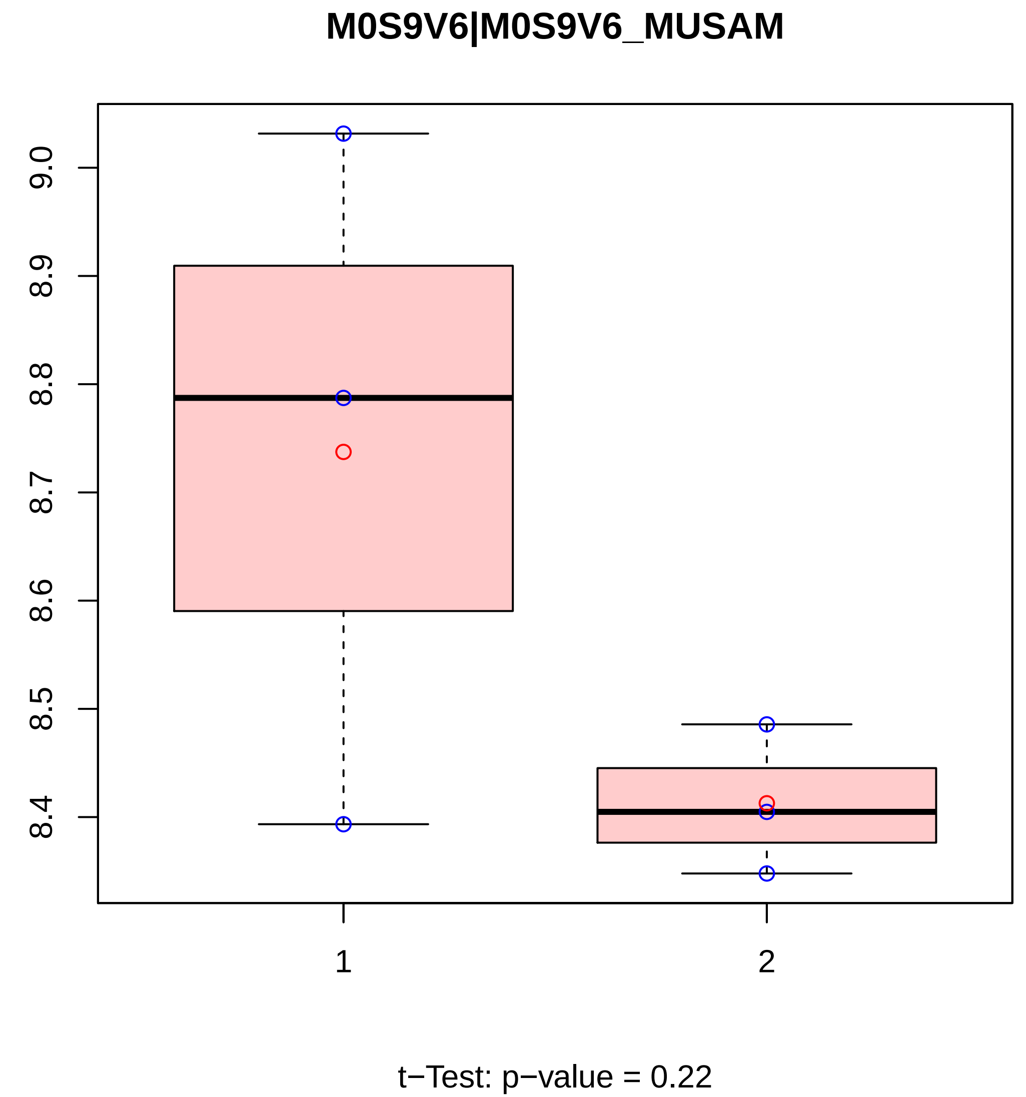
M0S9V6|M0S9V6_MUSAM
9.0
8.9
8.8
8.7
8.6
8.5
8.4
1
2
t−Test: p−value = 0.22

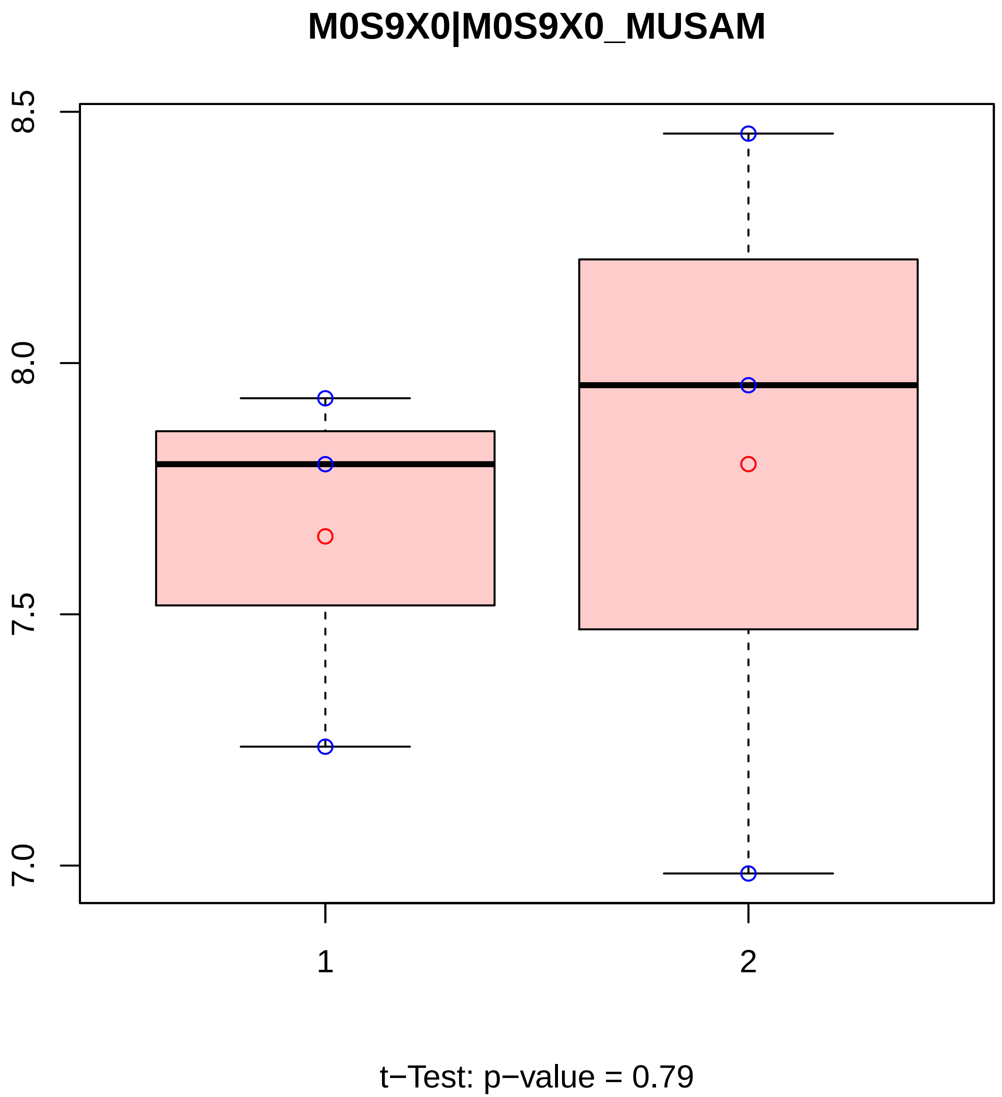

M0S9X0|M0S9X0_MUSAM
8.5
8.0
7.5
7.0
1
2
t−Test: p−value = 0.79

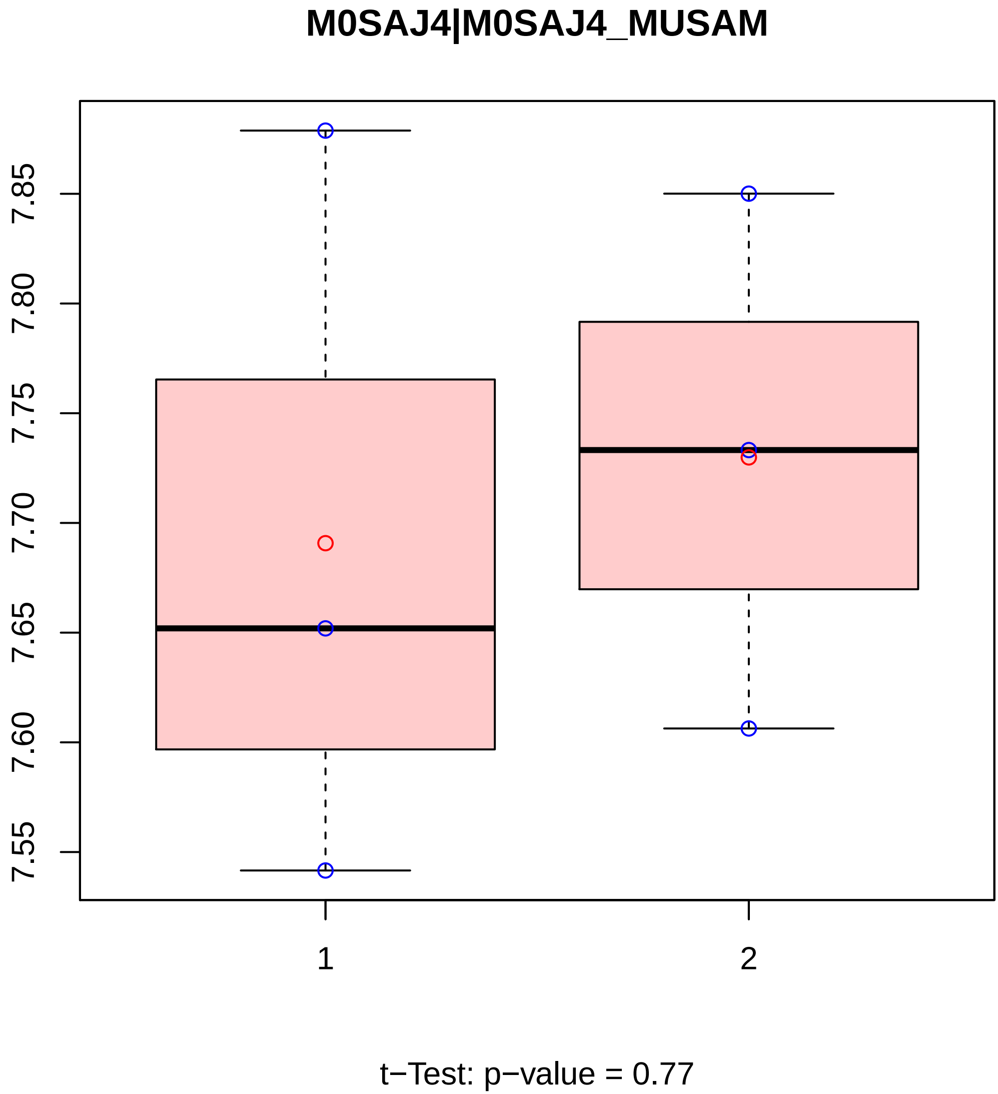
M0SAJ4|M0SAJ4_MUSAM
7.55
7.60
7.65
7.70
7.75
7.80
7.85
1
2
t−Test: p−value = 0.77

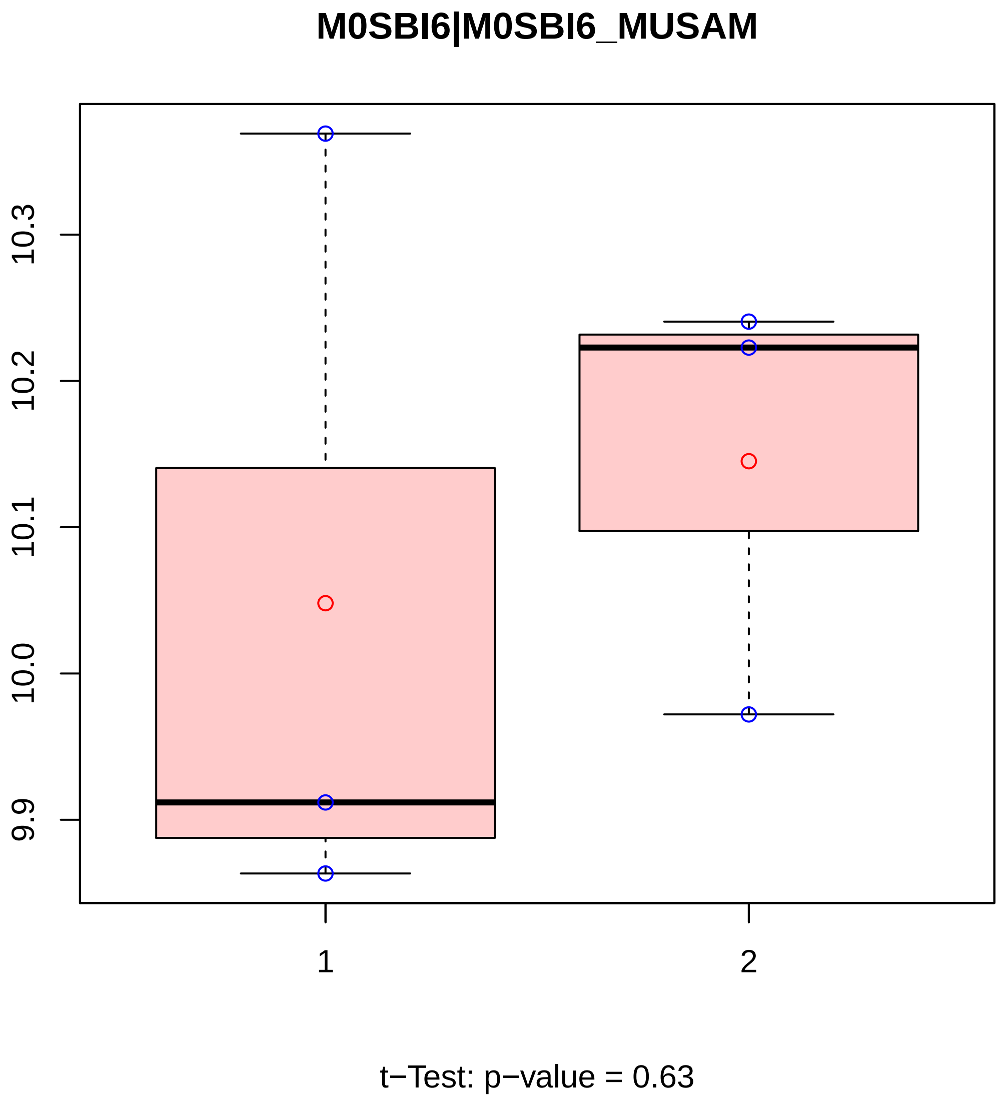
M0SBI6|M0SBI6_MUSAM
10.3
10.2
10.1
10.0
9.9
1
2
t−Test: p−value = 0.63

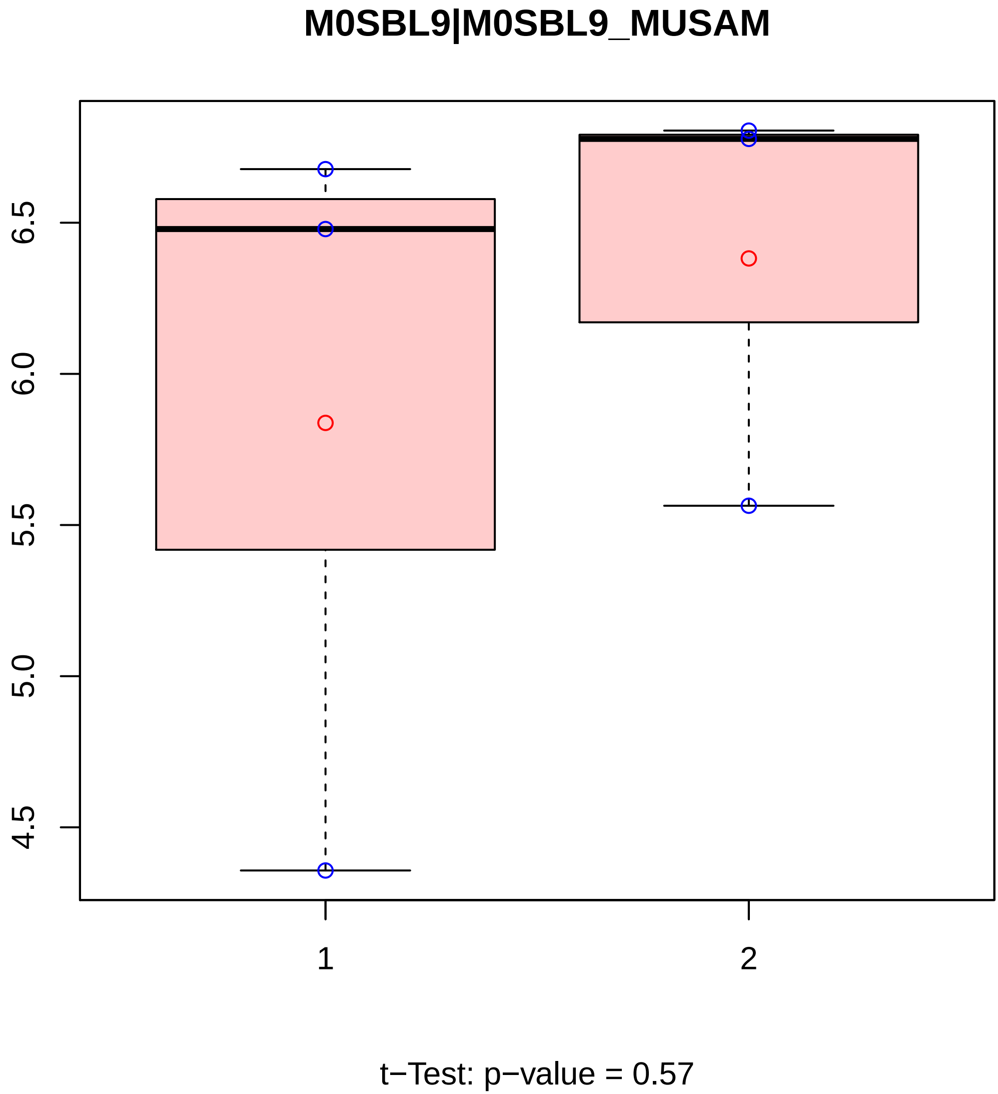

M0SBL9|M0SBL9_MUSAM
4.5
5.0
5.5
6.0
6.5
1
2
t−Test: p−value = 0.57

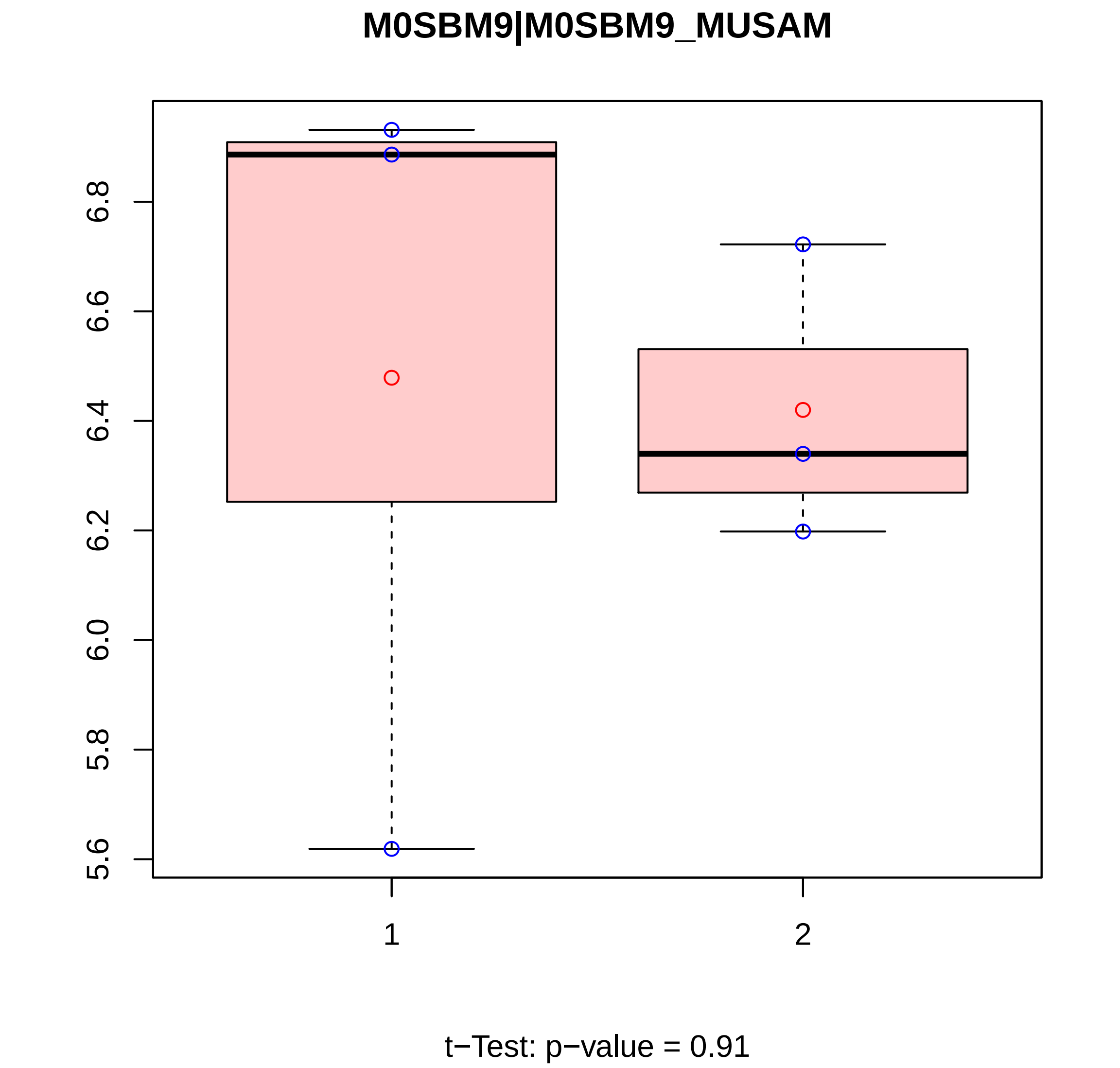
M0SBM9|M0SBM9_MUSAM
6.8
6.6
6.4
6.2
6.0
5.8
5.6
1
2
t−Test: p−value = 0.91

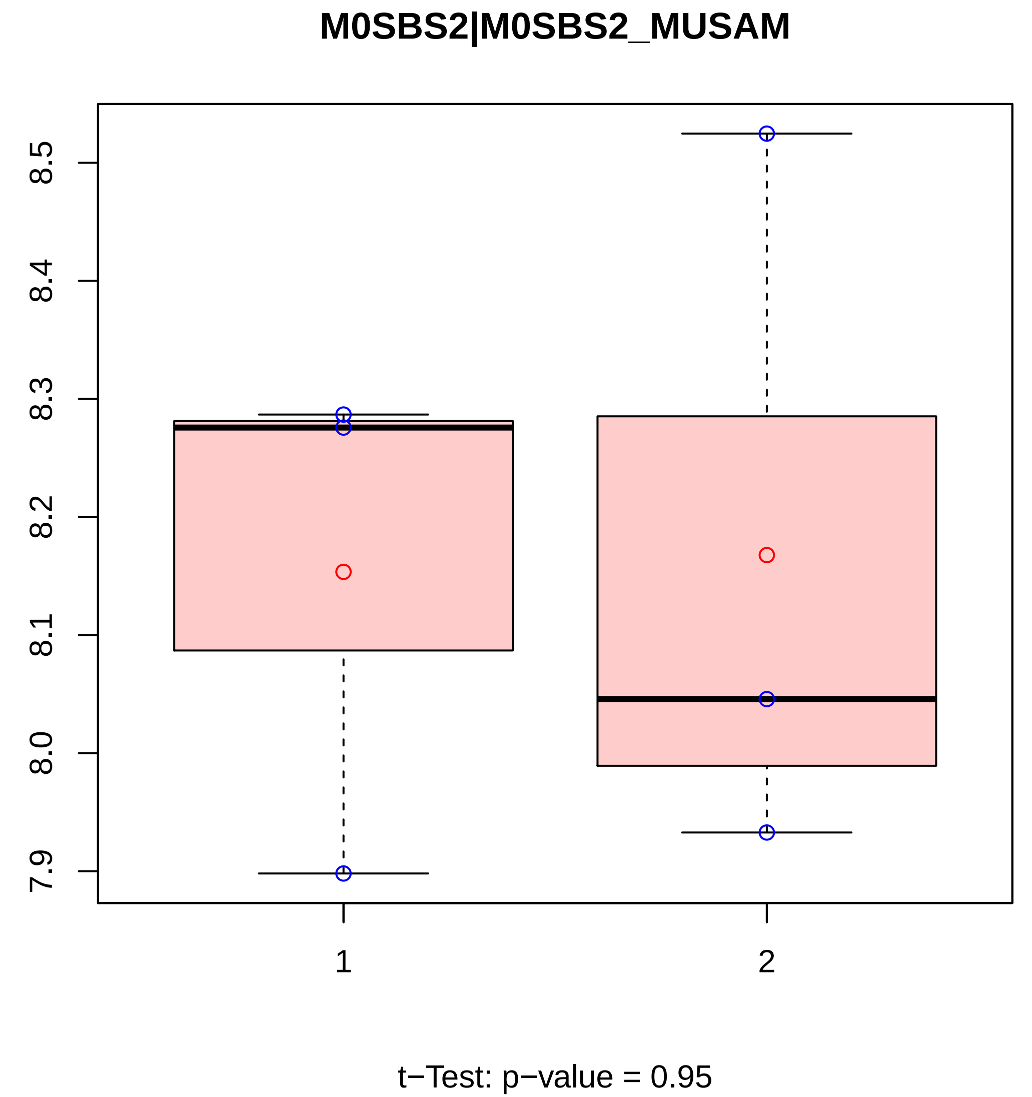
M0SBS2|M0SBS2_MUSAM
8.5
8.4
8.3
8.2
8.1
8.0
7.9
1
2
t−Test: p−value = 0.95

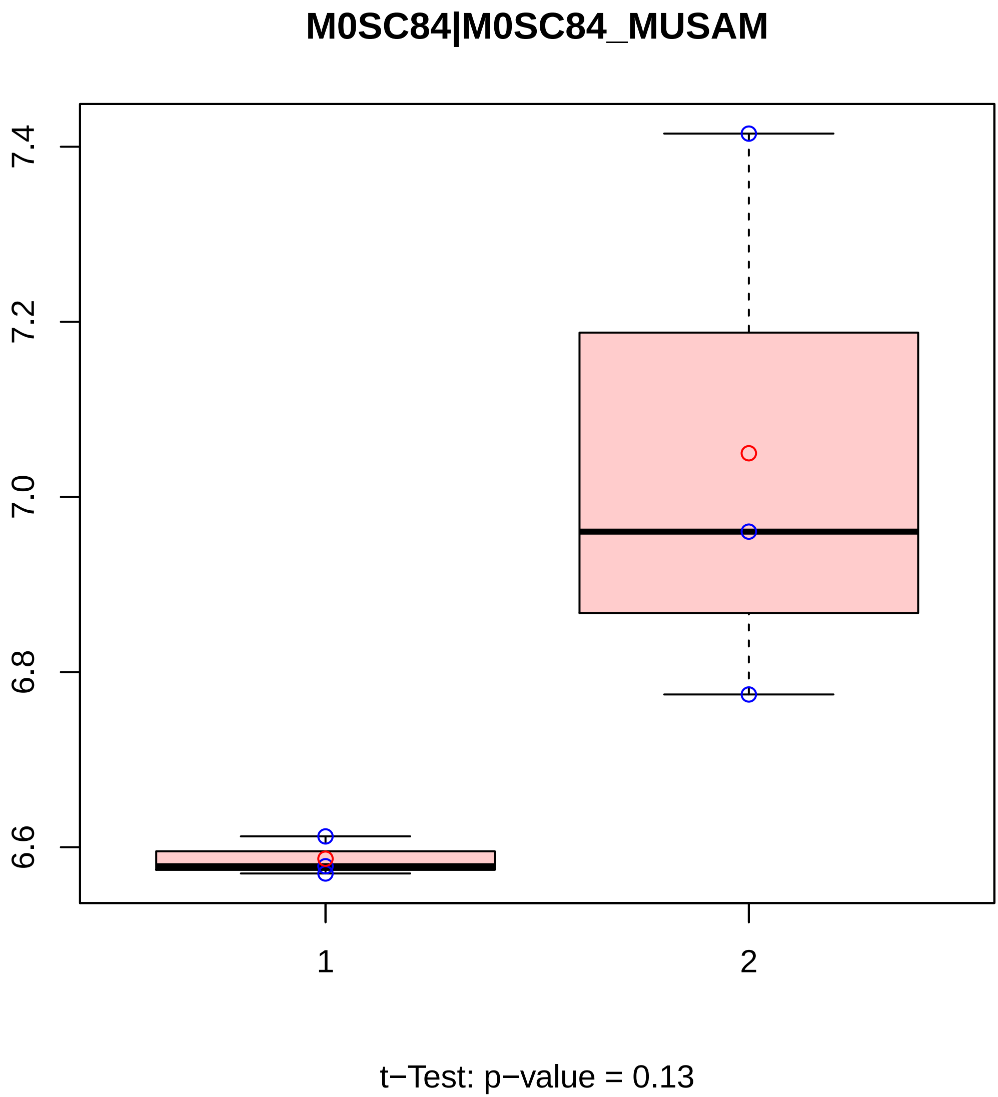

M0SC84|M0SC84_MUSAM
7.4
7.2
7.0
6.8
6.6
1
2
t−Test: p−value = 0.13

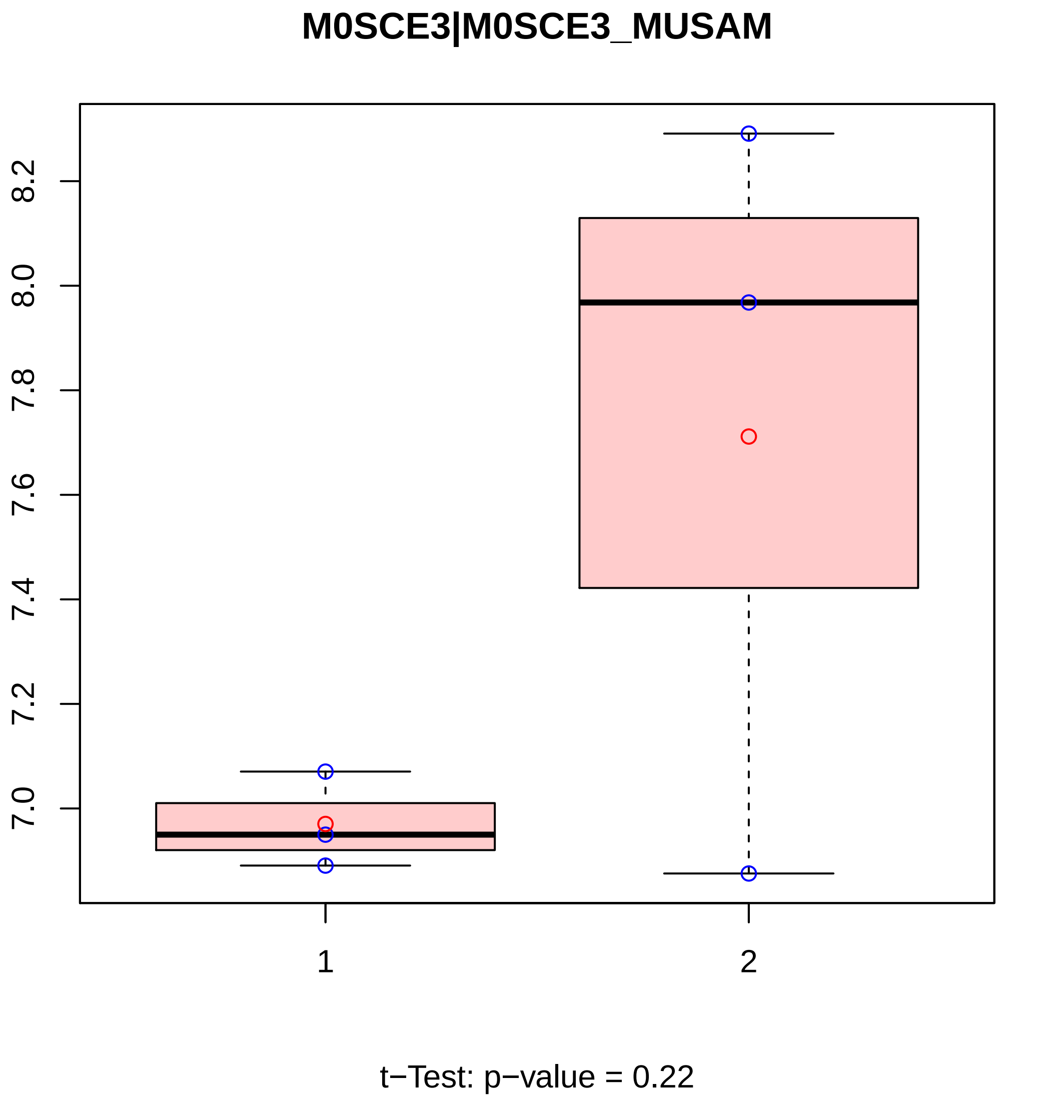

M0SCE3|M0SCE3_MUSAM
8.2
8.0
7.8
7.6
7.4
7.2
7.0
1
2
t−Test: p−value = 0.22

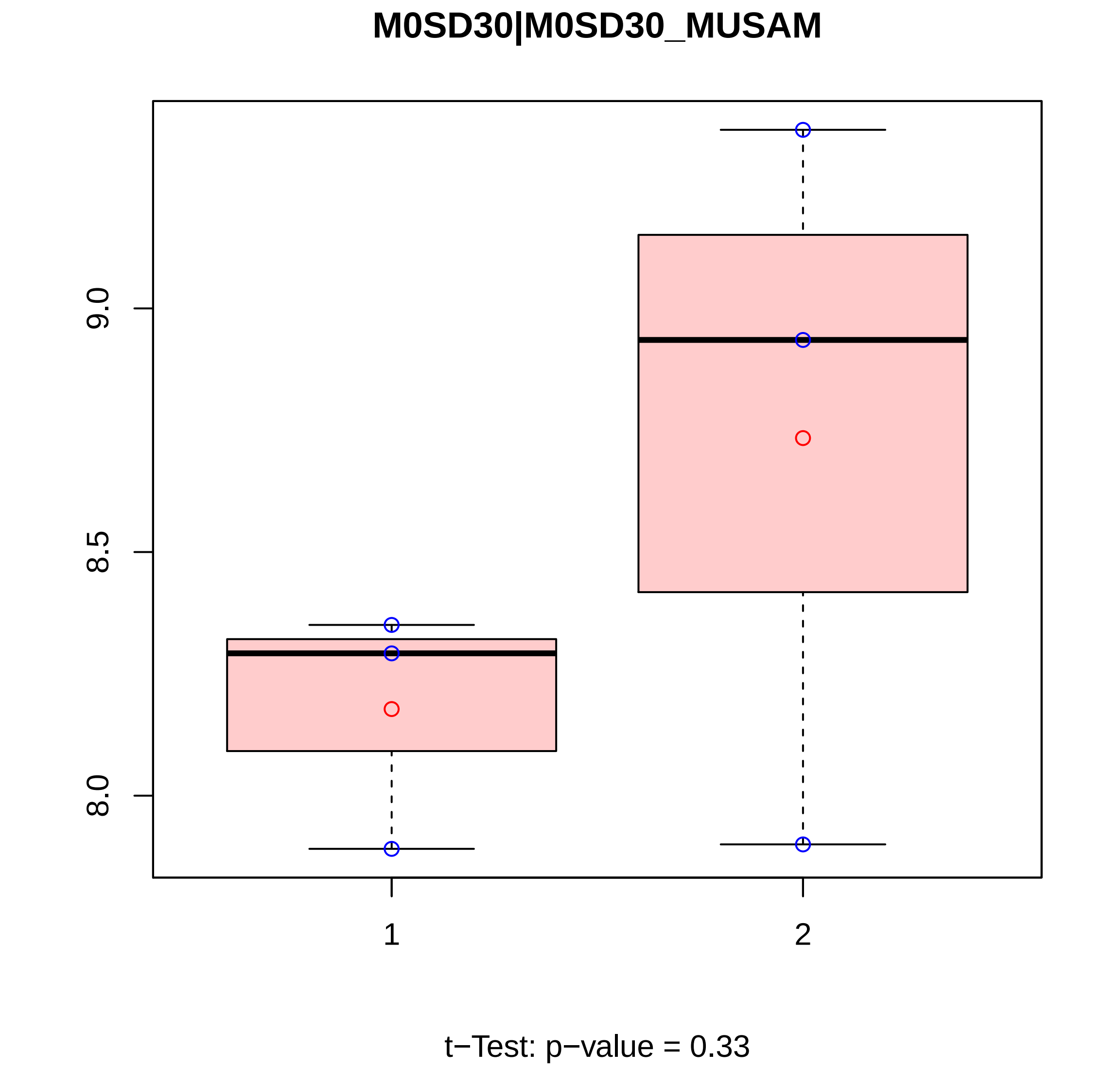

M0SD30|M0SD30_MUSAM
9.0
8.5
8.0
1
2
t−Test: p−value = 0.33

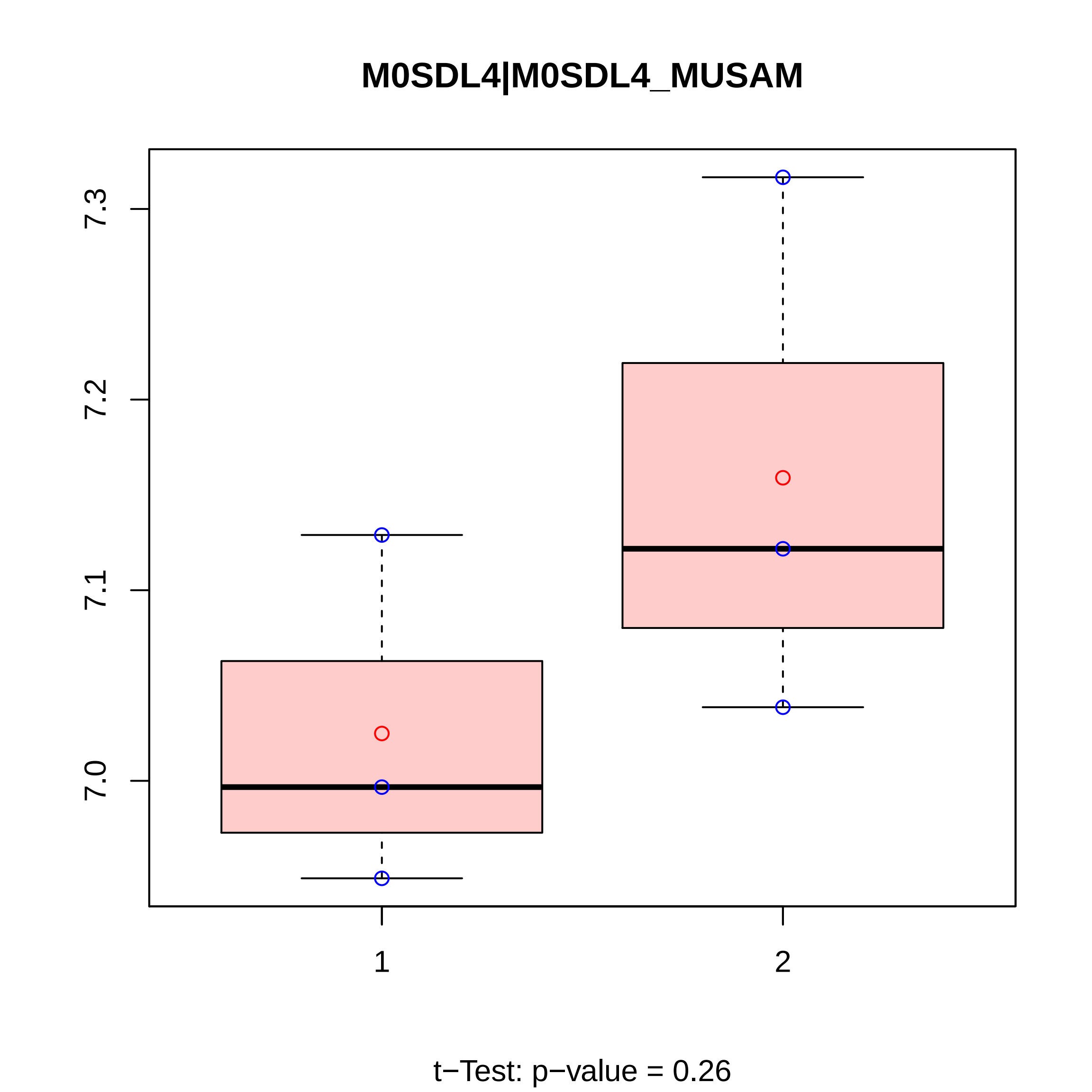

M0SDL4|M0SDL4_MUSAM
7.3
7.2
7.1
7.0
1
2
t−Test: p−value = 0.26

M0SDS2|M0SDS2_MUSAM

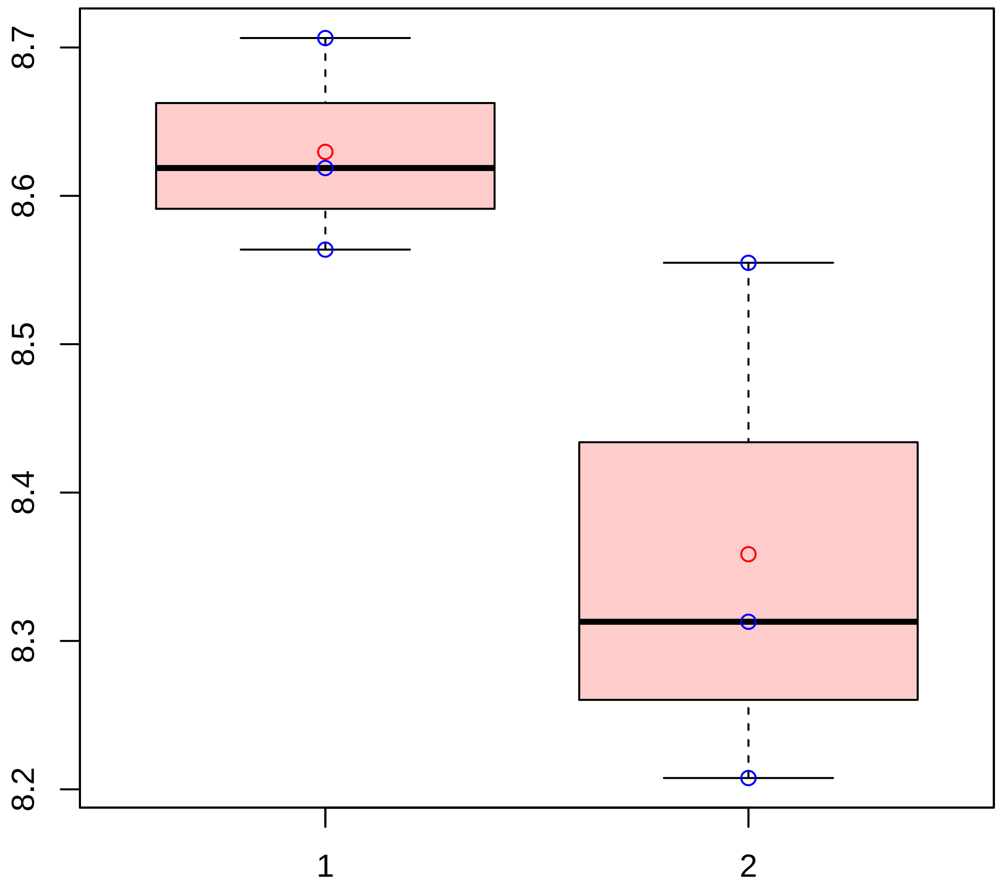

t−Test: p−value = 0.1

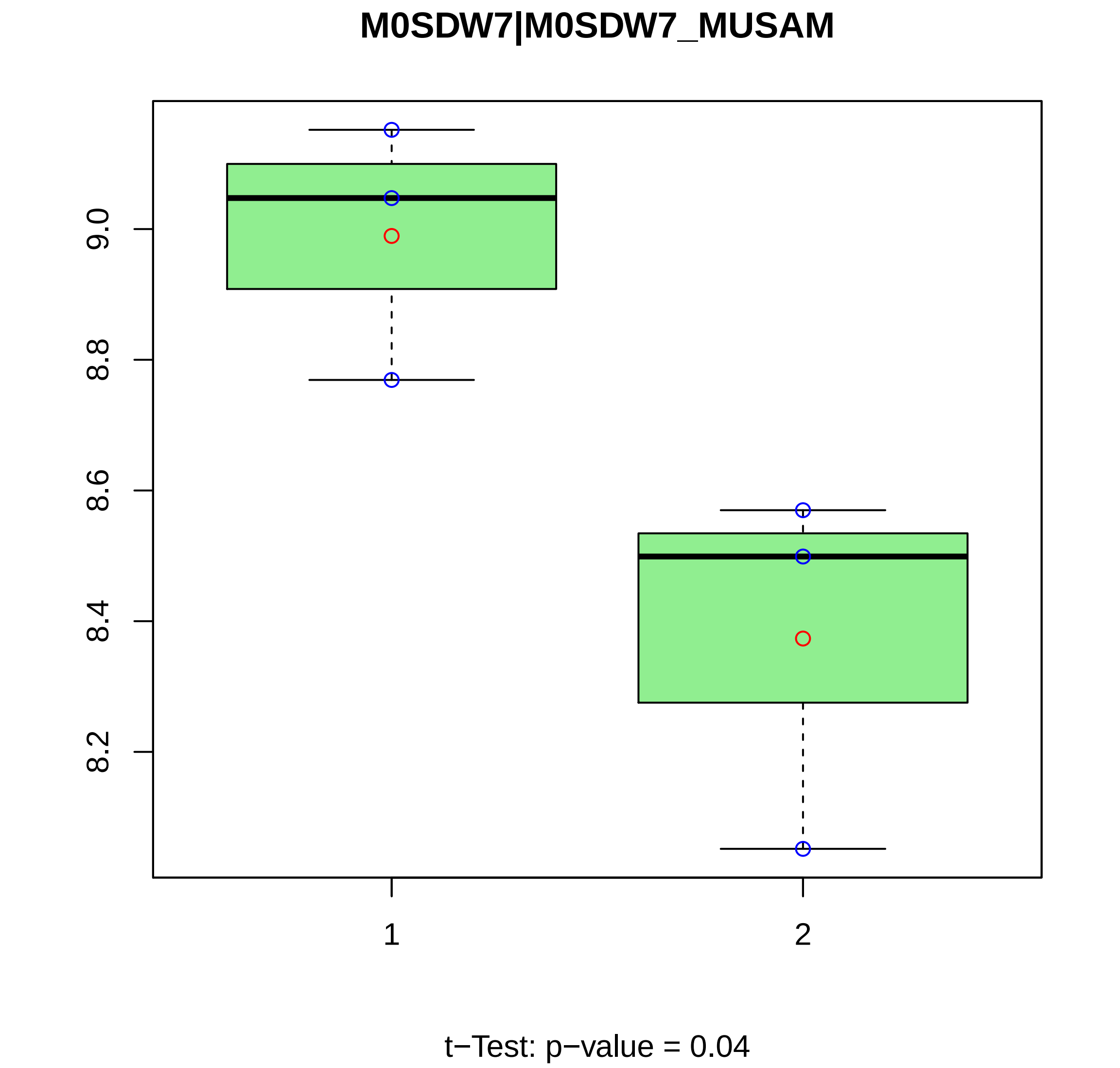
M0SDW7|M0SDW7_MUSAM
9.0
8.8
8.6
8.4
8.2
1
2
t−Test: p−value = 0.04

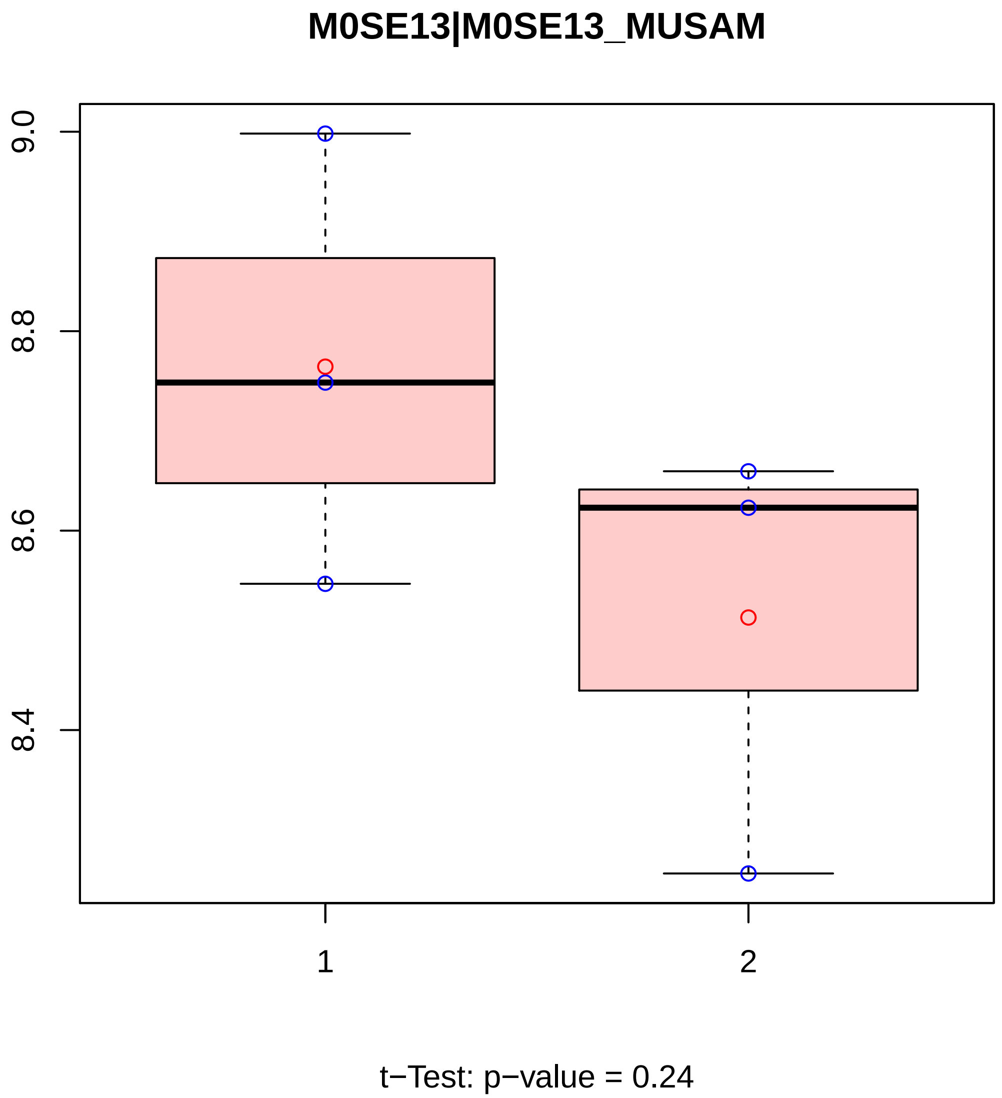
M0SE13|M0SE13_MUSAM
9.0
8.8
8.6
8.4
1
2
t−Test: p−value = 0.24

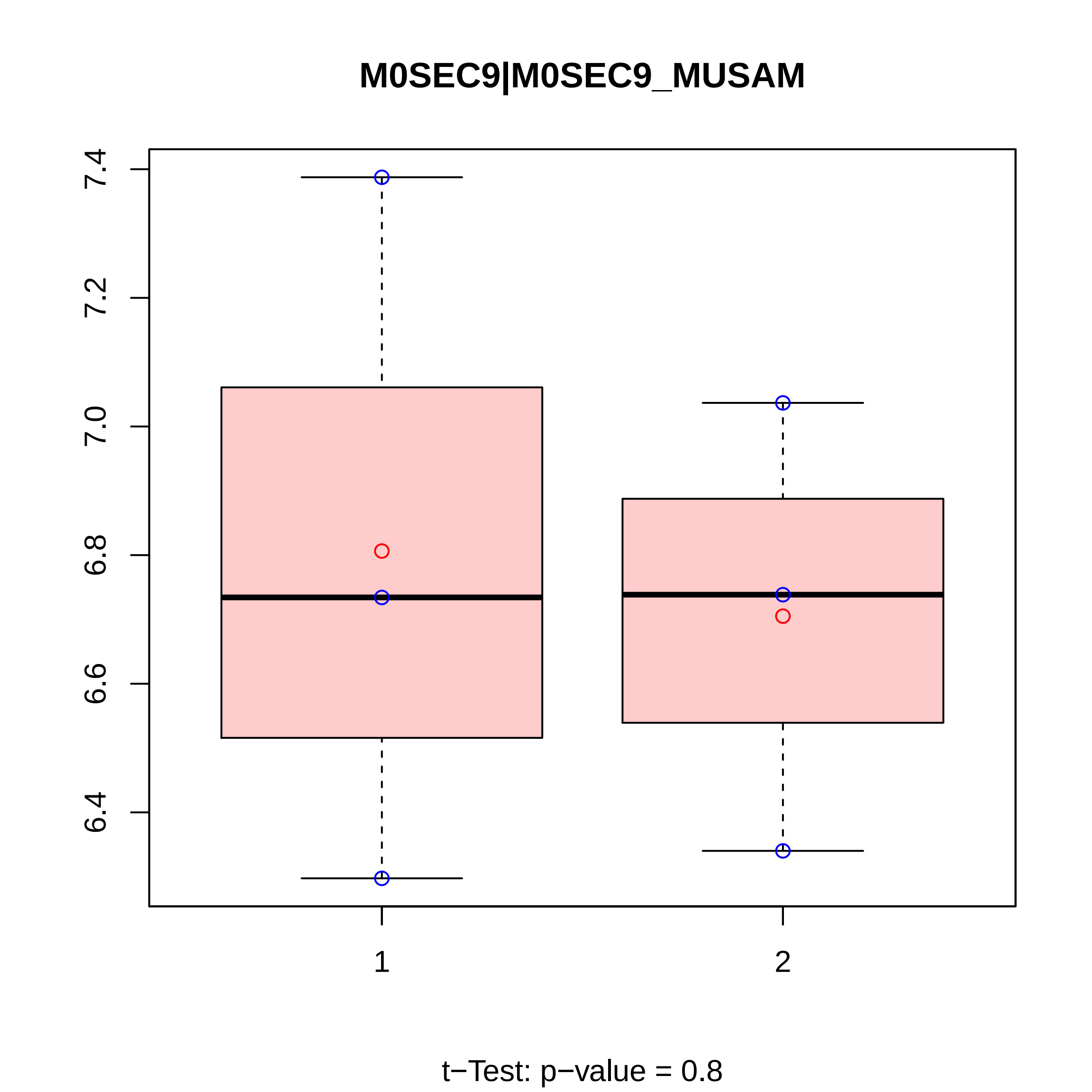
M0SEC9|M0SEC9_MUSAM
7.4
7.2
7.0
6.8
6.6
6.4
1
2
t−Test: p−value = 0.8

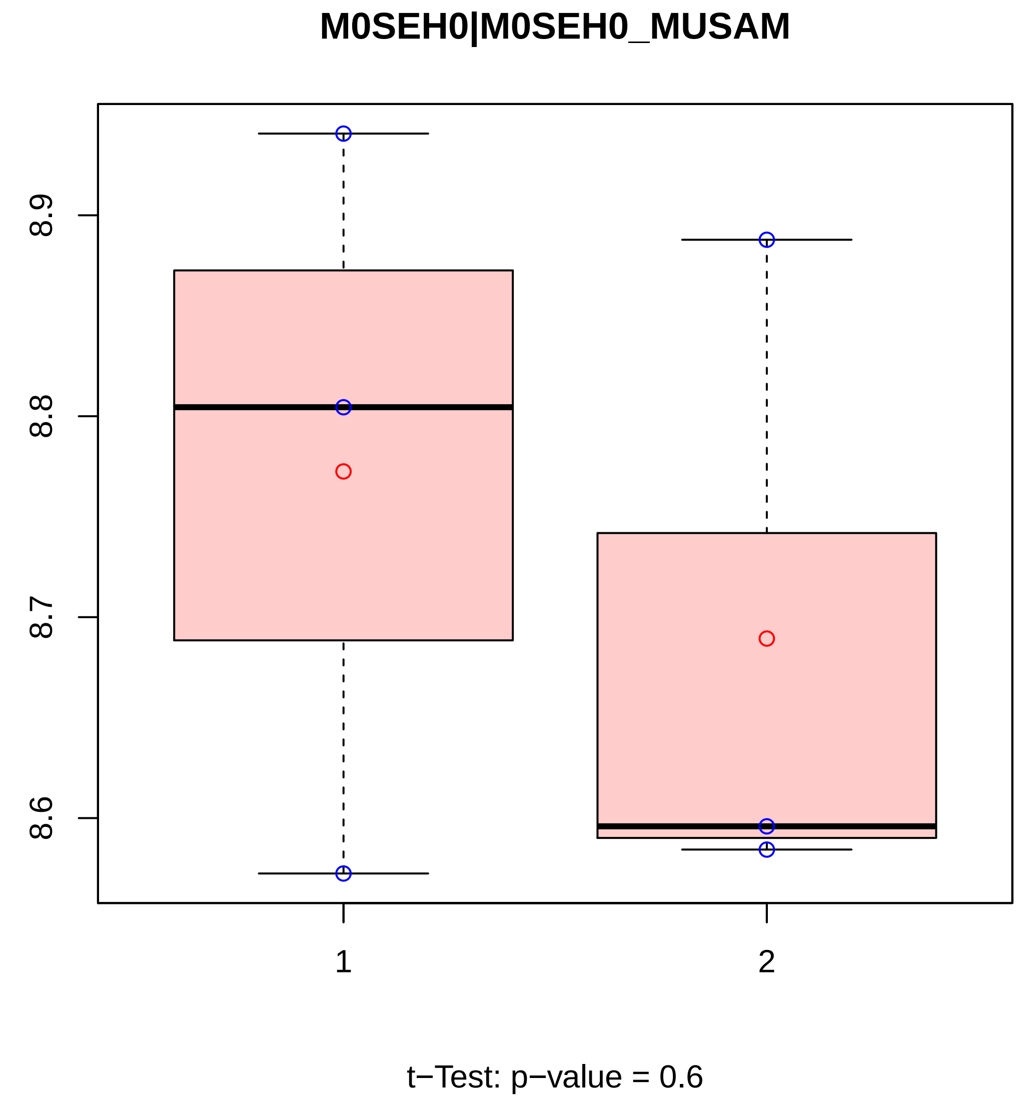
M0SEH0|M0SEH0_MUSAM
8.6
8.7
8.8
8.9
1
2
t−Test: p−value = 0.6

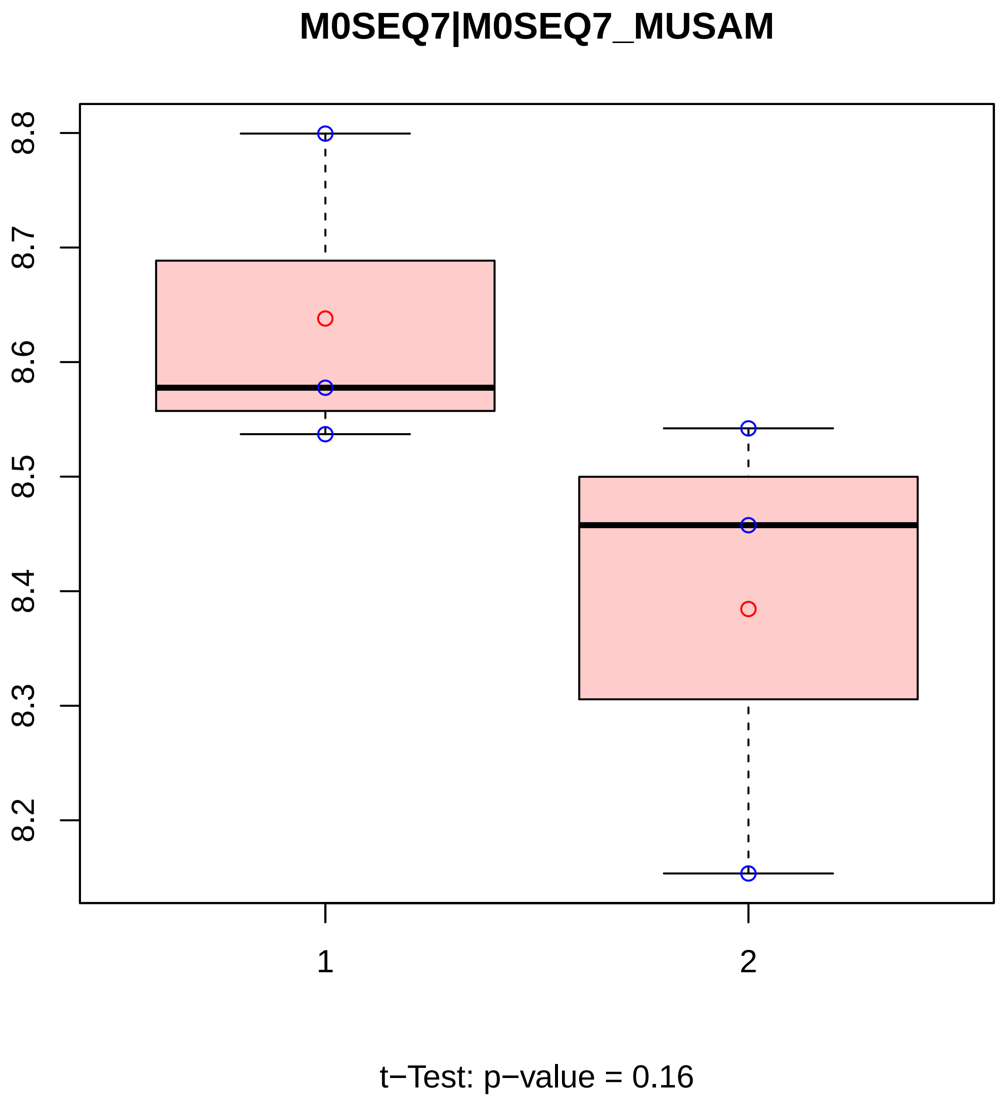
M0SEQ7|M0SEQ7_MUSAM
8.2
8.3
8.4
8.5
8.6
8.7
8.8
1
2
t−Test: p−value = 0.16

M0SFQ5|M0SFQ5_MUSAM

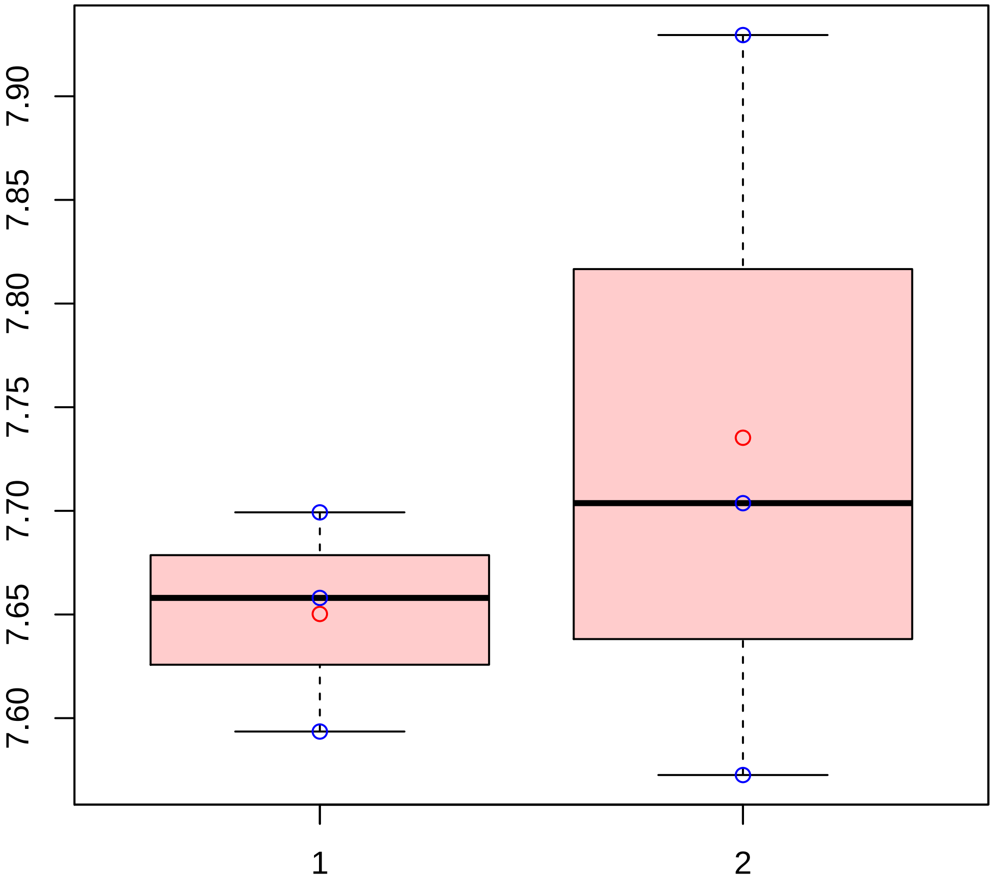

t−Test: p−value = 0.51

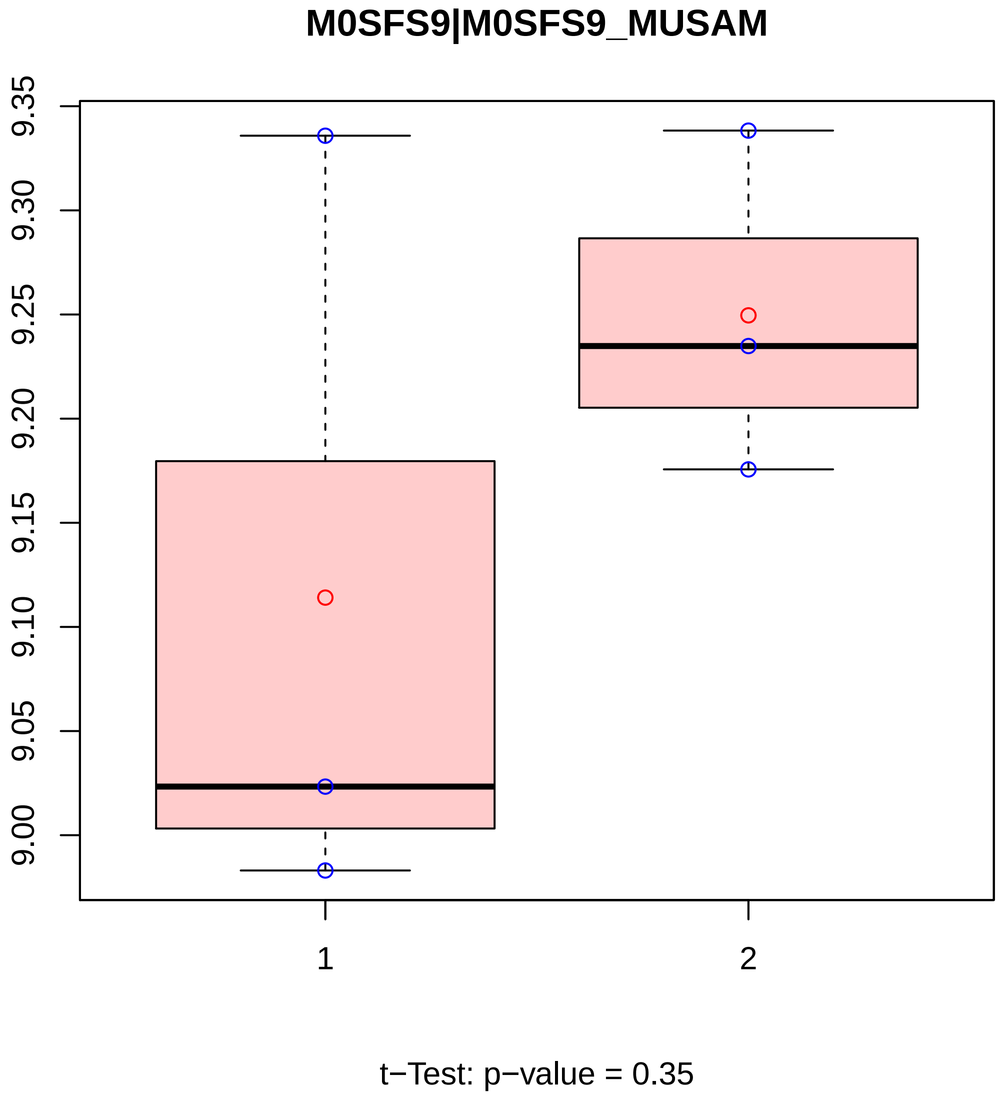
M0SFS9|M0SFS9_MUSAM
9.00
9.05
9.10
9.15
9.20
9.25
9.30
9.35
1
2
t−Test: p−value = 0.35

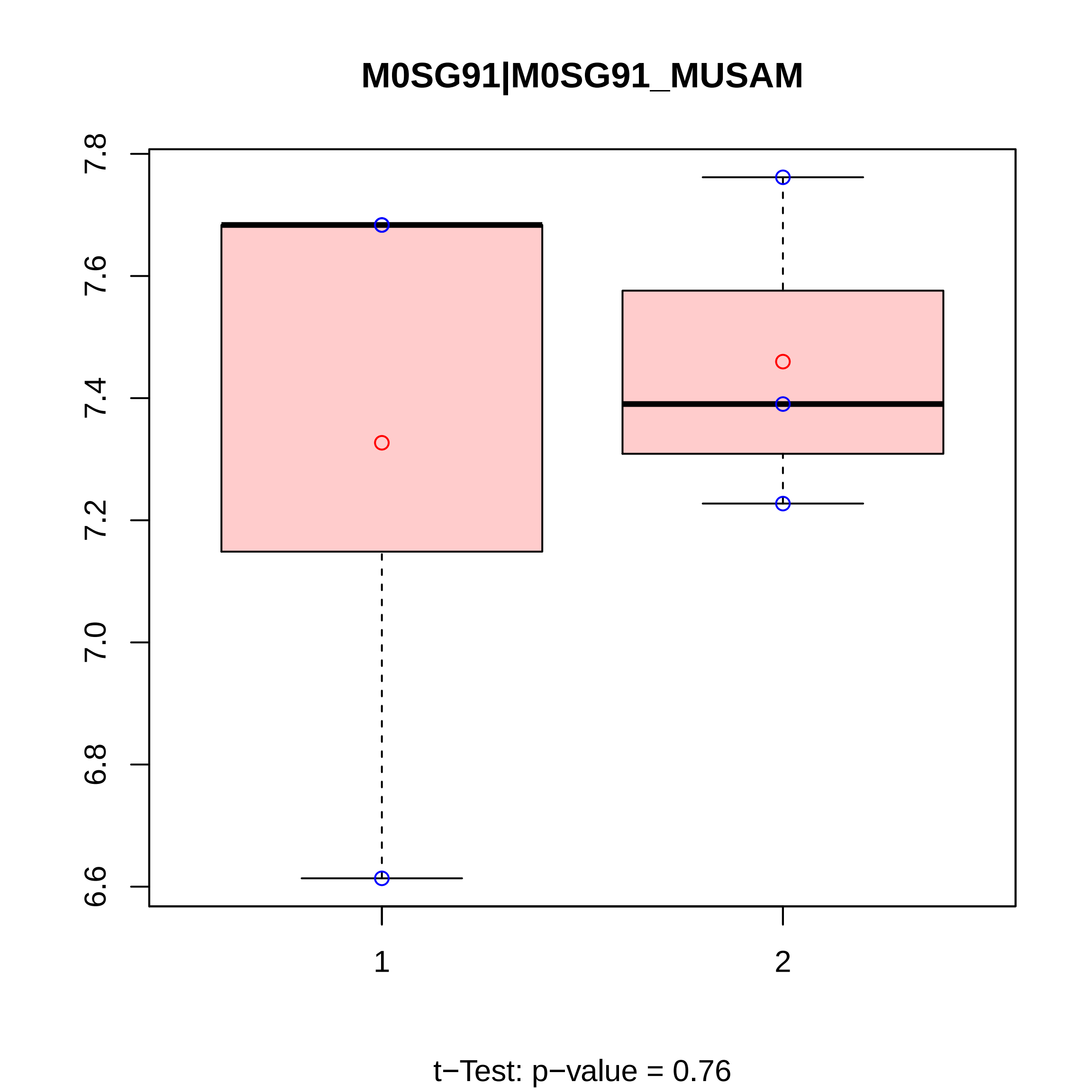
M0SG91|M0SG91_MUSAM
7.8
7.6
7.4
7.2
7.0
6.8
6.6
1
2
t−Test: p−value = 0.76

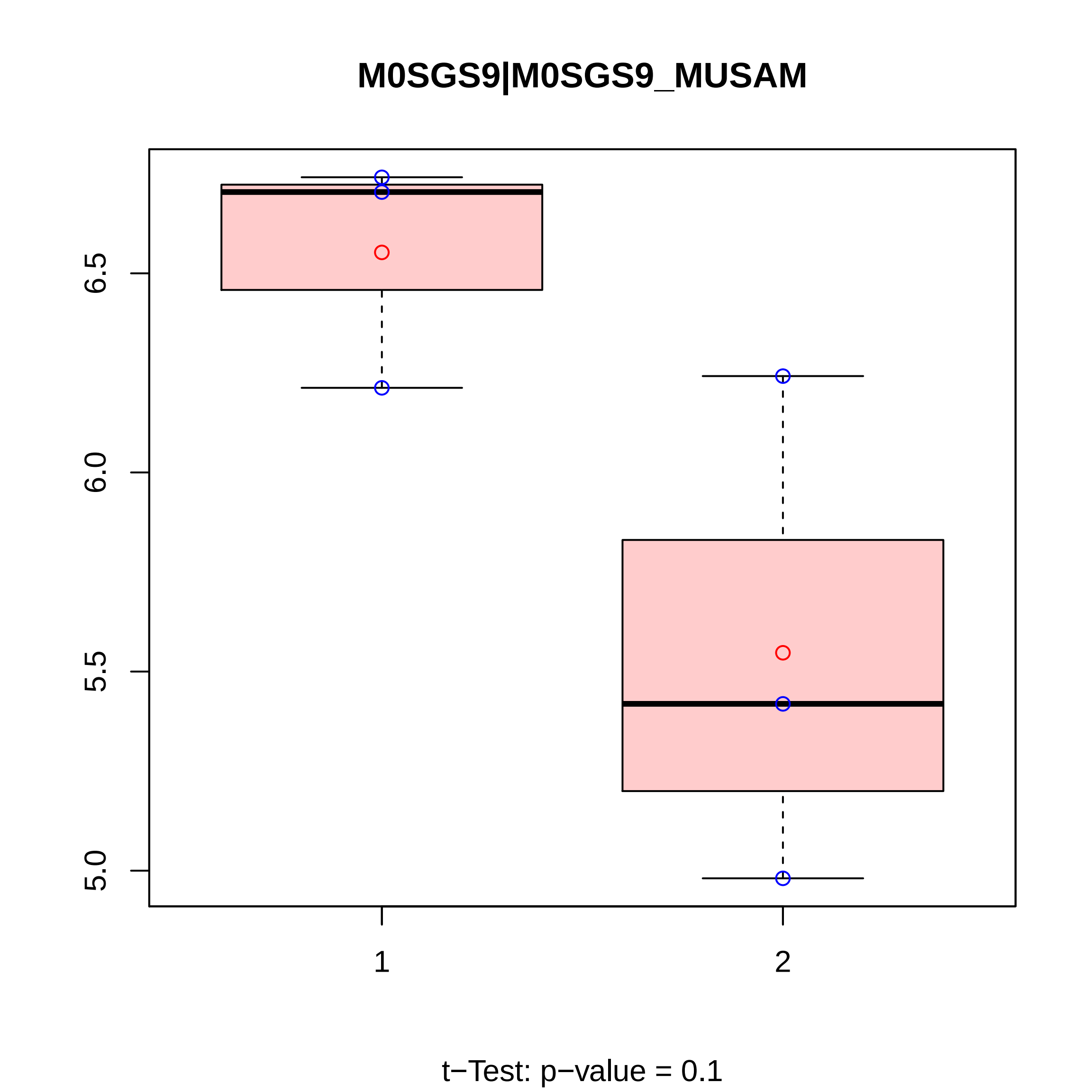
M0SGS9|M0SGS9_MUSAM
6.5
6.0
5.5
5.0
1
2
t−Test: p−value = 0.1

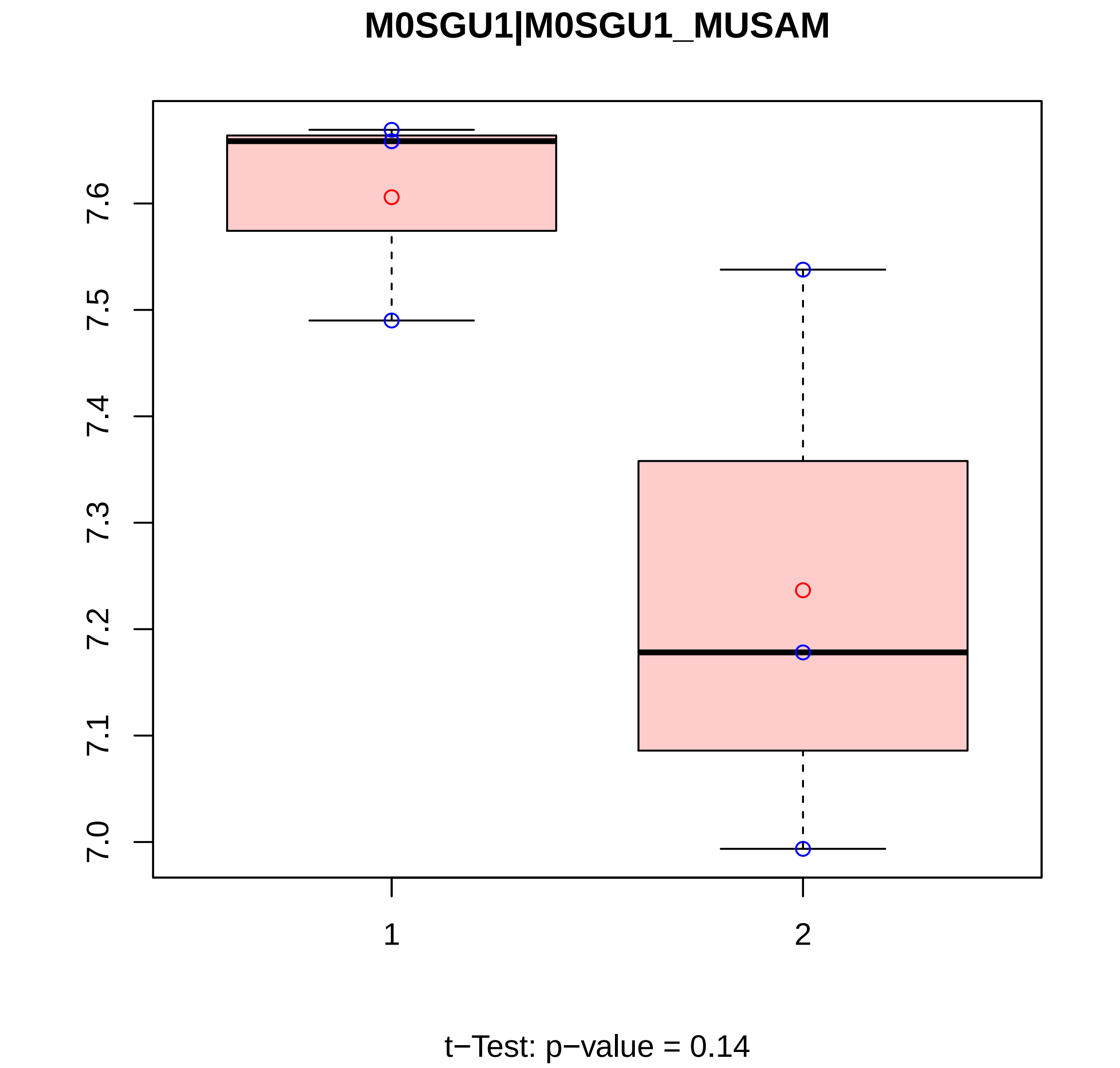
M0SGU1|M0SGU1_MUSAM
7.0
7.1
7.2
7.3
7.4
7.5
7.6
1
2
t−Test: p−value = 0.14

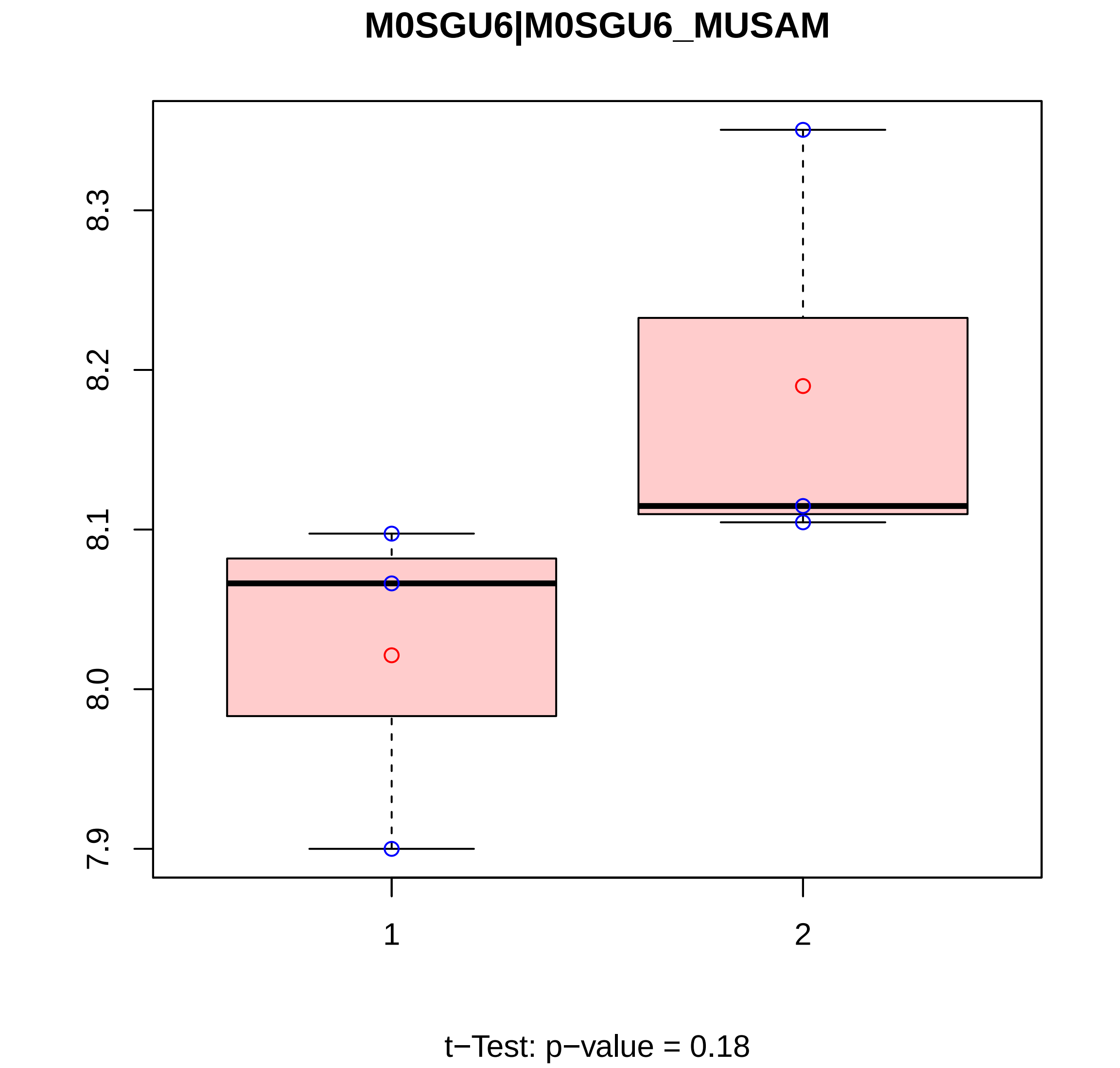
M0SGU6|M0SGU6_MUSAM
8.3
8.2
8.1
8.0
7.9
1
2
t−Test: p−value = 0.18

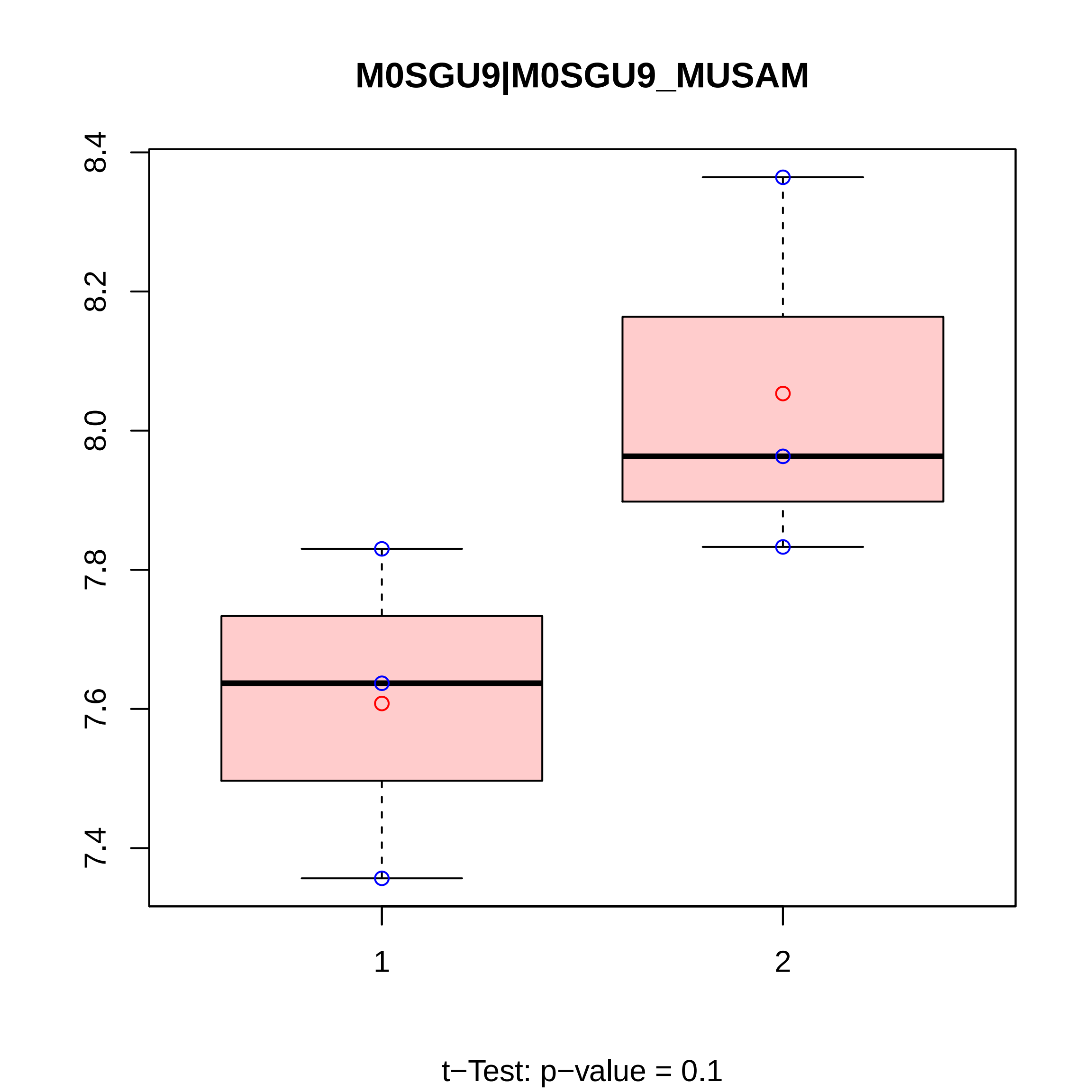
M0SGU9|M0SGU9_MUSAM
8.4
8.2
8.0
7.8
7.6
7.4
1
2
t−Test: p−value = 0.1

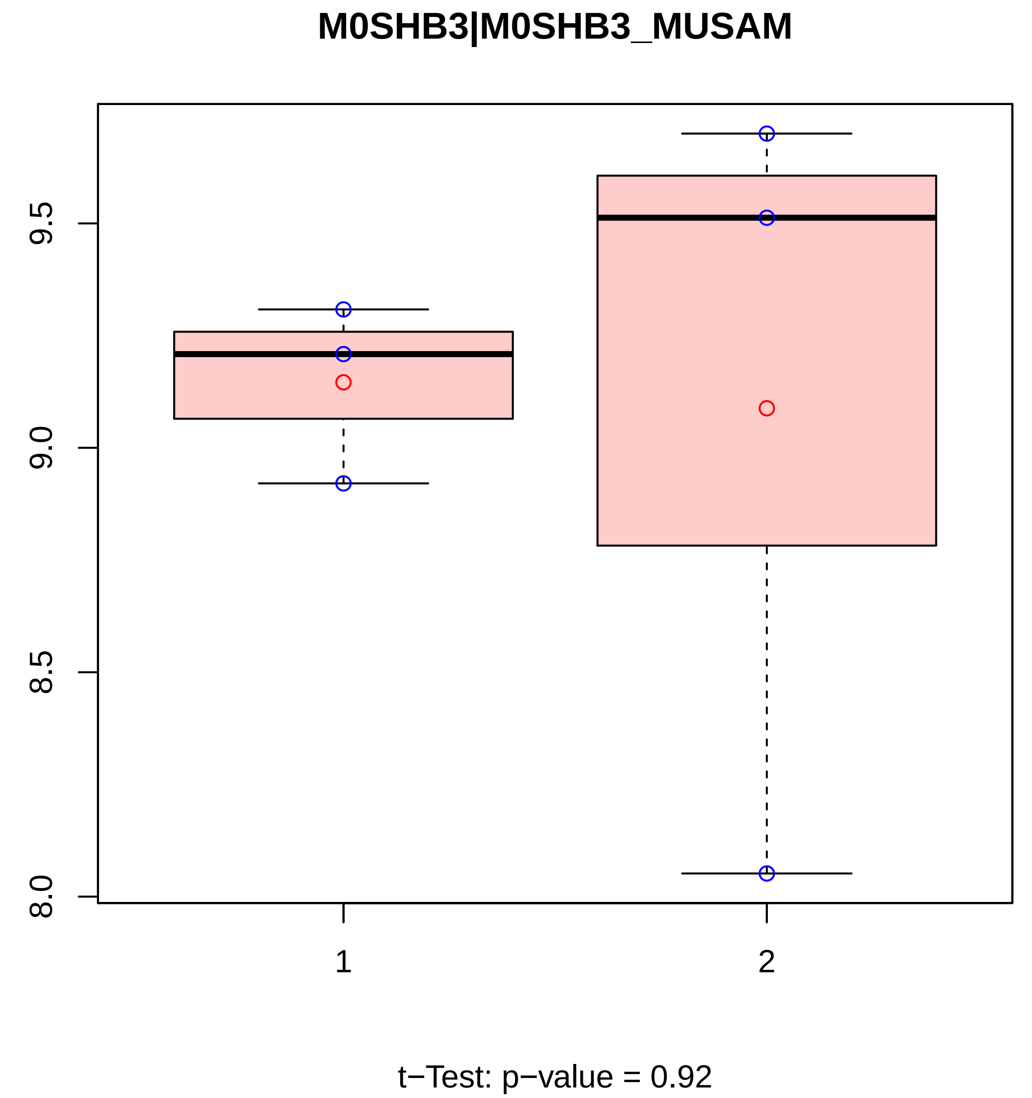

M0SHB3|M0SHB3_MUSAM
9.5
9.0
8.5
8.0
1
2
t−Test: p−value = 0.92

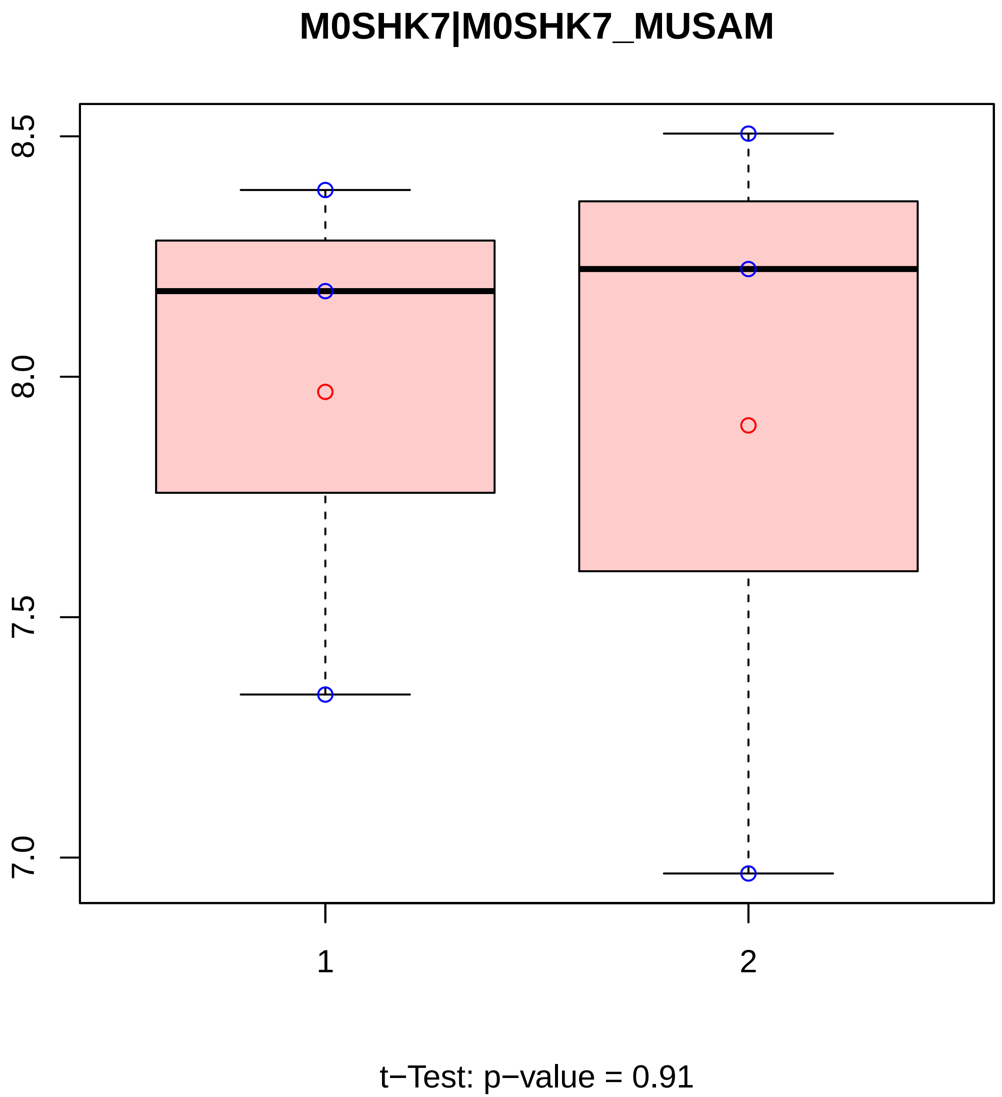
M0SHK7|M0SHK7_MUSAM
8.5
8.0
7.5
7.0
1
2
t−Test: p−value = 0.91

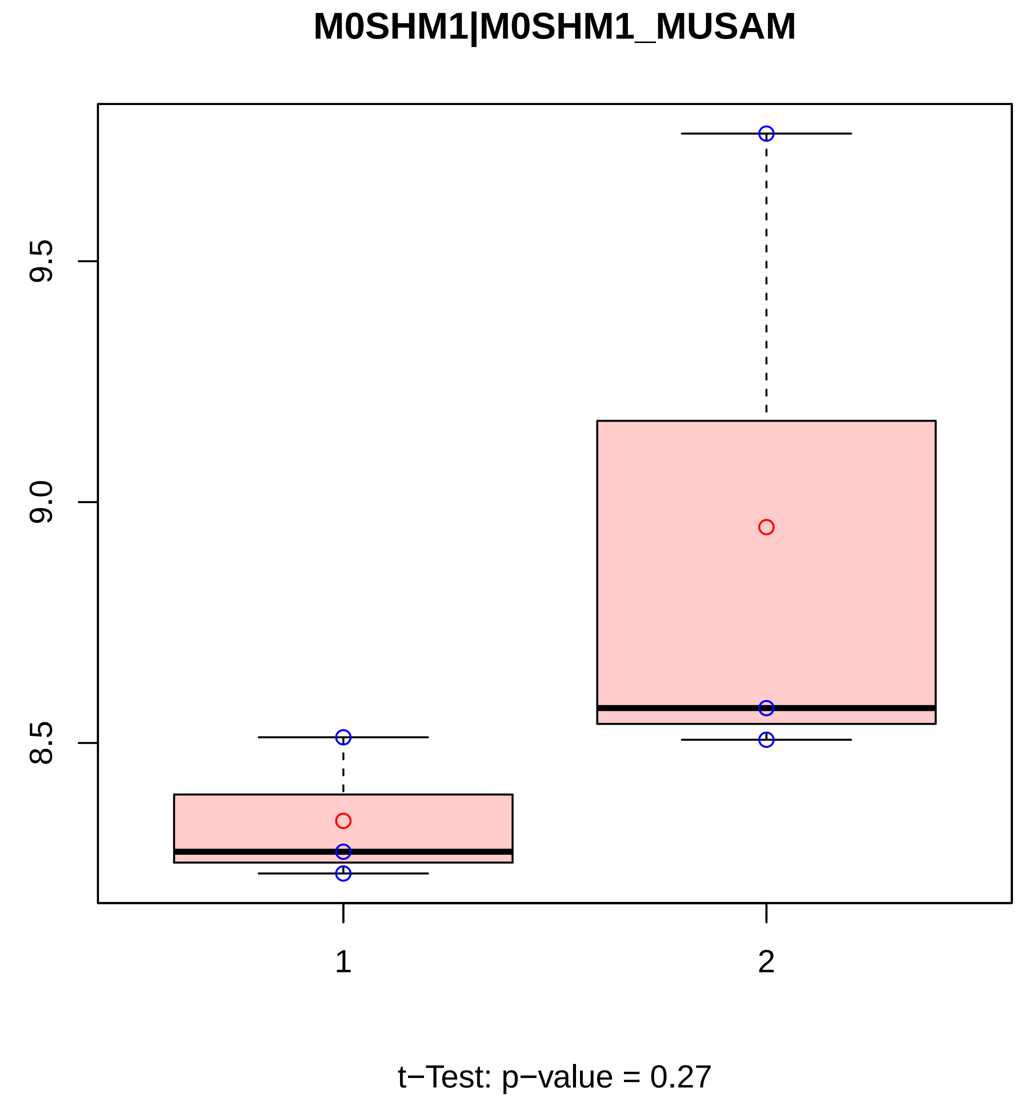
M0SHM1|M0SHM1_MUSAM
9.5
9.0
8.5
1
2
t−Test: p−value = 0.27

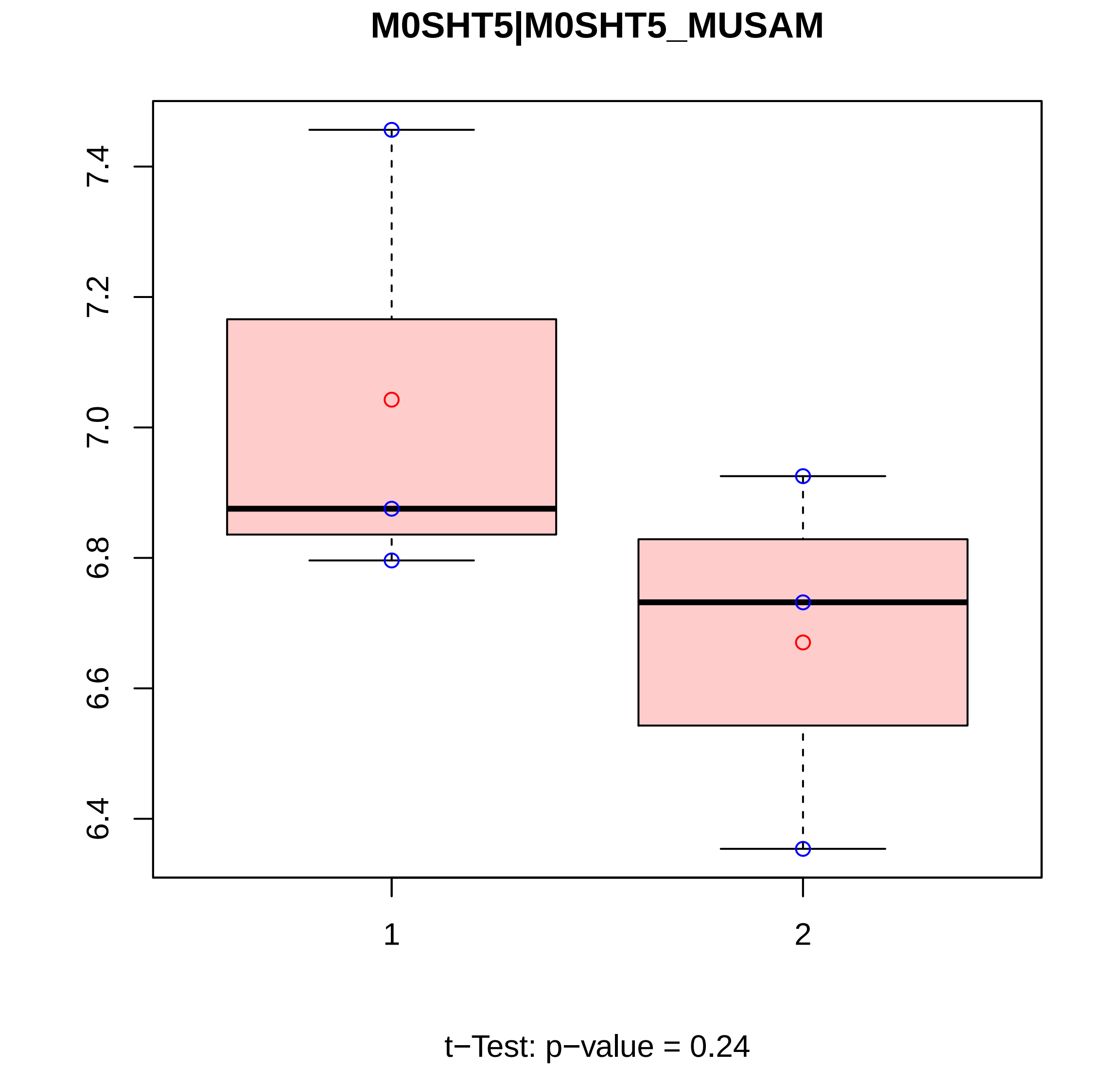
M0SHT5|M0SHT5_MUSAM
7.4
7.2
7.0
6.8
6.6
6.4
1
2
t−Test: p−value = 0.24

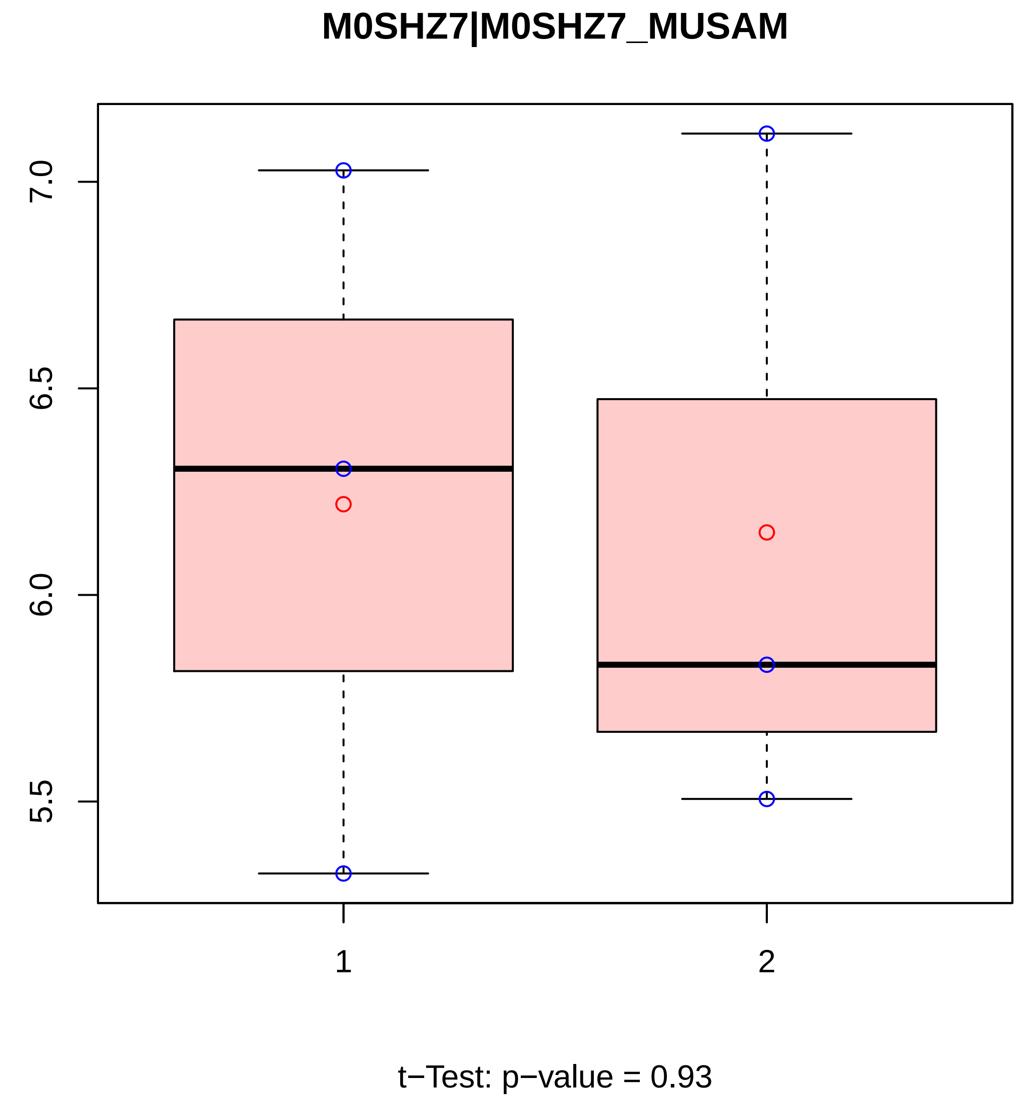
M0SHZ7|M0SHZ7_MUSAM
7.0
6.5
6.0
5.5
1
2
t−Test: p−value = 0.93

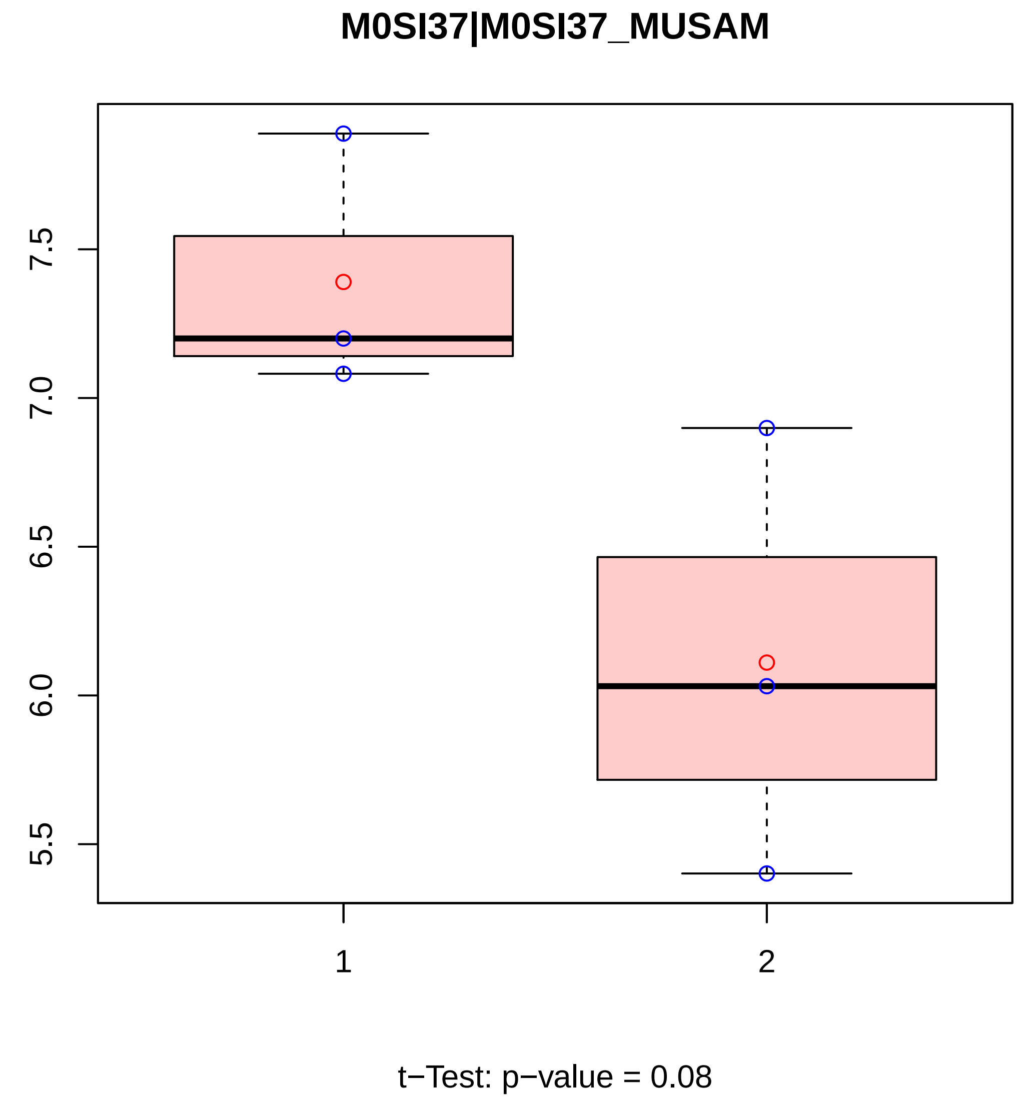
M0SI37|M0SI37_MUSAM
7.5
7.0
6.5
6.0
5.5
1
2
t−Test: p−value = 0.08

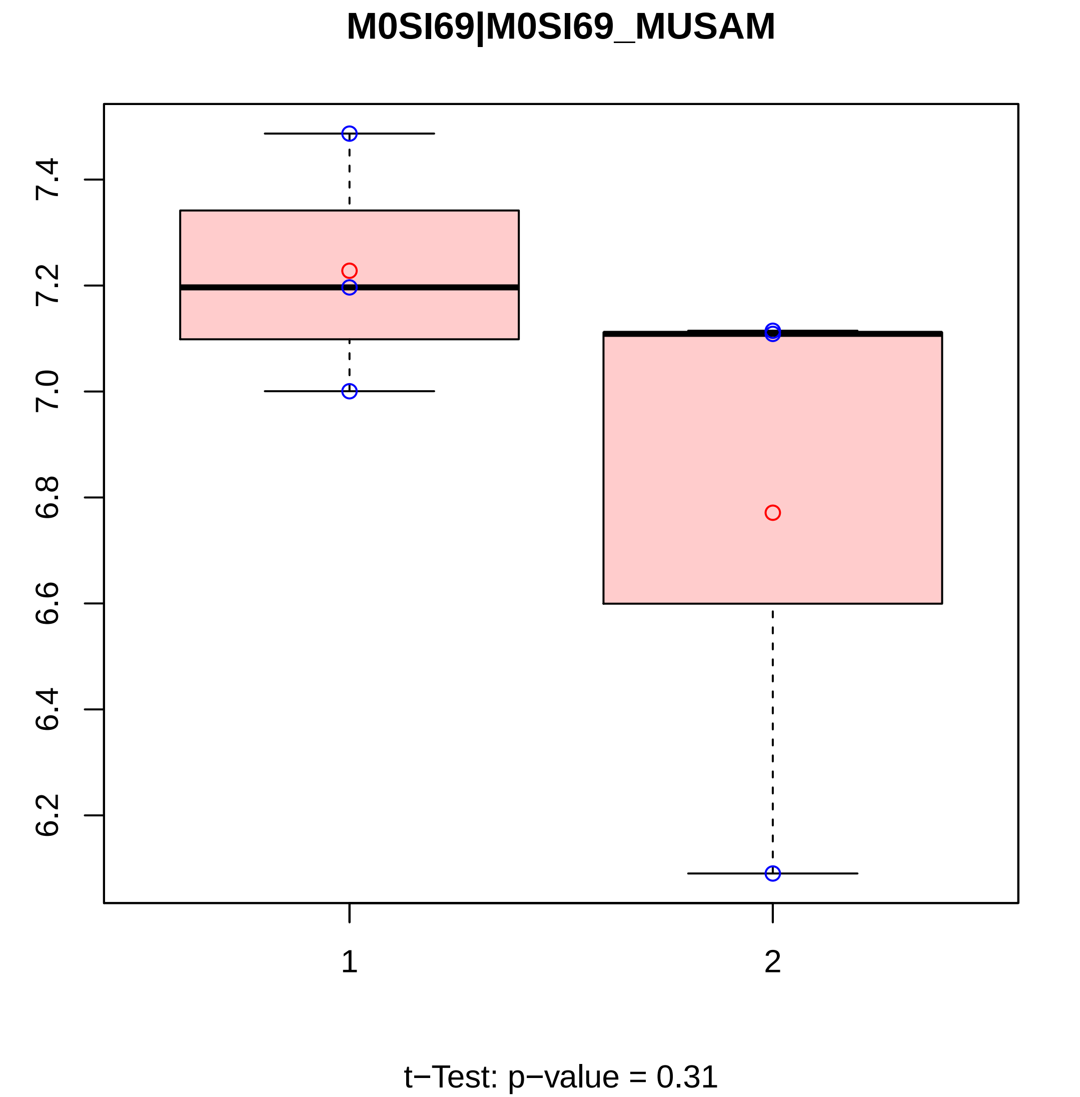

M0SI69|M0SI69_MUSAM
7.4
7.2
7.0
6.8
6.6
6.4
6.2
1
2
t−Test: p−value = 0.31

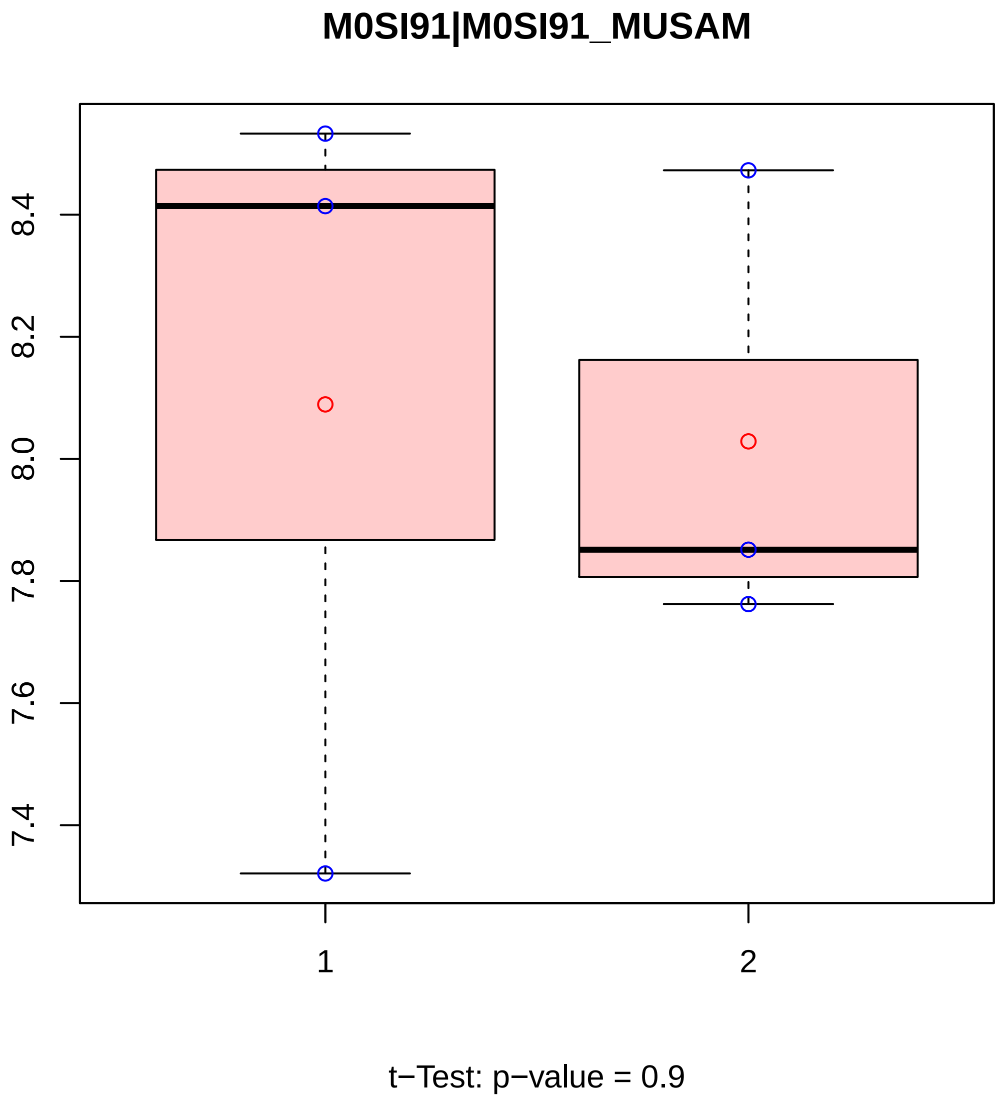
M0SI91|M0SI91_MUSAM
8.4
8.2
8.0
7.8
7.6
7.4
1
2
t−Test: p−value = 0.9

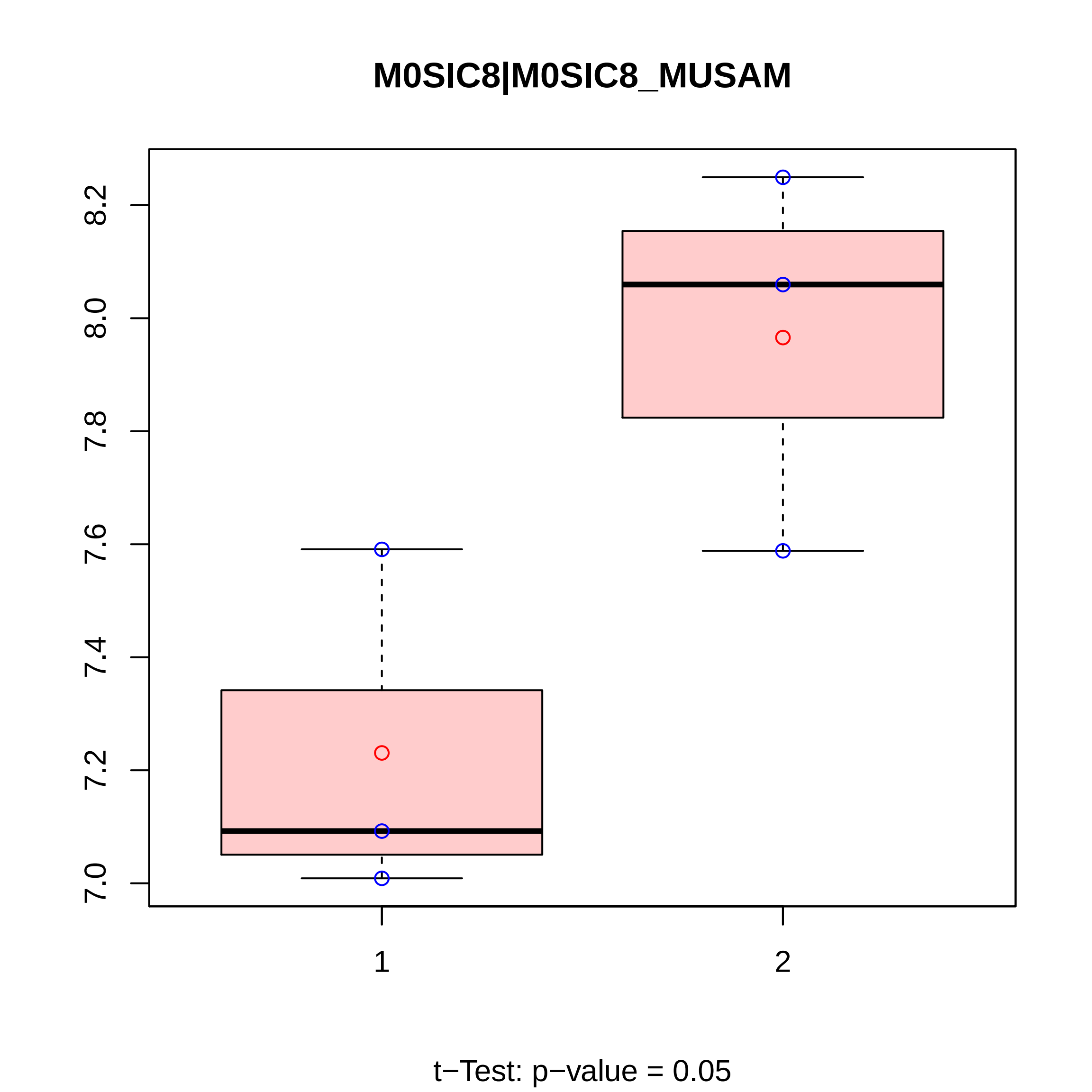
M0SIC8|M0SIC8_MUSAM
8.2
8.0
7.8
7.6
7.4
7.2
7.0
1
2
t−Test: p−value = 0.05

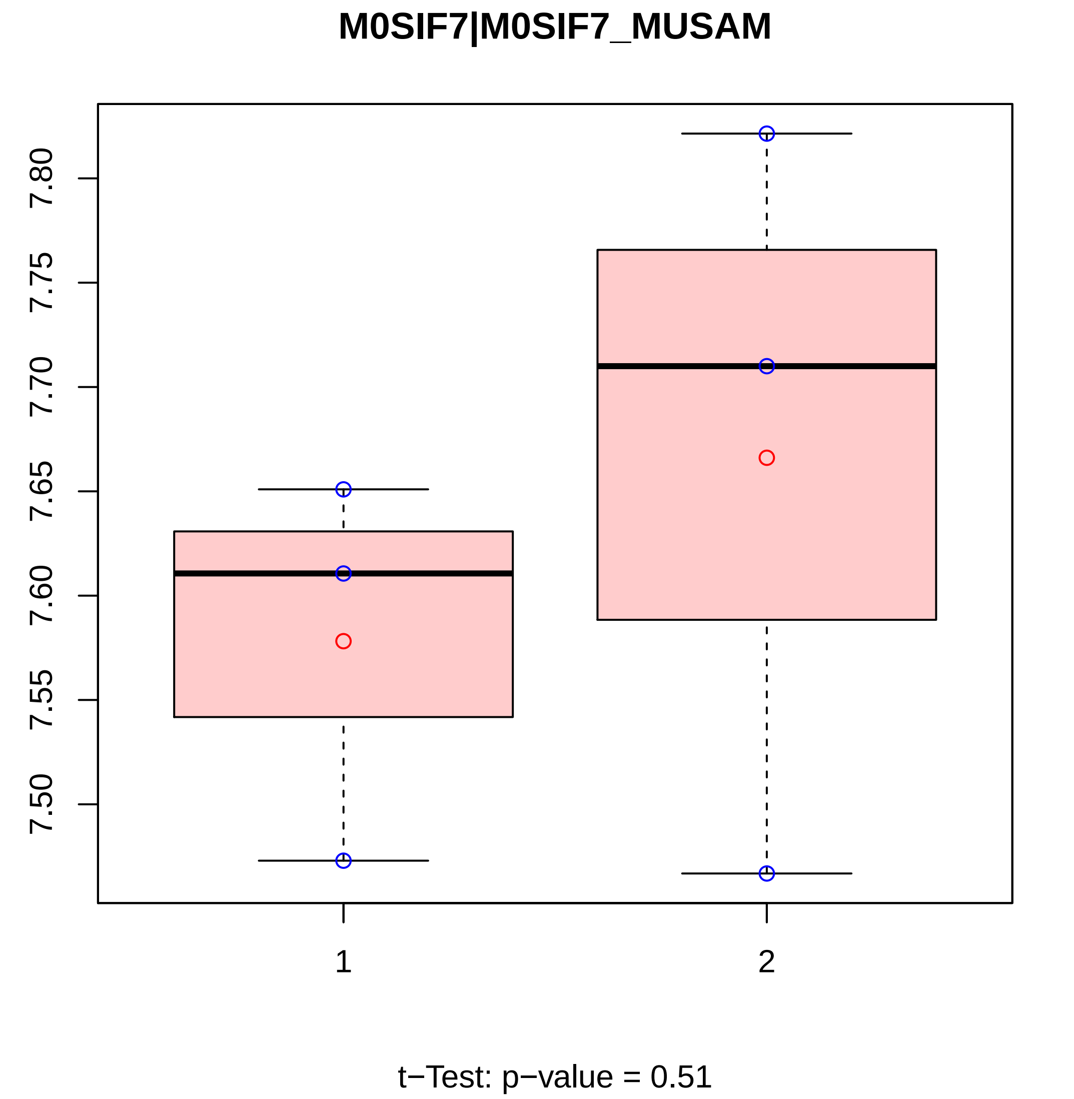
M0SIF7|M0SIF7_MUSAM
7.50
7.55
7.60
7.65
7.70
7.75
7.80
1
2
t−Test: p−value = 0.51

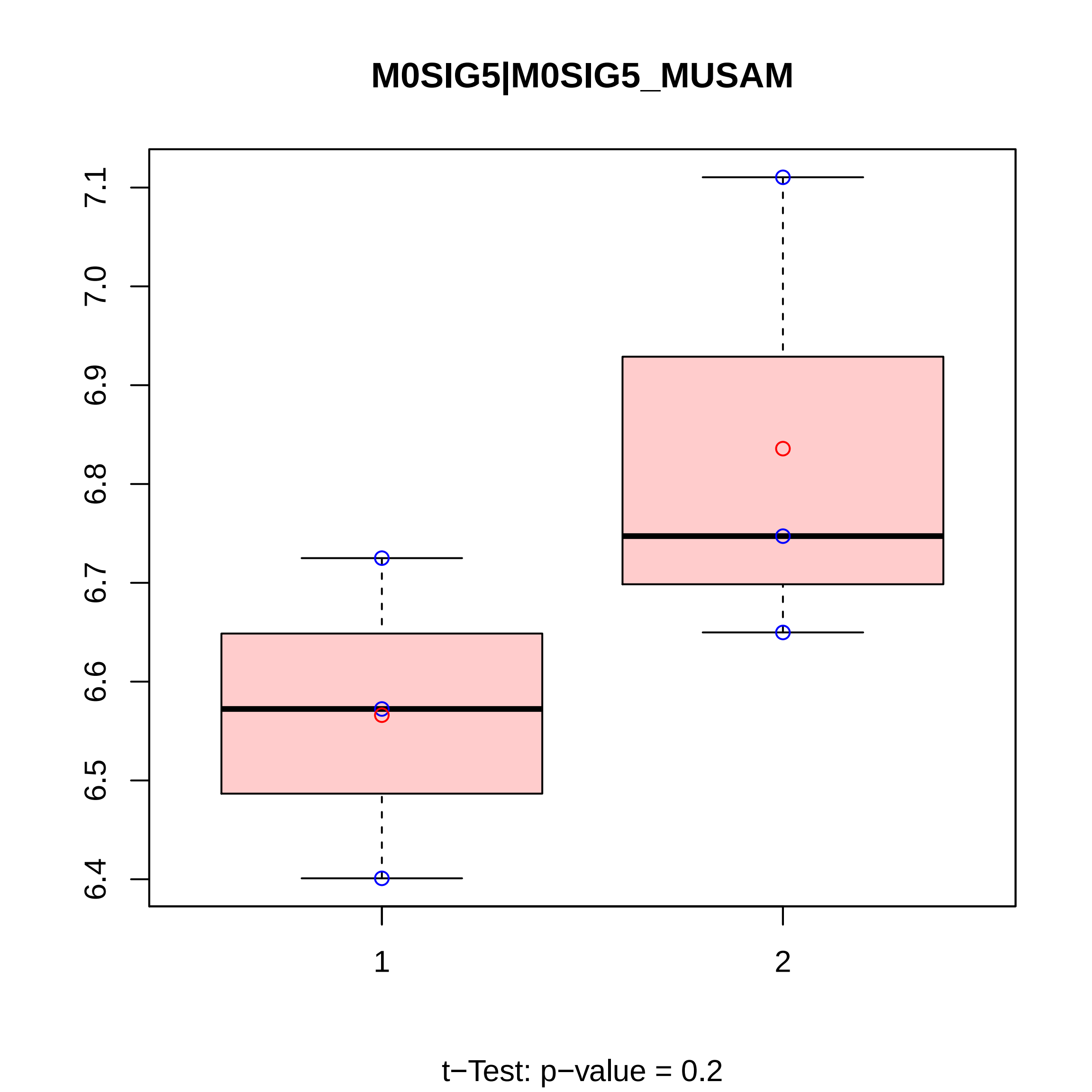
M0SIG5|M0SIG5_MUSAM
7.1
7.0
6.9
6.8
6.7
6.6
6.5
6.4
1
2
t−Test: p−value = 0.2

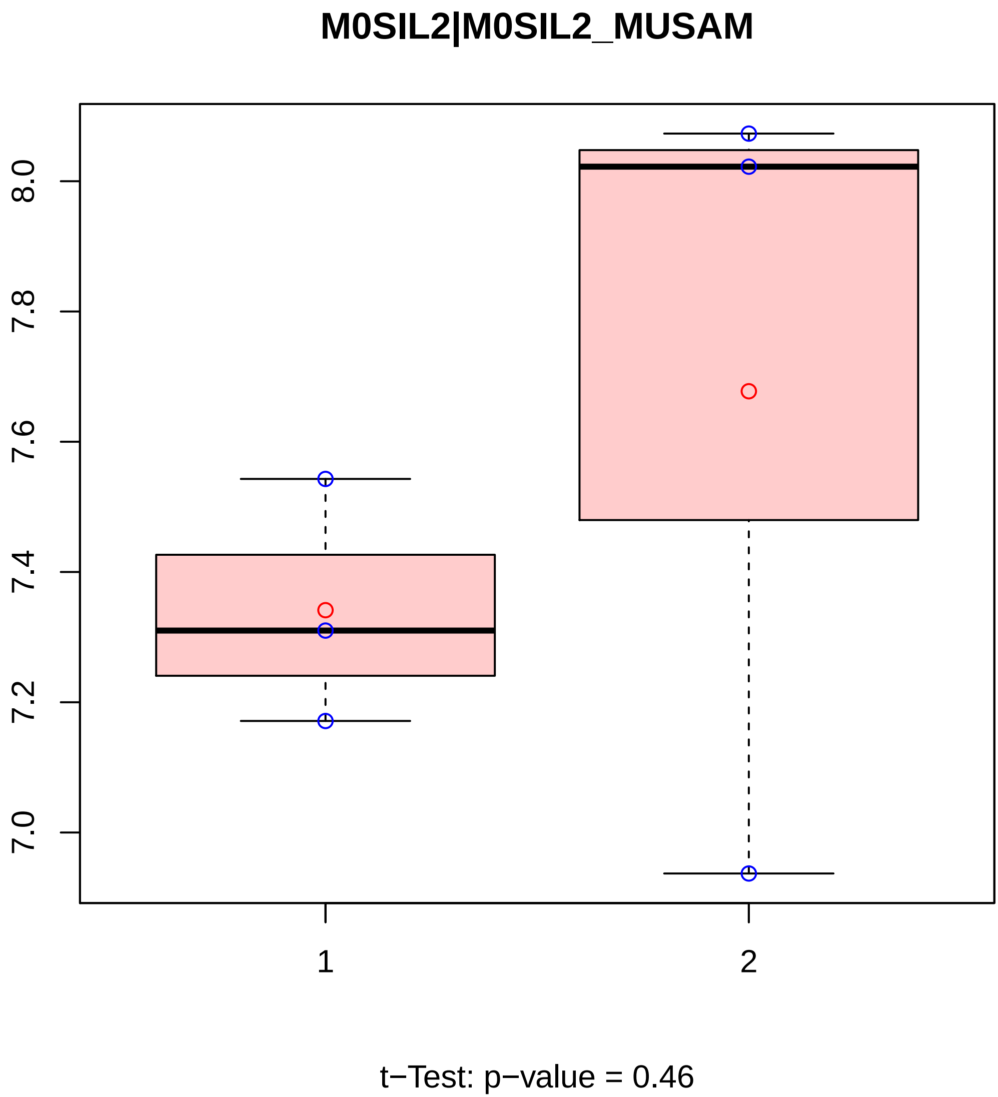
M0SIL2|M0SIL2_MUSAM
8.0
7.8
7.6
7.4
7.2
7.0
1
2
t−Test: p−value = 0.46

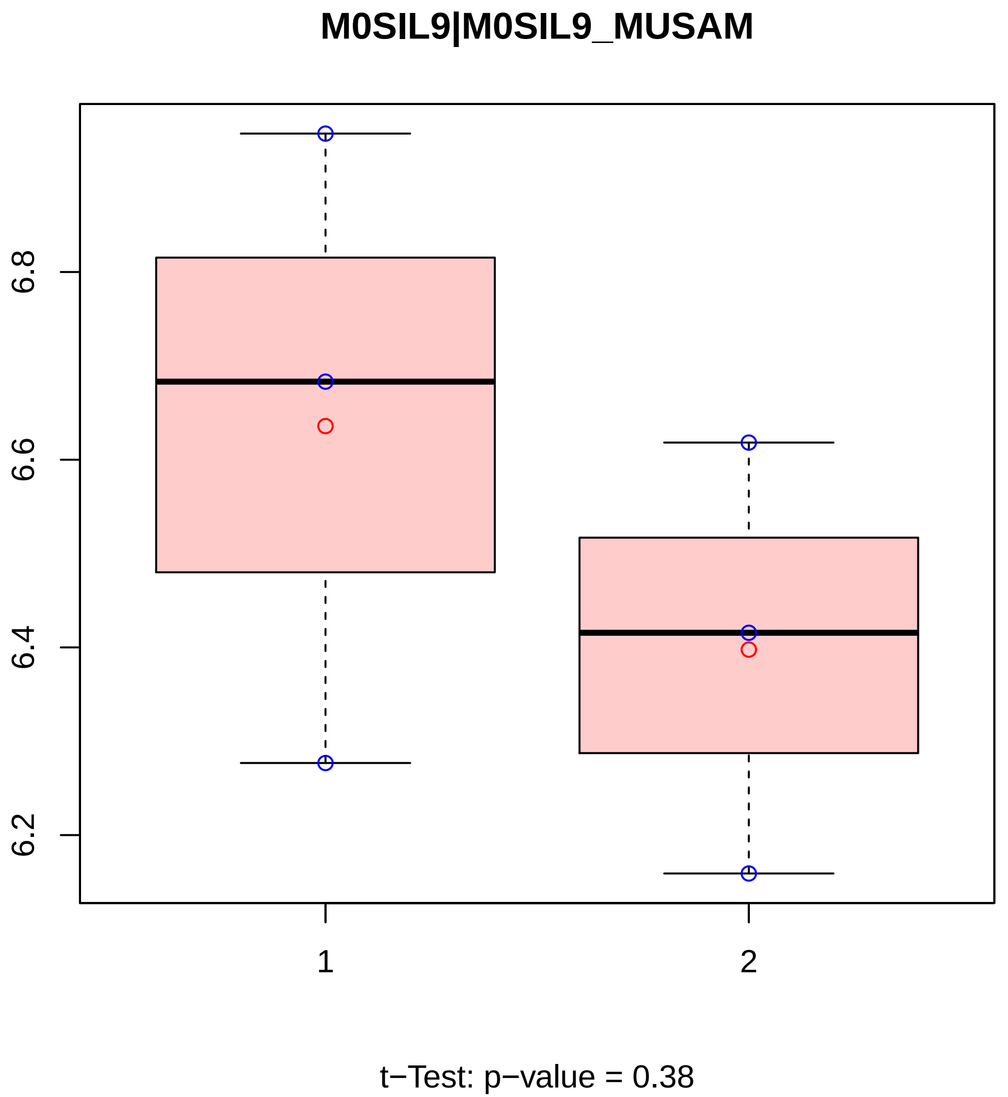
M0SIL9|M0SIL9_MUSAM
6.2
6.4
6.6
6.8
1
2
t−Test: p−value = 0.38

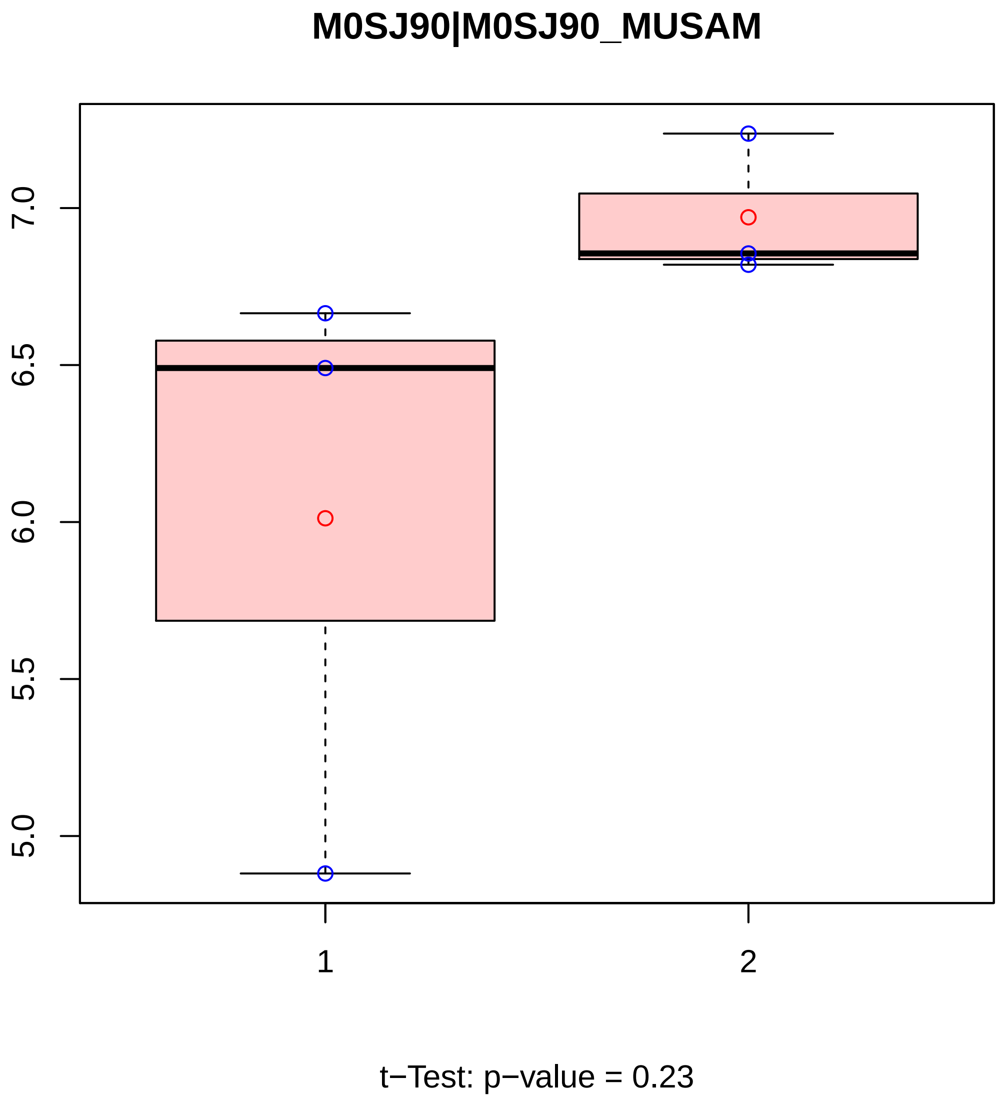
M0SJ90|M0SJ90_MUSAM
7.0
6.5
6.0
5.5
5.0
1
2
t−Test: p−value = 0.23

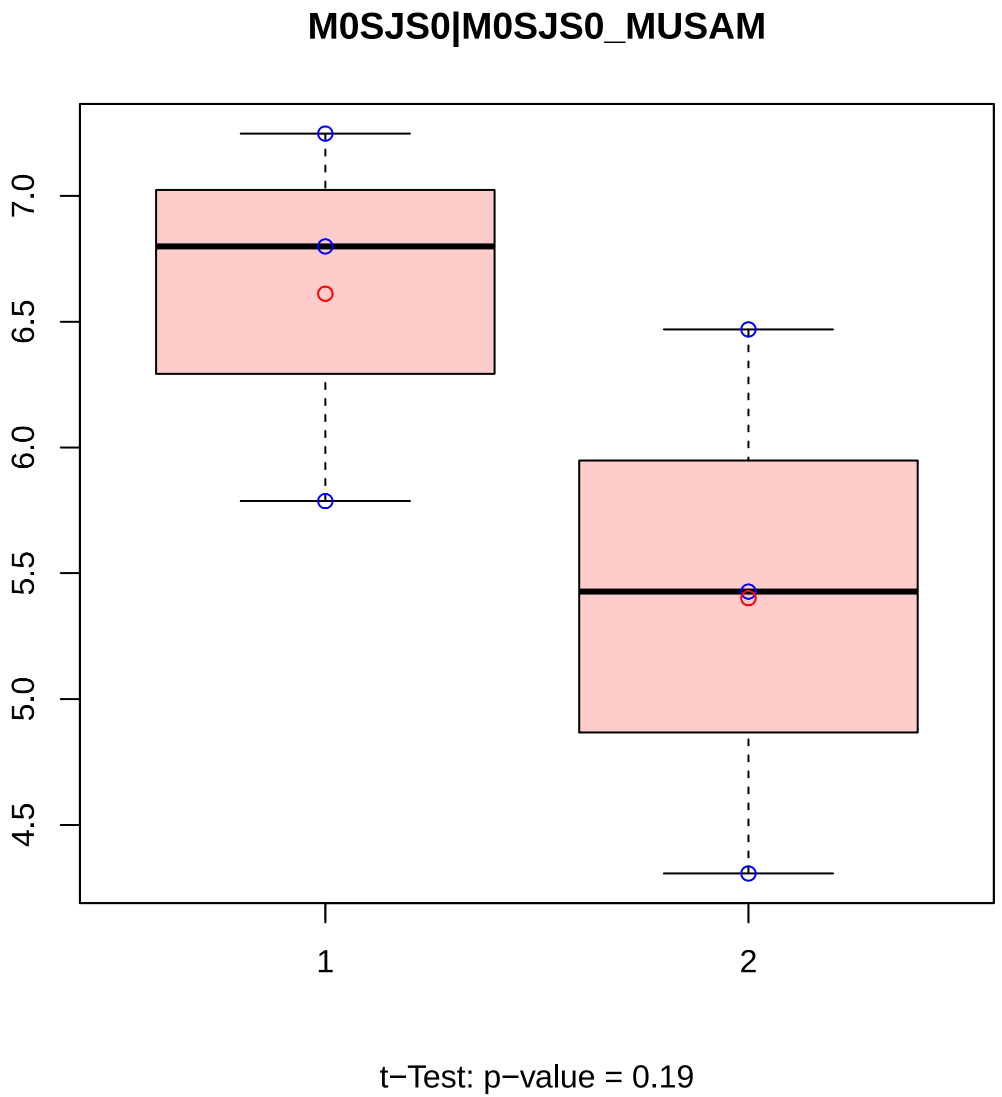
M0SJS0|M0SJS0_MUSAM
7.0
6.5
6.0
5.5
5.0
4.5
1
2
t−Test: p−value = 0.19

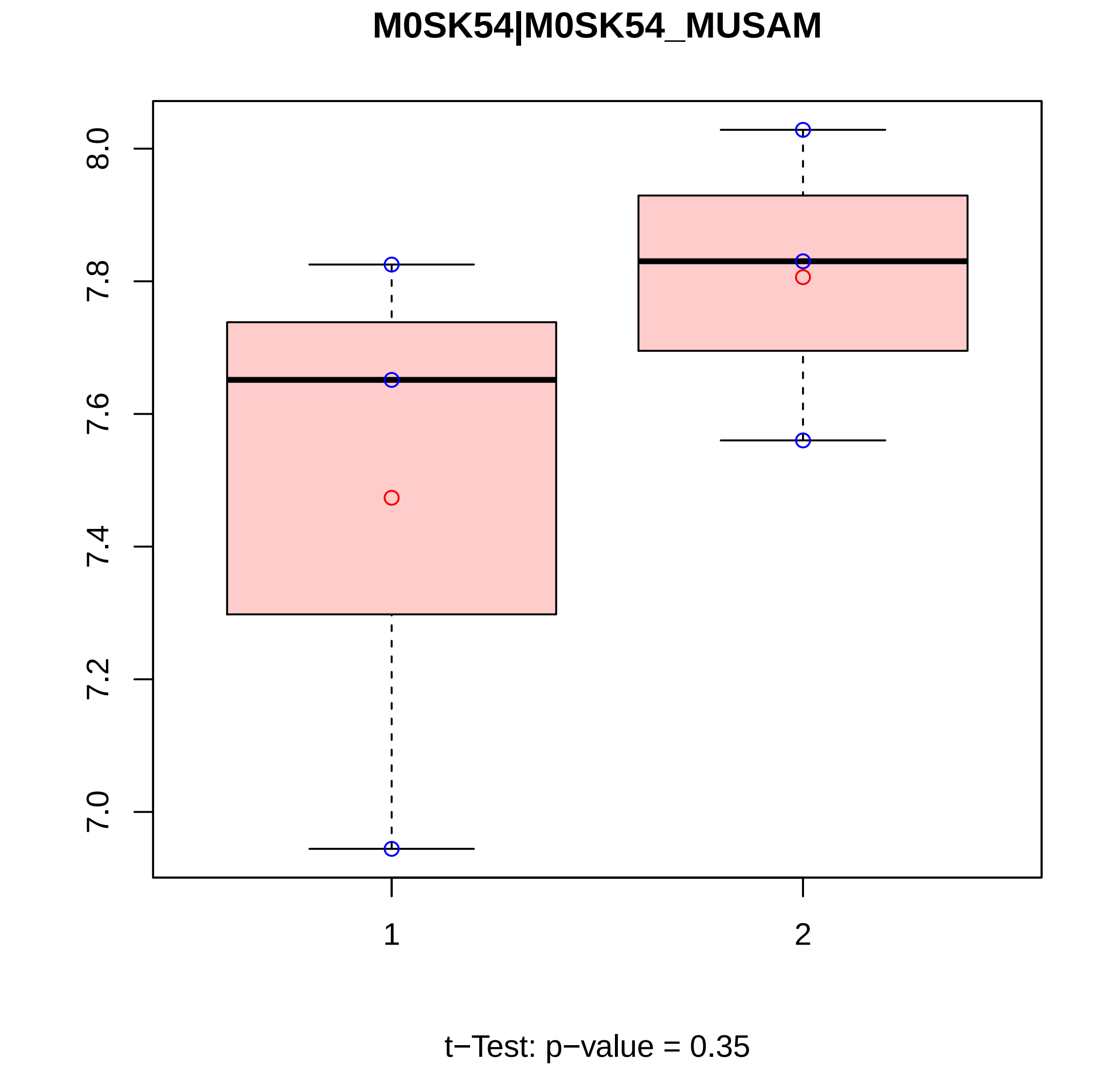
M0SK54|M0SK54_MUSAM
8.0
7.8
7.6
7.4
7.2
7.0
1
2
t−Test: p−value = 0.35

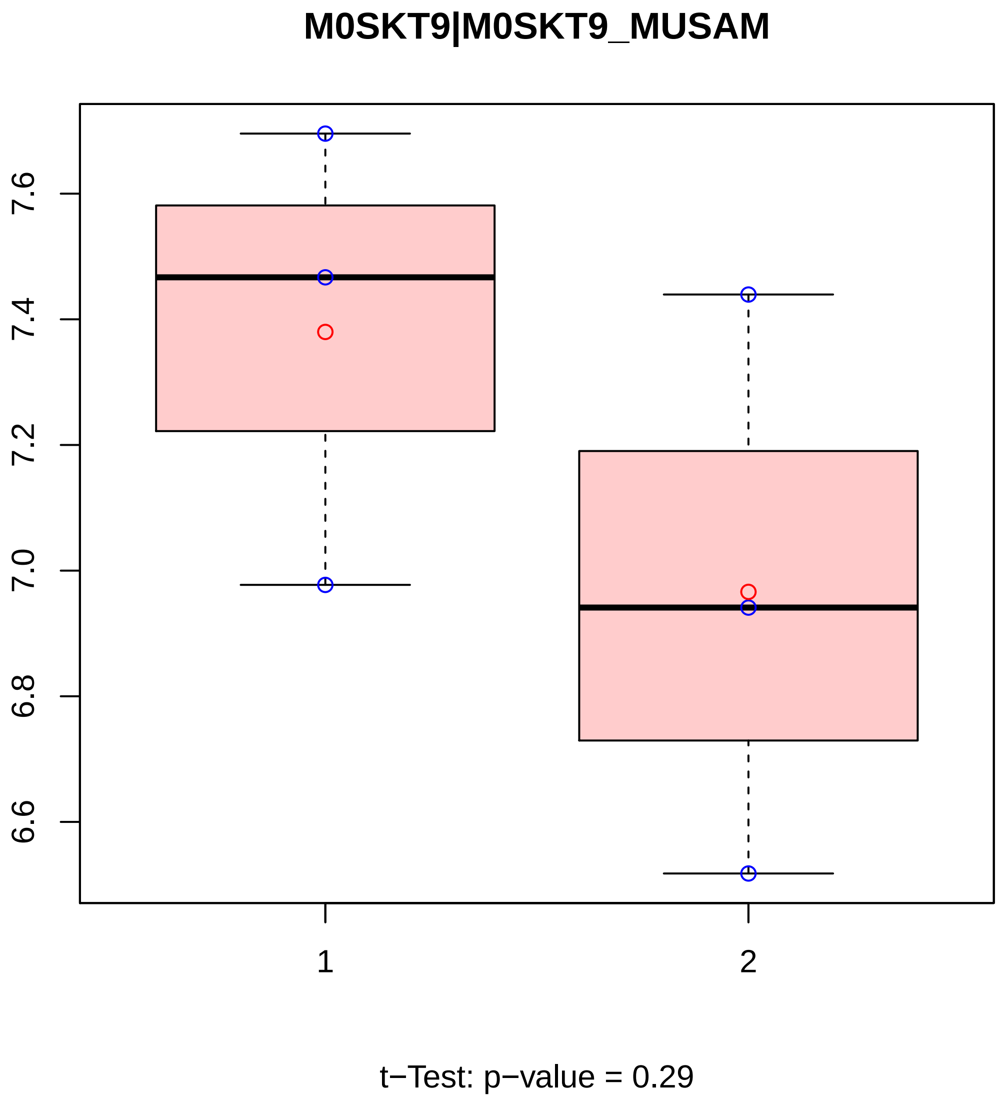

M0SKT9|M0SKT9_MUSAM
7.6
7.4
7.2
7.0
6.8
6.6
1
2
t−Test: p−value = 0.29

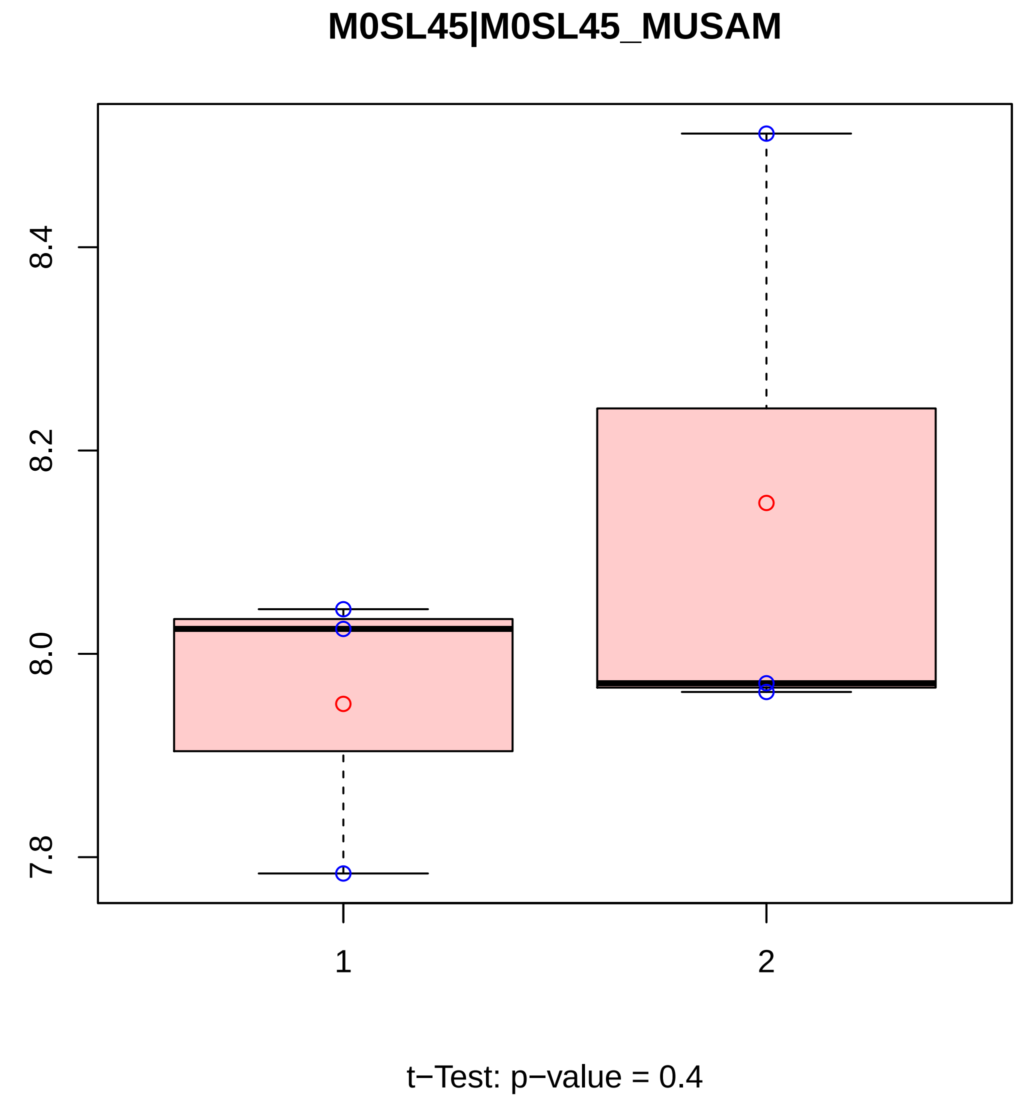
M0SL45|M0SL45_MUSAM
8.4
8.2
8.0
7.8
1
2
t−Test: p−value = 0.4

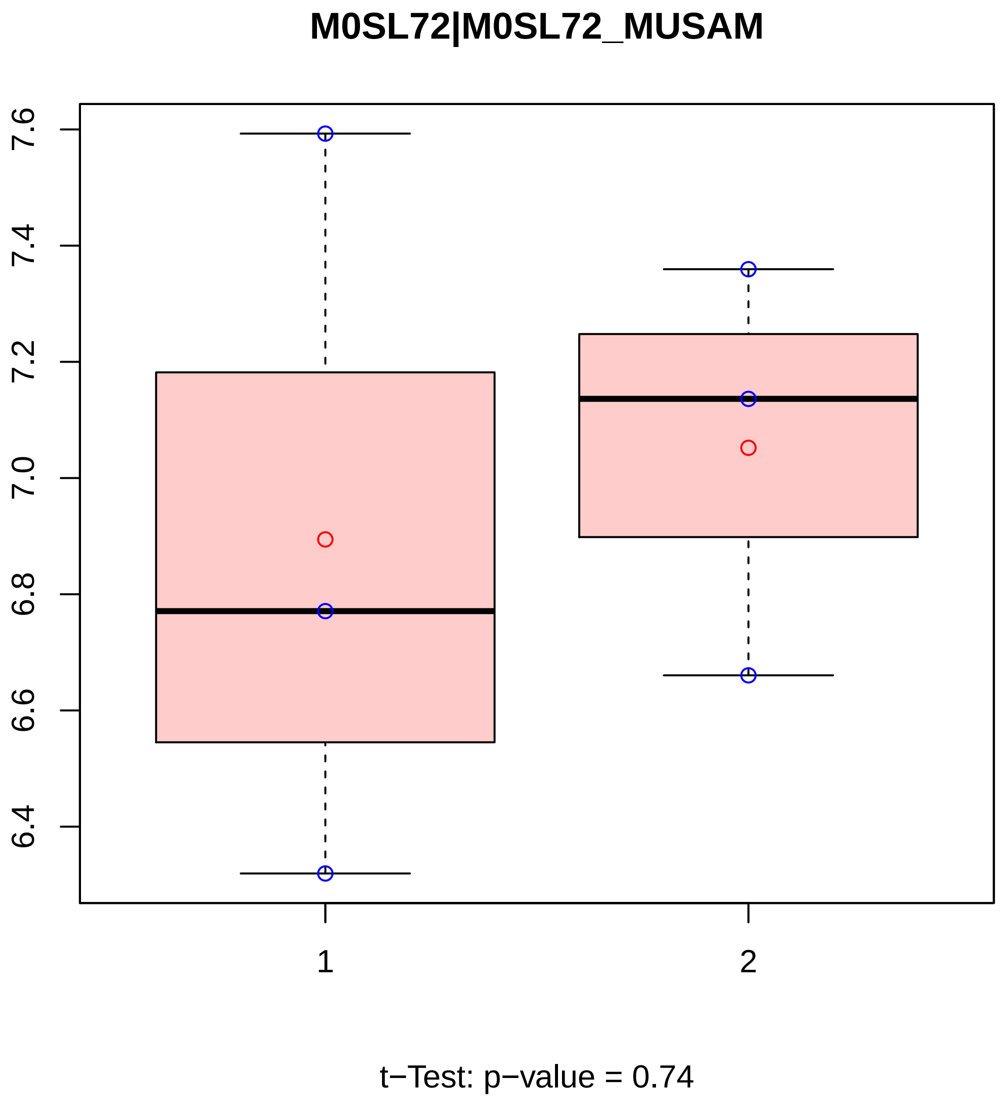
M0SL72|M0SL72_MUSAM
7.6
7.4
7.2
7.0
6.8
6.6
6.4
1
2
t−Test: p−value = 0.74

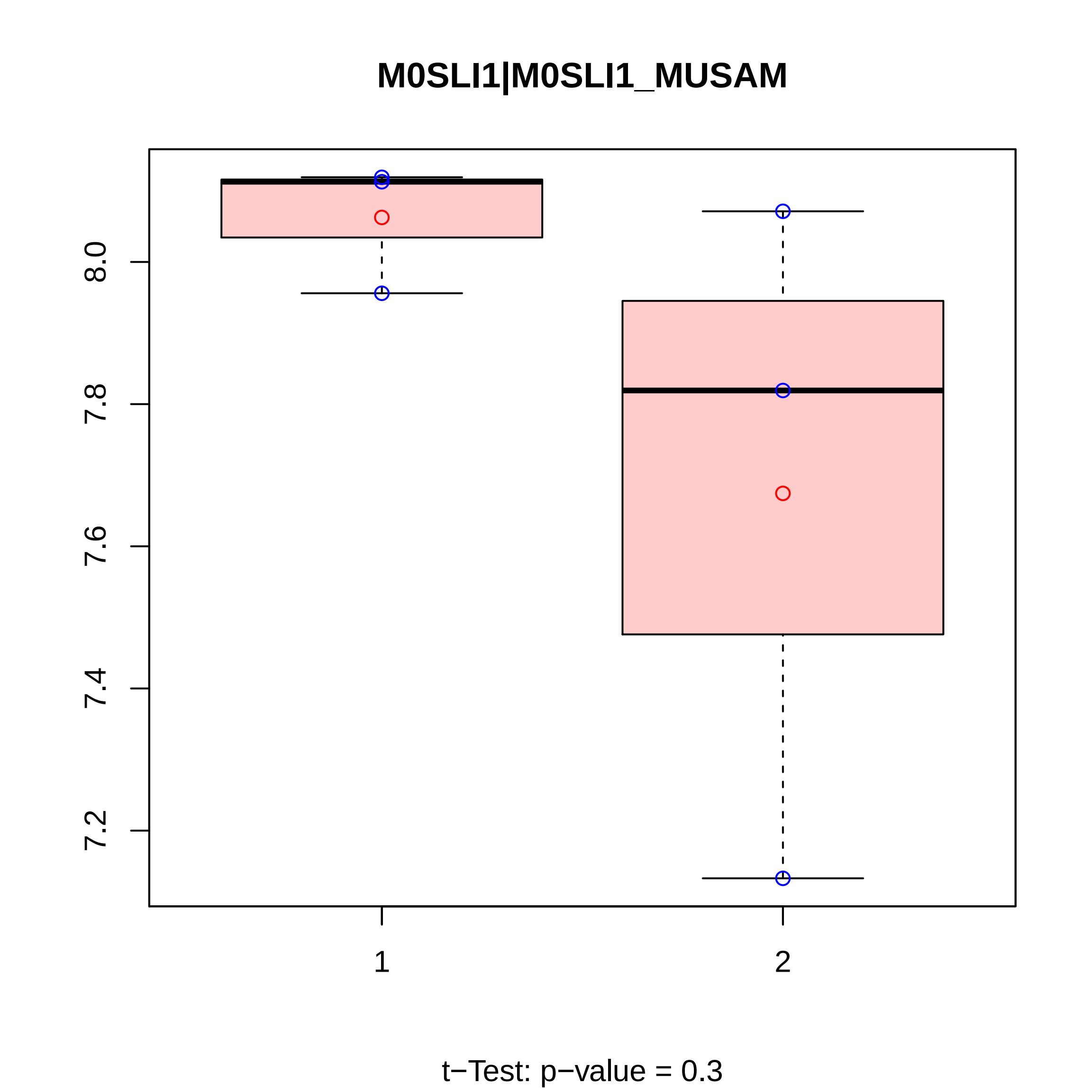
M0SLI1|M0SLI1_MUSAM
8.0
7.8
7.6
7.4
7.2
1
2
t−Test: p−value = 0.3

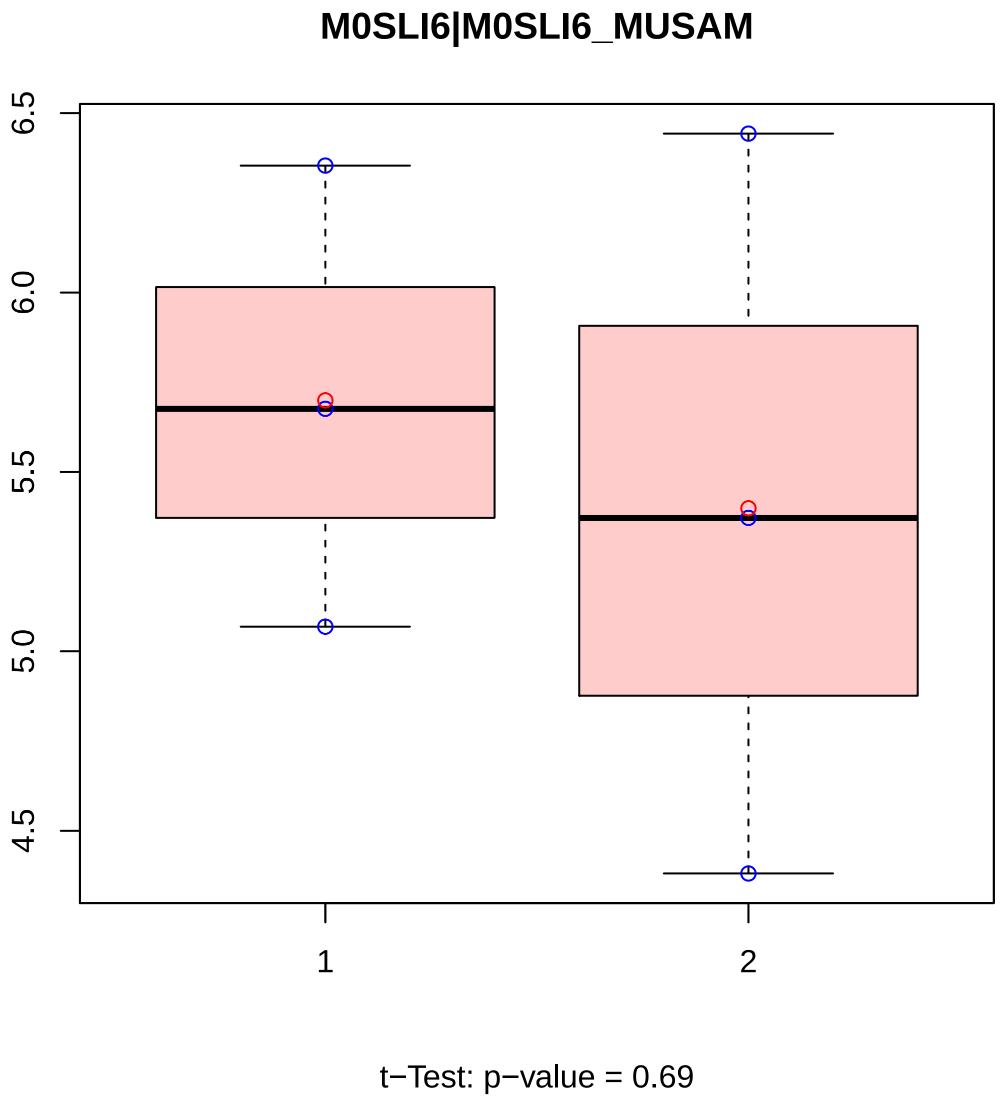

M0SLI6|M0SLI6_MUSAM
6.5
6.0
5.5
5.0
4.5
1
2
t−Test: p−value = 0.69

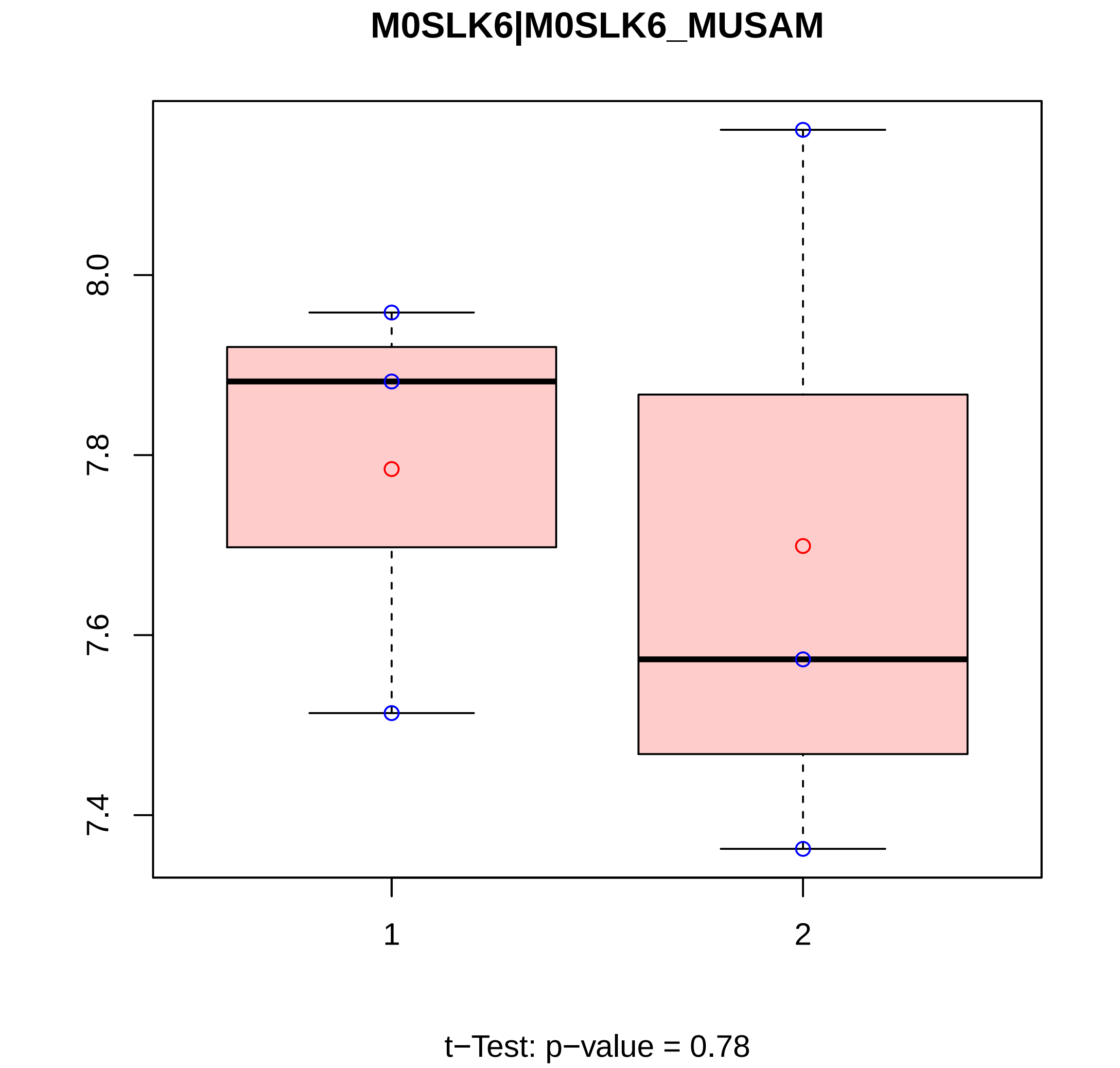
M0SLK6|M0SLK6_MUSAM
8.0
7.8
7.6
7.4
1
2
t−Test: p−value = 0.78

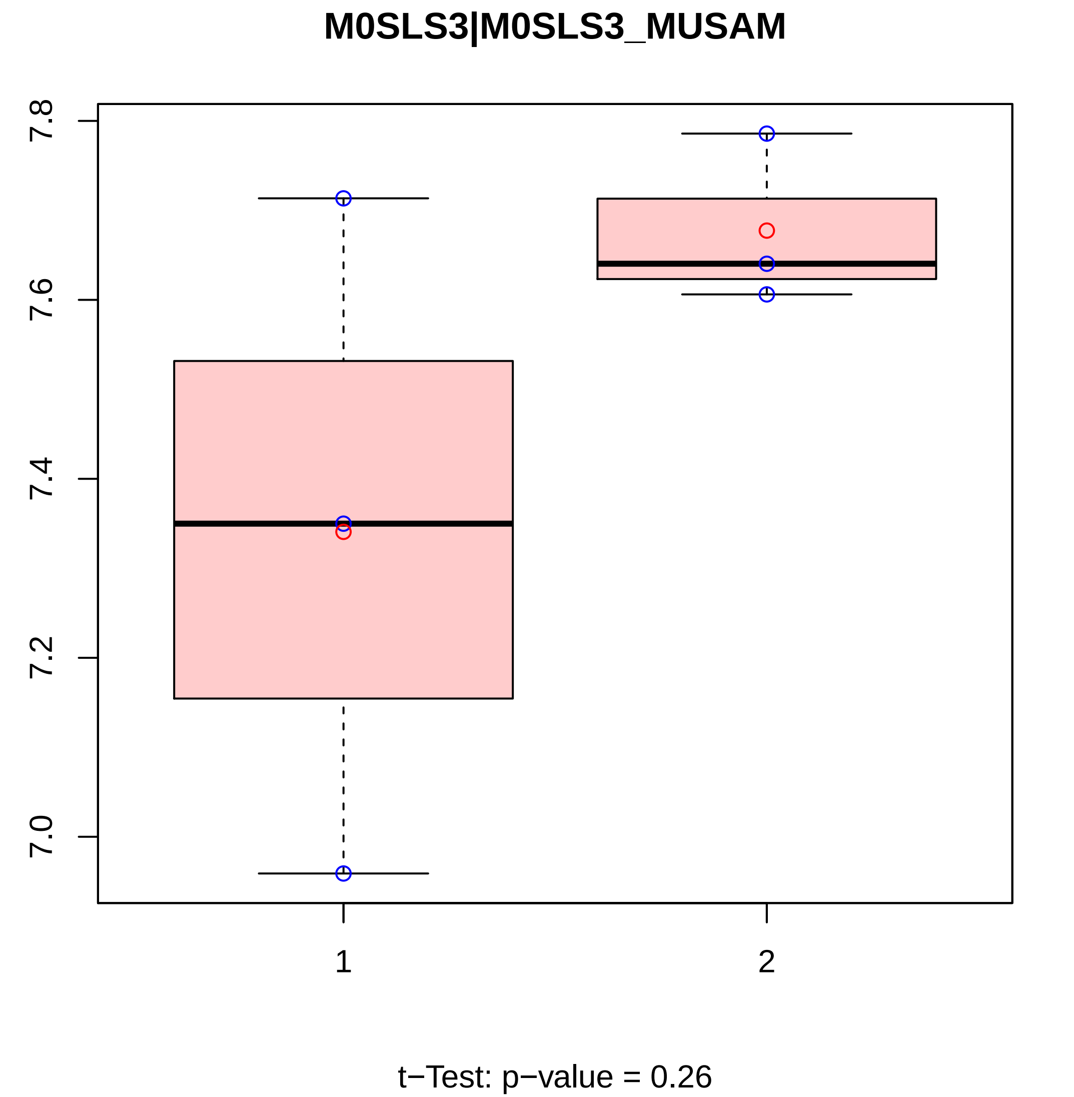
M0SLS3|M0SLS3_MUSAM
7.8
7.6
7.4
7.2
7.0
1
2
t−Test: p−value = 0.26

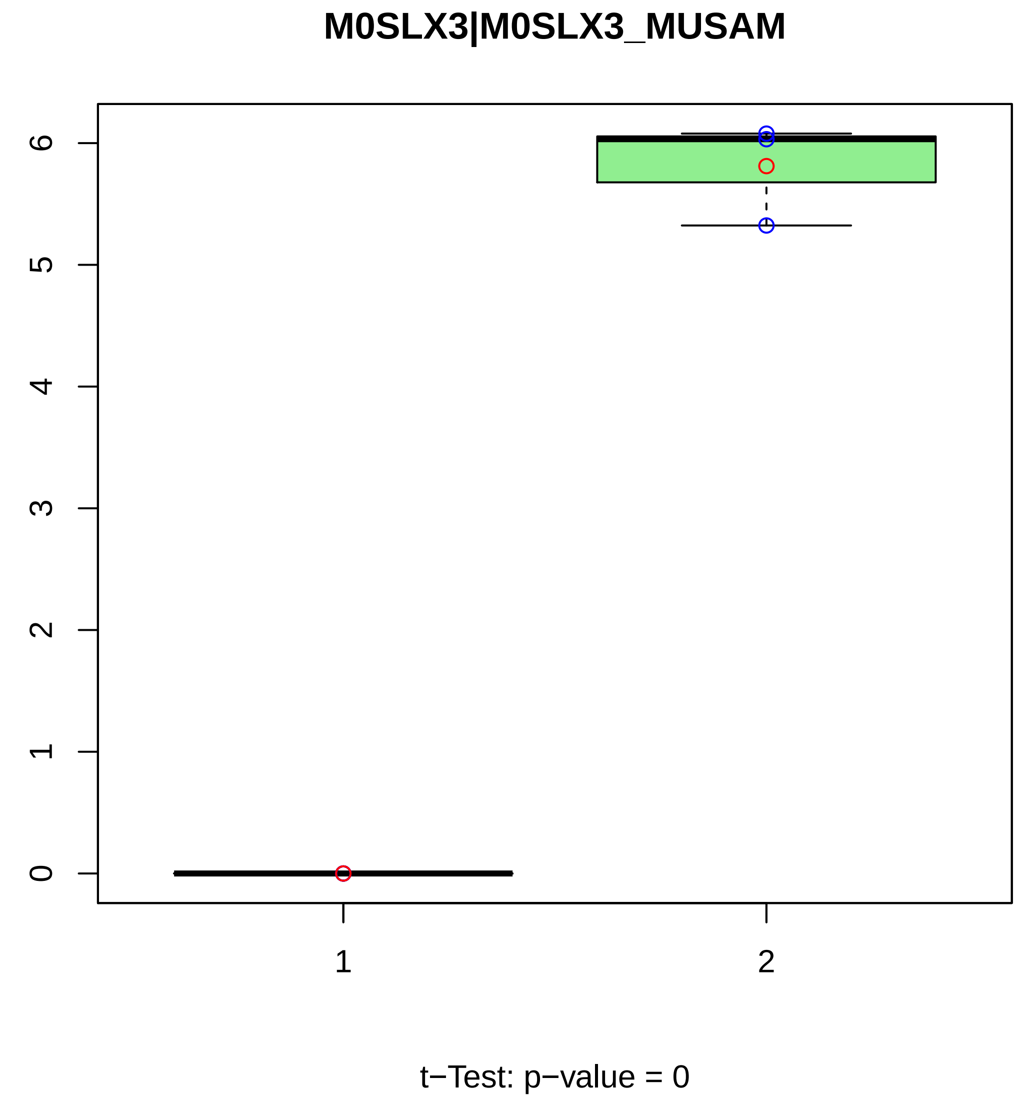
M0SLX3|M0SLX3_MUSAM
0
1
2
3
4
5
6
1
2
t−Test: p−value = 0

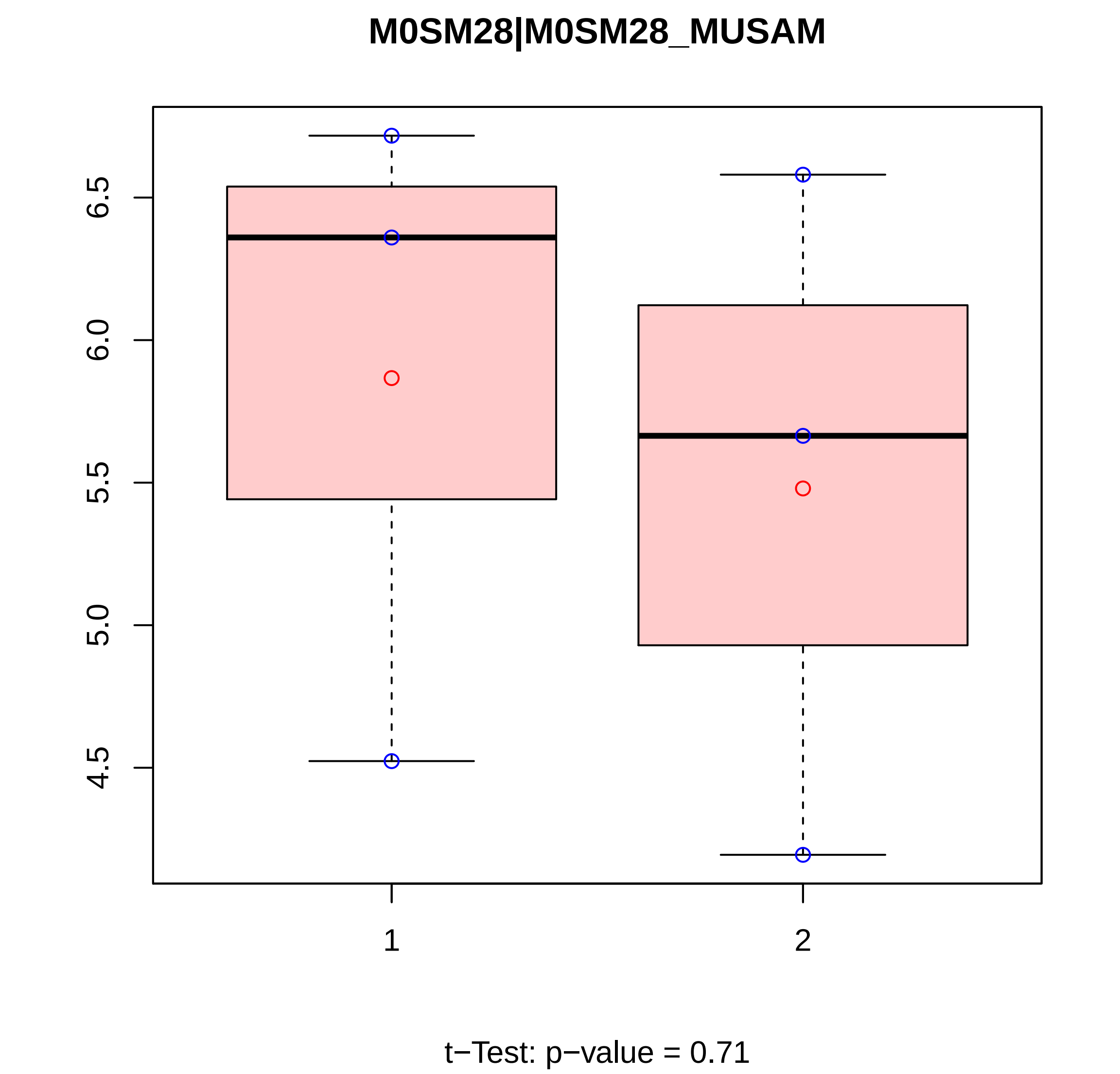
M0SM28|M0SM28_MUSAM
6.5
6.0
5.5
5.0
4.5
1
2
t−Test: p−value = 0.71

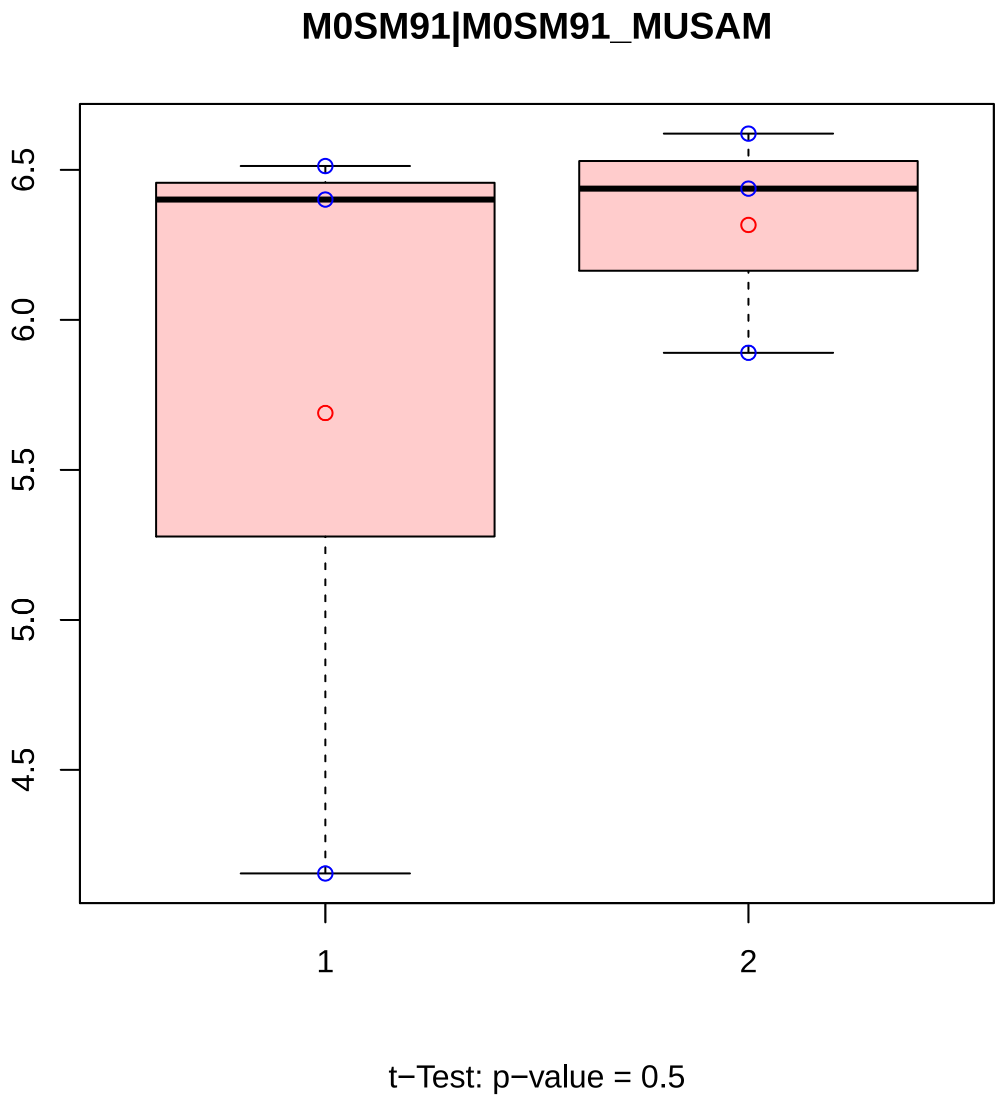

M0SM91|M0SM91_MUSAM
6.5
6.0
5.5
5.0
4.5
1
2
t−Test: p−value = 0.5

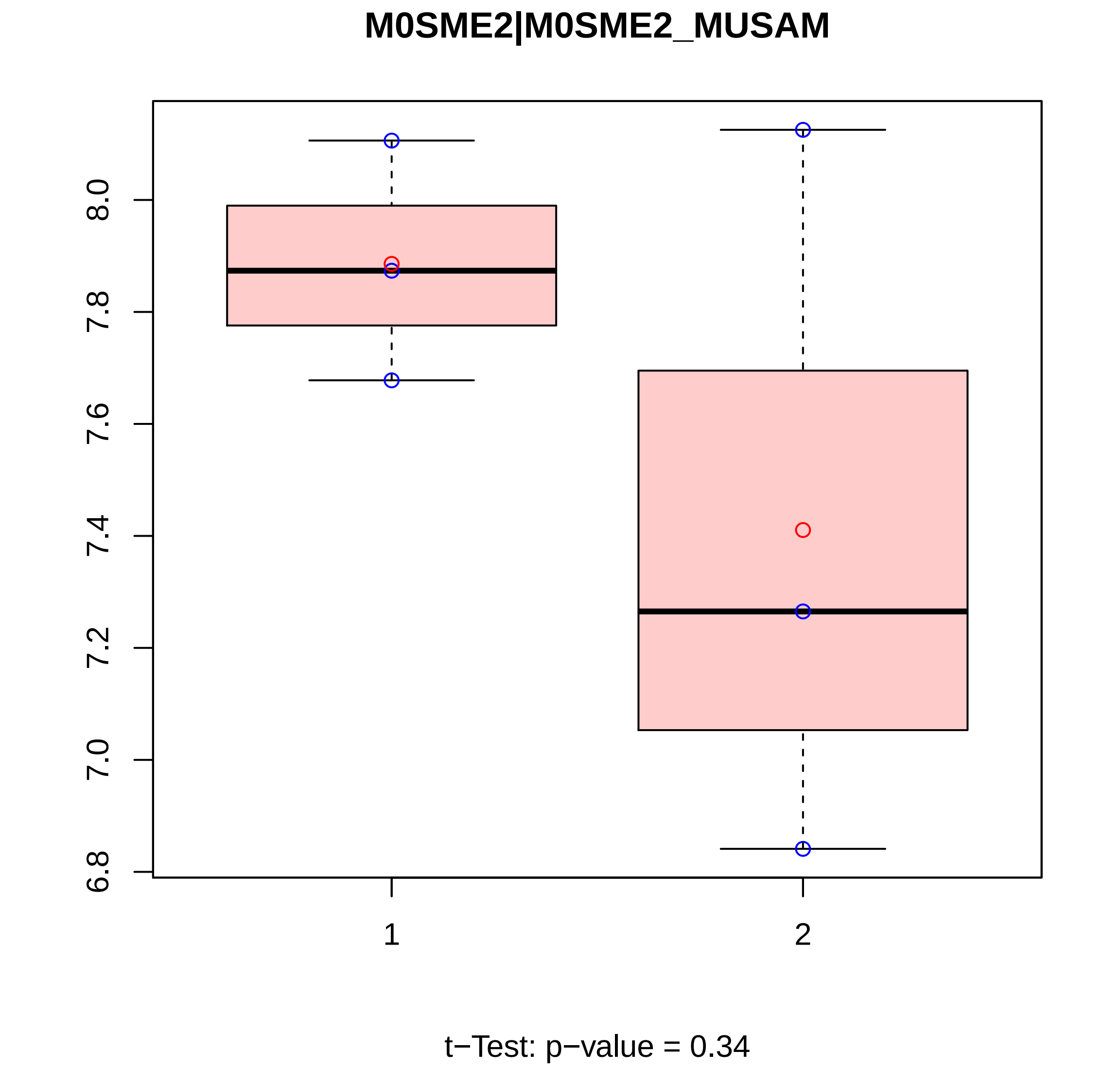
M0SME2|M0SME2_MUSAM
6.8
7.0
7.2
7.4
7.6
7.8
8.0
1
2
t−Test: p−value = 0.34

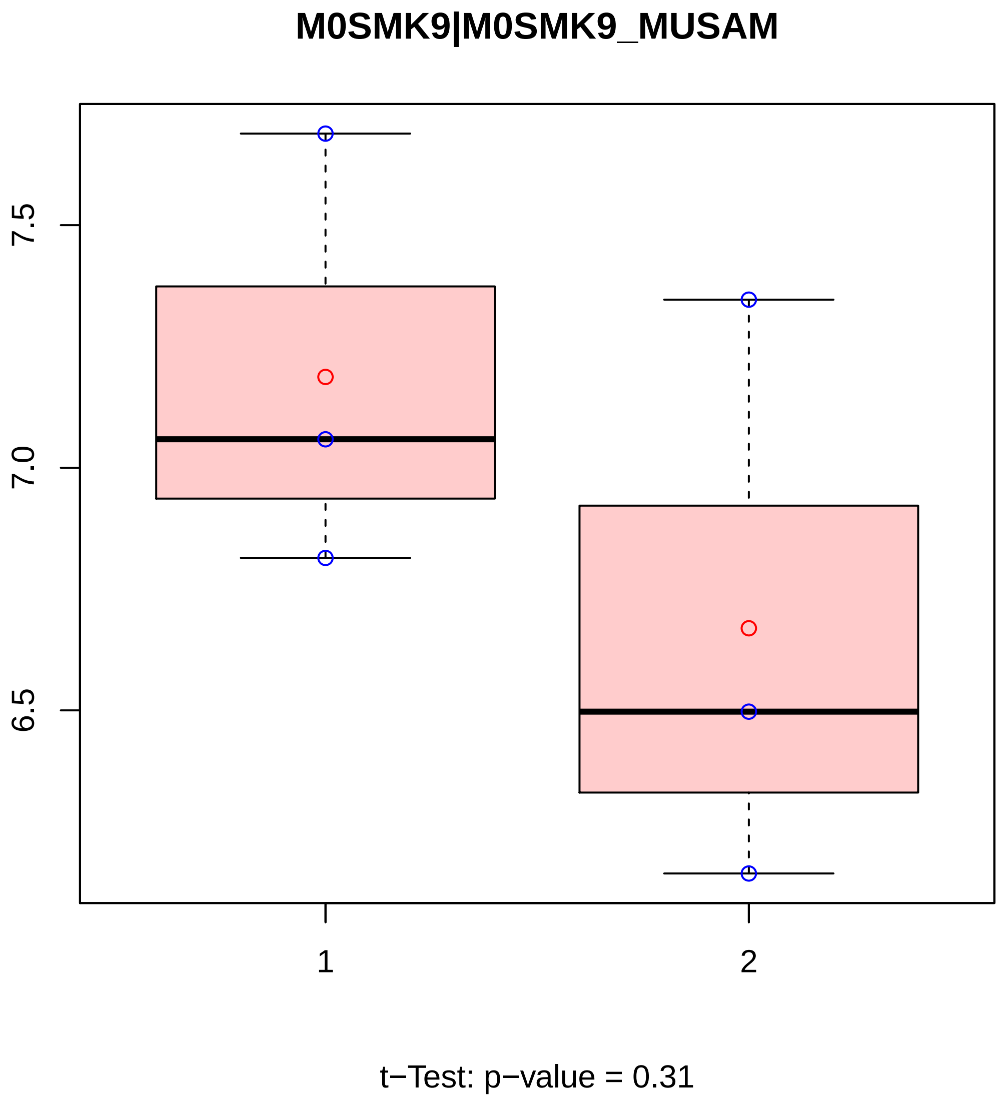
M0SMK9|M0SMK9_MUSAM
7.5
7.0
6.5
1
2
t−Test: p−value = 0.31

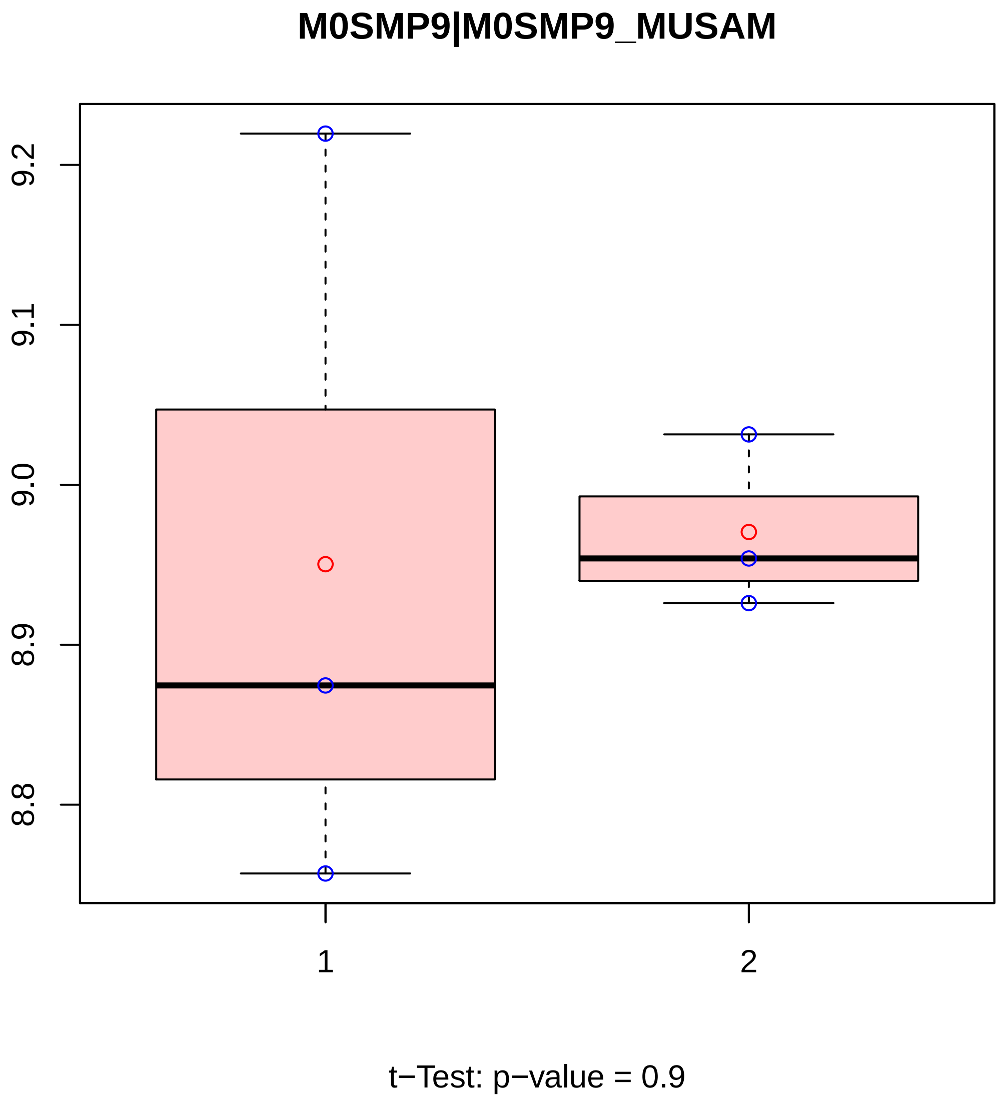
M0SMP9|M0SMP9_MUSAM
9.2
9.1
9.0
8.9
8.8
1
2
t−Test: p−value = 0.9

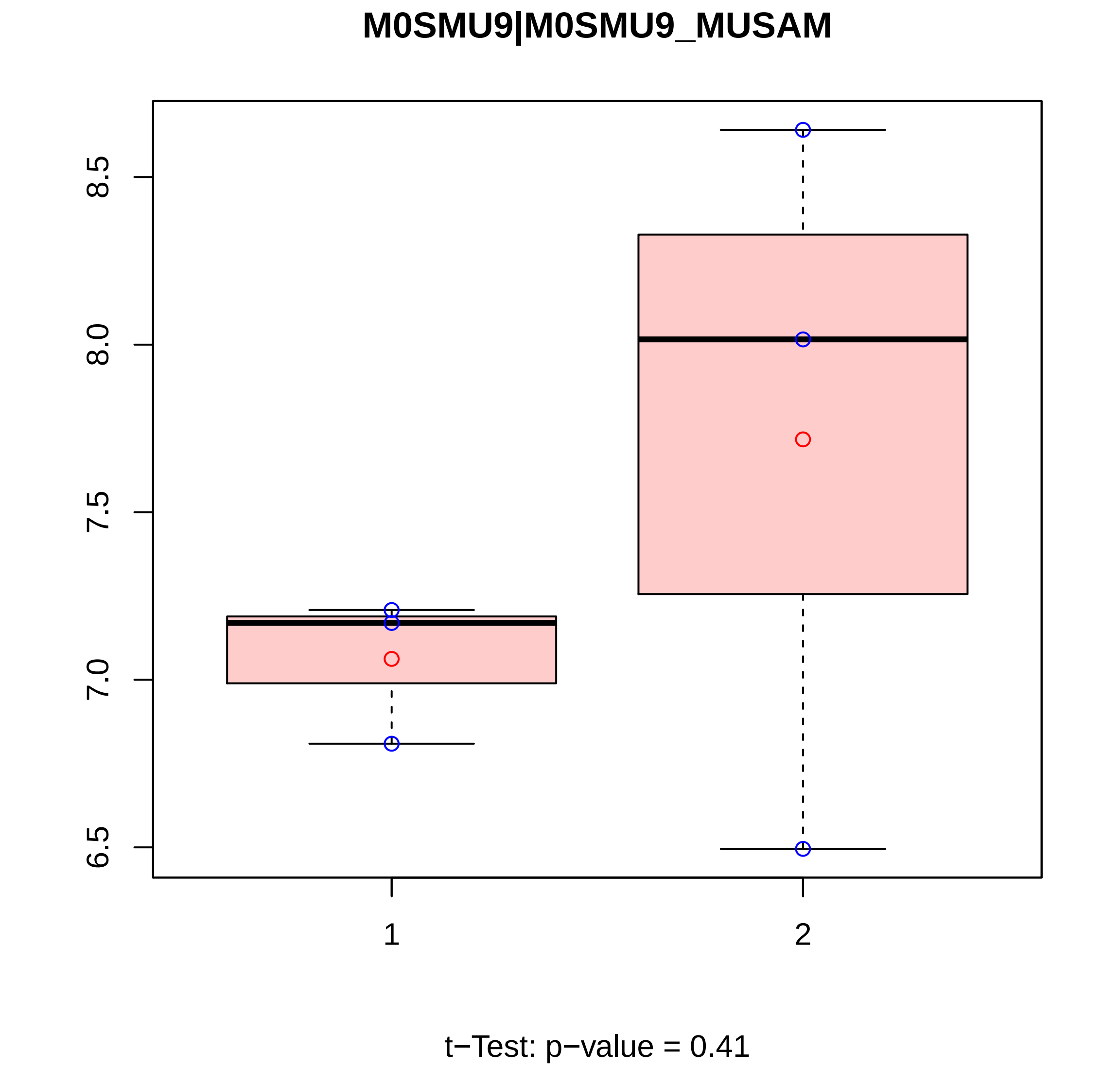
M0SMU9|M0SMU9_MUSAM
6.5
7.0
7.5
8.0
8.5
1
2
t−Test: p−value = 0.41

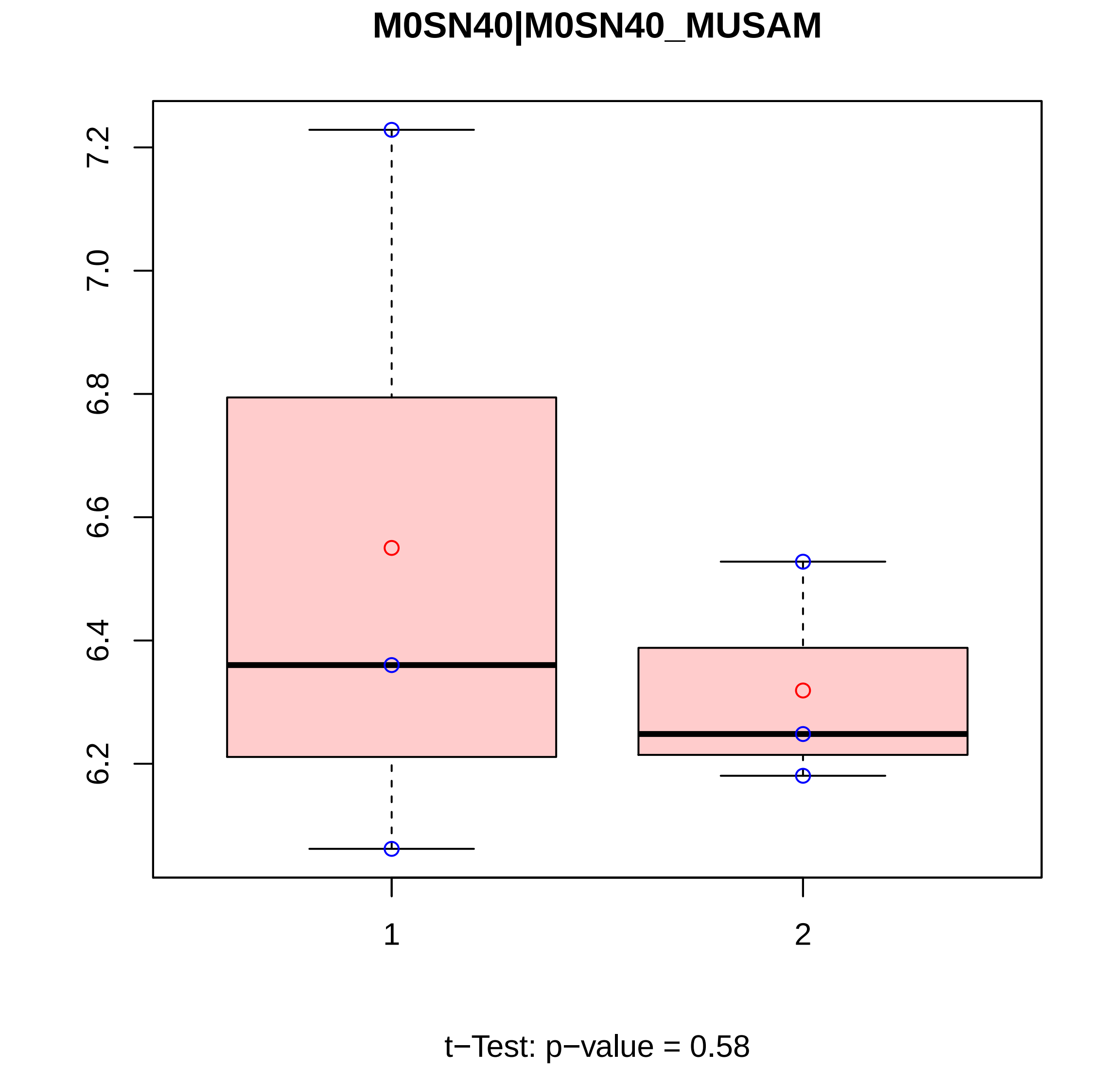
M0SN40|M0SN40_MUSAM
7.2
7.0
6.8
6.6
6.4
6.2
1
2
t−Test: p−value = 0.58

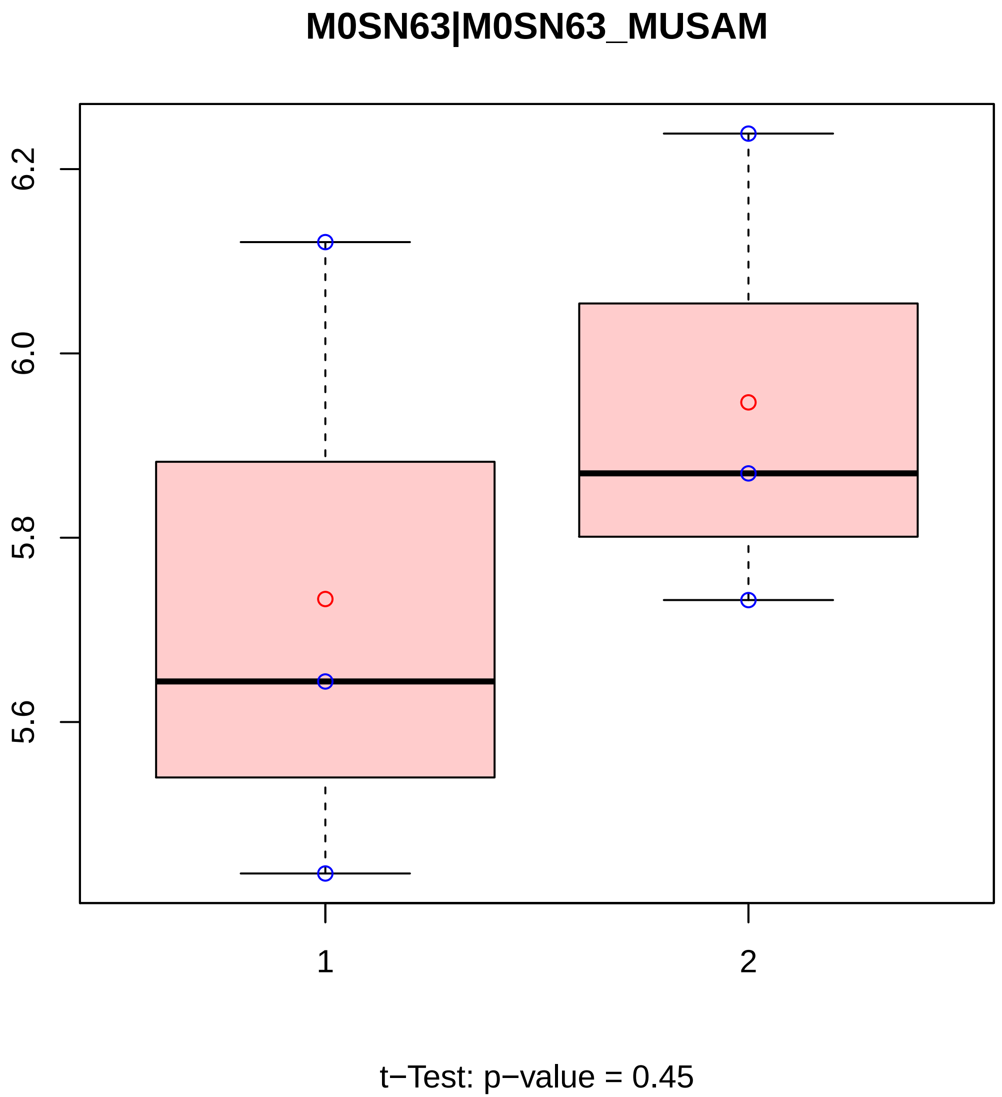

M0SN63|M0SN63_MUSAM
6.2
6.0
5.8
5.6
1
2
t−Test: p−value = 0.45

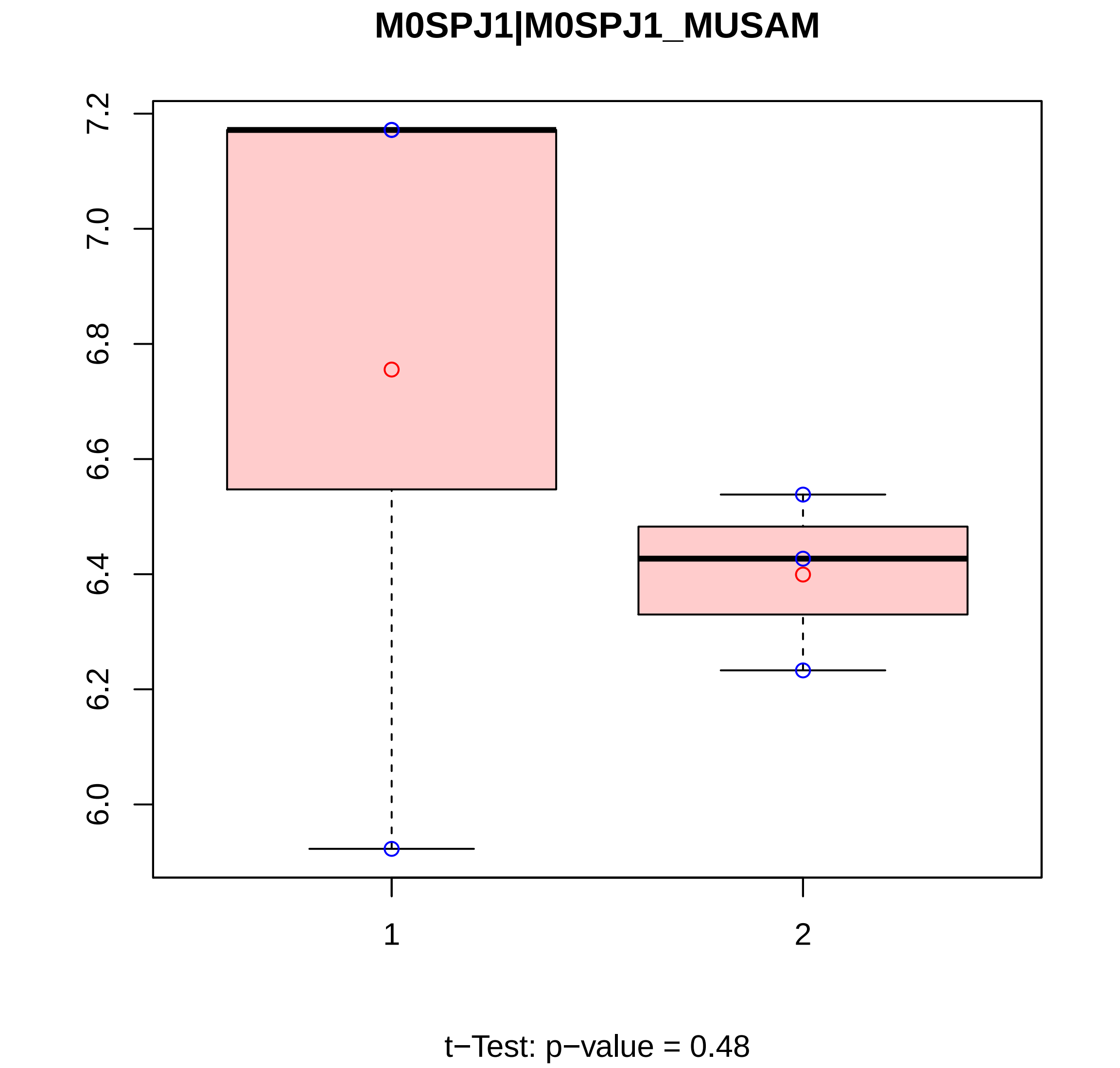

M0SPJ1|M0SPJ1_MUSAM
6.0
6.2
6.4
6.6
6.8
7.0
7.2
1
2
t−Test: p−value = 0.48

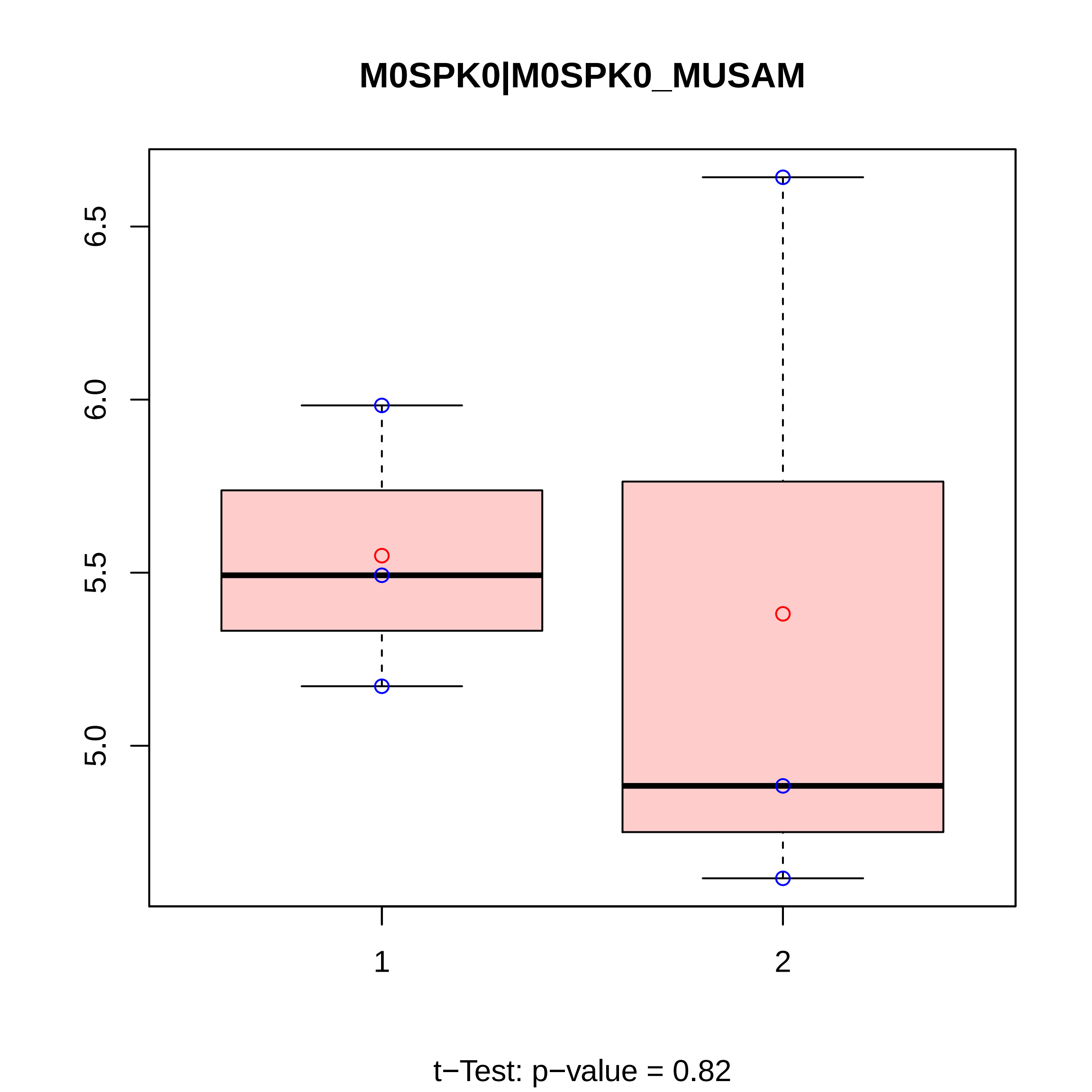
M0SPK0|M0SPK0_MUSAM
6.5
6.0
5.5
5.0
1
2
t−Test: p−value = 0.82

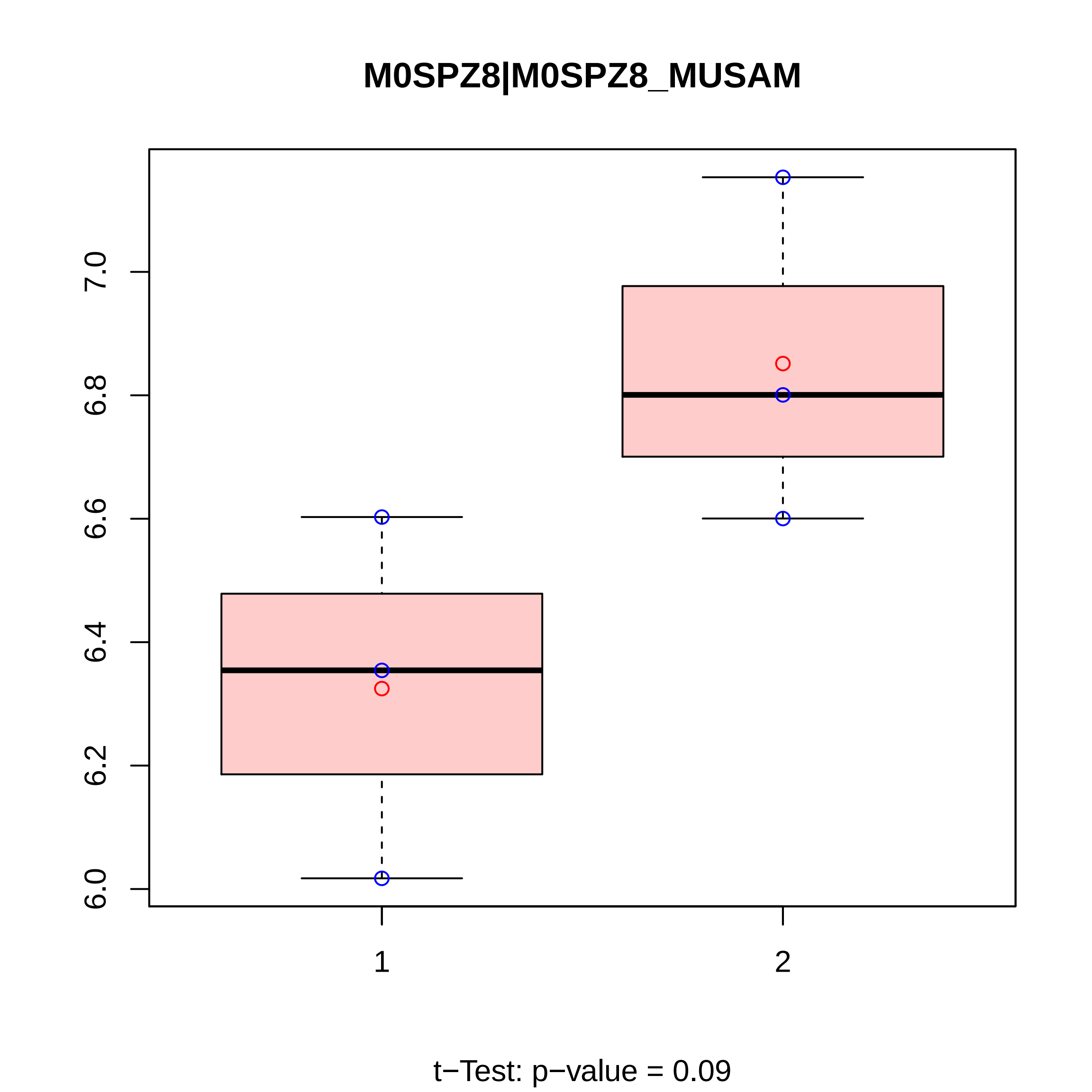
M0SPZ8|M0SPZ8_MUSAM
6.0
6.2
6.4
6.6
6.8
7.0
1
2
t−Test: p−value = 0.09

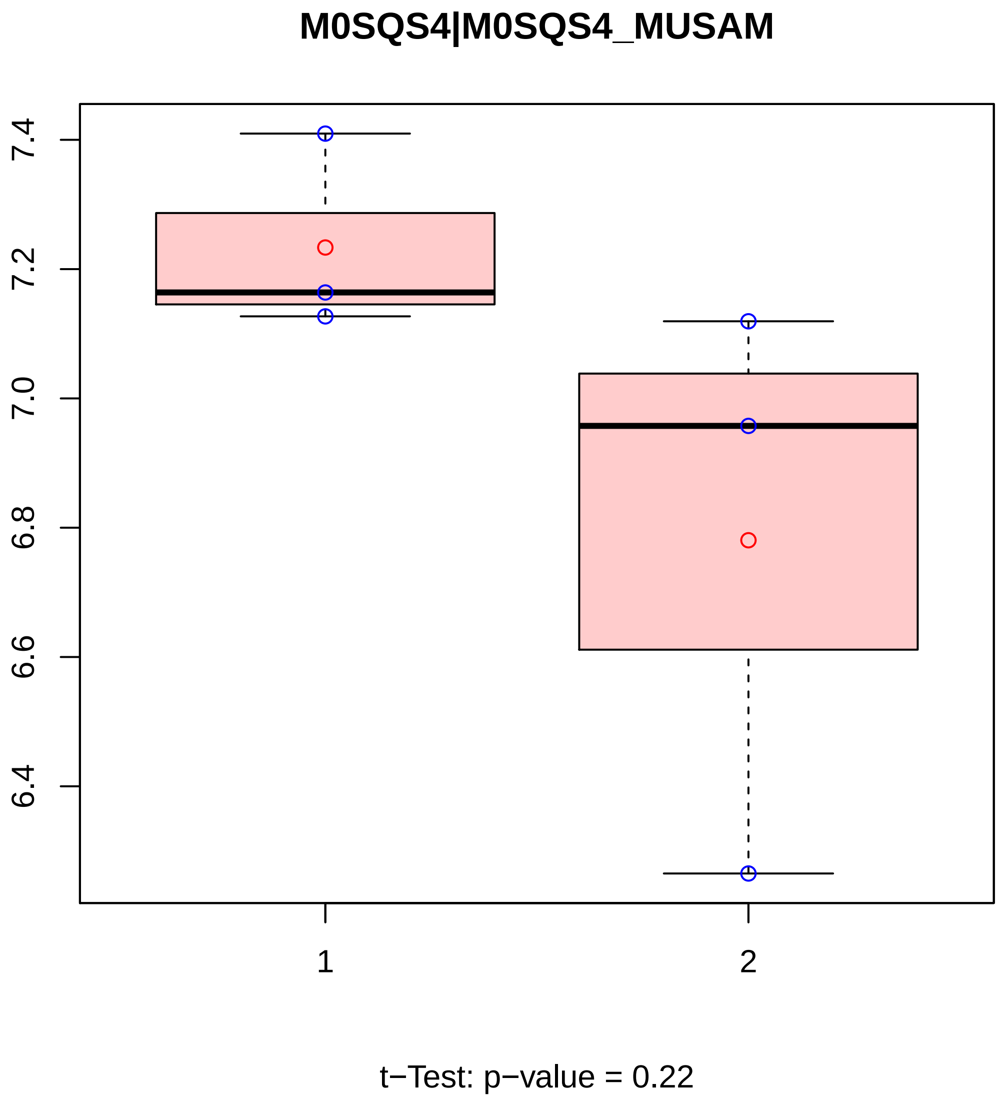
M0SQS4|M0SQS4_MUSAM
7.4
7.2
7.0
6.8
6.6
6.4
1
2
t−Test: p−value = 0.22

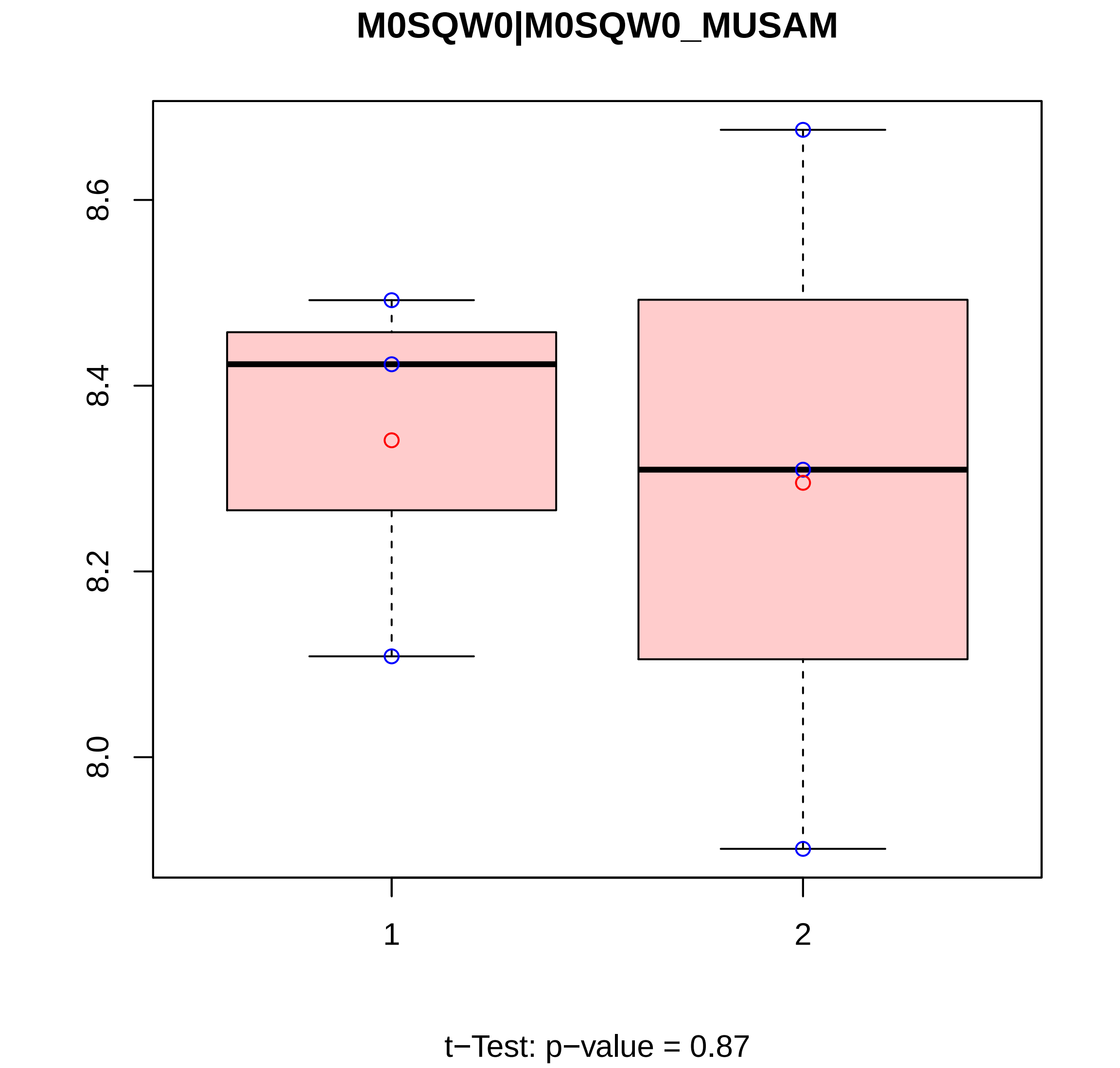
M0SQW0|M0SQW0_MUSAM
8.0
8.2
8.4
8.6
1
2
t−Test: p−value = 0.87

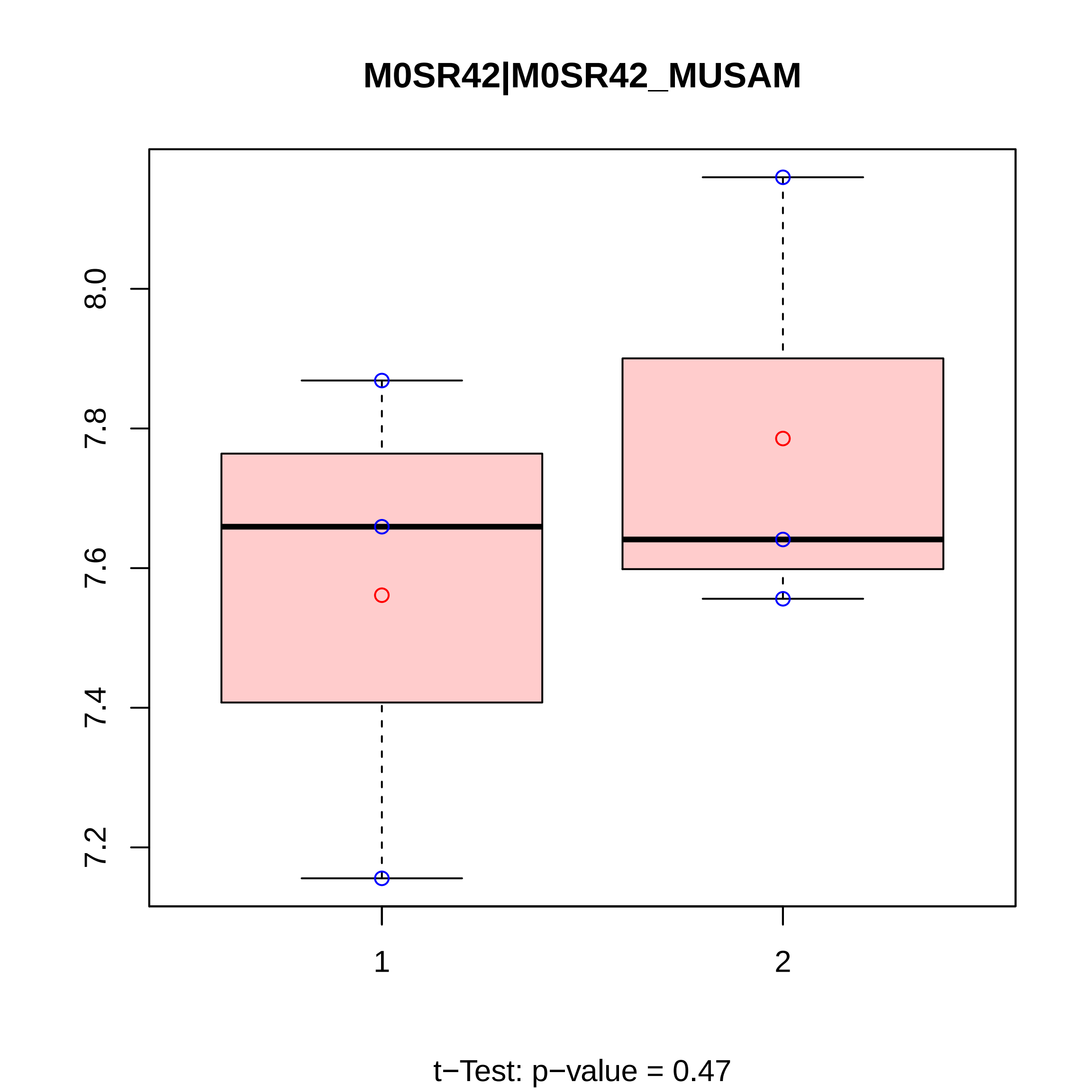
M0SR42|M0SR42_MUSAM
8.0
7.8
7.6
7.4
7.2
1
2
t−Test: p−value = 0.47

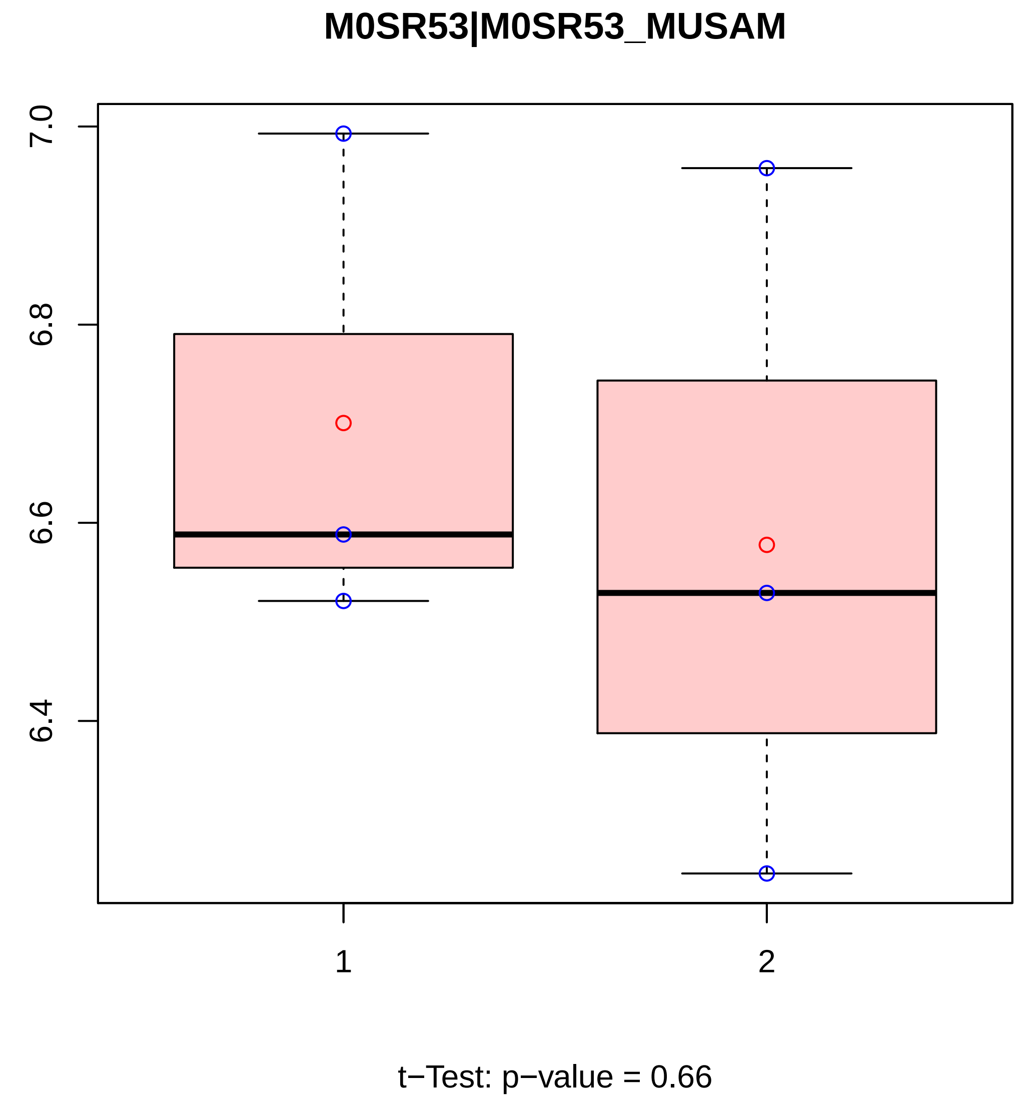
M0SR53|M0SR53_MUSAM
7.0
6.8
6.6
6.4
1
2
t−Test: p−value = 0.66

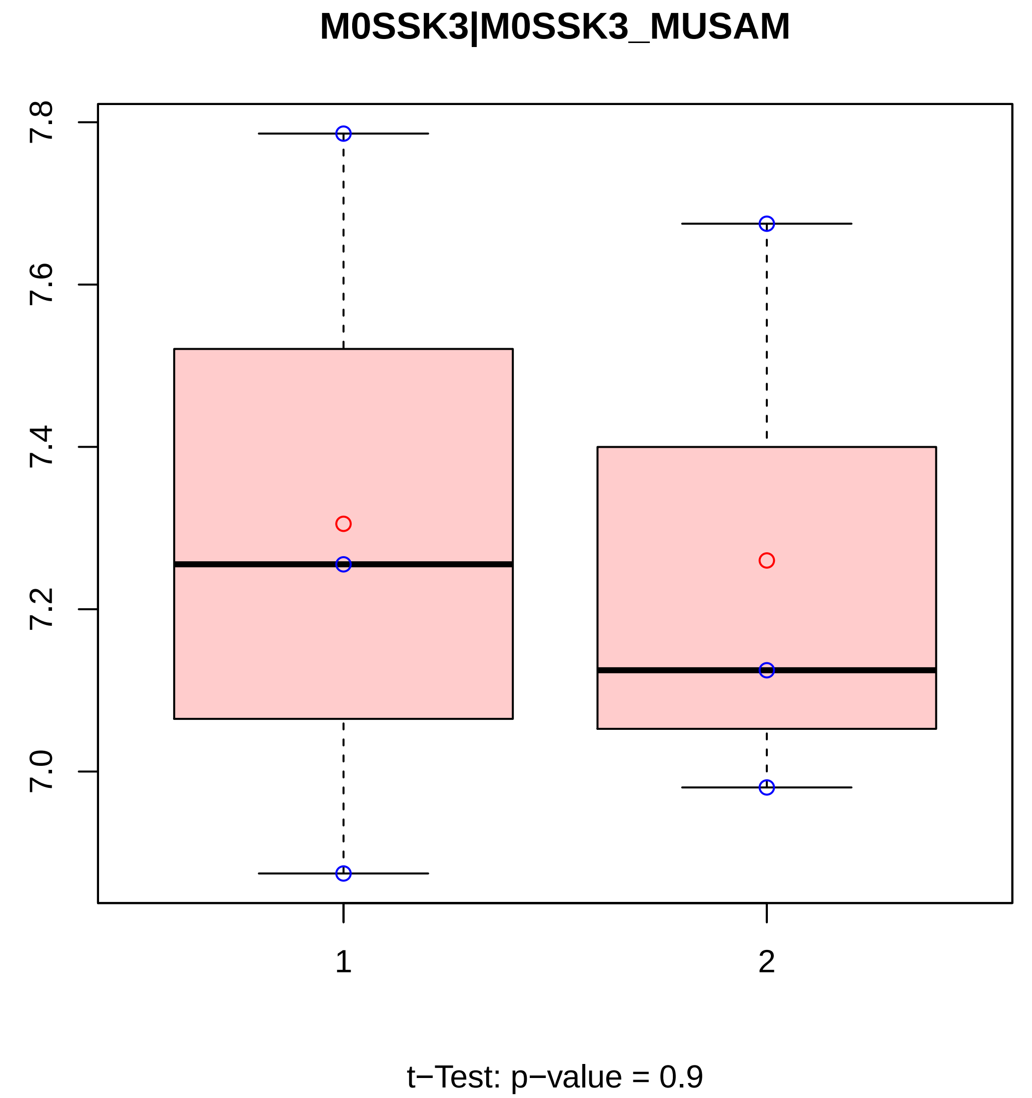

M0SSK3|M0SSK3_MUSAM
7.8
7.6
7.4
7.2
7.0
1
2
t−Test: p−value = 0.9

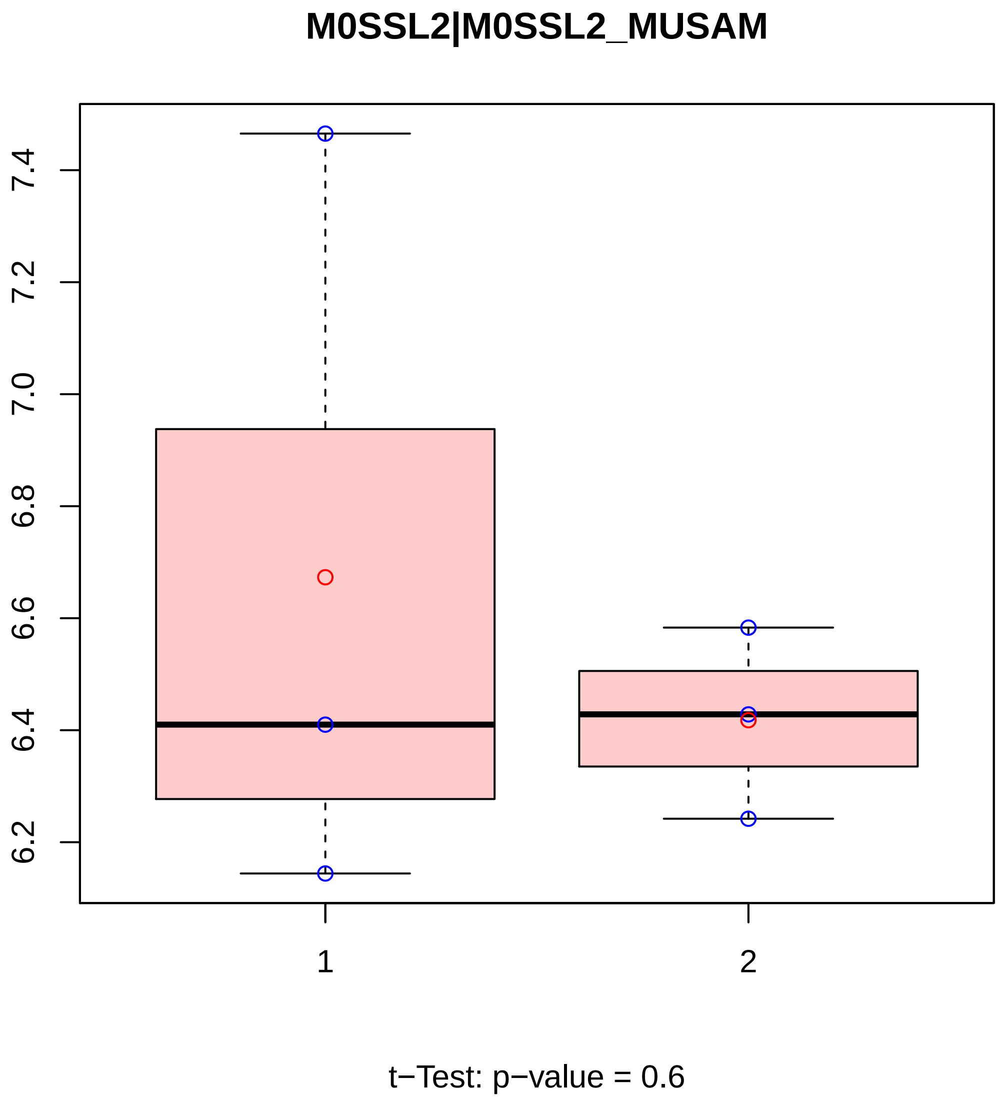
M0SSL2|M0SSL2_MUSAM
7.4
7.2
7.0
6.8
6.6
6.4
6.2
1
2
t−Test: p−value = 0.6

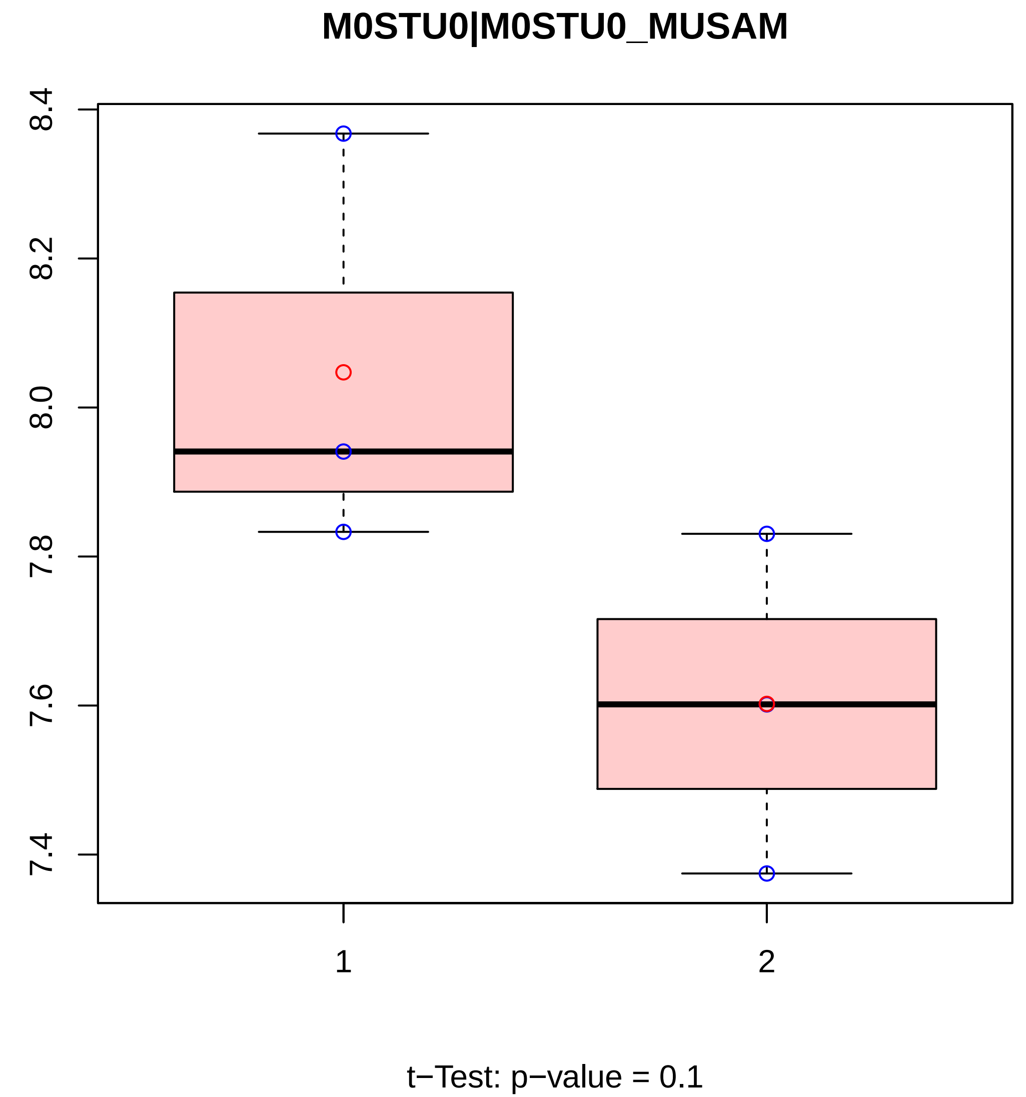
M0STU0|M0STU0_MUSAM
8.4
8.2
8.0
7.8
7.6
7.4
1
2
t−Test: p−value = 0.1

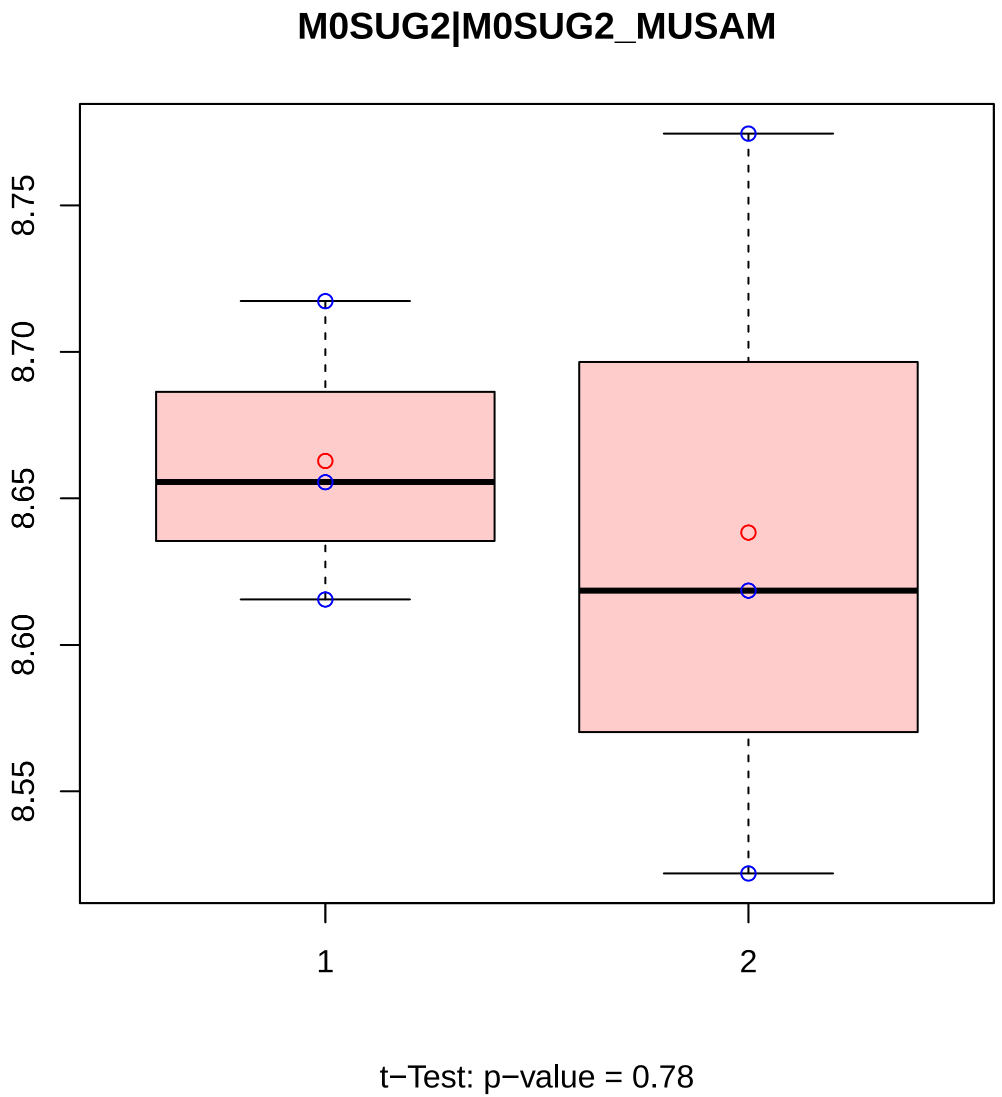

M0SUG2|M0SUG2_MUSAM
8.55
8.60
8.65
8.70
8.75
1
2
t−Test: p−value = 0.78

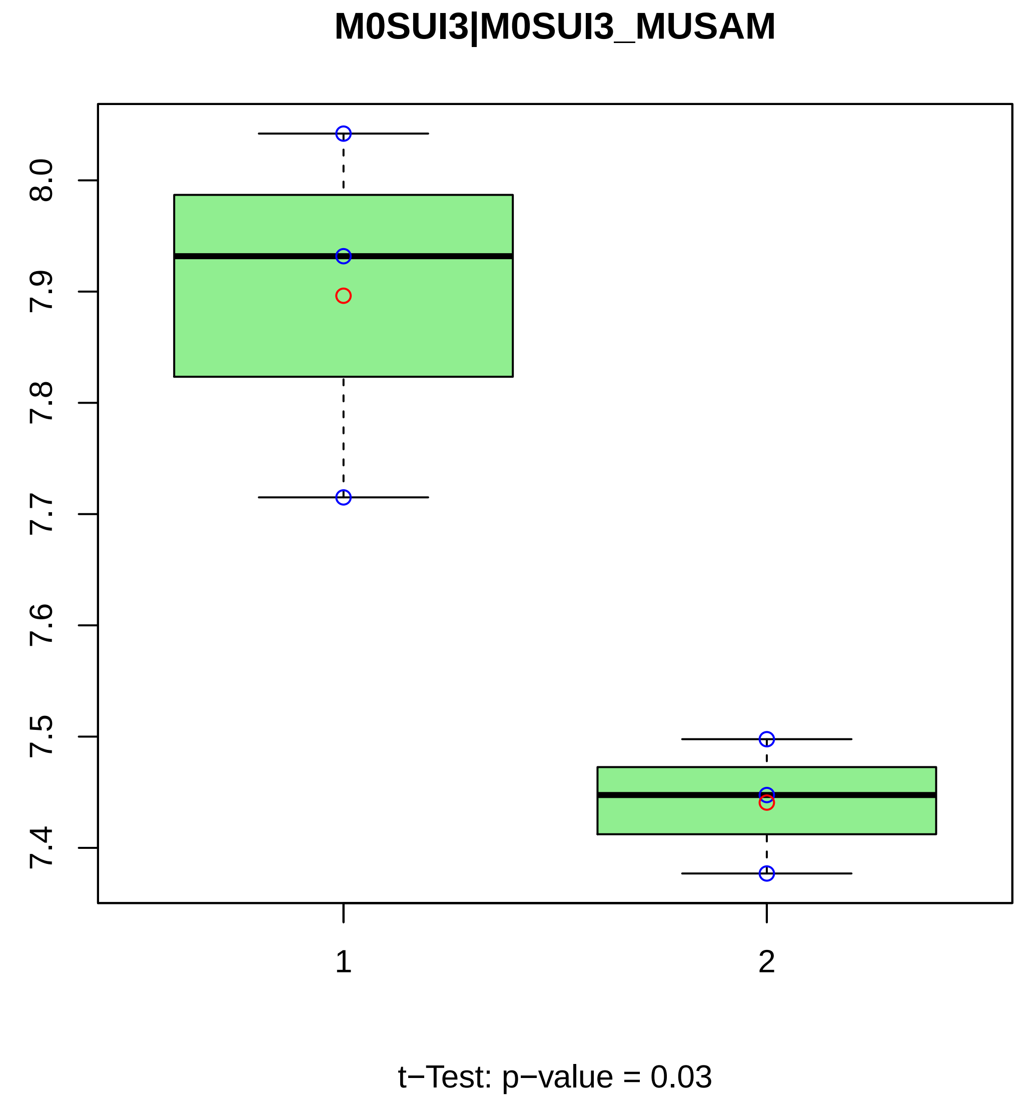
M0SUI3|M0SUI3_MUSAM
8.0
7.9
7.8
7.7
7.6
7.5
7.4
1
2
t−Test: p−value = 0.03

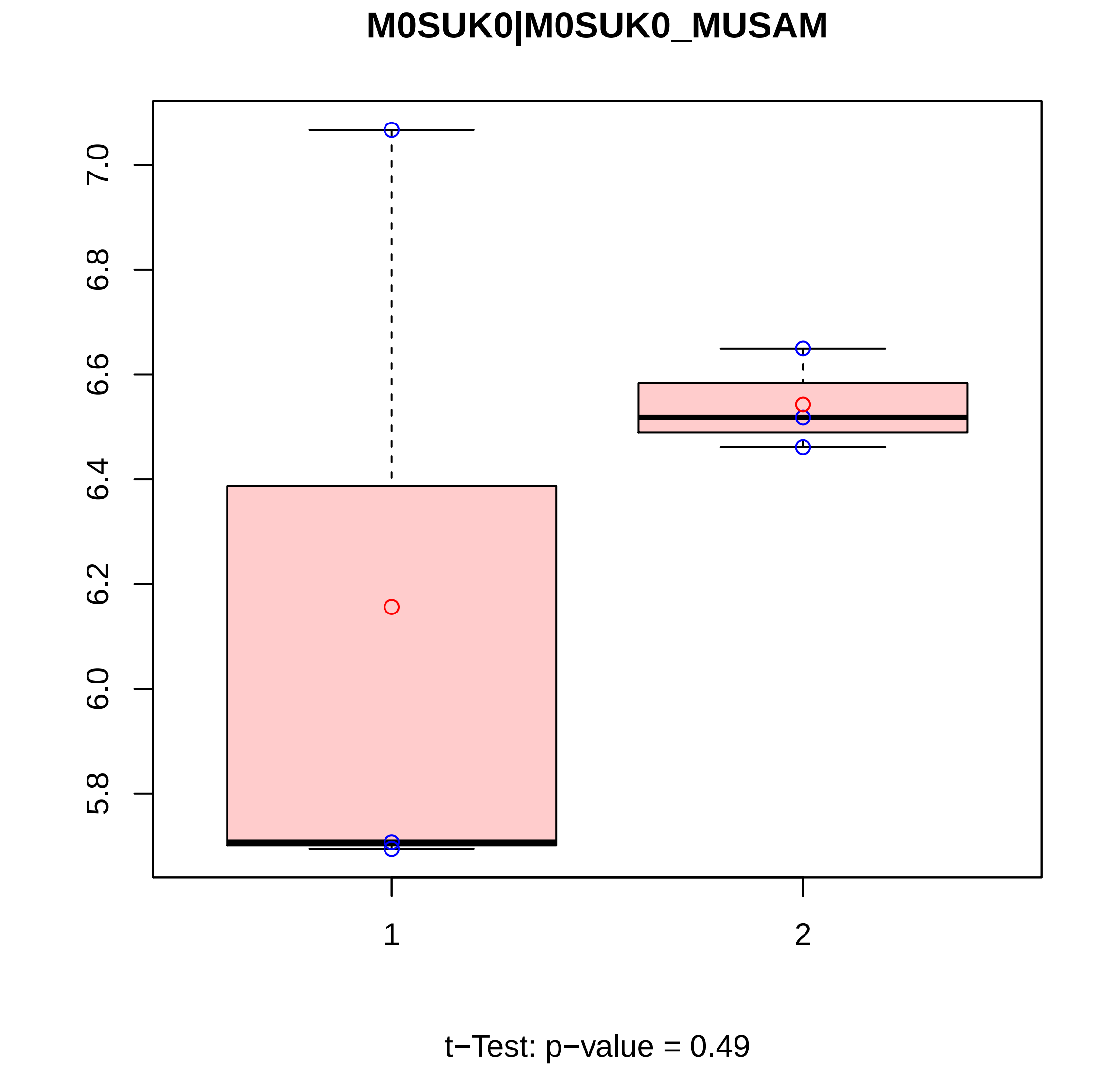

M0SUK0|M0SUK0_MUSAM
7.0
6.8
6.6
6.4
6.2
6.0
5.8
1
2
t−Test: p−value = 0.49

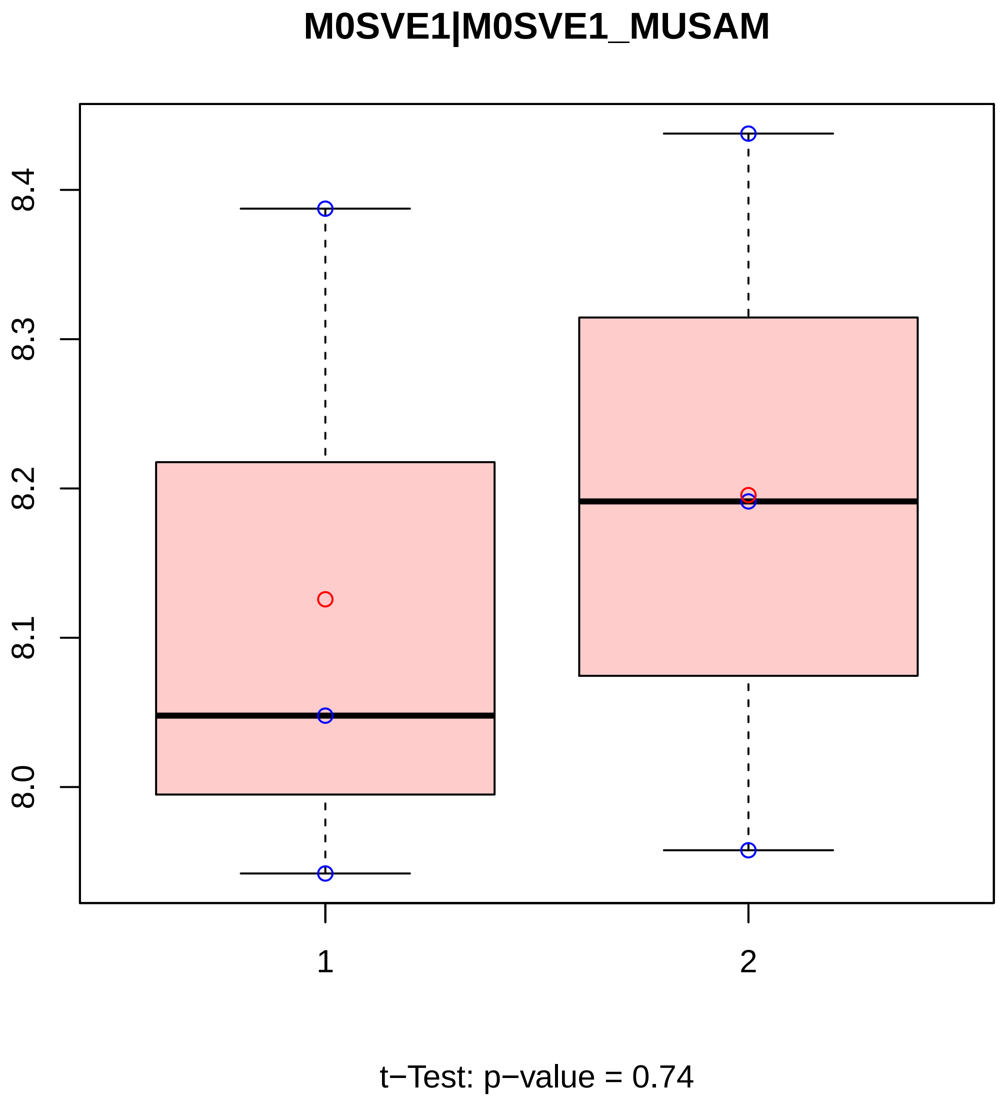
M0SVE1|M0SVE1_MUSAM
8.0
8.1
8.2
8.3
8.4
1
2
t−Test: p−value = 0.74

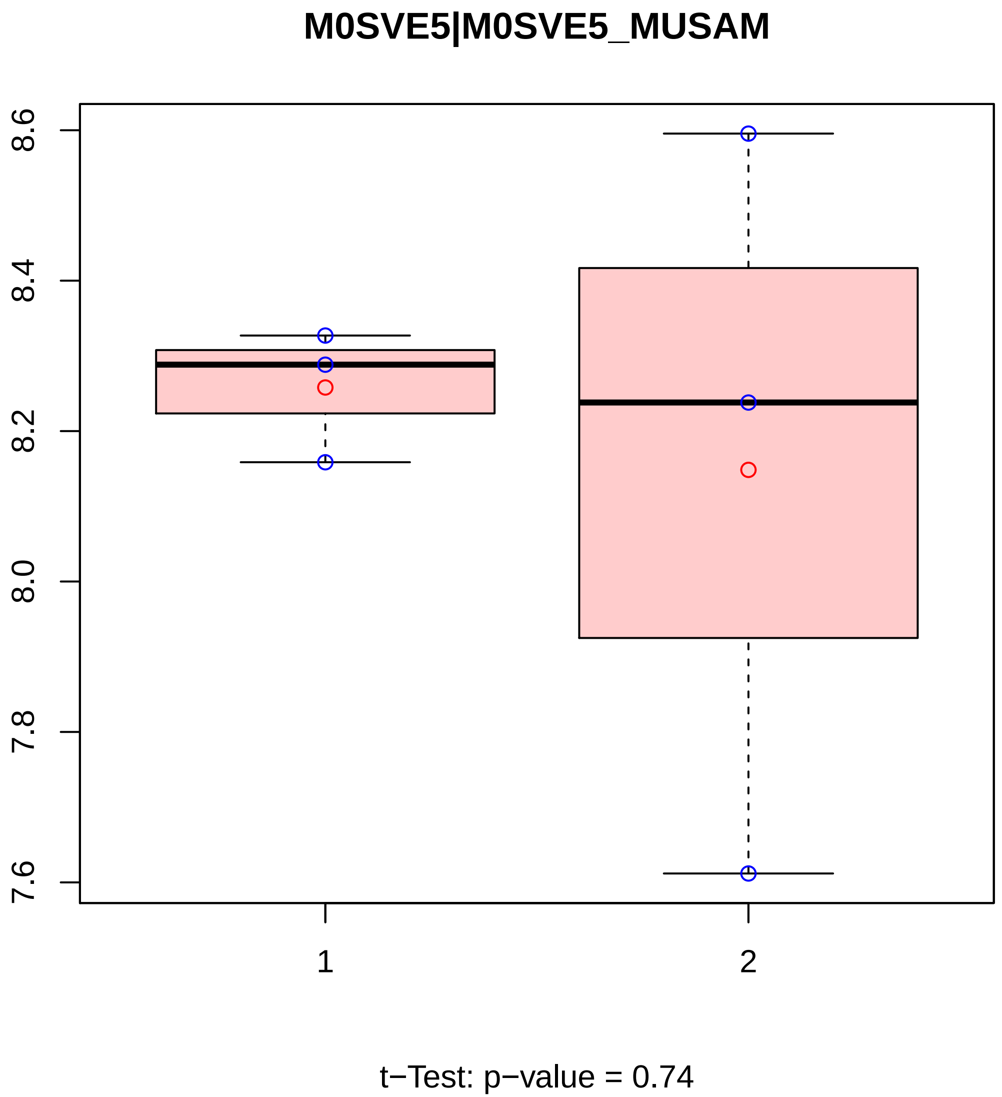

M0SVE5|M0SVE5_MUSAM
8.6
8.4
8.2
8.0
7.8
7.6
1
2
t−Test: p−value = 0.74

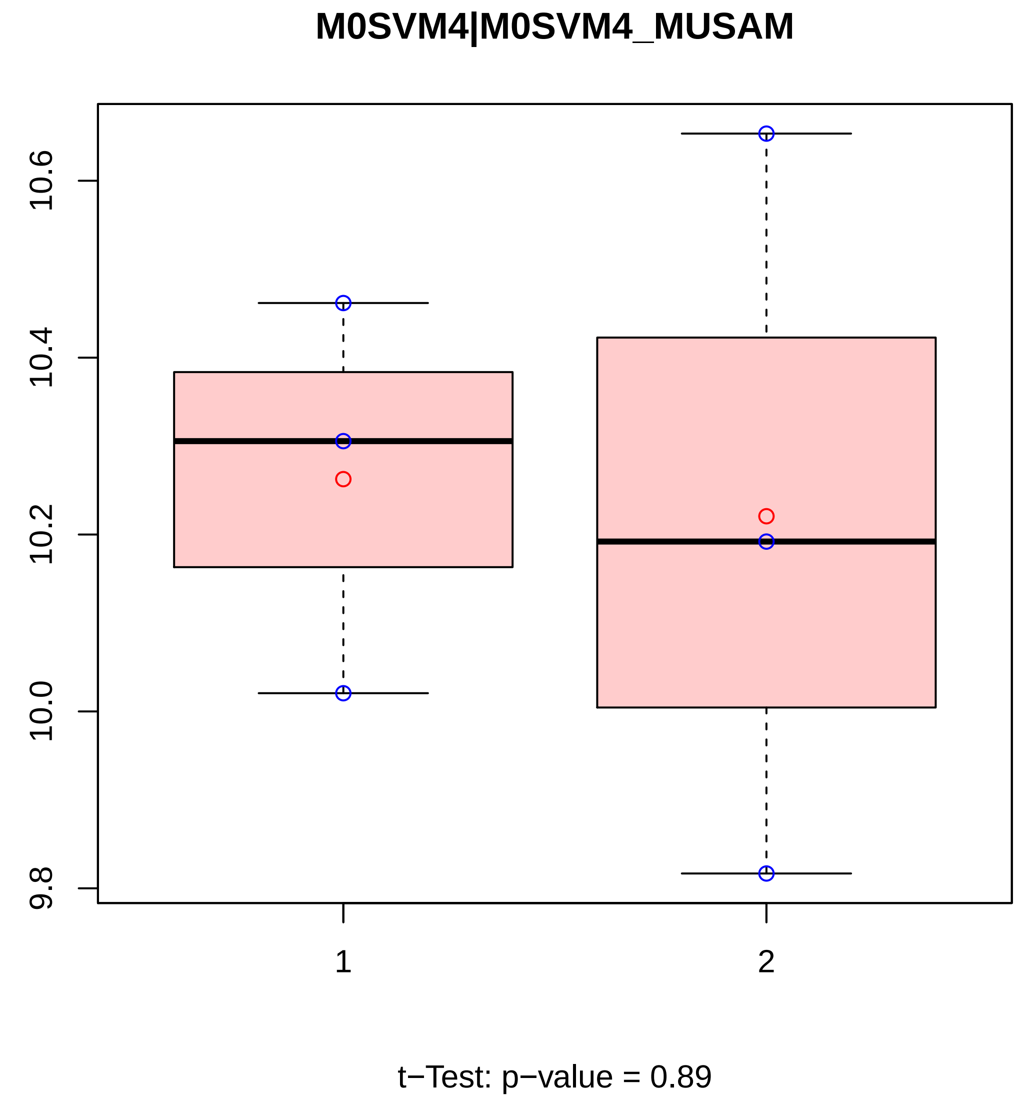
M0SVM4|M0SVM4_MUSAM
9.8
10.0
10.2
10.4
10.6
1
2
t−Test: p−value = 0.89

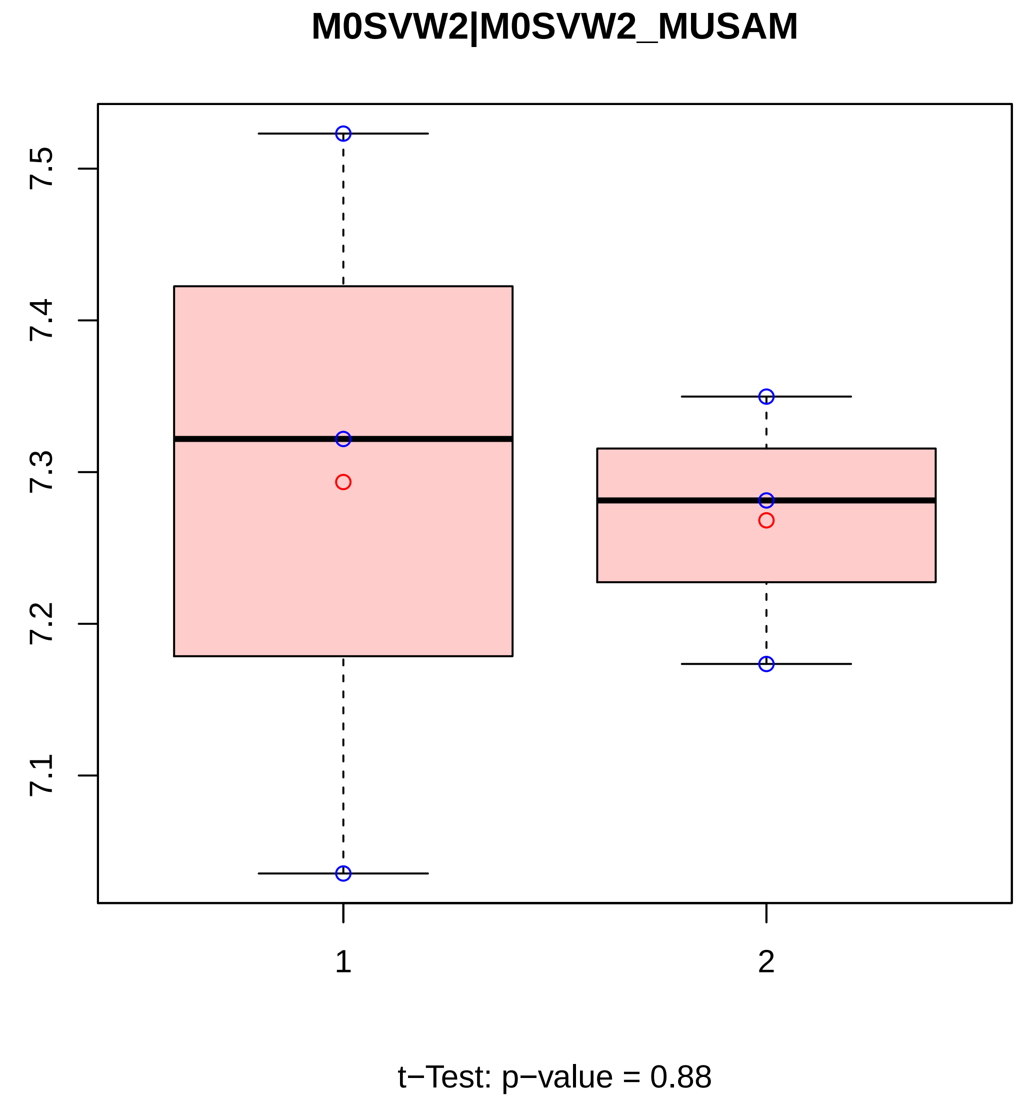
M0SVW2|M0SVW2_MUSAM
7.5
7.4
7.3
7.2
7.1
1
2
t−Test: p−value = 0.88

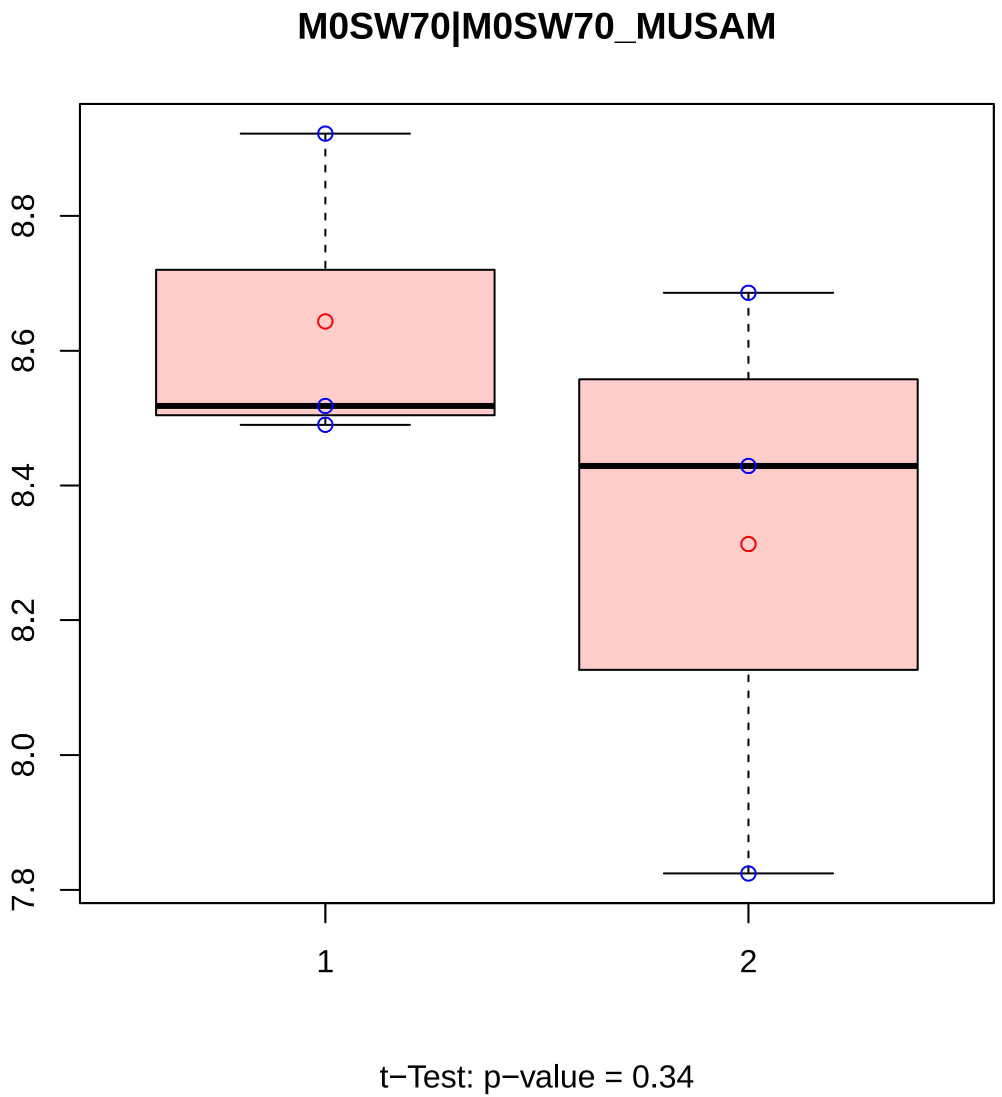
M0SW70|M0SW70_MUSAM
8.8
8.6
8.4
8.2
8.0
7.8
1
2
t−Test: p−value = 0.34

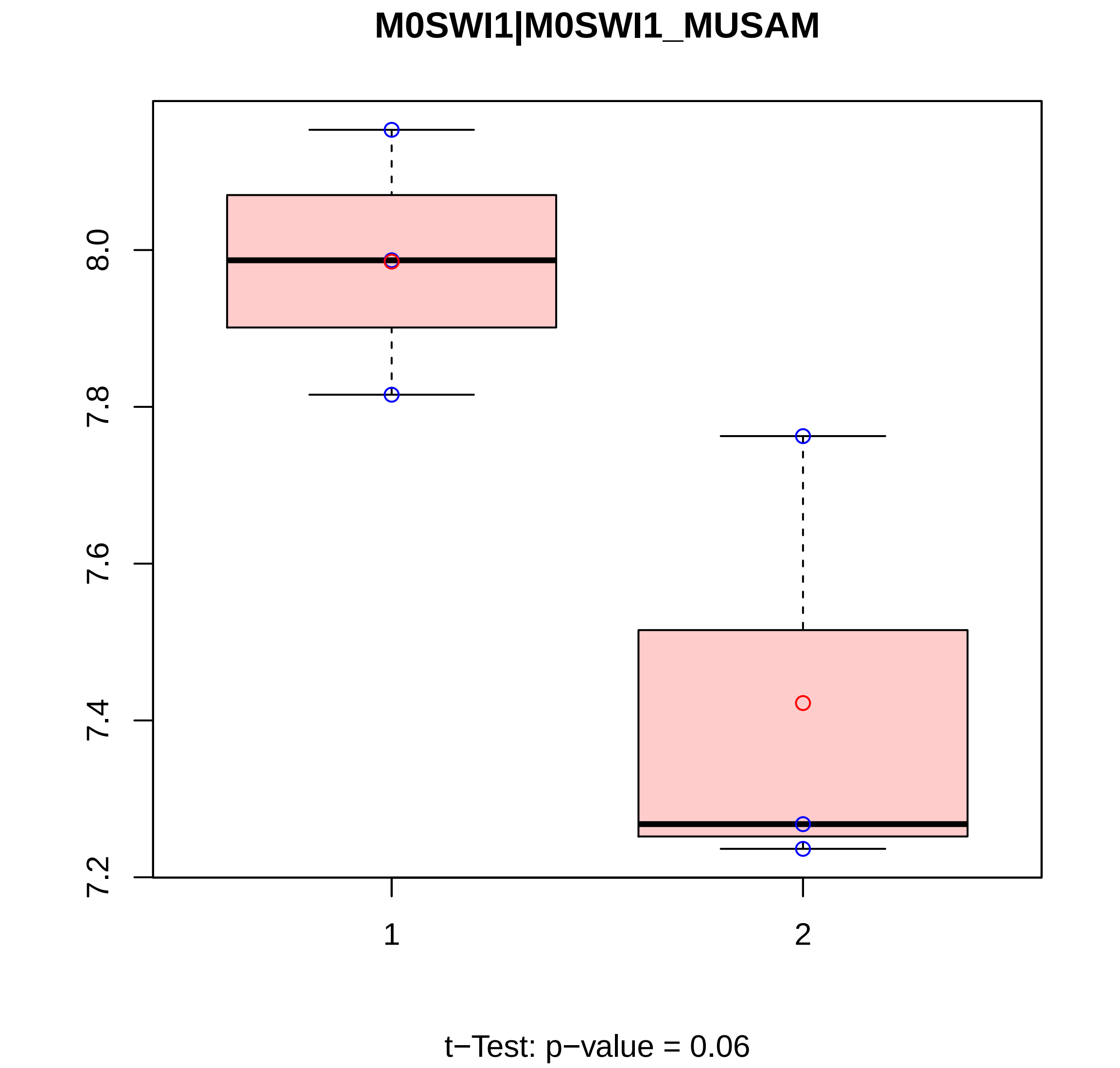
M0SWI1|M0SWI1_MUSAM
8.0
7.8
7.6
7.4
7.2
1
2
t−Test: p−value = 0.06

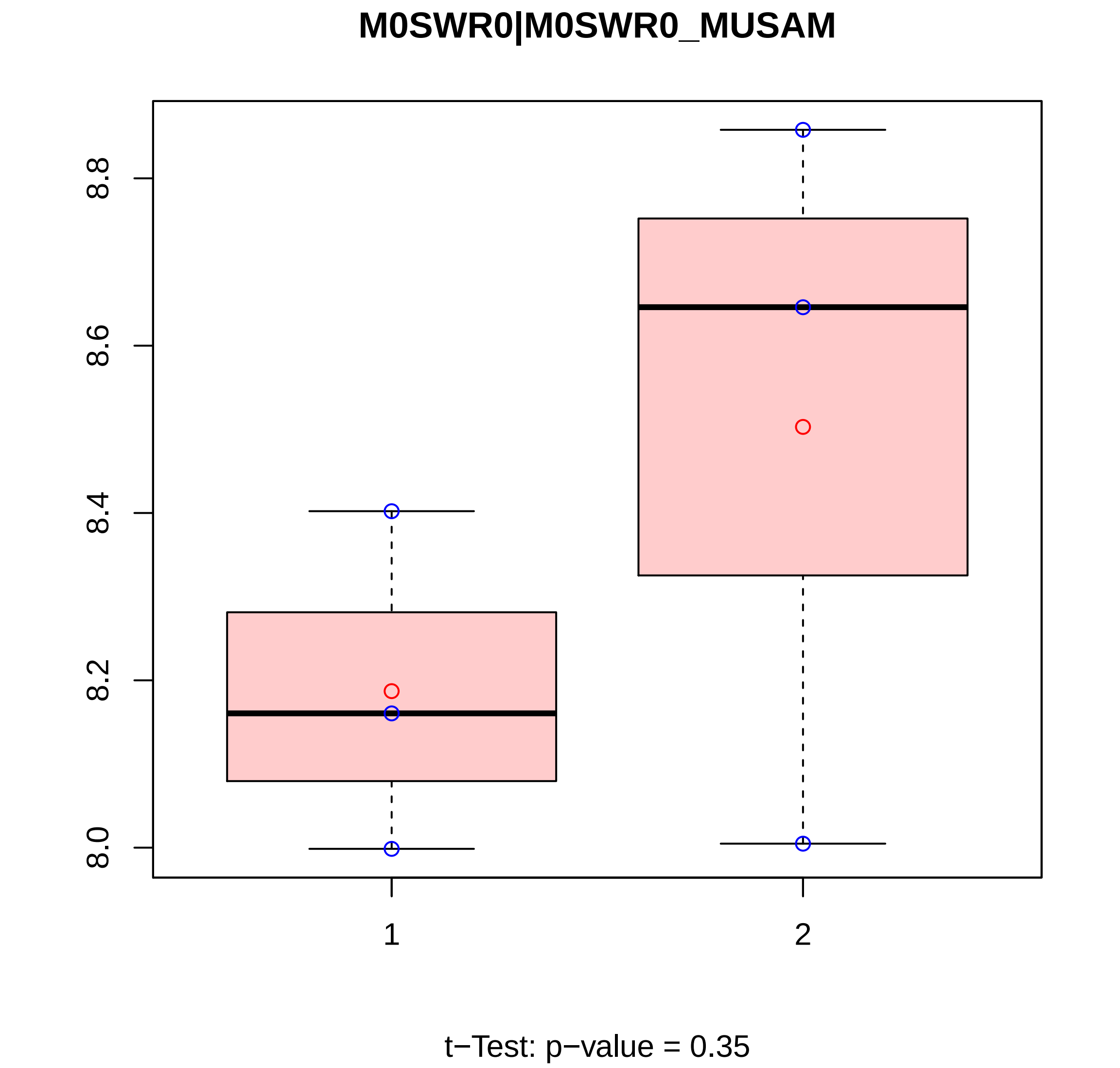
M0SWR0|M0SWR0_MUSAM
8.0
8.2
8.4
8.6
8.8
1
2
t−Test: p−value = 0.35

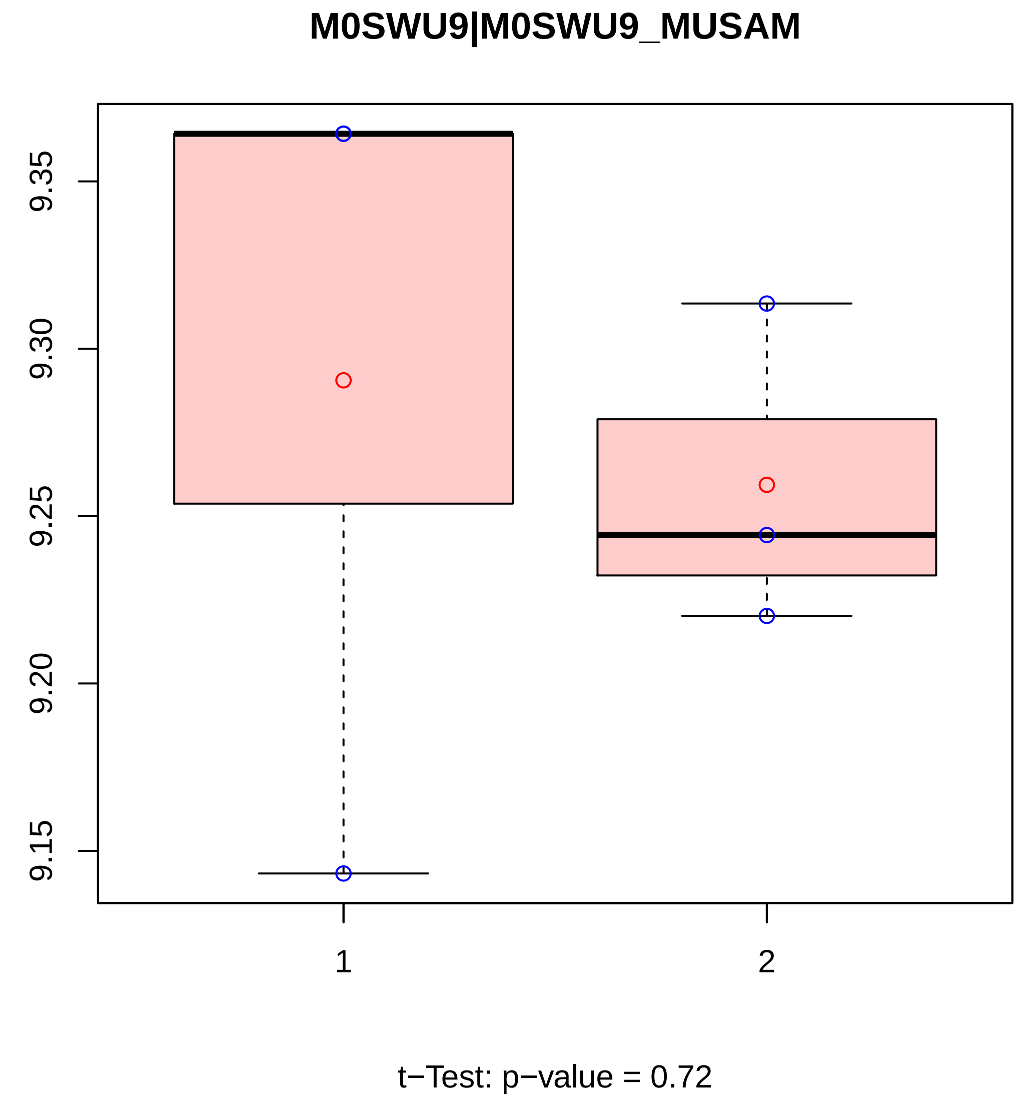
M0SWU9|M0SWU9_MUSAM
9.15
9.20
9.25
9.30
9.35
1
2
t−Test: p−value = 0.72

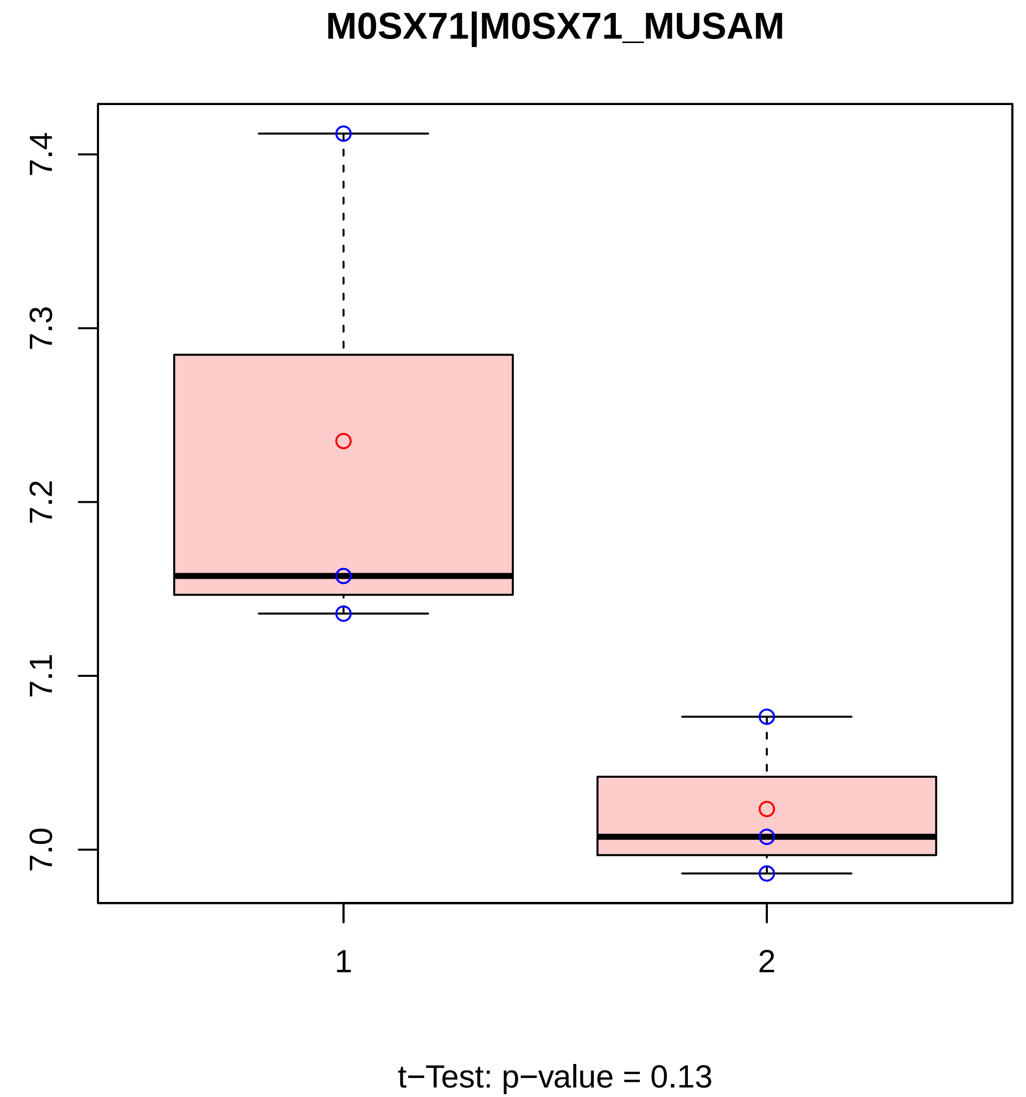
M0SX71|M0SX71_MUSAM
7.4
7.3
7.2
7.1
7.0
1
2
t−Test: p−value = 0.13

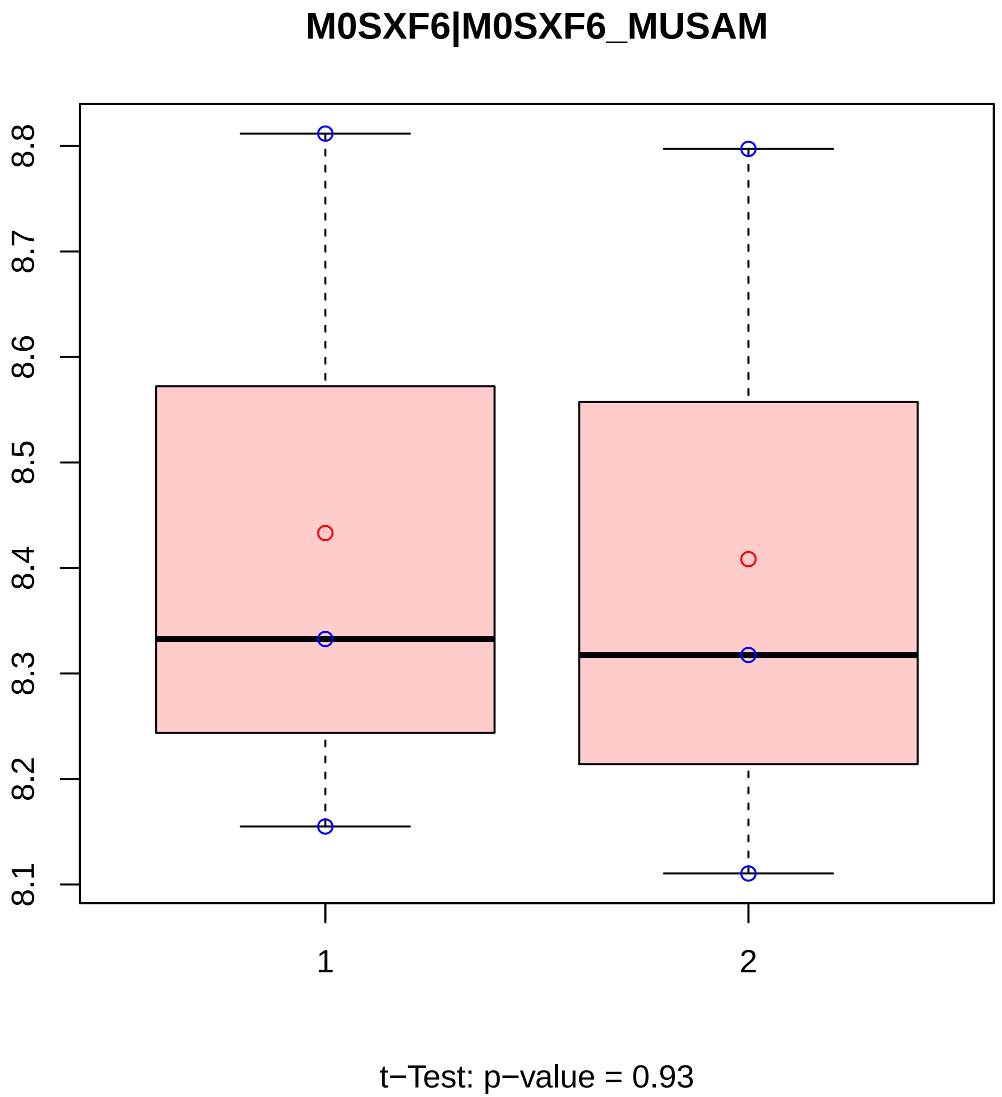

M0SXF6|M0SXF6_MUSAM
8.1
8.2
8.3
8.4
8.5
8.6
8.7
8.8
1
2
t−Test: p−value = 0.93

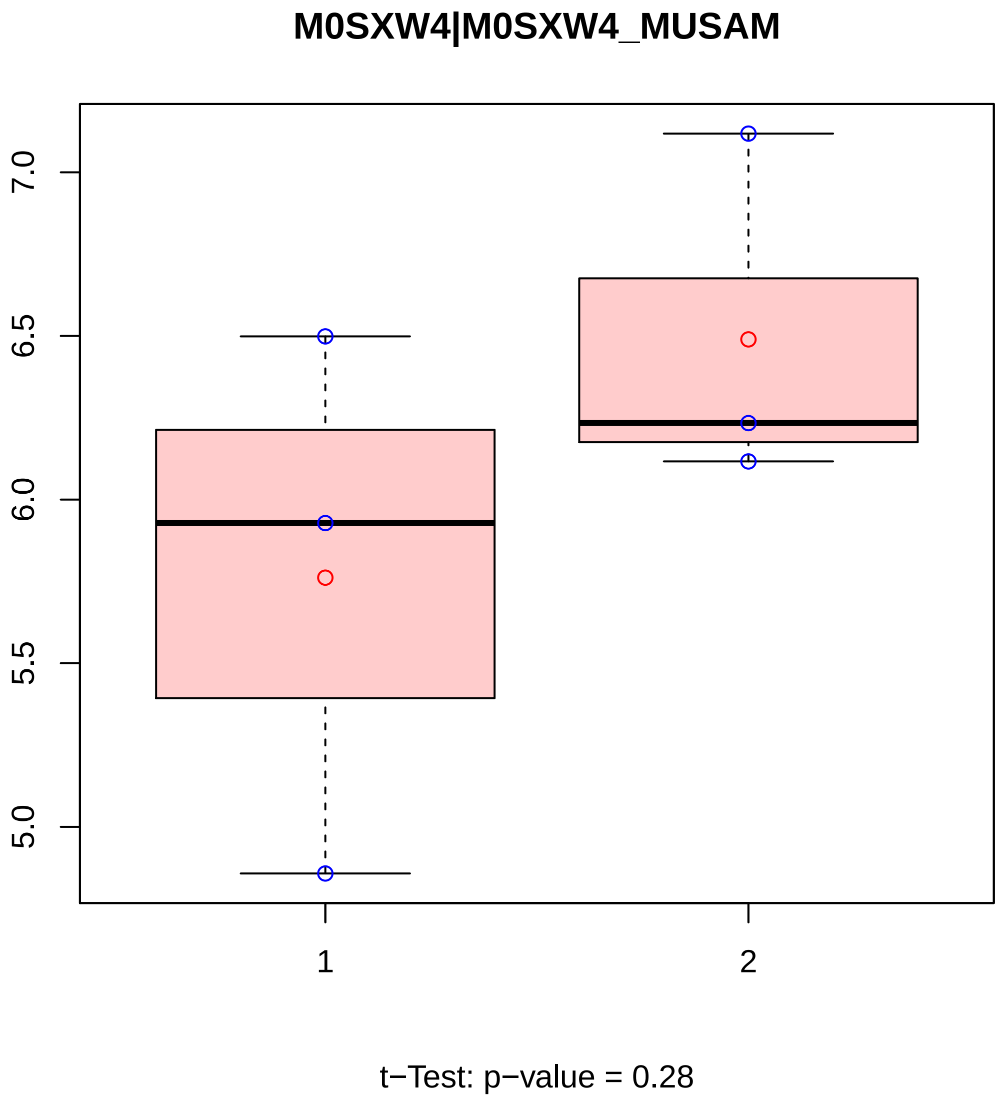
M0SXW4|M0SXW4_MUSAM
7.0
6.5
6.0
5.5
5.0
1
2
t−Test: p−value = 0.28

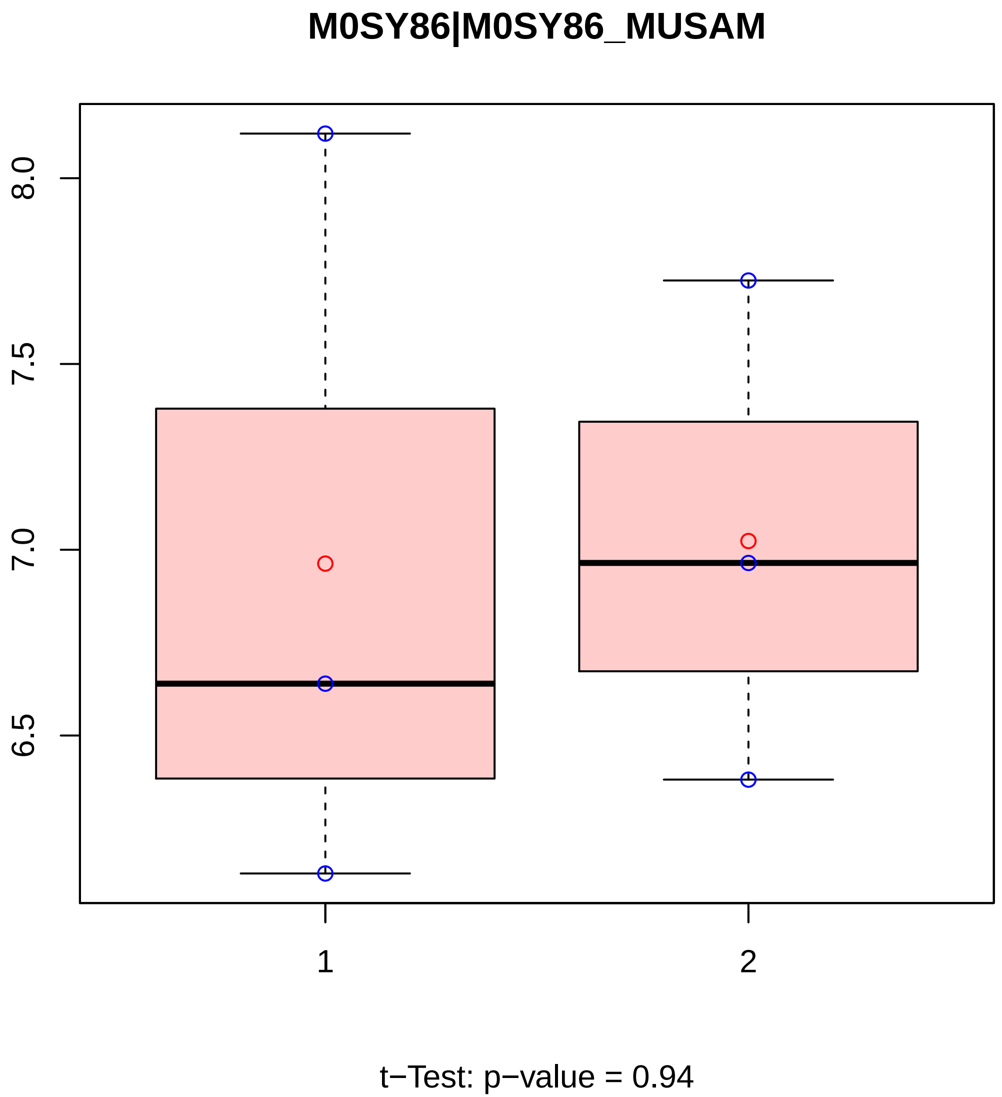
M0SY86|M0SY86_MUSAM
8.0
7.5
7.0
6.5
1
2
t−Test: p−value = 0.94

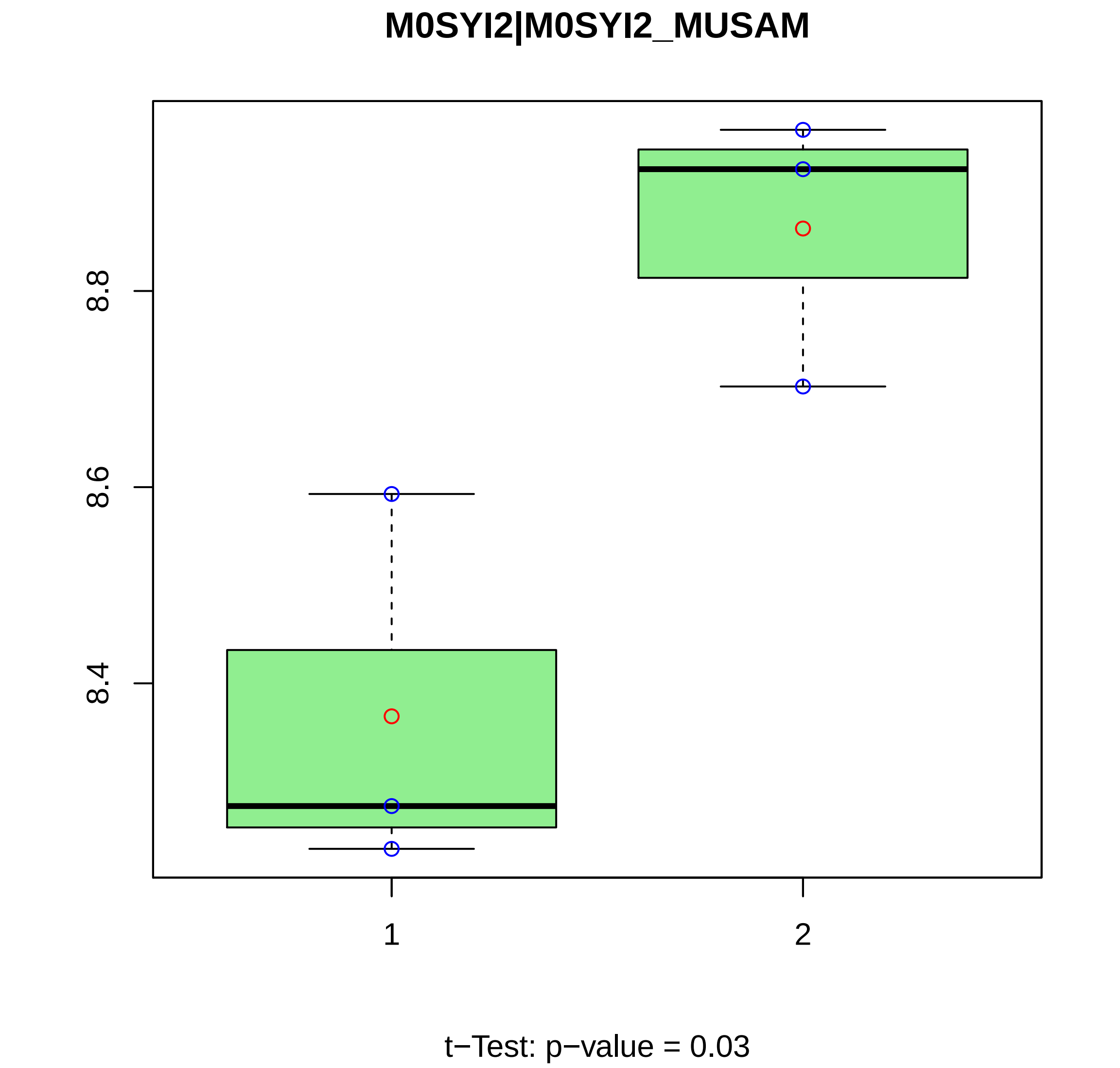

M0SYI2|M0SYI2_MUSAM
8.4
8.6
8.8
1
2
t−Test: p−value = 0.03

M0SYK7|M0SYK7_MUSAM

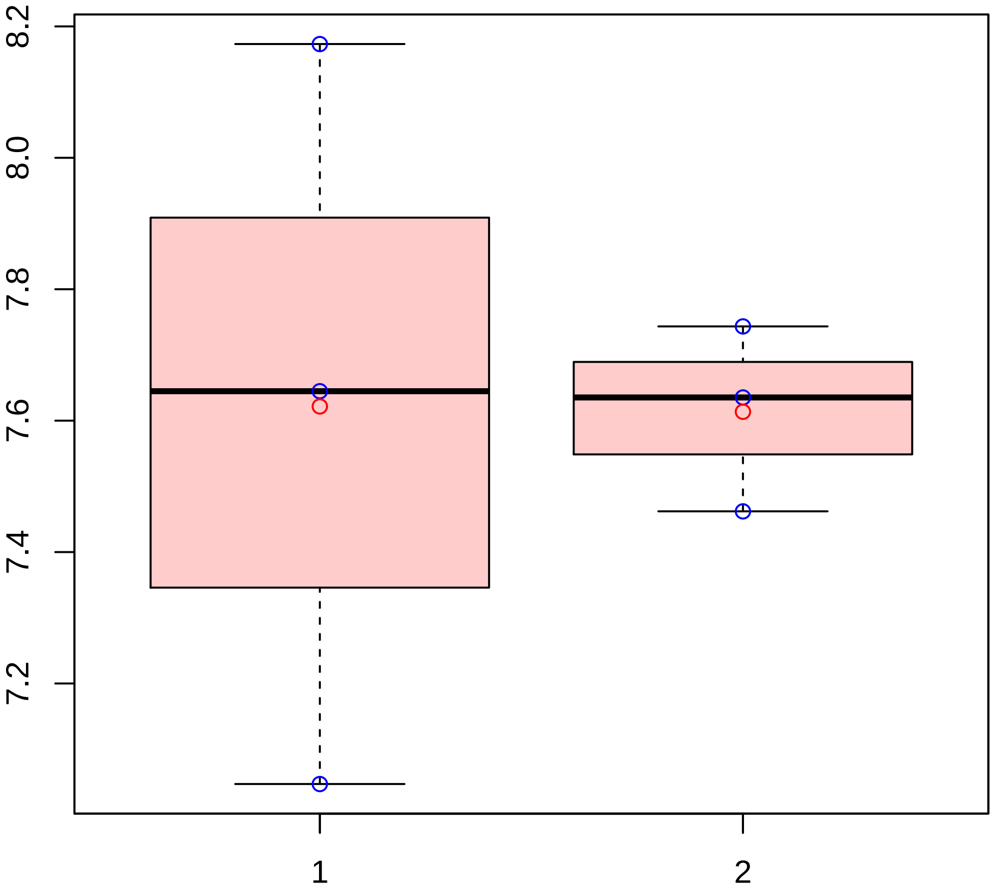

t−Test: p−value = 0.98

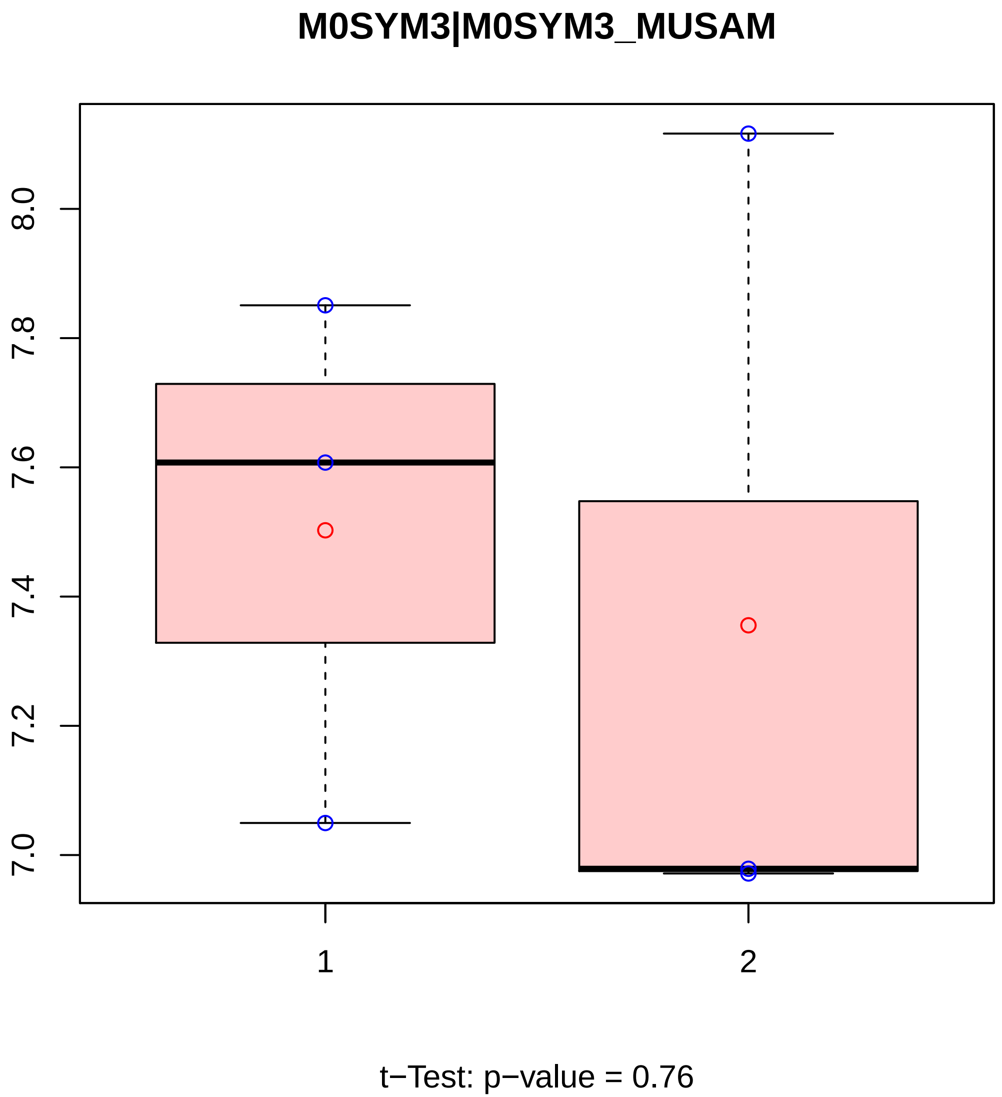
M0SYM3|M0SYM3_MUSAM
8.0
7.8
7.6
7.4
7.2
7.0
1
2
t−Test: p−value = 0.76

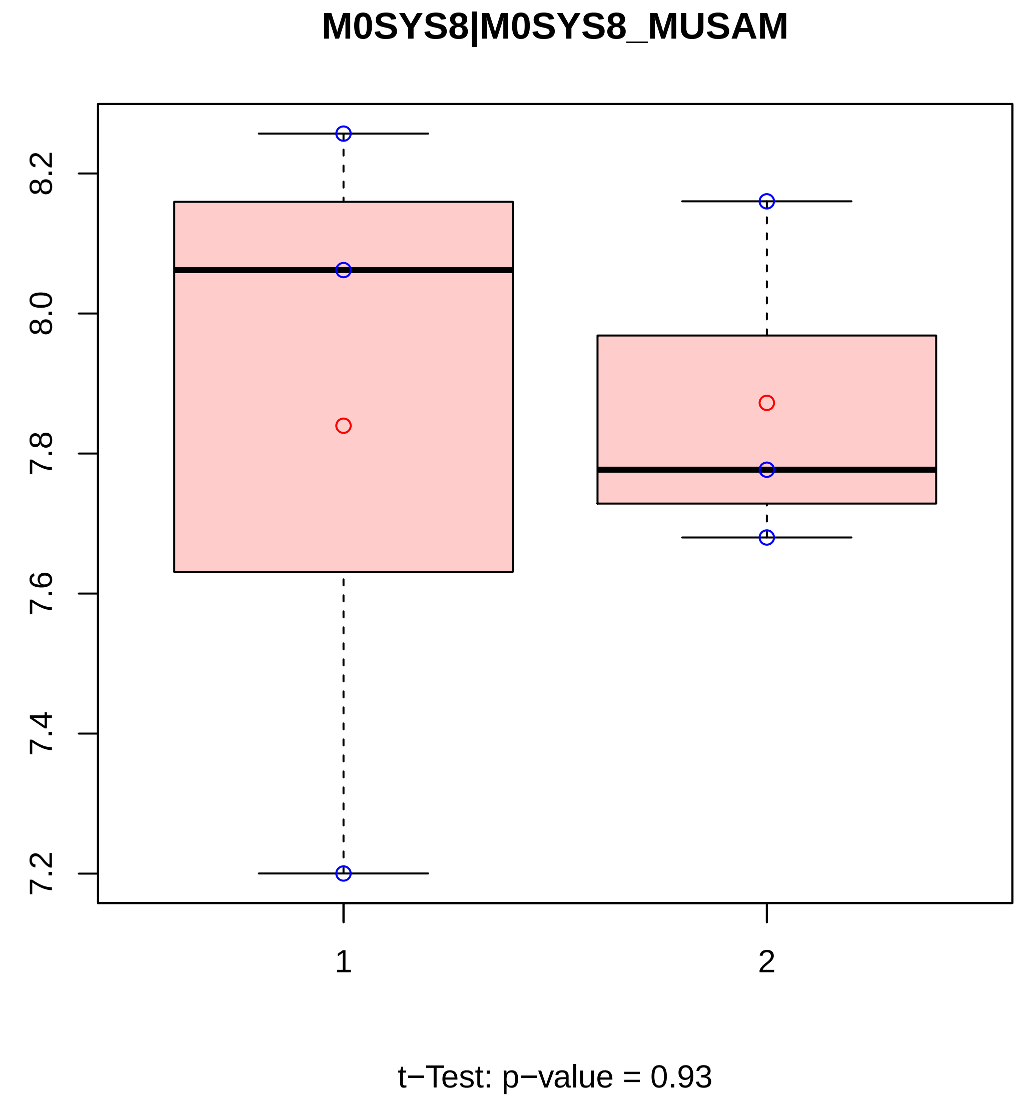
M0SYS8|M0SYS8_MUSAM
8.2
8.0
7.8
7.6
7.4
7.2
1
2
t−Test: p−value = 0.93

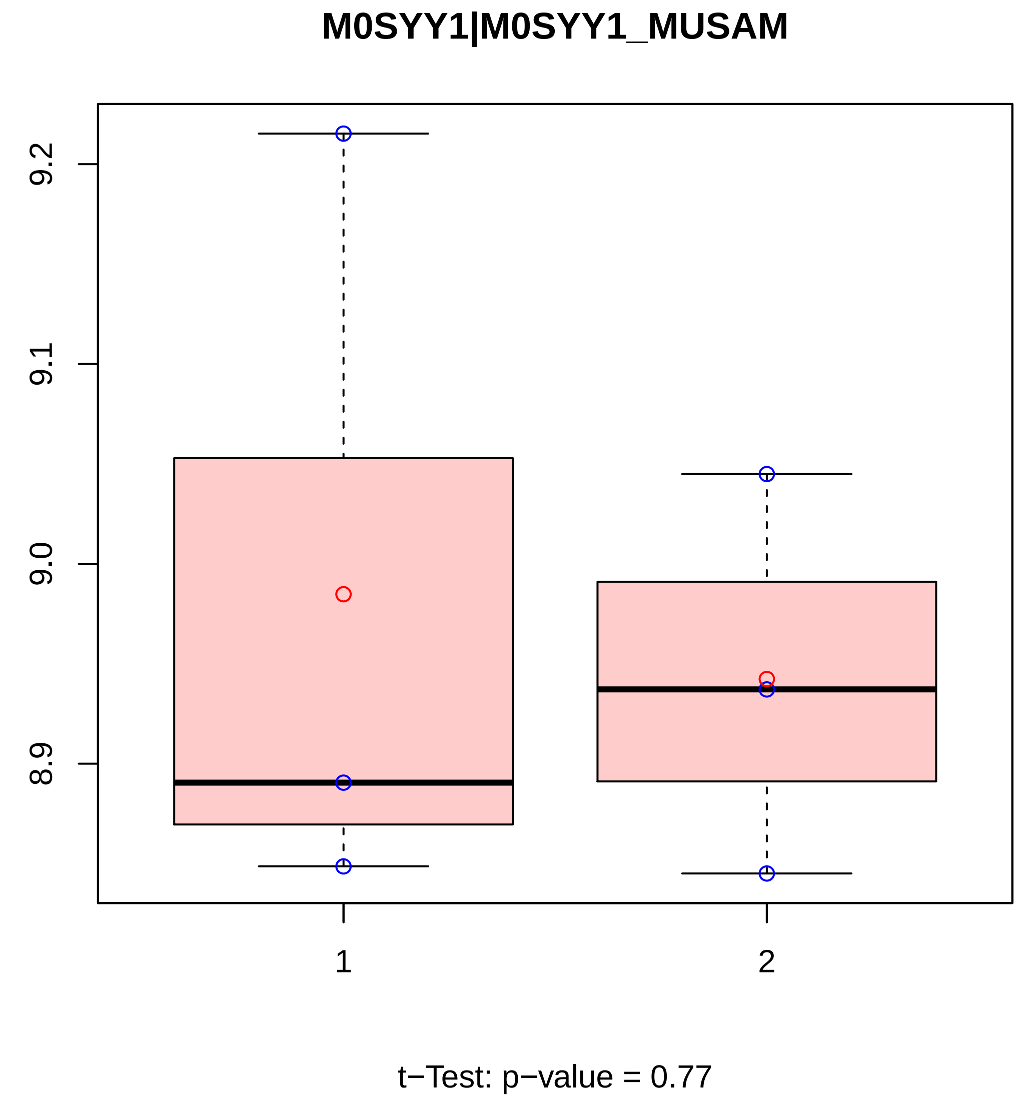
M0SYY1|M0SYY1_MUSAM
9.2
9.1
9.0
8.9
1
2
t−Test: p−value = 0.77

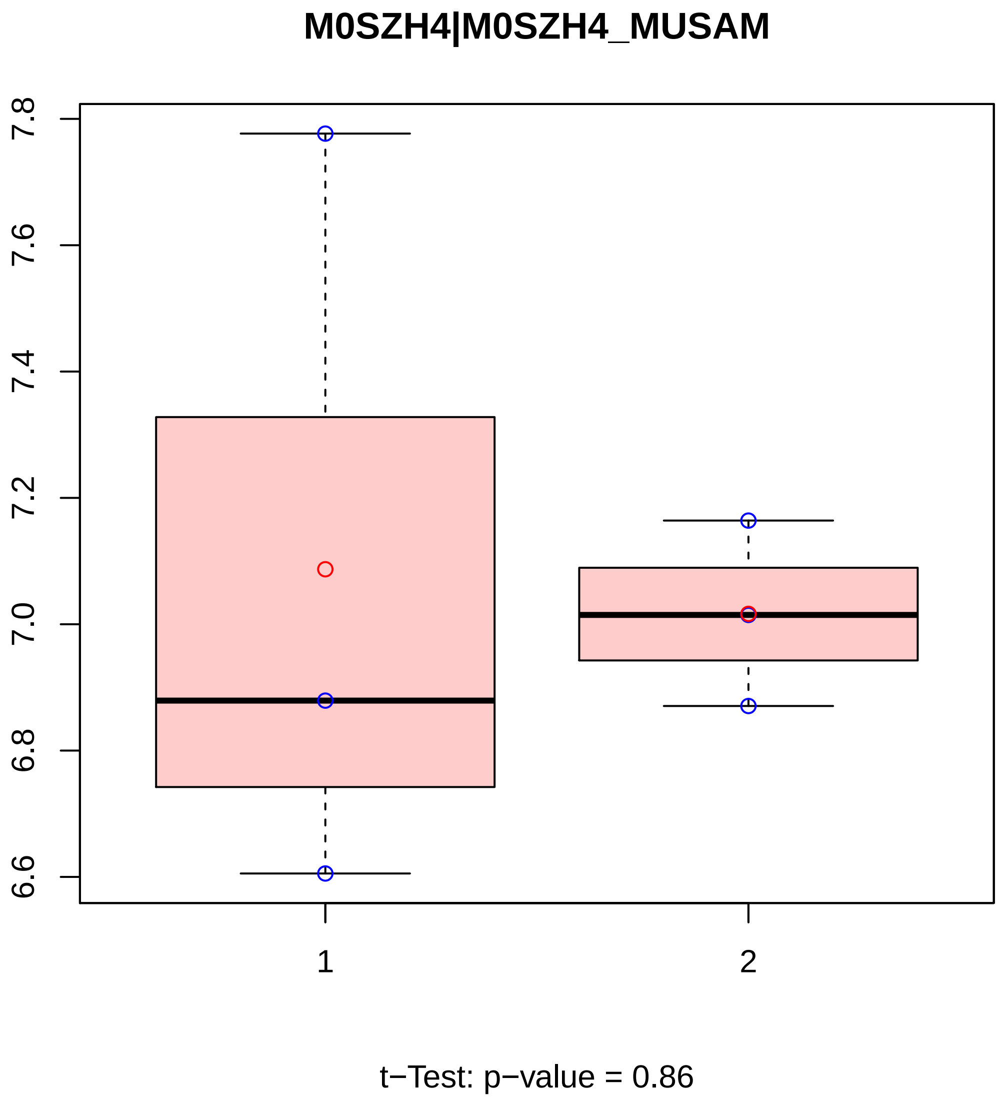
M0SZH4|M0SZH4_MUSAM
7.8
7.6
7.4
7.2
7.0
6.8
6.6
1
2
t−Test: p−value = 0.86

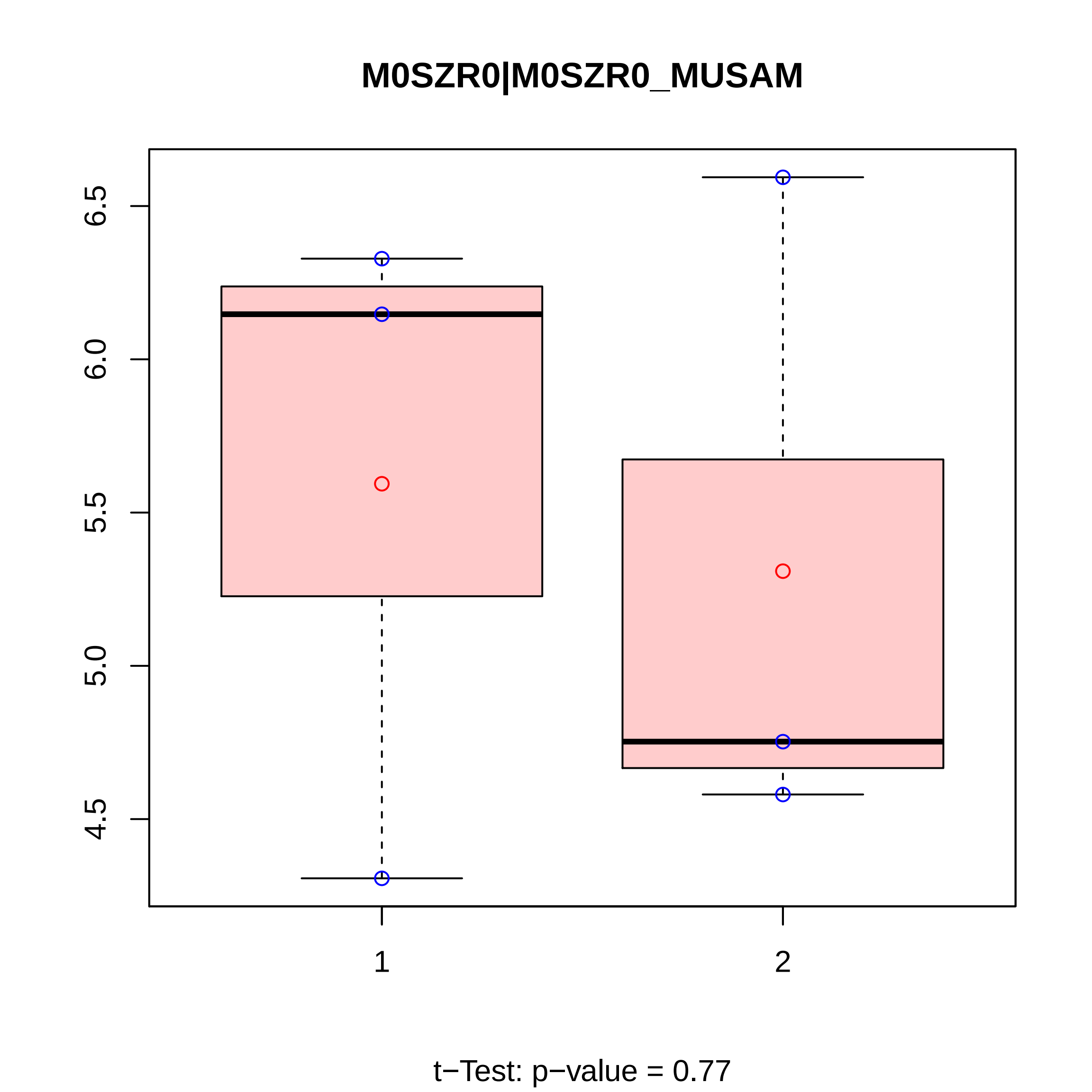

M0SZR0|M0SZR0_MUSAM
6.5
6.0
5.5
5.0
4.5
1
2
t−Test: p−value = 0.77

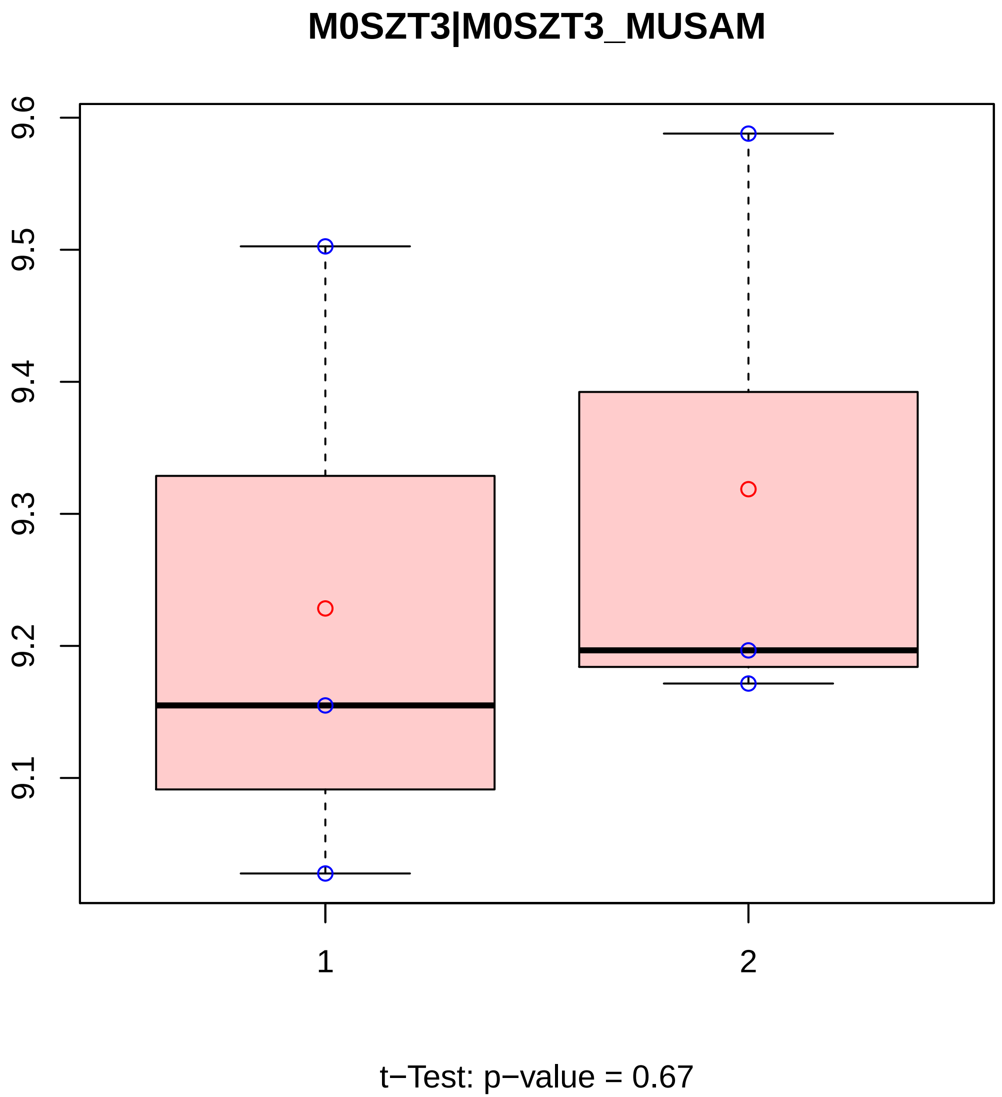
M0SZT3|M0SZT3_MUSAM
9.1
9.2
9.3
9.4
9.5
9.6
1
2
t−Test: p−value = 0.67

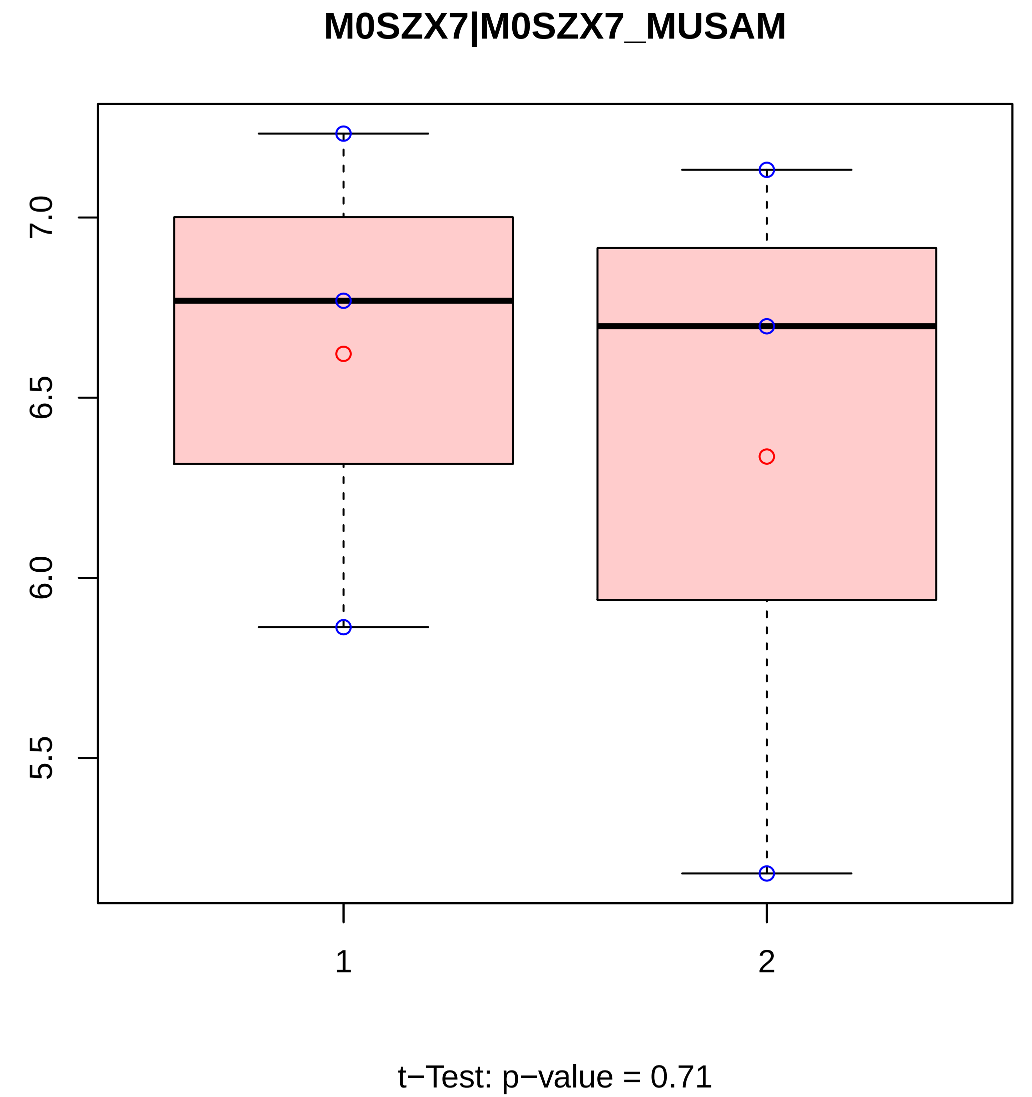
M0SZX7|M0SZX7_MUSAM
7.0
6.5
6.0
5.5
1
2
t−Test: p−value = 0.71

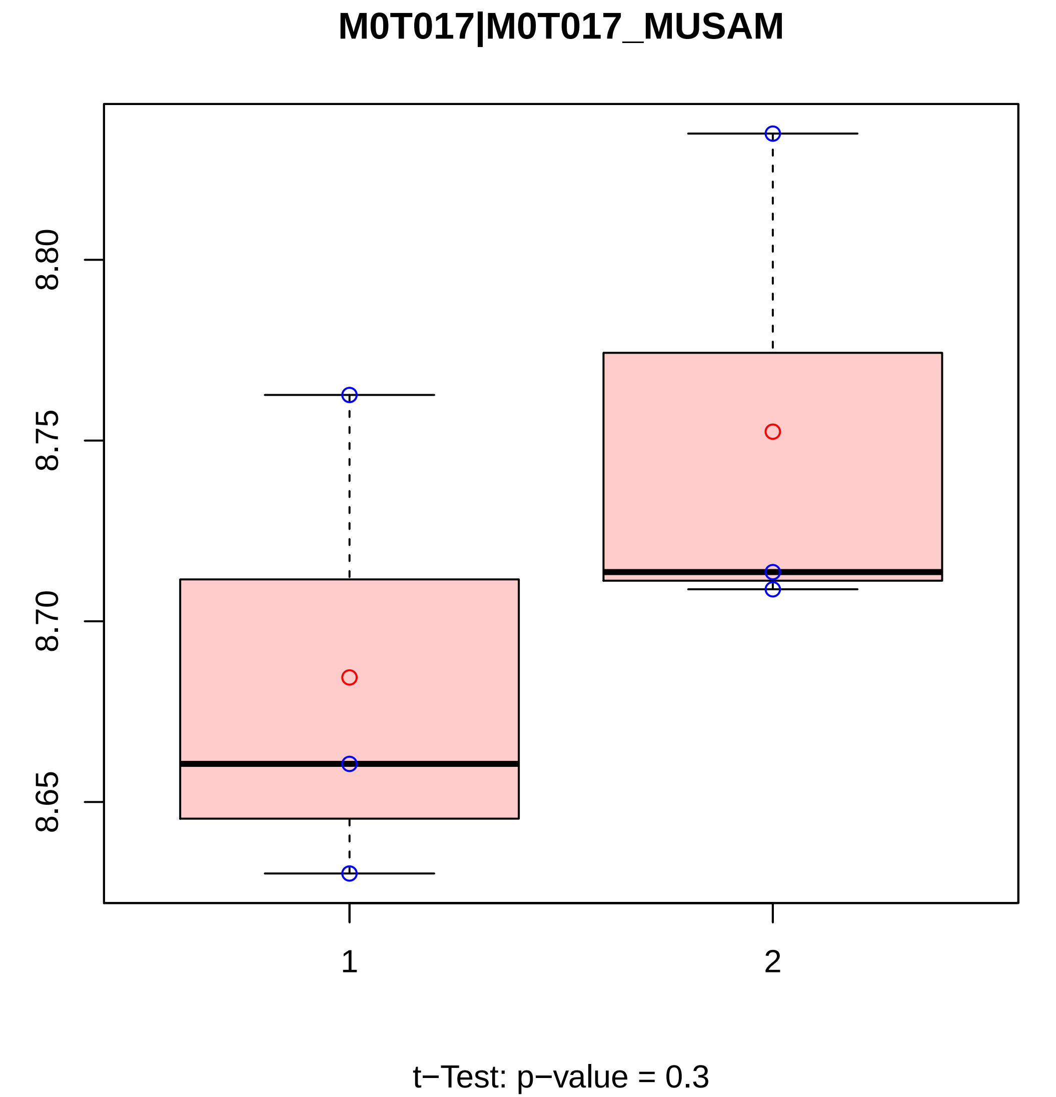
M0T017|M0T017_MUSAM
8.65
8.70
8.75
8.80
1
2
t−Test: p−value = 0.3

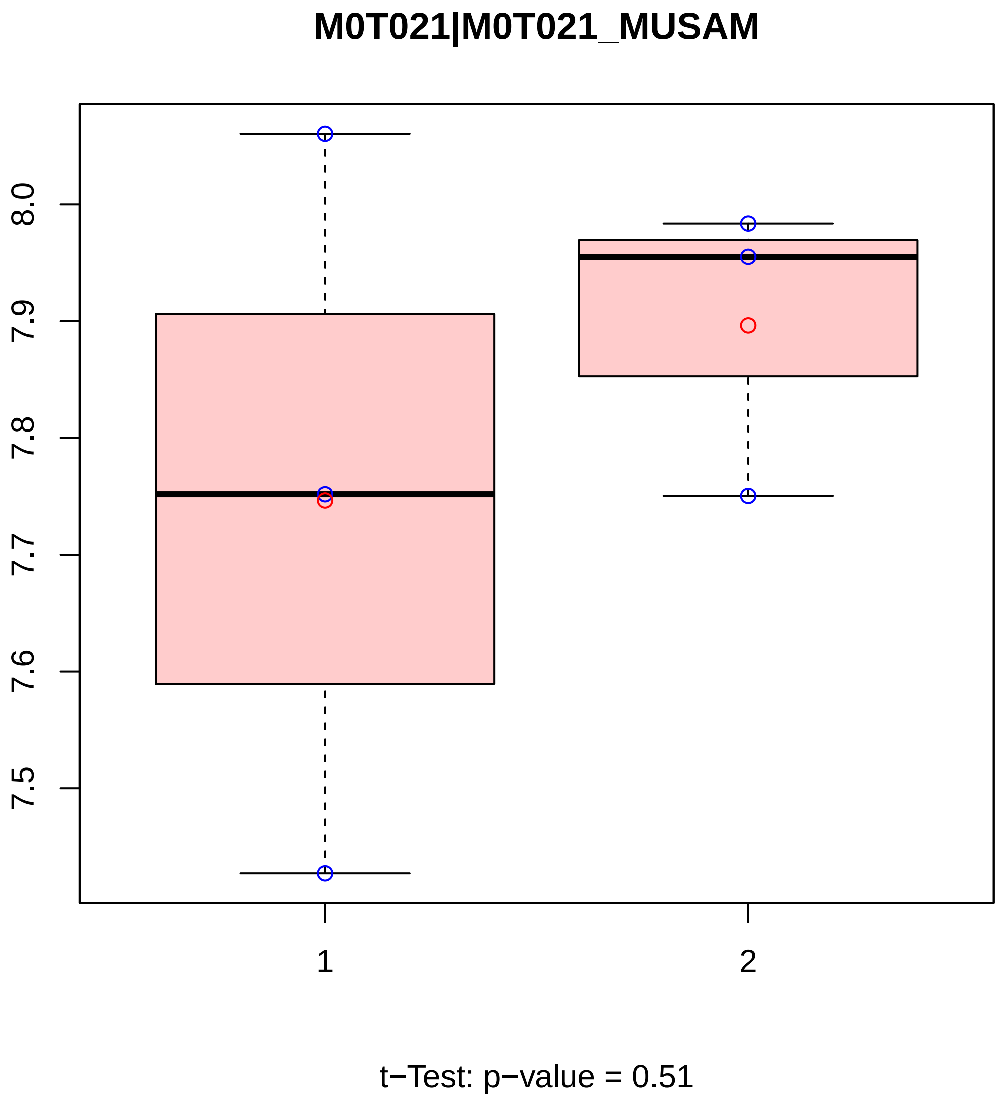

M0T021|M0T021_MUSAM
8.0
7.9
7.8
7.7
7.6
7.5
1
2
t−Test: p−value = 0.51

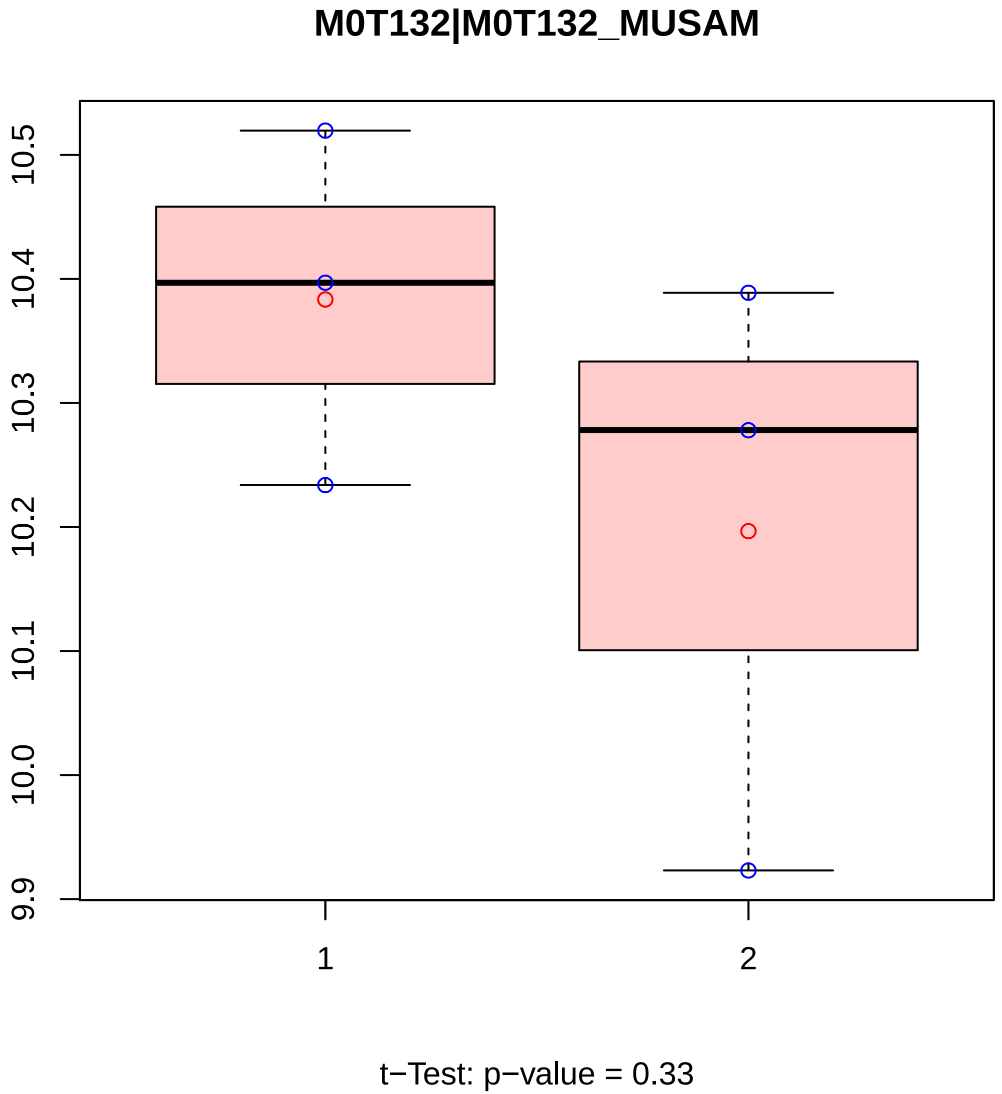
M0T132|M0T132_MUSAM
10.5
10.4
10.3
10.2
10.1
10.0
9.9
1
2
t−Test: p−value = 0.33

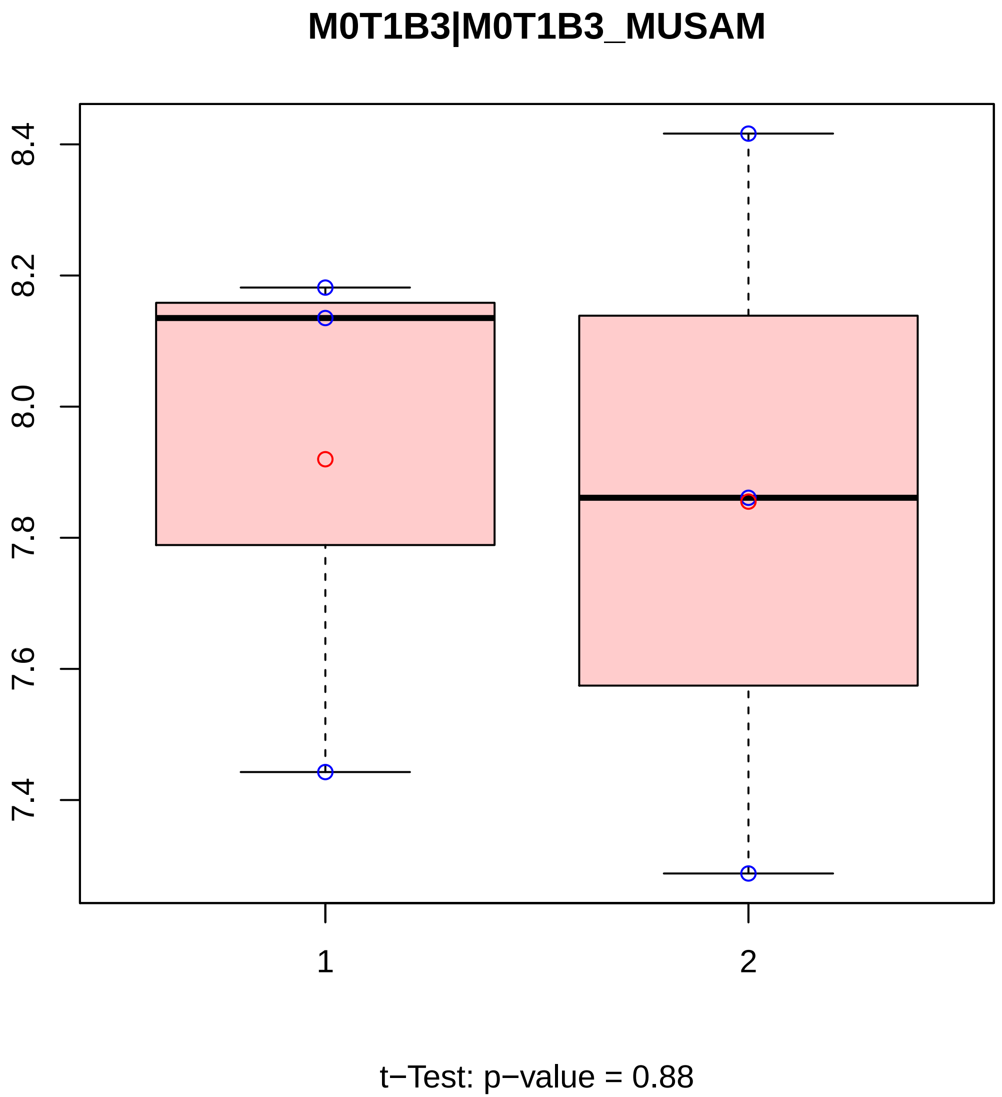
M0T1B3|M0T1B3_MUSAM
8.4
8.2
8.0
7.8
7.6
7.4
1
2
t−Test: p−value = 0.88

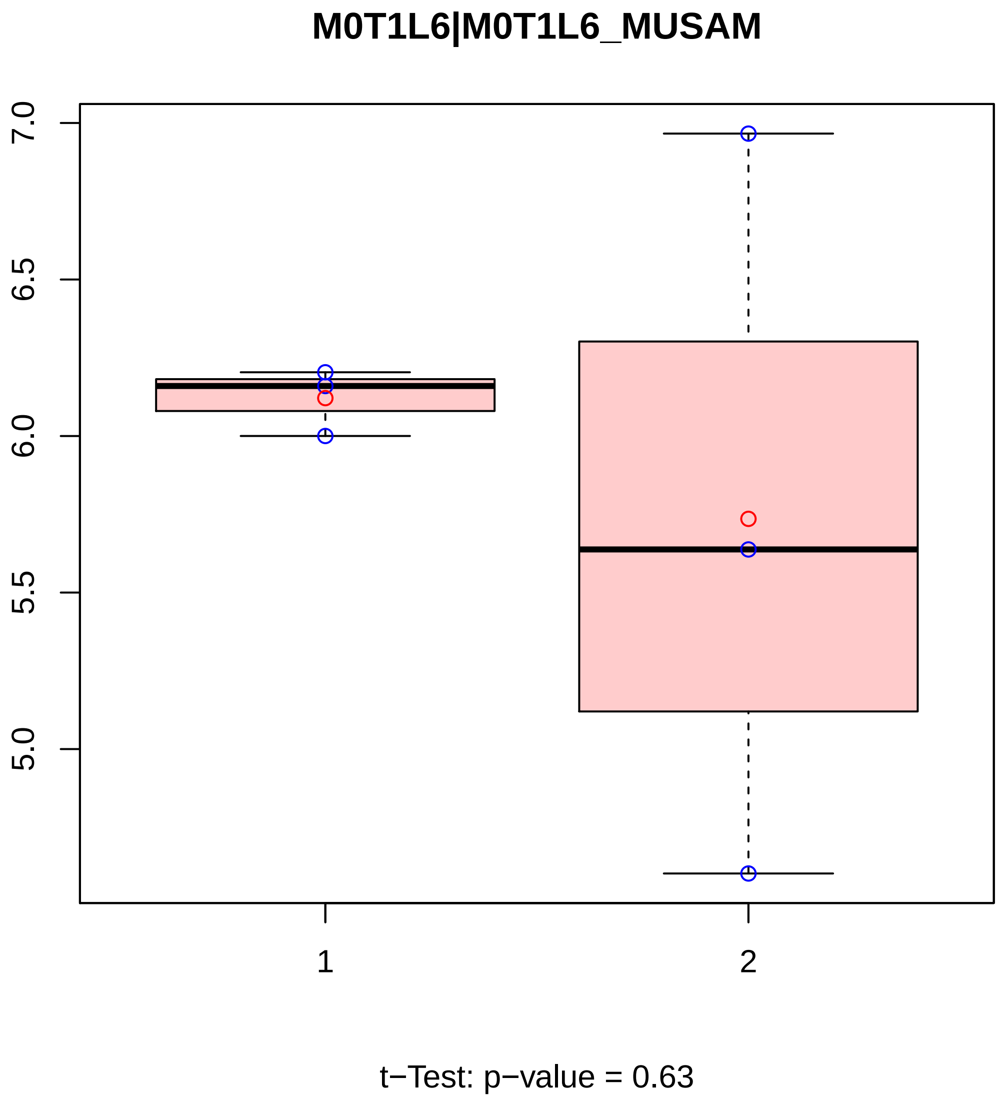
M0T1L6|M0T1L6_MUSAM
7.0
6.5
6.0
5.5
5.0
1
2
t−Test: p−value = 0.63

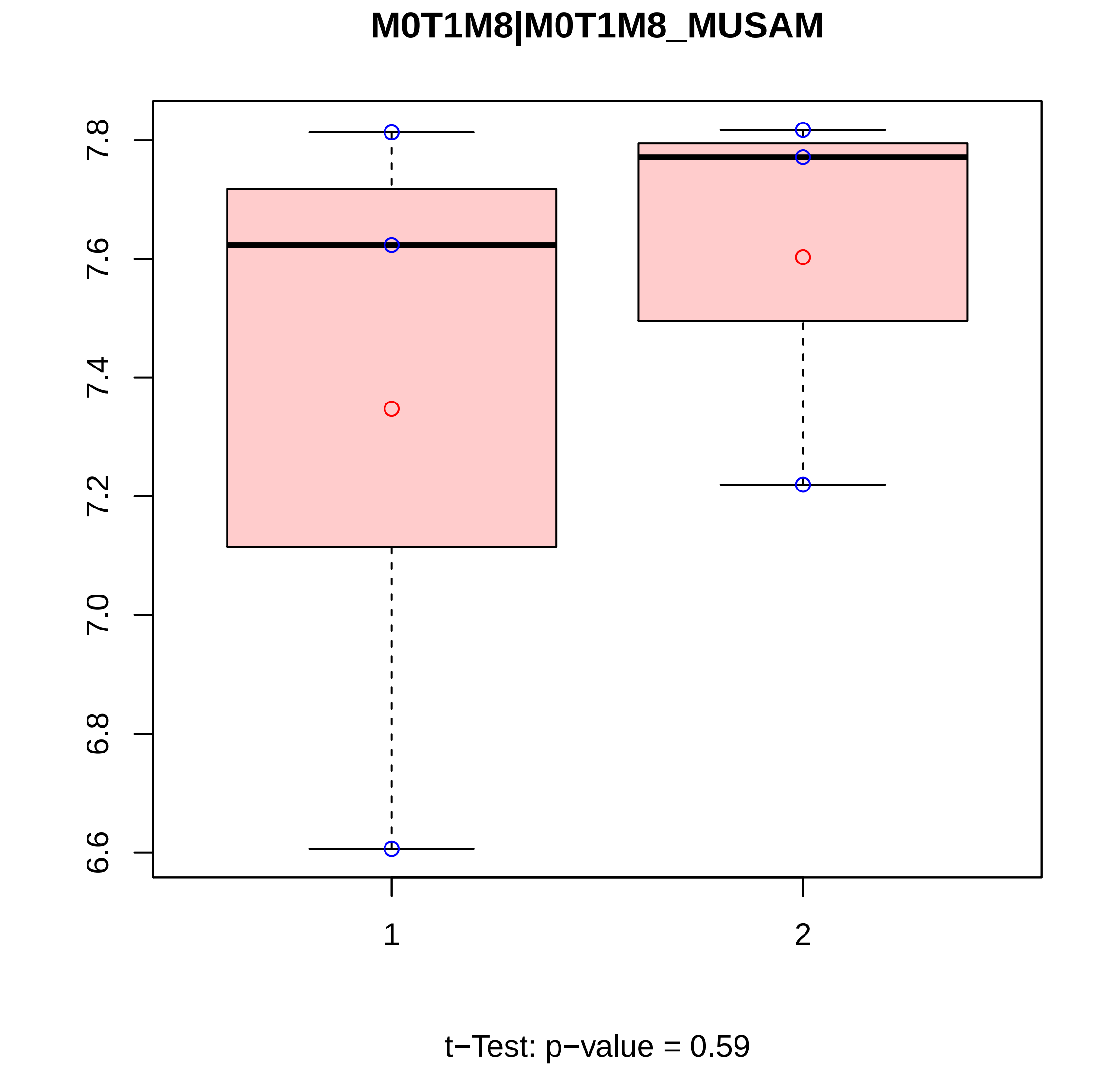

M0T1M8|M0T1M8_MUSAM
7.8
7.6
7.4
7.2
7.0
6.8
6.6
1
2
t−Test: p−value = 0.59

M0T1S1|M0T1S1_MUSAM

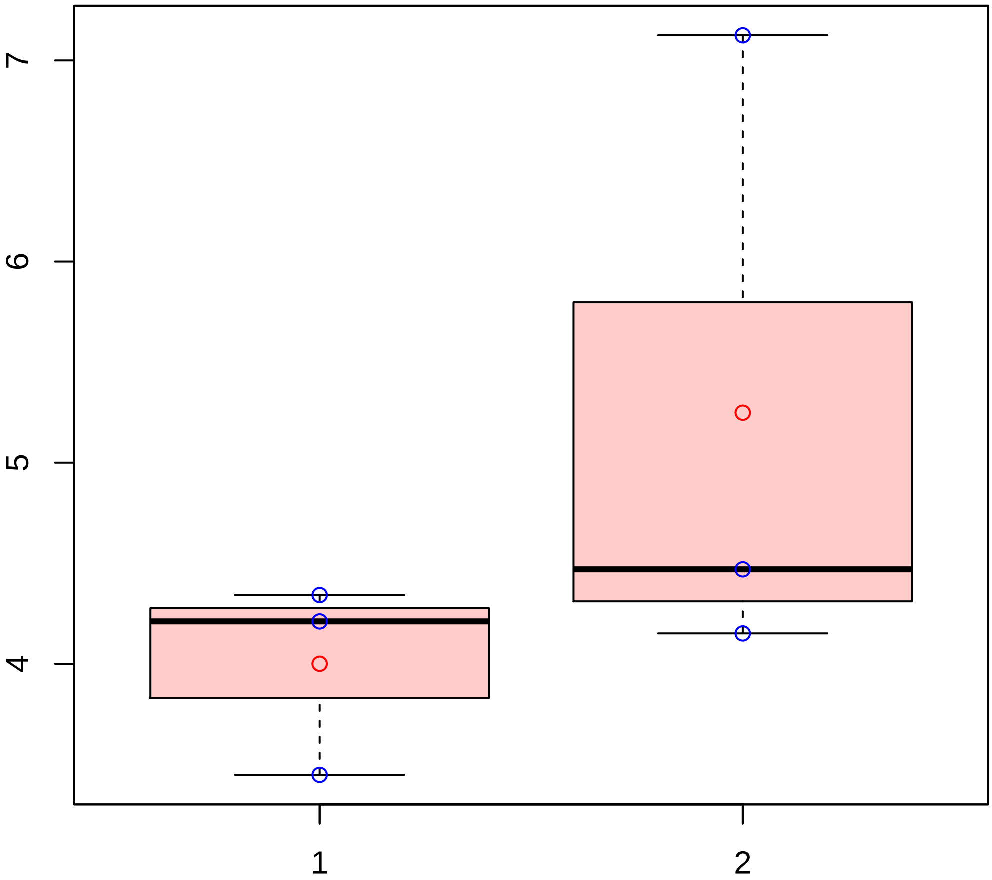

t−Test: p−value = 0.32

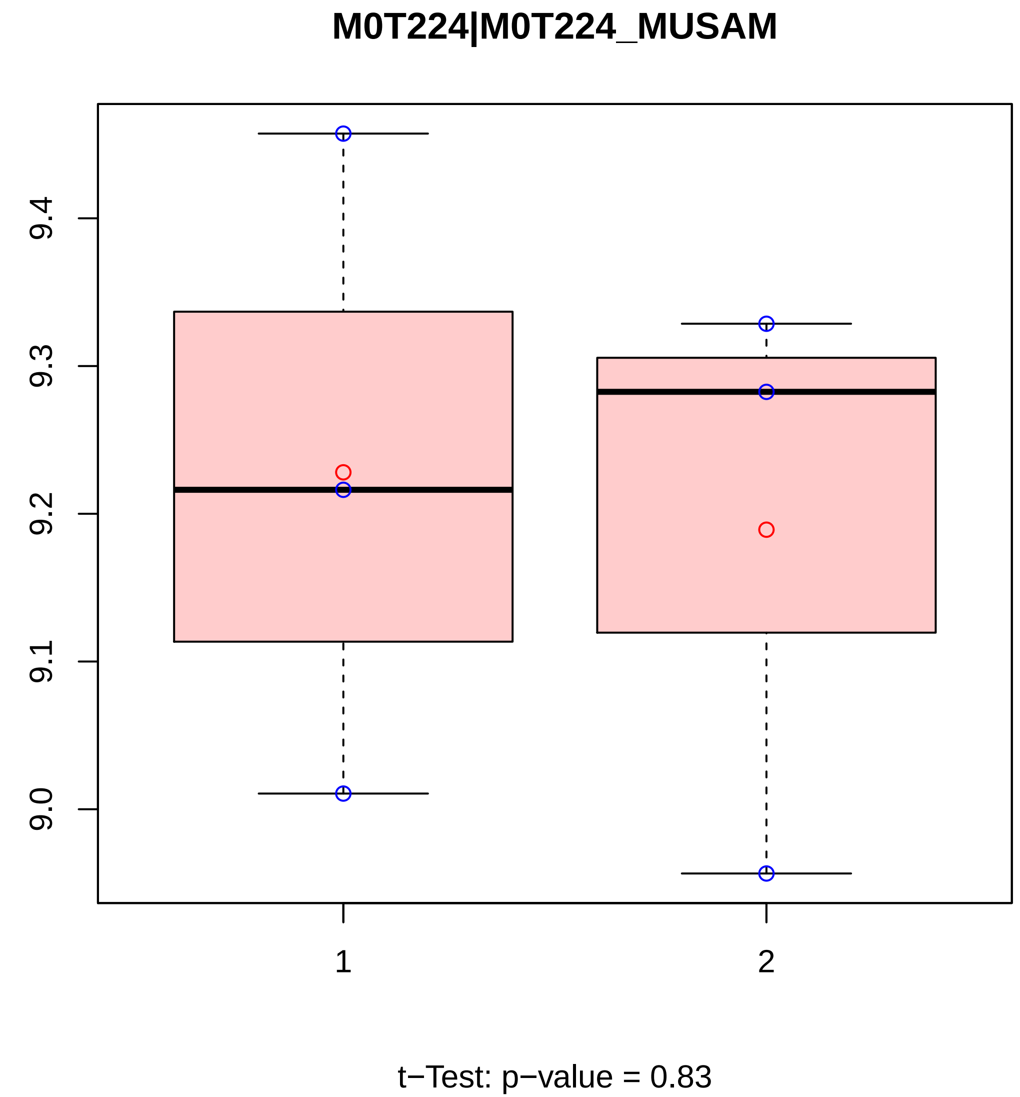
M0T224|M0T224_MUSAM
9.0
9.1
9.2
9.3
9.4
1
2
t−Test: p−value = 0.83

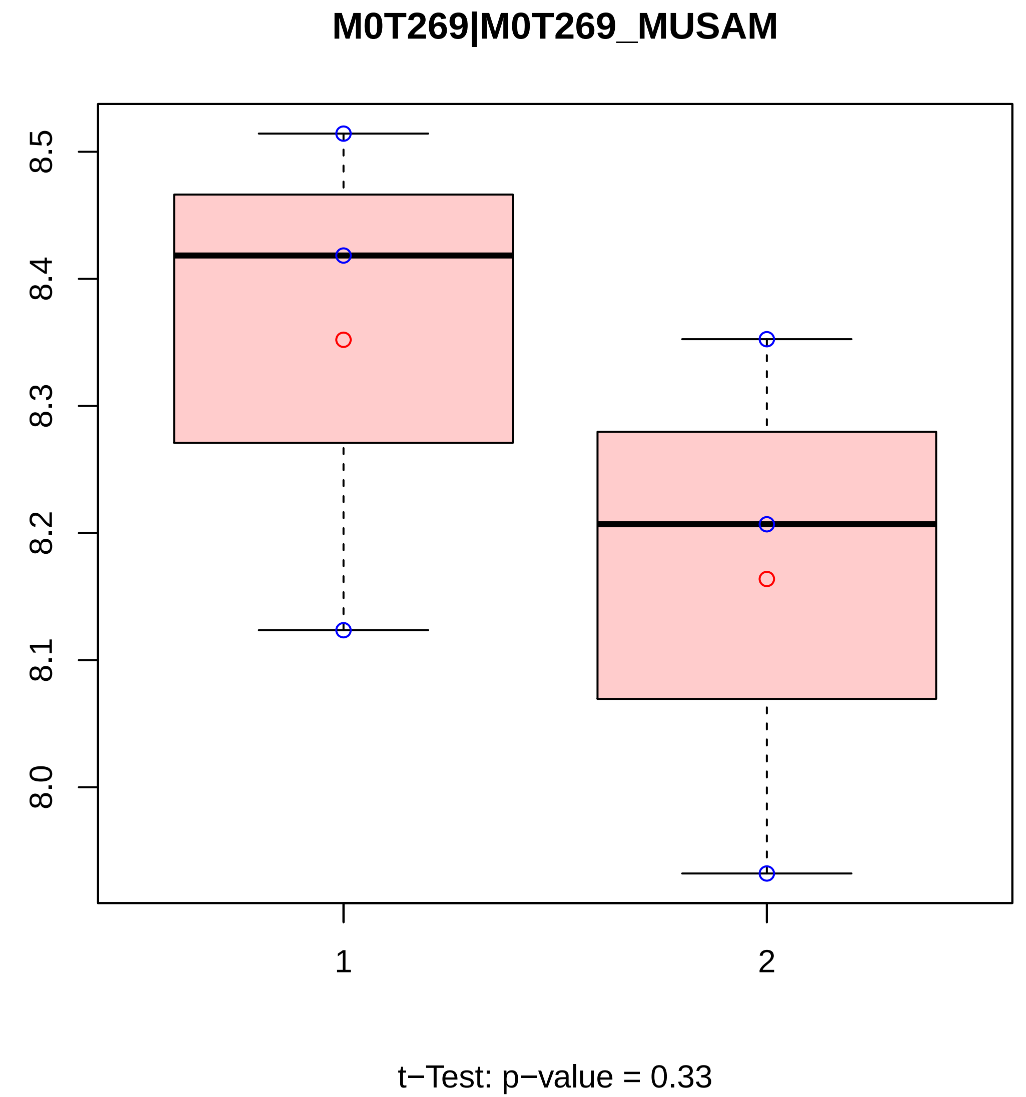
M0T269|M0T269_MUSAM
8.5
8.4
8.3
8.2
8.1
8.0
1
2
t−Test: p−value = 0.33

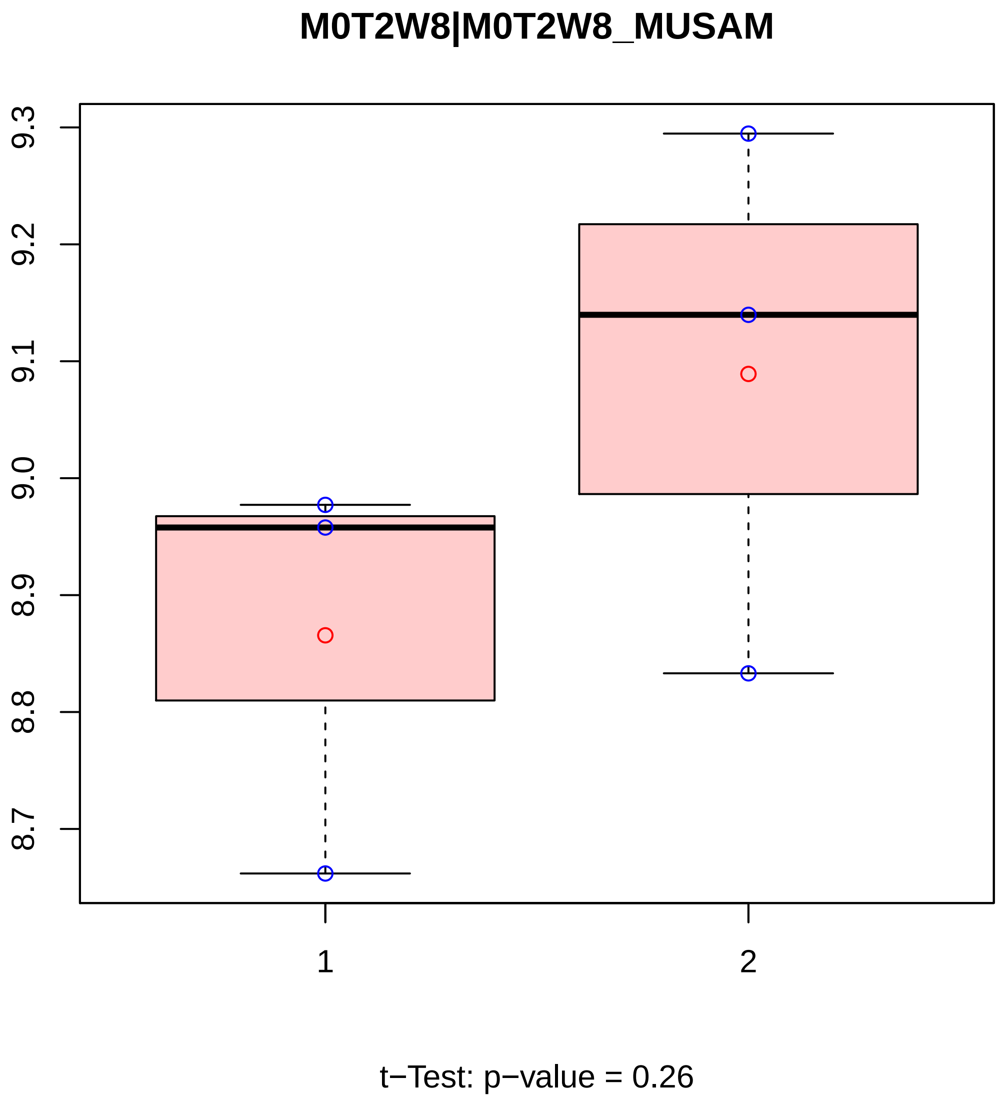
M0T2W8|M0T2W8_MUSAM
9.3
9.2
9.1
9.0
8.9
8.8
8.7
1
2
t−Test: p−value = 0.26

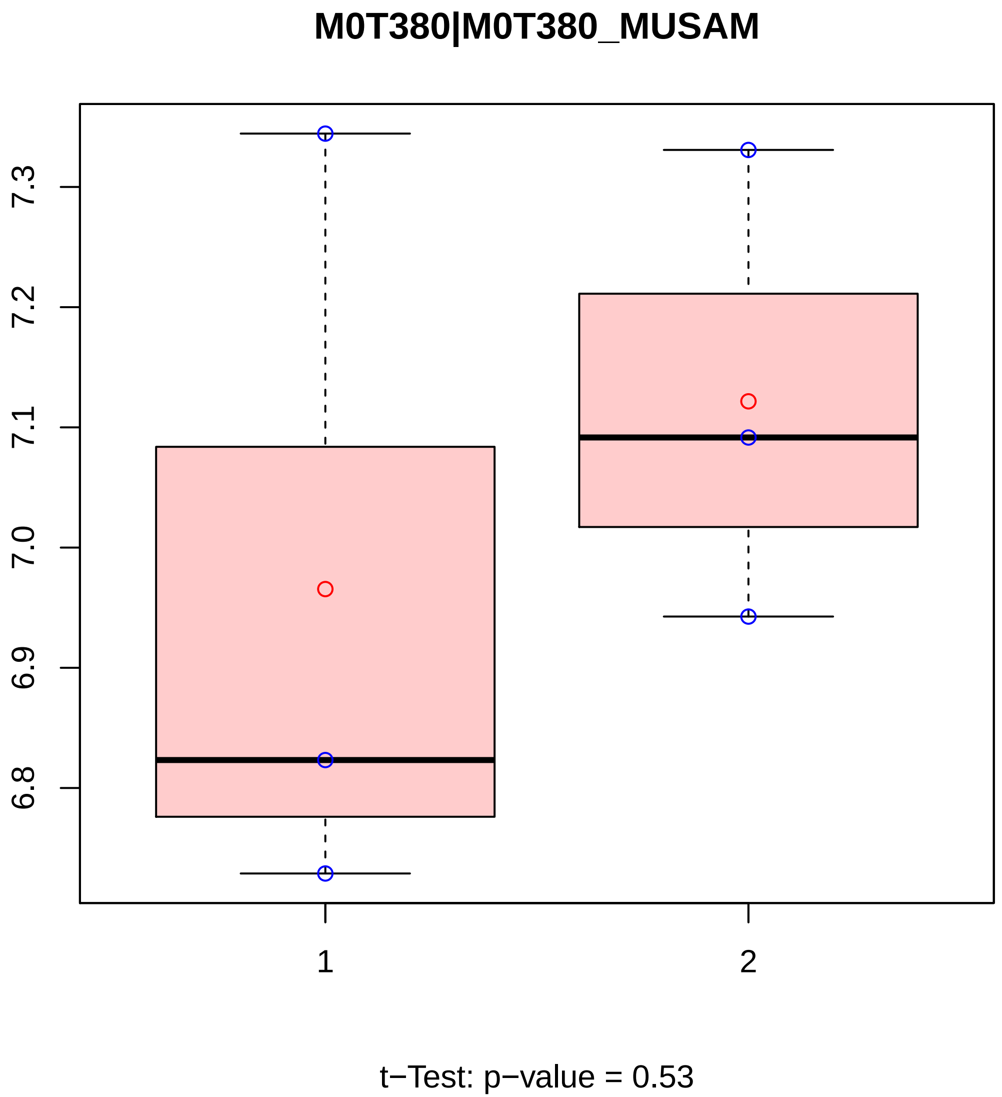
M0T380|M0T380_MUSAM
7.3
7.2
7.1
7.0
6.9
6.8
1
2
t−Test: p−value = 0.53

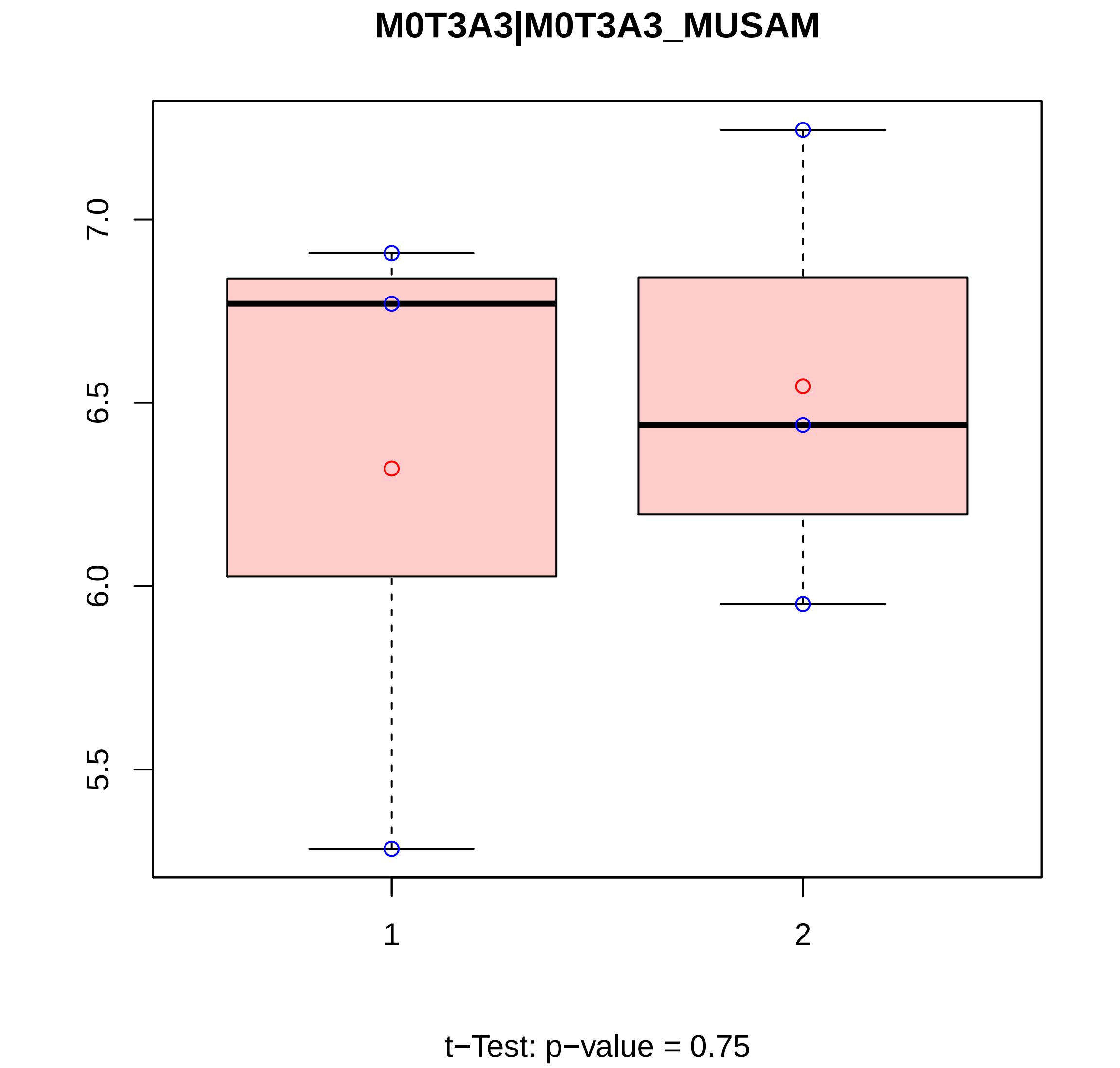
M0T3A3|M0T3A3_MUSAM
7.0
6.5
6.0
5.5
1
2
t−Test: p−value = 0.75

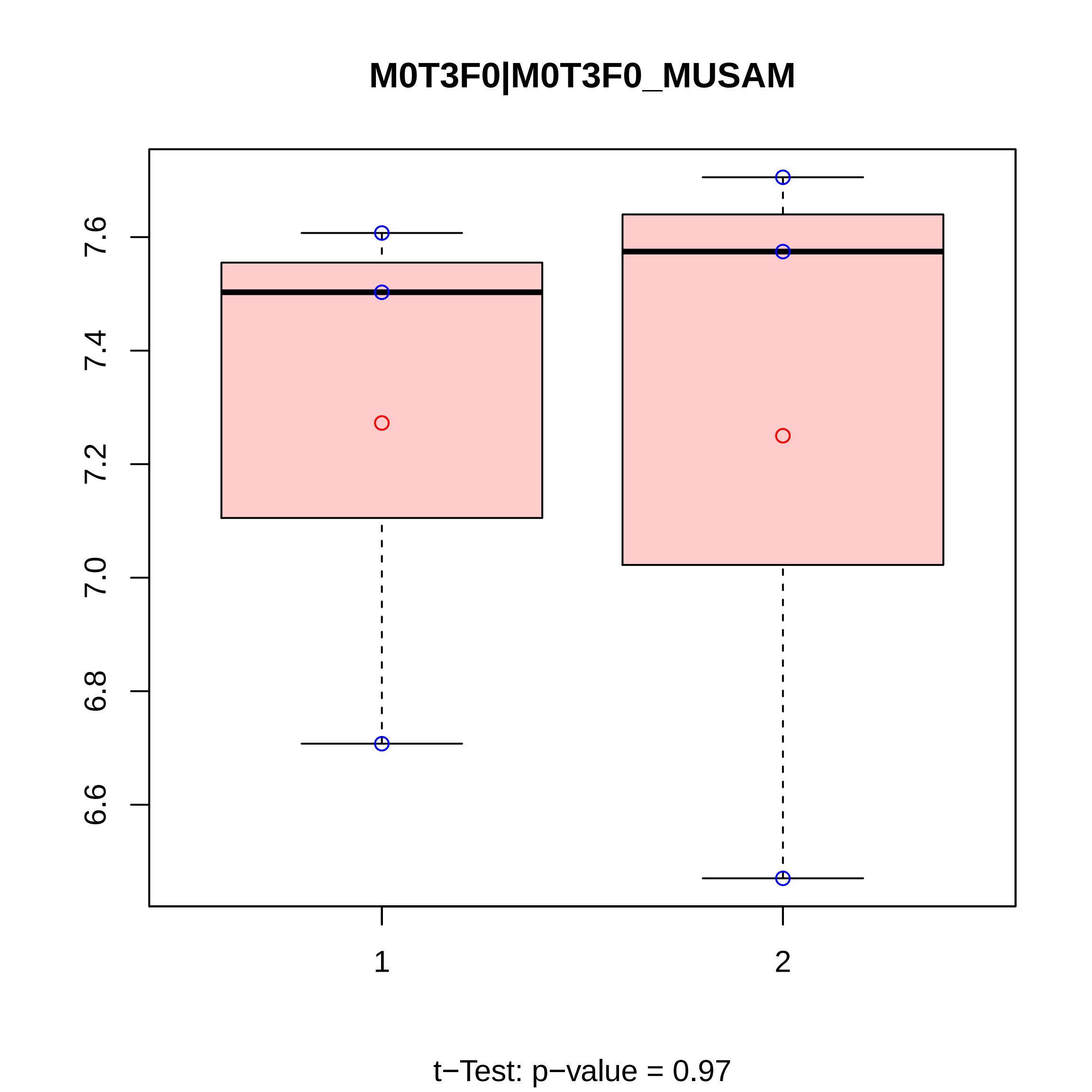
M0T3F0|M0T3F0_MUSAM
7.6
7.4
7.2
7.0
6.8
6.6
1
2
t−Test: p−value = 0.97

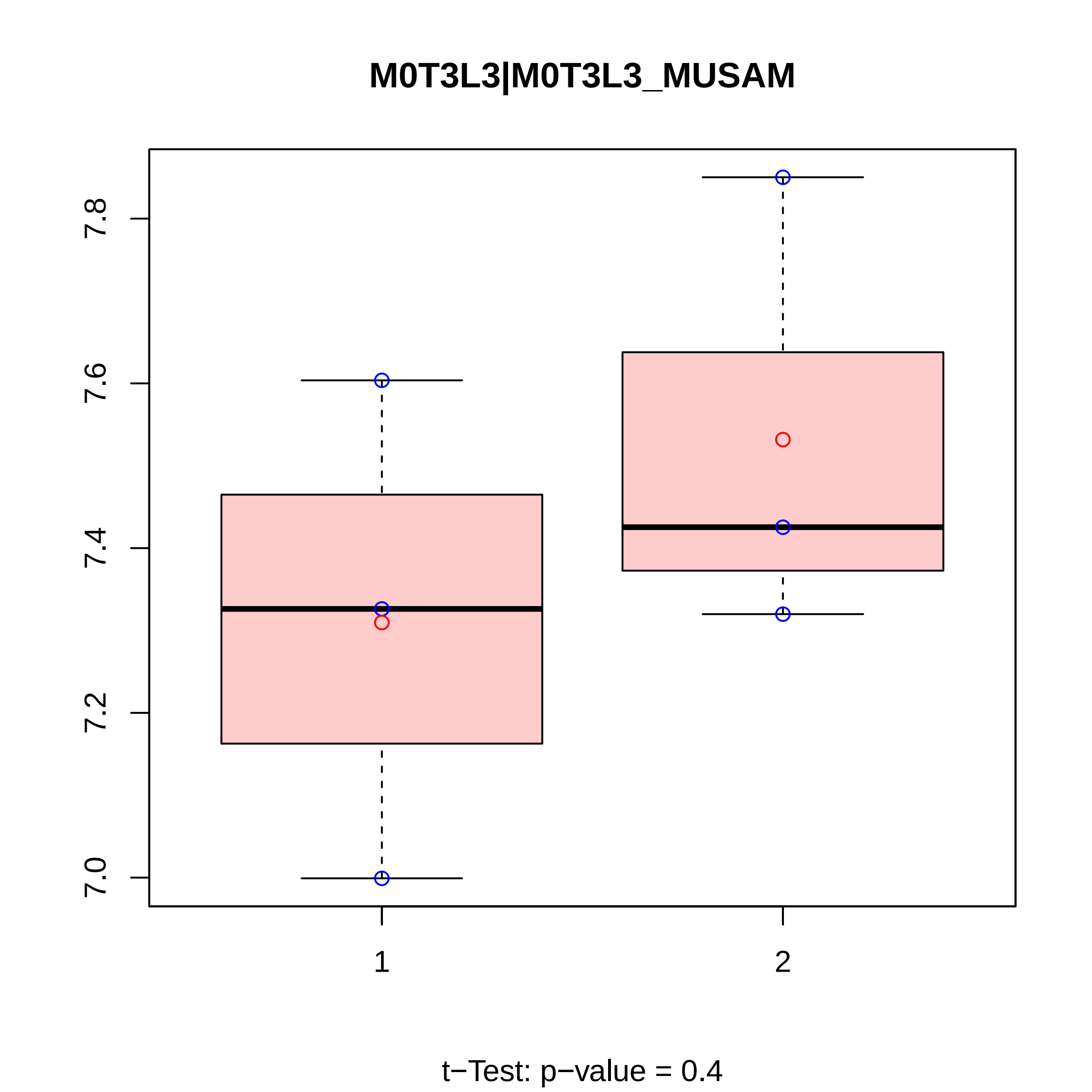
M0T3L3|M0T3L3_MUSAM
7.0
7.2
7.4
7.6
7.8
1
2
t−Test: p−value = 0.4

M0T3U9|M0T3U9_MUSAM

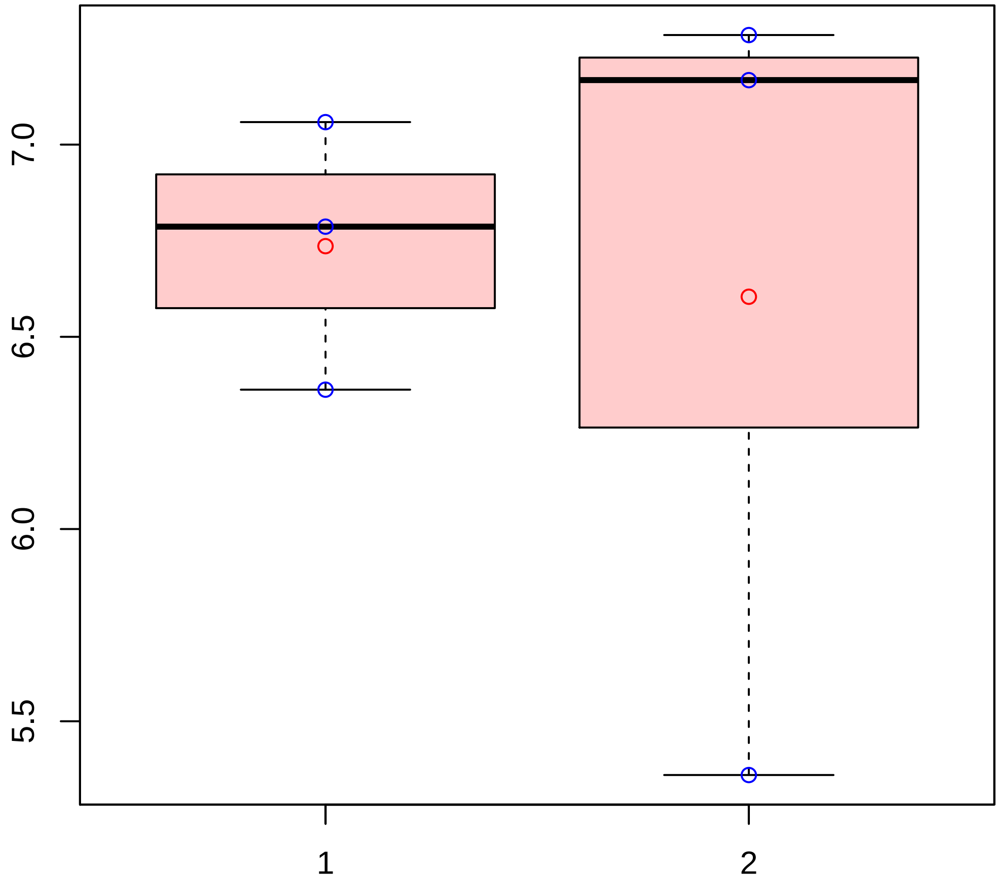

t−Test: p−value = 0.86

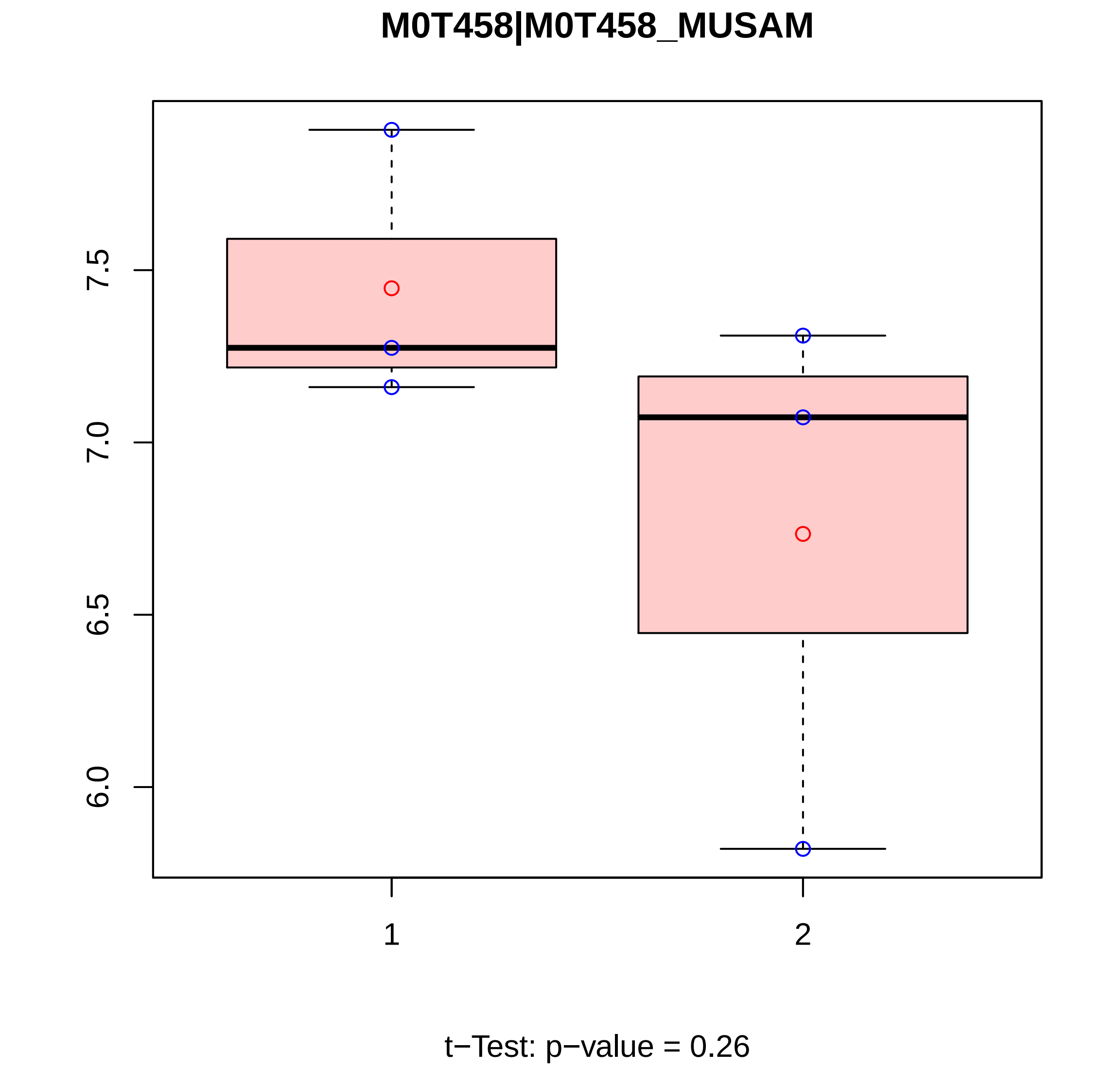
M0T458|M0T458_MUSAM
7.5
7.0
6.5
6.0
1
2
t−Test: p−value = 0.26

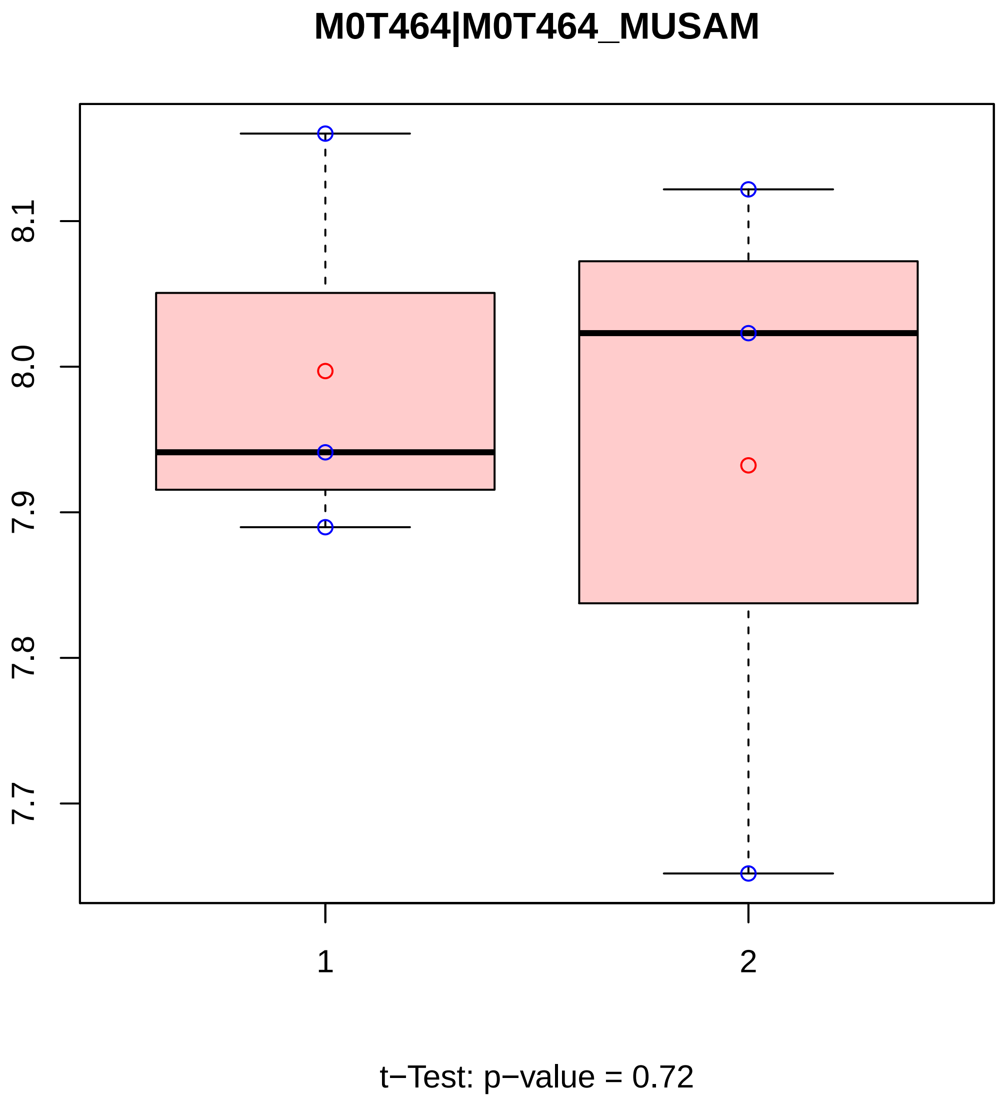
M0T464|M0T464_MUSAM
8.1
8.0
7.9
7.8
7.7
1
2
t−Test: p−value = 0.72

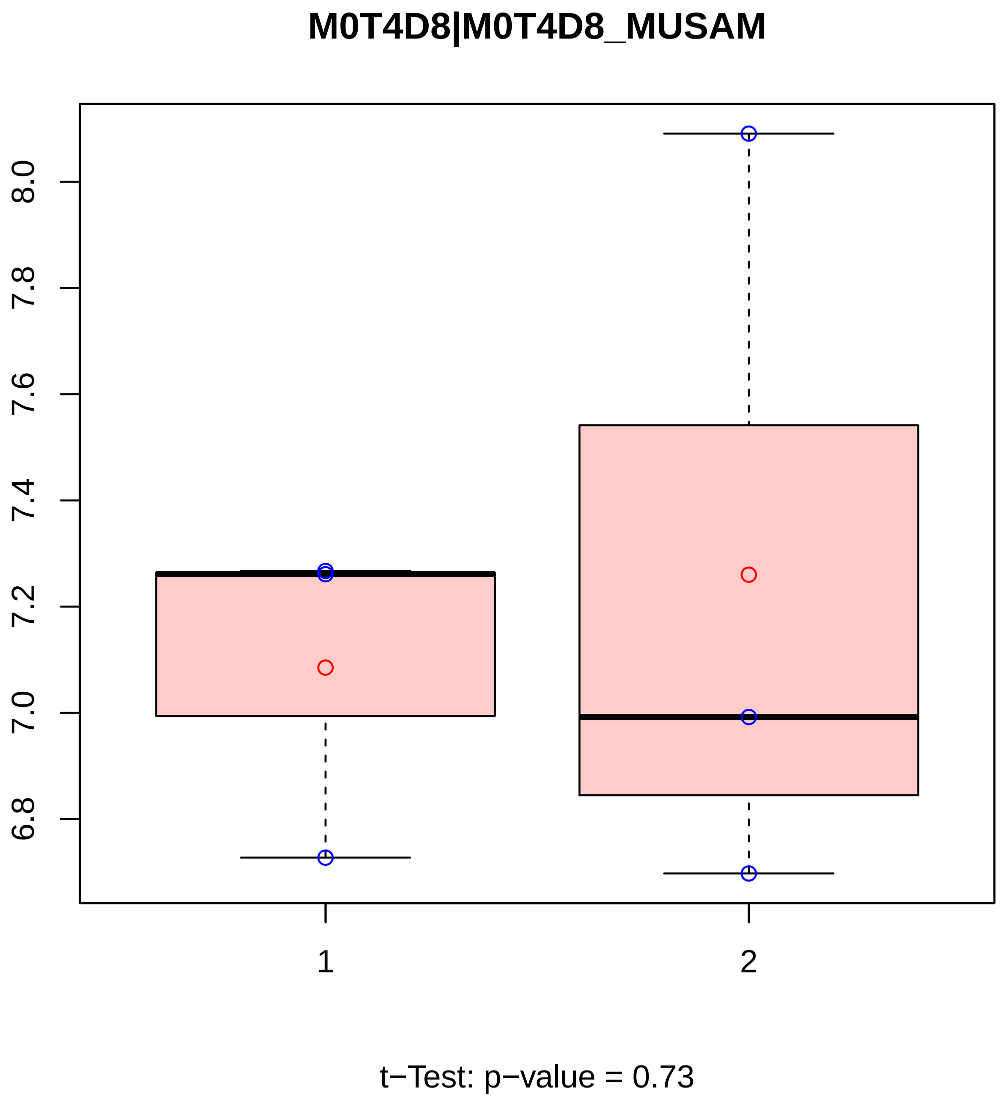
M0T4D8|M0T4D8_MUSAM
8.0
7.8
7.6
7.4
7.2
7.0
6.8
1
2
t−Test: p−value = 0.73

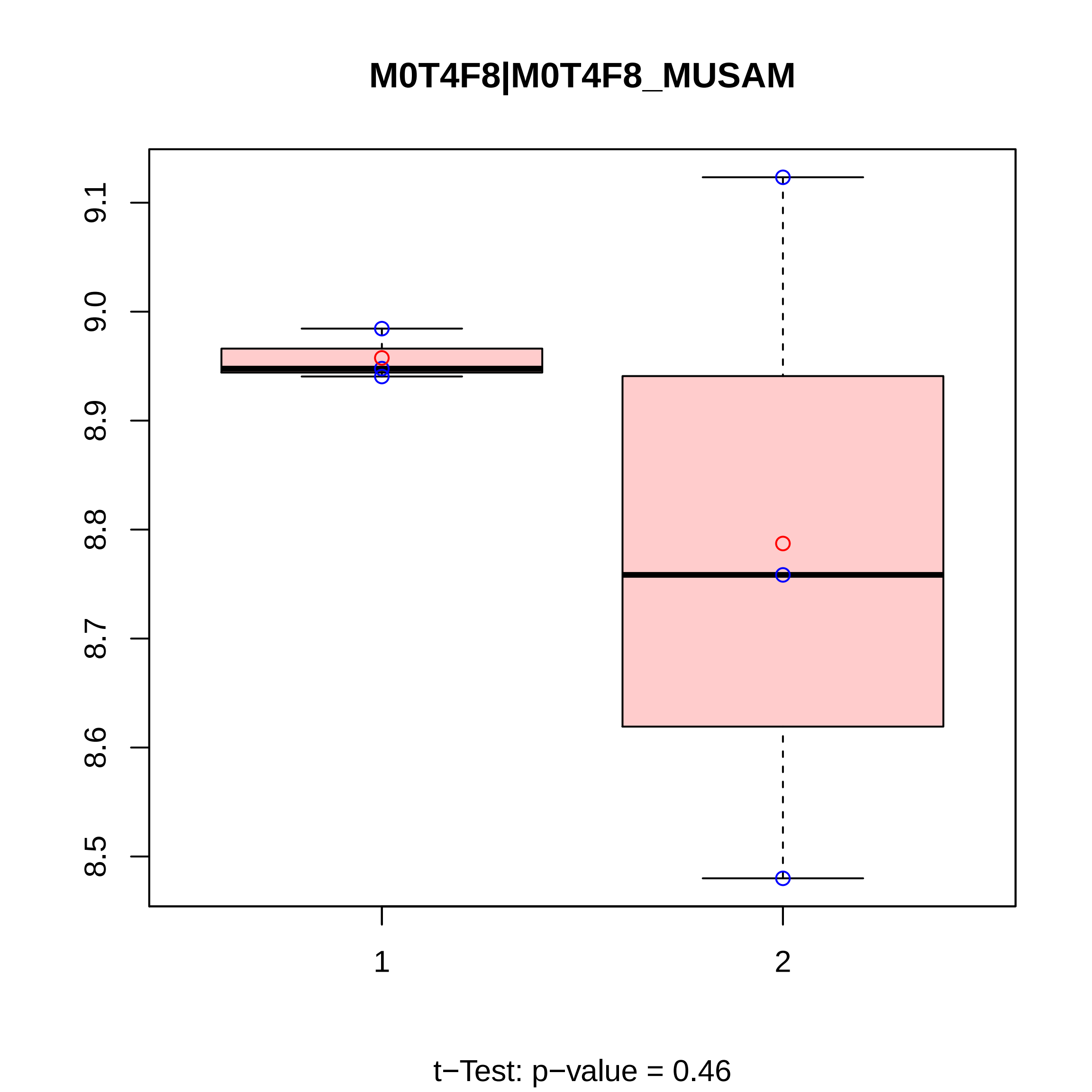

M0T4F8|M0T4F8_MUSAM
9.1
9.0
8.9
8.8
8.7
8.6
8.5
1
2
t−Test: p−value = 0.46

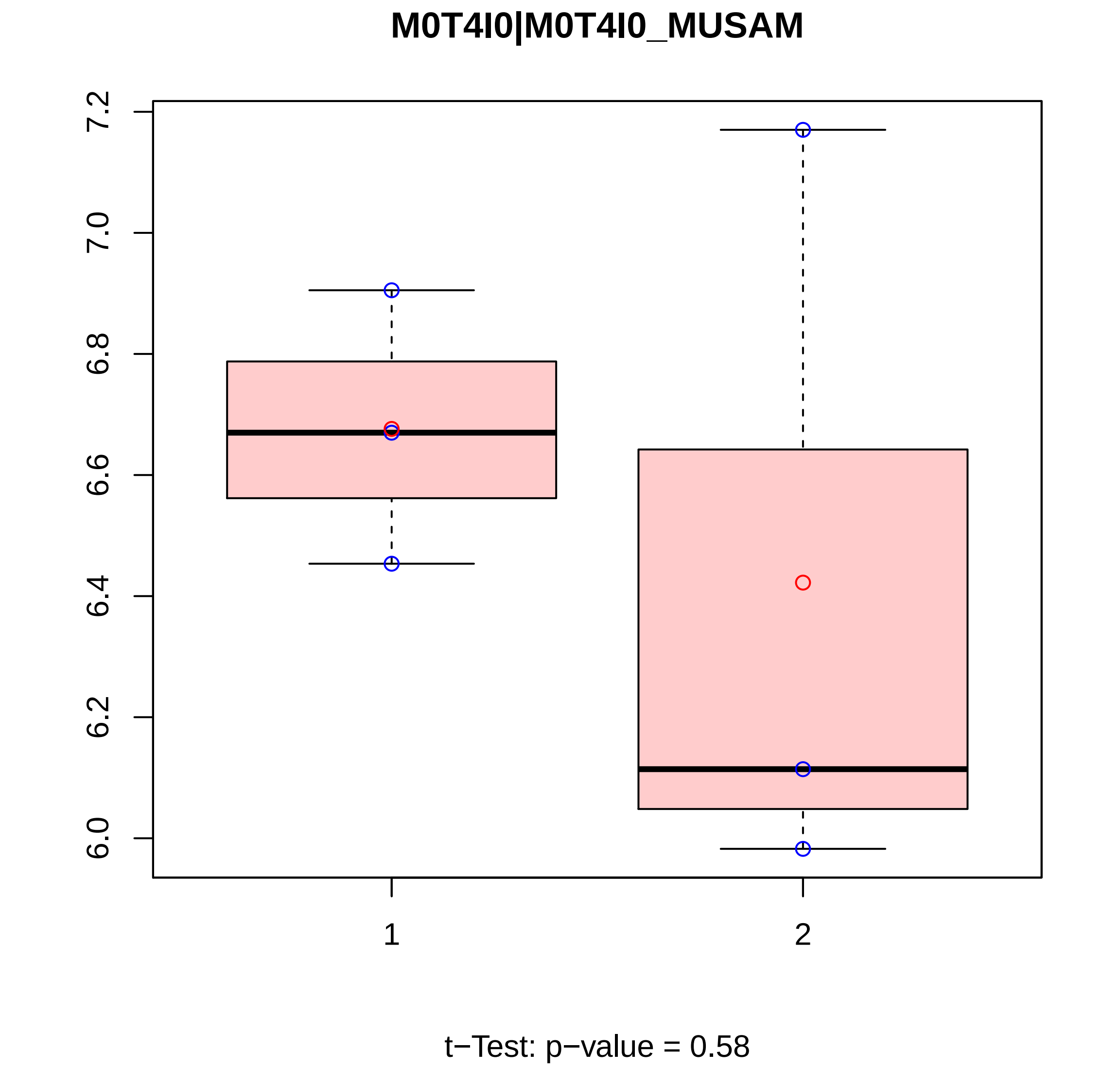

M0T4I0|M0T4I0_MUSAM
7.2
7.0
6.8
6.6
6.4
6.2
6.0
1
2
t−Test: p−value = 0.58

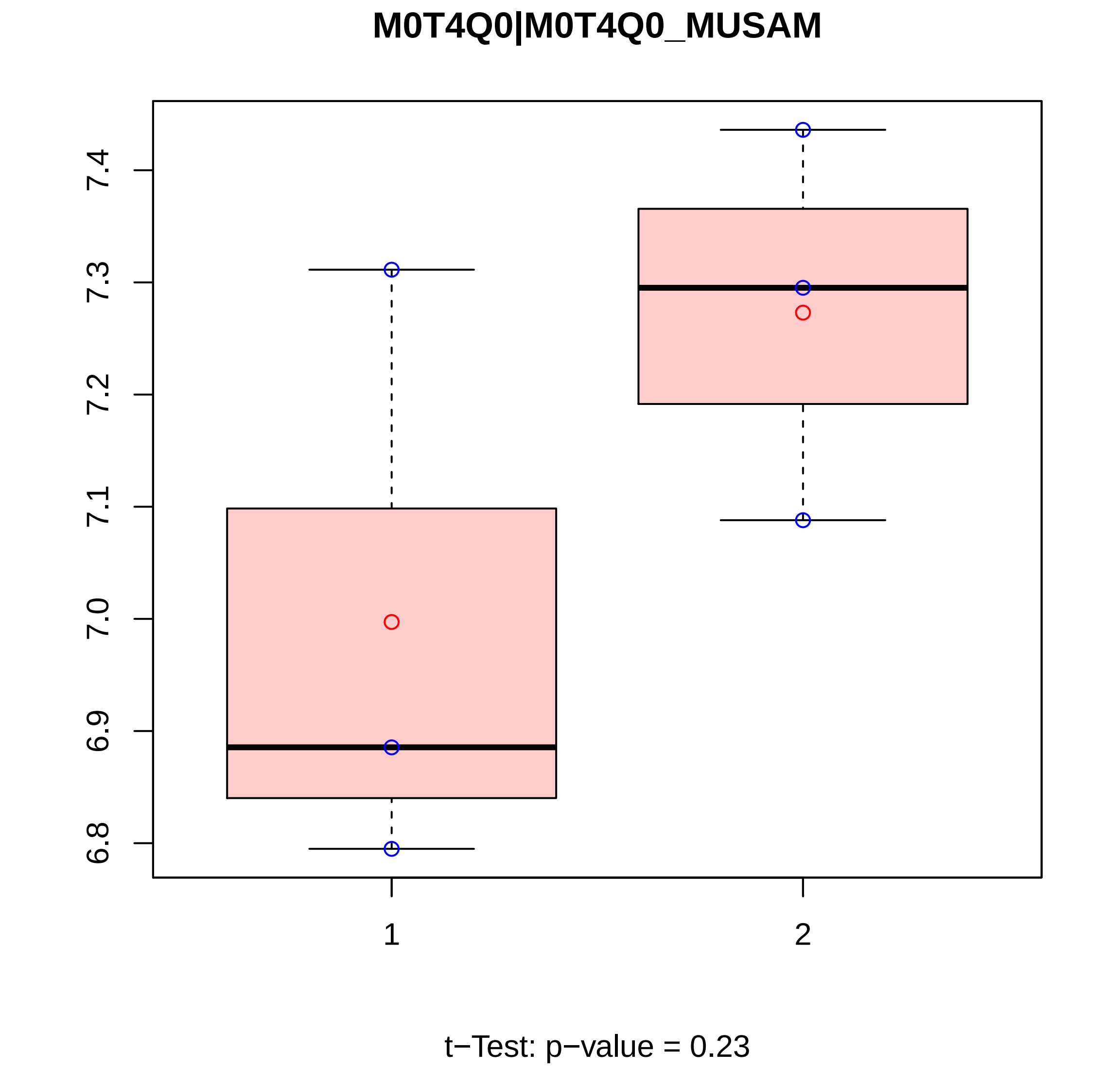

M0T4Q0|M0T4Q0_MUSAM
7.4
7.3
7.2
7.1
7.0
6.9
6.8
1
2
t−Test: p−value = 0.23

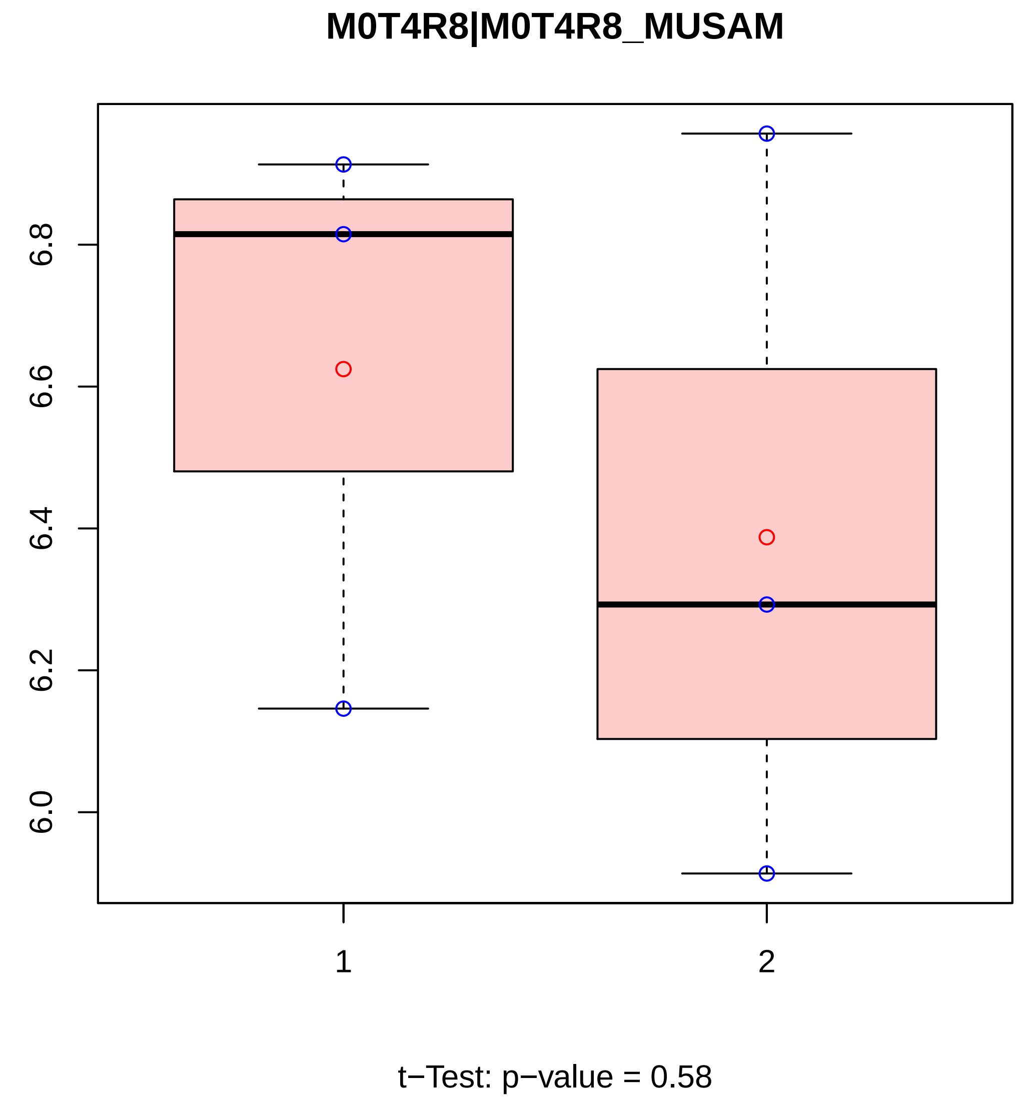
M0T4R8|M0T4R8_MUSAM
6.0
6.2
6.4
6.6
6.8
1
2
t−Test: p−value = 0.58

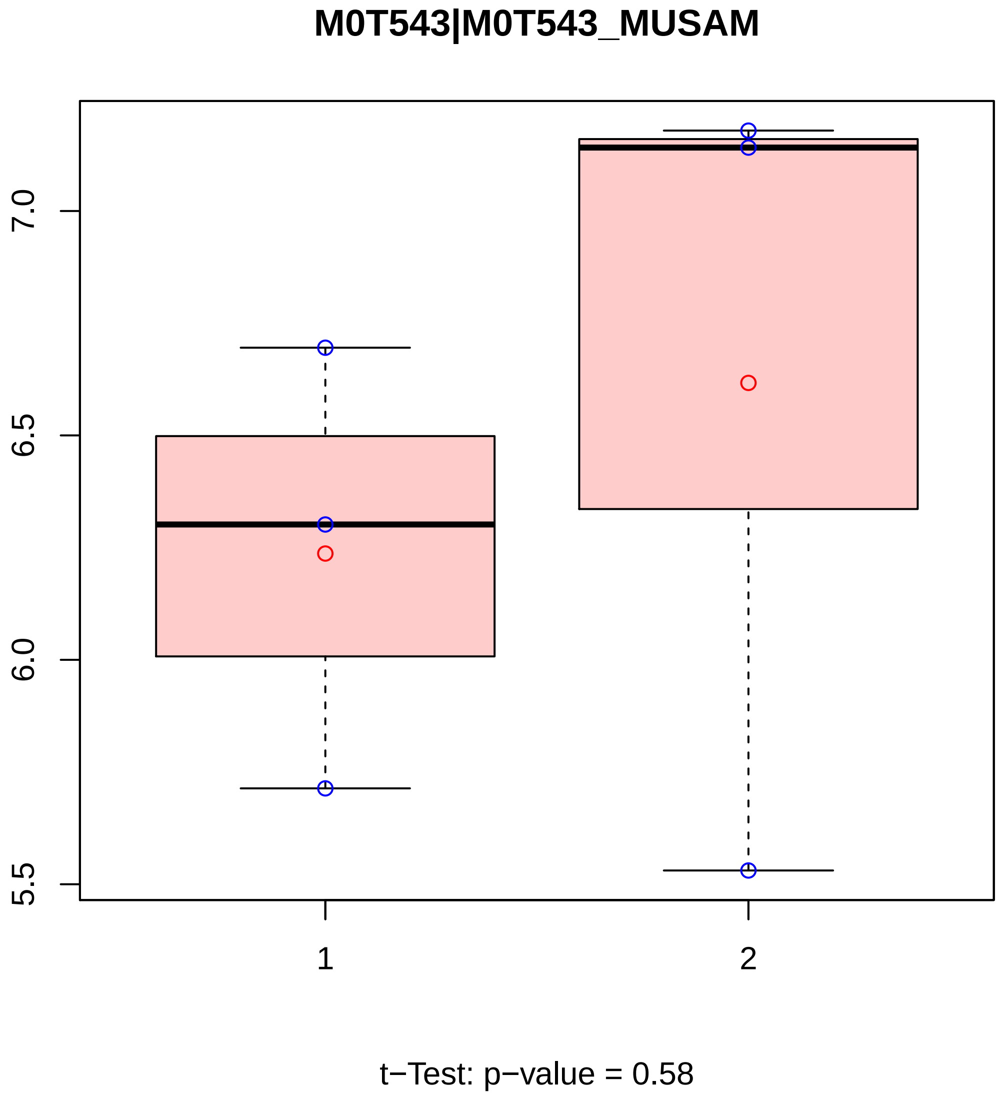
M0T543|M0T543_MUSAM
7.0
6.5
6.0
5.5
1
2
t−Test: p−value = 0.58

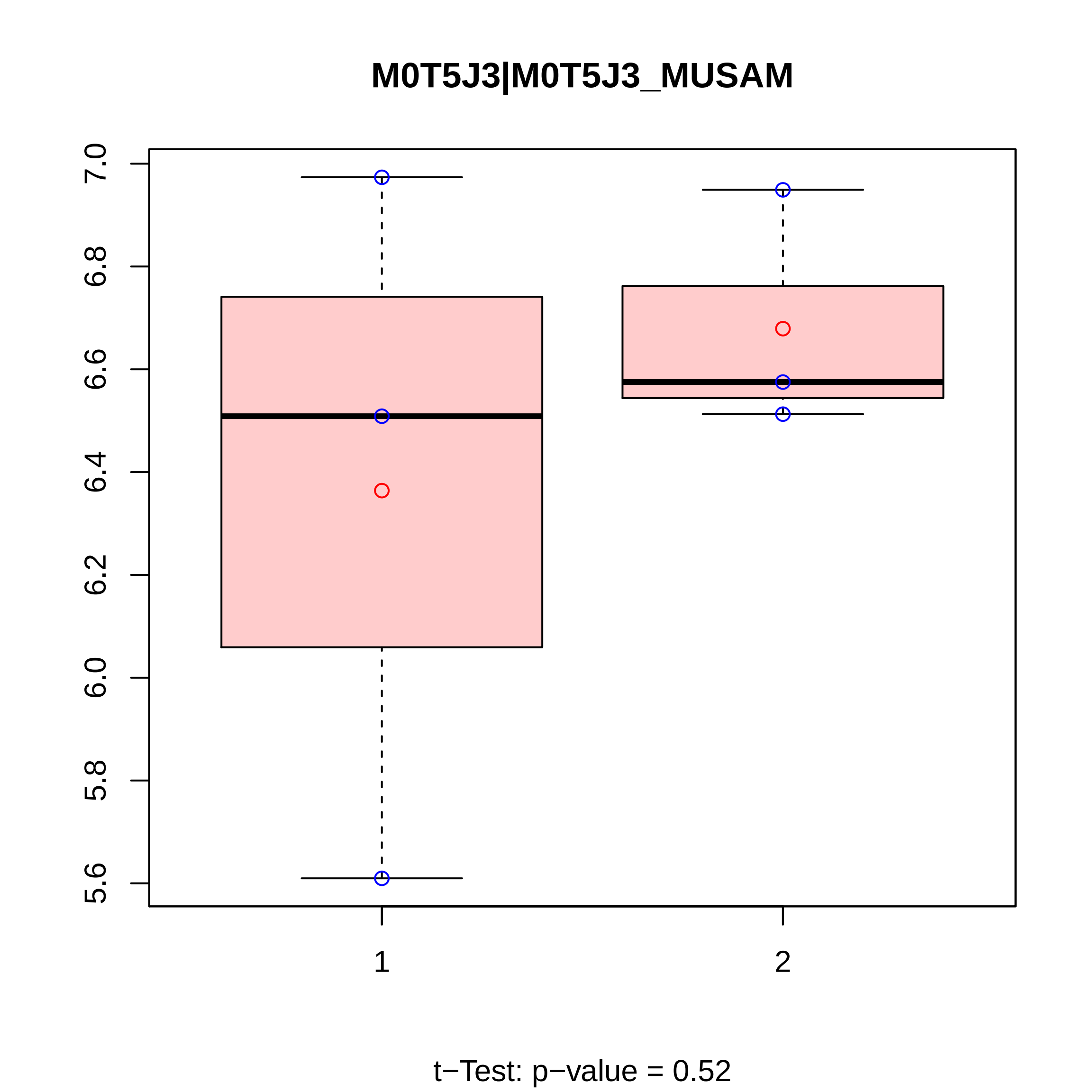
M0T5J3|M0T5J3_MUSAM
7.0
6.8
6.6
6.4
6.2
6.0
5.8
5.6
1
2
t−Test: p−value = 0.52

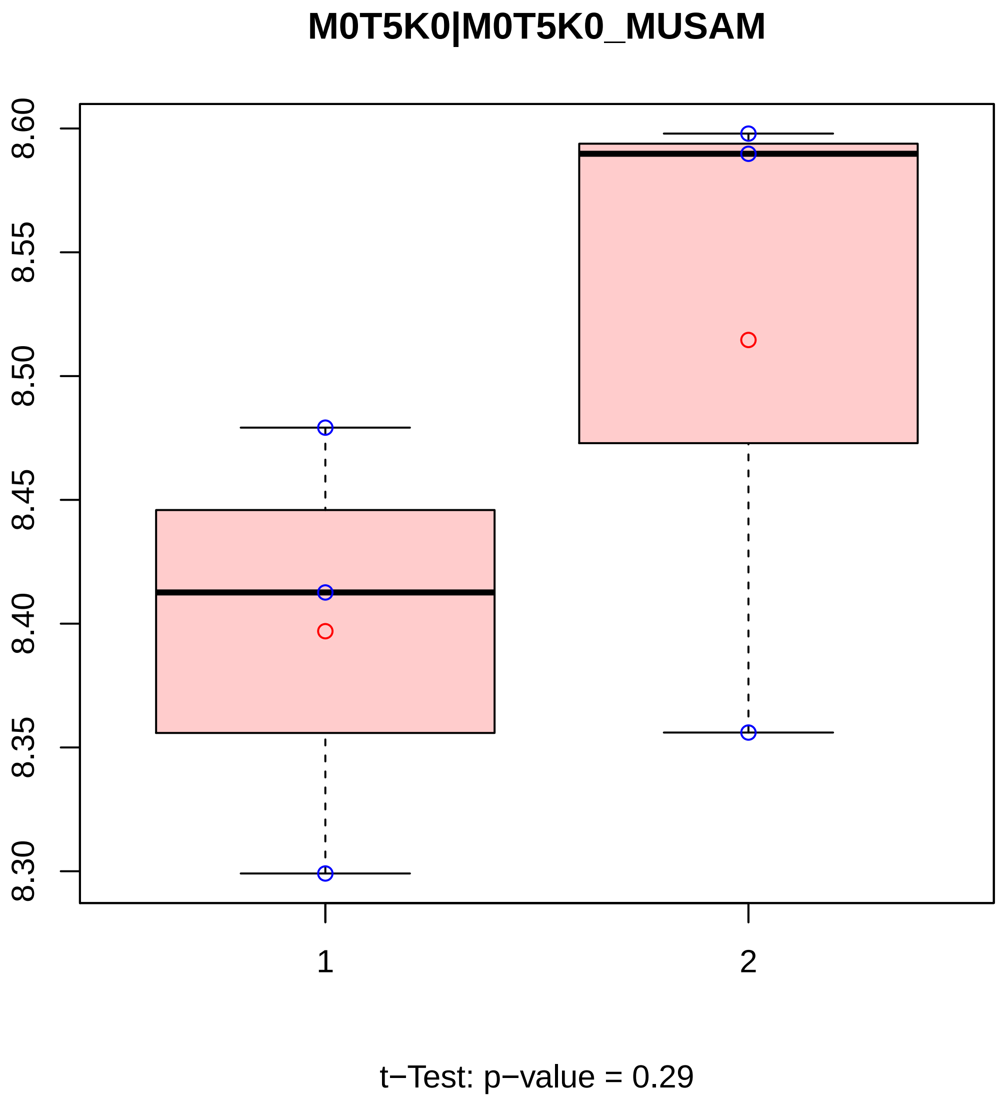
M0T5K0|M0T5K0_MUSAM
8.30
8.35
8.40
8.45
8.50
8.55
8.60
1
2
t−Test: p−value = 0.29

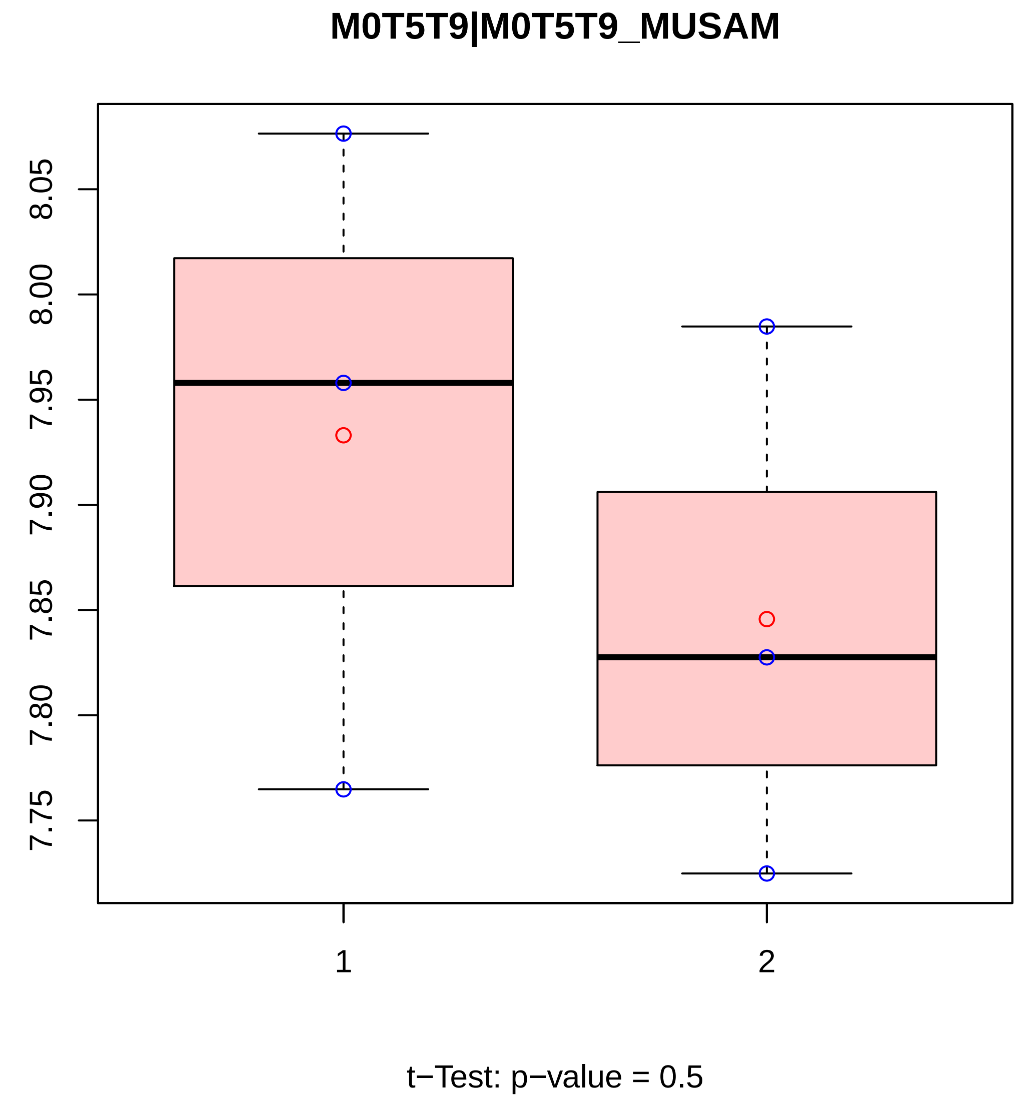

M0T5T9|M0T5T9_MUSAM
8.05
8.00
7.95
7.90
7.85
7.80
7.75
1
2
t−Test: p−value = 0.5

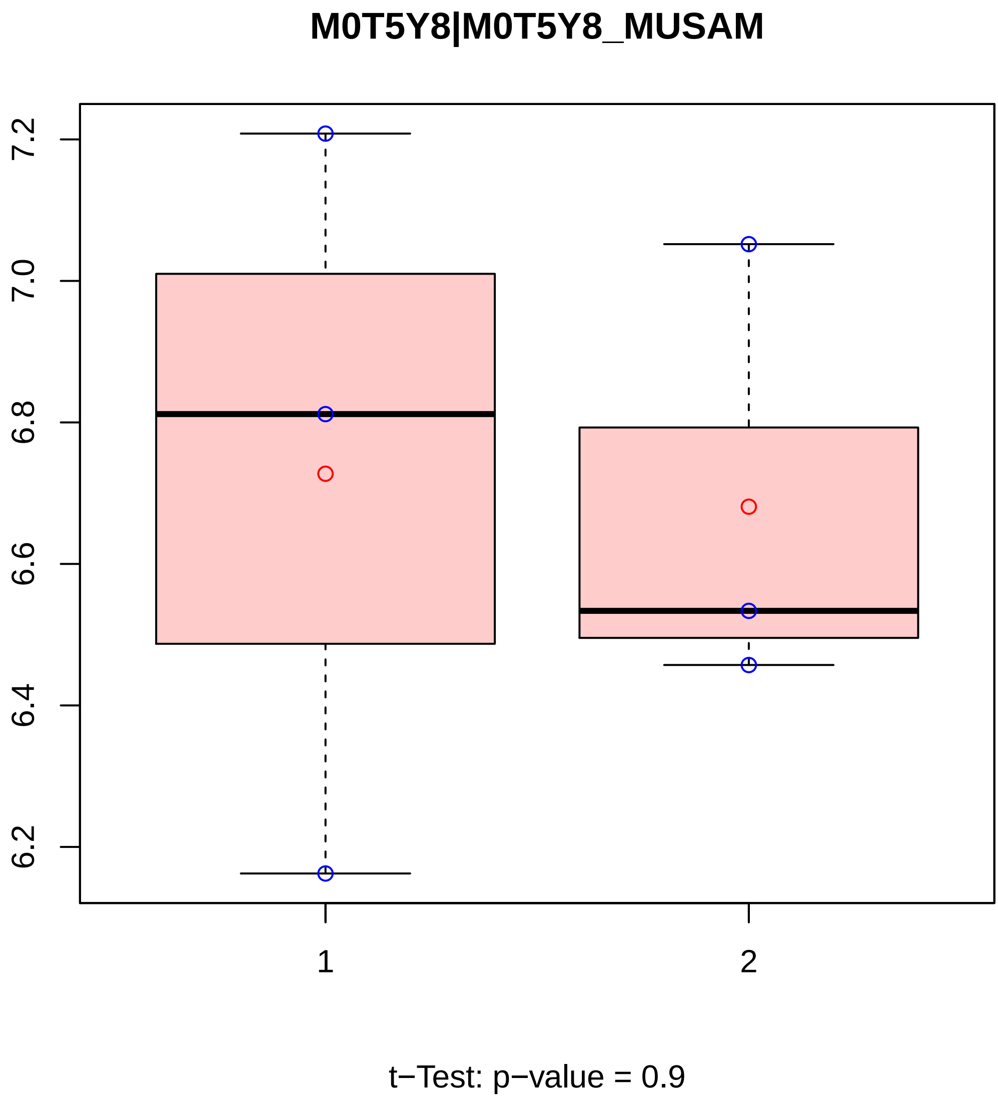
M0T5Y8|M0T5Y8_MUSAM
7.2
7.0
6.8
6.6
6.4
6.2
1
2
t−Test: p−value = 0.9

M0T641|M0T641_MUSAM

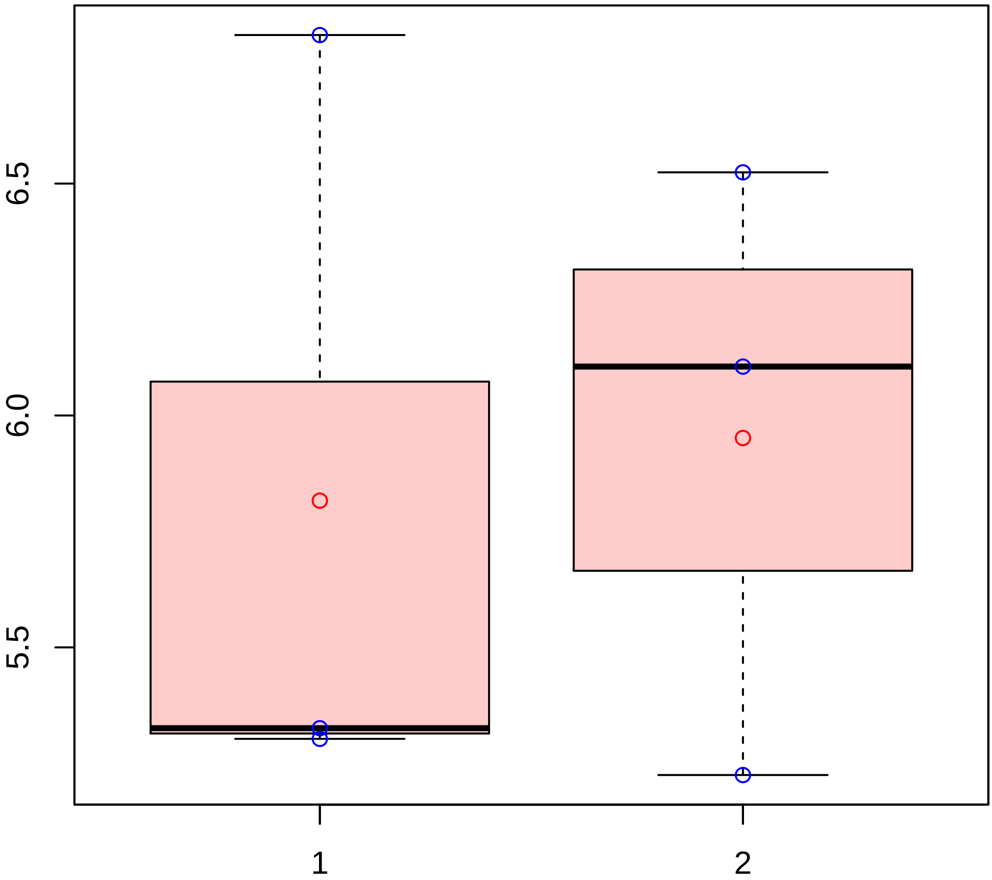

t−Test: p−value = 0.84

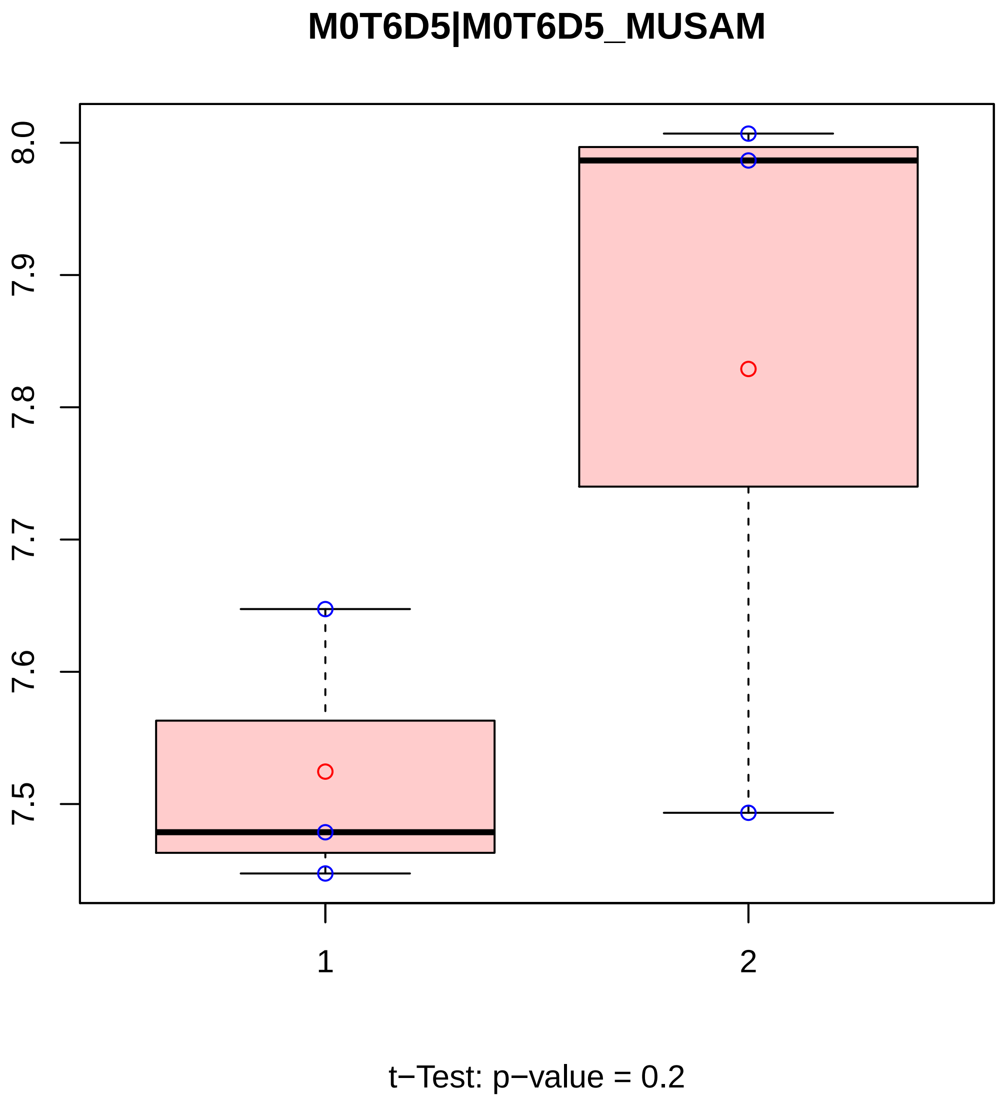

M0T6D5|M0T6D5_MUSAM
8.0
7.9
7.8
7.7
7.6
7.5
1
2
t−Test: p−value = 0.2

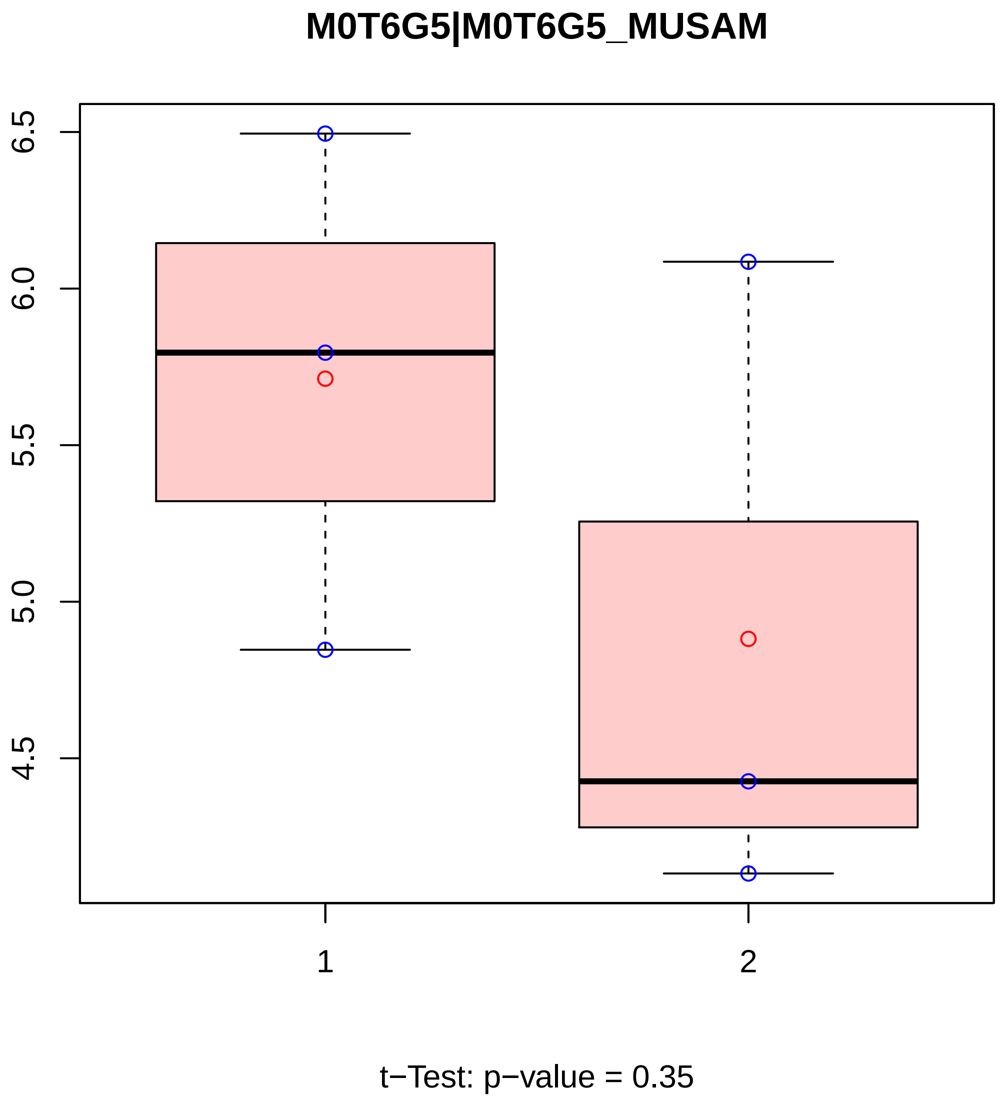

M0T6G5|M0T6G5_MUSAM
6.5
6.0
5.5
5.0
4.5
1
2
t−Test: p−value = 0.35

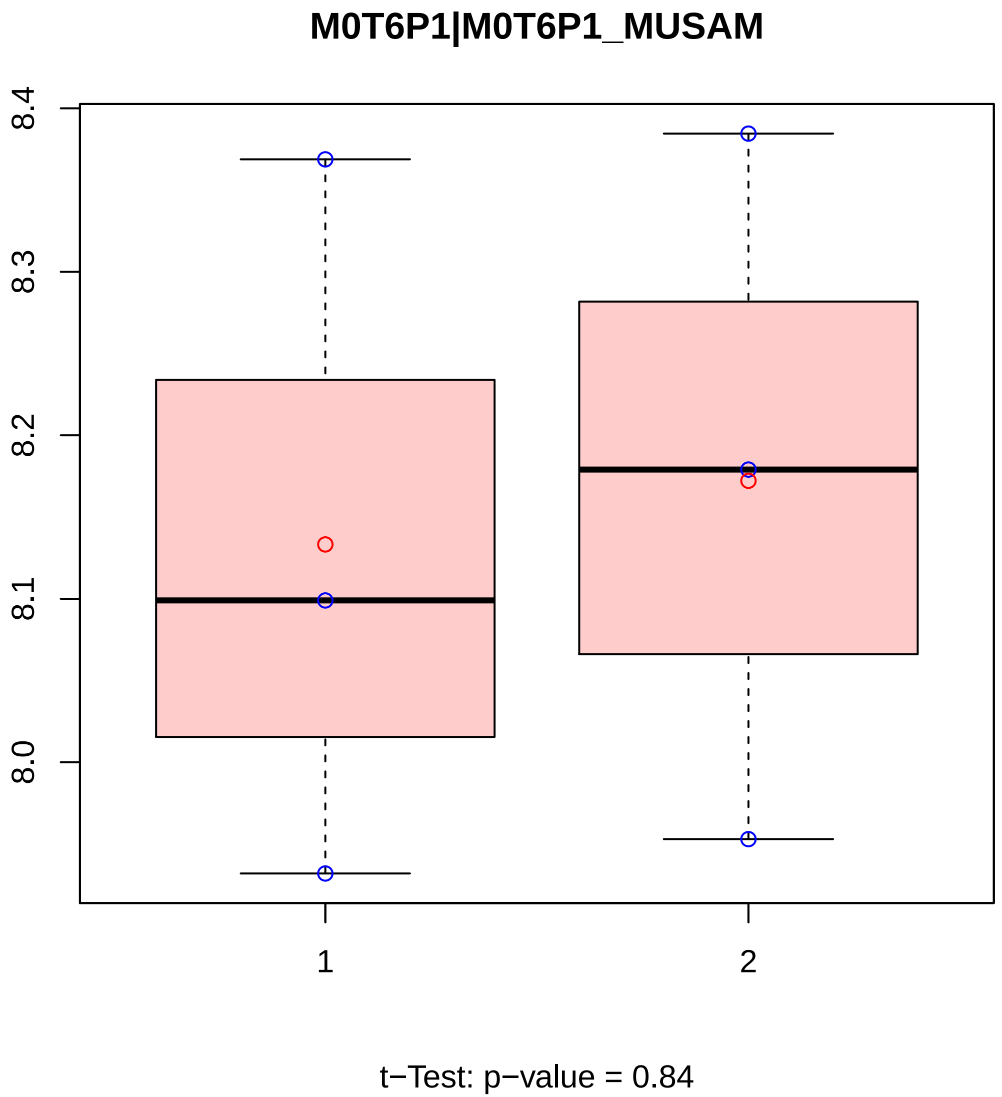
M0T6P1|M0T6P1_MUSAM
8.4
8.3
8.2
8.1
8.0
1
2
t−Test: p−value = 0.84

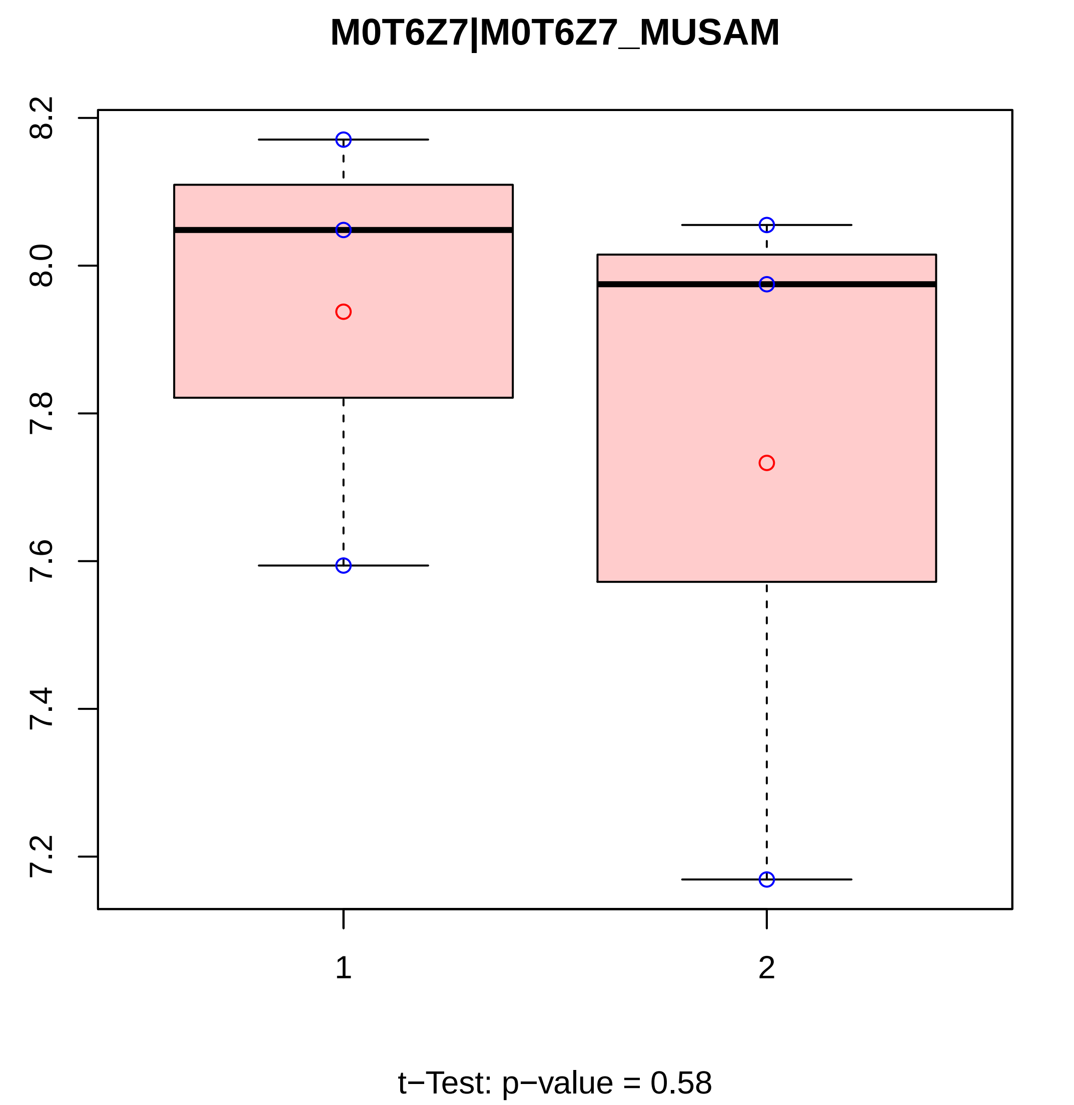
M0T6Z7|M0T6Z7_MUSAM
8.2
8.0
7.8
7.6
7.4
7.2
1
2
t−Test: p−value = 0.58

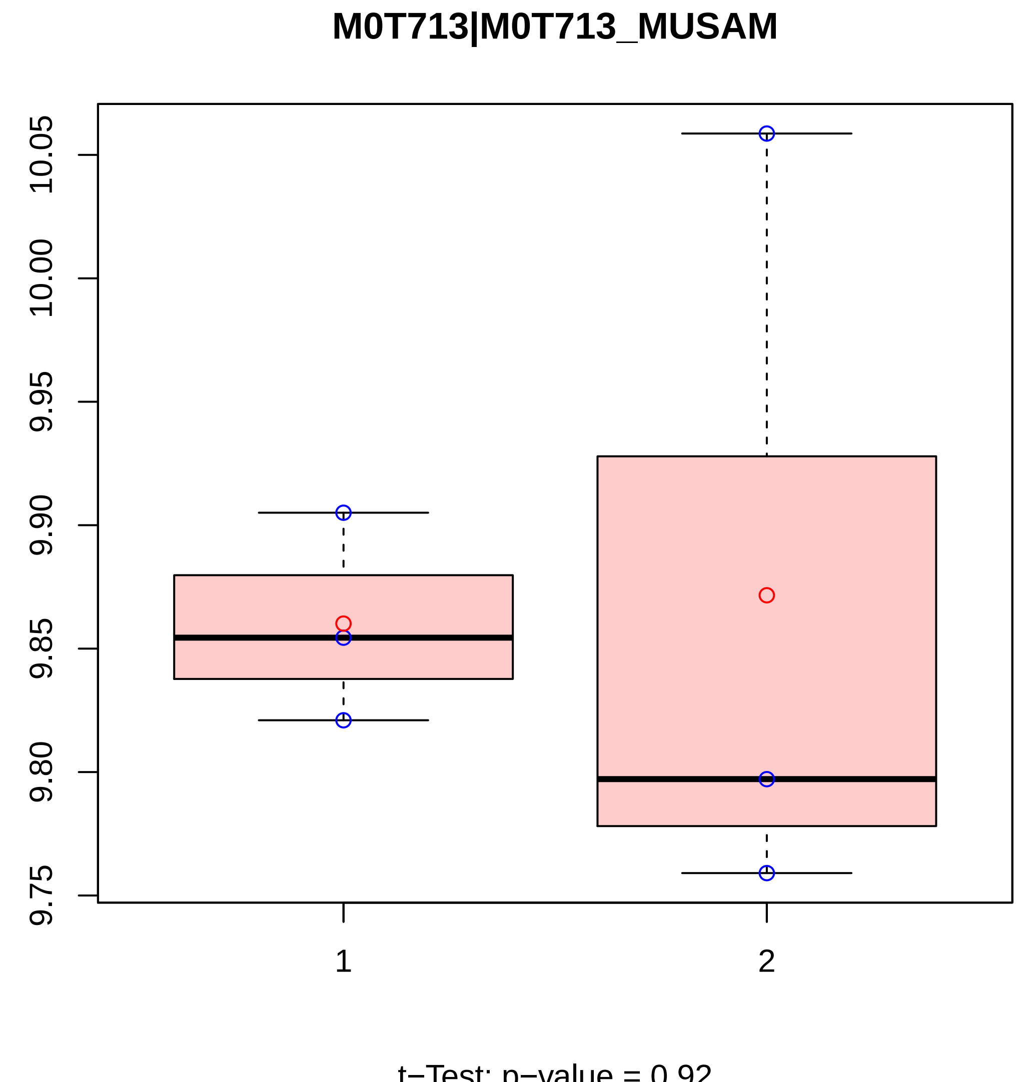
M0T713|M0T713_MUSAM
10.05
10.00
9.95
9.90
9.85
9.80
9.75
1
2
t−Test: p−value = 0.92

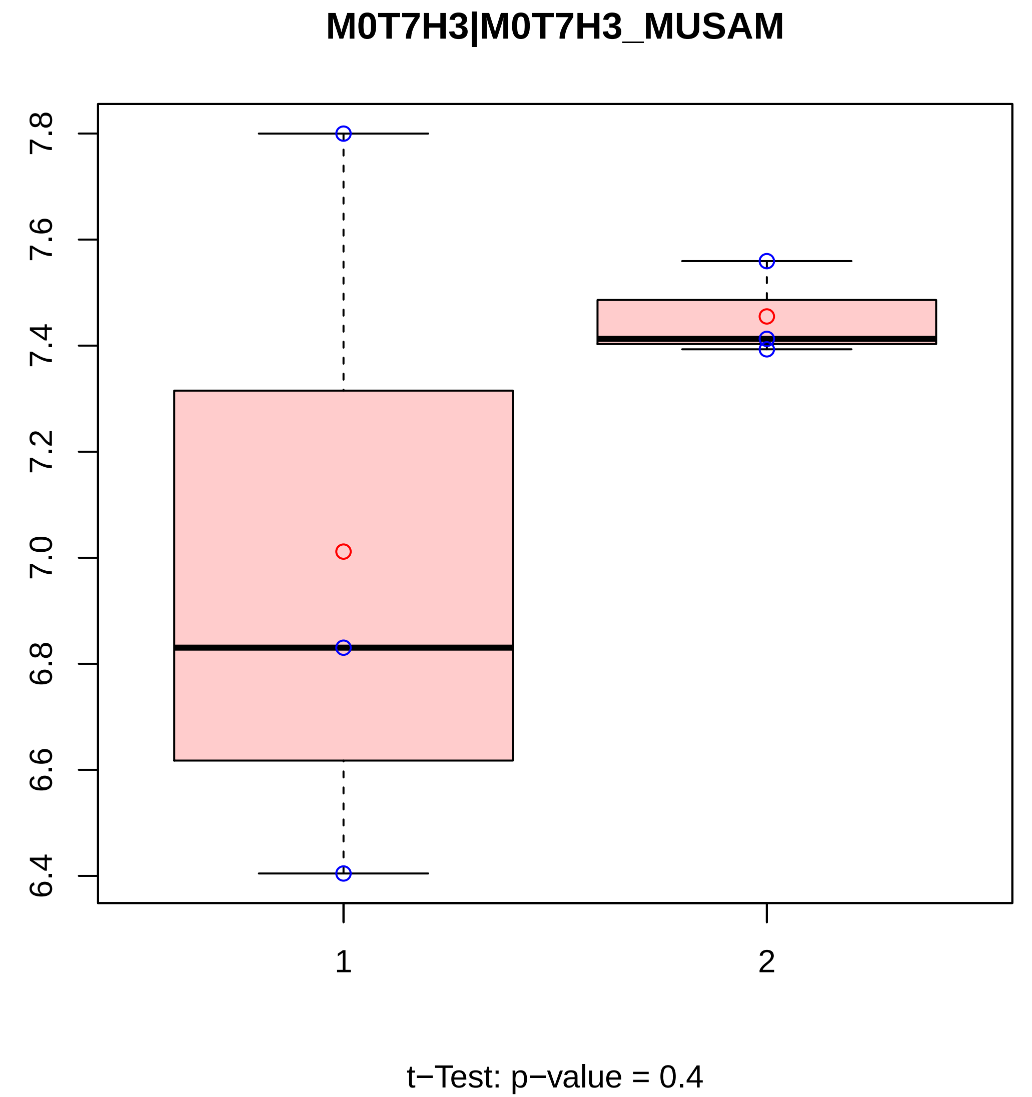
M0T7H3|M0T7H3_MUSAM
7.8
7.6
7.4
7.2
7.0
6.8
6.6
6.4
1
2
t−Test: p−value = 0.4

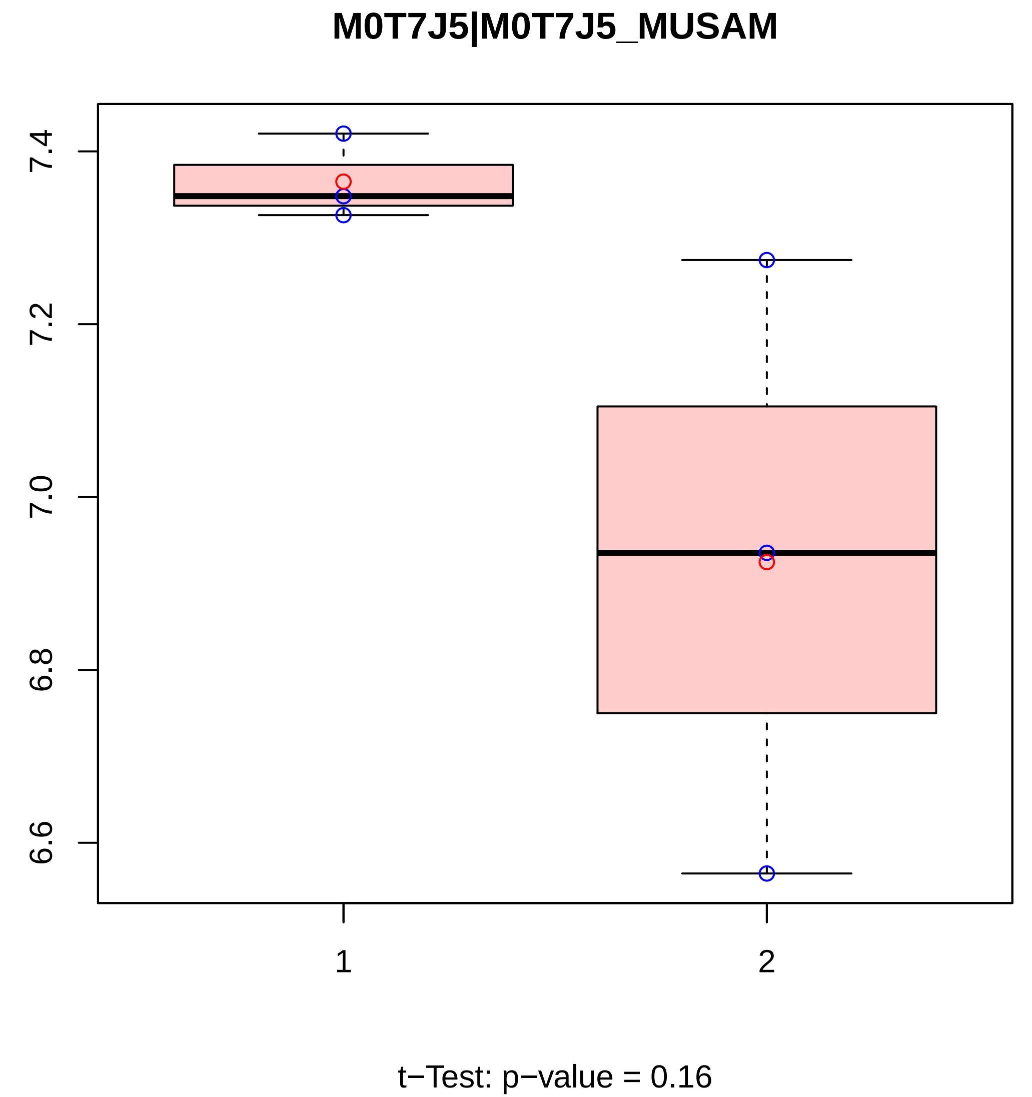
M0T7J5|M0T7J5_MUSAM
7.4
7.2
7.0
6.8
6.6
1
2
t−Test: p−value = 0.16

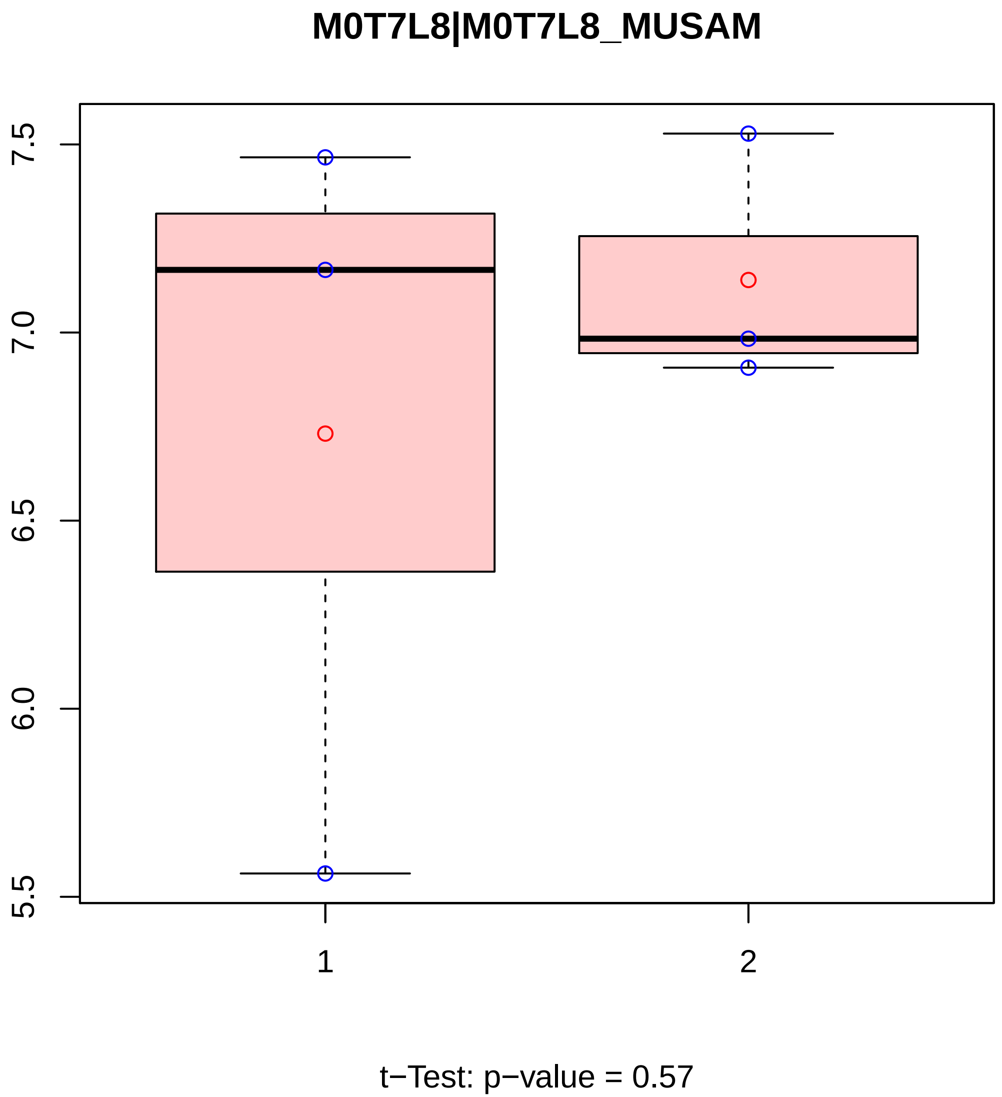
M0T7L8|M0T7L8_MUSAM
7.5
7.0
6.5
6.0
5.5
1
2
t−Test: p−value = 0.57

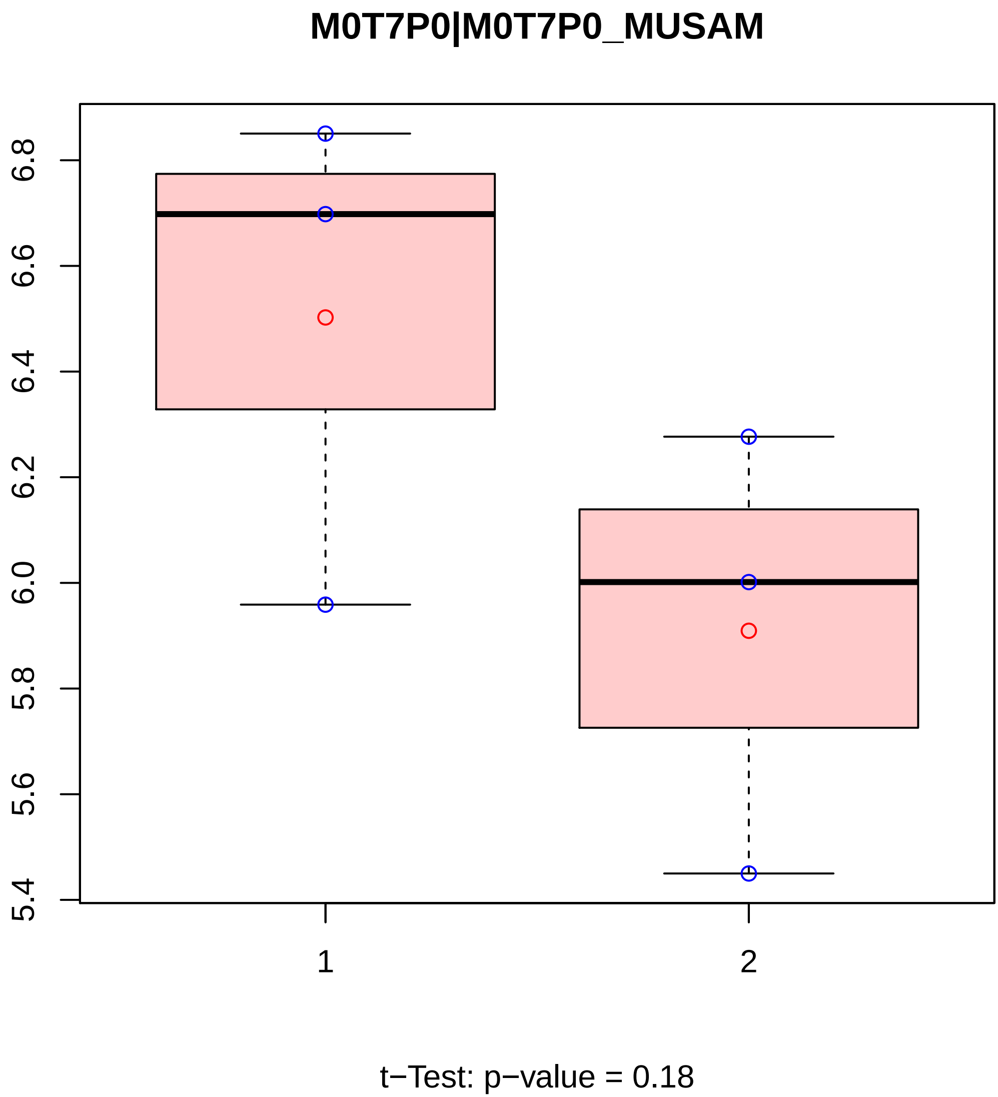
M0T7P0|M0T7P0_MUSAM
5.4
5.6
5.8
6.0
6.2
6.4
6.6
6.8
1
2
t−Test: p−value = 0.18

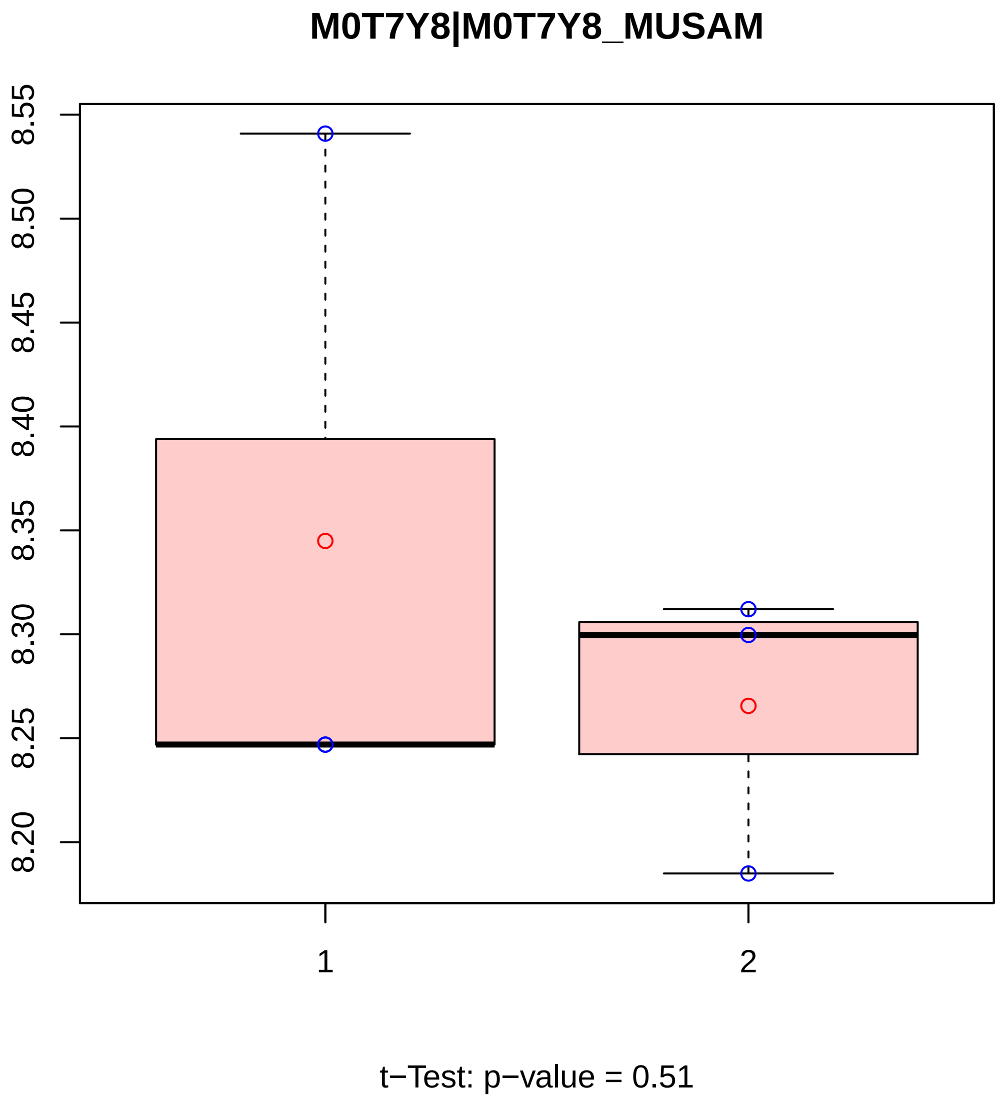

M0T7Y8|M0T7Y8_MUSAM
8.55
8.50
8.45
8.40
8.35
8.30
8.25
8.20
1
2
t−Test: p−value = 0.51

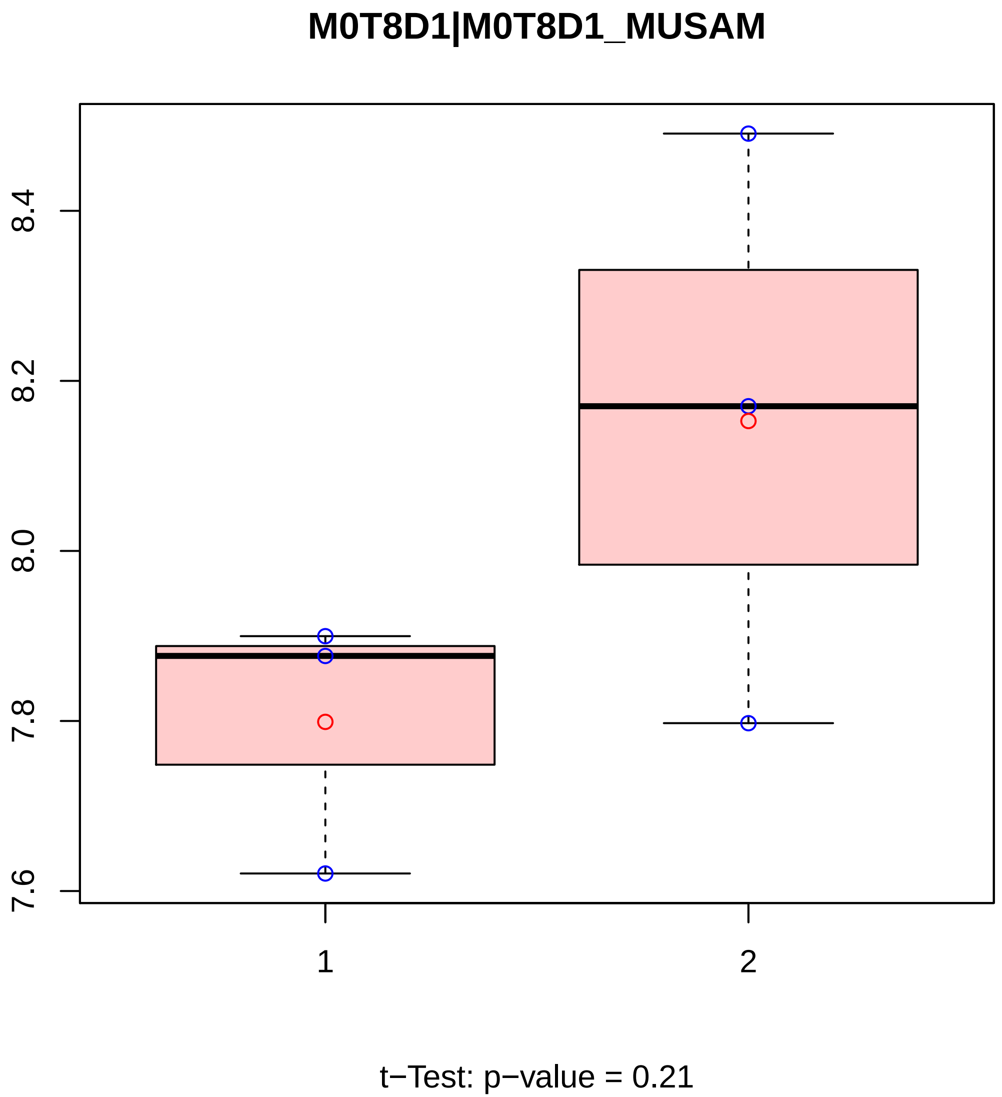

M0T8D1|M0T8D1_MUSAM
7.6
7.8
8.0
8.2
8.4
1
2
t−Test: p−value = 0.21

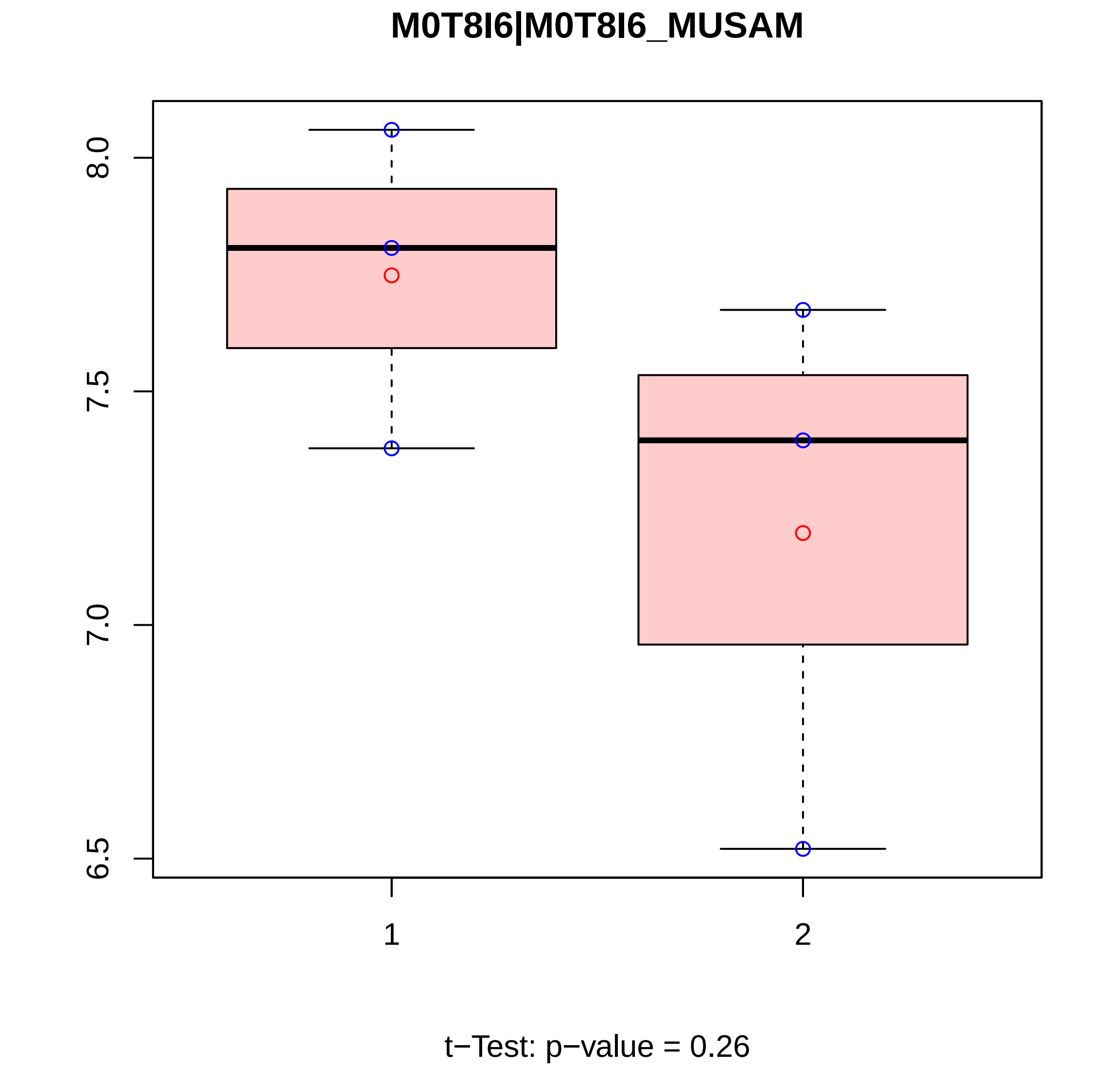
M0T8I6|M0T8I6_MUSAM
8.0
7.5
7.0
6.5
1
2
t−Test: p−value = 0.26

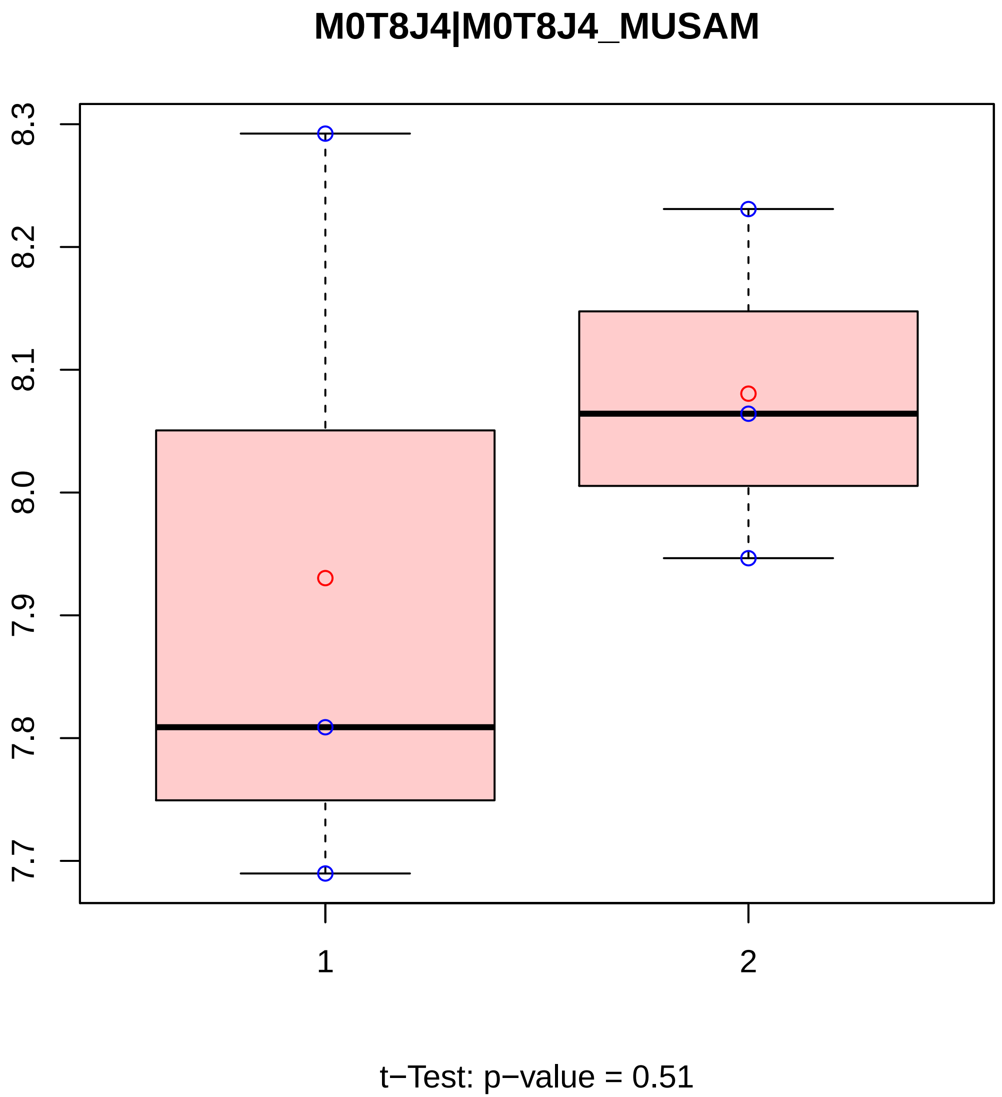
M0T8J4|M0T8J4_MUSAM
8.3
8.2
8.1
8.0
7.9
7.8
7.7
1
2
t−Test: p−value = 0.51

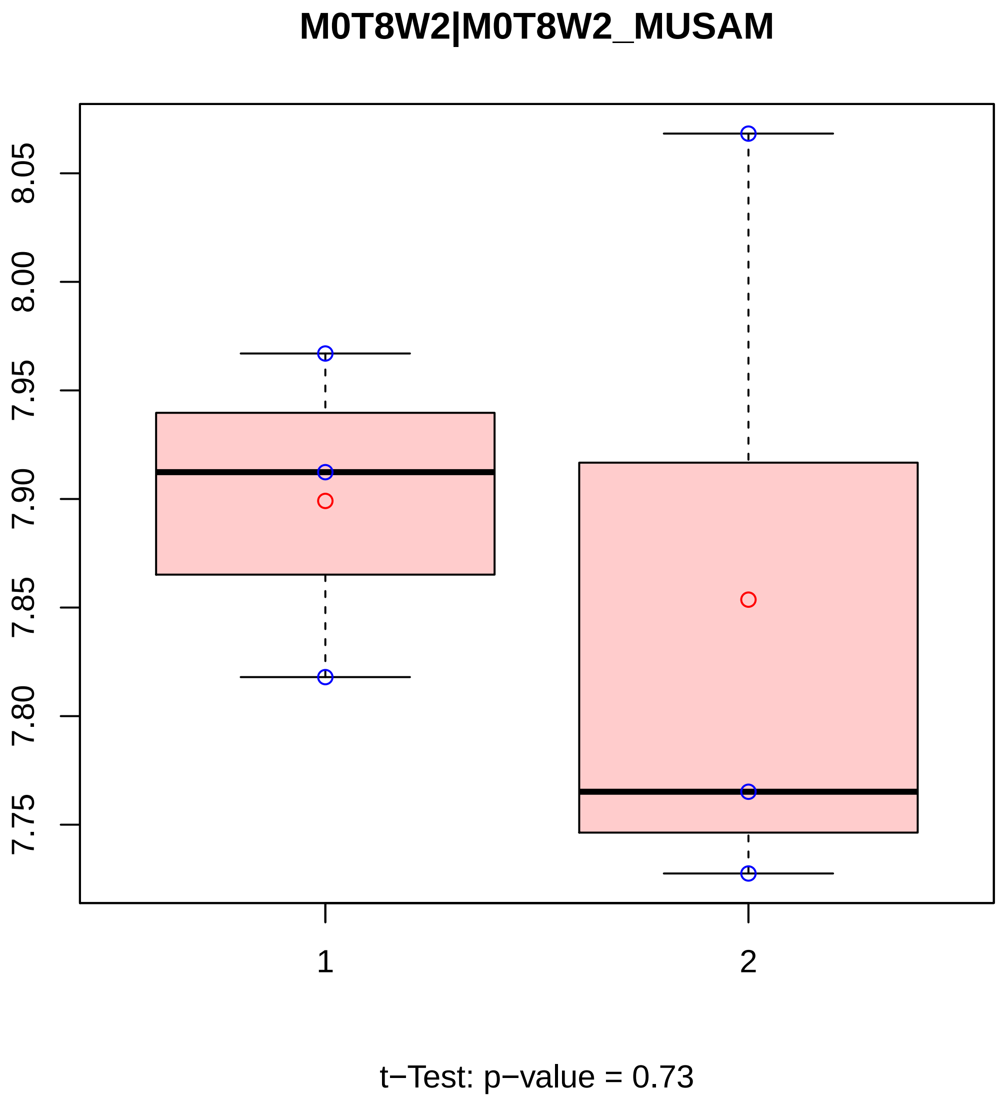

M0T8W2|M0T8W2_MUSAM
8.05
8.00
7.95
7.90
7.85
7.80
7.75
1
2
t−Test: p−value = 0.73

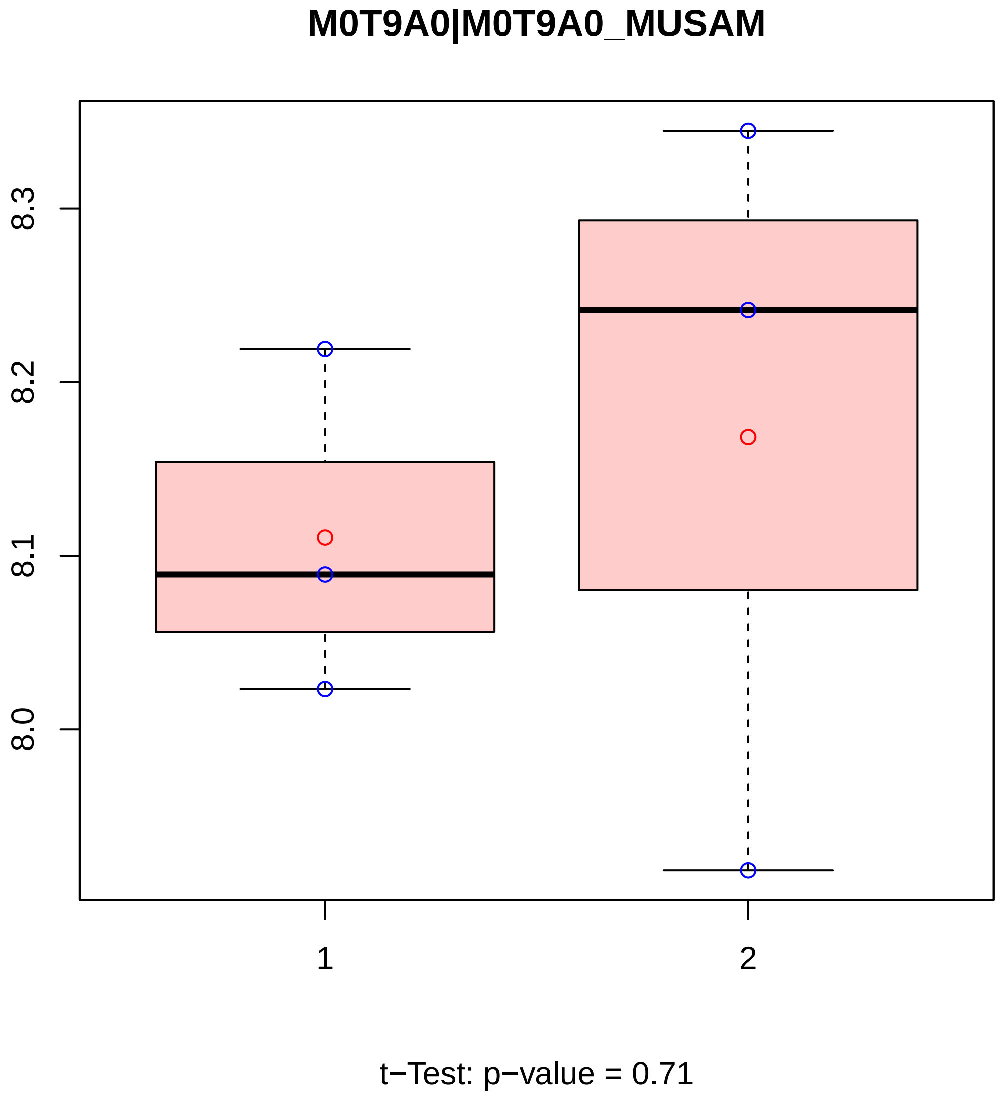
M0T9A0|M0T9A0_MUSAM
8.0
8.1
8.2
8.3
1
2
t−Test: p−value = 0.71

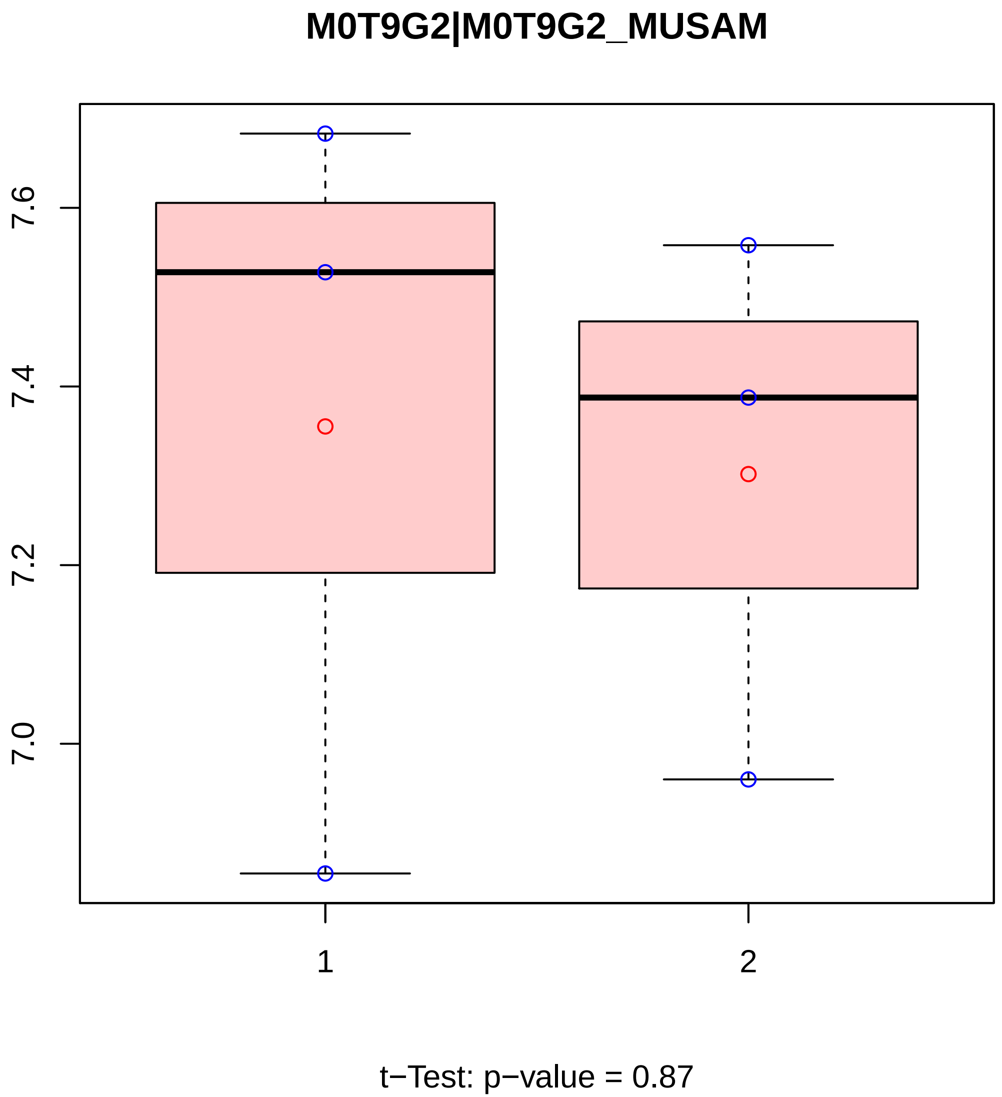
M0T9G2|M0T9G2_MUSAM
7.6
7.4
7.2
7.0
1
2
t−Test: p−value = 0.87

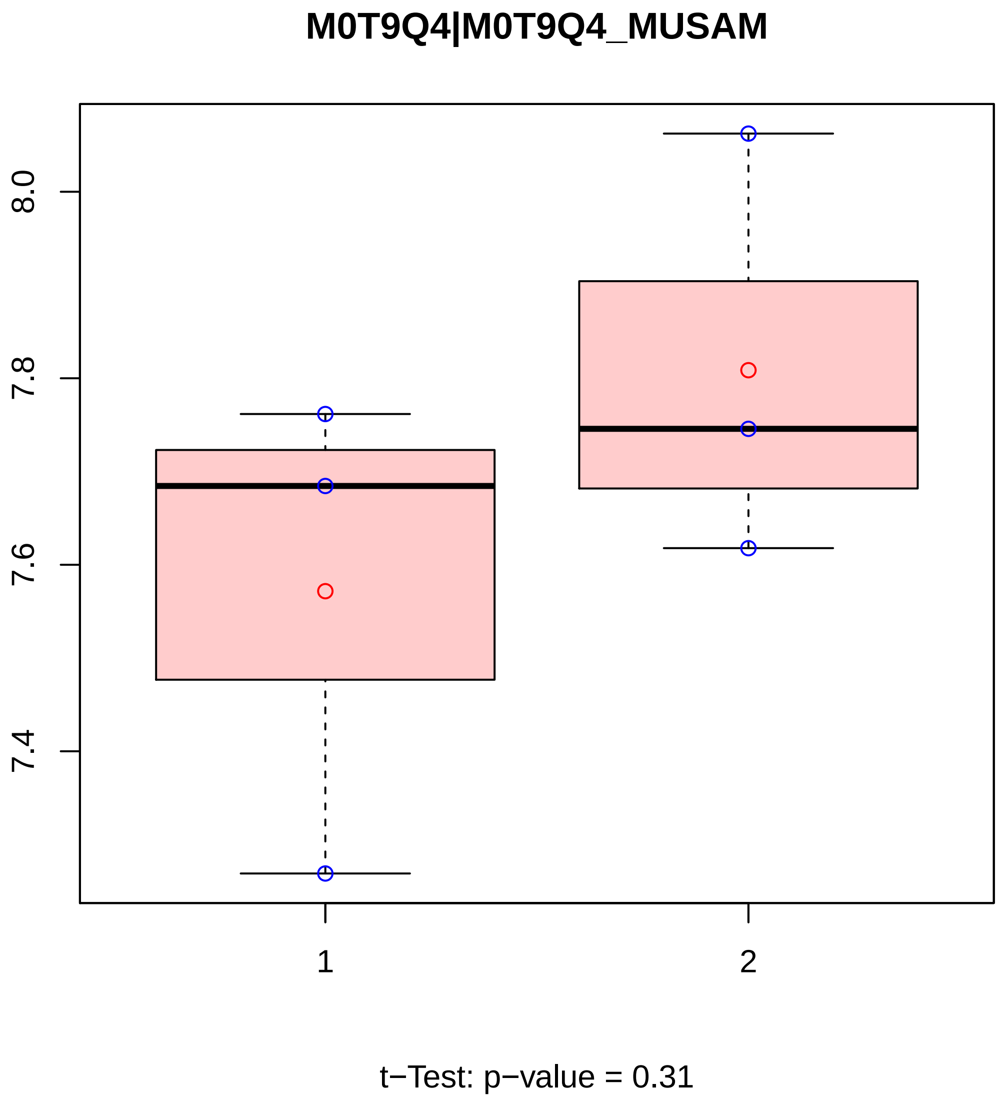

M0T9Q4|M0T9Q4_MUSAM
8.0
7.8
7.6
7.4
1
2
t−Test: p−value = 0.31

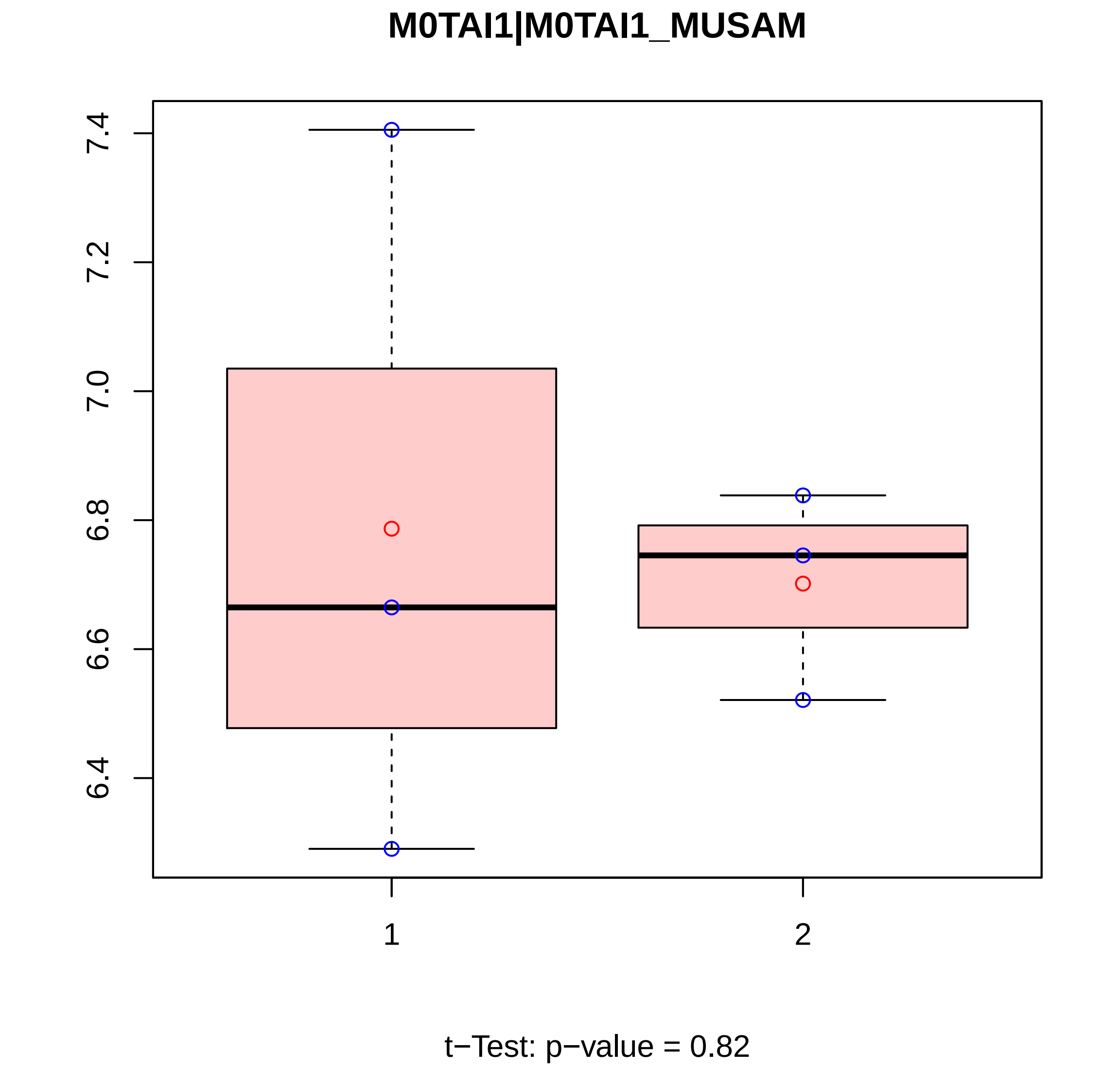
M0TAI1|M0TAI1_MUSAM
7.4
7.2
7.0
6.8
6.6
6.4
1
2
t−Test: p−value = 0.82

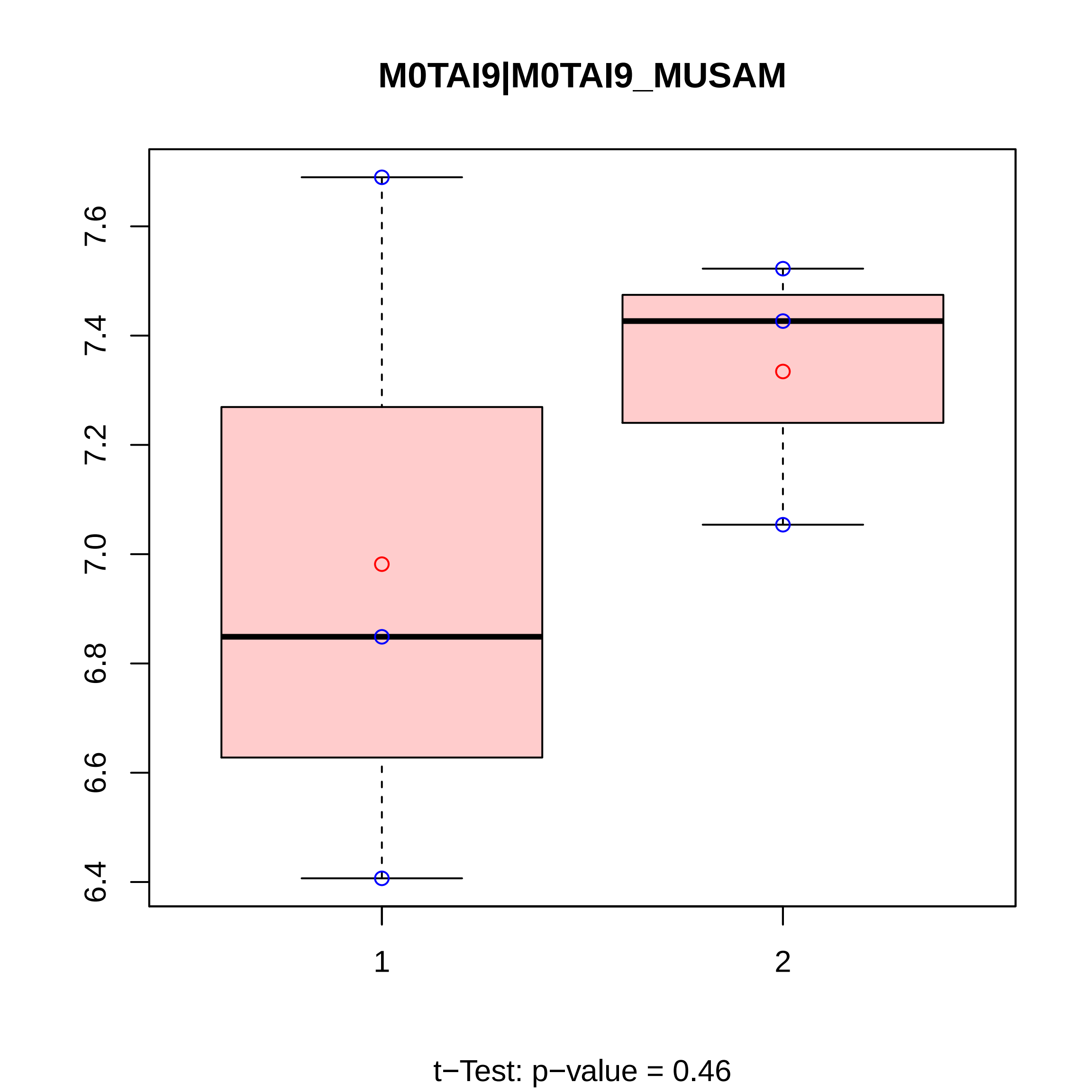
M0TAI9|M0TAI9_MUSAM
7.6
7.4
7.2
7.0
6.8
6.6
6.4
1
2
t−Test: p−value = 0.46

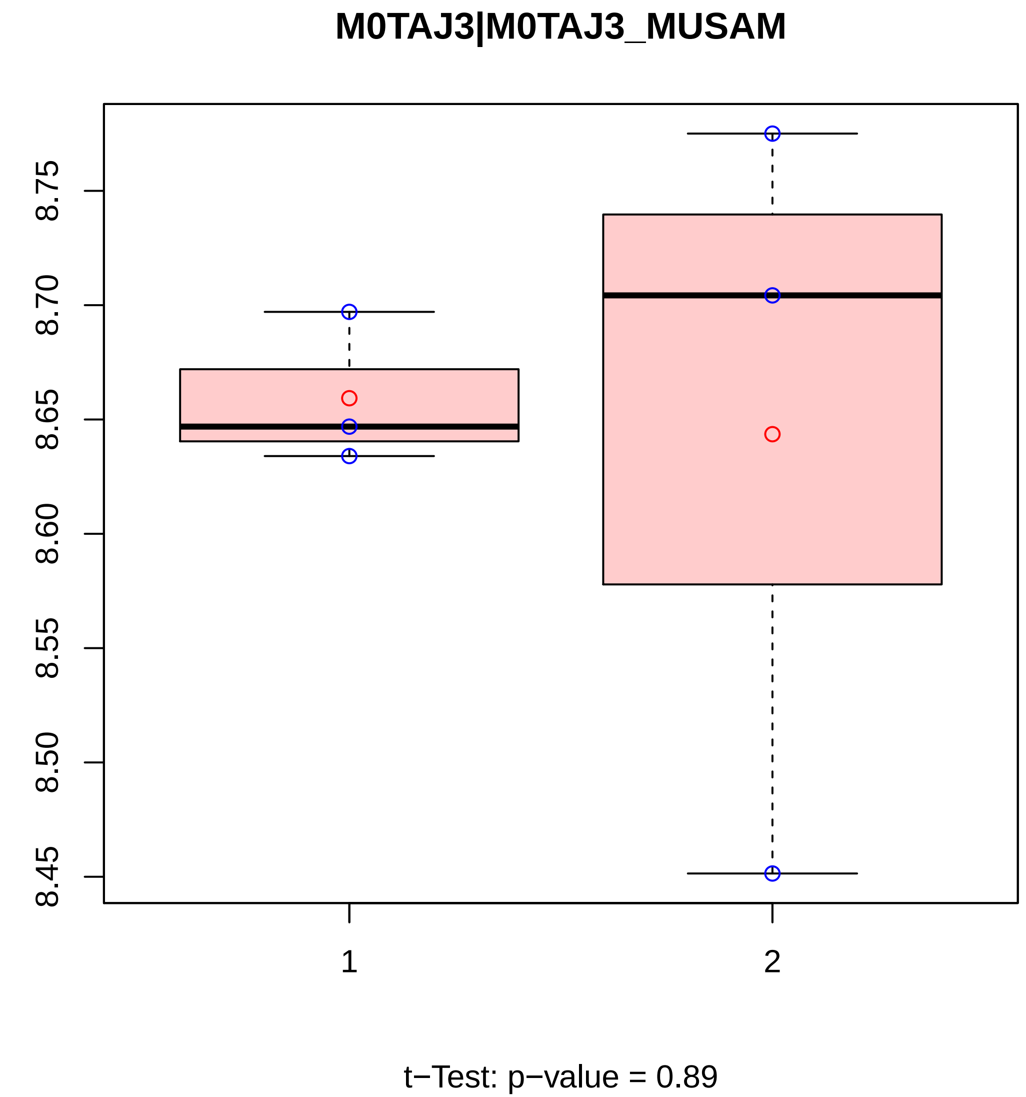

M0TAJ3|M0TAJ3_MUSAM
8.45
8.50
8.55
8.60
8.65
8.70
8.75
1
2
t−Test: p−value = 0.89

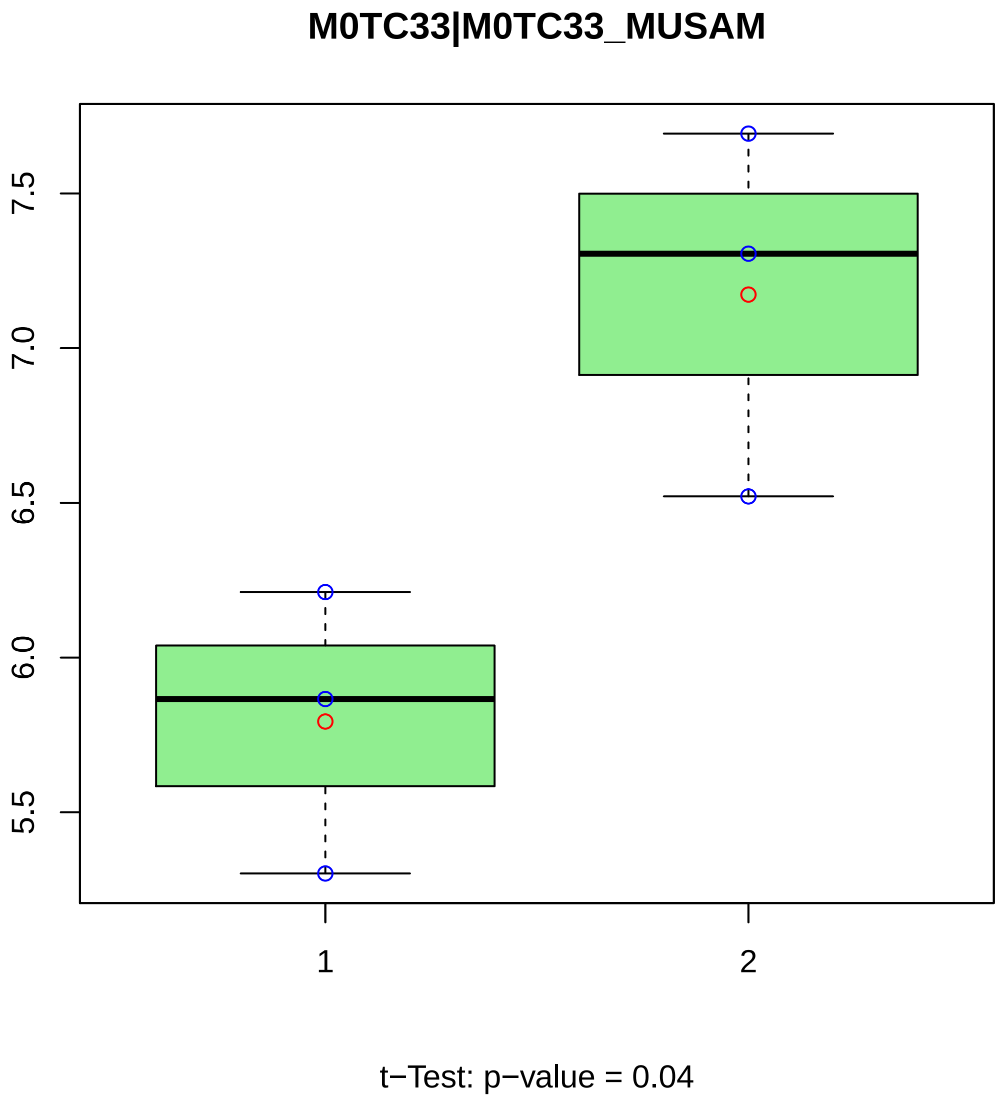
M0TC33|M0TC33_MUSAM
7.5
7.0
6.5
6.0
5.5
1
2
t−Test: p−value = 0.04

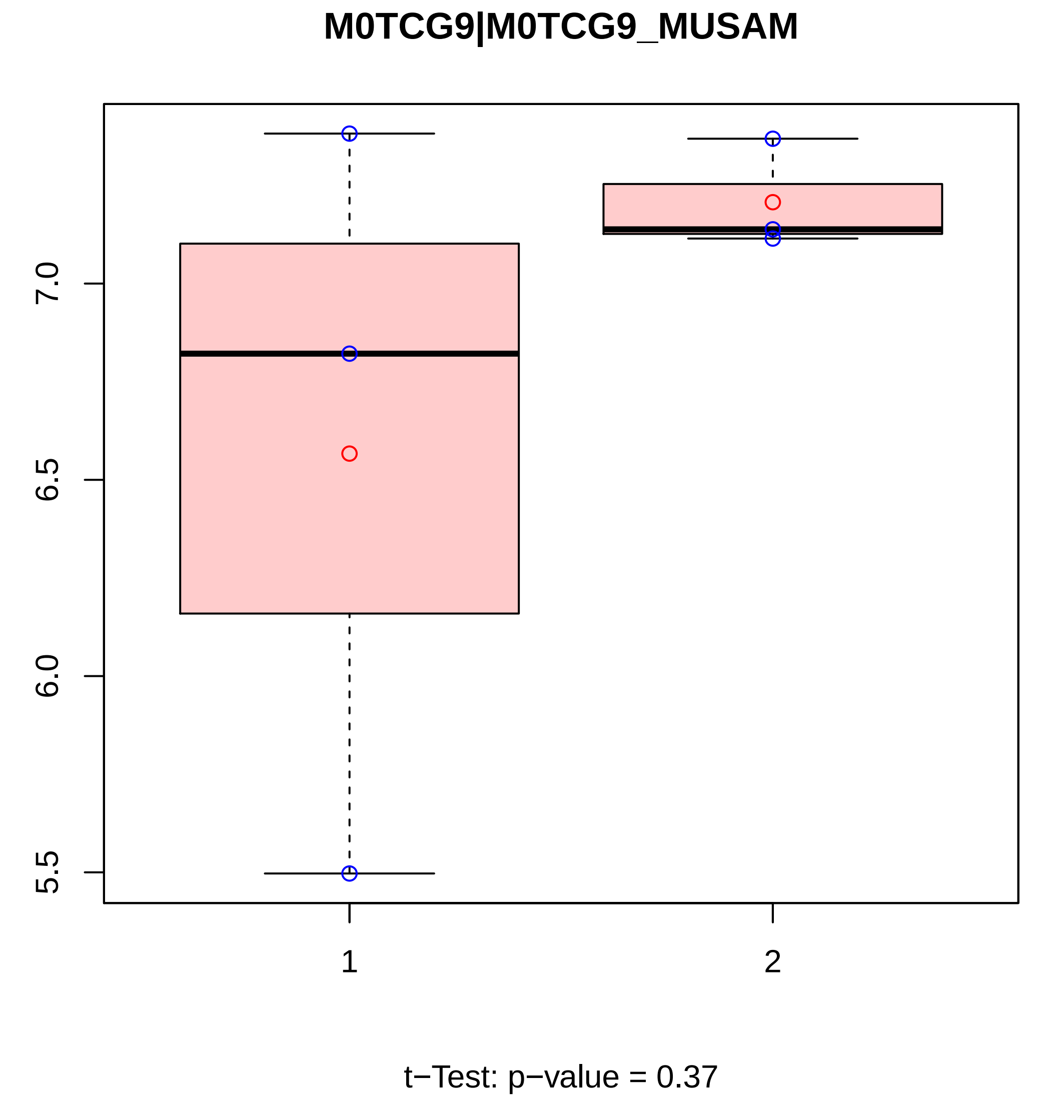
M0TCG9|M0TCG9_MUSAM
7.0
6.5
6.0
5.5
1
2
t−Test: p−value = 0.37

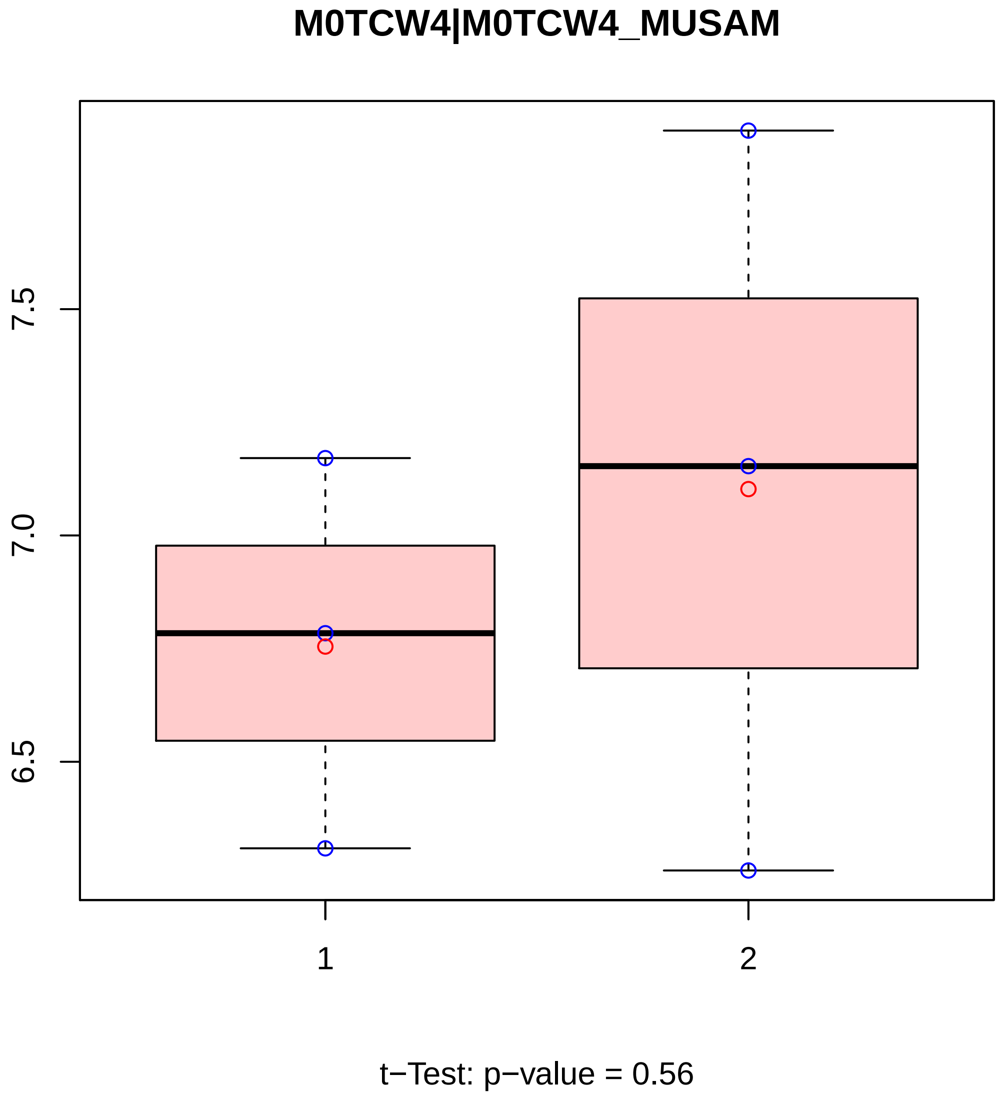
M0TCW4|M0TCW4_MUSAM
7.5
7.0
6.5
1
2
t−Test: p−value = 0.56

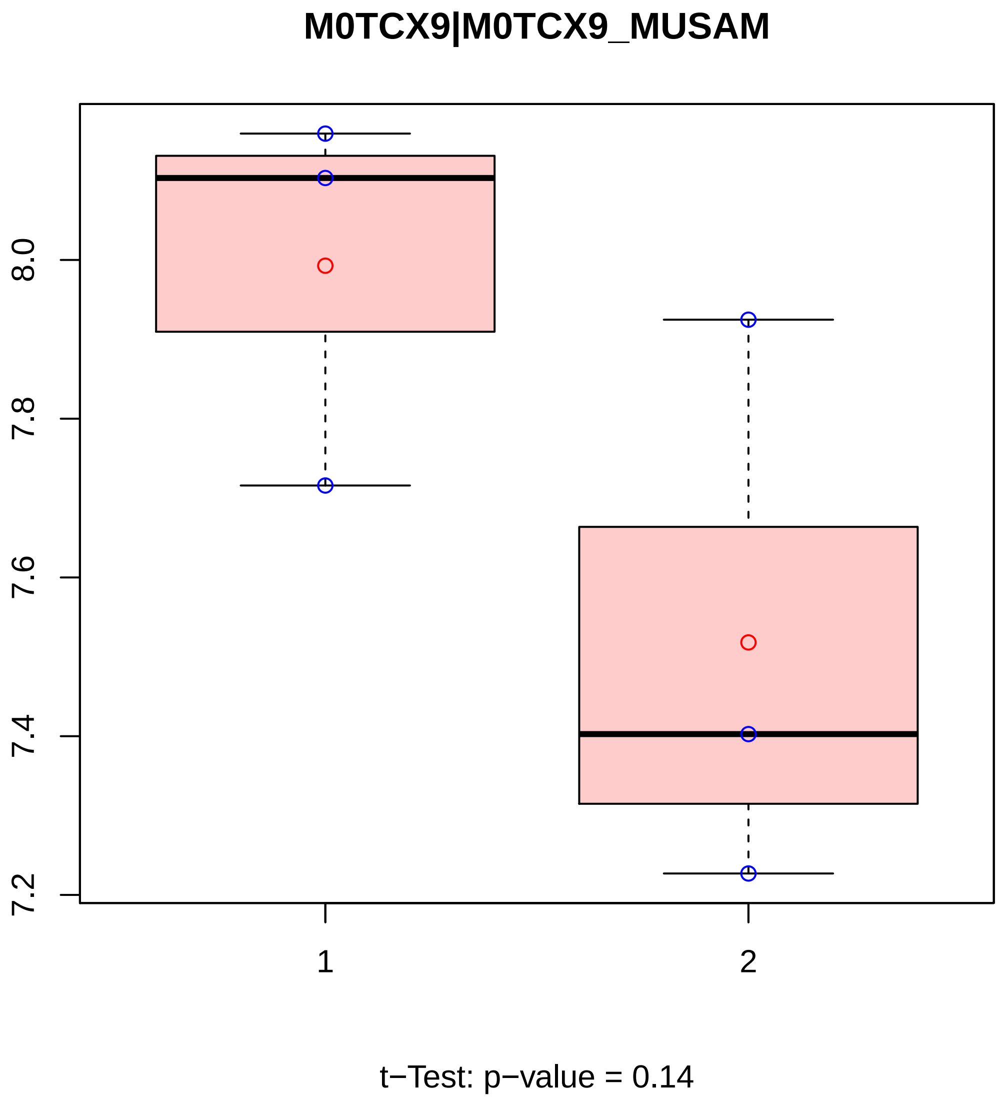
M0TCX9|M0TCX9_MUSAM
8.0
7.8
7.6
7.4
7.2
1
2
t−Test: p−value = 0.14

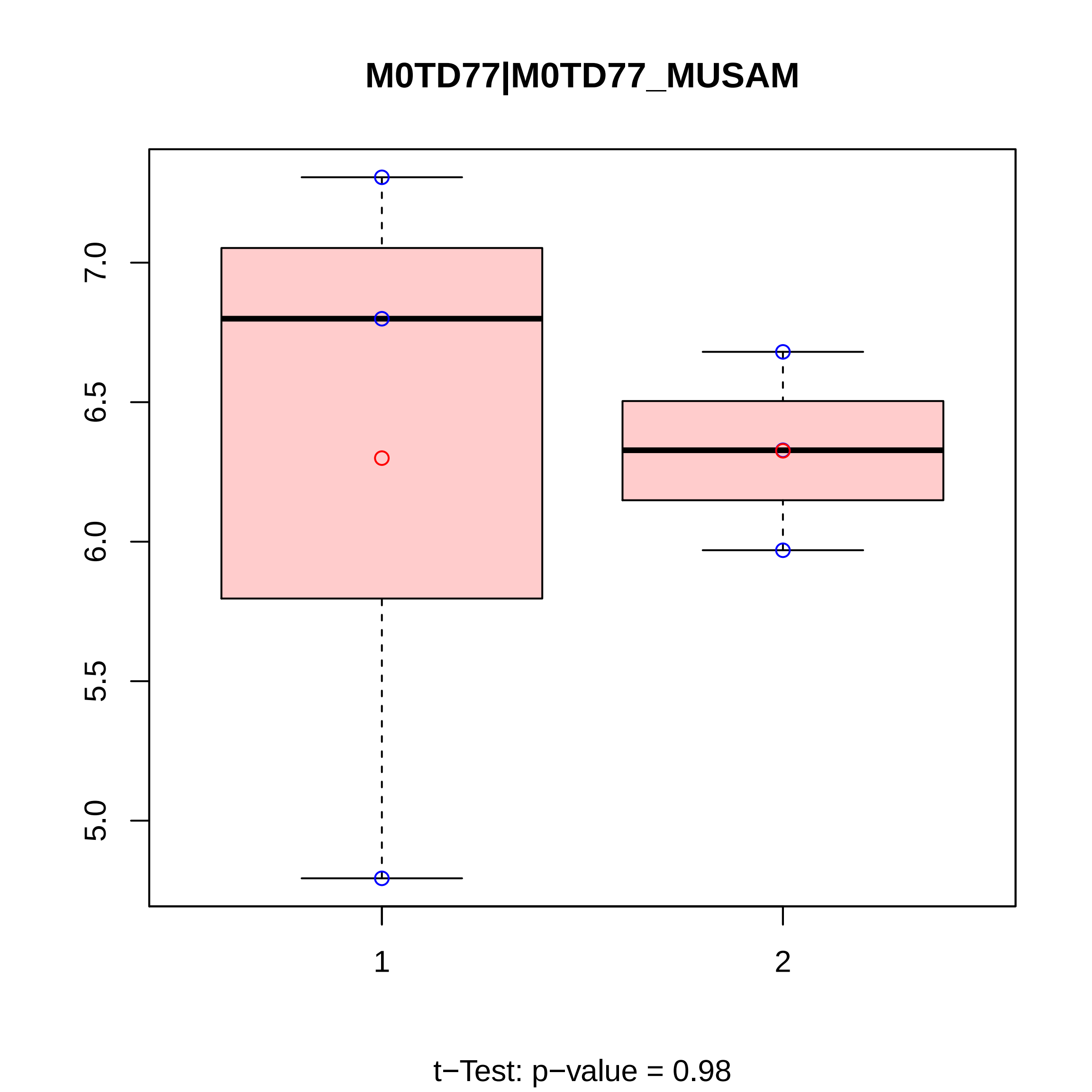
M0TD77|M0TD77_MUSAM
7.0
6.5
6.0
5.5
5.0
1
2
t−Test: p−value = 0.98

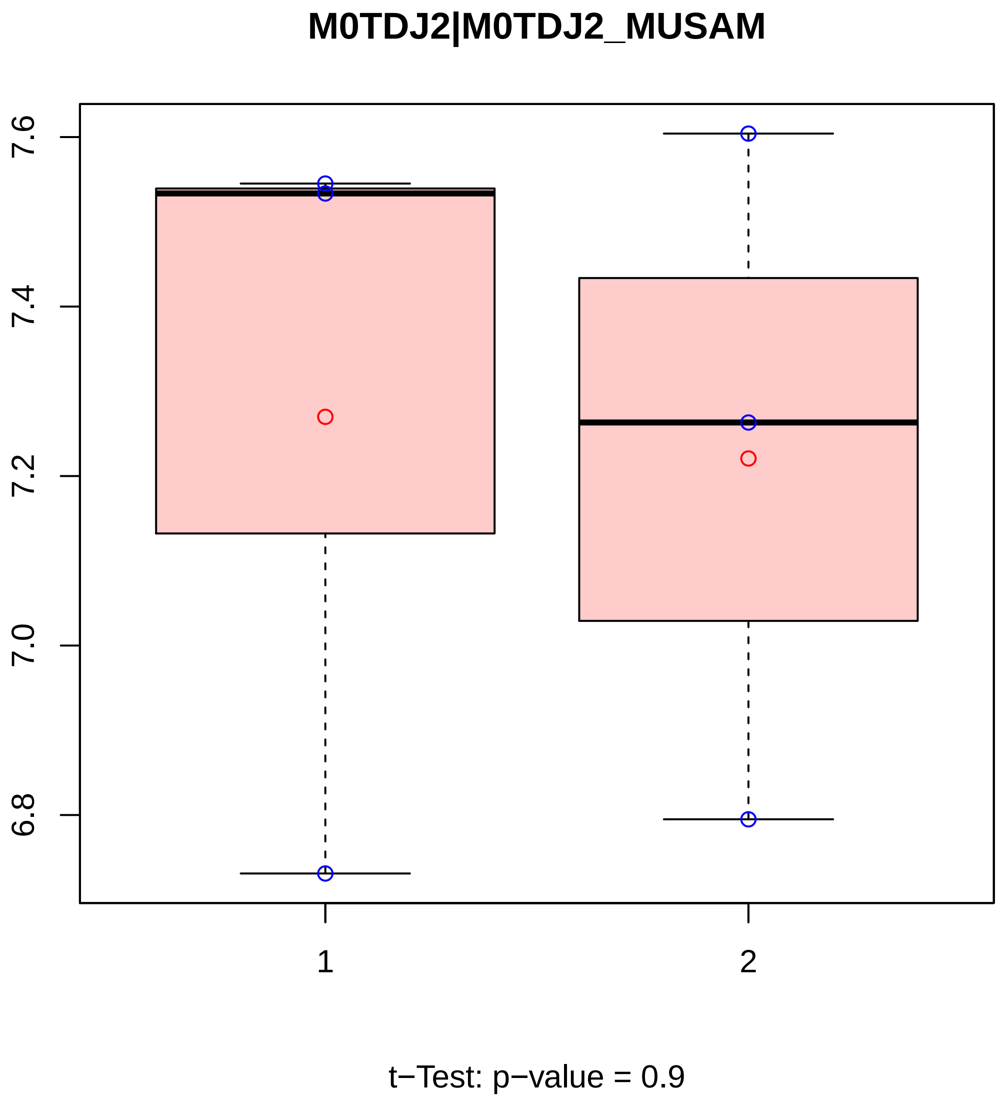
M0TDJ2|M0TDJ2_MUSAM
7.6
7.4
7.2
7.0
6.8
1
2
t−Test: p−value = 0.9

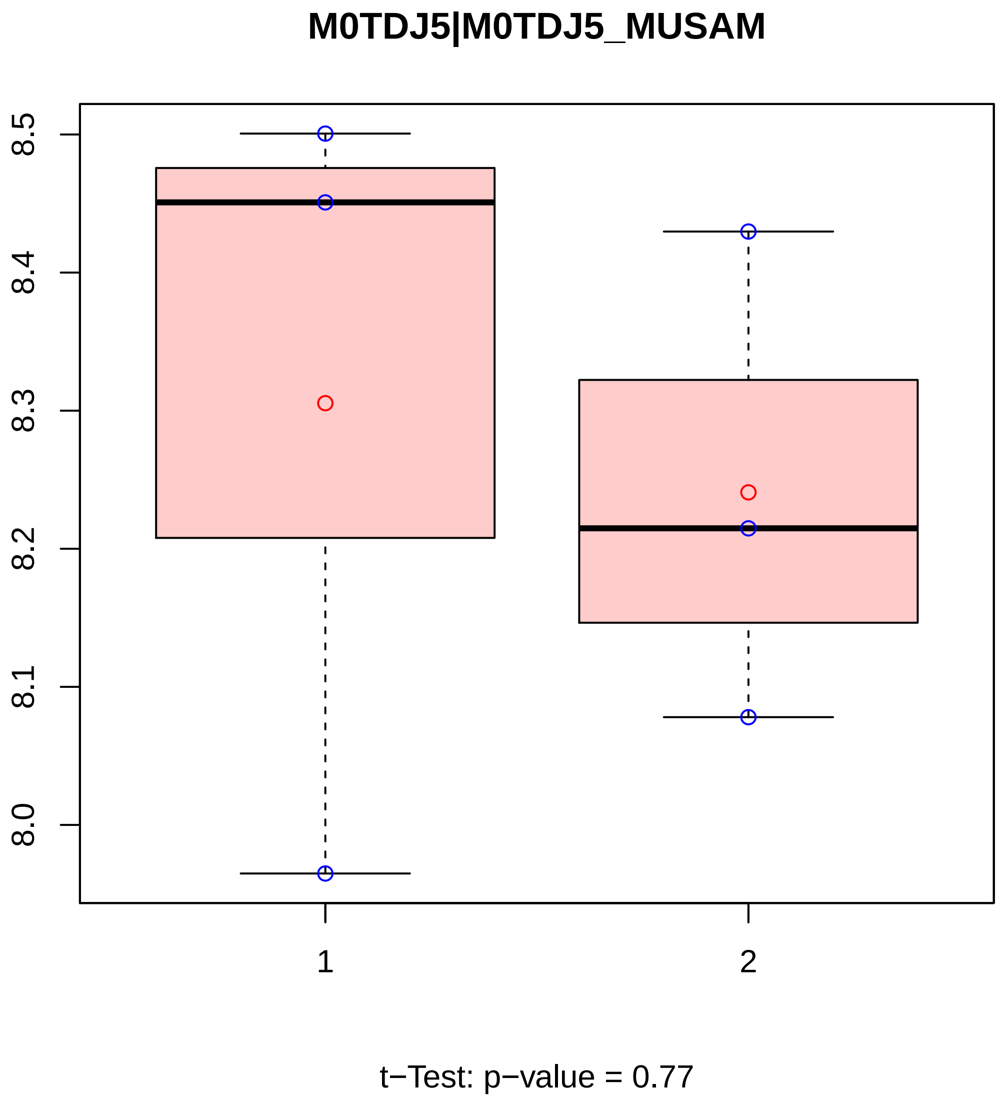
M0TDJ5|M0TDJ5_MUSAM
8.0
8.1
8.2
8.3
8.4
8.5
1
2
t−Test: p−value = 0.77

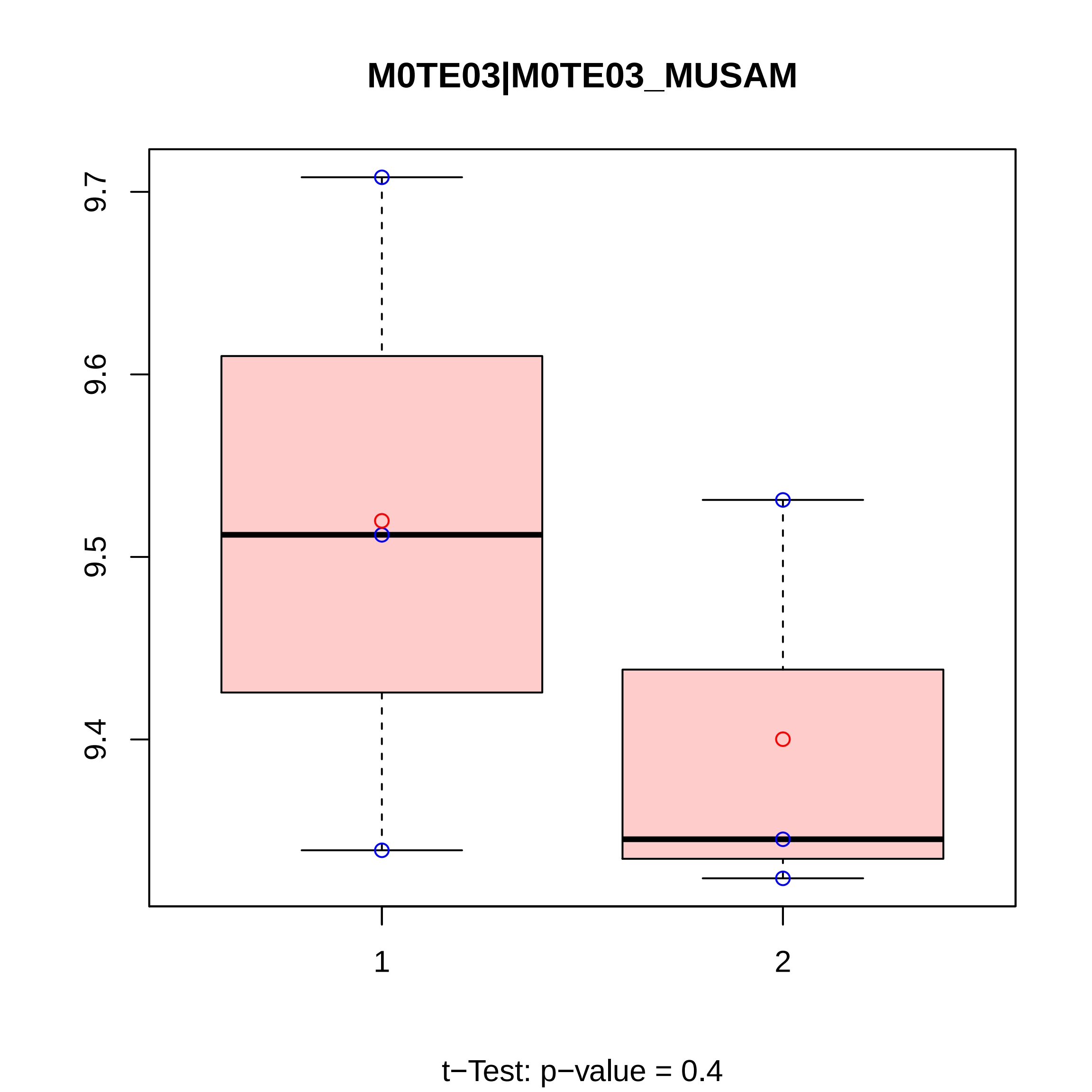
M0TE03|M0TE03_MUSAM
9.7
9.6
9.5
9.4
1
2
t−Test: p−value = 0.4

M0TEP4|M0TEP4_MUSAM

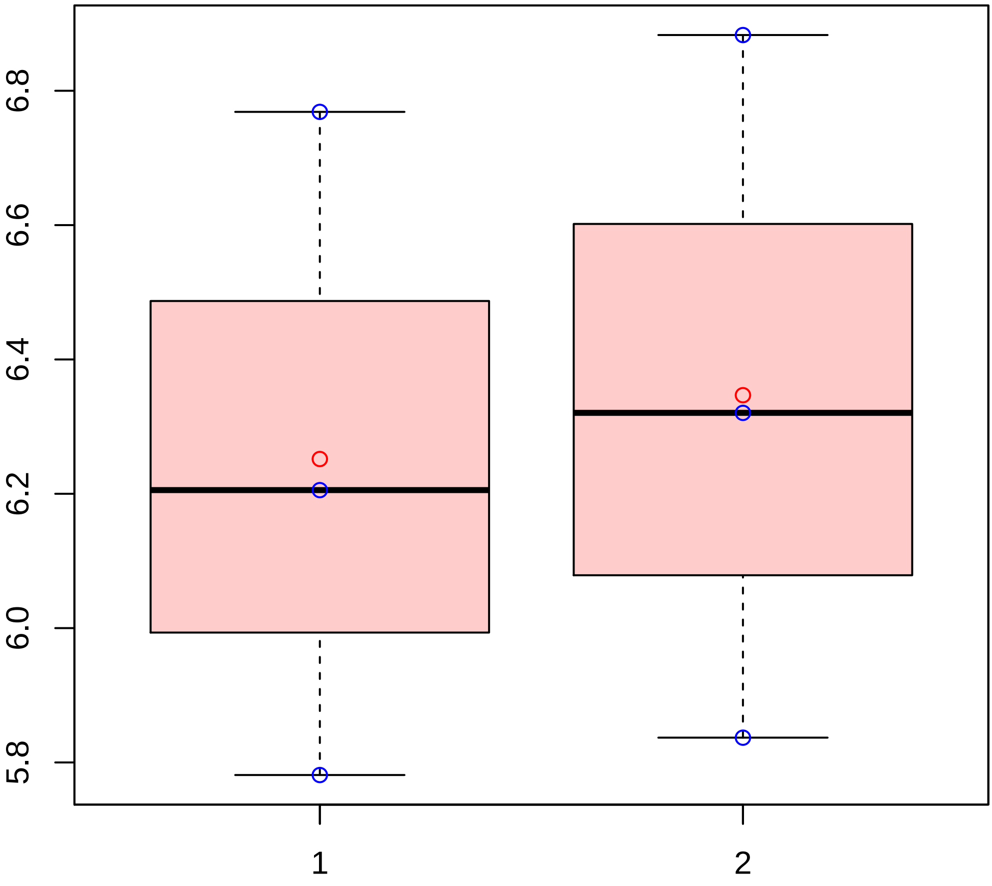

t−Test: p−value = 0.83

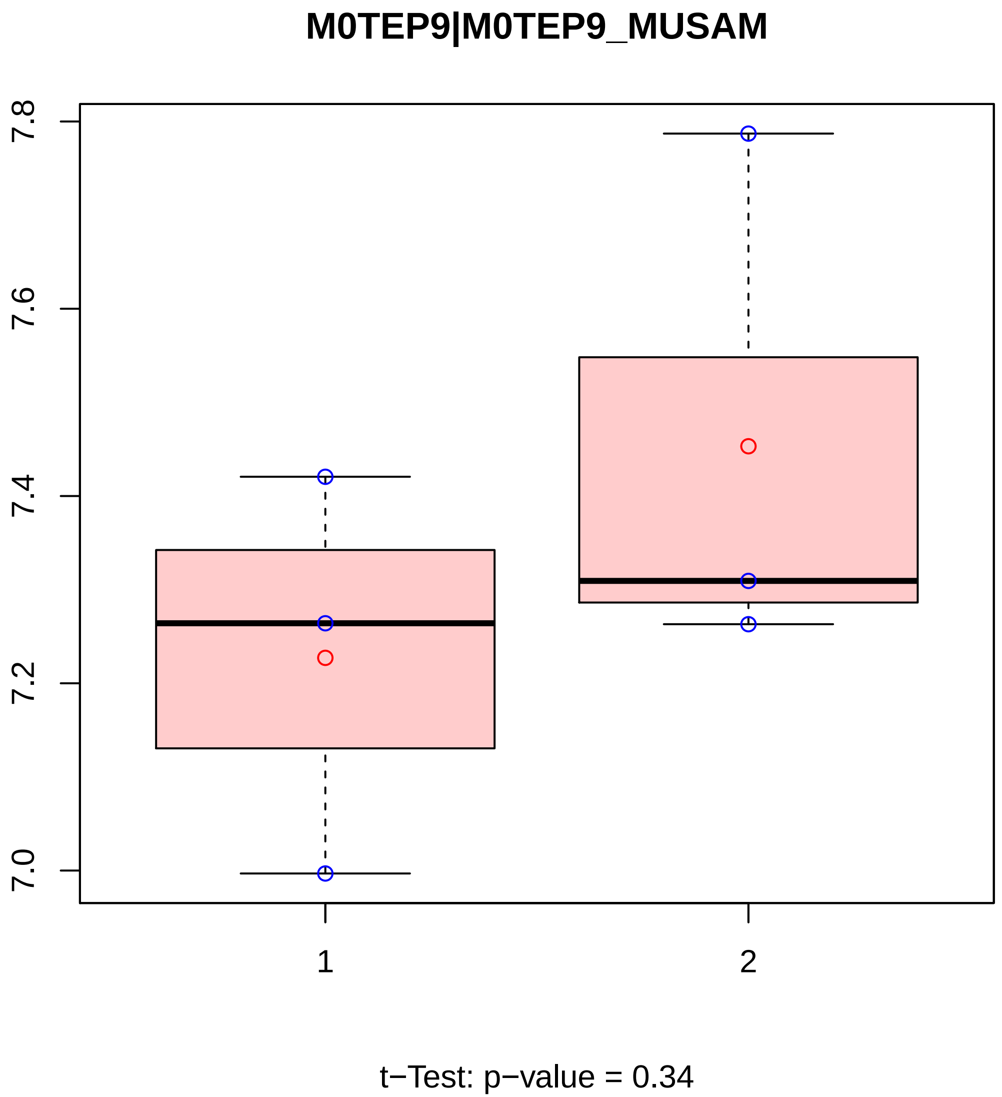

M0TEP9|M0TEP9_MUSAM
7.0
7.2
7.4
7.6
7.8
1
2
t−Test: p−value = 0.34

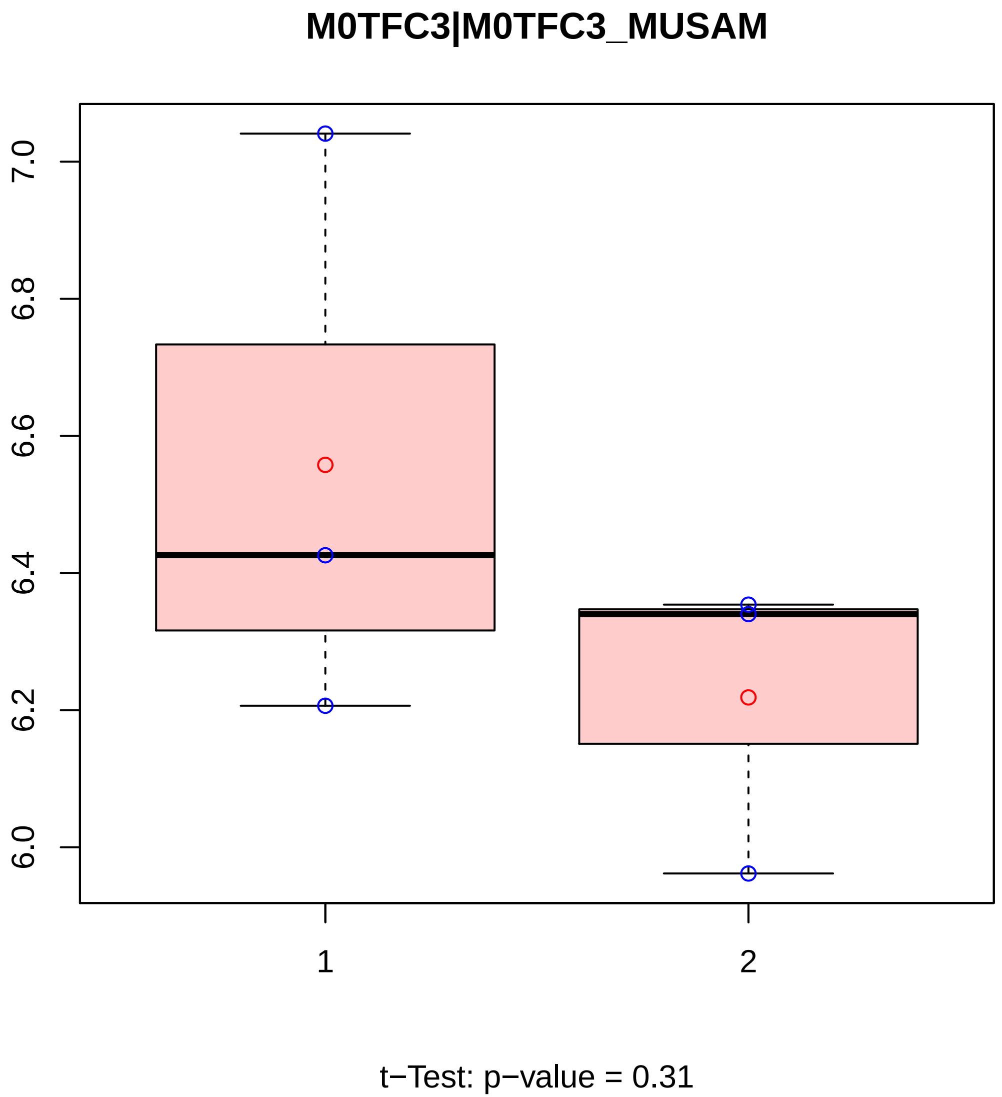
M0TFC3|M0TFC3_MUSAM
7.0
6.8
6.6
6.4
6.2
6.0
1
2
t−Test: p−value = 0.31

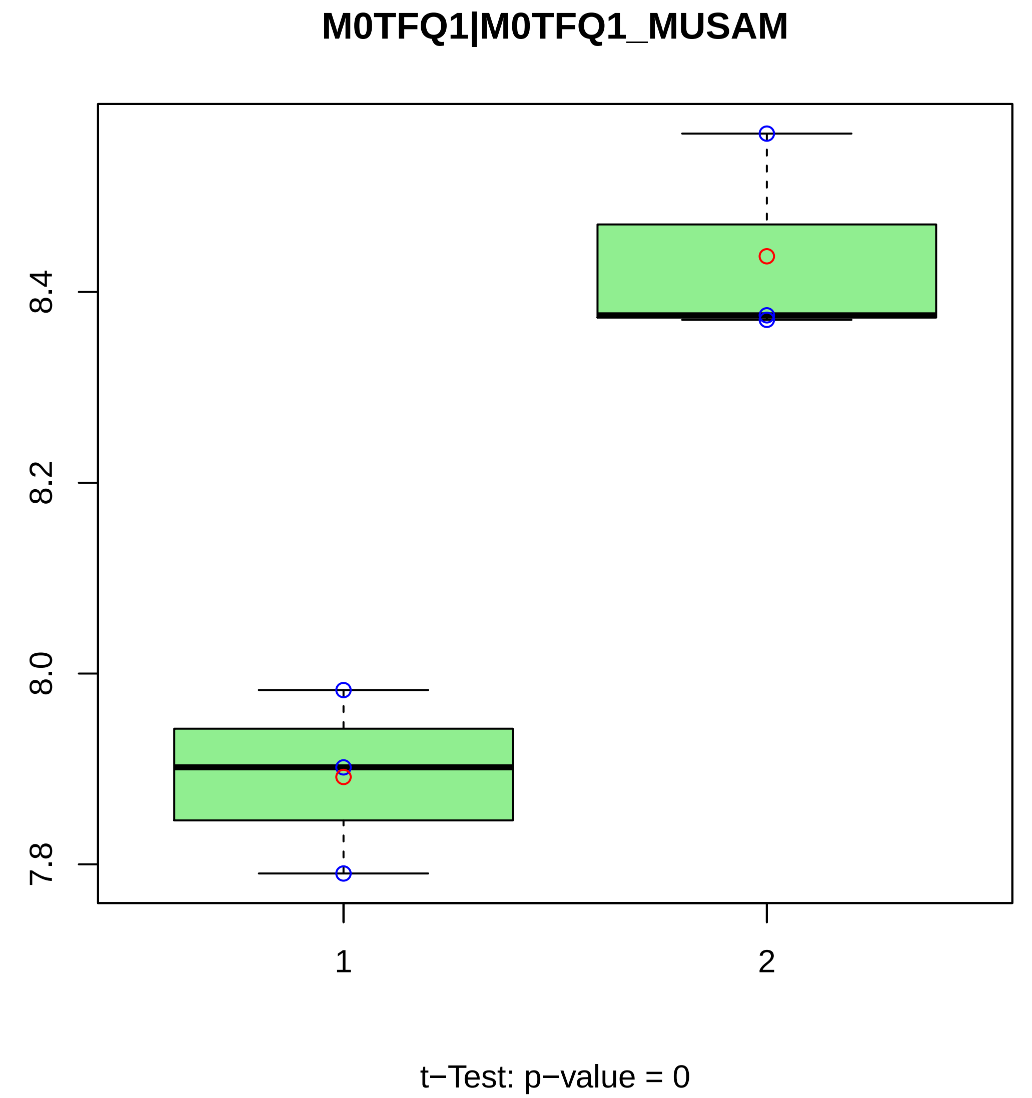
M0TFQ1|M0TFQ1_MUSAM
7.8
8.0
8.2
8.4
1
2
t−Test: p−value = 0

M0TG28|M0TG28_MUSAM

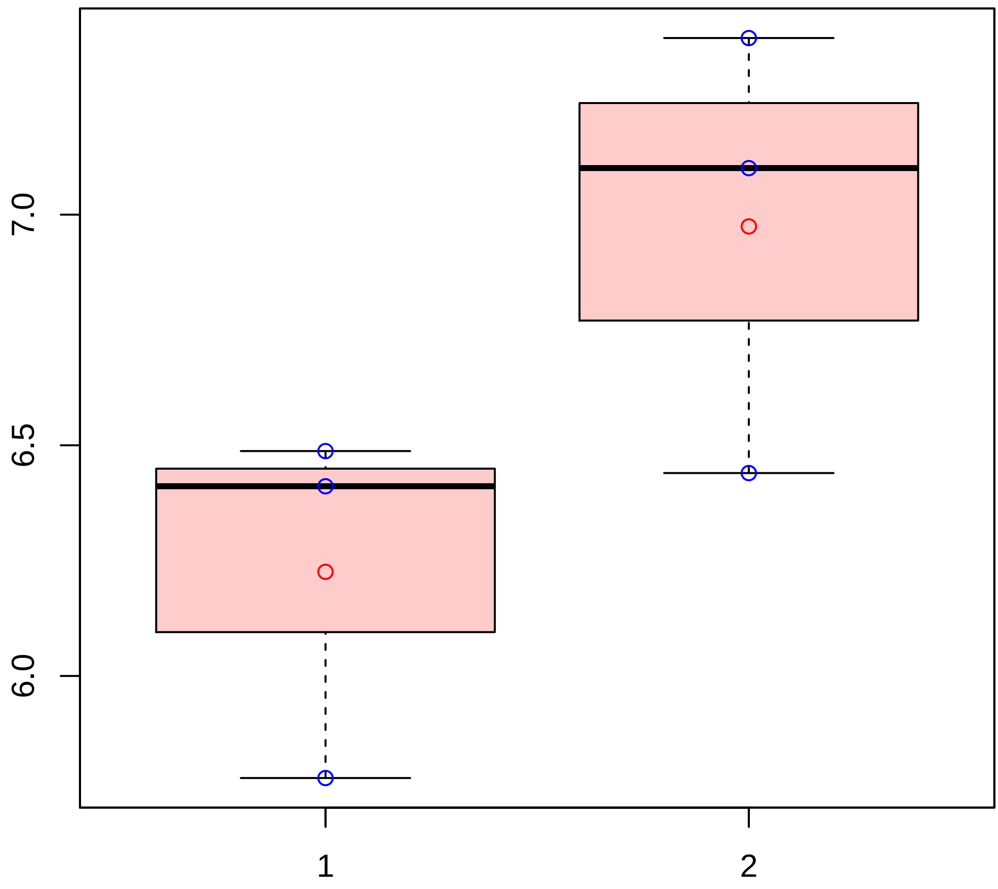

t−Test: p−value = 0.11

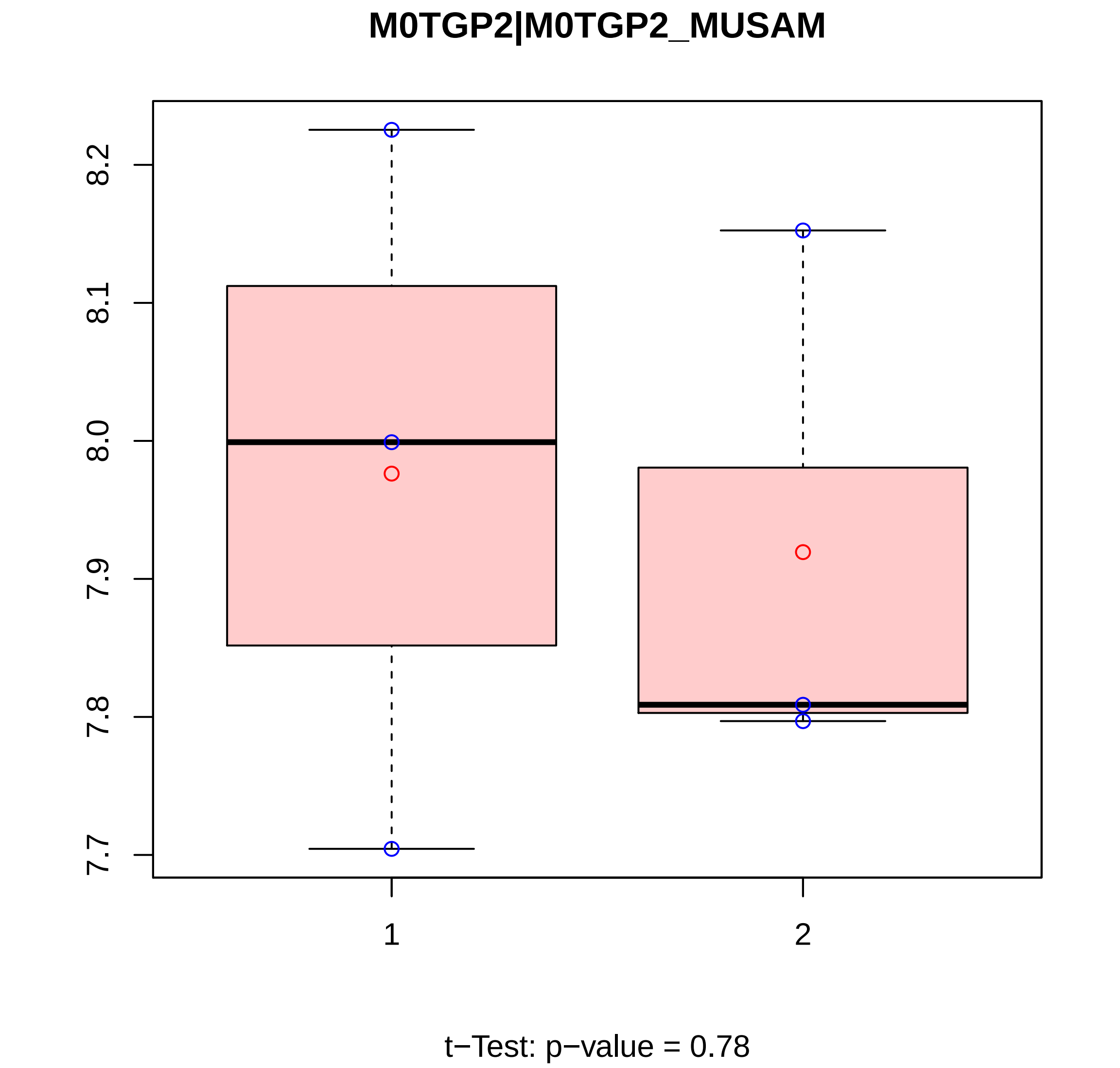
M0TGP2|M0TGP2_MUSAM
8.2
8.1
8.0
7.9
7.8
7.7
1
2
t−Test: p−value = 0.78

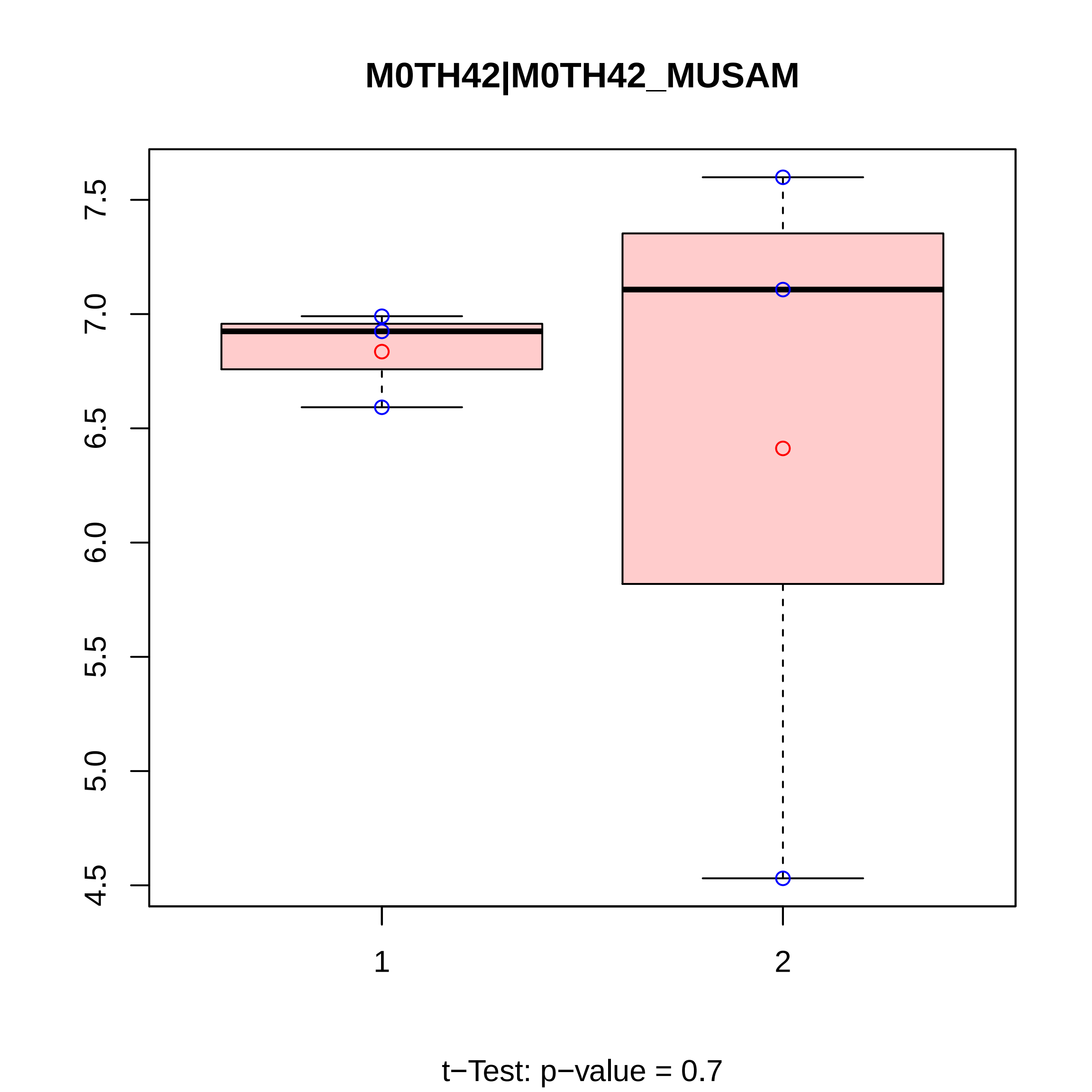

M0TH42|M0TH42_MUSAM
7.5
7.0
6.5
6.0
5.5
5.0
4.5
1
2
t−Test: p−value = 0.7

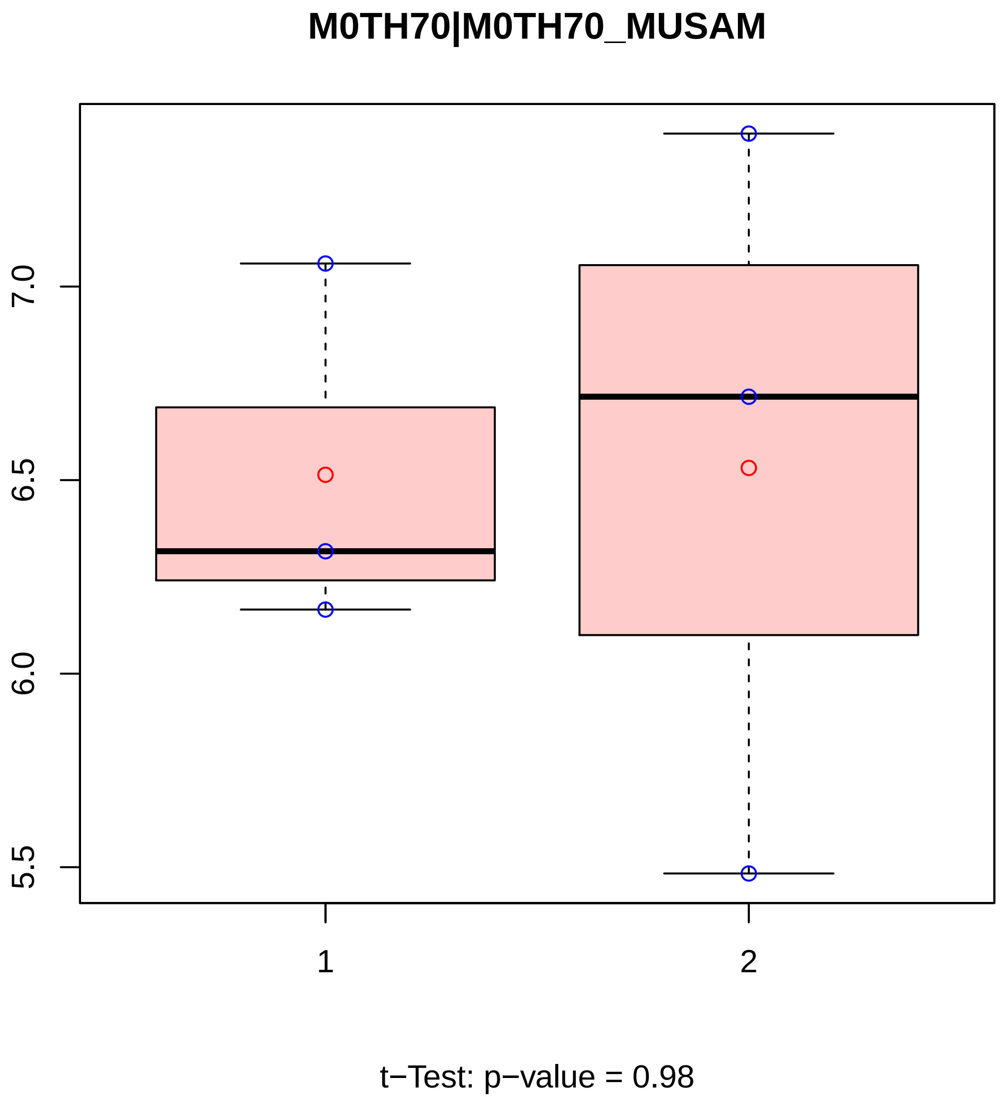
M0TH70|M0TH70_MUSAM
7.0
6.5
6.0
5.5
1
2
t−Test: p−value = 0.98

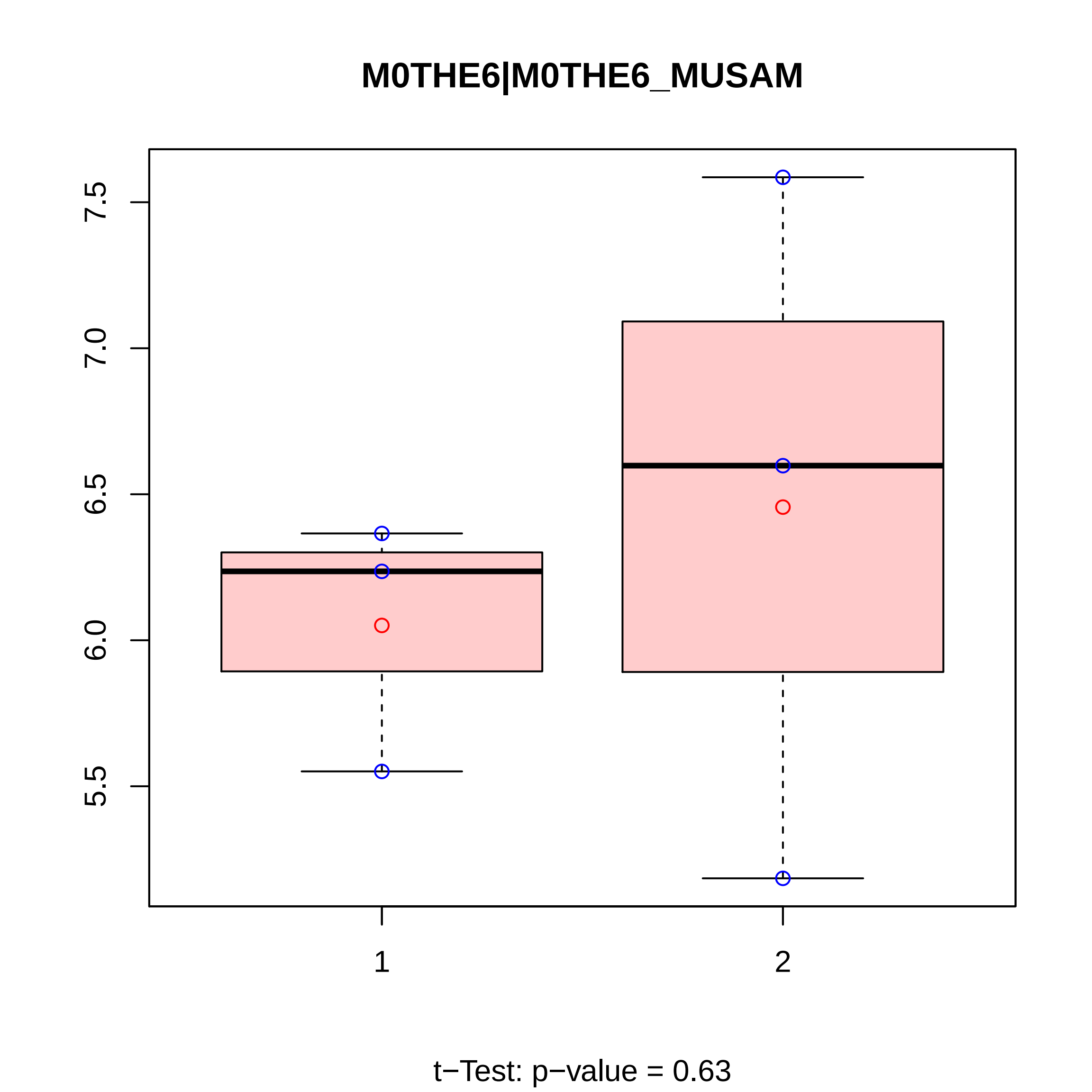
M0THE6|M0THE6_MUSAM
7.5
7.0
6.5
6.0
5.5
1
2
t−Test: p−value = 0.63

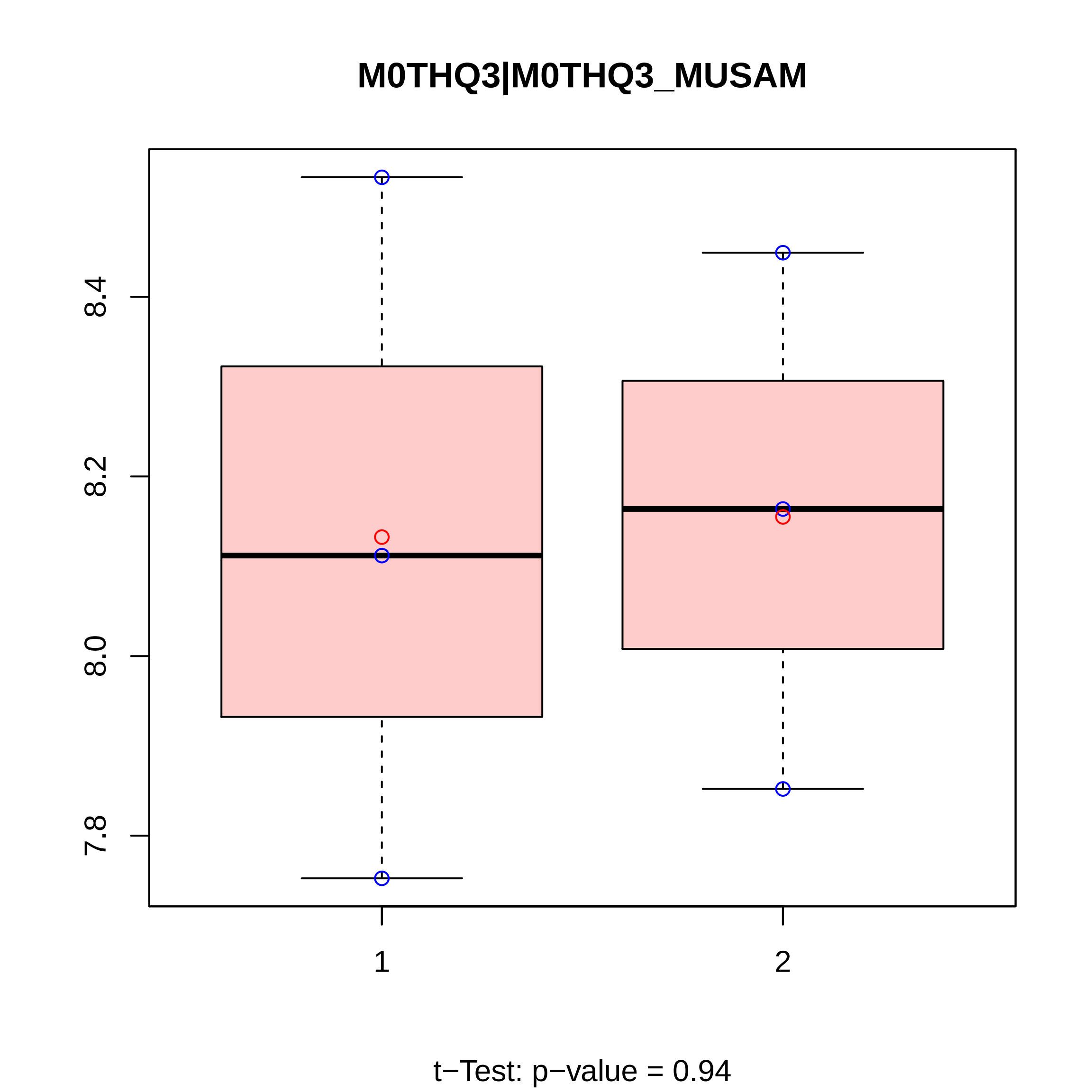
M0THQ3|M0THQ3_MUSAM
8.4
8.2
8.0
7.8
1
2
t−Test: p−value = 0.94

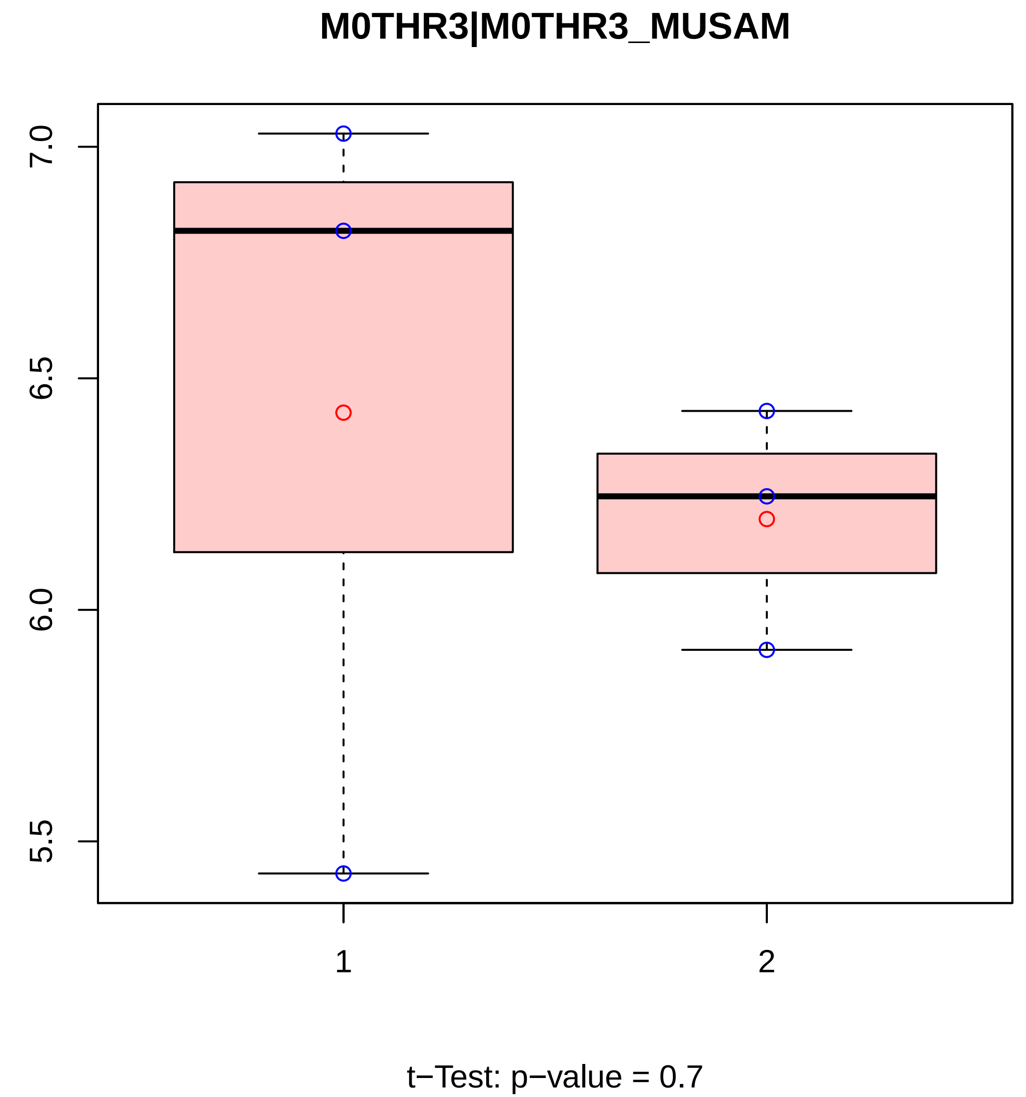
M0THR3|M0THR3_MUSAM
7.0
6.5
6.0
5.5
1
2
t−Test: p−value = 0.7

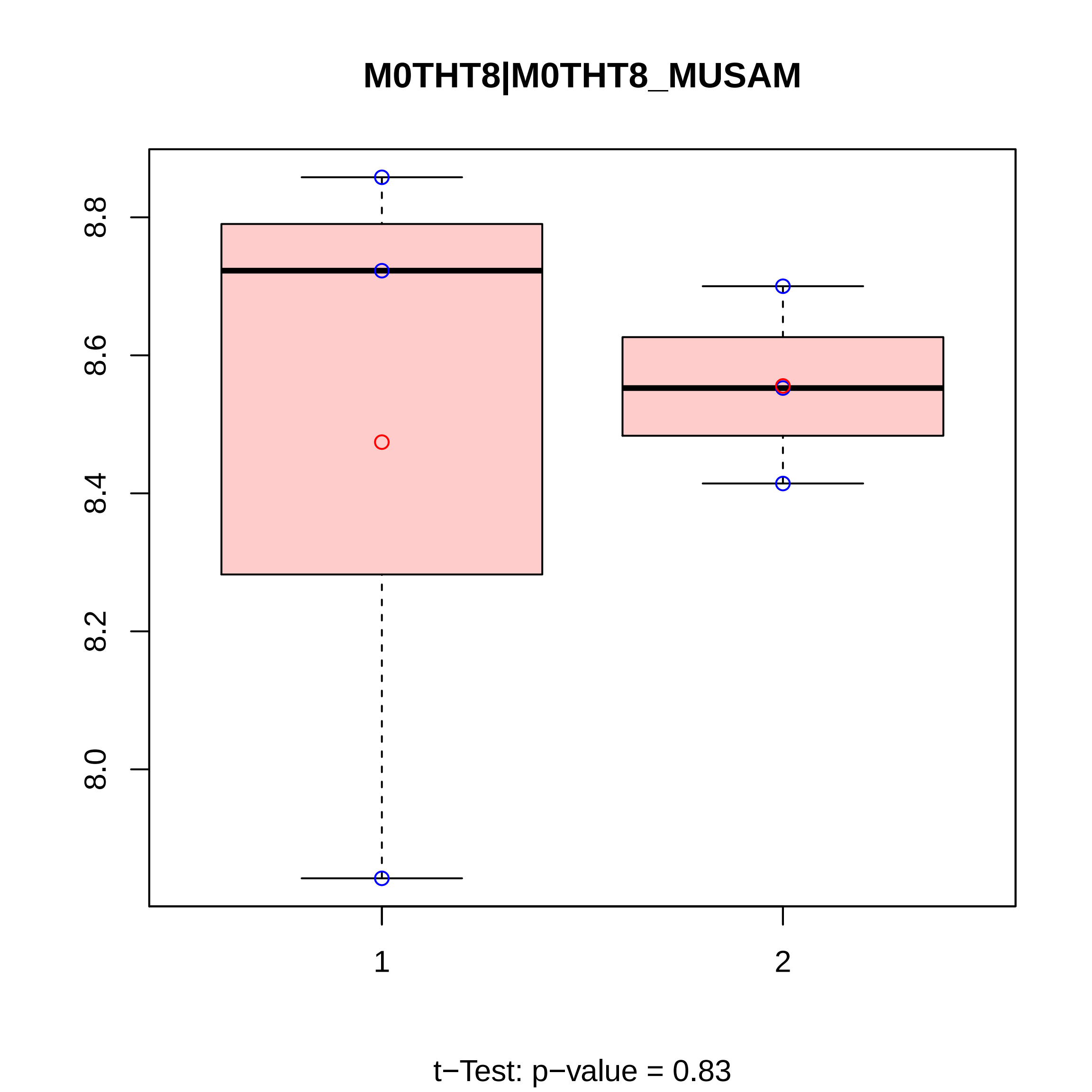
M0THT8|M0THT8_MUSAM
8.8
8.6
8.4
8.2
8.0
1
2
t−Test: p−value = 0.83

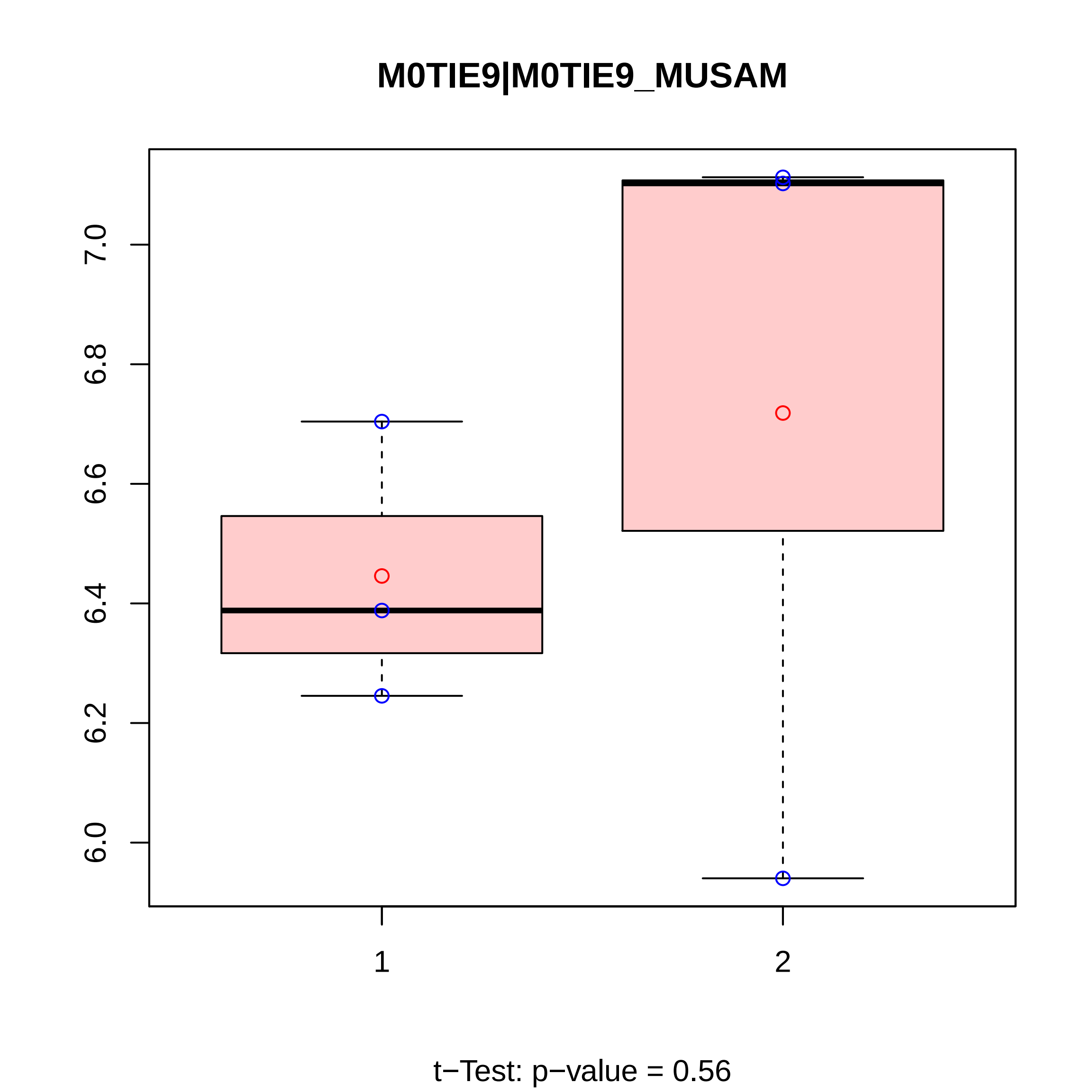

M0TIE9|M0TIE9_MUSAM
7.0
6.8
6.6
6.4
6.2
6.0
1
2
t−Test: p−value = 0.56

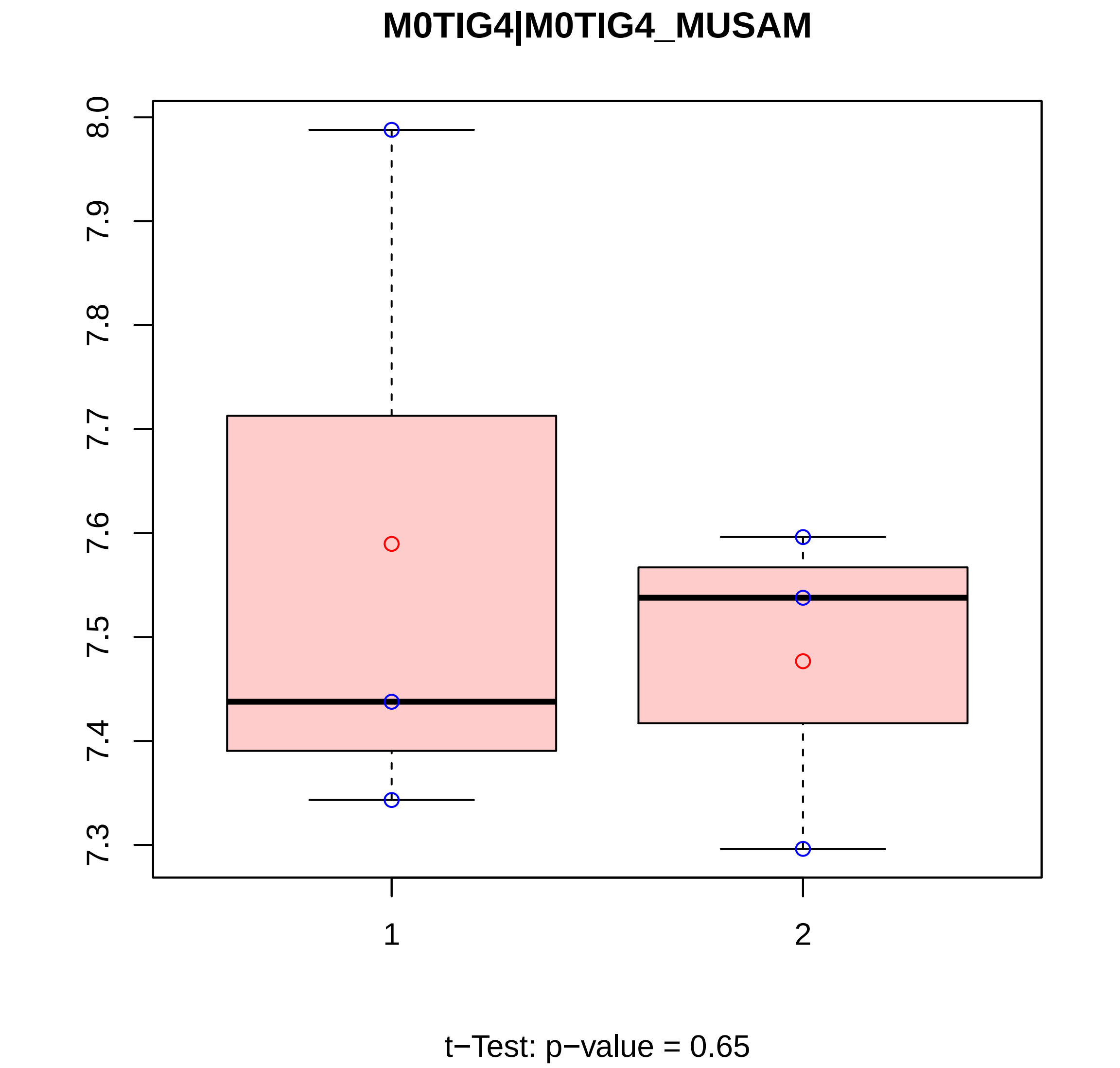
M0TIG4|M0TIG4_MUSAM
8.0
7.9
7.8
7.7
7.6
7.5
7.4
7.3
1
2
t−Test: p−value = 0.65

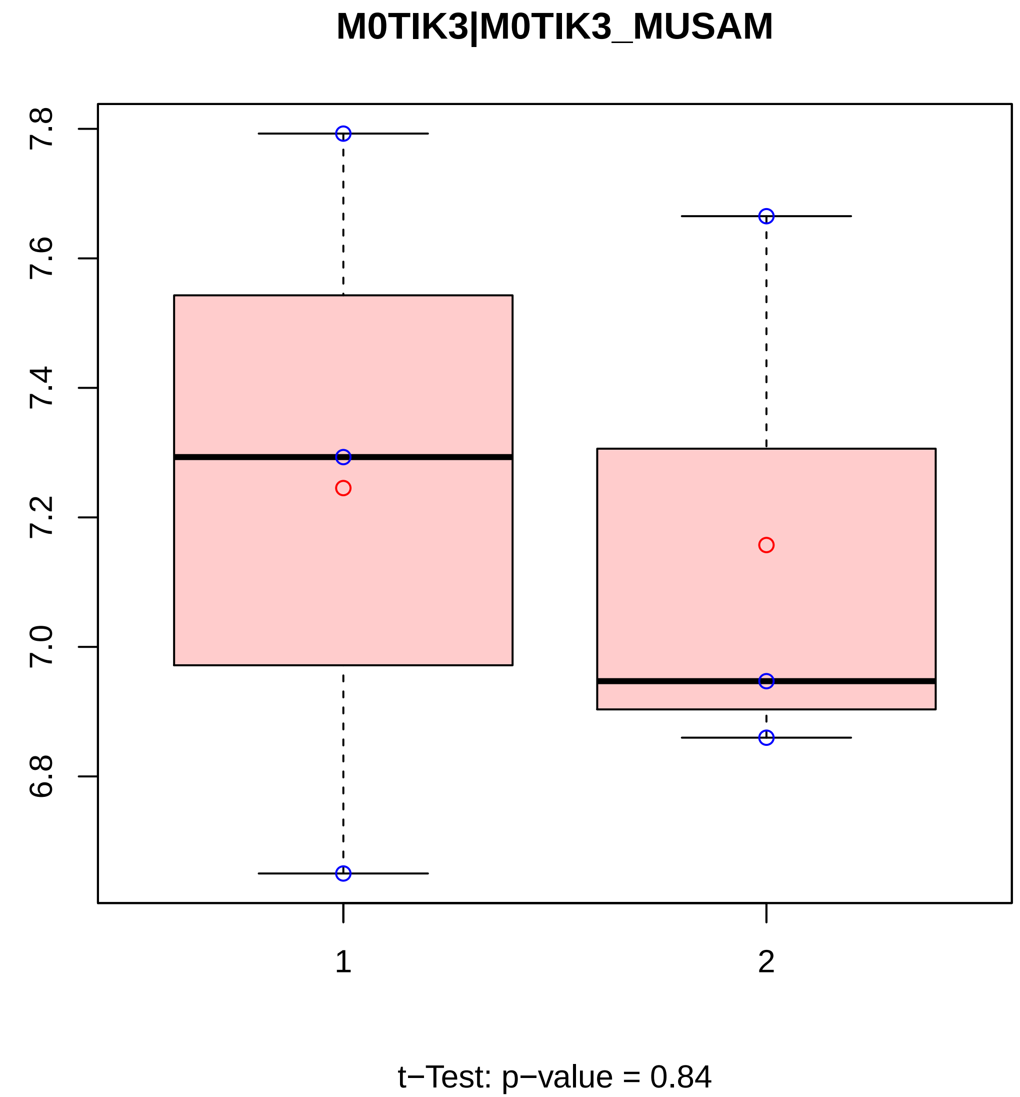
M0TIK3|M0TIK3_MUSAM
7.8
7.6
7.4
7.2
7.0
6.8
1
2
t−Test: p−value = 0.84

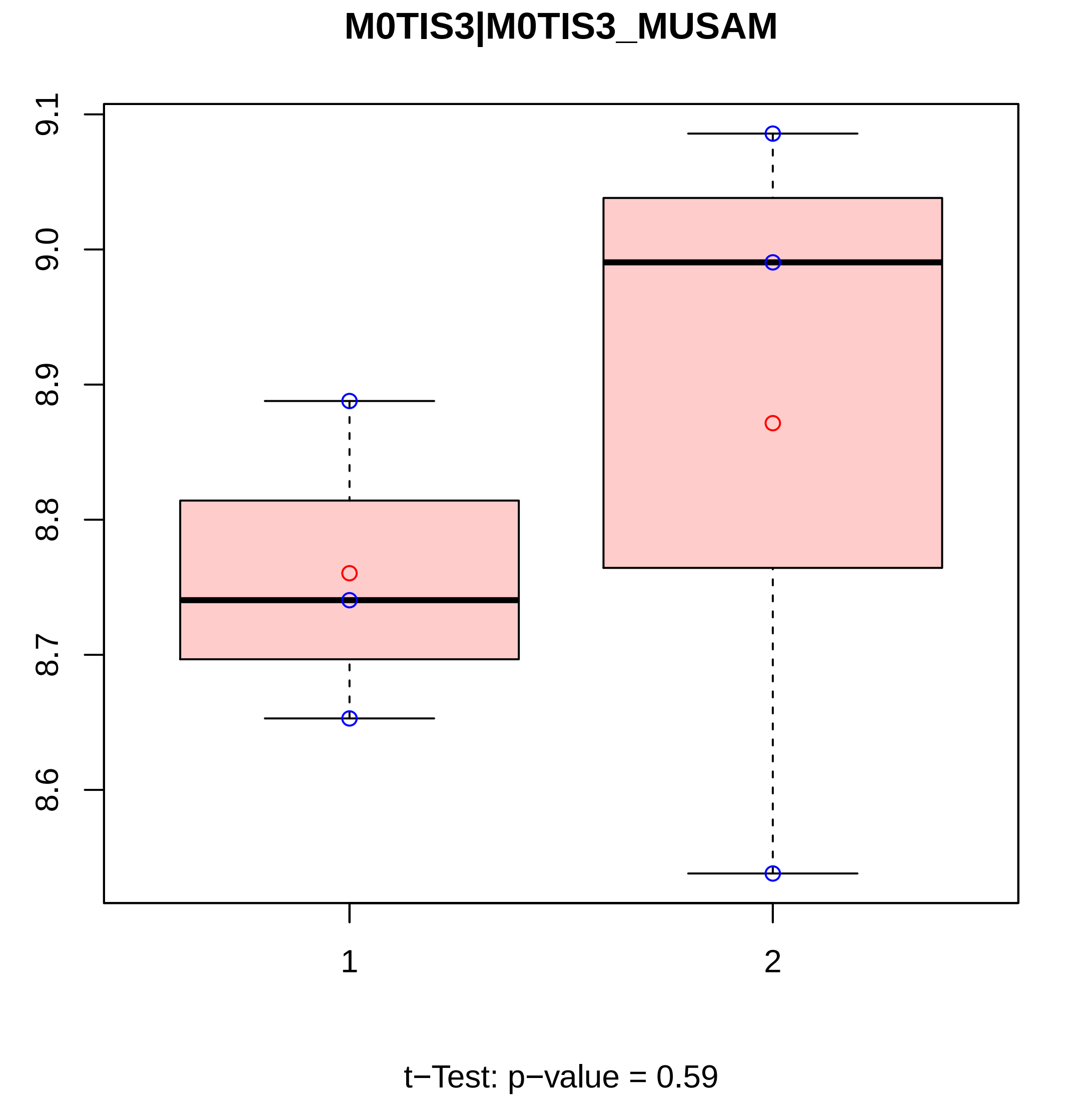

M0TIS3|M0TIS3_MUSAM
9.1
9.0
8.9
8.8
8.7
8.6
1
2
t−Test: p−value = 0.59

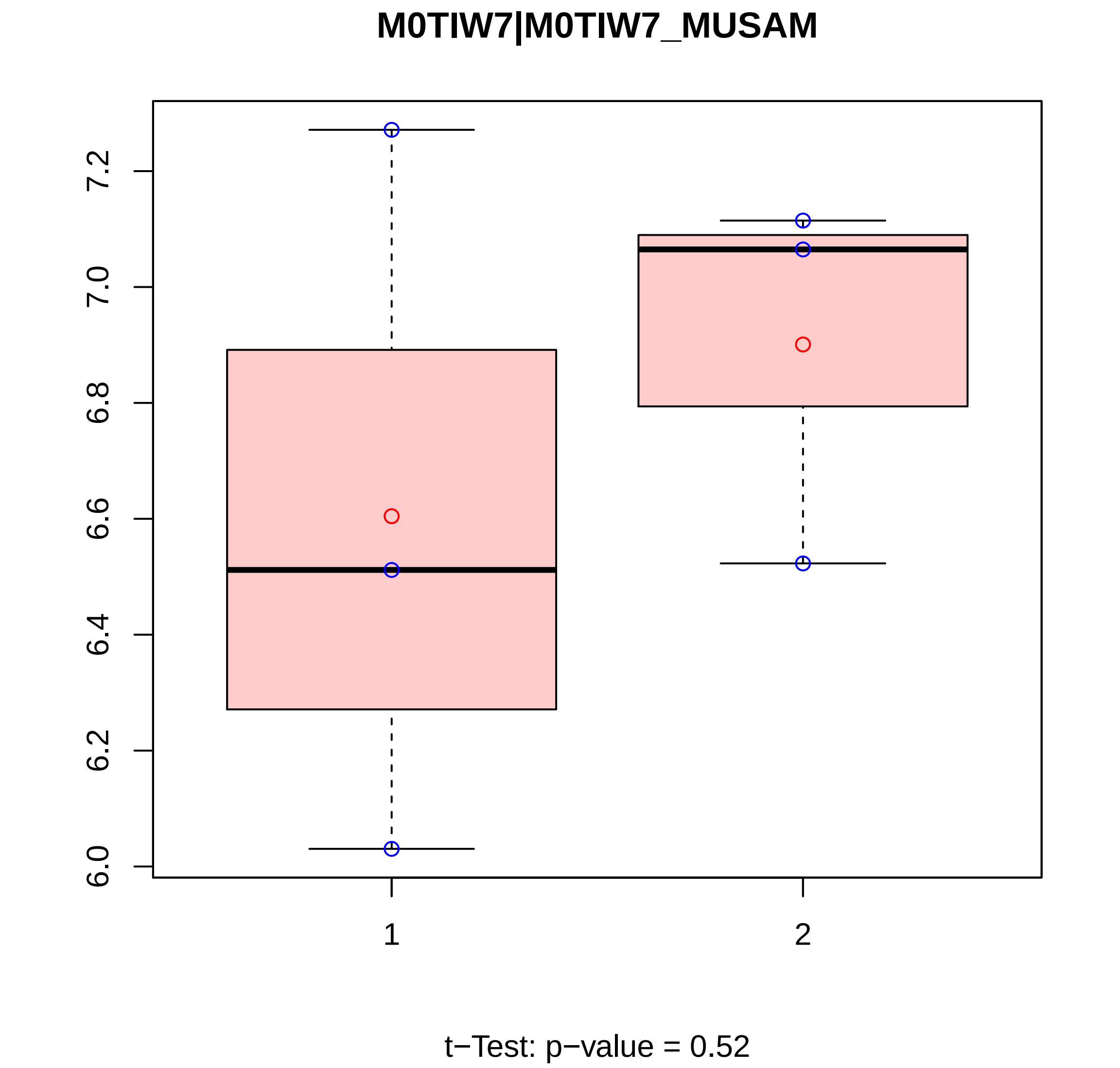
M0TIW7|M0TIW7_MUSAM
6.0
6.2
6.4
6.6
6.8
7.0
7.2
1
2
t−Test: p−value = 0.52

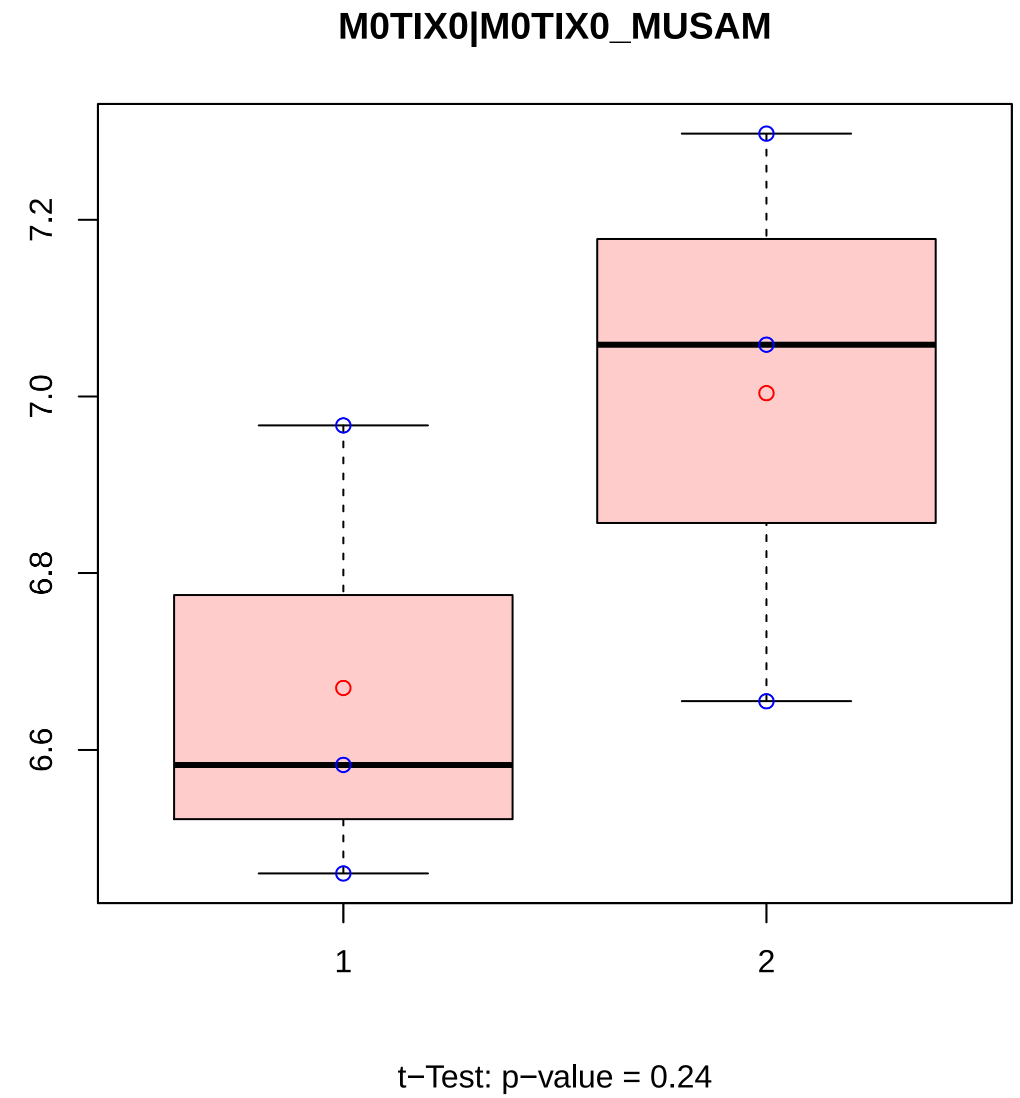
M0TIX0|M0TIX0_MUSAM
7.2
7.0
6.8
6.6
1
2
t−Test: p−value = 0.24

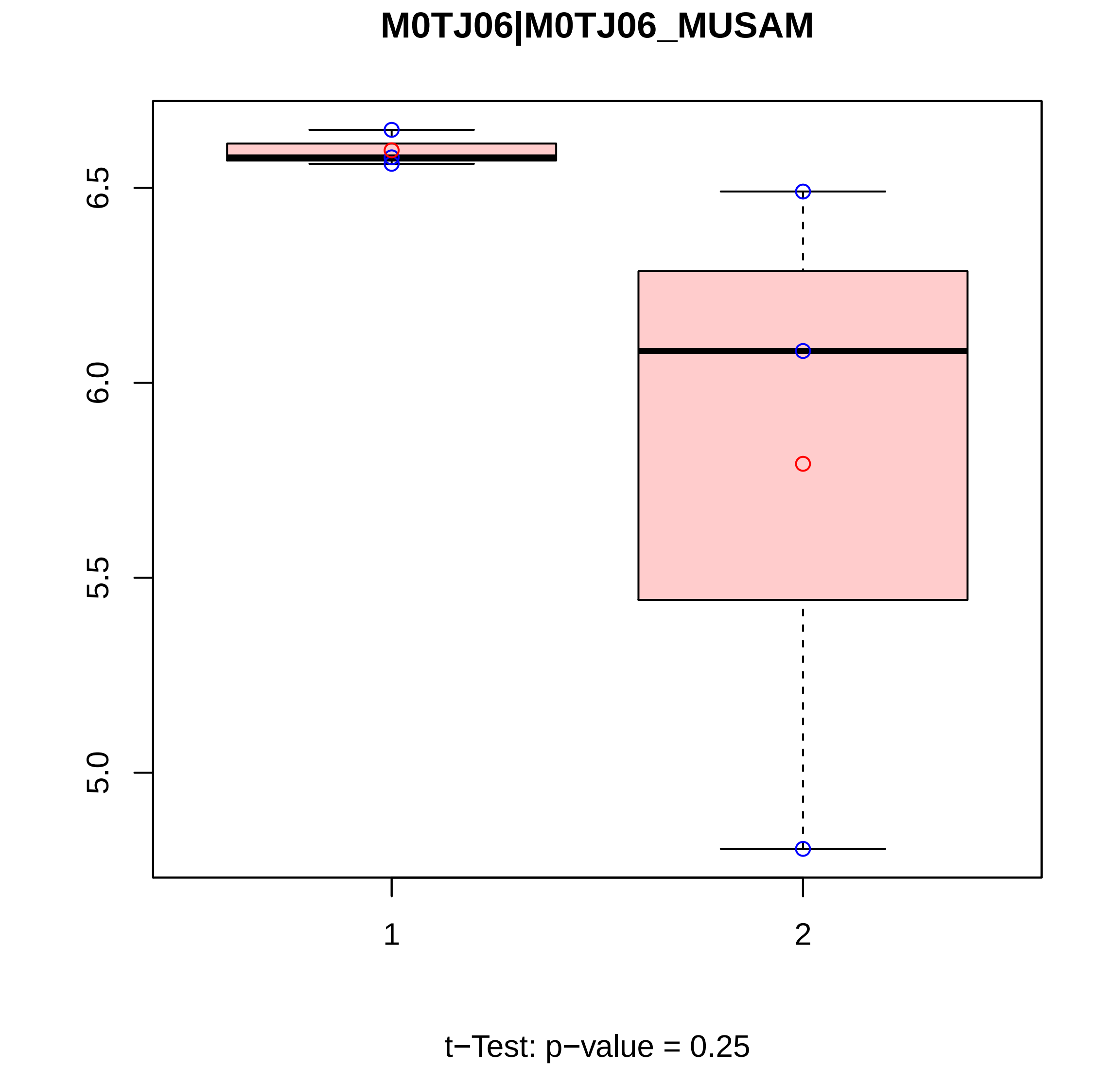

M0TJ06|M0TJ06_MUSAM
6.5
6.0
5.5
5.0
1
2
t−Test: p−value = 0.25

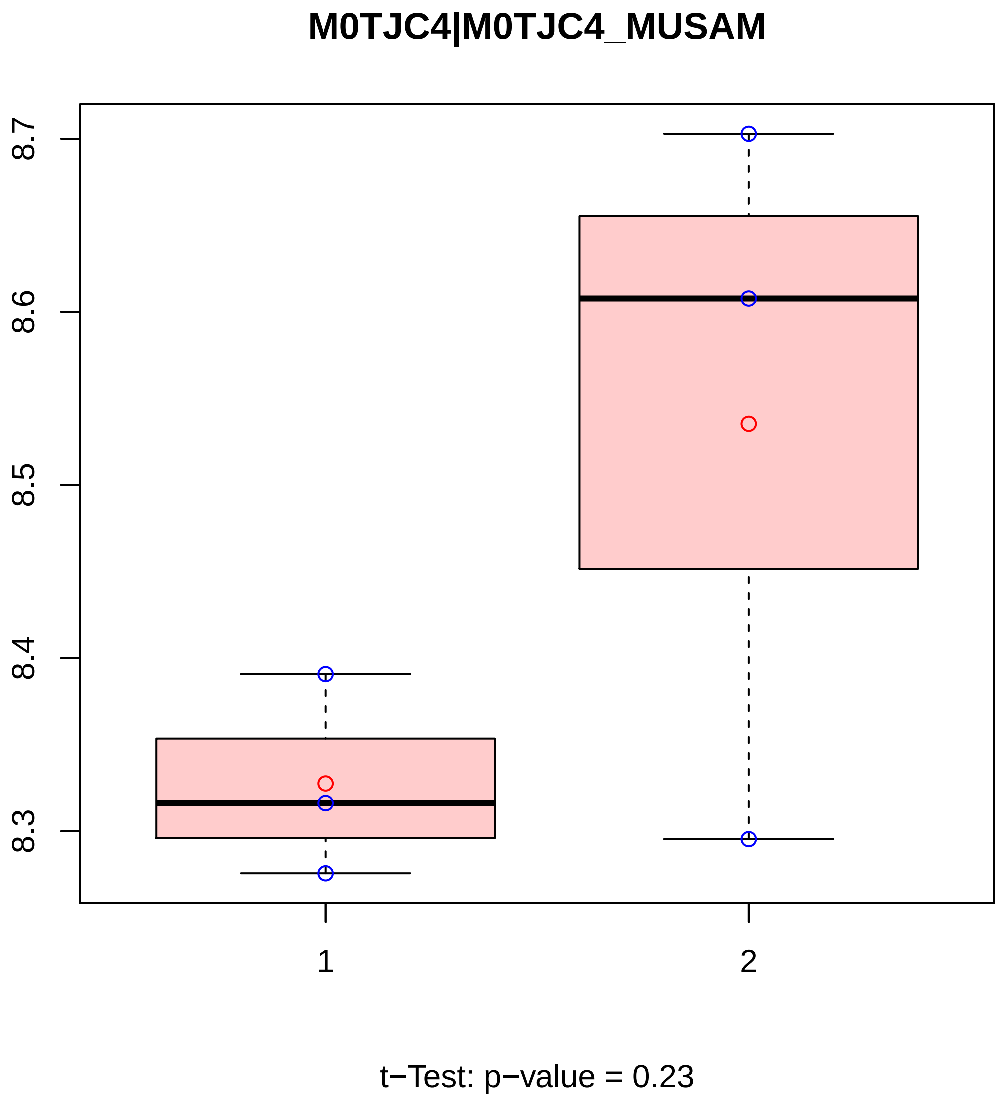

M0TJC4|M0TJC4_MUSAM
8.3
8.4
8.5
8.6
8.7
1
2
t−Test: p−value = 0.23

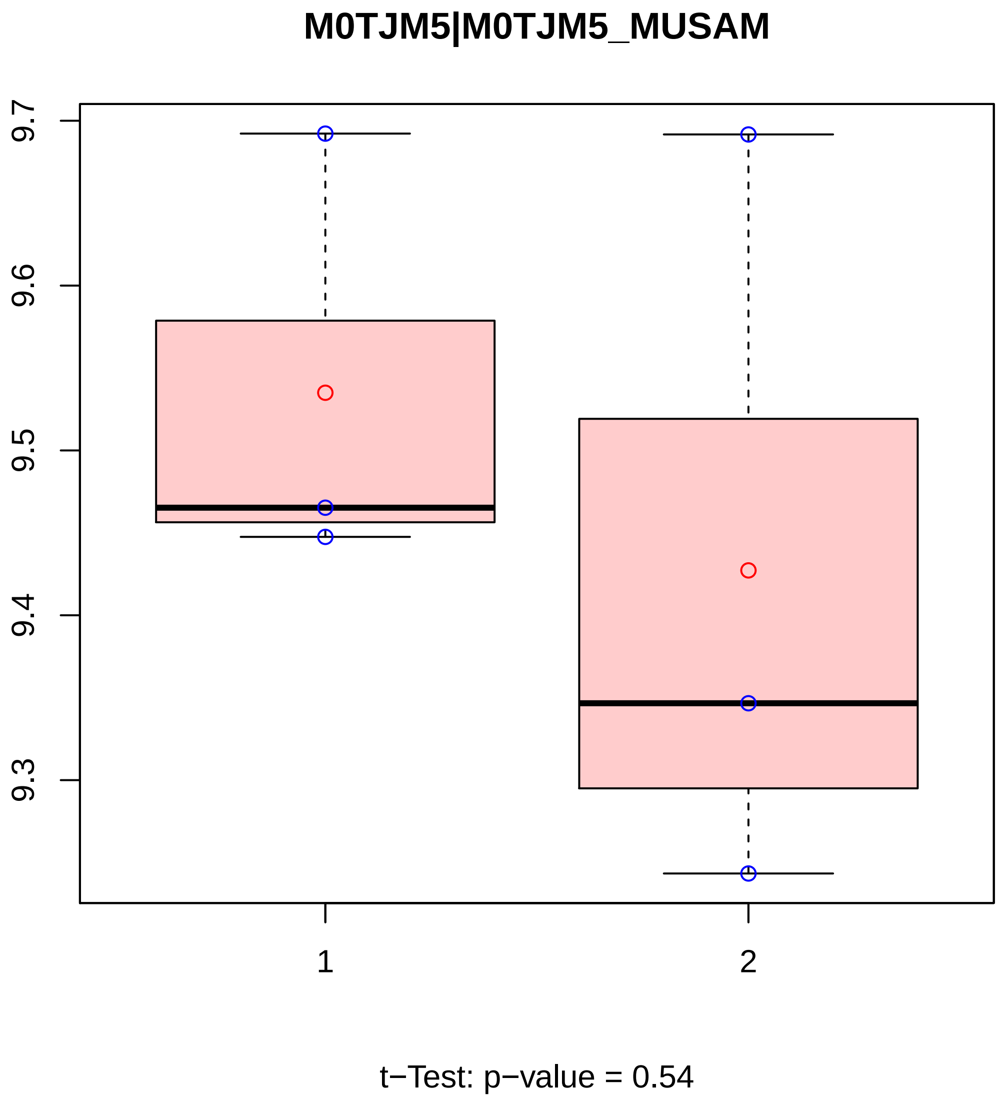
M0TJM5|M0TJM5_MUSAM
9.3
9.4
9.5
9.6
9.7
1
2
t−Test: p−value = 0.54

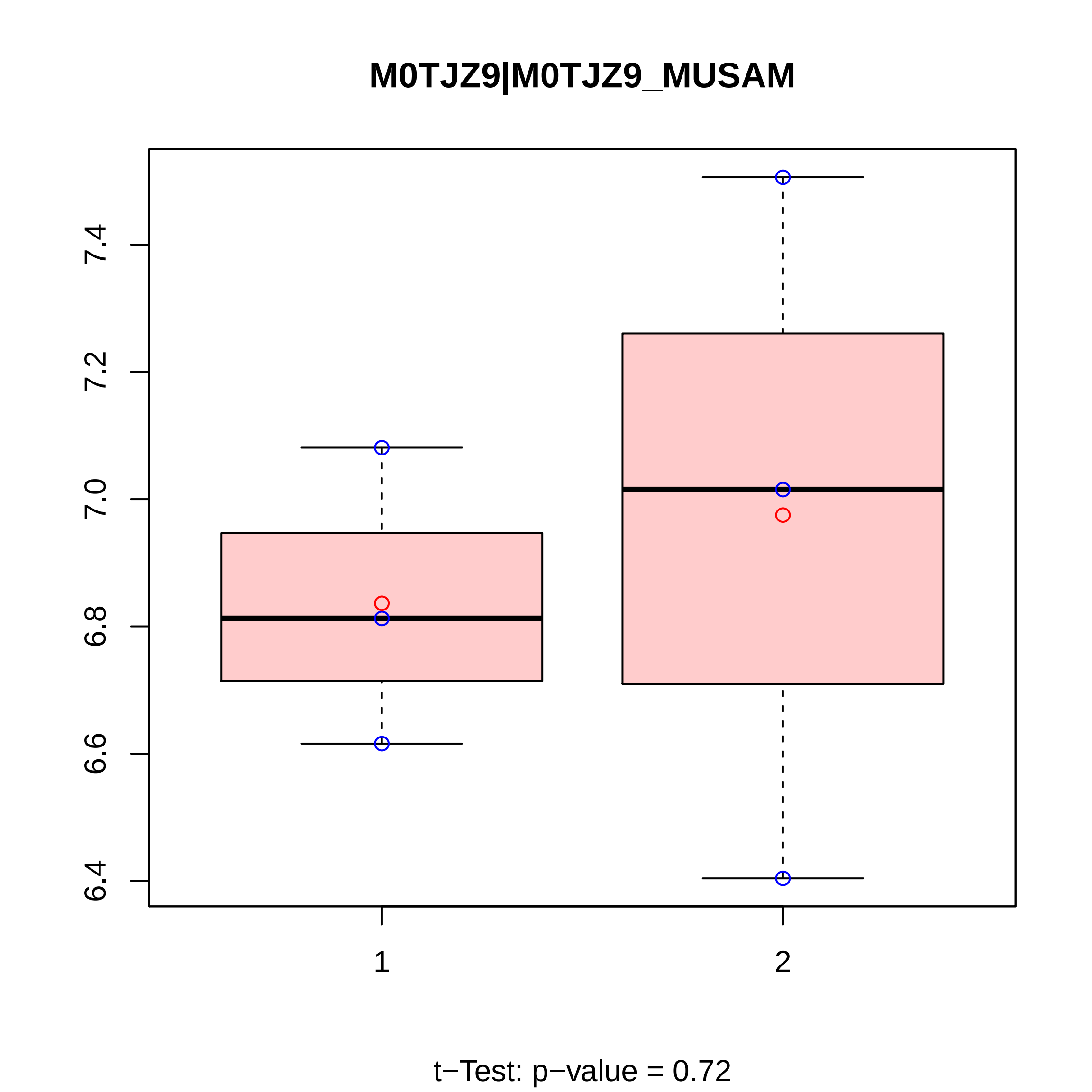
M0TJZ9|M0TJZ9_MUSAM
7.4
7.2
7.0
6.8
6.6
6.4
1
2
t−Test: p−value = 0.72

M0TK35|M0TK35_MUSAM

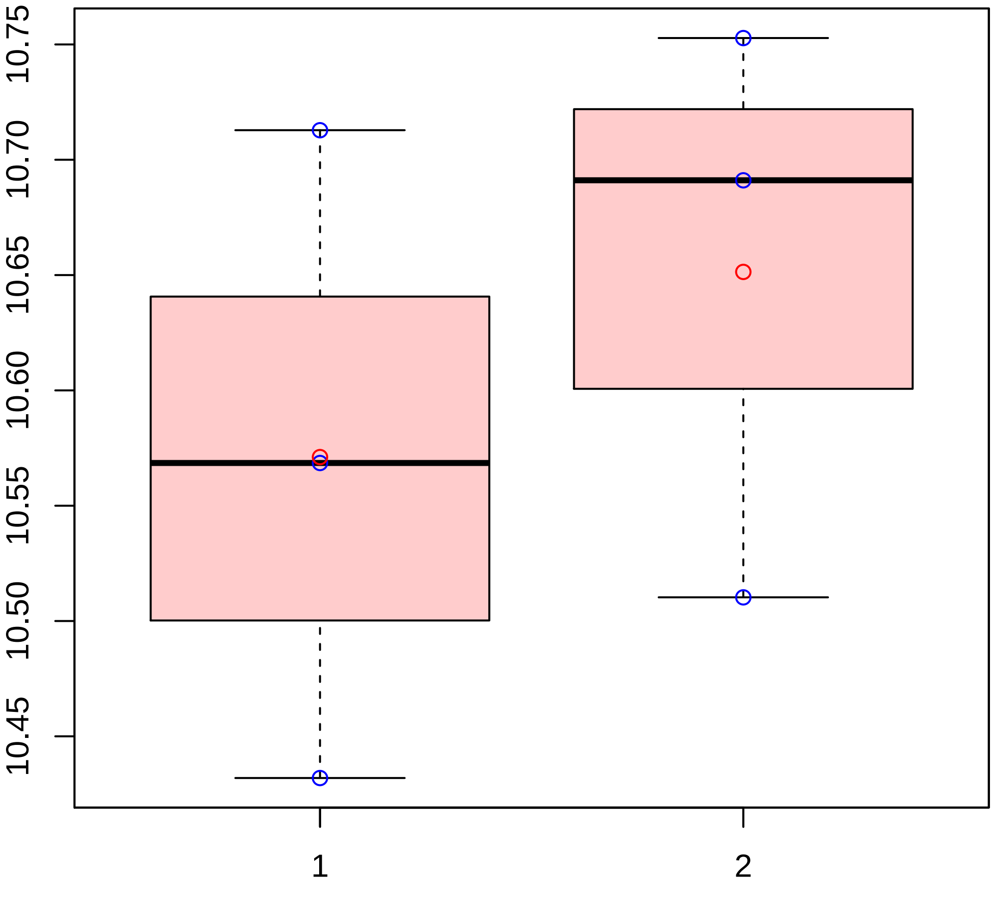

t−Test: p−value = 0.5

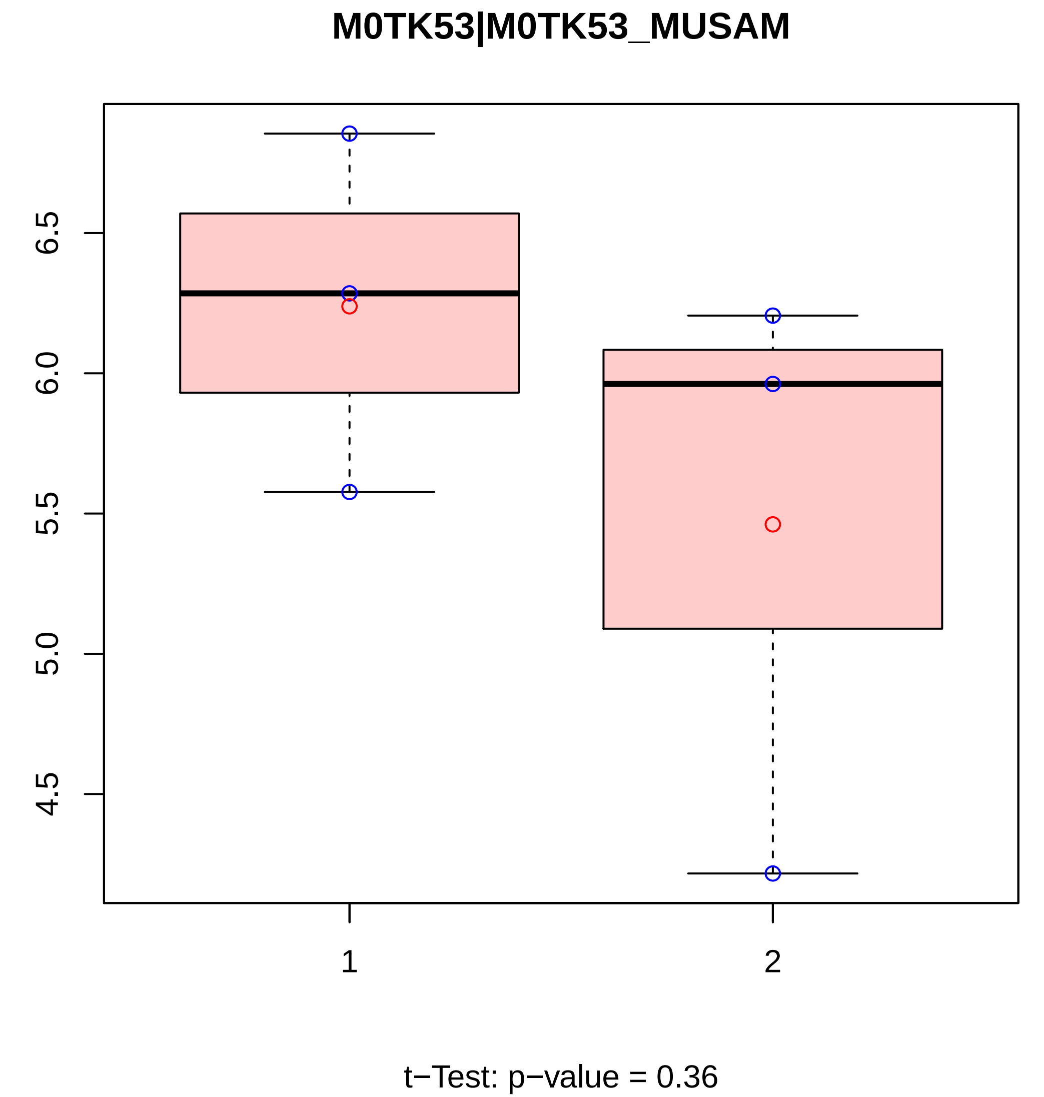
M0TK53|M0TK53_MUSAM
6.5
6.0
5.5
5.0
4.5
1
2
t−Test: p−value = 0.36

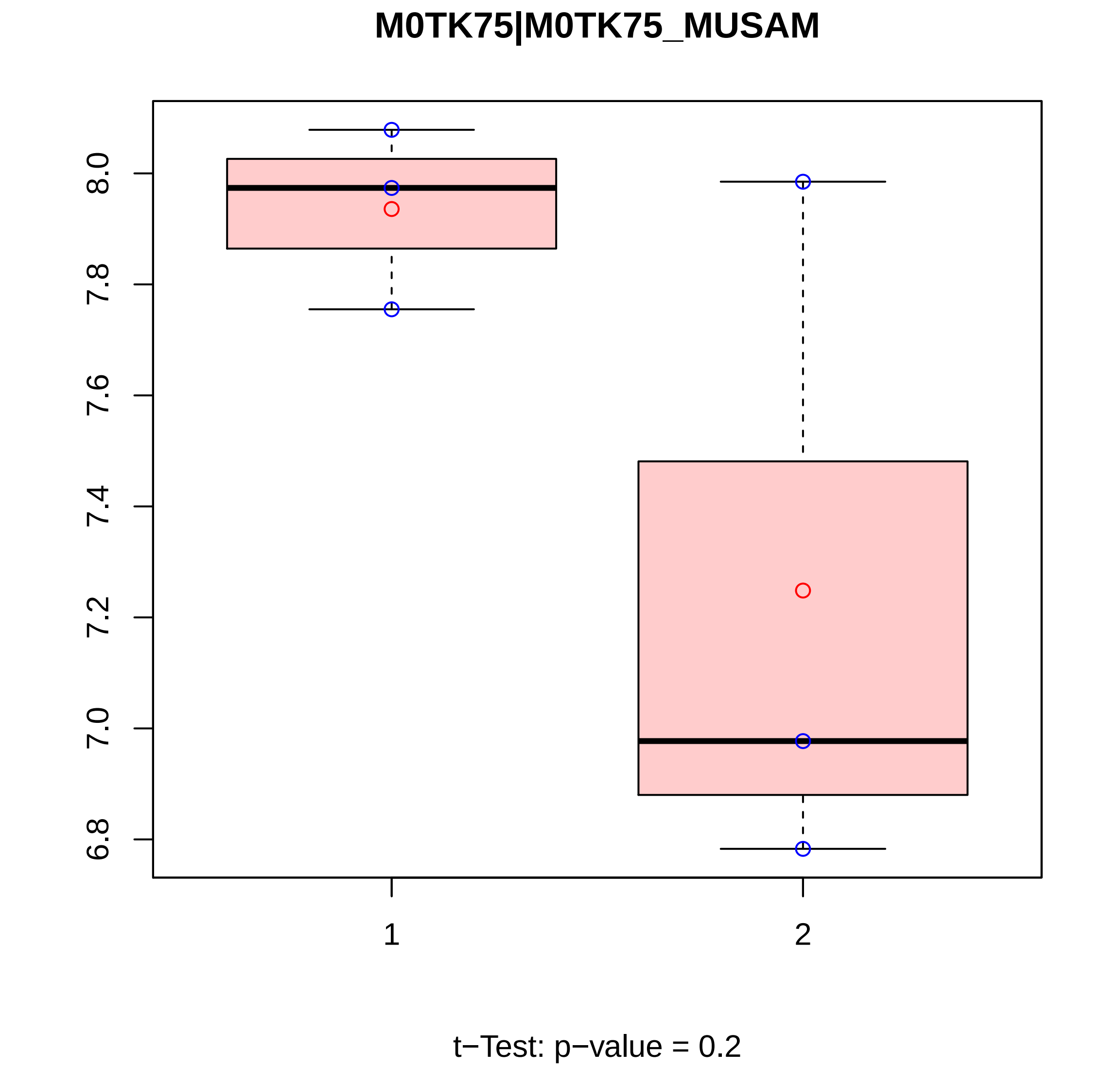
M0TK75|M0TK75_MUSAM
8.0
7.8
7.6
7.4
7.2
7.0
6.8
1
2
t−Test: p−value = 0.2

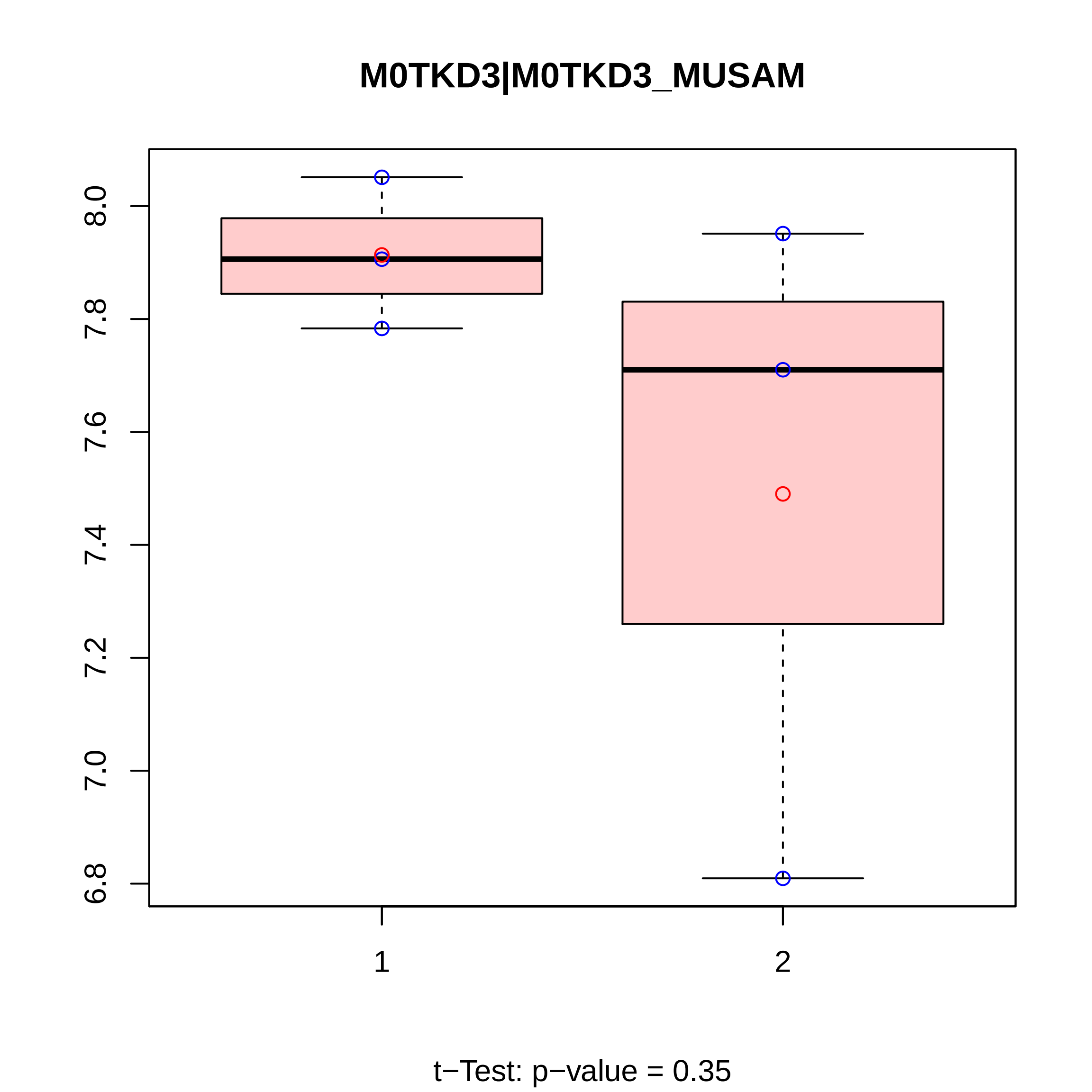
M0TKD3|M0TKD3_MUSAM
8.0
7.8
7.6
7.4
7.2
7.0
6.8
1
2
t−Test: p−value = 0.35

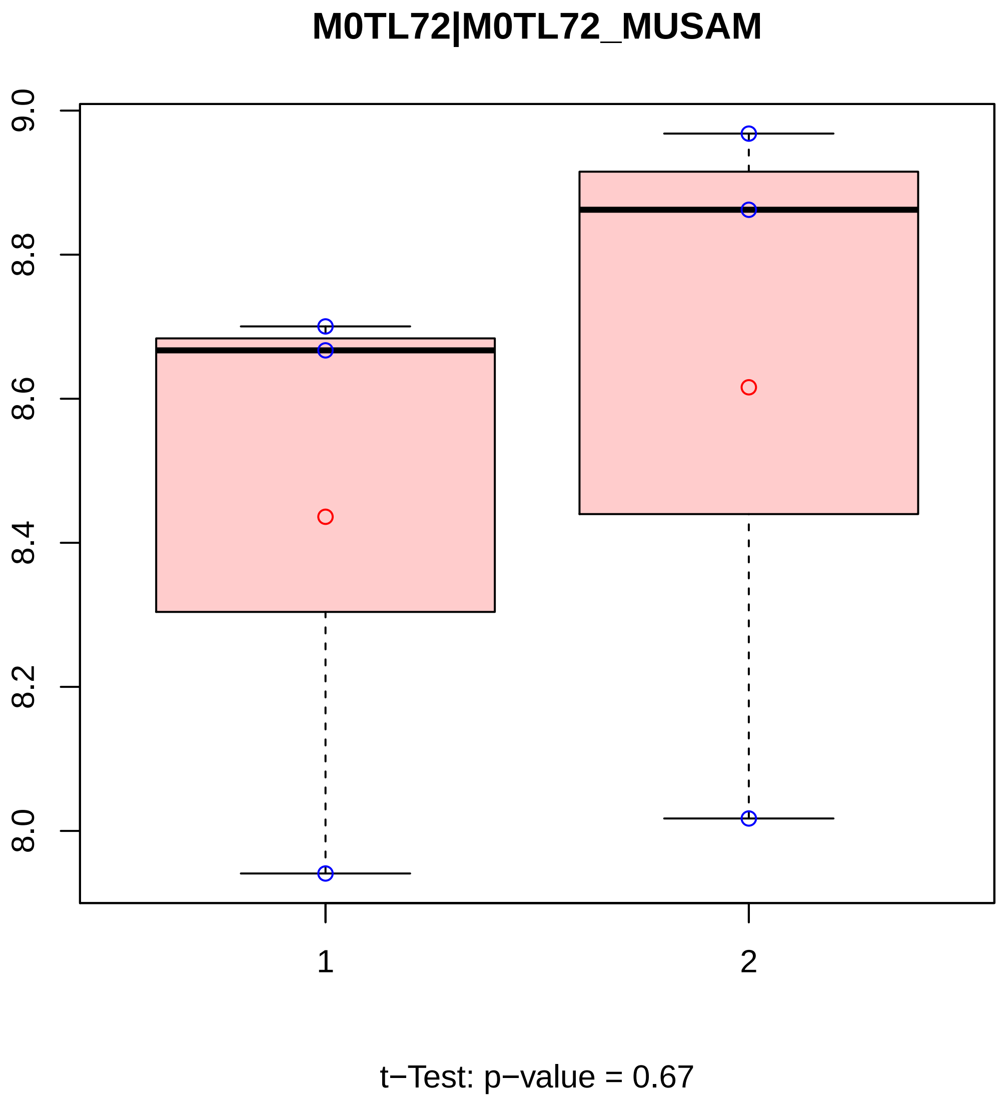
M0TL72|M0TL72_MUSAM
9.0
8.8
8.6
8.4
8.2
8.0
1
2
t−Test: p−value = 0.67

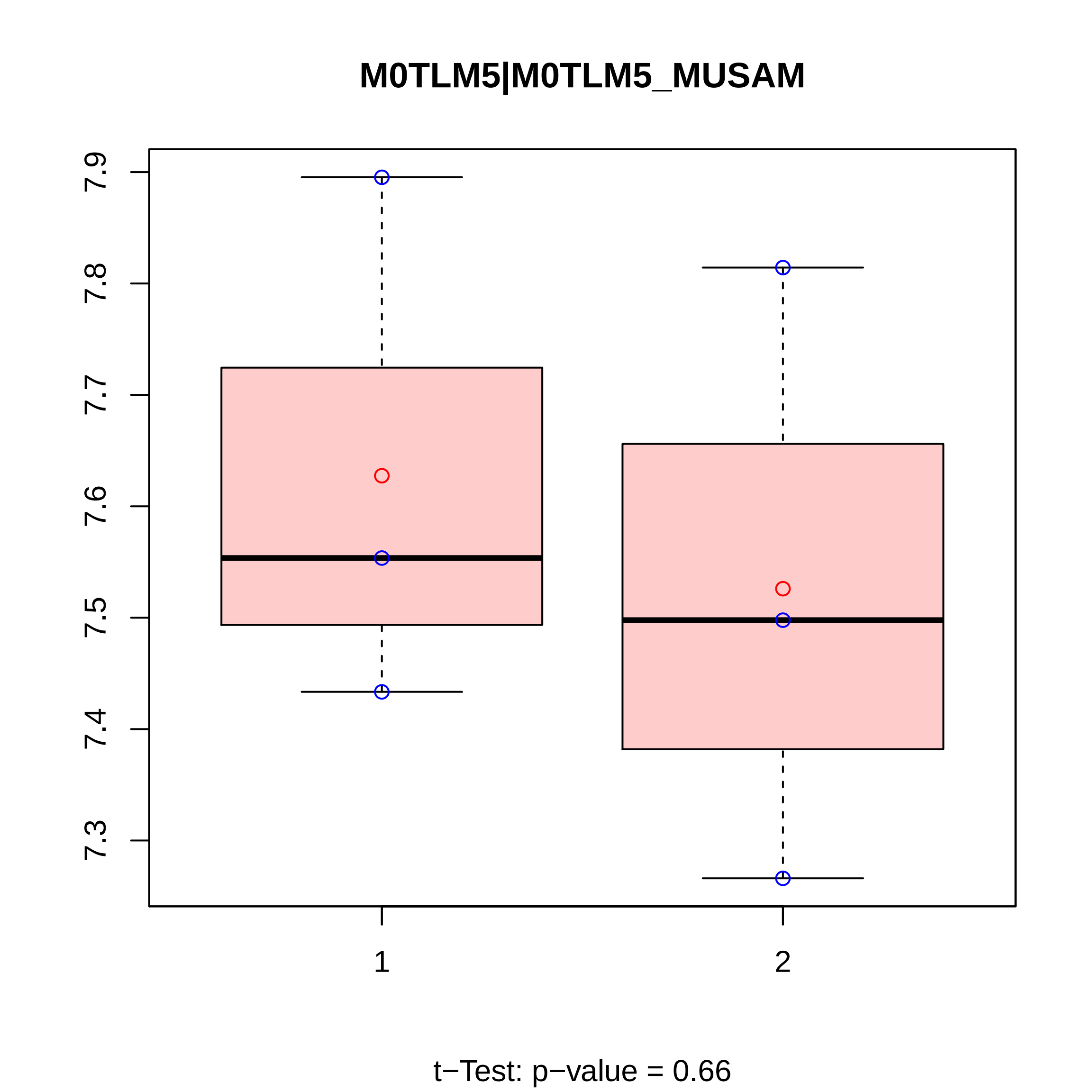
M0TLM5|M0TLM5_MUSAM
7.3
7.4
7.5
7.6
7.7
7.8
7.9
1
2
t−Test: p−value = 0.66

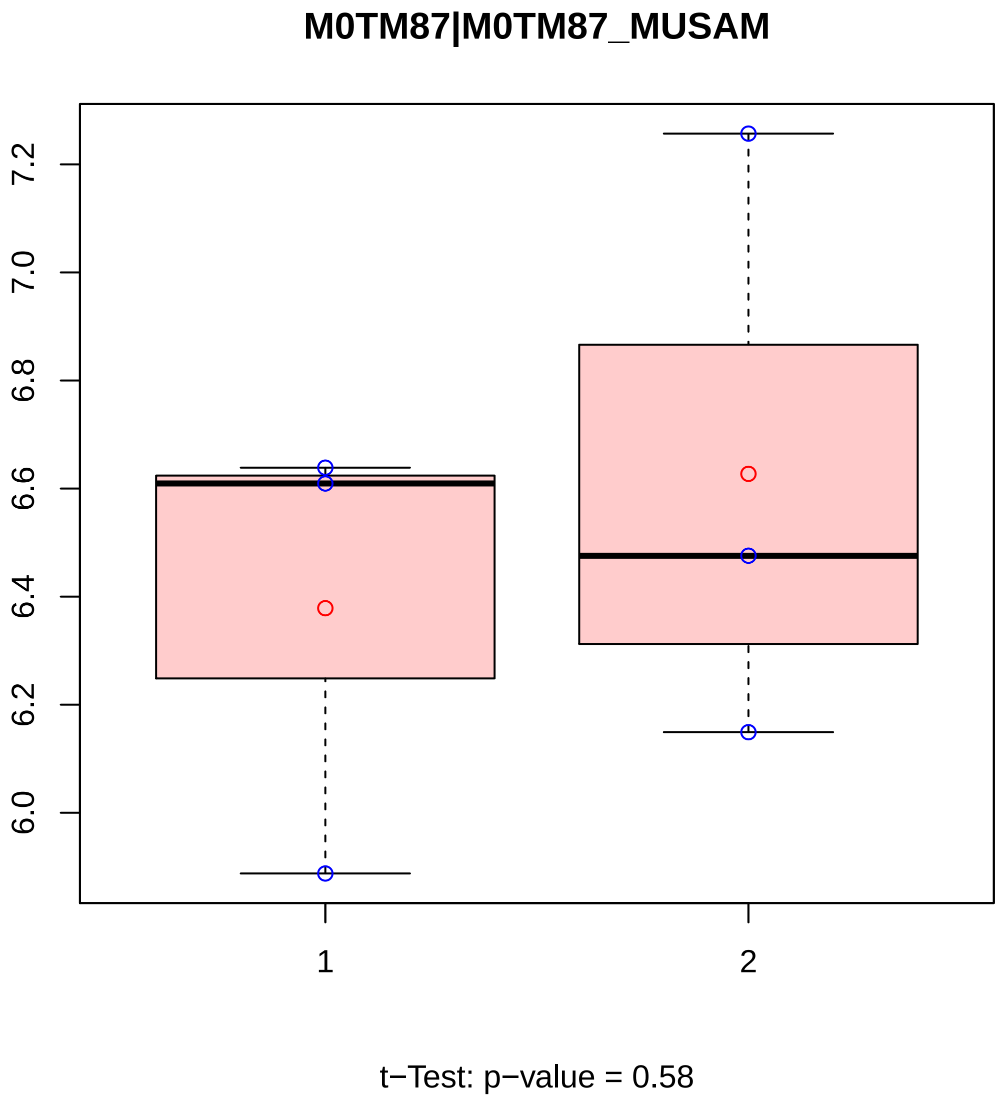
M0TM87|M0TM87_MUSAM
7.2
7.0
6.8
6.6
6.4
6.2
6.0
1
2
t−Test: p−value = 0.58

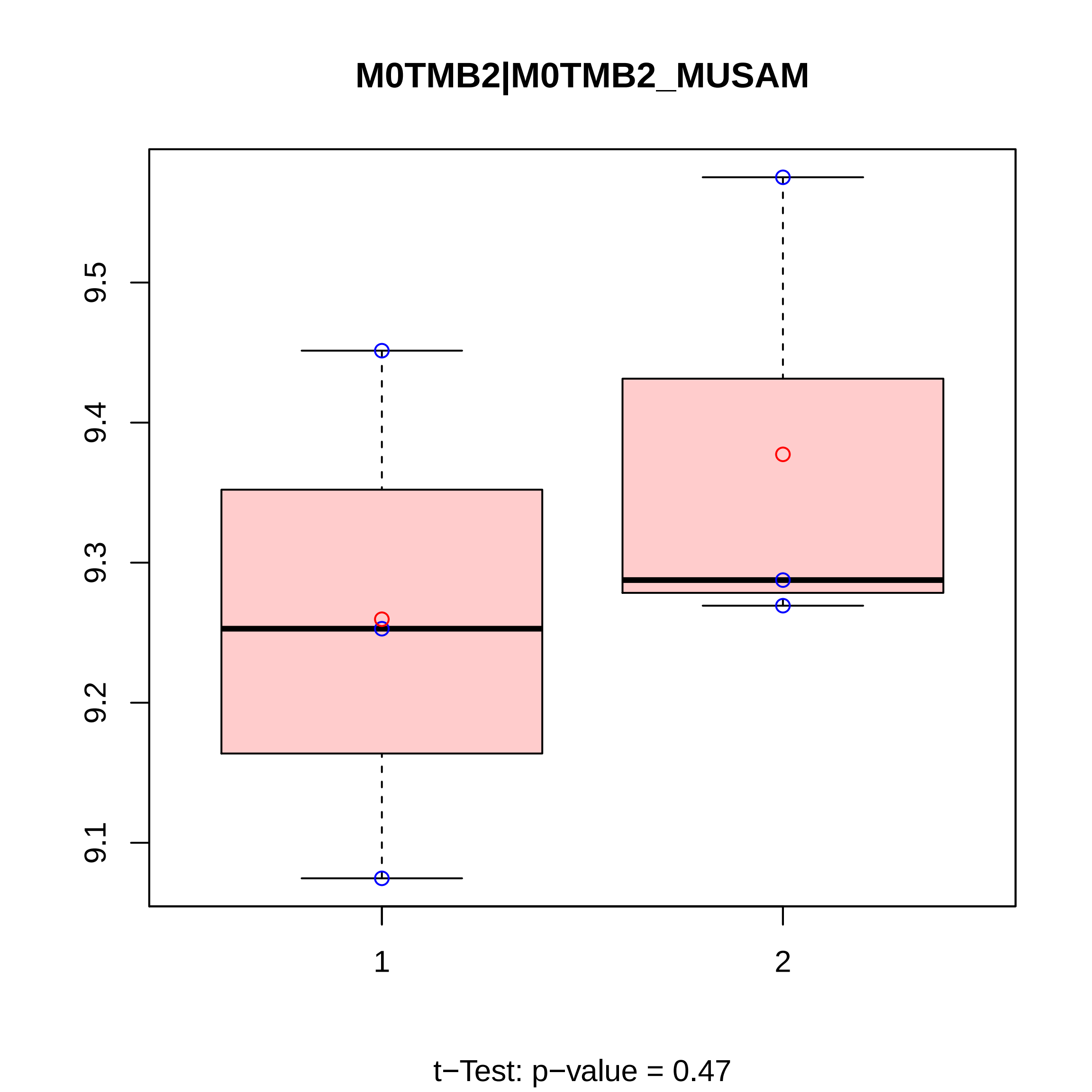

M0TMB2|M0TMB2_MUSAM
9.1
9.2
9.3
9.4
9.5
1
2
t−Test: p−value = 0.47

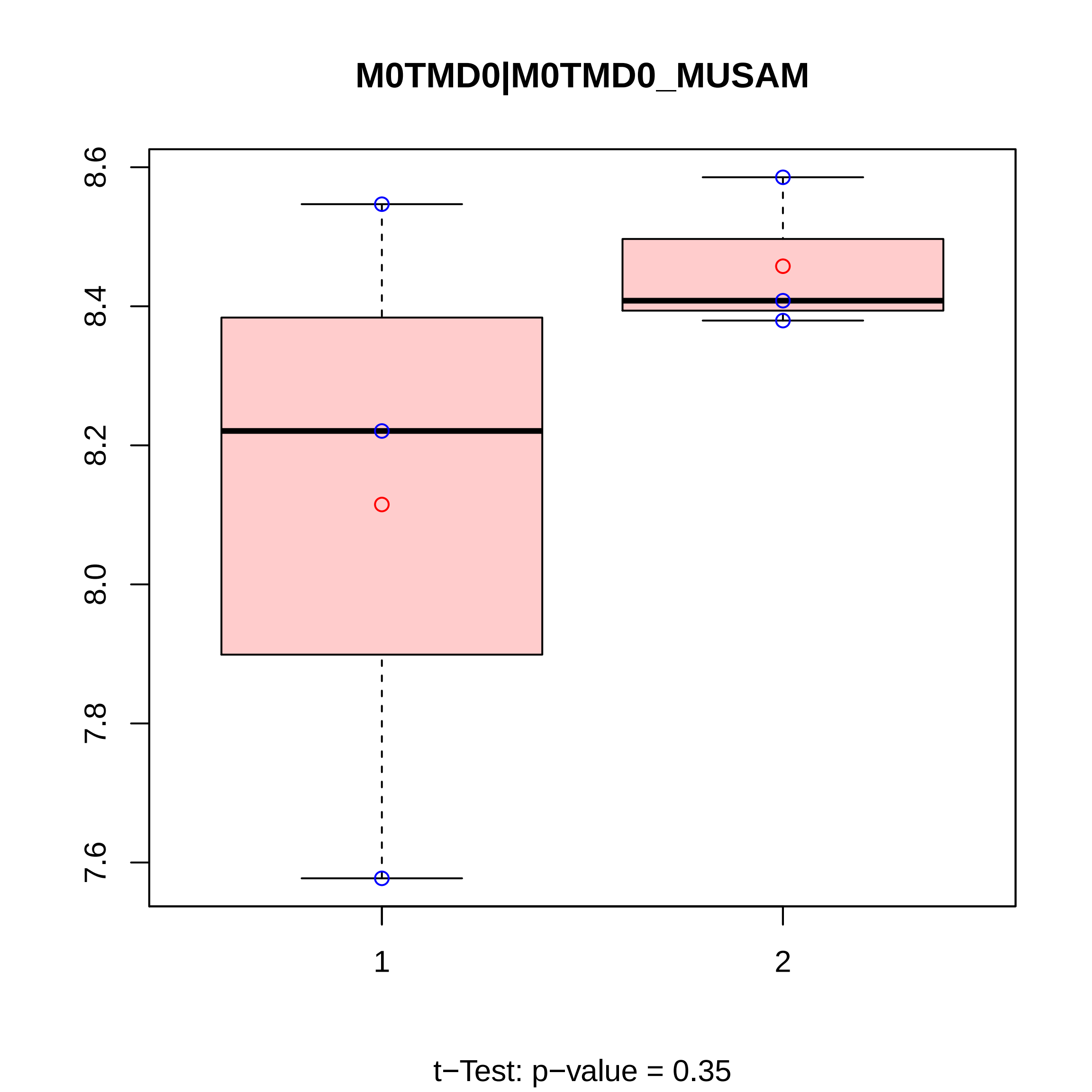

M0TMD0|M0TMD0_MUSAM
8.6
8.4
8.2
8.0
7.8
7.6
1
2
t−Test: p−value = 0.35

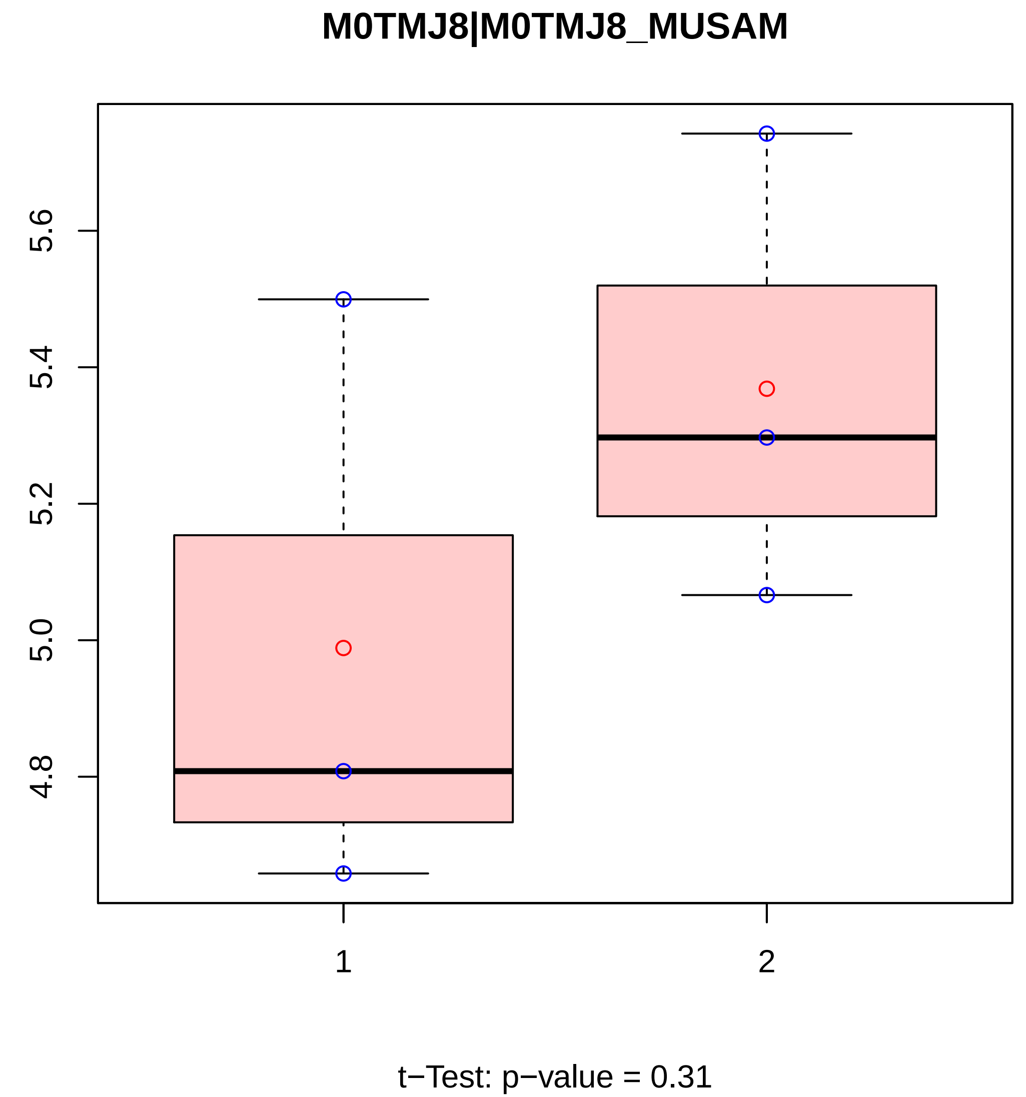
M0TMJ8|M0TMJ8_MUSAM
5.6
5.4
5.2
5.0
4.8
1
2
t−Test: p−value = 0.31

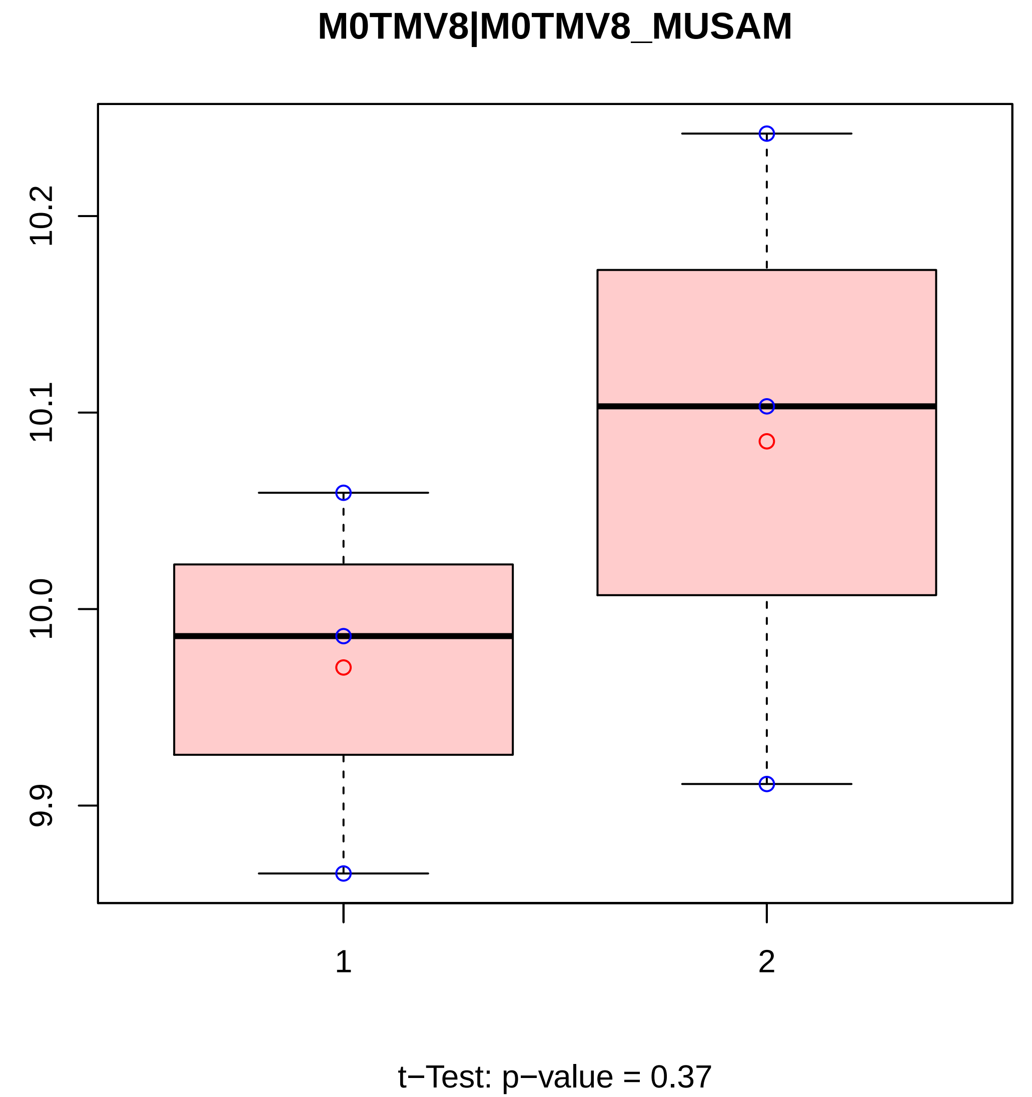
M0TMV8|M0TMV8_MUSAM
10.2
10.1
10.0
9.9
1
2
t−Test: p−value = 0.37

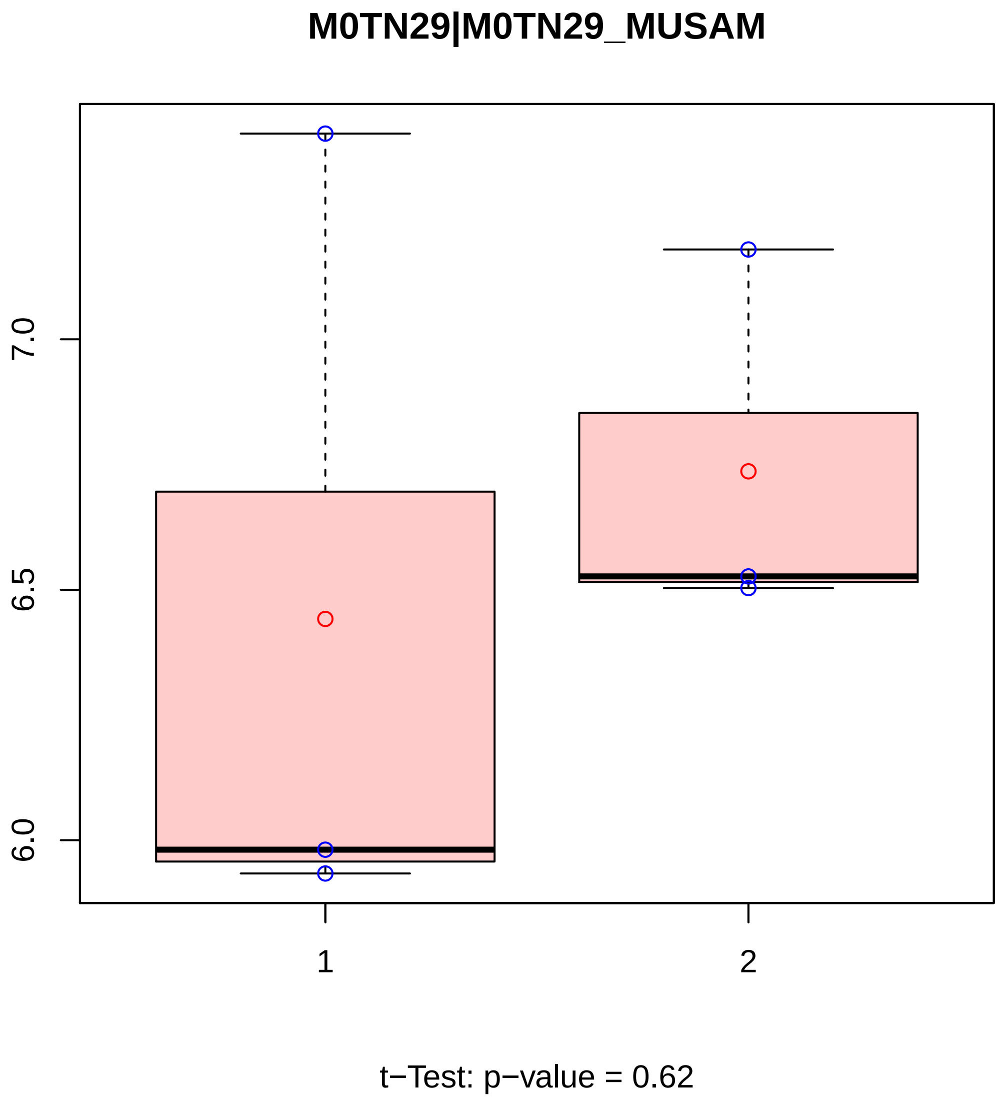

M0TN29|M0TN29_MUSAM
7.0
6.5
6.0
1
2
t−Test: p−value = 0.62

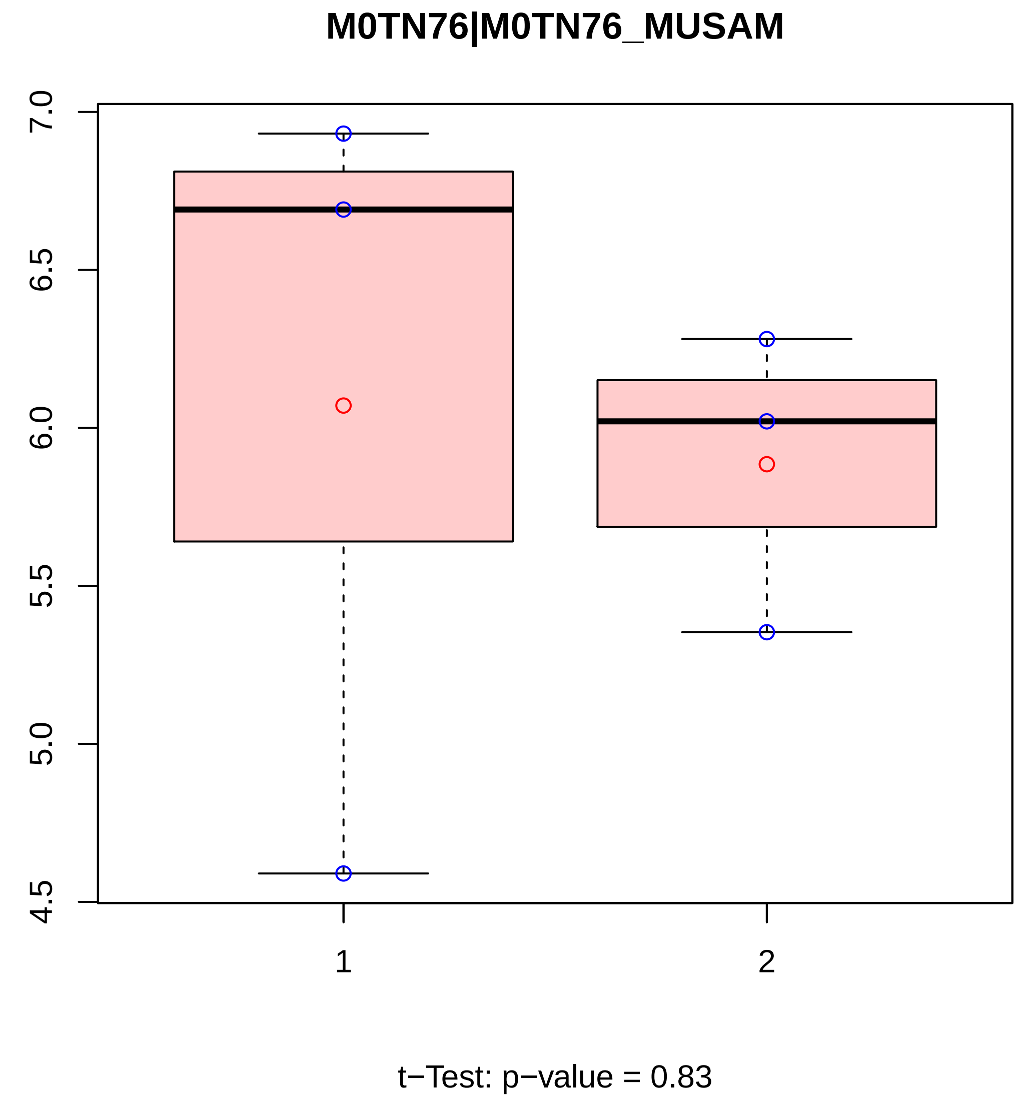
M0TN76|M0TN76_MUSAM
7.0
6.5
6.0
5.5
5.0
4.5
1
2
t−Test: p−value = 0.83

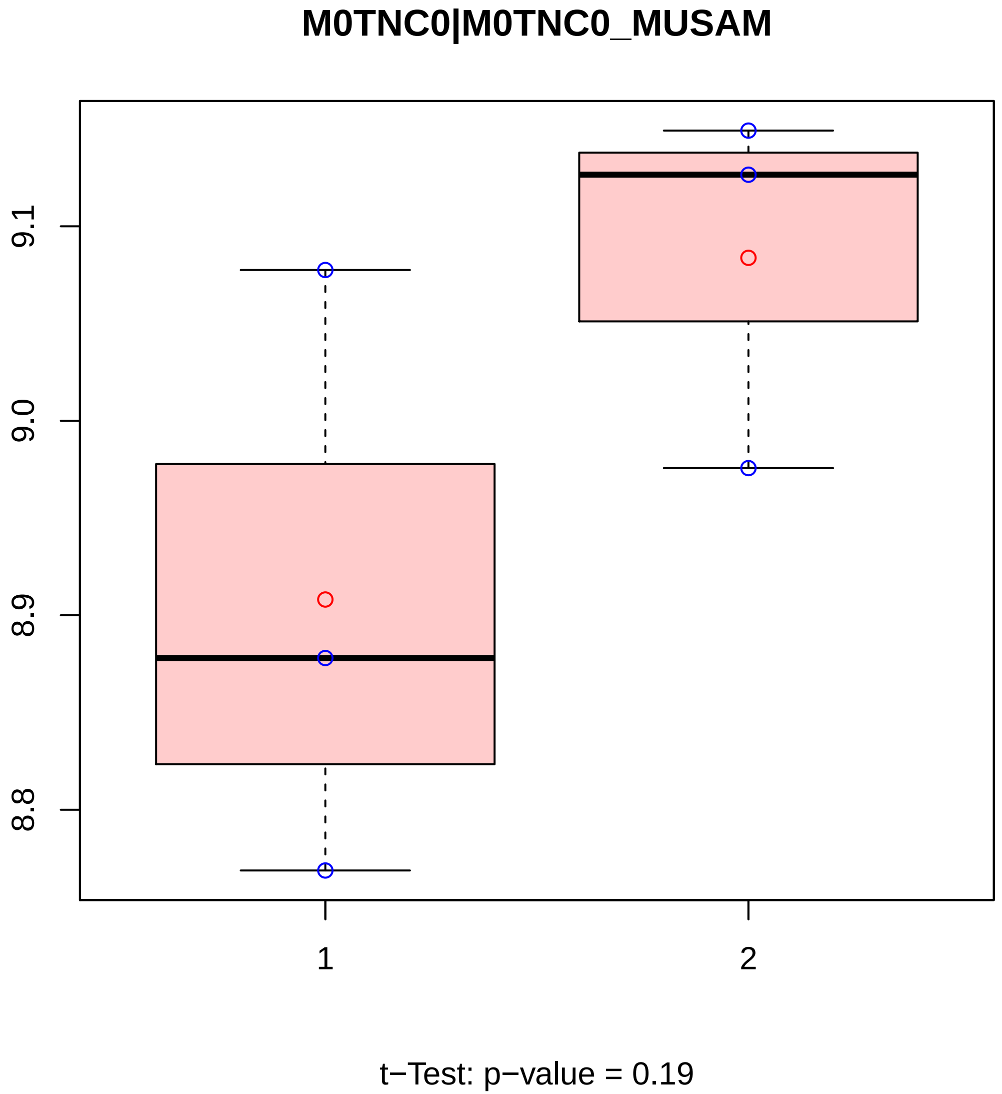
M0TNC0|M0TNC0_MUSAM
9.1
9.0
8.9
8.8
1
2
t−Test: p−value = 0.19

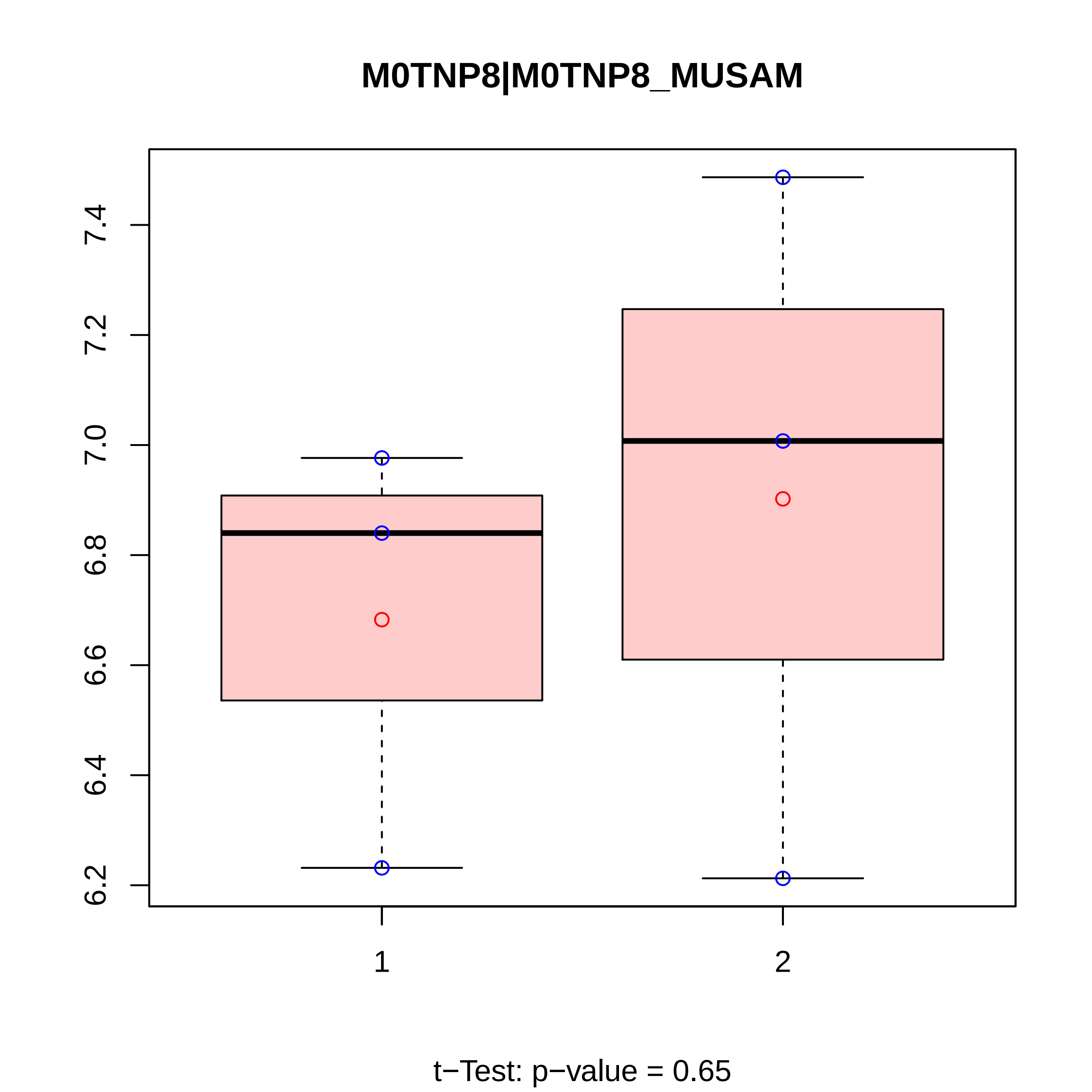
M0TNP8|M0TNP8_MUSAM
7.4
7.2
7.0
6.8
6.6
6.4
6.2
1
2
t−Test: p−value = 0.65

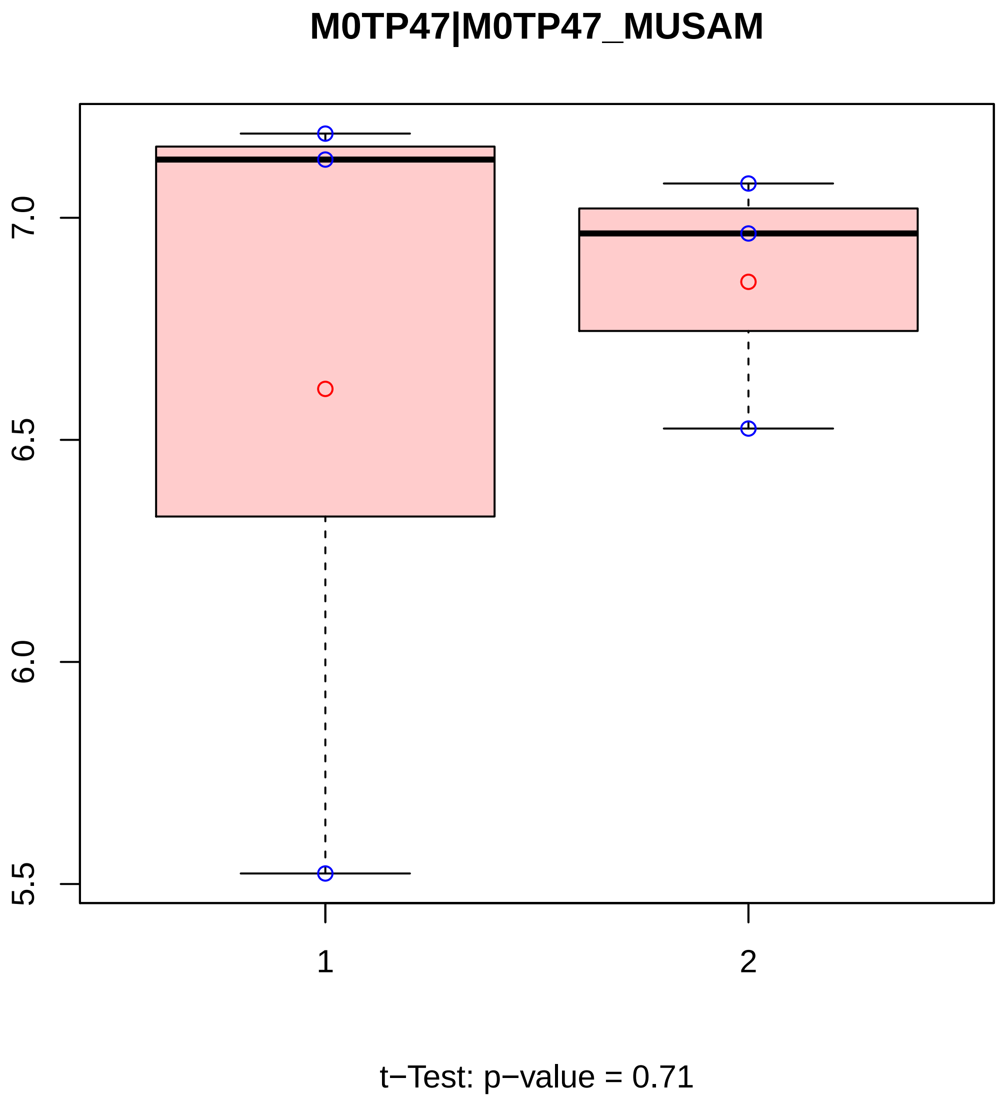
M0TP47|M0TP47_MUSAM
7.0
6.5
6.0
5.5
1
2
t−Test: p−value = 0.71

M0TPB5|M0TPB5_MUSAM

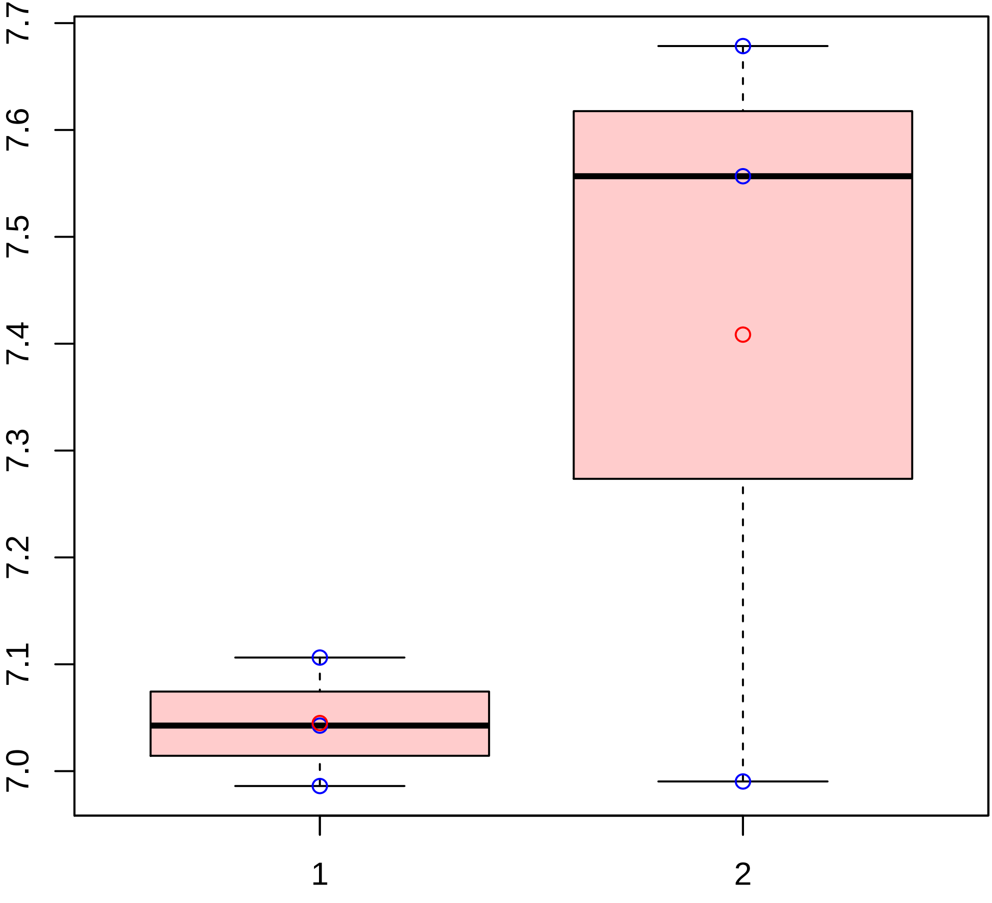

t−Test: p−value = 0.23

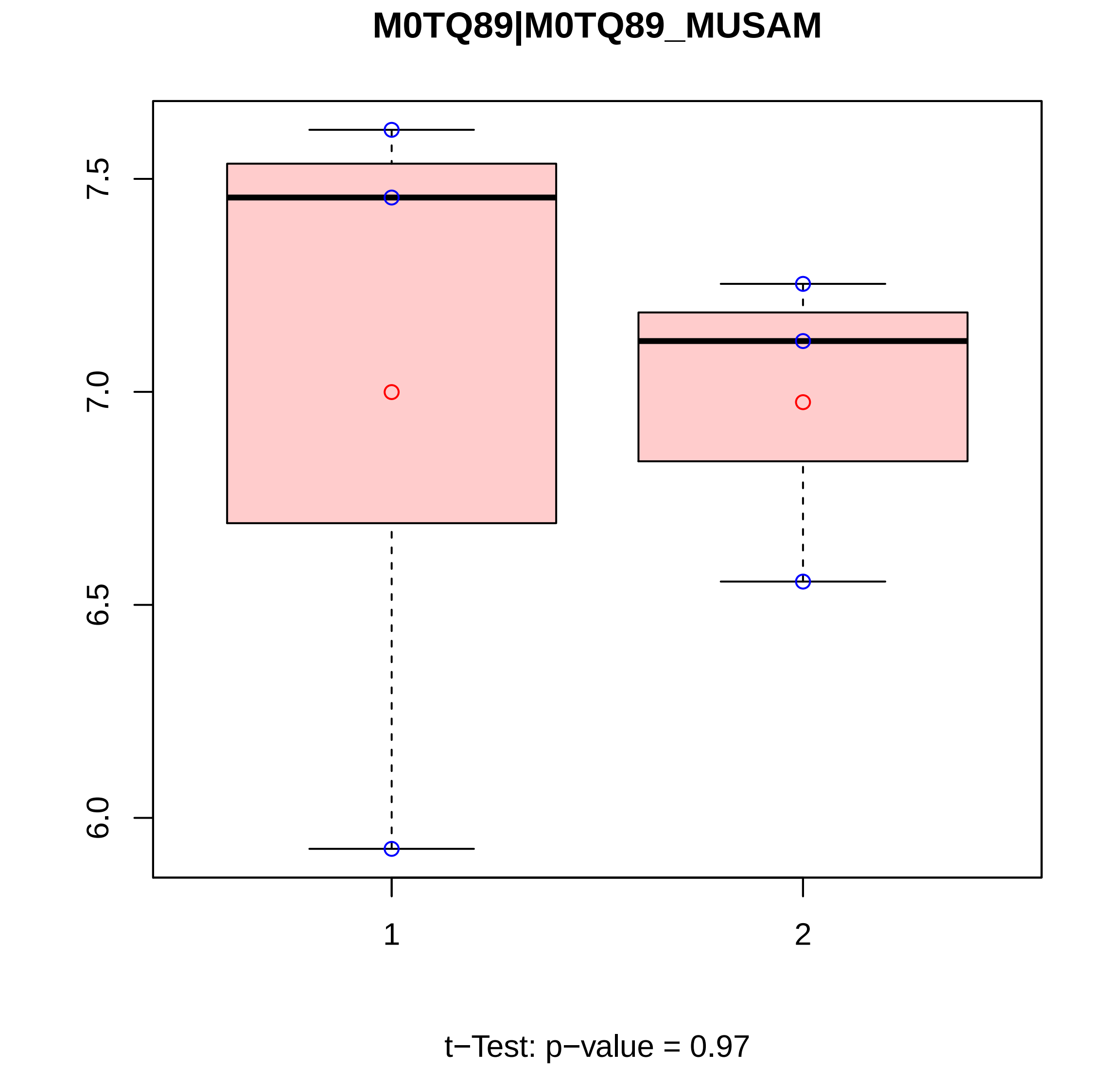
M0TQ89|M0TQ89_MUSAM
7.5
7.0
6.5
6.0
1
2
t−Test: p−value = 0.97

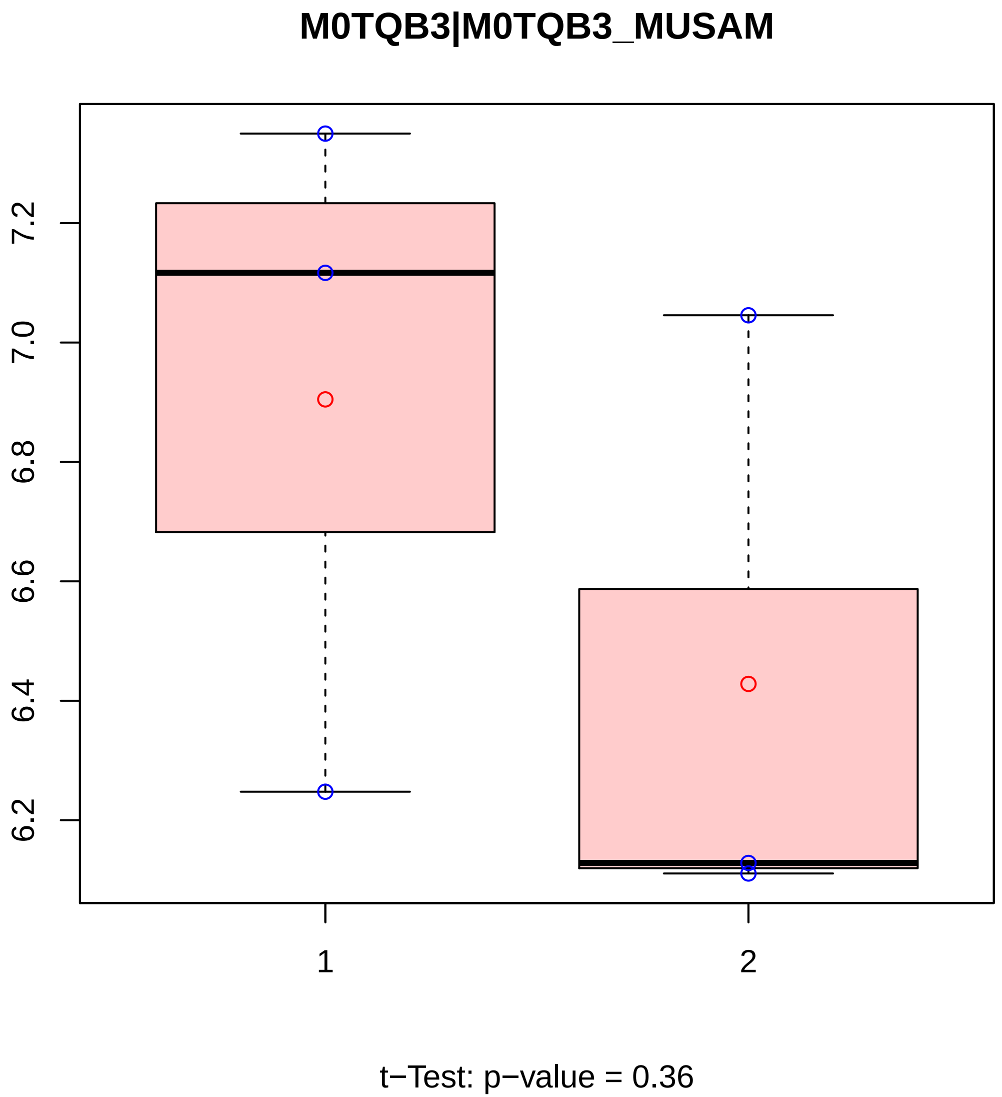
M0TQB3|M0TQB3_MUSAM
7.2
7.0
6.8
6.6
6.4
6.2
1
2
t−Test: p−value = 0.36

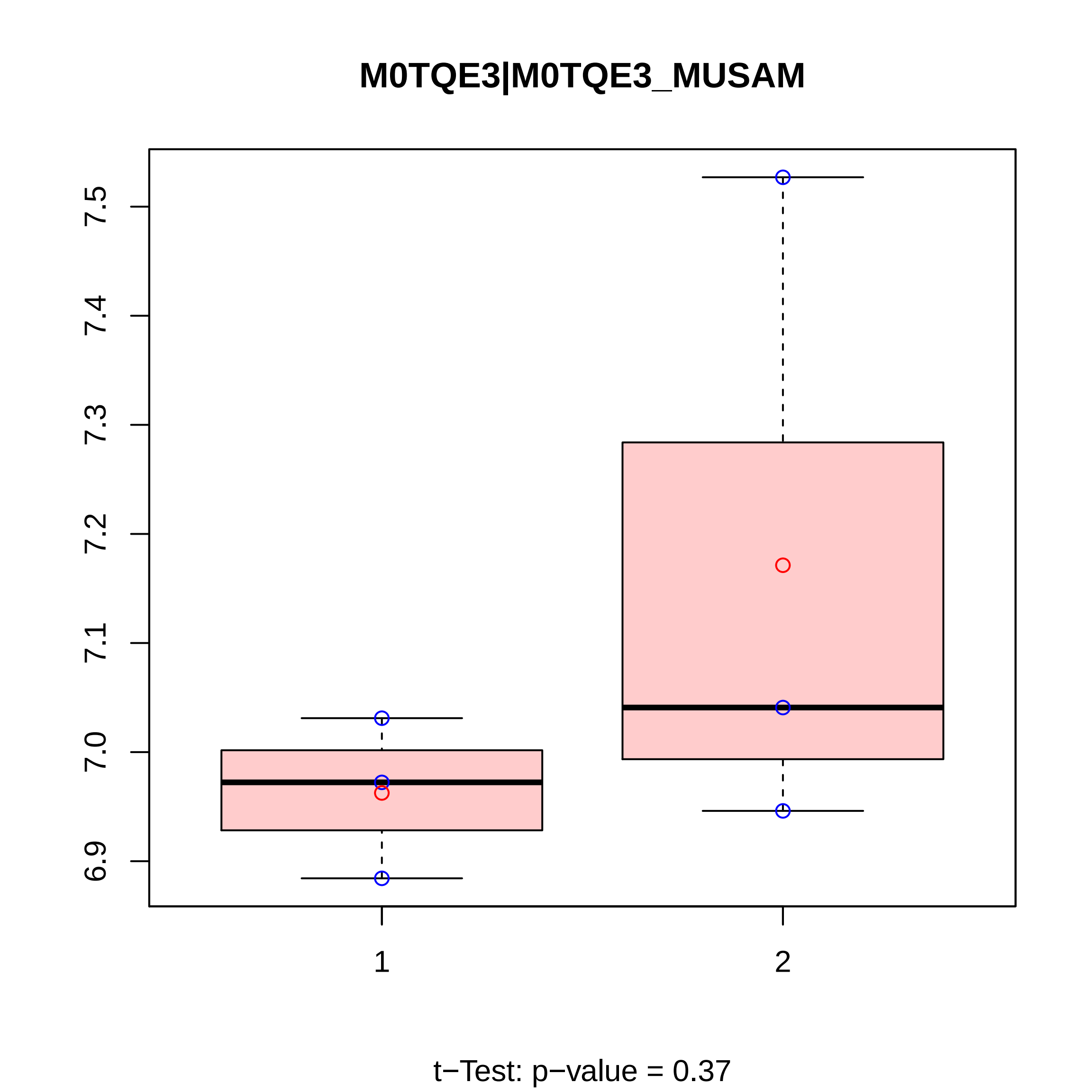
M0TQE3|M0TQE3_MUSAM
7.5
7.4
7.3
7.2
7.1
7.0
6.9
1
2
t−Test: p−value = 0.37

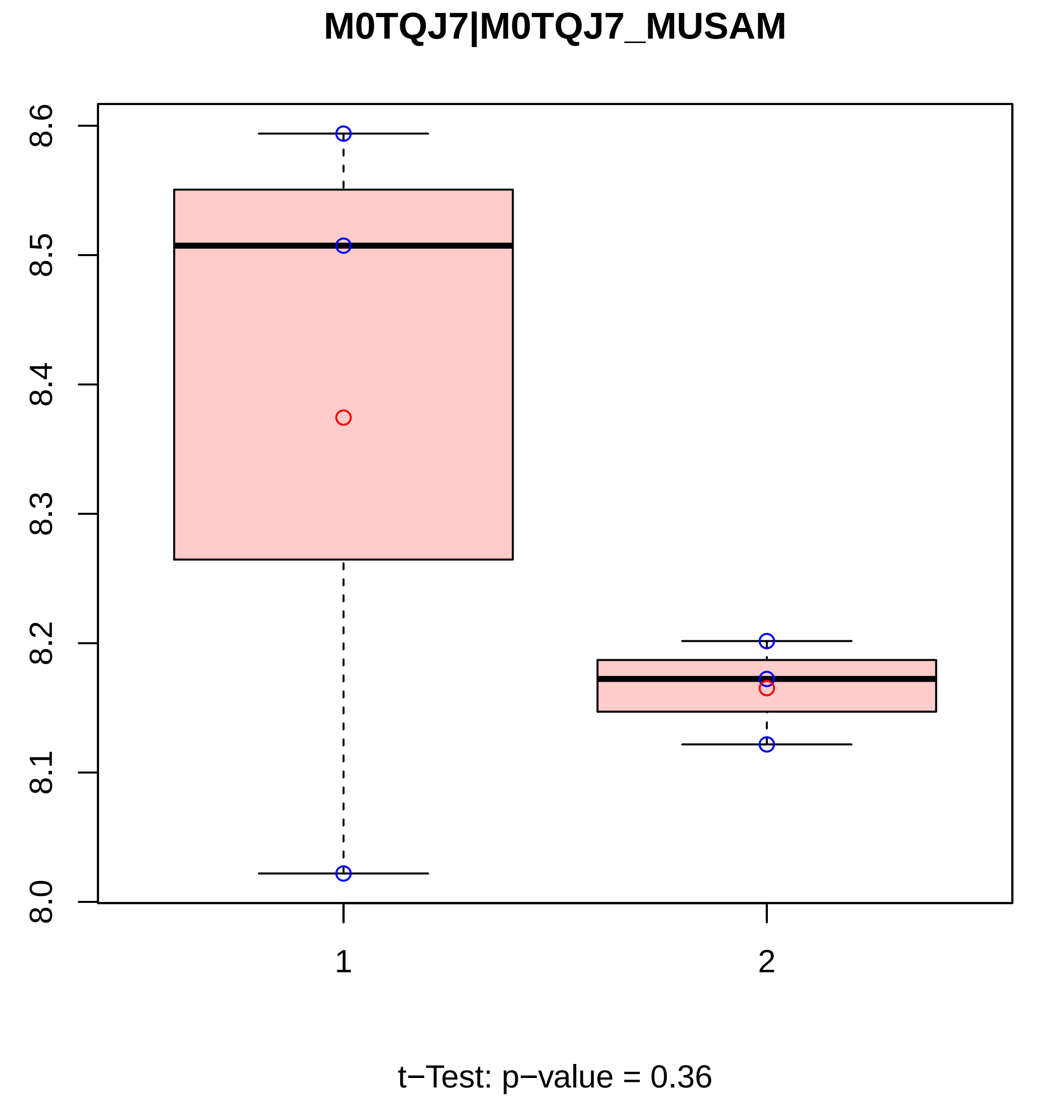

M0TQJ7|M0TQJ7_MUSAM
8.0
8.1
8.2
8.3
8.4
8.5
8.6
1
2
t−Test: p−value = 0.36

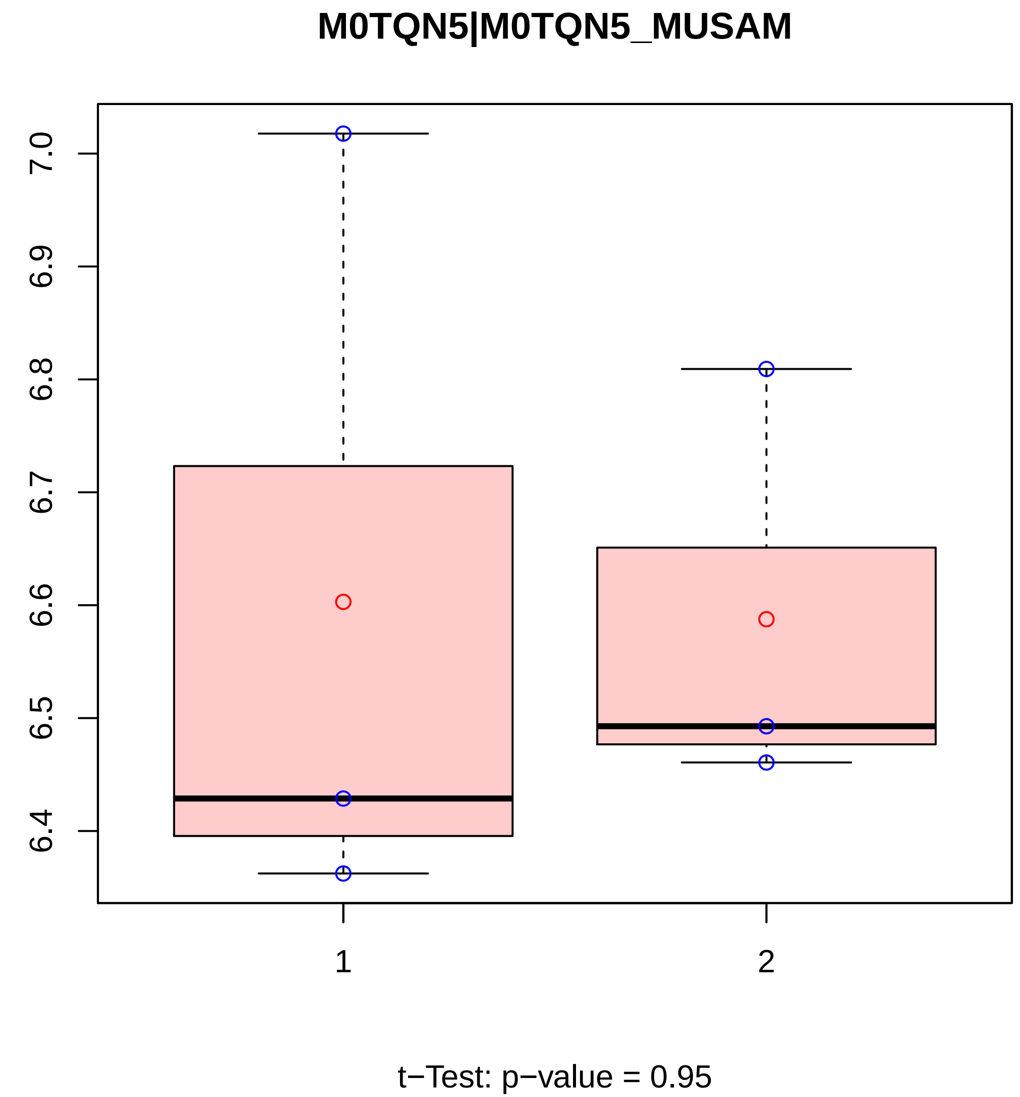

M0TQN5|M0TQN5_MUSAM
7.0
6.9
6.8
6.7
6.6
6.5
6.4
1
2
t−Test: p−value = 0.95

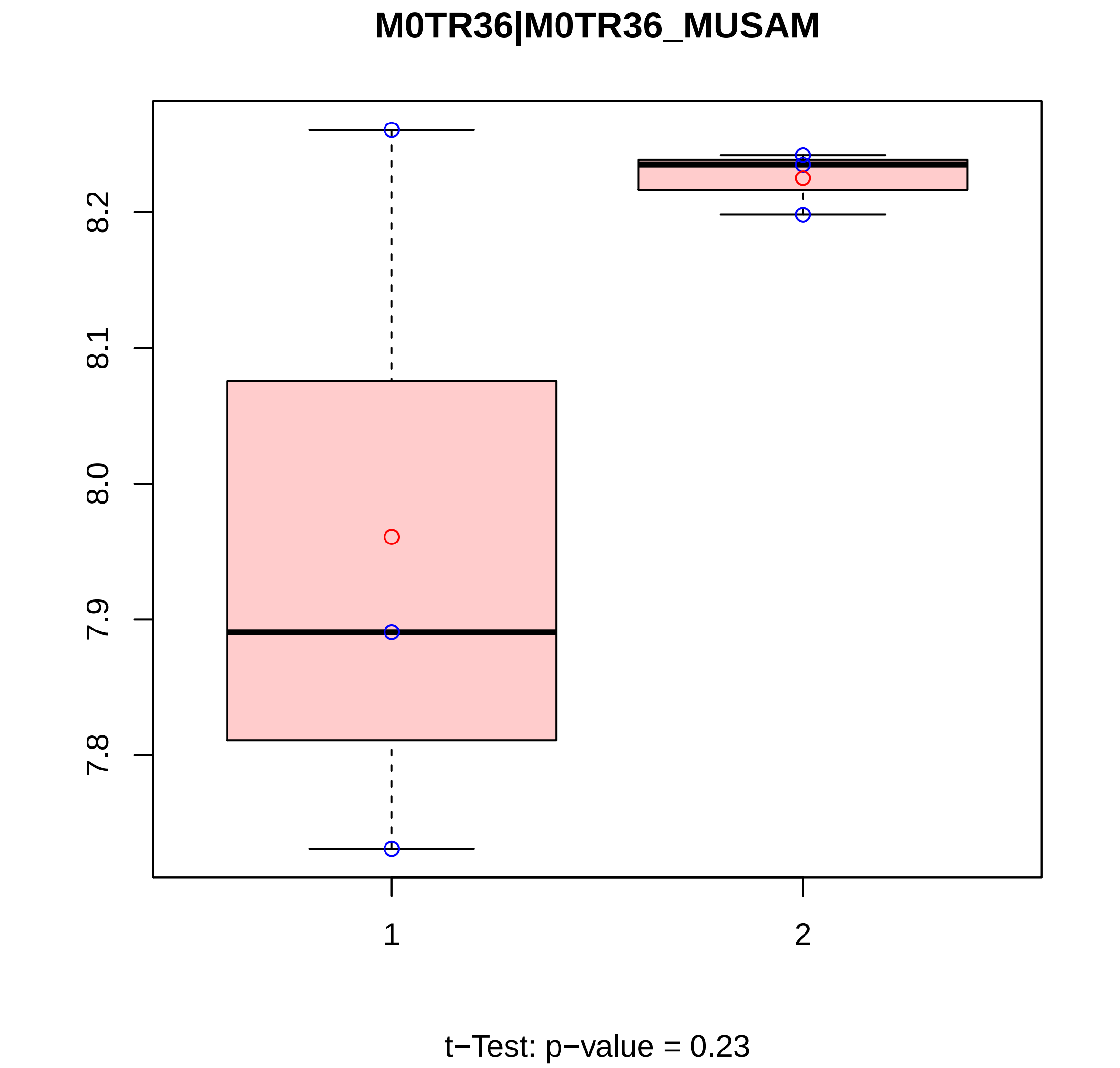

M0TR36|M0TR36_MUSAM
8.2
8.1
8.0
7.9
7.8
1
2
t−Test: p−value = 0.23

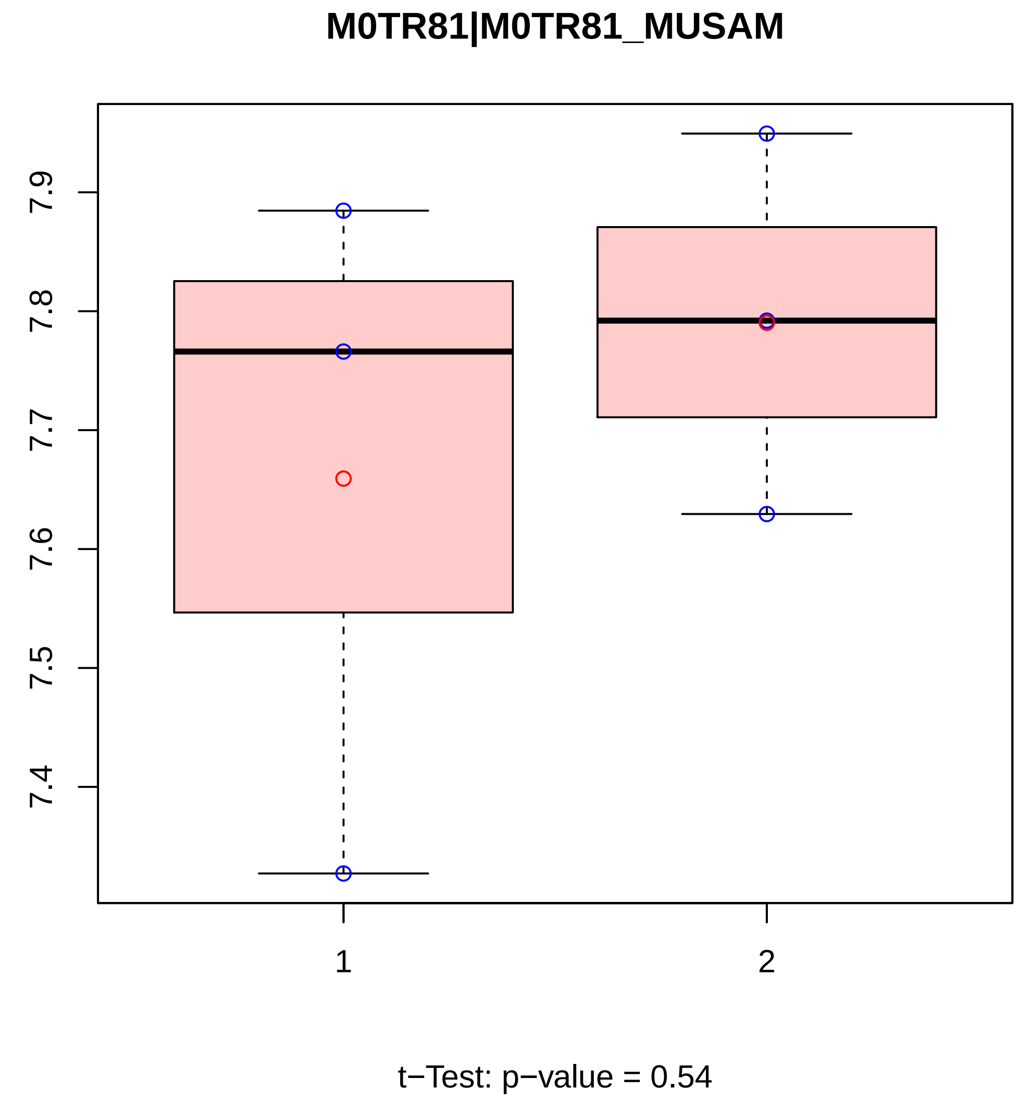
M0TR81|M0TR81_MUSAM
7.4
7.5
7.6
7.7
7.8
7.9
1
2
t−Test: p−value = 0.54

M0TRB6|M0TRB6_MUSAM

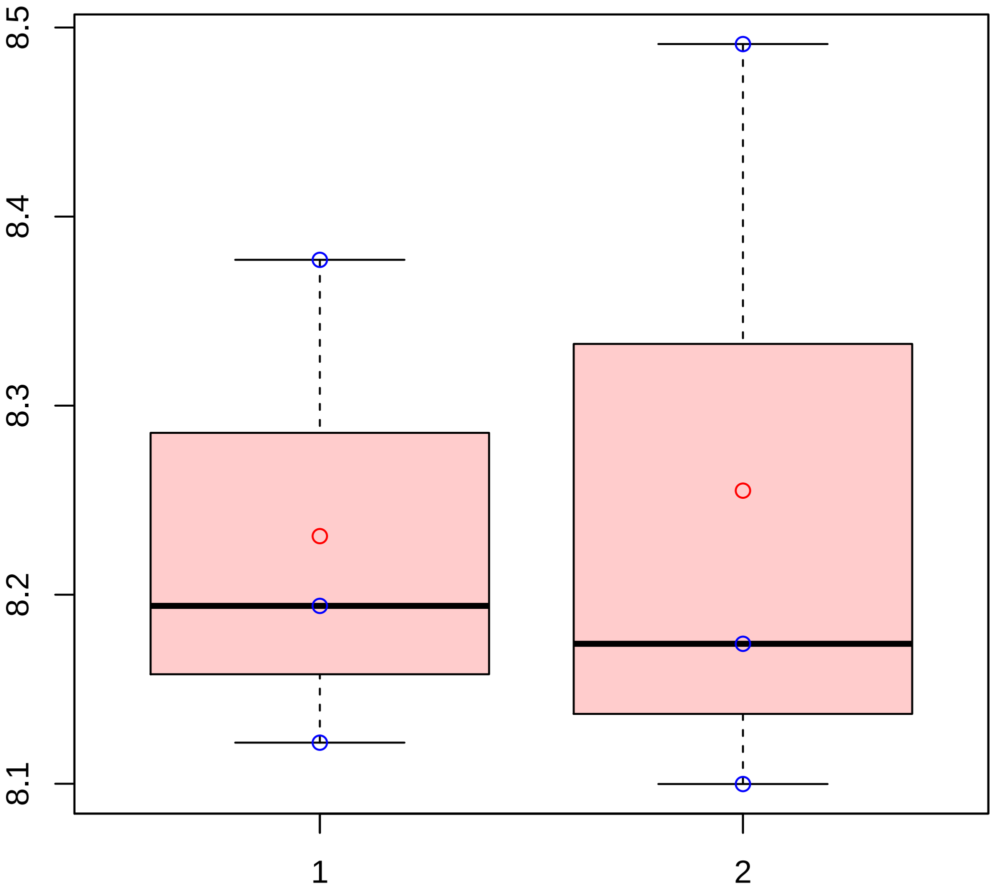

t−Test: p−value = 0.88

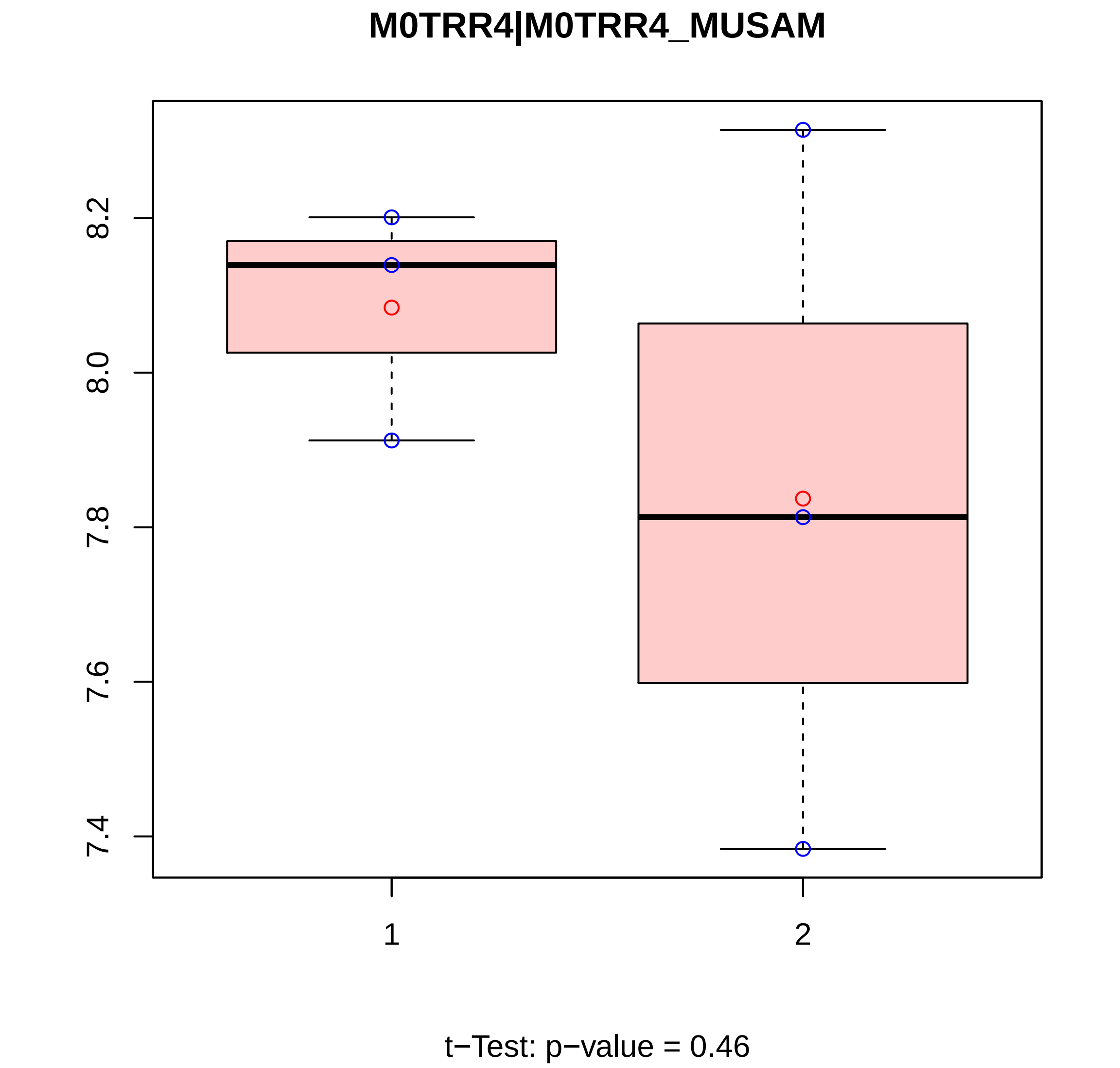
M0TRR4|M0TRR4_MUSAM
8.2
8.0
7.8
7.6
7.4
1
2
t−Test: p−value = 0.46

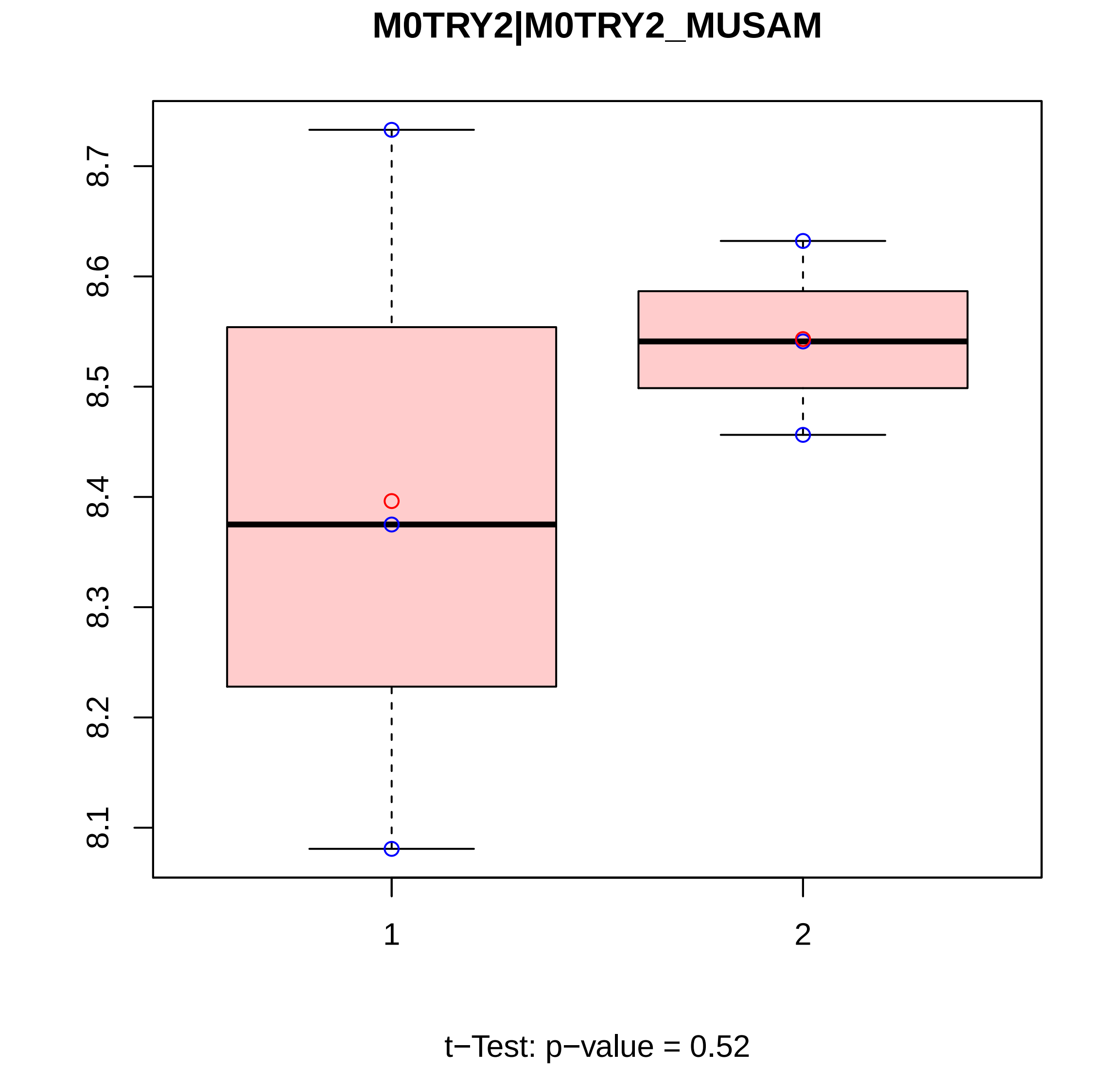
M0TRY2|M0TRY2_MUSAM
8.1
8.2
8.3
8.4
8.5
8.6
8.7
1
2
t−Test: p−value = 0.52

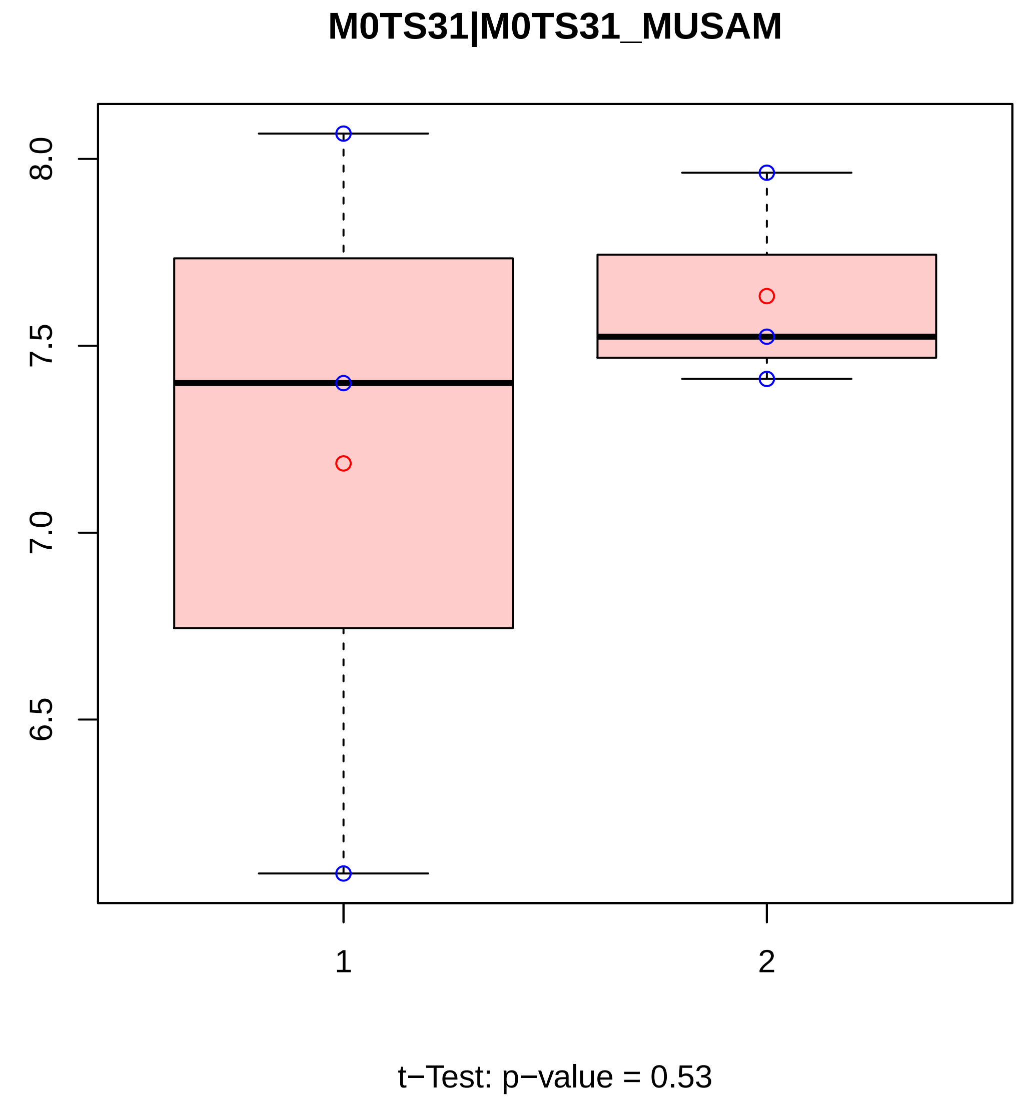

M0TS31|M0TS31_MUSAM
8.0
7.5
7.0
6.5
1
2
t−Test: p−value = 0.53

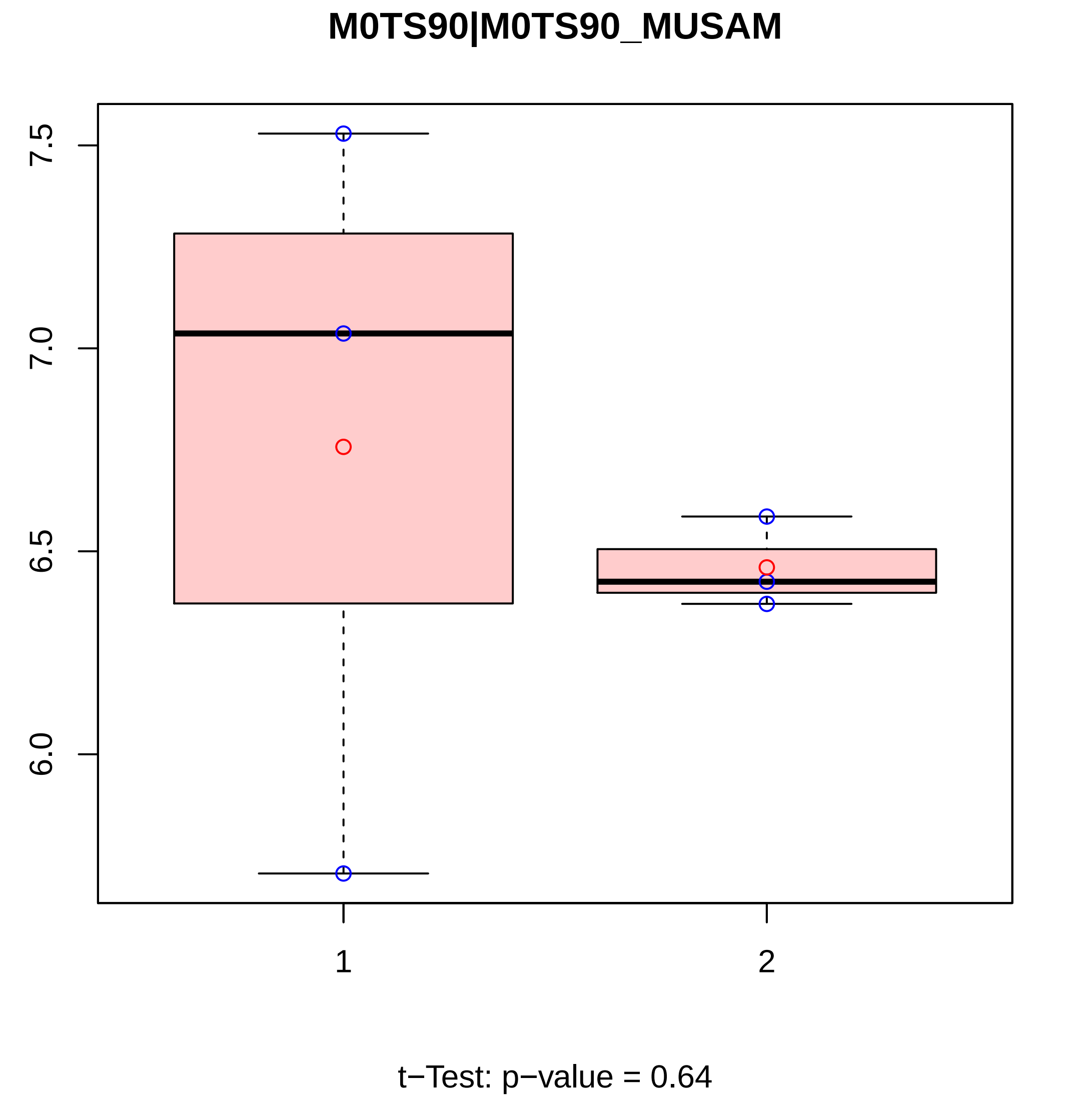
M0TS90|M0TS90_MUSAM
7.5
7.0
6.5
6.0
1
2
t−Test: p−value = 0.64

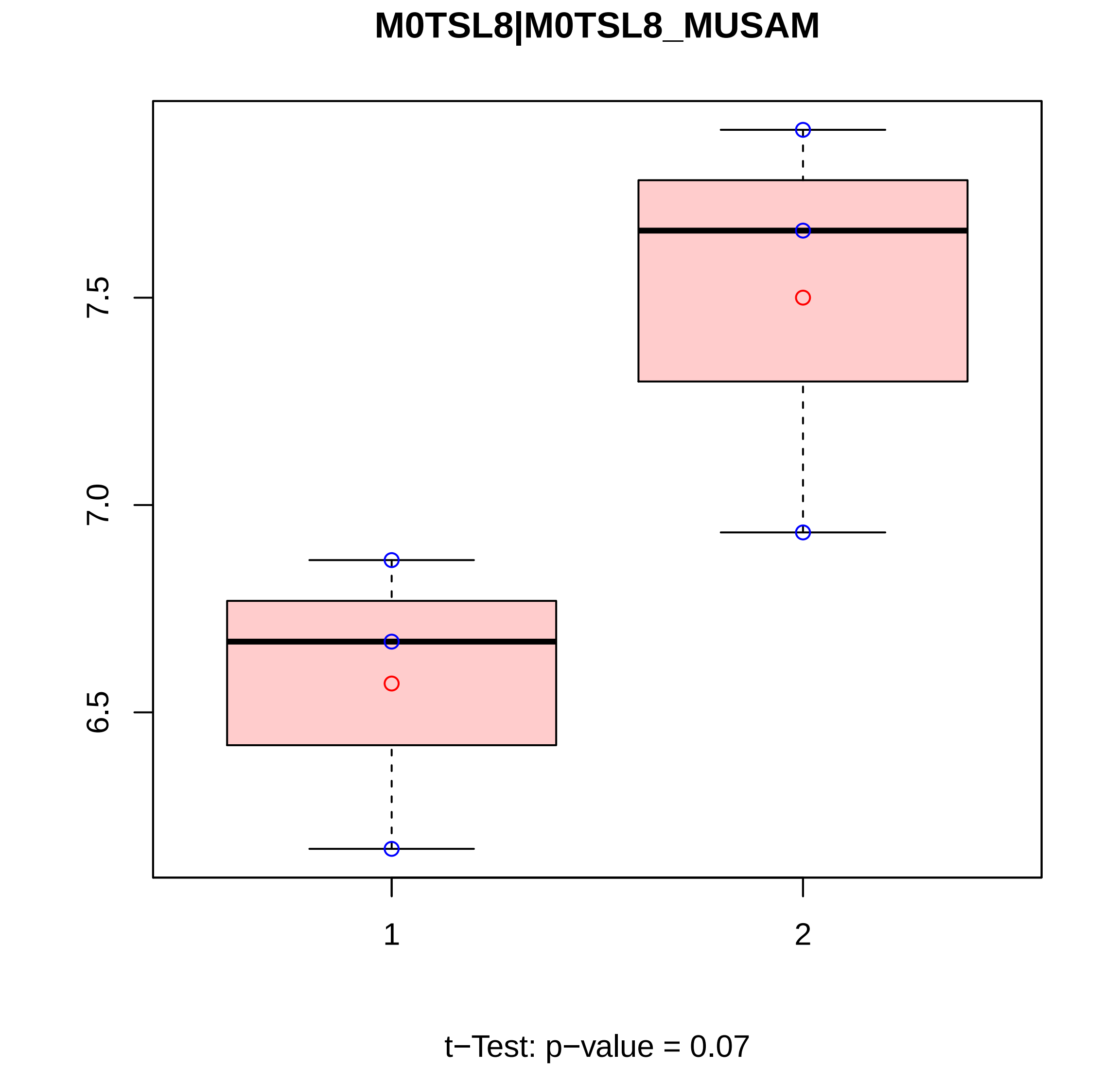
M0TSL8|M0TSL8_MUSAM
6.5
7.0
7.5
1
2
t−Test: p−value = 0.07

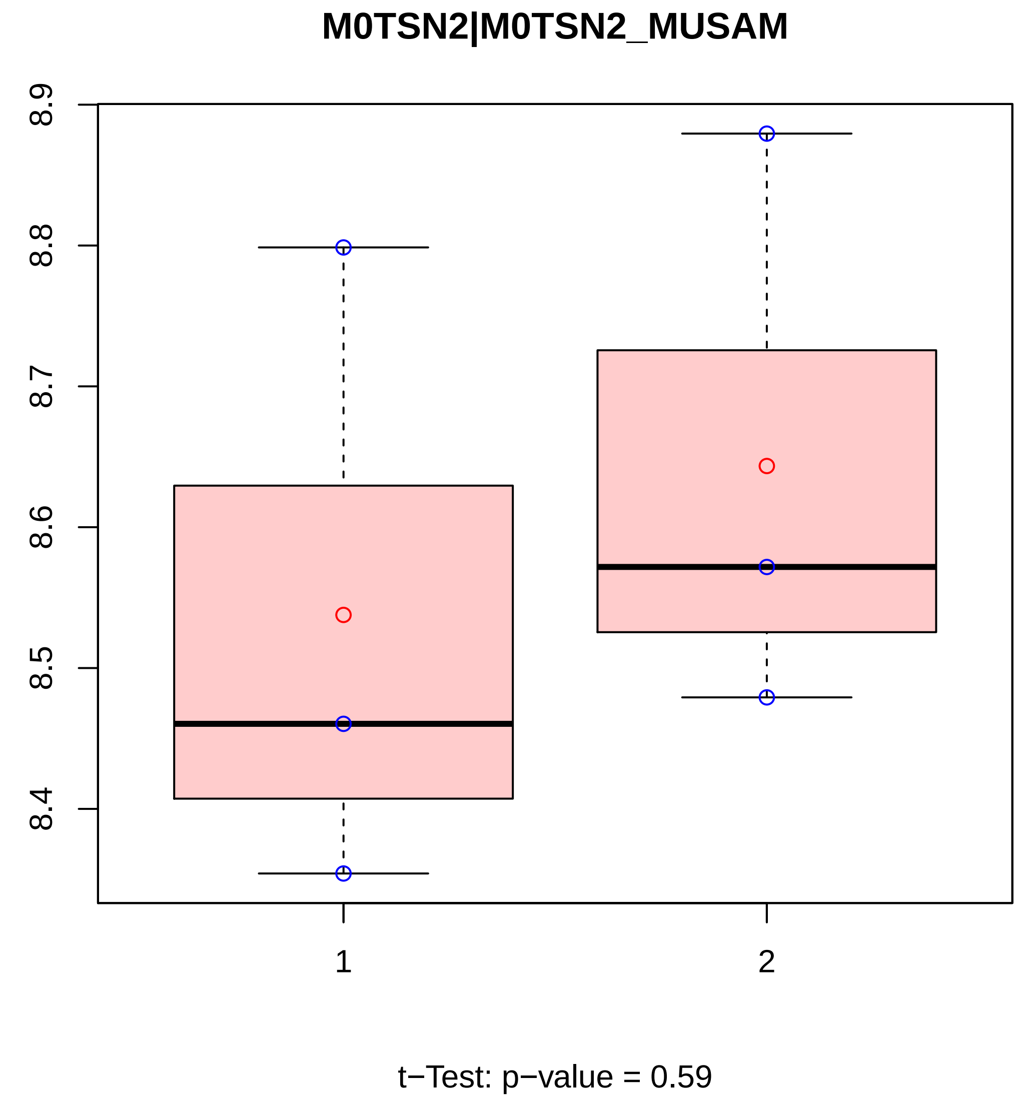
M0TSN2|M0TSN2_MUSAM
8.4
8.5
8.6
8.7
8.8
8.9
1
2
t−Test: p−value = 0.59

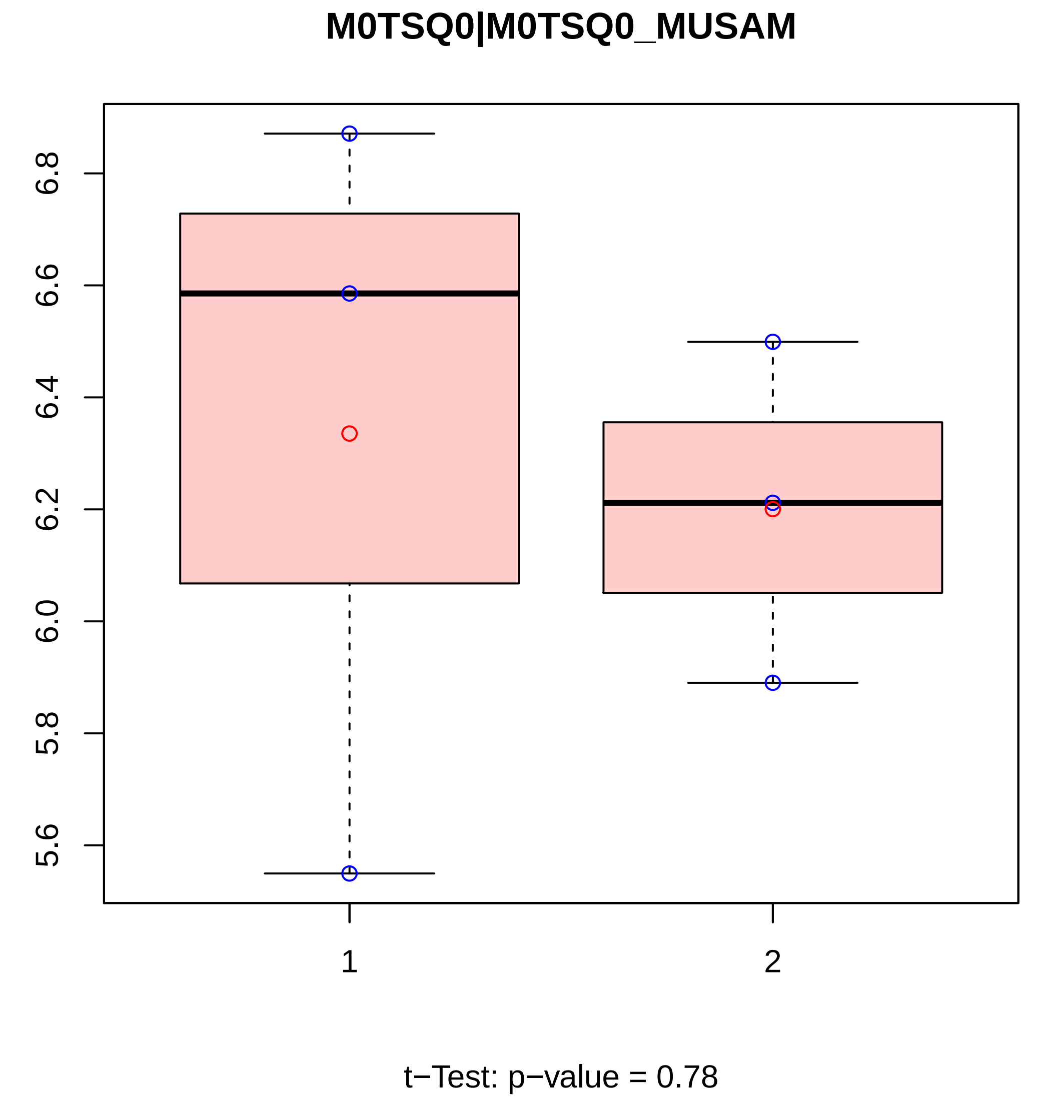
M0TSQ0|M0TSQ0_MUSAM
6.8
6.6
6.4
6.2
6.0
5.8
5.6
1
2
t−Test: p−value = 0.78

M0TT66|M0TT66_MUSAM

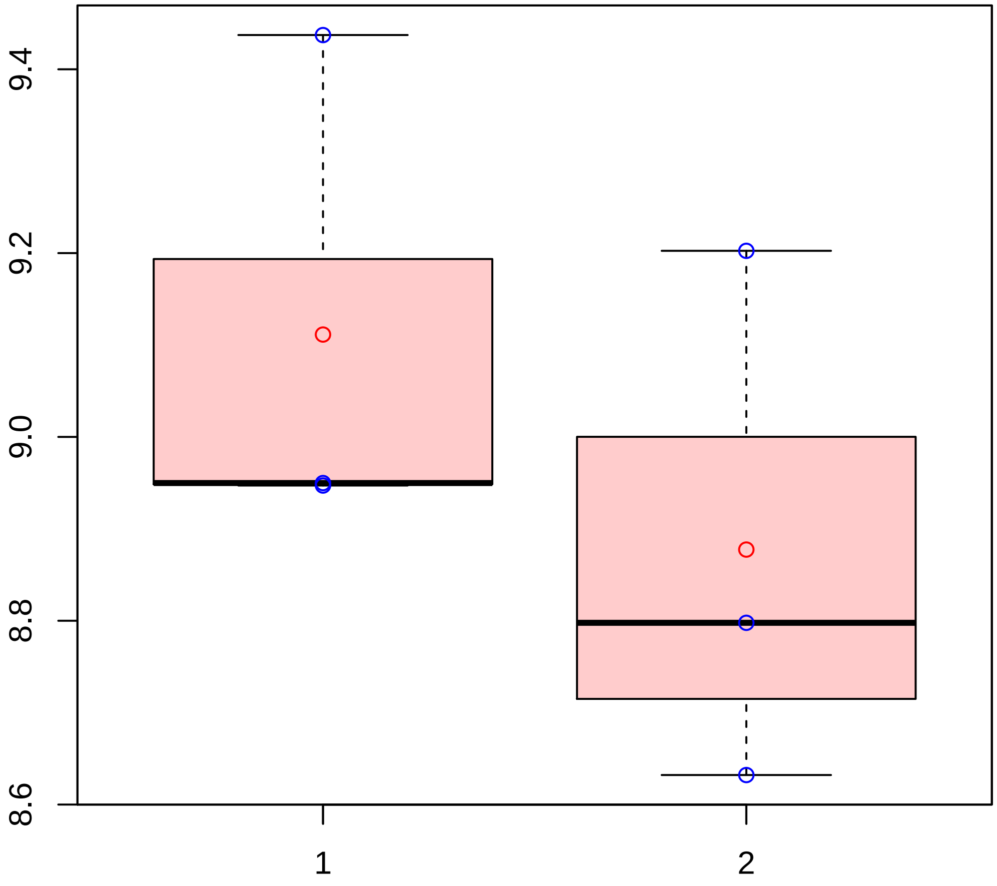

t−Test: p−value = 0.38

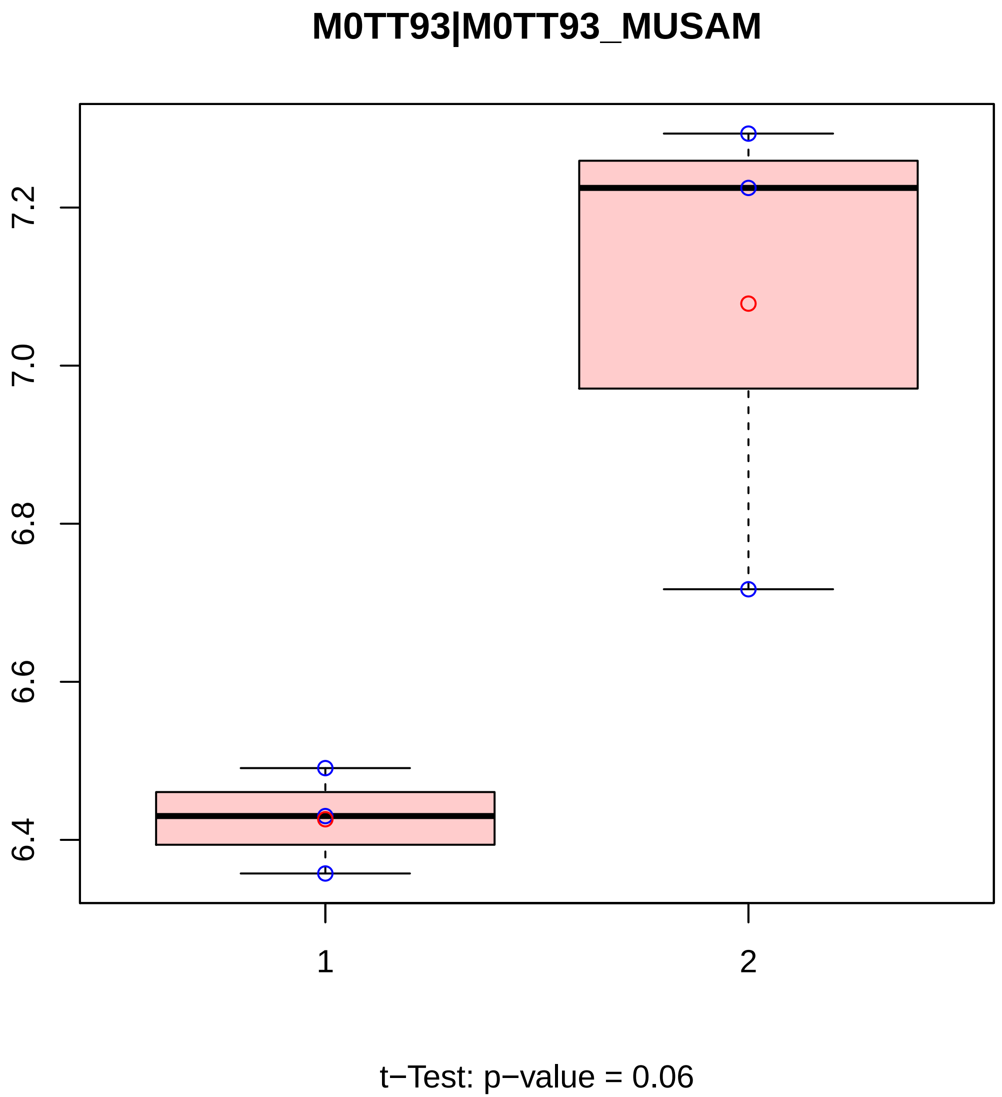
M0TT93|M0TT93_MUSAM
7.2
7.0
6.8
6.6
6.4
1
2
t−Test: p−value = 0.06

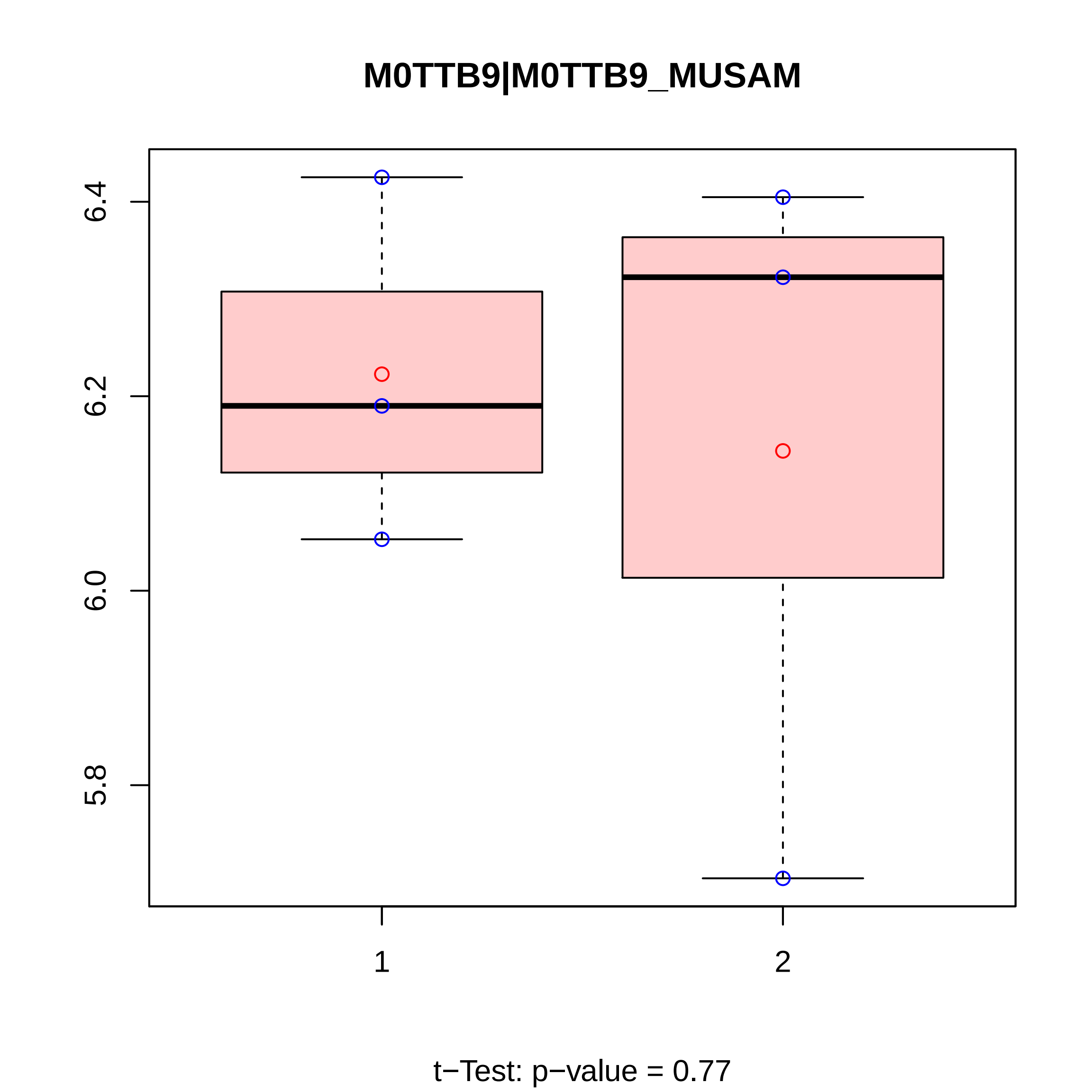
M0TTB9|M0TTB9_MUSAM
6.4
6.2
6.0
5.8
1
2
t−Test: p−value = 0.77

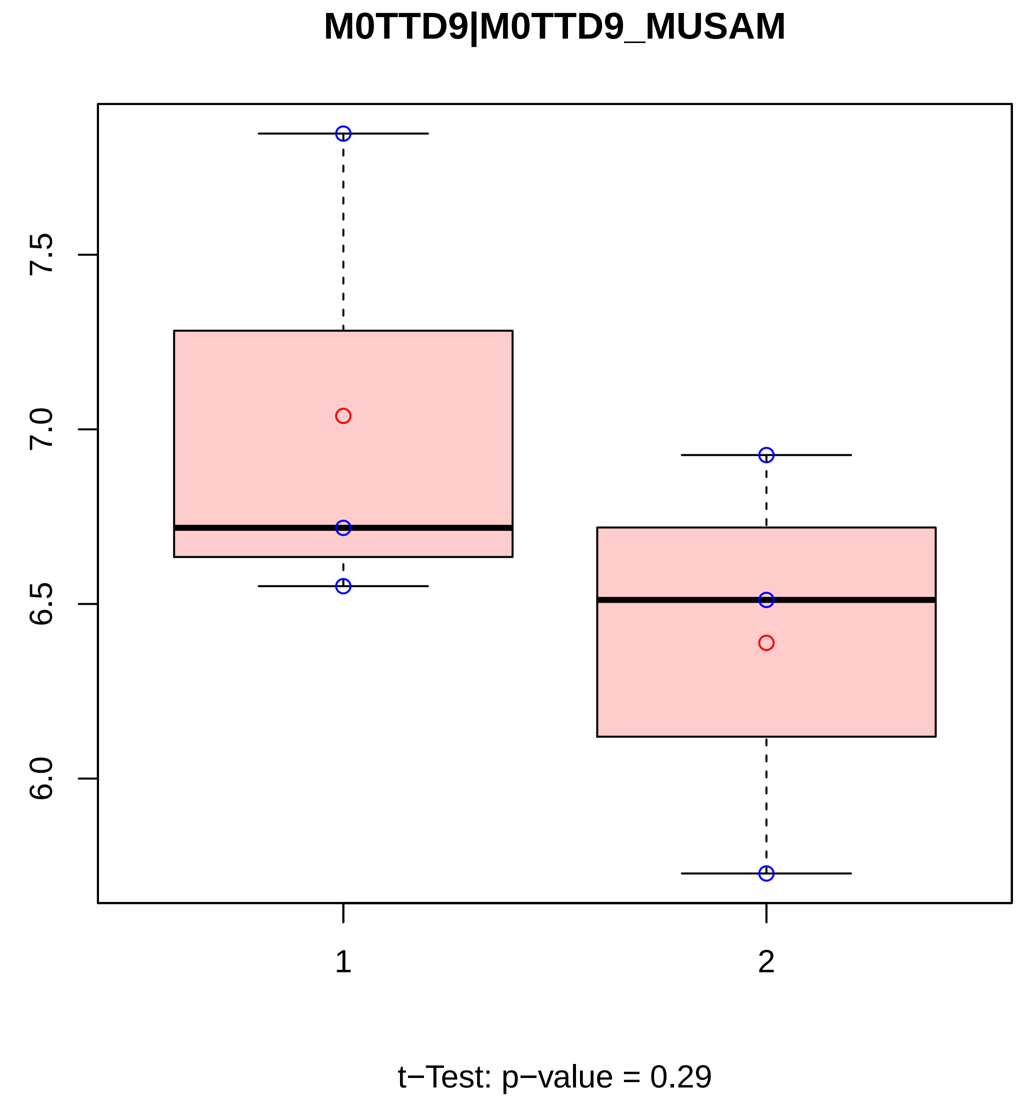
M0TTD9|M0TTD9_MUSAM
7.5
7.0
6.5
6.0
1
2
t−Test: p−value = 0.29

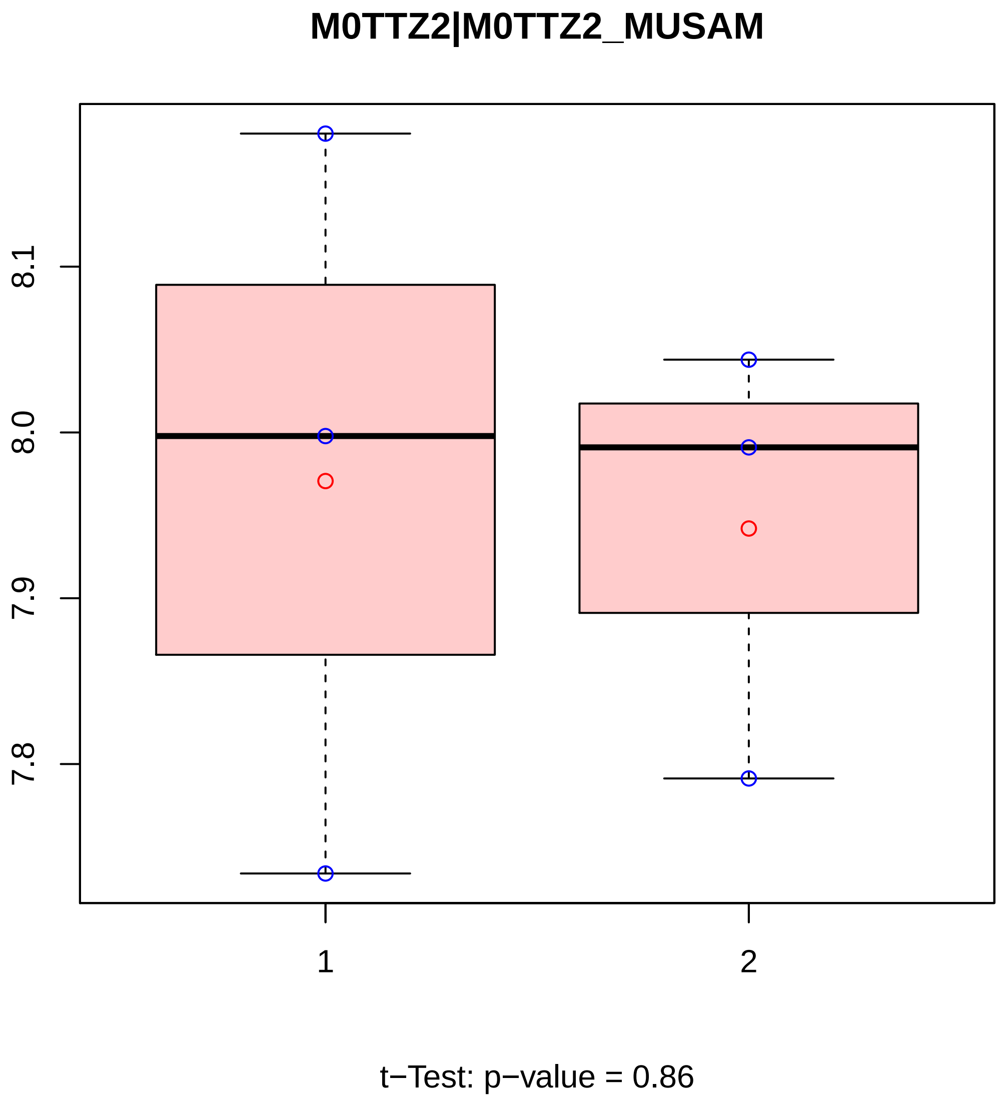

M0TTZ2|M0TTZ2_MUSAM
8.1
8.0
7.9
7.8
1
2
t−Test: p−value = 0.86

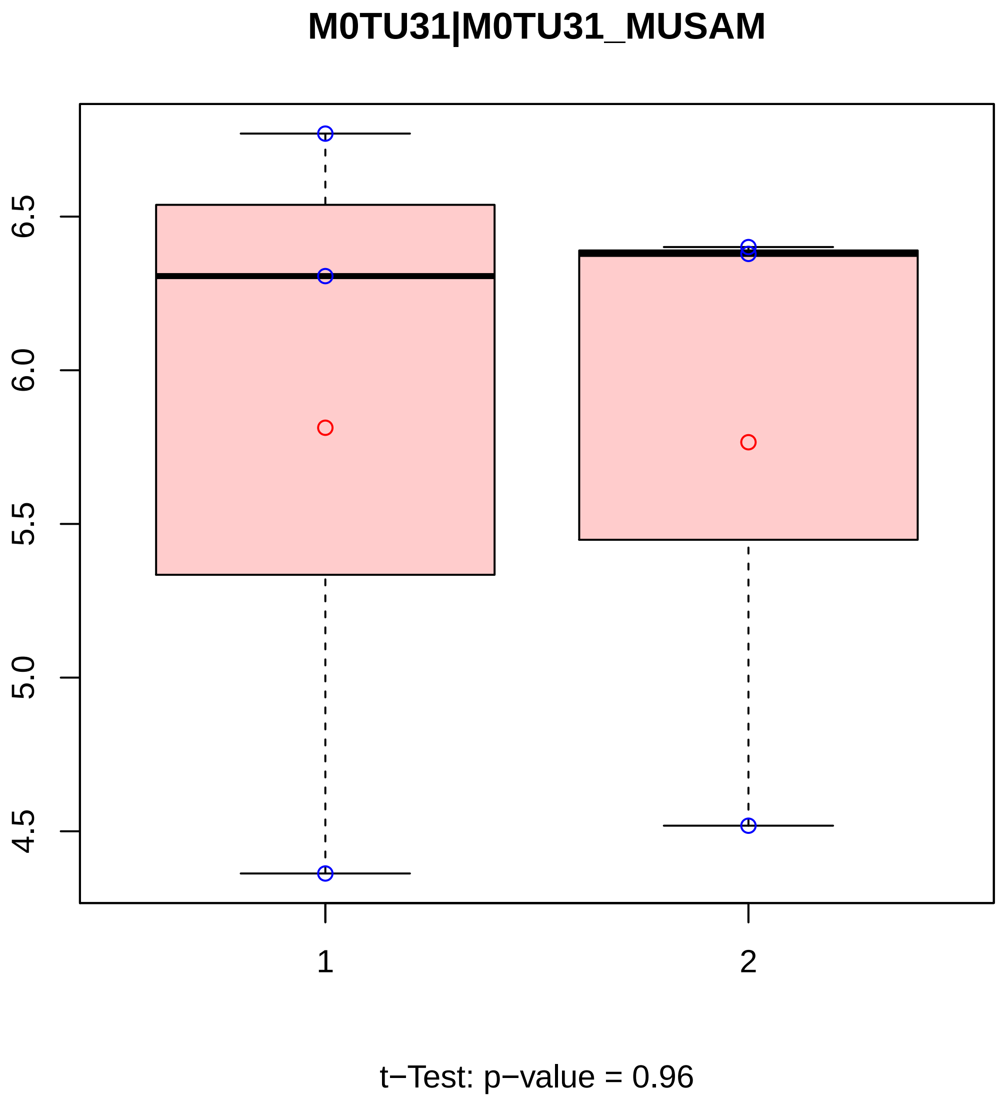

M0TU31|M0TU31_MUSAM
6.5
6.0
5.5
5.0
4.5
1
2
t−Test: p−value = 0.96

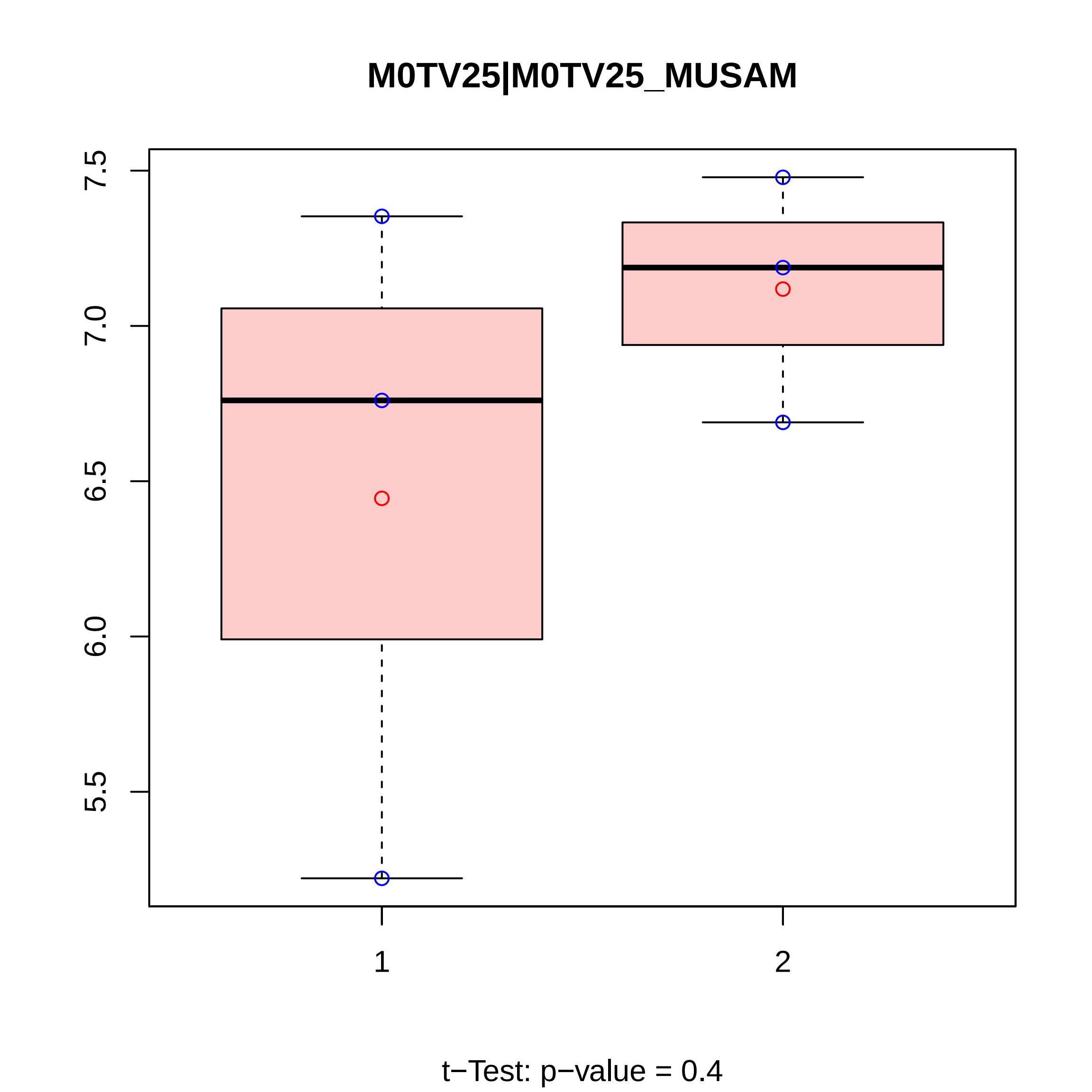

M0TV25|M0TV25_MUSAM
7.5
7.0
6.5
6.0
5.5
1
2
t−Test: p−value = 0.4

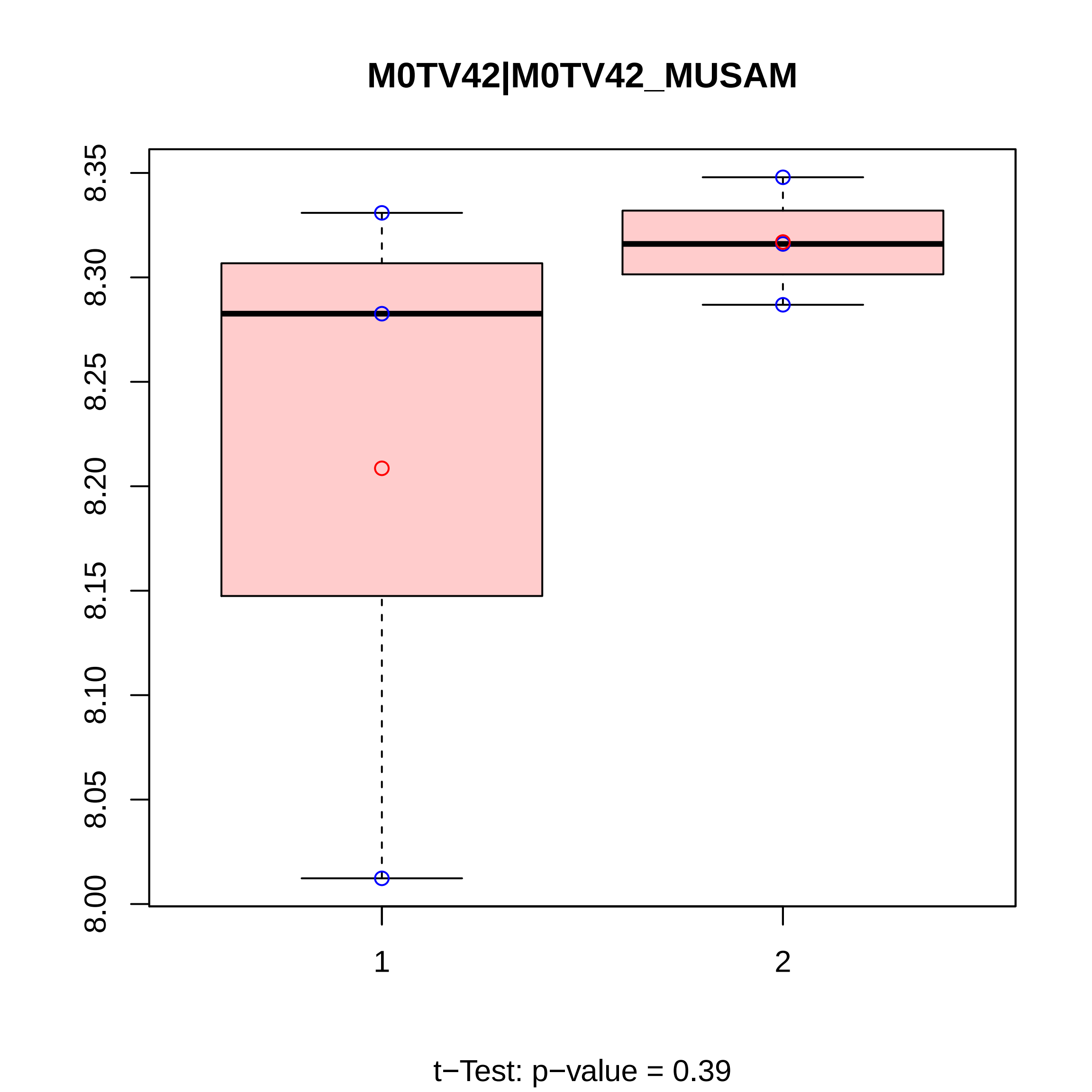
M0TV42|M0TV42_MUSAM
8.00
8.05
8.10
8.15
8.20
8.25
8.30
8.35
1
2
t−Test: p−value = 0.39

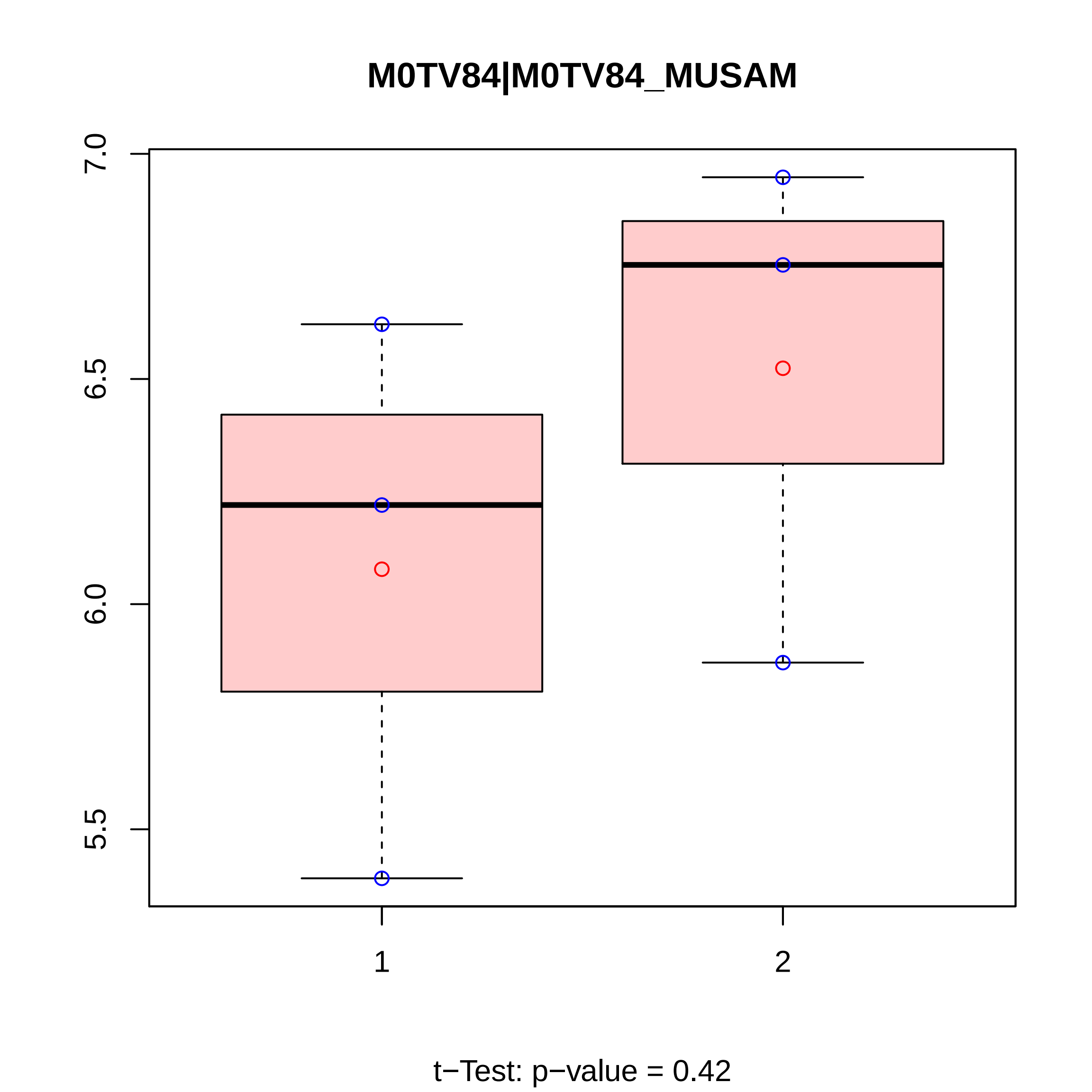

M0TV84|M0TV84_MUSAM
7.0
6.5
6.0
5.5
1
2
t−Test: p−value = 0.42

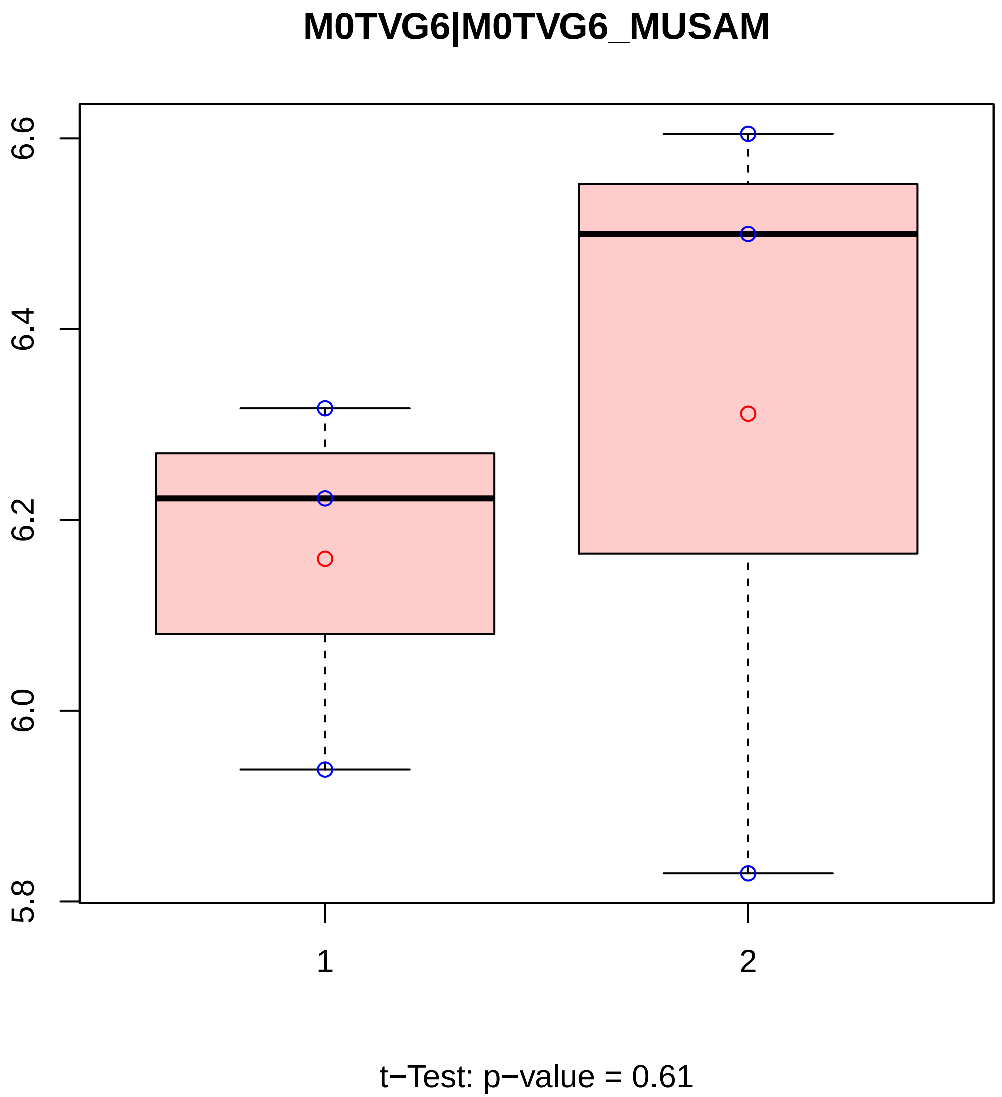

M0TVG6|M0TVG6_MUSAM
6.6
6.4
6.2
6.0
5.8
1
2
t−Test: p−value = 0.61

M0TVU9|M0TVU9_MUSAM

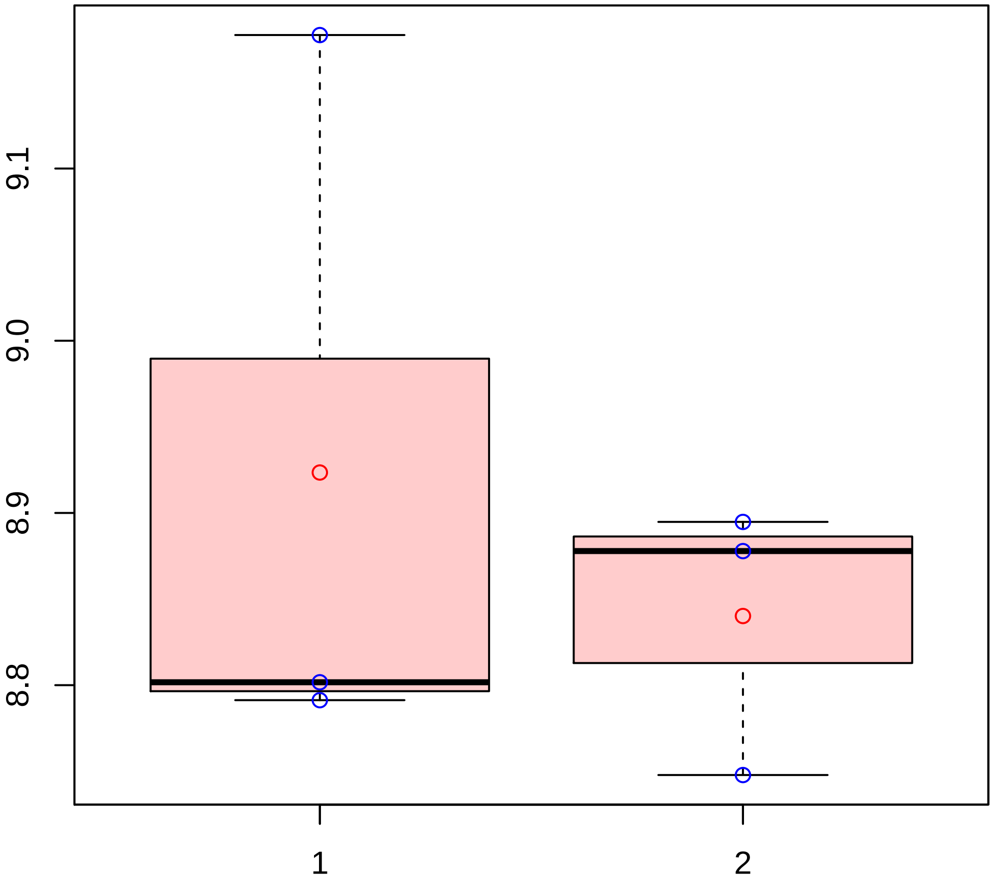

t−Test: p−value = 0.59

M0TVY0|M0TVY0_MUSAM

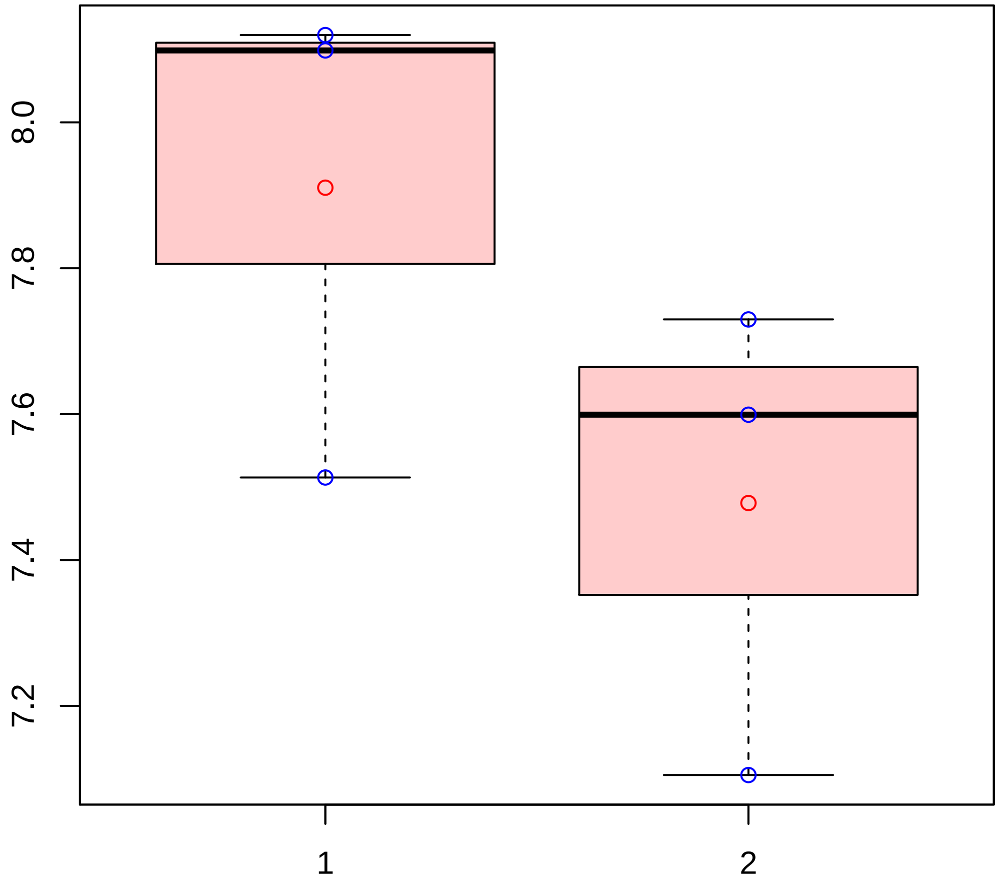

t−Test: p−value = 0.19

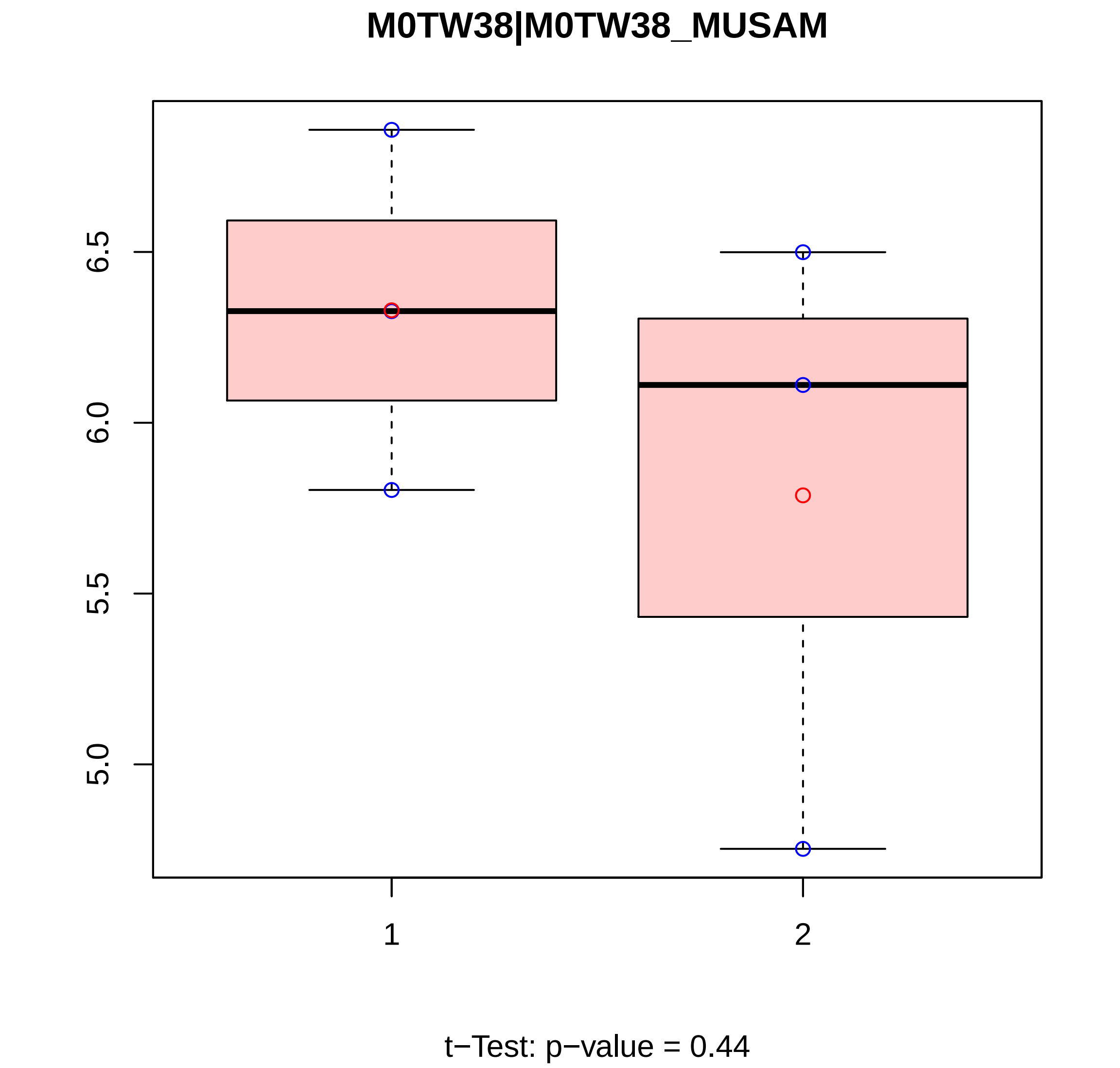
M0TW38|M0TW38_MUSAM
5.0
5.5
6.0
6.5
1
2
t−Test: p−value = 0.44

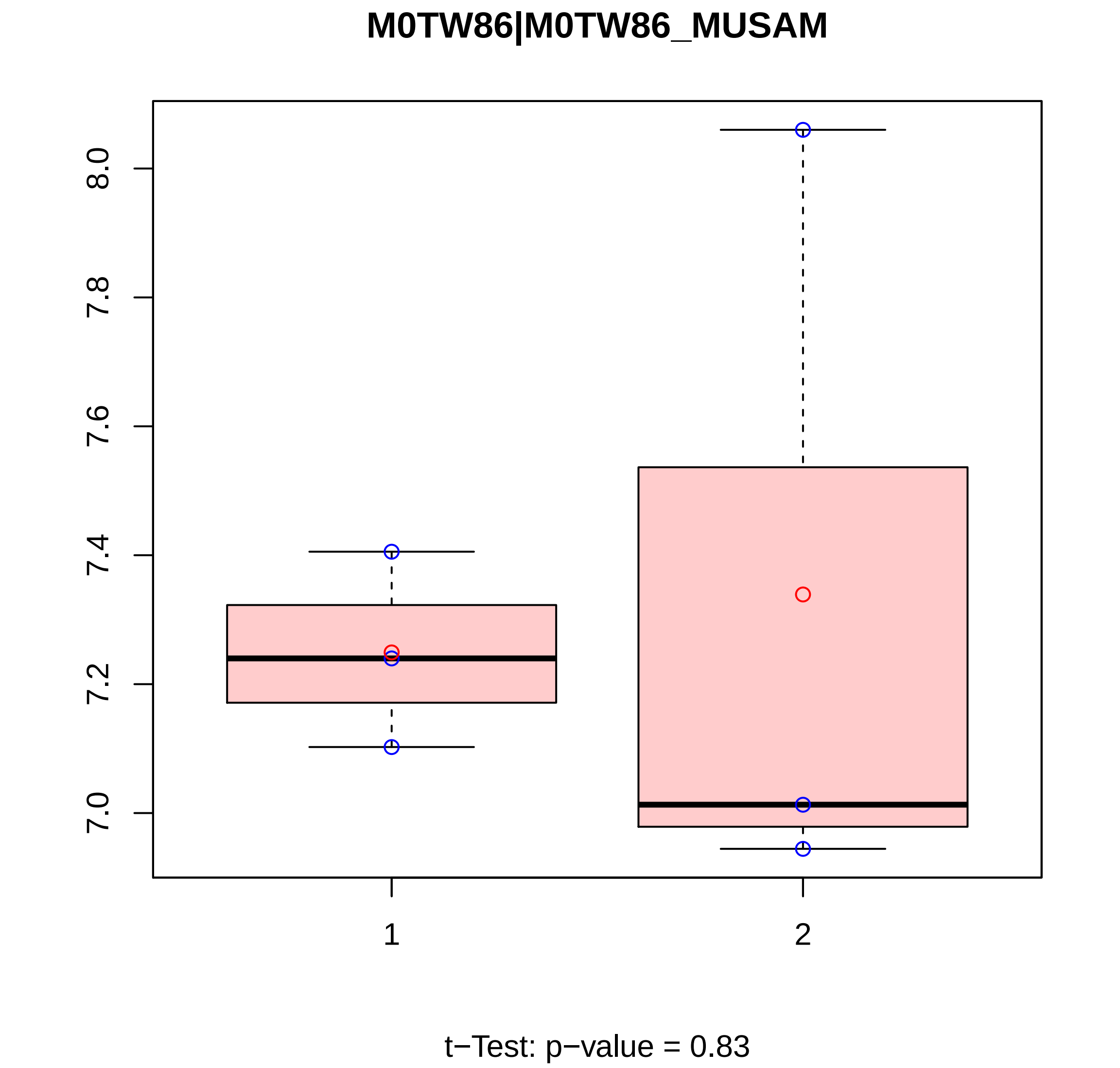
M0TW86|M0TW86_MUSAM
8.0
7.8
7.6
7.4
7.2
7.0
1
2
t−Test: p−value = 0.83

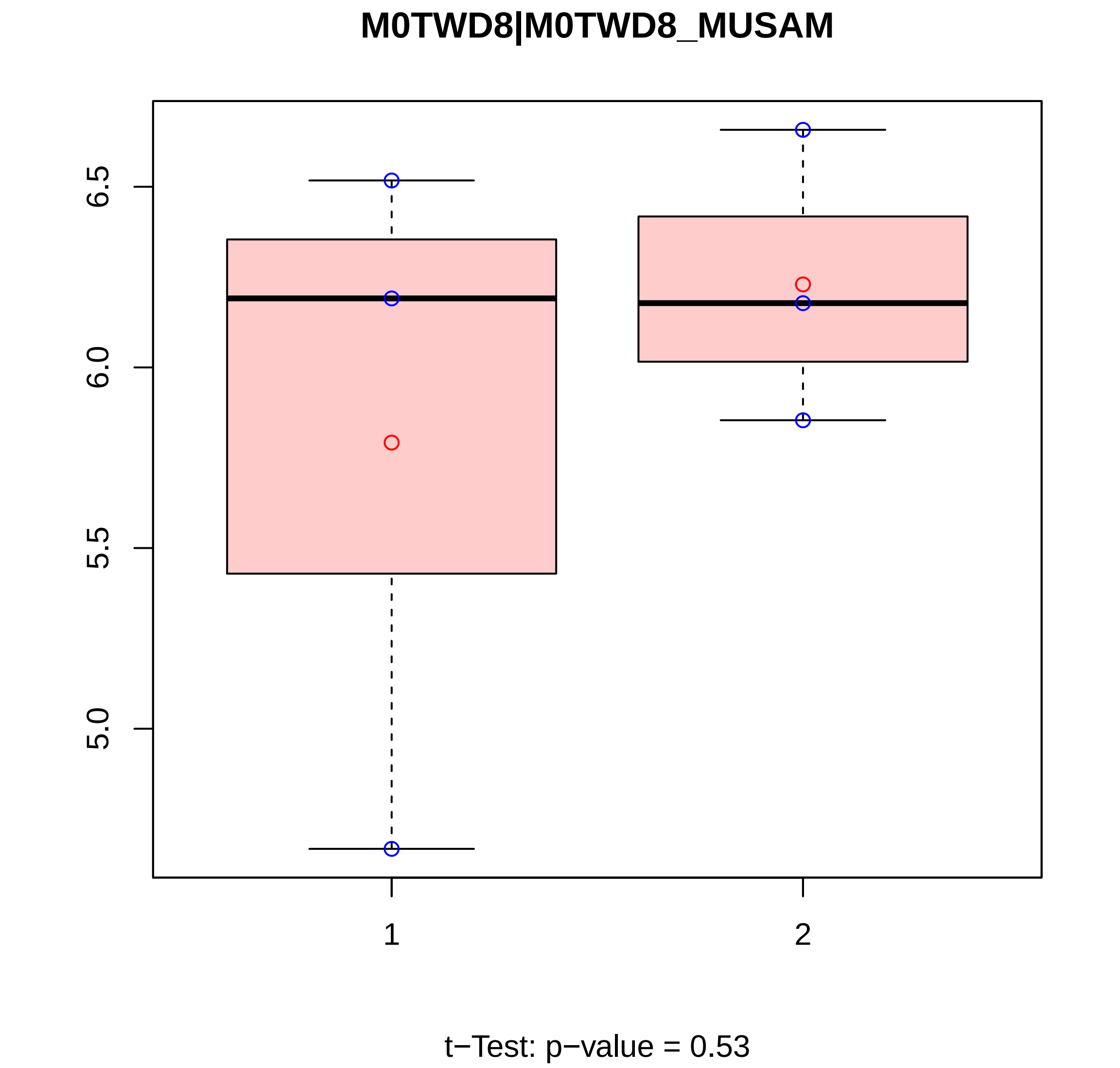

M0TWD8|M0TWD8_MUSAM
6.5
6.0
5.5
5.0
1
2
t−Test: p−value = 0.53

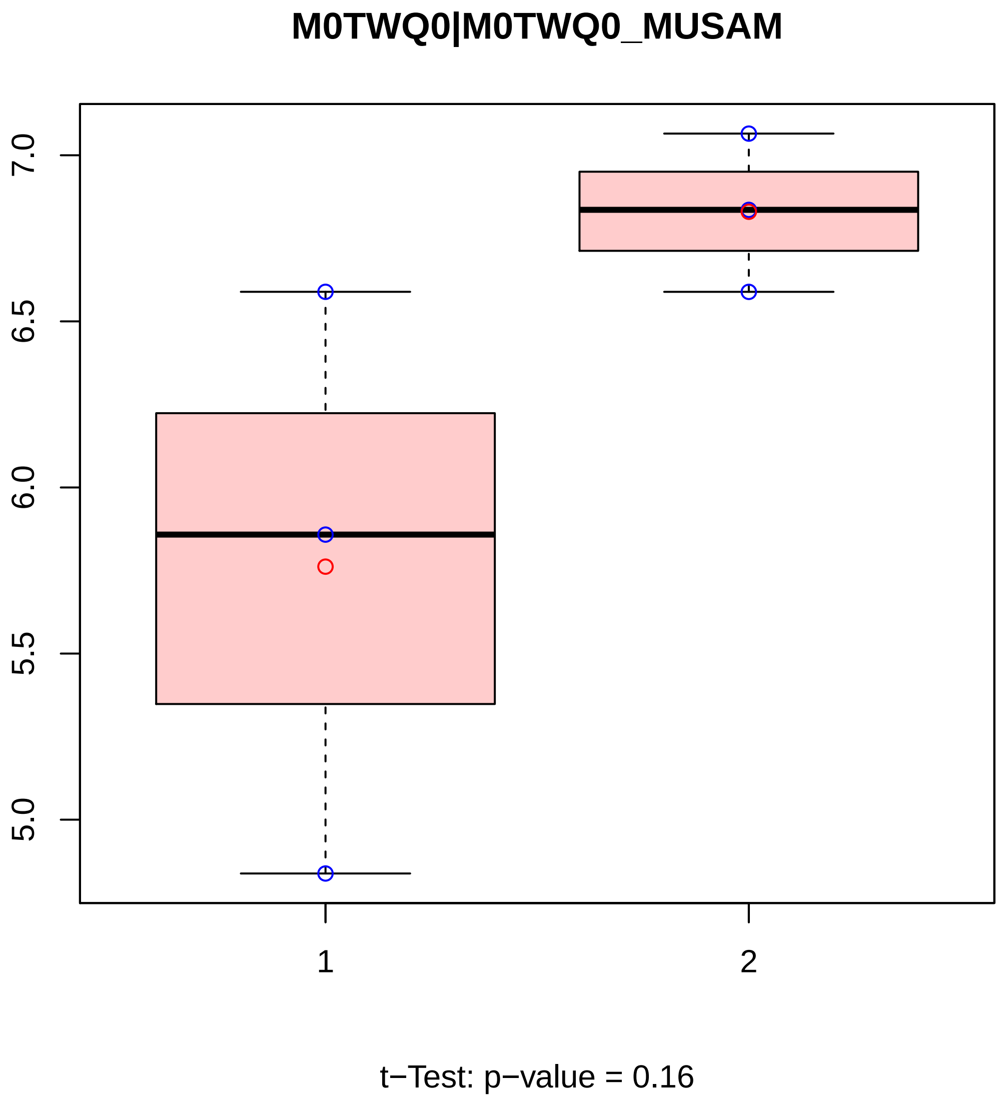

M0TWQ0|M0TWQ0_MUSAM
7.0
6.5
6.0
5.5
5.0
1
2
t−Test: p−value = 0.16

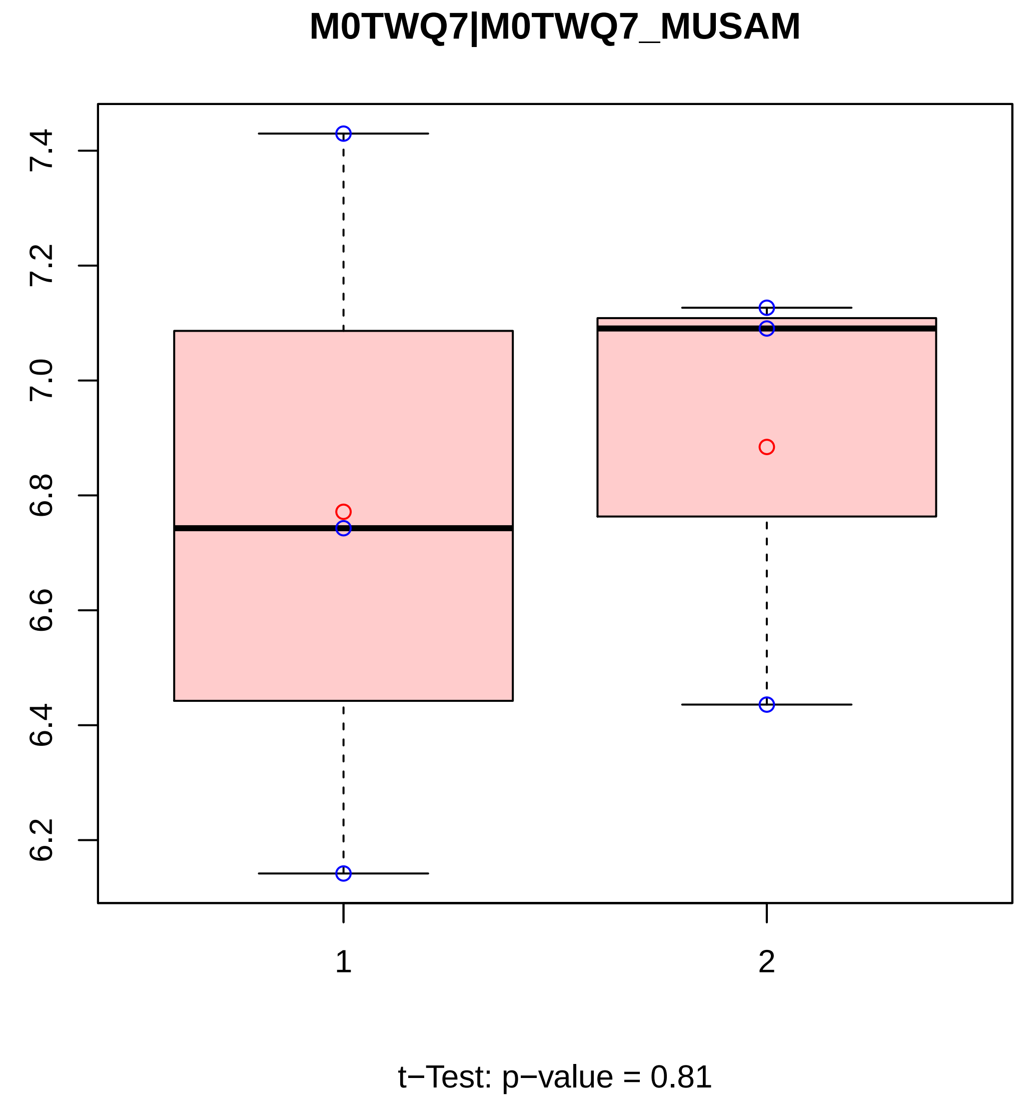
M0TWQ7|M0TWQ7_MUSAM
7.4
7.2
7.0
6.8
6.6
6.4
6.2
1
2
t−Test: p−value = 0.81

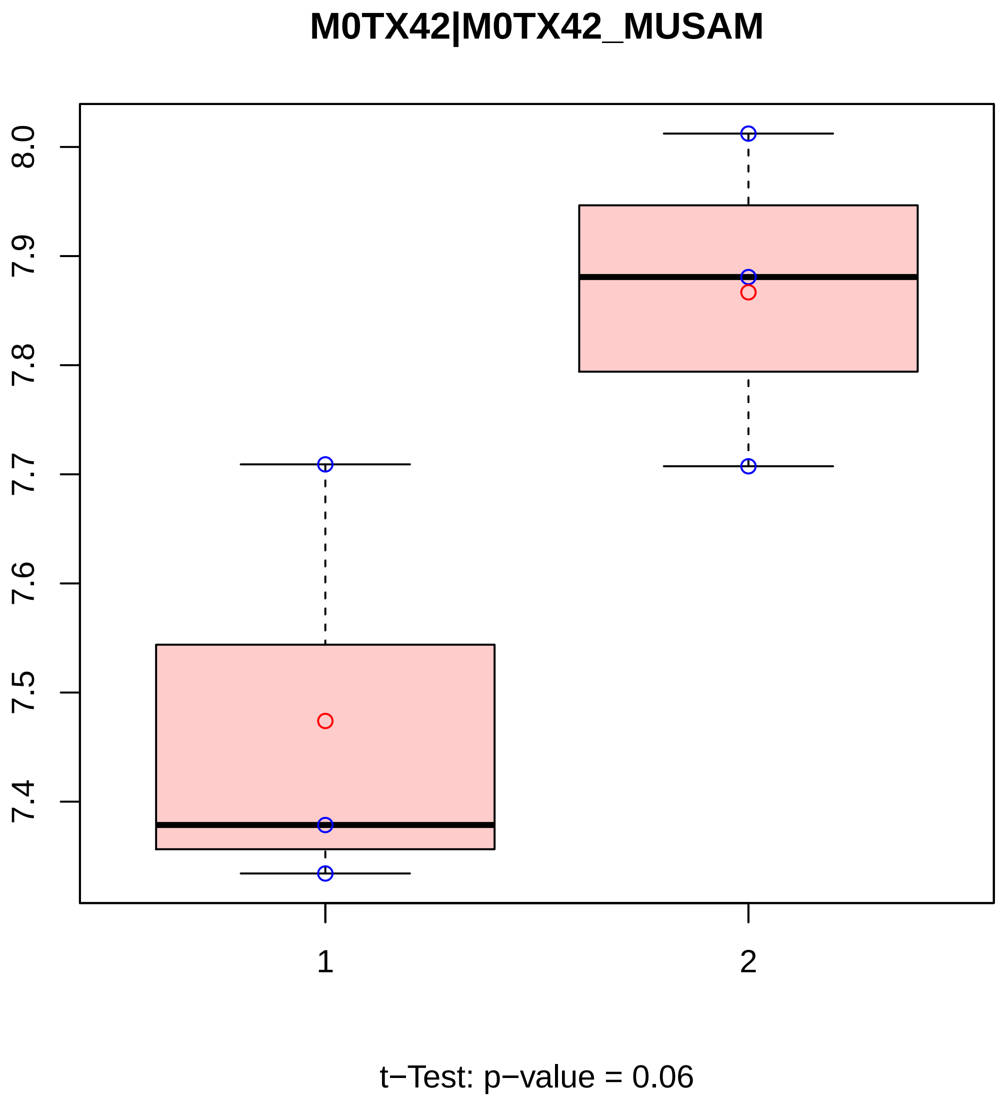
M0TX42|M0TX42_MUSAM
8.0
7.9
7.8
7.7
7.6
7.5
7.4
1
2
t−Test: p−value = 0.06

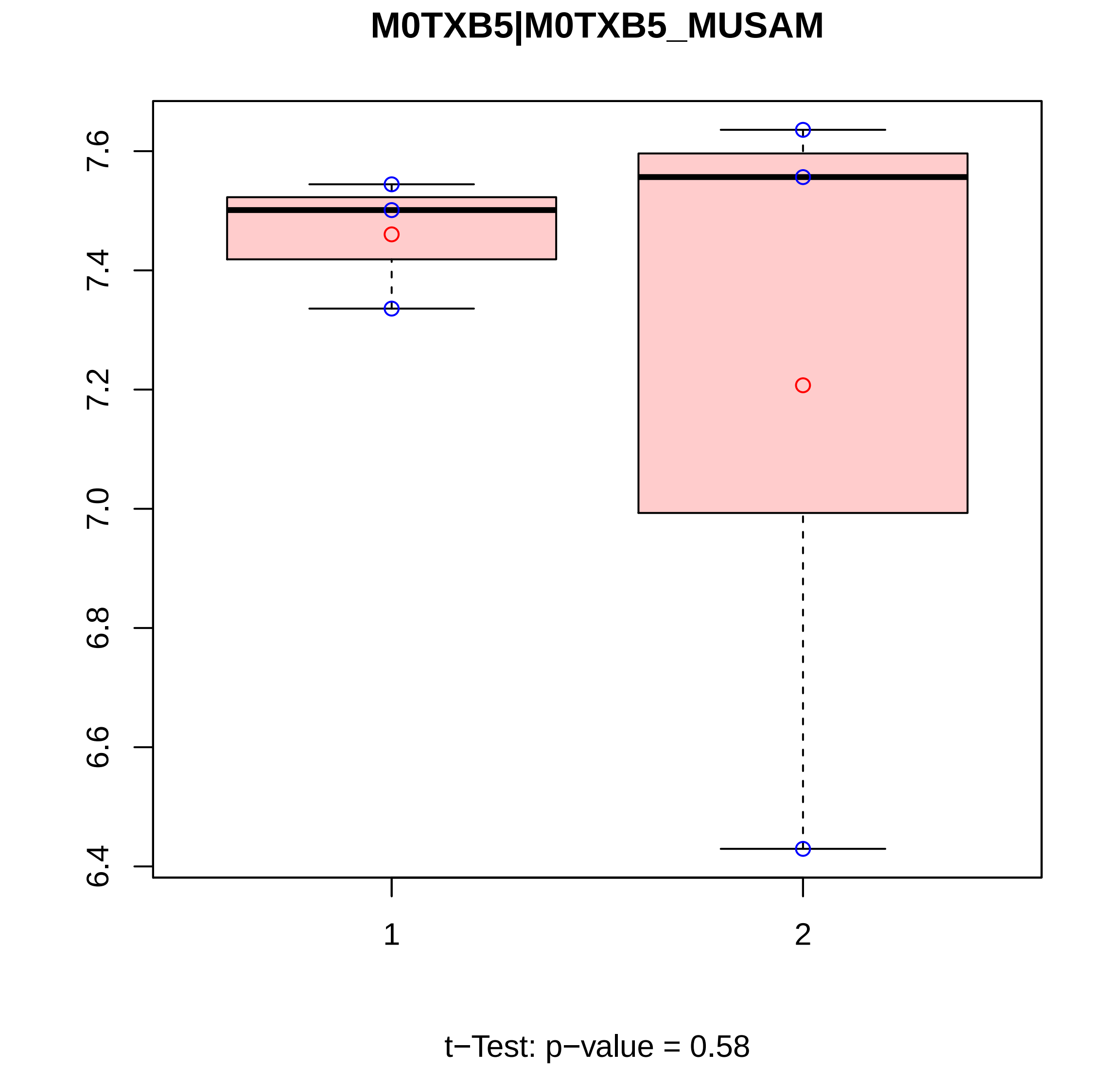
M0TXB5|M0TXB5_MUSAM
7.6
7.4
7.2
7.0
6.8
6.6
6.4
1
2
t−Test: p−value = 0.58

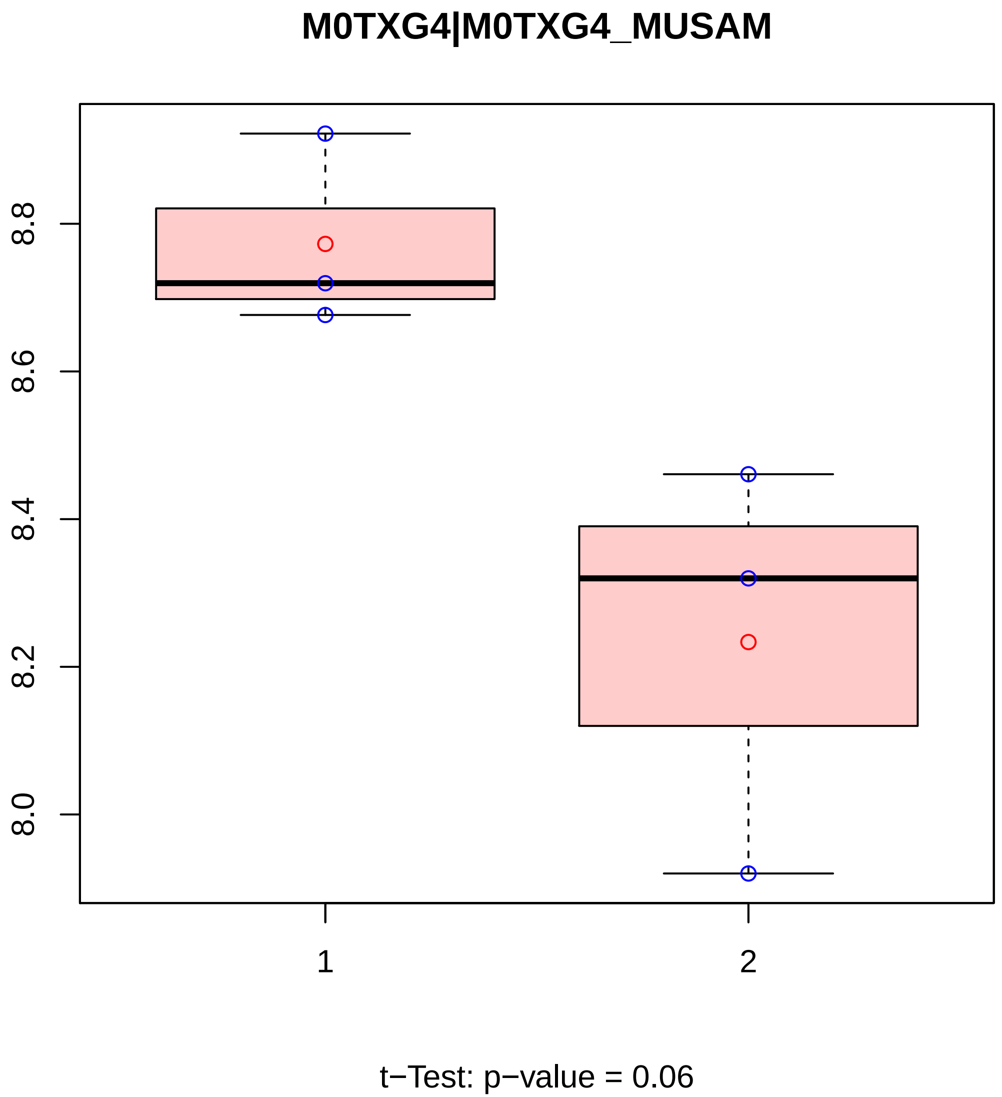

M0TXG4|M0TXG4_MUSAM
8.0
8.2
8.4
8.6
8.8
1
2
t−Test: p−value = 0.06

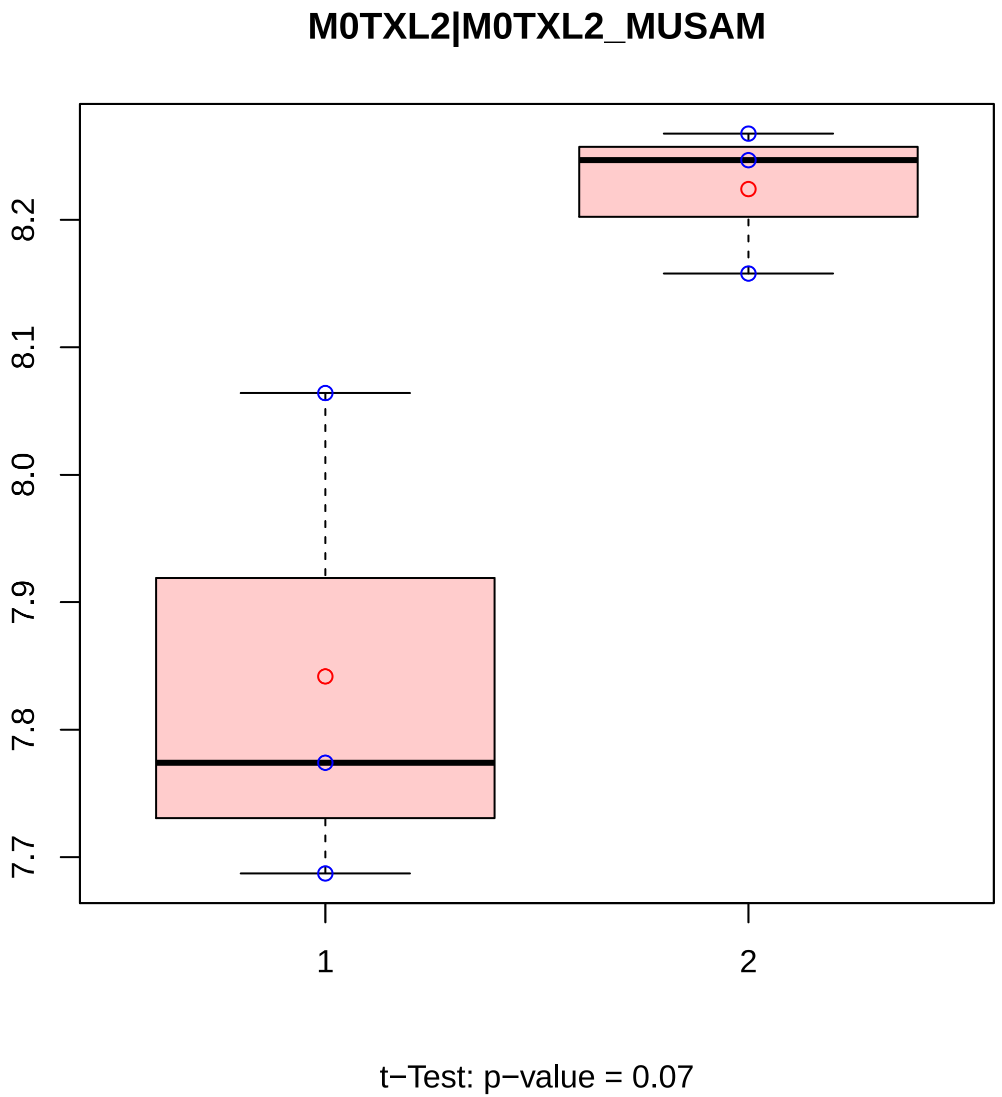
M0TXL2|M0TXL2_MUSAM
8.2
8.1
8.0
7.9
7.8
7.7
1
2
t−Test: p−value = 0.07

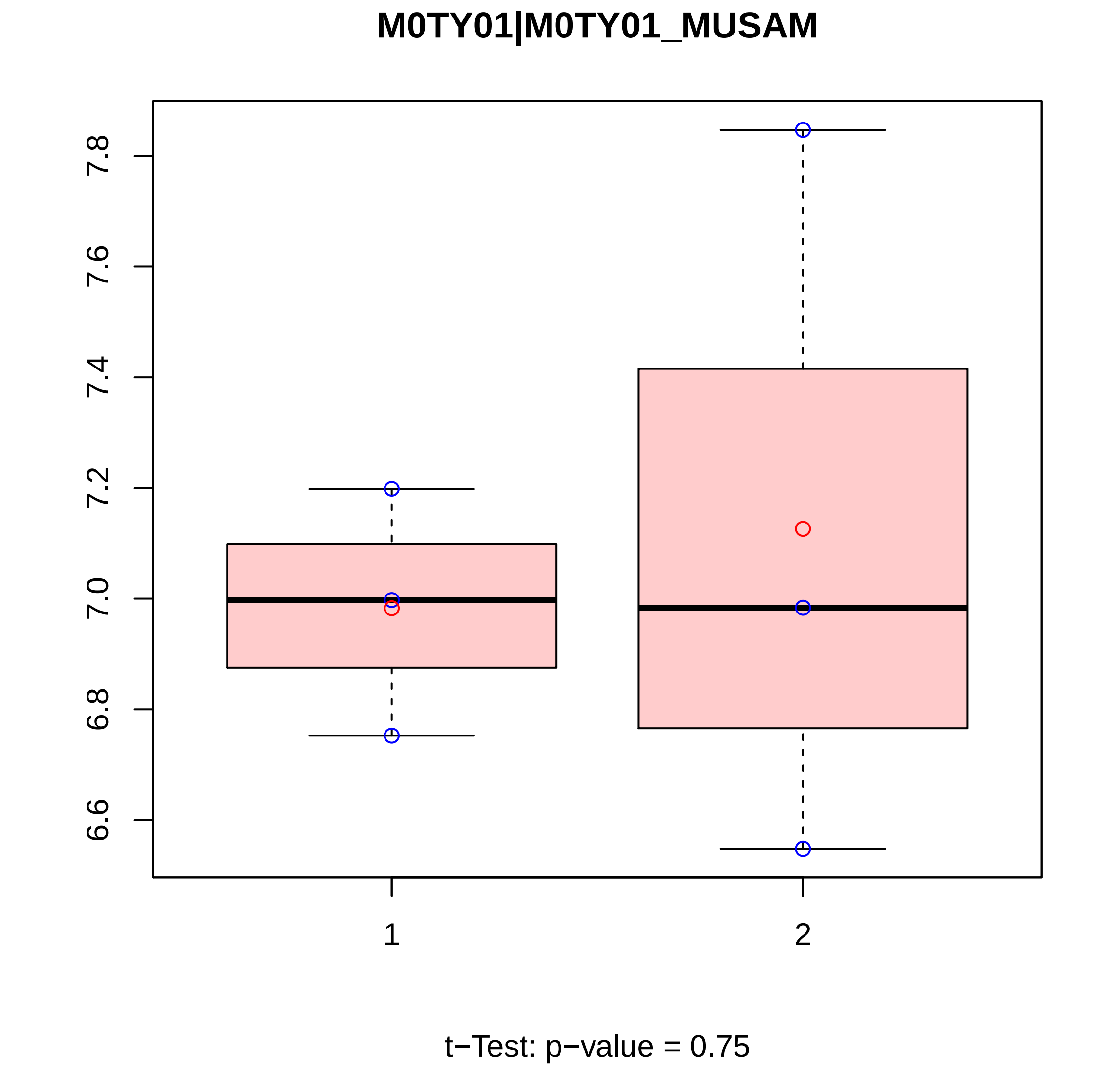
M0TY01|M0TY01_MUSAM
7.8
7.6
7.4
7.2
7.0
6.8
6.6
1
2
t−Test: p−value = 0.75

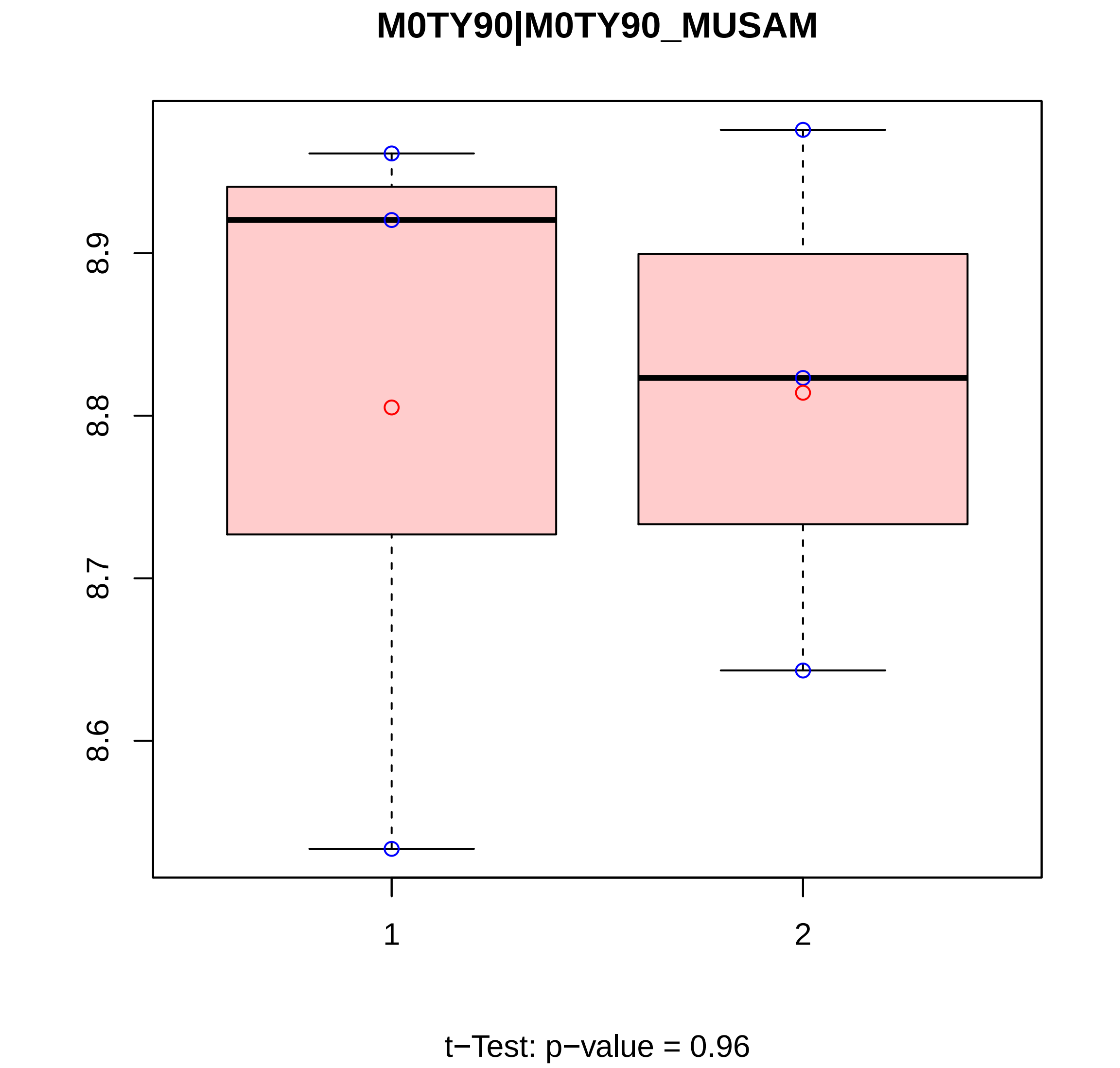
M0TY90|M0TY90_MUSAM
8.9
8.8
8.7
8.6
1
2
t−Test: p−value = 0.96

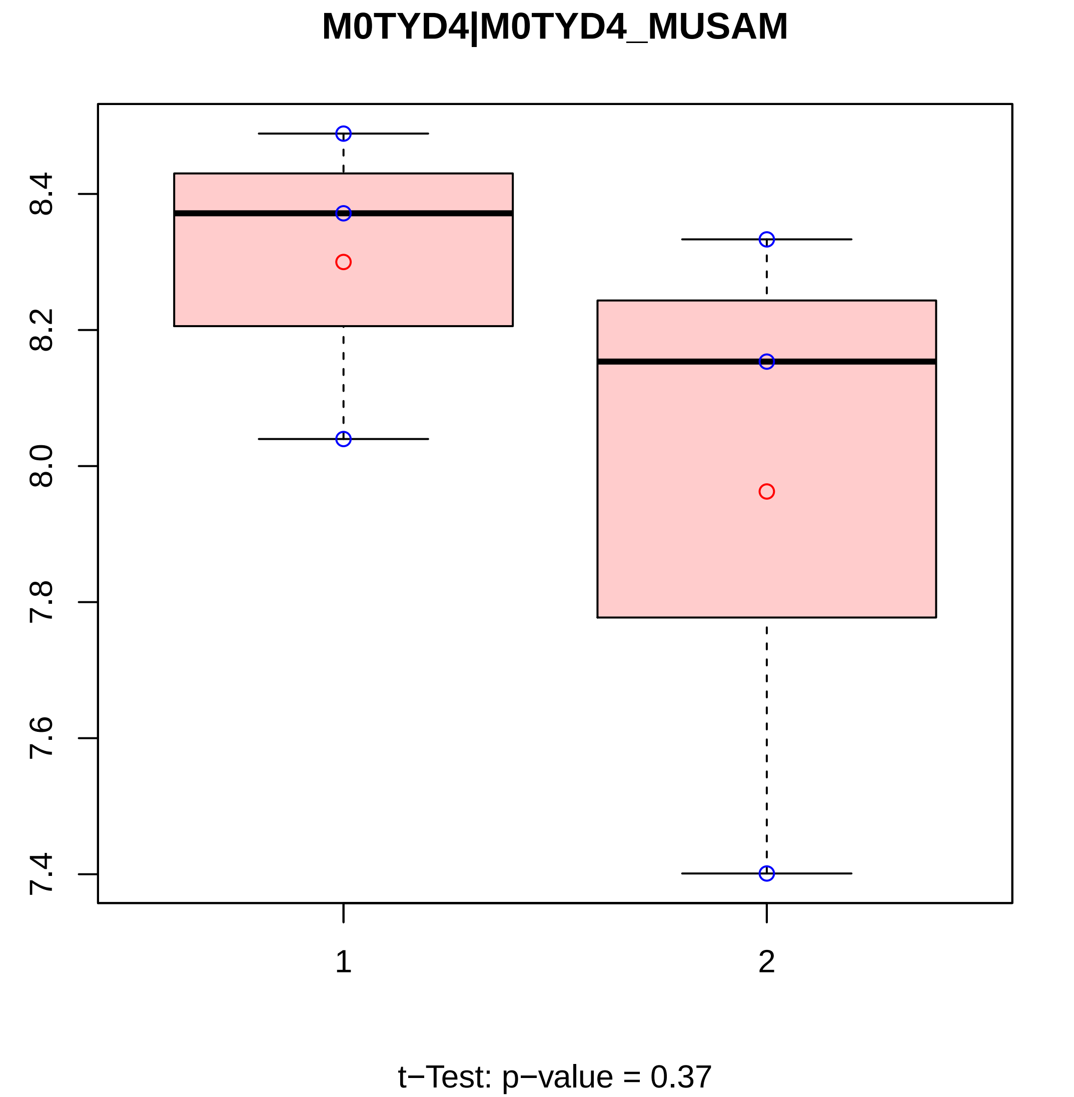
M0TYD4|M0TYD4_MUSAM
8.4
8.2
8.0
7.8
7.6
7.4
1
2
t−Test: p−value = 0.37

M0TZA6|M0TZA6_MUSAM

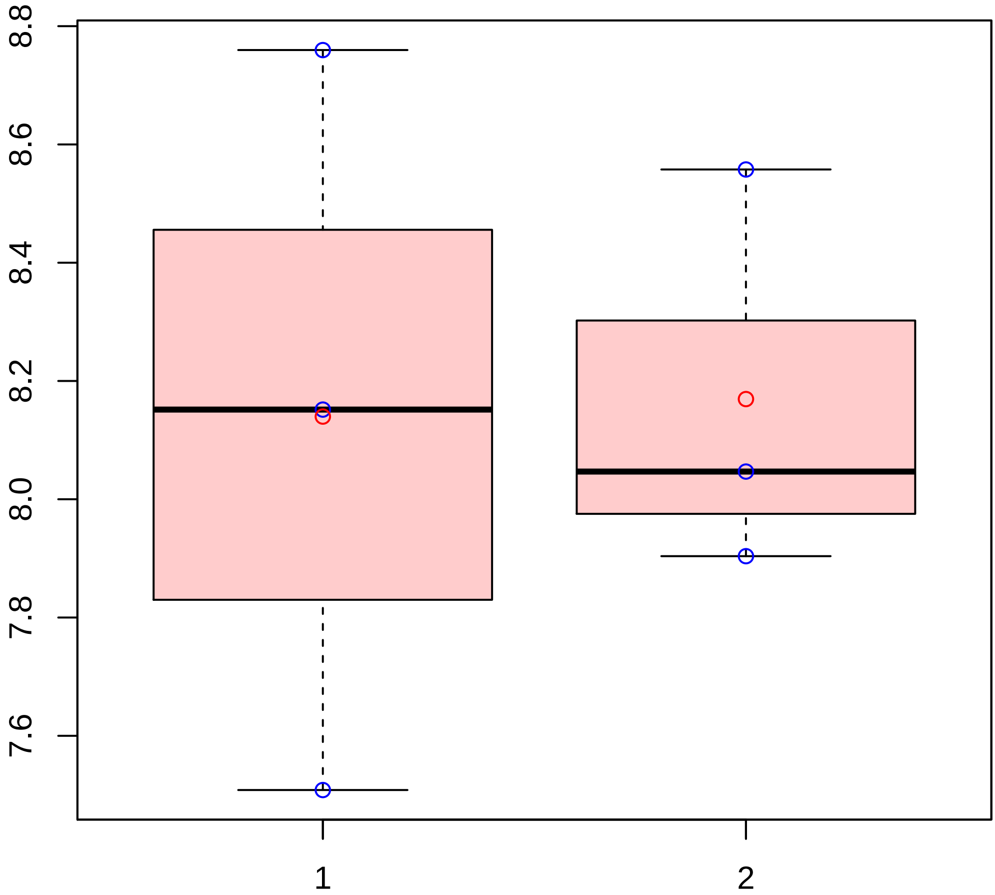

t−Test: p−value = 0.95

M0TZE8|M0TZE8_MUSAM

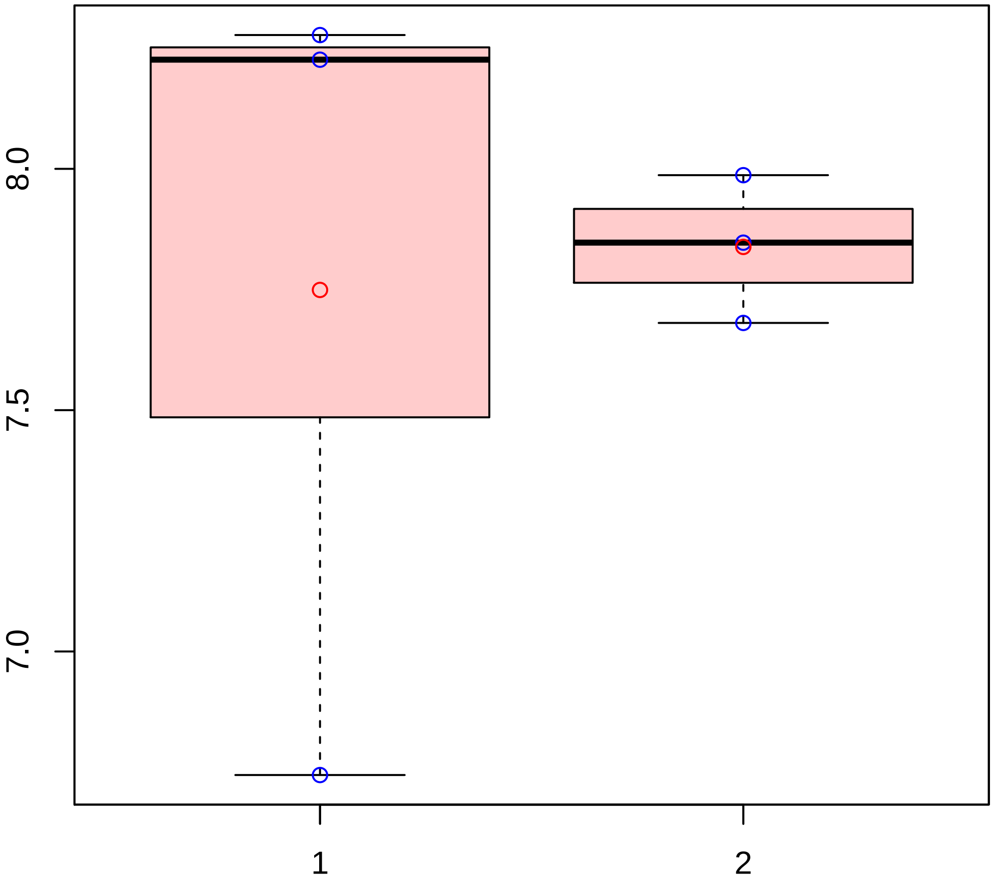

t−Test: p−value = 0.88

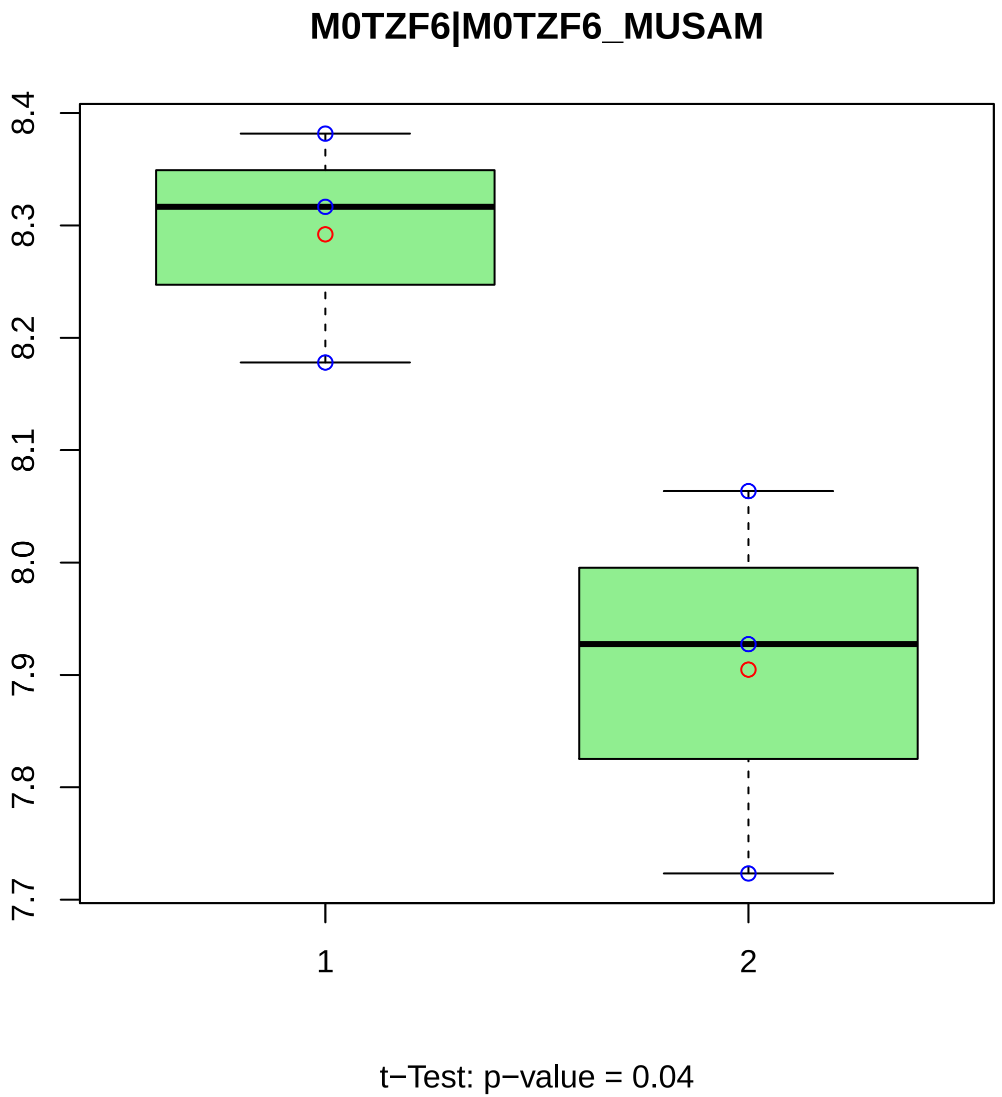
M0TZF6|M0TZF6_MUSAM
8.4
8.3
8.2
8.1
8.0
7.9
7.8
7.7
1
2
t−Test: p−value = 0.04

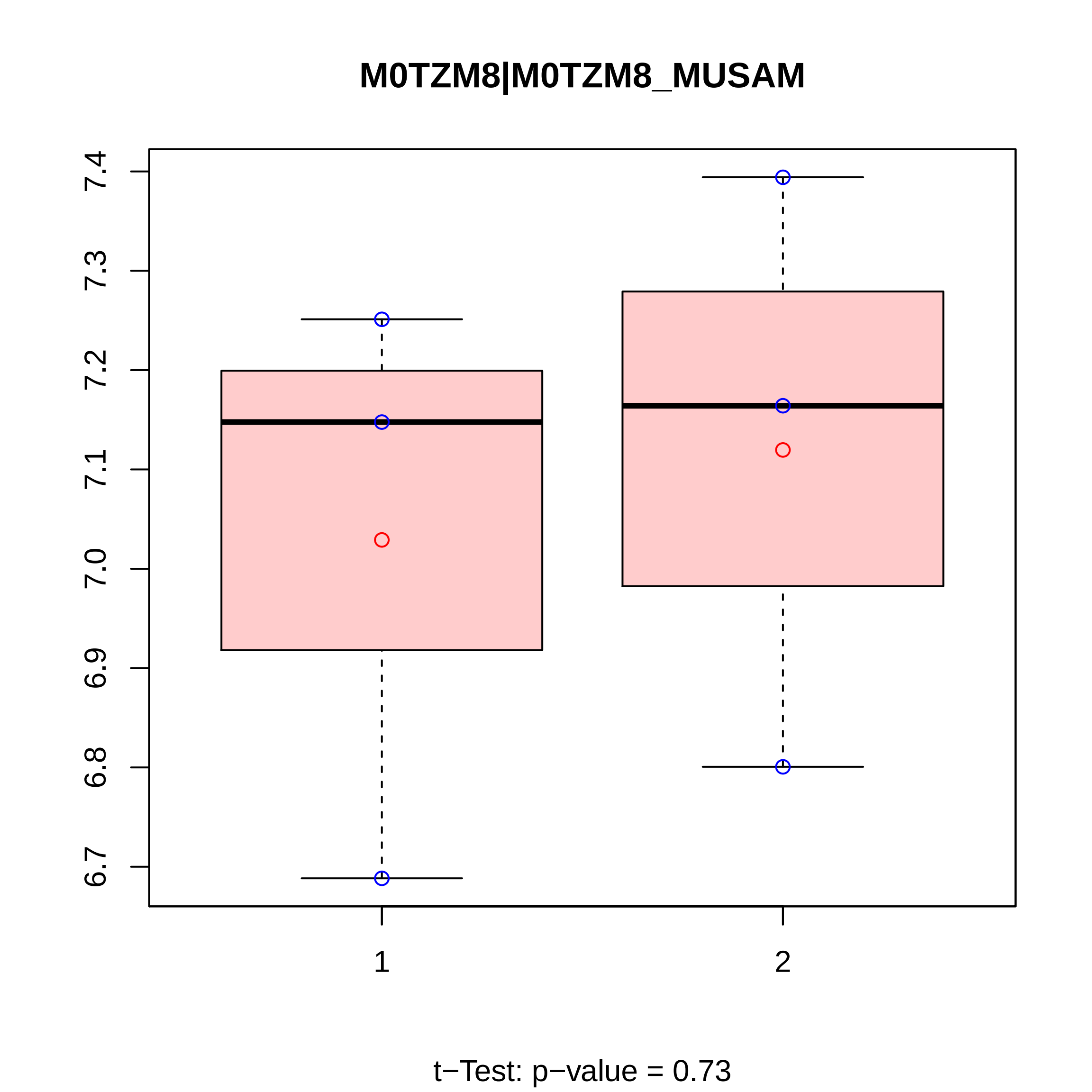
M0TZM8|M0TZM8_MUSAM
7.4
7.3
7.2
7.1
7.0
6.9
6.8
6.7
1
2
t−Test: p−value = 0.73

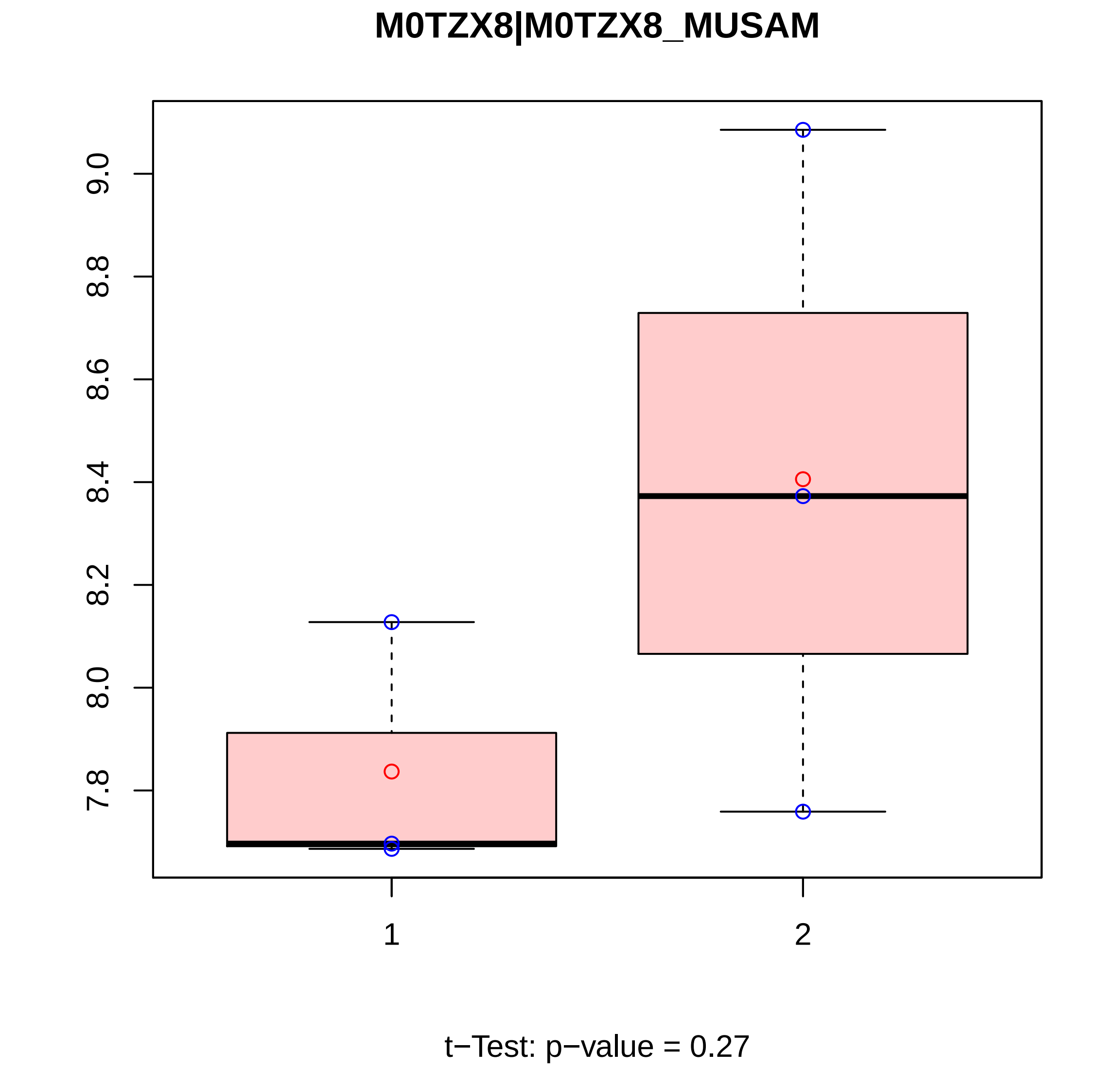
M0TZX8|M0TZX8_MUSAM
9.0
8.8
8.6
8.4
8.2
8.0
7.8
1
2
t−Test: p−value = 0.27

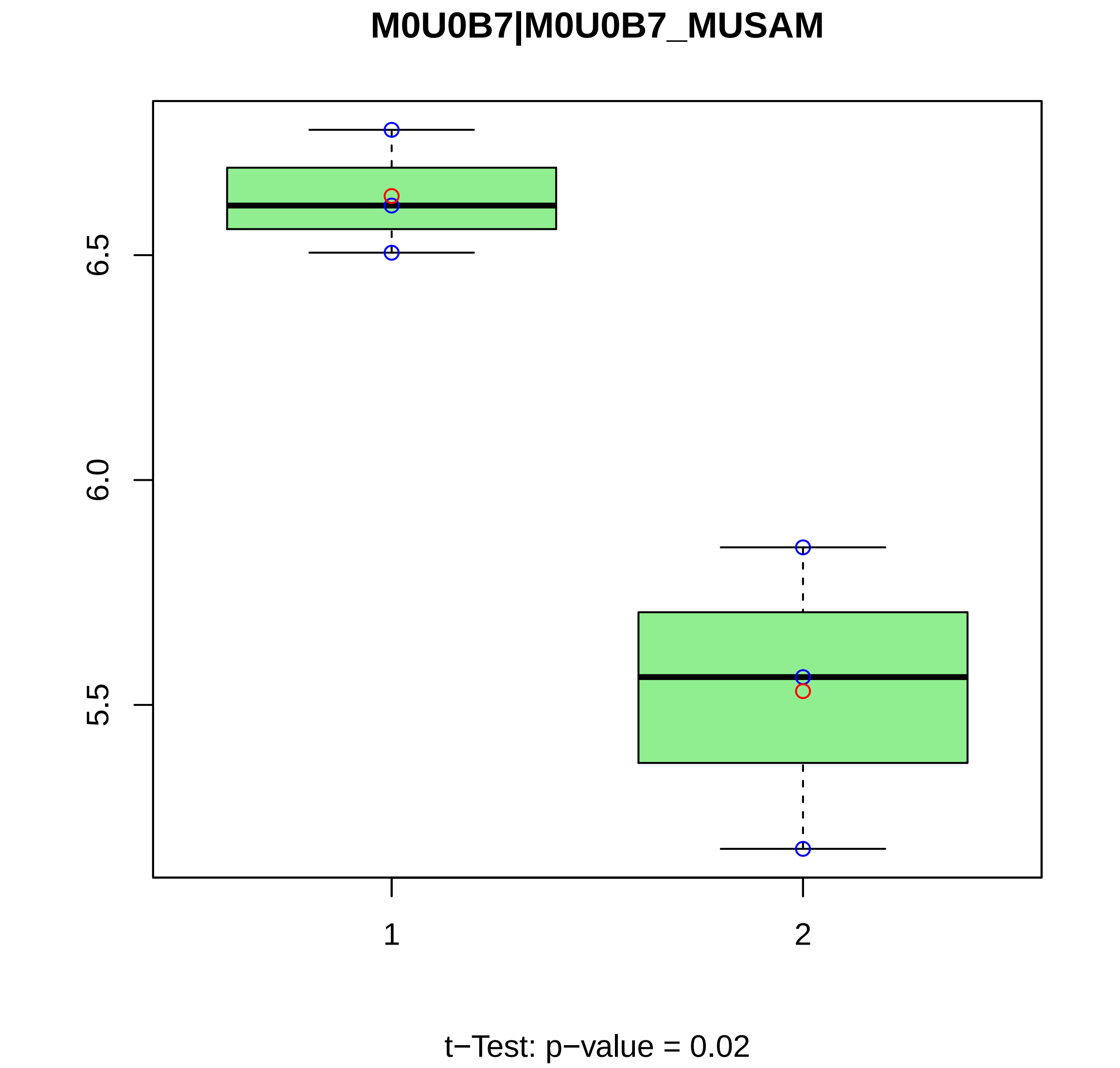
M0U0B7|M0U0B7_MUSAM
6.5
6.0
5.5
1
2
t−Test: p−value = 0.02

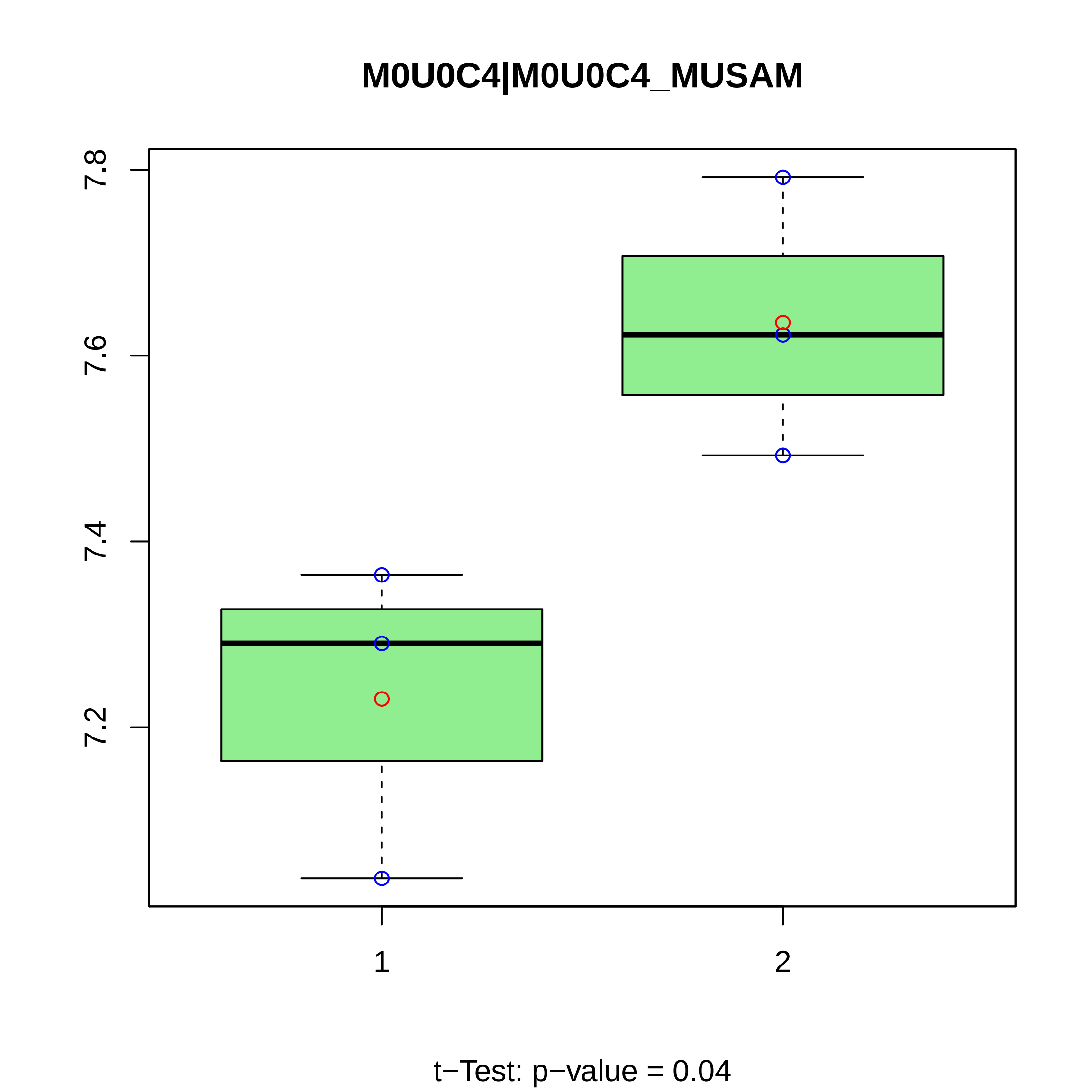
M0U0C4|M0U0C4_MUSAM
7.8
7.6
7.4
7.2
1
2
t−Test: p−value = 0.04

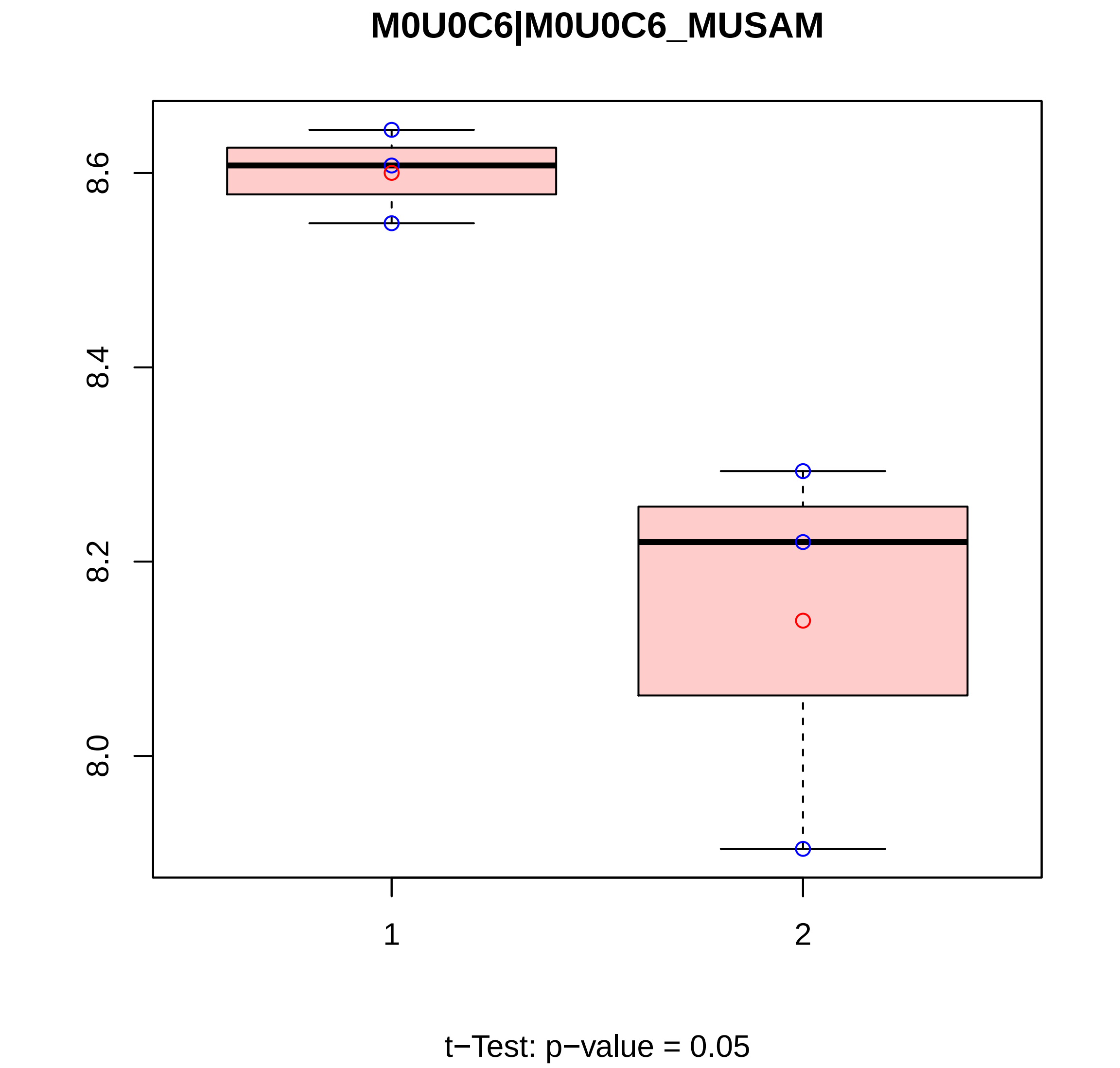
M0U0C6|M0U0C6_MUSAM
8.6
8.4
8.2
8.0
1
2
t−Test: p−value = 0.05

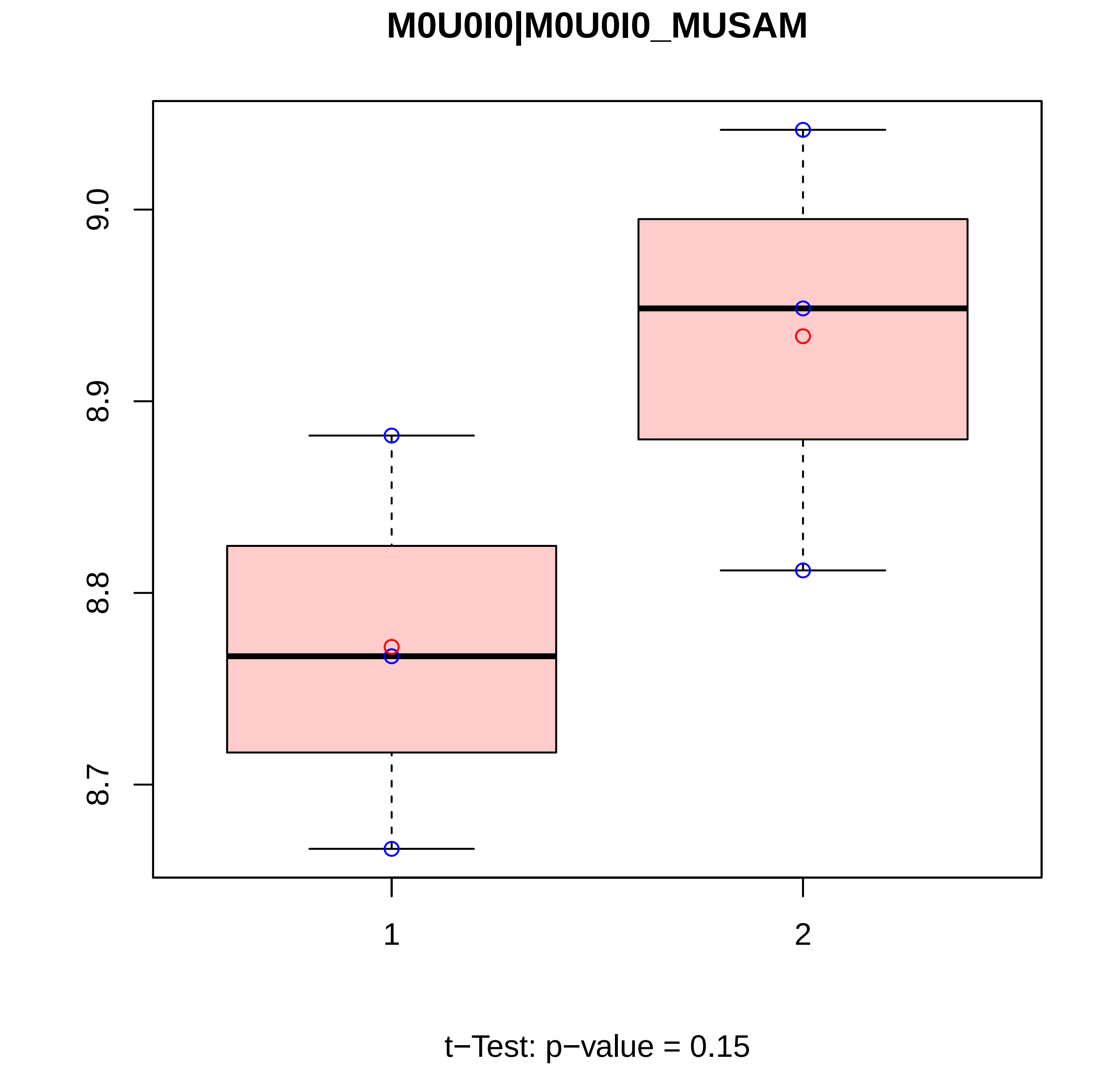
M0U0I0|M0U0I0_MUSAM
9.0
8.9
8.8
8.7
1
2
t−Test: p−value = 0.15

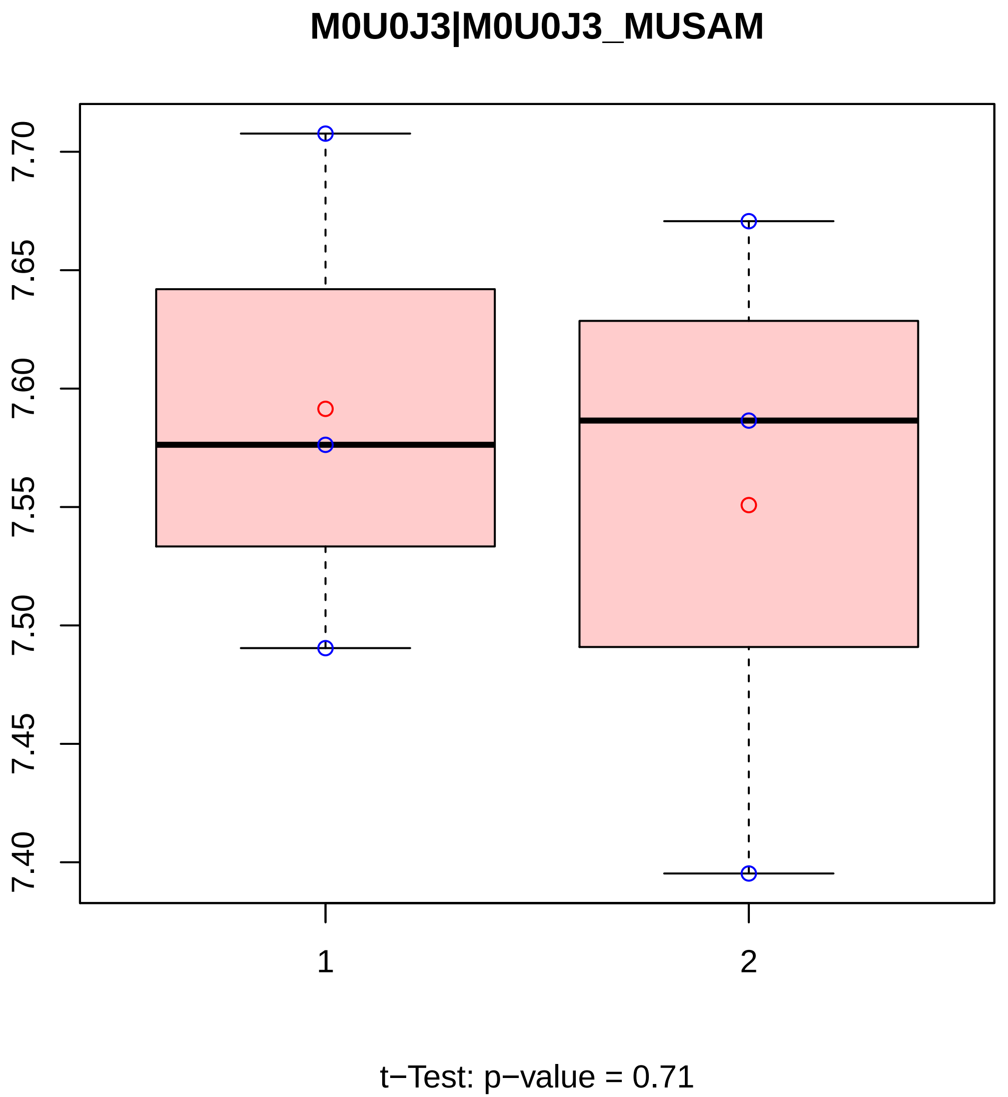
M0U0J3|M0U0J3_MUSAM
7.70
7.65
7.60
7.55
7.50
7.45
7.40
1
2
t−Test: p−value = 0.71

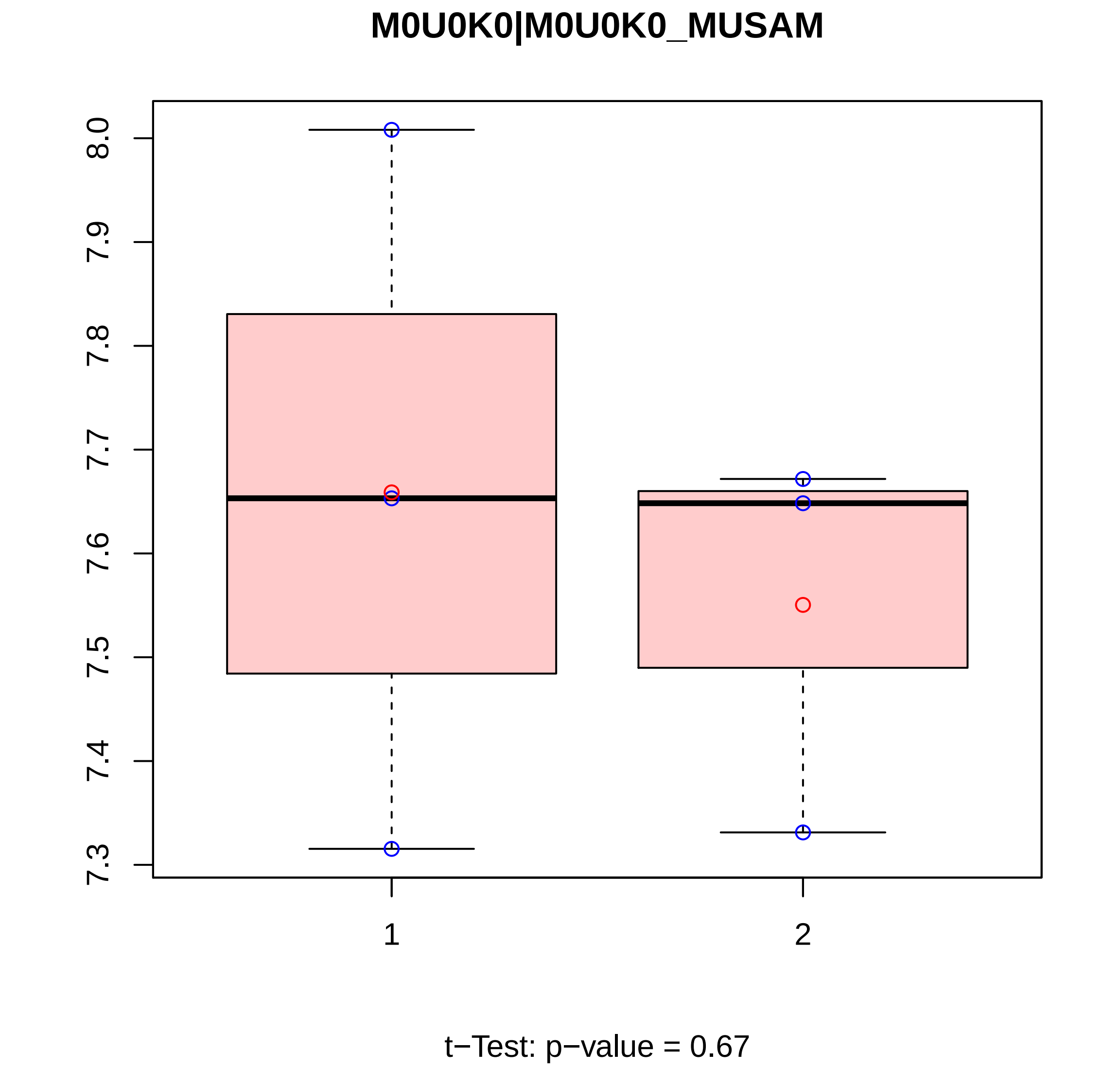
M0U0K0|M0U0K0_MUSAM
8.0
7.9
7.8
7.7
7.6
7.5
7.4
7.3
1
2
t−Test: p−value = 0.67

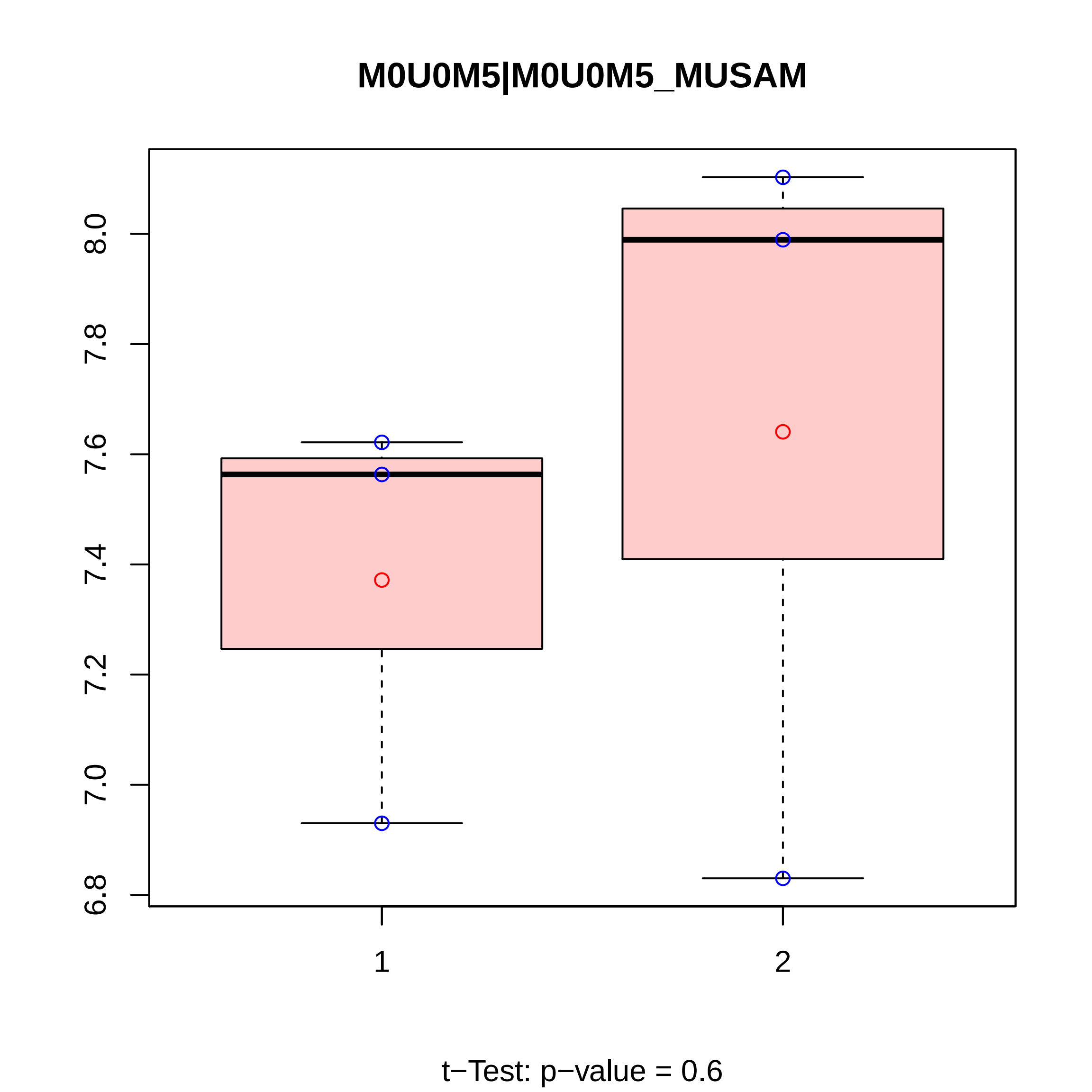

M0U0M5|M0U0M5_MUSAM
8.0
7.8
7.6
7.4
7.2
7.0
6.8
1
2
t−Test: p−value = 0.6

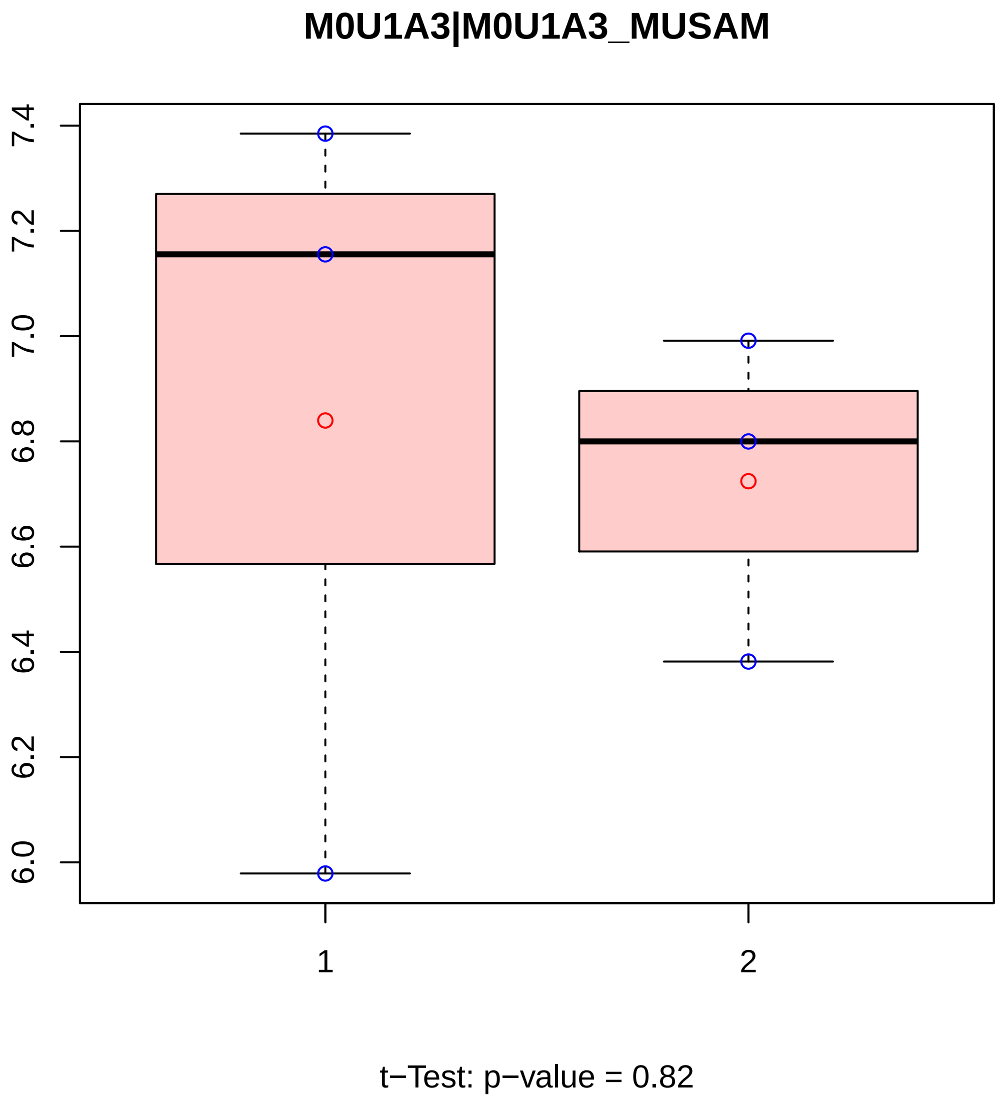
M0U1A3|M0U1A3_MUSAM
7.4
7.2
7.0
6.8
6.6
6.4
6.2
6.0
1
2
t−Test: p−value = 0.82

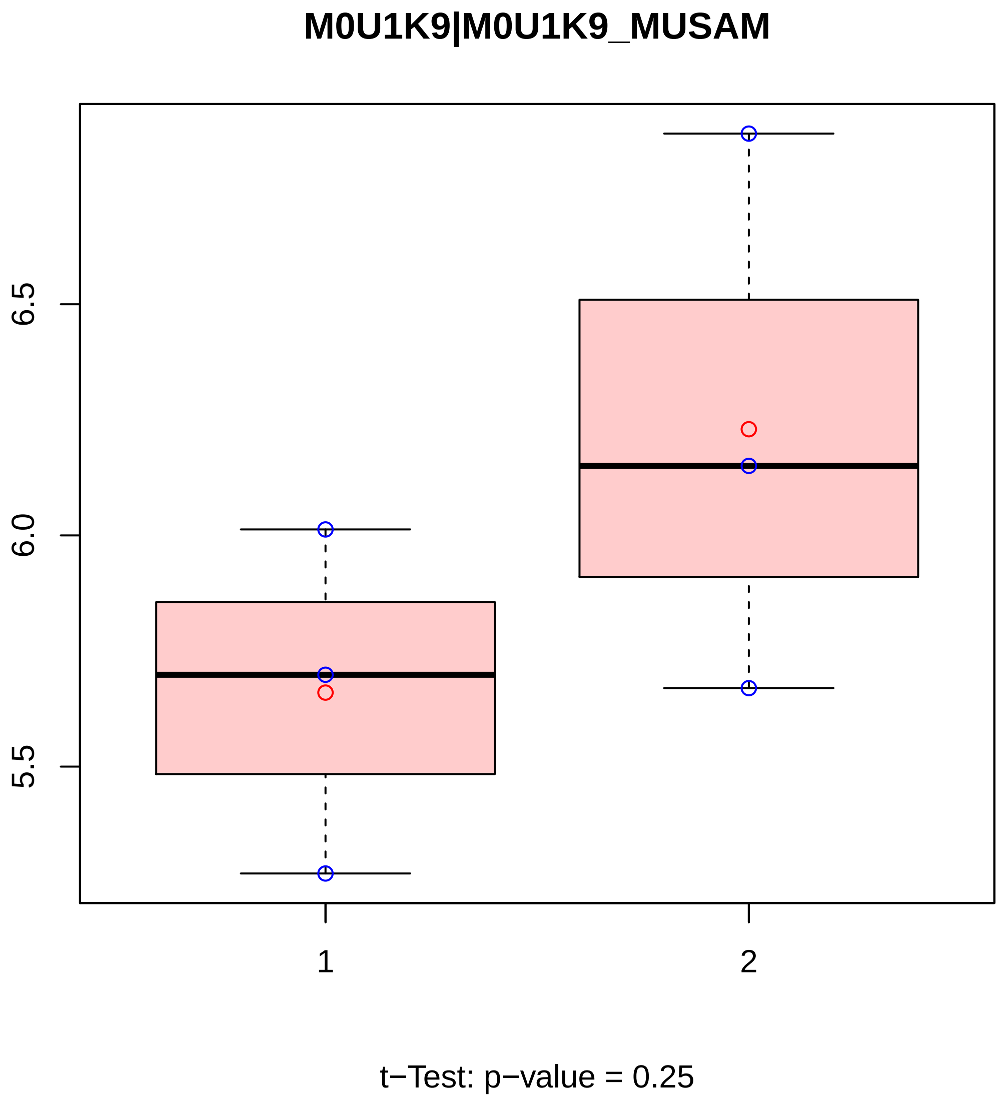
M0U1K9|M0U1K9_MUSAM
6.5
6.0
5.5
1
2
t−Test: p−value = 0.25

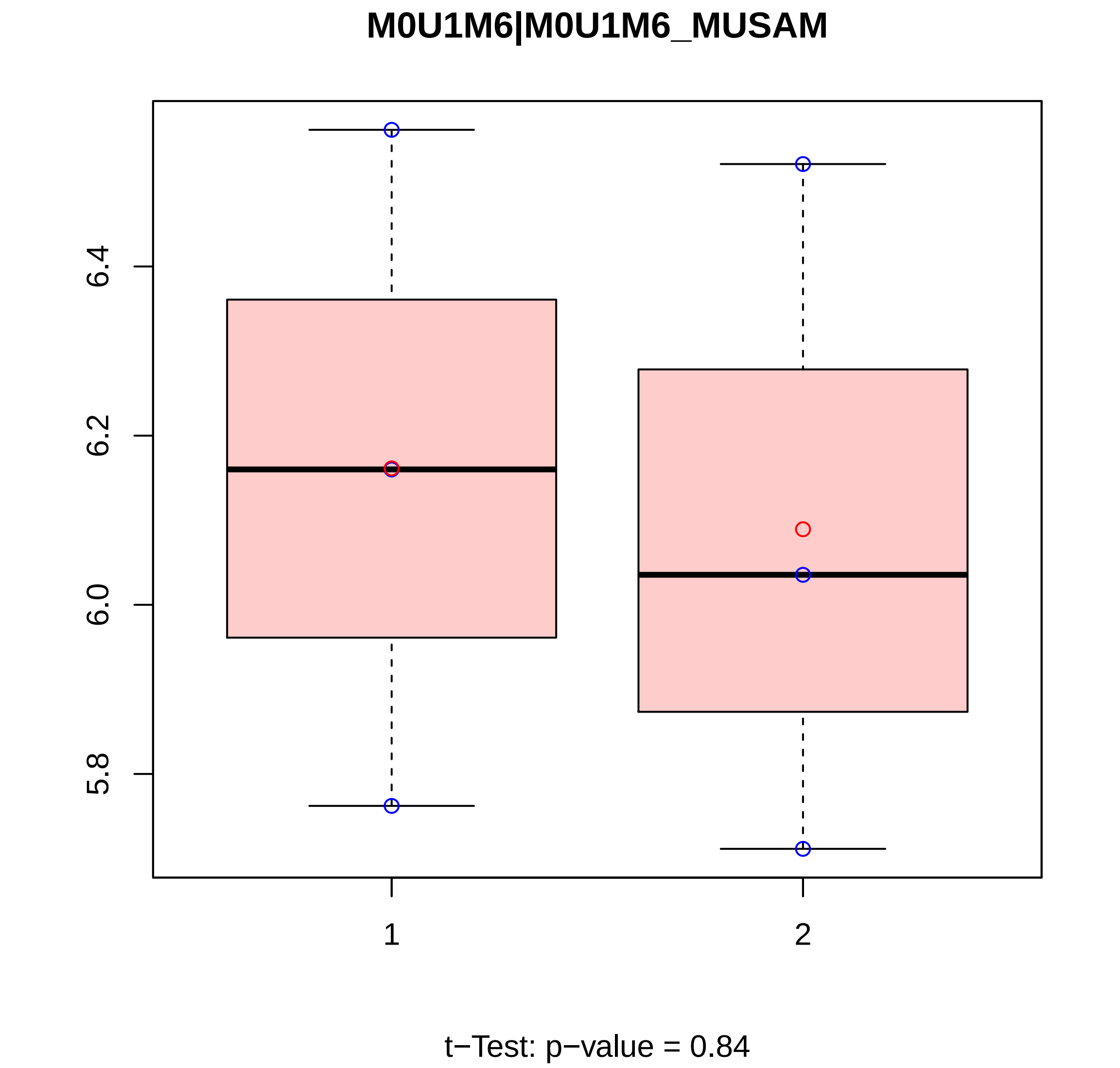
M0U1M6|M0U1M6_MUSAM
6.4
6.2
6.0
5.8
1
2
t−Test: p−value = 0.84

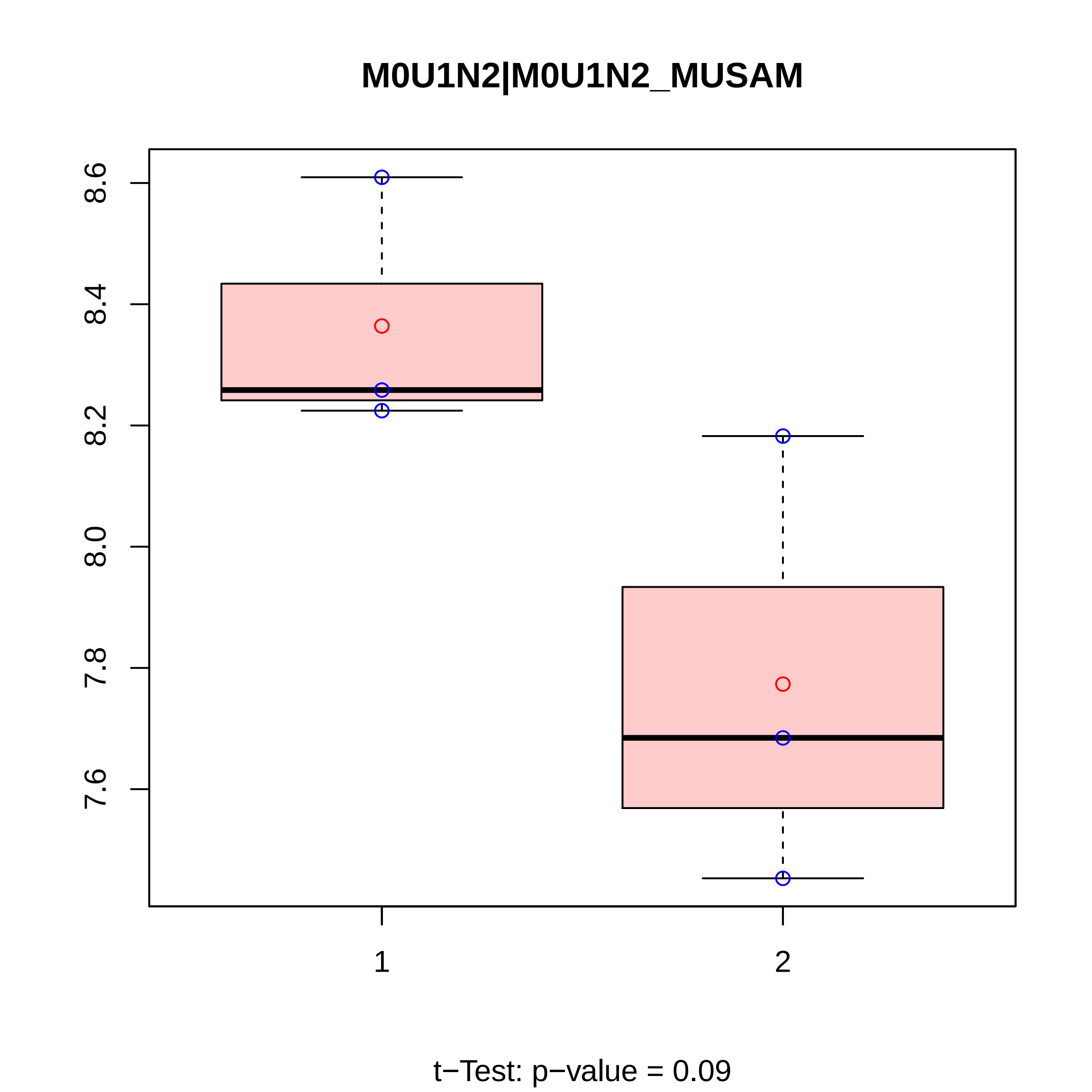
M0U1N2|M0U1N2_MUSAM
8.6
8.4
8.2
8.0
7.8
7.6
1
2
t−Test: p−value = 0.09

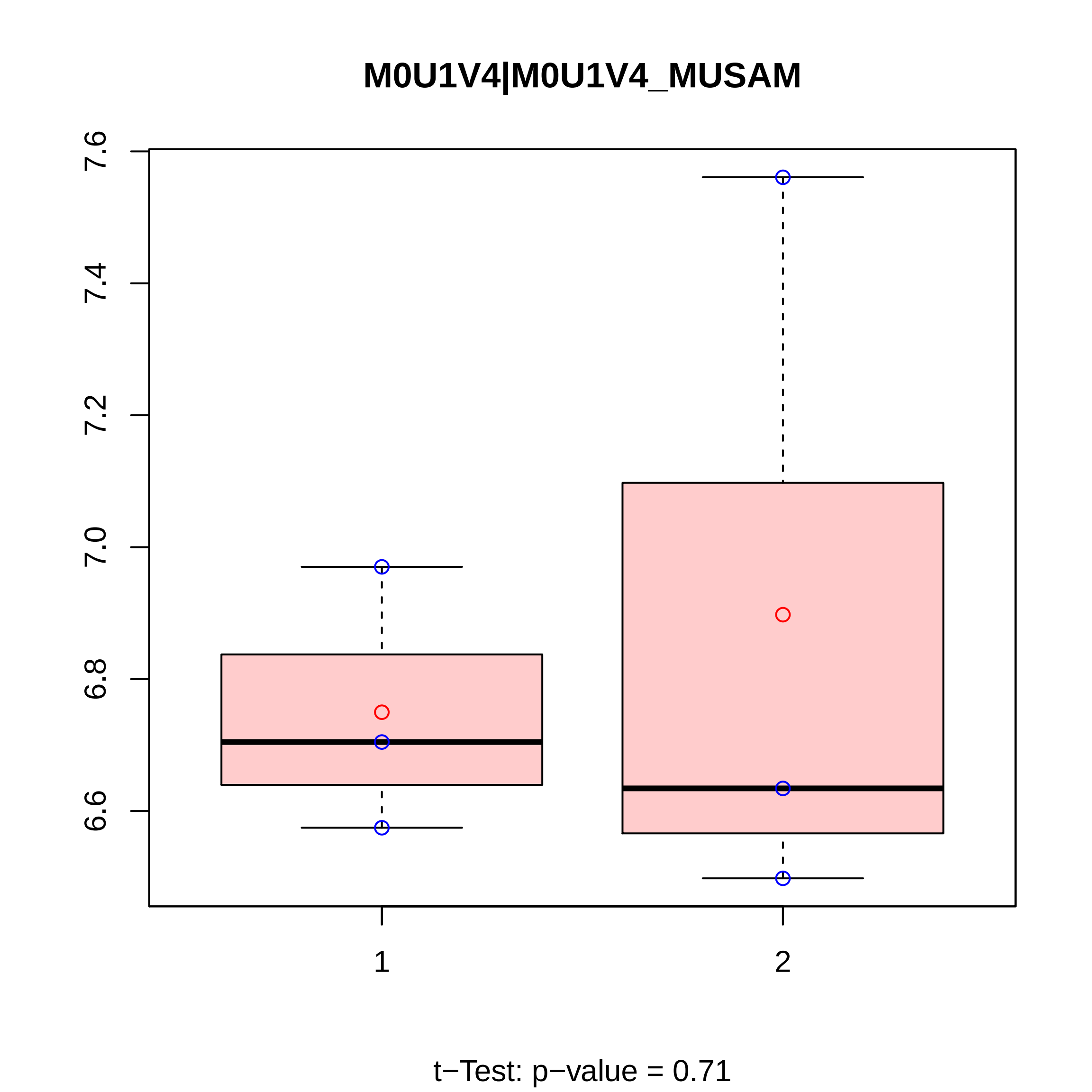

M0U1V4|M0U1V4_MUSAM
7.6
7.4
7.2
7.0
6.8
6.6
1
2
t−Test: p−value = 0.71

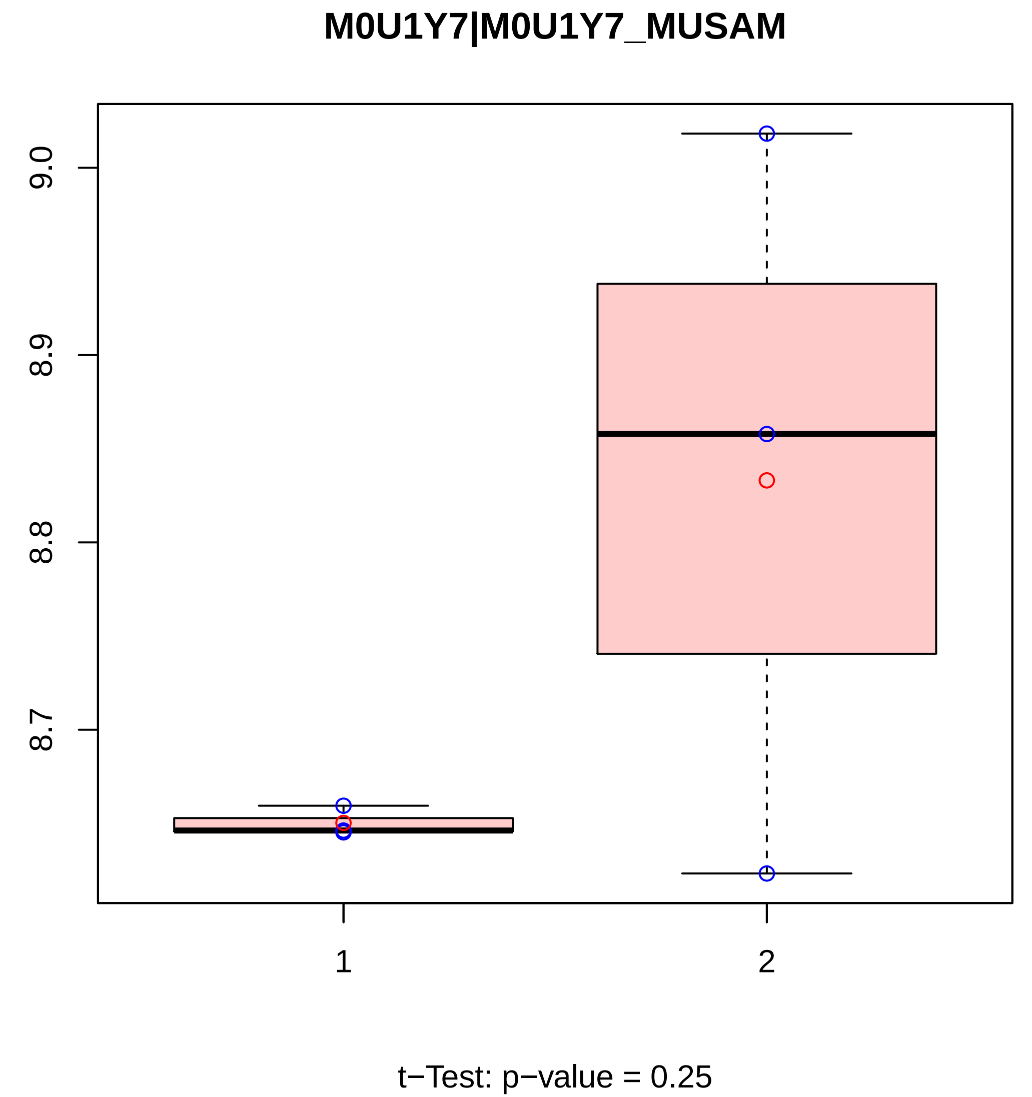
M0U1Y7|M0U1Y7_MUSAM
9.0
8.9
8.8
8.7
1
2
t−Test: p−value = 0.25

M0U2D0|M0U2D0_MUSAM

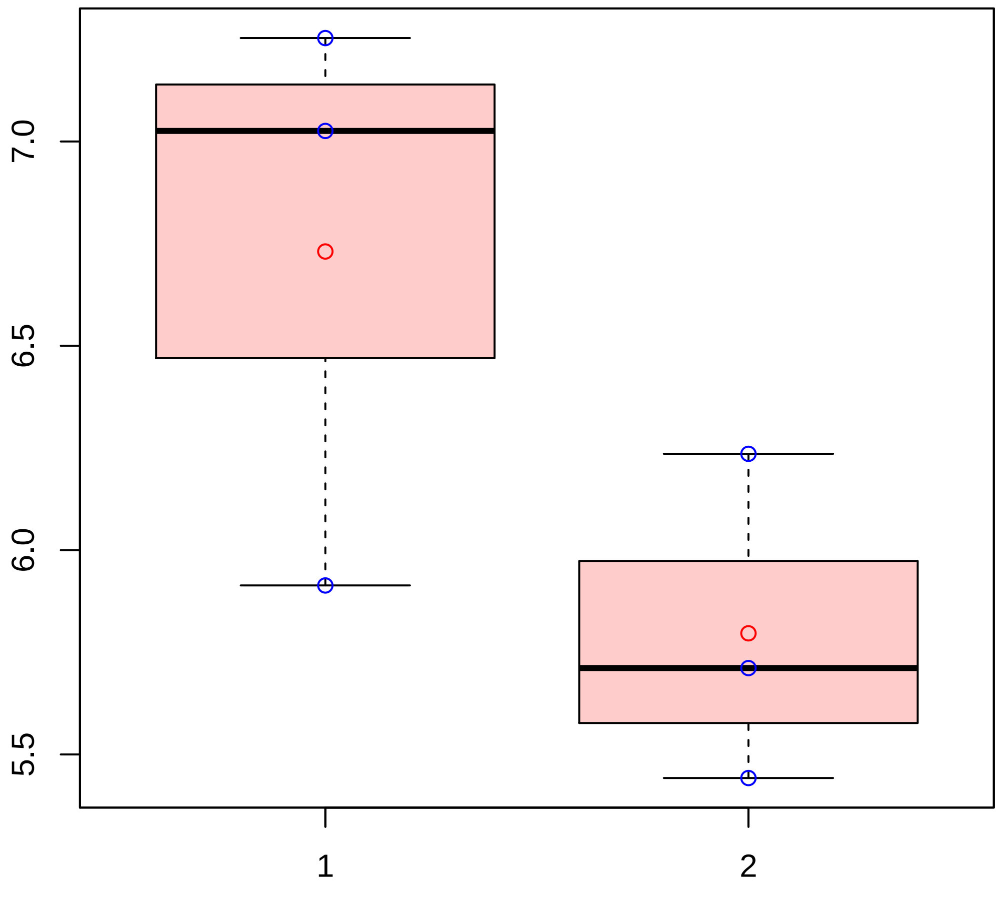

t−Test: p−value = 0.14

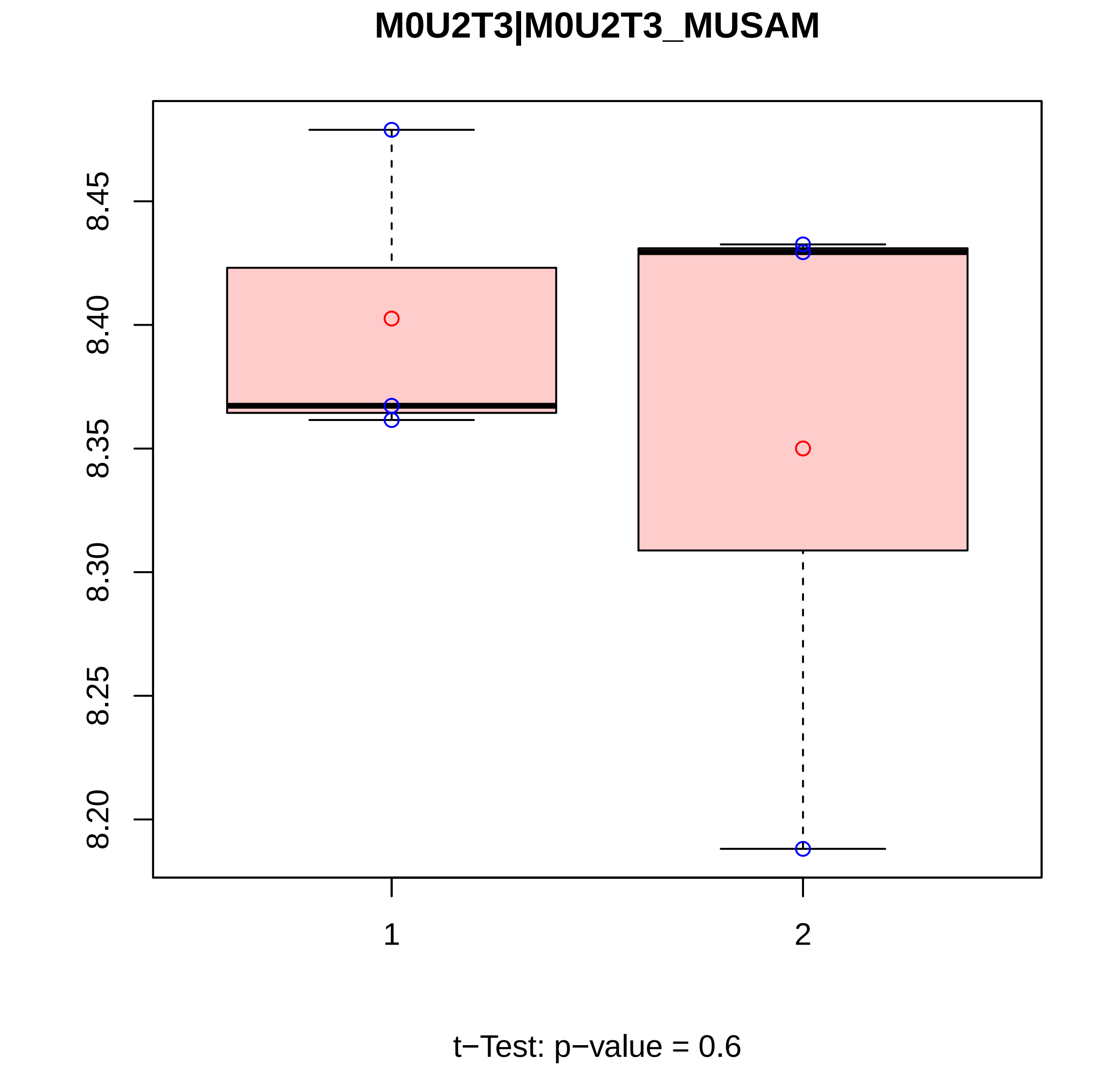

M0U2T3|M0U2T3_MUSAM
8.45
8.40
8.35
8.30
8.25
8.20
1
2
t−Test: p−value = 0.6

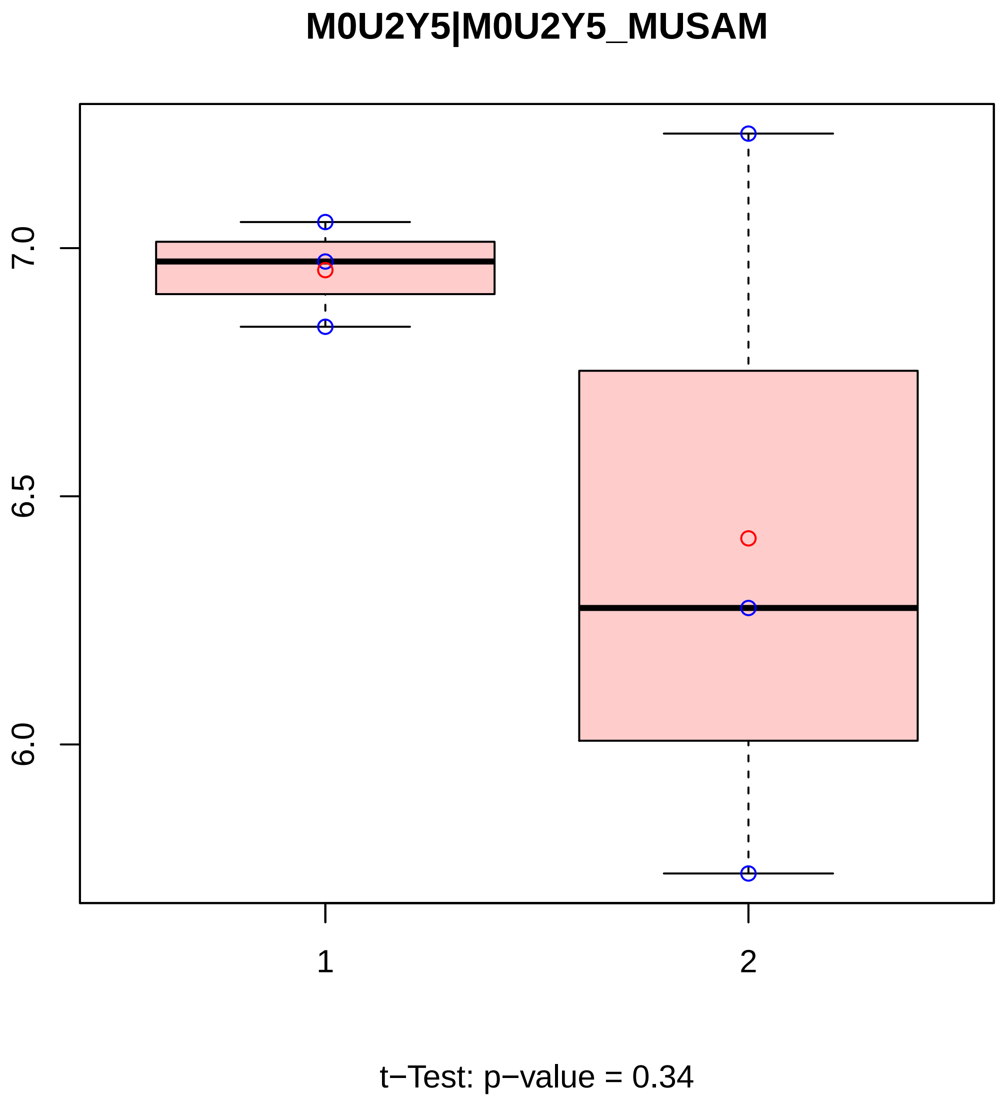

M0U2Y5|M0U2Y5_MUSAM
7.0
6.5
6.0
1
2
t−Test: p−value = 0.34

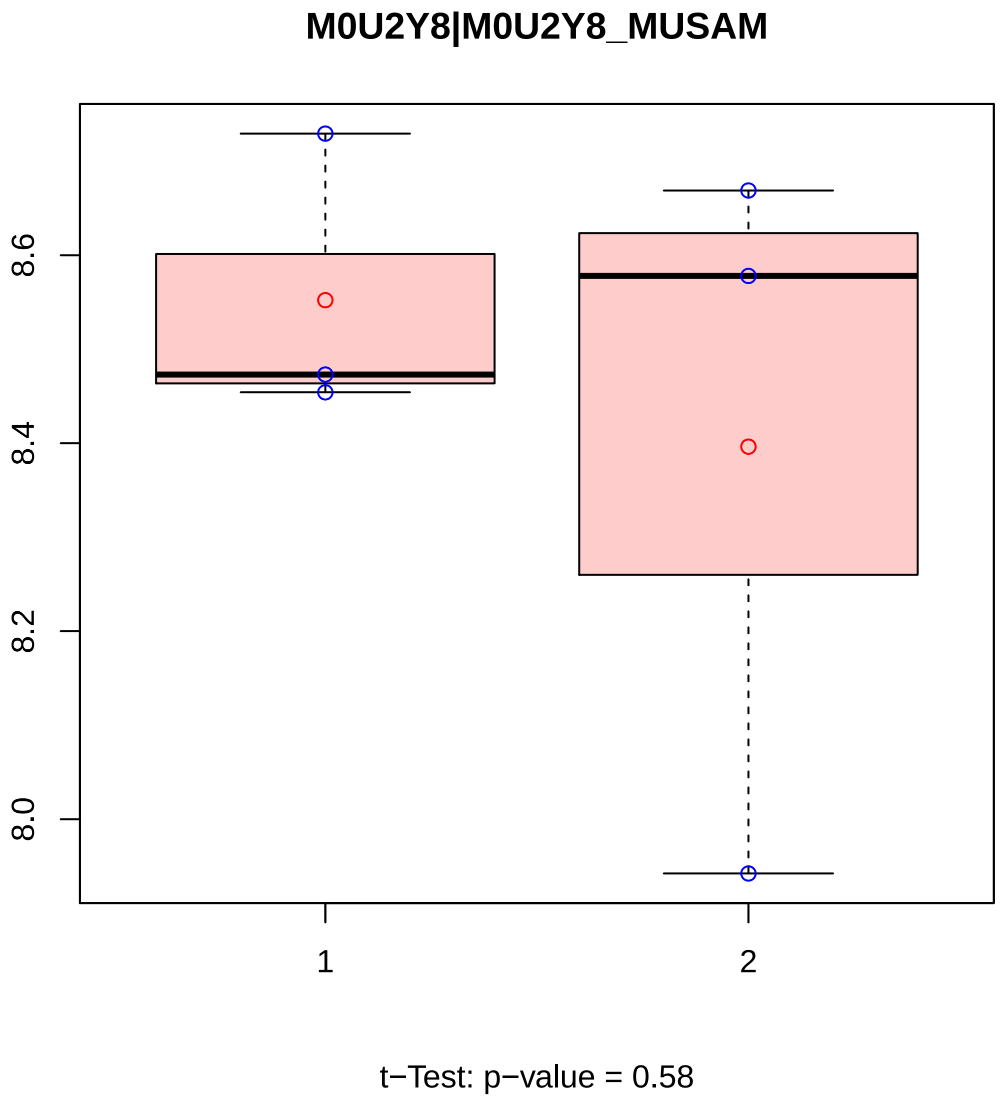

M0U2Y8|M0U2Y8_MUSAM
8.0
8.2
8.4
8.6
1
2
t−Test: p−value = 0.58

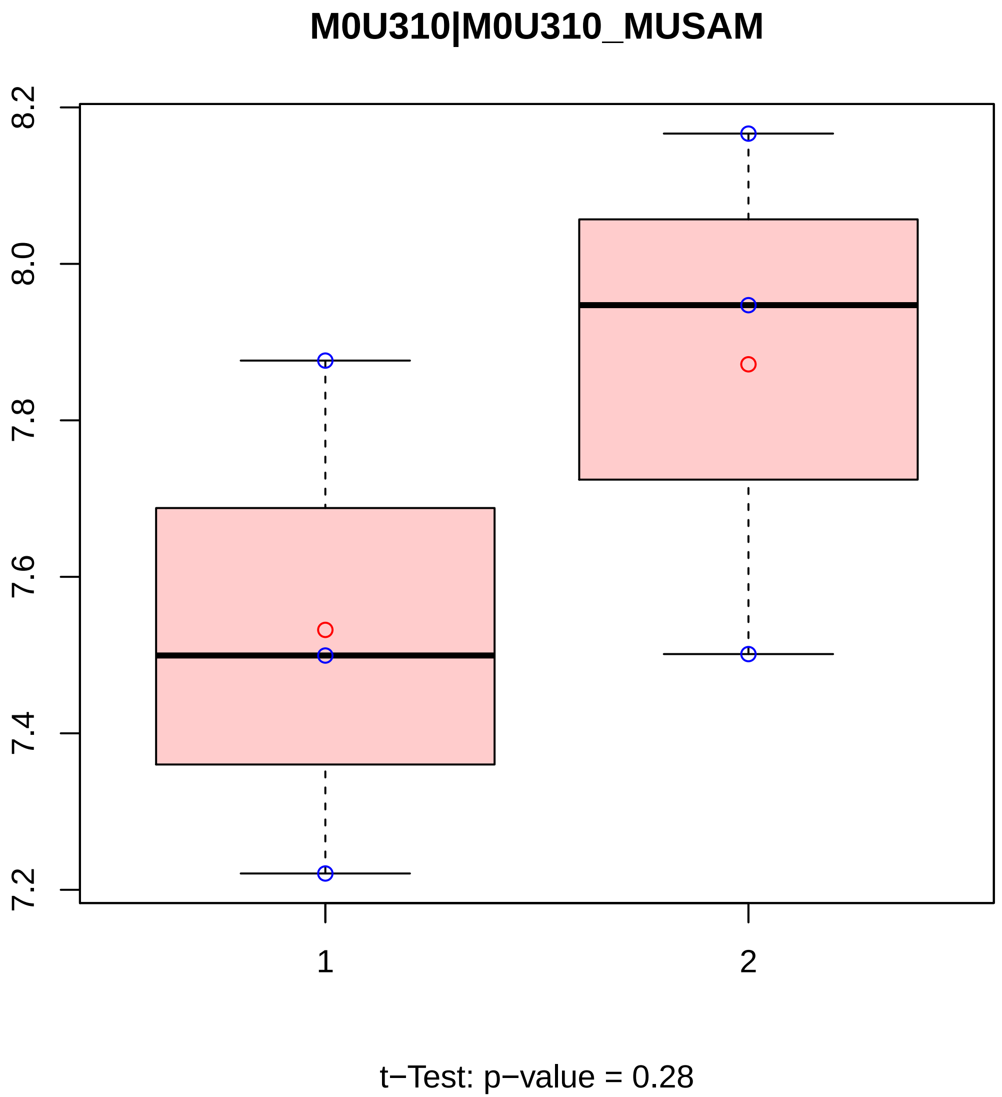
M0U310|M0U310_MUSAM
8.2
8.0
7.8
7.6
7.4
7.2
1
2
t−Test: p−value = 0.28

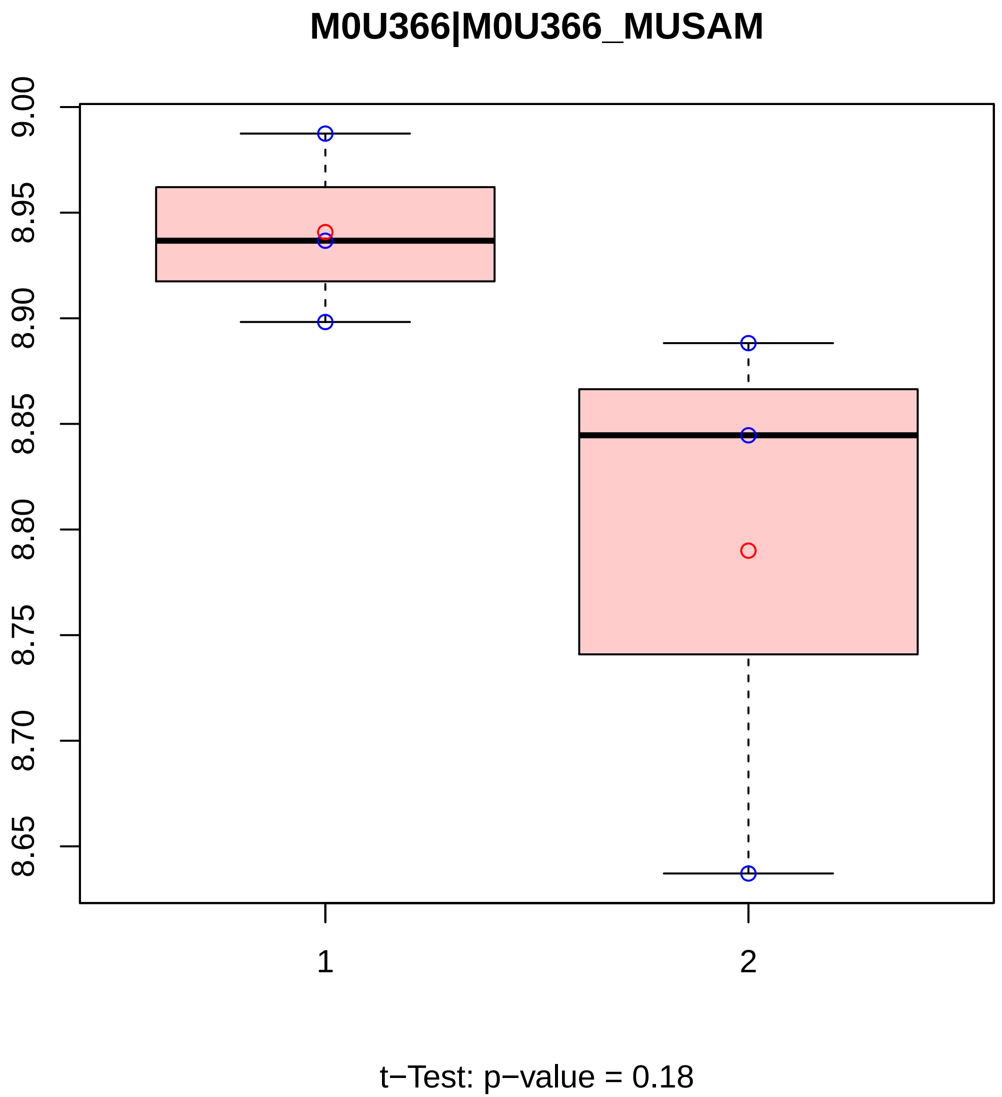

M0U366|M0U366_MUSAM
9.00
8.95
8.90
8.85
8.80
8.75
8.70
8.65
1
2
t−Test: p−value = 0.18

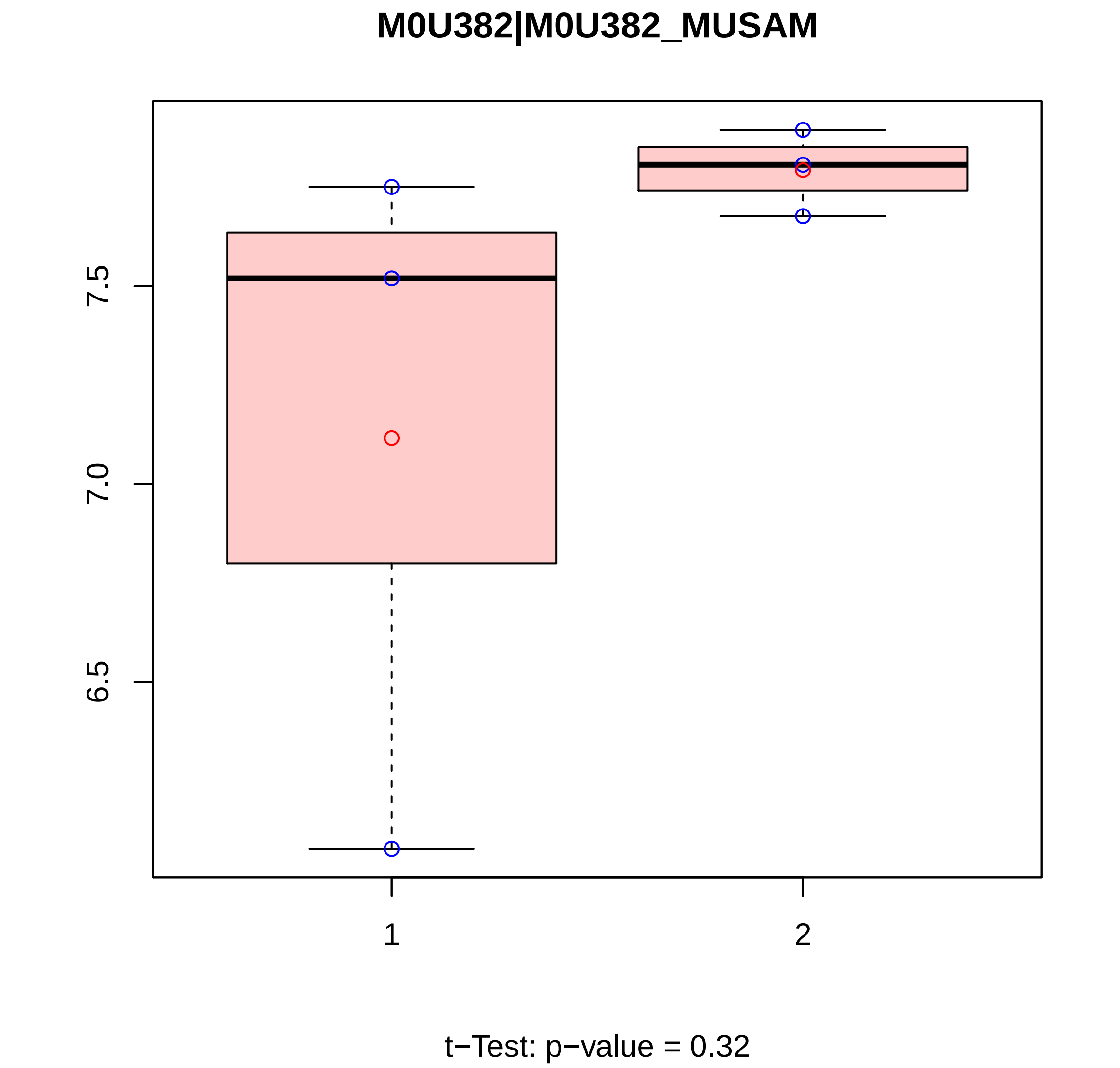
M0U382|M0U382_MUSAM
7.5
7.0
6.5
1
2
t−Test: p−value = 0.32

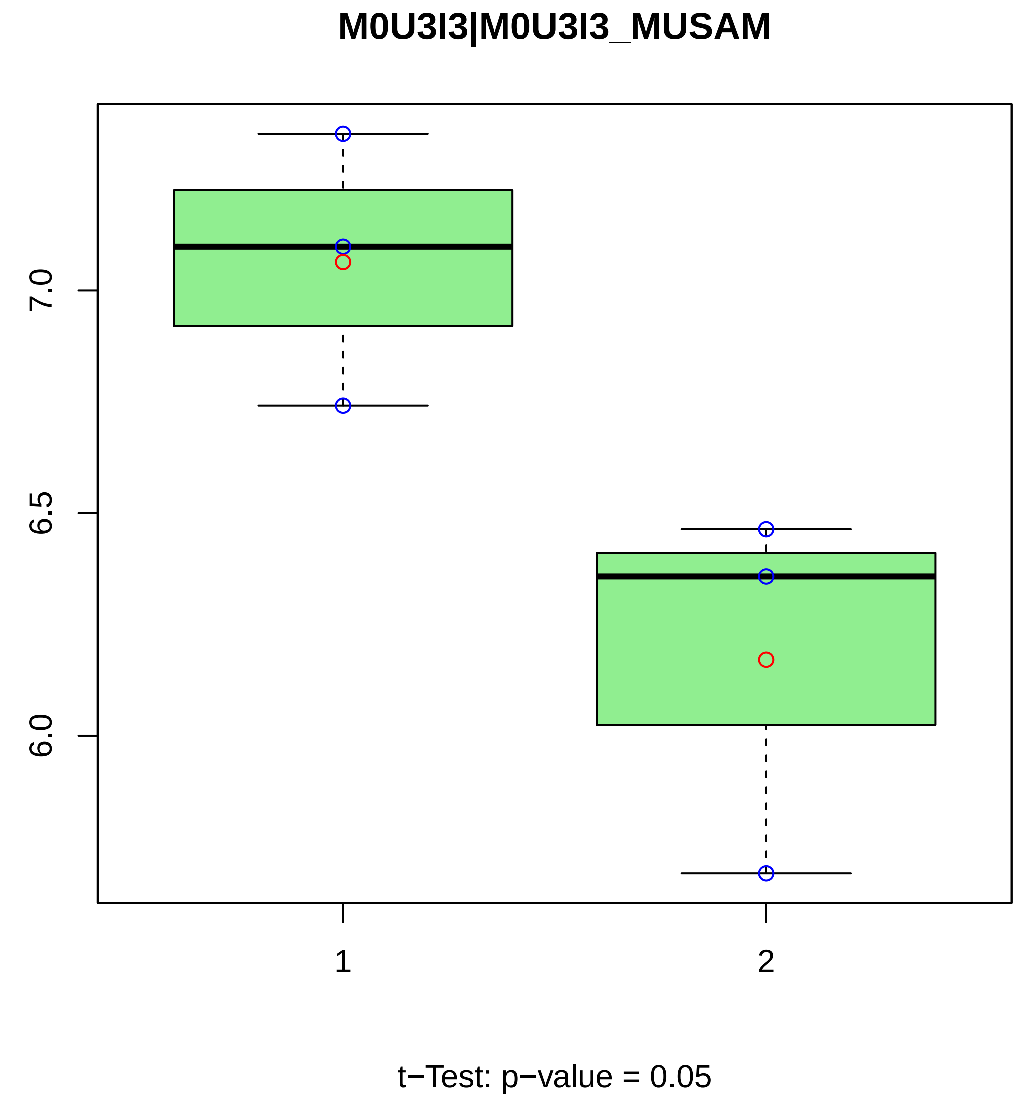
M0U3I3|M0U3I3_MUSAM
7.0
6.5
6.0
1
2
t−Test: p−value = 0.05

M0U3M2|M0U3M2_MUSAM

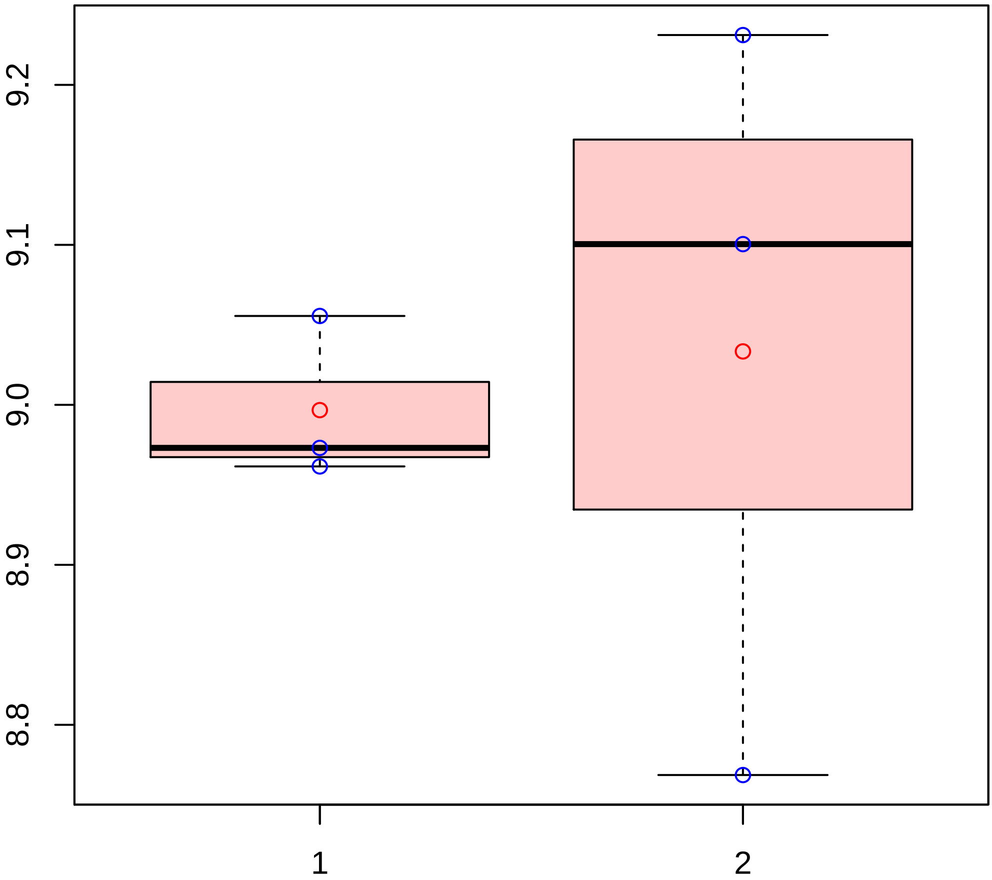

t−Test: p−value = 0.82

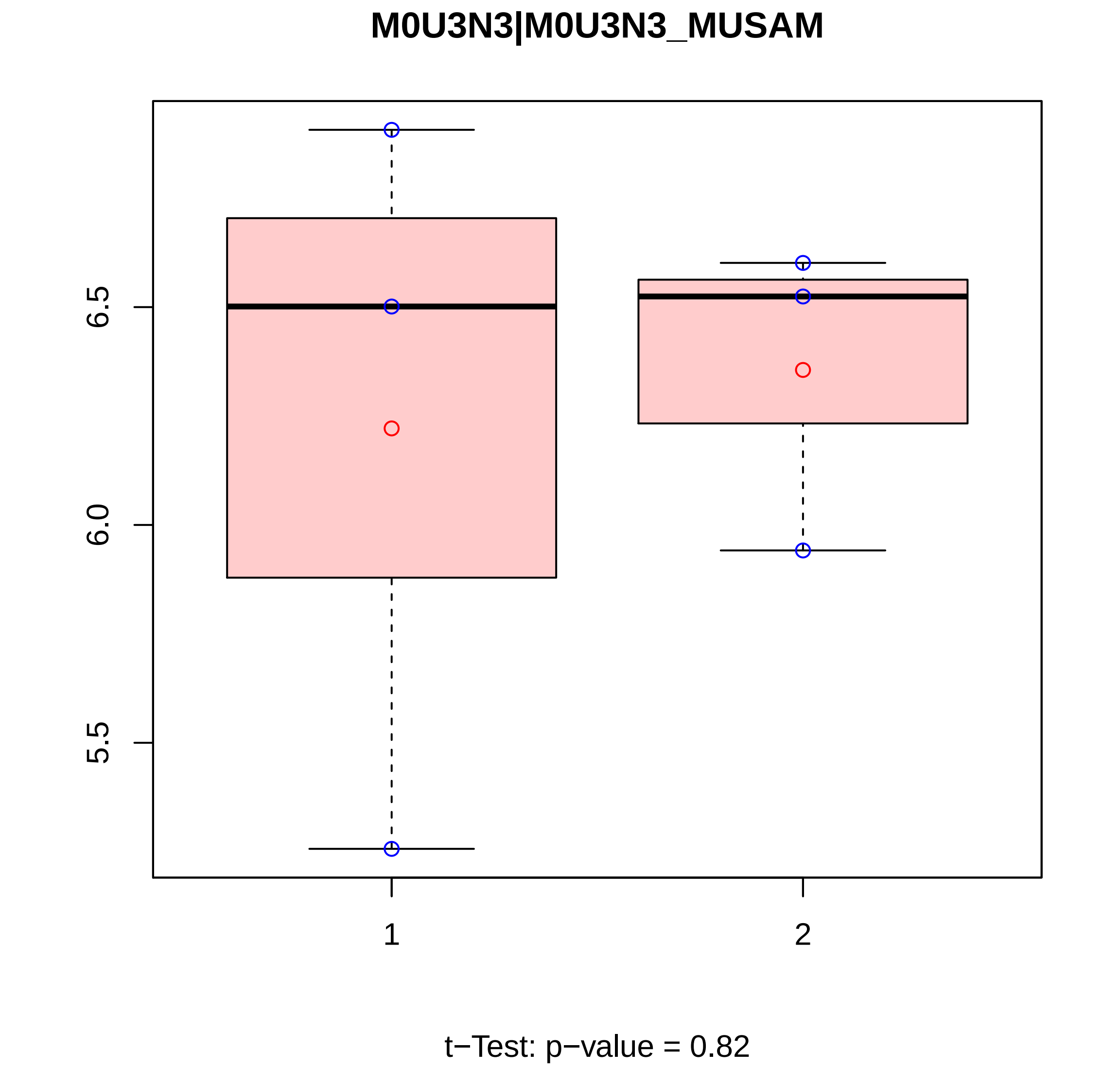

M0U3N3|M0U3N3_MUSAM
6.5
6.0
5.5
1
2
t−Test: p−value = 0.82

M0U3W7|M0U3W7_MUSAM

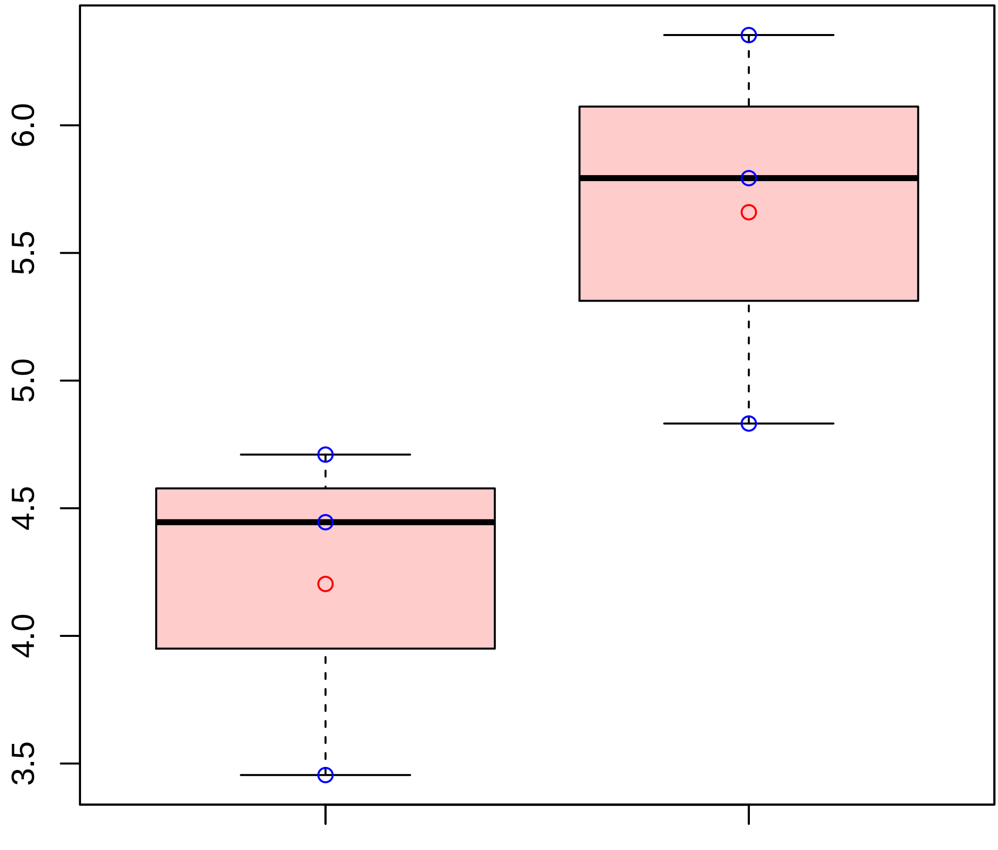

t−Test: p−value = 0.07

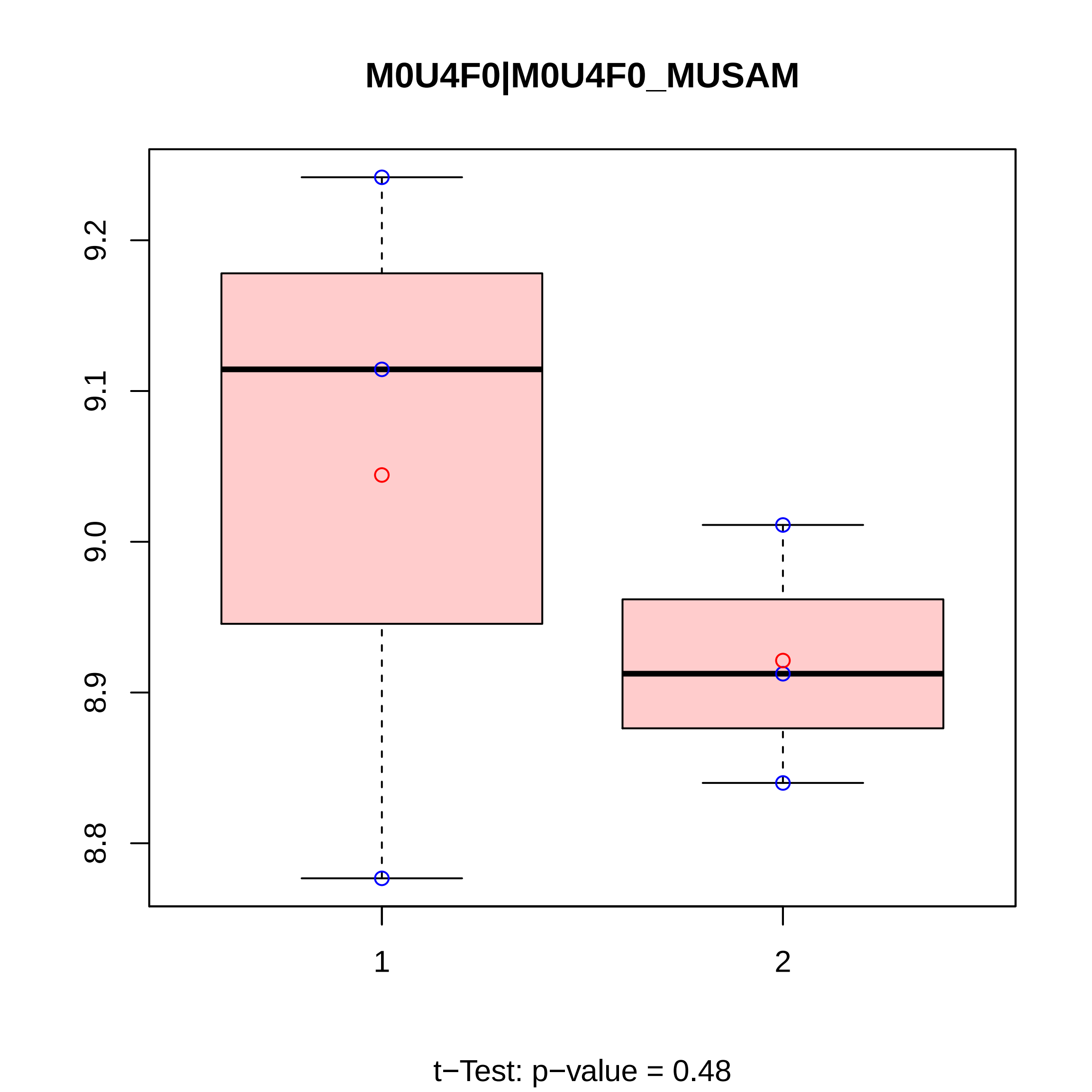
M0U4F0|M0U4F0_MUSAM
9.2
9.1
9.0
8.9
8.8
1
2
t−Test: p−value = 0.48

M0U4I4|M0U4I4_MUSAM

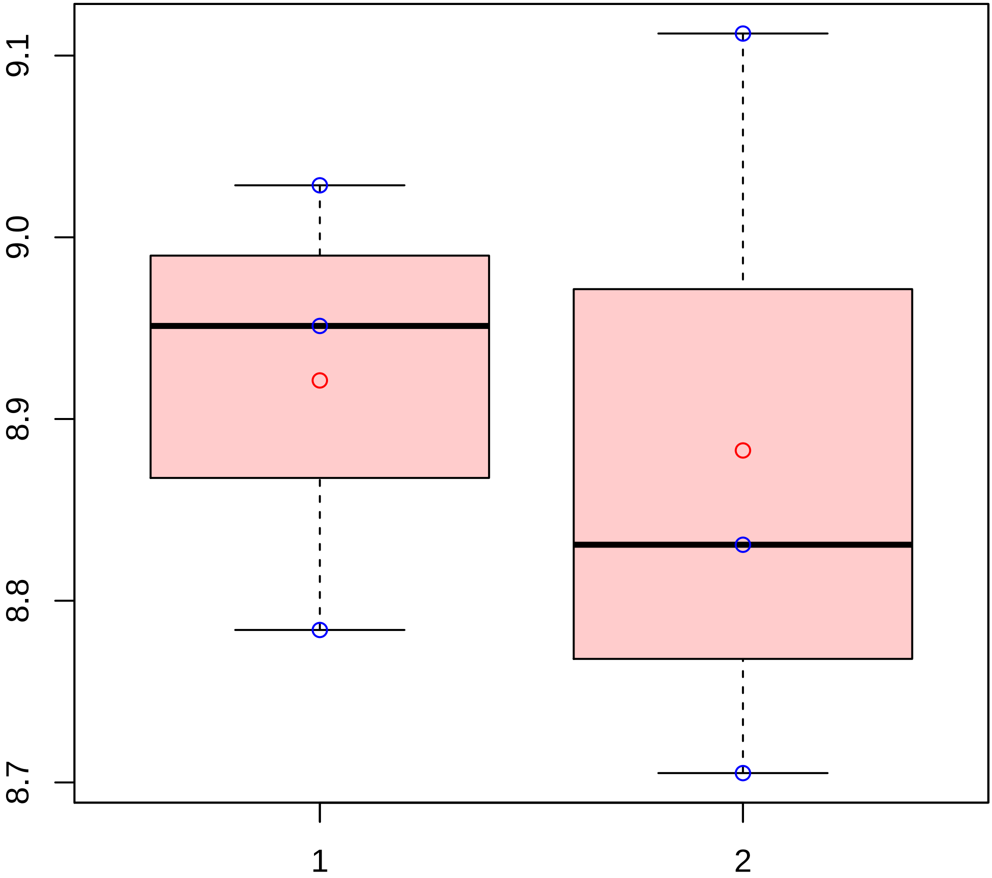

t−Test: p−value = 0.8

M0U4U2|M0U4U2_MUSAM

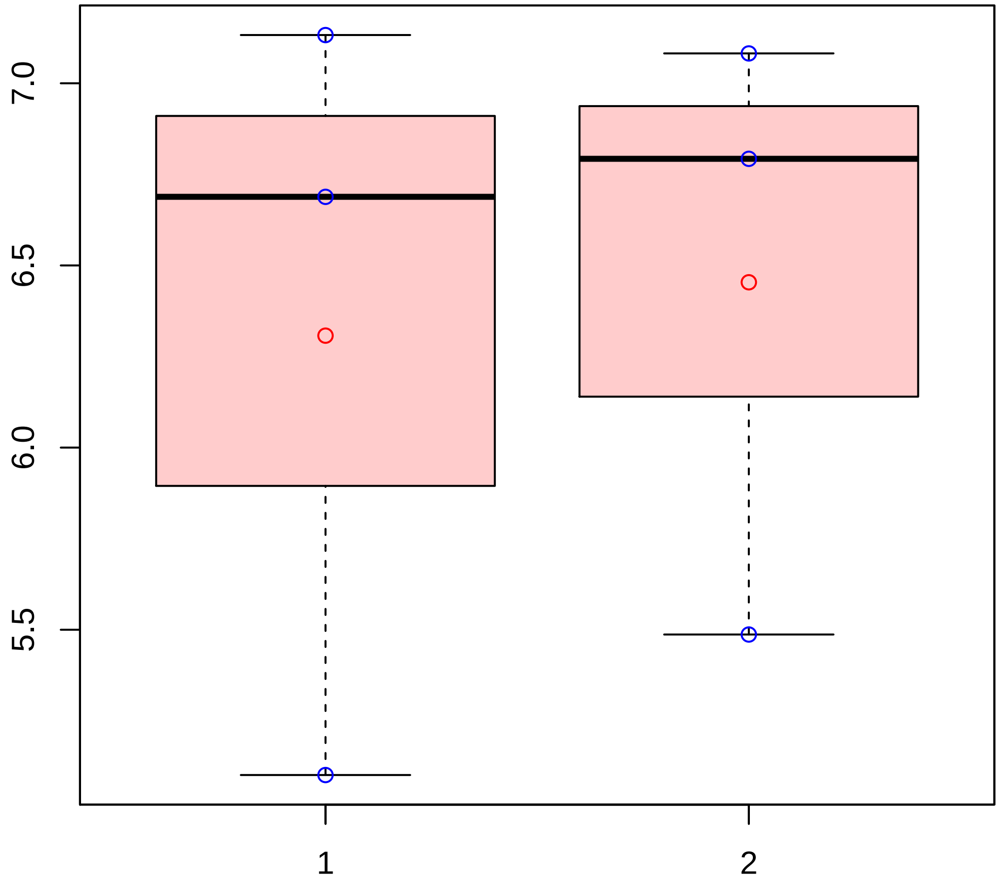

t−Test: p−value = 0.86

M0U511|M0U511_MUSAM

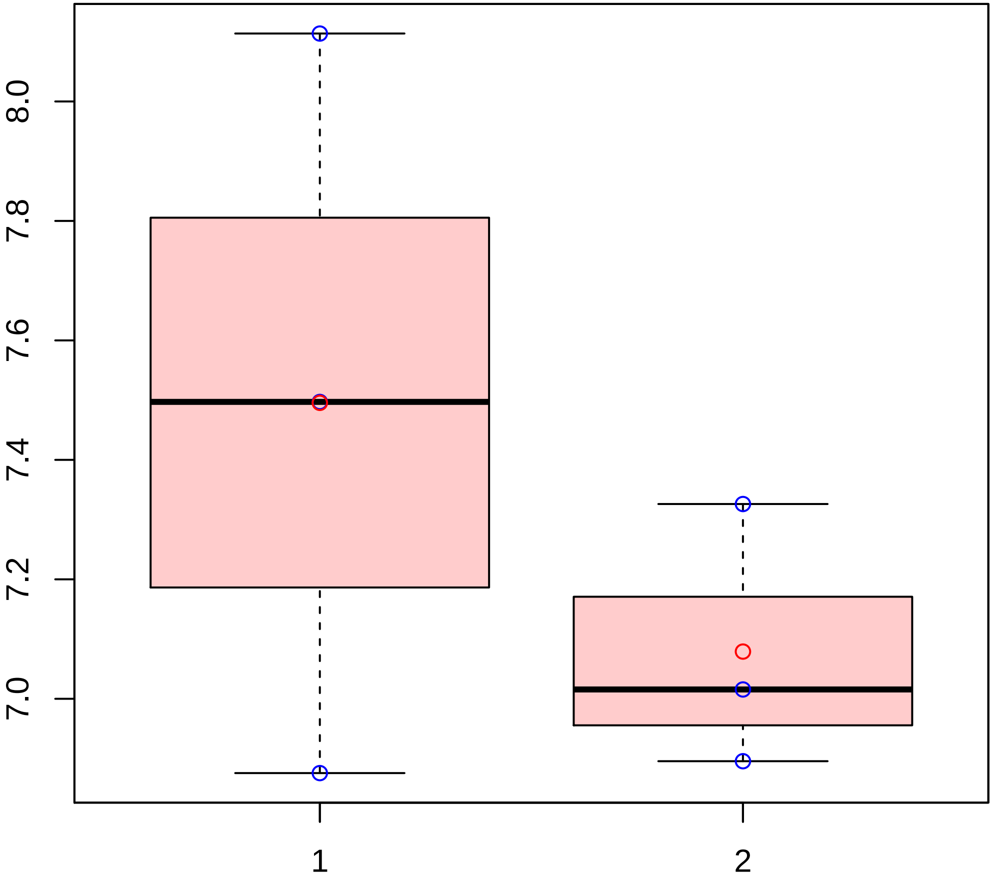

t−Test: p−value = 0.37

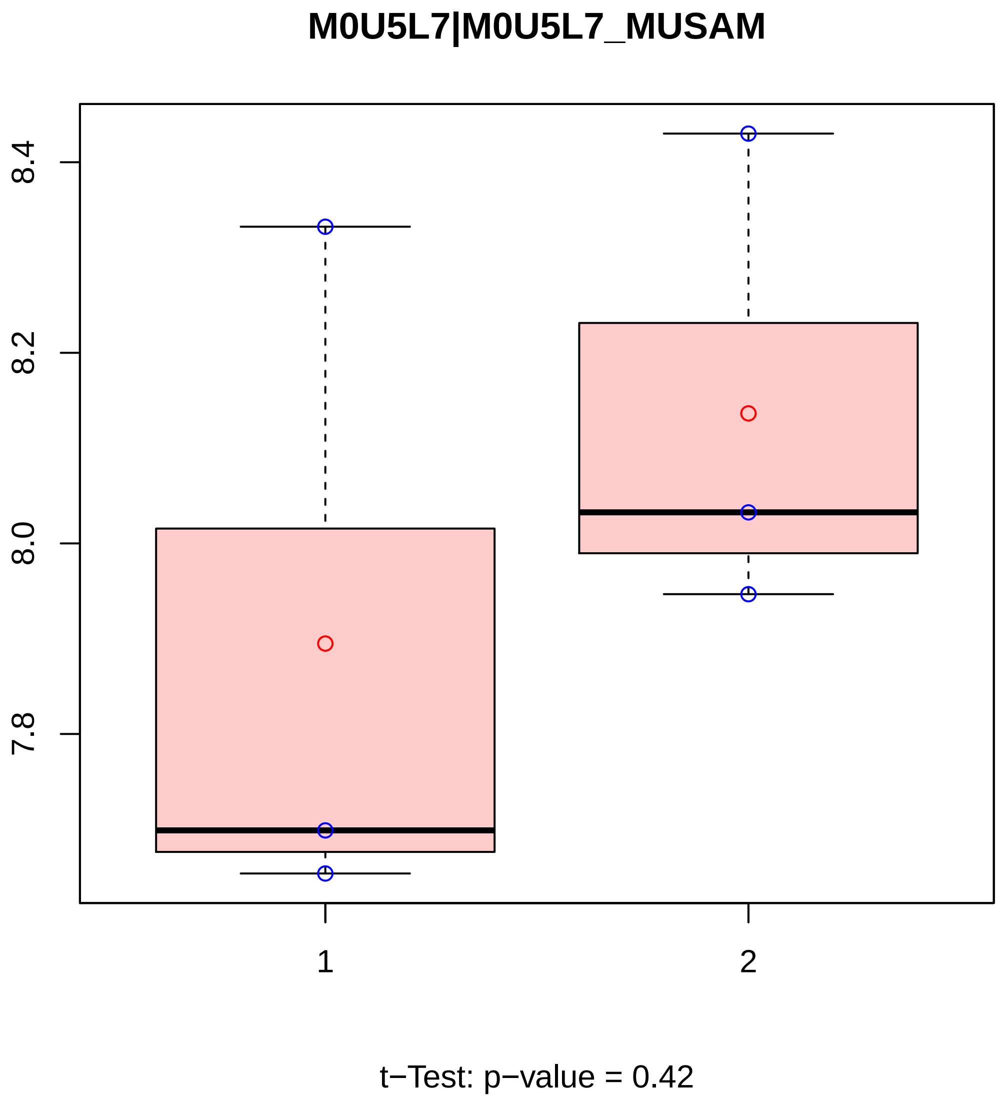
M0U5L7|M0U5L7_MUSAM
8.4
8.2
8.0
7.8
1
2
t−Test: p−value = 0.42

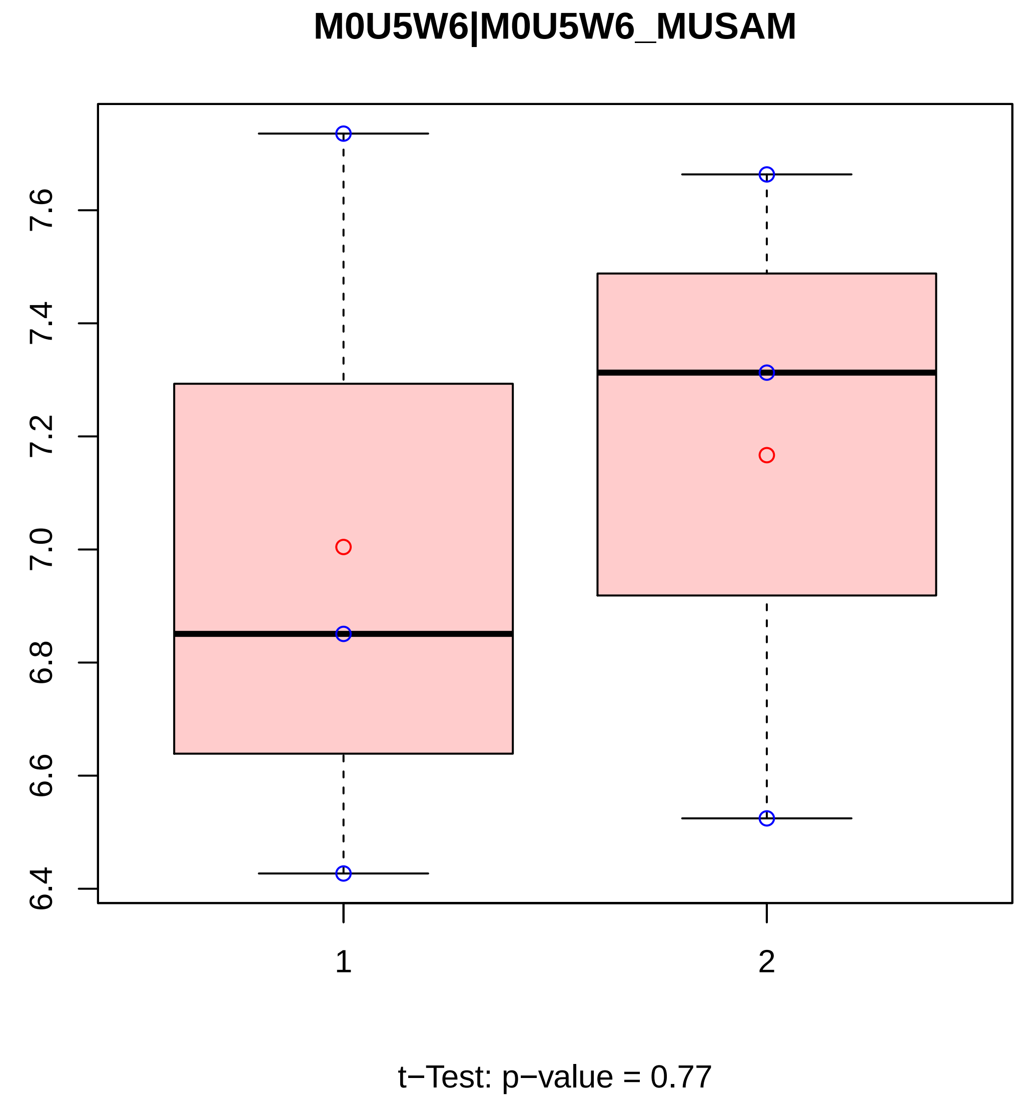
M0U5W6|M0U5W6_MUSAM
7.6
7.4
7.2
7.0
6.8
6.6
6.4
1
2
t−Test: p−value = 0.77

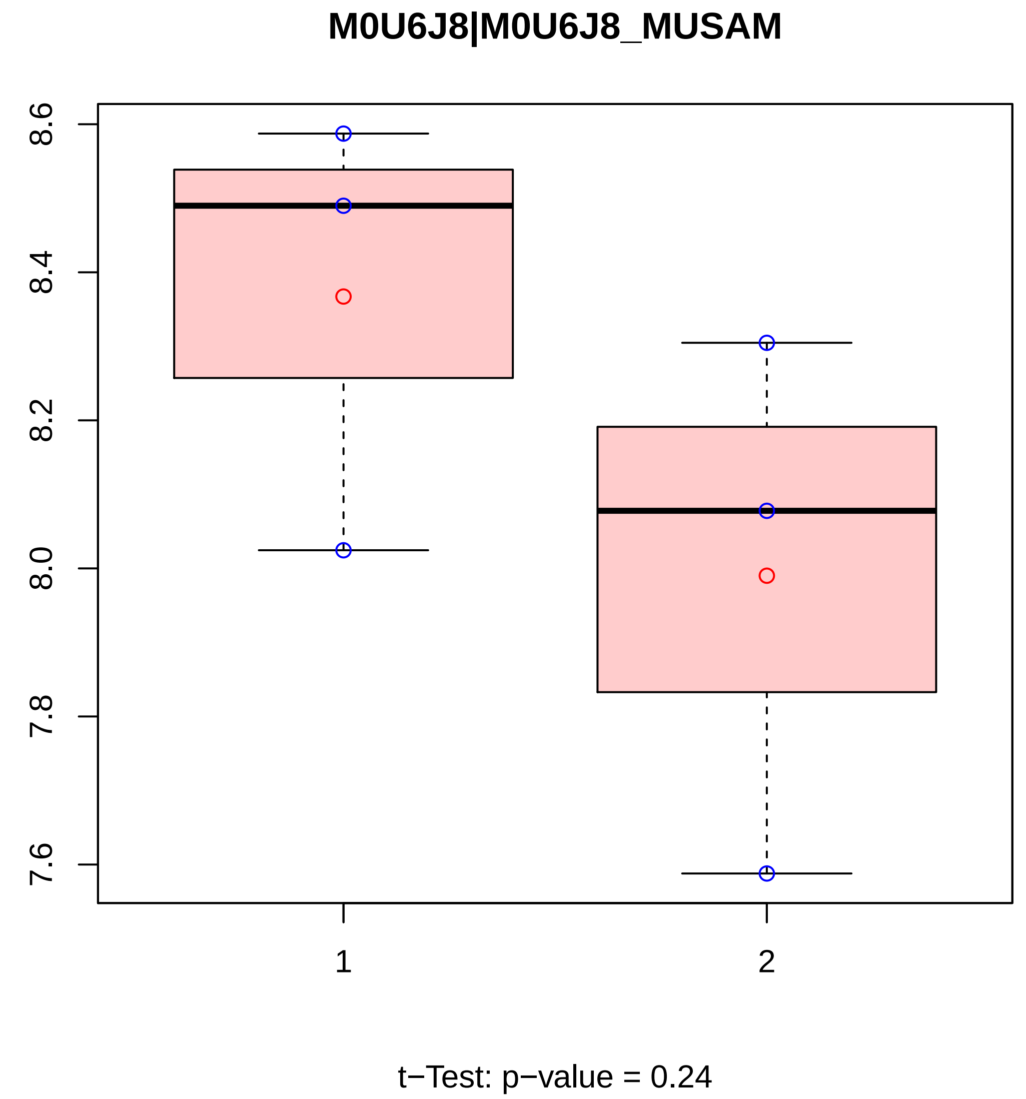

M0U6J8|M0U6J8_MUSAM
8.6
8.4
8.2
8.0
7.8
7.6
1
2
t−Test: p−value = 0.24

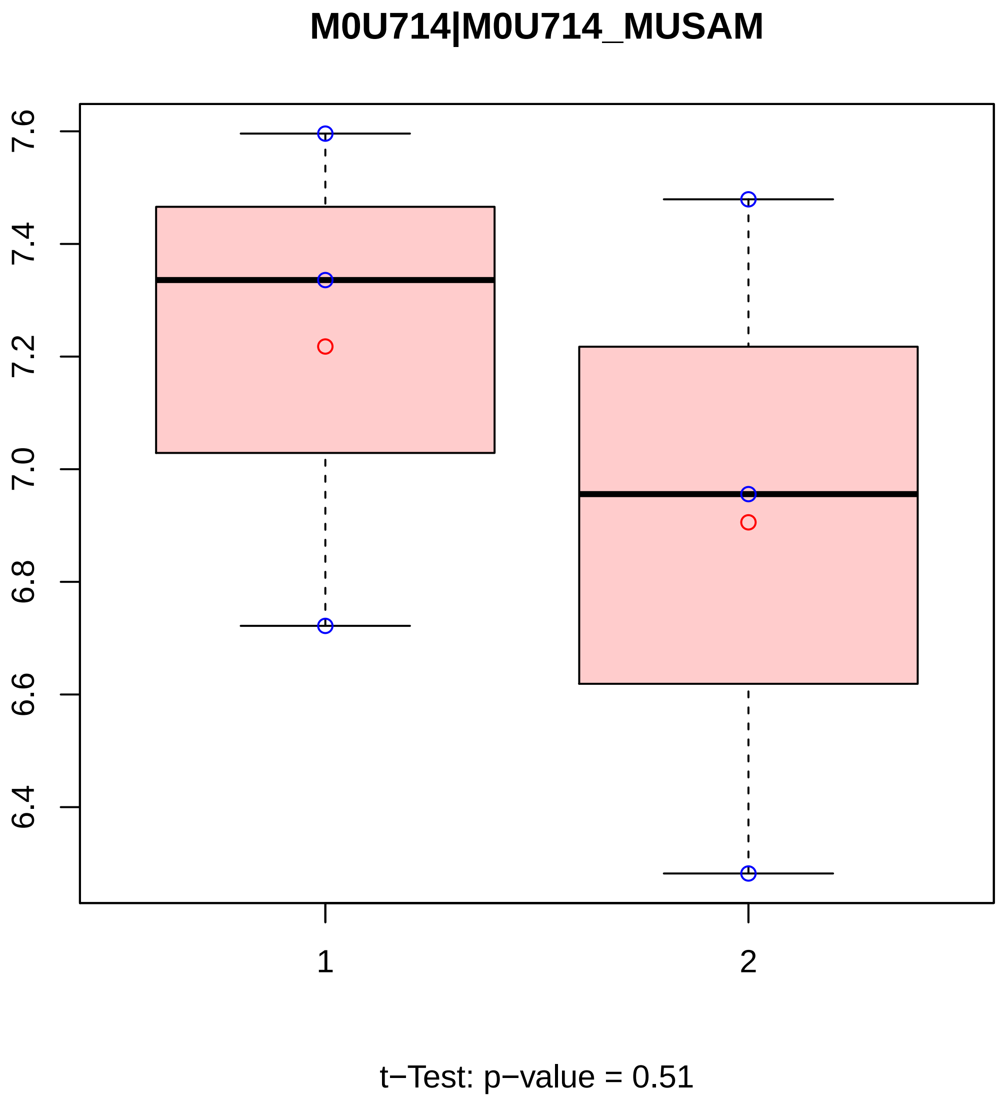
M0U714|M0U714_MUSAM
7.6
7.4
7.2
7.0
6.8
6.6
6.4
1
2
t−Test: p−value = 0.51

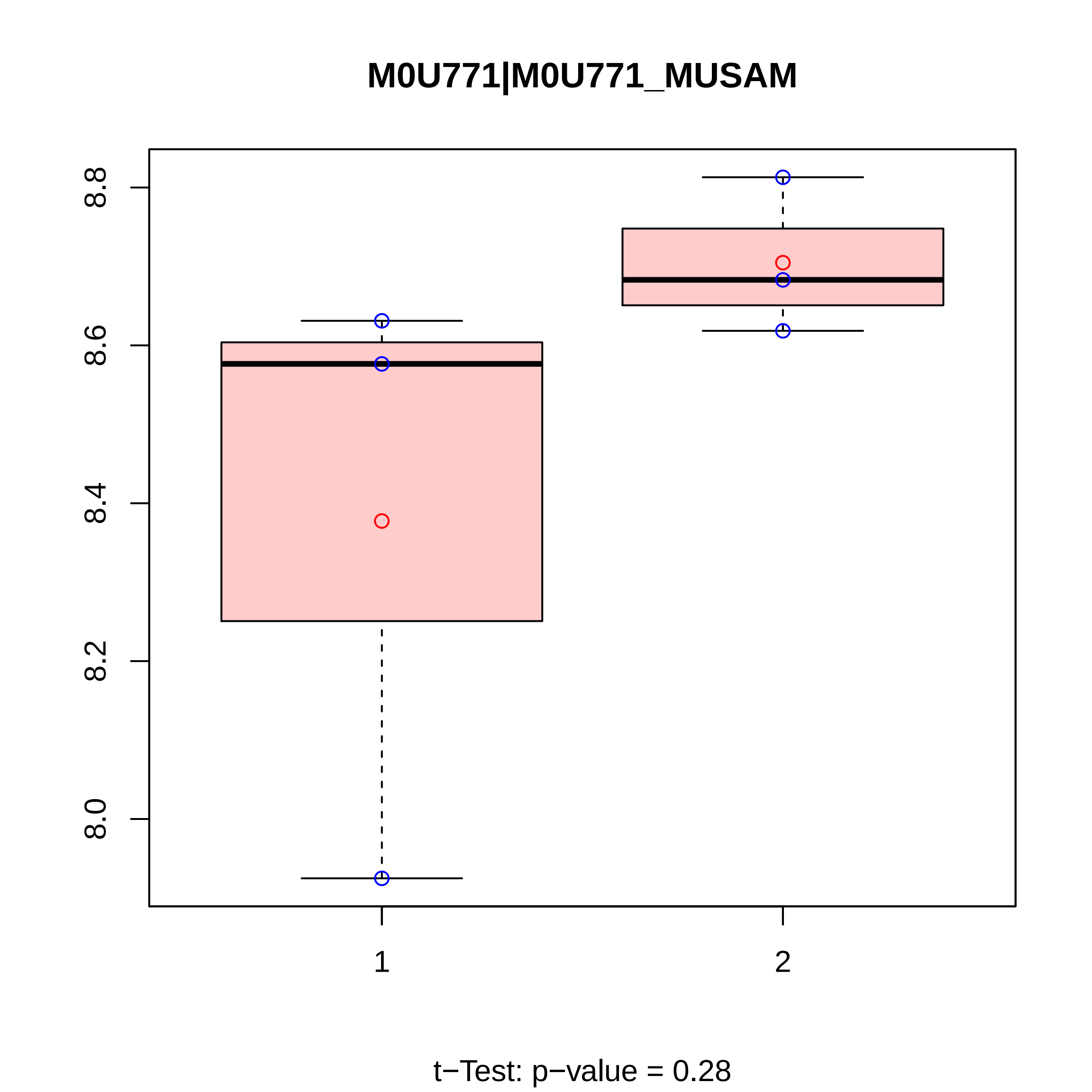
M0U771|M0U771_MUSAM
8.0
8.2
8.4
8.6
8.8
1
2
t−Test: p−value = 0.28

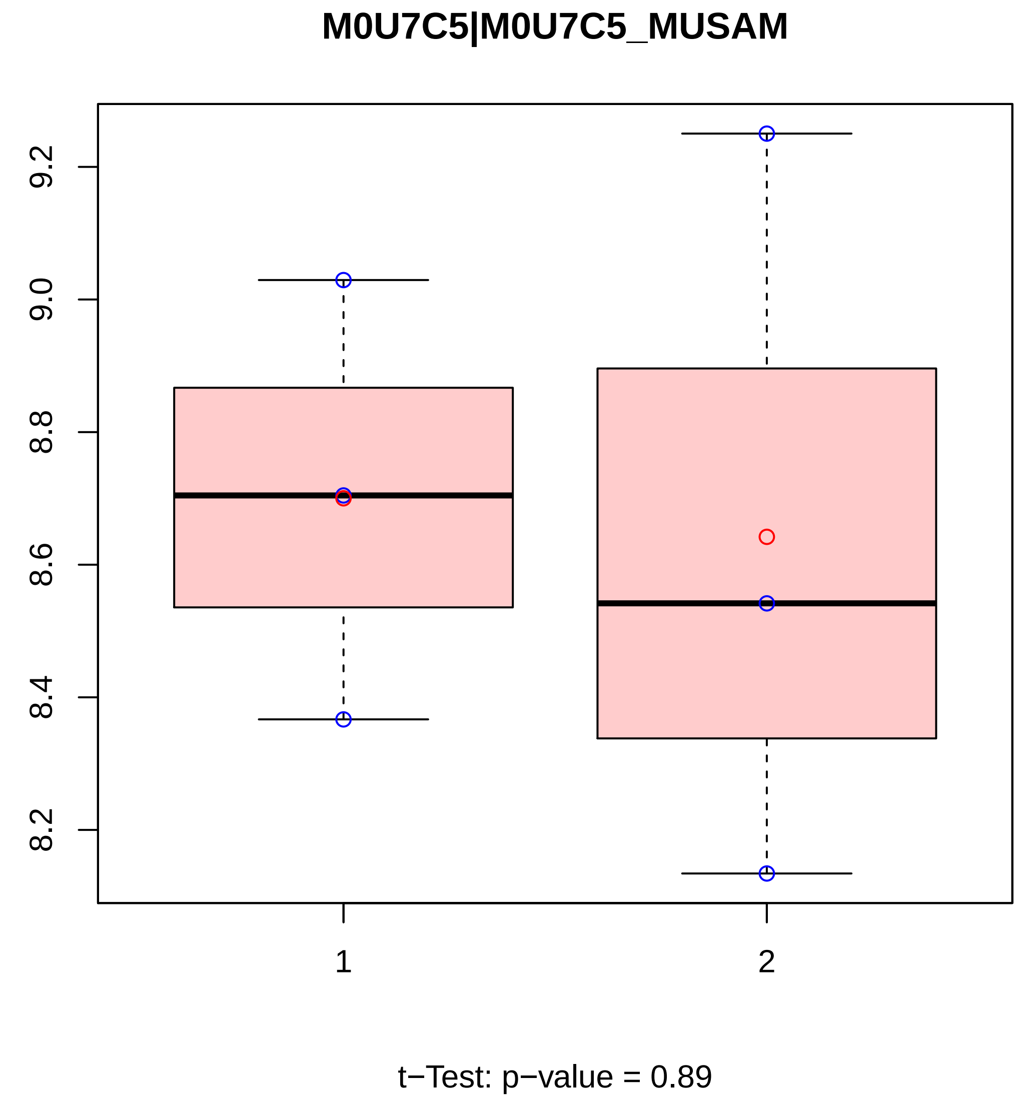
M0U7C5|M0U7C5_MUSAM
9.2
9.0
8.8
8.6
8.4
8.2
1
2
t−Test: p−value = 0.89

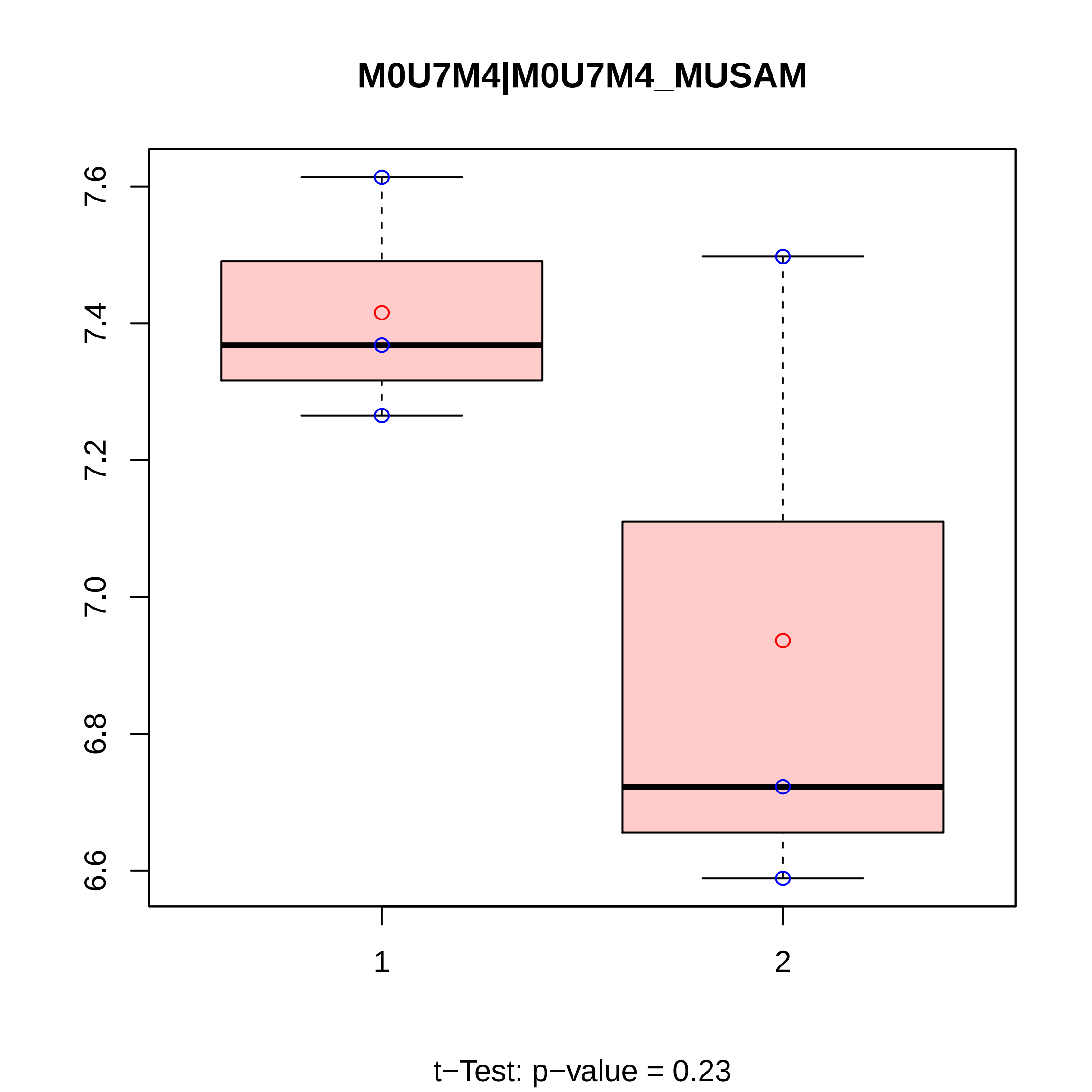
M0U7M4|M0U7M4_MUSAM
7.6
7.4
7.2
7.0
6.8
6.6
1
2
t−Test: p−value = 0.23

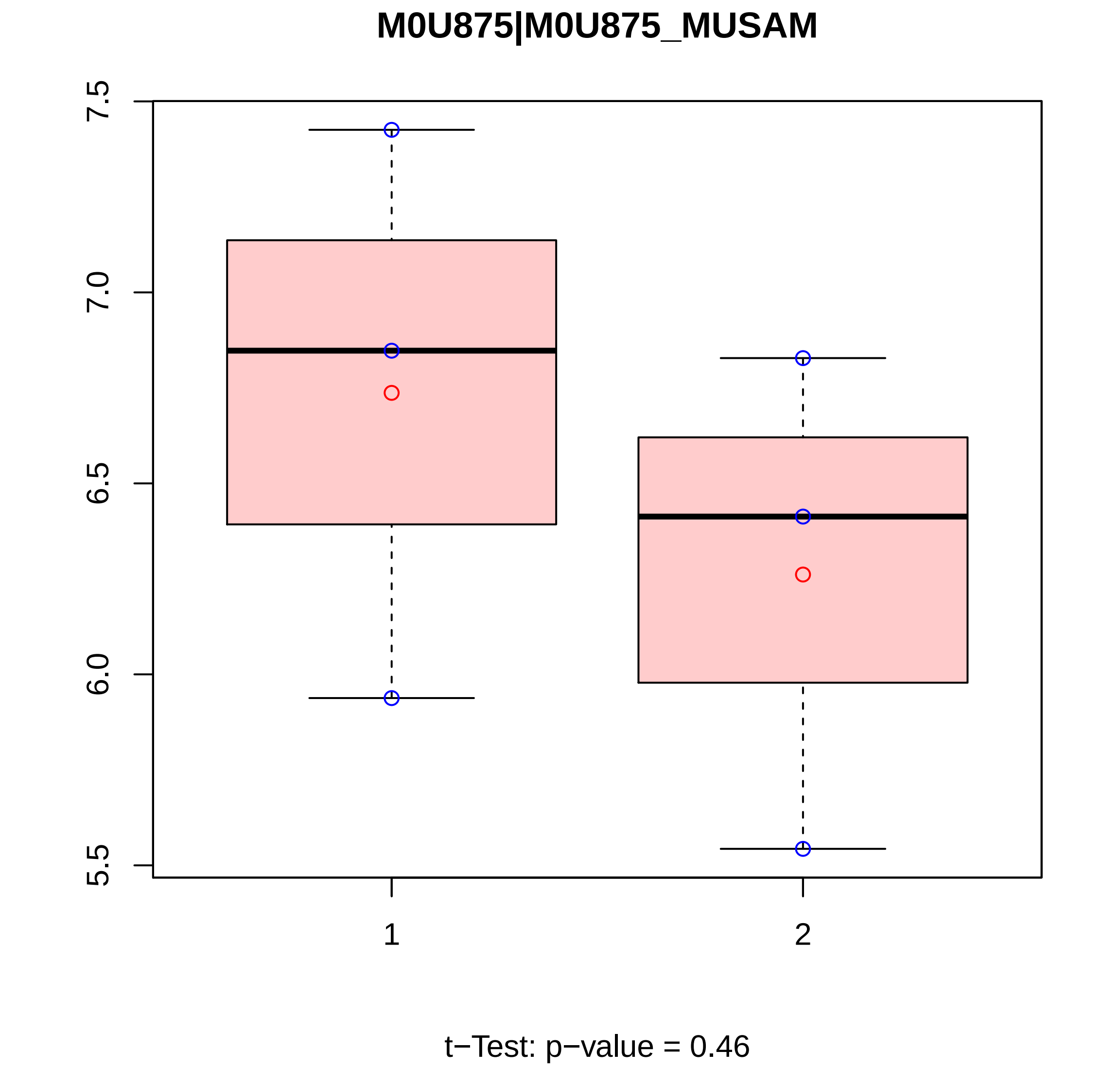
M0U875|M0U875_MUSAM
7.5
7.0
6.5
6.0
5.5
1
2
t−Test: p−value = 0.46

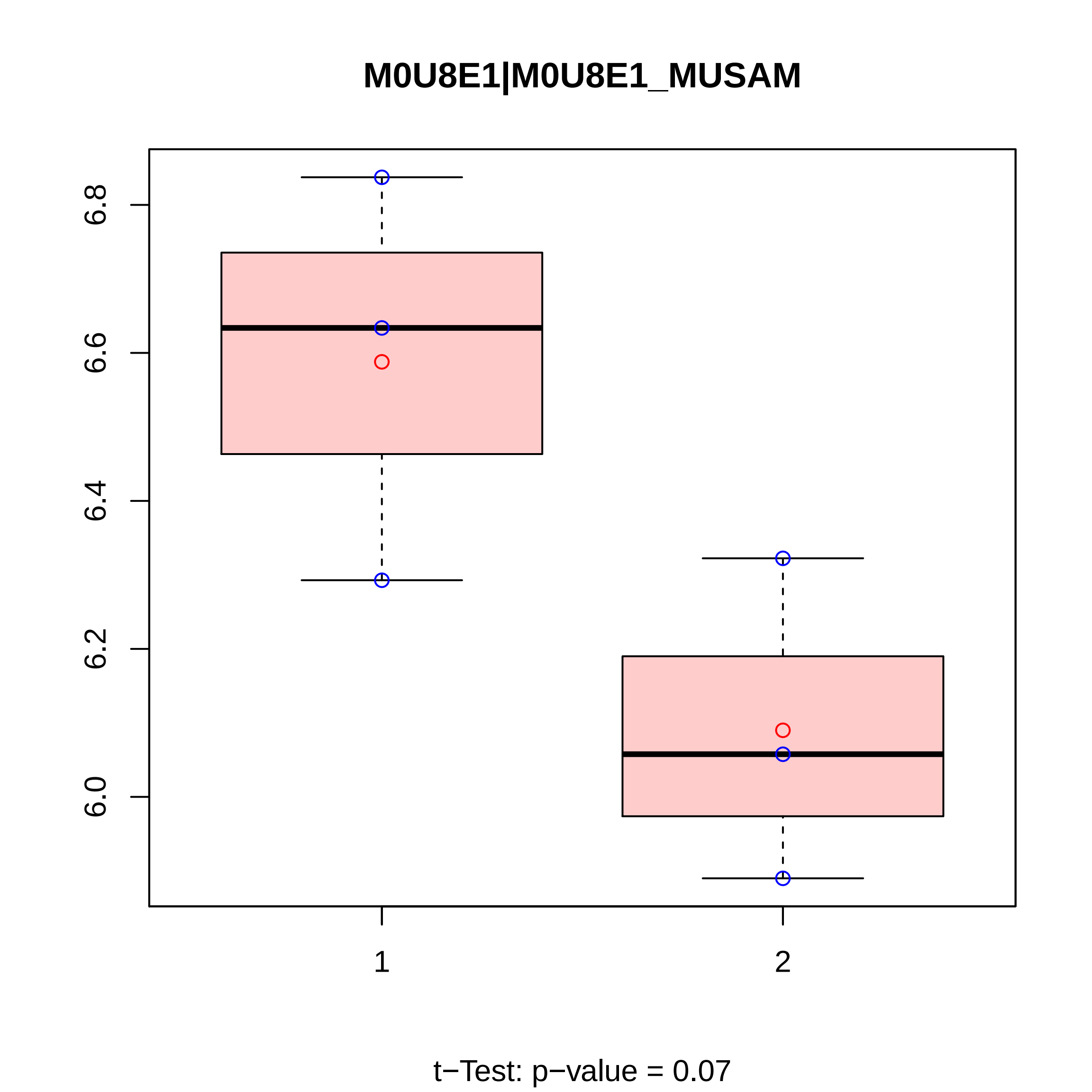

M0U8E1|M0U8E1_MUSAM
6.0
6.2
6.4
6.6
6.8
1
2
t−Test: p−value = 0.07

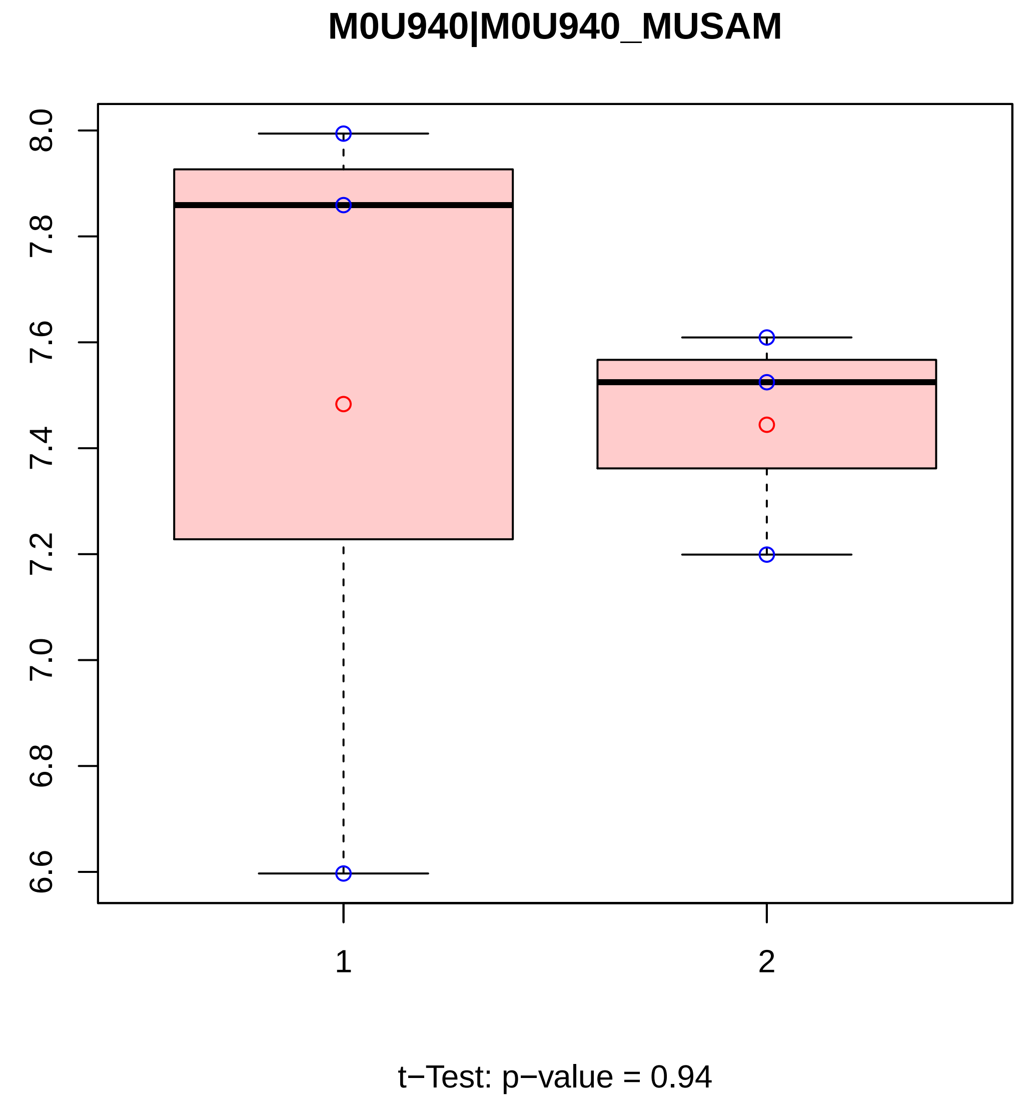
M0U940|M0U940_MUSAM
8.0
7.8
7.6
7.4
7.2
7.0
6.8
6.6
1
2
t−Test: p−value = 0.94

M0U9D2|M0U9D2_MUSAM

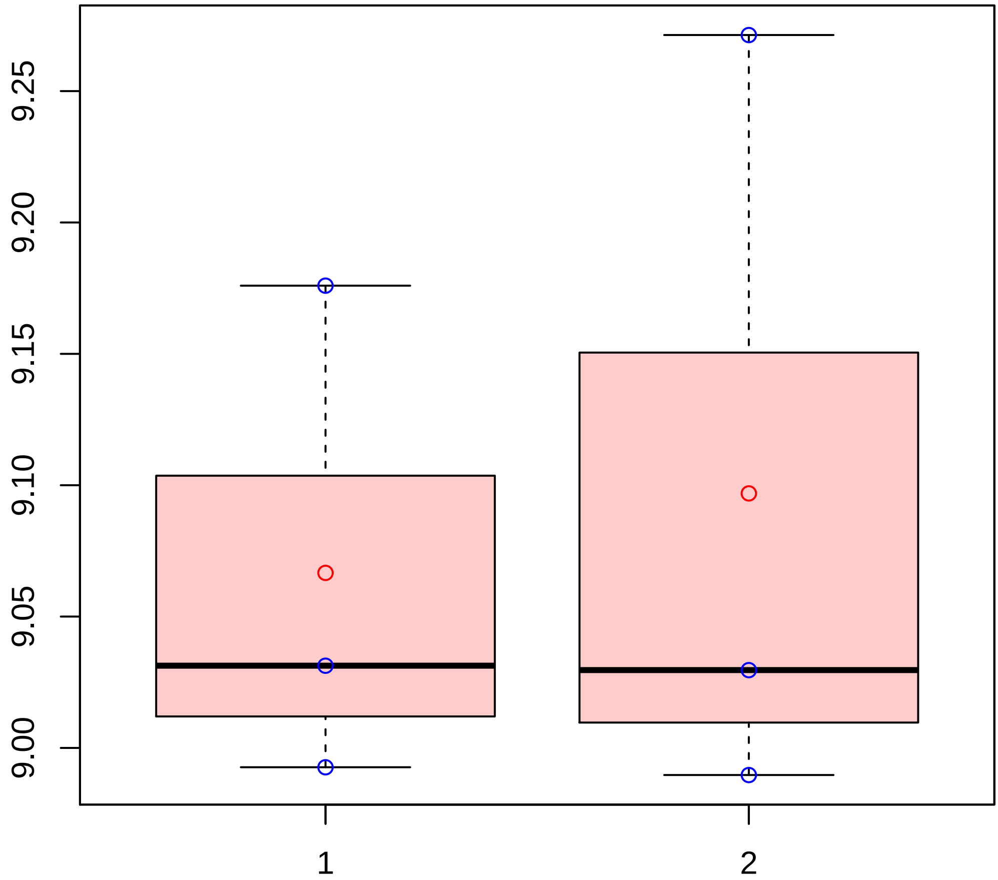

t−Test: p−value = 0.79

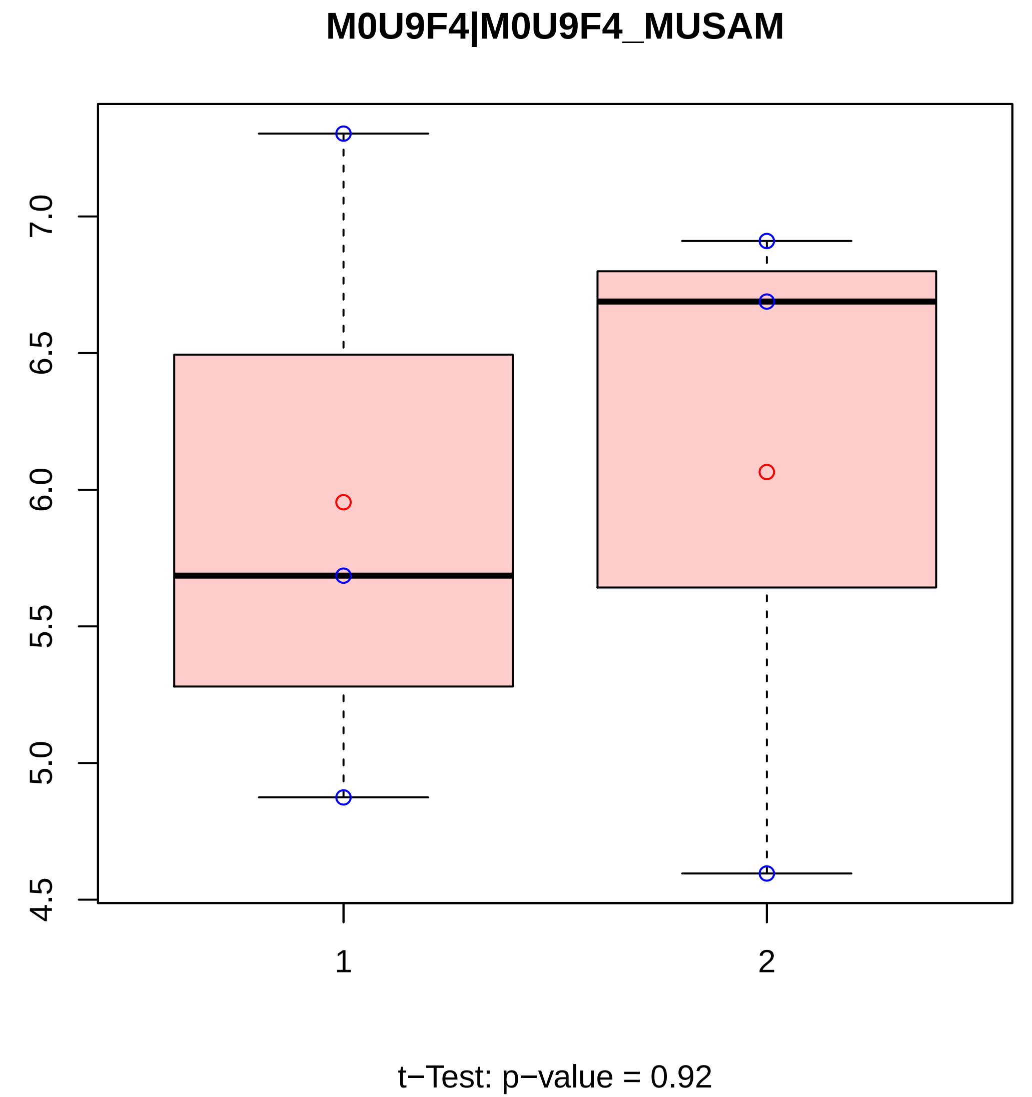
M0U9F4|M0U9F4_MUSAM
4.5
5.0
5.5
6.0
6.5
7.0
1
2
t−Test: p−value = 0.92

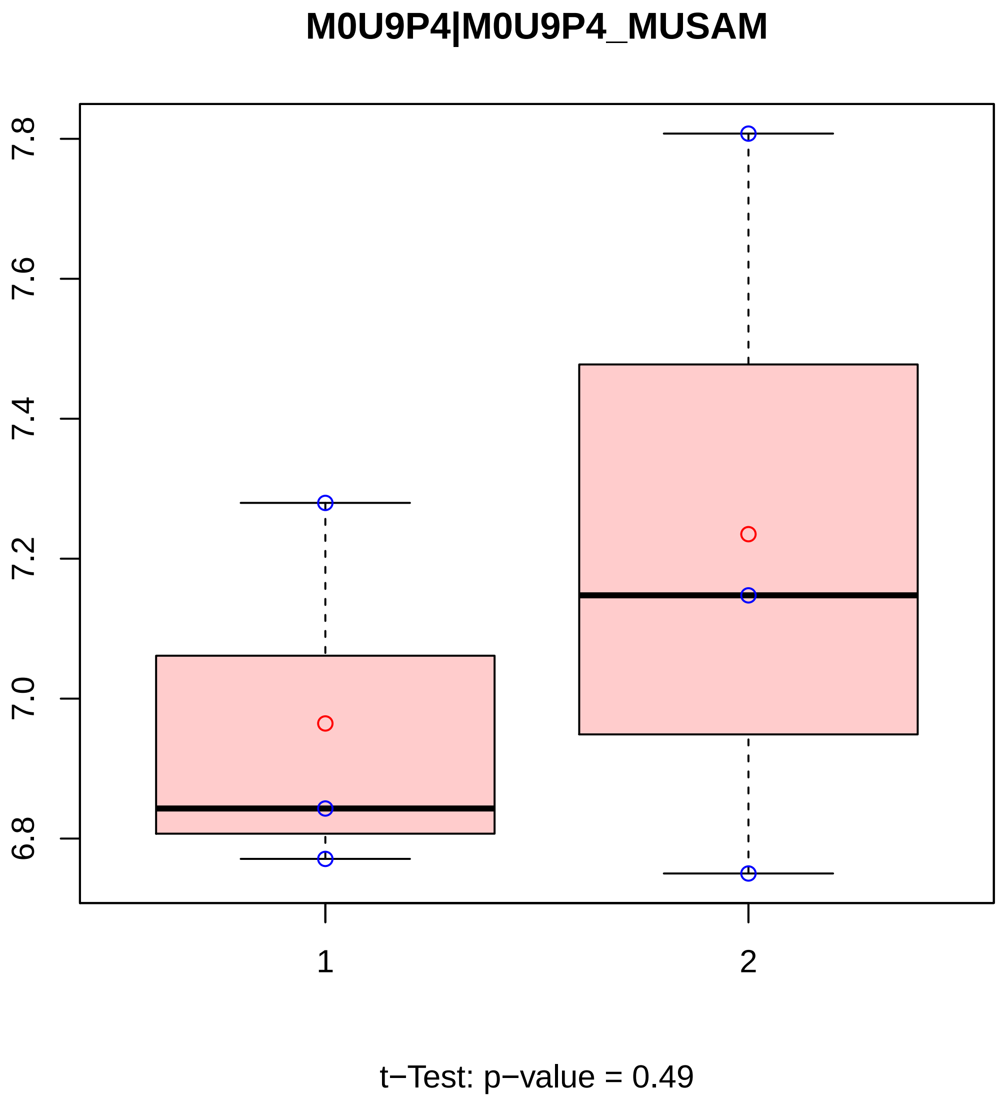
M0U9P4|M0U9P4_MUSAM
7.8
7.6
7.4
7.2
7.0
6.8
1
2
t−Test: p−value = 0.49

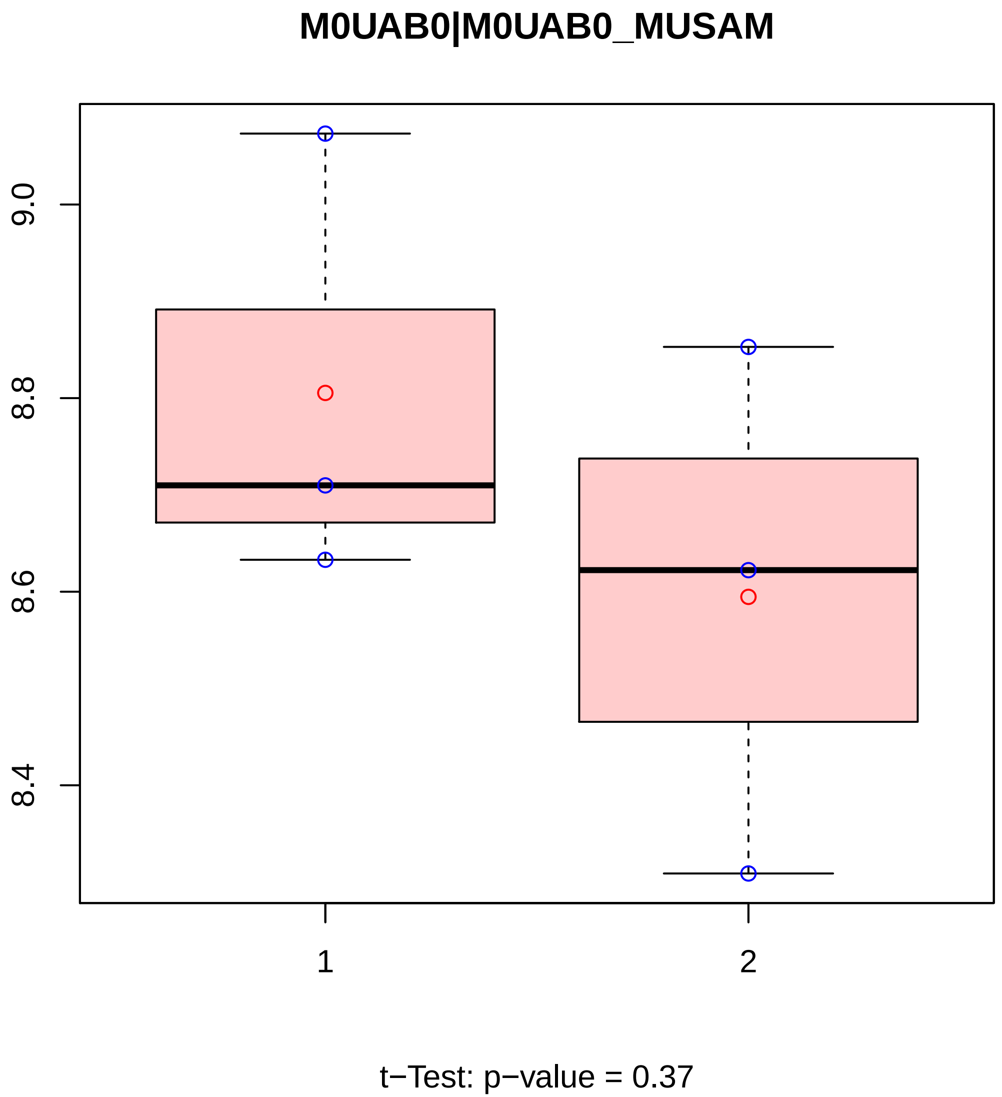
M0UAB0|M0UAB0_MUSAM
9.0
8.8
8.6
8.4
1
2
t−Test: p−value = 0.37

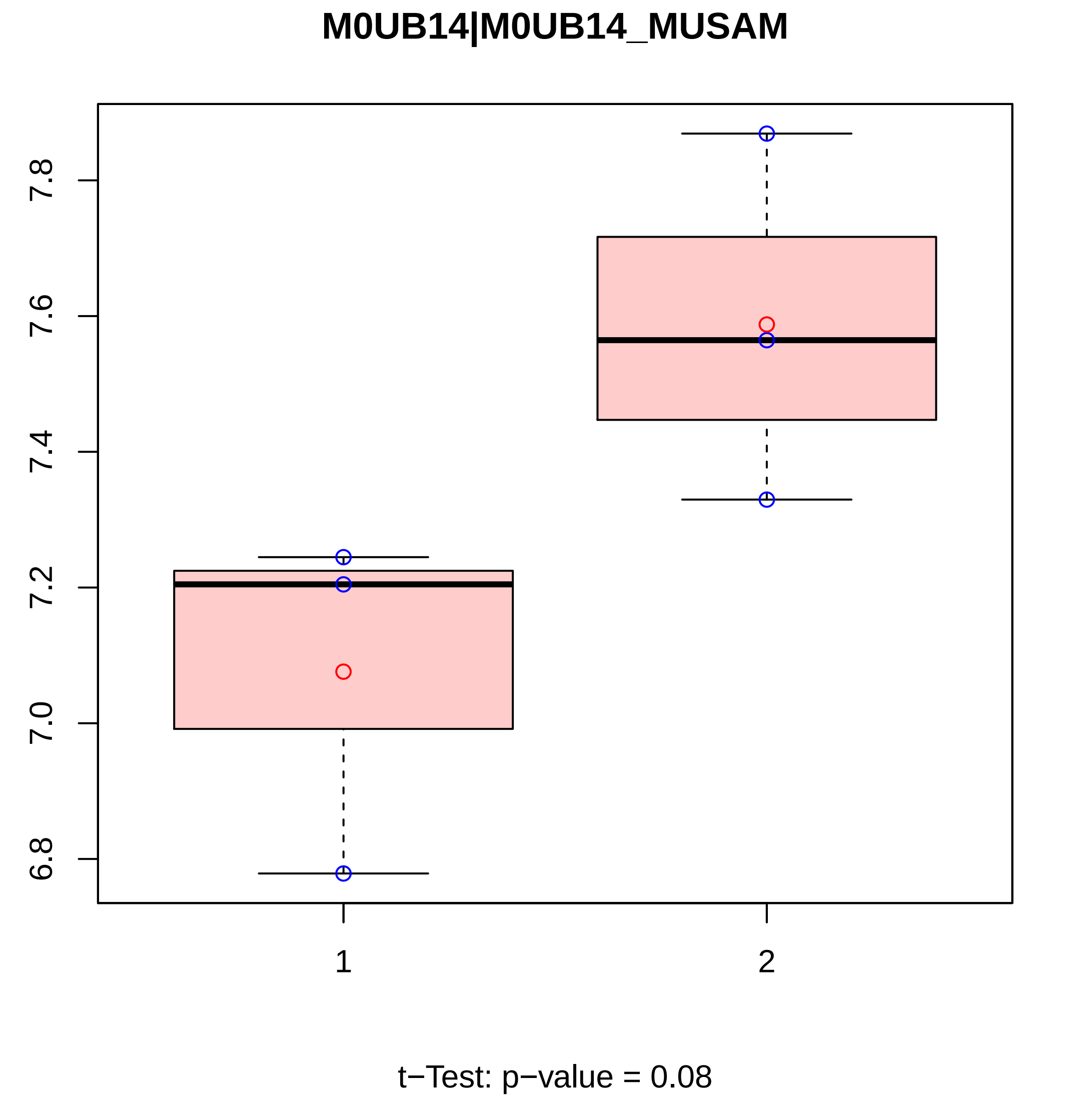
M0UB14|M0UB14_MUSAM
6.8
7.0
7.2
7.4
7.6
7.8
1
2
t−Test: p−value = 0.08

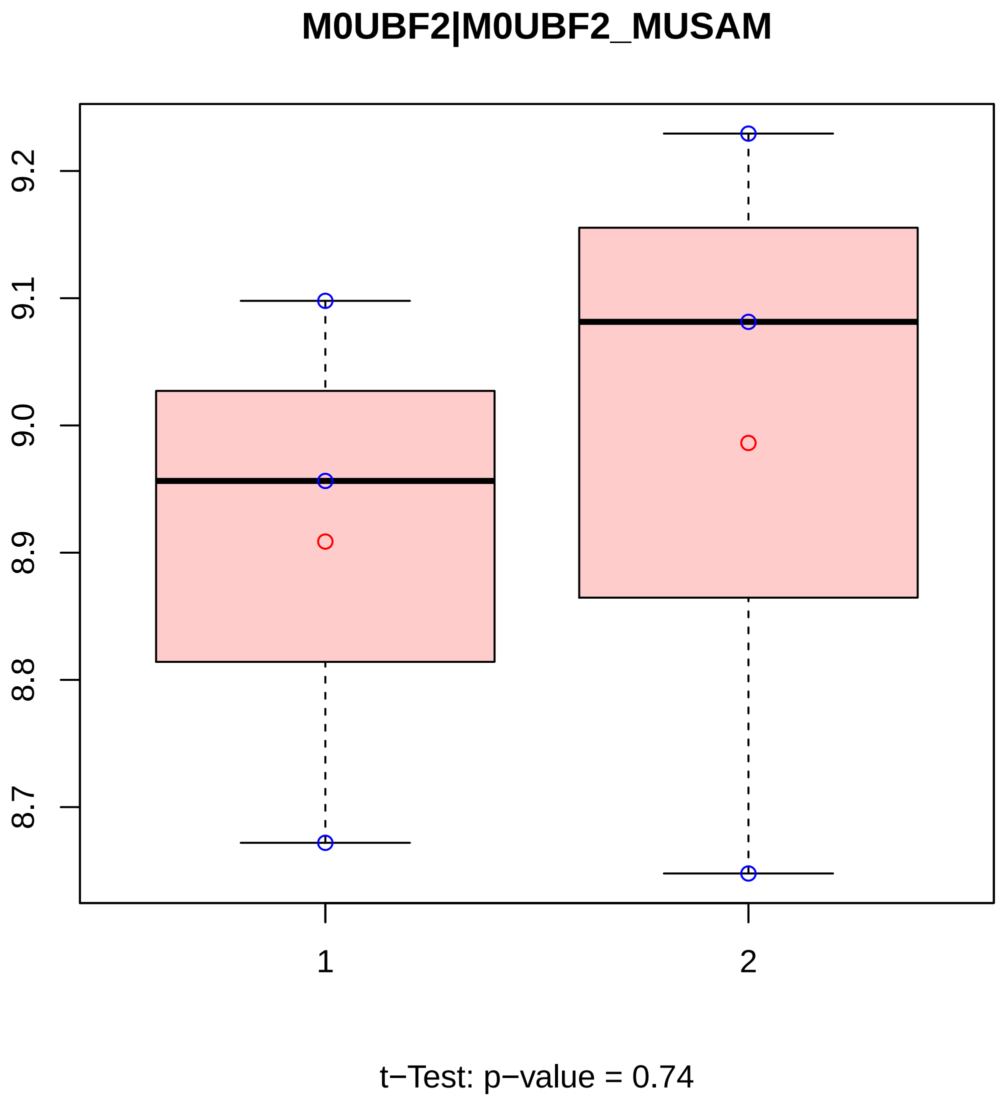
M0UBF2|M0UBF2_MUSAM
9.2
9.1
9.0
8.9
8.8
8.7
1
2
t−Test: p−value = 0.74

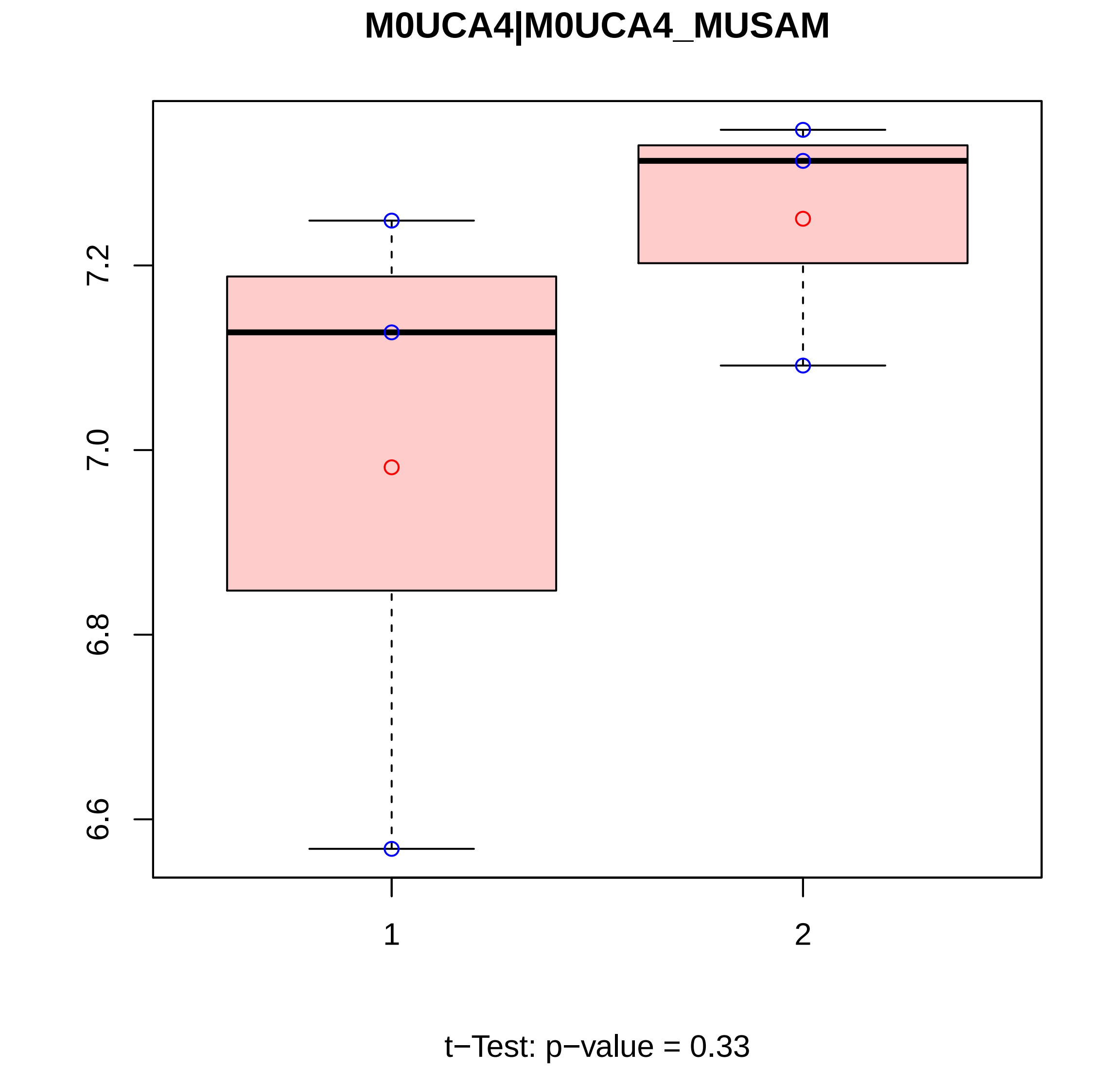
M0UCA4|M0UCA4_MUSAM
7.2
7.0
6.8
6.6
1
2
t−Test: p−value = 0.33

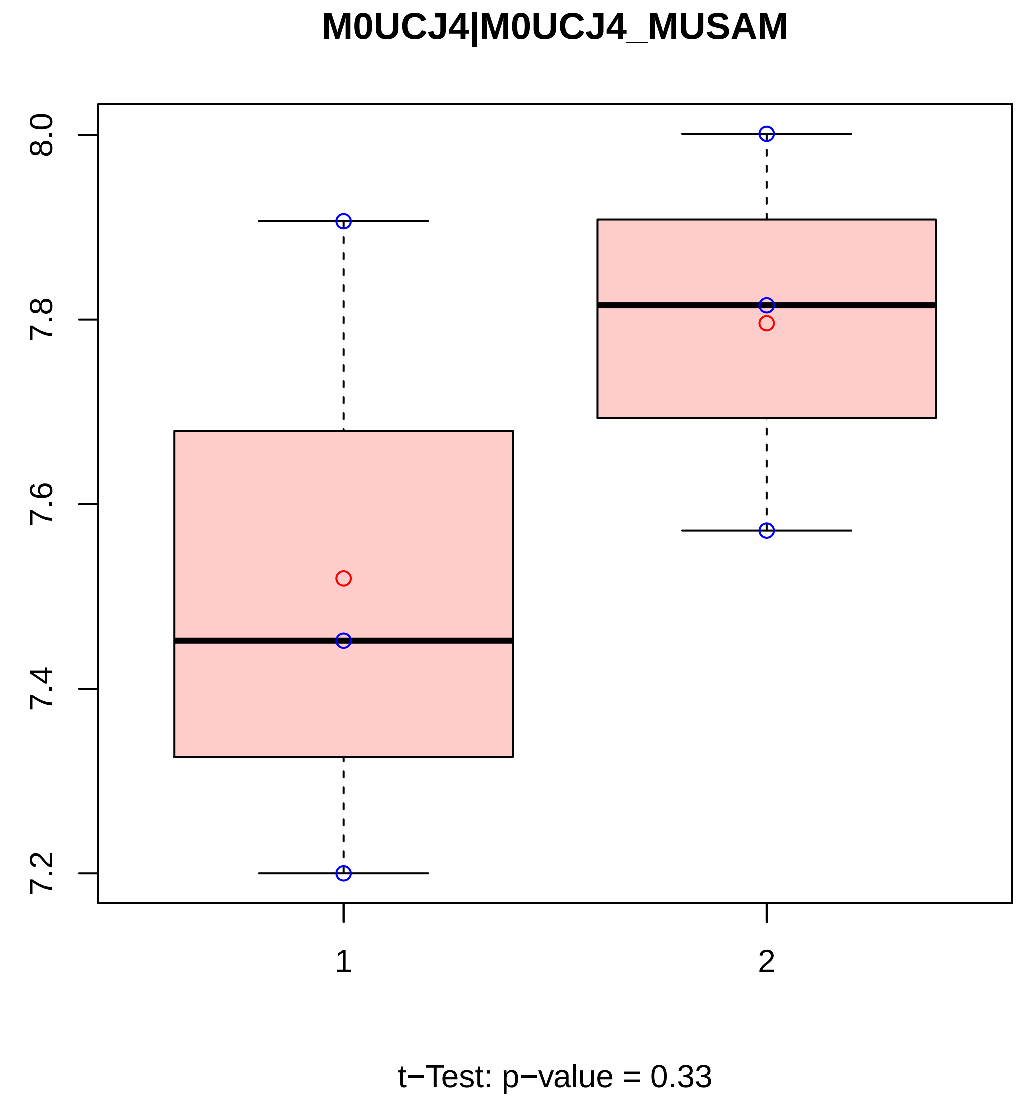
M0UCJ4|M0UCJ4_MUSAM
8.0
7.8
7.6
7.4
7.2
1
2
t−Test: p−value = 0.33

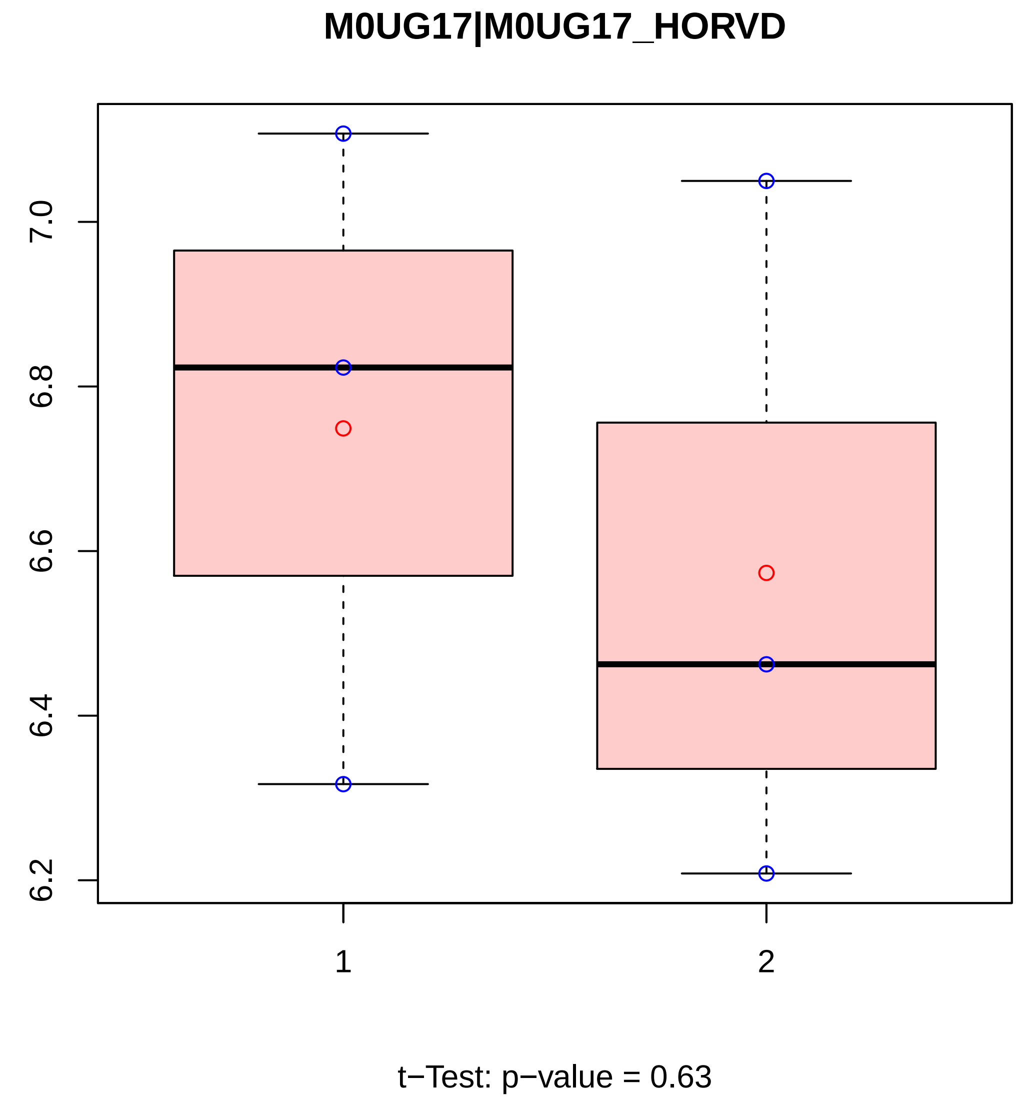
M0UG17|M0UG17_HORVD
7.0
6.8
6.6
6.4
6.2
1
2
t−Test: p−value = 0.63

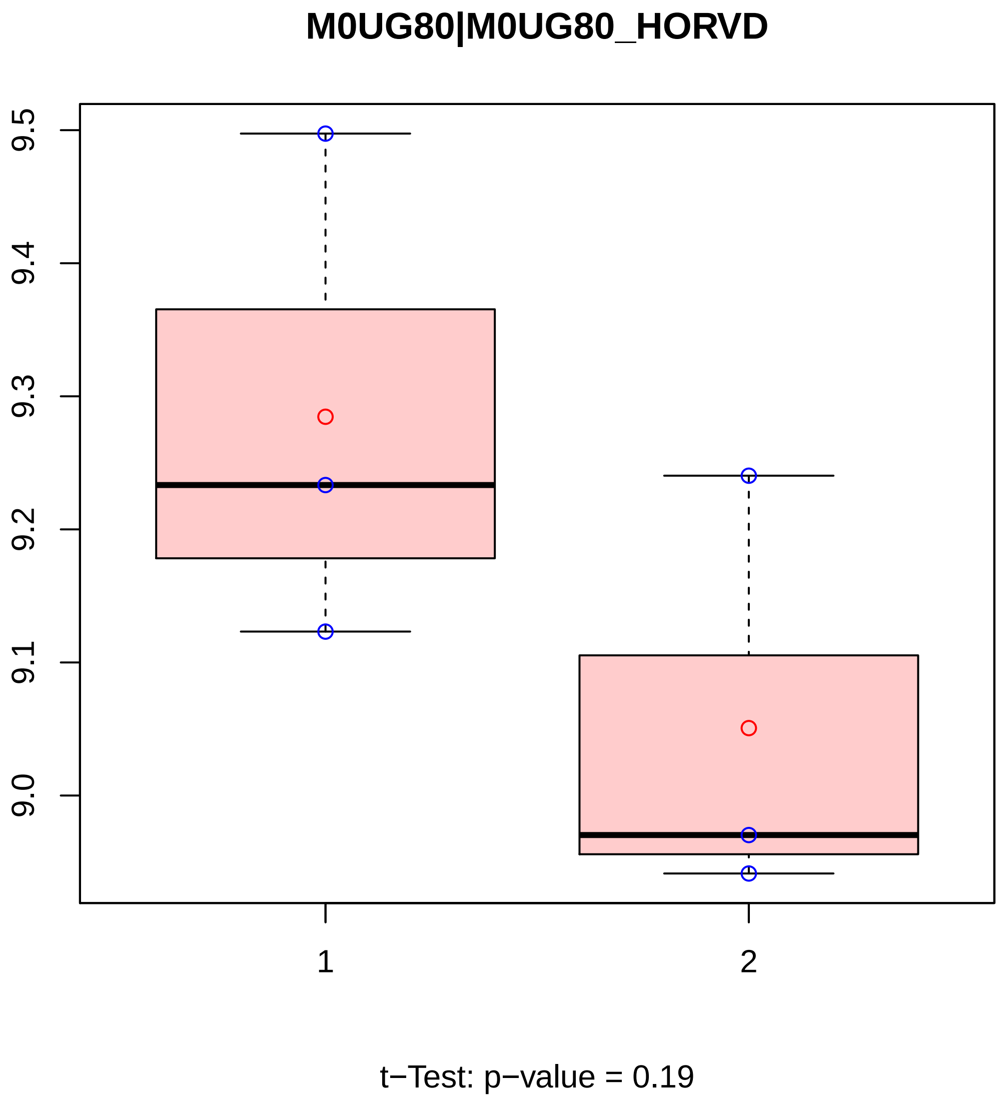

M0UG80|M0UG80_HORVD
9.0
9.1
9.2
9.3
9.4
9.5
1
2
t−Test: p−value = 0.19

M0UHI0|M0UHI0_HORVD

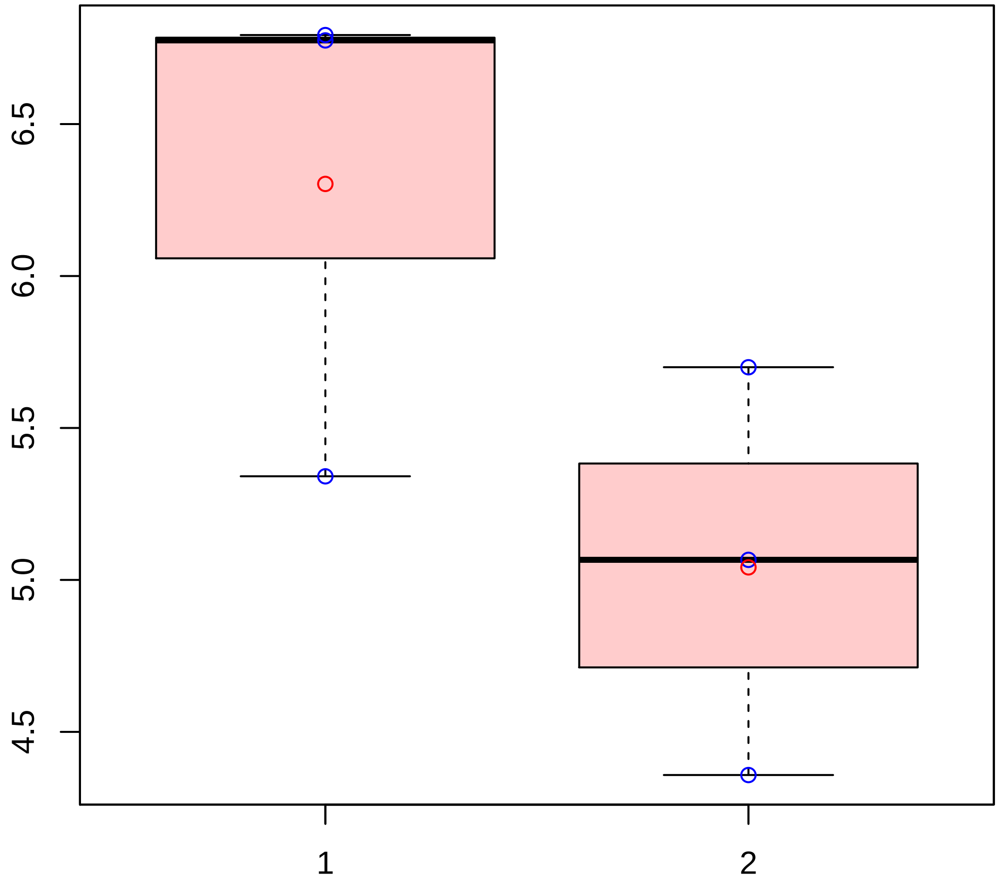

t−Test: p−value = 0.11

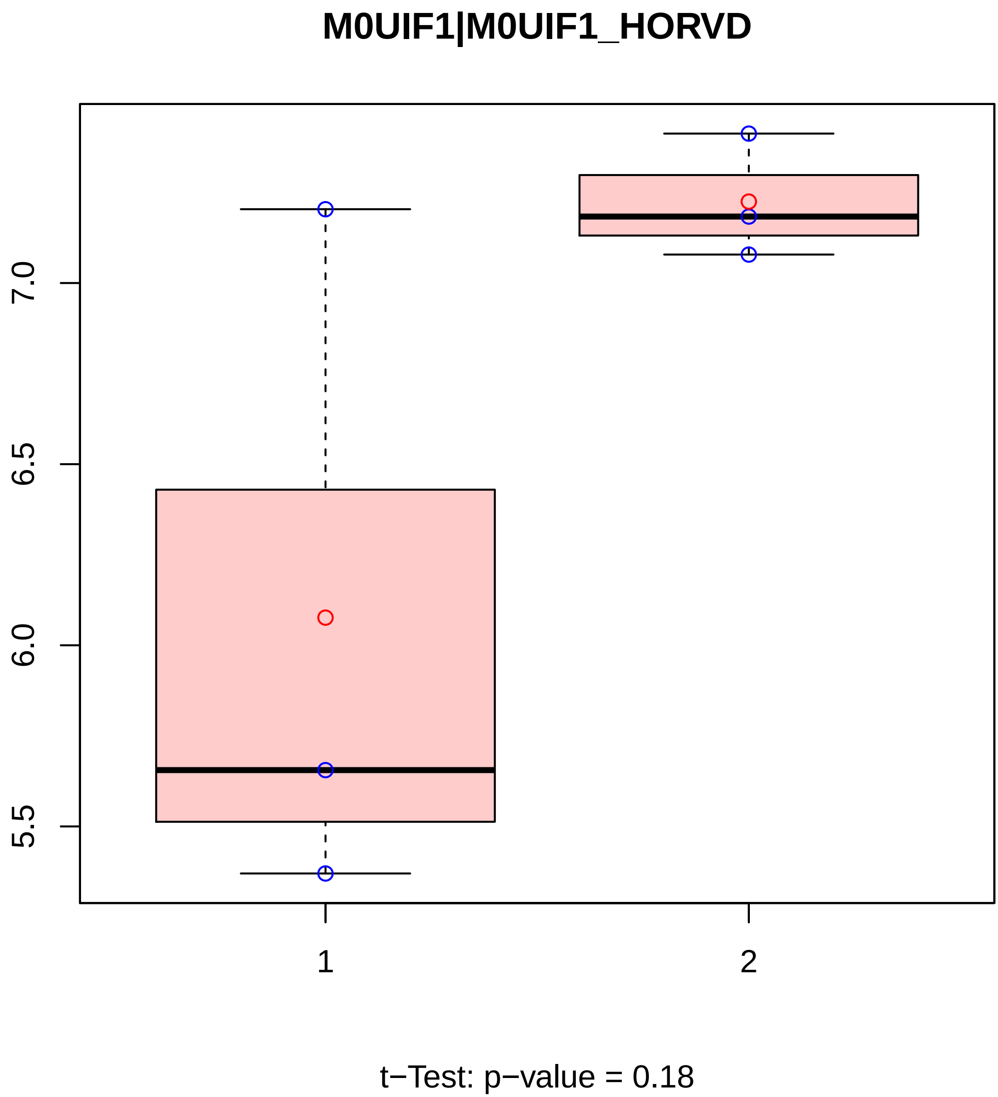
M0UIF1|M0UIF1_HORVD
7.0
6.5
6.0
5.5
1
2
t−Test: p−value = 0.18

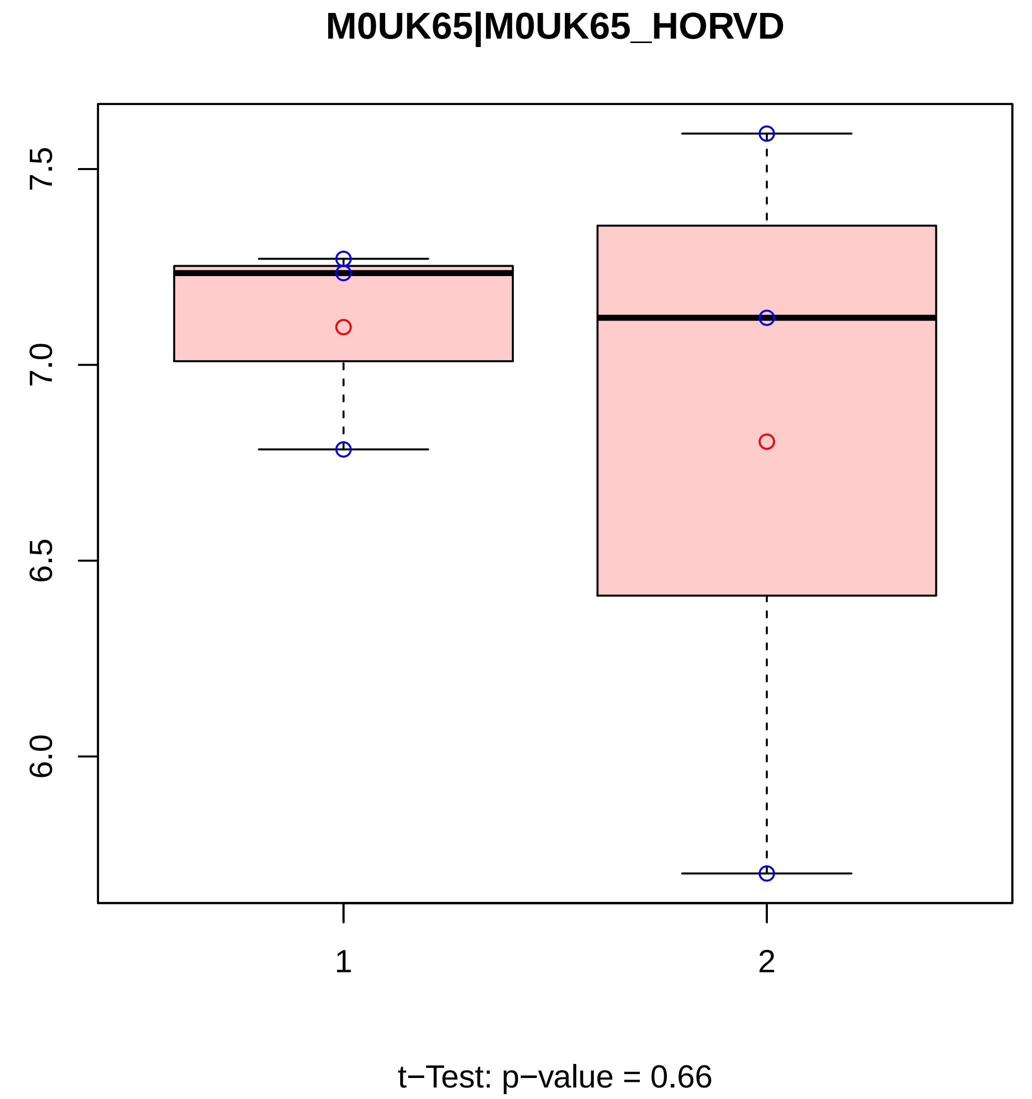
M0UK65|M0UK65_HORVD
7.5
7.0
6.5
6.0
1
2
t−Test: p−value = 0.66

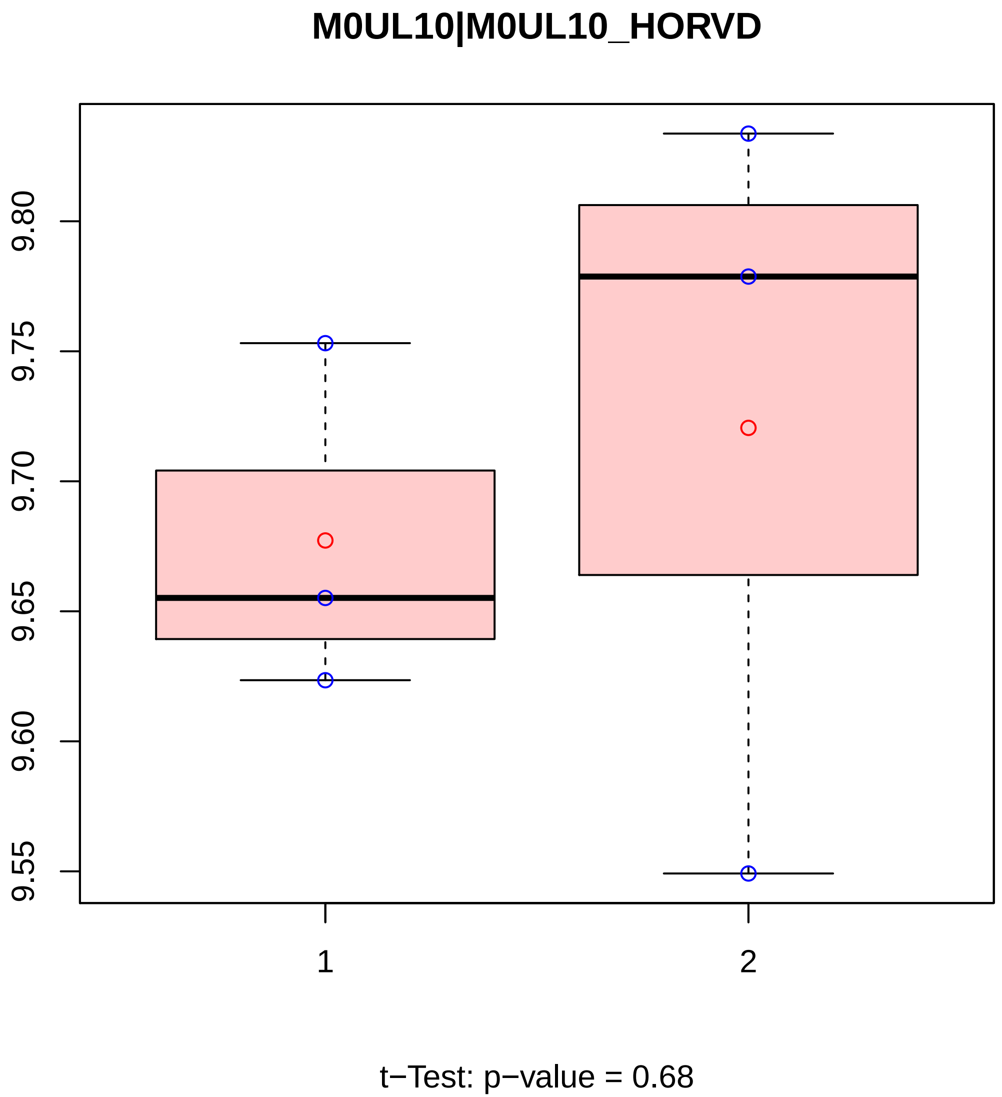
M0UL10|M0UL10_HORVD
9.55
9.60
9.65
9.70
9.75
9.80
1
2
t−Test: p−value = 0.68

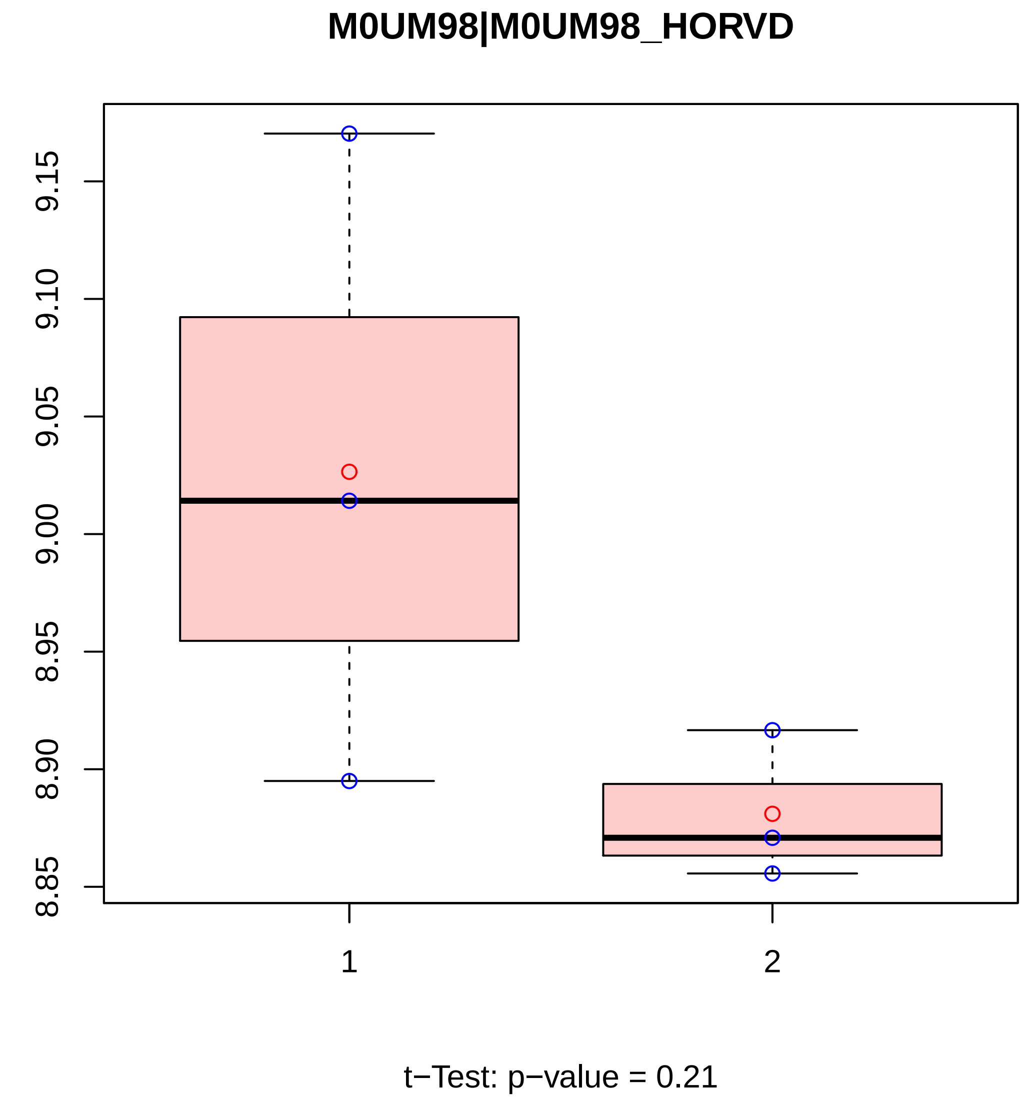
M0UM98|M0UM98_HORVD
9.15
9.10
9.05
9.00
8.95
8.90
8.85
1
2
t−Test: p−value = 0.21

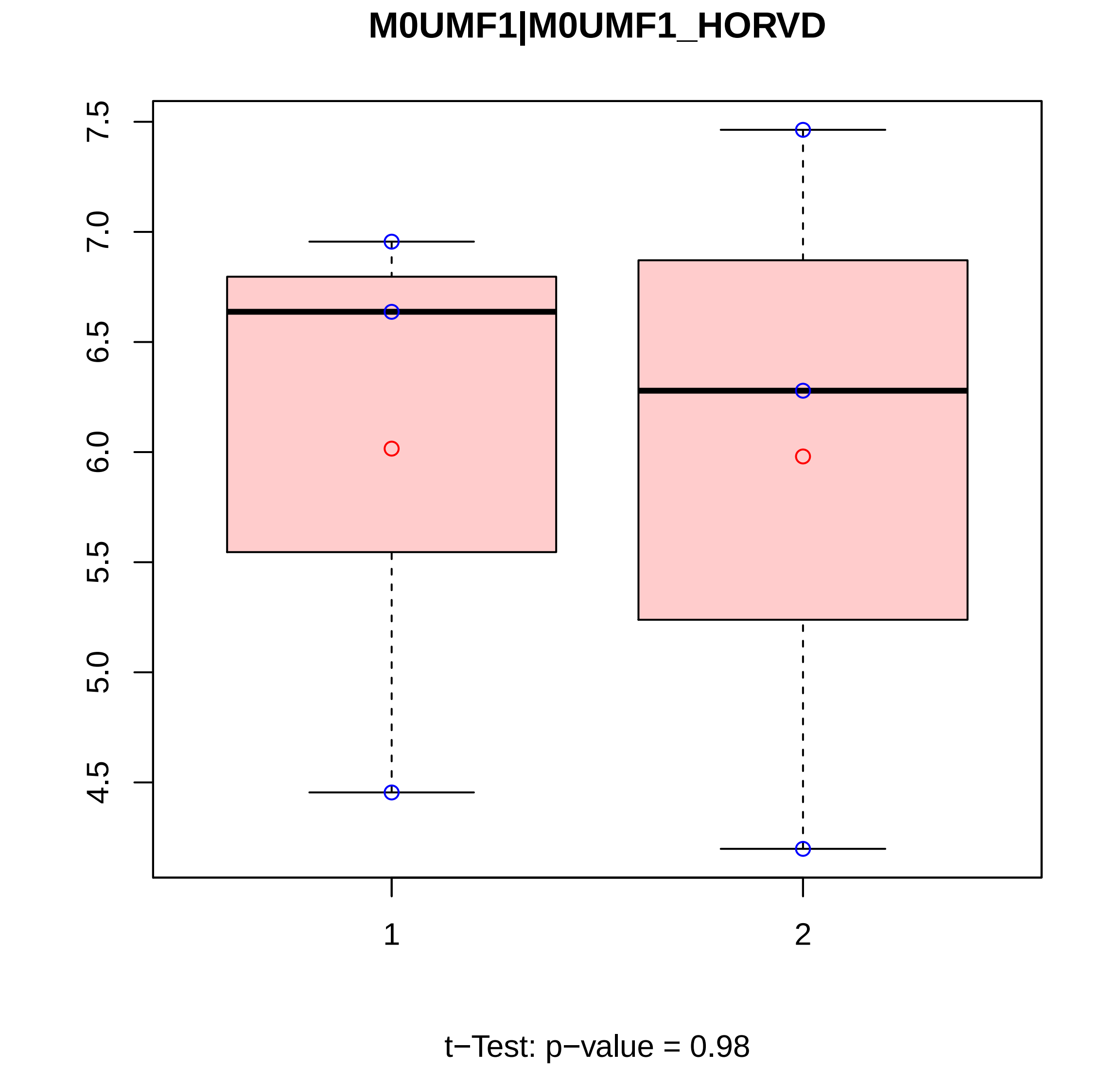

M0UMF1|M0UMF1_HORVD
7.5
7.0
6.5
6.0
5.5
5.0
4.5
1
2
t−Test: p−value = 0.98

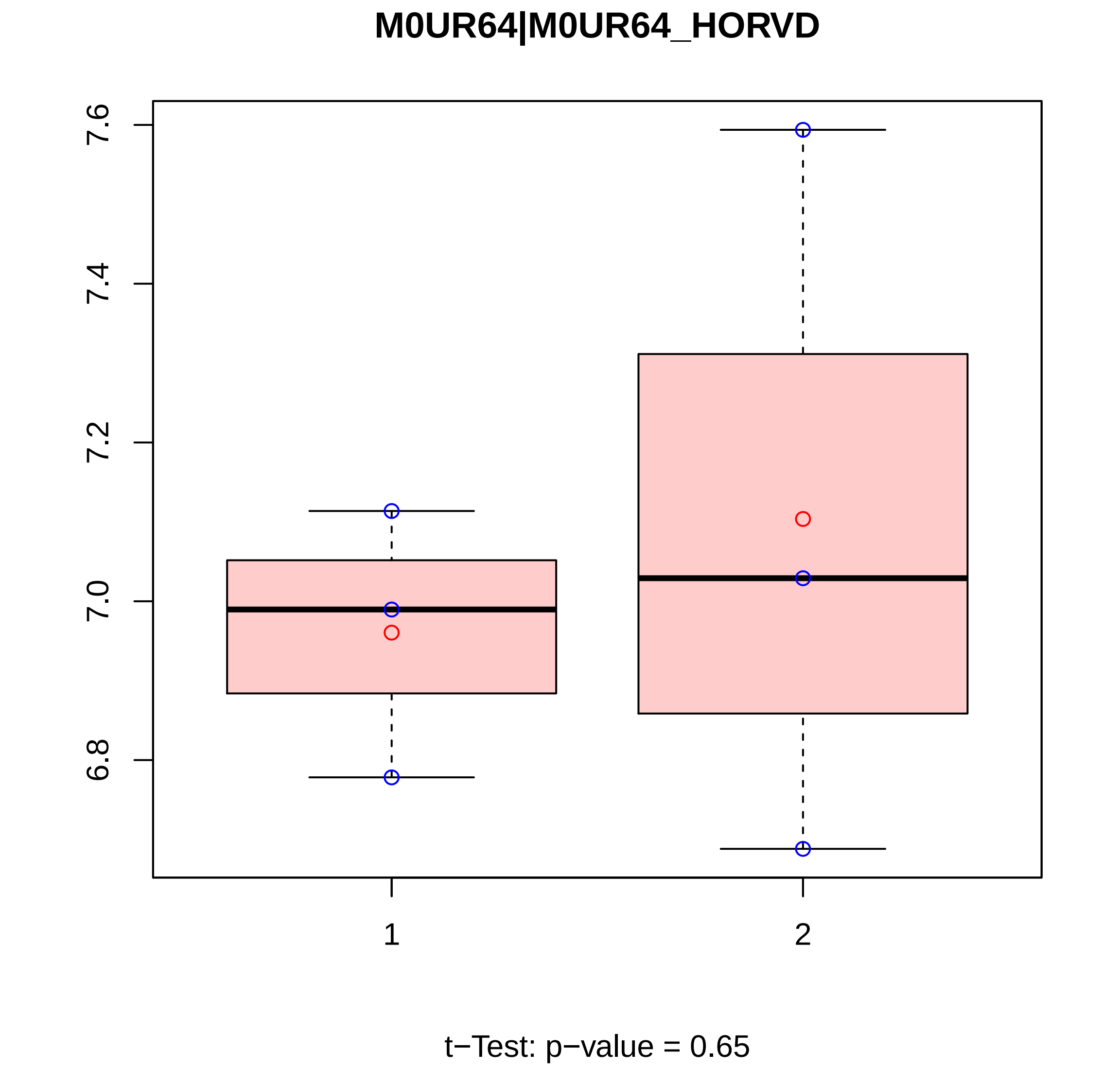
M0UR64|M0UR64_HORVD
7.6
7.4
7.2
7.0
6.8
1
2
t−Test: p−value = 0.65

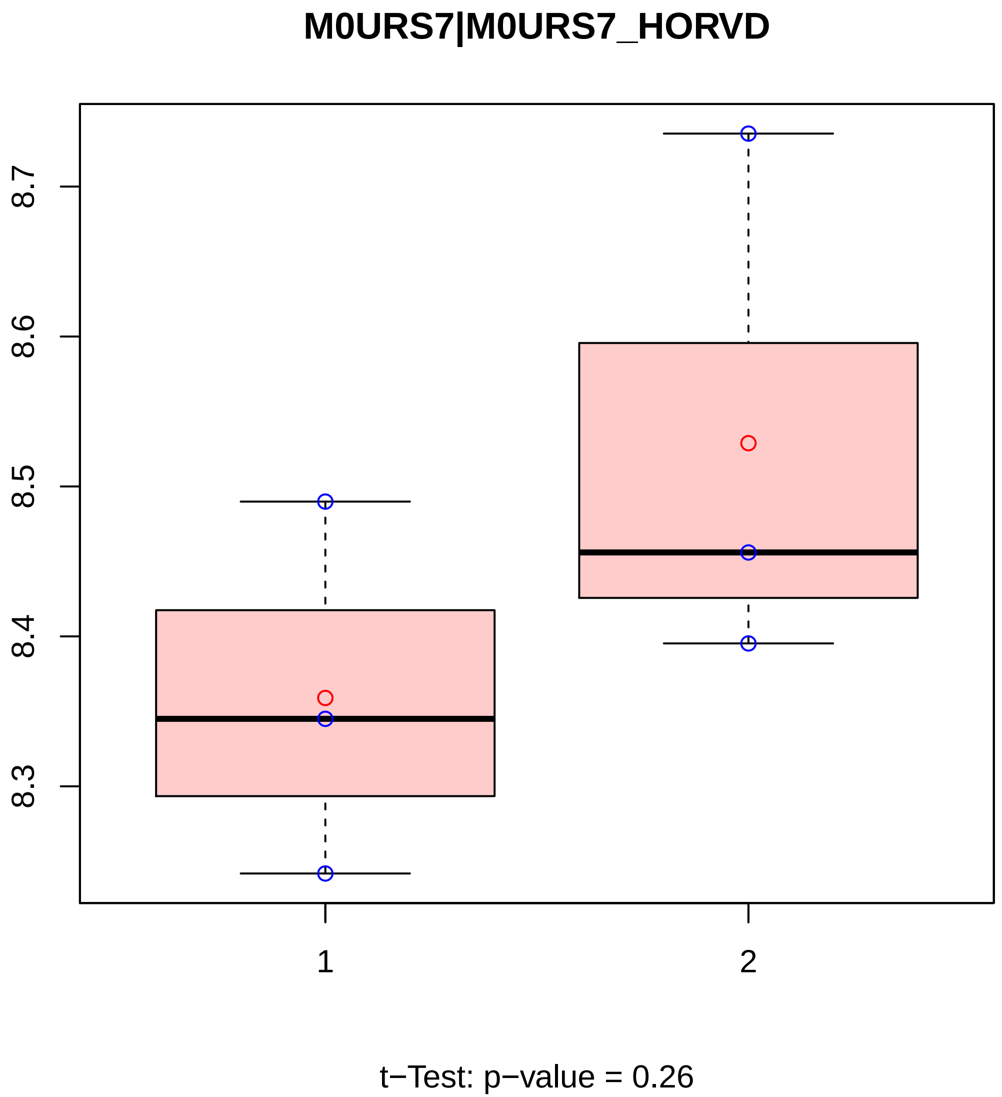
M0URS7|M0URS7_HORVD
8.3
8.4
8.5
8.6
8.7
1
2
t−Test: p−value = 0.26

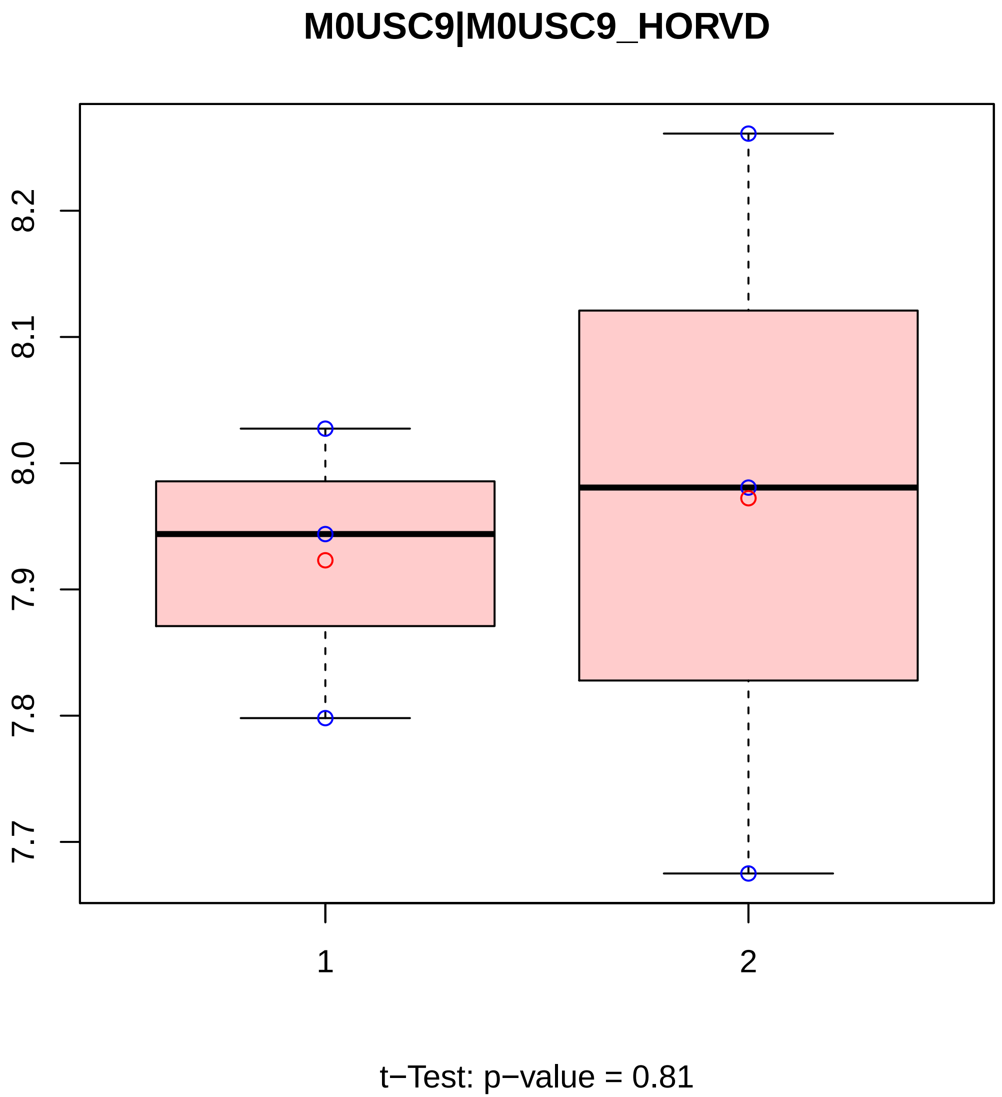
M0USC9|M0USC9_HORVD
8.2
8.1
8.0
7.9
7.8
7.7
1
2
t−Test: p−value = 0.81

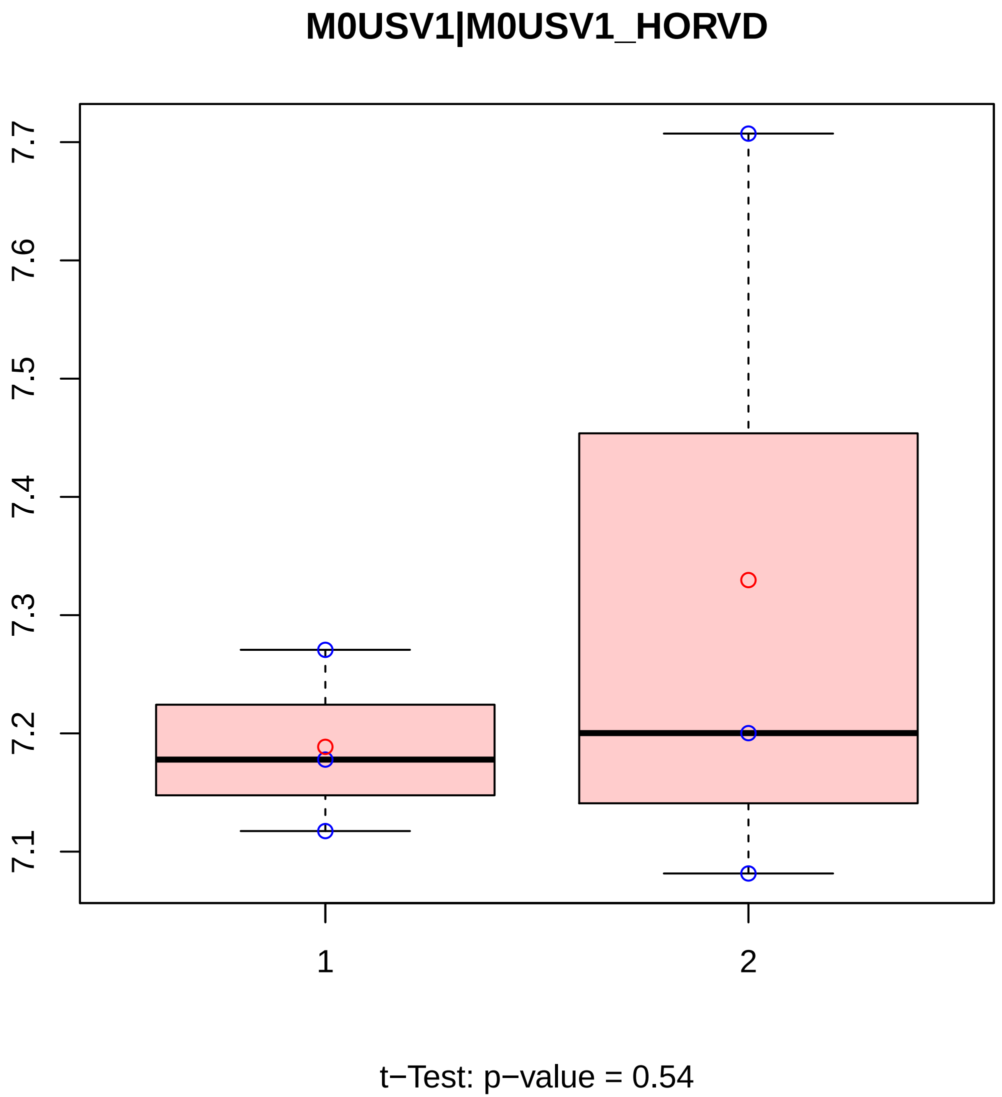

M0USV1|M0USV1_HORVD
7.1
7.2
7.3
7.4
7.5
7.6
7.7
1
2
t−Test: p−value = 0.54

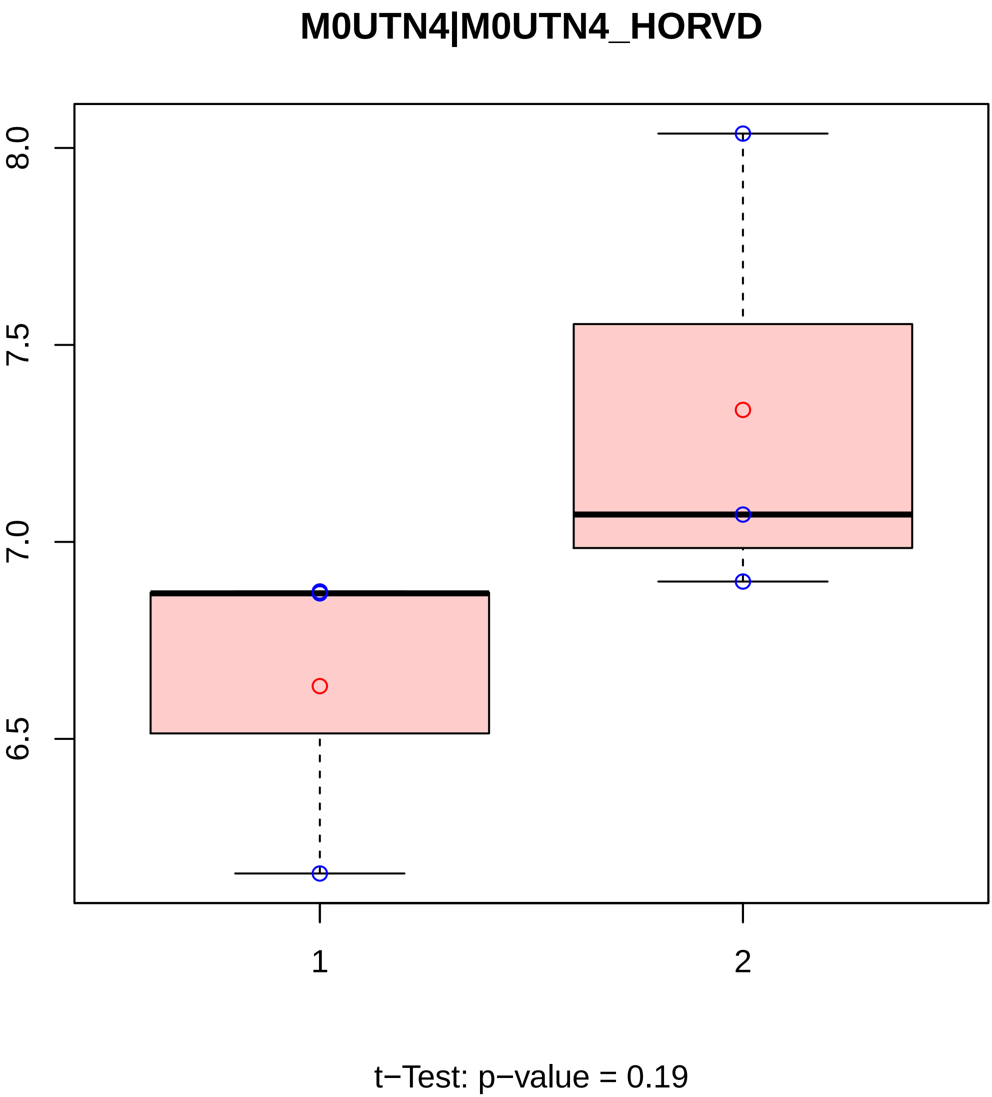
M0UTN4|M0UTN4_HORVD
8.0
7.5
7.0
6.5
1
2
t−Test: p−value = 0.19

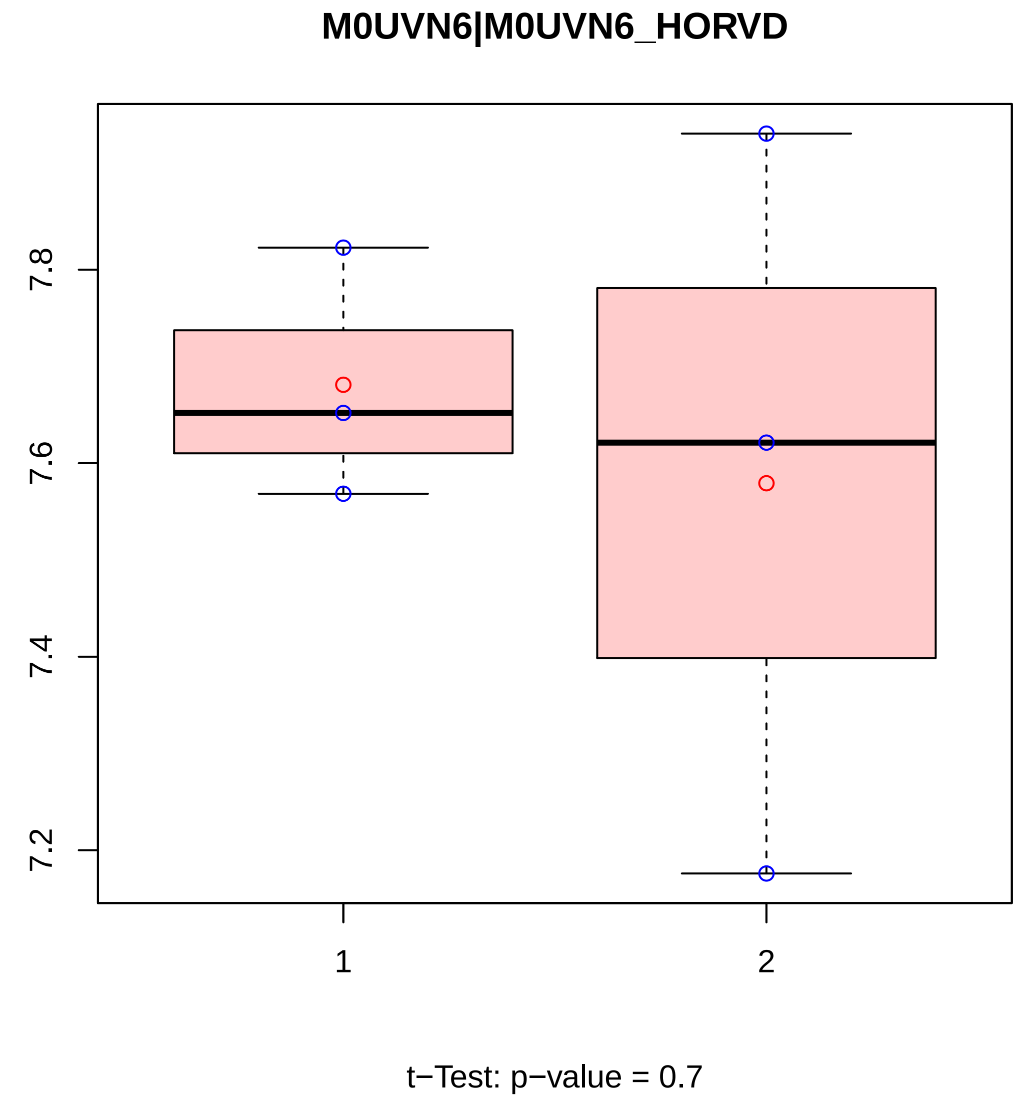
M0UVN6|M0UVN6_HORVD
7.2
7.4
7.6
7.8
1
2
t−Test: p−value = 0.7

M0UWD2|M0UWD2_HORVD

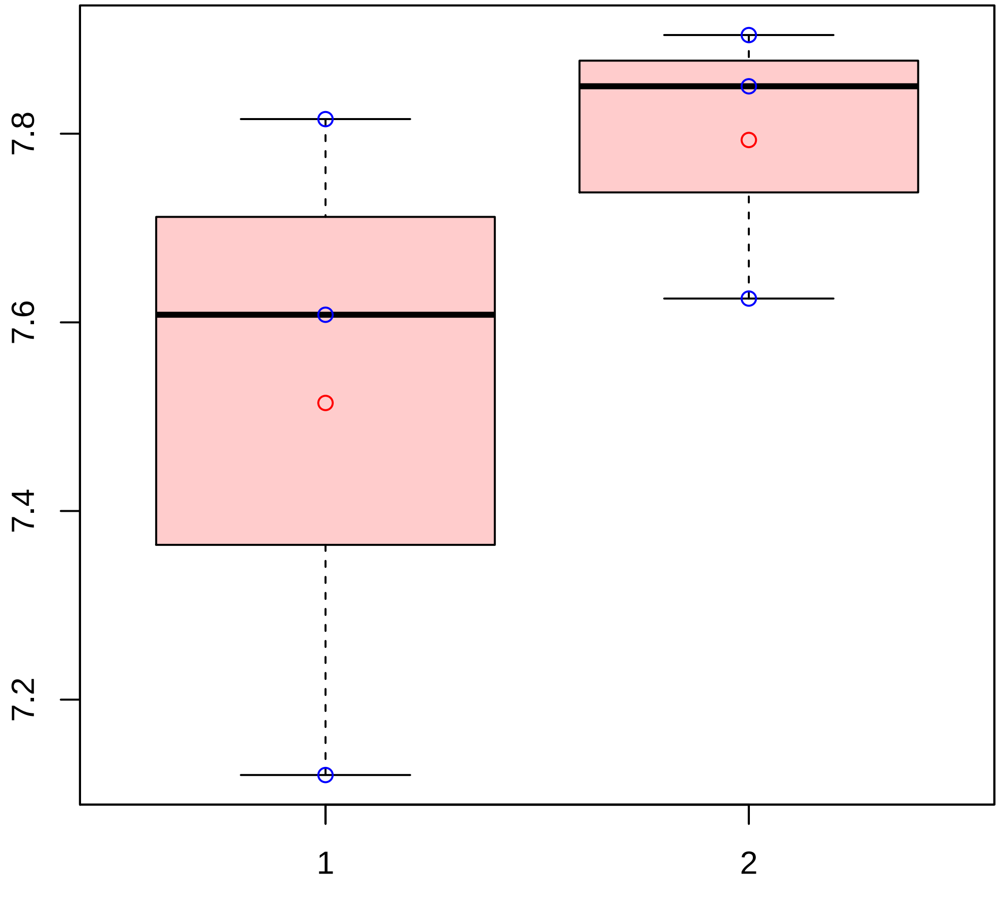

t−Test: p−value = 0.31

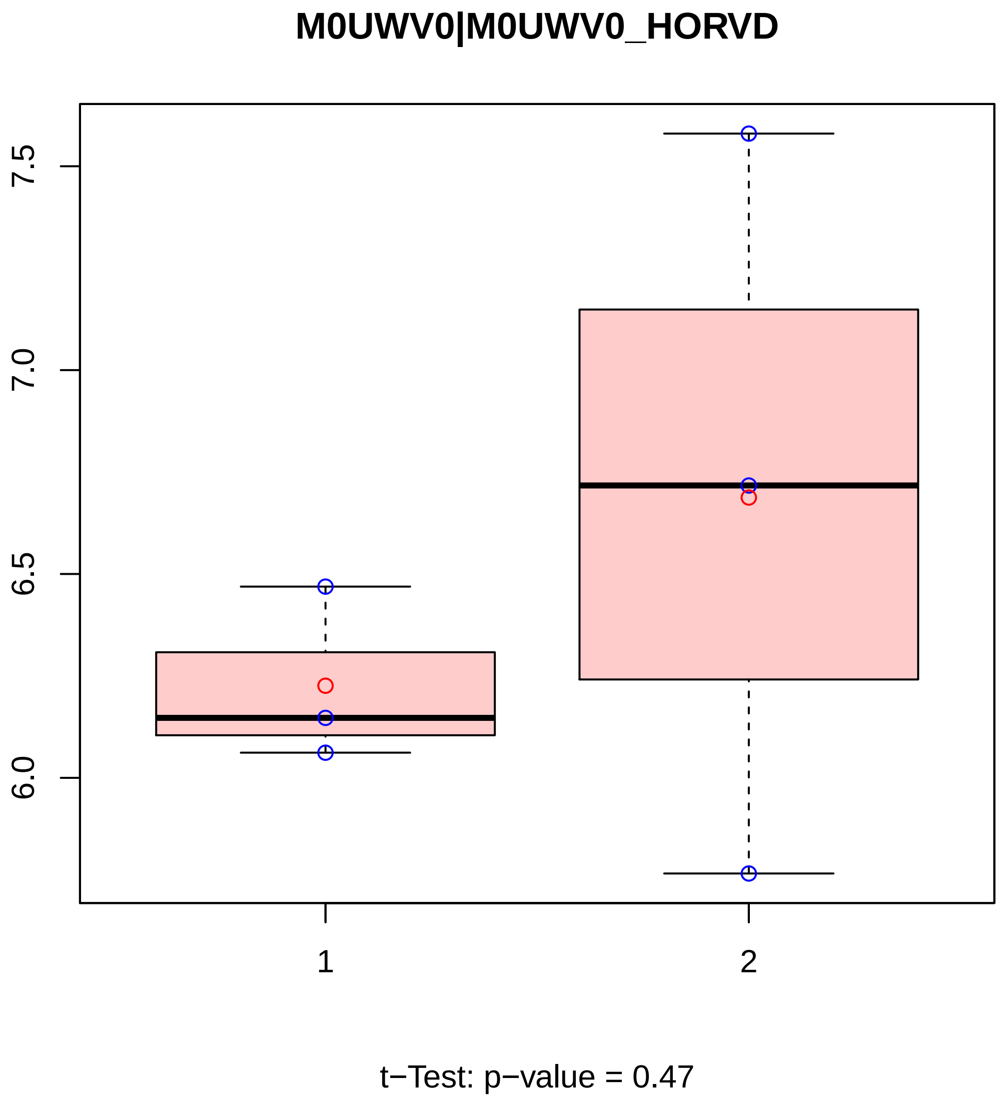

M0UWV0|M0UWV0_HORVD
6.0
6.5
7.0
7.5
1
2
t−Test: p−value = 0.47

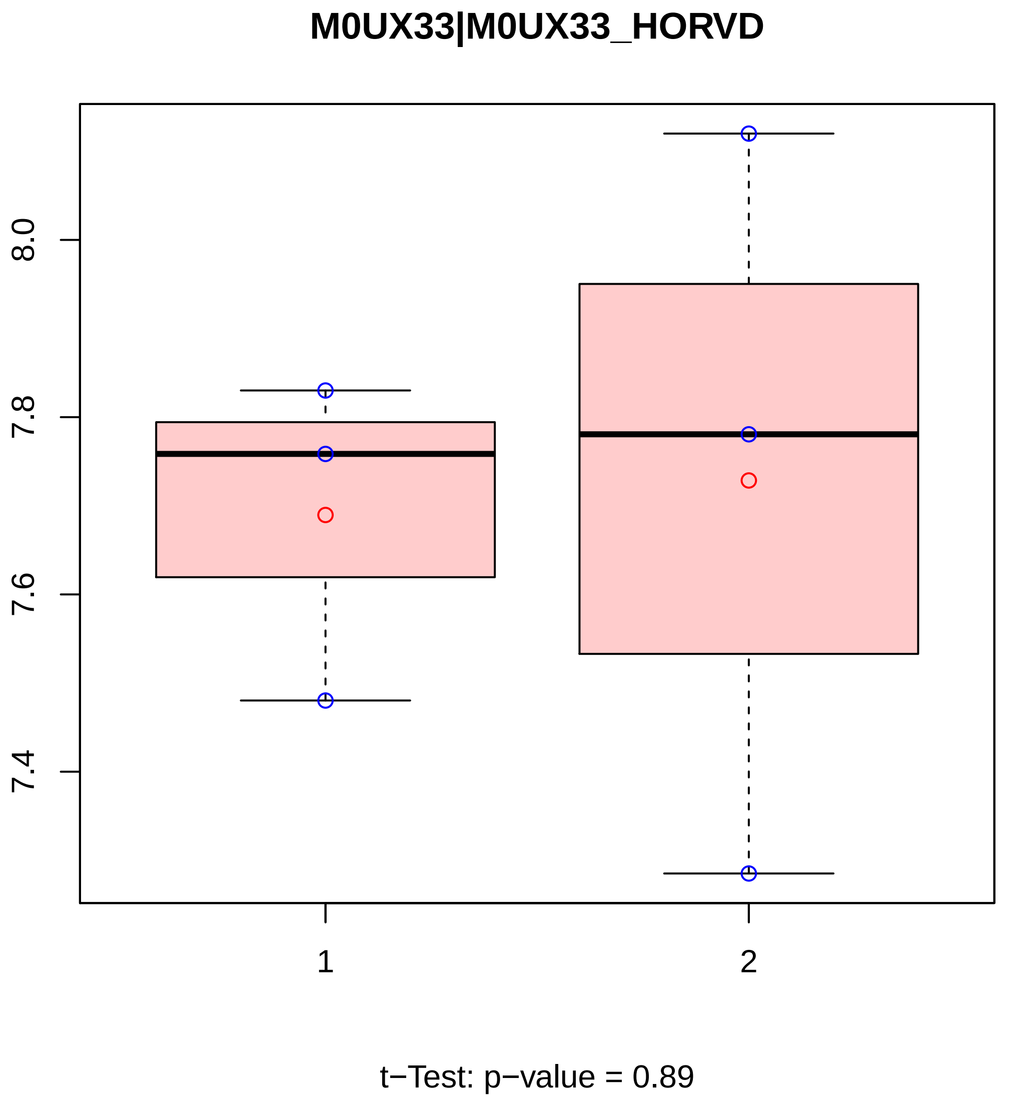

M0UX33|M0UX33_HORVD
8.0
7.8
7.6
7.4
1
2
t−Test: p−value = 0.89

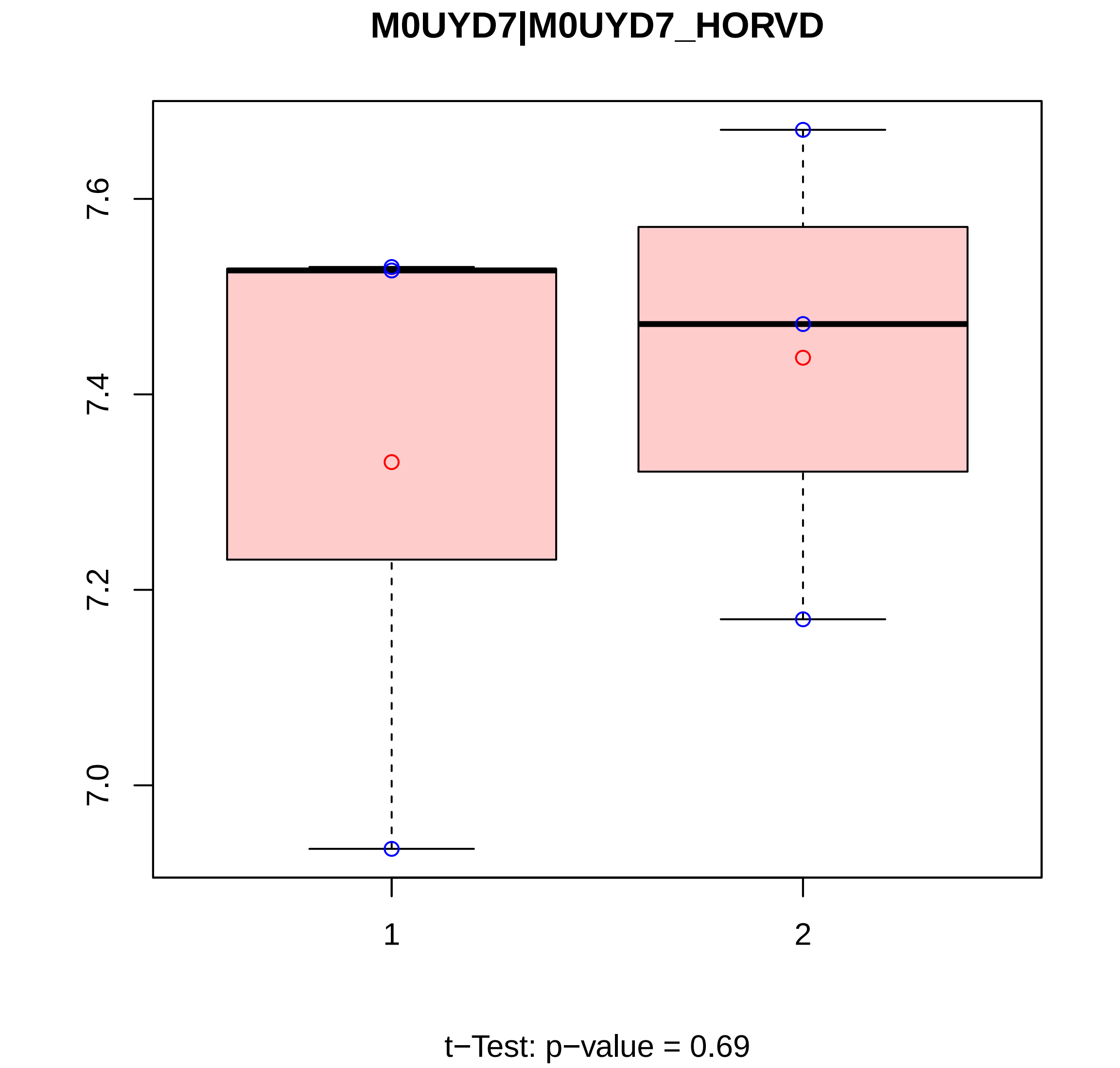

M0UYD7|M0UYD7_HORVD
7.0
7.2
7.4
7.6
1
2
t−Test: p−value = 0.69

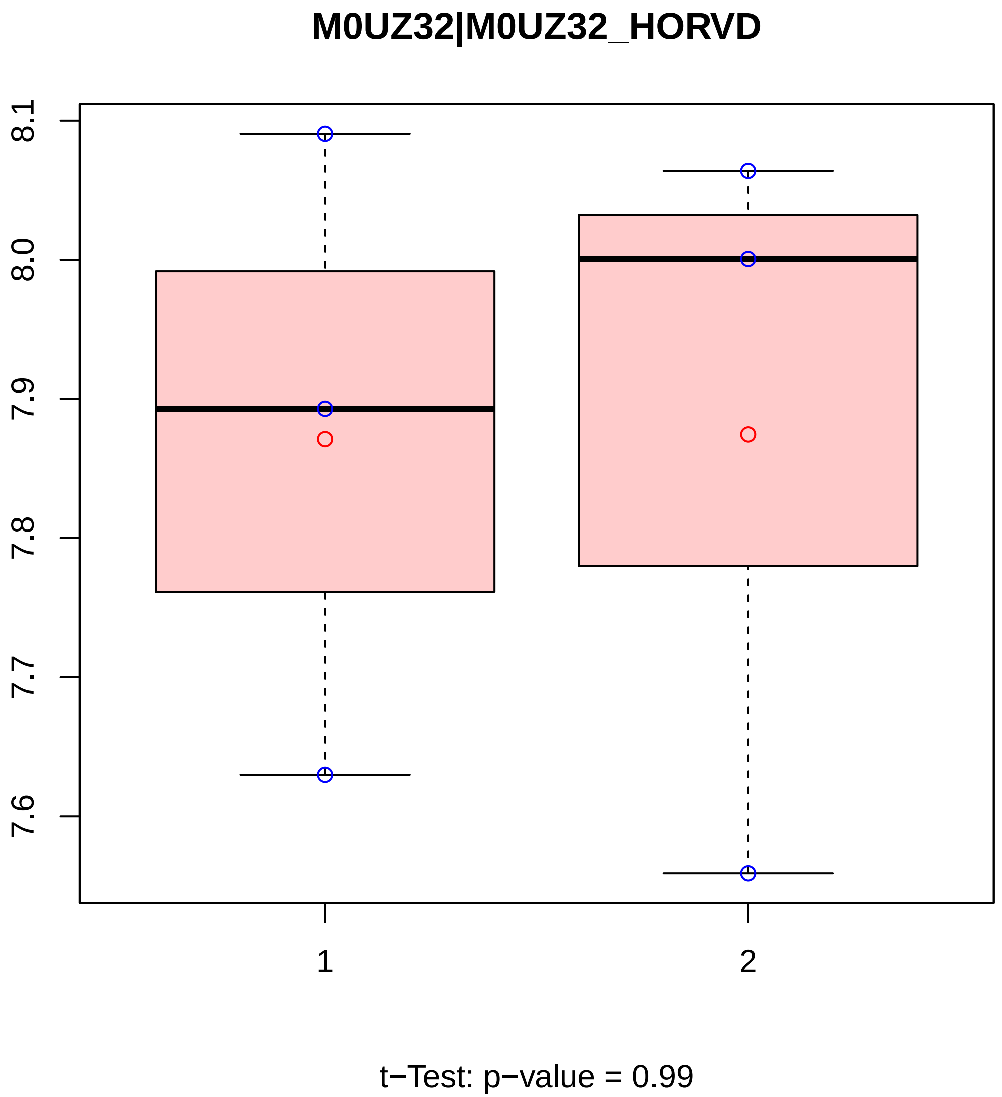
M0UZ32|M0UZ32_HORVD
8.1
8.0
7.9
7.8
7.7
7.6
1
2
t−Test: p−value = 0.99

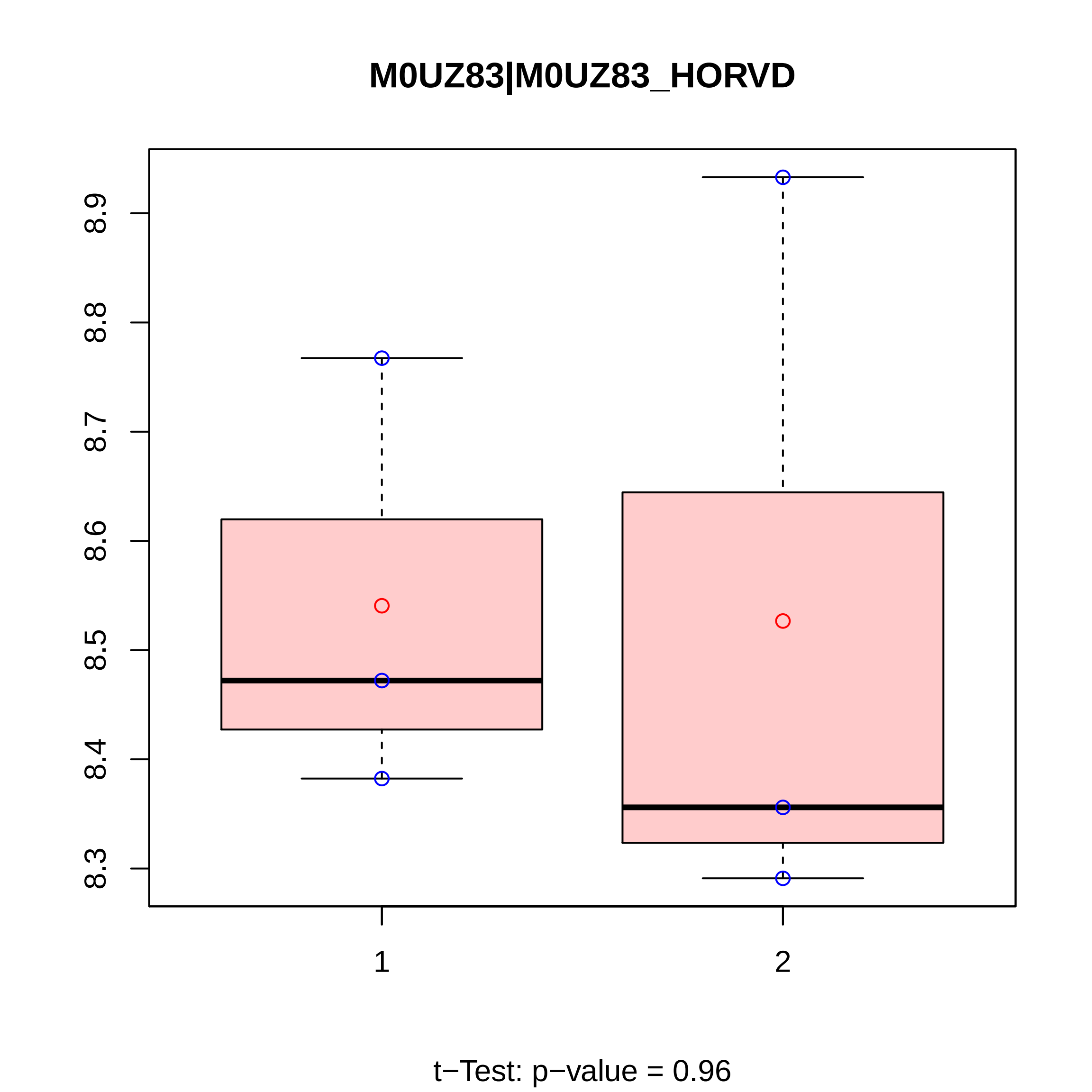
M0UZ83|M0UZ83_HORVD
8.3
8.4
8.5
8.6
8.7
8.8
8.9
1
2
t−Test: p−value = 0.96

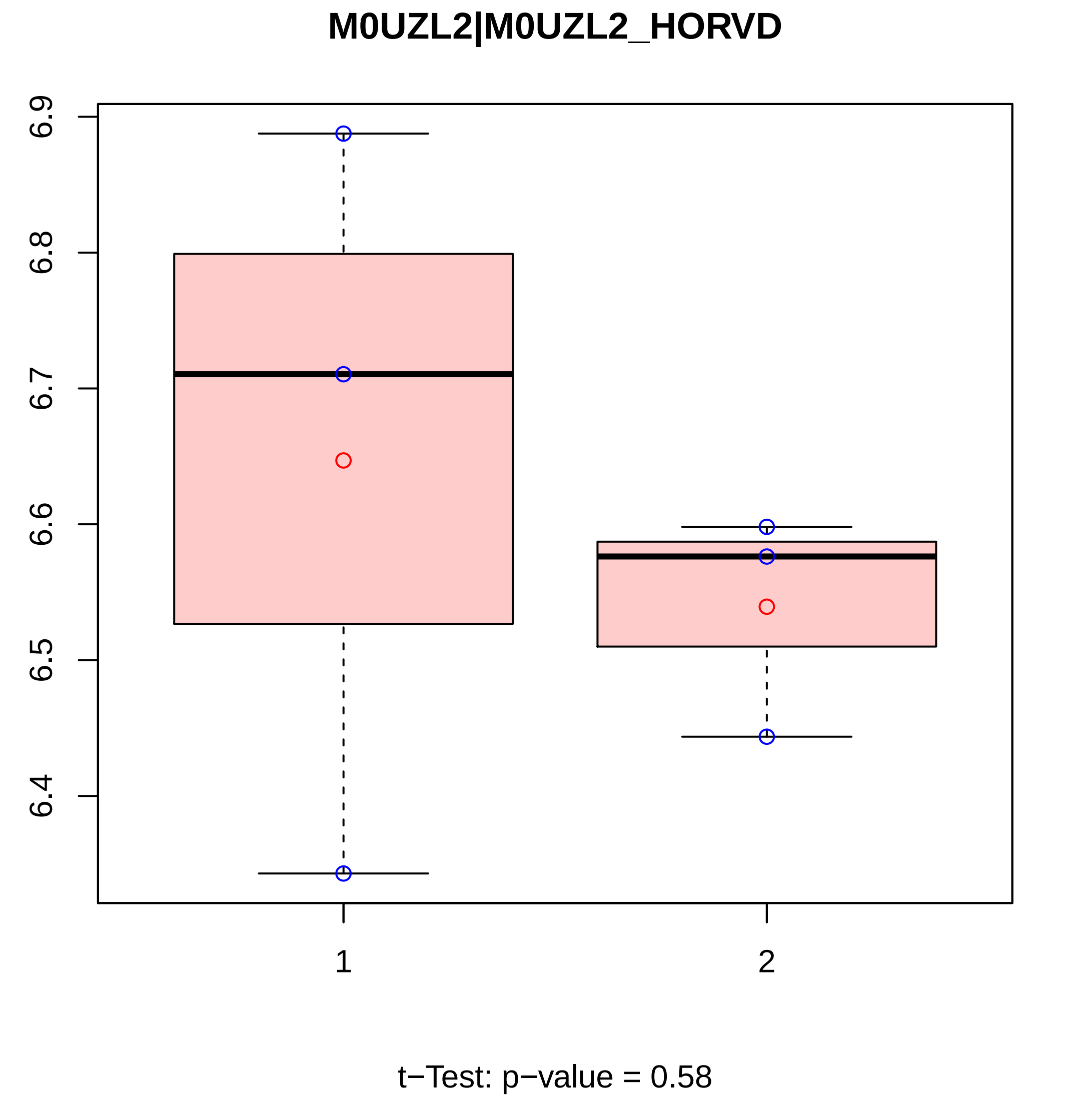
M0UZL2|M0UZL2_HORVD
6.9
6.8
6.7
6.6
6.5
6.4
1
2
t−Test: p−value = 0.58

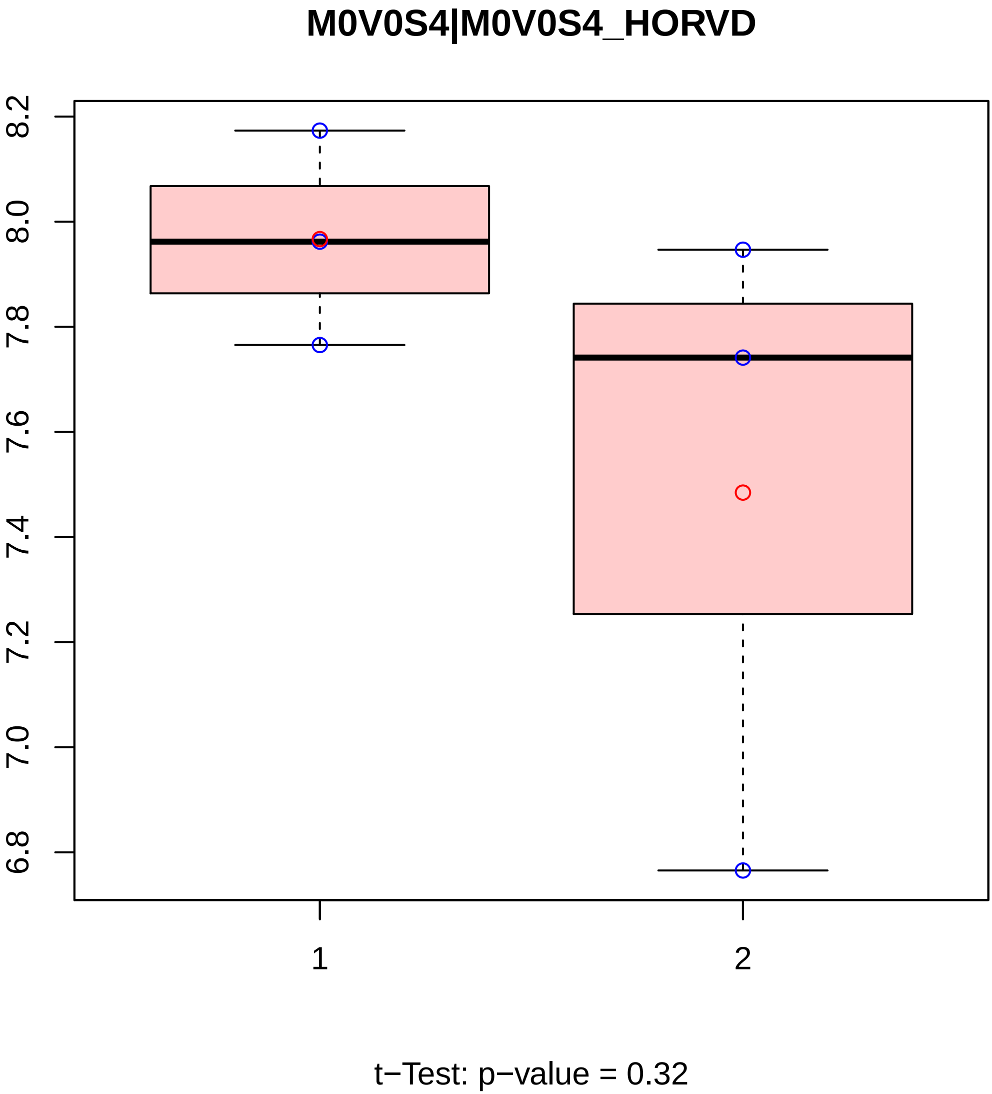
M0V0S4|M0V0S4_HORVD
8.2
8.0
7.8
7.6
7.4
7.2
7.0
6.8
1
2
t−Test: p−value = 0.32

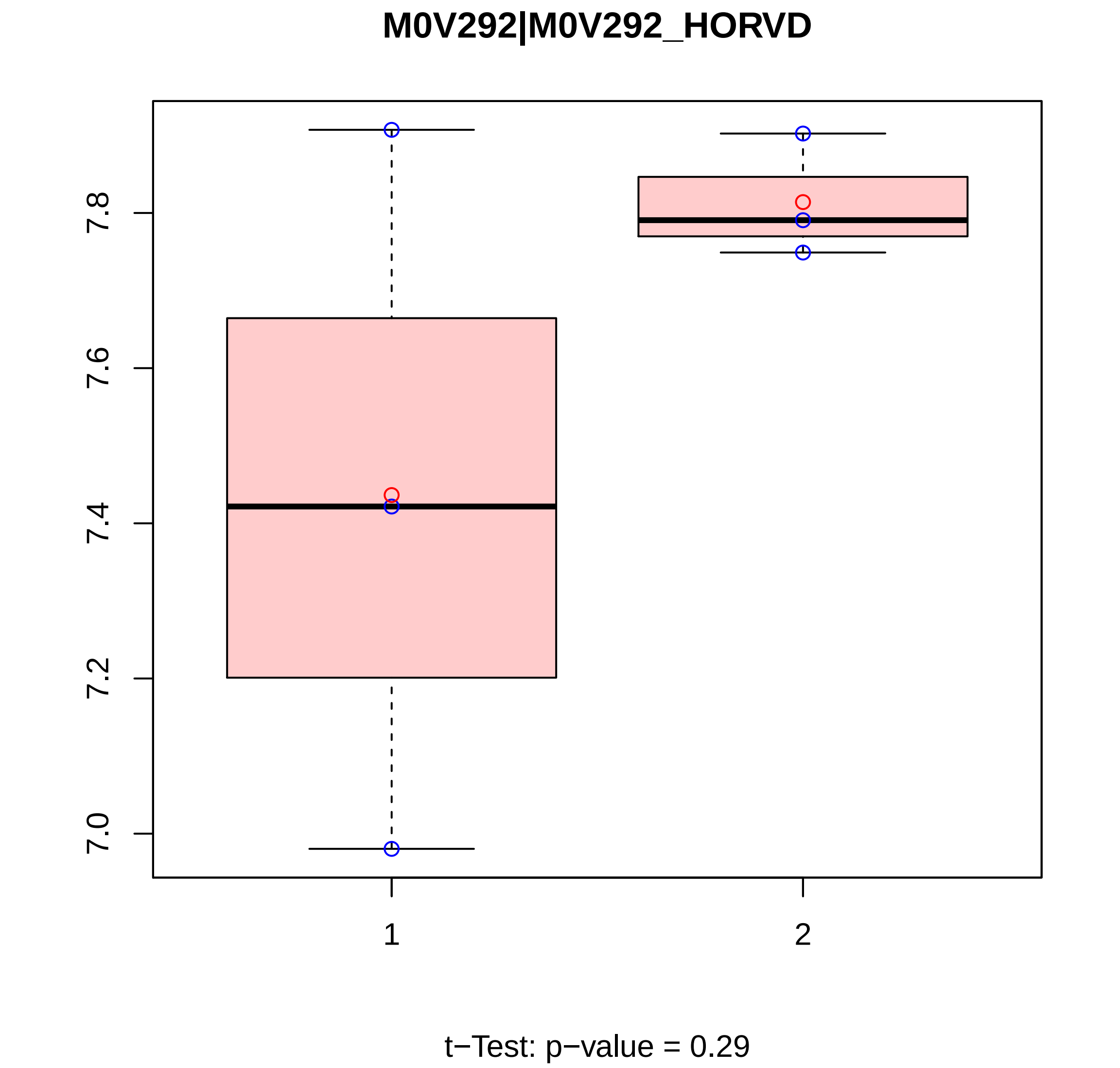
M0V292|M0V292_HORVD
7.0
7.2
7.4
7.6
7.8
1
2
t−Test: p−value = 0.29

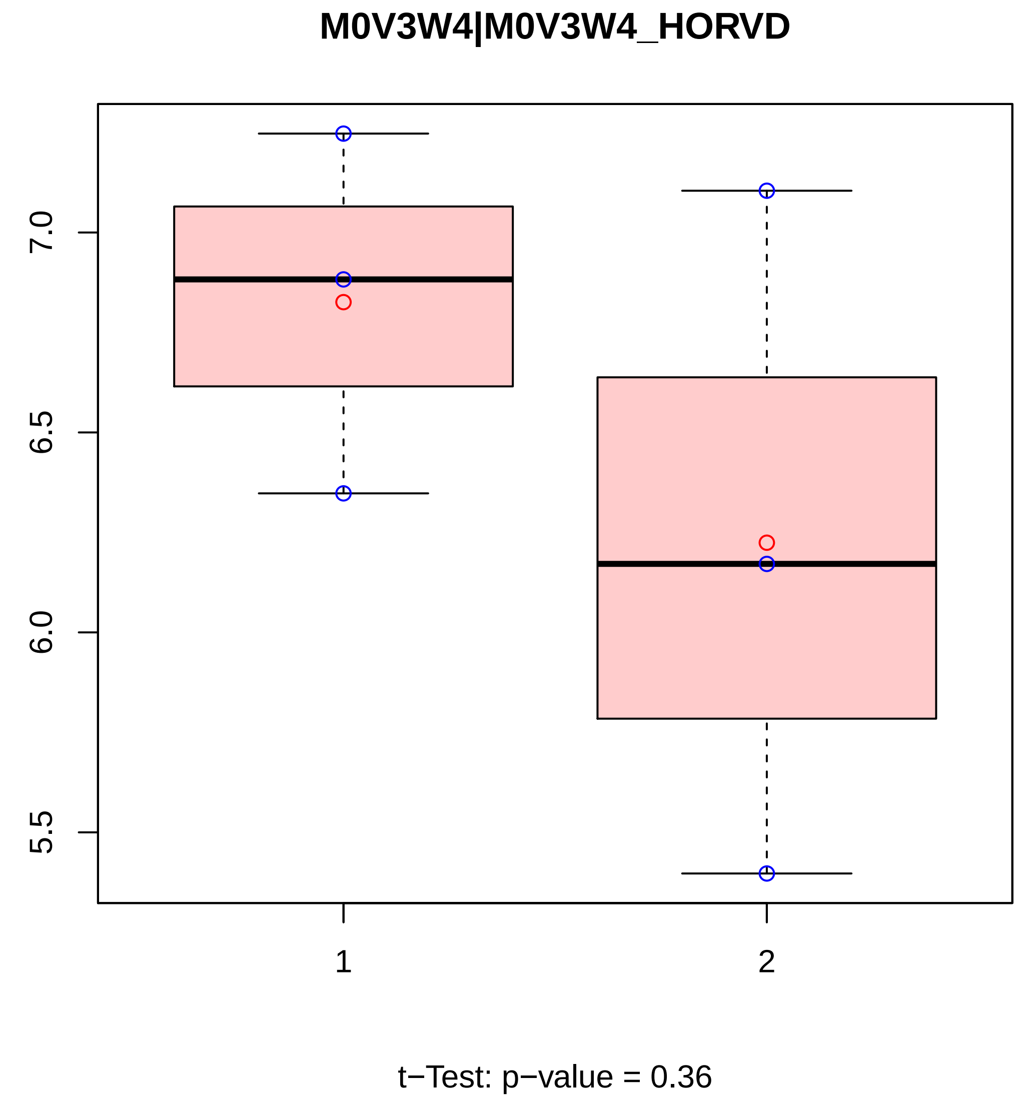
M0V3W4|M0V3W4_HORVD
7.0
6.5
6.0
5.5
1
2
t−Test: p−value = 0.36

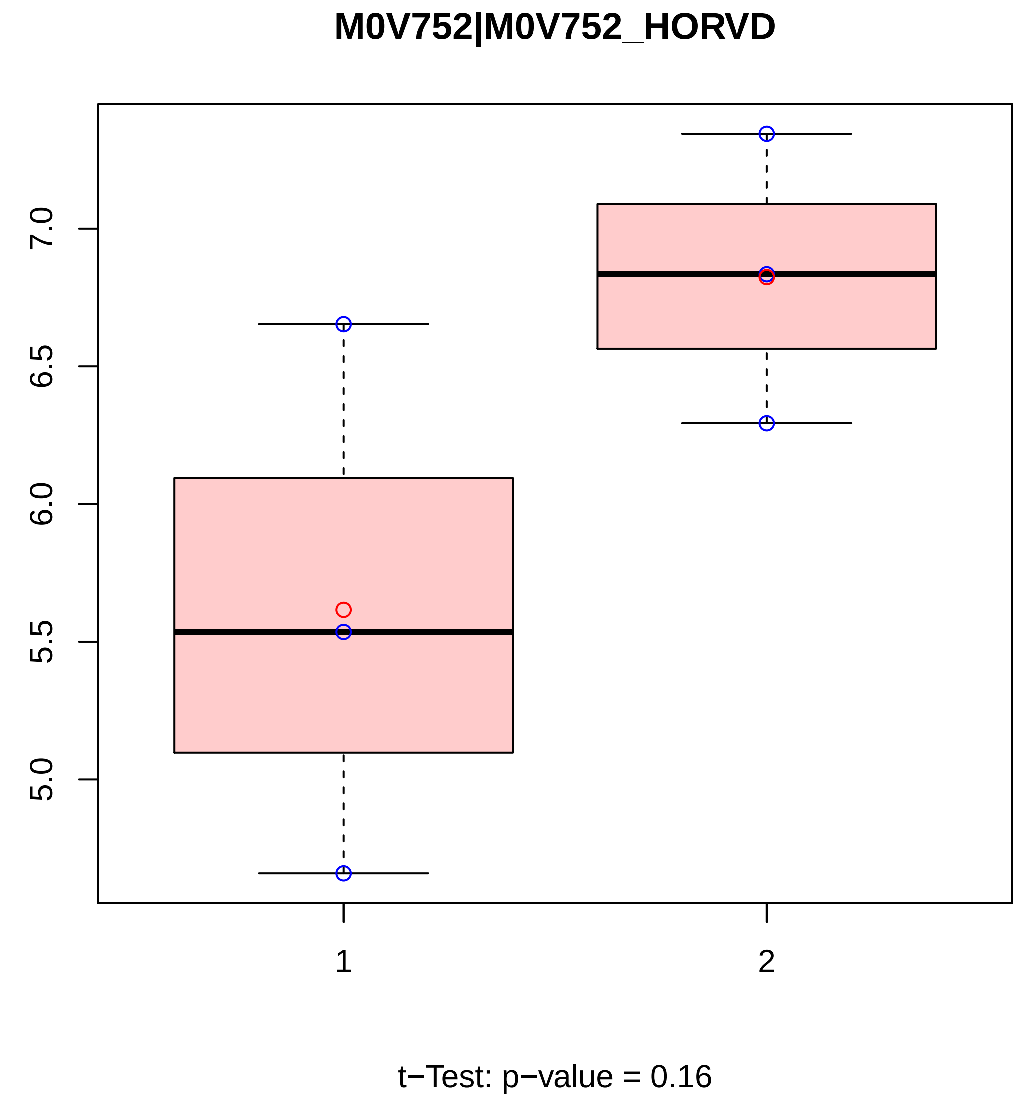
M0V752|M0V752_HORVD
5.0
5.5
6.0
6.5
7.0
1
2
t−Test: p−value = 0.16

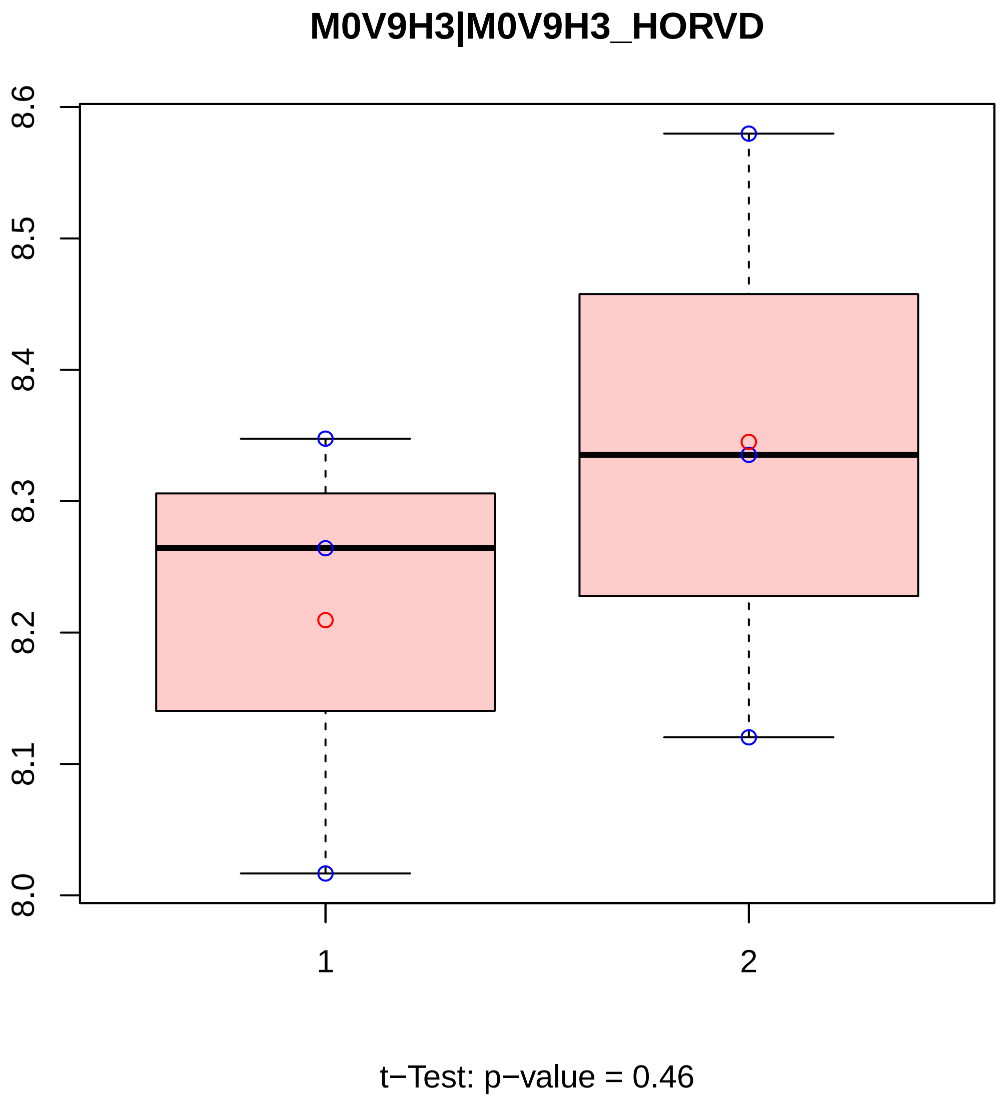
M0V9H3|M0V9H3_HORVD
8.0
8.1
8.2
8.3
8.4
8.5
8.6
1
2
t−Test: p−value = 0.46

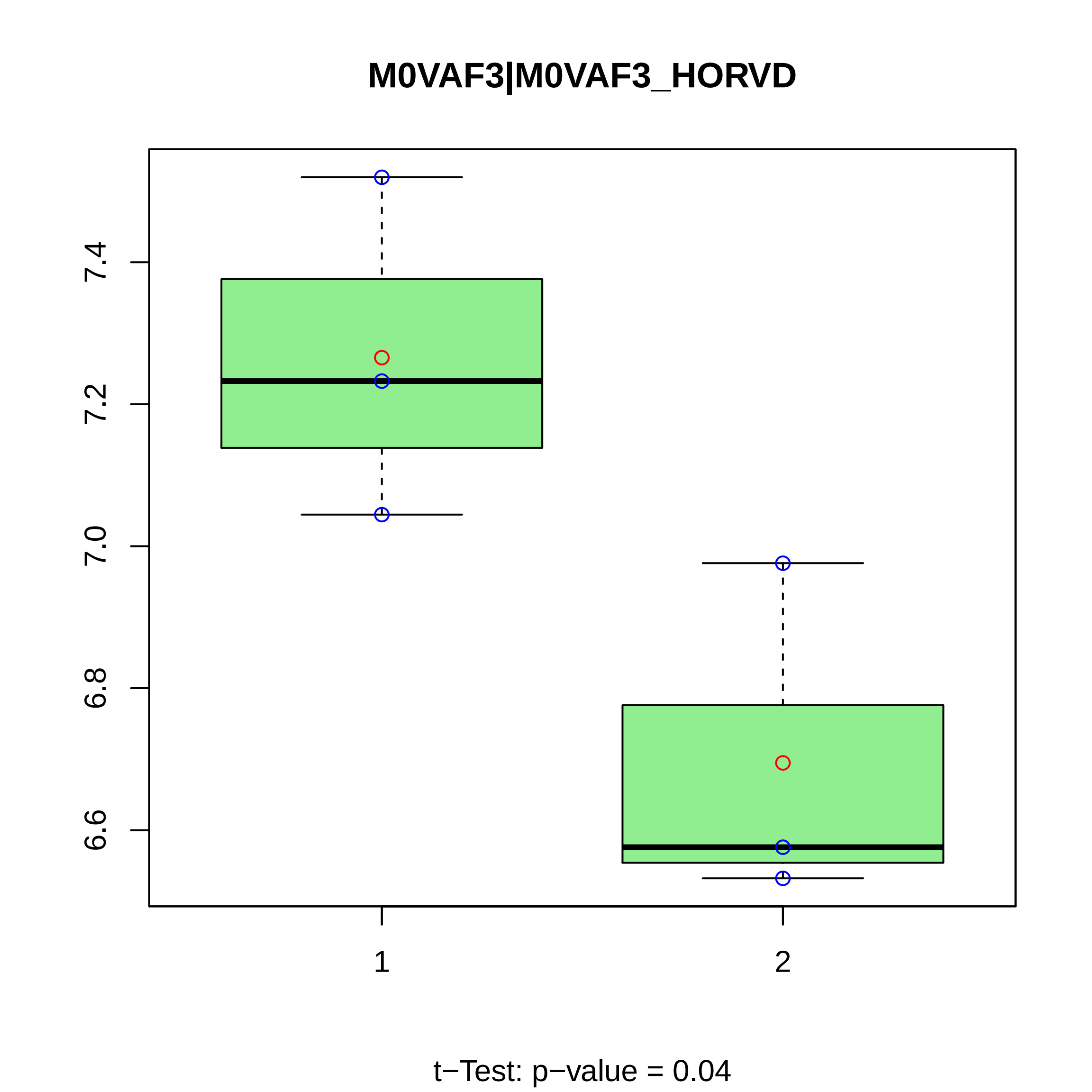
M0VAF3|M0VAF3_HORVD
7.4
7.2
7.0
6.8
6.6
1
2
t−Test: p−value = 0.04

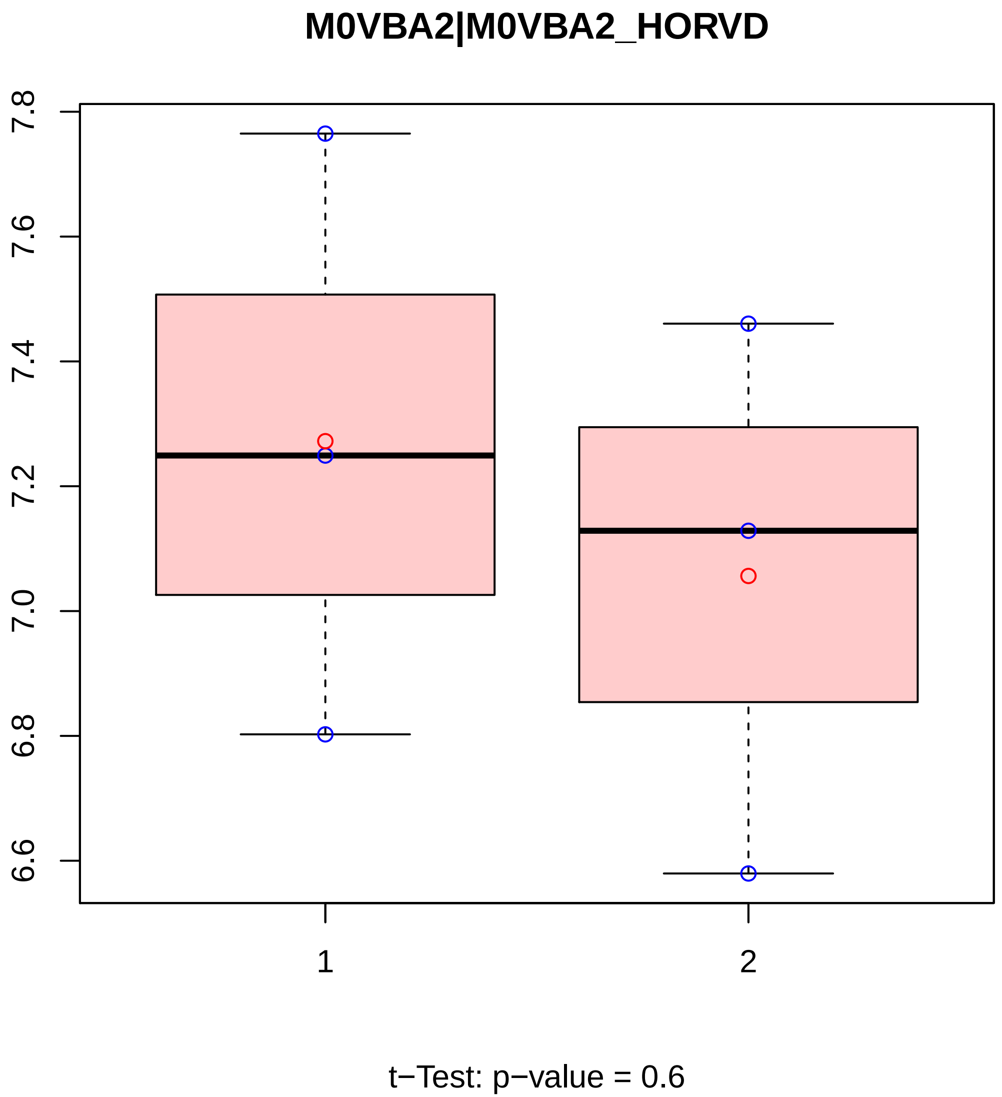
M0VBA2|M0VBA2_HORVD
7.8
7.6
7.4
7.2
7.0
6.8
6.6
1
2
t−Test: p−value = 0.6

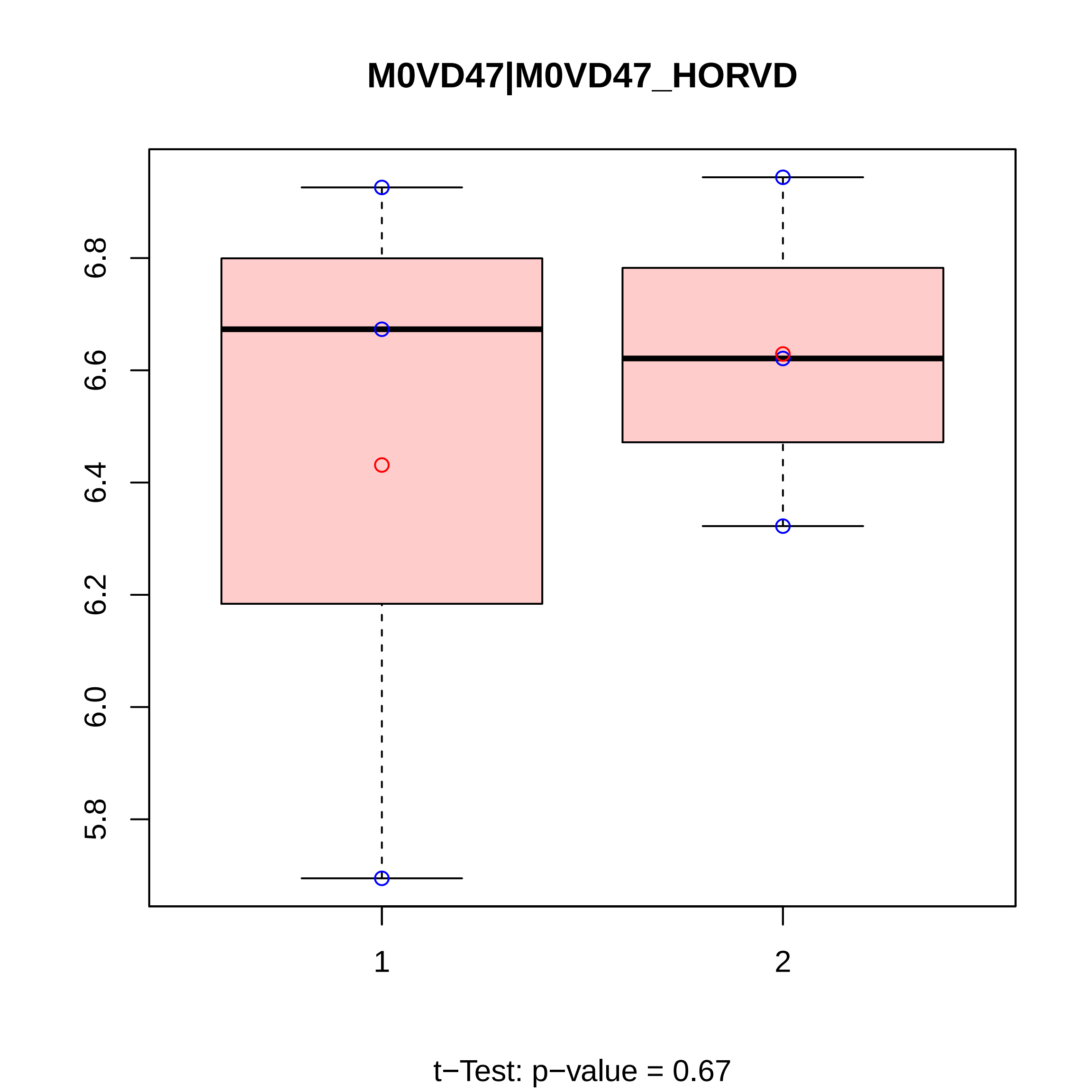
M0VD47|M0VD47_HORVD
6.8
6.6
6.4
6.2
6.0
5.8
1
2
t−Test: p−value = 0.67

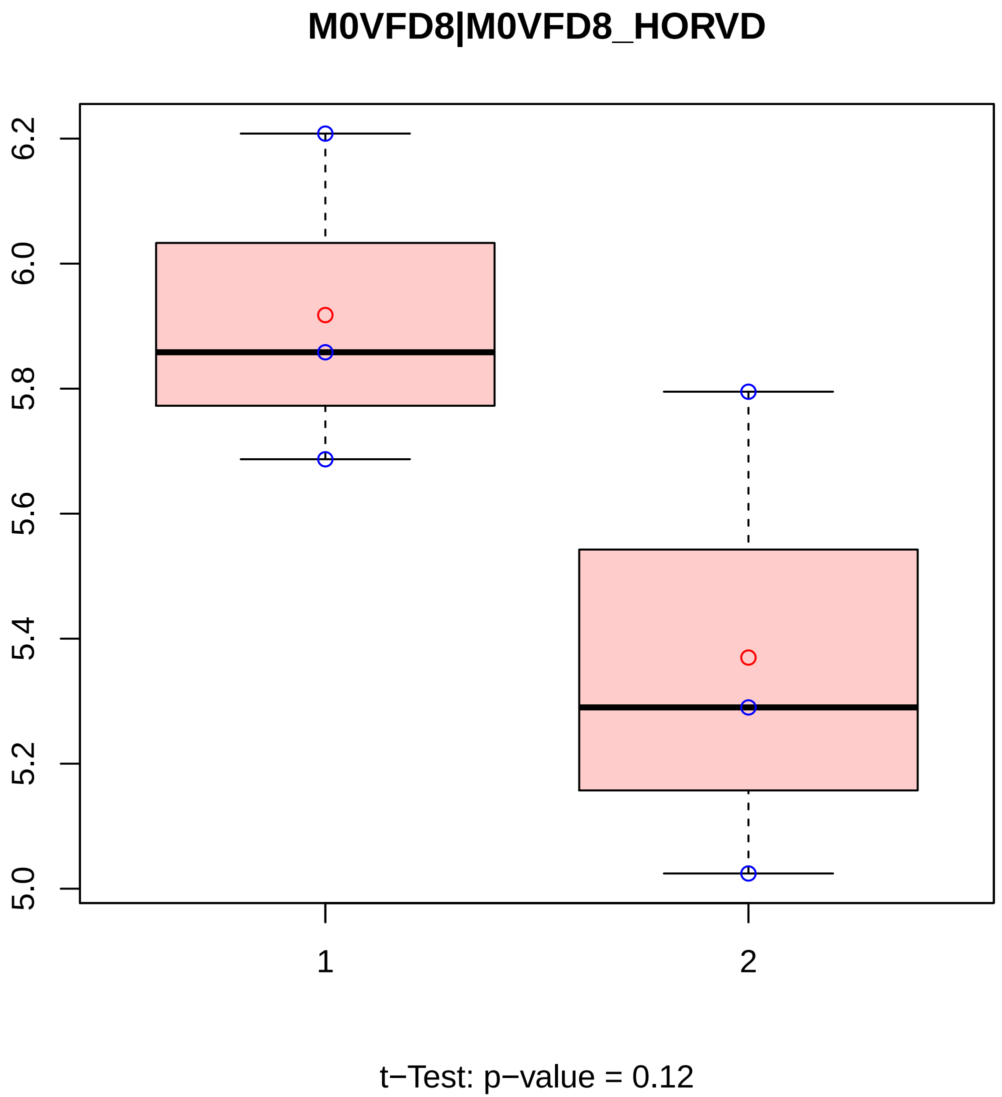
M0VFD8|M0VFD8_HORVD
6.2
6.0
5.8
5.6
5.4
5.2
5.0
1
2
t−Test: p−value = 0.12

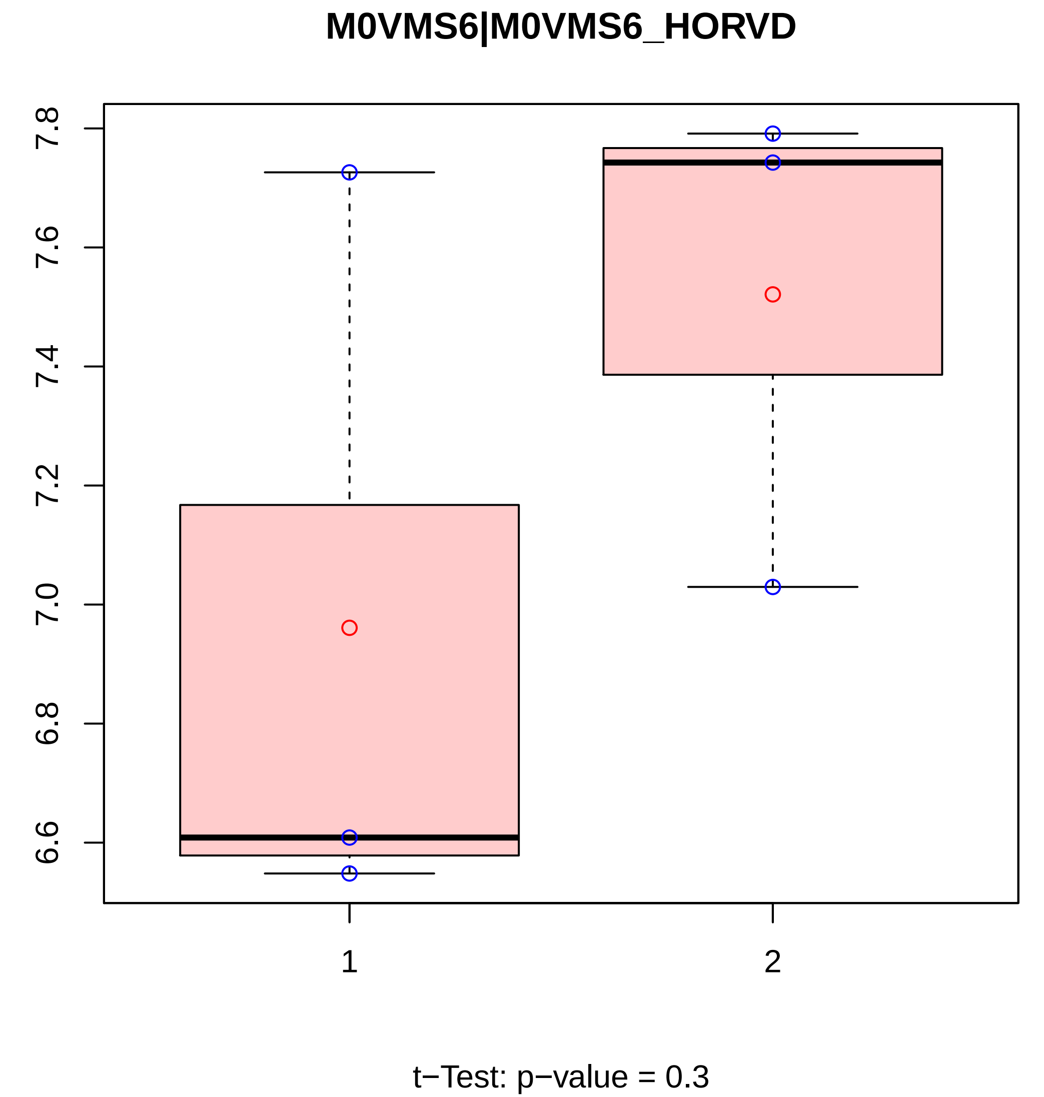
M0VMS6|M0VMS6_HORVD
7.8
7.6
7.4
7.2
7.0
6.8
6.6
1
2
t−Test: p−value = 0.3

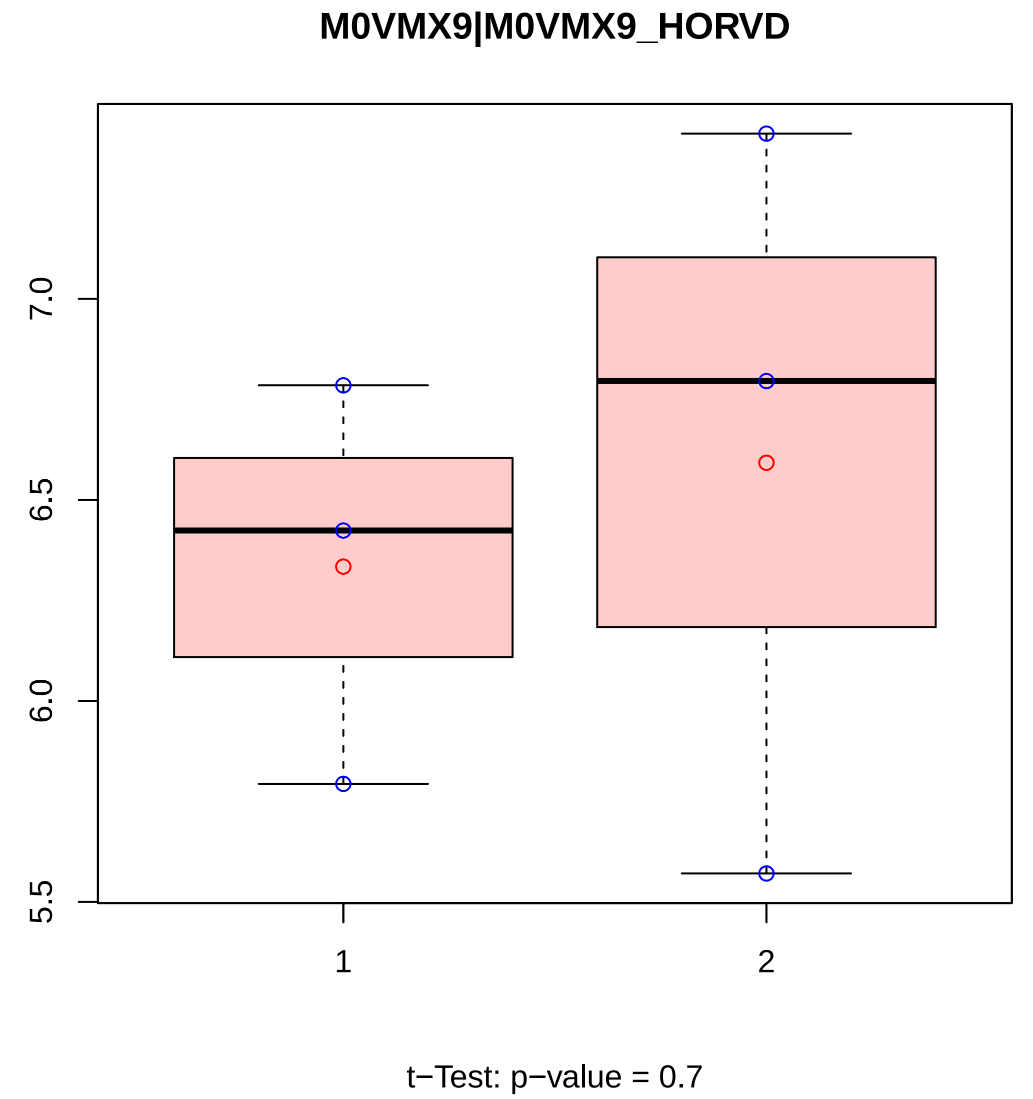
M0VMX9|M0VMX9_HORVD
7.0
6.5
6.0
5.5
1
2
t−Test: p−value = 0.7

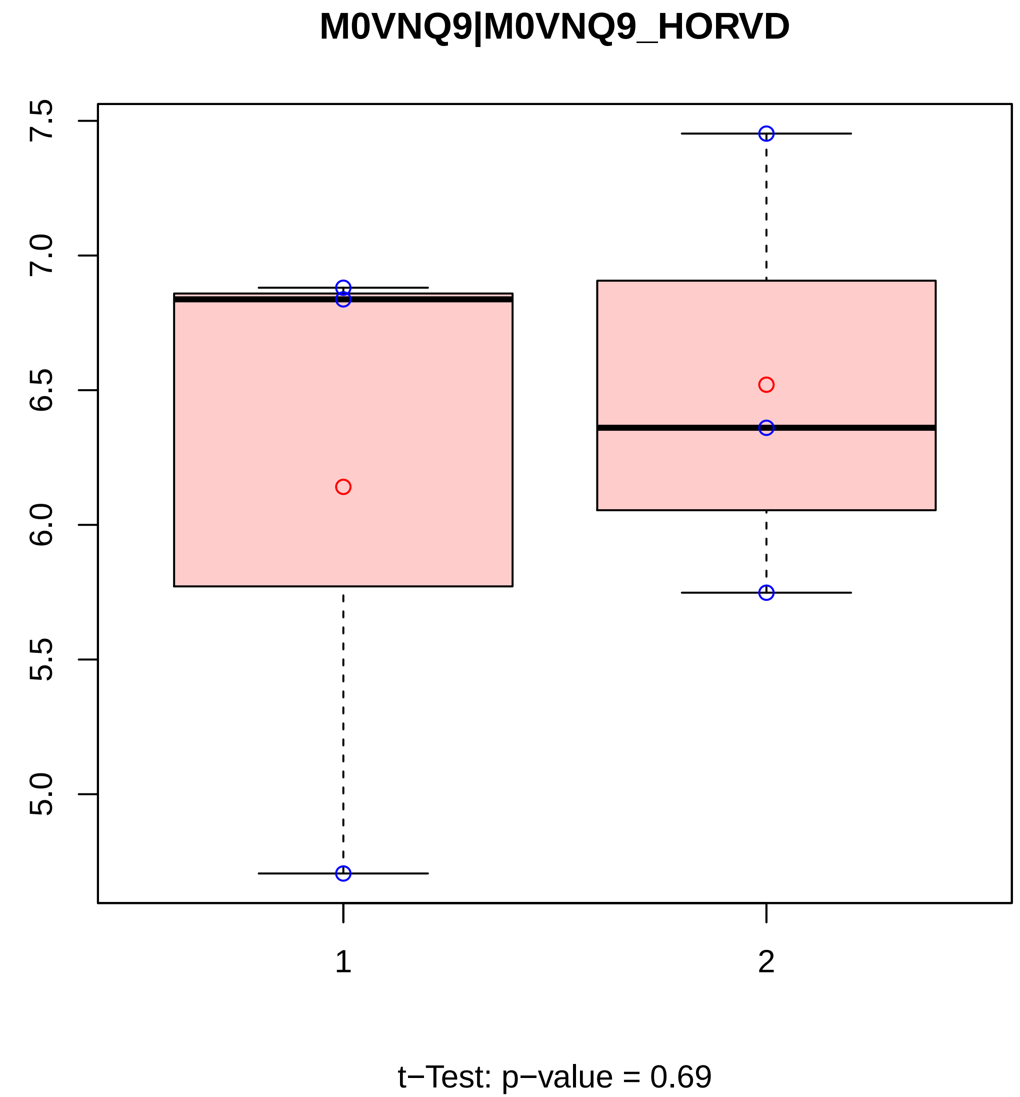
M0VNQ9|M0VNQ9_HORVD
7.5
7.0
6.5
6.0
5.5
5.0
1
2
t−Test: p−value = 0.69

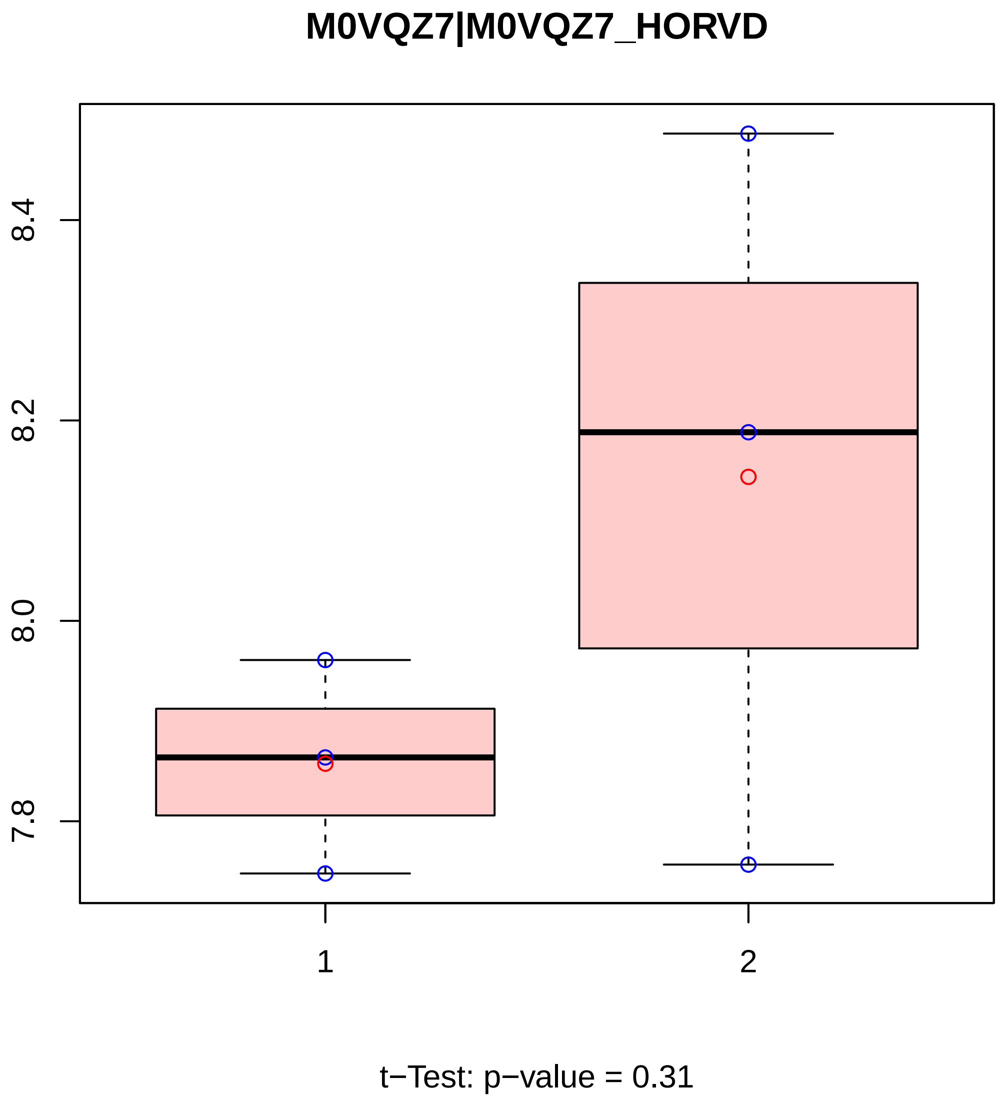
M0VQZ7|M0VQZ7_HORVD
8.4
8.2
8.0
7.8
1
2
t−Test: p−value = 0.31

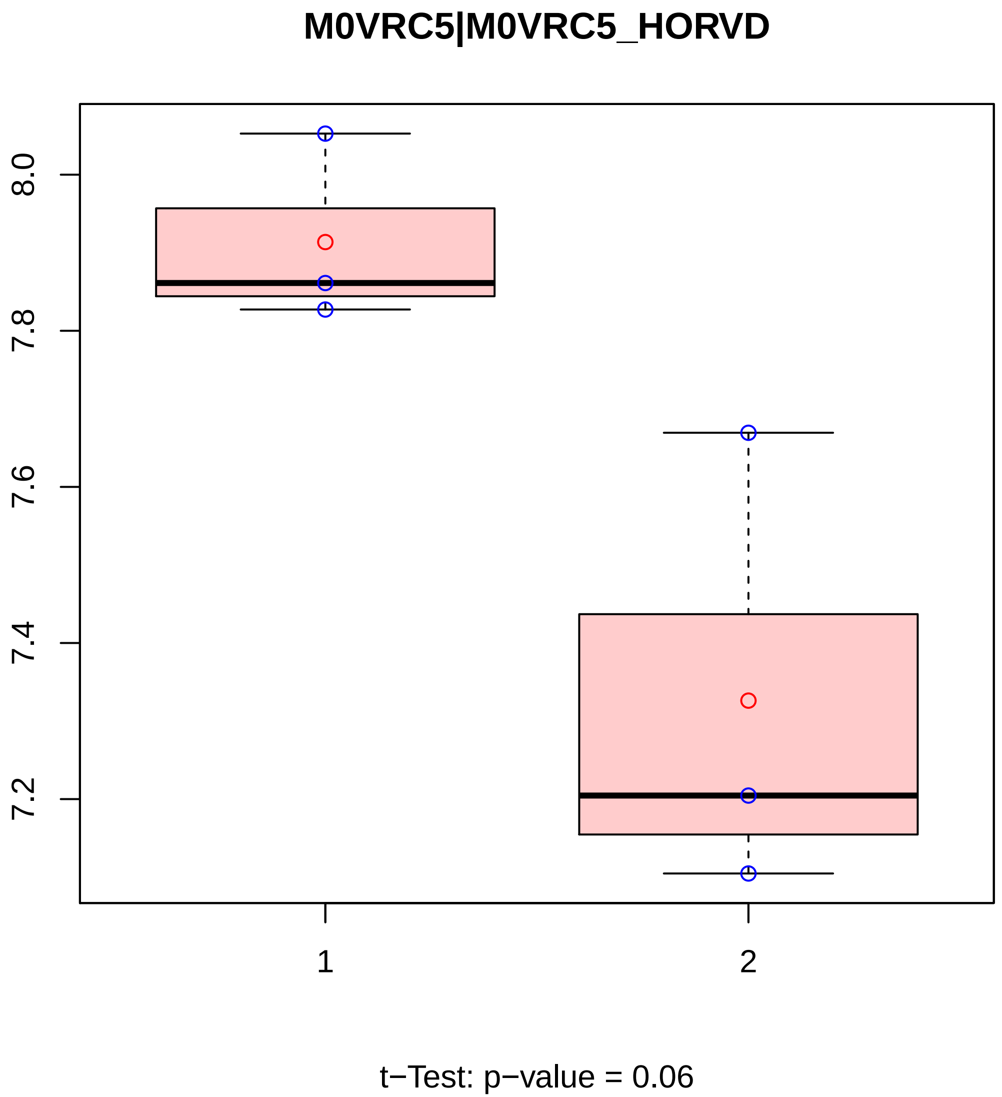

M0VRC5|M0VRC5_HORVD
8.0
7.8
7.6
7.4
7.2
1
2
t−Test: p−value = 0.06

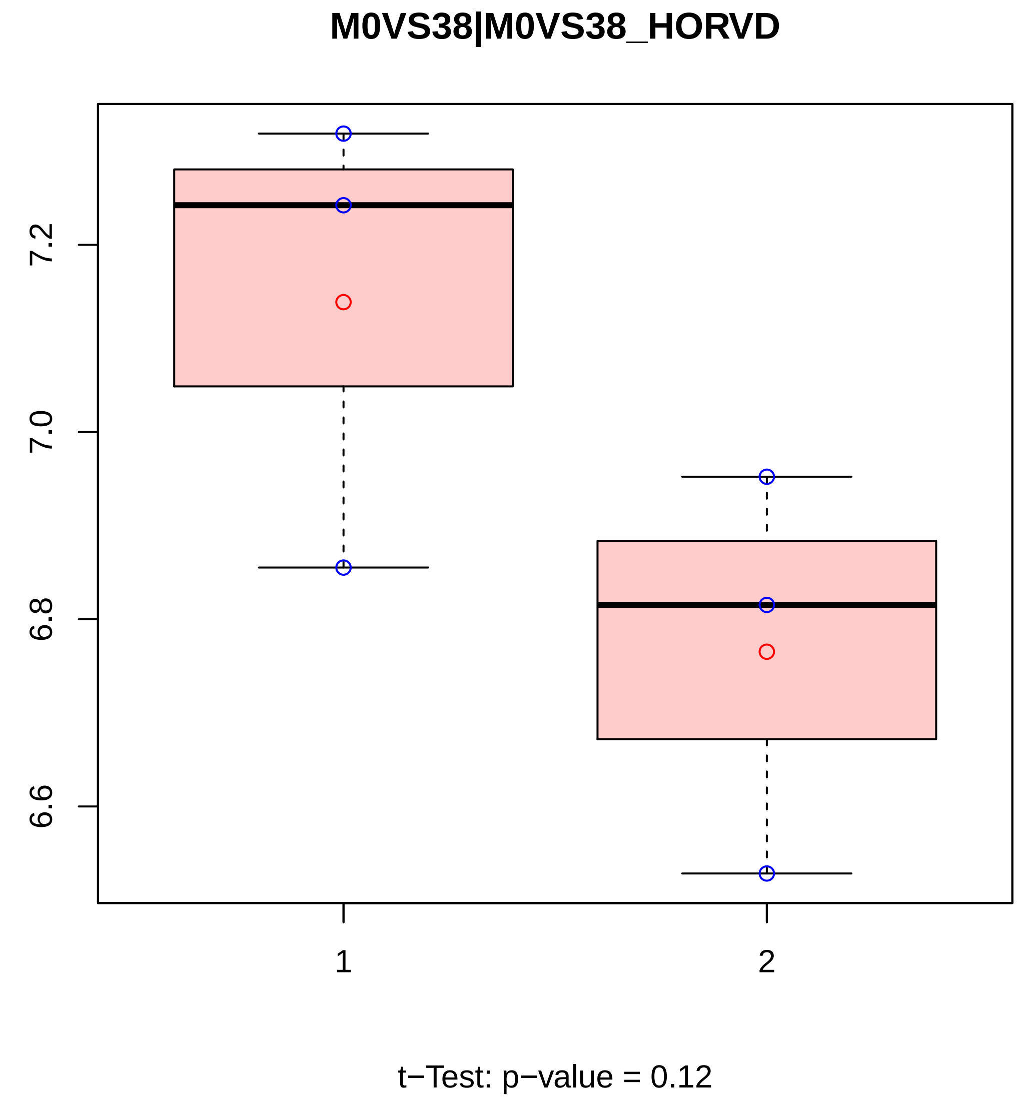
M0VS38|M0VS38_HORVD
7.2
7.0
6.8
6.6
1
2
t−Test: p−value = 0.12

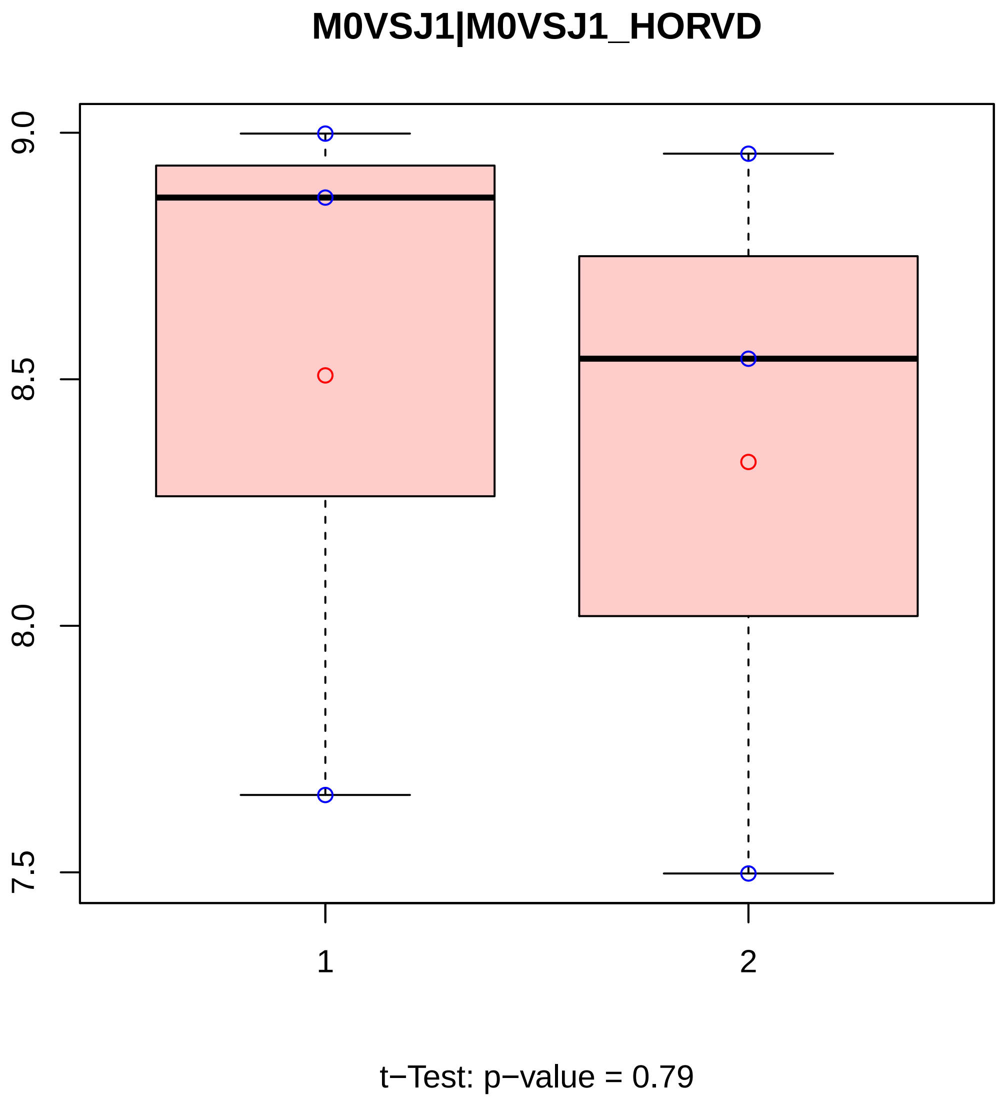
M0VSJ1|M0VSJ1_HORVD
9.0
8.5
8.0
7.5
1
2
t−Test: p−value = 0.79

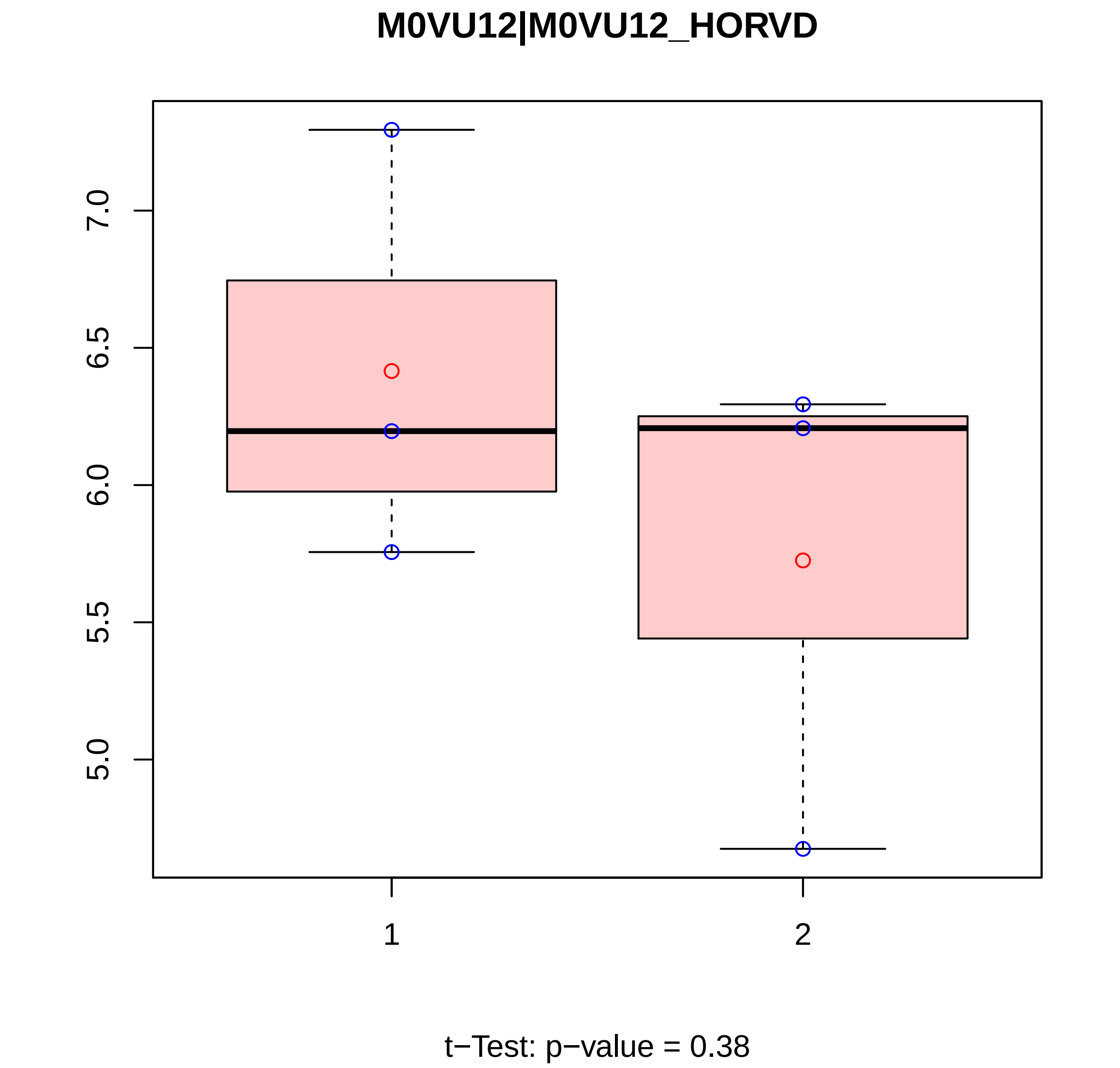

M0VU12|M0VU12_HORVD
7.0
6.5
6.0
5.5
5.0
1
2
t−Test: p−value = 0.38

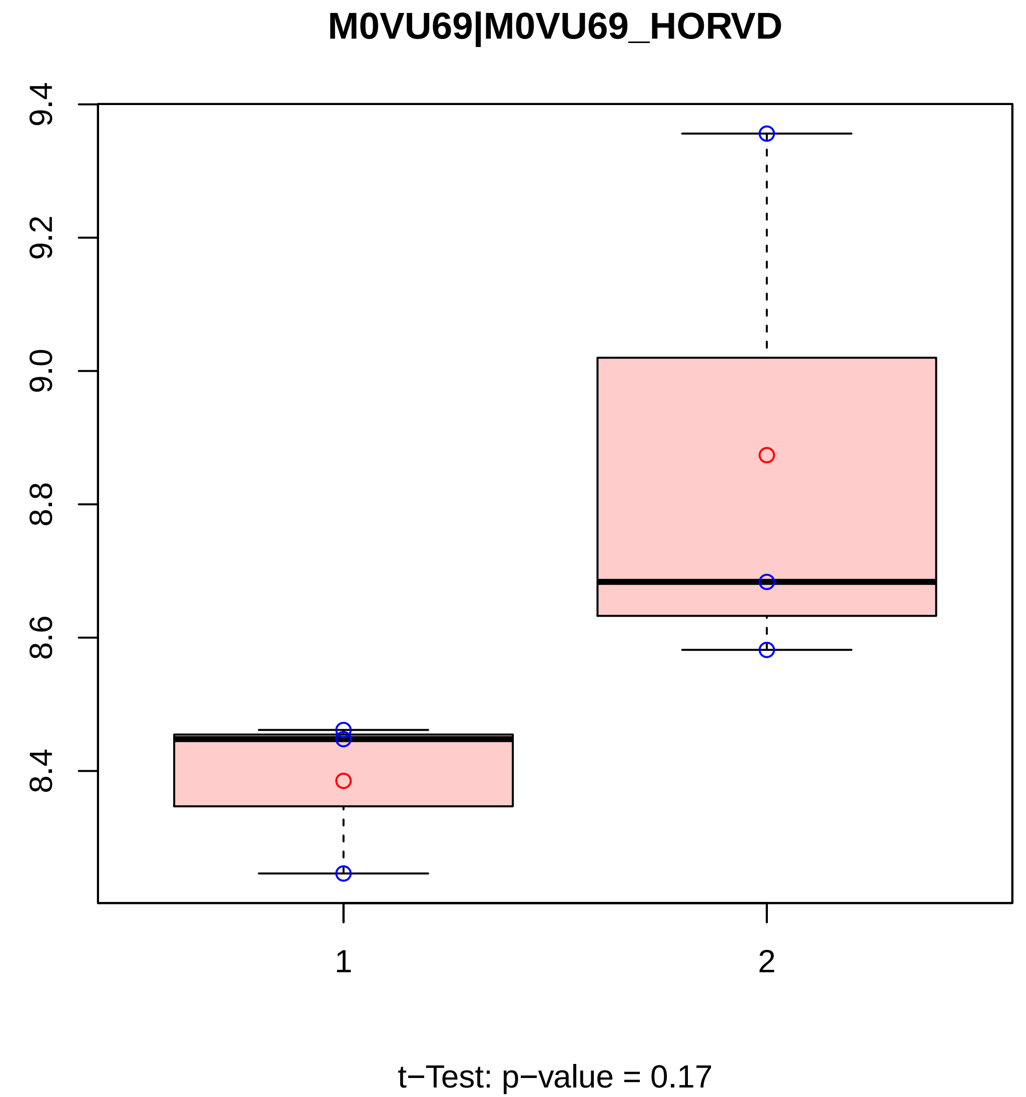
M0VU69|M0VU69_HORVD
9.4
9.2
9.0
8.8
8.6
8.4
1
2
t−Test: p−value = 0.17

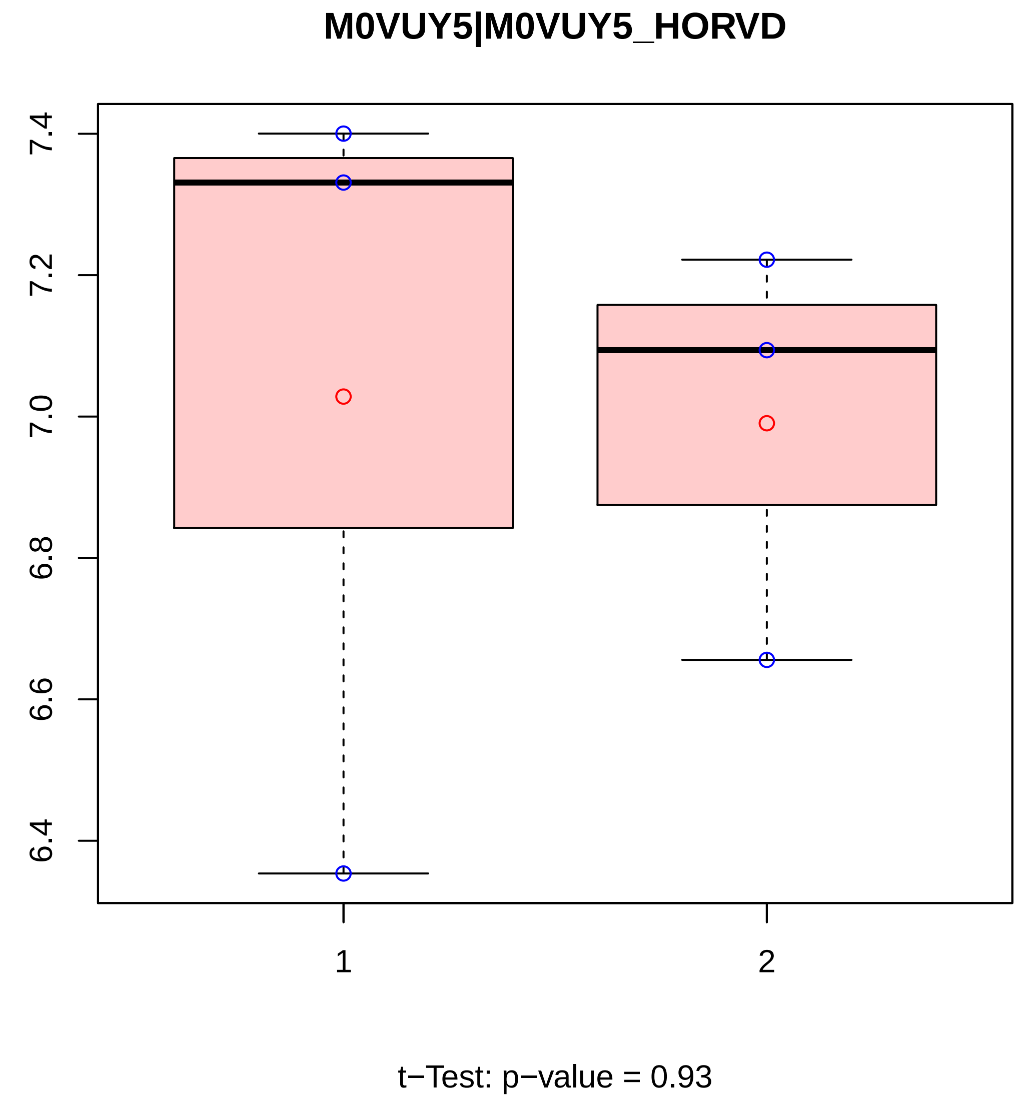
M0VUY5|M0VUY5_HORVD
7.4
7.2
7.0
6.8
6.6
6.4
1
2
t−Test: p−value = 0.93

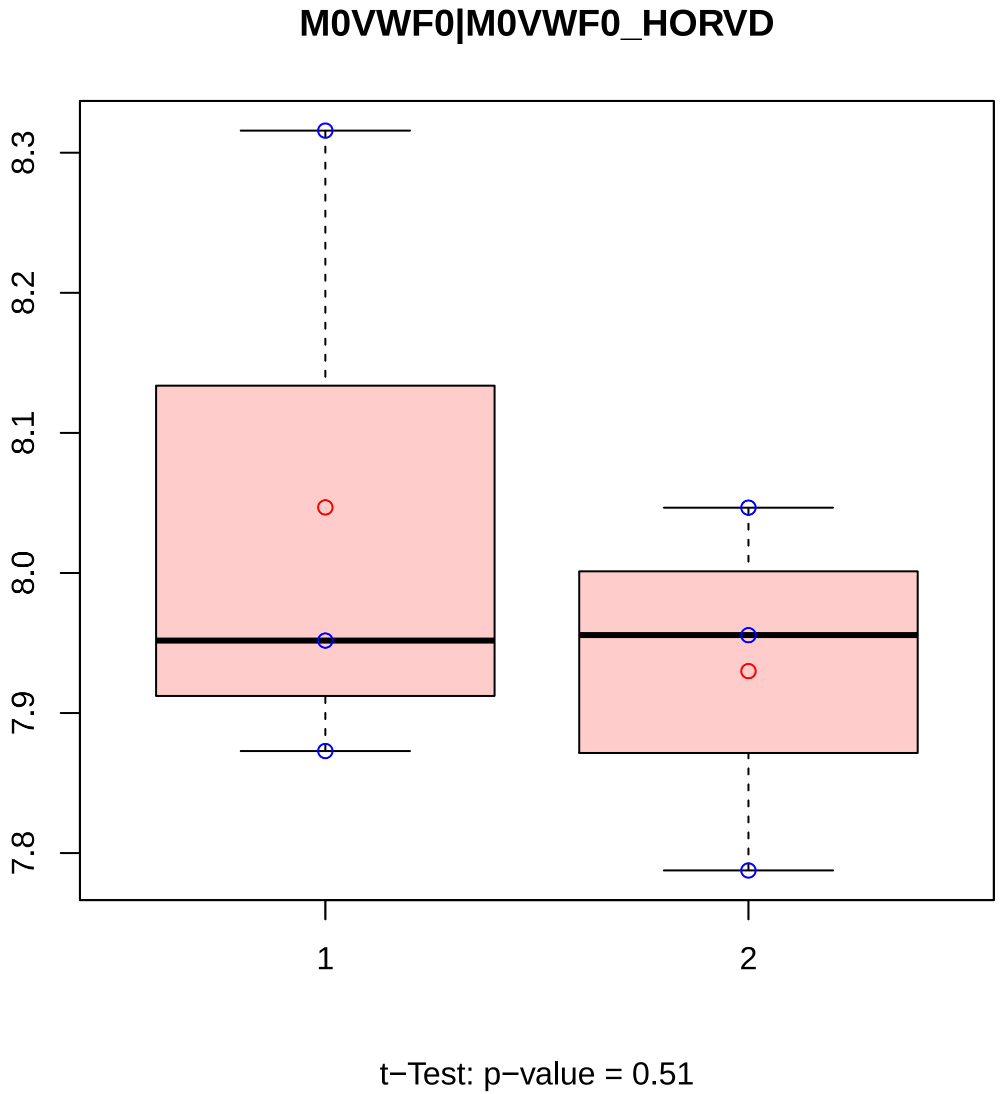
M0VWF0|M0VWF0_HORVD
8.3
8.2
8.1
8.0
7.9
7.8
1
2
t−Test: p−value = 0.51

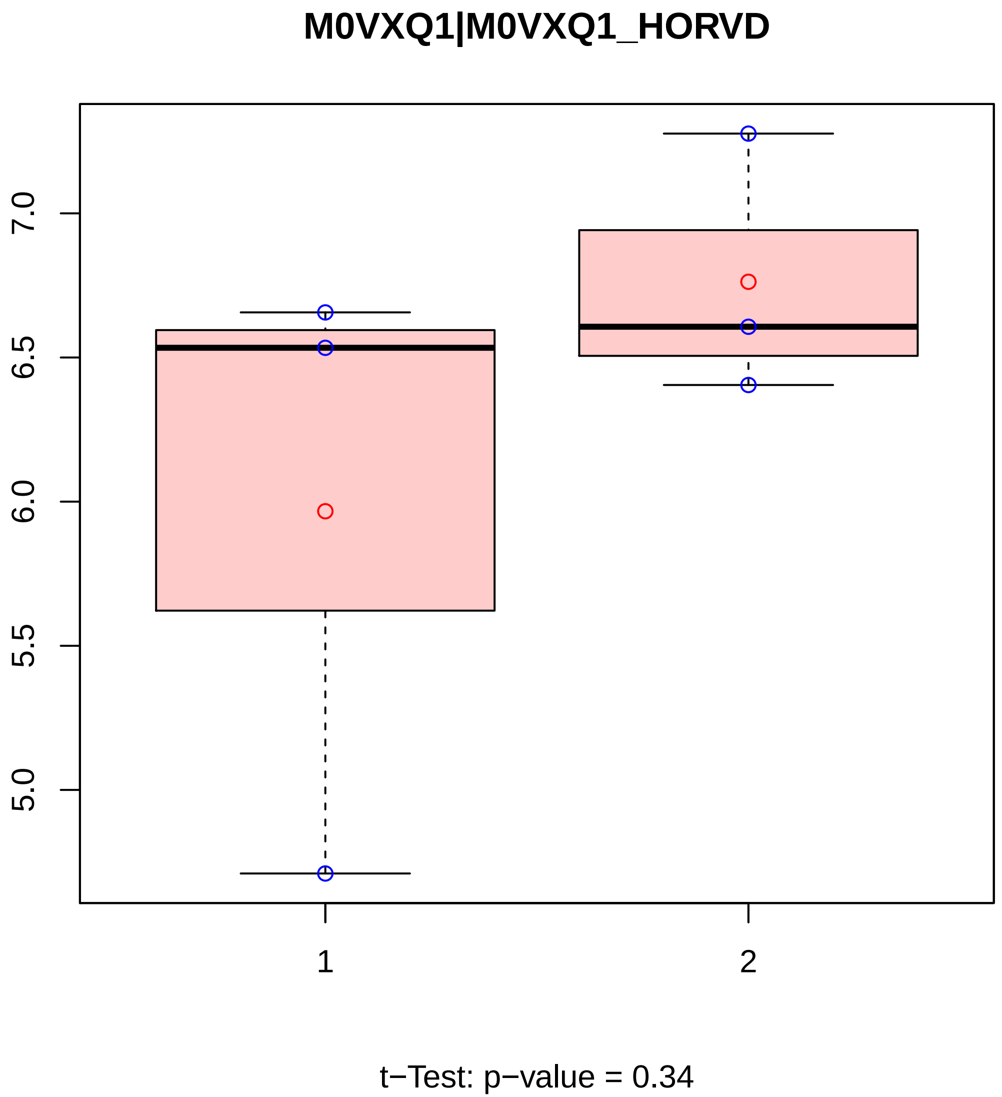
M0VXQ1|M0VXQ1_HORVD
7.0
6.5
6.0
5.5
5.0
1
2
t−Test: p−value = 0.34

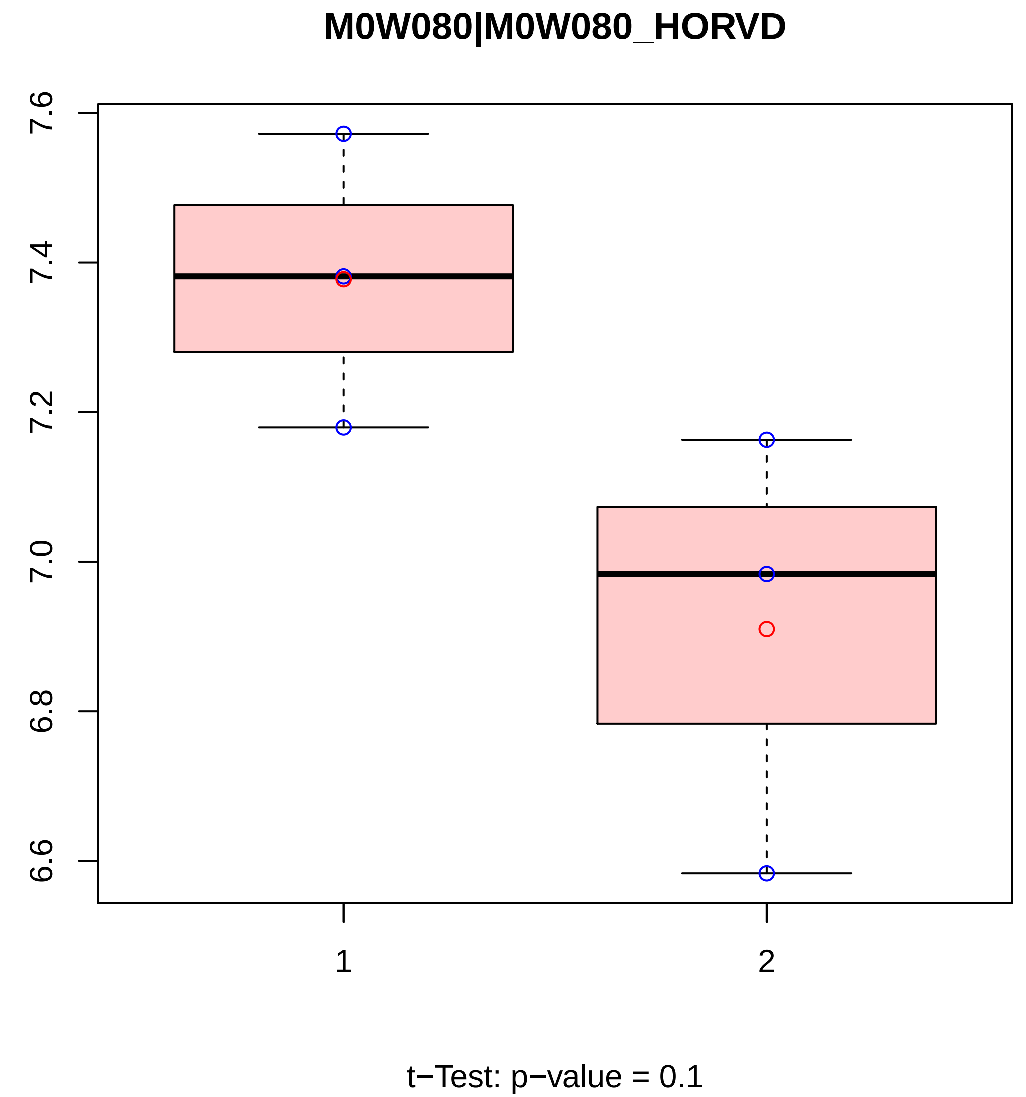
M0W080|M0W080_HORVD
7.6
7.4
7.2
7.0
6.8
6.6
1
2
t−Test: p−value = 0.1

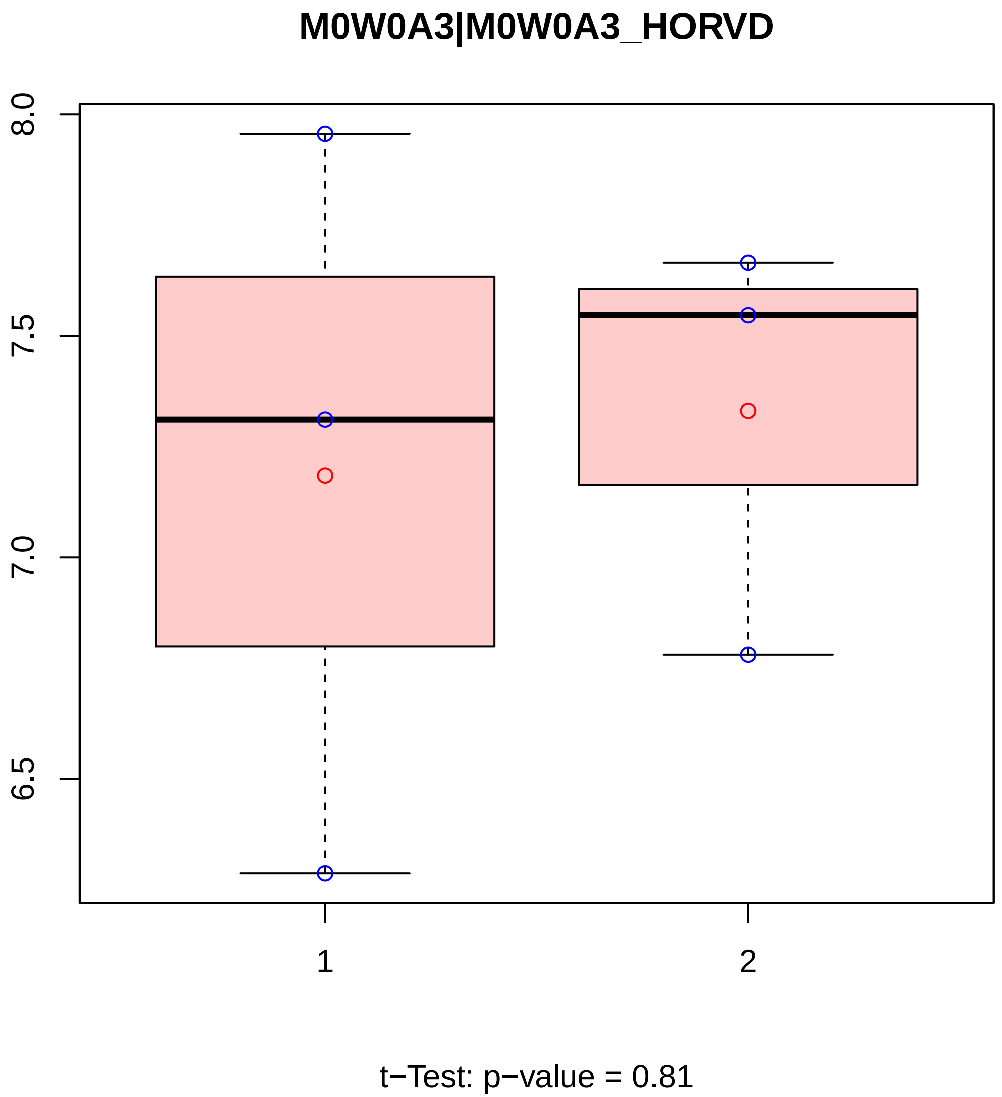
M0W0A3|M0W0A3_HORVD
8.0
7.5
7.0
6.5
1
2
t−Test: p−value = 0.81

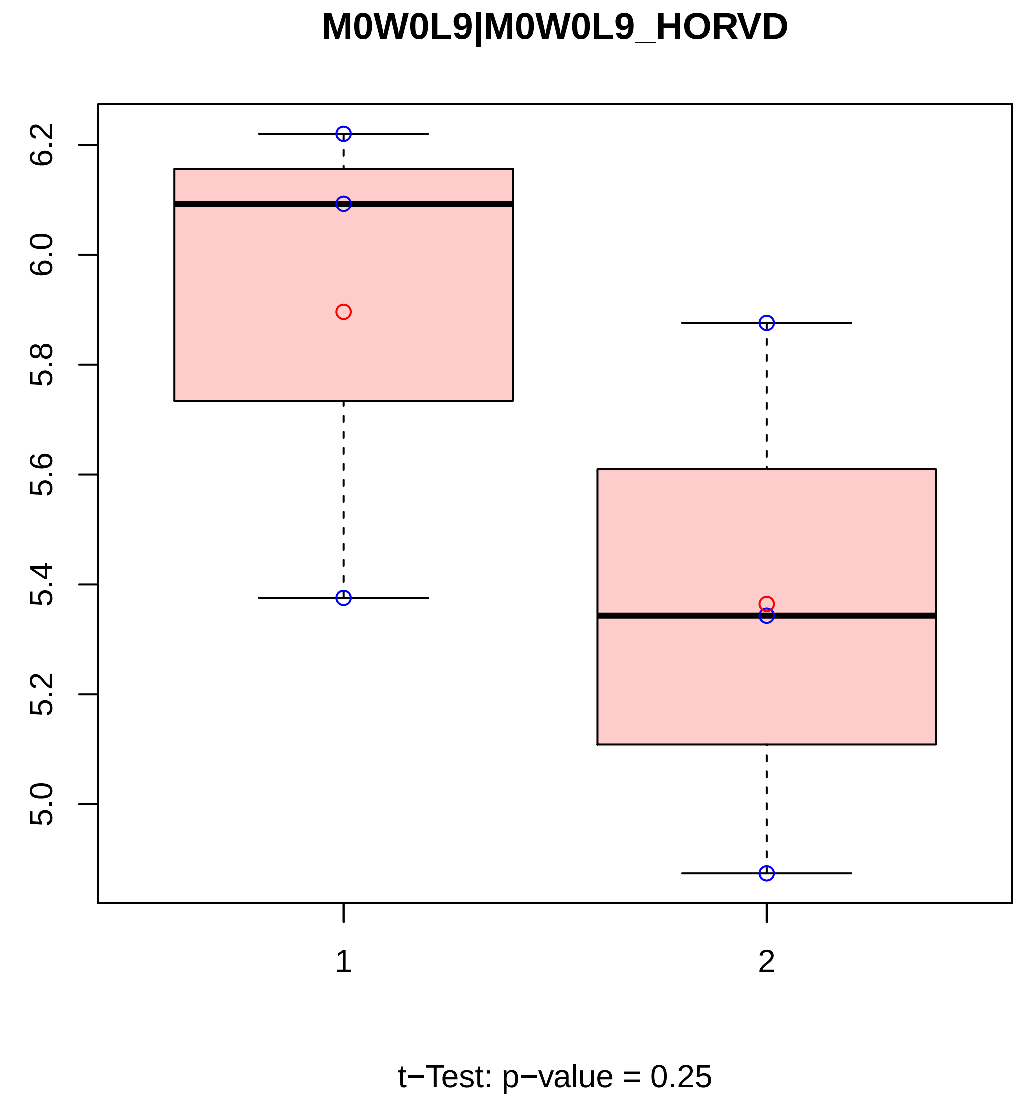
M0W0L9|M0W0L9_HORVD
6.2
6.0
5.8
5.6
5.4
5.2
5.0
1
2
t−Test: p−value = 0.25

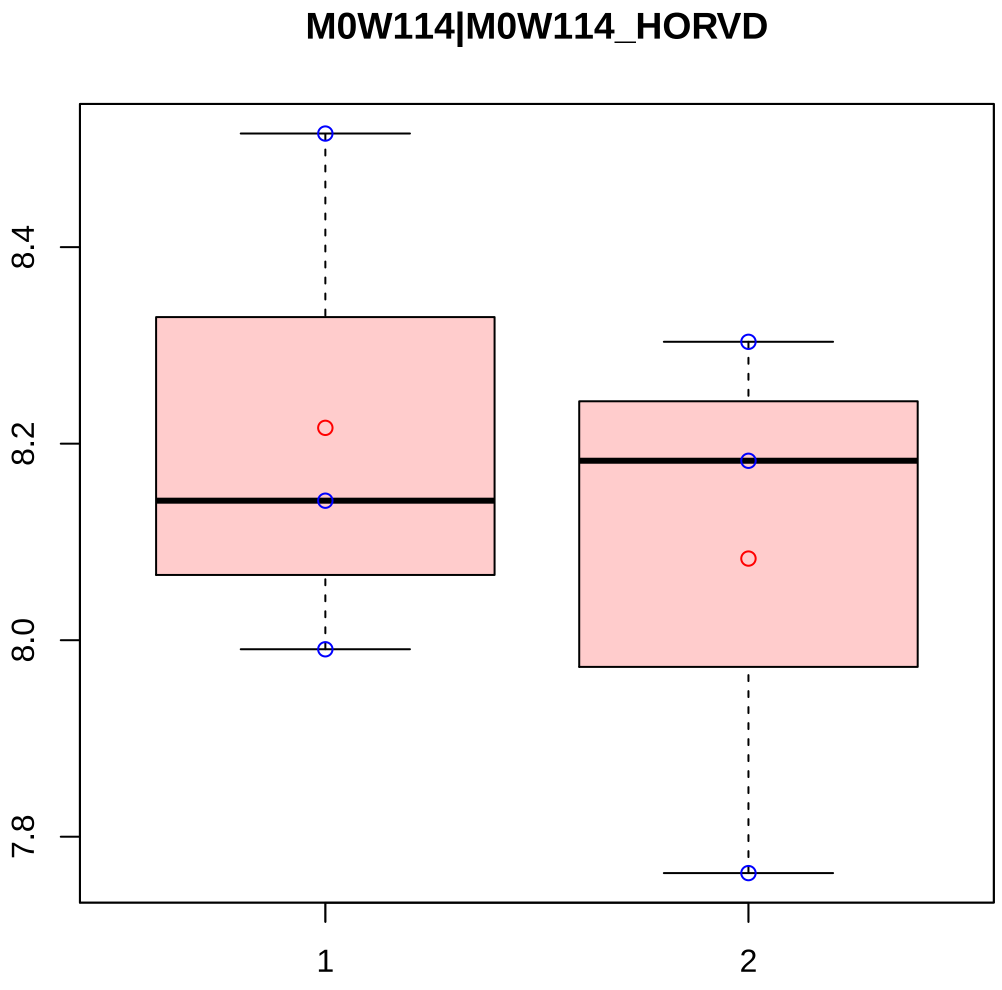

t−Test: p−value = 0.59

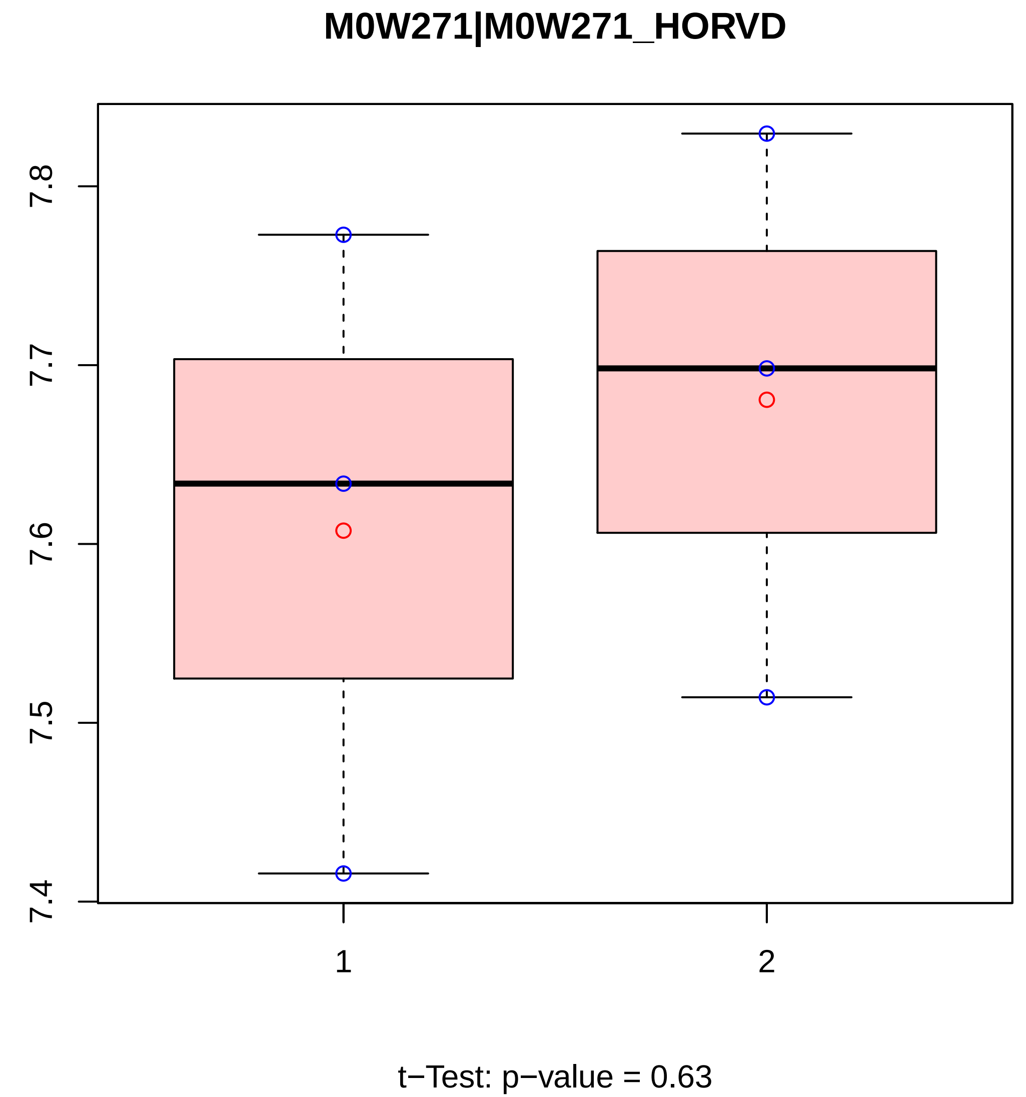
M0W271|M0W271_HORVD
7.4
7.5
7.6
7.7
7.8
1
2
t−Test: p−value = 0.63

M0W276|M0W276_HORVD

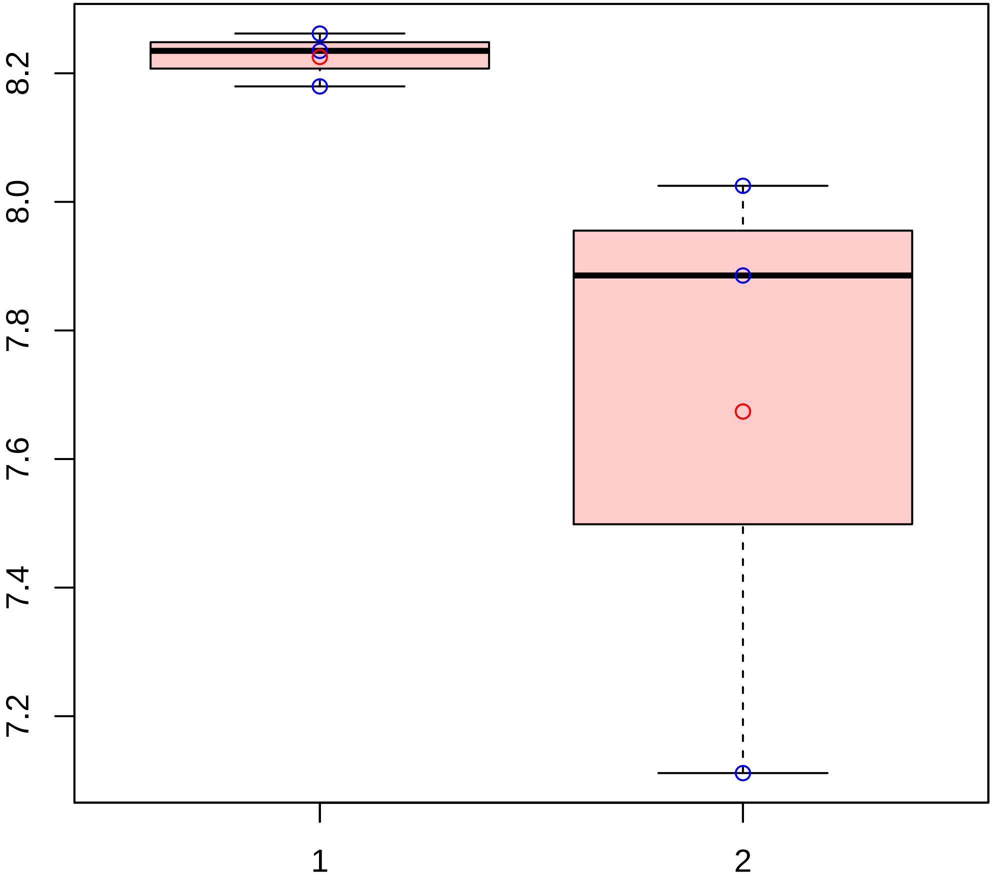

t−Test: p−value = 0.19

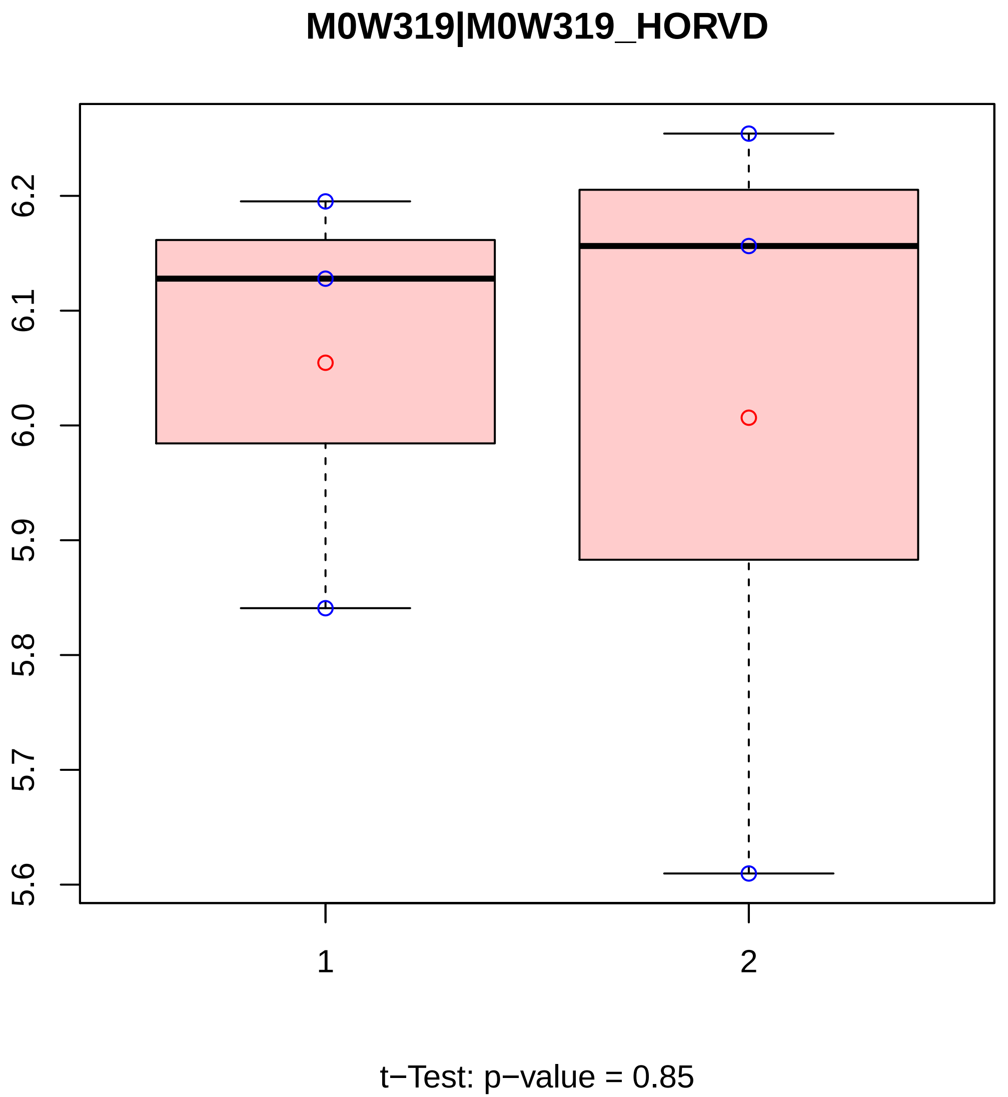
M0W319|M0W319_HORVD
6.2
6.1
6.0
5.9
5.8
5.7
5.6
1
2
t−Test: p−value = 0.85

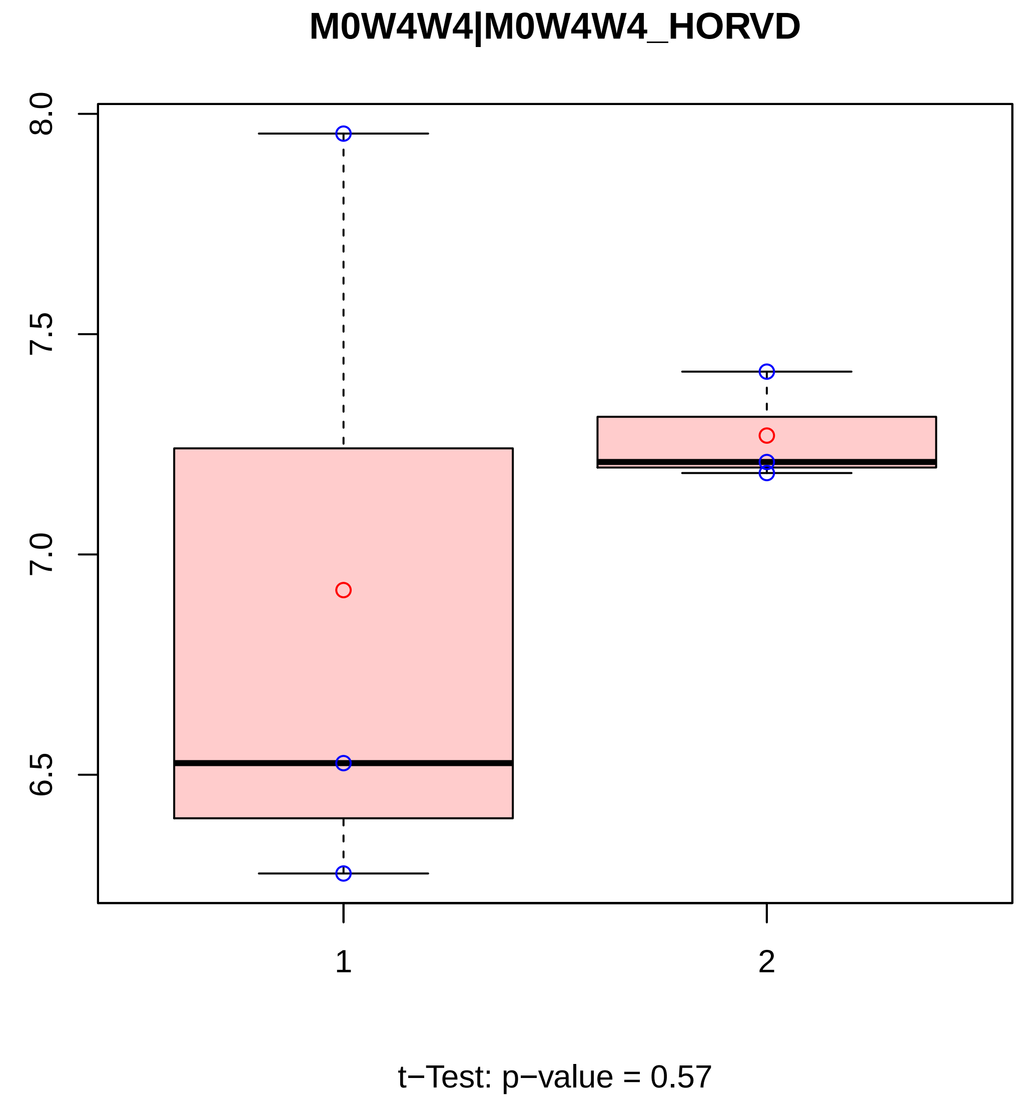
M0W4W4|M0W4W4_HORVD
8.0
7.5
7.0
6.5
1
2
t−Test: p−value = 0.57

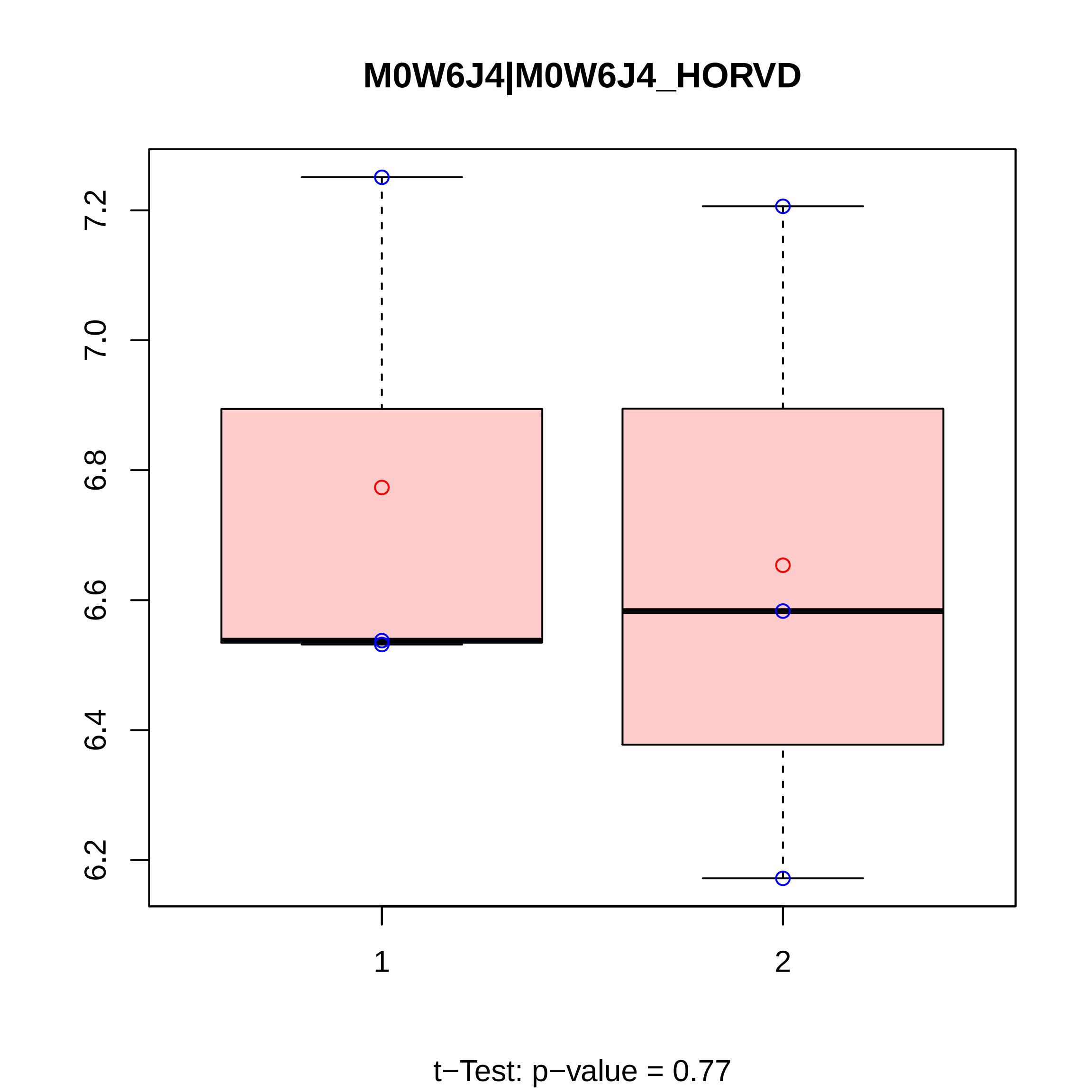

M0W6J4|M0W6J4_HORVD
7.2
7.0
6.8
6.6
6.4
6.2
1
2
t−Test: p−value = 0.77

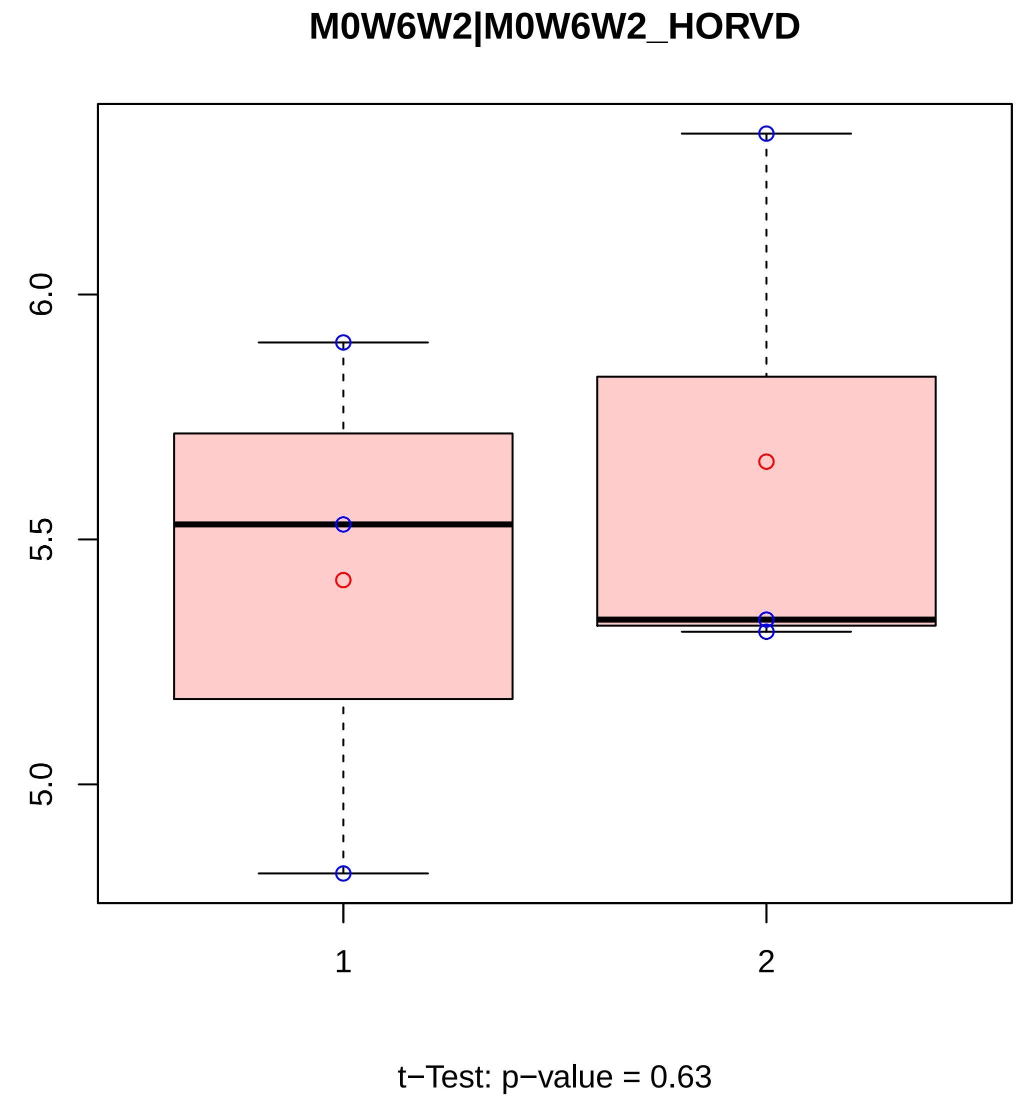
M0W6W2|M0W6W2_HORVD
6.0
5.5
5.0
1
2
t−Test: p−value = 0.63

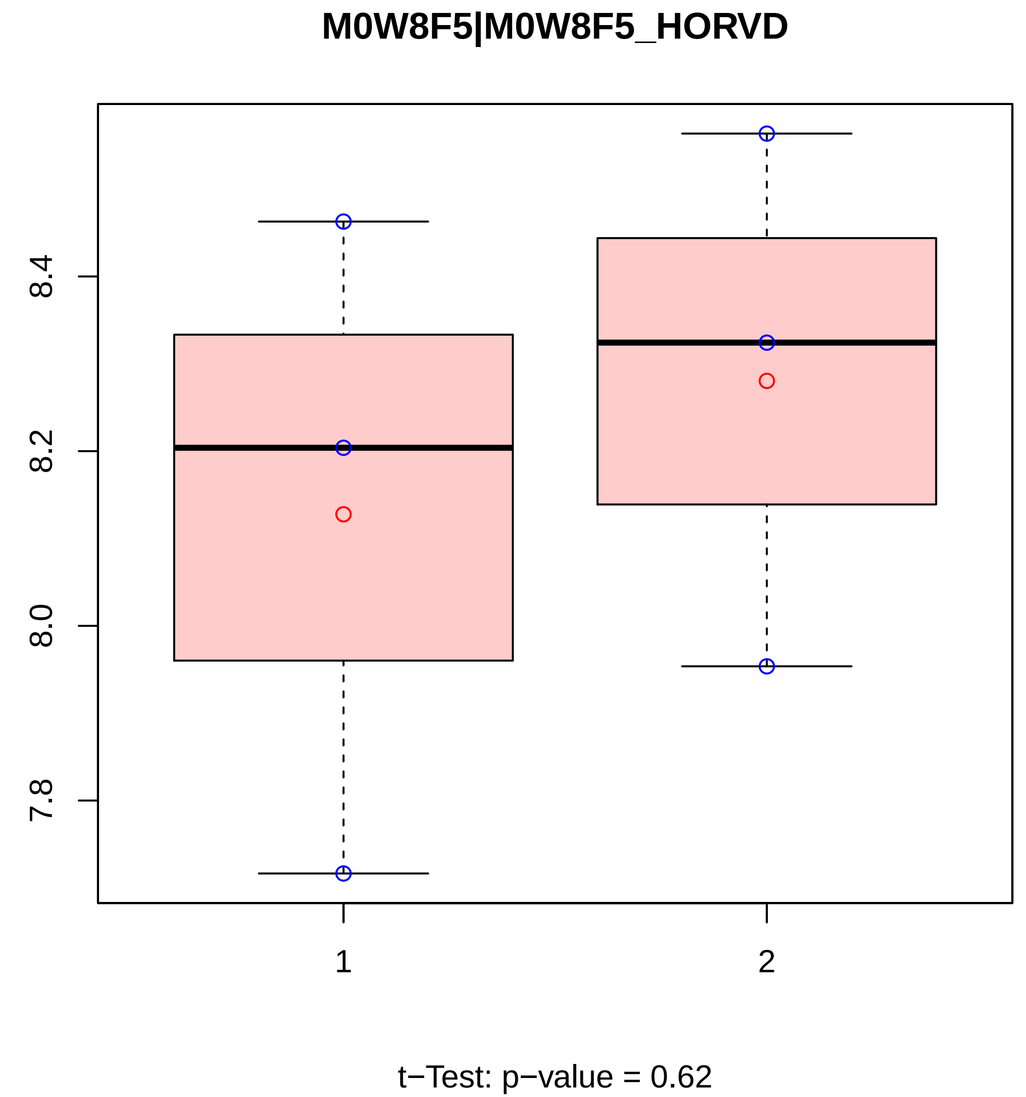
M0W8F5|M0W8F5_HORVD
8.4
8.2
8.0
7.8
1
2
t−Test: p−value = 0.62

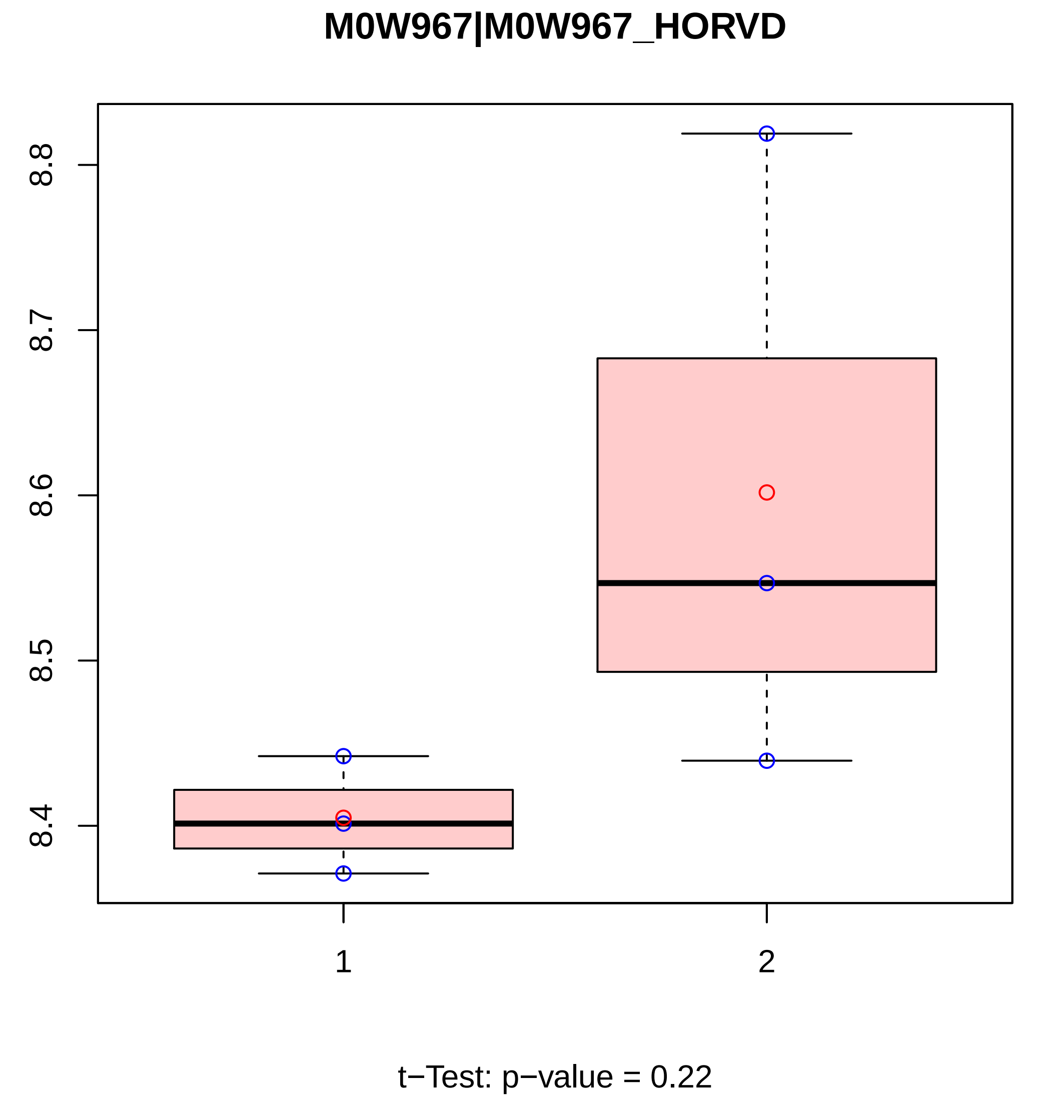
M0W967|M0W967_HORVD
8.4
8.5
8.6
8.7
8.8
1
2
t−Test: p−value = 0.22

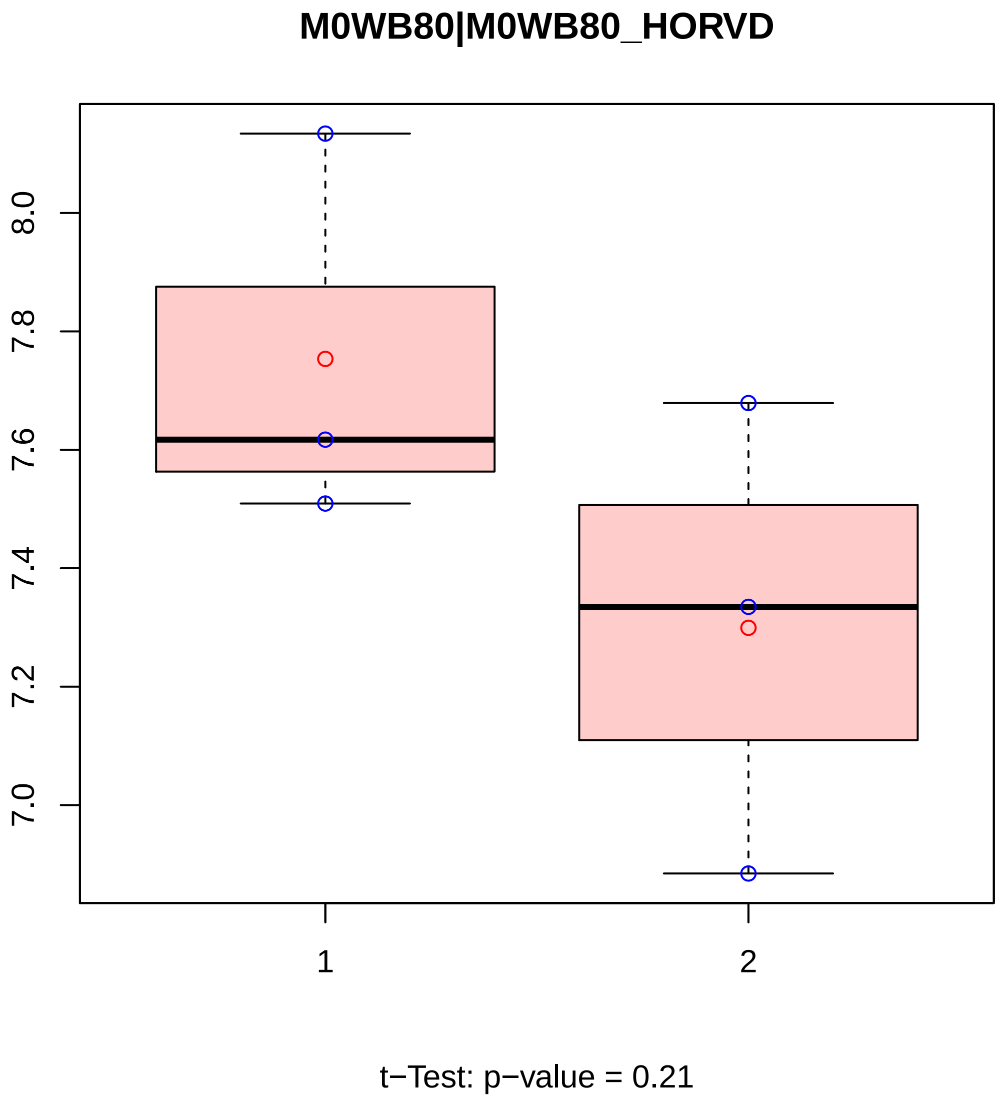

M0WB80|M0WB80_HORVD
8.0
7.8
7.6
7.4
7.2
7.0
1
2
t−Test: p−value = 0.21

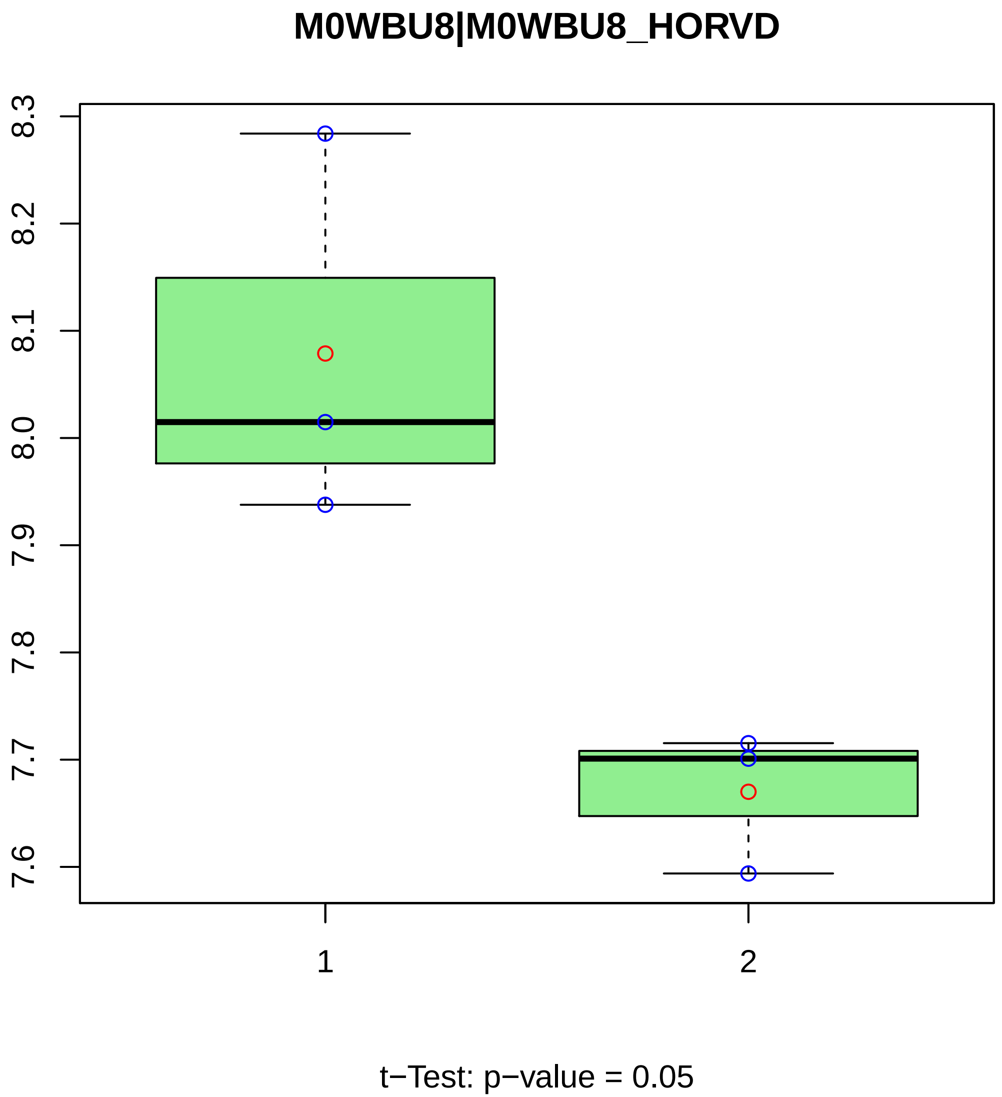
M0WBU8|M0WBU8_HORVD
8.3
8.2
8.1
8.0
7.9
7.8
7.7
7.6
1
2
t−Test: p−value = 0.05

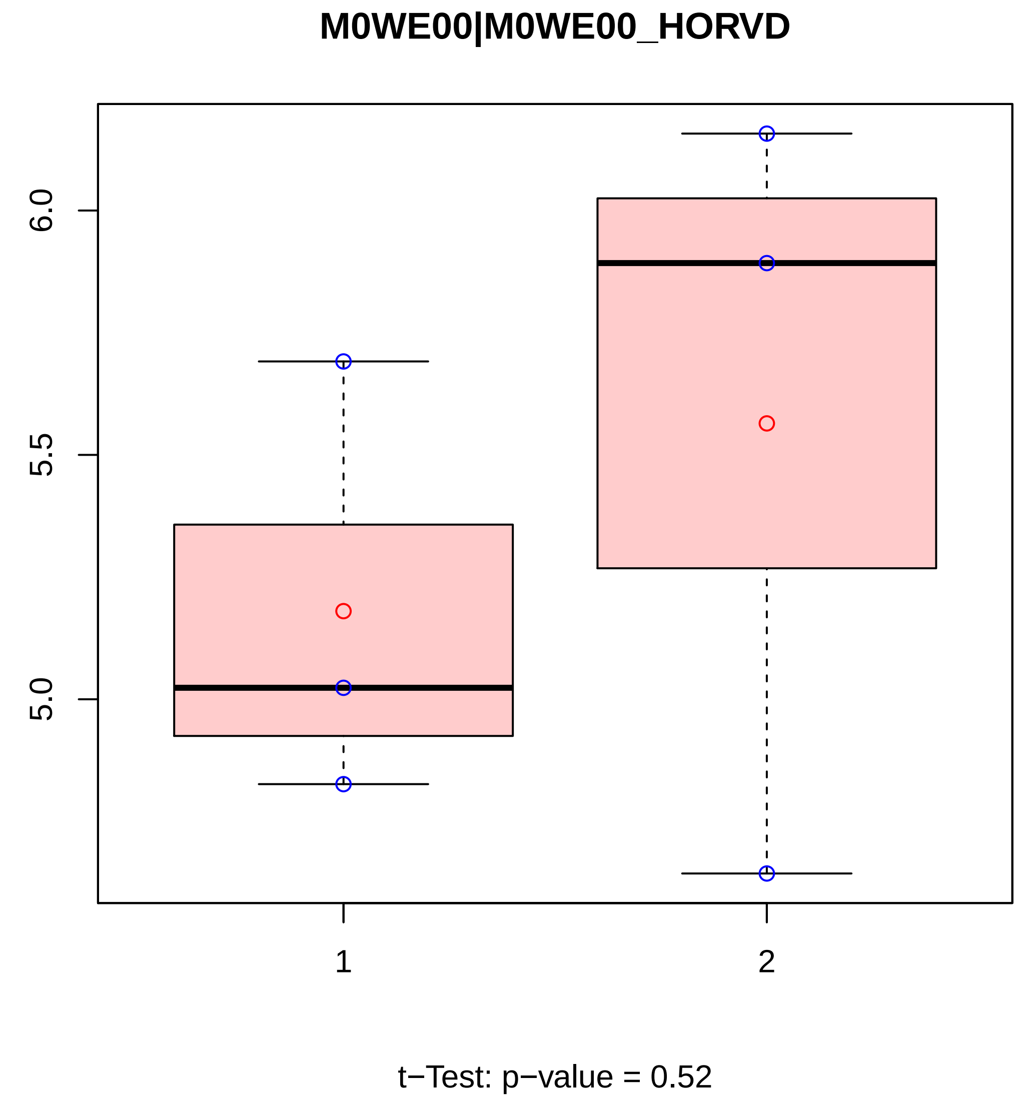
M0WE00|M0WE00_HORVD
6.0
5.5
5.0
1
2
t−Test: p−value = 0.52

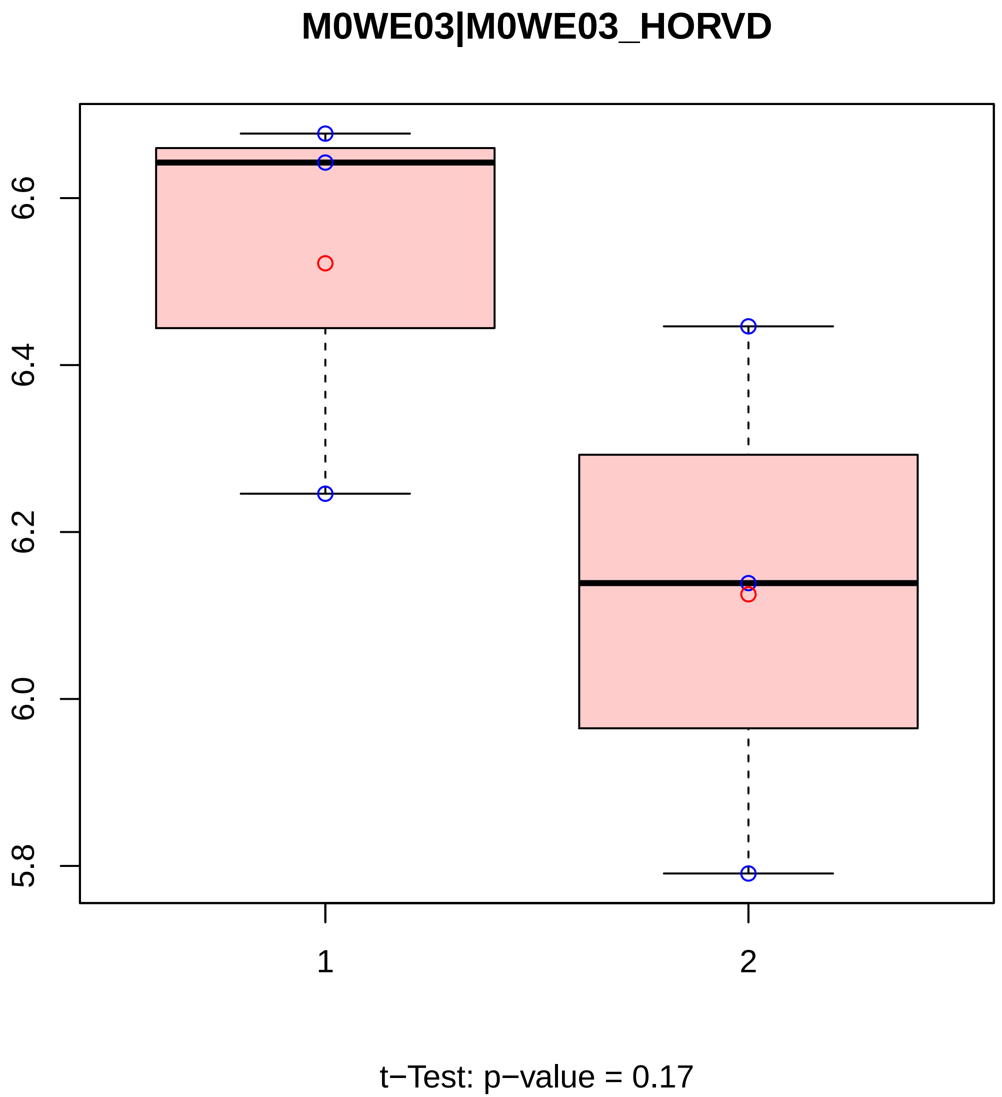
M0WE03|M0WE03_HORVD
6.6
6.4
6.2
6.0
5.8
1
2
t−Test: p−value = 0.17

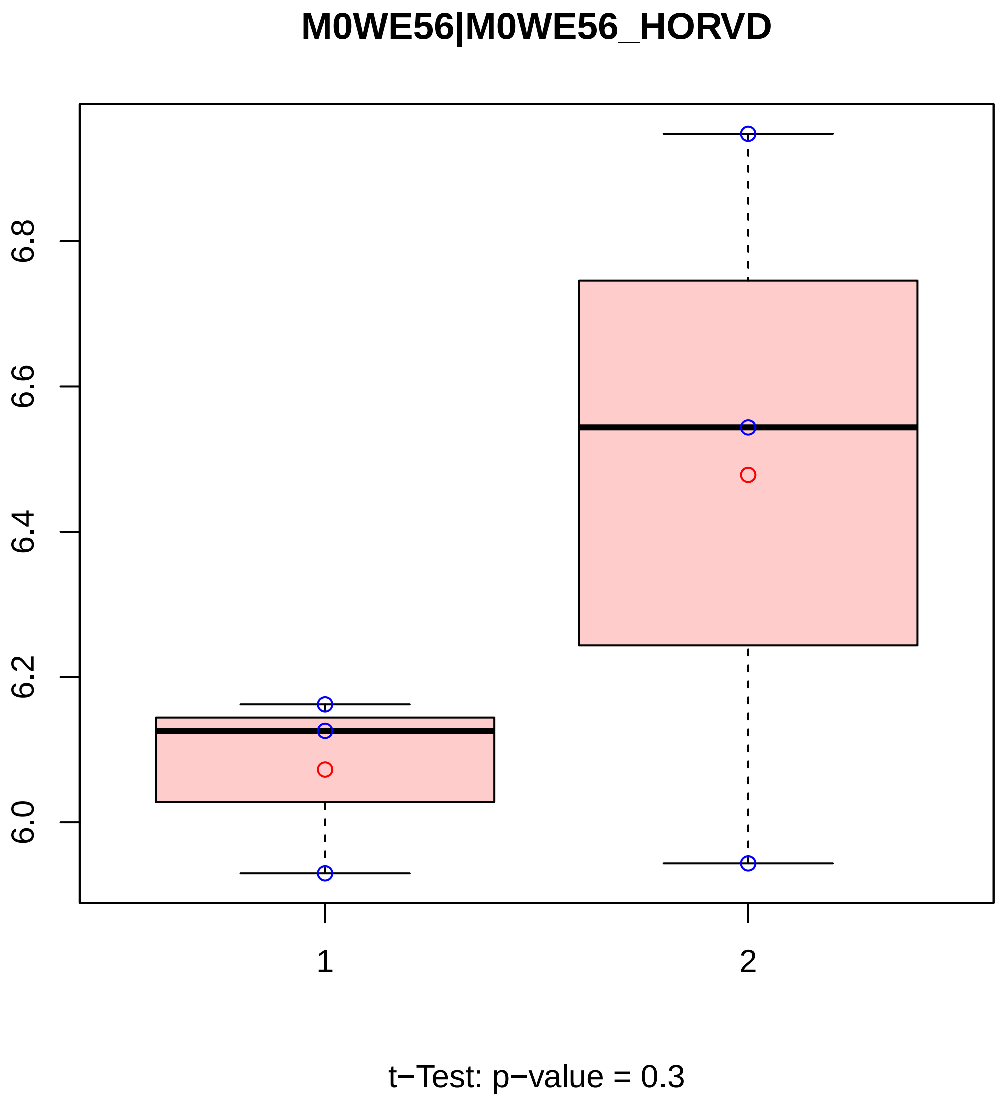
M0WE56|M0WE56_HORVD
6.0
6.2
6.4
6.6
6.8
1
2
t−Test: p−value = 0.3

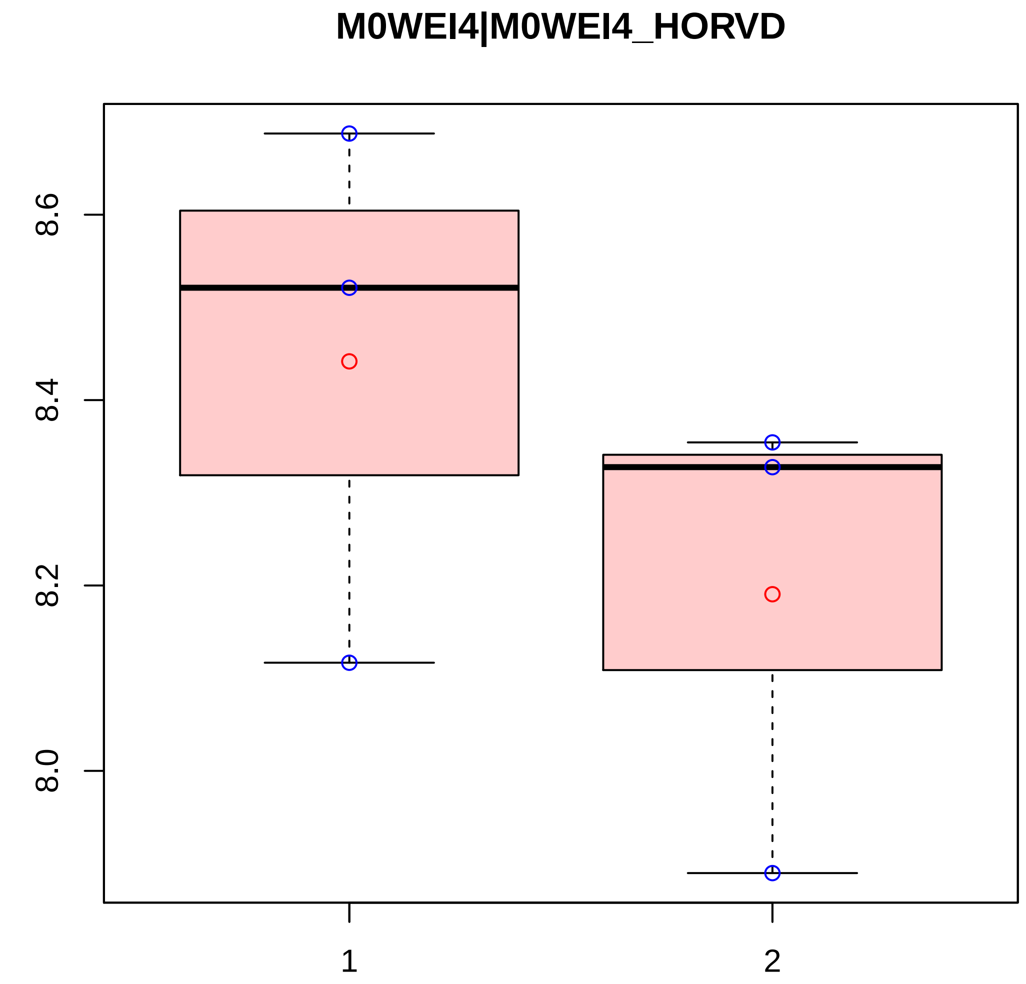

t−Test: p−value = 0.33

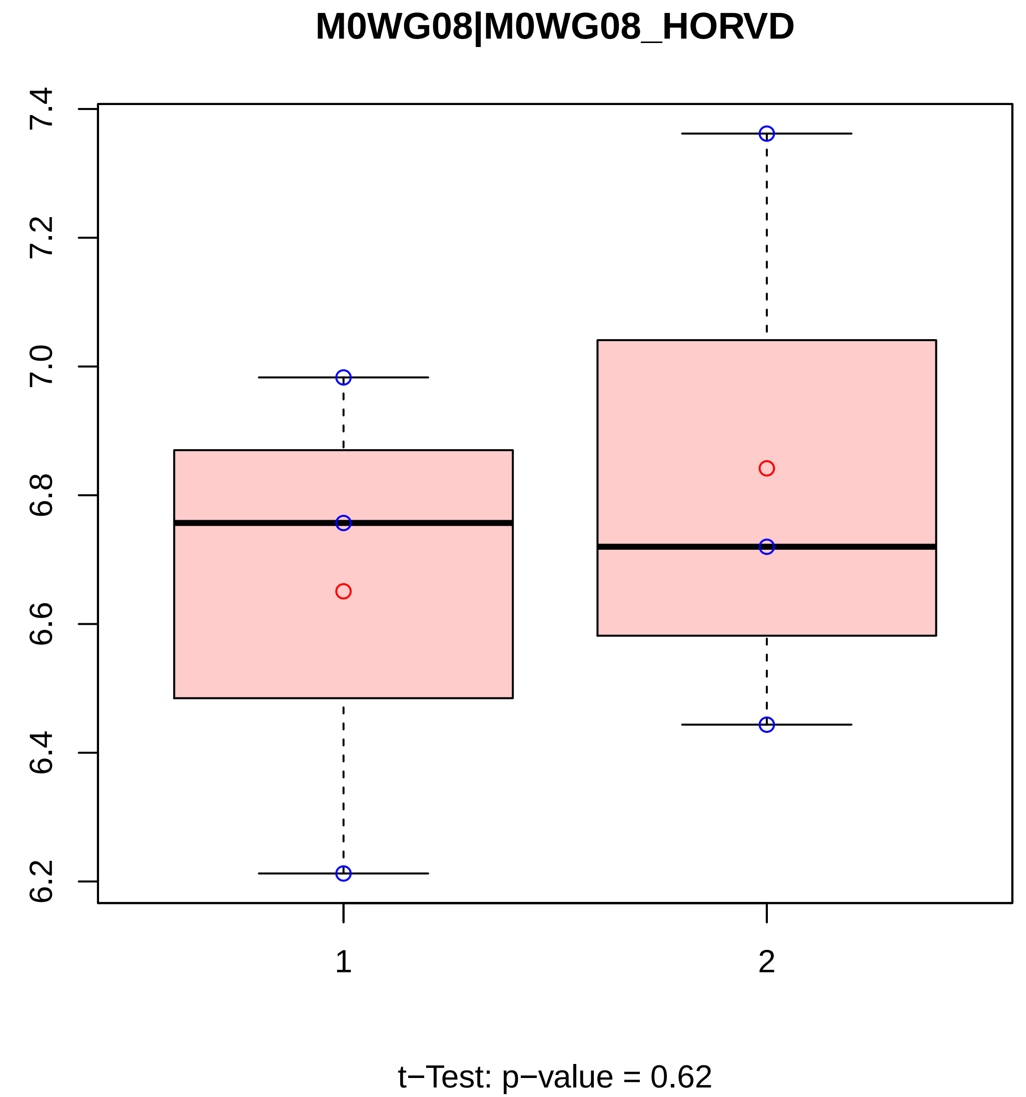
M0WG08|M0WG08_HORVD
7.4
7.2
7.0
6.8
6.6
6.4
6.2
1
2
t−Test: p−value = 0.62

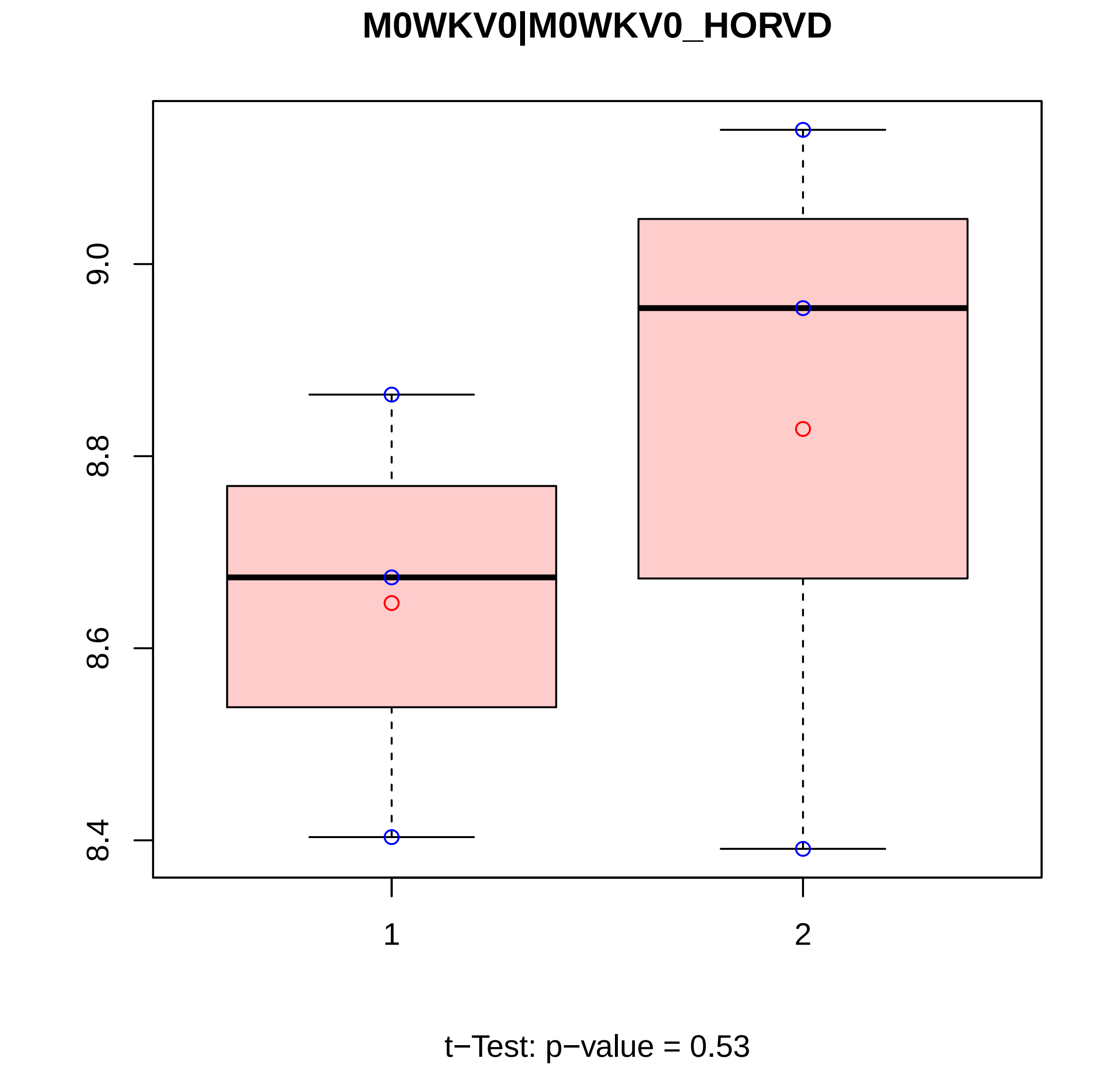
M0WKV0|M0WKV0_HORVD
9.0
8.8
8.6
8.4
1
2
t−Test: p−value = 0.53

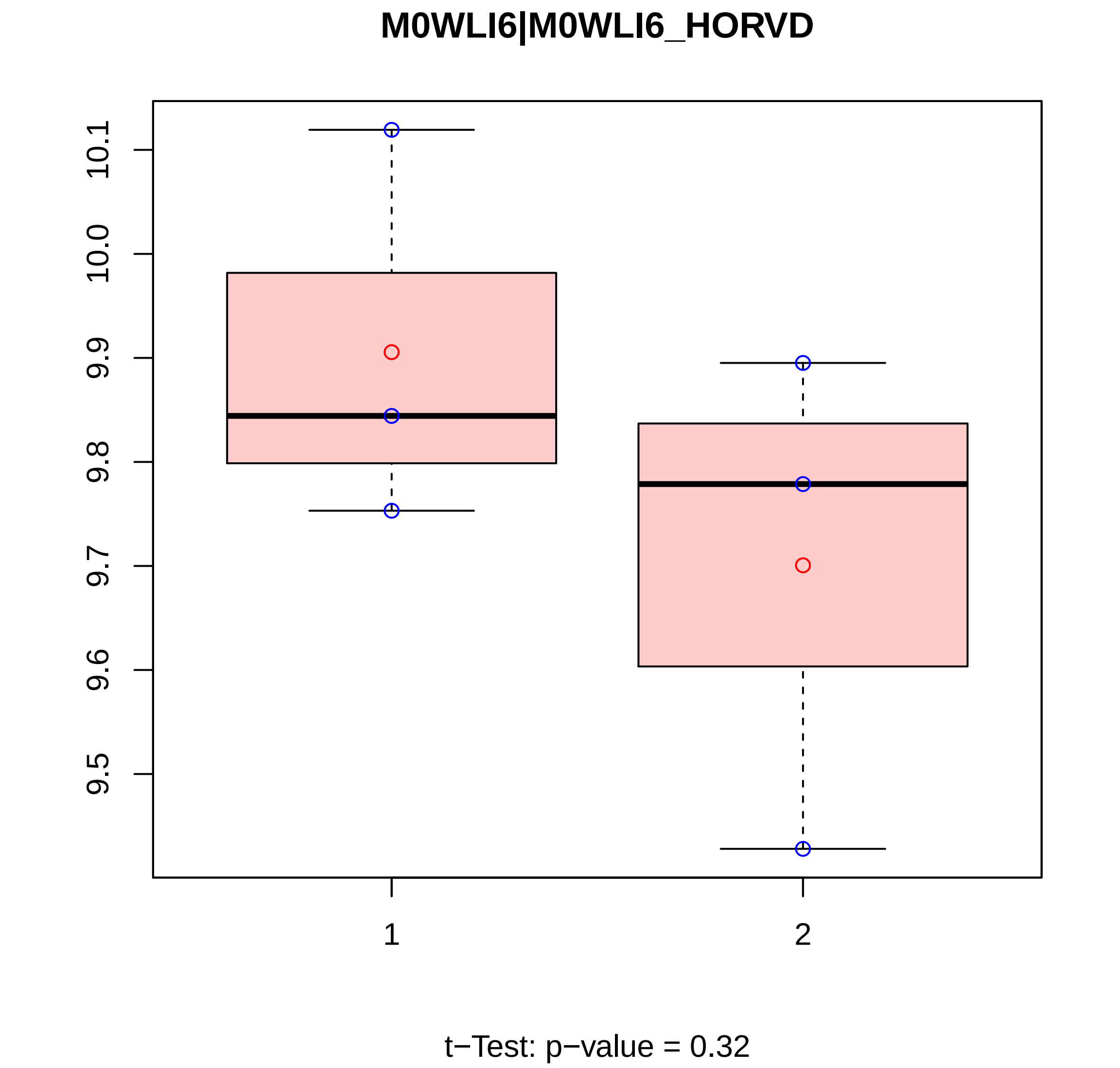
M0WLI6|M0WLI6_HORVD
10.1
10.0
9.9
9.8
9.7
9.6
9.5
1
2
t−Test: p−value = 0.32

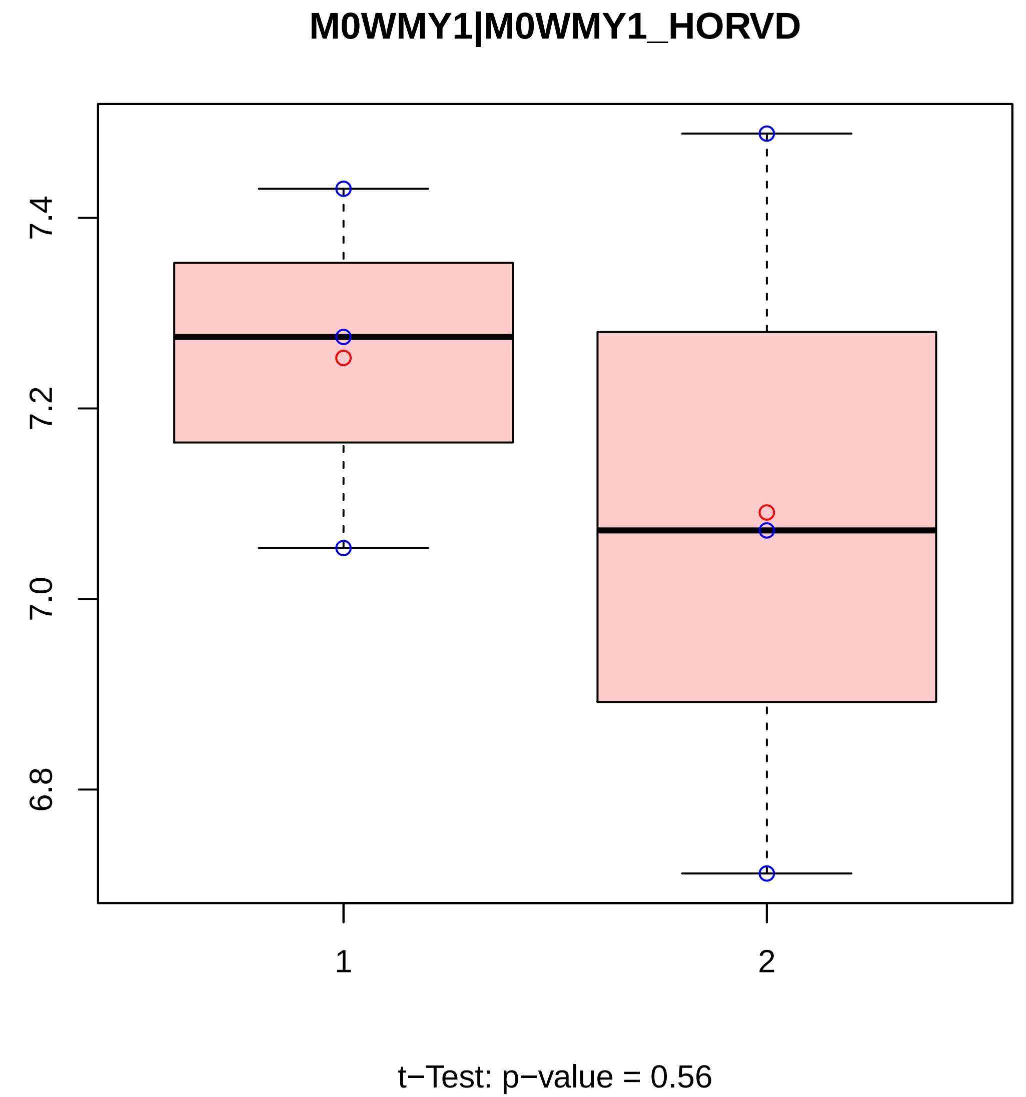
M0WMY1|M0WMY1_HORVD
7.4
7.2
7.0
6.8
1
2
t−Test: p−value = 0.56

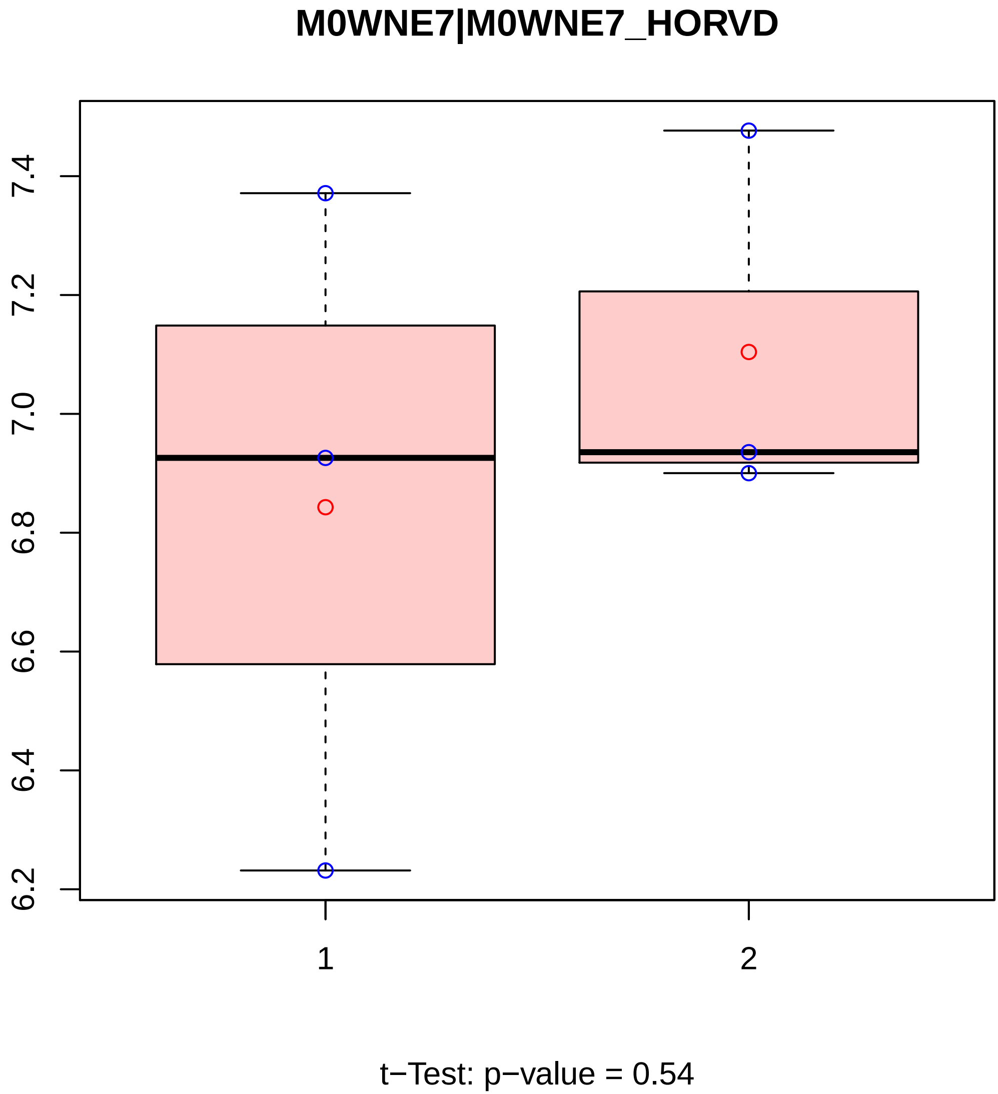

M0WNE7|M0WNE7_HORVD
6.2
6.4
6.6
6.8
7.0
7.2
7.4
1
2
t−Test: p−value = 0.54

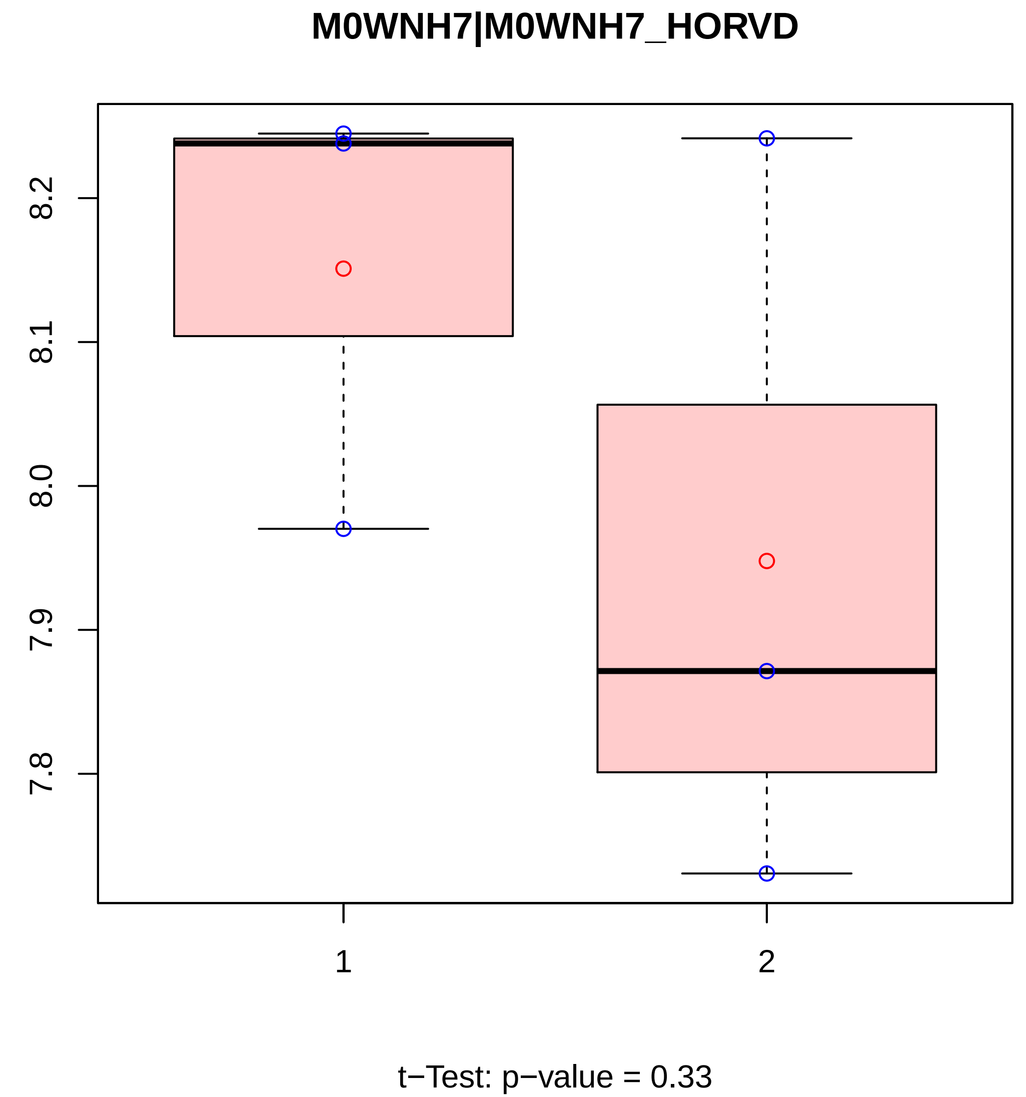
M0WNH7|M0WNH7_HORVD
8.2
8.1
8.0
7.9
7.8
1
2
t−Test: p−value = 0.33

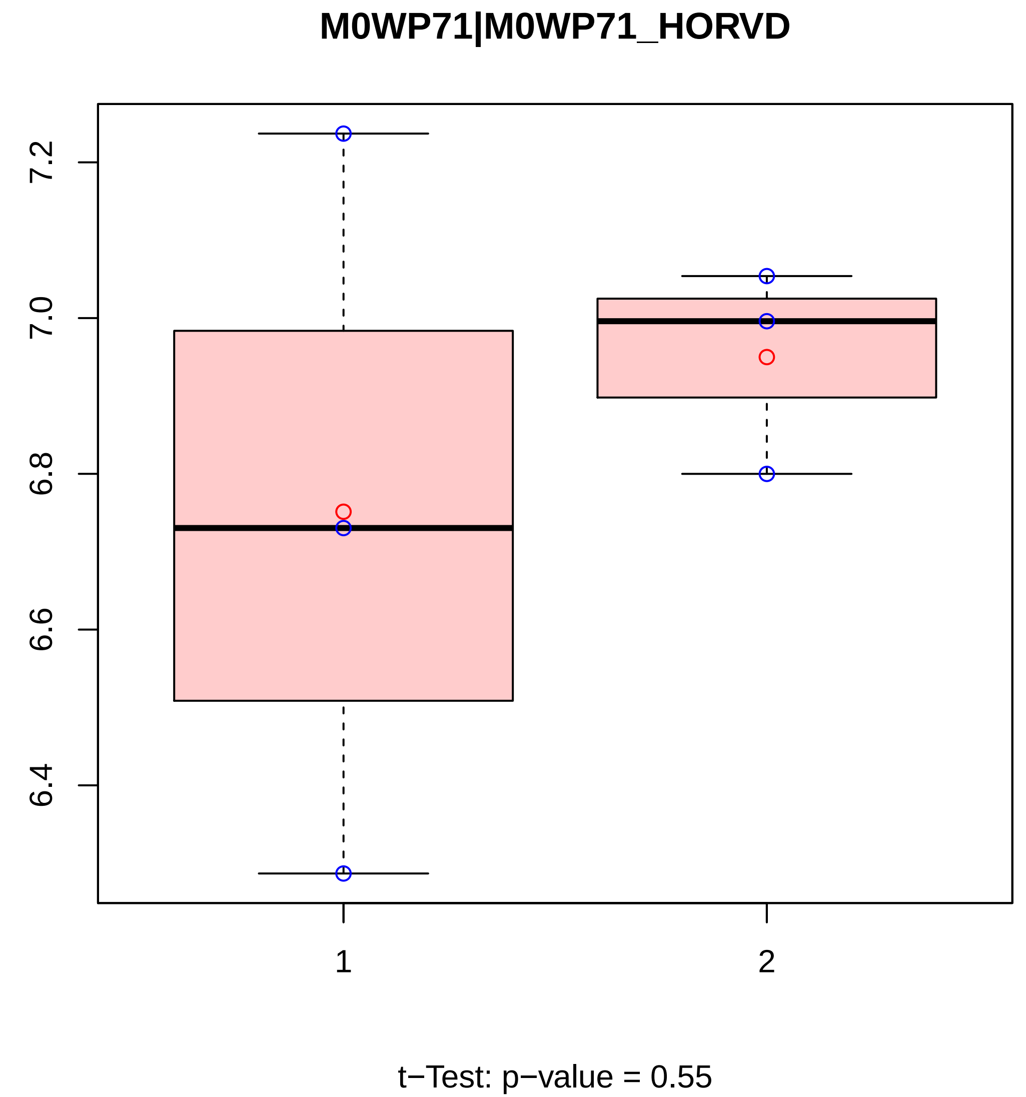
M0WP71|M0WP71_HORVD
7.2
7.0
6.8
6.6
6.4
1
2
t−Test: p−value = 0.55

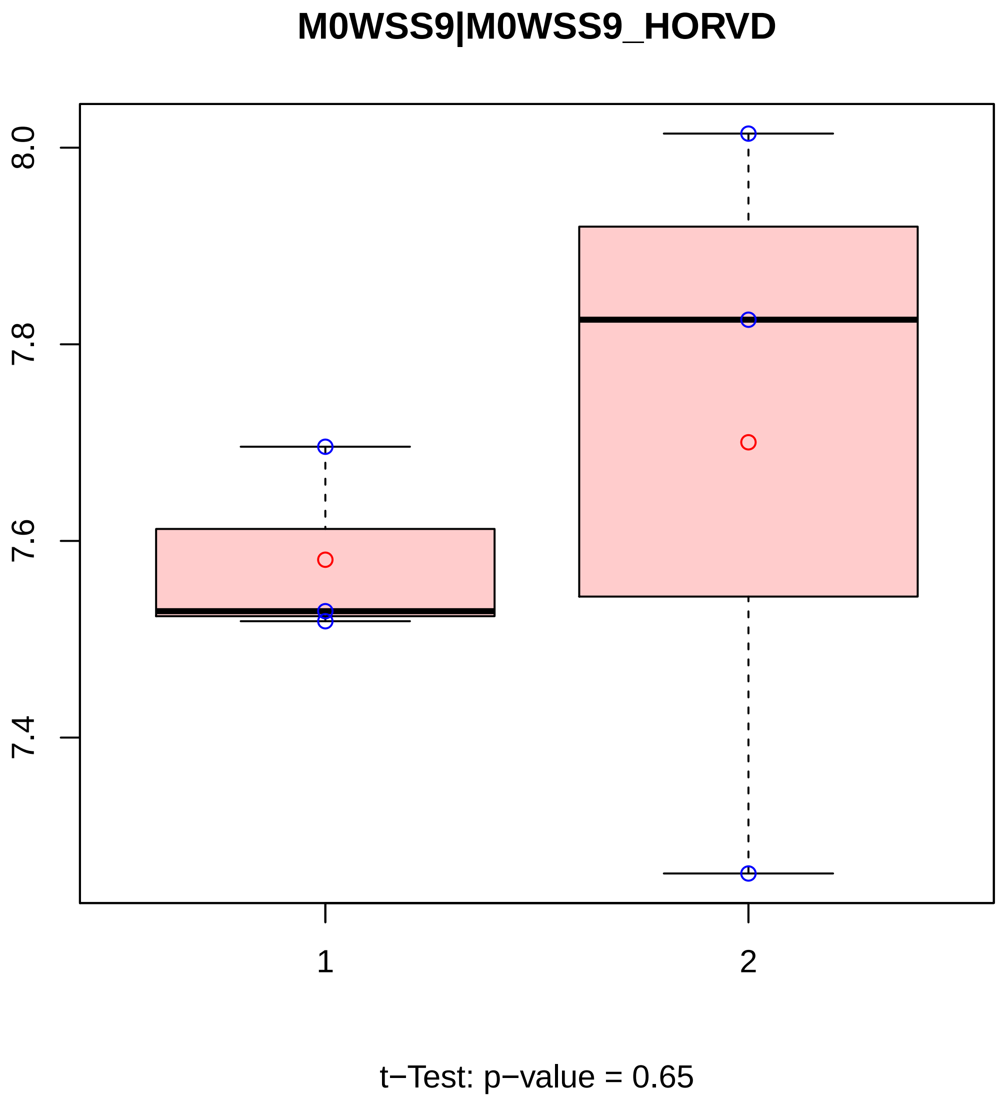
M0WSS9|M0WSS9_HORVD
8.0
7.8
7.6
7.4
1
2
t−Test: p−value = 0.65

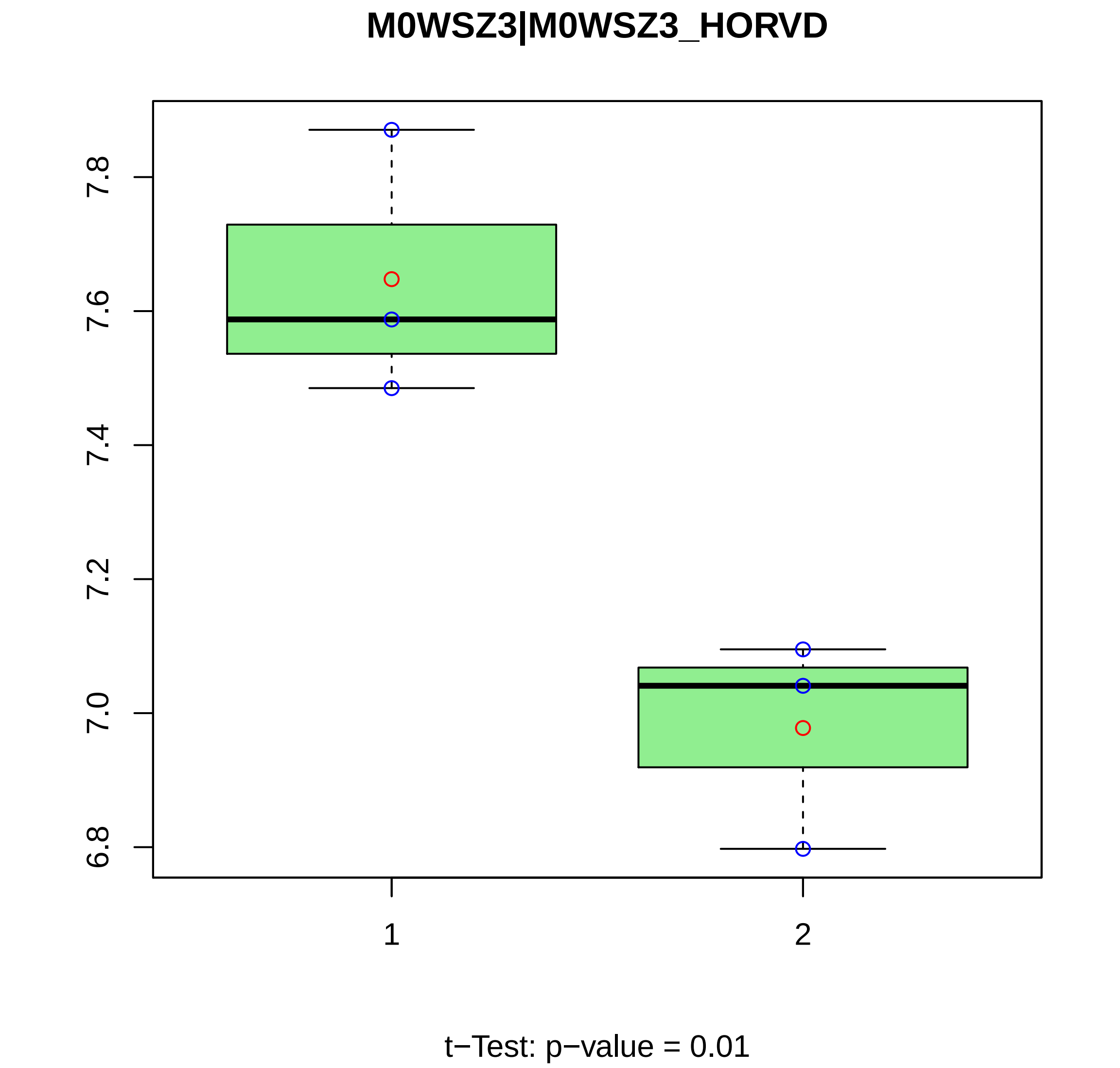
M0WSZ3|M0WSZ3_HORVD
7.8
7.6
7.4
7.2
7.0
6.8
1
2
t−Test: p−value = 0.01

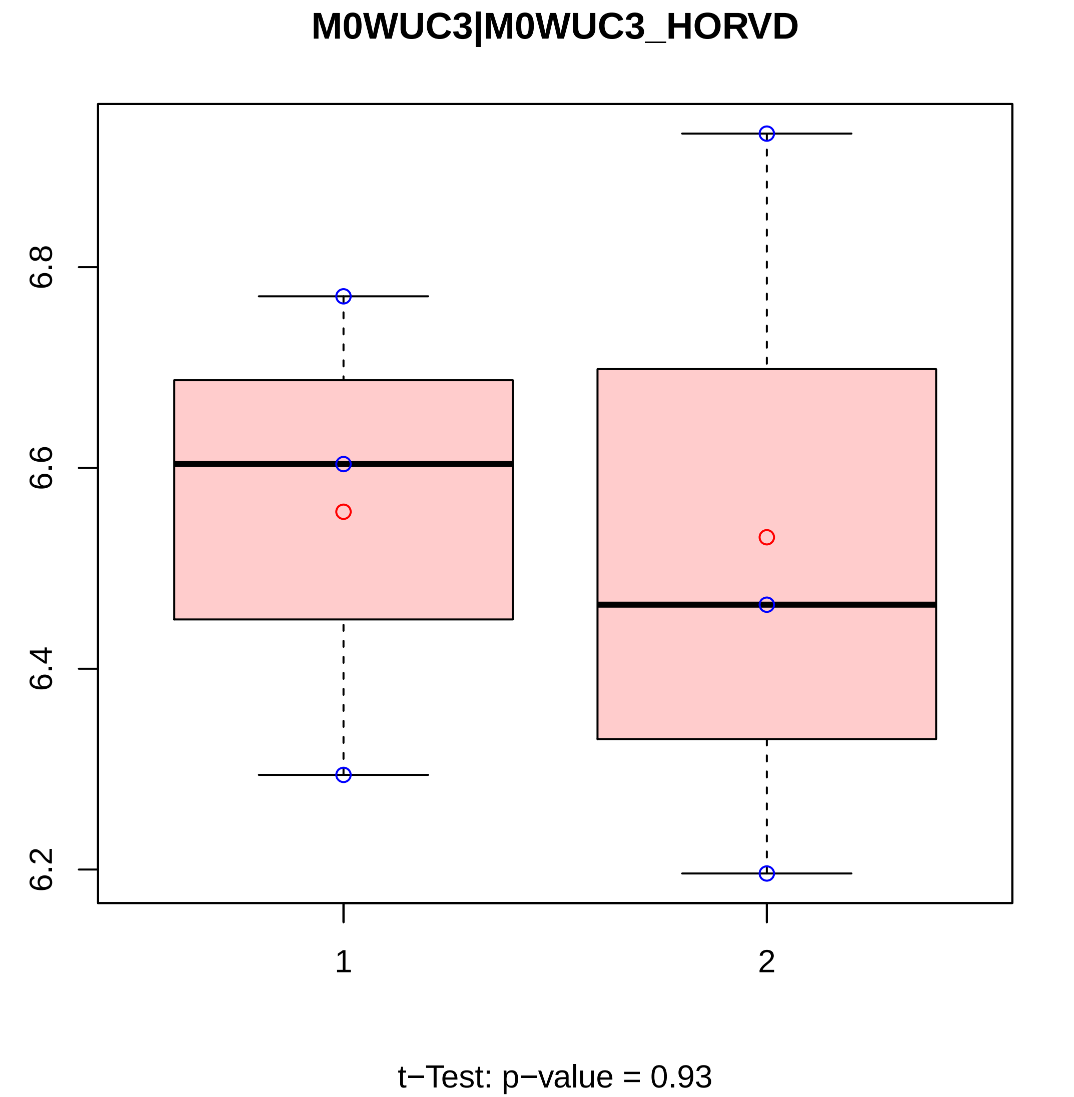
M0WUC3|M0WUC3_HORVD
6.2
6.4
6.6
6.8
1
2
t−Test: p−value = 0.93

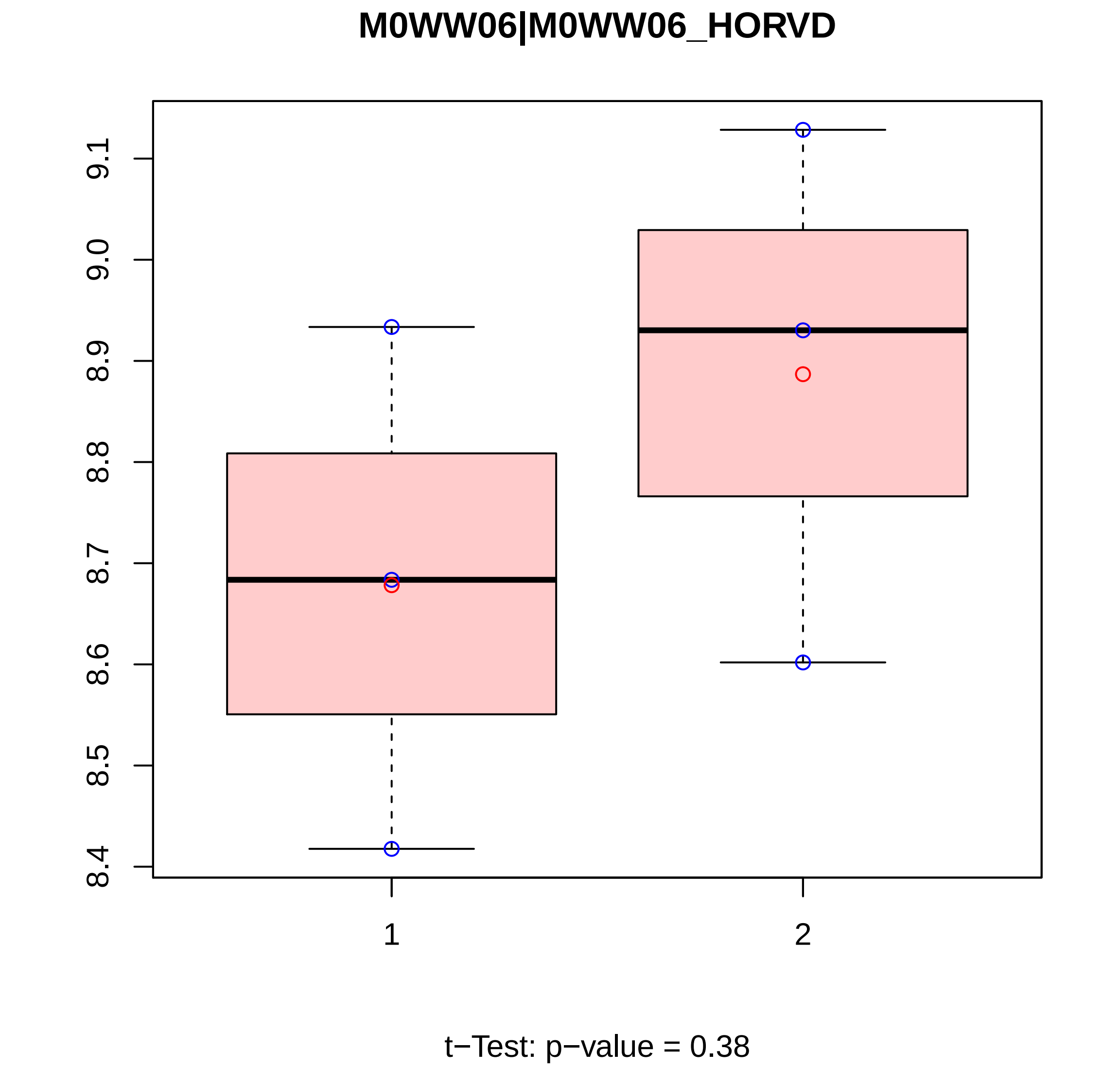
M0WW06|M0WW06_HORVD
9.1
9.0
8.9
8.8
8.7
8.6
8.5
8.4
1
2
t−Test: p−value = 0.38

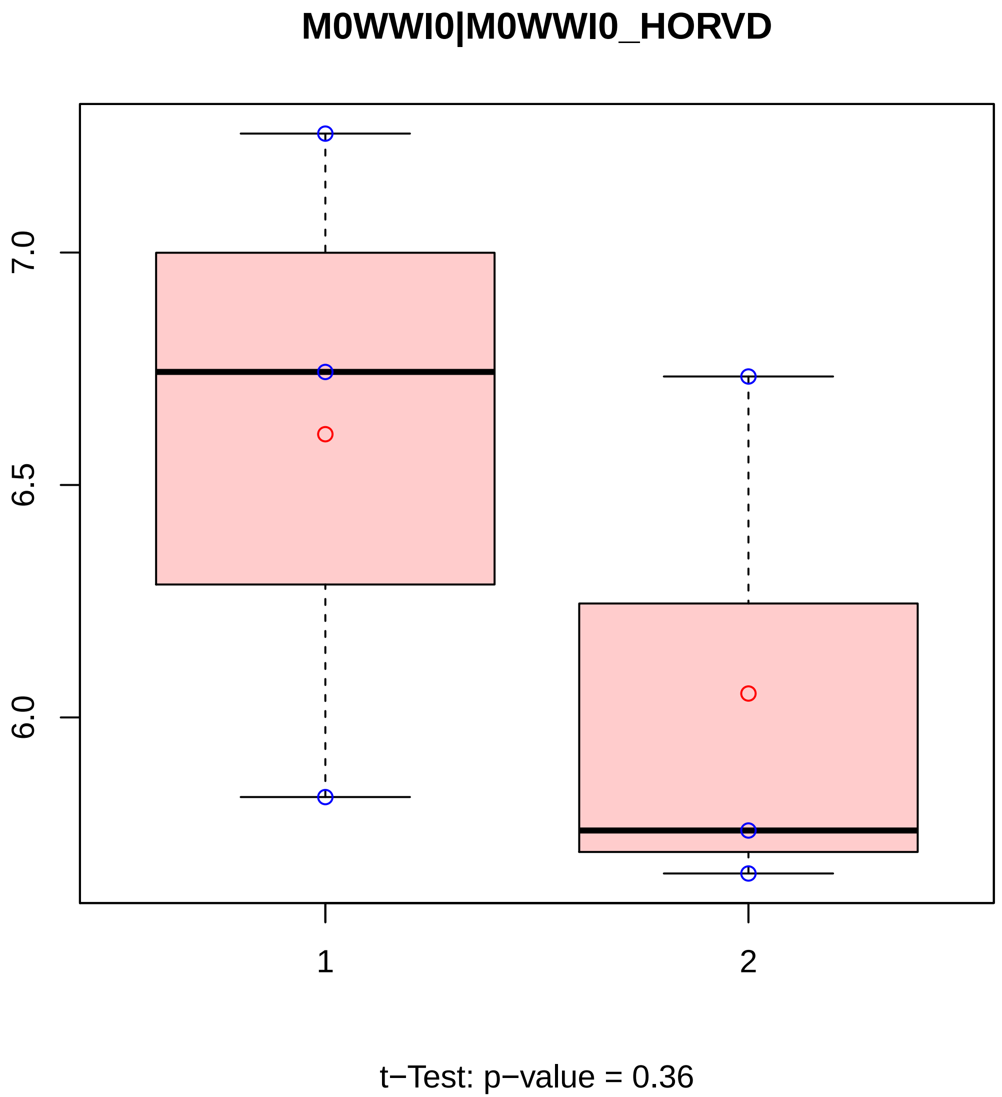
M0WWI0|M0WWI0_HORVD
7.0
6.5
6.0
1
2
t−Test: p−value = 0.36

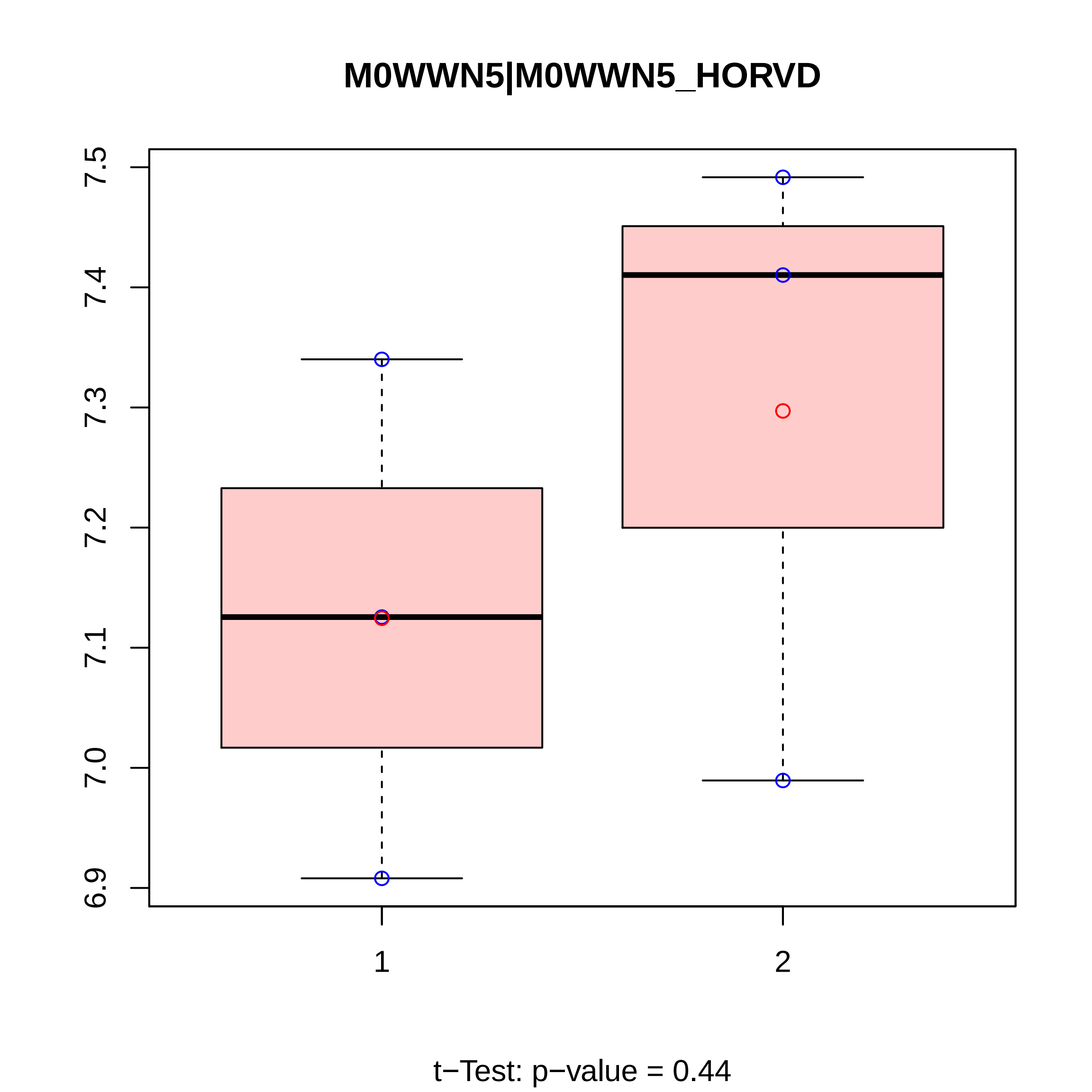
M0WWN5|M0WWN5_HORVD
7.5
7.4
7.3
7.2
7.1
7.0
6.9
1
2
t−Test: p−value = 0.44

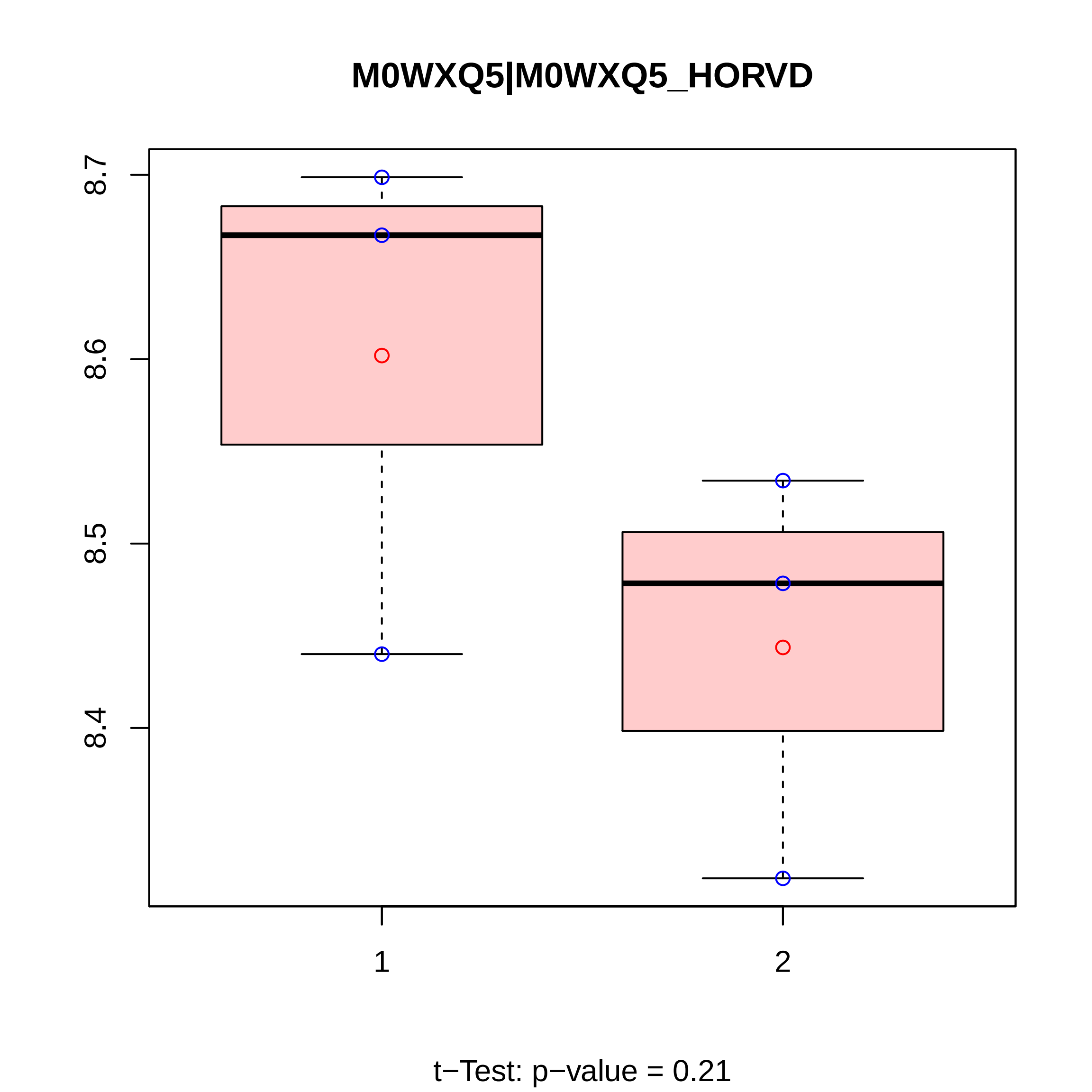
M0WXQ5|M0WXQ5_HORVD
8.4
8.5
8.6
8.7
1
2
t−Test: p−value = 0.21

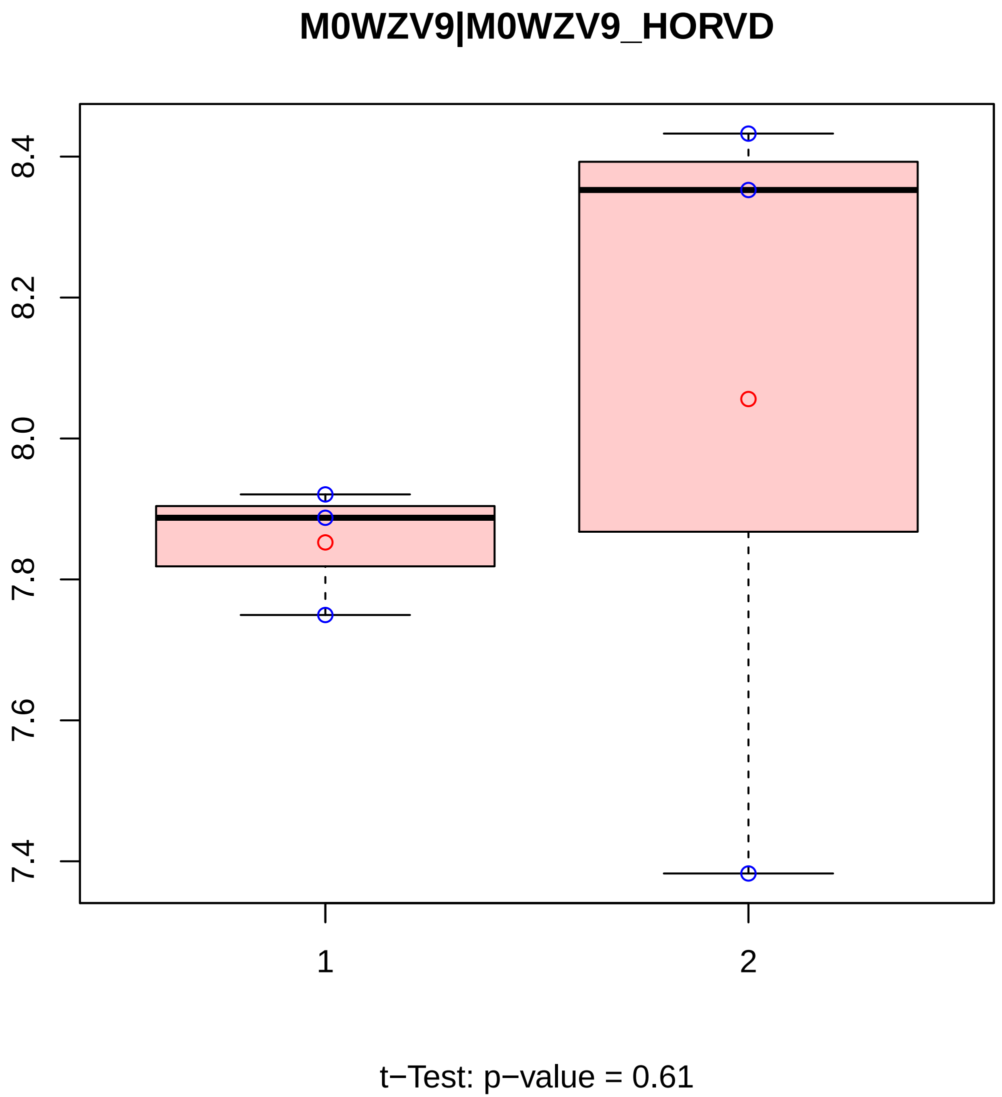
M0WZV9|M0WZV9_HORVD
8.4
8.2
8.0
7.8
7.6
7.4
1
2
t−Test: p−value = 0.61

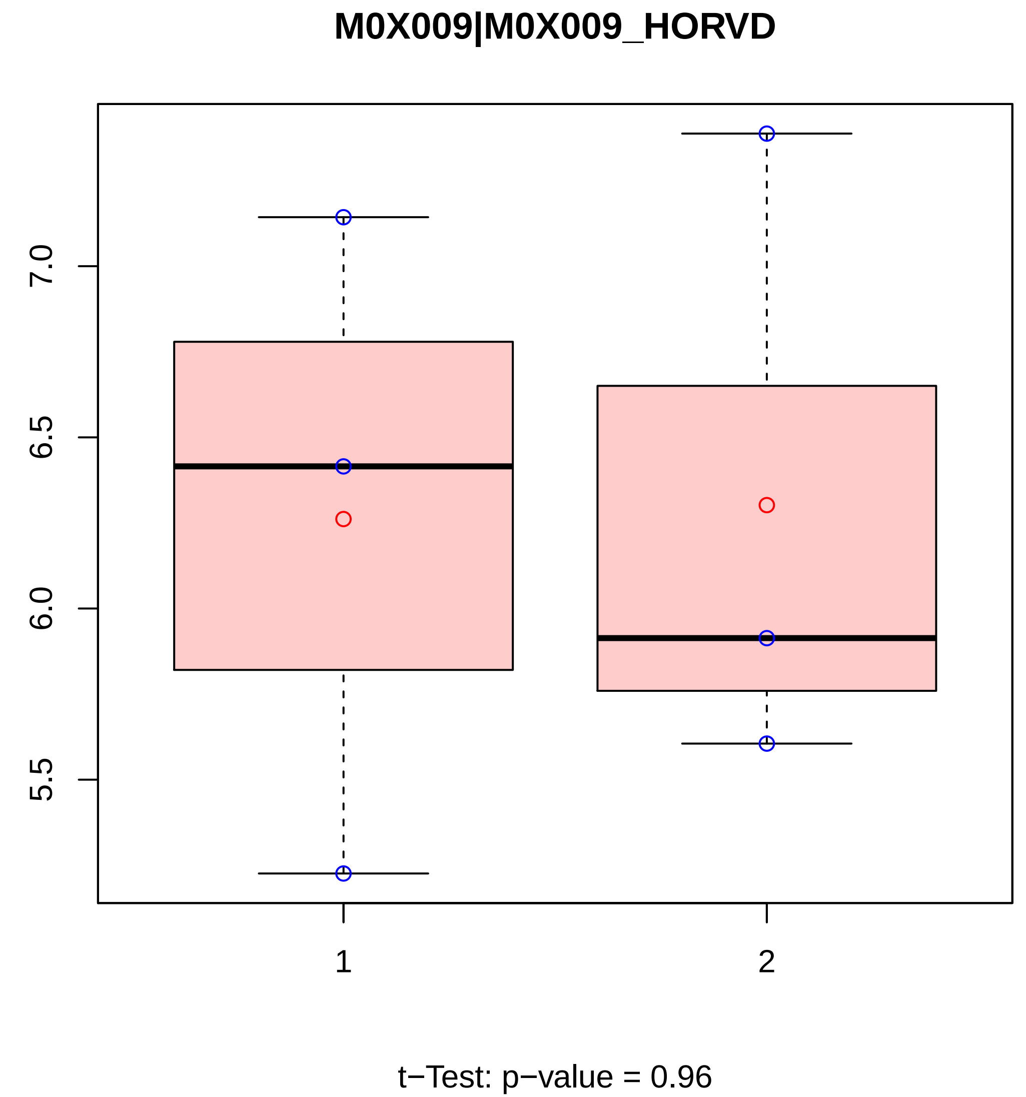

M0X009|M0X009_HORVD
7.0
6.5
6.0
5.5
1
2
t−Test: p−value = 0.96

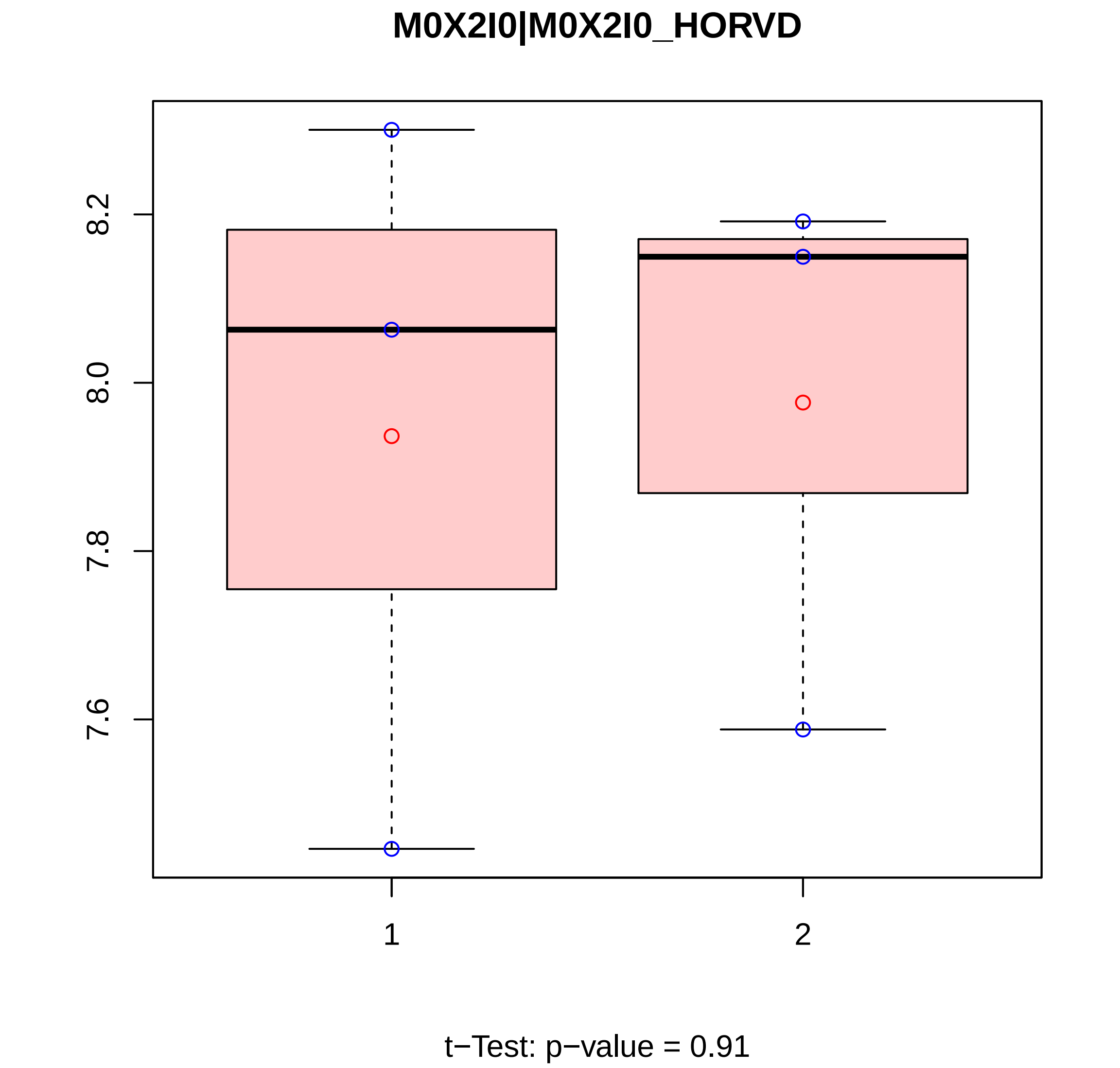
M0X2I0|M0X2I0_HORVD
8.2
8.0
7.8
7.6
1
2
t−Test: p−value = 0.91

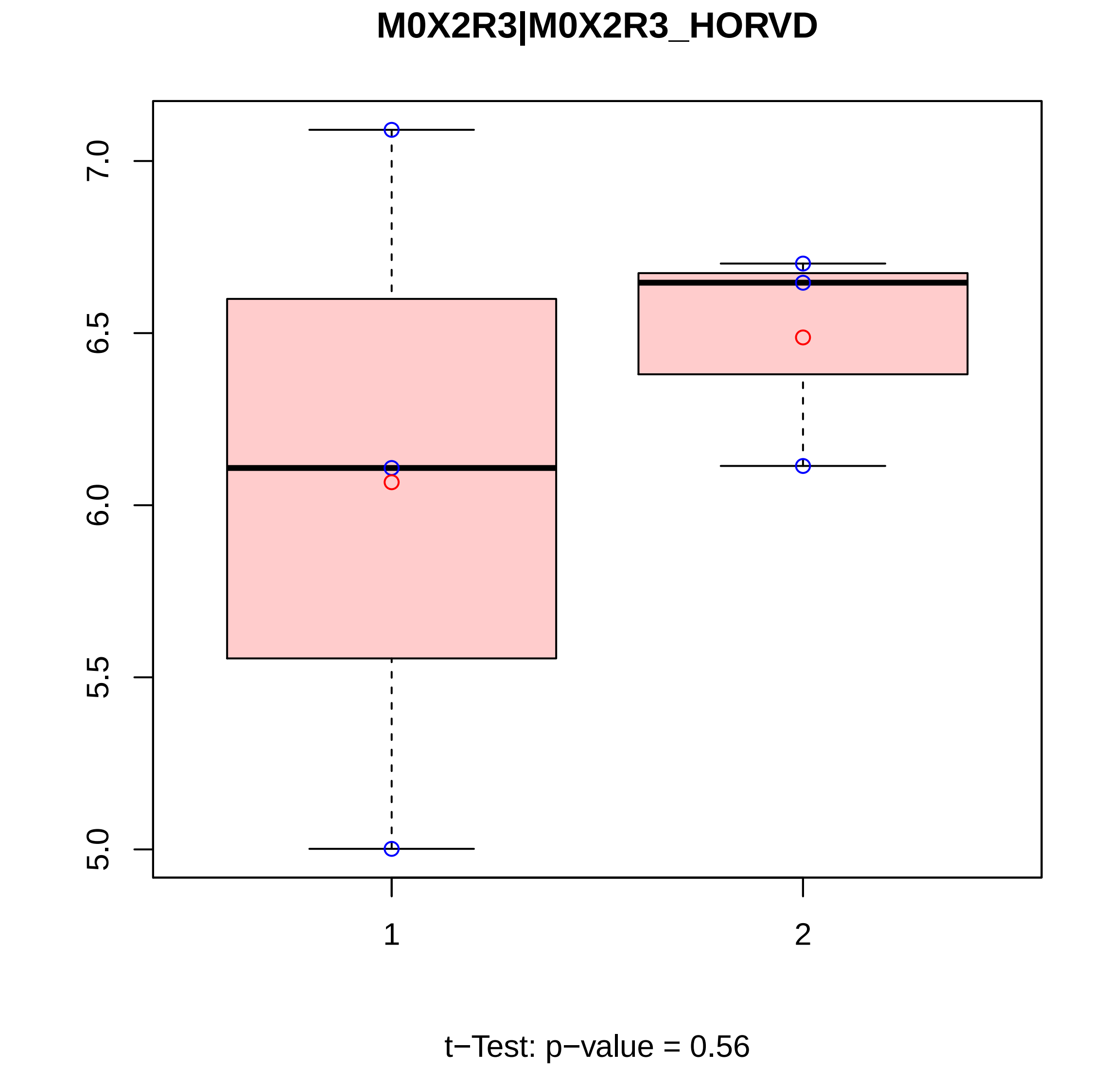
M0X2R3|M0X2R3_HORVD
7.0
6.5
6.0
5.5
5.0
1
2
t−Test: p−value = 0.56

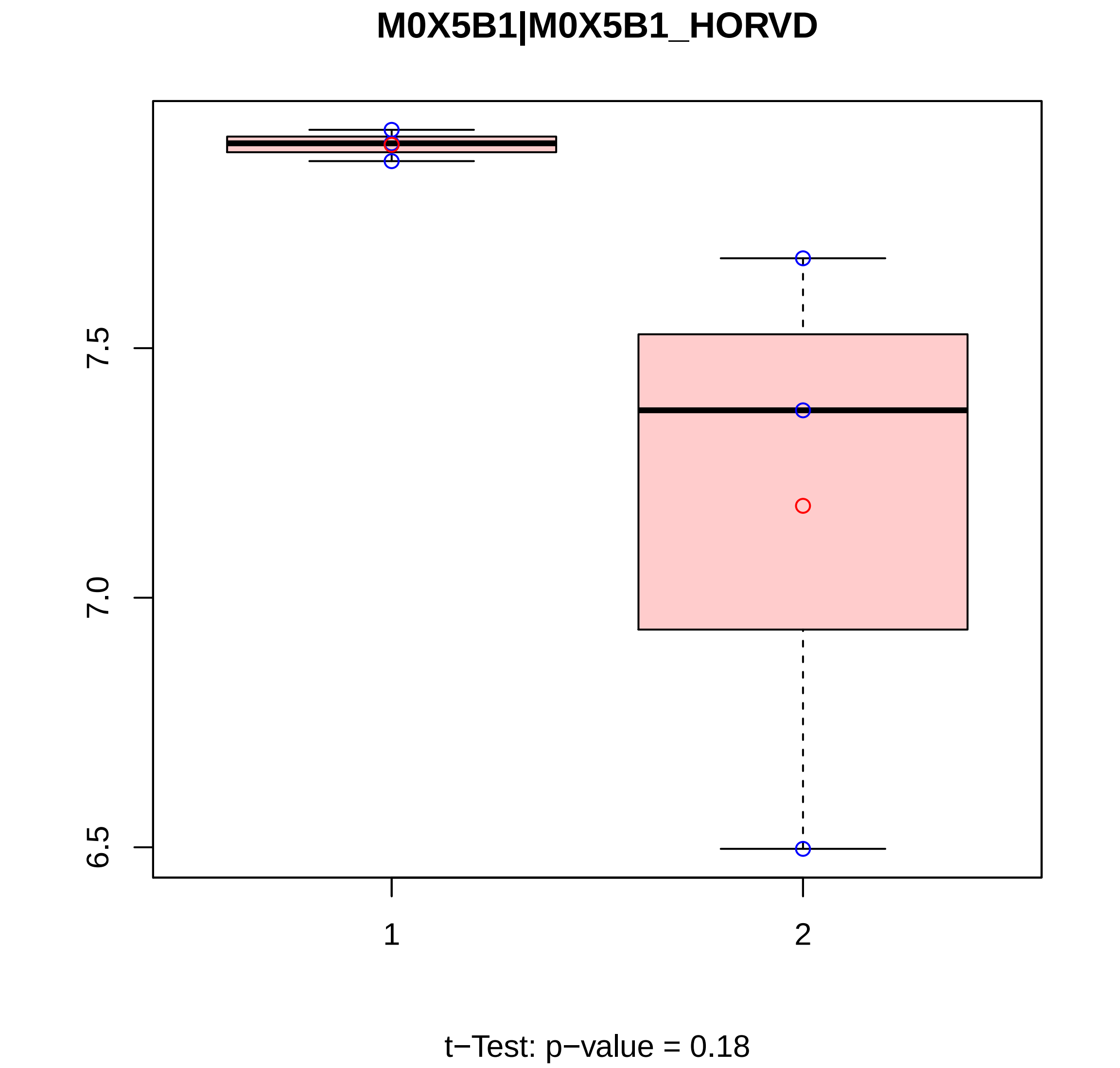
M0X5B1|M0X5B1_HORVD
7.5
7.0
6.5
1
2
t−Test: p−value = 0.18

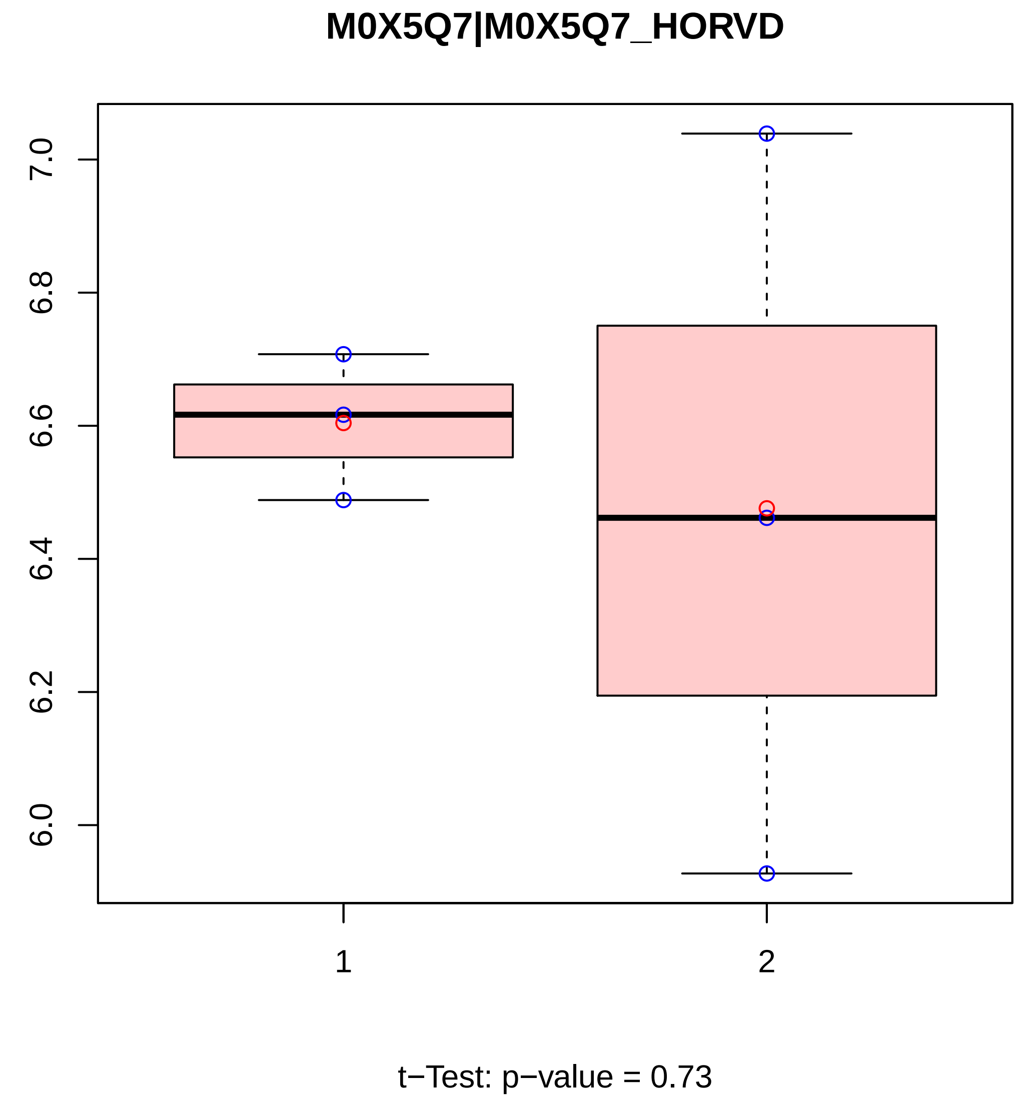

M0X5Q7|M0X5Q7_HORVD
7.0
6.8
6.6
6.4
6.2
6.0
1
2
t−Test: p−value = 0.73

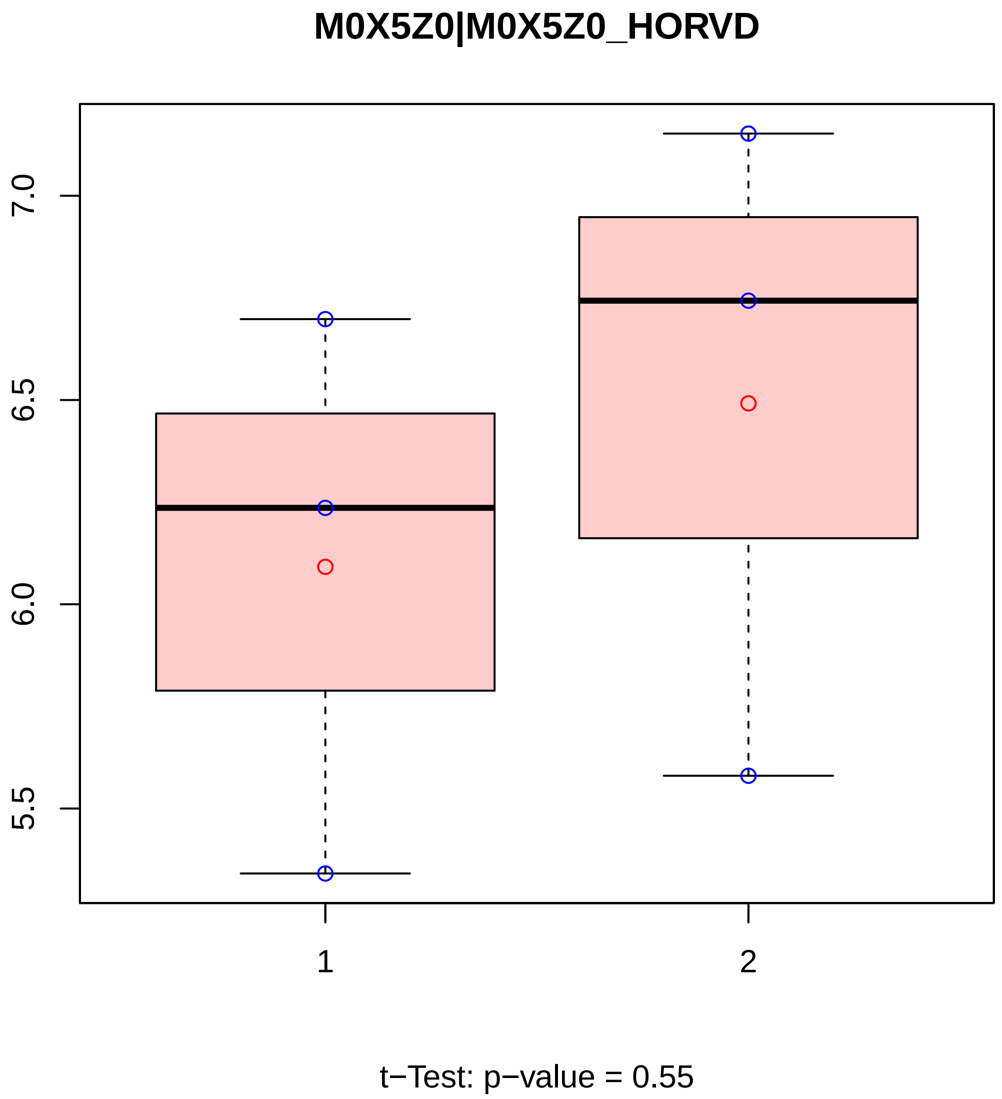
M0X5Z0|M0X5Z0_HORVD
7.0
6.5
6.0
5.5
1
2
t−Test: p−value = 0.55

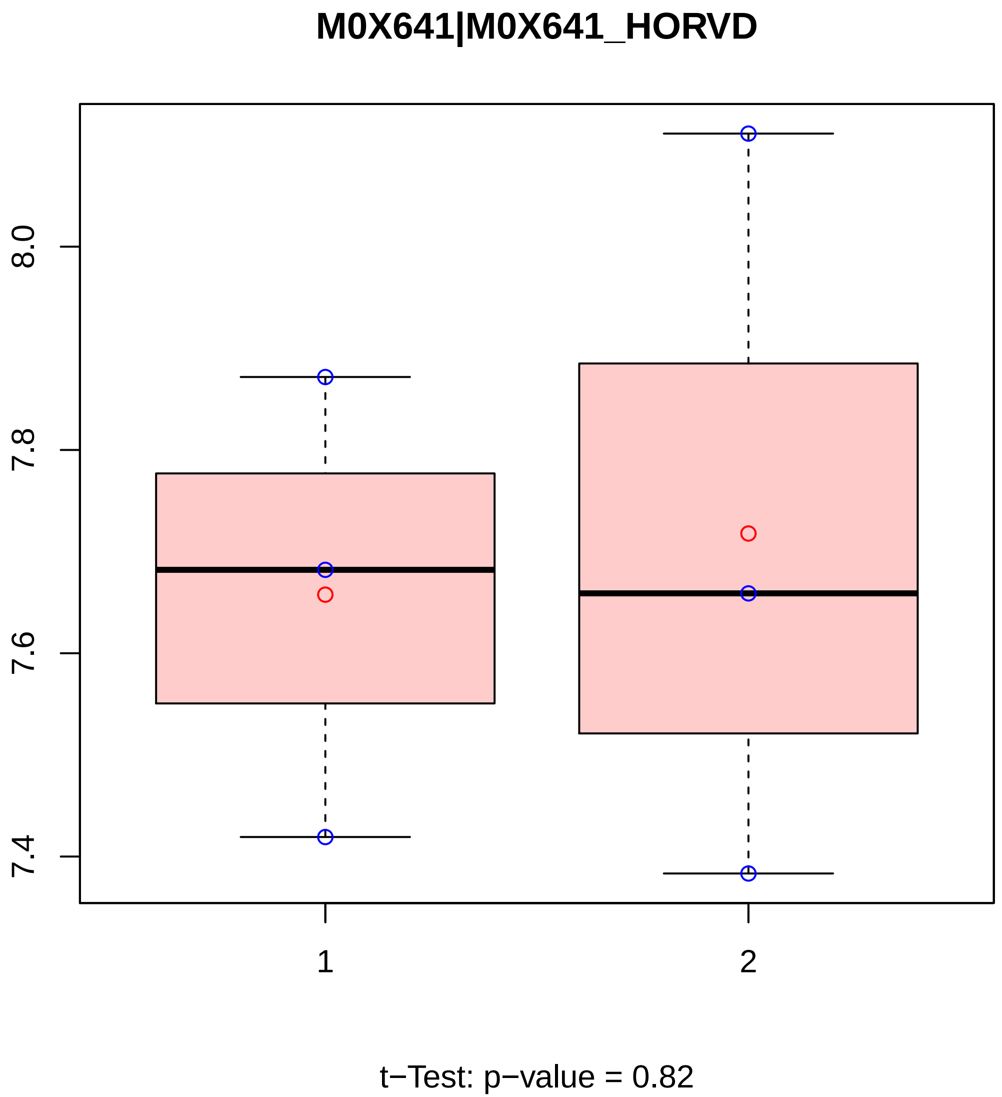
M0X641|M0X641_HORVD
7.4
7.6
7.8
8.0
1
2
t−Test: p−value = 0.82

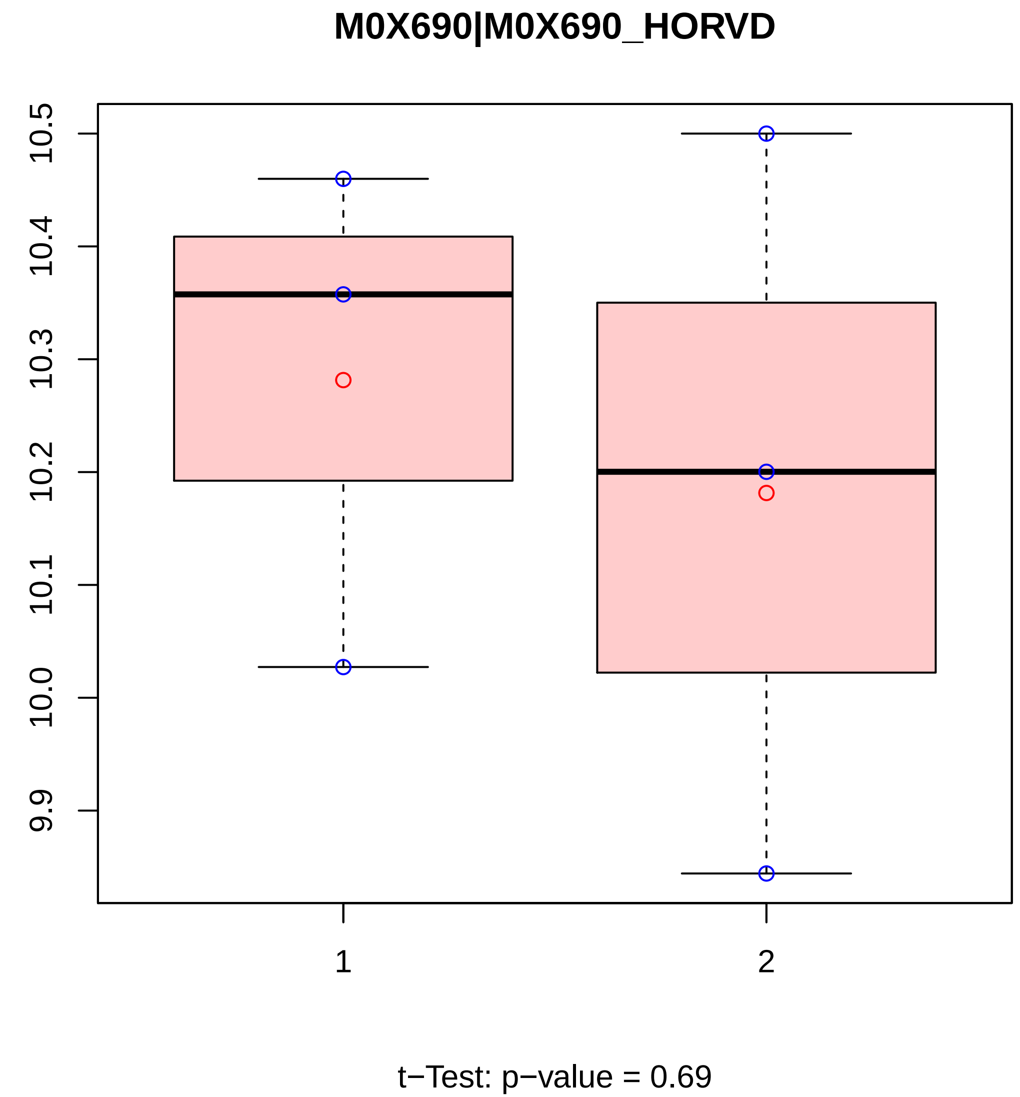
M0X690|M0X690_HORVD
10.5
10.4
10.3
10.2
10.1
10.0
9.9
1
2
t−Test: p−value = 0.69

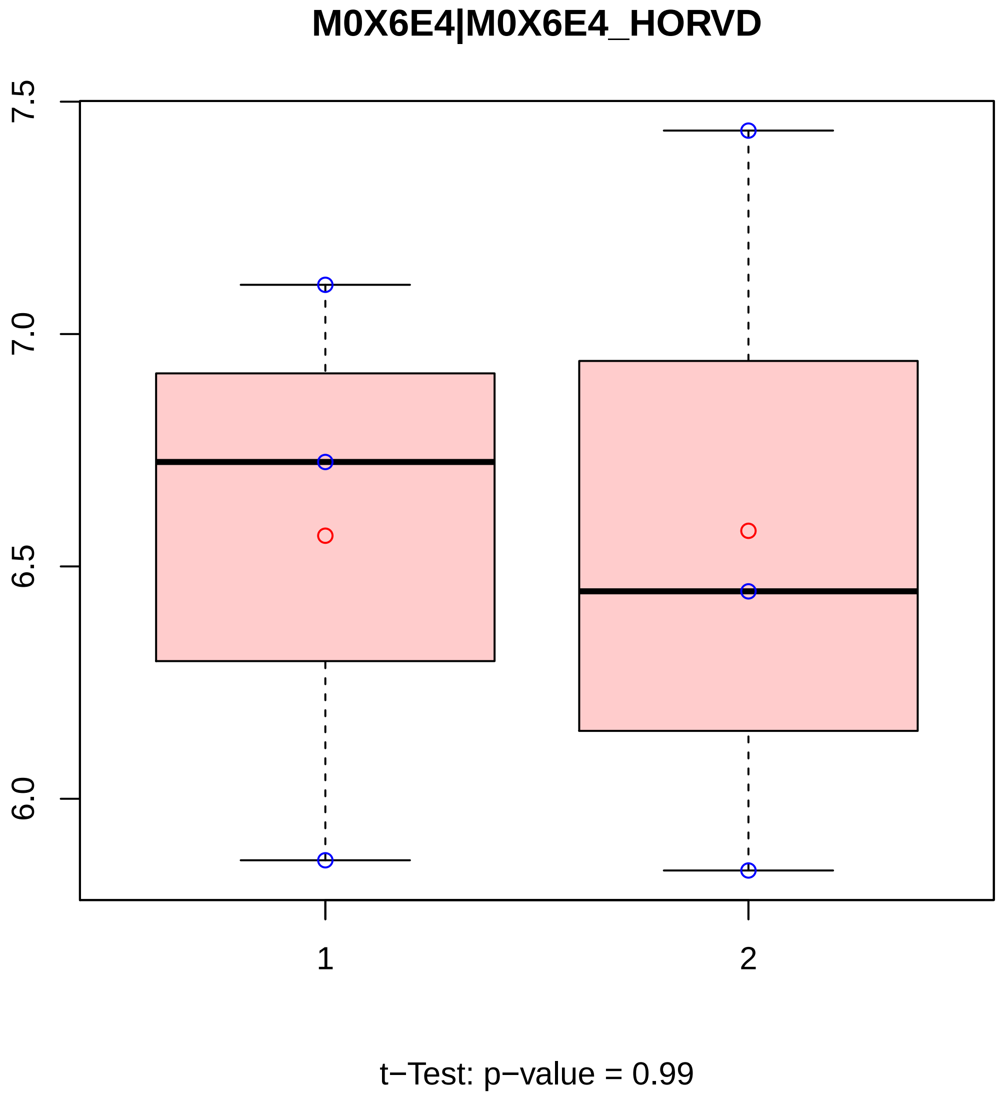
M0X6E4|M0X6E4_HORVD
7.5
7.0
6.5
6.0
1
2
t−Test: p−value = 0.99

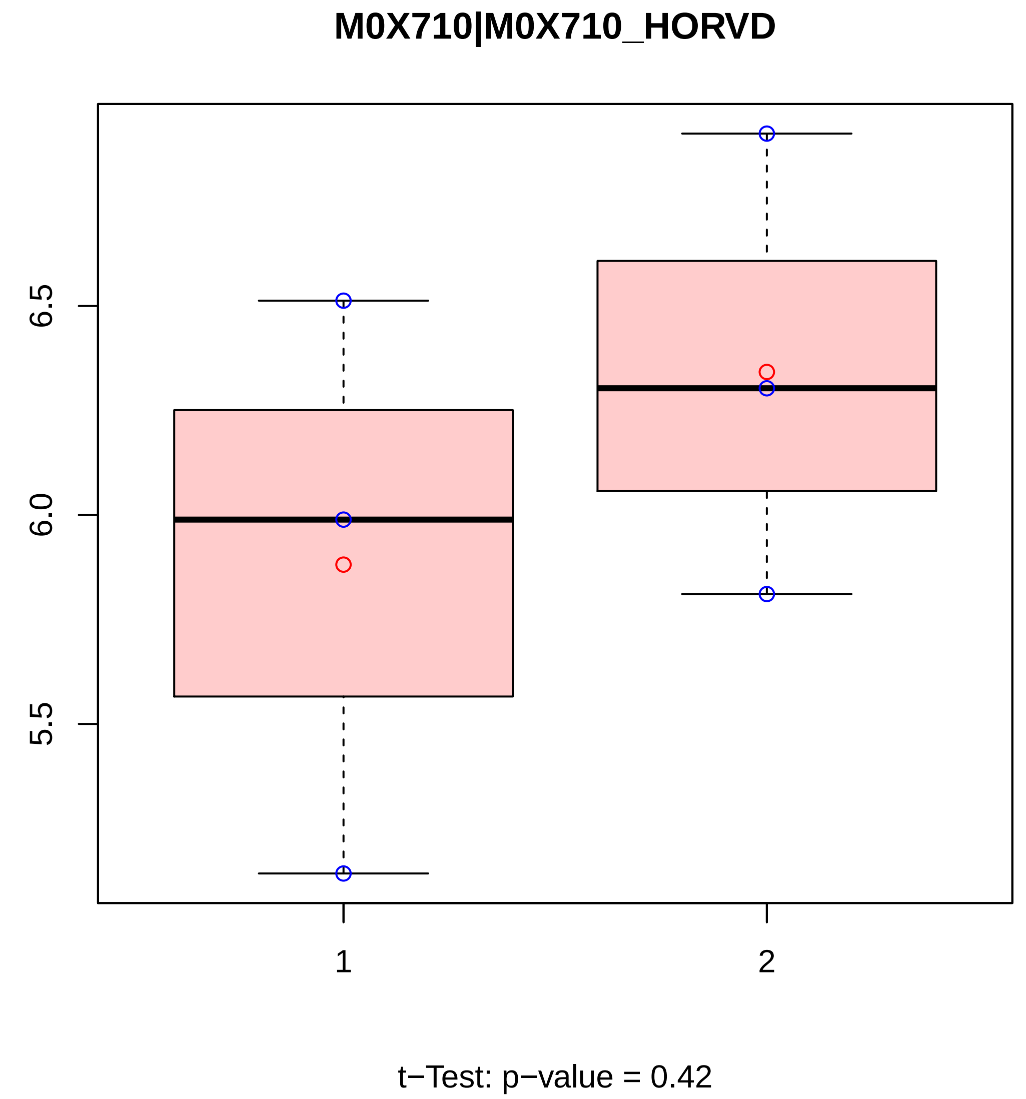
M0X710|M0X710_HORVD
6.5
6.0
5.5
1
2
t−Test: p−value = 0.42

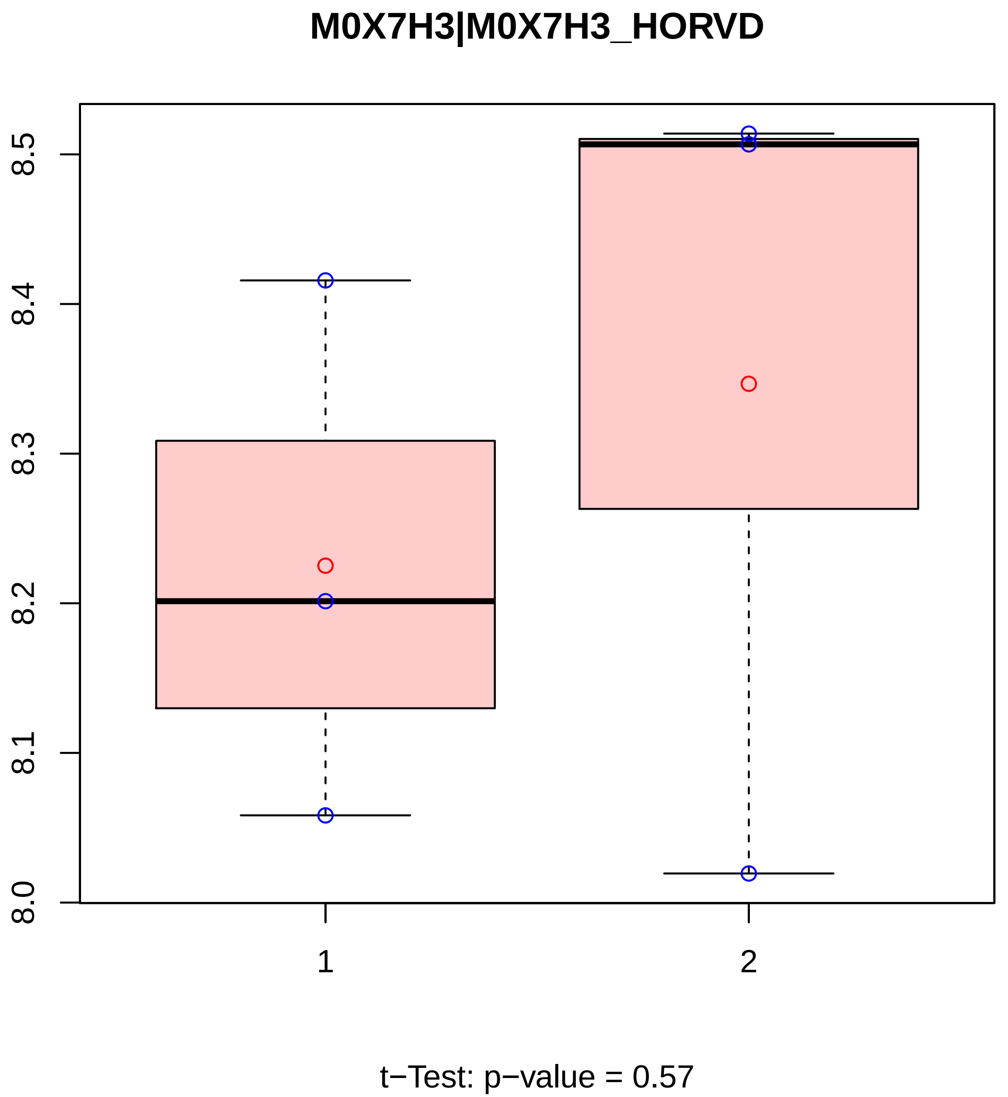

M0X7H3|M0X7H3_HORVD
8.0
8.1
8.2
8.3
8.4
8.5
1
2
t−Test: p−value = 0.57

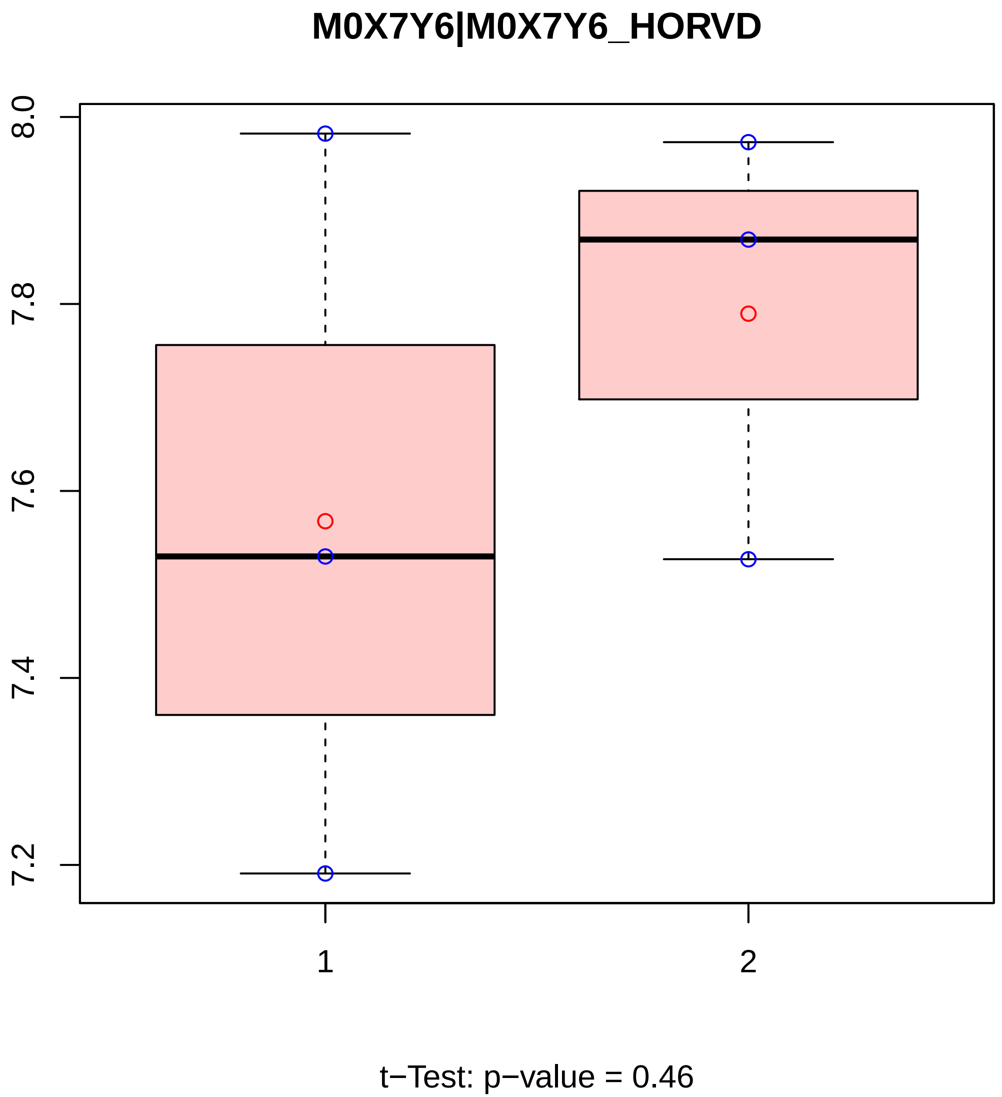
M0X7Y6|M0X7Y6_HORVD
8.0
7.8
7.6
7.4
7.2
1
2
t−Test: p−value = 0.46

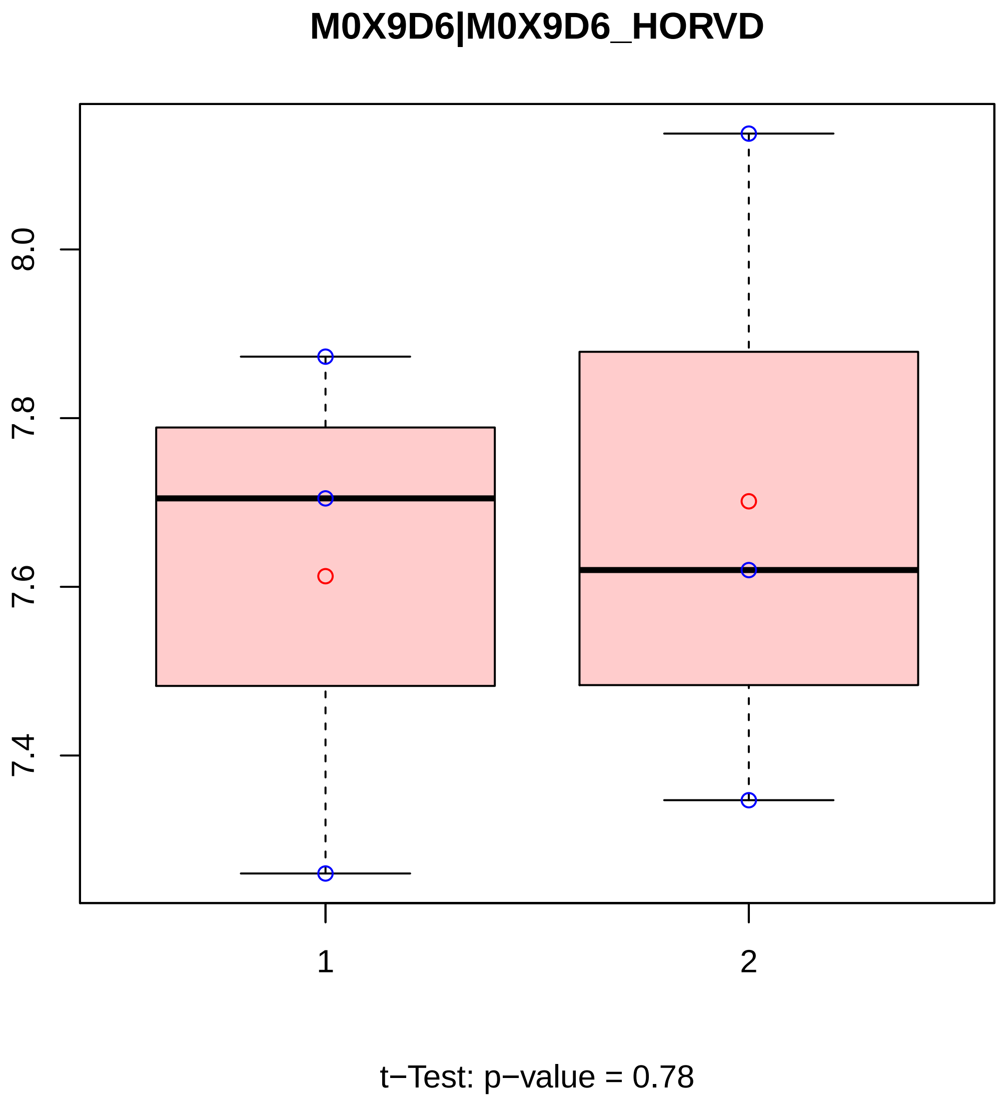
M0X9D6|M0X9D6_HORVD
8.0
7.8
7.6
7.4
1
2
t−Test: p−value = 0.78

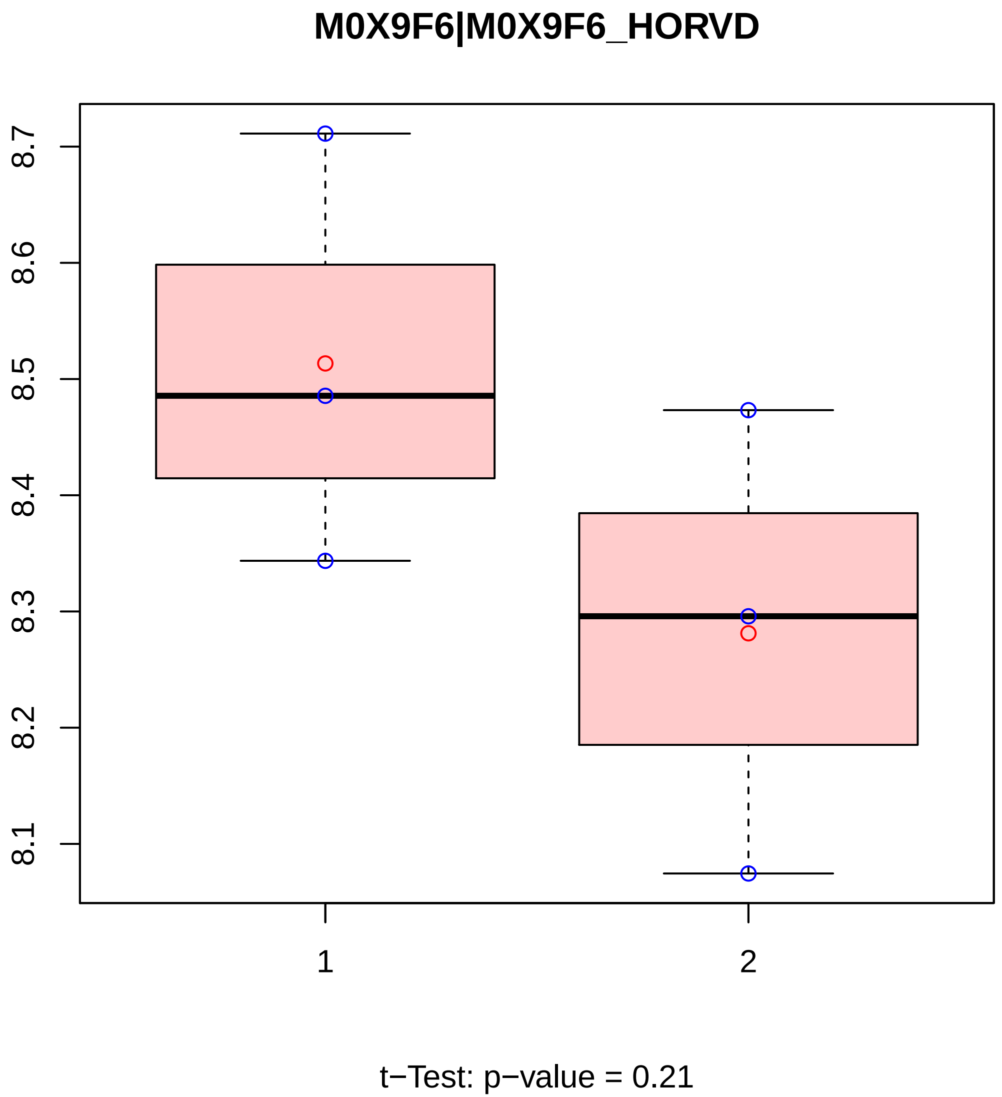
M0X9F6|M0X9F6_HORVD
8.1
8.2
8.3
8.4
8.5
8.6
8.7
1
2
t−Test: p−value = 0.21

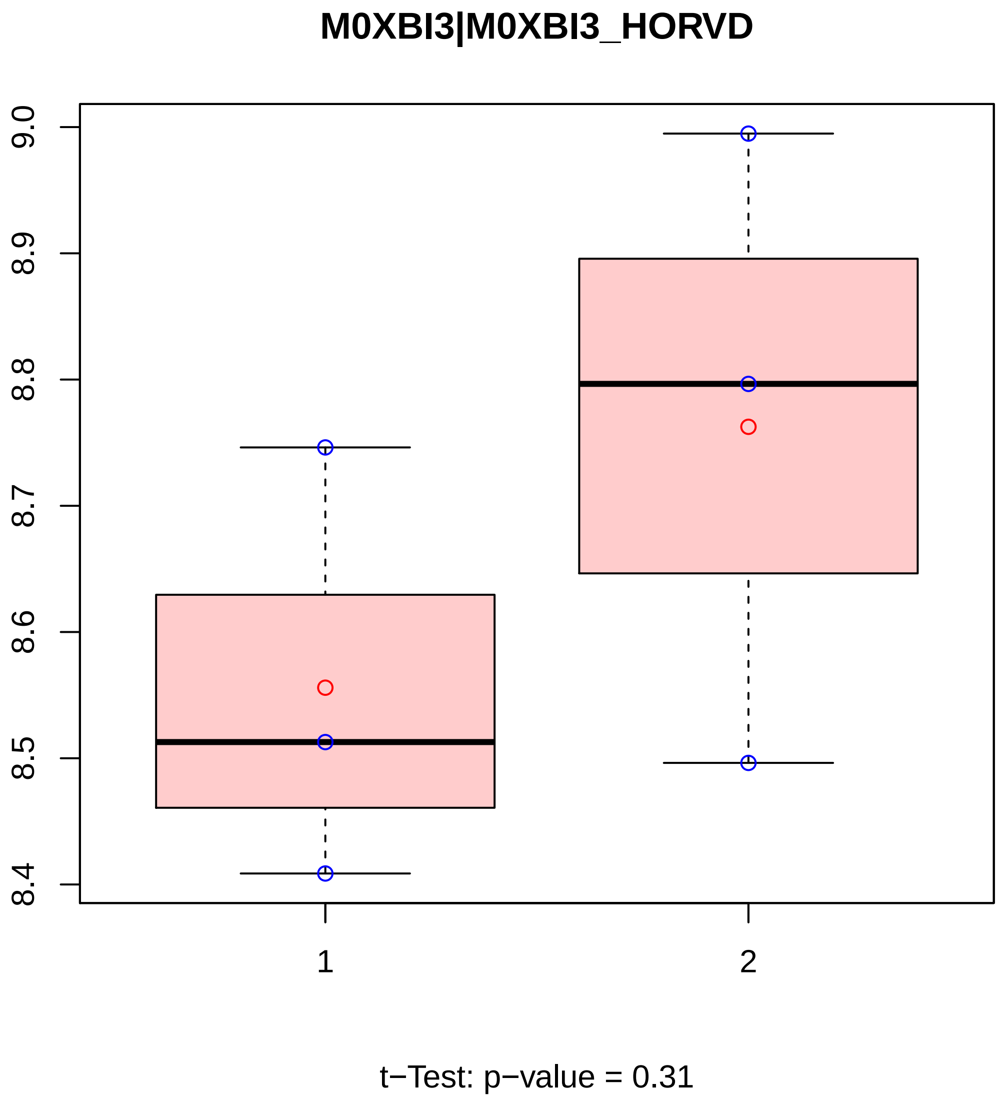
M0XBI3|M0XBI3_HORVD
9.0
8.9
8.8
8.7
8.6
8.5
8.4
1
2
t−Test: p−value = 0.31

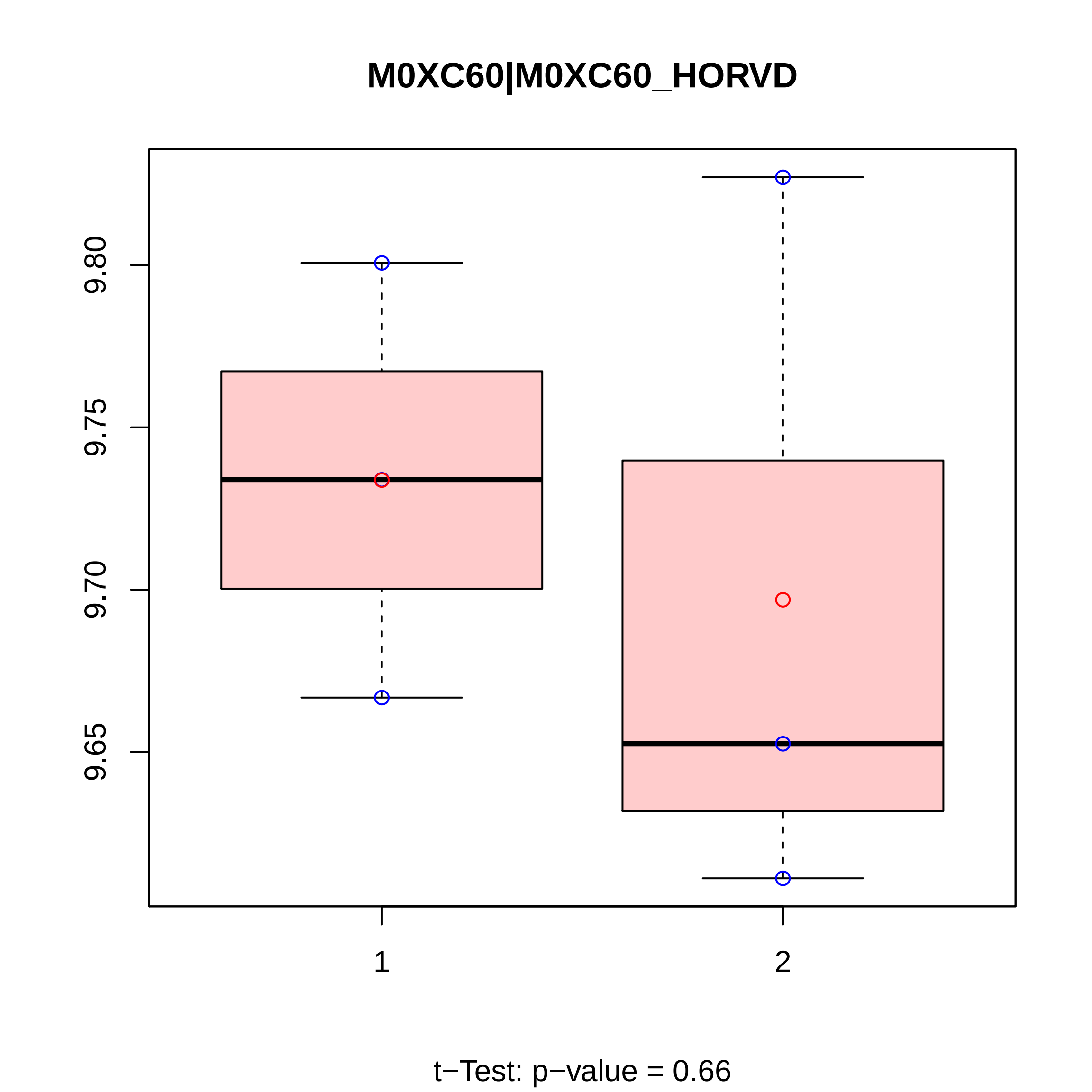
M0XC60|M0XC60_HORVD
9.65
9.70
9.75
9.80
1
2
t−Test: p−value = 0.66

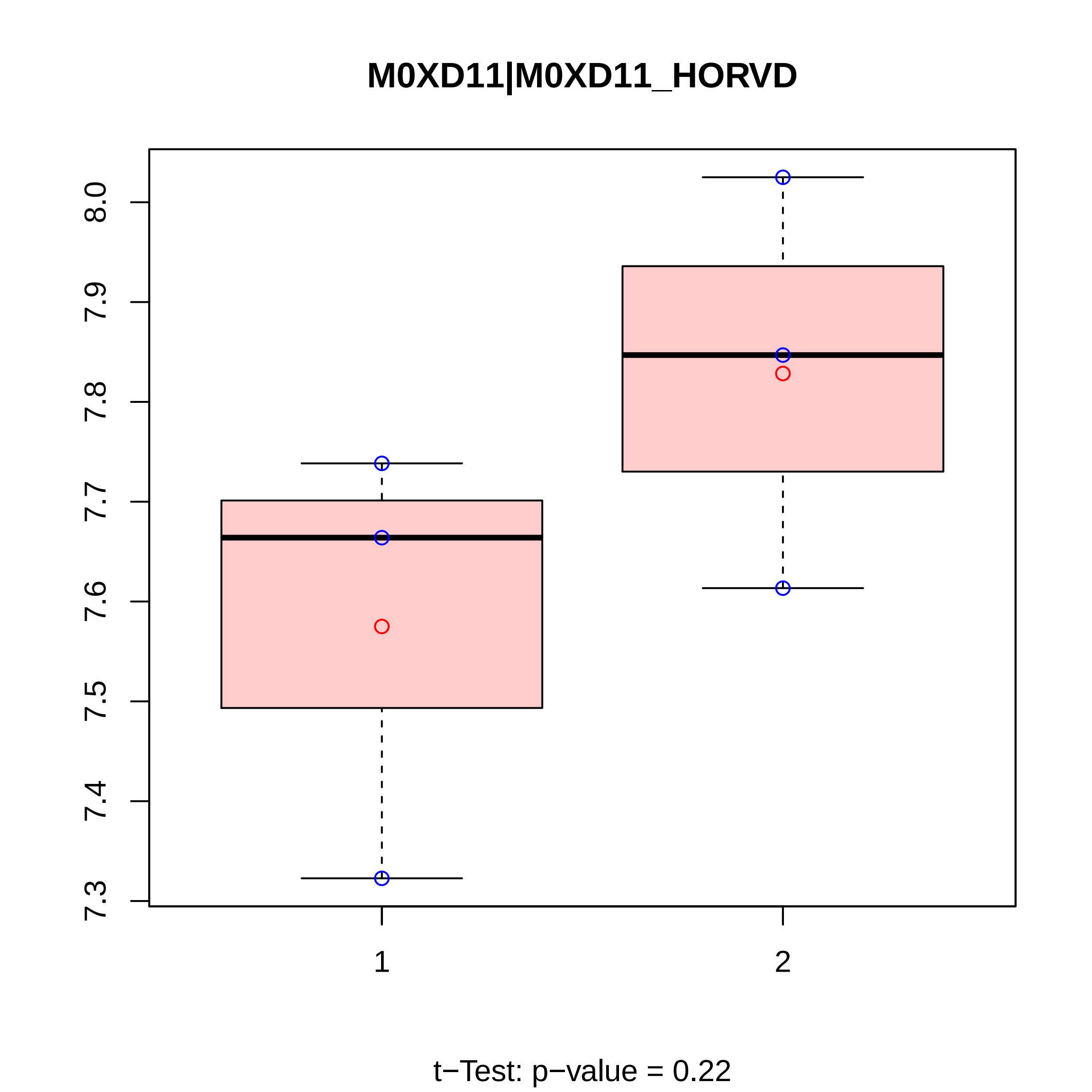
M0XD11|M0XD11_HORVD
8.0
7.9
7.8
7.7
7.6
7.5
7.4
7.3
1
2
t−Test: p−value = 0.22

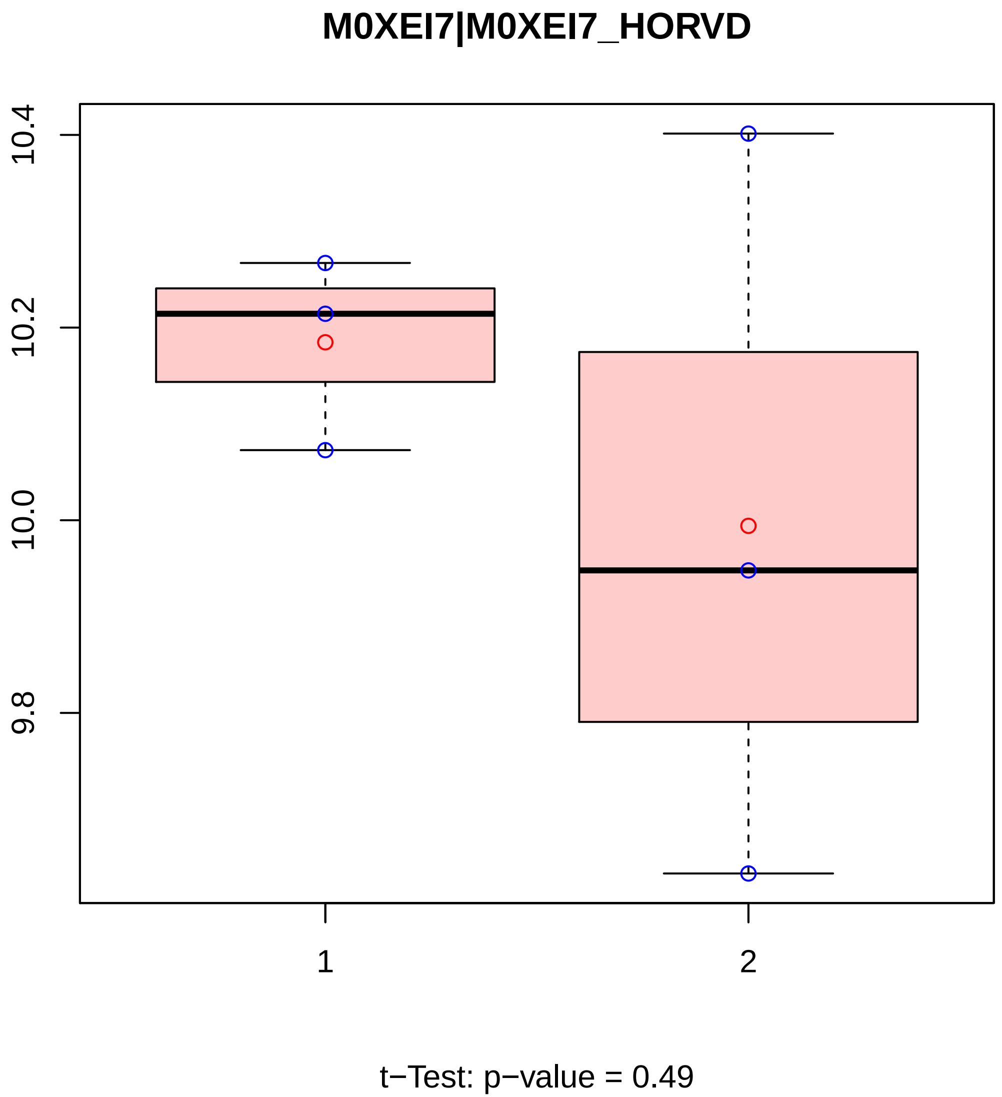
M0XEI7|M0XEI7_HORVD
10.4
10.2
10.0
9.8
1
2
t−Test: p−value = 0.49

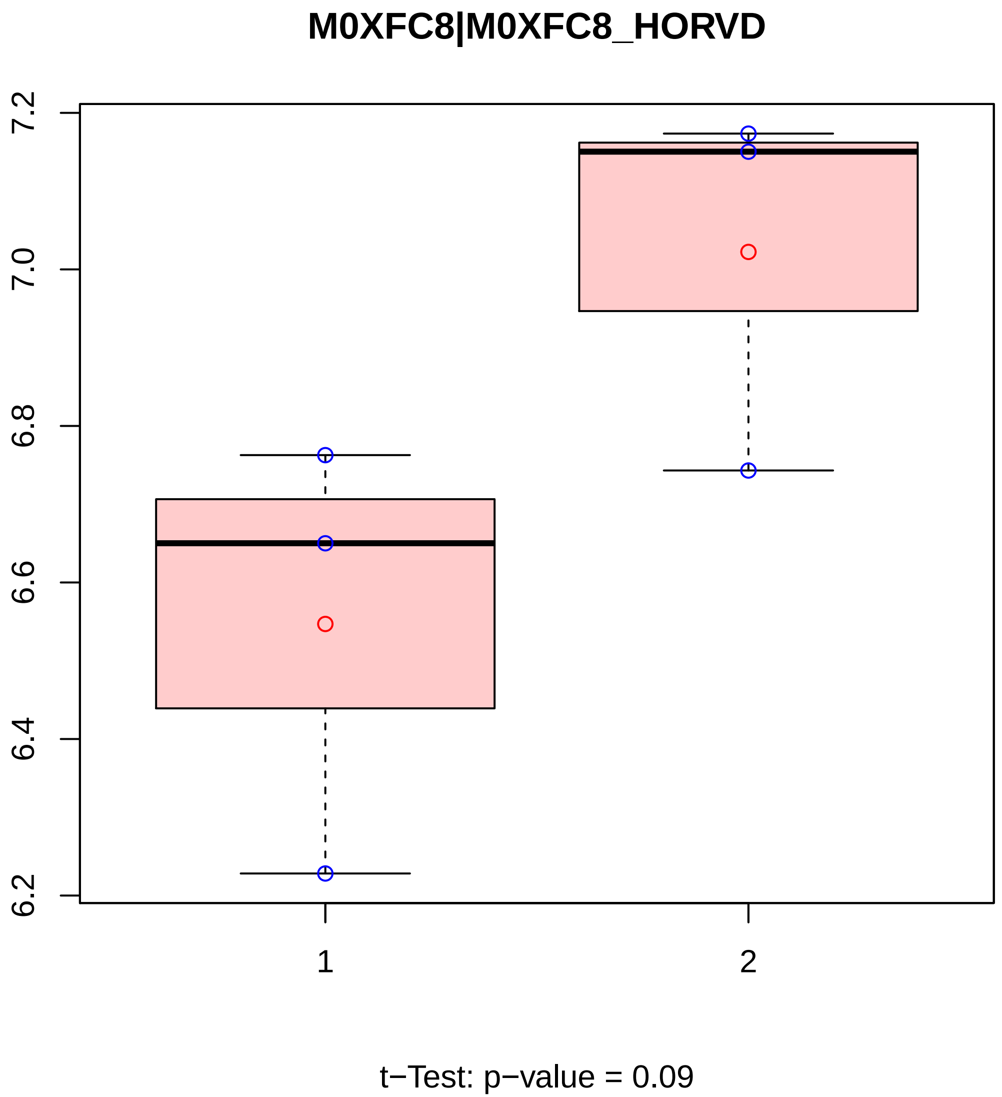
M0XFC8|M0XFC8_HORVD
7.2
7.0
6.8
6.6
6.4
6.2
1
2
t−Test: p−value = 0.09

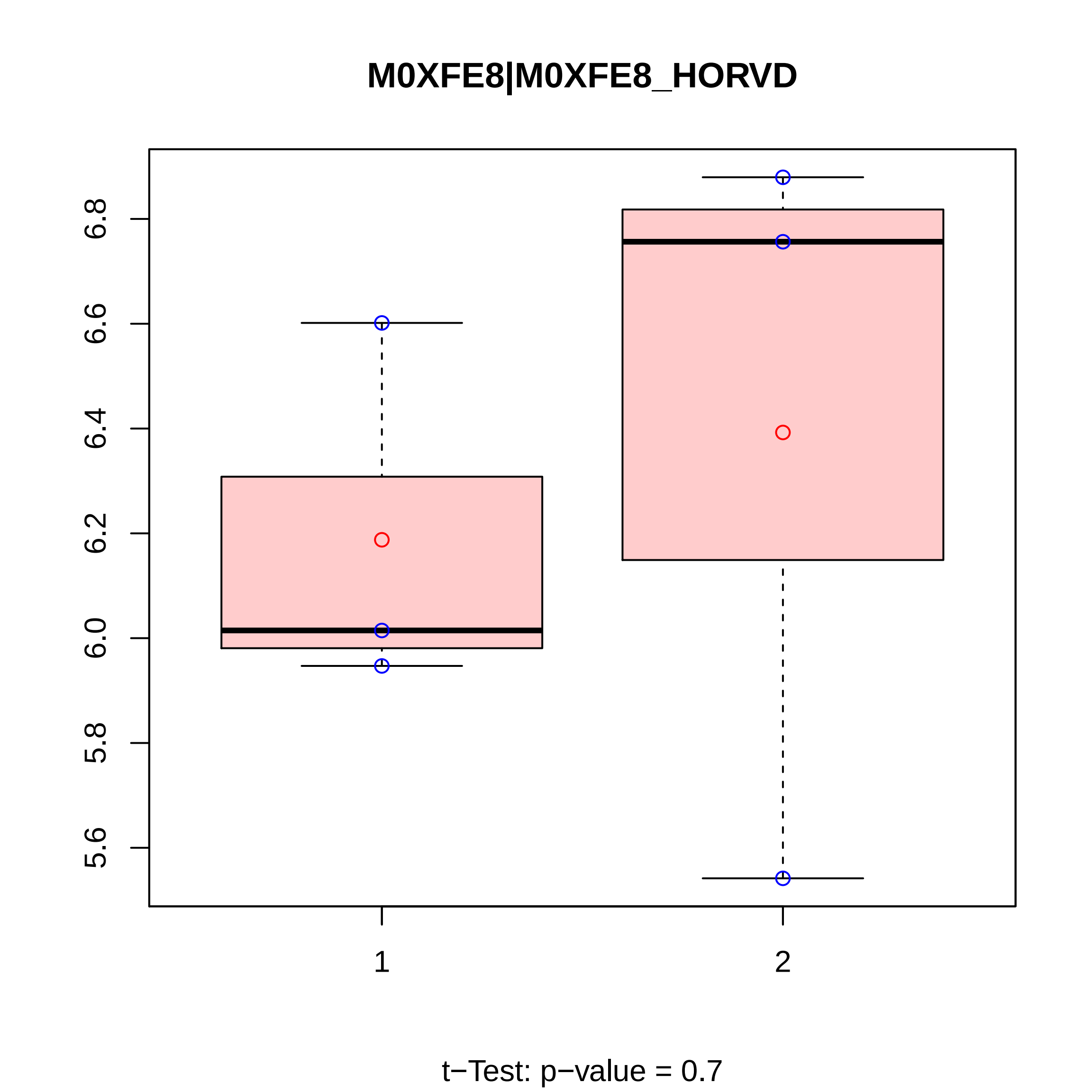
M0XFE8|M0XFE8_HORVD
6.8
6.6
6.4
6.2
6.0
5.8
5.6
1
2
t−Test: p−value = 0.7

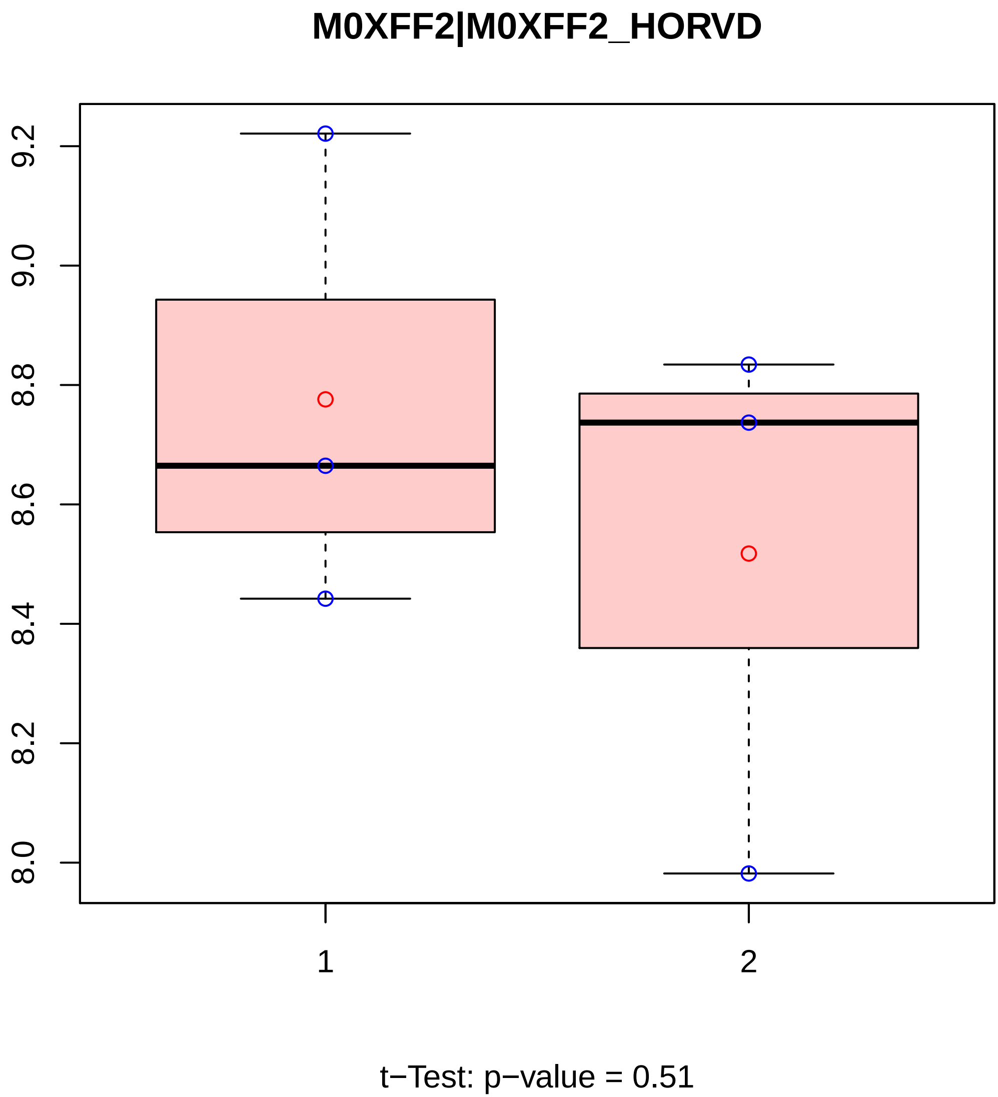
M0XFF2|M0XFF2_HORVD
9.2
9.0
8.8
8.6
8.4
8.2
8.0
1
2
t−Test: p−value = 0.51

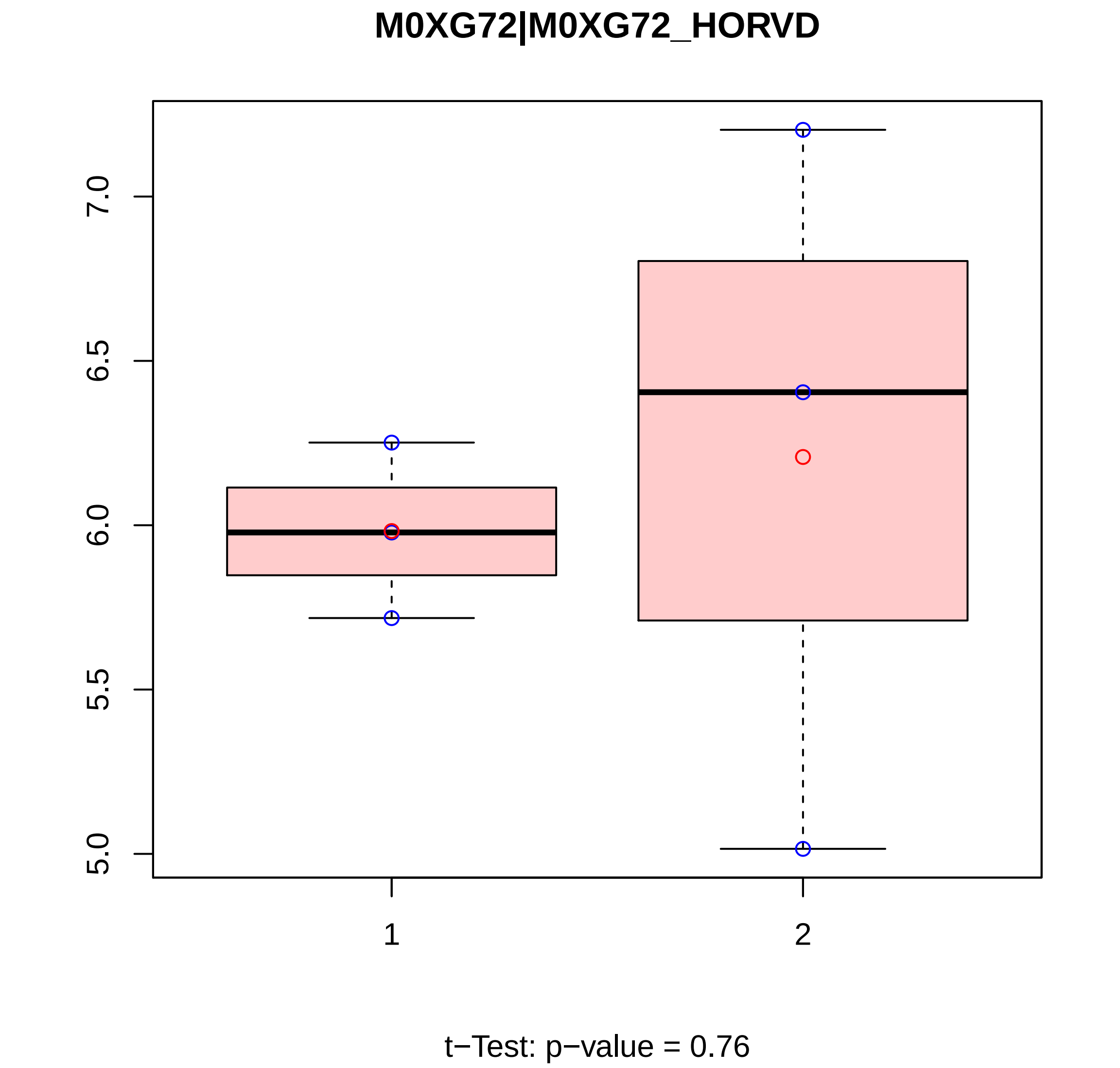
M0XG72|M0XG72_HORVD
7.0
6.5
6.0
5.5
5.0
1
2
t−Test: p−value = 0.76

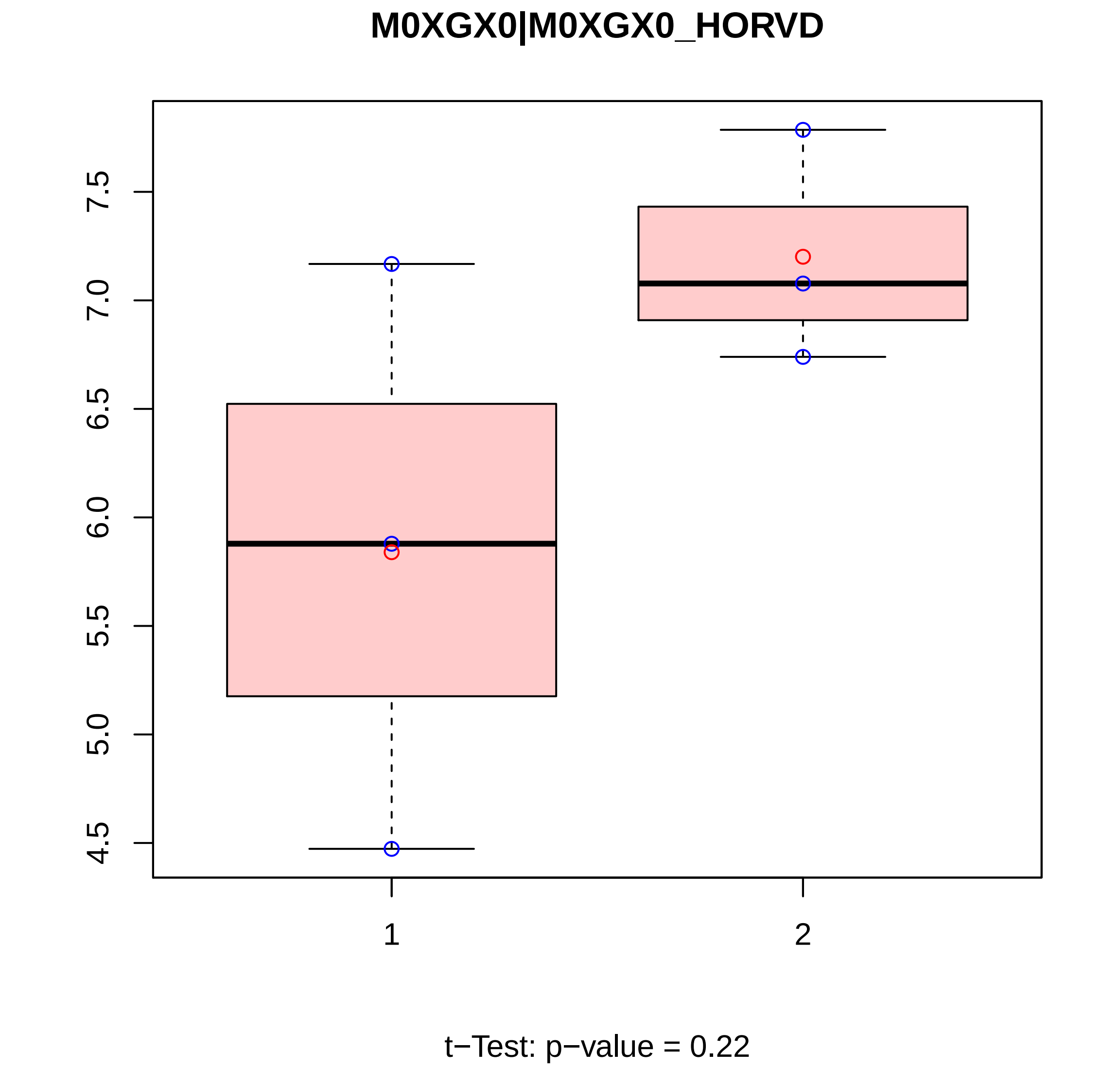

M0XGX0|M0XGX0_HORVD
7.5
7.0
6.5
6.0
5.5
5.0
4.5
1
2
t−Test: p−value = 0.22

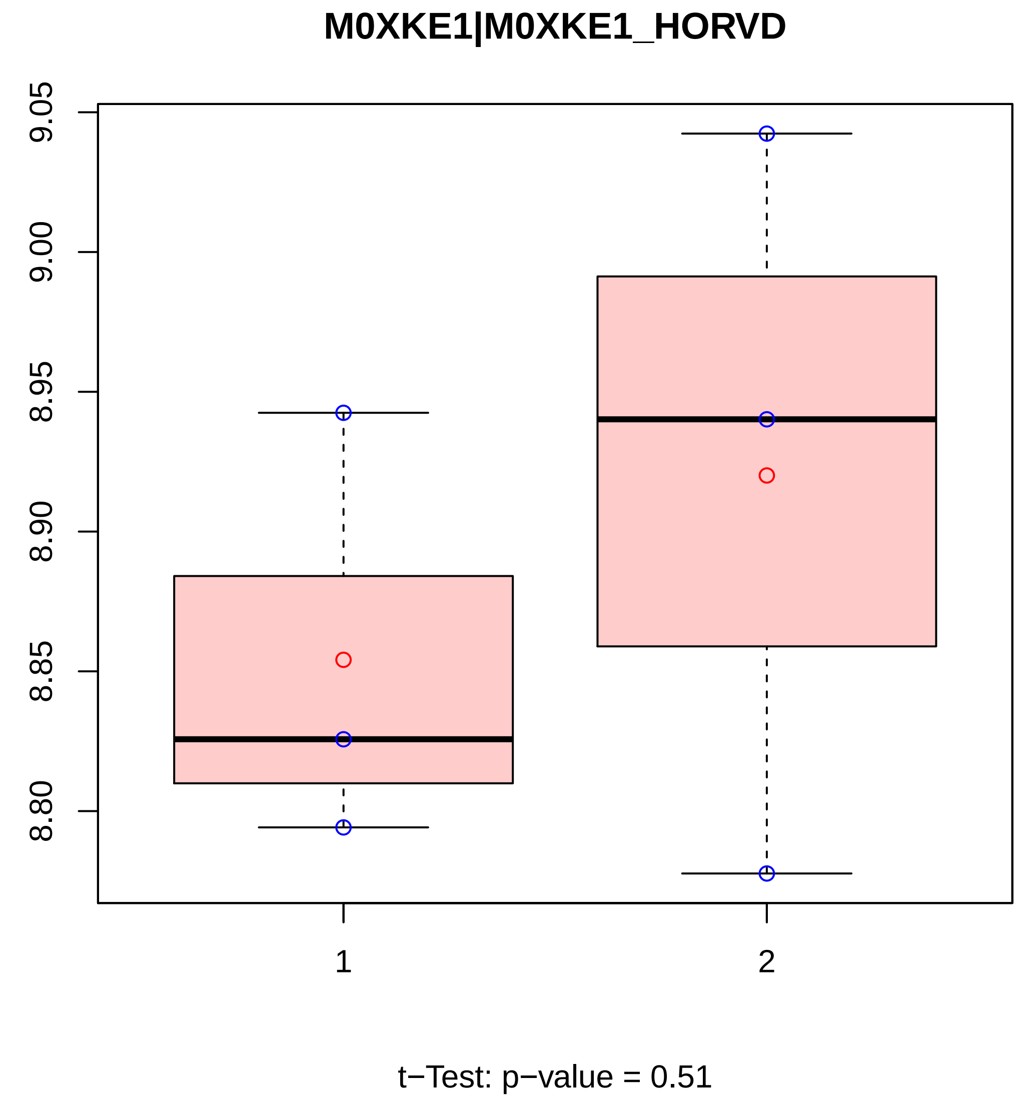
M0XKE1|M0XKE1_HORVD
9.05
9.00
8.95
8.90
8.85
8.80
1
2
t−Test: p−value = 0.51

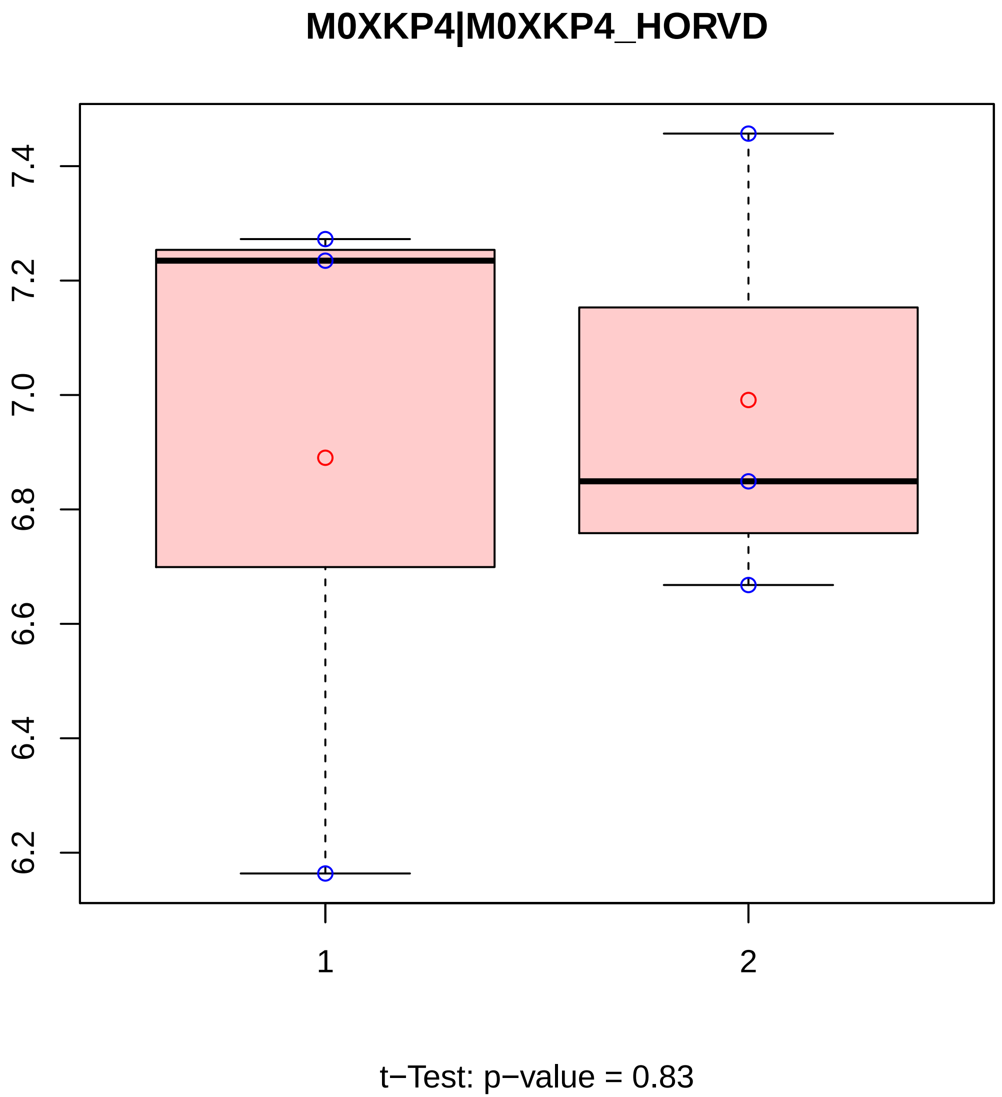
M0XKP4|M0XKP4_HORVD
7.4
7.2
7.0
6.8
6.6
6.4
6.2
1
2
t−Test: p−value = 0.83

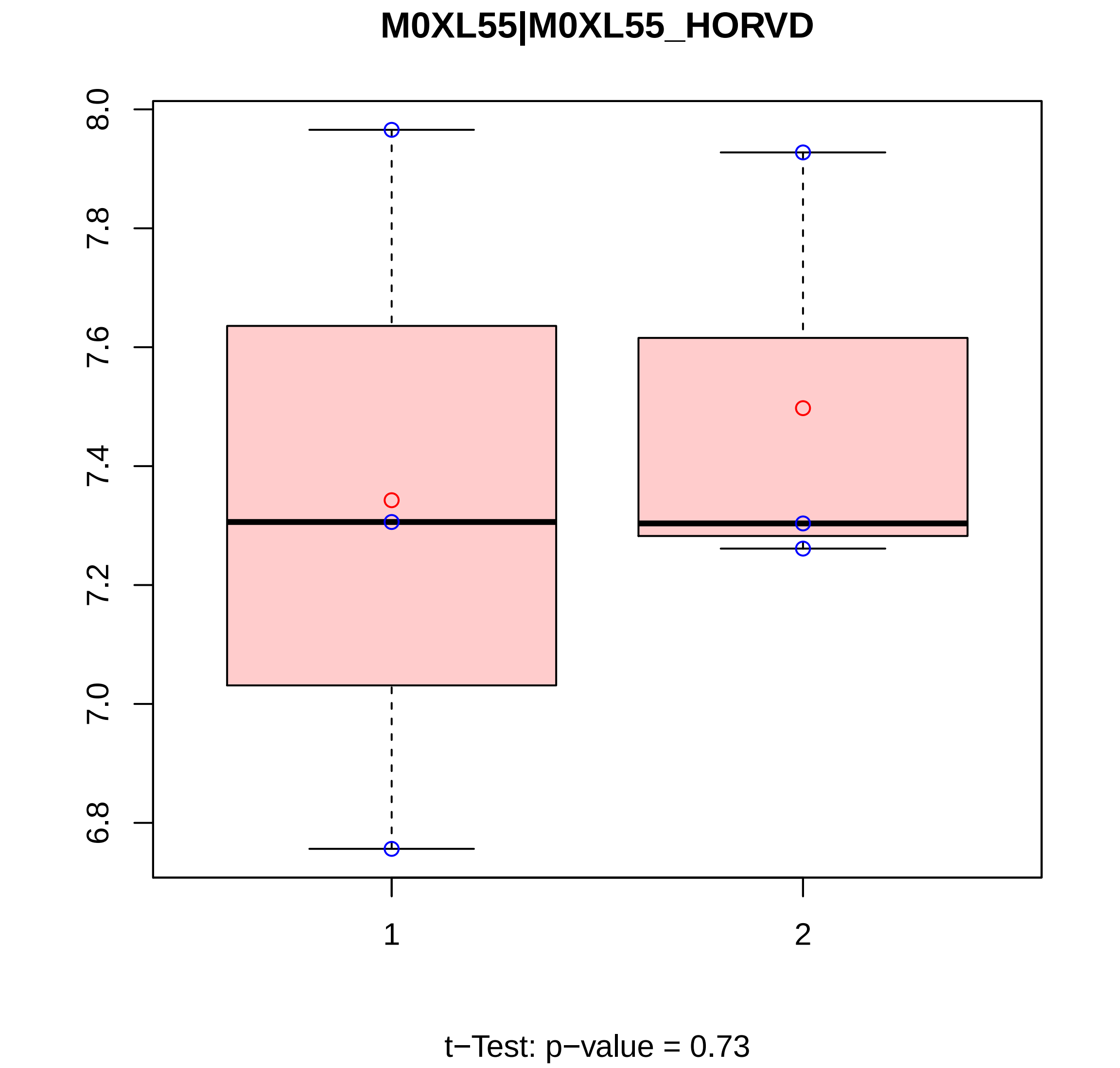
M0XL55|M0XL55_HORVD
8.0
7.8
7.6
7.4
7.2
7.0
6.8
1
2
t−Test: p−value = 0.73

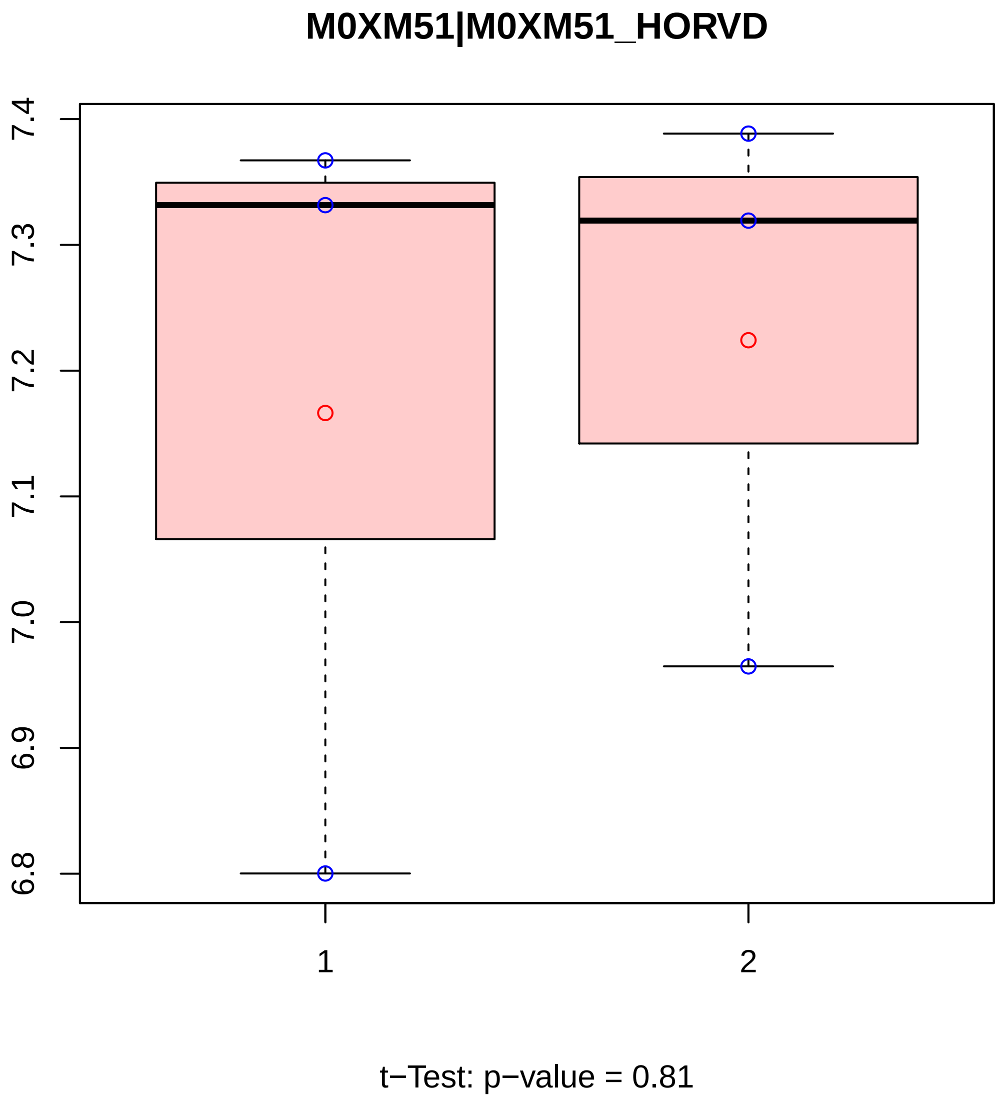
M0XM51|M0XM51_HORVD
7.4
7.3
7.2
7.1
7.0
6.9
6.8
1
2
t−Test: p−value = 0.81

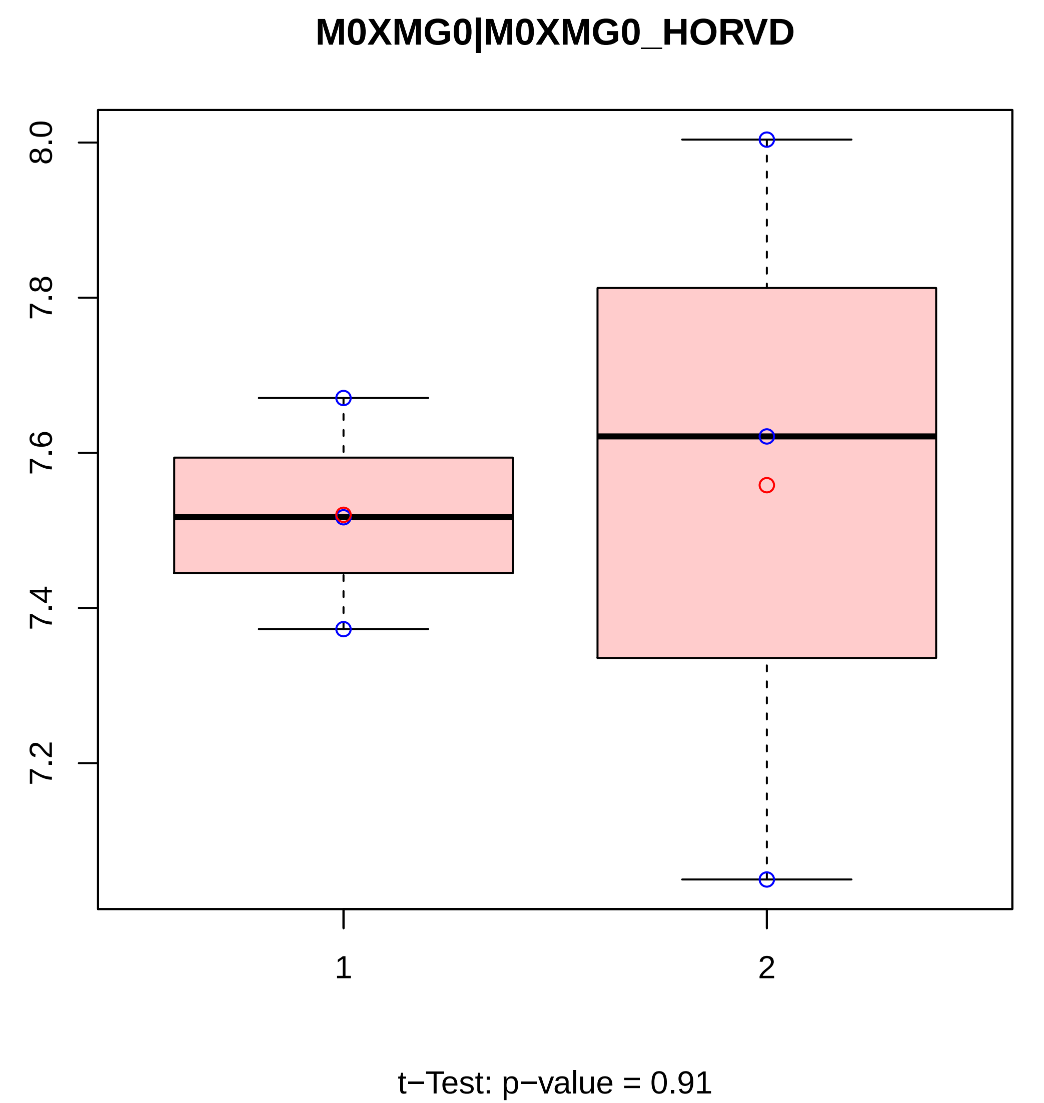

M0XMG0|M0XMG0_HORVD
8.0
7.8
7.6
7.4
7.2
1
2
t−Test: p−value = 0.91

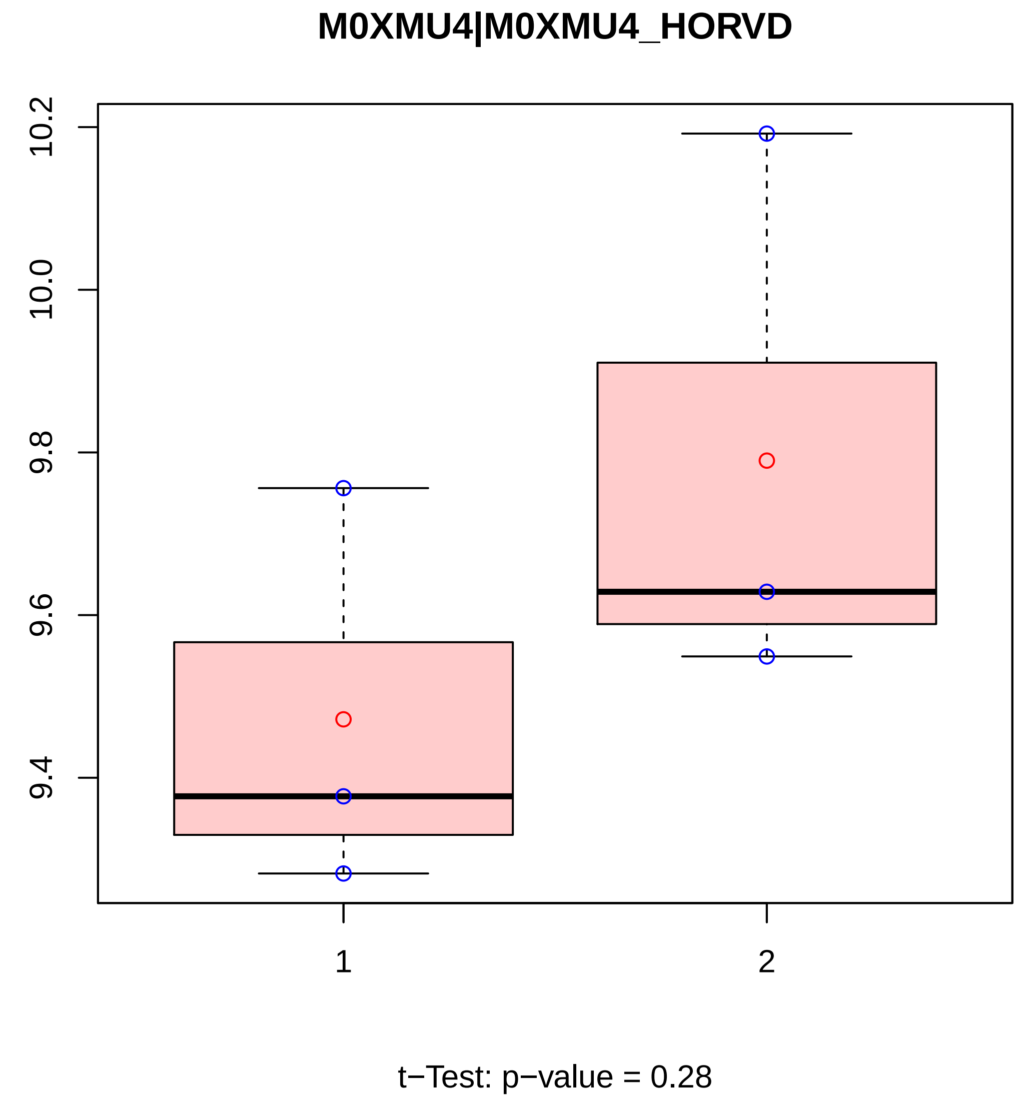
M0XMU4|M0XMU4_HORVD
10.2
10.0
9.8
9.6
9.4
1
2
t−Test: p−value = 0.28

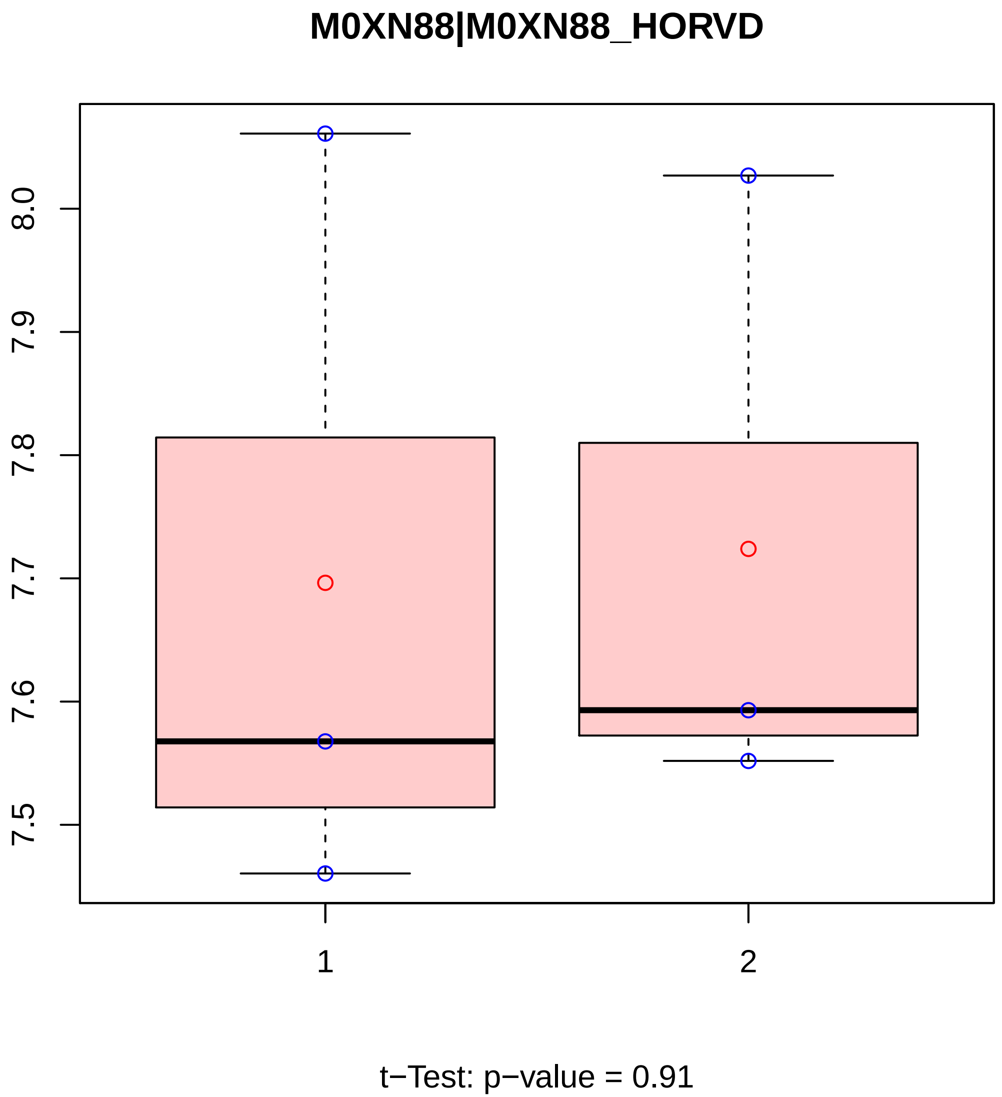
M0XN88|M0XN88_HORVD
8.0
7.9
7.8
7.7
7.6
7.5
1
2
t−Test: p−value = 0.91

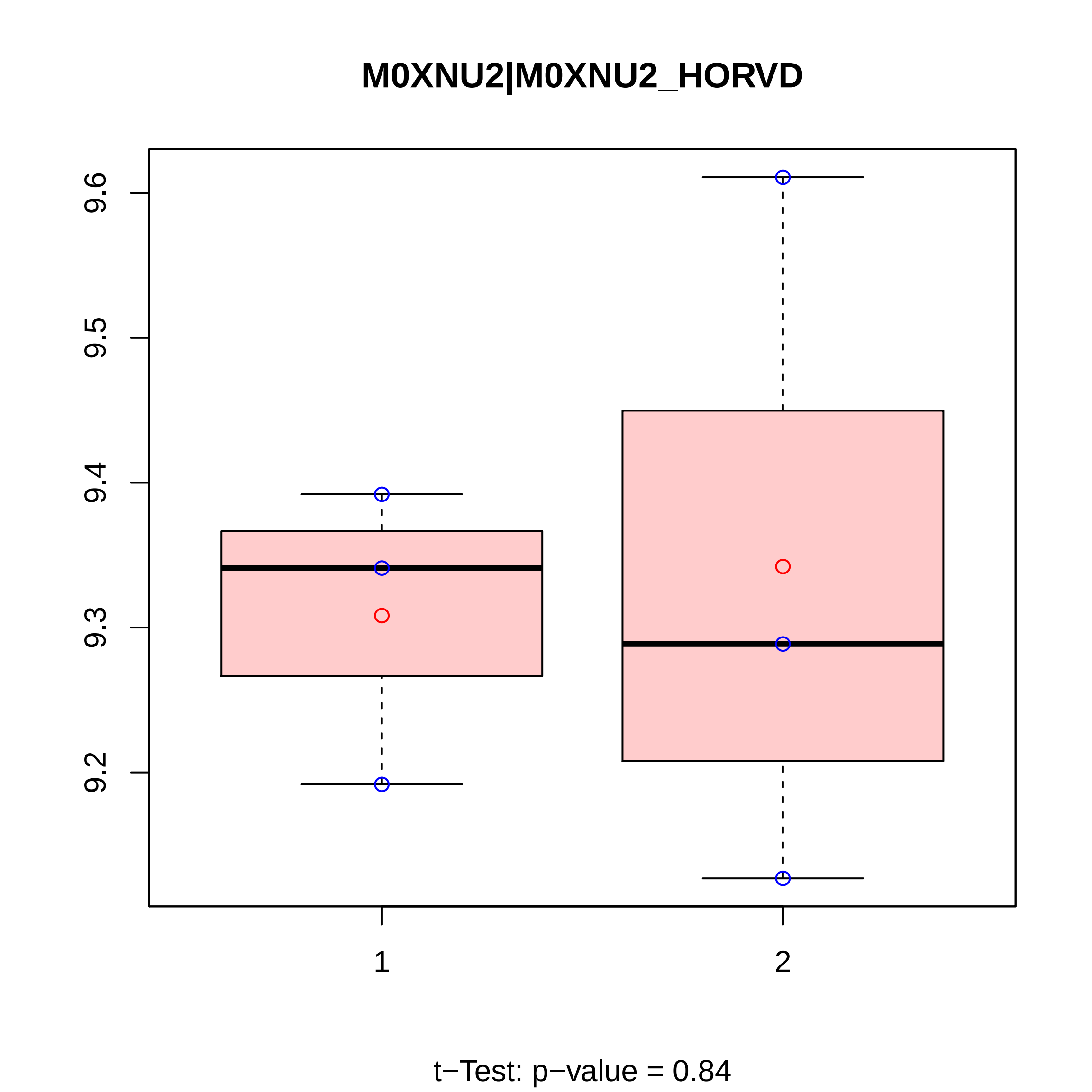
M0XNU2|M0XNU2_HORVD
9.6
9.5
9.4
9.3
9.2
1
2
t−Test: p−value = 0.84

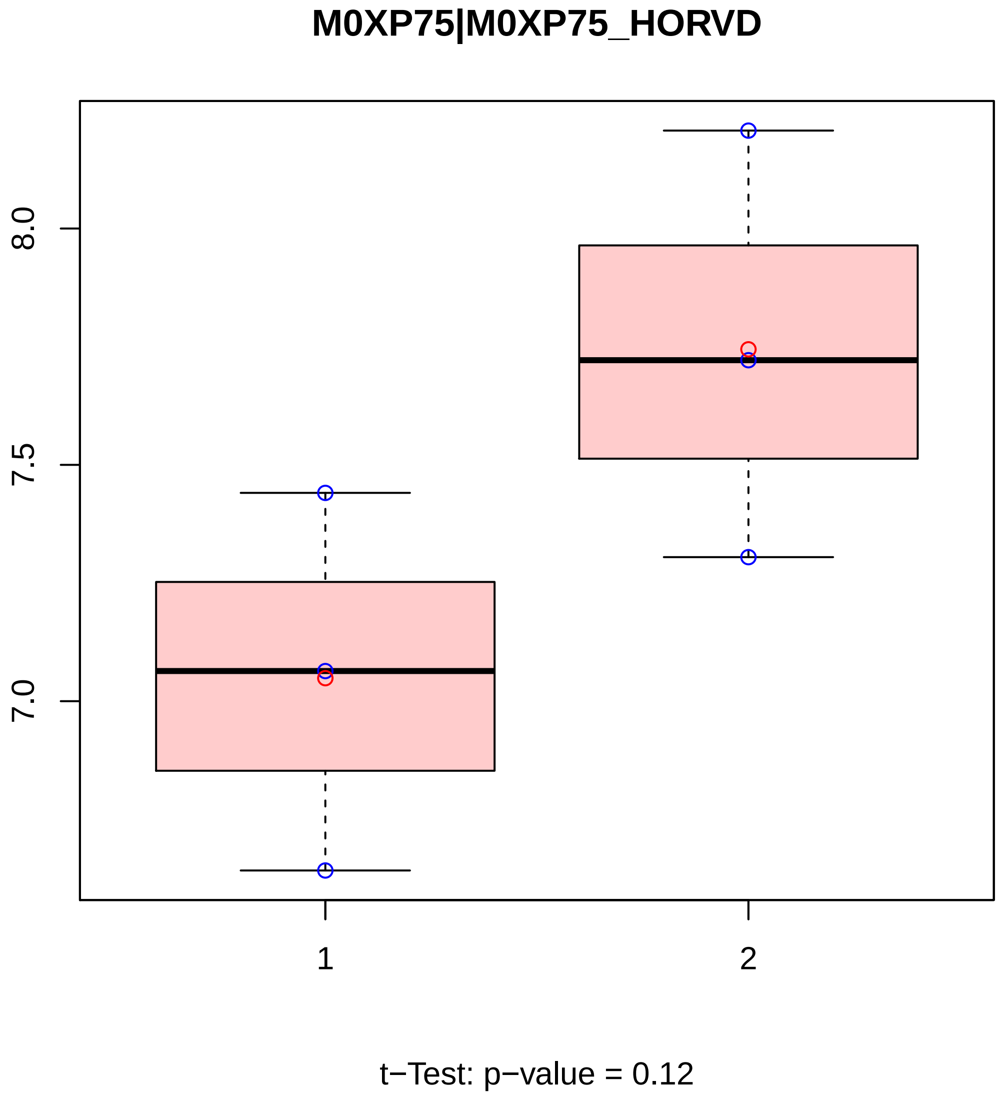

M0XP75|M0XP75_HORVD
8.0
7.5
7.0
1
2
t−Test: p−value = 0.12

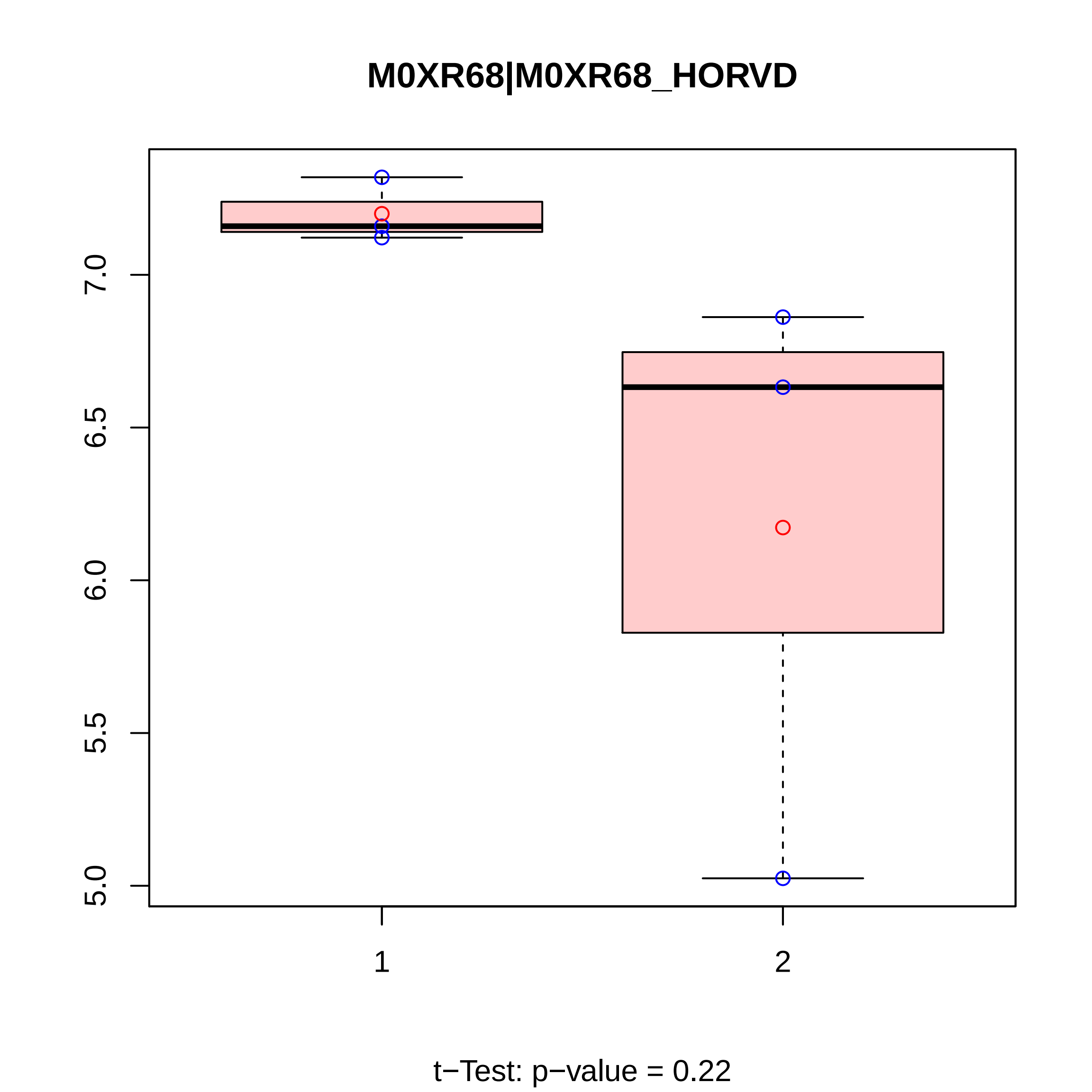
M0XR68|M0XR68_HORVD
5.0
5.5
6.0
6.5
7.0
1
2
t−Test: p−value = 0.22

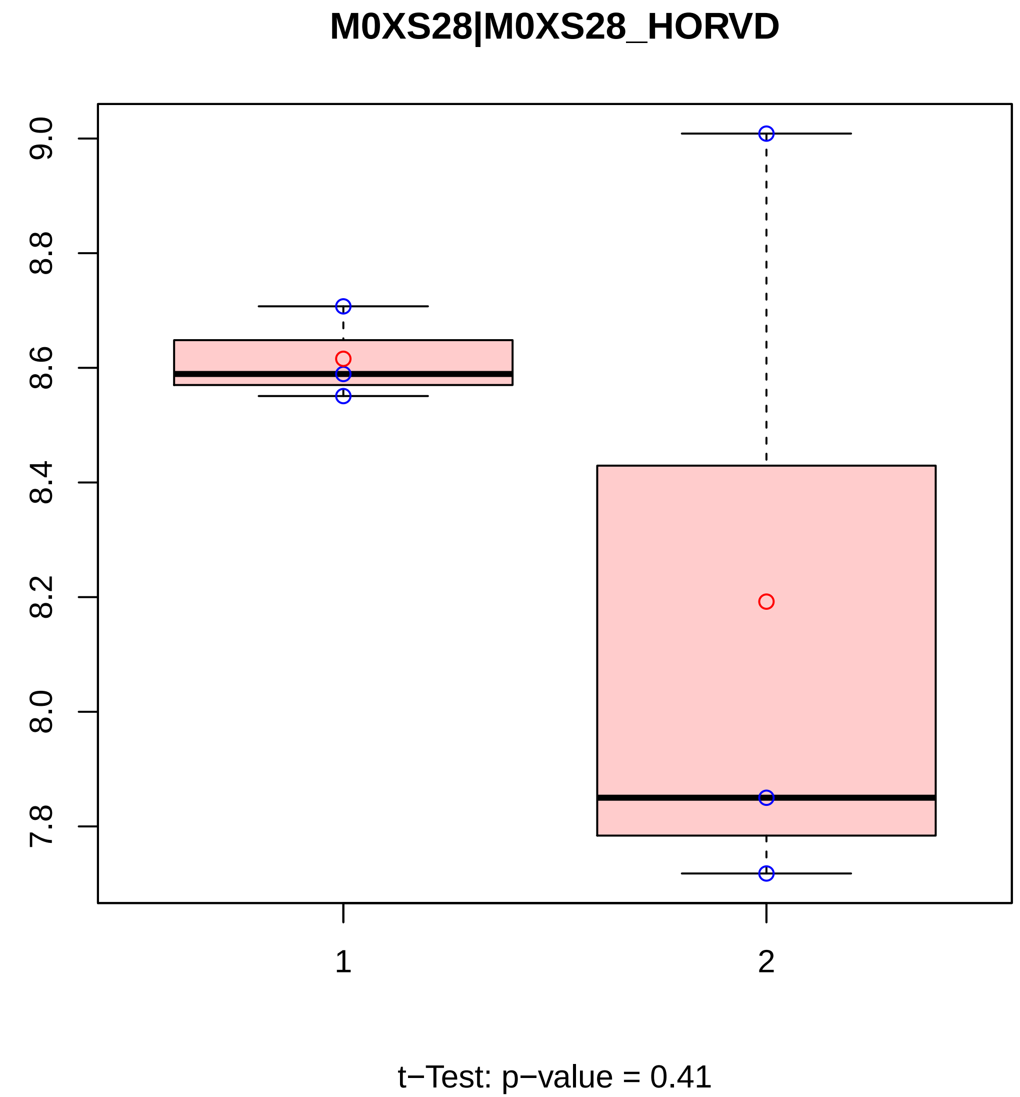
M0XS28|M0XS28_HORVD
9.0
8.8
8.6
8.4
8.2
8.0
7.8
1
2
t−Test: p−value = 0.41

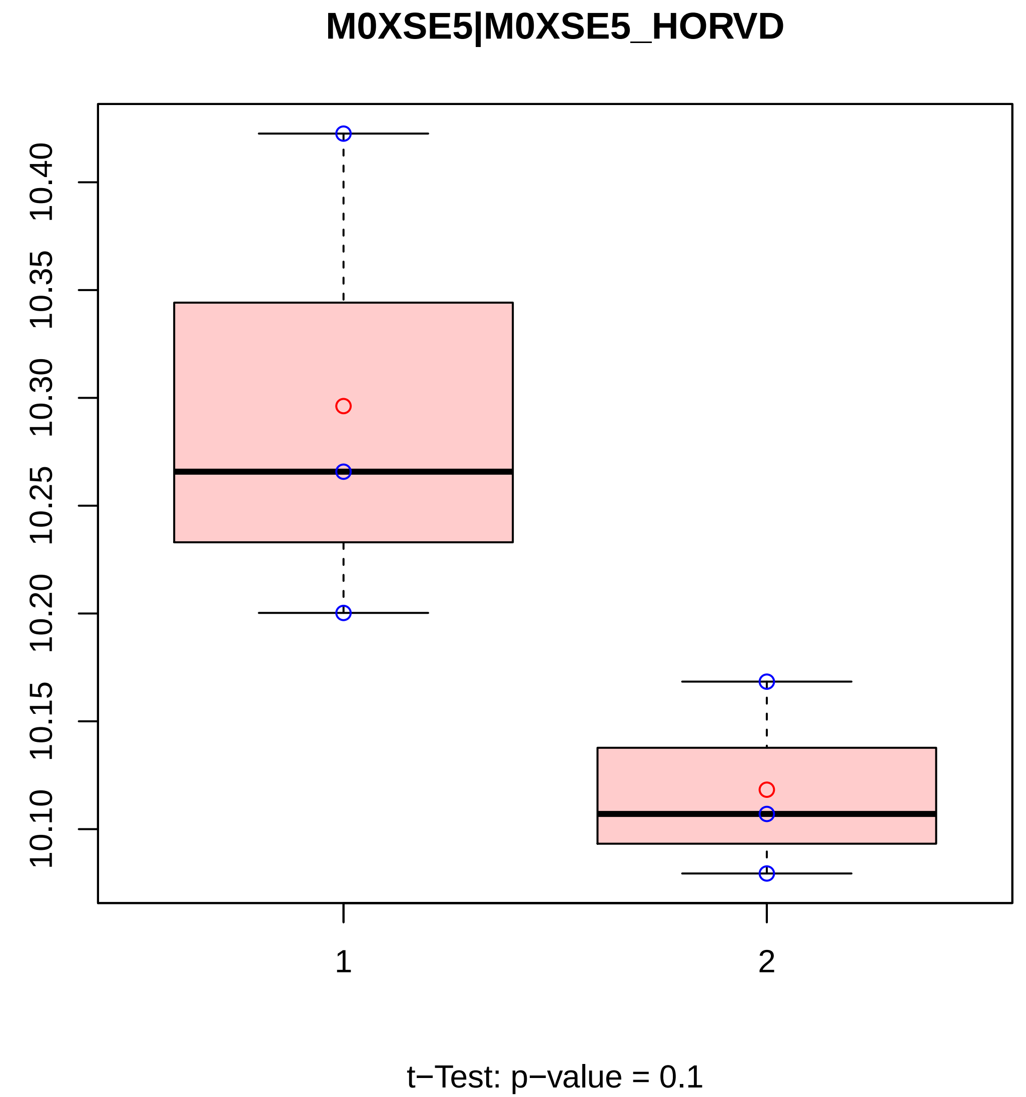
M0XSE5|M0XSE5_HORVD
10.10
10.15
10.20
10.25
10.30
10.35
10.40
1
2
t−Test: p−value = 0.1

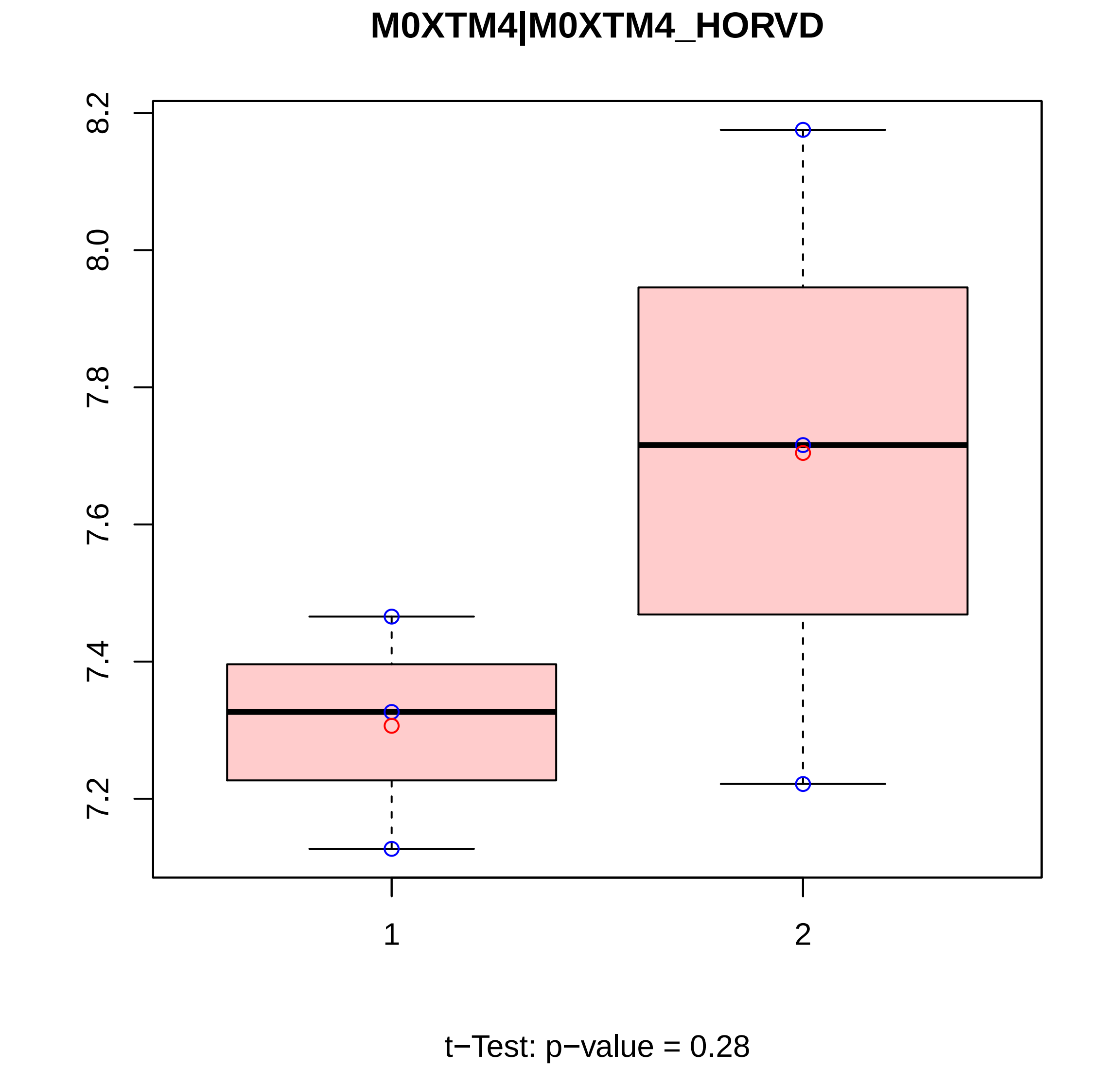
M0XTM4|M0XTM4_HORVD
8.2
8.0
7.8
7.6
7.4
7.2
1
2
t−Test: p−value = 0.28

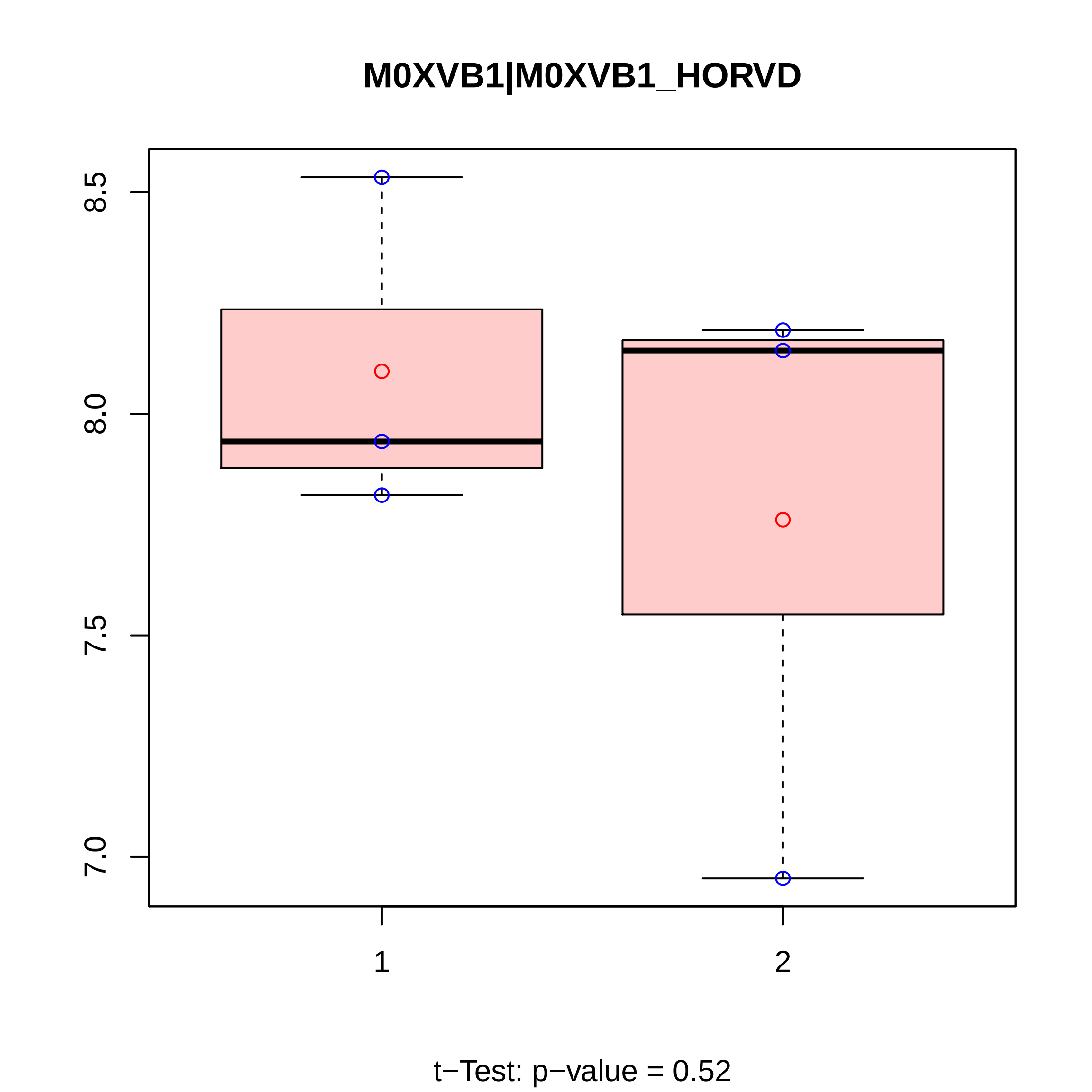
M0XVB1|M0XVB1_HORVD
8.5
8.0
7.5
7.0
1
2
t−Test: p−value = 0.52

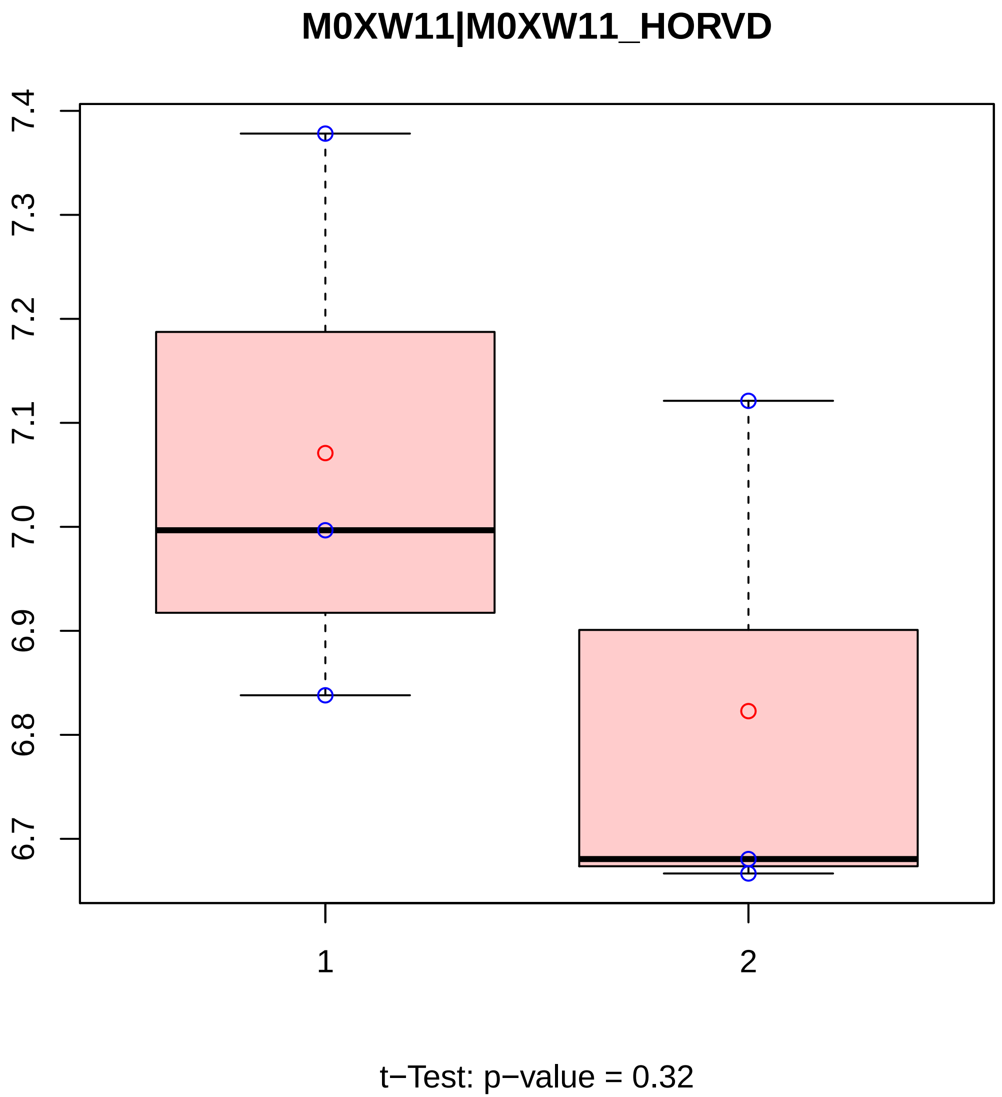
M0XW11|M0XW11_HORVD
7.4
7.3
7.2
7.1
7.0
6.9
6.8
6.7
1
2
t−Test: p−value = 0.32

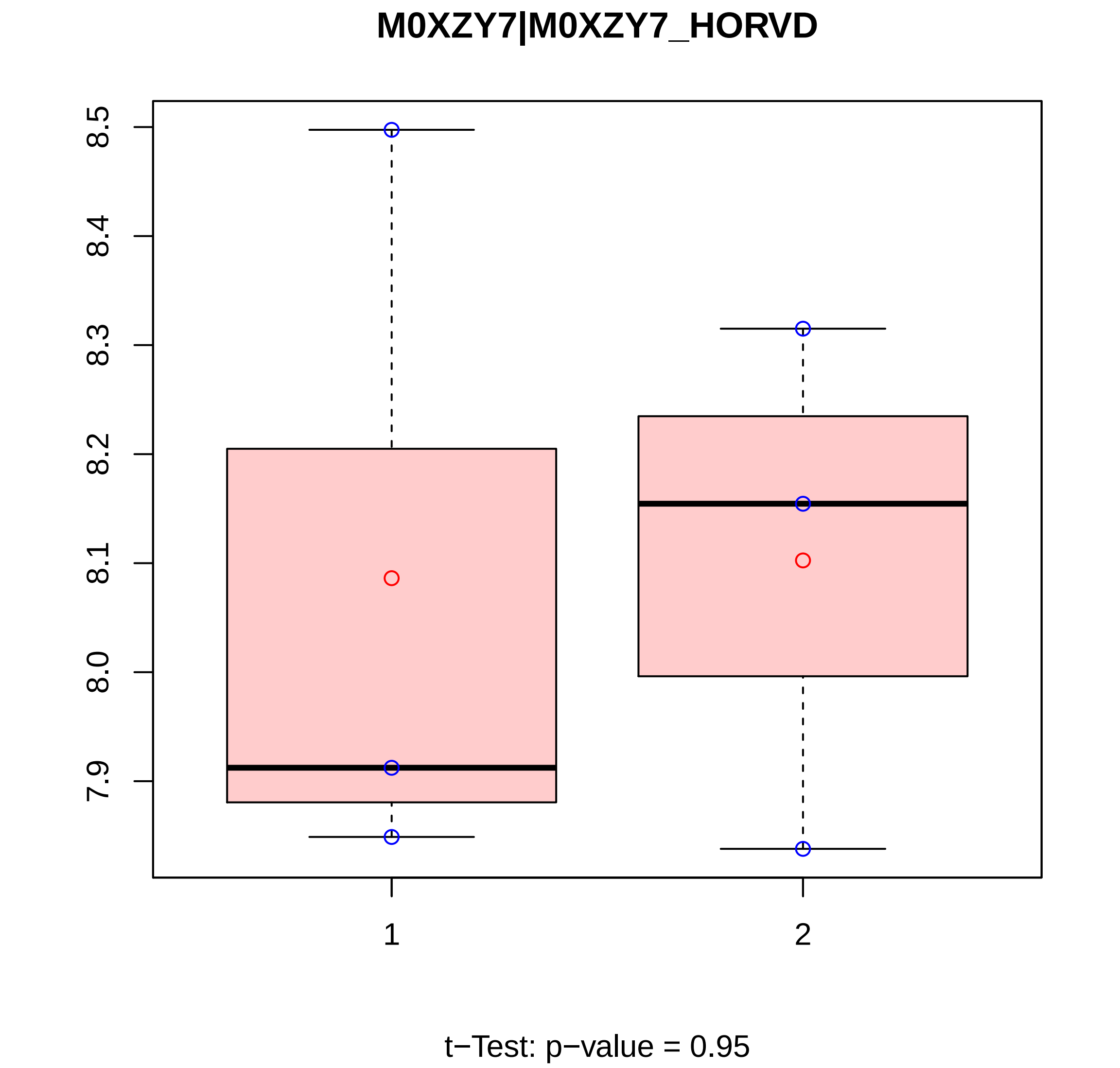
M0XZY7|M0XZY7_HORVD
8.5
8.4
8.3
8.2
8.1
8.0
7.9
1
2
t−Test: p−value = 0.95

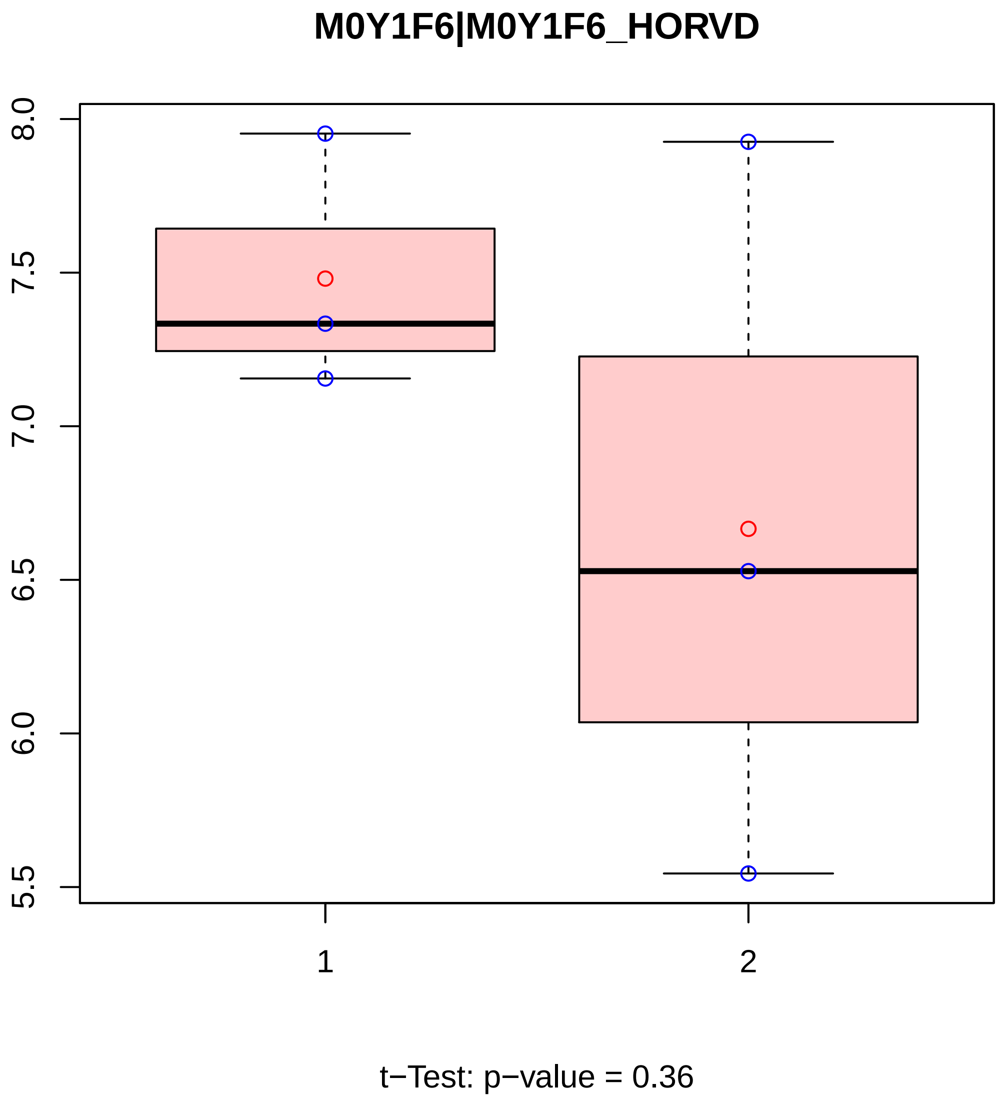
M0Y1F6|M0Y1F6_HORVD
8.0
7.5
7.0
6.5
6.0
5.5
1
2
t−Test: p−value = 0.36

M0Y1G8|M0Y1G8_HORVD

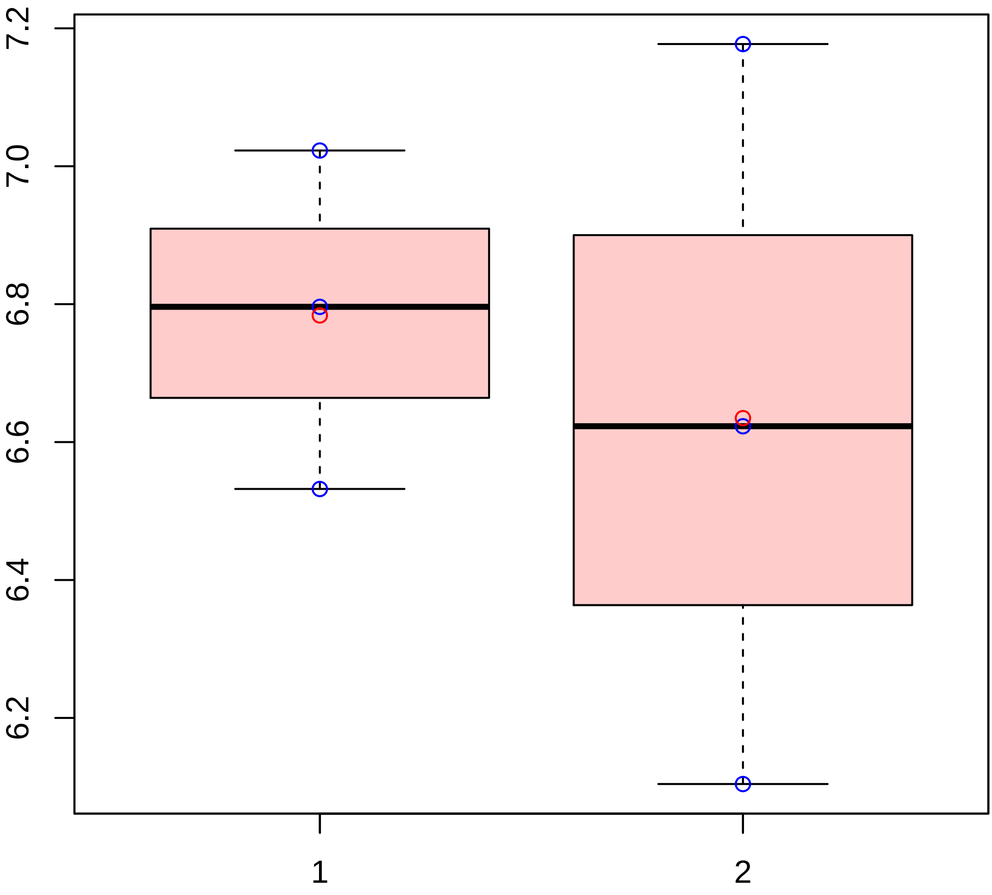

t−Test: p−value = 0.69

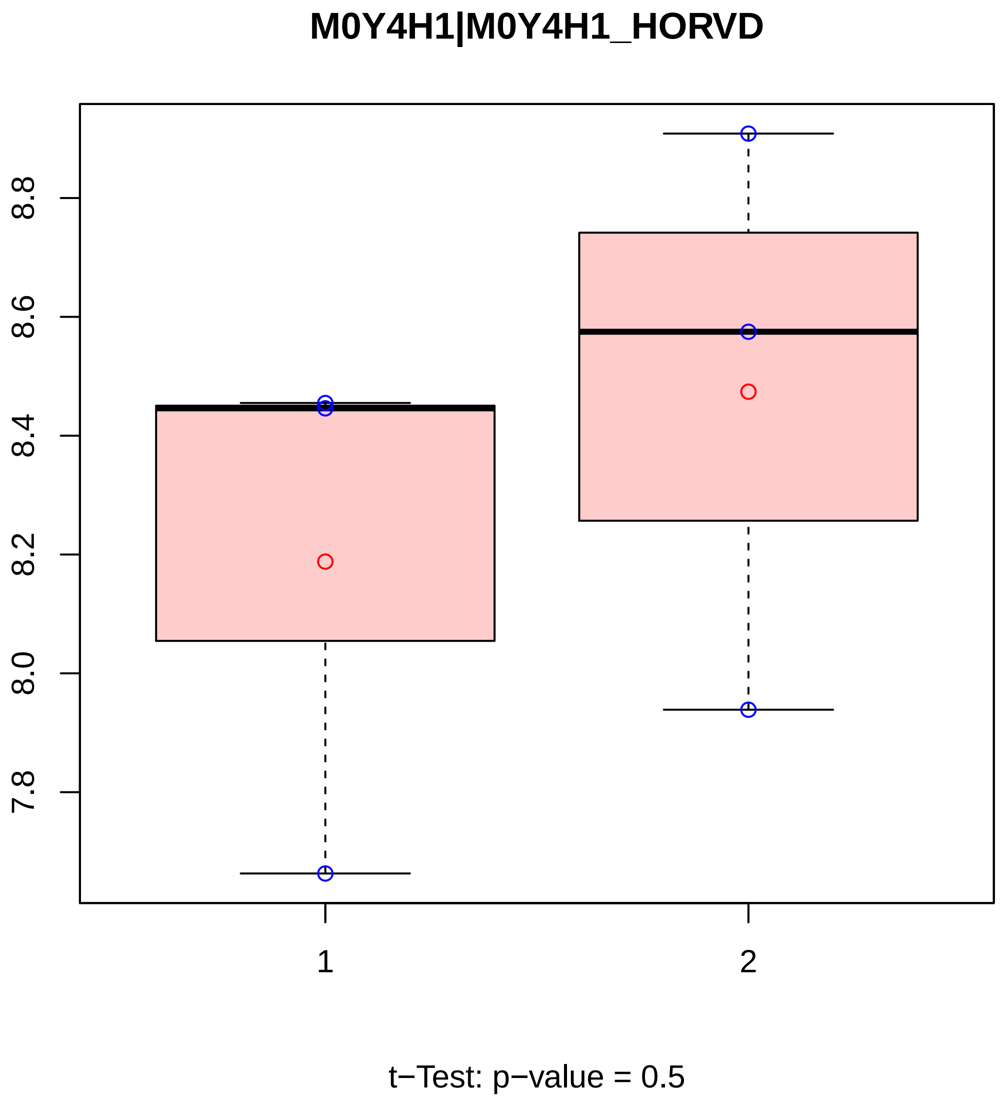
M0Y4H1|M0Y4H1_HORVD
7.8
8.0
8.2
8.4
8.6
8.8
1
2
t−Test: p−value = 0.5

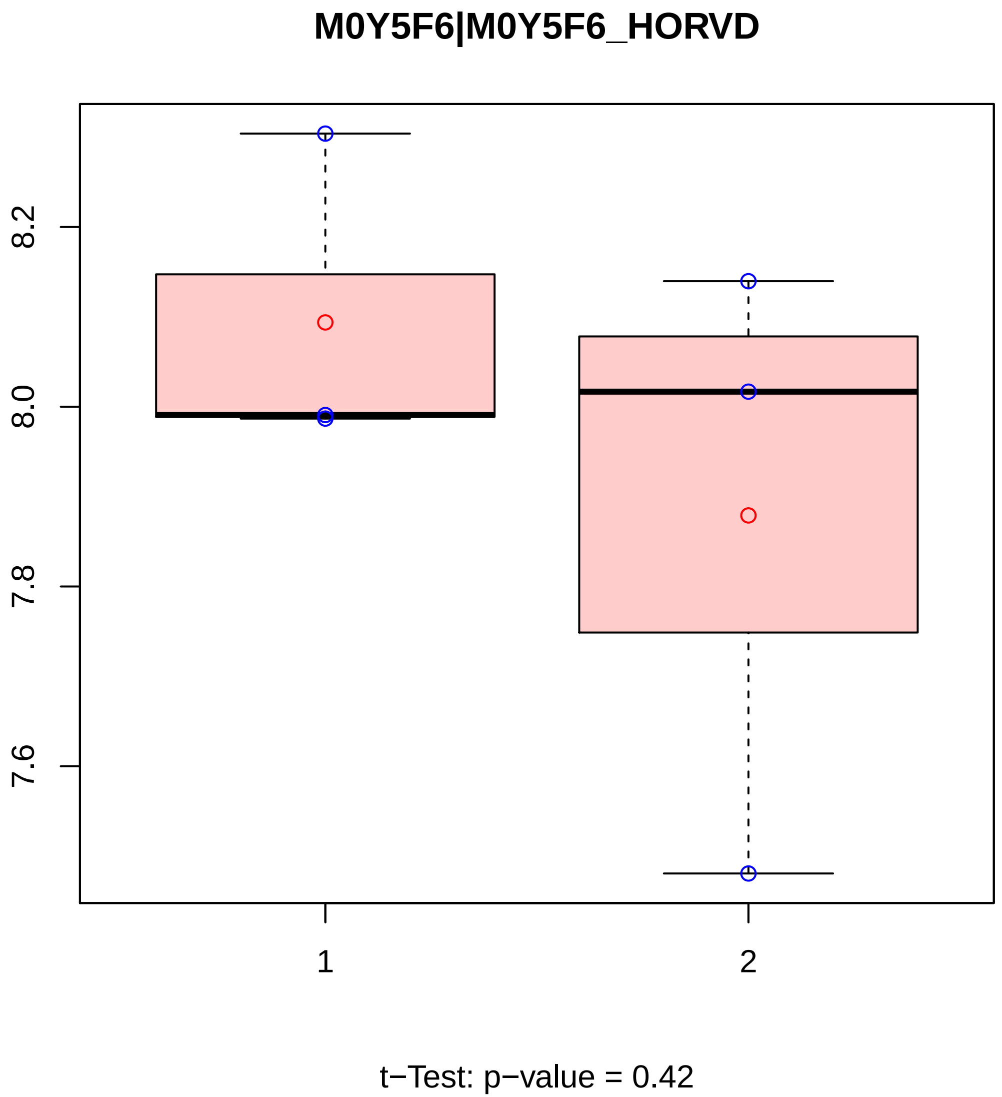
M0Y5F6|M0Y5F6_HORVD
8.2
8.0
7.8
7.6
1
2
t−Test: p−value = 0.42

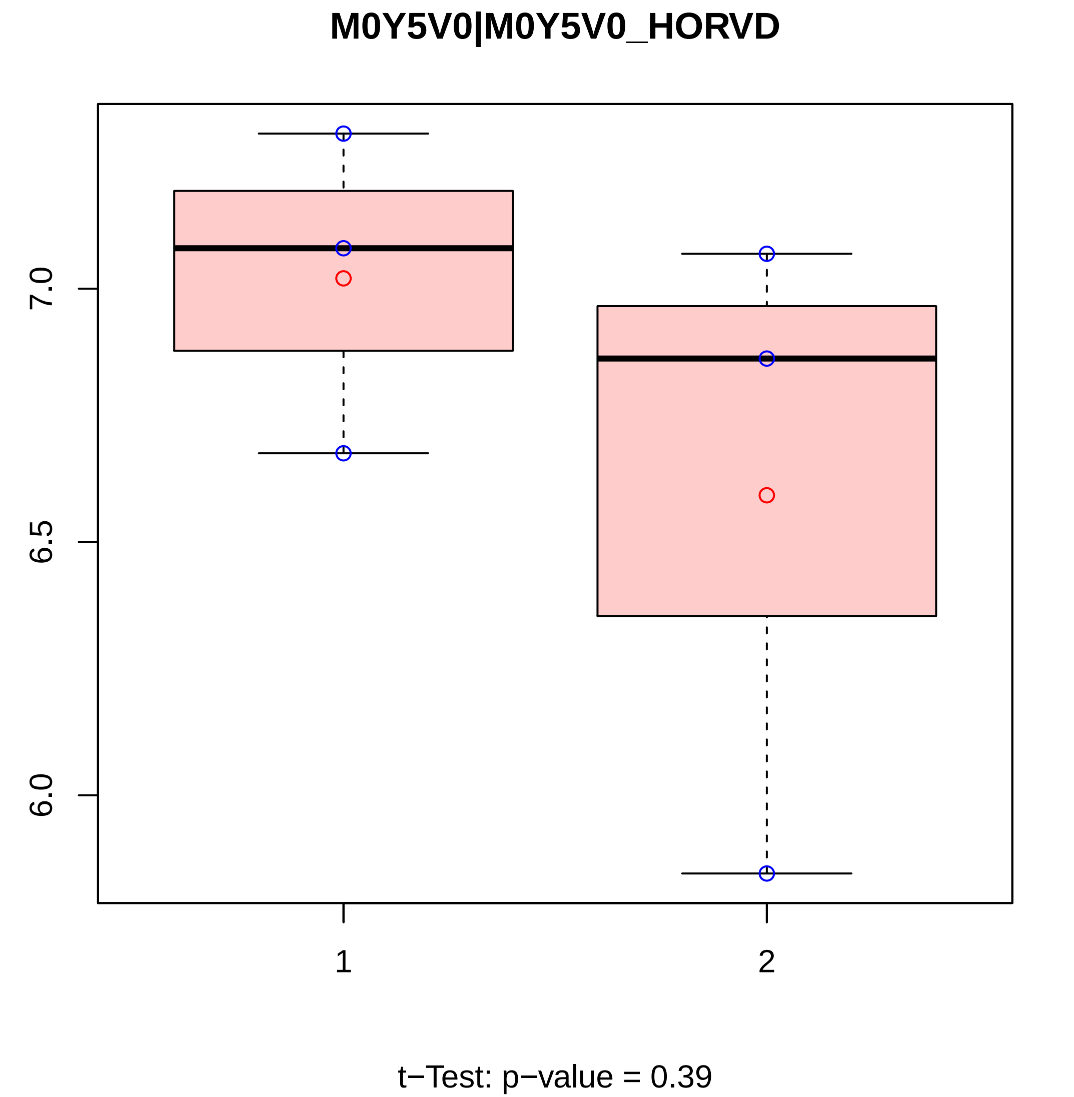
M0Y5V0|M0Y5V0_HORVD
7.0
6.5
6.0
1
2
t−Test: p−value = 0.39

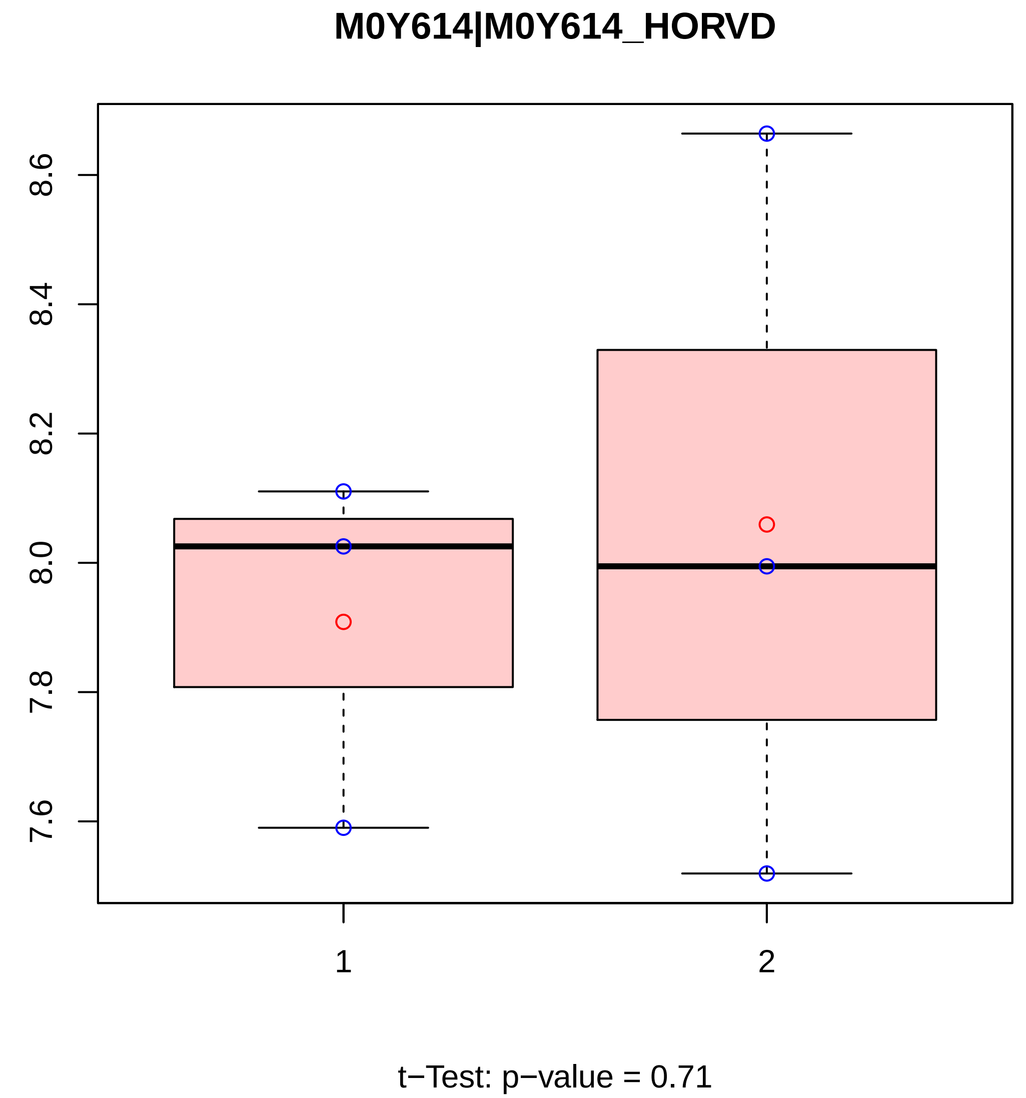
M0Y614|M0Y614_HORVD
8.6
8.4
8.2
8.0
7.8
7.6
1
2
t−Test: p−value = 0.71

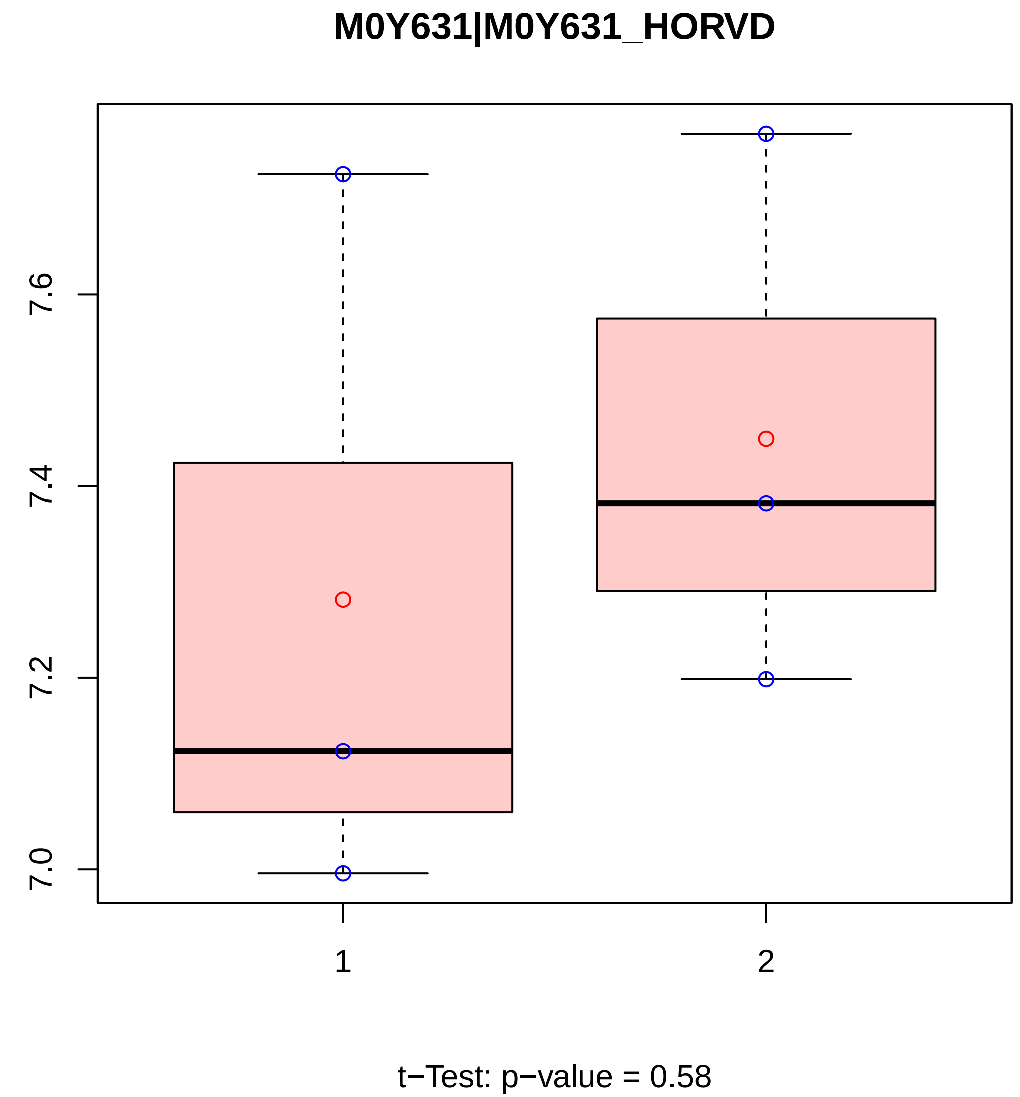
M0Y631|M0Y631_HORVD
7.0
7.2
7.4
7.6
1
2
t−Test: p−value = 0.58

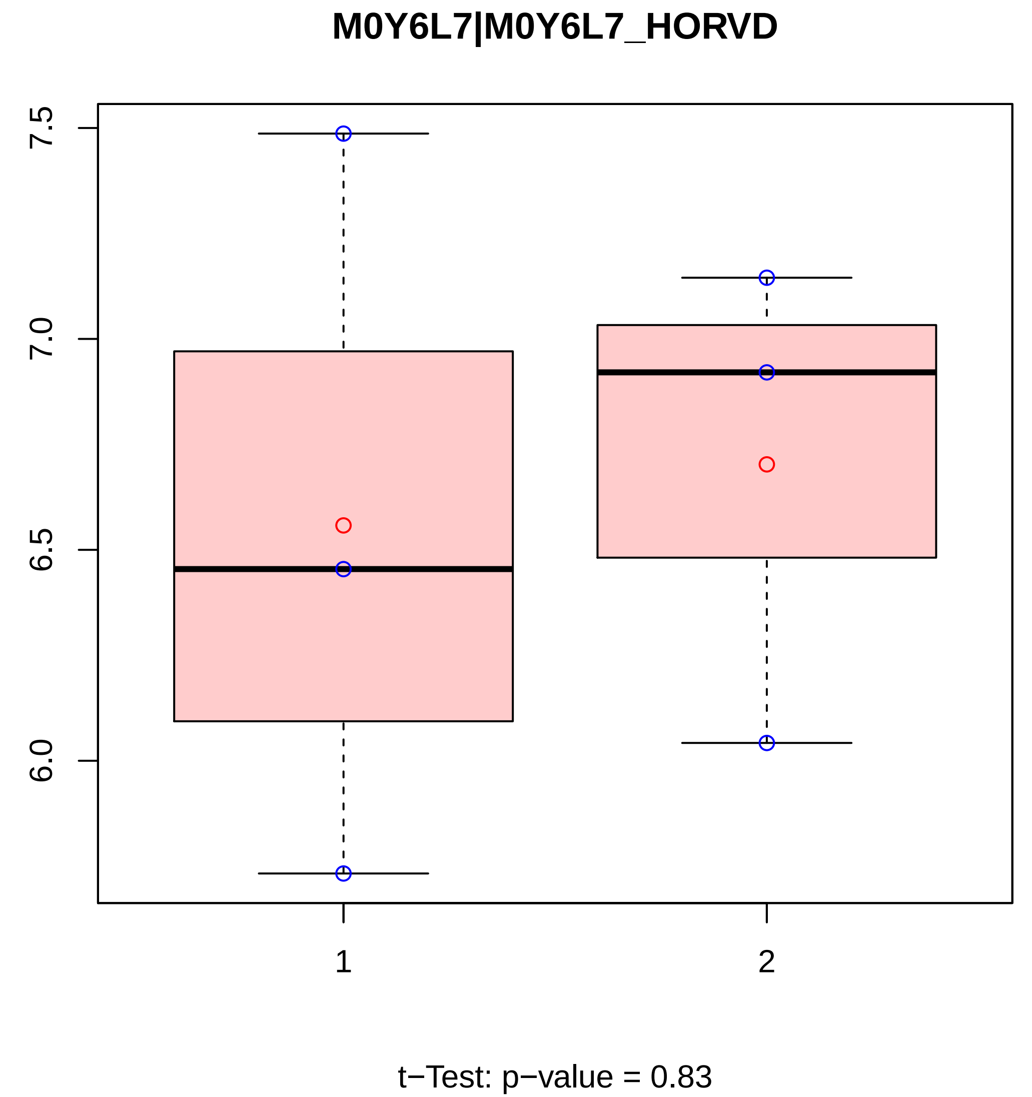
M0Y6L7|M0Y6L7_HORVD
7.5
7.0
6.5
6.0
1
2
t−Test: p−value = 0.83

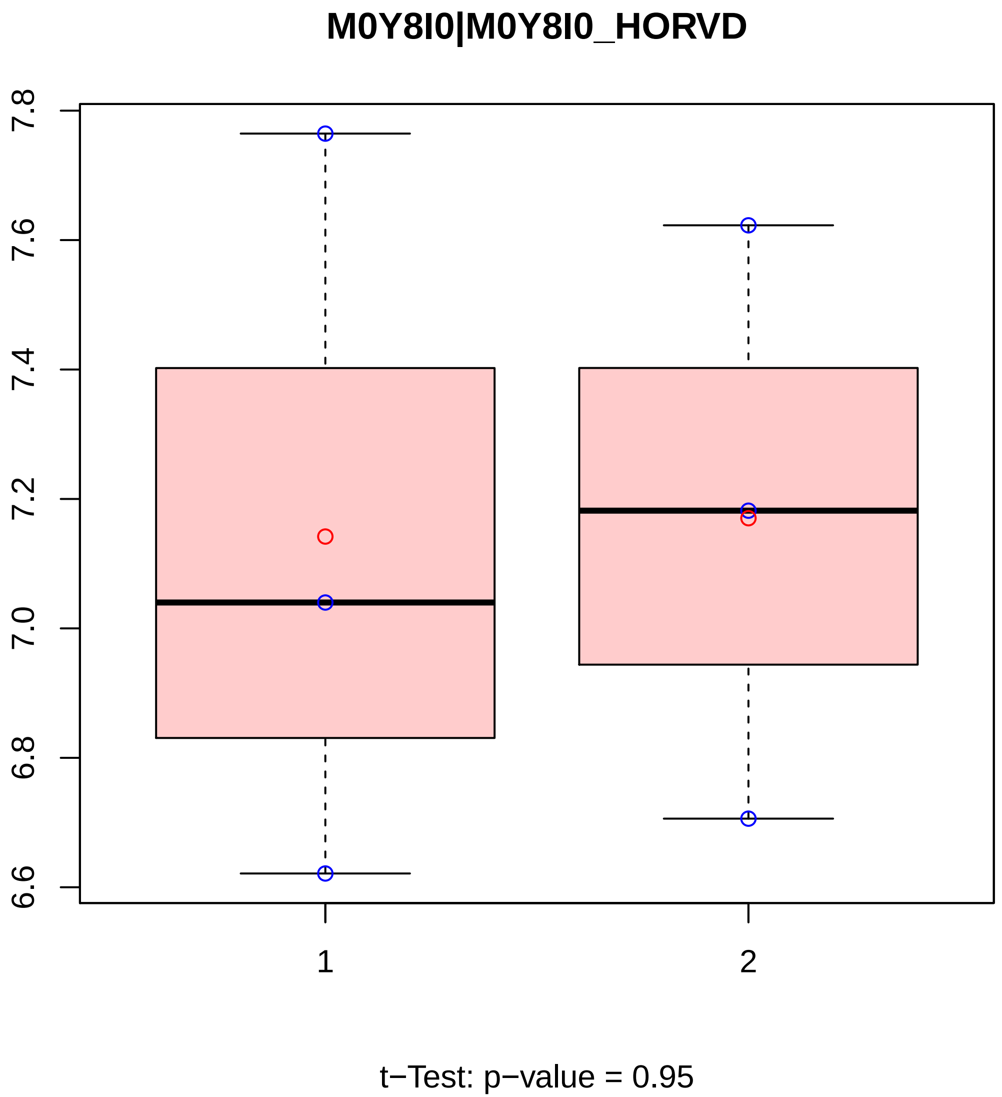
M0Y8I0|M0Y8I0_HORVD
7.8
7.6
7.4
7.2
7.0
6.8
6.6
1
2
t−Test: p−value = 0.95

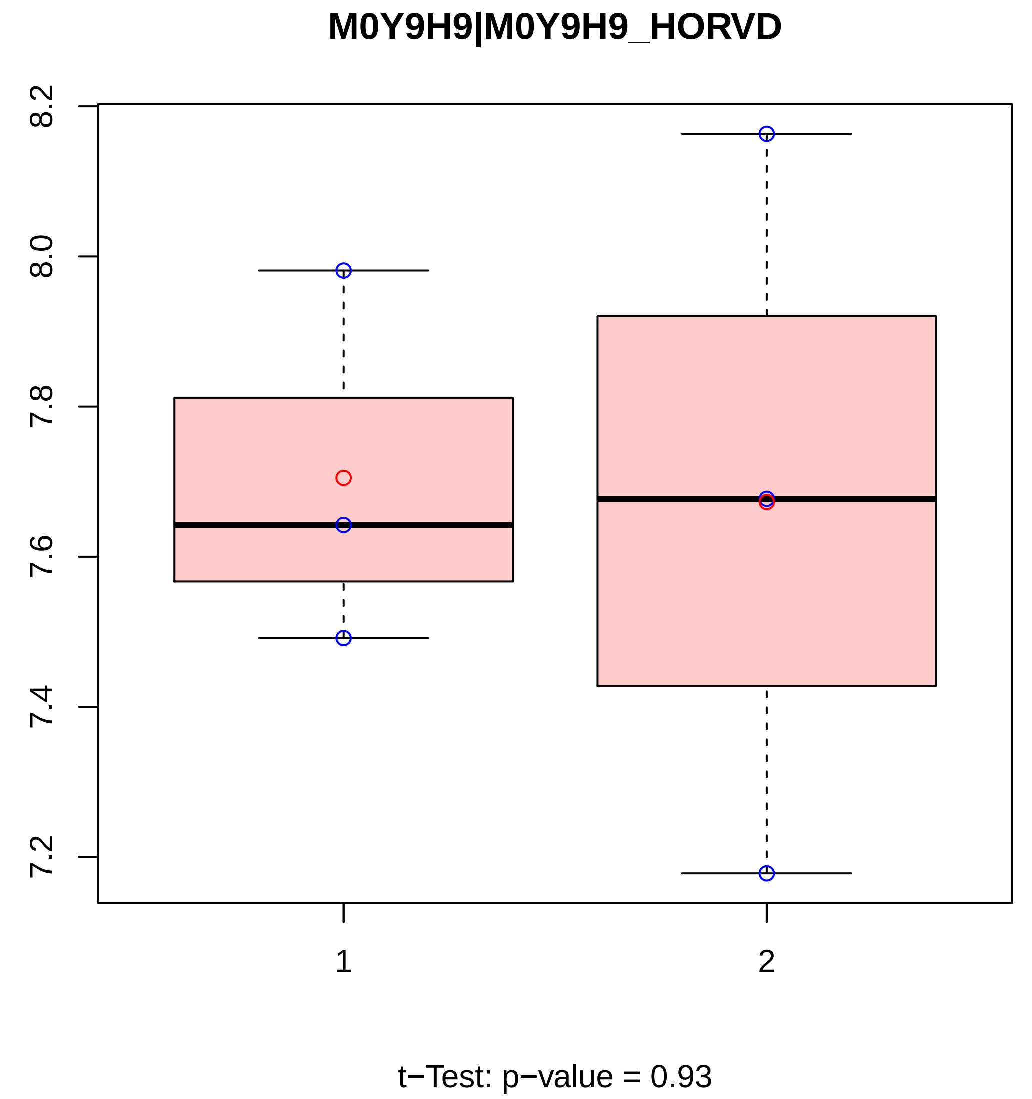
M0Y9H9|M0Y9H9_HORVD
8.2
8.0
7.8
7.6
7.4
7.2
1
2
t−Test: p−value = 0.93

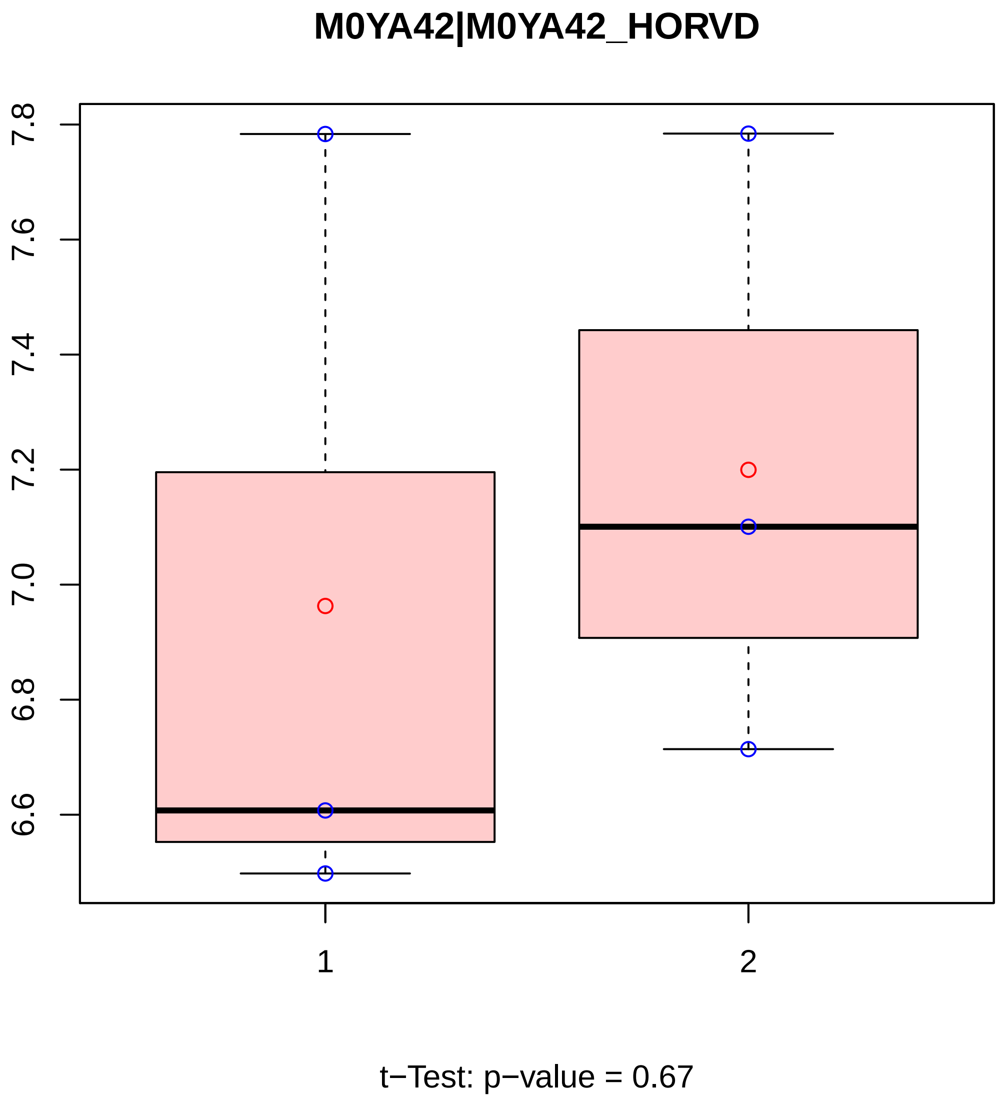

M0YA42|M0YA42_HORVD
7.8
7.6
7.4
7.2
7.0
6.8
6.6
1
2
t−Test: p−value = 0.67

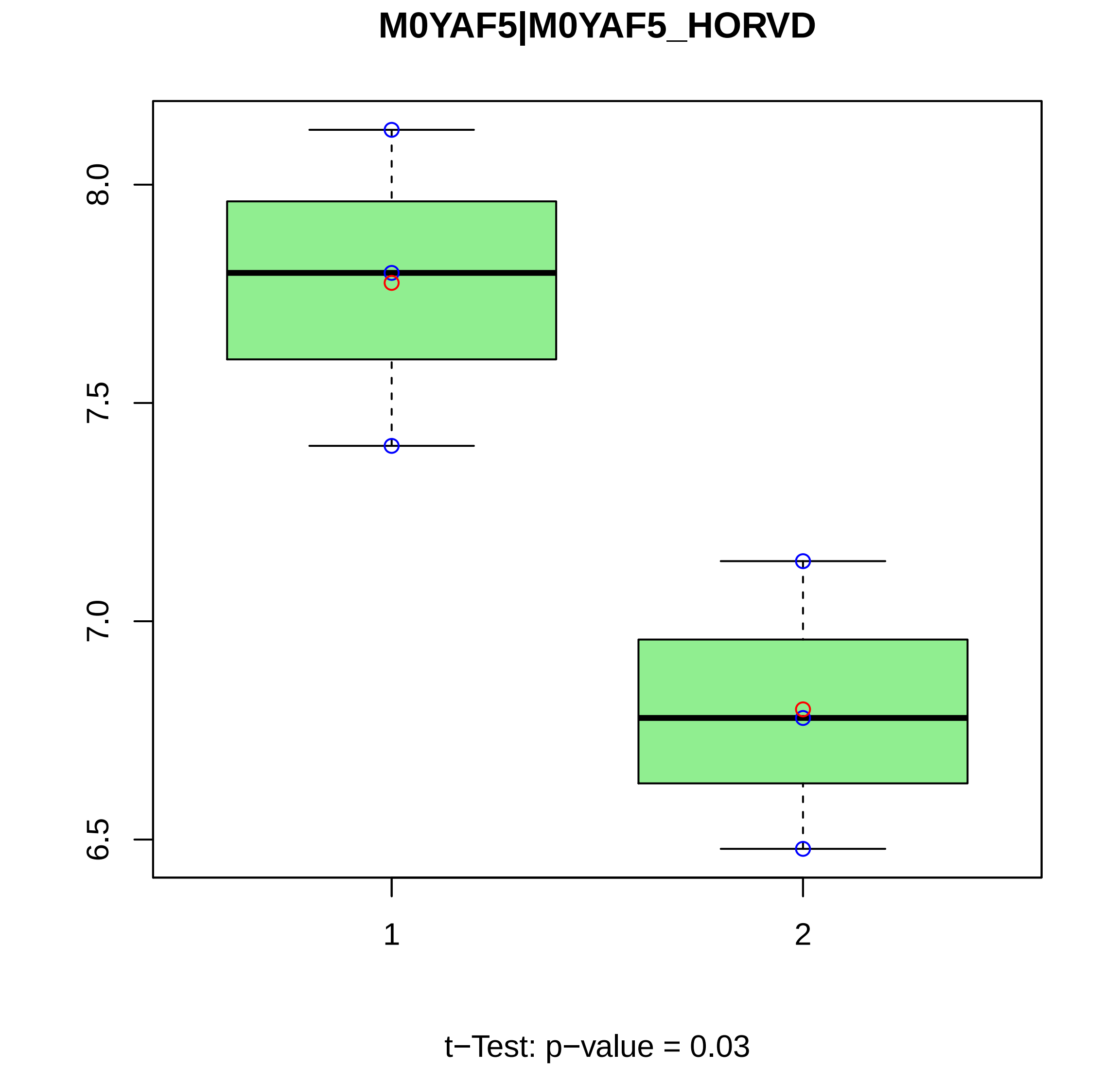
M0YAF5|M0YAF5_HORVD
8.0
7.5
7.0
6.5
1
2
t−Test: p−value = 0.03

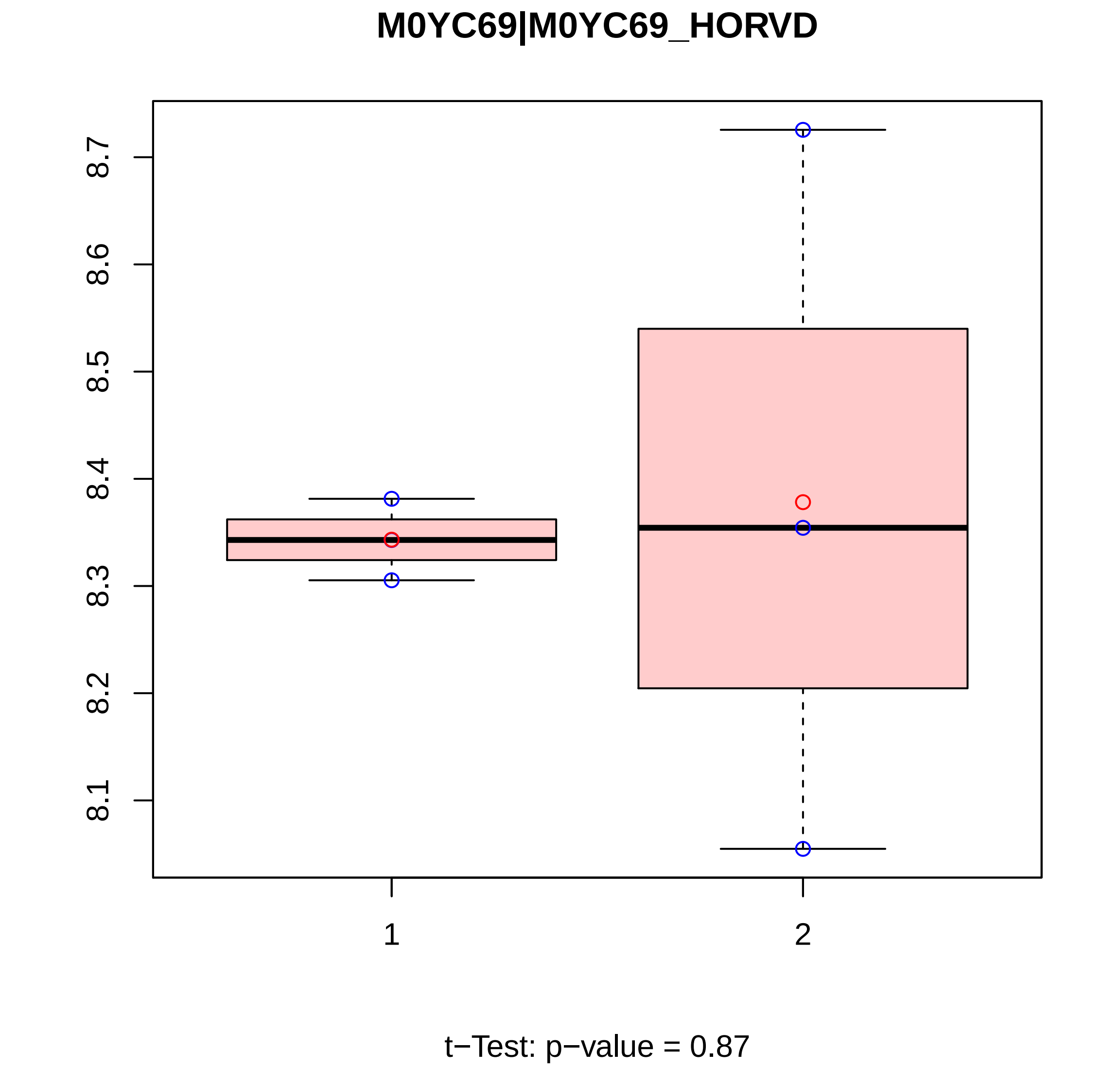
M0YC69|M0YC69_HORVD
8.1
8.2
8.3
8.4
8.5
8.6
8.7
1
2
t−Test: p−value = 0.87

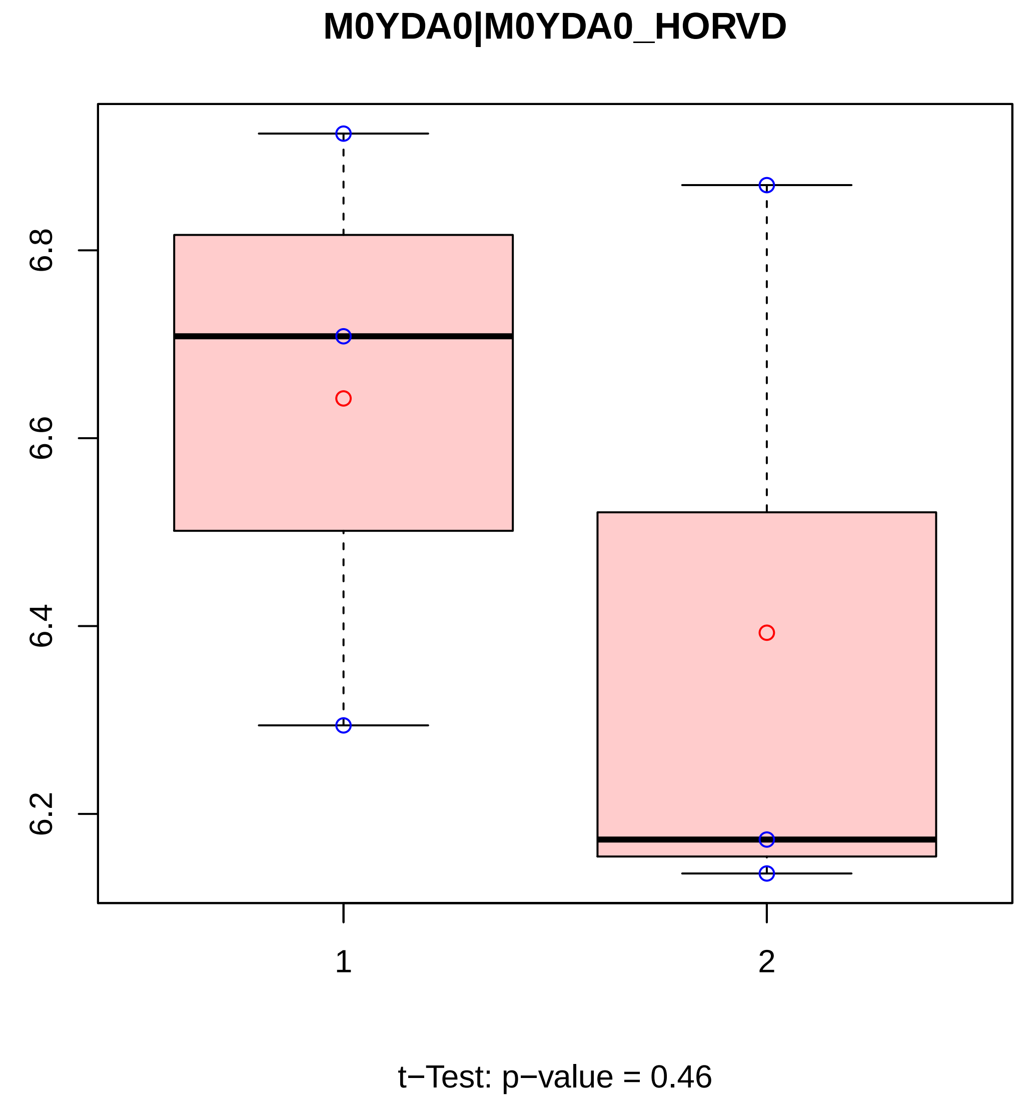
M0YDA0|M0YDA0_HORVD
6.2
6.4
6.6
6.8
1
2
t−Test: p−value = 0.46

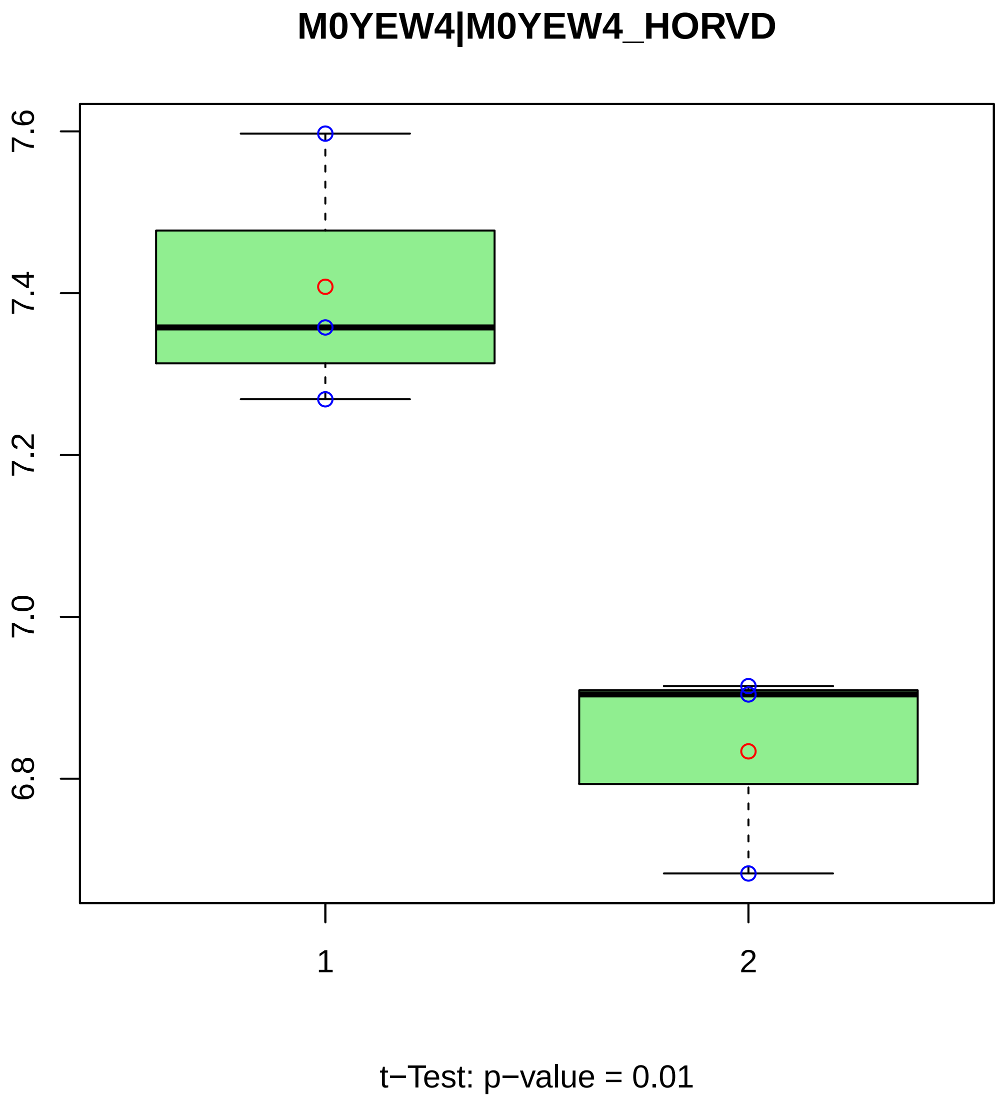
M0YEW4|M0YEW4_HORVD
7.6
7.4
7.2
7.0
6.8
1
2
t−Test: p−value = 0.01

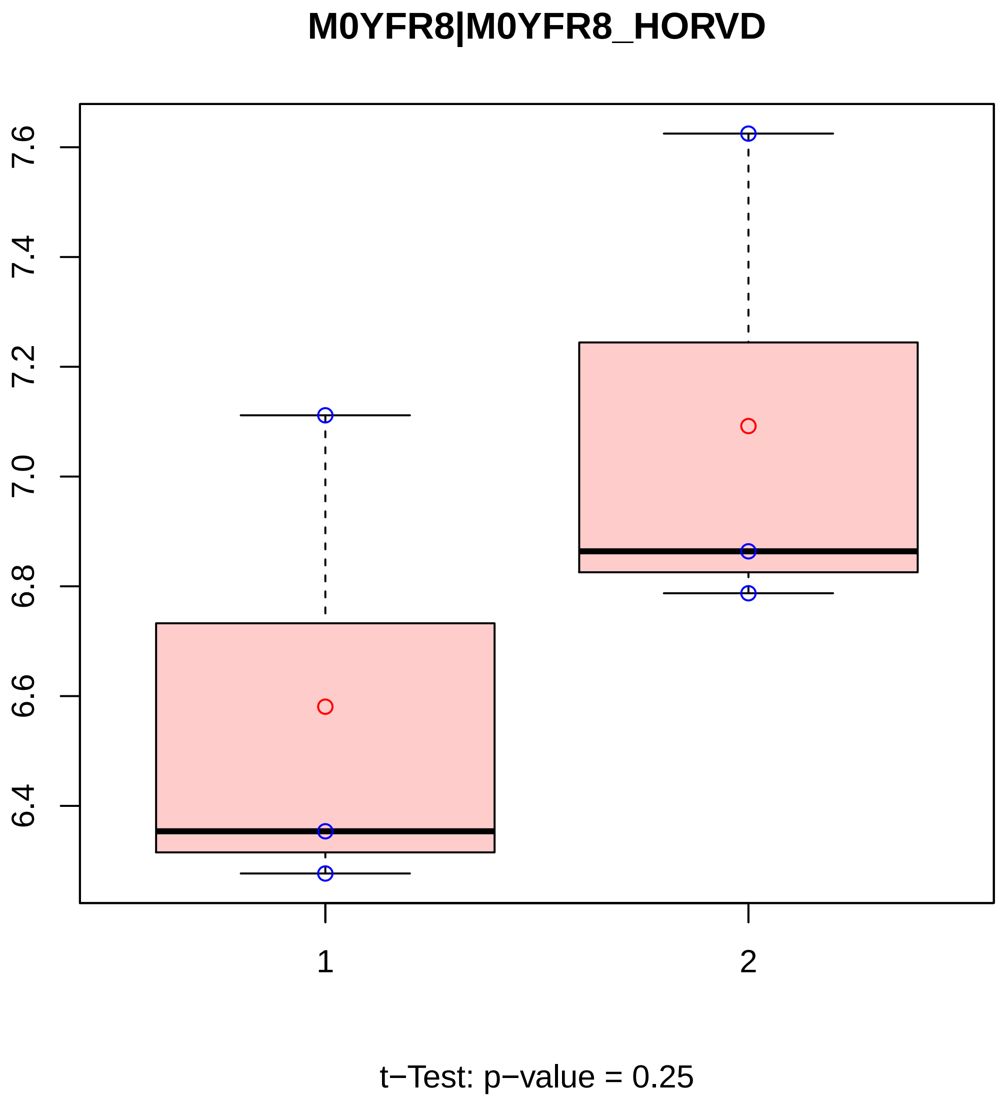
M0YFR8|M0YFR8_HORVD
7.6
7.4
7.2
7.0
6.8
6.6
6.4
1
2
t−Test: p−value = 0.25

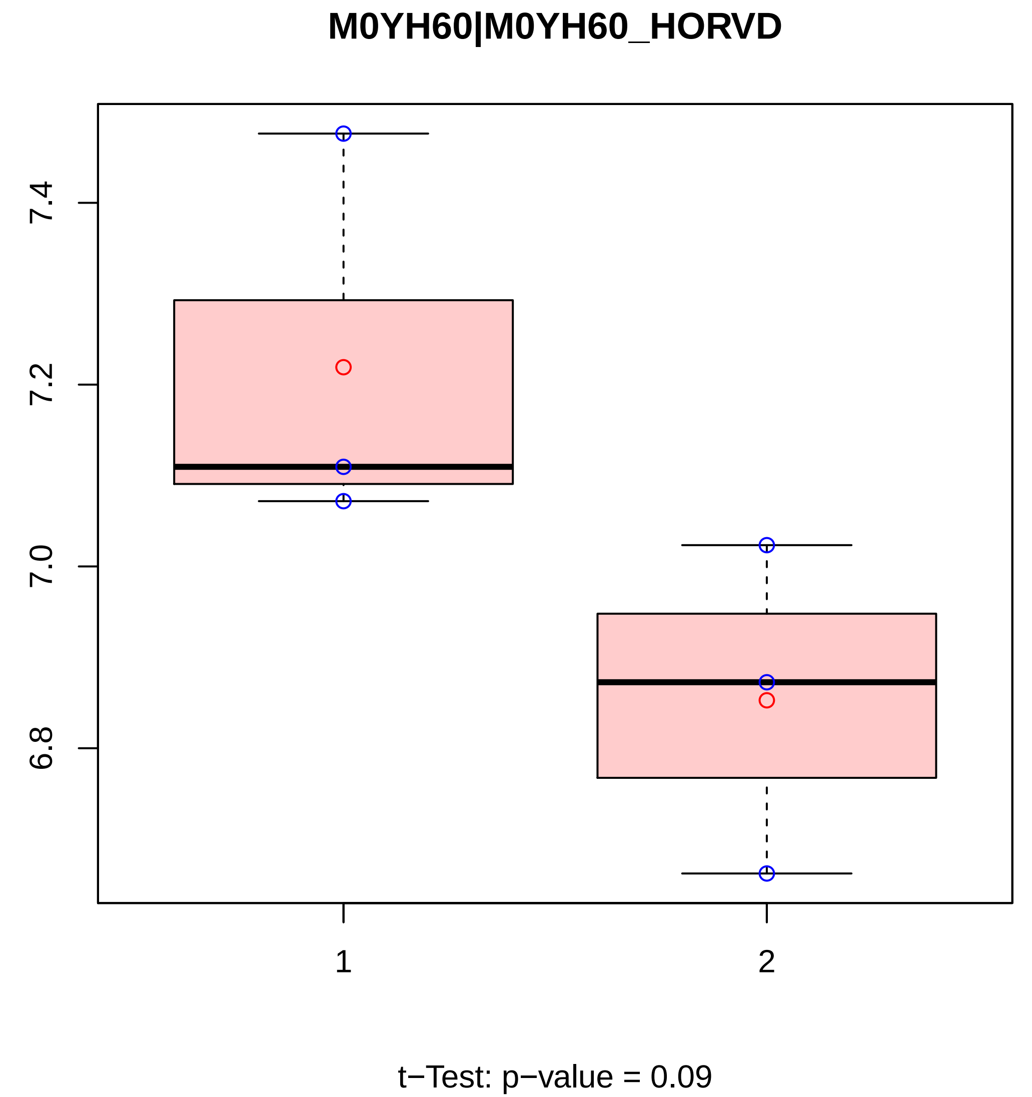
M0YH60|M0YH60_HORVD
7.4
7.2
7.0
6.8
1
2
t−Test: p−value = 0.09

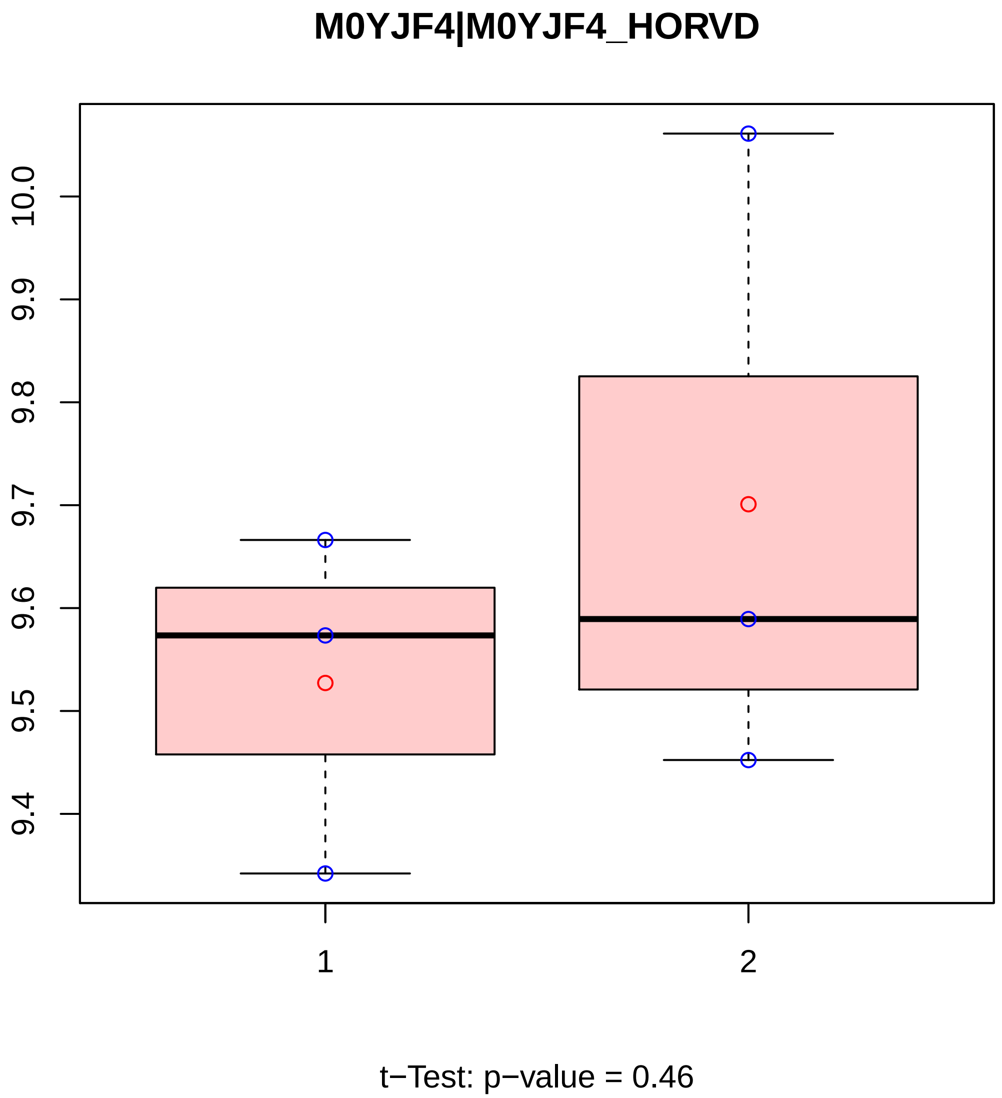
M0YJF4|M0YJF4_HORVD
9.4
9.5
9.6
9.7
9.8
9.9
10.0
1
2
t−Test: p−value = 0.46

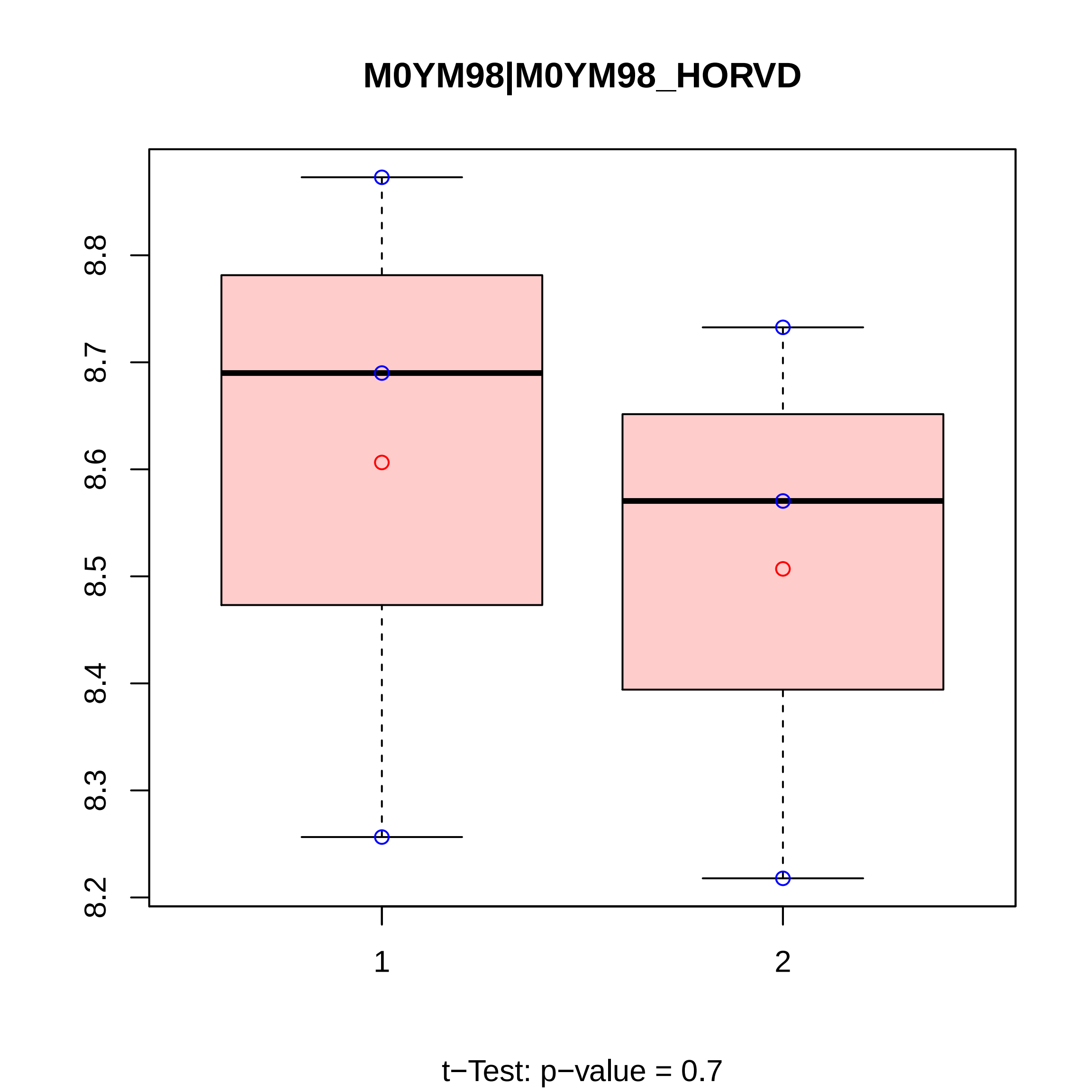
M0YM98|M0YM98_HORVD
8.2
8.3
8.4
8.5
8.6
8.7
8.8
1
2
t−Test: p−value = 0.7

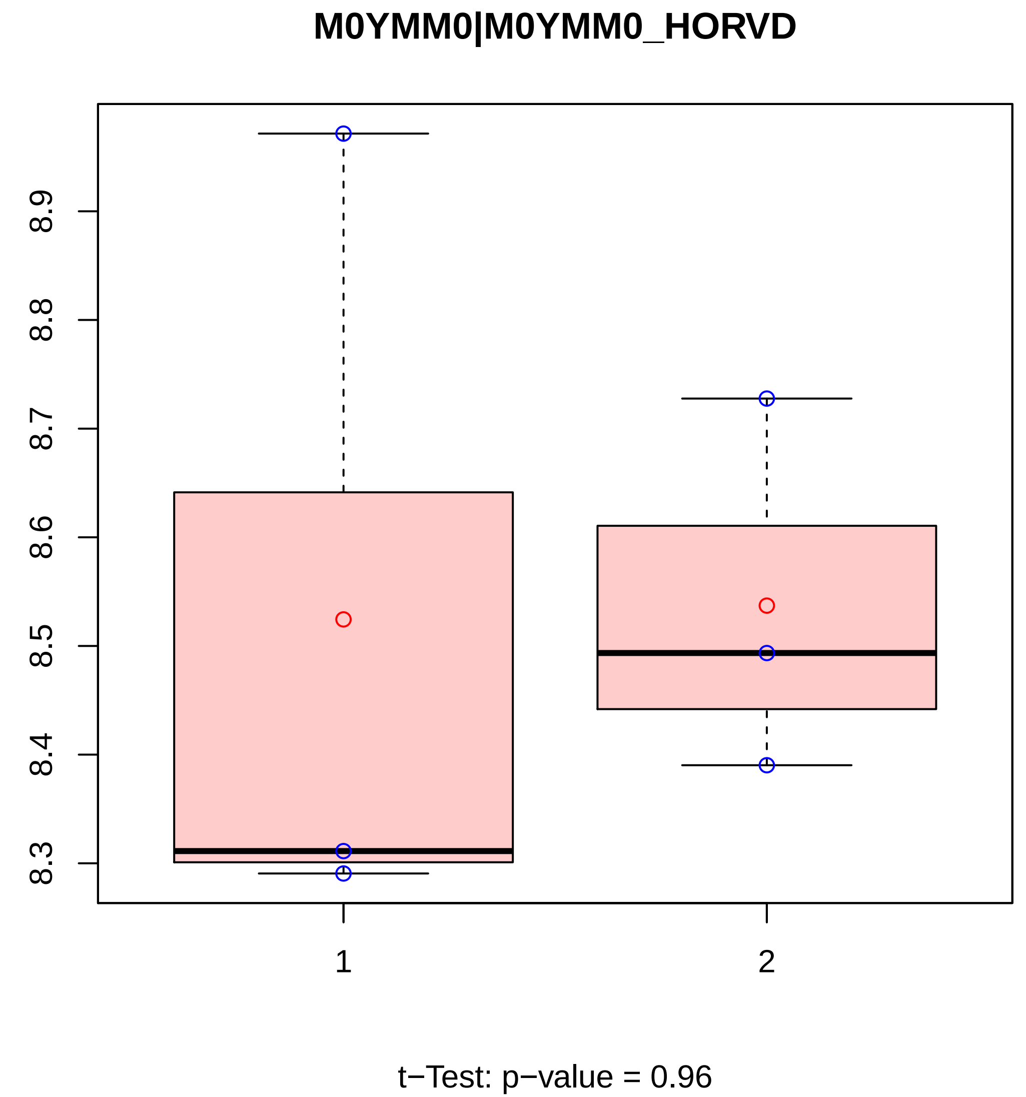
M0YMM0|M0YMM0_HORVD
8.3
8.4
8.5
8.6
8.7
8.8
8.9
1
2
t−Test: p−value = 0.96

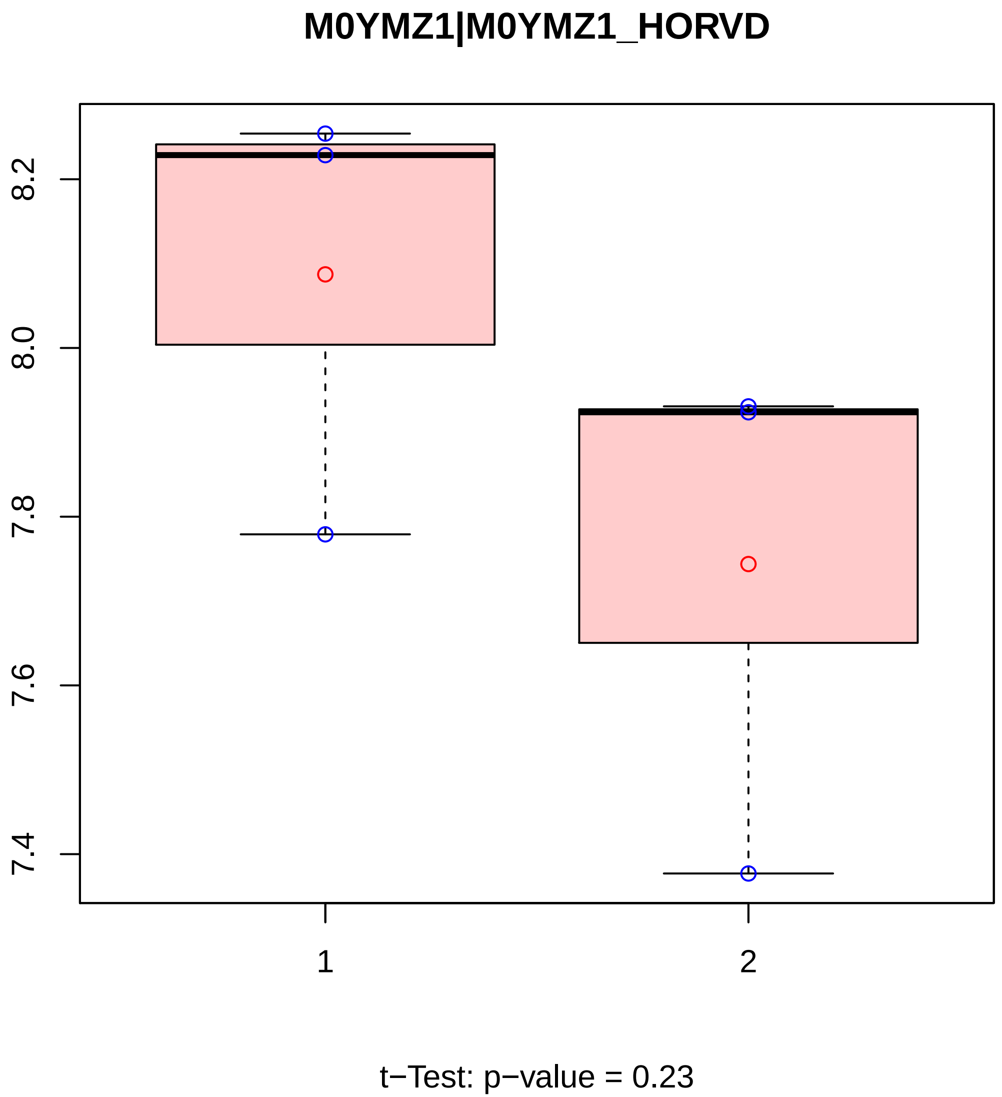
M0YMZ1|M0YMZ1_HORVD
7.4
7.6
7.8
8.0
8.2
1
2
t−Test: p−value = 0.23

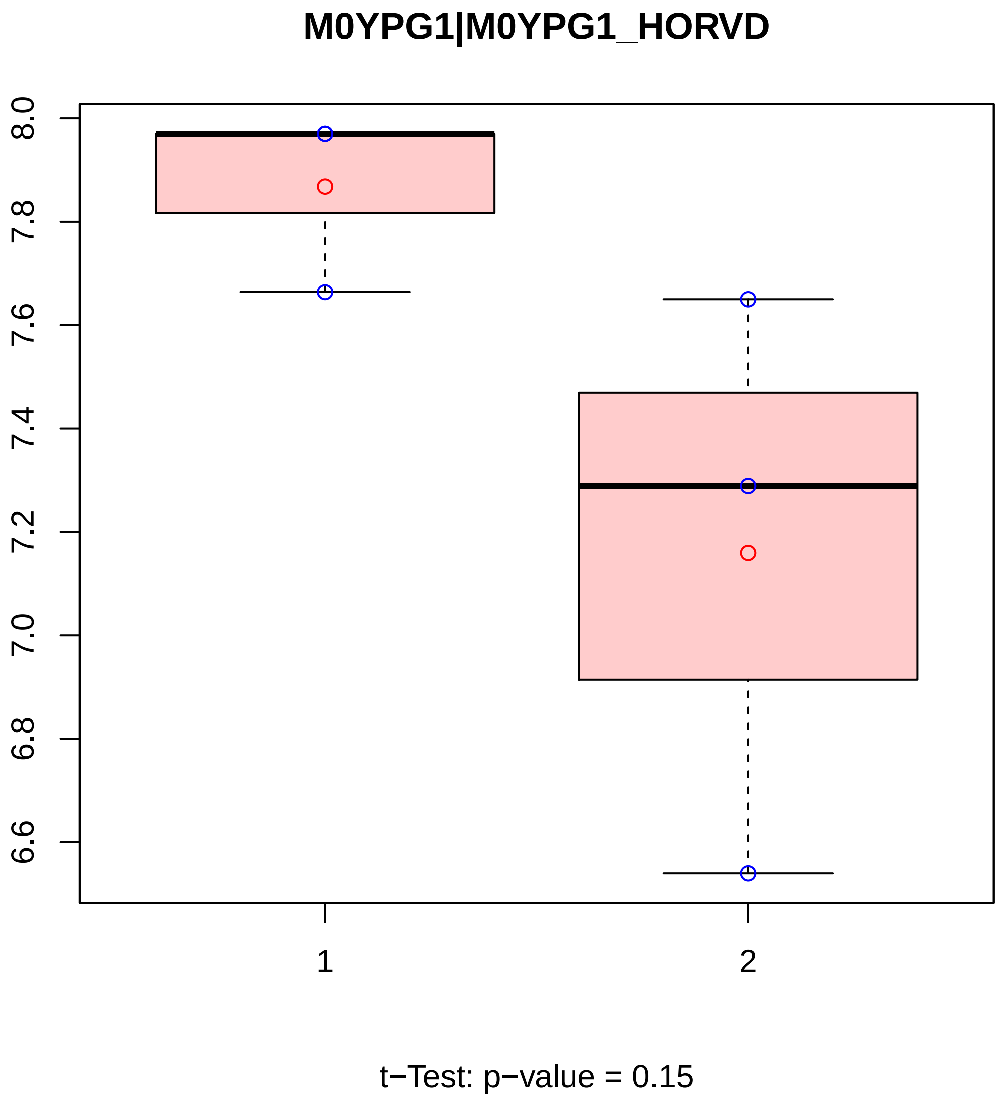
M0YPG1|M0YPG1_HORVD
8.0
7.8
7.6
7.4
7.2
7.0
6.8
6.6
1
2
t−Test: p−value = 0.15

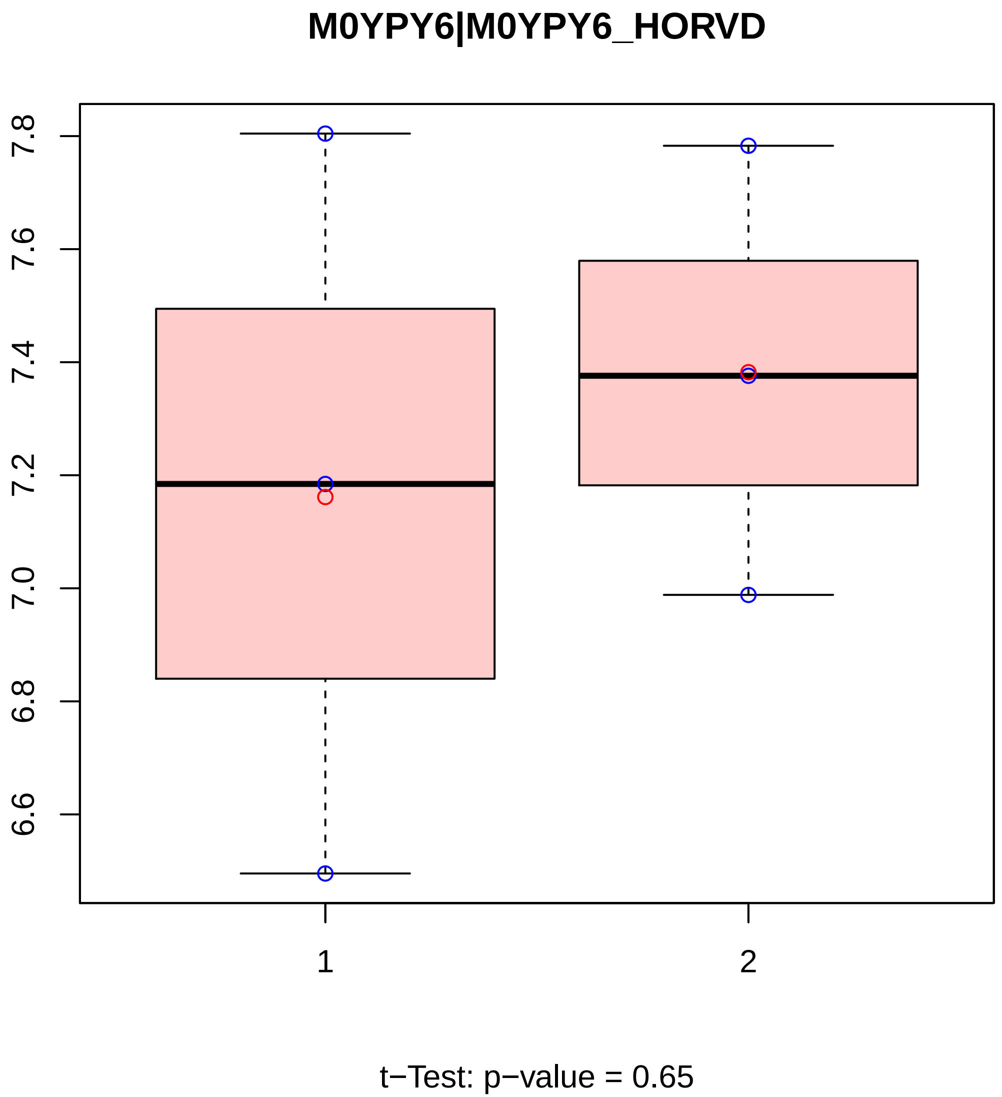
M0YPY6|M0YPY6_HORVD
7.8
7.6
7.4
7.2
7.0
6.8
6.6
1
2
t−Test: p−value = 0.65

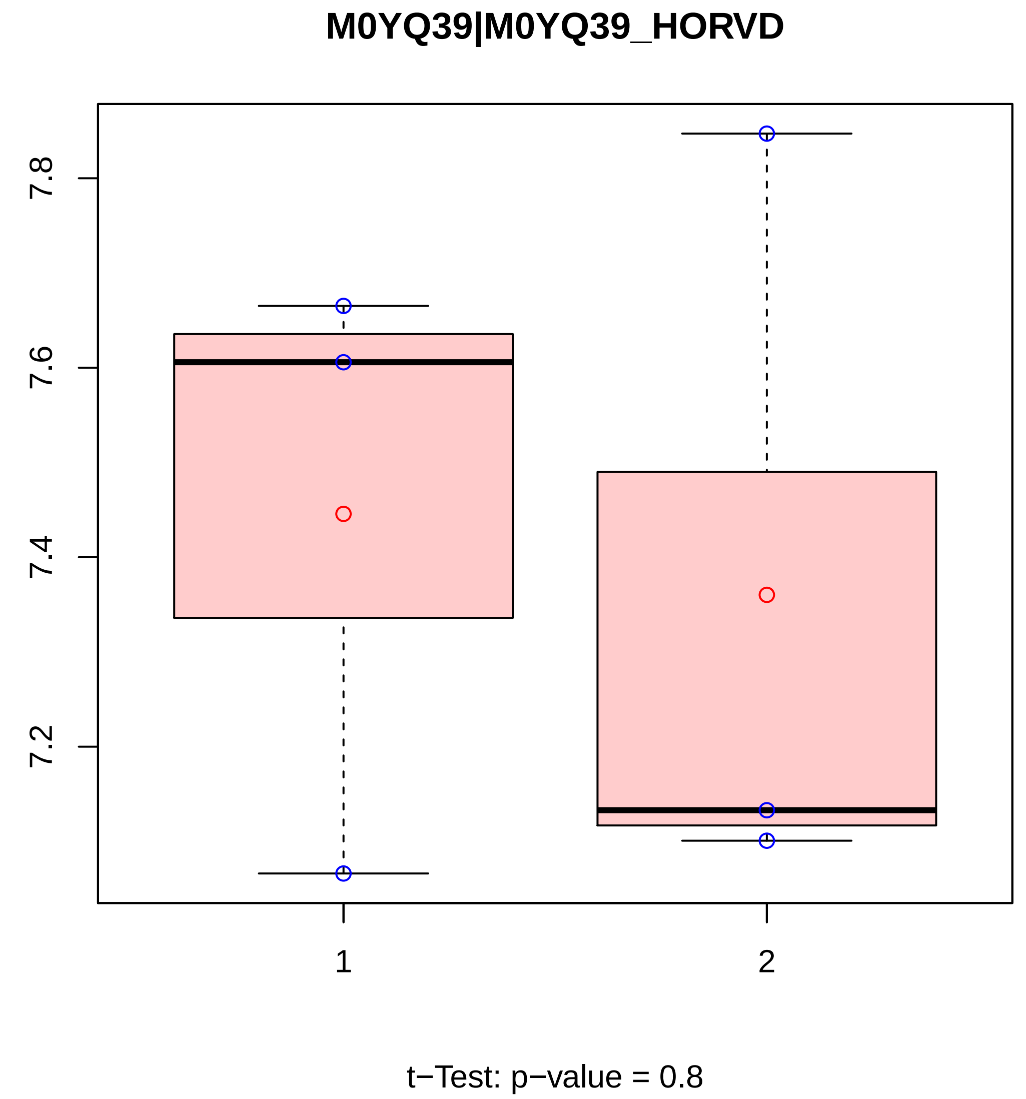
M0YQ39|M0YQ39_HORVD
7.2
7.4
7.6
7.8
1
2
t−Test: p−value = 0.8

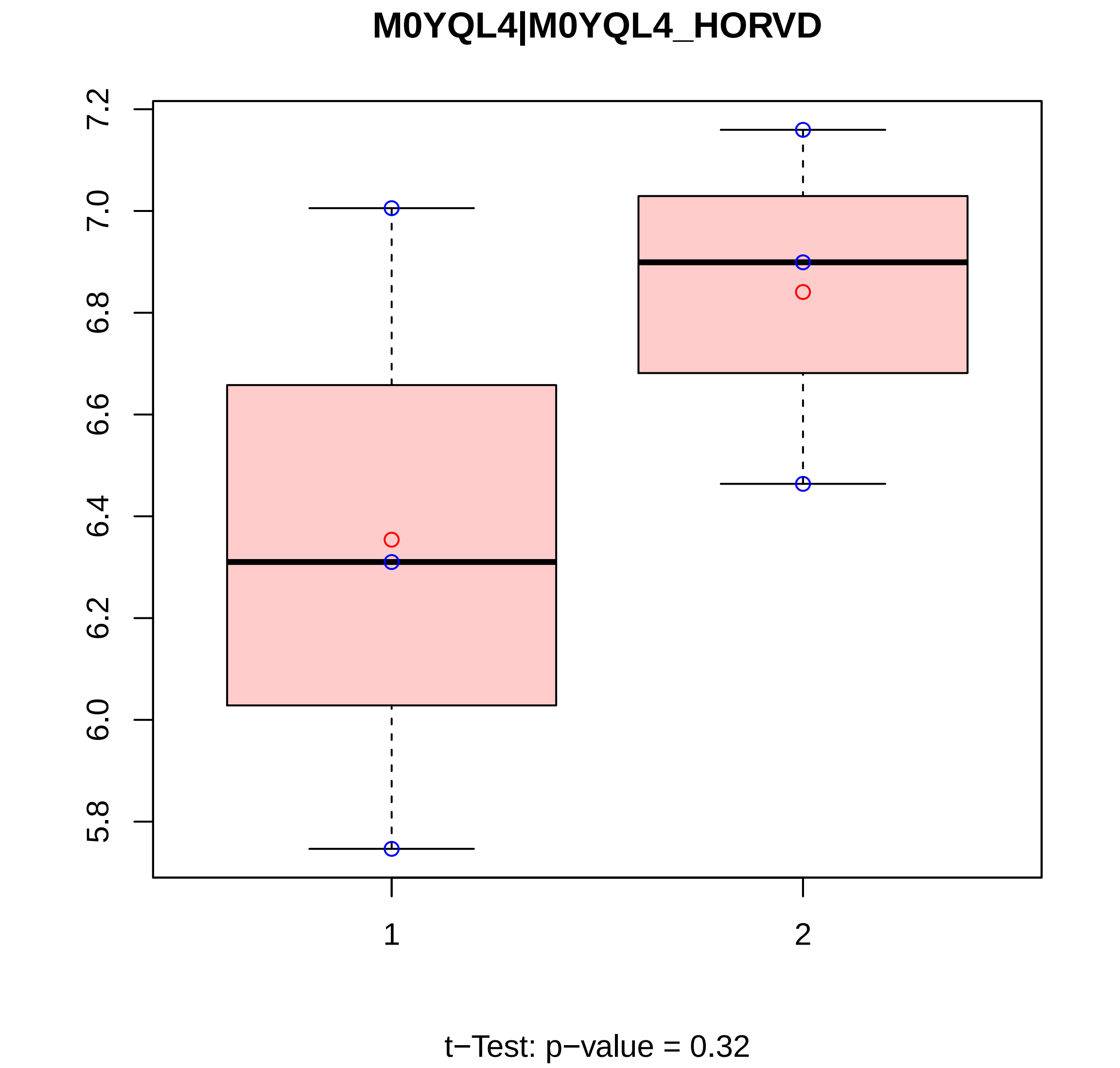
M0YQL4|M0YQL4_HORVD
7.2
7.0
6.8
6.6
6.4
6.2
6.0
5.8
1
2
t−Test: p−value = 0.32

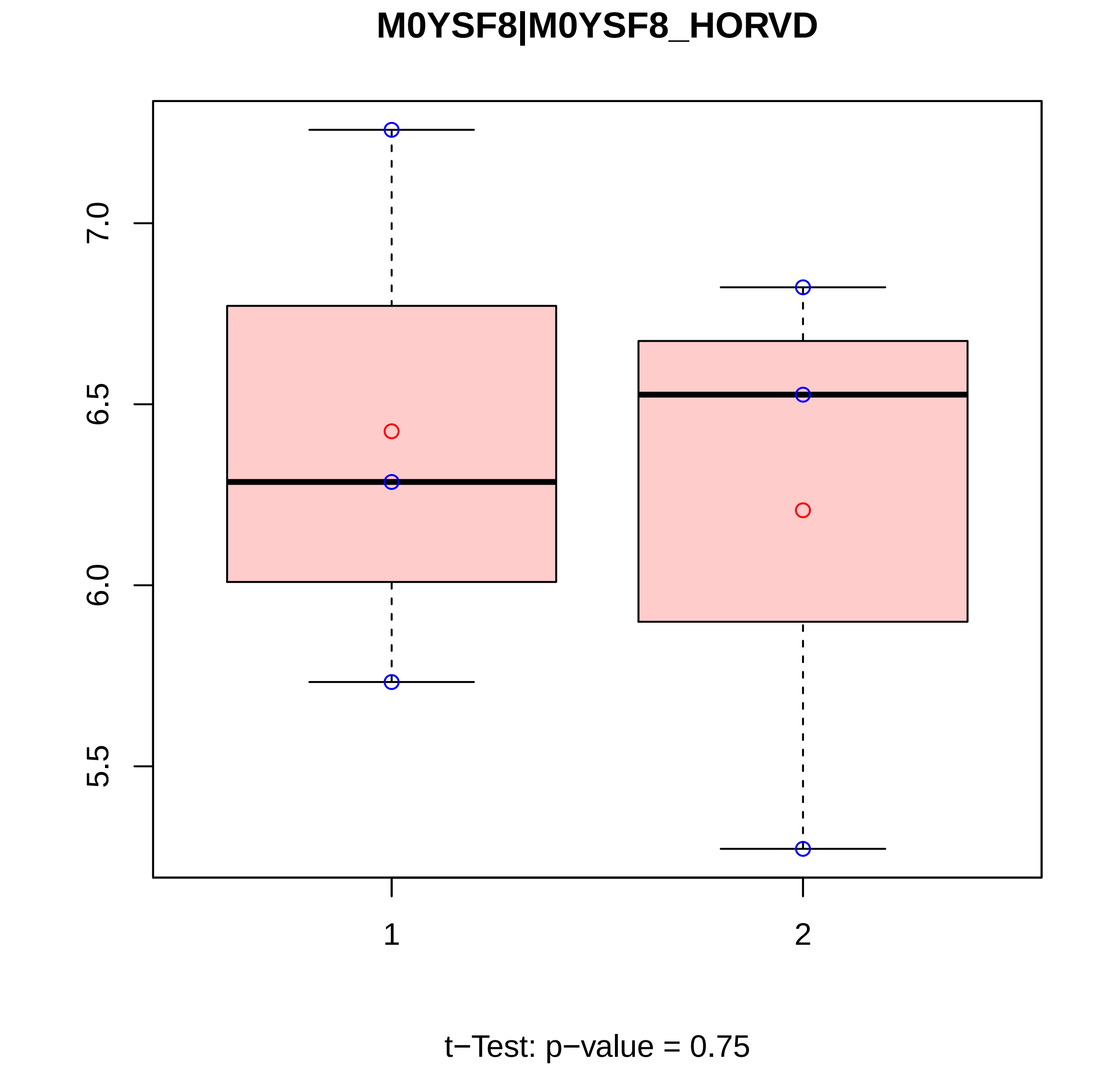
M0YSF8|M0YSF8_HORVD
7.0
6.5
6.0
5.5
1
2
t−Test: p−value = 0.75

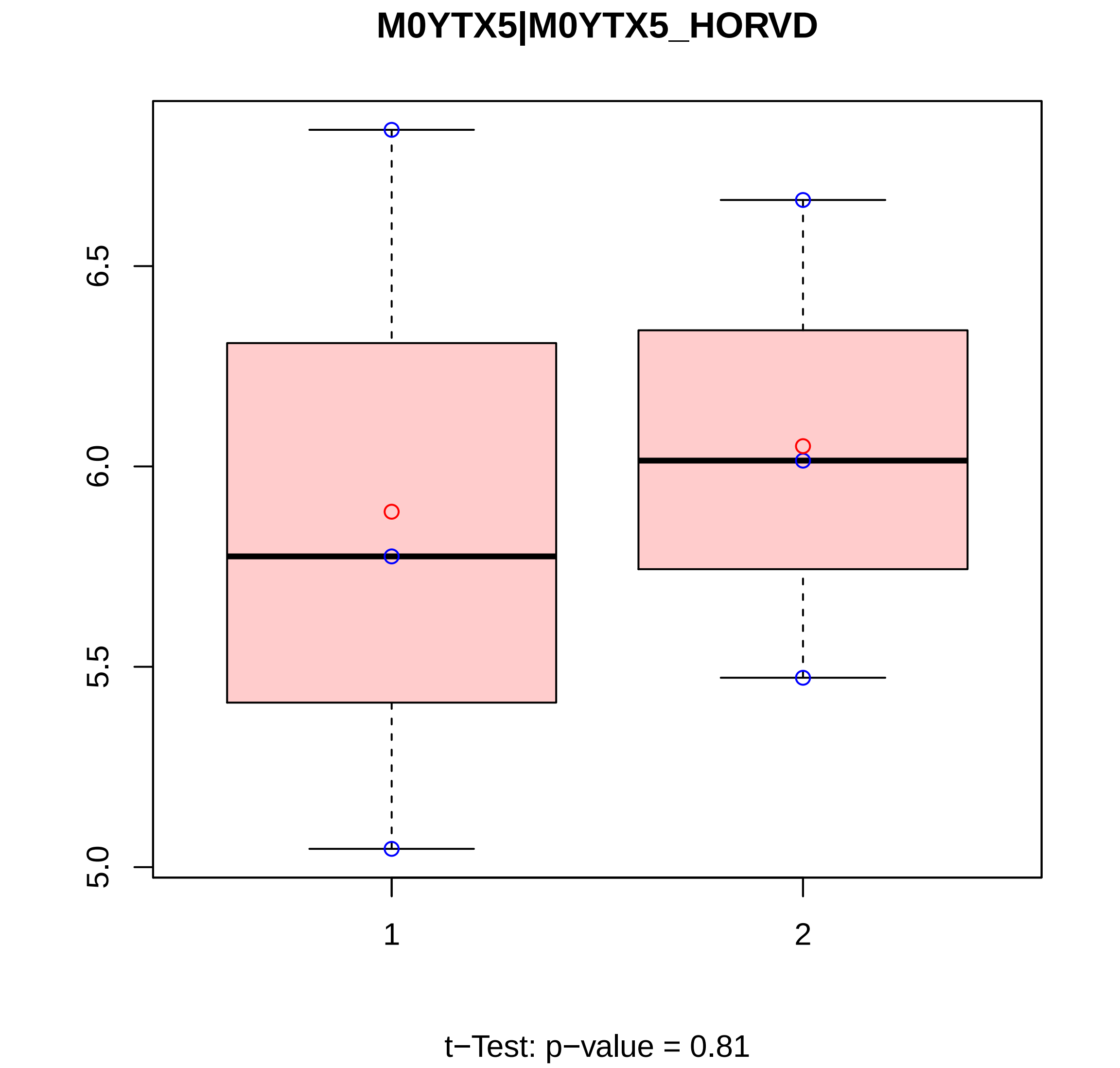
M0YTX5|M0YTX5_HORVD
6.5
6.0
5.5
5.0
1
2
t−Test: p−value = 0.81

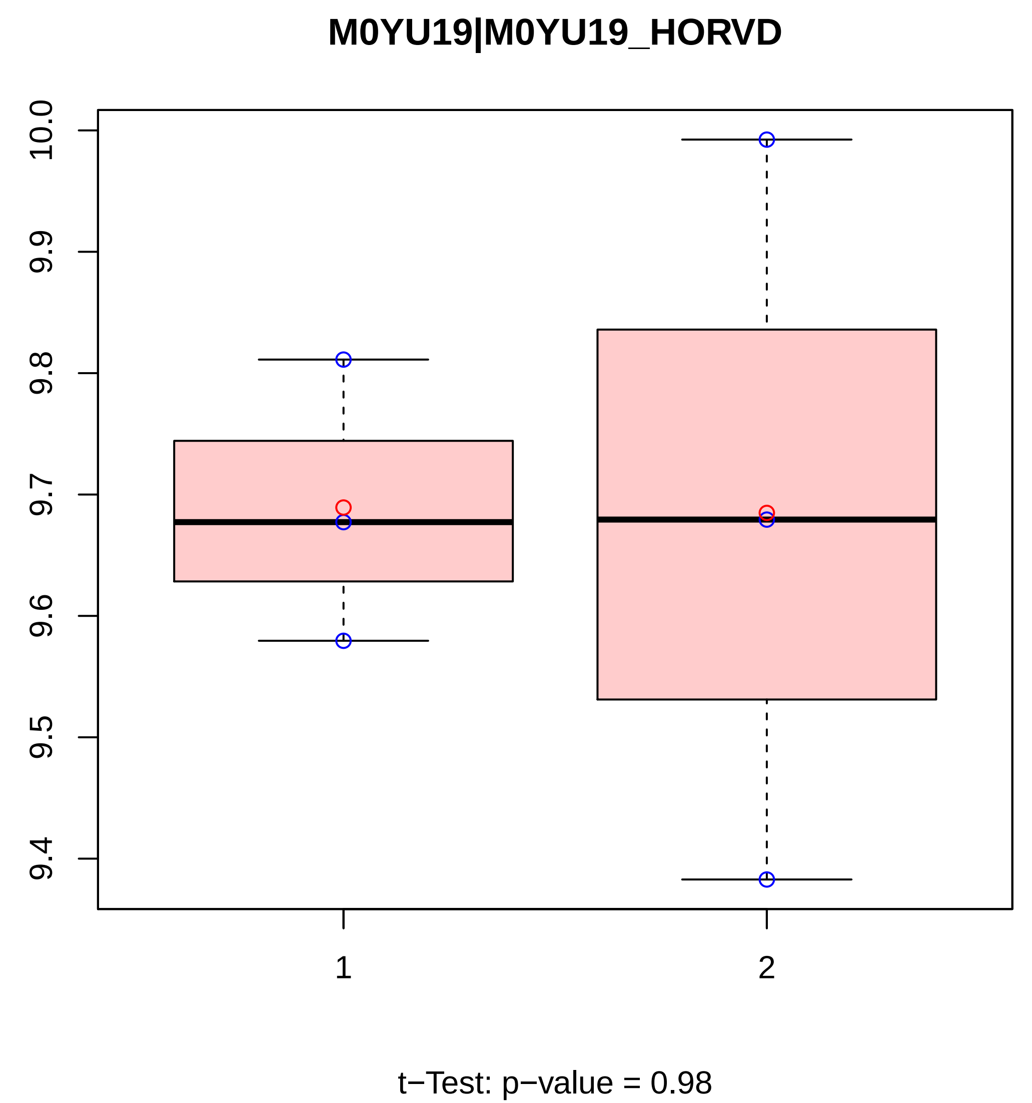

M0YU19|M0YU19_HORVD
10.0
9.9
9.8
9.7
9.6
9.5
9.4
1
2
t−Test: p−value = 0.98

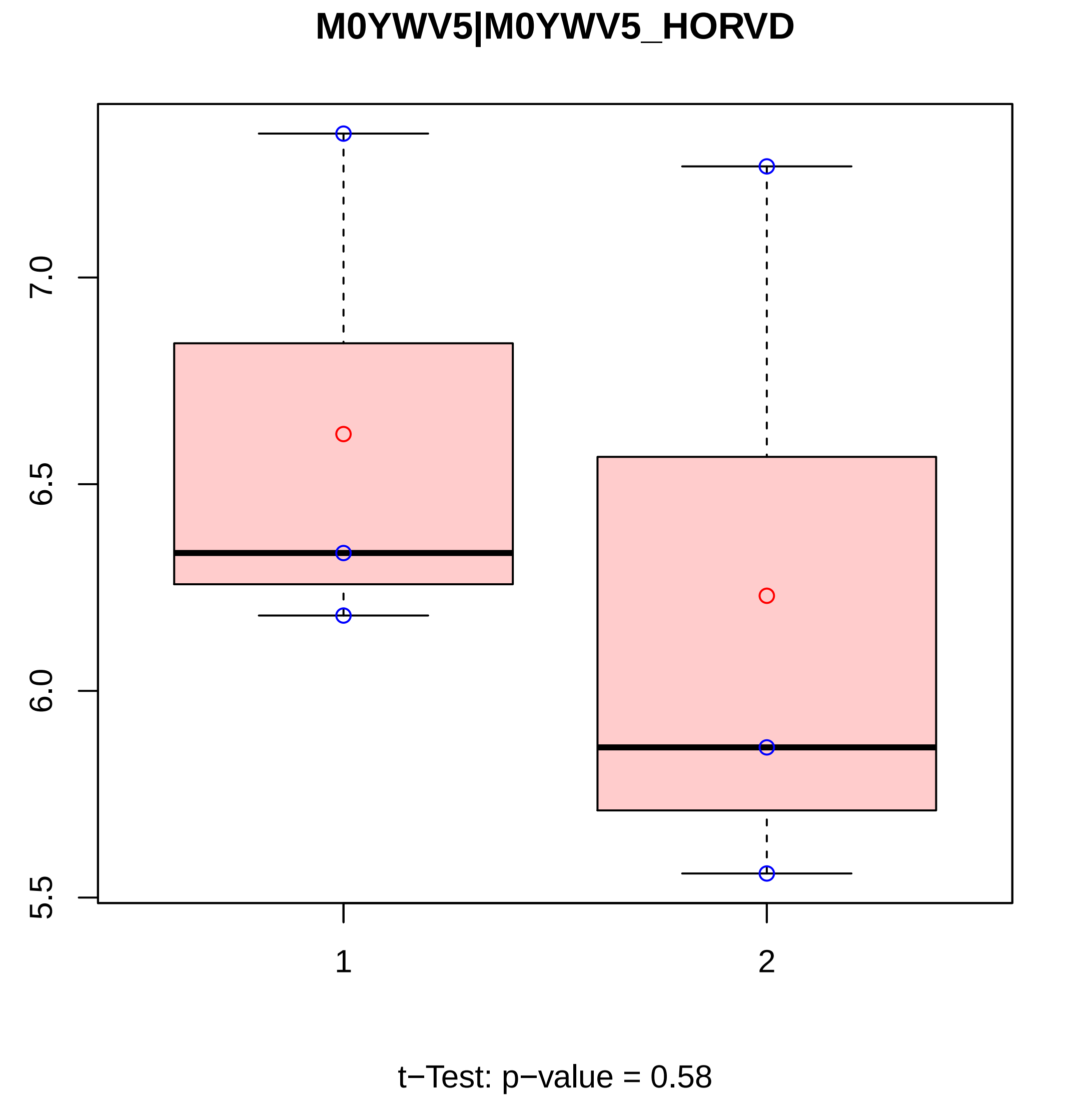
M0YWV5|M0YWV5_HORVD
7.0
6.5
6.0
5.5
1
2
t−Test: p−value = 0.58

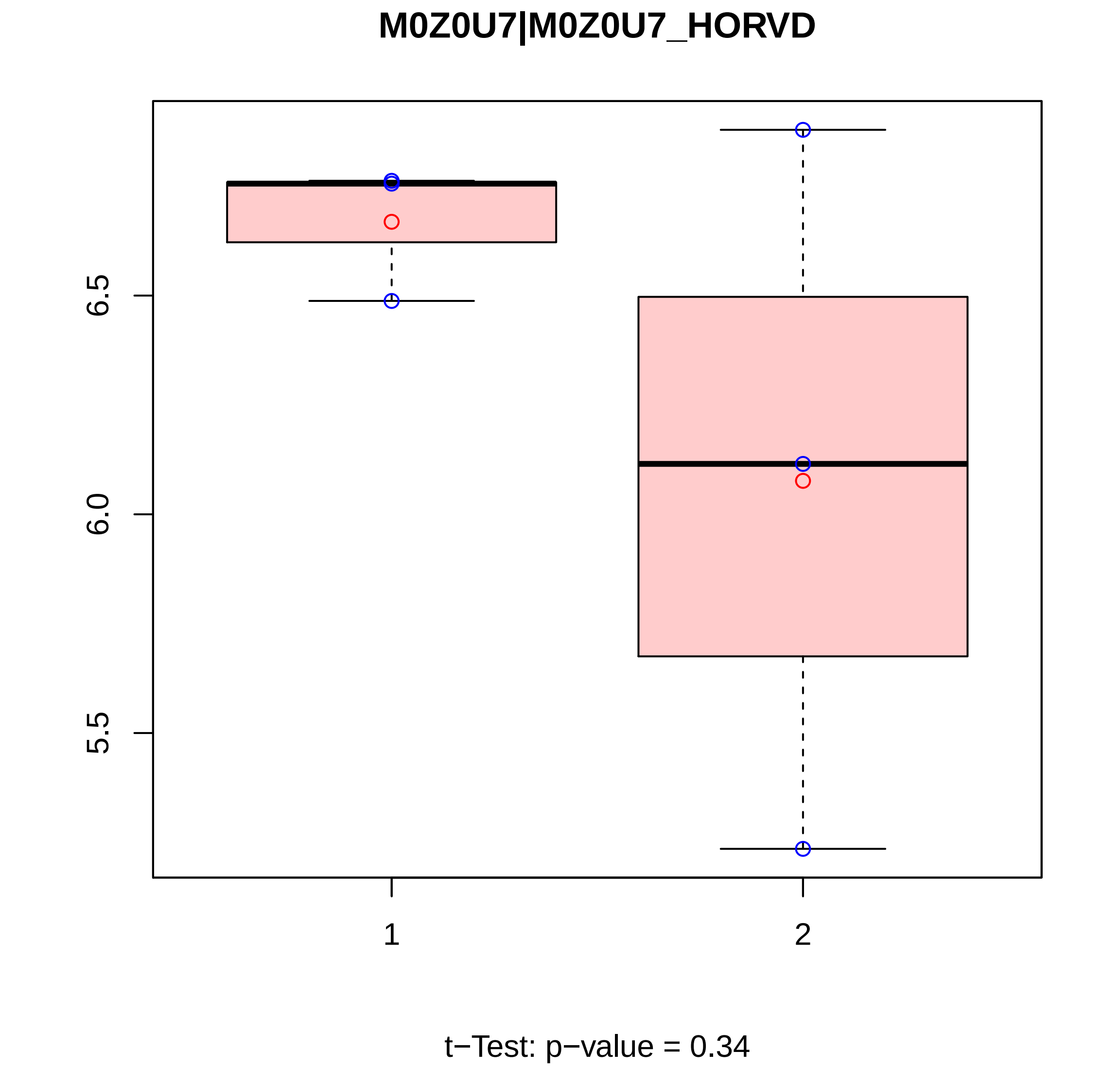
M0Z0U7|M0Z0U7_HORVD
6.5
6.0
5.5
1
2
t−Test: p−value = 0.34

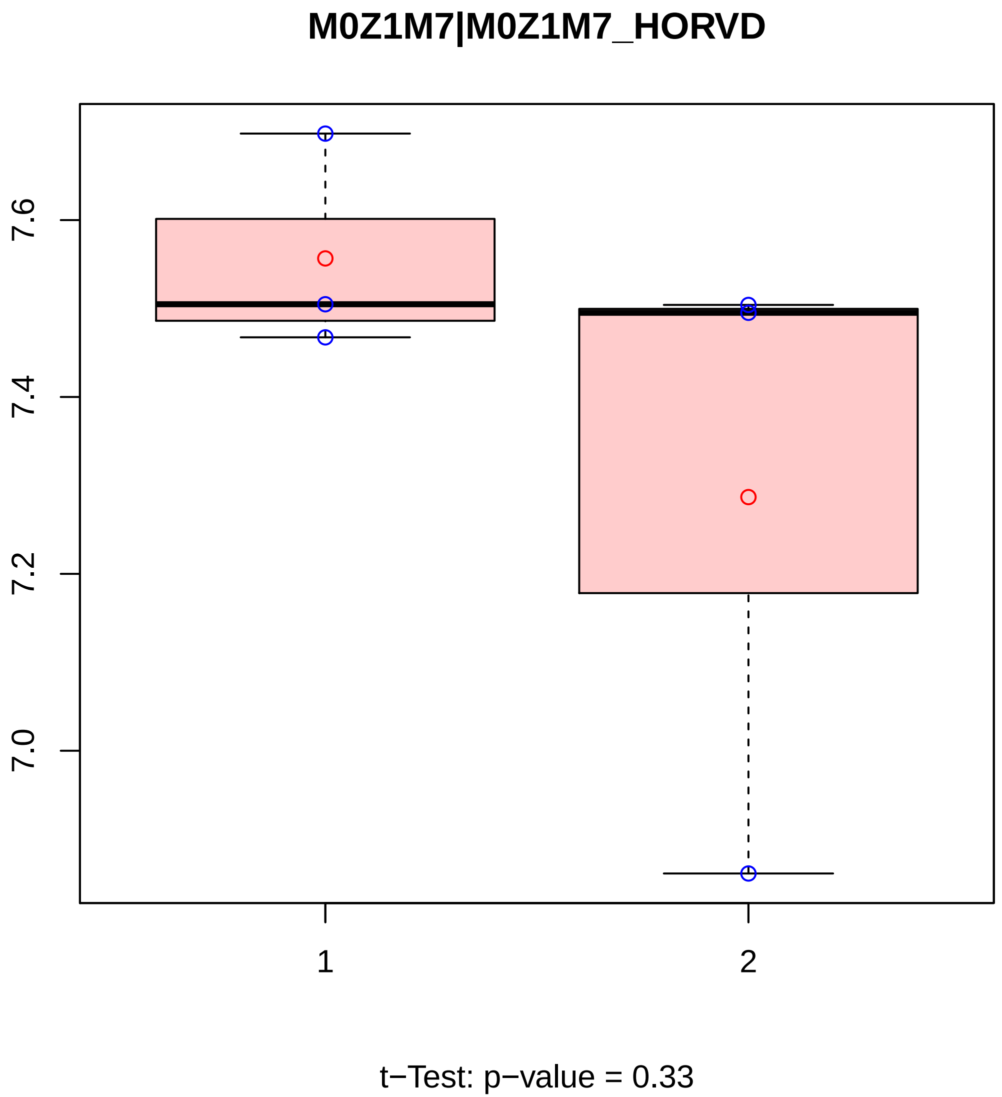

M0Z1M7|M0Z1M7_HORVD
7.6
7.4
7.2
7.0
1
2
t−Test: p−value = 0.33

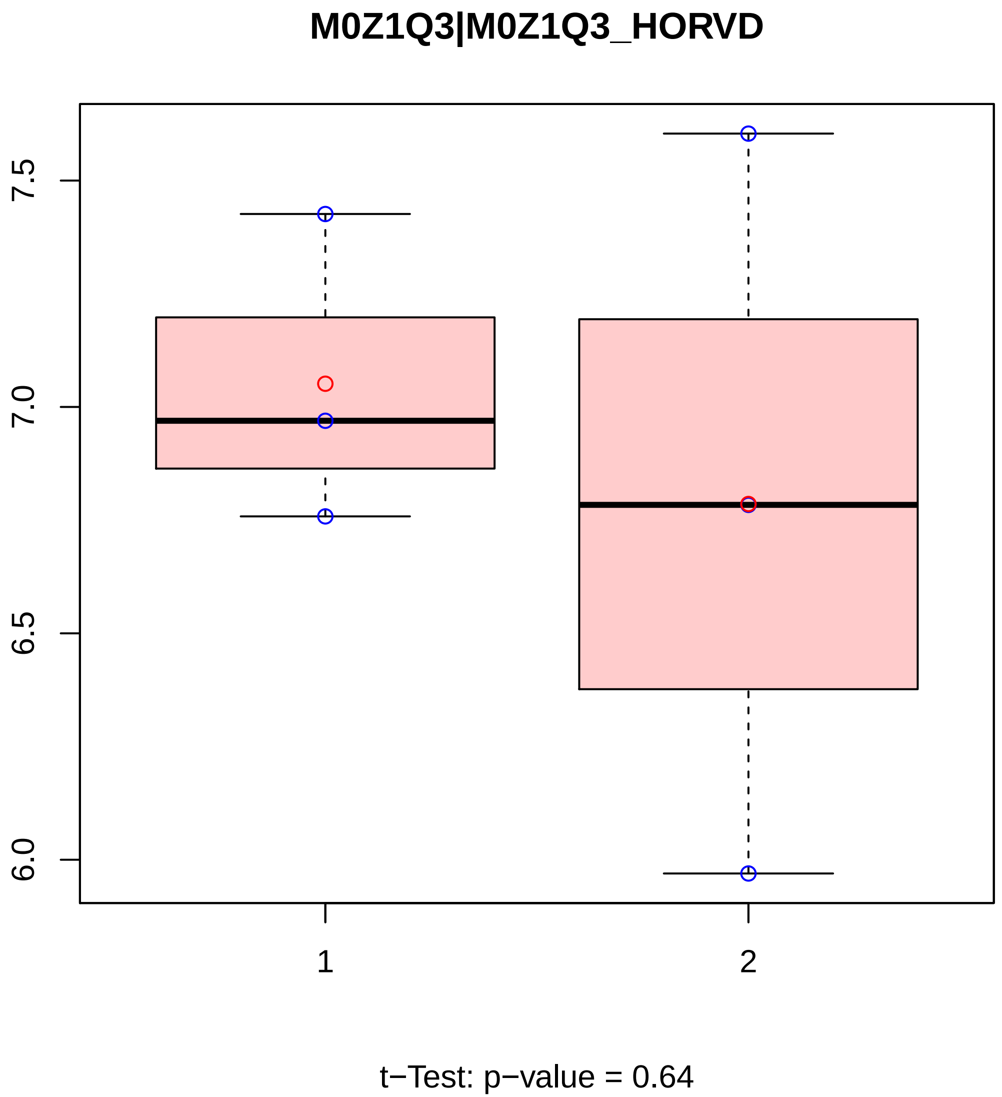
M0Z1Q3|M0Z1Q3_HORVD
7.5
7.0
6.5
6.0
1
2
t−Test: p−value = 0.64

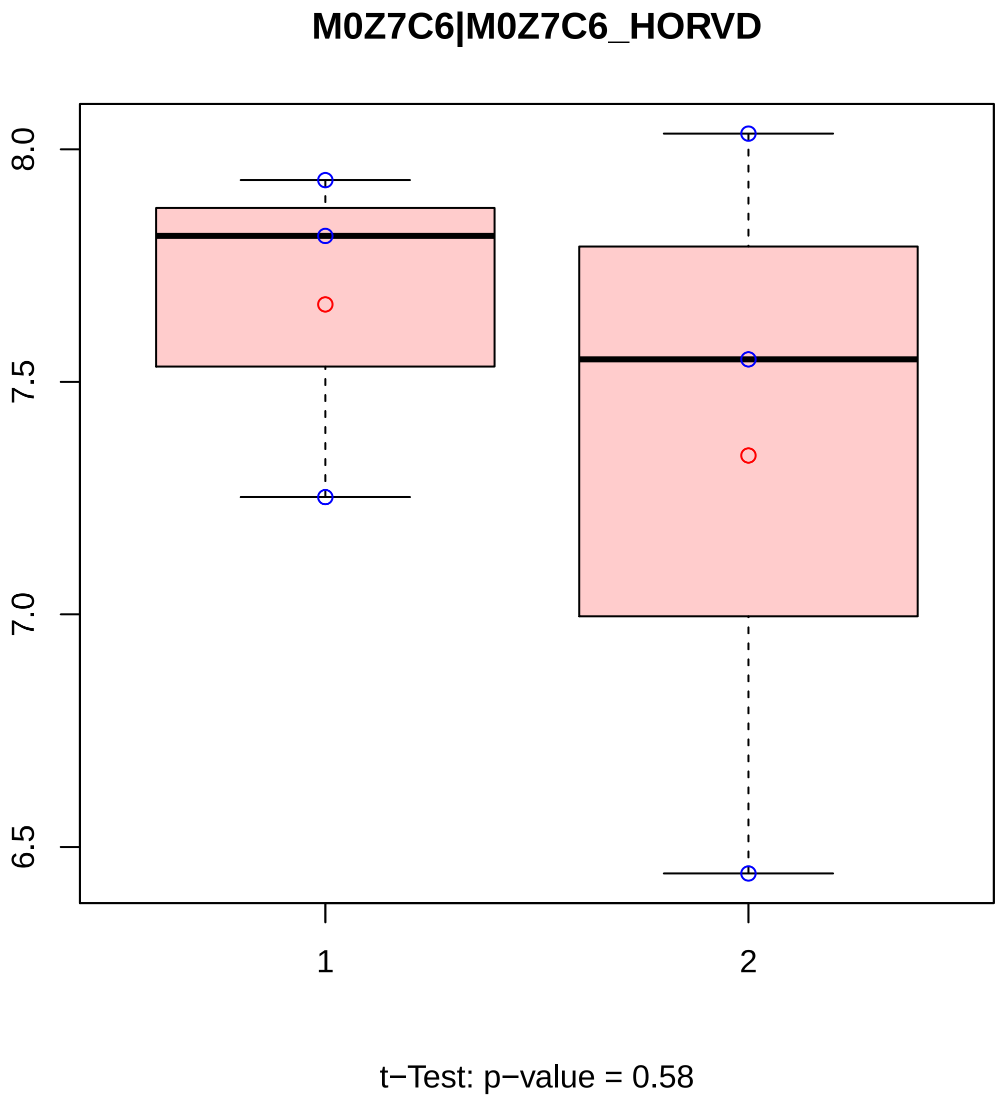

M0Z7C6|M0Z7C6_HORVD
8.0
7.5
7.0
6.5
1
2
t−Test: p−value = 0.58

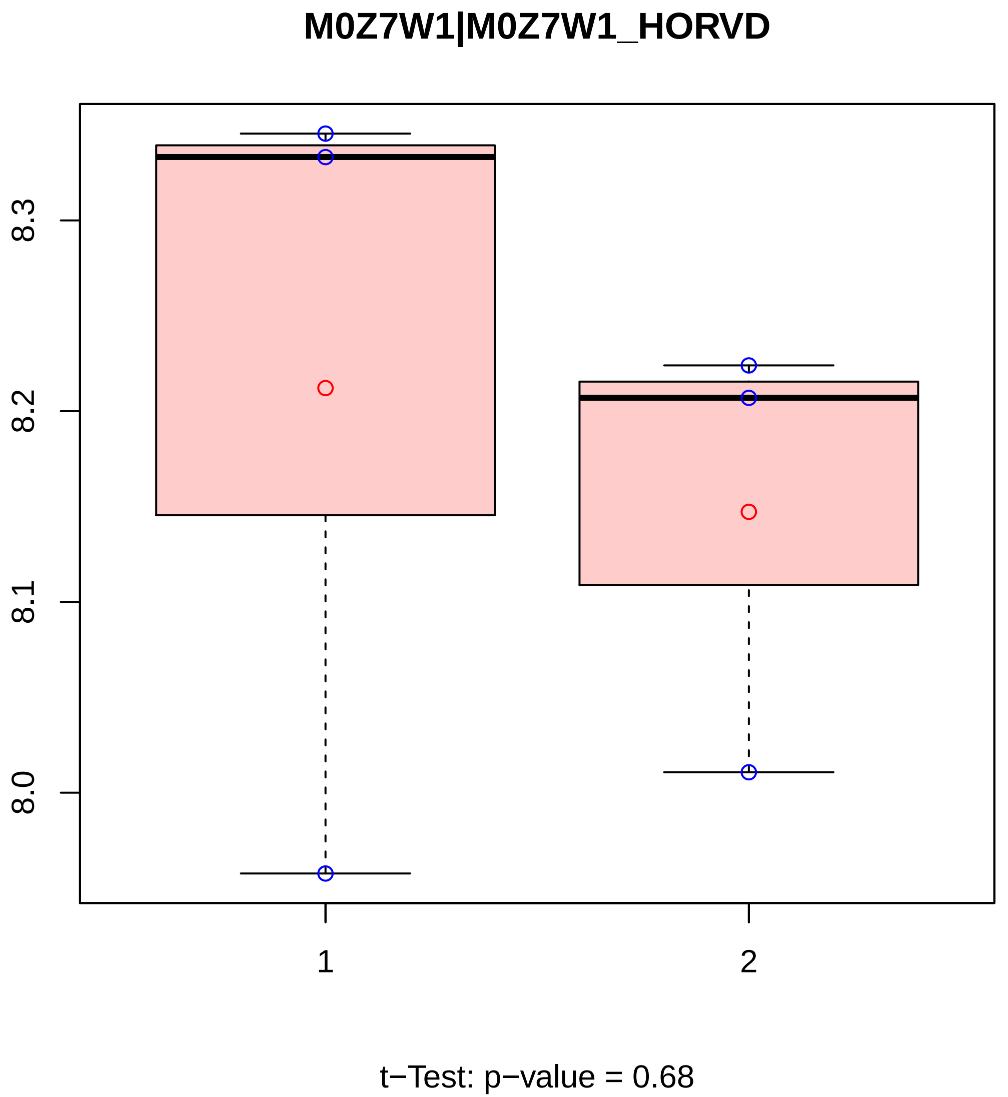
M0Z7W1|M0Z7W1_HORVD
8.0
8.1
8.2
8.3
1
2
t−Test: p−value = 0.68

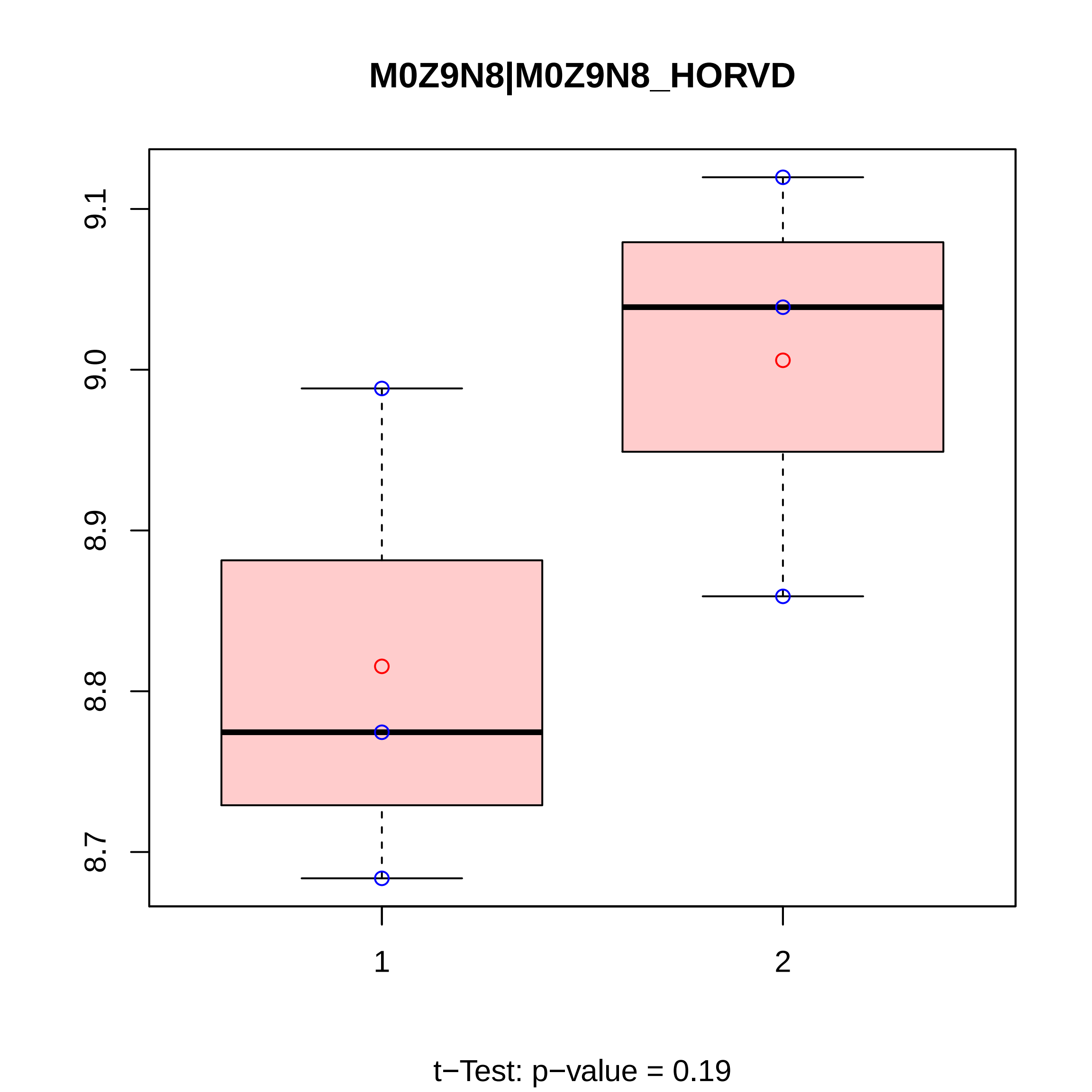

M0Z9N8|M0Z9N8_HORVD
9.1
9.0
8.9
8.8
8.7
1
2
t−Test: p−value = 0.19

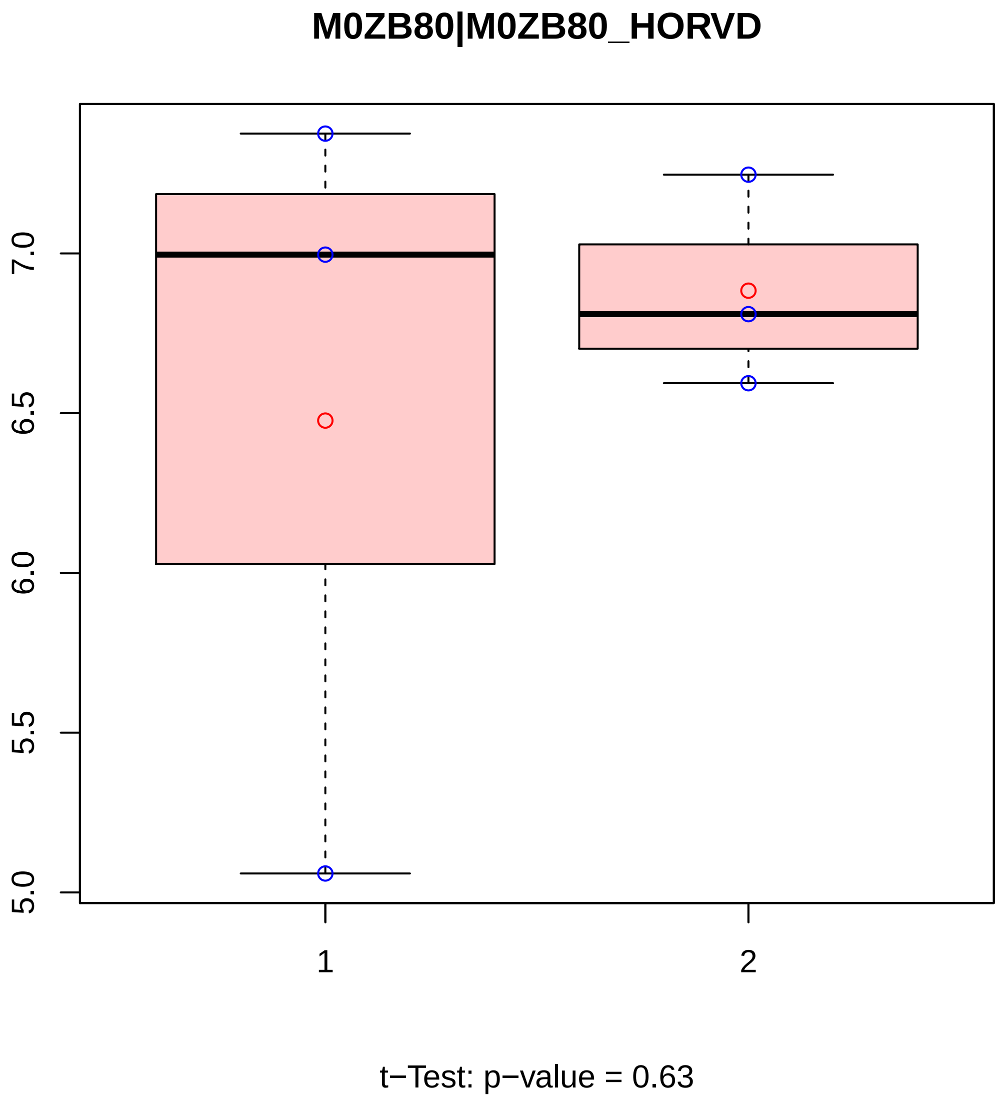
M0ZB80|M0ZB80_HORVD
7.0
6.5
6.0
5.5
5.0
1
2
t−Test: p−value = 0.63

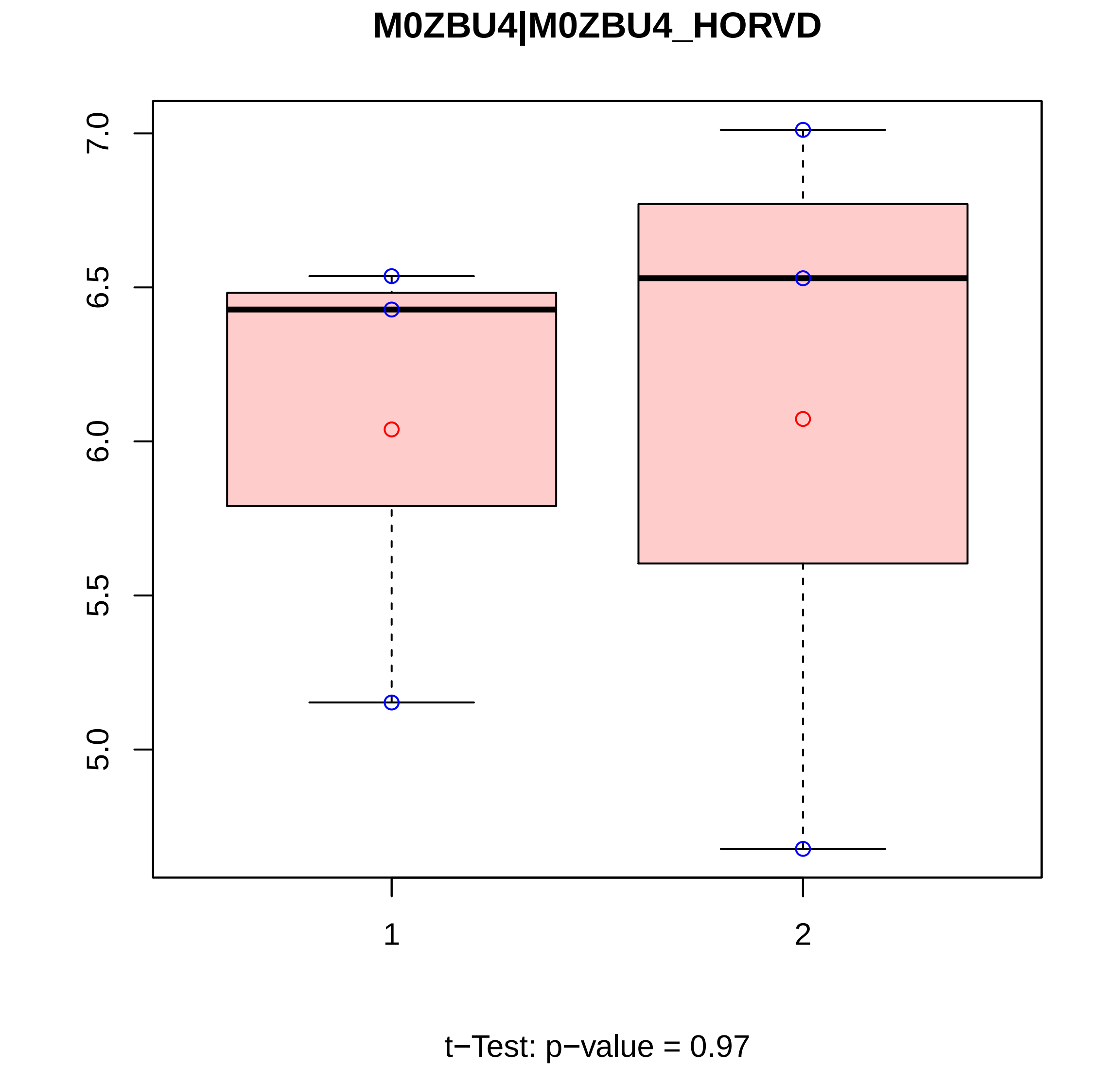
M0ZBU4|M0ZBU4_HORVD
7.0
6.5
6.0
5.5
5.0
1
2
t−Test: p−value = 0.97

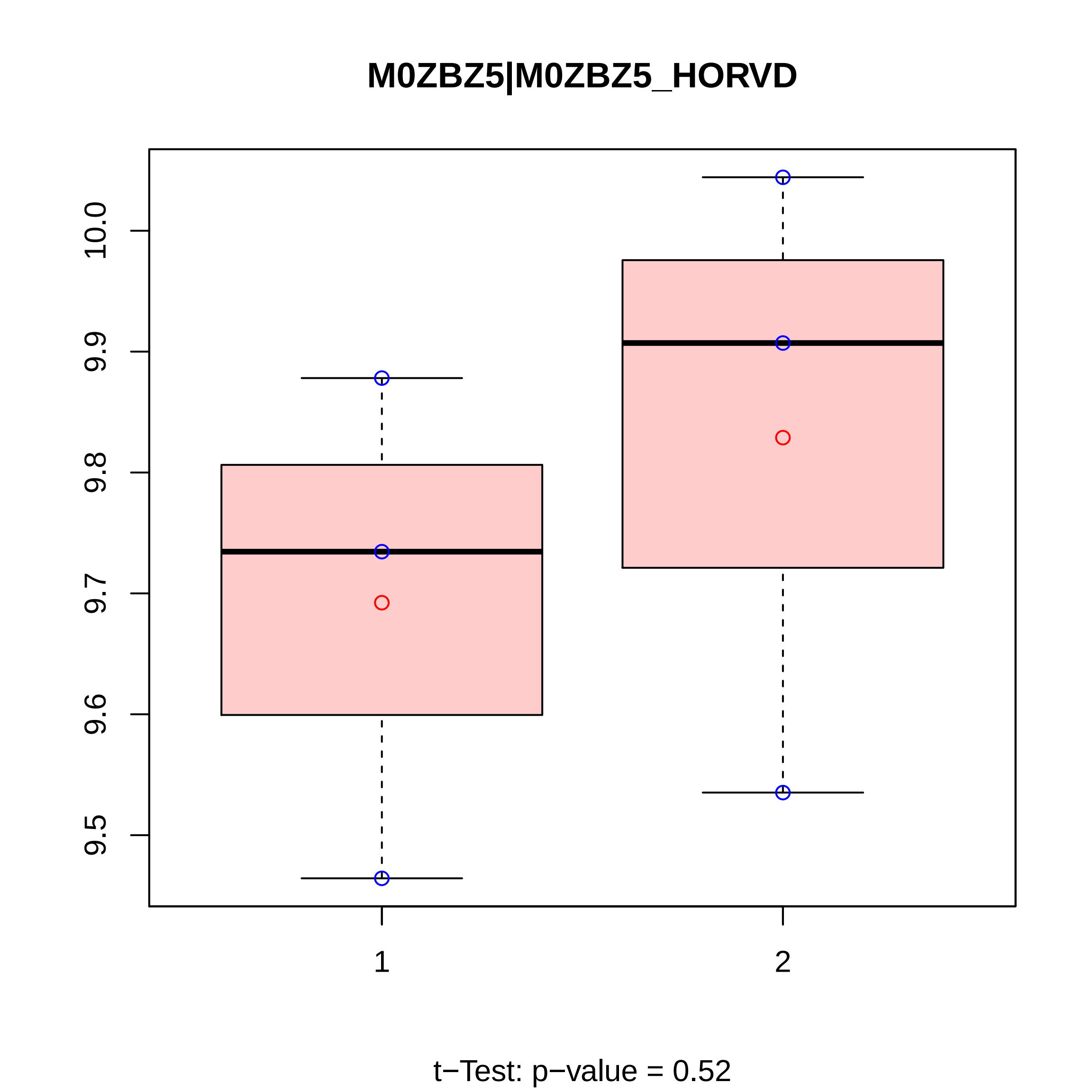

M0ZBZ5|M0ZBZ5_HORVD
10.0
9.9
9.8
9.7
9.6
9.5
1
2
t−Test: p−value = 0.52

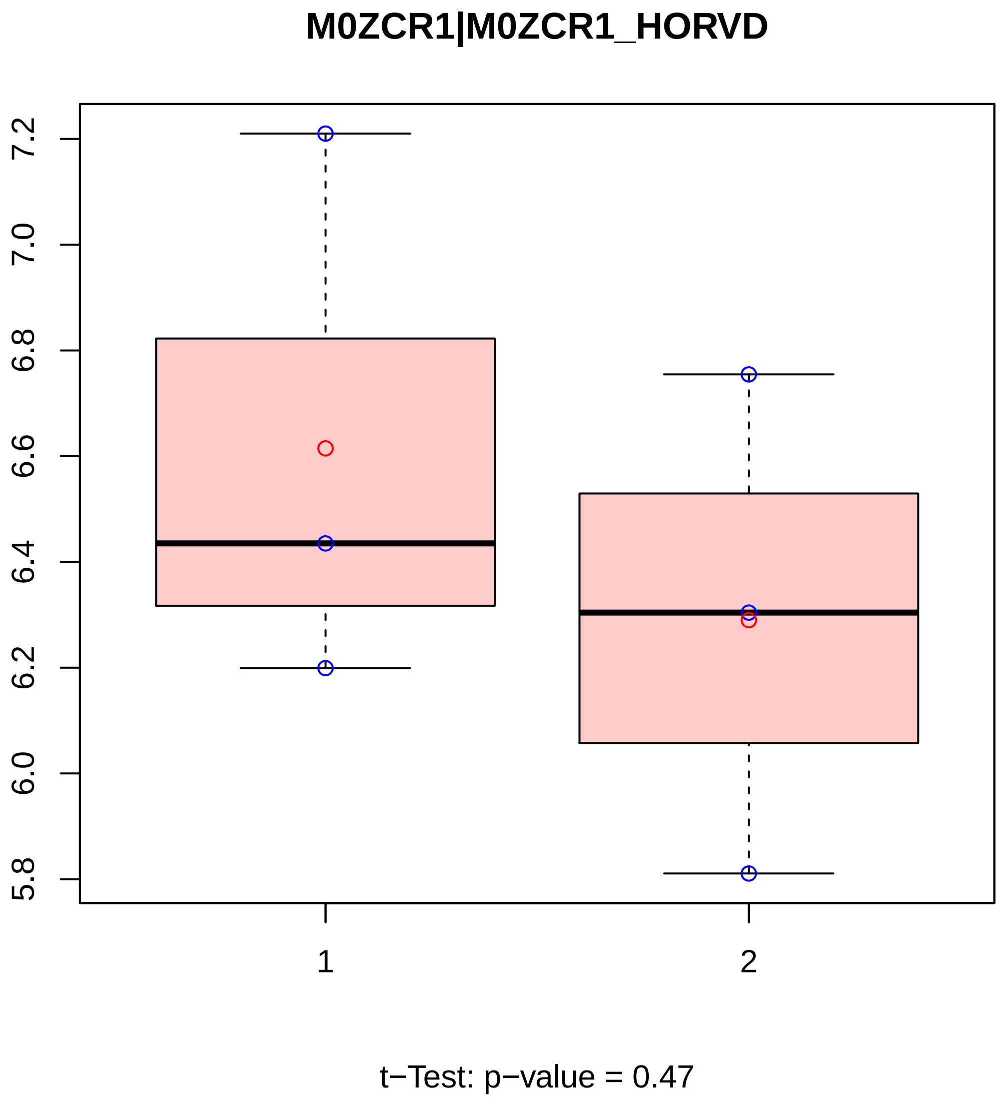
M0ZCR1|M0ZCR1_HORVD
7.2
7.0
6.8
6.6
6.4
6.2
6.0
5.8
1
2
t−Test: p−value = 0.47

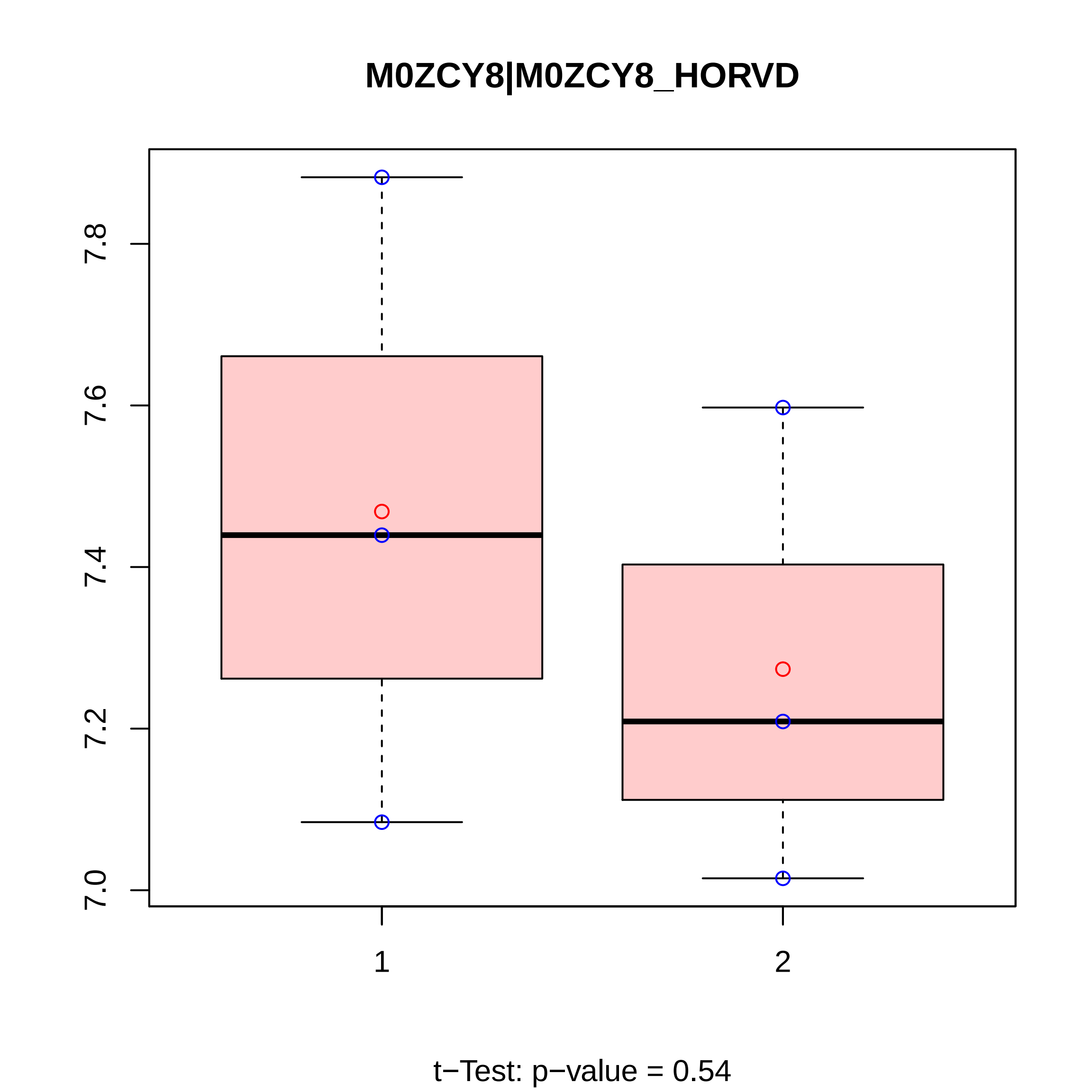

M0ZCY8|M0ZCY8_HORVD
7.0
7.2
7.4
7.6
7.8
1
2
t−Test: p−value = 0.54

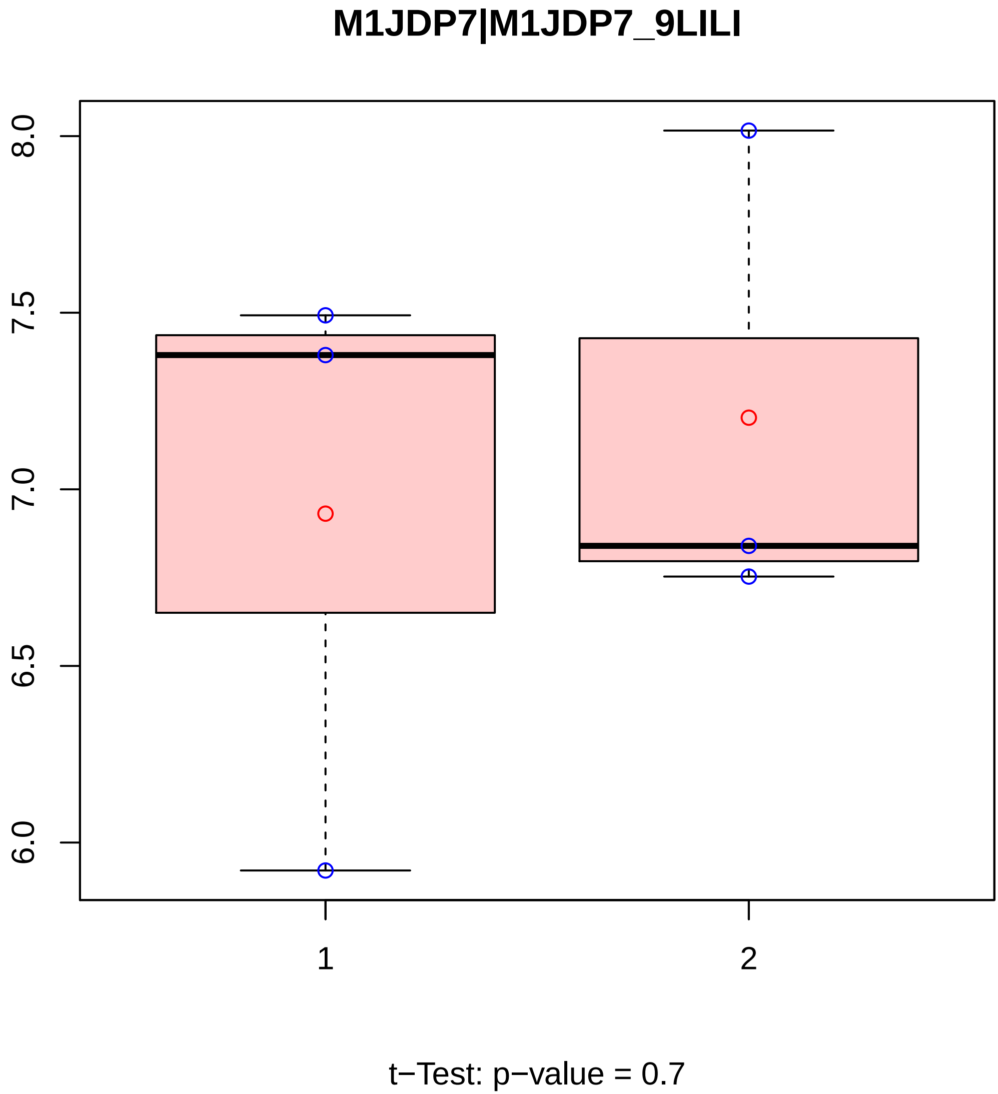
M1JDP7|M1JDP7_9LILI
8.0
7.5
7.0
6.5
6.0
1
2
t−Test: p−value = 0.7

M1JYJ5|M1JYJ5_9LILI

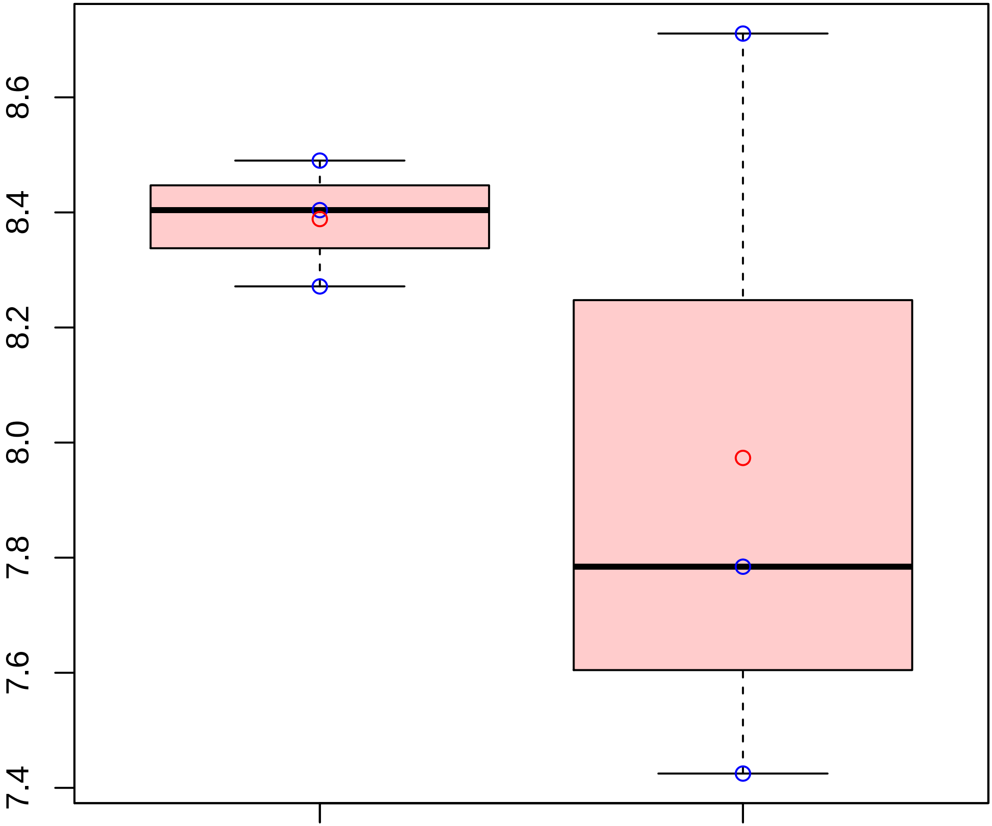

t−Test: p−value = 0.39

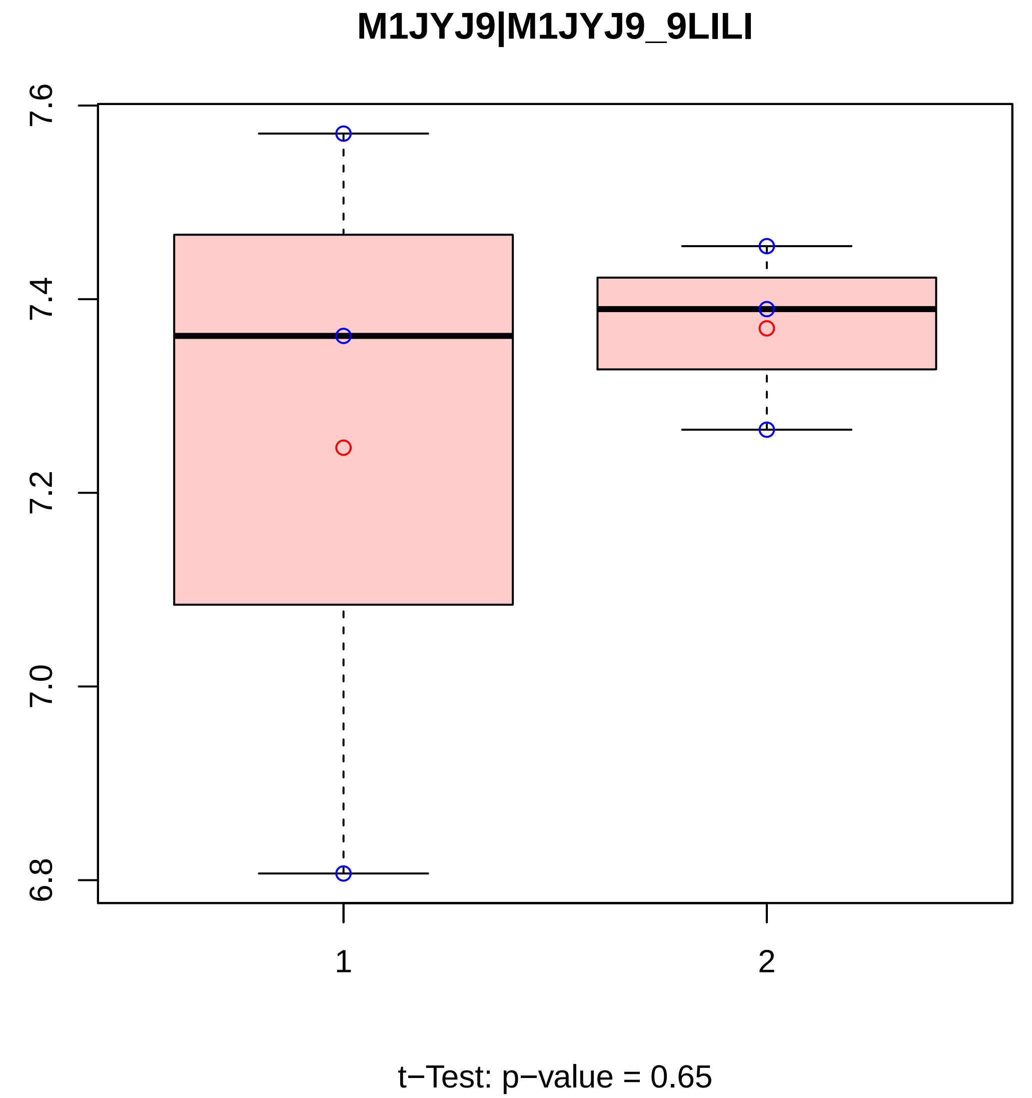
M1JYJ9|M1JYJ9_9LILI
7.6
7.4
7.2
7.0
6.8
1
2
t−Test: p−value = 0.65

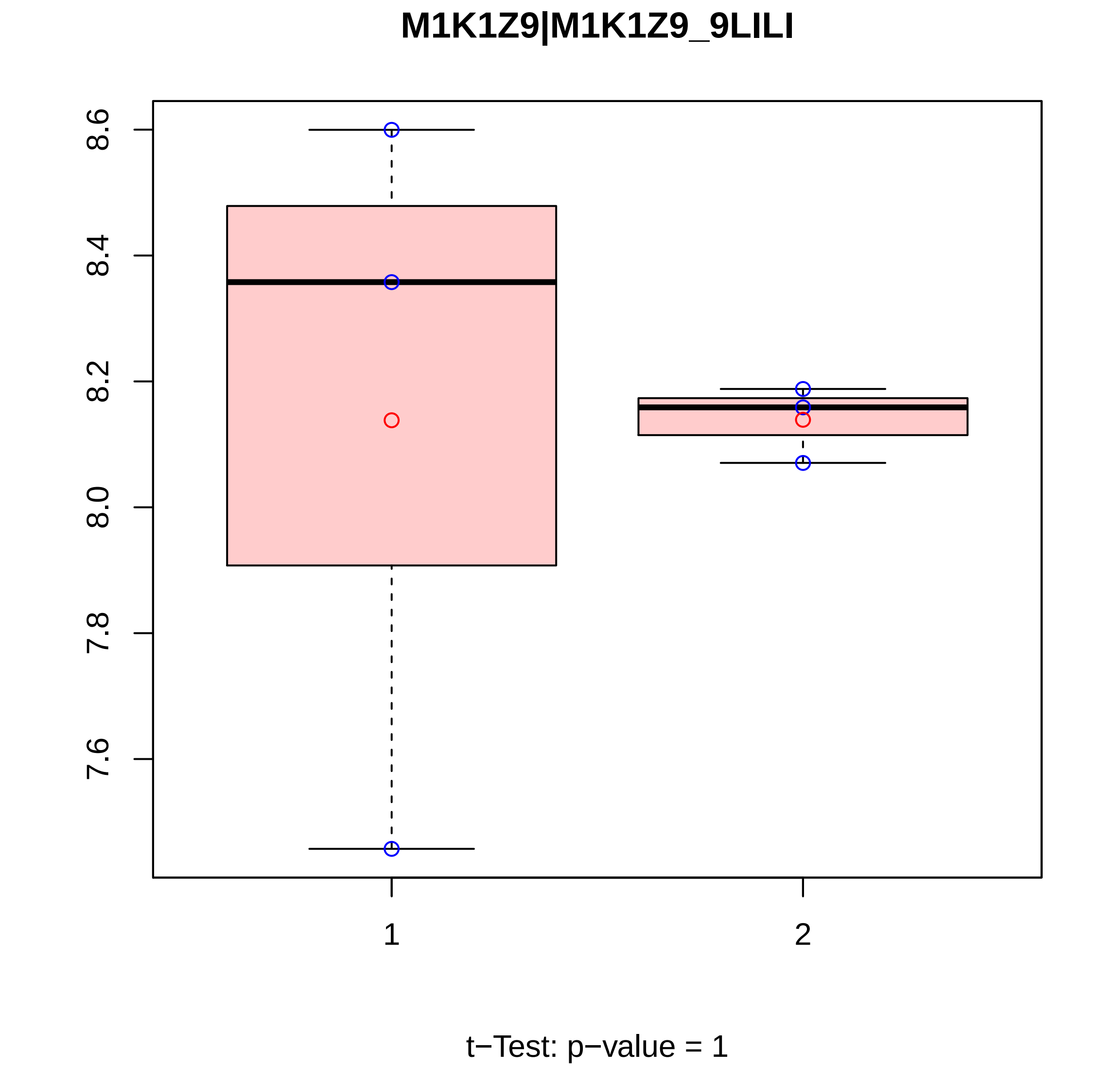
M1K1Z9|M1K1Z9_9LILI
8.6
8.4
8.2
8.0
7.8
7.6
1
2
t−Test: p−value = 1

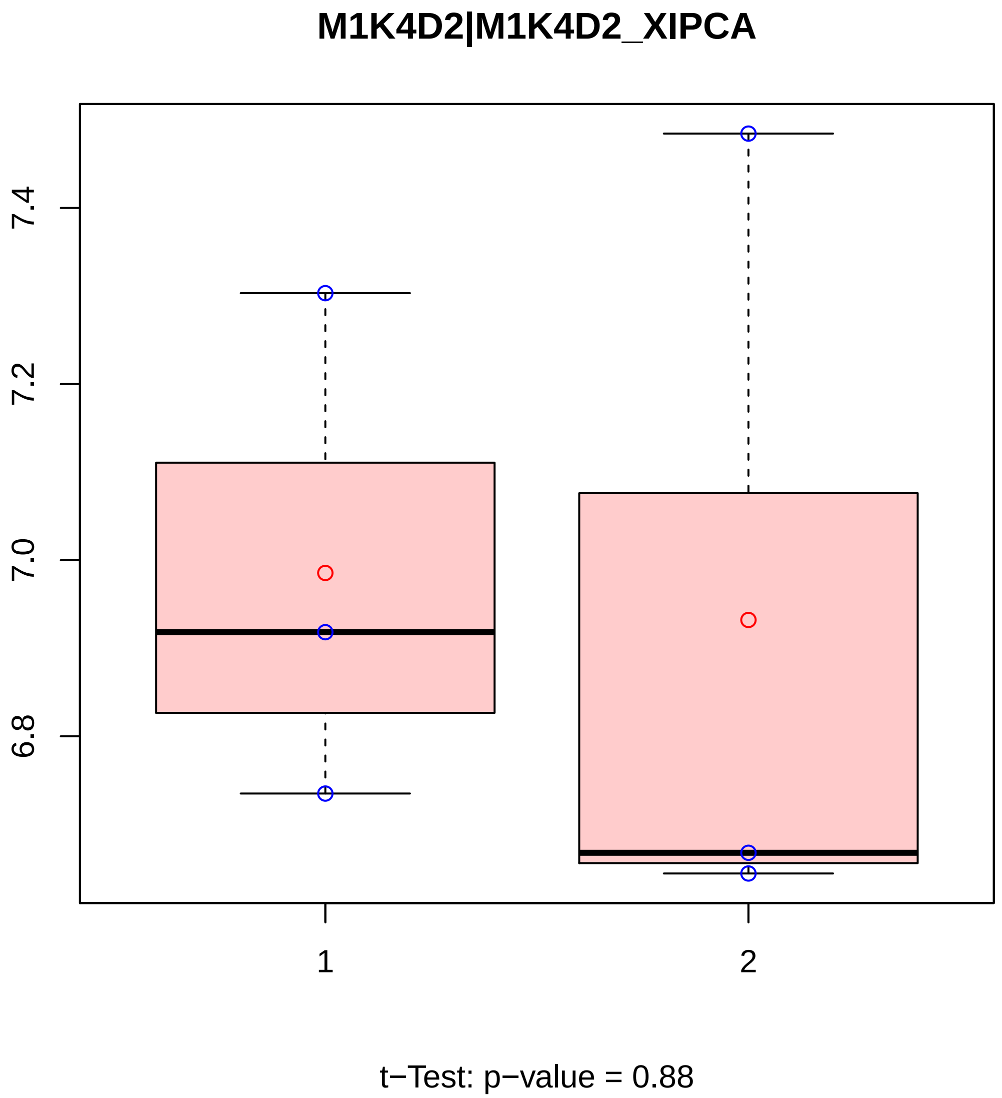

M1K4D2|M1K4D2_XIPCA
7.4
7.2
7.0
6.8
1
2
t−Test: p−value = 0.88

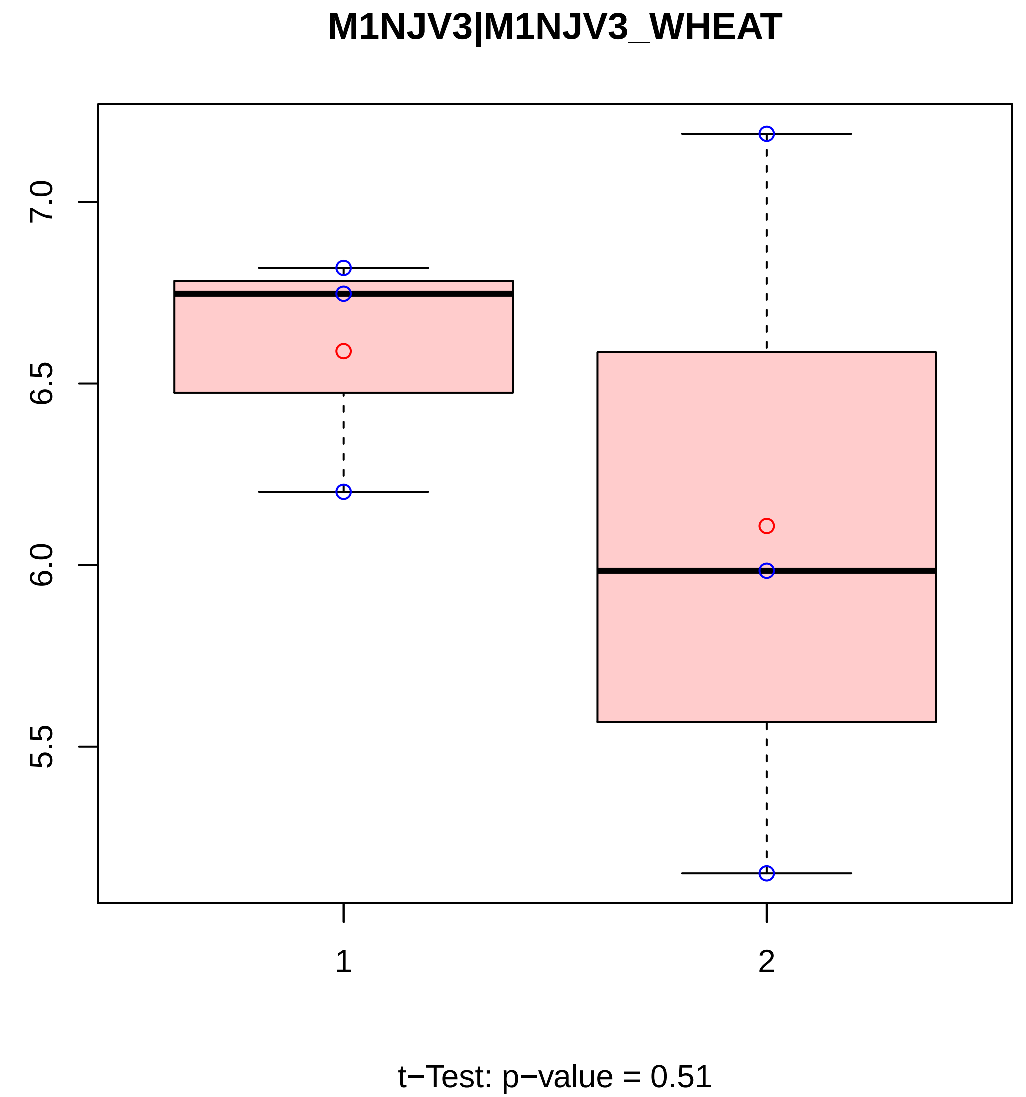
M1NJV3|M1NJV3_WHEAT
7.0
6.5
6.0
5.5
1
2
t−Test: p−value = 0.51

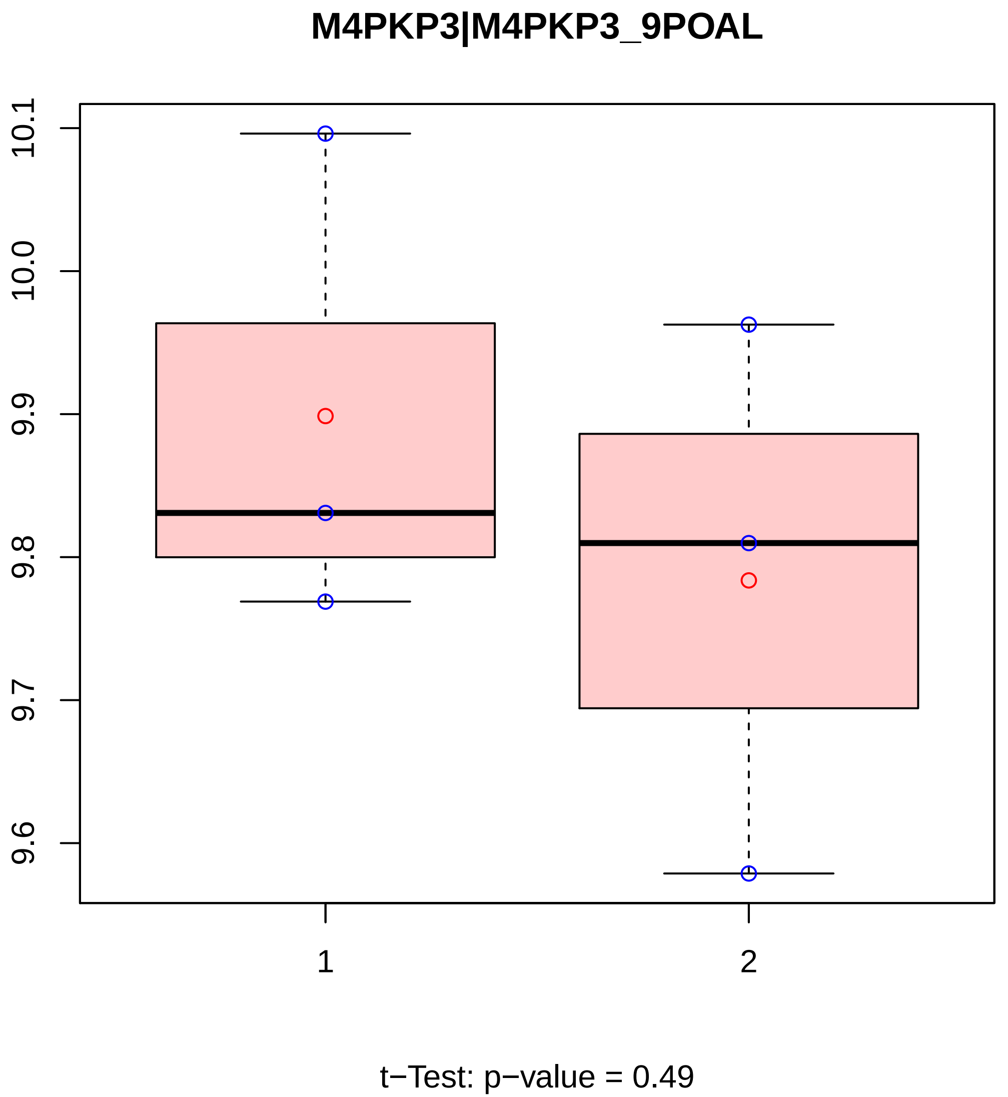

M4PKP3|M4PKP3_9POAL
10.1
10.0
9.9
9.8
9.7
9.6
1
2
t−Test: p−value = 0.49

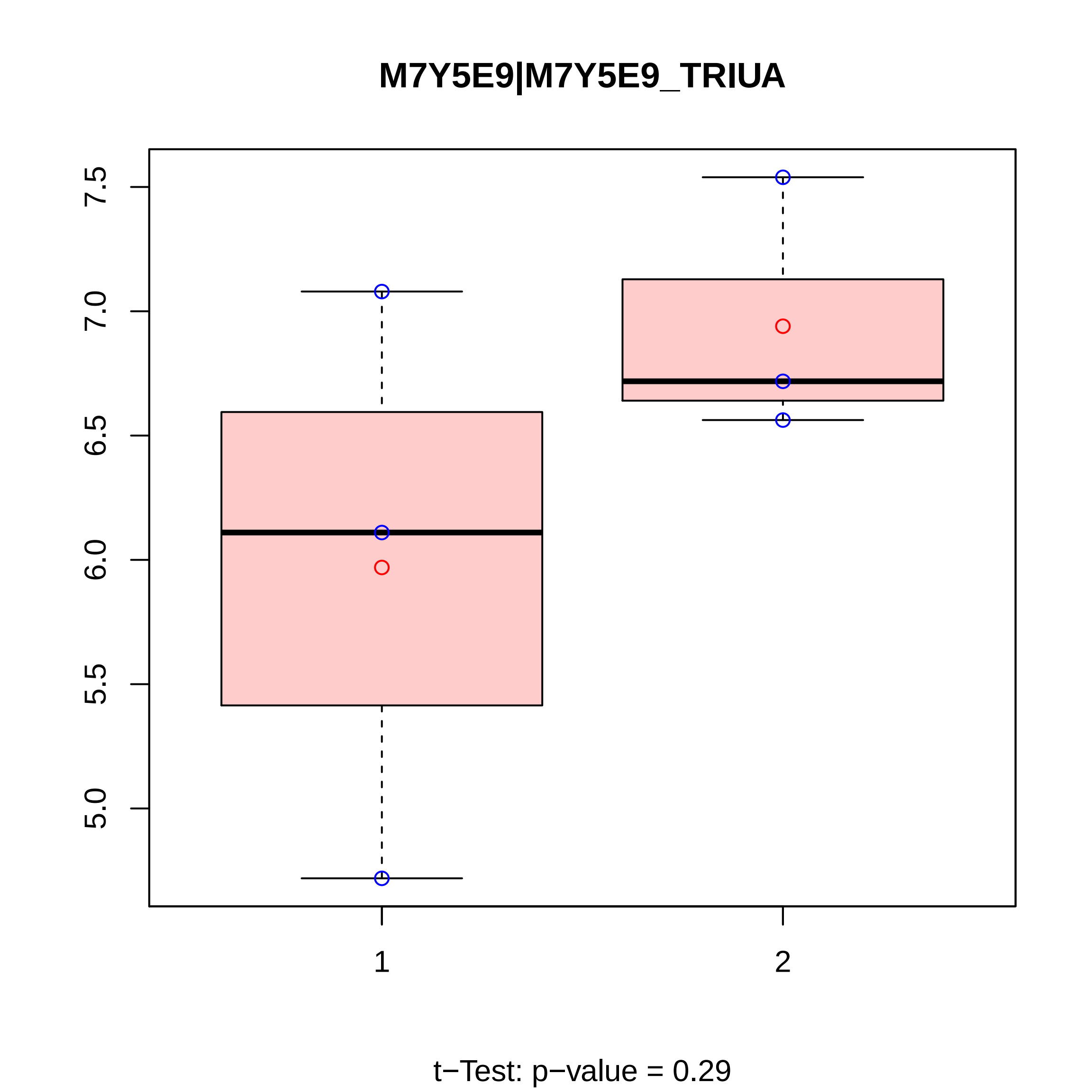
M7Y5E9|M7Y5E9_TRIUA
7.5
7.0
6.5
6.0
5.5
5.0
1
2
t−Test: p−value = 0.29

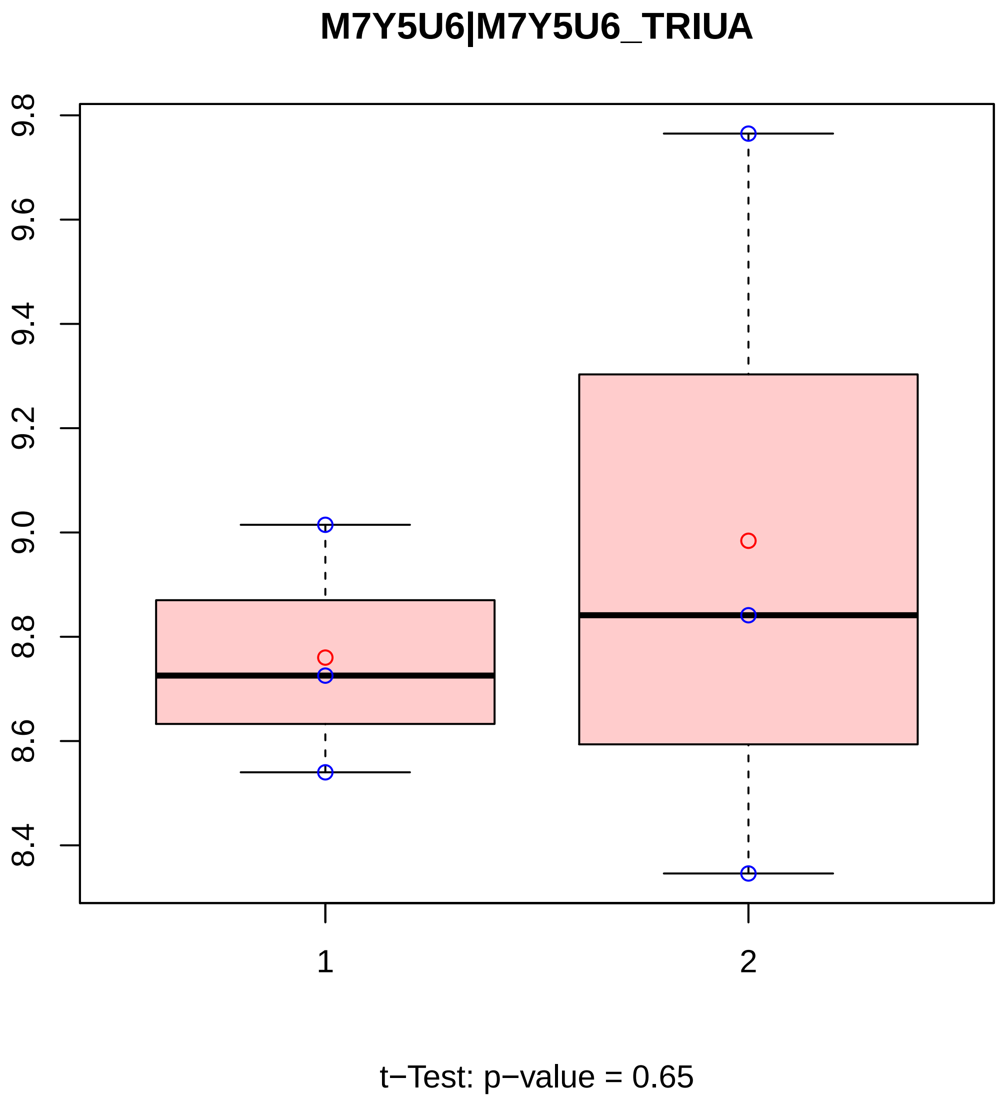
M7Y5U6|M7Y5U6_TRIUA
9.8
9.6
9.4
9.2
9.0
8.8
8.6
8.4
1
2
t−Test: p−value = 0.65

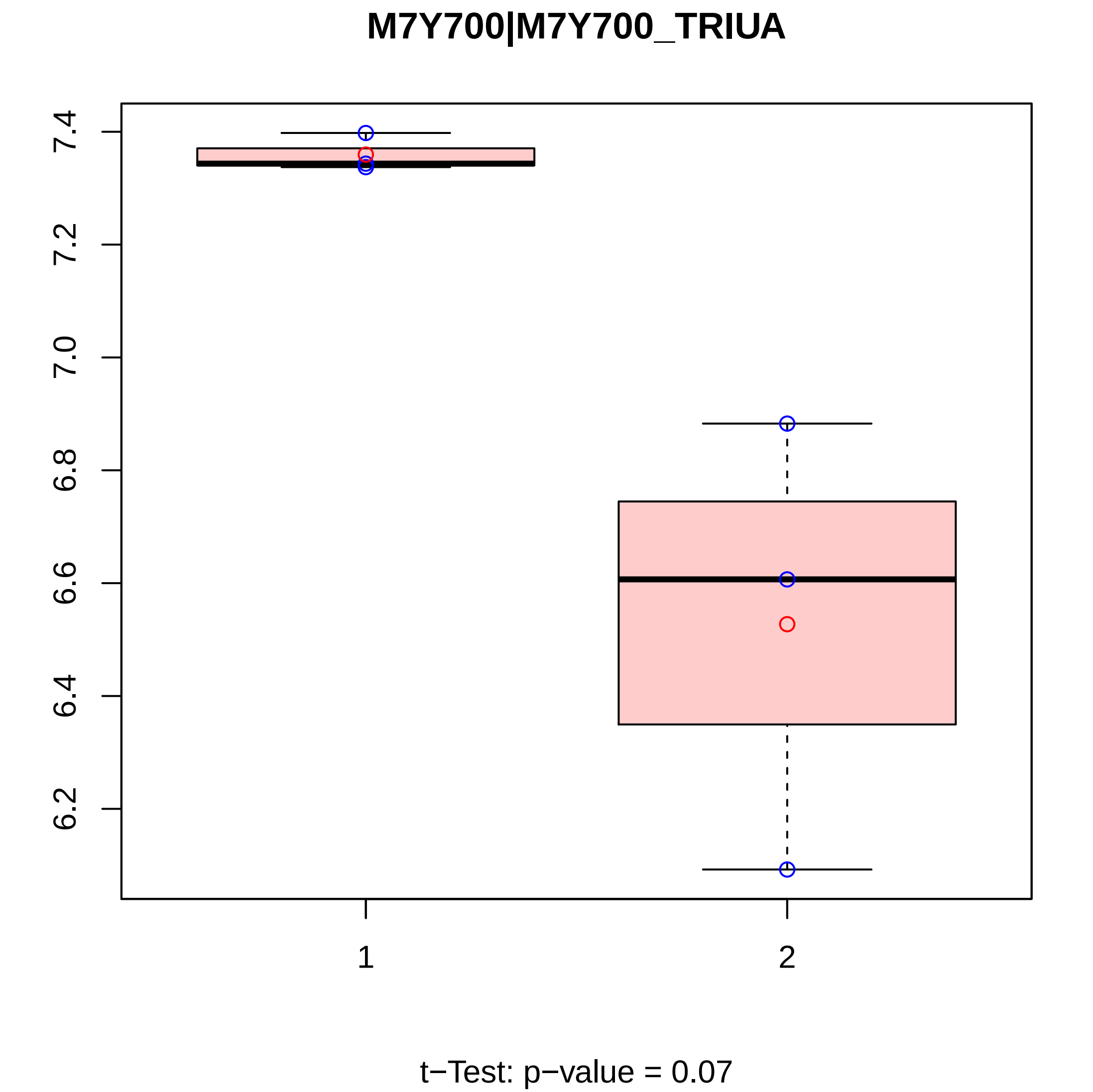
M7Y700|M7Y700_TRIUA
7.4
7.2
7.0
6.8
6.6
6.4
6.2
1
2
t−Test: p−value = 0.07

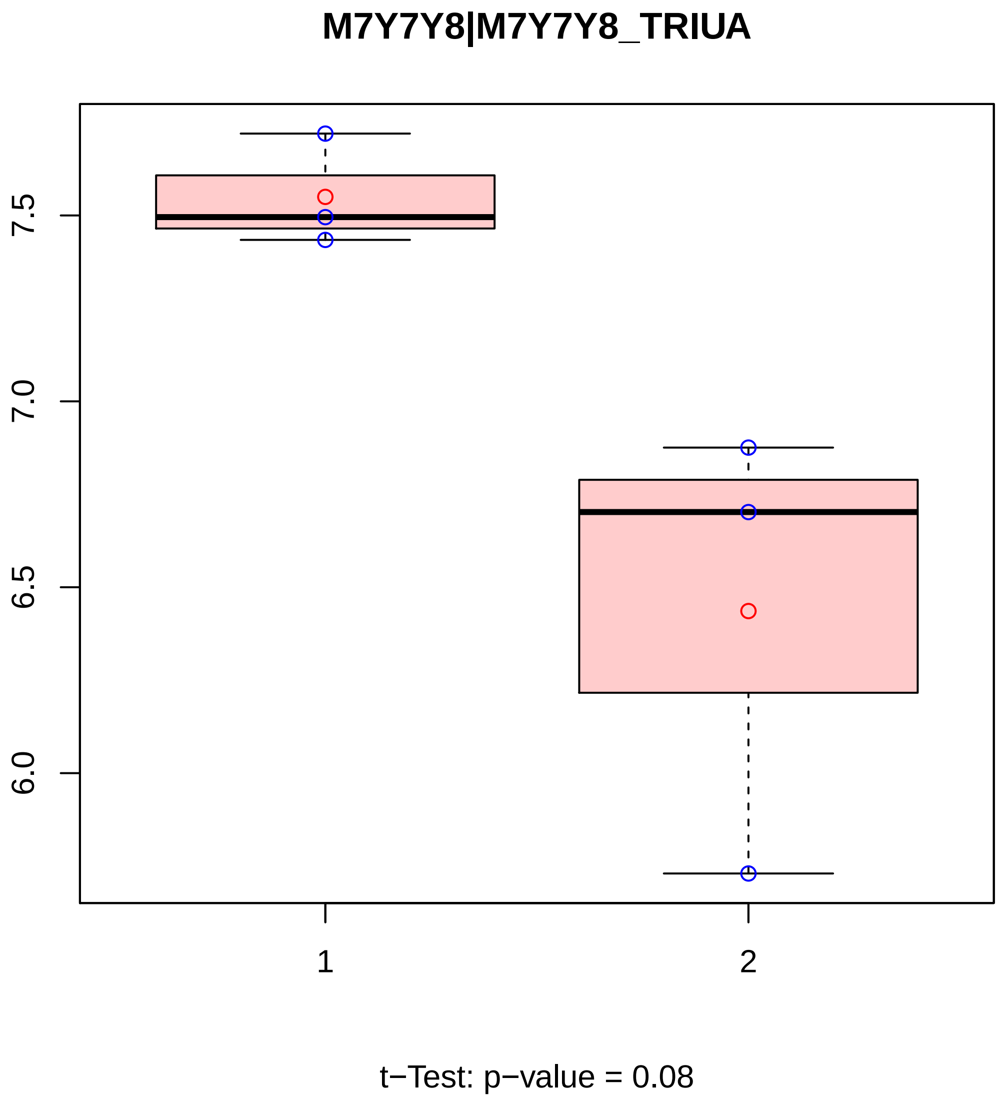
M7Y7Y8|M7Y7Y8_TRIUA
7.5
7.0
6.5
6.0
1
2
t−Test: p−value = 0.08

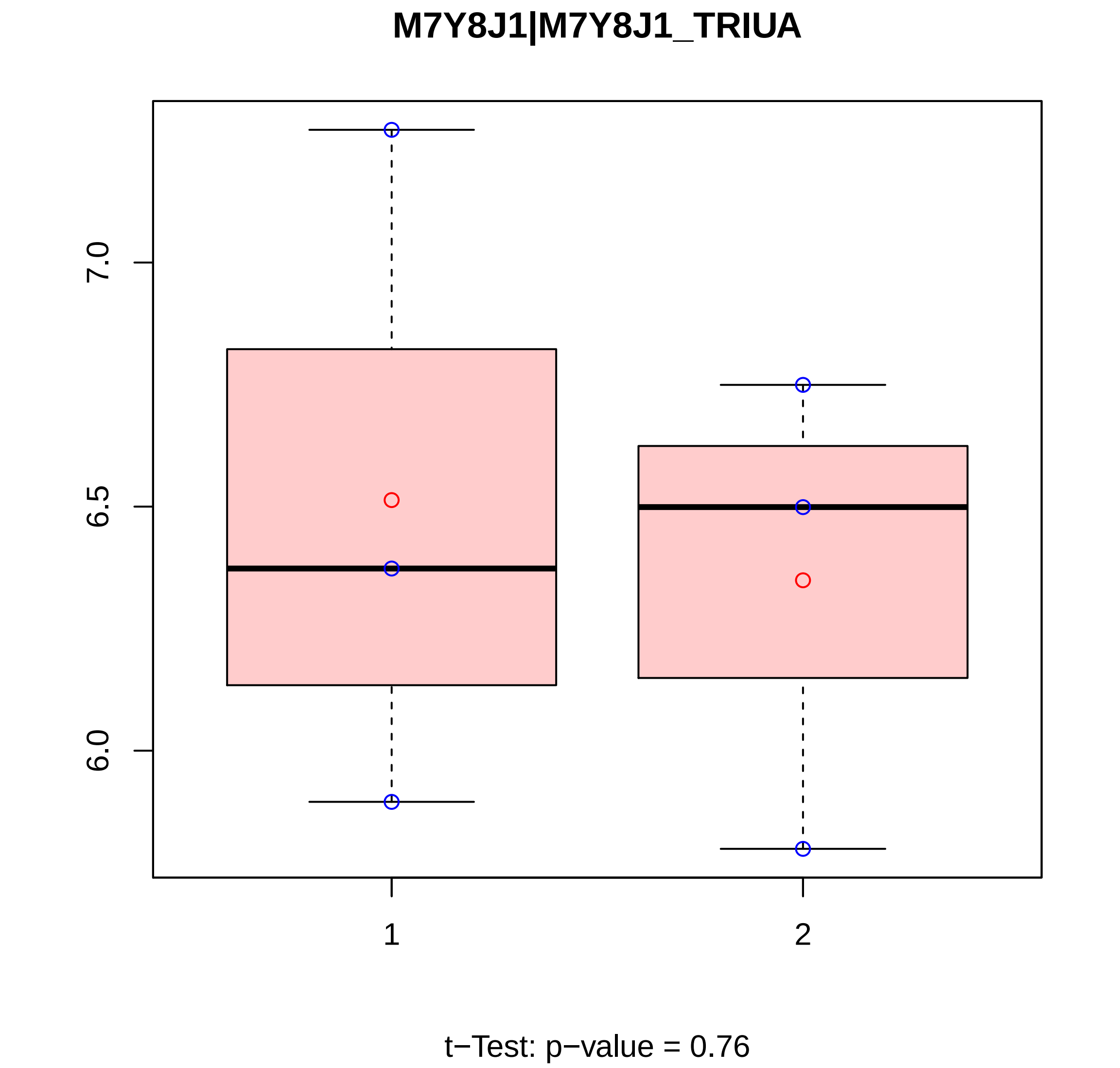
M7Y8J1|M7Y8J1_TRIUA
7.0
6.5
6.0
1
2
t−Test: p−value = 0.76

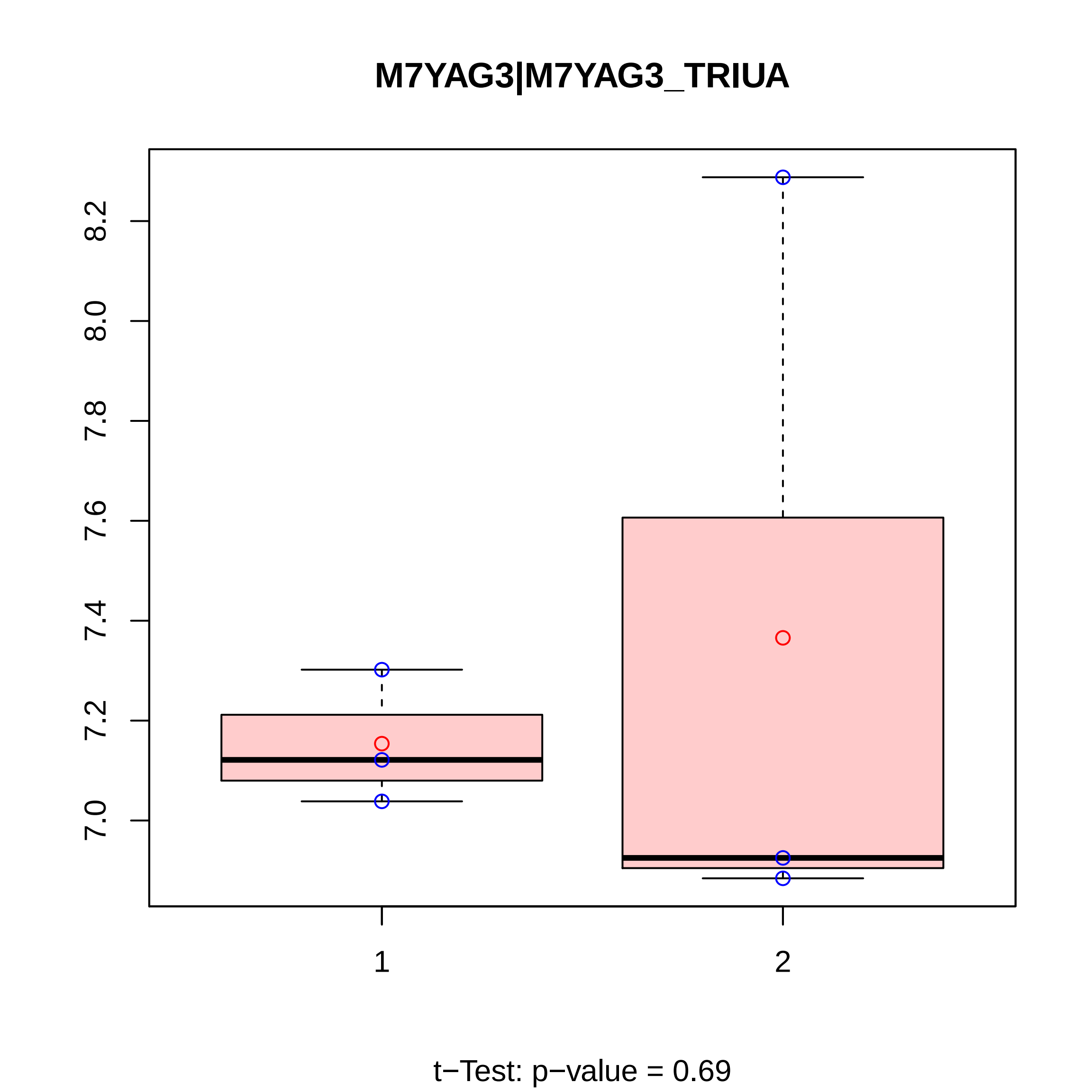
M7YAG3|M7YAG3_TRIUA
8.2
8.0
7.8
7.6
7.4
7.2
7.0
1
2
t−Test: p−value = 0.69

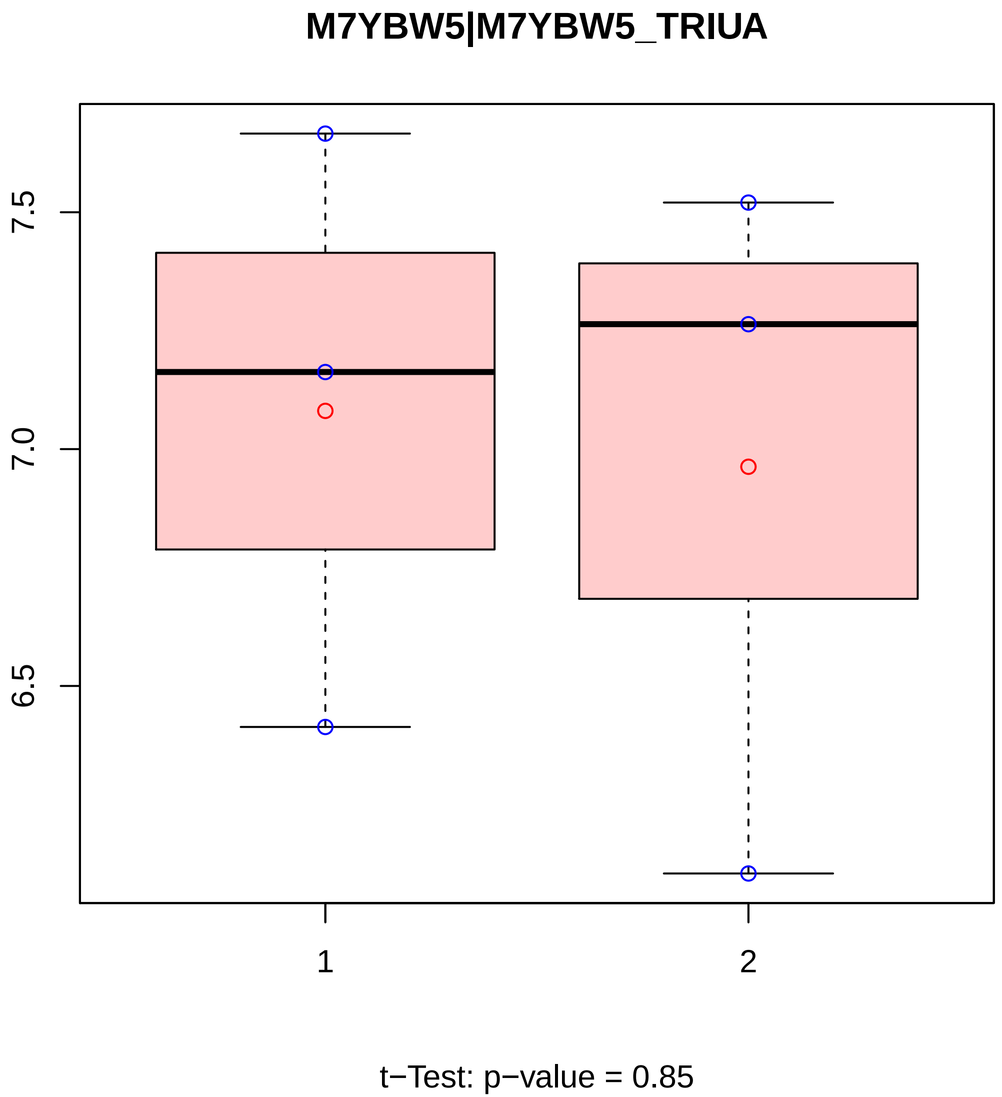
M7YBW5|M7YBW5_TRIUA
7.5
7.0
6.5
1
2
t−Test: p−value = 0.85

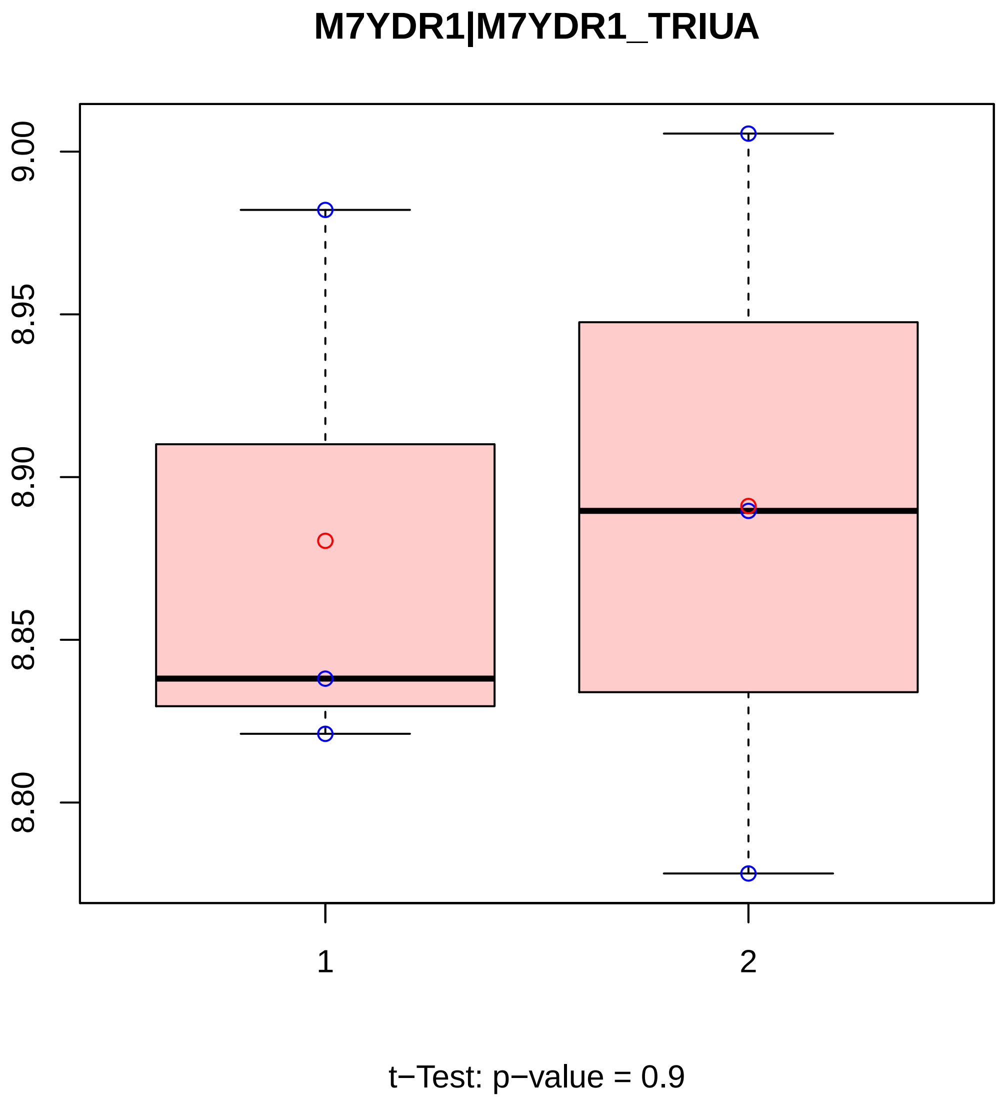
M7YDR1|M7YDR1_TRIUA
9.00
8.95
8.90
8.85
8.80
1
2
t−Test: p−value = 0.9

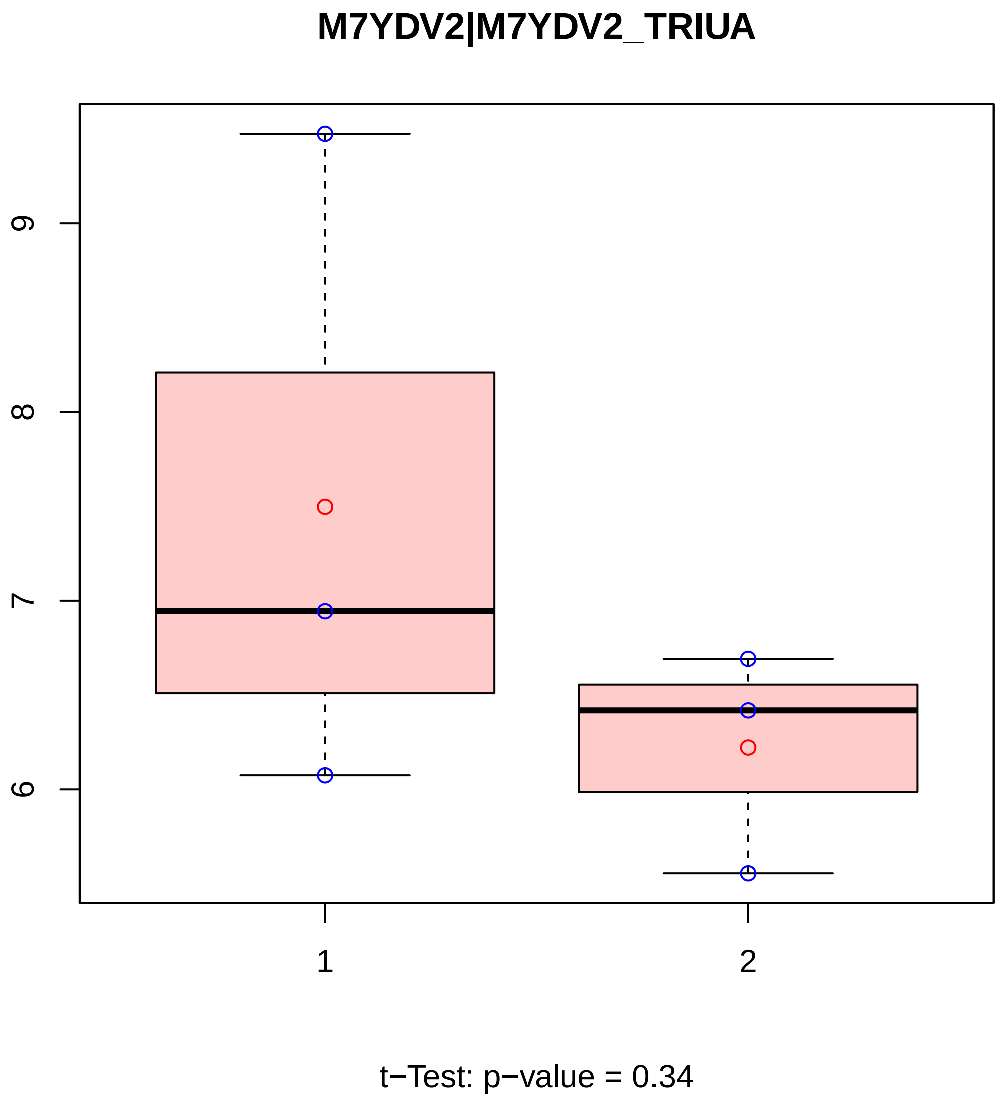
M7YDV2|M7YDV2_TRIUA
9
8
7
6
1
2
t−Test: p−value = 0.34

M7YE69|M7YE69_TRIUA

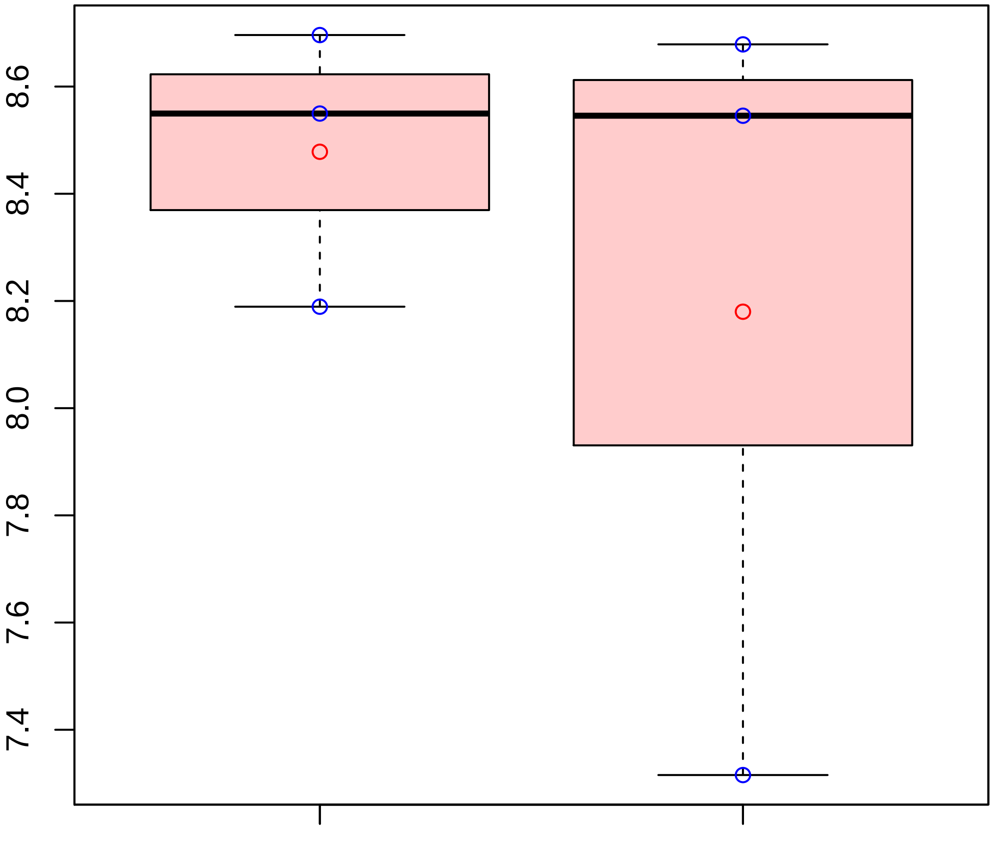

t−Test: p−value = 0.57

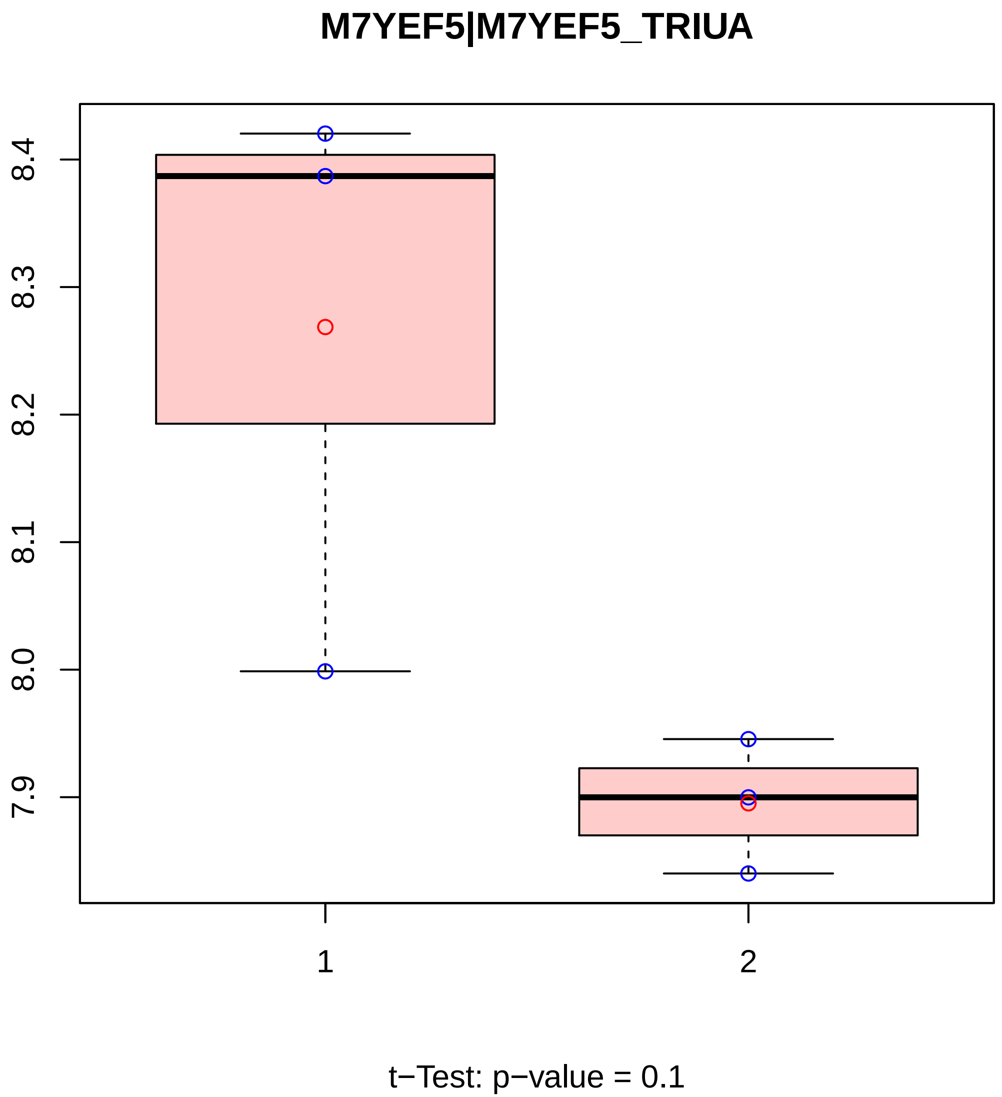
M7YEF5|M7YEF5_TRIUA
8.4
8.3
8.2
8.1
8.0
7.9
1
2
t−Test: p−value = 0.1

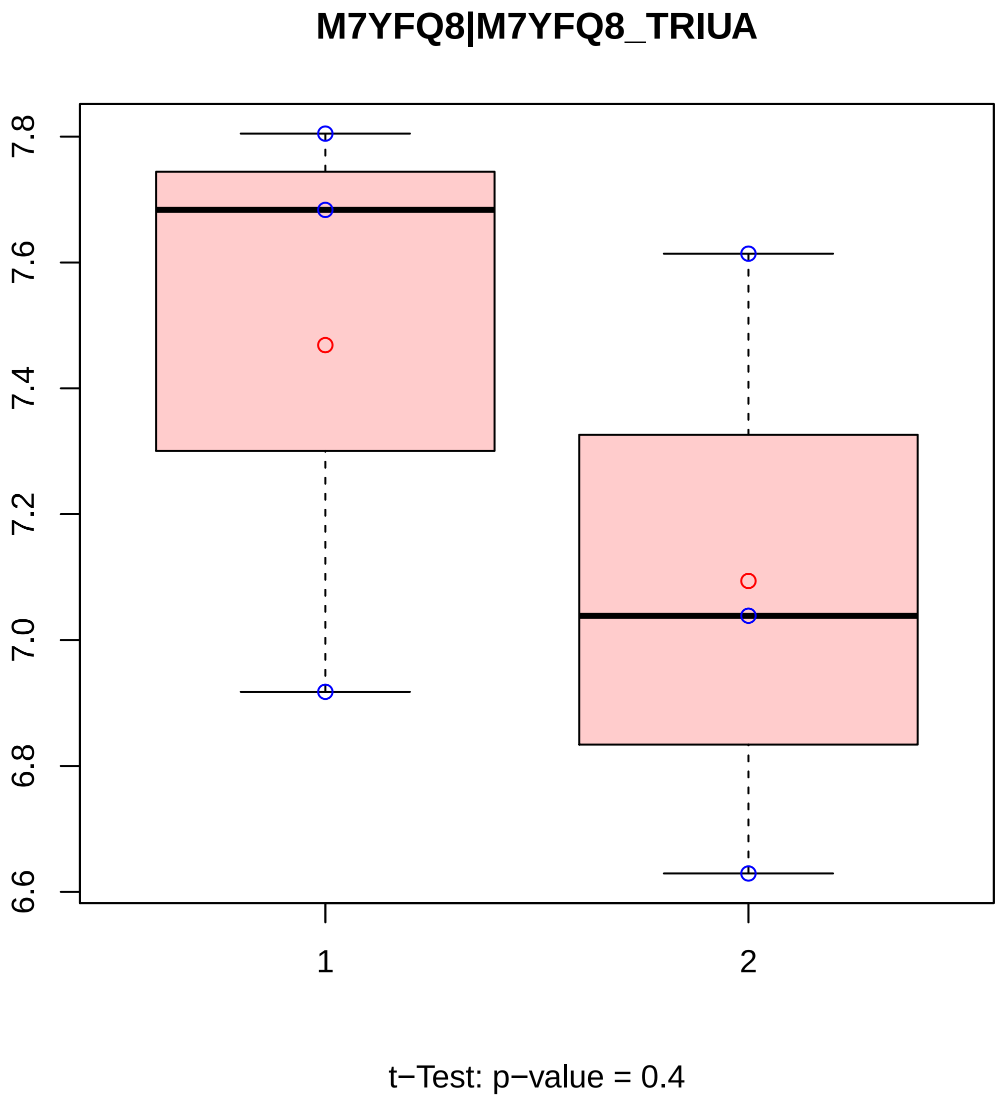

M7YFQ8|M7YFQ8_TRIUA
7.8
7.6
7.4
7.2
7.0
6.8
6.6
1
2
t−Test: p−value = 0.4

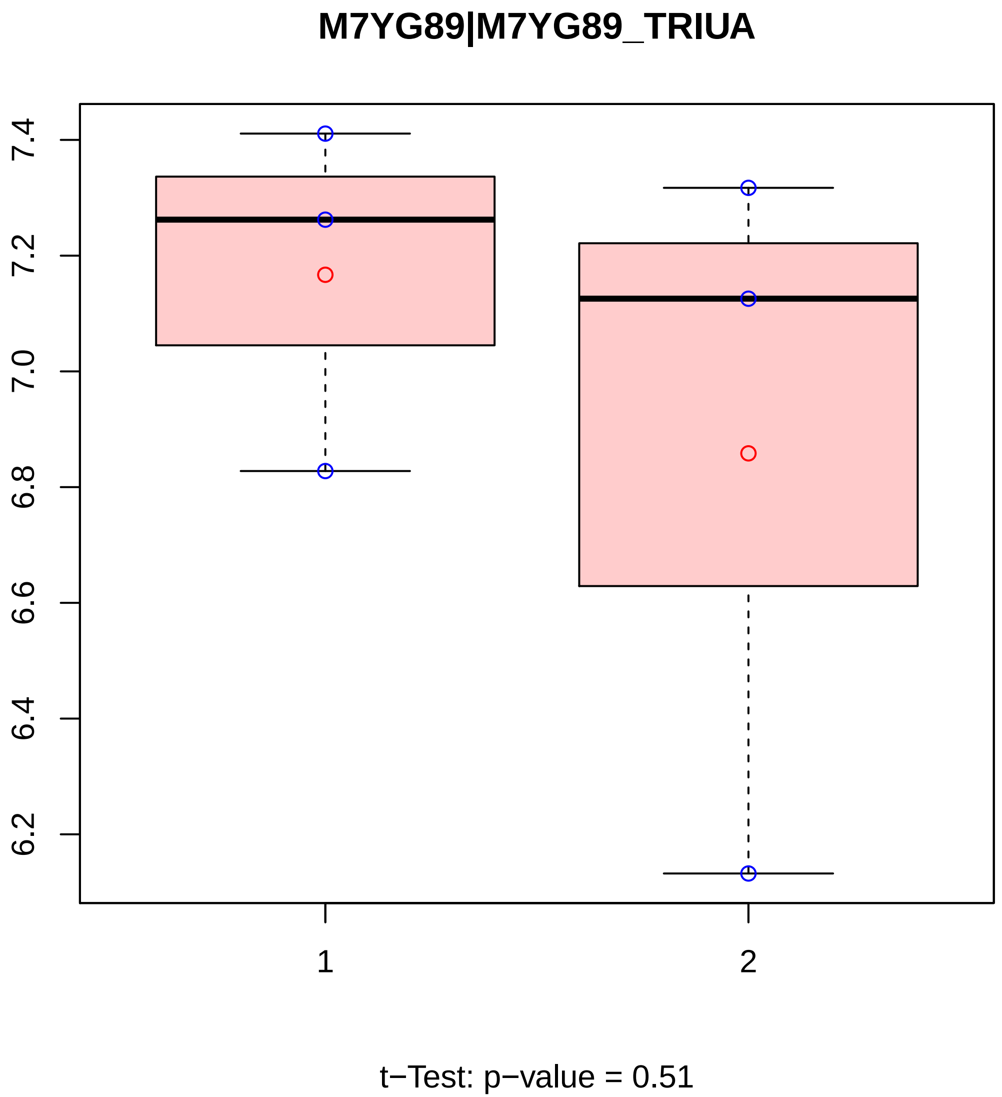
M7YG89|M7YG89_TRIUA
7.4
7.2
7.0
6.8
6.6
6.4
6.2
1
2
t−Test: p−value = 0.51

M7YGC6|M7YGC6_TRIUA

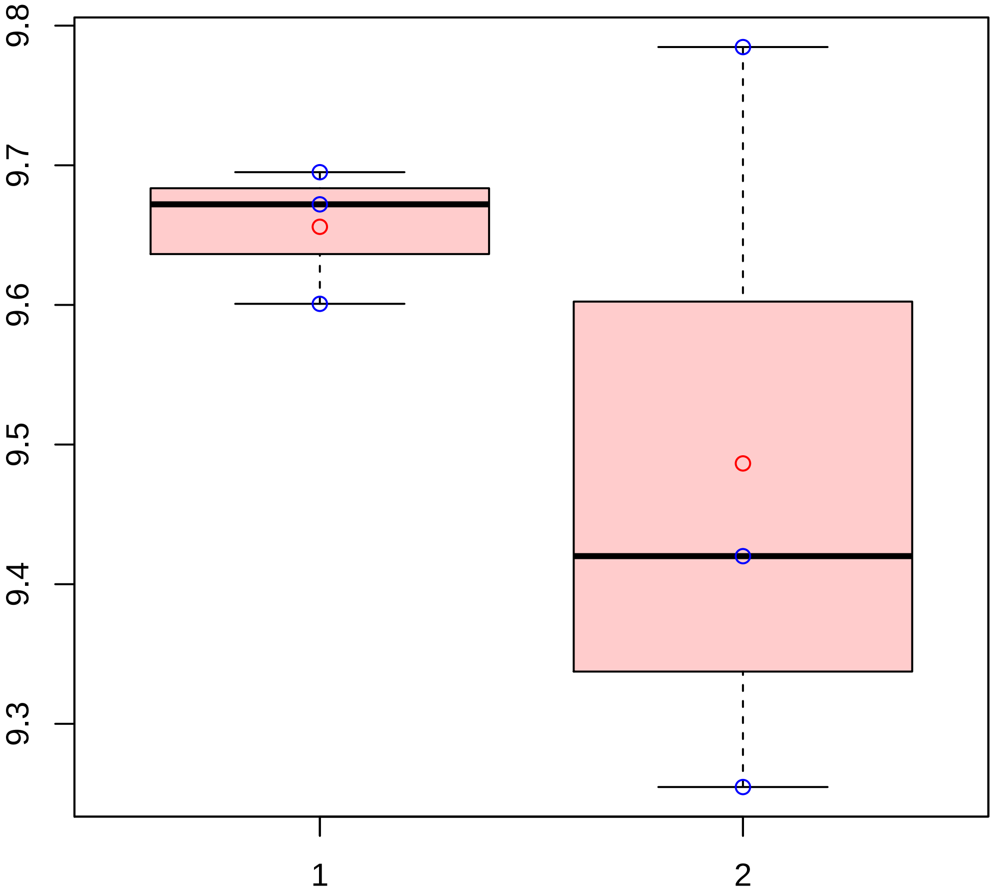

t−Test: p−value = 0.39

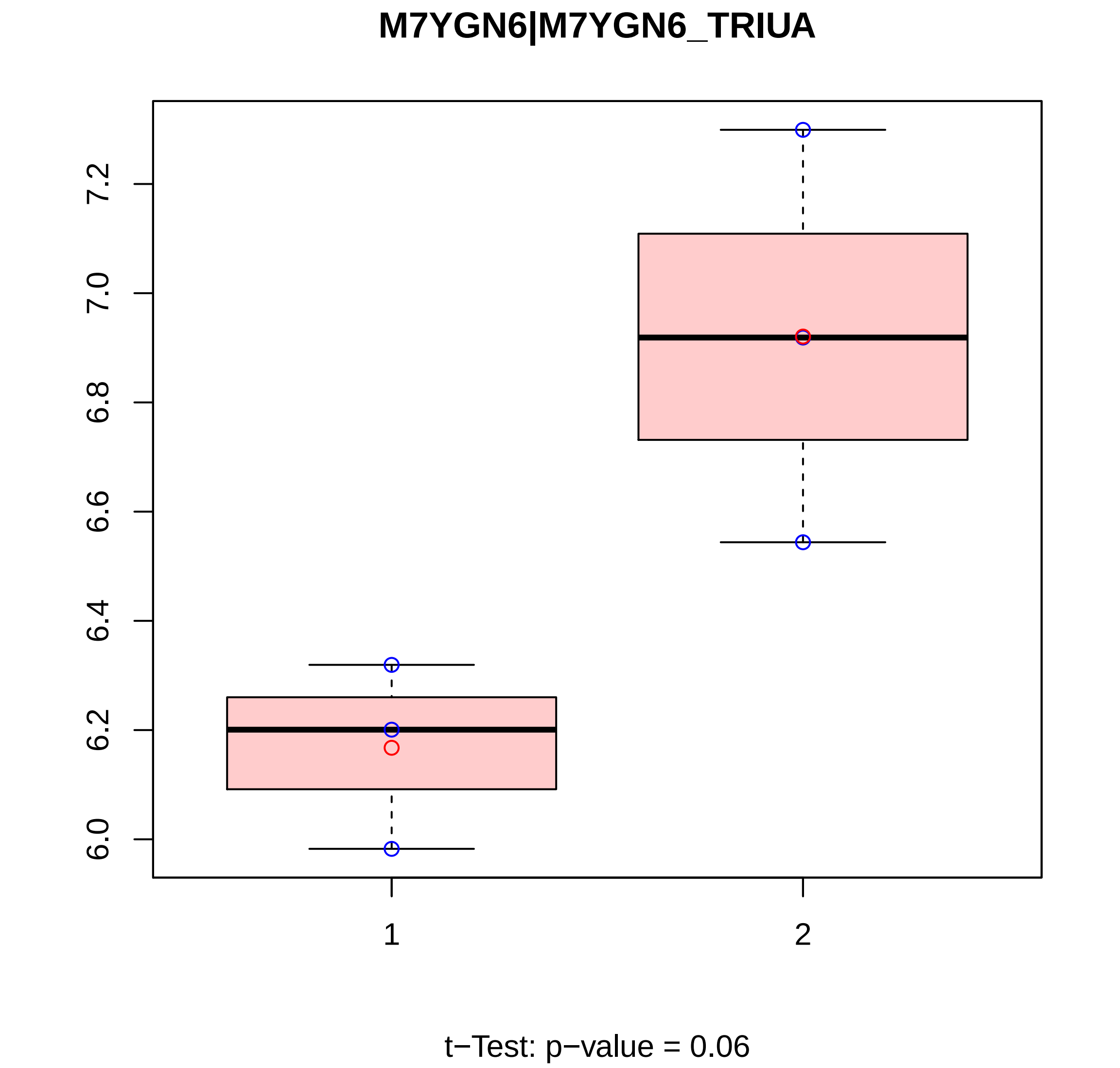

M7YGN6|M7YGN6_TRIUA
7.2
7.0
6.8
6.6
6.4
6.2
6.0
1
2
t−Test: p−value = 0.06

M7YGZ5|M7YGZ5_TRIUA

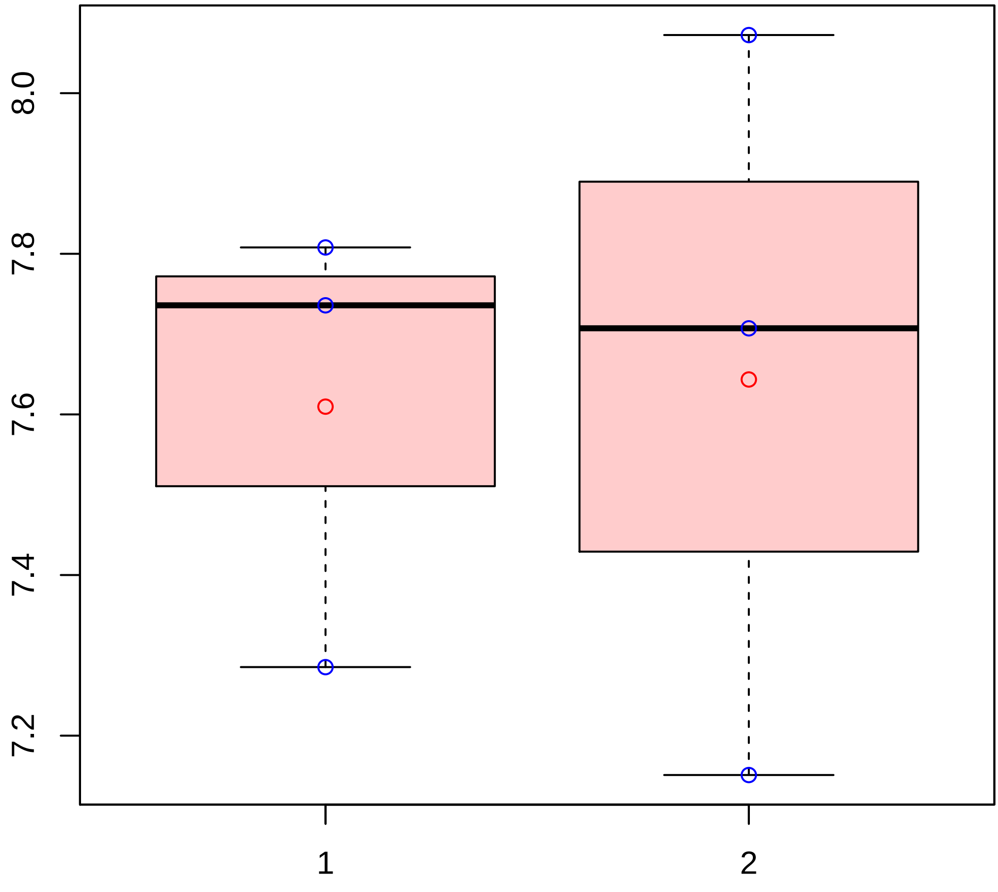

t−Test: p−value = 0.92

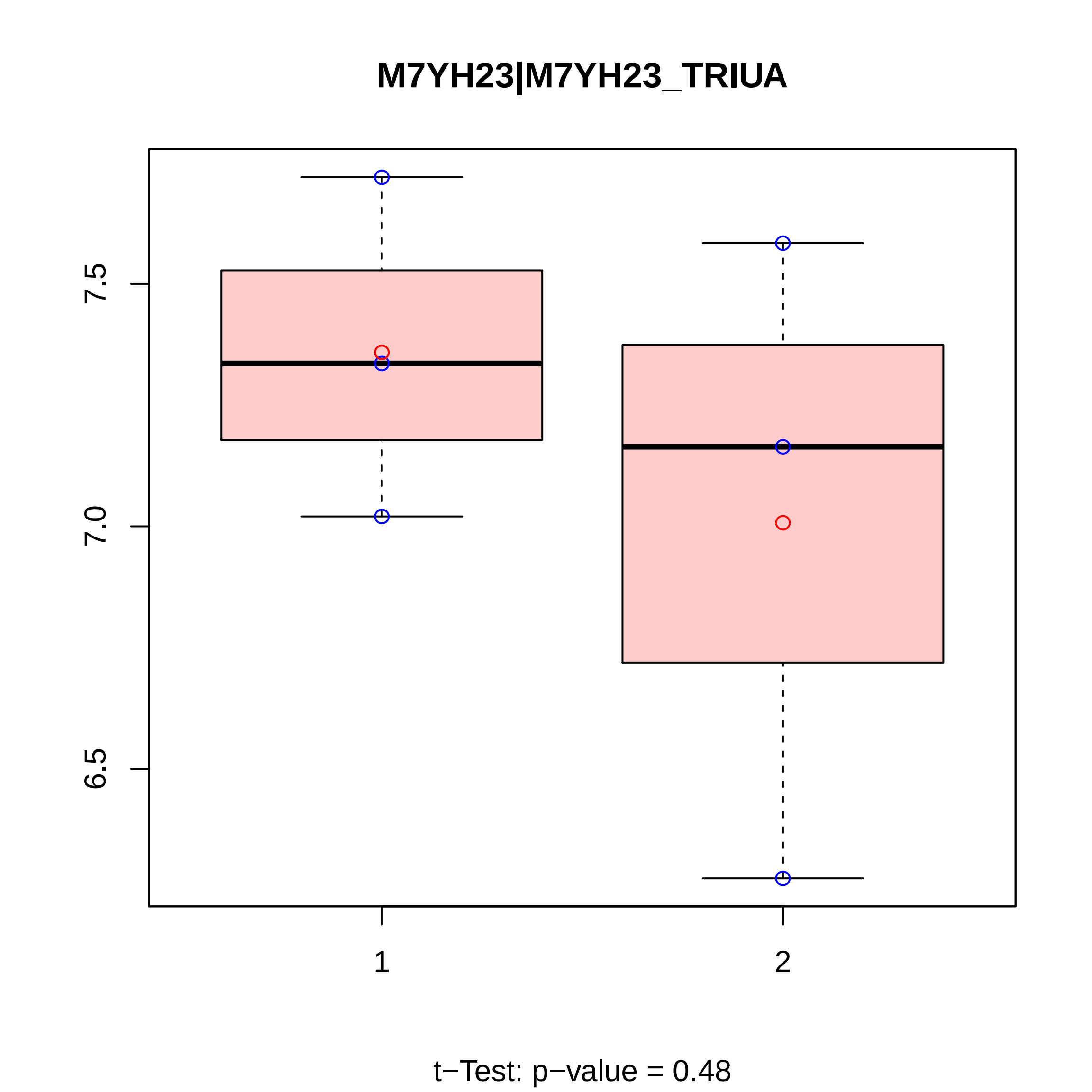

M7YH23|M7YH23_TRIUA
7.5
7.0
6.5
1
2
t−Test: p−value = 0.48

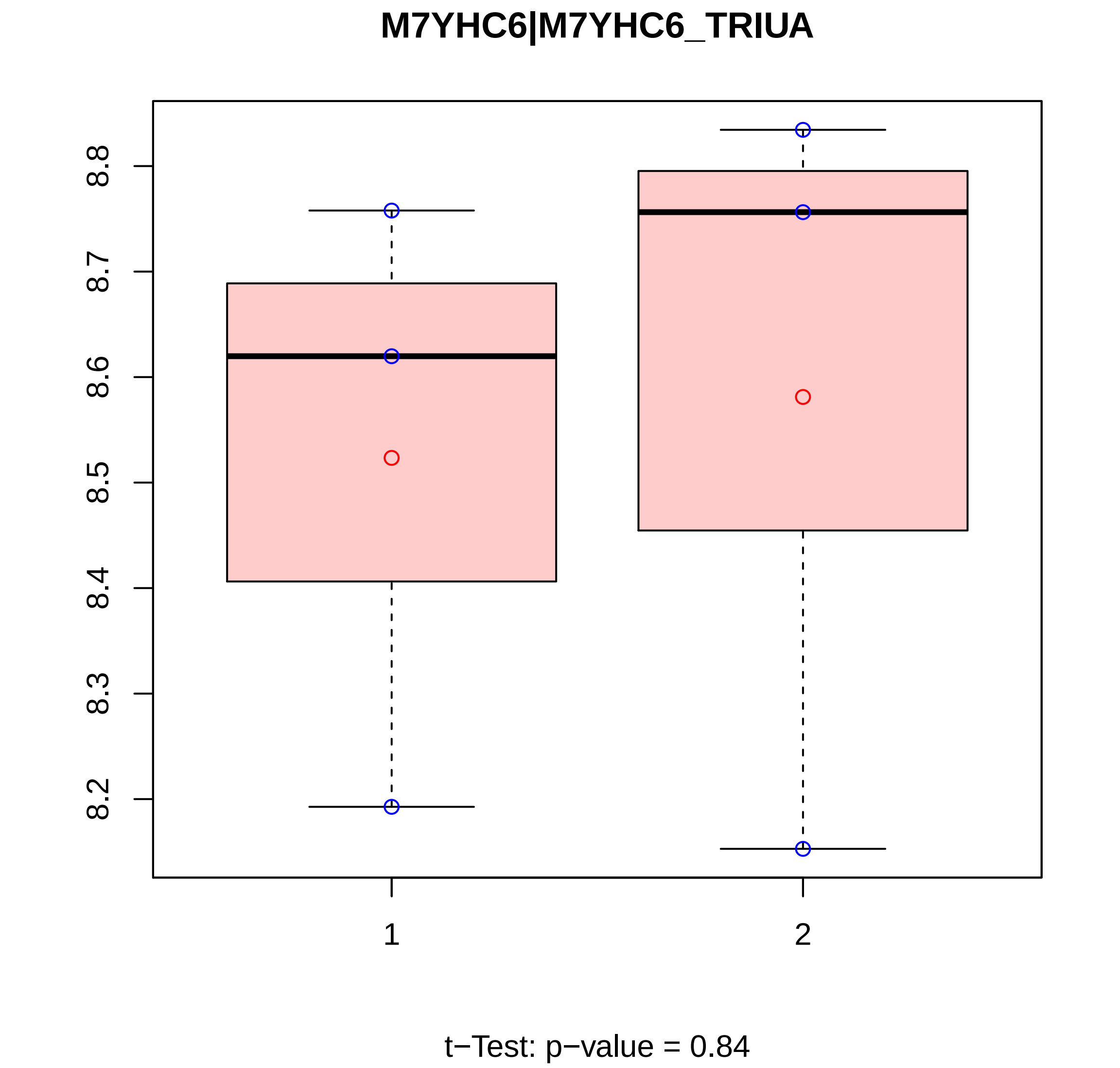
M7YHC6|M7YHC6_TRIUA
8.2
8.3
8.4
8.5
8.6
8.7
8.8
1
2
t−Test: p−value = 0.84

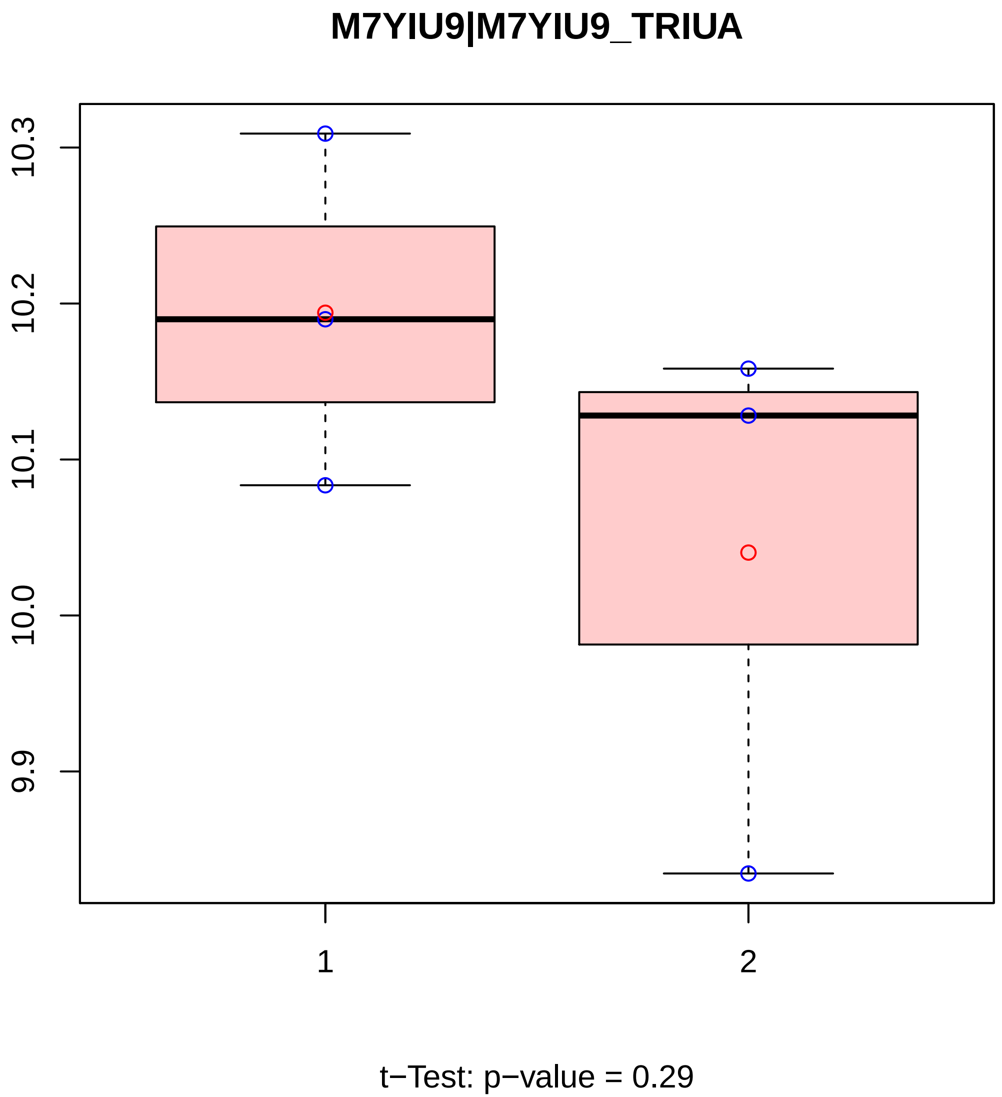
M7YIU9|M7YIU9_TRIUA
10.3
10.2
10.1
10.0
9.9
1
2
t−Test: p−value = 0.29

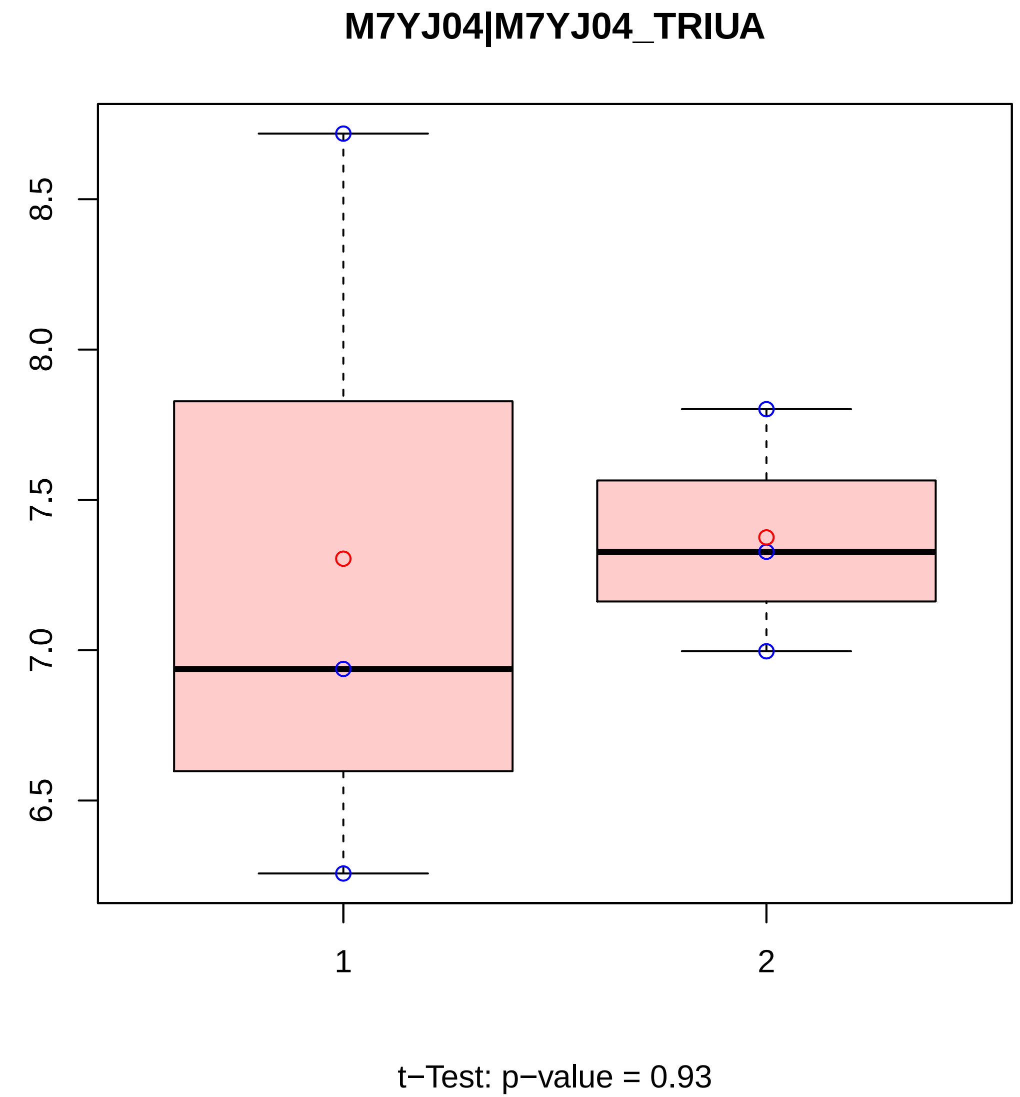
M7YJ04|M7YJ04_TRIUA
8.5
8.0
7.5
7.0
6.5
1
2
t−Test: p−value = 0.93

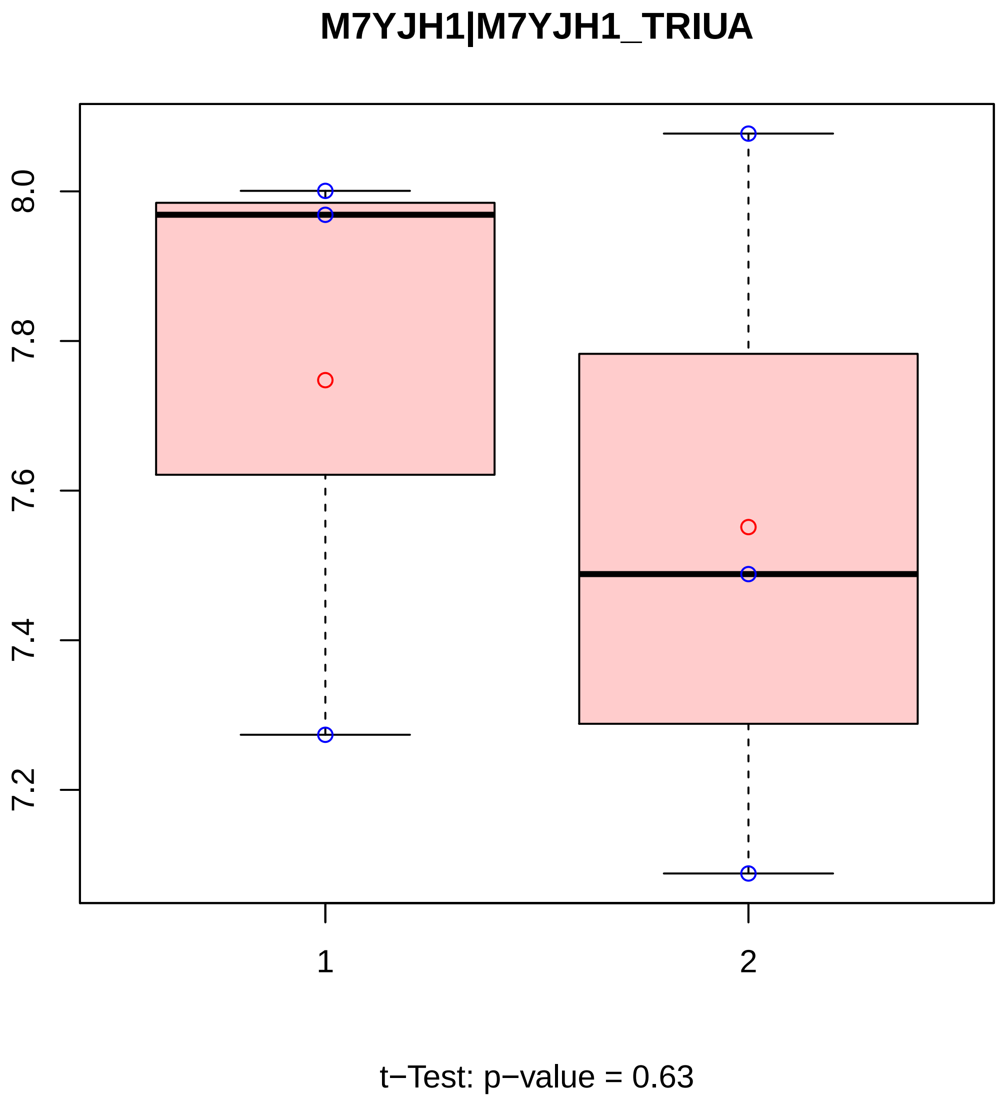
M7YJH1|M7YJH1_TRIUA
8.0
7.8
7.6
7.4
7.2
1
2
t−Test: p−value = 0.63

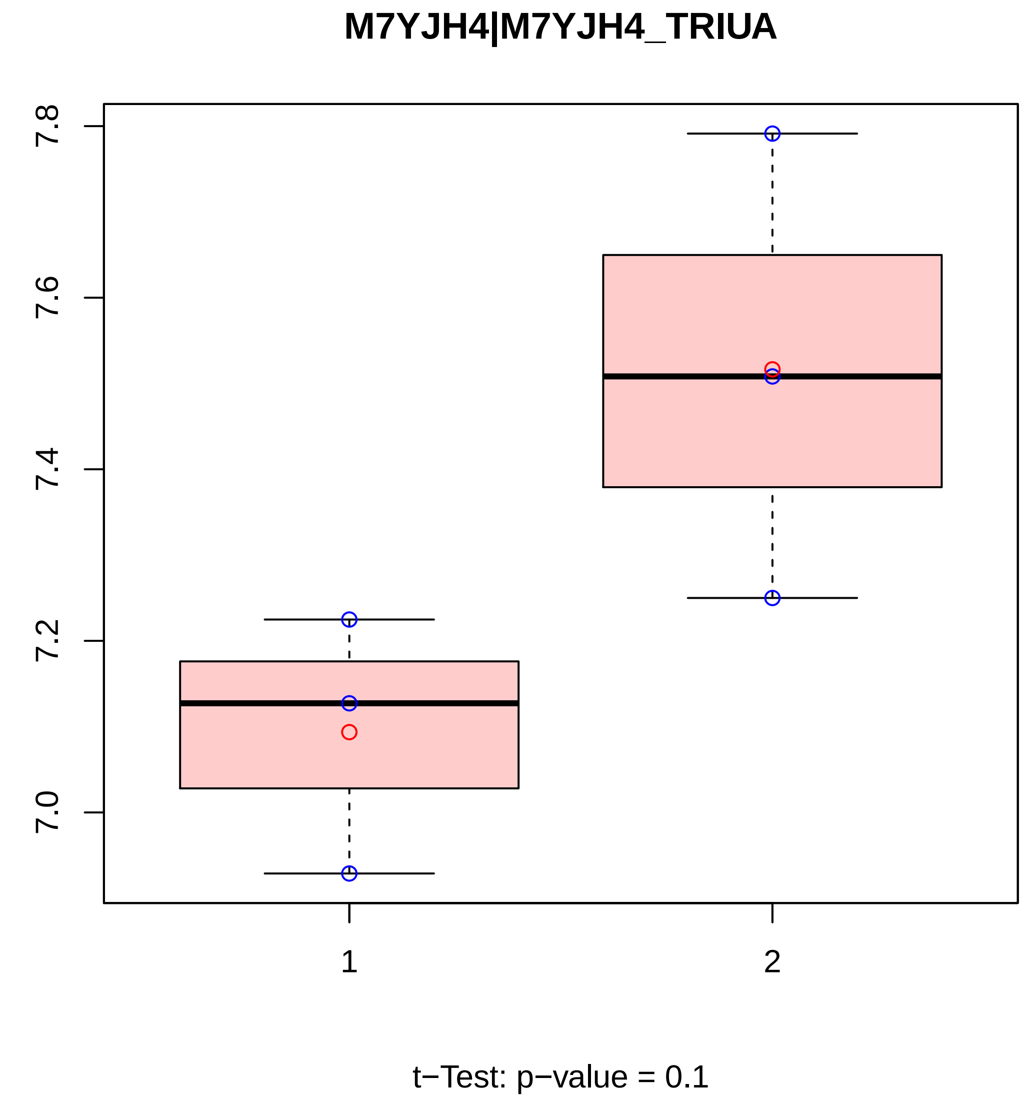
M7YJH4|M7YJH4_TRIUA
7.0
7.2
7.4
7.6
7.8
1
2
t−Test: p−value = 0.1

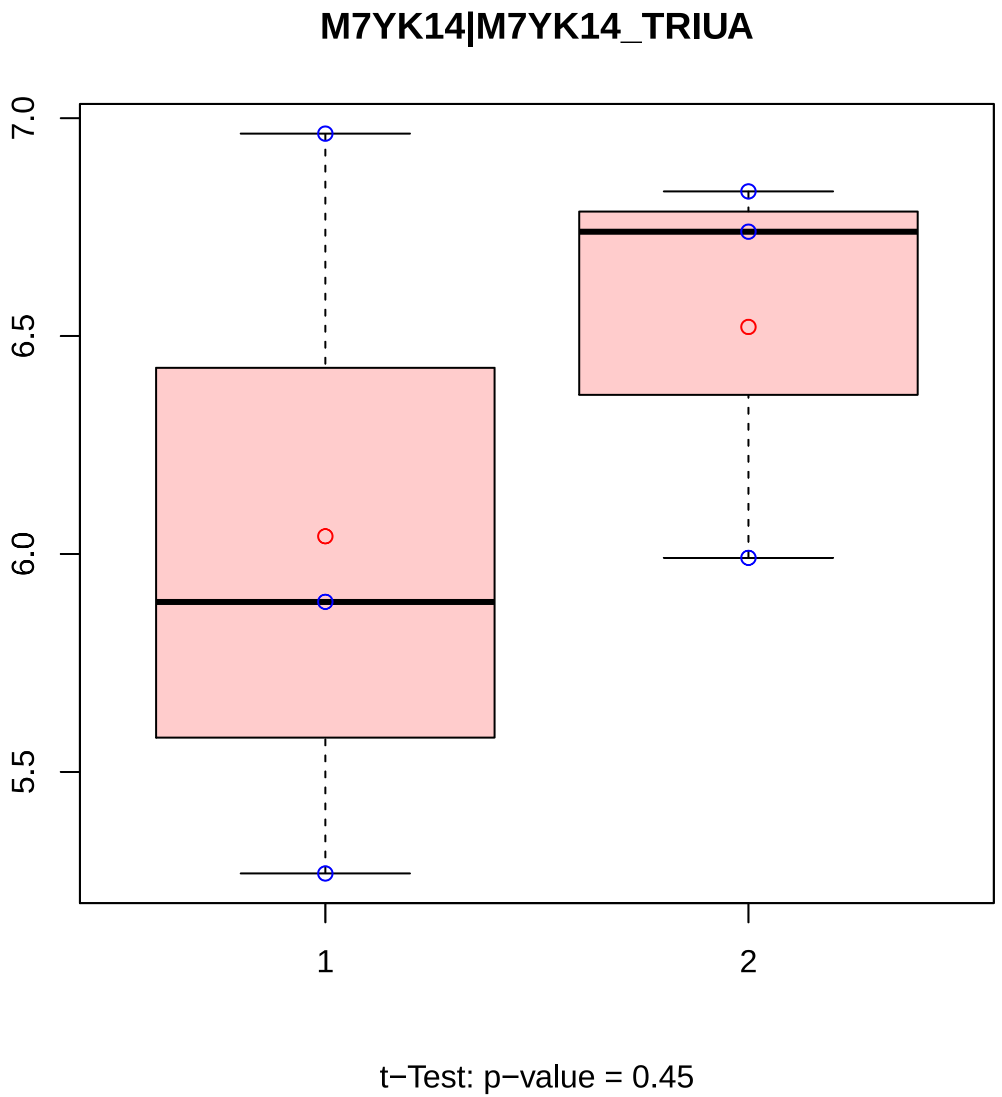
M7YK14|M7YK14_TRIUA
7.0
6.5
6.0
5.5
1
2
t−Test: p−value = 0.45

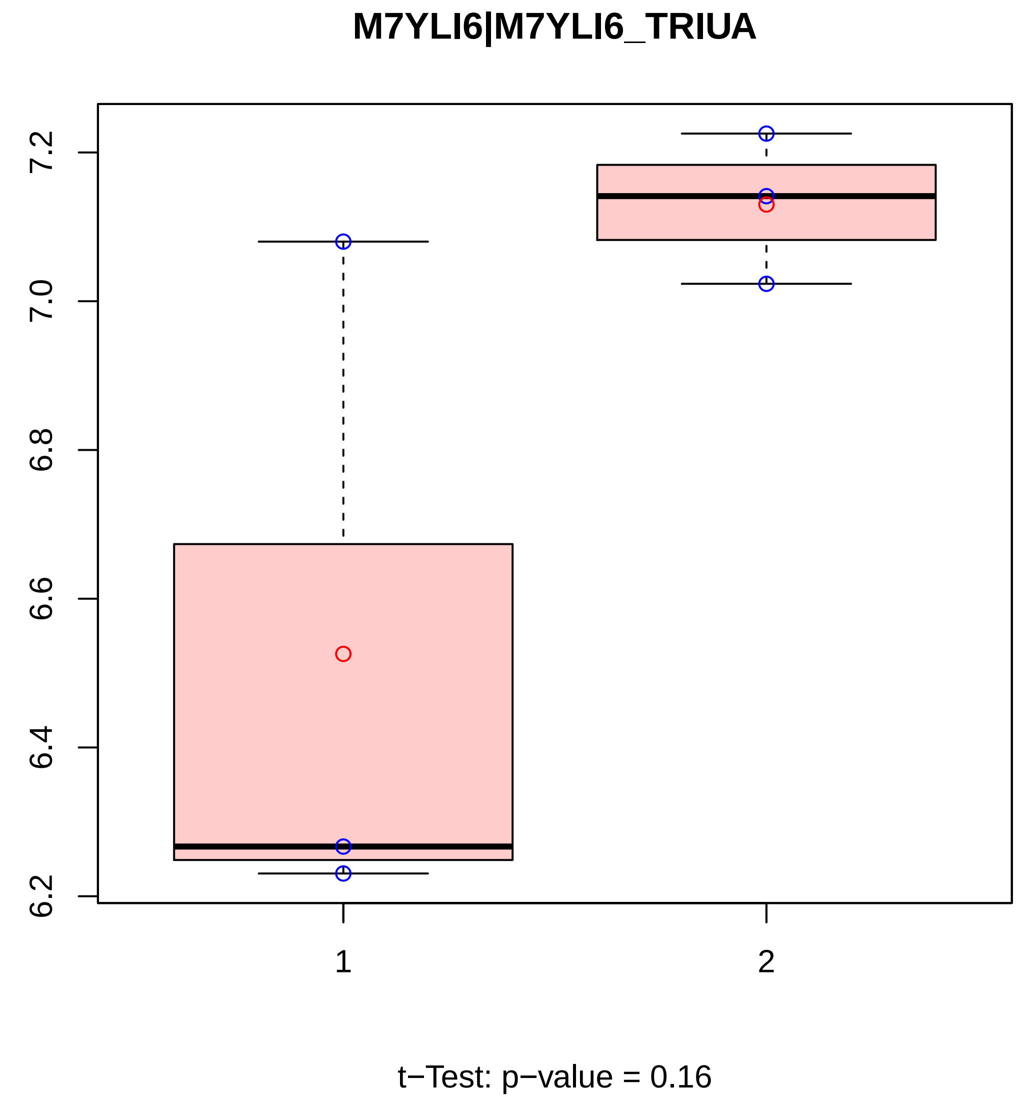
M7YLI6|M7YLI6_TRIUA
7.2
7.0
6.8
6.6
6.4
6.2
1
2
t−Test: p−value = 0.16

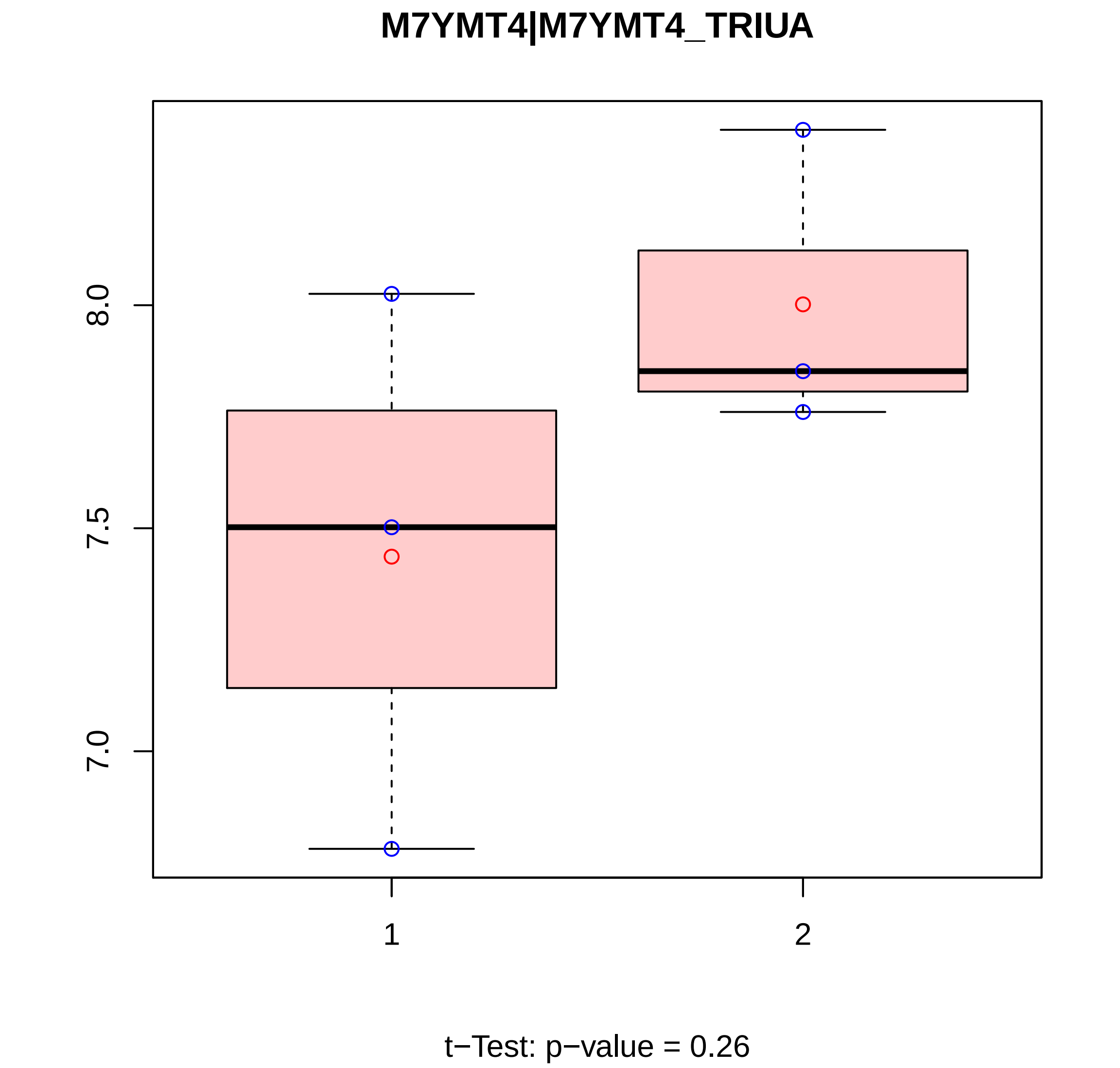

M7YMT4|M7YMT4_TRIUA
8.0
7.5
7.0
1
2
t−Test: p−value = 0.26

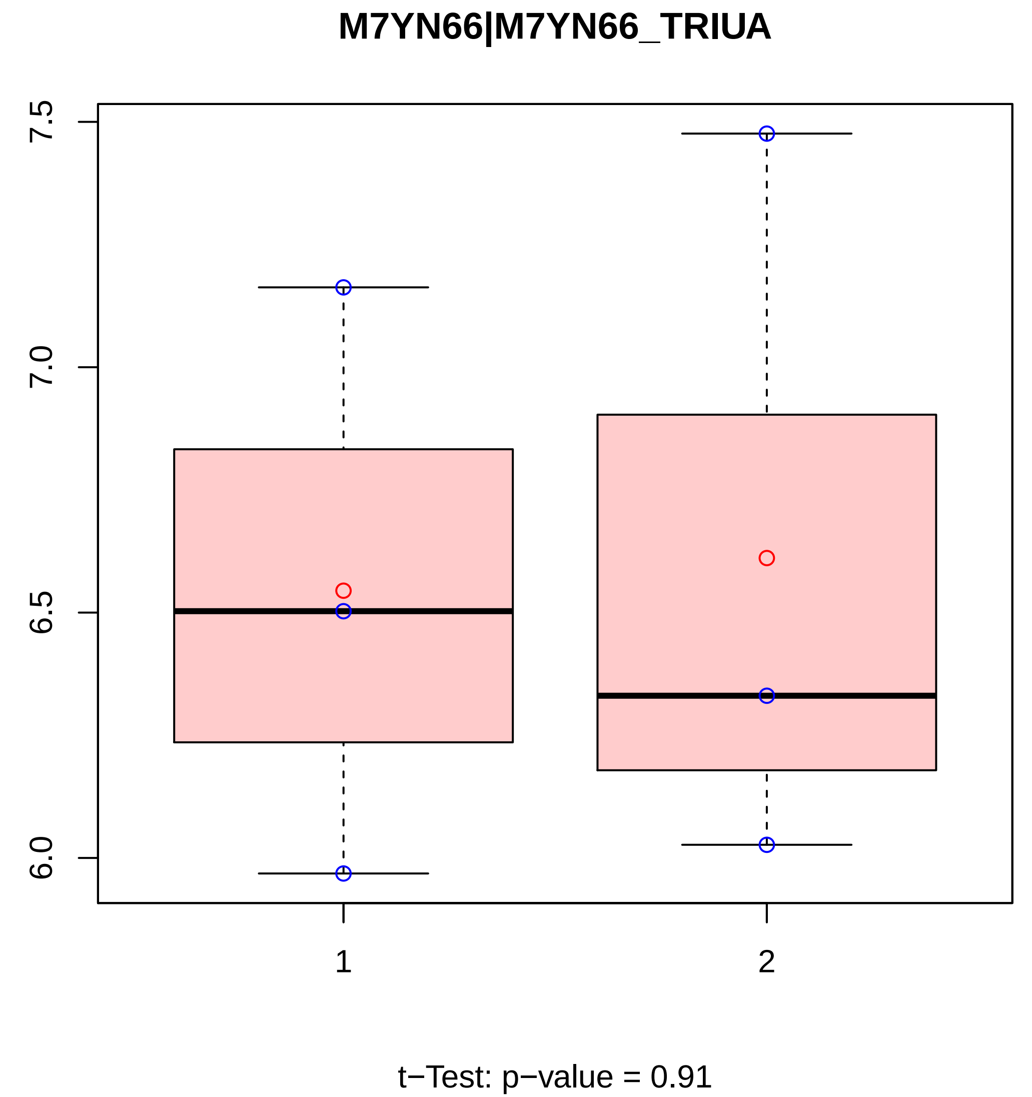

M7YN66|M7YN66_TRIUA
7.5
7.0
6.5
6.0
1
2
t−Test: p−value = 0.91

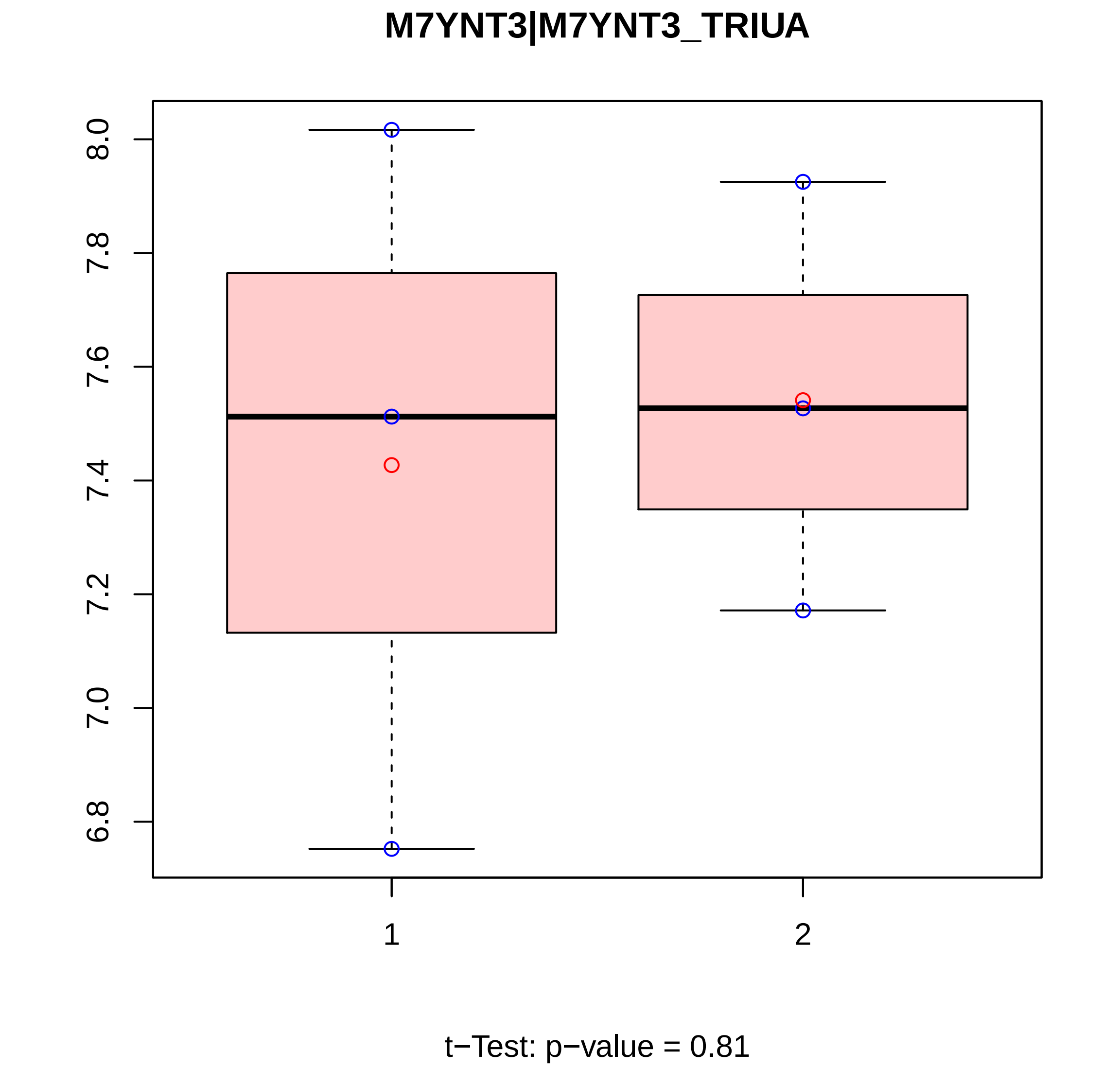

M7YNT3|M7YNT3_TRIUA
8.0
7.8
7.6
7.4
7.2
7.0
6.8
1
2
t−Test: p−value = 0.81

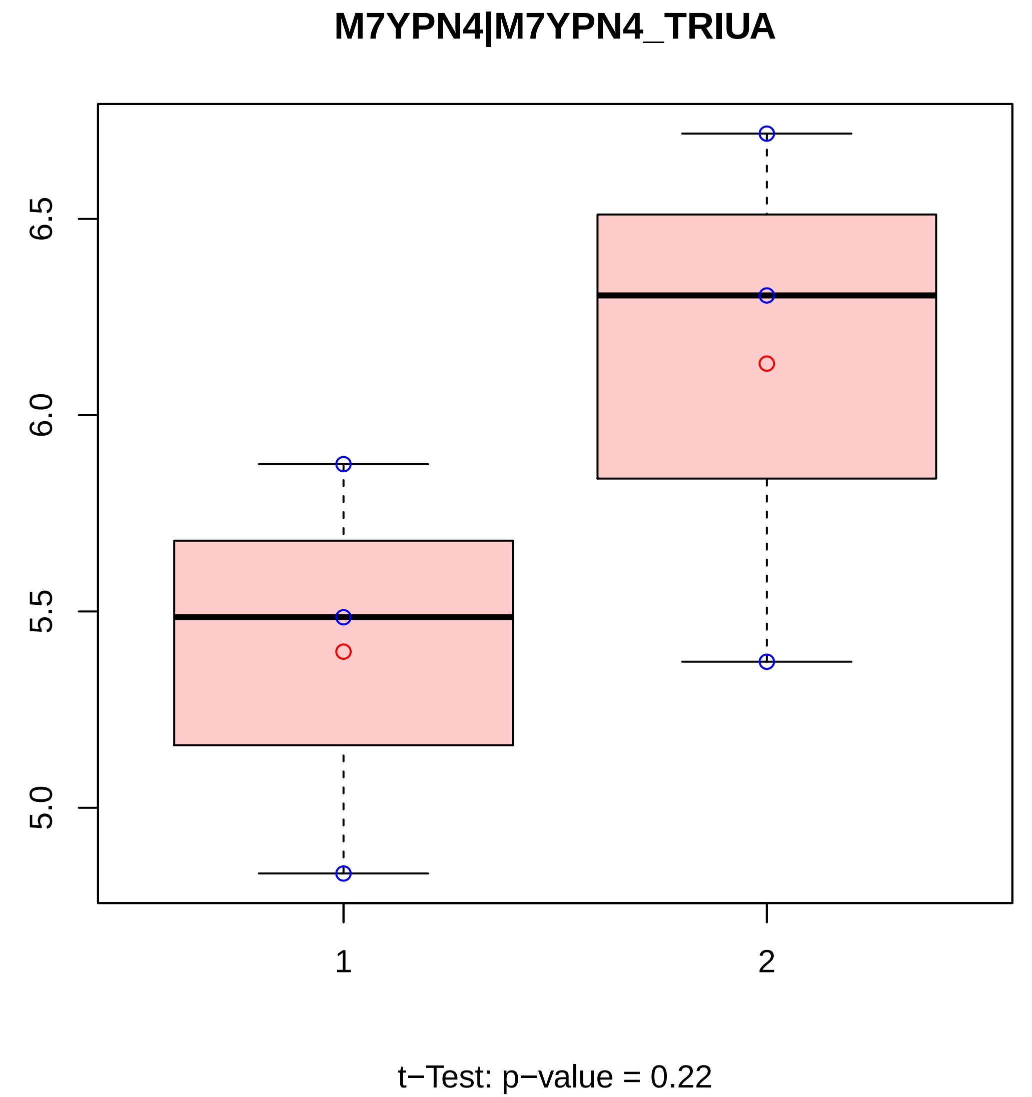
M7YPN4|M7YPN4_TRIUA
6.5
6.0
5.5
5.0
1
2
t−Test: p−value = 0.22

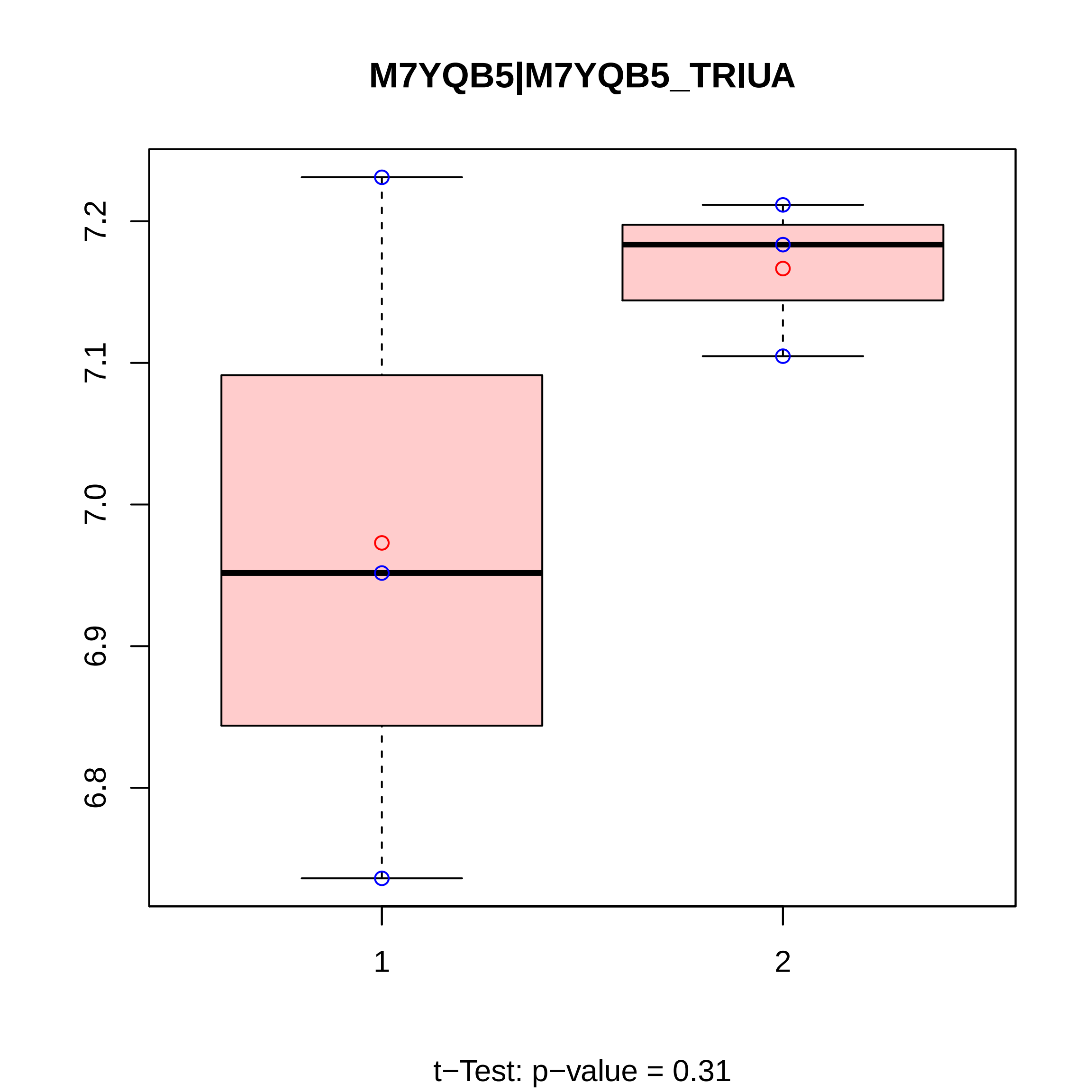
M7YQB5|M7YQB5_TRIUA
7.2
7.1
7.0
6.9
6.8
1
2
t−Test: p−value = 0.31

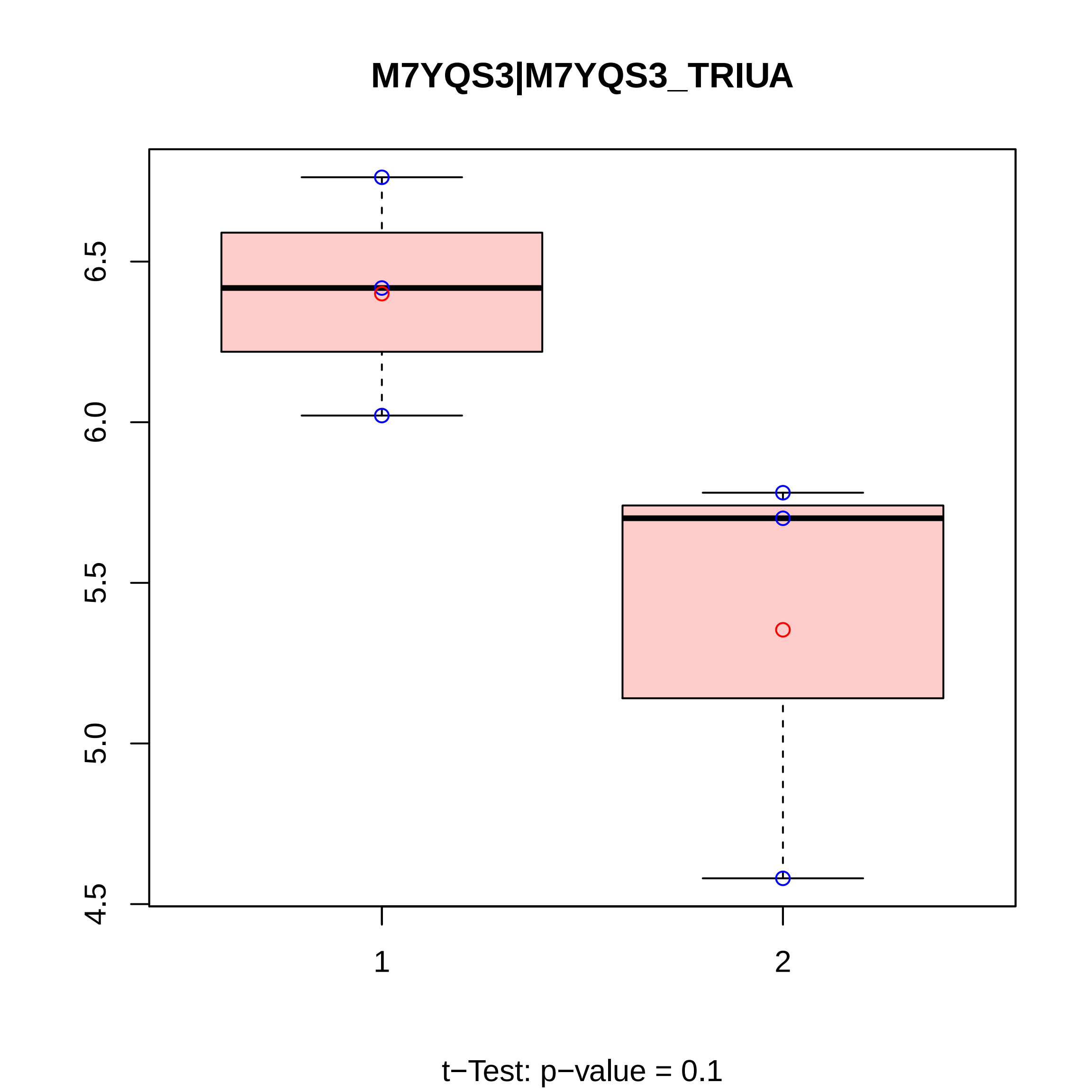
M7YQS3|M7YQS3_TRIUA
6.5
6.0
5.5
5.0
4.5
1
2
t−Test: p−value = 0.1

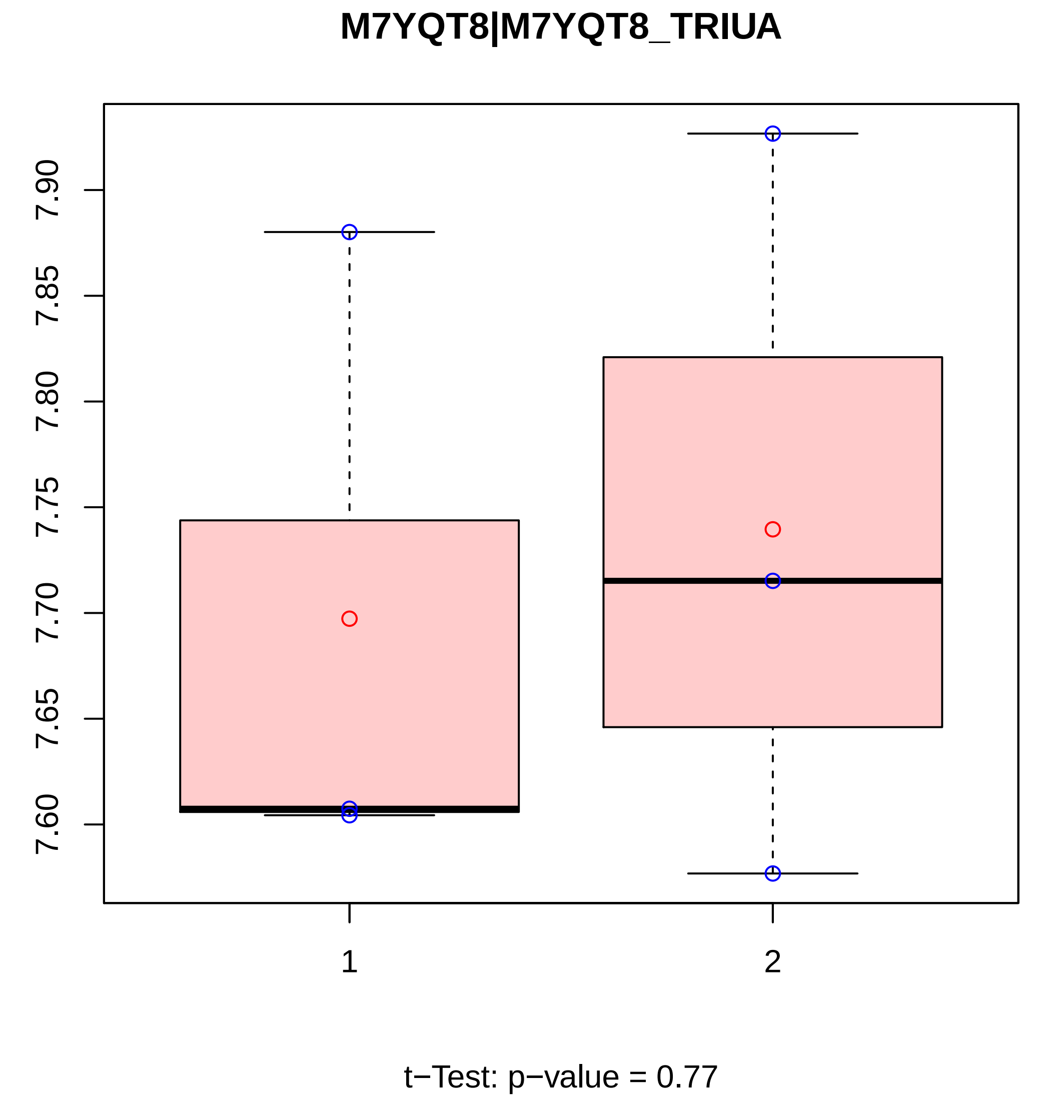
M7YQT8|M7YQT8_TRIUA
7.60
7.65
7.70
7.75
7.80
7.85
7.90
1
2
t−Test: p−value = 0.77

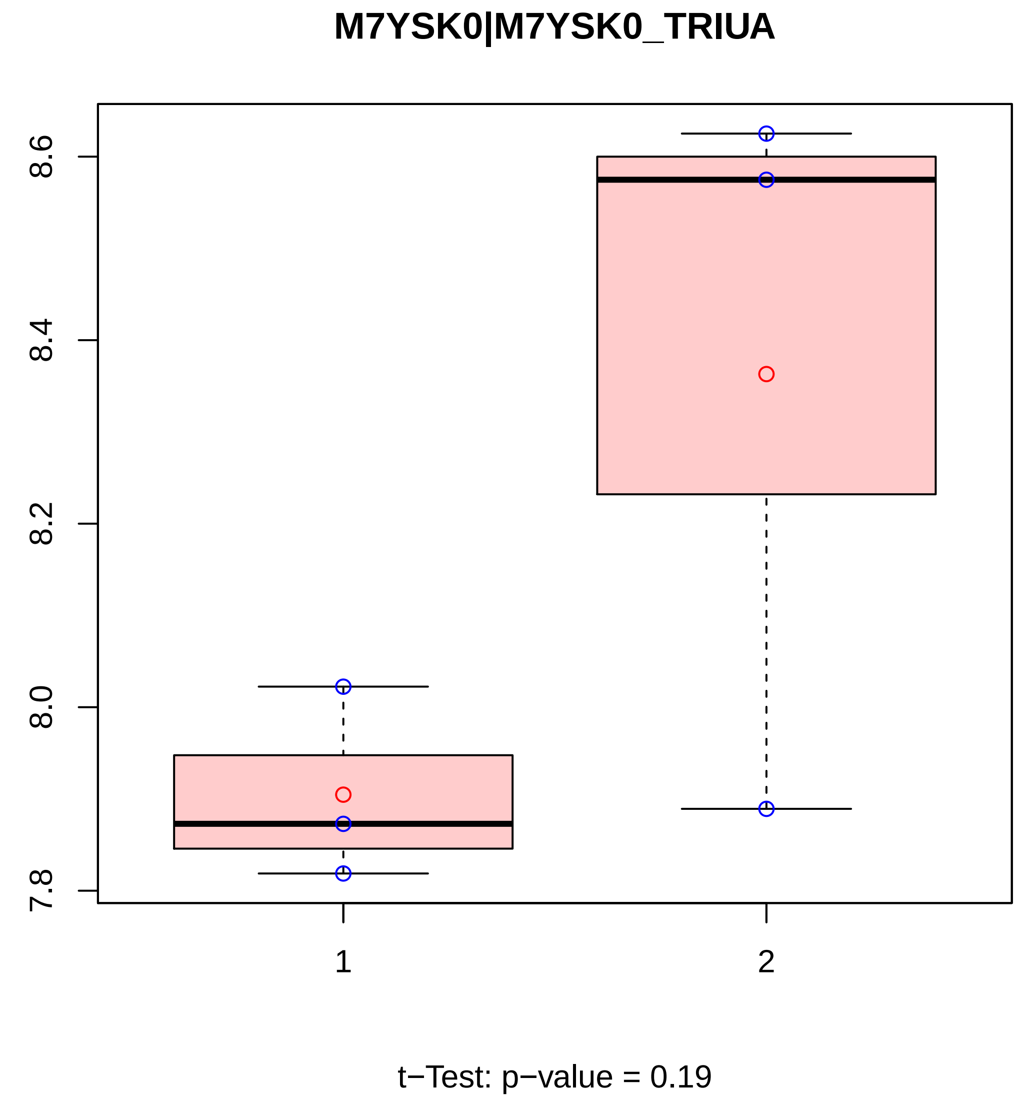
M7YSK0|M7YSK0_TRIUA
8.6
8.4
8.2
8.0
7.8
1
2
t−Test: p−value = 0.19

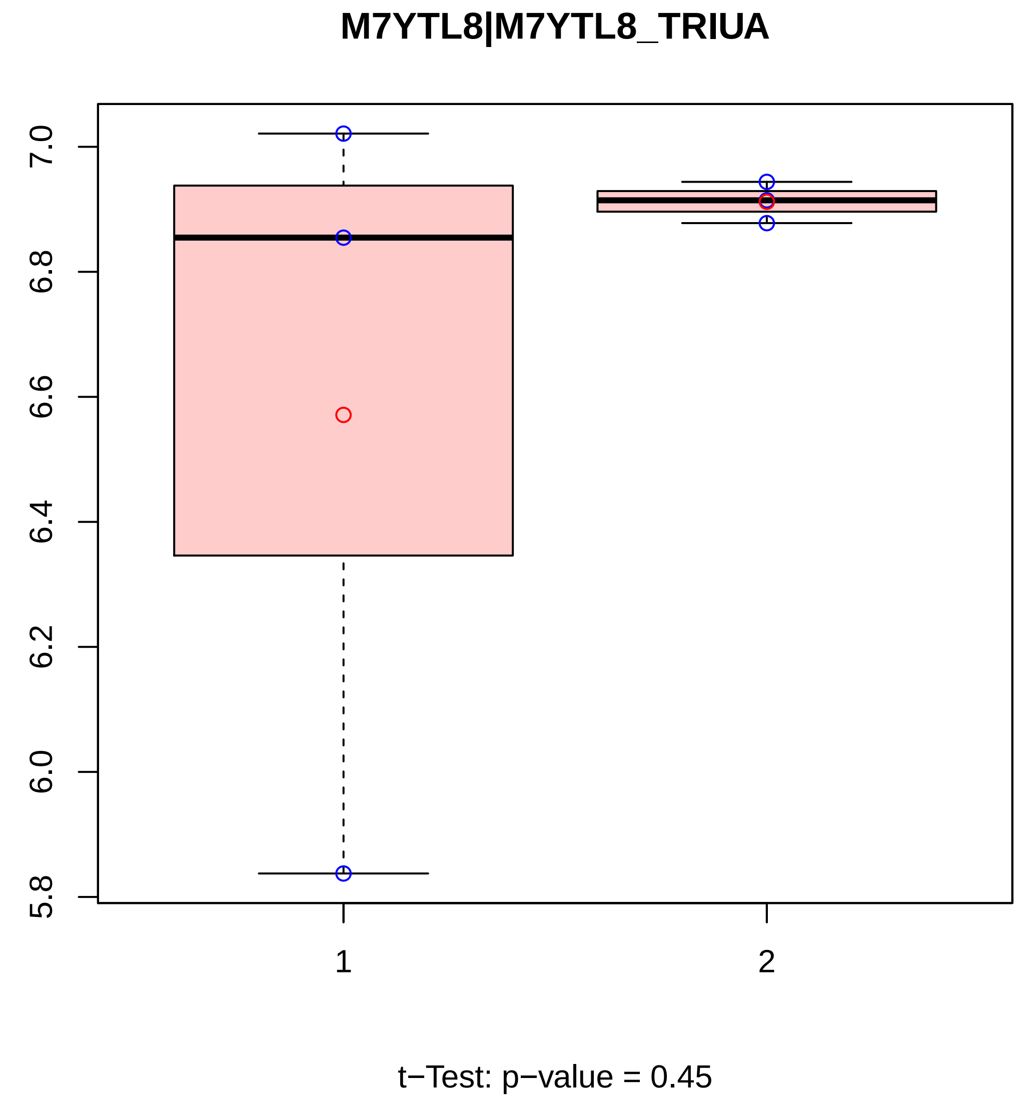

M7YTL8|M7YTL8_TRIUA
7.0
6.8
6.6
6.4
6.2
6.0
5.8
1
2
t−Test: p−value = 0.45

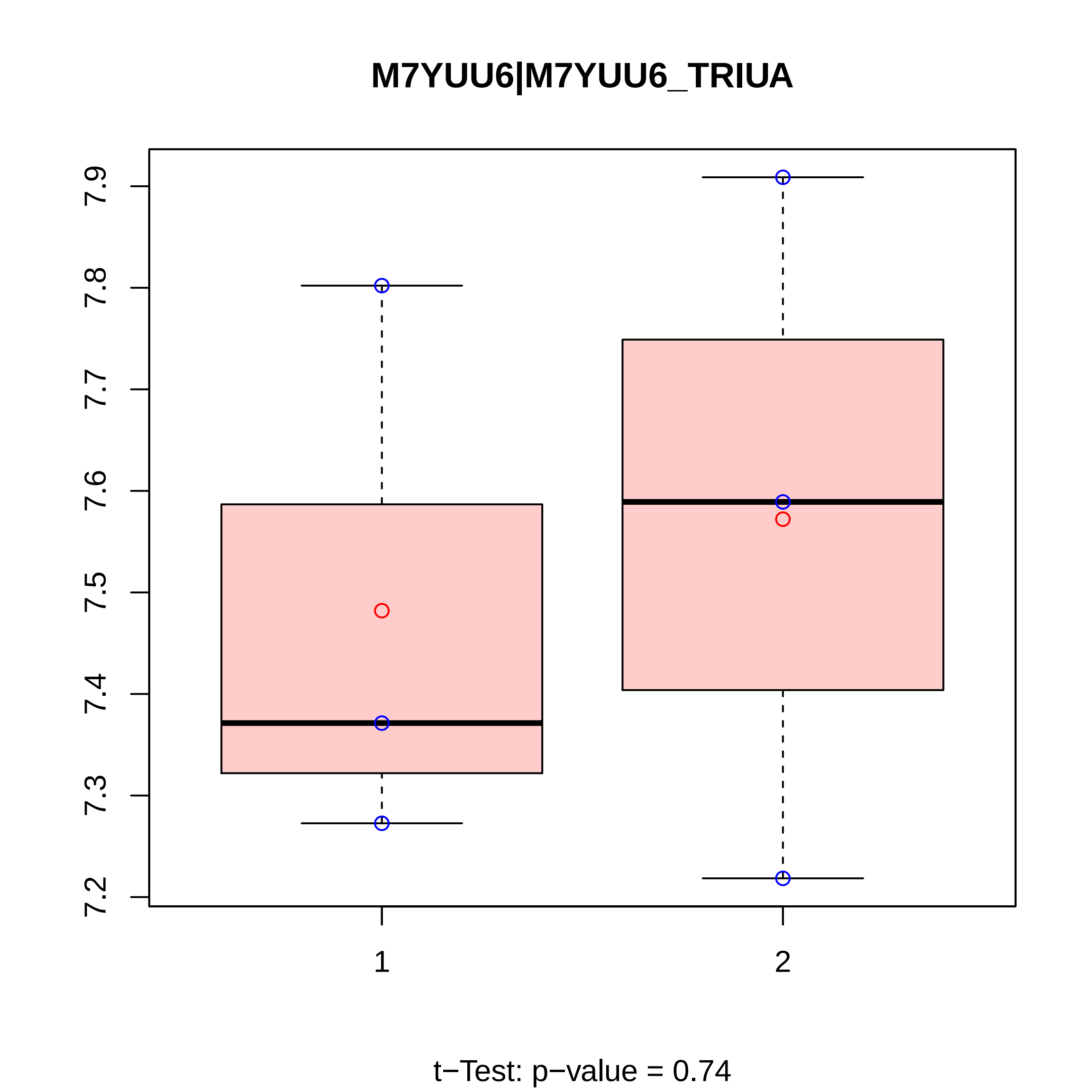
M7YUU6|M7YUU6_TRIUA
7.2
7.3
7.4
7.5
7.6
7.7
7.8
7.9
1
2
t−Test: p−value = 0.74

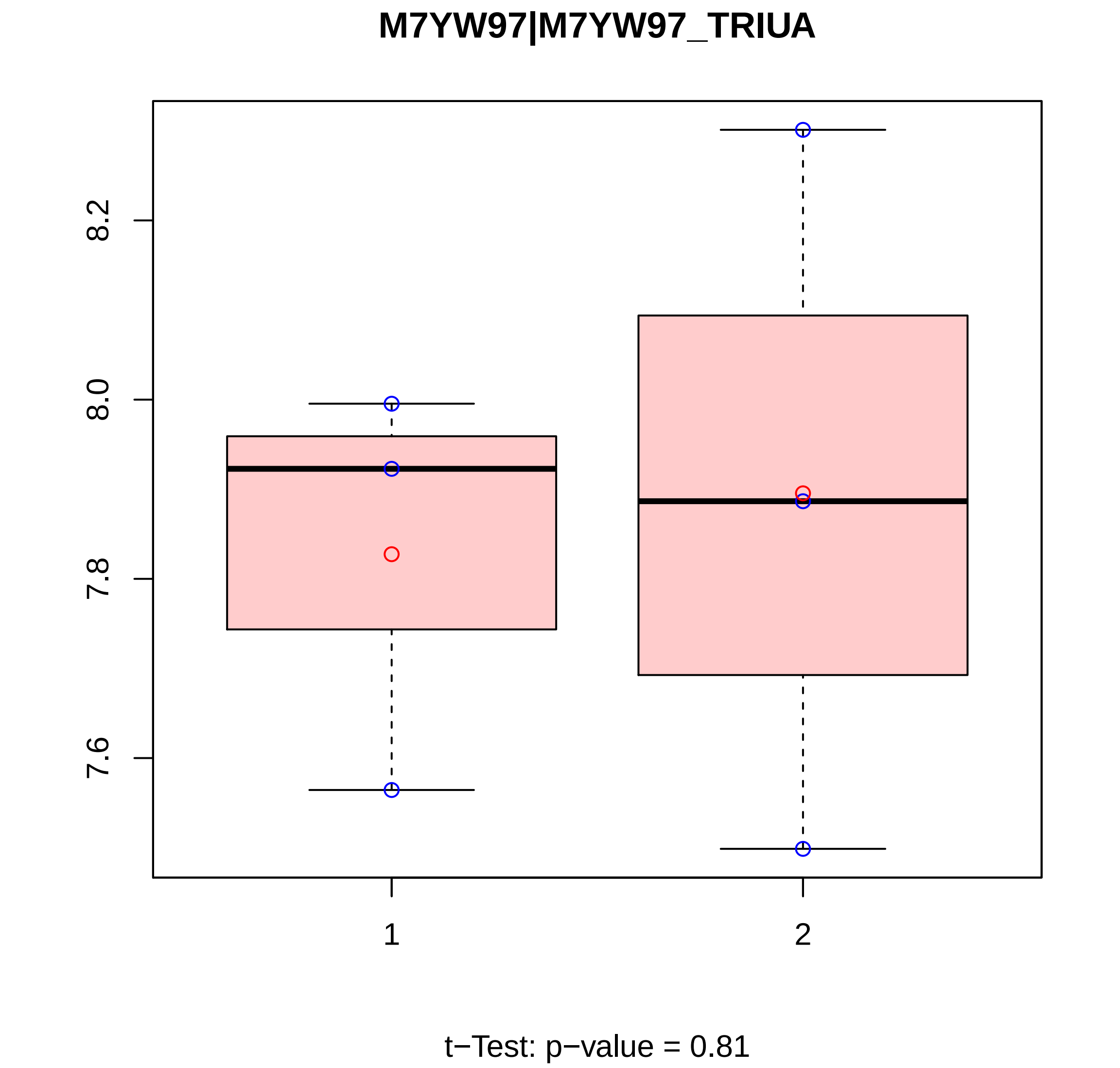
M7YW97|M7YW97_TRIUA
8.2
8.0
7.8
7.6
1
2
t−Test: p−value = 0.81

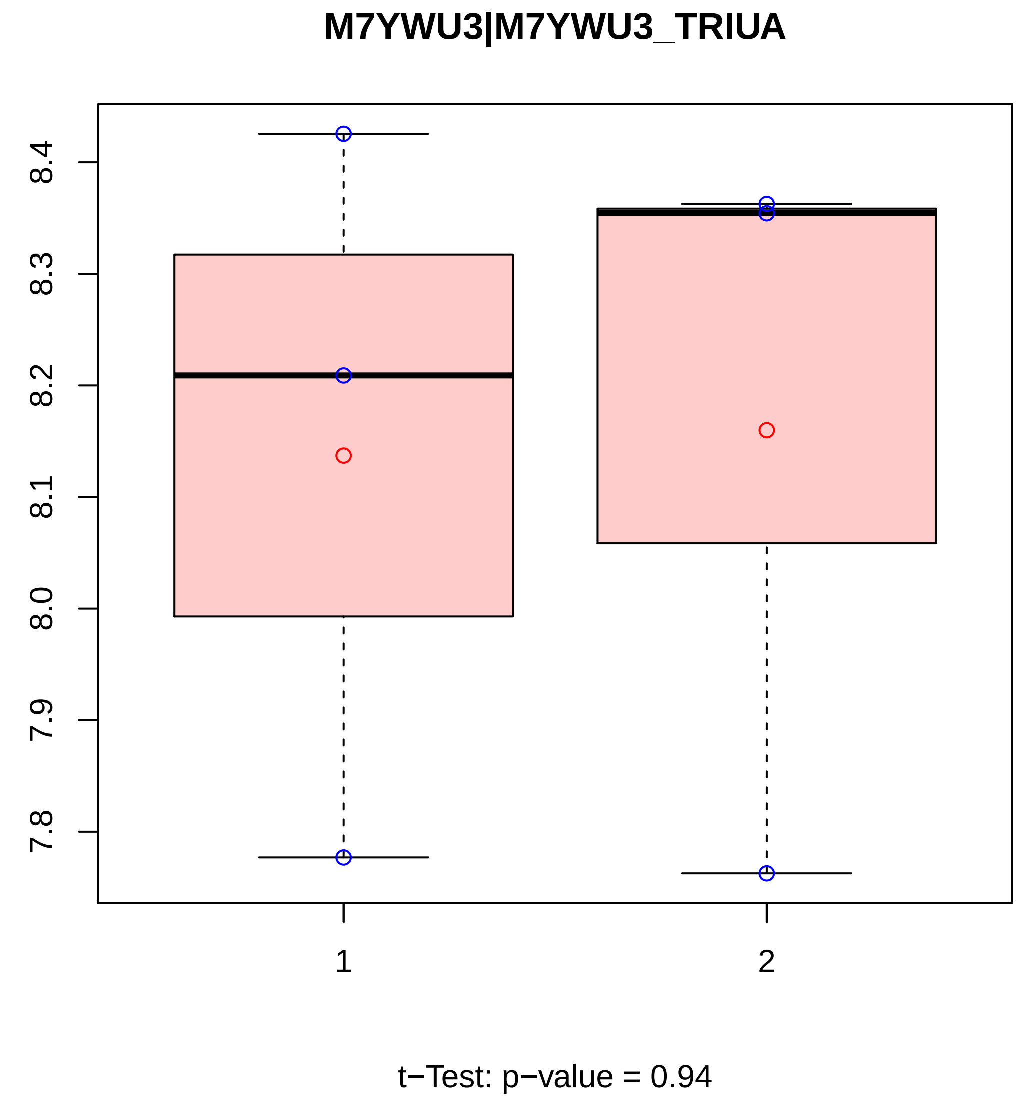
M7YWU3|M7YWU3_TRIUA
8.4
8.3
8.2
8.1
8.0
7.9
7.8
1
2
t−Test: p−value = 0.94

M7YXE0|M7YXE0_TRIUA

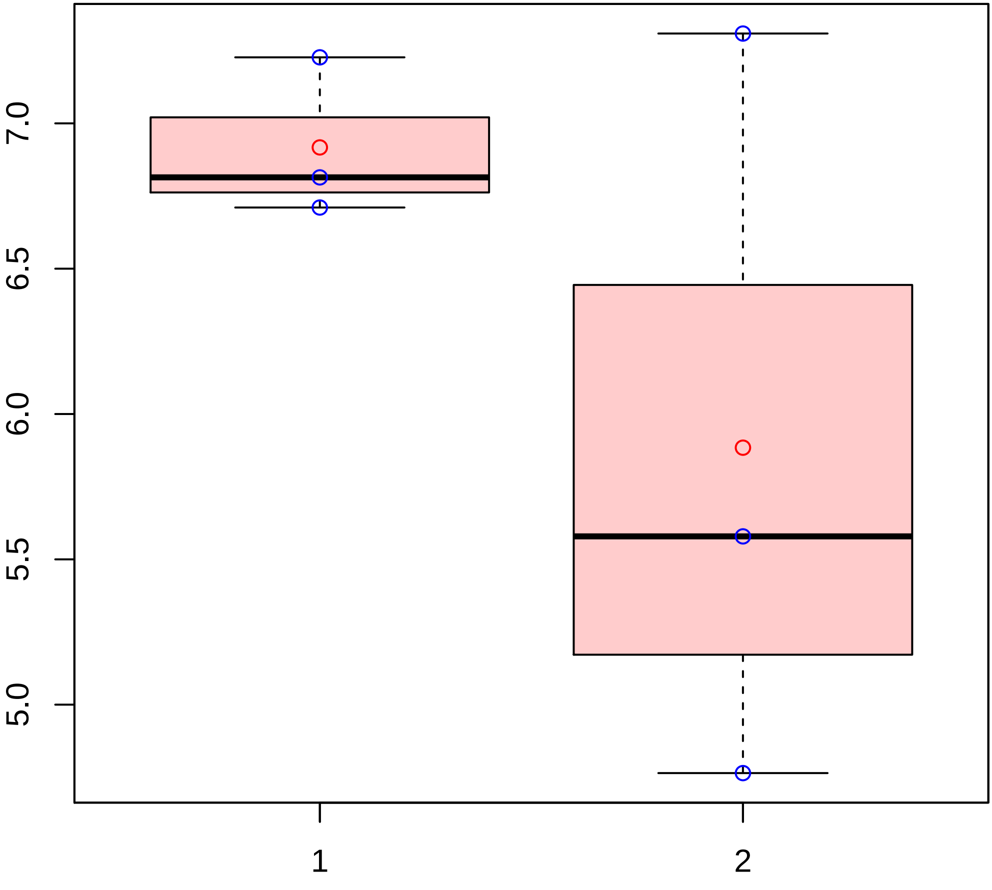

t−Test: p−value = 0.3

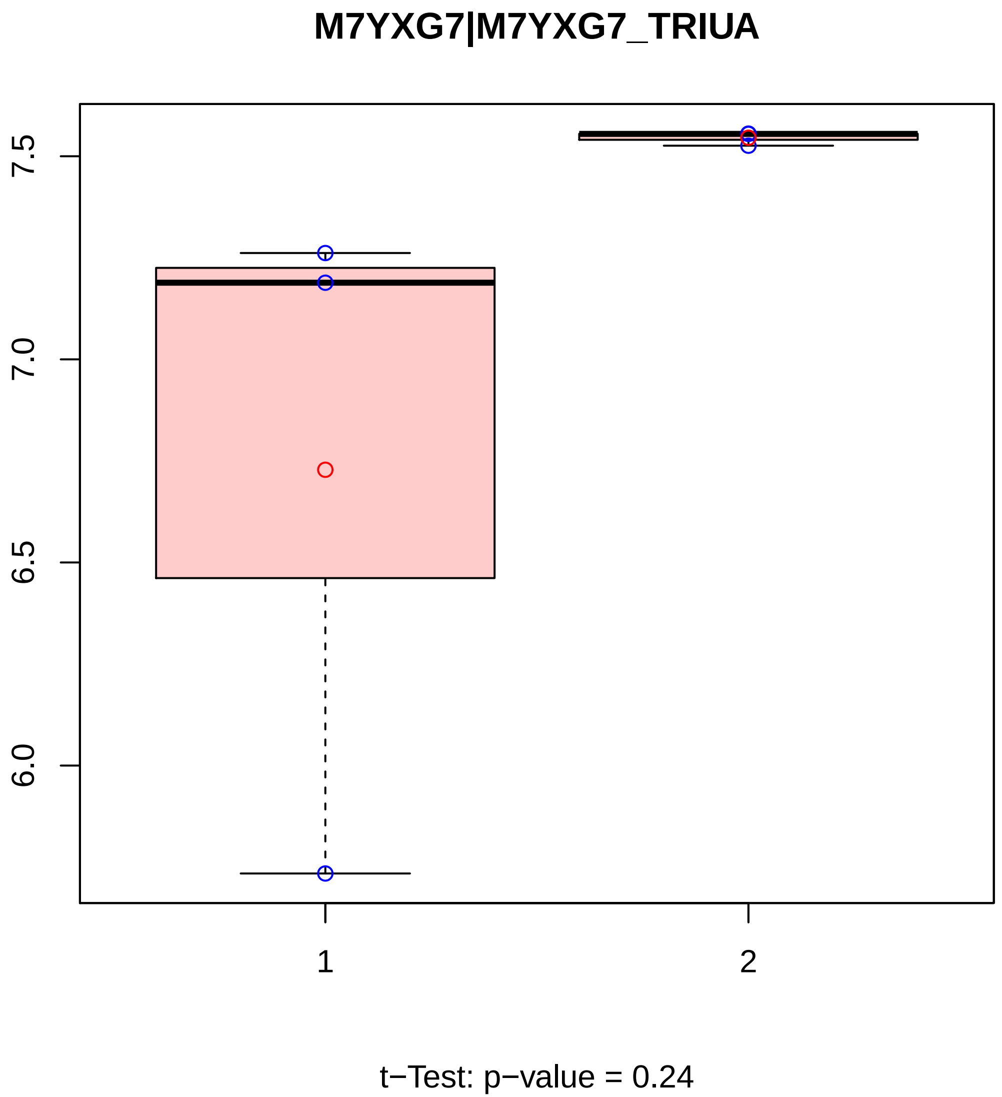

M7YXG7|M7YXG7_TRIUA
7.5
7.0
6.5
6.0
1
2
t−Test: p−value = 0.24

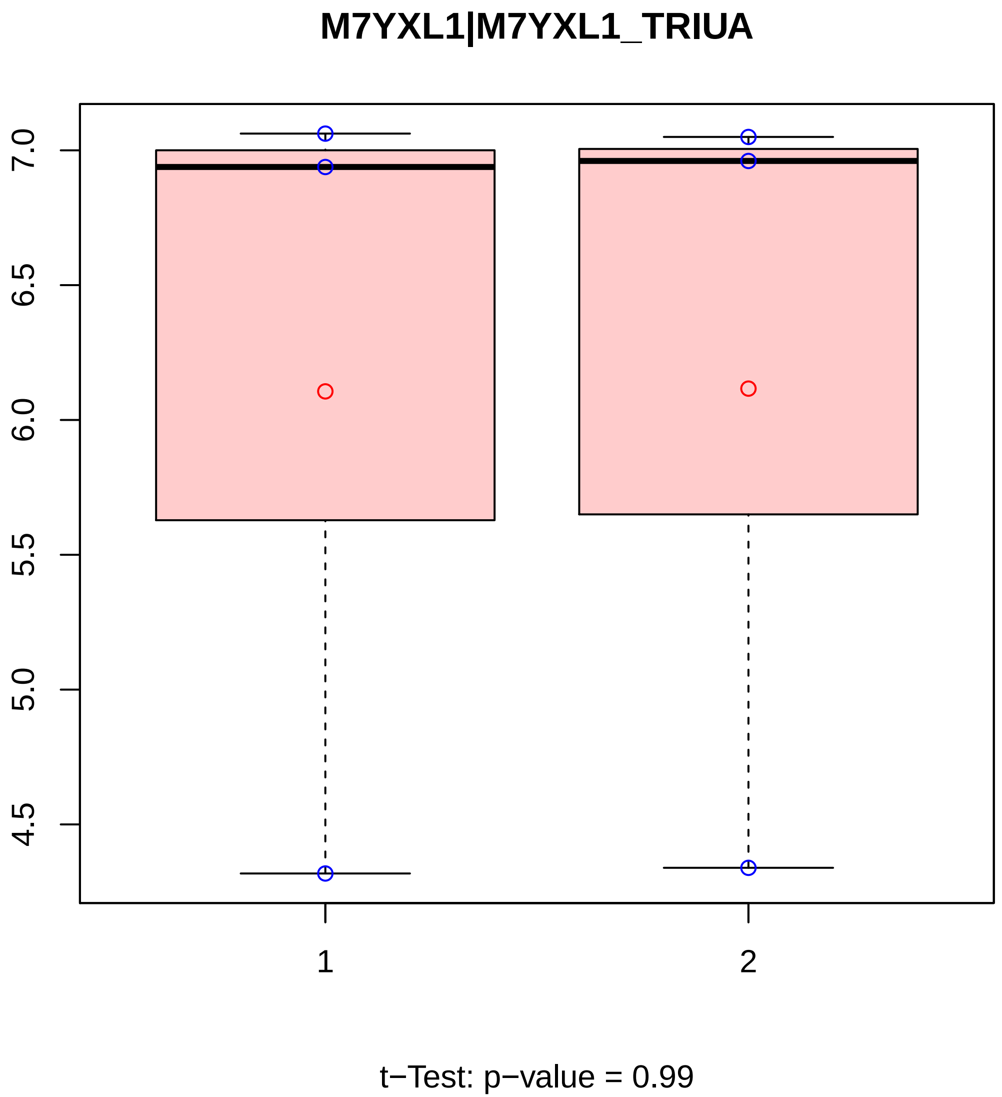

M7YXL1|M7YXL1_TRIUA
7.0
6.5
6.0
5.5
5.0
4.5
1
2
t−Test: p−value = 0.99

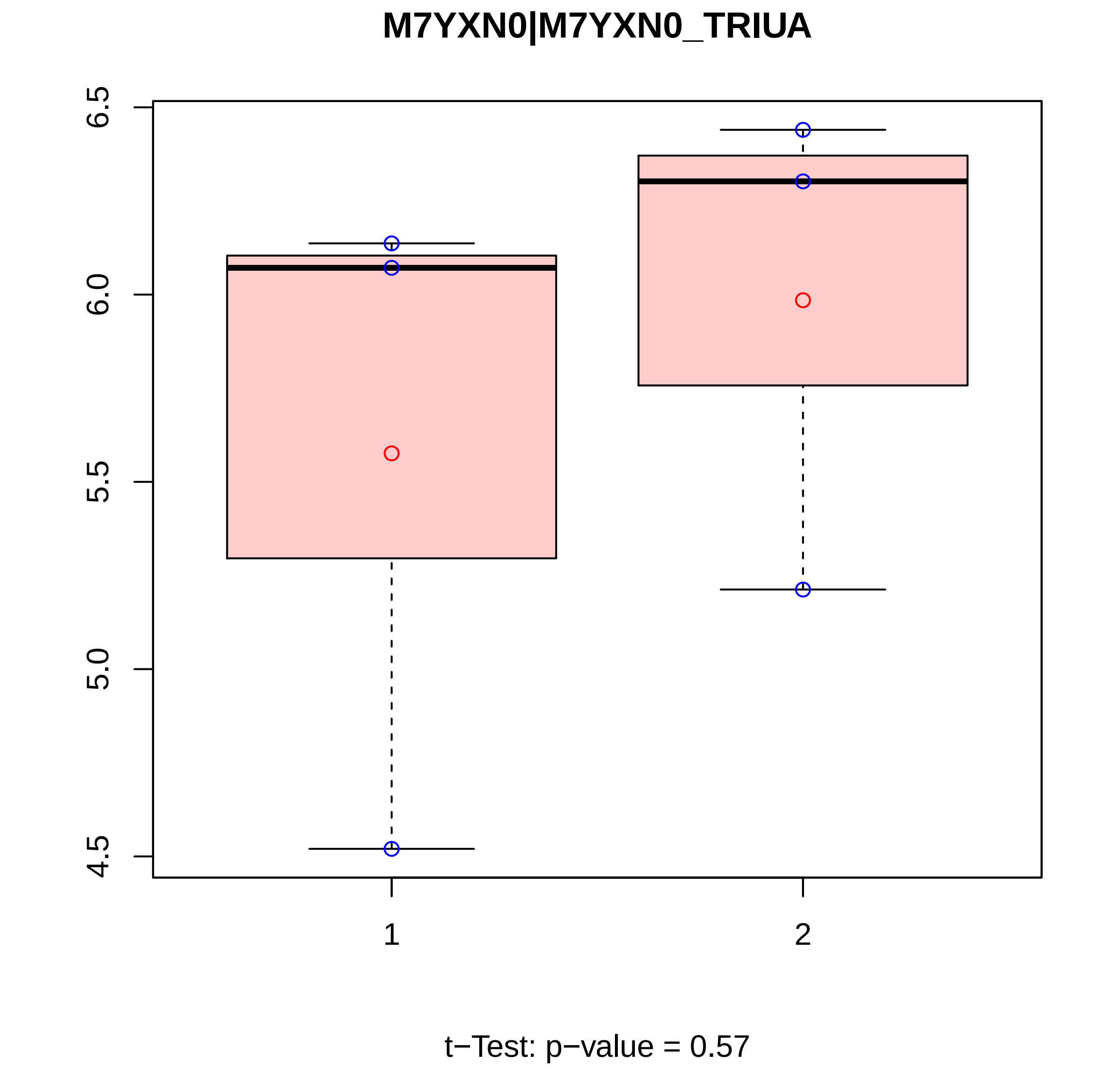
M7YXN0|M7YXN0_TRIUA
6.5
6.0
5.5
5.0
4.5
1
2
t−Test: p−value = 0.57

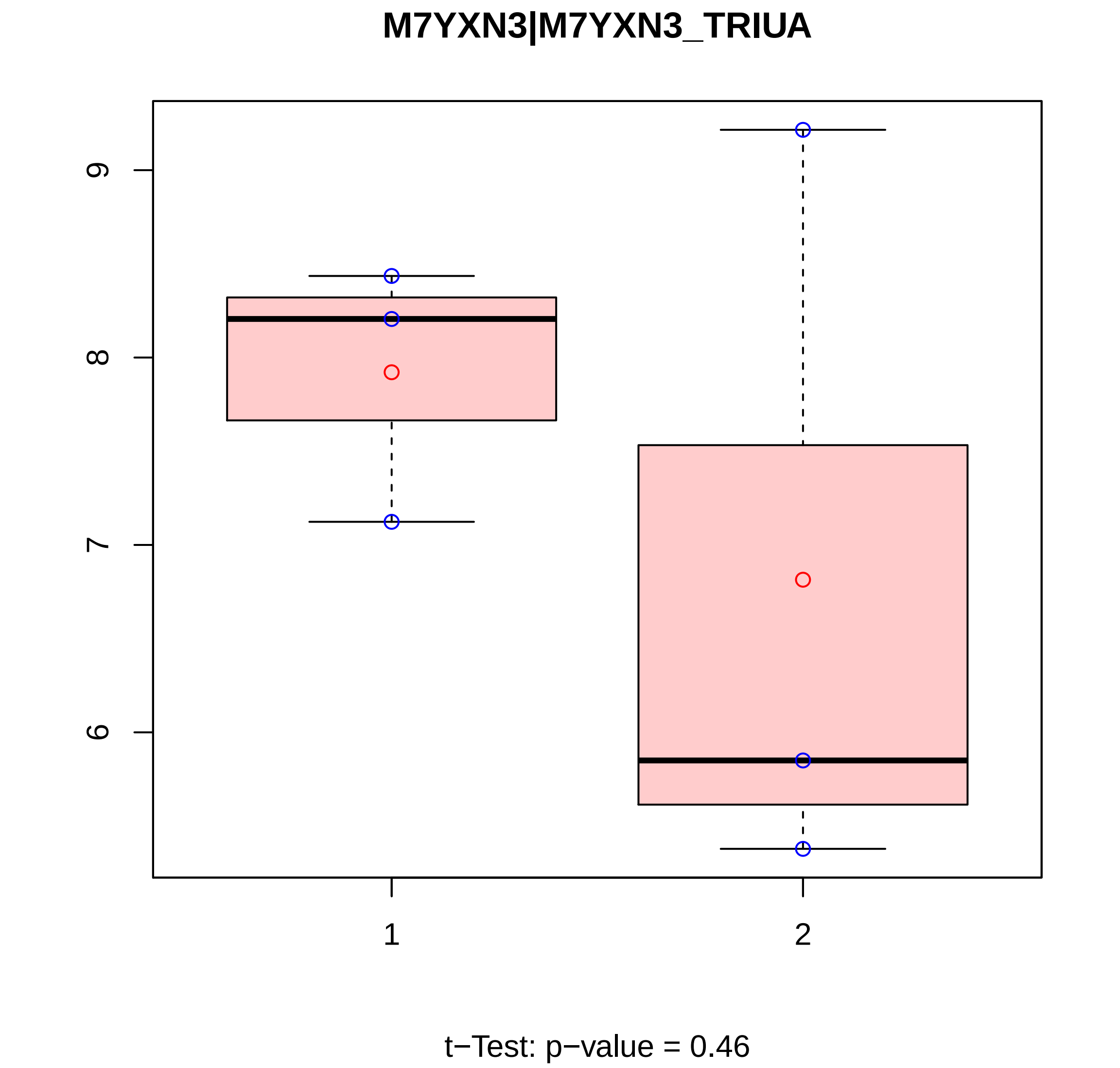
M7YXN3|M7YXN3_TRIUA
9
8
7
6
1
2
t−Test: p−value = 0.46

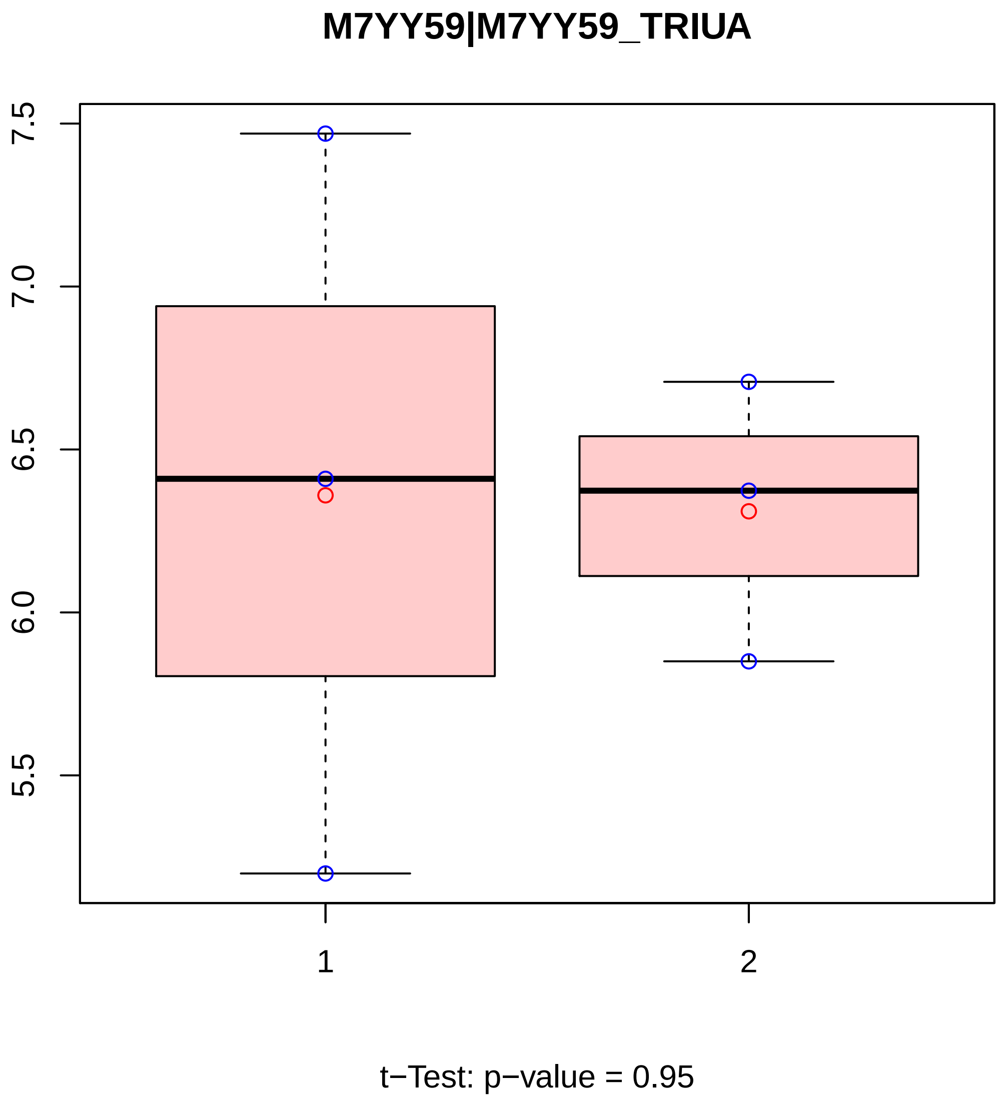
M7YY59|M7YY59_TRIUA
7.5
7.0
6.5
6.0
5.5
1
2
t−Test: p−value = 0.95

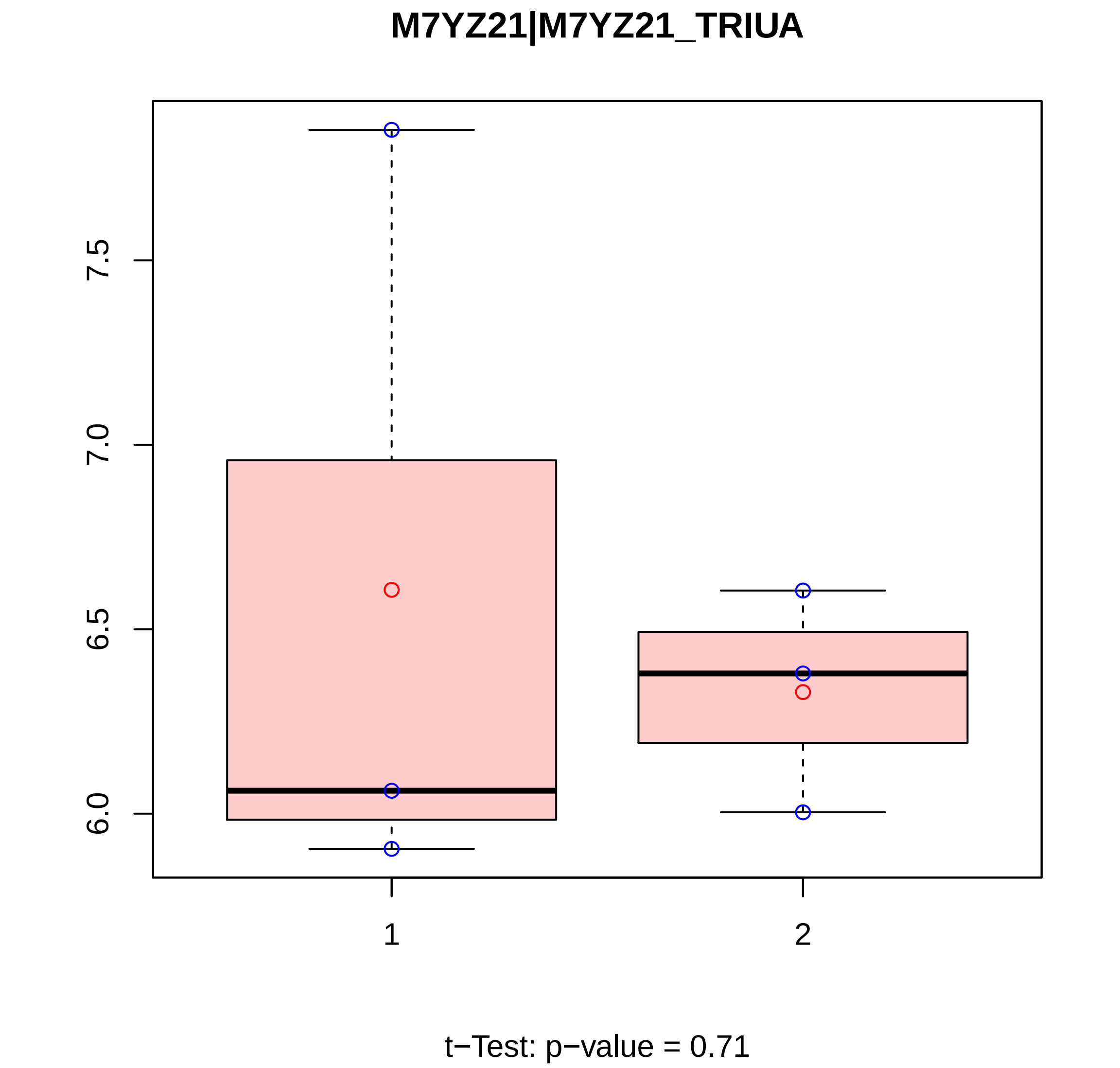
M7YZ21|M7YZ21_TRIUA
6.0
6.5
7.0
7.5
1
2
t−Test: p−value = 0.71

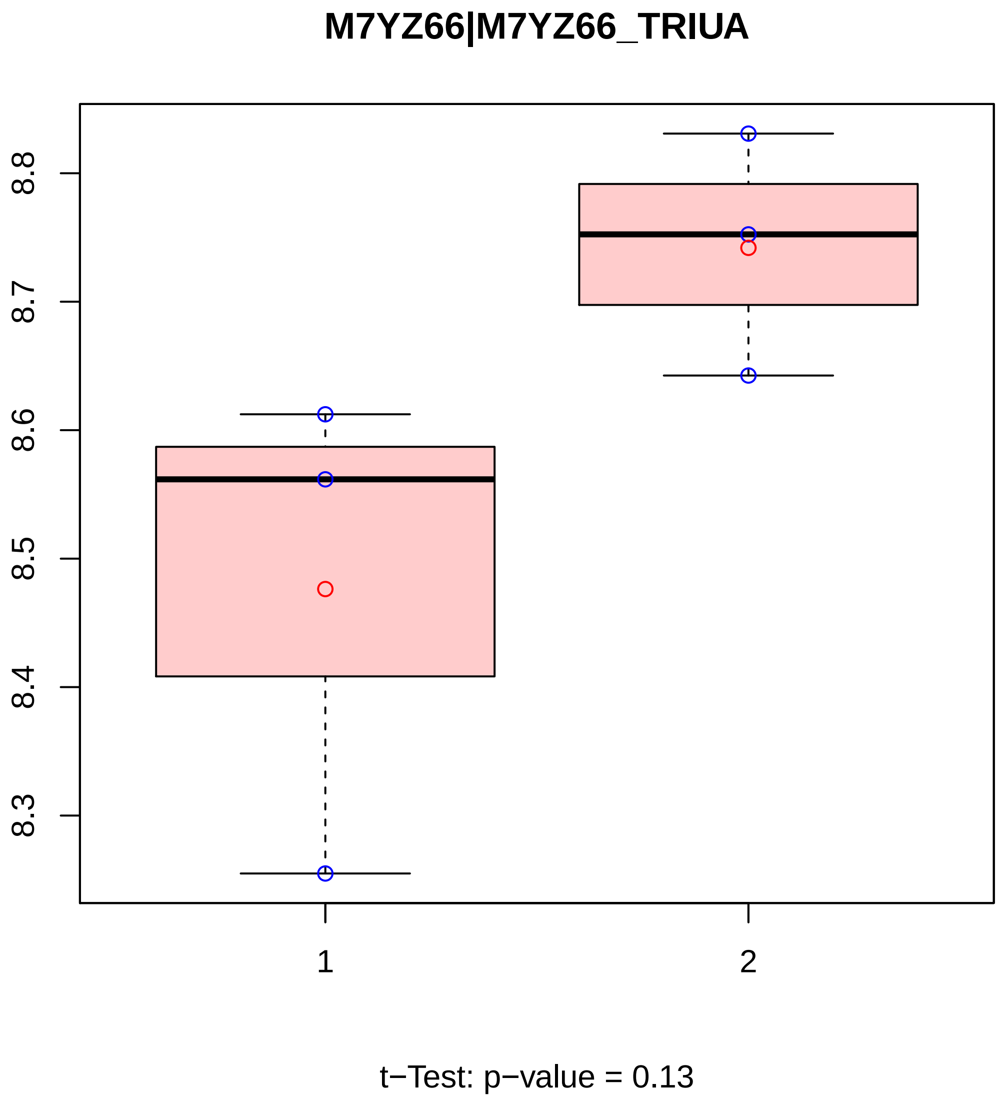
M7YZ66|M7YZ66_TRIUA
8.3
8.4
8.5
8.6
8.7
8.8
1
2
t−Test: p−value = 0.13

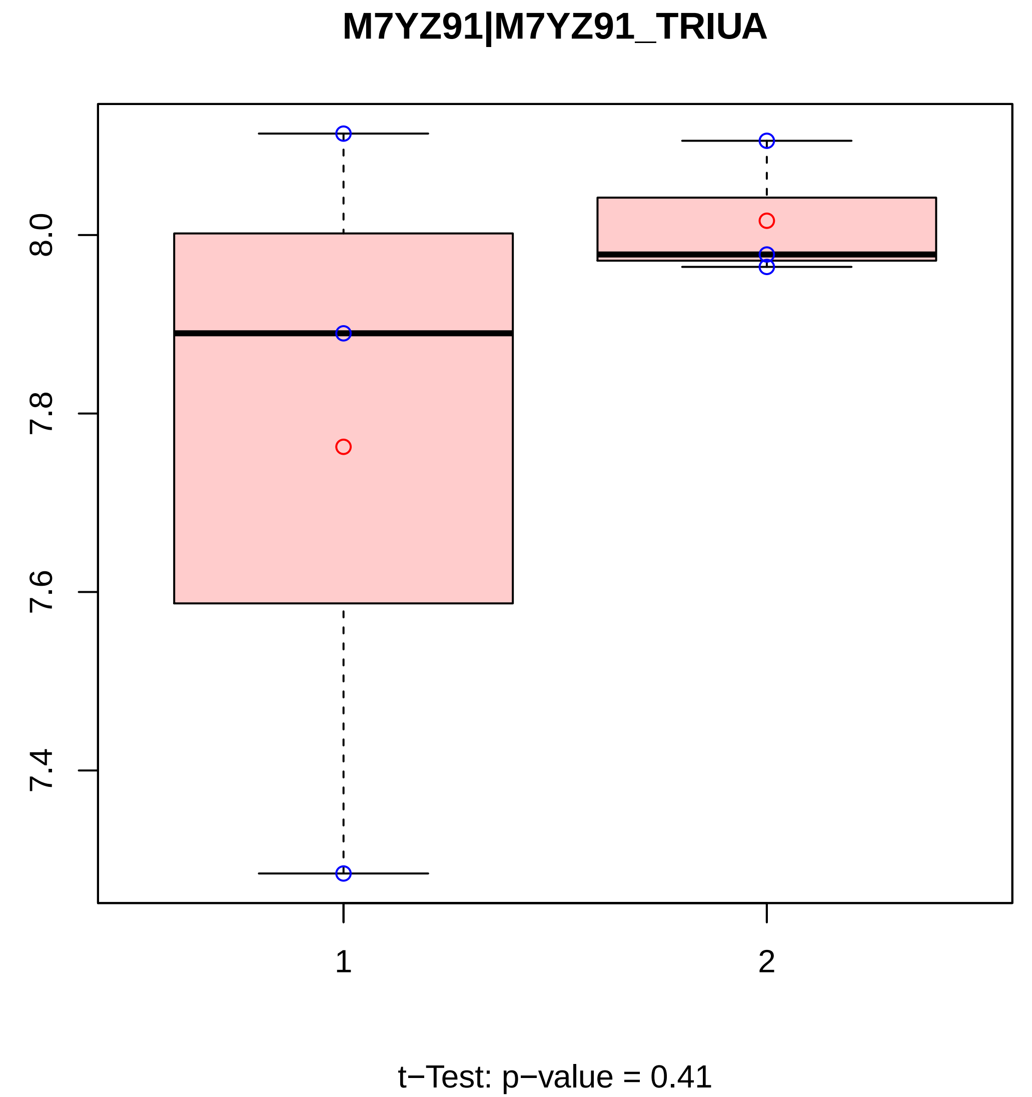
M7YZ91|M7YZ91_TRIUA
7.4
7.6
7.8
8.0
1
2
t−Test: p−value = 0.41

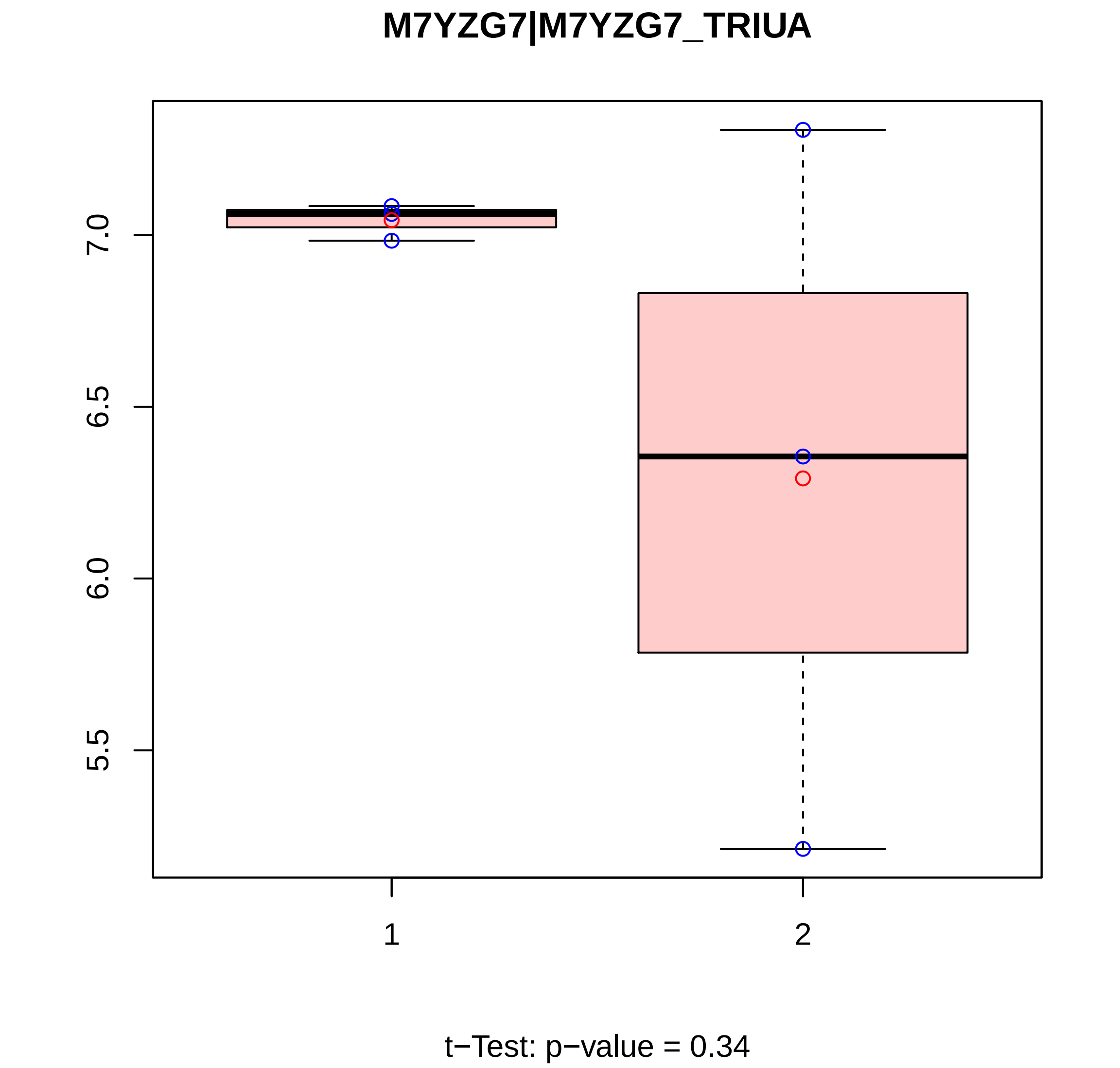

M7YZG7|M7YZG7_TRIUA
7.0
6.5
6.0
5.5
1
2
t−Test: p−value = 0.34

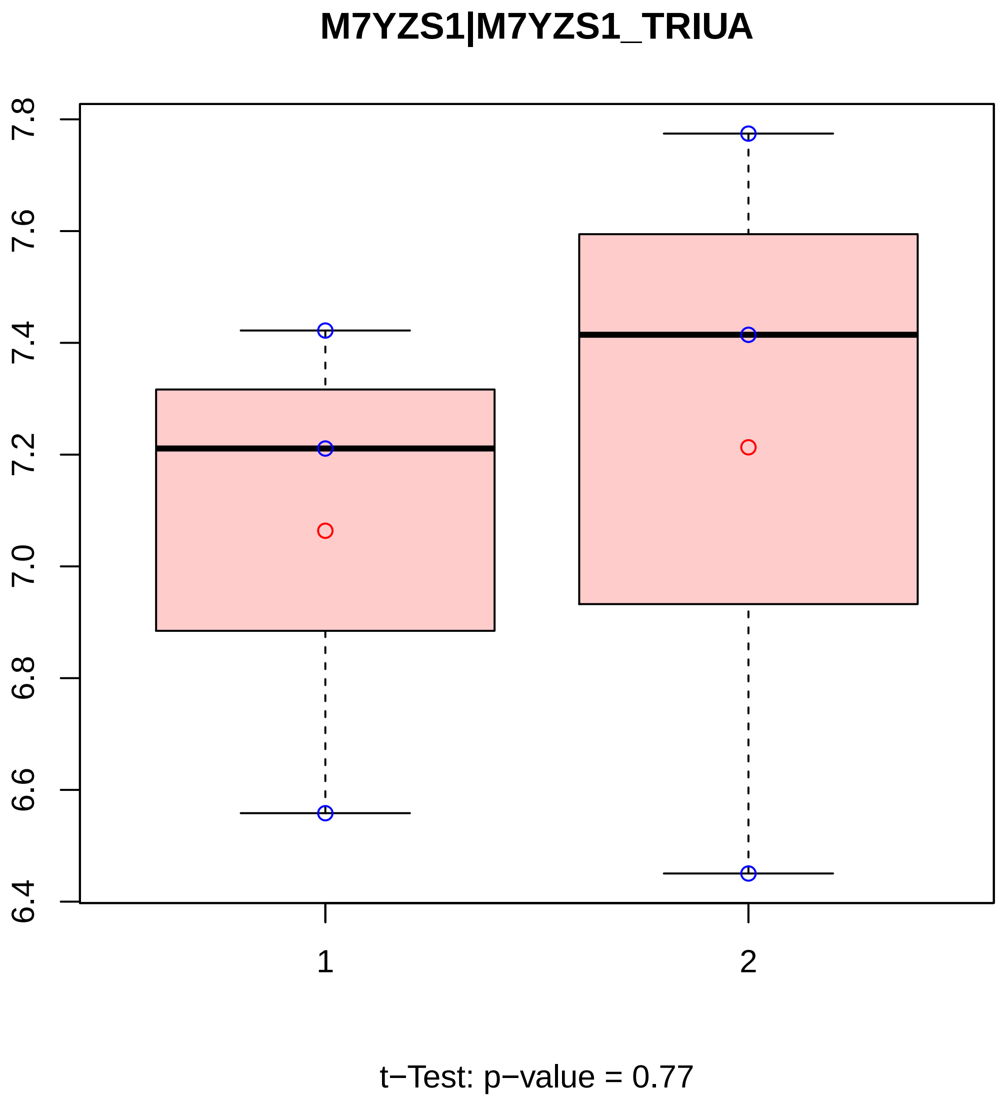
M7YZS1|M7YZS1_TRIUA
7.8
7.6
7.4
7.2
7.0
6.8
6.6
6.4
1
2
t−Test: p−value = 0.77

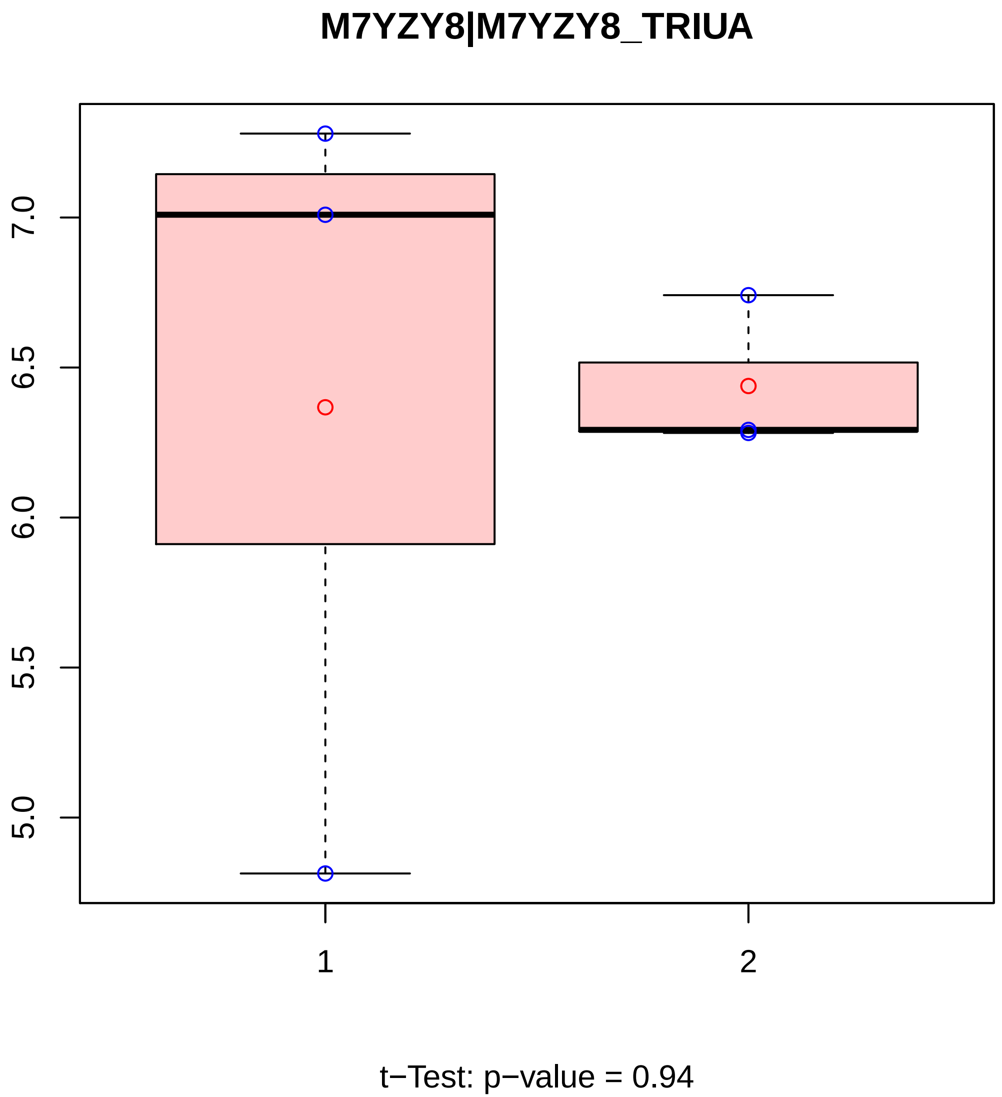
M7YZY8|M7YZY8_TRIUA
7.0
6.5
6.0
5.5
5.0
1
2
t−Test: p−value = 0.94

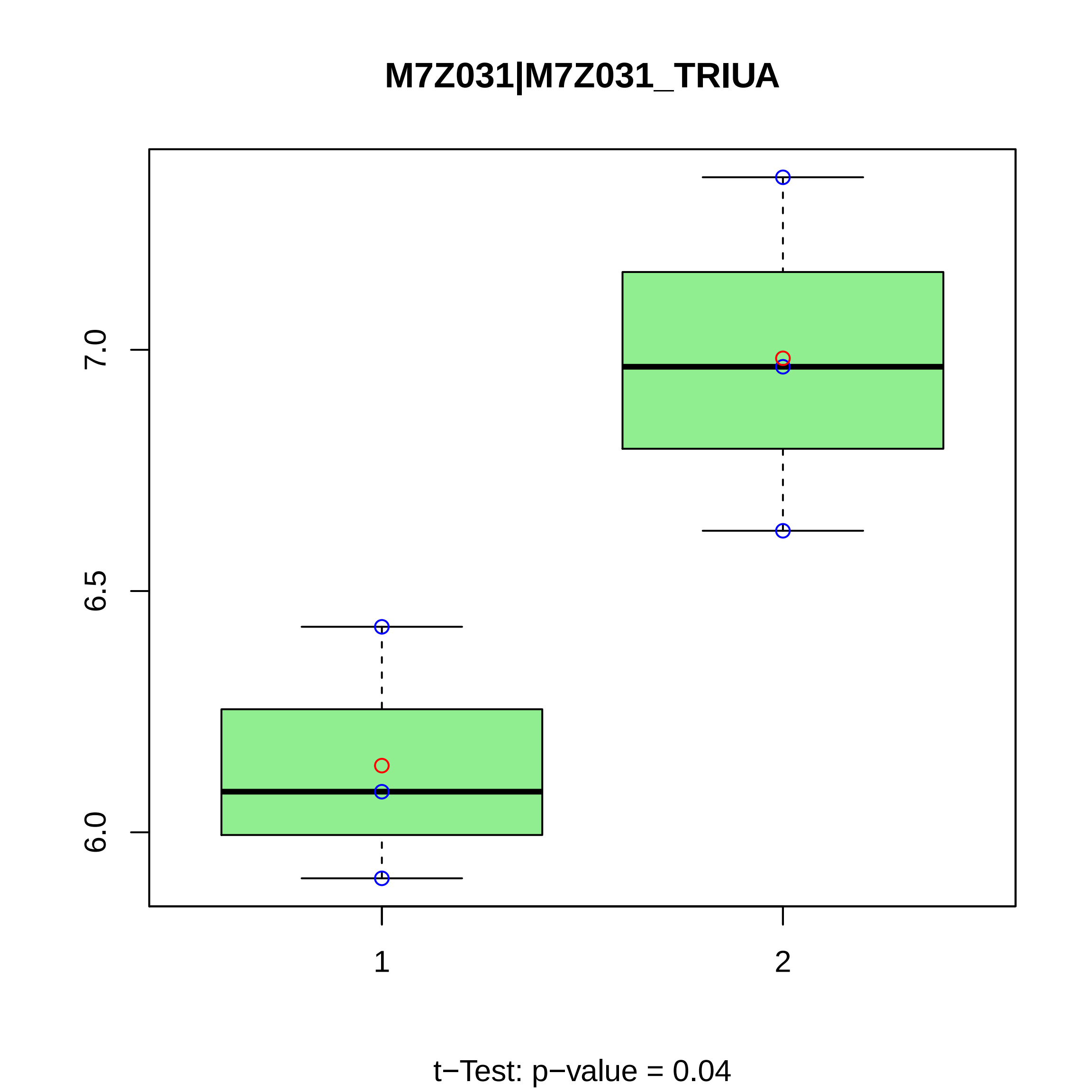

M7Z031|M7Z031_TRIUA
7.0
6.5
6.0
1
2
t−Test: p−value = 0.04

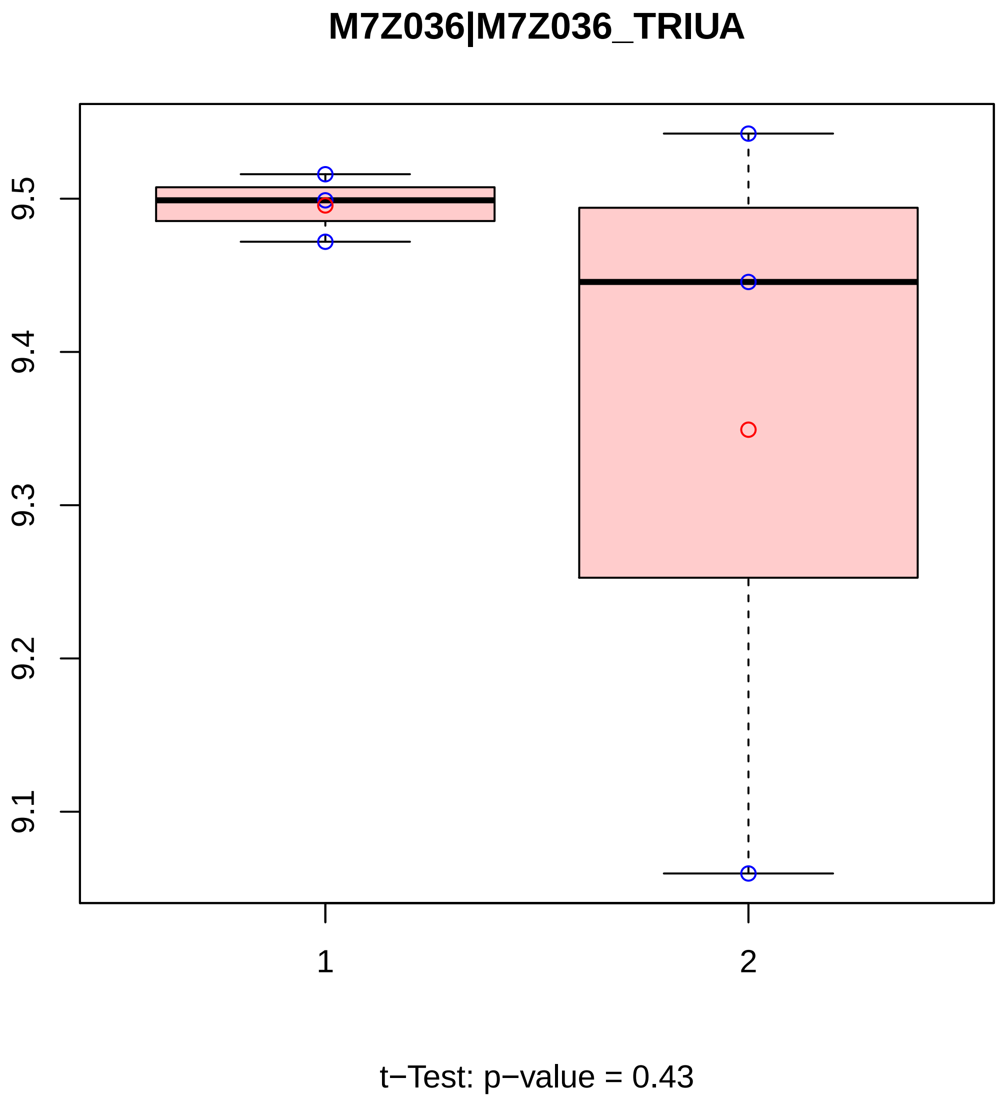
M7Z036|M7Z036_TRIUA
9.1
9.2
9.3
9.4
9.5
1
2
t−Test: p−value = 0.43

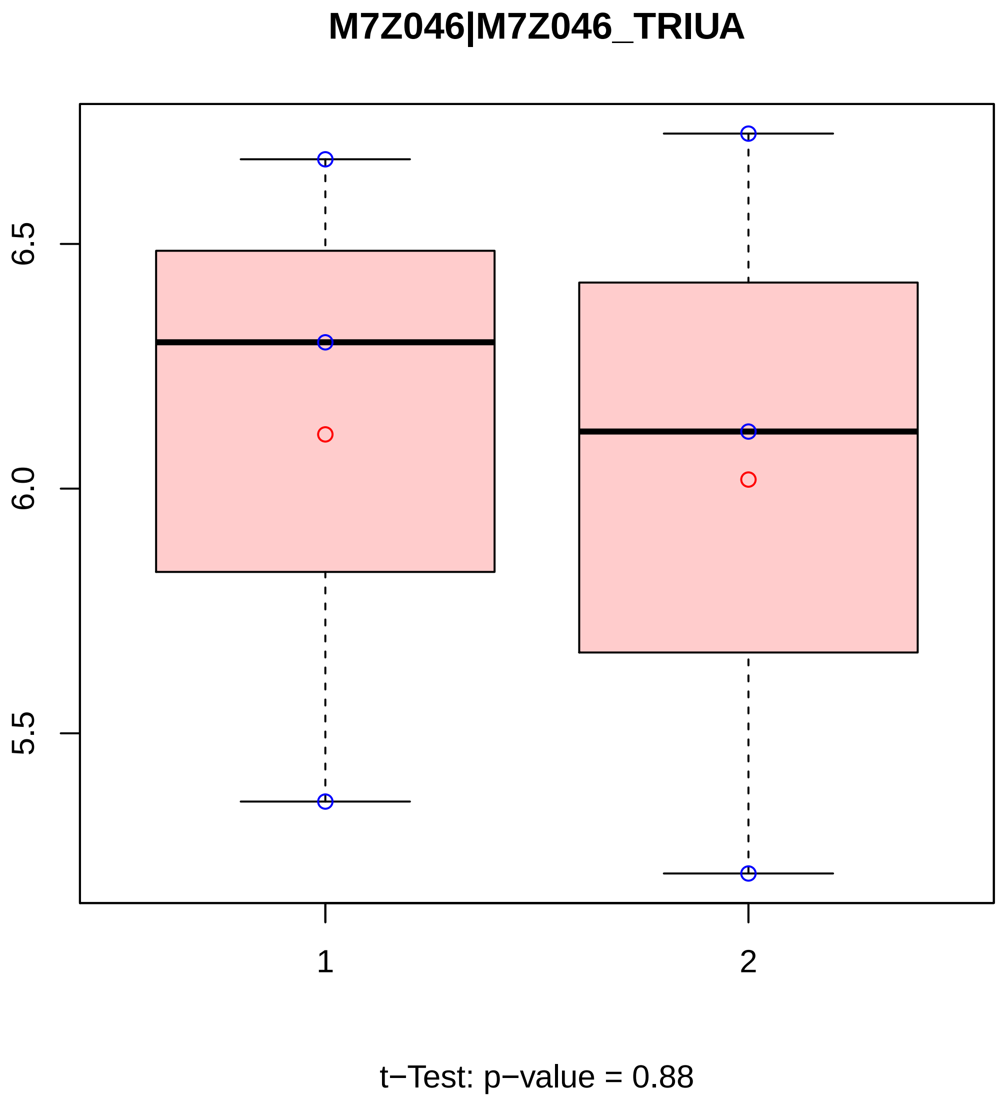
M7Z046|M7Z046_TRIUA
6.5
6.0
5.5
1
2
t−Test: p−value = 0.88

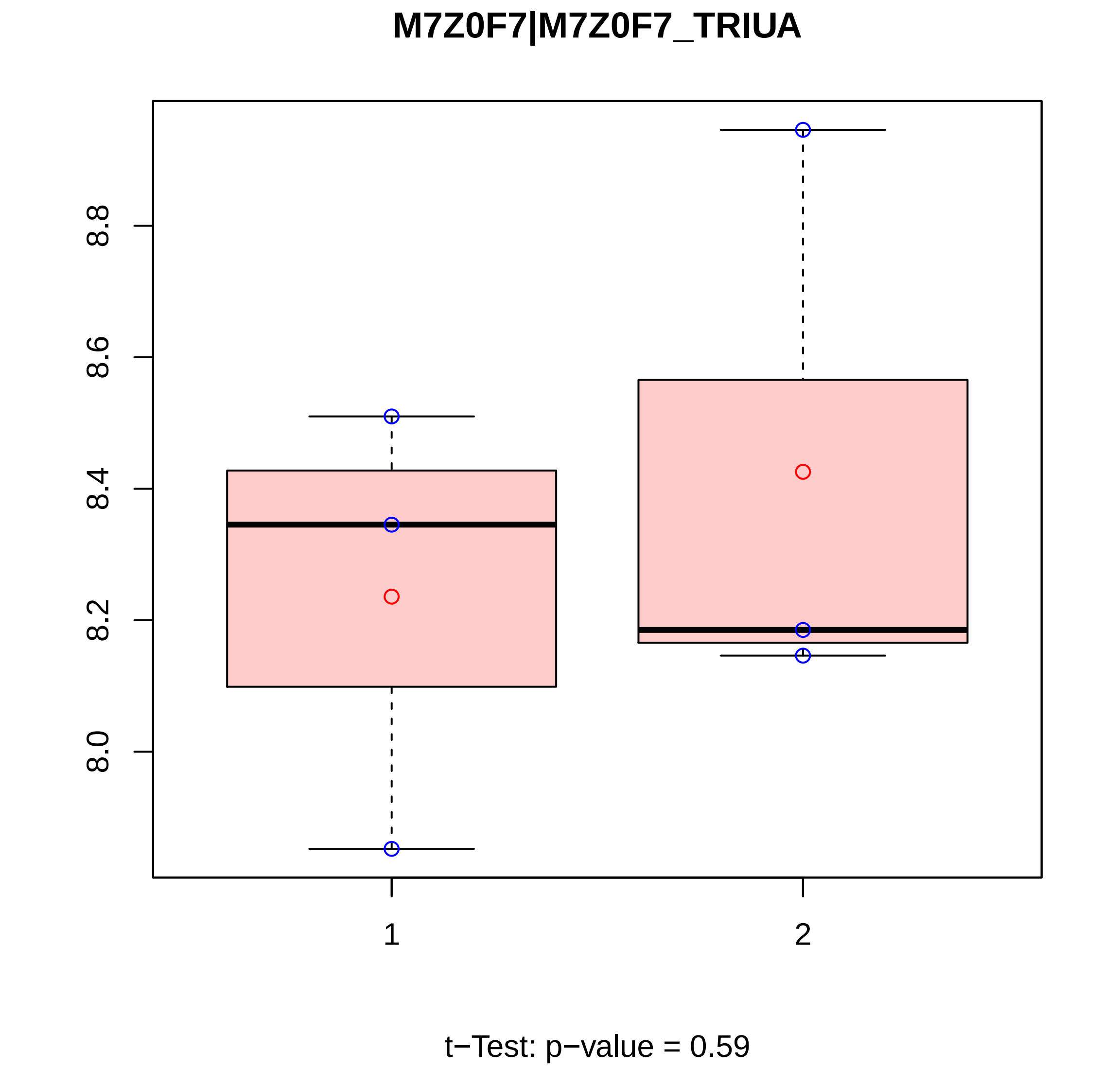
M7Z0F7|M7Z0F7_TRIUA
8.0
8.2
8.4
8.6
8.8
1
2
t−Test: p−value = 0.59

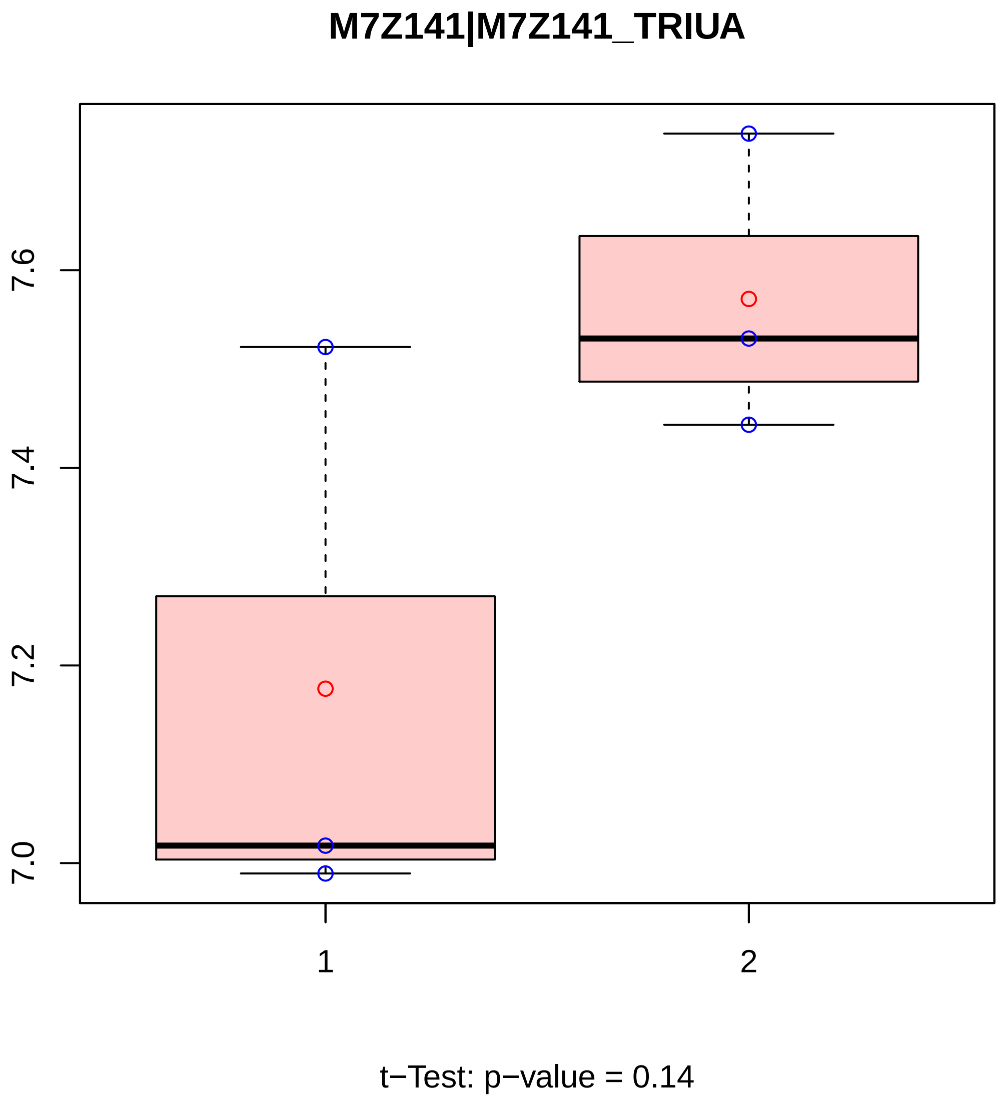
M7Z141|M7Z141_TRIUA
7.0
7.2
7.4
7.6
1
2
t−Test: p−value = 0.14

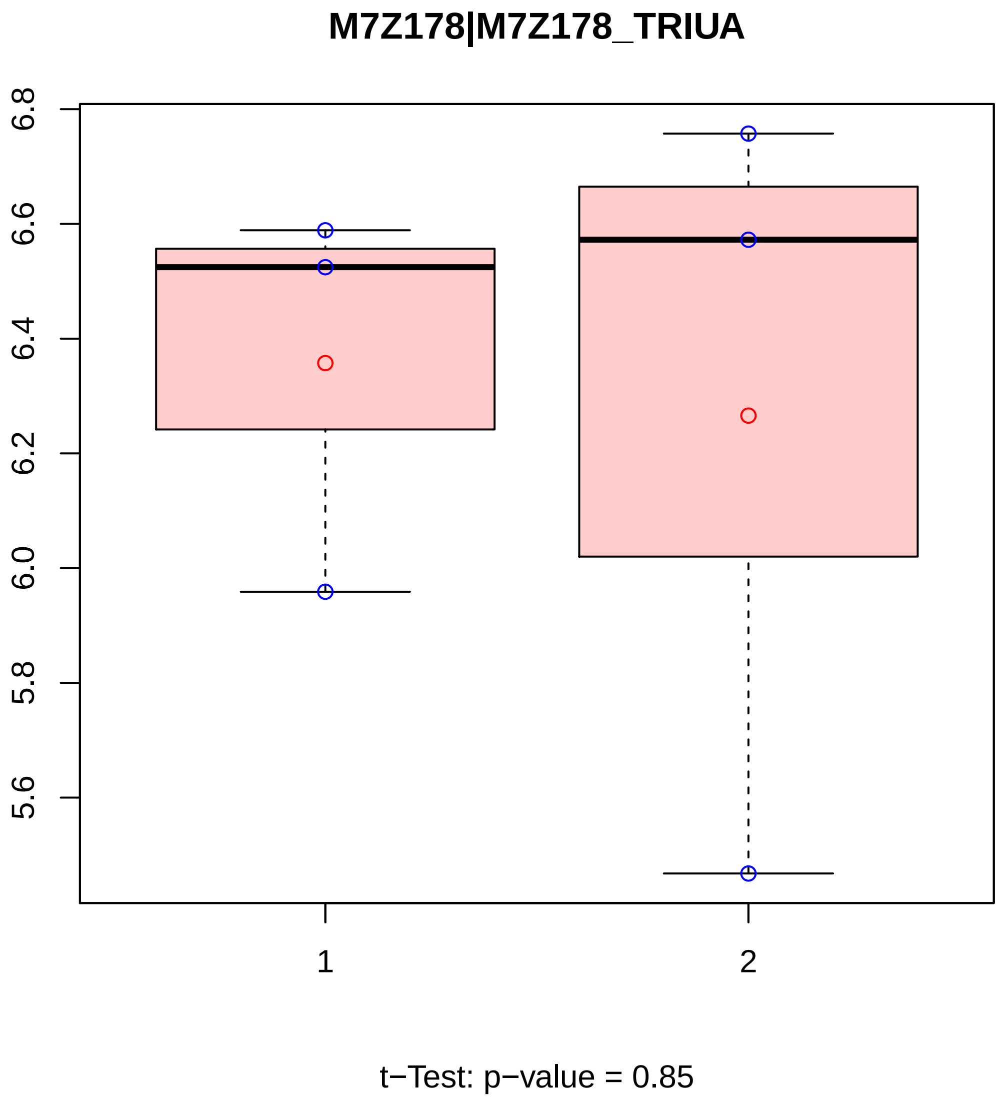
M7Z178|M7Z178_TRIUA
6.8
6.6
6.4
6.2
6.0
5.8
5.6
1
2
t−Test: p−value = 0.85

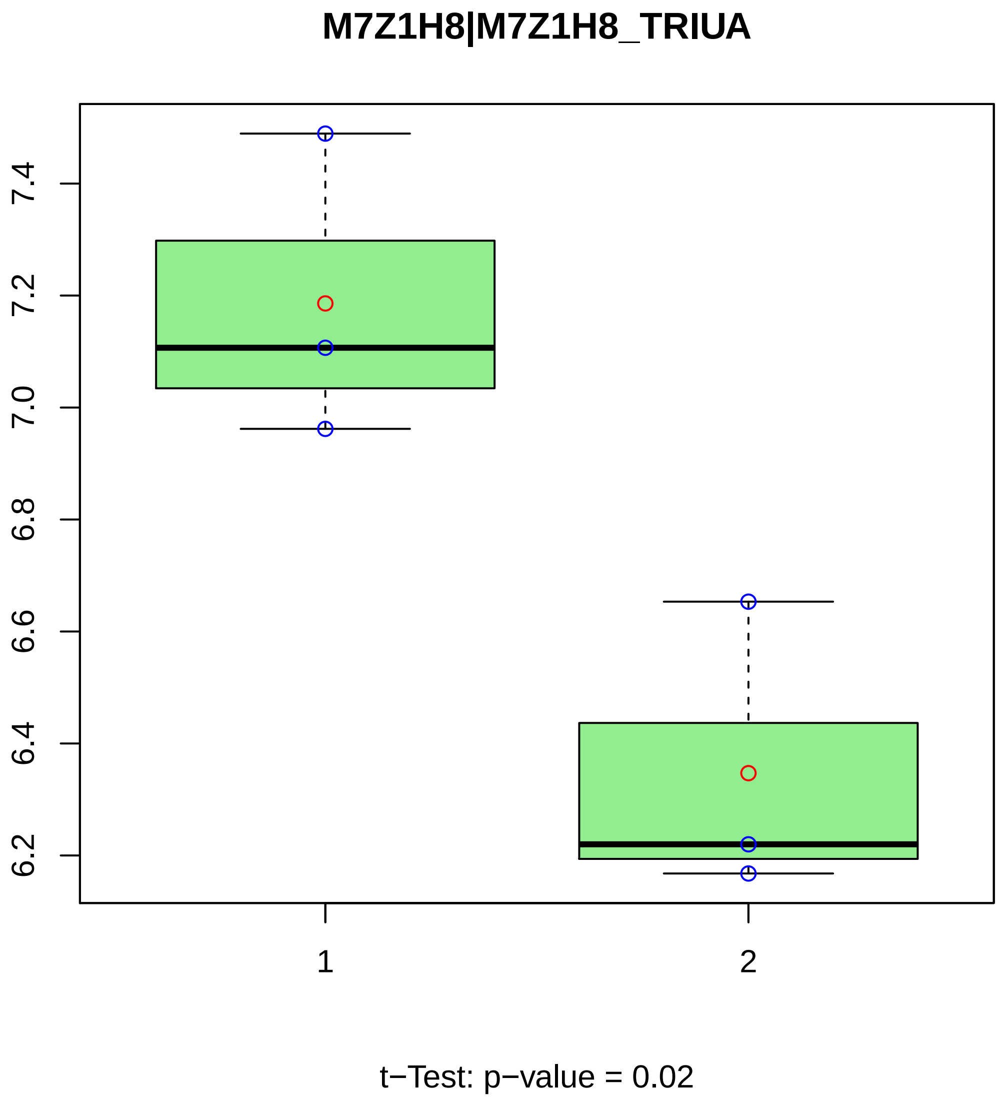
M7Z1H8|M7Z1H8_TRIUA
7.4
7.2
7.0
6.8
6.6
6.4
6.2
1
2
t−Test: p−value = 0.02

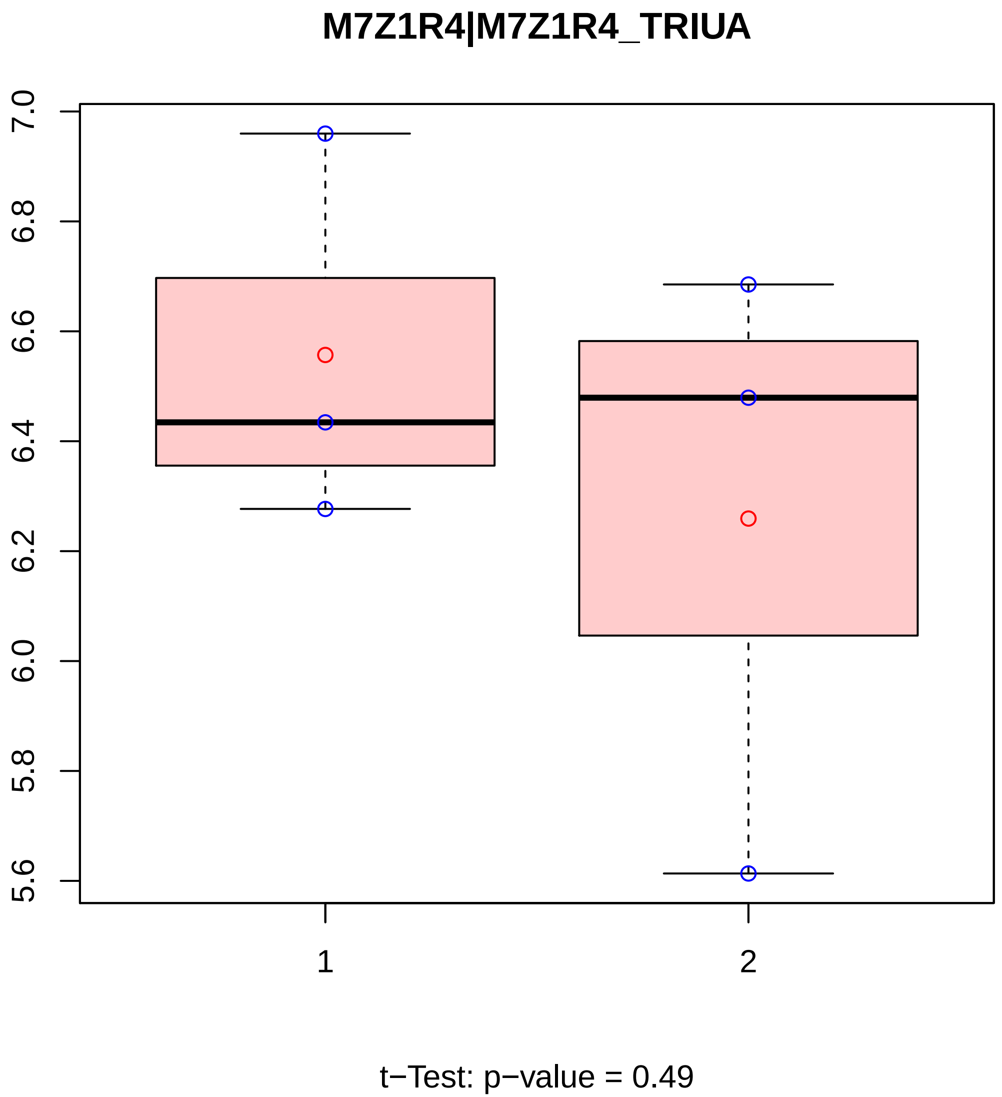
M7Z1R4|M7Z1R4_TRIUA
7.0
6.8
6.6
6.4
6.2
6.0
5.8
5.6
1
2
t−Test: p−value = 0.49

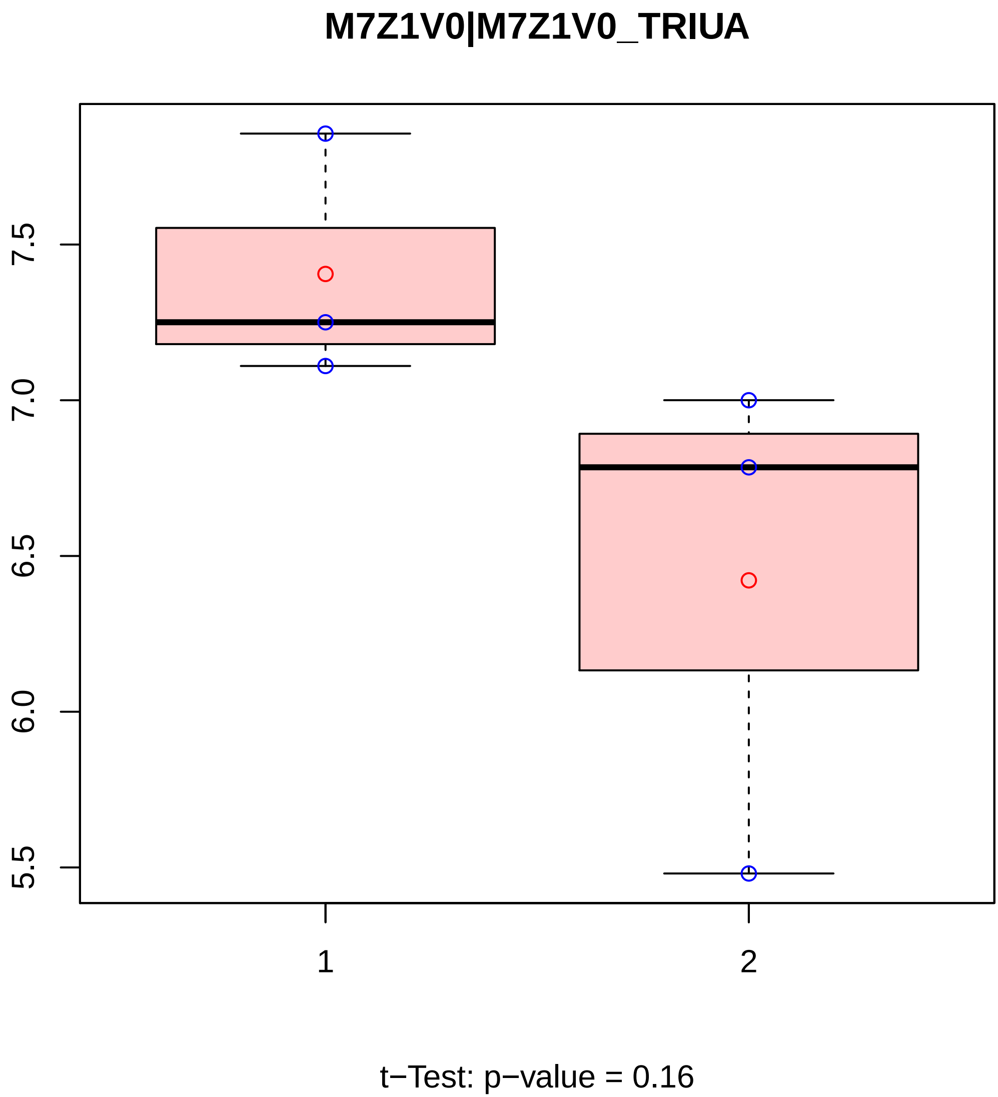

M7Z1V0|M7Z1V0_TRIUA
7.5
7.0
6.5
6.0
5.5
1
2
t−Test: p−value = 0.16

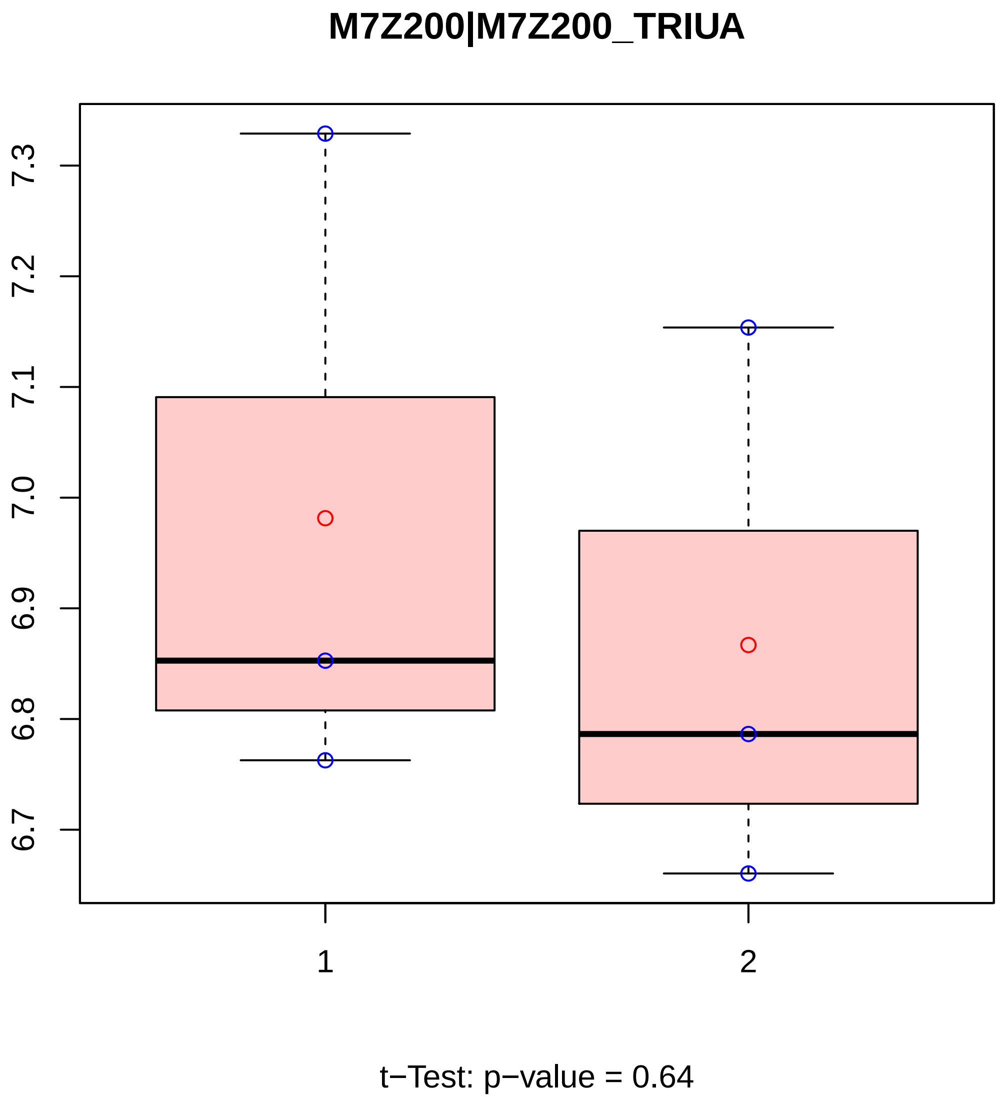

M7Z200|M7Z200_TRIUA
7.3
7.2
7.1
7.0
6.9
6.8
6.7
1
2
t−Test: p−value = 0.64

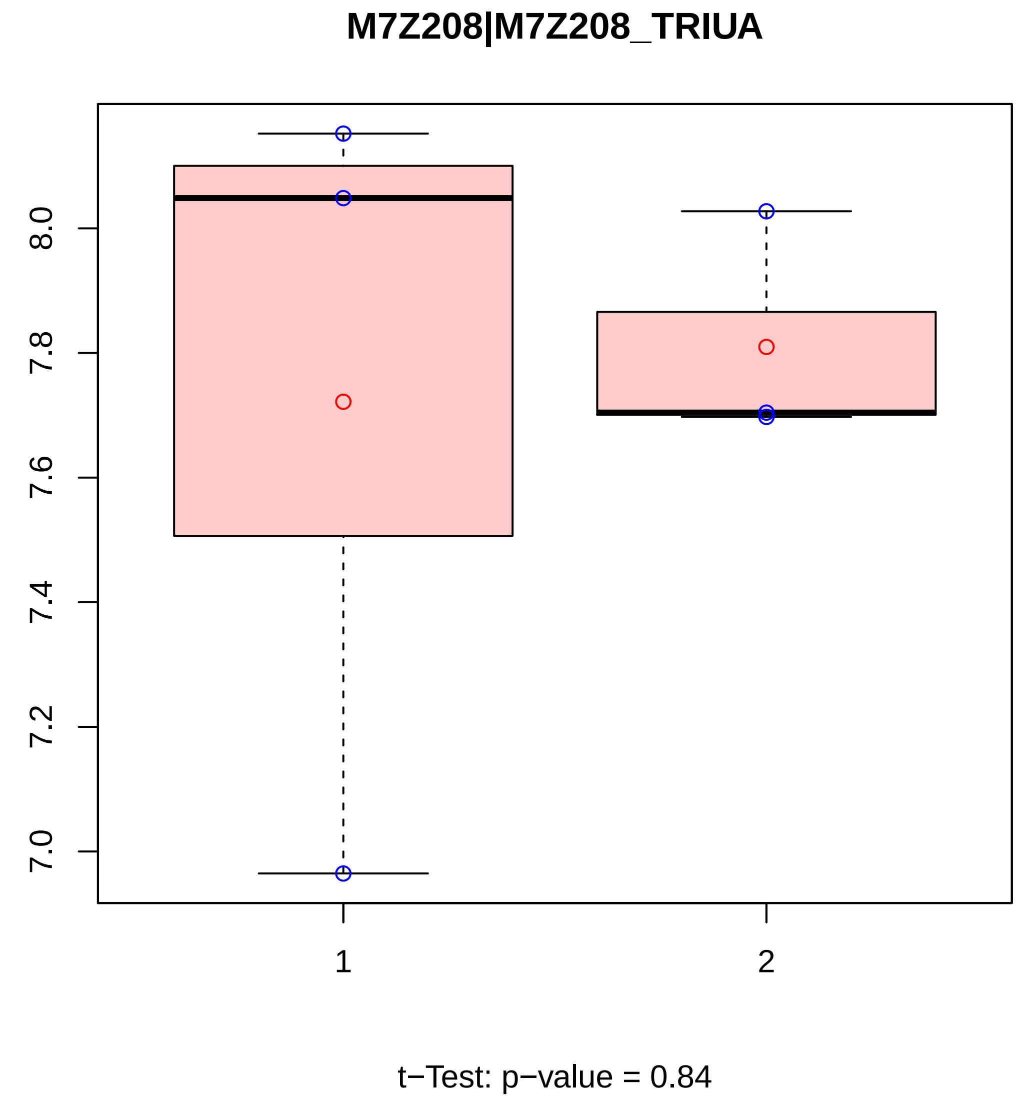
M7Z208|M7Z208_TRIUA
8.0
7.8
7.6
7.4
7.2
7.0
1
2
t−Test: p−value = 0.84

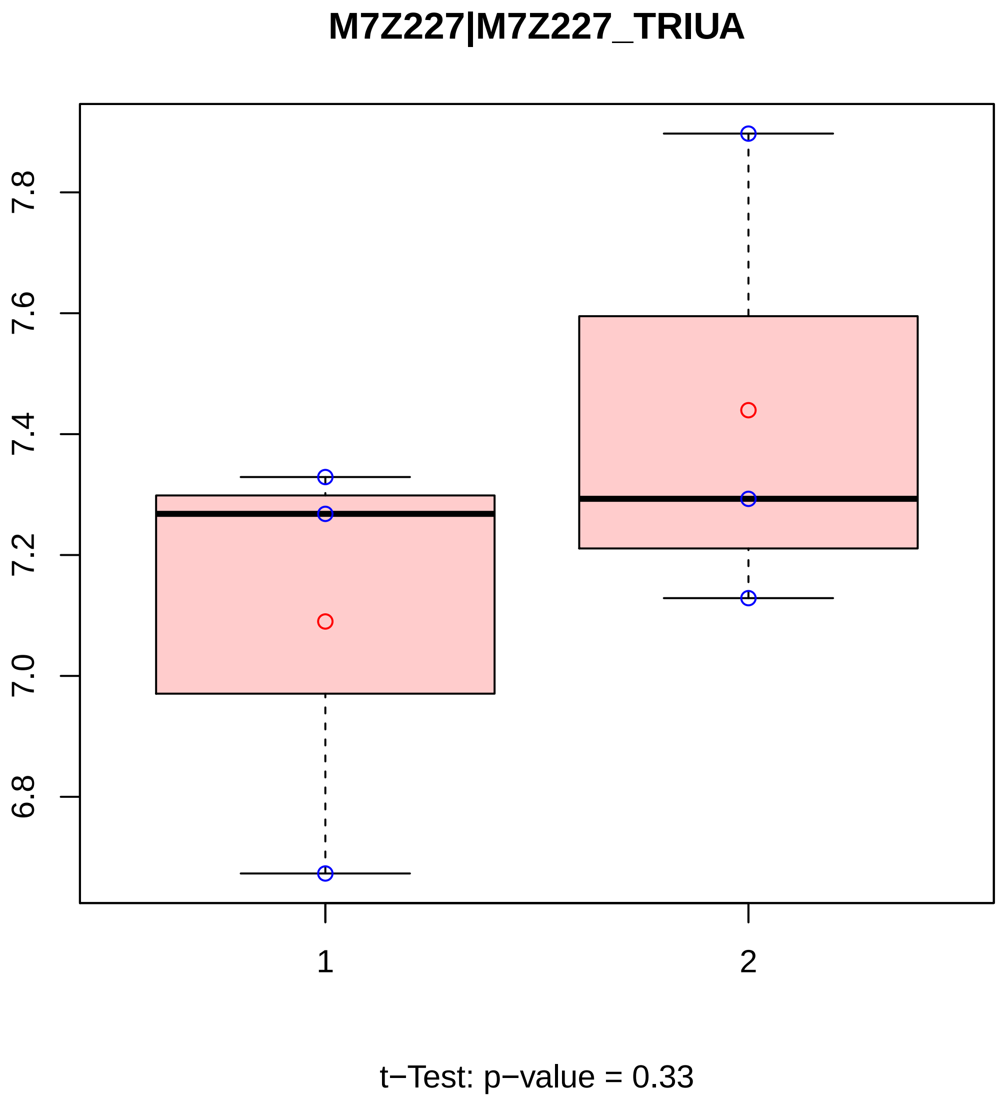
M7Z227|M7Z227_TRIUA
7.8
7.6
7.4
7.2
7.0
6.8
1
2
t−Test: p−value = 0.33

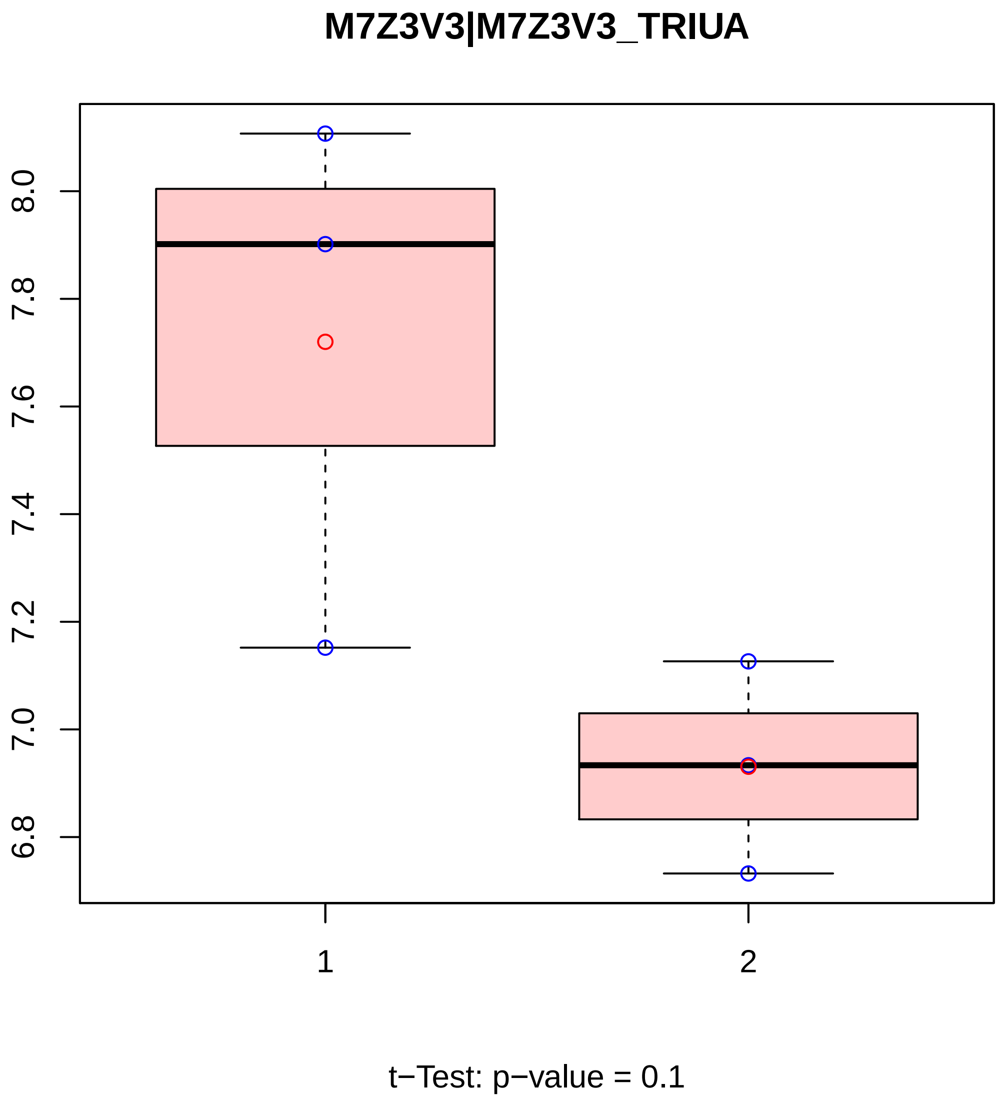

M7Z3V3|M7Z3V3_TRIUA
8.0
7.8
7.6
7.4
7.2
7.0
6.8
1
2
t−Test: p−value = 0.1

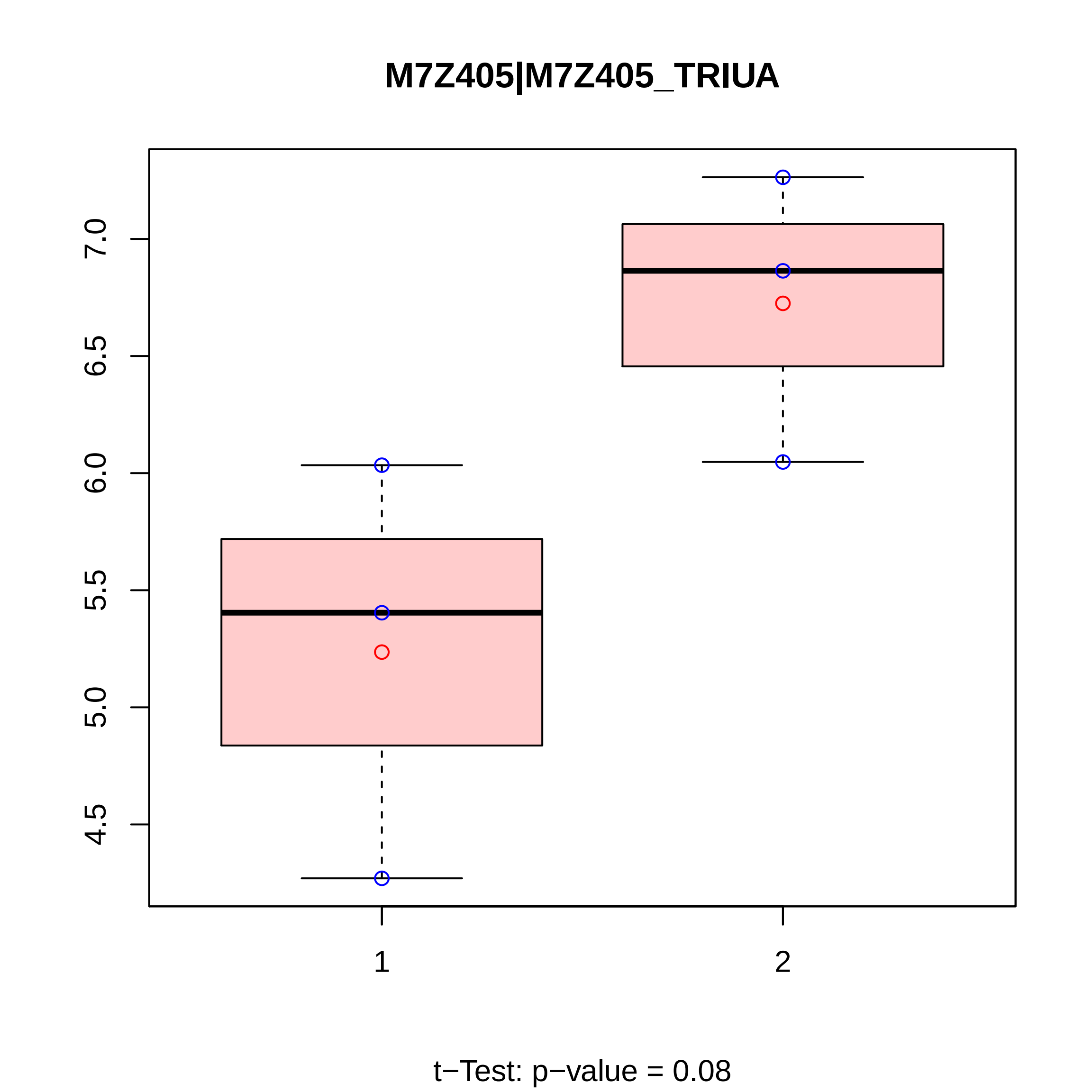
M7Z405|M7Z405_TRIUA
7.0
6.5
6.0
5.5
5.0
4.5
1
2
t−Test: p−value = 0.08

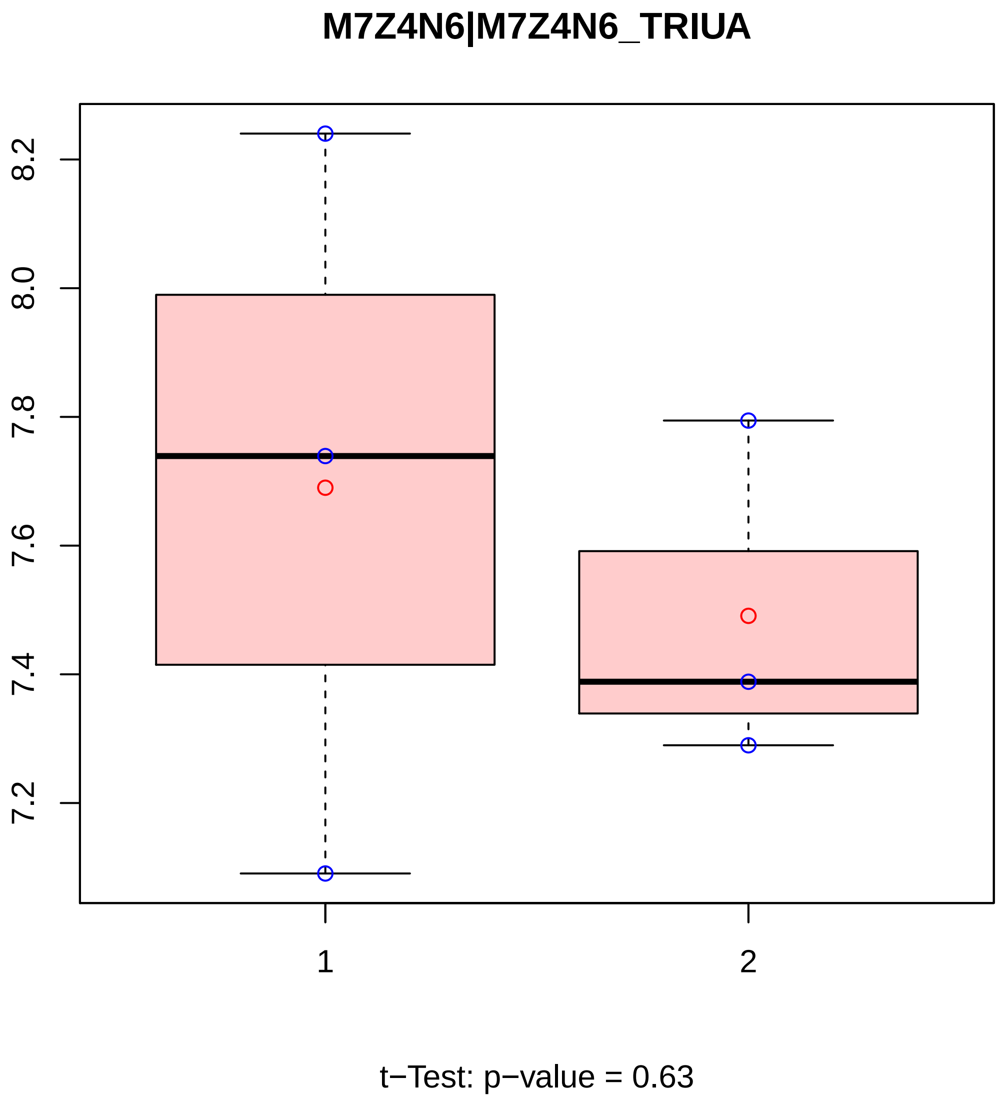
M7Z4N6|M7Z4N6_TRIUA
8.2
8.0
7.8
7.6
7.4
7.2
1
2
t−Test: p−value = 0.63

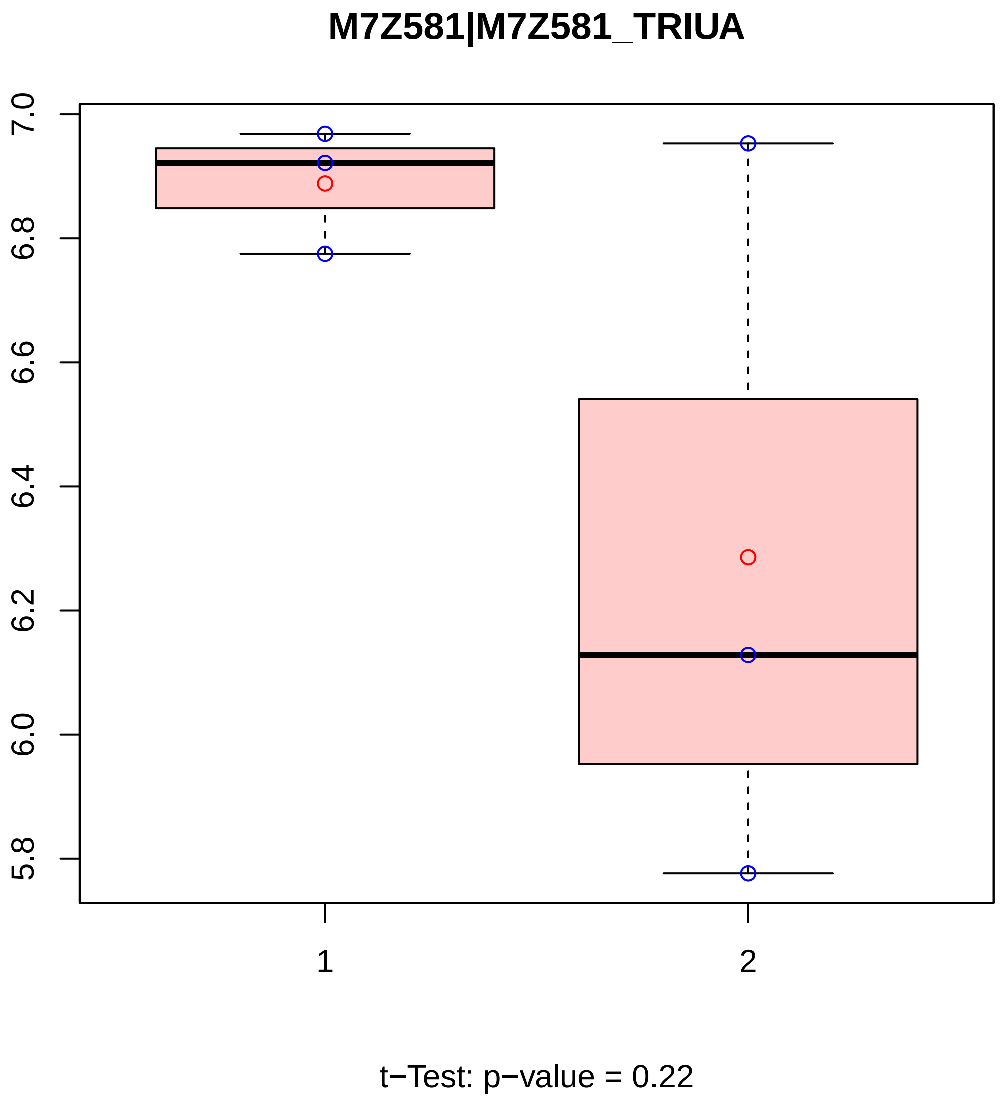
M7Z581|M7Z581_TRIUA
7.0
6.8
6.6
6.4
6.2
6.0
5.8
1
2
t−Test: p−value = 0.22

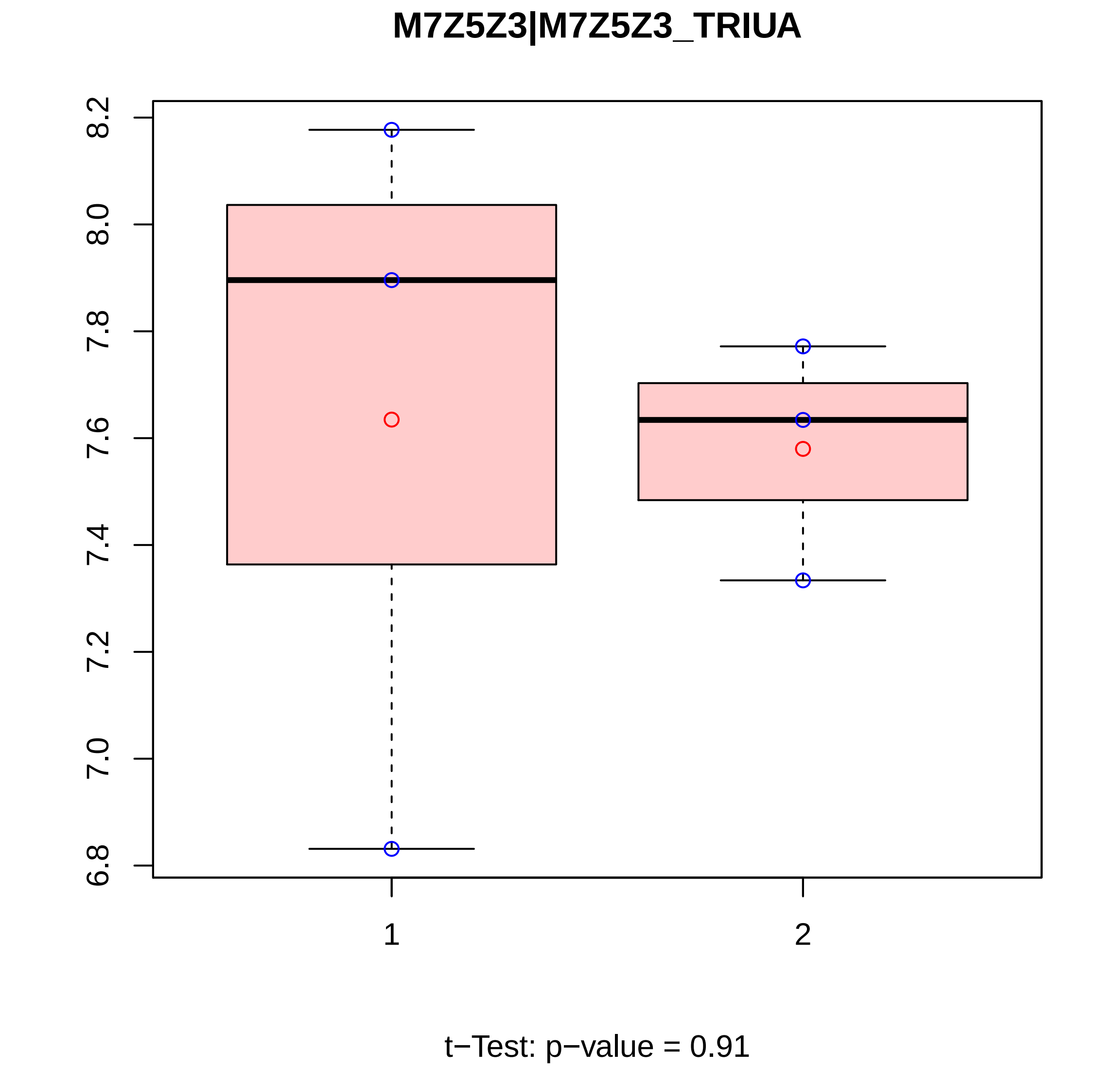
M7Z5Z3|M7Z5Z3_TRIUA
8.2
8.0
7.8
7.6
7.4
7.2
7.0
6.8
1
2
t−Test: p−value = 0.91

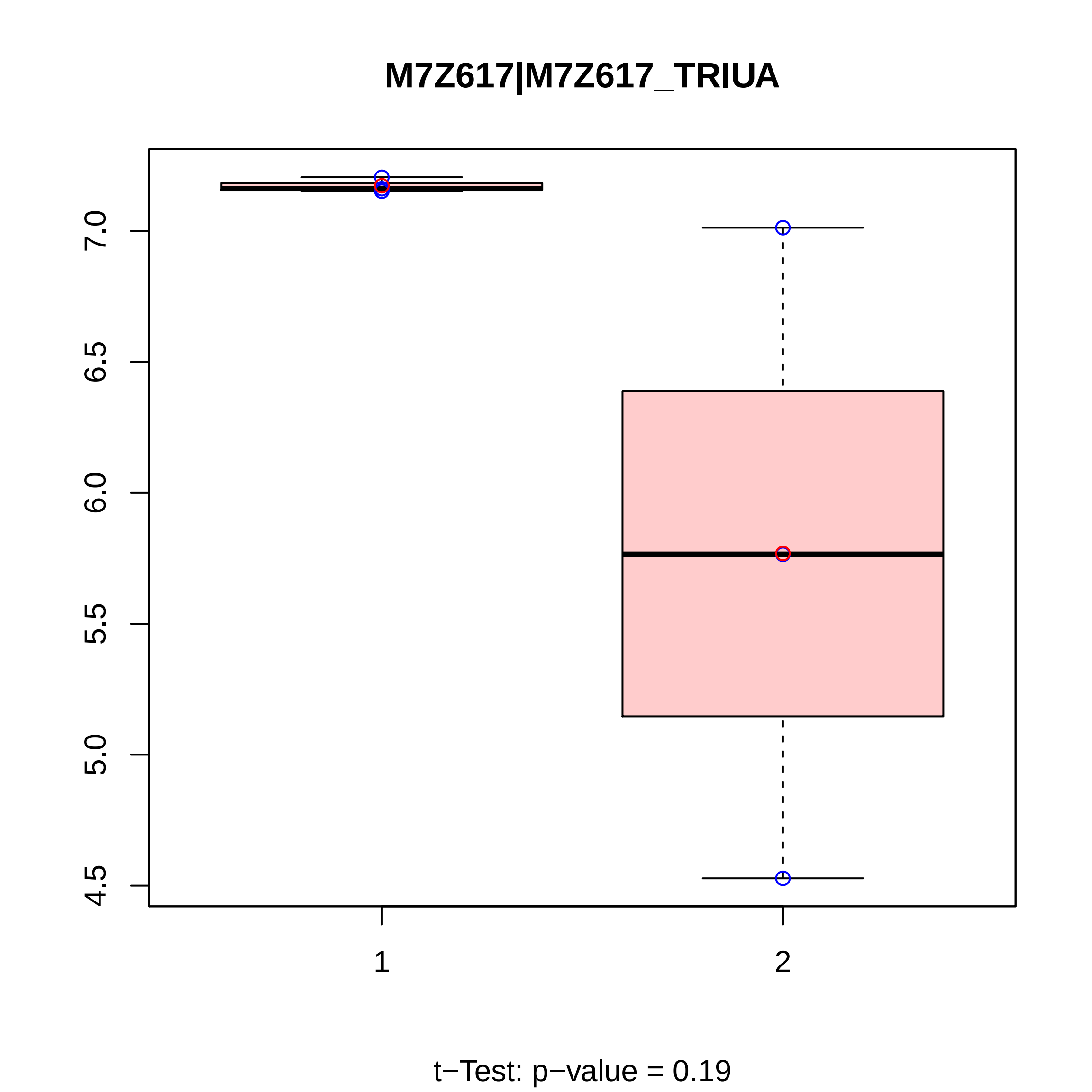

M7Z617|M7Z617_TRIUA
7.0
6.5
6.0
5.5
5.0
4.5
1
2
t−Test: p−value = 0.19

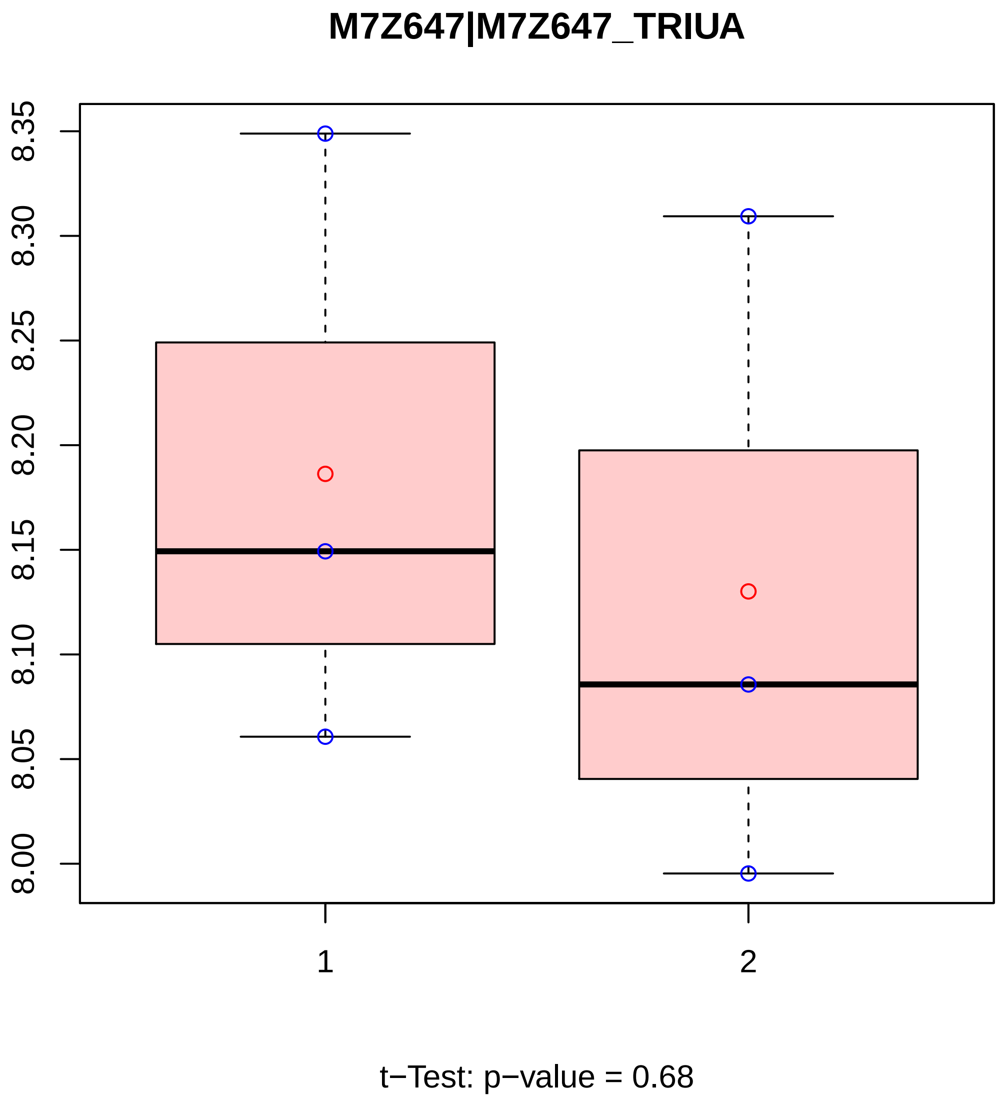
M7Z647|M7Z647_TRIUA
8.00
8.05
8.10
8.15
8.20
8.25
8.30
8.35
1
2
t−Test: p−value = 0.68

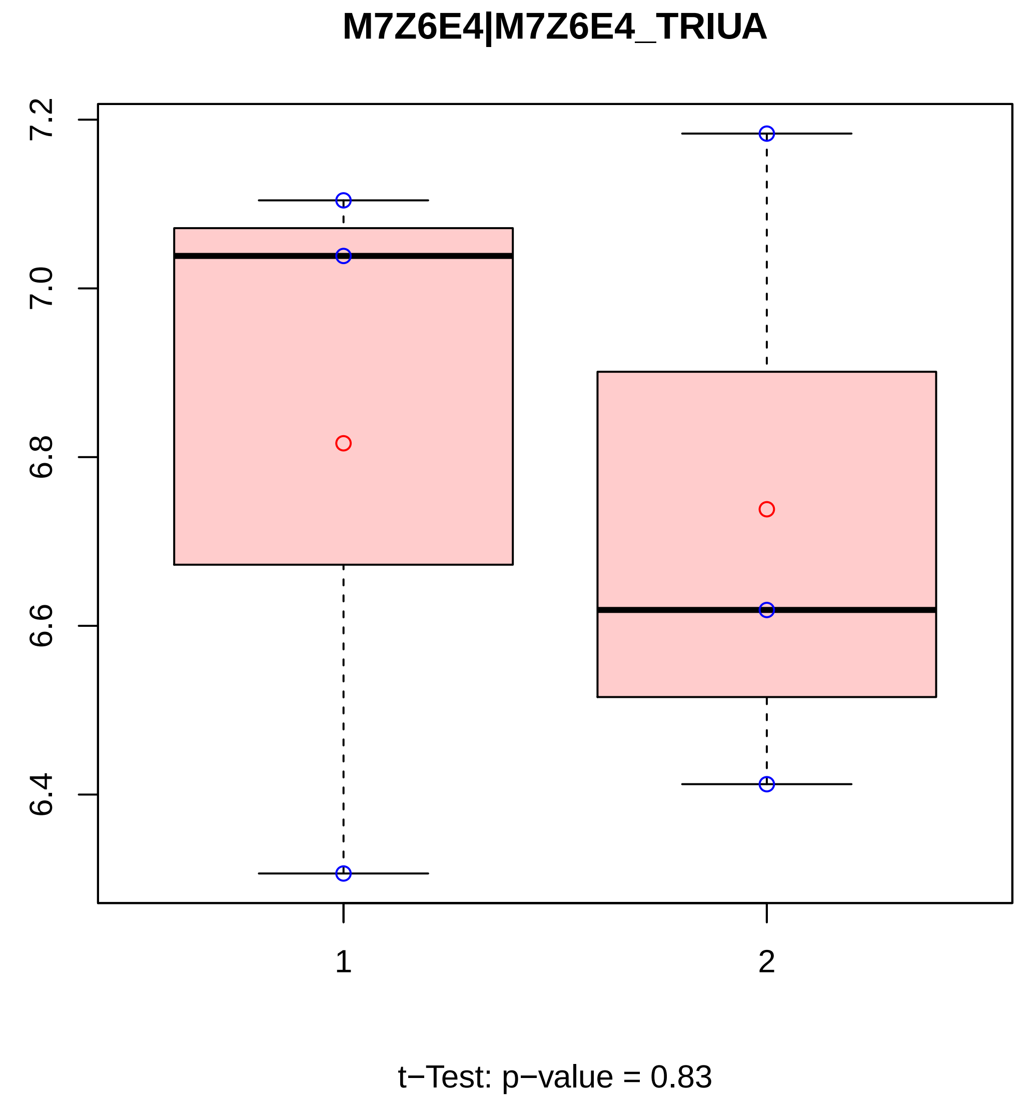
M7Z6E4|M7Z6E4_TRIUA
7.2
7.0
6.8
6.6
6.4
1
2
t−Test: p−value = 0.83

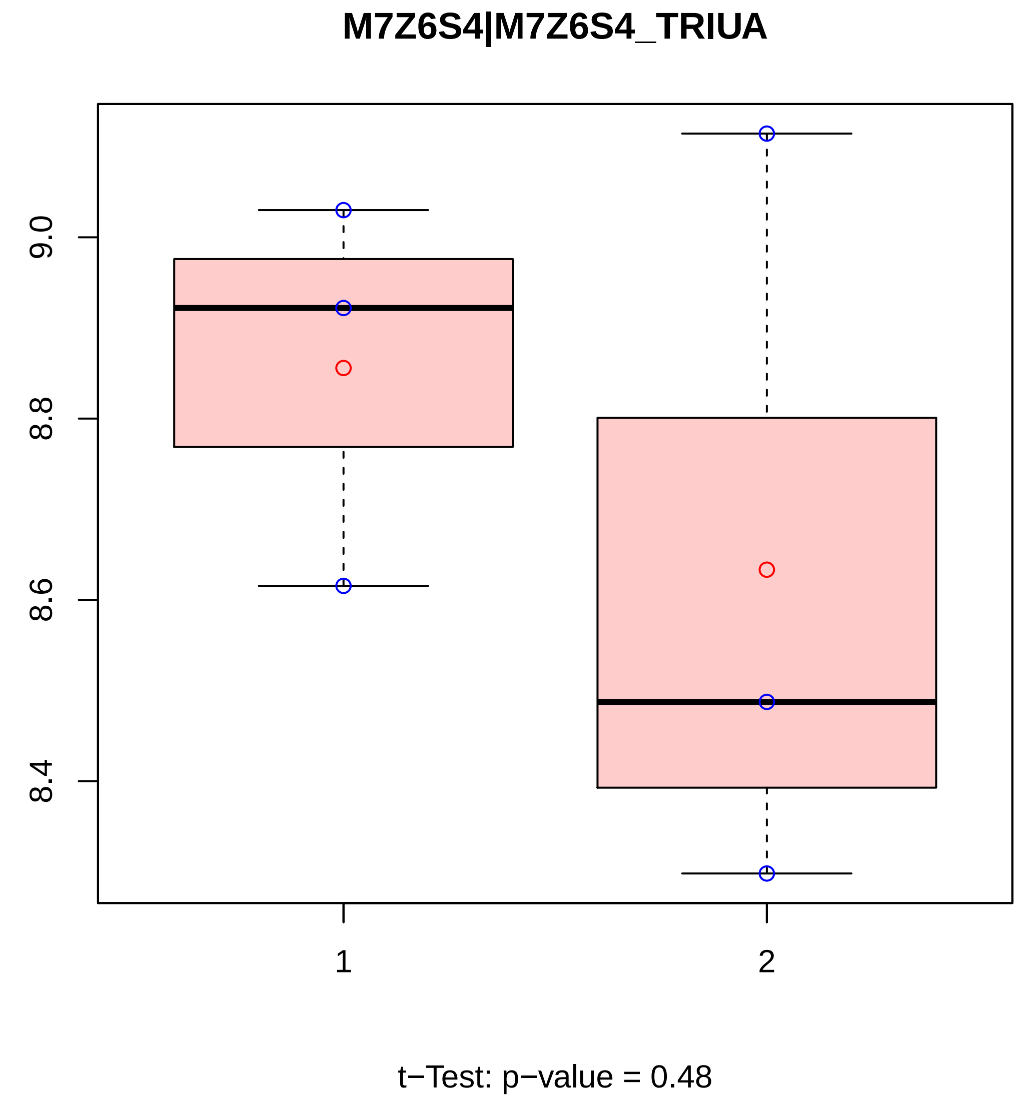
M7Z6S4|M7Z6S4_TRIUA
9.0
8.8
8.6
8.4
1
2
t−Test: p−value = 0.48

M7Z6W6|M7Z6W6_TRIUA

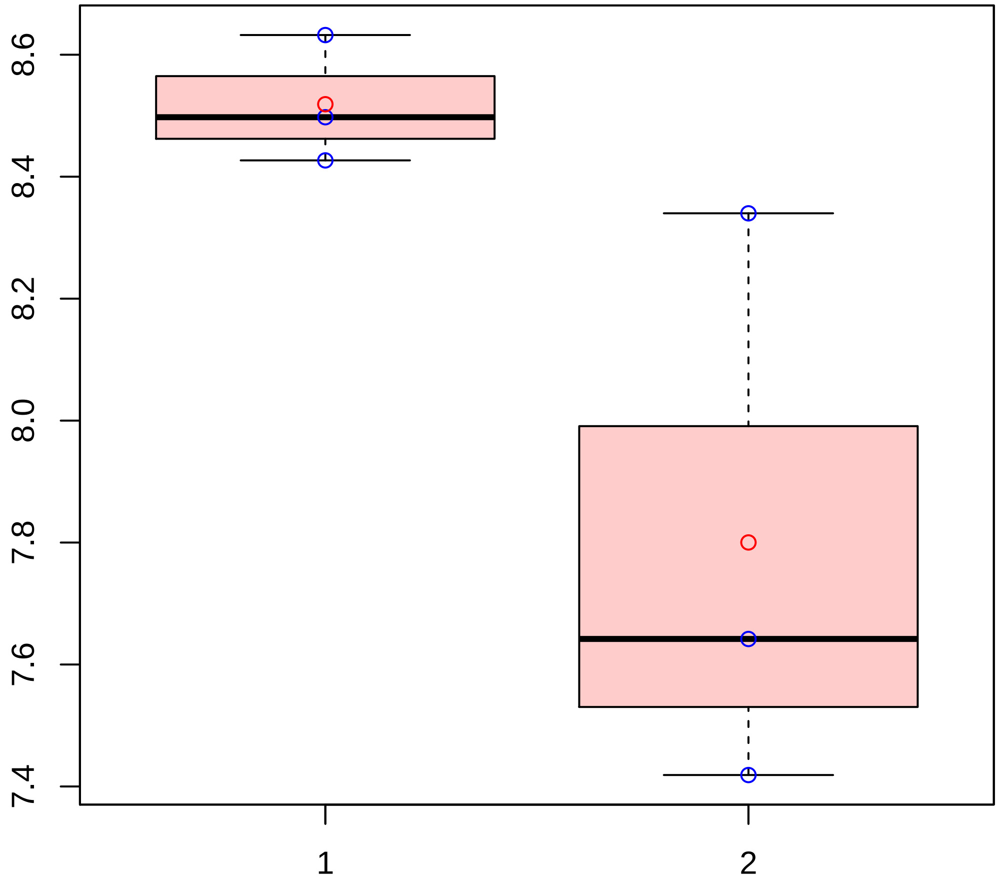

t−Test: p−value = 0.12

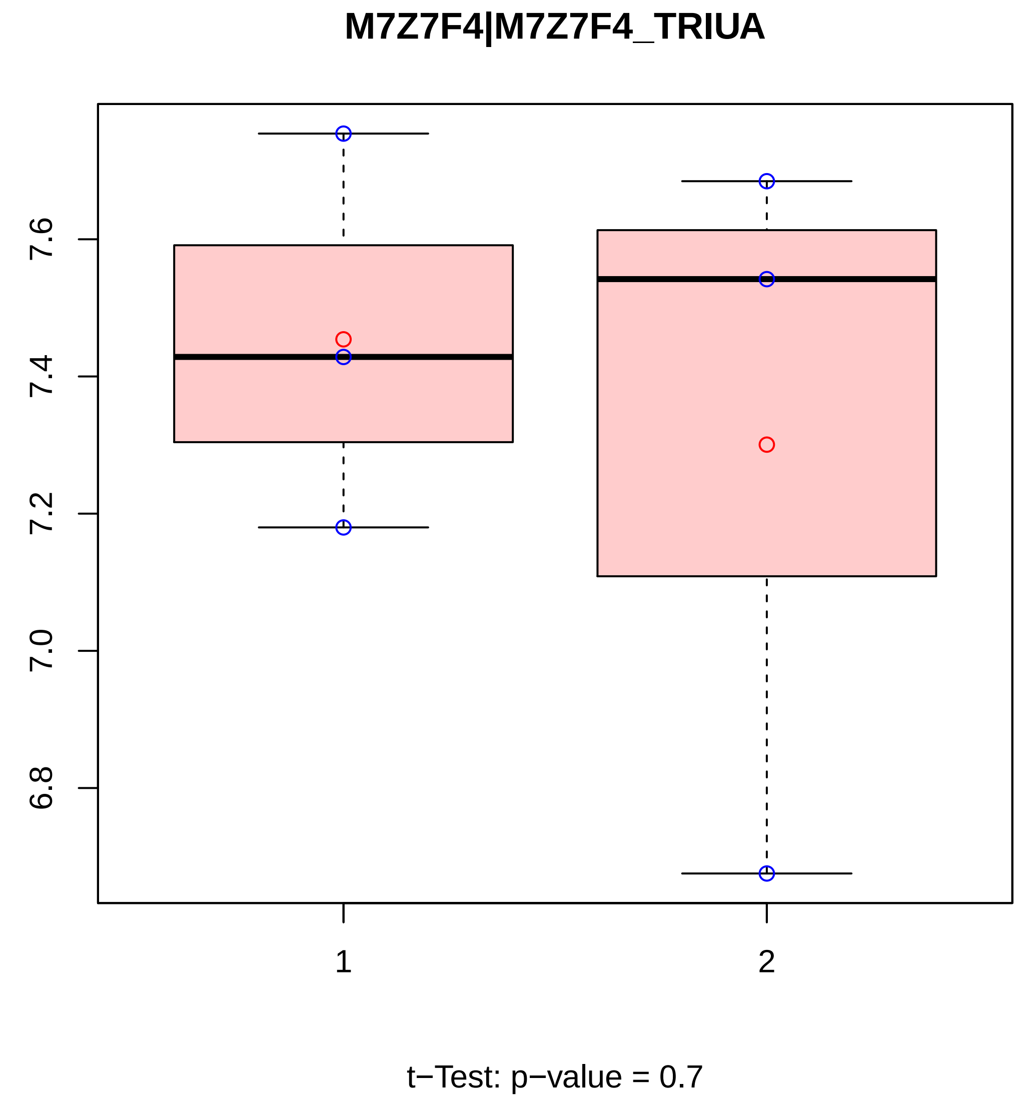
M7Z7F4|M7Z7F4_TRIUA
7.6
7.4
7.2
7.0
6.8
1
2
t−Test: p−value = 0.7

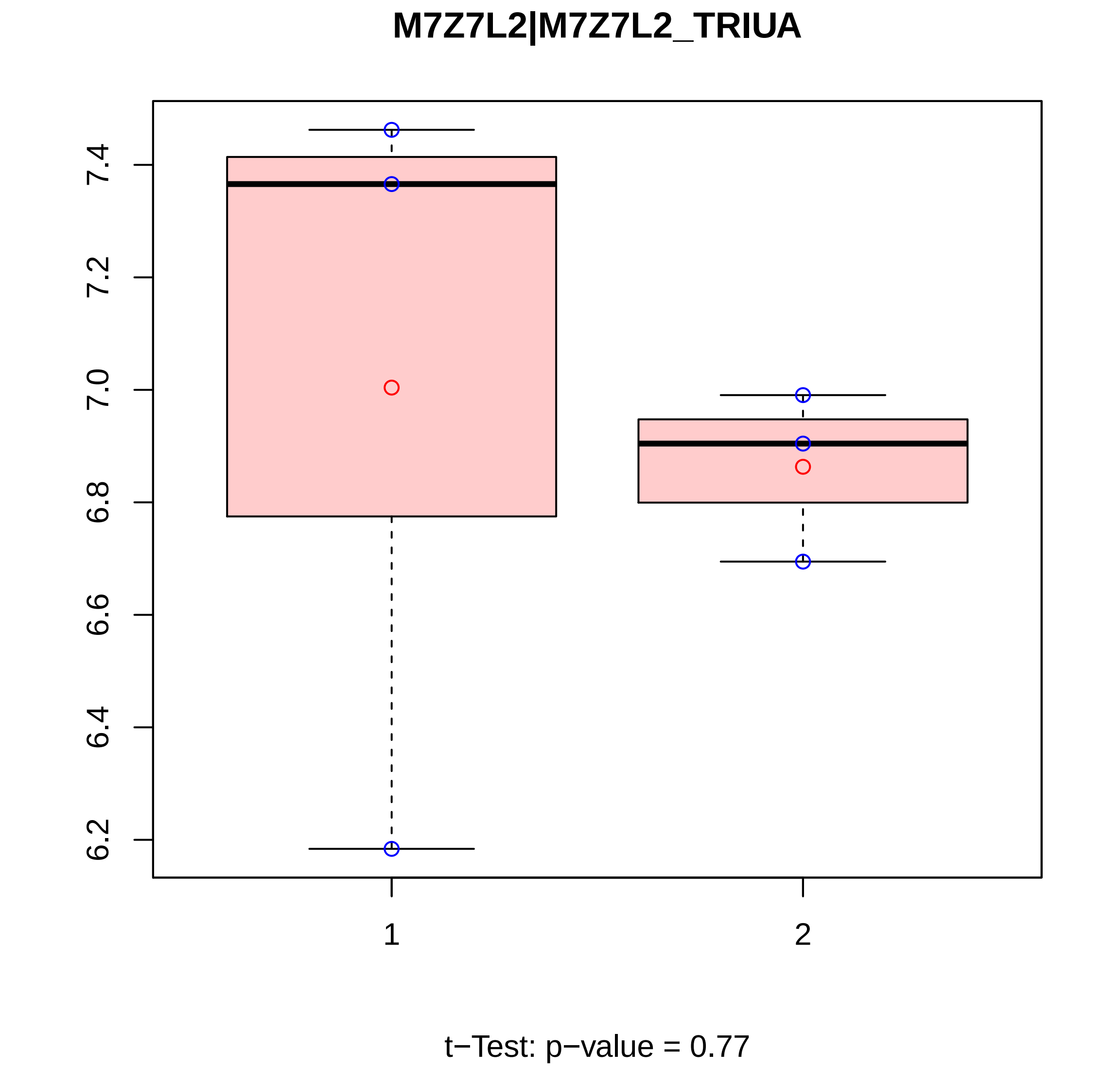
M7Z7L2|M7Z7L2_TRIUA
7.4
7.2
7.0
6.8
6.6
6.4
6.2
1
2
t−Test: p−value = 0.77

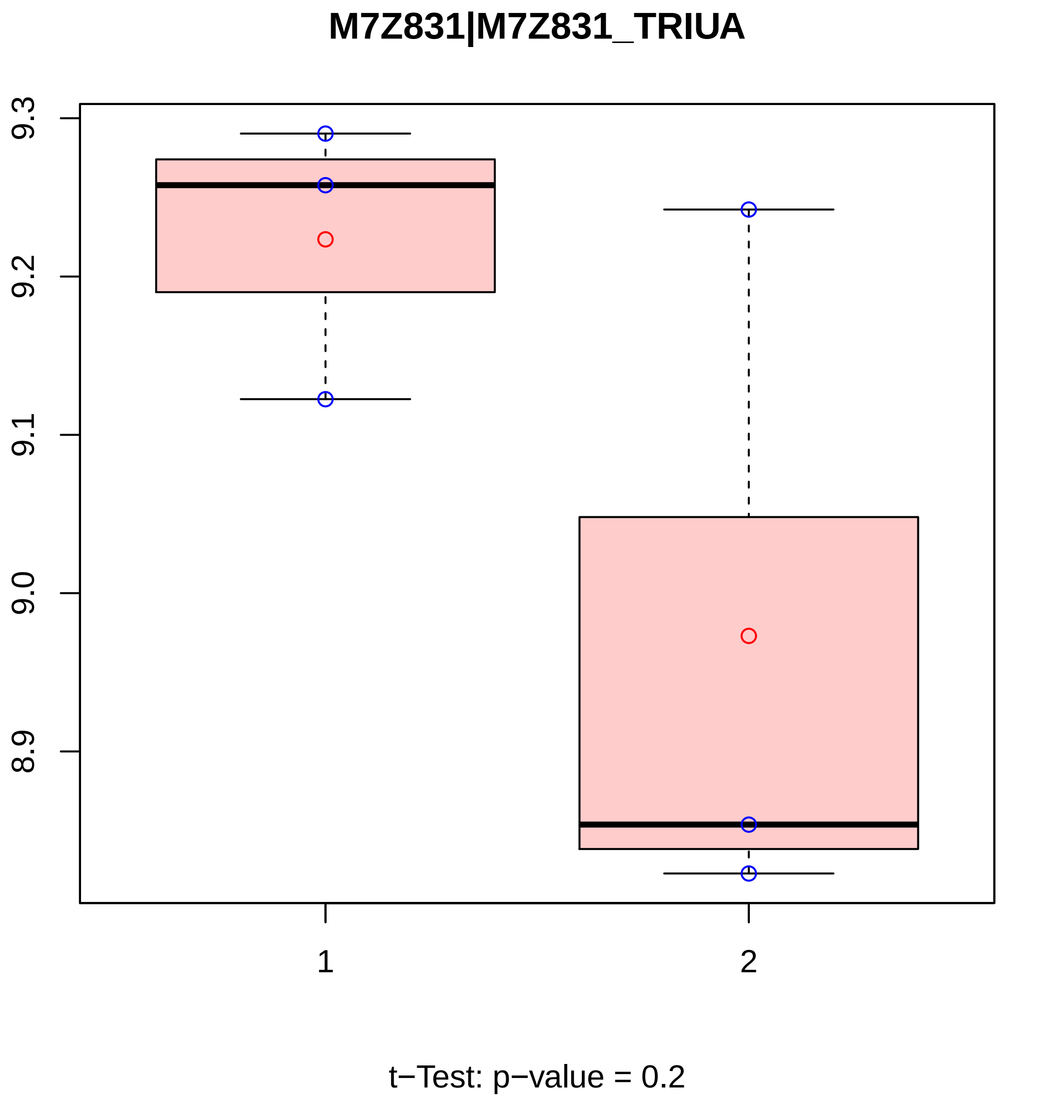
M7Z831|M7Z831_TRIUA
9.3
9.2
9.1
9.0
8.9
1
2
t−Test: p−value = 0.2

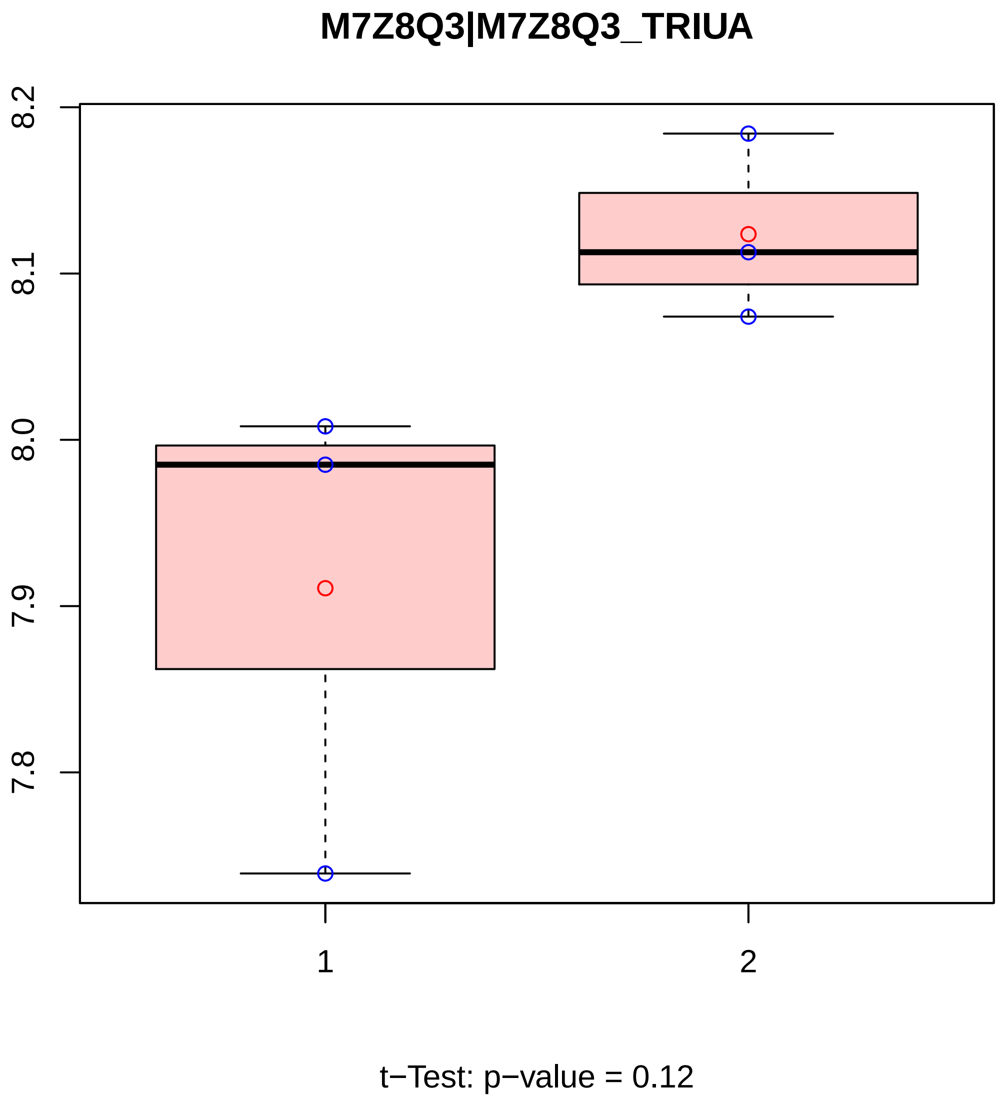

M7Z8Q3|M7Z8Q3_TRIUA
8.2
8.1
8.0
7.9
7.8
1
2
t−Test: p−value = 0.12

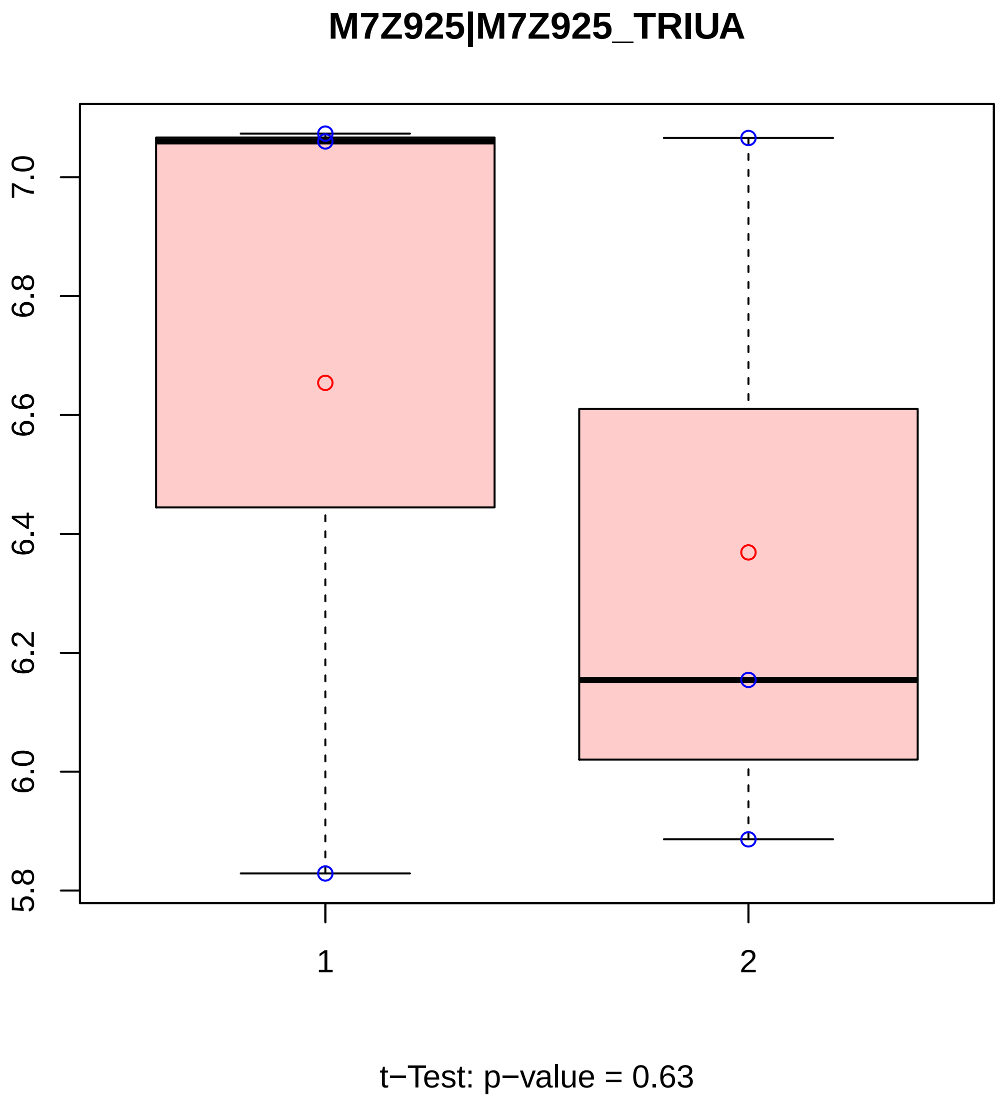

M7Z925|M7Z925_TRIUA
5.8
6.0
6.2
6.4
6.6
6.8
7.0
1
2
t−Test: p−value = 0.63

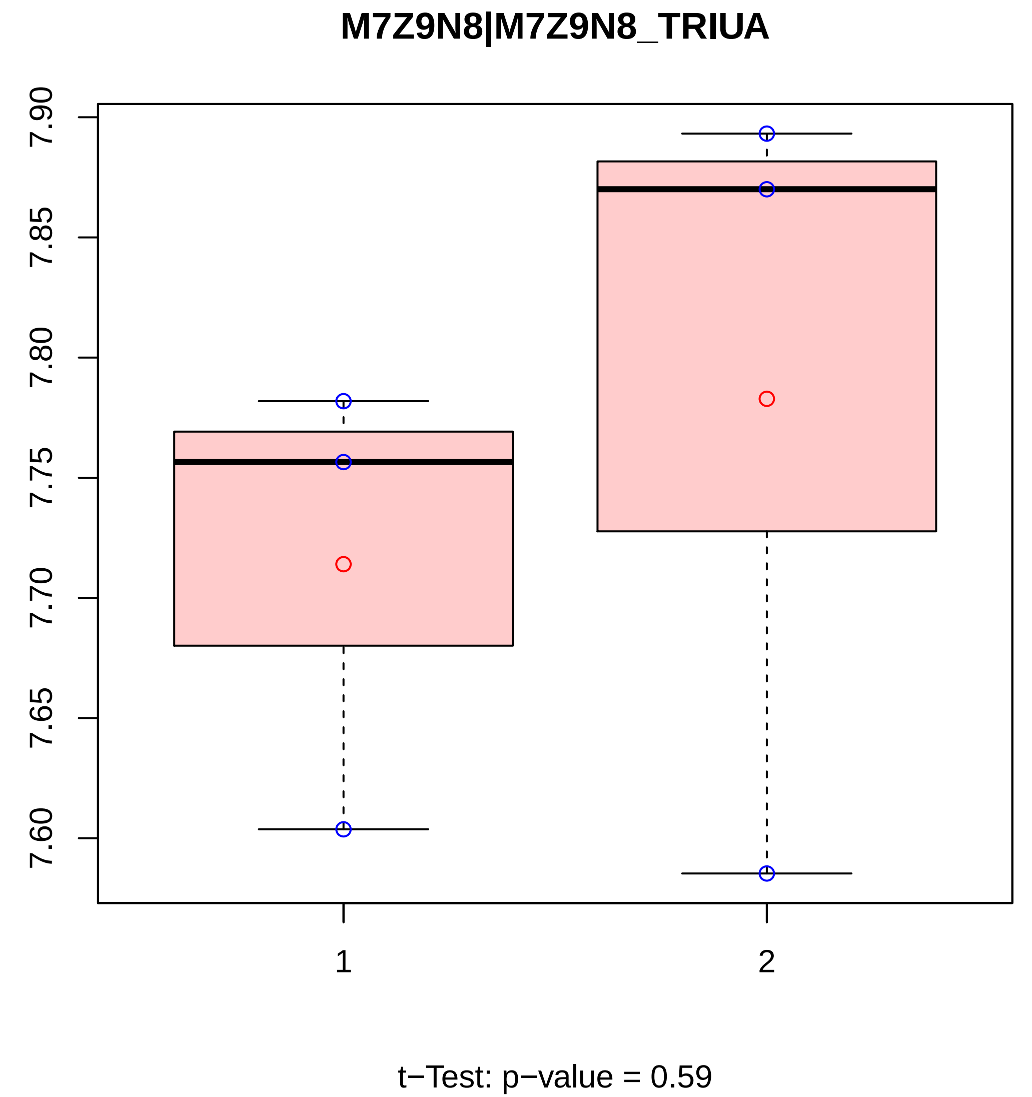
M7Z9N8|M7Z9N8_TRIUA
7.90
7.85
7.80
7.75
7.70
7.65
7.60
1
2
t−Test: p−value = 0.59

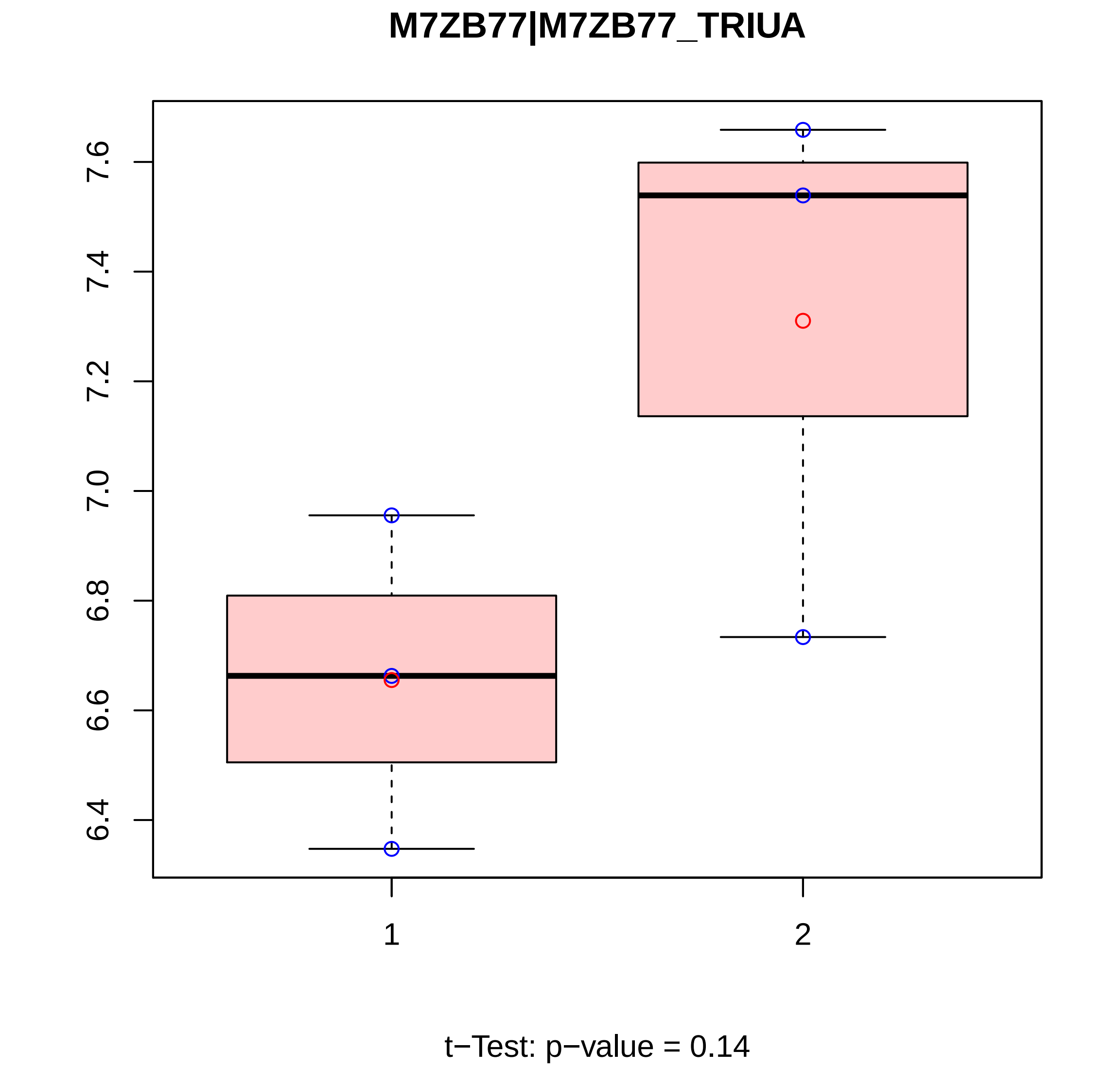
M7ZB77|M7ZB77_TRIUA
7.6
7.4
7.2
7.0
6.8
6.6
6.4
1
2
t−Test: p−value = 0.14

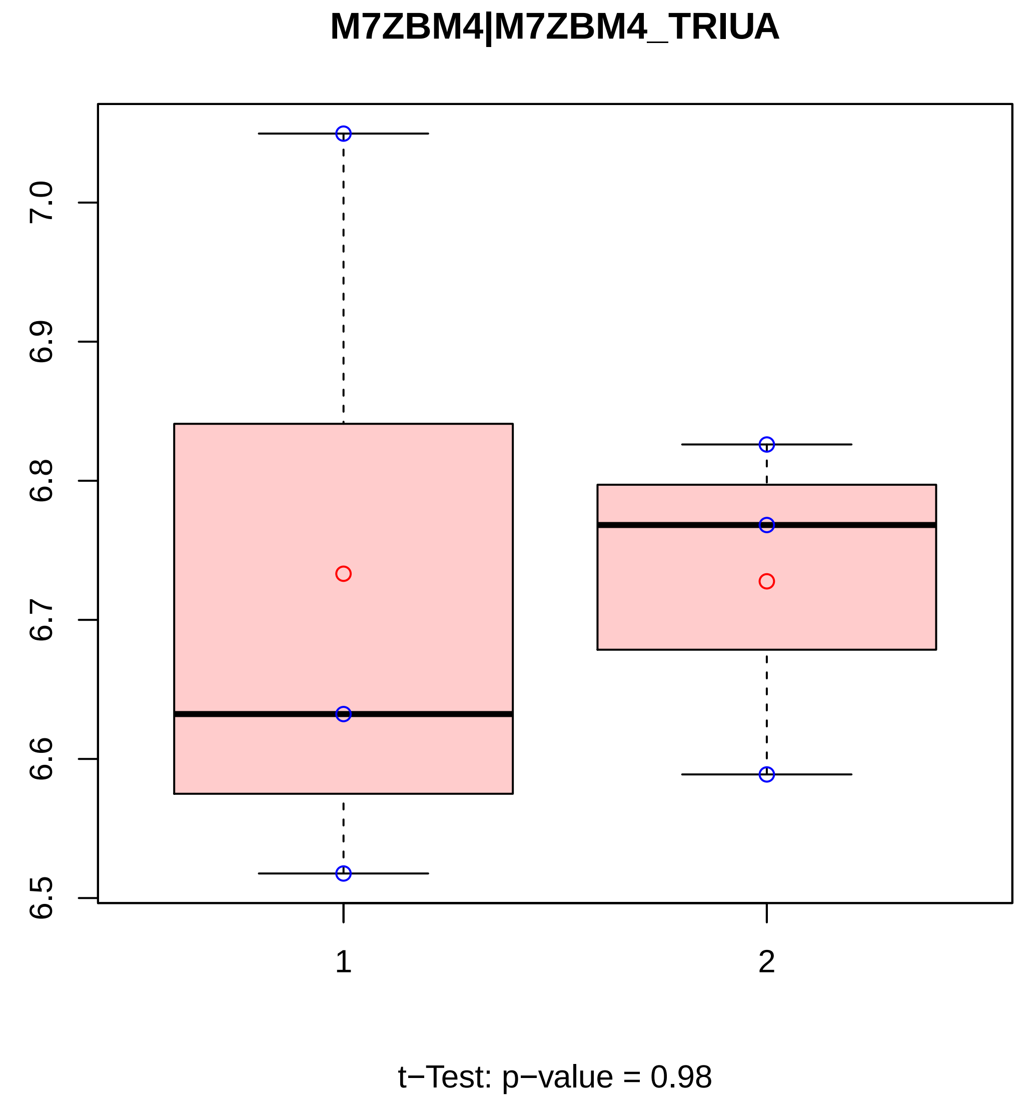
M7ZBM4|M7ZBM4_TRIUA
6.5
6.6
6.7
6.8
6.9
7.0
1
2
t−Test: p−value = 0.98

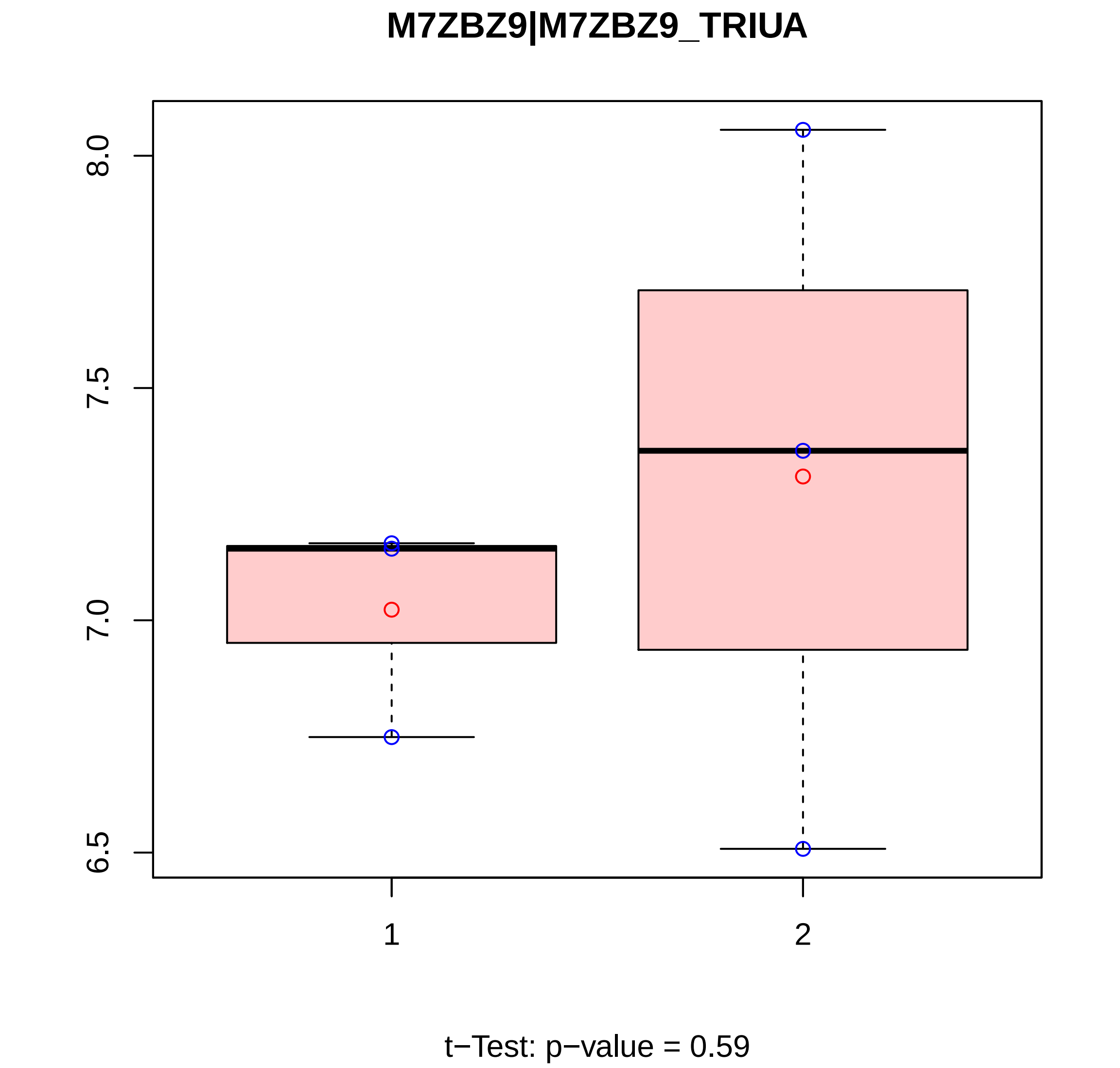
M7ZBZ9|M7ZBZ9_TRIUA
8.0
7.5
7.0
6.5
1
2
t−Test: p−value = 0.59

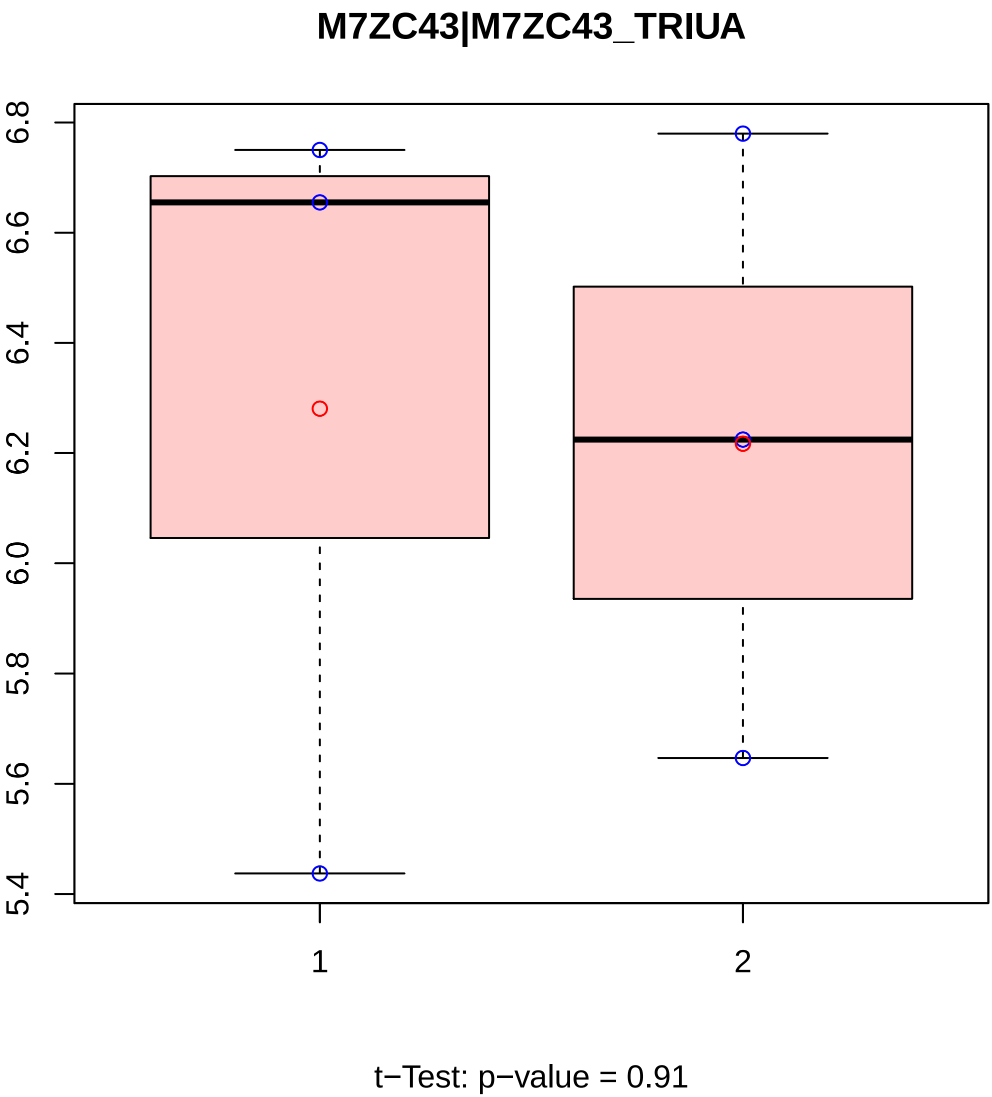
M7ZC43|M7ZC43_TRIUA
6.8
6.6
6.4
6.2
6.0
5.8
5.6
5.4
1
2
t−Test: p−value = 0.91

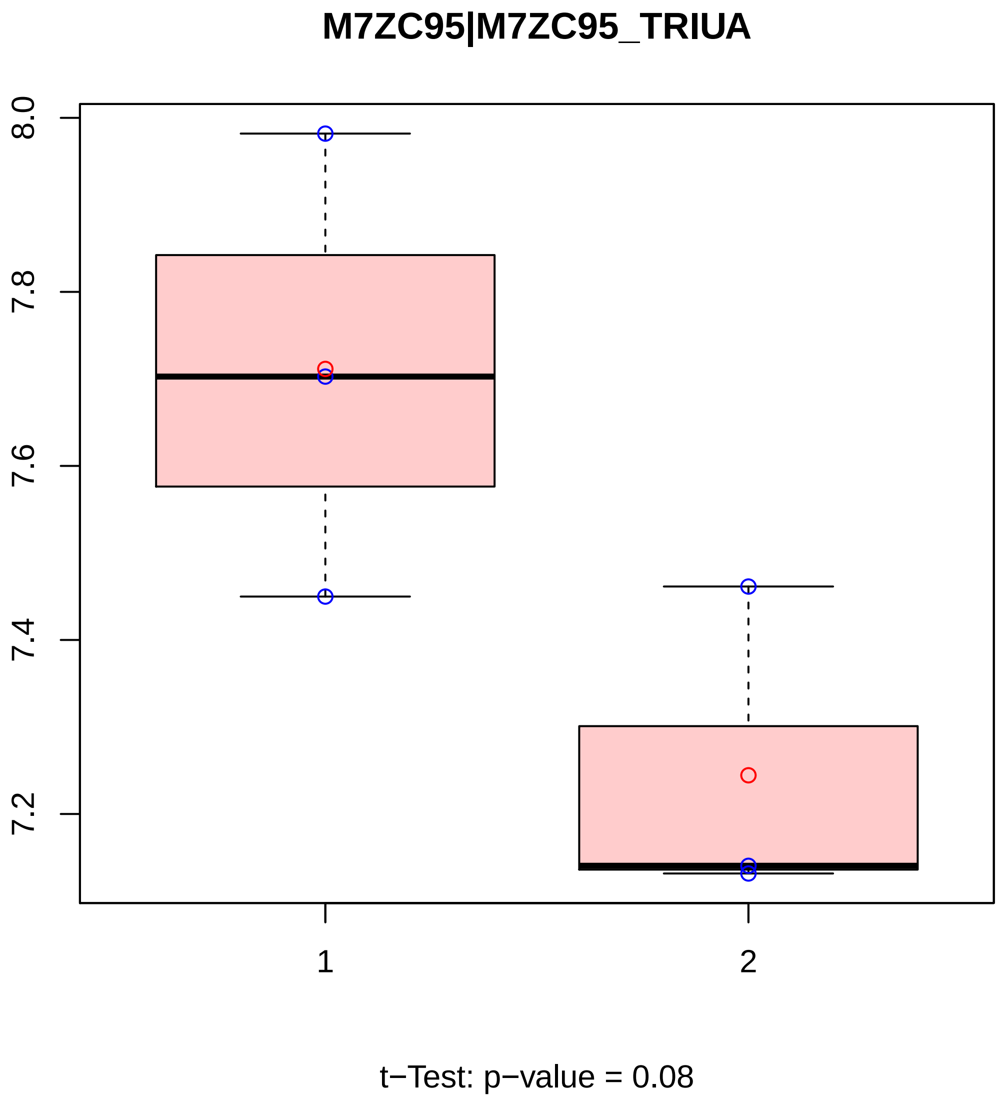
M7ZC95|M7ZC95_TRIUA
8.0
7.8
7.6
7.4
7.2
1
2
t−Test: p−value = 0.08

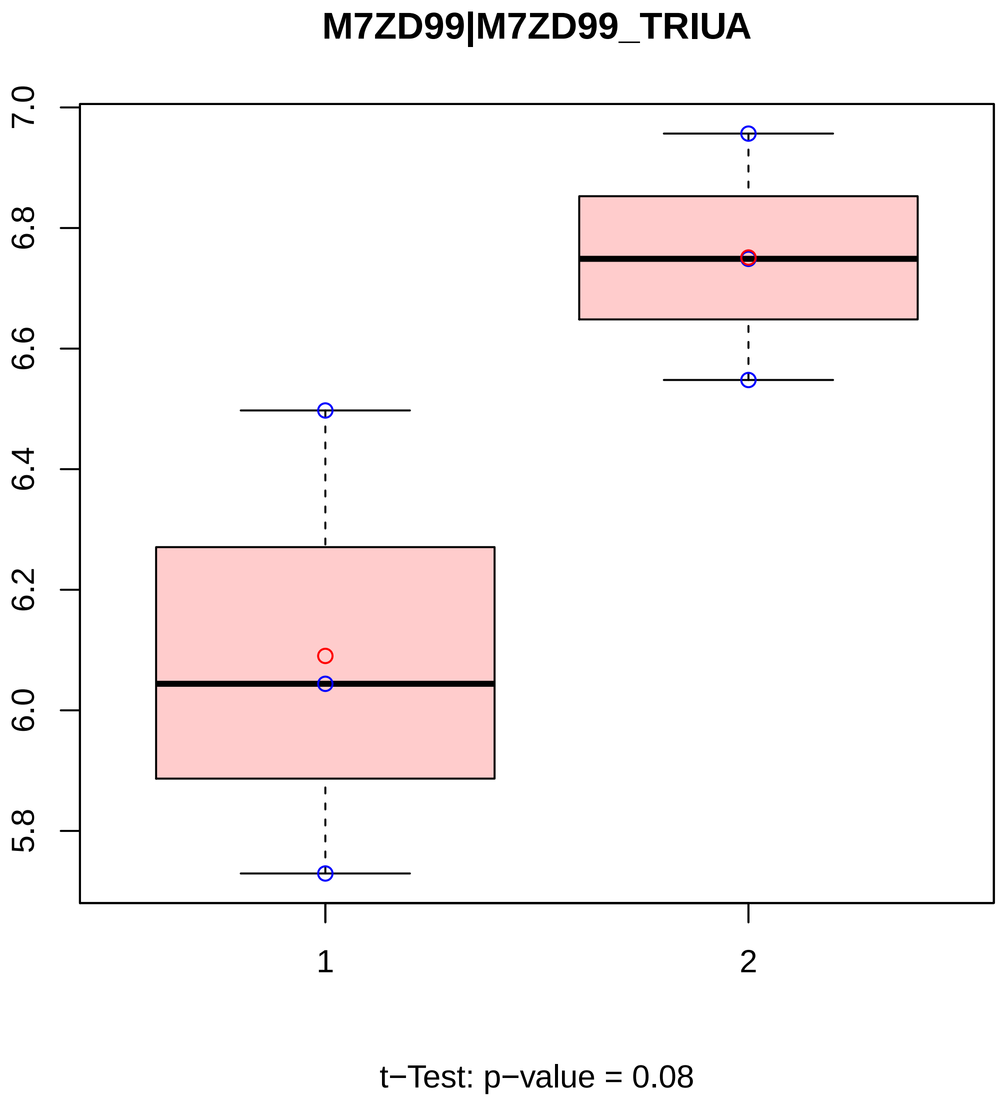
M7ZD99|M7ZD99_TRIUA
7.0
6.8
6.6
6.4
6.2
6.0
5.8
1
2
t−Test: p−value = 0.08

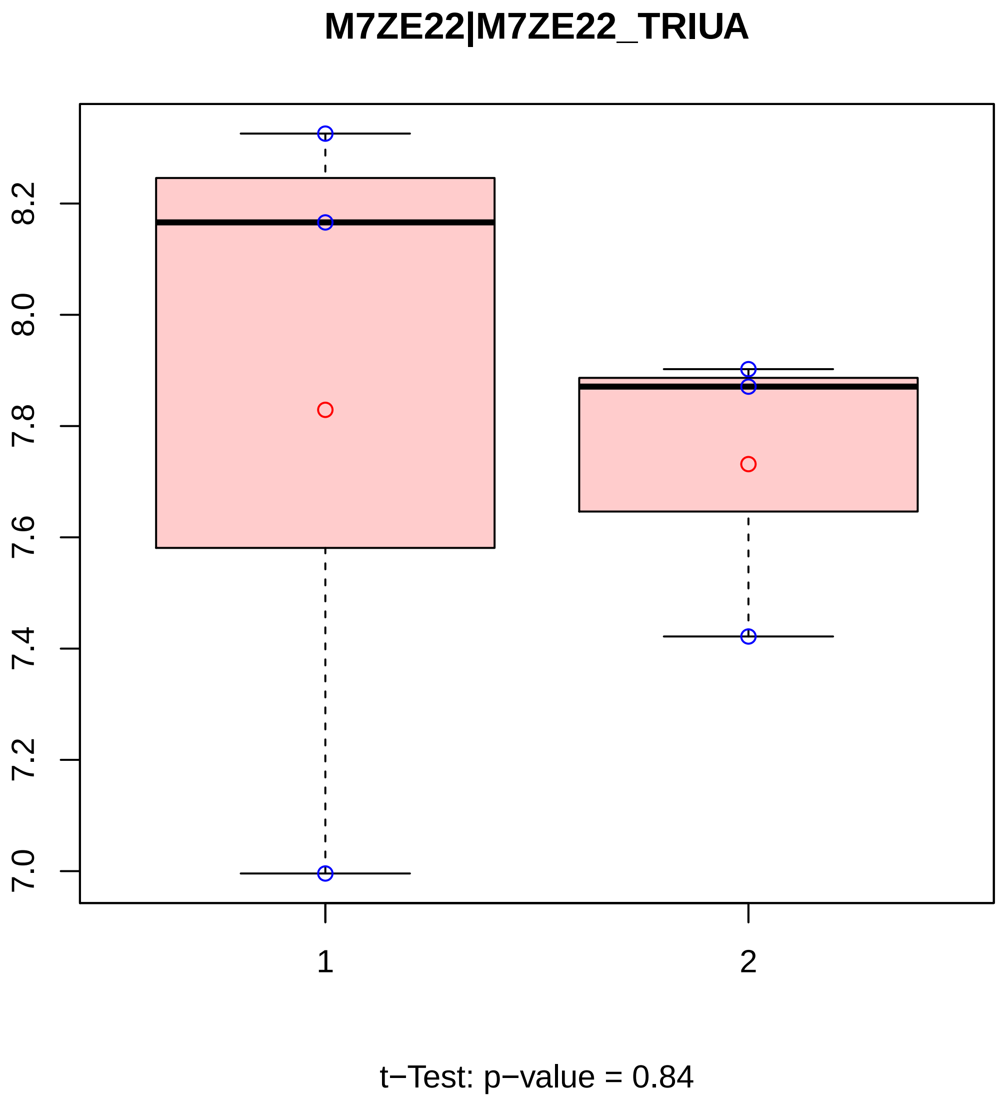
M7ZE22|M7ZE22_TRIUA
8.2
8.0
7.8
7.6
7.4
7.2
7.0
1
2
t−Test: p−value = 0.84

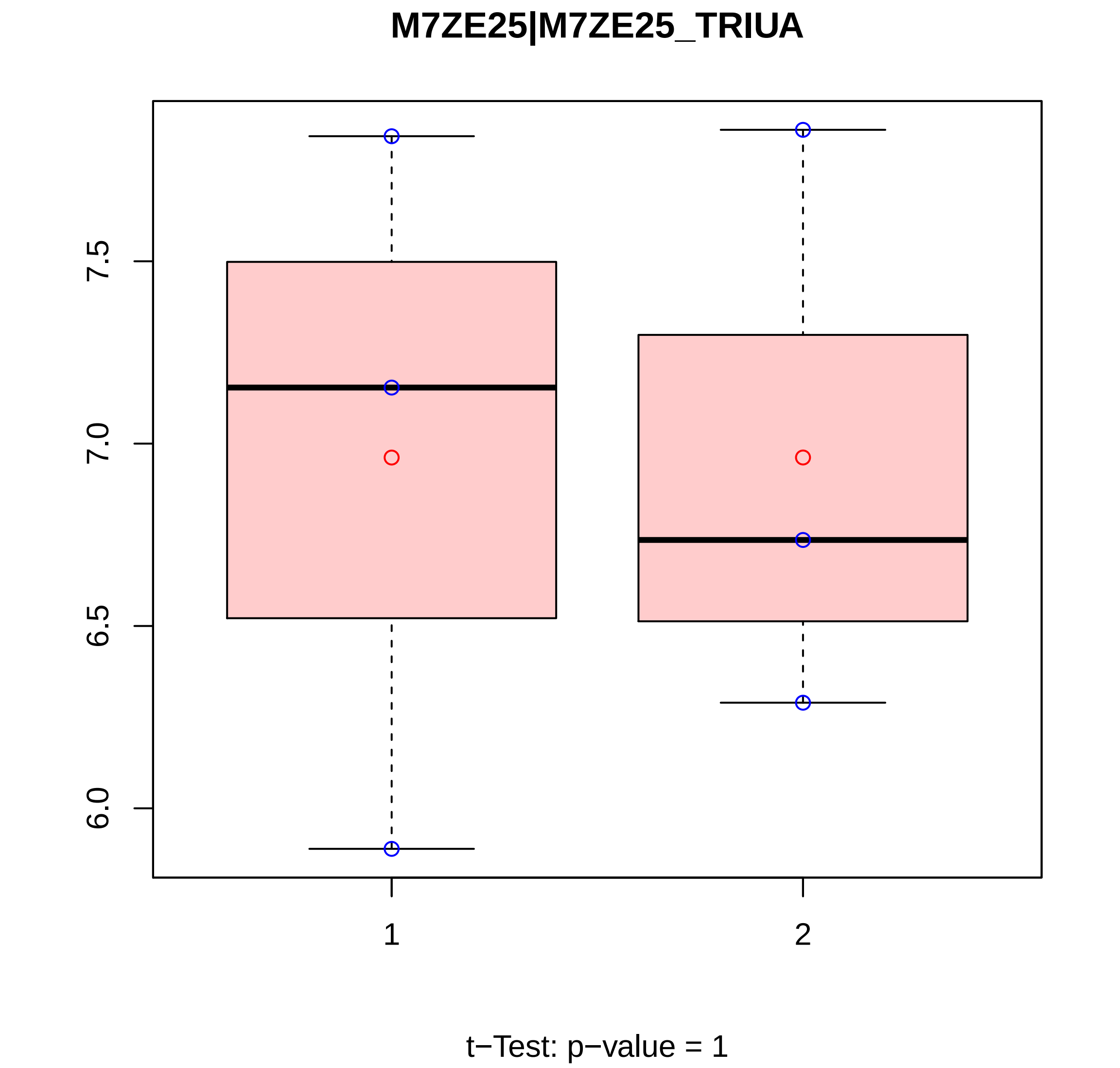
M7ZE25|M7ZE25_TRIUA
7.5
7.0
6.5
6.0
1
2
t−Test: p−value = 1

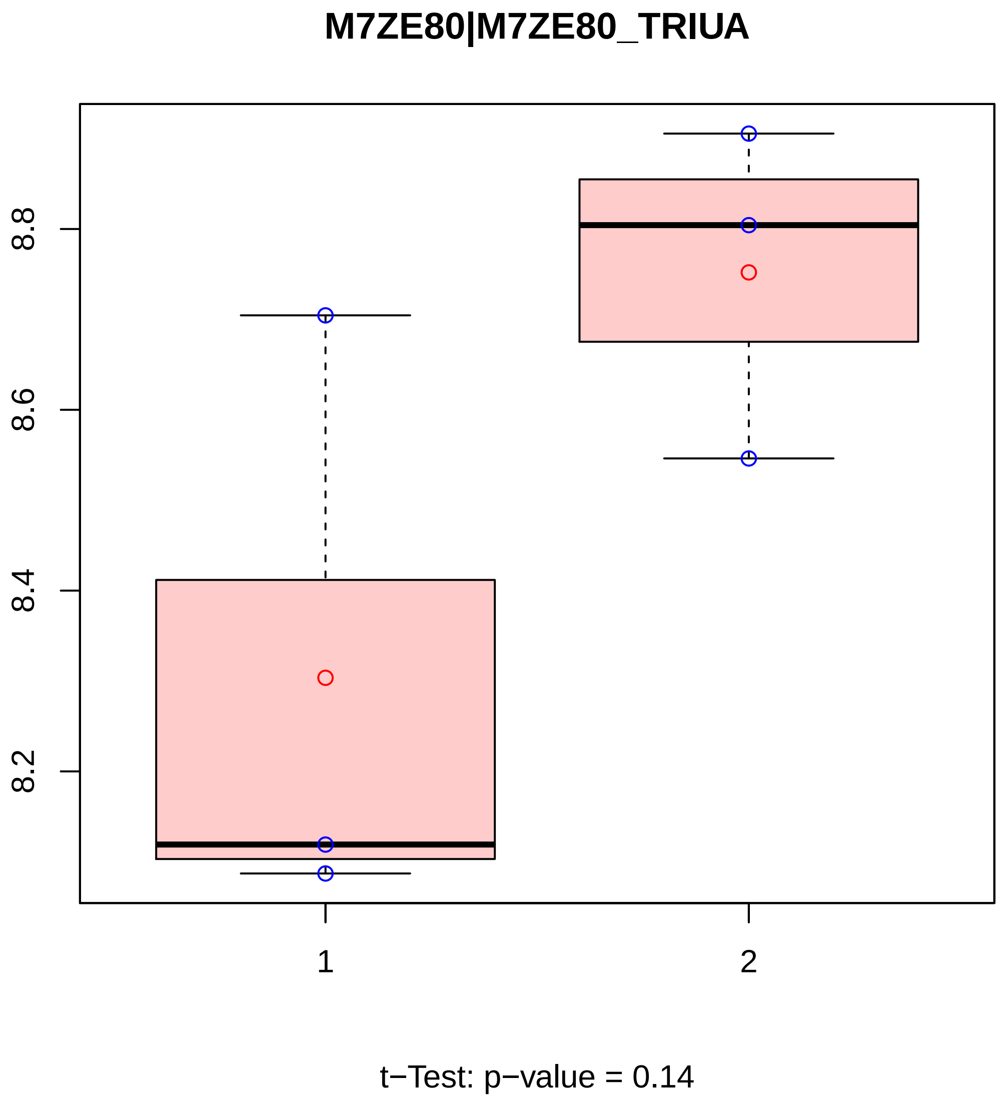

M7ZE80|M7ZE80_TRIUA
8.2
8.4
8.6
8.8
1
2
t−Test: p−value = 0.14

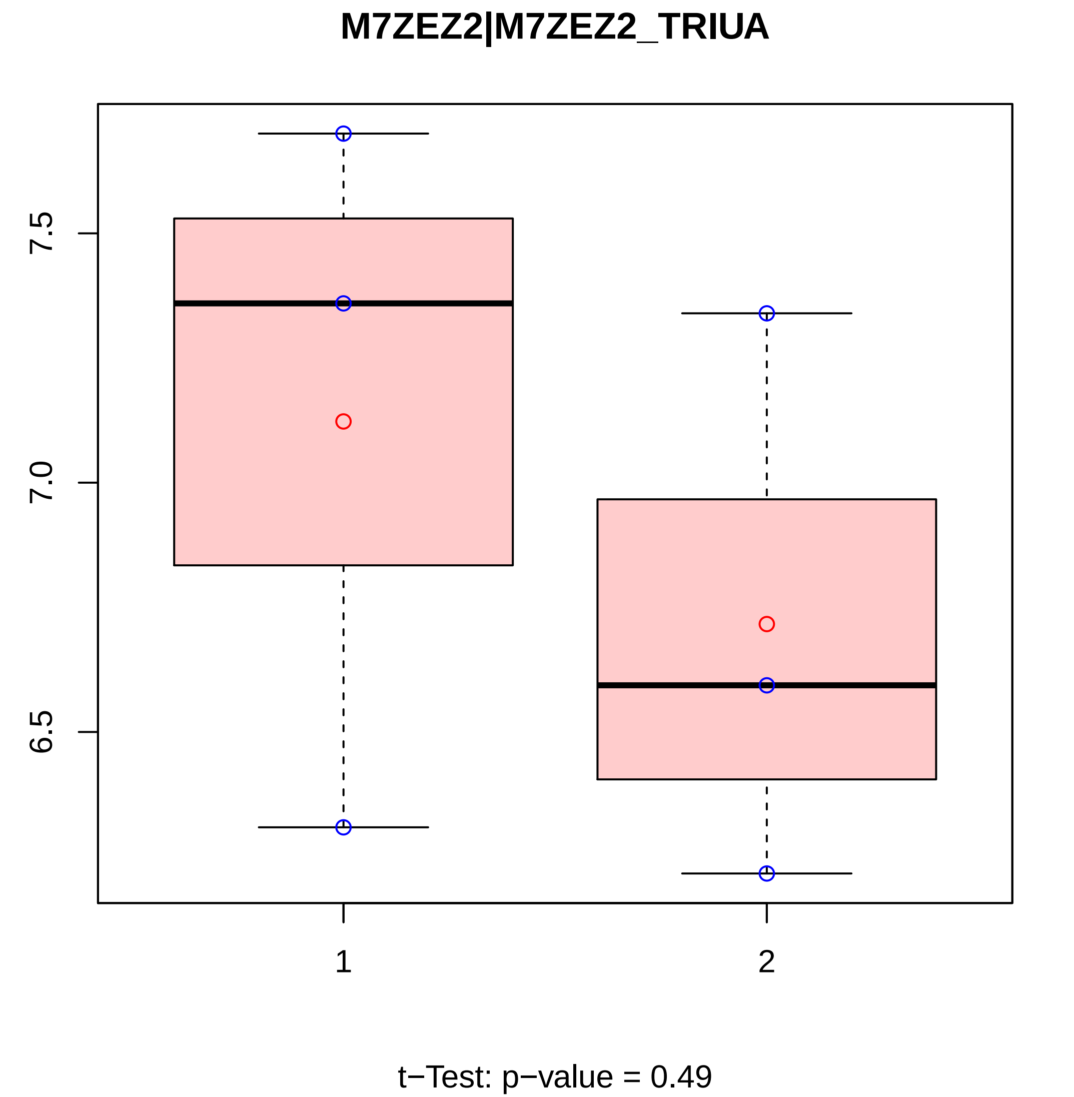
M7ZEZ2|M7ZEZ2_TRIUA
7.5
7.0
6.5
1
2
t−Test: p−value = 0.49

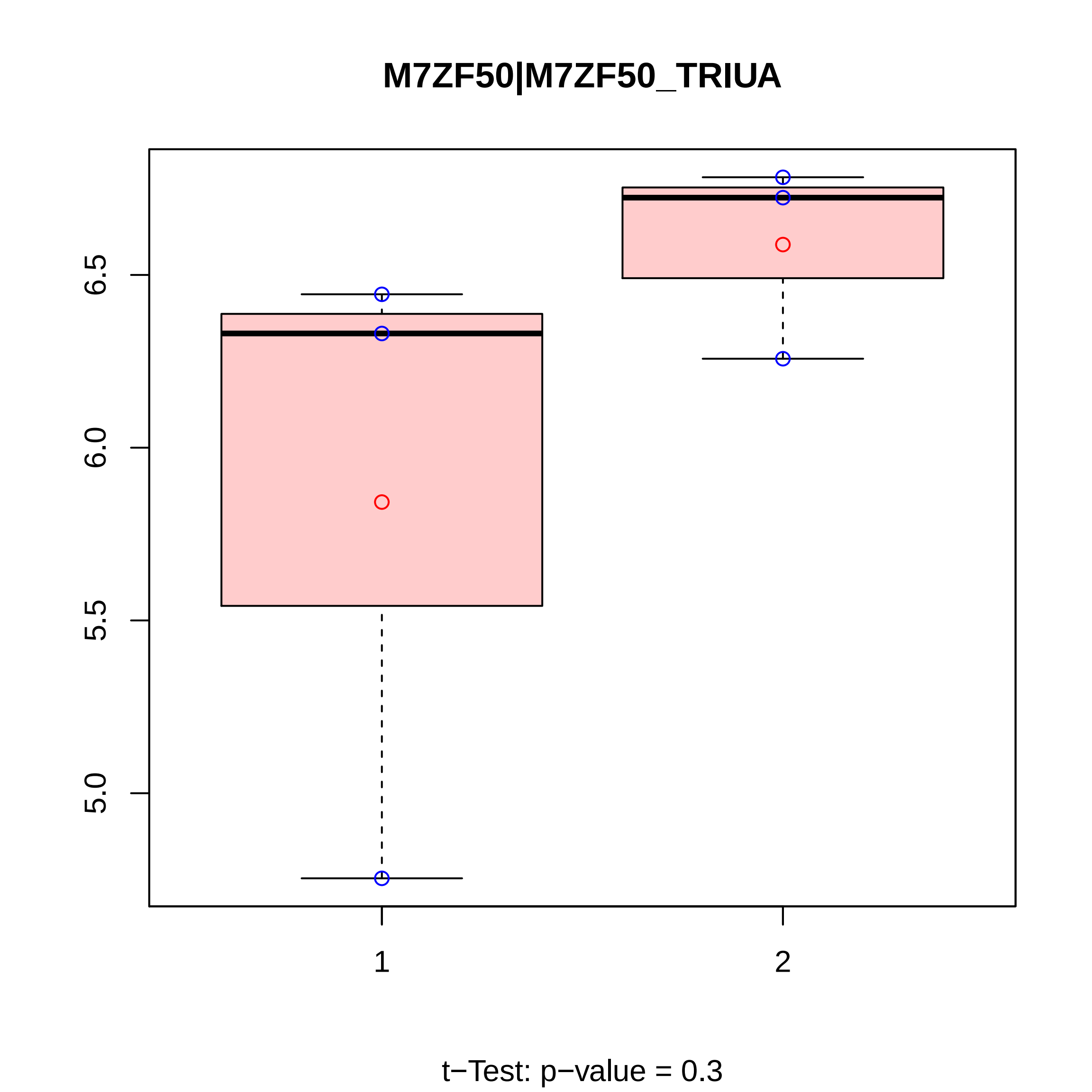
M7ZF50|M7ZF50_TRIUA
6.5
6.0
5.5
5.0
1
2
t−Test: p−value = 0.3

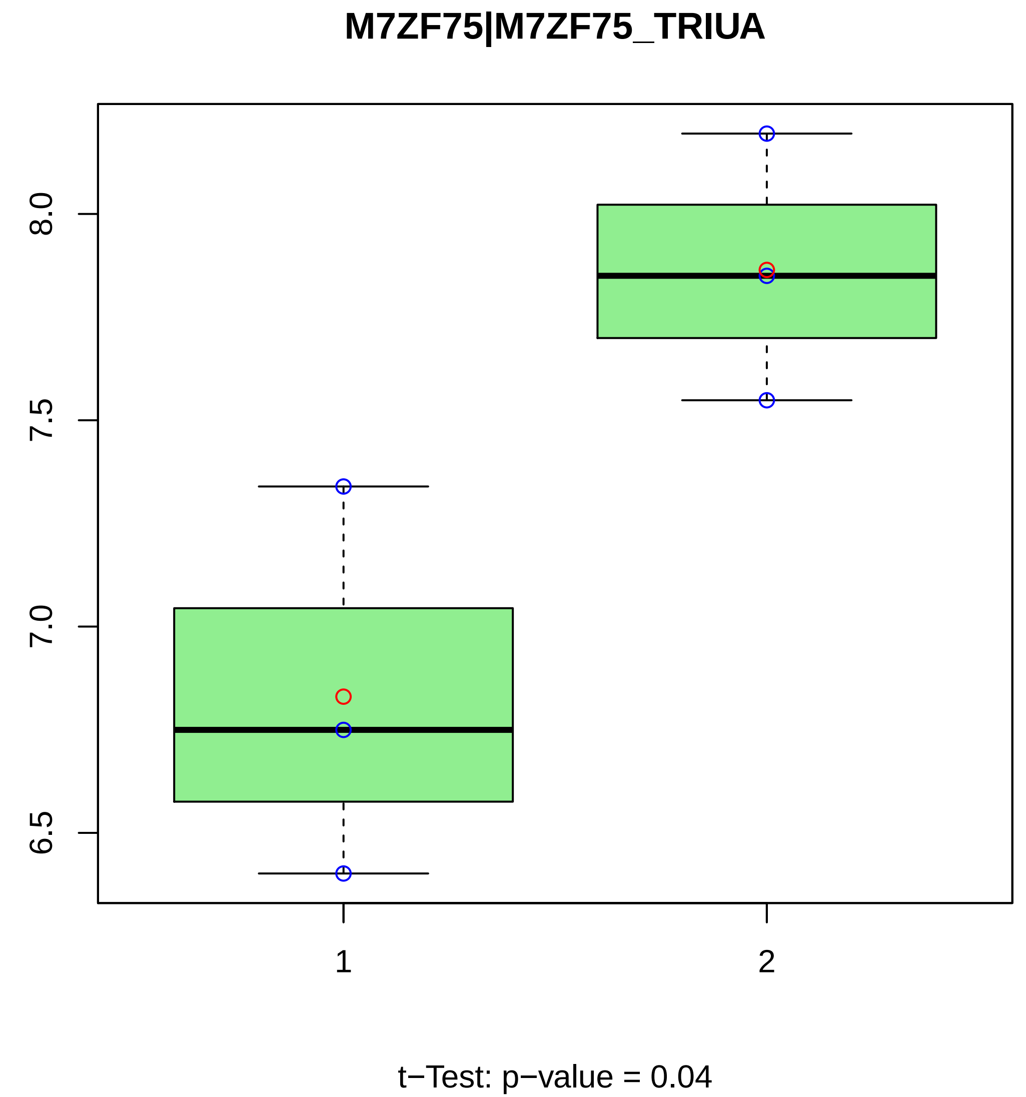
M7ZF75|M7ZF75_TRIUA
8.0
7.5
7.0
6.5
1
2
t−Test: p−value = 0.04

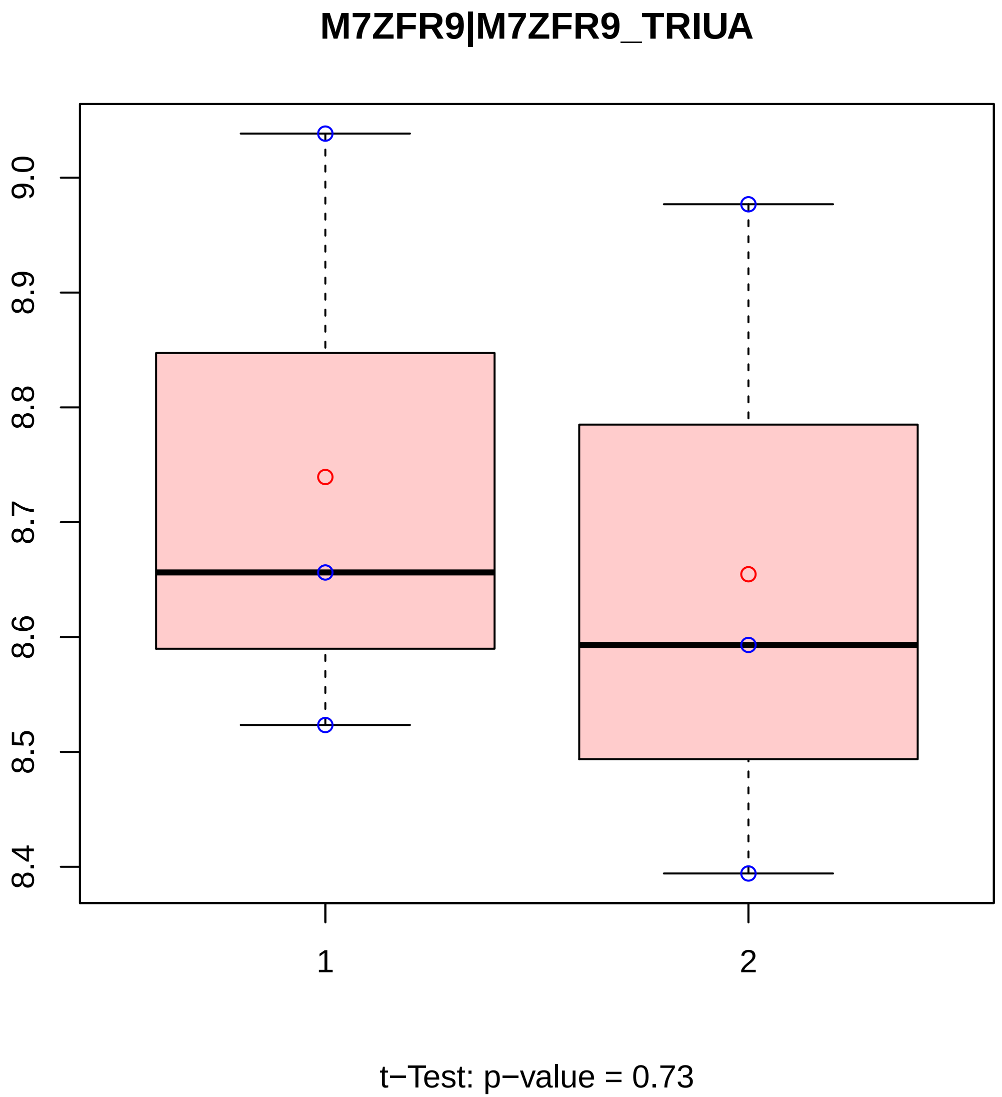

M7ZFR9|M7ZFR9_TRIUA
9.0
8.9
8.8
8.7
8.6
8.5
8.4
1
2
t−Test: p−value = 0.73

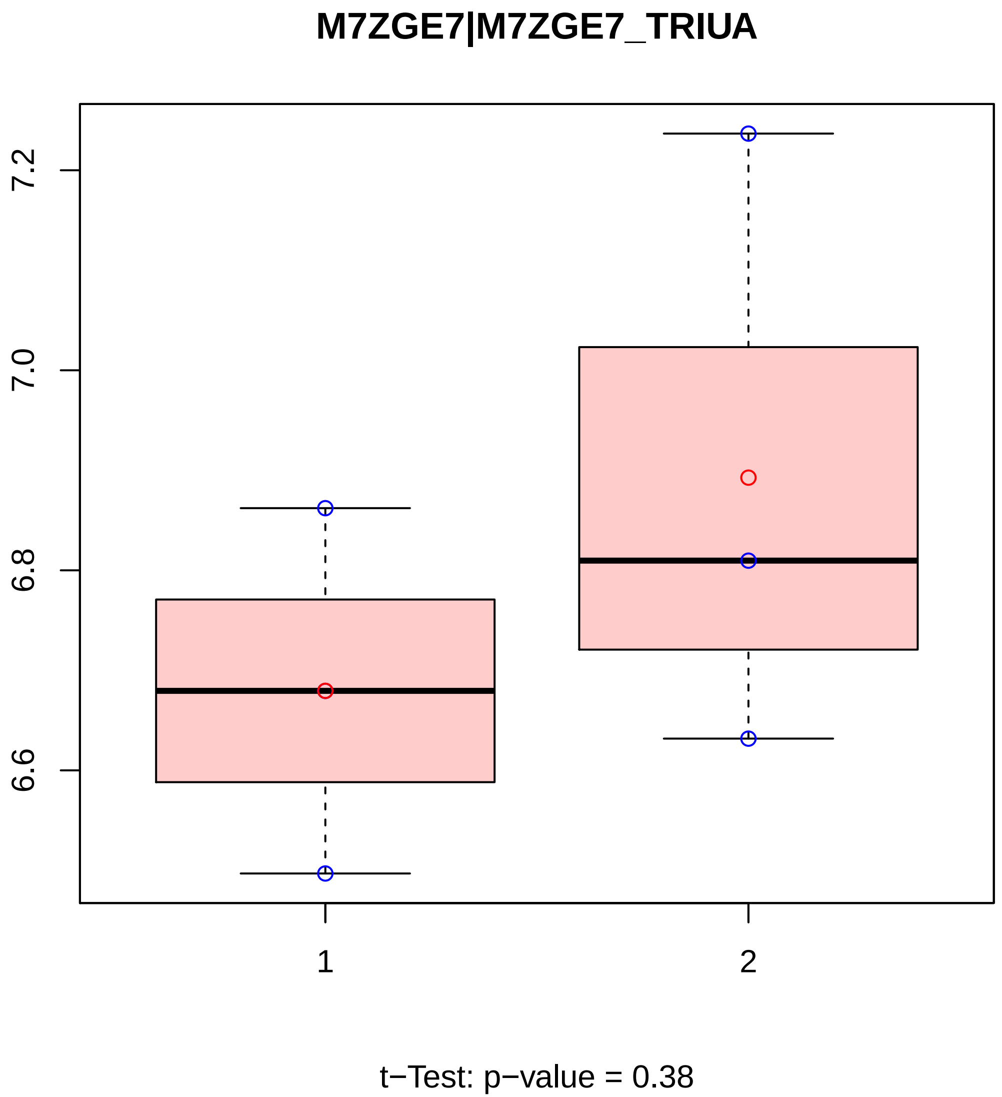
M7ZGE7|M7ZGE7_TRIUA
7.2
7.0
6.8
6.6
1
2
t−Test: p−value = 0.38

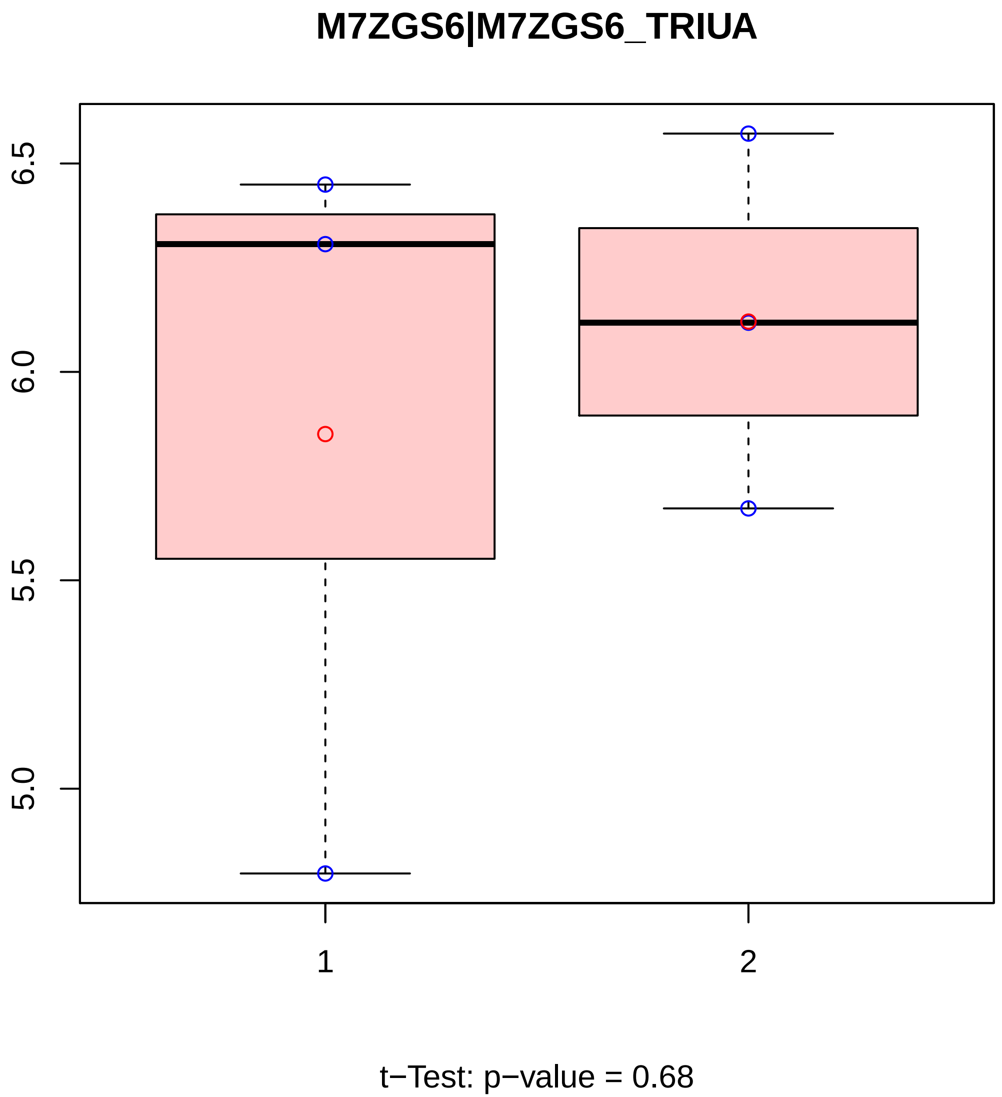
M7ZGS6|M7ZGS6_TRIUA
6.5
6.0
5.5
5.0
1
2
t−Test: p−value = 0.68

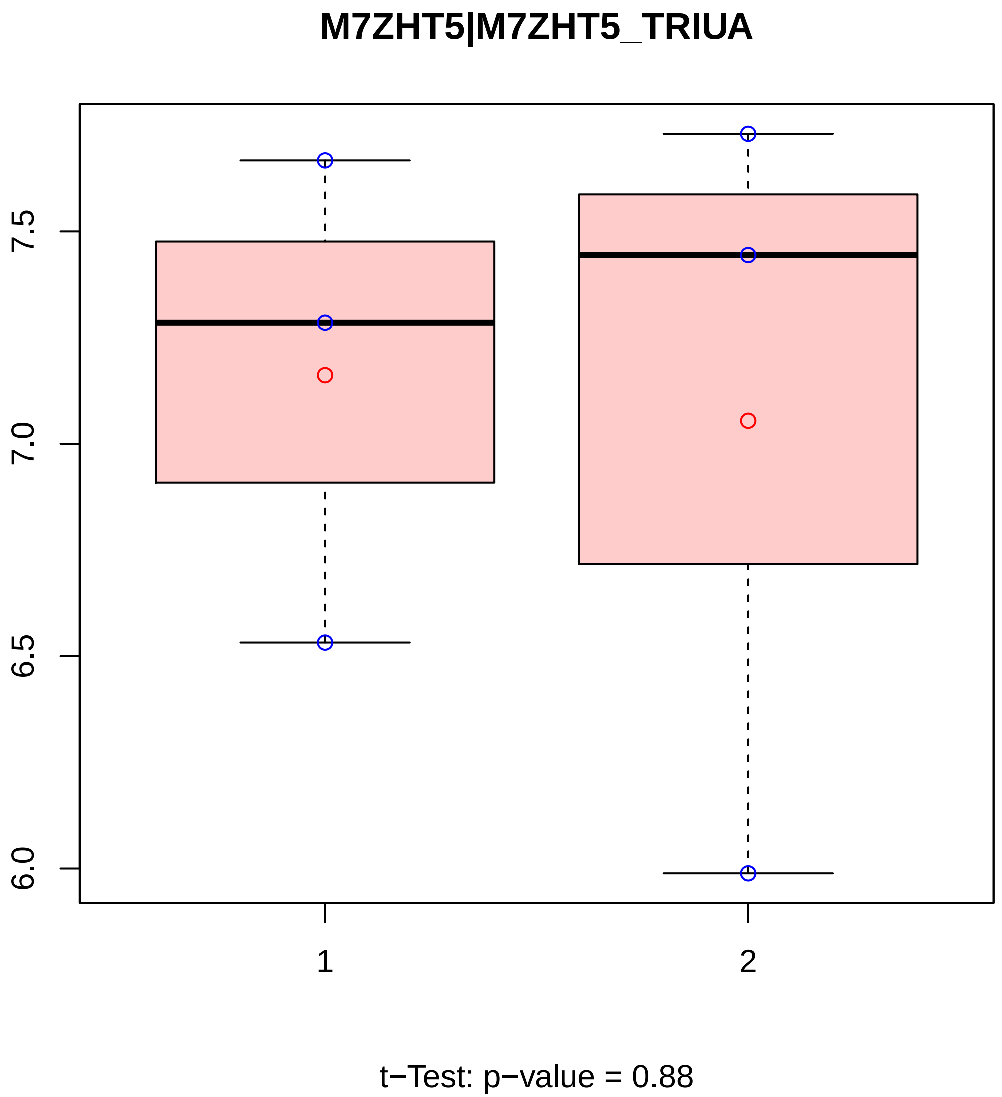
M7ZHT5|M7ZHT5_TRIUA
6.0
6.5
7.0
7.5
1
2
t−Test: p−value = 0.88

M7ZI02|M7ZI02_TRIUA

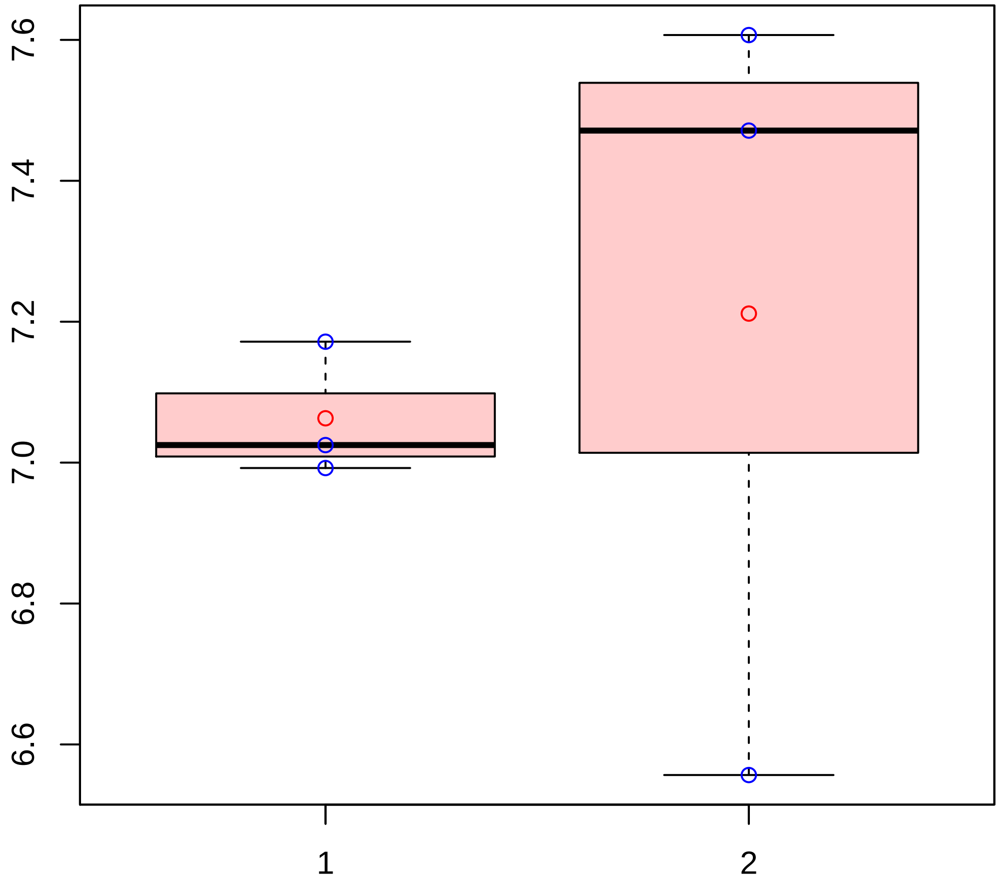

t−Test: p−value = 0.7

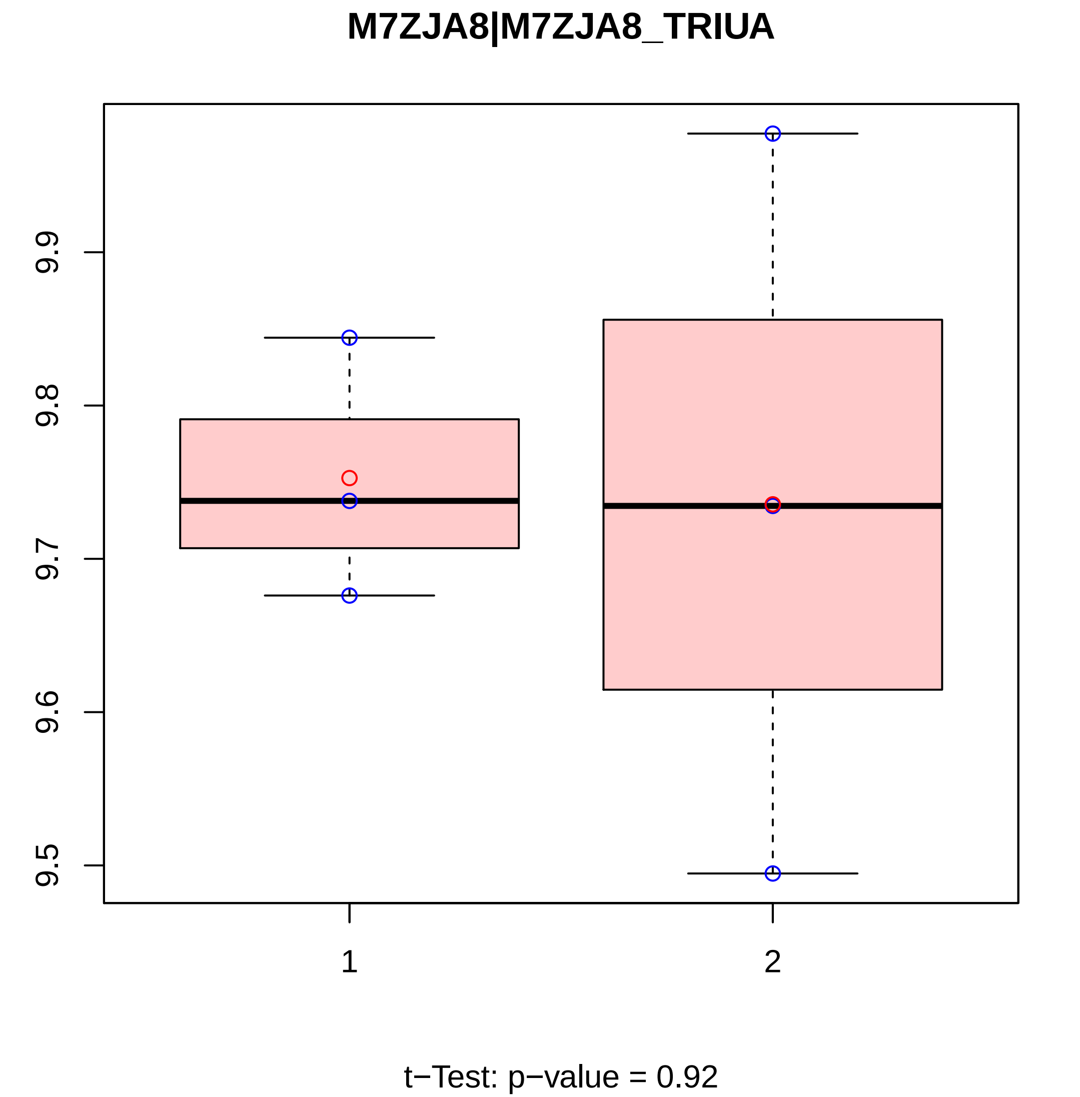
M7ZJA8|M7ZJA8_TRIUA
9.5
9.6
9.7
9.8
9.9
1
2
t−Test: p−value = 0.92

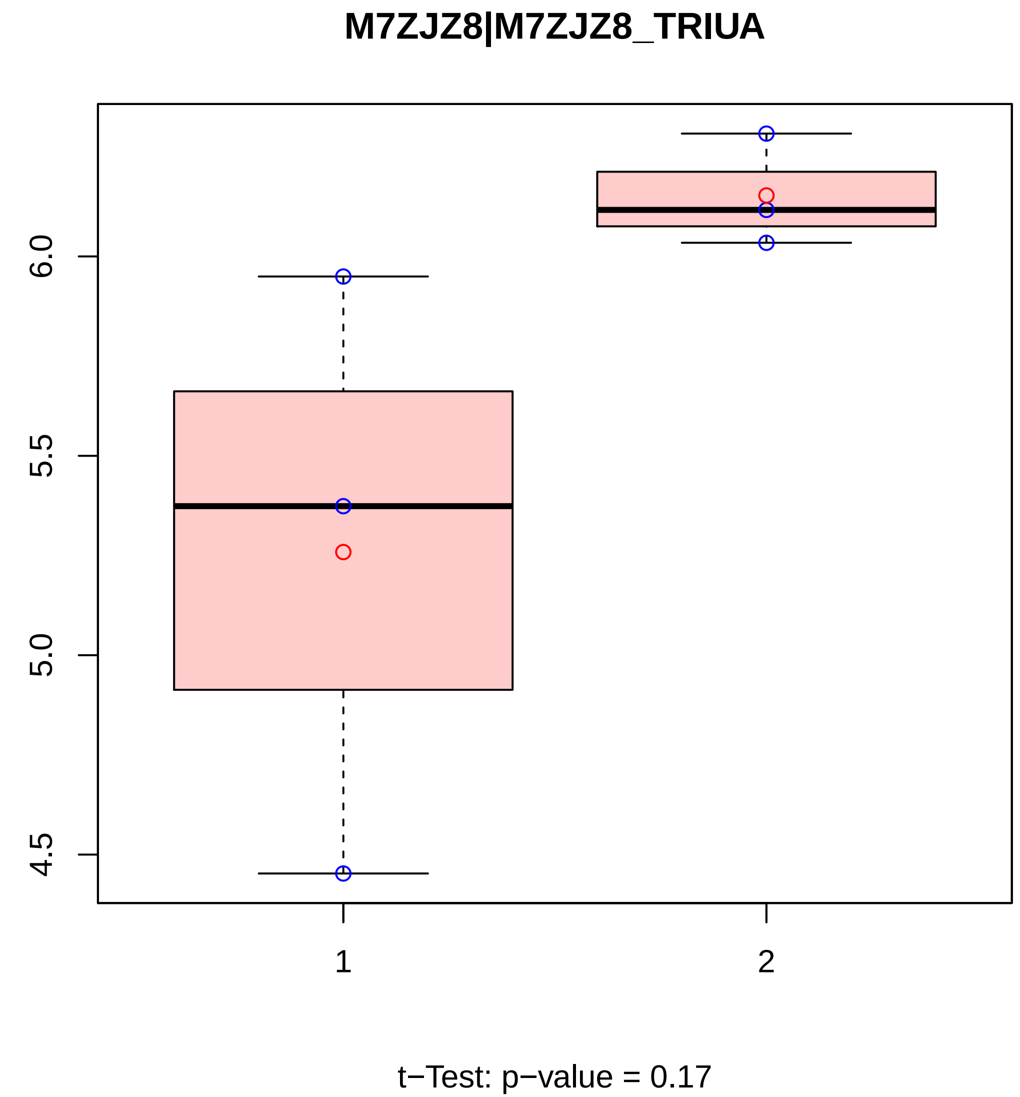
M7ZJZ8|M7ZJZ8_TRIUA
4.5
5.0
5.5
6.0
1
2
t−Test: p−value = 0.17

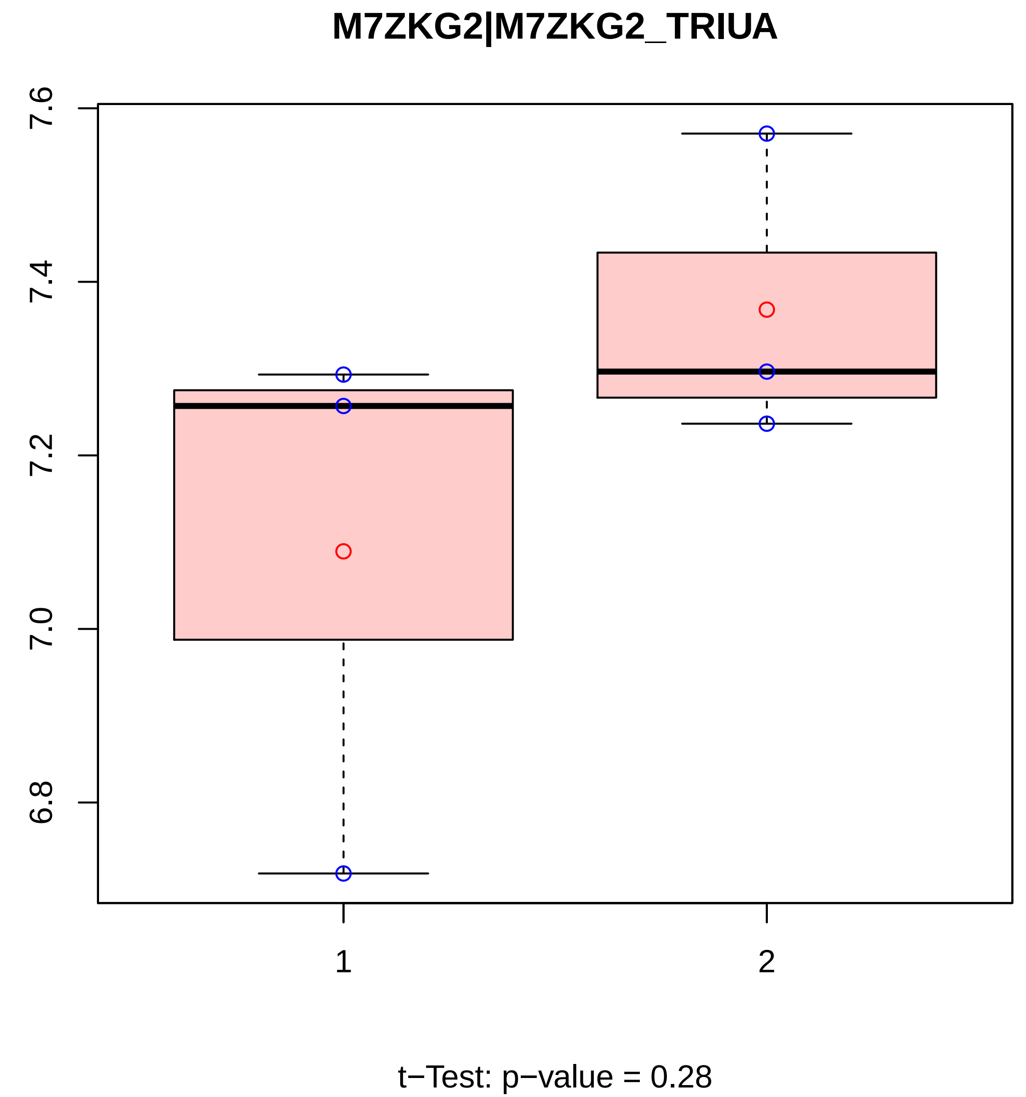
M7ZKG2|M7ZKG2_TRIUA
7.6
7.4
7.2
7.0
6.8
1
2
t−Test: p−value = 0.28

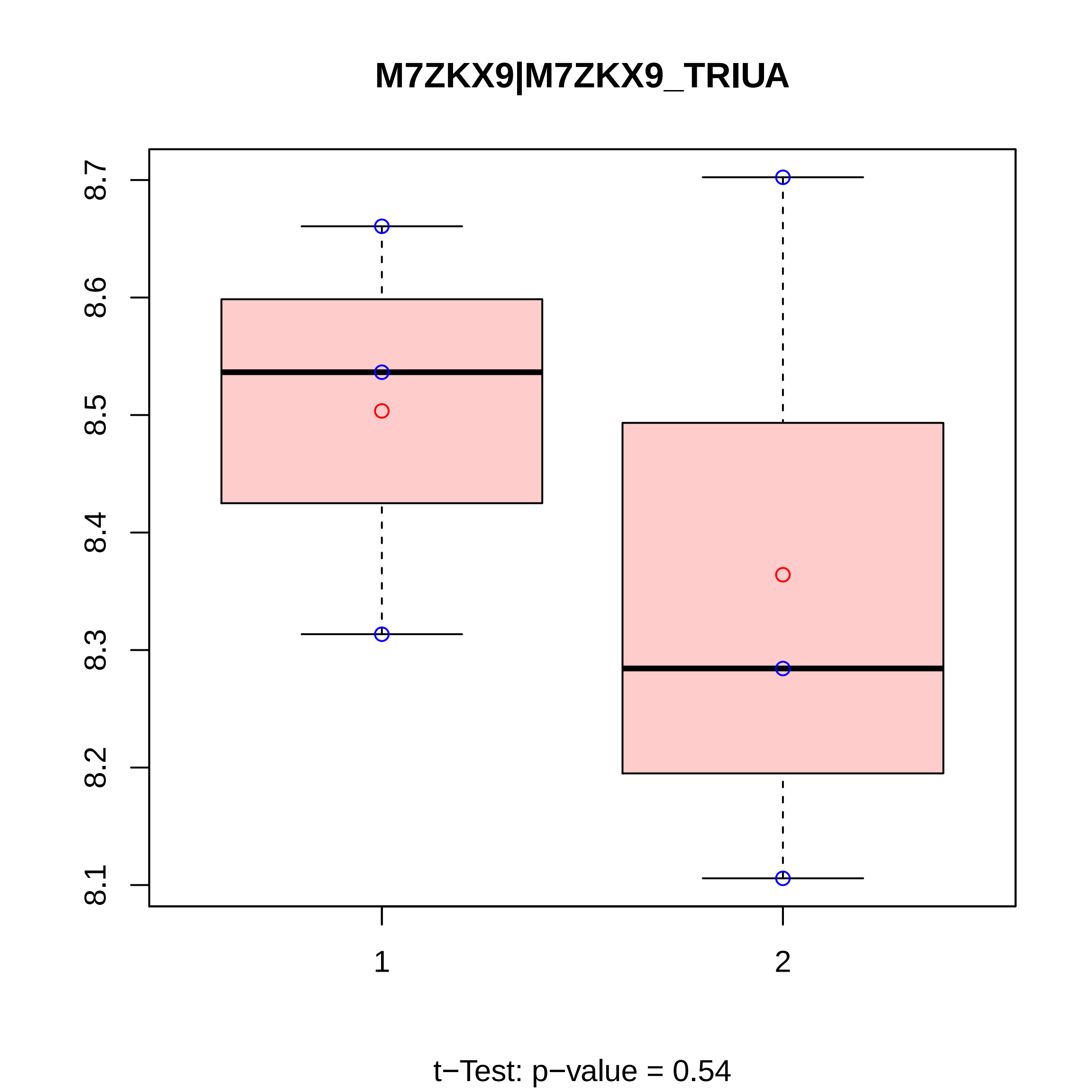
M7ZKX9|M7ZKX9_TRIUA
8.1
8.2
8.3
8.4
8.5
8.6
8.7
1
2
t−Test: p−value = 0.54

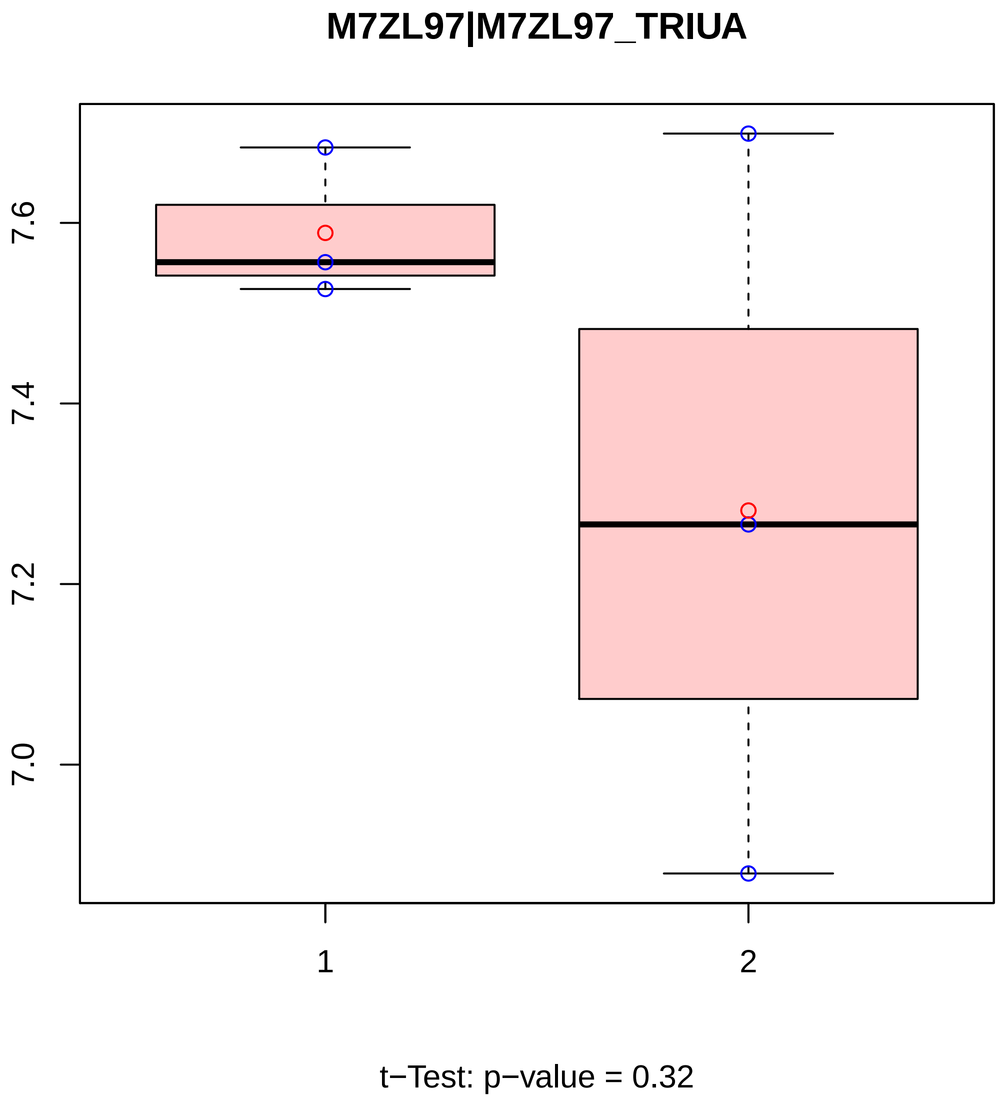
M7ZL97|M7ZL97_TRIUA
7.0
7.2
7.4
7.6
1
2
t−Test: p−value = 0.32

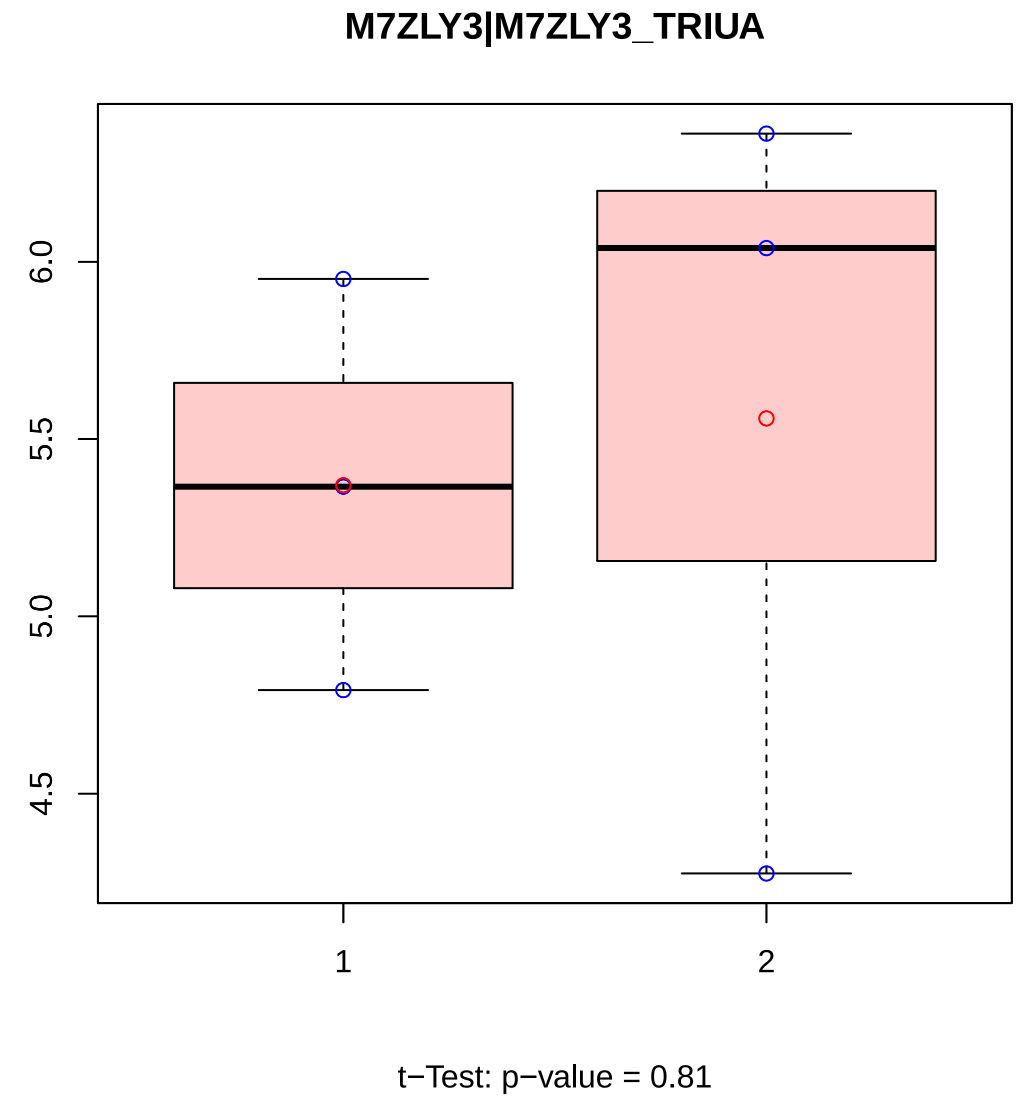
M7ZLY3|M7ZLY3_TRIUA
6.0
5.5
5.0
4.5
1
2
t−Test: p−value = 0.81

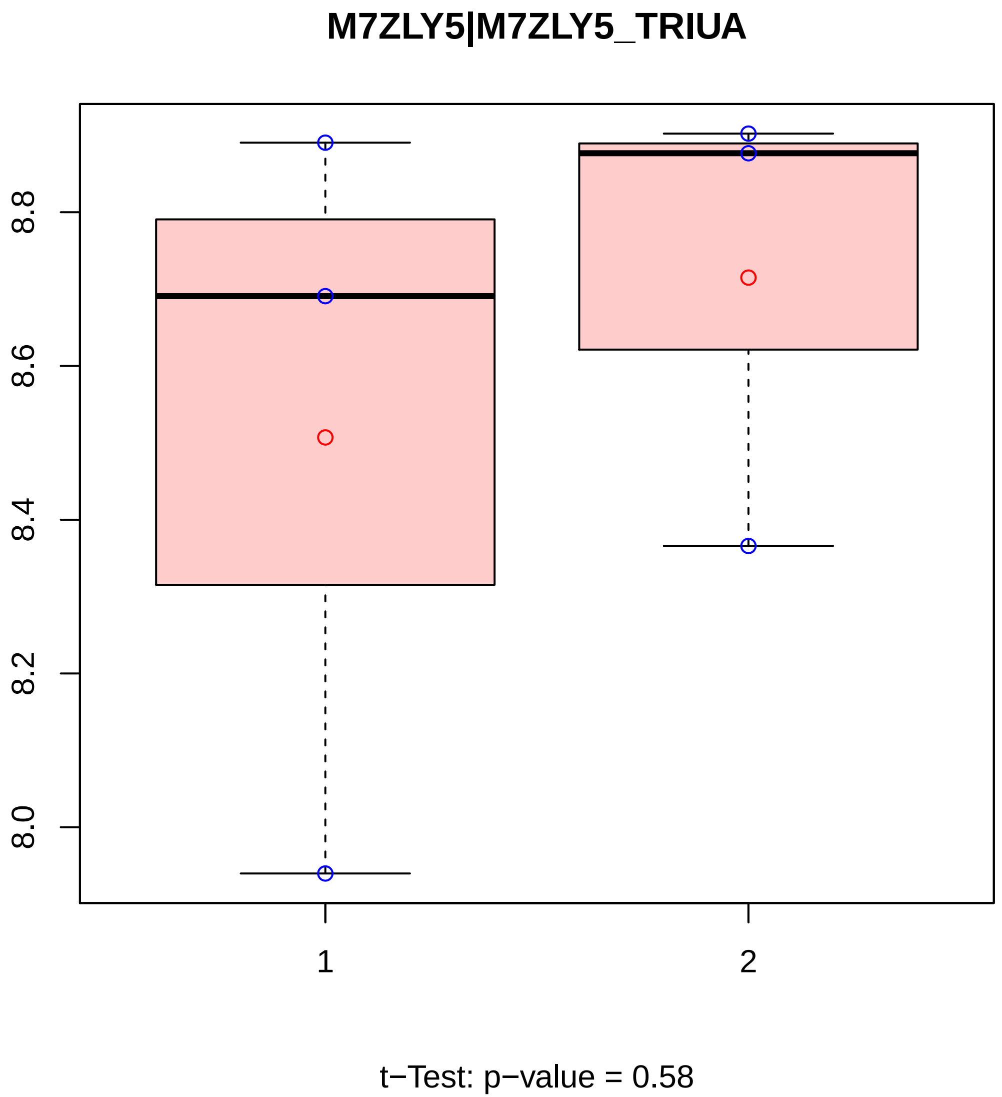
M7ZLY5|M7ZLY5_TRIUA
8.0
8.2
8.4
8.6
8.8
1
2
t−Test: p−value = 0.58

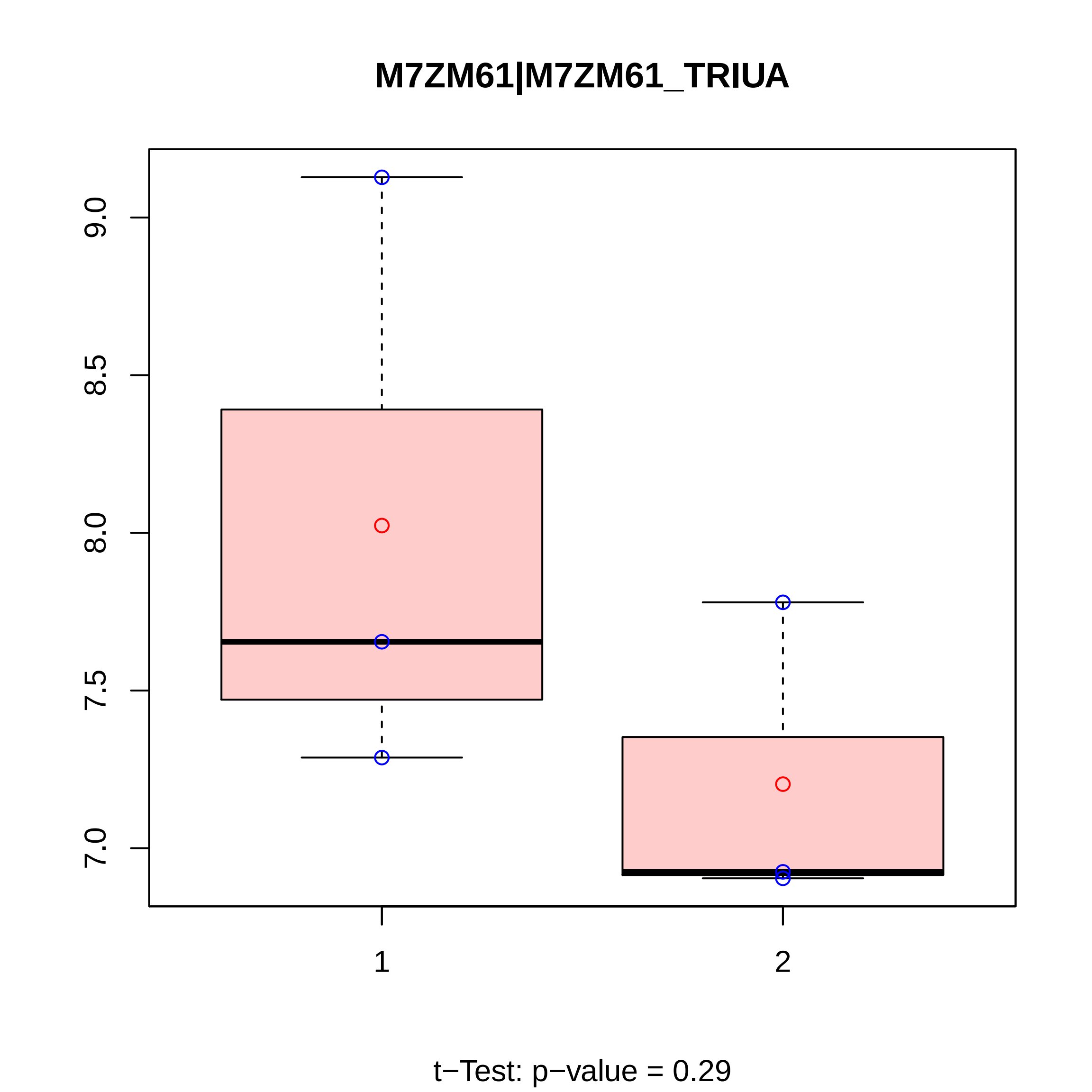
M7ZM61|M7ZM61_TRIUA
9.0
8.5
8.0
7.5
7.0
1
2
t−Test: p−value = 0.29

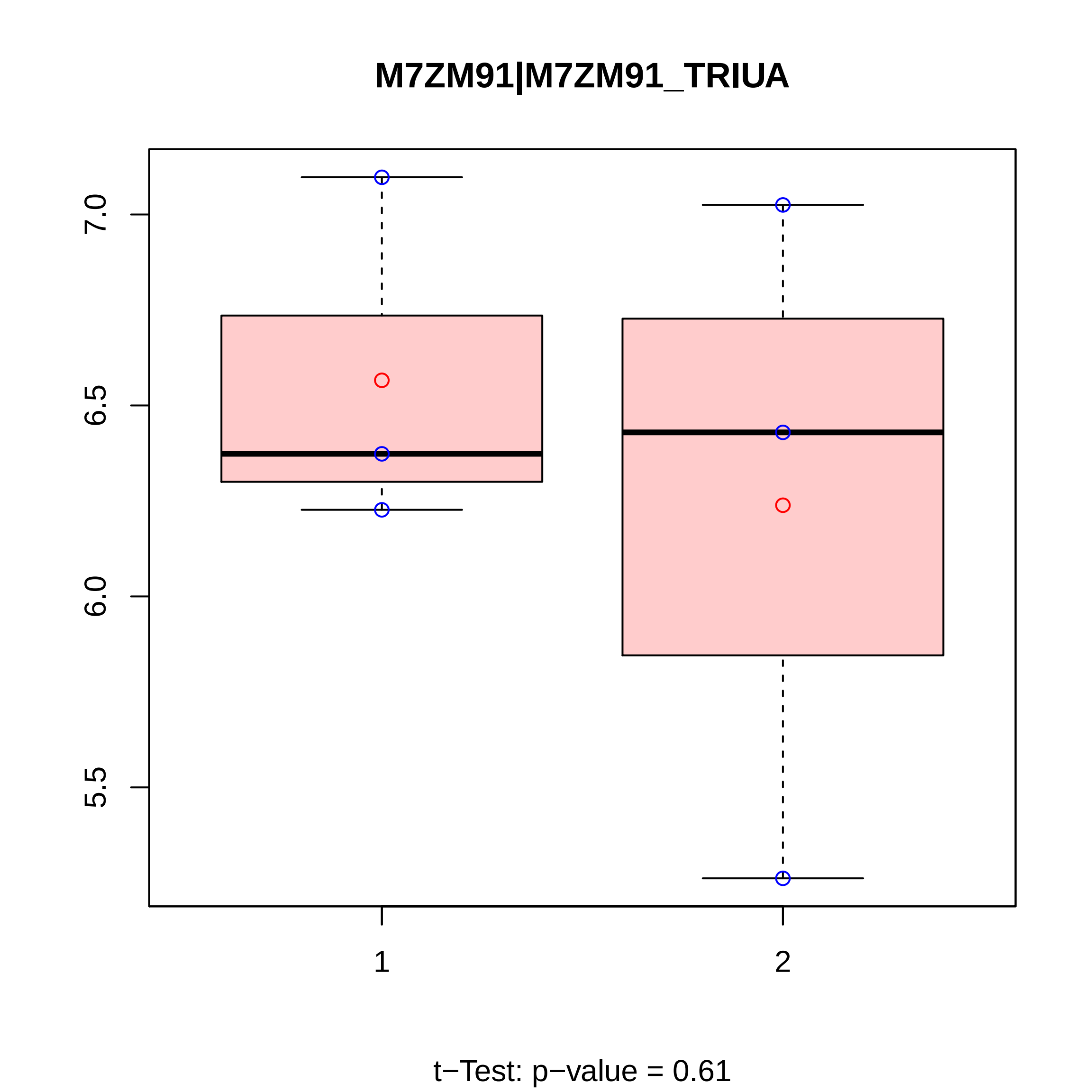

M7ZM91|M7ZM91_TRIUA
7.0
6.5
6.0
5.5
1
2
t−Test: p−value = 0.61

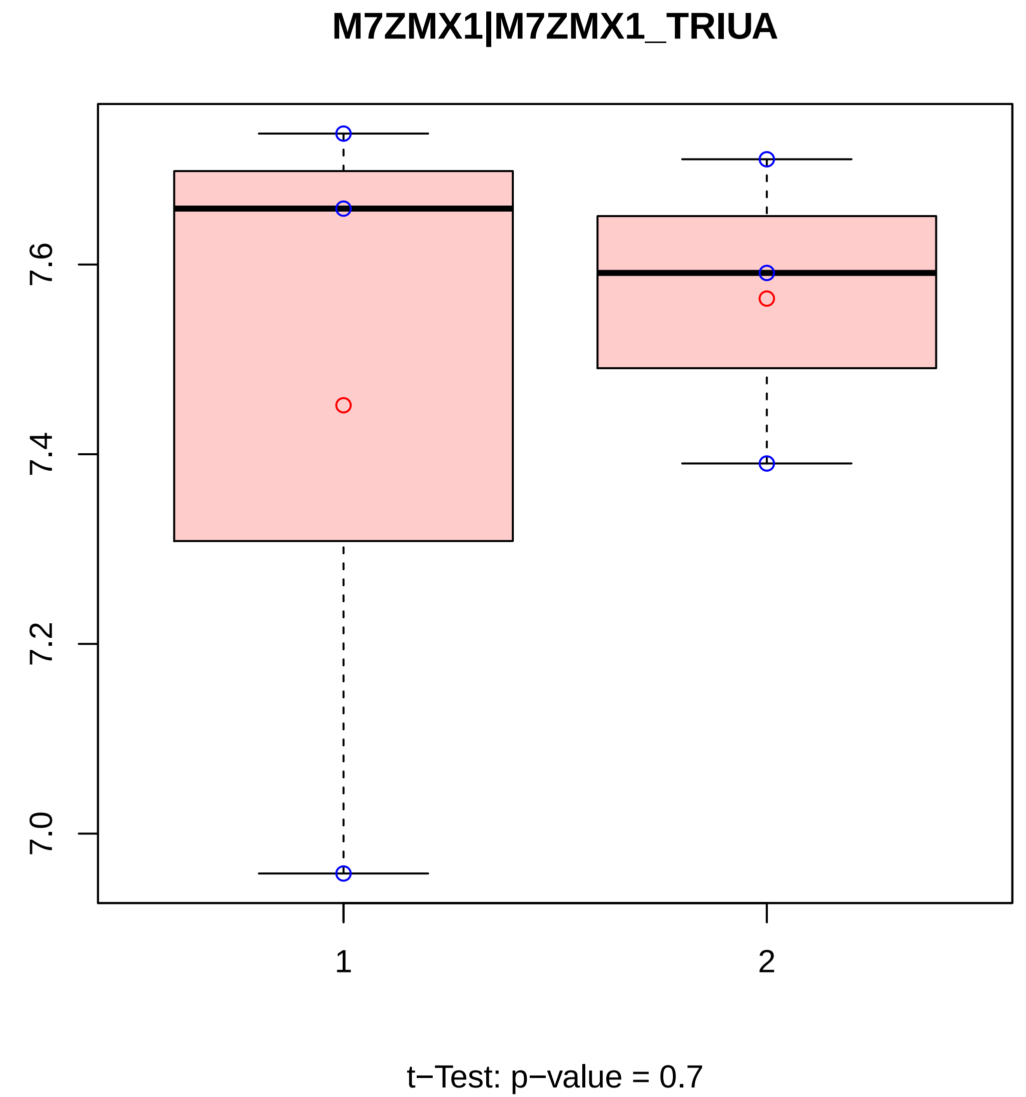
M7ZMX1|M7ZMX1_TRIUA
7.0
7.2
7.4
7.6
1
2
t−Test: p−value = 0.7

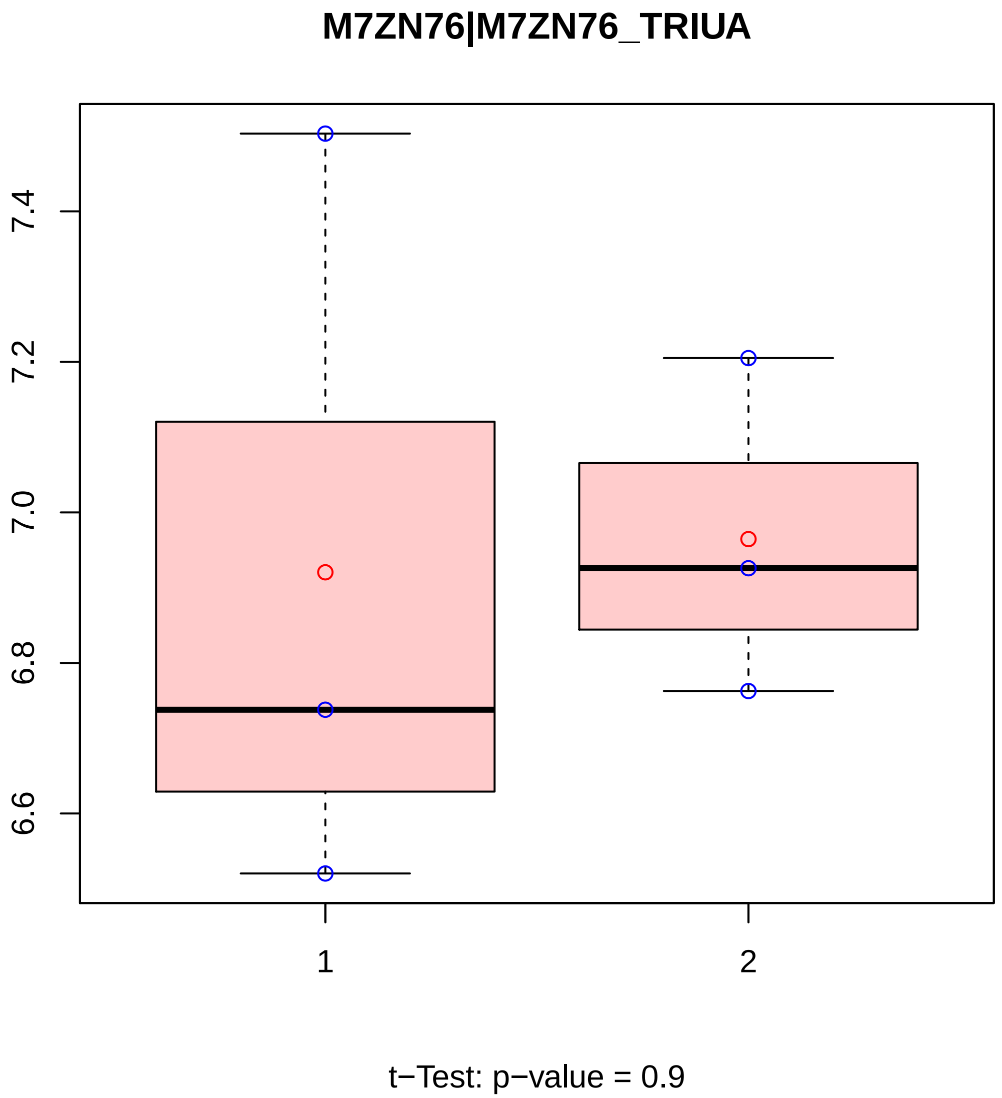

M7ZN76|M7ZN76_TRIUA
7.4
7.2
7.0
6.8
6.6
1
2
t−Test: p−value = 0.9

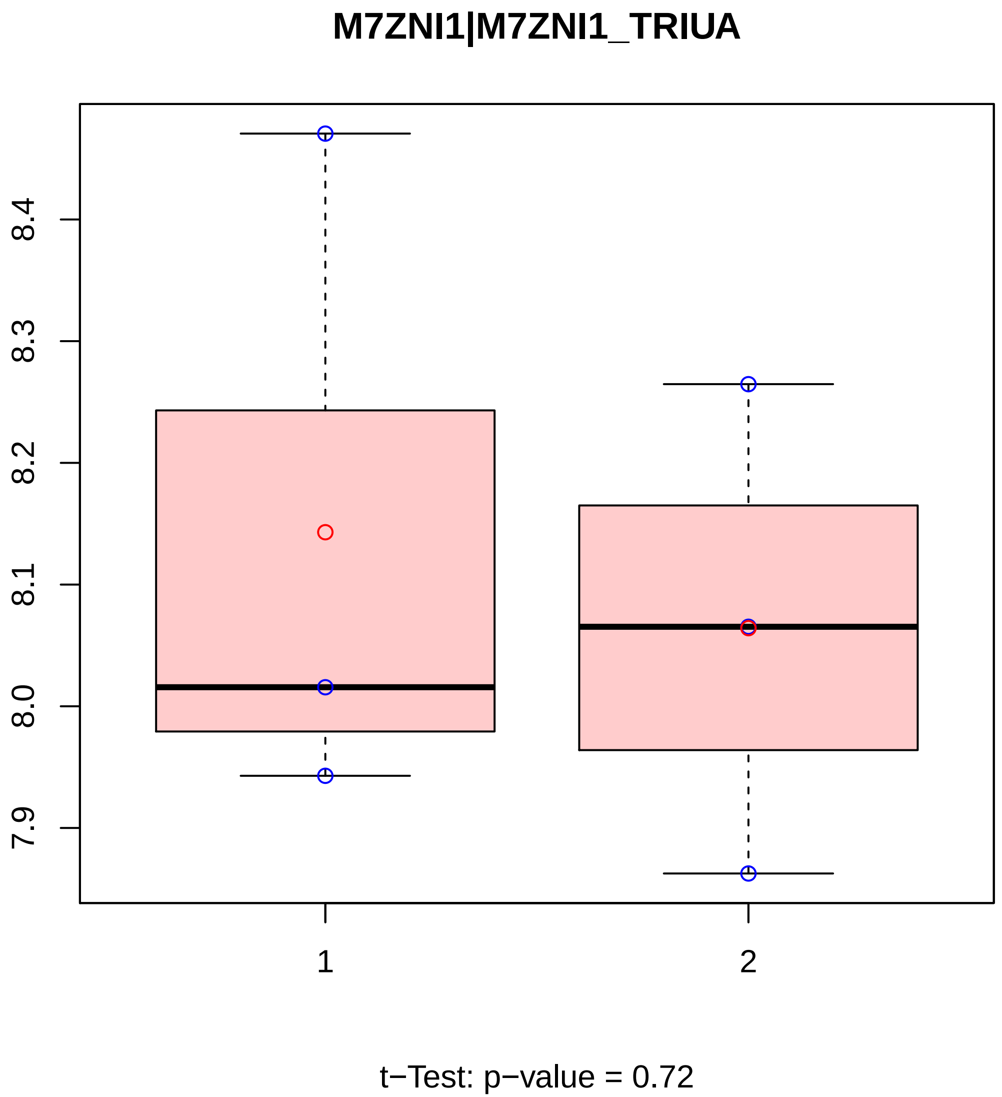
M7ZNI1|M7ZNI1_TRIUA
7.9
8.0
8.1
8.2
8.3
8.4
1
2
t−Test: p−value = 0.72

M7ZPN9|M7ZPN9_TRIUA

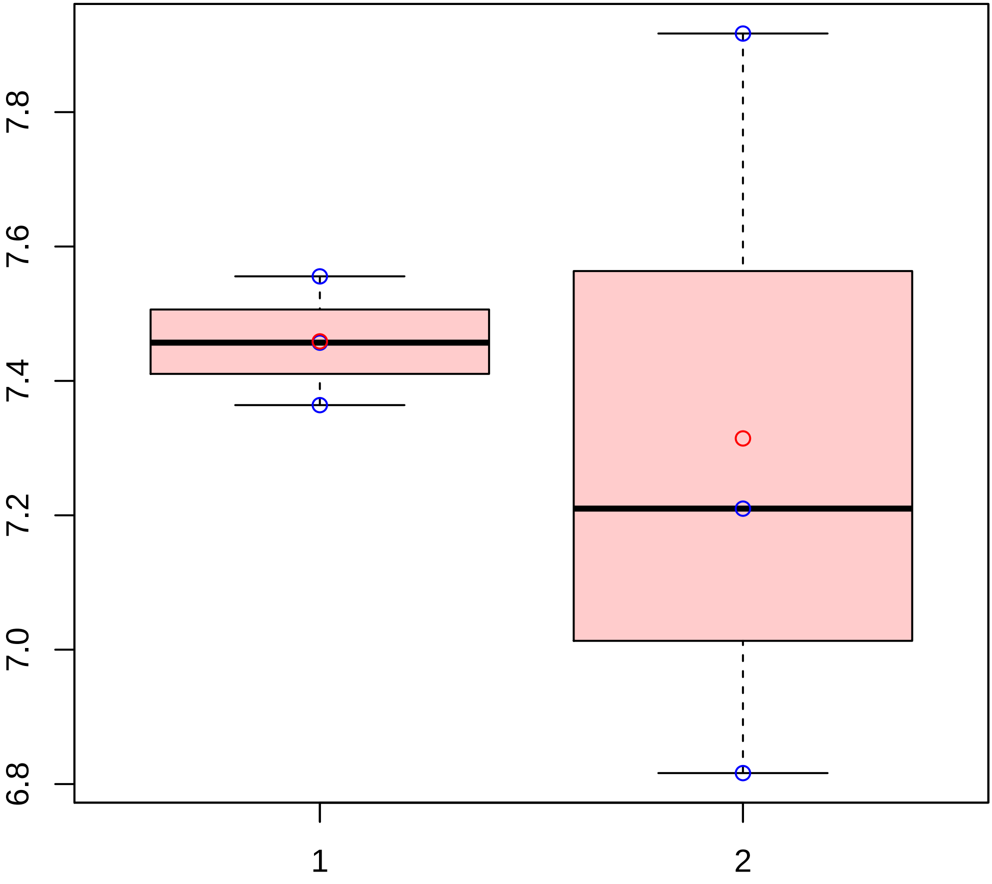

t−Test: p−value = 0.7

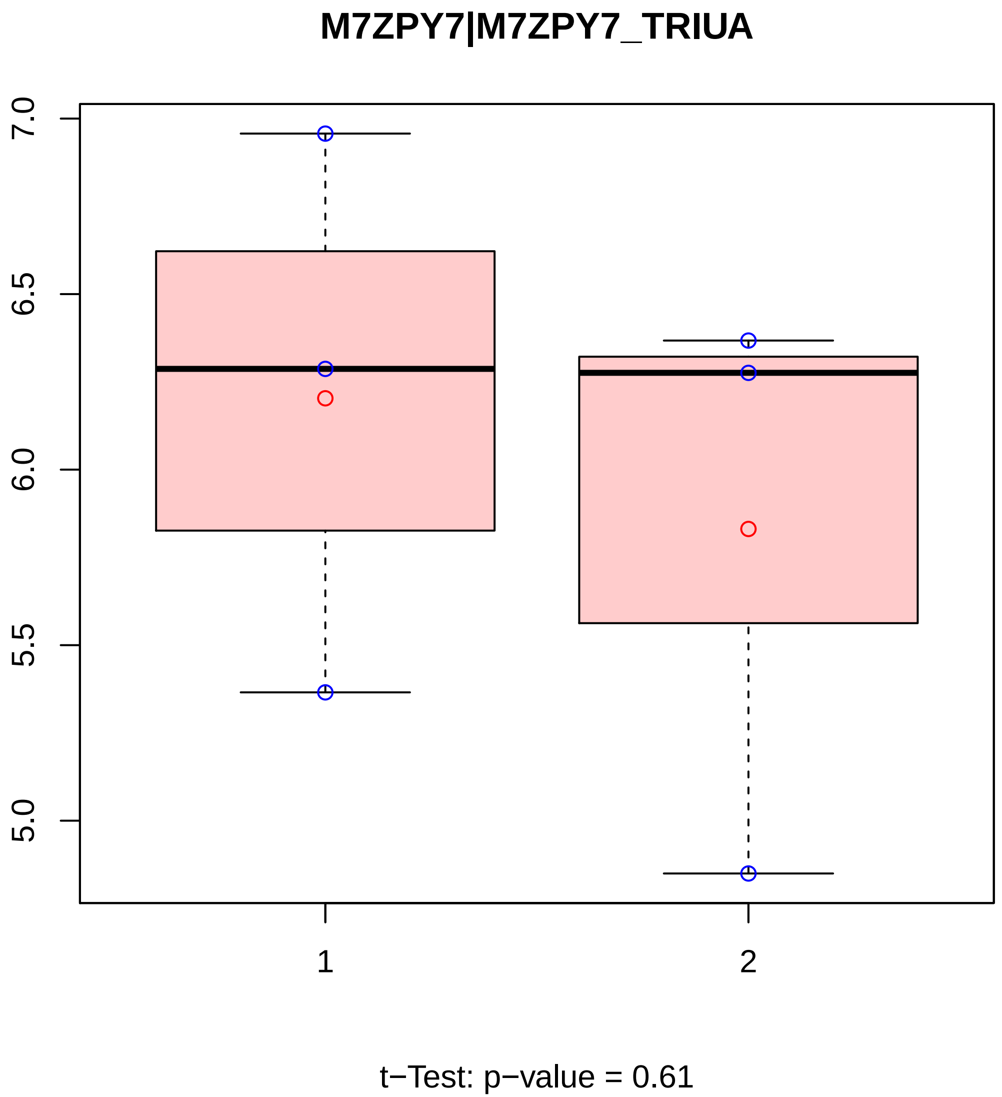
M7ZPY7|M7ZPY7_TRIUA
7.0
6.5
6.0
5.5
5.0
1
2
t−Test: p−value = 0.61

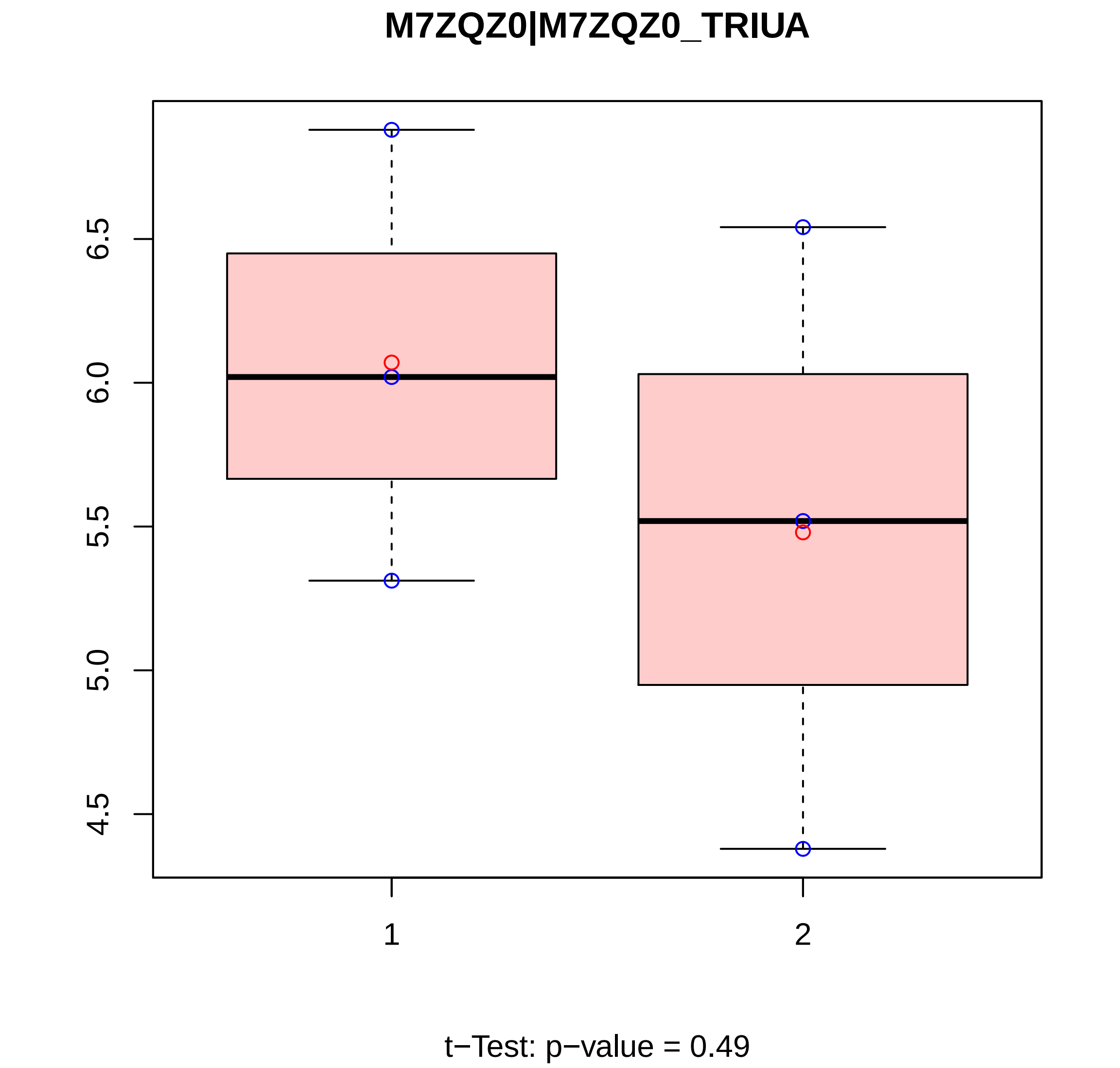
M7ZQZ0|M7ZQZ0_TRIUA
4.5
5.0
5.5
6.0
6.5
1
2
t−Test: p−value = 0.49

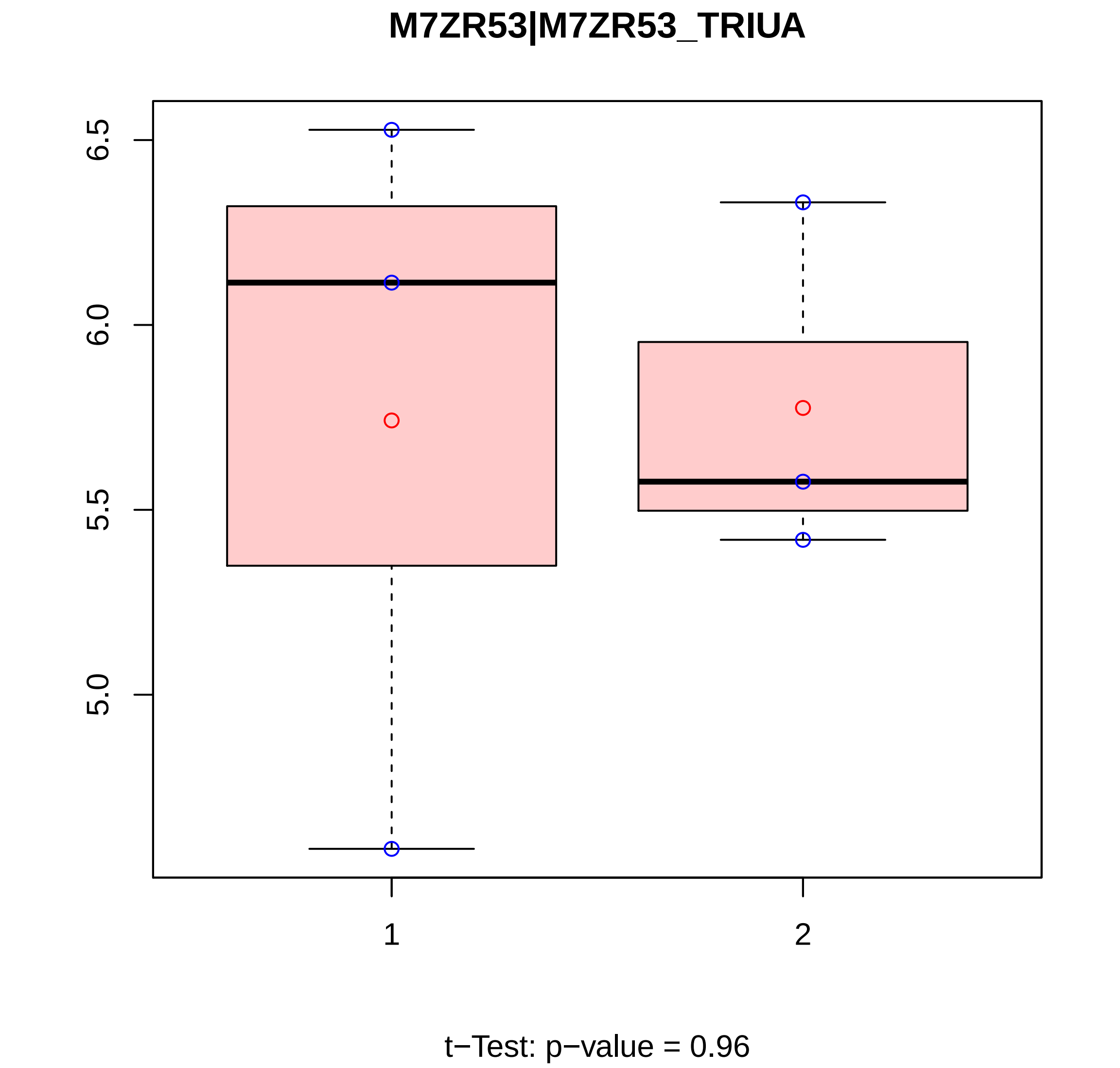
M7ZR53|M7ZR53_TRIUA
6.5
6.0
5.5
5.0
1
2
t−Test: p−value = 0.96

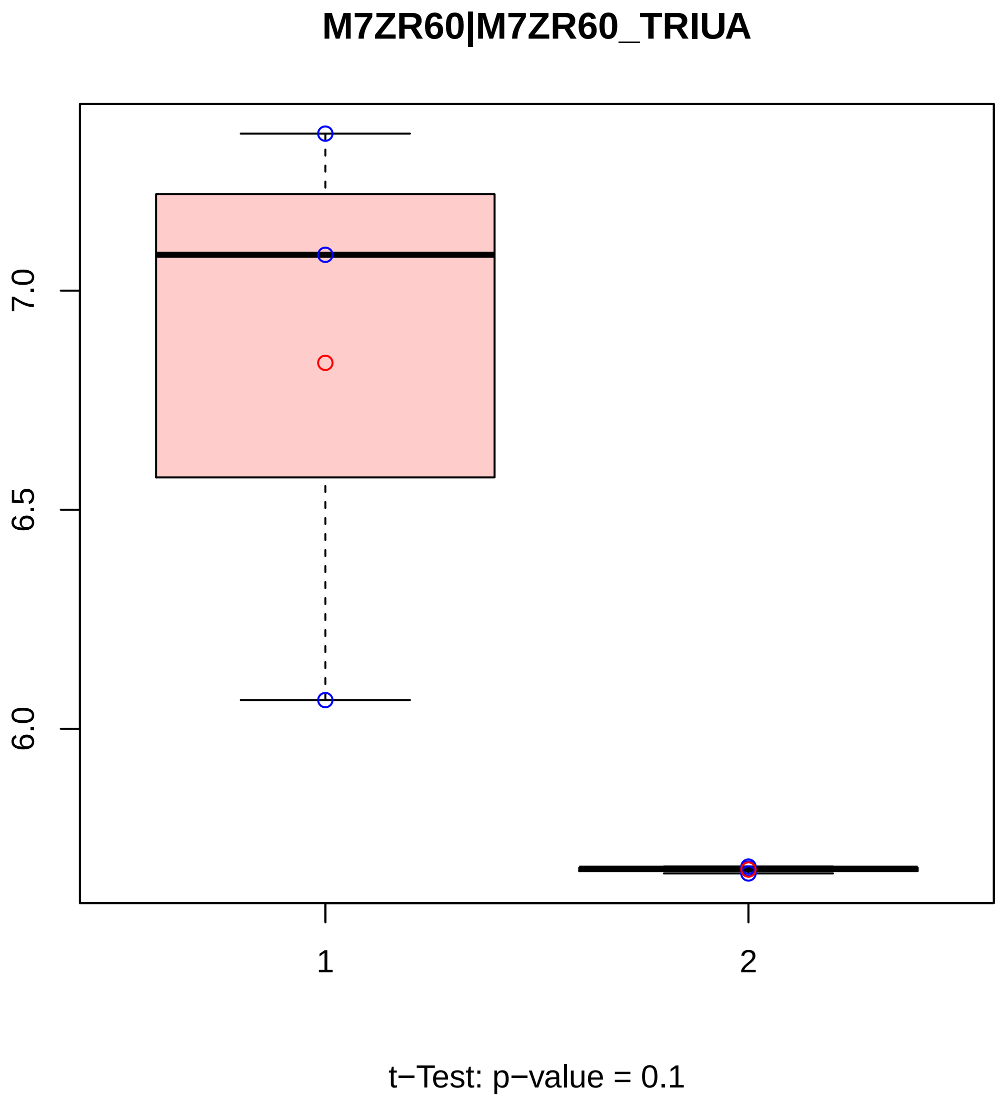

M7ZR60|M7ZR60_TRIUA
7.0
6.5
6.0
1
2
t−Test: p−value = 0.1

M7ZSN5|M7ZSN5_TRIUA

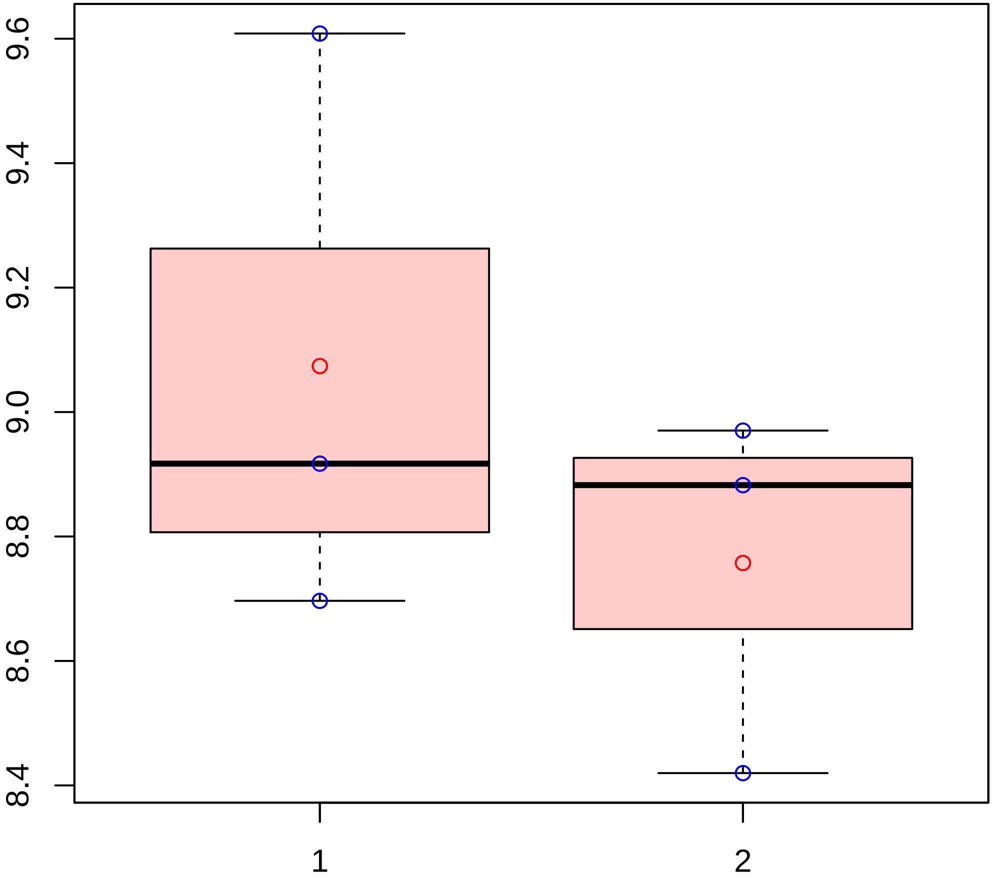

t−Test: p−value = 0.39

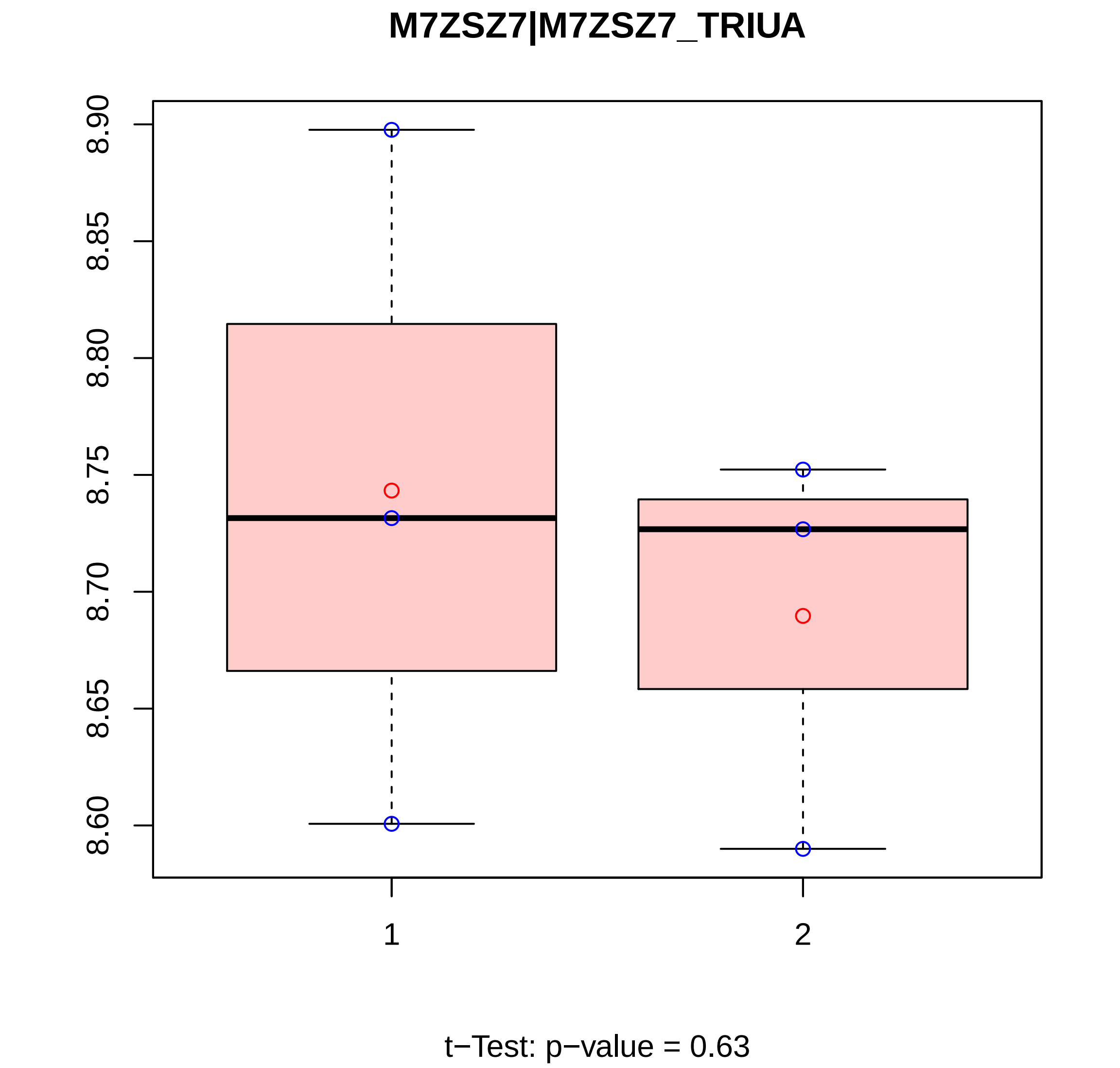

M7ZSZ7|M7ZSZ7_TRIUA
8.60
8.65
8.70
8.75
8.80
8.85
8.90
1
2
t−Test: p−value = 0.63

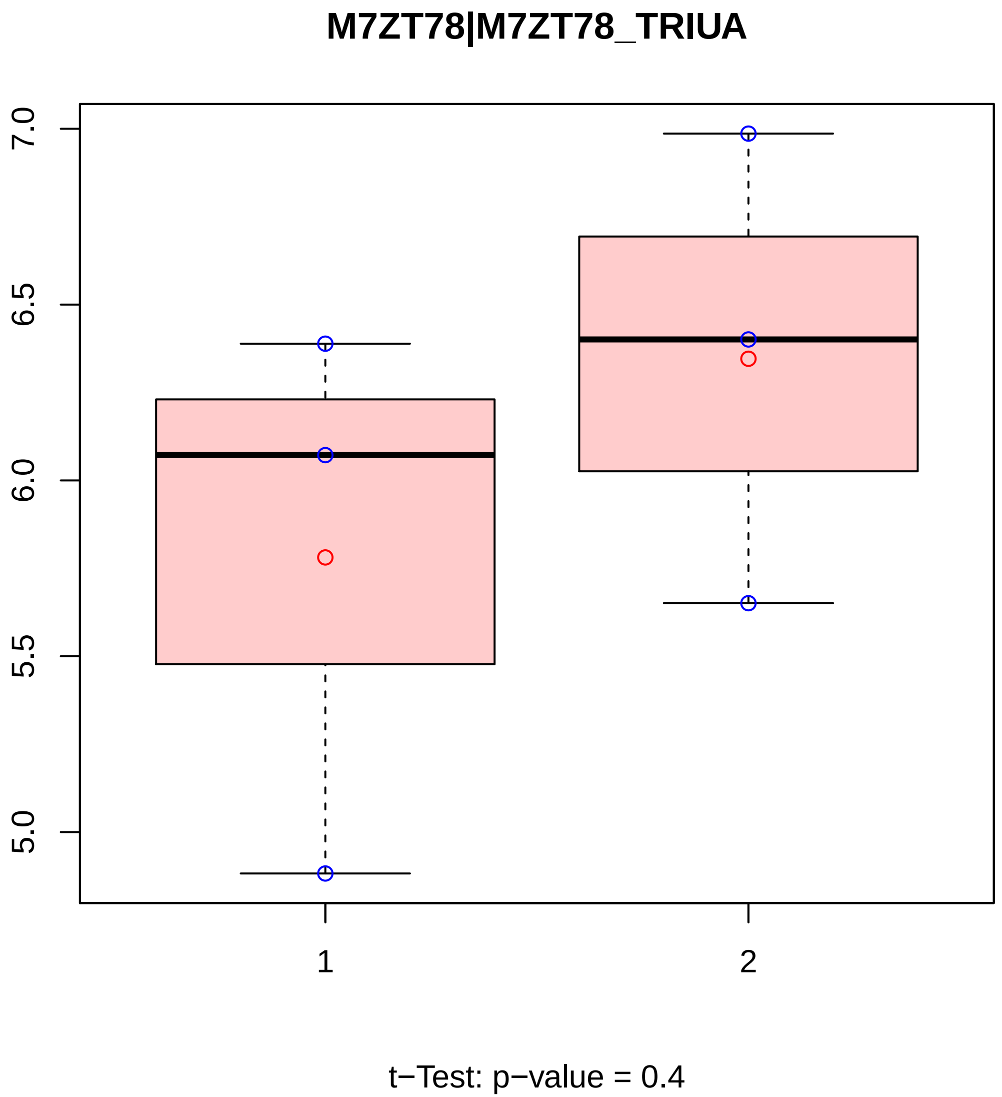

M7ZT78|M7ZT78_TRIUA
7.0
6.5
6.0
5.5
5.0
1
2
t−Test: p−value = 0.4

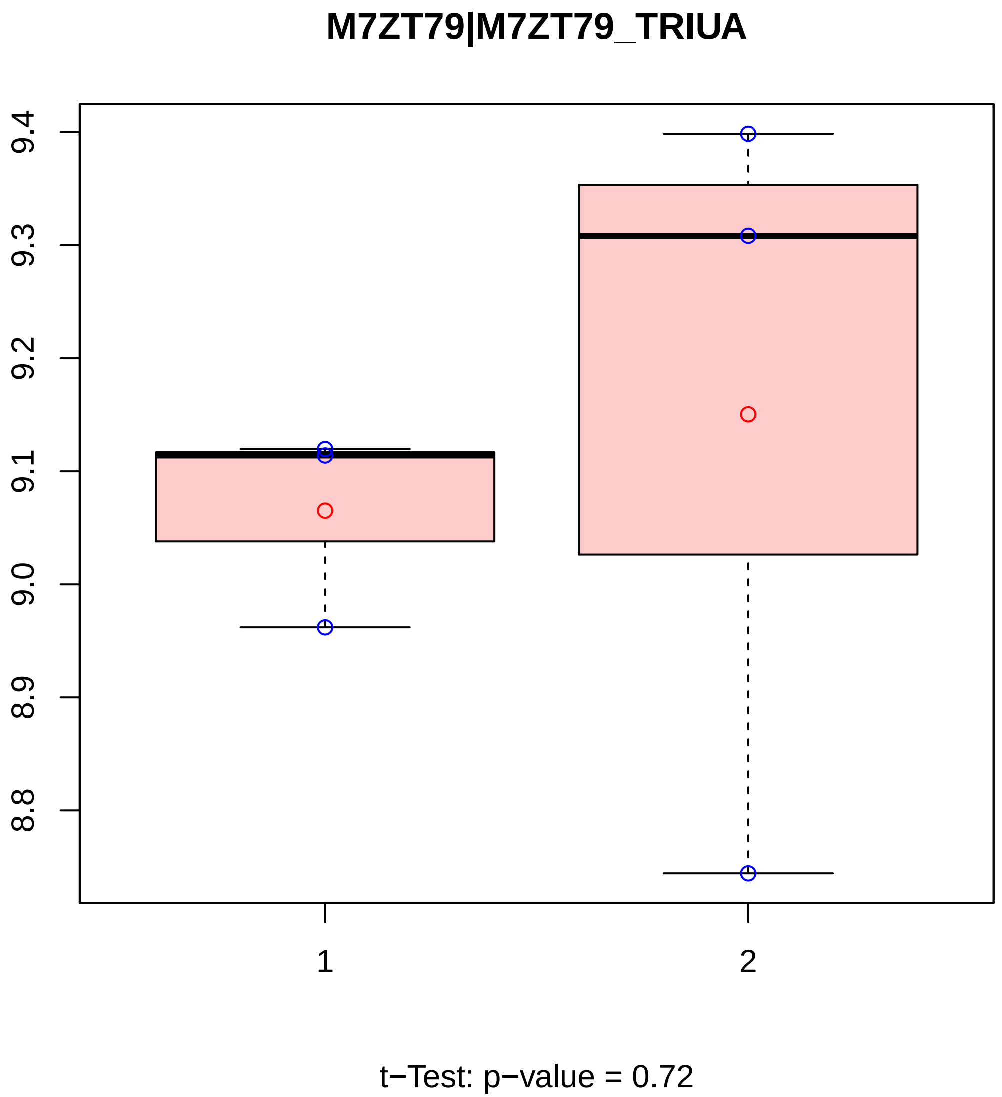
M7ZT79|M7ZT79_TRIUA
9.4
9.3
9.2
9.1
9.0
8.9
8.8
1
2
t−Test: p−value = 0.72

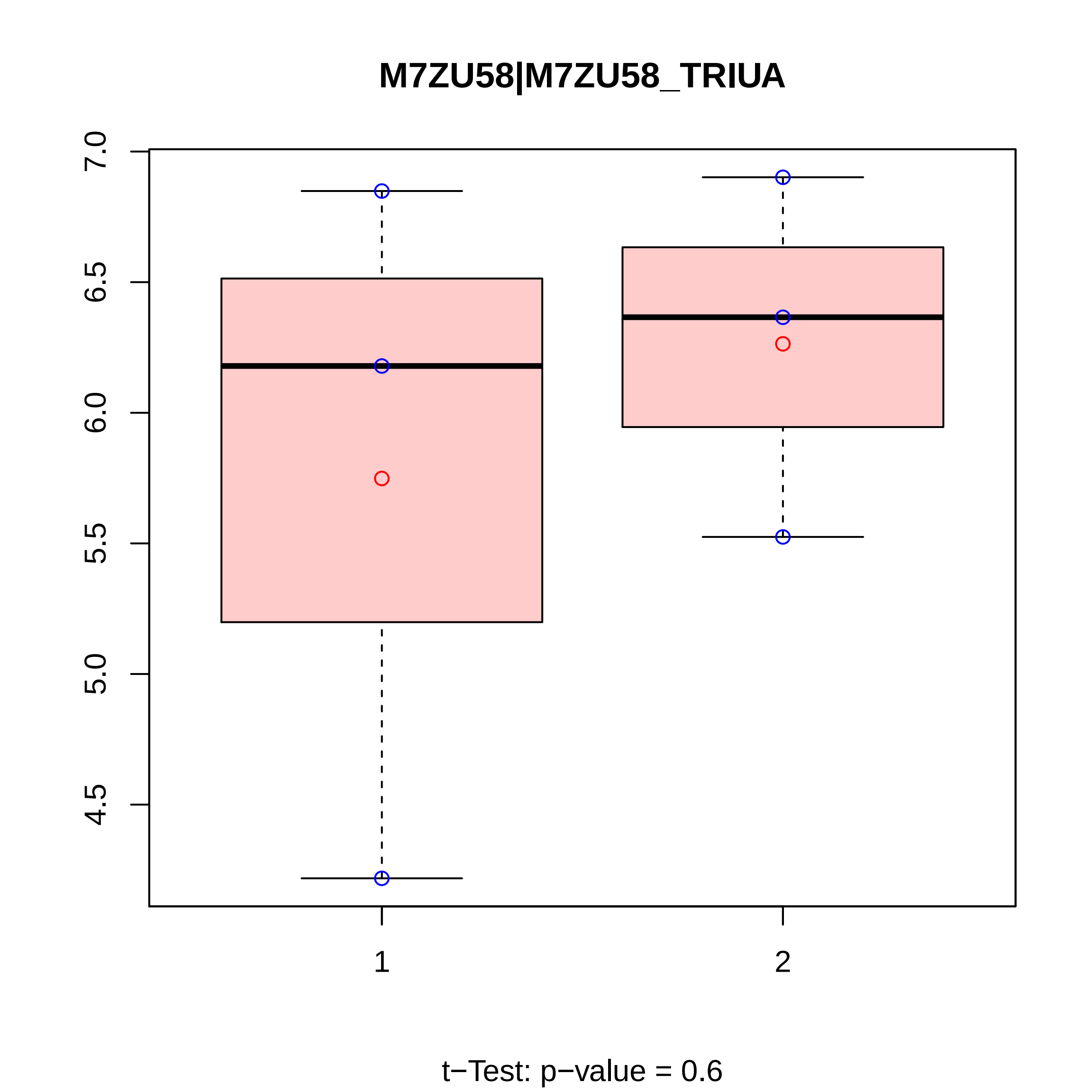

M7ZU58|M7ZU58_TRIUA
7.0
6.5
6.0
5.5
5.0
4.5
1
2
t−Test: p−value = 0.6

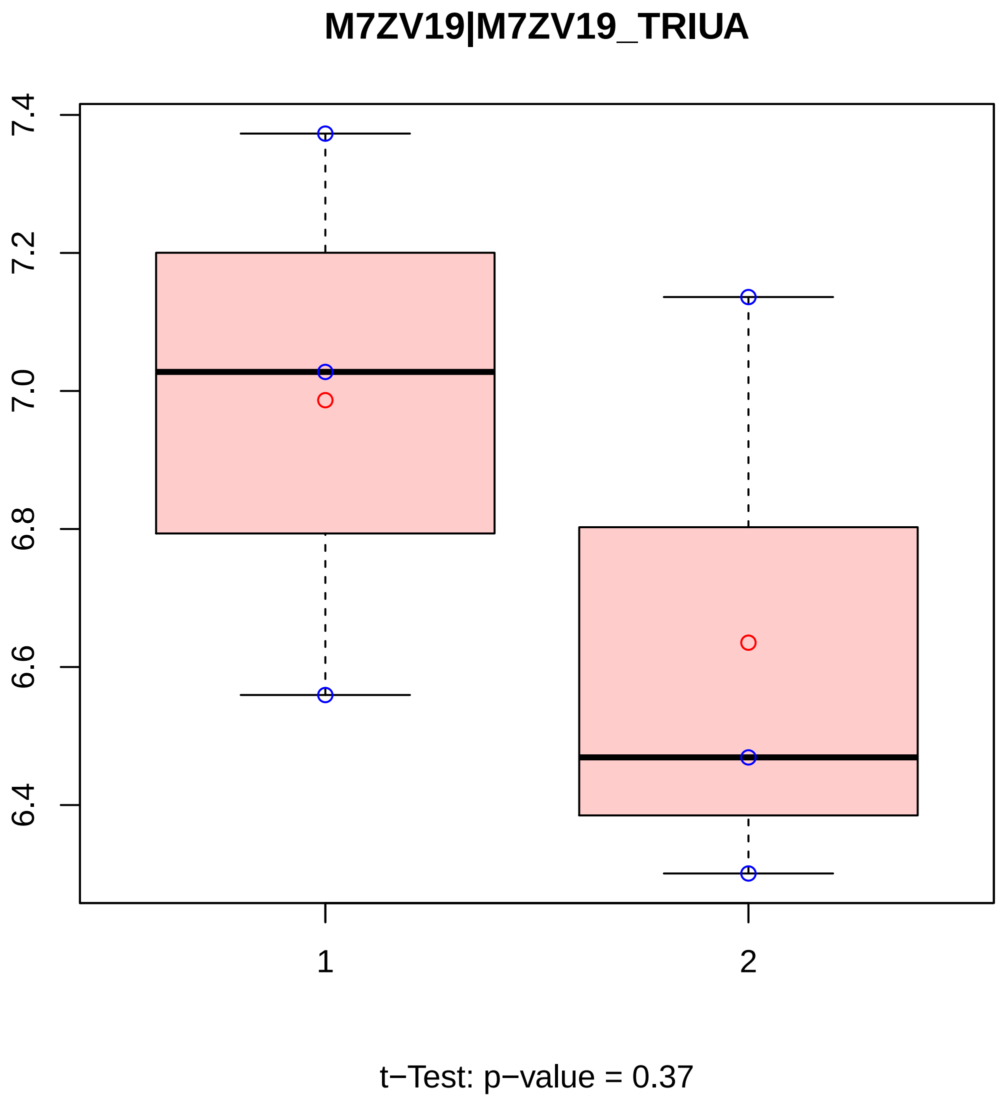

M7ZV19|M7ZV19_TRIUA
7.4
7.2
7.0
6.8
6.6
6.4
1
2
t−Test: p−value = 0.37

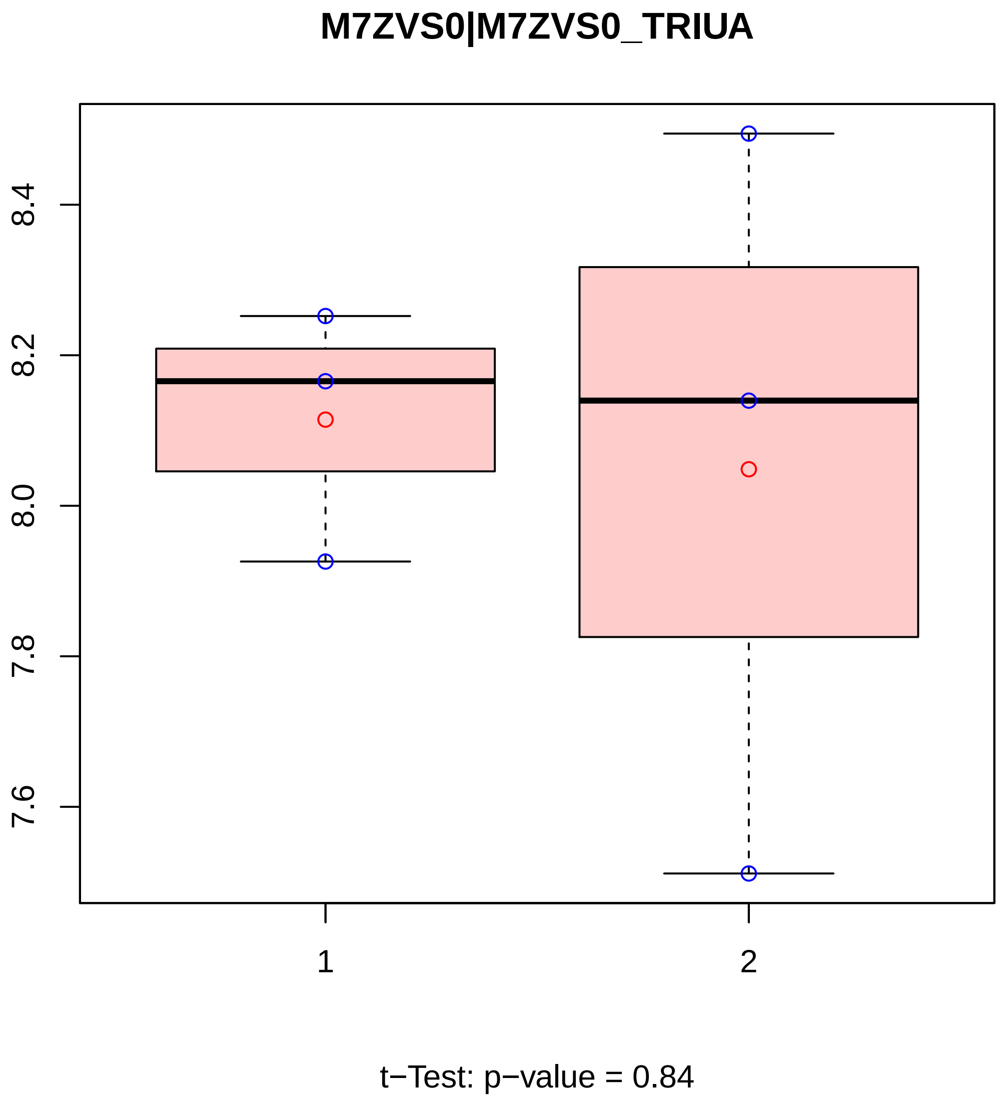

M7ZVS0|M7ZVS0_TRIUA
8.4
8.2
8.0
7.8
7.6
1
2
t−Test: p−value = 0.84

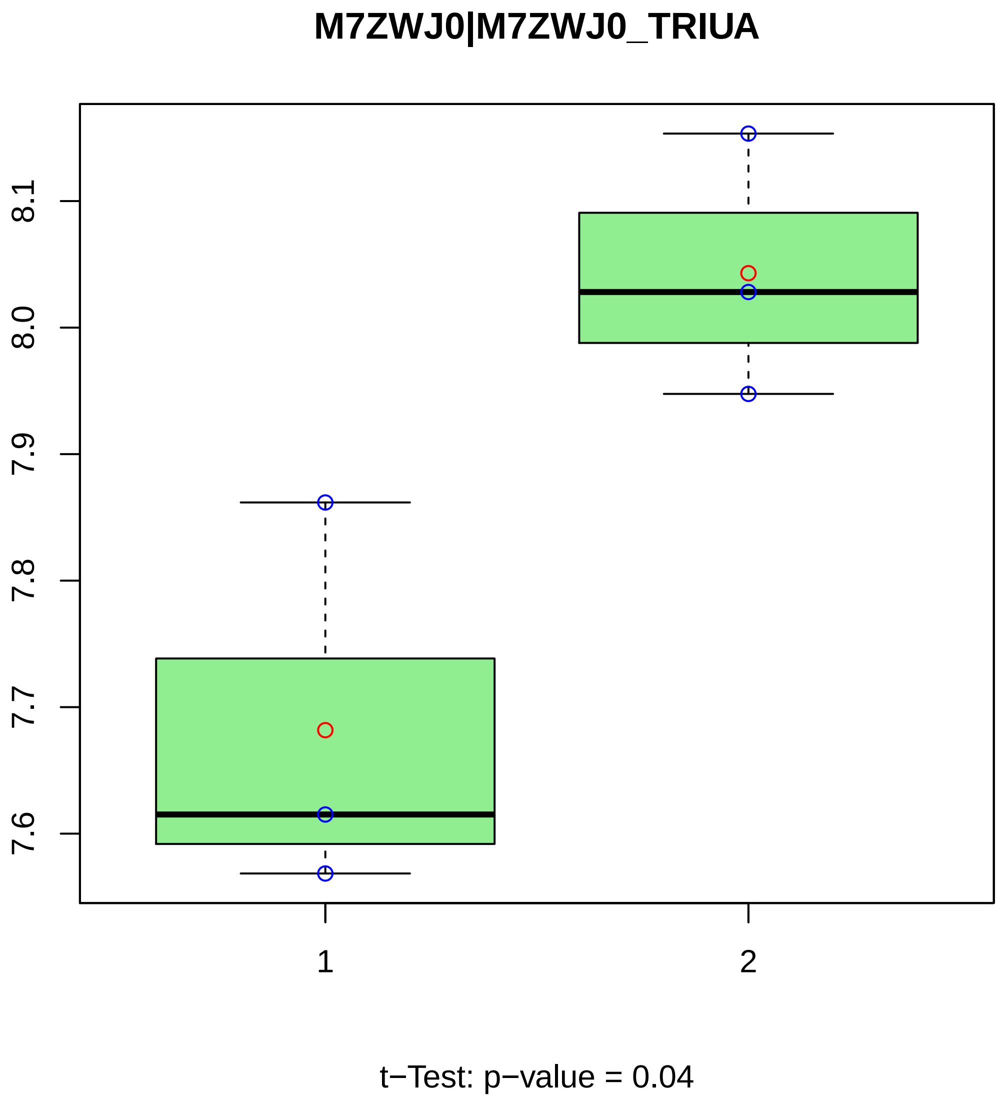
M7ZWJ0|M7ZWJ0_TRIUA
8.1
8.0
7.9
7.8
7.7
7.6
1
2
t−Test: p−value = 0.04

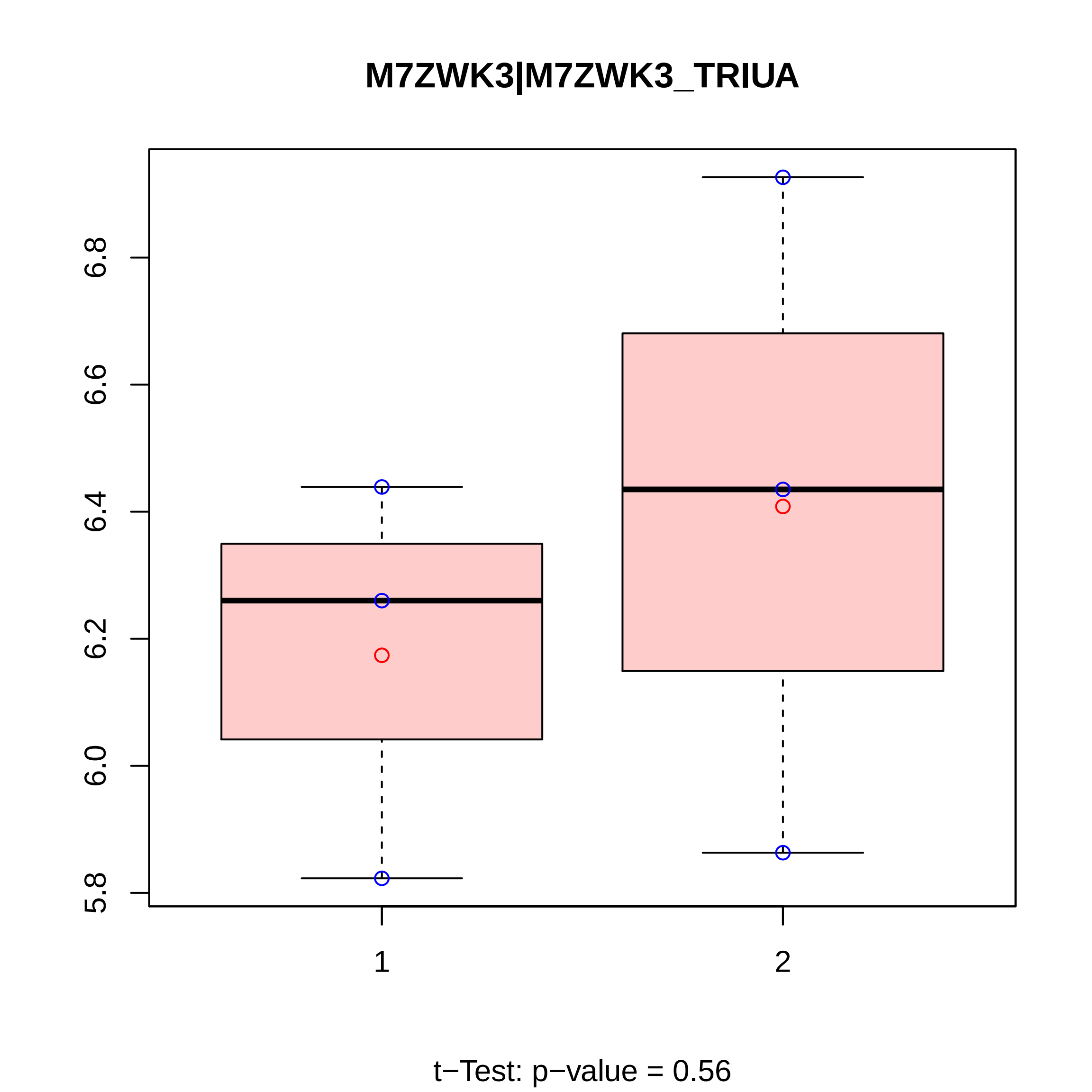
M7ZWK3|M7ZWK3_TRIUA
6.8
6.6
6.4
6.2
6.0
5.8
1
2
t−Test: p−value = 0.56

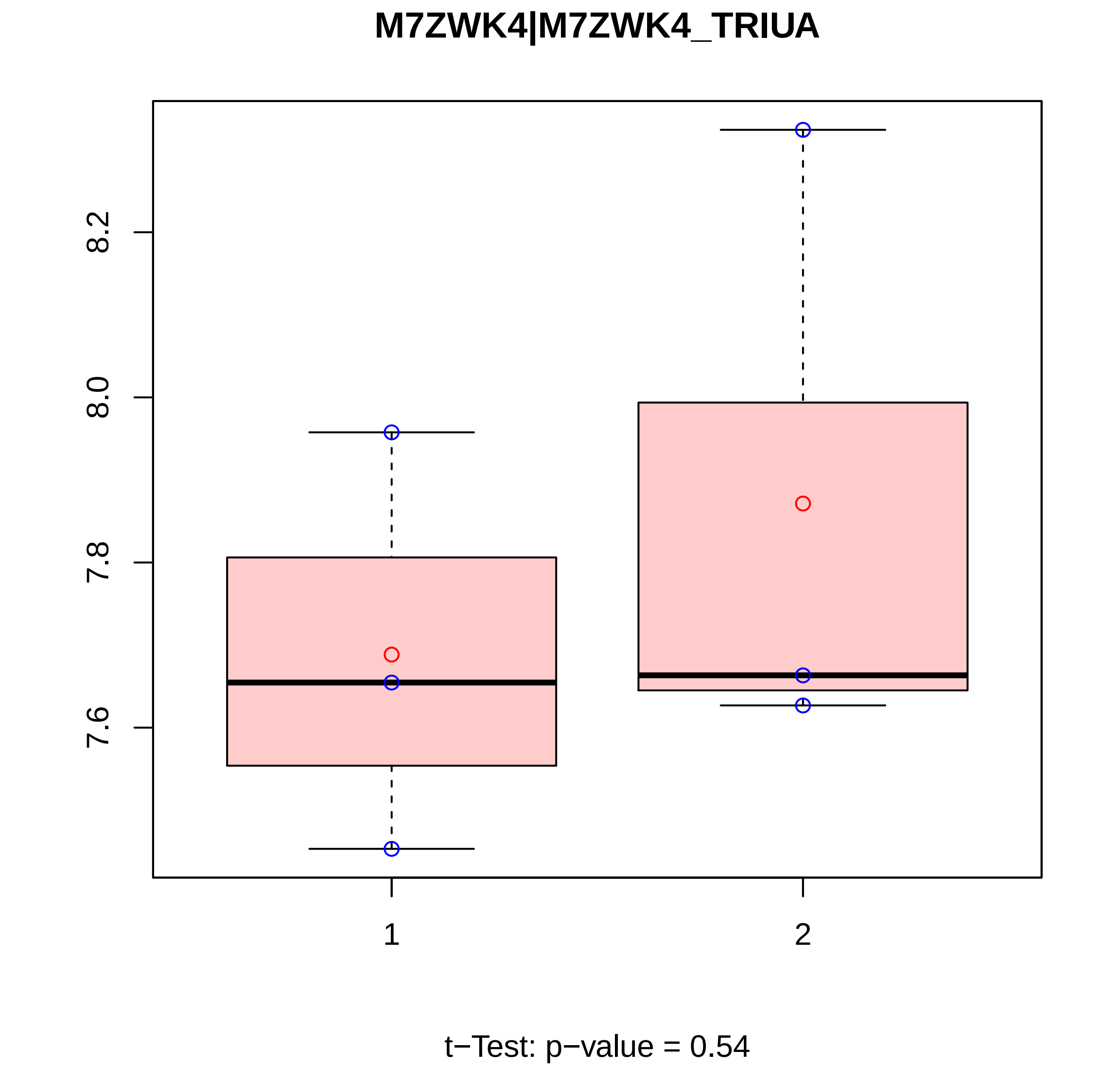
M7ZWK4|M7ZWK4_TRIUA
8.2
8.0
7.8
7.6
1
2
t−Test: p−value = 0.54

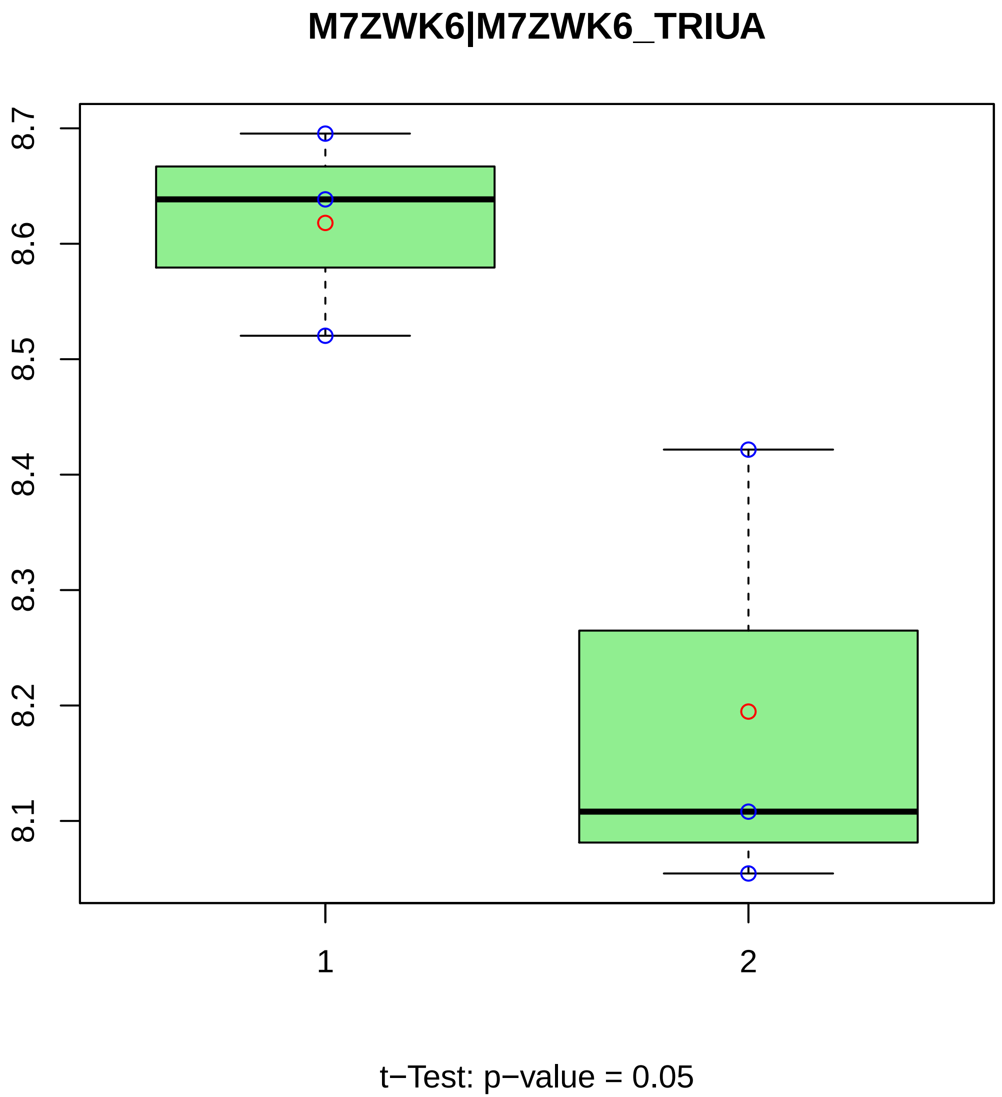

M7ZWK6|M7ZWK6_TRIUA
8.1
8.2
8.3
8.4
8.5
8.6
8.7
1
2
t−Test: p−value = 0.05

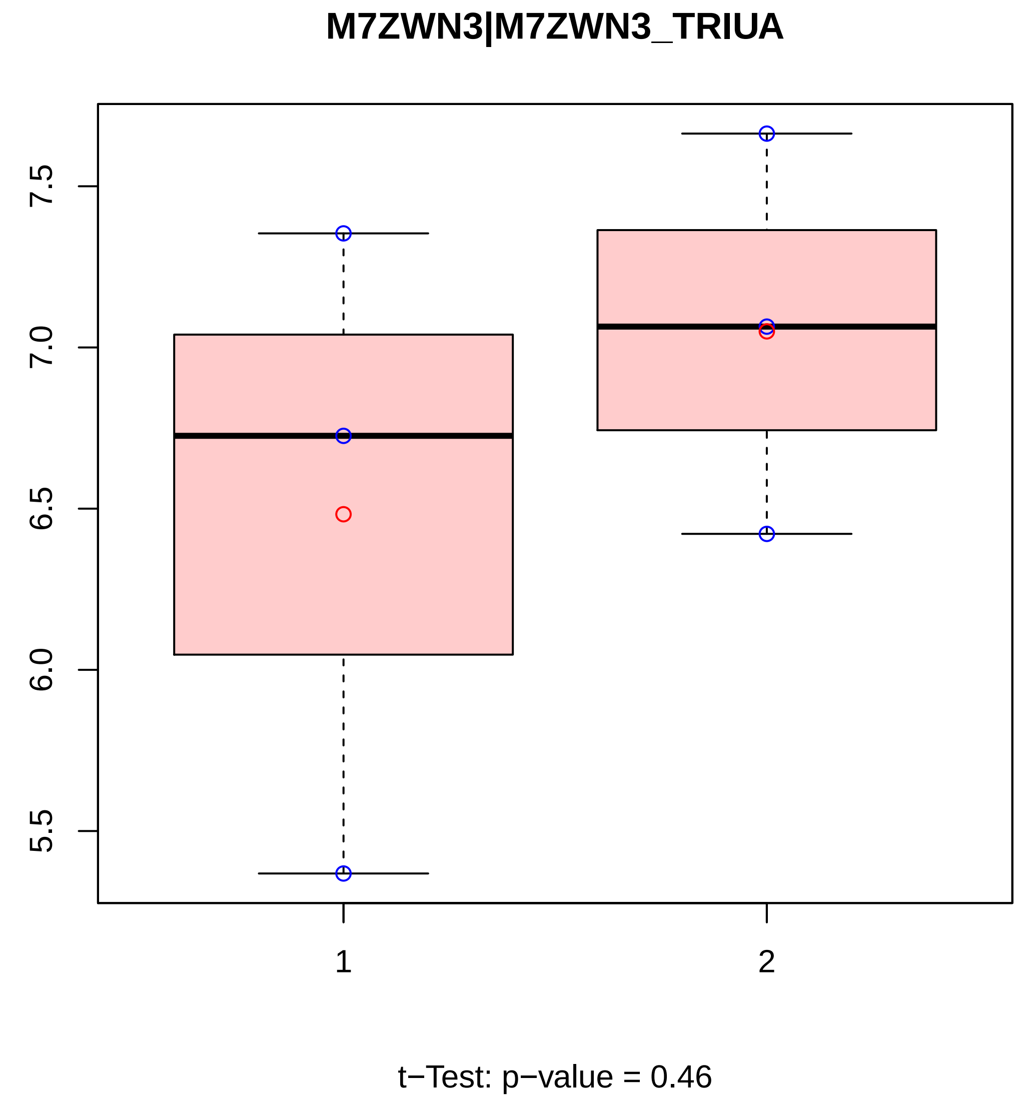
M7ZWN3|M7ZWN3_TRIUA
7.5
7.0
6.5
6.0
5.5
1
2
t−Test: p−value = 0.46

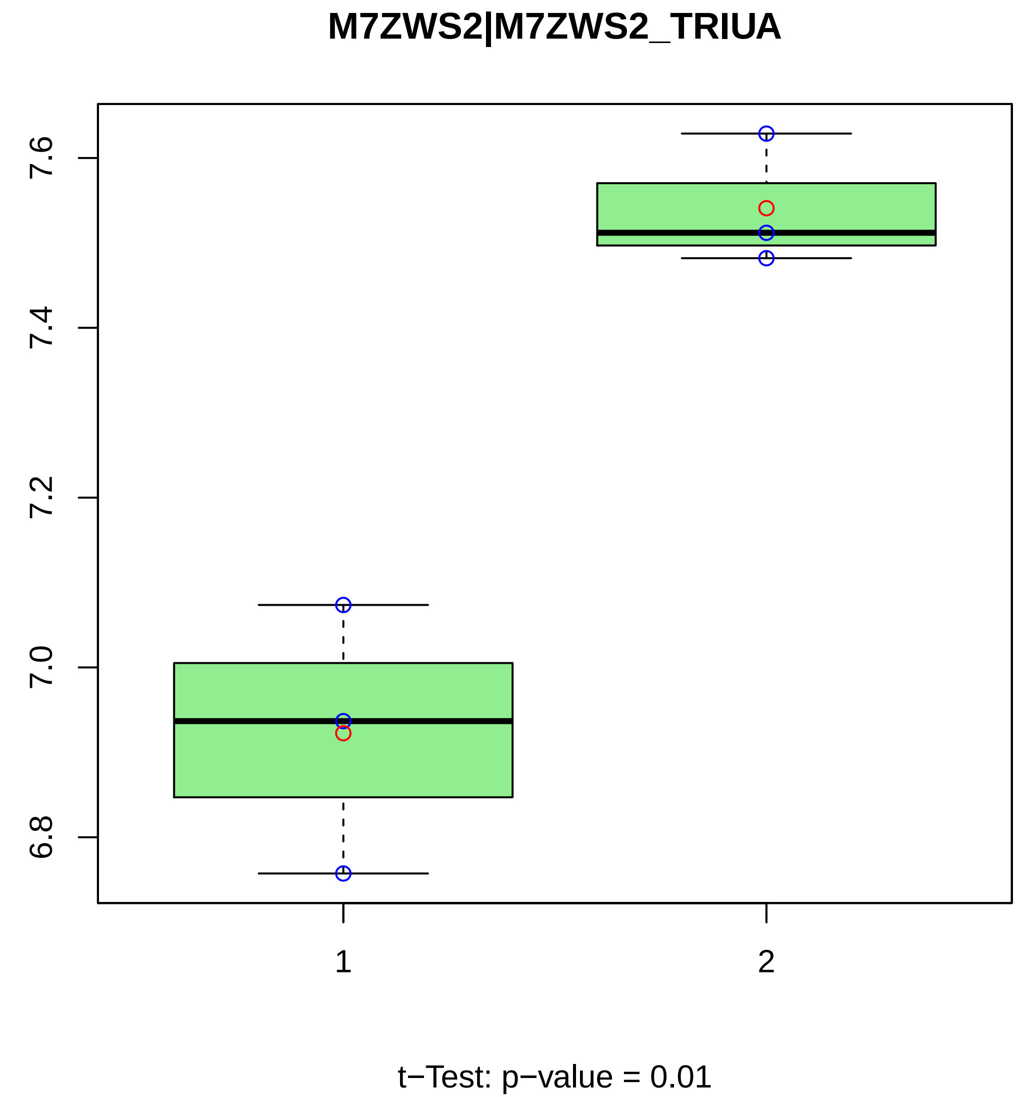
M7ZWS2|M7ZWS2_TRIUA
7.6
7.4
7.2
7.0
6.8
1
2
t−Test: p−value = 0.01

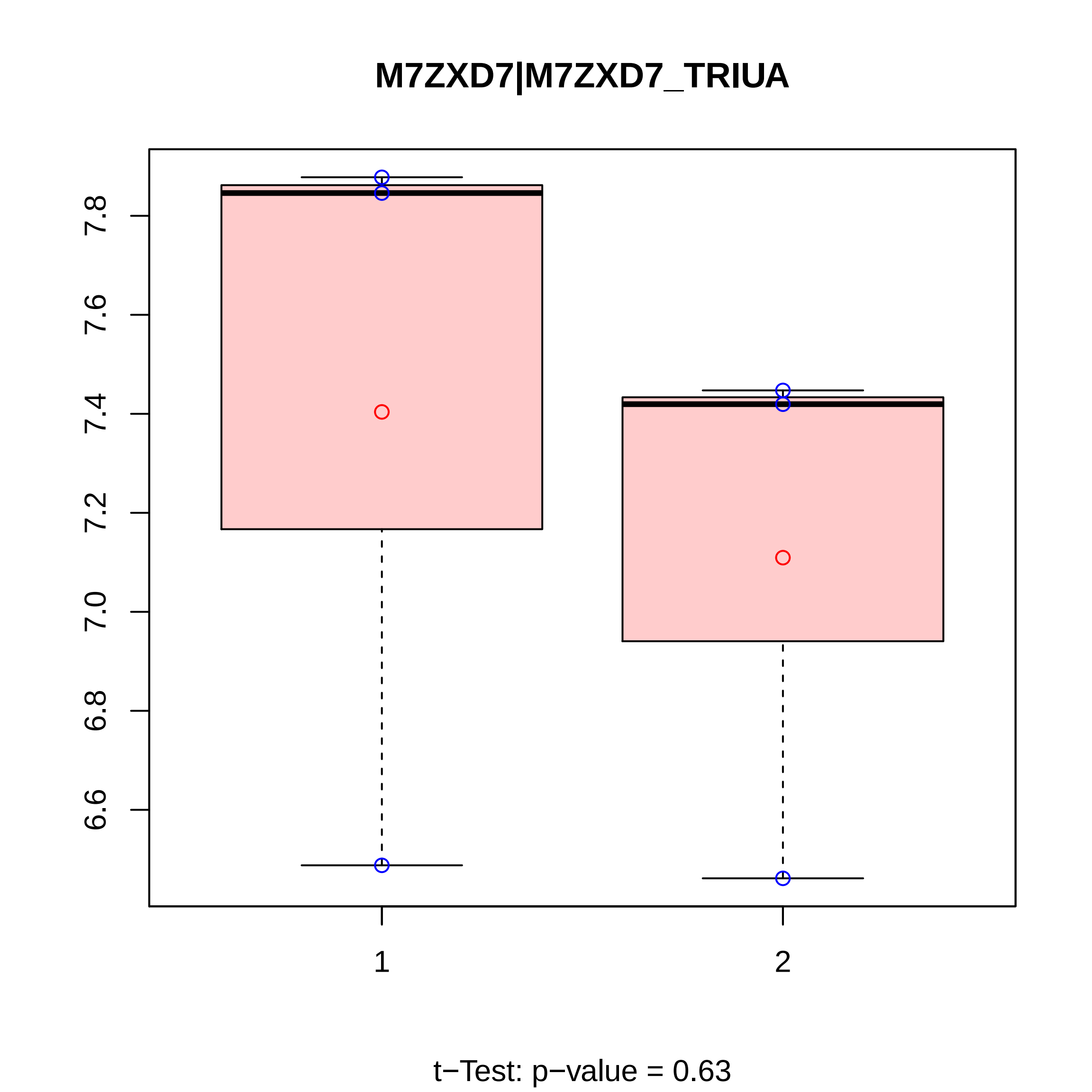
M7ZXD7|M7ZXD7_TRIUA
7.8
7.6
7.4
7.2
7.0
6.8
6.6
1
2
t−Test: p−value = 0.63

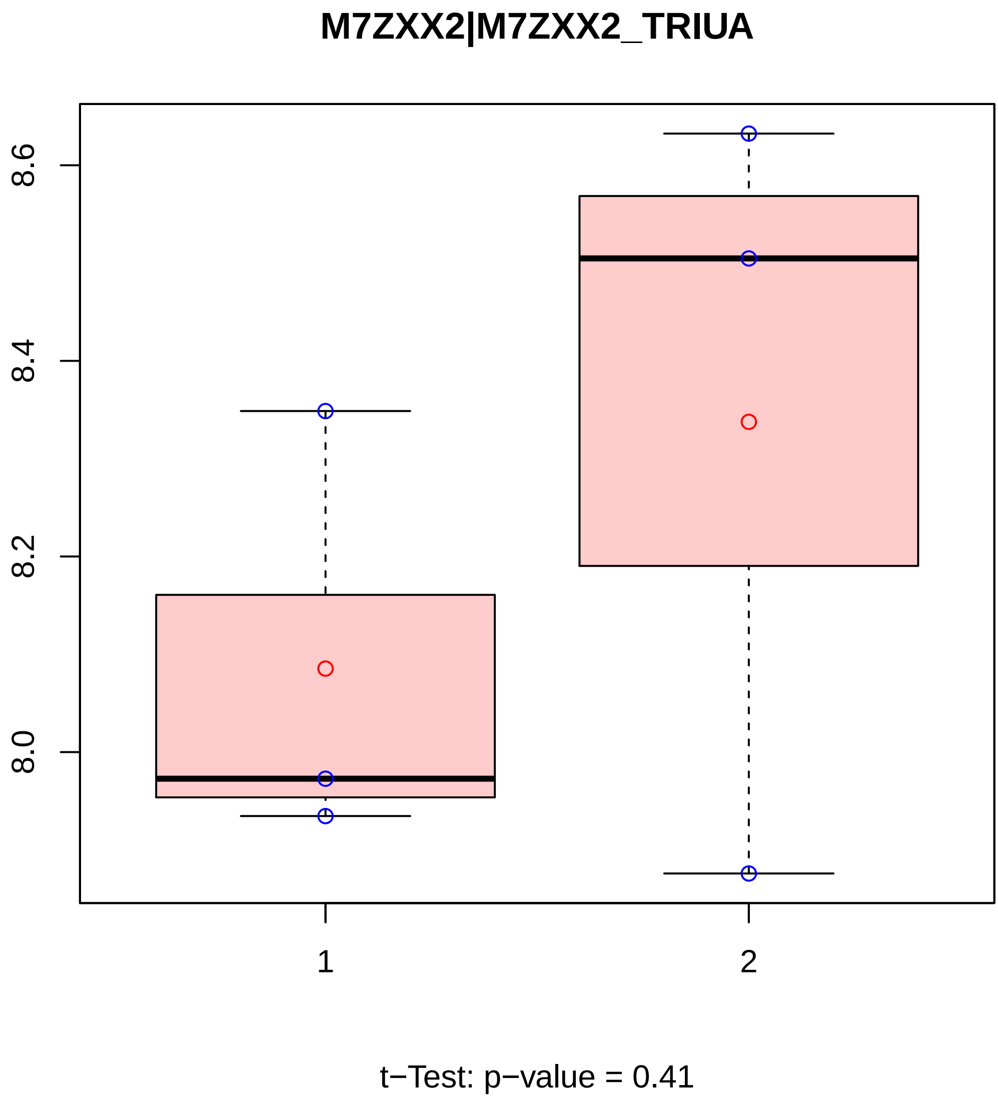
M7ZXX2|M7ZXX2_TRIUA
8.0
8.2
8.4
8.6
1
2
t−Test: p−value = 0.41

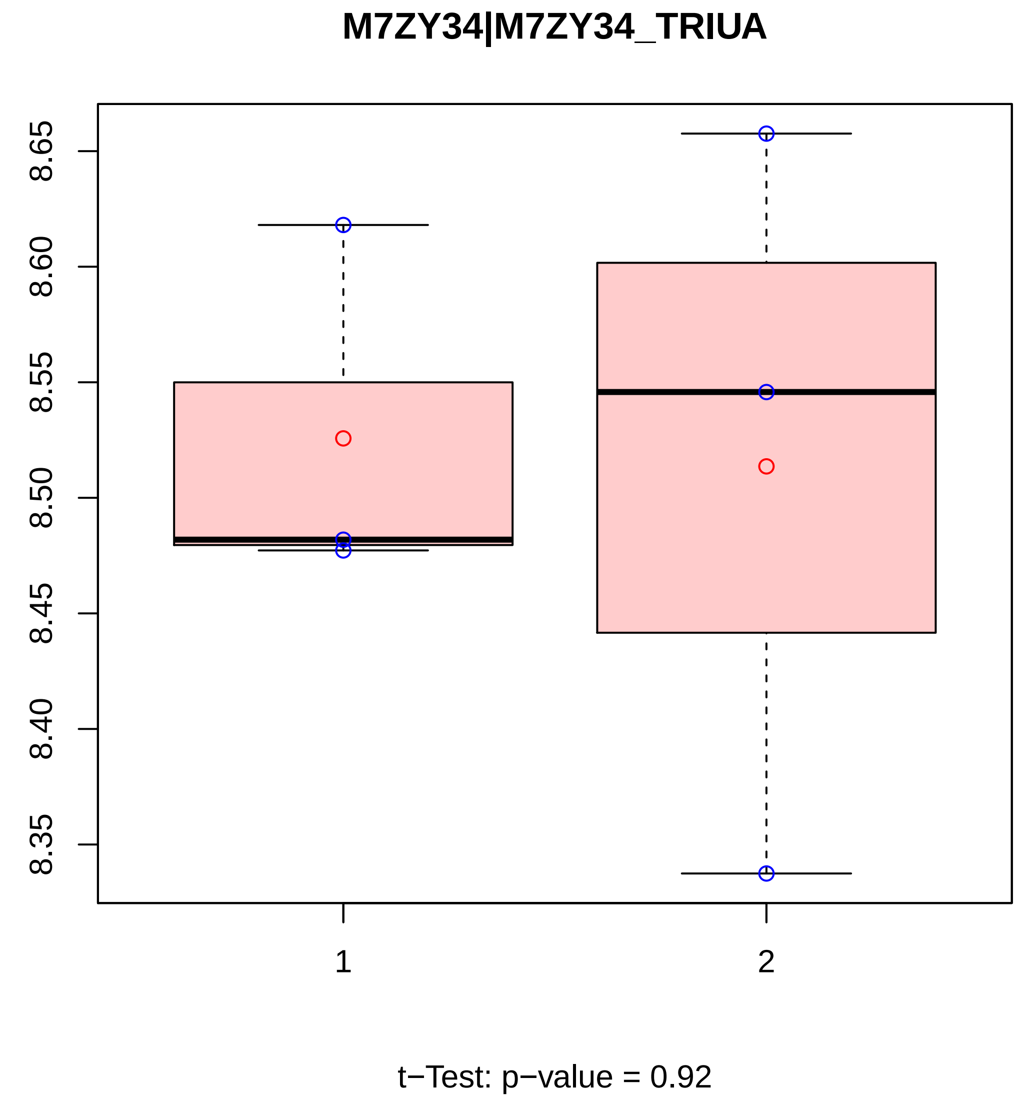

M7ZY34|M7ZY34_TRIUA
8.35
8.40
8.45
8.50
8.55
8.60
8.65
1
2
t−Test: p−value = 0.92

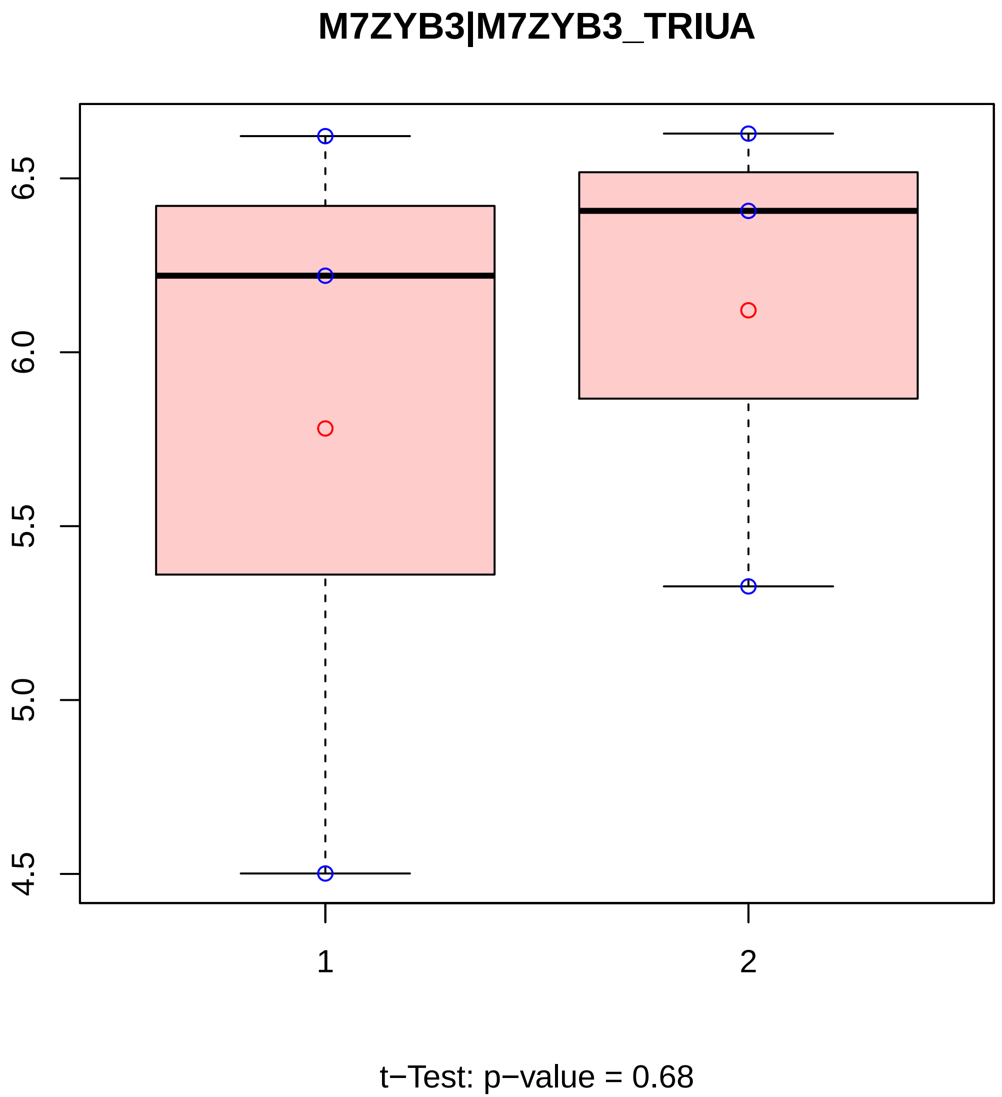
M7ZYB3|M7ZYB3_TRIUA
6.5
6.0
5.5
5.0
4.5
1
2
t−Test: p−value = 0.68

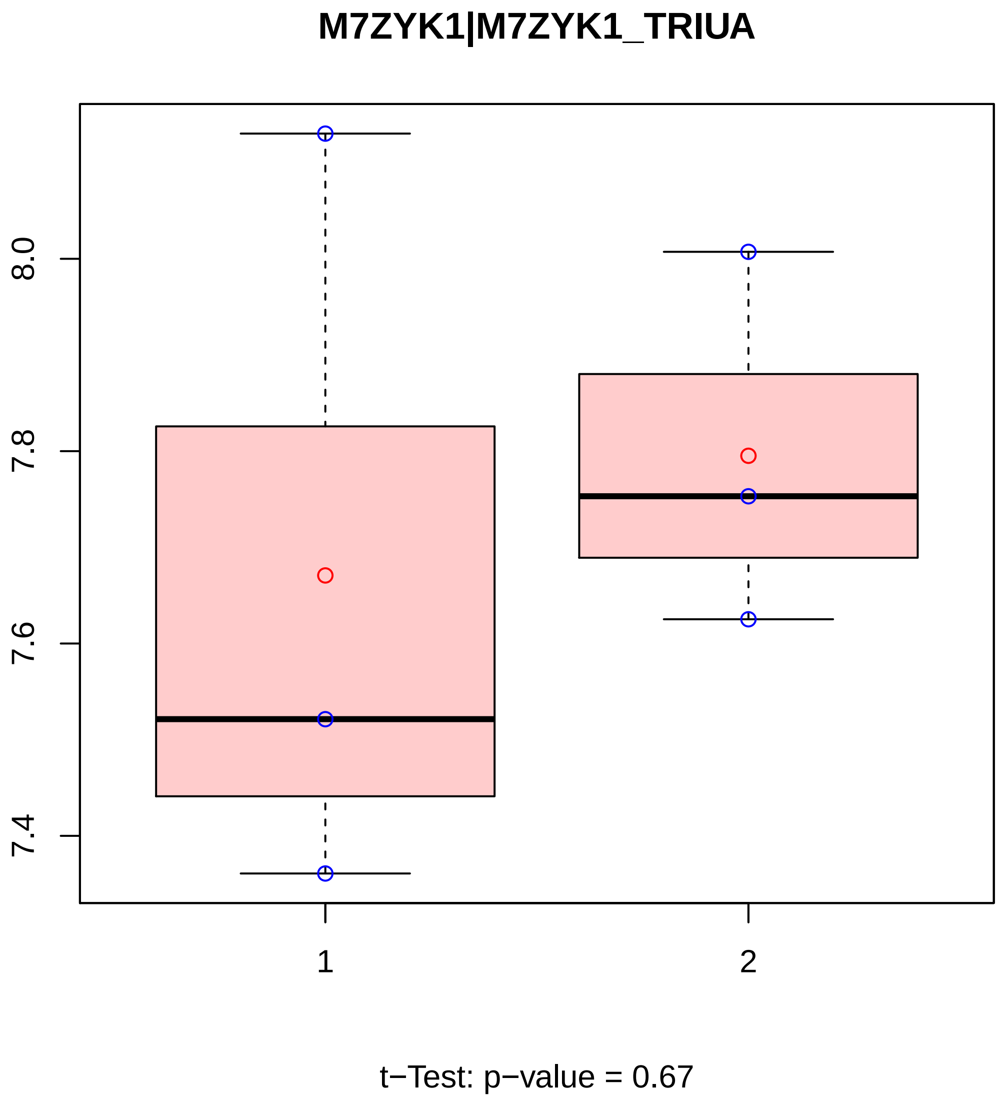
M7ZYK1|M7ZYK1_TRIUA
8.0
7.8
7.6
7.4
1
2
t−Test: p−value = 0.67

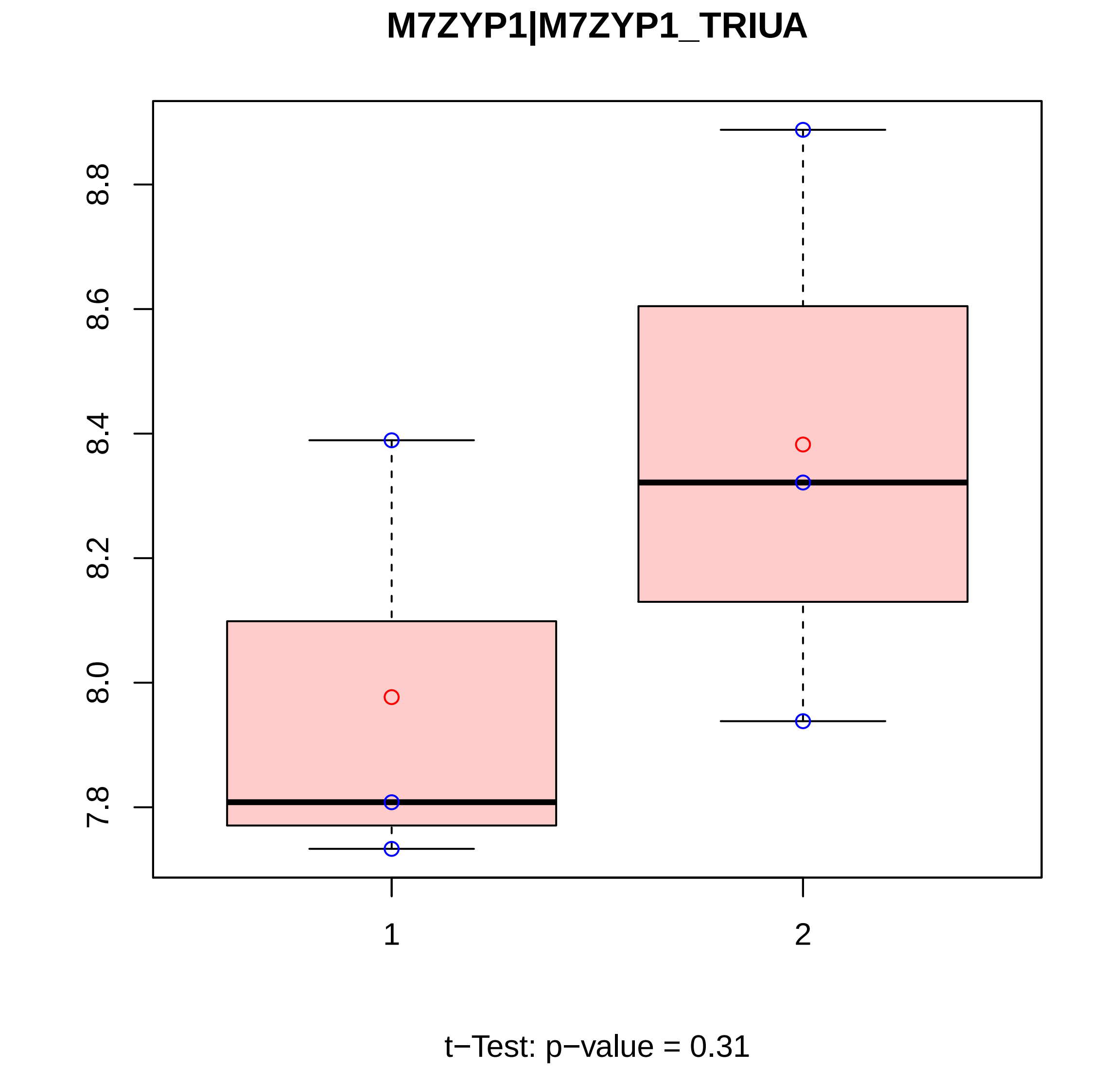

M7ZYP1|M7ZYP1_TRIUA
7.8
8.0
8.2
8.4
8.6
8.8
1
2
t−Test: p−value = 0.31

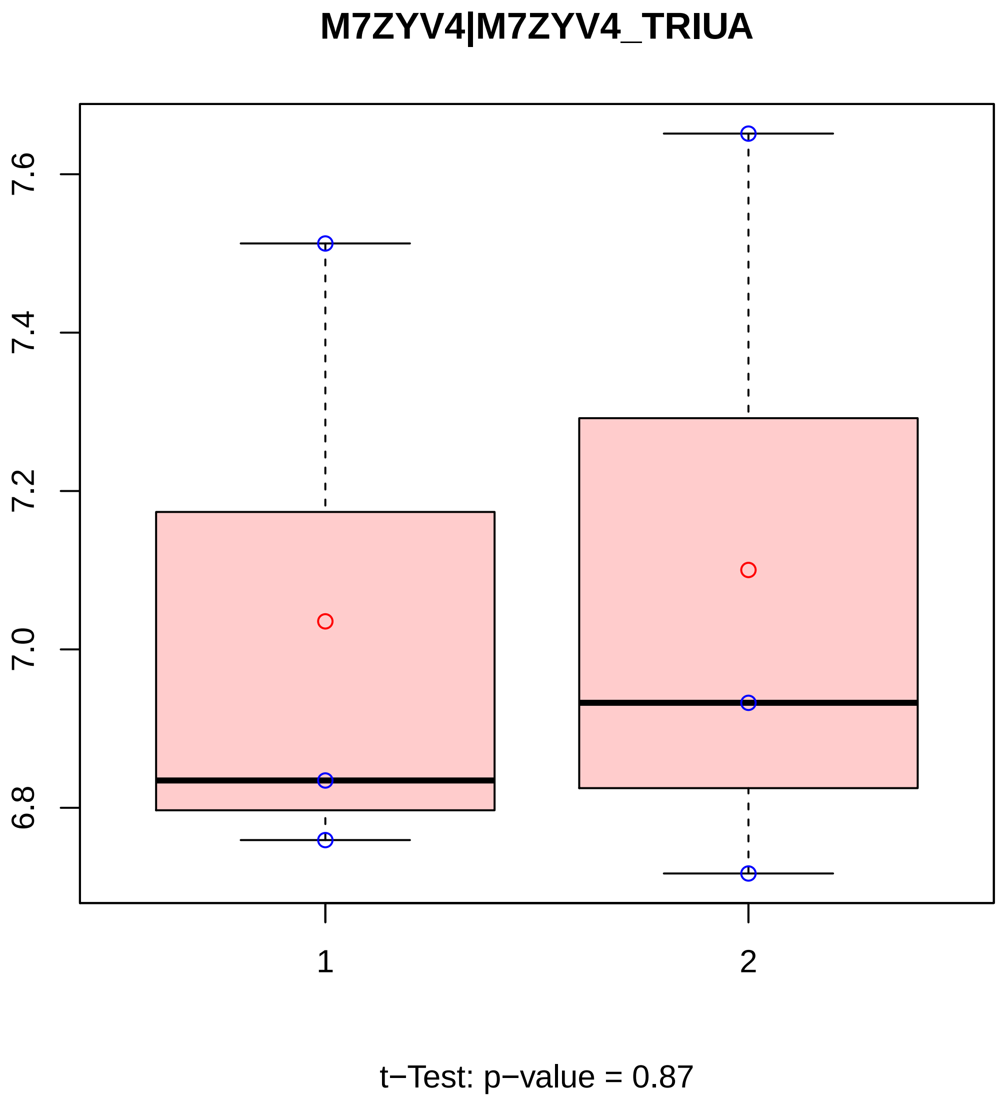
M7ZYV4|M7ZYV4_TRIUA
7.6
7.4
7.2
7.0
6.8
1
2
t−Test: p−value = 0.87

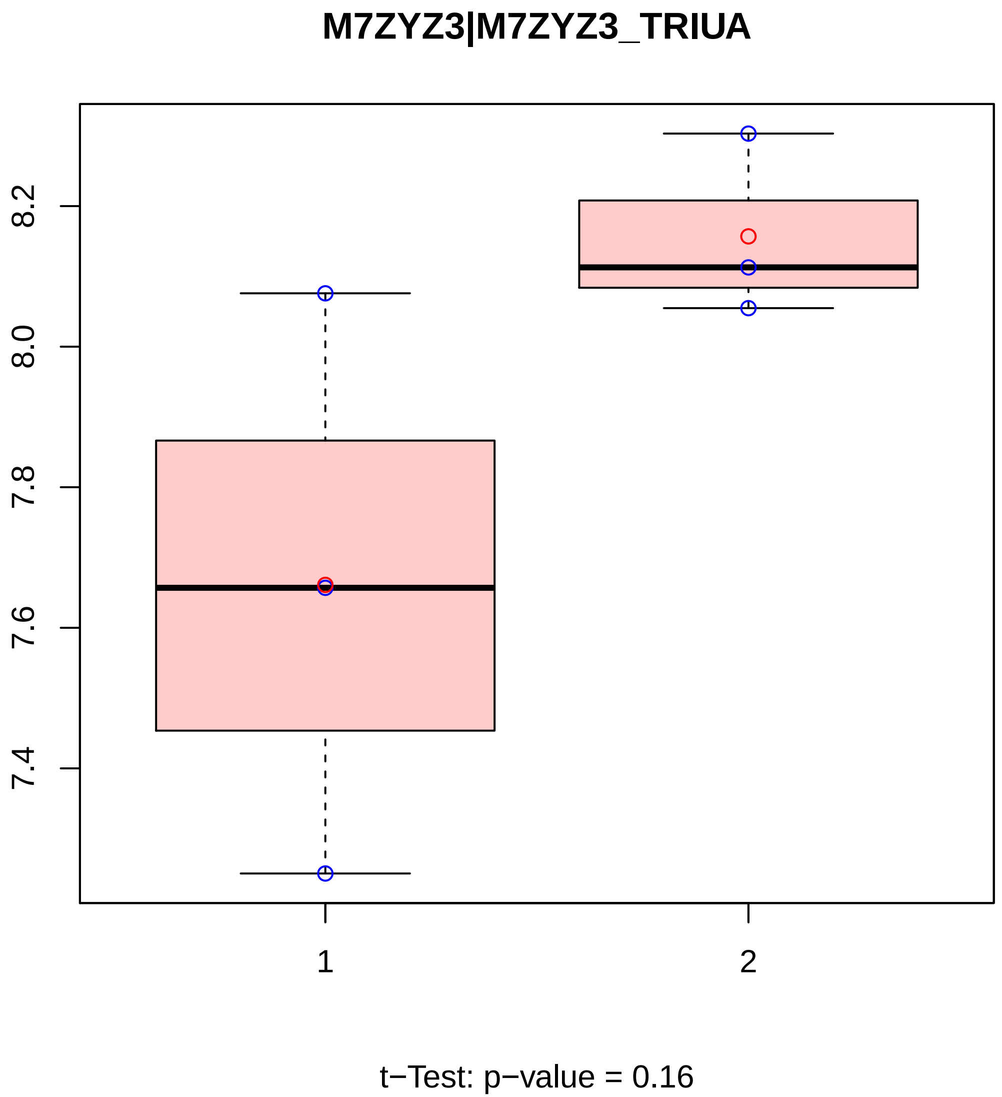
M7ZYZ3|M7ZYZ3_TRIUA
8.2
8.0
7.8
7.6
7.4
1
2
t−Test: p−value = 0.16

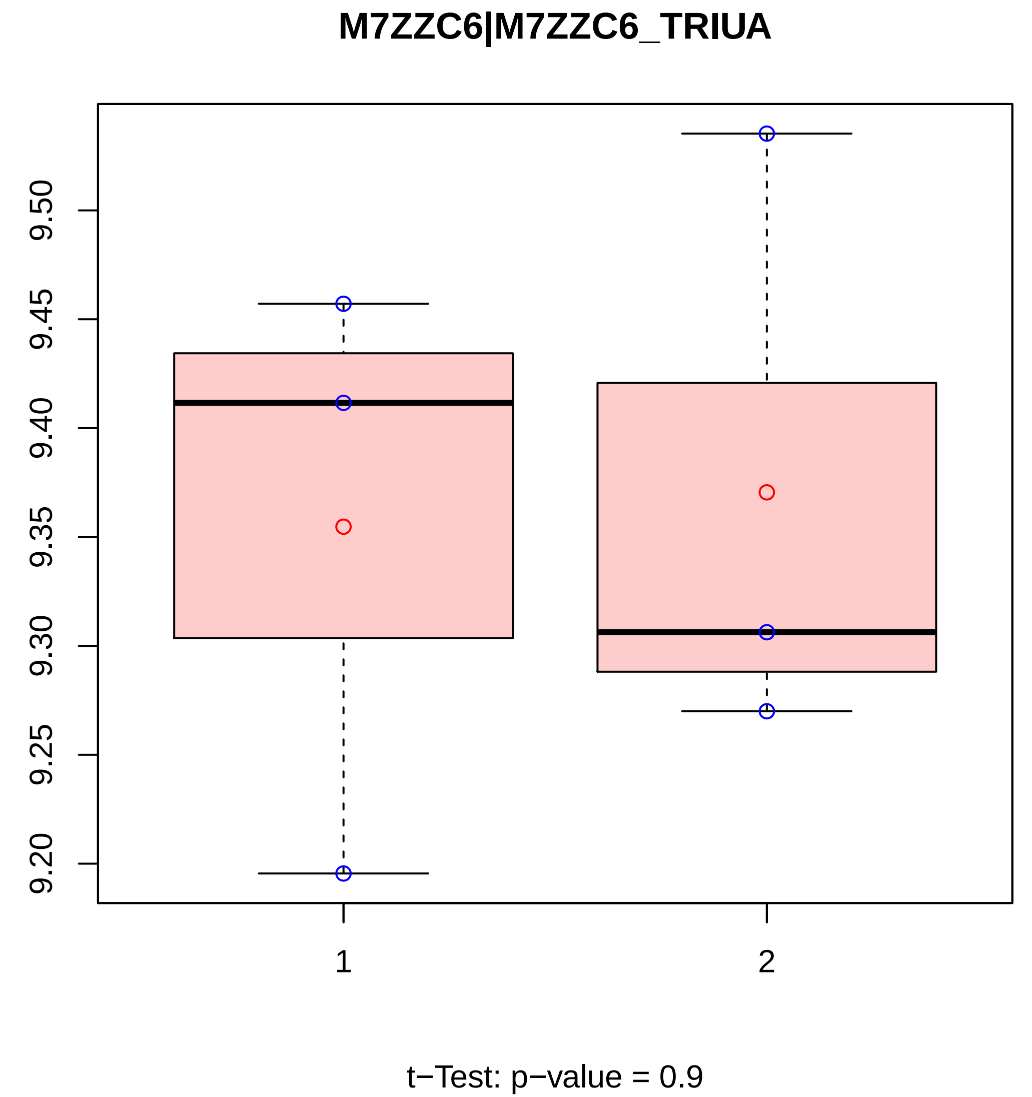
M7ZZC6|M7ZZC6_TRIUA
9.20
9.25
9.30
9.35
9.40
9.45
9.50
1
2
t−Test: p−value = 0.9

M7ZZK7|M7ZZK7_TRIUA

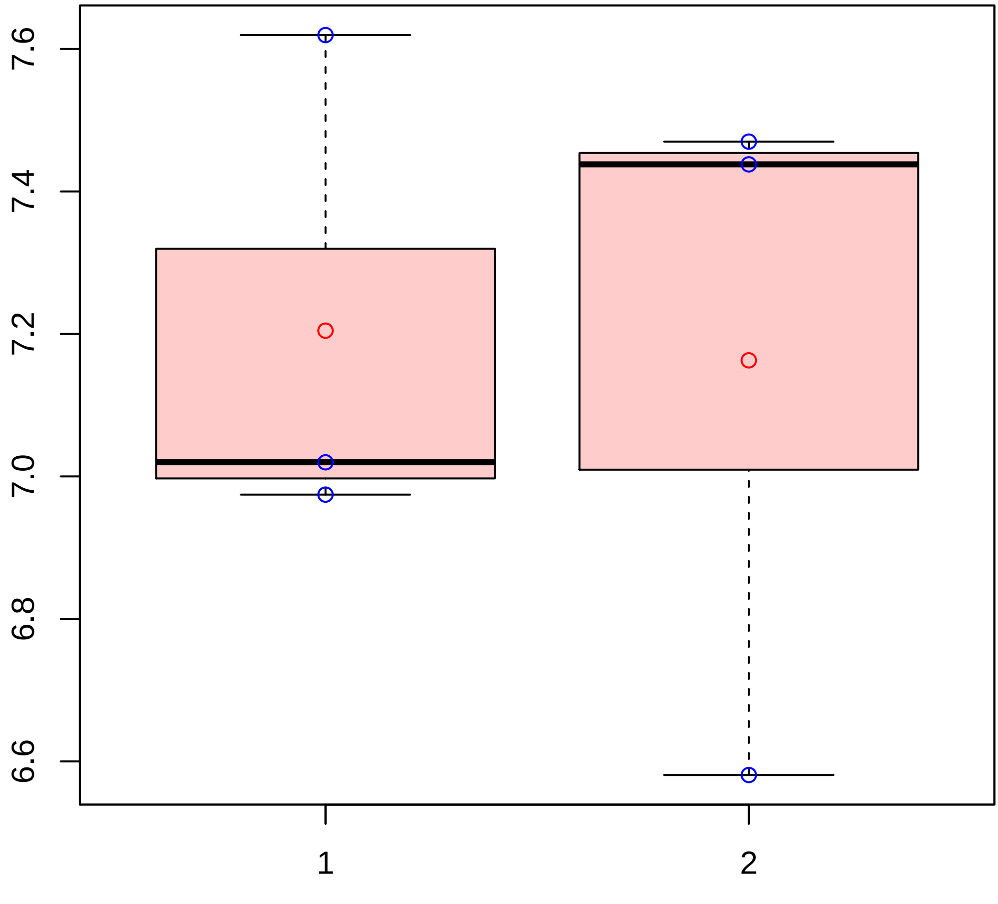

t−Test: p−value = 0.91

M8A0S1|M8A0S1_TRIUA

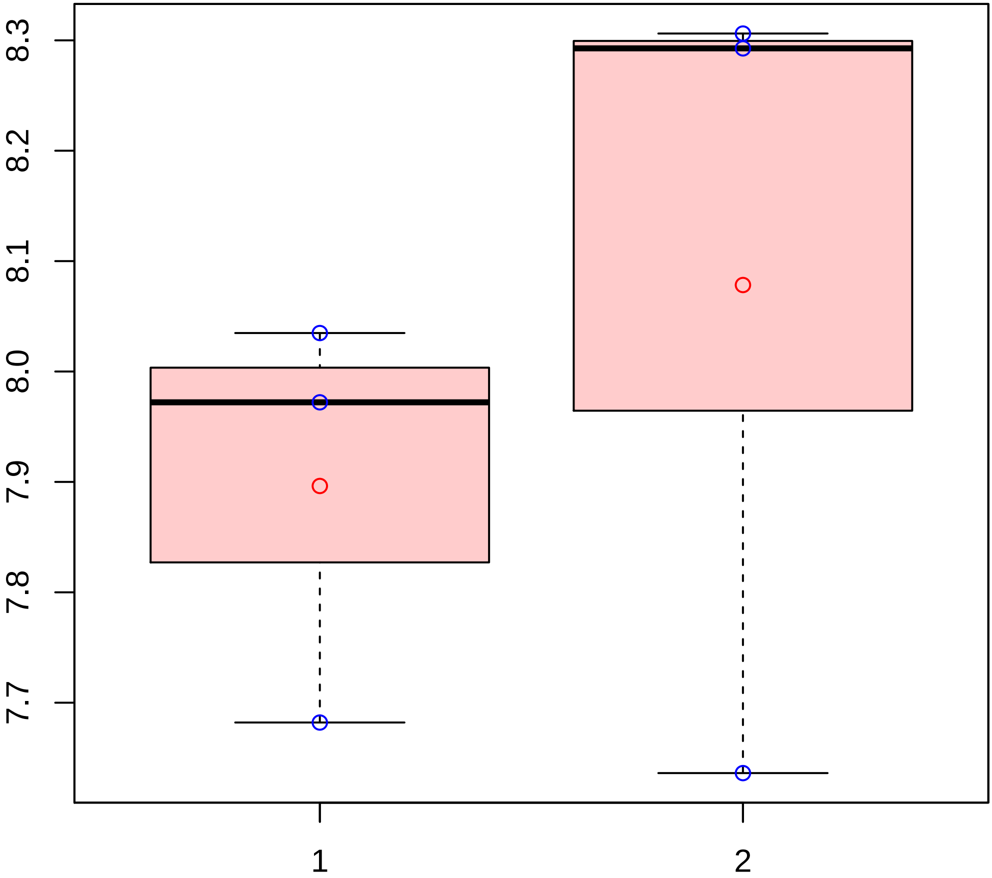

t−Test: p−value = 0.51

M8A257|M8A257_TRIUA

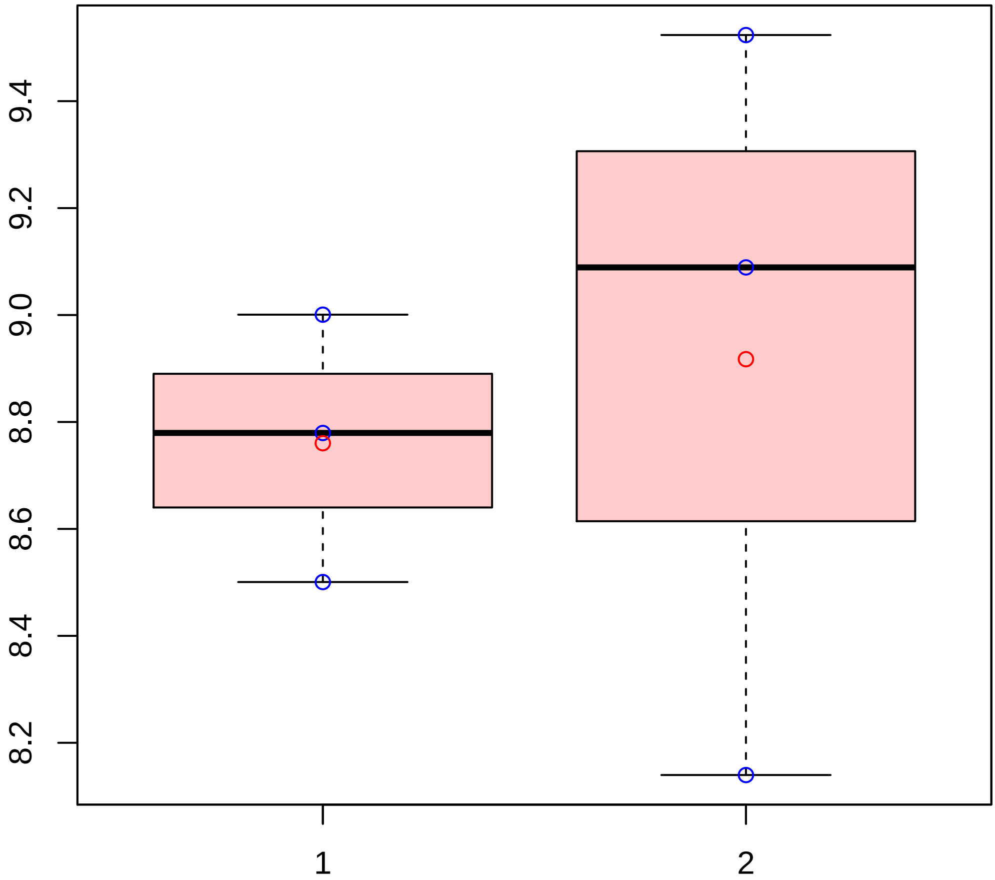

t−Test: p−value = 0.75

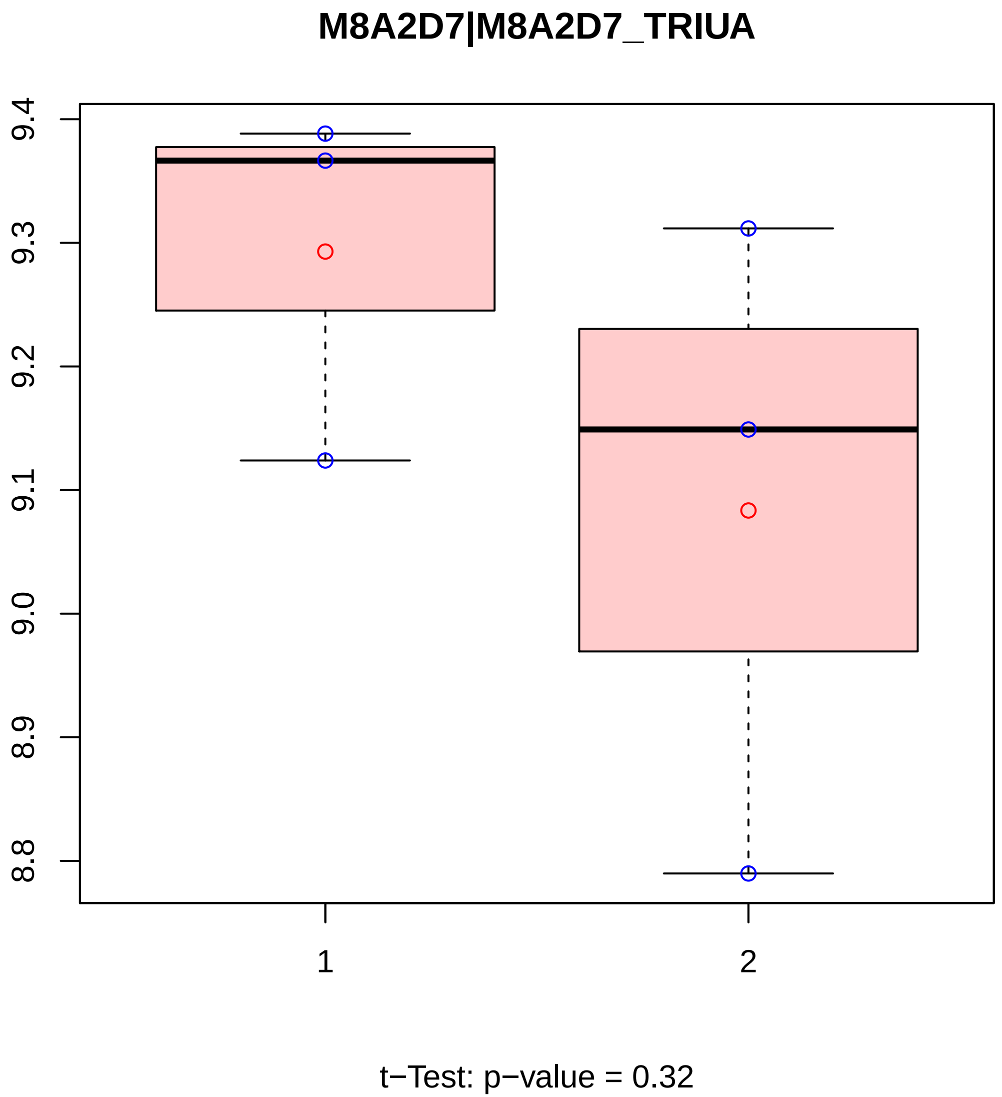

M8A2D7|M8A2D7_TRIUA
9.4
9.3
9.2
9.1
9.0
8.9
8.8
1
2
t−Test: p−value = 0.32

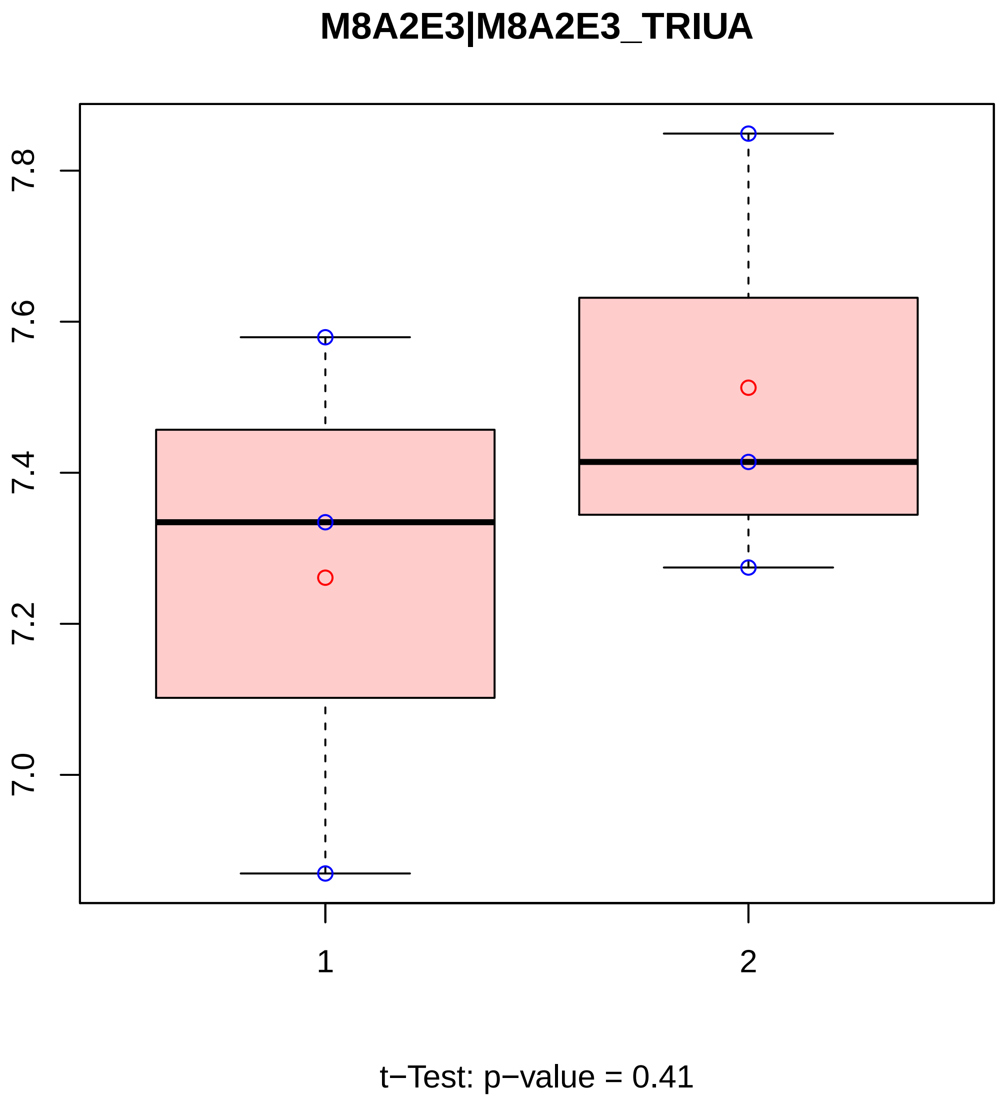

M8A2E3|M8A2E3_TRIUA
7.0
7.2
7.4
7.6
7.8
1
2
t−Test: p−value = 0.41

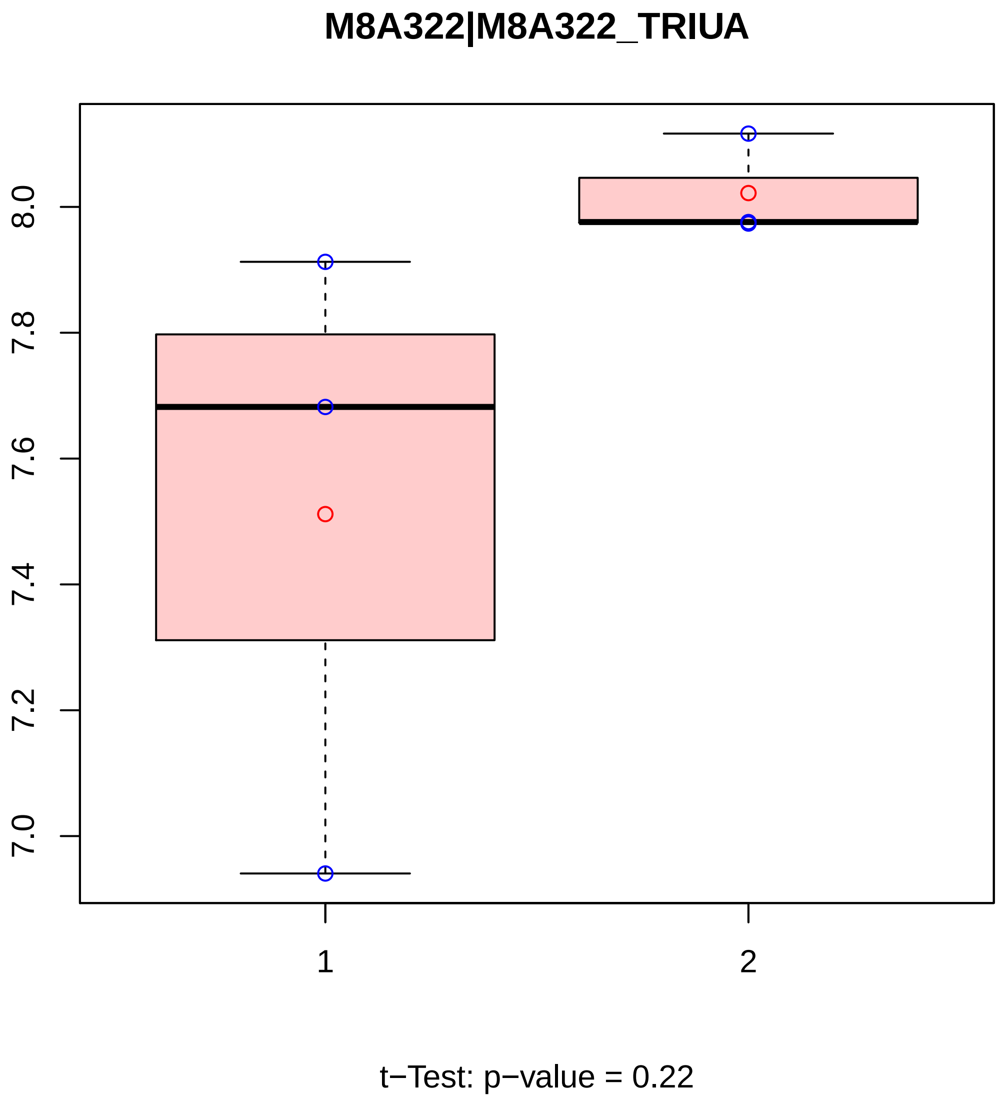
M8A322|M8A322_TRIUA
7.0
7.2
7.4
7.6
7.8
8.0
1
2
t−Test: p−value = 0.22

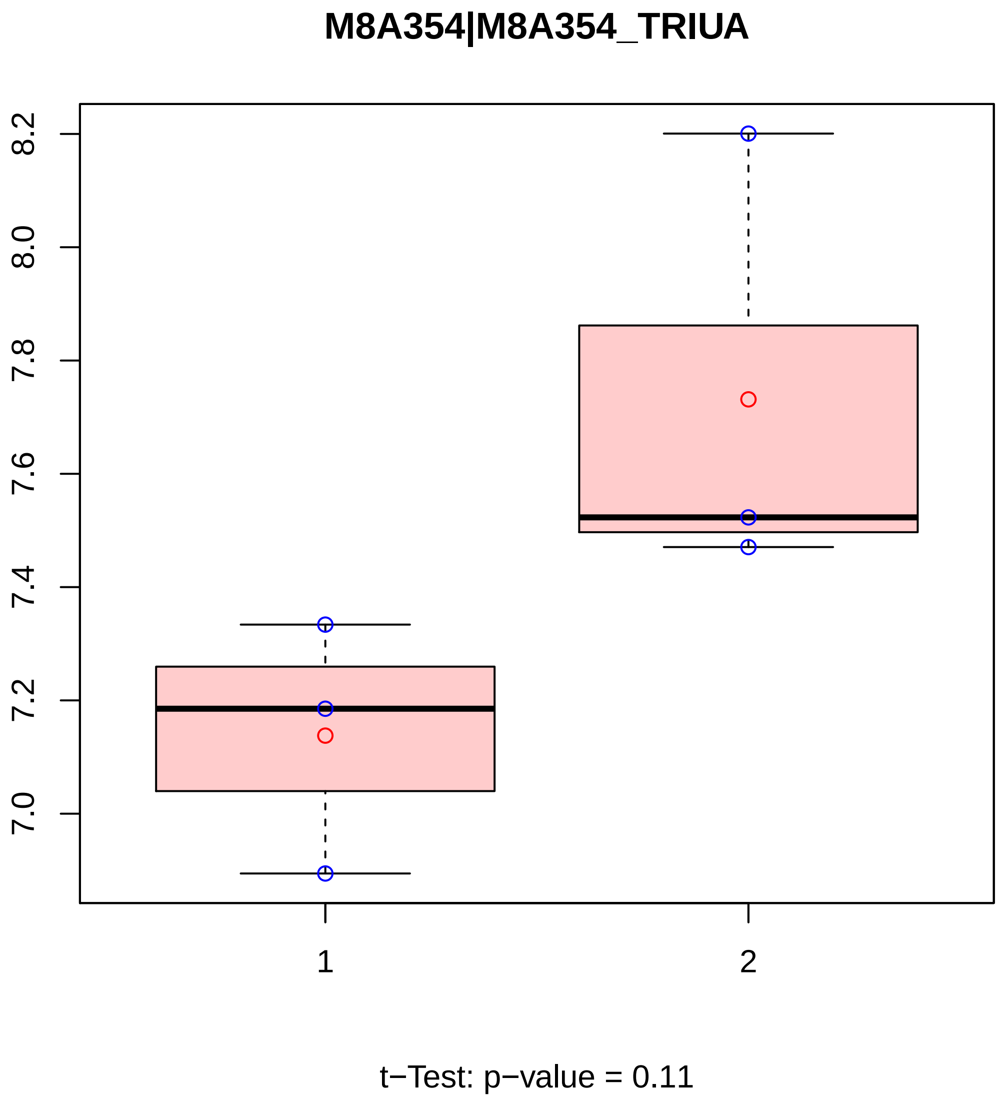
M8A354|M8A354_TRIUA
8.2
8.0
7.8
7.6
7.4
7.2
7.0
1
2
t−Test: p−value = 0.11

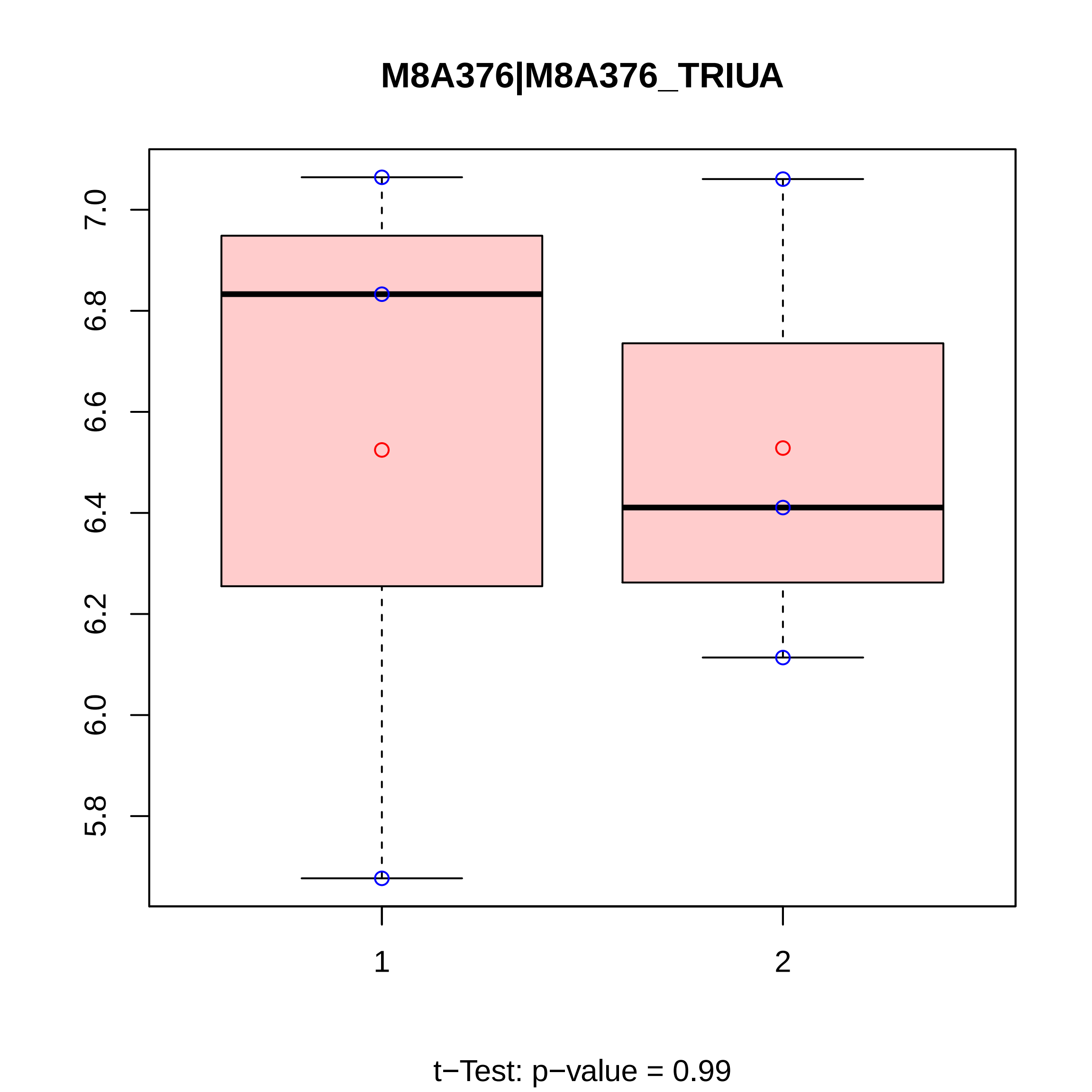
M8A376|M8A376_TRIUA
7.0
6.8
6.6
6.4
6.2
6.0
5.8
1
2
t−Test: p−value = 0.99

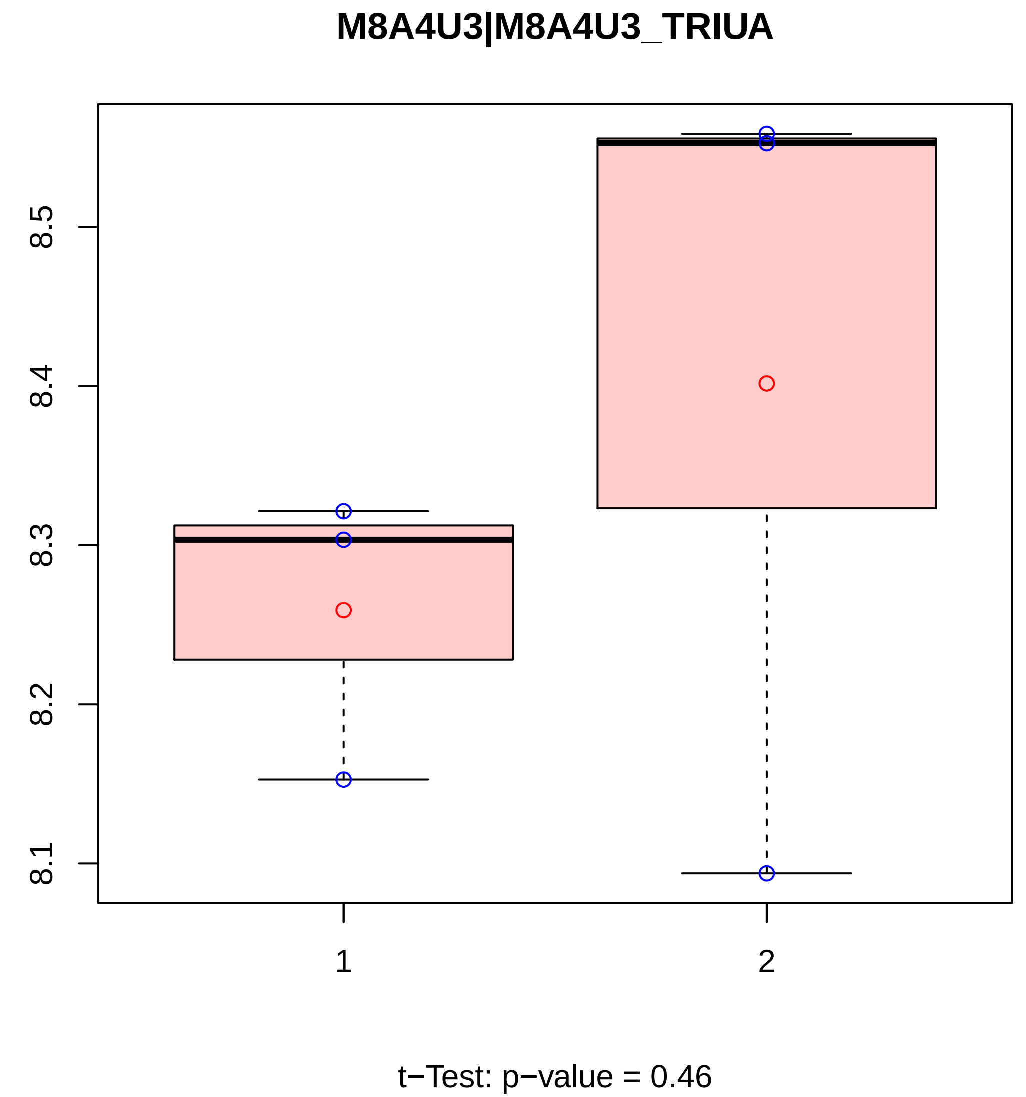

M8A4U3|M8A4U3_TRIUA
8.1
8.2
8.3
8.4
8.5
1
2
t−Test: p−value = 0.46

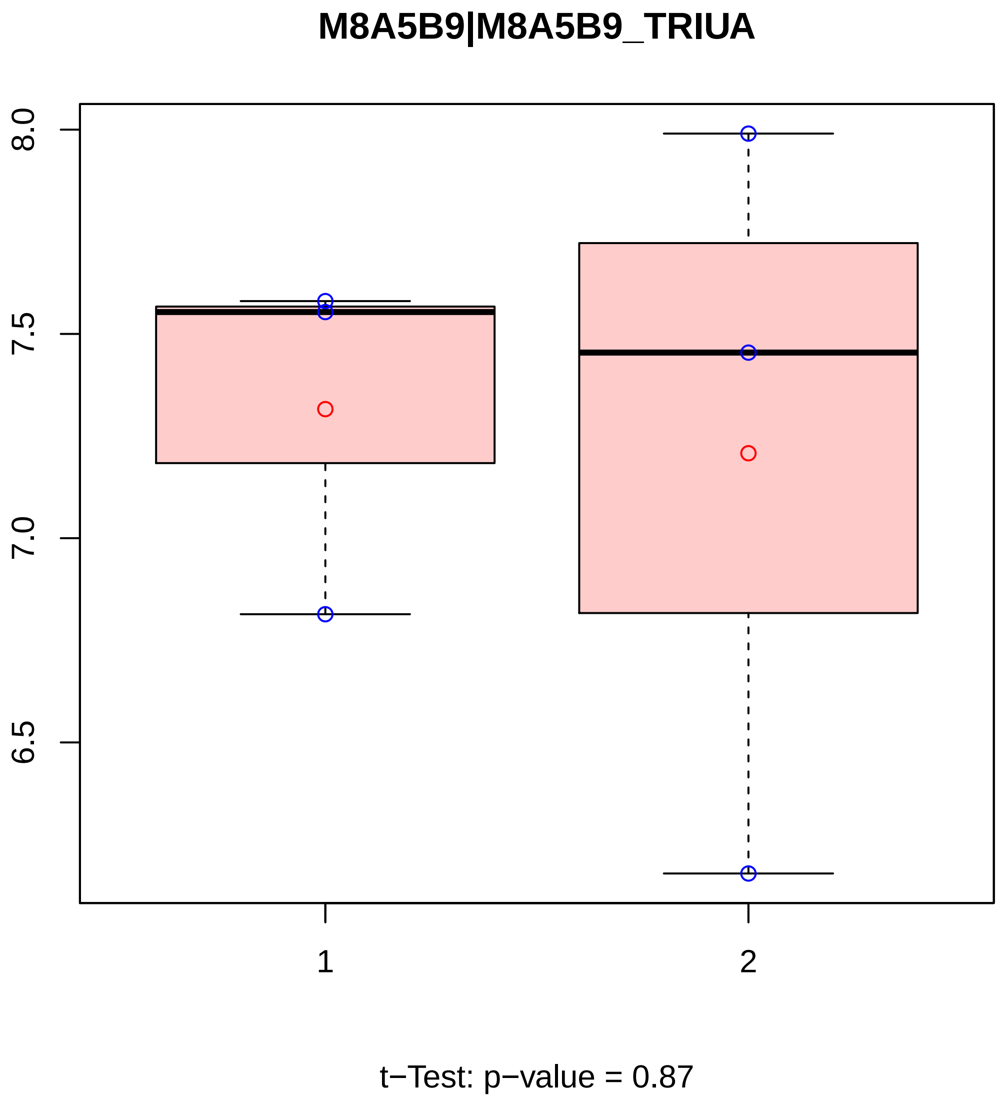
M8A5B9|M8A5B9_TRIUA
8.0
7.5
7.0
6.5
1
2
t−Test: p−value = 0.87

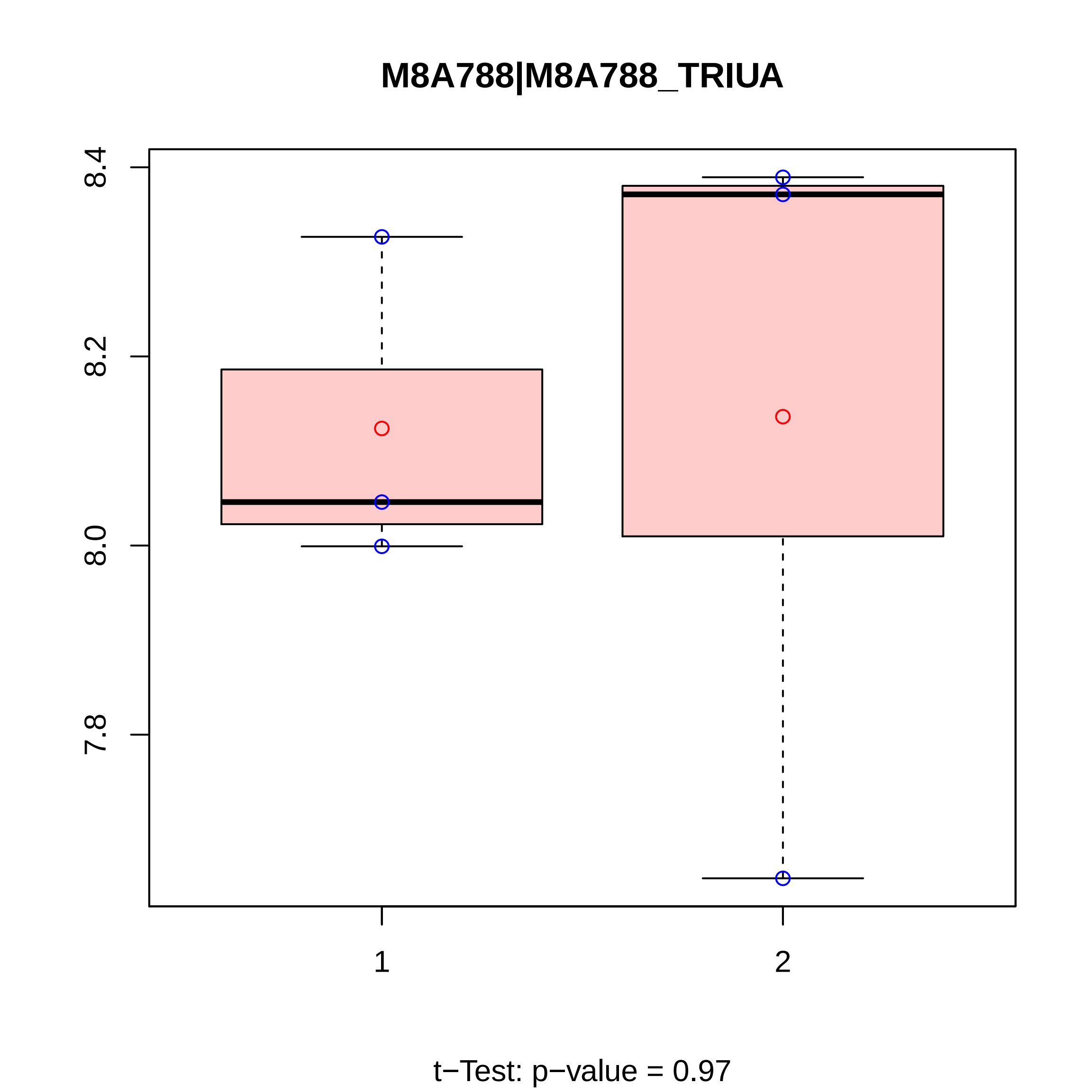
M8A788|M8A788_TRIUA
8.4
8.2
8.0
7.8
1
2
t−Test: p−value = 0.97

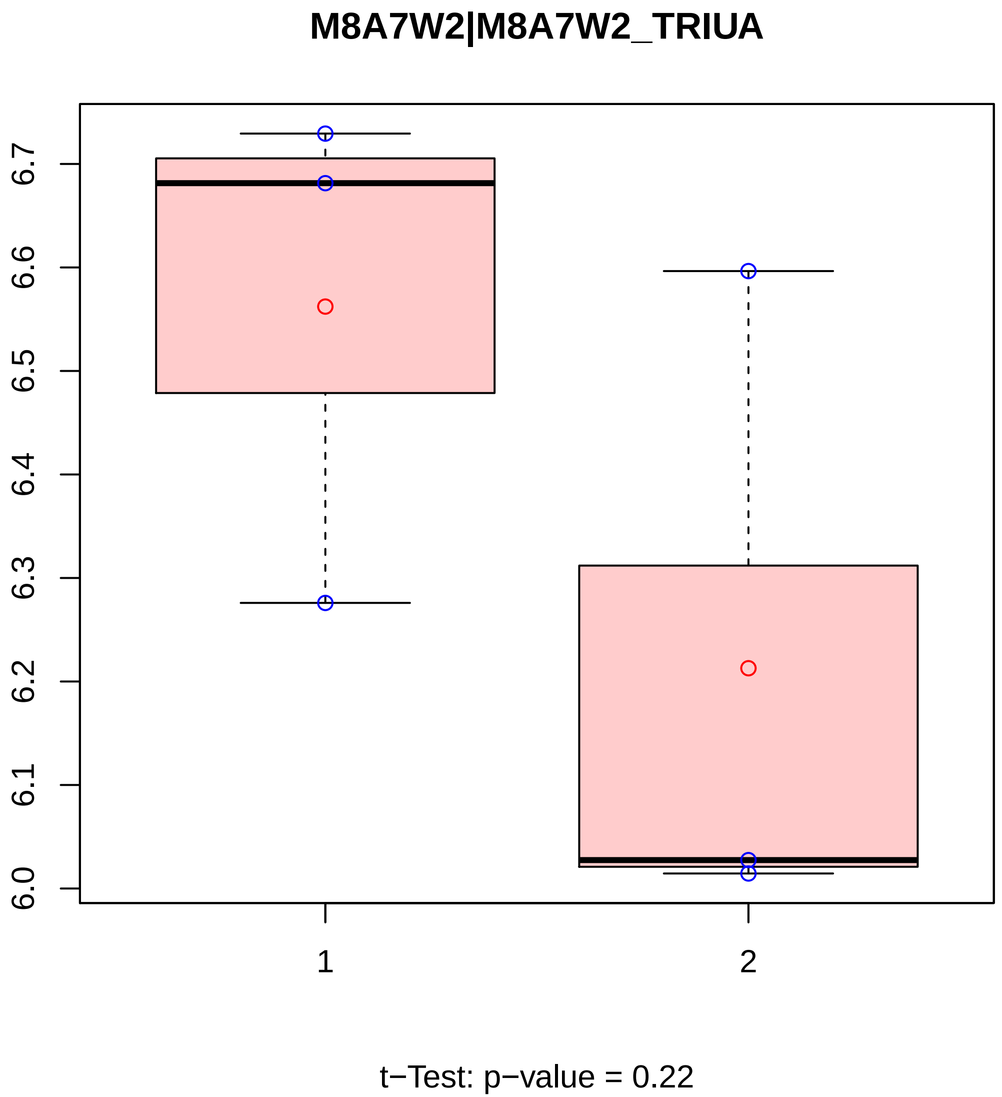

M8A7W2|M8A7W2_TRIUA
6.0
6.1
6.2
6.3
6.4
6.5
6.6
6.7
1
2
t−Test: p−value = 0.22

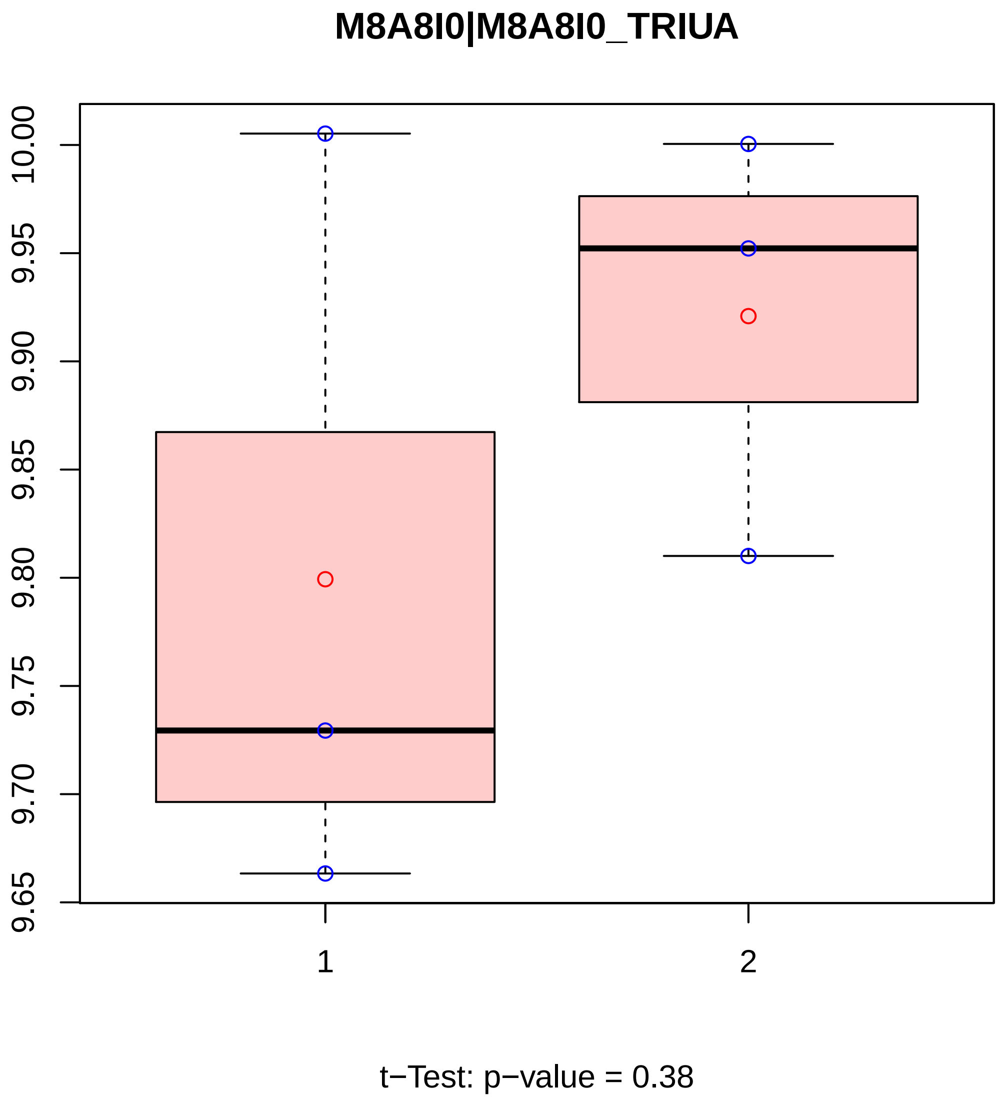
M8A8I0|M8A8I0_TRIUA
10.00
9.95
9.90
9.85
9.80
9.75
9.70
9.65
1
2
t−Test: p−value = 0.38

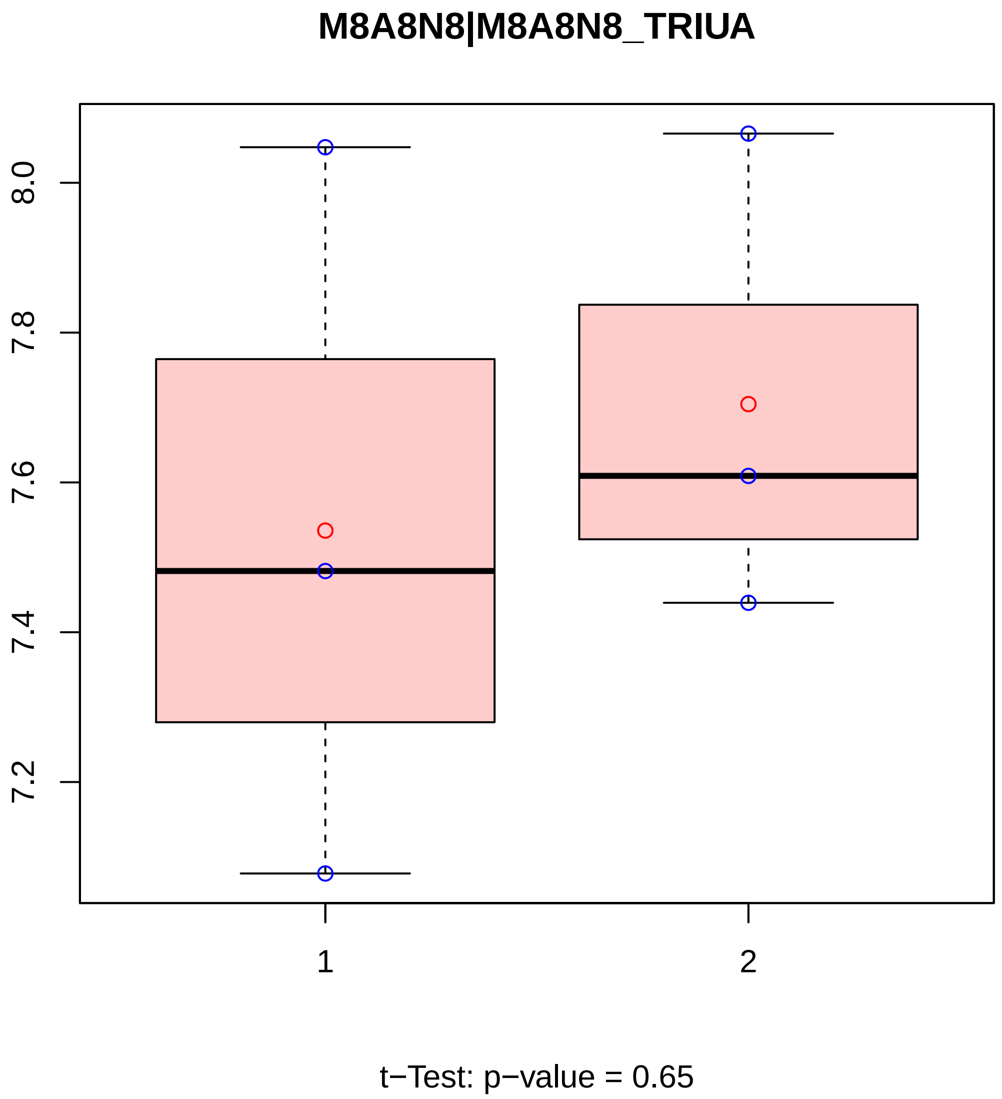
M8A8N8|M8A8N8_TRIUA
8.0
7.8
7.6
7.4
7.2
1
2
t−Test: p−value = 0.65

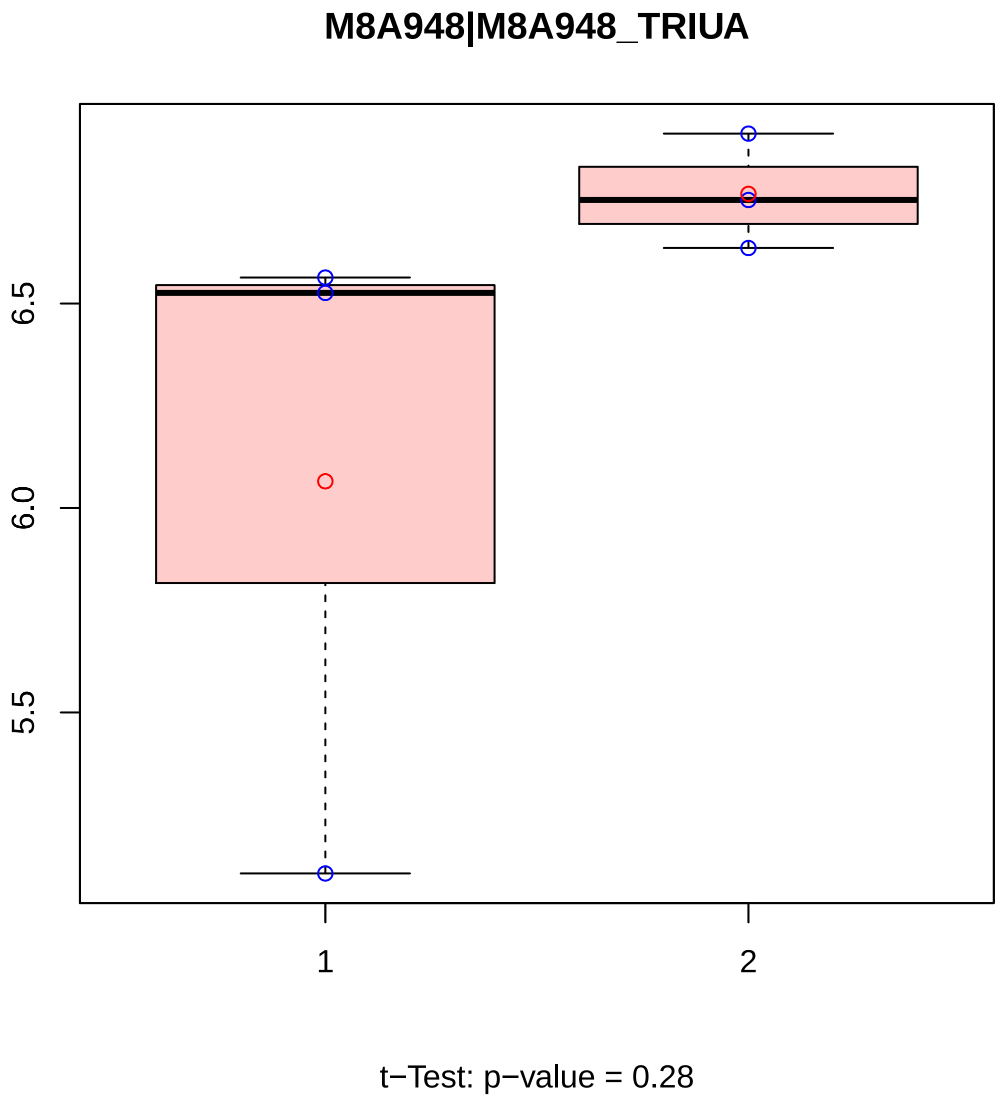

M8A948|M8A948_TRIUA
6.5
6.0
5.5
1
2
t−Test: p−value = 0.28

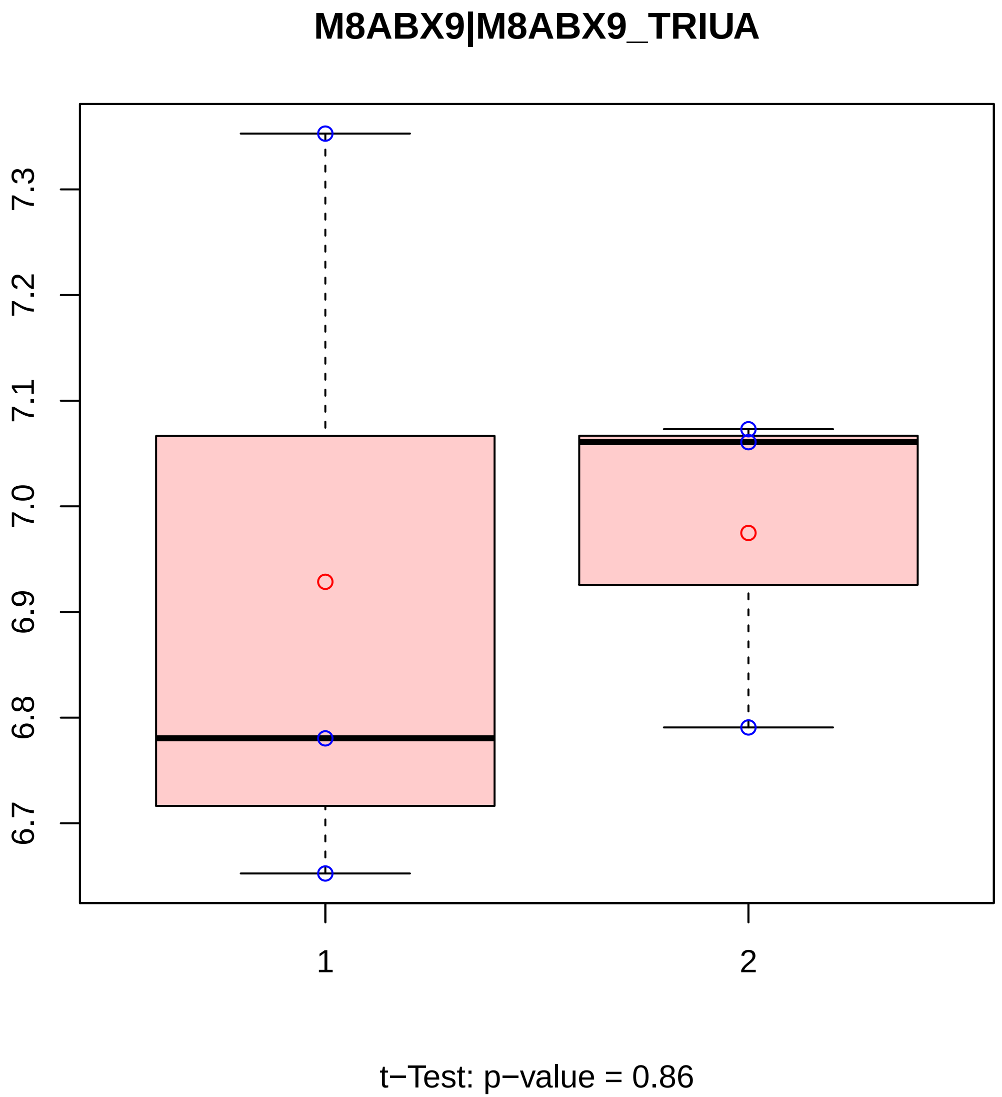
M8ABX9|M8ABX9_TRIUA
7.3
7.2
7.1
7.0
6.9
6.8
6.7
1
2
t−Test: p−value = 0.86

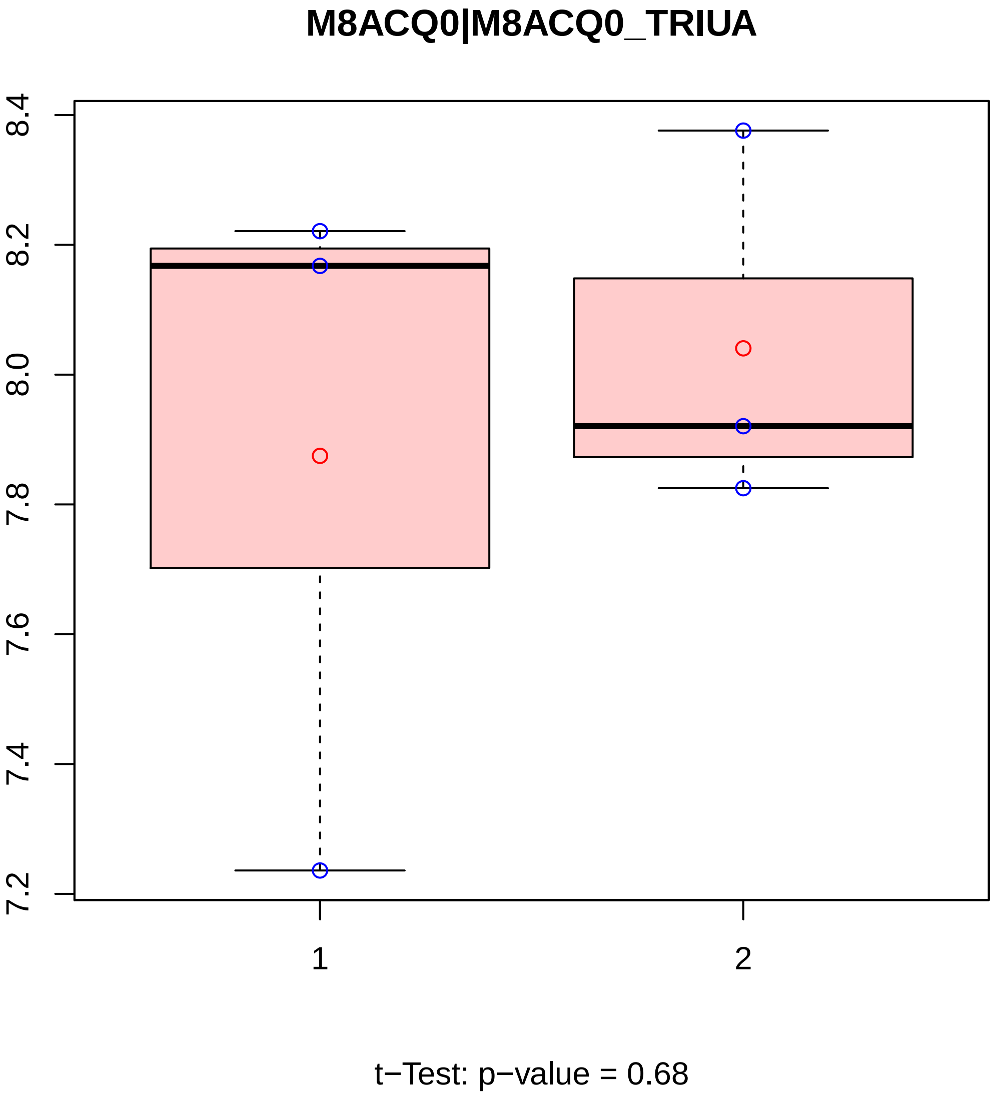
M8ACQ0|M8ACQ0_TRIUA
8.4
8.2
8.0
7.8
7.6
7.4
7.2
1
2
t−Test: p−value = 0.68

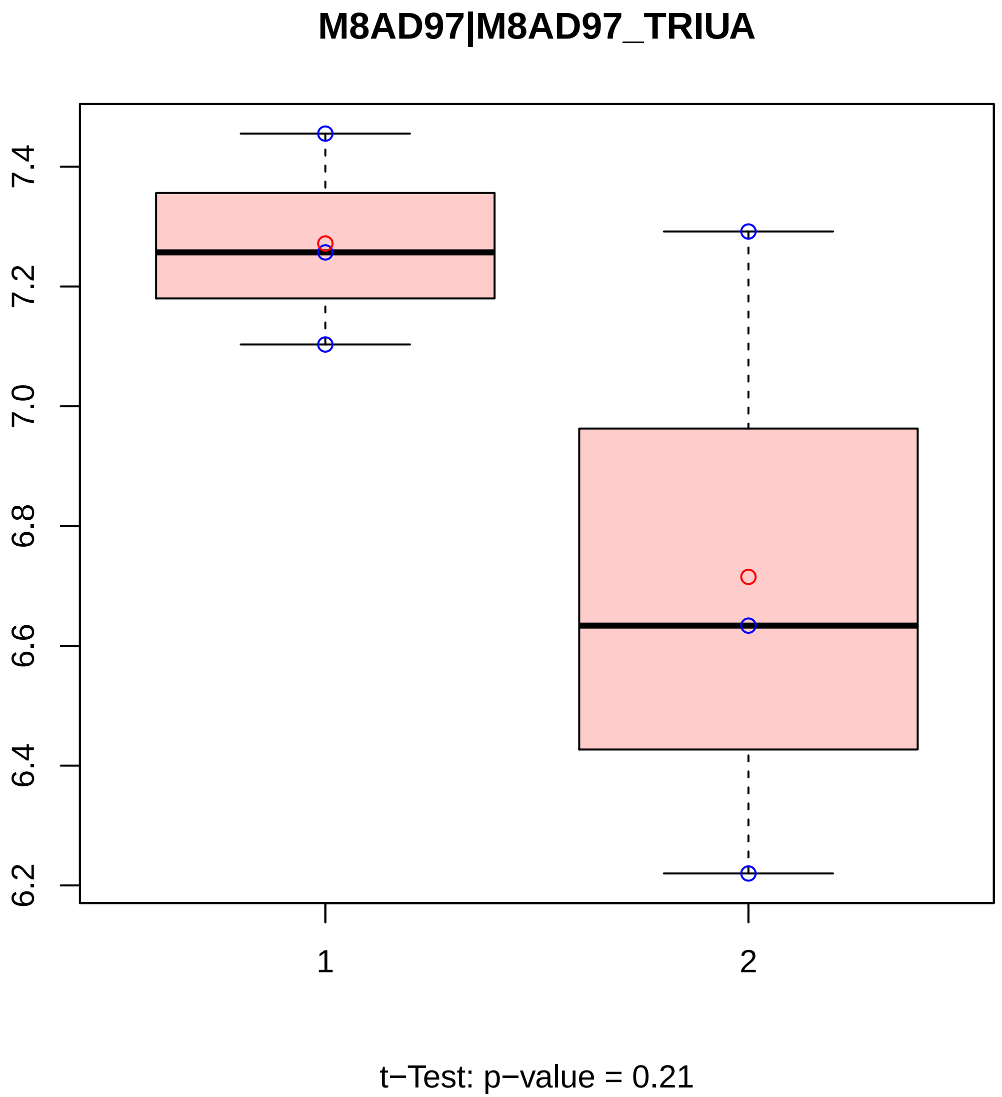
M8AD97|M8AD97_TRIUA
7.4
7.2
7.0
6.8
6.6
6.4
6.2
1
2
t−Test: p−value = 0.21

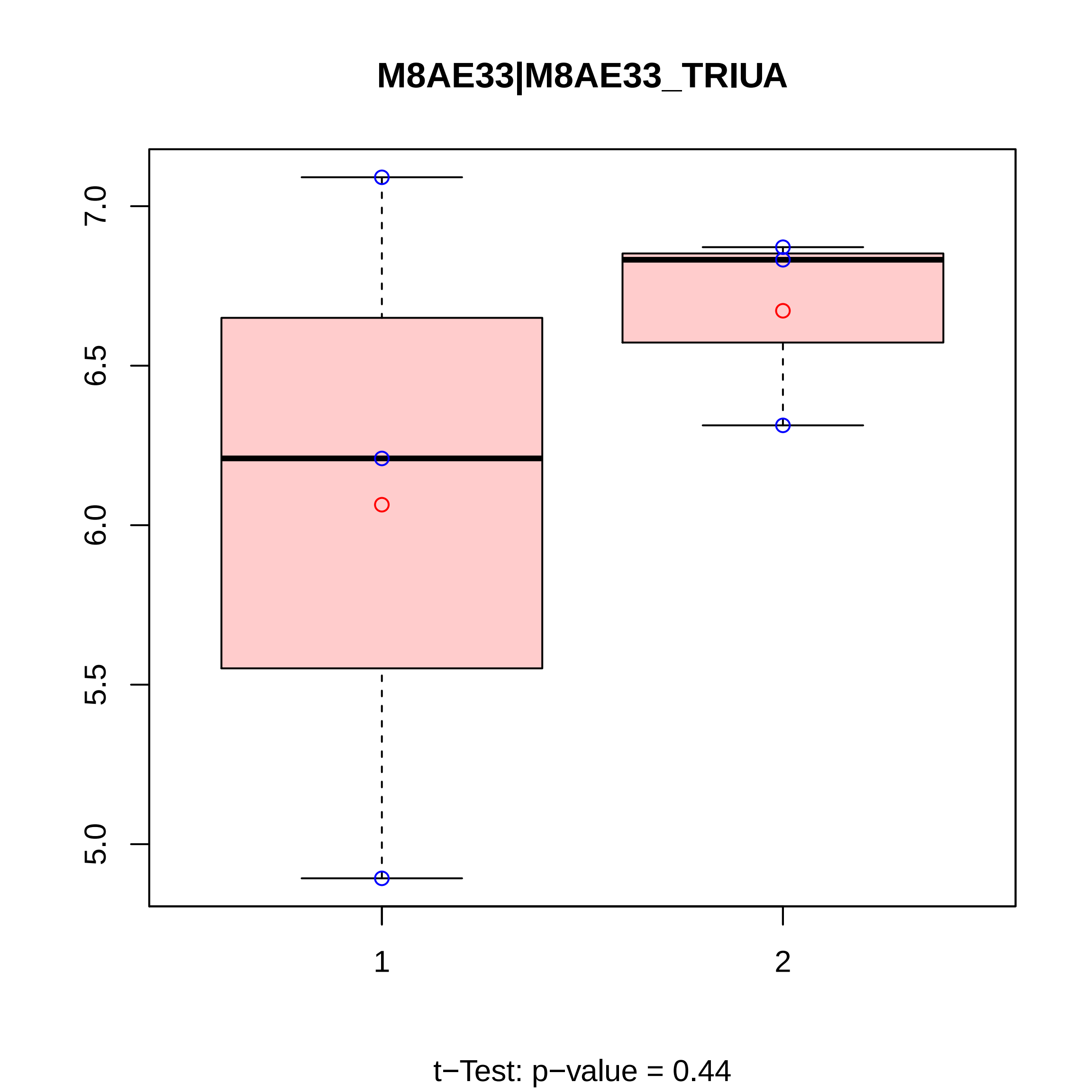
M8AE33|M8AE33_TRIUA
7.0
6.5
6.0
5.5
5.0
1
2
t−Test: p−value = 0.44

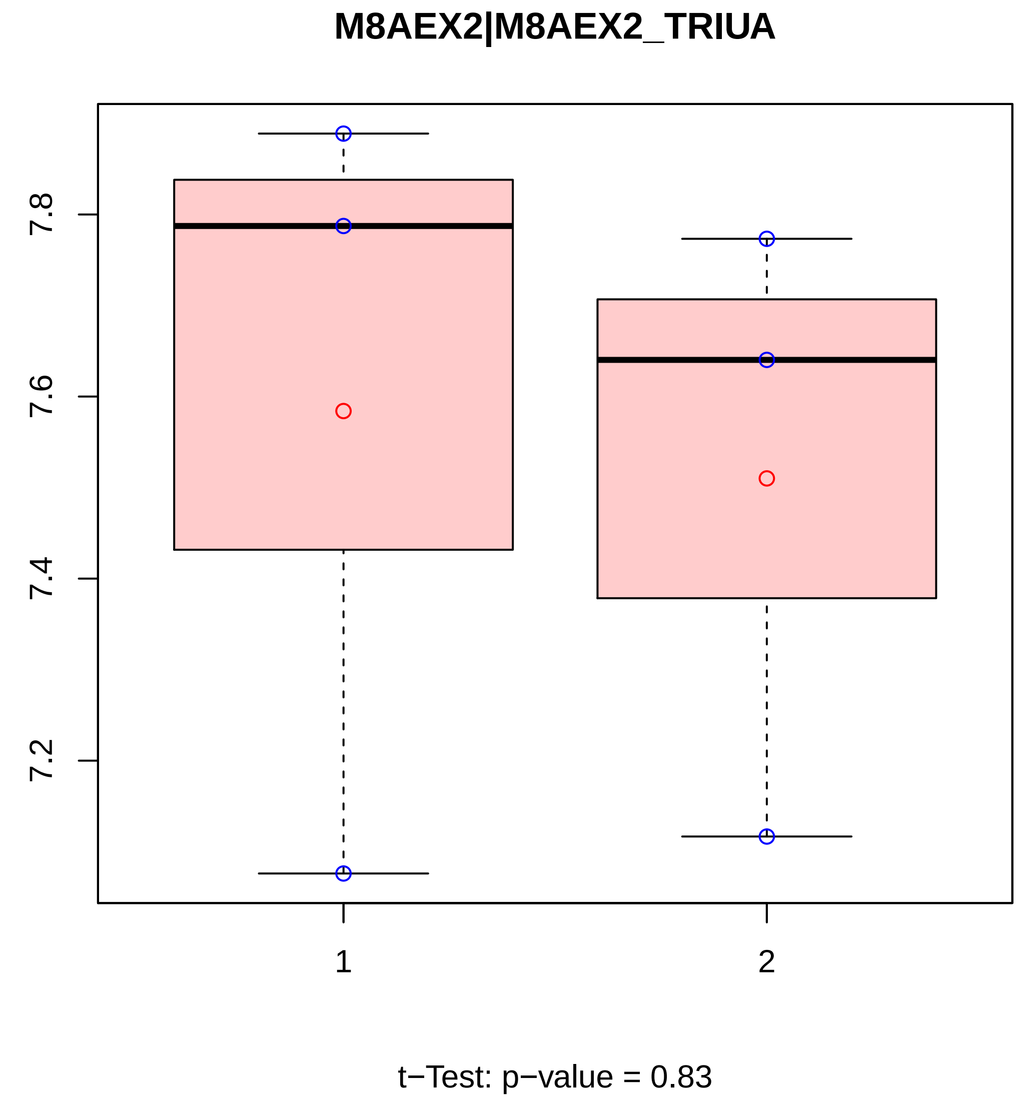
M8AEX2|M8AEX2_TRIUA
7.2
7.4
7.6
7.8
1
2
t−Test: p−value = 0.83

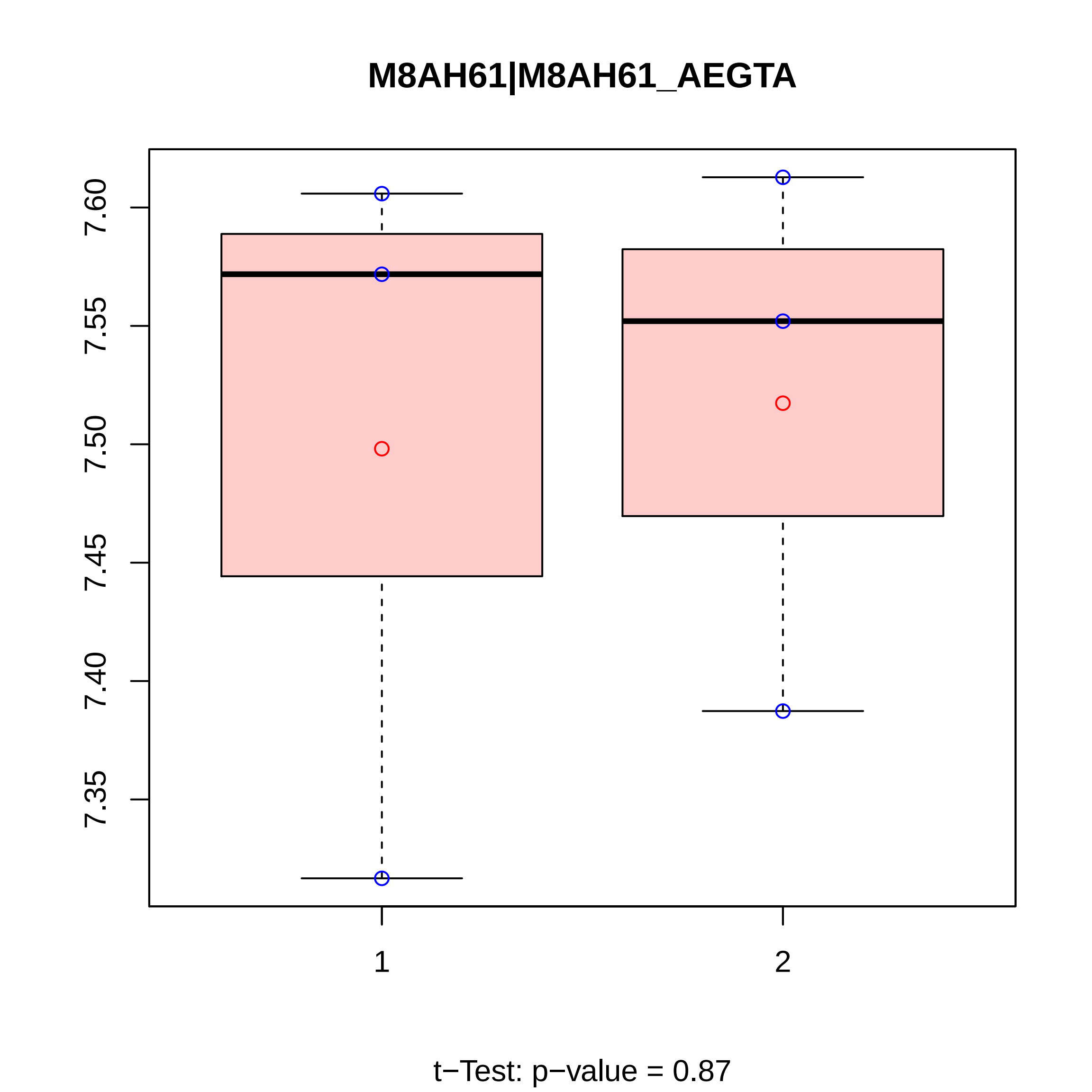

M8AH61|M8AH61_AEGTA
7.60
7.55
7.50
7.45
7.40
7.35
1
2
t−Test: p−value = 0.87

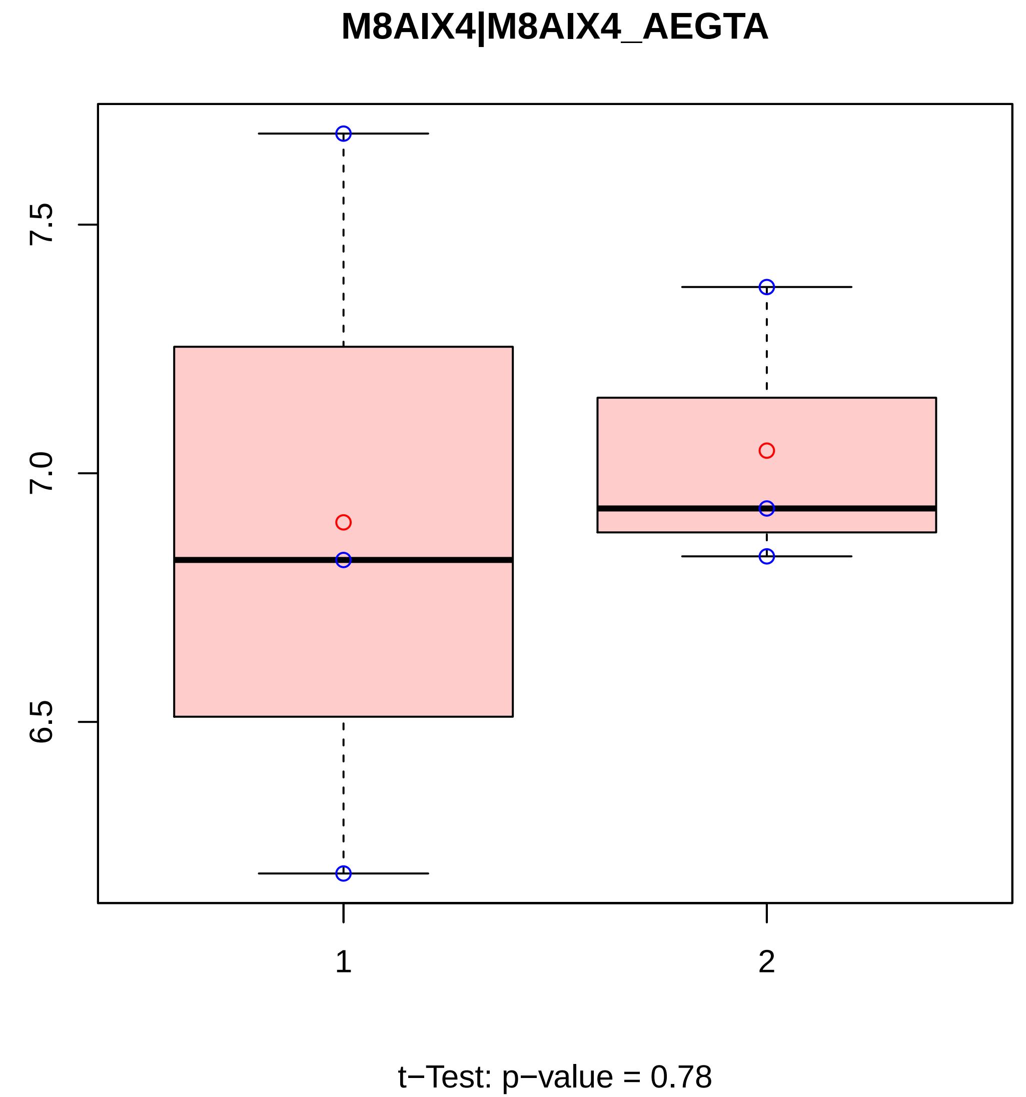

M8AIX4|M8AIX4_AEGTA
7.5
7.0
6.5
1
2
t−Test: p−value = 0.78

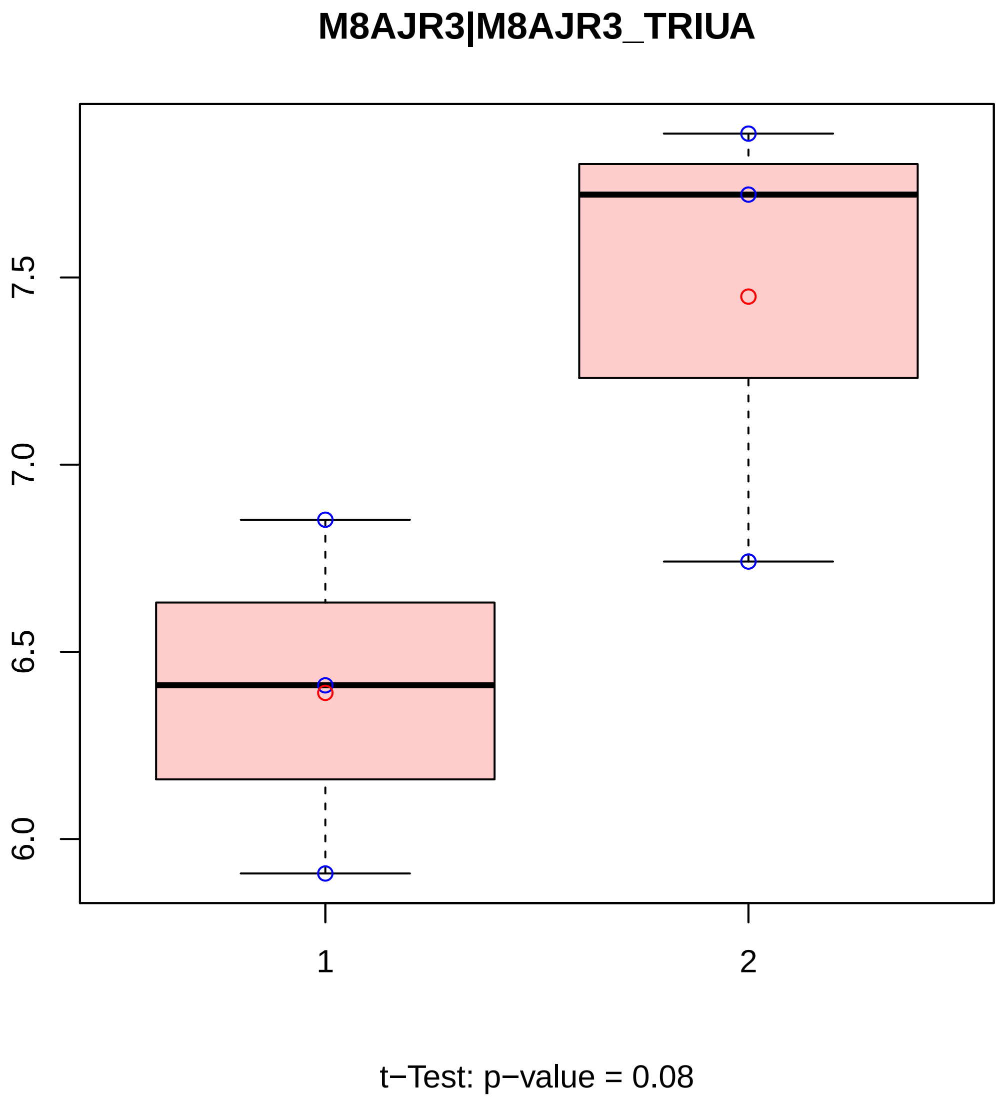

M8AJR3|M8AJR3_TRIUA
7.5
7.0
6.5
6.0
1
2
t−Test: p−value = 0.08

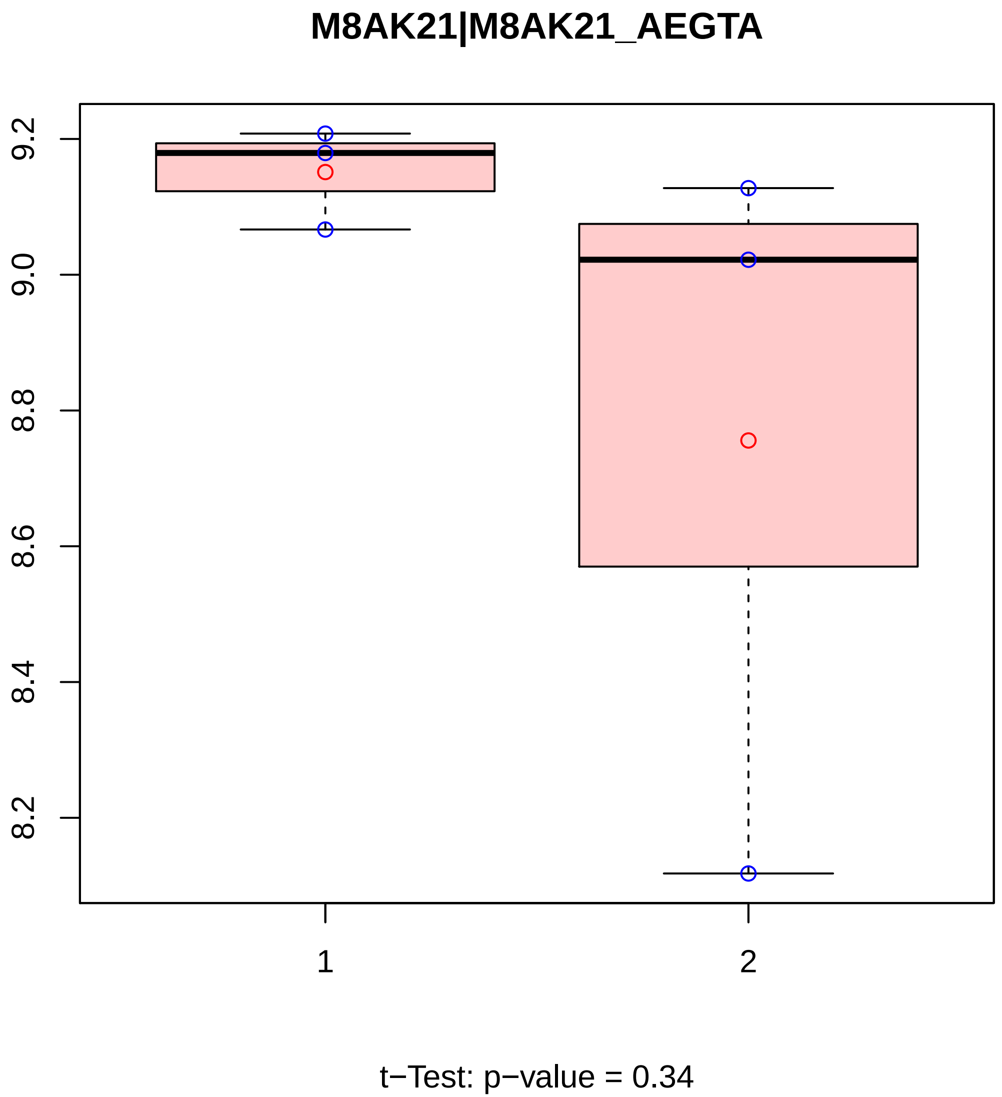
M8AK21|M8AK21_AEGTA
9.2
9.0
8.8
8.6
8.4
8.2
1
2
t−Test: p−value = 0.34

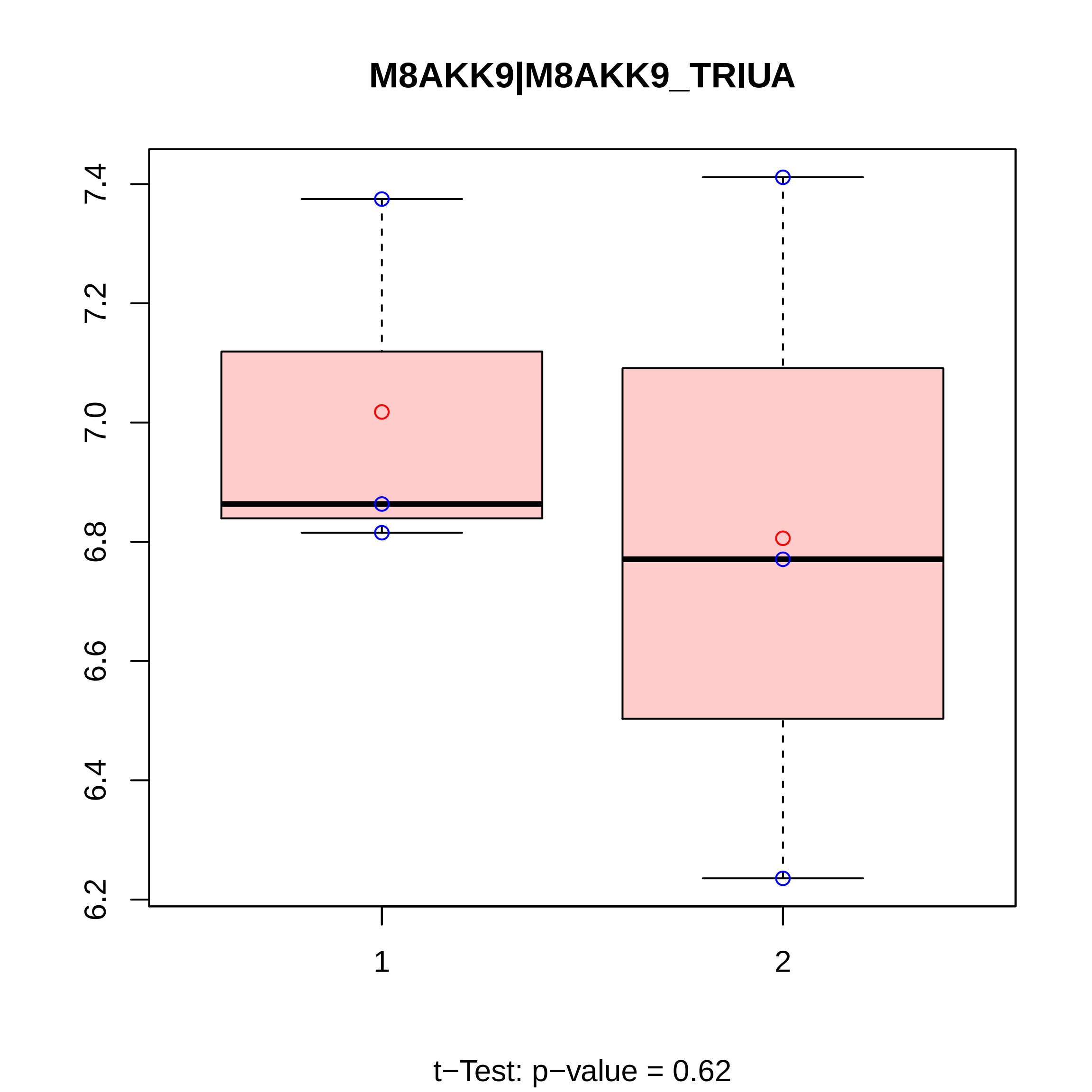
M8AKK9|M8AKK9_TRIUA
7.4
7.2
7.0
6.8
6.6
6.4
6.2
1
2
t−Test: p−value = 0.62

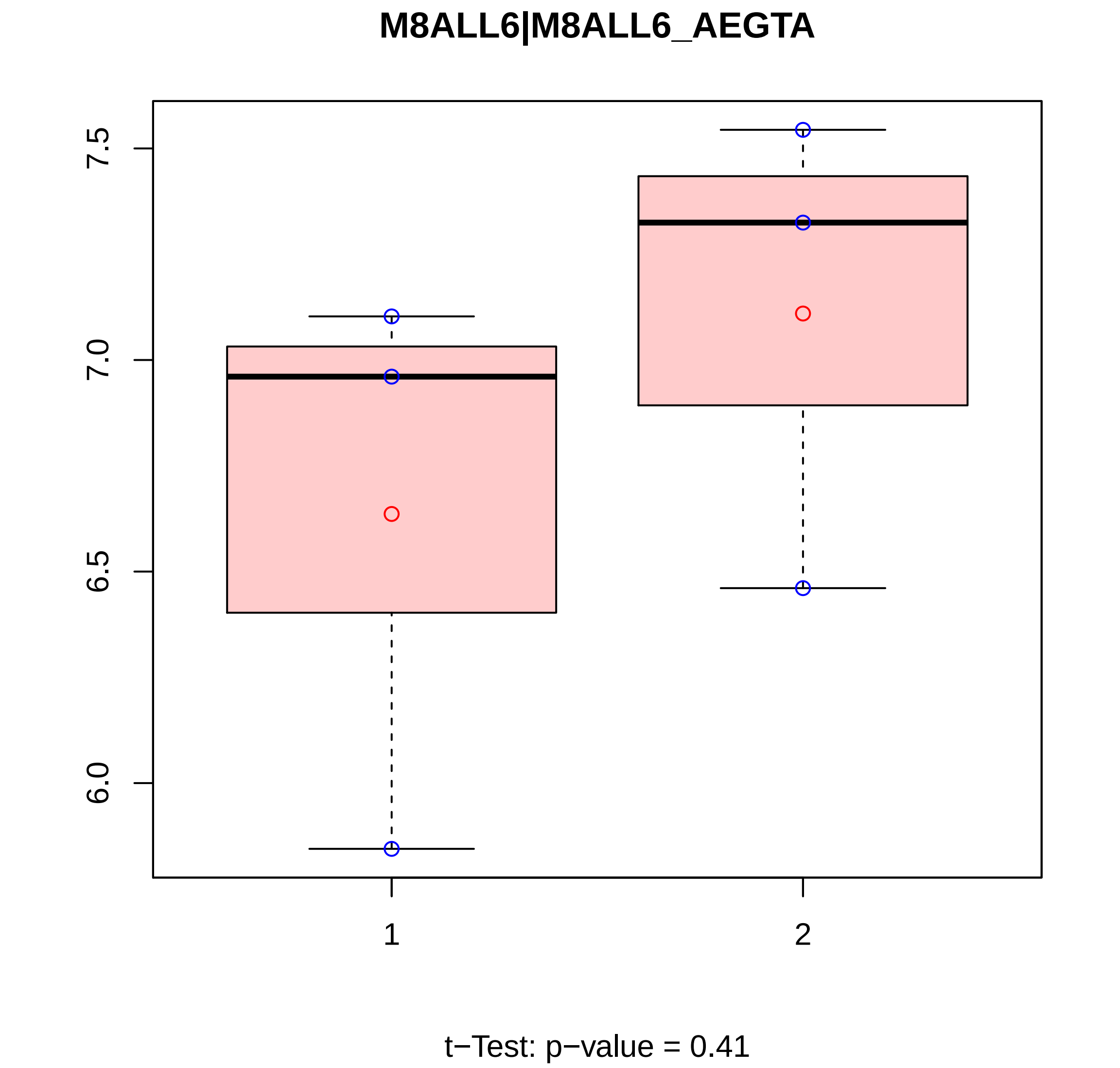
M8ALL6|M8ALL6_AEGTA
7.5
7.0
6.5
6.0
1
2
t−Test: p−value = 0.41

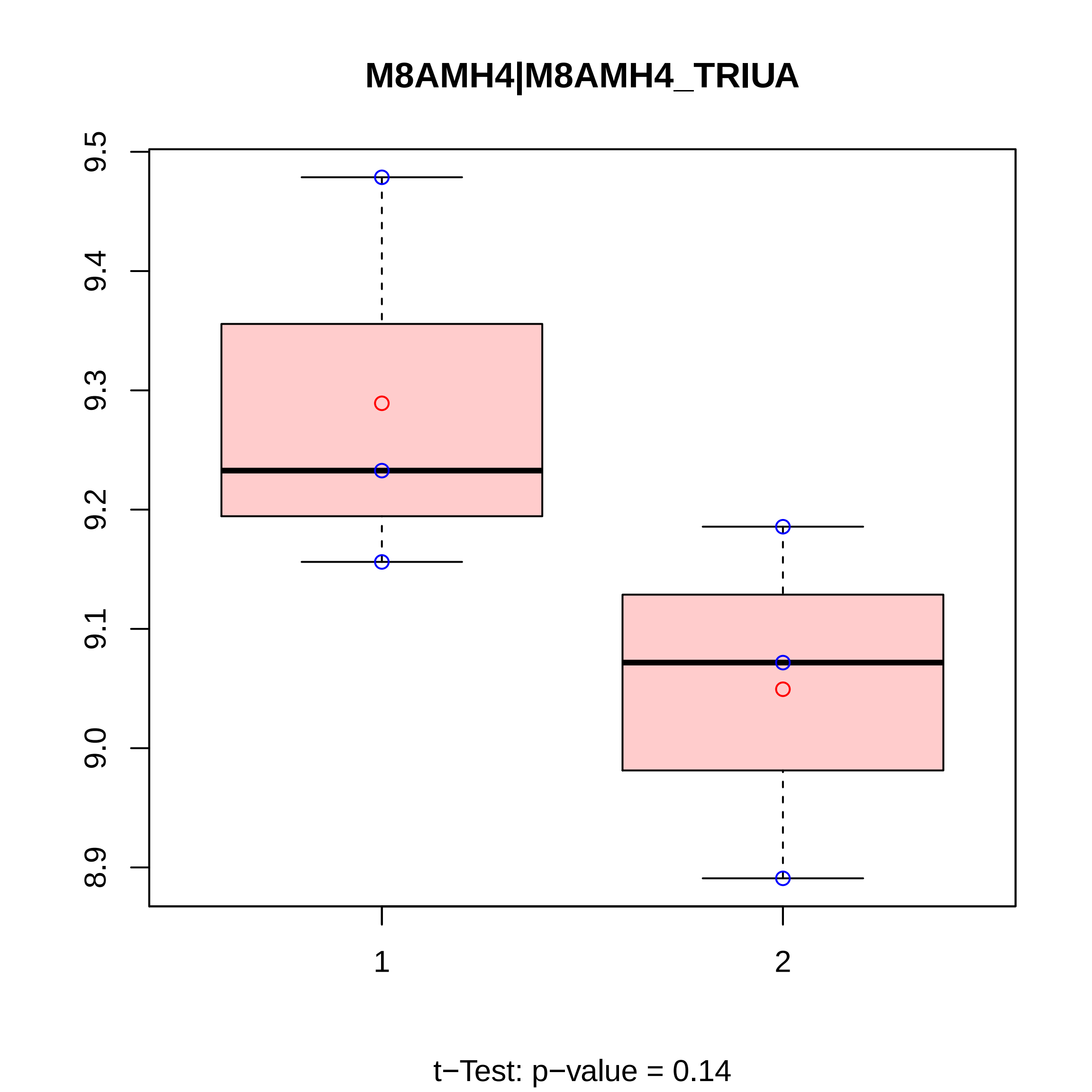
M8AMH4|M8AMH4_TRIUA
9.5
9.4
9.3
9.2
9.1
9.0
8.9
1
2
t−Test: p−value = 0.14

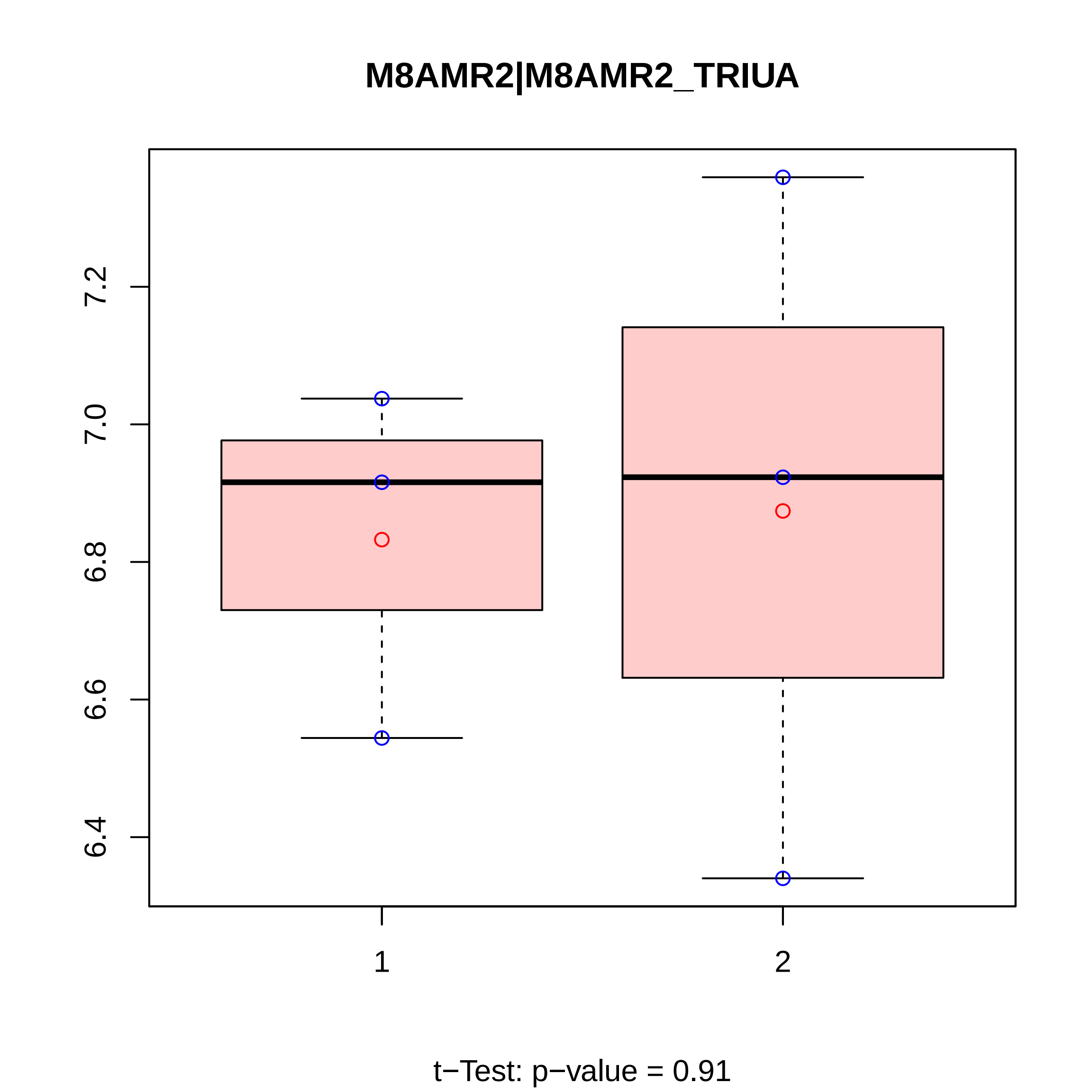
M8AMR2|M8AMR2_TRIUA
7.2
7.0
6.8
6.6
6.4
1
2
t−Test: p−value = 0.91

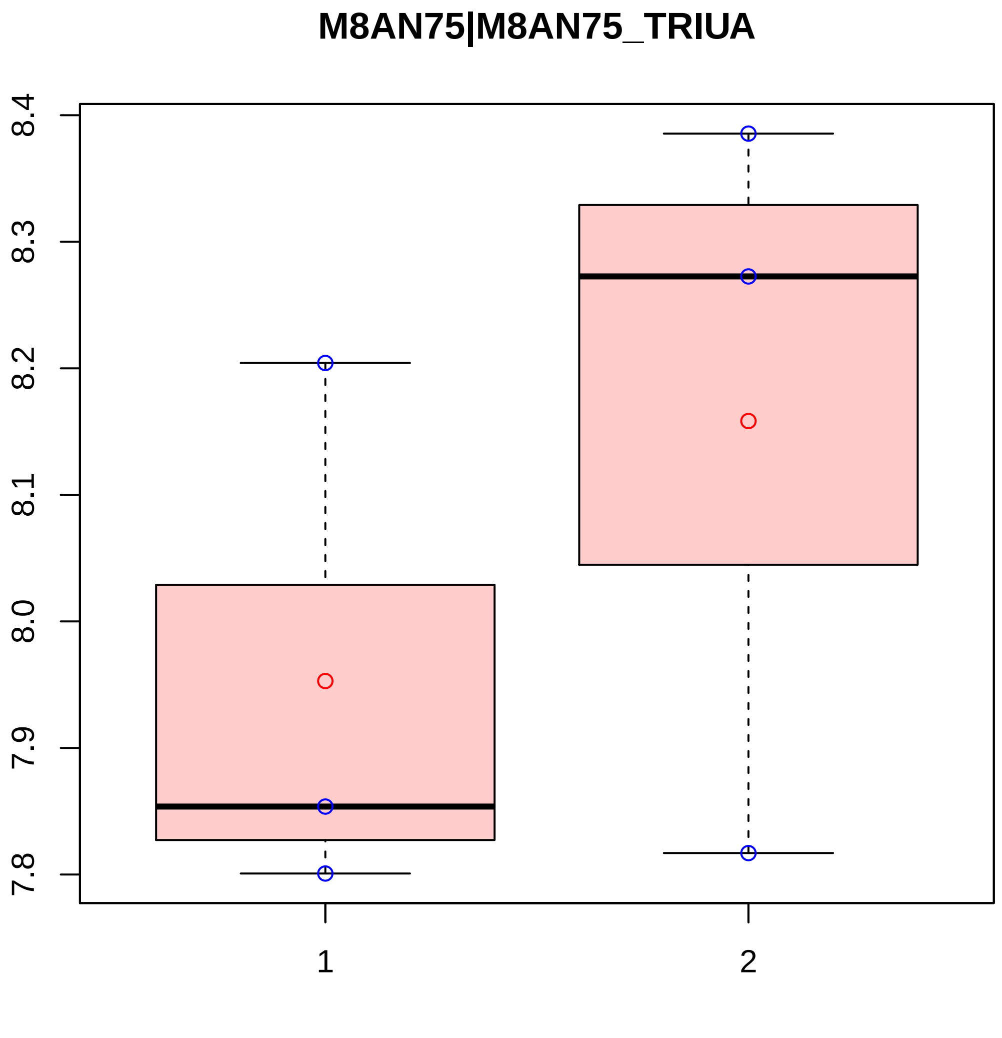

M8AN75|M8AN75_TRIUA
8.4
8.3
8.2
8.1
8.0
7.9
7.8
1
2
t−Test: p−value = 0.4

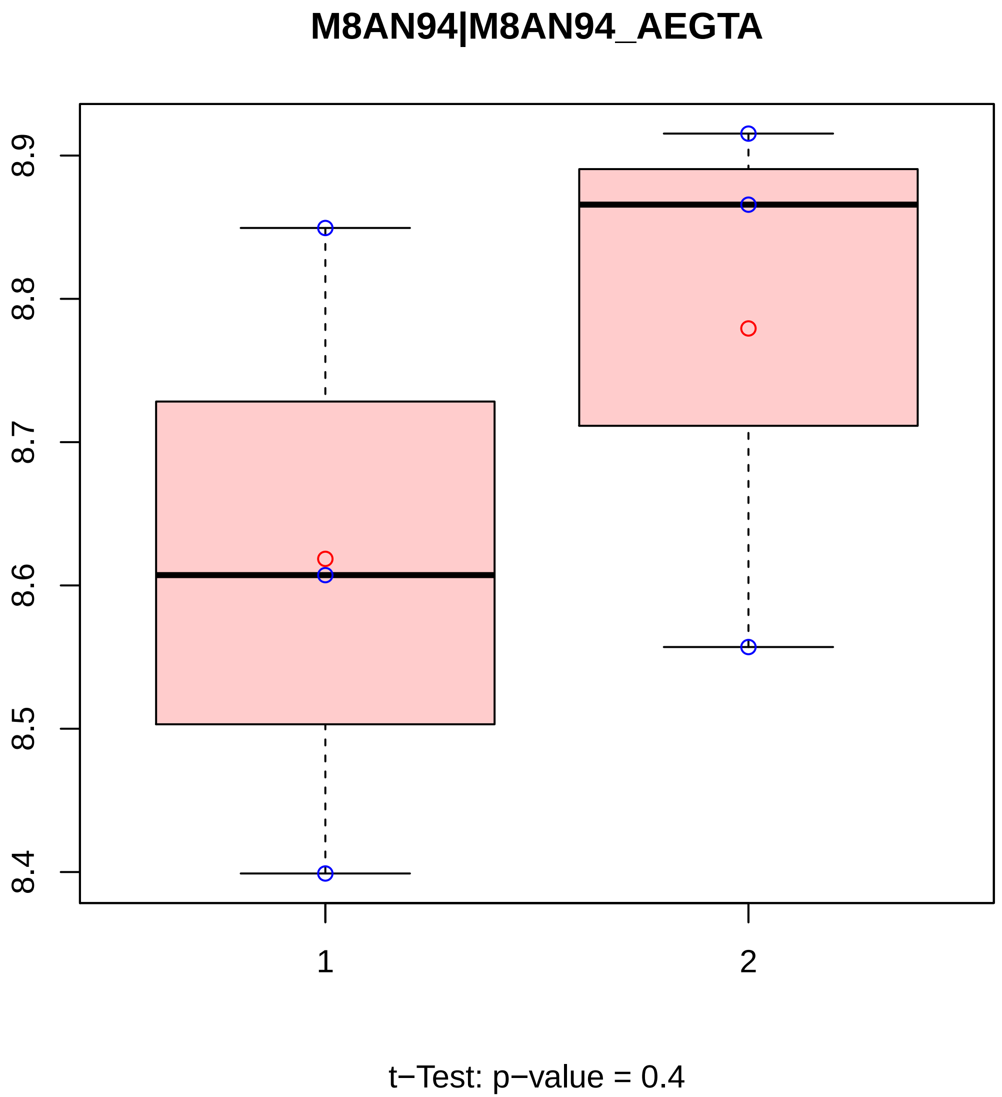
M8AN94|M8AN94_AEGTA
8.4
8.5
8.6
8.7
8.8
8.9
1
2
t−Test: p−value = 0.4

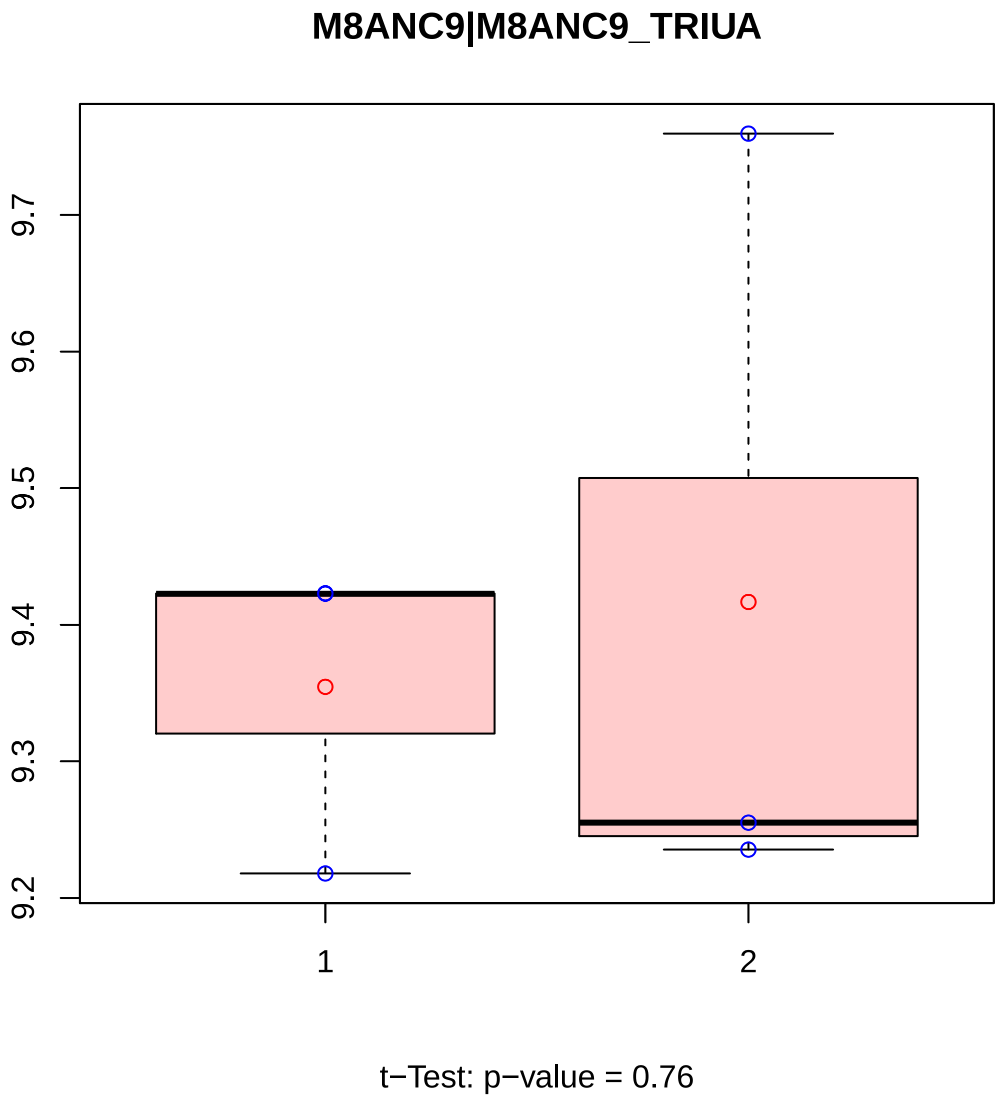

M8ANC9|M8ANC9_TRIUA
9.2
9.3
9.4
9.5
9.6
9.7
1
2
t−Test: p−value = 0.76

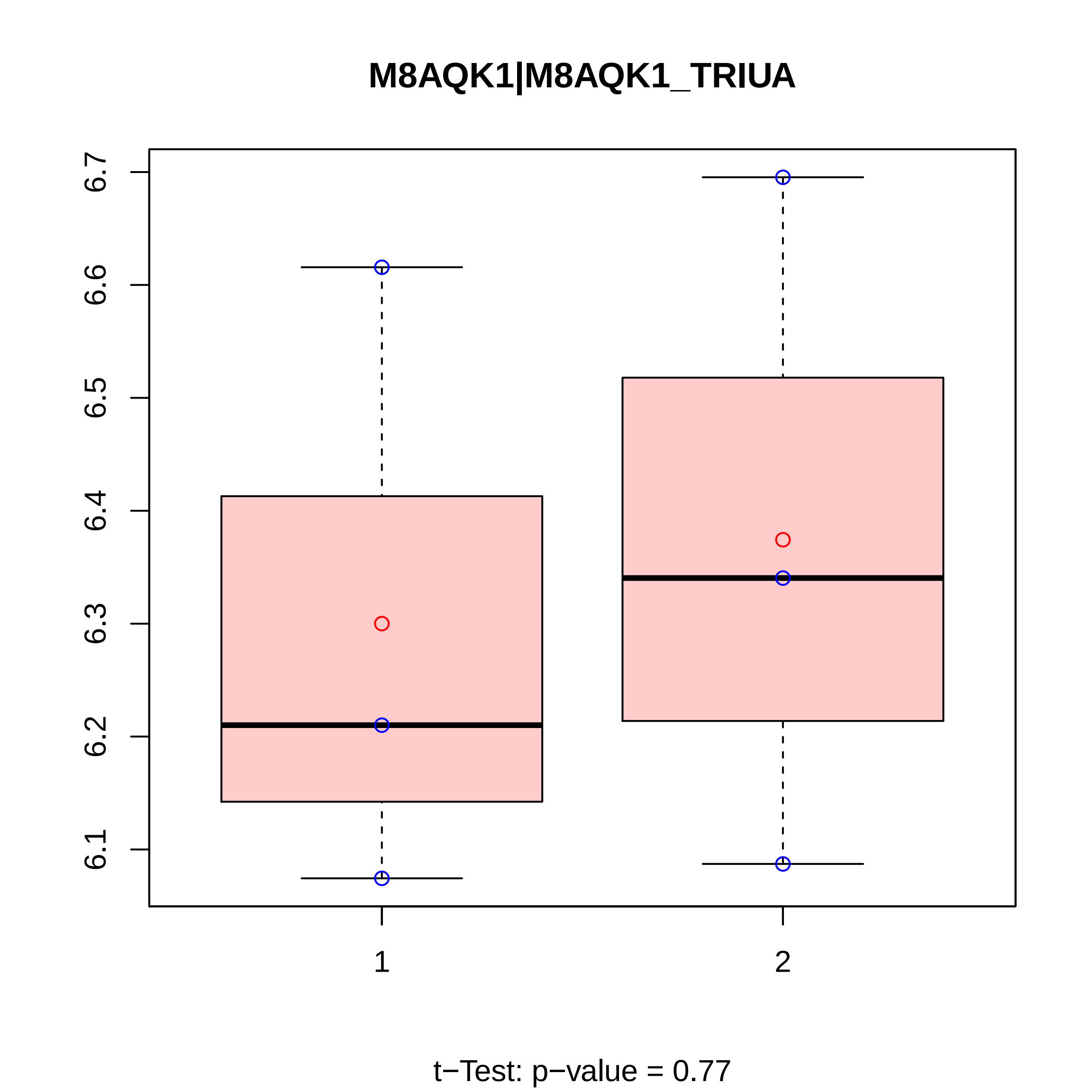
M8AQK1|M8AQK1_TRIUA
6.1
6.2
6.3
6.4
6.5
6.6
6.7
1
2
t−Test: p−value = 0.77

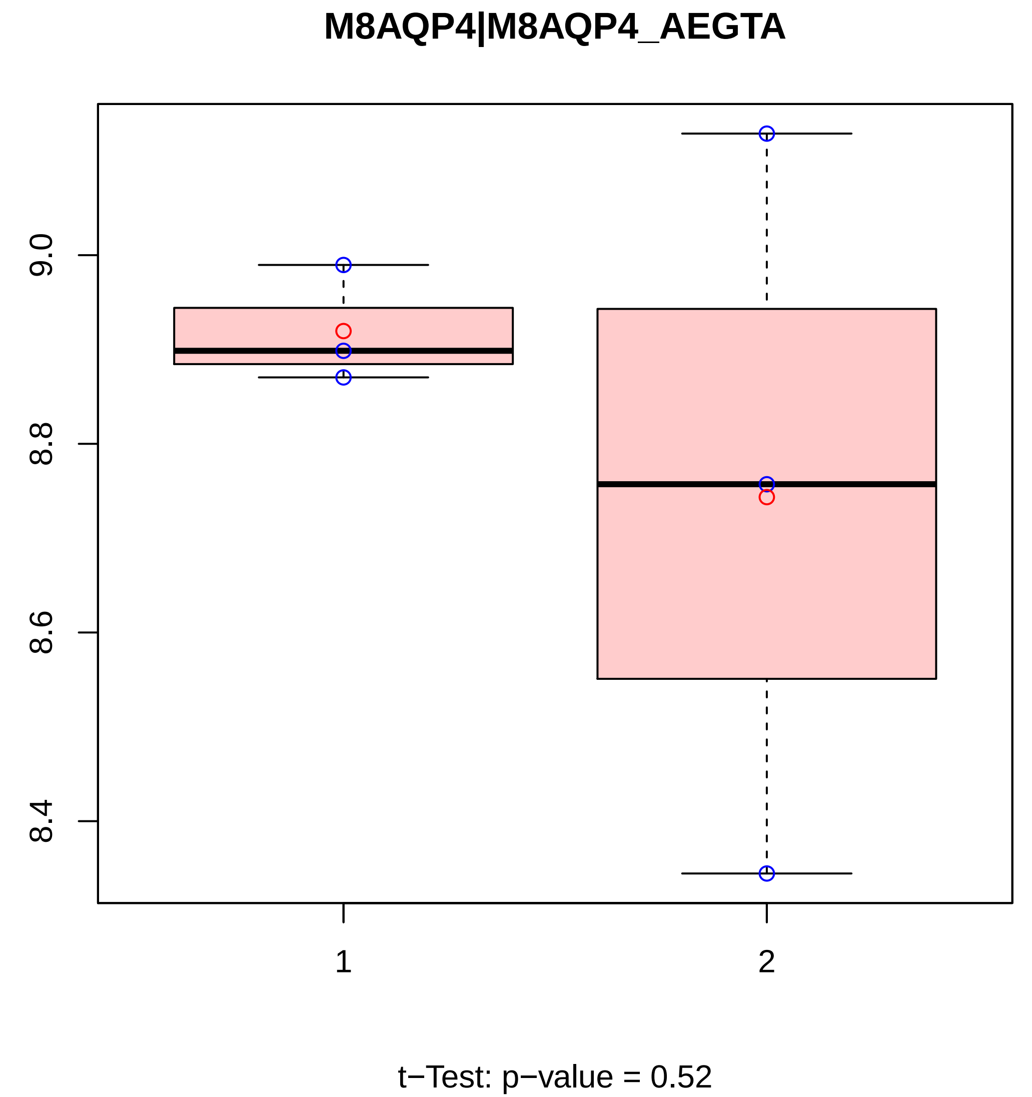
M8AQP4|M8AQP4_AEGTA
9.0
8.8
8.6
8.4
1
2
t−Test: p−value = 0.52

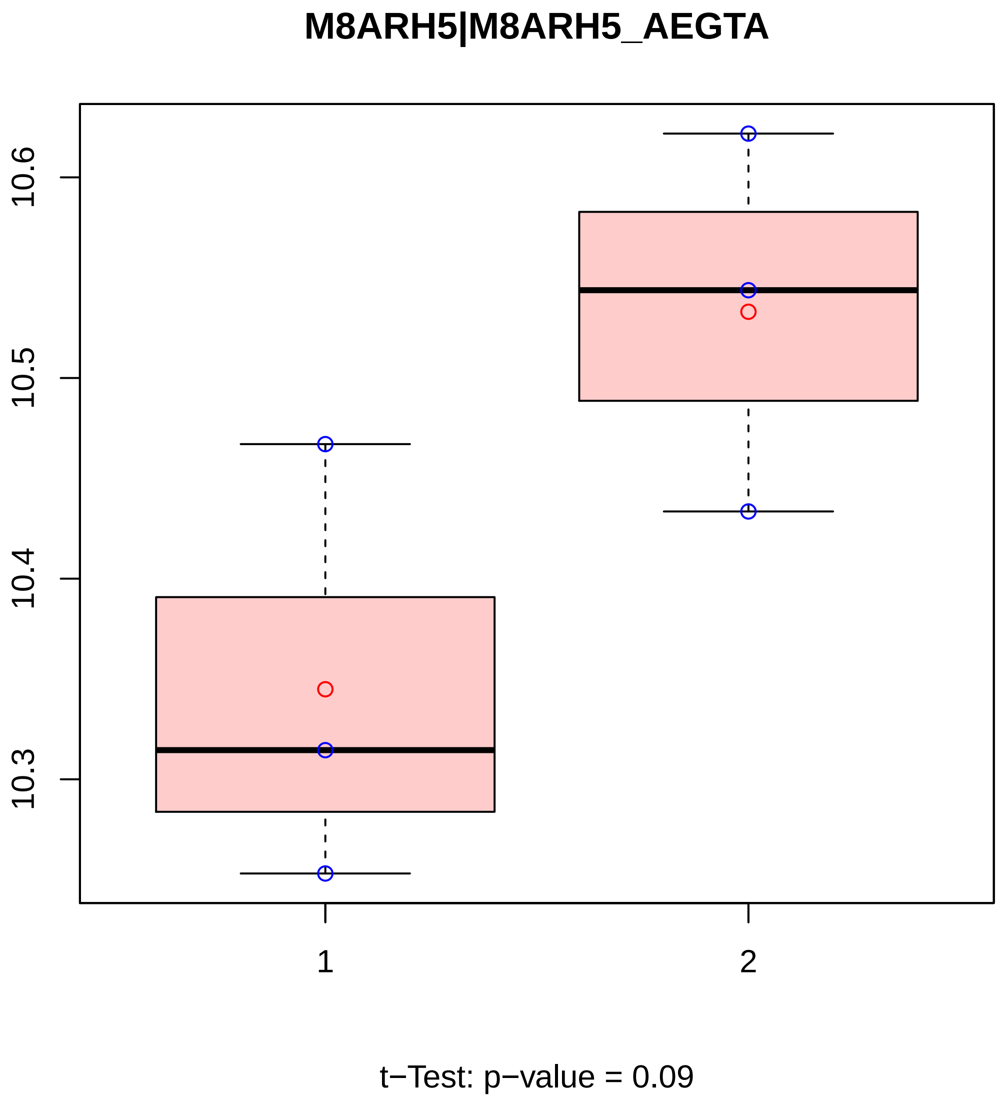
M8ARH5|M8ARH5_AEGTA
10.6
10.5
10.4
10.3
1
2
t−Test: p−value = 0.09

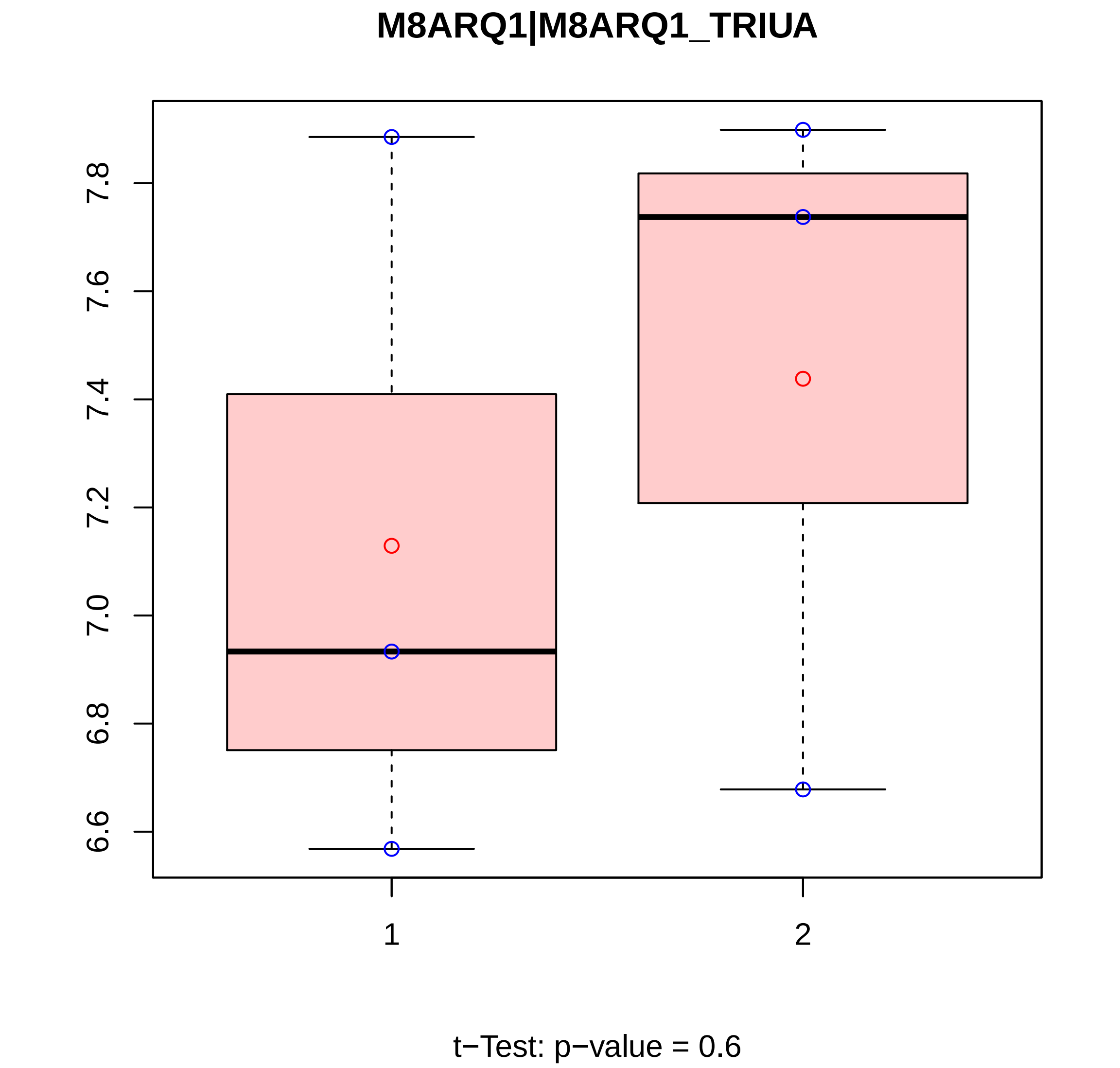
M8ARQ1|M8ARQ1_TRIUA
6.6
6.8
7.0
7.2
7.4
7.6
7.8
1
2
t−Test: p−value = 0.6

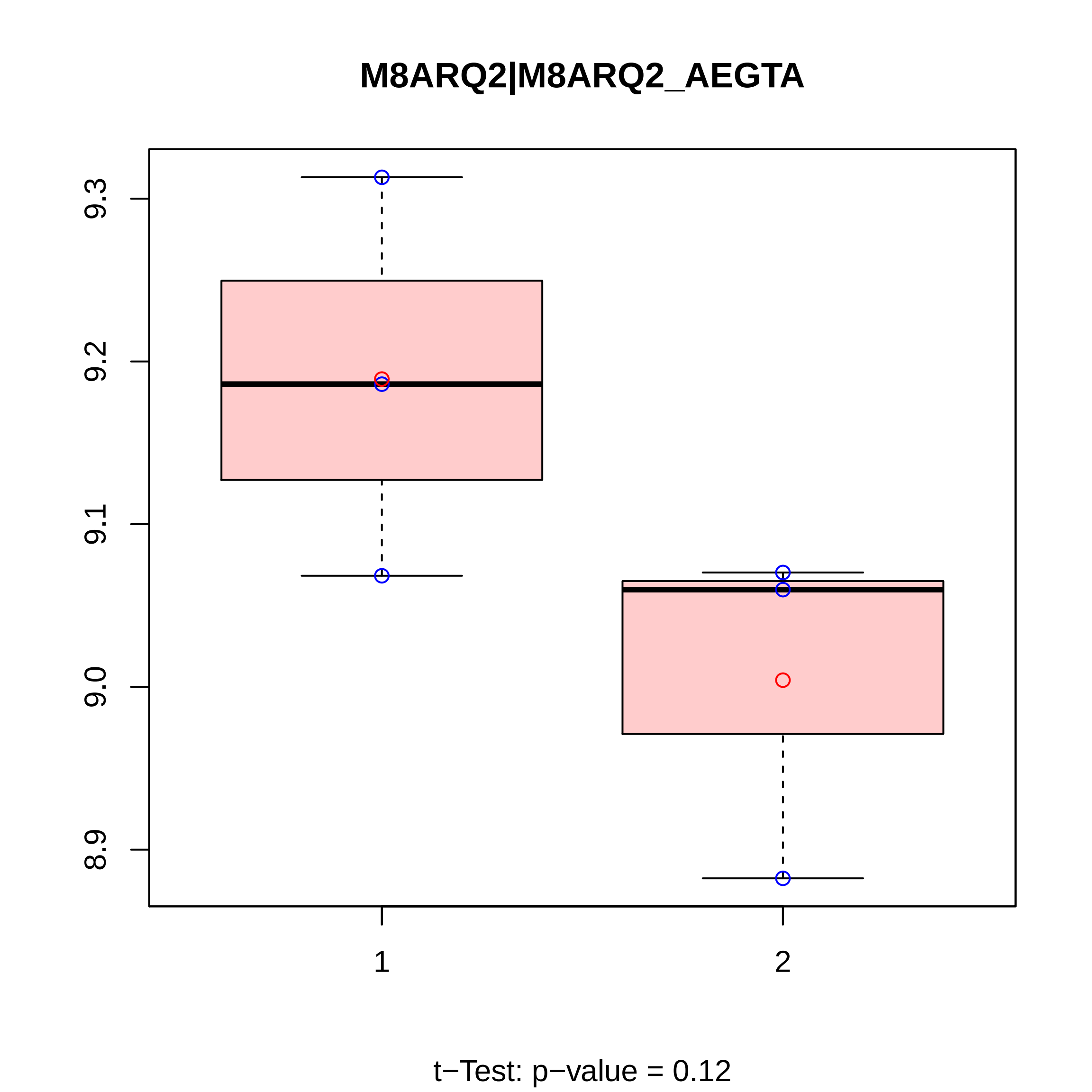
M8ARQ2|M8ARQ2_AEGTA
9.3
9.2
9.1
9.0
8.9
1
2
t−Test: p−value = 0.12

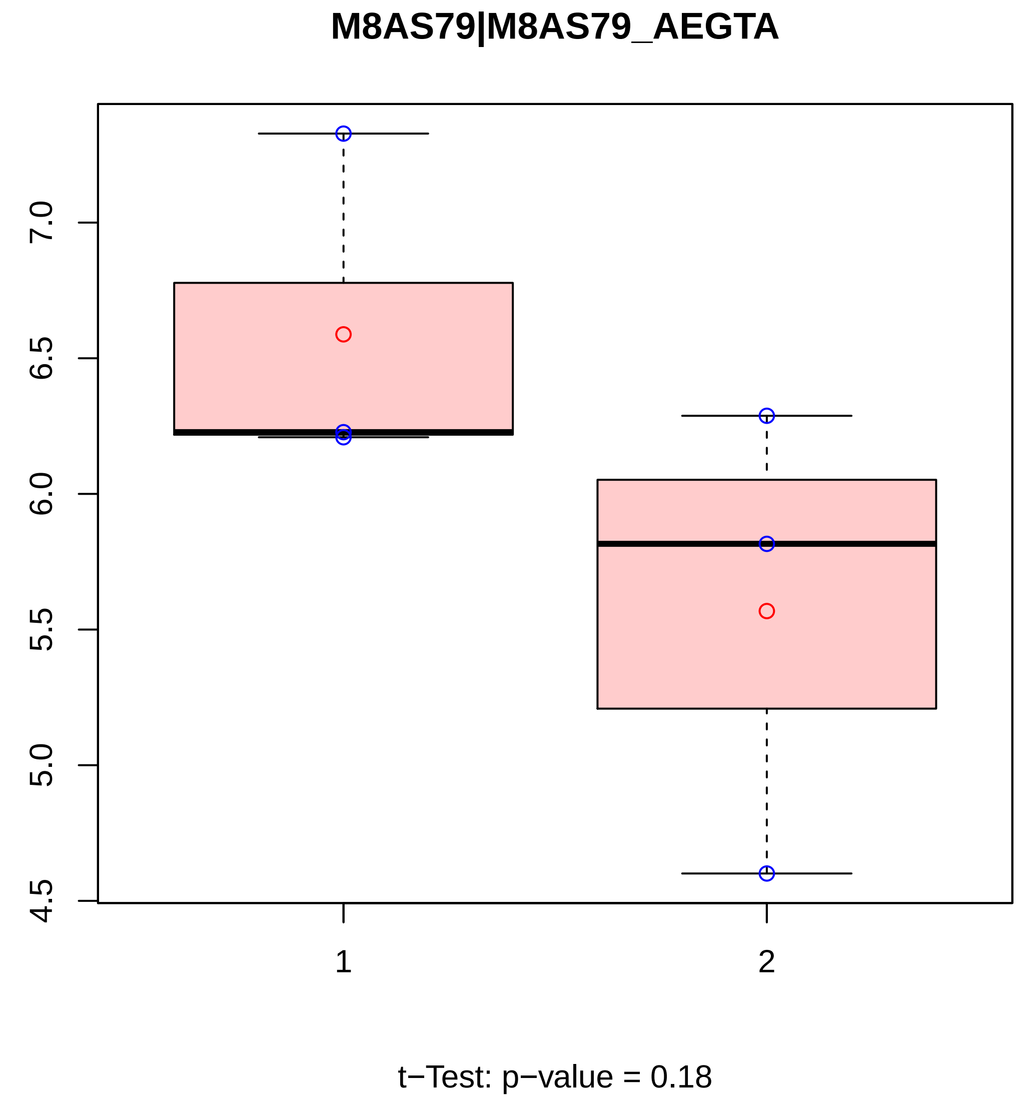

M8AS79|M8AS79_AEGTA
4.5
5.0
5.5
6.0
6.5
7.0
1
2
t−Test: p−value = 0.18

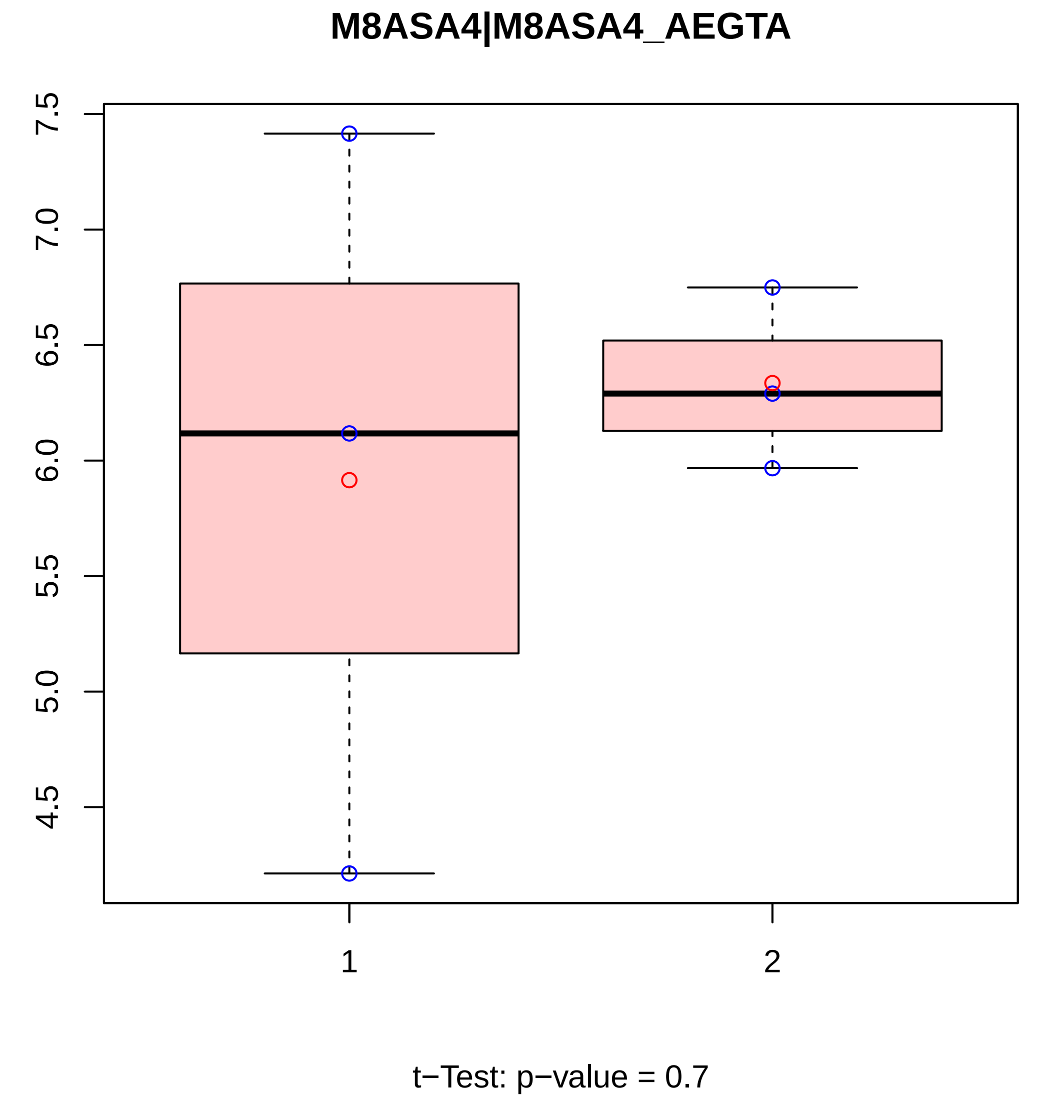
M8ASA4|M8ASA4_AEGTA
7.5
7.0
6.5
6.0
5.5
5.0
4.5
1
2
t−Test: p−value = 0.7

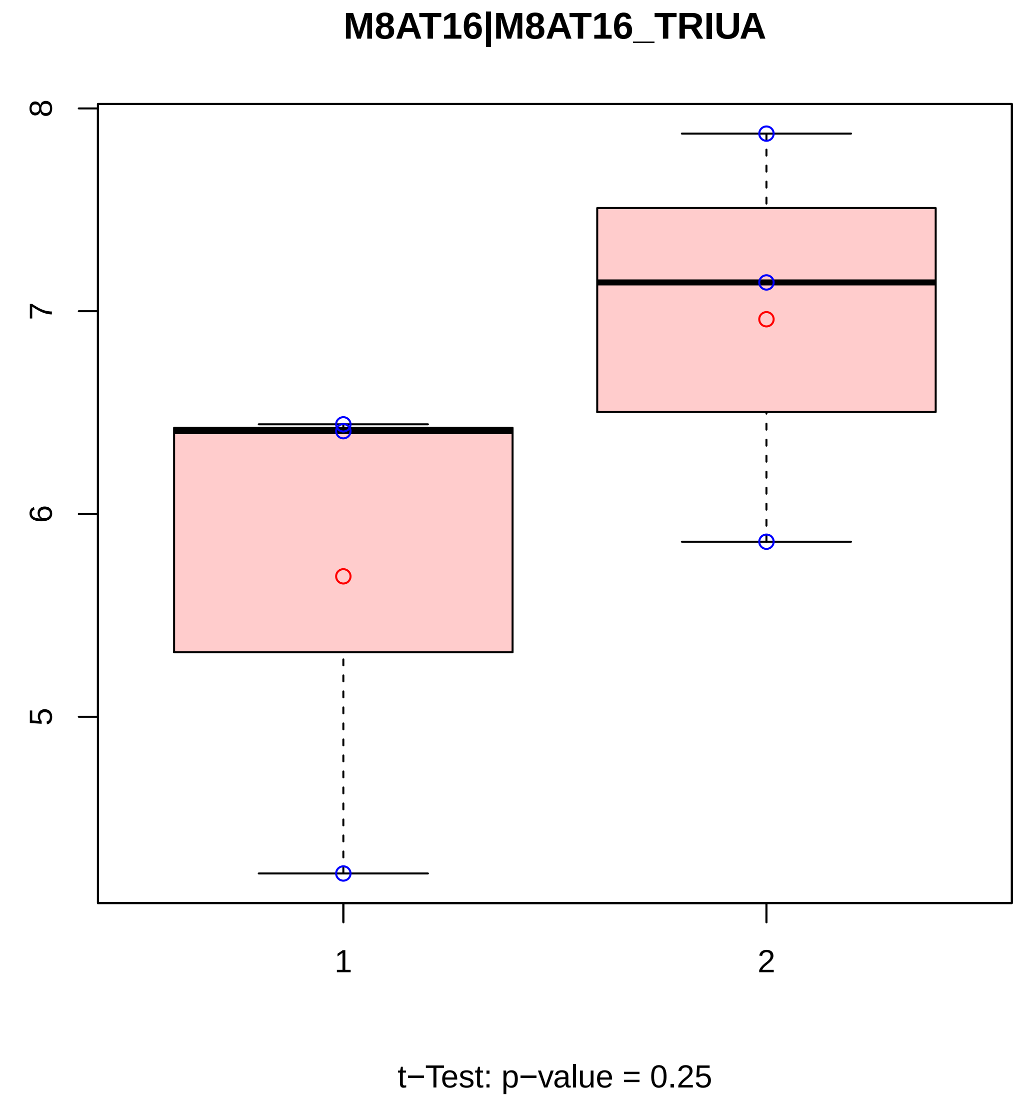
M8AT16|M8AT16_TRIUA
8
7
6
5
1
2
t−Test: p−value = 0.25

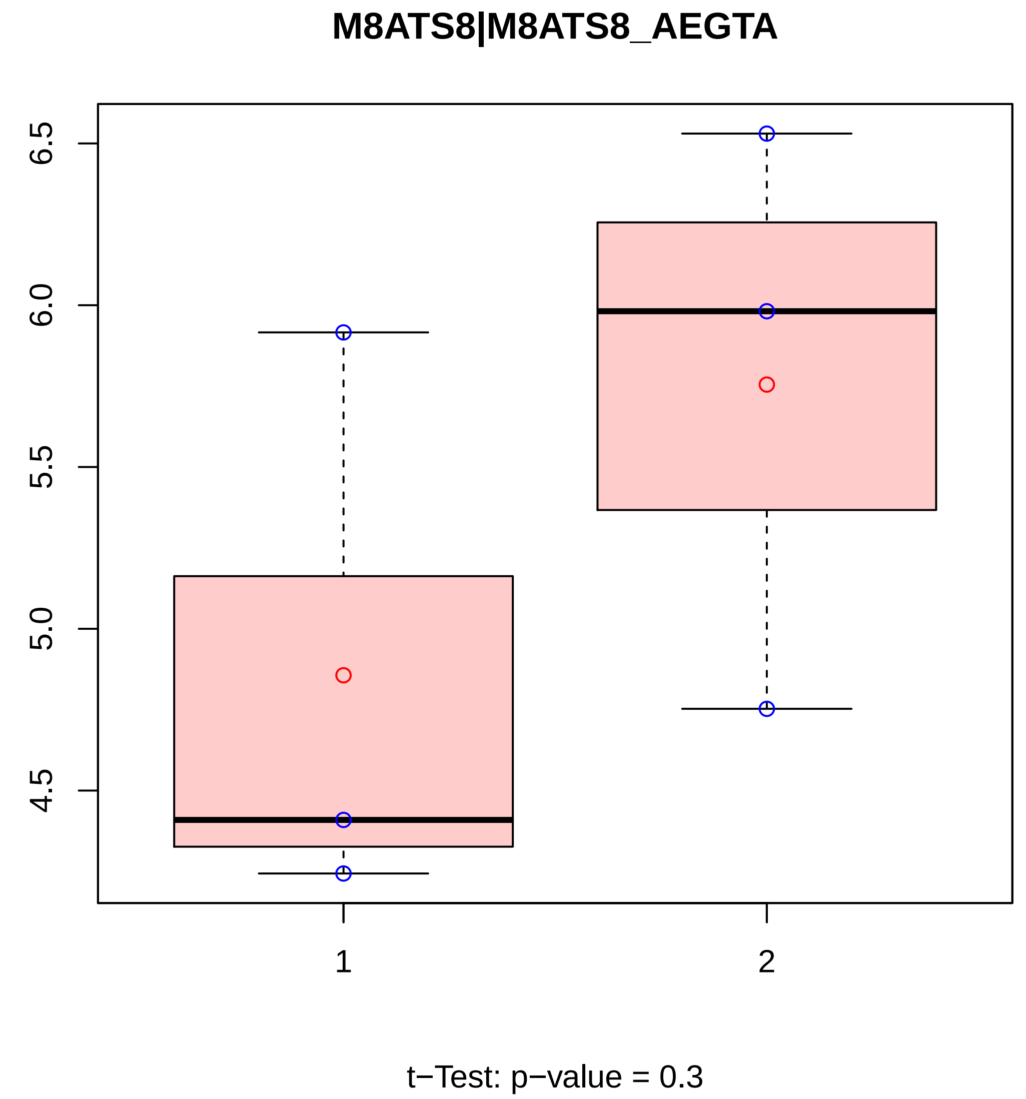
M8ATS8|M8ATS8_AEGTA
6.5
6.0
5.5
5.0
4.5
1
2
t−Test: p−value = 0.3

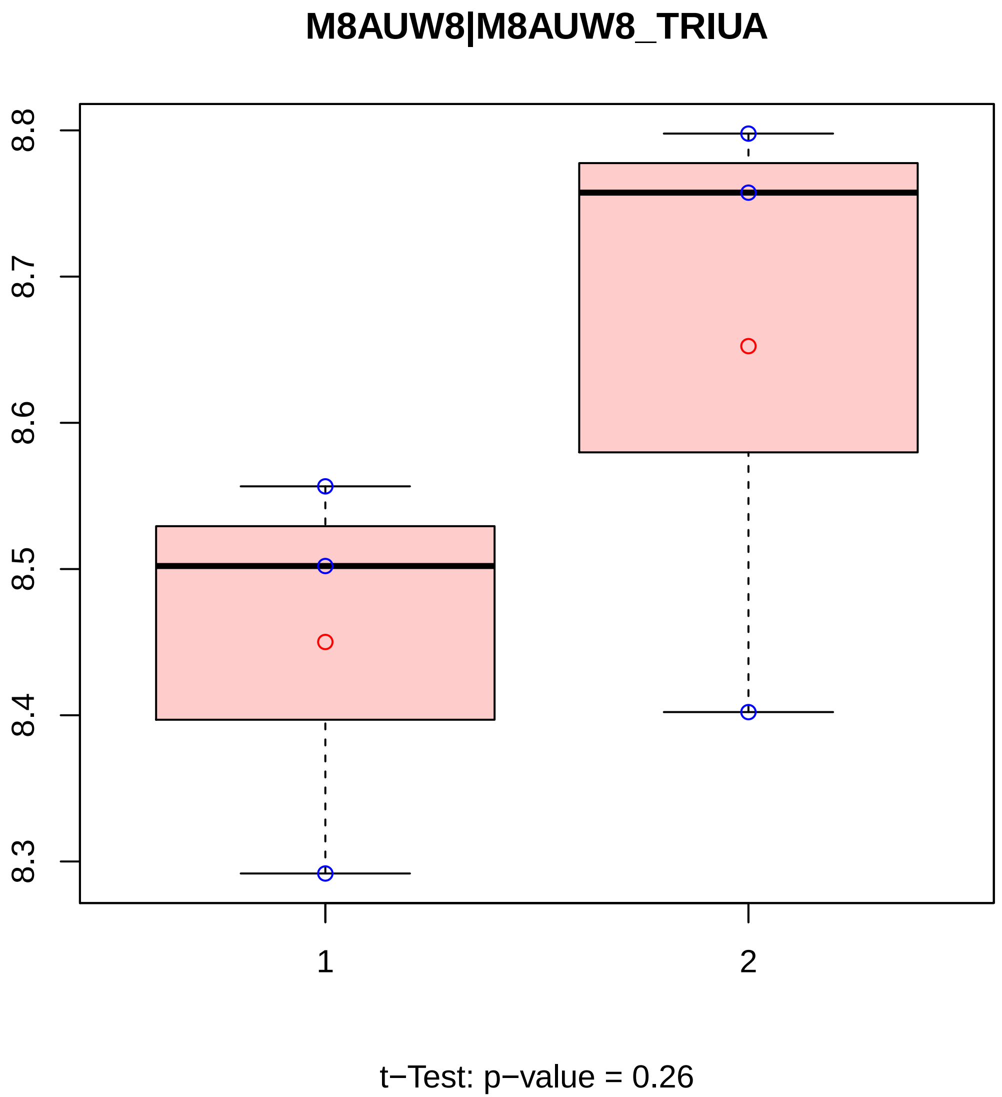
M8AUW8|M8AUW8_TRIUA
8.8
8.7
8.6
8.5
8.4
8.3
1
2
t−Test: p−value = 0.26

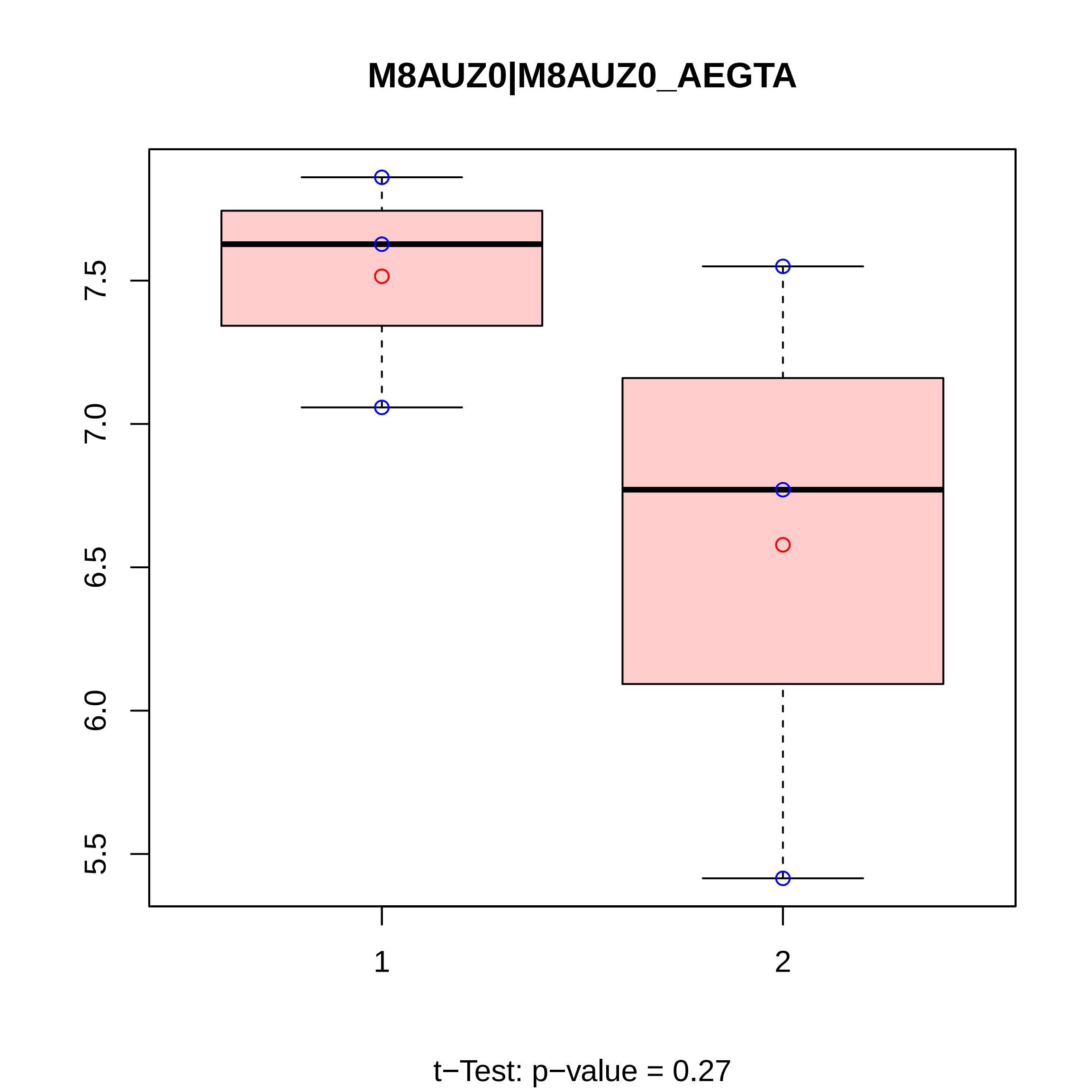
M8AUZ0|M8AUZ0_AEGTA
7.5
7.0
6.5
6.0
5.5
1
2
t−Test: p−value = 0.27

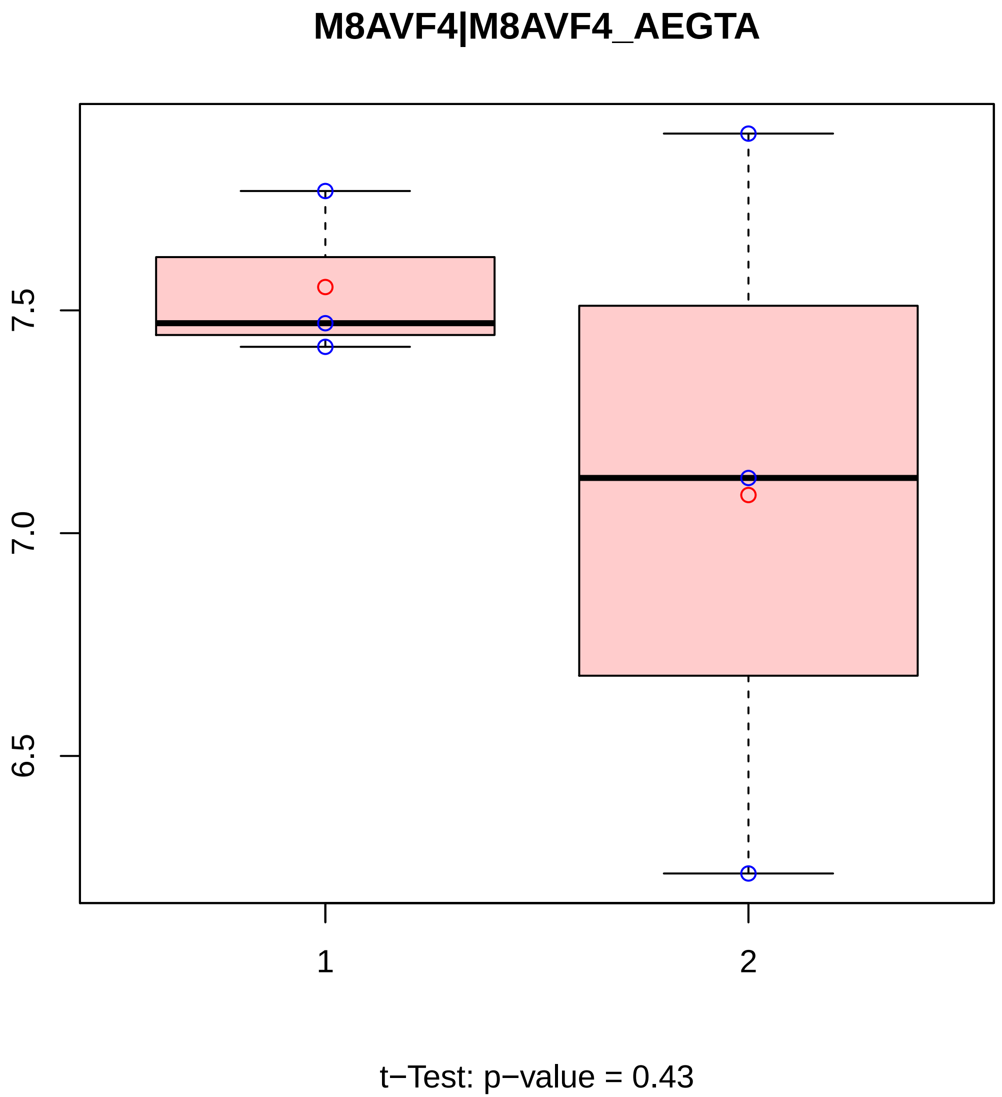

M8AVF4|M8AVF4_AEGTA
7.5
7.0
6.5
1
2
t−Test: p−value = 0.43

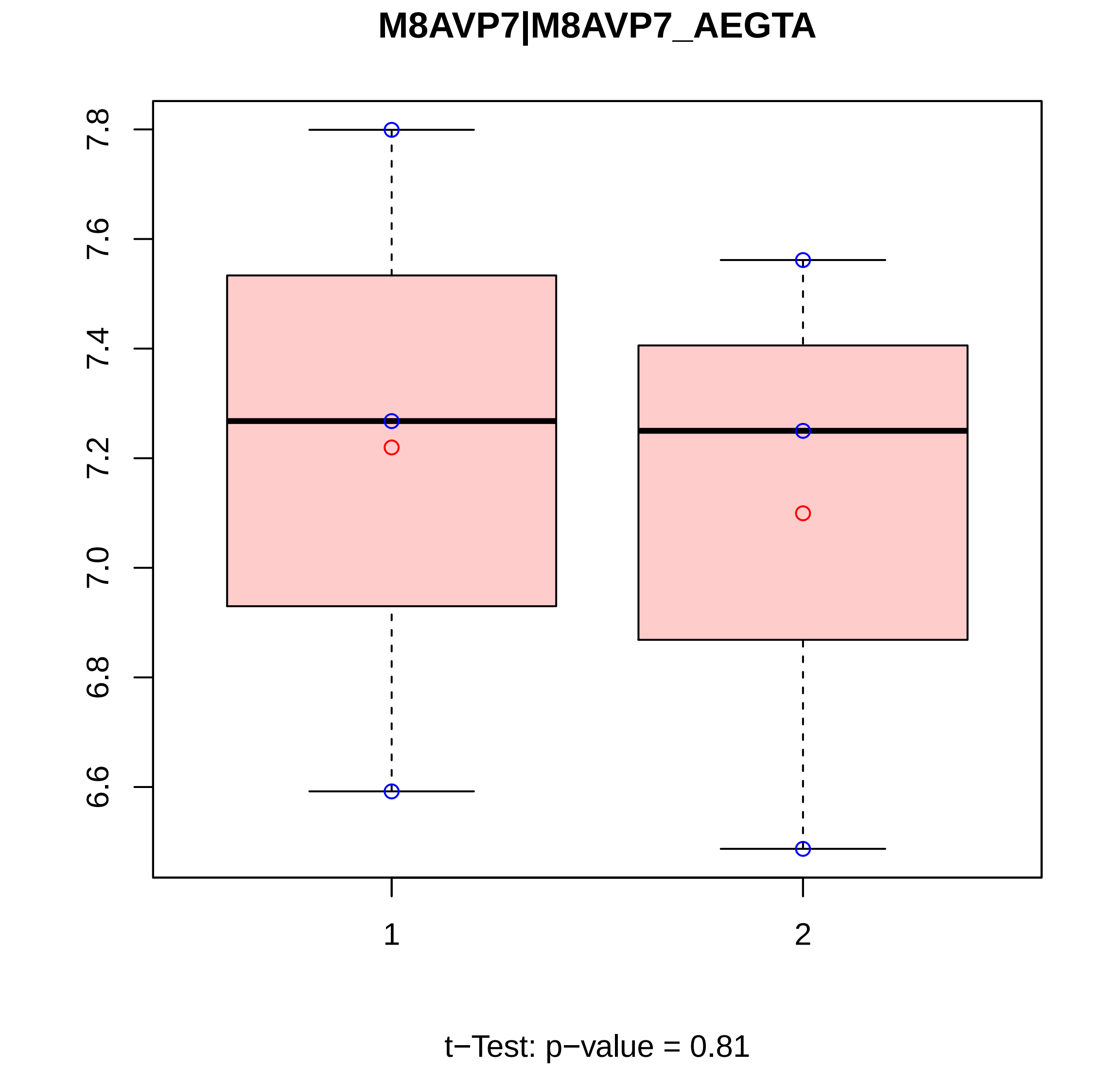

M8AVP7|M8AVP7_AEGTA
7.8
7.6
7.4
7.2
7.0
6.8
6.6
1
2
t−Test: p−value = 0.81

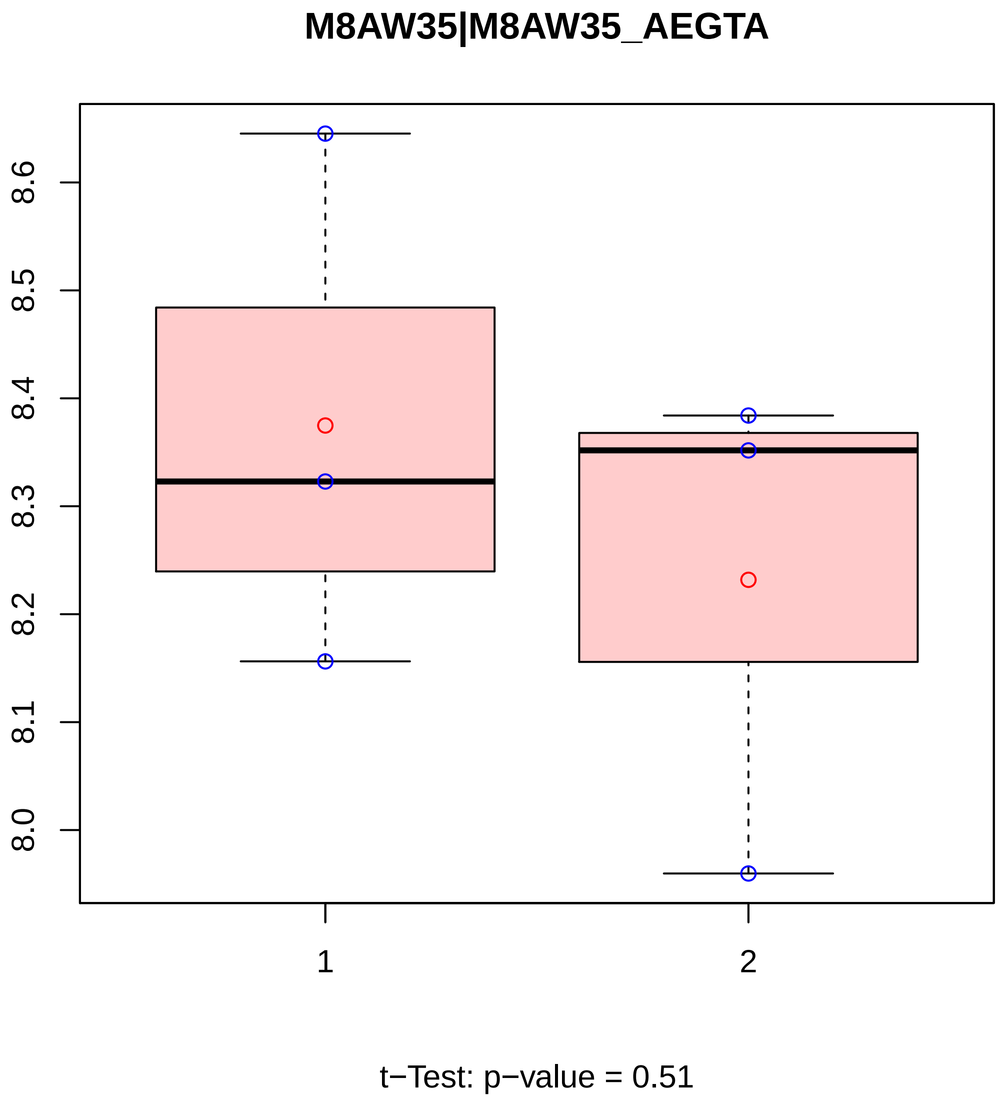
M8AW35|M8AW35_AEGTA
8.0
8.1
8.2
8.3
8.4
8.5
8.6
1
2
t−Test: p−value = 0.51

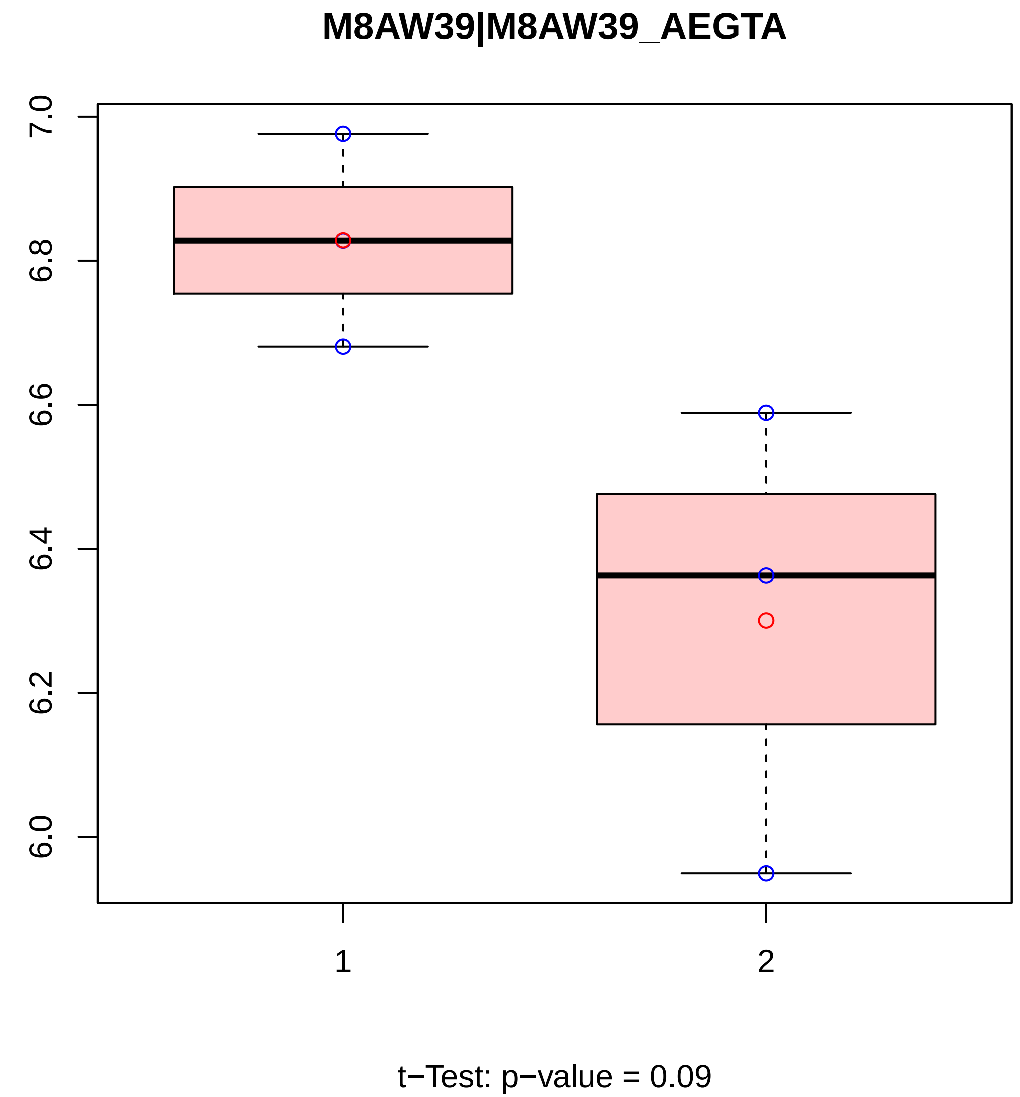
M8AW39|M8AW39_AEGTA
7.0
6.8
6.6
6.4
6.2
6.0
1
2
t−Test: p−value = 0.09

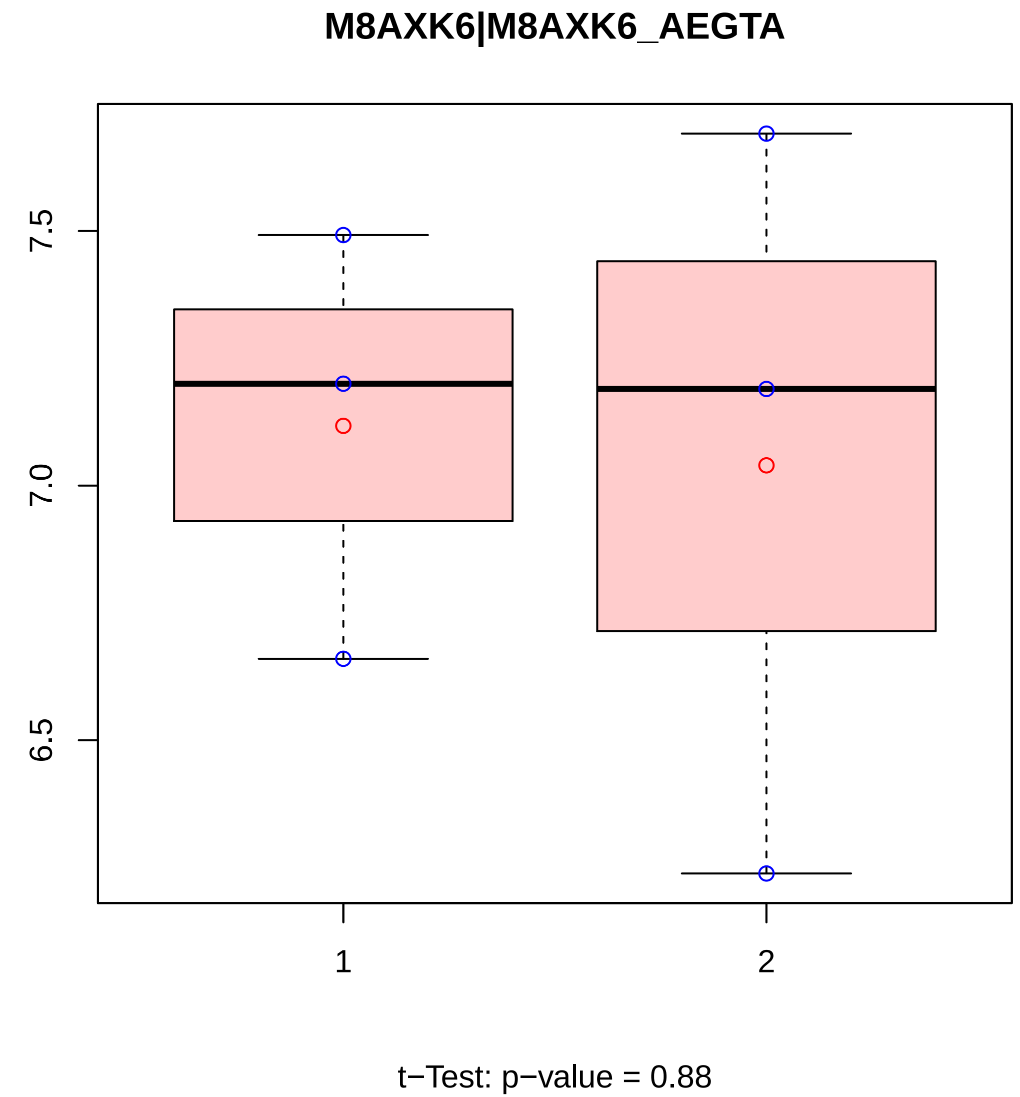
M8AXK6|M8AXK6_AEGTA
7.5
7.0
6.5
1
2
t−Test: p−value = 0.88

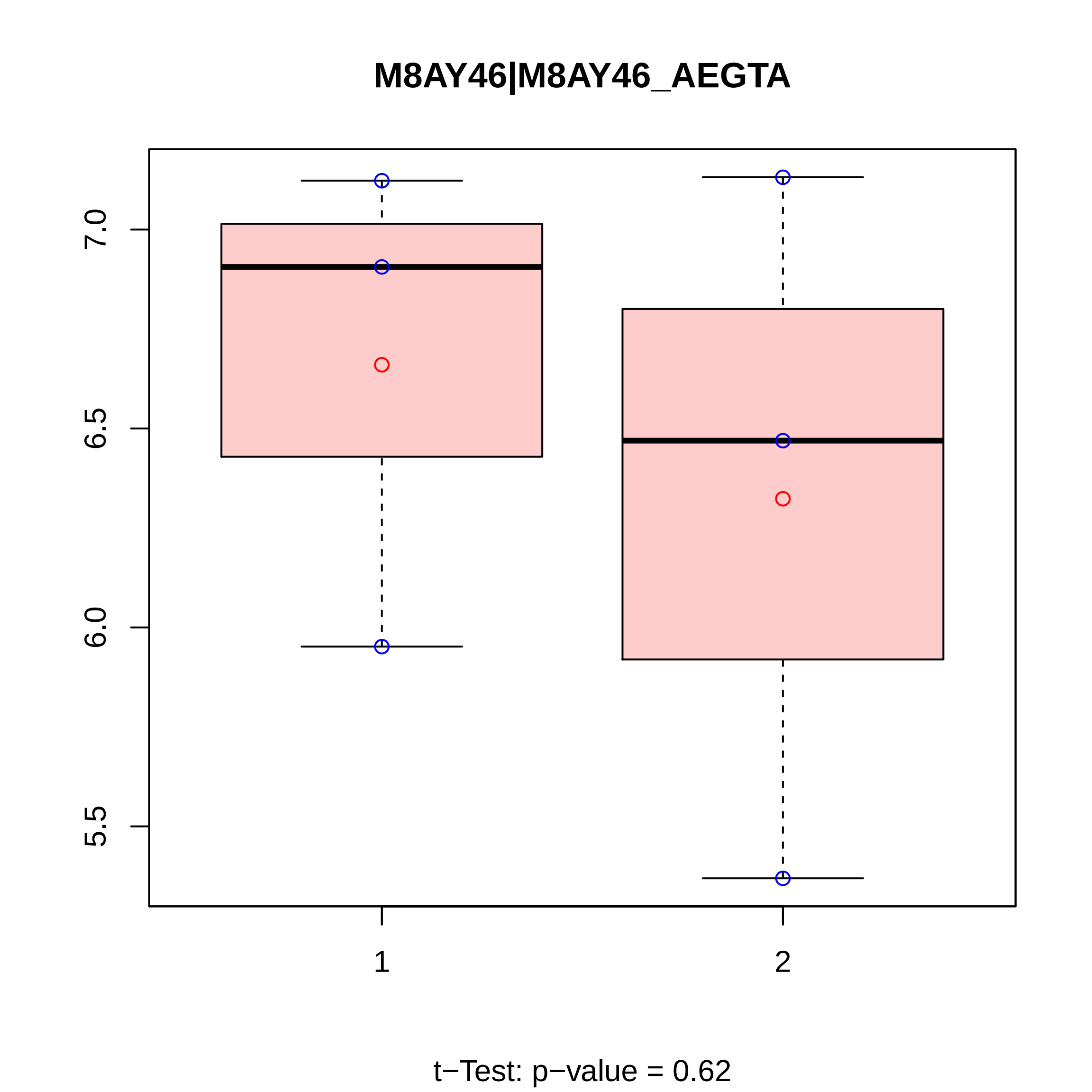
M8AY46|M8AY46_AEGTA
7.0
6.5
6.0
5.5
1
2
t−Test: p−value = 0.62

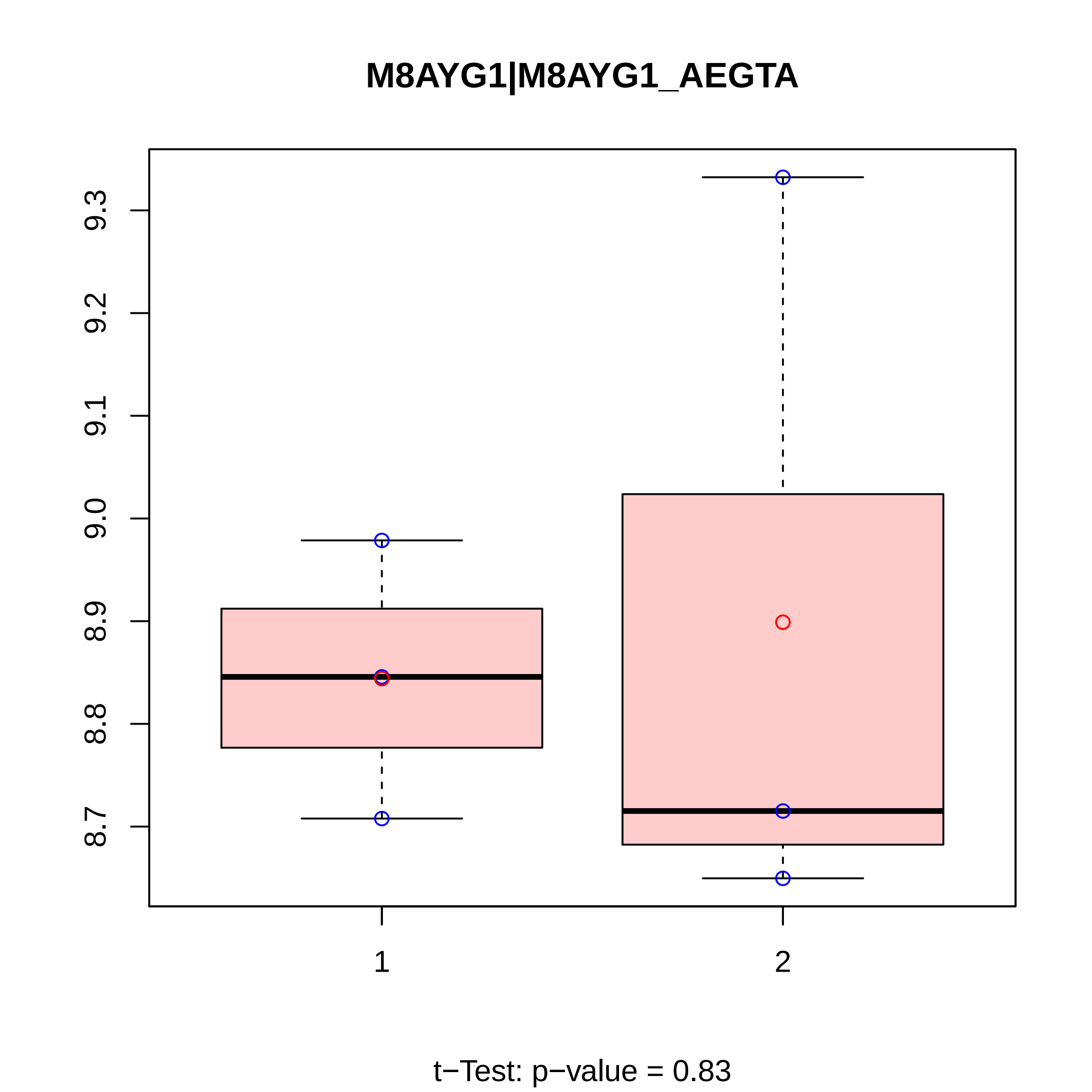
M8AYG1|M8AYG1_AEGTA
9.3
9.2
9.1
9.0
8.9
8.8
8.7
1
2
t−Test: p−value = 0.83

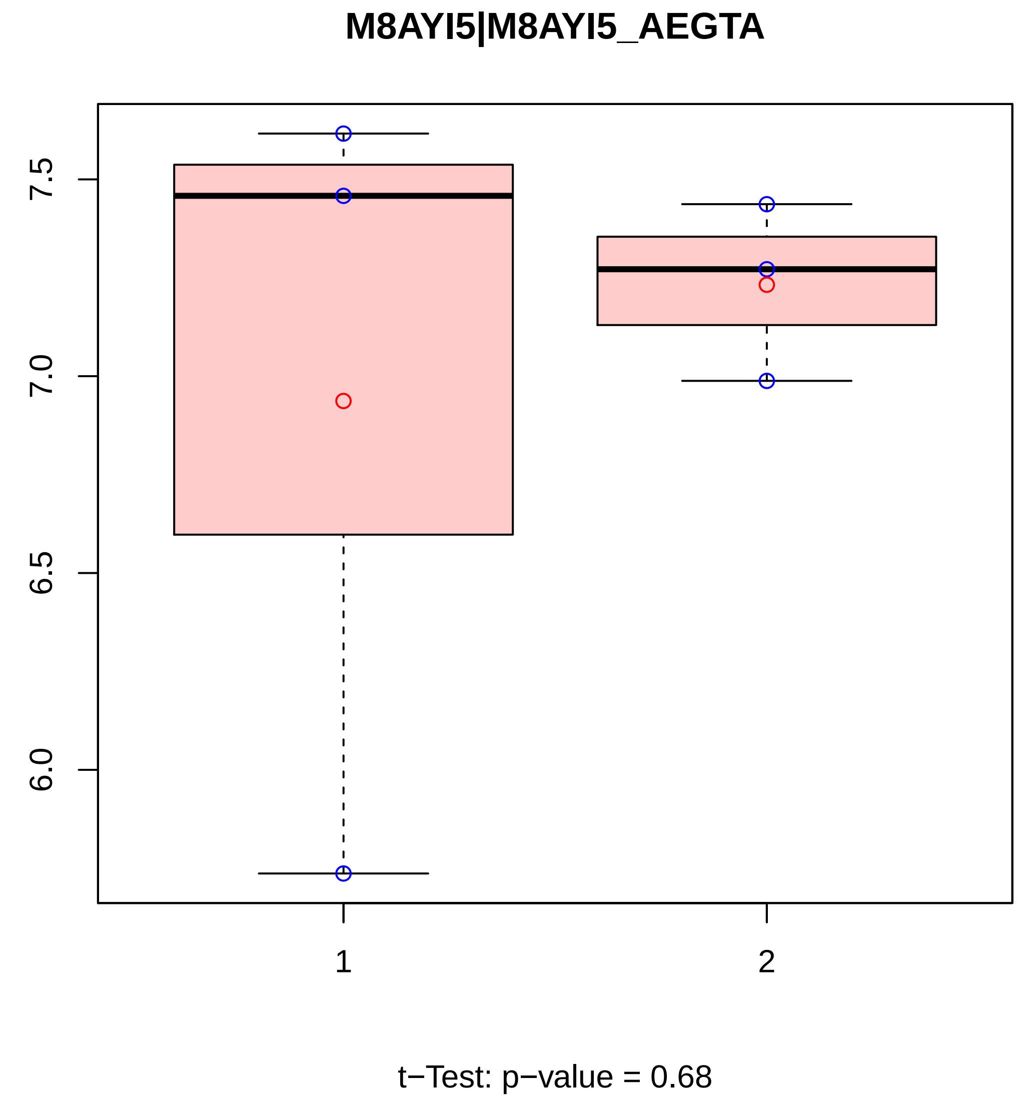
M8AYI5|M8AYI5_AEGTA
7.5
7.0
6.5
6.0
1
2
t−Test: p−value = 0.68

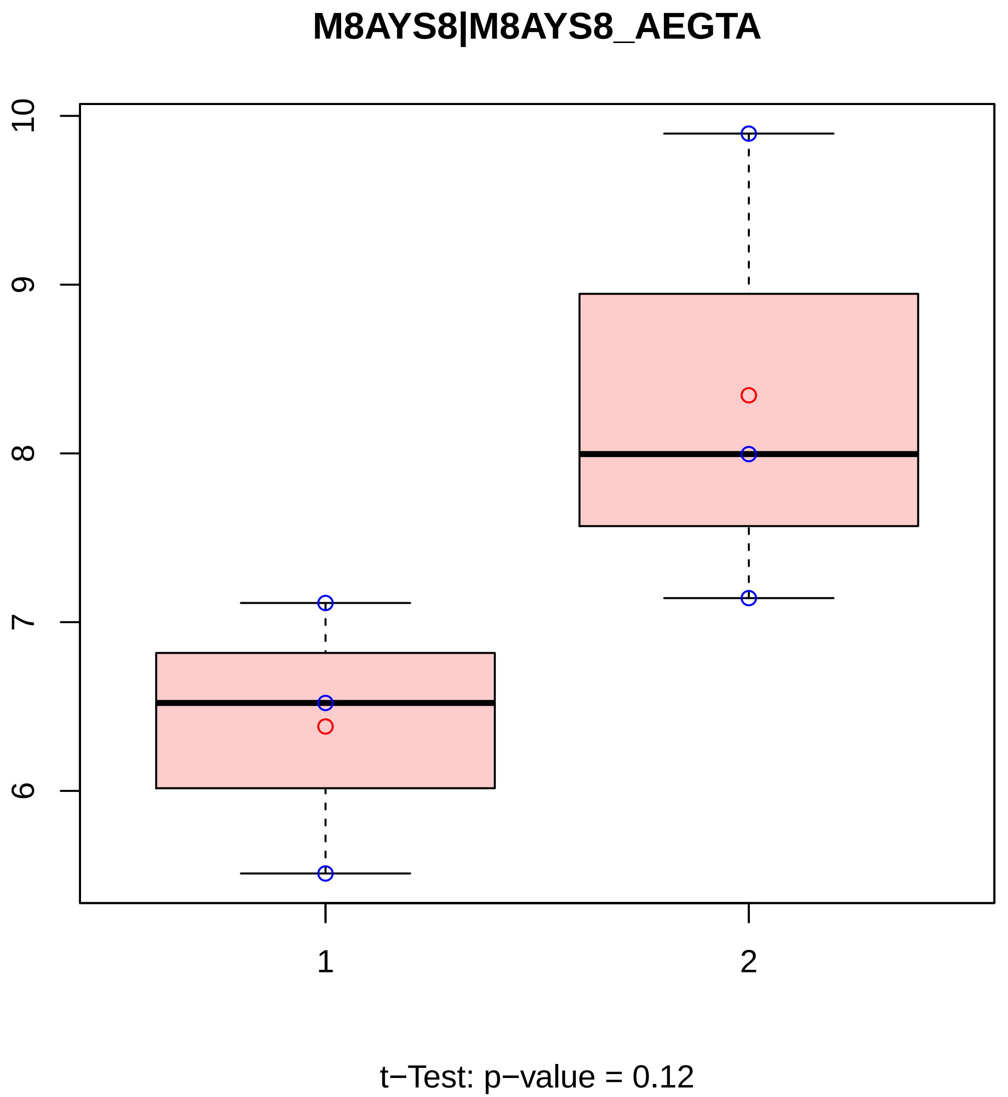
M8AYS8|M8AYS8_AEGTA
10
9
8
7
6
1
2
t−Test: p−value = 0.12

M8AZ01|M8AZ01_AEGTA

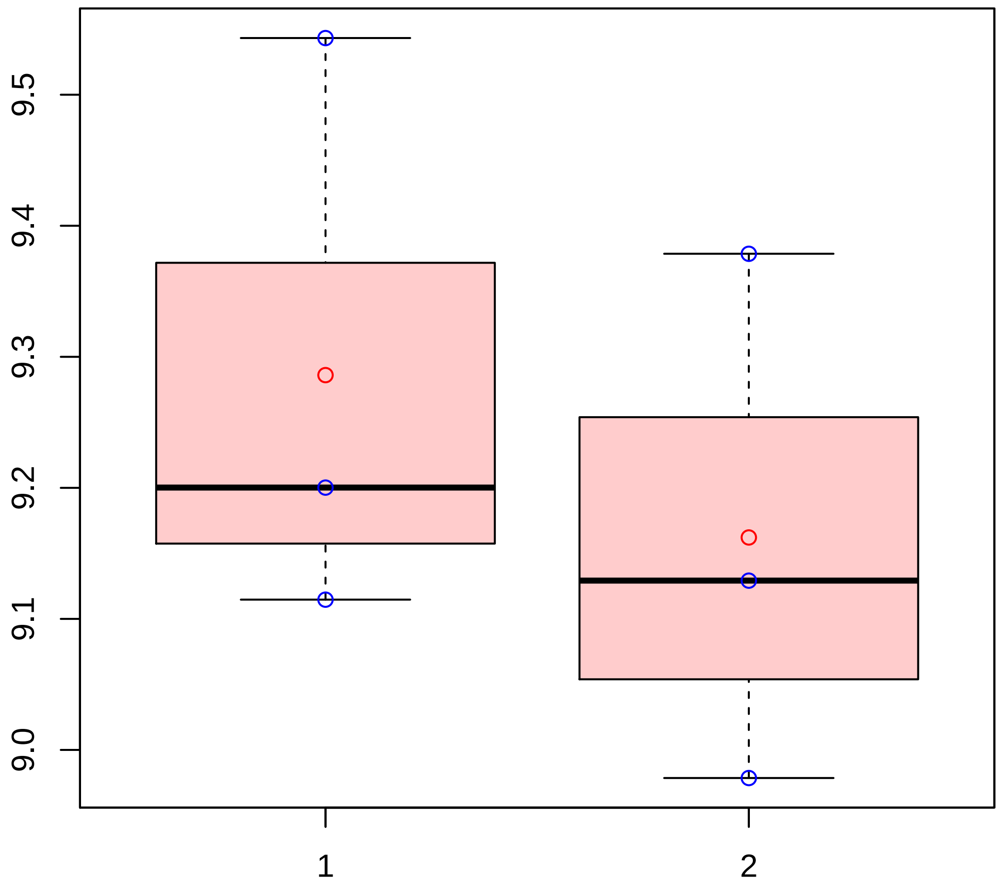

t−Test: p−value = 0.52

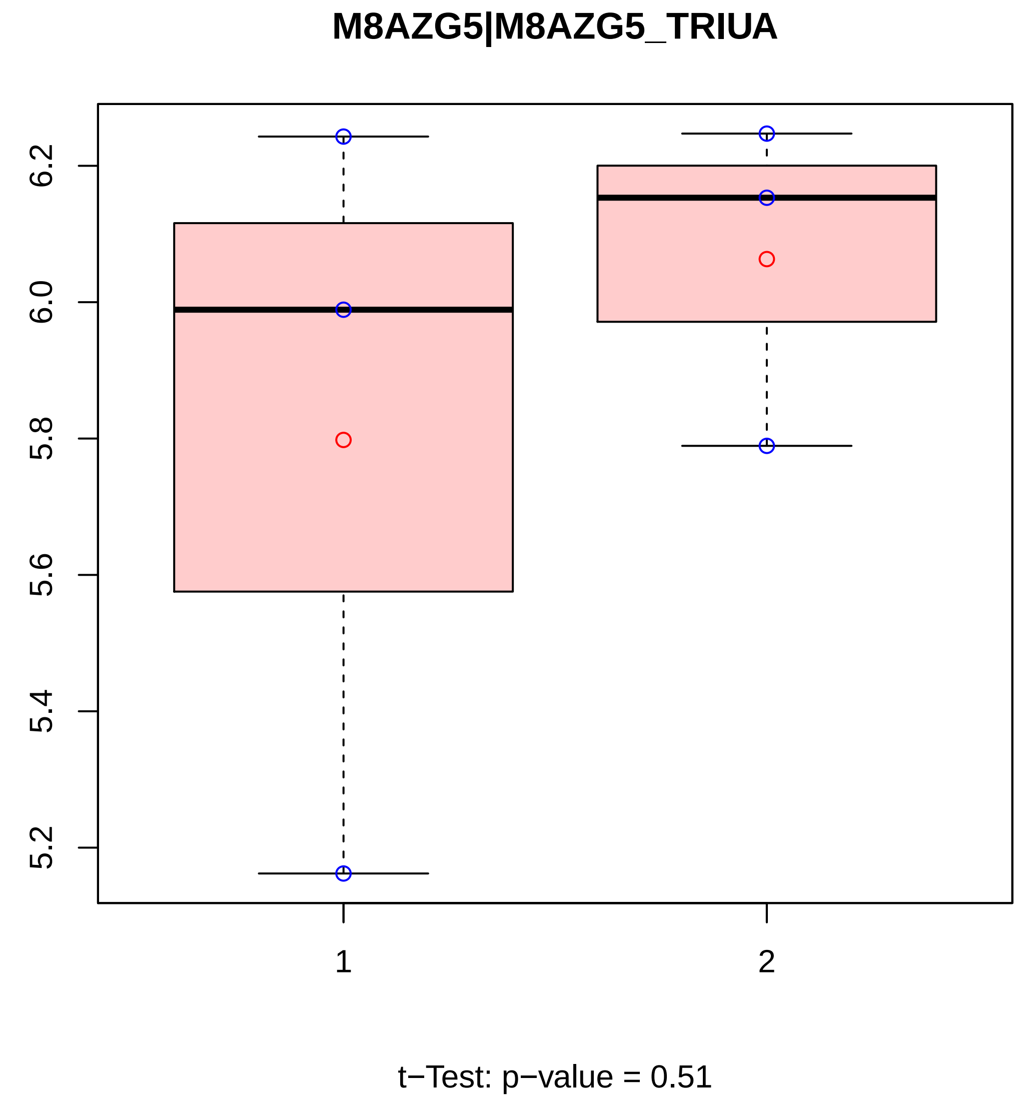
M8AZG5|M8AZG5_TRIUA
6.2
6.0
5.8
5.6
5.4
5.2
1
2
t−Test: p−value = 0.51

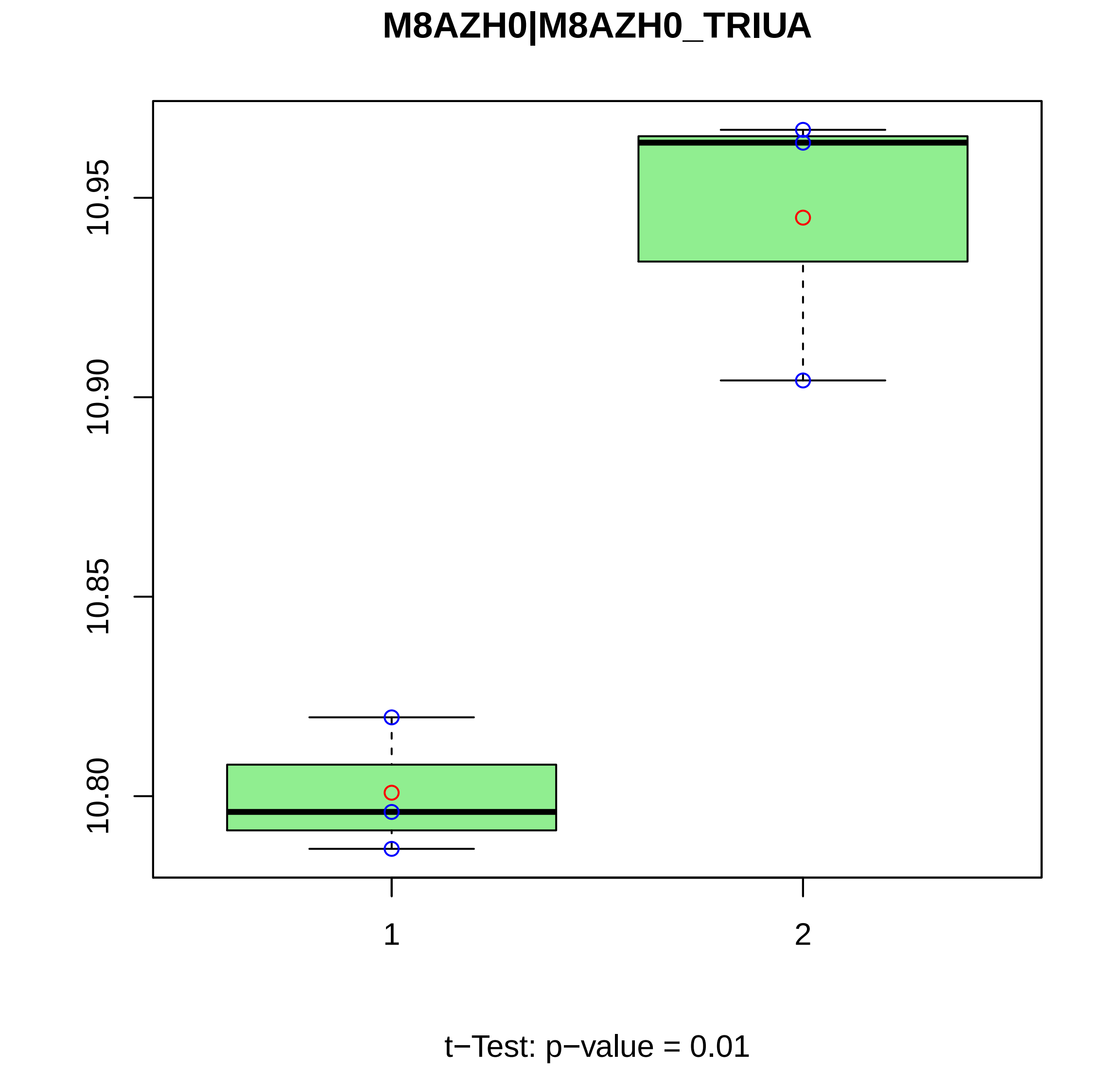
M8AZH0|M8AZH0_TRIUA
10.80
10.85
10.90
10.95
1
2
t−Test: p−value = 0.01

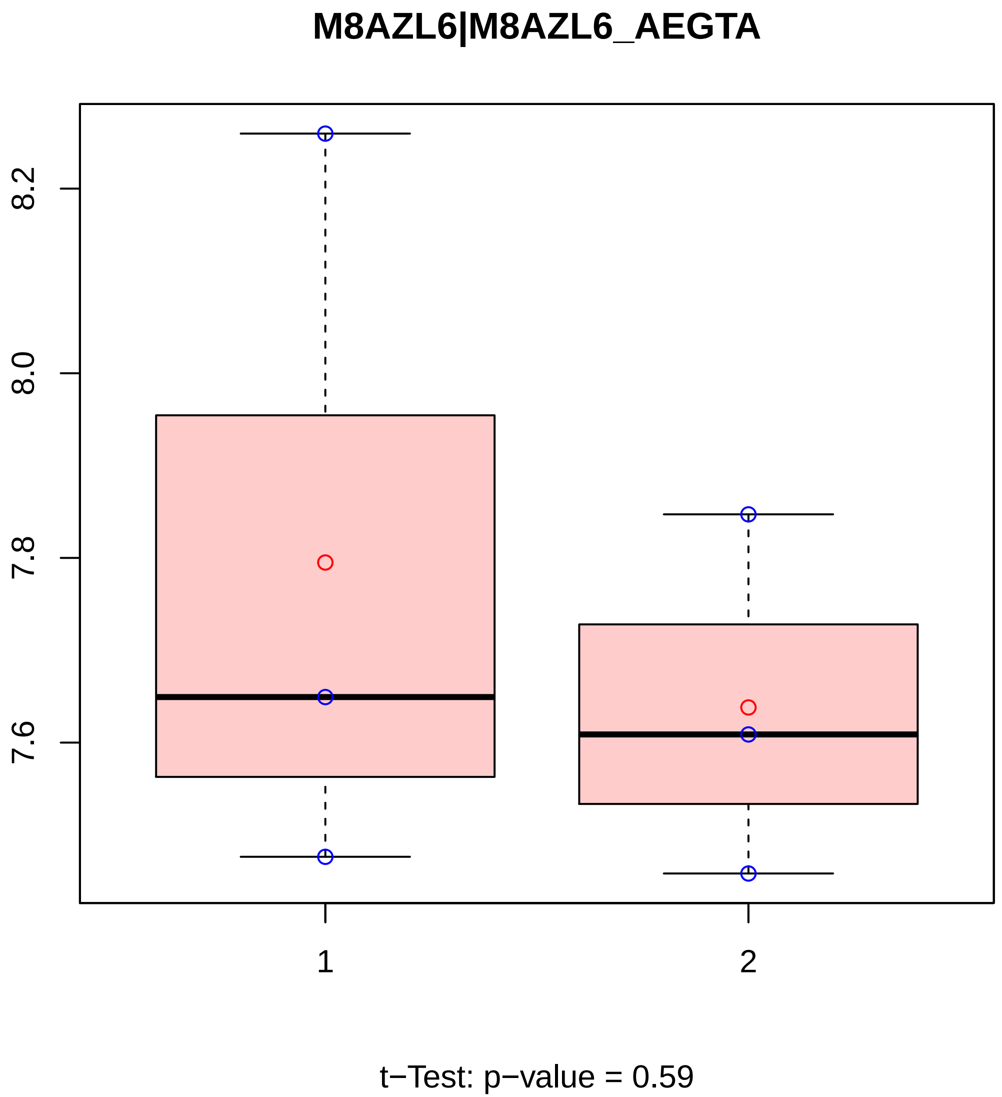

M8AZL6|M8AZL6_AEGTA
8.2
8.0
7.8
7.6
1
2
t−Test: p−value = 0.59

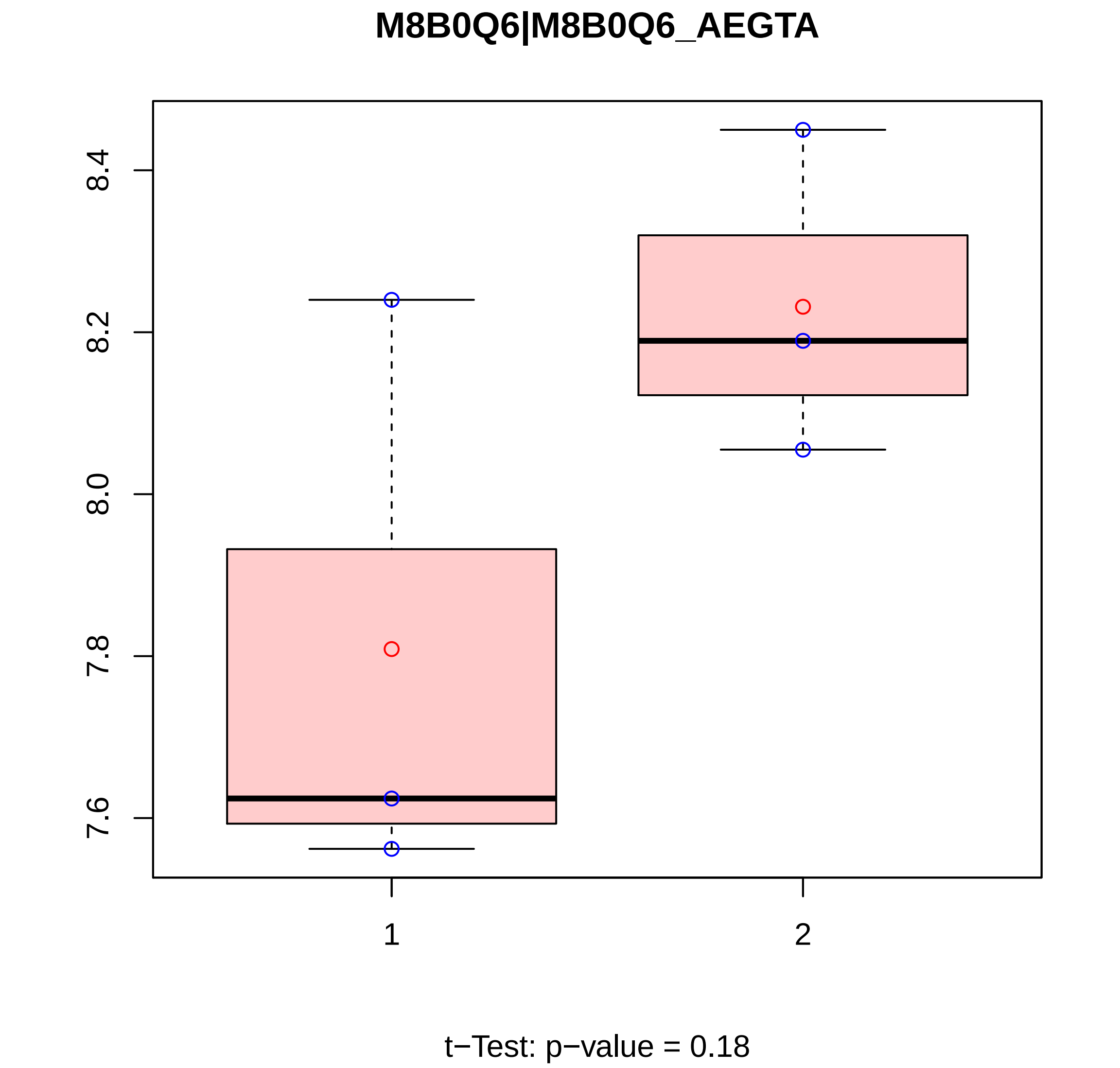
M8B0Q6|M8B0Q6_AEGTA
8.4
8.2
8.0
7.8
7.6
1
2
t−Test: p−value = 0.18

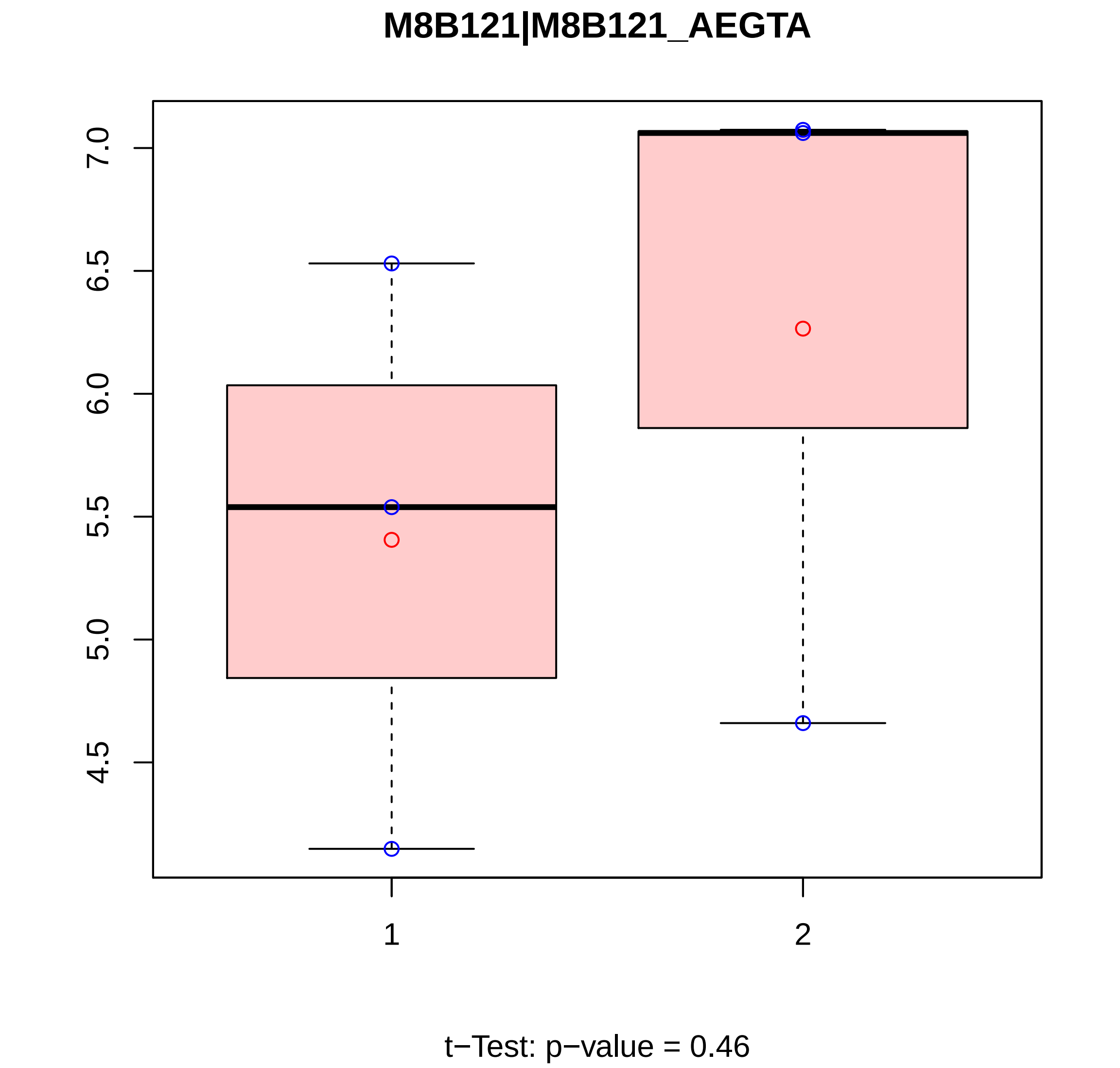

M8B121|M8B121_AEGTA
7.0
6.5
6.0
5.5
5.0
4.5
1
2
t−Test: p−value = 0.46

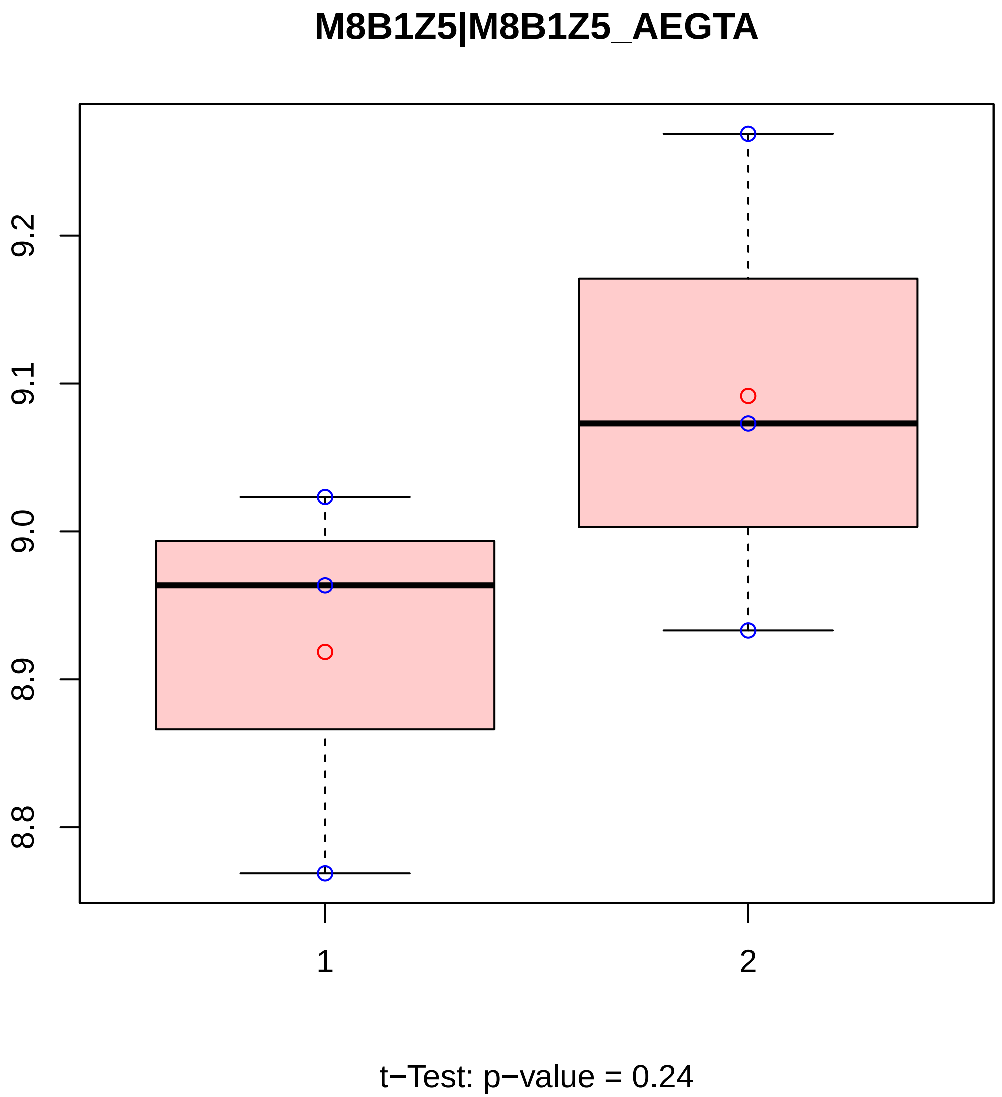

M8B1Z5|M8B1Z5_AEGTA
9.2
9.1
9.0
8.9
8.8
1
2
t−Test: p−value = 0.24

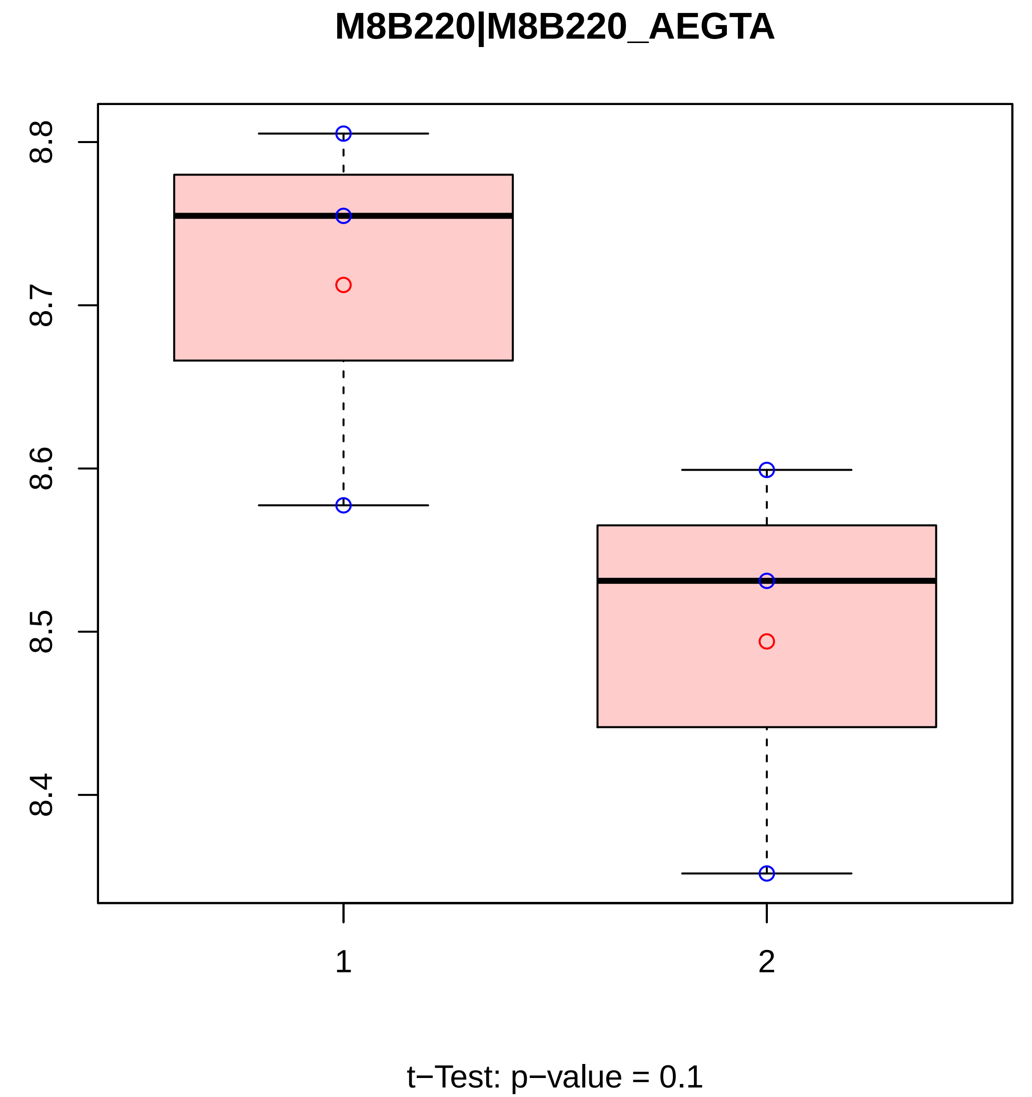
M8B220|M8B220_AEGTA
8.8
8.7
8.6
8.5
8.4
1
2
t−Test: p−value = 0.1

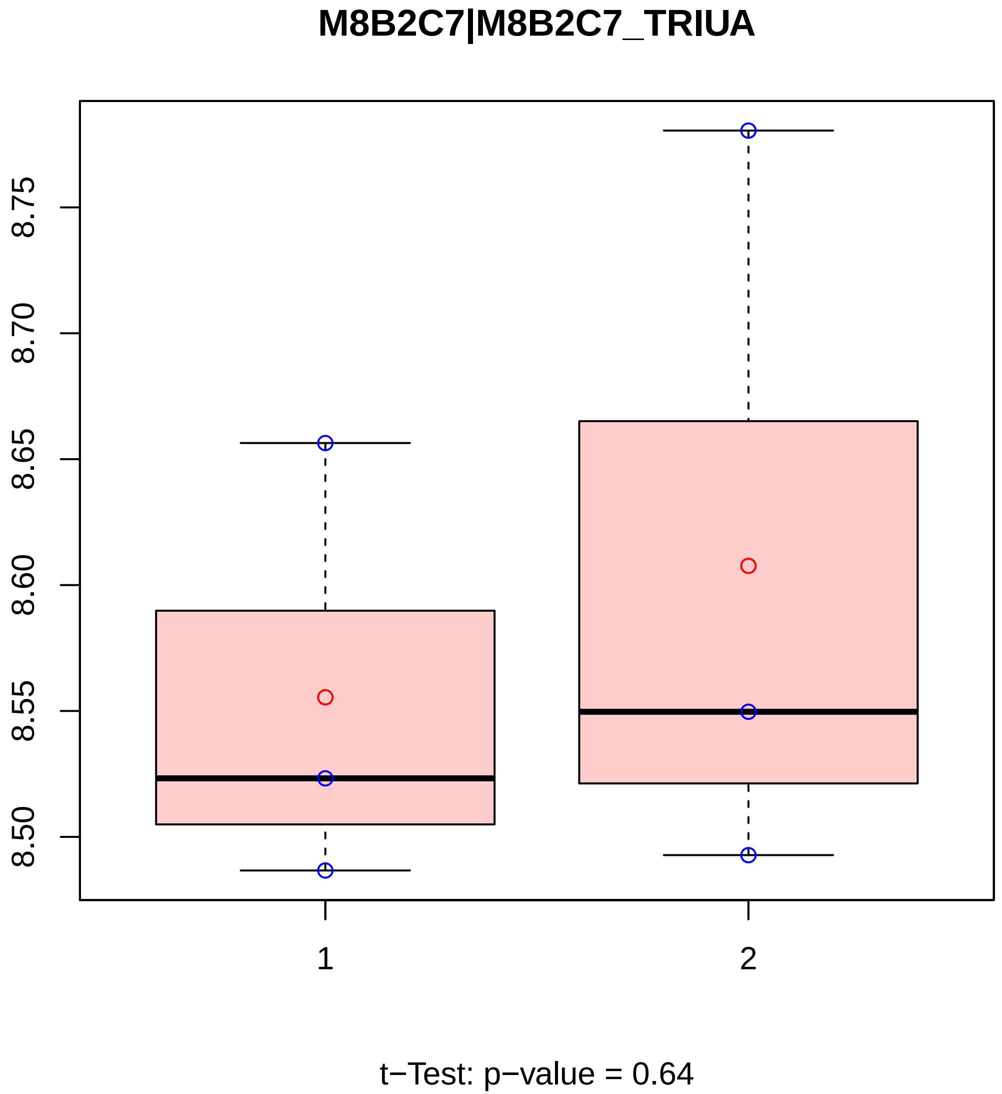
M8B2C7|M8B2C7_TRIUA
8.50
8.55
8.60
8.65
8.70
8.75
1
2
t−Test: p−value = 0.64

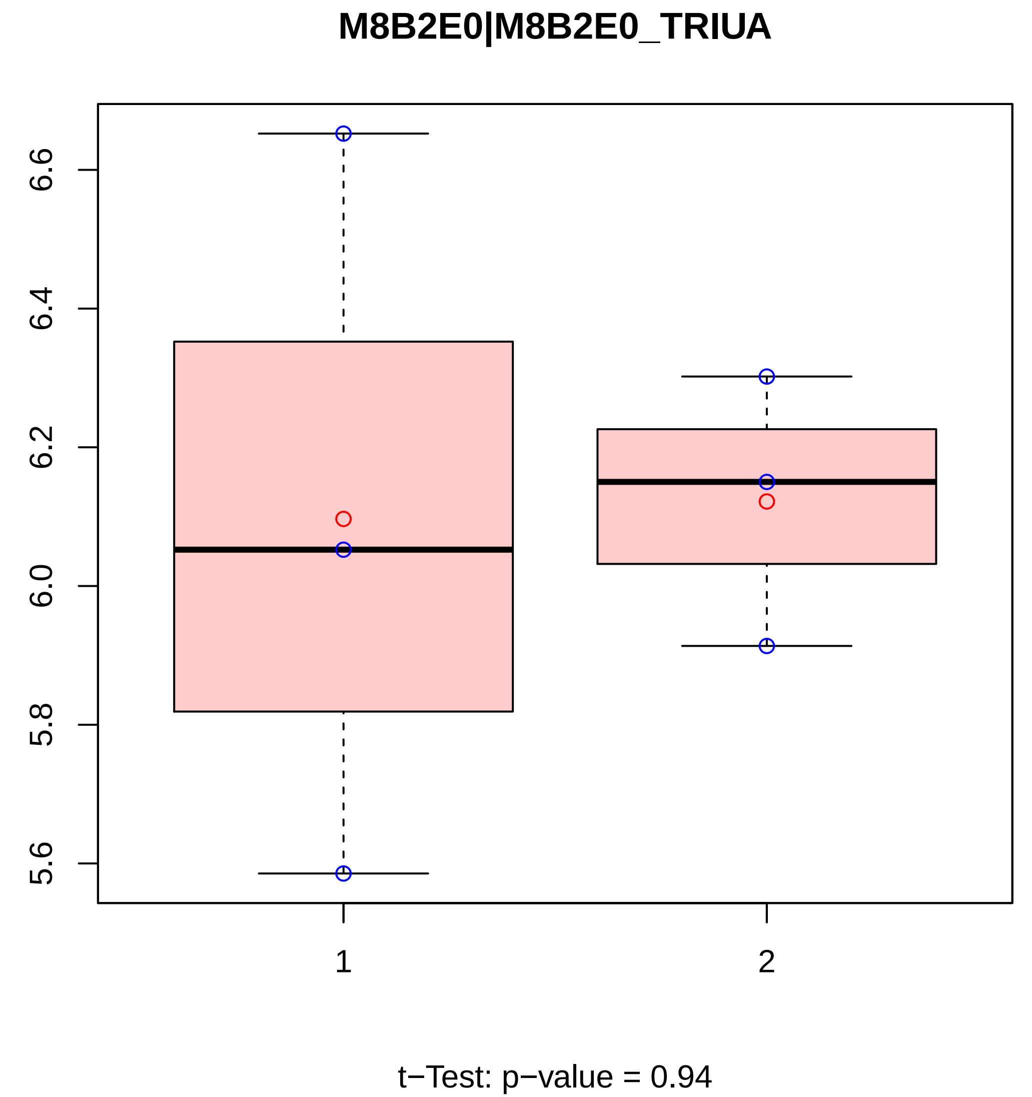

M8B2E0|M8B2E0_TRIUA
6.6
6.4
6.2
6.0
5.8
5.6
1
2
t−Test: p−value = 0.94

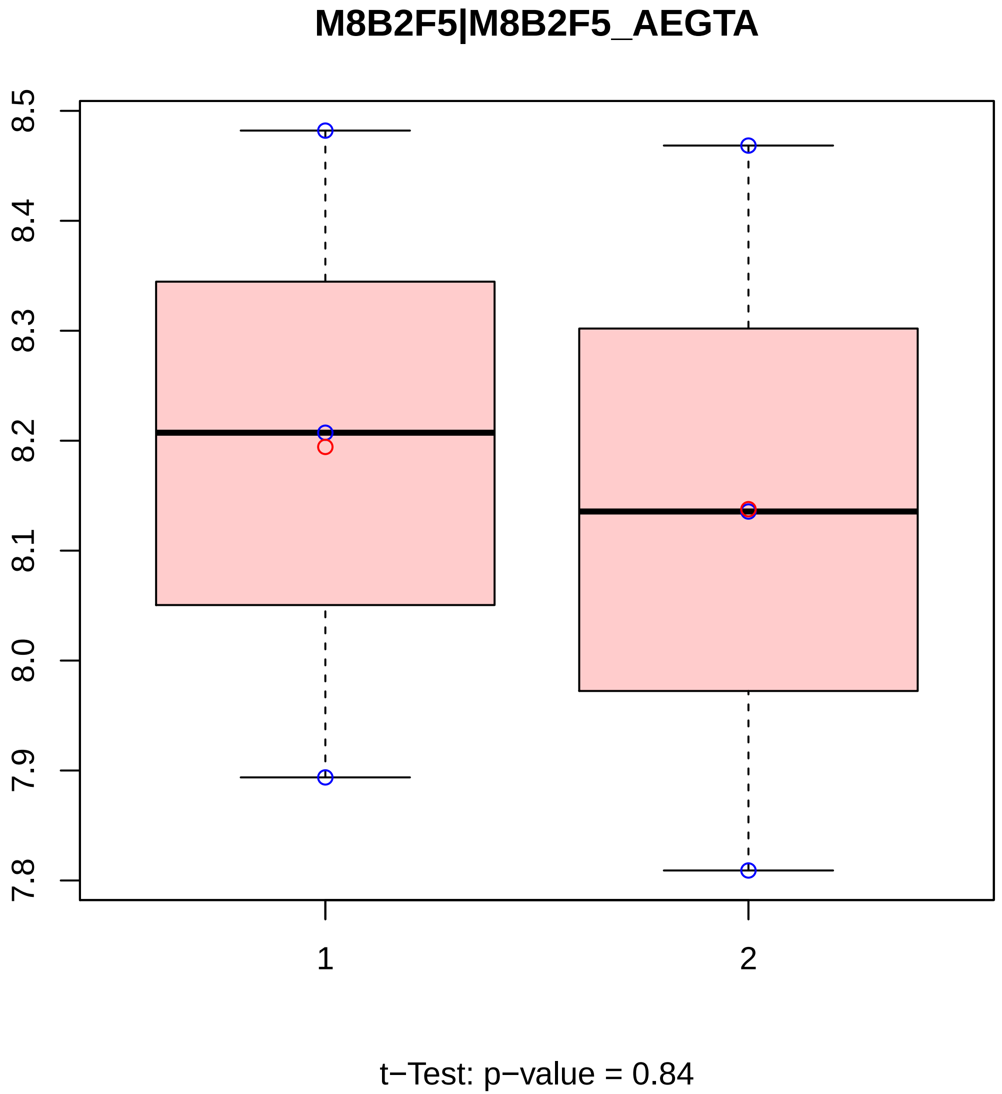
M8B2F5|M8B2F5_AEGTA
8.5
8.4
8.3
8.2
8.1
8.0
7.9
7.8
1
2
t−Test: p−value = 0.84

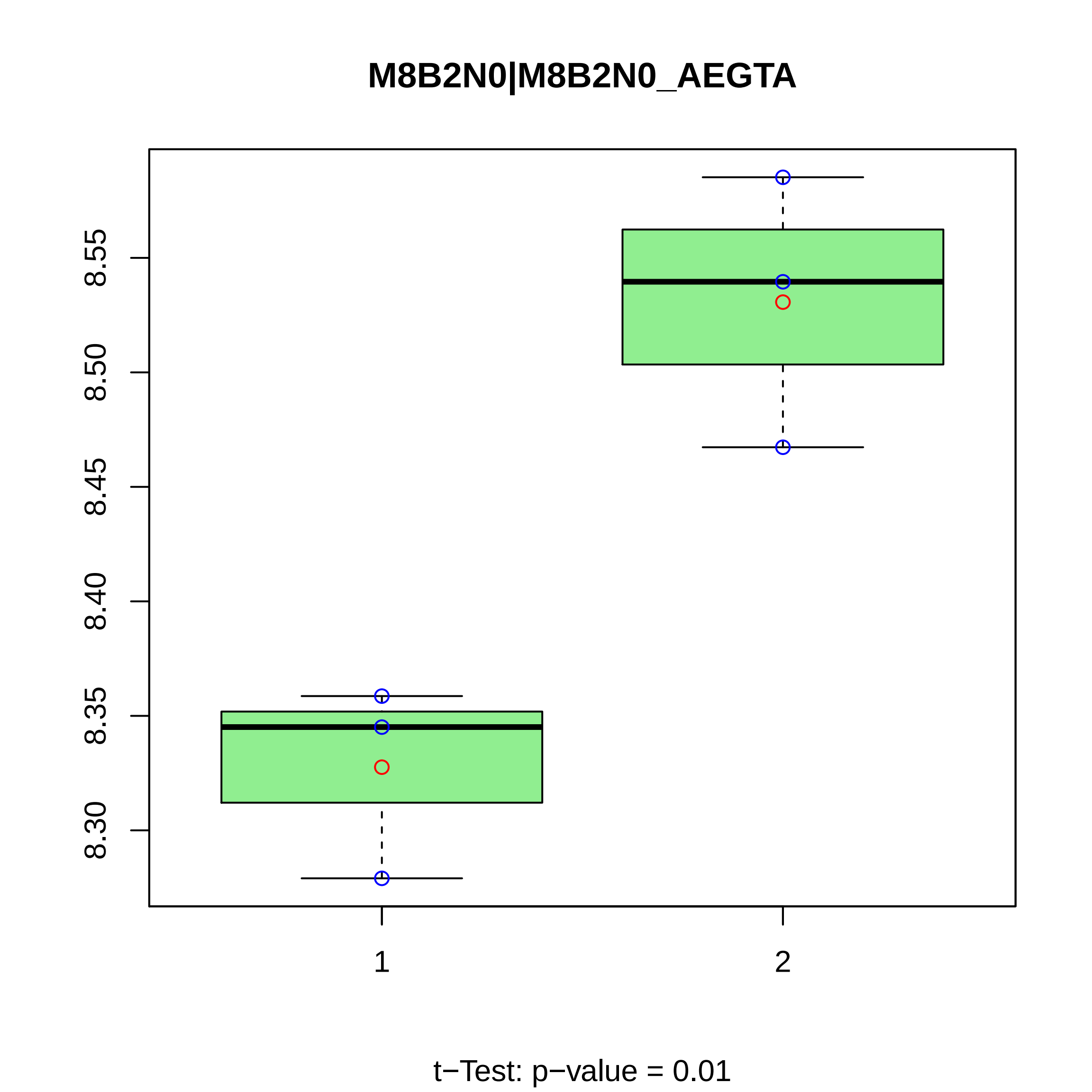
M8B2N0|M8B2N0_AEGTA
8.30
8.35
8.40
8.45
8.50
8.55
1
2
t−Test: p−value = 0.01

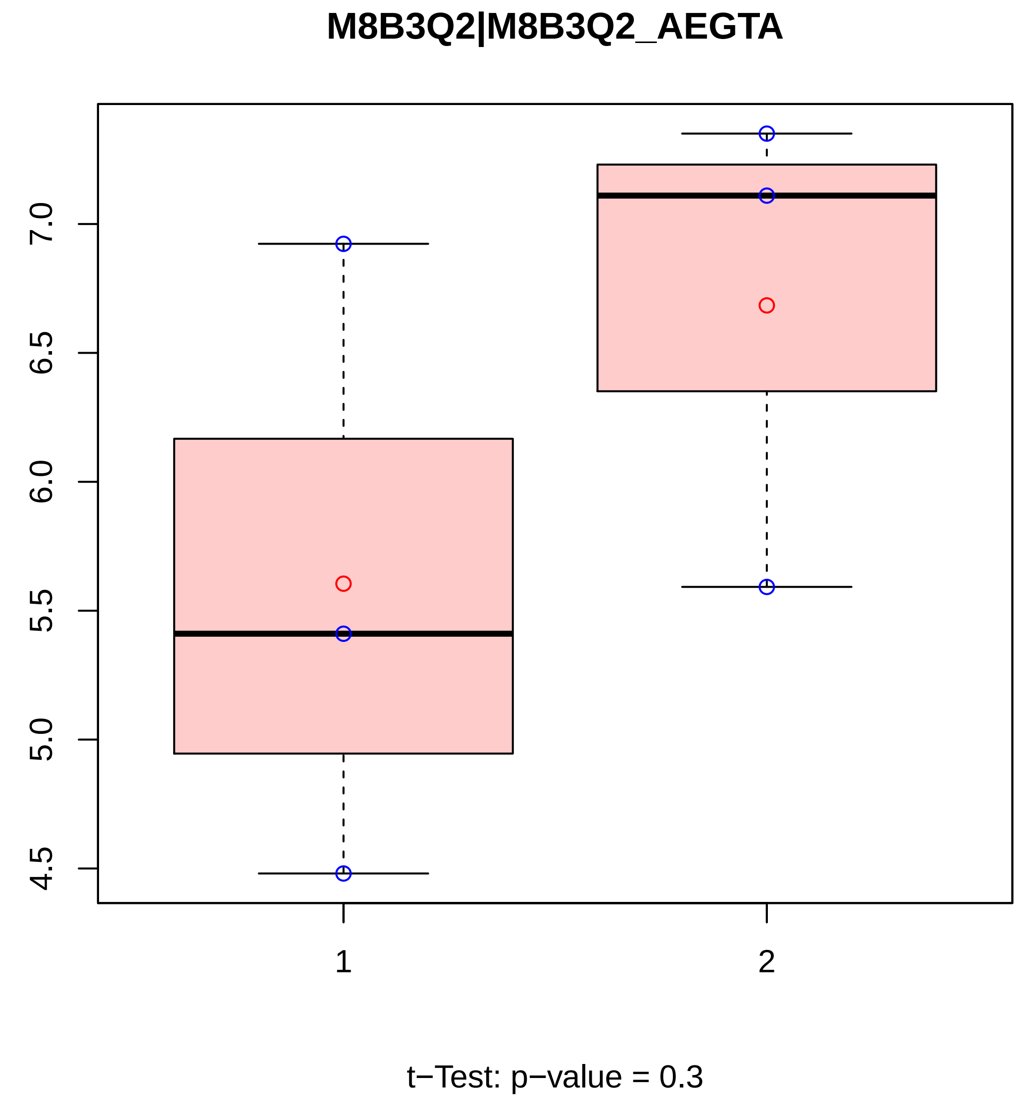

M8B3Q2|M8B3Q2_AEGTA
7.0
6.5
6.0
5.5
5.0
4.5
1
2
t−Test: p−value = 0.3

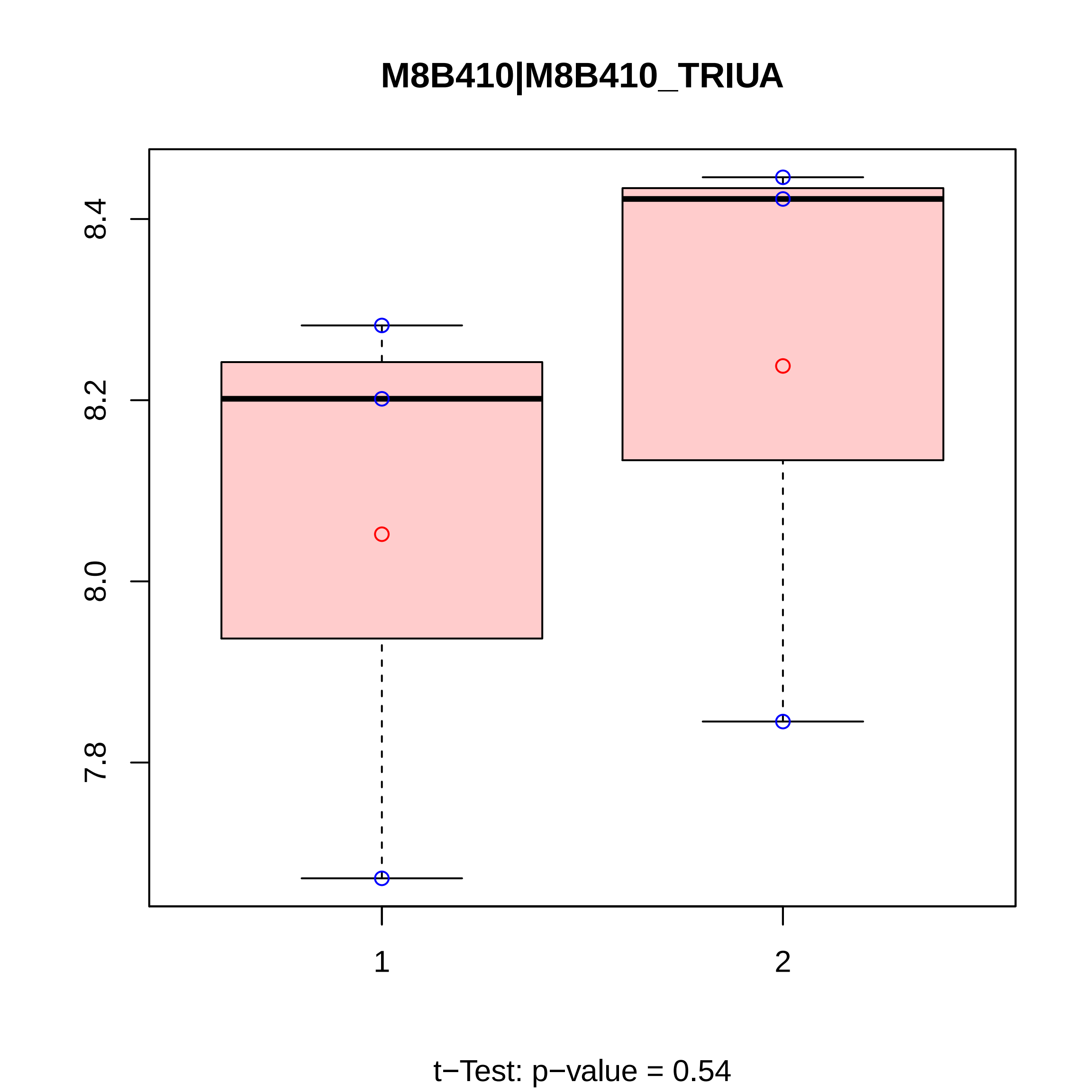
M8B410|M8B410_TRIUA
8.4
8.2
8.0
7.8
1
2
t−Test: p−value = 0.54

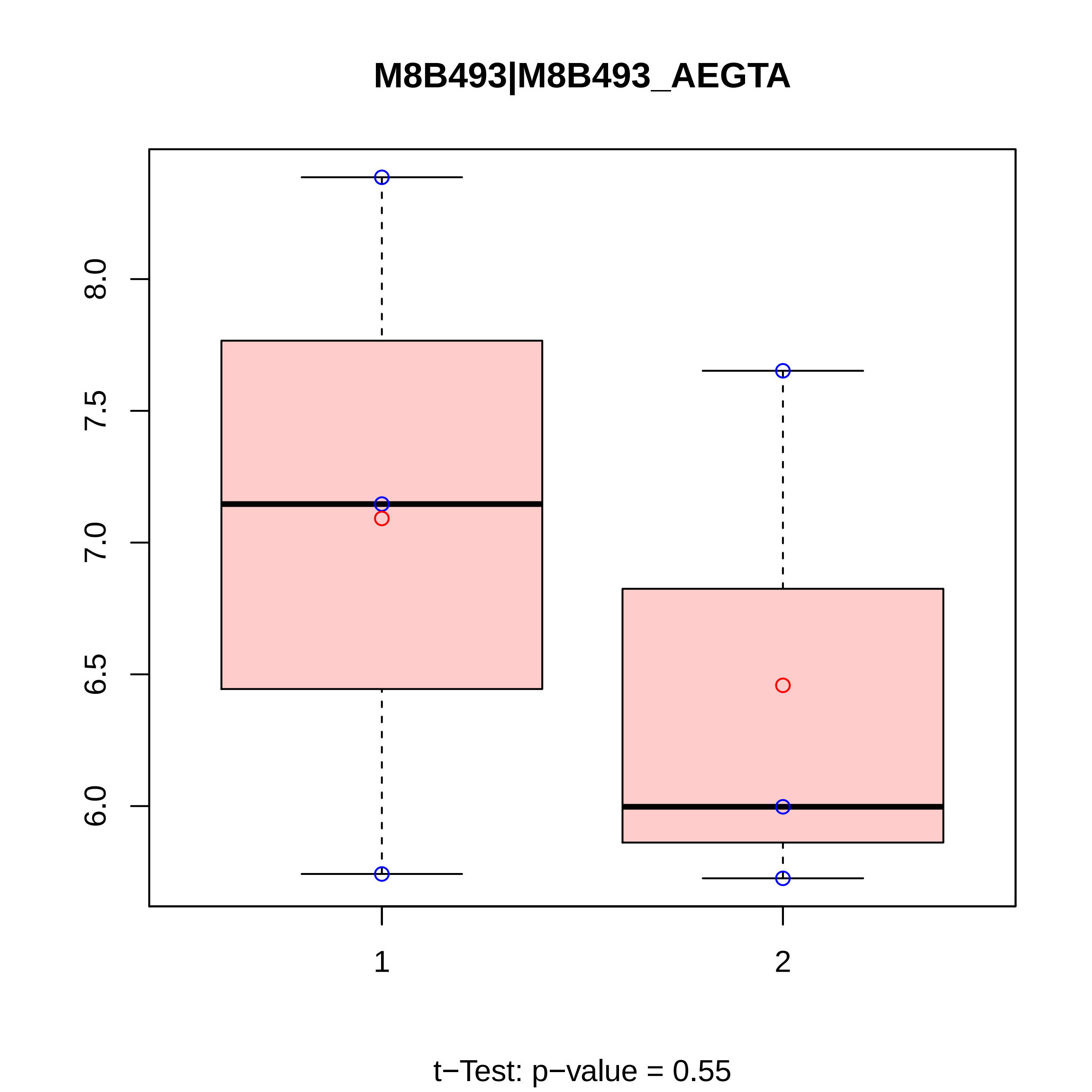

M8B493|M8B493_AEGTA
8.0
7.5
7.0
6.5
6.0
1
2
t−Test: p−value = 0.55

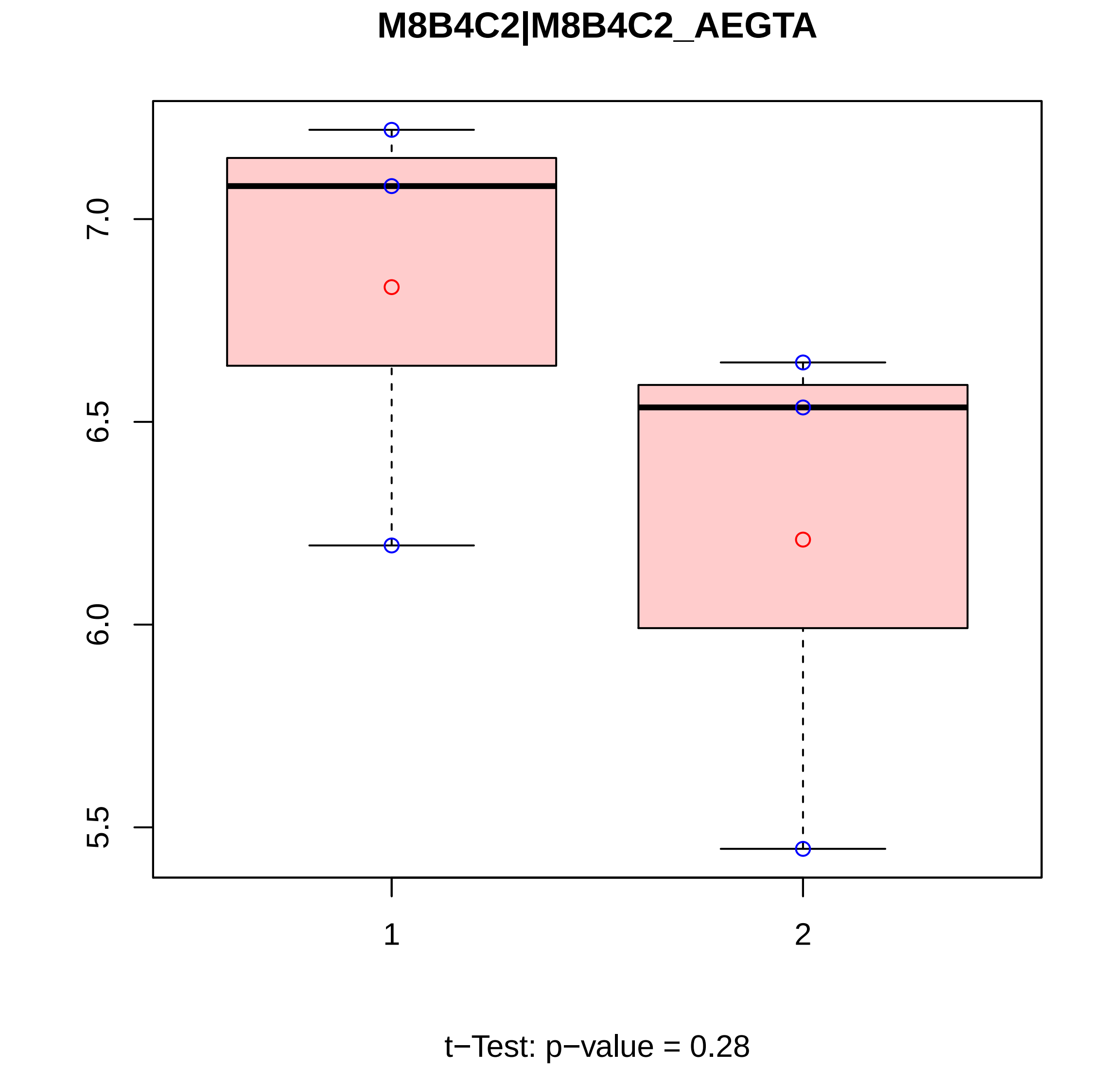
M8B4C2|M8B4C2_AEGTA
7.0
6.5
6.0
5.5
1
2
t−Test: p−value = 0.28

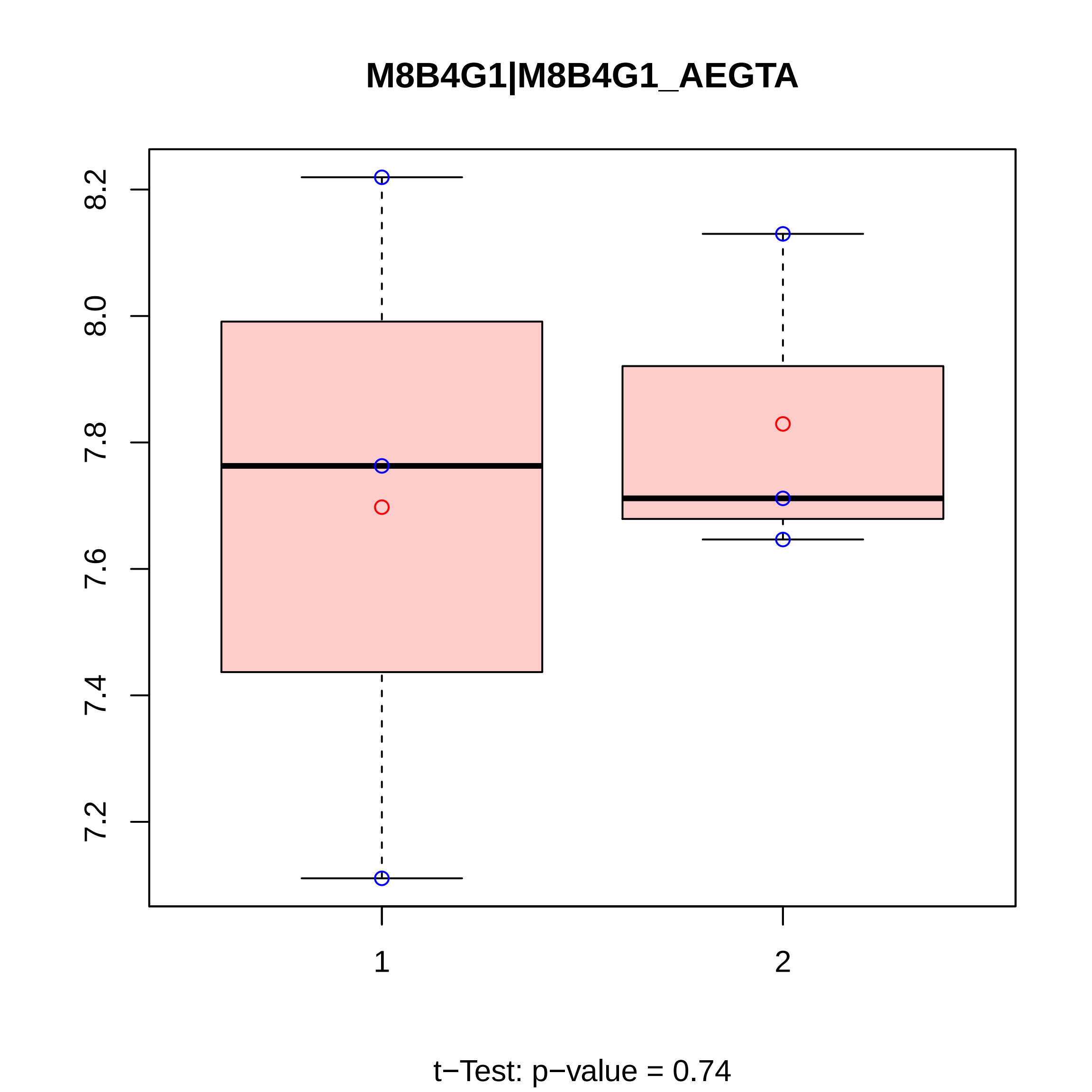

M8B4G1|M8B4G1_AEGTA
8.2
8.0
7.8
7.6
7.4
7.2
1
2
t−Test: p−value = 0.74

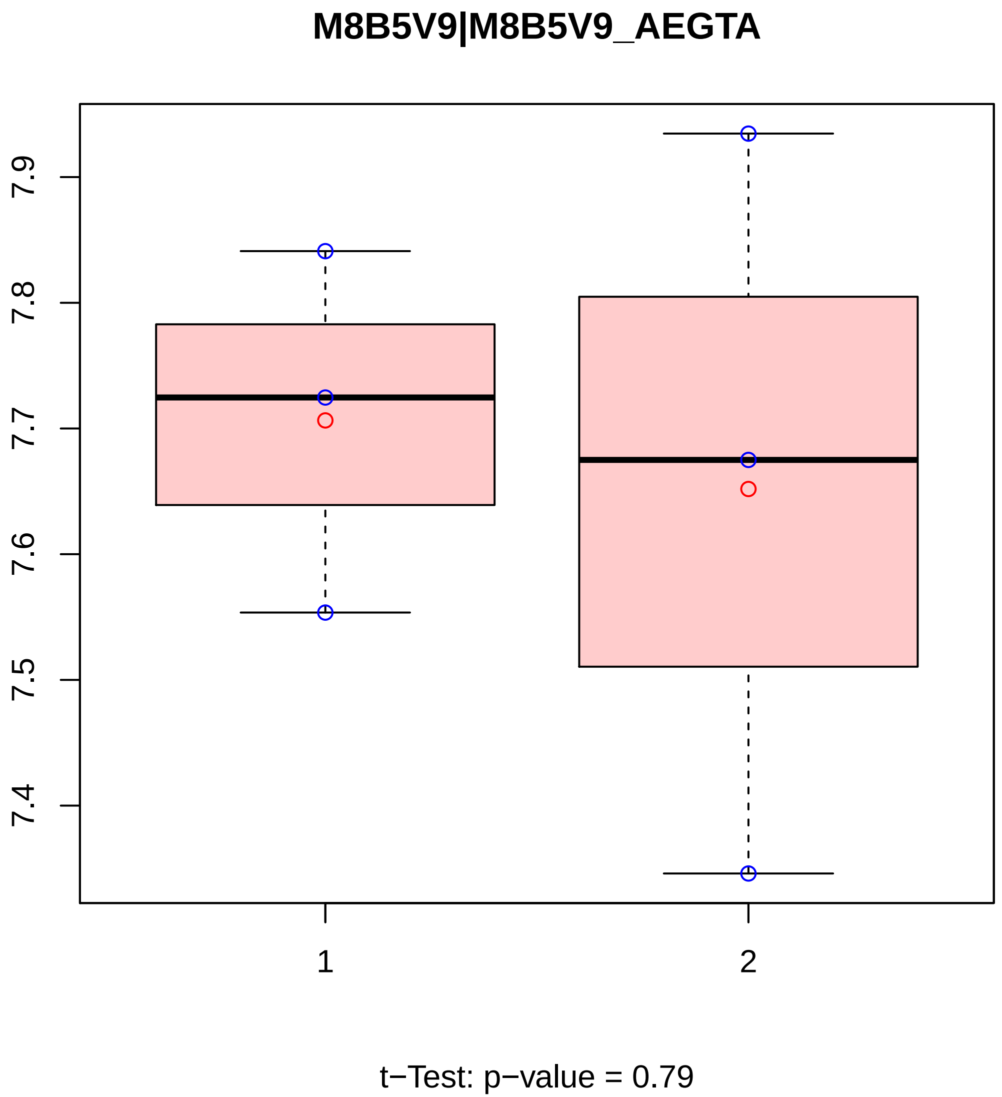
M8B5V9|M8B5V9_AEGTA
7.9
7.8
7.7
7.6
7.5
7.4
1
2
t−Test: p−value = 0.79

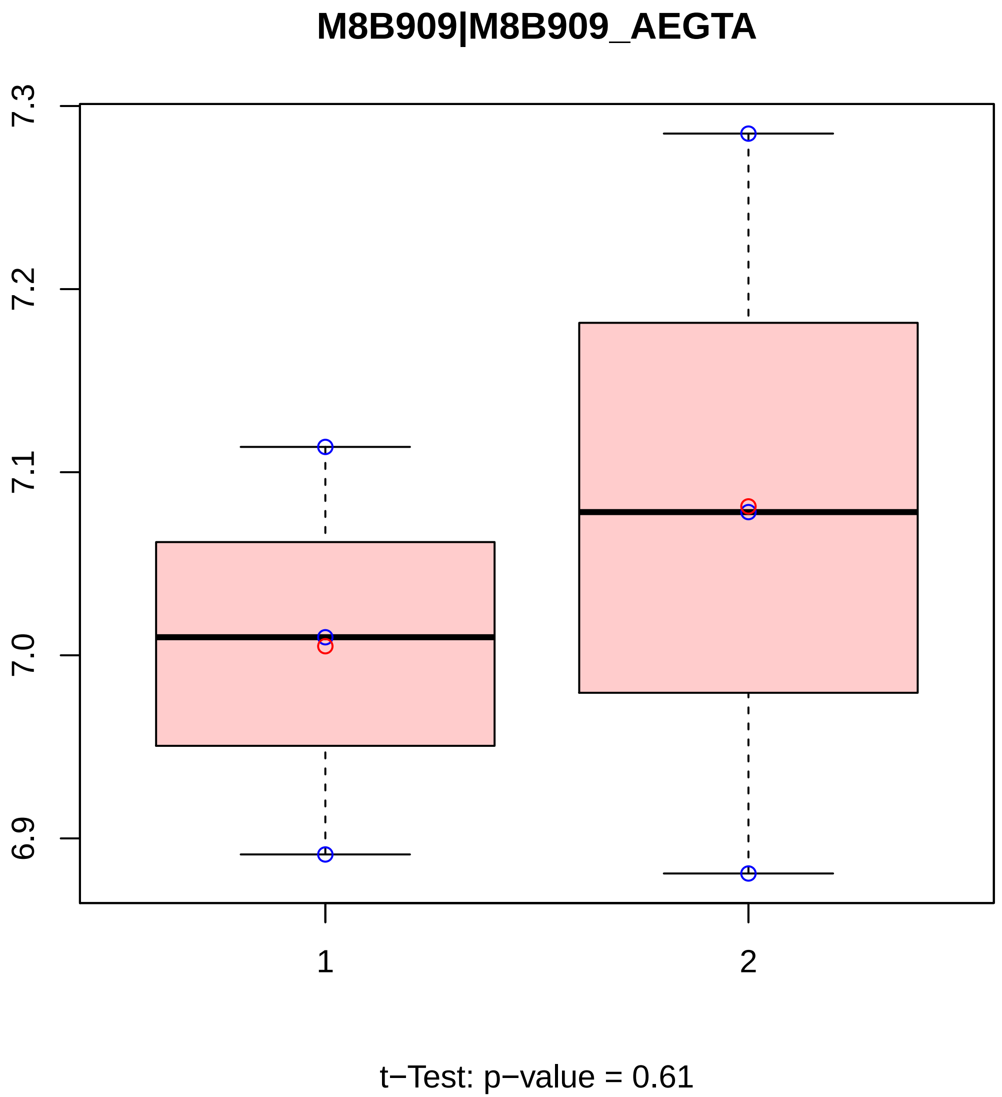

M8B909|M8B909_AEGTA
7.3
7.2
7.1
7.0
6.9
1
2
t−Test: p−value = 0.61

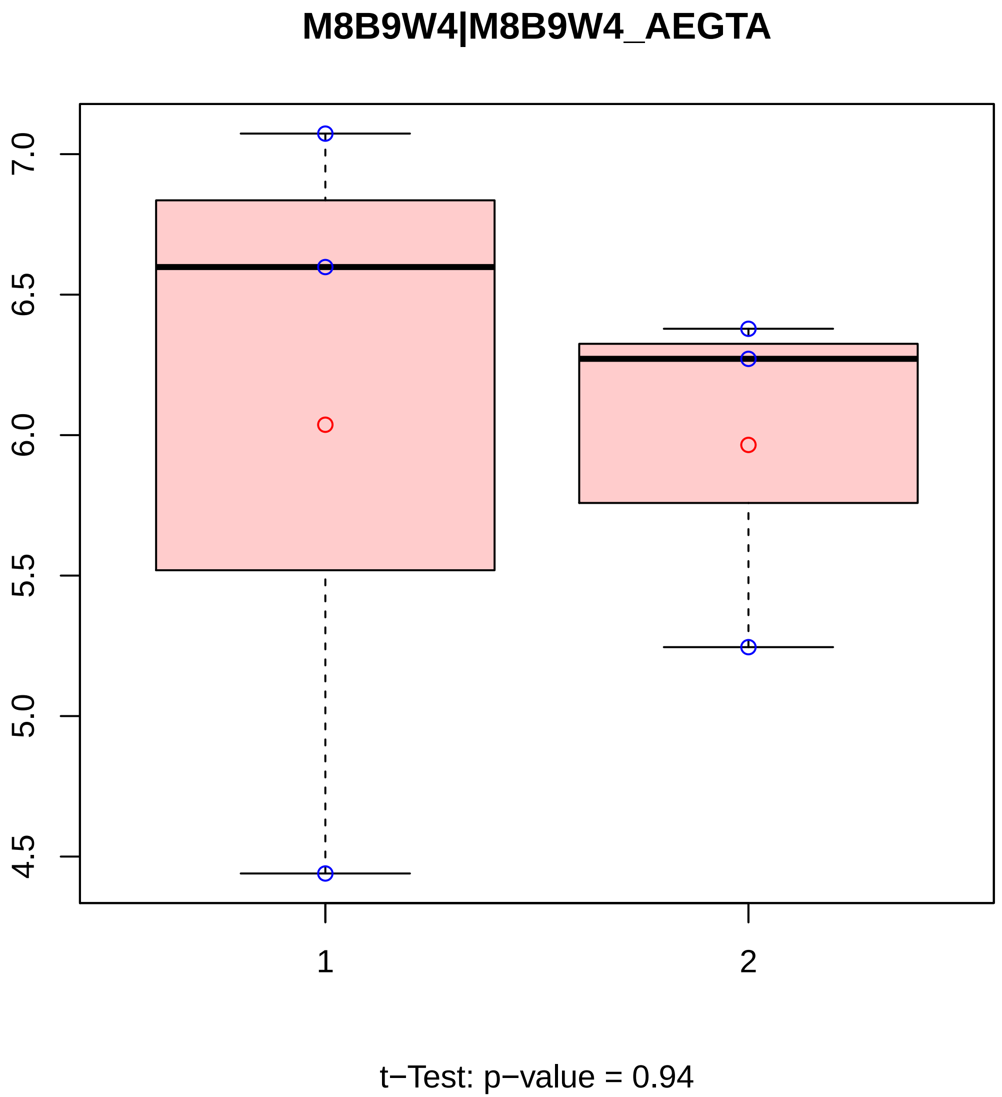
M8B9W4|M8B9W4_AEGTA
7.0
6.5
6.0
5.5
5.0
4.5
1
2
t−Test: p−value = 0.94

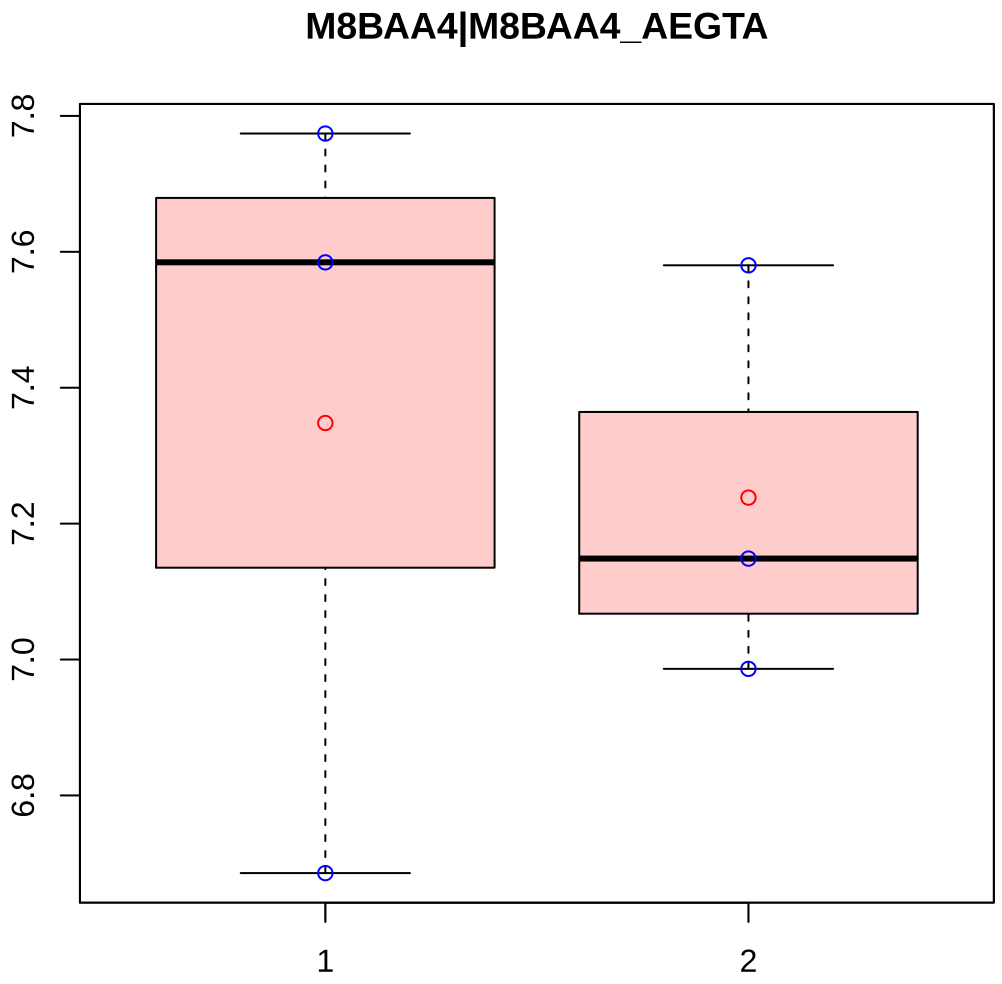

t−Test: p−value = 0.79

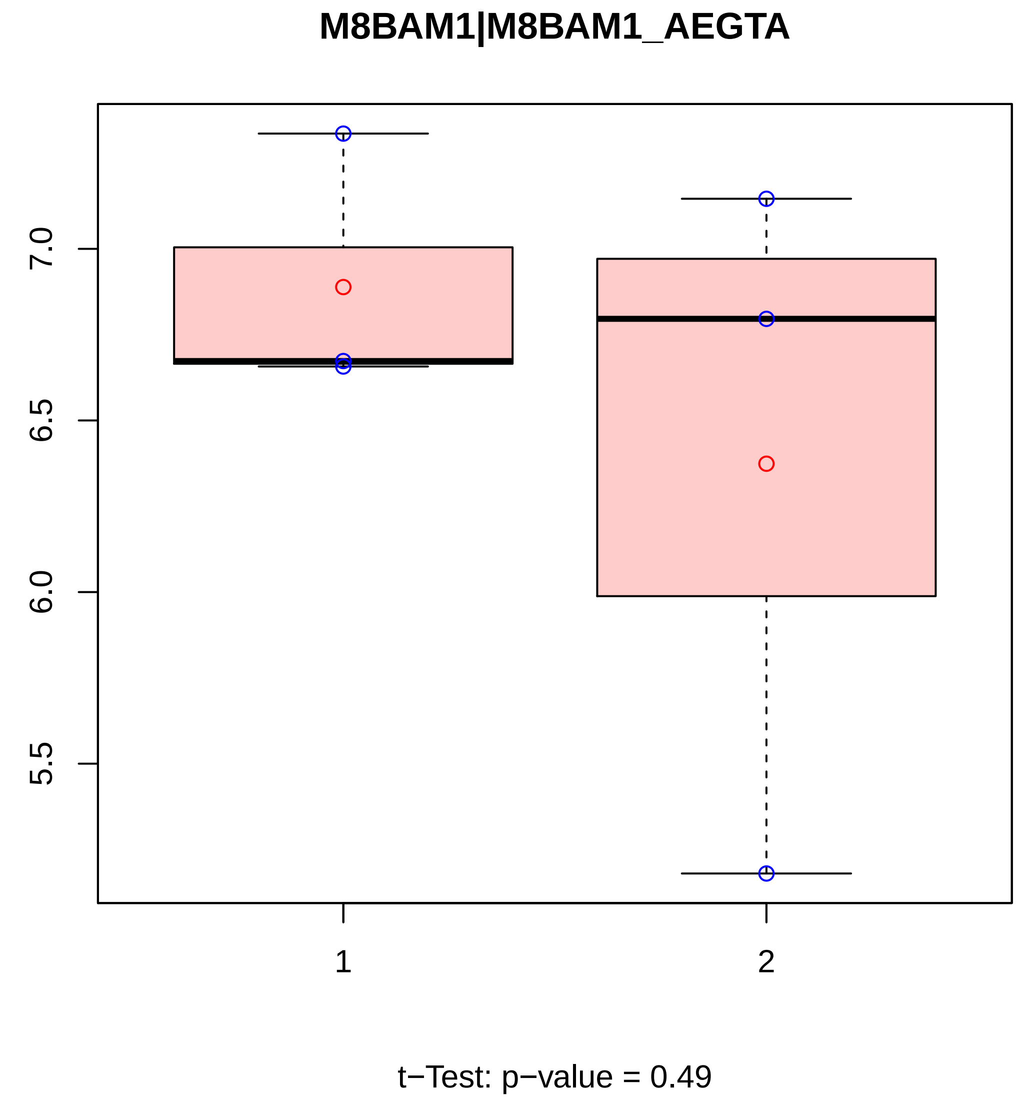
M8BAM1|M8BAM1_AEGTA
7.0
6.5
6.0
5.5
1
2
t−Test: p−value = 0.49

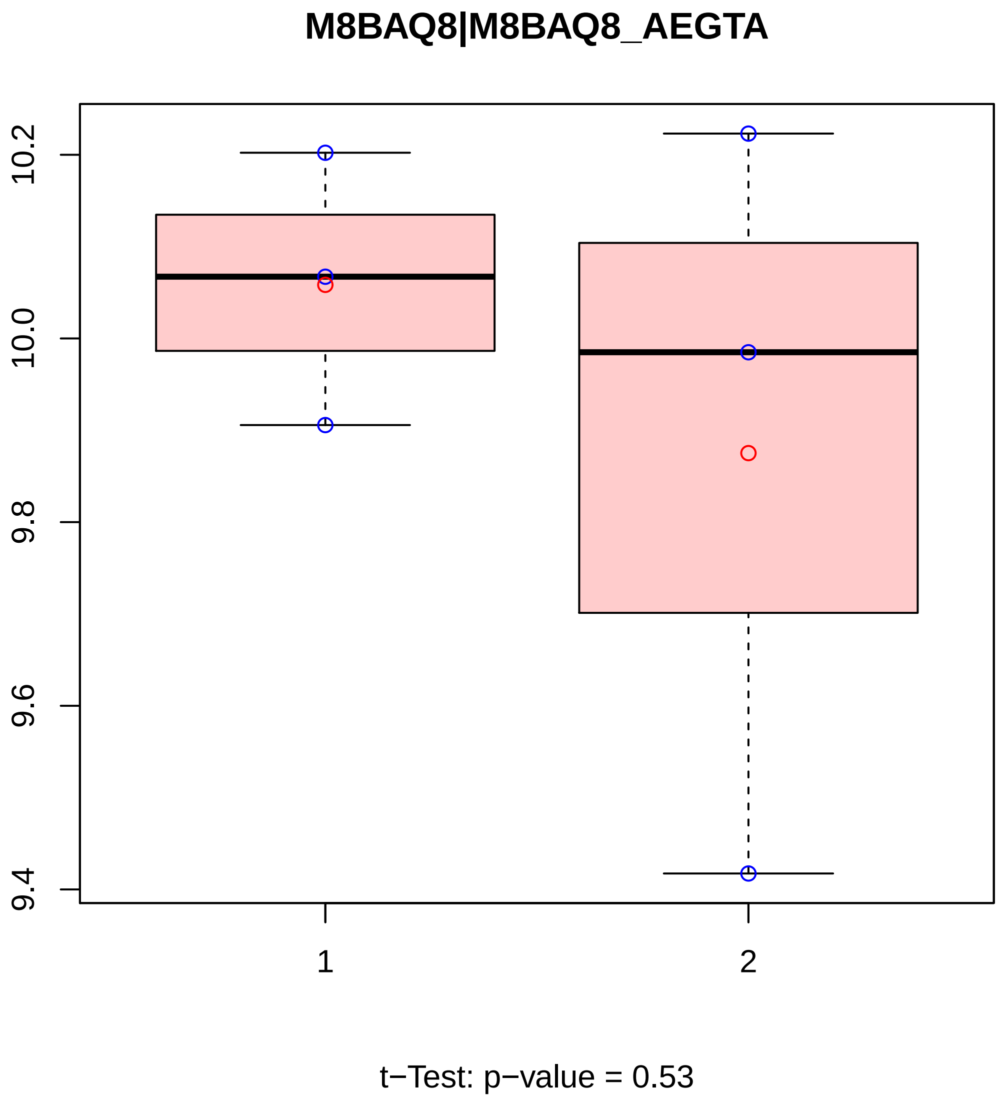
M8BAQ8|M8BAQ8_AEGTA
10.2
10.0
9.8
9.6
9.4
1
2
t−Test: p−value = 0.53

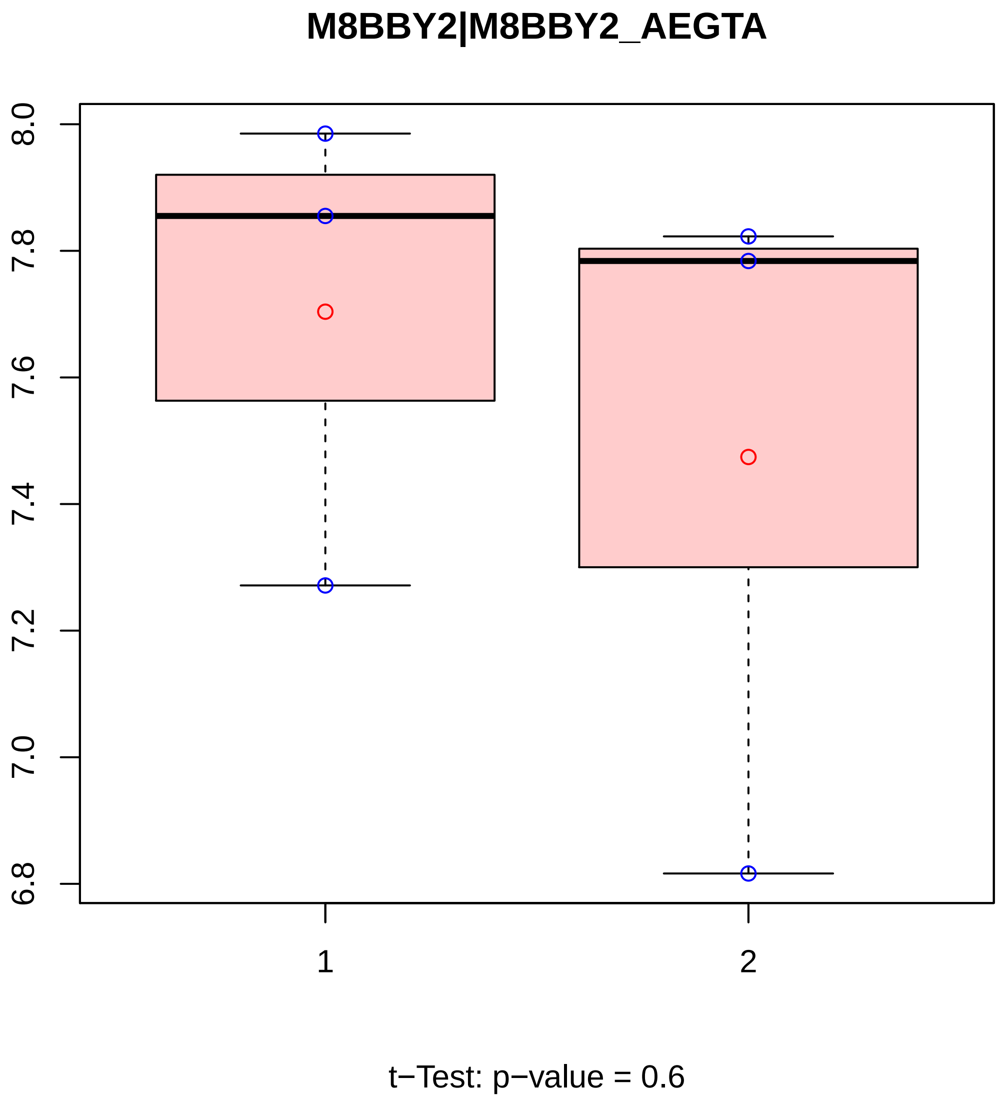
M8BBY2|M8BBY2_AEGTA
8.0
7.8
7.6
7.4
7.2
7.0
6.8
1
2
t−Test: p−value = 0.6

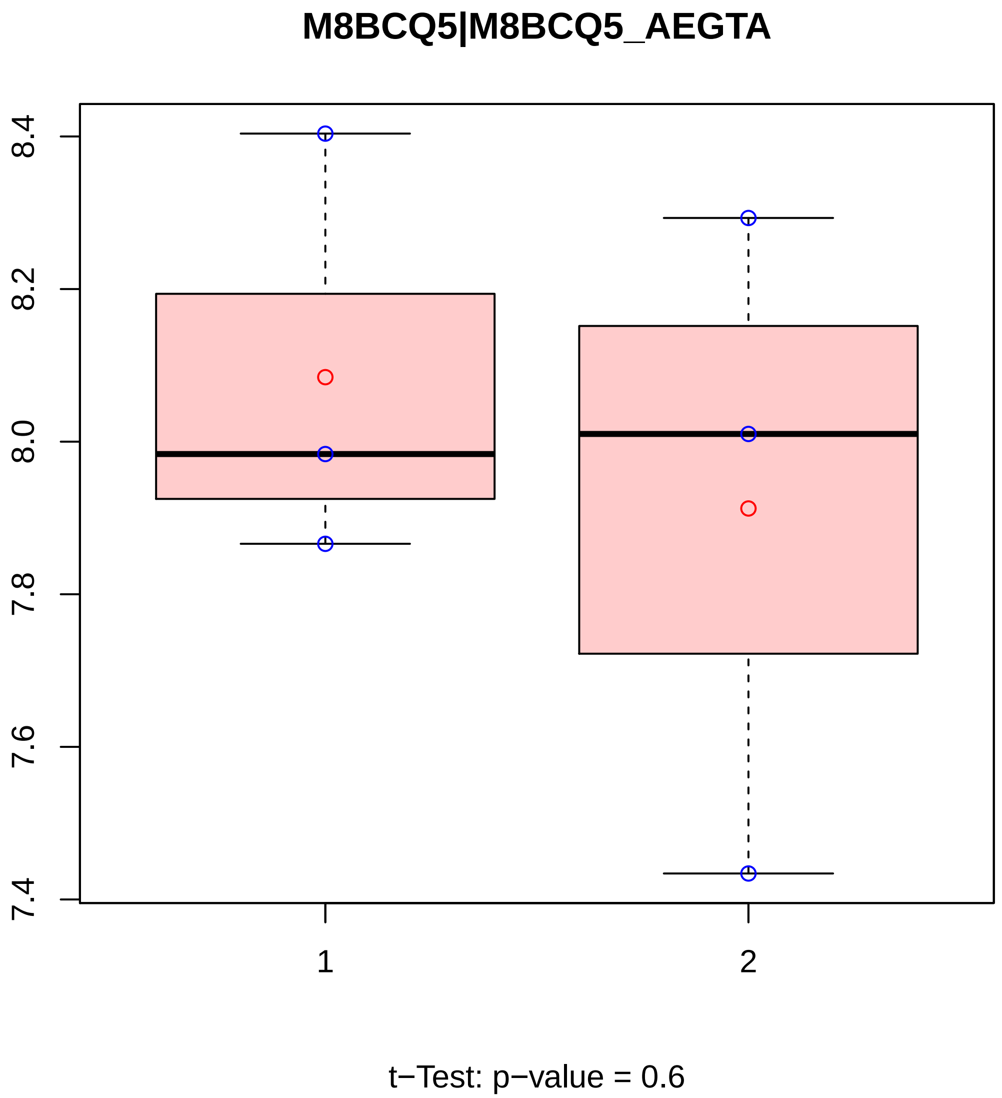

M8BCQ5|M8BCQ5_AEGTA
8.4
8.2
8.0
7.8
7.6
7.4
1
2
t−Test: p−value = 0.6

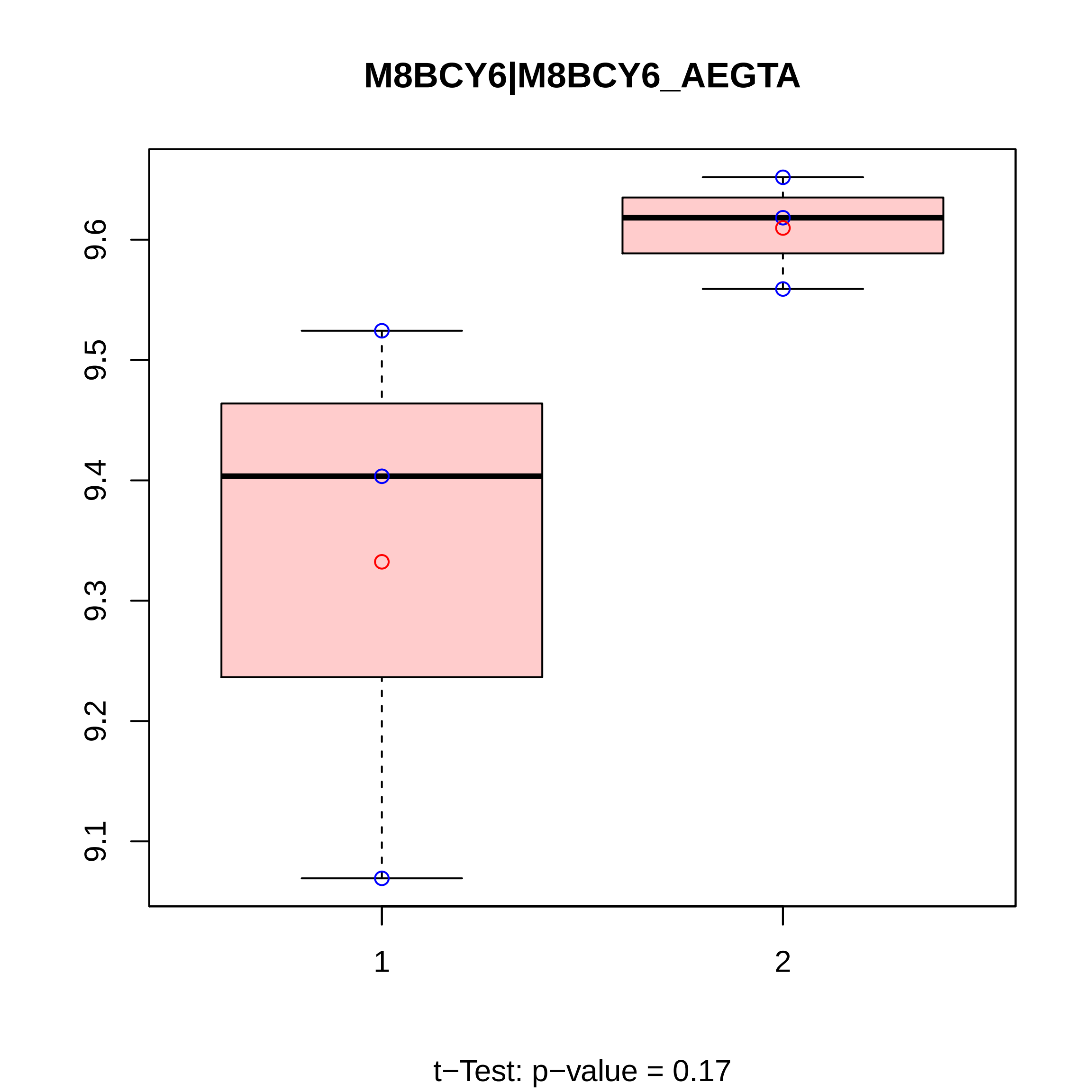

M8BCY6|M8BCY6_AEGTA
9.1
9.2
9.3
9.4
9.5
9.6
1
2
t−Test: p−value = 0.17

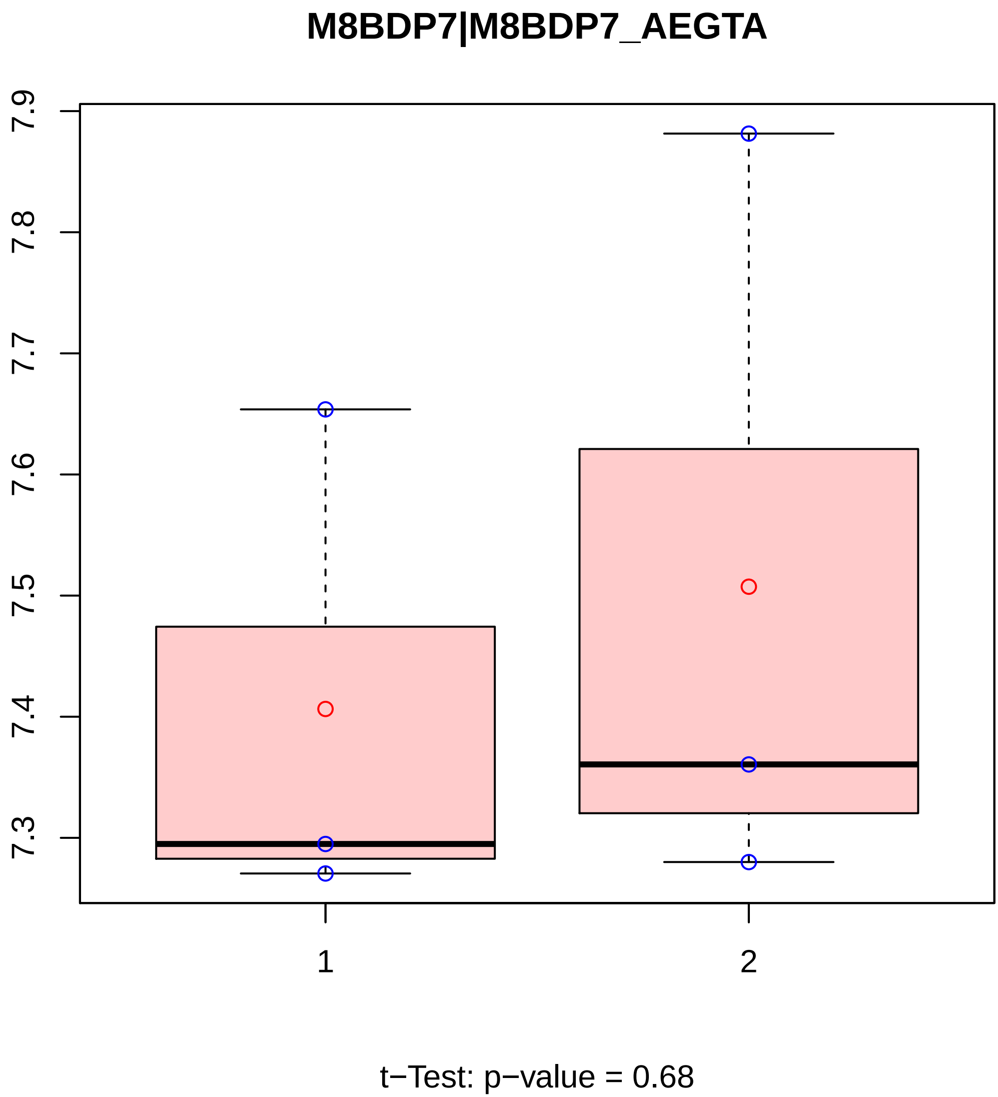

M8BDP7|M8BDP7_AEGTA
7.9
7.8
7.7
7.6
7.5
7.4
7.3
1
2
t−Test: p−value = 0.68

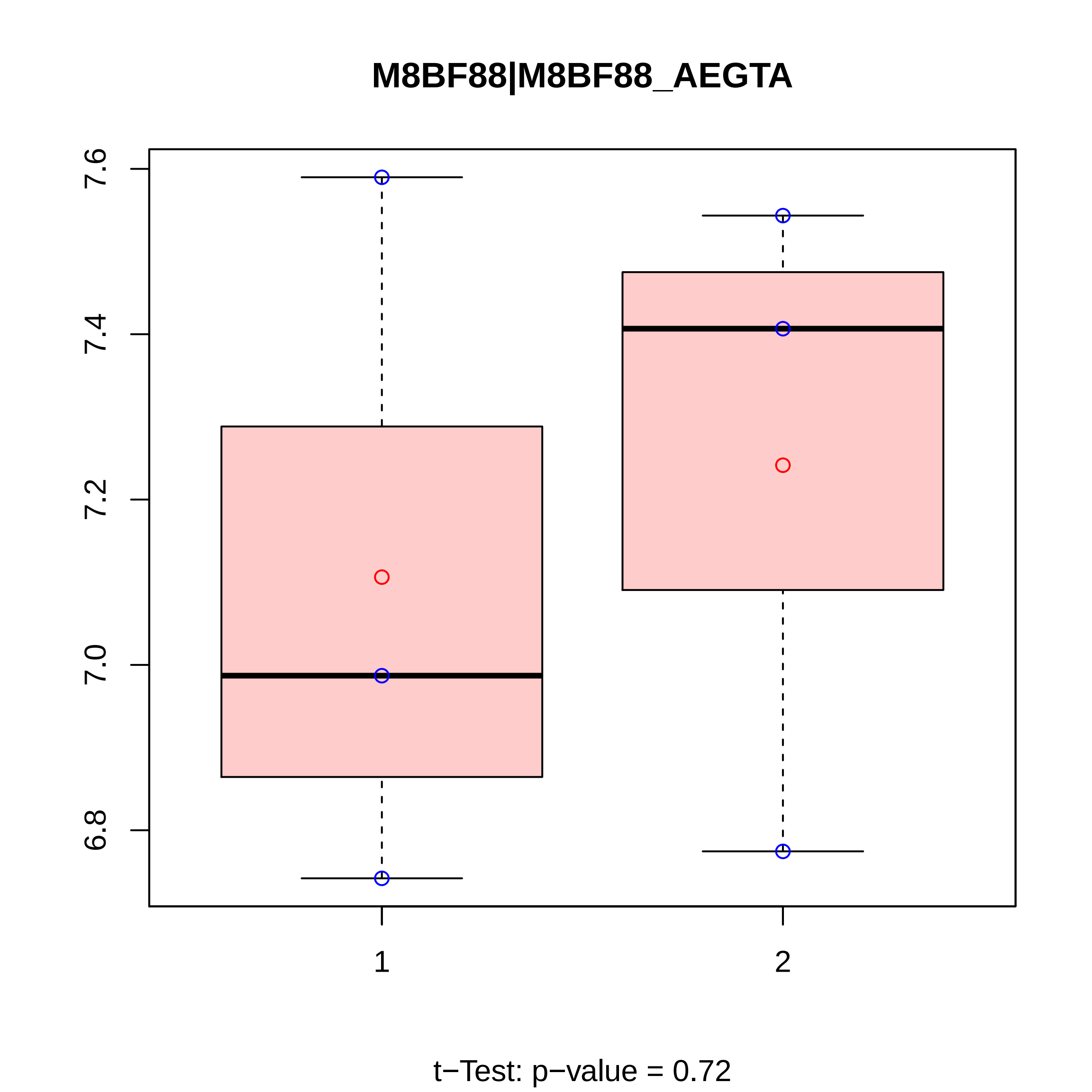

M8BF88|M8BF88_AEGTA
7.6
7.4
7.2
7.0
6.8
1
2
t−Test: p−value = 0.72

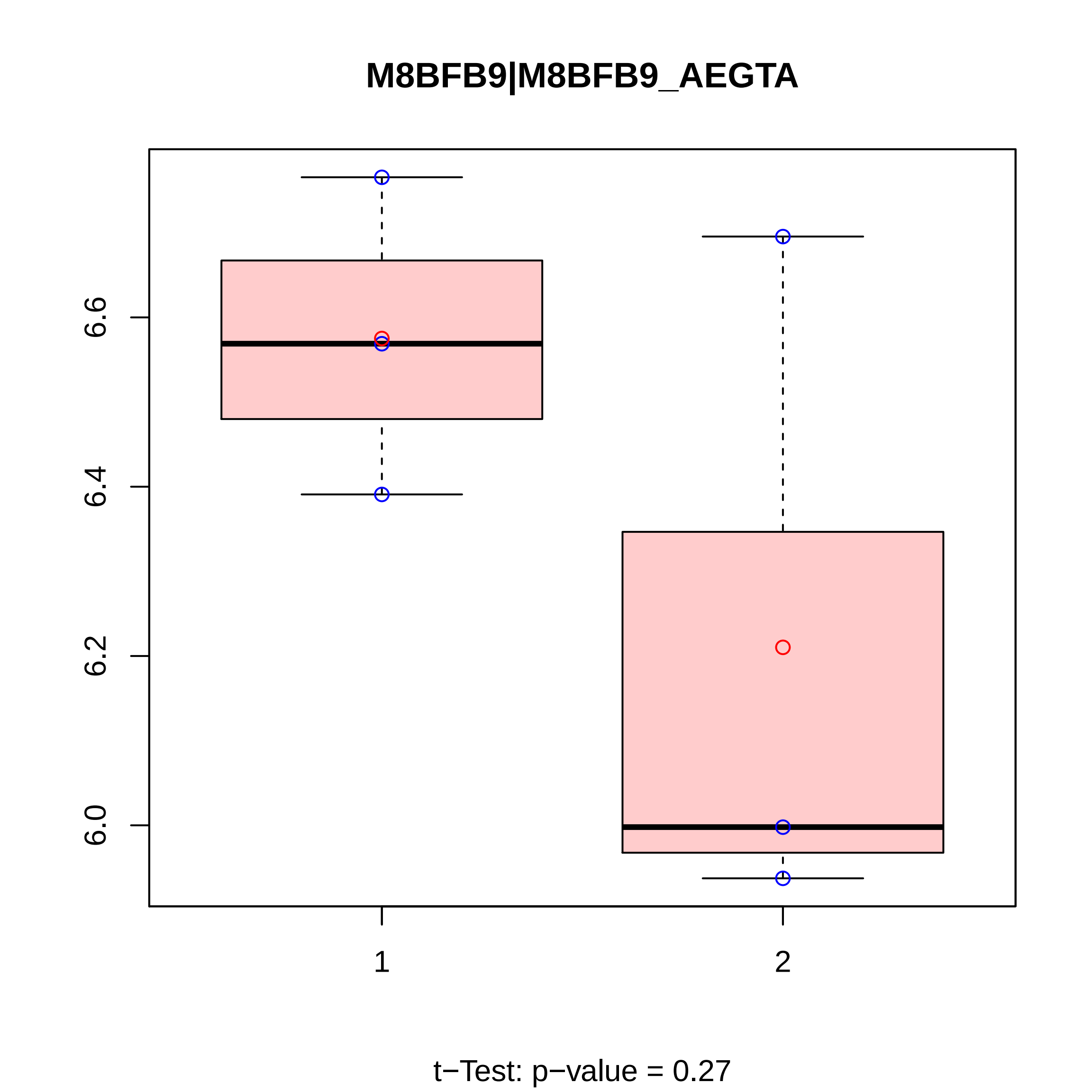
M8BFB9|M8BFB9_AEGTA
6.0
6.2
6.4
6.6
1
2
t−Test: p−value = 0.27

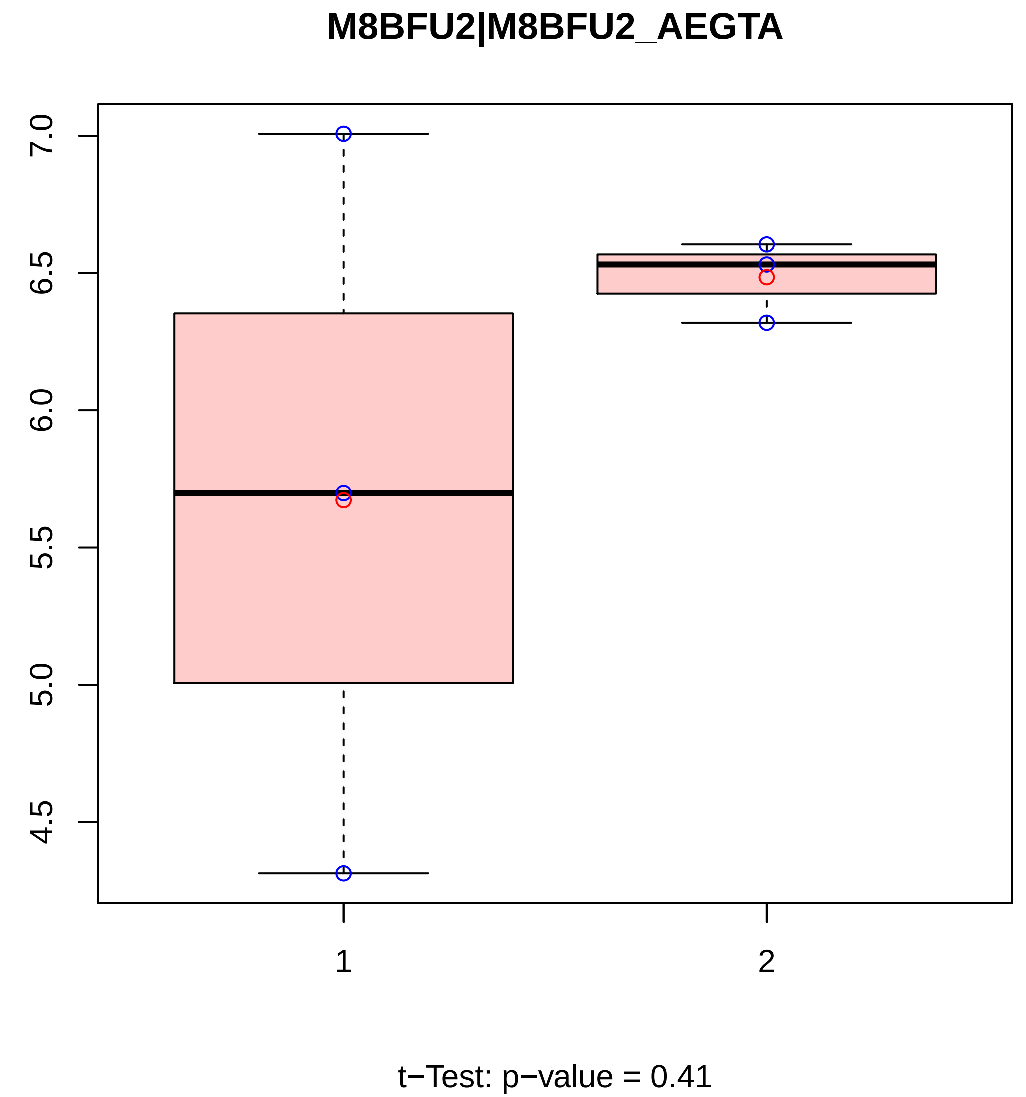
M8BFU2|M8BFU2_AEGTA
7.0
6.5
6.0
5.5
5.0
4.5
1
2
t−Test: p−value = 0.41

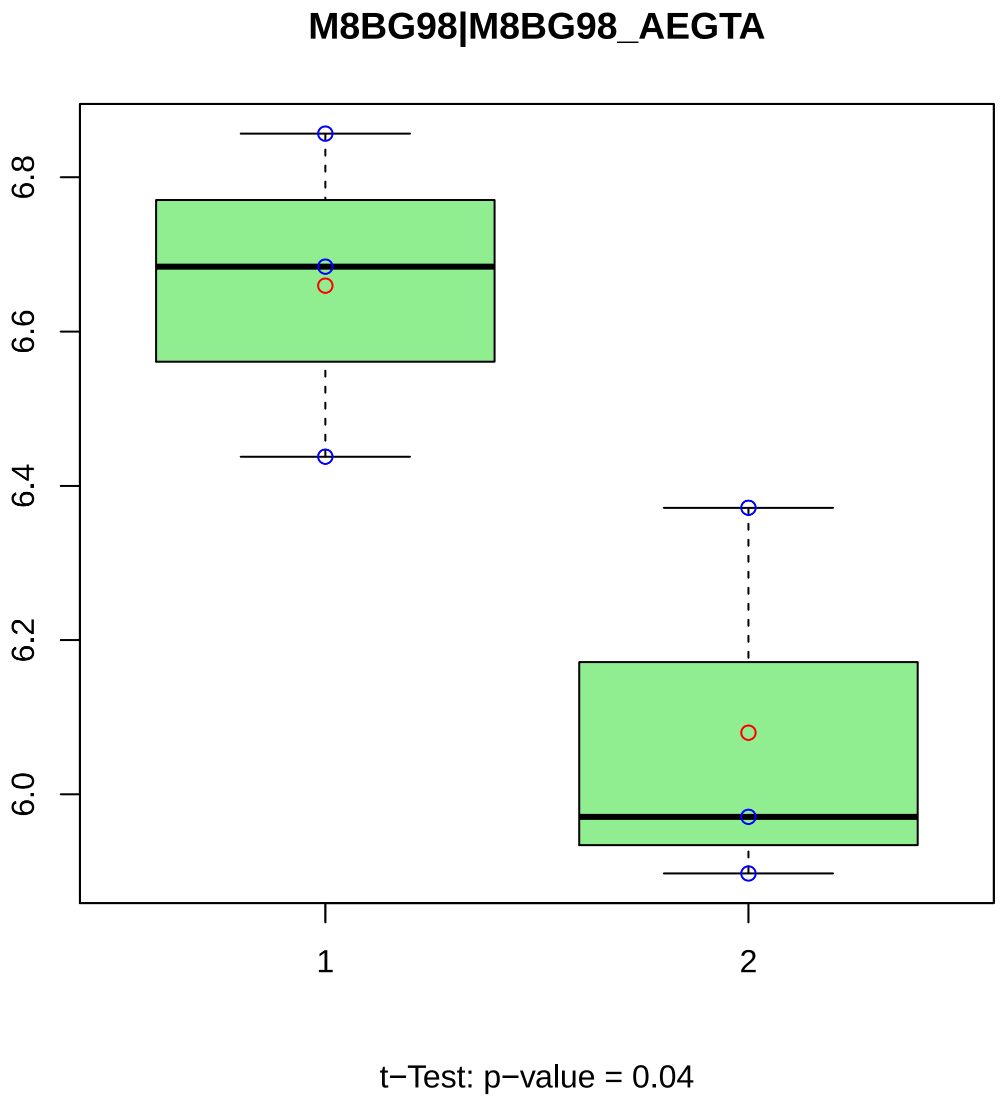
M8BG98|M8BG98_AEGTA
6.8
6.6
6.4
6.2
6.0
1
2
t−Test: p−value = 0.04

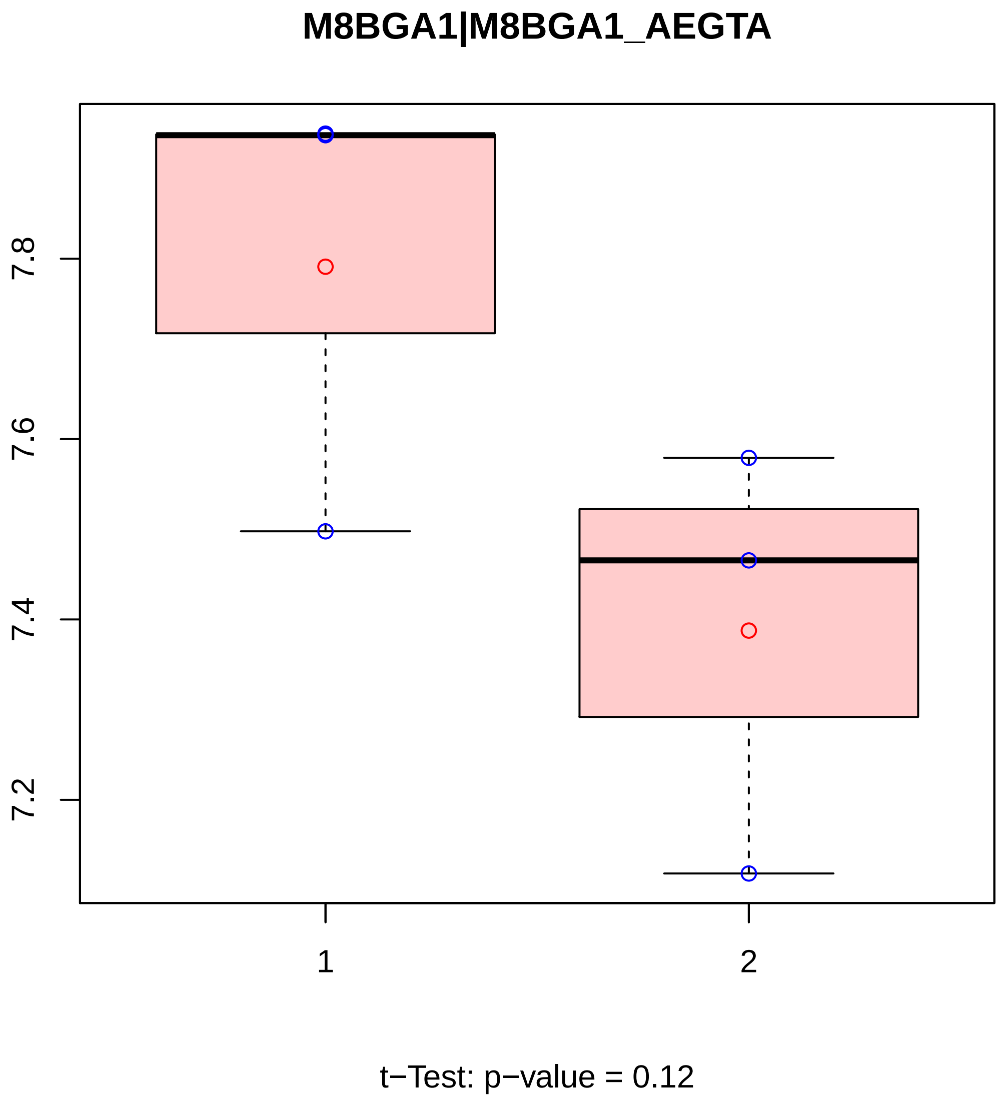
M8BGA1|M8BGA1_AEGTA
7.2
7.4
7.6
7.8
1
2
t−Test: p−value = 0.12

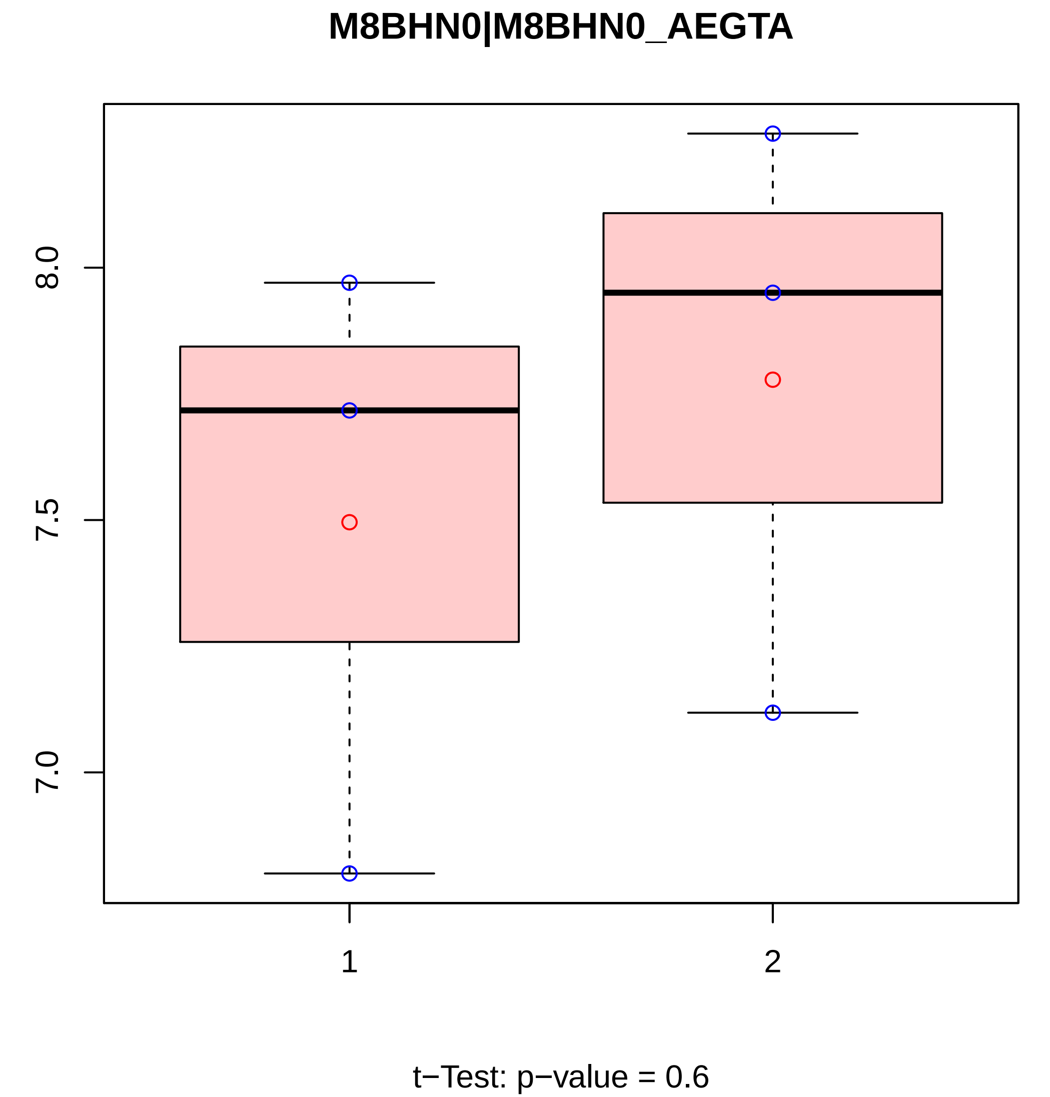
M8BHN0|M8BHN0_AEGTA
8.0
7.5
7.0
1
2
t−Test: p−value = 0.6

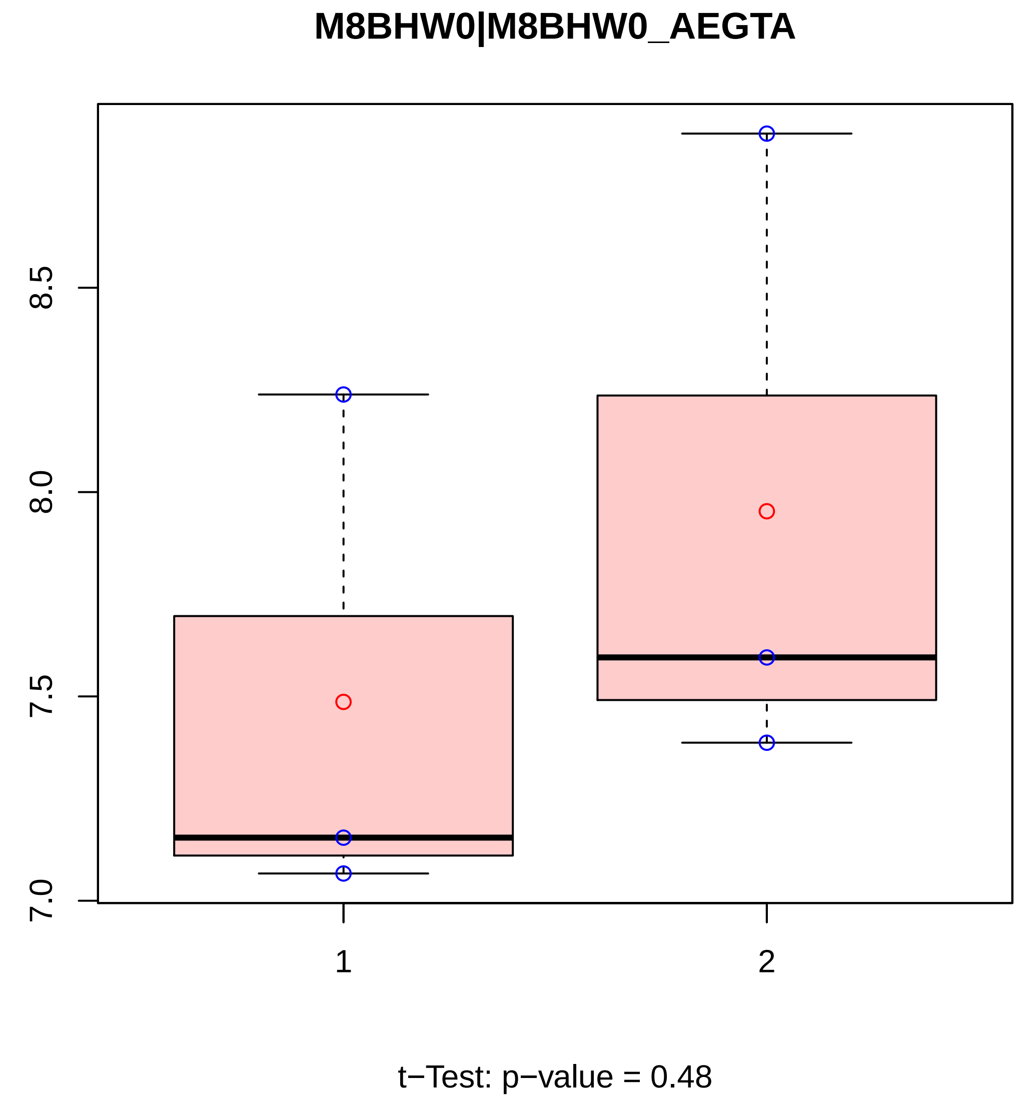
M8BHW0|M8BHW0_AEGTA
7.0
7.5
8.0
8.5
1
2
t−Test: p−value = 0.48

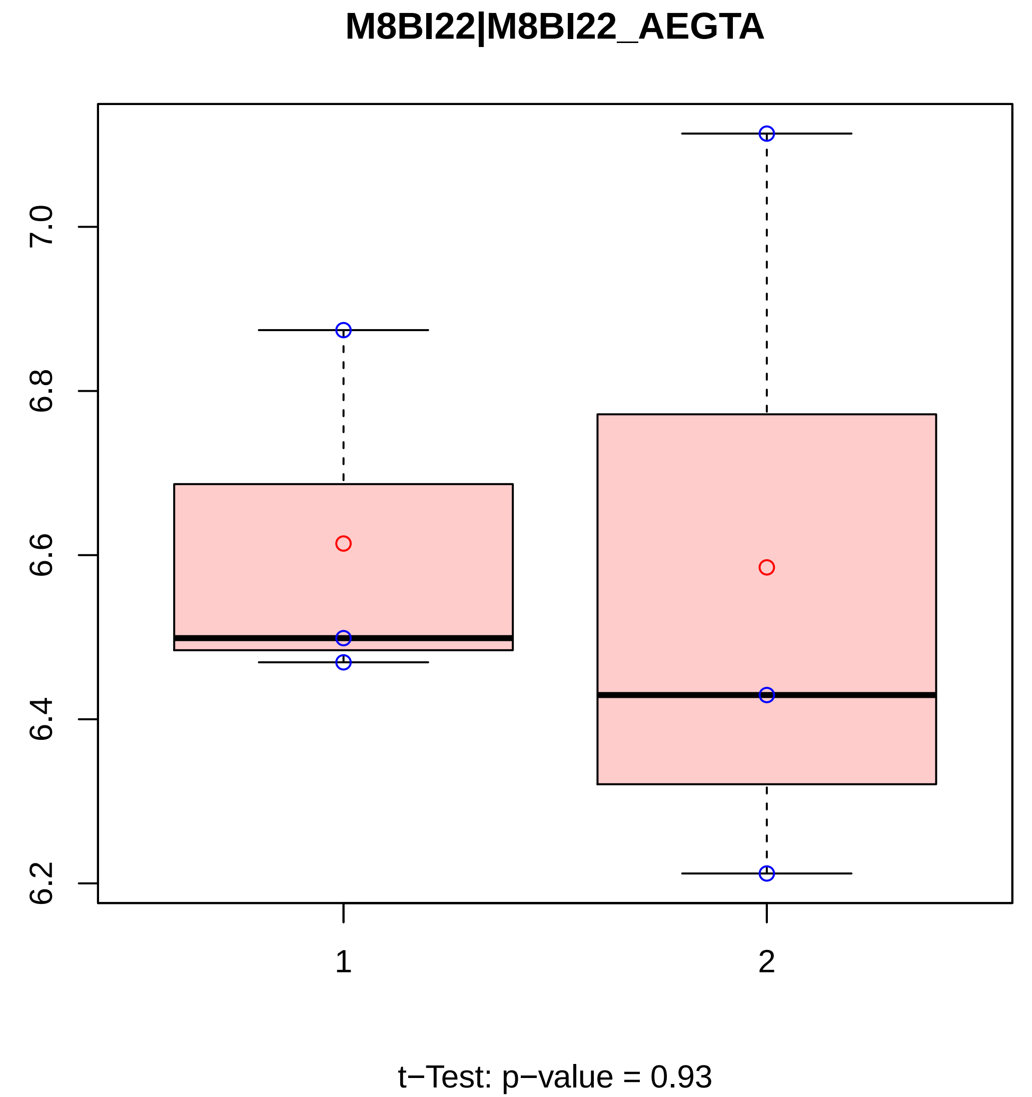
M8BI22|M8BI22_AEGTA
7.0
6.8
6.6
6.4
6.2
1
2
t−Test: p−value = 0.93

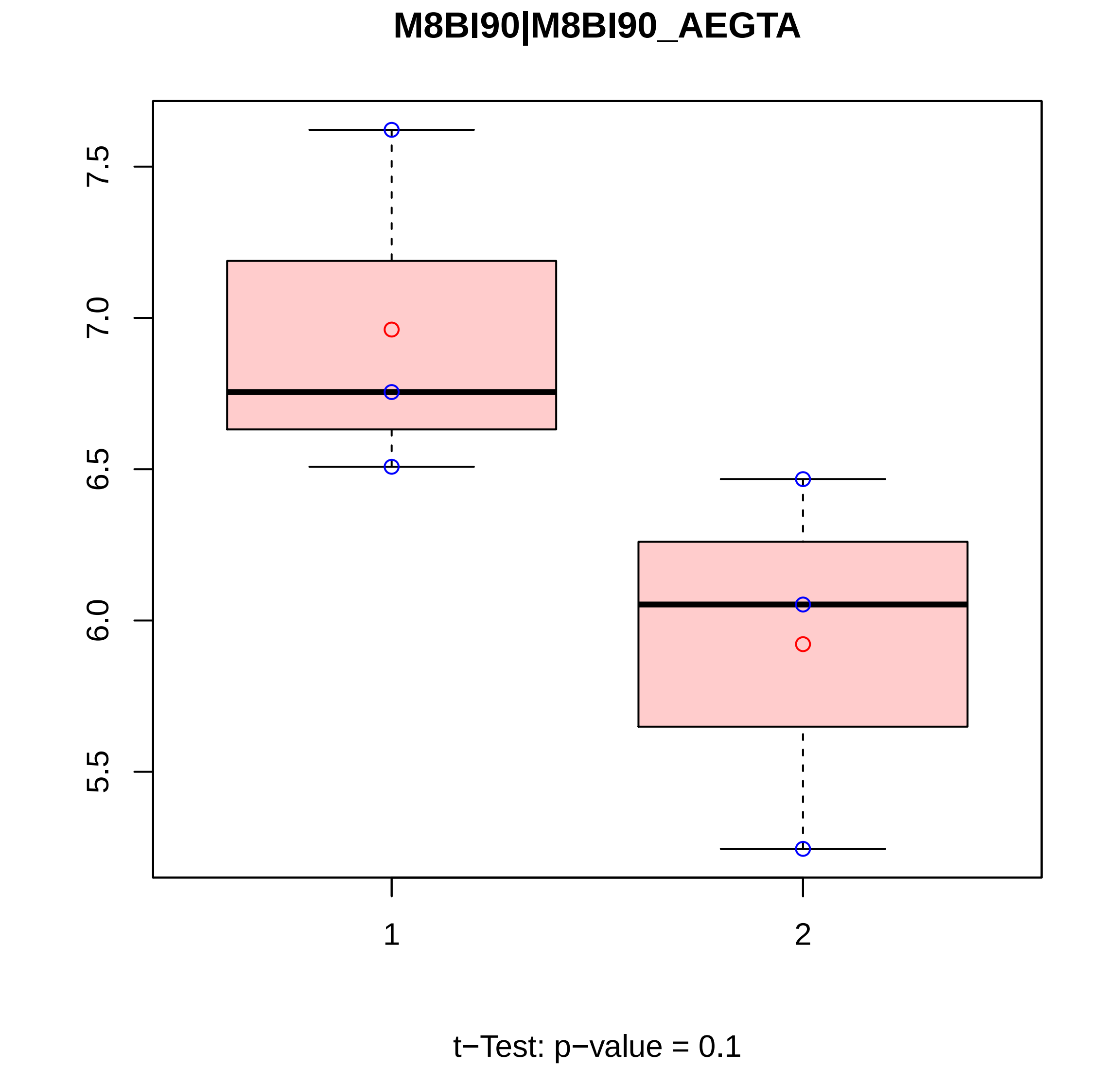
M8BI90|M8BI90_AEGTA
7.5
7.0
6.5
6.0
5.5
1
2
t−Test: p−value = 0.1

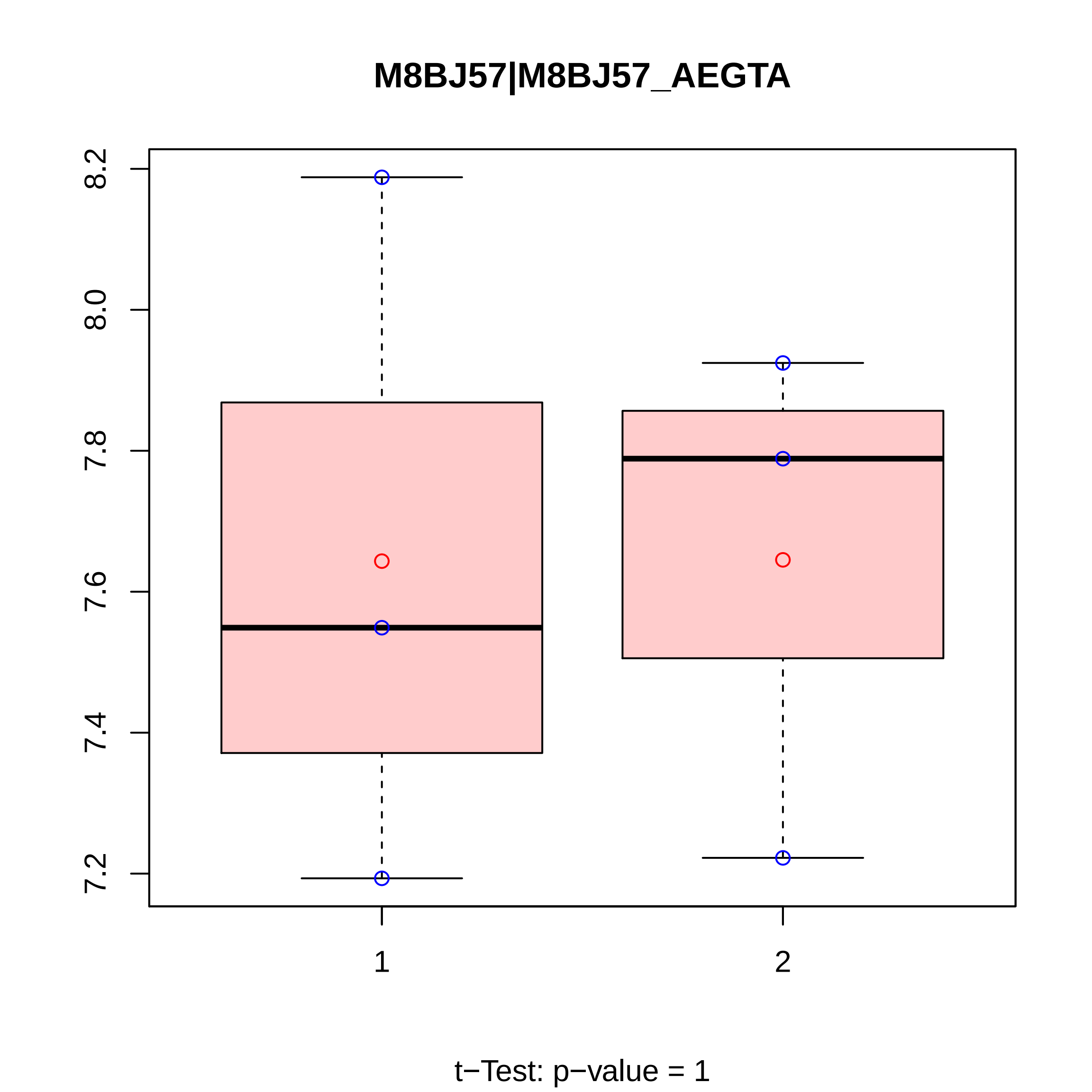

M8BJ57|M8BJ57_AEGTA
8.2
8.0
7.8
7.6
7.4
7.2
1
2
t−Test: p−value = 1

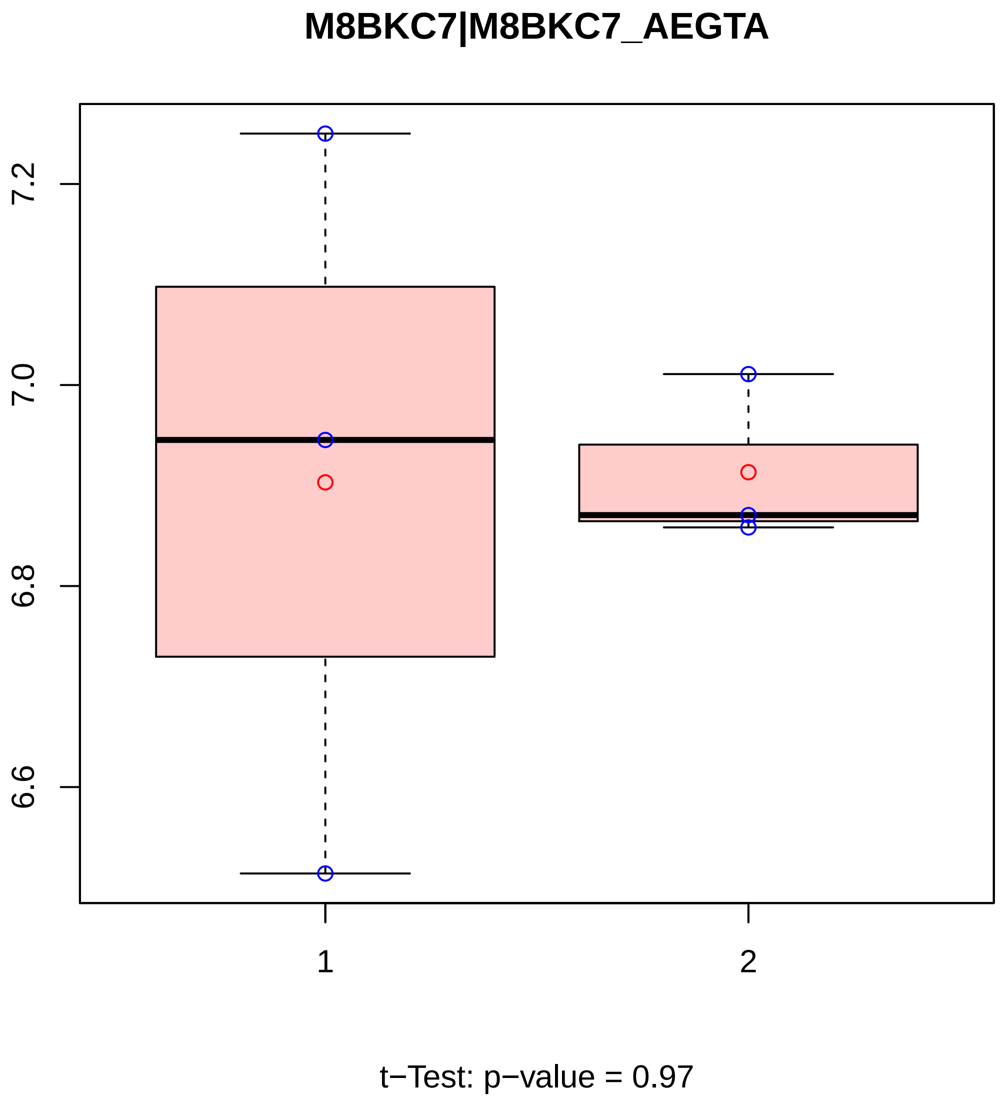
M8BKC7|M8BKC7_AEGTA
7.2
7.0
6.8
6.6
1
2
t−Test: p−value = 0.97

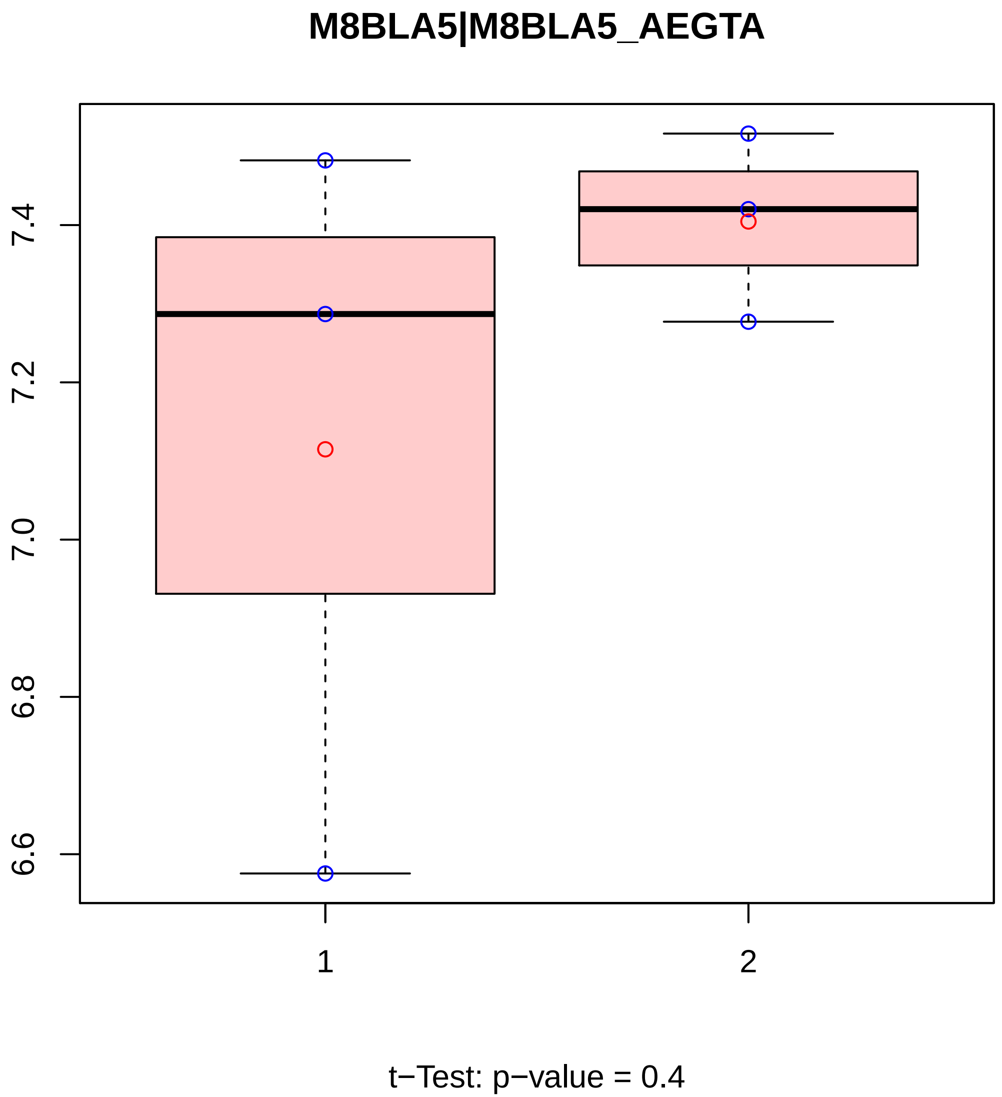
M8BLA5|M8BLA5_AEGTA
7.4
7.2
7.0
6.8
6.6
1
2
t−Test: p−value = 0.4

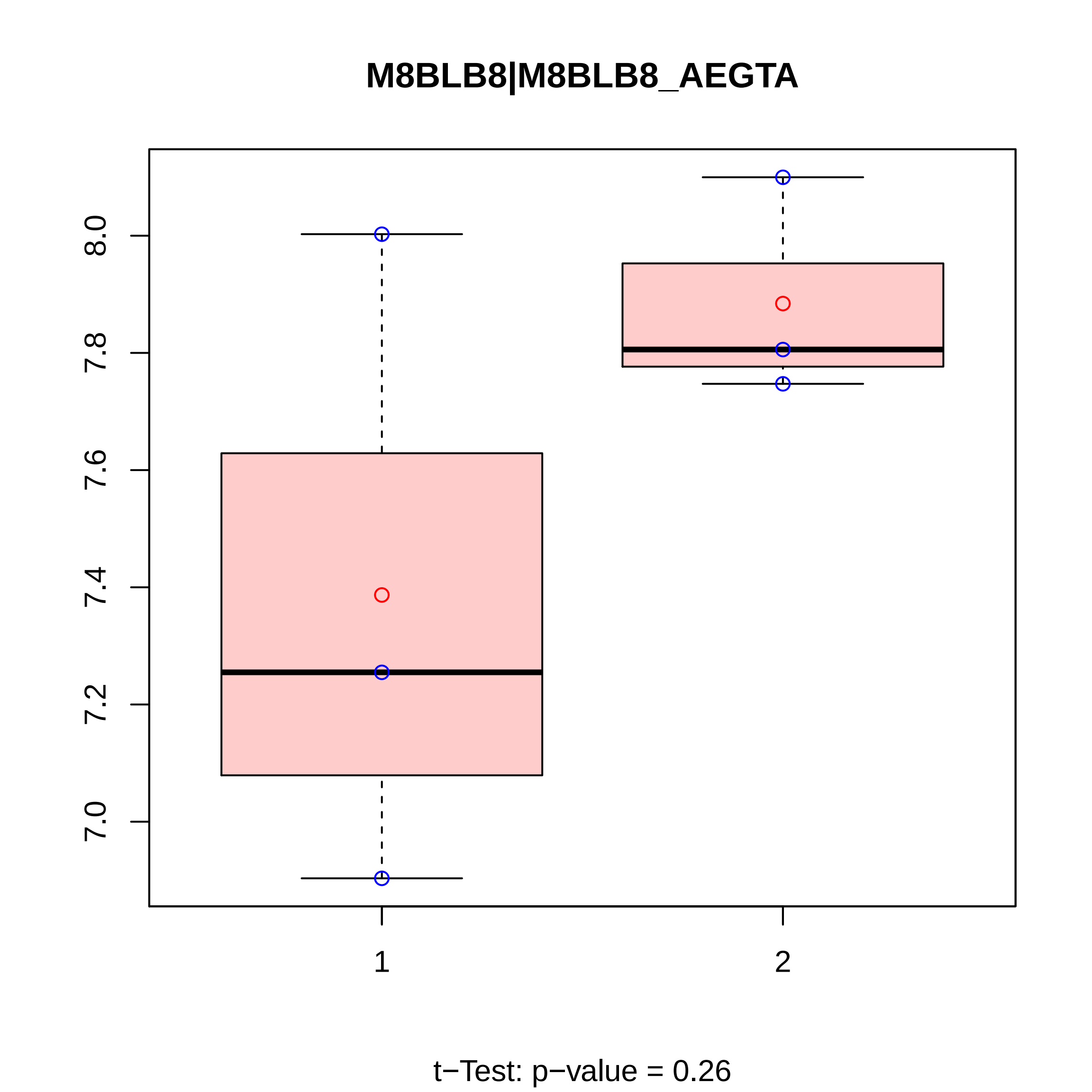
M8BLB8|M8BLB8_AEGTA
8.0
7.8
7.6
7.4
7.2
7.0
1
2
t−Test: p−value = 0.26

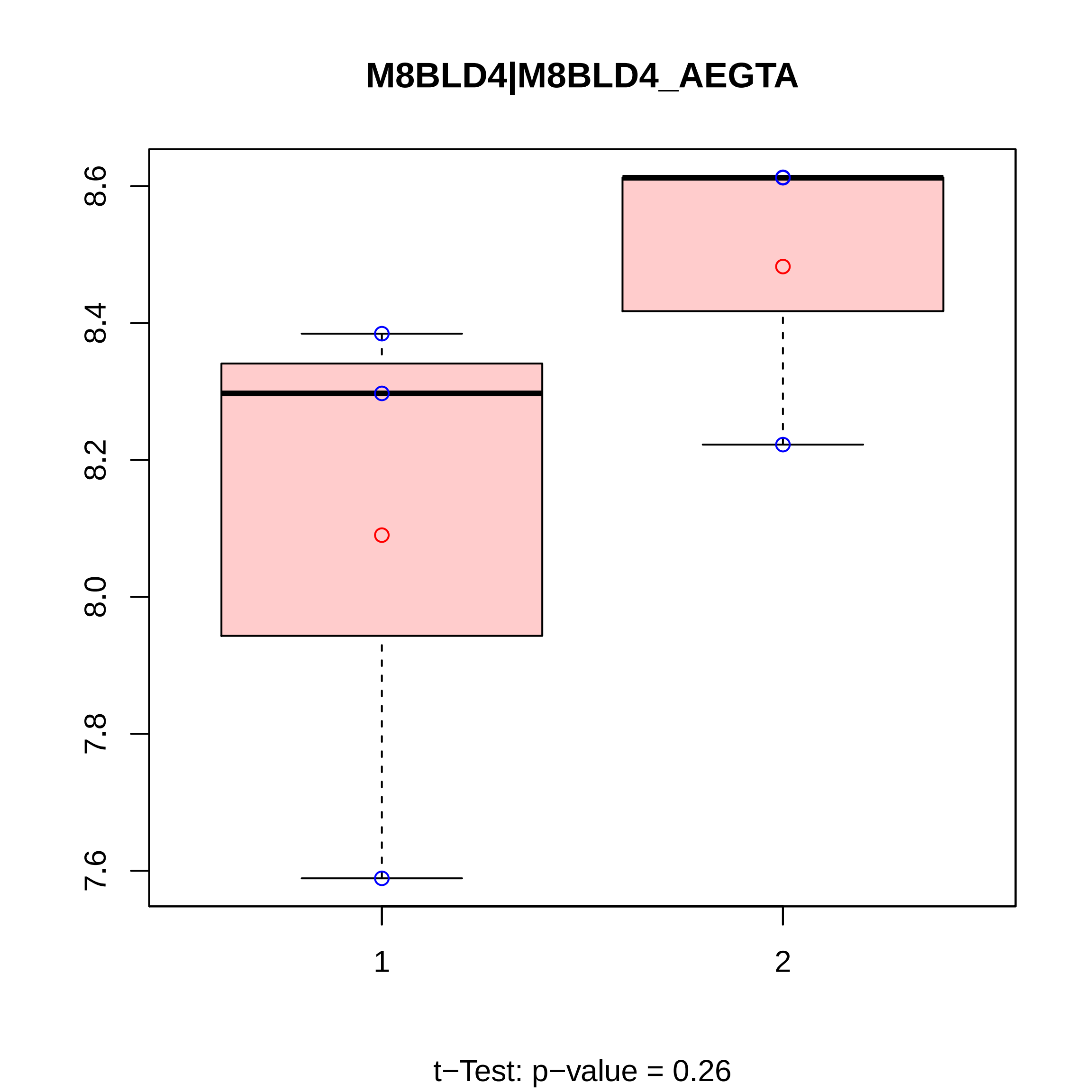

M8BLD4|M8BLD4_AEGTA
8.6
8.4
8.2
8.0
7.8
7.6
1
2
t−Test: p−value = 0.26

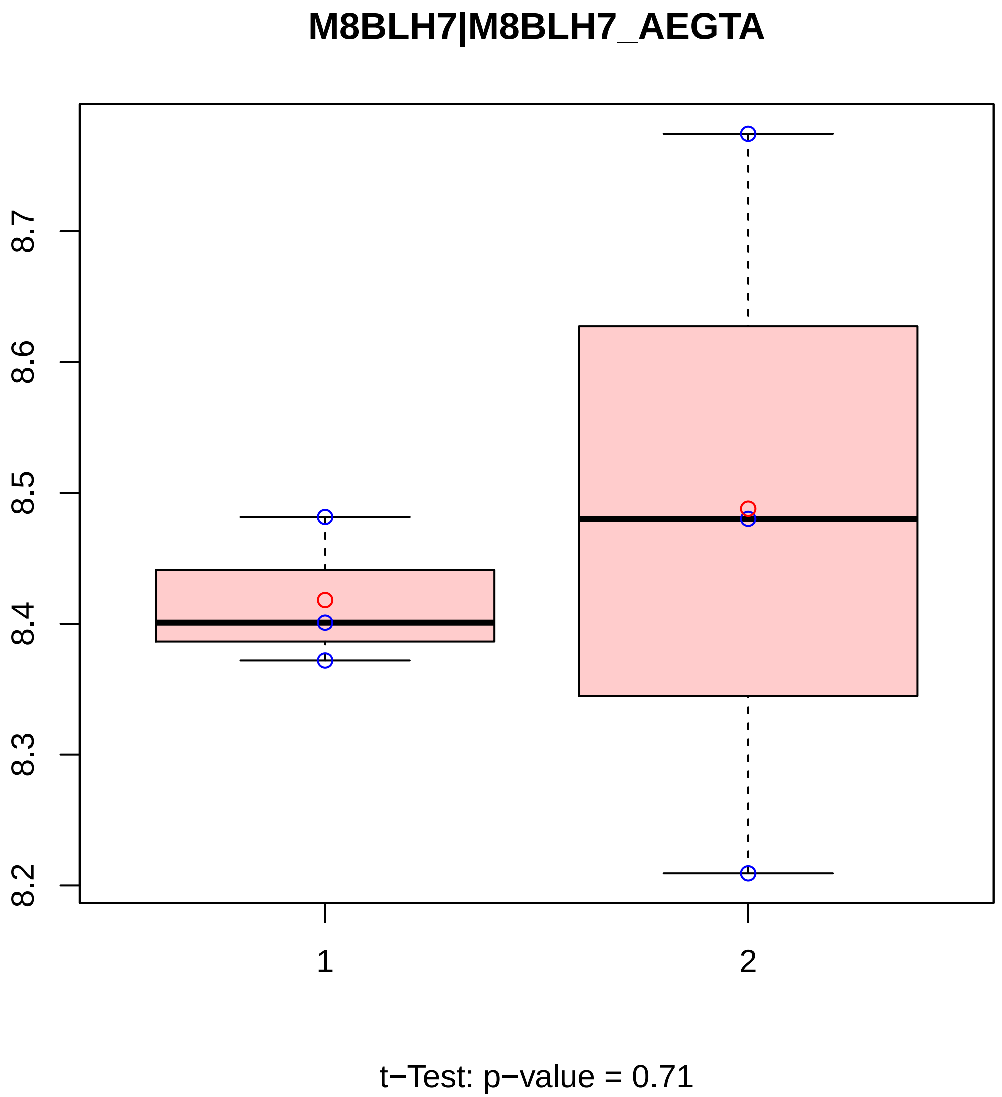
M8BLH7|M8BLH7_AEGTA
8.2
8.3
8.4
8.5
8.6
8.7
1
2
t−Test: p−value = 0.71

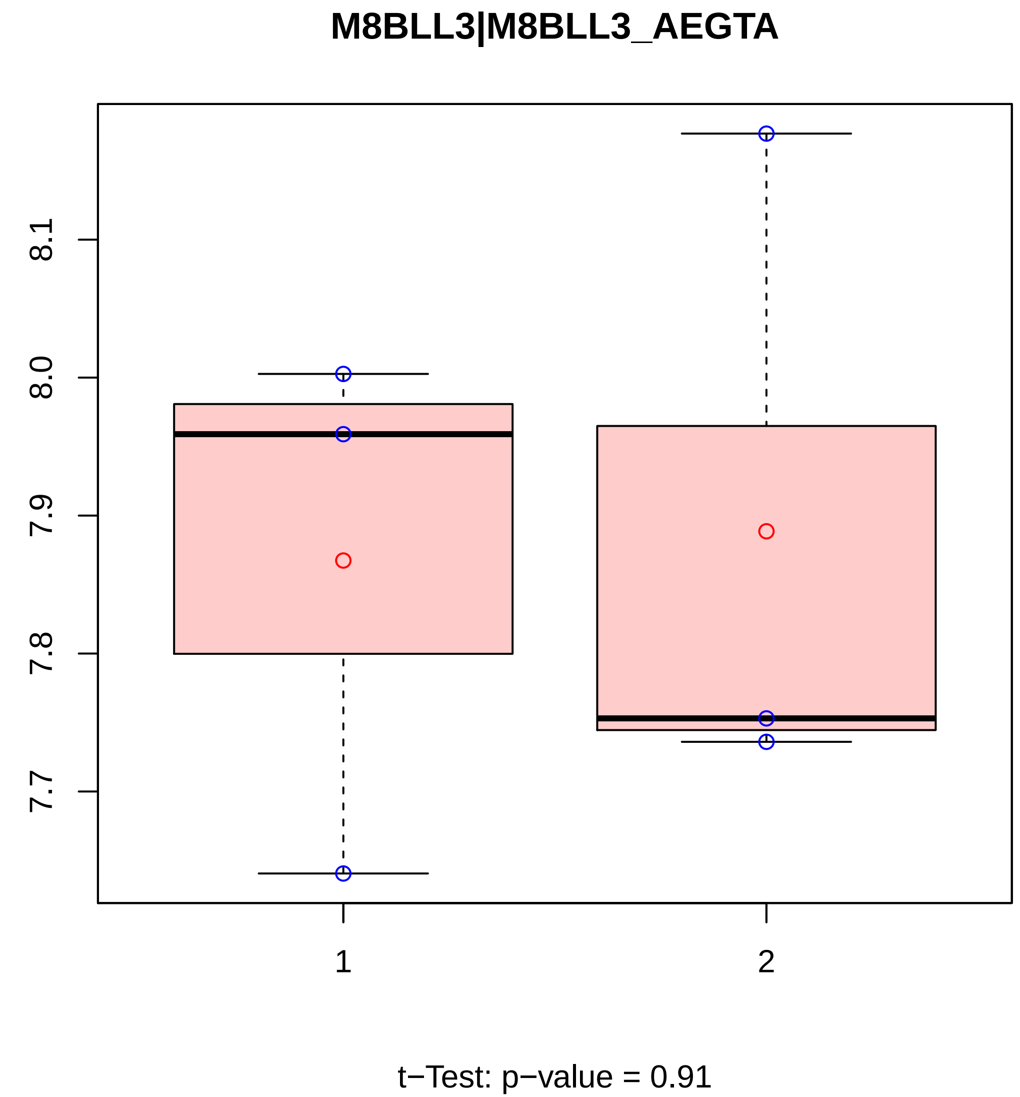
M8BLL3|M8BLL3_AEGTA
8.1
8.0
7.9
7.8
7.7
1
2
t−Test: p−value = 0.91

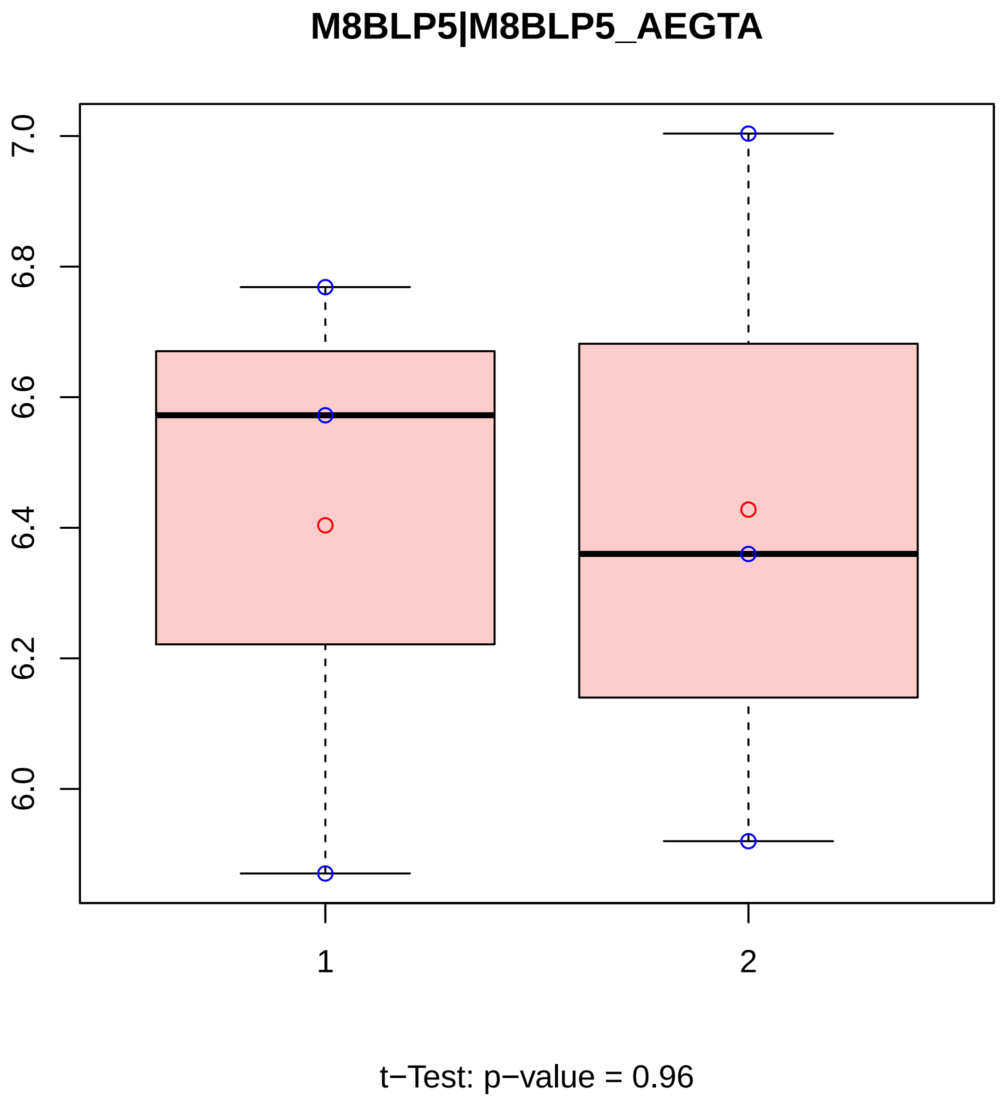
M8BLP5|M8BLP5_AEGTA
7.0
6.8
6.6
6.4
6.2
6.0
1
2
t−Test: p−value = 0.96

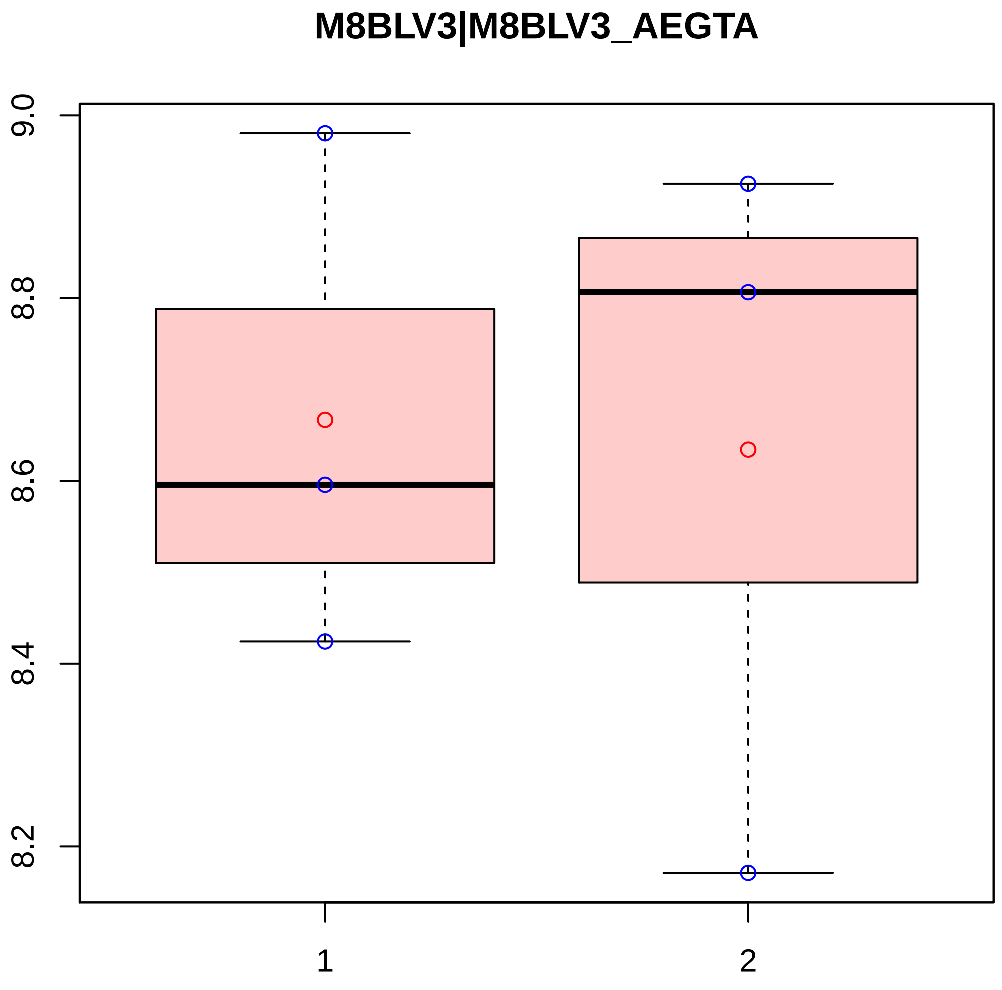

t−Test: p−value = 0.92

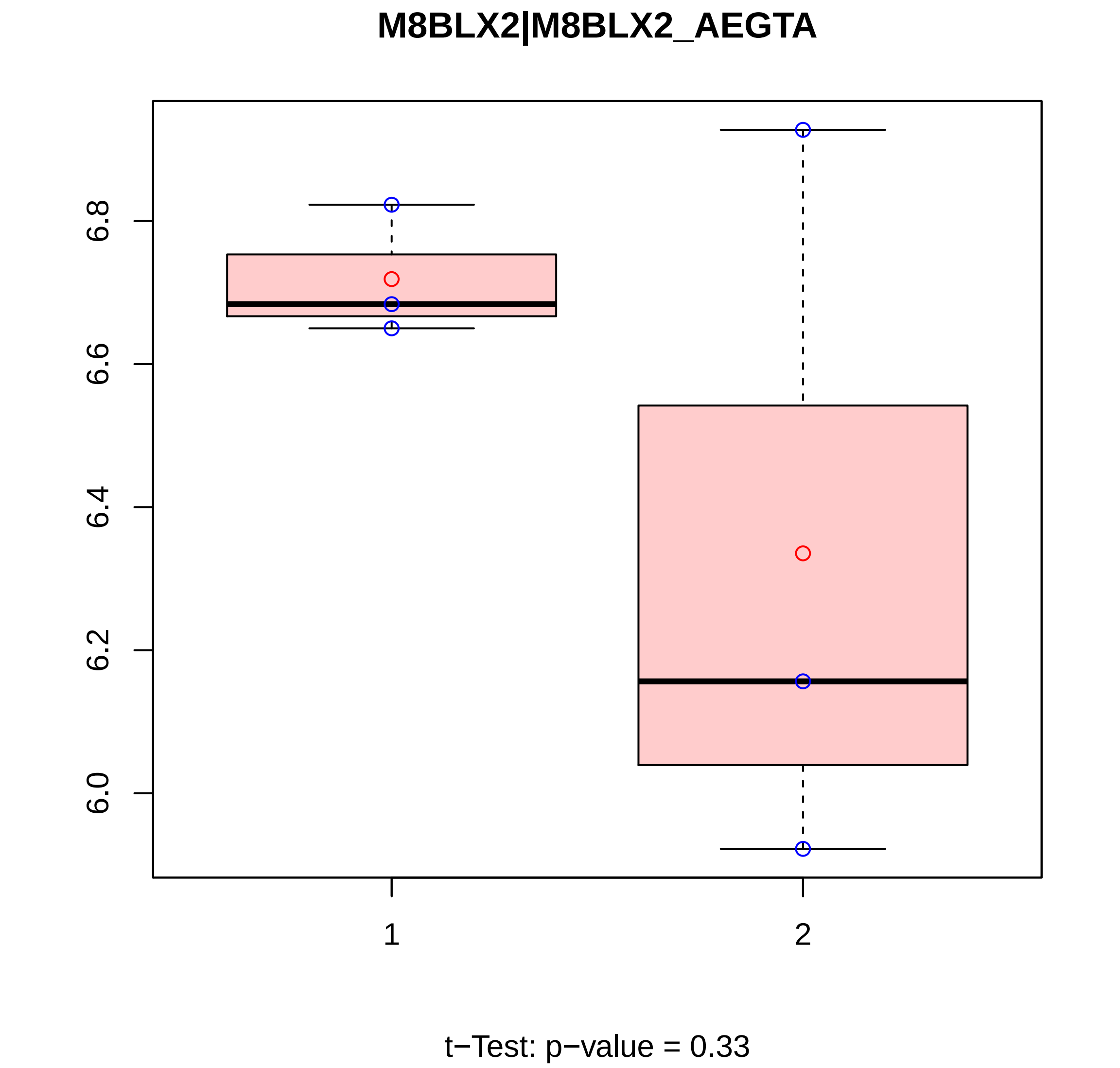
M8BLX2|M8BLX2_AEGTA
6.0
6.2
6.4
6.6
6.8
1
2
t−Test: p−value = 0.33

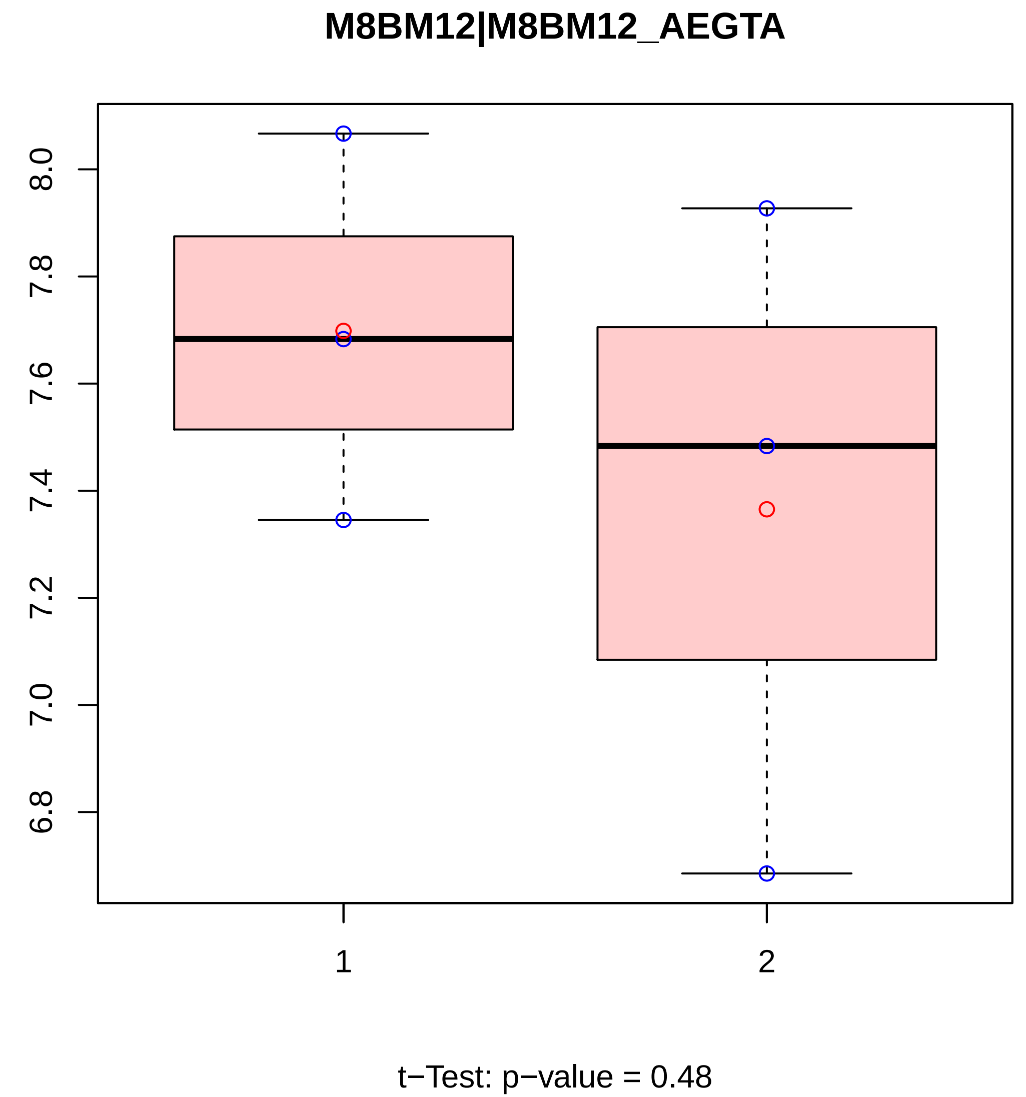
M8BM12|M8BM12_AEGTA
8.0
7.8
7.6
7.4
7.2
7.0
6.8
1
2
t−Test: p−value = 0.48

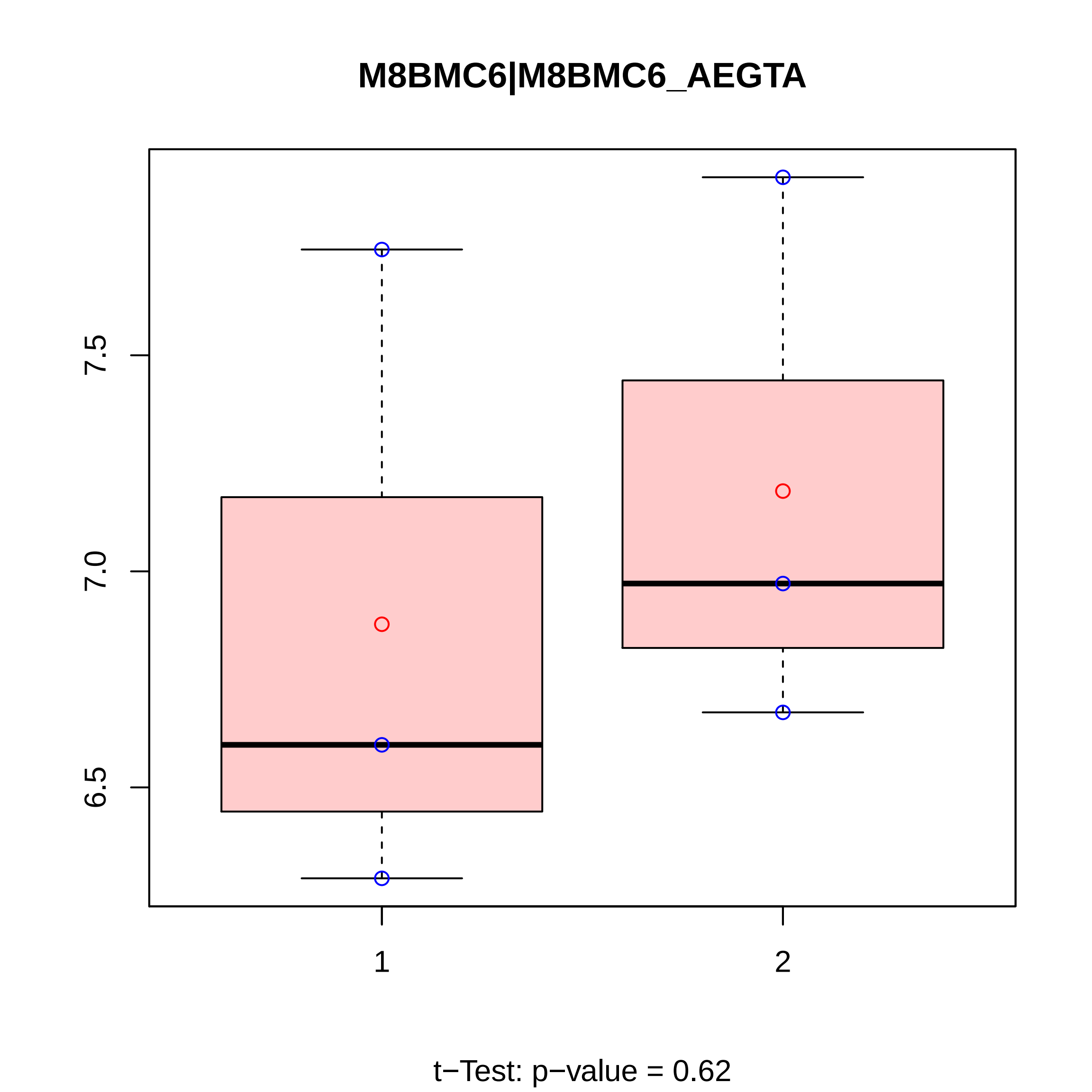
M8BMC6|M8BMC6_AEGTA
7.5
7.0
6.5
1
2
t−Test: p−value = 0.62

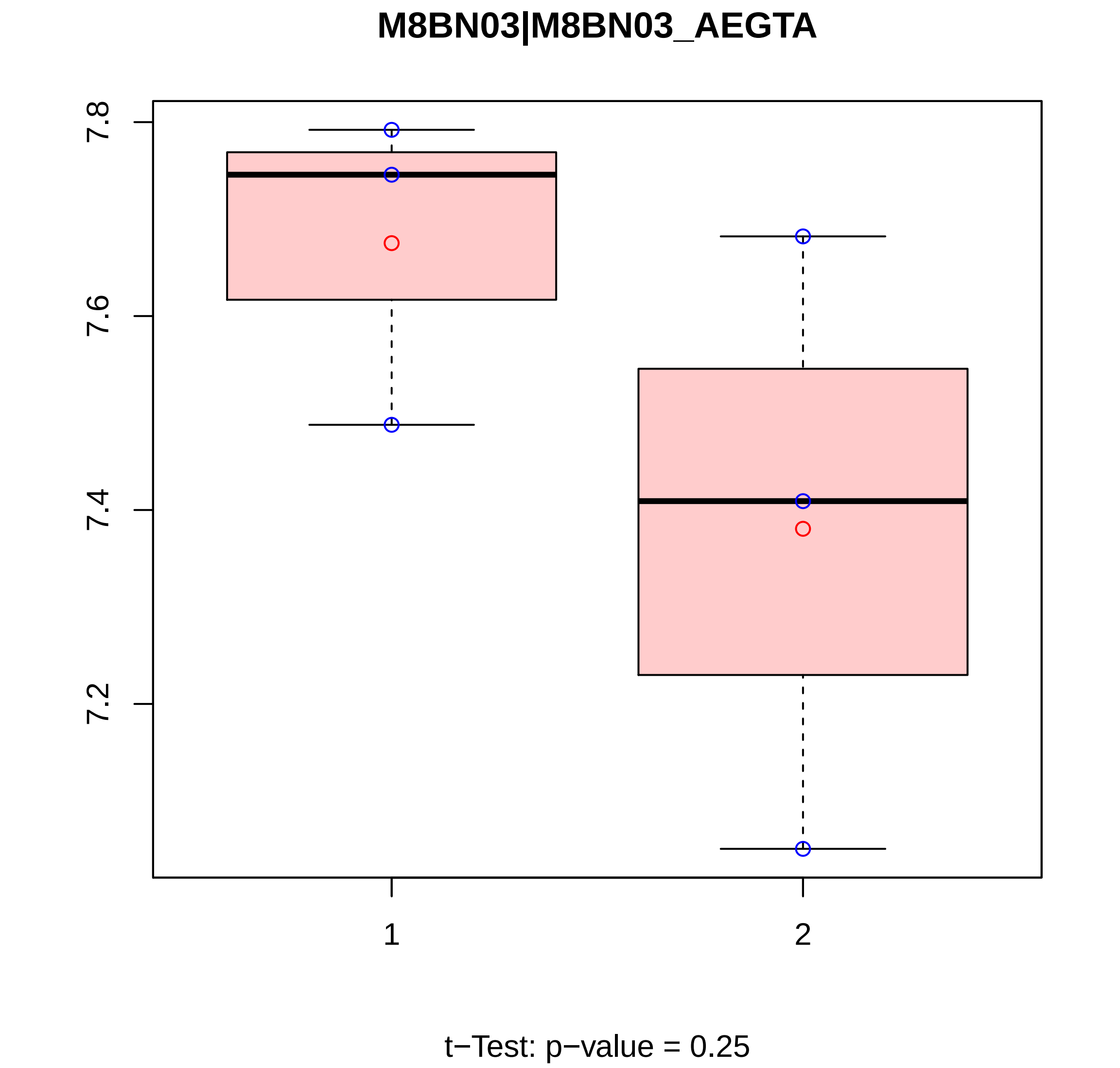
M8BN03|M8BN03_AEGTA
7.8
7.6
7.4
7.2
1
2
t−Test: p−value = 0.25

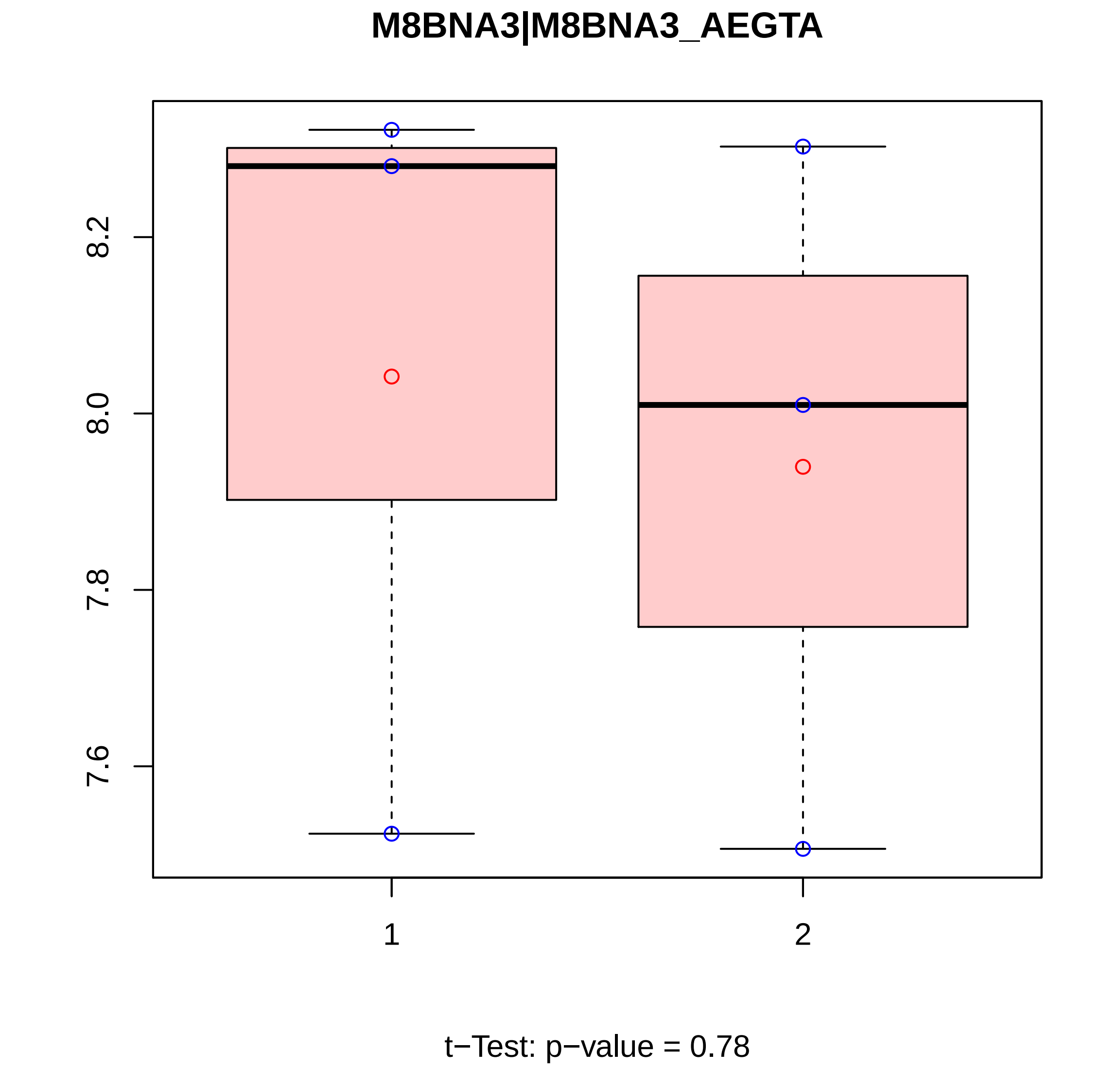
M8BNA3|M8BNA3_AEGTA
8.2
8.0
7.8
7.6
1
2
t−Test: p−value = 0.78

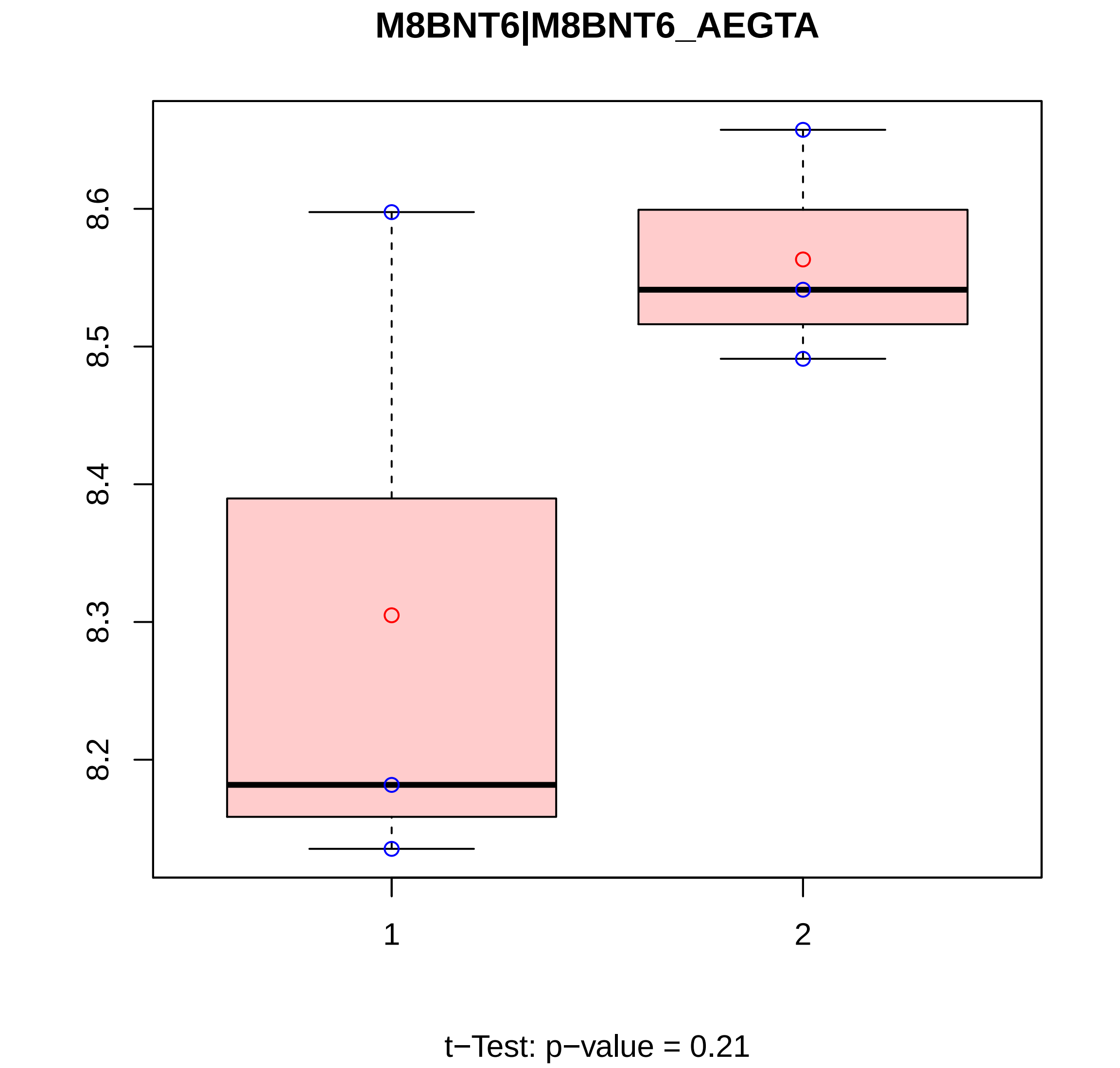
M8BNT6|M8BNT6_AEGTA
8.2
8.3
8.4
8.5
8.6
1
2
t−Test: p−value = 0.21

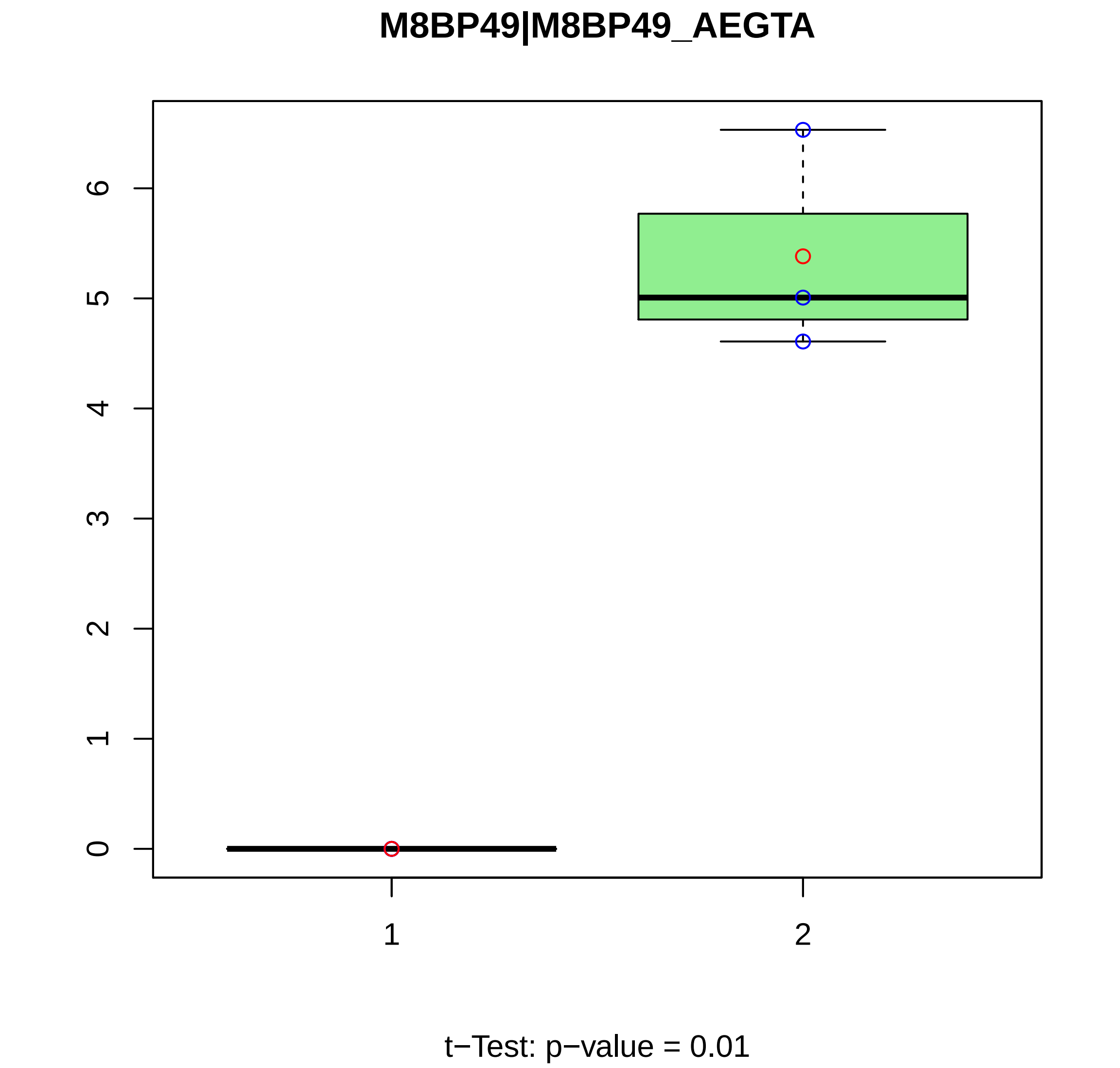
M8BP49|M8BP49_AEGTA
0
1
2
3
4
5
6
1
2
t−Test: p−value = 0.01

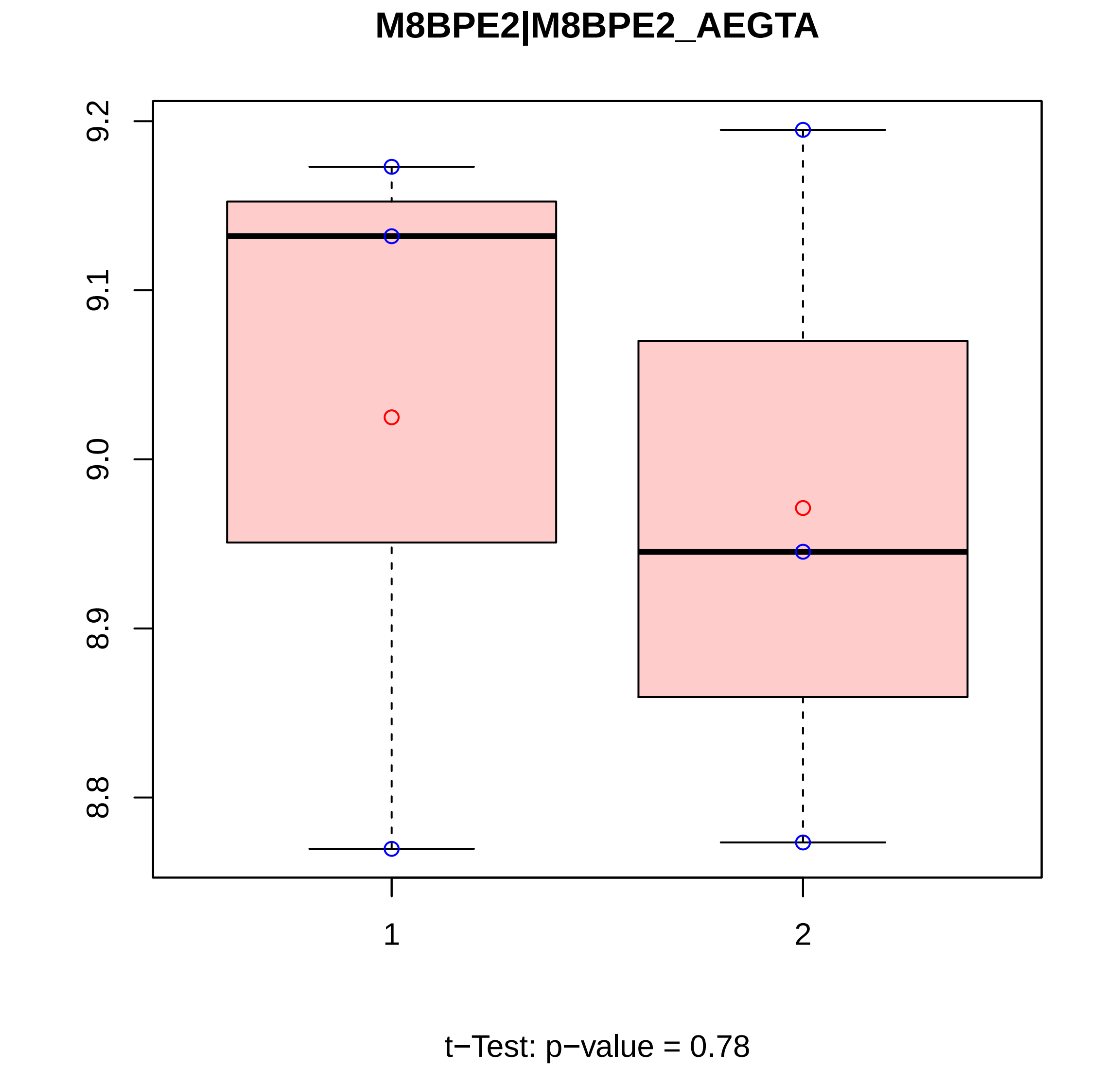
M8BPE2|M8BPE2_AEGTA
9.2
9.1
9.0
8.9
8.8
1
2
t−Test: p−value = 0.78

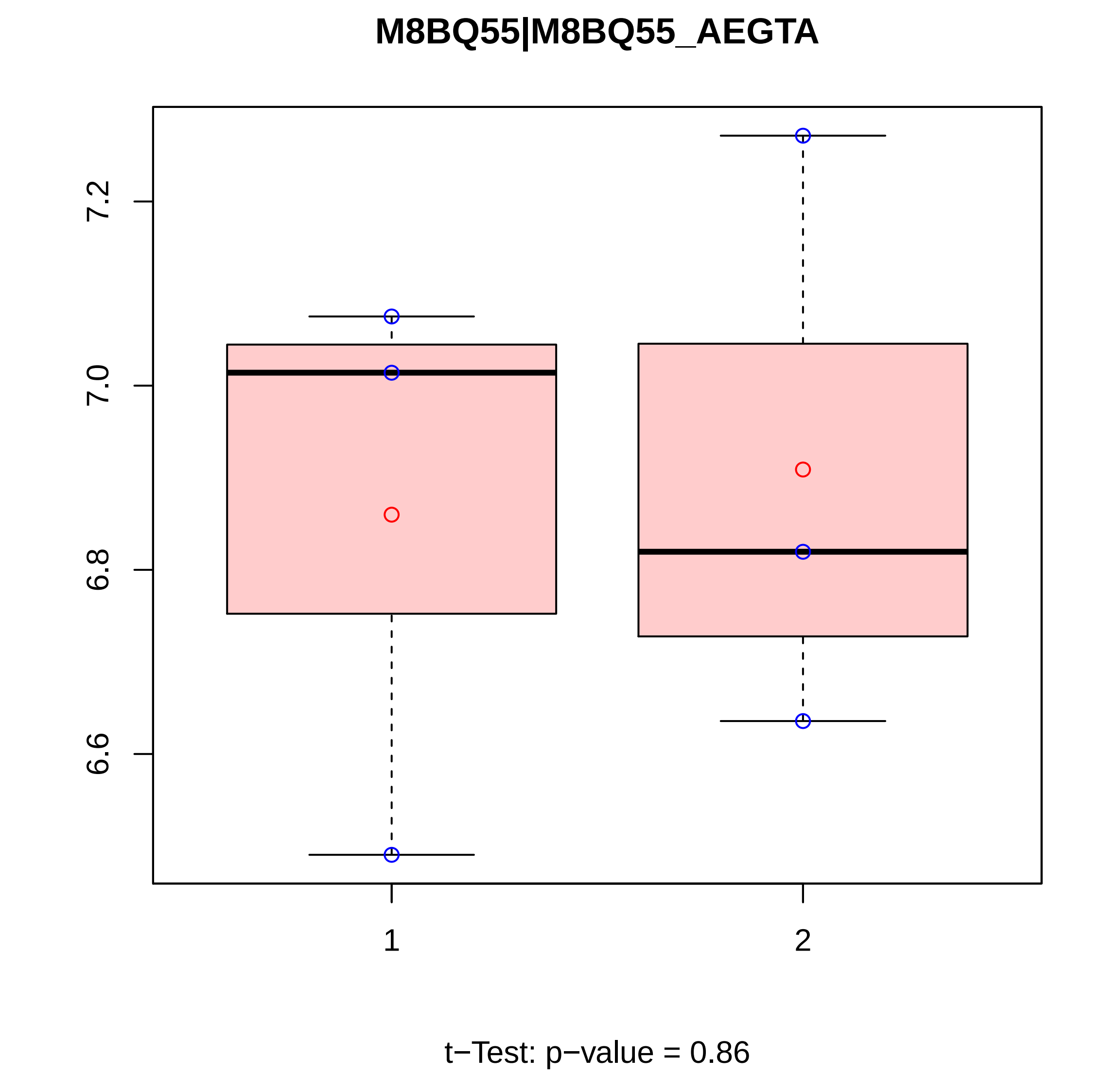
M8BQ55|M8BQ55_AEGTA
7.2
7.0
6.8
6.6
1
2
t−Test: p−value = 0.86

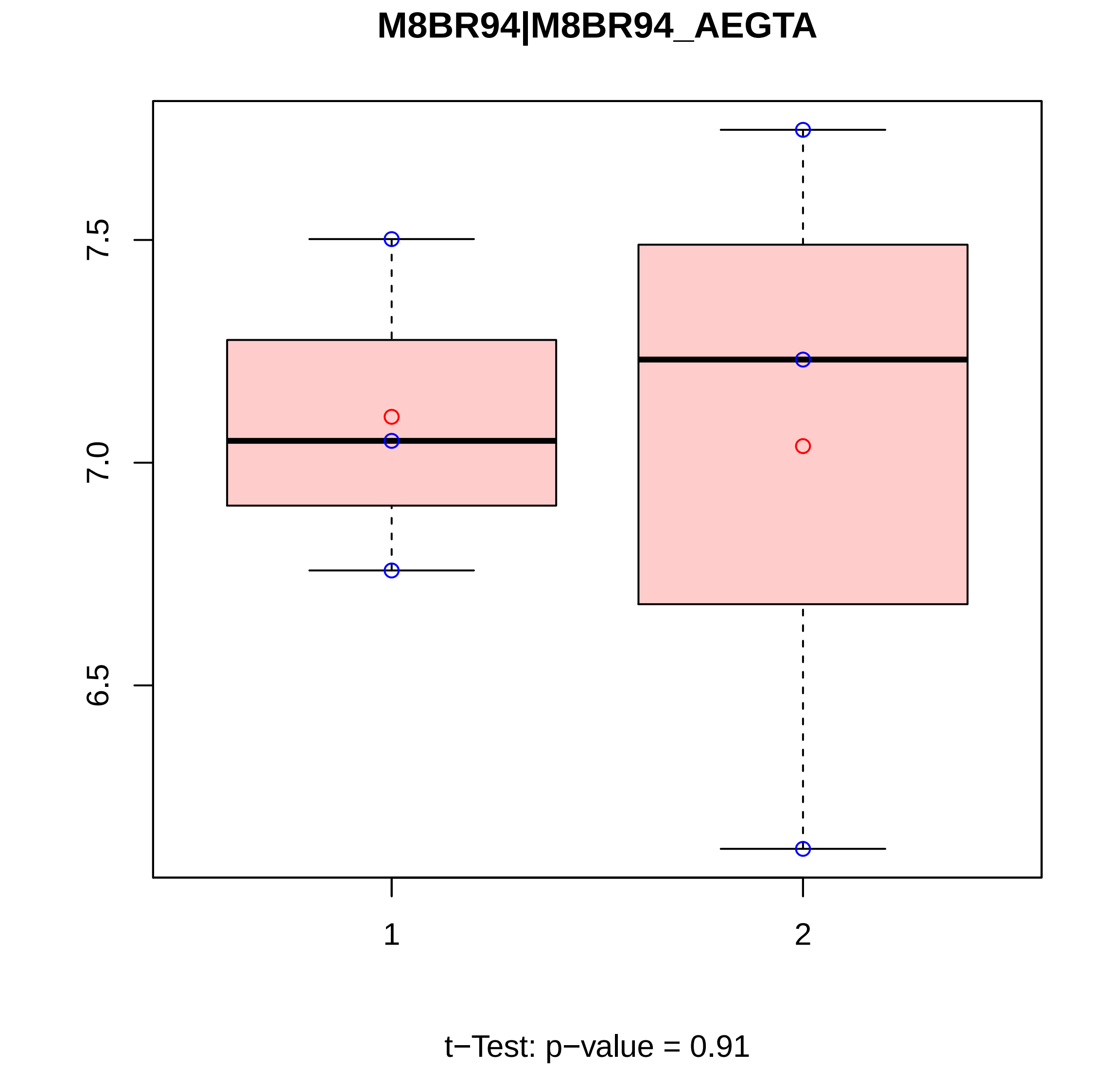
M8BR94|M8BR94_AEGTA
7.5
7.0
6.5
1
2
t−Test: p−value = 0.91

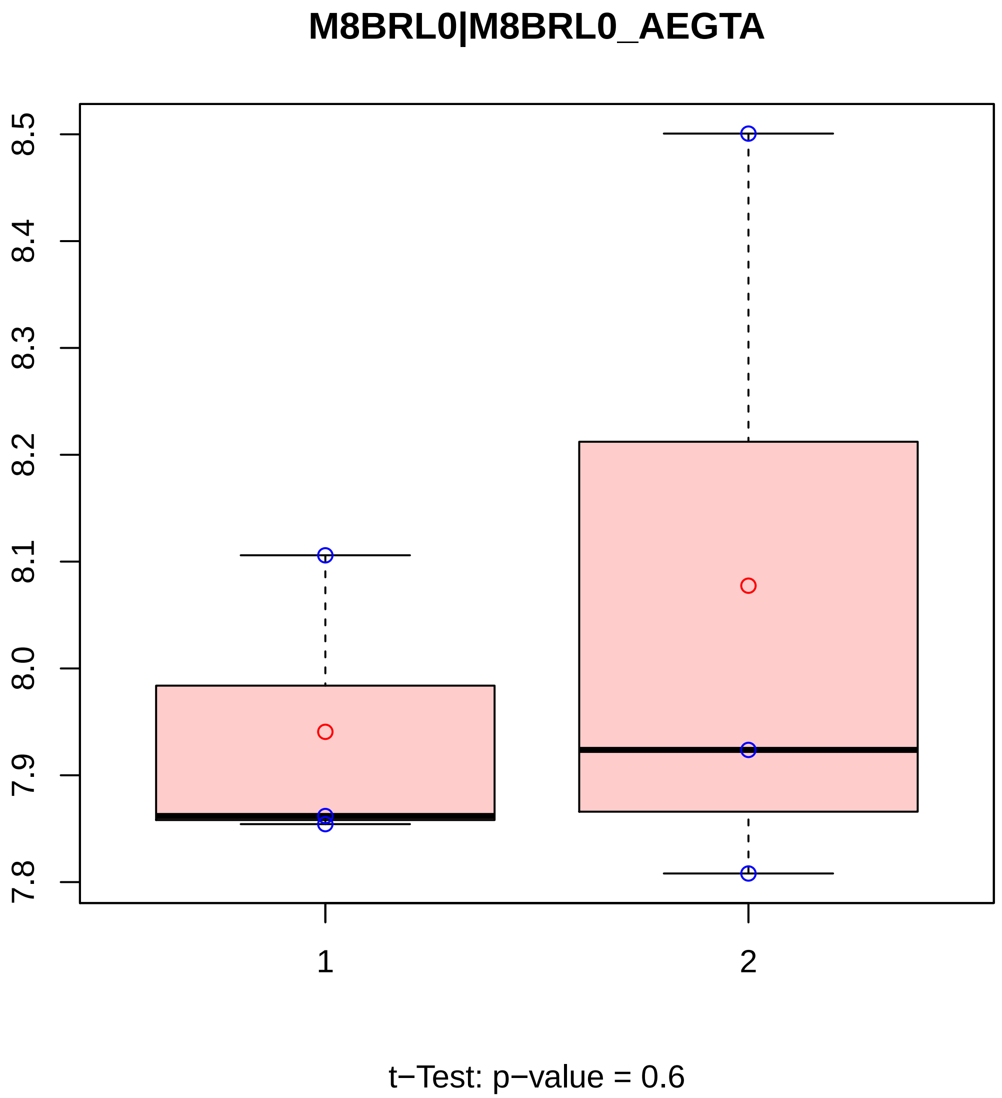
M8BRL0|M8BRL0_AEGTA
8.5
8.4
8.3
8.2
8.1
8.0
7.9
7.8
1
2
t−Test: p−value = 0.6

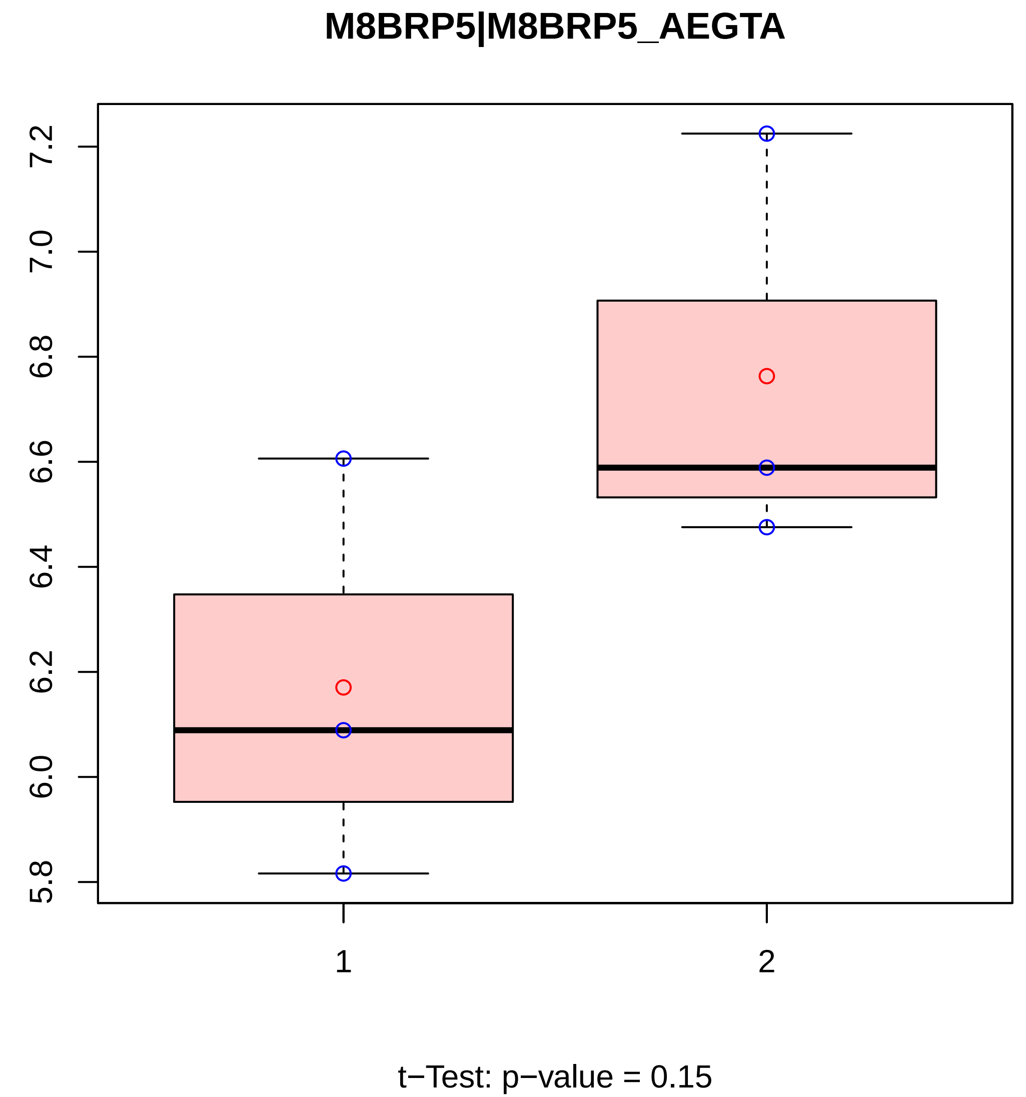
M8BRP5|M8BRP5_AEGTA
7.2
7.0
6.8
6.6
6.4
6.2
6.0
5.8
1
2
t−Test: p−value = 0.15

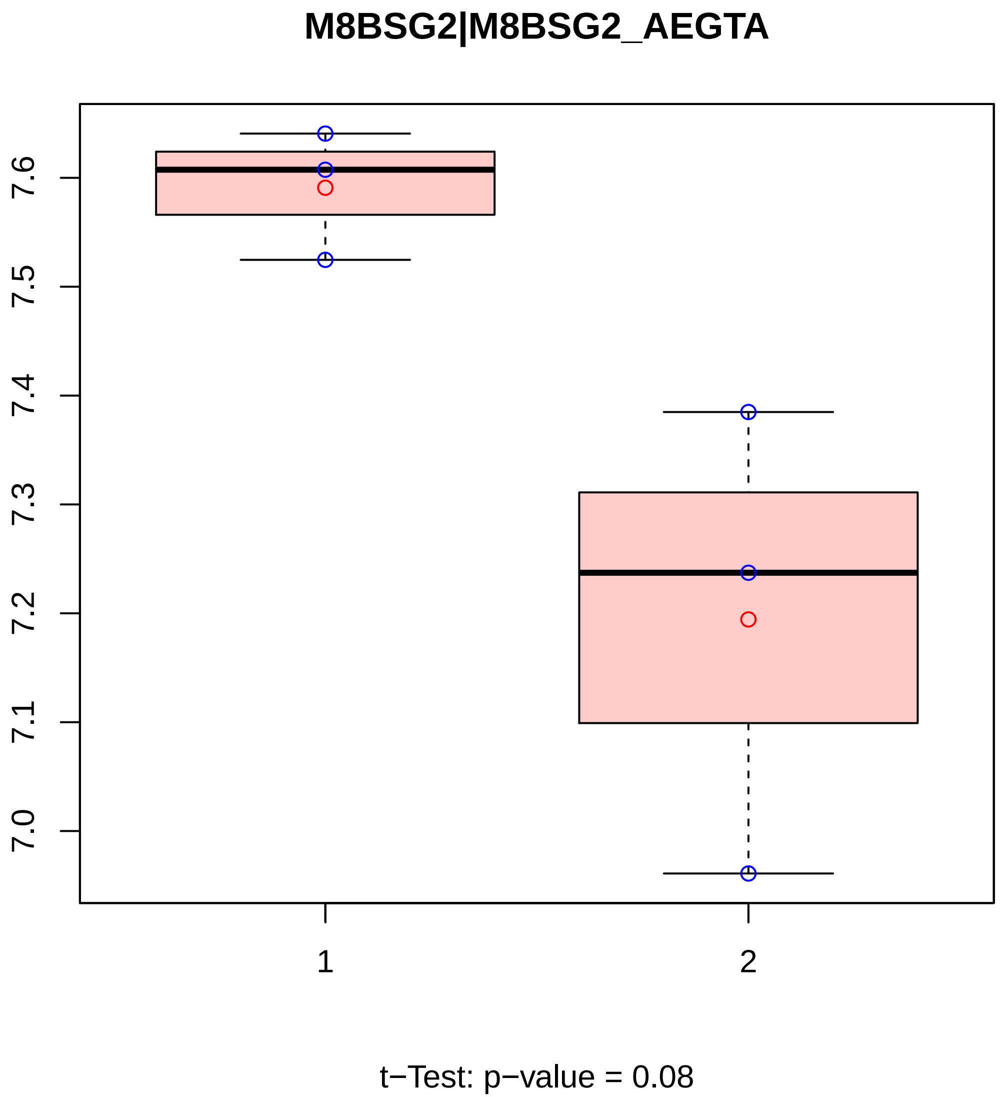
M8BSG2|M8BSG2_AEGTA
7.0
7.1
7.2
7.3
7.4
7.5
7.6
1
2
t−Test: p−value = 0.08

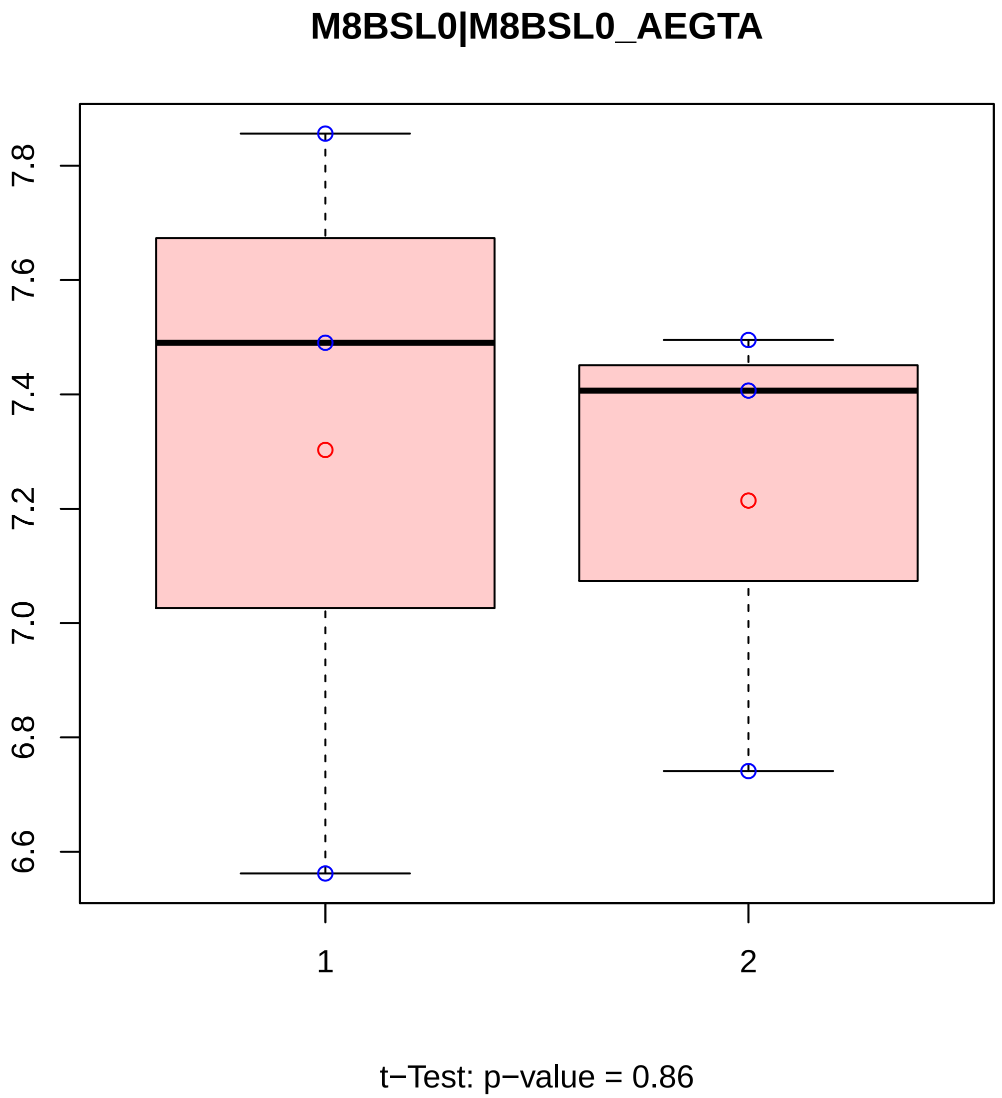
M8BSL0|M8BSL0_AEGTA
6.6
6.8
7.0
7.2
7.4
7.6
7.8
1
2
t−Test: p−value = 0.86

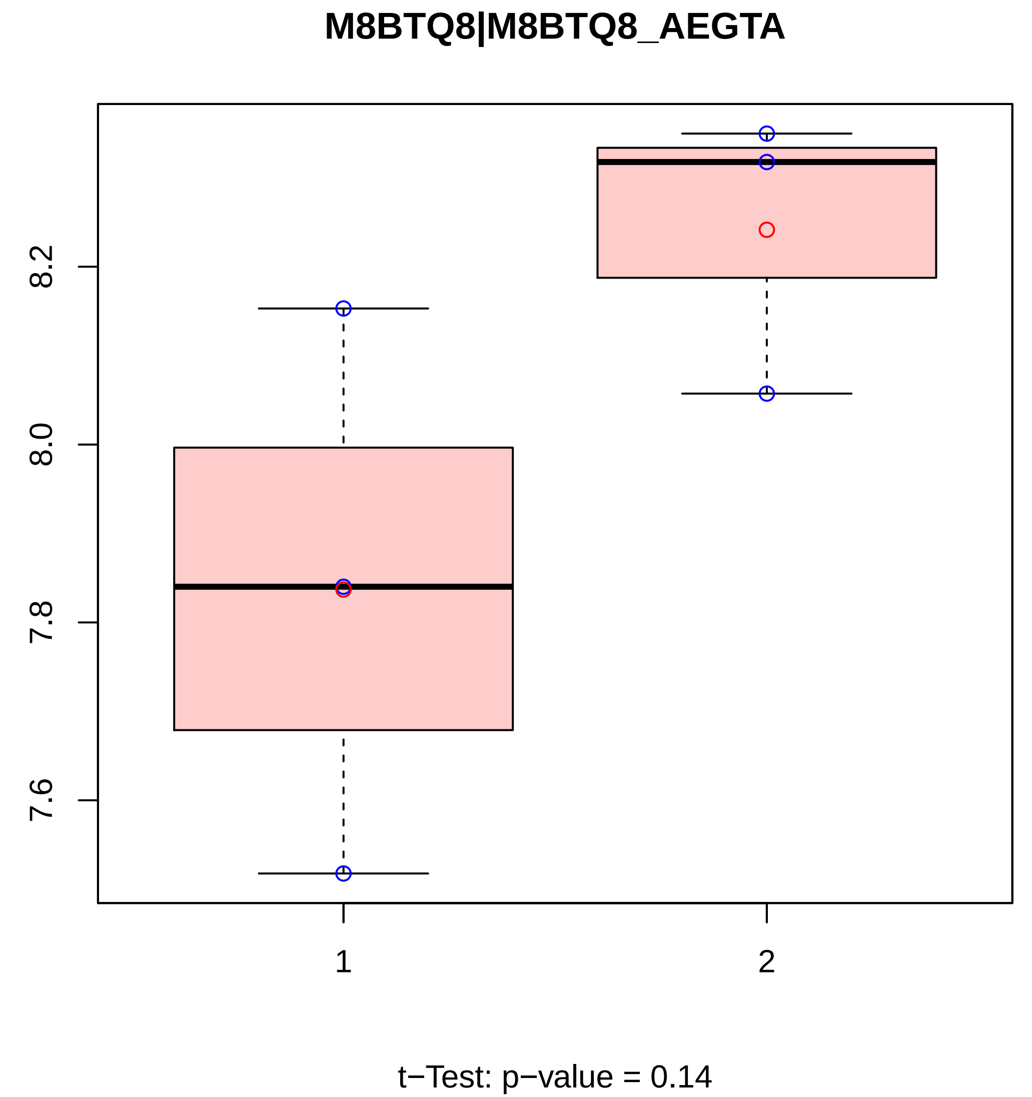
M8BTQ8|M8BTQ8_AEGTA
8.2
8.0
7.8
7.6
1
2
t−Test: p−value = 0.14

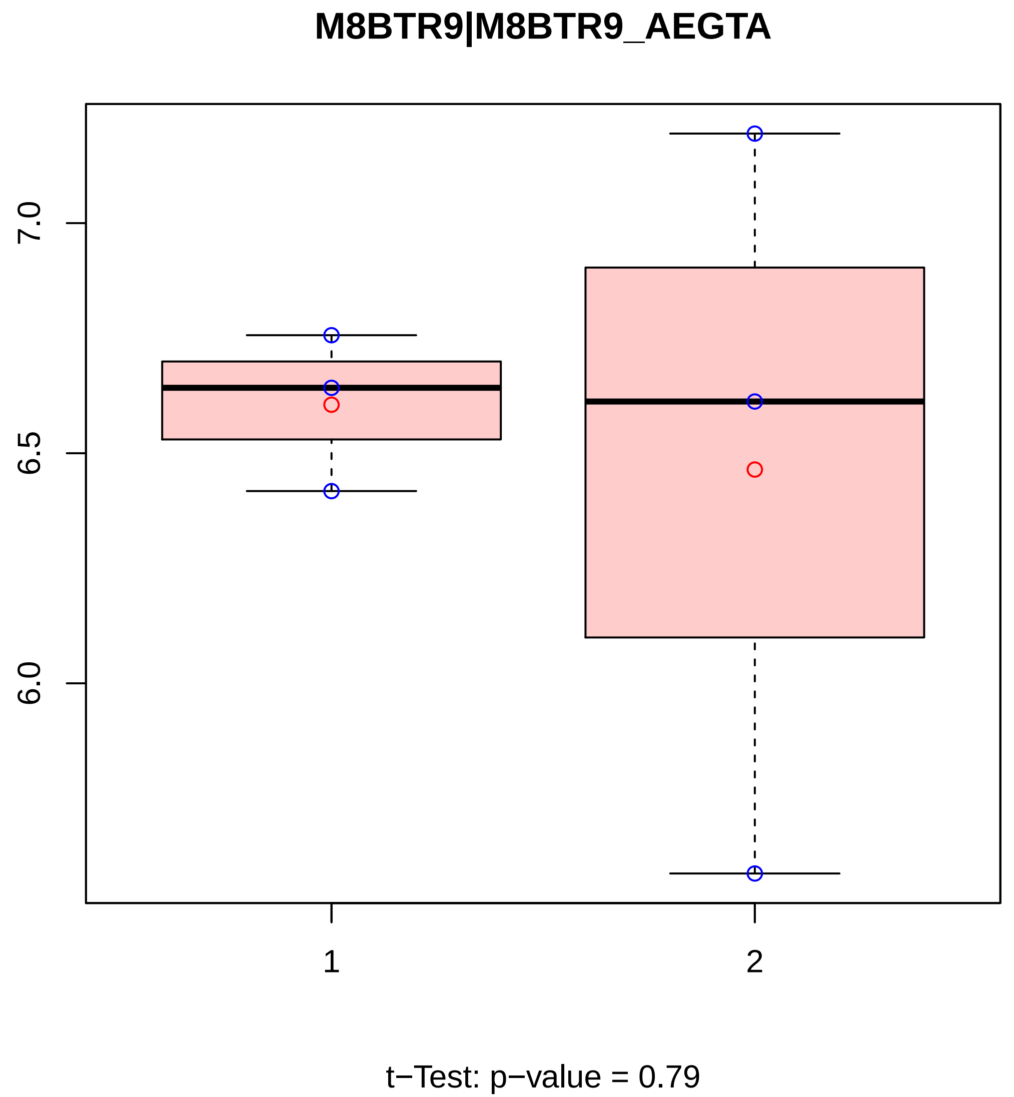
M8BTR9|M8BTR9_AEGTA
7.0
6.5
6.0
1
2
t−Test: p−value = 0.79

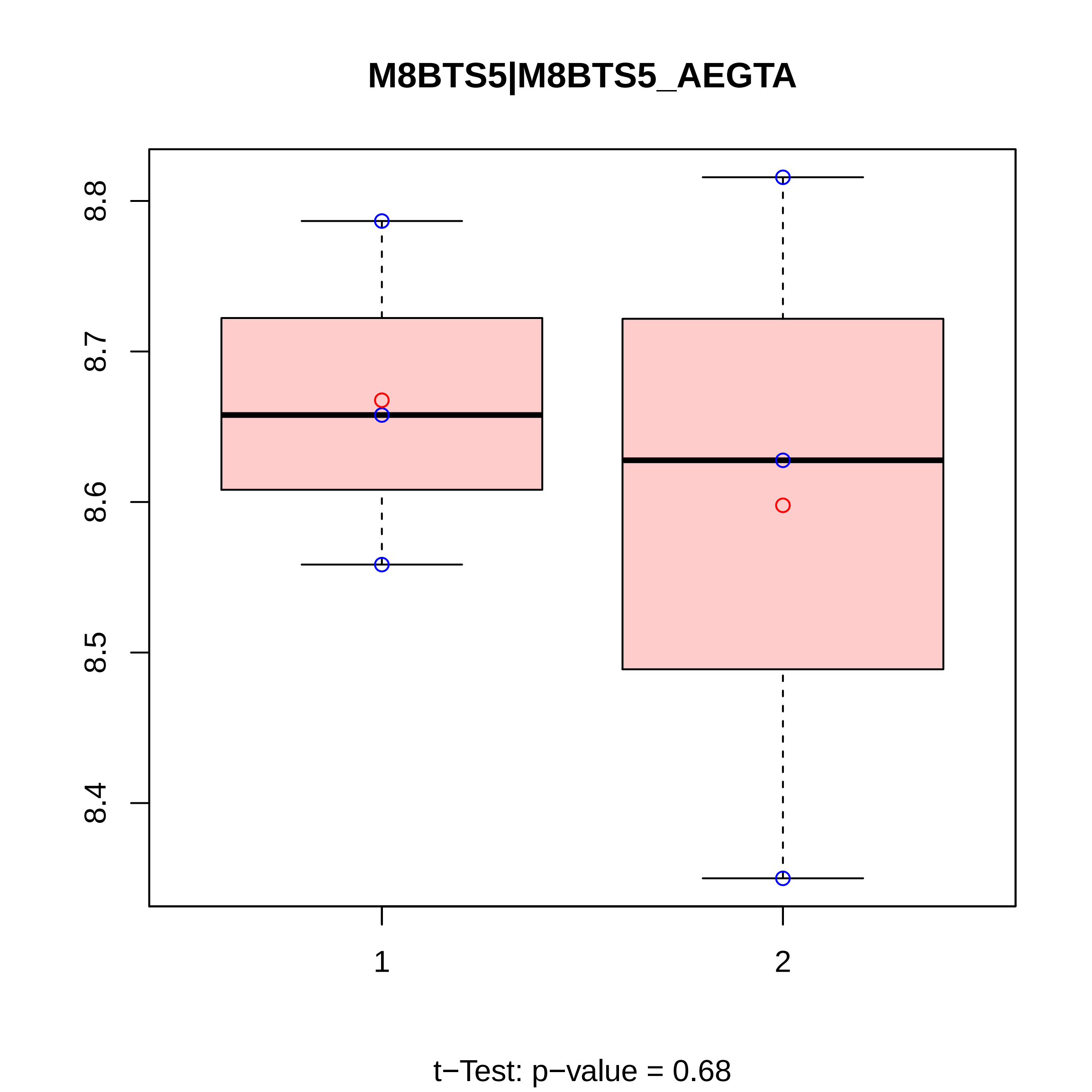
M8BTS5|M8BTS5_AEGTA
8.4
8.5
8.6
8.7
8.8
1
2
t−Test: p−value = 0.68

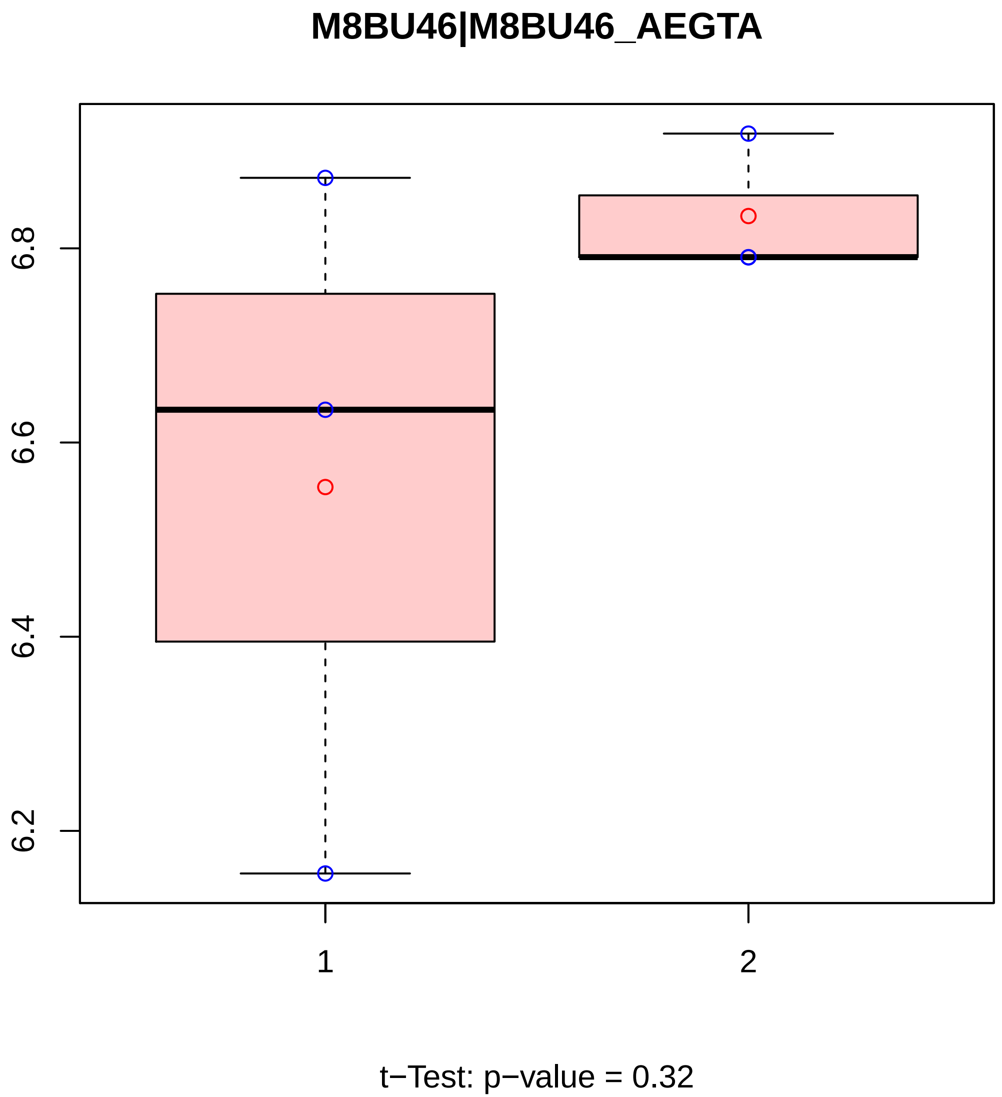
M8BU46|M8BU46_AEGTA
6.2
6.4
6.6
6.8
1
2
t−Test: p−value = 0.32

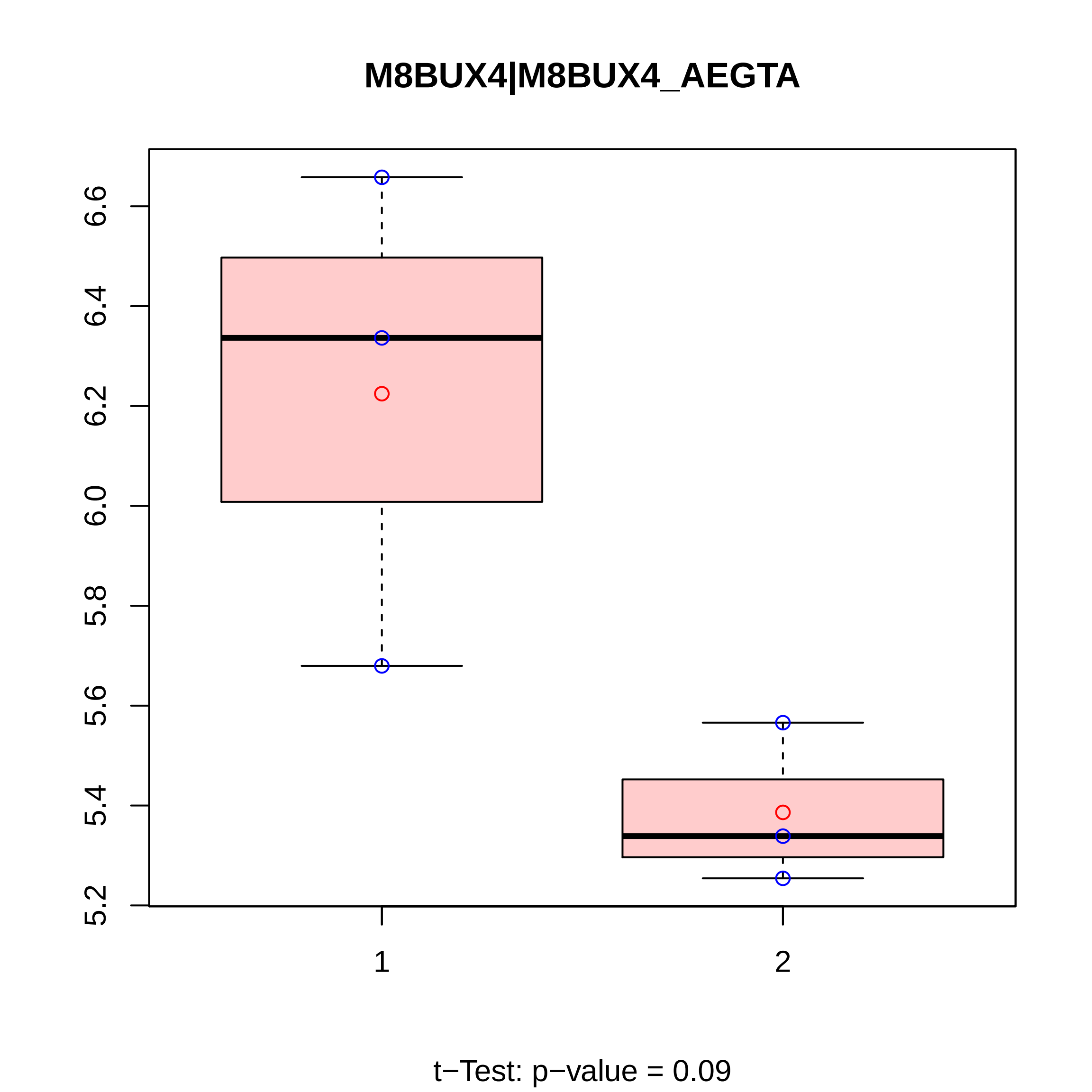

M8BUX4|M8BUX4_AEGTA
6.6
6.4
6.2
6.0
5.8
5.6
5.4
5.2
1
2
t−Test: p−value = 0.09

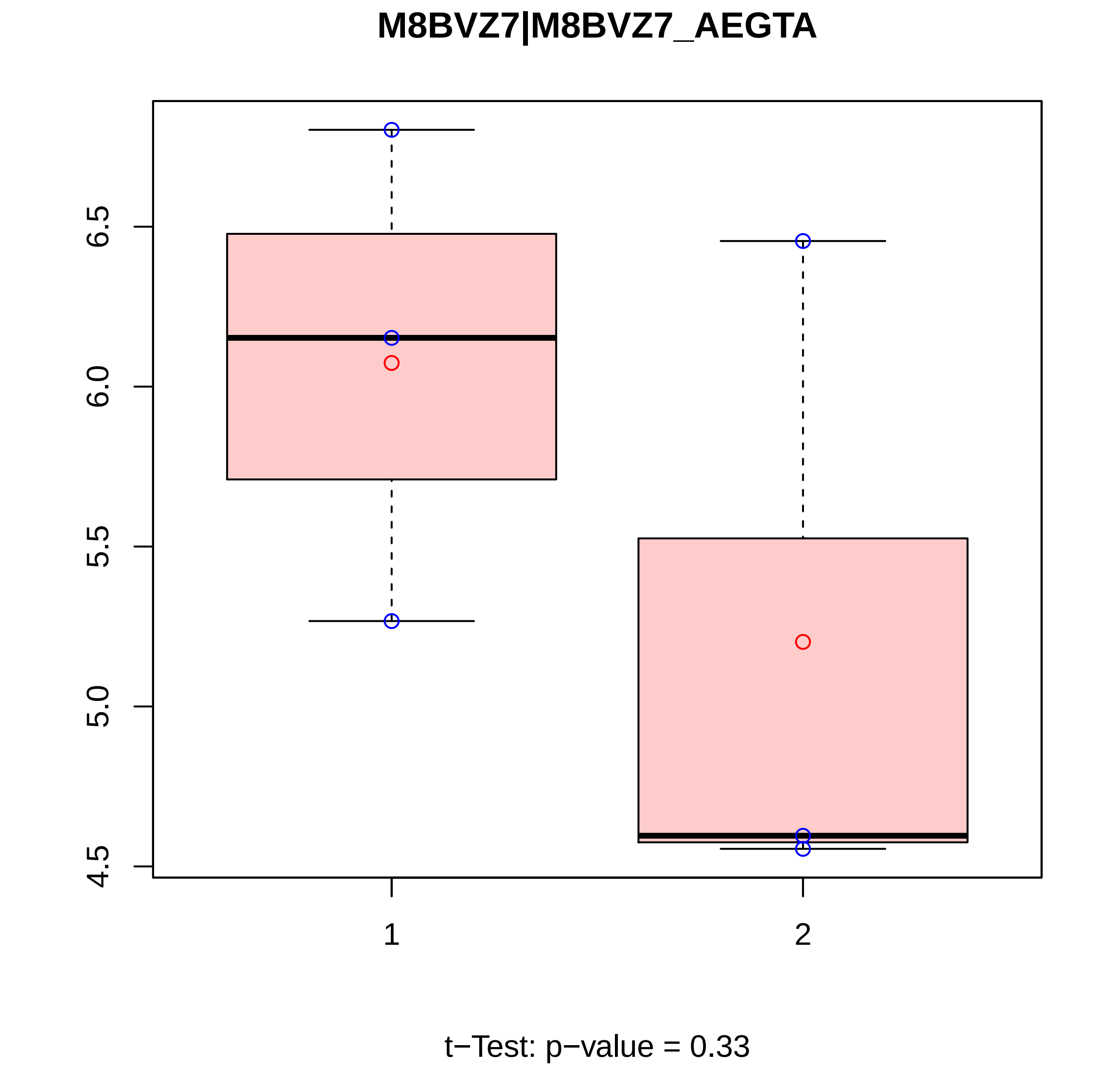
M8BVZ7|M8BVZ7_AEGTA
6.5
6.0
5.5
5.0
4.5
1
2
t−Test: p−value = 0.33

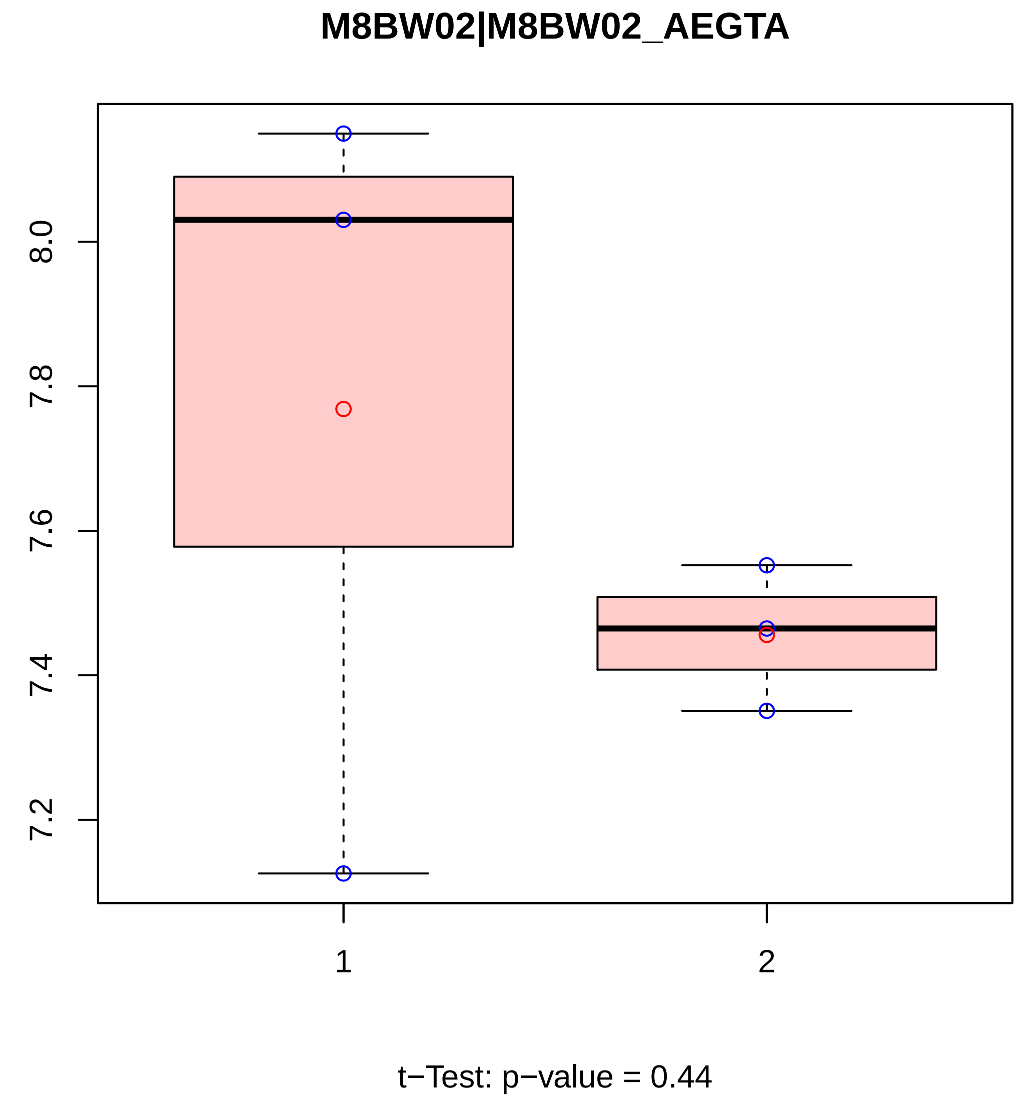
M8BW02|M8BW02_AEGTA
8.0
7.8
7.6
7.4
7.2
1
2
t−Test: p−value = 0.44

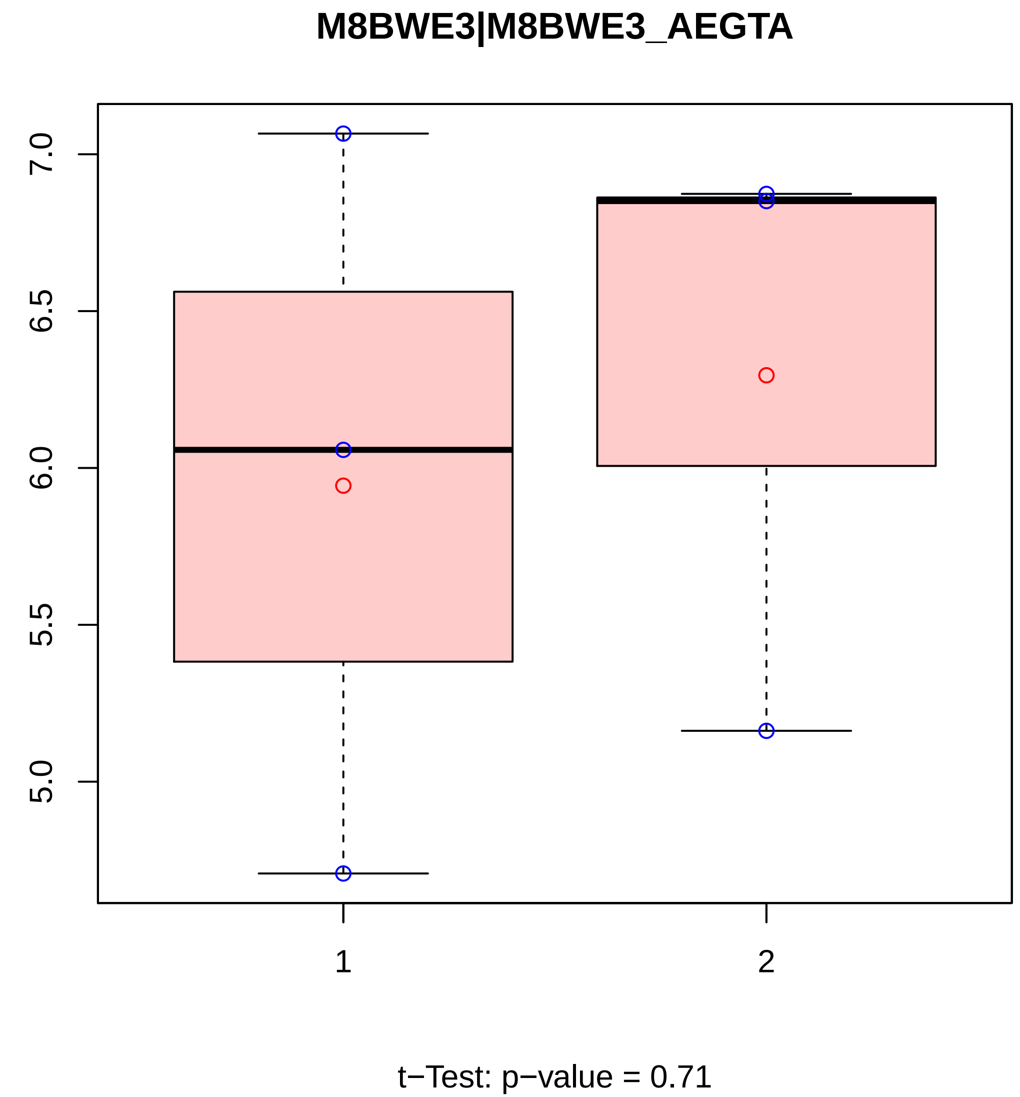
M8BWE3|M8BWE3_AEGTA
7.0
6.5
6.0
5.5
5.0
1
2
t−Test: p−value = 0.71

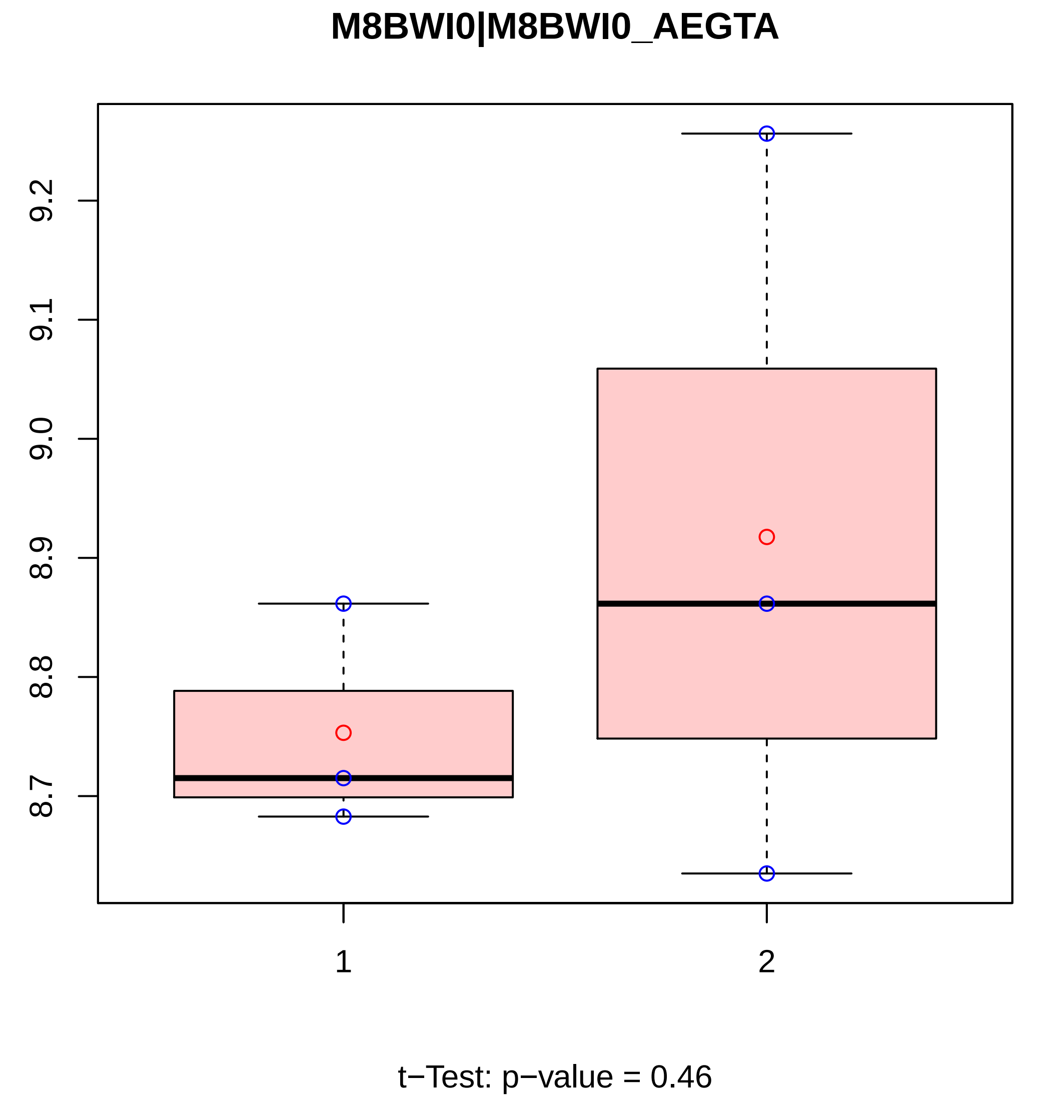
M8BWI0|M8BWI0_AEGTA
8.7
8.8
8.9
9.0
9.1
9.2
1
2
t−Test: p−value = 0.46

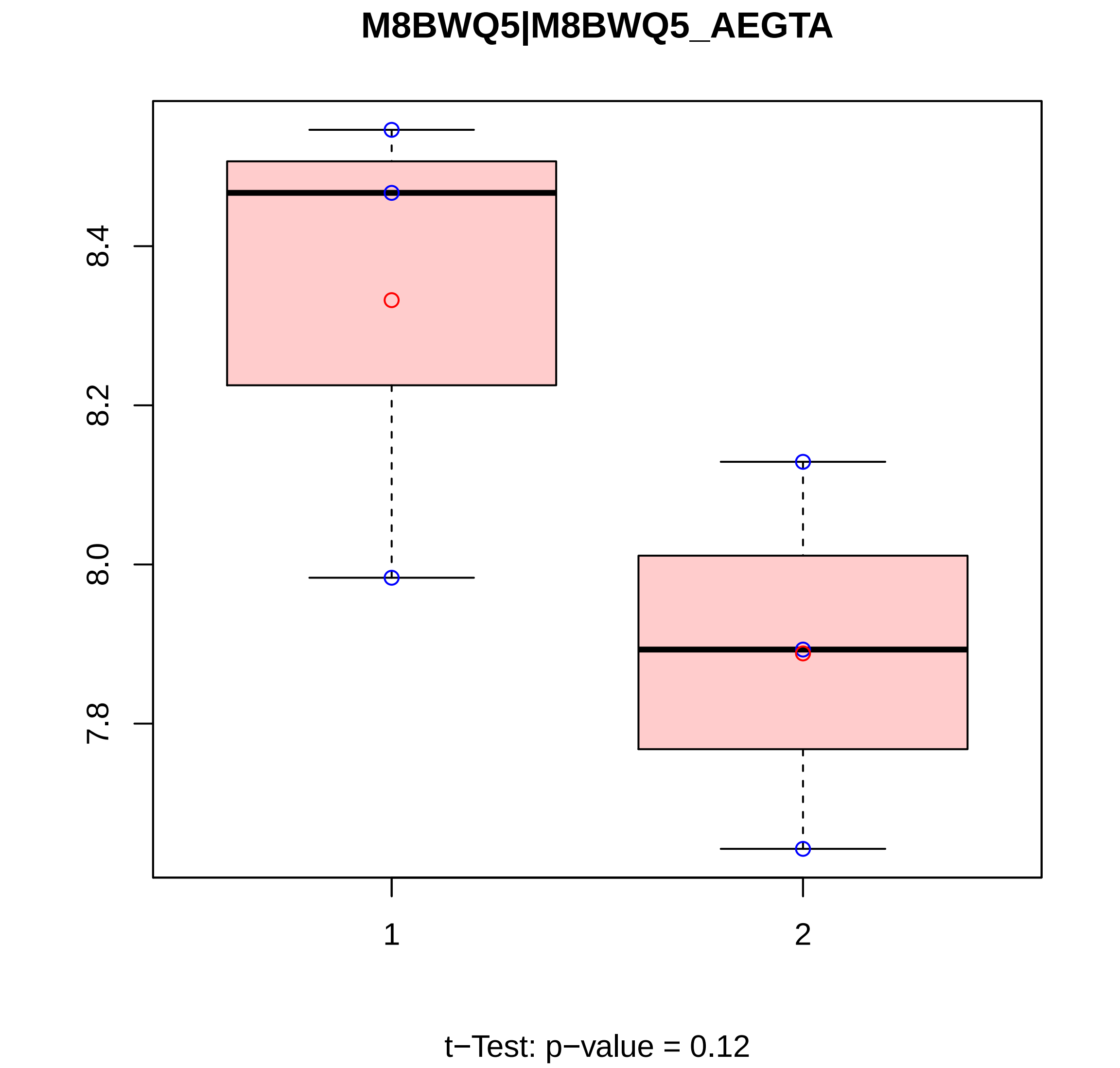

M8BWQ5|M8BWQ5_AEGTA
7.8
8.0
8.2
8.4
1
2
t−Test: p−value = 0.12

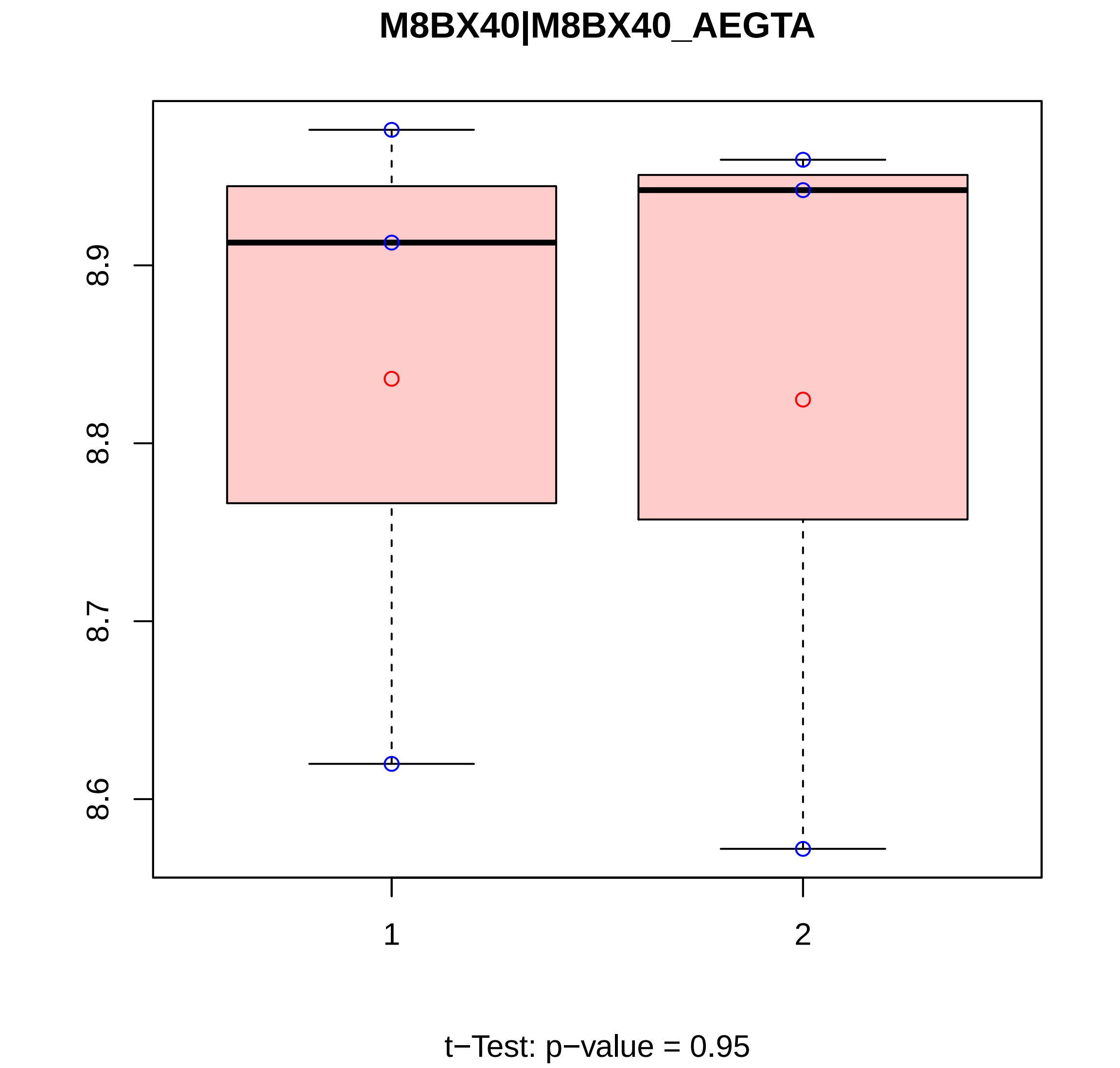
M8BX40|M8BX40_AEGTA
8.6
8.7
8.8
8.9
1
2
t−Test: p−value = 0.95

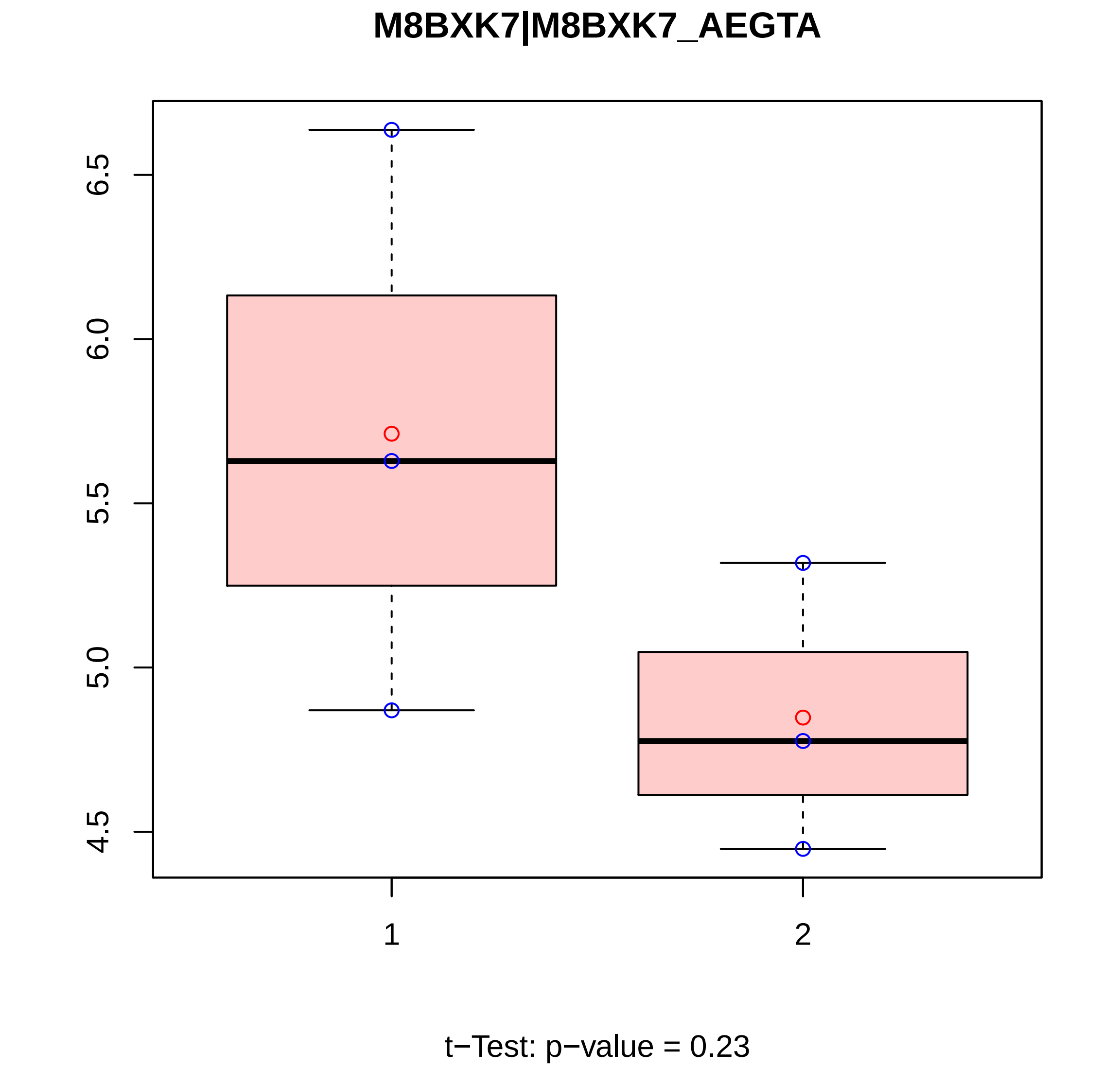
M8BXK7|M8BXK7_AEGTA
6.5
6.0
5.5
5.0
4.5
1
2
t−Test: p−value = 0.23

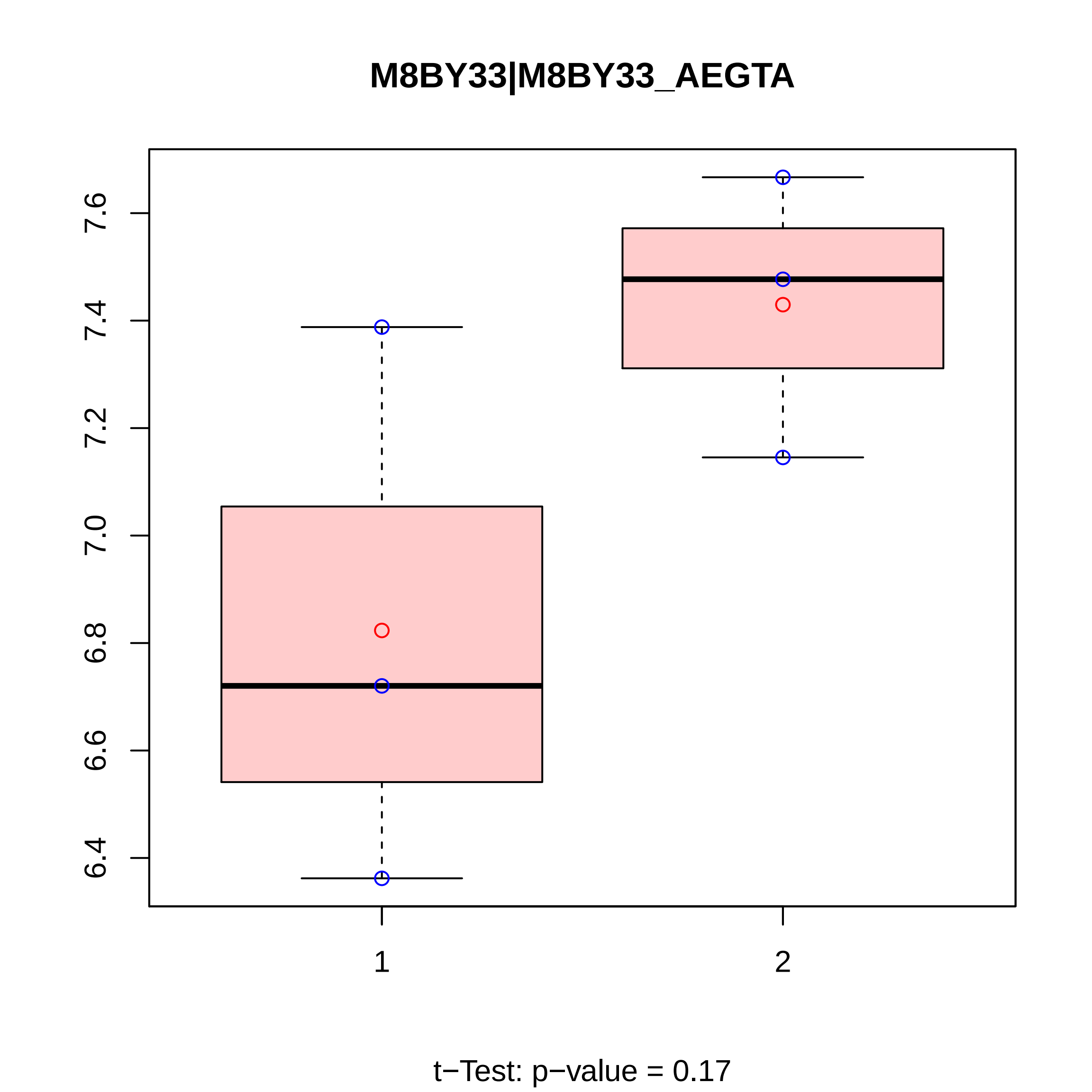

M8BY33|M8BY33_AEGTA
7.6
7.4
7.2
7.0
6.8
6.6
6.4
1
2
t−Test: p−value = 0.17

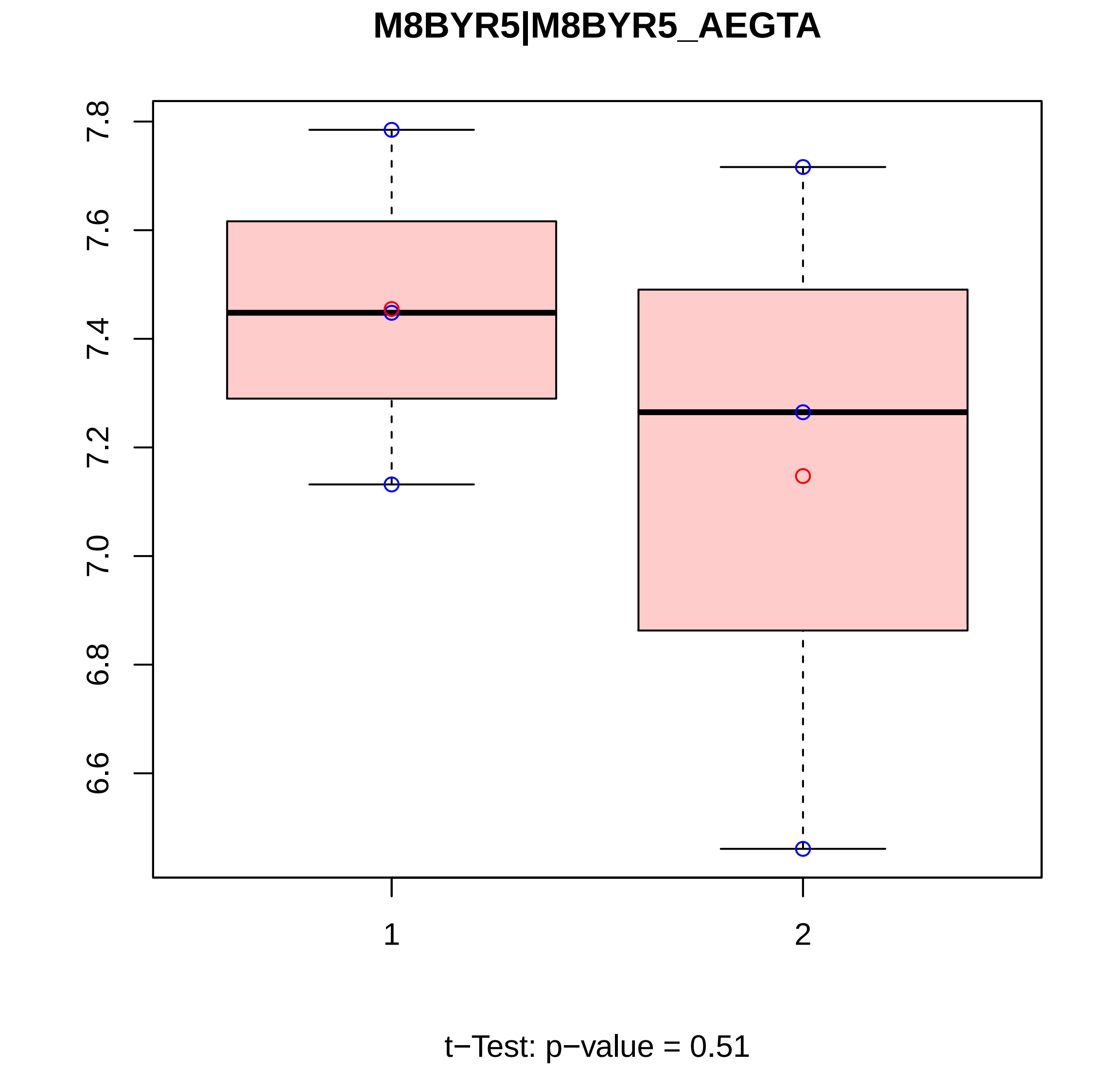
M8BYR5|M8BYR5_AEGTA
7.8
7.6
7.4
7.2
7.0
6.8
6.6
1
2
t−Test: p−value = 0.51

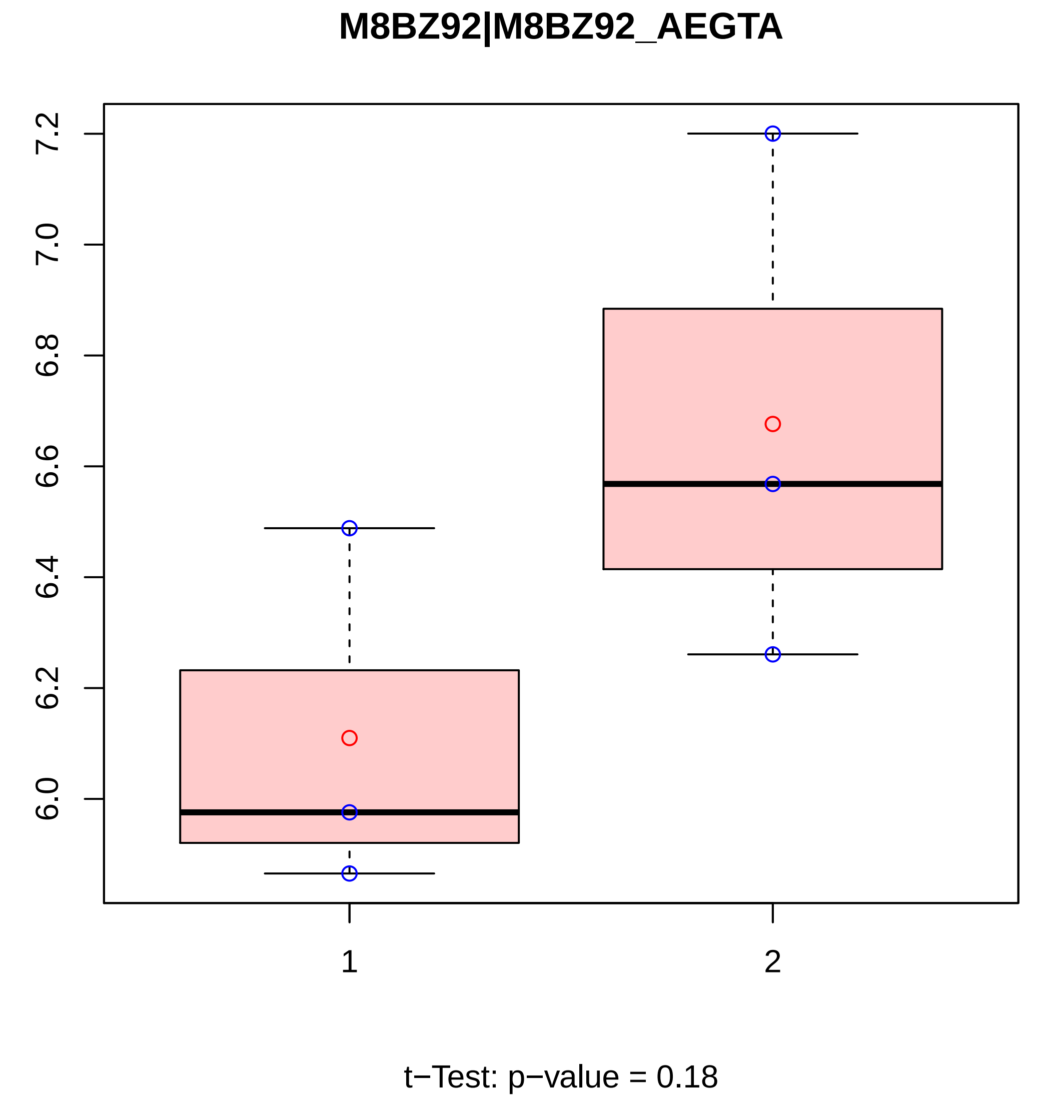
M8BZ92|M8BZ92_AEGTA
7.2
7.0
6.8
6.6
6.4
6.2
6.0
1
2
t−Test: p−value = 0.18

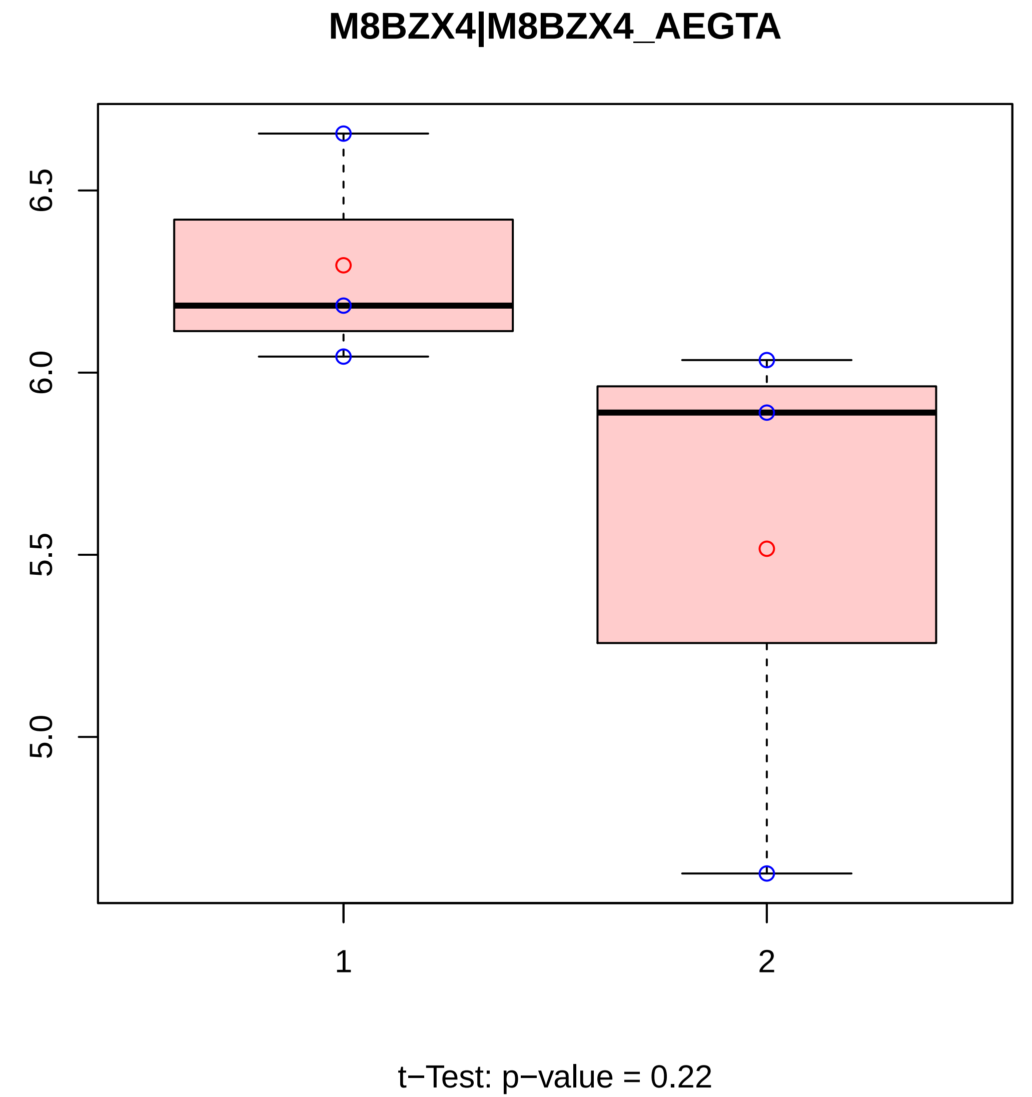
M8BZX4|M8BZX4_AEGTA
6.5
6.0
5.5
5.0
1
2
t−Test: p−value = 0.22

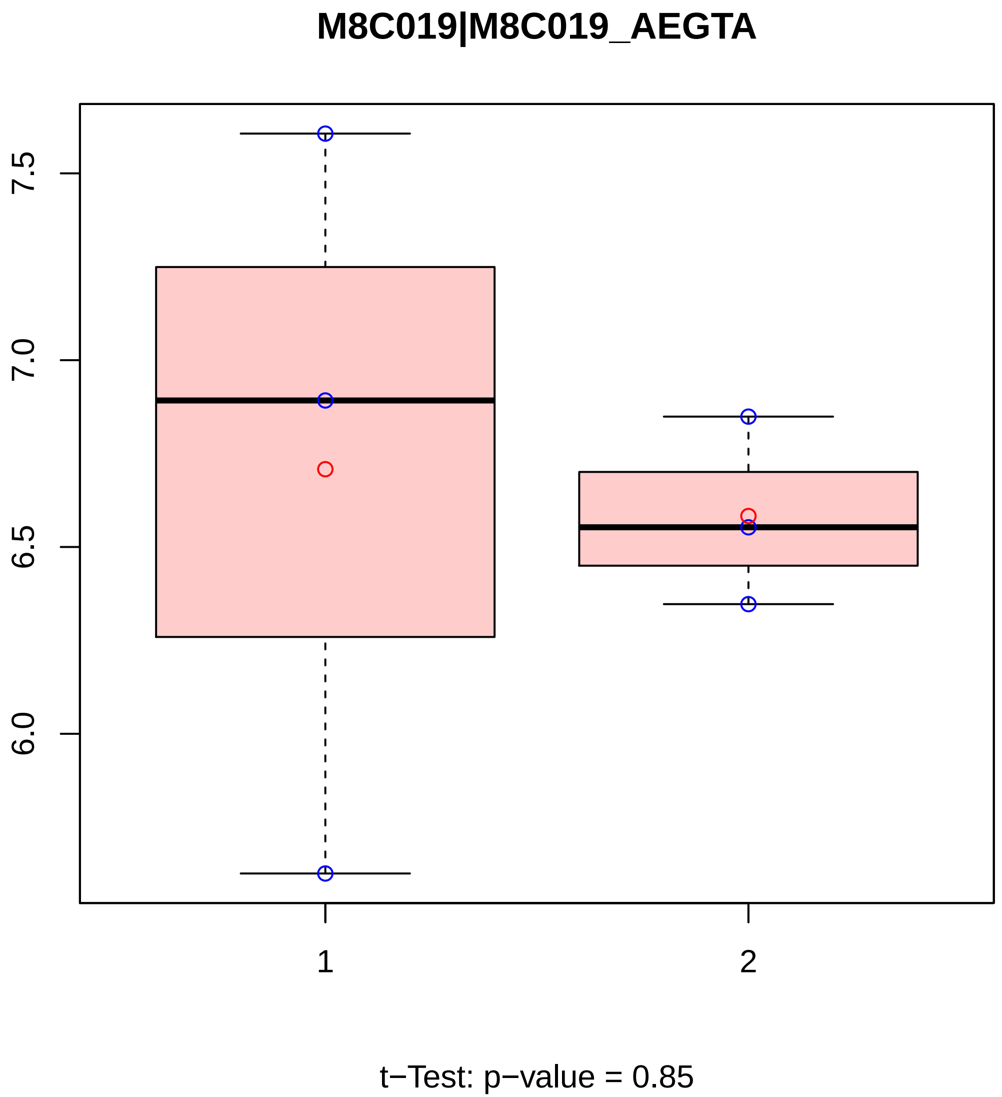
M8C019|M8C019_AEGTA
7.5
7.0
6.5
6.0
1
2
t−Test: p−value = 0.85

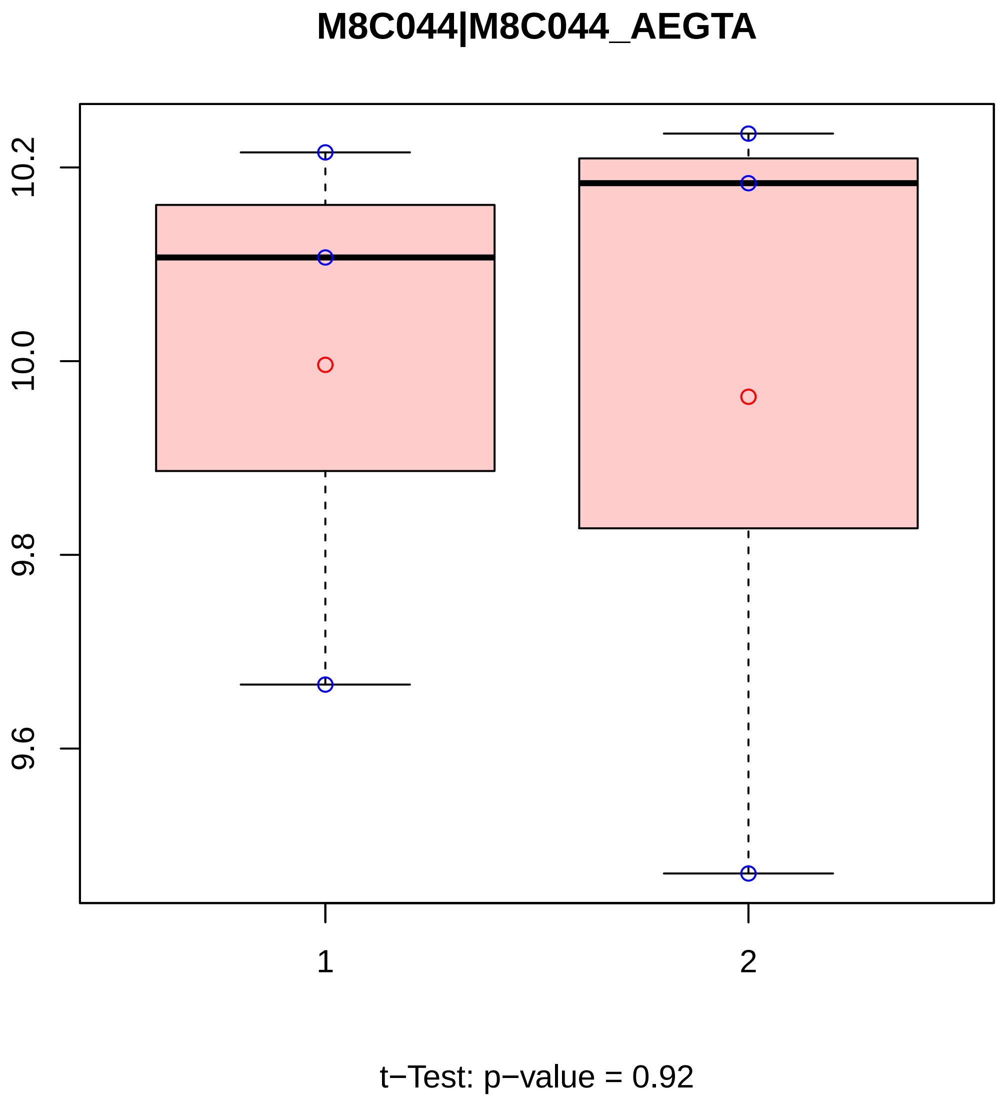
M8C044|M8C044_AEGTA
10.2
10.0
9.8
9.6
1
2
t−Test: p−value = 0.92

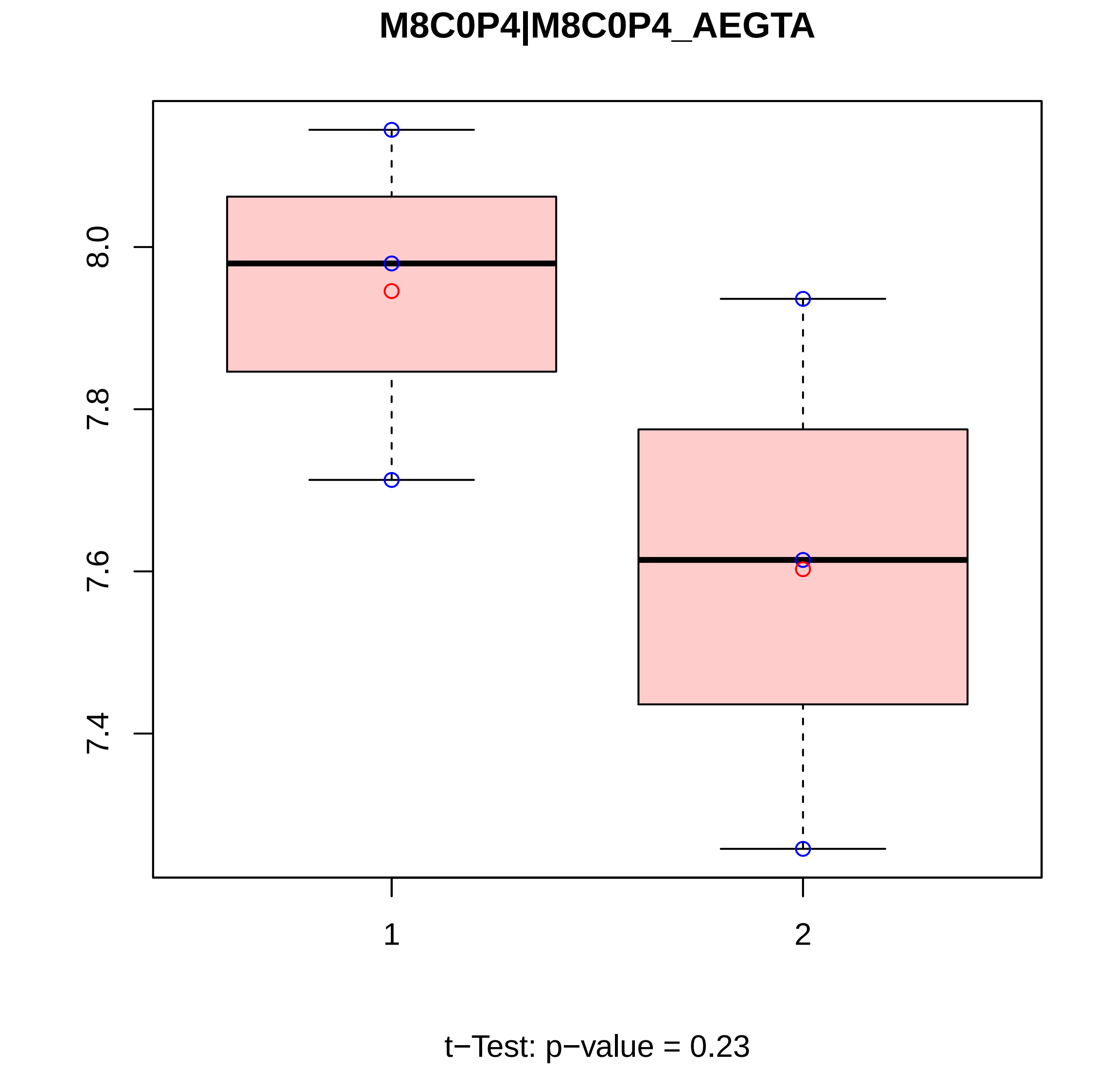
M8C0P4|M8C0P4_AEGTA
8.0
7.8
7.6
7.4
1
2
t−Test: p−value = 0.23

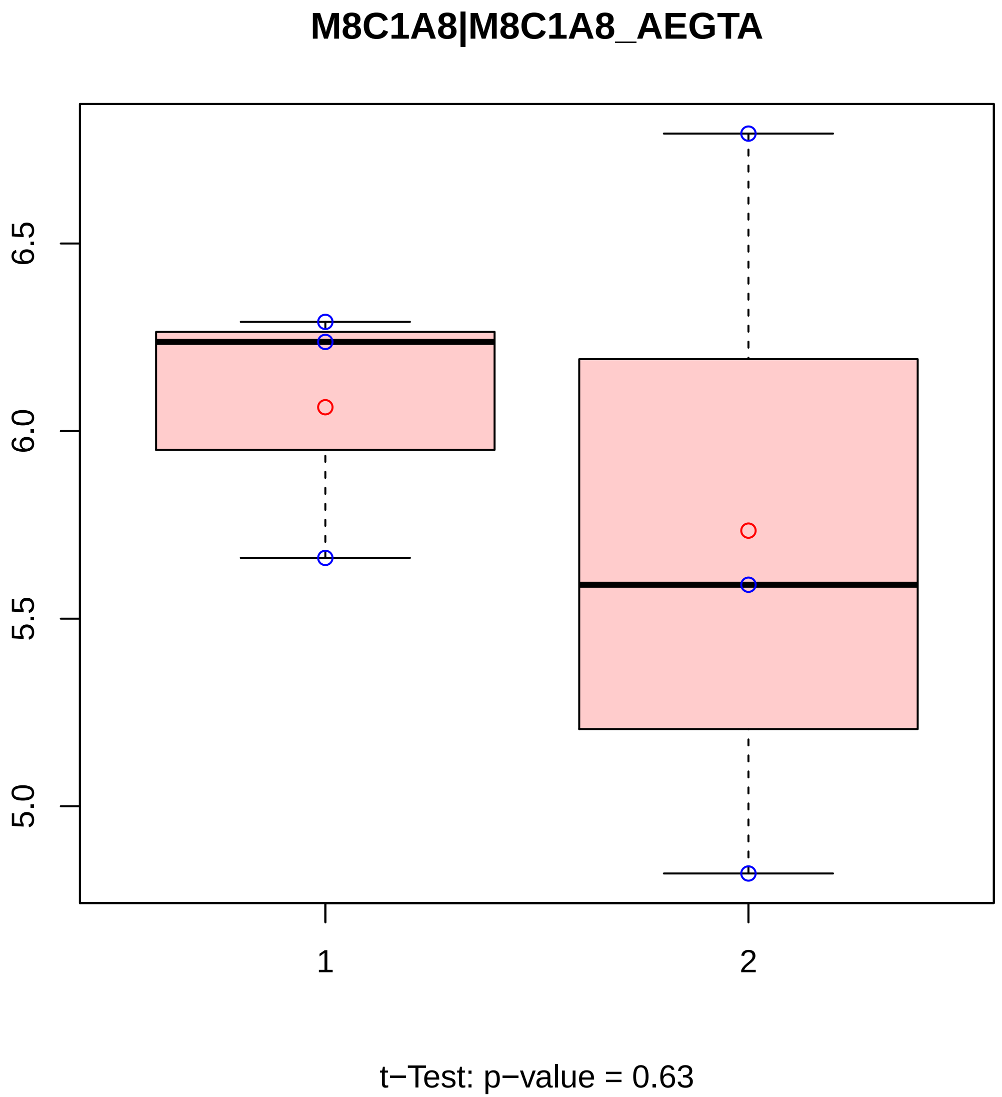
M8C1A8|M8C1A8_AEGTA
6.5
6.0
5.5
5.0
1
2
t−Test: p−value = 0.63

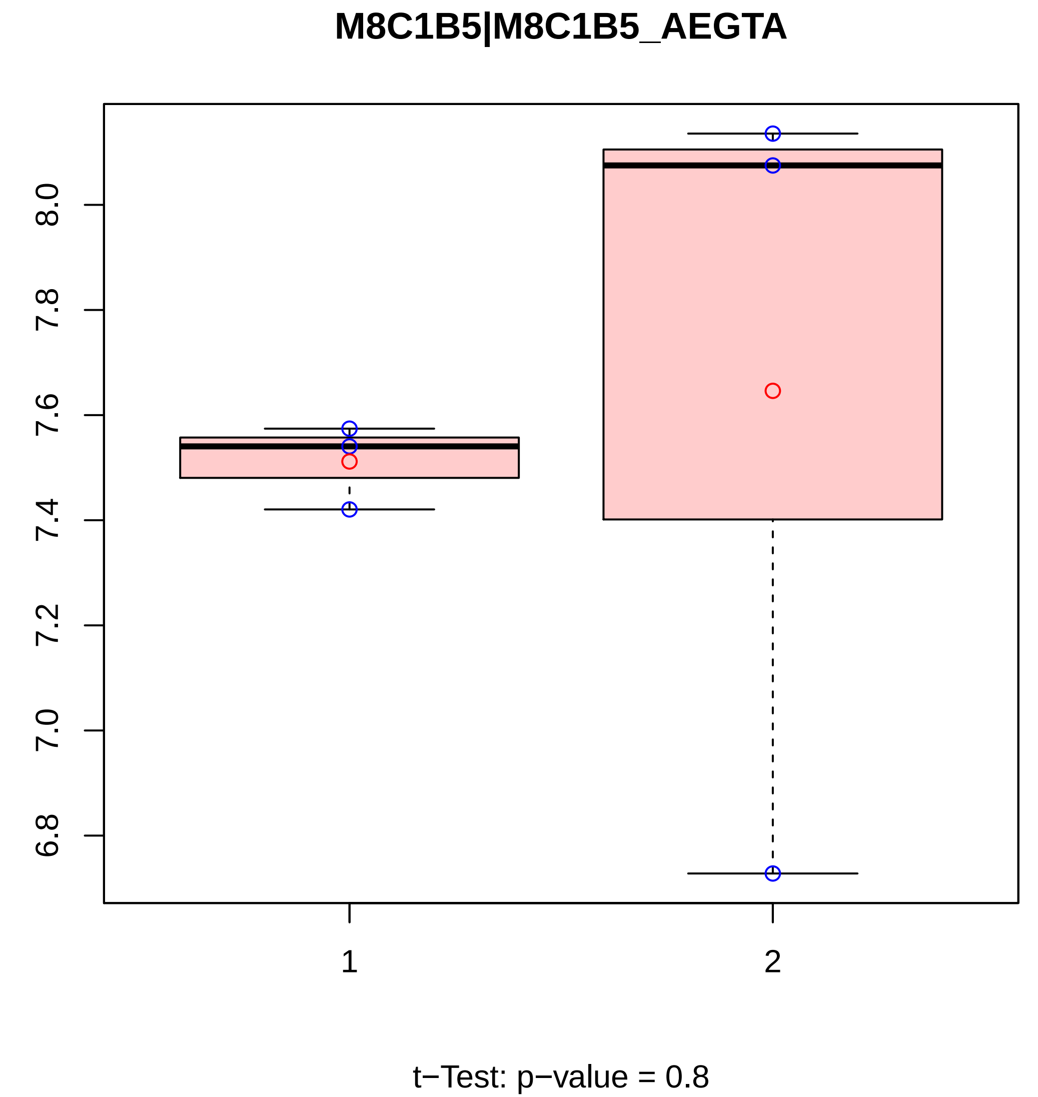
M8C1B5|M8C1B5_AEGTA
6.8
7.0
7.2
7.4
7.6
7.8
8.0
1
2
t−Test: p−value = 0.8

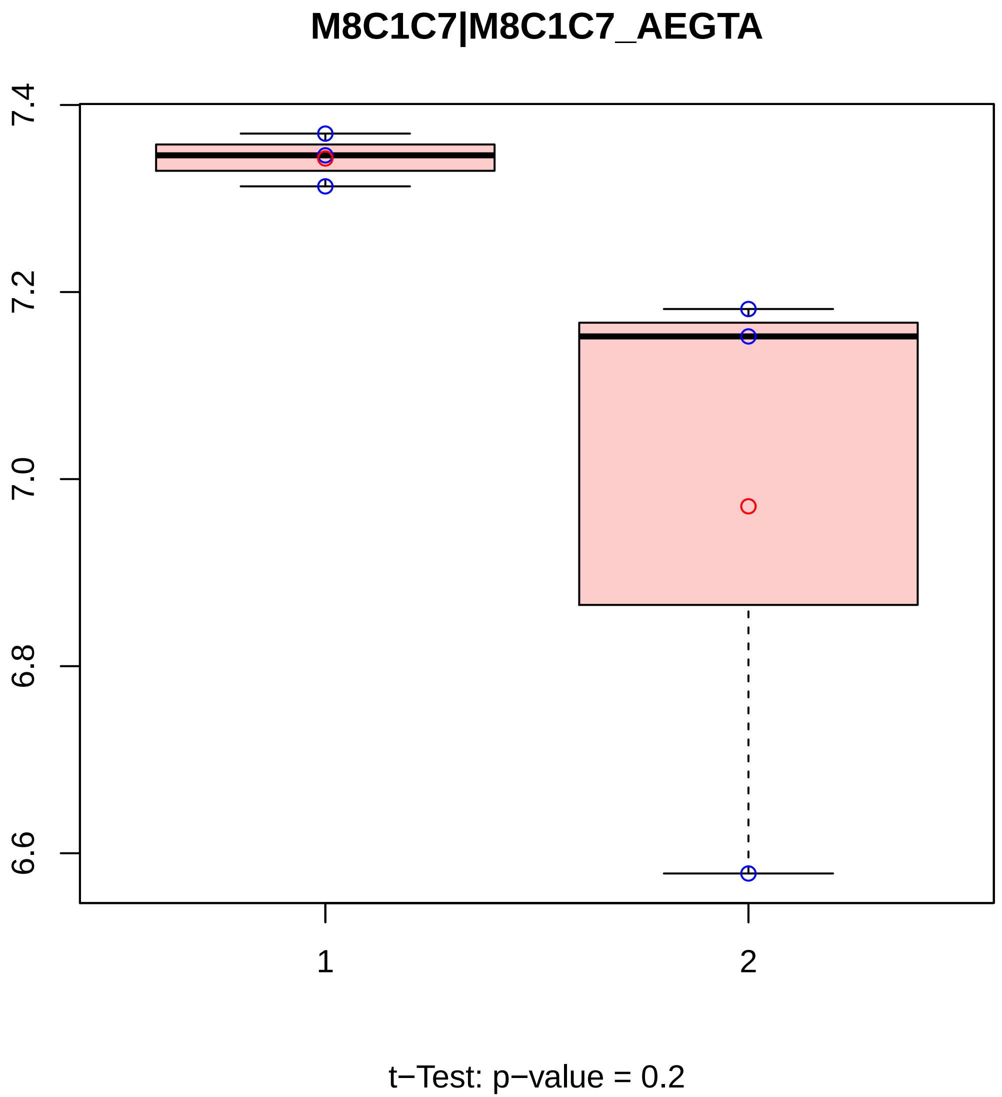
M8C1C7|M8C1C7_AEGTA
7.4
7.2
7.0
6.8
6.6
1
2
t−Test: p−value = 0.2

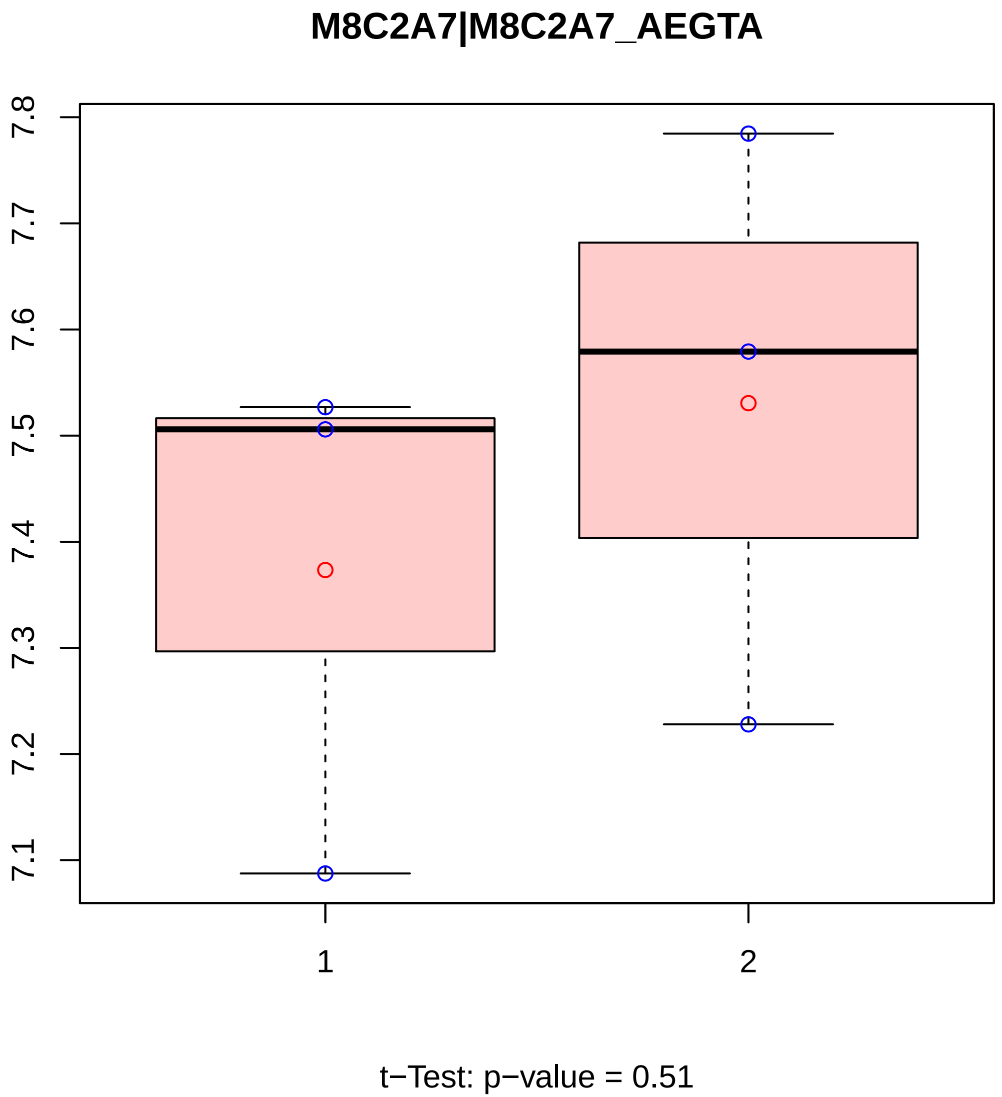
M8C2A7|M8C2A7_AEGTA
7.1
7.2
7.3
7.4
7.5
7.6
7.7
7.8
1
2
t−Test: p−value = 0.51

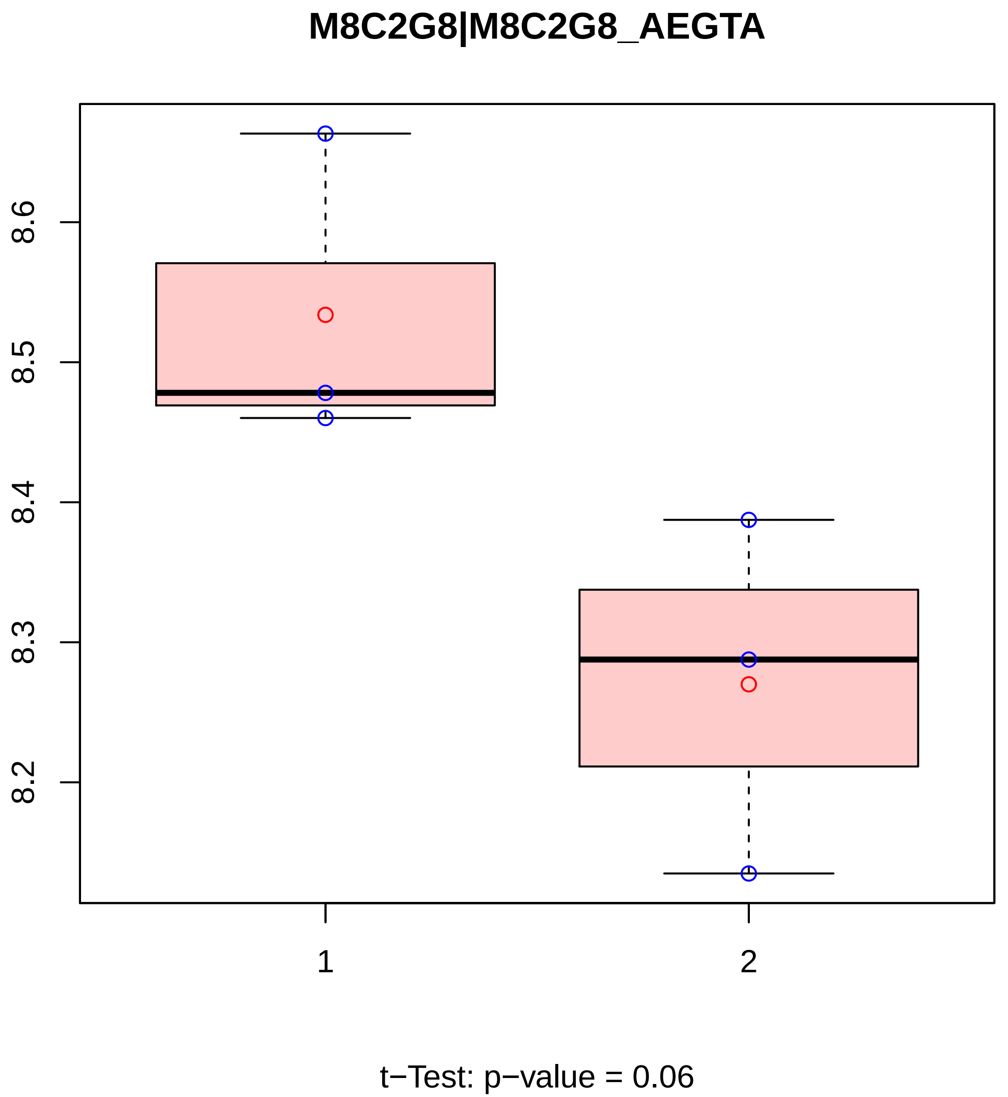
M8C2G8|M8C2G8_AEGTA
8.2
8.3
8.4
8.5
8.6
1
2
t−Test: p−value = 0.06

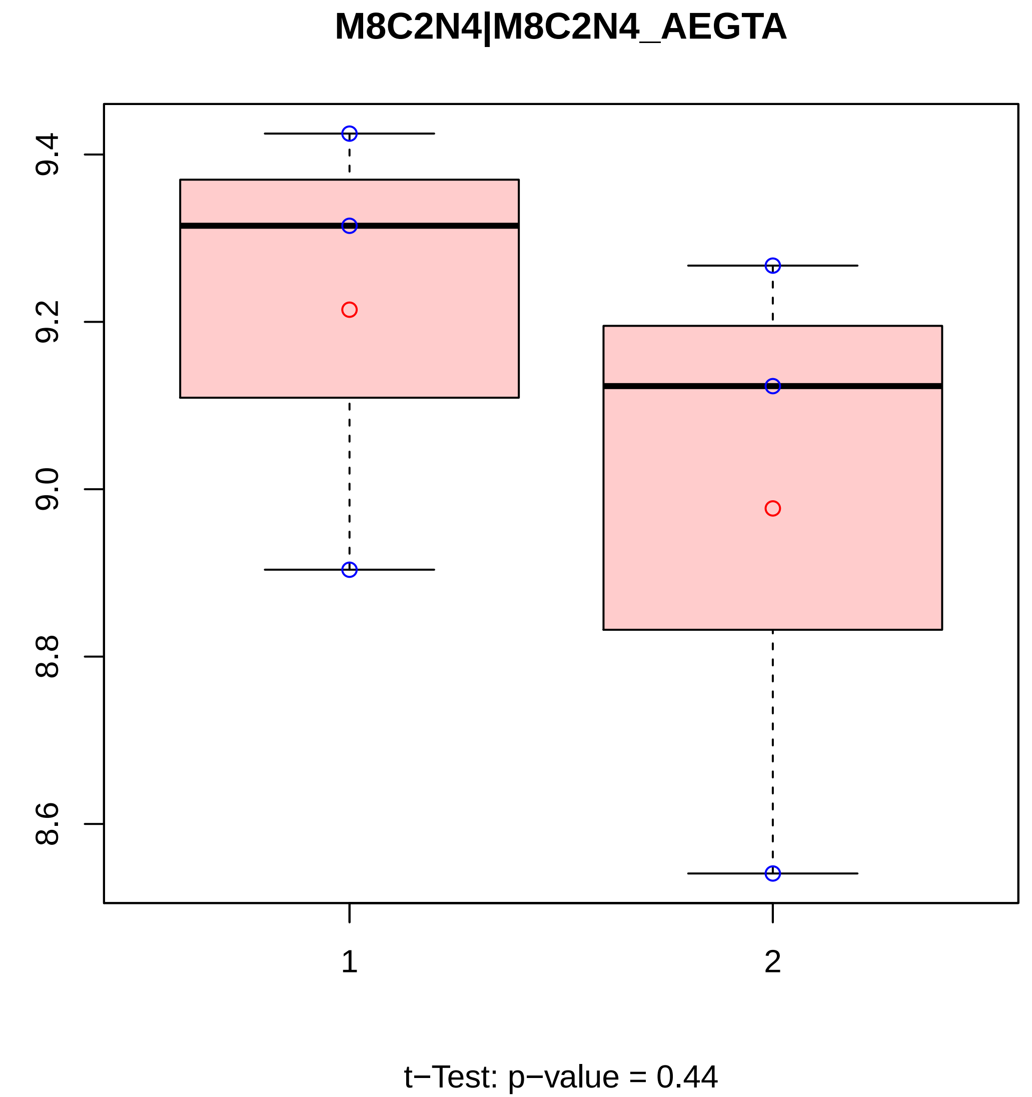
M8C2N4|M8C2N4_AEGTA
9.4
9.2
9.0
8.8
8.6
1
2
t−Test: p−value = 0.44

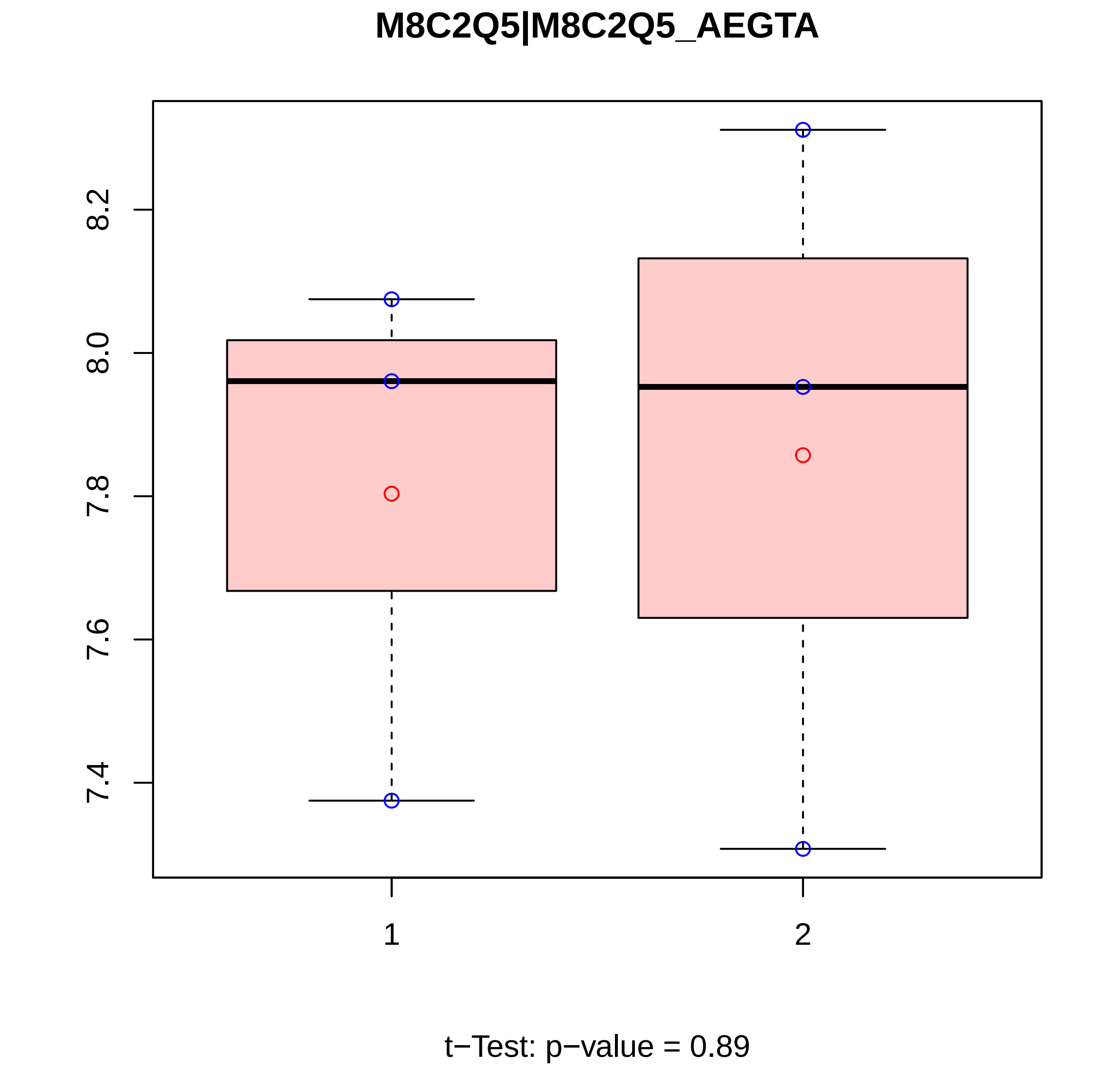
M8C2Q5|M8C2Q5_AEGTA
8.2
8.0
7.8
7.6
7.4
1
2
t−Test: p−value = 0.89

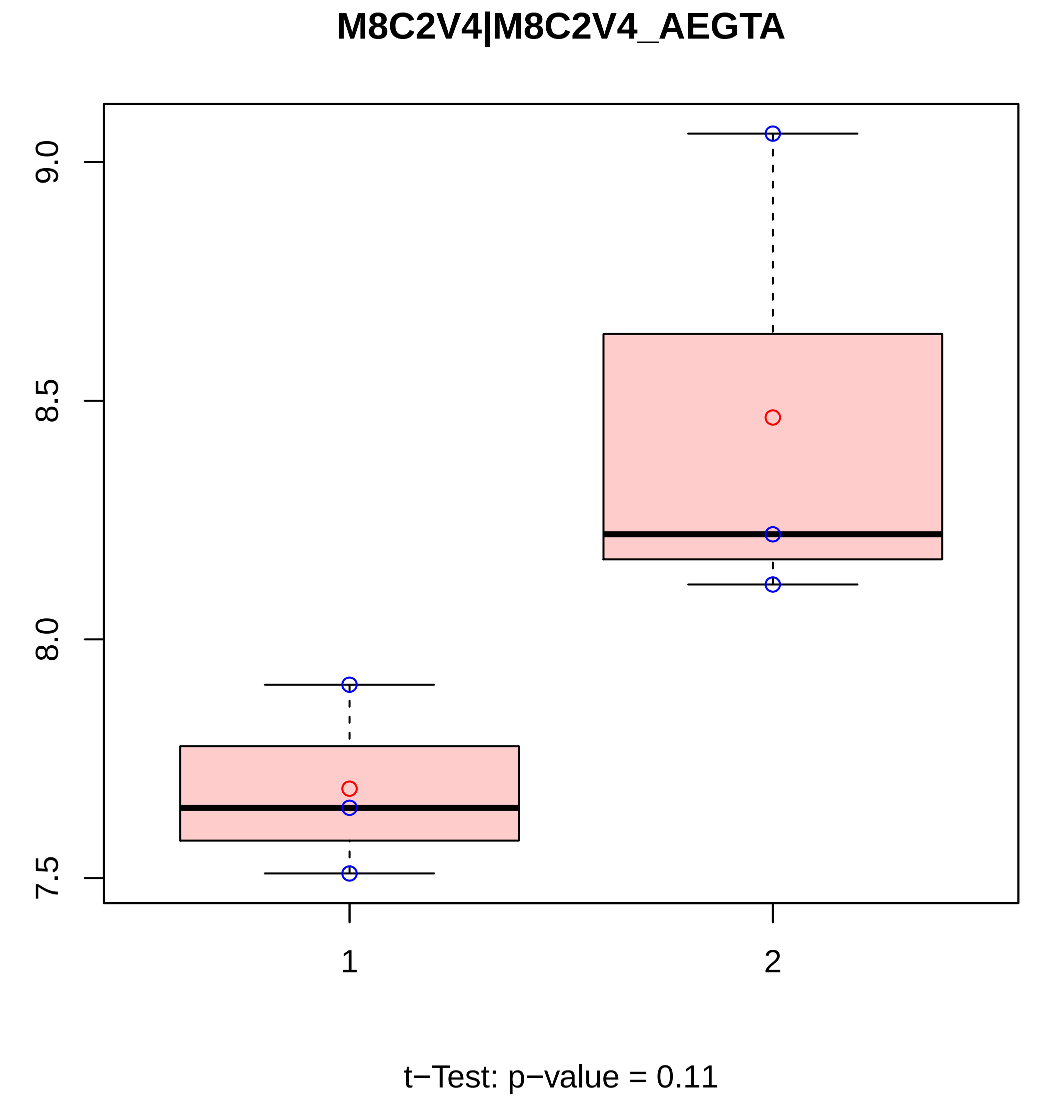
M8C2V4|M8C2V4_AEGTA
9.0
8.5
8.0
7.5
1
2
t−Test: p−value = 0.11

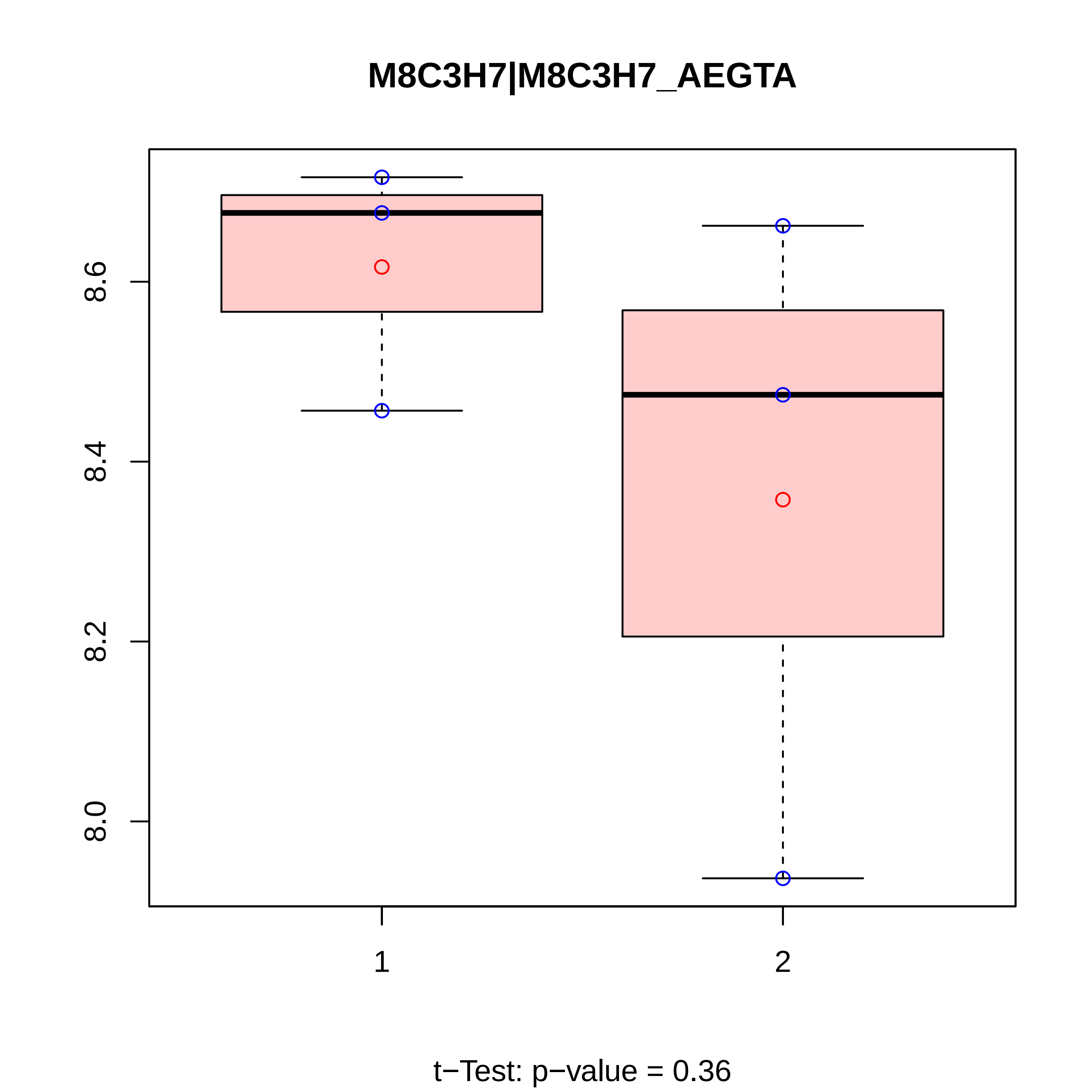

M8C3H7|M8C3H7_AEGTA
8.0
8.2
8.4
8.6
1
2
t−Test: p−value = 0.36

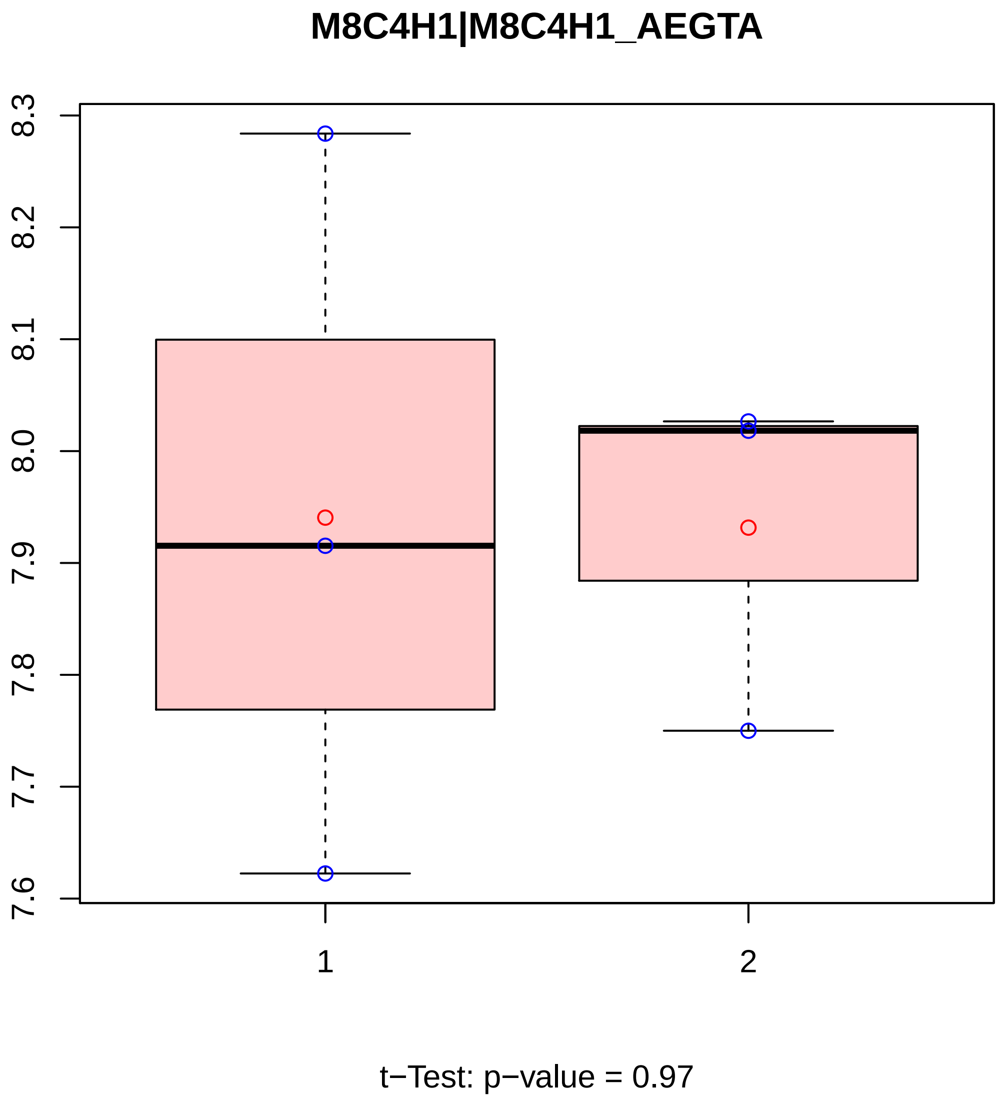

M8C4H1|M8C4H1_AEGTA
8.3
8.2
8.1
8.0
7.9
7.8
7.7
7.6
1
2
t−Test: p−value = 0.97

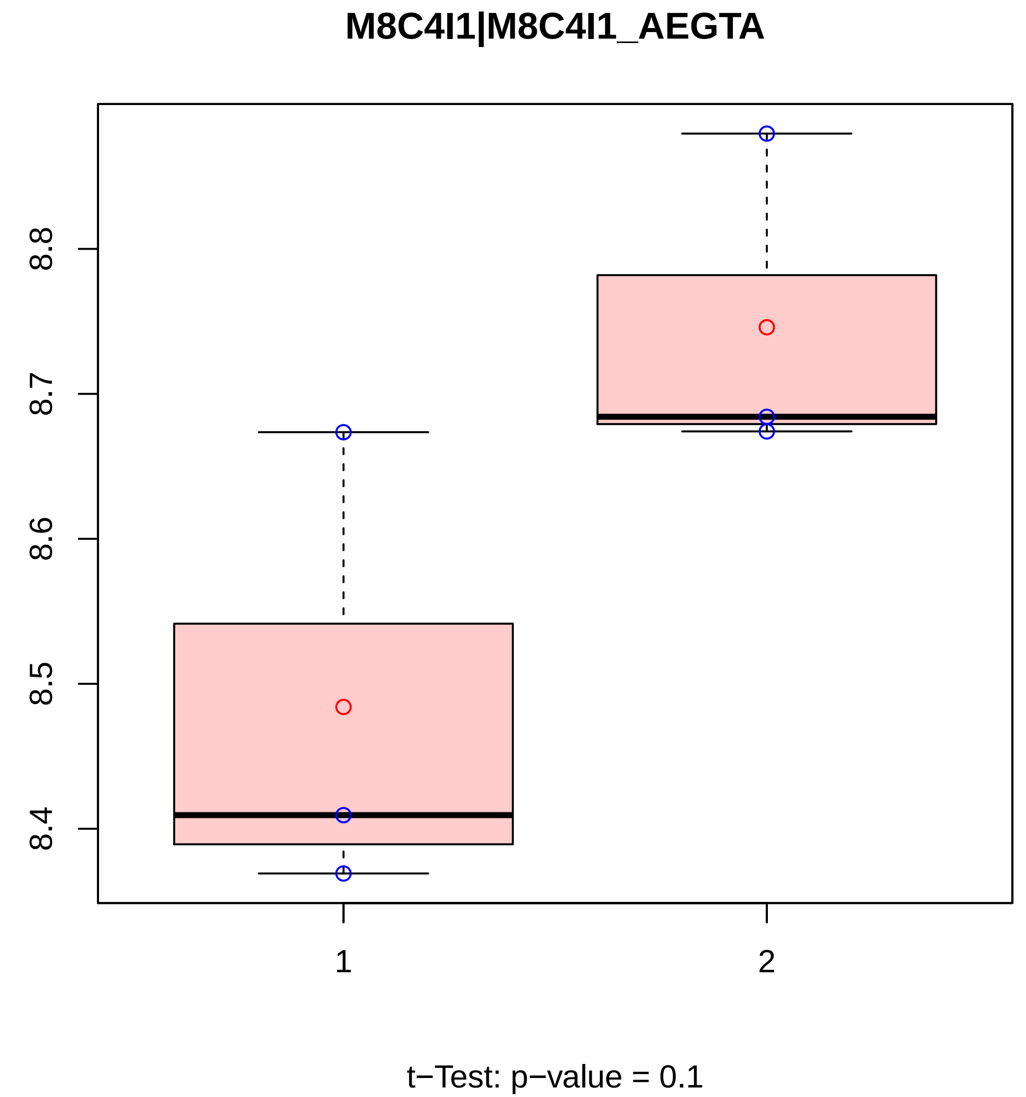
M8C4I1|M8C4I1_AEGTA
8.4
8.5
8.6
8.7
8.8
1
2
t−Test: p−value = 0.1

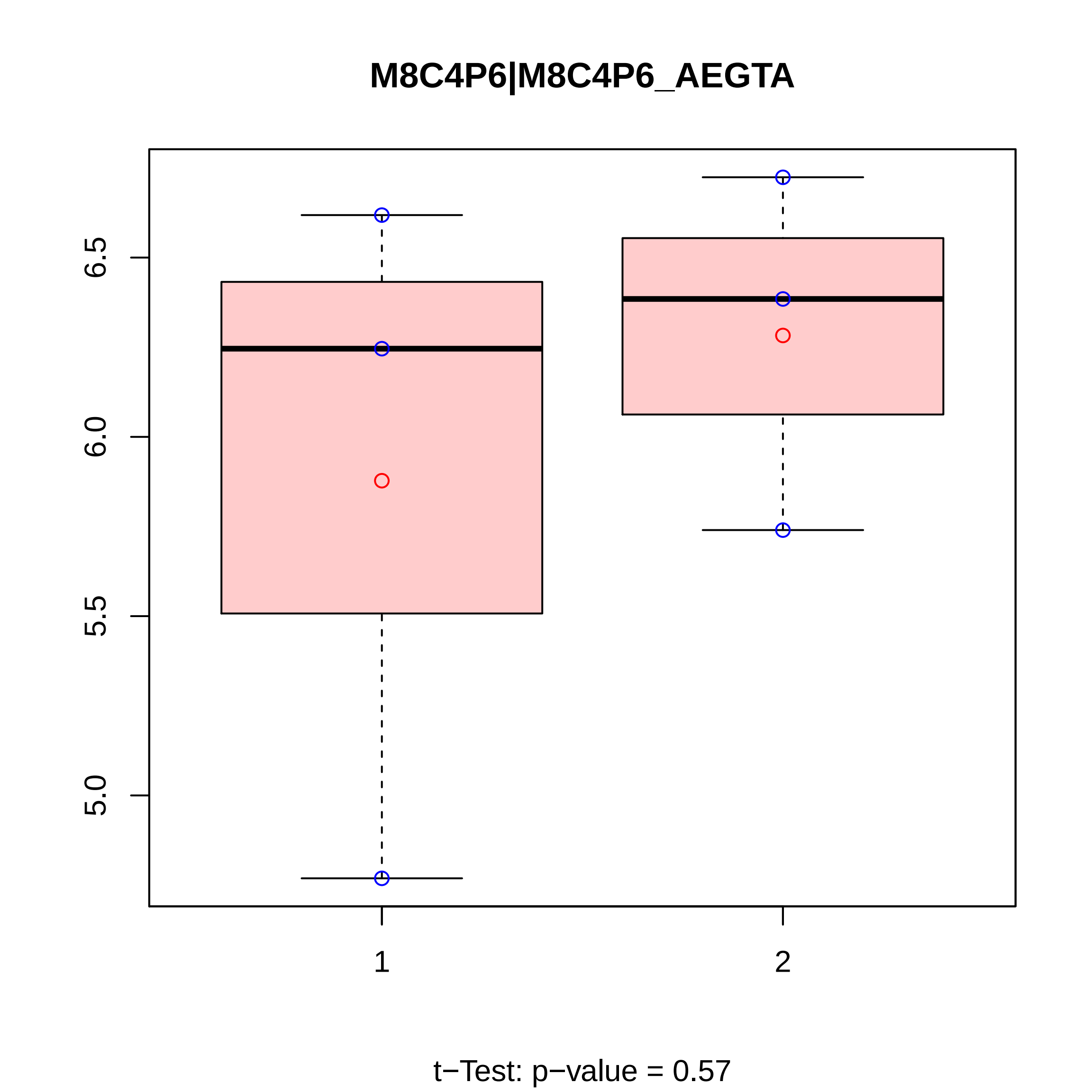

M8C4P6|M8C4P6_AEGTA
6.5
6.0
5.5
5.0
1
2
t−Test: p−value = 0.57

M8C564|M8C564_AEGTA

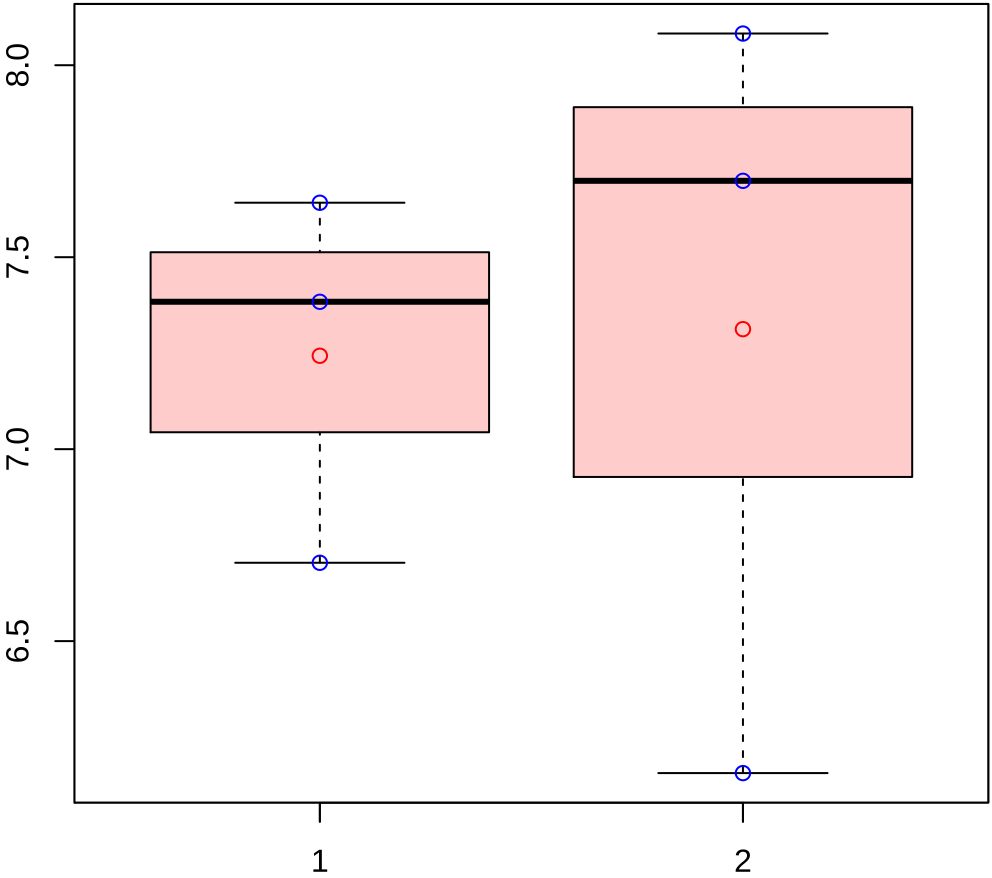

t−Test: p−value = 0.92

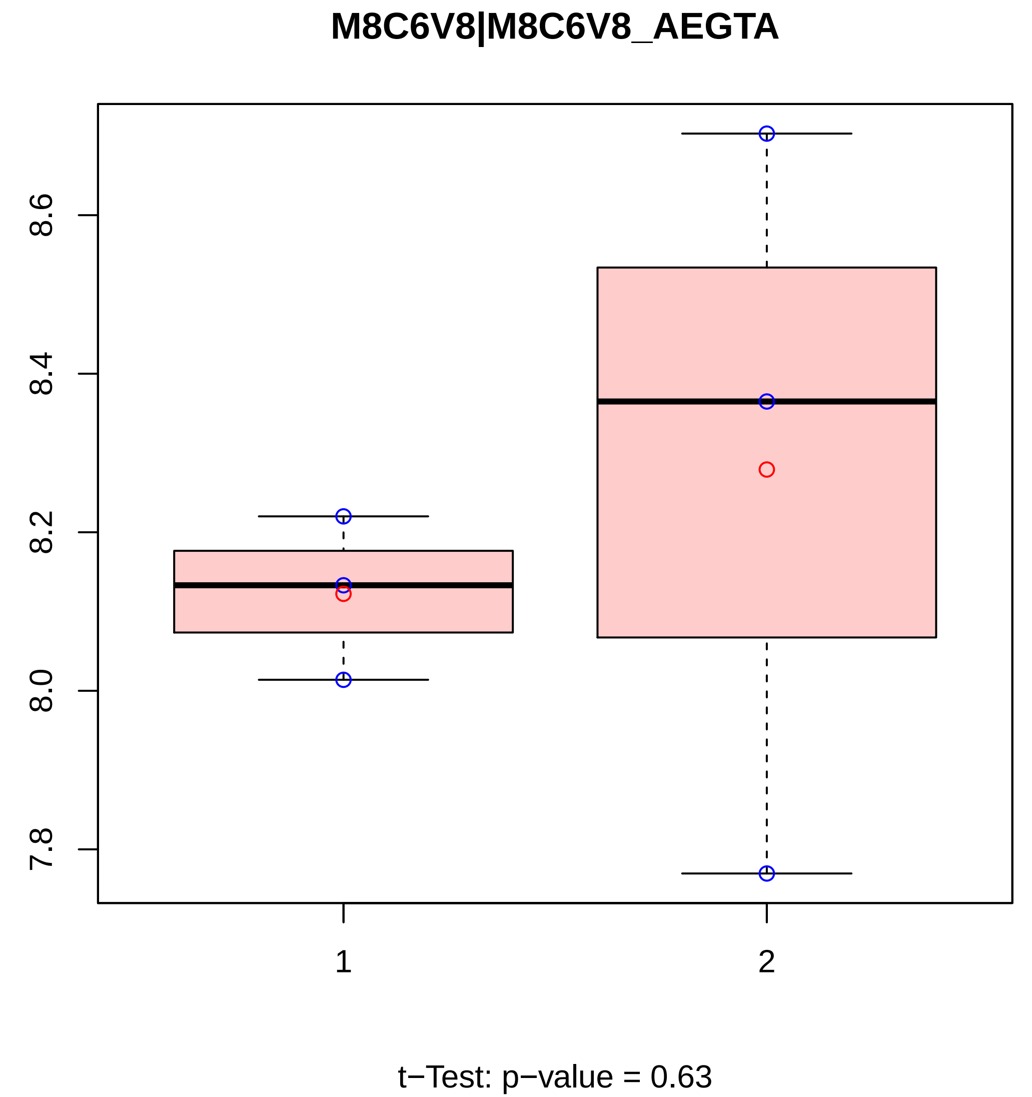

M8C6V8|M8C6V8_AEGTA
8.6
8.4
8.2
8.0
7.8
1
2
t−Test: p−value = 0.63

M8C7A2|M8C7A2_AEGTA

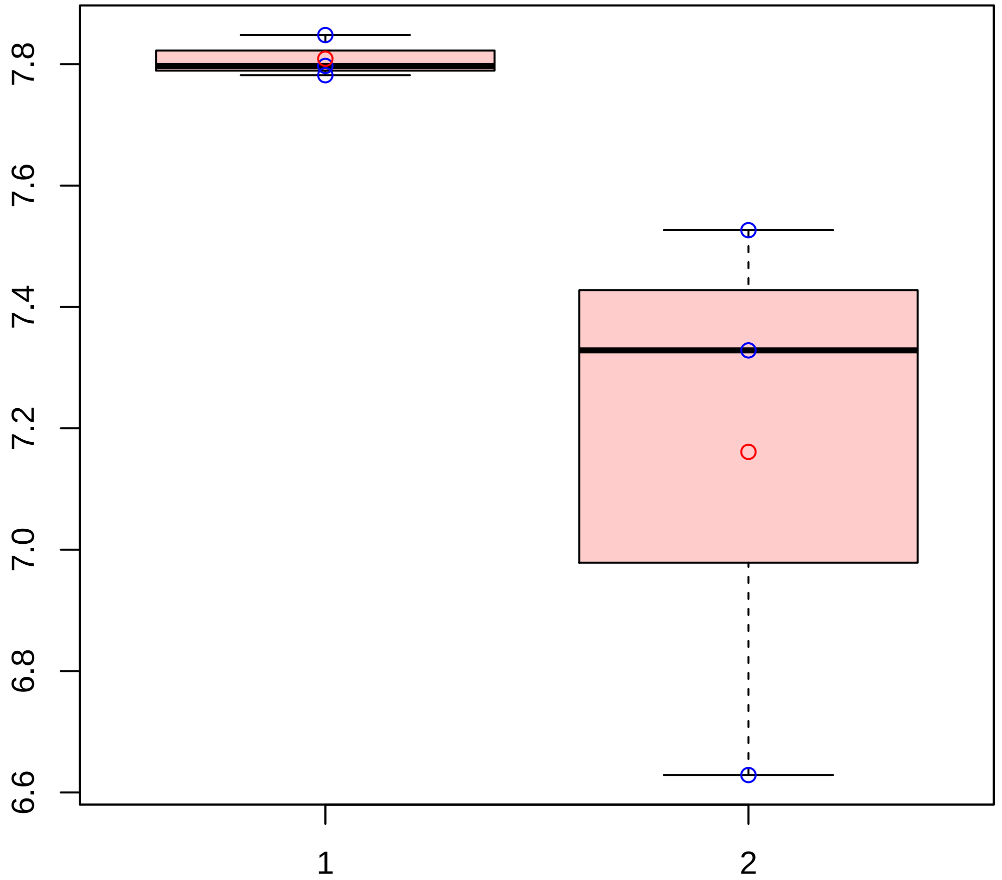

t−Test: p−value = 0.14

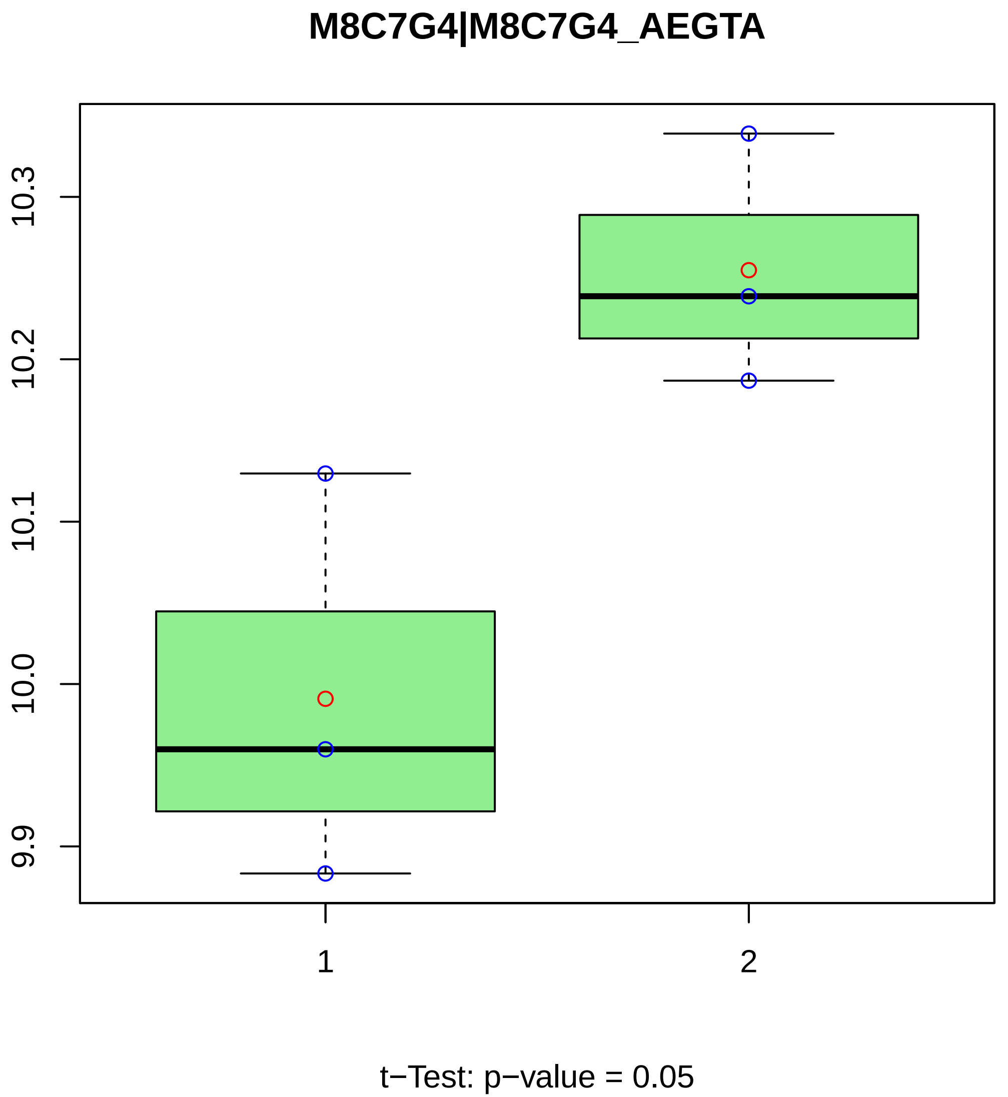

M8C7G4|M8C7G4_AEGTA
9.9
10.0
10.1
10.2
10.3
1
2
t−Test: p−value = 0.05

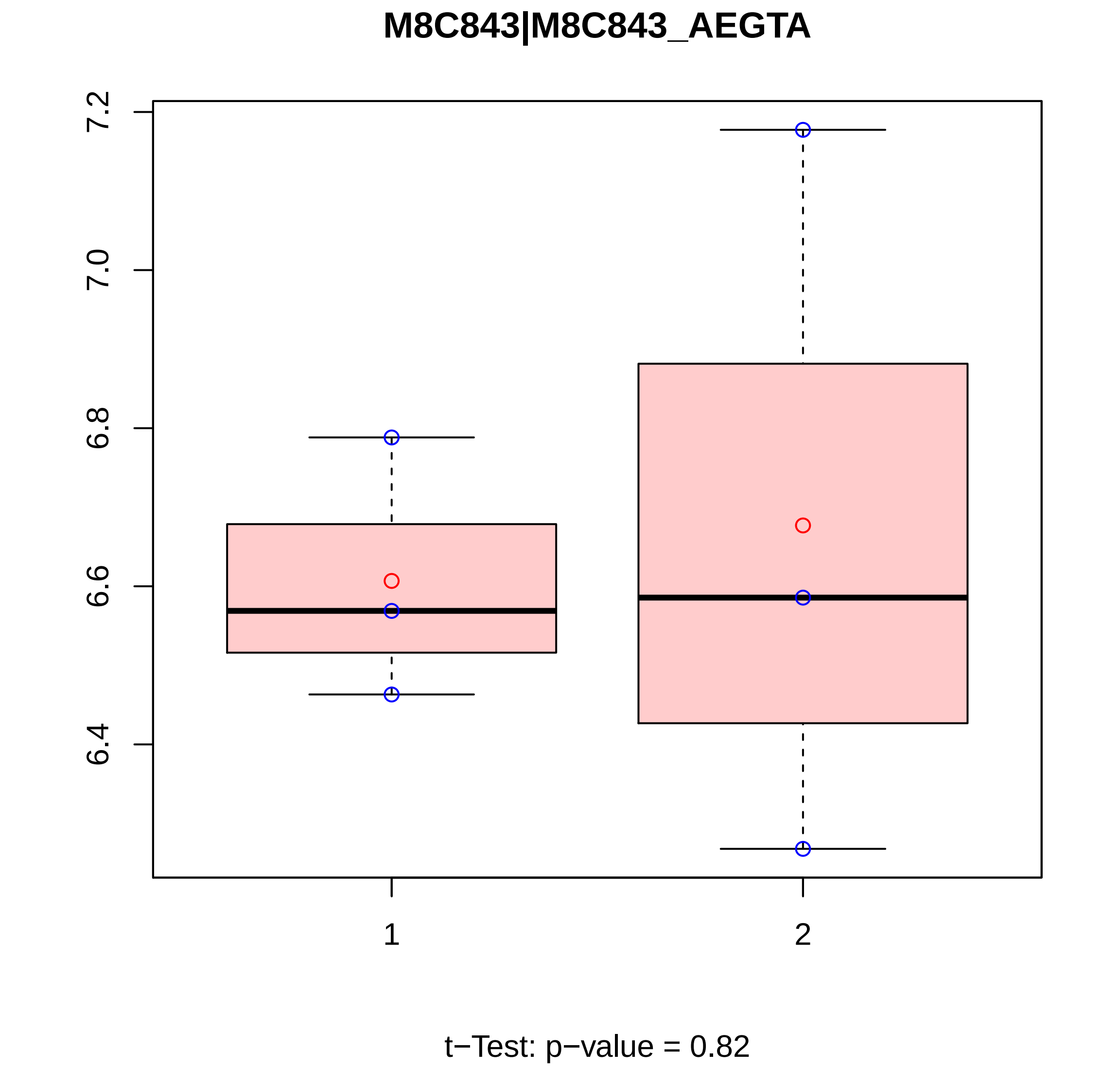

M8C843|M8C843_AEGTA
6.4
6.6
6.8
7.0
7.2
1
2
t−Test: p−value = 0.82

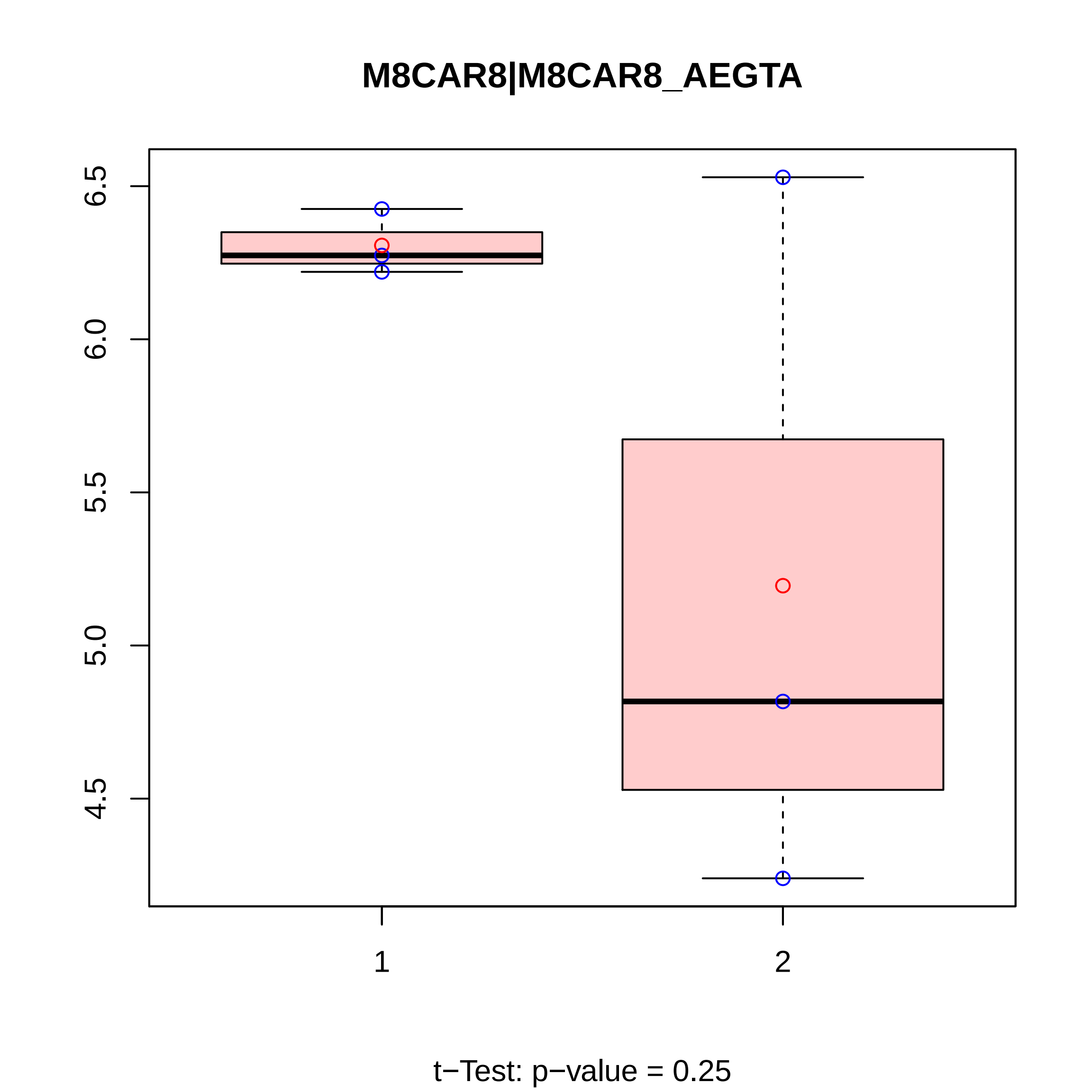
M8CAR8|M8CAR8_AEGTA
6.5
6.0
5.5
5.0
4.5
1
2
t−Test: p−value = 0.25

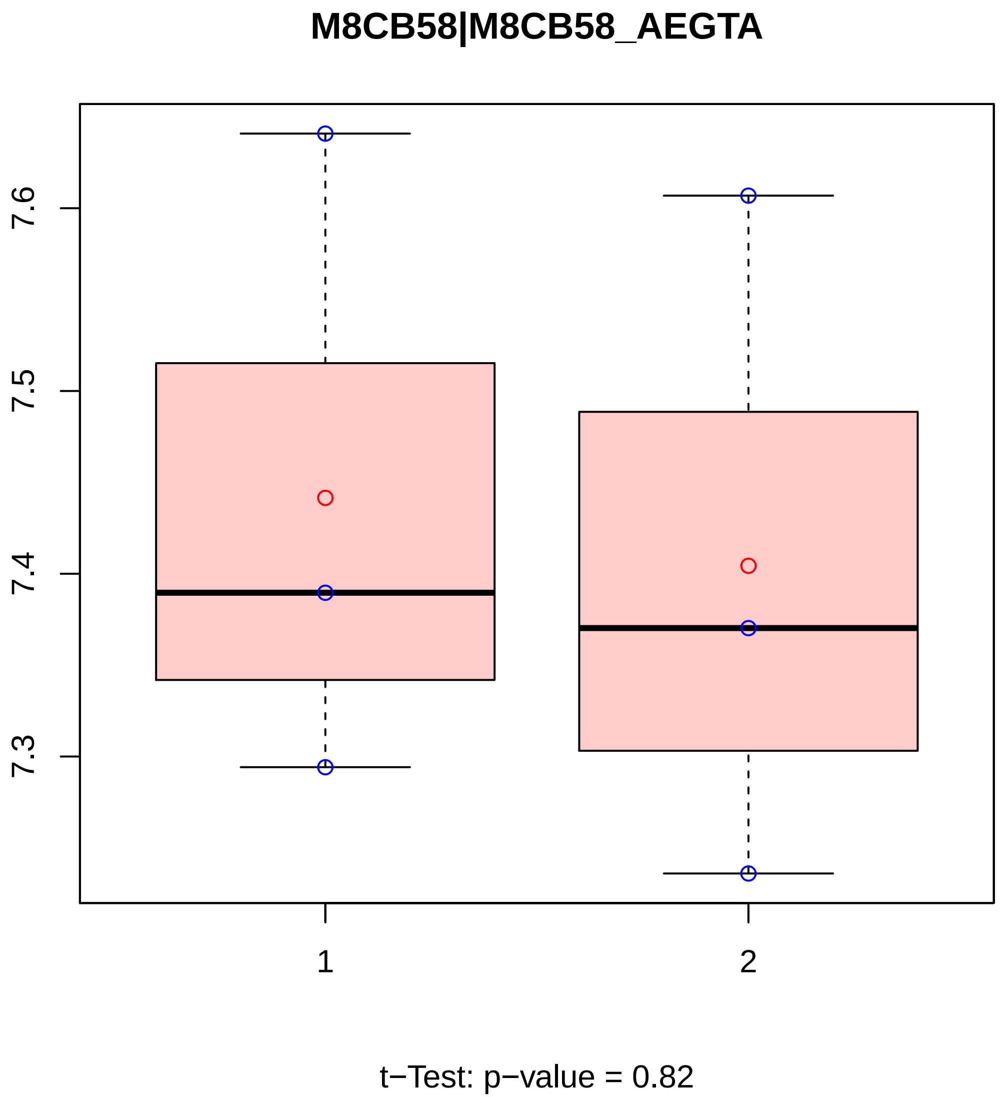
M8CB58|M8CB58_AEGTA
7.6
7.5
7.4
7.3
1
2
t−Test: p−value = 0.82

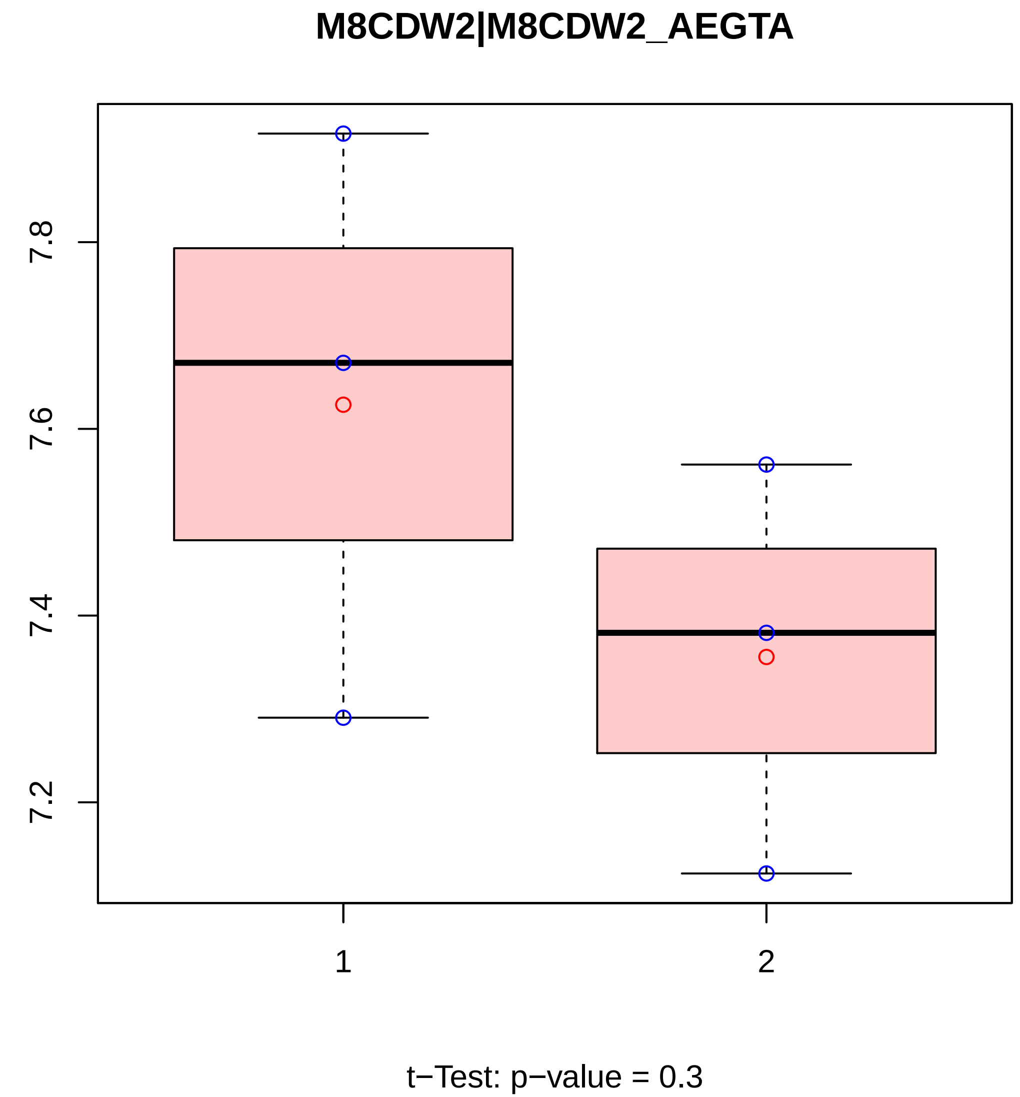
M8CDW2|M8CDW2_AEGTA
7.2
7.4
7.6
7.8
1
2
t−Test: p−value = 0.3

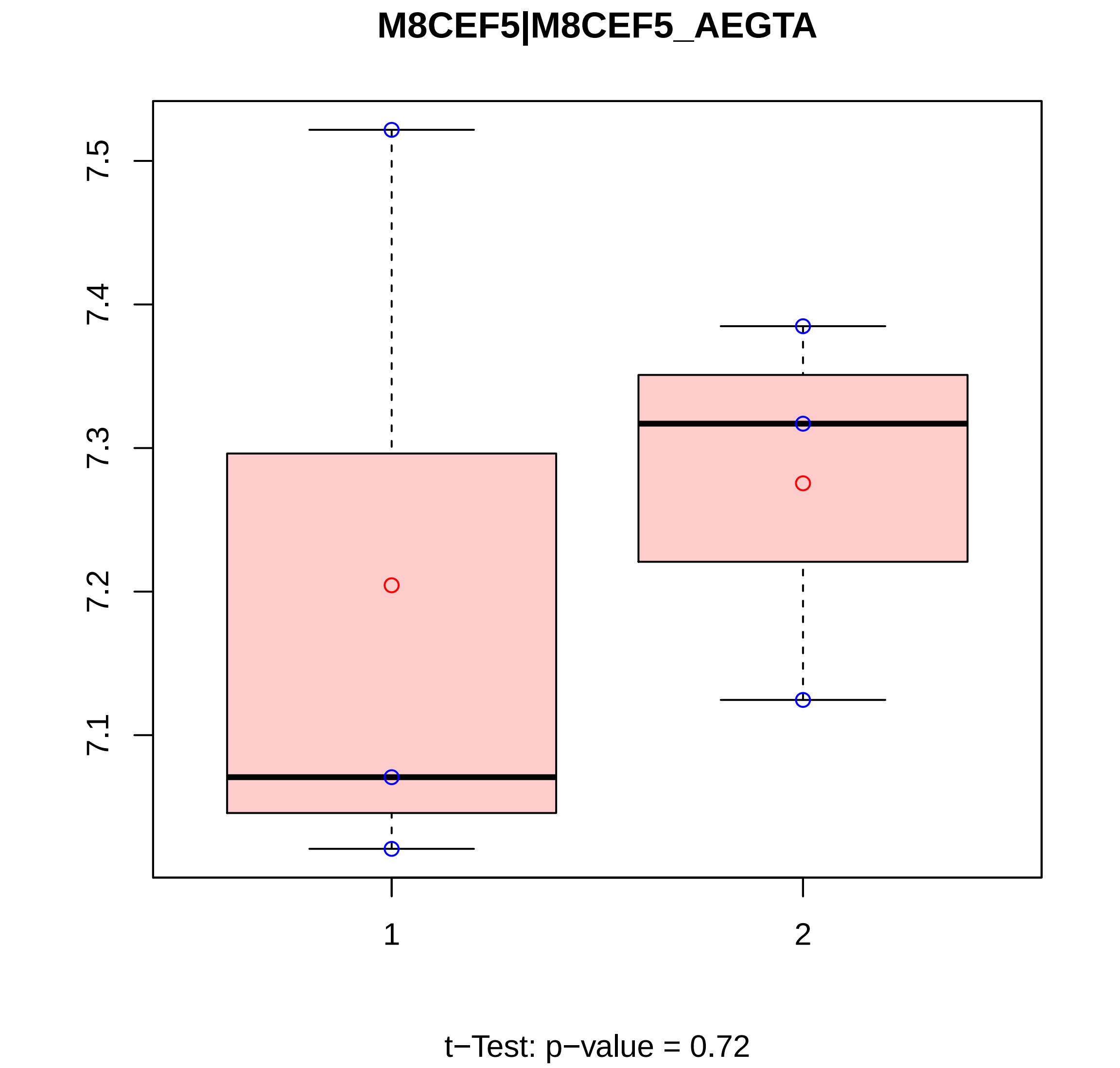
M8CEF5|M8CEF5_AEGTA
7.1
7.2
7.3
7.4
7.5
1
2
t−Test: p−value = 0.72

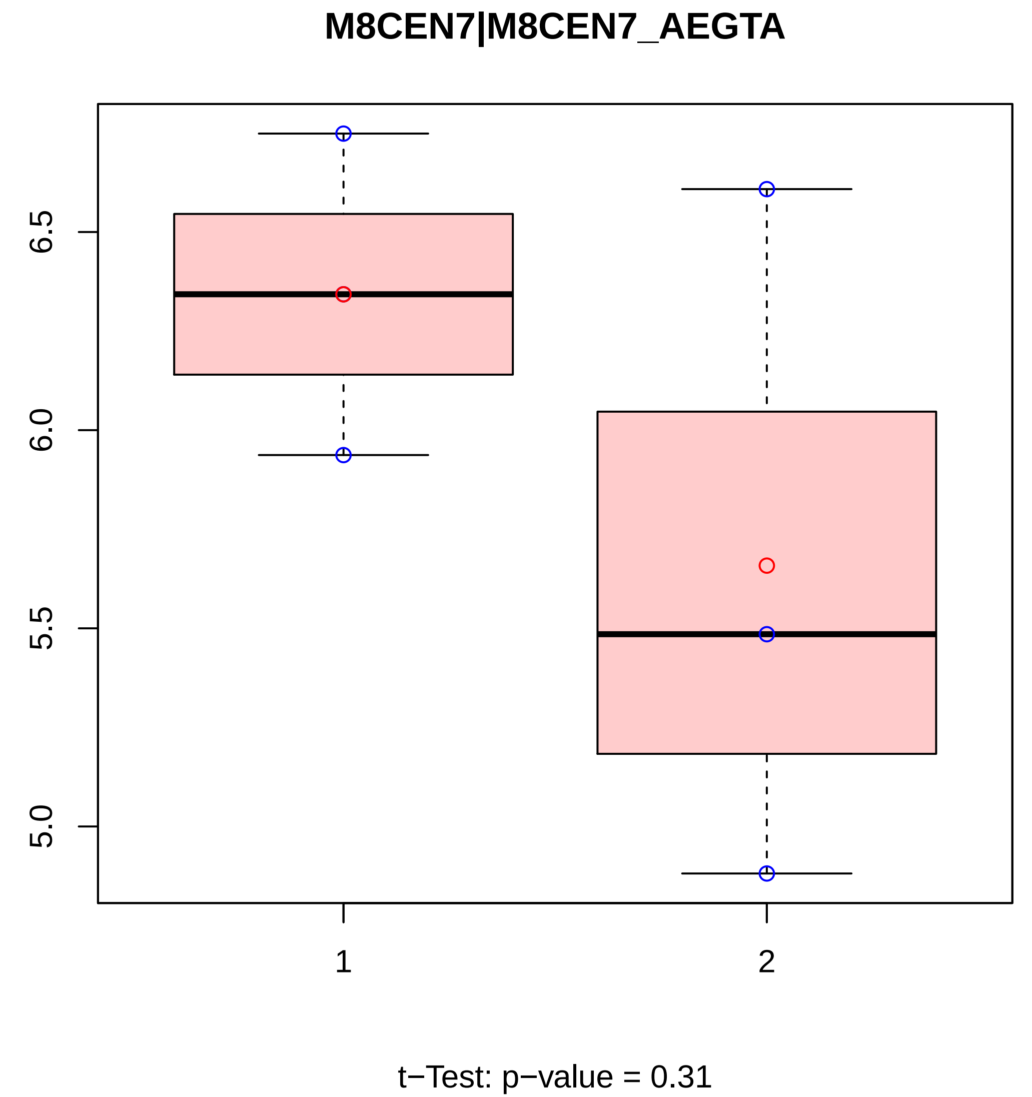
M8CEN7|M8CEN7_AEGTA
6.5
6.0
5.5
5.0
1
2
t−Test: p−value = 0.31

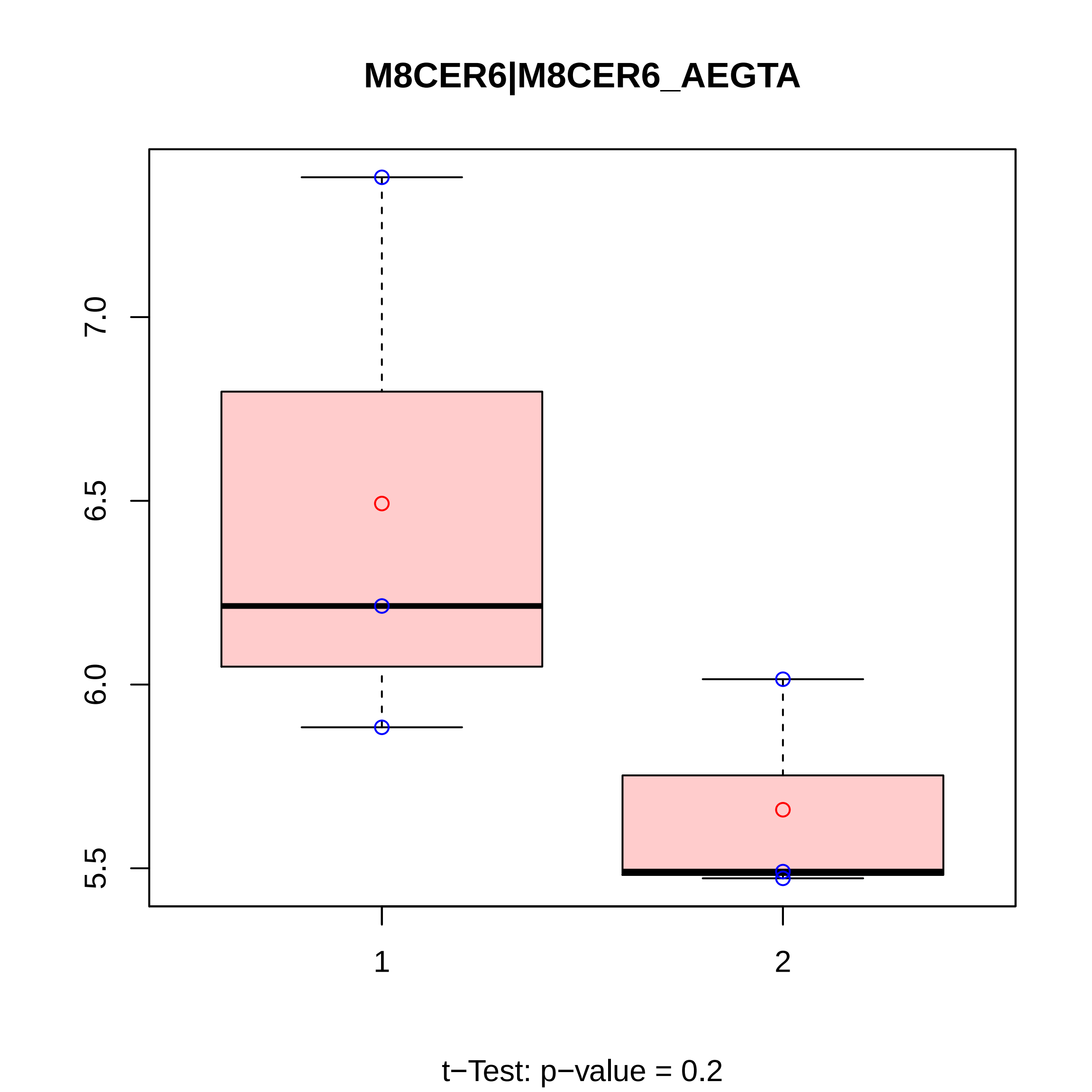

M8CER6|M8CER6_AEGTA
7.0
6.5
6.0
5.5
1
2
t−Test: p−value = 0.2

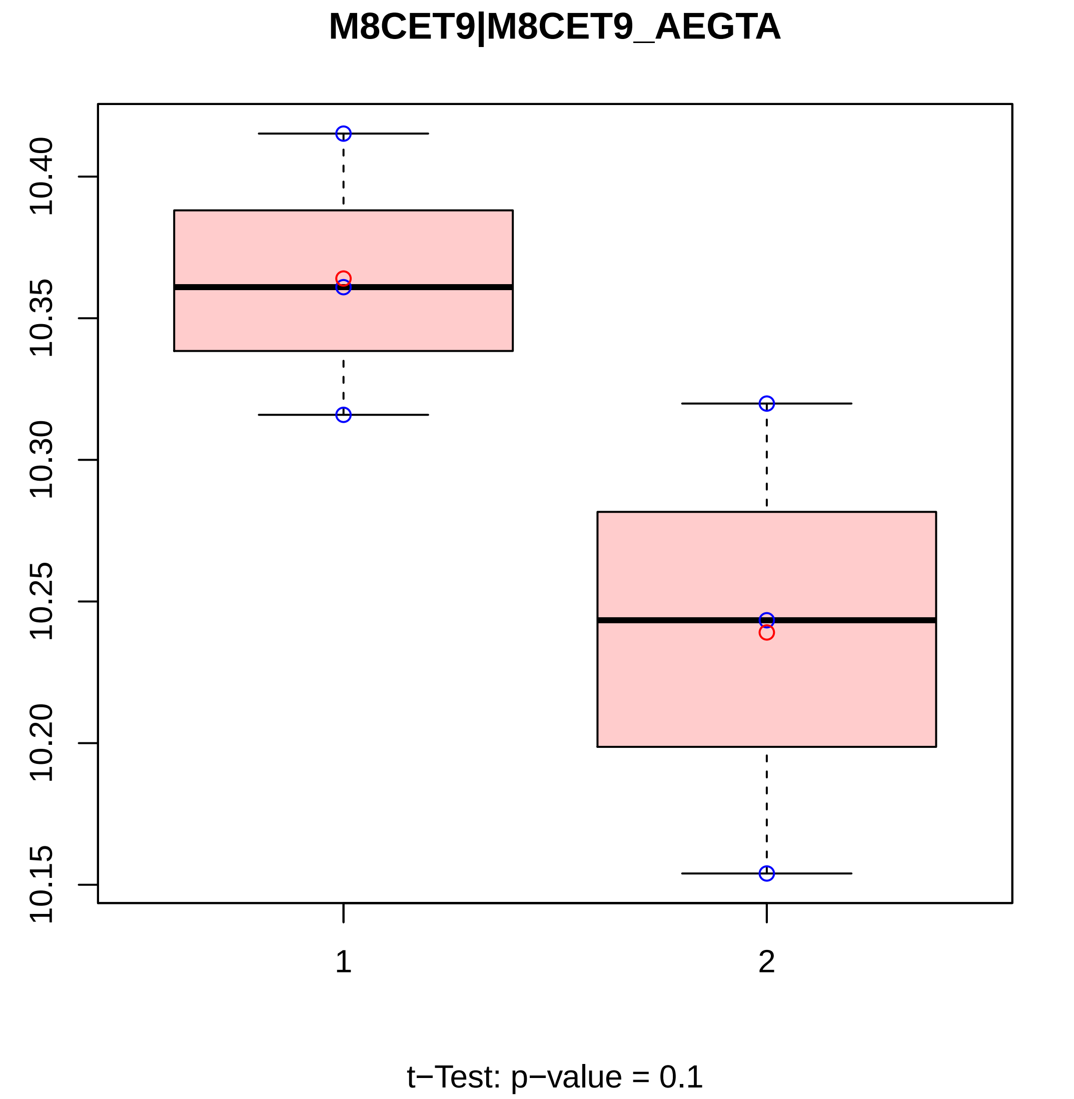
M8CET9|M8CET9_AEGTA
10.15
10.20
10.25
10.30
10.35
10.40
1
2
t−Test: p−value = 0.1

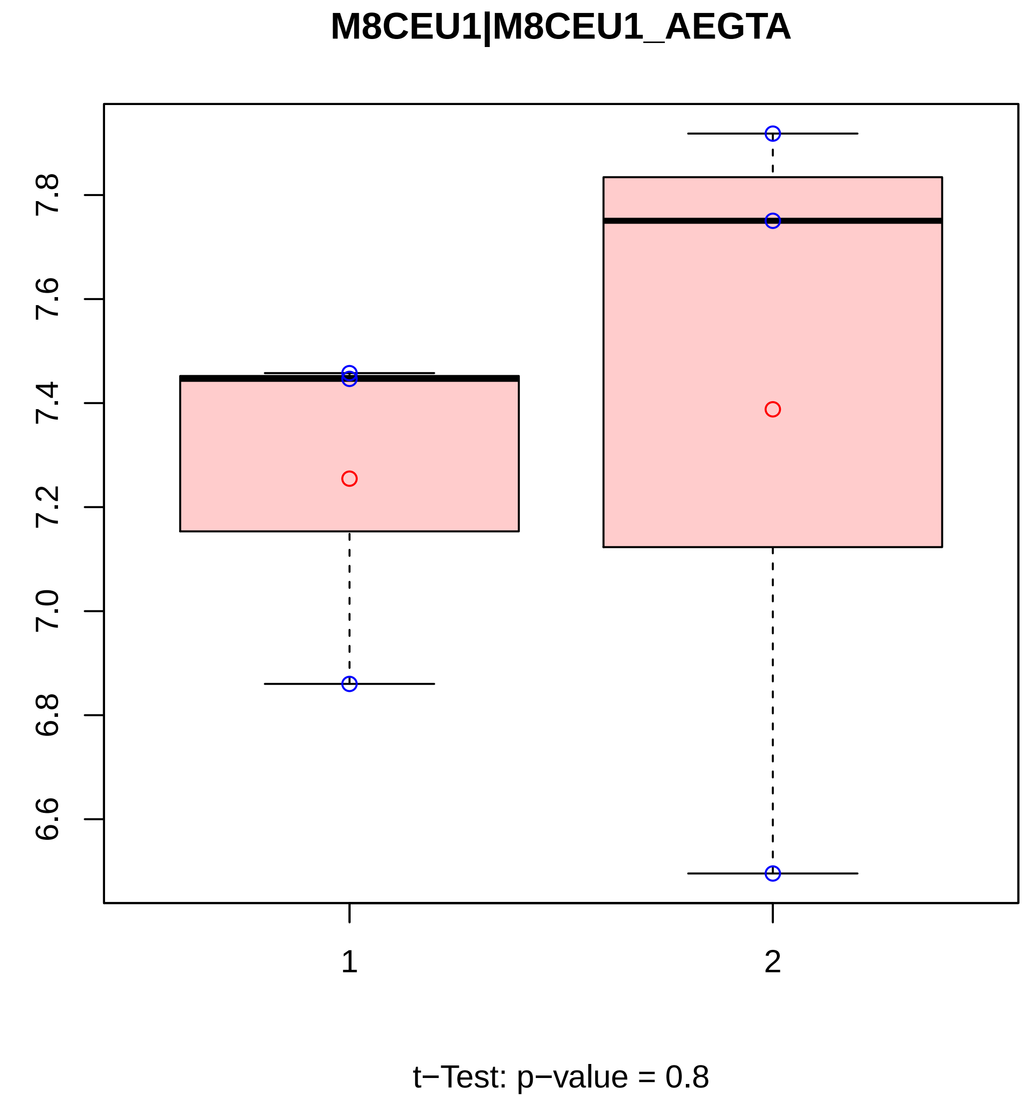
M8CEU1|M8CEU1_AEGTA
7.8
7.6
7.4
7.2
7.0
6.8
6.6
1
2
t−Test: p−value = 0.8

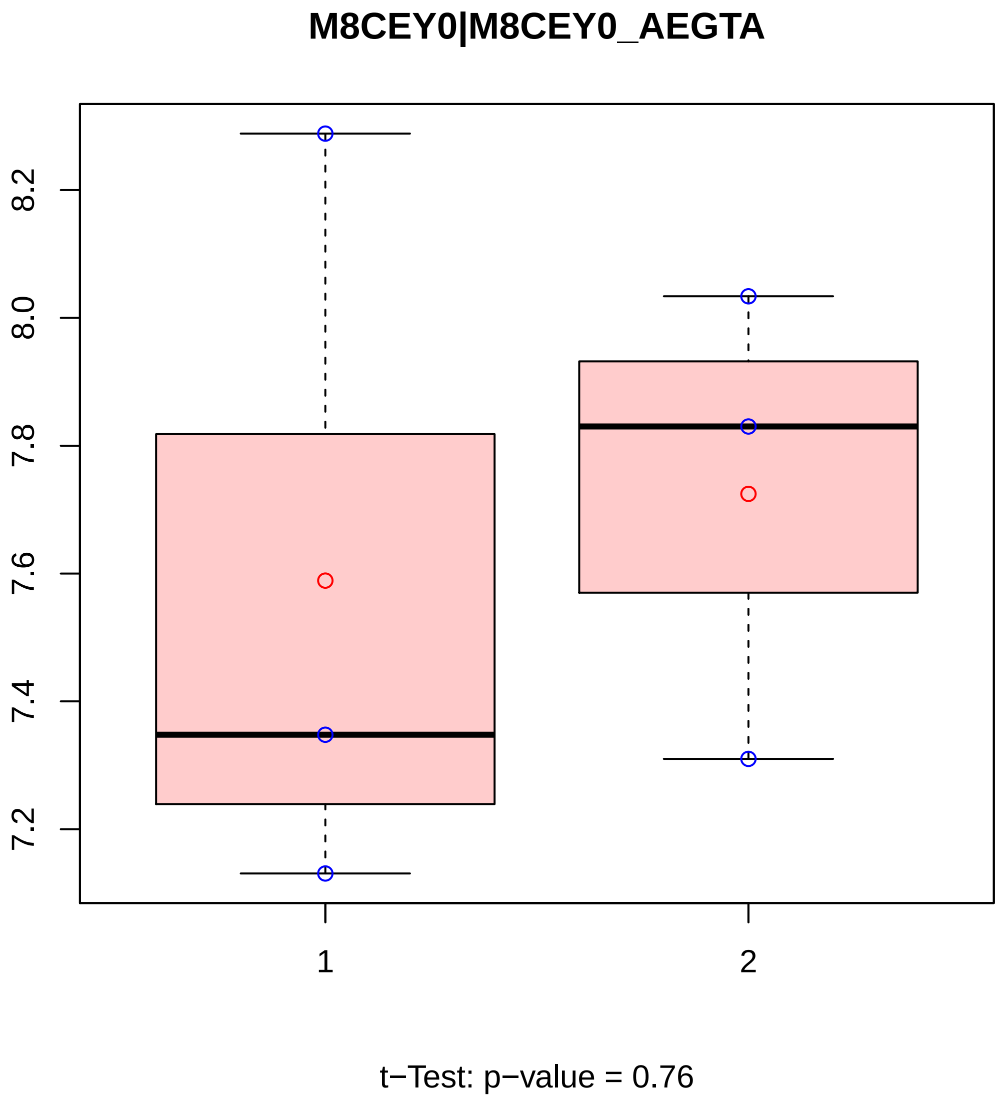

M8CEY0|M8CEY0_AEGTA
8.2
8.0
7.8
7.6
7.4
7.2
1
2
t−Test: p−value = 0.76

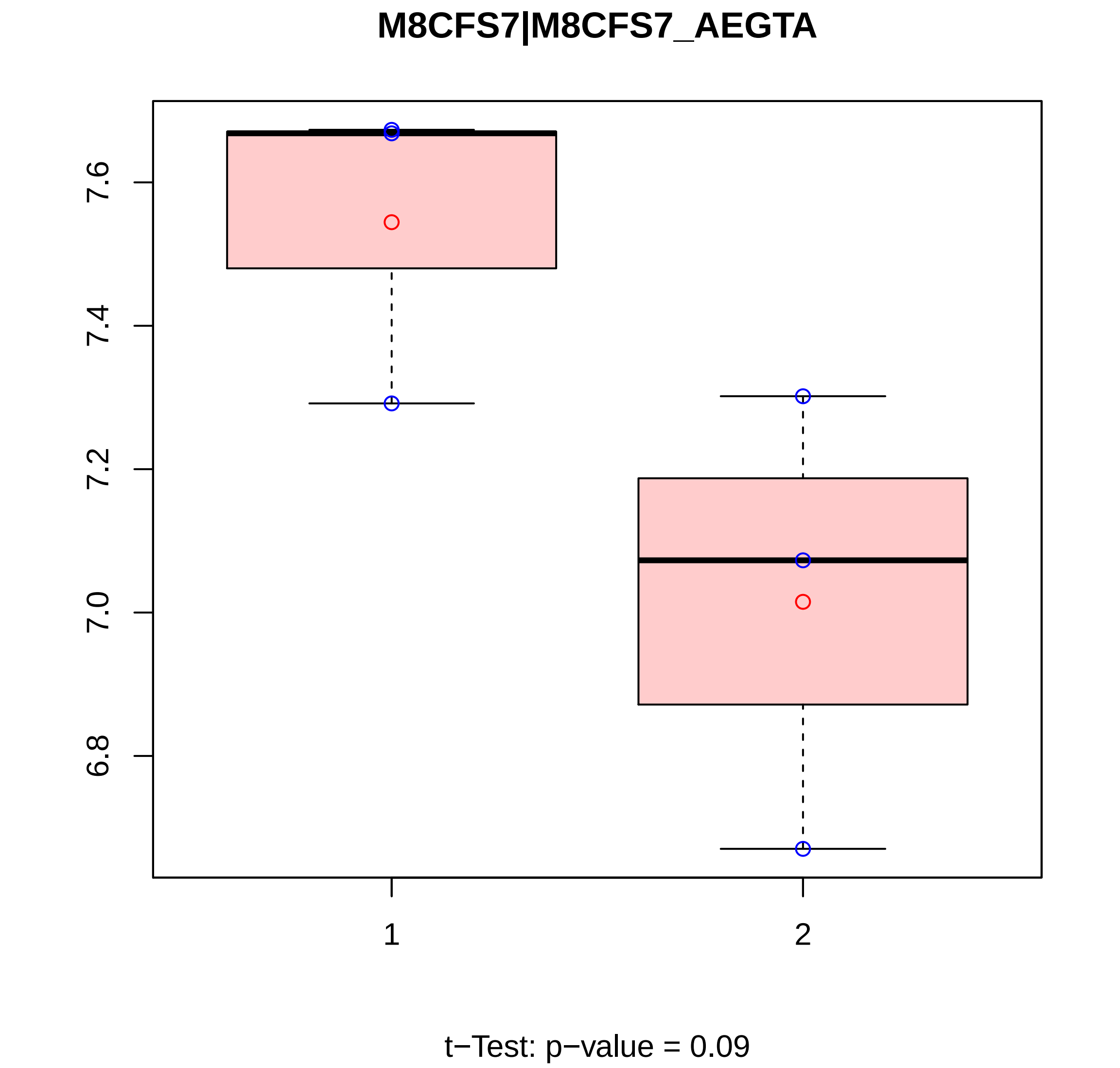

M8CFS7|M8CFS7_AEGTA
7.6
7.4
7.2
7.0
6.8
1
2
t−Test: p−value = 0.09

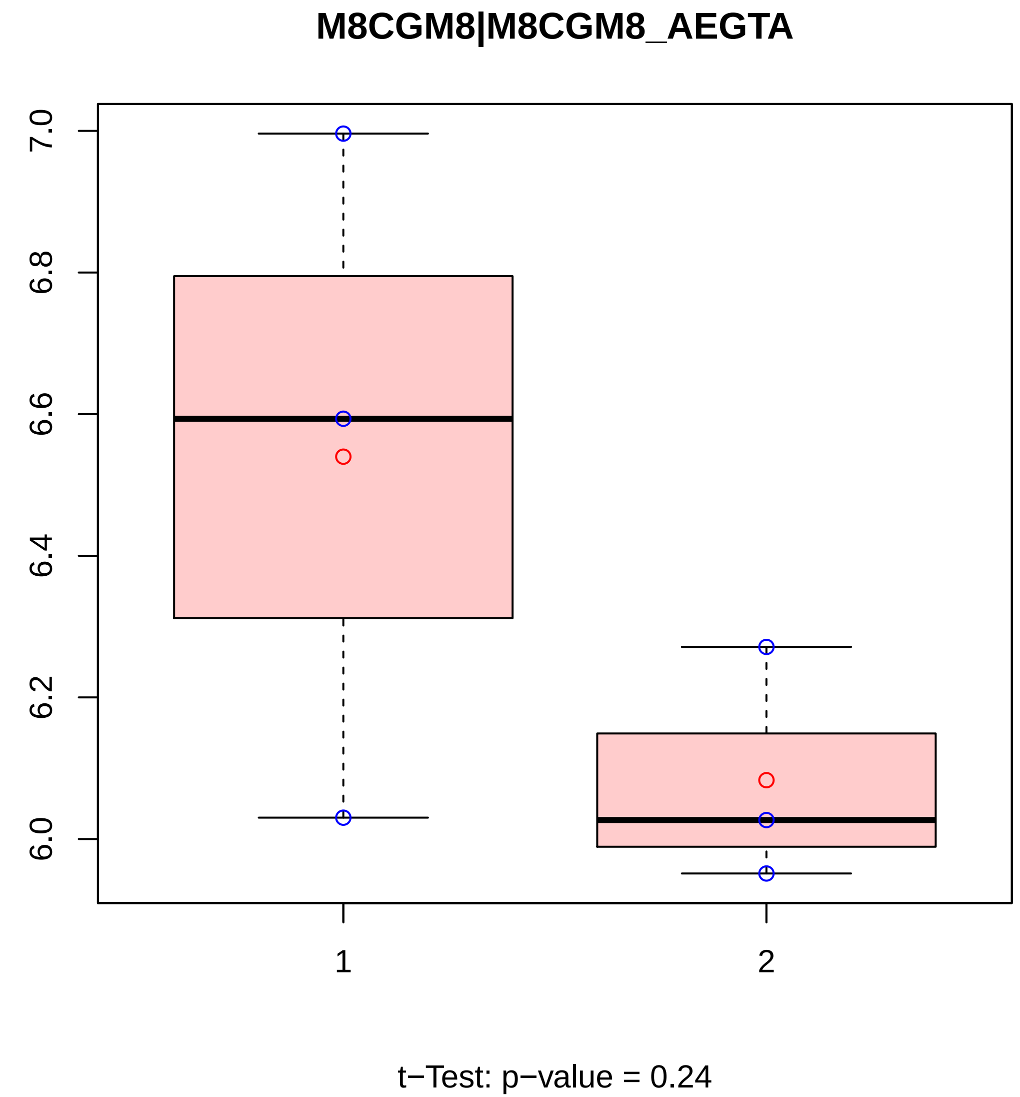
M8CGM8|M8CGM8_AEGTA
7.0
6.8
6.6
6.4
6.2
6.0
1
2
t−Test: p−value = 0.24

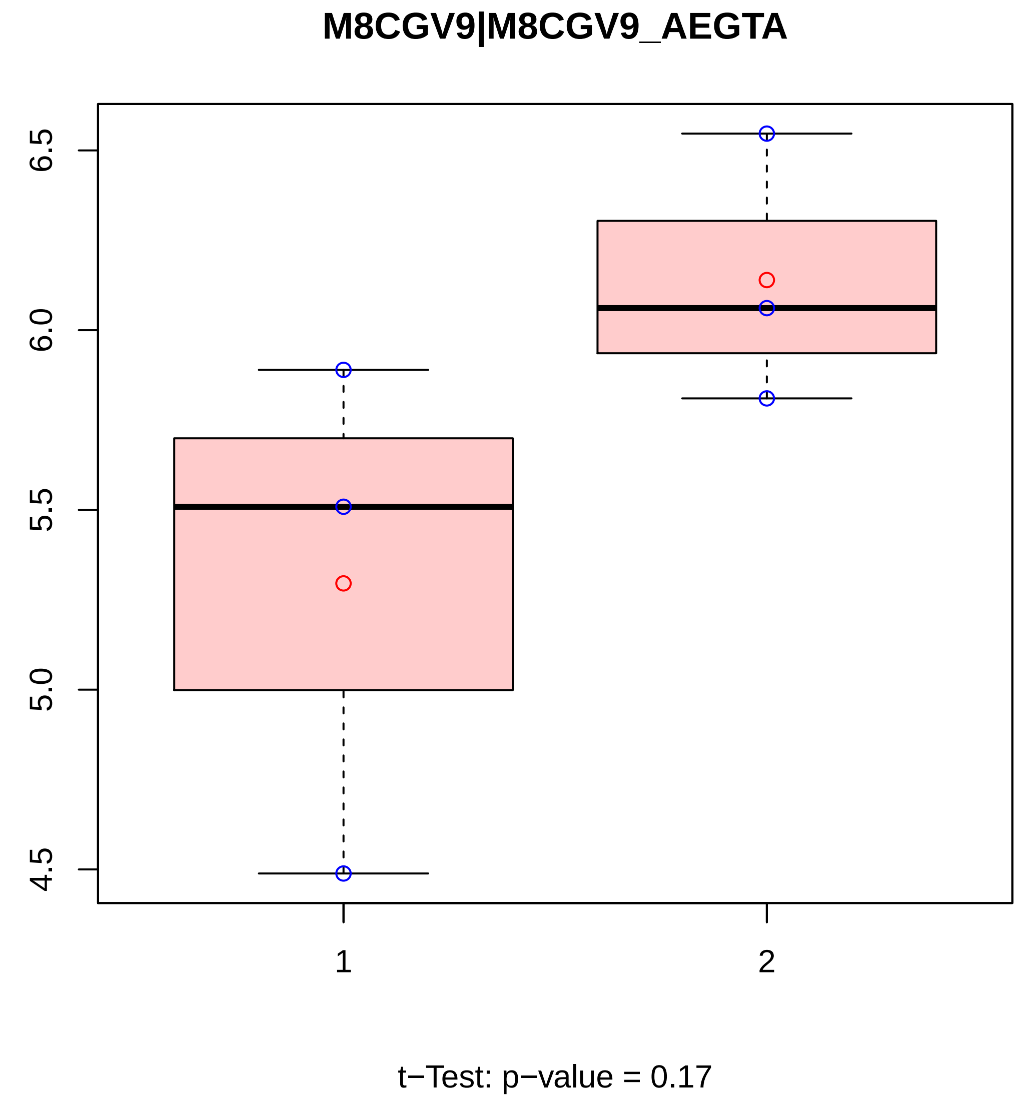
M8CGV9|M8CGV9_AEGTA
6.5
6.0
5.5
5.0
4.5
1
2
t−Test: p−value = 0.17

M8CI56|M8CI56_AEGTA

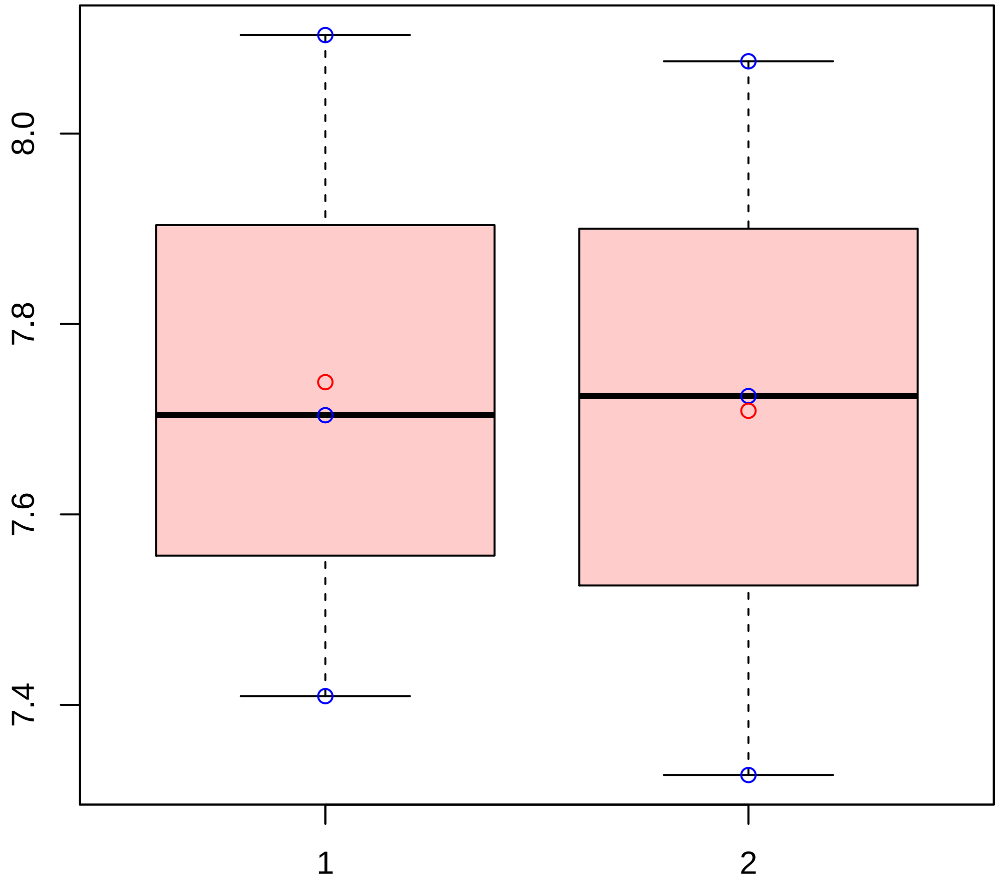

t−Test: p−value = 0.92

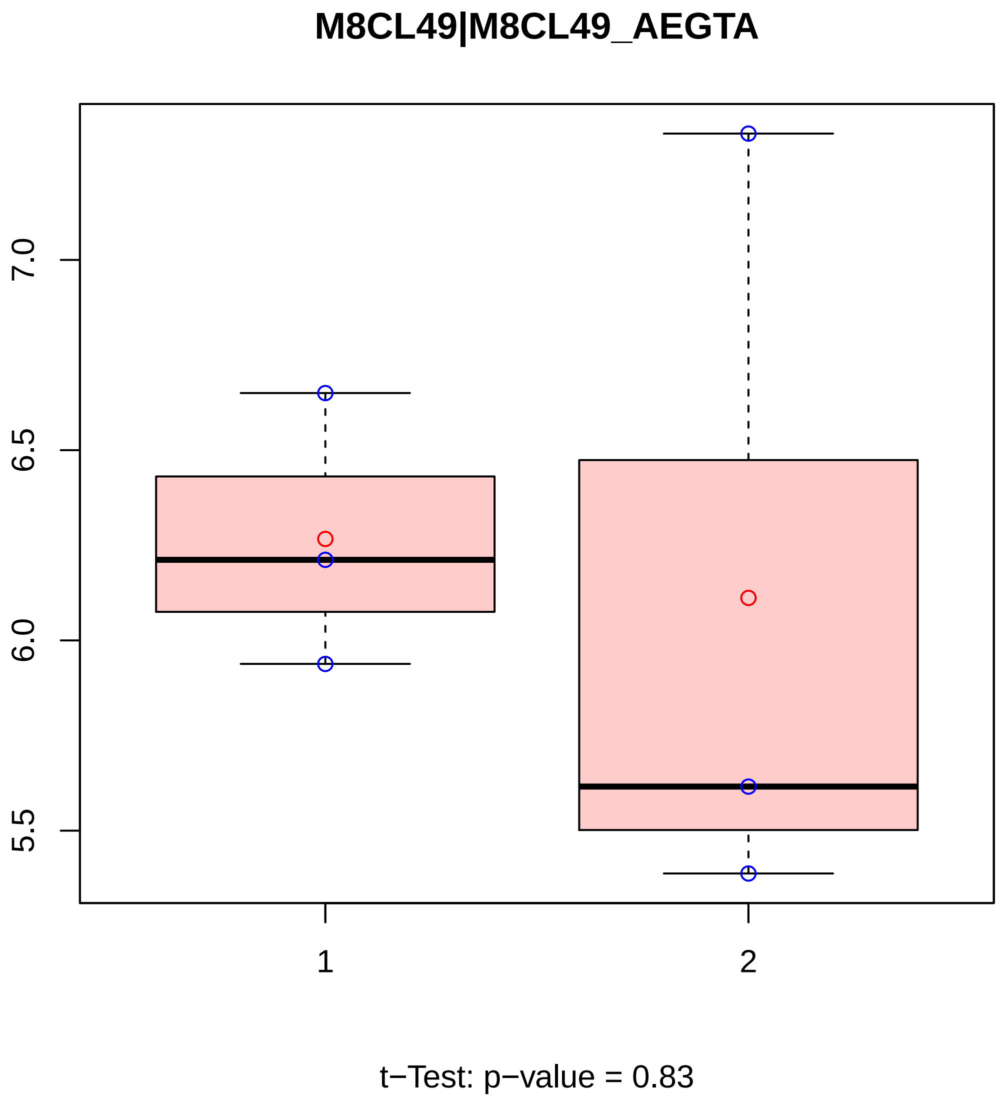

M8CL49|M8CL49_AEGTA
7.0
6.5
6.0
5.5
1
2
t−Test: p−value = 0.83

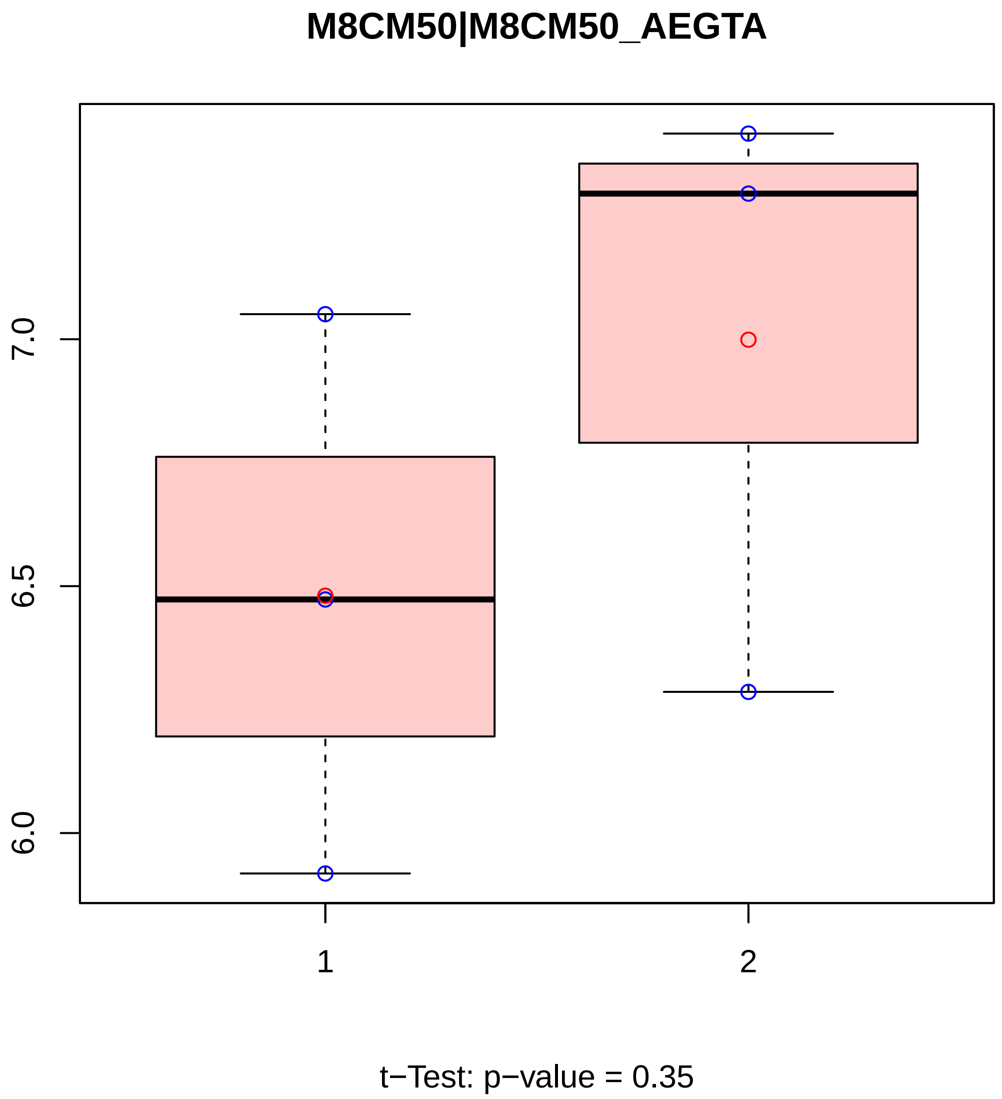
M8CM50|M8CM50_AEGTA
7.0
6.5
6.0
1
2
t−Test: p−value = 0.35

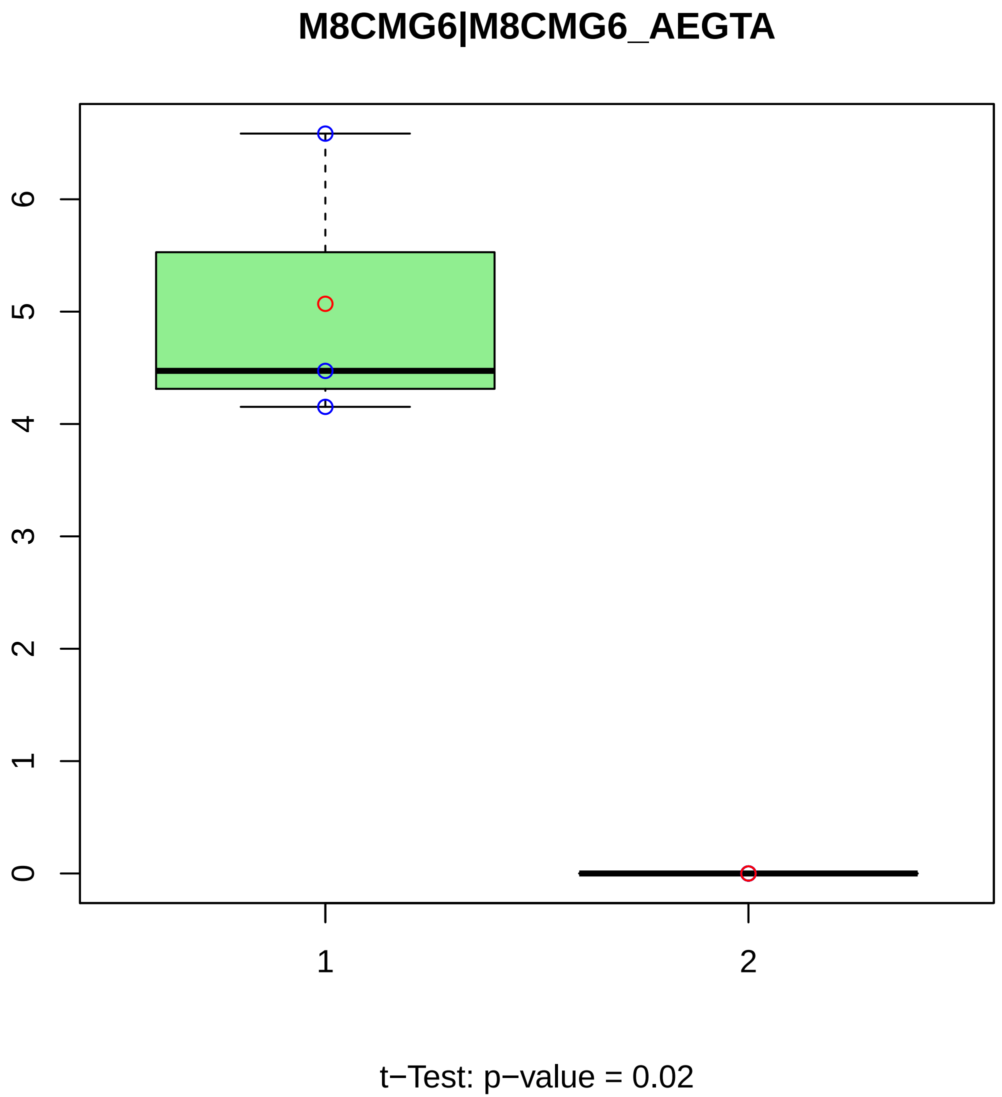
M8CMG6|M8CMG6_AEGTA
0
1
2
3
4
5
6
1
2
t−Test: p−value = 0.02

M8CNU2|M8CNU2_AEGTA

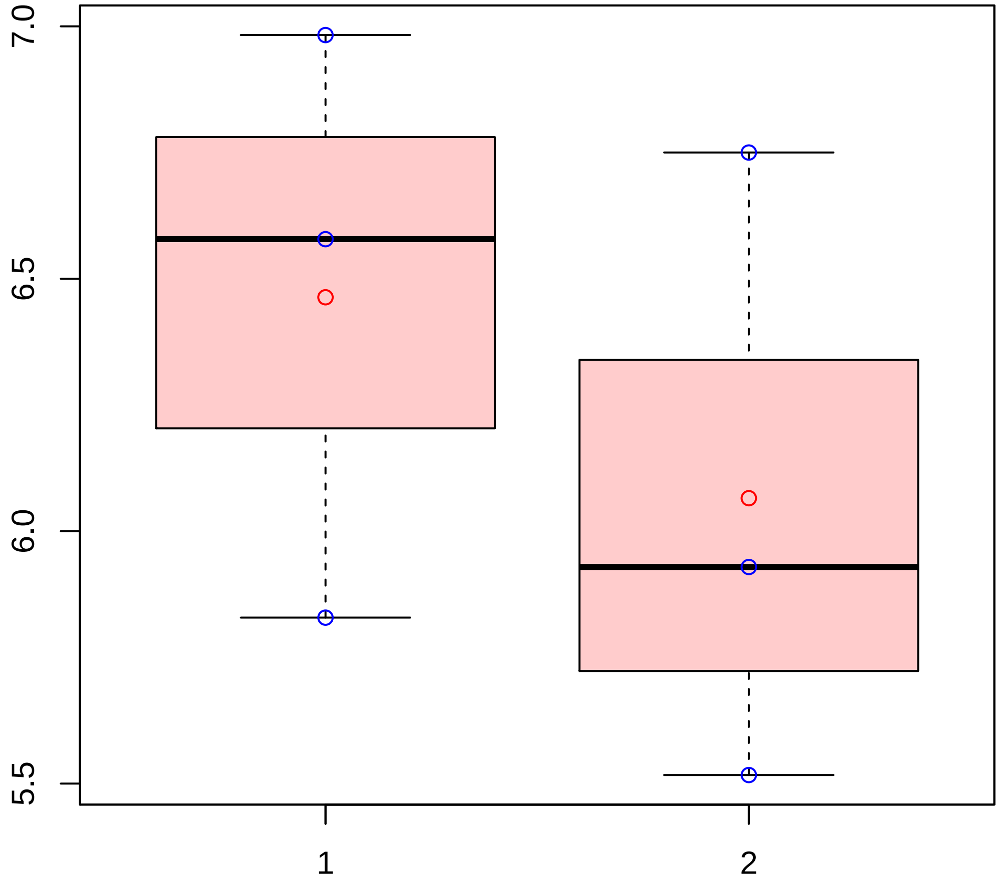

t−Test: p−value = 0.47

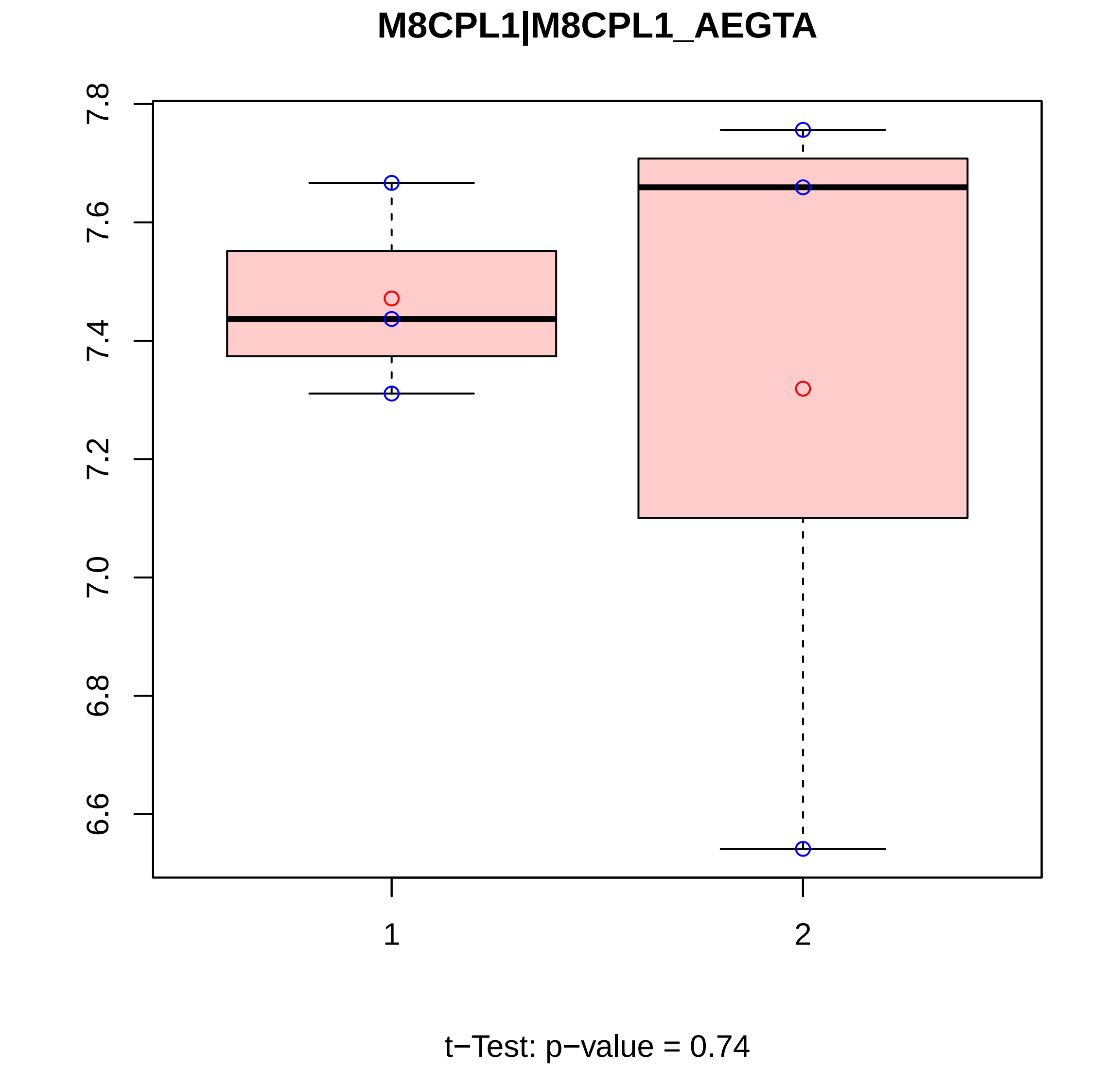
M8CPL1|M8CPL1_AEGTA
7.8
7.6
7.4
7.2
7.0
6.8
6.6
1
2
t−Test: p−value = 0.74

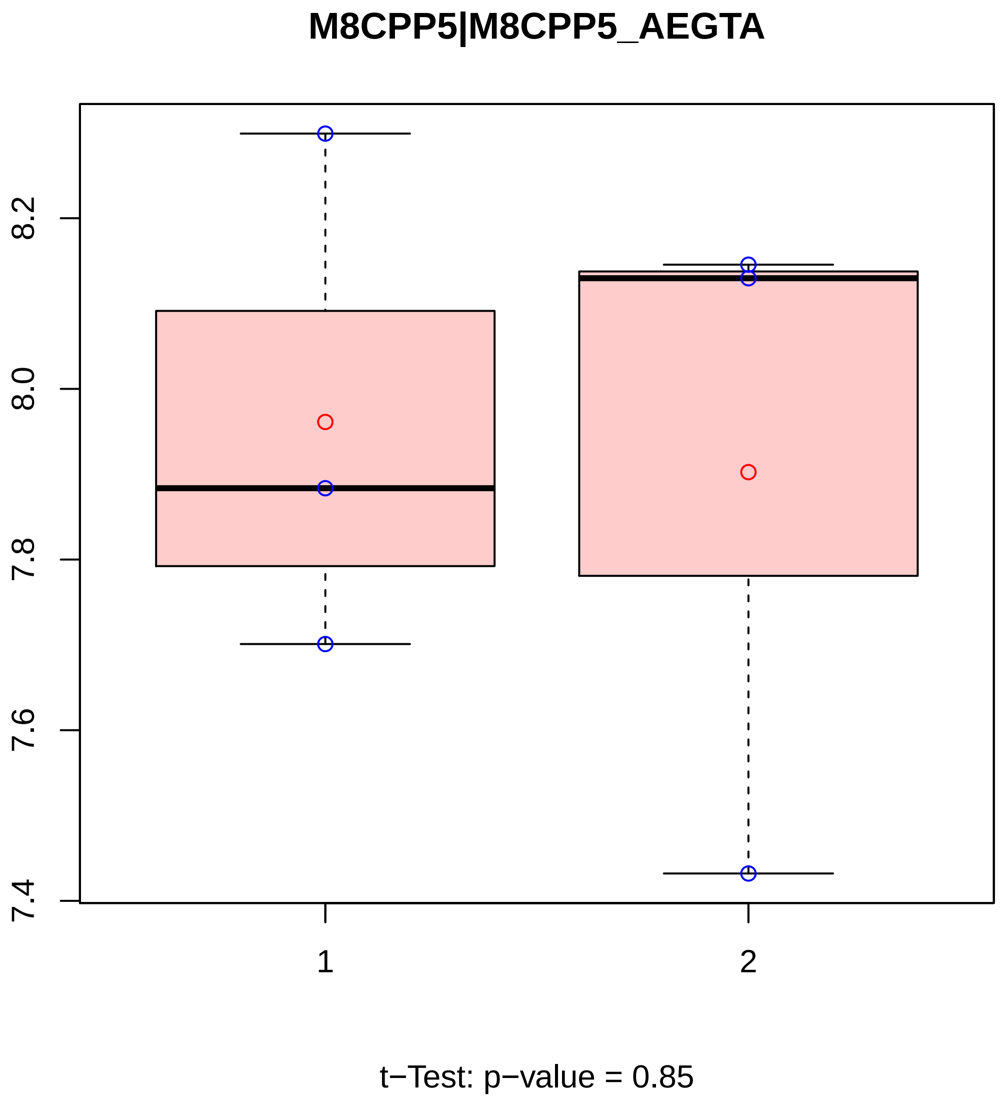

M8CPP5|M8CPP5_AEGTA
8.2
8.0
7.8
7.6
7.4
1
2
t−Test: p−value = 0.85

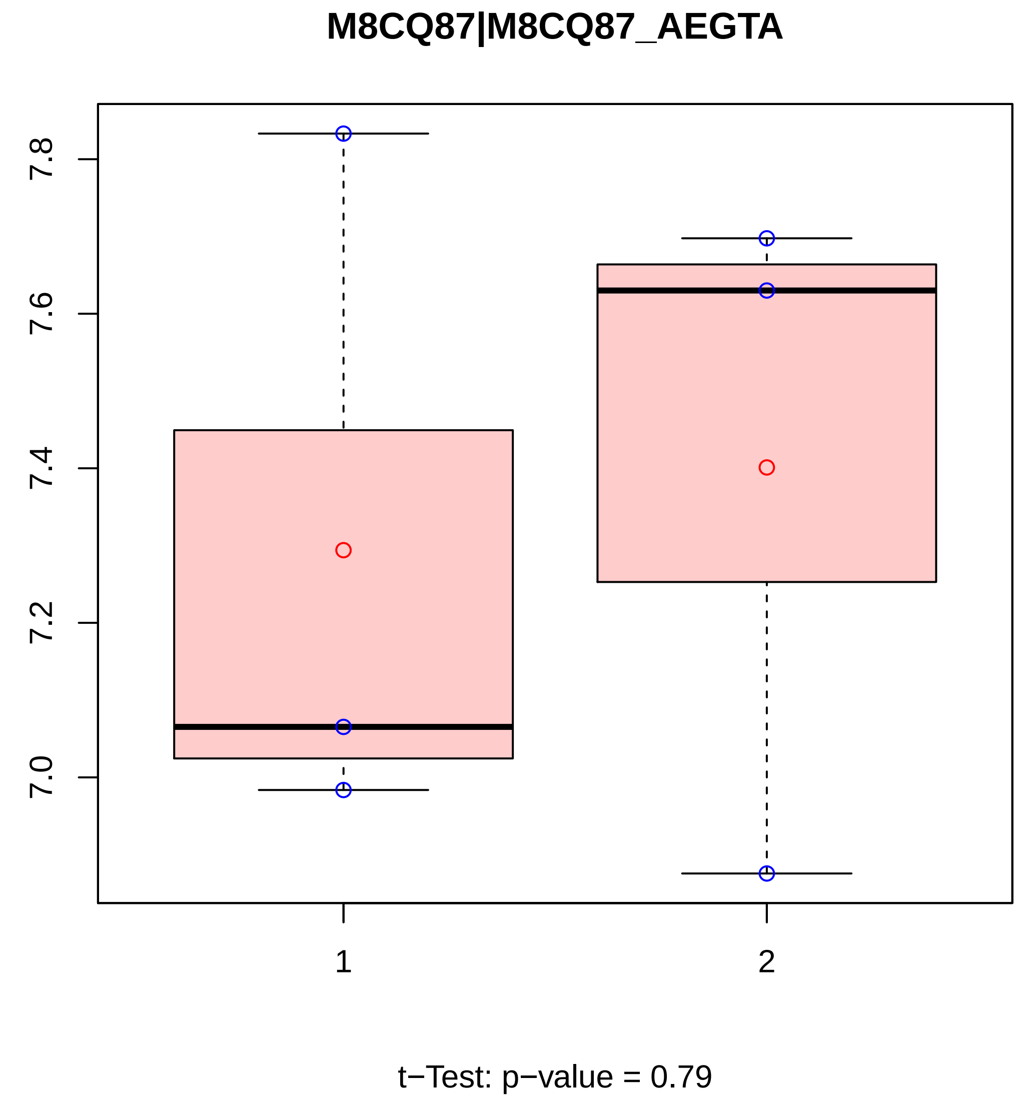
M8CQ87|M8CQ87_AEGTA
7.0
7.2
7.4
7.6
7.8
1
2
t−Test: p−value = 0.79

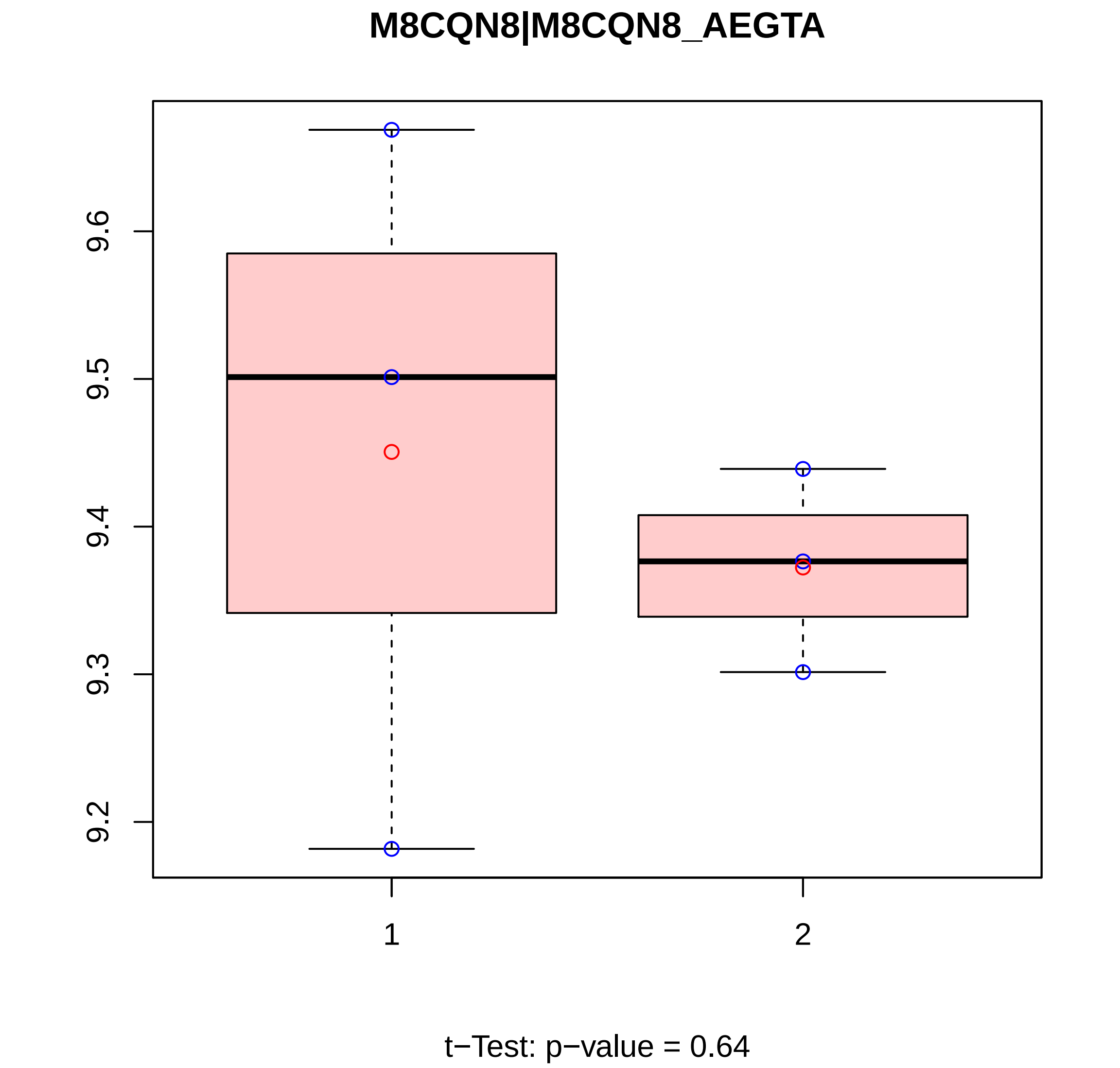
M8CQN8|M8CQN8_AEGTA
9.2
9.3
9.4
9.5
9.6
1
2
t−Test: p−value = 0.64

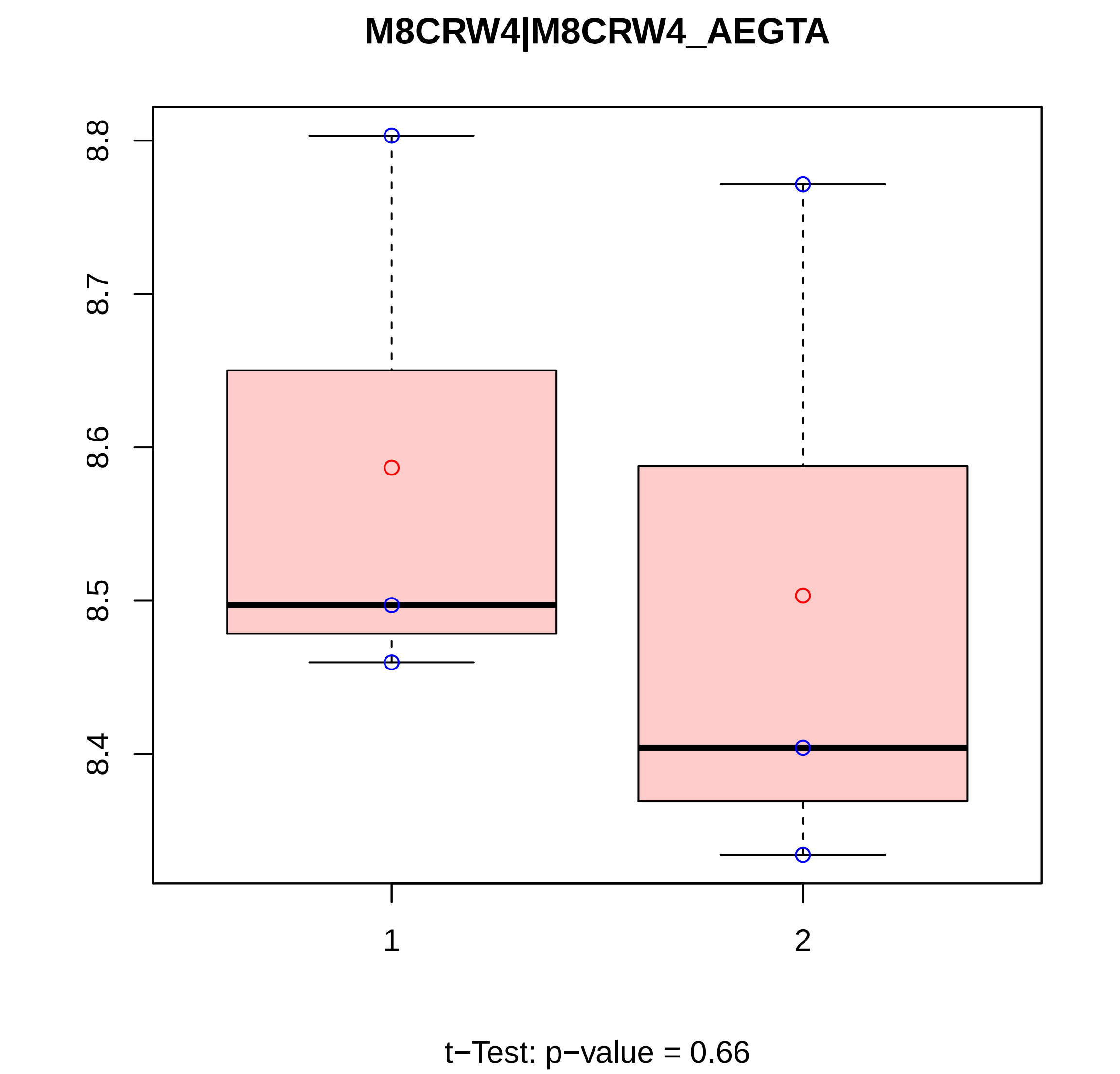
M8CRW4|M8CRW4_AEGTA
8.4
8.5
8.6
8.7
8.8
1
2
t−Test: p−value = 0.66

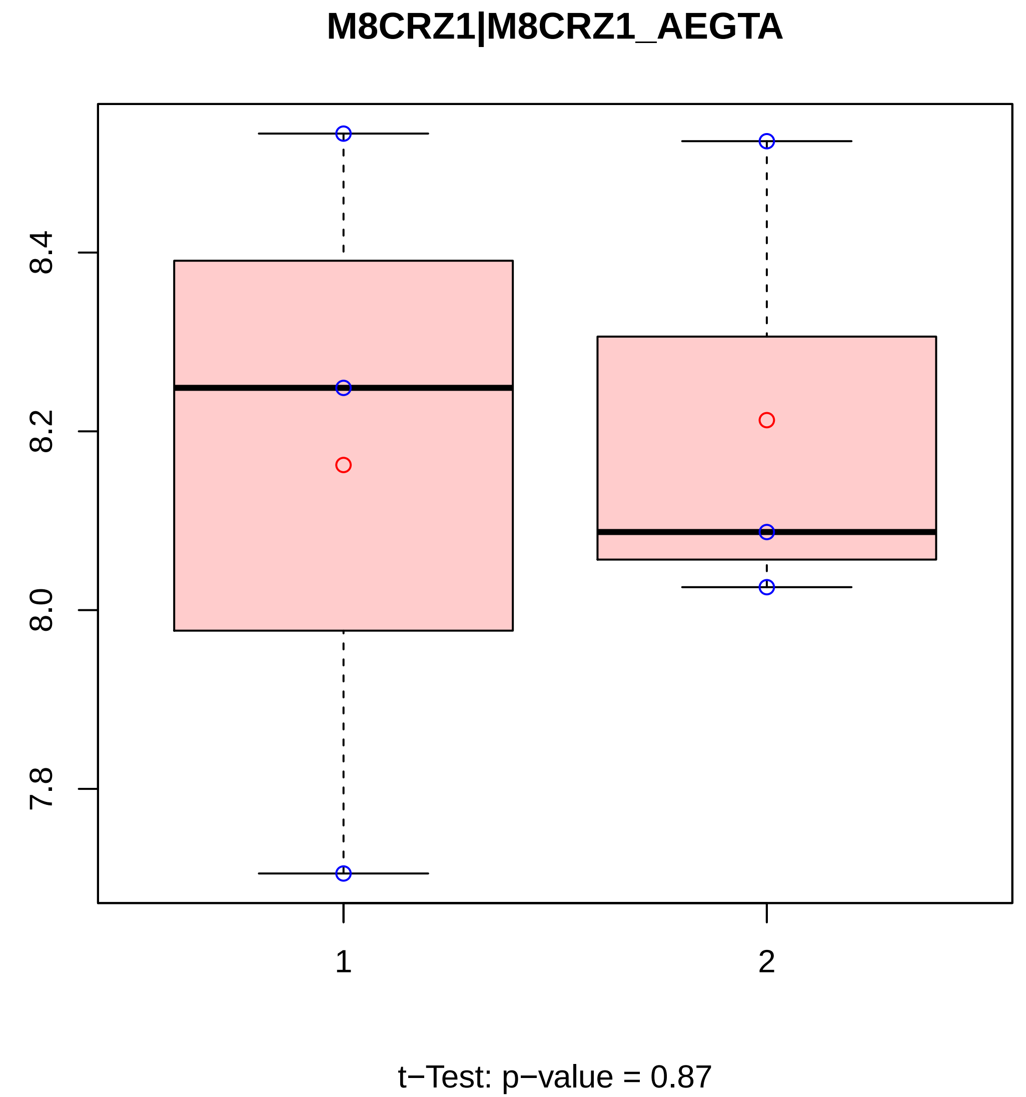

M8CRZ1|M8CRZ1_AEGTA
8.4
8.2
8.0
7.8
1
2
t−Test: p−value = 0.87

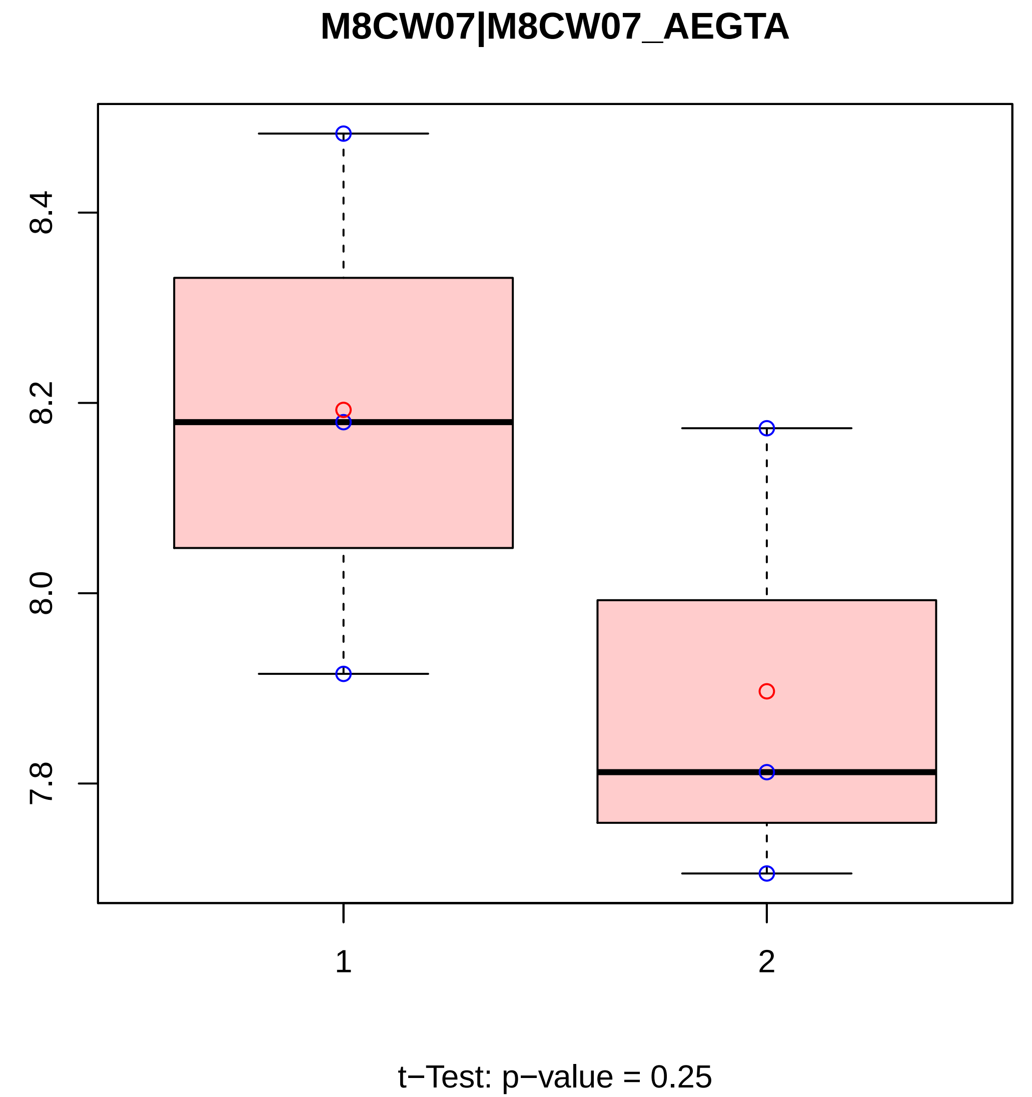
M8CW07|M8CW07_AEGTA
8.4
8.2
8.0
7.8
1
2
t−Test: p−value = 0.25

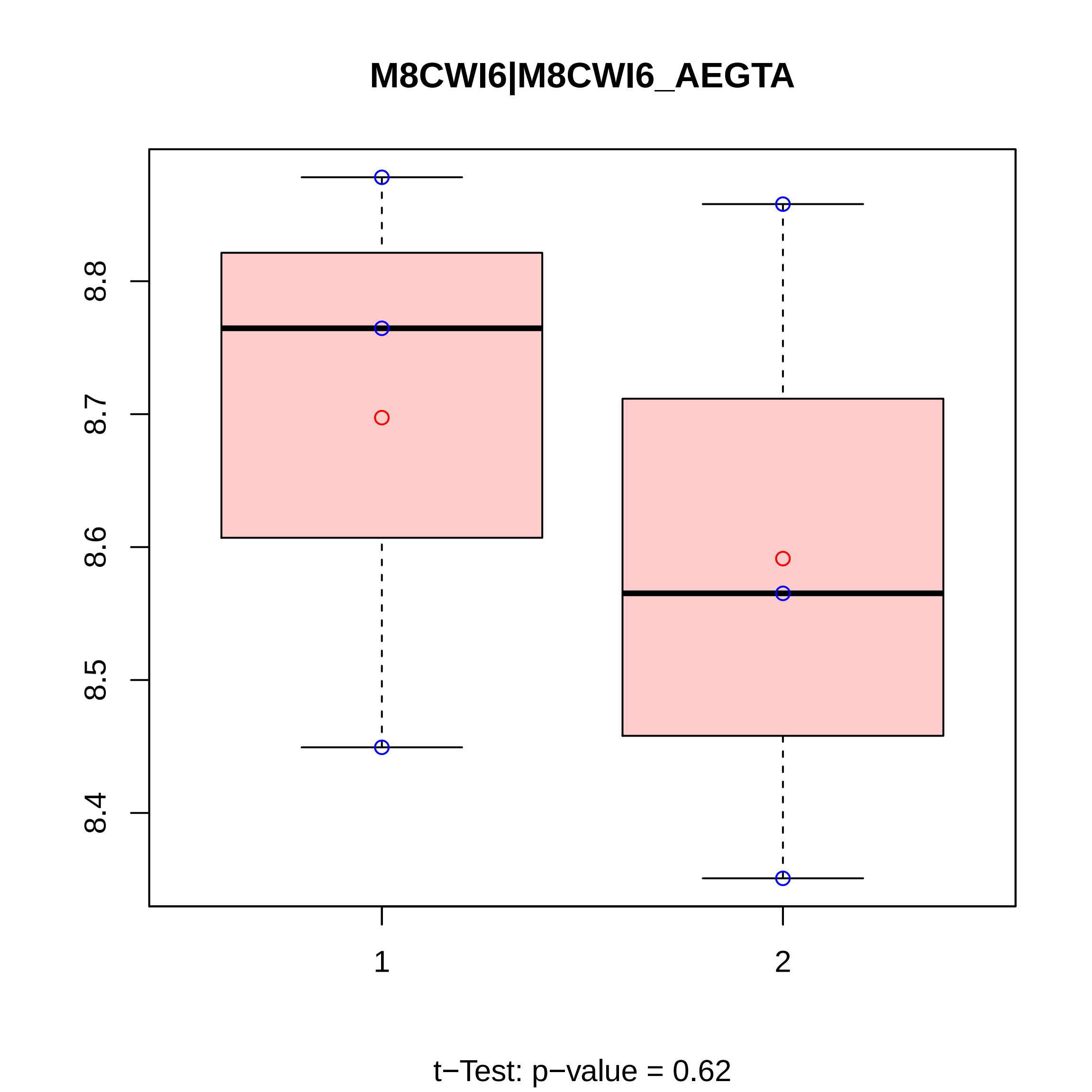

M8CWI6|M8CWI6_AEGTA
8.4
8.5
8.6
8.7
8.8
1
2
t−Test: p−value = 0.62

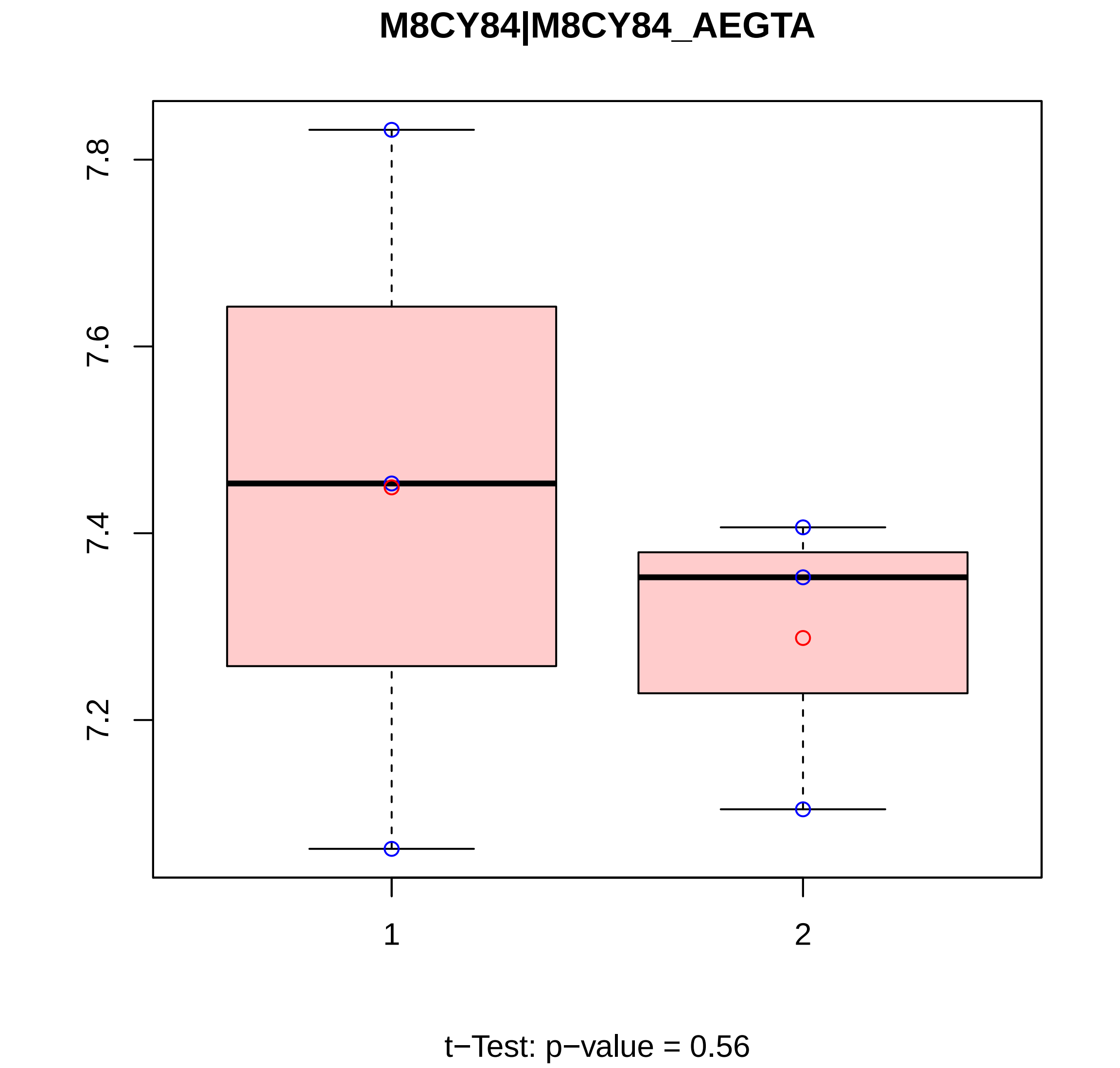

M8CY84|M8CY84_AEGTA
7.8
7.6
7.4
7.2
1
2
t−Test: p−value = 0.56

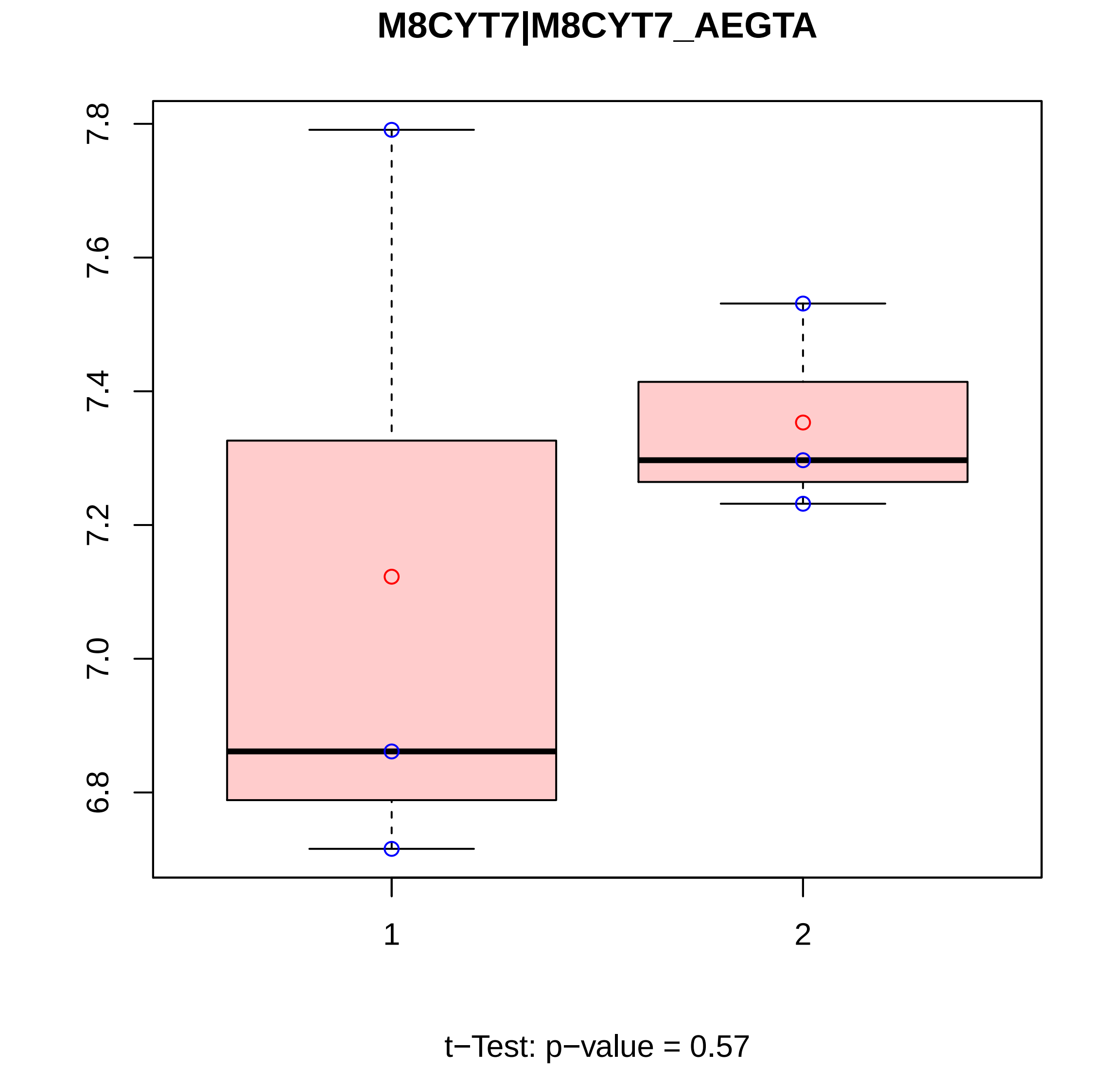
M8CYT7|M8CYT7_AEGTA
7.8
7.6
7.4
7.2
7.0
6.8
1
2
t−Test: p−value = 0.57

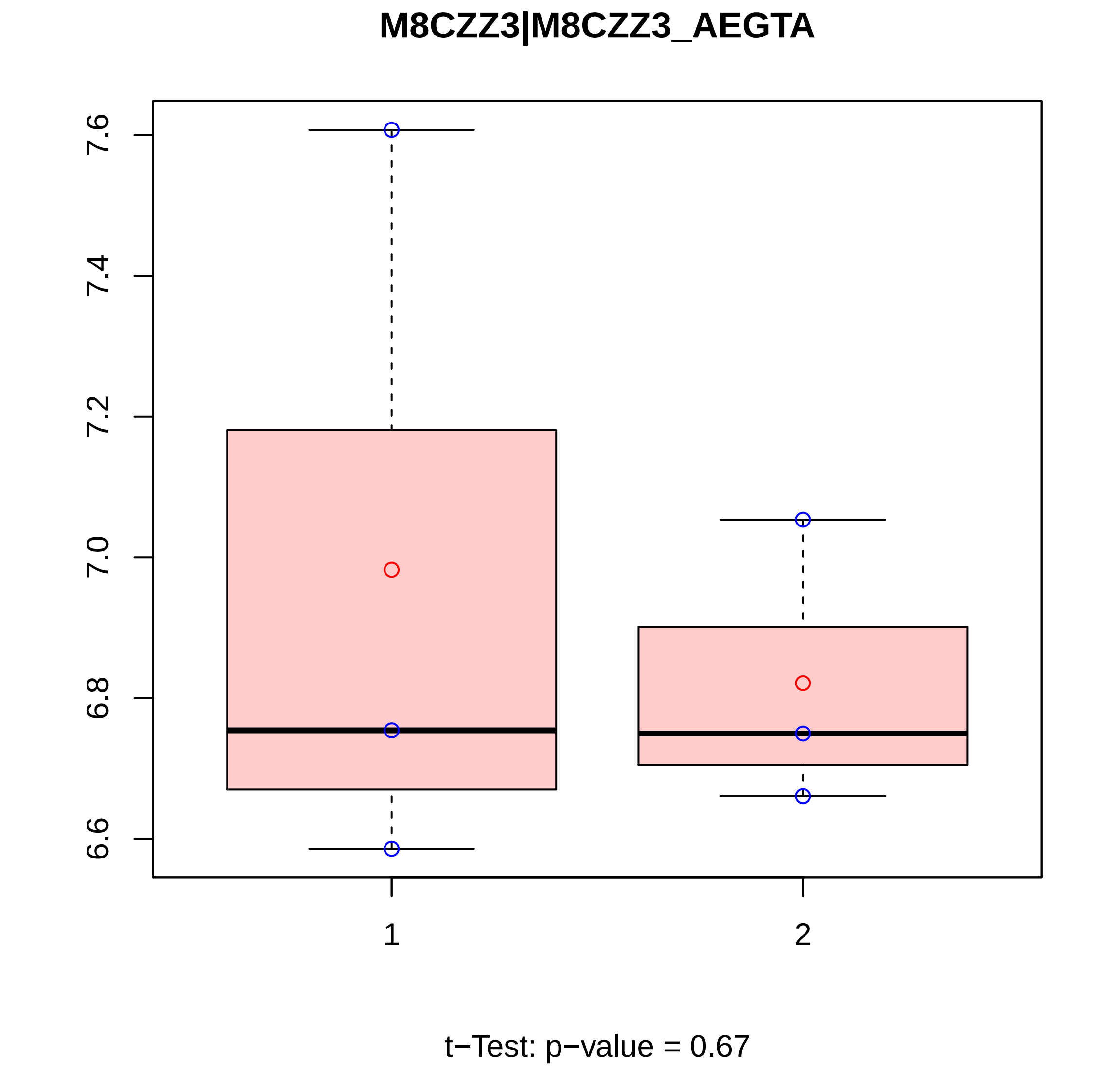
M8CZZ3|M8CZZ3_AEGTA
7.6
7.4
7.2
7.0
6.8
6.6
1
2
t−Test: p−value = 0.67

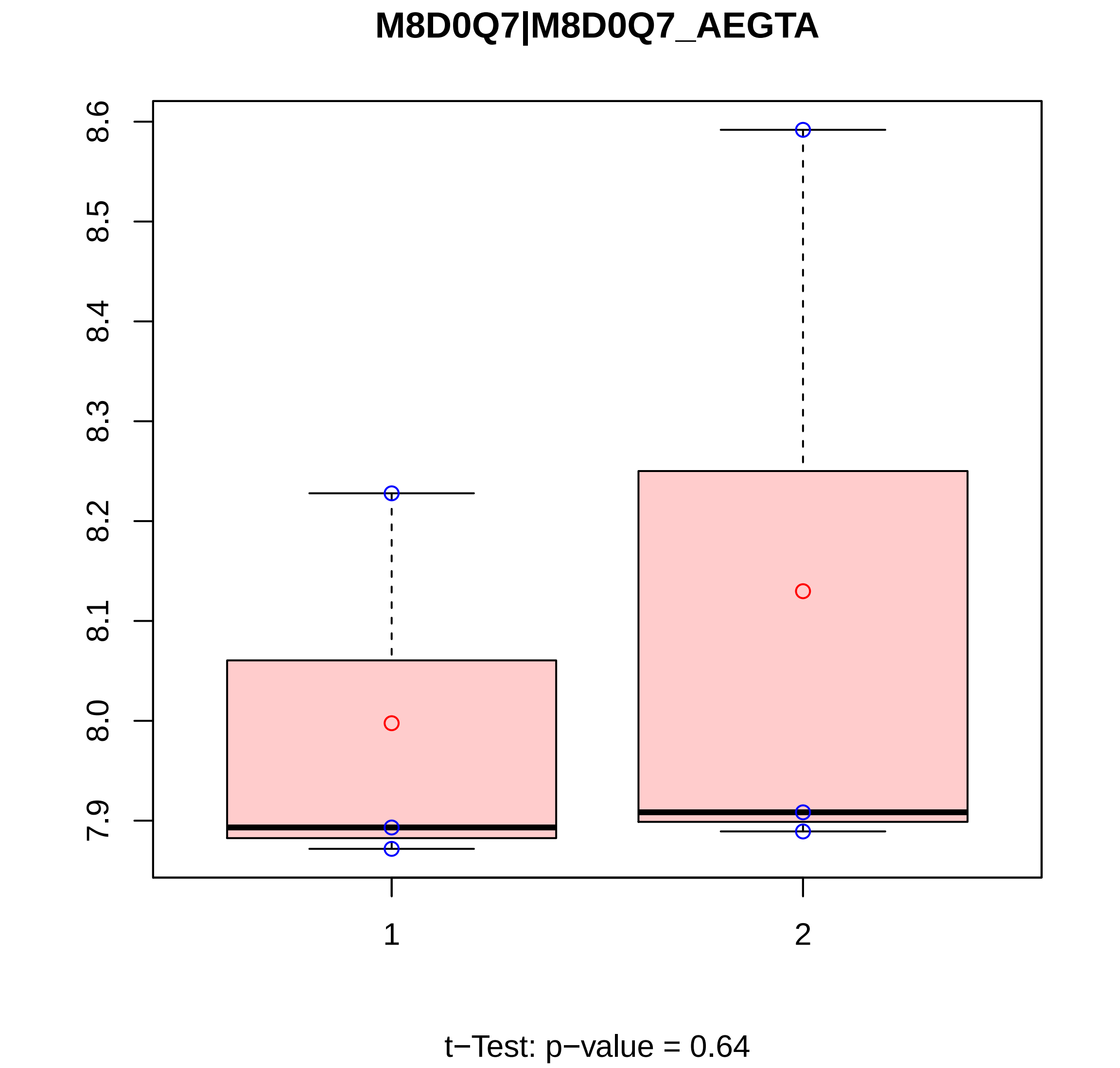
M8D0Q7|M8D0Q7_AEGTA
8.6
8.5
8.4
8.3
8.2
8.1
8.0
7.9
1
2
t−Test: p−value = 0.64

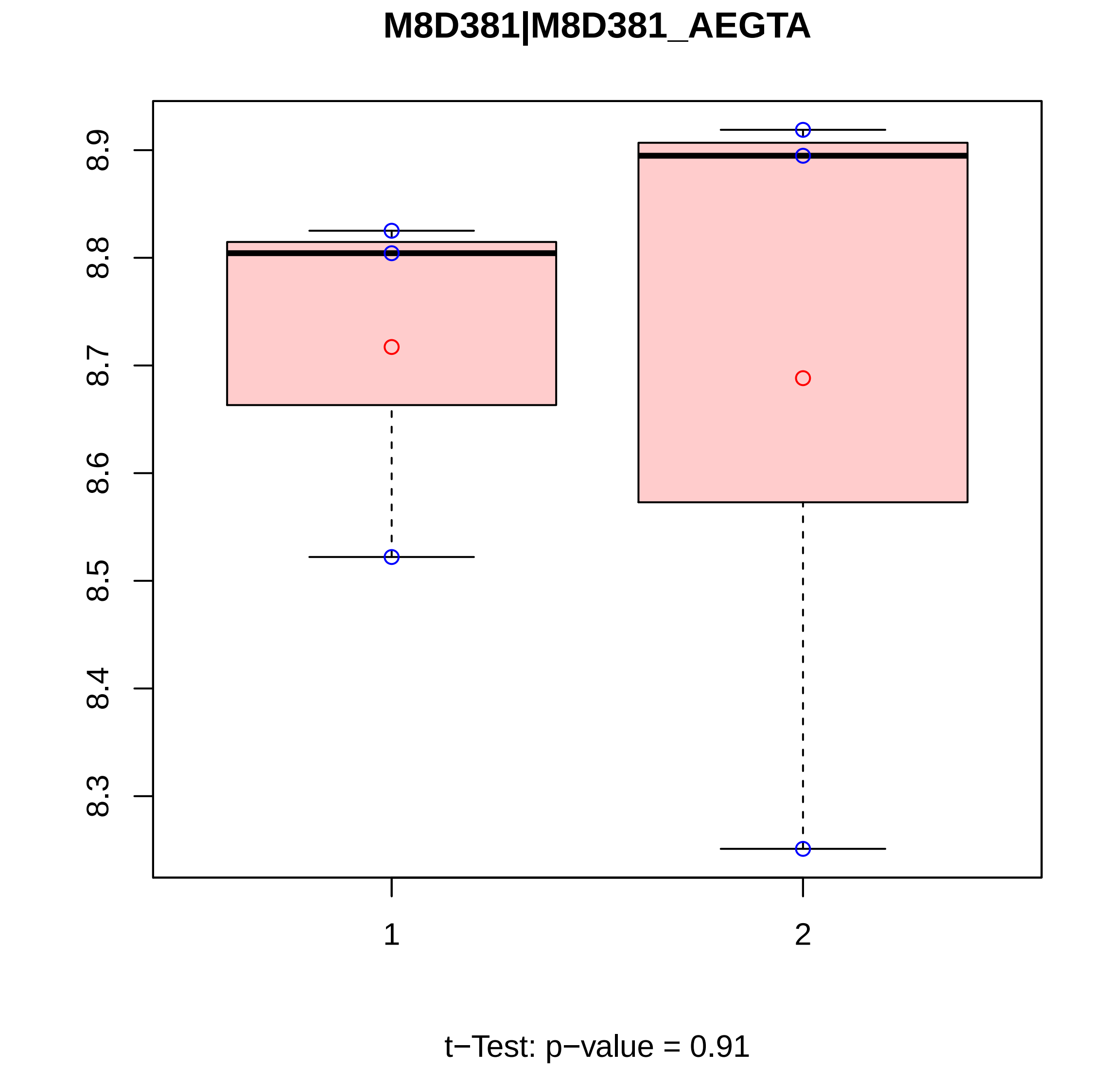

M8D381|M8D381_AEGTA
8.9
8.8
8.7
8.6
8.5
8.4
8.3
1
2
t−Test: p−value = 0.91

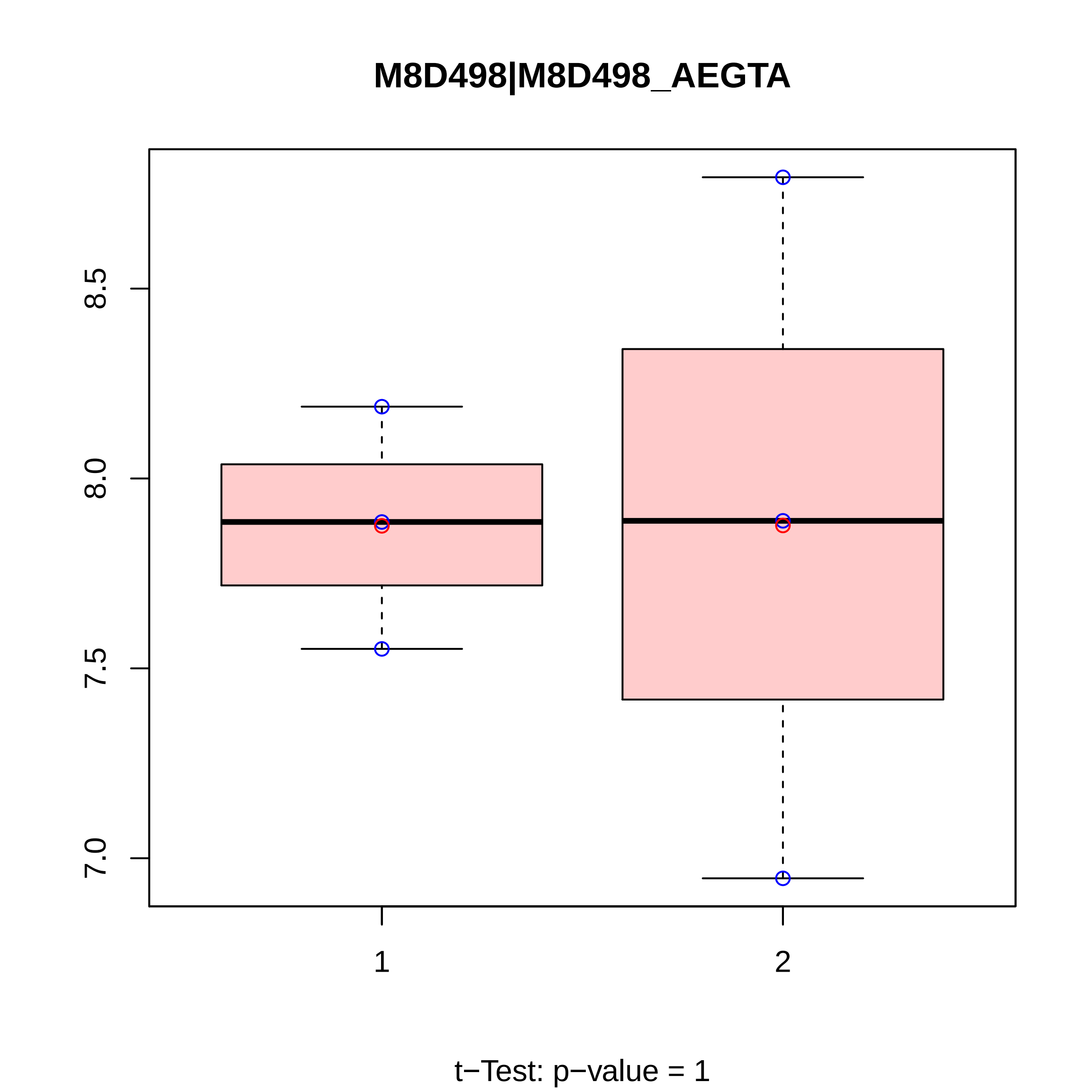

M8D498|M8D498_AEGTA
8.5
8.0
7.5
7.0
1
2
t−Test: p−value = 1

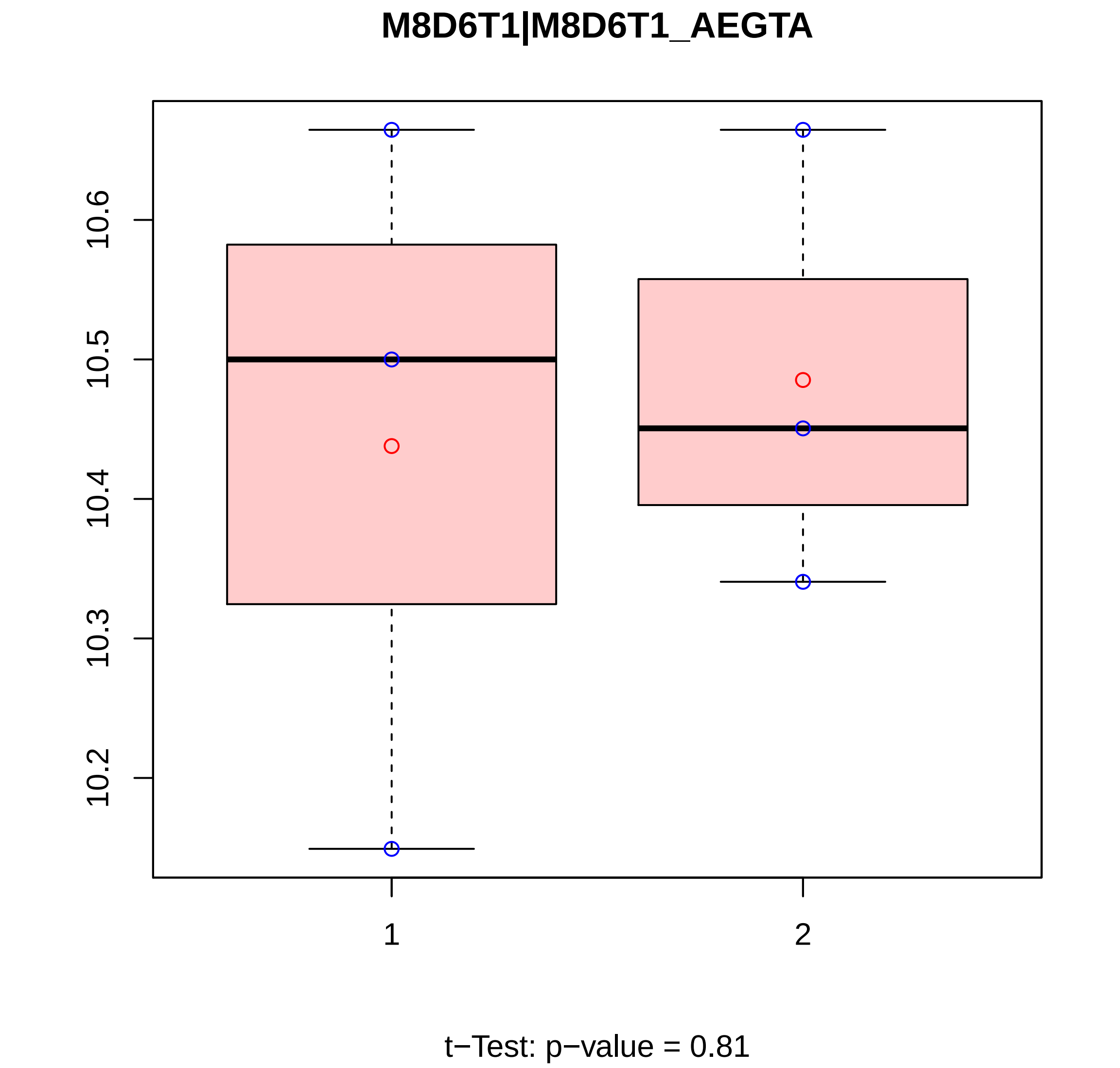
M8D6T1|M8D6T1_AEGTA
10.2
10.3
10.4
10.5
10.6
1
2
t−Test: p−value = 0.81

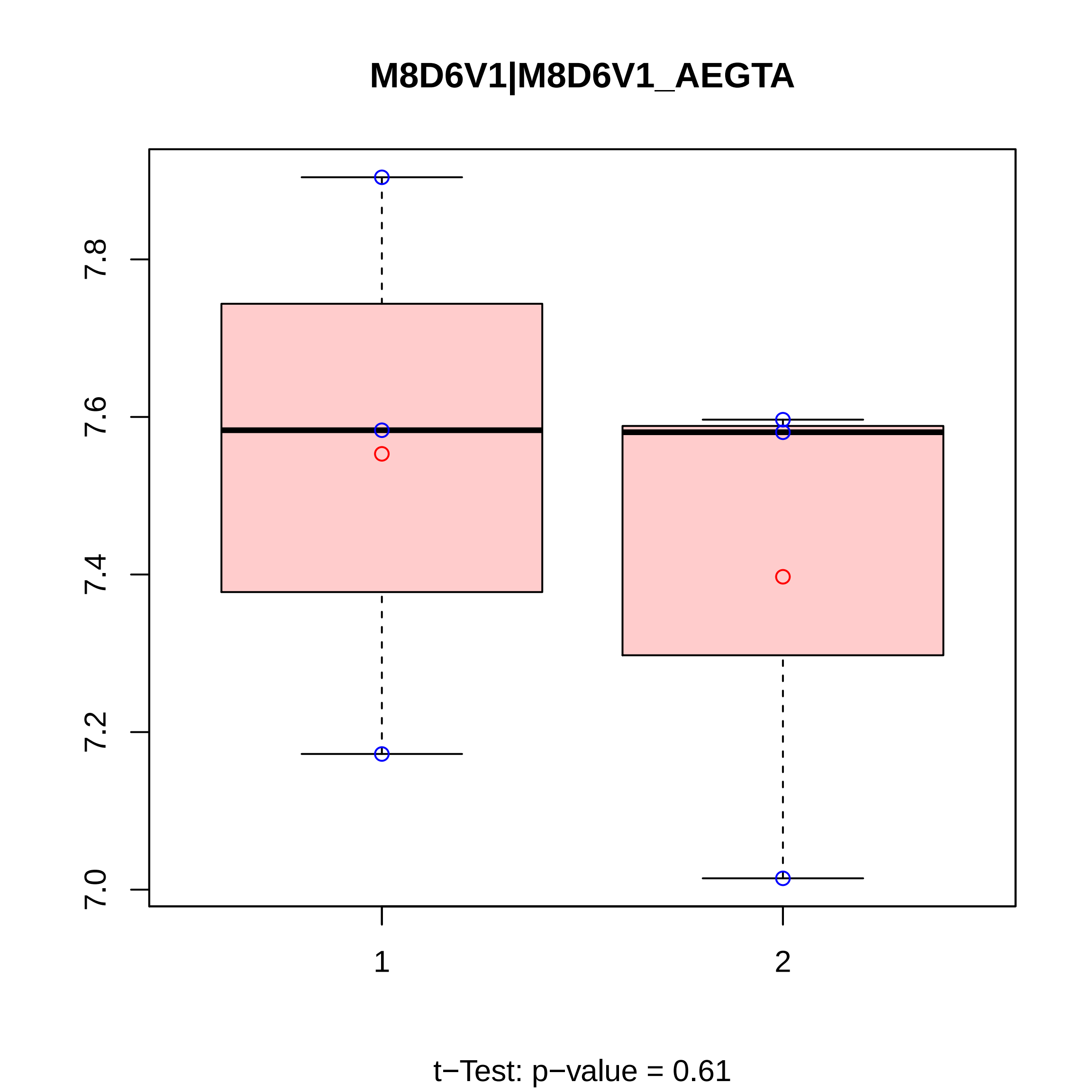

M8D6V1|M8D6V1_AEGTA
7.0
7.2
7.4
7.6
7.8
1
2
t−Test: p−value = 0.61

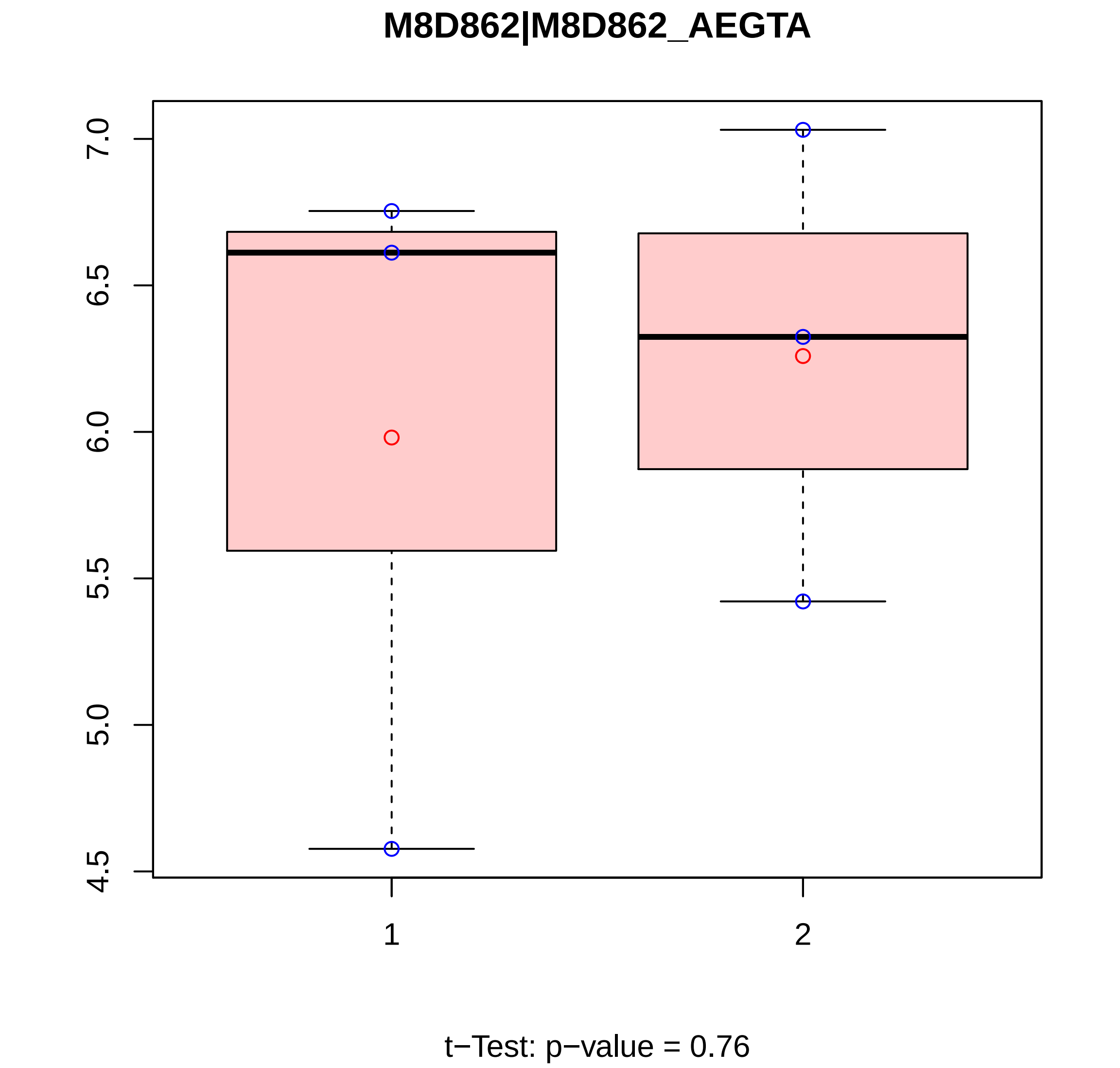

M8D862|M8D862_AEGTA
7.0
6.5
6.0
5.5
5.0
4.5
1
2
t−Test: p−value = 0.76

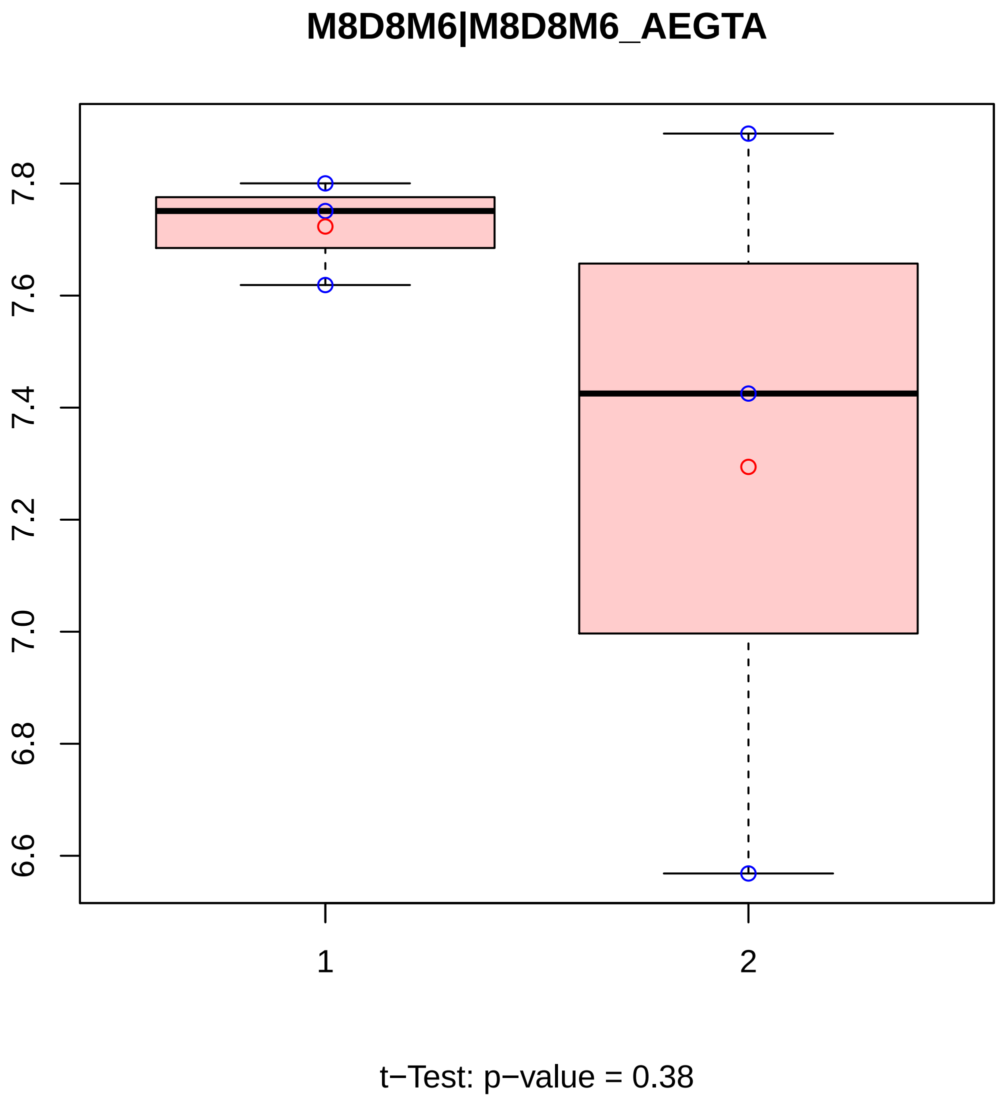
M8D8M6|M8D8M6_AEGTA
7.8
7.6
7.4
7.2
7.0
6.8
6.6
1
2
t−Test: p−value = 0.38

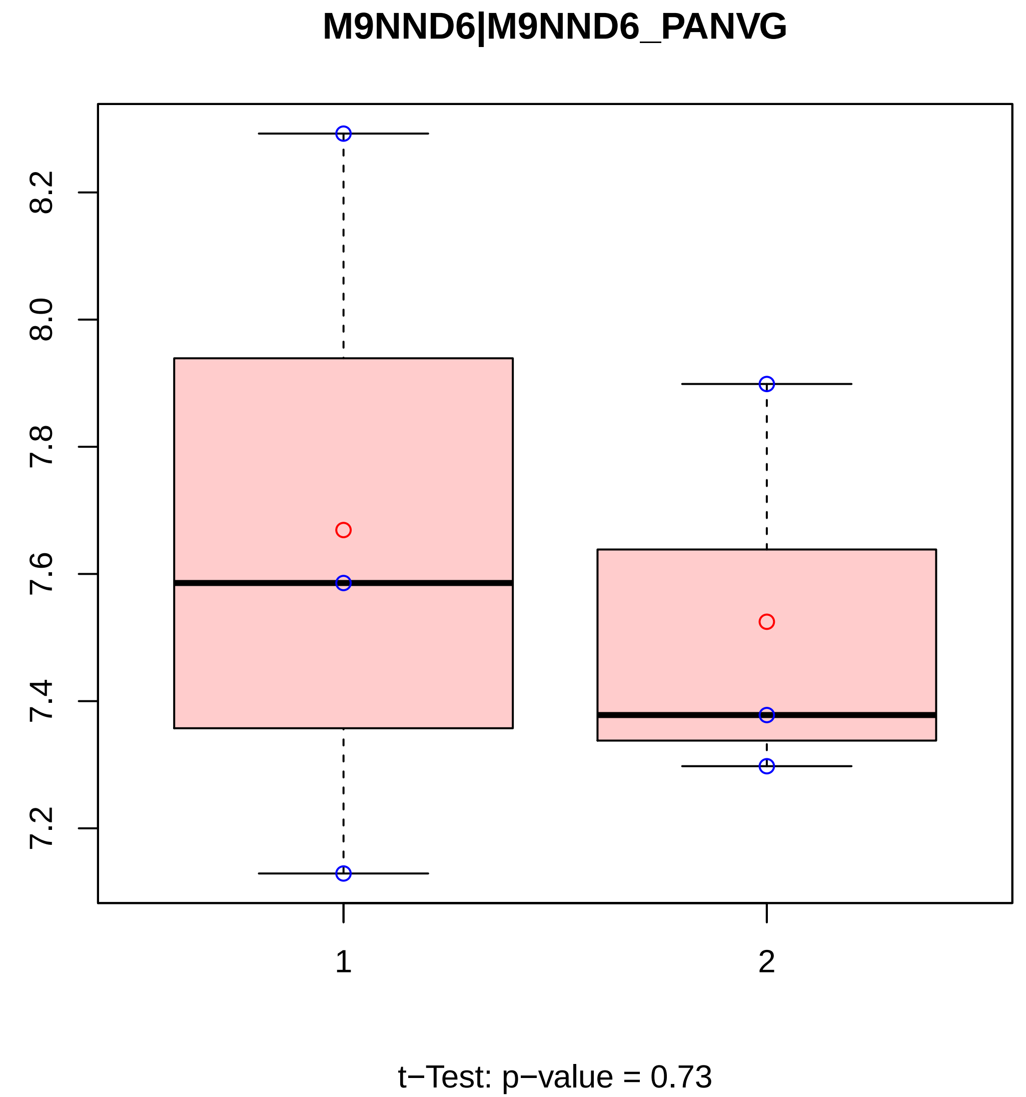

M9NND6|M9NND6_PANVG
8.2
8.0
7.8
7.6
7.4
7.2
1
2
t−Test: p−value = 0.73

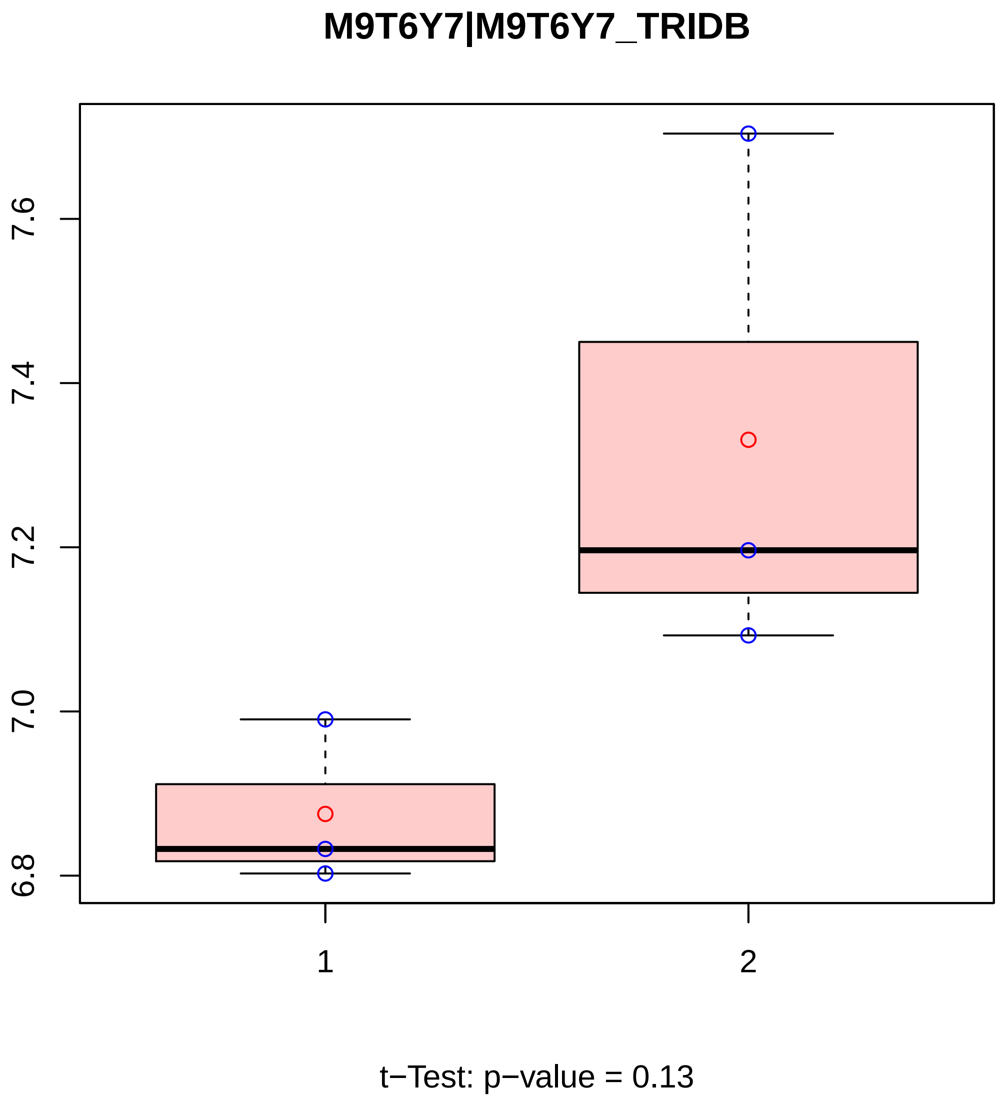
M9T6Y7|M9T6Y7_TRIDB
7.6
7.4
7.2
7.0
6.8
1
2
t−Test: p−value = 0.13

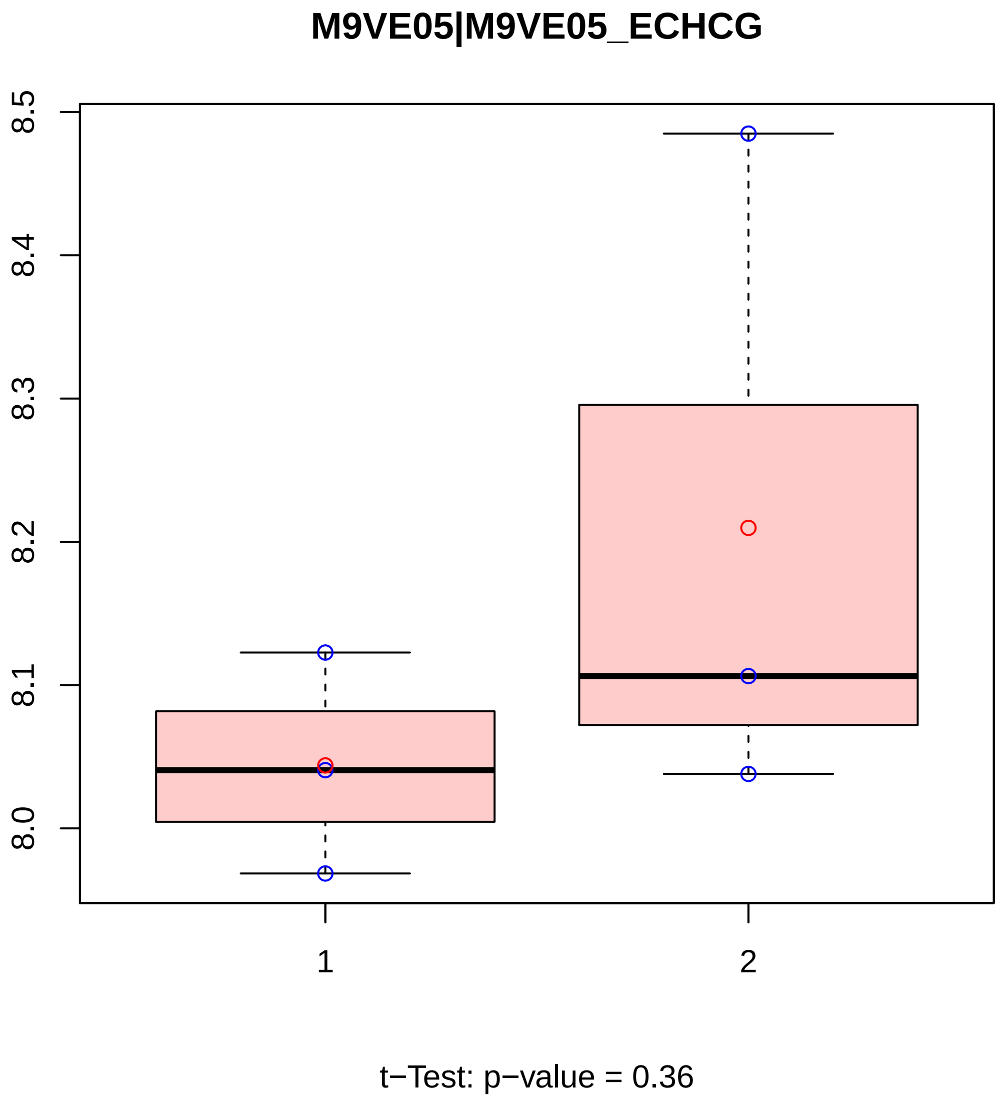
M9VE05|M9VE05_ECHCG
8.0
8.1
8.2
8.3
8.4
8.5
1
2
t−Test: p−value = 0.36

N1QQ42|N1QQ42_AEGTA

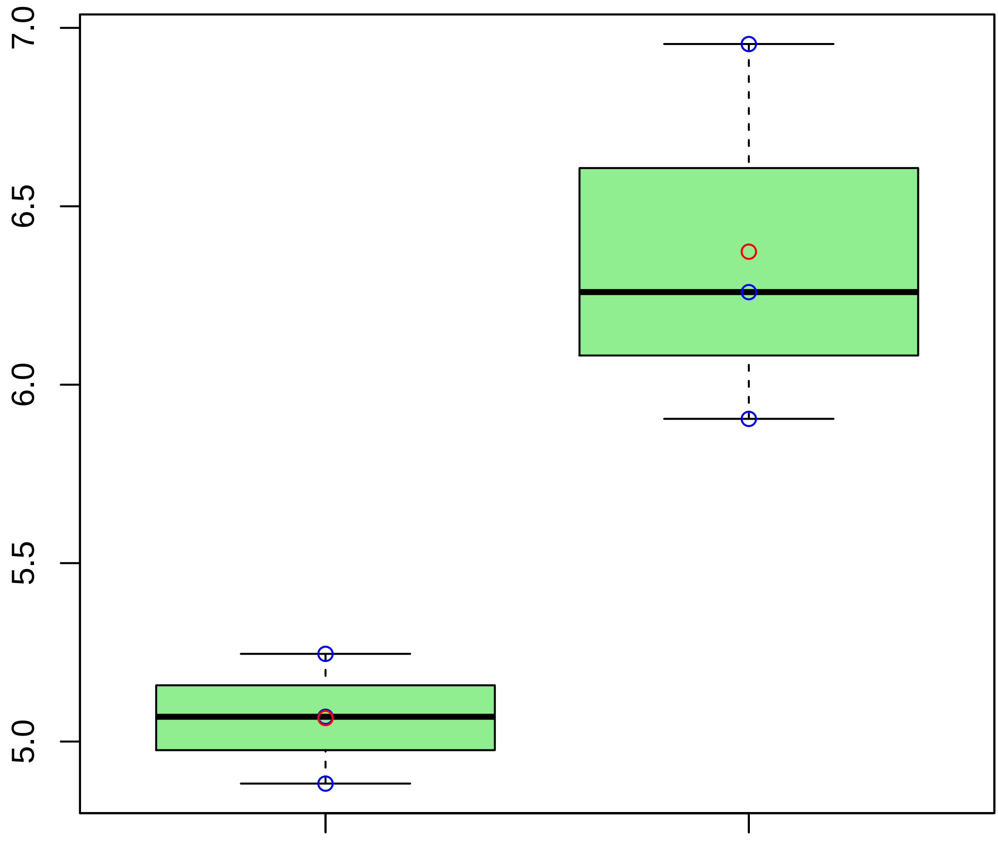

t−Test: p−value = 0.04

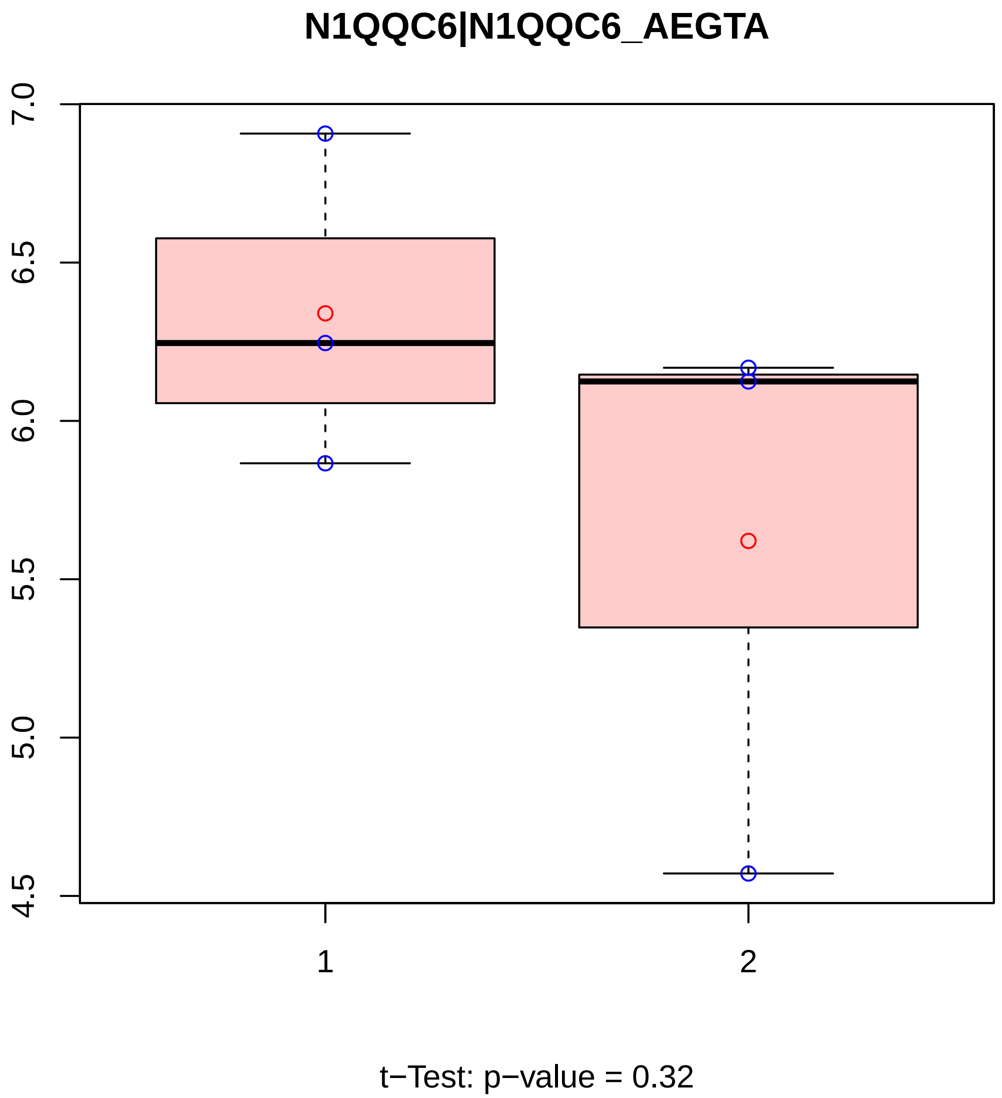

N1QQC6|N1QQC6_AEGTA
7.0
6.5
6.0
5.5
5.0
4.5
1
2
t−Test: p−value = 0.32

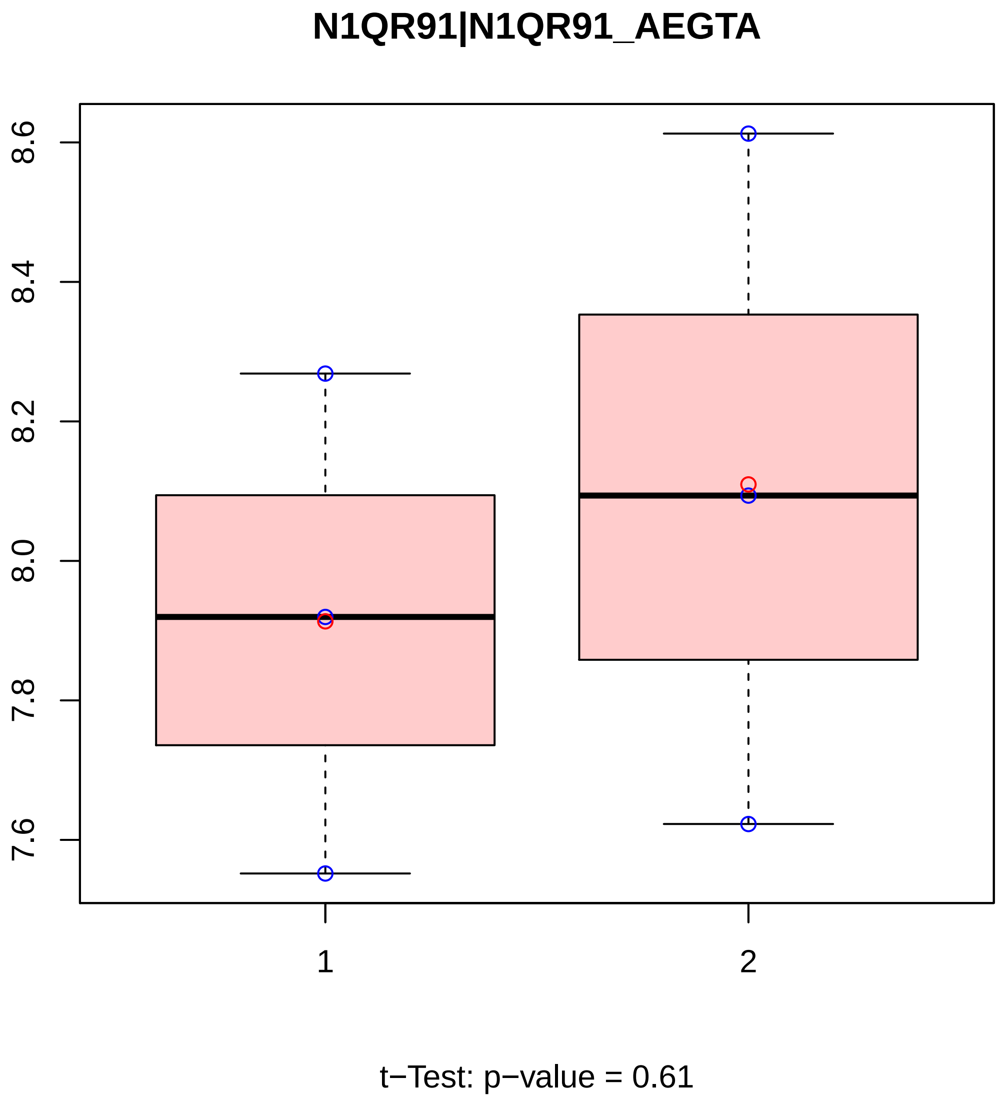

N1QR91|N1QR91_AEGTA
8.6
8.4
8.2
8.0
7.8
7.6
1
2
t−Test: p−value = 0.61

N1QSL7|N1QSL7_AEGTA

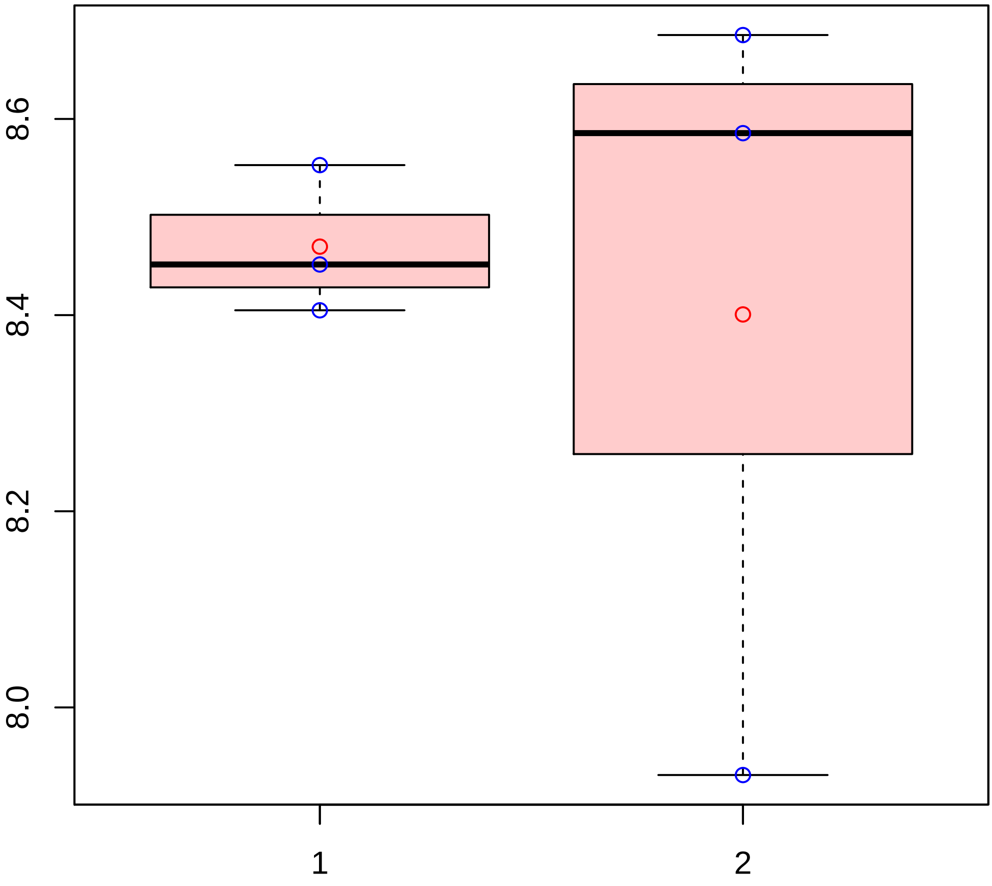

t−Test: p−value = 0.8

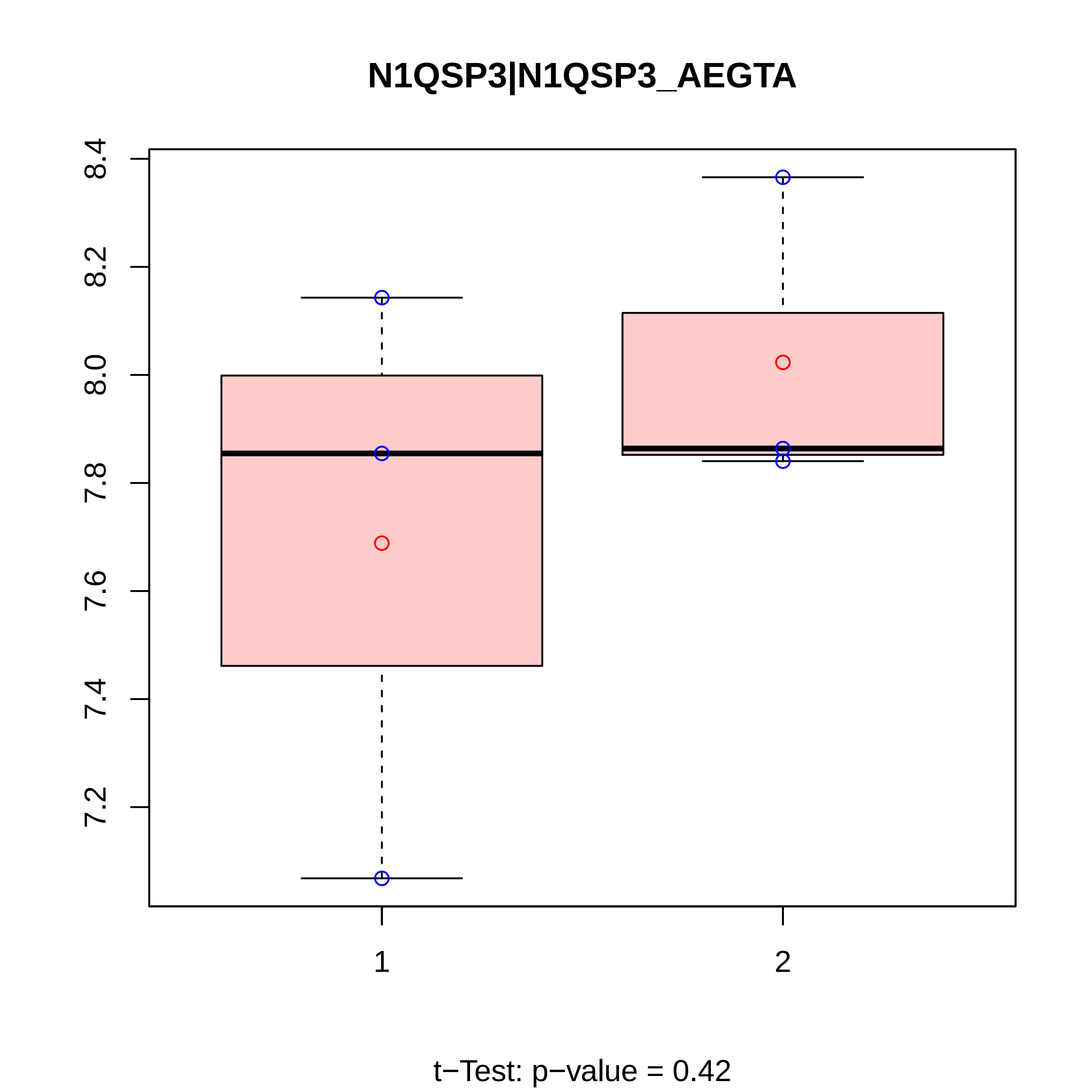

N1QSP3|N1QSP3_AEGTA
8.4
8.2
8.0
7.8
7.6
7.4
7.2
1
2
t−Test: p−value = 0.42

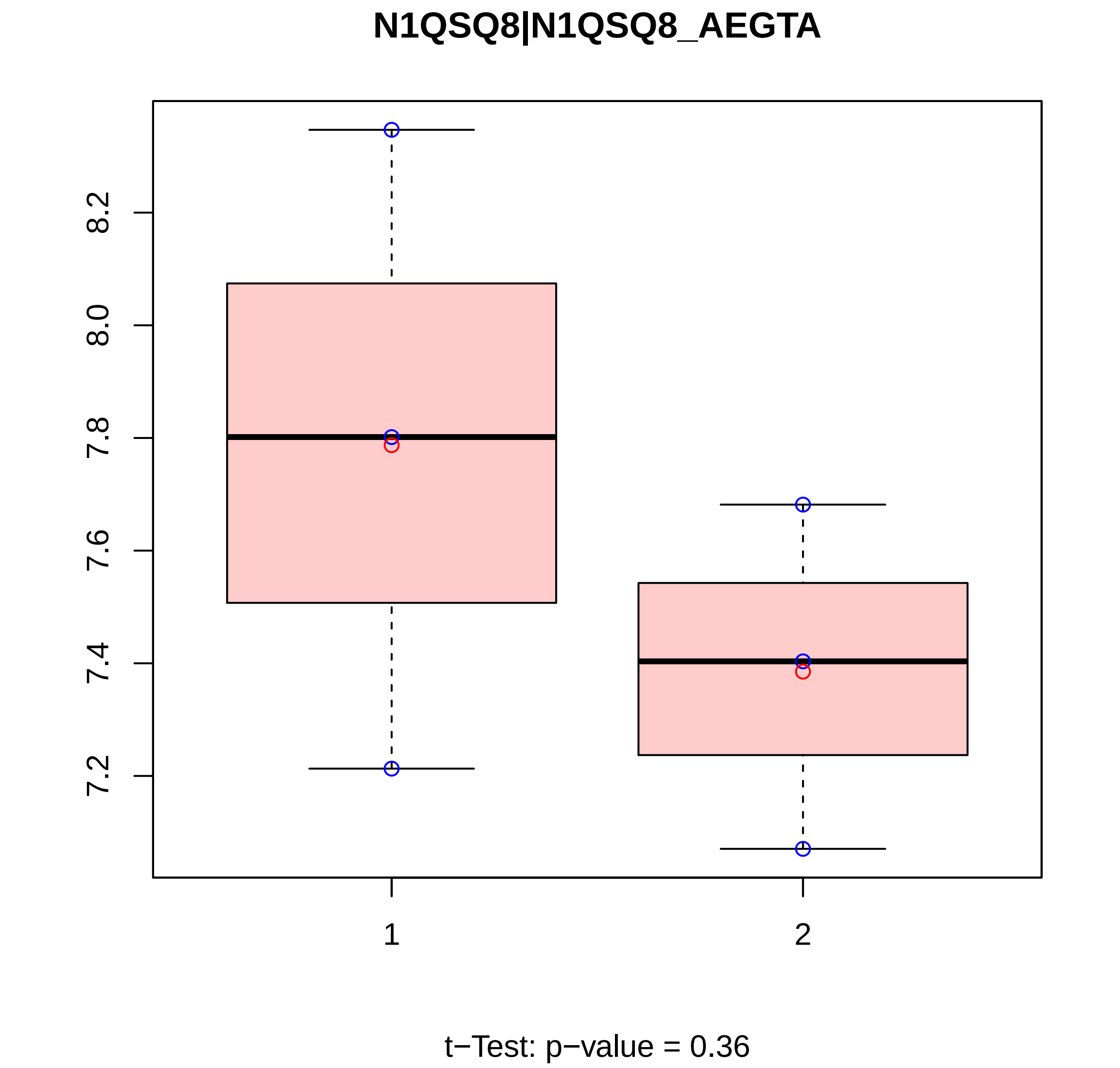
N1QSQ8|N1QSQ8_AEGTA
7.2
7.4
7.6
7.8
8.0
8.2
1
2
t−Test: p−value = 0.36

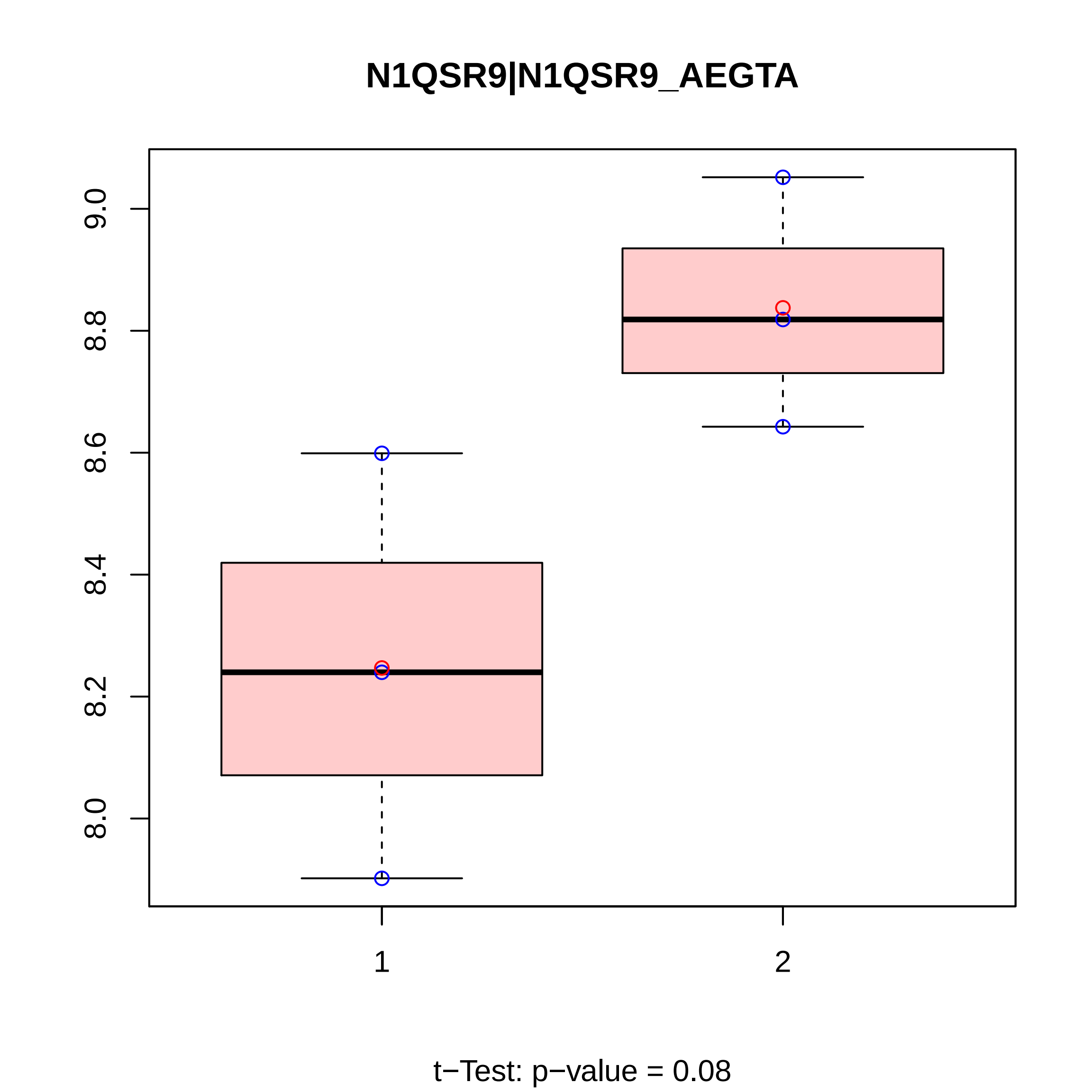
N1QSR9|N1QSR9_AEGTA
9.0
8.8
8.6
8.4
8.2
8.0
1
2
t−Test: p−value = 0.08

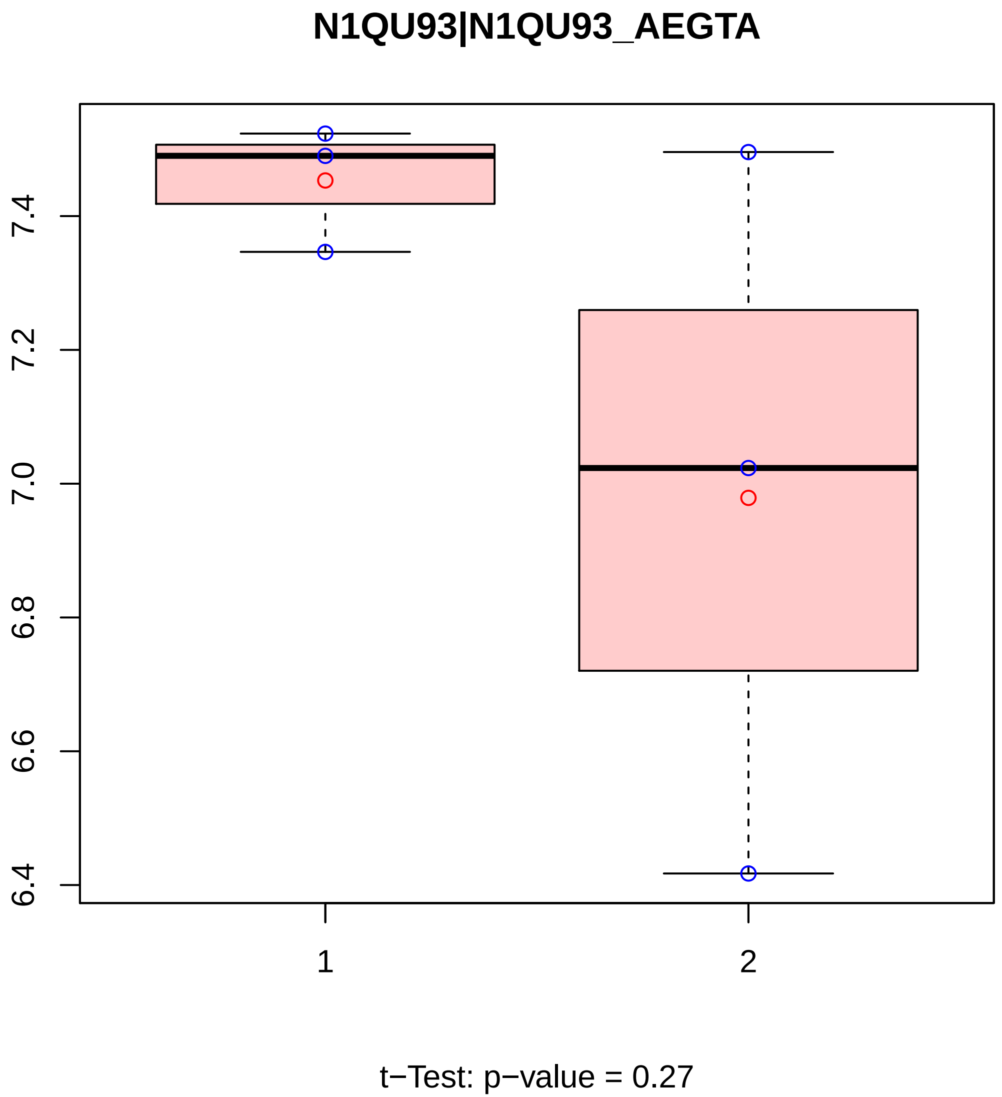
N1QU93|N1QU93_AEGTA
7.4
7.2
7.0
6.8
6.6
6.4
1
2
t−Test: p−value = 0.27

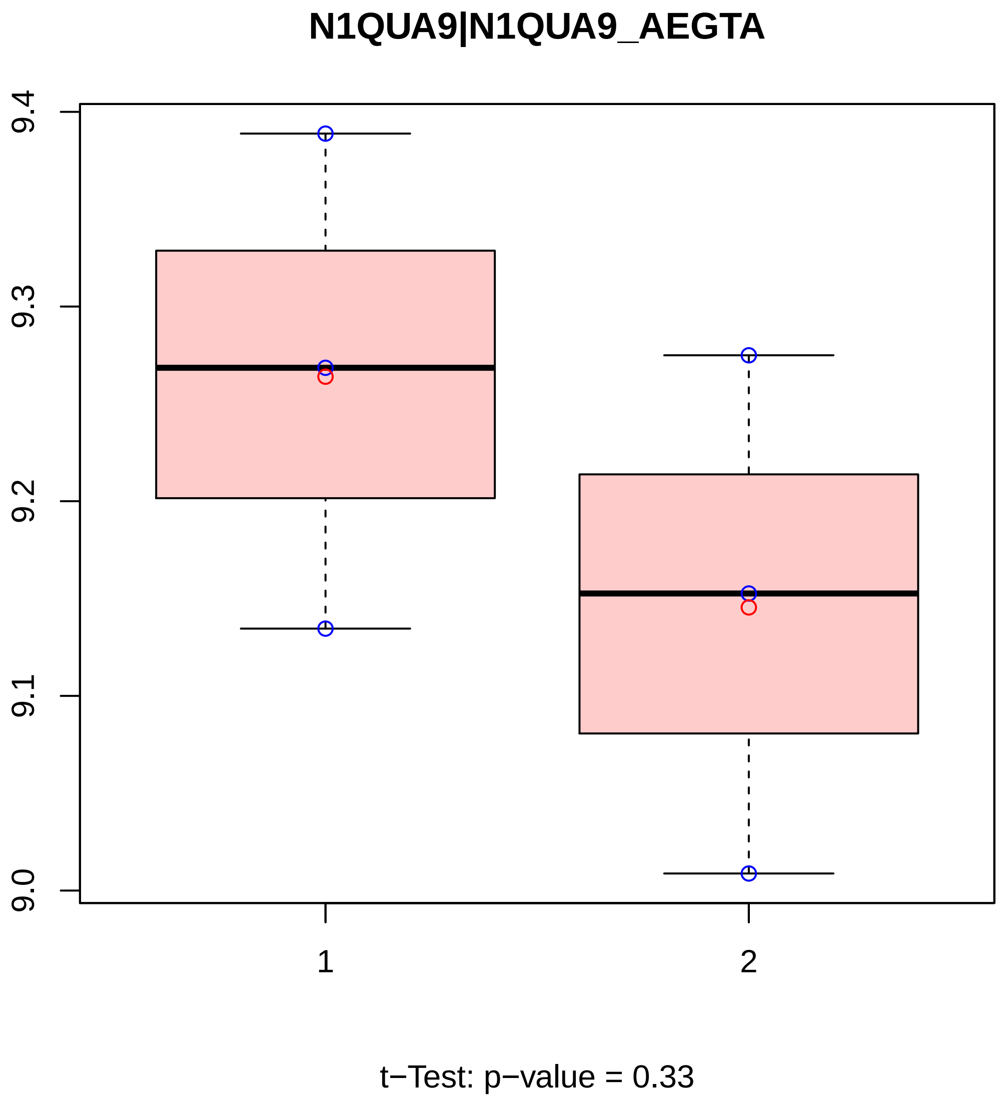

N1QUA9|N1QUA9_AEGTA
9.0
9.1
9.2
9.3
9.4
1
2
t−Test: p−value = 0.33

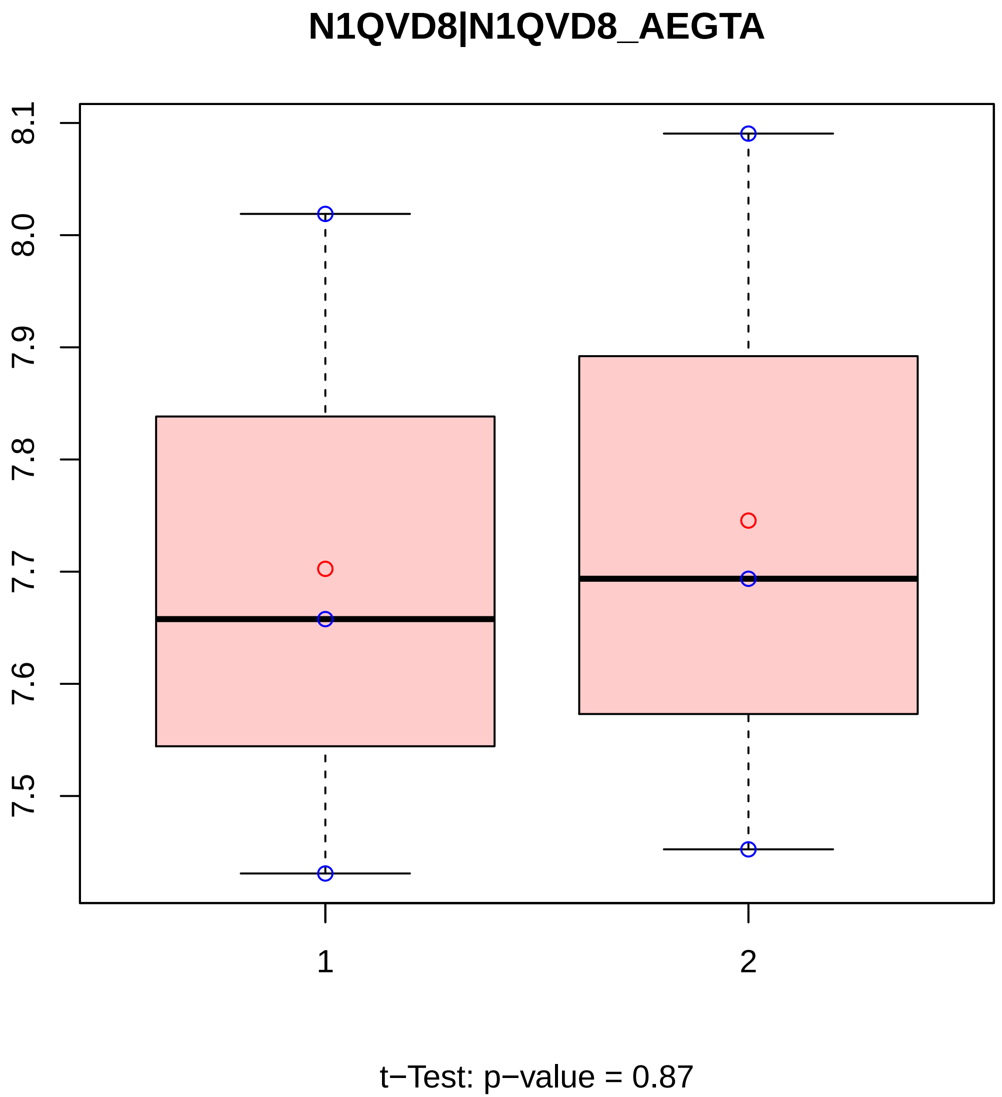
N1QVD8|N1QVD8_AEGTA
8.1
8.0
7.9
7.8
7.7
7.6
7.5
1
2
t−Test: p−value = 0.87

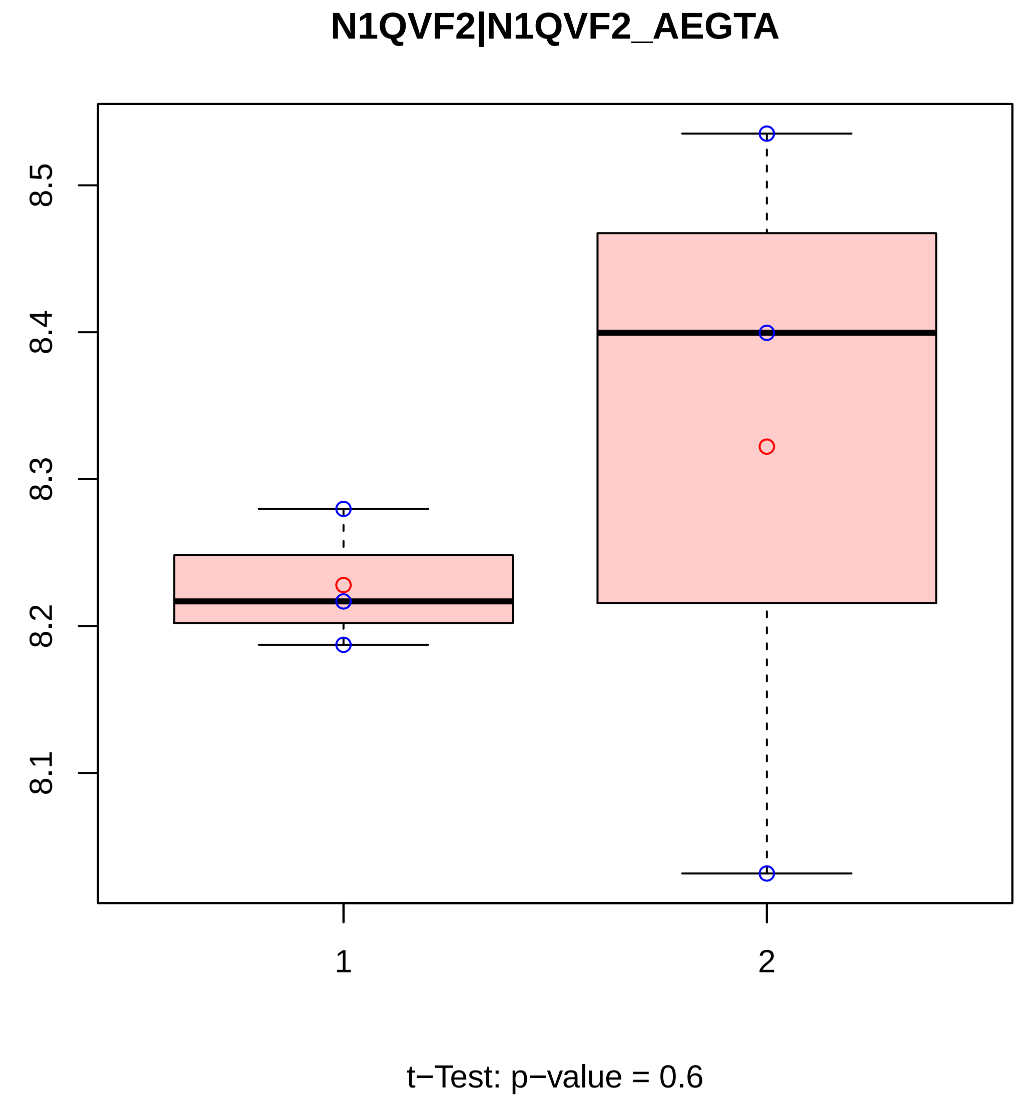
N1QVF2|N1QVF2_AEGTA
8.1
8.2
8.3
8.4
8.5
1
2
t−Test: p−value = 0.6

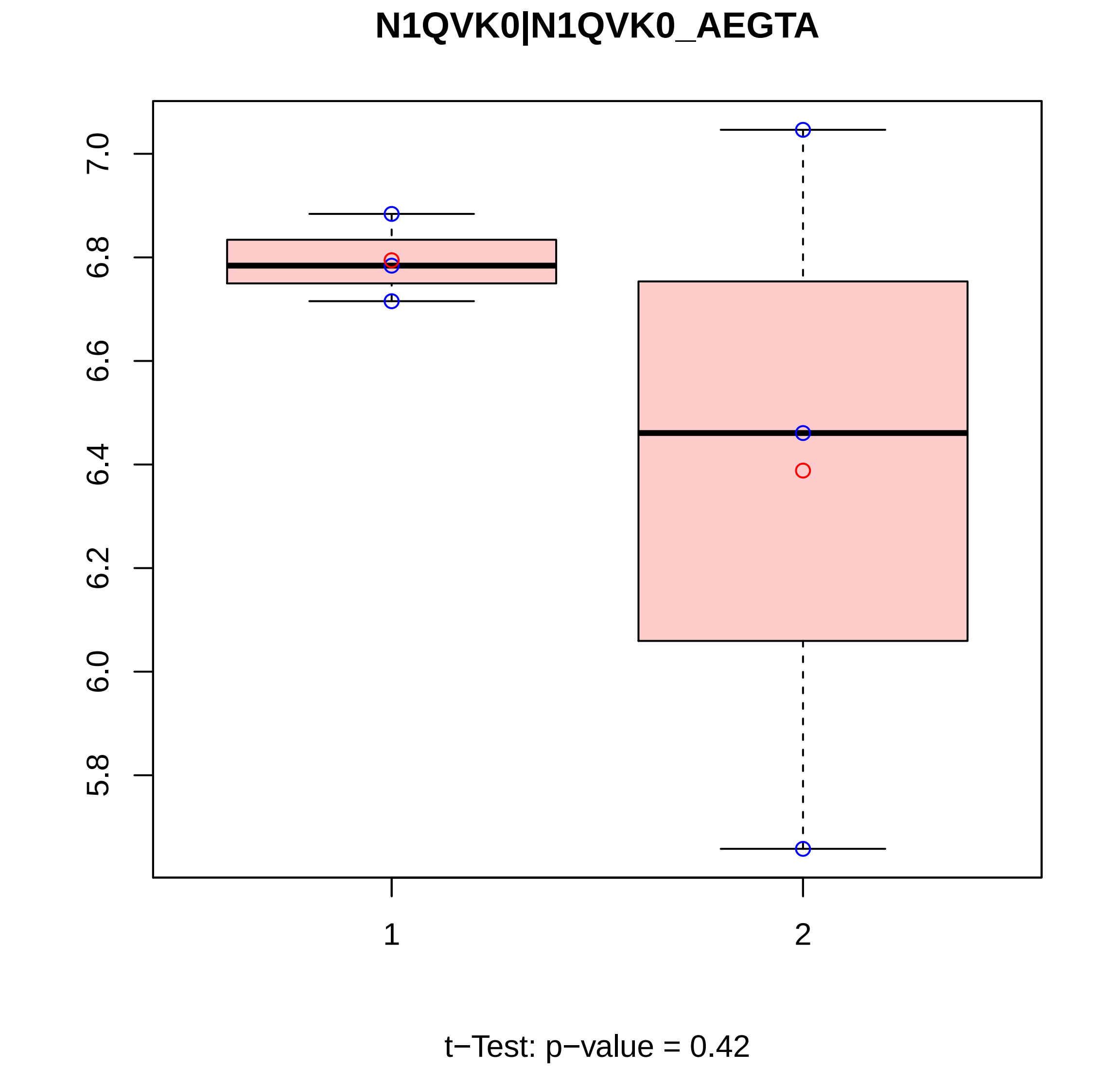
N1QVK0|N1QVK0_AEGTA
7.0
6.8
6.6
6.4
6.2
6.0
5.8
1
2
t−Test: p−value = 0.42

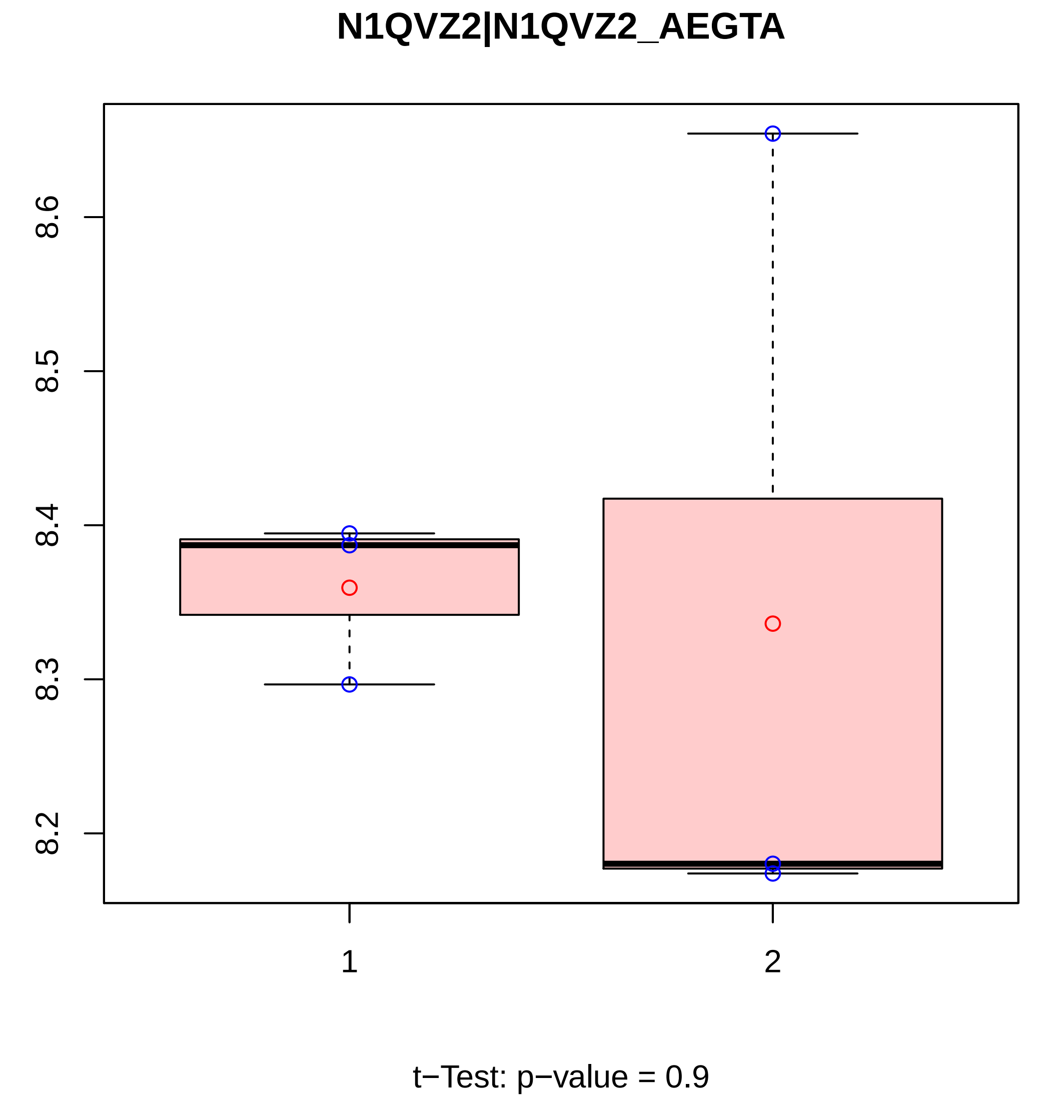

N1QVZ2|N1QVZ2_AEGTA
8.2
8.3
8.4
8.5
8.6
1
2
t−Test: p−value = 0.9

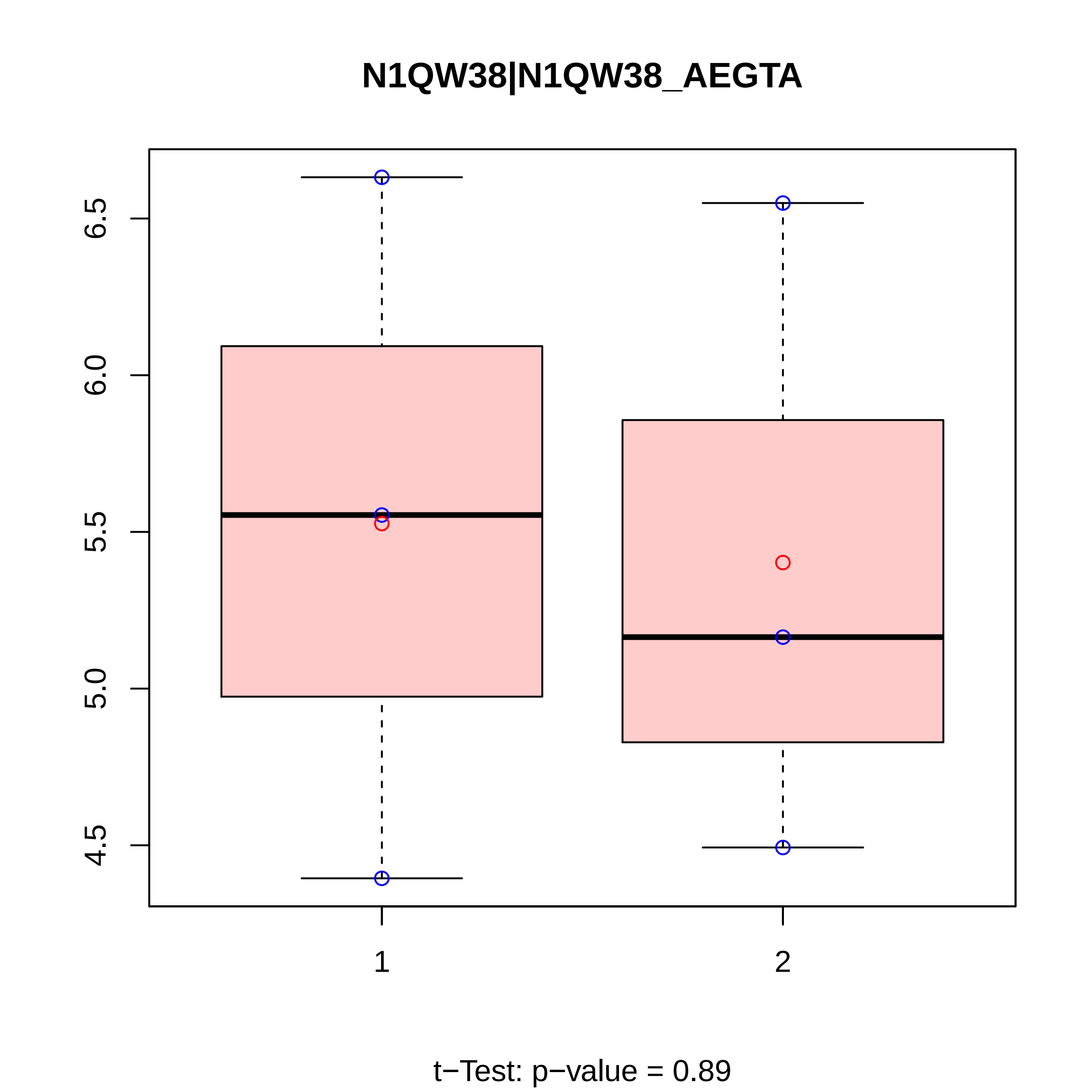
N1QW38|N1QW38_AEGTA
6.5
6.0
5.5
5.0
4.5
1
2
t−Test: p−value = 0.89

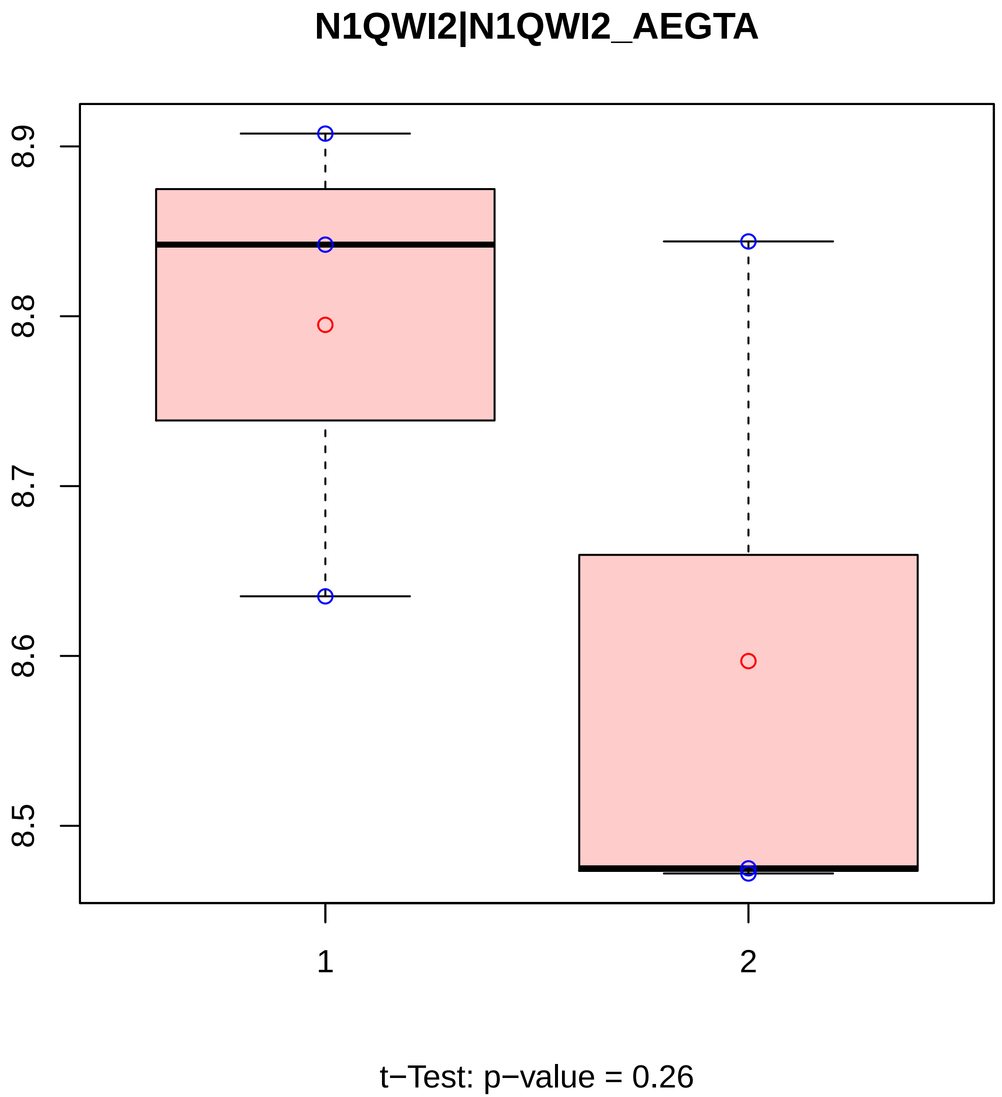
N1QWI2|N1QWI2_AEGTA
8.5
8.6
8.7
8.8
8.9
1
2
t−Test: p−value = 0.26

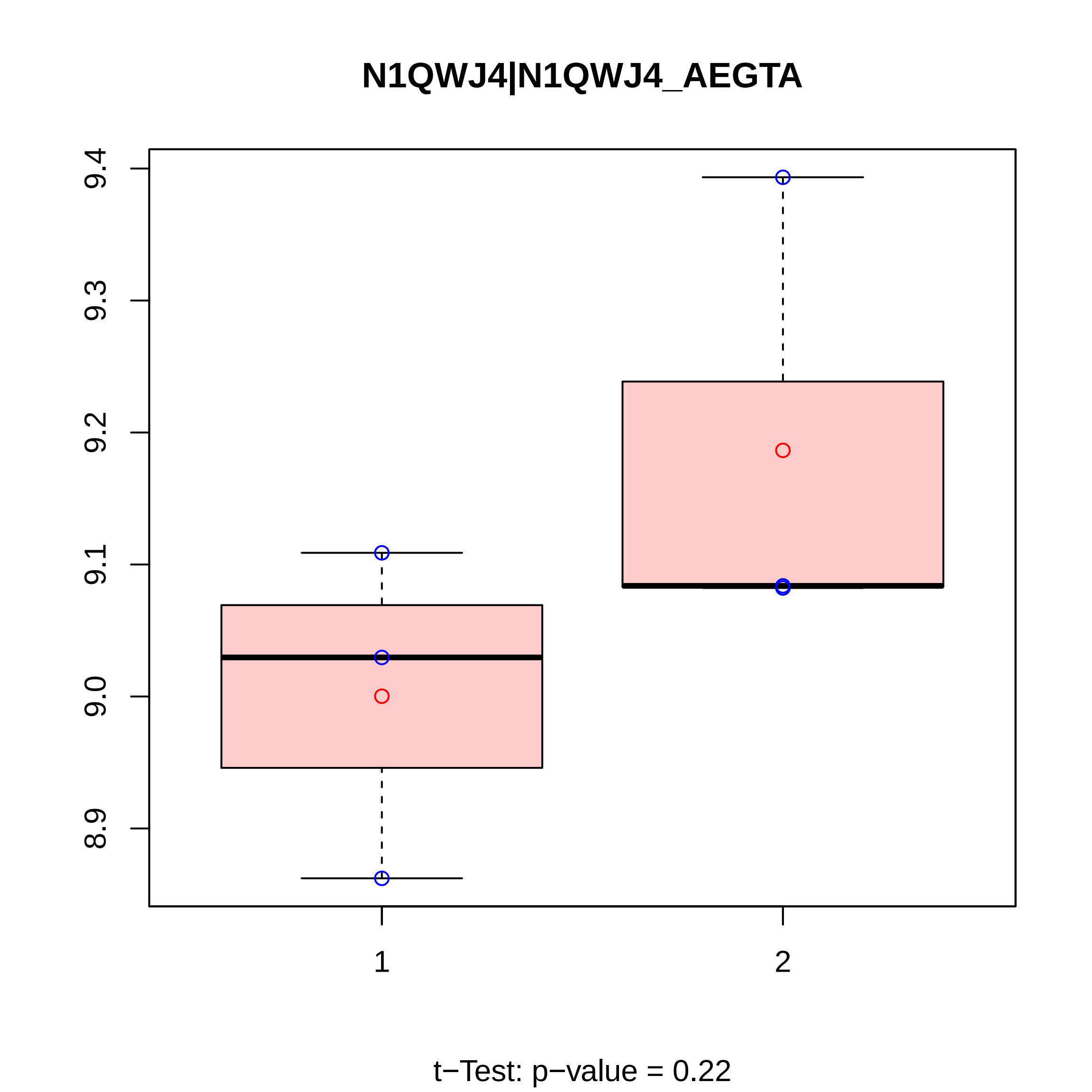

N1QWJ4|N1QWJ4_AEGTA
9.4
9.3
9.2
9.1
9.0
8.9
1
2
t−Test: p−value = 0.22

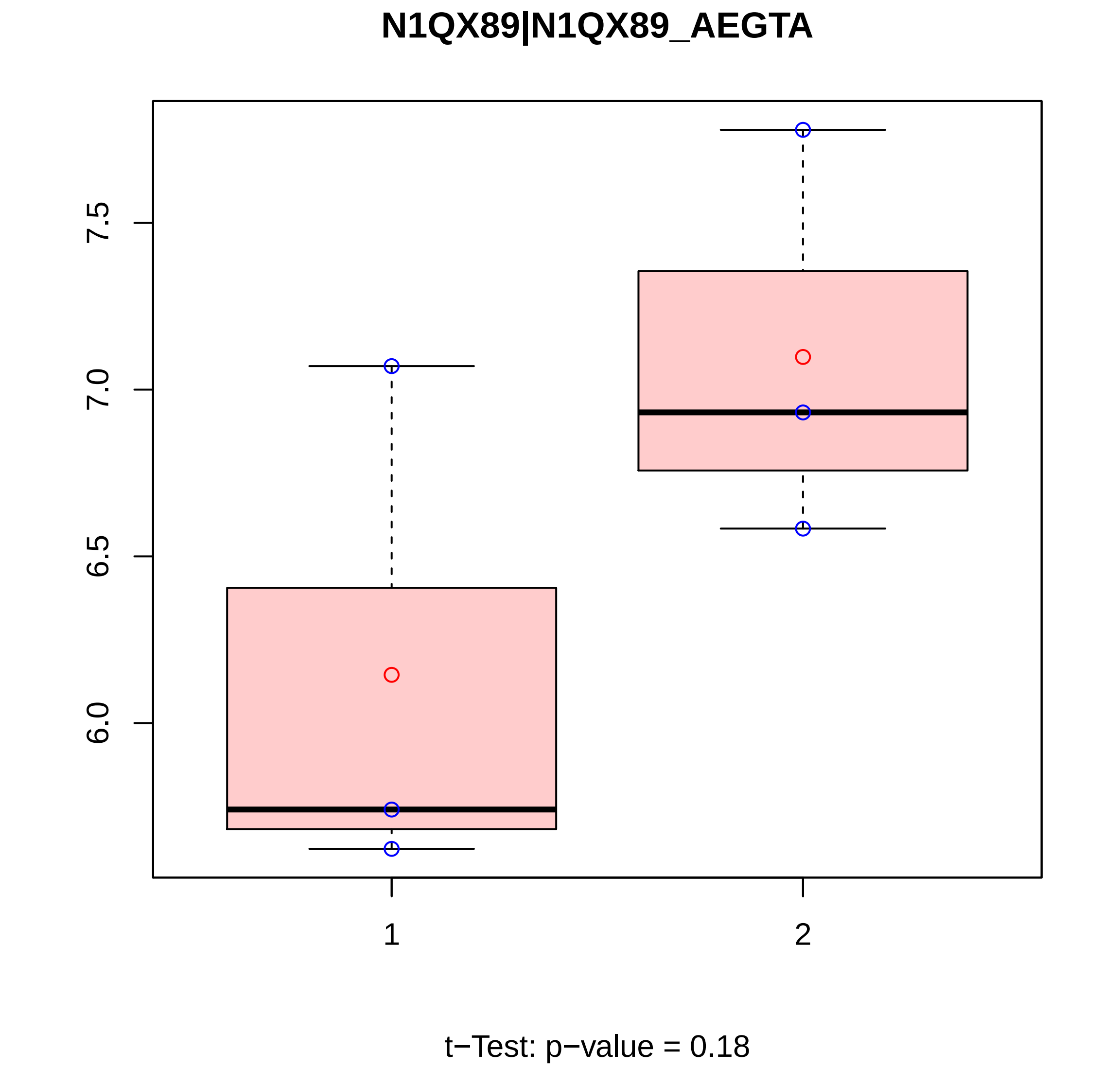
N1QX89|N1QX89_AEGTA
7.5
7.0
6.5
6.0
1
2
t−Test: p−value = 0.18

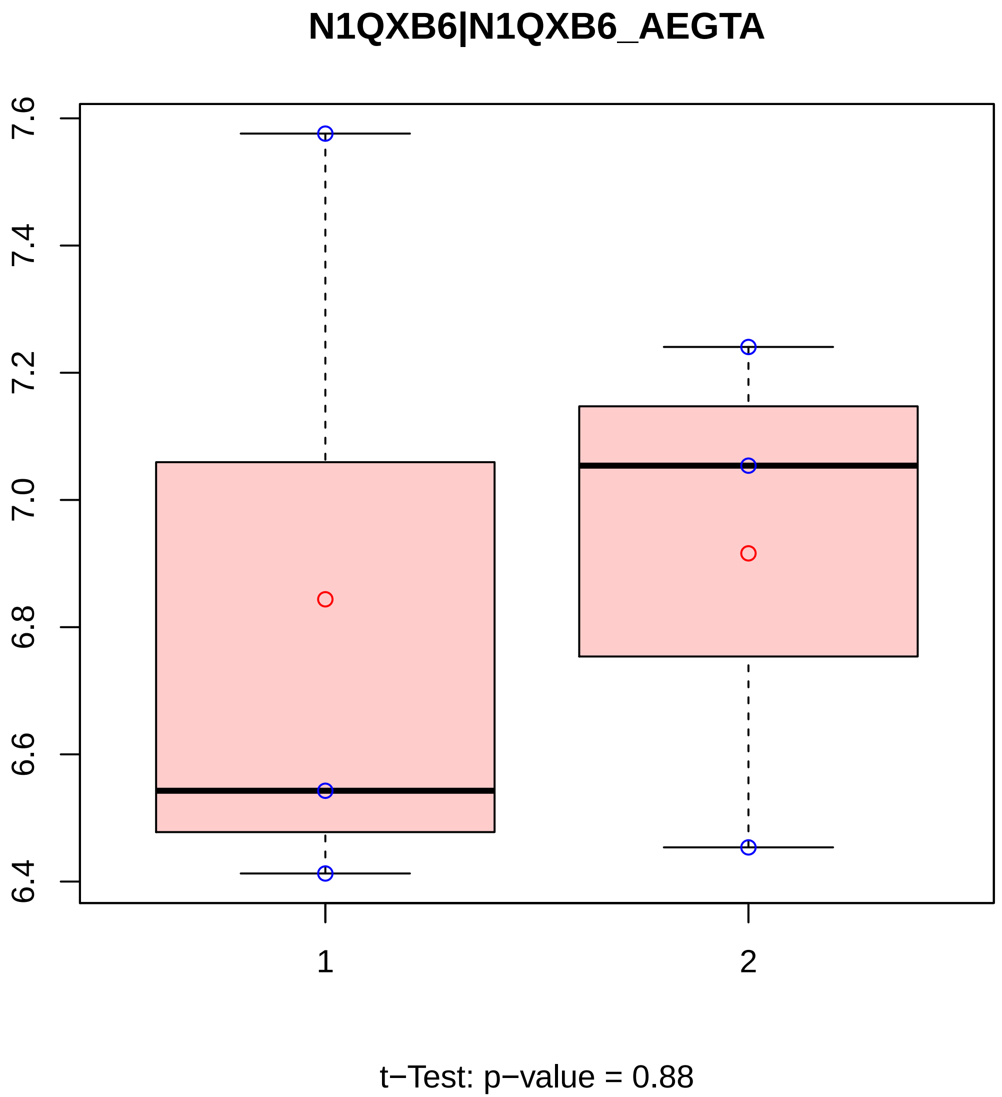
N1QXB6|N1QXB6_AEGTA
7.6
7.4
7.2
7.0
6.8
6.6
6.4
1
2
t−Test: p−value = 0.88

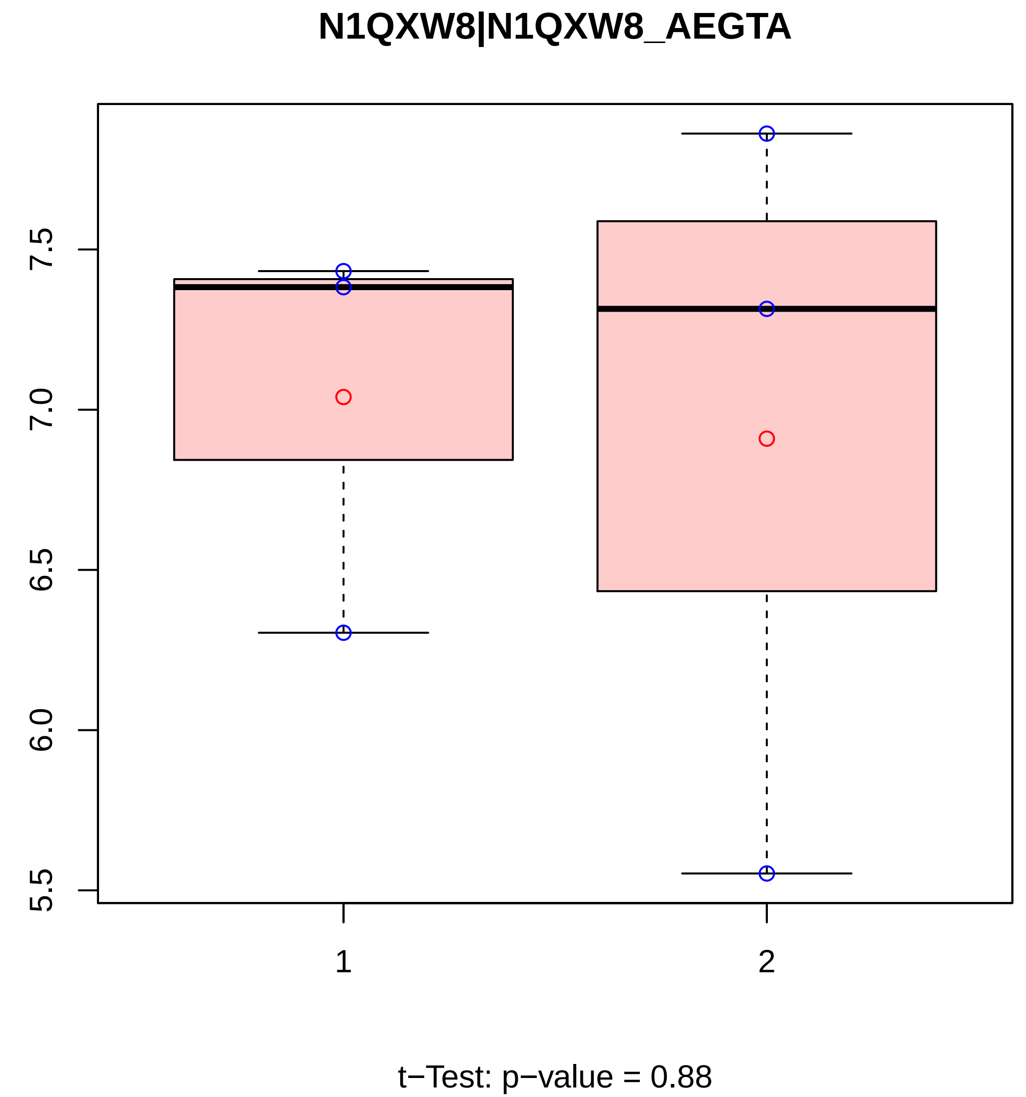

N1QXW8|N1QXW8_AEGTA
7.5
7.0
6.5
6.0
5.5
1
2
t−Test: p−value = 0.88

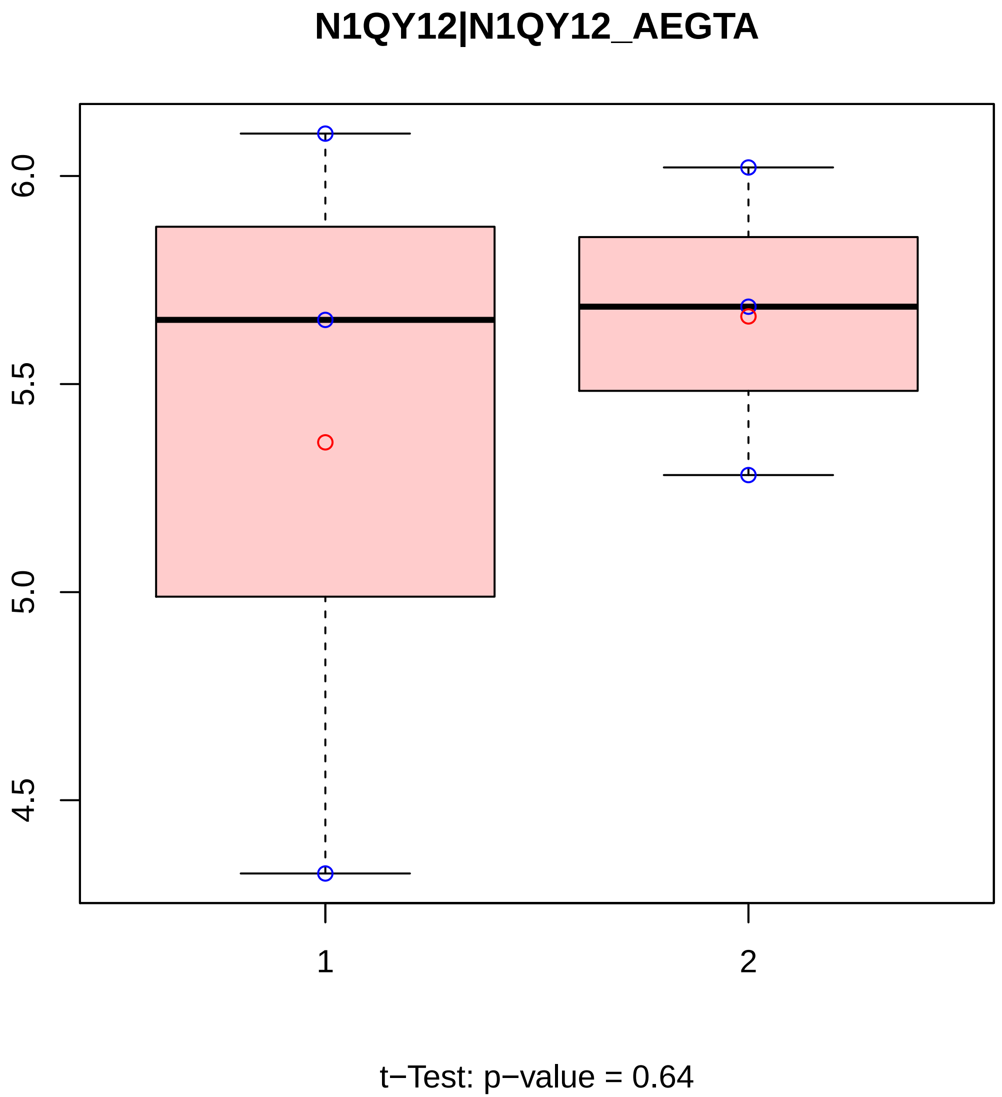

N1QY12|N1QY12_AEGTA
6.0
5.5
5.0
4.5
1
2
t−Test: p−value = 0.64

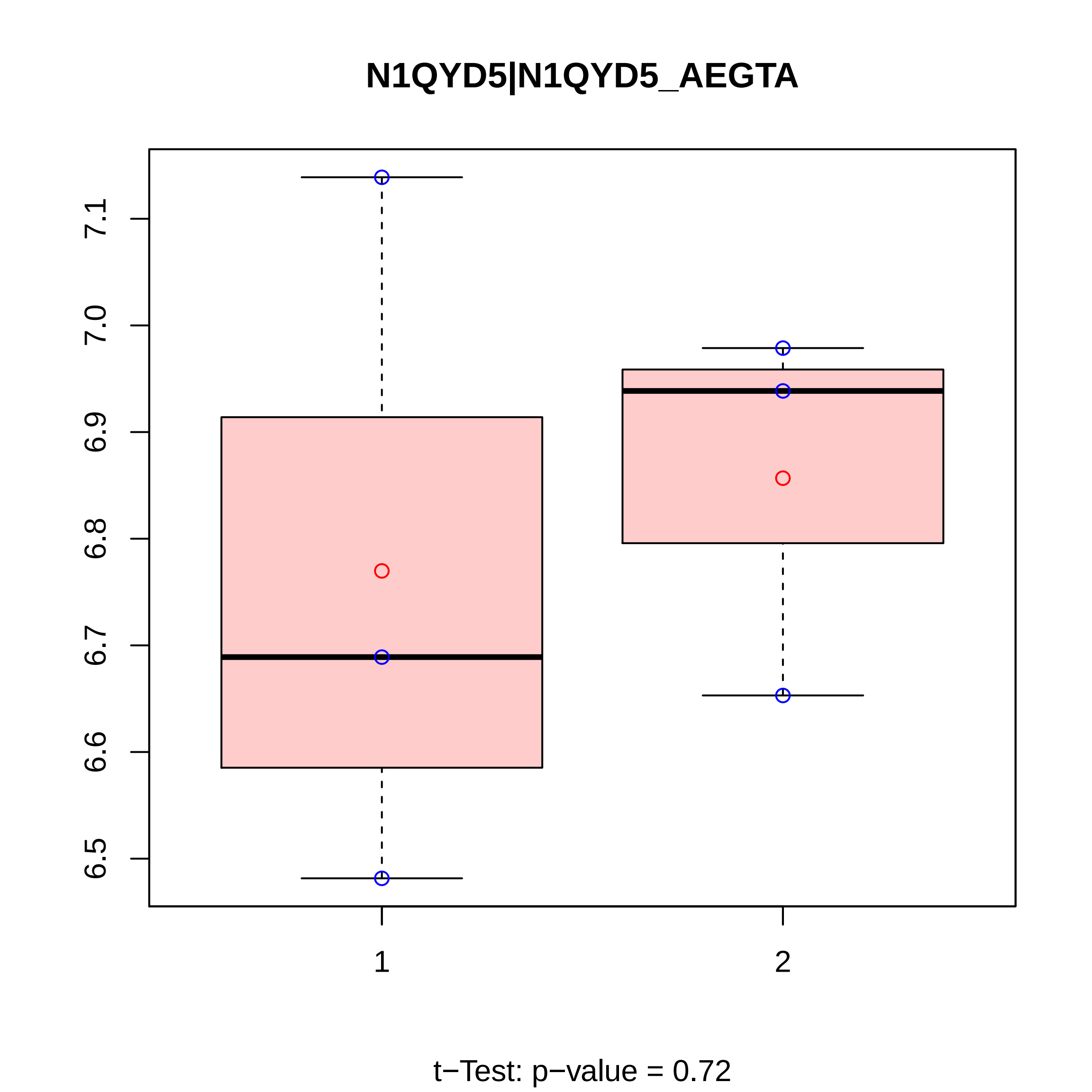
N1QYD5|N1QYD5_AEGTA
7.1
7.0
6.9
6.8
6.7
6.6
6.5
1
2
t−Test: p−value = 0.72

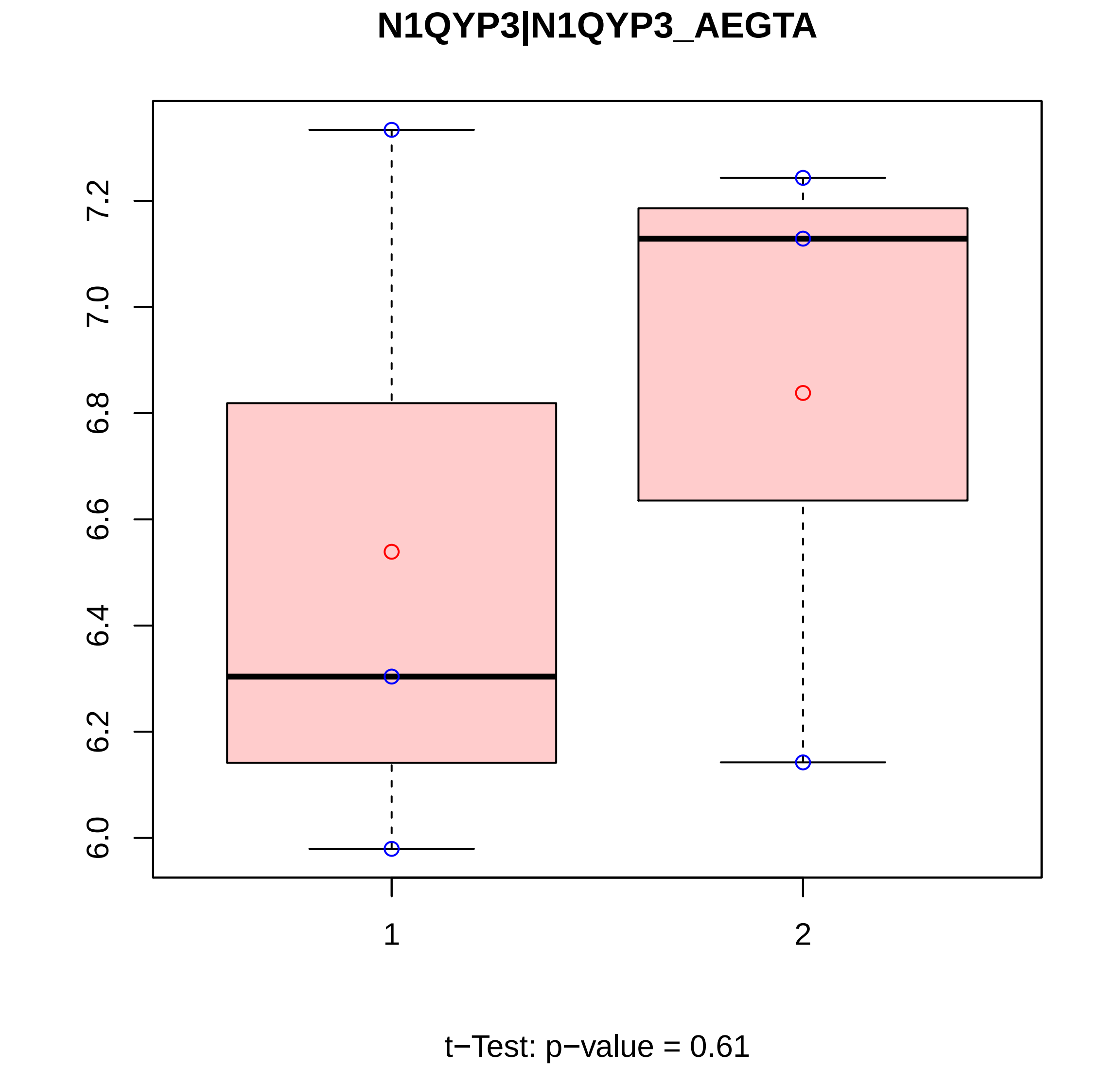

N1QYP3|N1QYP3_AEGTA
6.0
6.2
6.4
6.6
6.8
7.0
7.2
1
2
t−Test: p−value = 0.61

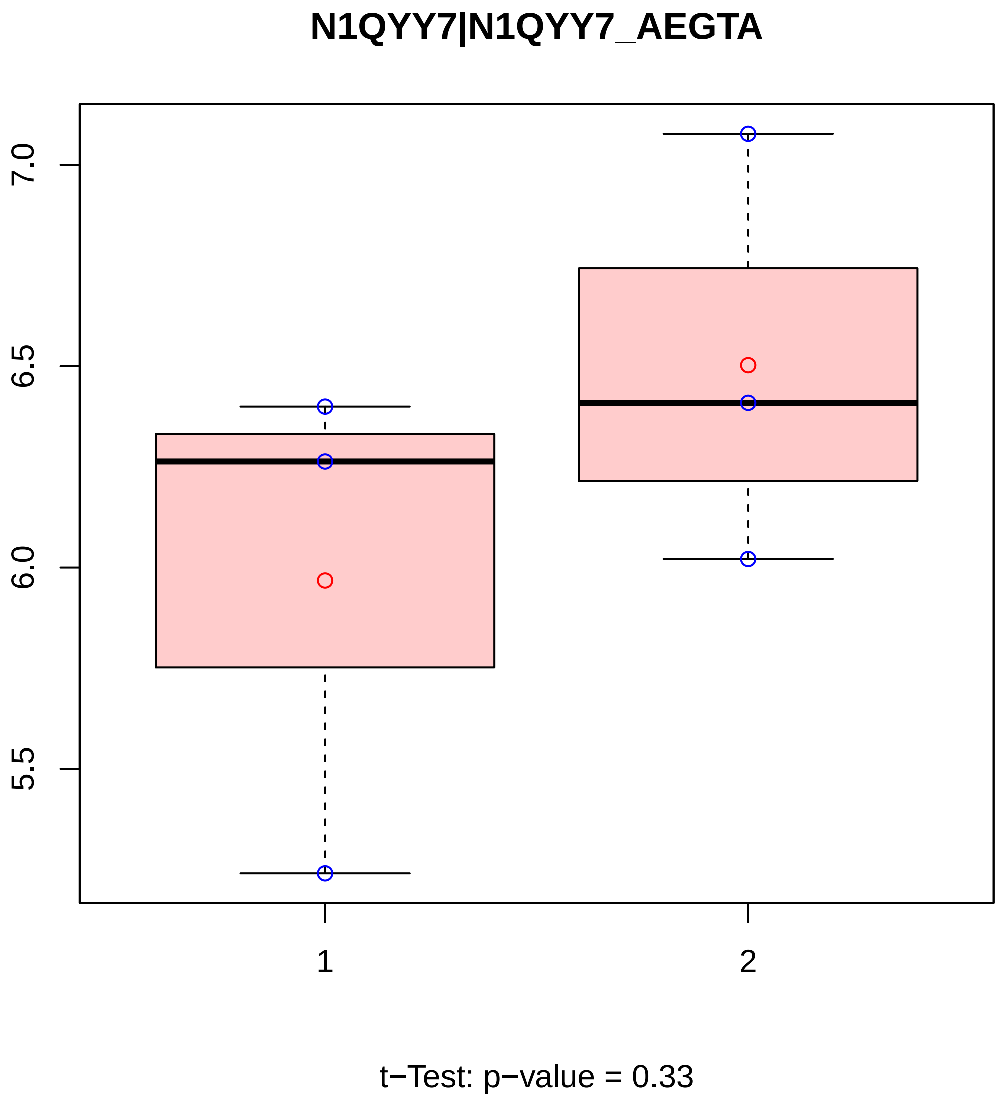
N1QYY7|N1QYY7_AEGTA
7.0
6.5
6.0
5.5
1
2
t−Test: p−value = 0.33

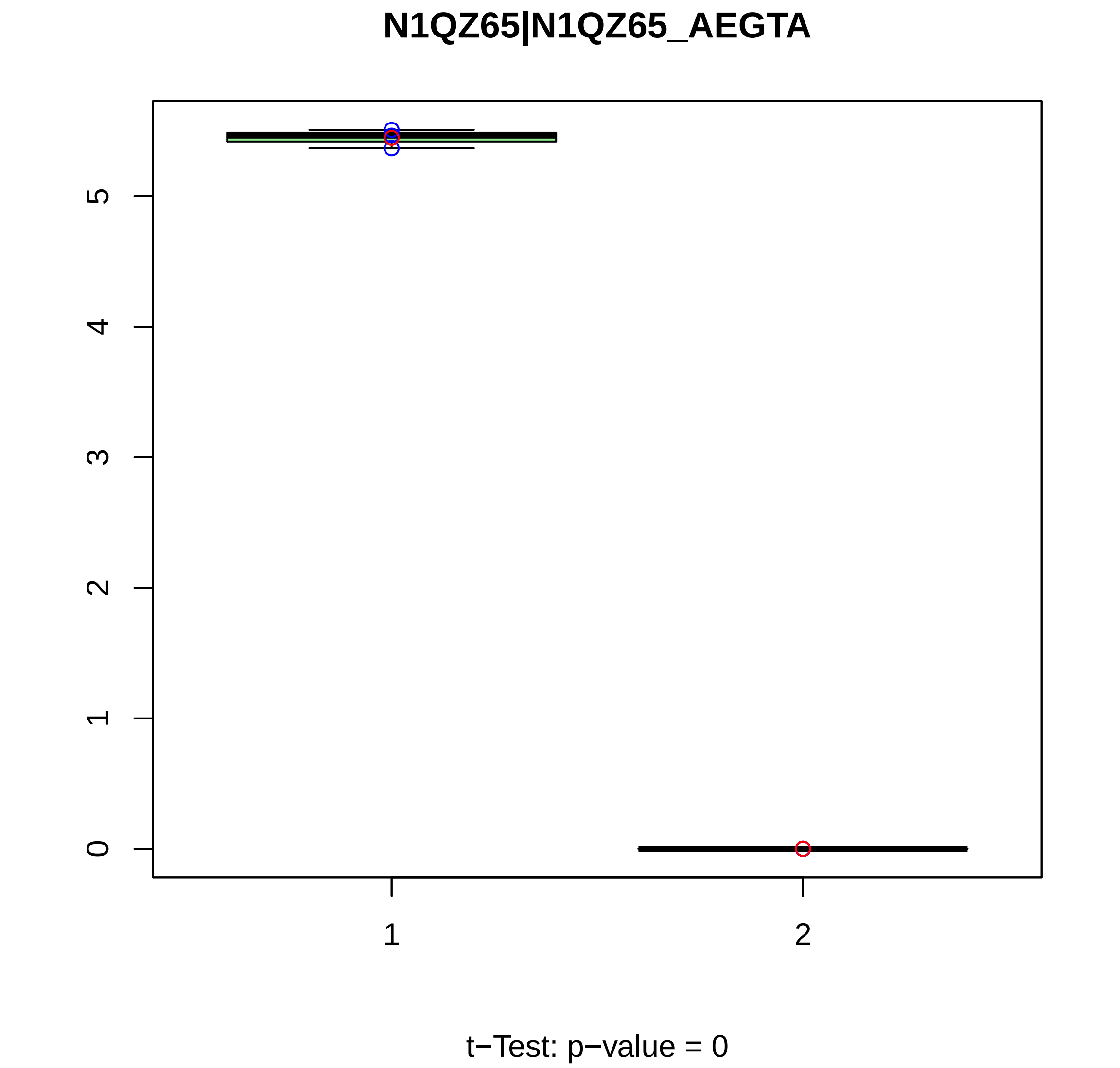
N1QZ65|N1QZ65_AEGTA
0
1
2
3
4
5
1
2
t−Test: p−value = 0

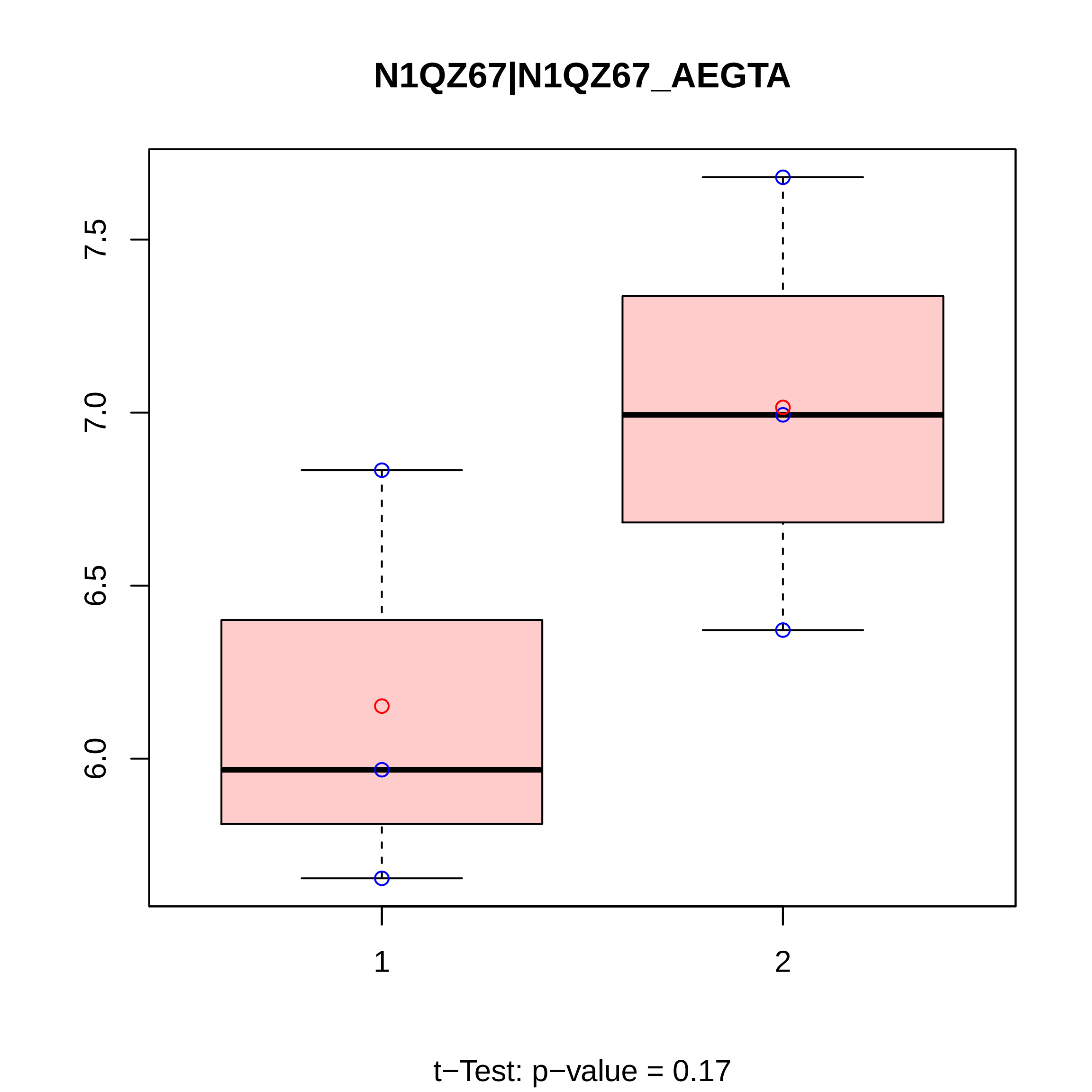
N1QZ67|N1QZ67_AEGTA
7.5
7.0
6.5
6.0
1
2
t−Test: p−value = 0.17

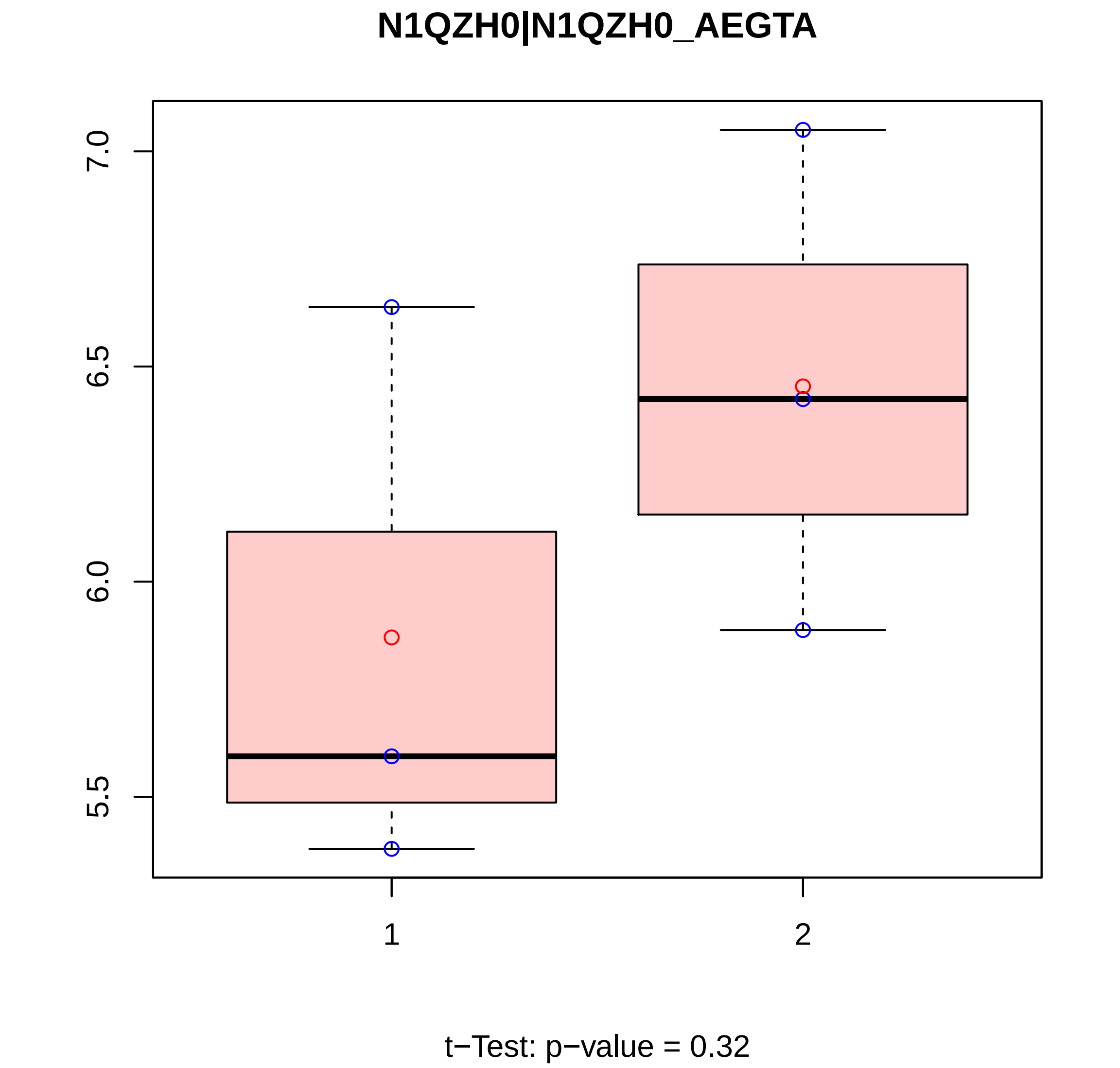
N1QZH0|N1QZH0_AEGTA
7.0
6.5
6.0
5.5
1
2
t−Test: p−value = 0.32

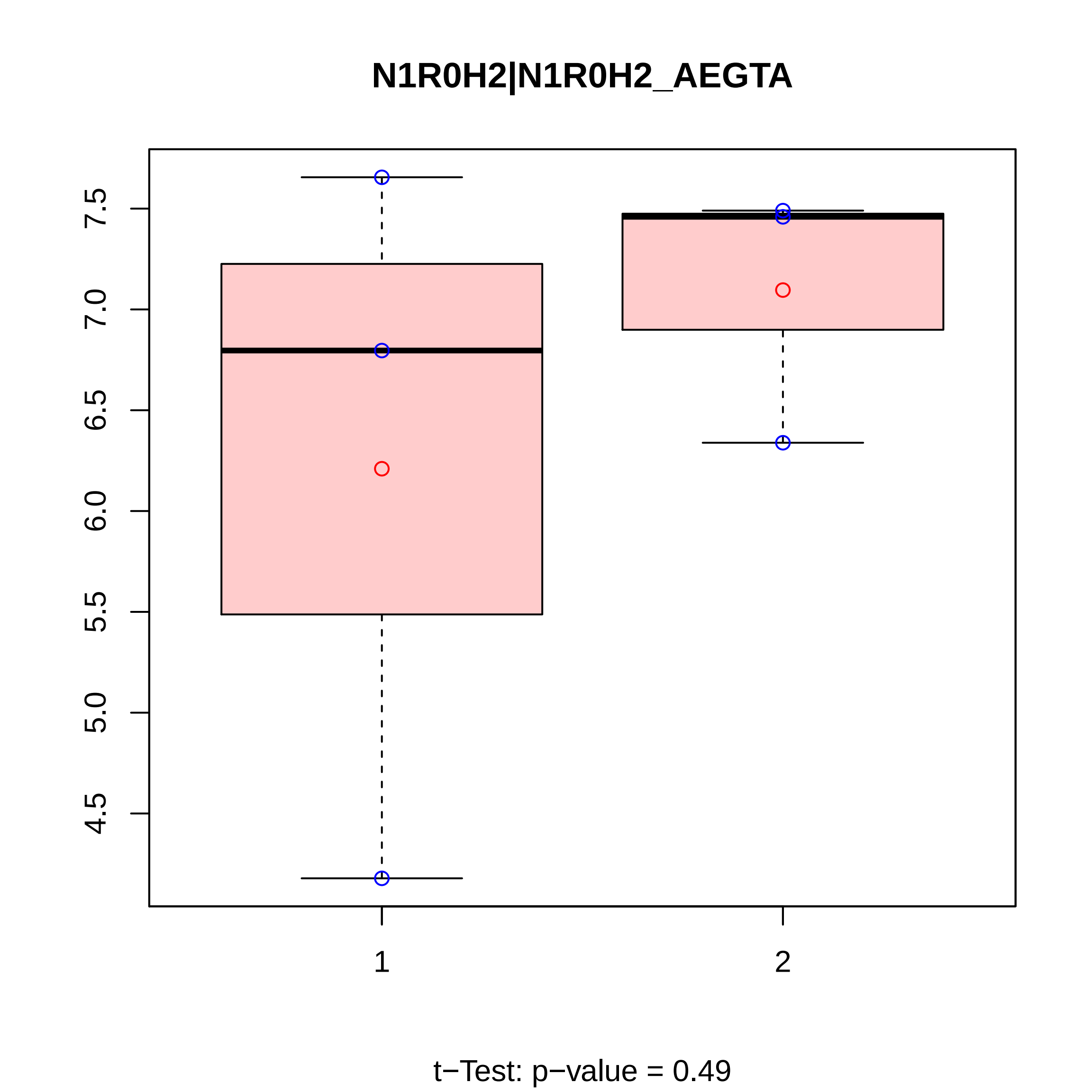

N1R0H2|N1R0H2_AEGTA
7.5
7.0
6.5
6.0
5.5
5.0
4.5
1
2
t−Test: p−value = 0.49

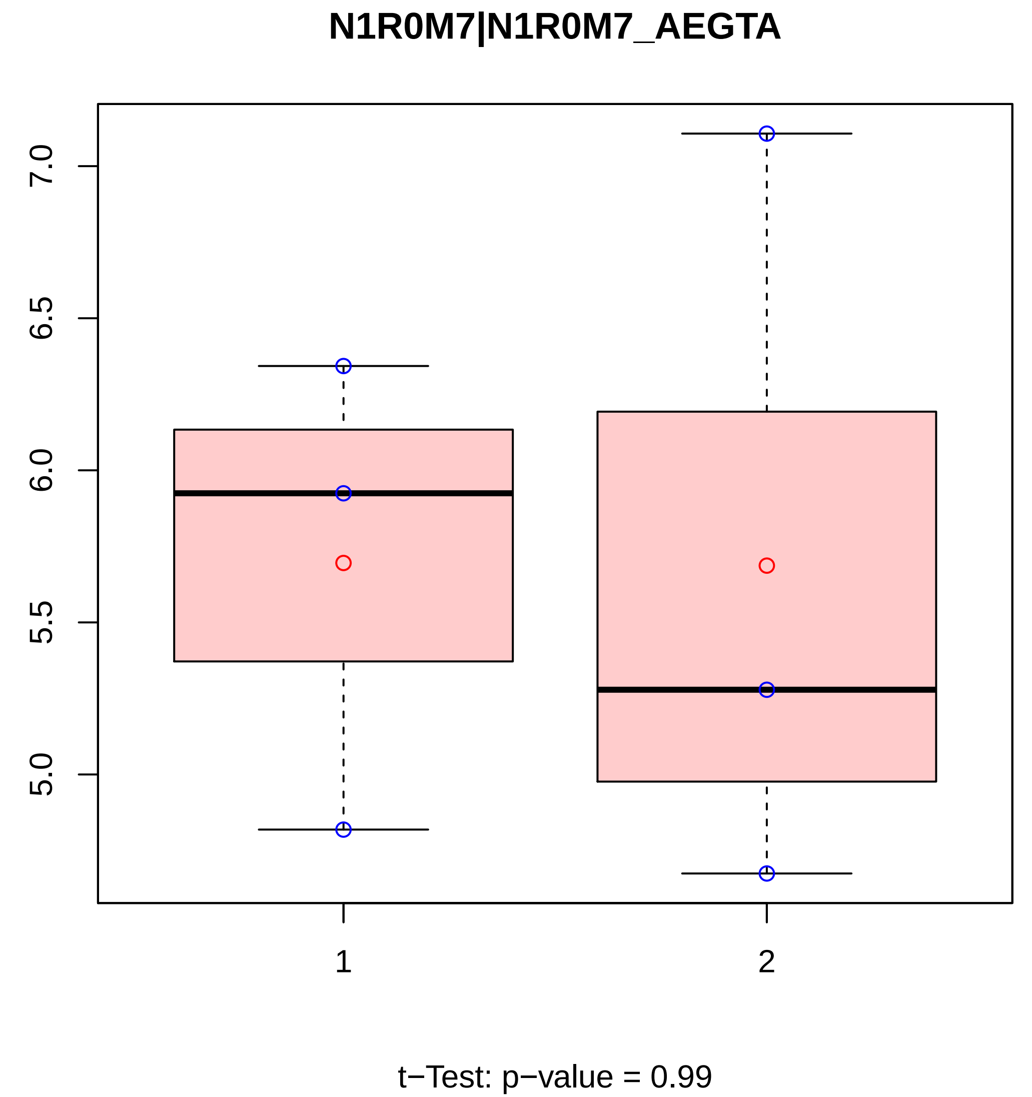
N1R0M7|N1R0M7_AEGTA
7.0
6.5
6.0
5.5
5.0
1
2
t−Test: p−value = 0.99

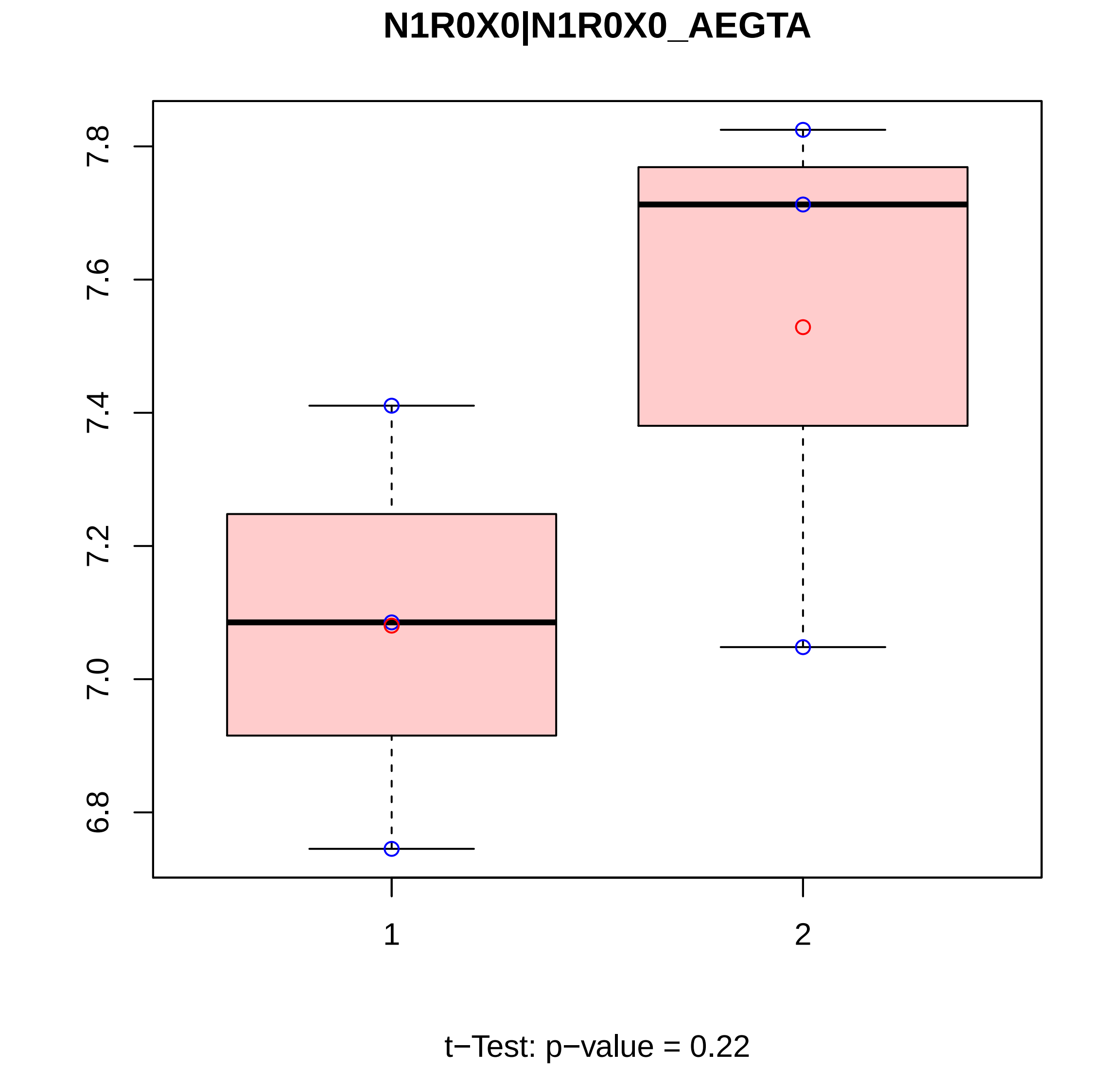

N1R0X0|N1R0X0_AEGTA
6.8
7.0
7.2
7.4
7.6
7.8
1
2
t−Test: p−value = 0.22

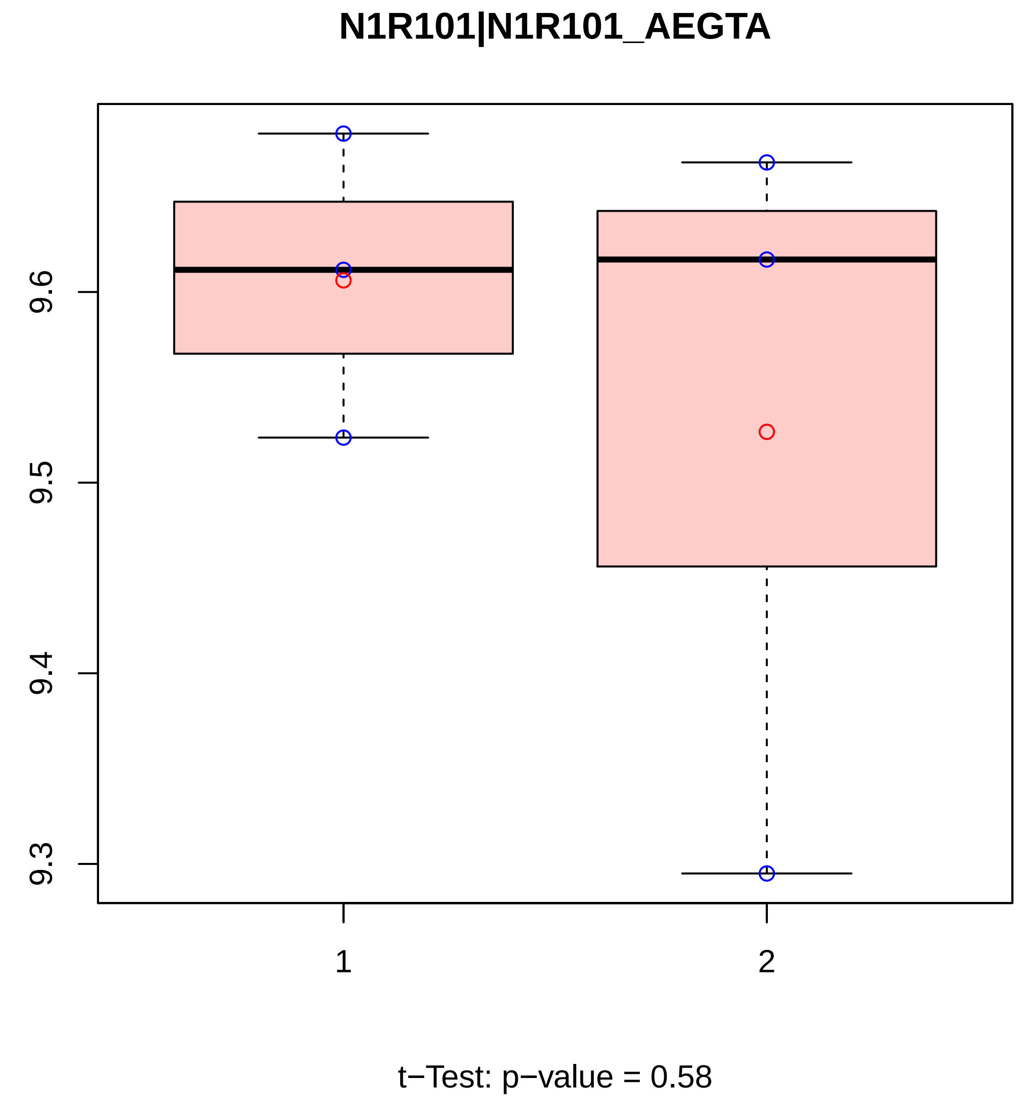
N1R101|N1R101_AEGTA
9.3
9.4
9.5
9.6
1
2
t−Test: p−value = 0.58

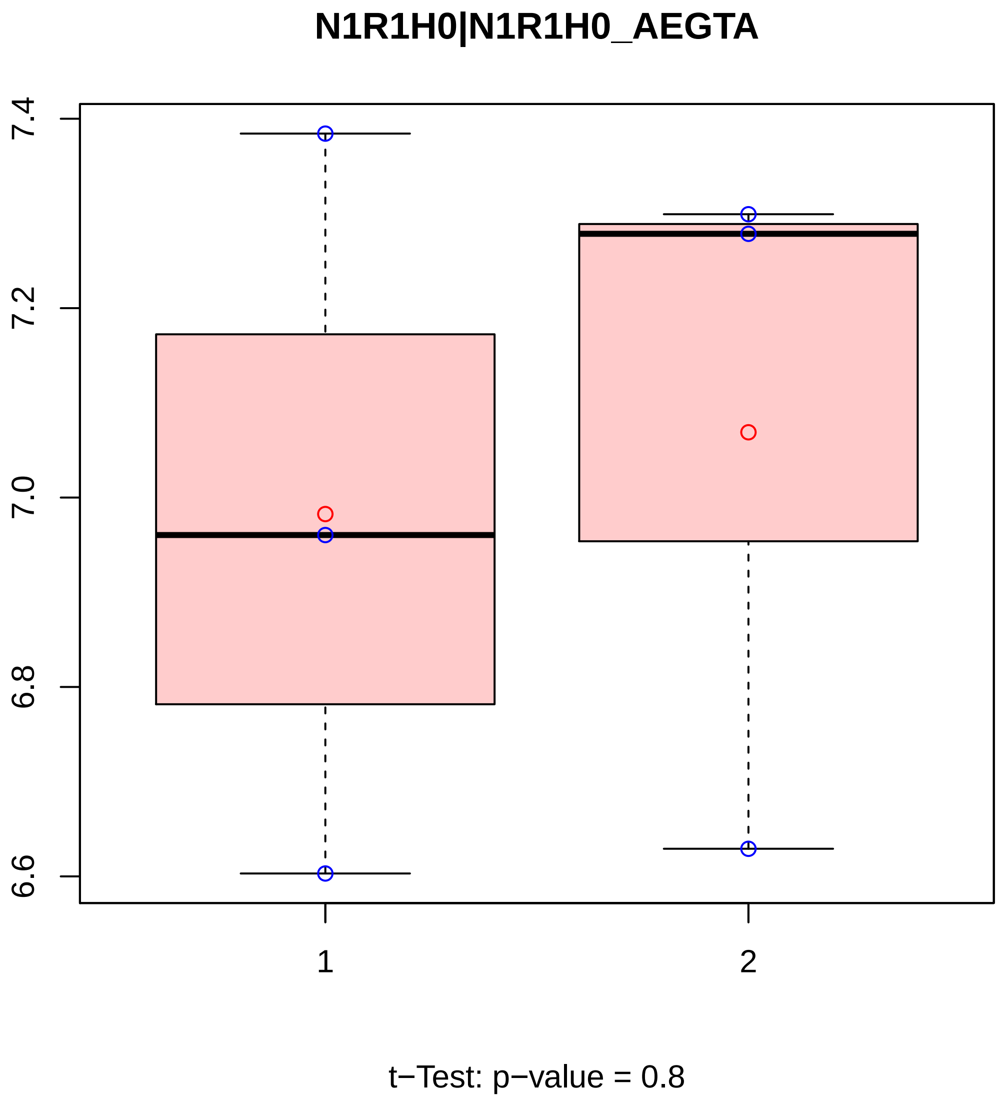
N1R1H0|N1R1H0_AEGTA
7.4
7.2
7.0
6.8
6.6
1
2
t−Test: p−value = 0.8

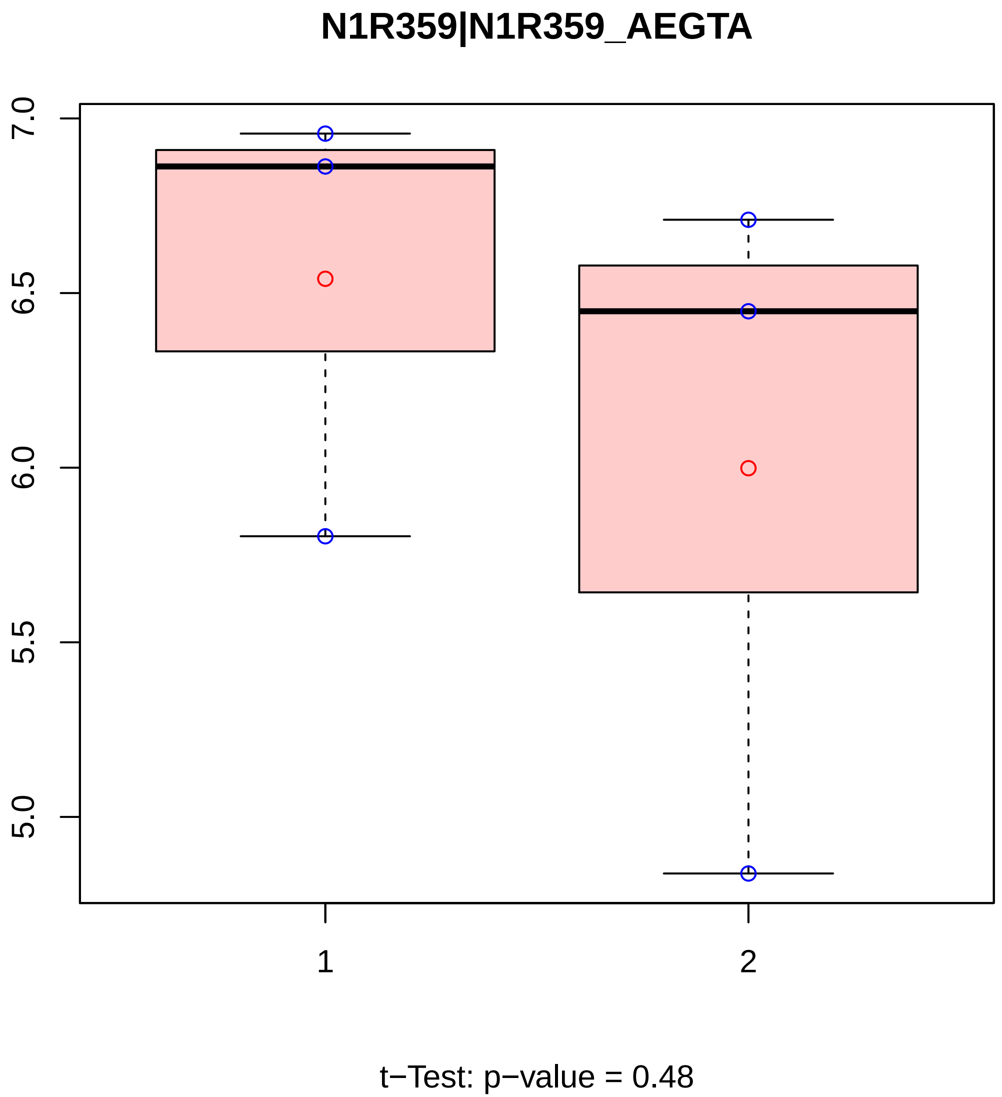
N1R359|N1R359_AEGTA
7.0
6.5
6.0
5.5
5.0
1
2
t−Test: p−value = 0.48

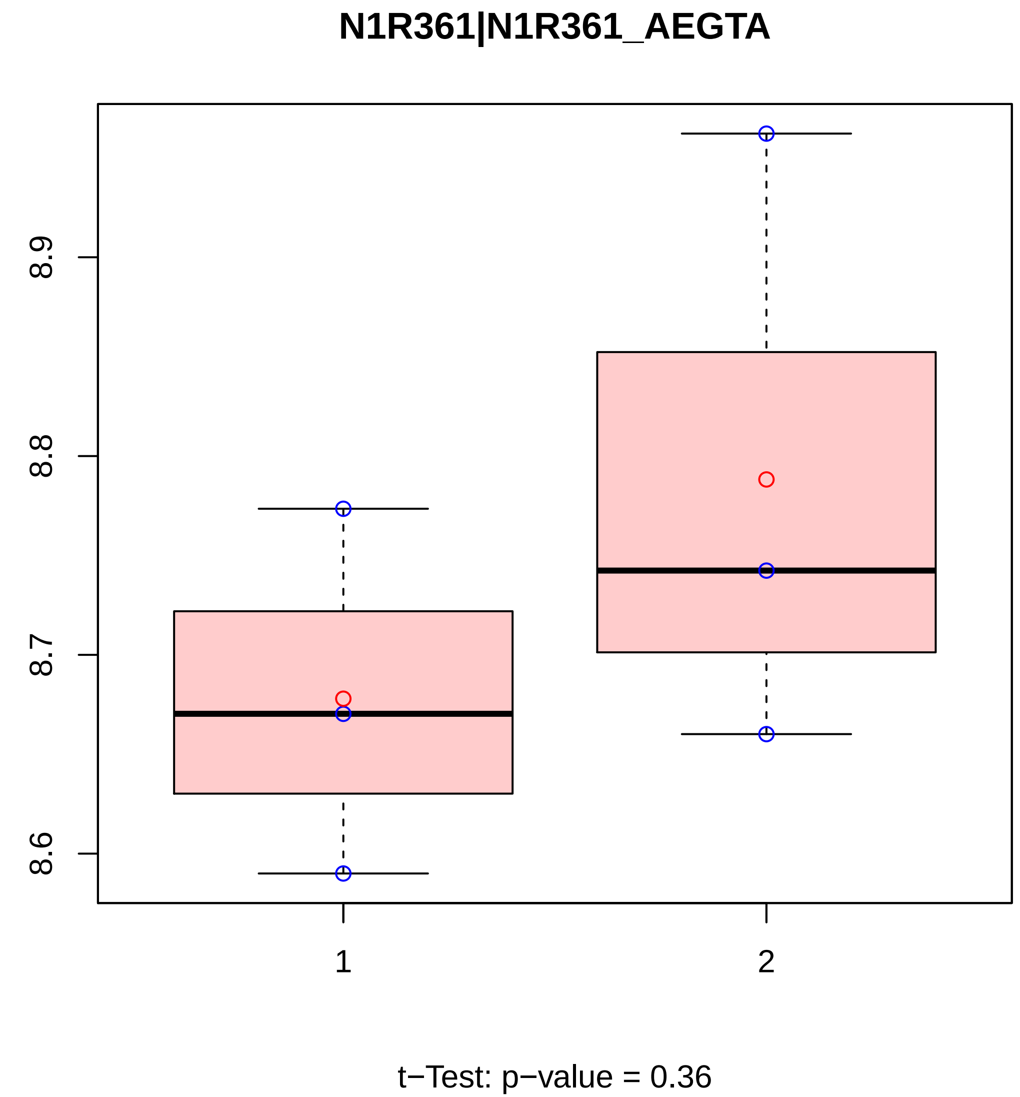
N1R361|N1R361_AEGTA
8.6
8.7
8.8
8.9
1
2
t−Test: p−value = 0.36

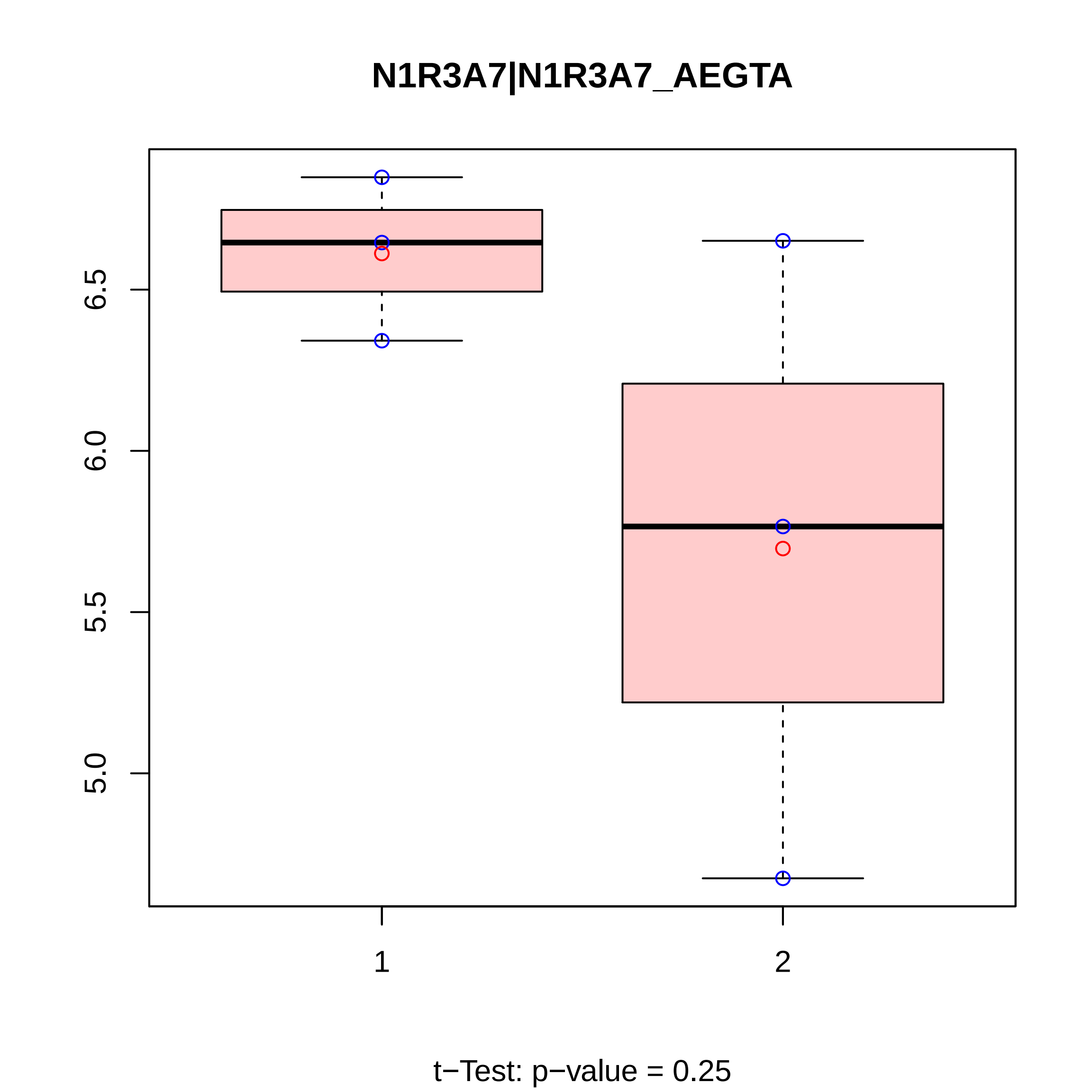
N1R3A7|N1R3A7_AEGTA
6.5
6.0
5.5
5.0
1
2
t−Test: p−value = 0.25

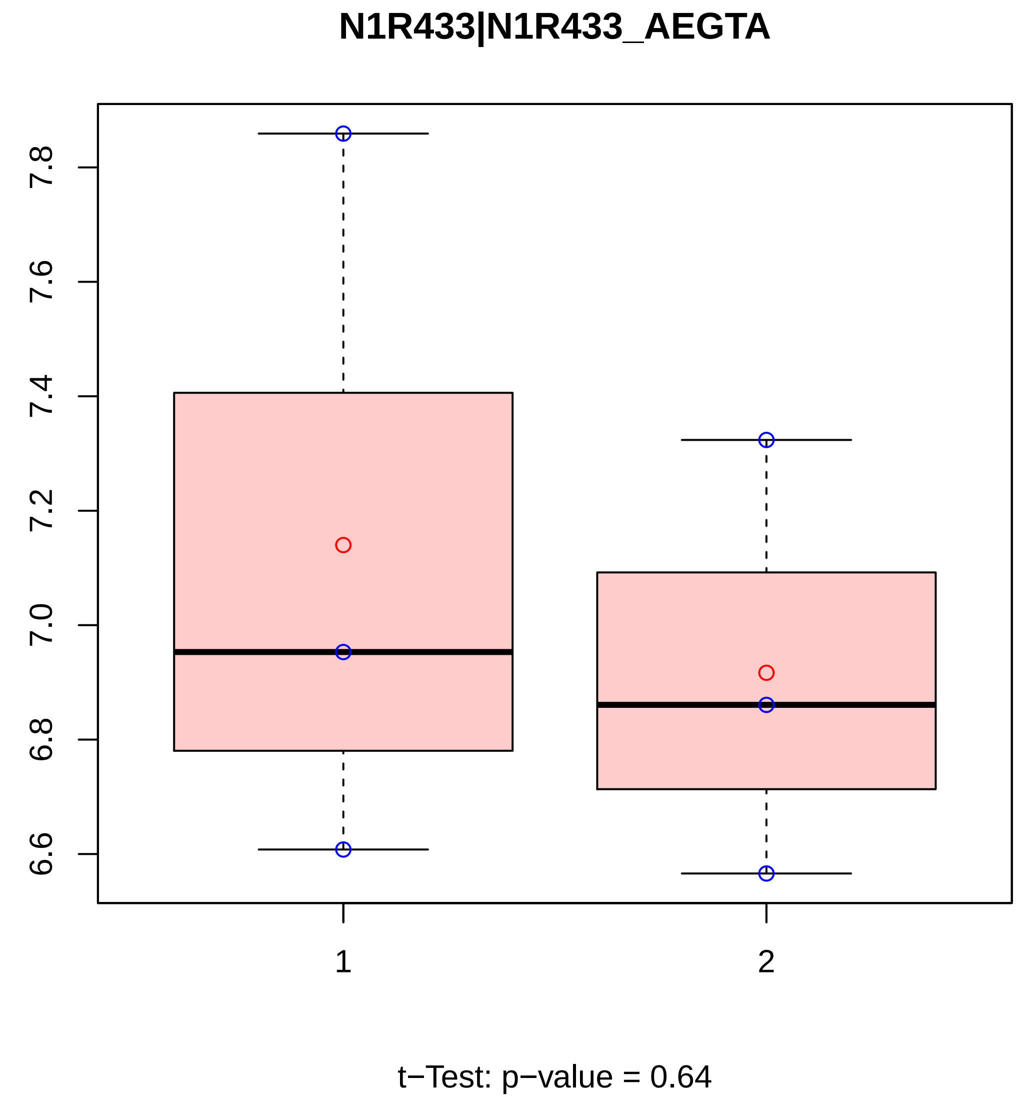
N1R433|N1R433_AEGTA
7.8
7.6
7.4
7.2
7.0
6.8
6.6
1
2
t−Test: p−value = 0.64

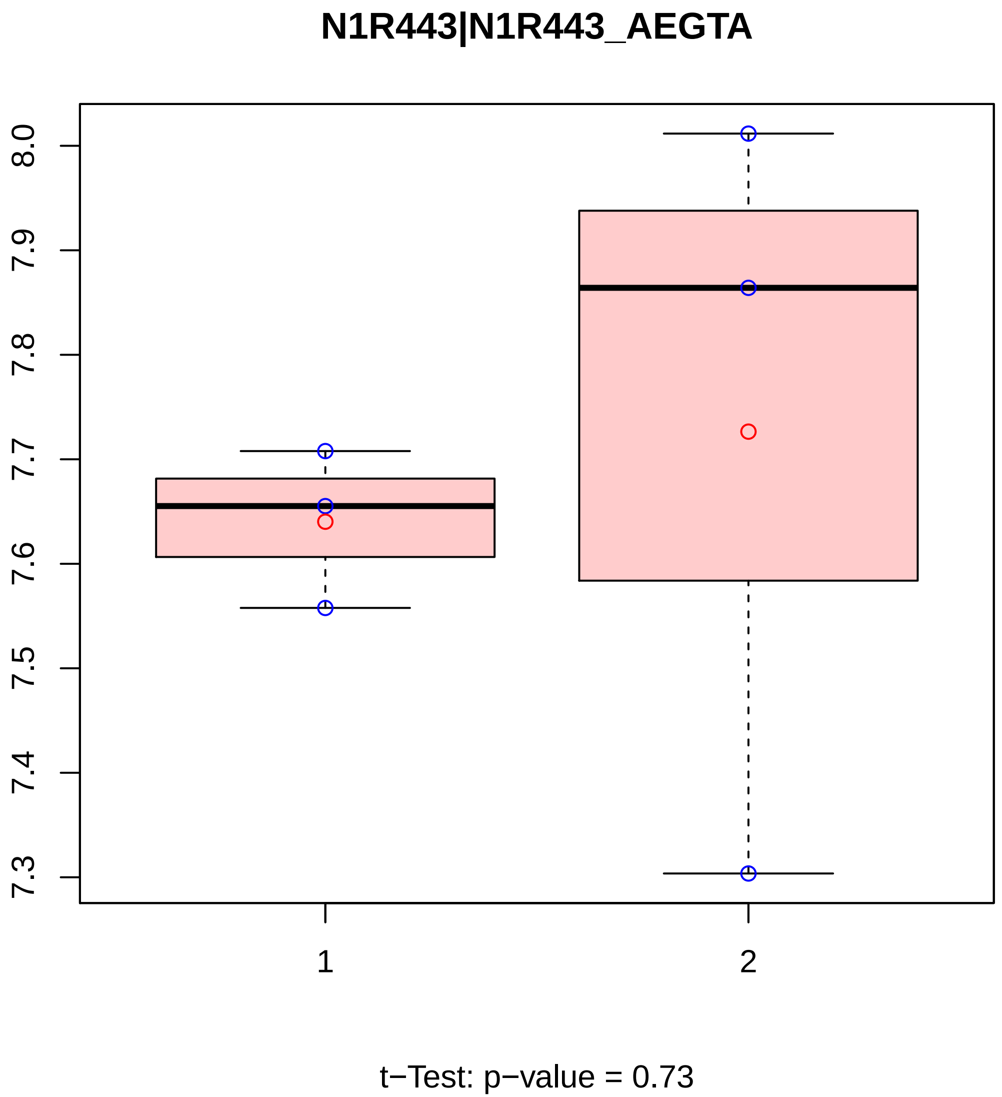
N1R443|N1R443_AEGTA
8.0
7.9
7.8
7.7
7.6
7.5
7.4
7.3
1
2
t−Test: p−value = 0.73

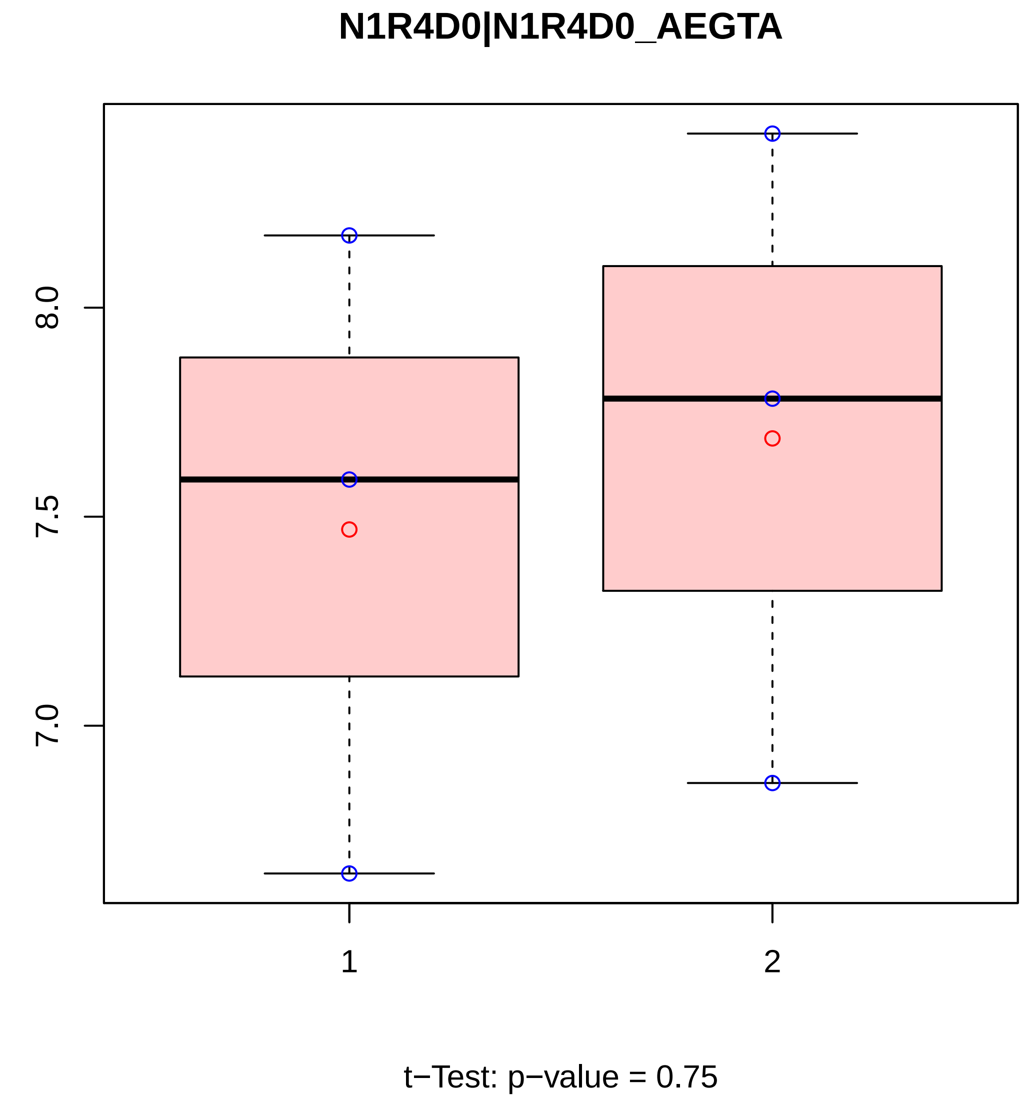
N1R4D0|N1R4D0_AEGTA
8.0
7.5
7.0
1
2
t−Test: p−value = 0.75

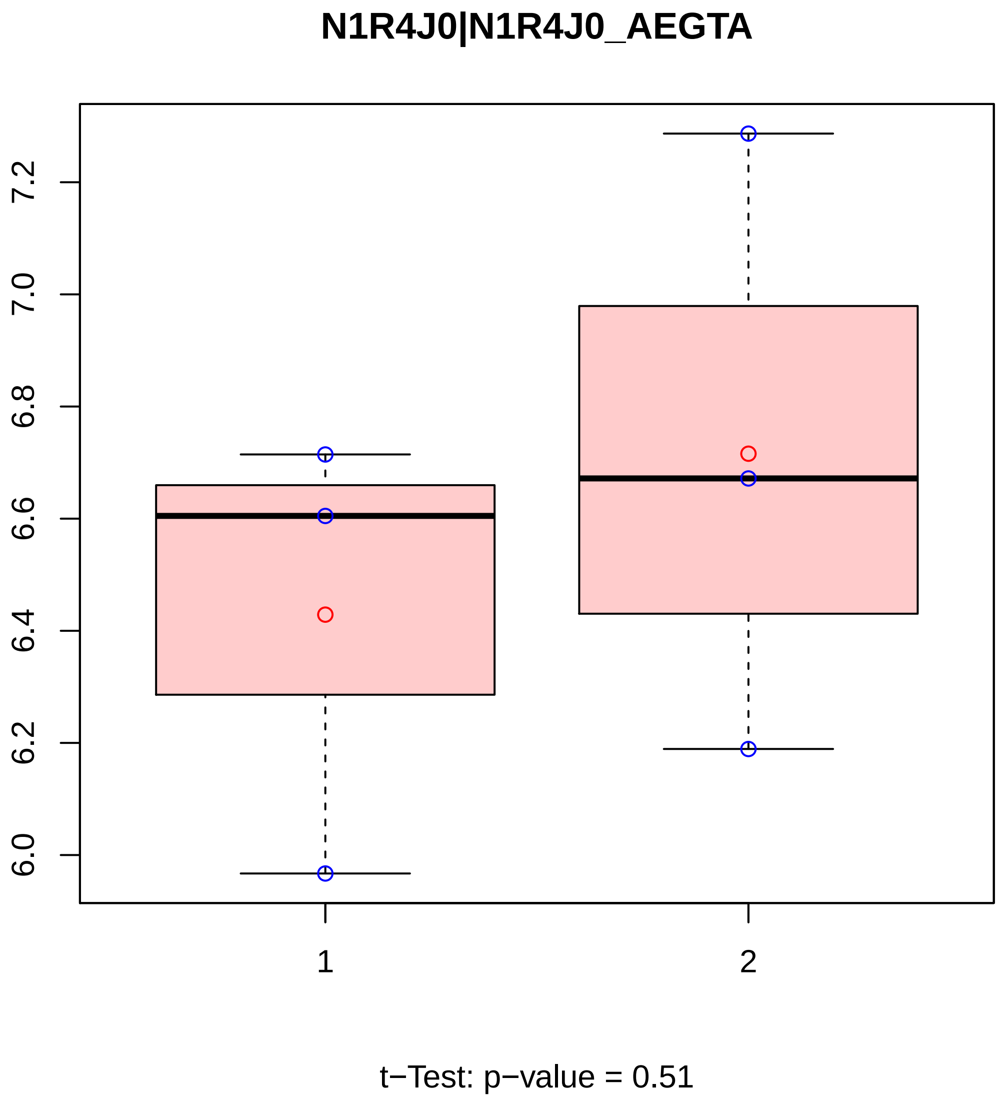
N1R4J0|N1R4J0_AEGTA
7.2
7.0
6.8
6.6
6.4
6.2
6.0
1
2
t−Test: p−value = 0.51

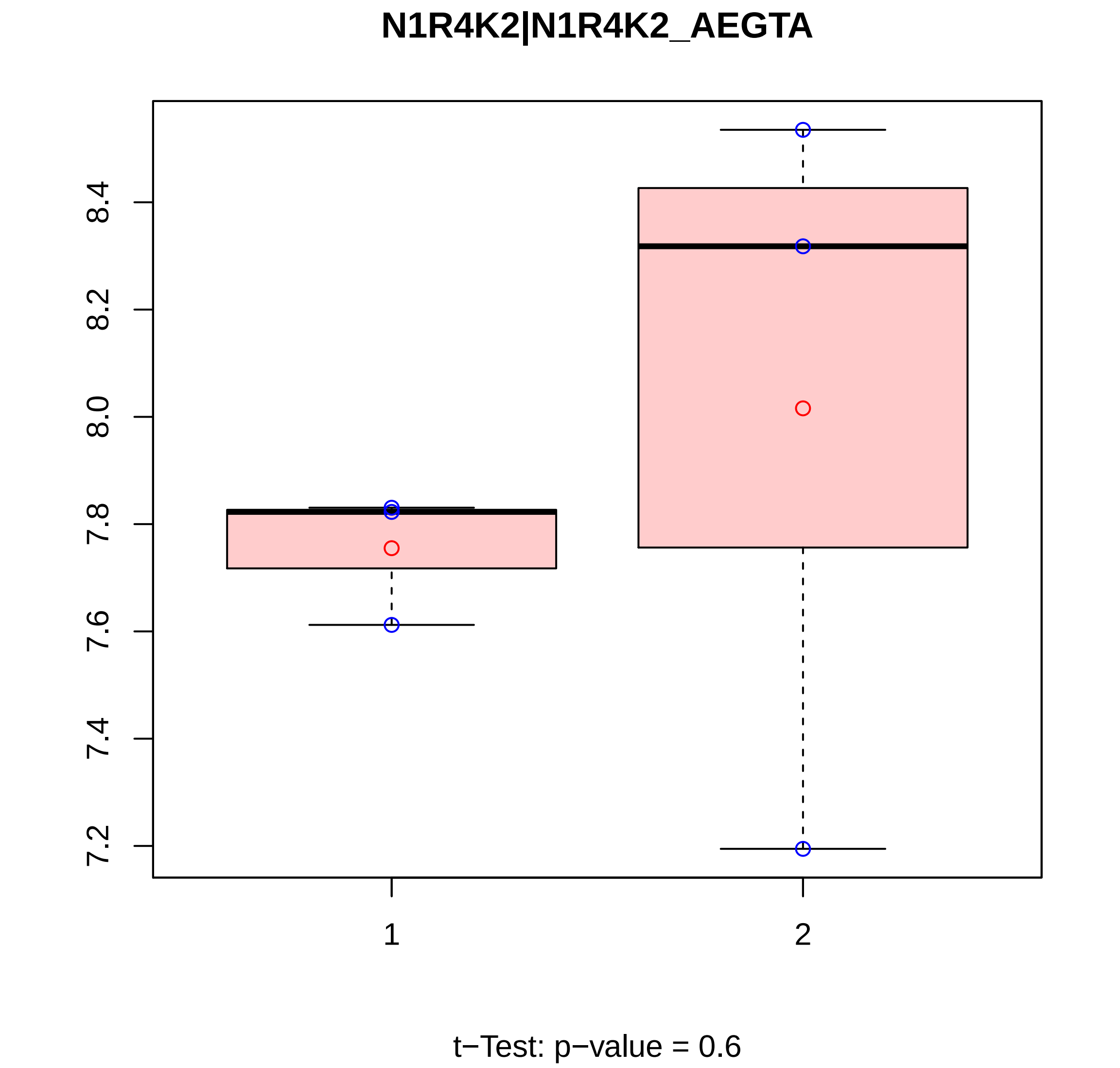
N1R4K2|N1R4K2_AEGTA
8.4
8.2
8.0
7.8
7.6
7.4
7.2
1
2
t−Test: p−value = 0.6

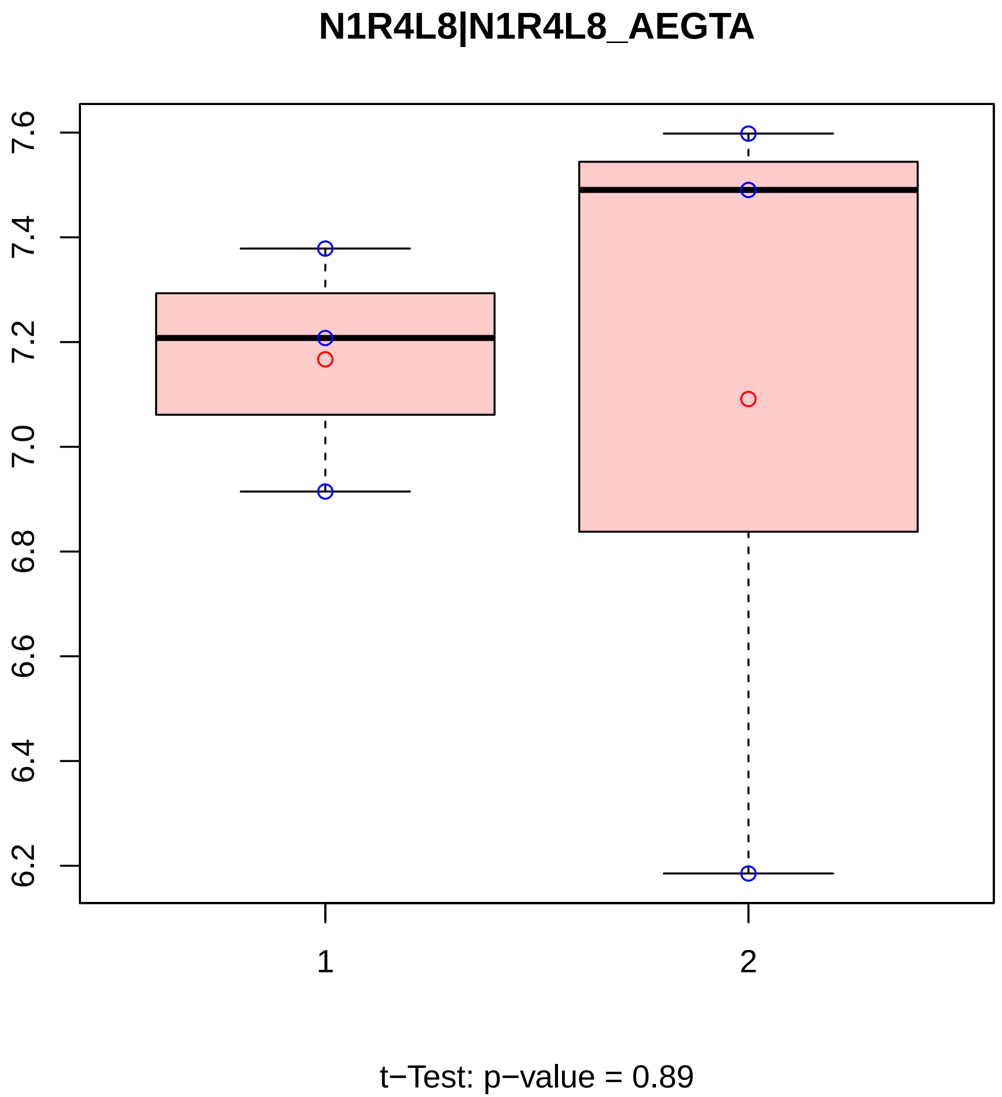

N1R4L8|N1R4L8_AEGTA
7.6
7.4
7.2
7.0
6.8
6.6
6.4
6.2
1
2
t−Test: p−value = 0.89

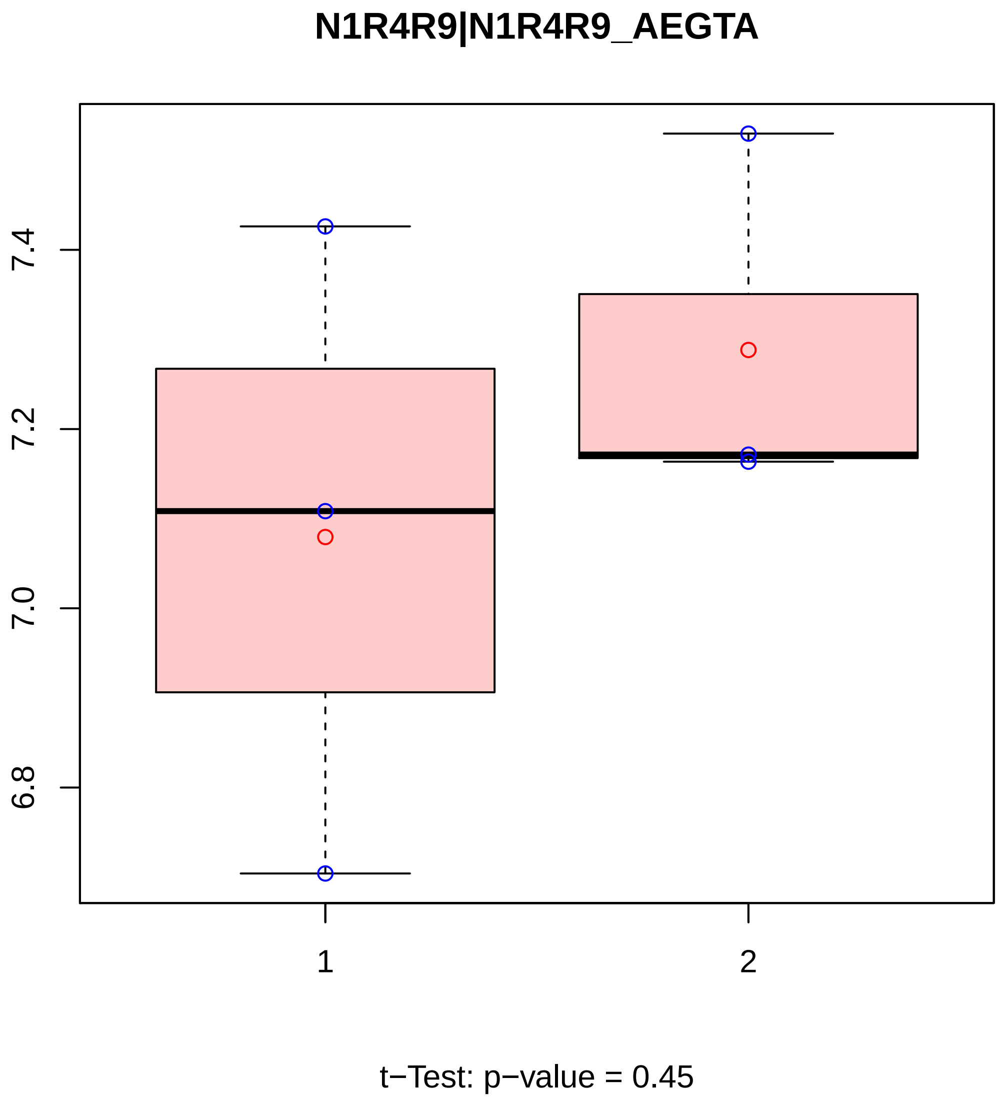

N1R4R9|N1R4R9_AEGTA
7.4
7.2
7.0
6.8
1
2
t−Test: p−value = 0.45

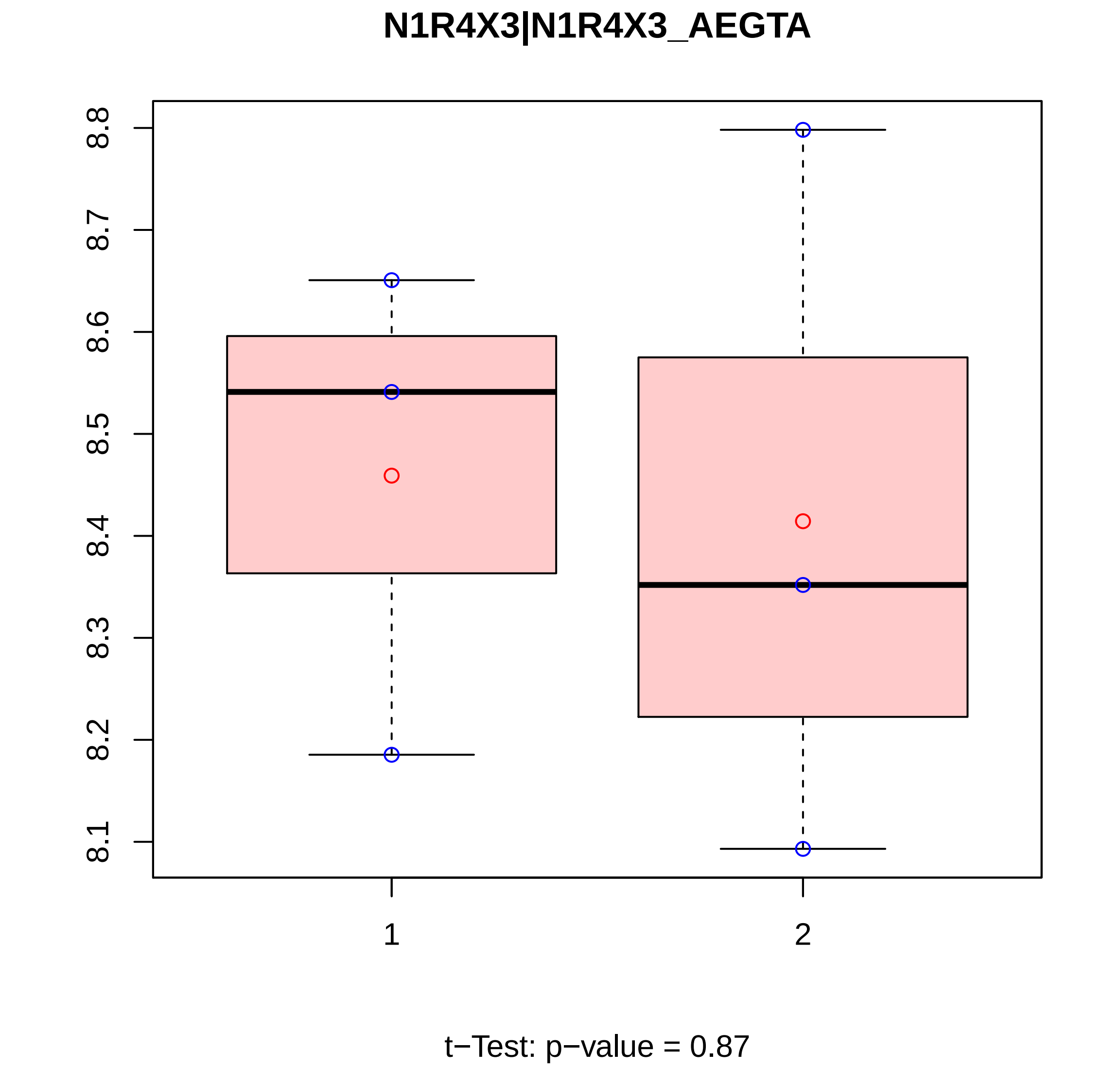

N1R4X3|N1R4X3_AEGTA
8.1
8.2
8.3
8.4
8.5
8.6
8.7
8.8
1
2
t−Test: p−value = 0.87

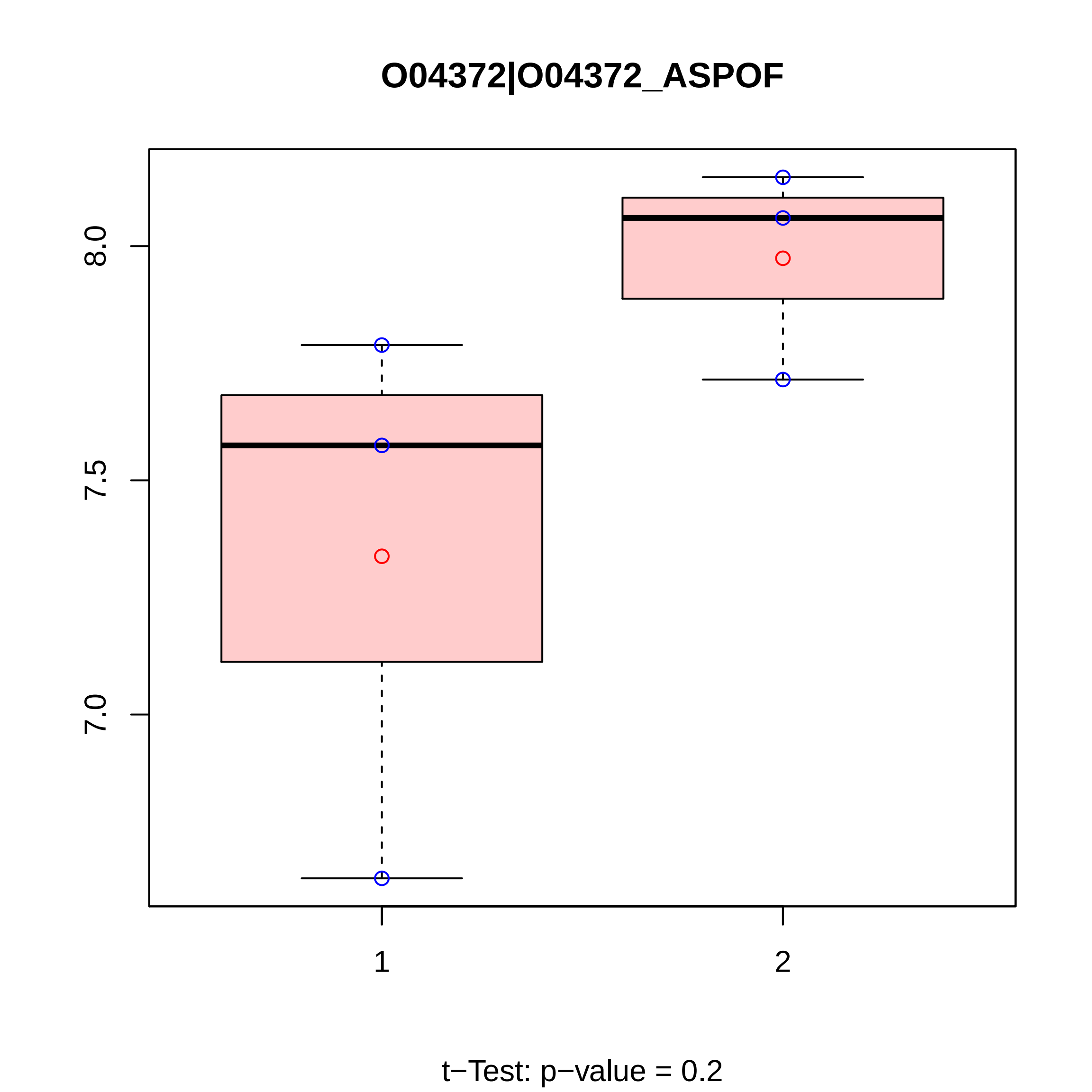
O04372|O04372_ASPOF
8.0
7.5
7.0
1
2
t−Test: p−value = 0.2

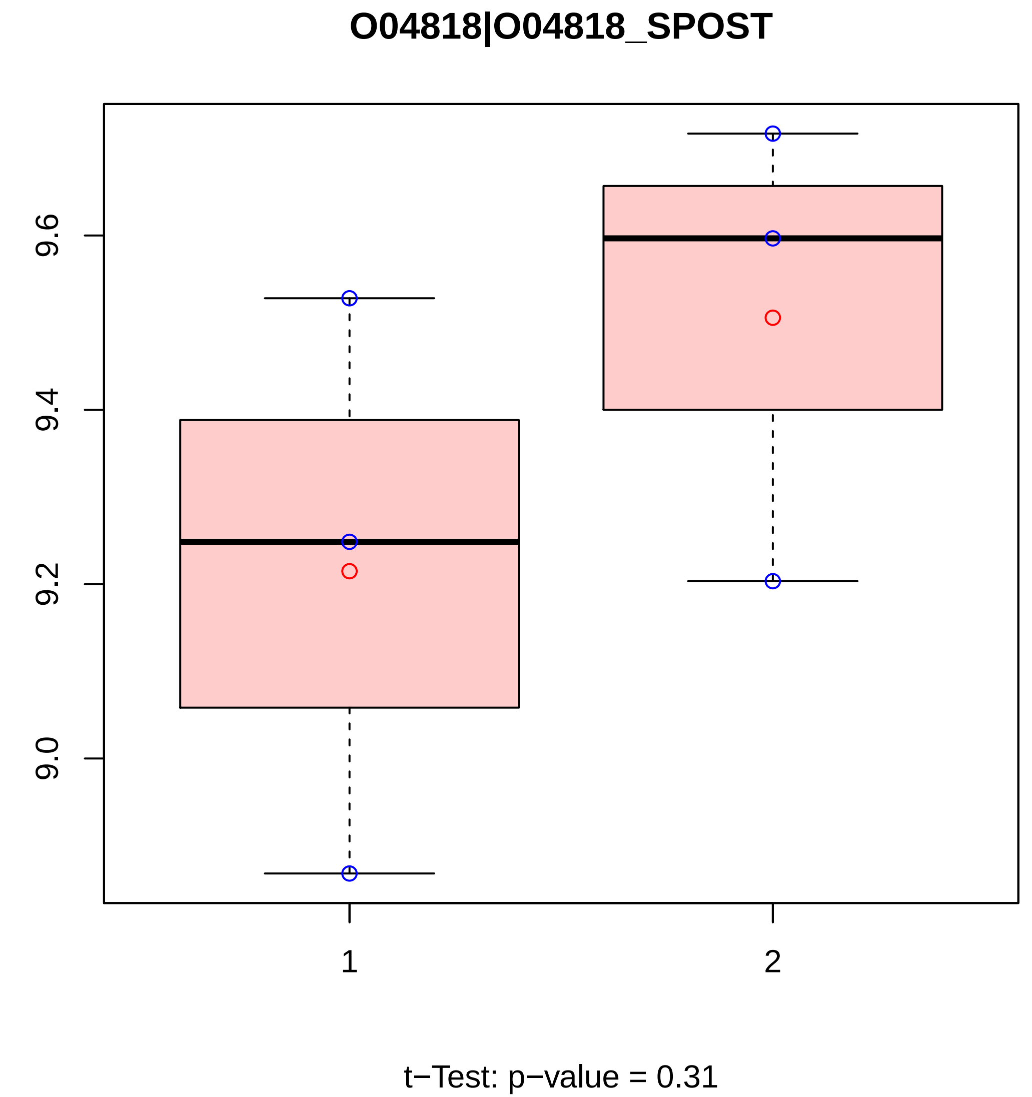
O04818|O04818_SPOST
9.0
9.2
9.4
9.6
1
2
t−Test: p−value = 0.31

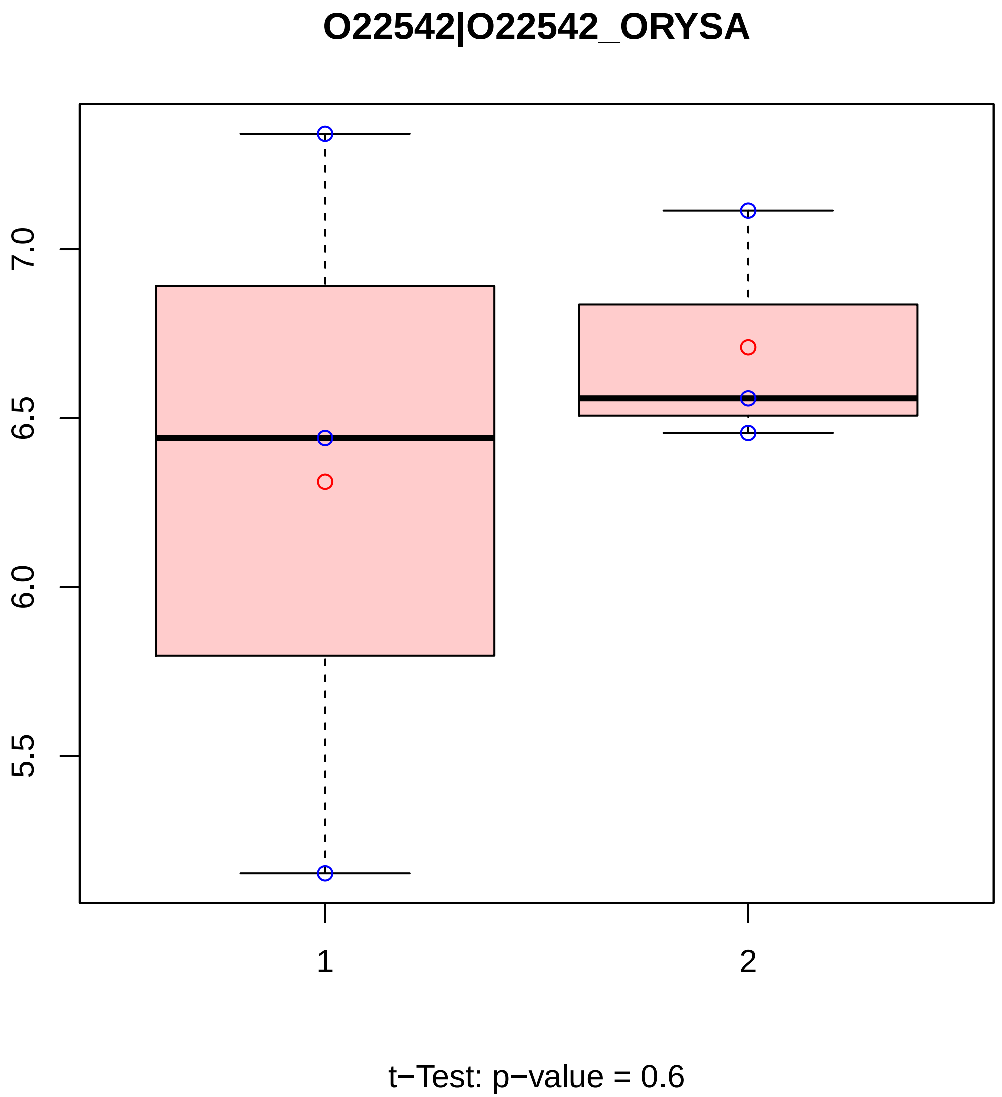
O22542|O22542_ORYSA
7.0
6.5
6.0
5.5
1
2
t−Test: p−value = 0.6

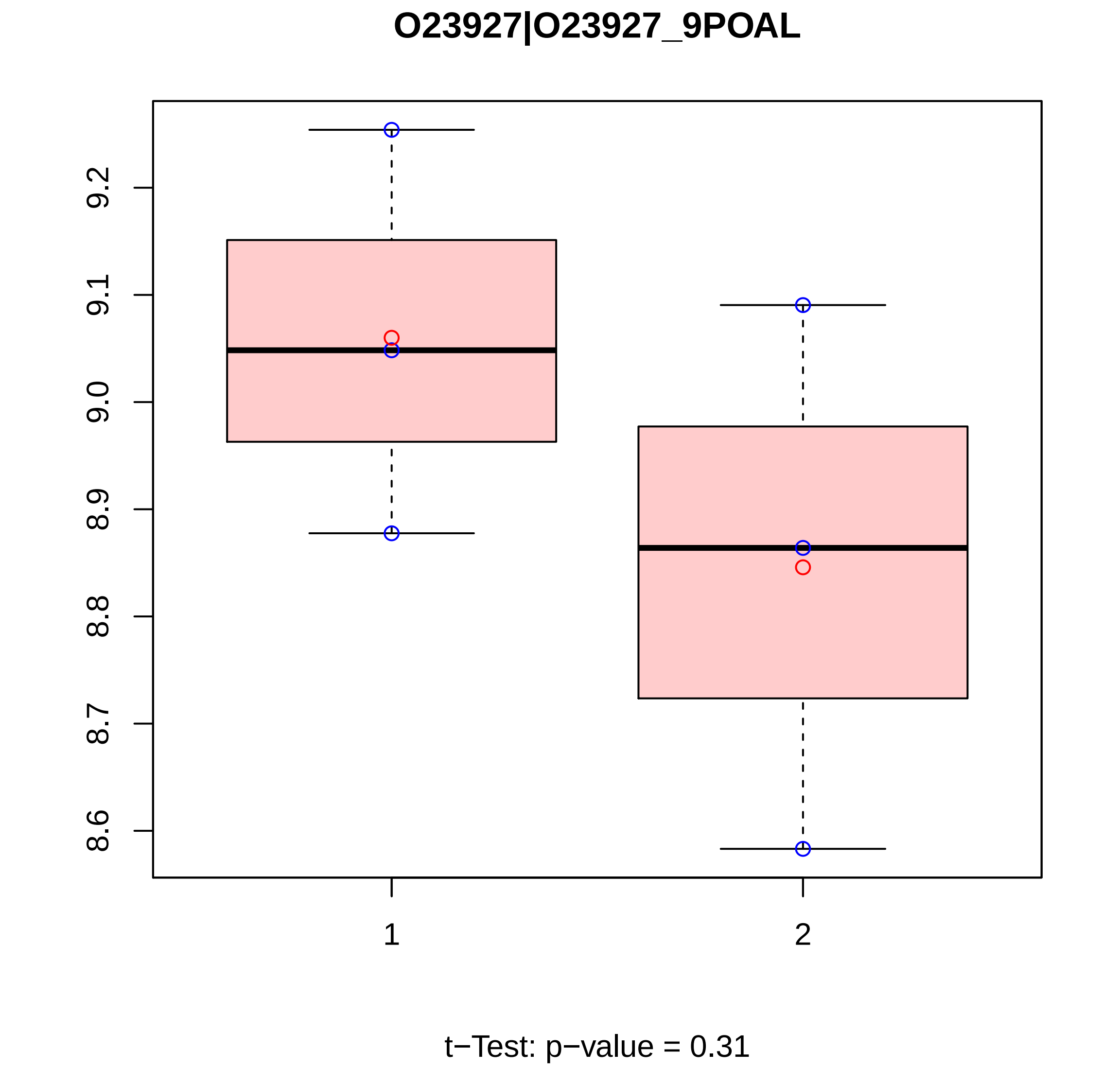
O23927|O23927_9POAL
9.2
9.1
9.0
8.9
8.8
8.7
8.6
1
2
t−Test: p−value = 0.31

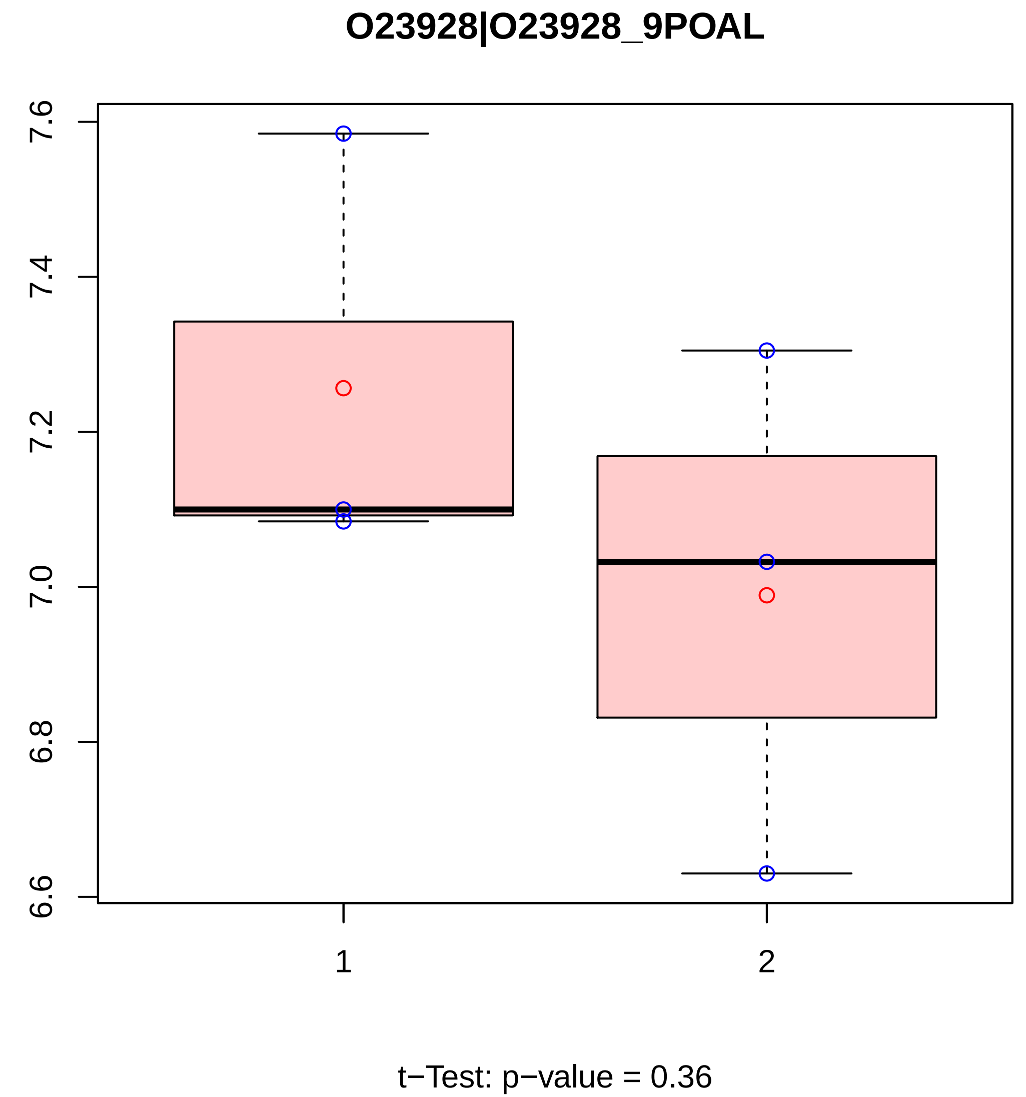
O23928|O23928_9POAL
7.6
7.4
7.2
7.0
6.8
6.6
1
2
t−Test: p−value = 0.36

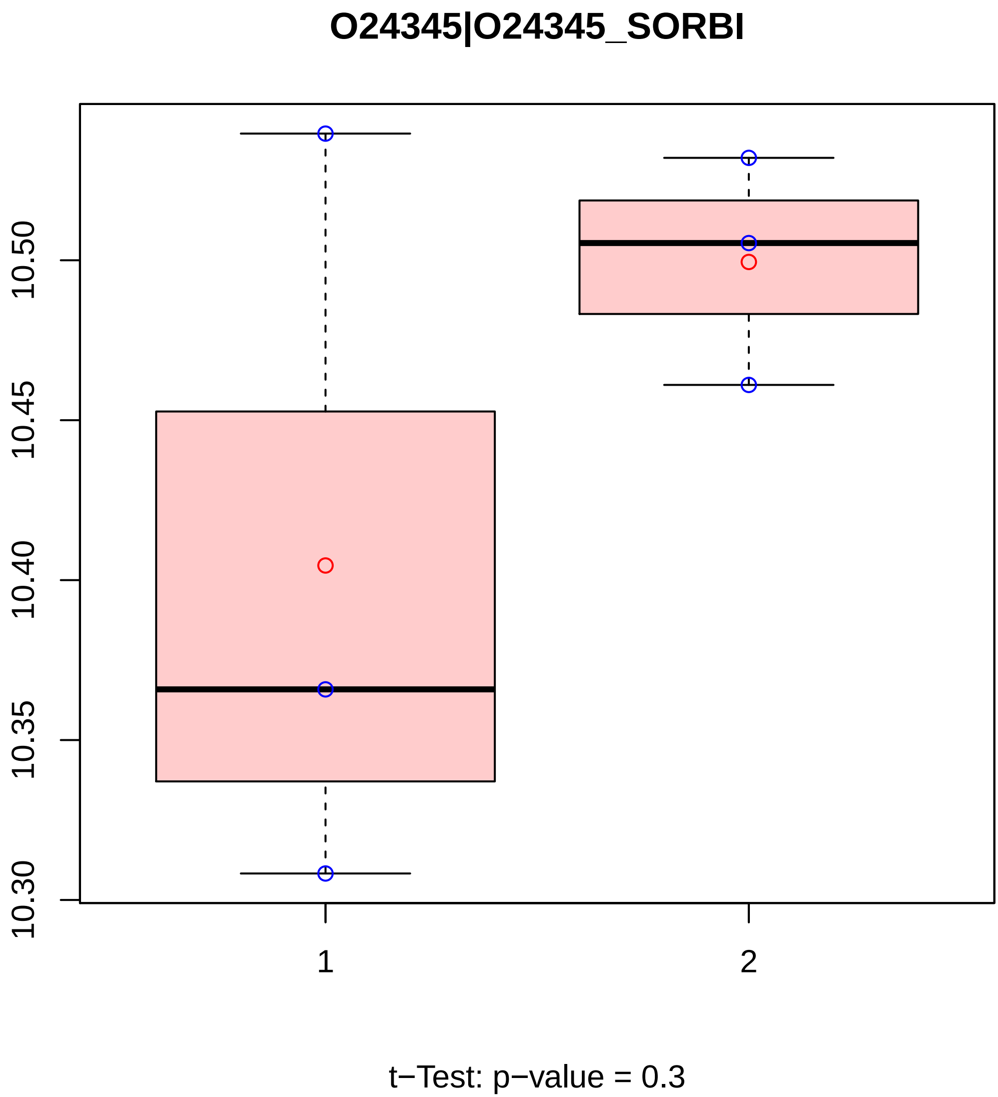
O24345|O24345_SORBI
10.30
10.35
10.40
10.45
10.50
1
2
t−Test: p−value = 0.3

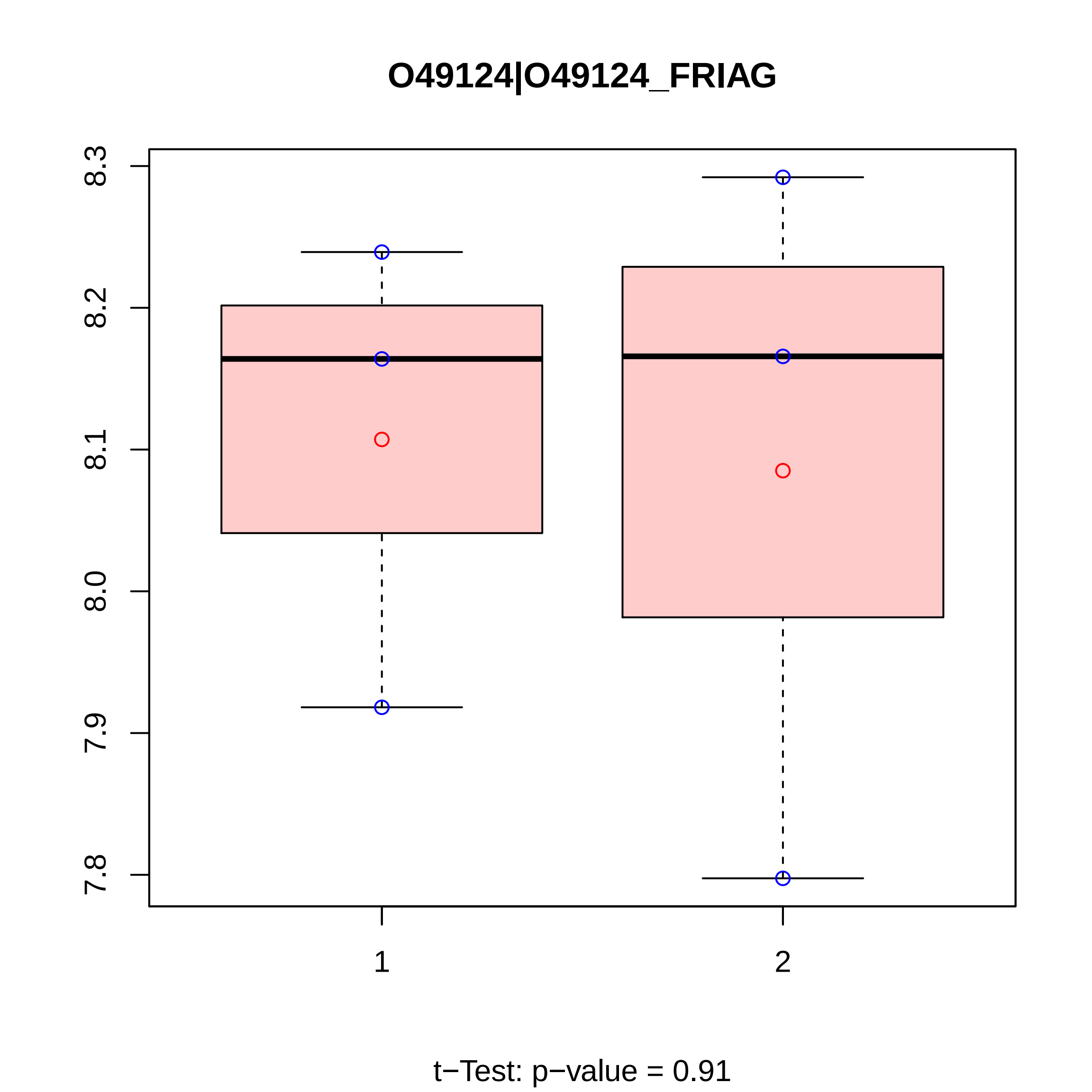
O49124|O49124_FRIAG
8.3
8.2
8.1
8.0
7.9
7.8
1
2
t−Test: p−value = 0.91

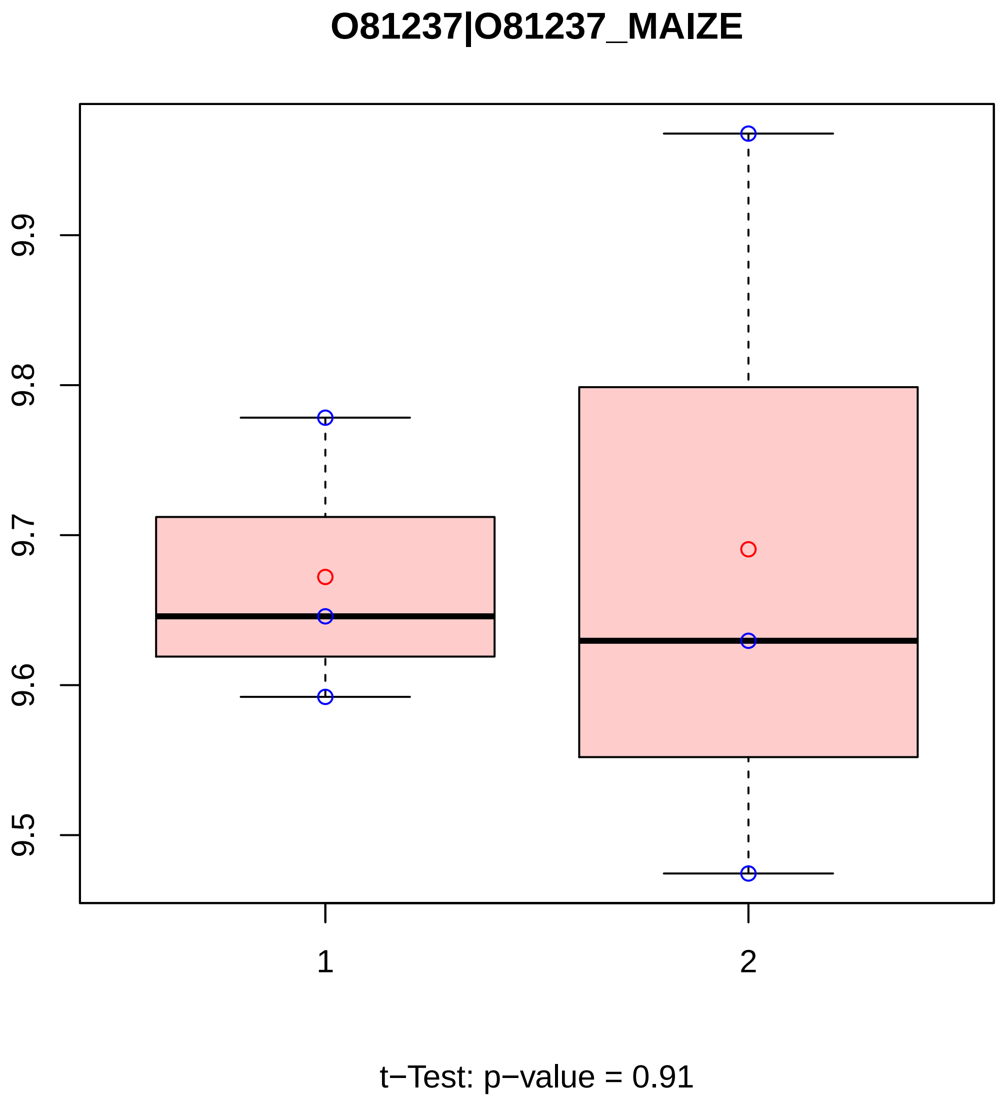
O81237|O81237_MAIZE
9.5
9.6
9.7
9.8
9.9
1
2
t−Test: p−value = 0.91

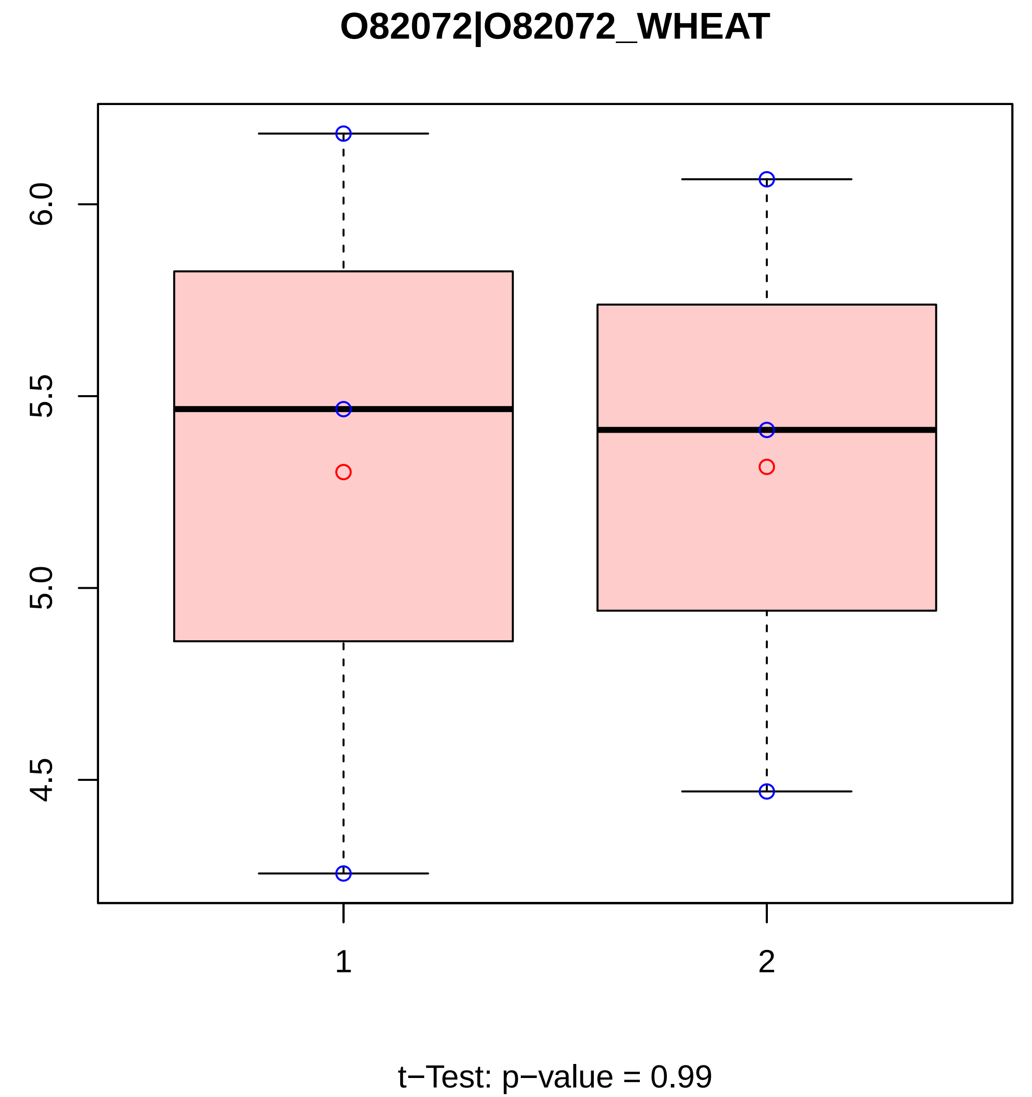
O82072|O82072_WHEAT
6.0
5.5
5.0
4.5
1
2
t−Test: p−value = 0.99

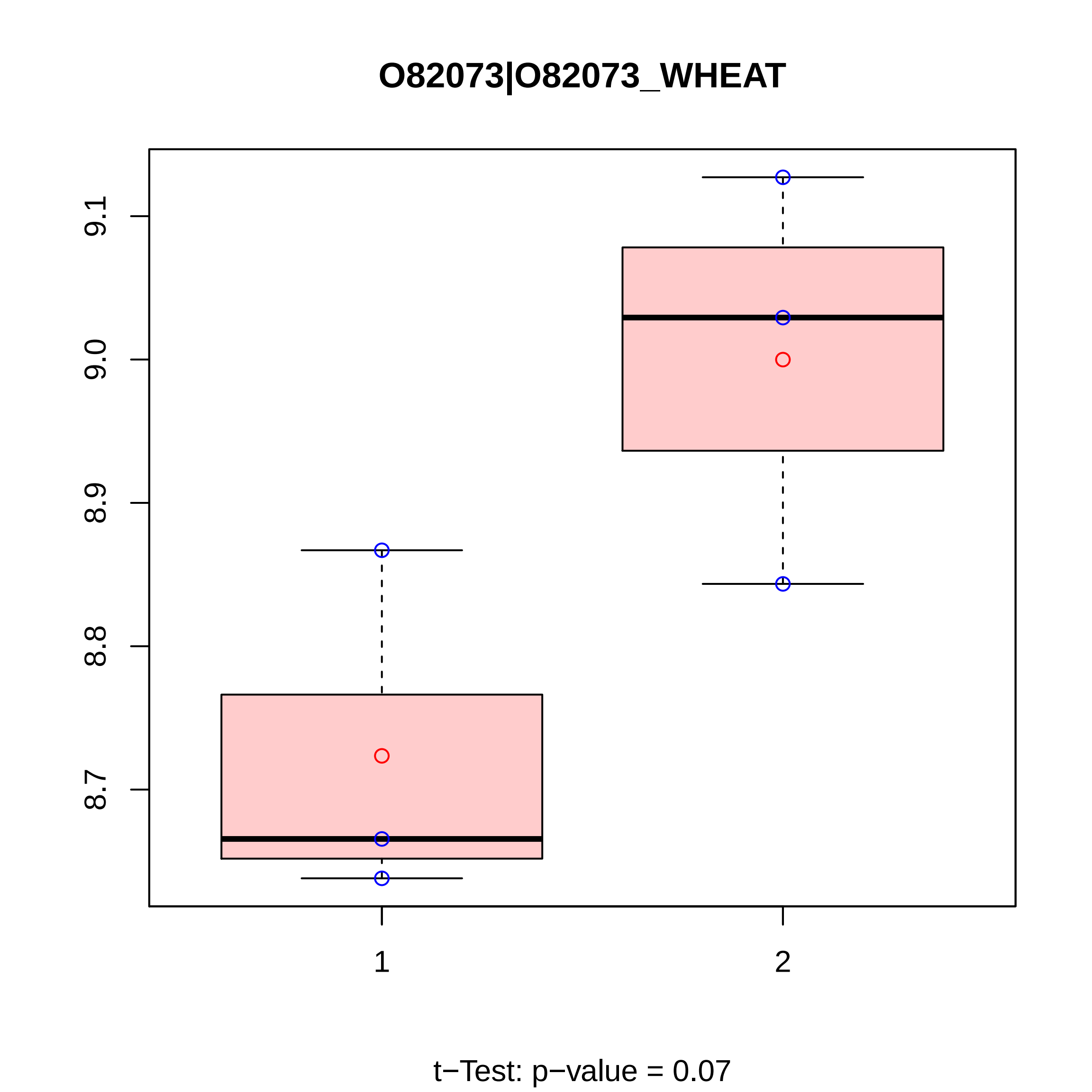

O82073|O82073_WHEAT
9.1
9.0
8.9
8.8
8.7
1
2
t−Test: p−value = 0.07

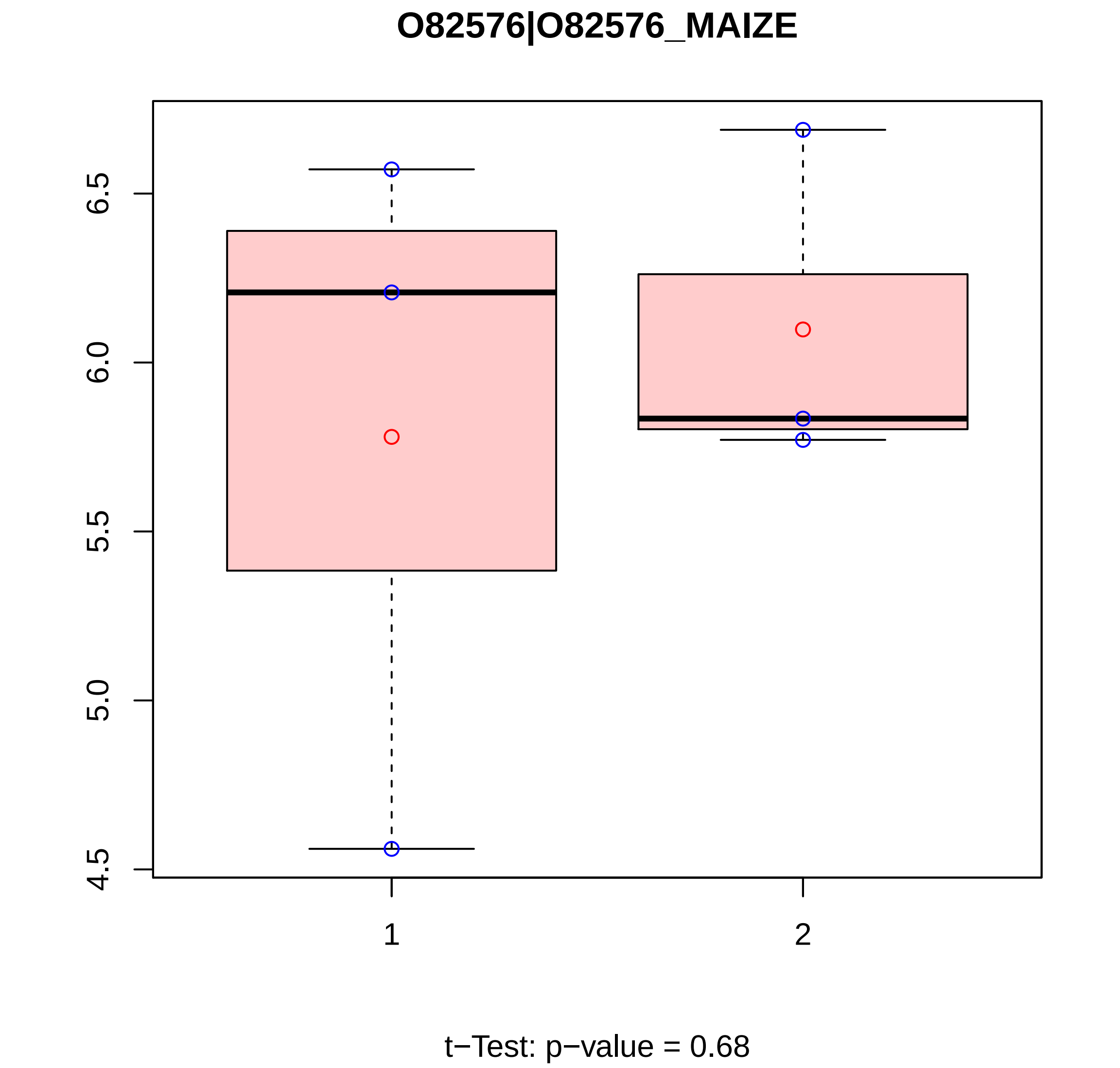
O82576|O82576_MAIZE
6.5
6.0
5.5
5.0
4.5
1
2
t−Test: p−value = 0.68

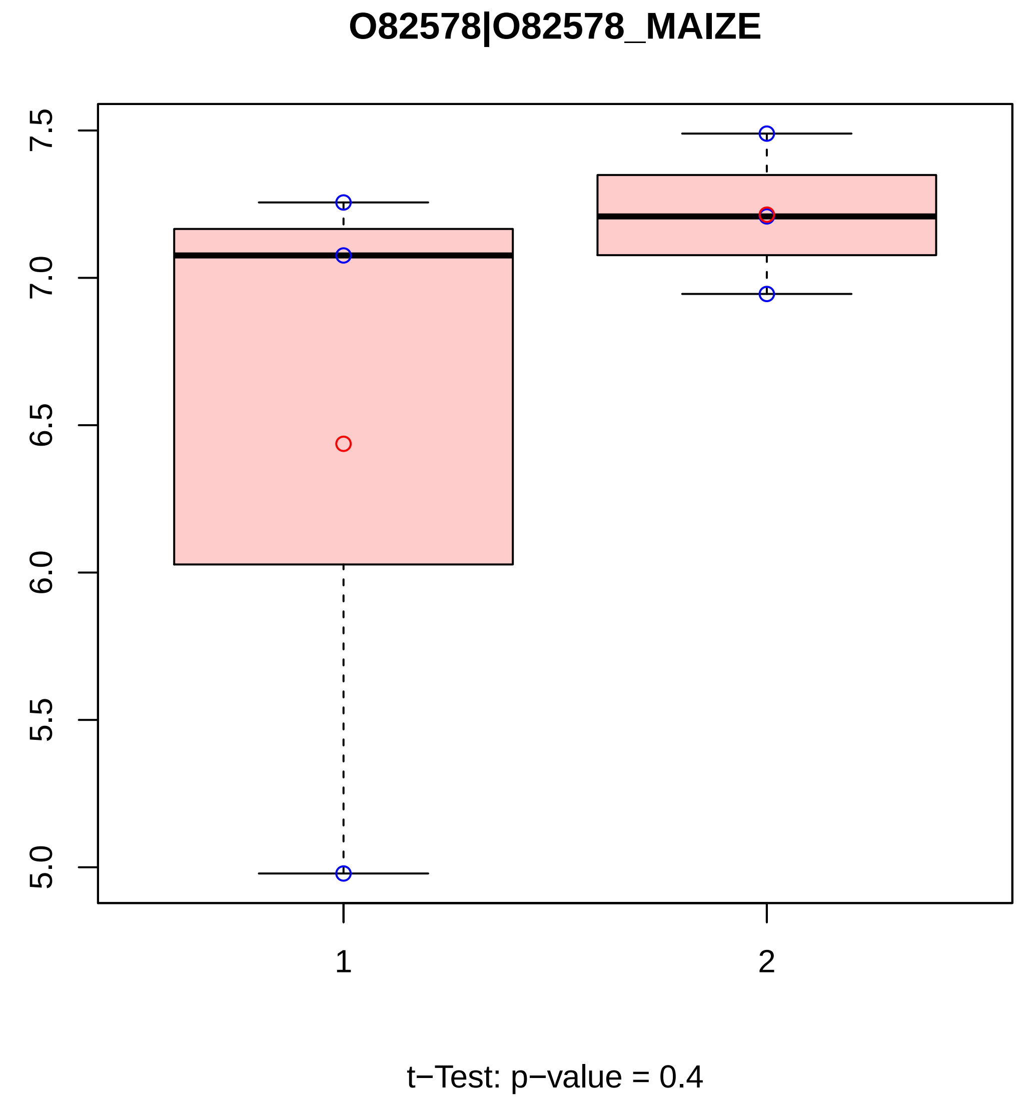
O82578|O82578_MAIZE
7.5
7.0
6.5
6.0
5.5
5.0
1
2
t−Test: p−value = 0.4

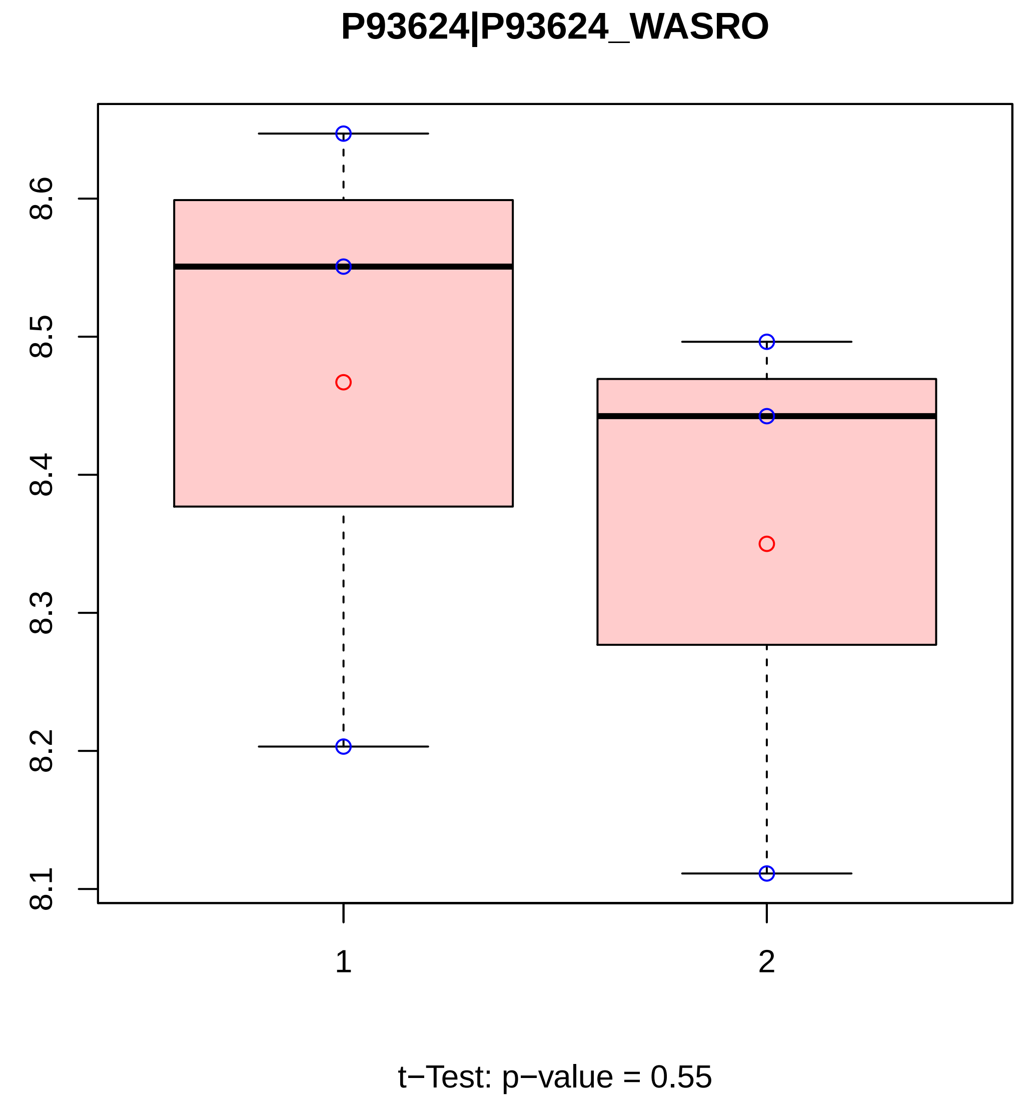
P93624|P93624_WASRO
8.1
8.2
8.3
8.4
8.5
8.6
1
2
t−Test: p−value = 0.55

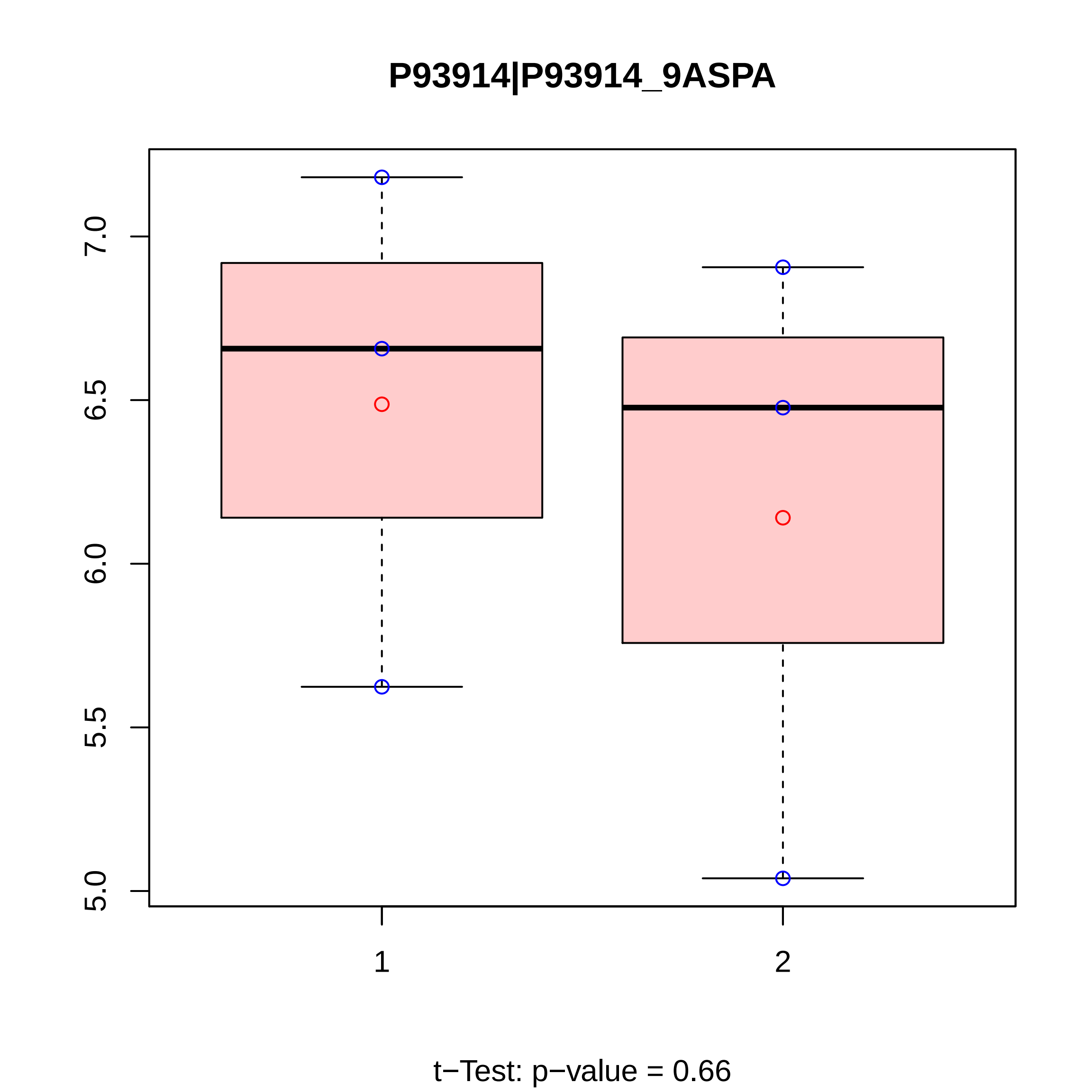
P93914|P93914_9ASPA
7.0
6.5
6.0
5.5
5.0
1
2
t−Test: p−value = 0.66

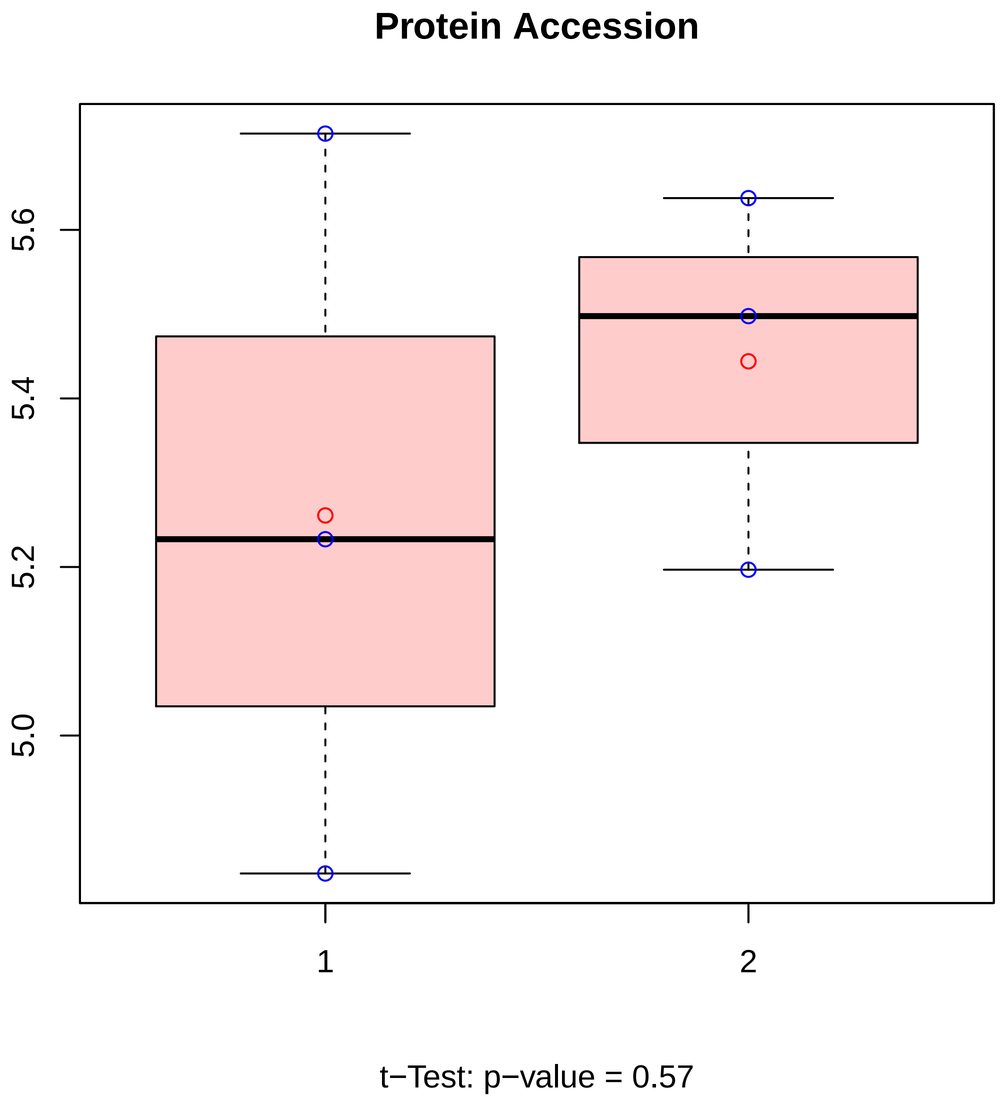

Protein Accession
5.0
5.2
5.4
5.6
1
2
t−Test: p−value = 0.57

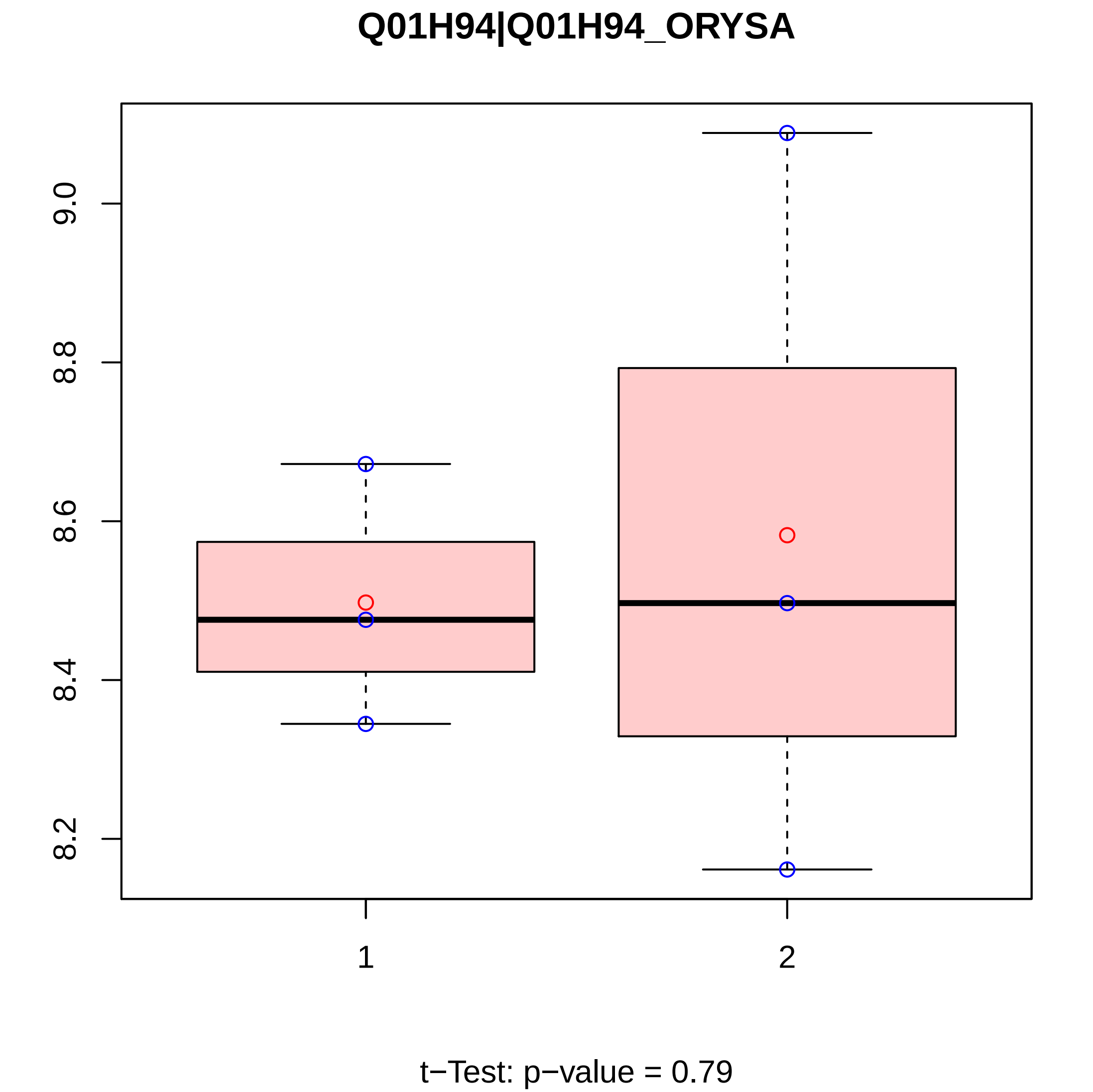
Q01H94|Q01H94_ORYSA
9.0
8.8
8.6
8.4
8.2
1
2
t−Test: p−value = 0.79

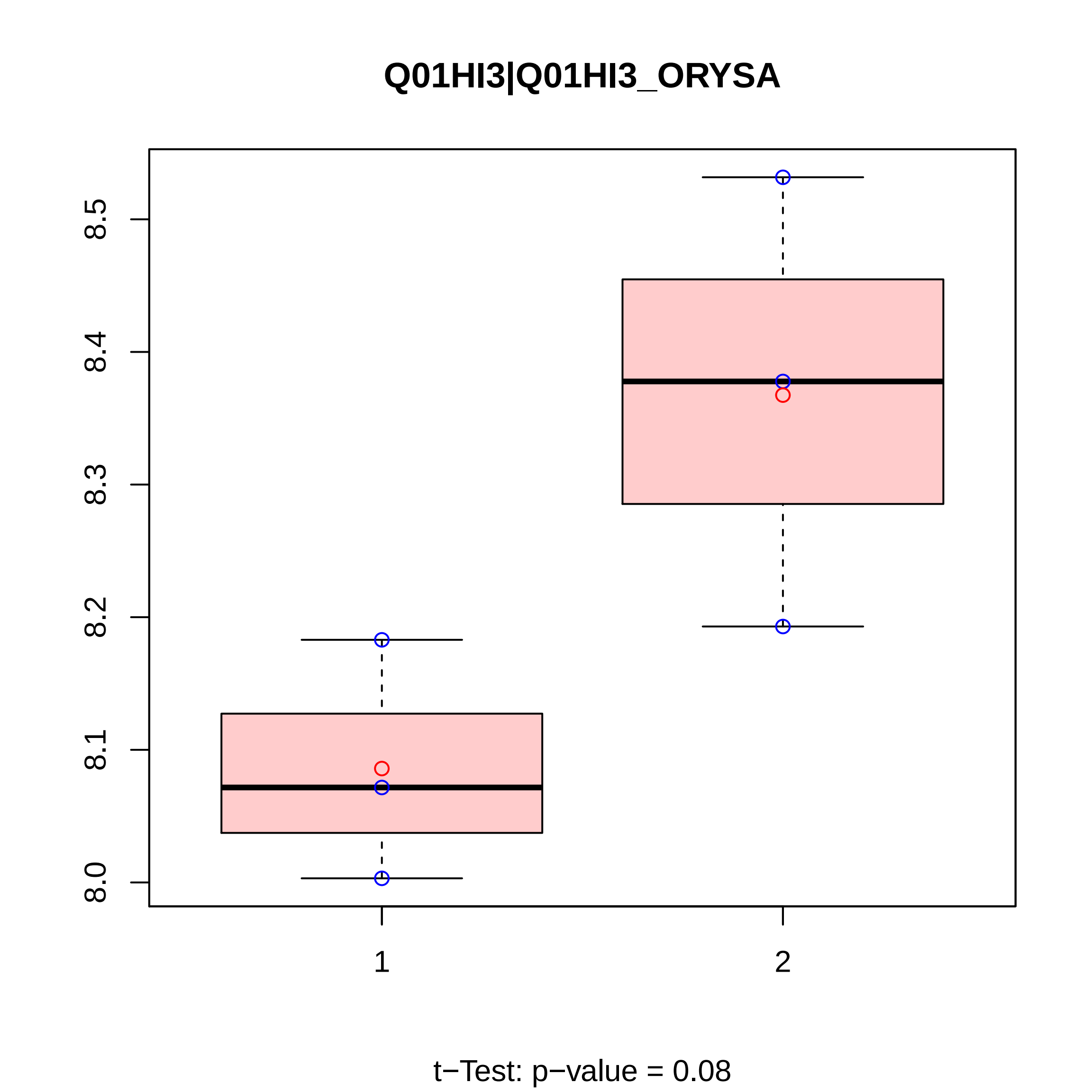
Q01HI3|Q01HI3_ORYSA
8.0
8.1
8.2
8.3
8.4
8.5
1
2
t−Test: p−value = 0.08

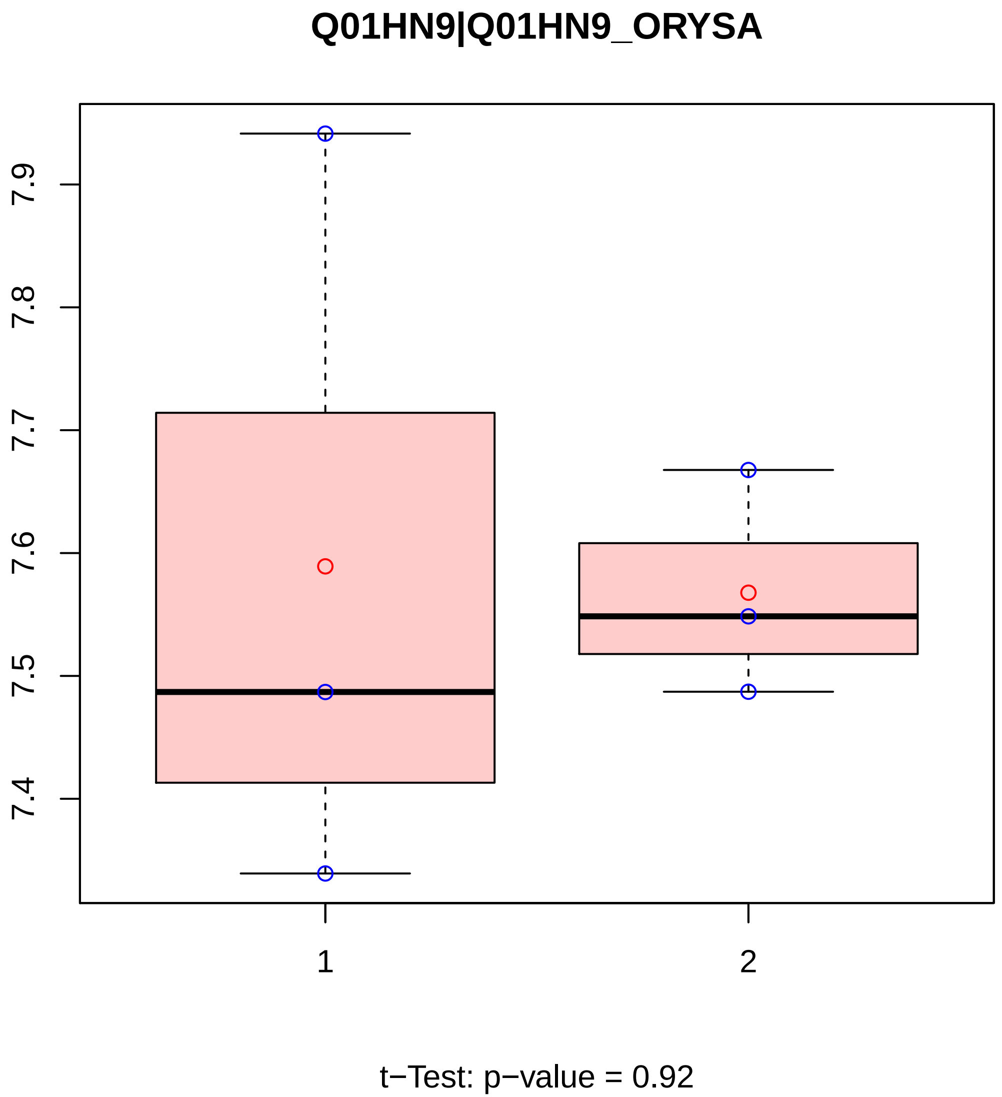
Q01HN9|Q01HN9_ORYSA
7.4
7.5
7.6
7.7
7.8
7.9
1
2
t−Test: p−value = 0.92

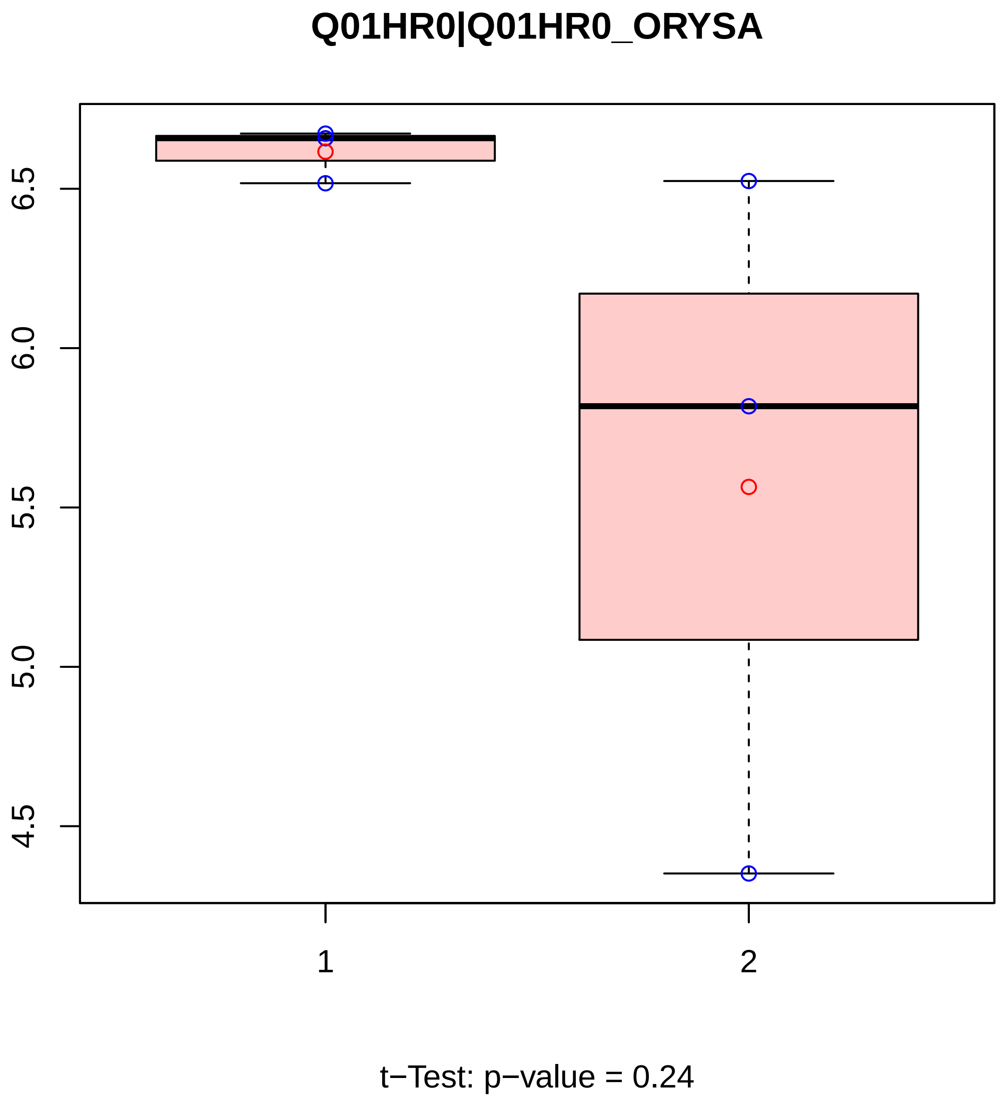
Q01HR0|Q01HR0_ORYSA
6.5
6.0
5.5
5.0
4.5
1
2
t−Test: p−value = 0.24

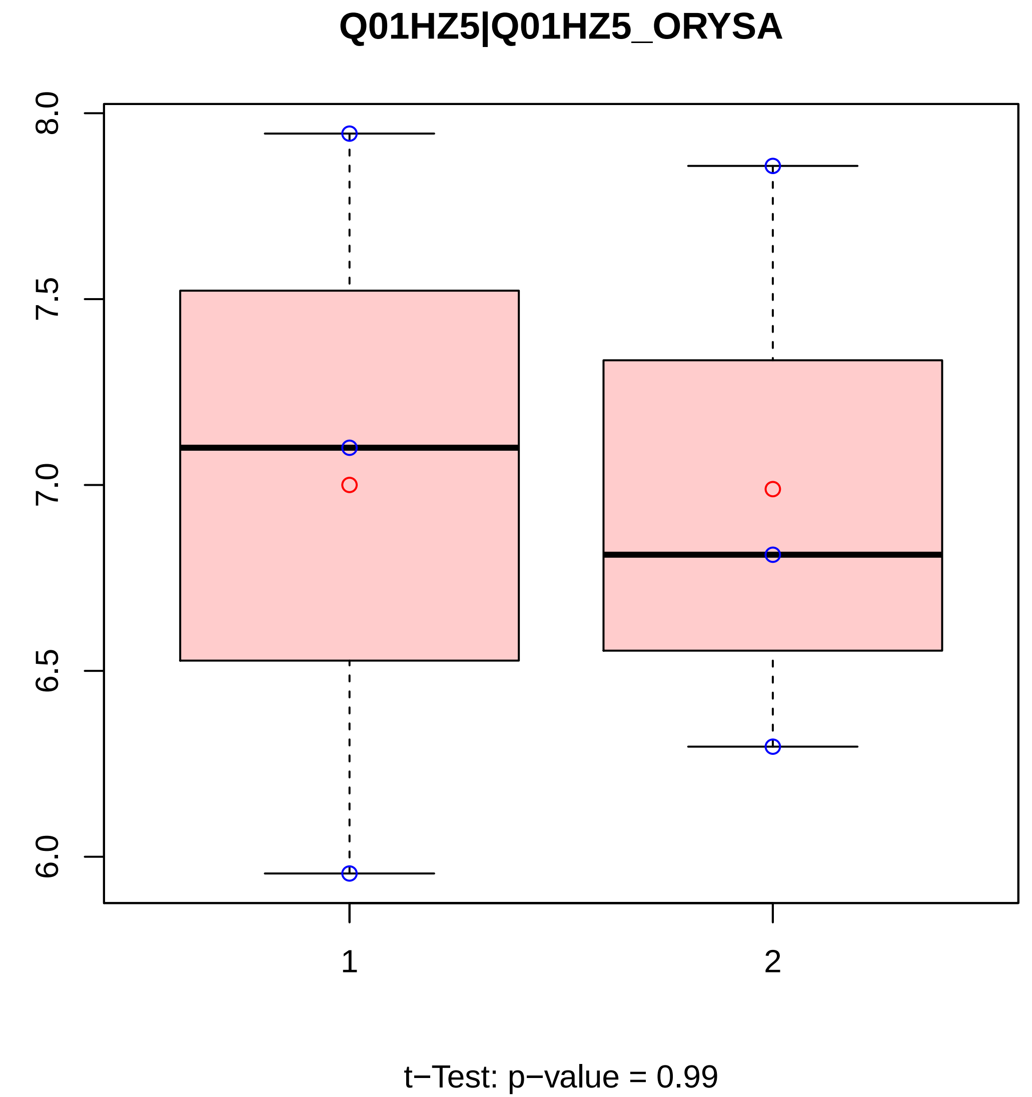
Q01HZ5|Q01HZ5_ORYSA
8.0
7.5
7.0
6.5
6.0
1
2
t−Test: p−value = 0.99

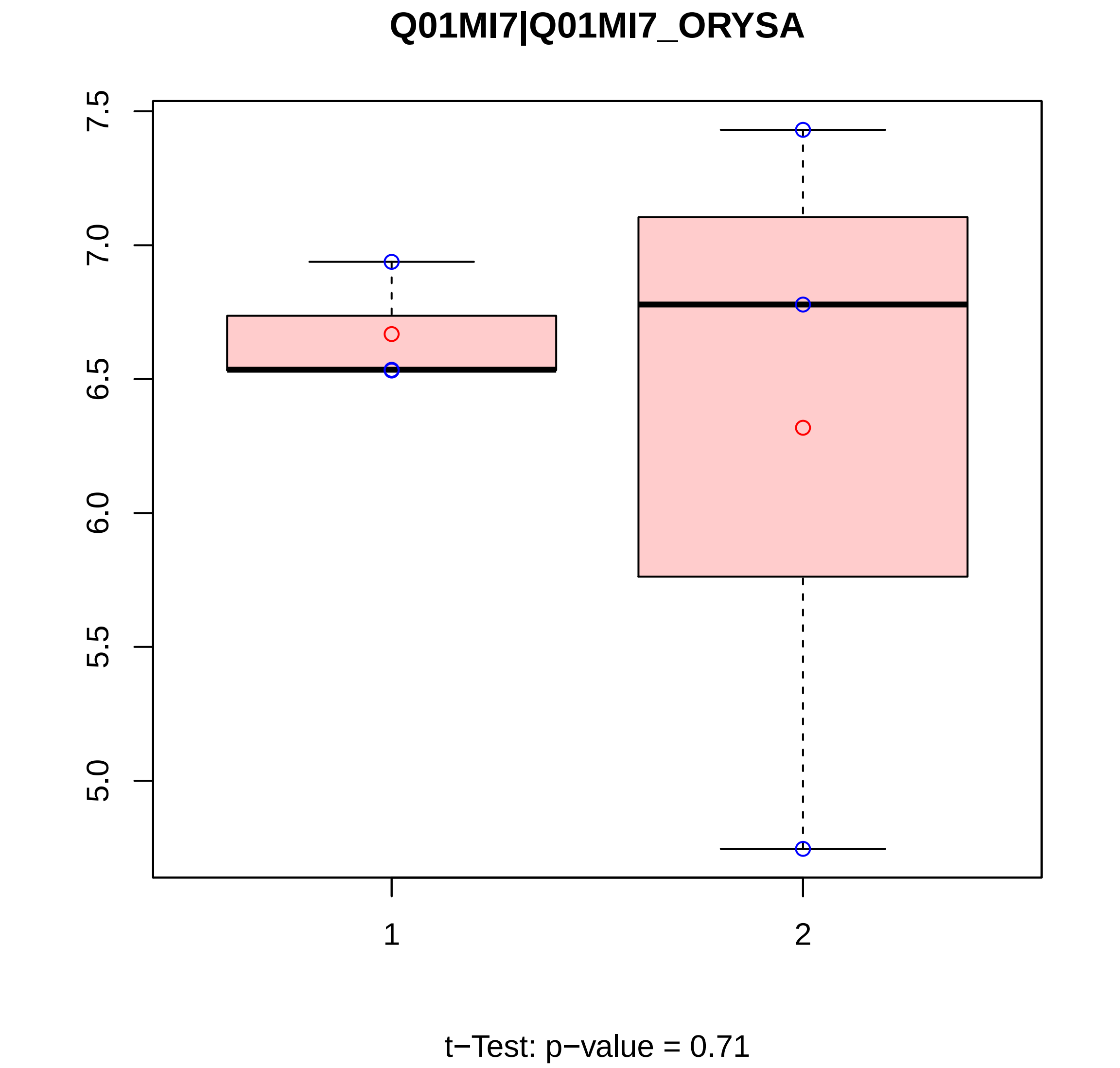
Q01MI7|Q01MI7_ORYSA
7.5
7.0
6.5
6.0
5.5
5.0
1
2
t−Test: p−value = 0.71

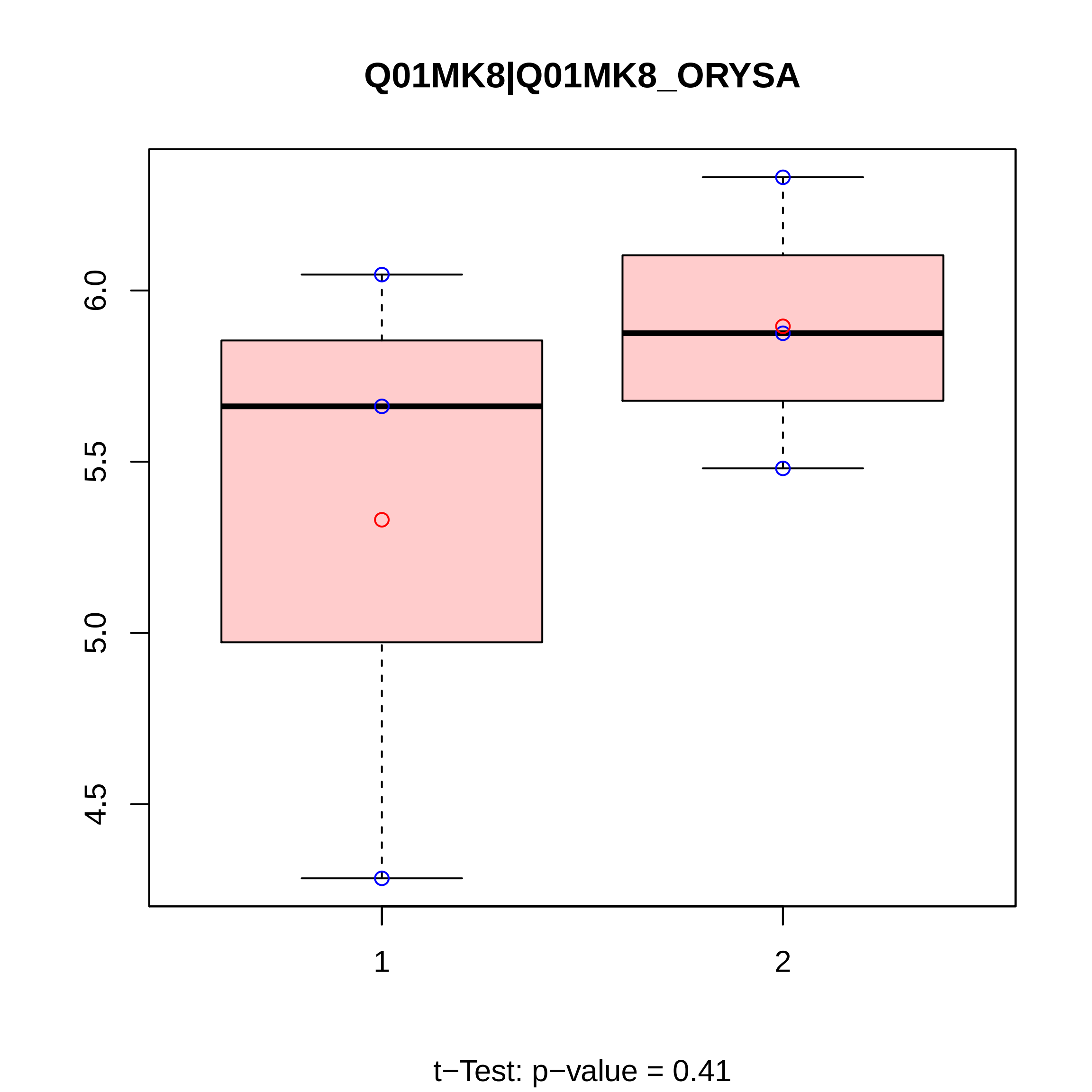
Q01MK8|Q01MK8_ORYSA
6.0
5.5
5.0
4.5
1
2
t−Test: p−value = 0.41

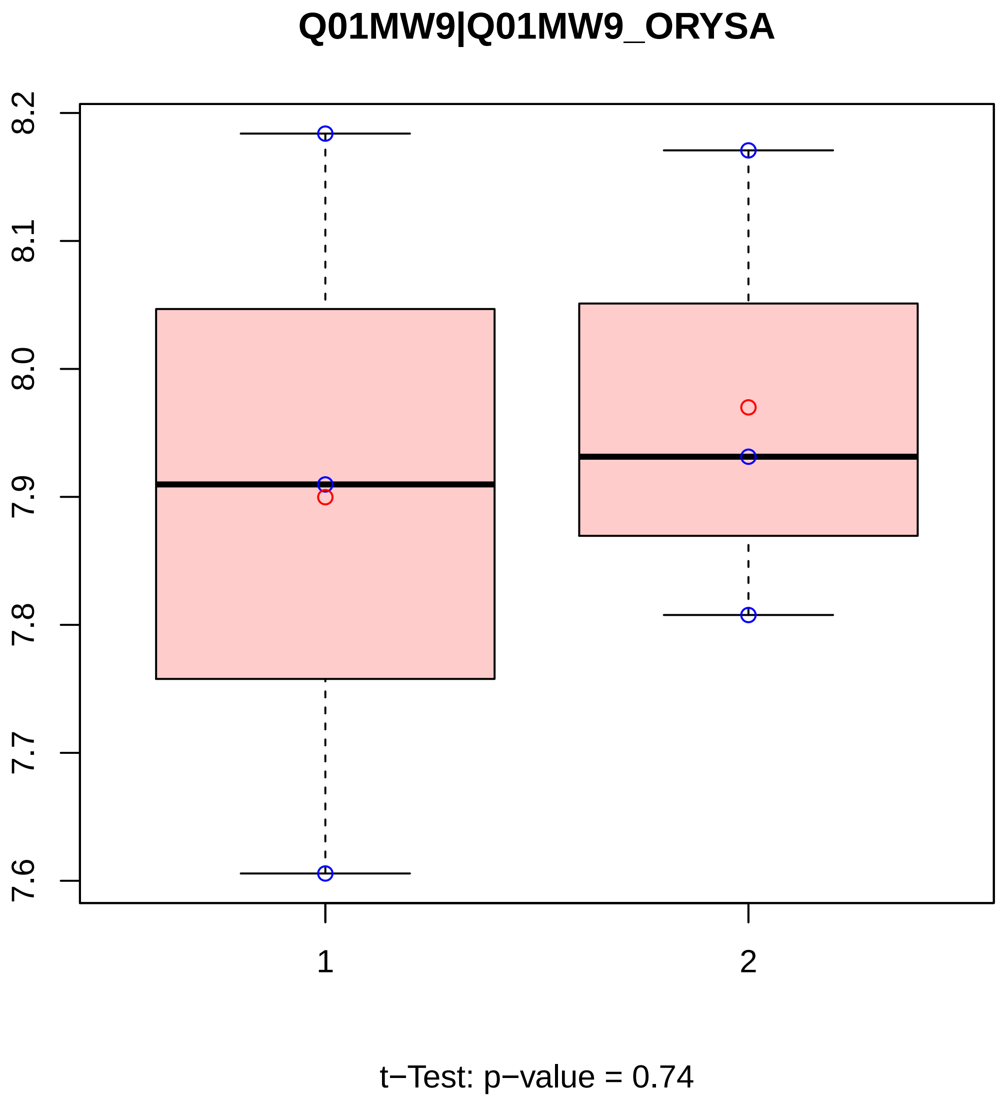
Q01MW9|Q01MW9_ORYSA
8.2
8.1
8.0
7.9
7.8
7.7
7.6
1
2
t−Test: p−value = 0.74

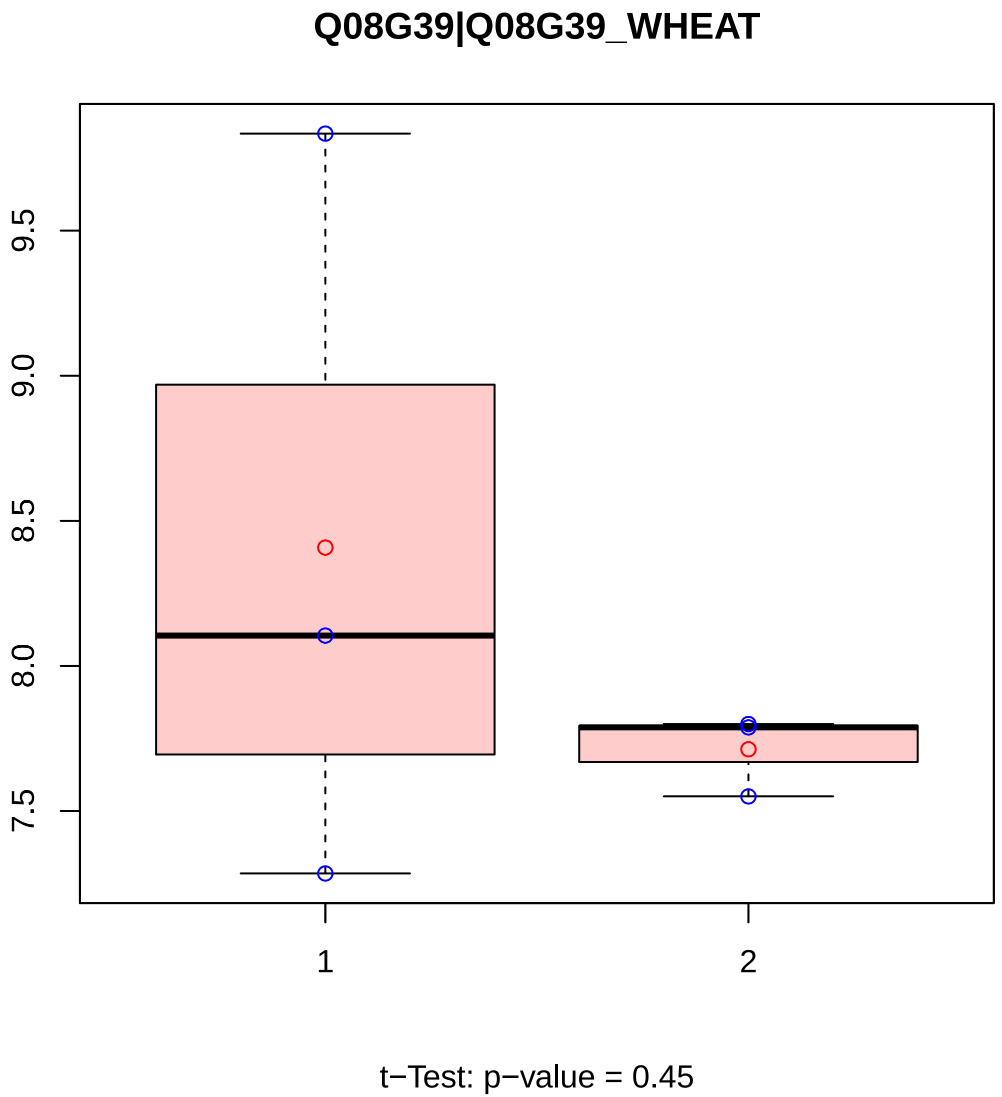
Q08G39|Q08G39_WHEAT
9.5
9.0
8.5
8.0
7.5
1
2
t−Test: p−value = 0.45

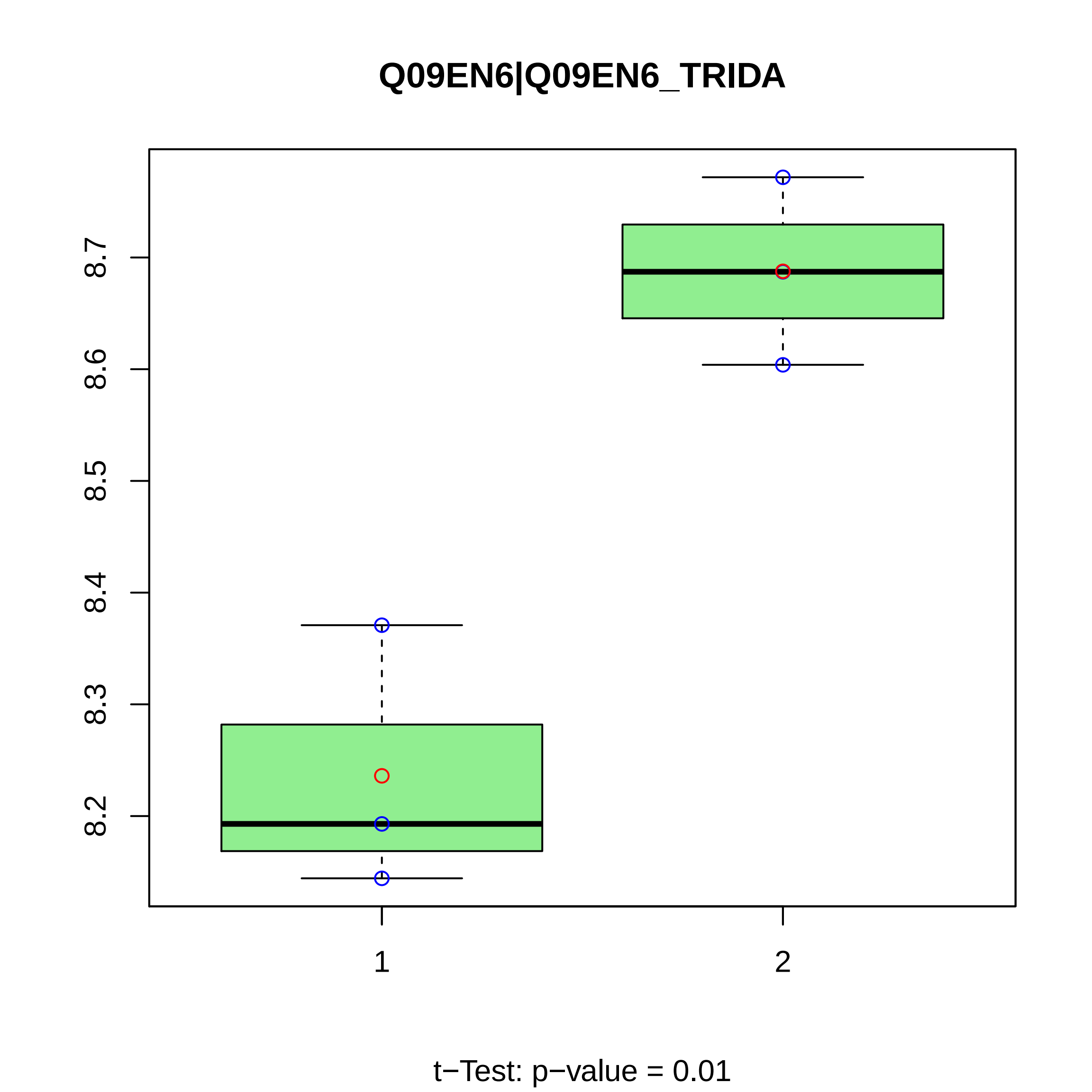
Q09EN6|Q09EN6_TRIDA
8.2
8.3
8.4
8.5
8.6
8.7
1
2
t−Test: p−value = 0.01

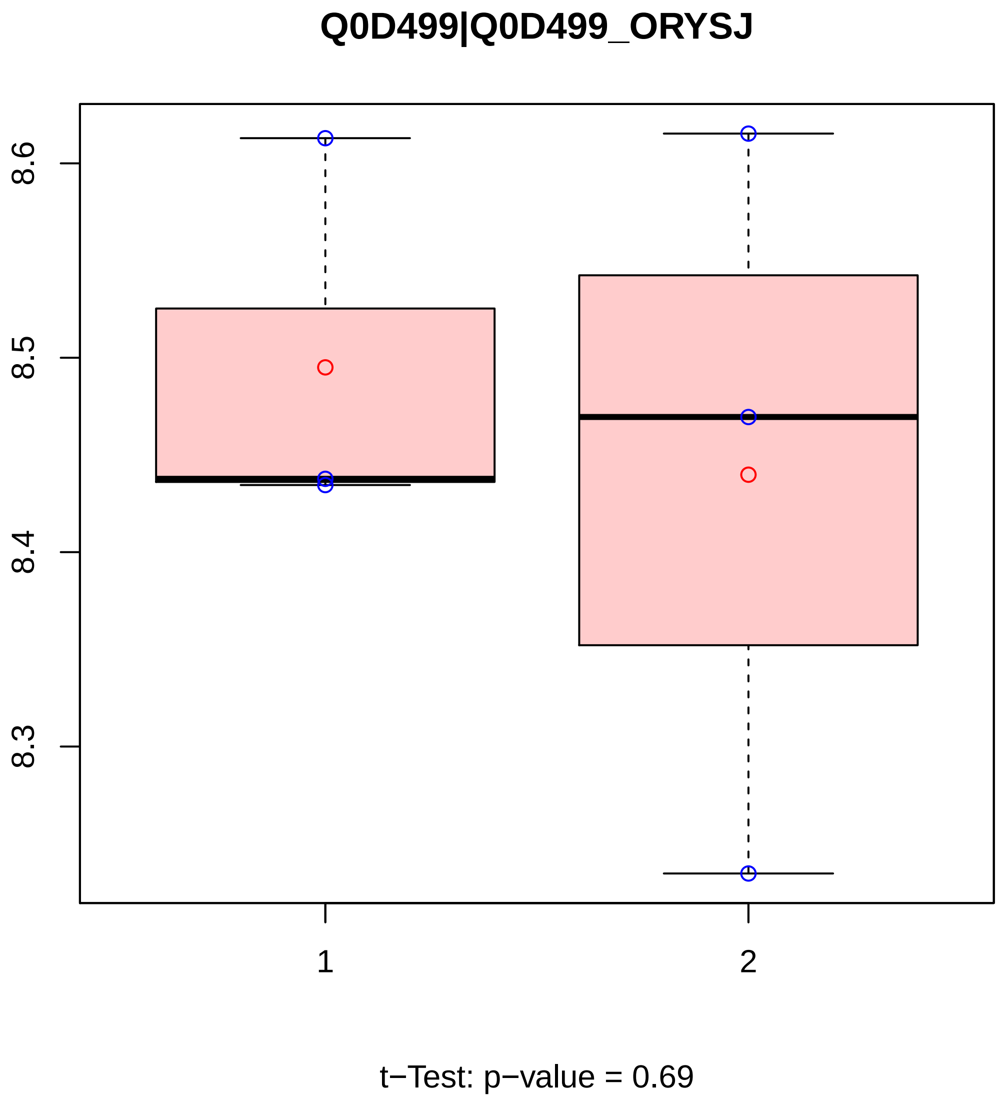
Q0D499|Q0D499_ORYSJ
8.6
8.5
8.4
8.3
1
2
t−Test: p−value = 0.69

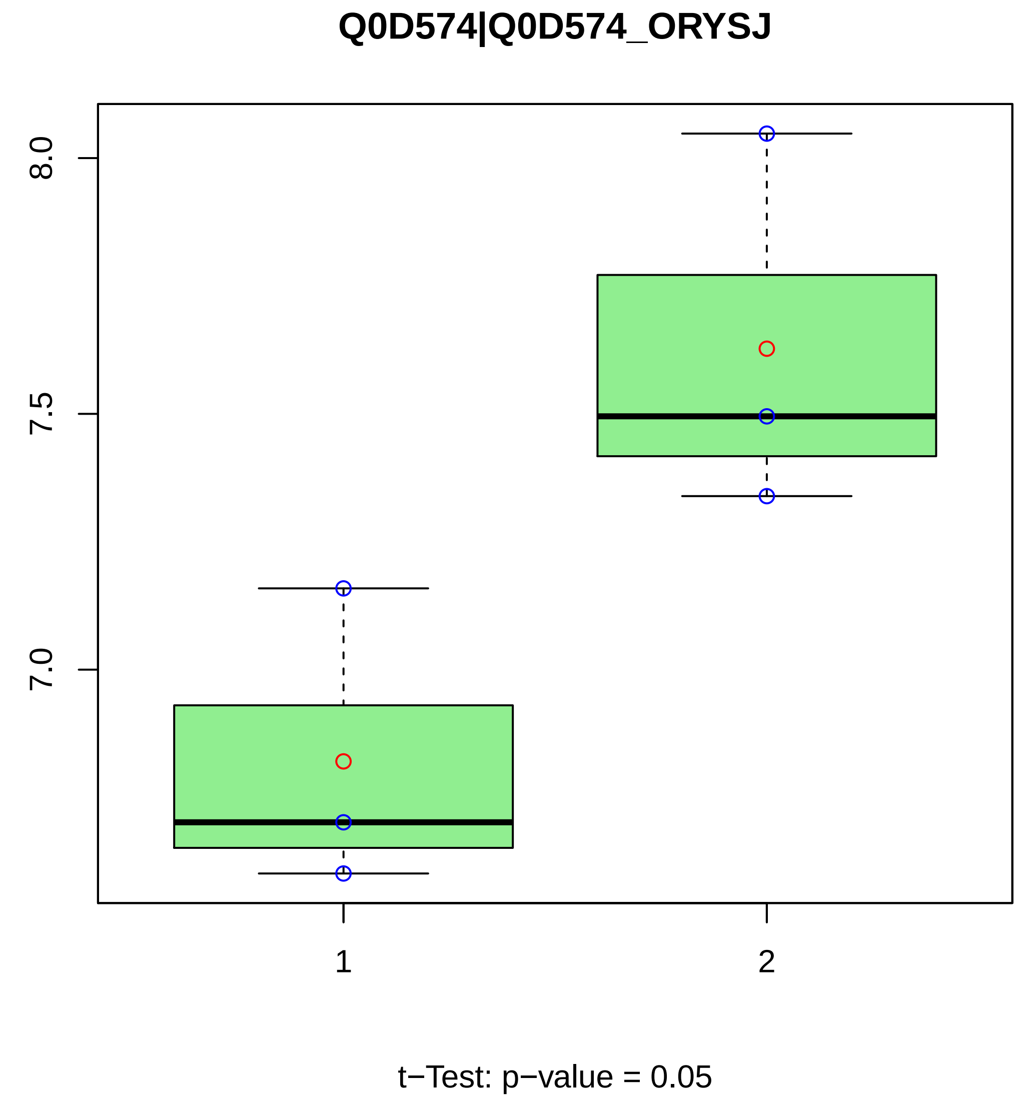
Q0D574|Q0D574_ORYSJ
8.0
7.5
7.0
1
2
t−Test: p−value = 0.05

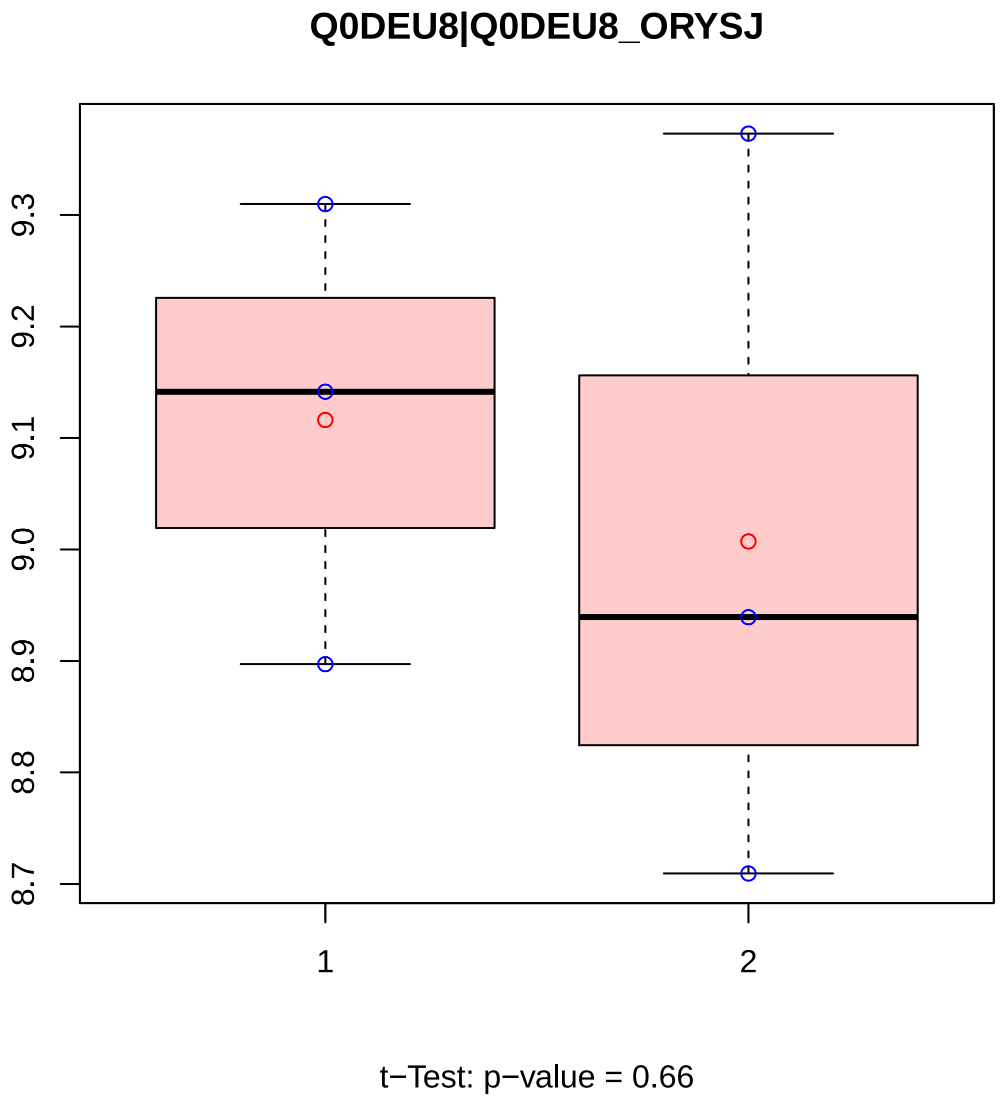
Q0DEU8|Q0DEU8_ORYSJ
9.3
9.2
9.1
9.0
8.9
8.8
8.7
1
2
t−Test: p−value = 0.66

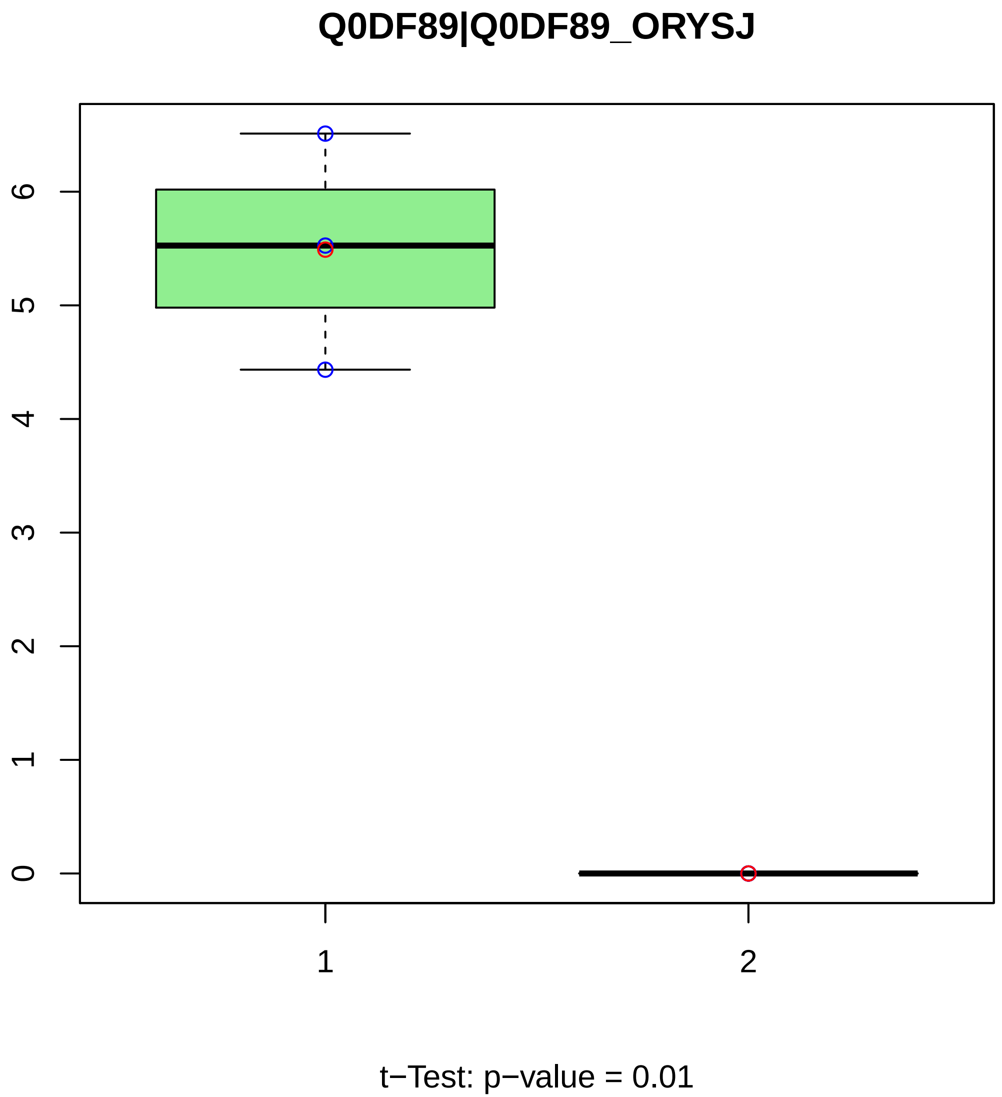
Q0DF89|Q0DF89_ORYSJ
0
1
2
3
4
5
6
1
2
t−Test: p−value = 0.01

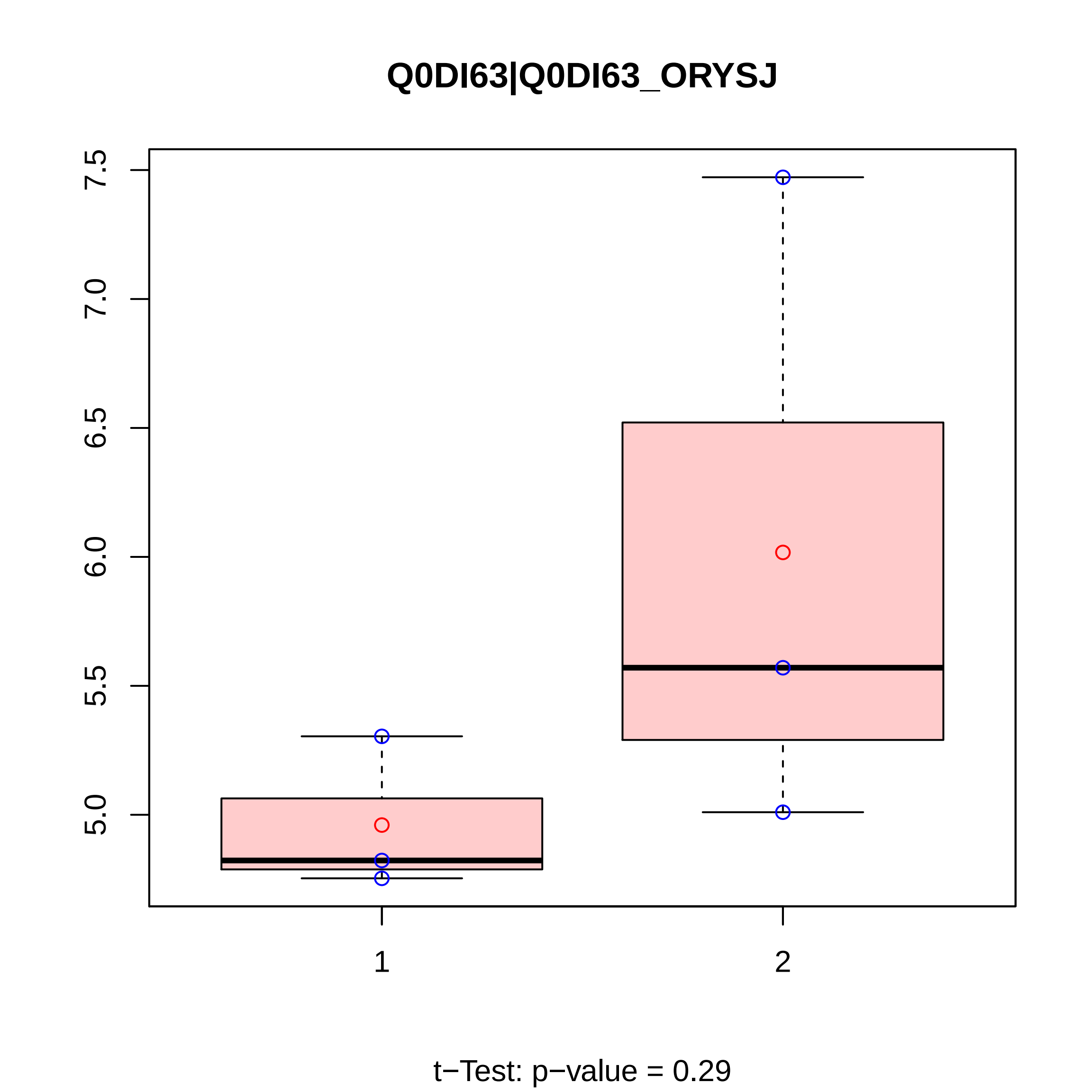
Q0DI63|Q0DI63_ORYSJ
7.5
7.0
6.5
6.0
5.5
5.0
1
2
t−Test: p−value = 0.29

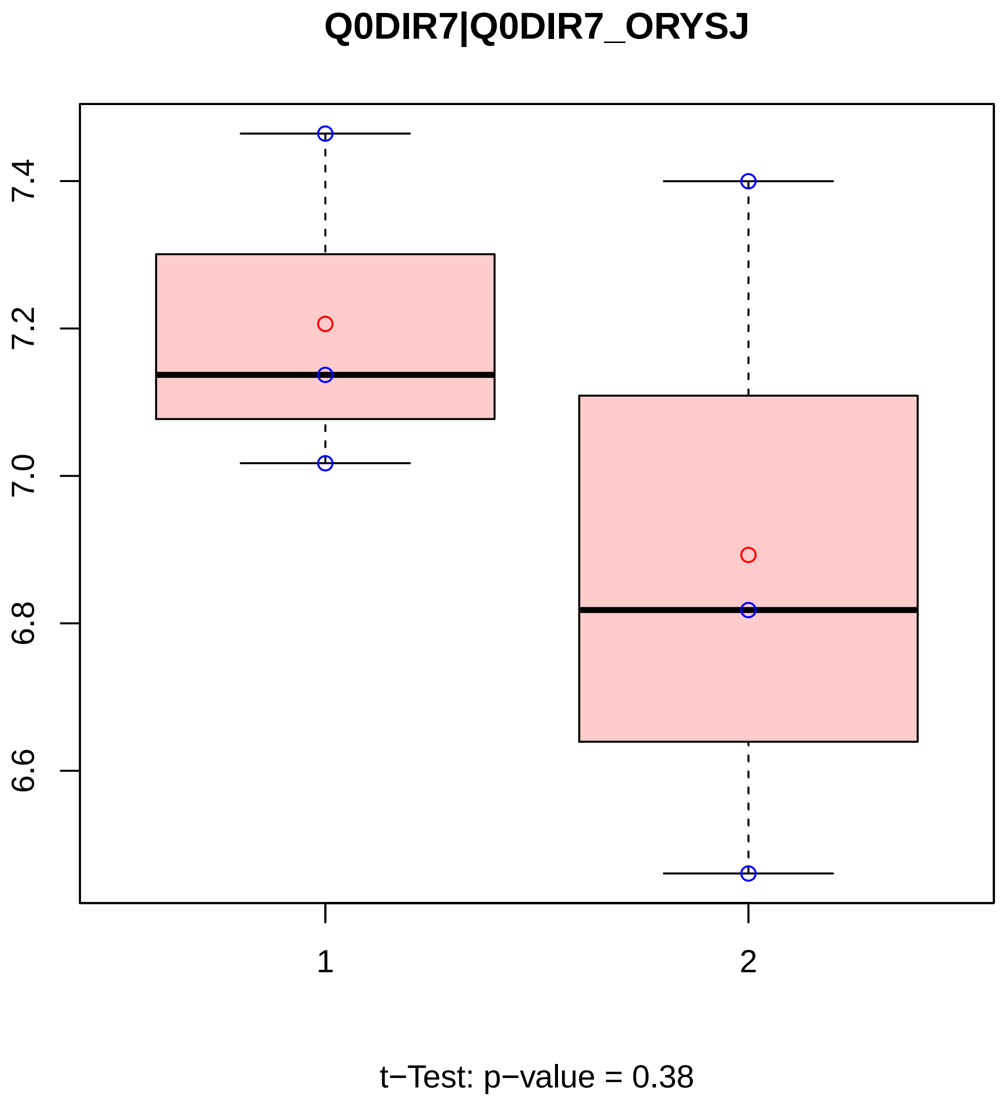
Q0DIR7|Q0DIR7_ORYSJ
7.4
7.2
7.0
6.8
6.6
1
2
t−Test: p−value = 0.38

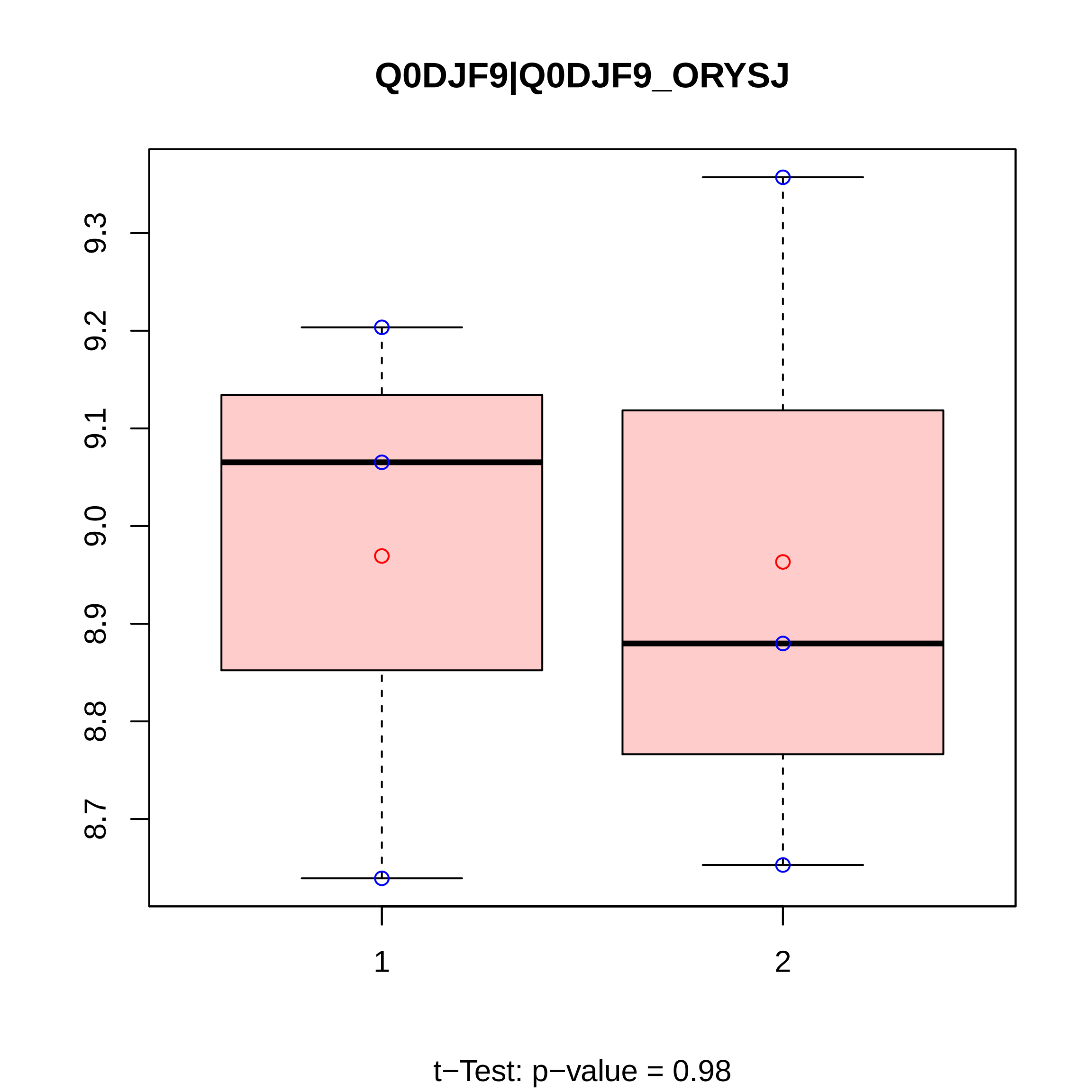
Q0DJF9|Q0DJF9_ORYSJ
9.3
9.2
9.1
9.0
8.9
8.8
8.7
1
2
t−Test: p−value = 0.98

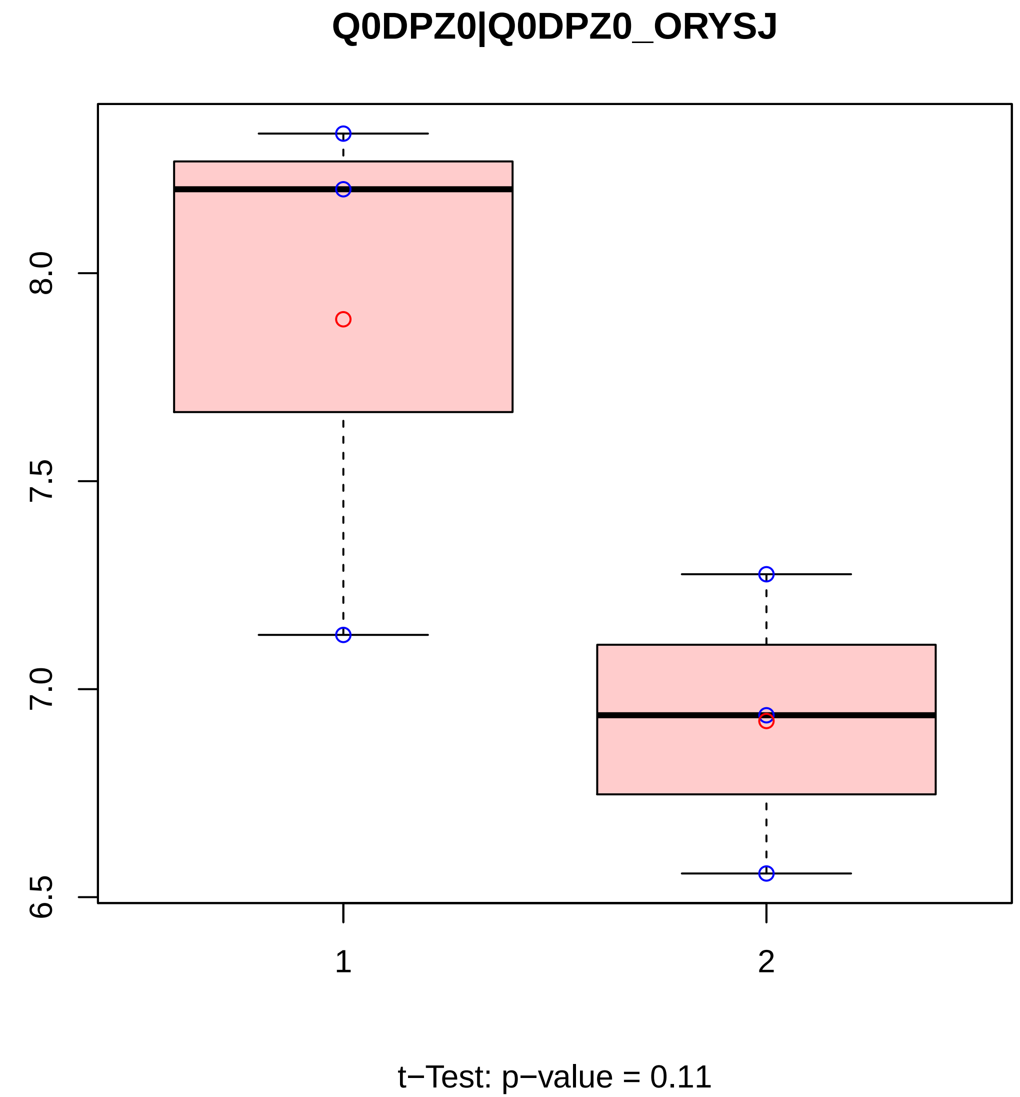

Q0DPZ0|Q0DPZ0_ORYSJ
8.0
7.5
7.0
6.5
1
2
t−Test: p−value = 0.11

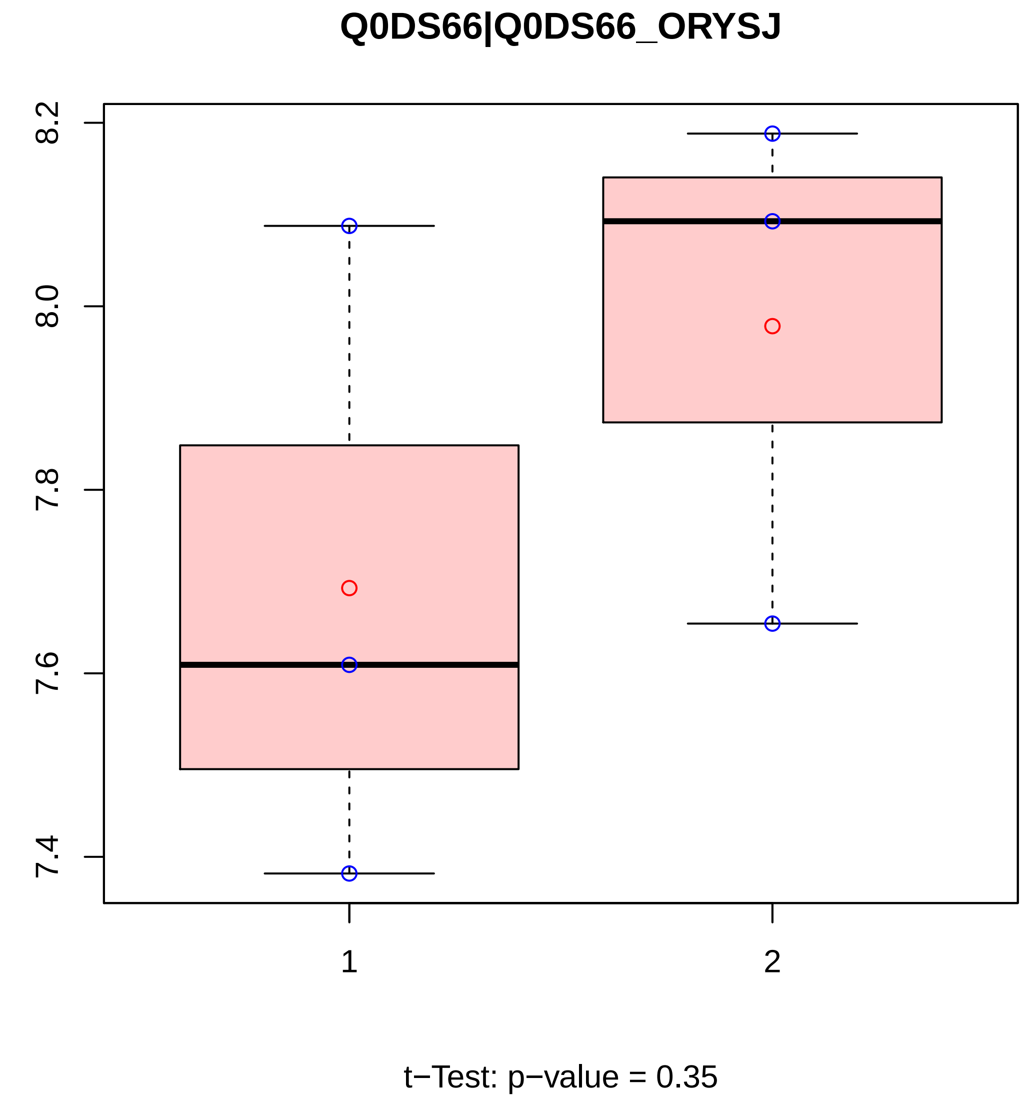

Q0DS66|Q0DS66_ORYSJ
8.2
8.0
7.8
7.6
7.4
1
2
t−Test: p−value = 0.35

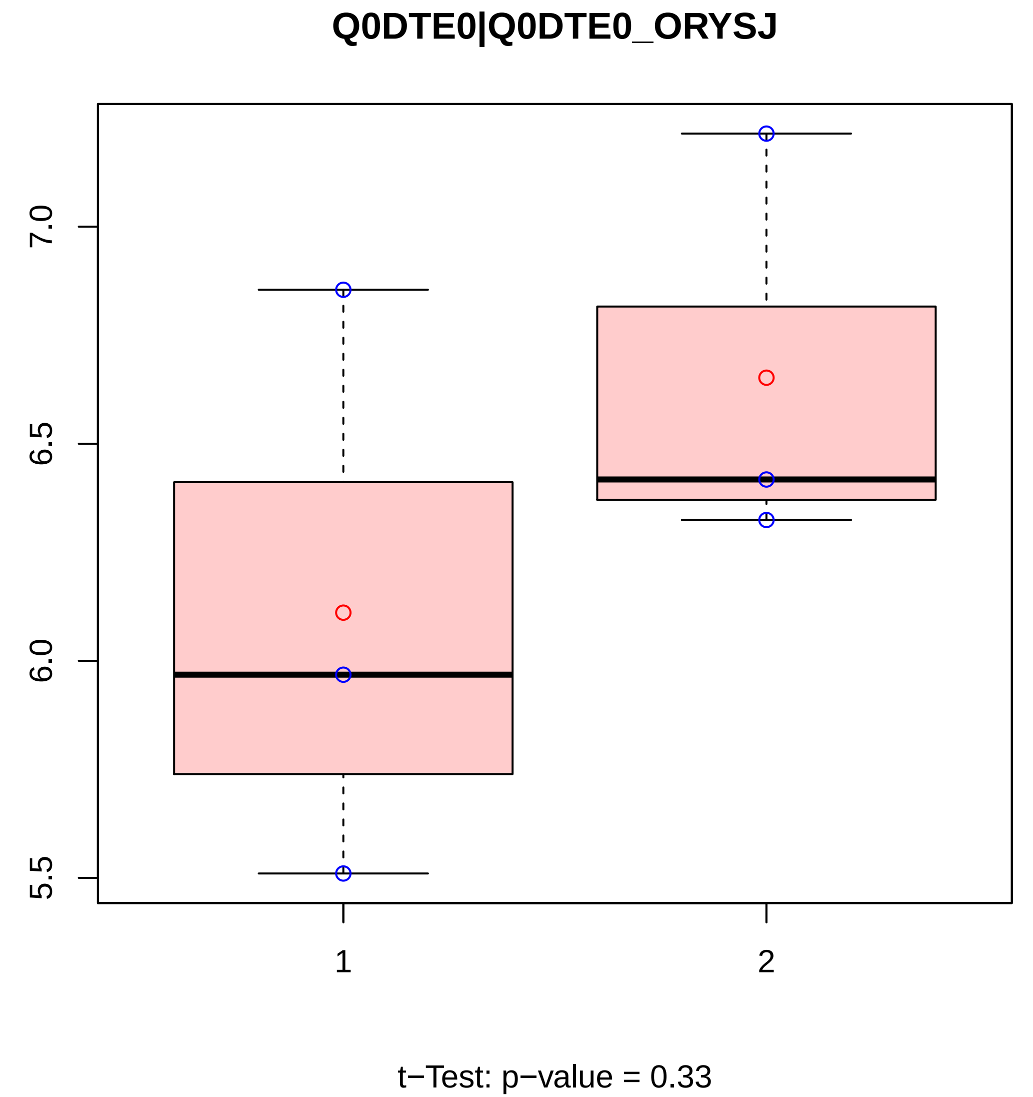
Q0DTE0|Q0DTE0_ORYSJ
7.0
6.5
6.0
5.5
1
2
t−Test: p−value = 0.33

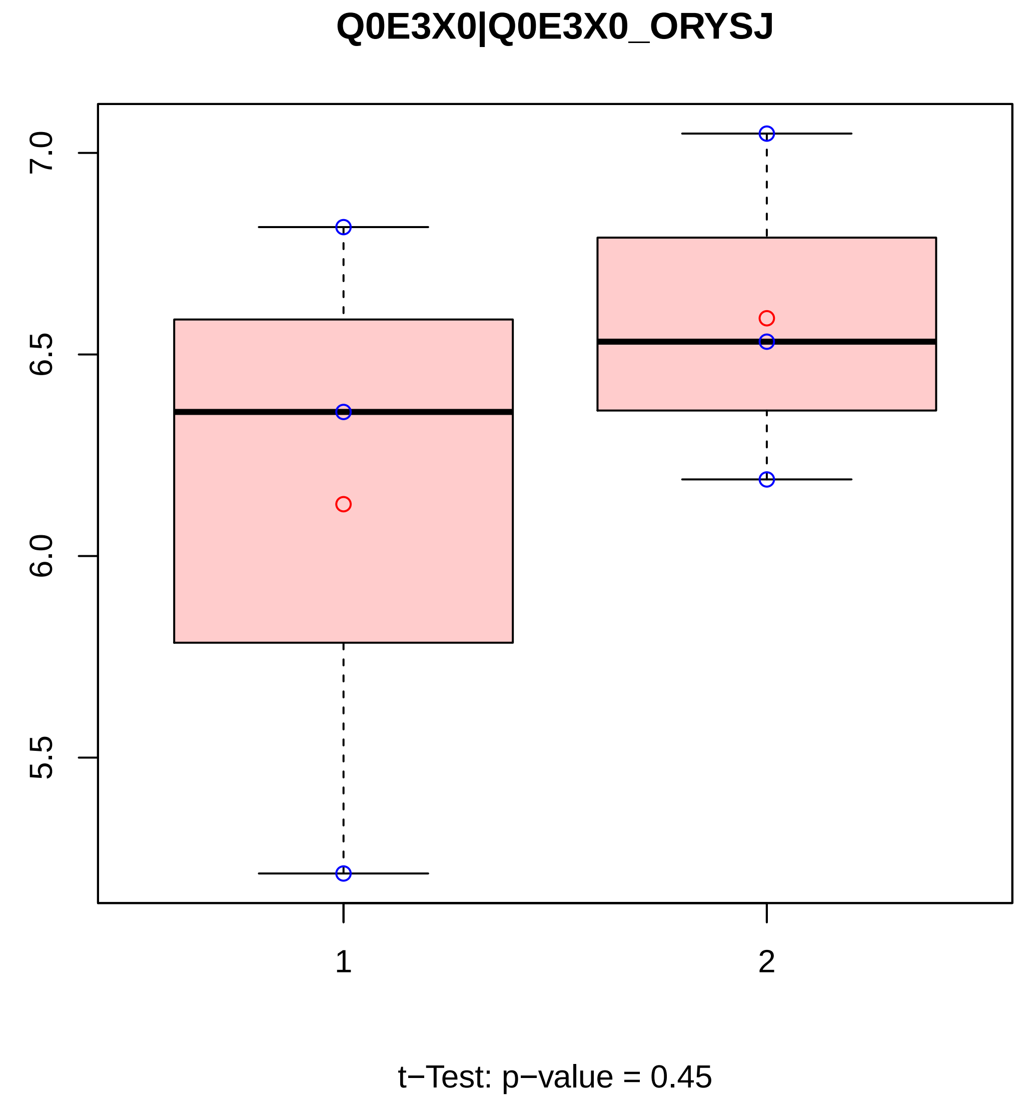
Q0E3X0|Q0E3X0_ORYSJ
7.0
6.5
6.0
5.5
1
2
t−Test: p−value = 0.45

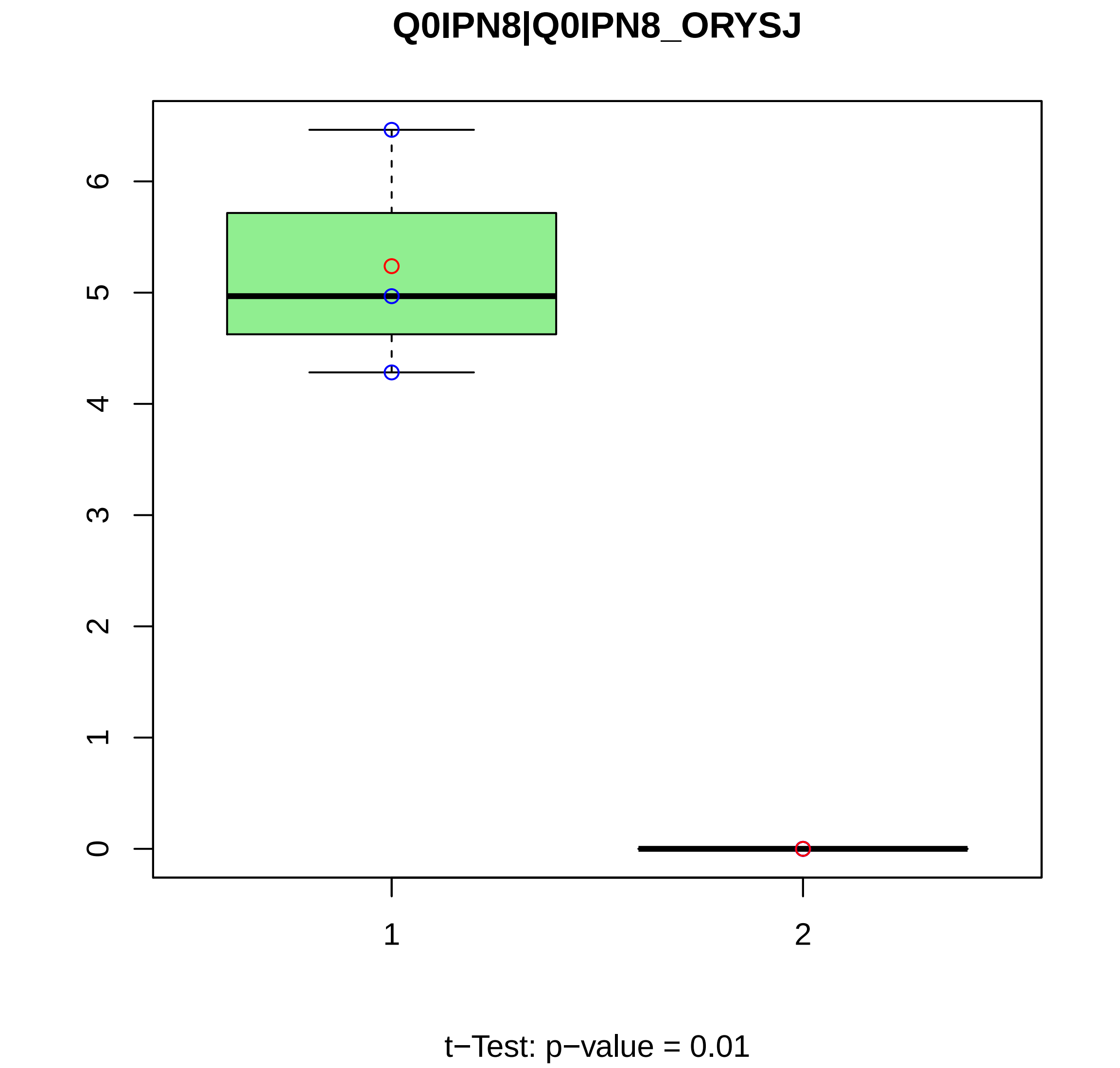
Q0IPN8|Q0IPN8_ORYSJ
0
1
2
3
4
5
6
1
2
t−Test: p−value = 0.01

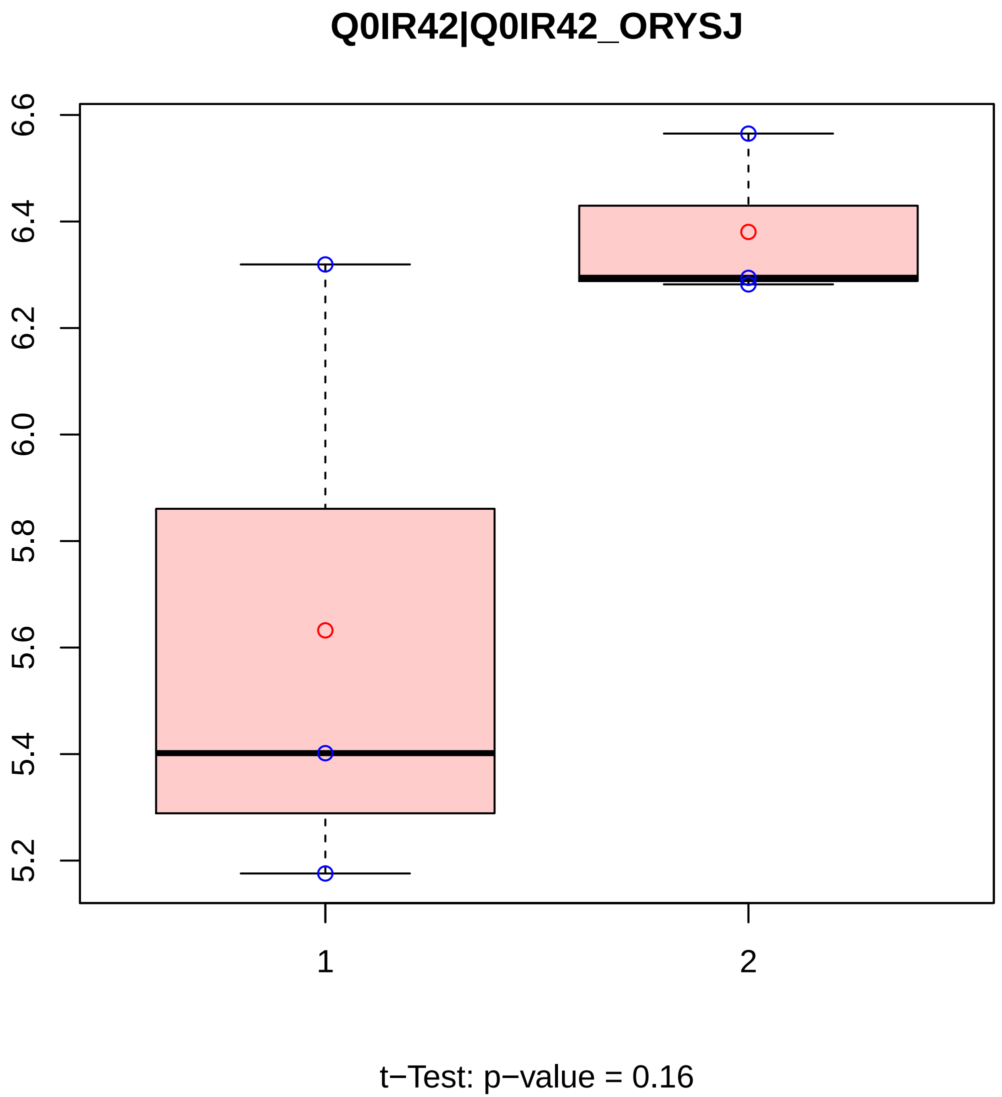
Q0IR42|Q0IR42_ORYSJ
6.6
6.4
6.2
6.0
5.8
5.6
5.4
5.2
1
2
t−Test: p−value = 0.16

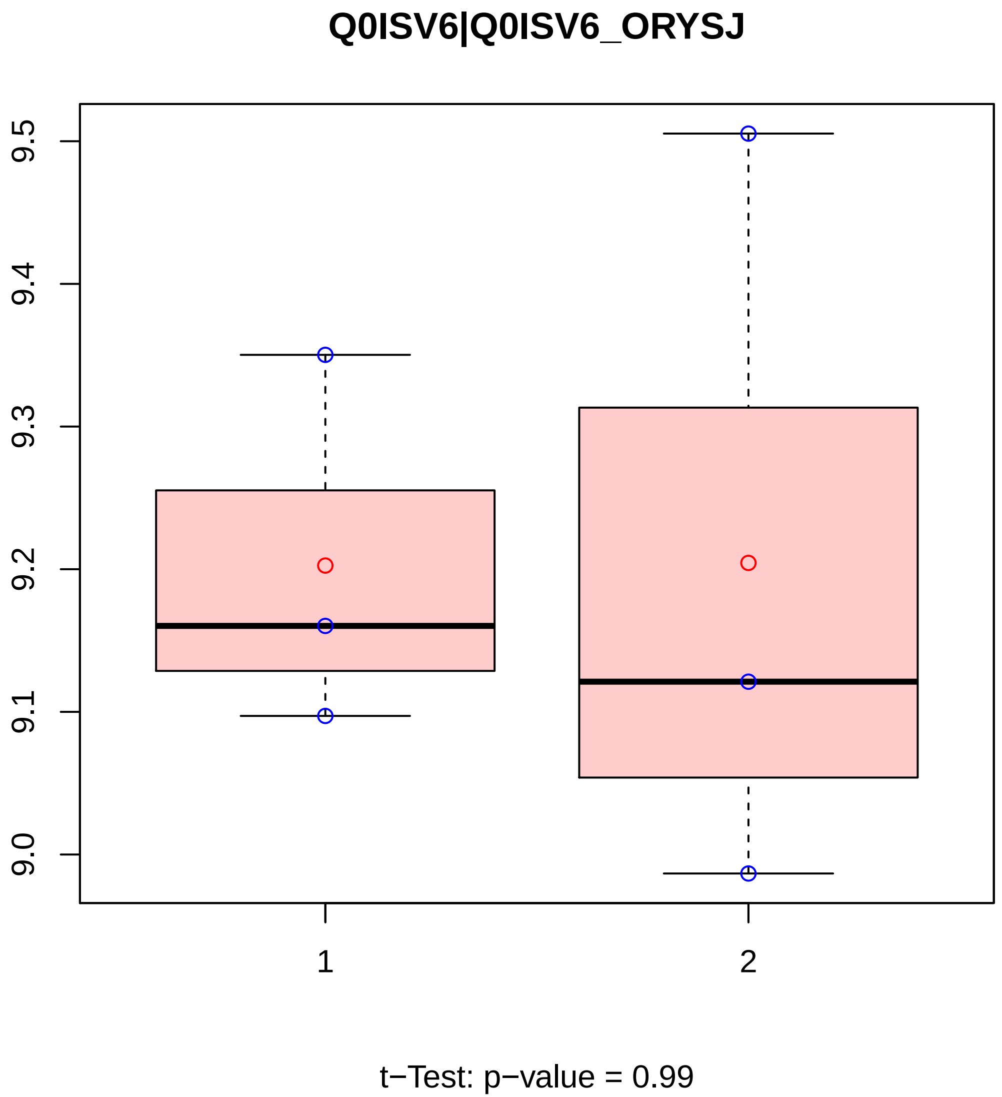

Q0ISV6|Q0ISV6_ORYSJ
9.0
9.1
9.2
9.3
9.4
9.5
1
2
t−Test: p−value = 0.99

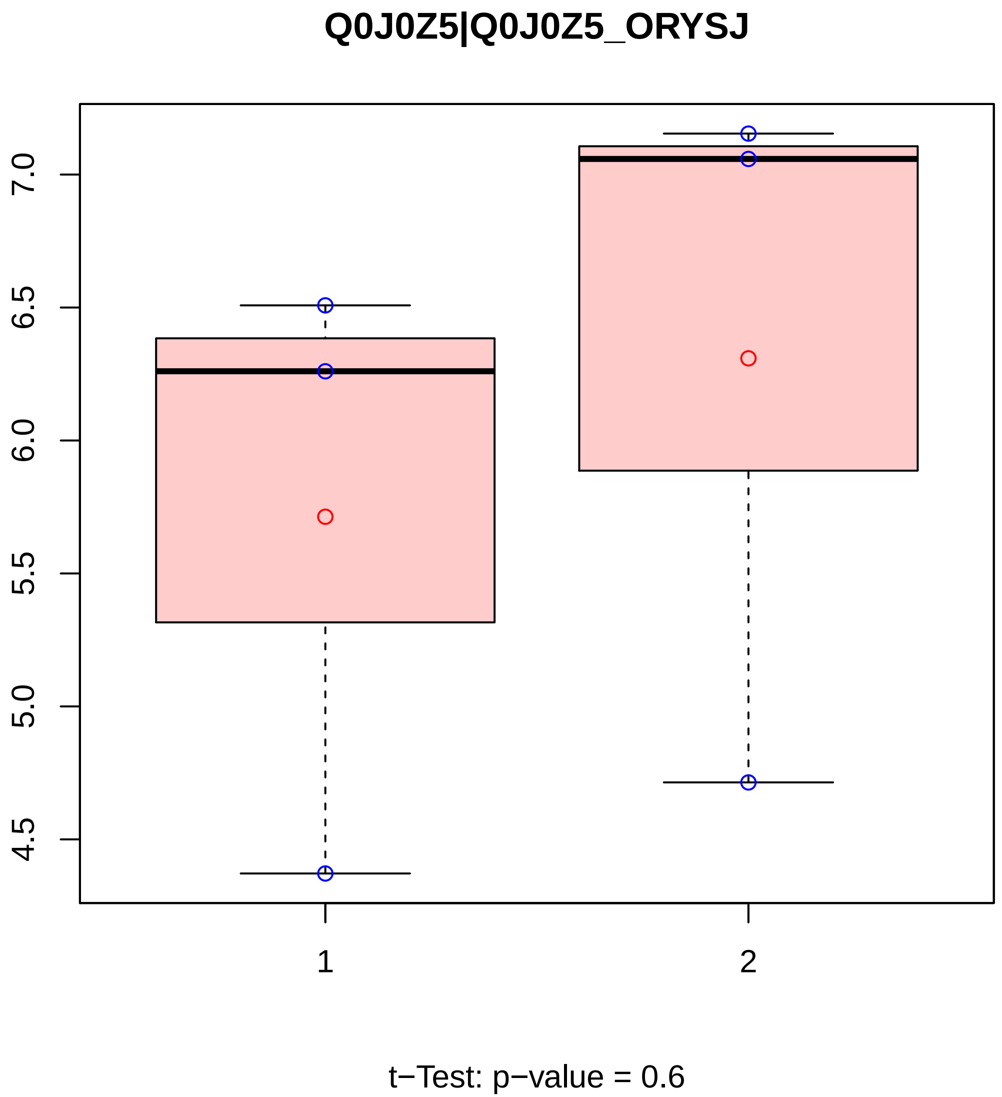

Q0J0Z5|Q0J0Z5_ORYSJ
7.0
6.5
6.0
5.5
5.0
4.5
1
2
t−Test: p−value = 0.6

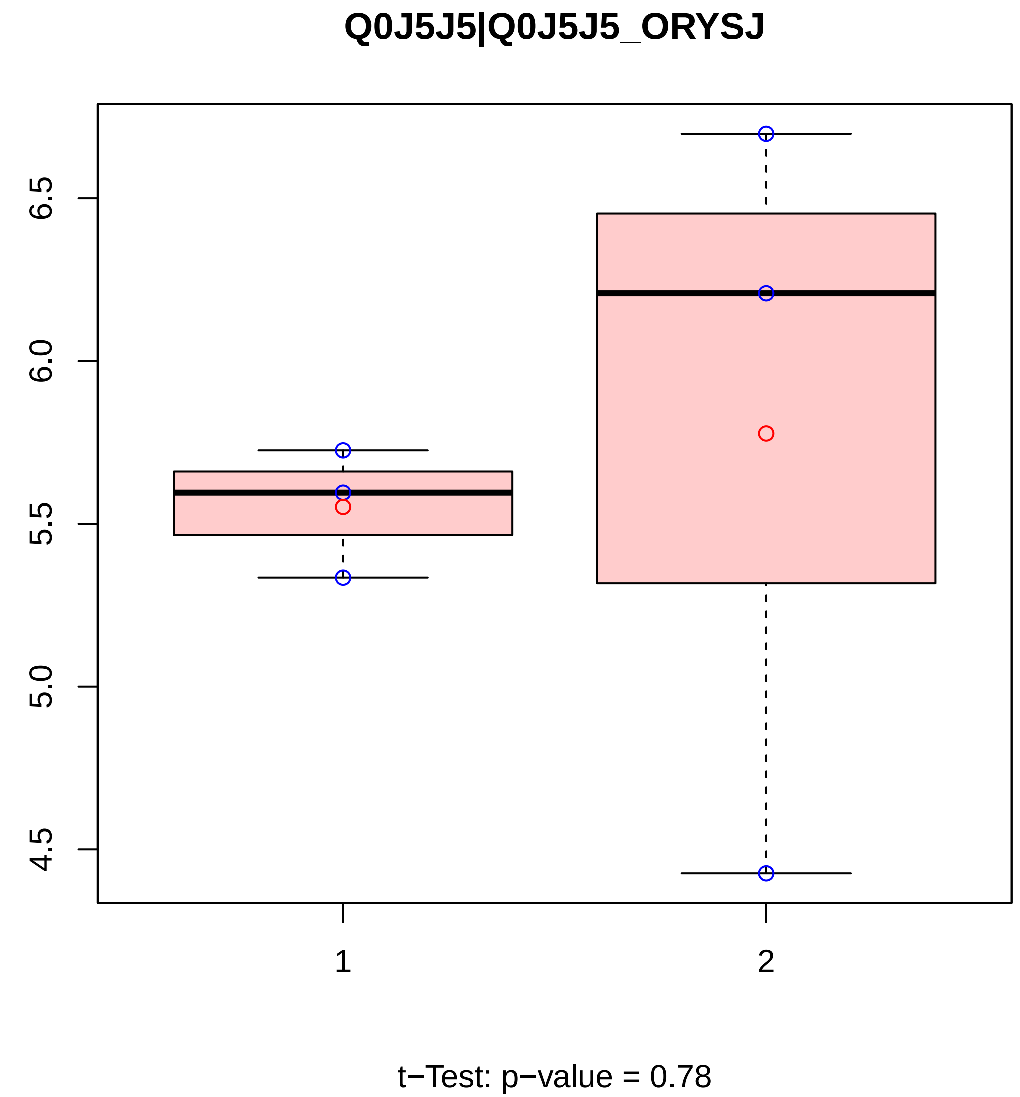
Q0J5J5|Q0J5J5_ORYSJ
6.5
6.0
5.5
5.0
4.5
1
2
t−Test: p−value = 0.78

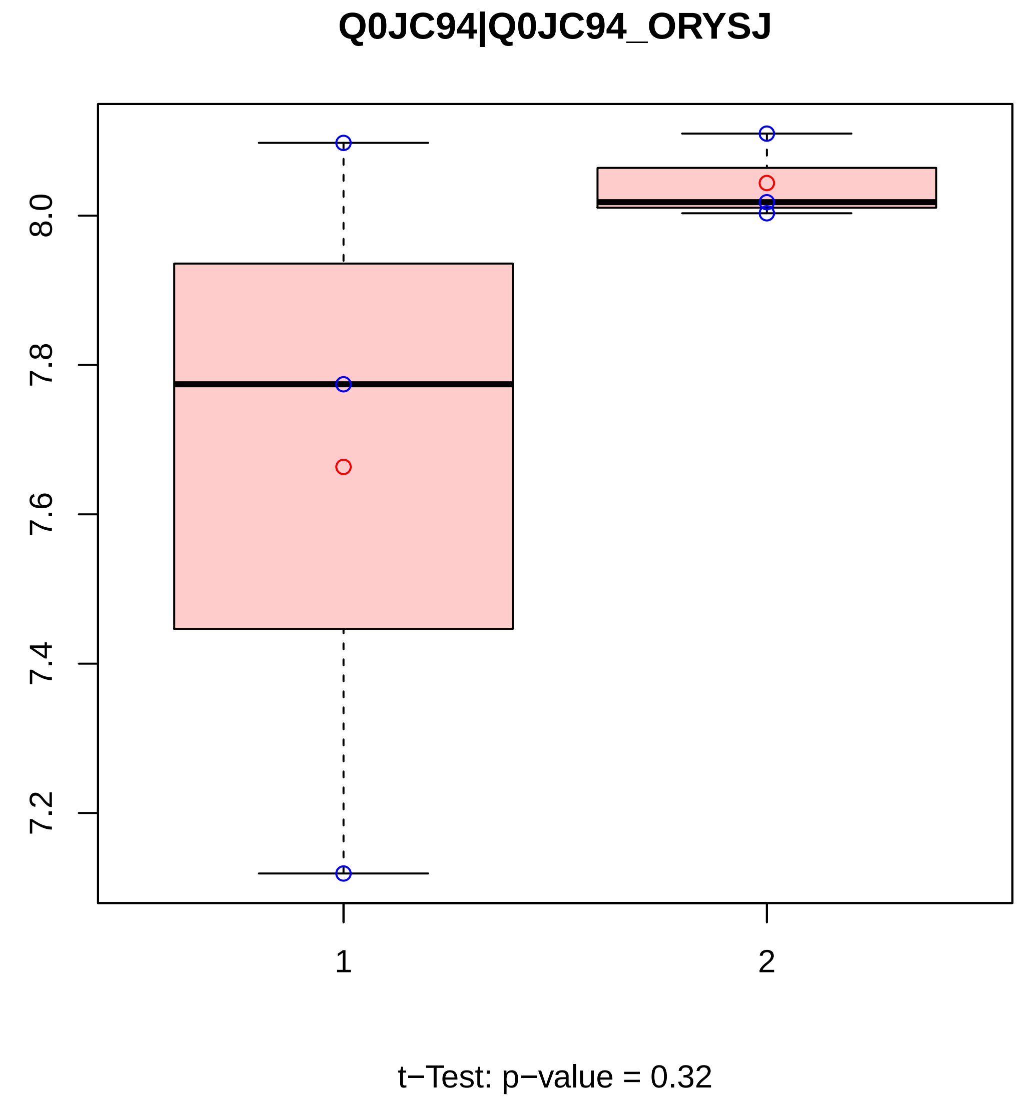
Q0JC94|Q0JC94_ORYSJ
8.0
7.8
7.6
7.4
7.2
1
2
t−Test: p−value = 0.32

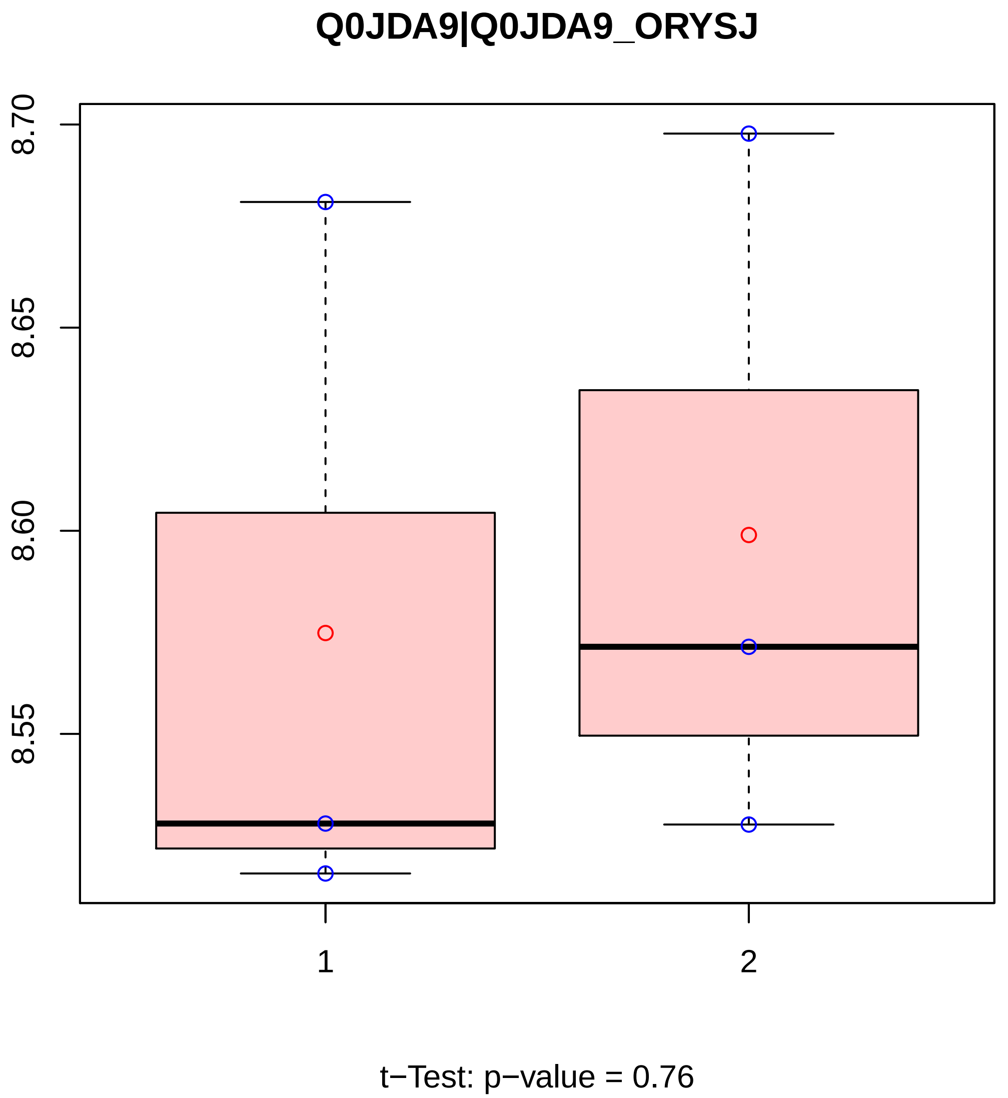

Q0JDA9|Q0JDA9_ORYSJ
8.70
8.65
8.60
8.55
1
2
t−Test: p−value = 0.76

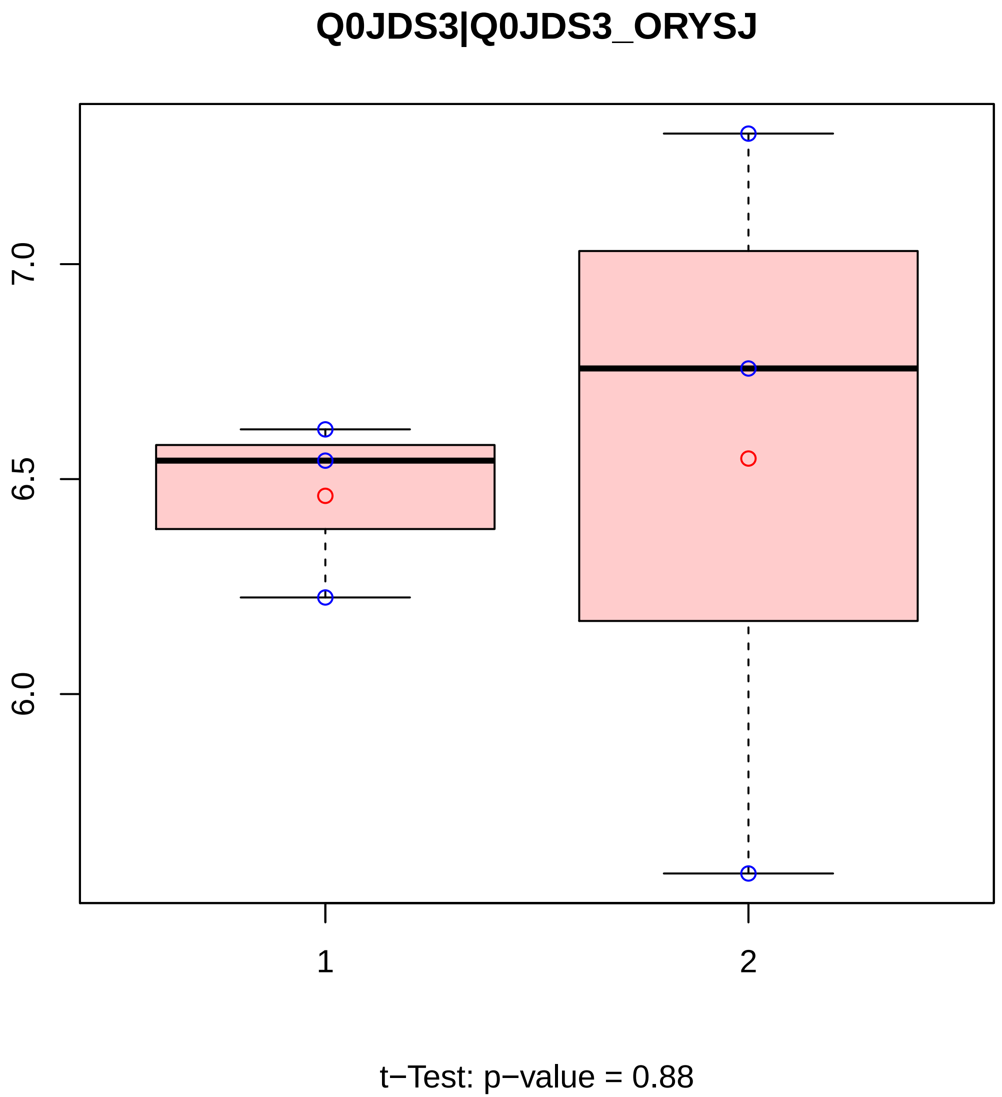
Q0JDS3|Q0JDS3_ORYSJ
7.0
6.5
6.0
1
2
t−Test: p−value = 0.88

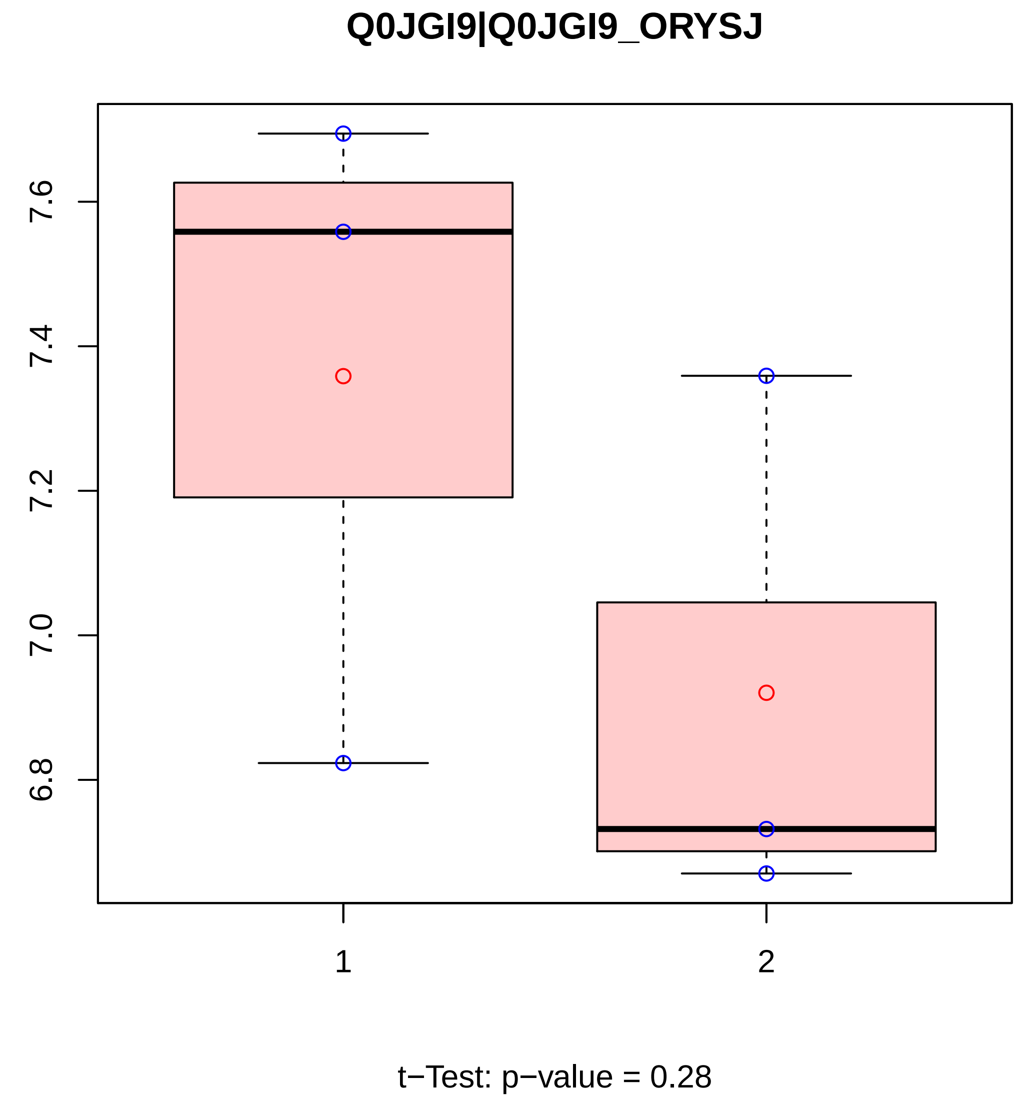
Q0JGI9|Q0JGI9_ORYSJ
7.6
7.4
7.2
7.0
6.8
1
2
t−Test: p−value = 0.28

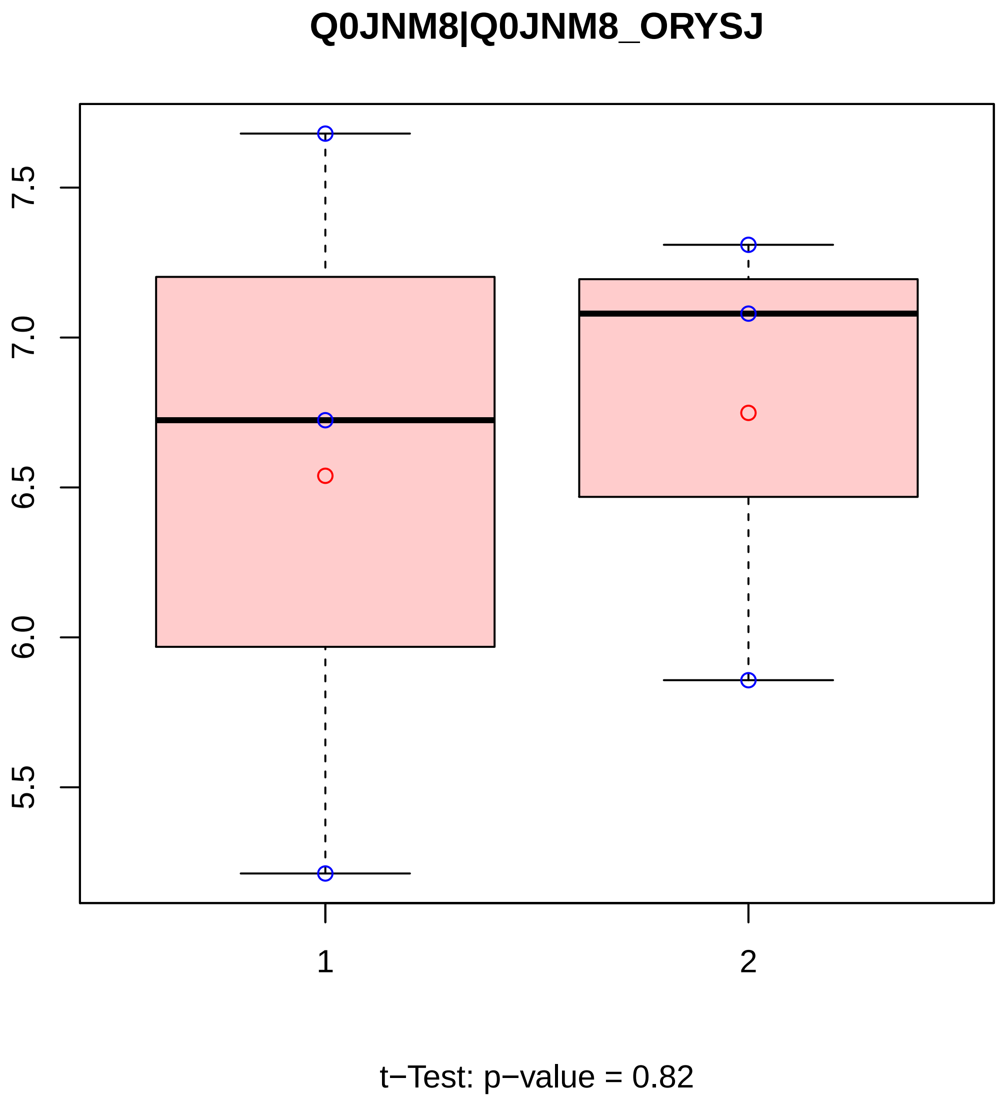
Q0JNM8|Q0JNM8_ORYSJ
7.5
7.0
6.5
6.0
5.5
1
2
t−Test: p−value = 0.82

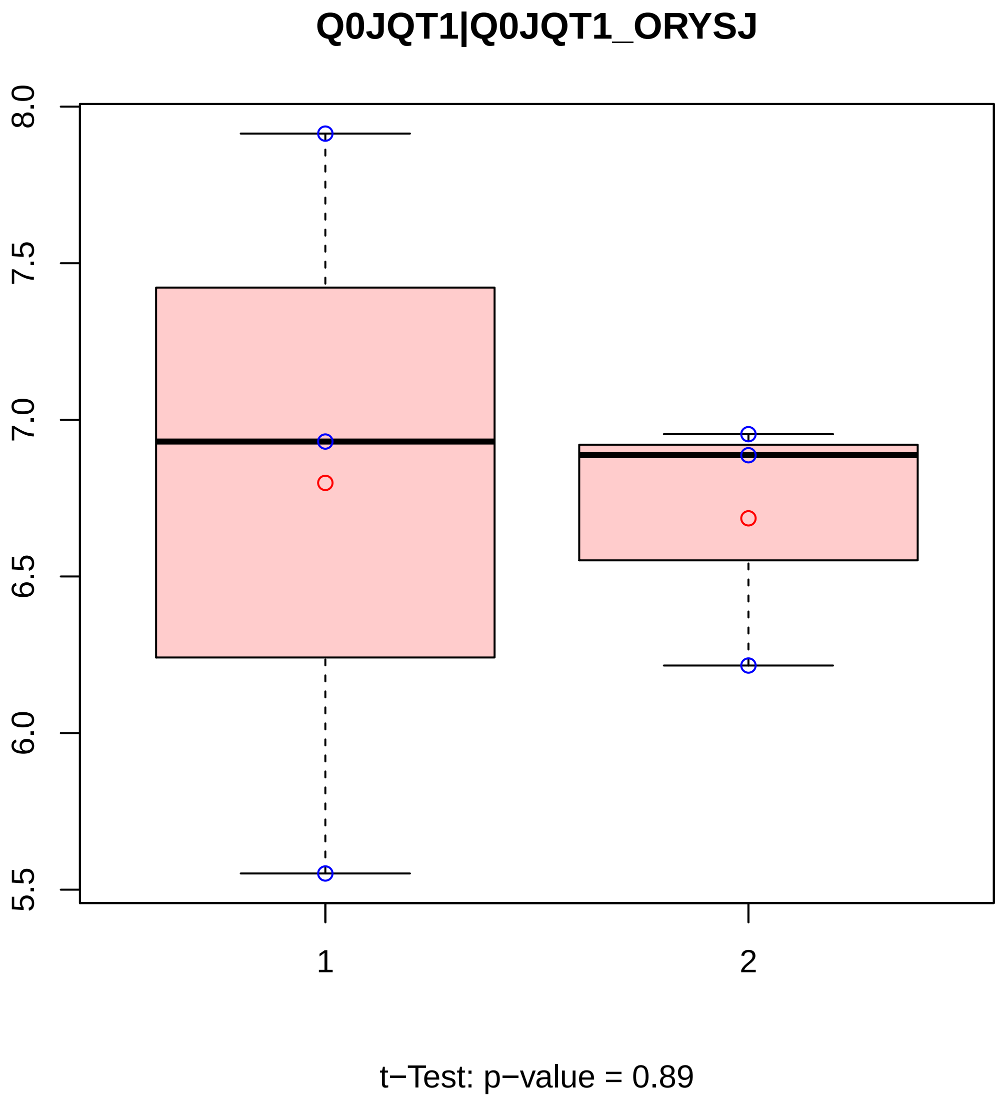
Q0JQT1|Q0JQT1_ORYSJ
8.0
7.5
7.0
6.5
6.0
5.5
1
2
t−Test: p−value = 0.89

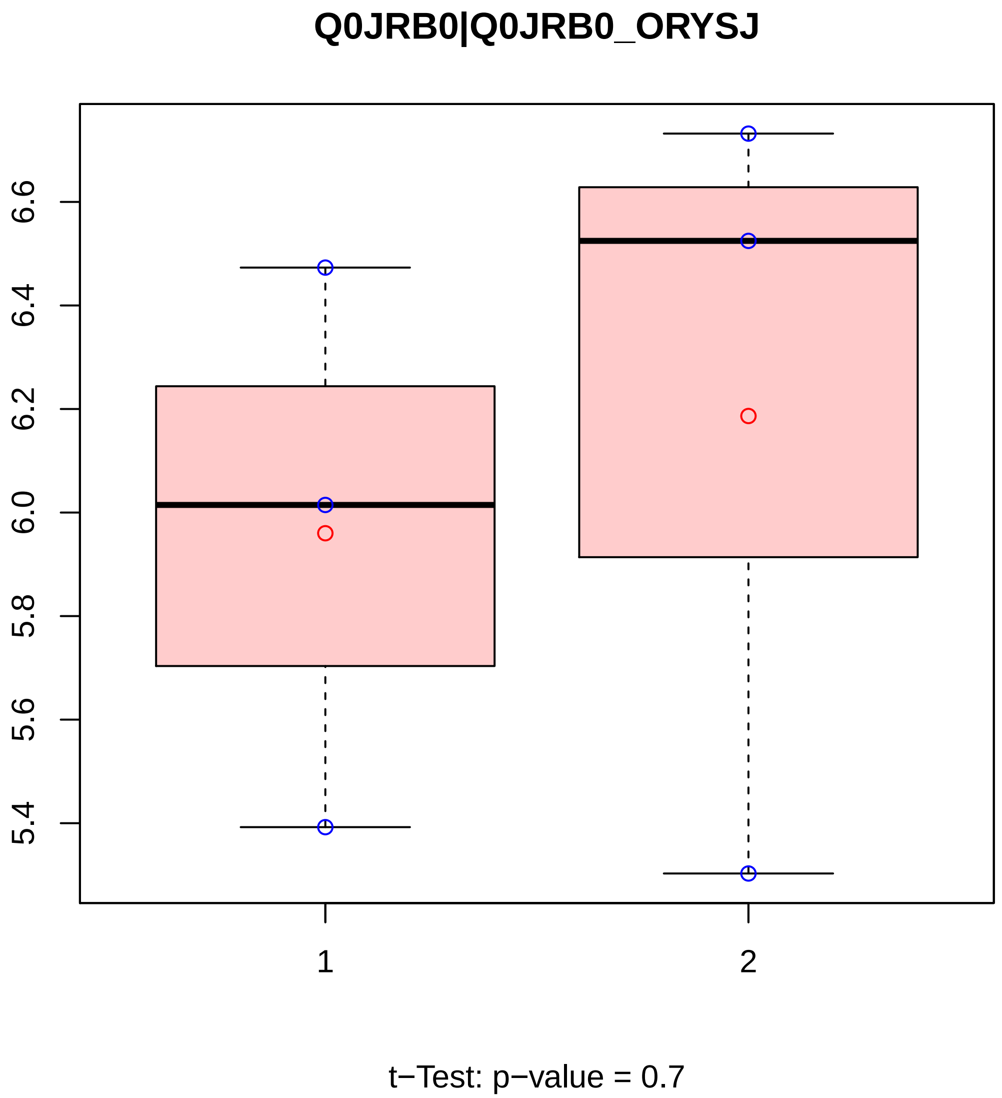

Q0JRB0|Q0JRB0_ORYSJ
6.6
6.4
6.2
6.0
5.8
5.6
5.4
1
2
t−Test: p−value = 0.7

Q0KKZ5|Q0KKZ5_ZOSMR

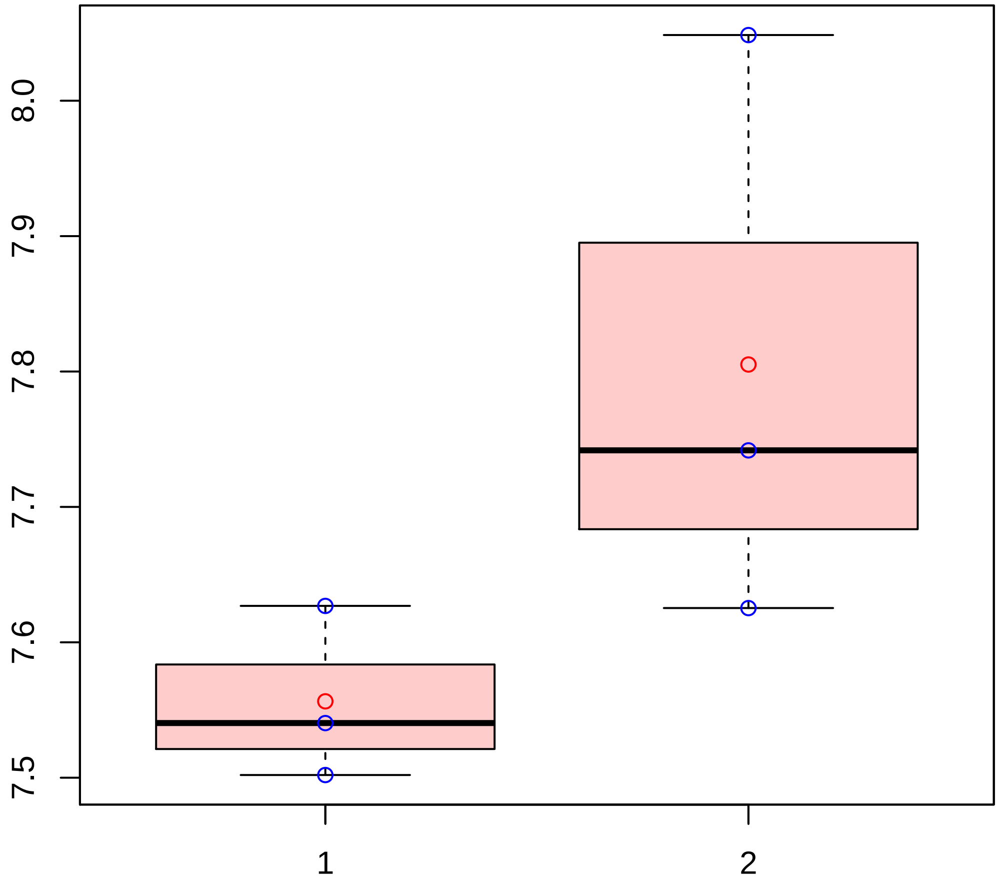

t−Test: p−value = 0.18

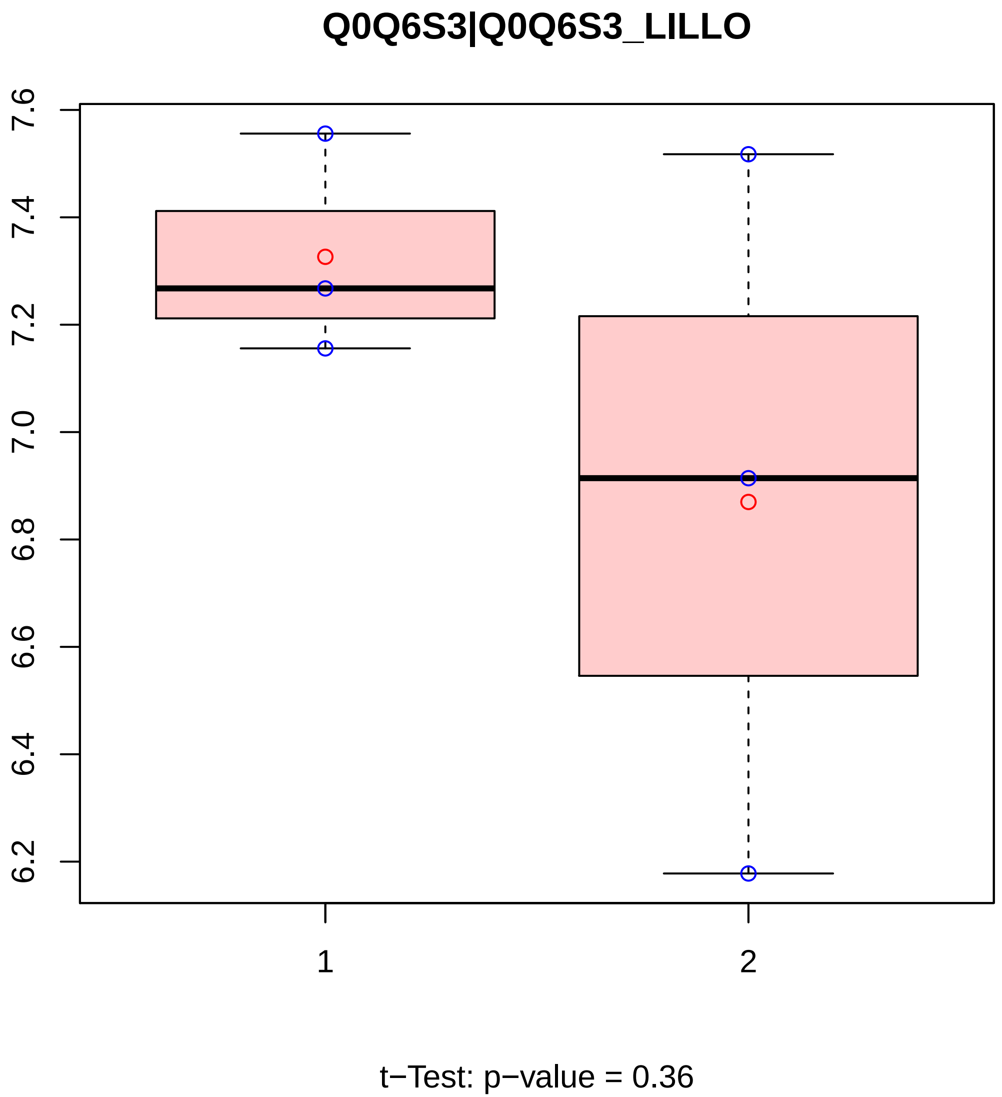

Q0Q6S3|Q0Q6S3_LILLO
7.6
7.4
7.2
7.0
6.8
6.6
6.4
6.2
1
2
t−Test: p−value = 0.36

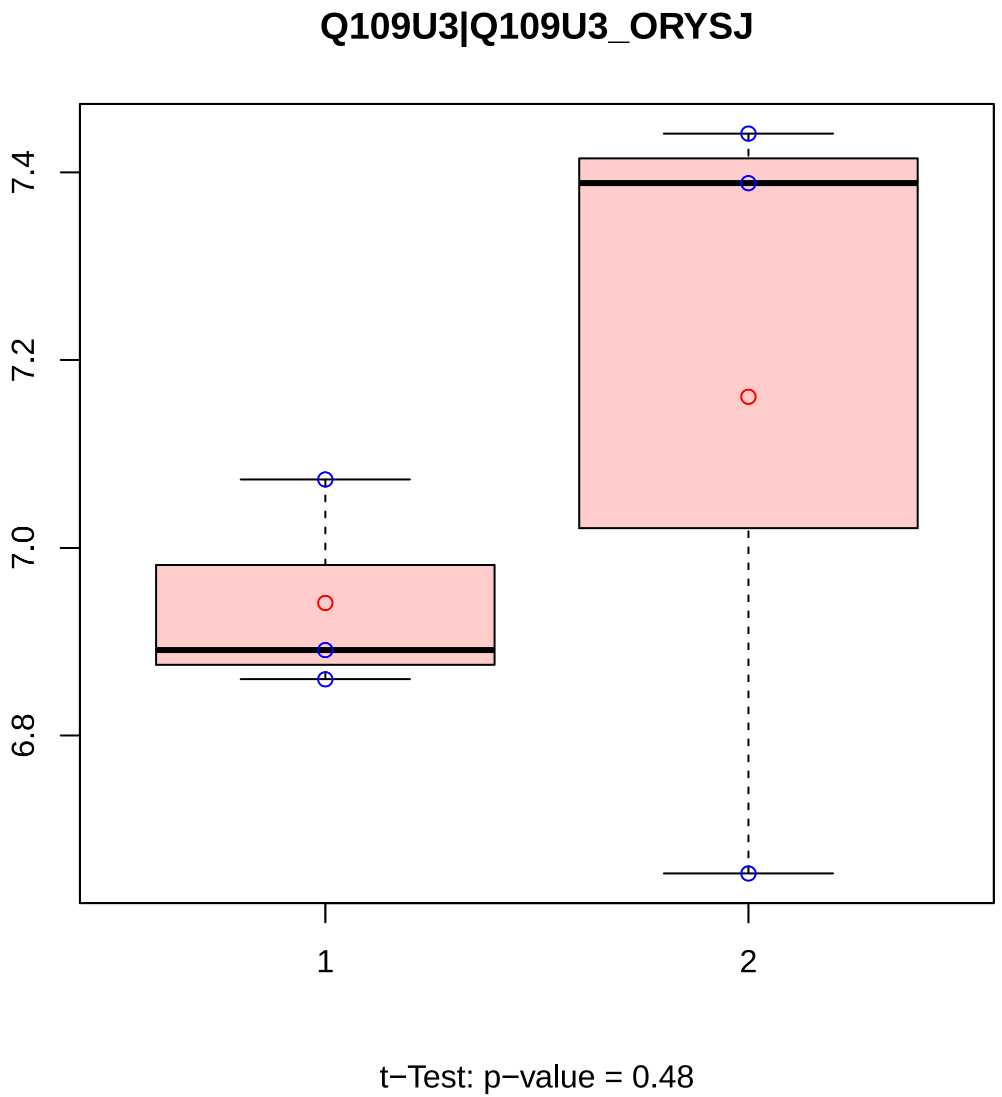
Q109U3|Q109U3_ORYSJ
7.4
7.2
7.0
6.8
1
2
t−Test: p−value = 0.48

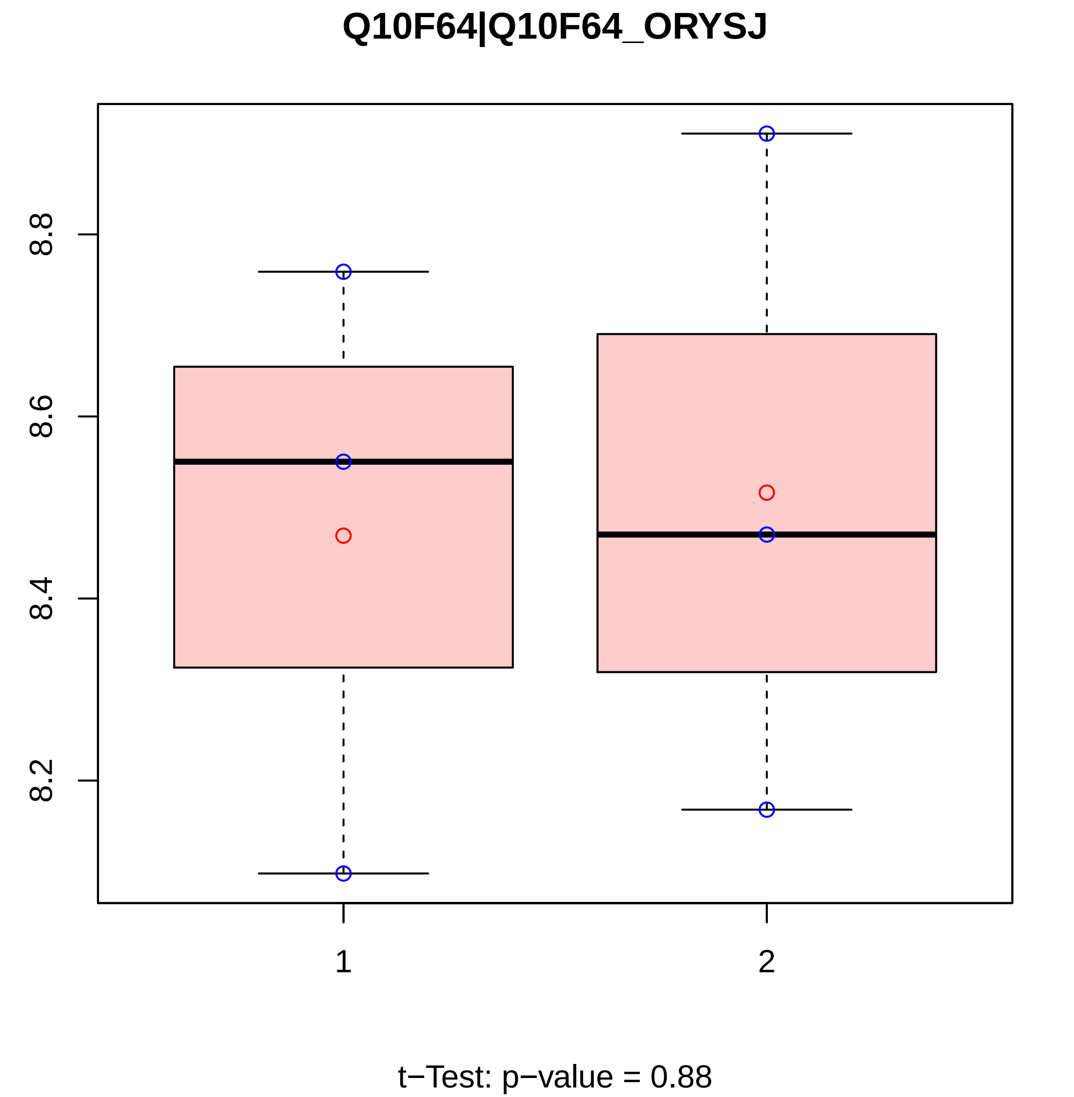
Q10F64|Q10F64_ORYSJ
8.2
8.4
8.6
8.8
1
2
t−Test: p−value = 0.88

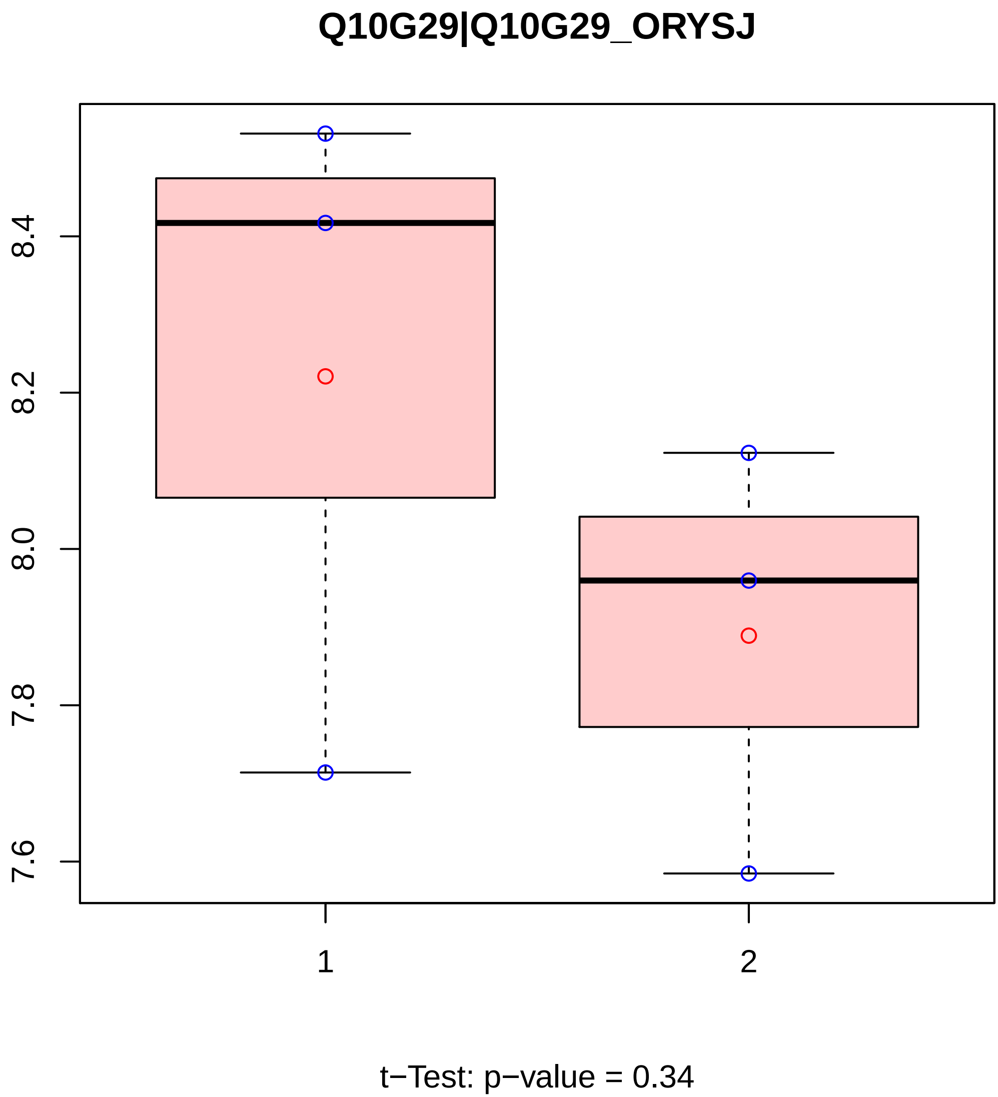

Q10G29|Q10G29_ORYSJ
8.4
8.2
8.0
7.8
7.6
1
2
t−Test: p−value = 0.34

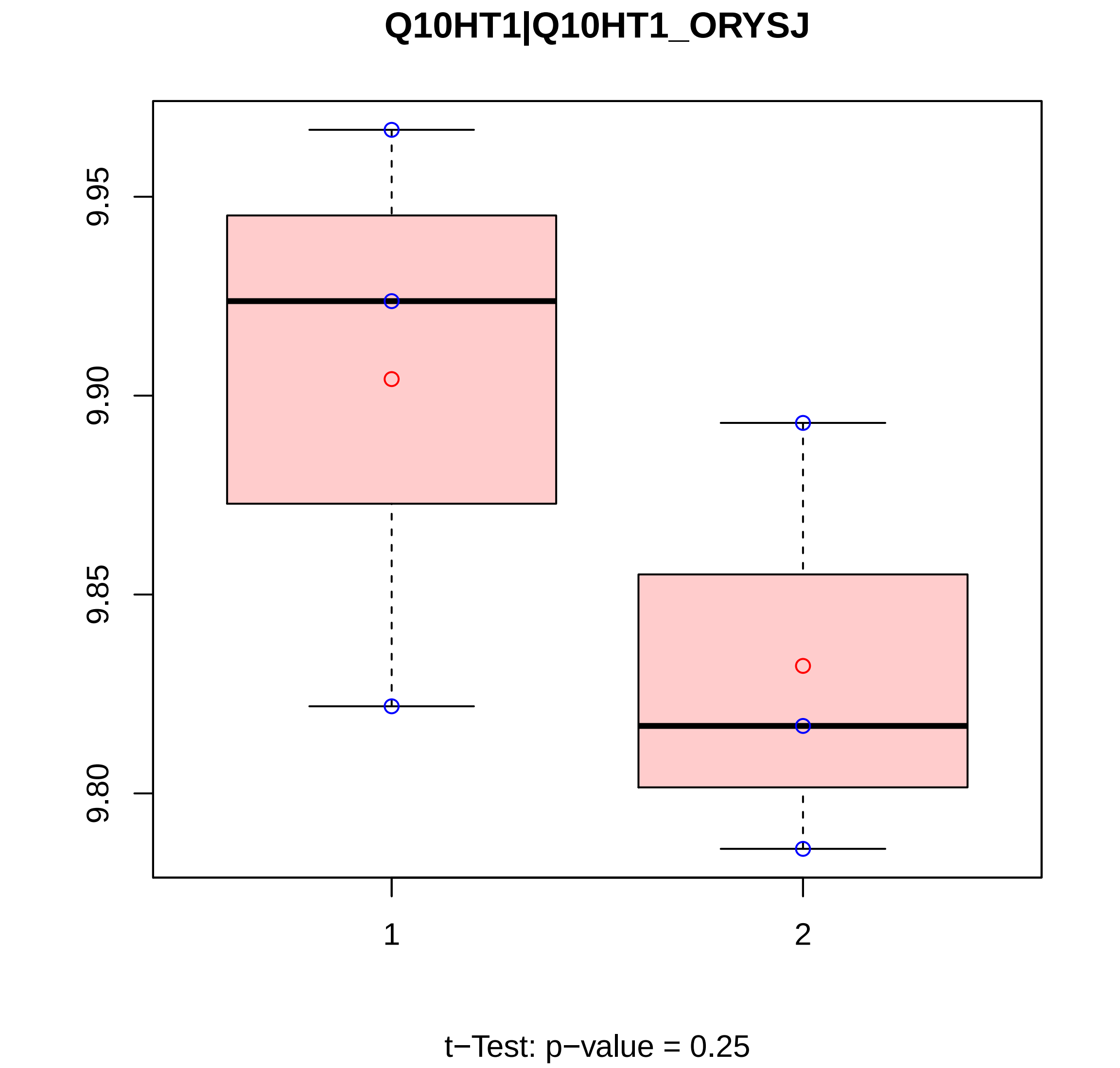
Q10HT1|Q10HT1_ORYSJ
9.80
9.85
9.90
9.95
1
2
t−Test: p−value = 0.25

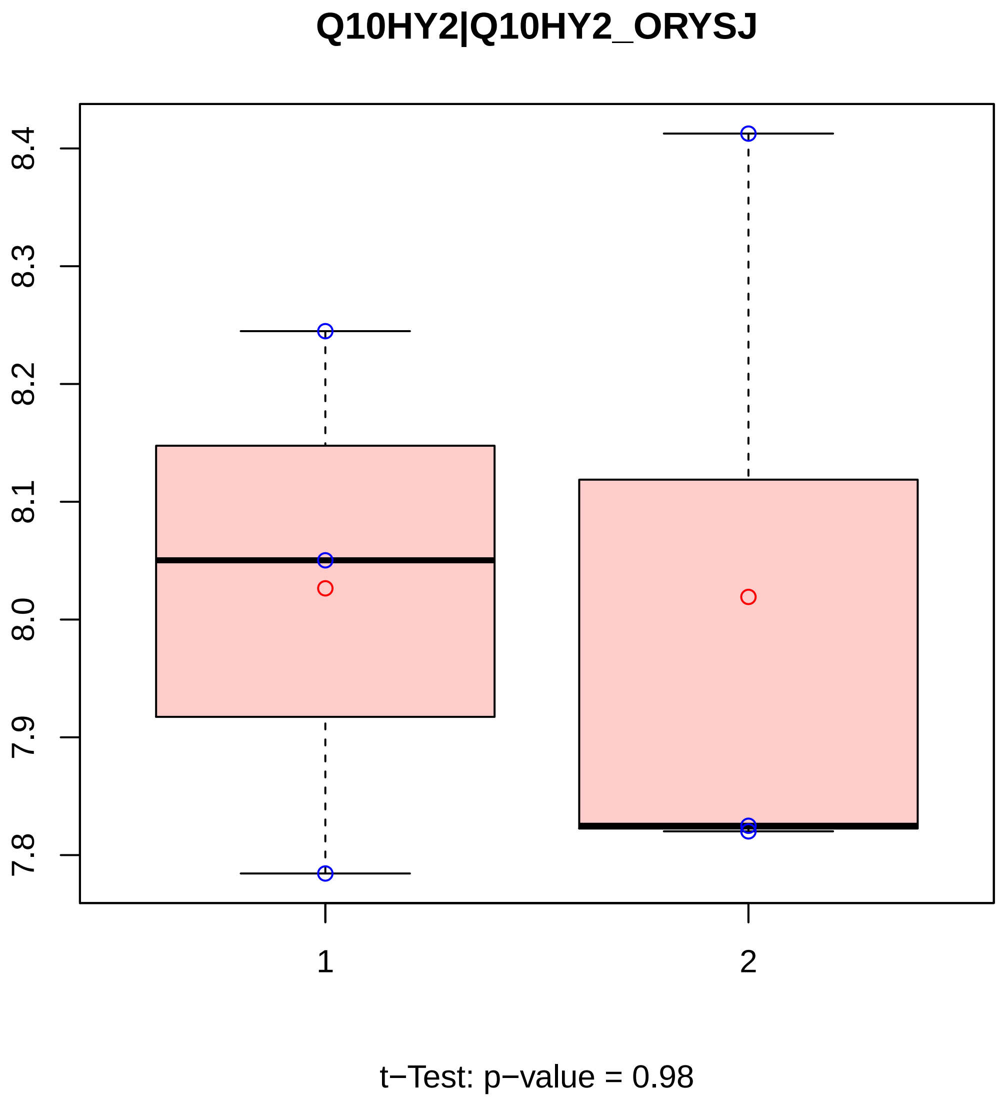

Q10HY2|Q10HY2_ORYSJ
8.4
8.3
8.2
8.1
8.0
7.9
7.8
1
2
t−Test: p−value = 0.98

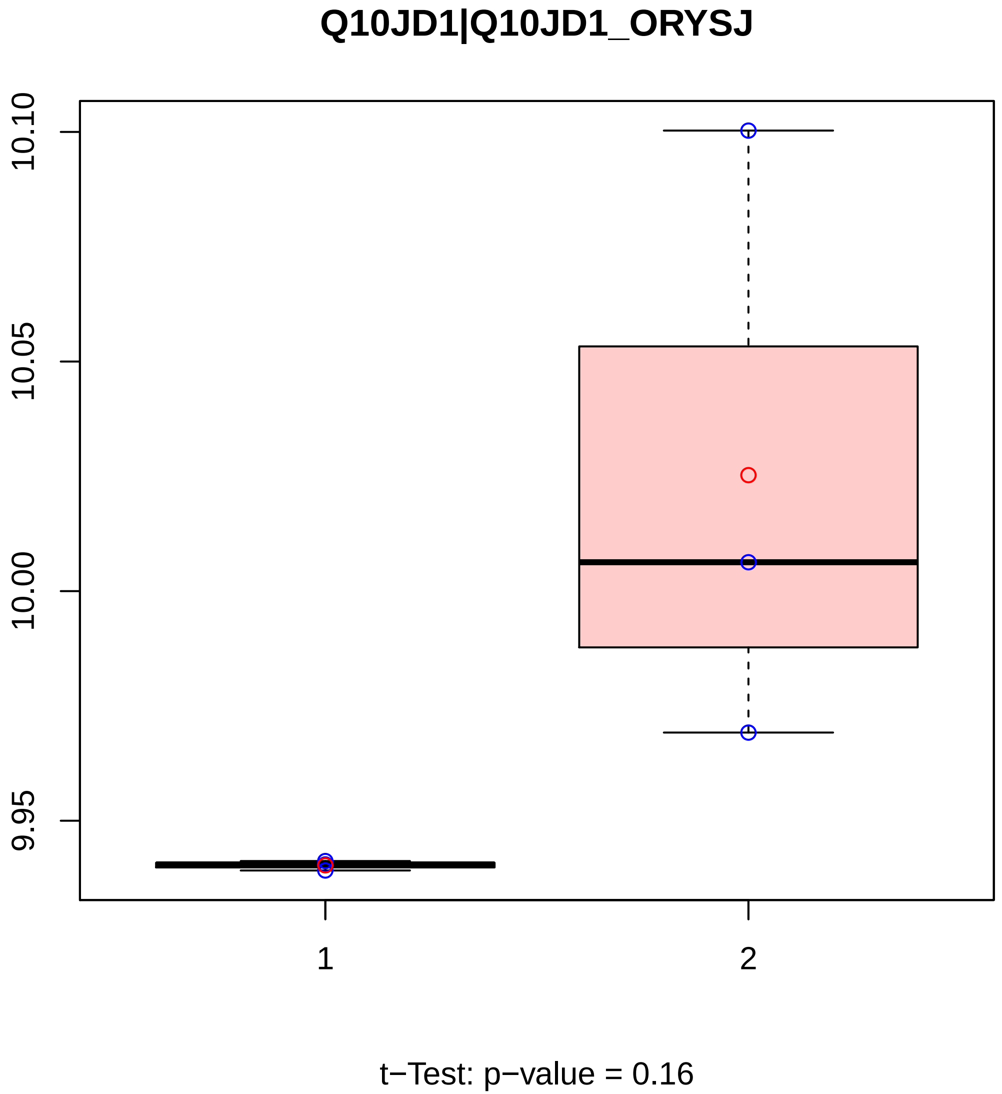
Q10JD1|Q10JD1_ORYSJ
10.10
10.05
10.00
9.95
1
2
t−Test: p−value = 0.16

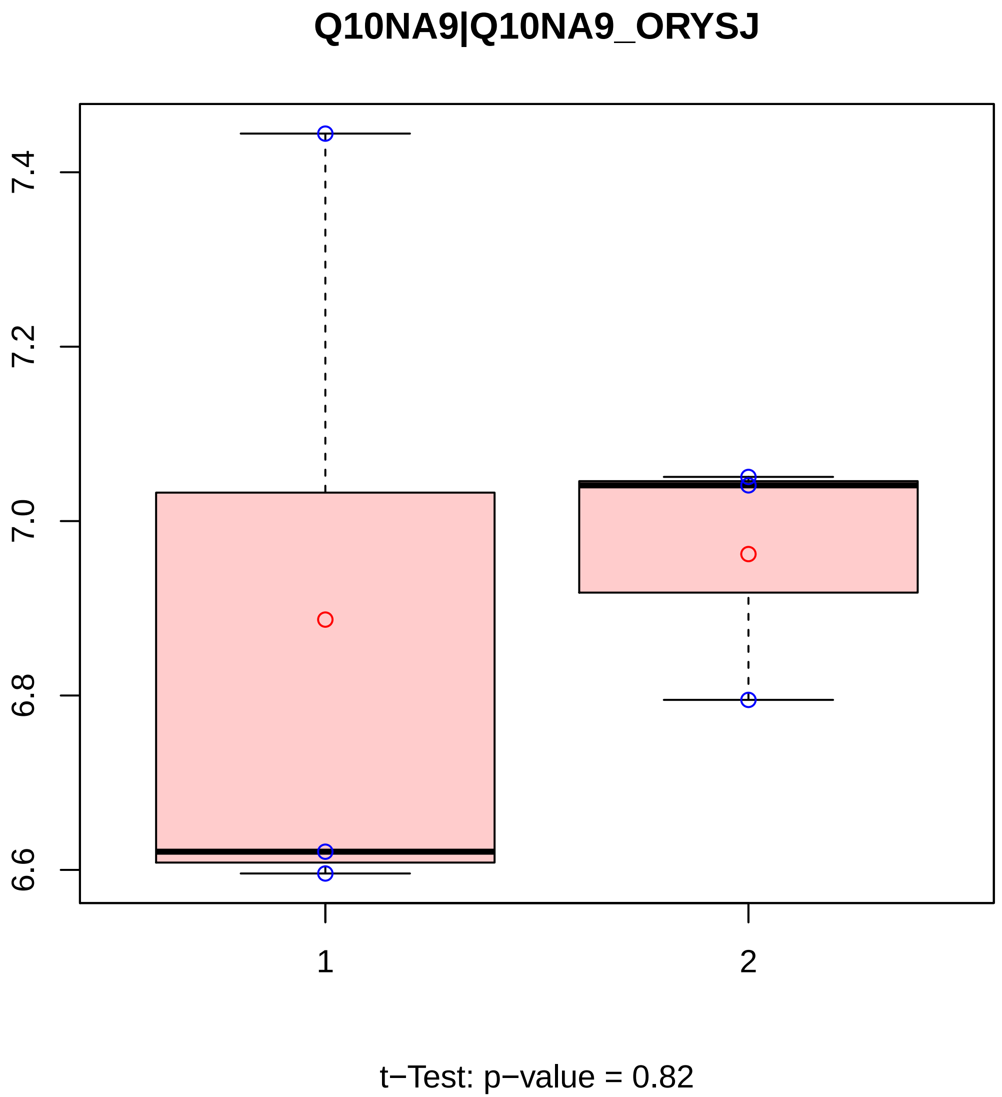

Q10NA9|Q10NA9_ORYSJ
7.4
7.2
7.0
6.8
6.6
1
2
t−Test: p−value = 0.82

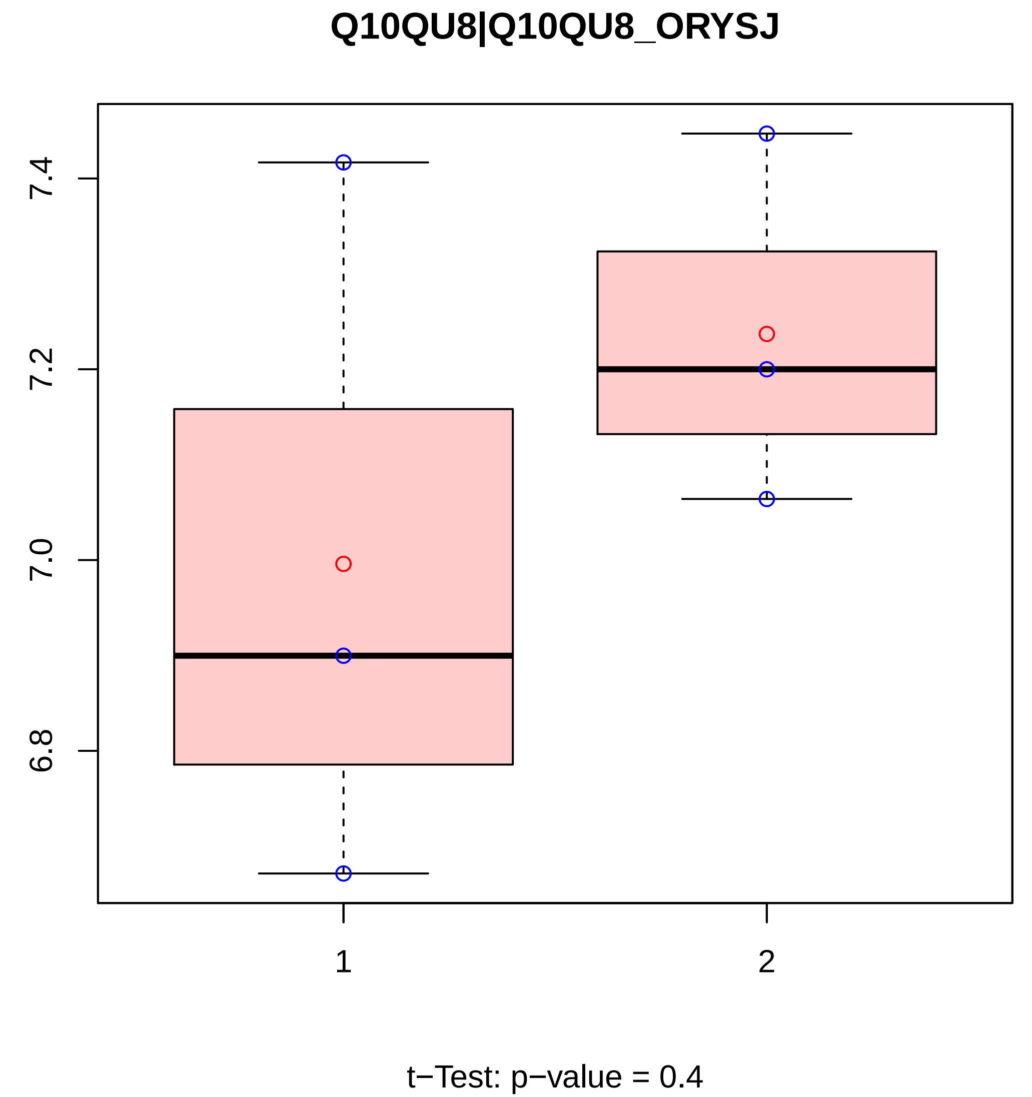
Q10QU8|Q10QU8_ORYSJ
7.4
7.2
7.0
6.8
1
2
t−Test: p−value = 0.4

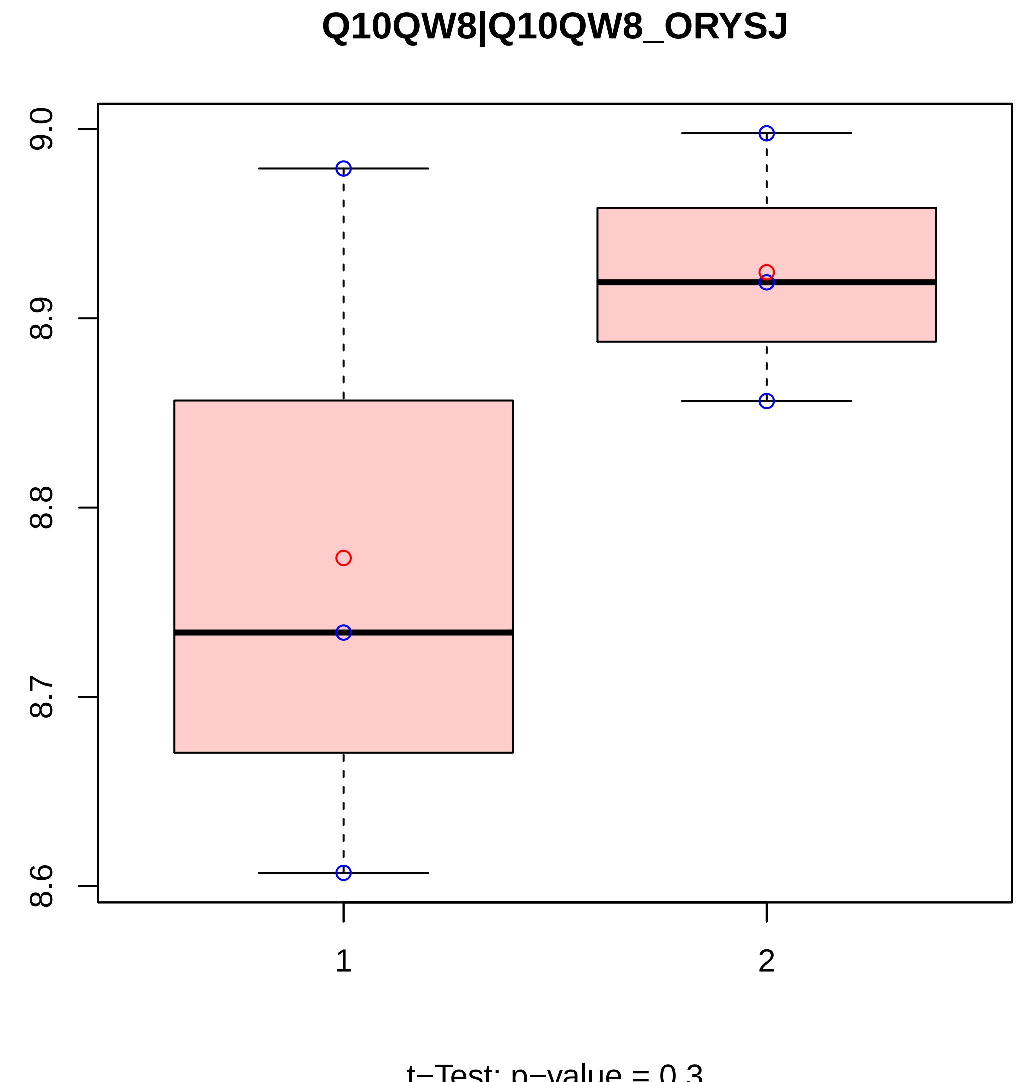
Q10QW8|Q10QW8_ORYSJ
9.0
8.9
8.8
8.7
8.6
1
2
t−Test: p−value = 0.3

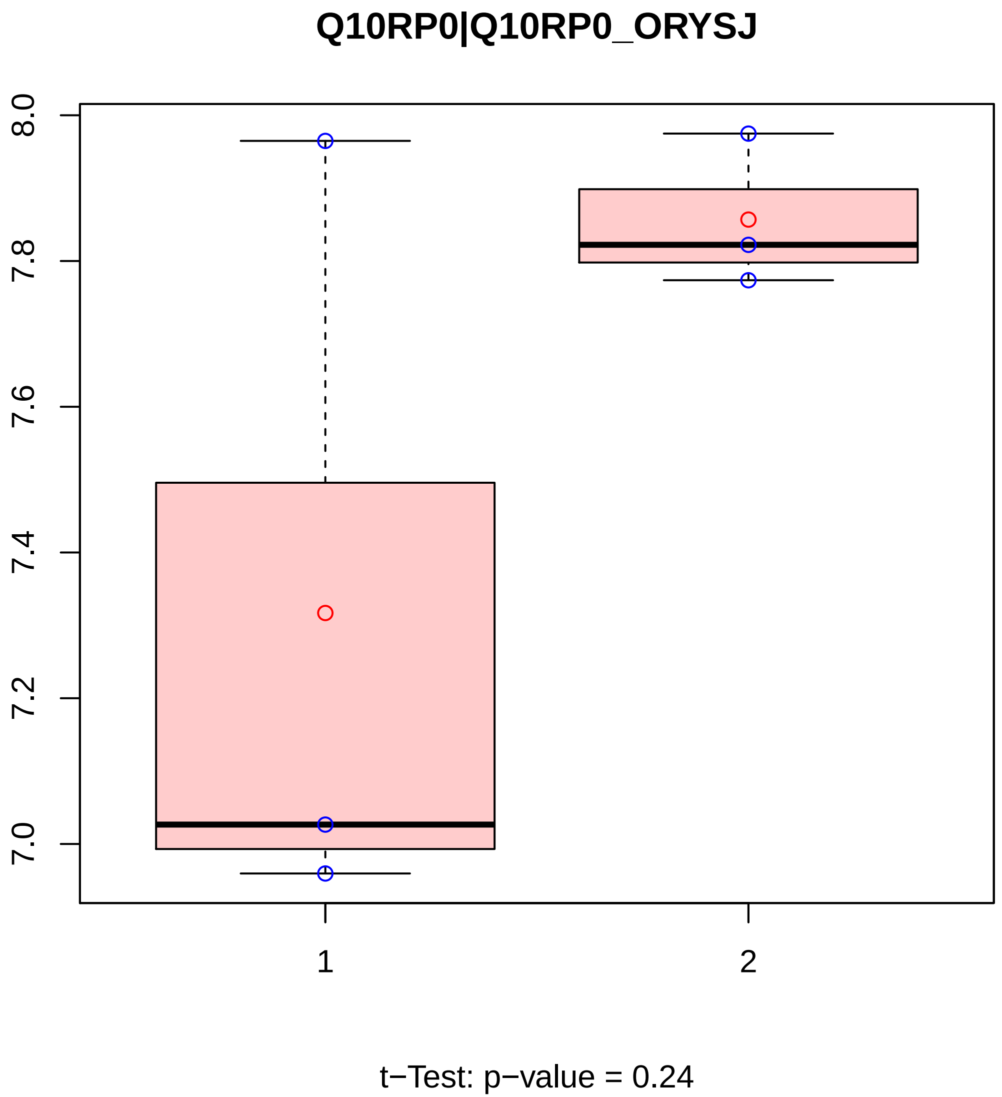

Q10RP0|Q10RP0_ORYSJ
8.0
7.8
7.6
7.4
7.2
7.0
1
2
t−Test: p−value = 0.24

Q10SM8|Q10SM8_ORYSJ

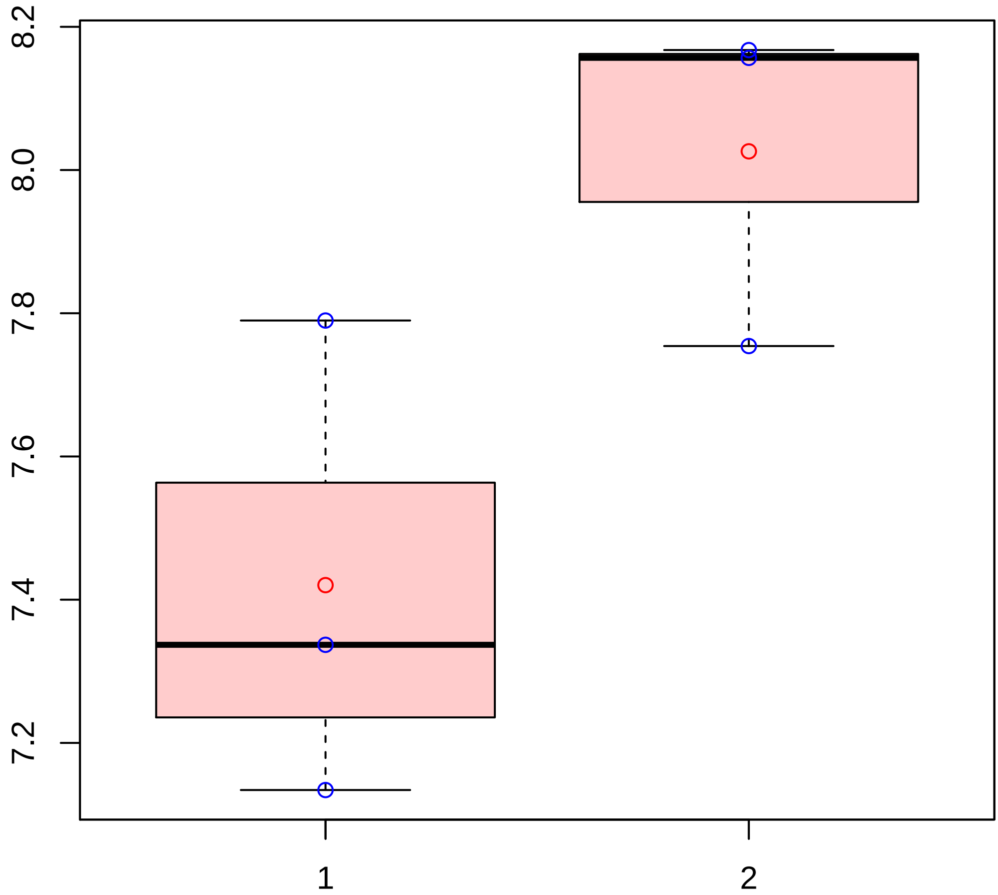

t−Test: p−value = 0.07

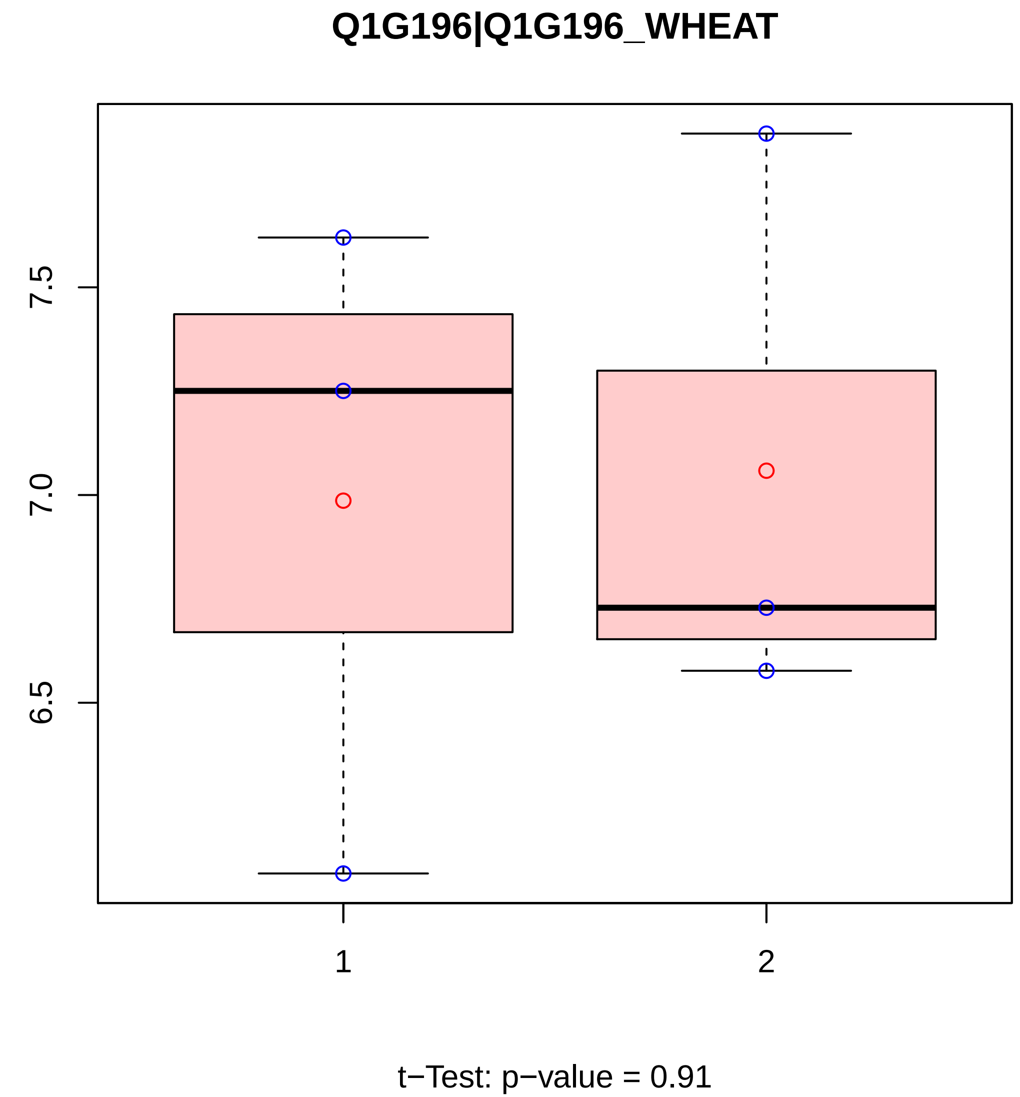
Q1G196|Q1G196_WHEAT
7.5
7.0
6.5
1
2
t−Test: p−value = 0.91

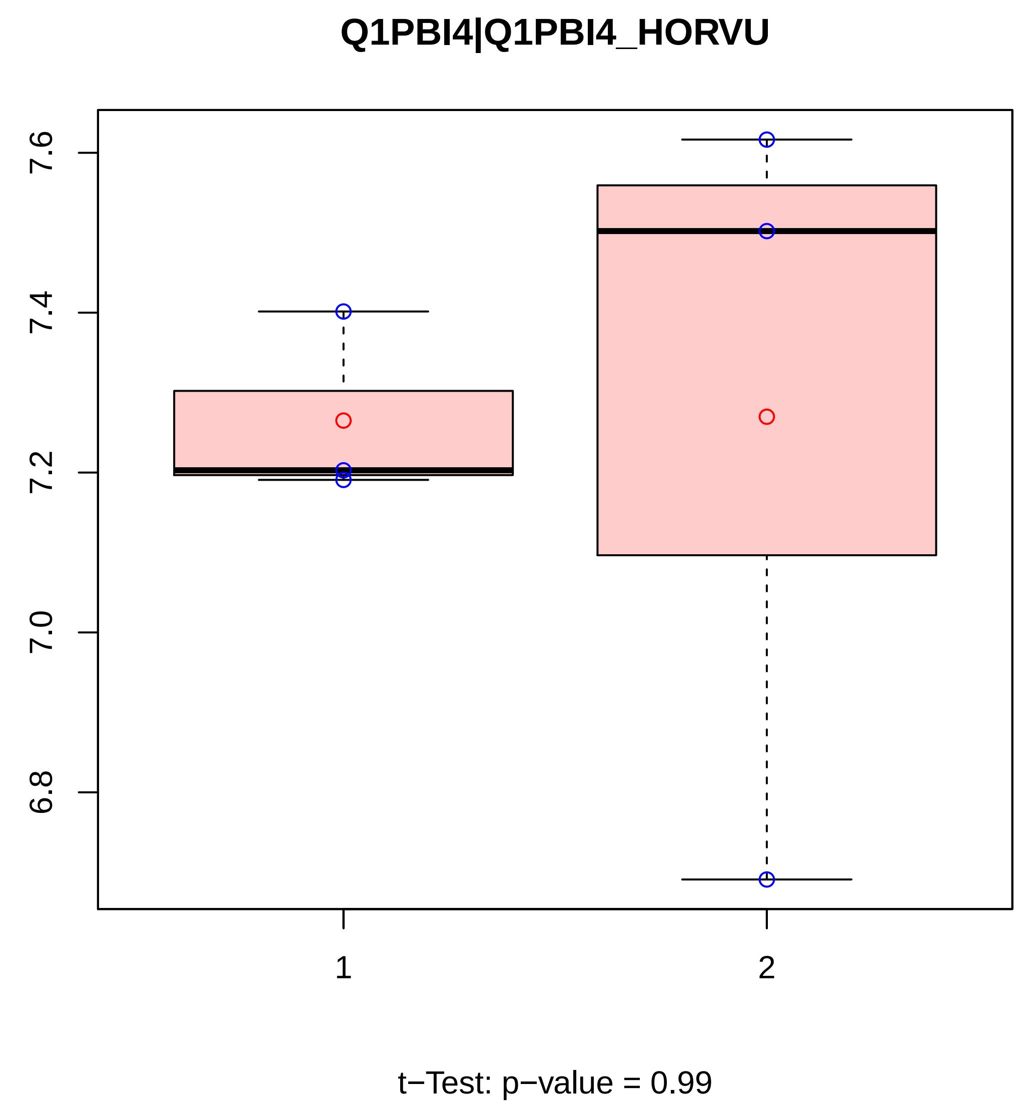
Q1PBI4|Q1PBI4_HORVU
7.6
7.4
7.2
7.0
6.8
1
2
t−Test: p−value = 0.99

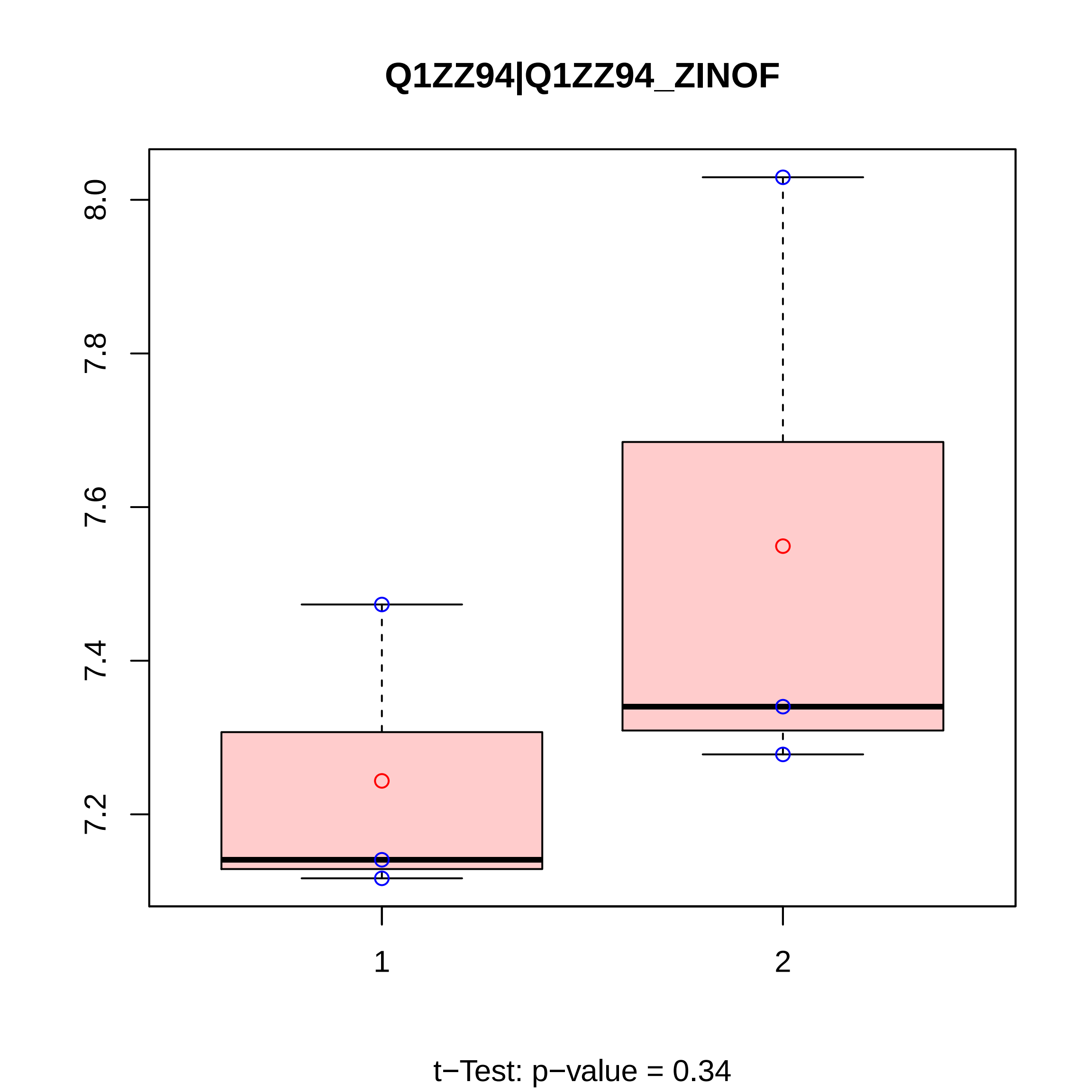
Q1ZZ94|Q1ZZ94_ZINOF
8.0
7.8
7.6
7.4
7.2
1
2
t−Test: p−value = 0.34

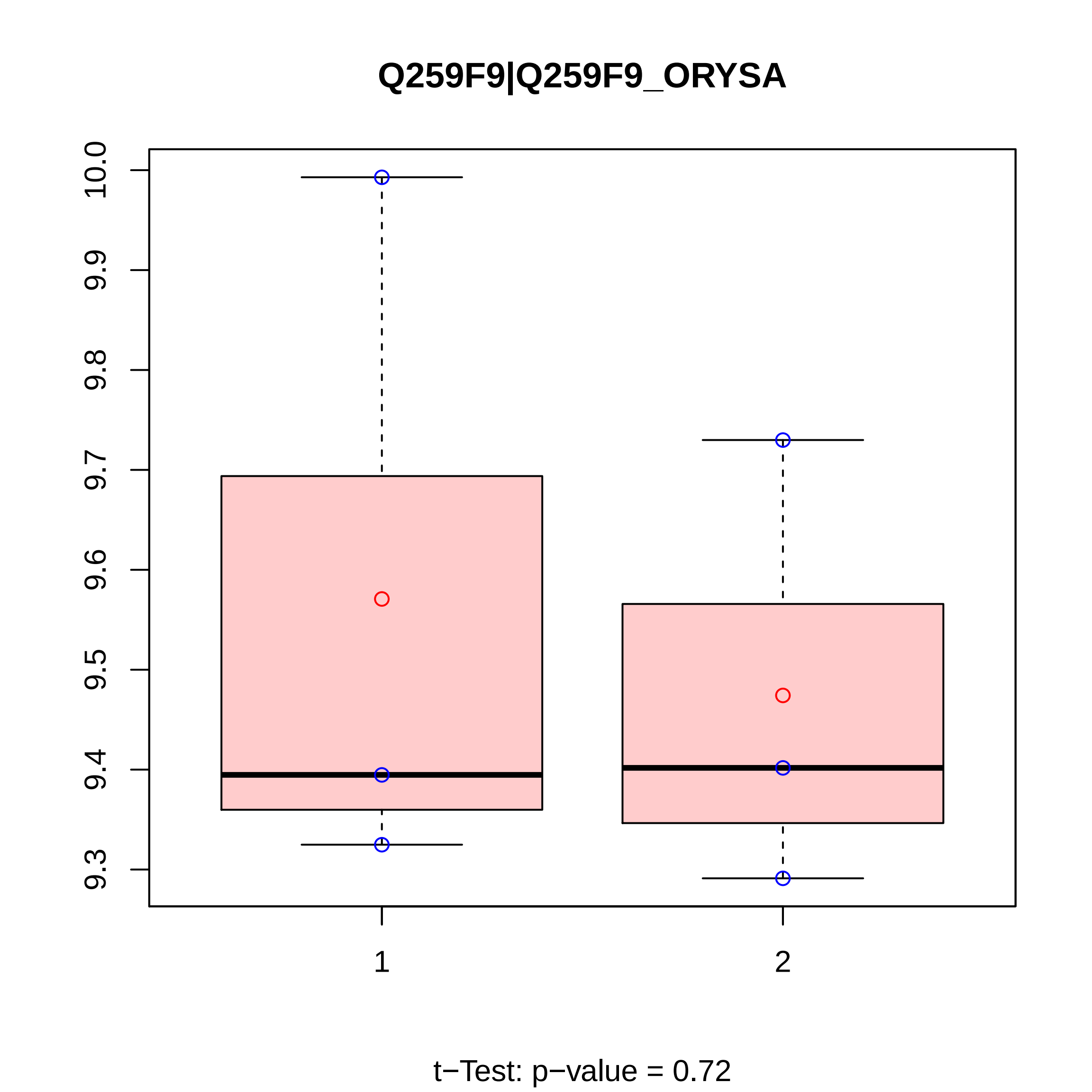
Q259F9|Q259F9_ORYSA
10.0
9.9
9.8
9.7
9.6
9.5
9.4
9.3
1
2
t−Test: p−value = 0.72

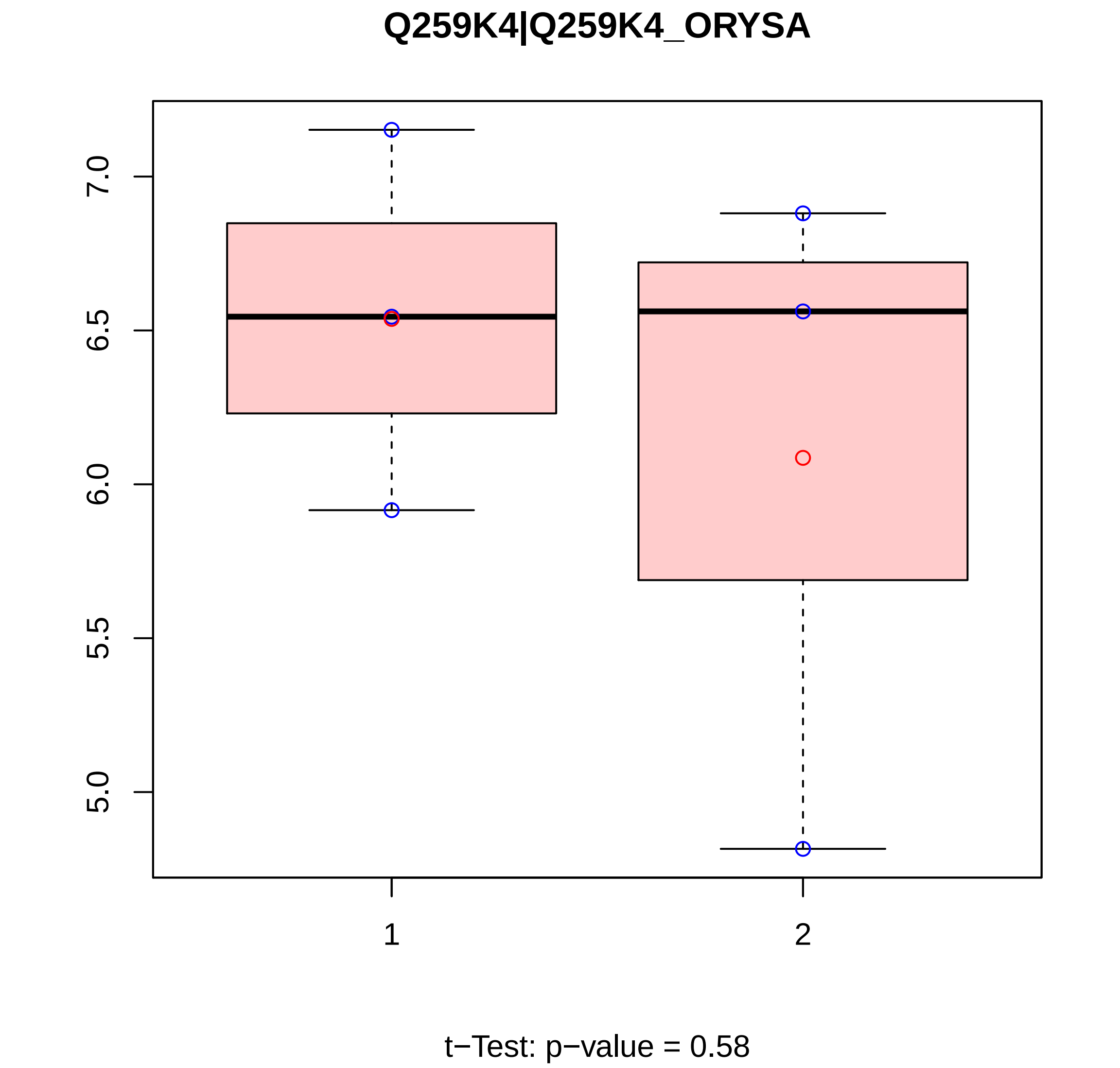
Q259K4|Q259K4_ORYSA
7.0
6.5
6.0
5.5
5.0
1
2
t−Test: p−value = 0.58

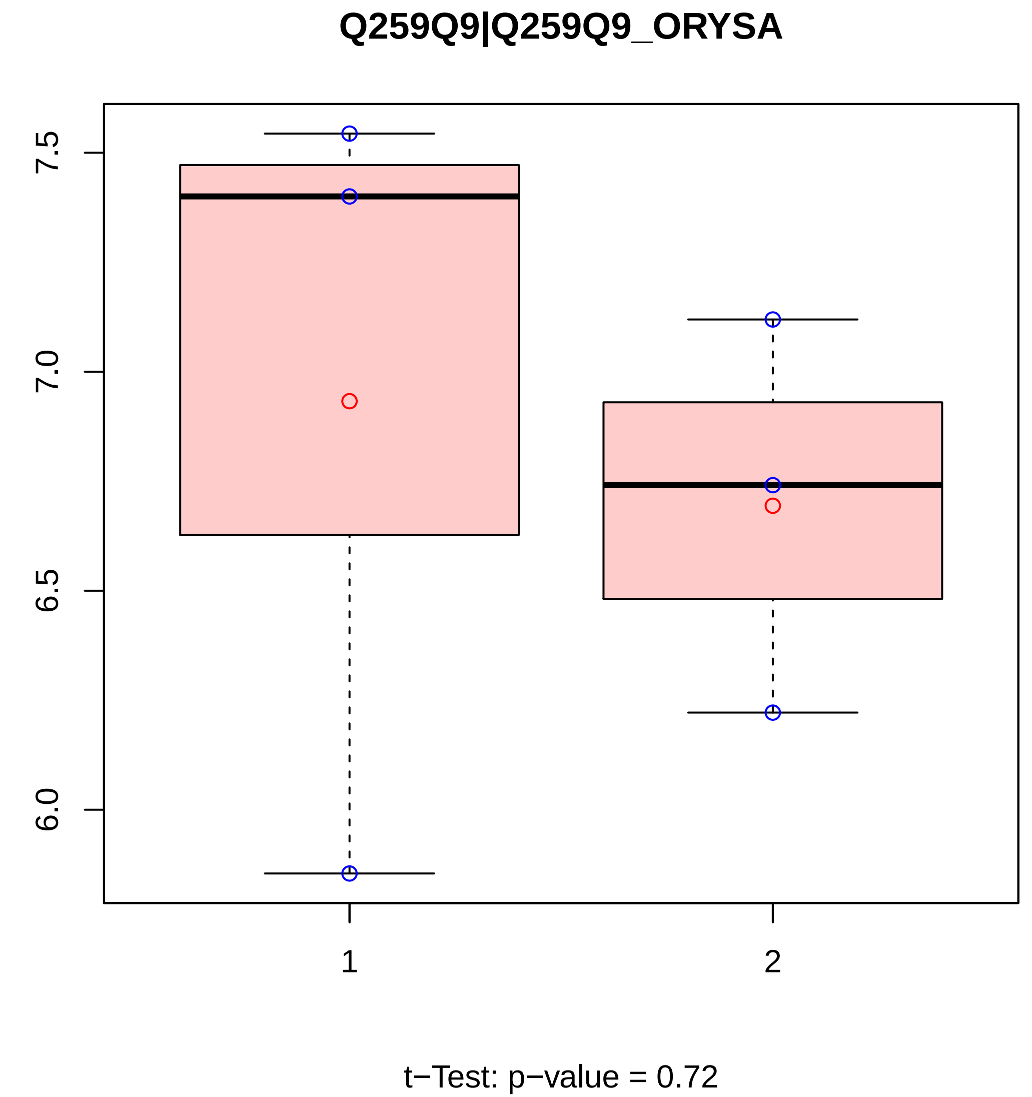
Q259Q9|Q259Q9_ORYSA
7.5
7.0
6.5
6.0
1
2
t−Test: p−value = 0.72

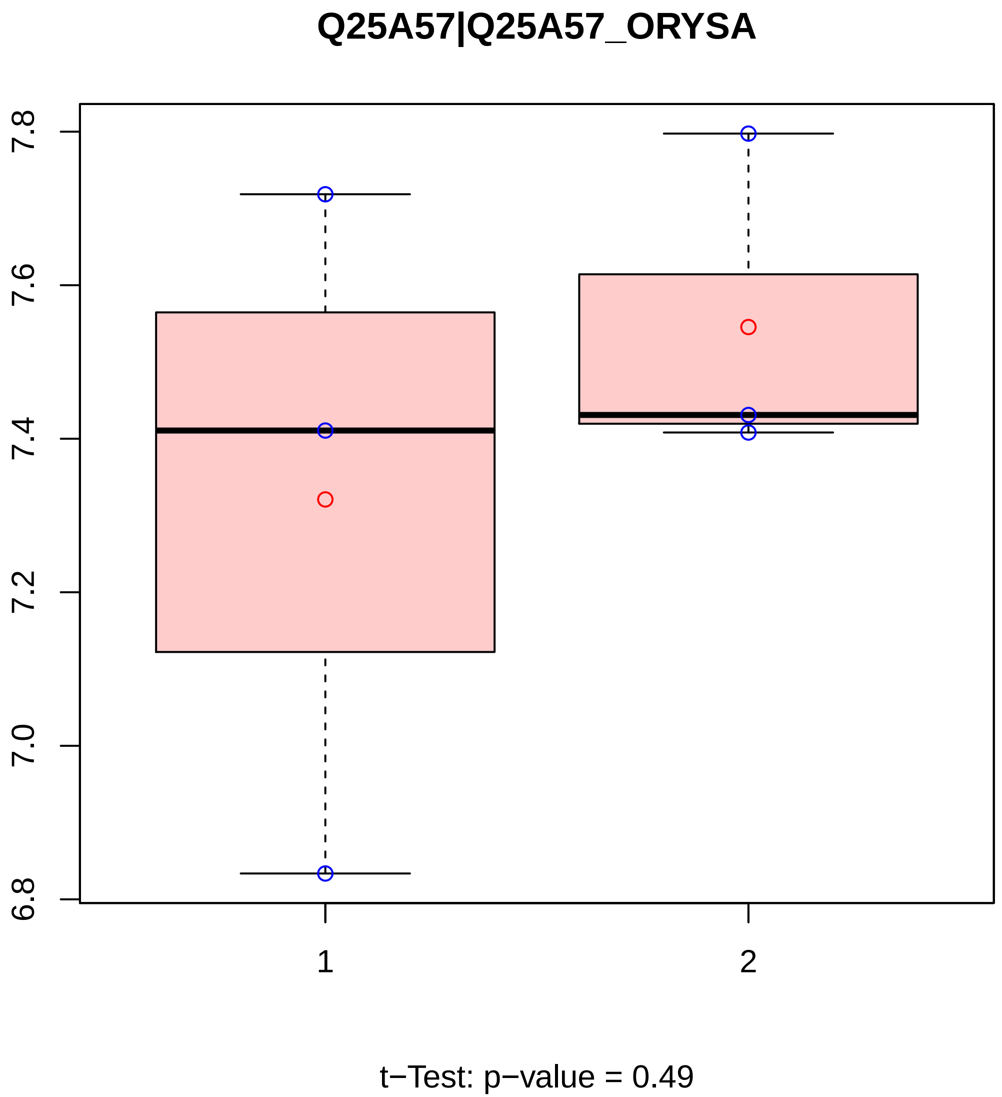
Q25A57|Q25A57_ORYSA
7.8
7.6
7.4
7.2
7.0
6.8
1
2
t−Test: p−value = 0.49

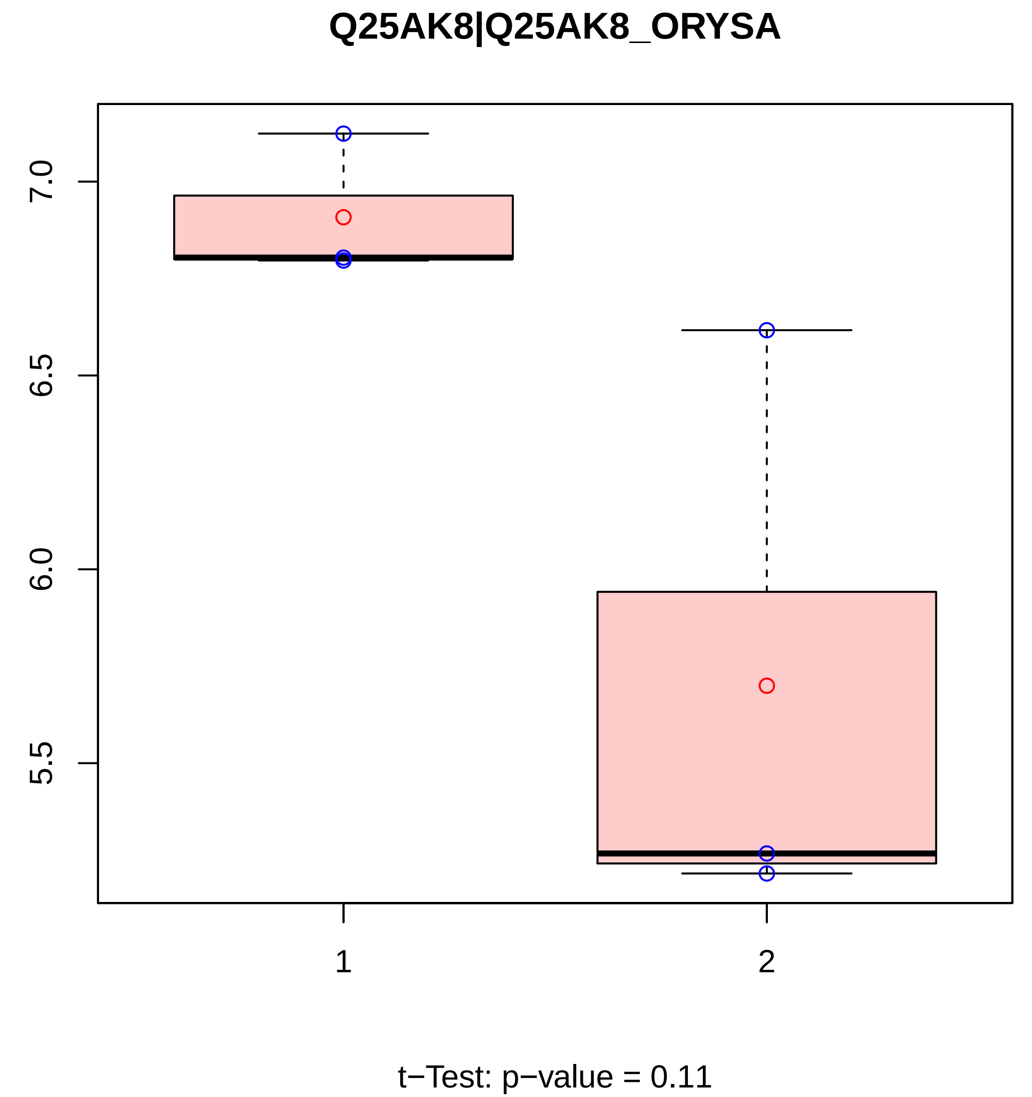

Q25AK8|Q25AK8_ORYSA
7.0
6.5
6.0
5.5
1
2
t−Test: p−value = 0.11

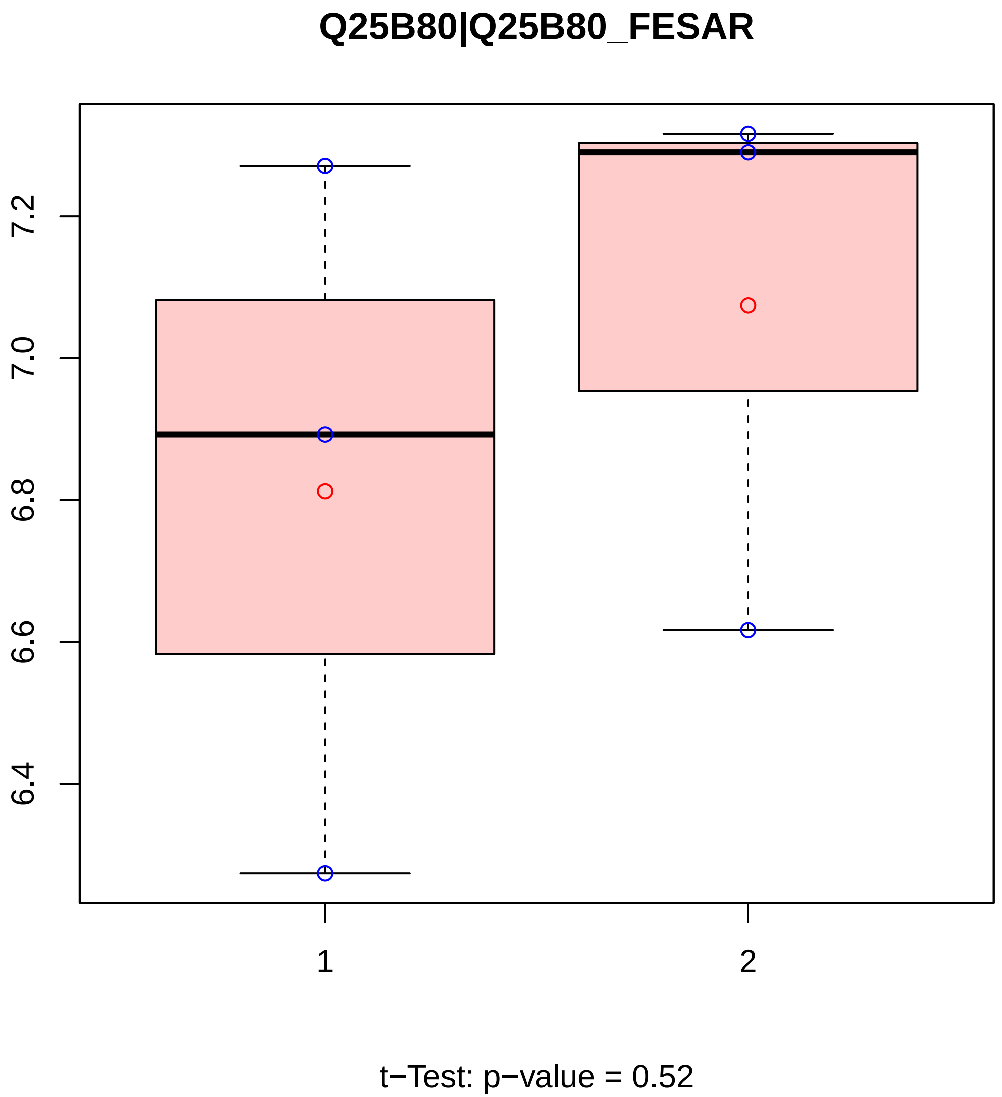

Q25B80|Q25B80_FESAR
7.2
7.0
6.8
6.6
6.4
1
2
t−Test: p−value = 0.52

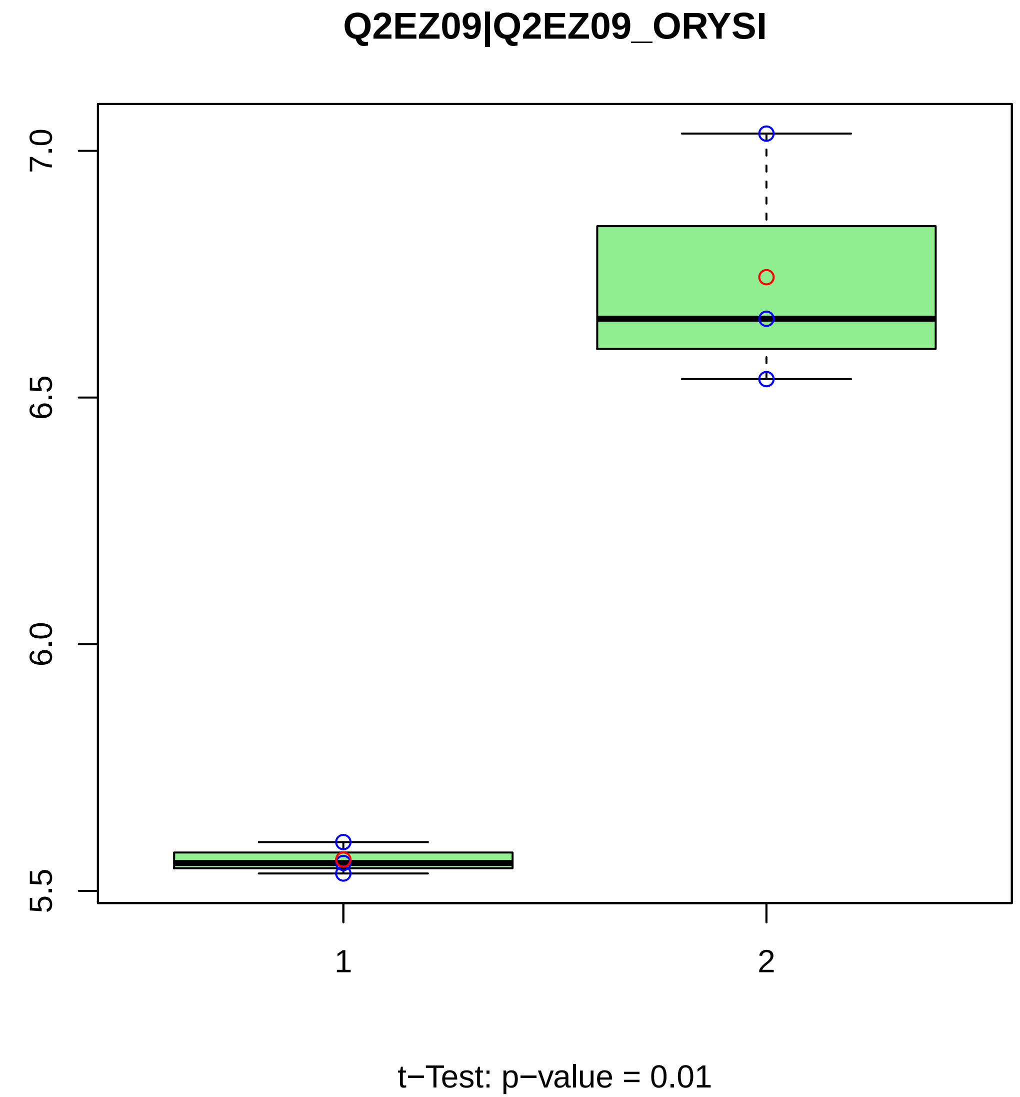
Q2EZ09|Q2EZ09_ORYSI
7.0
6.5
6.0
5.5
1
2
t−Test: p−value = 0.01

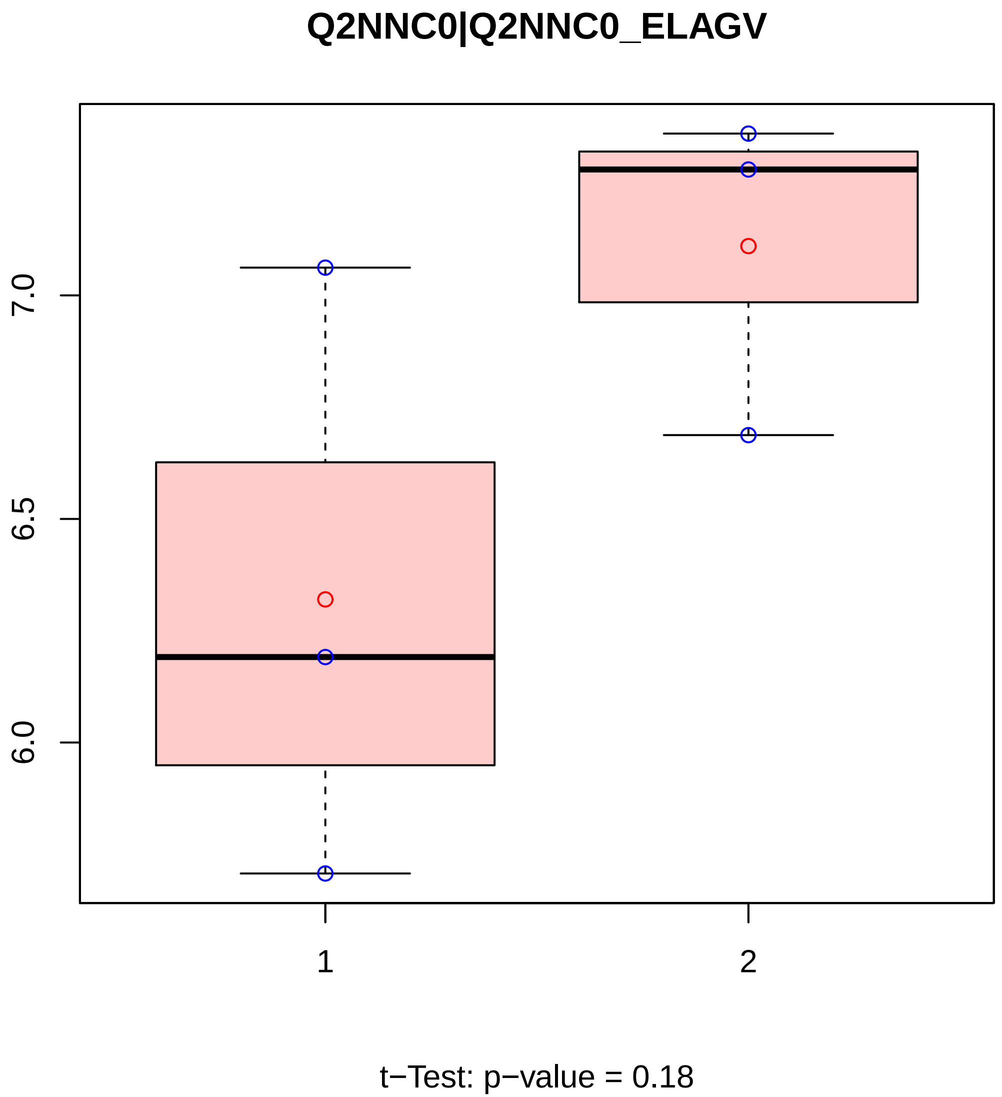
Q2NNC0|Q2NNC0_ELAGV
7.0
6.5
6.0
1
2
t−Test: p−value = 0.18

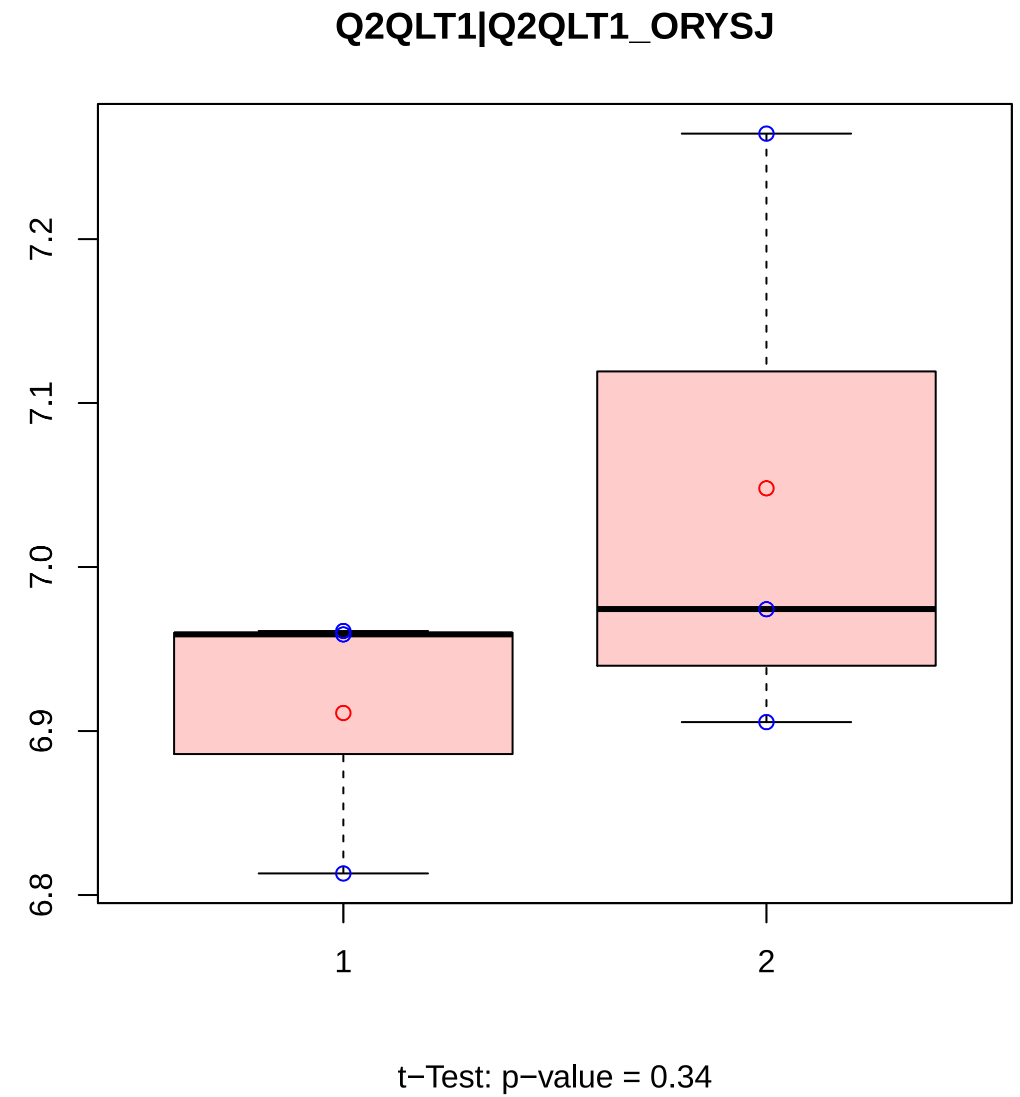
Q2QLT1|Q2QLT1_ORYSJ
7.2
7.1
7.0
6.9
6.8
1
2
t−Test: p−value = 0.34

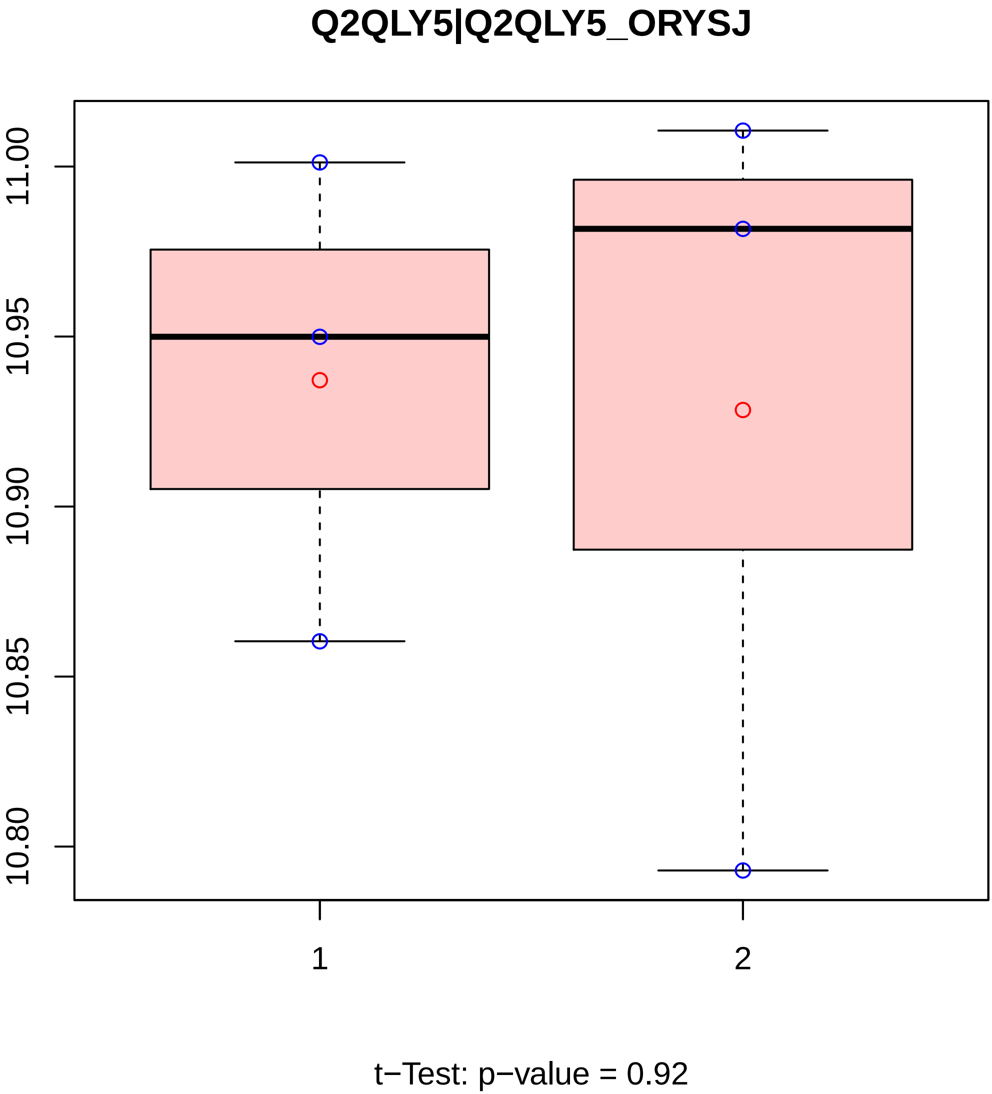

Q2QLY5|Q2QLY5_ORYSJ
11.00
10.95
10.90
10.85
10.80
1
2
t−Test: p−value = 0.92

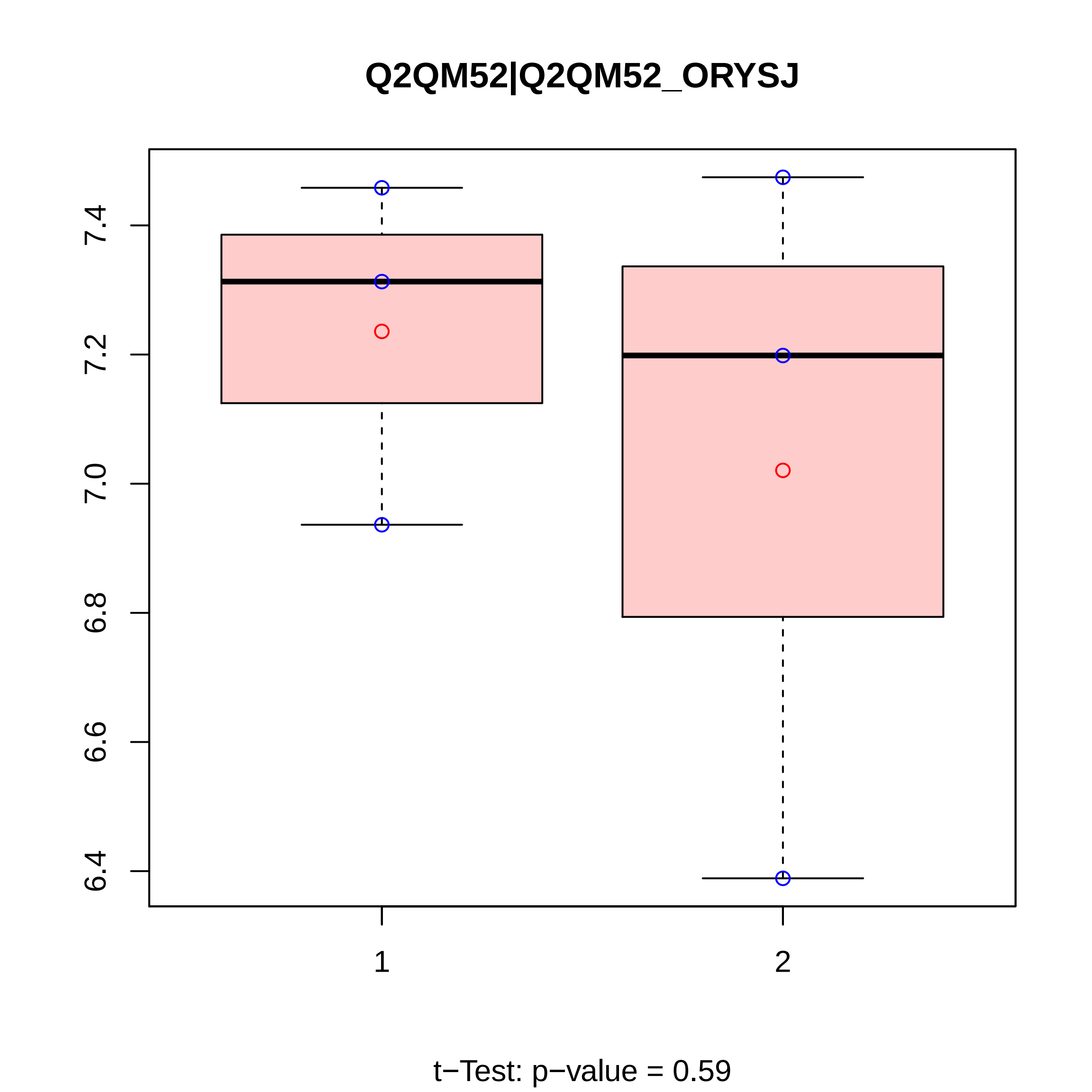
Q2QM52|Q2QM52_ORYSJ
7.4
7.2
7.0
6.8
6.6
6.4
1
2
t−Test: p−value = 0.59

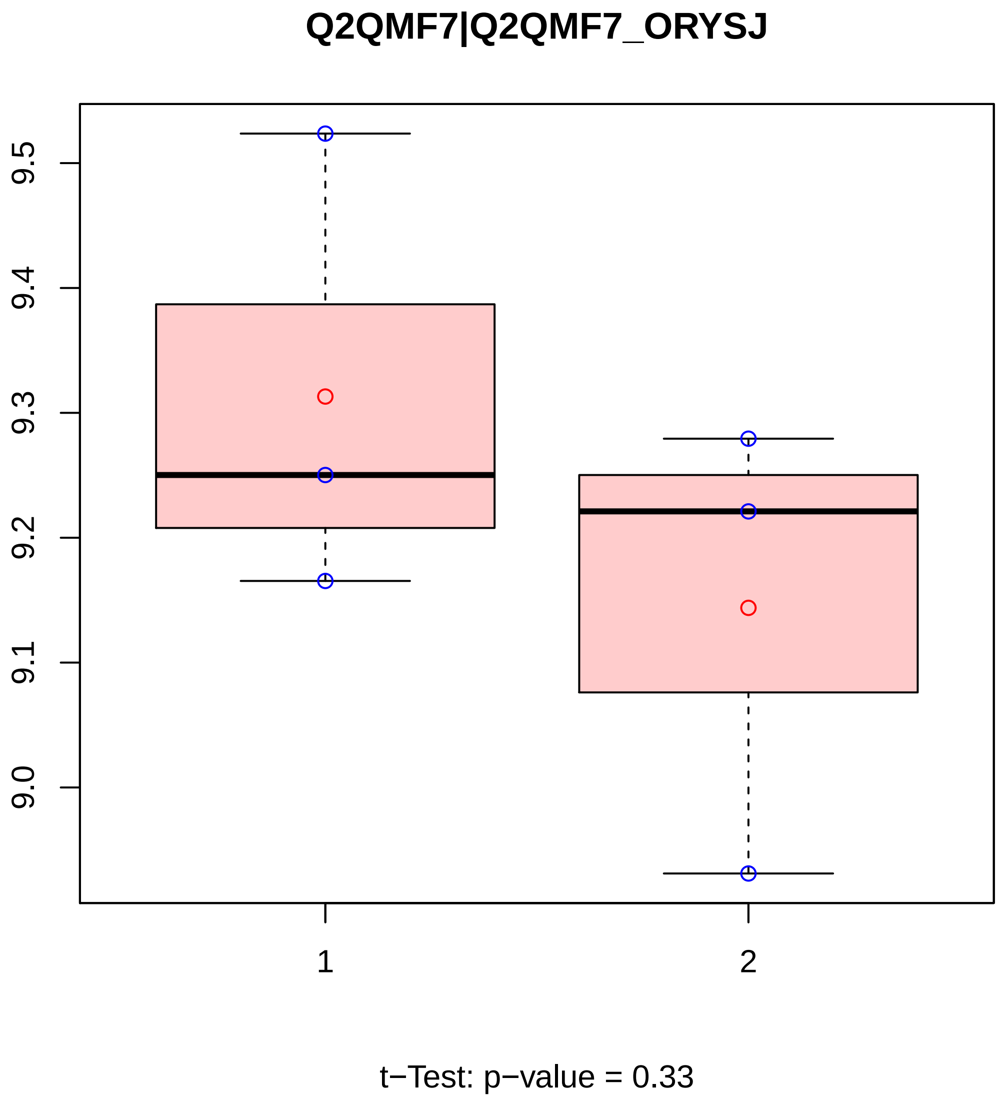
Q2QMF7|Q2QMF7_ORYSJ
9.0
9.1
9.2
9.3
9.4
9.5
1
2
t−Test: p−value = 0.33

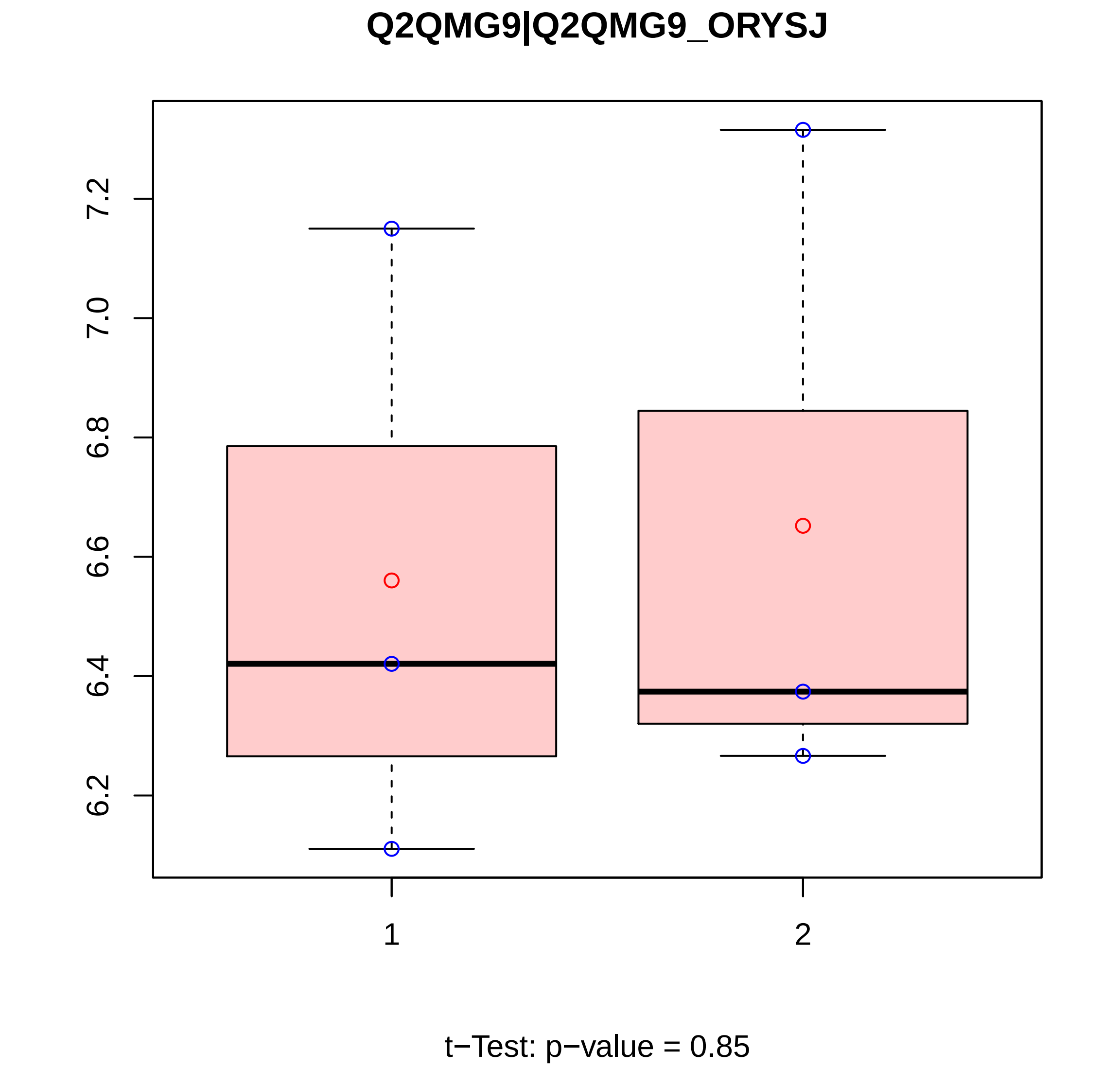
Q2QMG9|Q2QMG9_ORYSJ
6.2
6.4
6.6
6.8
7.0
7.2
1
2
t−Test: p−value = 0.85

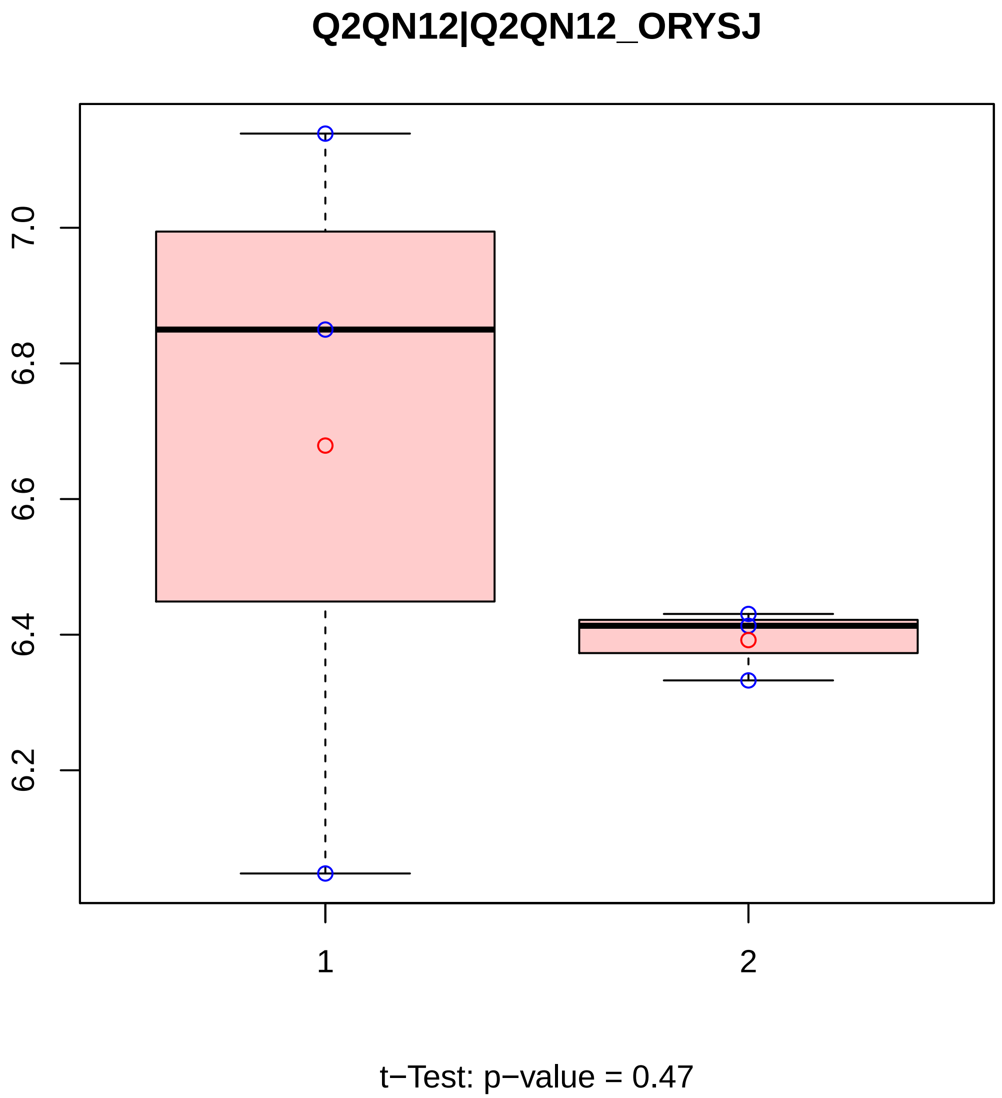
Q2QN12|Q2QN12_ORYSJ
7.0
6.8
6.6
6.4
6.2
1
2
t−Test: p−value = 0.47

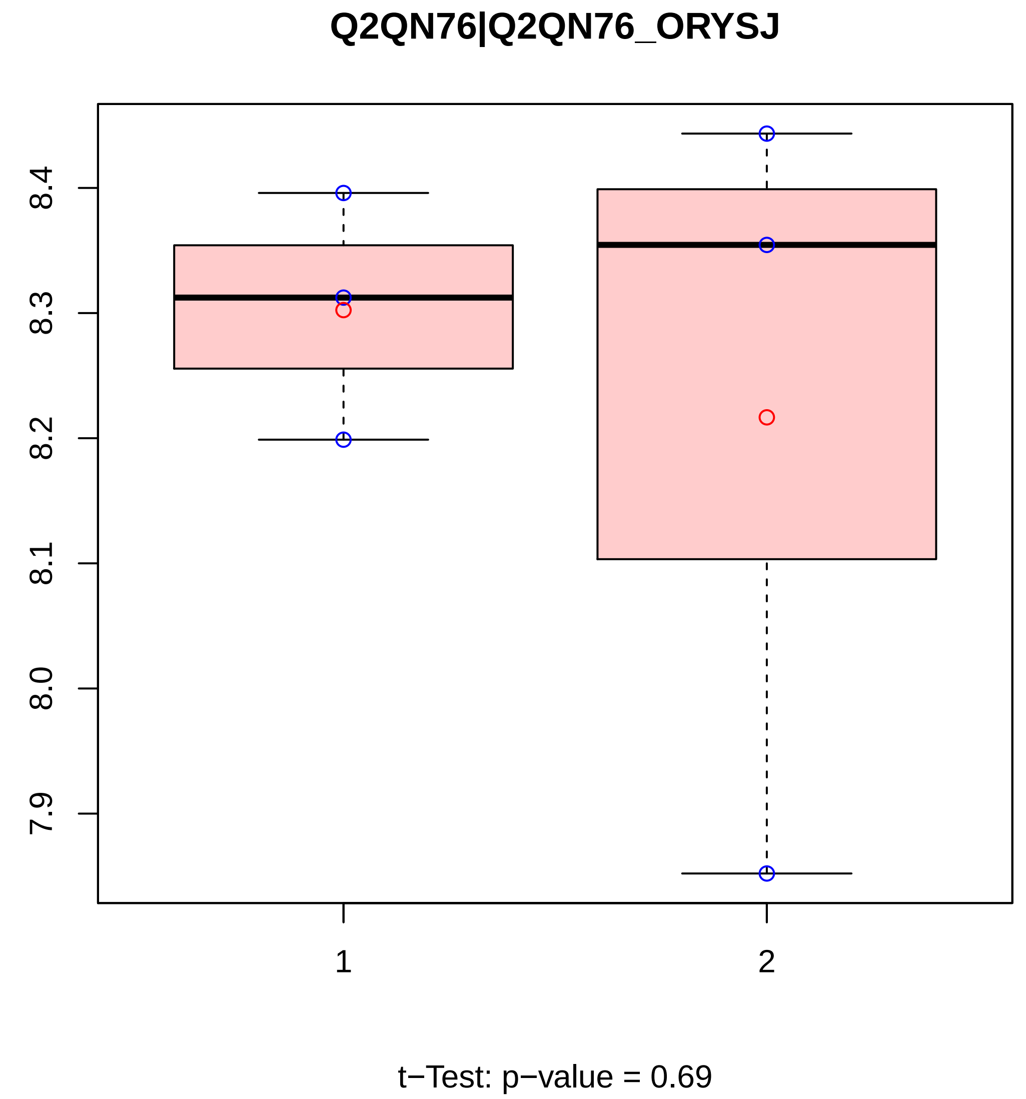
Q2QN76|Q2QN76_ORYSJ
8.4
8.3
8.2
8.1
8.0
7.9
1
2
t−Test: p−value = 0.69

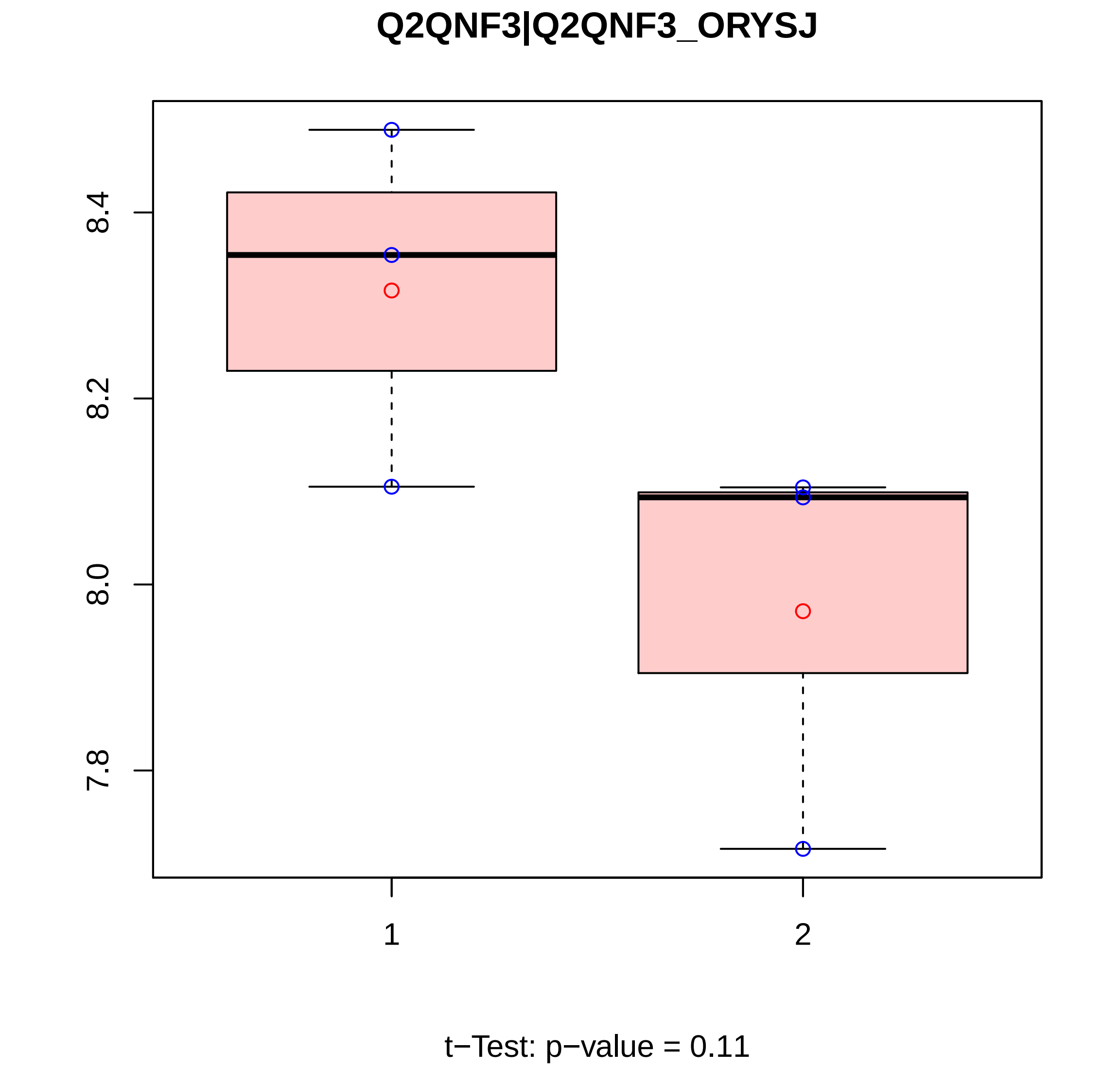
Q2QNF3|Q2QNF3_ORYSJ
8.4
8.2
8.0
7.8
1
2
t−Test: p−value = 0.11

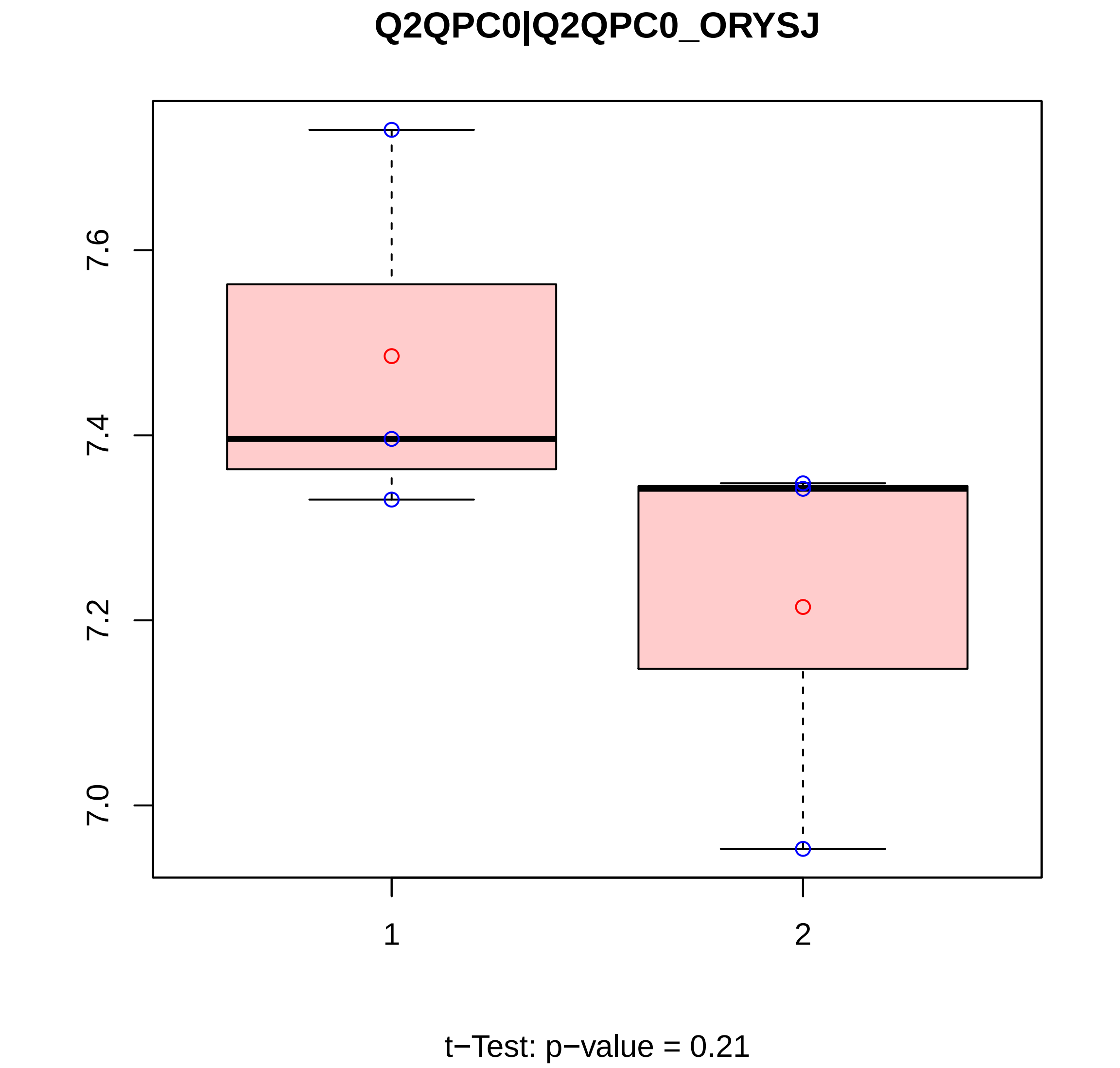
Q2QPC0|Q2QPC0_ORYSJ
7.0
7.2
7.4
7.6
1
2
t−Test: p−value = 0.21

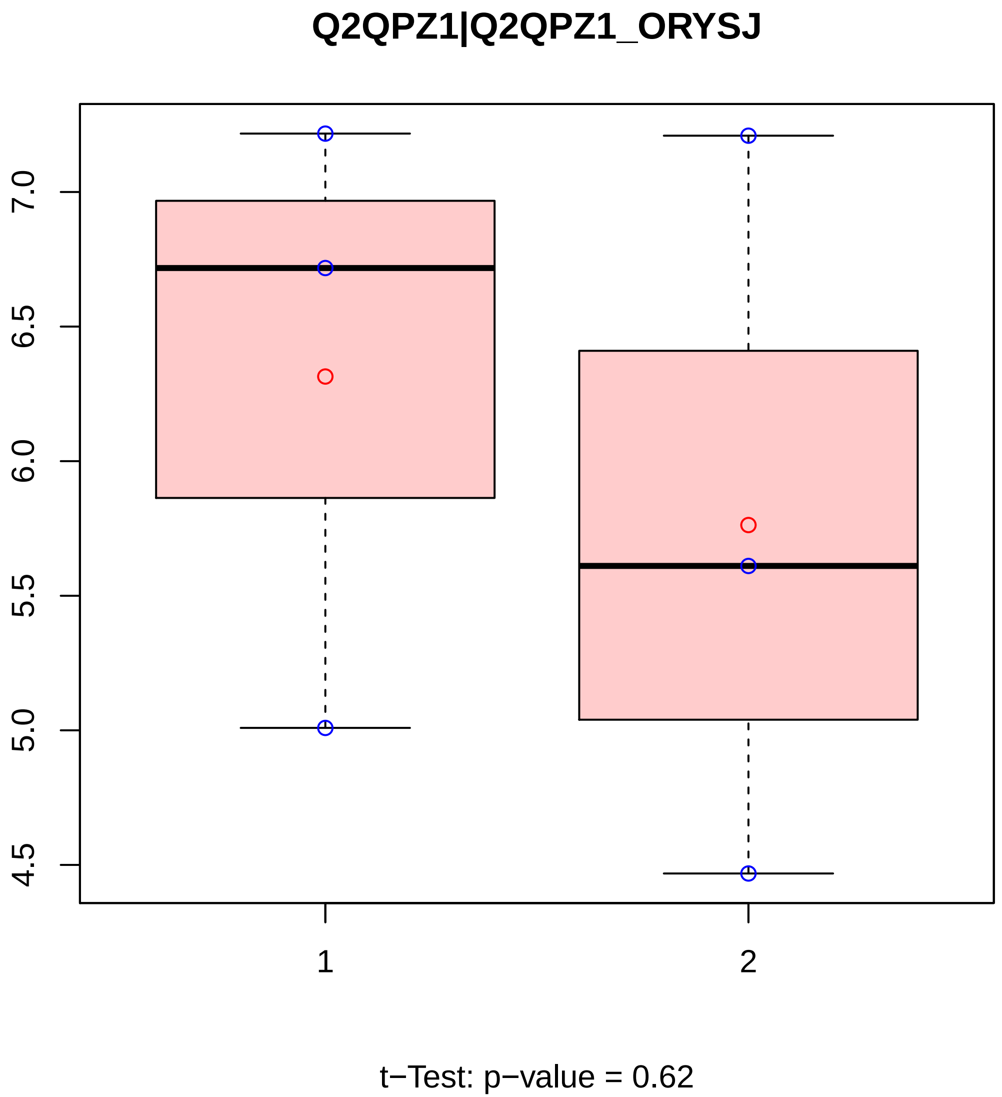
Q2QPZ1|Q2QPZ1_ORYSJ
7.0
6.5
6.0
5.5
5.0
4.5
1
2
t−Test: p−value = 0.62

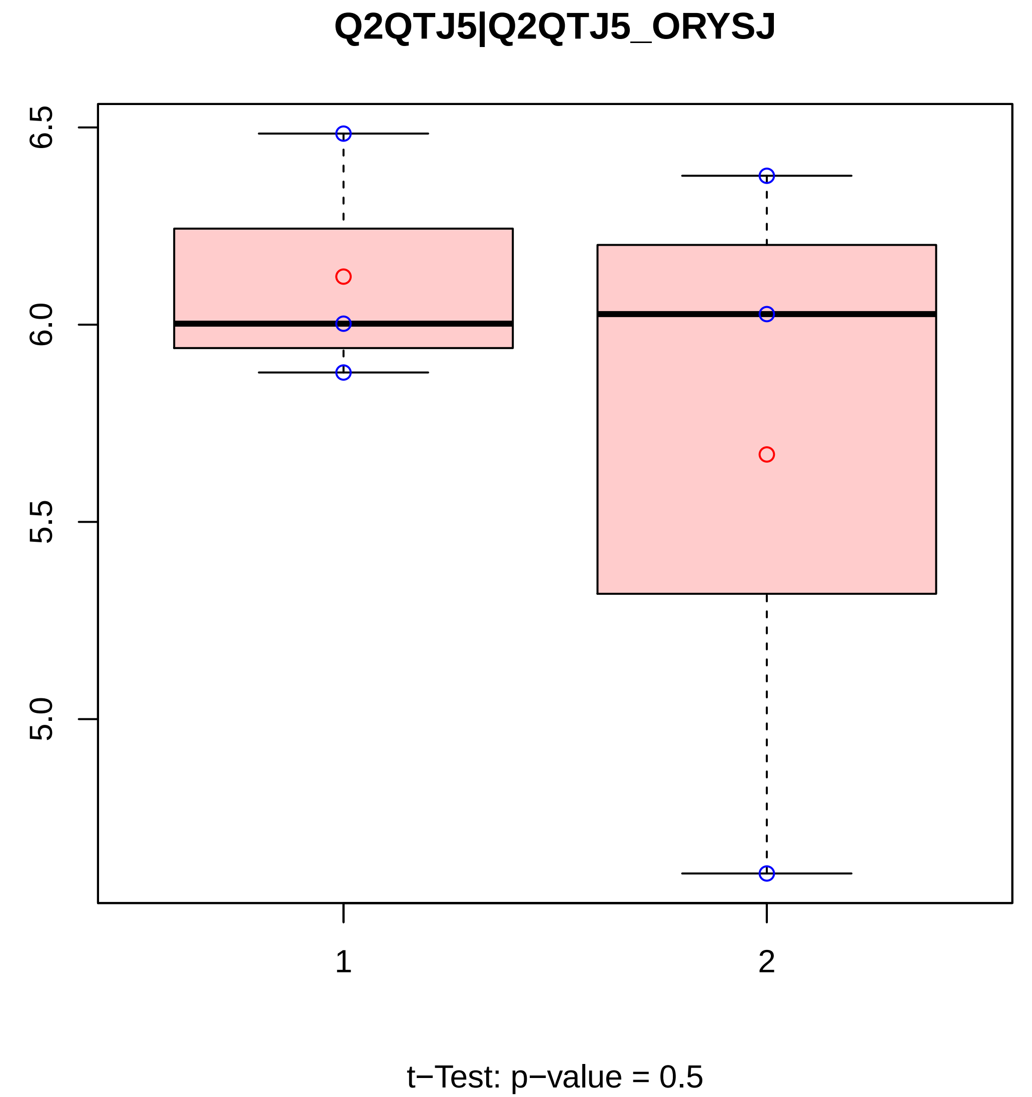
Q2QTJ5|Q2QTJ5_ORYSJ
6.5
6.0
5.5
5.0
1
2
t−Test: p−value = 0.5

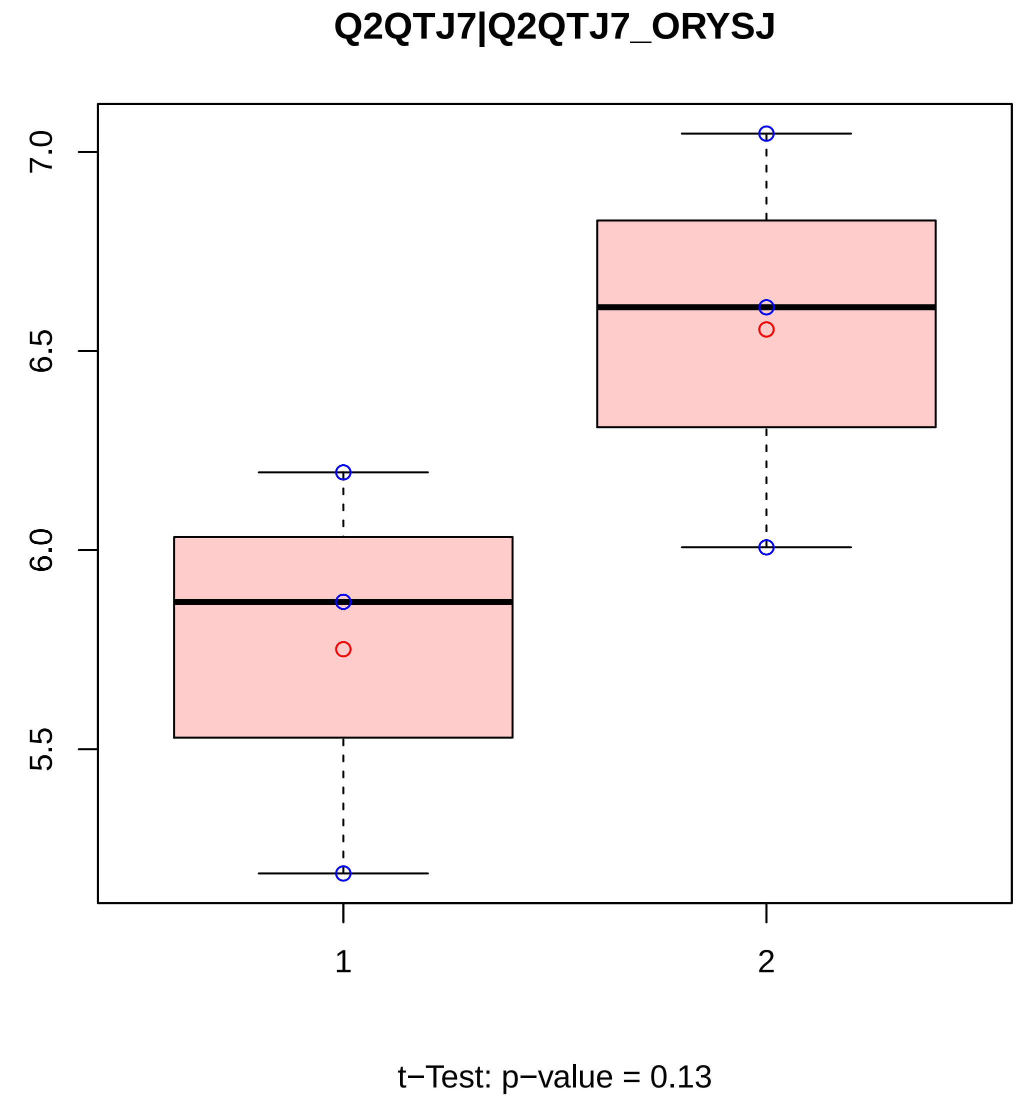
Q2QTJ7|Q2QTJ7_ORYSJ
7.0
6.5
6.0
5.5
1
2
t−Test: p−value = 0.13

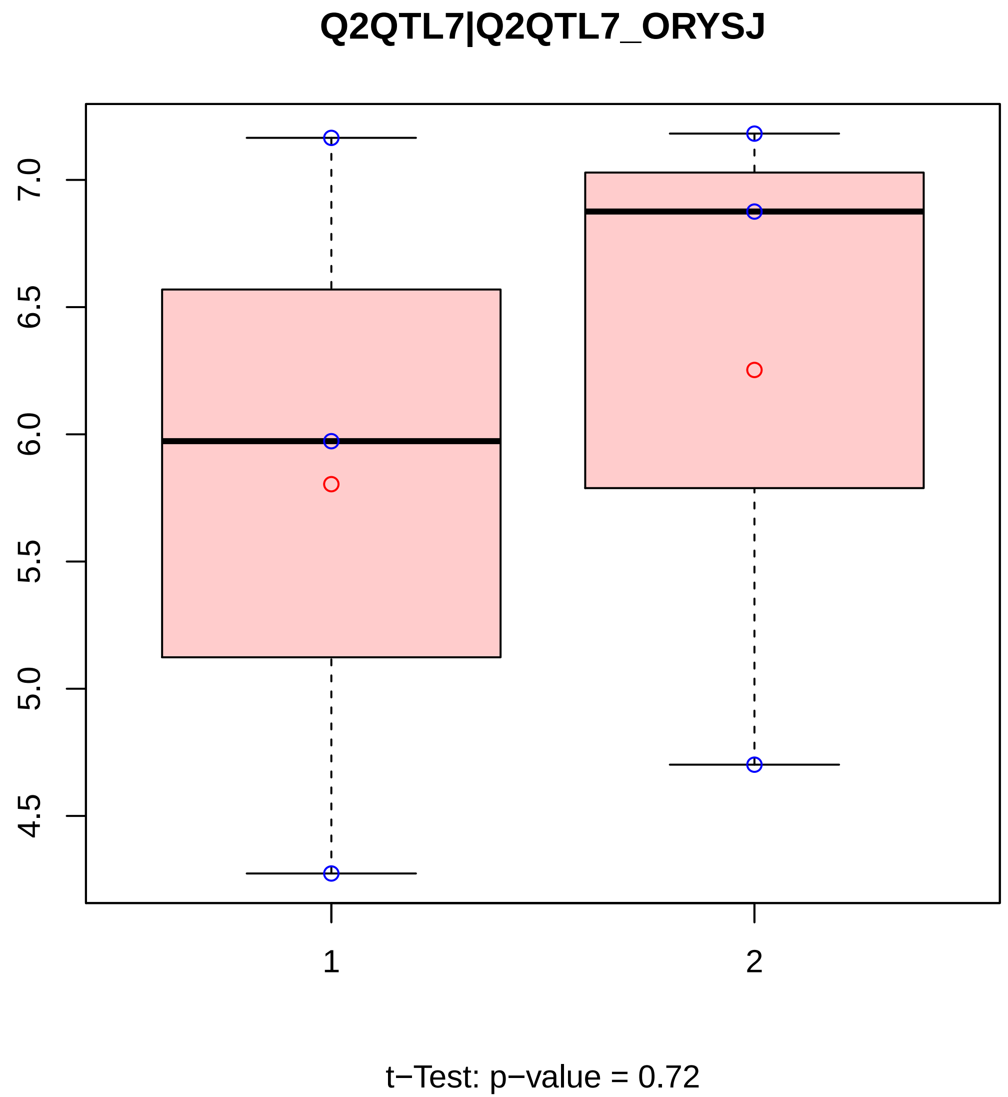
Q2QTL7|Q2QTL7_ORYSJ
7.0
6.5
6.0
5.5
5.0
4.5
1
2
t−Test: p−value = 0.72

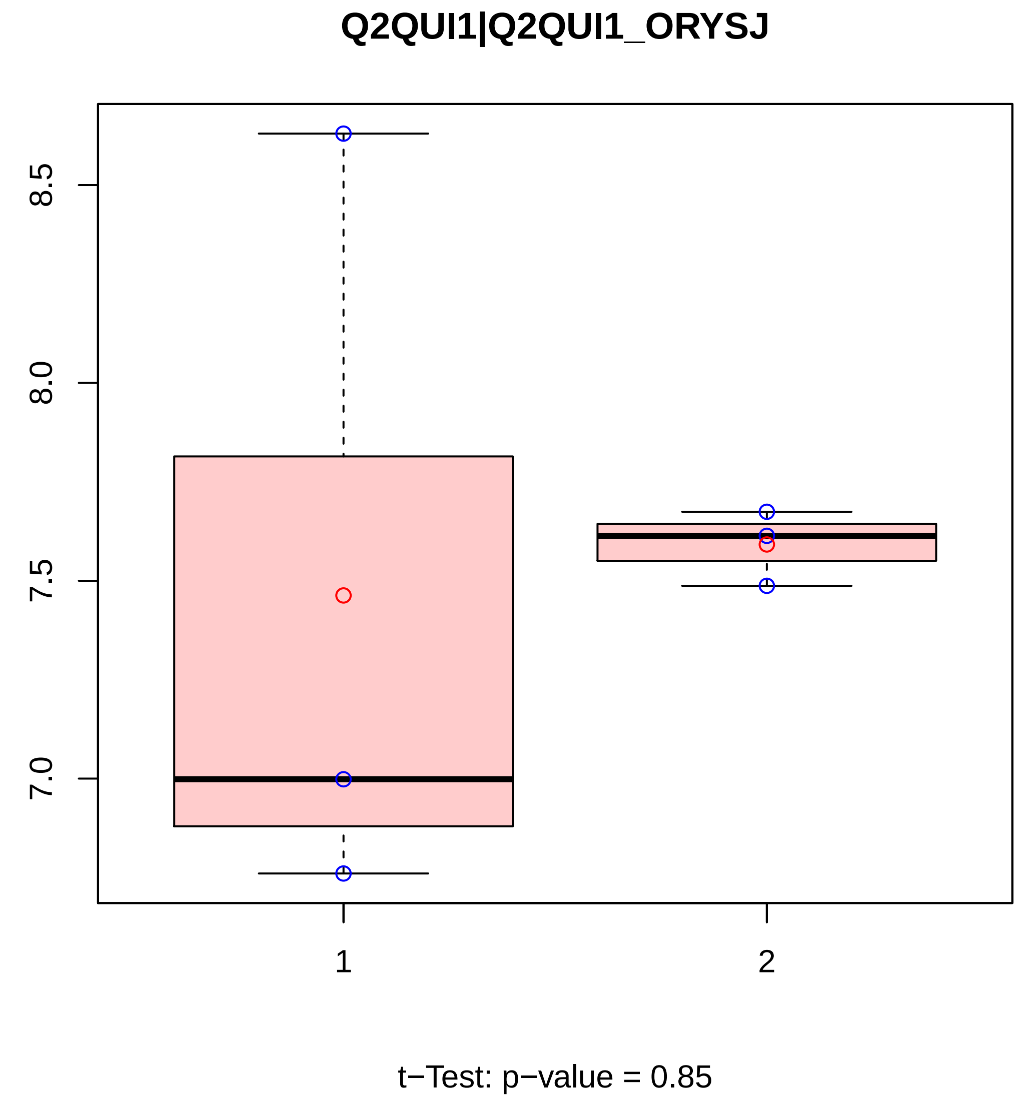
Q2QUI1|Q2QUI1_ORYSJ
8.5
8.0
7.5
7.0
1
2
t−Test: p−value = 0.85

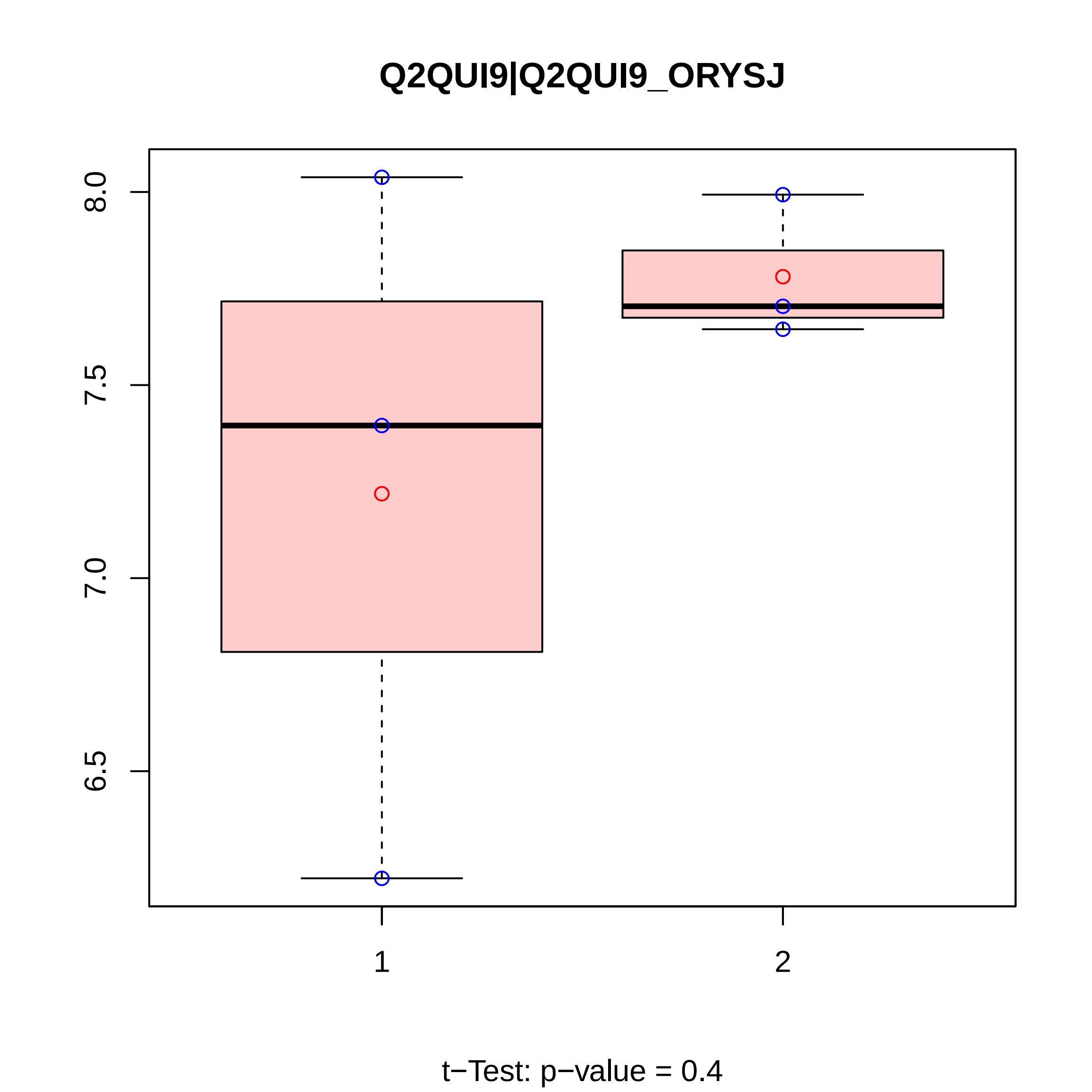

Q2QUI9|Q2QUI9_ORYSJ
8.0
7.5
7.0
6.5
1
2
t−Test: p−value = 0.4

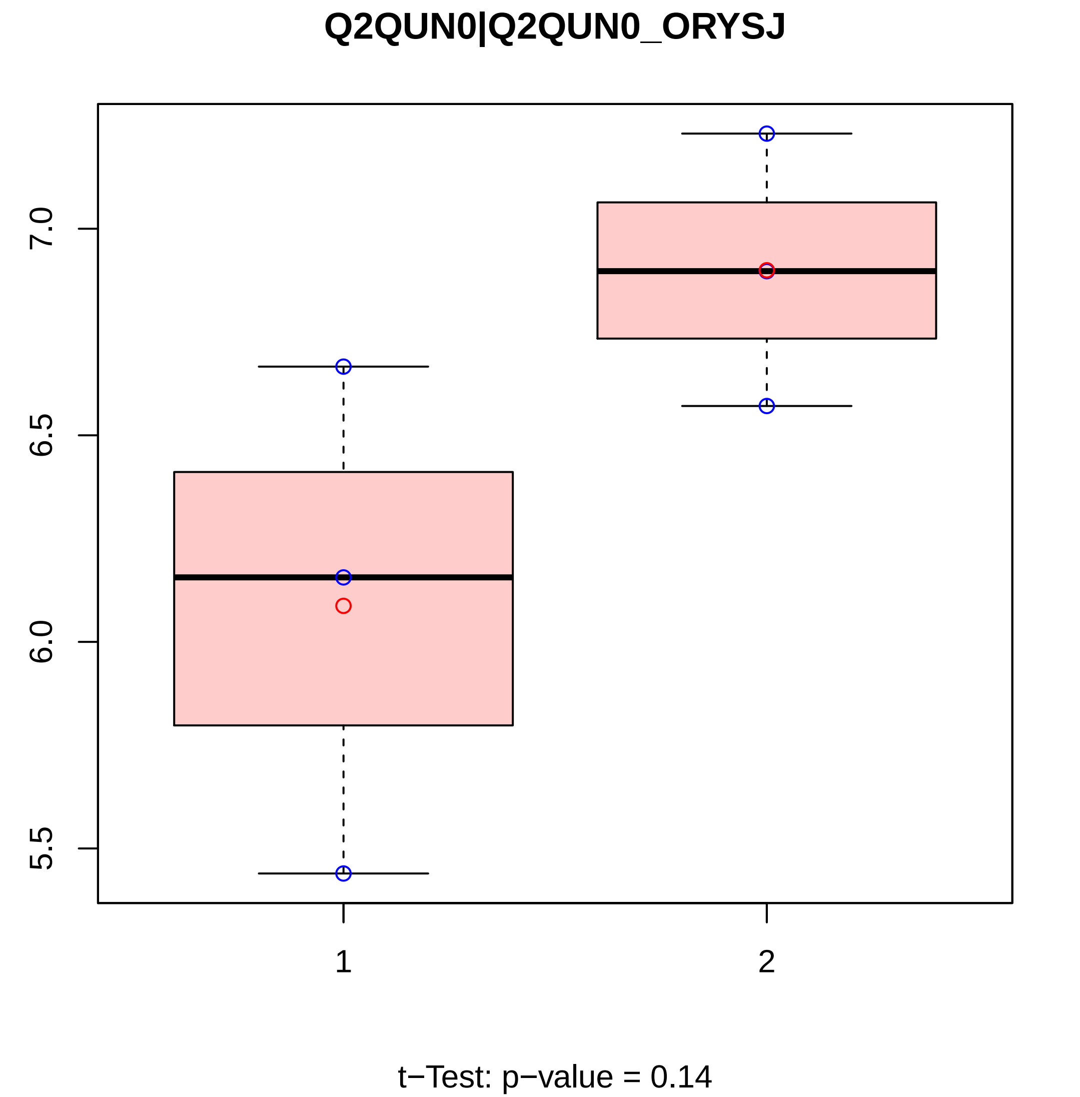
Q2QUN0|Q2QUN0_ORYSJ
7.0
6.5
6.0
5.5
1
2
t−Test: p−value = 0.14

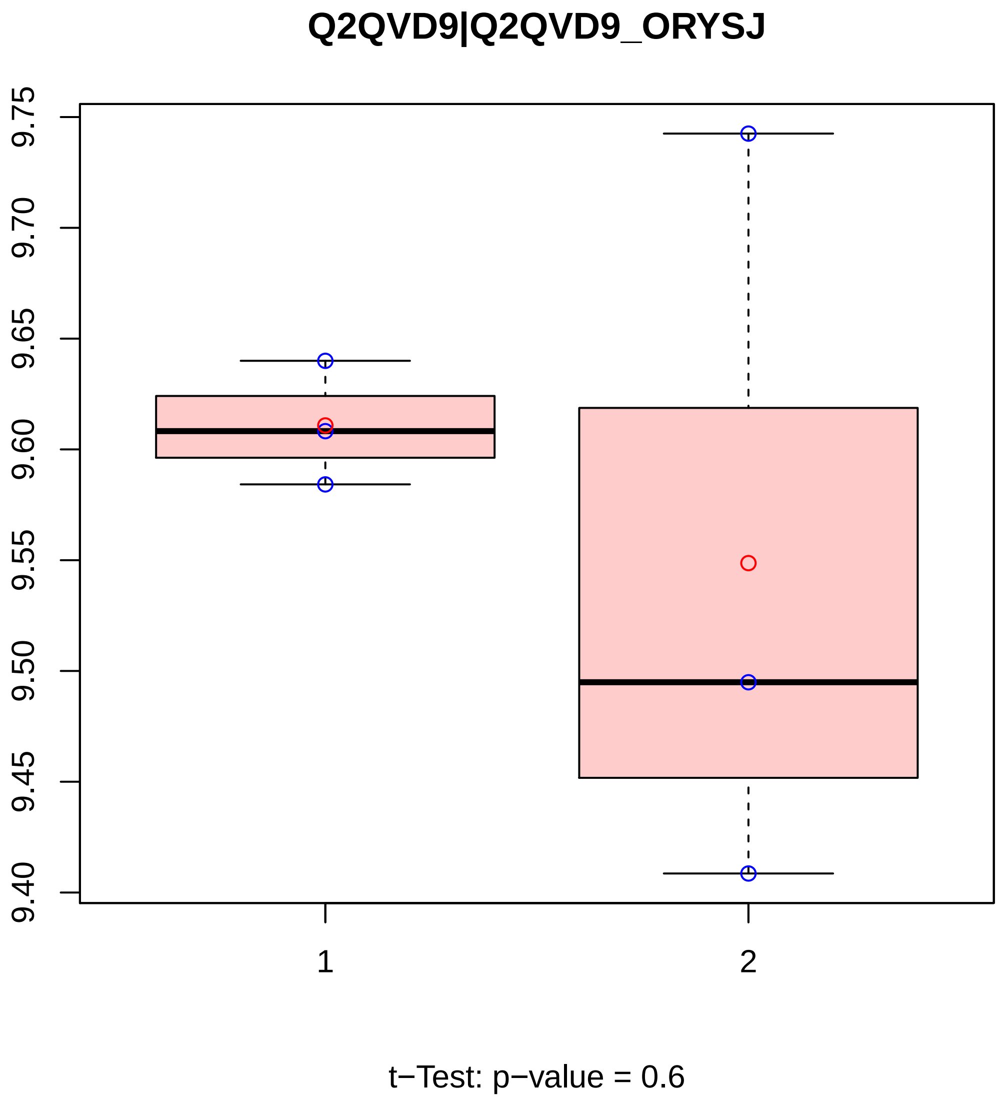
Q2QVD9|Q2QVD9_ORYSJ
9.40
9.45
9.50
9.55
9.60
9.65
9.70
9.75
1
2
t−Test: p−value = 0.6

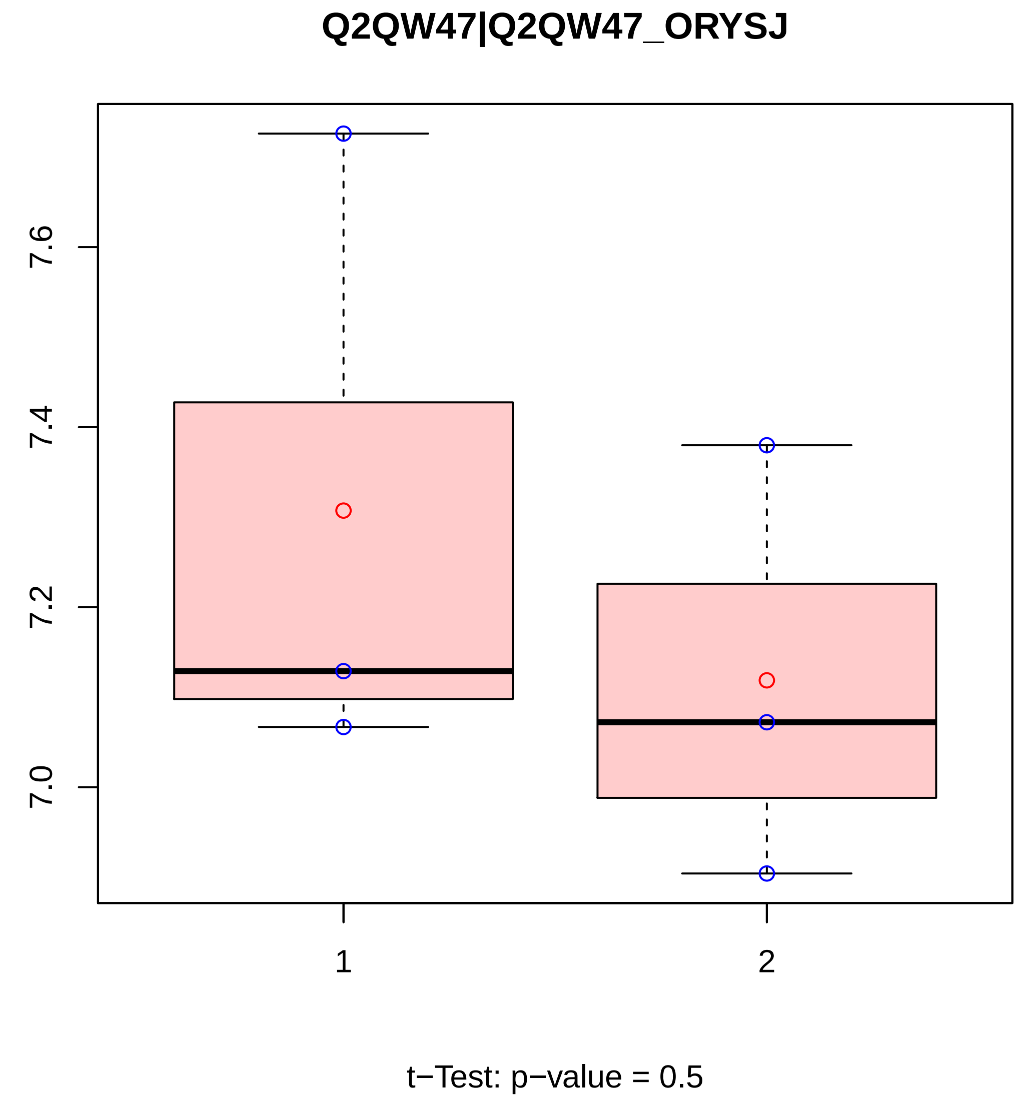
Q2QW47|Q2QW47_ORYSJ
7.0
7.2
7.4
7.6
1
2
t−Test: p−value = 0.5

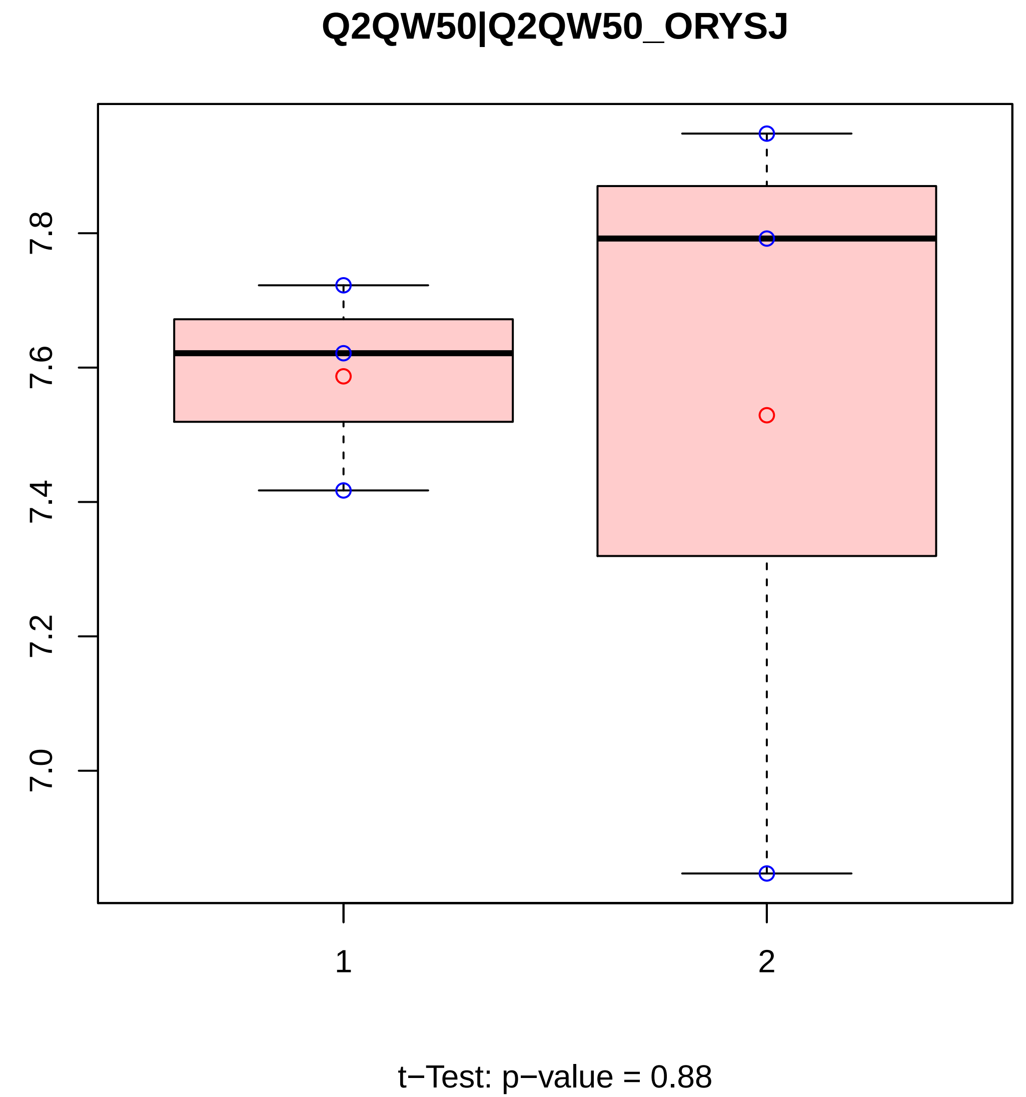
Q2QW50|Q2QW50_ORYSJ
7.0
7.2
7.4
7.6
7.8
1
2
t−Test: p−value = 0.88

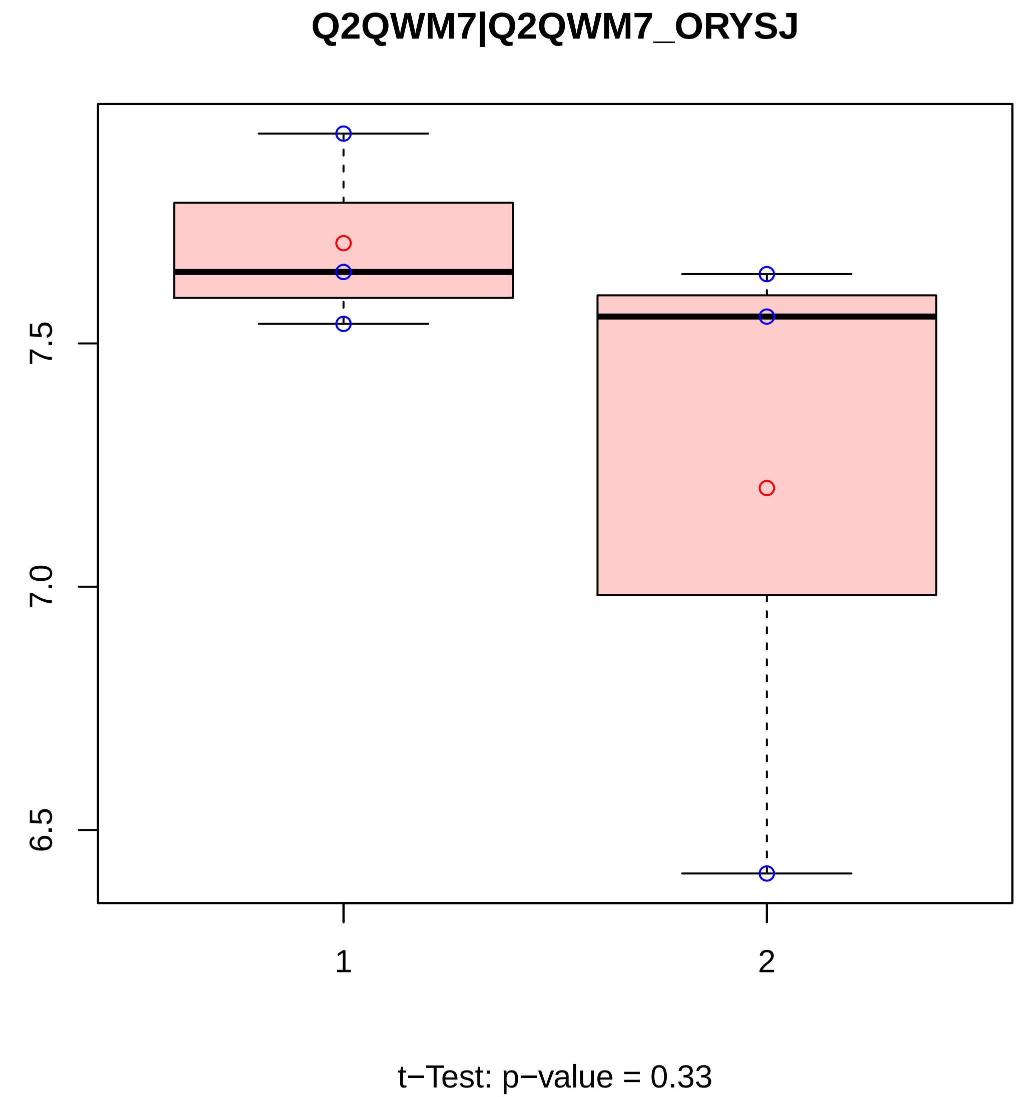
Q2QWM7|Q2QWM7_ORYSJ
7.5
7.0
6.5
1
2
t−Test: p−value = 0.33

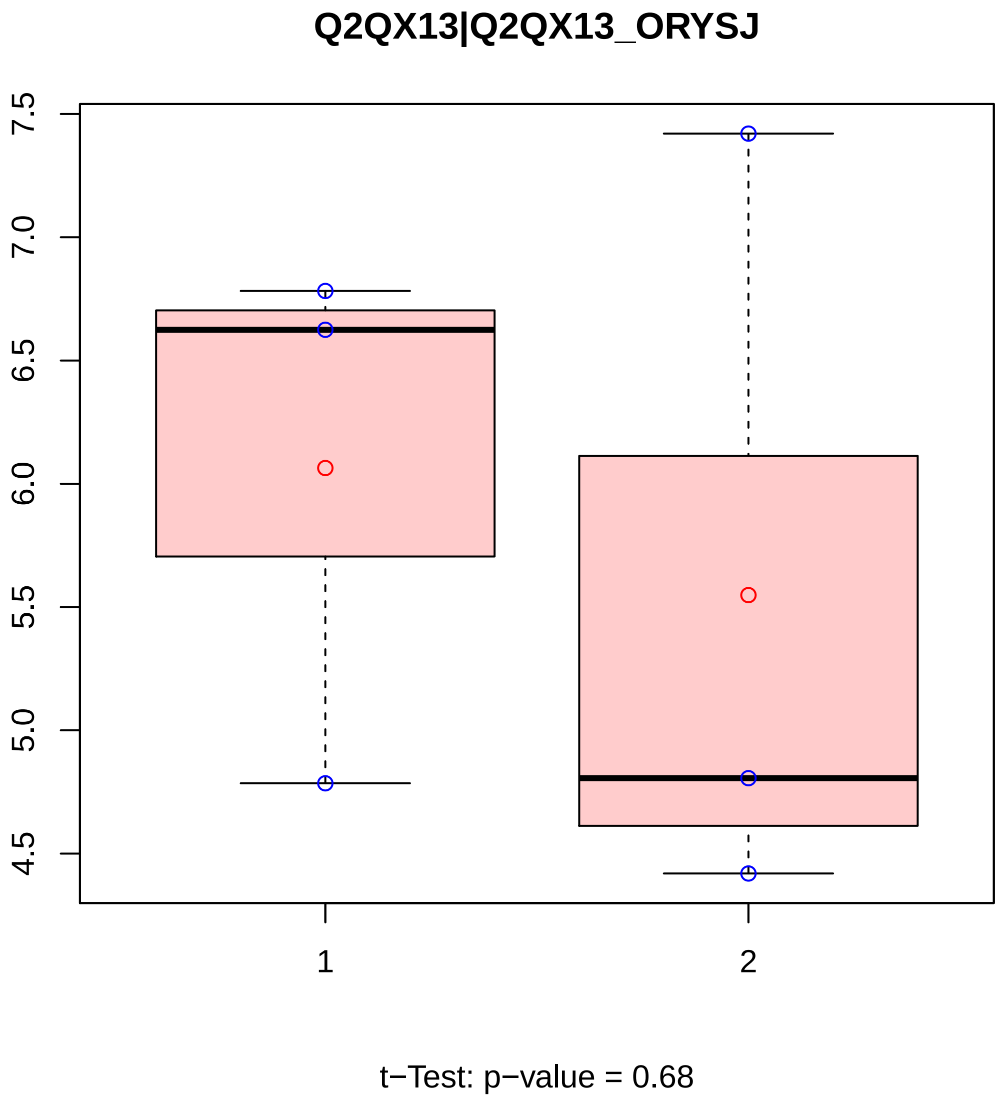

Q2QX13|Q2QX13_ORYSJ
7.5
7.0
6.5
6.0
5.5
5.0
4.5
1
2
t−Test: p−value = 0.68

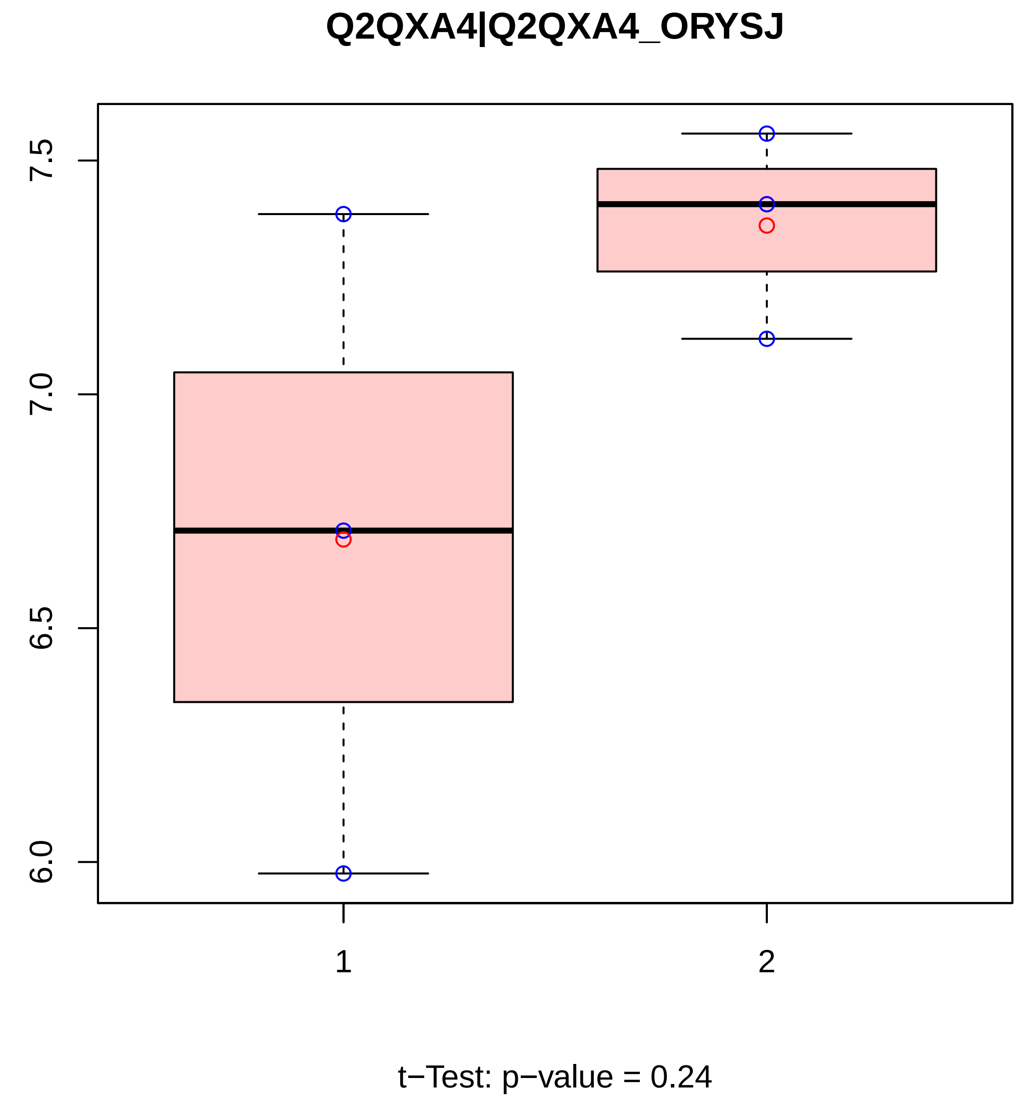
Q2QXA4|Q2QXA4_ORYSJ
7.5
7.0
6.5
6.0
1
2
t−Test: p−value = 0.24

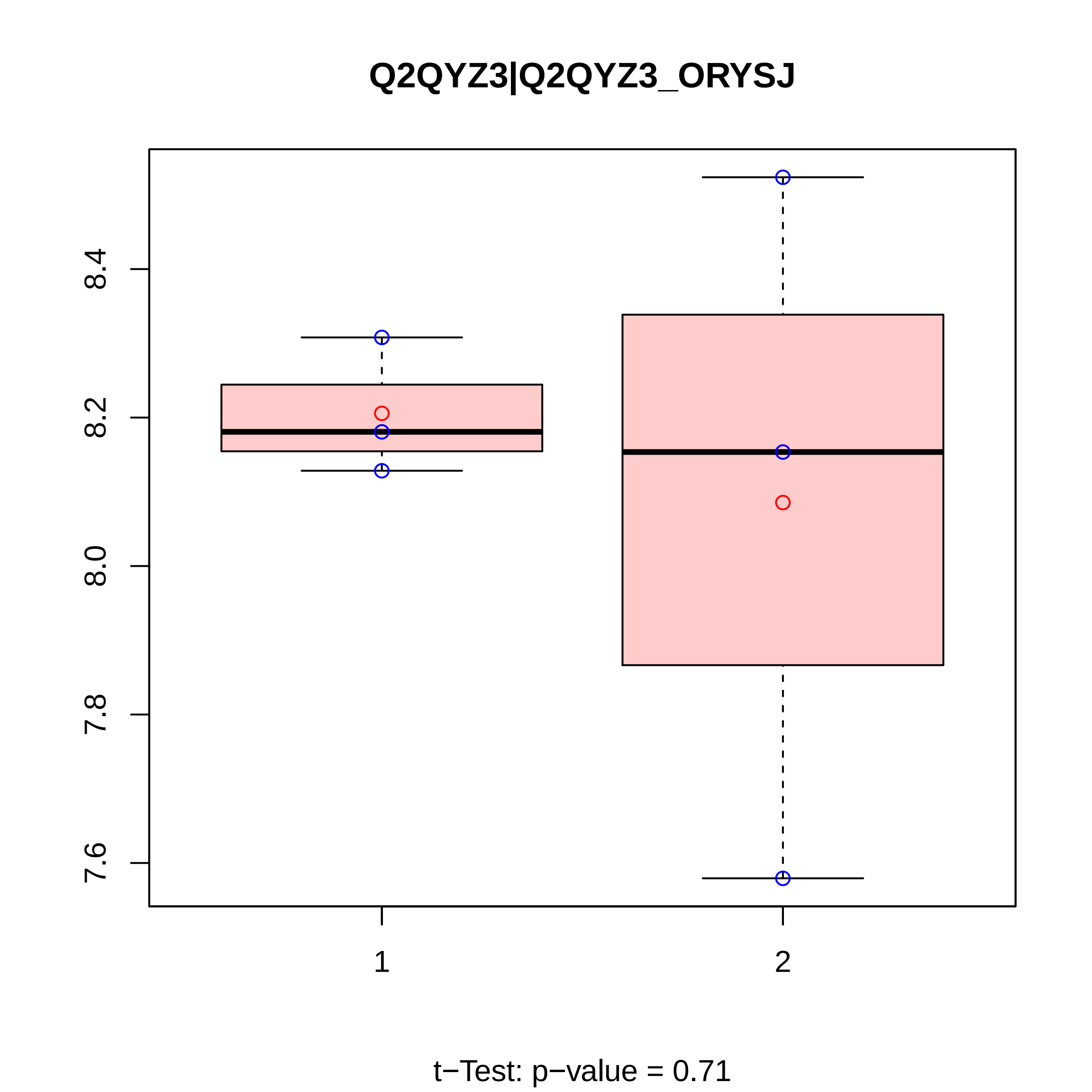

Q2QYZ3|Q2QYZ3_ORYSJ
8.4
8.2
8.0
7.8
7.6
1
2
t−Test: p−value = 0.71

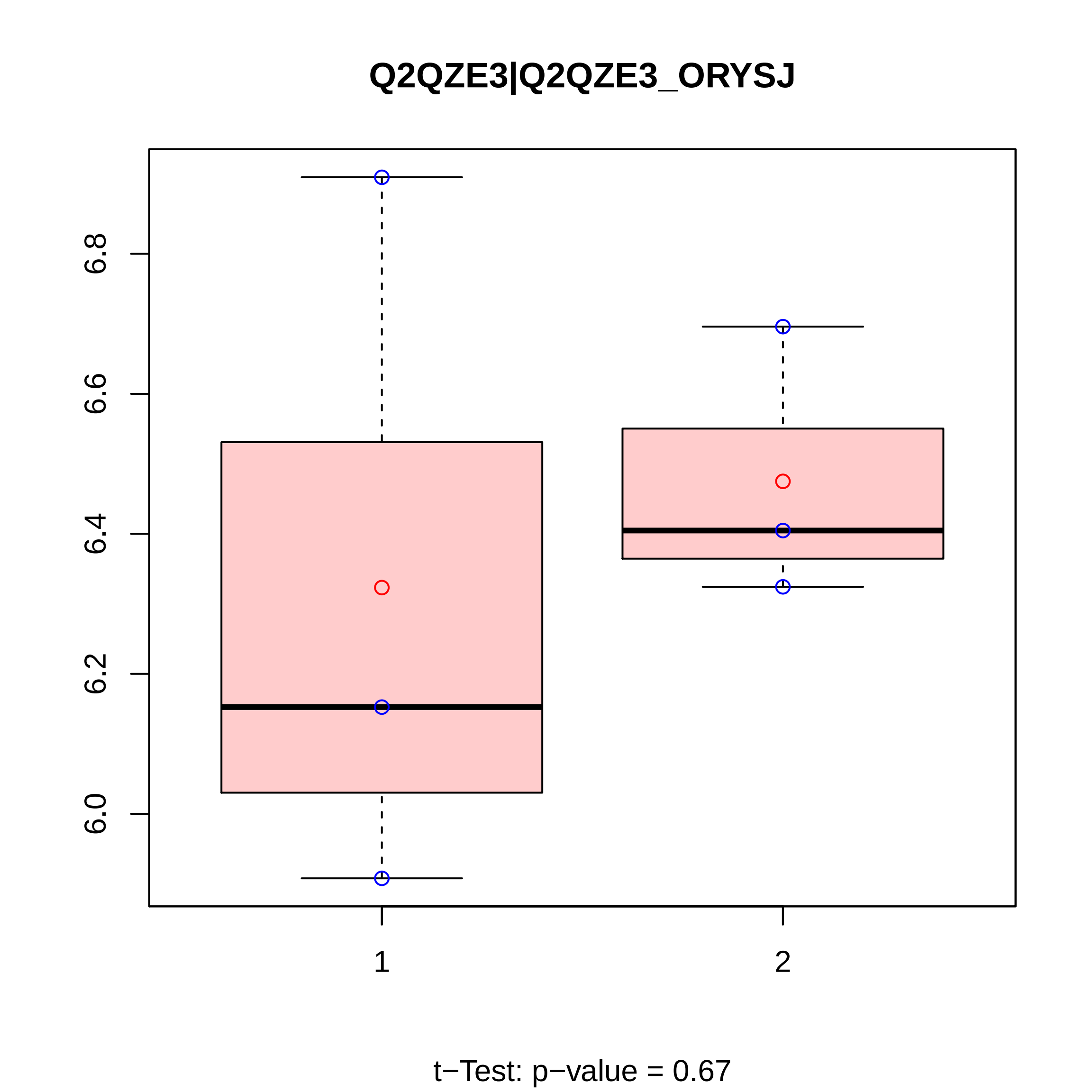
Q2QZE3|Q2QZE3_ORYSJ
6.0
6.2
6.4
6.6
6.8
1
2
t−Test: p−value = 0.67

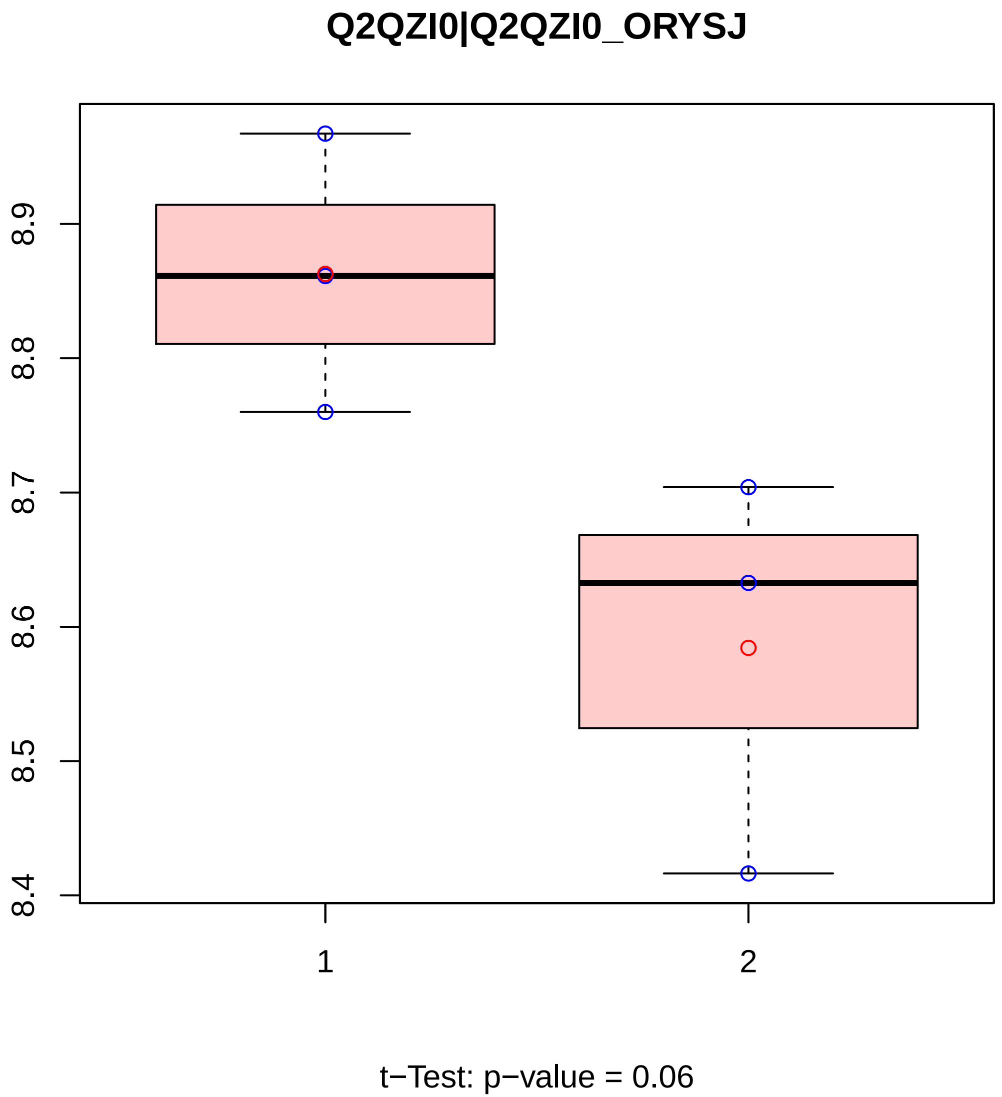

Q2QZI0|Q2QZI0_ORYSJ
8.4
8.5
8.6
8.7
8.8
8.9
1
2
t−Test: p−value = 0.06

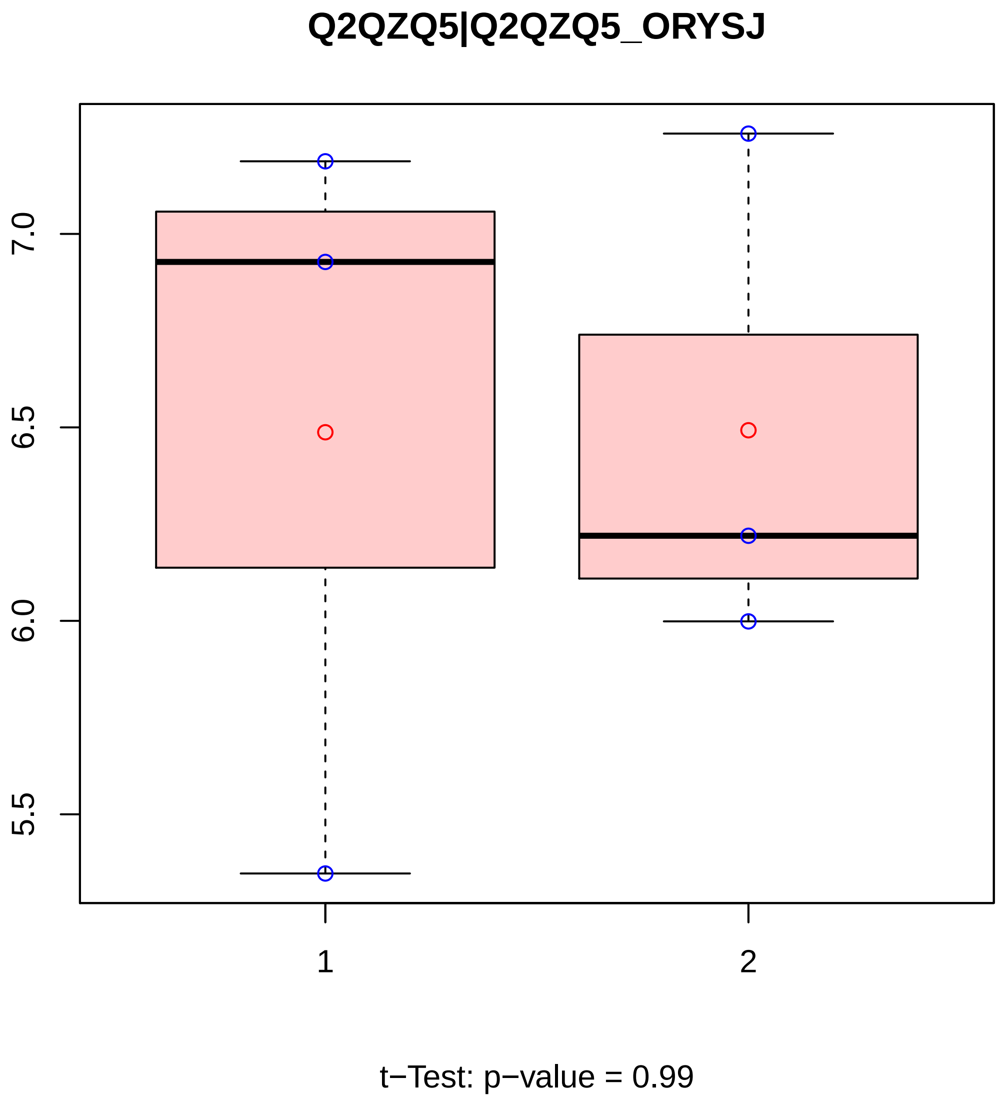

Q2QZQ5|Q2QZQ5_ORYSJ
7.0
6.5
6.0
5.5
1
2
t−Test: p−value = 0.99

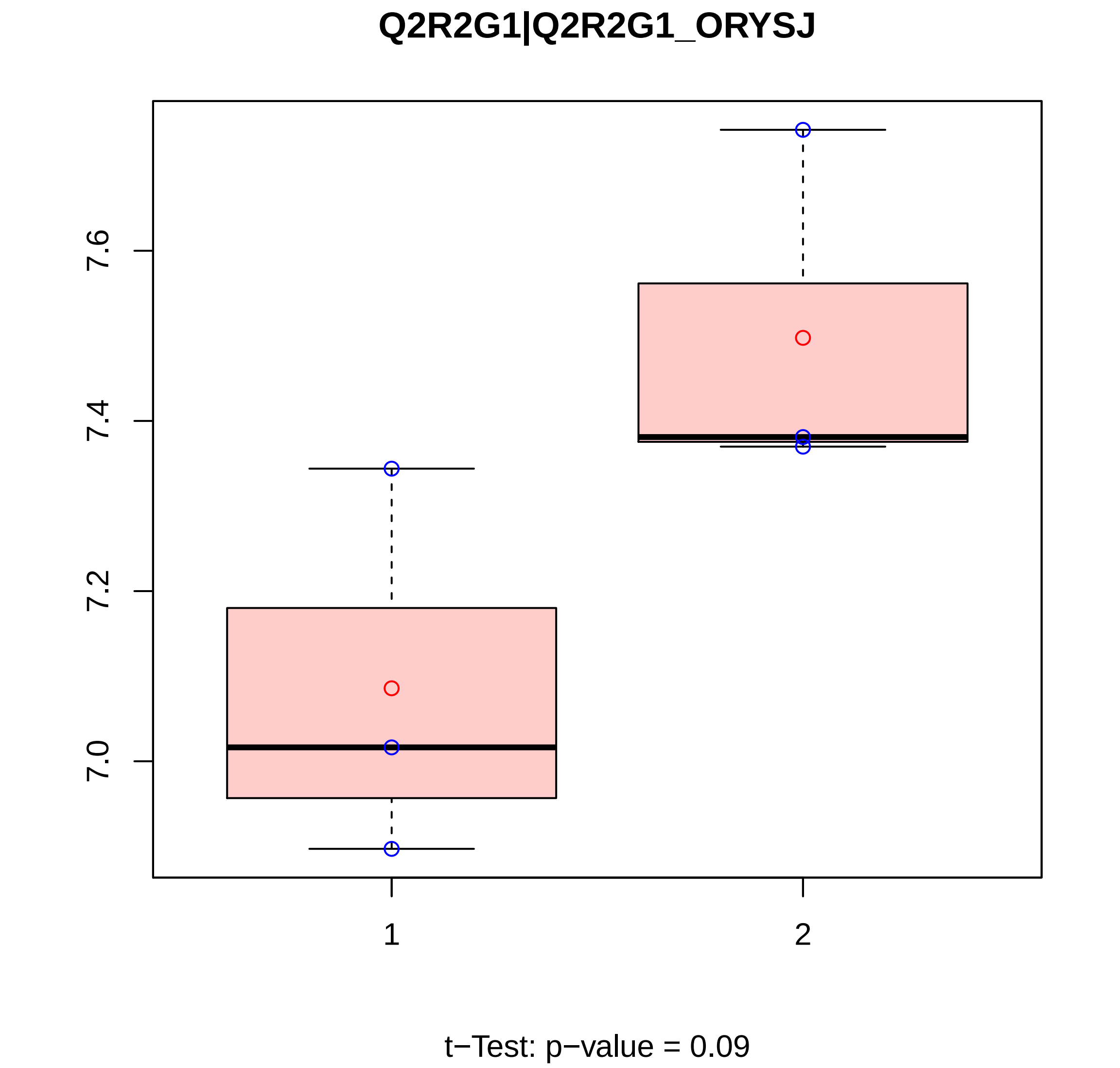
Q2R2G1|Q2R2G1_ORYSJ
7.0
7.2
7.4
7.6
1
2
t−Test: p−value = 0.09

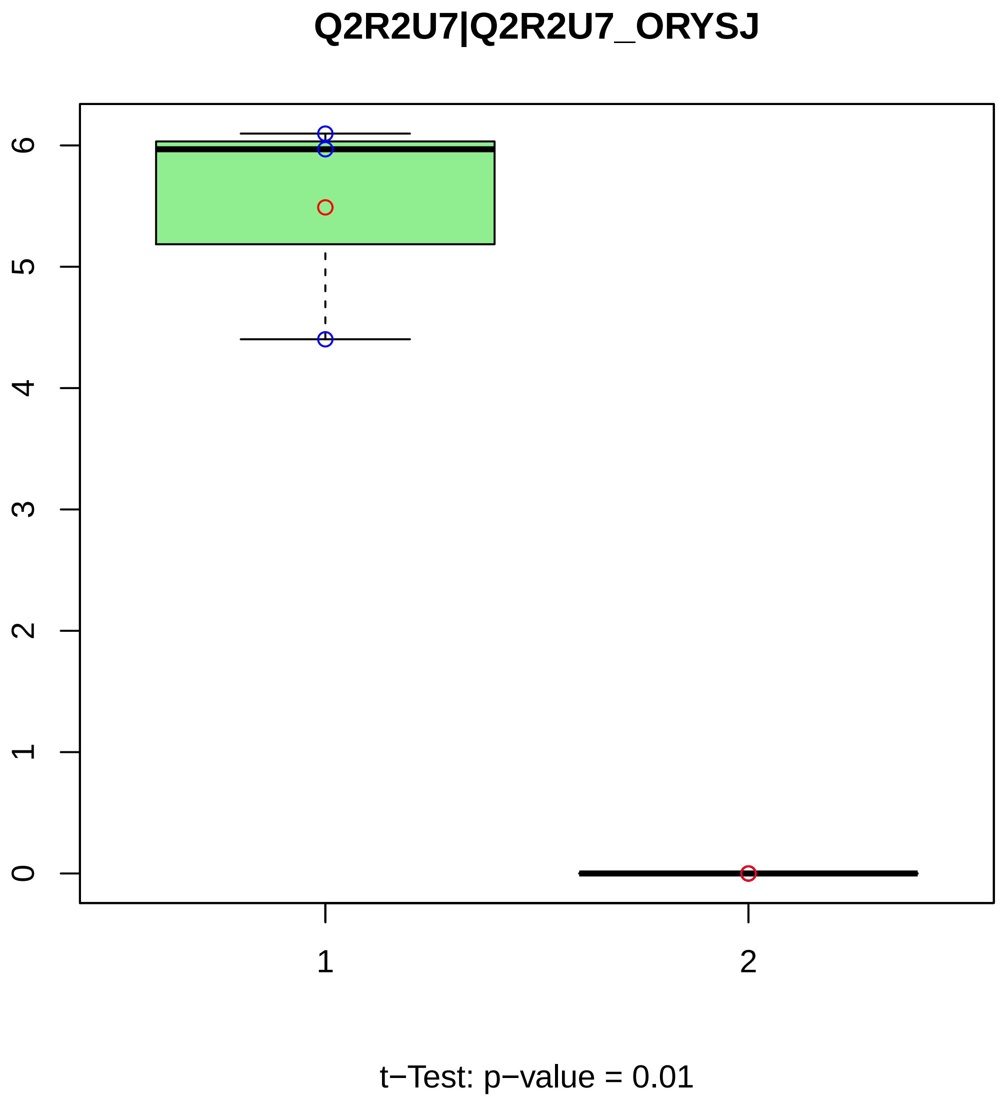
Q2R2U7|Q2R2U7_ORYSJ
0
1
2
3
4
5
6
1
2
t−Test: p−value = 0.01

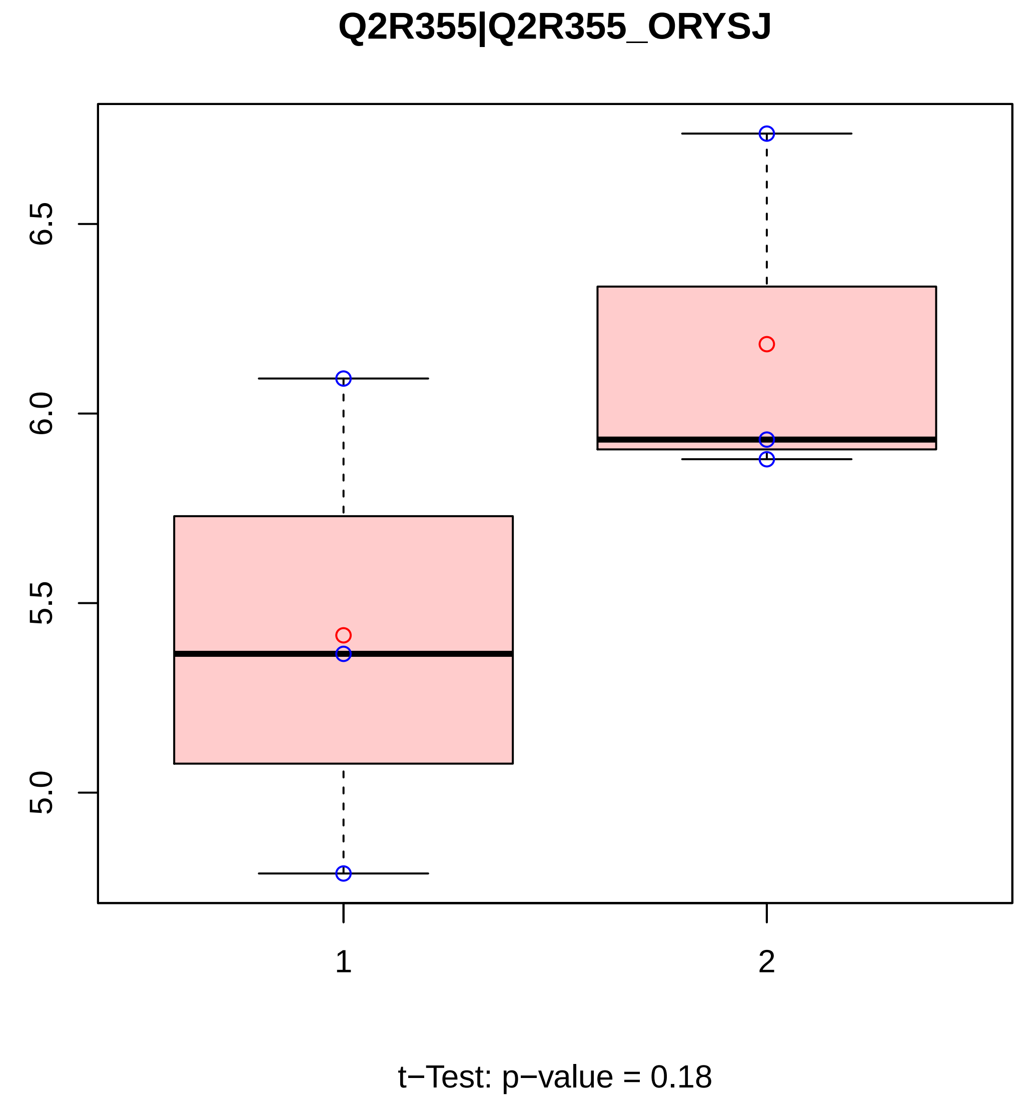
Q2R355|Q2R355_ORYSJ
6.5
6.0
5.5
5.0
1
2
t−Test: p−value = 0.18

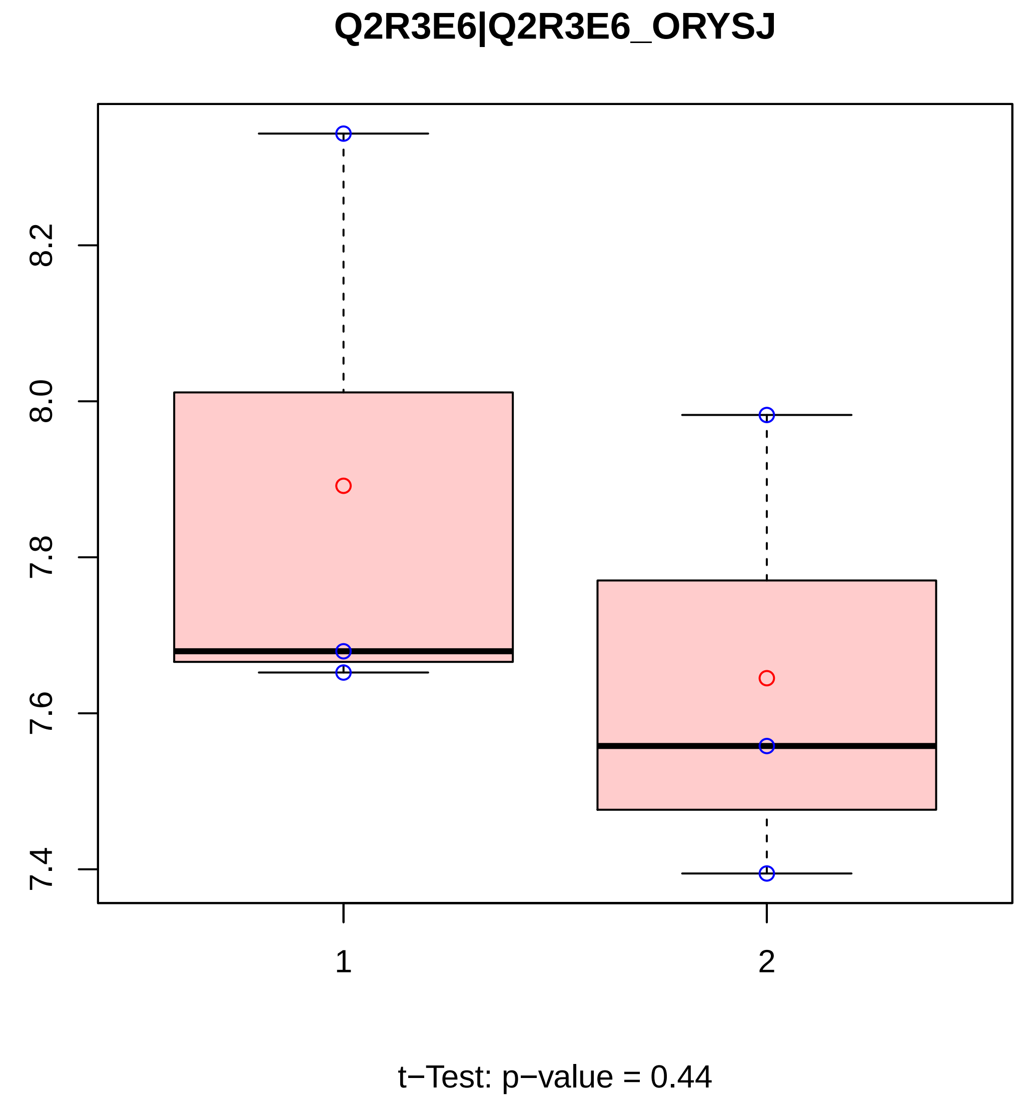
Q2R3E6|Q2R3E6_ORYSJ
8.2
8.0
7.8
7.6
7.4
1
2
t−Test: p−value = 0.44

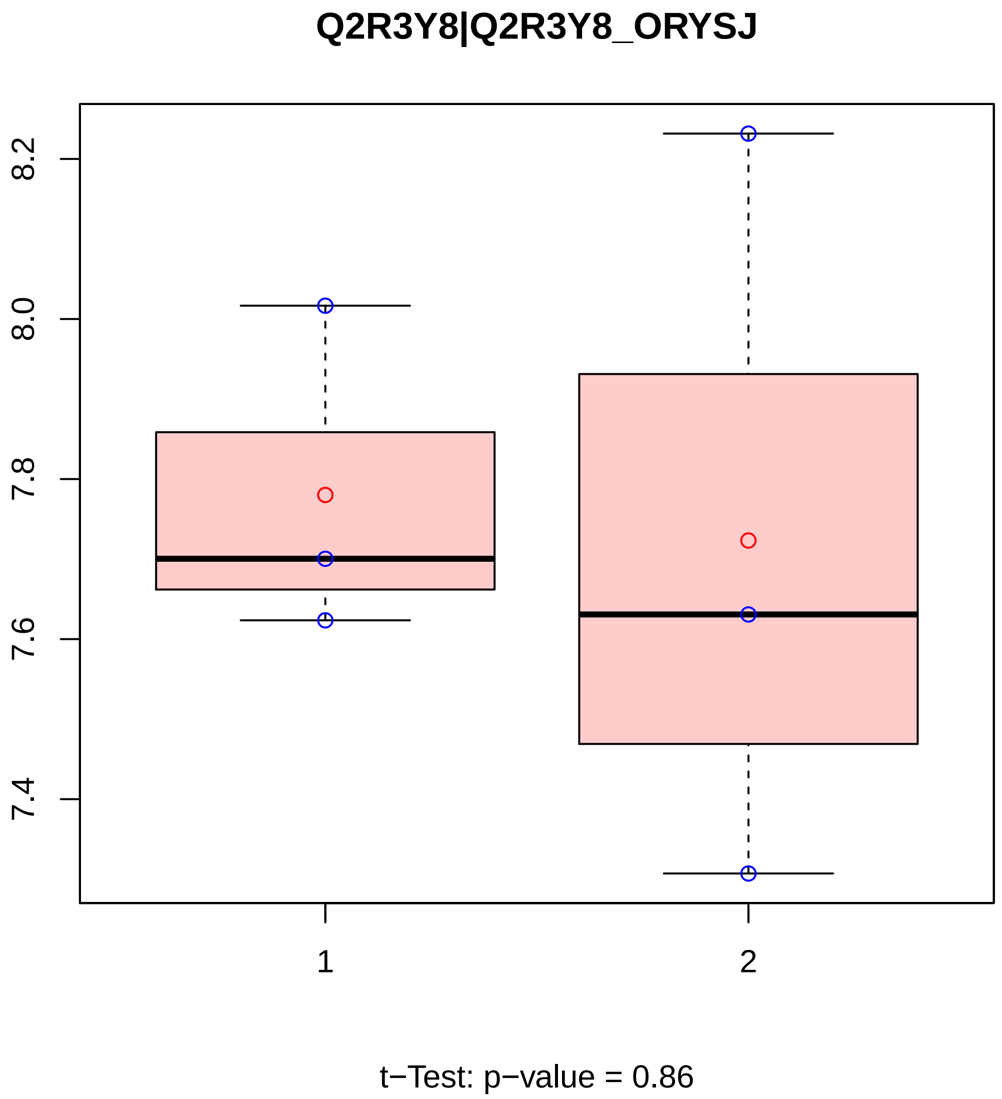
Q2R3Y8|Q2R3Y8_ORYSJ
8.2
8.0
7.8
7.6
7.4
1
2
t−Test: p−value = 0.86

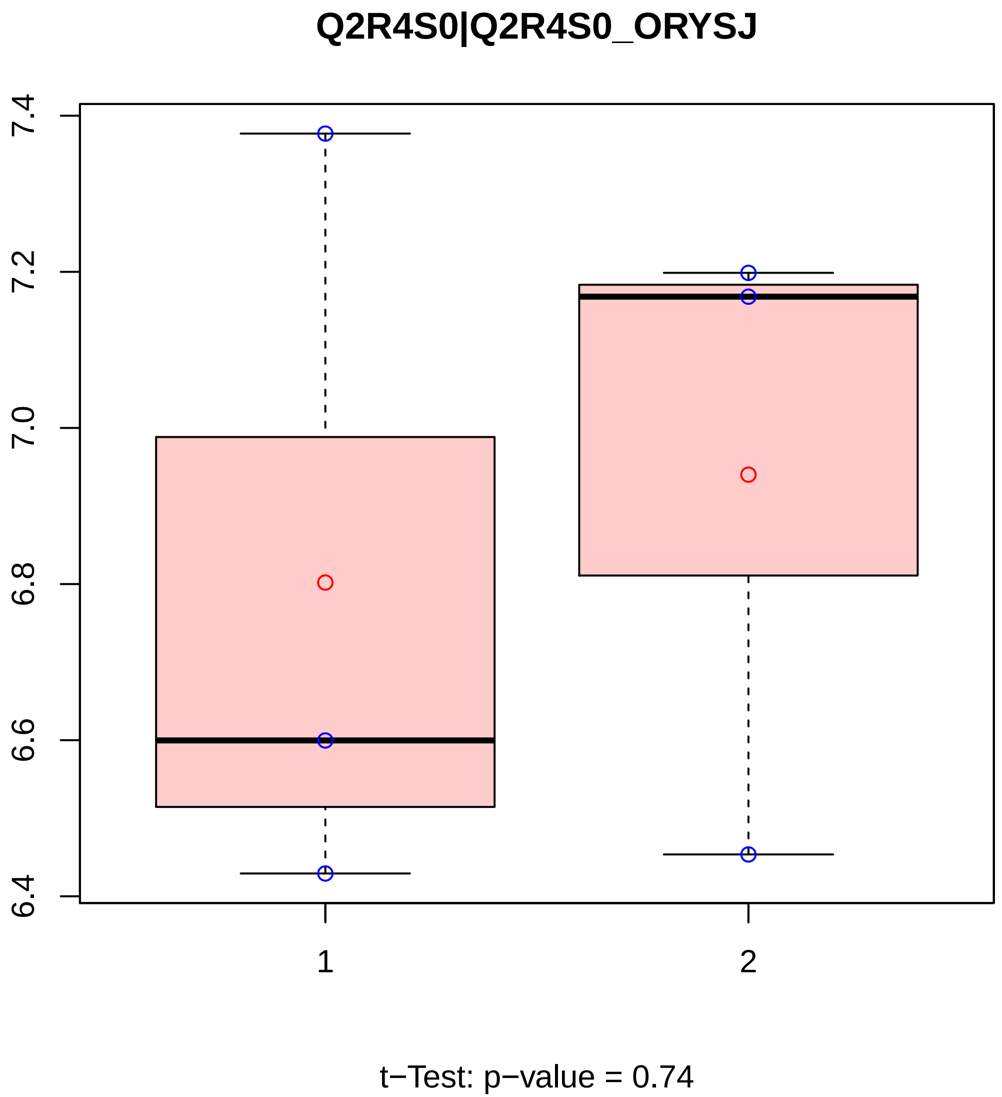

Q2R4S0|Q2R4S0_ORYSJ
7.4
7.2
7.0
6.8
6.6
6.4
1
2
t−Test: p−value = 0.74

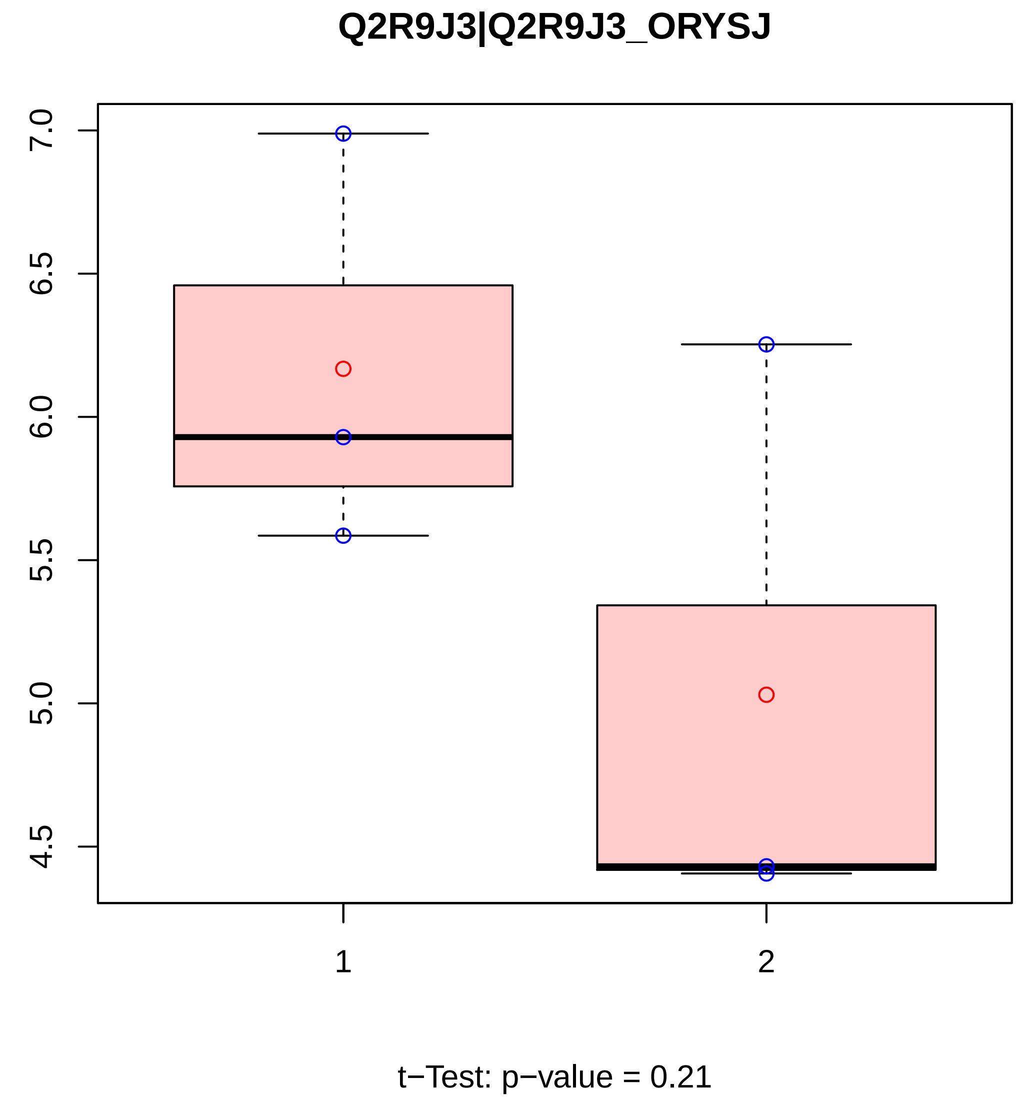
Q2R9J3|Q2R9J3_ORYSJ
7.0
6.5
6.0
5.5
5.0
4.5
1
2
t−Test: p−value = 0.21

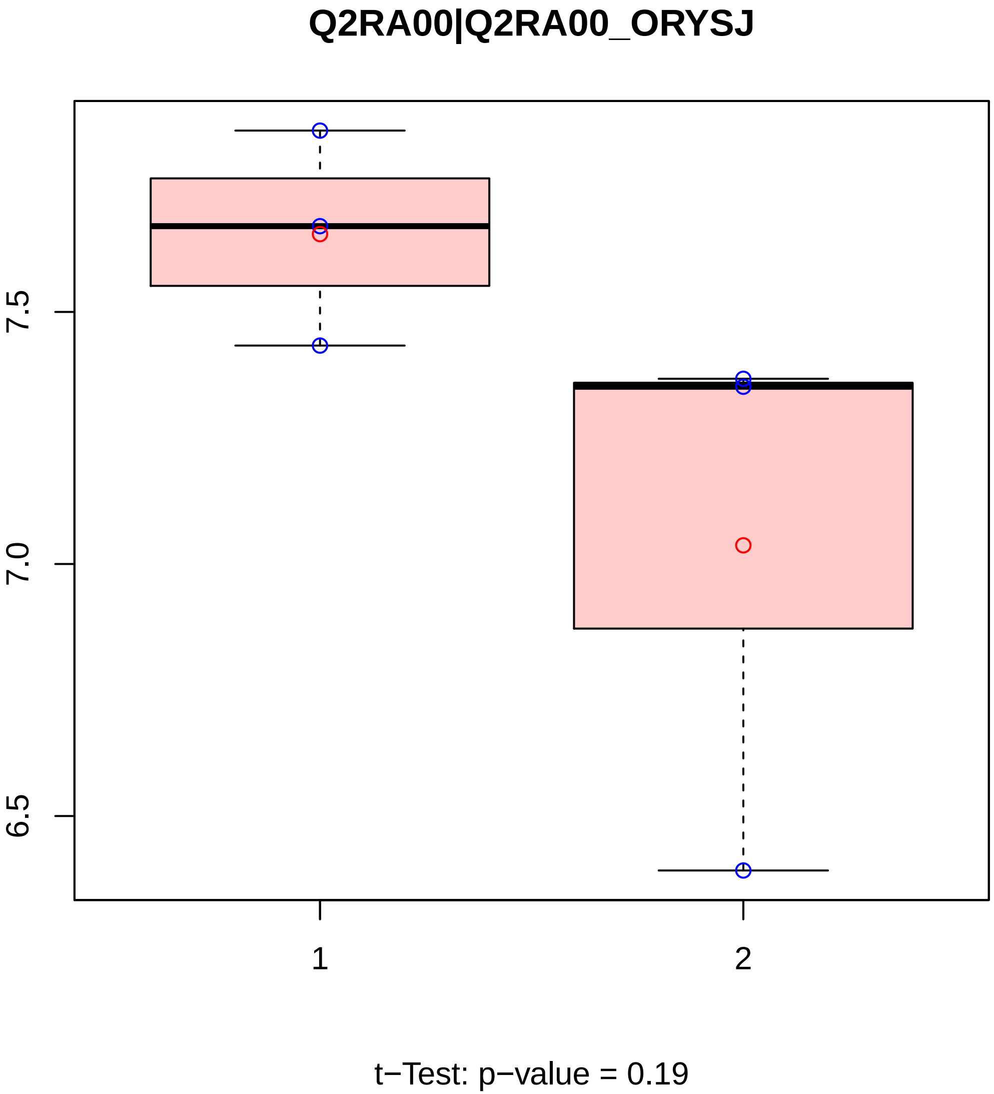

Q2RA00|Q2RA00_ORYSJ
7.5
7.0
6.5
1
2
t−Test: p−value = 0.19

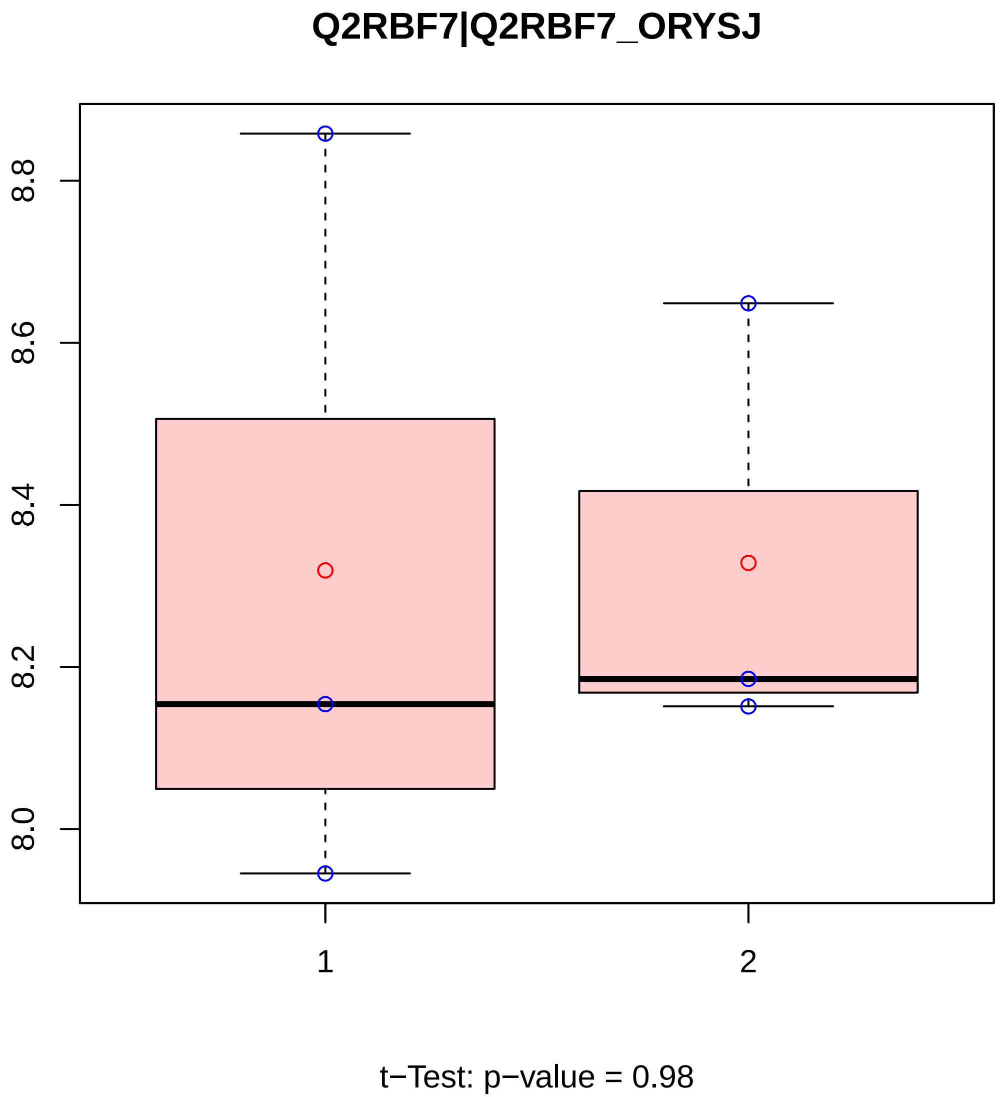
Q2RBF7|Q2RBF7_ORYSJ
8.0
8.2
8.4
8.6
8.8
1
2
t−Test: p−value = 0.98

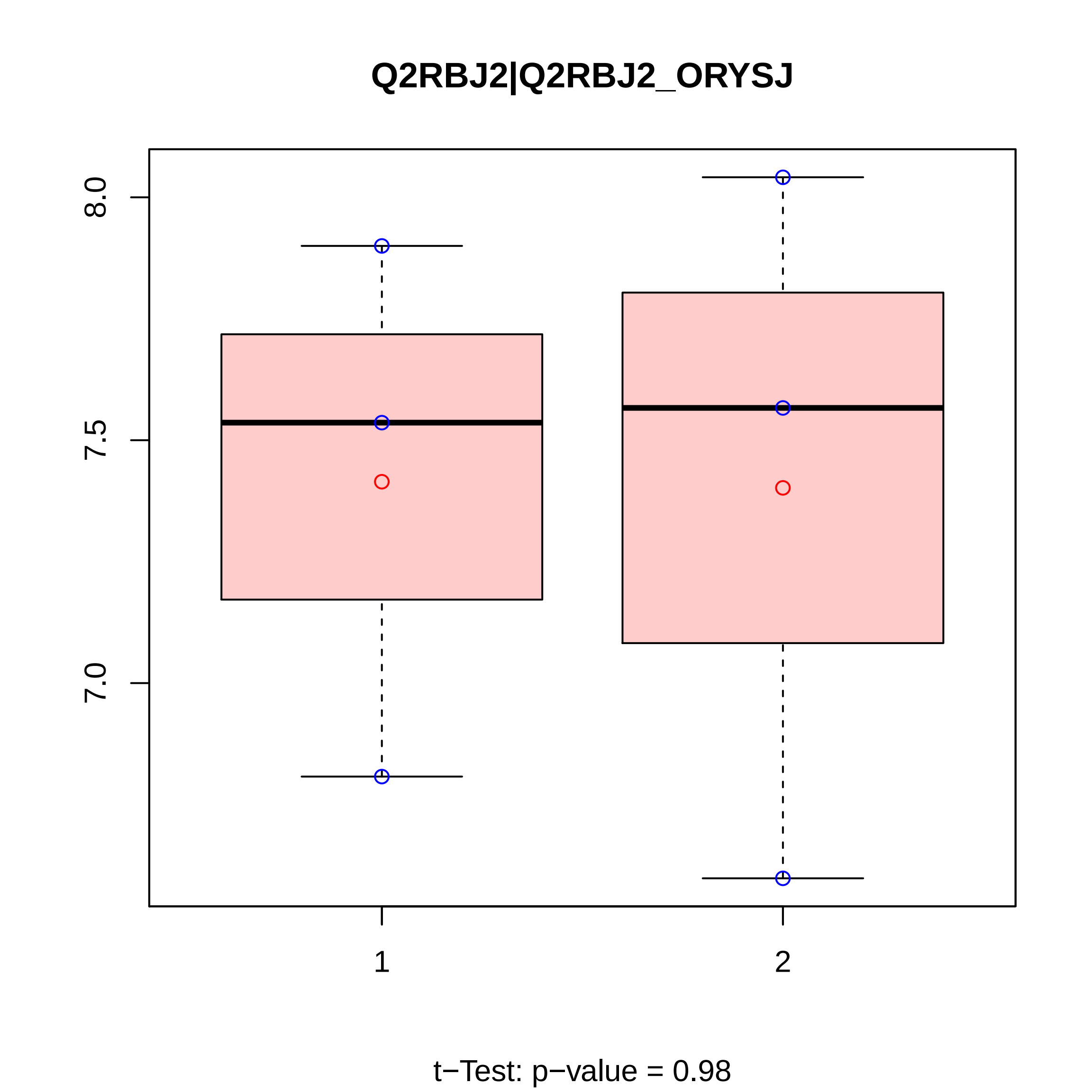
Q2RBJ2|Q2RBJ2_ORYSJ
8.0
7.5
7.0
1
2
t−Test: p−value = 0.98

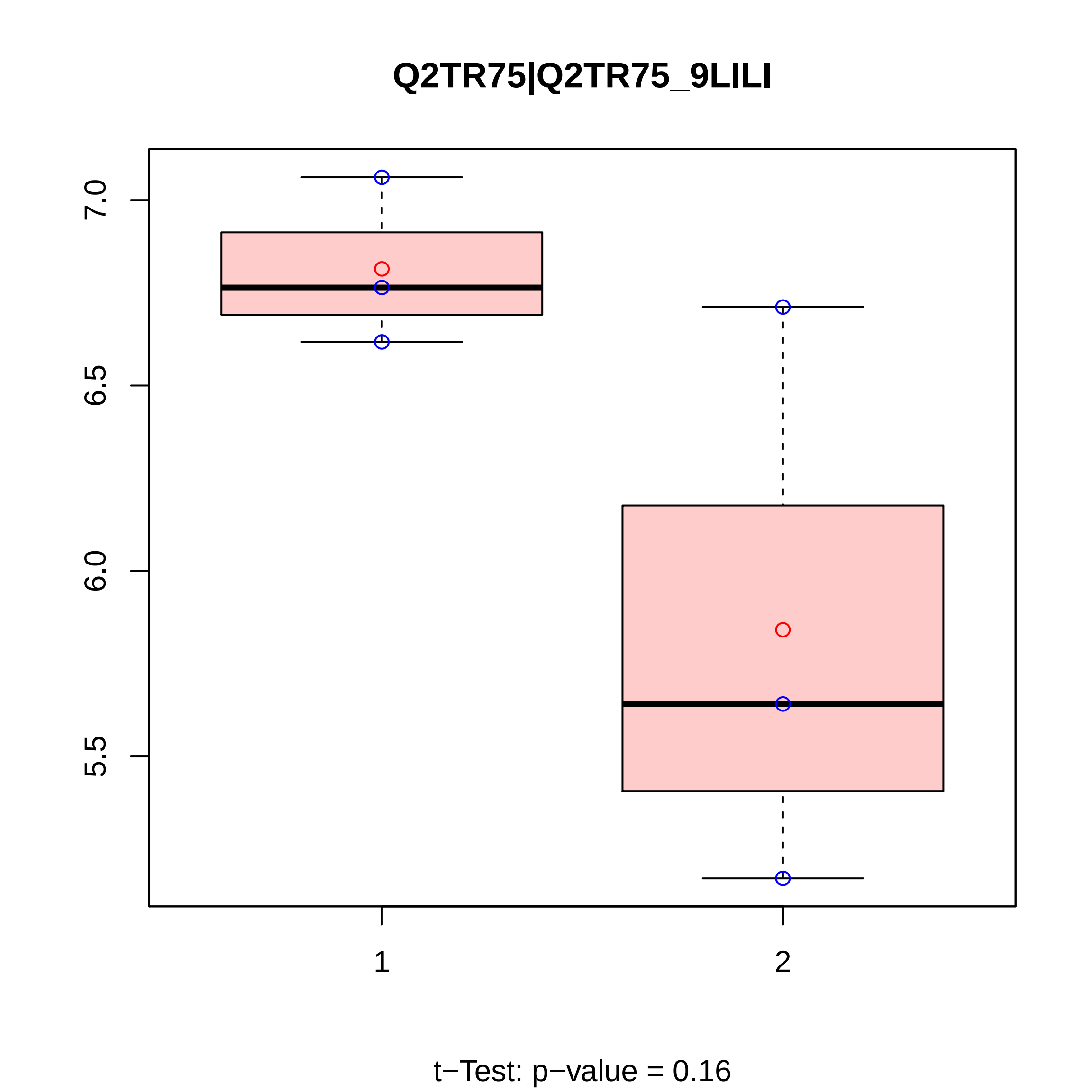
Q2TR75|Q2TR75_9LILI
7.0
6.5
6.0
5.5
1
2
t−Test: p−value = 0.16

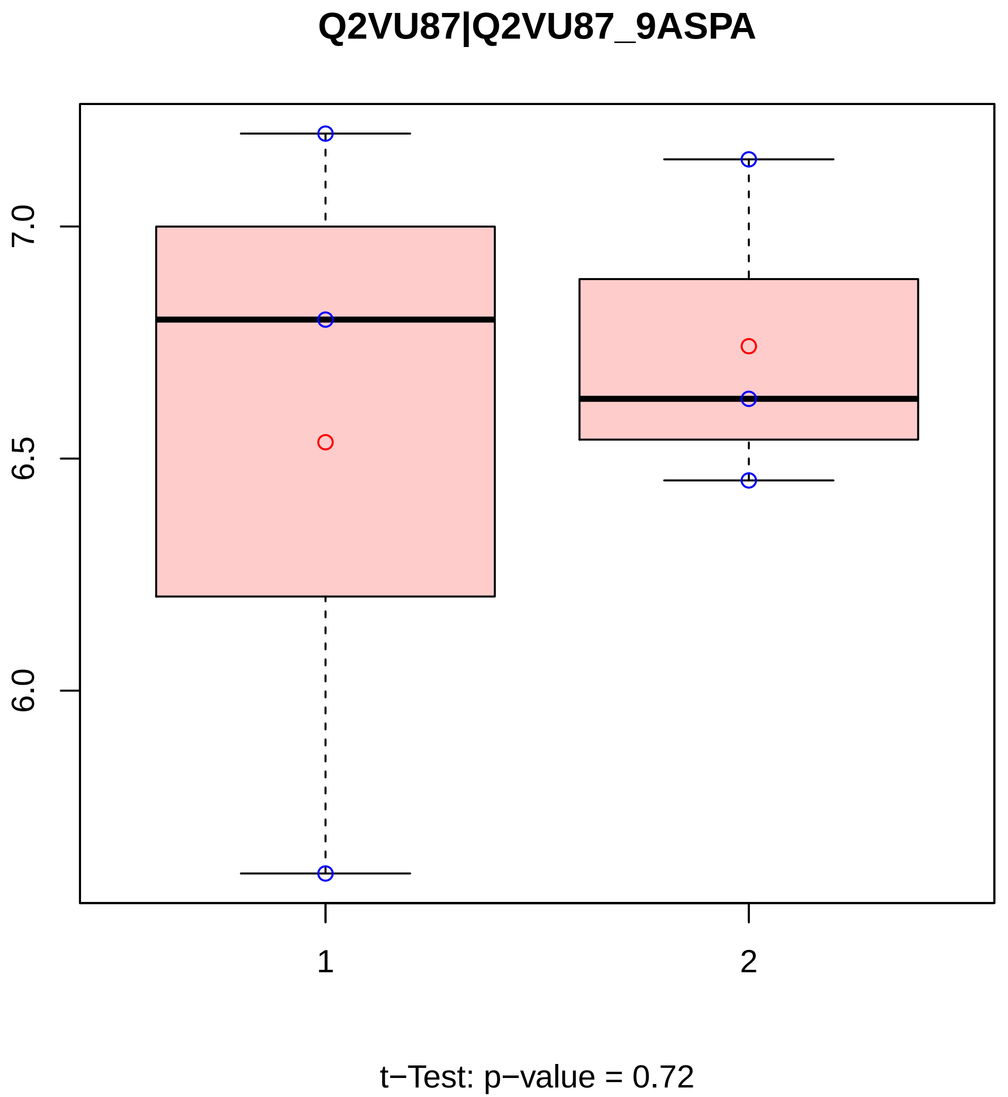
Q2VU87|Q2VU87_9ASPA
7.0
6.5
6.0
1
2
t−Test: p−value = 0.72

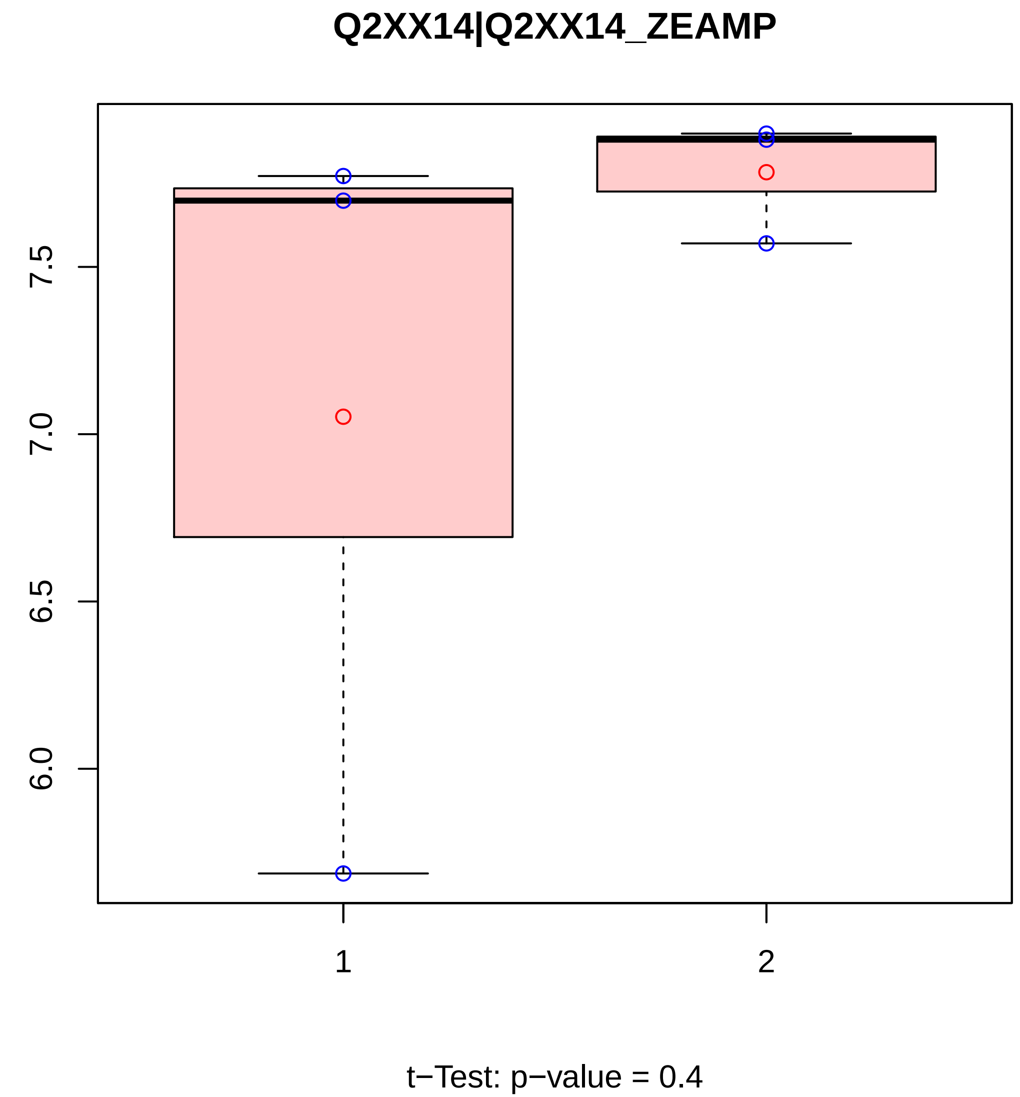
Q2XX14|Q2XX14_ZEAMP
7.5
7.0
6.5
6.0
1
2
t−Test: p−value = 0.4

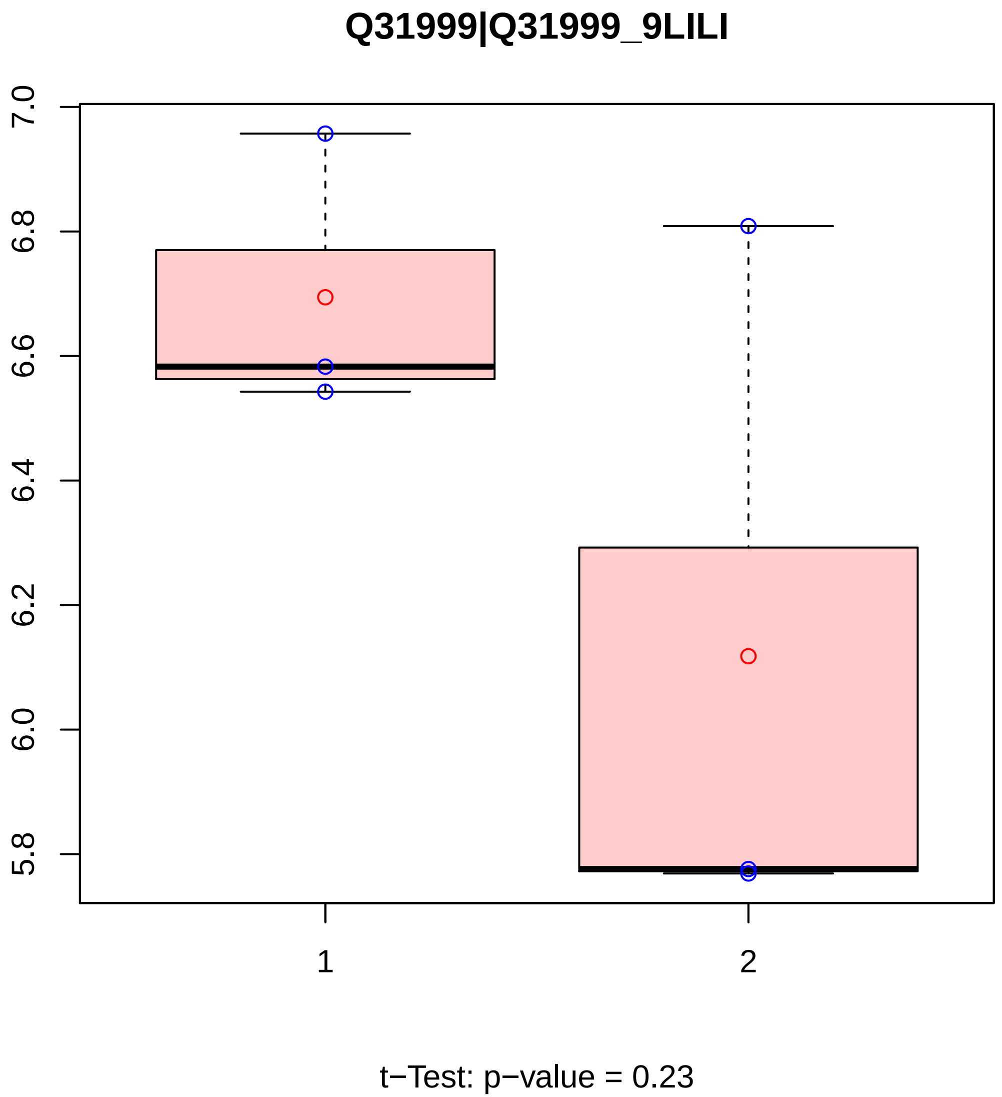
Q31999|Q31999_9LILI
7.0
6.8
6.6
6.4
6.2
6.0
5.8
1
2
t−Test: p−value = 0.23

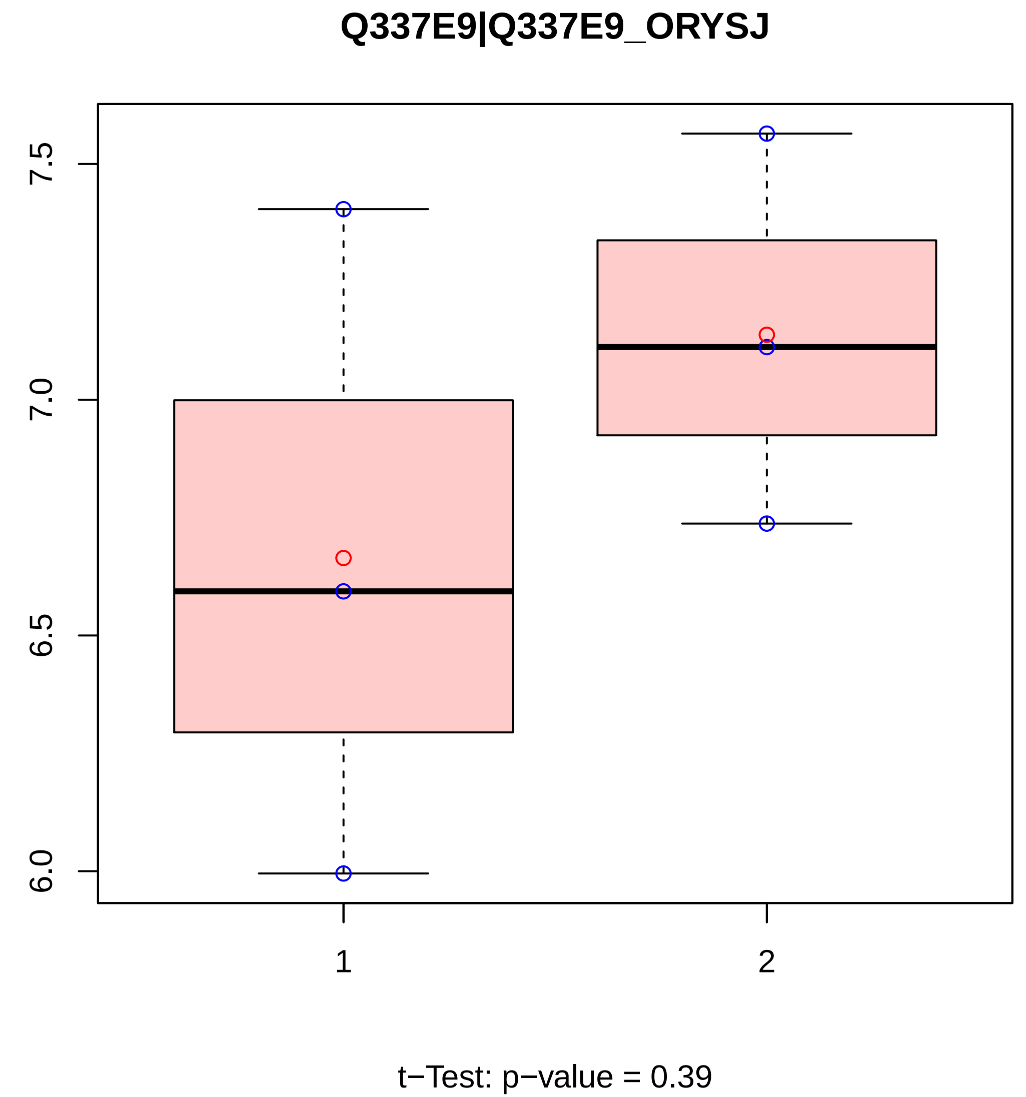
Q337E9|Q337E9_ORYSJ
7.5
7.0
6.5
6.0
1
2
t−Test: p−value = 0.39

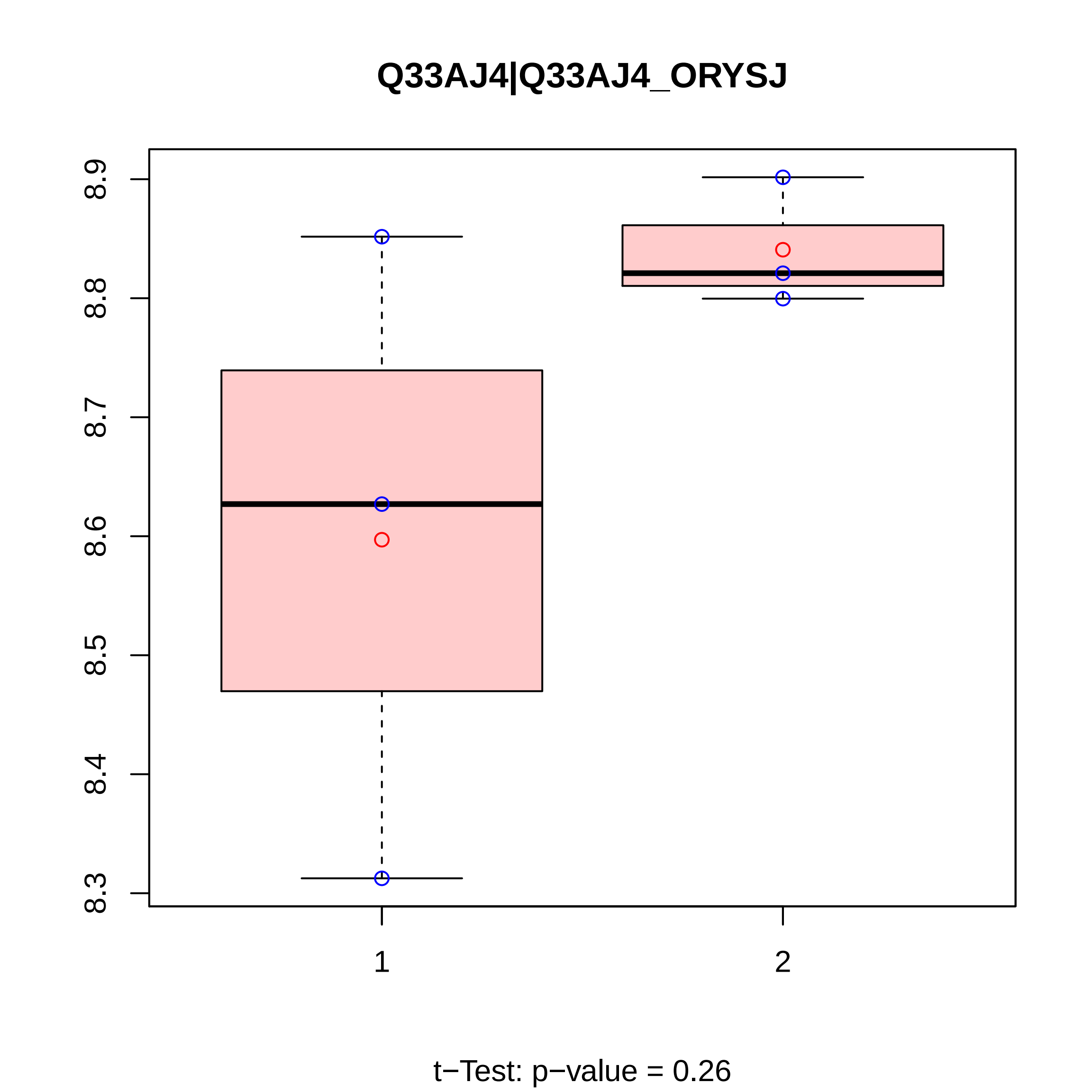
Q33AJ4|Q33AJ4_ORYSJ
8.3
8.4
8.5
8.6
8.7
8.8
8.9
1
2
t−Test: p−value = 0.26

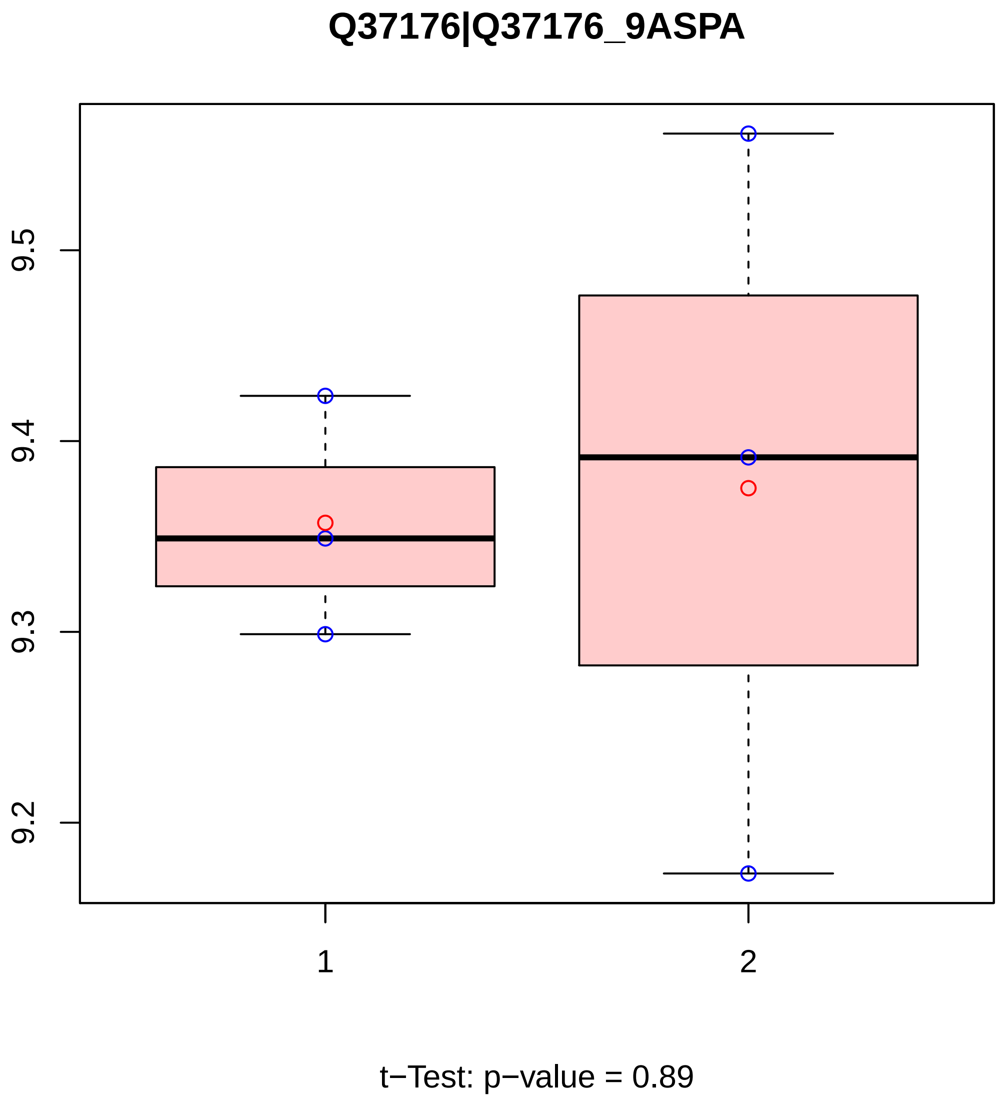
Q37176|Q37176_9ASPA
9.2
9.3
9.4
9.5
1
2
t−Test: p−value = 0.89

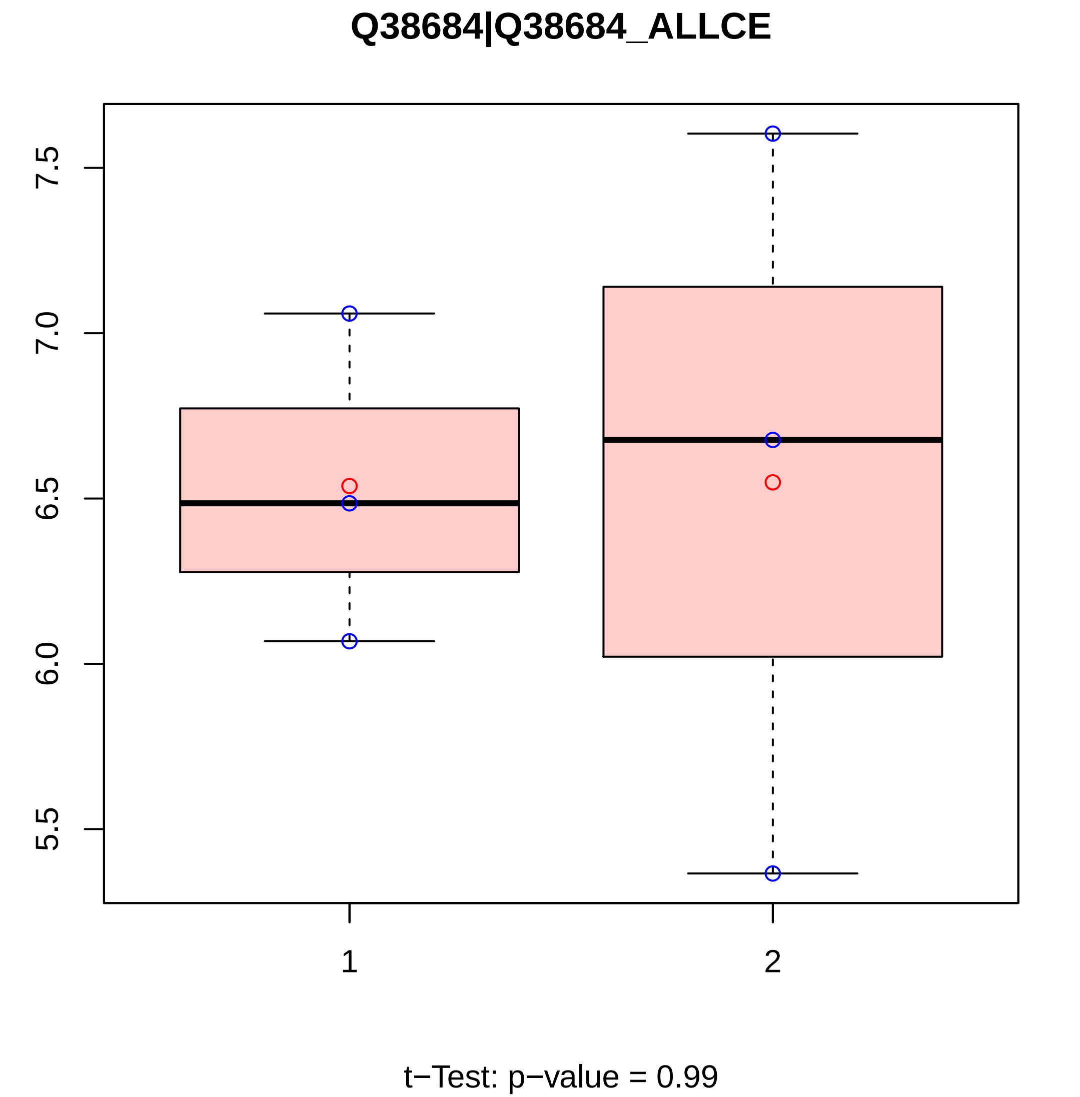
Q38684|Q38684_ALLCE
7.5
7.0
6.5
6.0
5.5
1
2
t−Test: p−value = 0.99

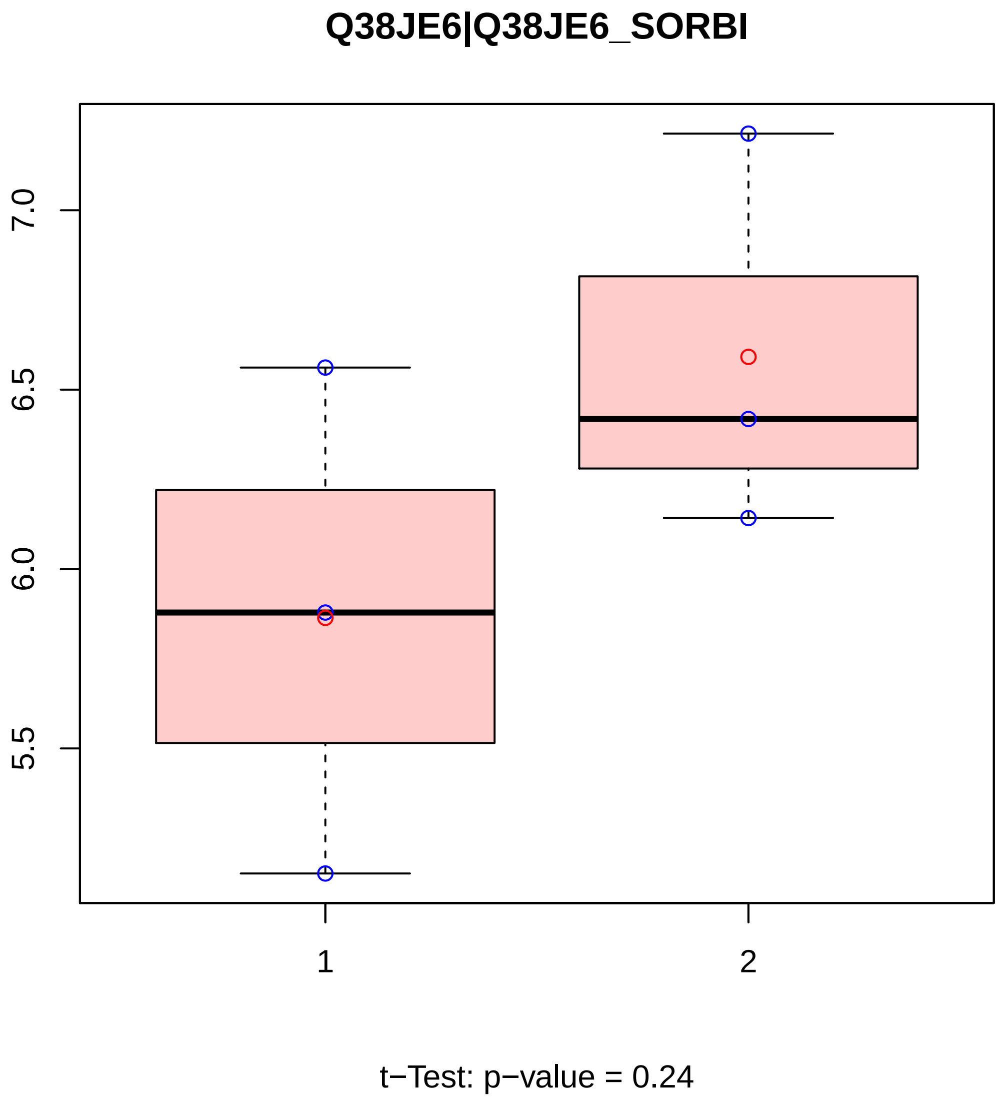
Q38JE6|Q38JE6_SORBI
7.0
6.5
6.0
5.5
1
2
t−Test: p−value = 0.24

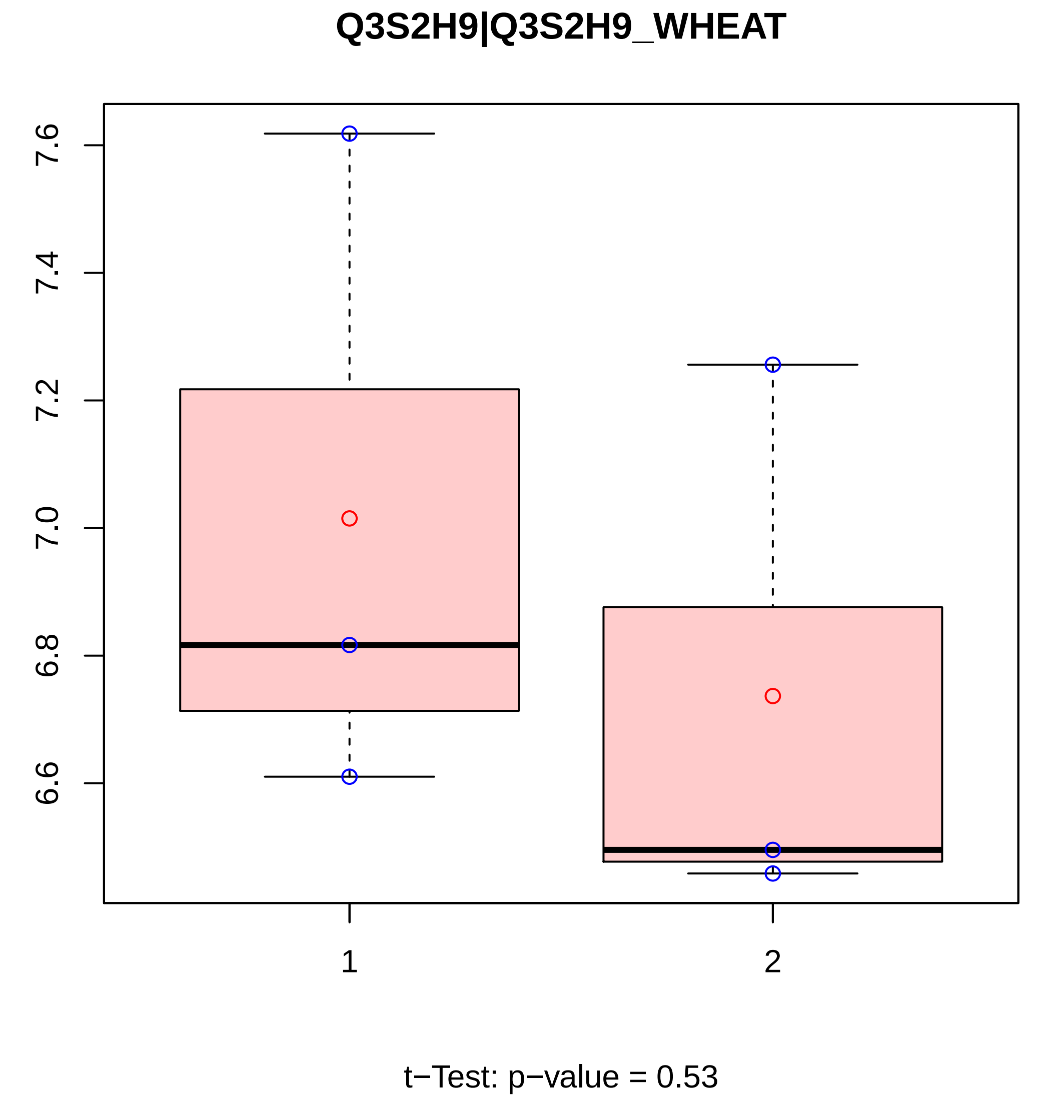
Q3S2H9|Q3S2H9_WHEAT
7.6
7.4
7.2
7.0
6.8
6.6
1
2
t−Test: p−value = 0.53

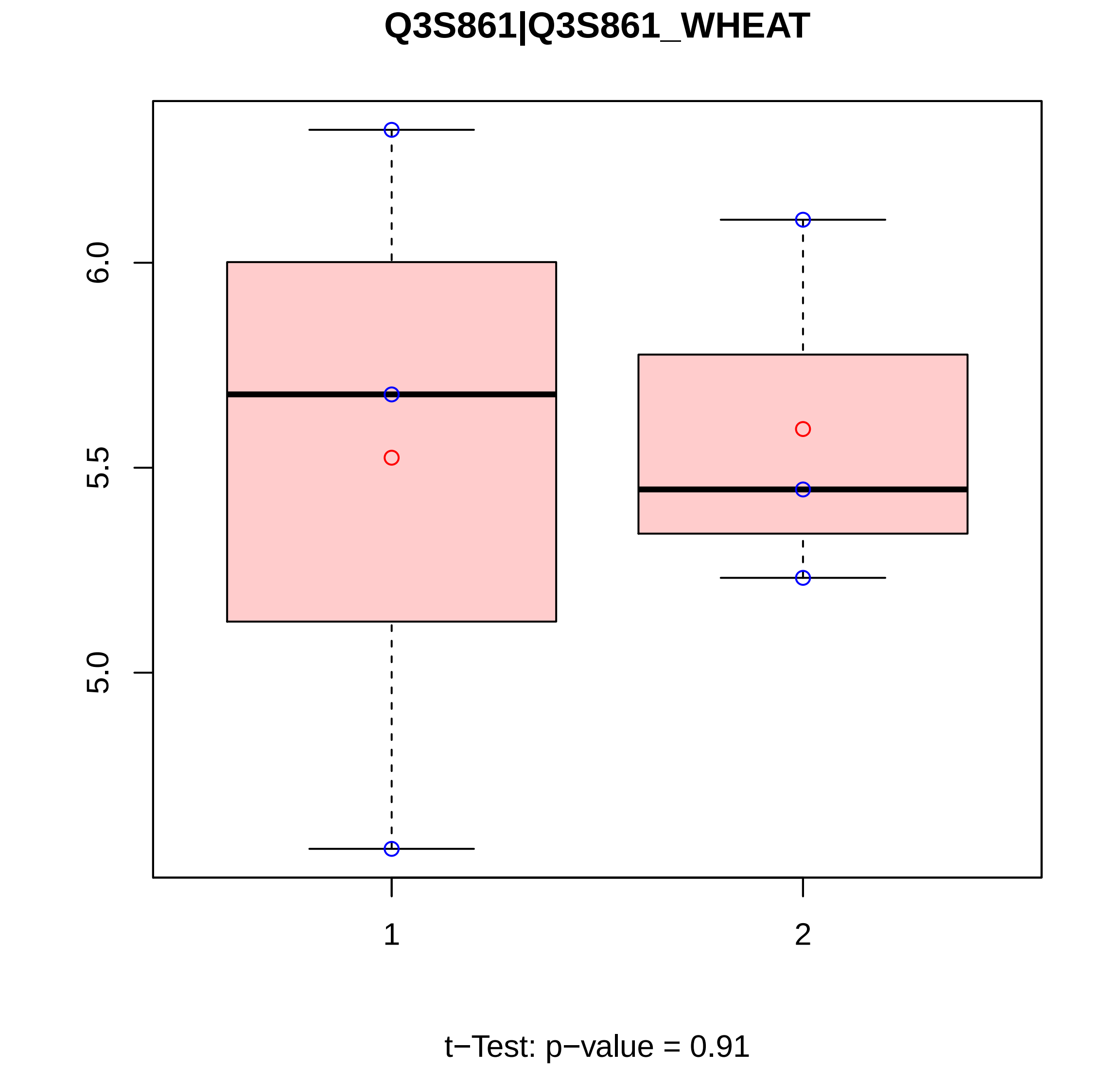
Q3S861|Q3S861_WHEAT
6.0
5.5
5.0
1
2
t−Test: p−value = 0.91

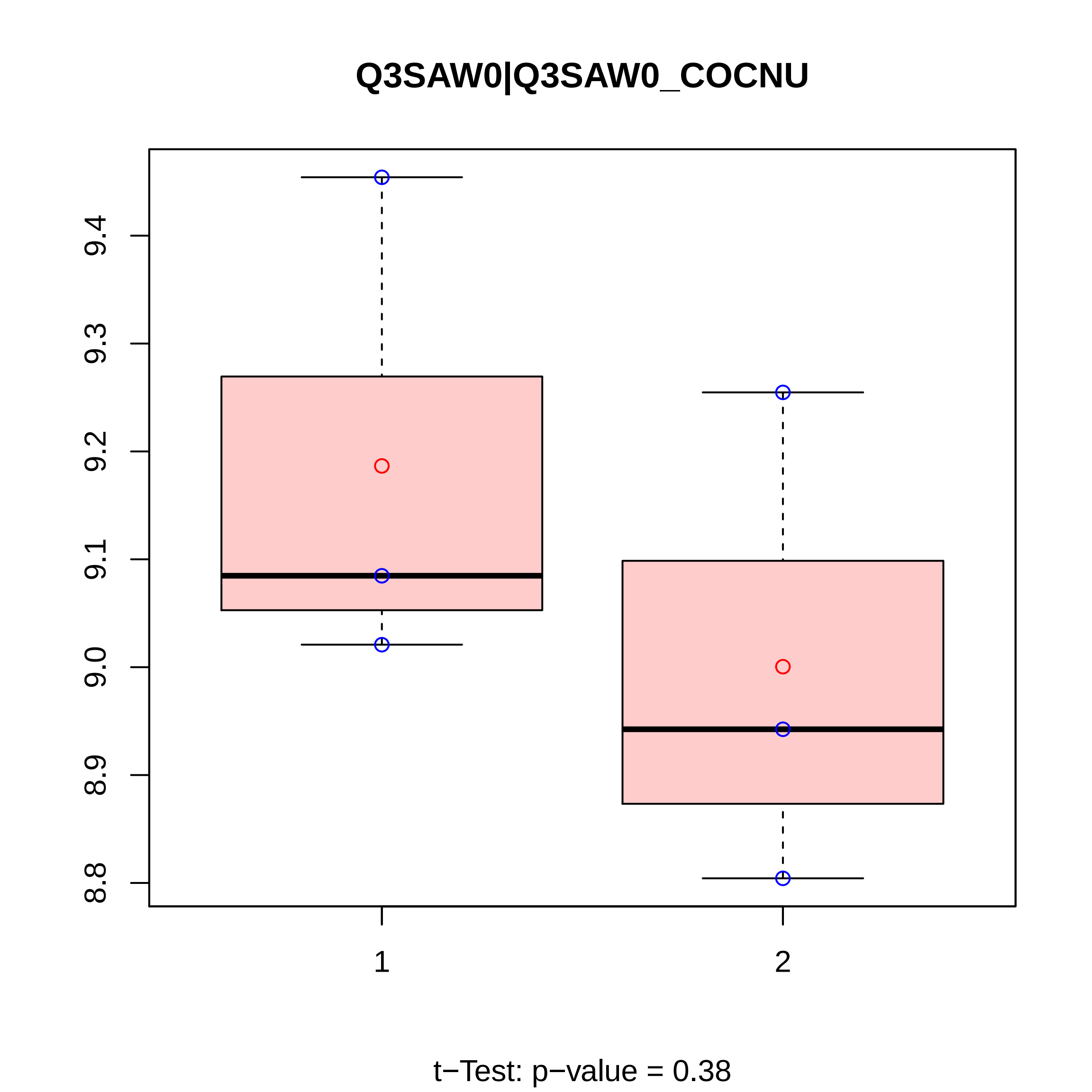
Q3SAW0|Q3SAW0_COCNU
9.4
9.3
9.2
9.1
9.0
8.9
8.8
1
2
t−Test: p−value = 0.38

Q3ZPP0|Q3ZPP0_9ASPA

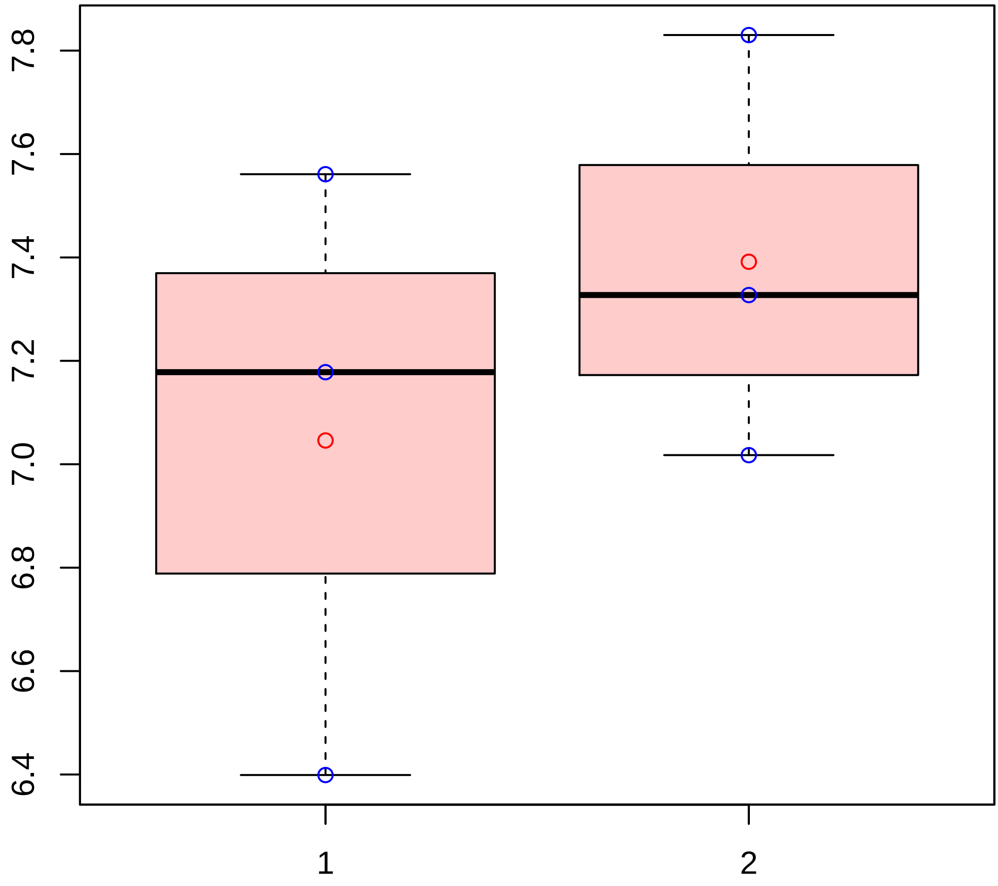

t−Test: p−value = 0.46

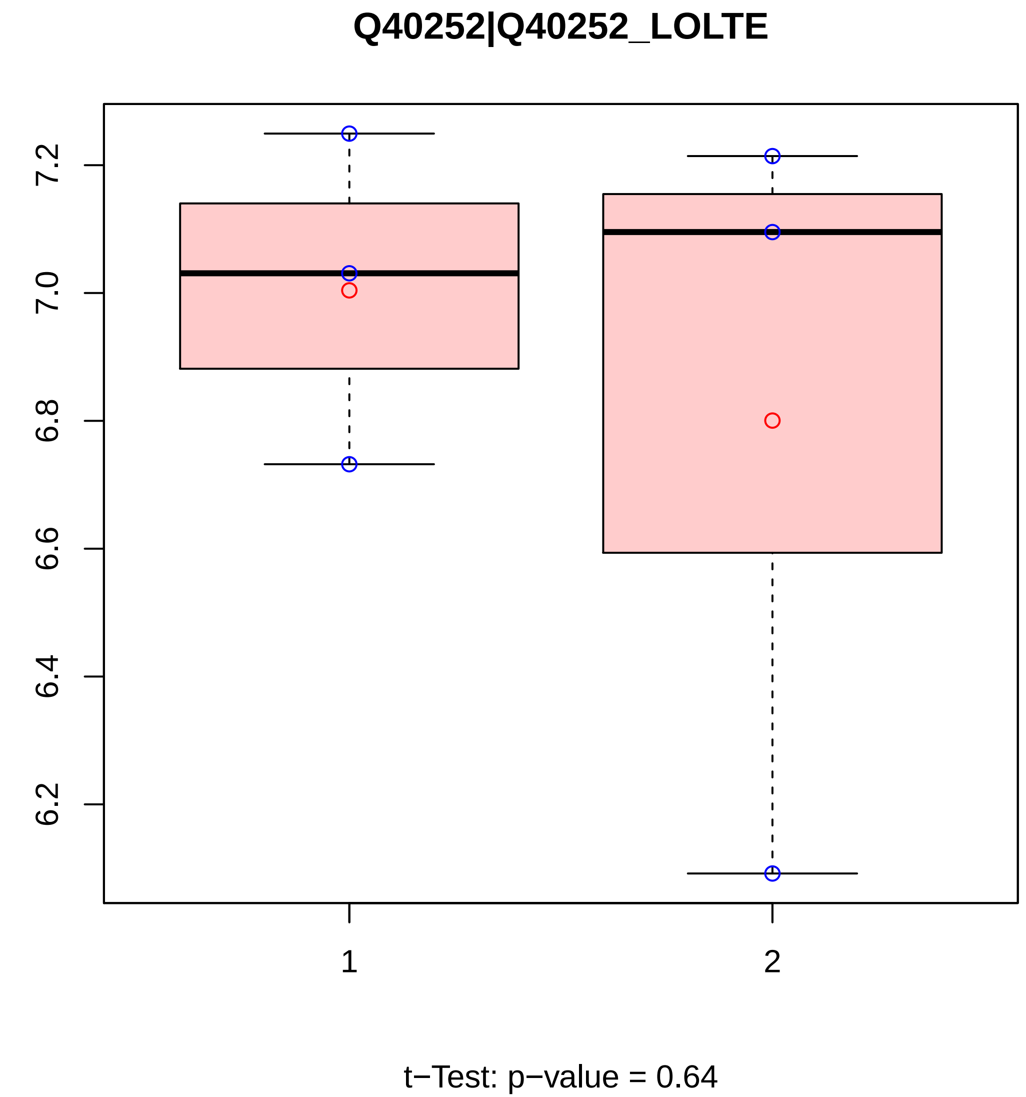
Q40252|Q40252_LOLTE
7.2
7.0
6.8
6.6
6.4
6.2
1
2
t−Test: p−value = 0.64

Q40693|Q40693_ORYSA

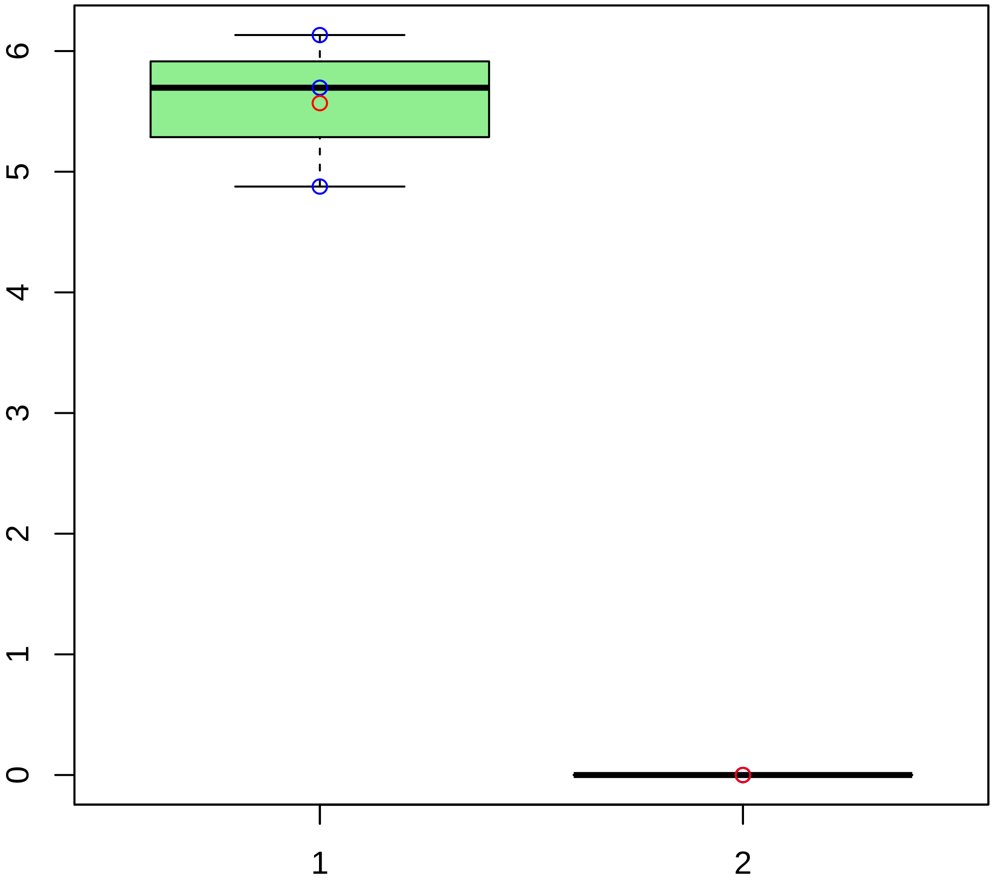

t−Test: p−value = 0

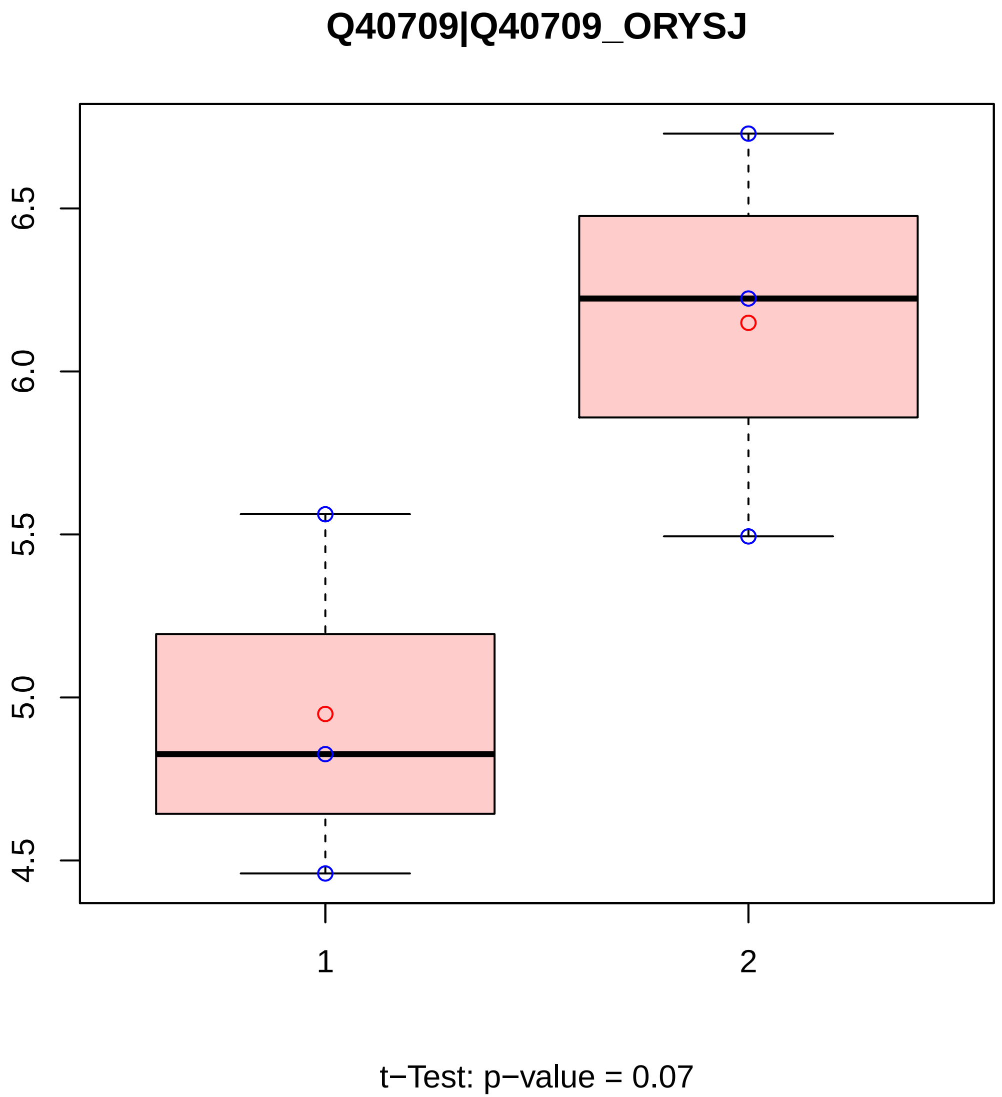
Q40709|Q40709_ORYSJ
6.5
6.0
5.5
5.0
4.5
1
2
t−Test: p−value = 0.07

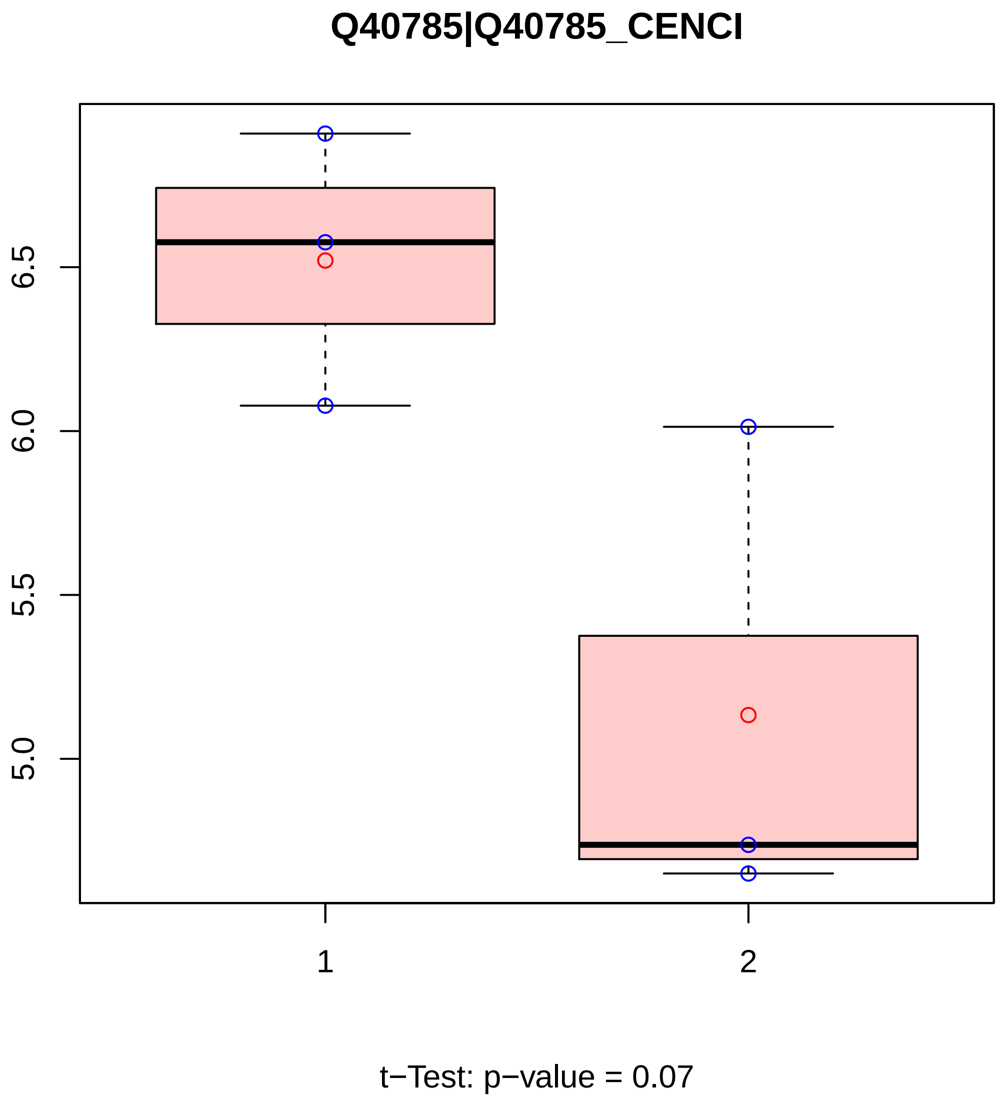
Q40785|Q40785_CENCI
5.0
5.5
6.0
6.5
1
2
t−Test: p−value = 0.07

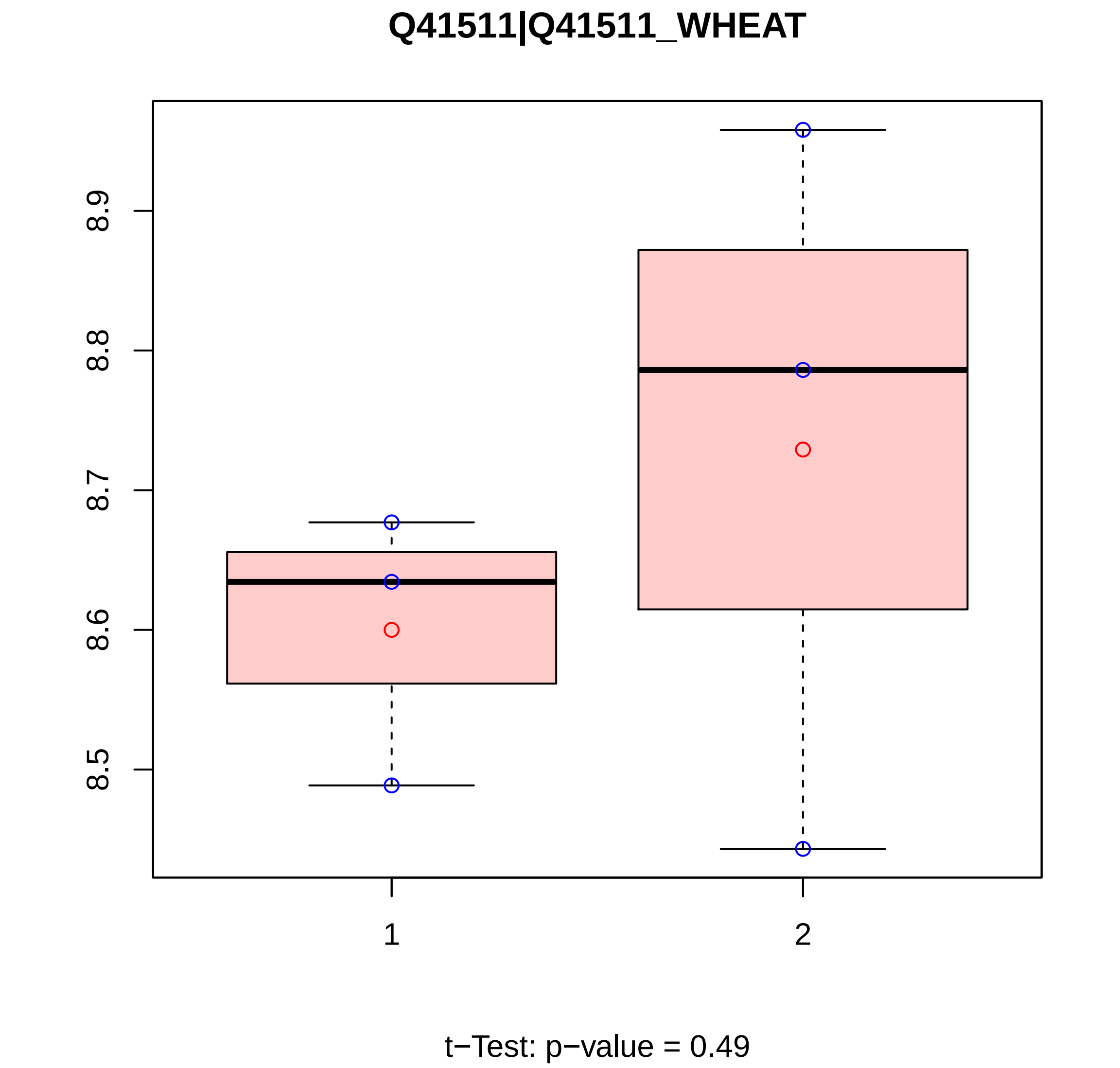
Q41511|Q41511_WHEAT
8.5
8.6
8.7
8.8
8.9
1
2
t−Test: p−value = 0.49

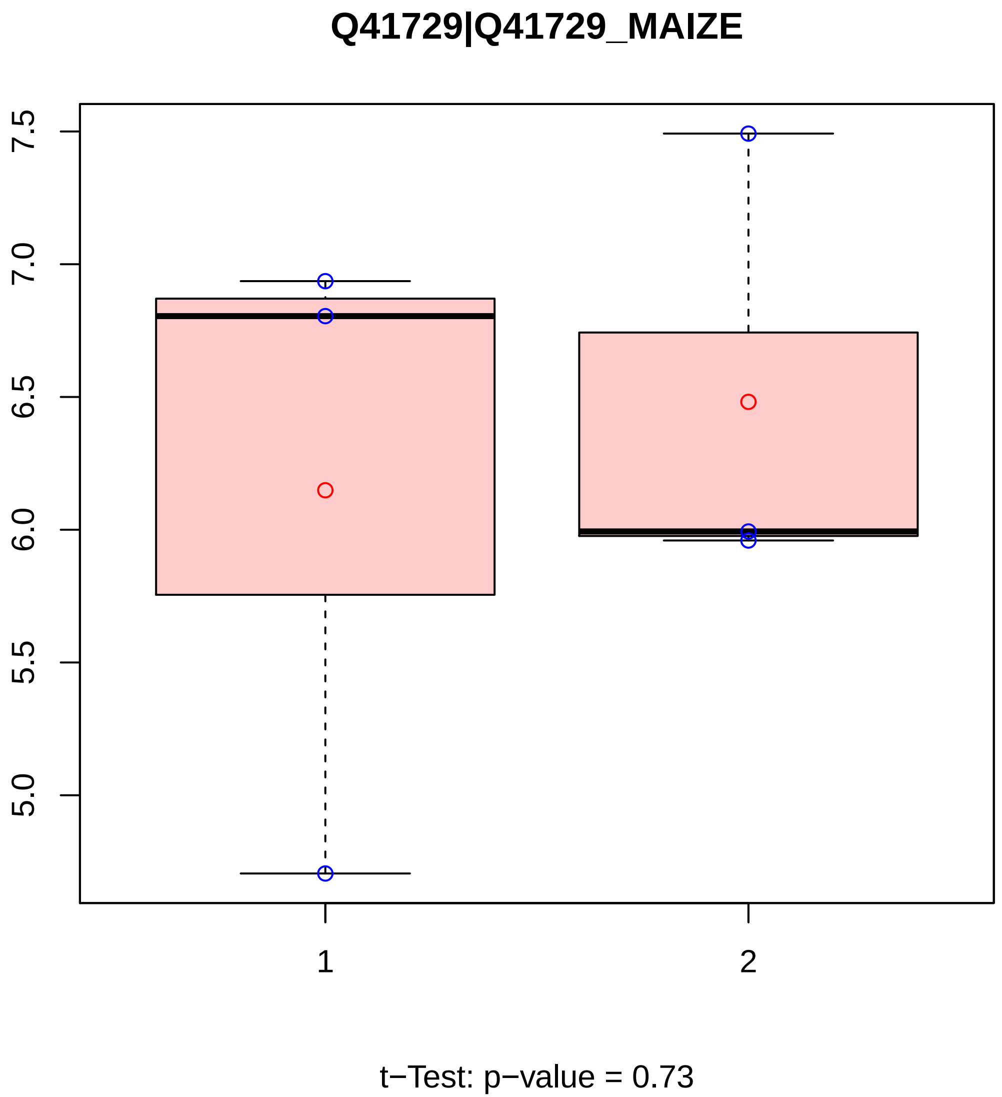
Q41729|Q41729_MAIZE
7.5
7.0
6.5
6.0
5.5
5.0
1
2
t−Test: p−value = 0.73

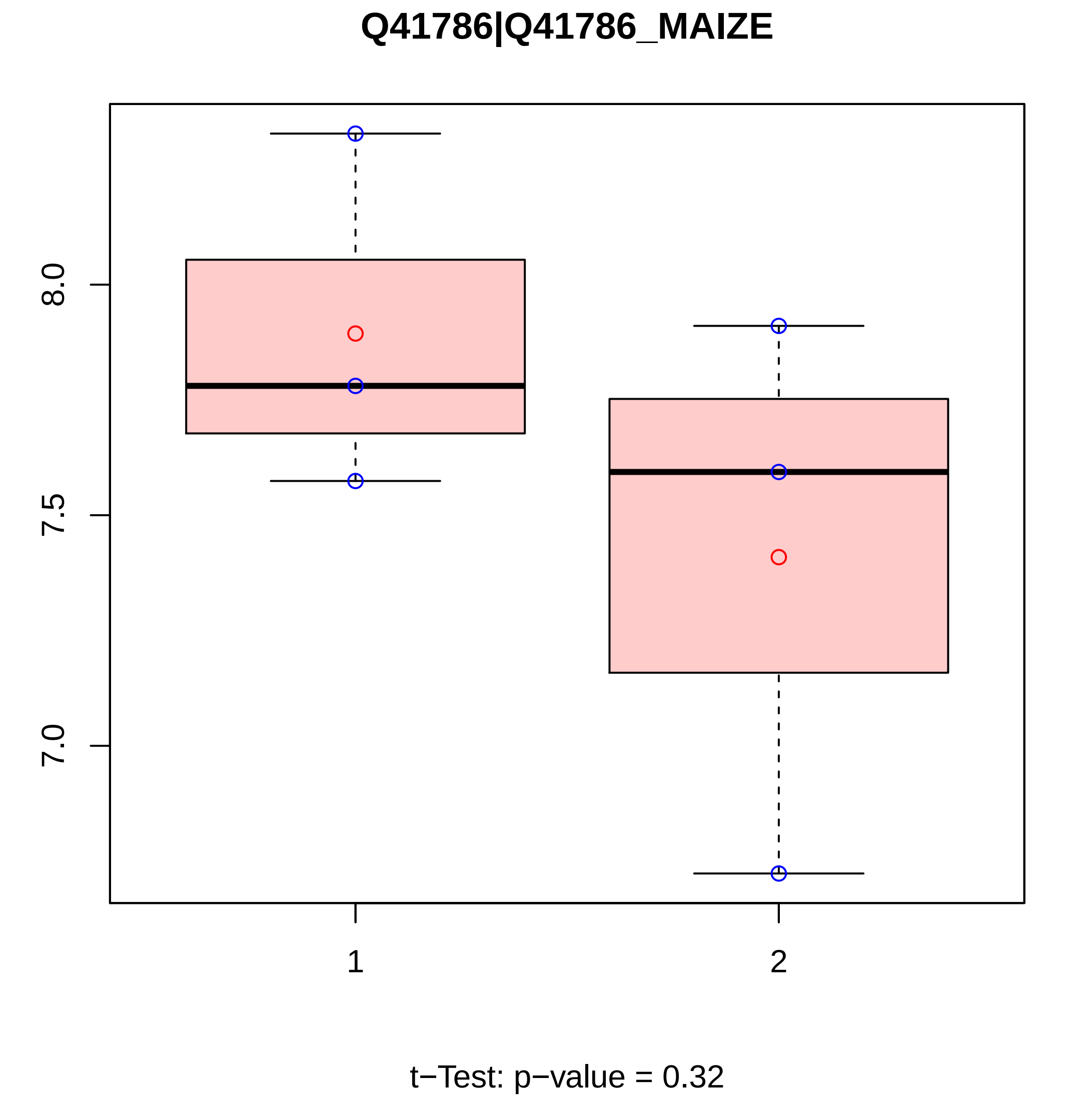
Q41786|Q41786_MAIZE
8.0
7.5
7.0
1
2
t−Test: p−value = 0.32

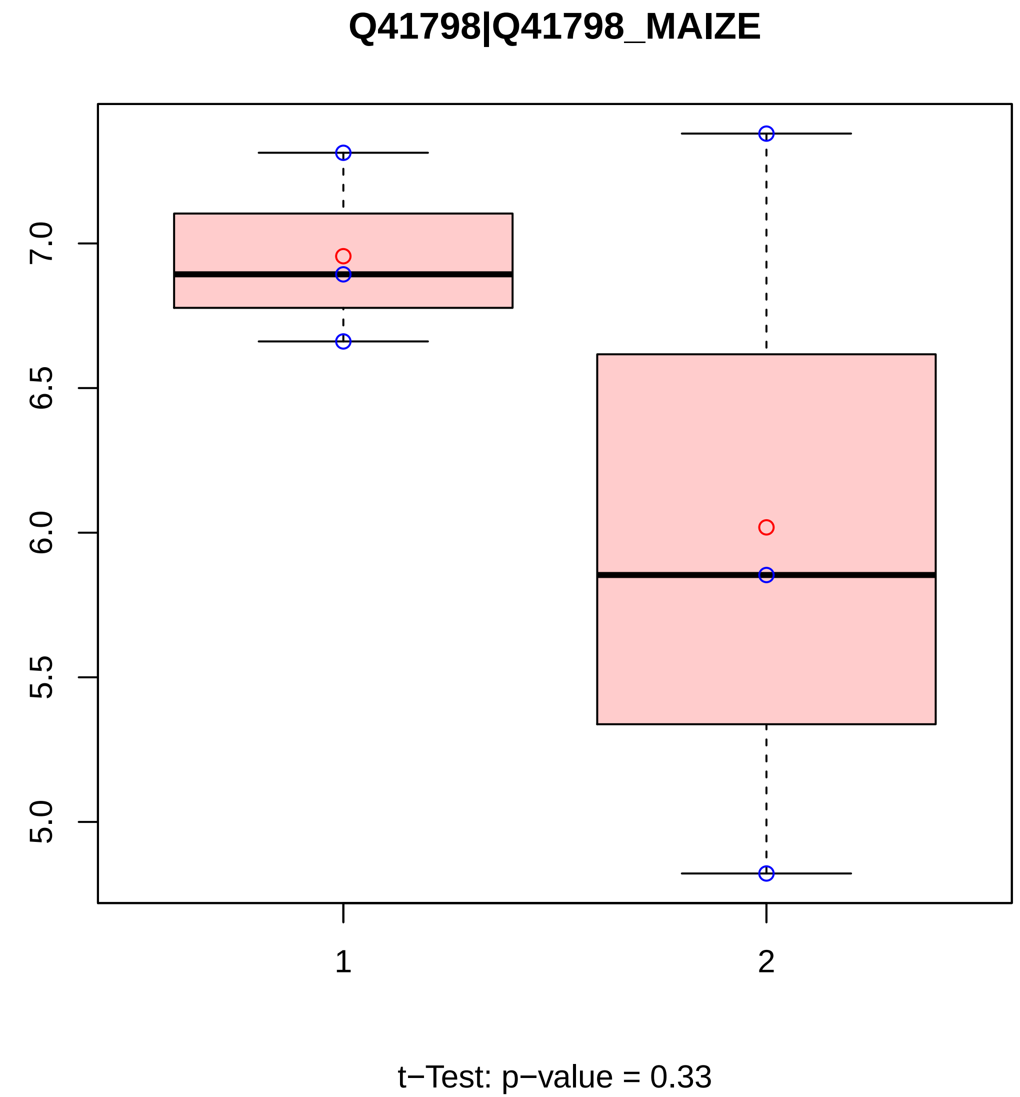
Q41798|Q41798_MAIZE
7.0
6.5
6.0
5.5
5.0
1
2
t−Test: p−value = 0.33

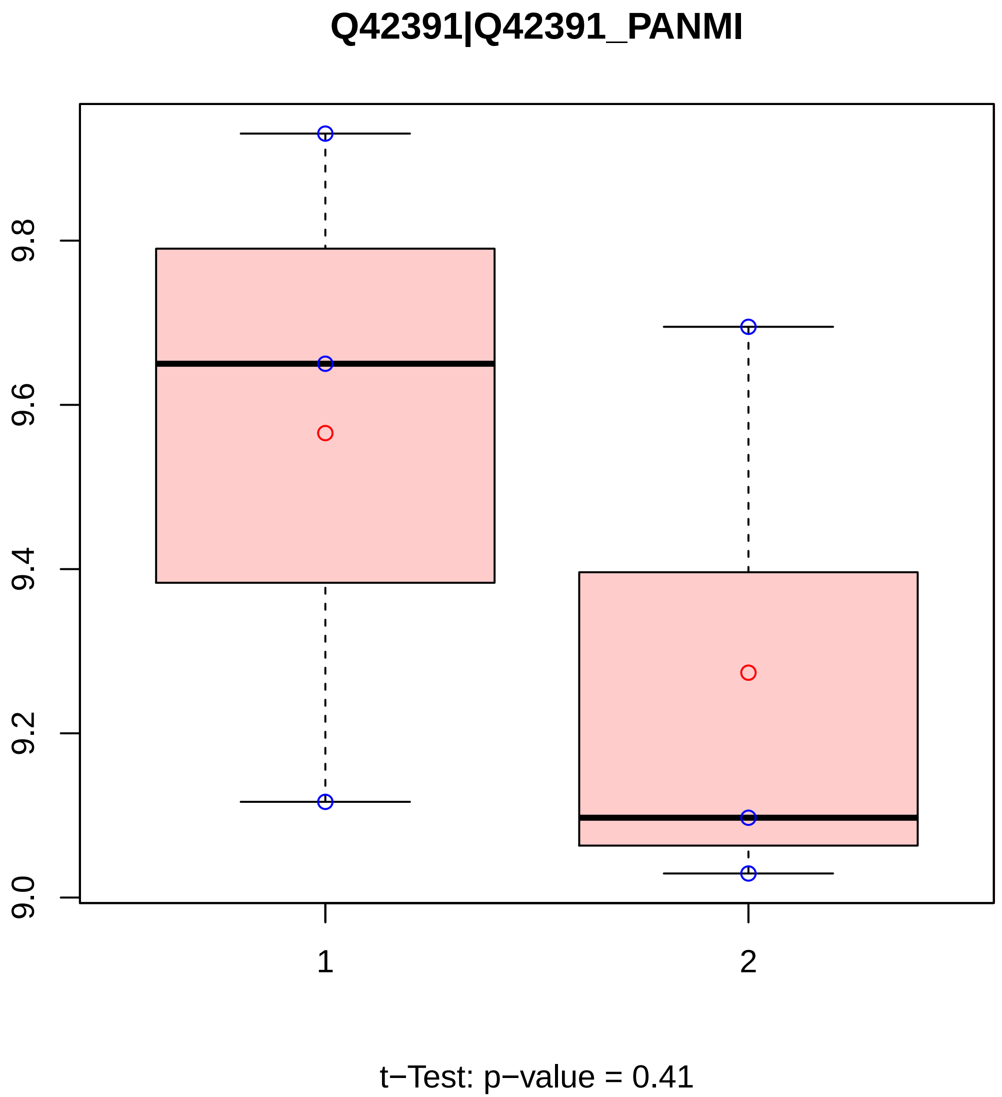

Q42391|Q42391_PANMI
9.0
9.2
9.4
9.6
9.8
1
2
t−Test: p−value = 0.41

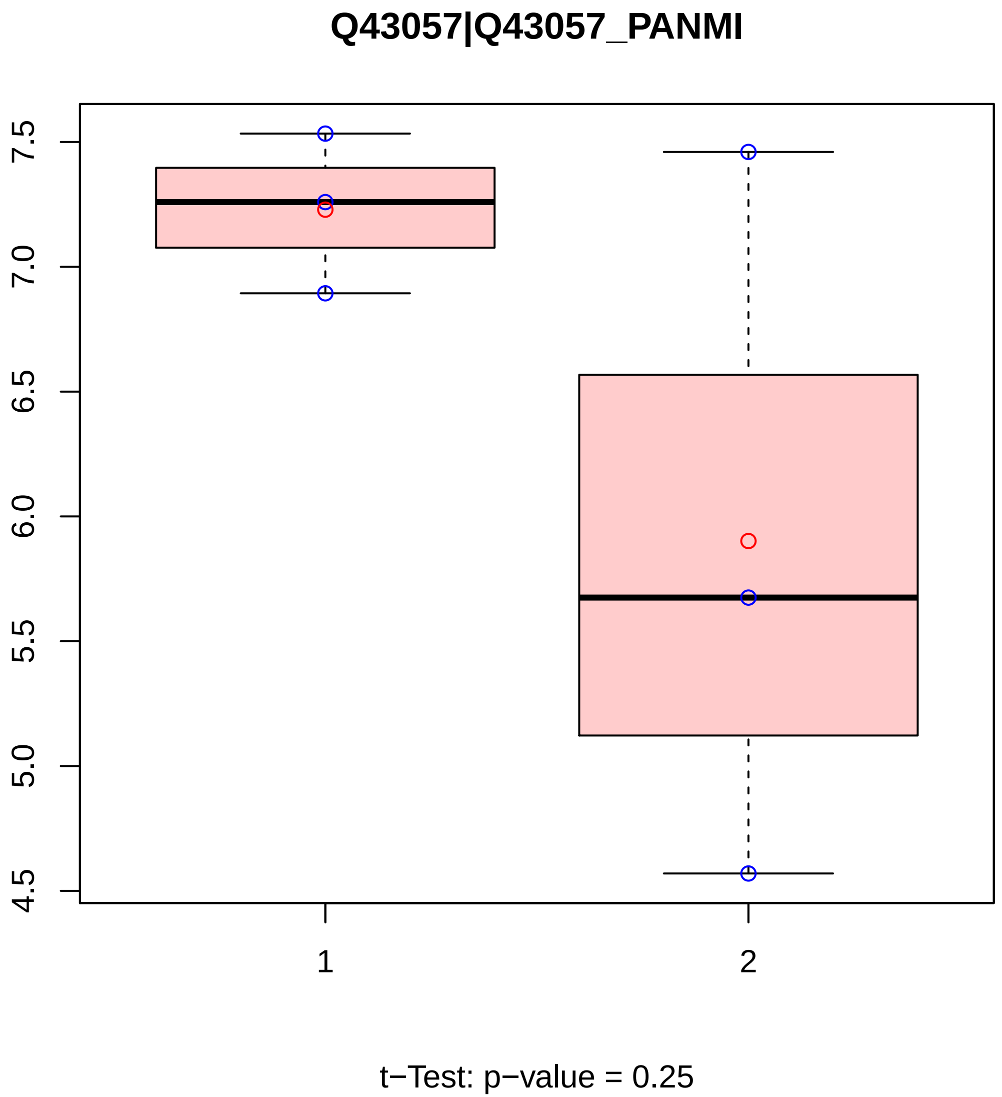
Q43057|Q43057_PANMI
7.5
7.0
6.5
6.0
5.5
5.0
4.5
1
2
t−Test: p−value = 0.25

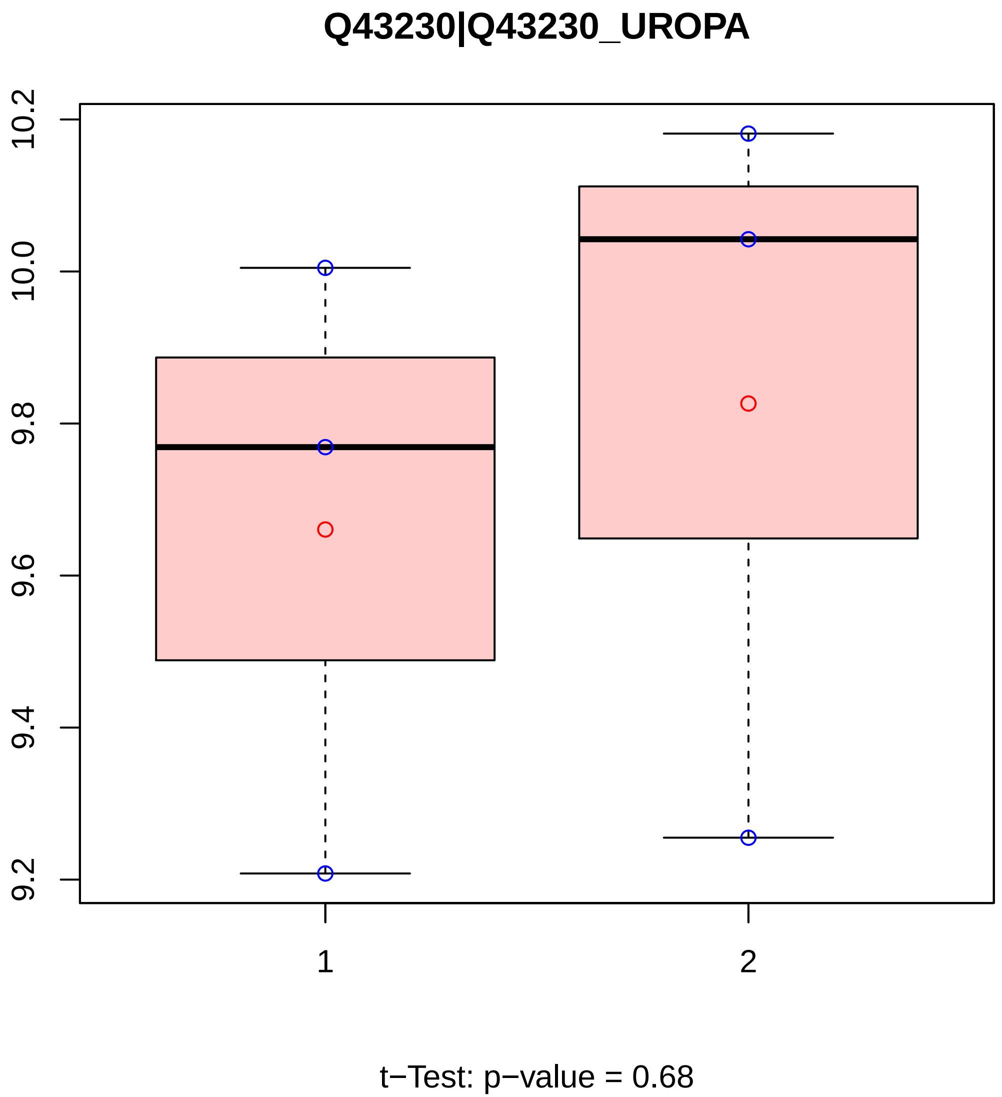
Q43230|Q43230_UROPA
10.2
10.0
9.8
9.6
9.4
9.2
1
2
t−Test: p−value = 0.68

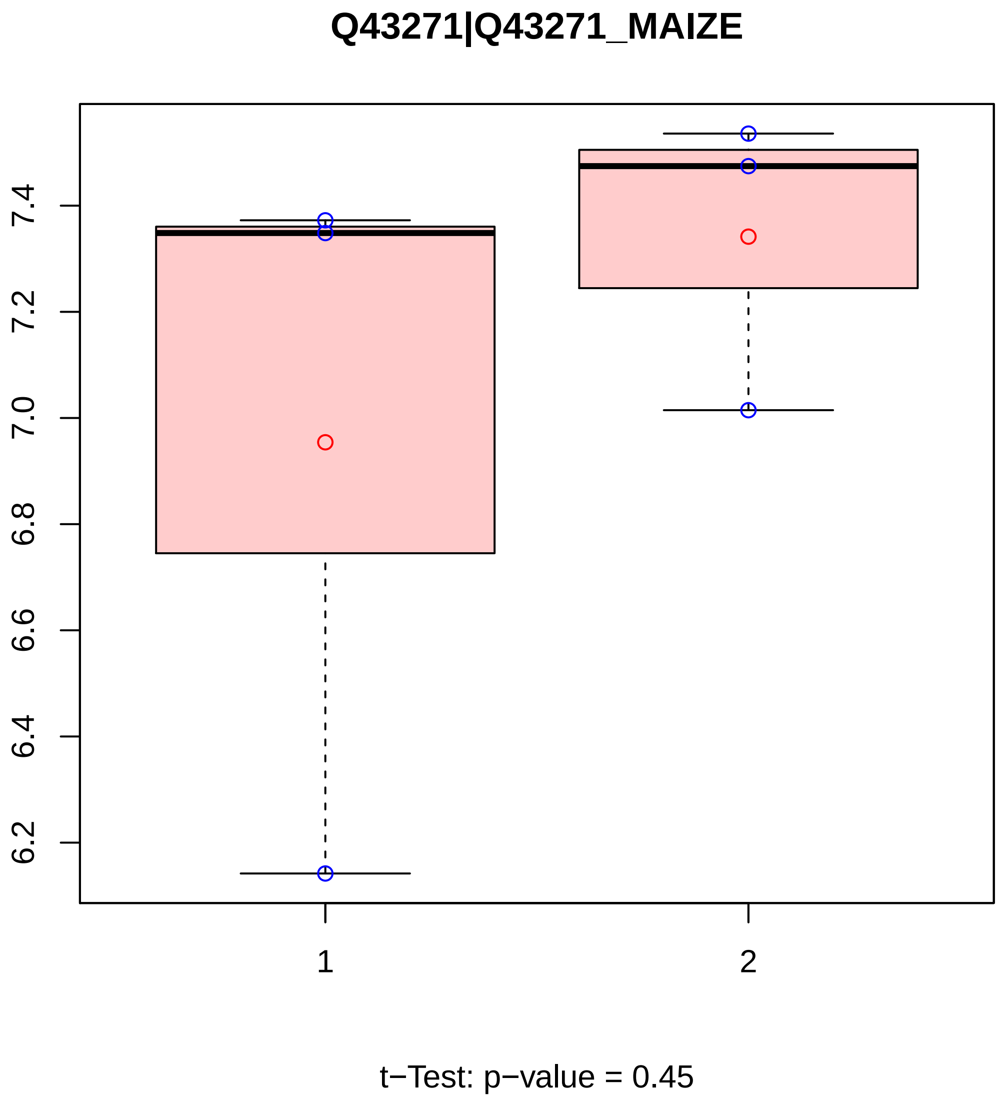
Q43271|Q43271_MAIZE
7.4
7.2
7.0
6.8
6.6
6.4
6.2
1
2
t−Test: p−value = 0.45

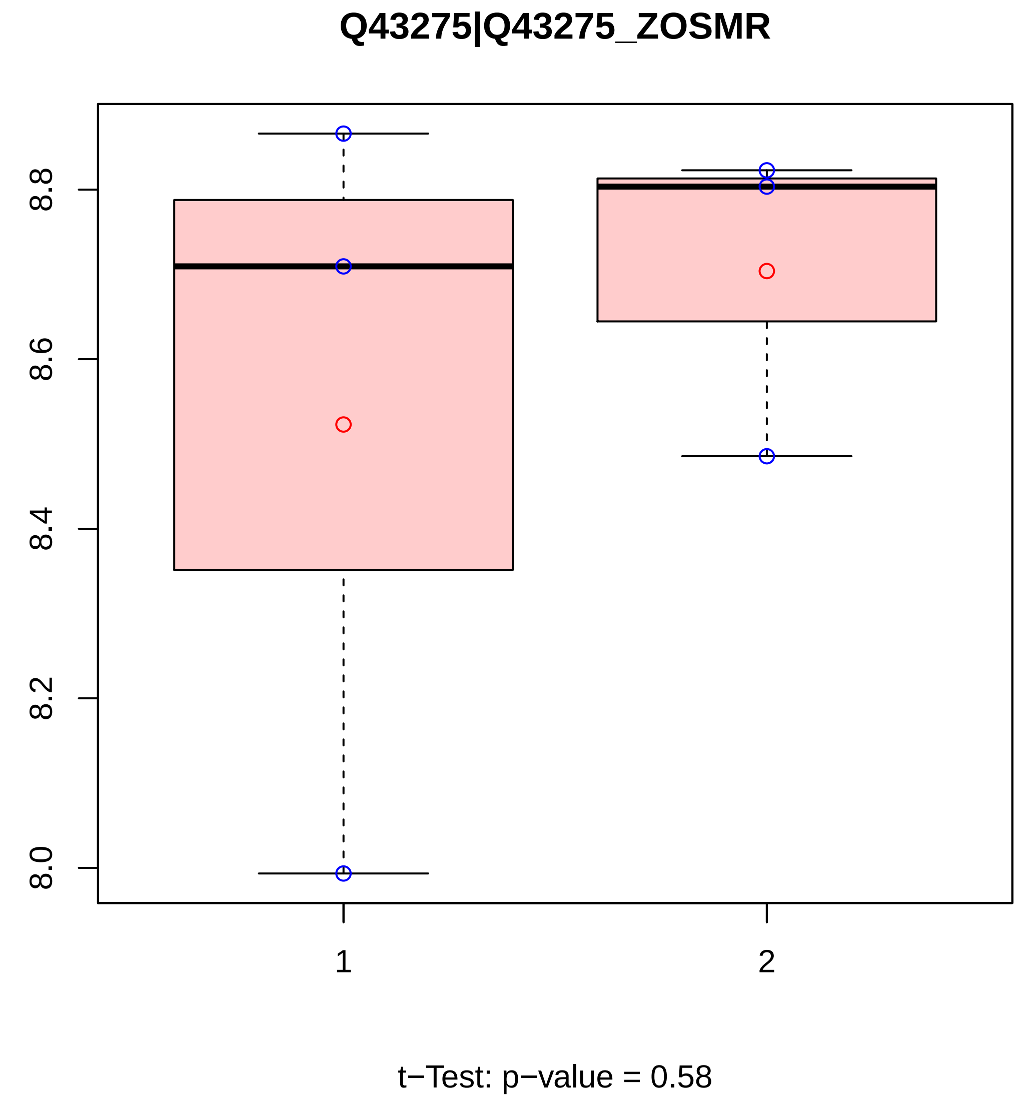
Q43275|Q43275_ZOSMR
8.0
8.2
8.4
8.6
8.8
1
2
t−Test: p−value = 0.58

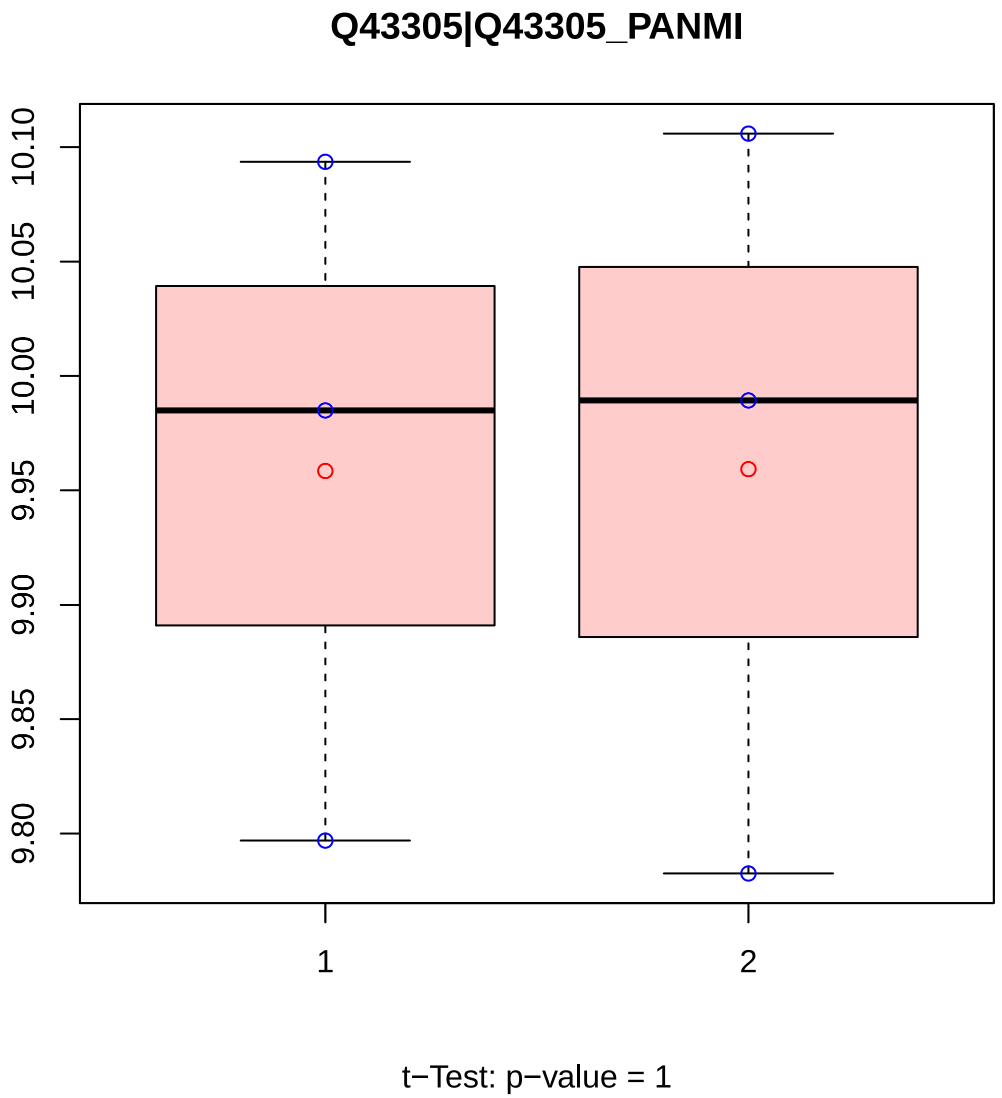

Q43305|Q43305_PANMI
10.10
10.05
10.00
9.95
9.90
9.85
9.80
1
2
t−Test: p−value = 1

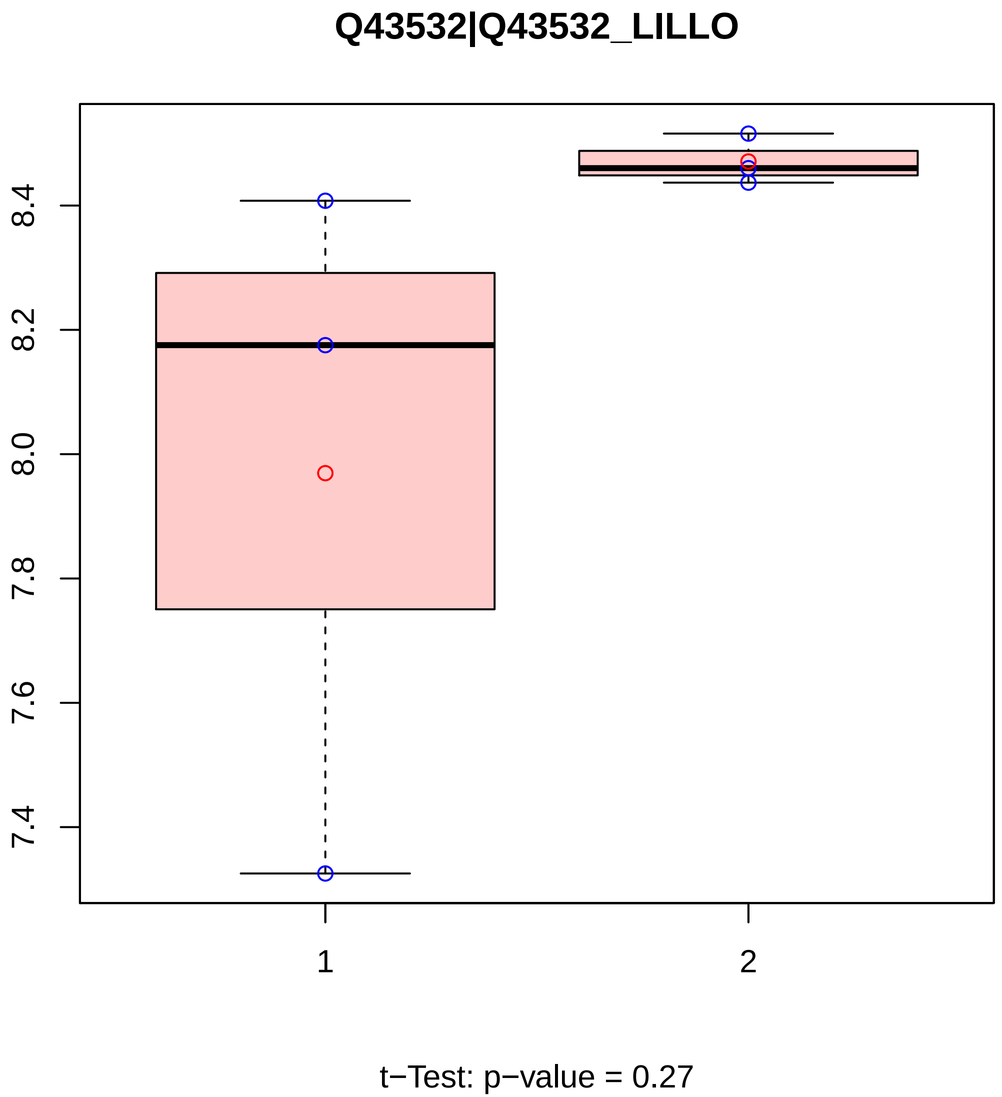
Q43532|Q43532_LILLO
8.4
8.2
8.0
7.8
7.6
7.4
1
2
t−Test: p−value = 0.27

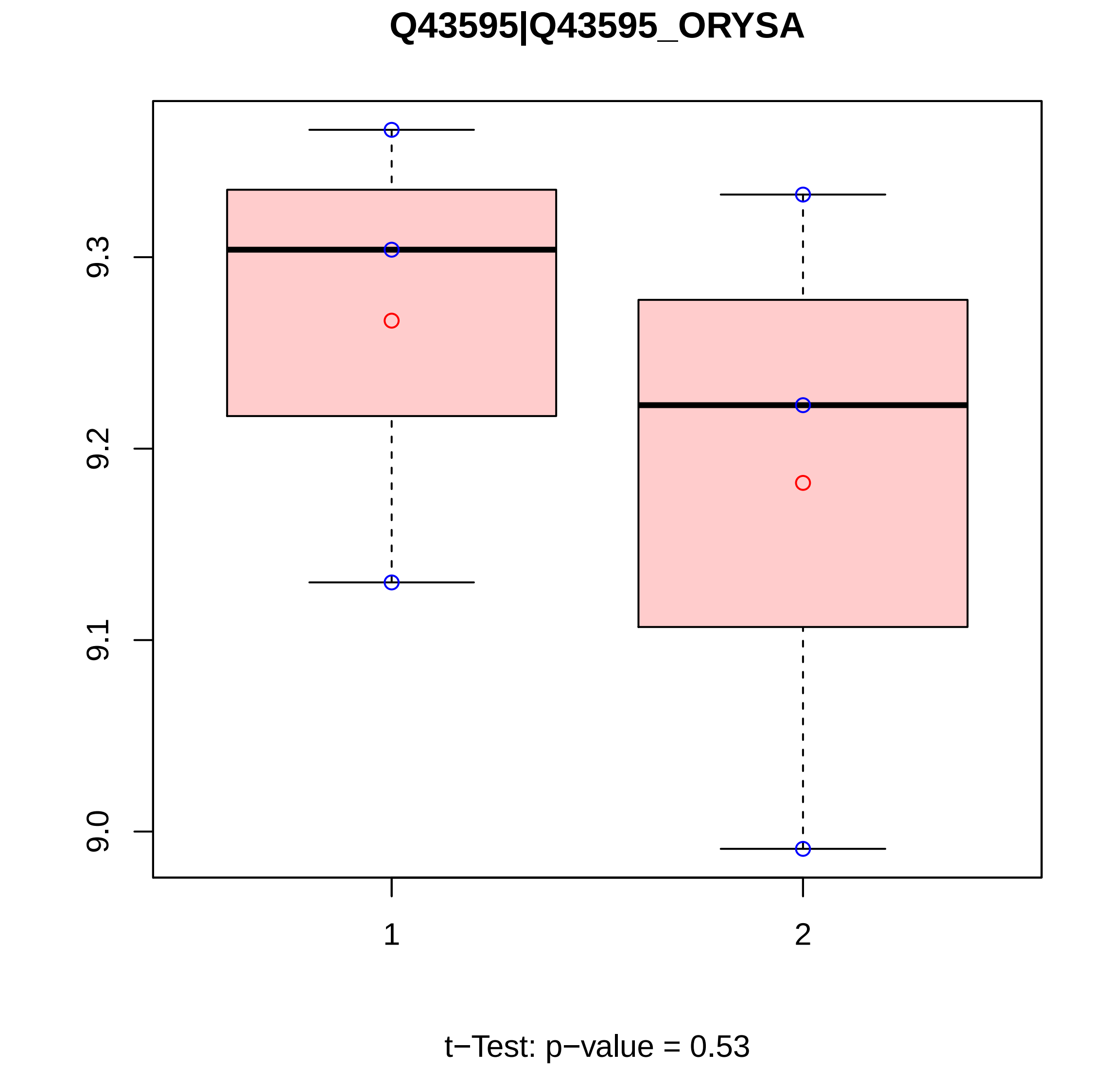
Q43595|Q43595_ORYSA
9.0
9.1
9.2
9.3
1
2
t−Test: p−value = 0.53

Q49HE4|Q49HE4_MAIZE

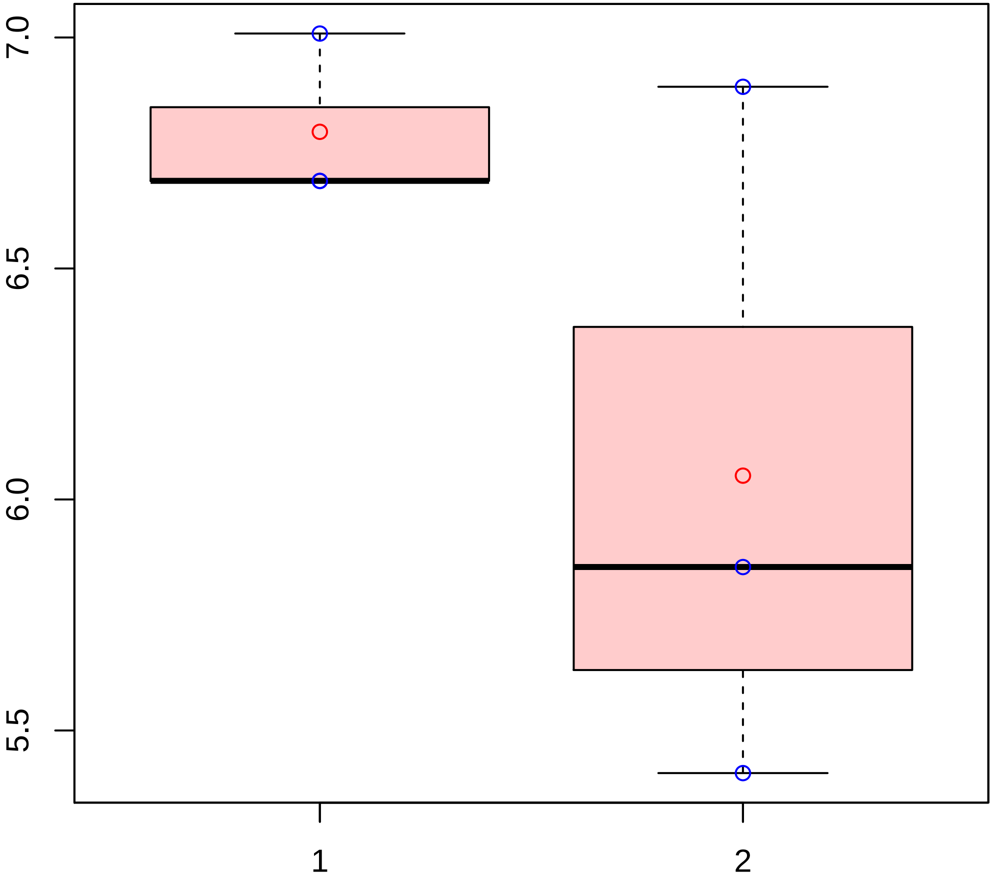

t−Test: p−value = 0.23

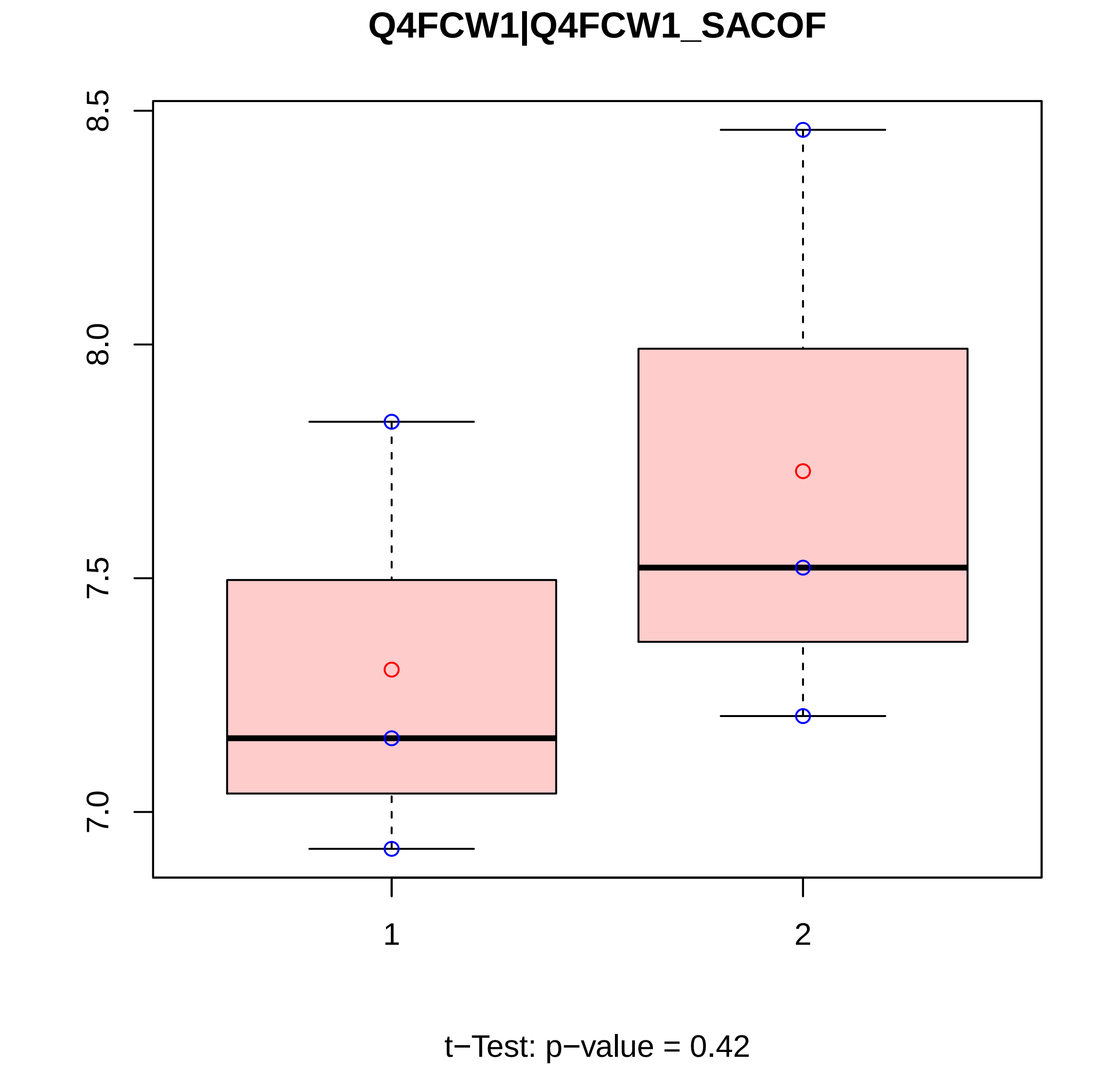
Q4FCW1|Q4FCW1_SACOF
8.5
8.0
7.5
7.0
1
2
t−Test: p−value = 0.42

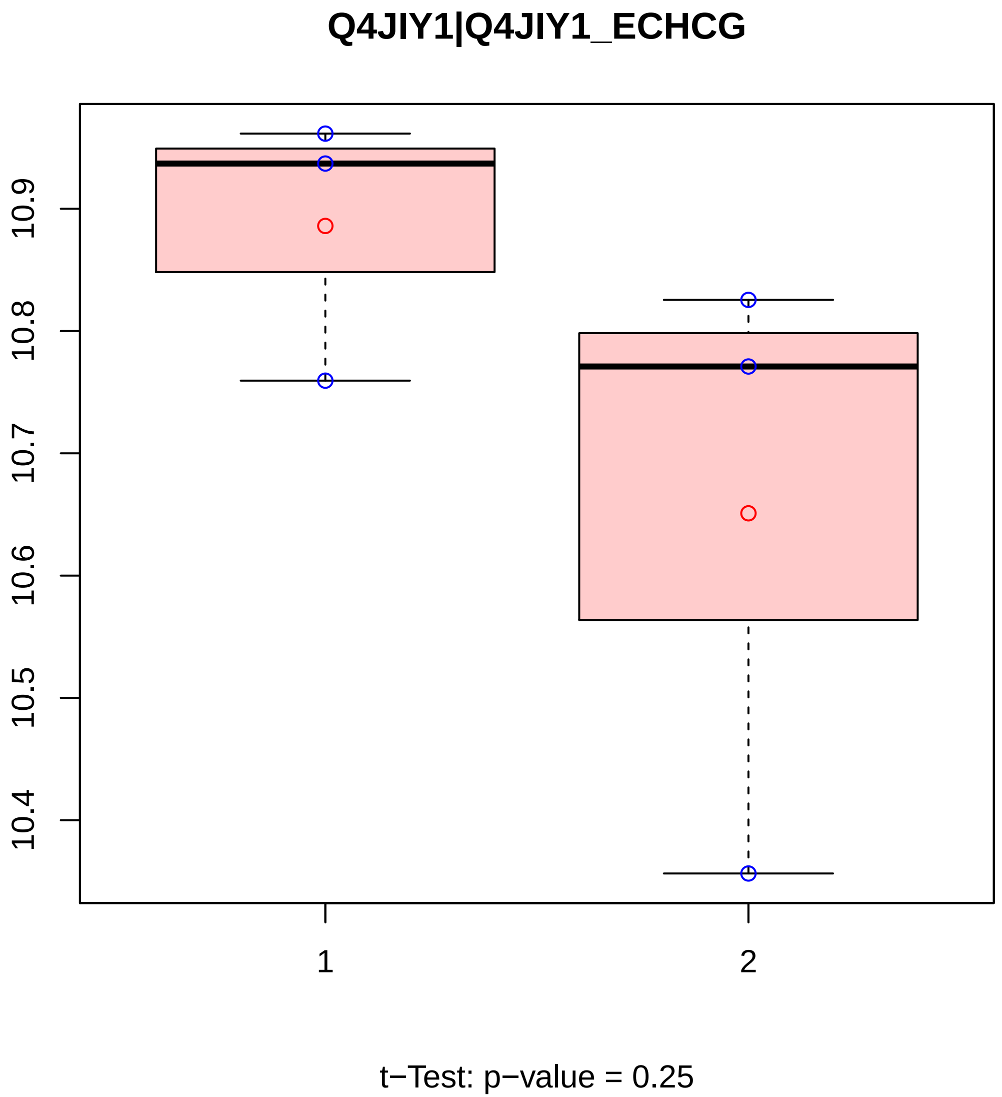
Q4JIY1|Q4JIY1_ECHCG
10.4
10.5
10.6
10.7
10.8
10.9
1
2
t−Test: p−value = 0.25

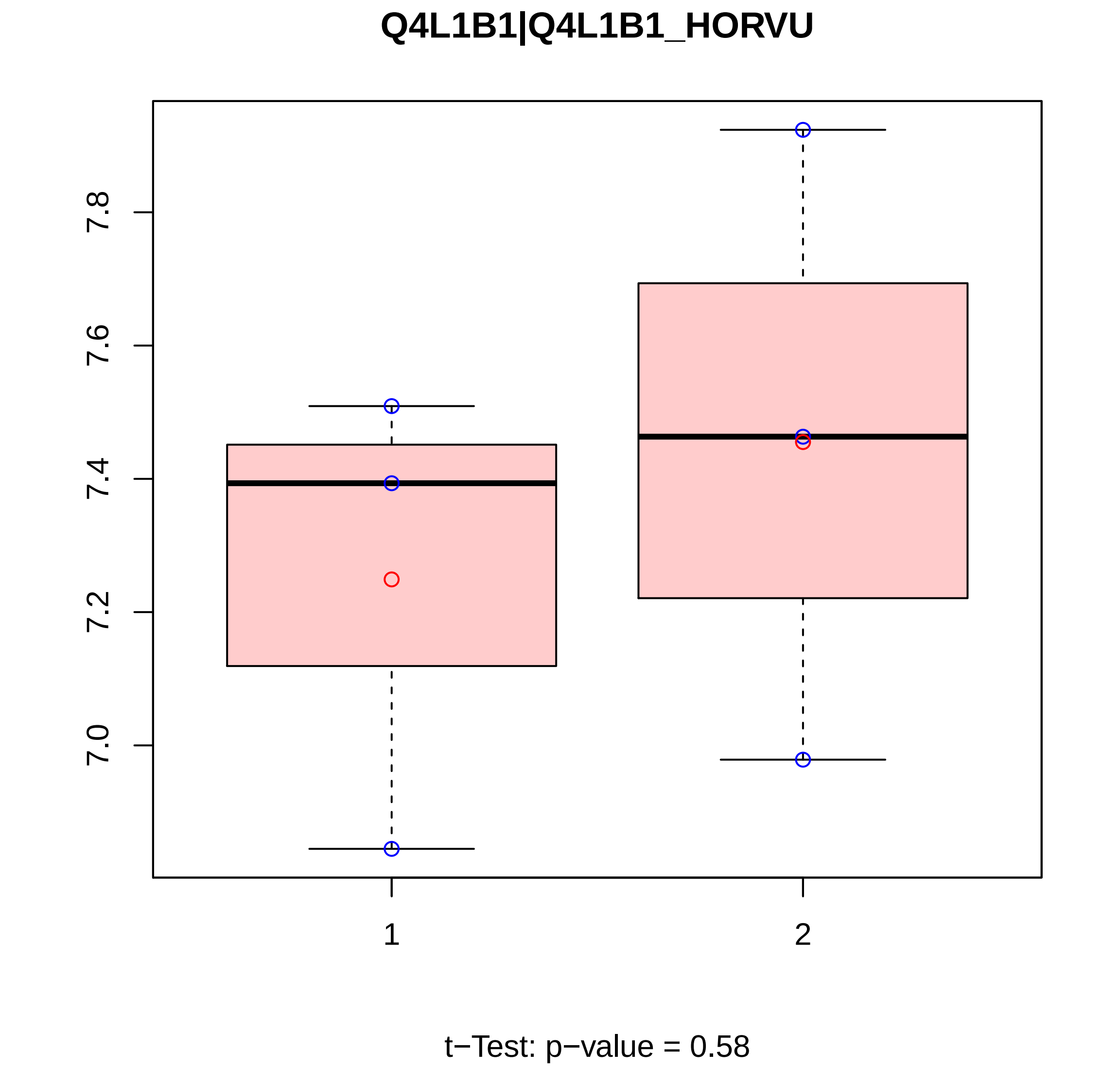
Q4L1B1|Q4L1B1_HORVU
7.0
7.2
7.4
7.6
7.8
1
2
t−Test: p−value = 0.58

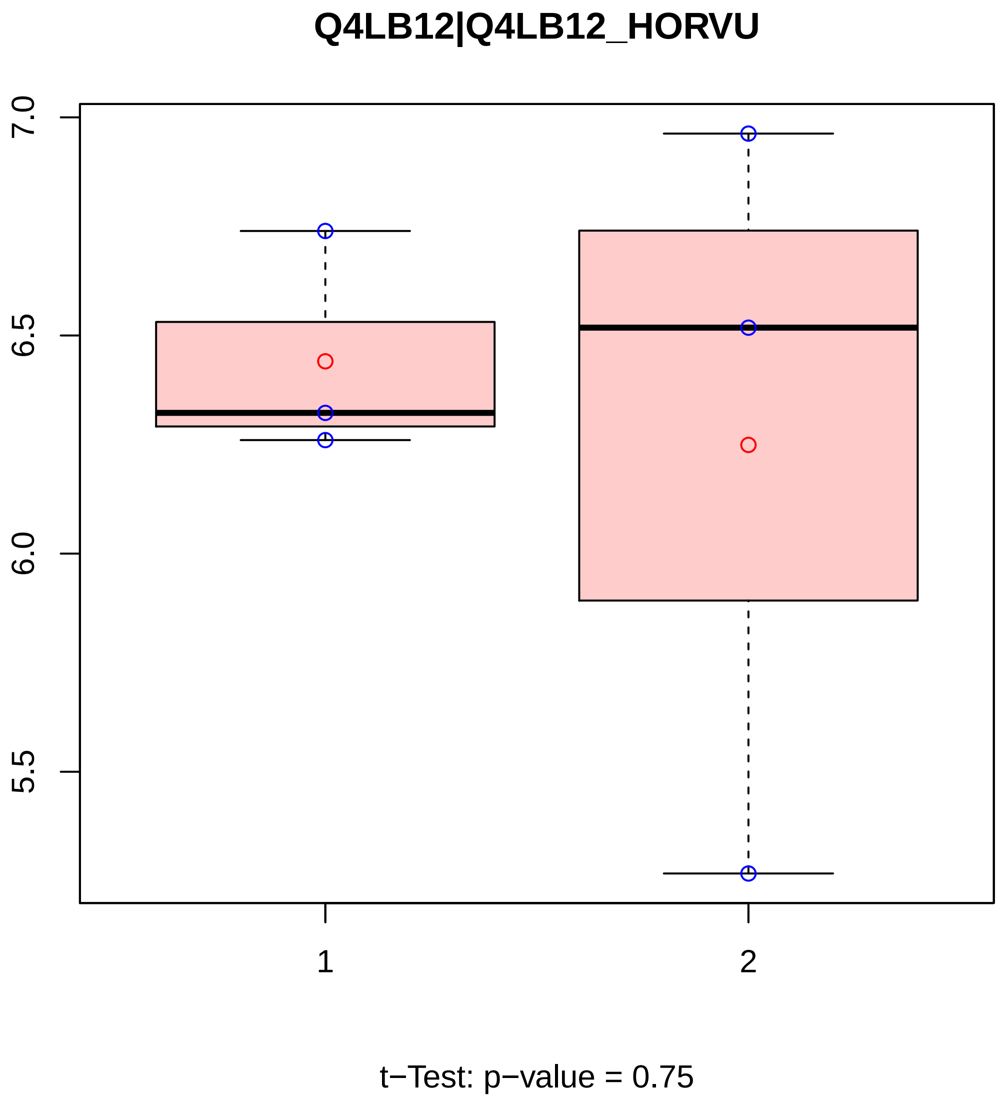
Q4LB12|Q4LB12_HORVU
7.0
6.5
6.0
5.5
1
2
t−Test: p−value = 0.75

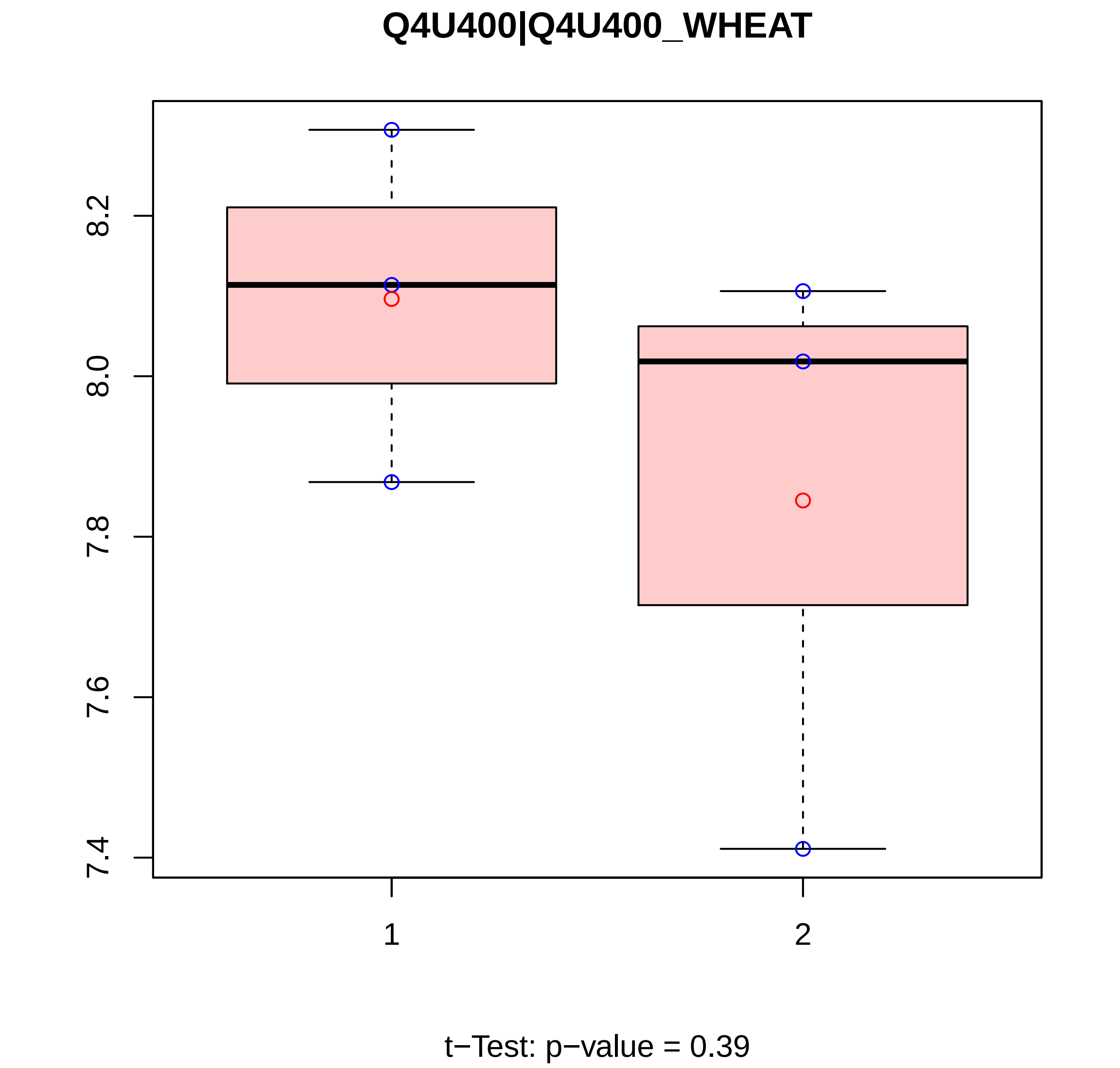

Q4U400|Q4U400_WHEAT
8.2
8.0
7.8
7.6
7.4
1
2
t−Test: p−value = 0.39

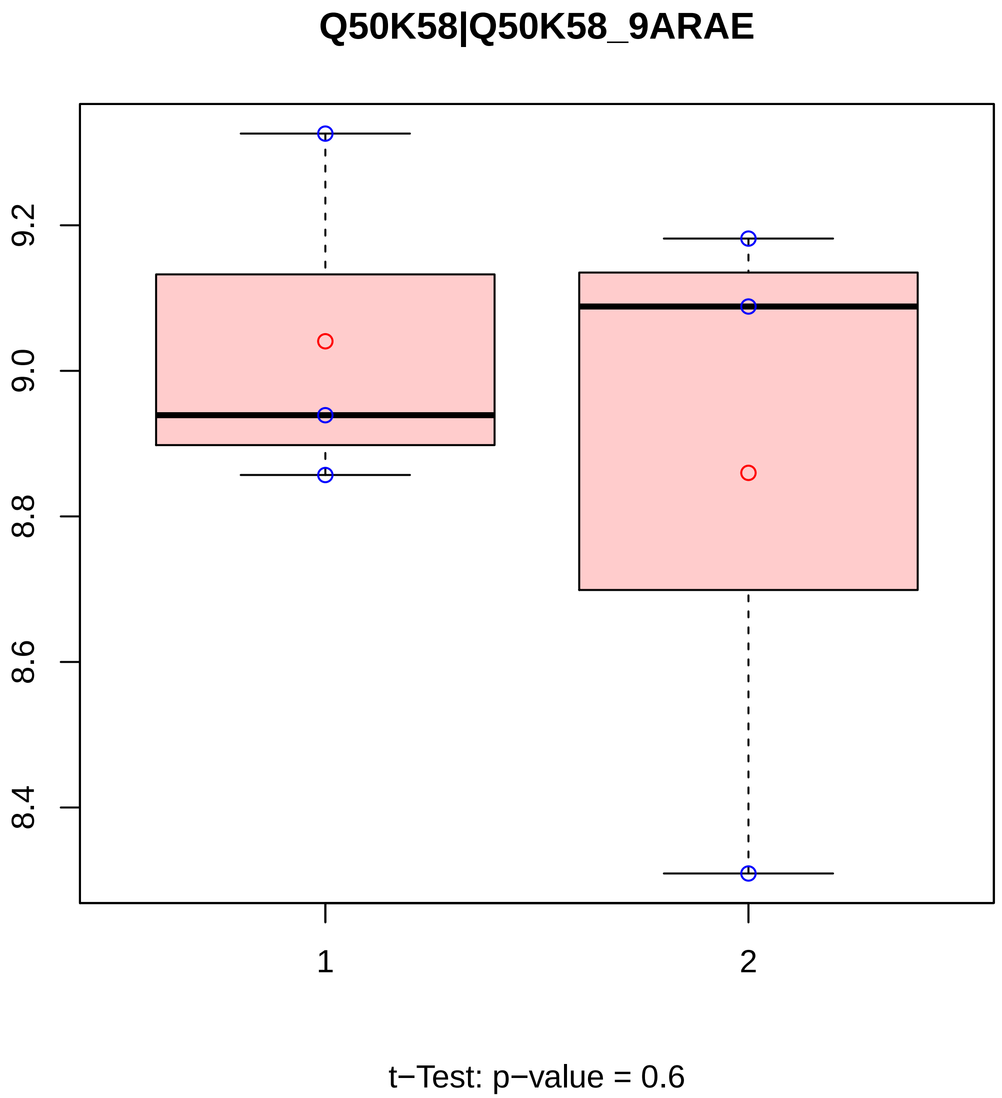
Q50K58|Q50K58_9ARAE
9.2
9.0
8.8
8.6
8.4
1
2
t−Test: p−value = 0.6

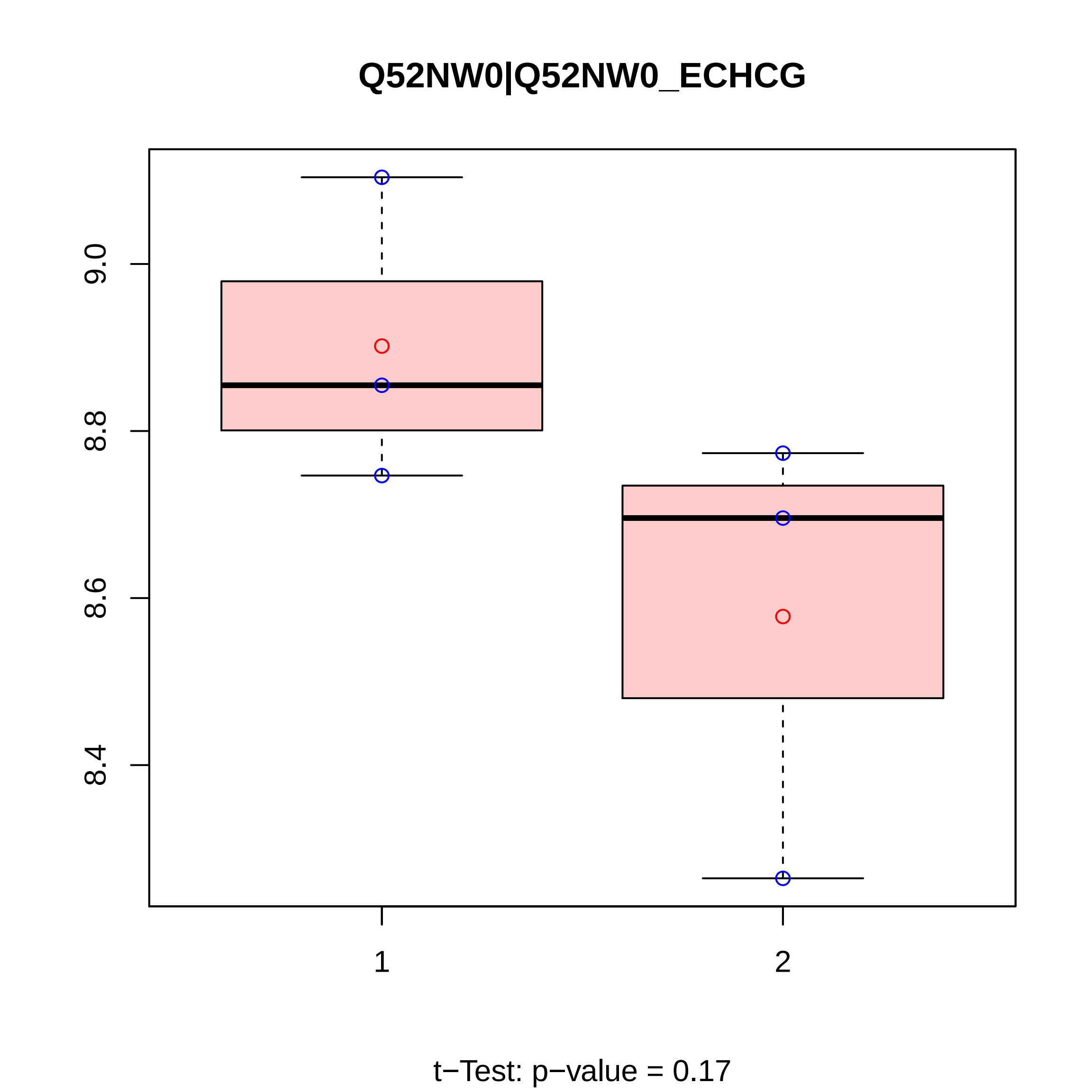

Q52NW0|Q52NW0_ECHCG
9.0
8.8
8.6
8.4
1
2
t−Test: p−value = 0.17

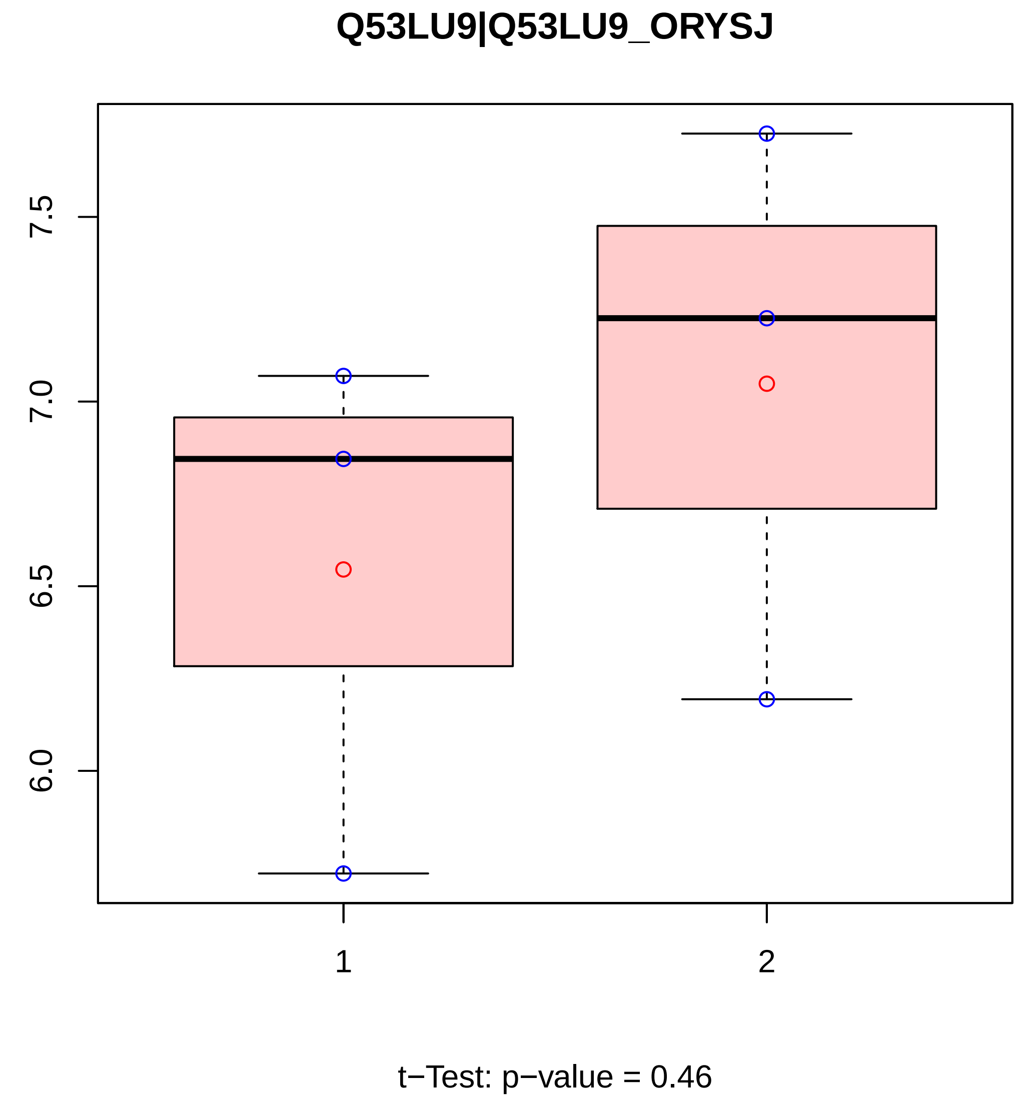
Q53LU9|Q53LU9_ORYSJ
7.5
7.0
6.5
6.0
1
2
t−Test: p−value = 0.46

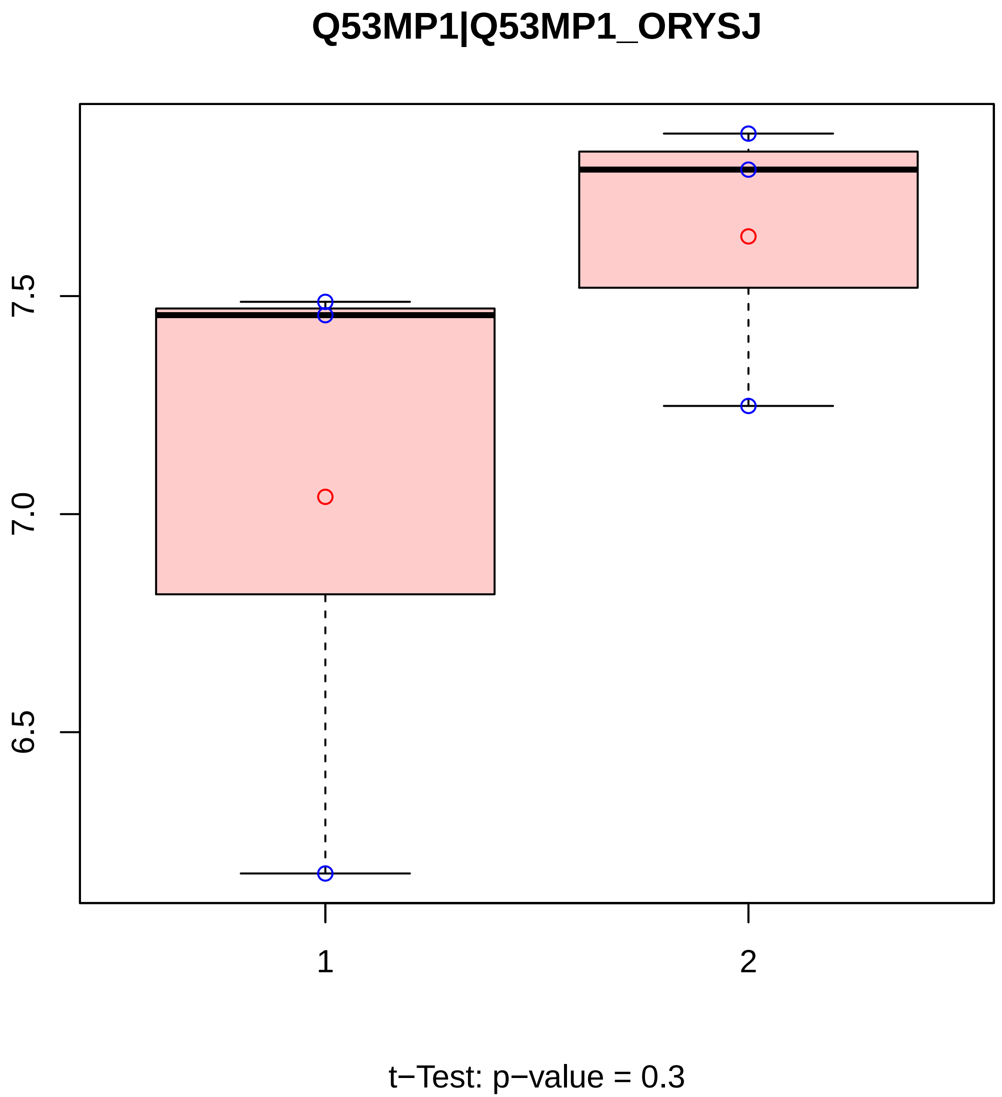
Q53MP1|Q53MP1_ORYSJ
7.5
7.0
6.5
1
2
t−Test: p−value = 0.3

Q53NB5|Q53NB5_ORYSJ

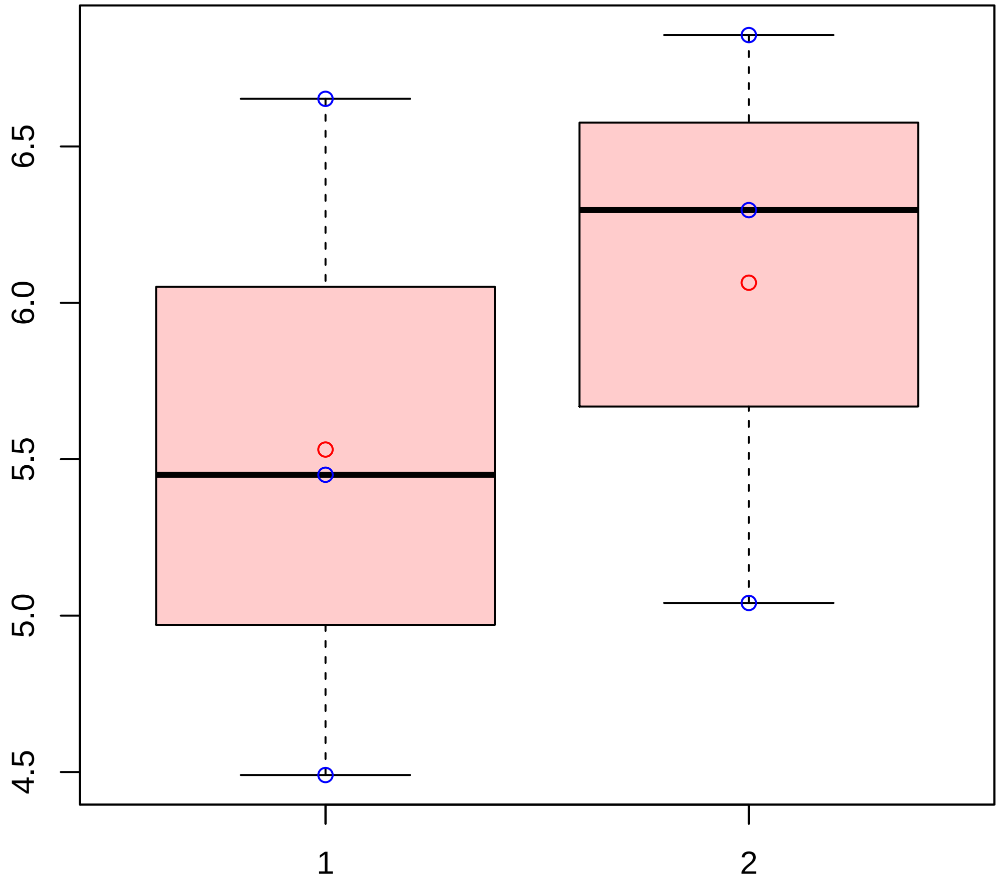

t−Test: p−value = 0.55

Q53NQ5|Q53NQ5_ORYSJ

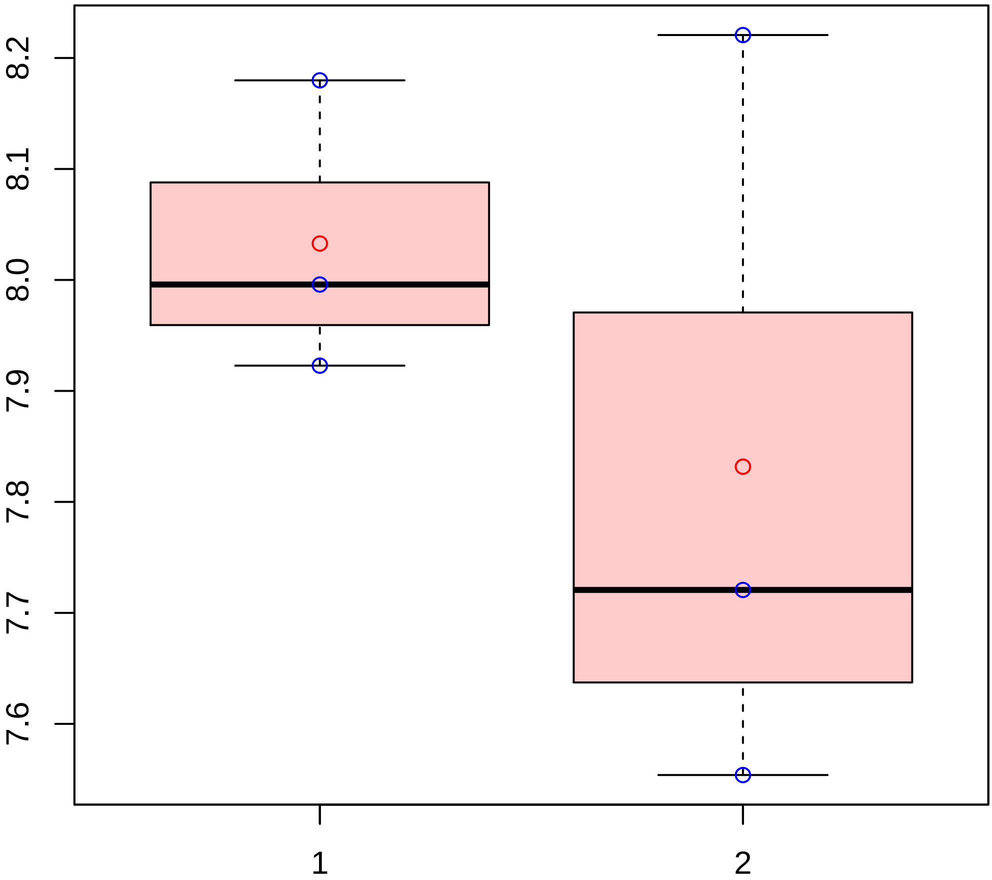

t−Test: p−value = 0.43

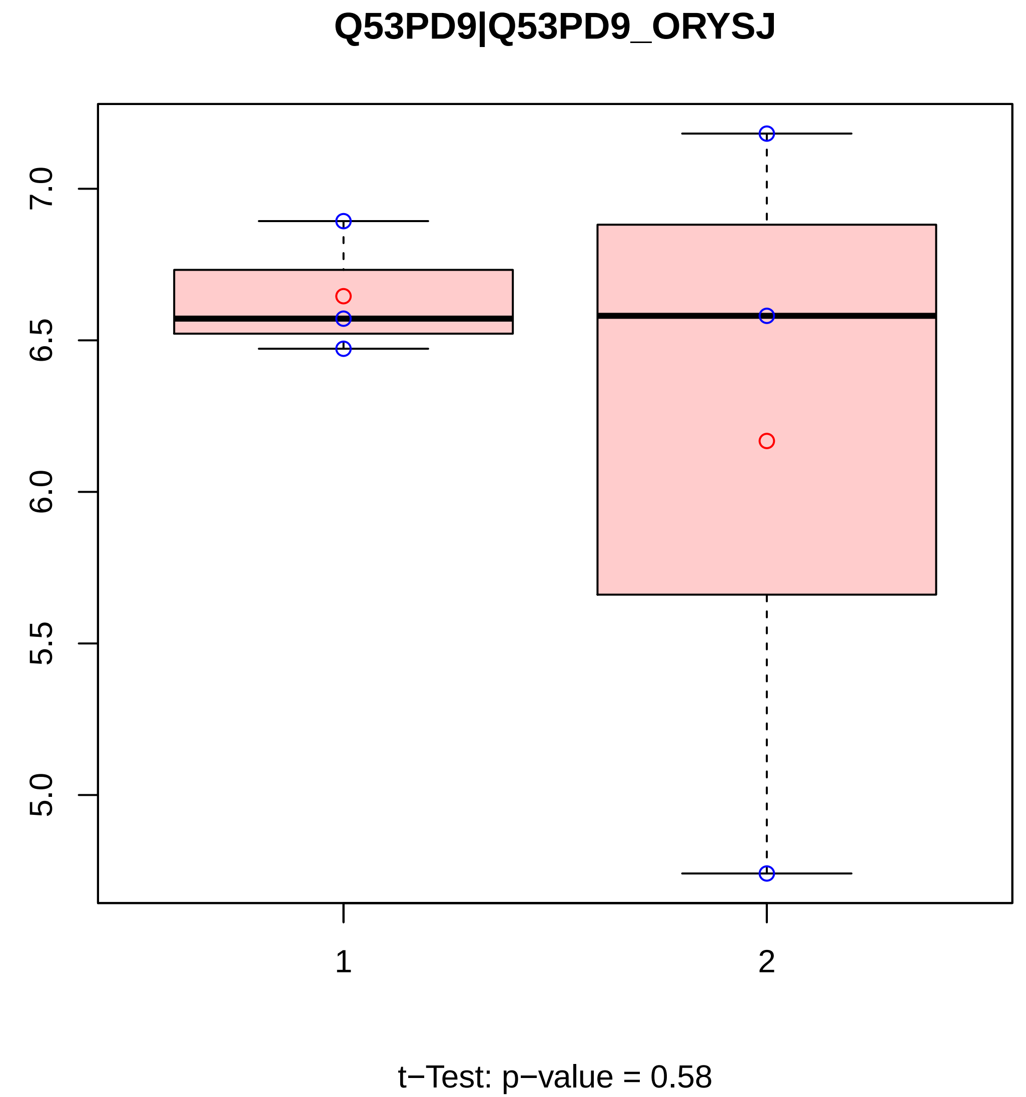

Q53PD9|Q53PD9_ORYSJ
7.0
6.5
6.0
5.5
5.0
1
2
t−Test: p−value = 0.58

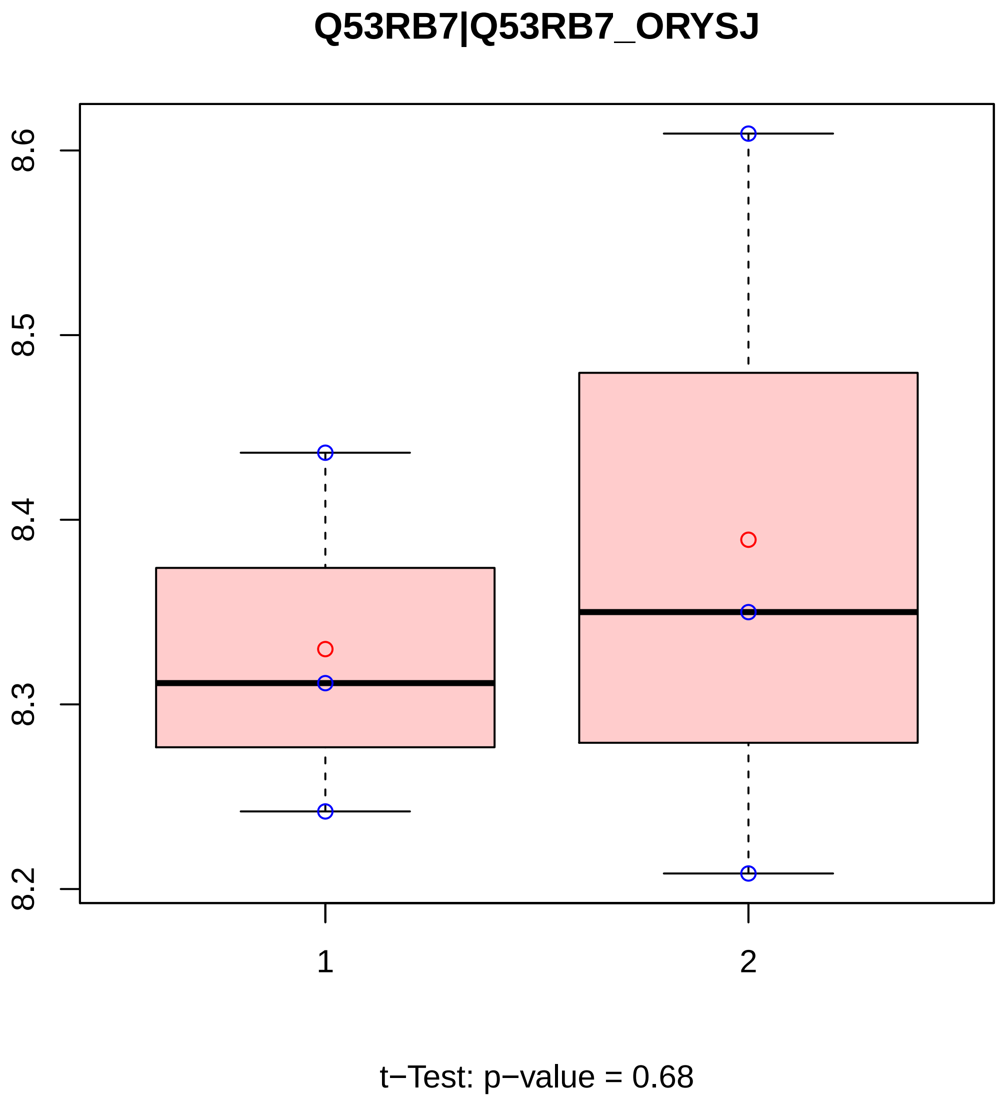
Q53RB7|Q53RB7_ORYSJ
8.2
8.3
8.4
8.5
8.6
1
2
t−Test: p−value = 0.68

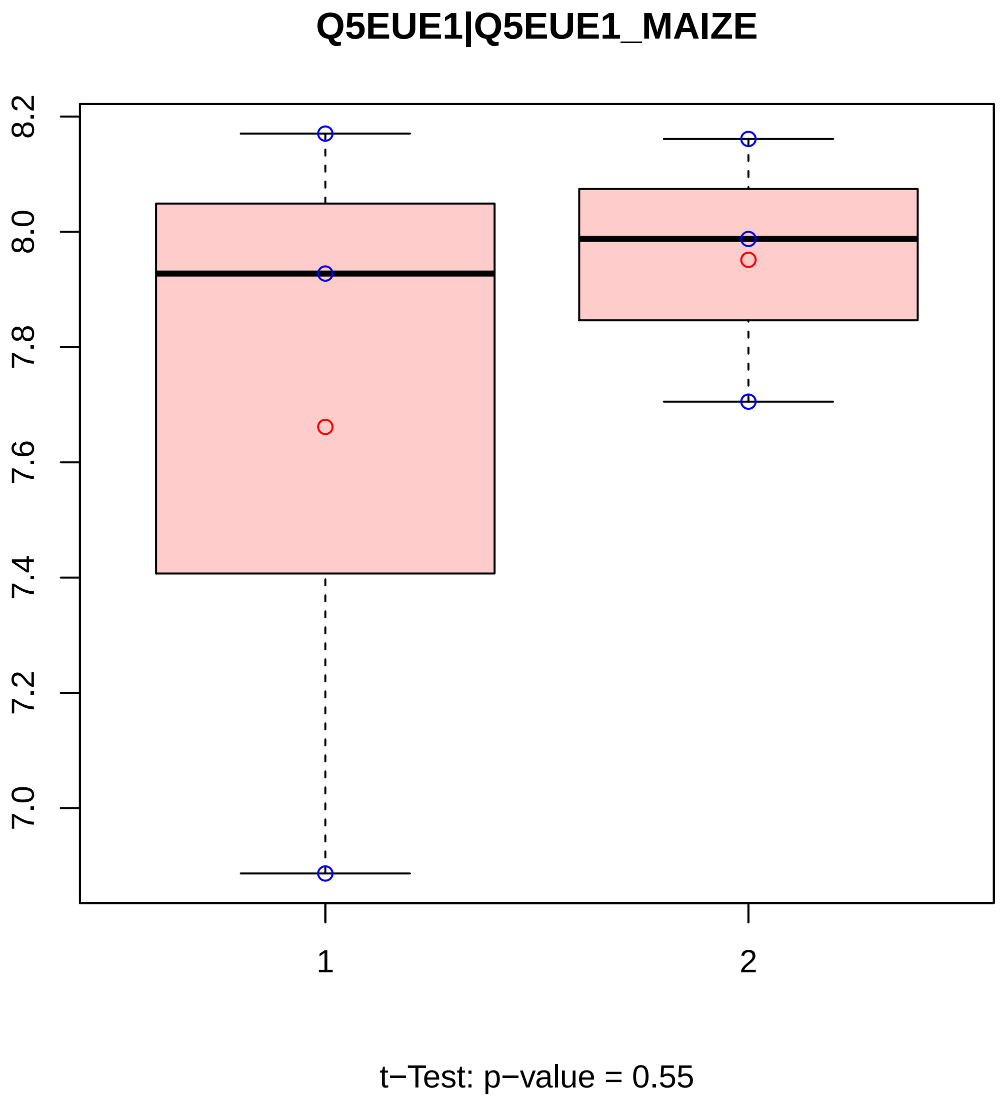
Q5EUE1|Q5EUE1_MAIZE
8.2
8.0
7.8
7.6
7.4
7.2
7.0
1
2
t−Test: p−value = 0.55

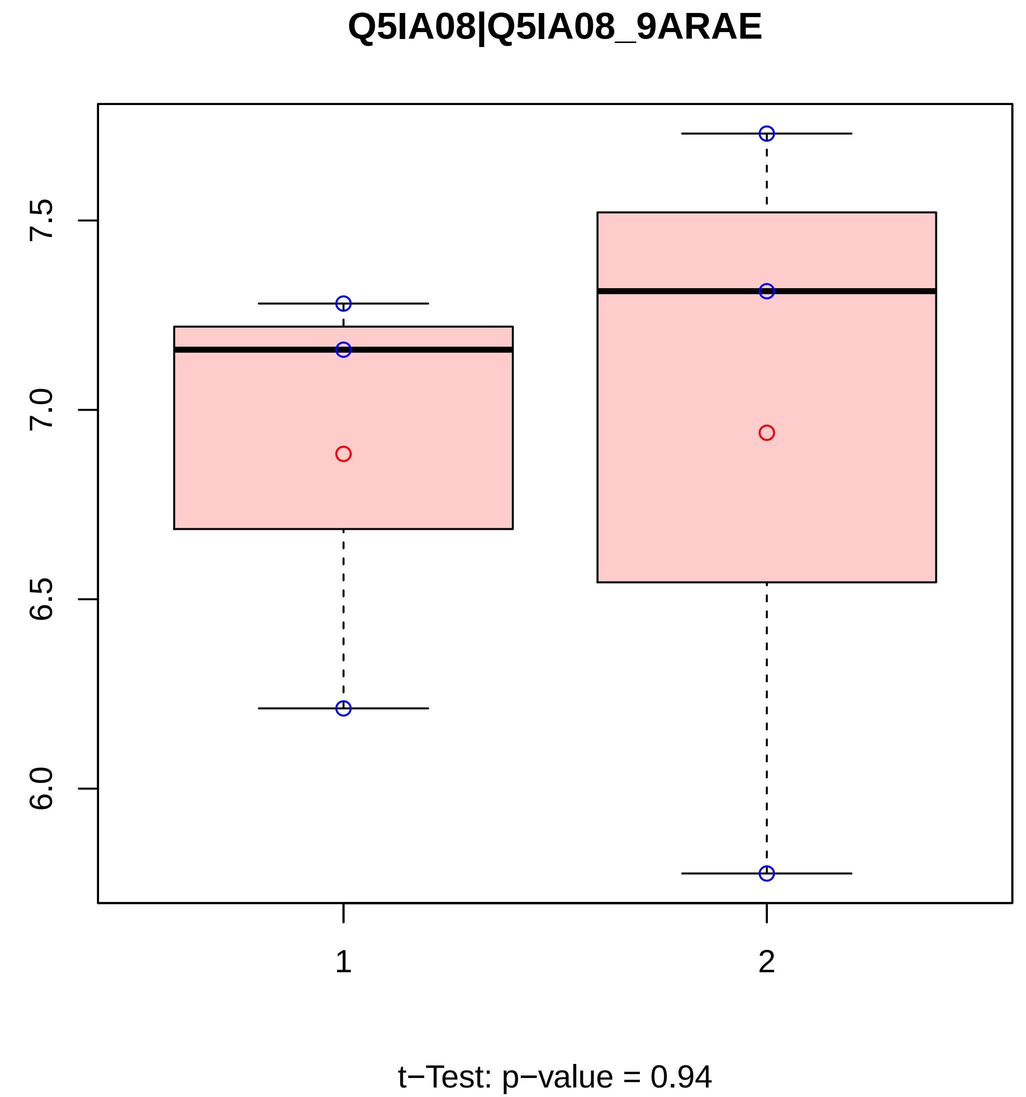

Q5IA08|Q5IA08_9ARAE
7.5
7.0
6.5
6.0
1
2
t−Test: p−value = 0.94

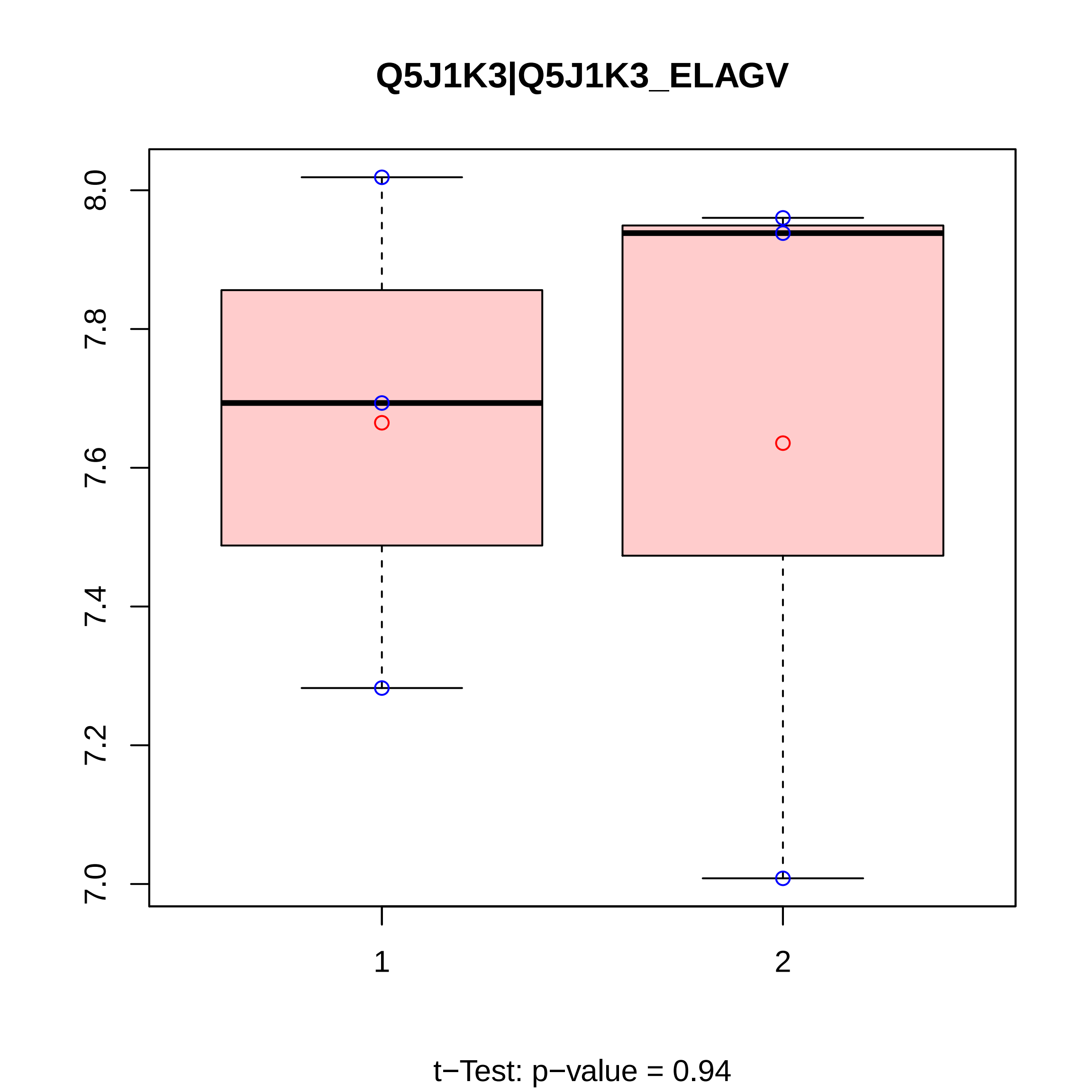

Q5J1K3|Q5J1K3_ELAGV
8.0
7.8
7.6
7.4
7.2
7.0
1
2
t−Test: p−value = 0.94

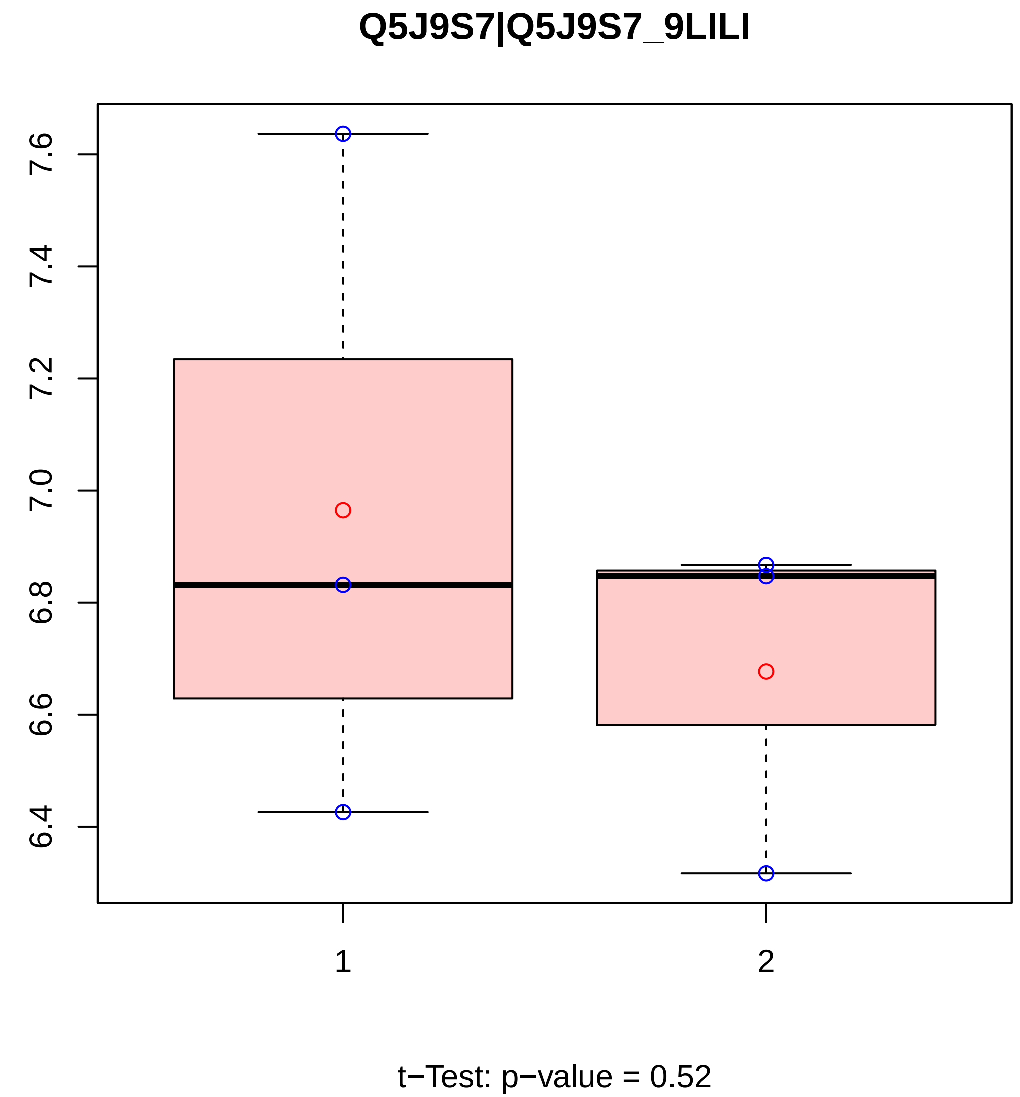
Q5J9S7|Q5J9S7_9LILI
7.6
7.4
7.2
7.0
6.8
6.6
6.4
1
2
t−Test: p−value = 0.52

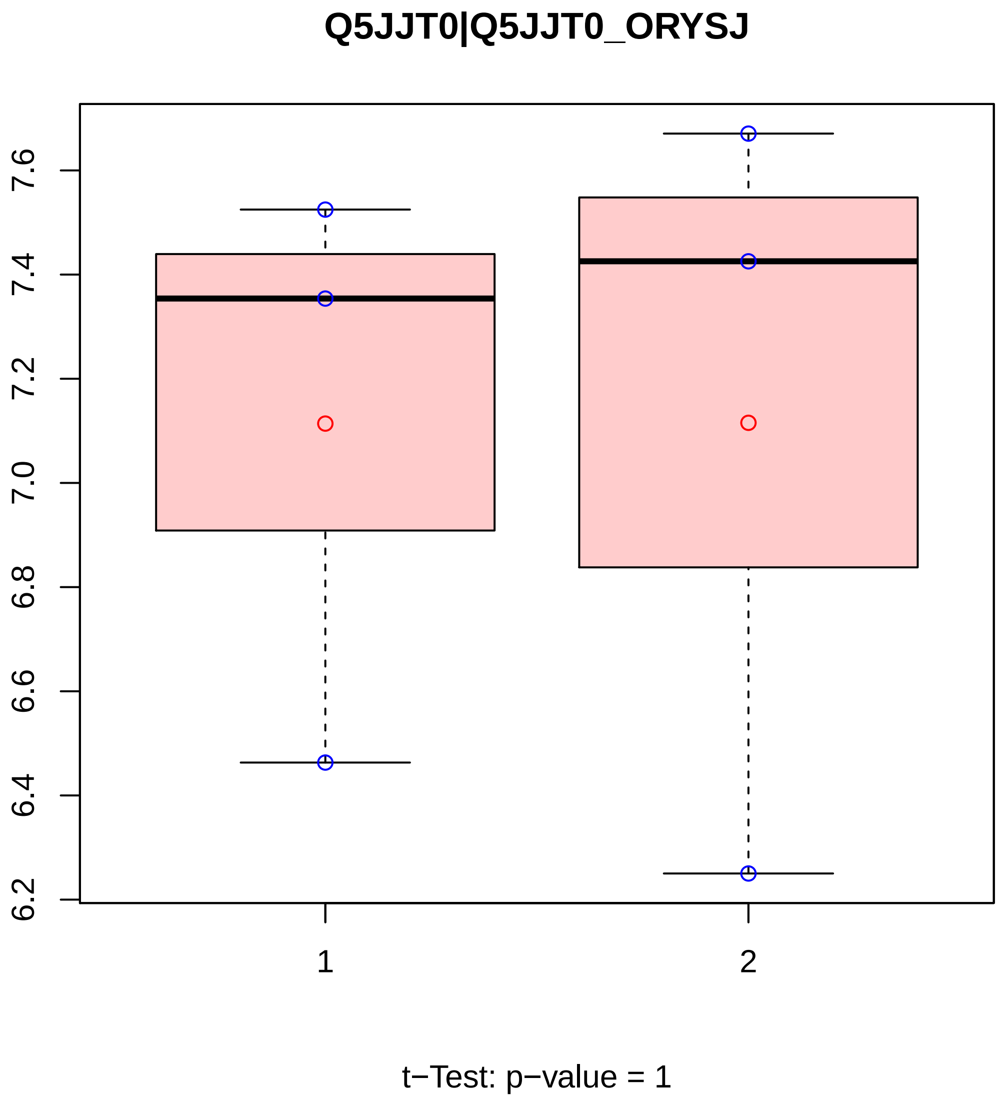

Q5JJT0|Q5JJT0_ORYSJ
7.6
7.4
7.2
7.0
6.8
6.6
6.4
6.2
1
2
t−Test: p−value = 1

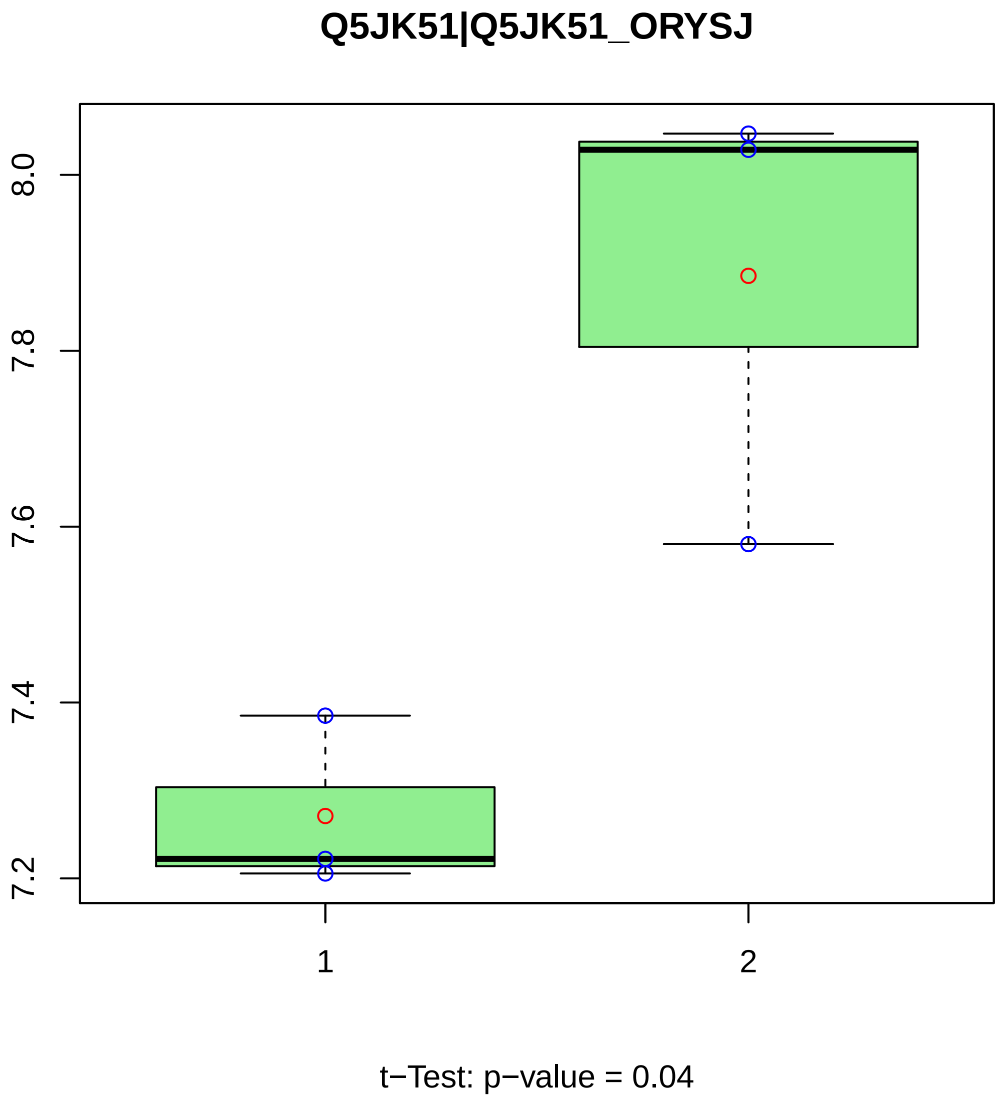
Q5JK51|Q5JK51_ORYSJ
8.0
7.8
7.6
7.4
7.2
1
2
t−Test: p−value = 0.04

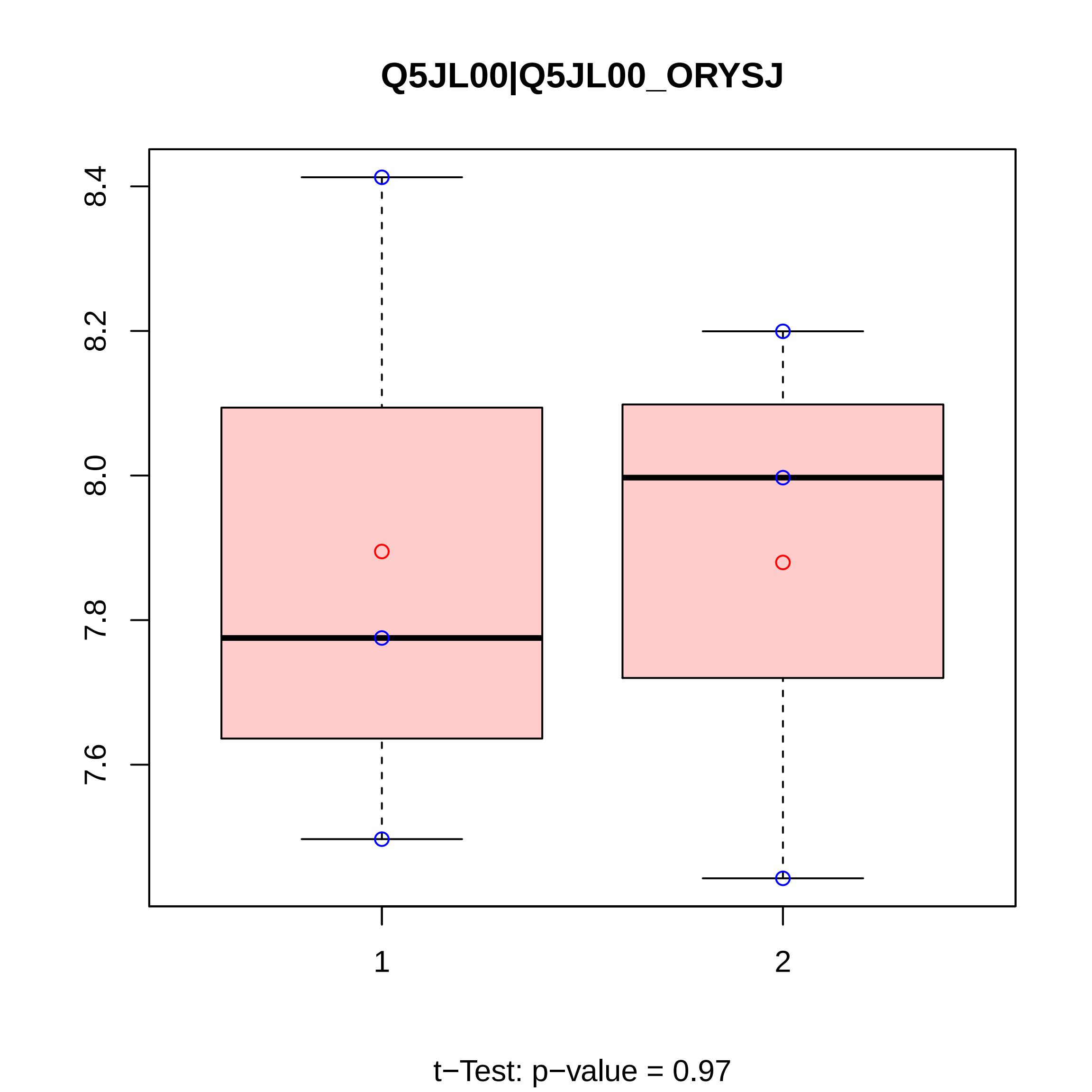

Q5JL00|Q5JL00_ORYSJ
8.4
8.2
8.0
7.8
7.6
1
2
t−Test: p−value = 0.97

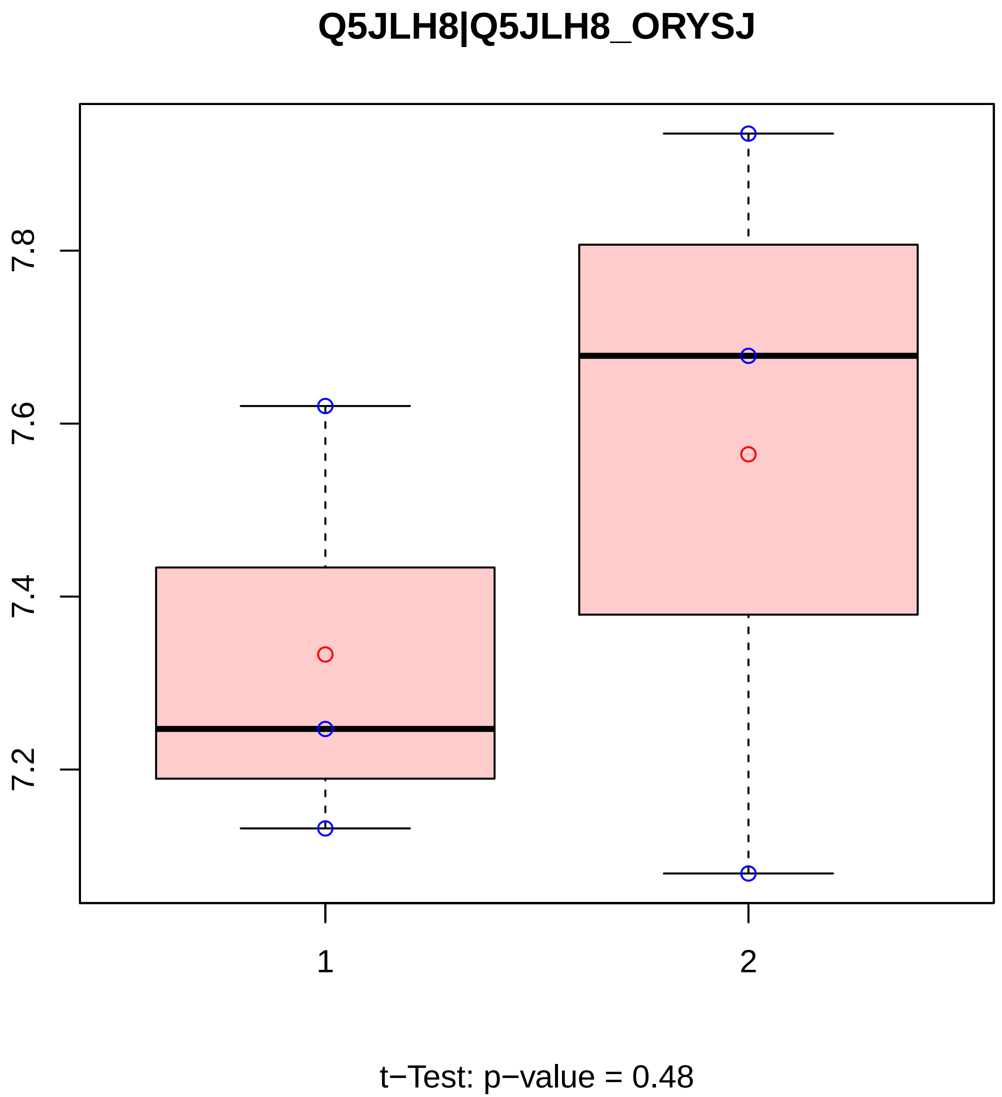
Q5JLH8|Q5JLH8_ORYSJ
7.2
7.4
7.6
7.8
1
2
t−Test: p−value = 0.48

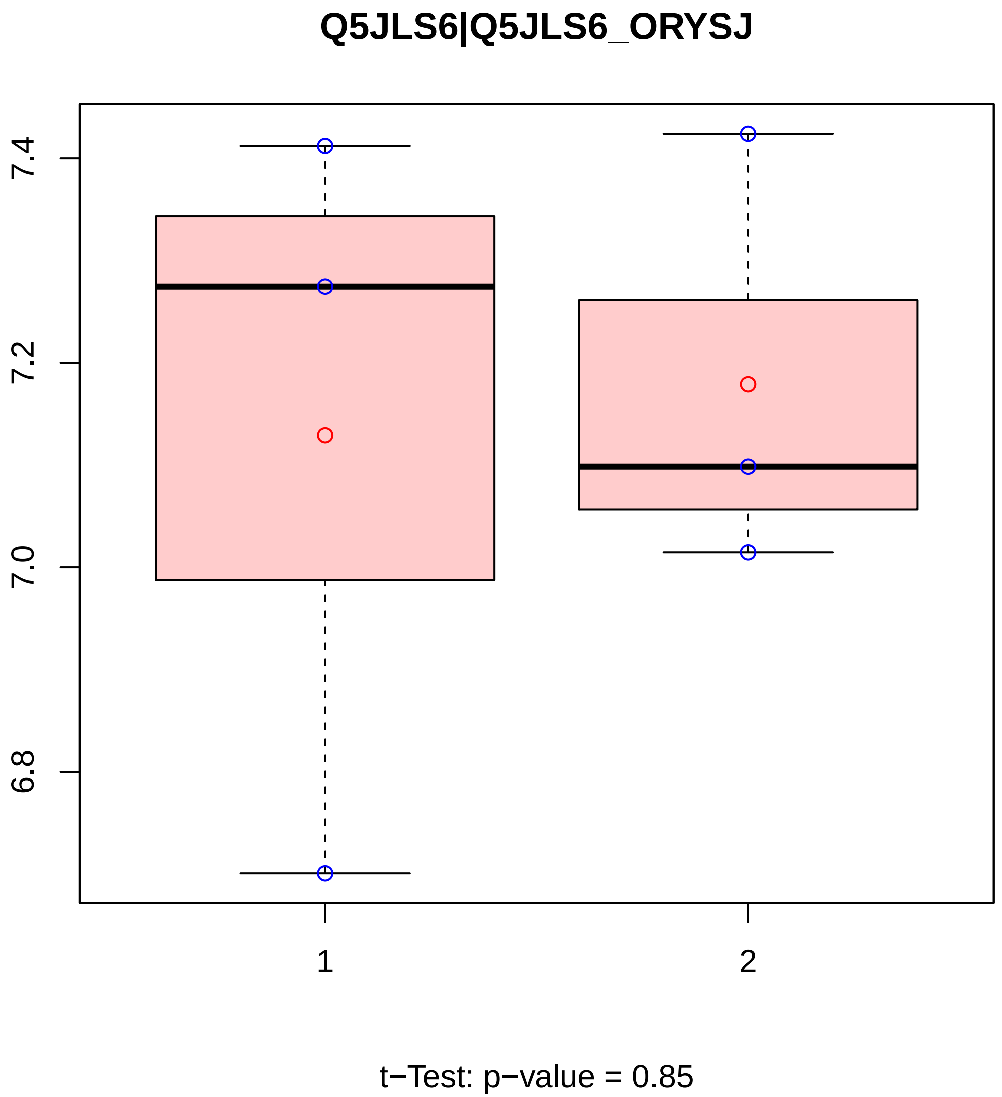
Q5JLS6|Q5JLS6_ORYSJ
7.4
7.2
7.0
6.8
1
2
t−Test: p−value = 0.85

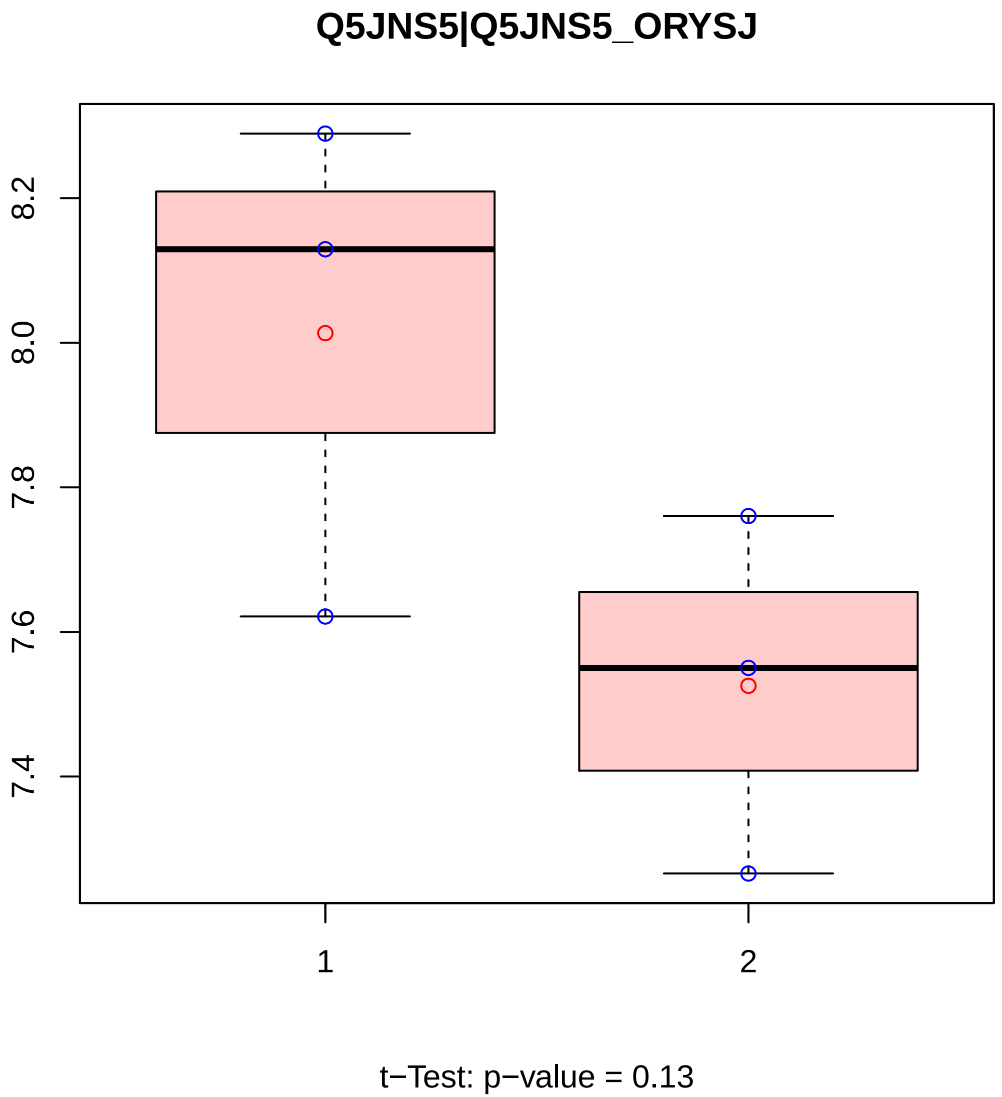

Q5JNS5|Q5JNS5_ORYSJ
8.2
8.0
7.8
7.6
7.4
1
2
t−Test: p−value = 0.13

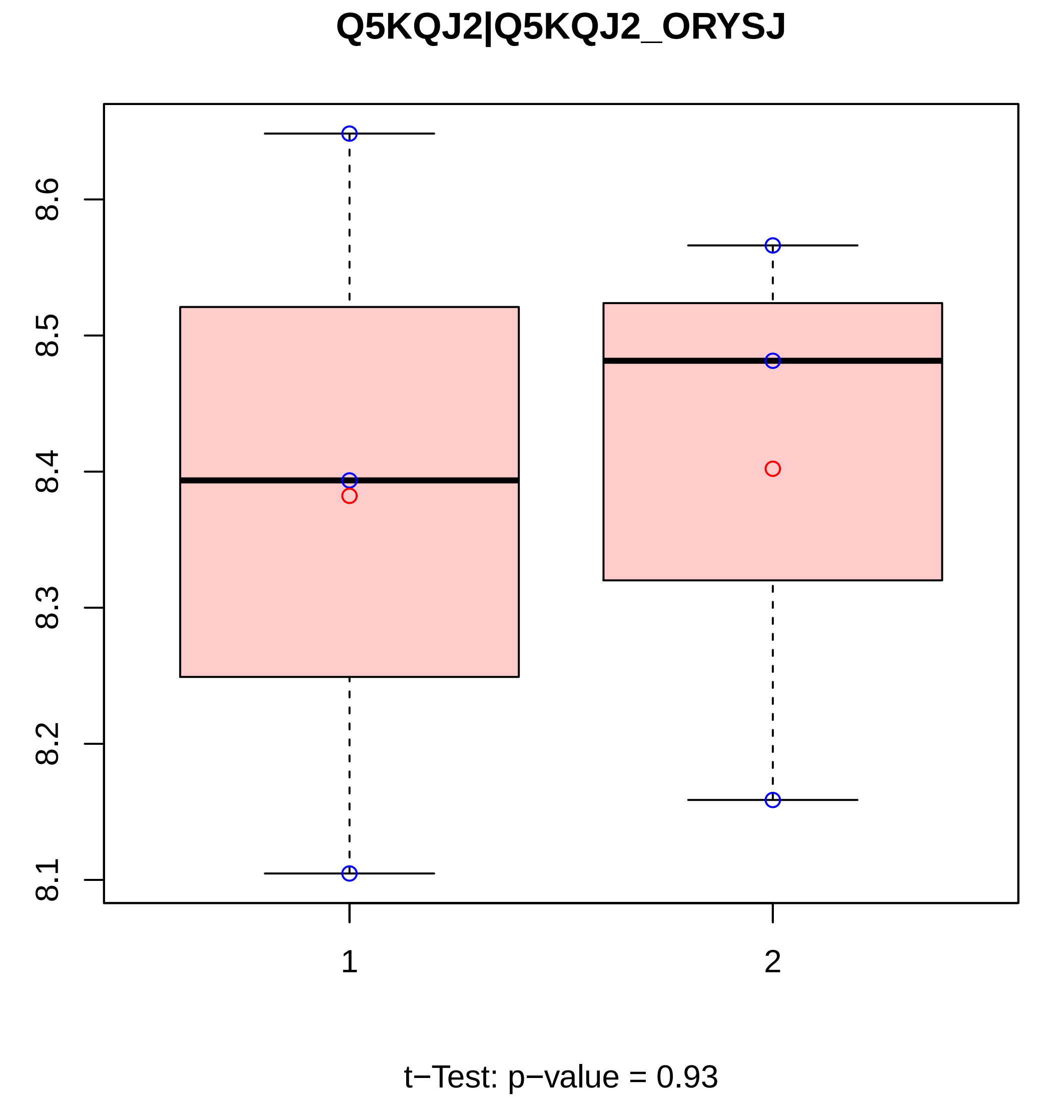
Q5KQJ2|Q5KQJ2_ORYSJ
8.1
8.2
8.3
8.4
8.5
8.6
1
2
t−Test: p−value = 0.93

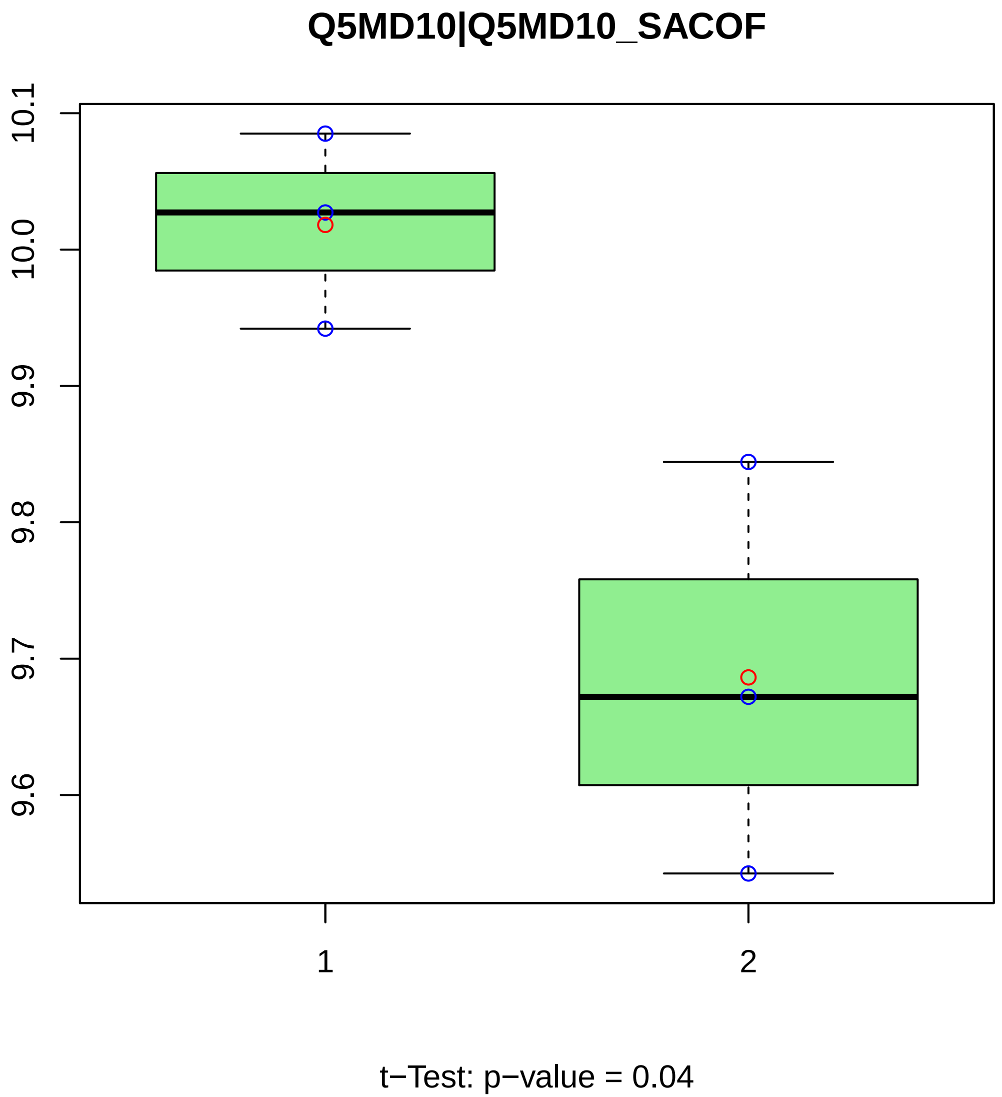
Q5MD10|Q5MD10_SACOF
10.1
10.0
9.9
9.8
9.7
9.6
1
2
t−Test: p−value = 0.04

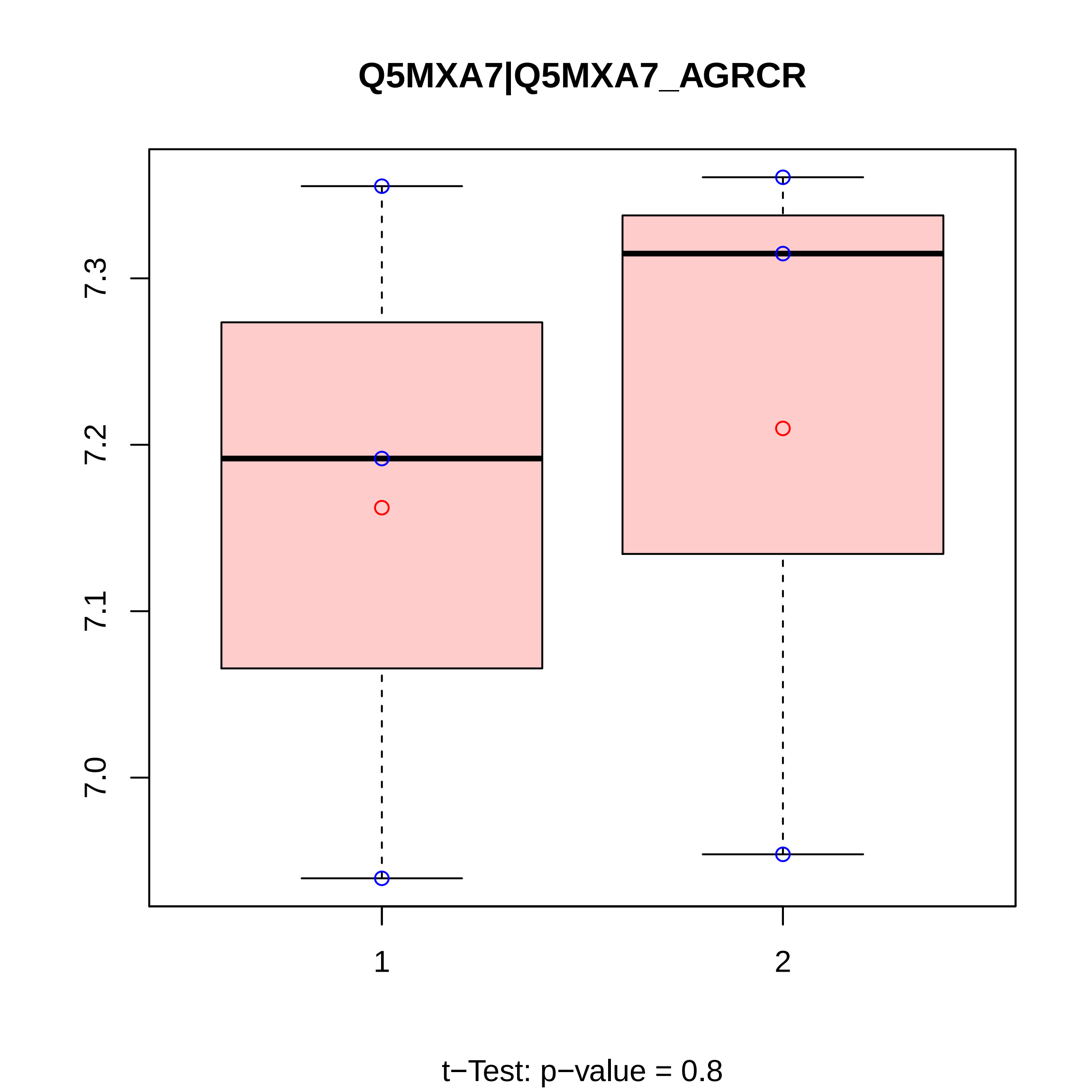

Q5MXA7|Q5MXA7_AGRCR
7.0
7.1
7.2
7.3
1
2
t−Test: p−value = 0.8

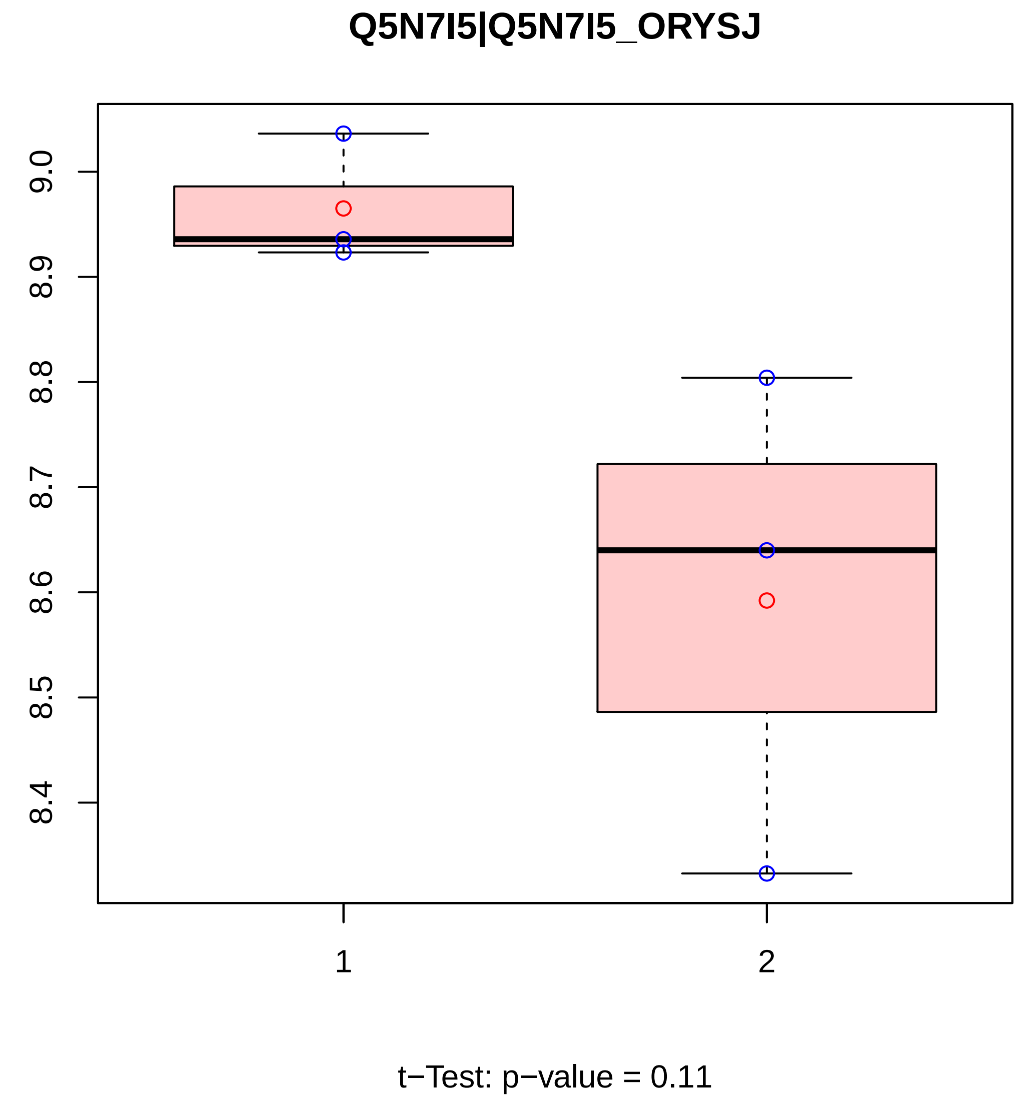
Q5N7I5|Q5N7I5_ORYSJ
9.0
8.9
8.8
8.7
8.6
8.5
8.4
1
2
t−Test: p−value = 0.11

Q5N884|Q5N884_ORYSJ

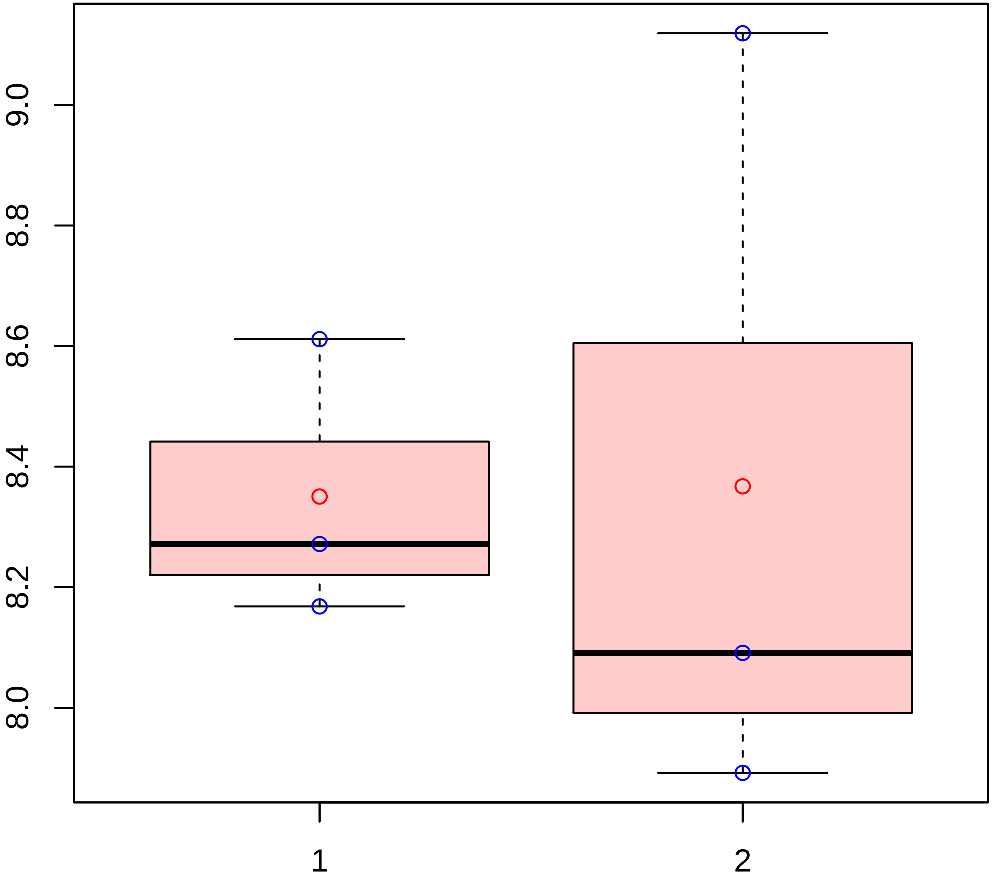

t−Test: p−value = 0.97

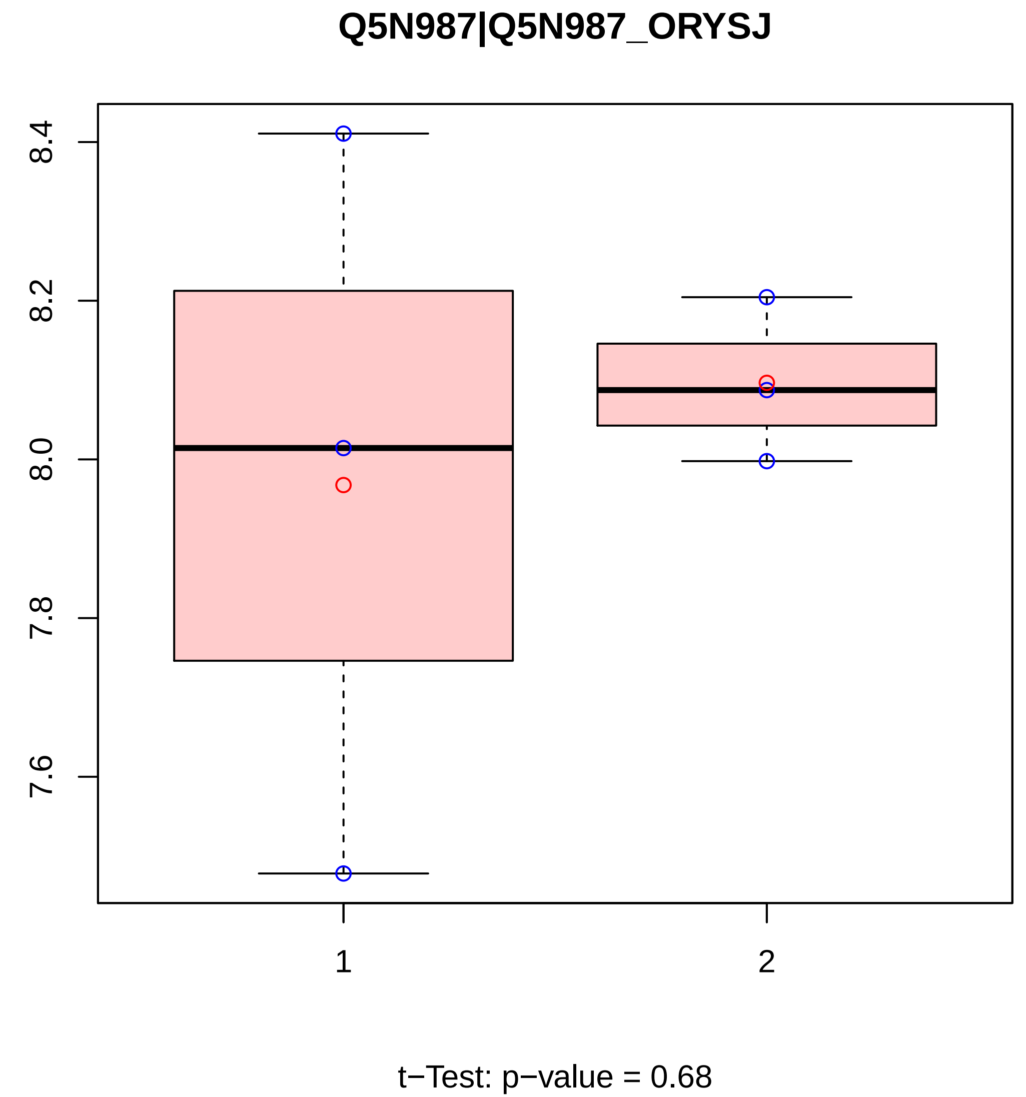
Q5N987|Q5N987_ORYSJ
8.4
8.2
8.0
7.8
7.6
1
2
t−Test: p−value = 0.68

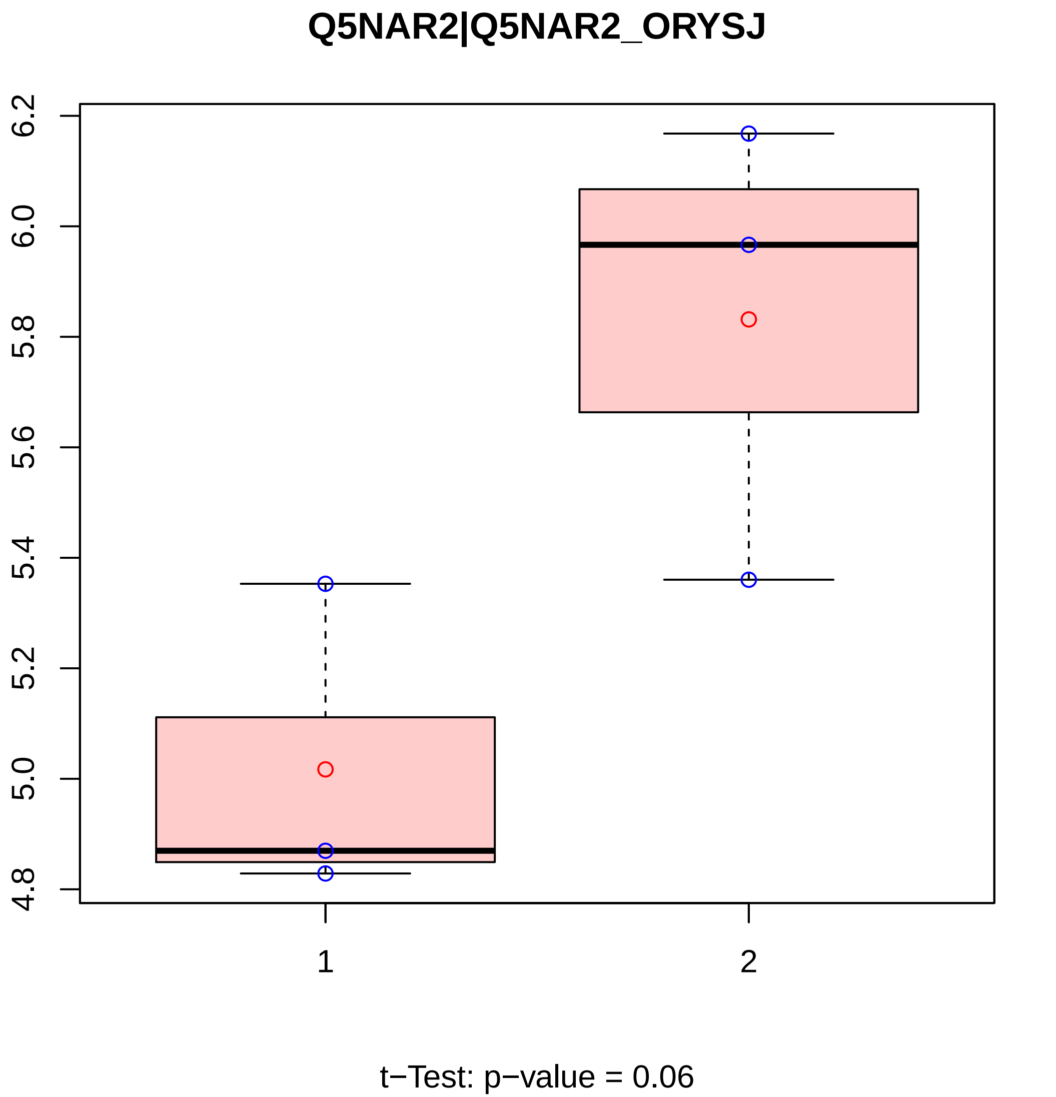
Q5NAR2|Q5NAR2_ORYSJ
6.2
6.0
5.8
5.6
5.4
5.2
5.0
4.8
1
2
t−Test: p−value = 0.06

Q5Q0X7|Q5Q0X7_SACOF

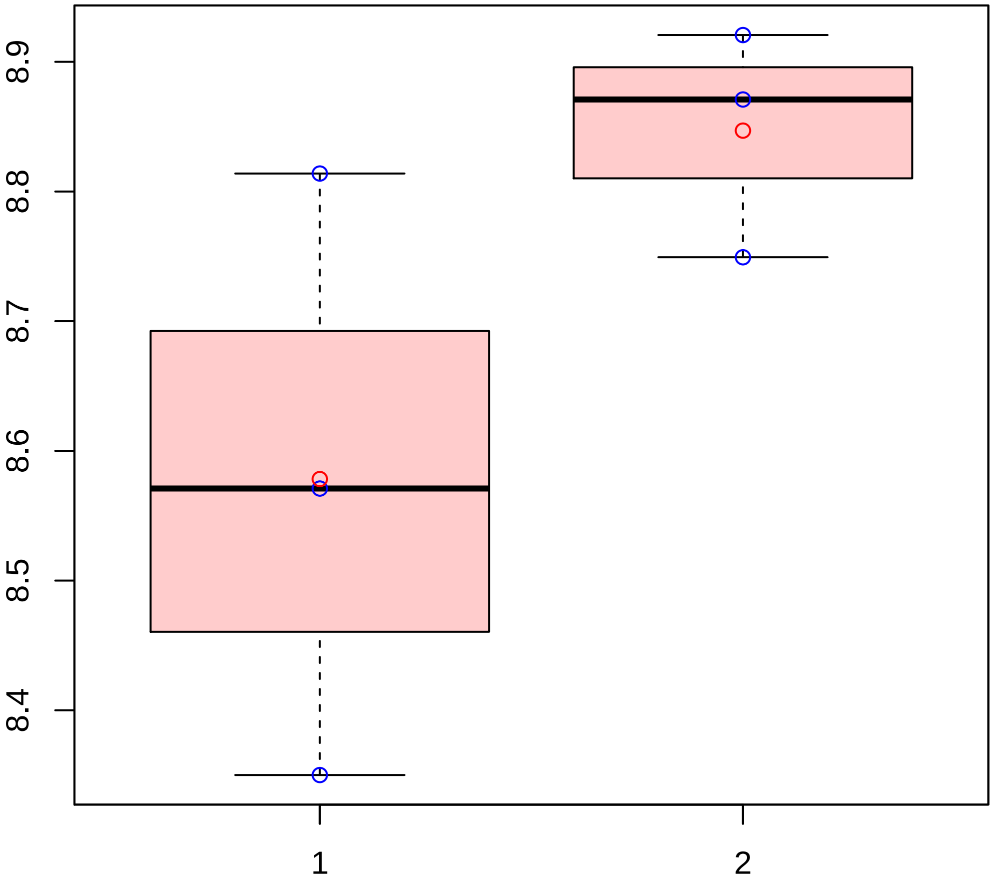

t−Test: p−value = 0.17

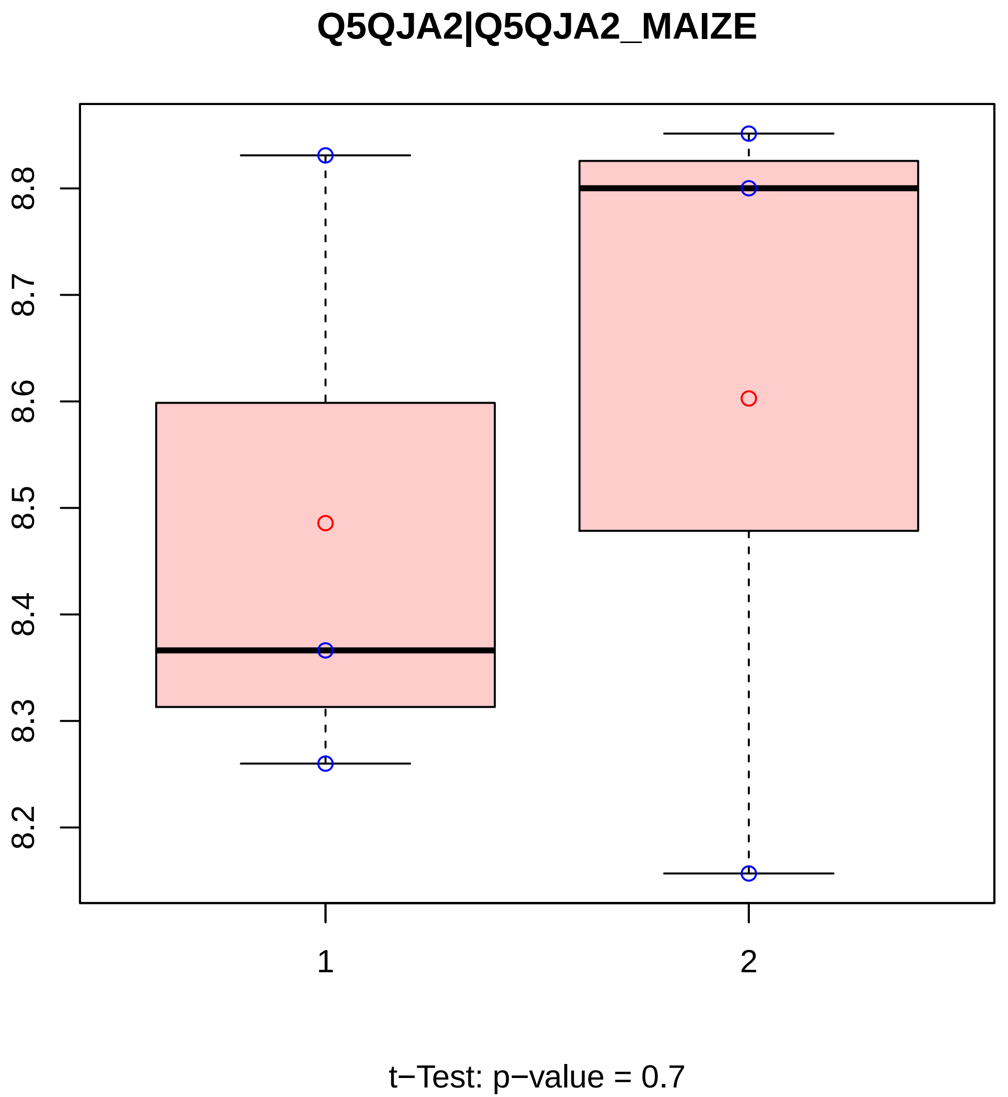
Q5QJA2|Q5QJA2_MAIZE
8.2
8.3
8.4
8.5
8.6
8.7
8.8
1
2
t−Test: p−value = 0.7

Q5QJB5|Q5QJB5_HORVD

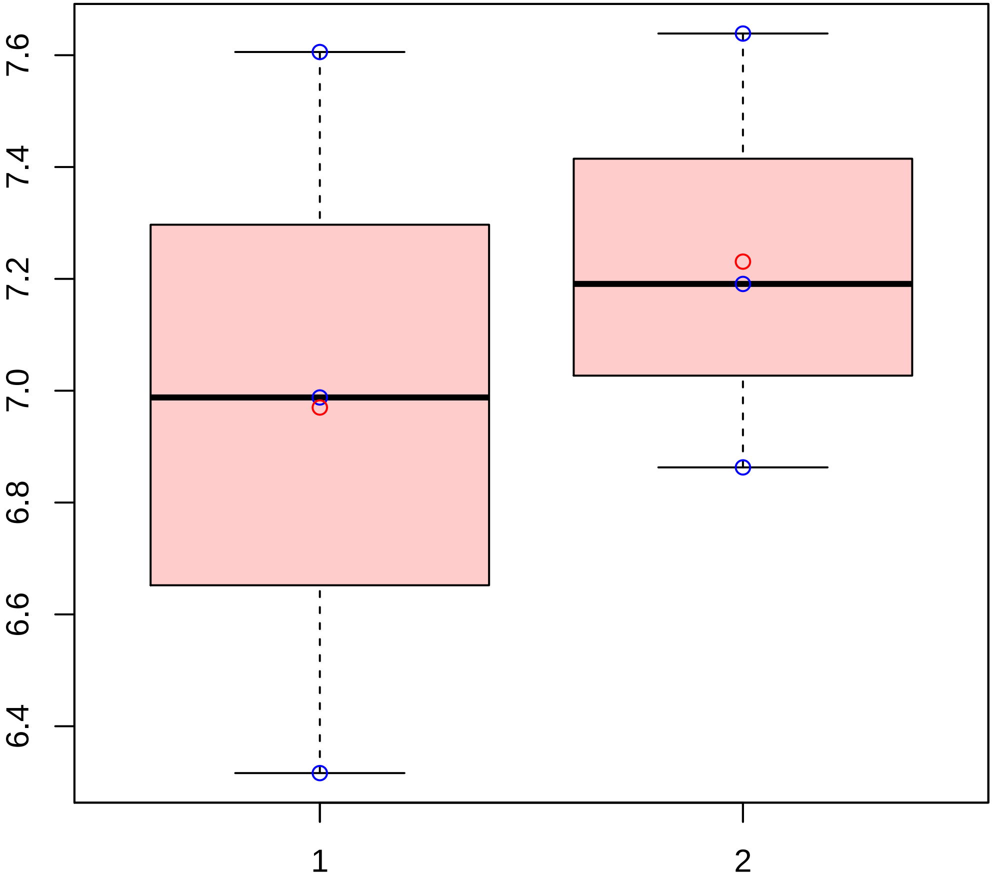

t−Test: p−value = 0.59

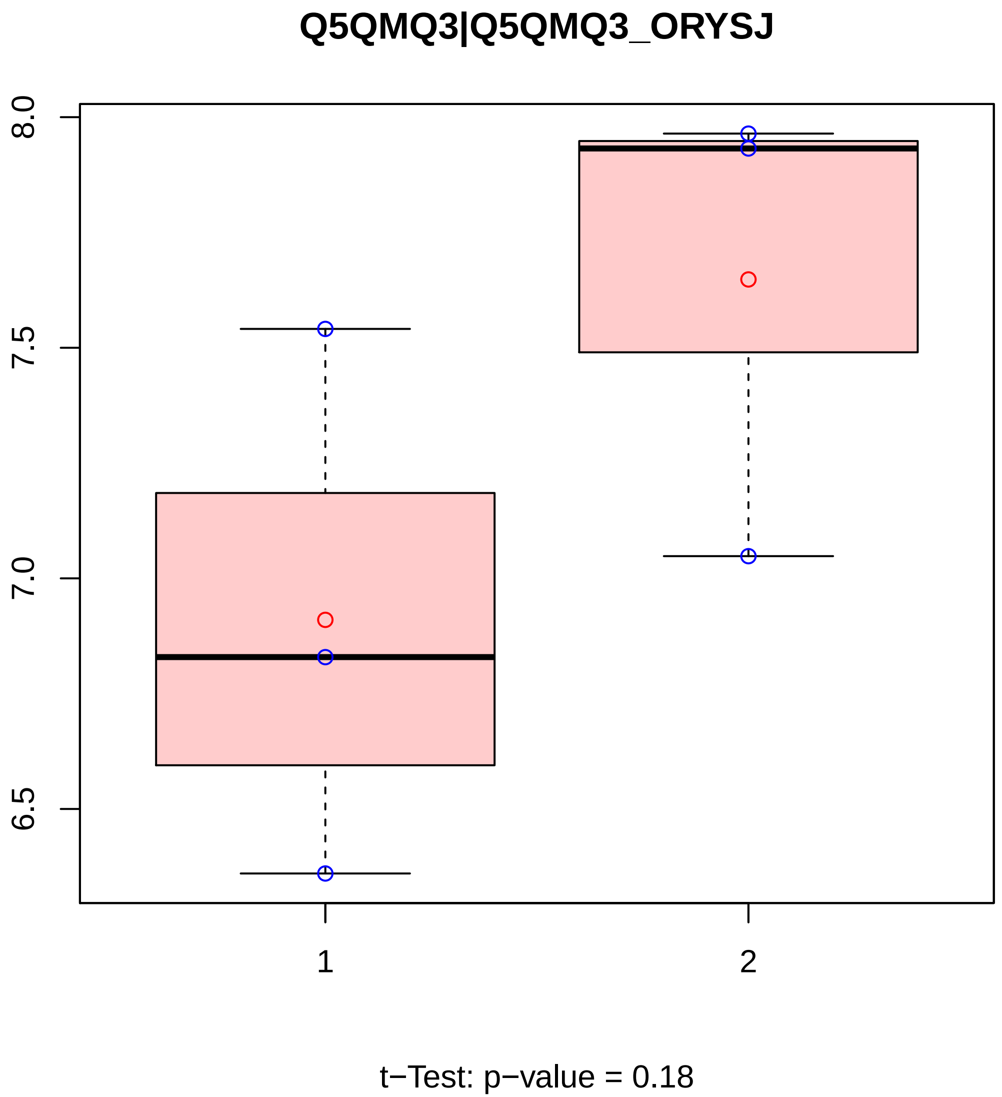
Q5QMQ3|Q5QMQ3_ORYSJ
8.0
7.5
7.0
6.5
1
2
t−Test: p−value = 0.18

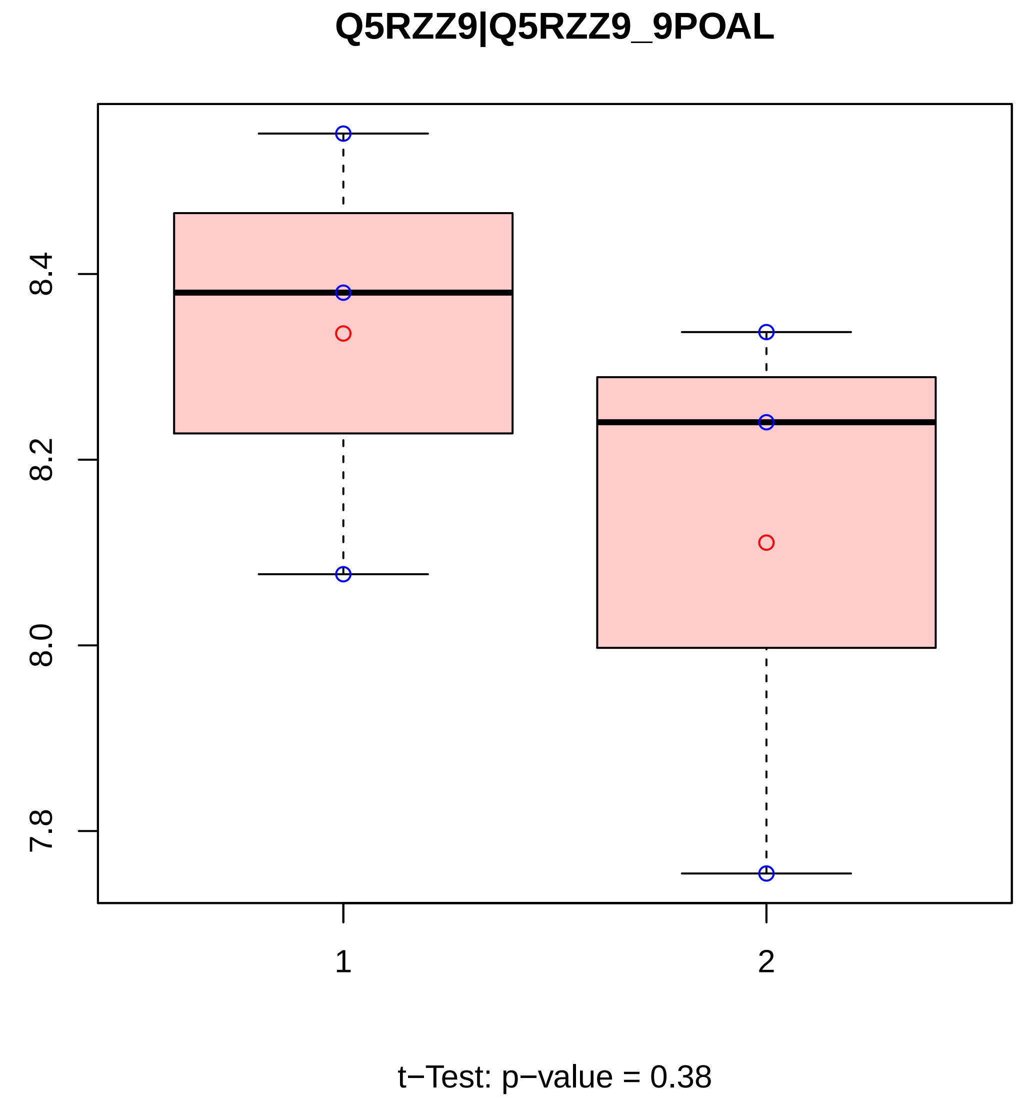

Q5RZZ9|Q5RZZ9_9POAL
8.4
8.2
8.0
7.8
1
2
t−Test: p−value = 0.38

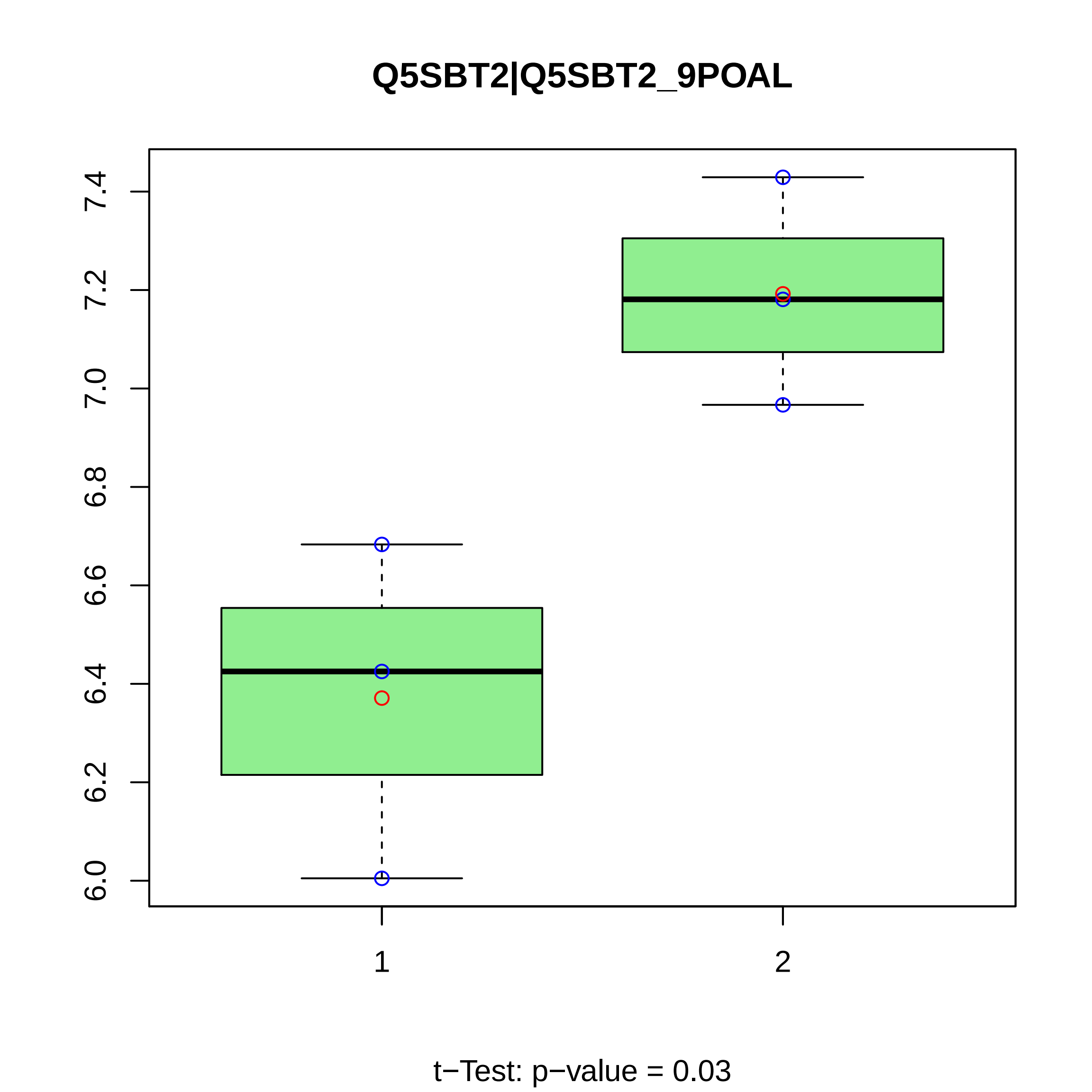

Q5SBT2|Q5SBT2_9POAL
7.4
7.2
7.0
6.8
6.6
6.4
6.2
6.0
1
2
t−Test: p−value = 0.03

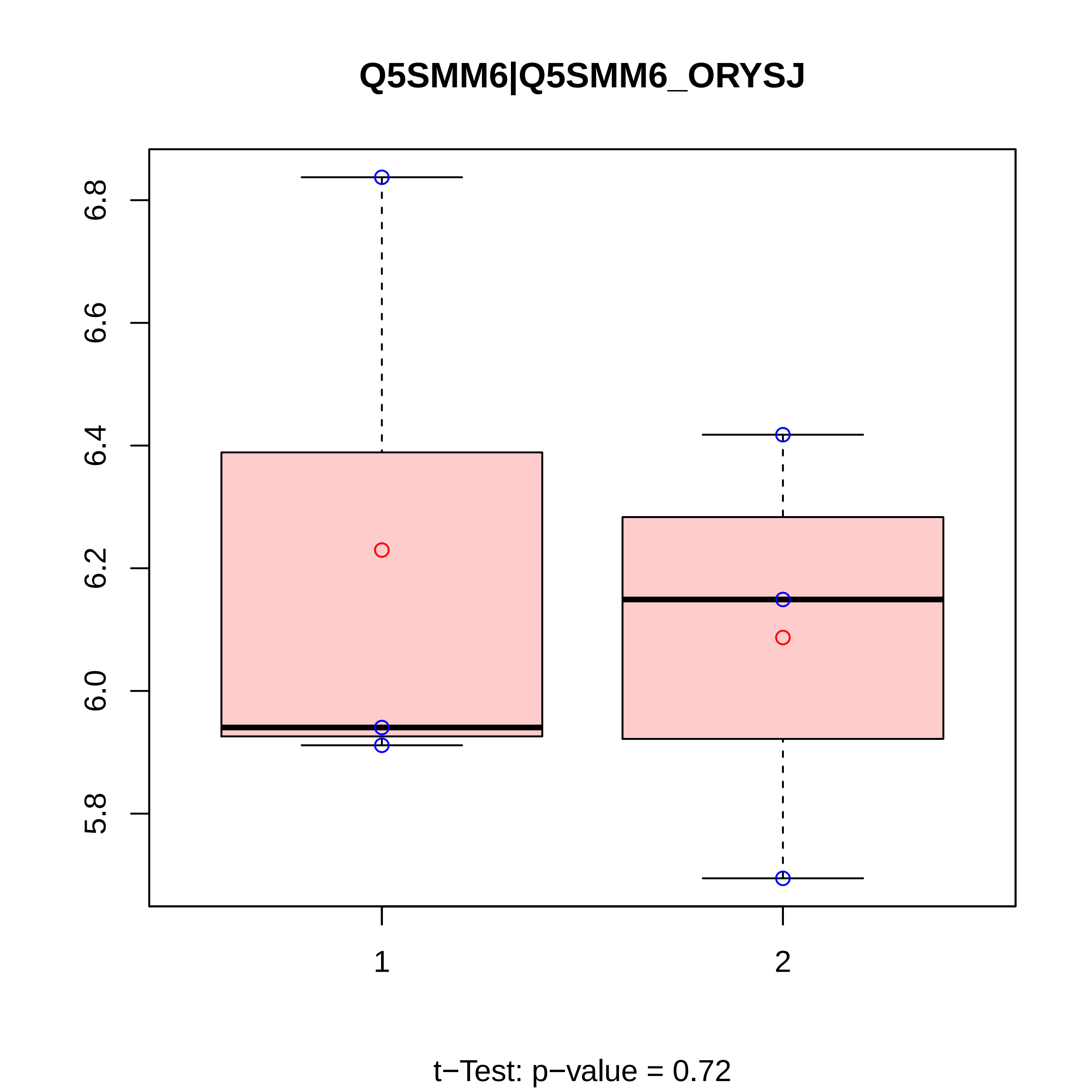

Q5SMM6|Q5SMM6_ORYSJ
6.8
6.6
6.4
6.2
6.0
5.8
1
2
t−Test: p−value = 0.72

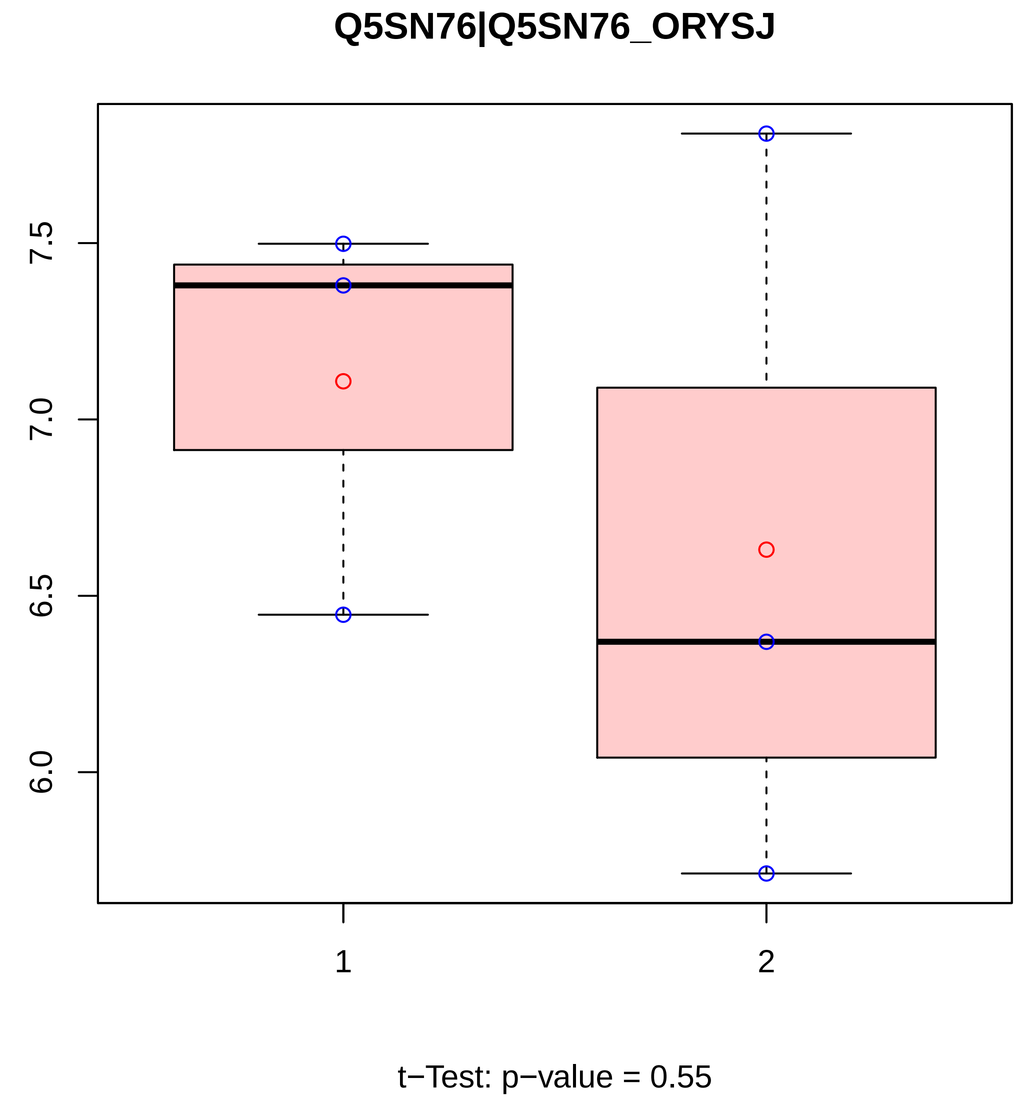
Q5SN76|Q5SN76_ORYSJ
7.5
7.0
6.5
6.0
1
2
t−Test: p−value = 0.55

Q5TK93|Q5TK93_BAMOL

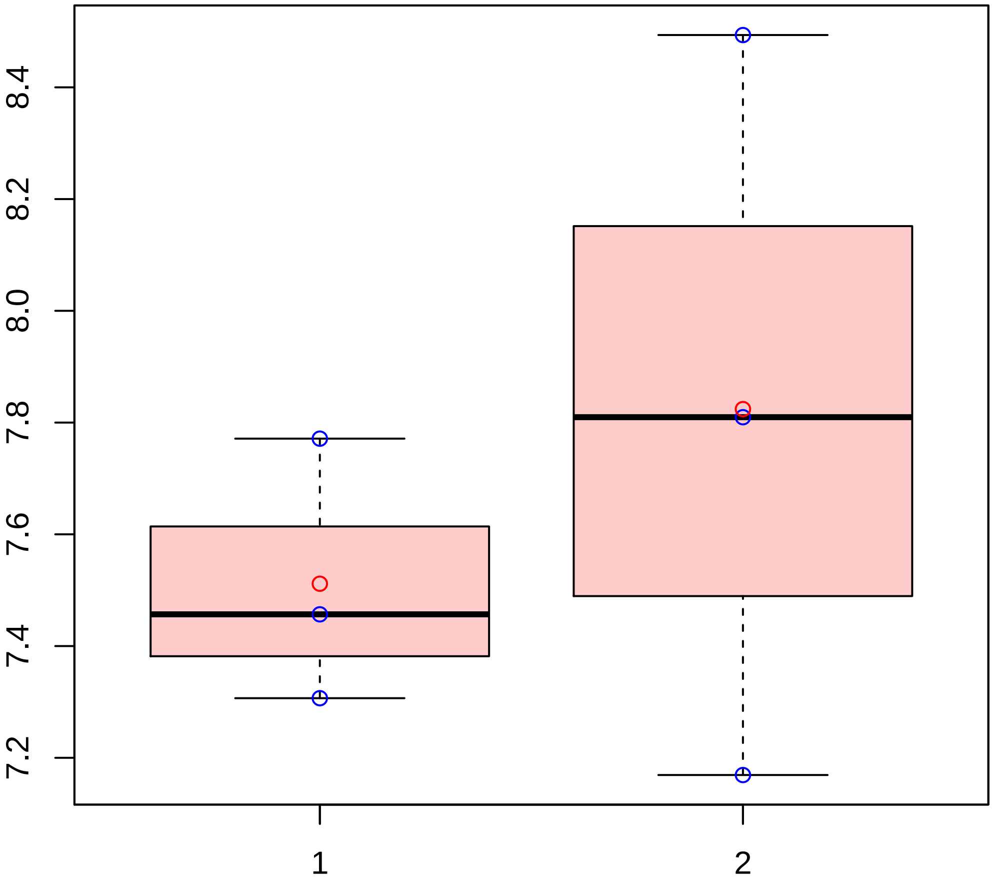

t−Test: p−value = 0.51

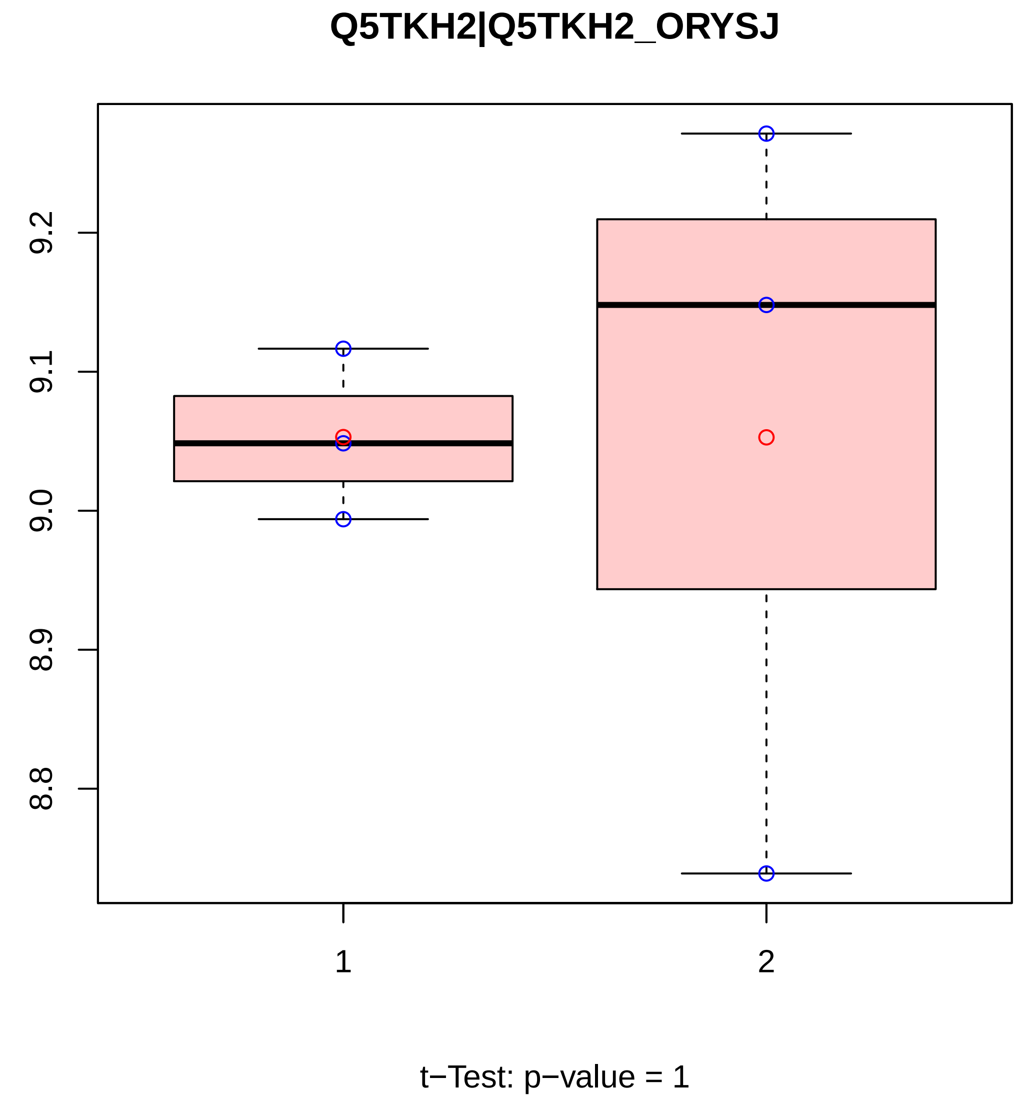
Q5TKH2|Q5TKH2_ORYSJ
9.2
9.1
9.0
8.9
8.8
1
2
t−Test: p−value = 1

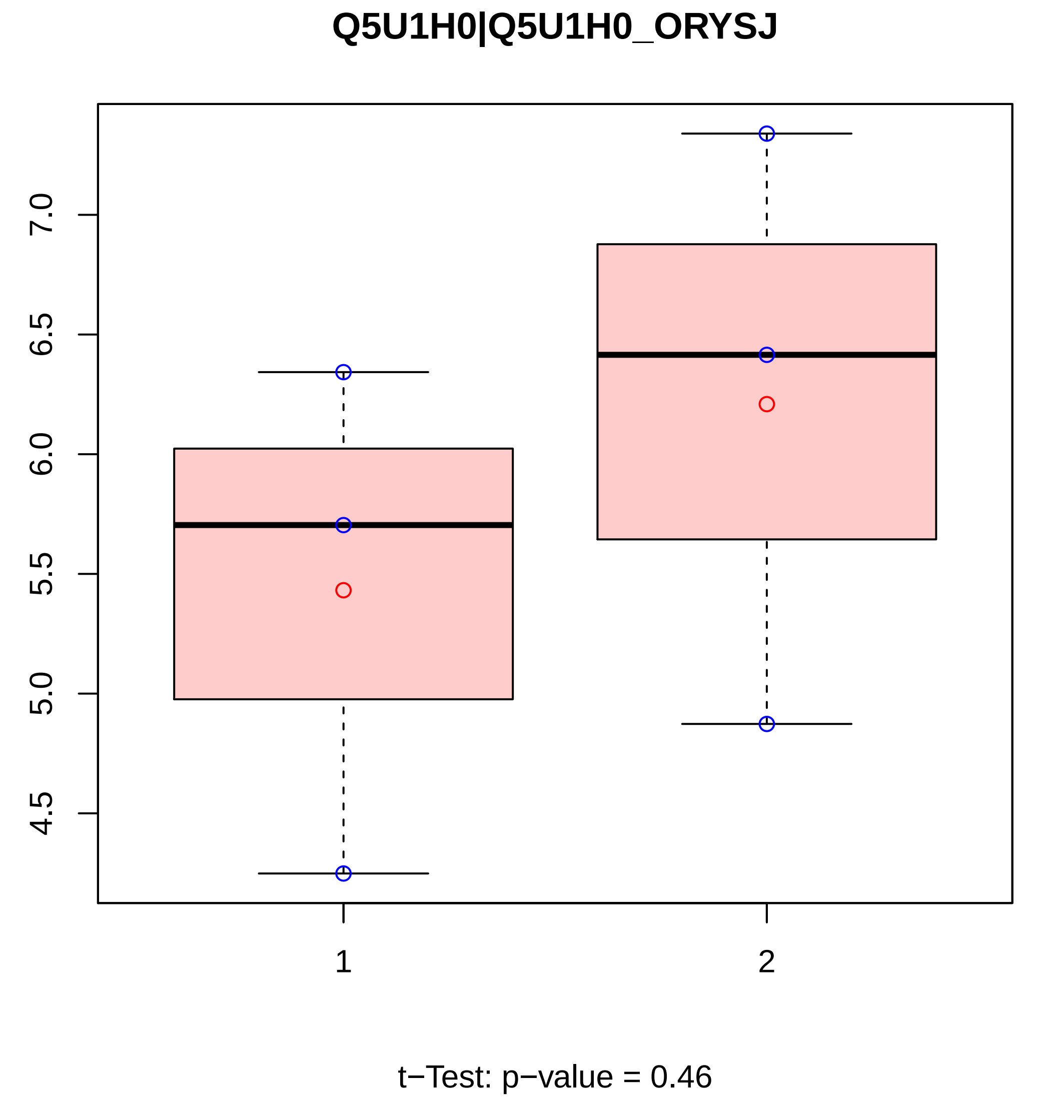
Q5U1H0|Q5U1H0_ORYSJ
4.5
5.0
5.5
6.0
6.5
7.0
1
2
t−Test: p−value = 0.46

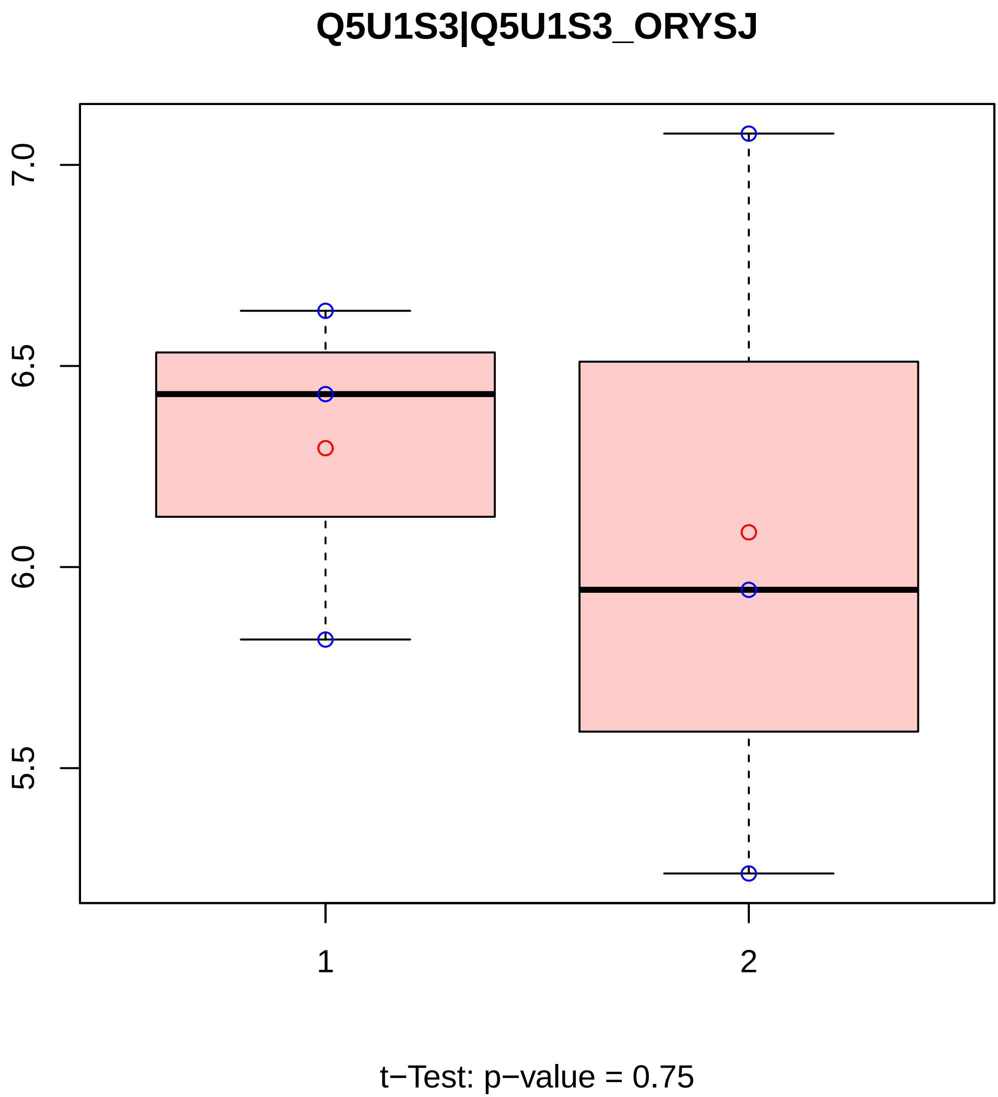
Q5U1S3|Q5U1S3_ORYSJ
7.0
6.5
6.0
5.5
1
2
t−Test: p−value = 0.75

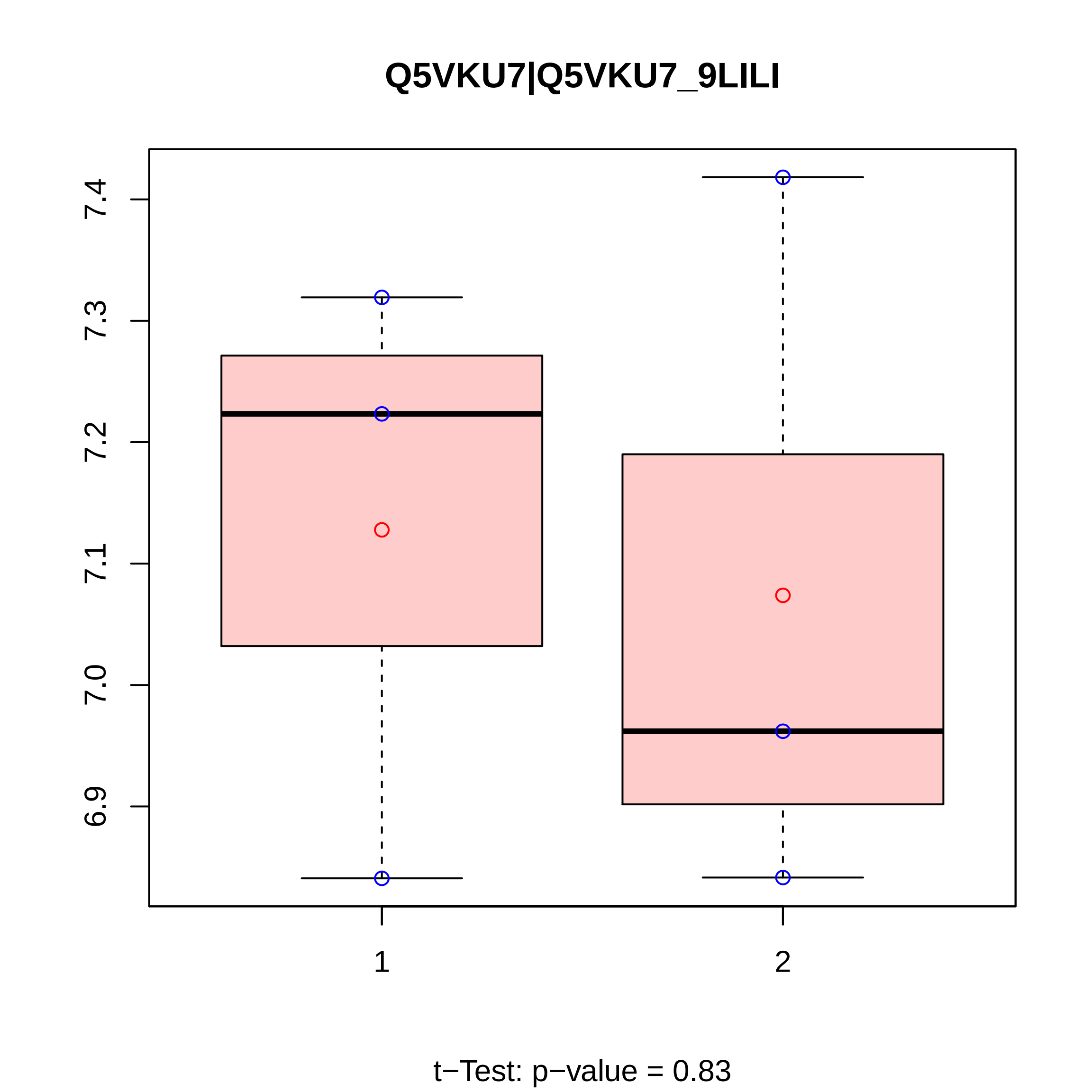

Q5VKU7|Q5VKU7_9LILI
7.4
7.3
7.2
7.1
7.0
6.9
1
2
t−Test: p−value = 0.83

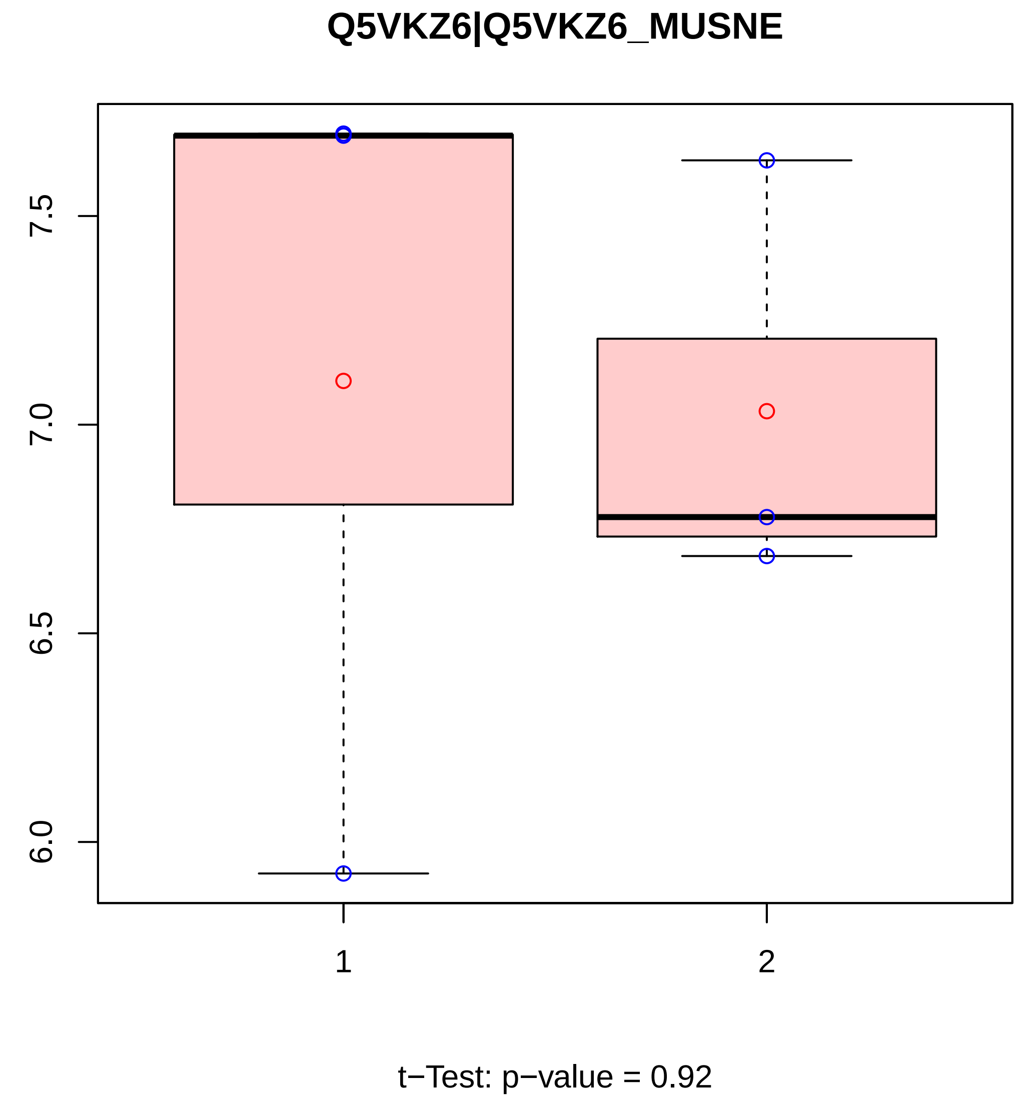
Q5VKZ6|Q5VKZ6_MUSNE
7.5
7.0
6.5
6.0
1
2
t−Test: p−value = 0.92

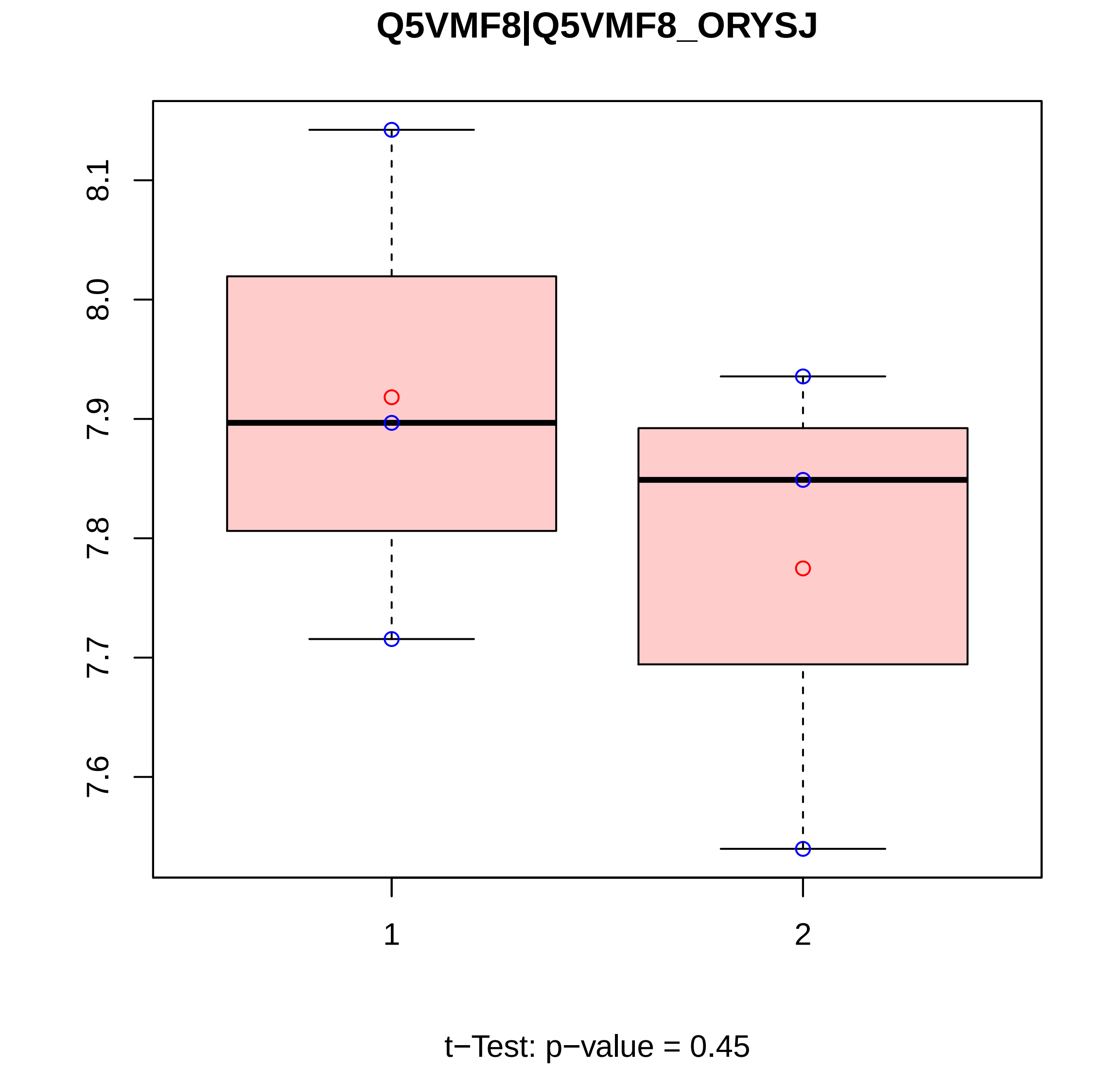
Q5VMF8|Q5VMF8_ORYSJ
8.1
8.0
7.9
7.8
7.7
7.6
1
2
t−Test: p−value = 0.45

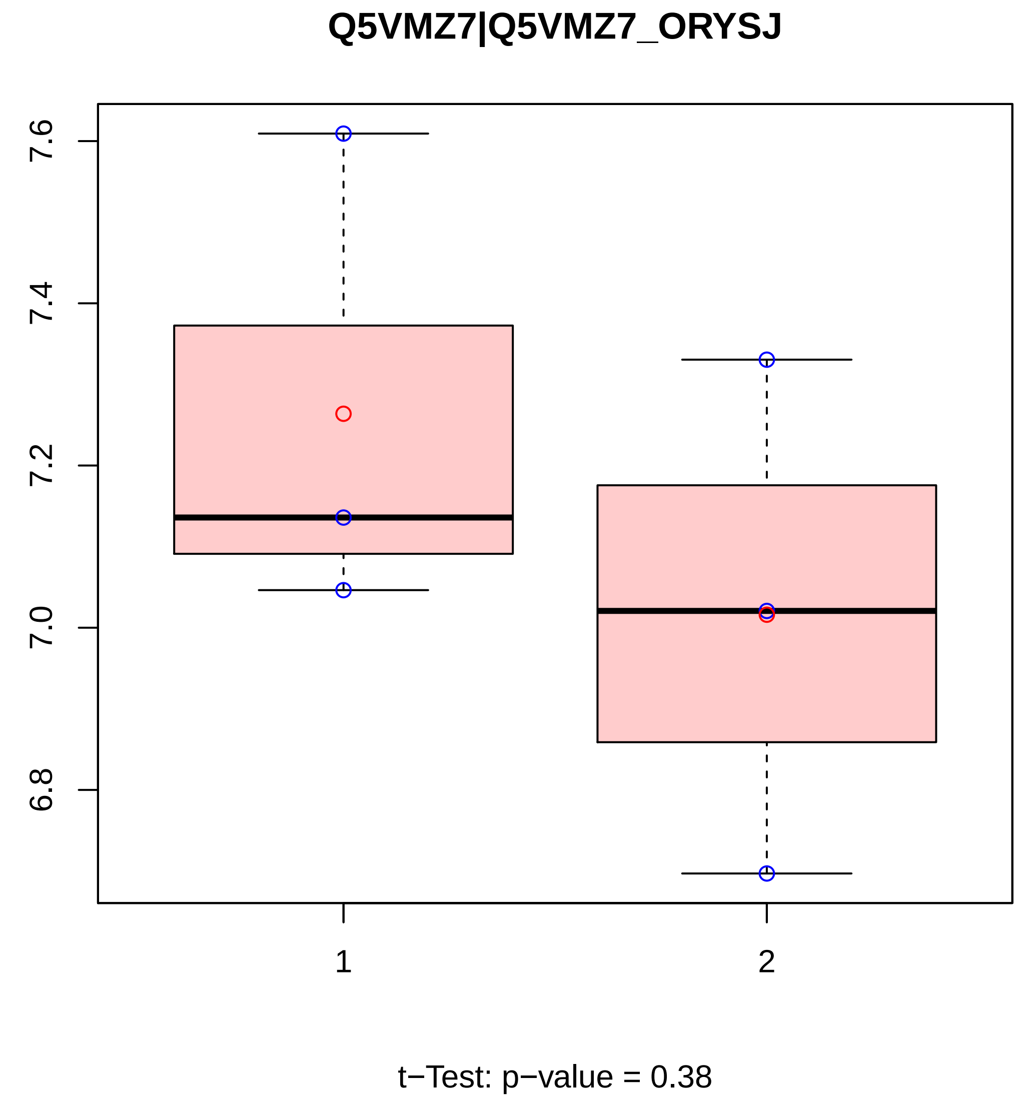

Q5VMZ7|Q5VMZ7_ORYSJ
7.6
7.4
7.2
7.0
6.8
1
2
t−Test: p−value = 0.38

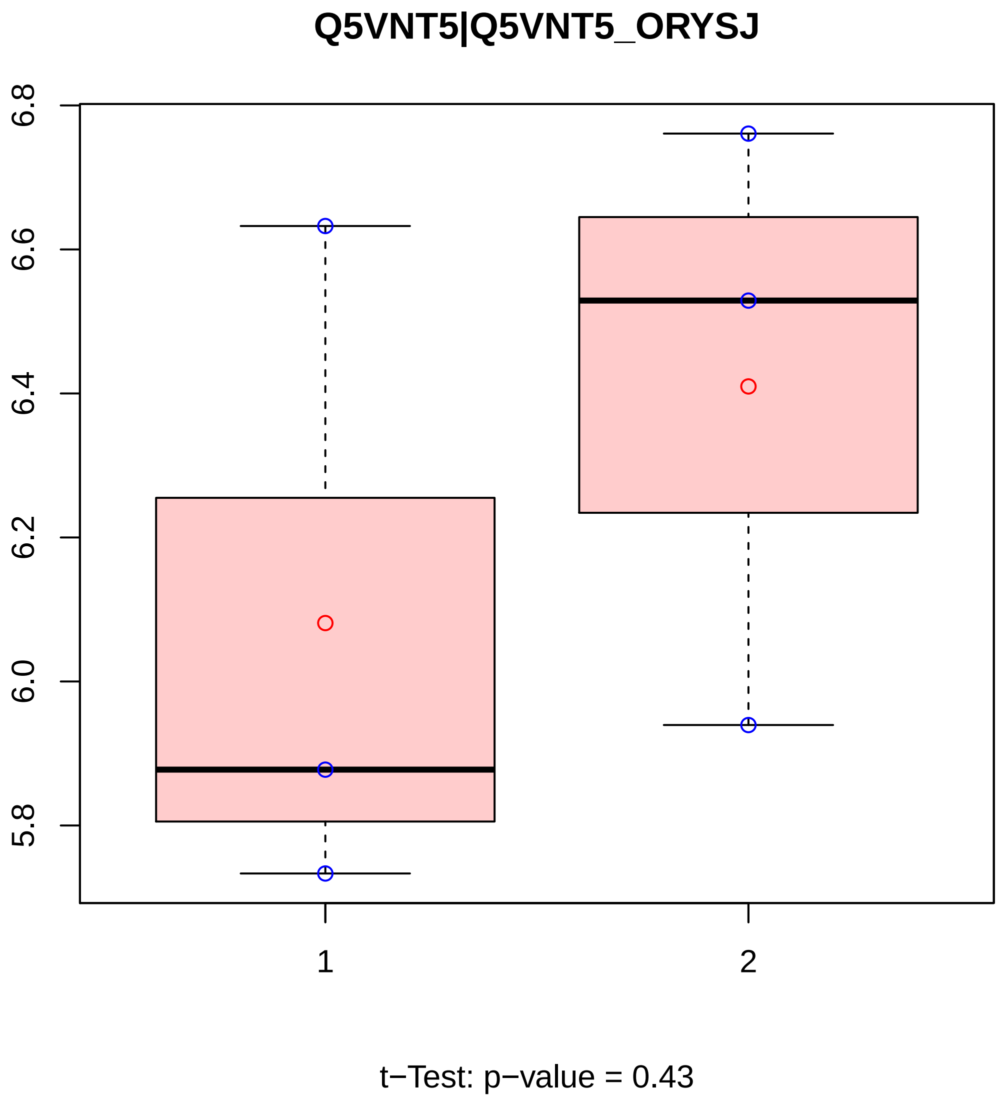
Q5VNT5|Q5VNT5_ORYSJ
6.8
6.6
6.4
6.2
6.0
5.8
1
2
t−Test: p−value = 0.43

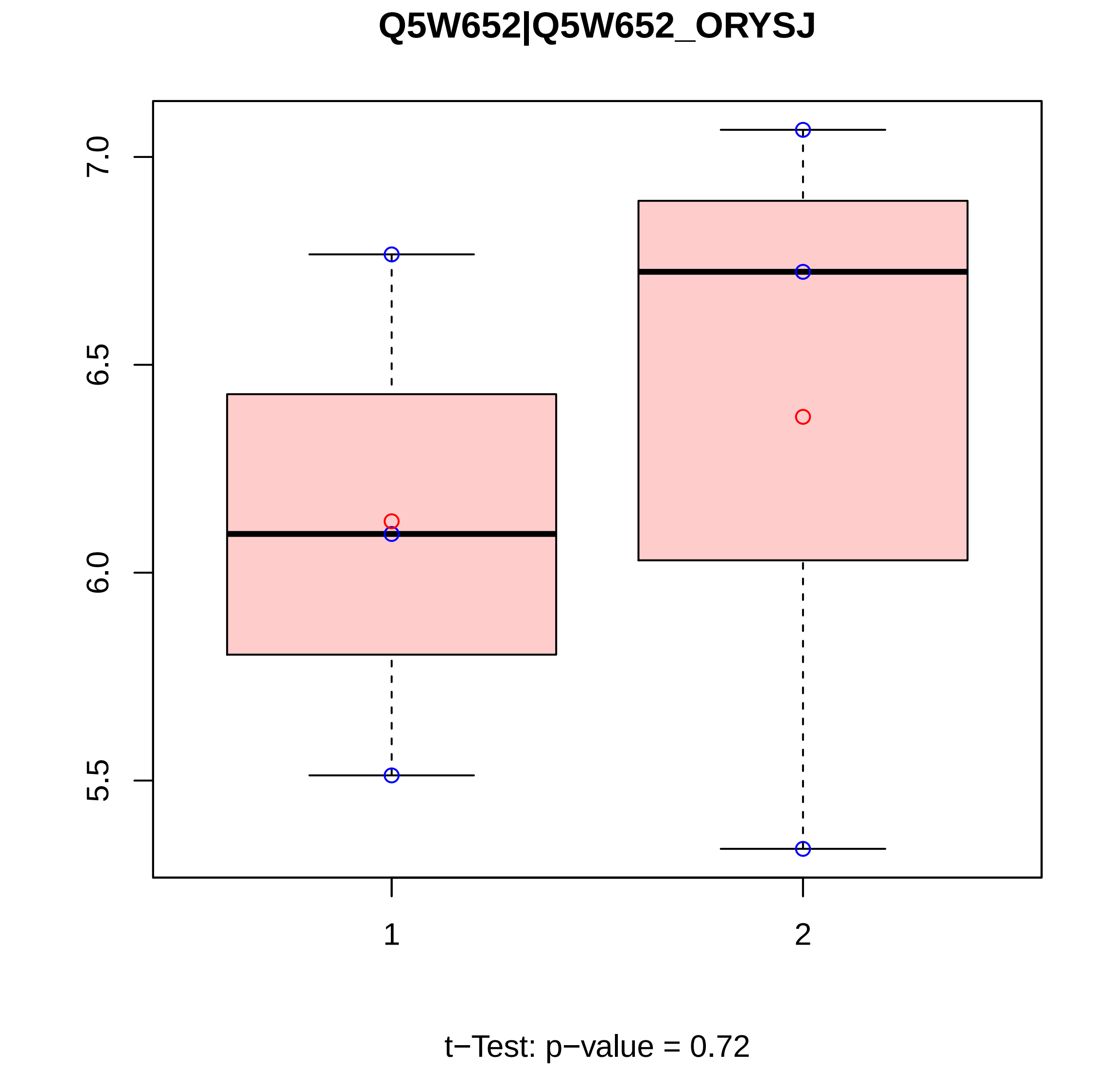
Q5W652|Q5W652_ORYSJ
7.0
6.5
6.0
5.5
1
2
t−Test: p−value = 0.72

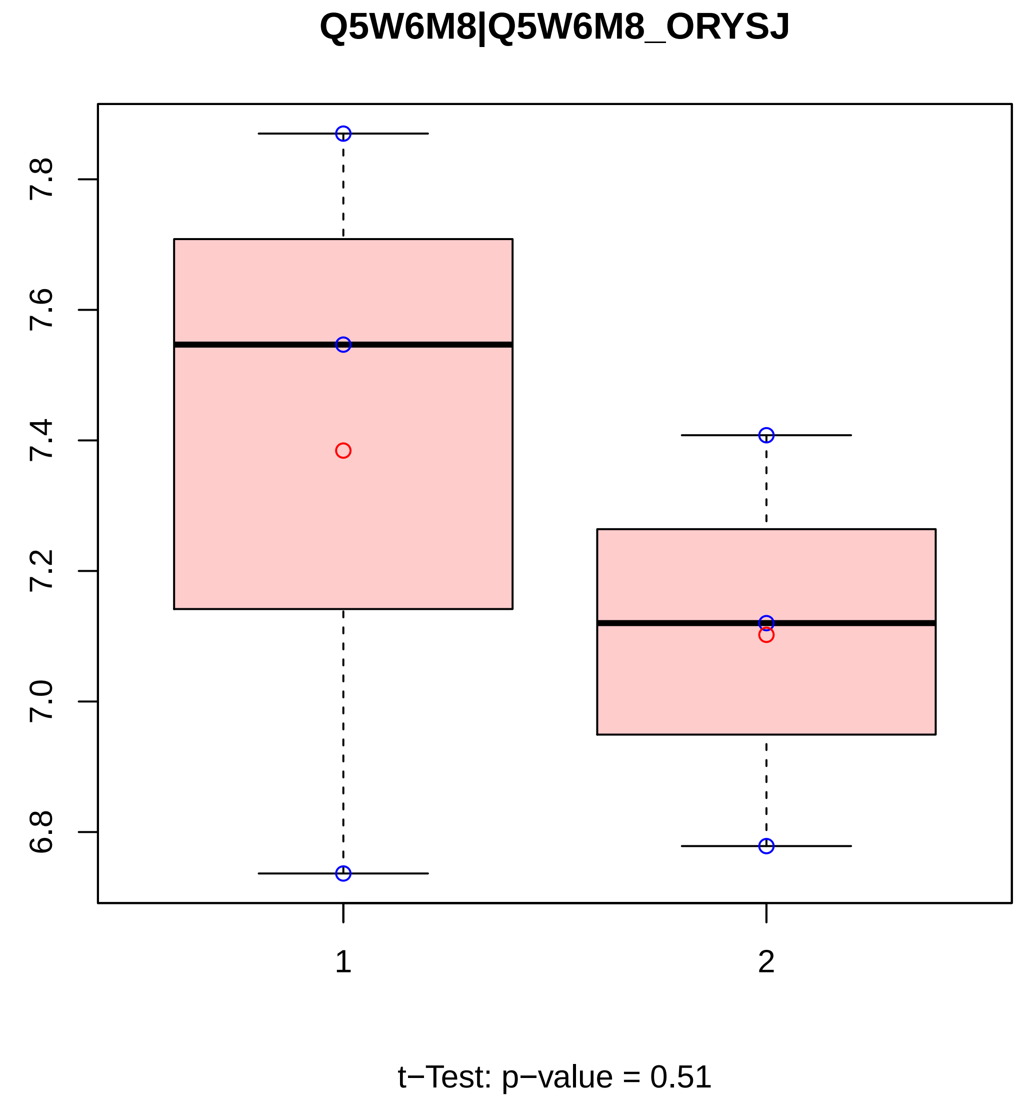

Q5W6M8|Q5W6M8_ORYSJ
7.8
7.6
7.4
7.2
7.0
6.8
1
2
t−Test: p−value = 0.51

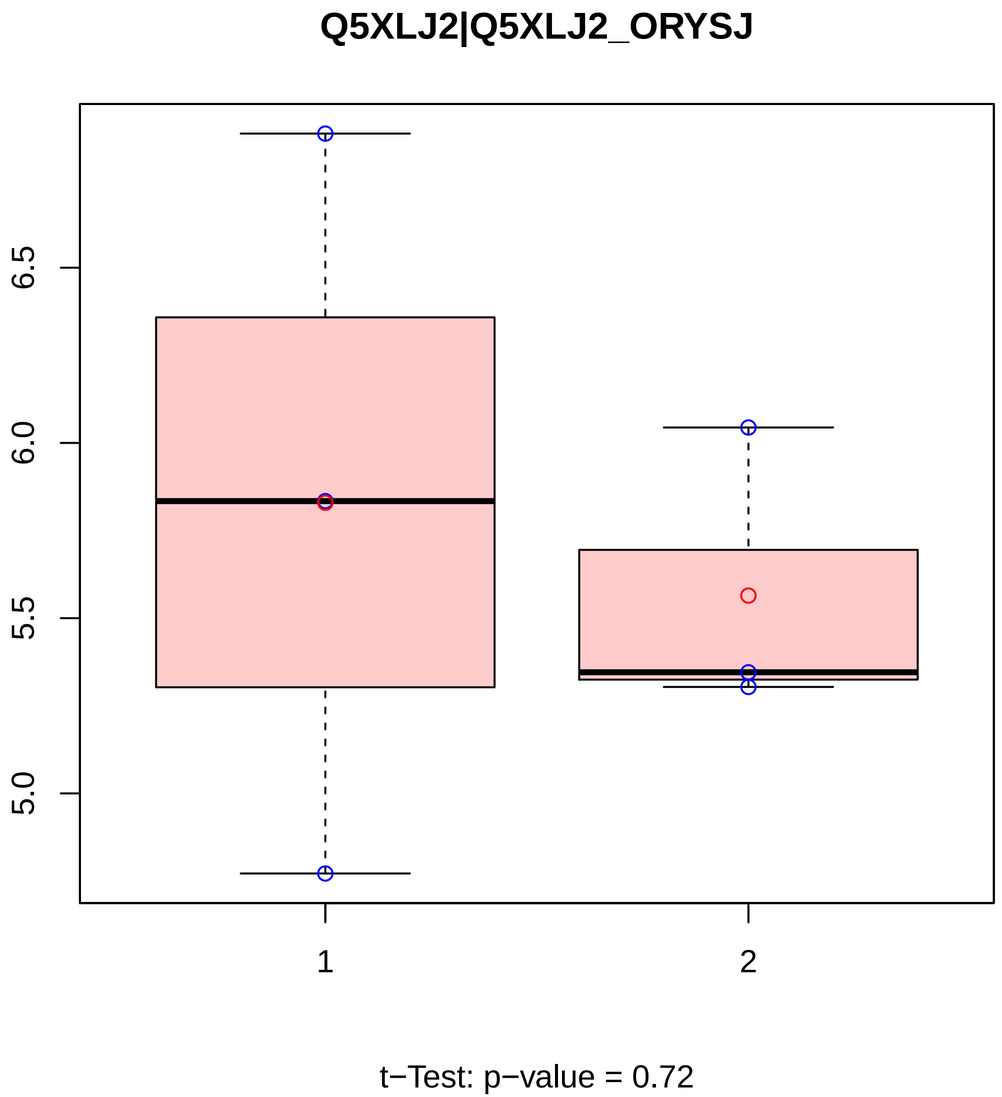

Q5XLJ2|Q5XLJ2_ORYSJ
5.0
5.5
6.0
6.5
1
2
t−Test: p−value = 0.72

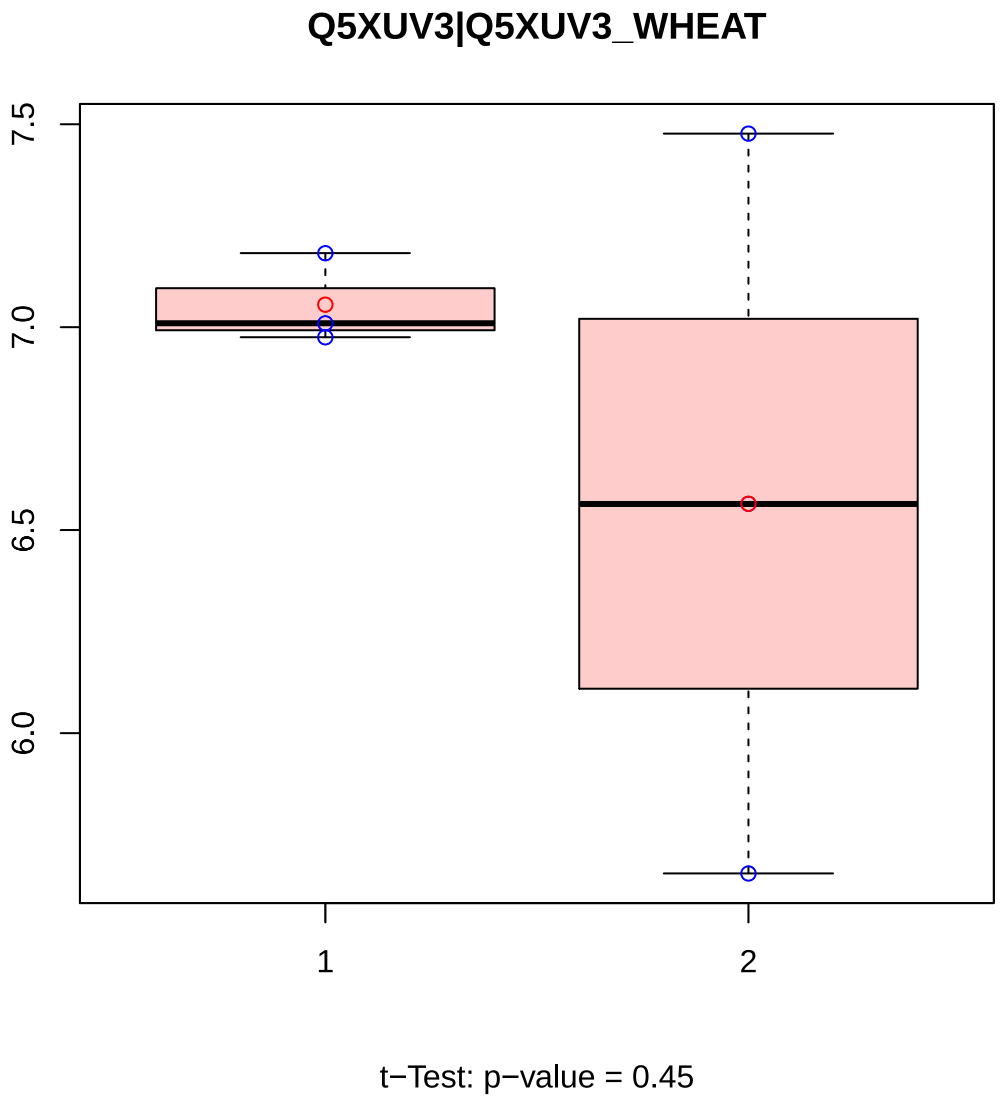
Q5XUV3|Q5XUV3_WHEAT
7.5
7.0
6.5
6.0
1
2
t−Test: p−value = 0.45

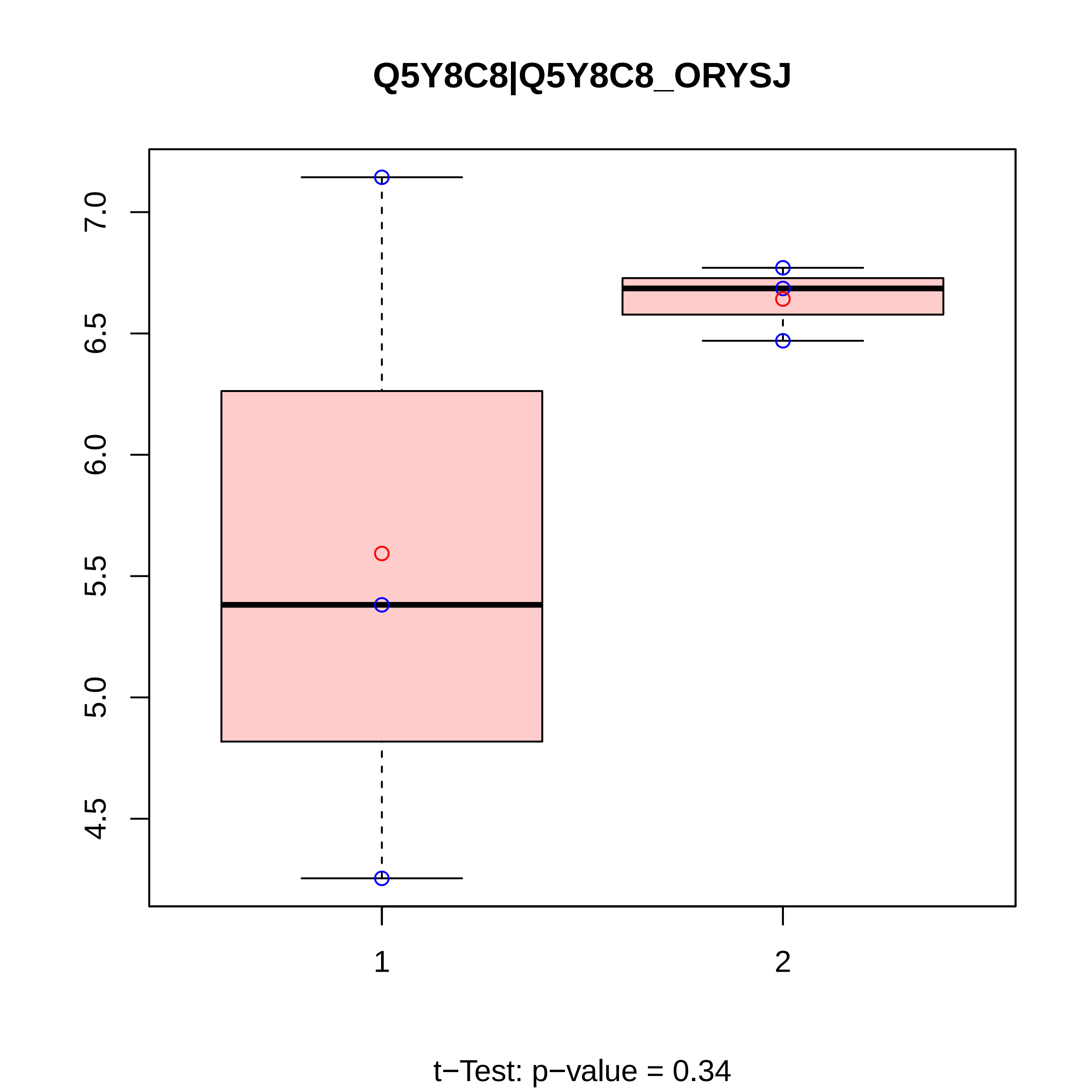
Q5Y8C8|Q5Y8C8_ORYSJ
7.0
6.5
6.0
5.5
5.0
4.5
1
2
t−Test: p−value = 0.34

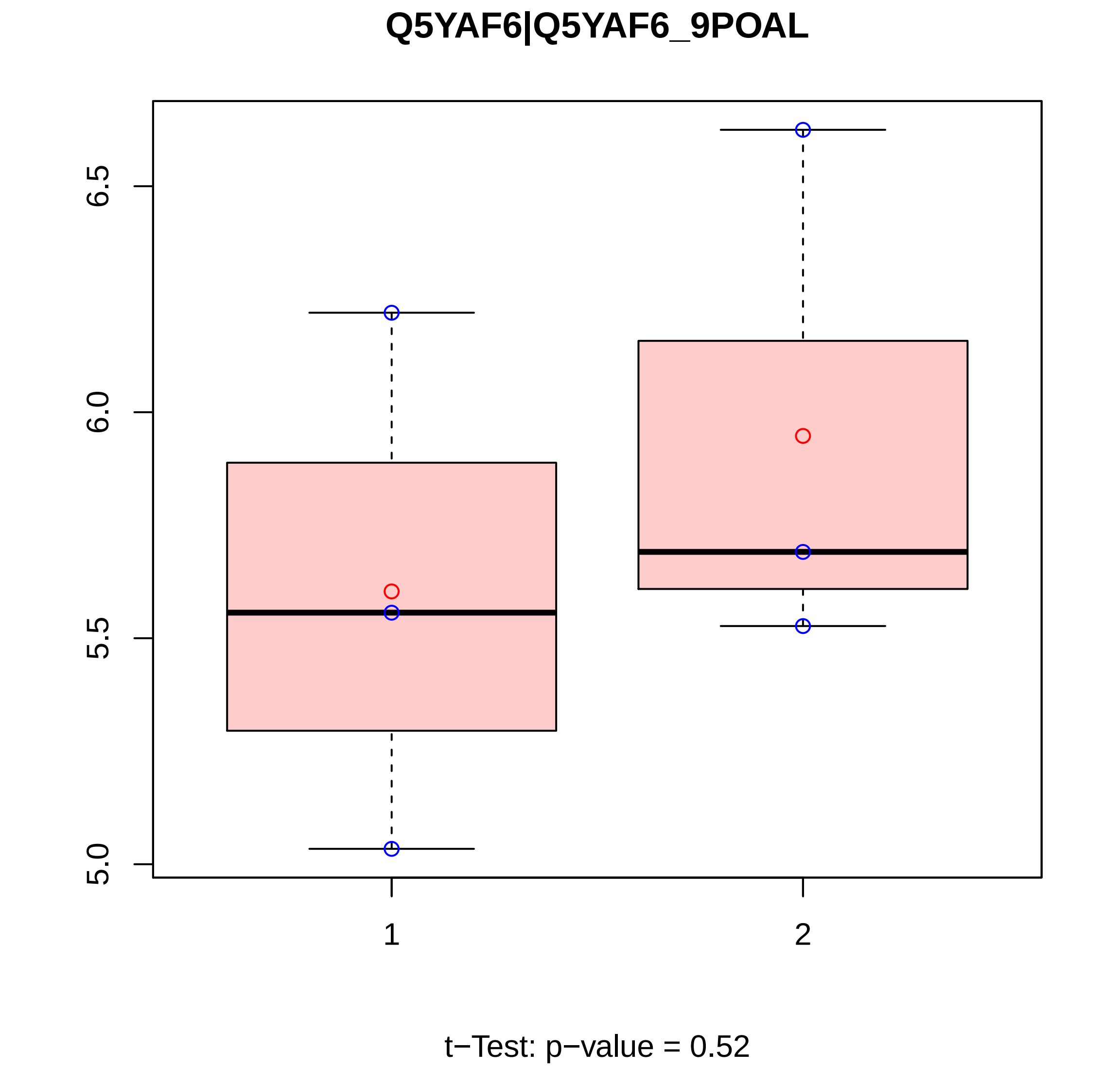
Q5YAF6|Q5YAF6_9POAL
5.0
5.5
6.0
6.5
1
2
t−Test: p−value = 0.52

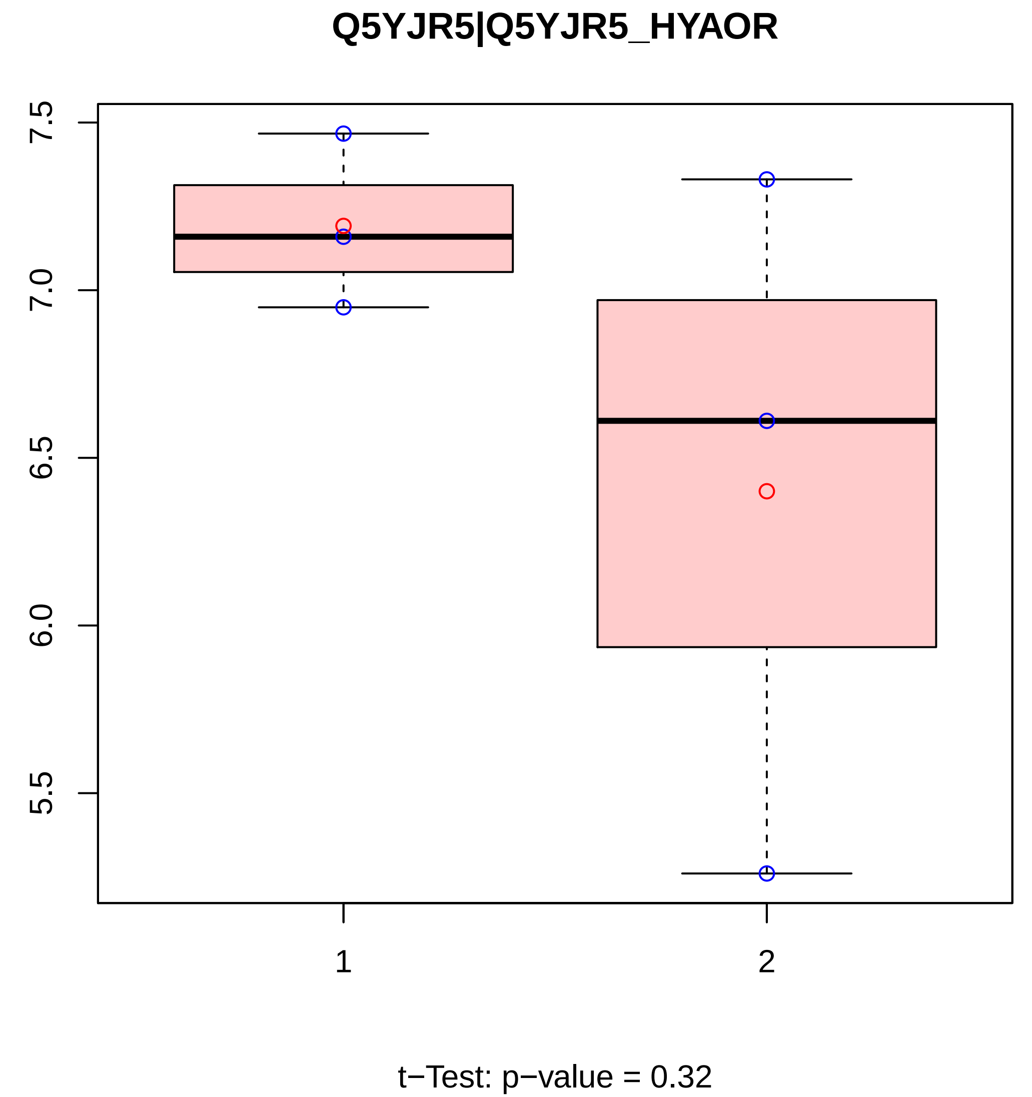
Q5YJR5|Q5YJR5_HYAOR
7.5
7.0
6.5
6.0
5.5
1
2
t−Test: p−value = 0.32

Q5ZAS4|Q5ZAS4_ORYSJ

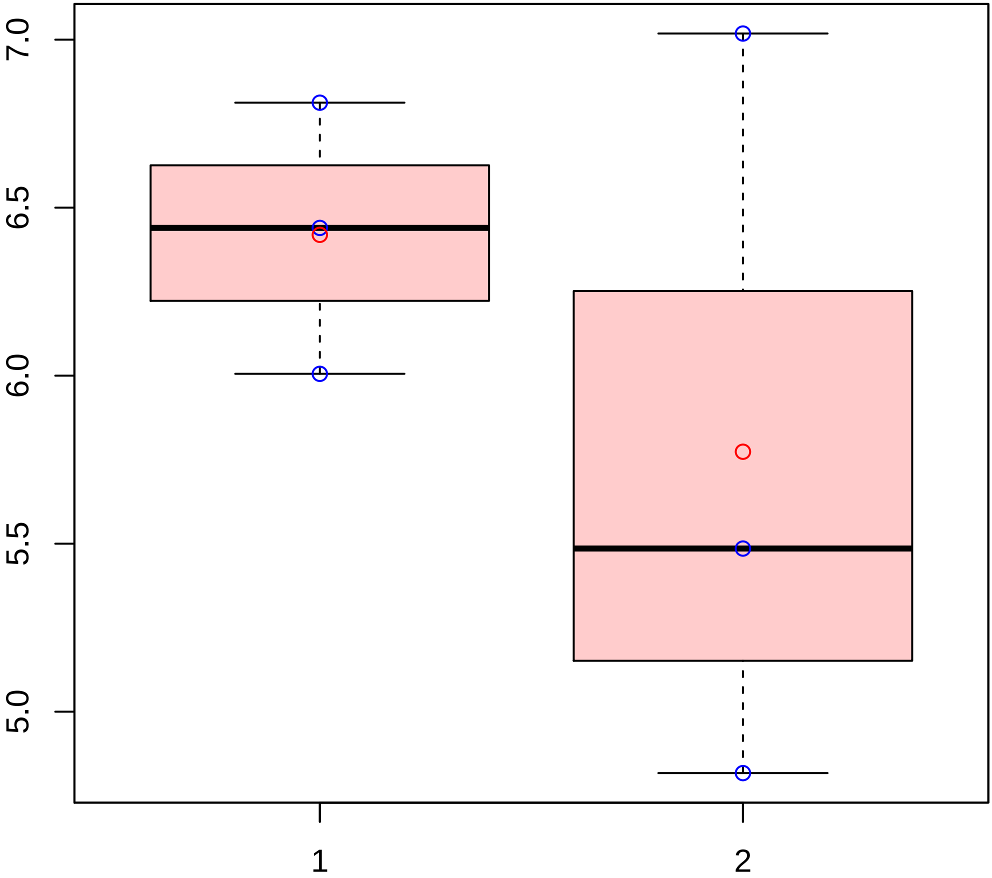

t−Test: p−value = 0.43

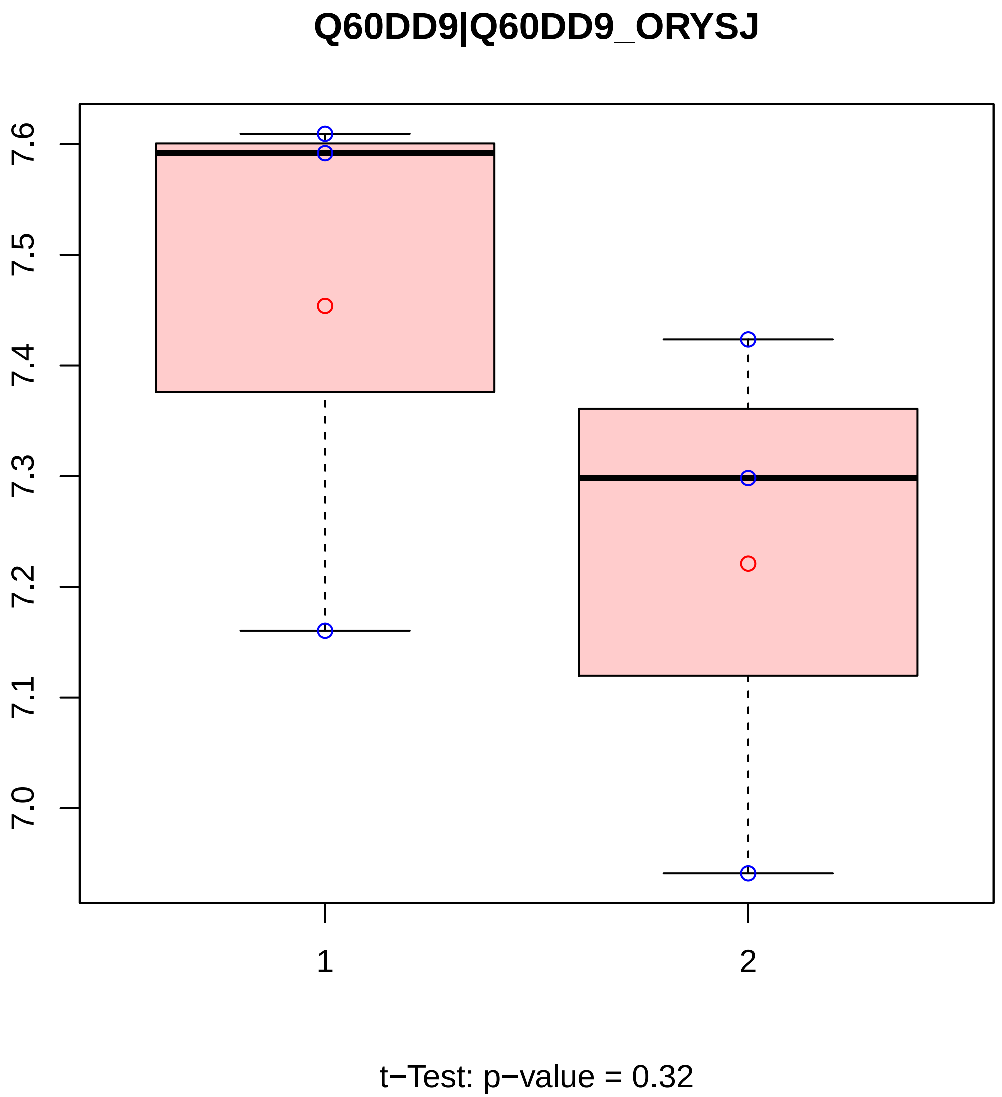

Q60DD9|Q60DD9_ORYSJ
7.0
7.1
7.2
7.3
7.4
7.5
7.6
1
2
t−Test: p−value = 0.32

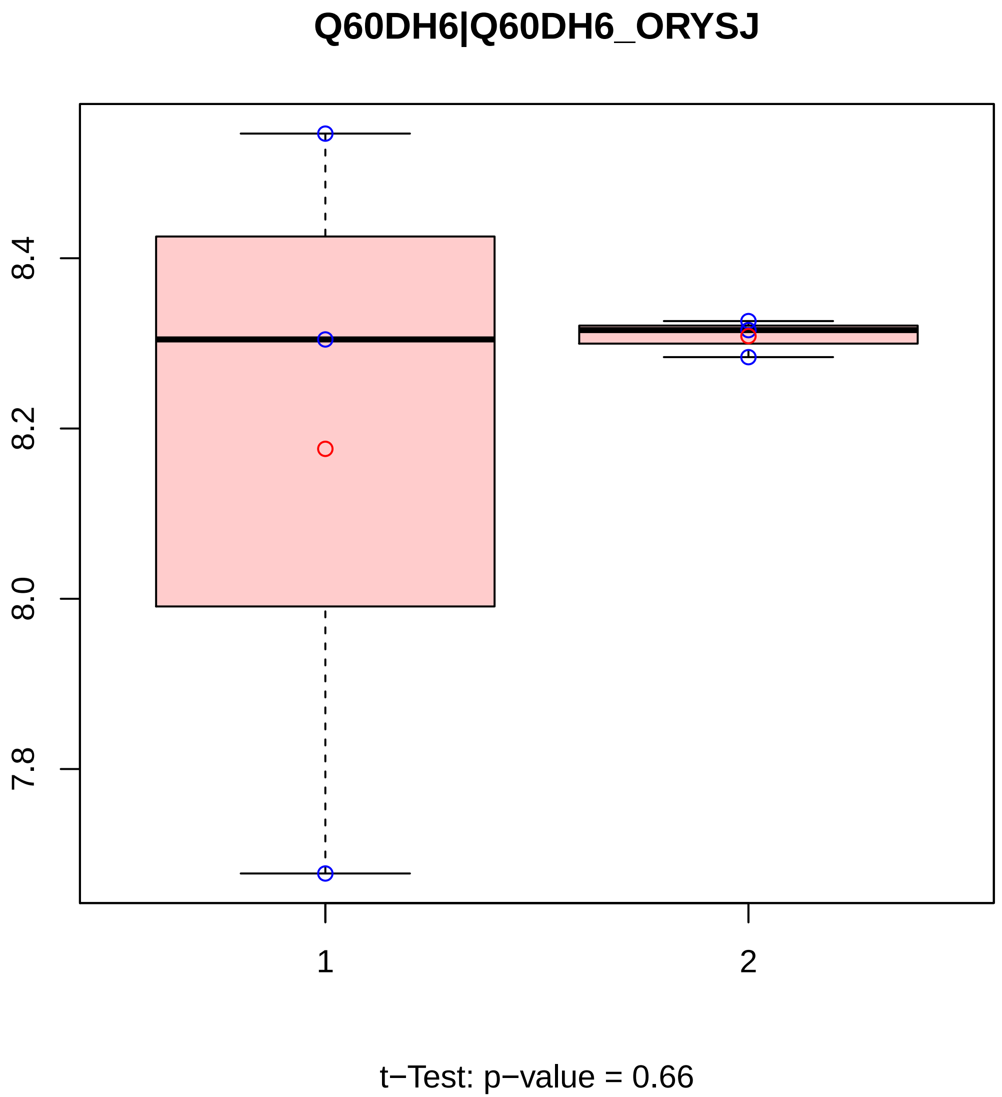
Q60DH6|Q60DH6_ORYSJ
8.4
8.2
8.0
7.8
1
2
t−Test: p−value = 0.66

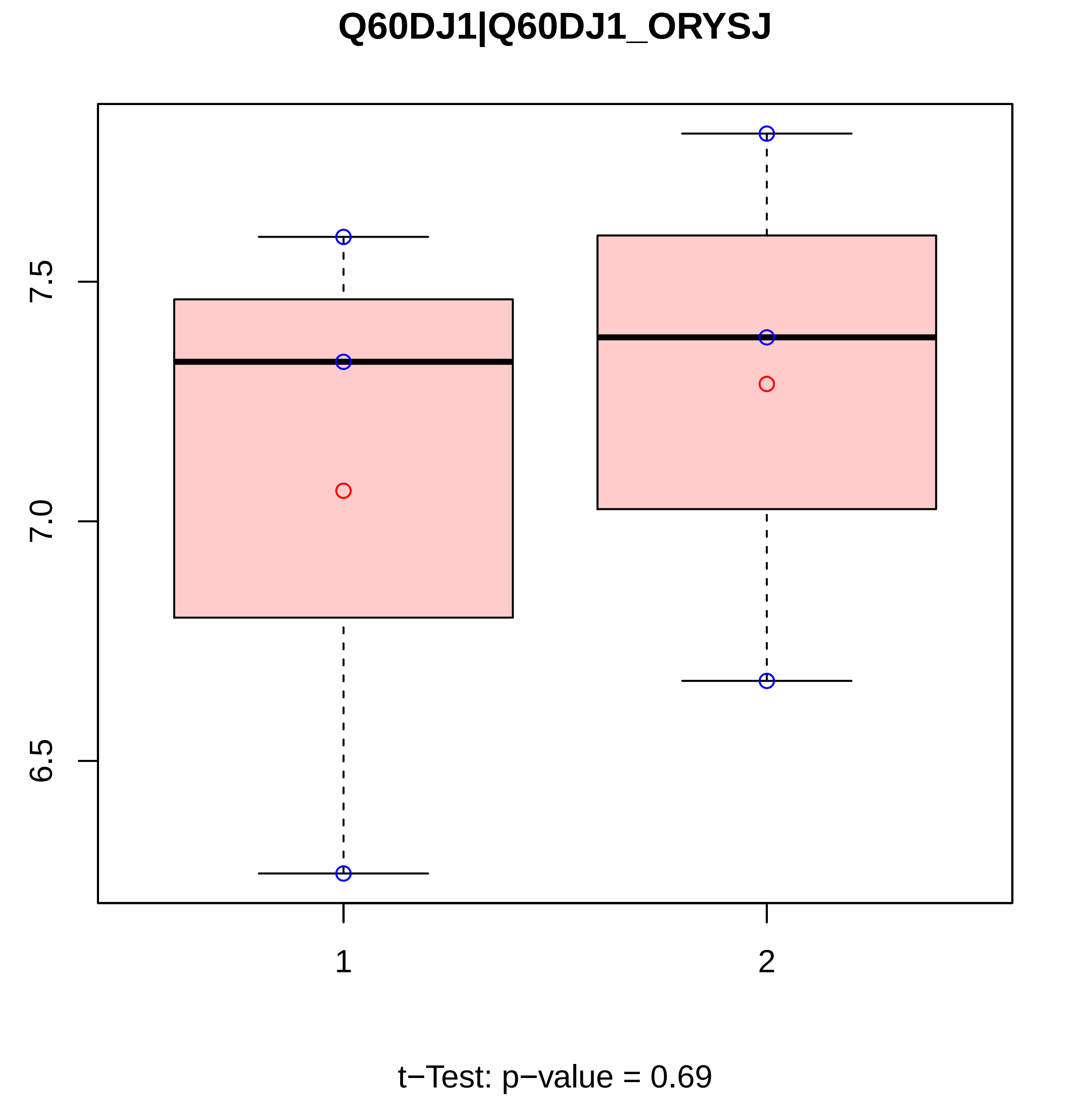

Q60DJ1|Q60DJ1_ORYSJ
7.5
7.0
6.5
1
2
t−Test: p−value = 0.69

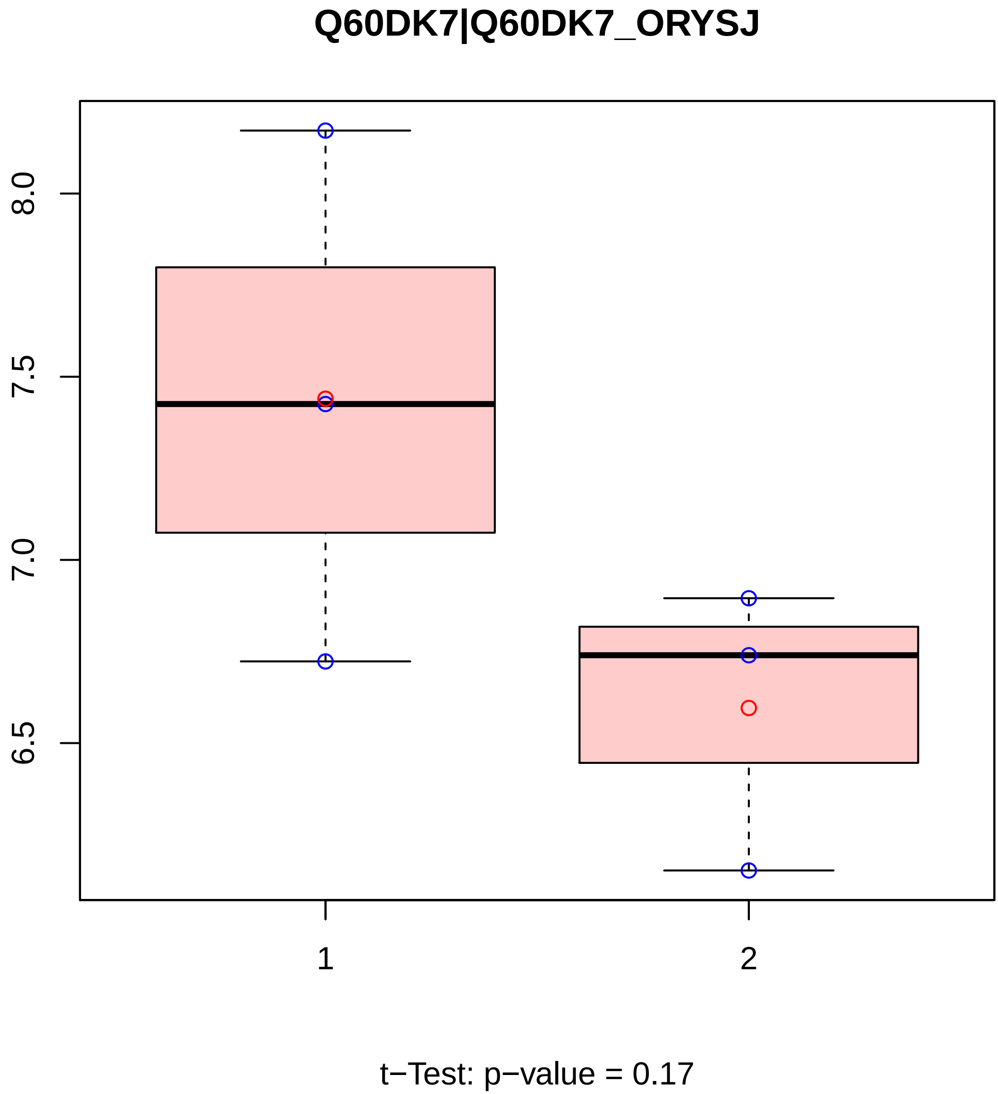

Q60DK7|Q60DK7_ORYSJ
8.0
7.5
7.0
6.5
1
2
t−Test: p−value = 0.17

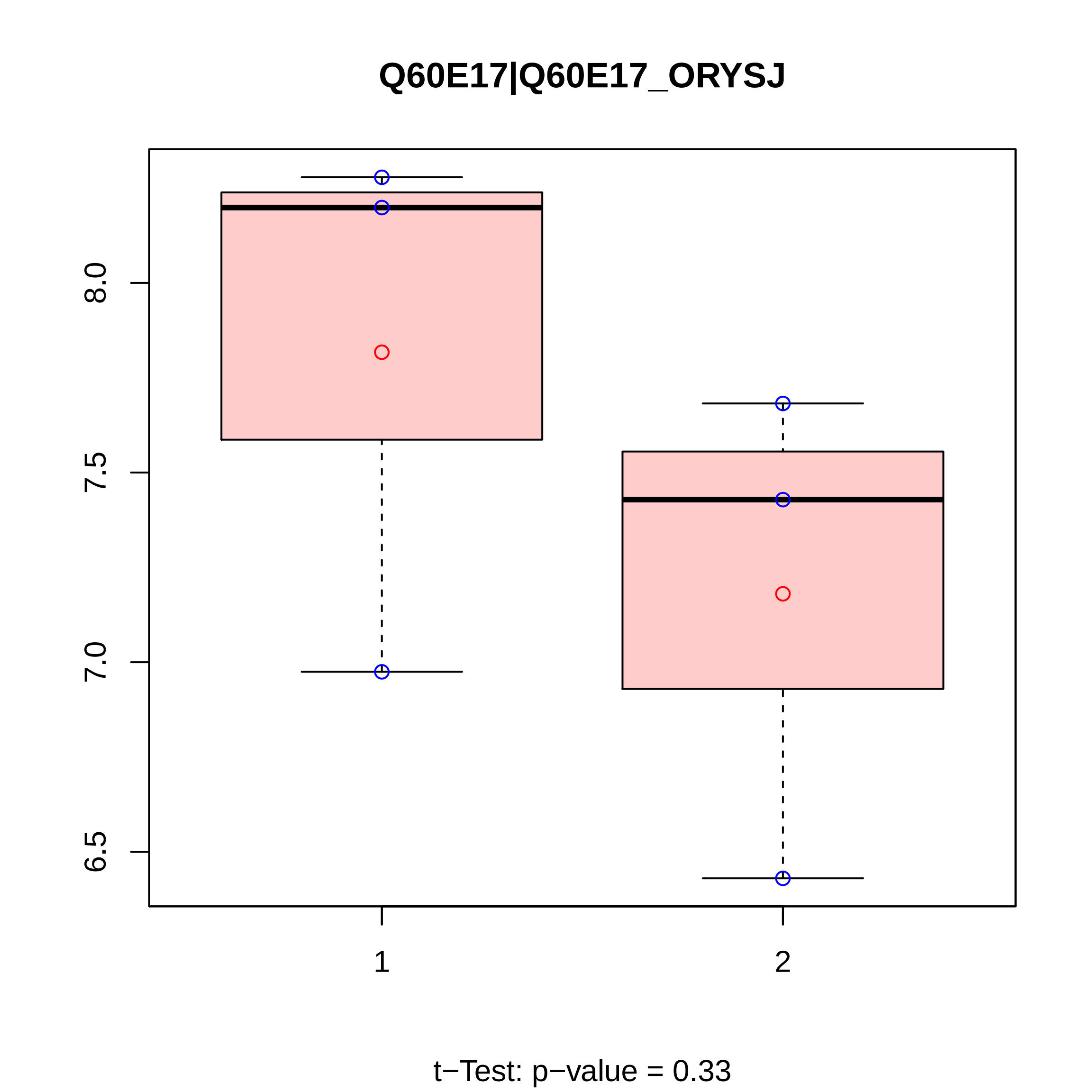

Q60E17|Q60E17_ORYSJ
8.0
7.5
7.0
6.5
1
2
t−Test: p−value = 0.33

Q60E25|Q60E25_ORYSJ

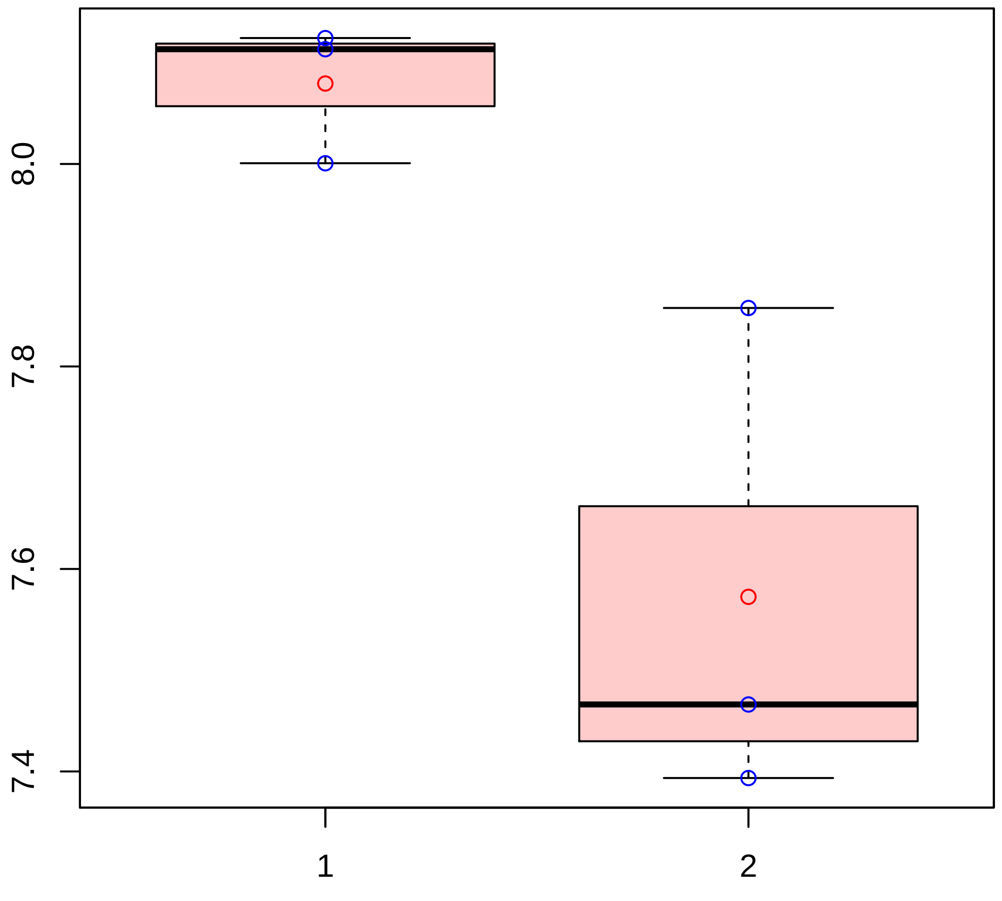

t−Test: p−value = 0.06

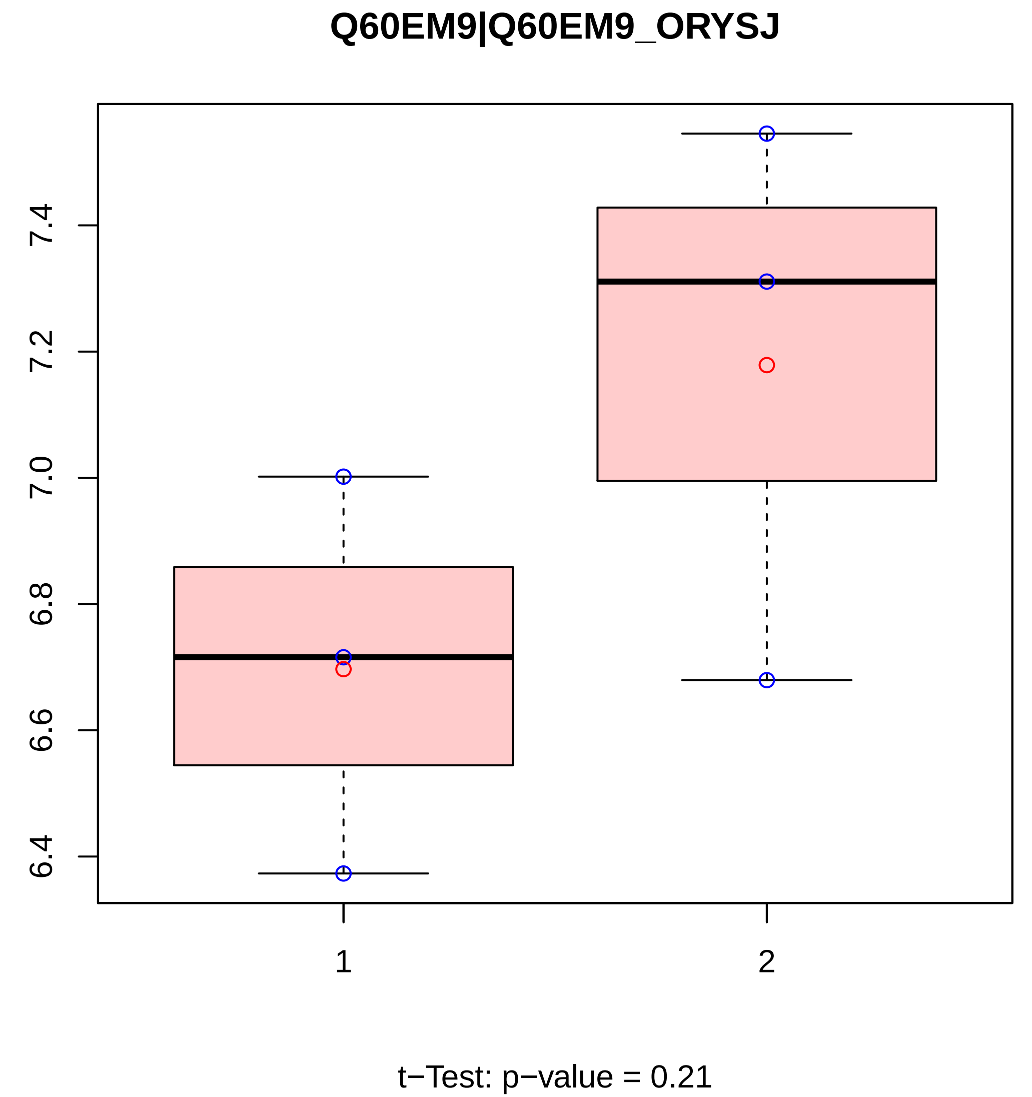

Q60EM9|Q60EM9_ORYSJ
6.4
6.6
6.8
7.0
7.2
7.4
1
2
t−Test: p−value = 0.21

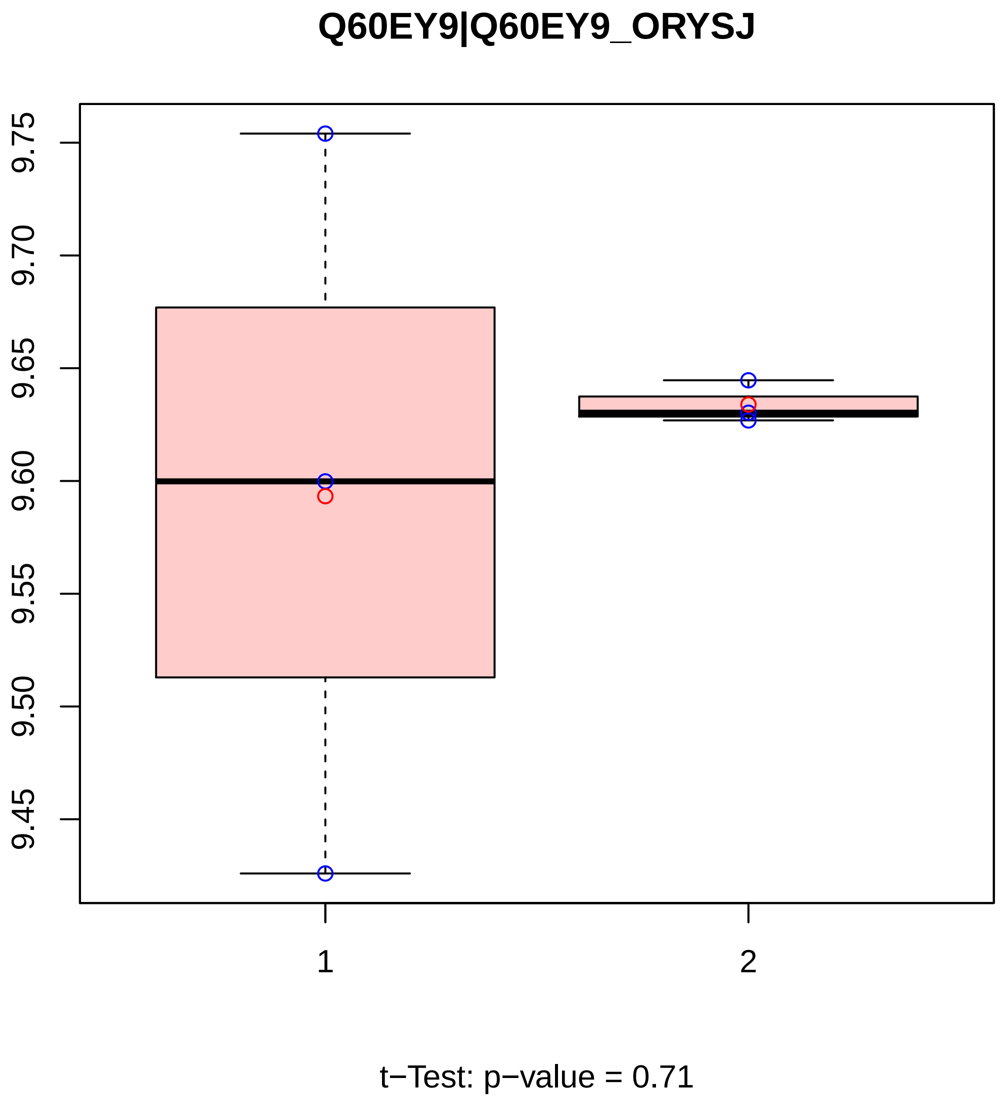

Q60EY9|Q60EY9_ORYSJ
9.75
9.70
9.65
9.60
9.55
9.50
9.45
1
2
t−Test: p−value = 0.71

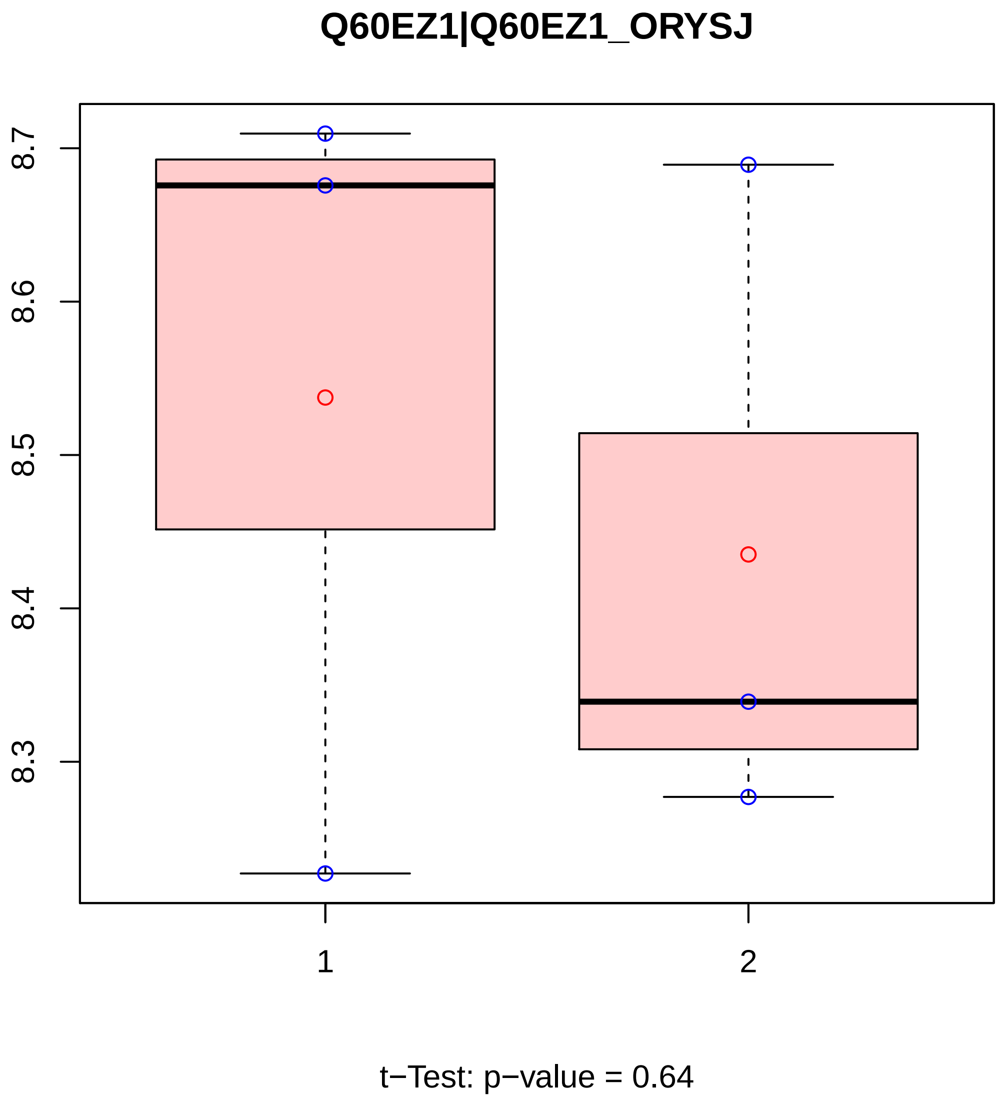
Q60EZ1|Q60EZ1_ORYSJ
8.3
8.4
8.5
8.6
8.7
1
2
t−Test: p−value = 0.64

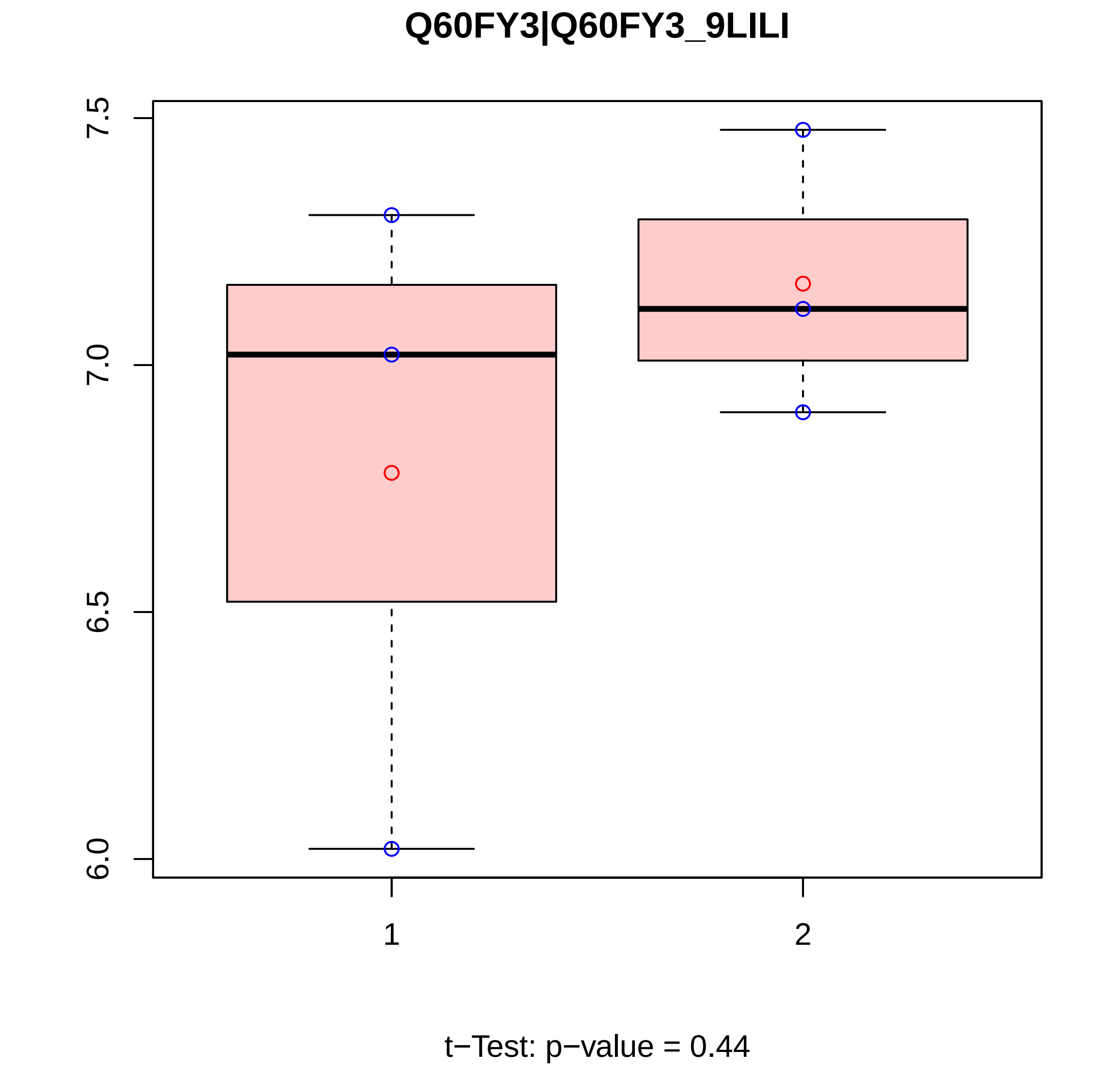

Q60FY3|Q60FY3_9LILI
7.5
7.0
6.5
6.0
1
2
t−Test: p−value = 0.44

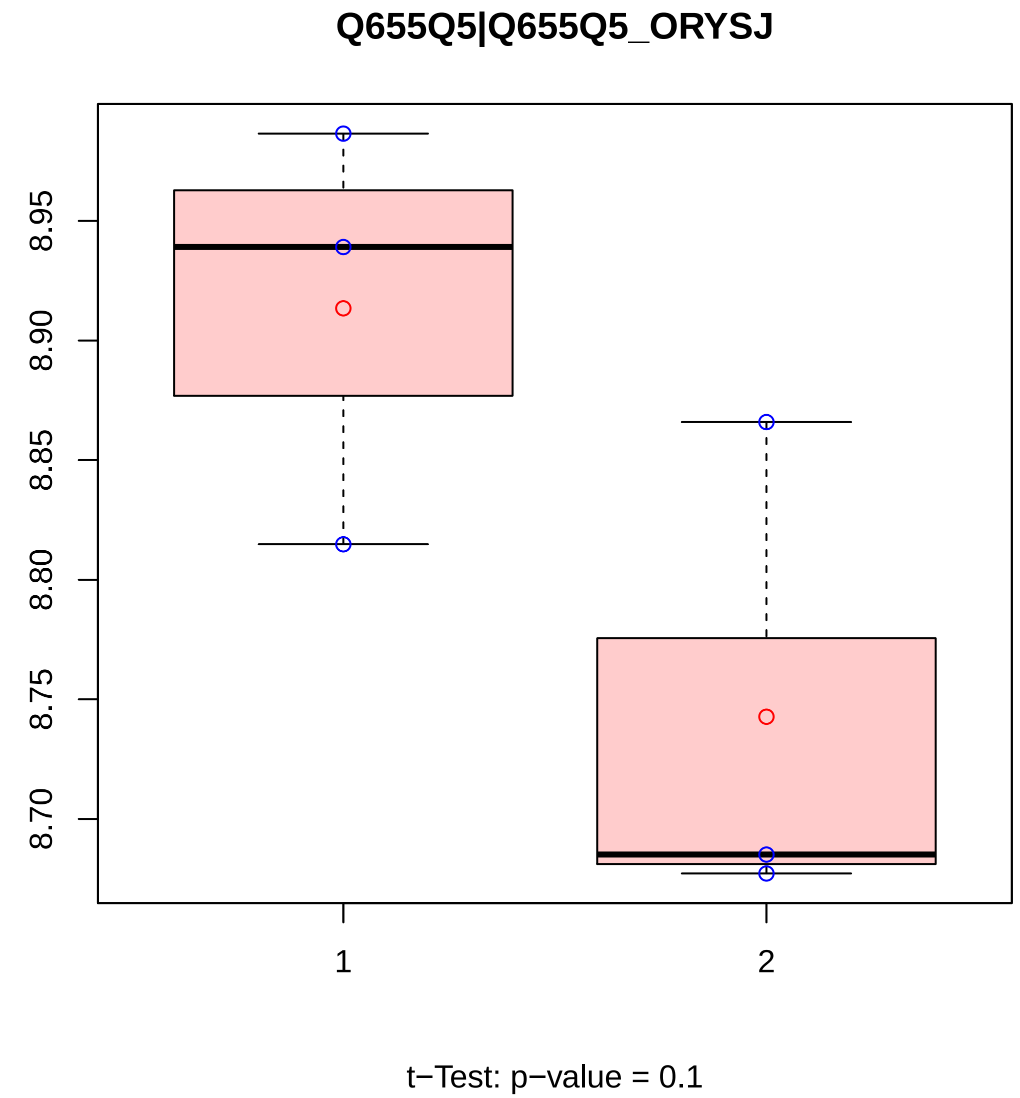
Q655Q5|Q655Q5_ORYSJ
8.70
8.75
8.80
8.85
8.90
8.95
1
2
t−Test: p−value = 0.1

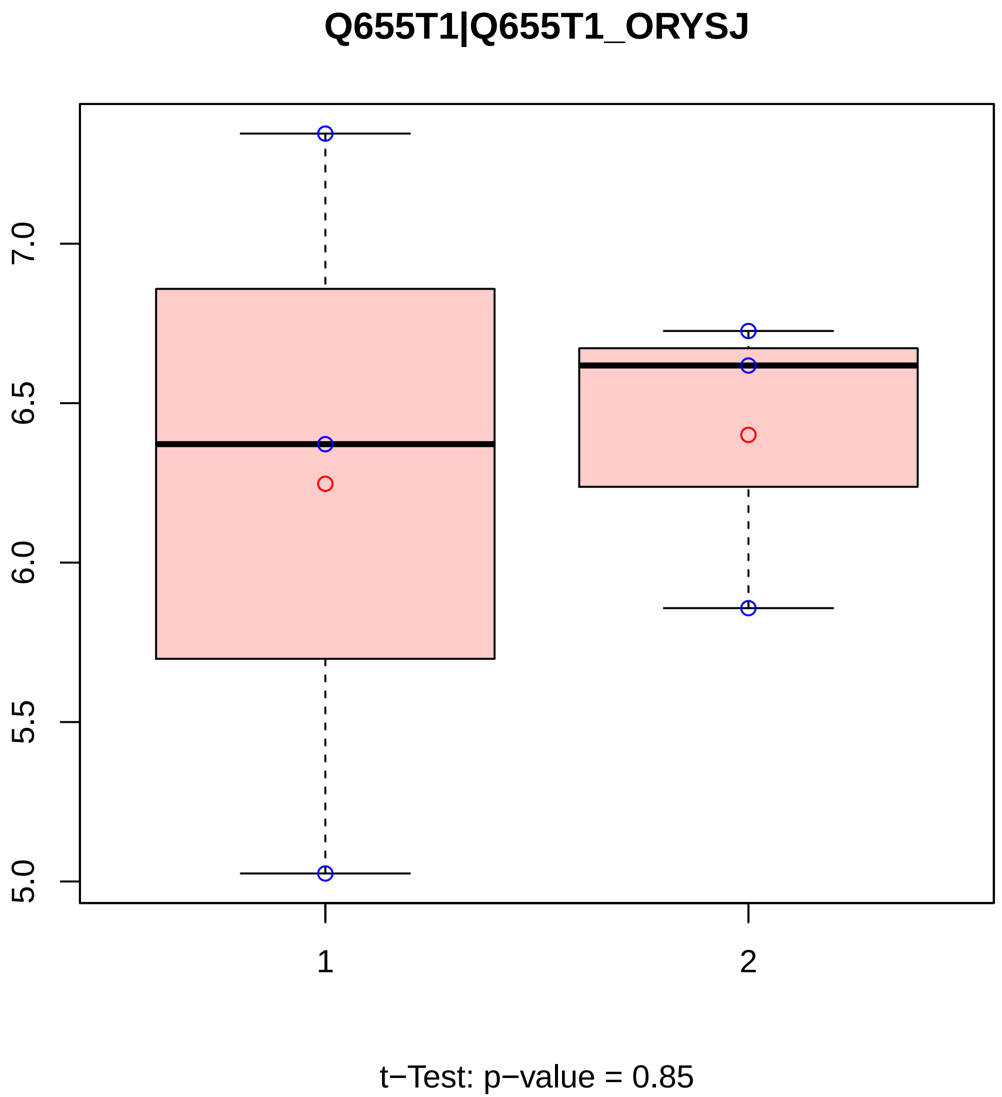
Q655T1|Q655T1_ORYSJ
5.0
5.5
6.0
6.5
7.0
1
2
t−Test: p−value = 0.85

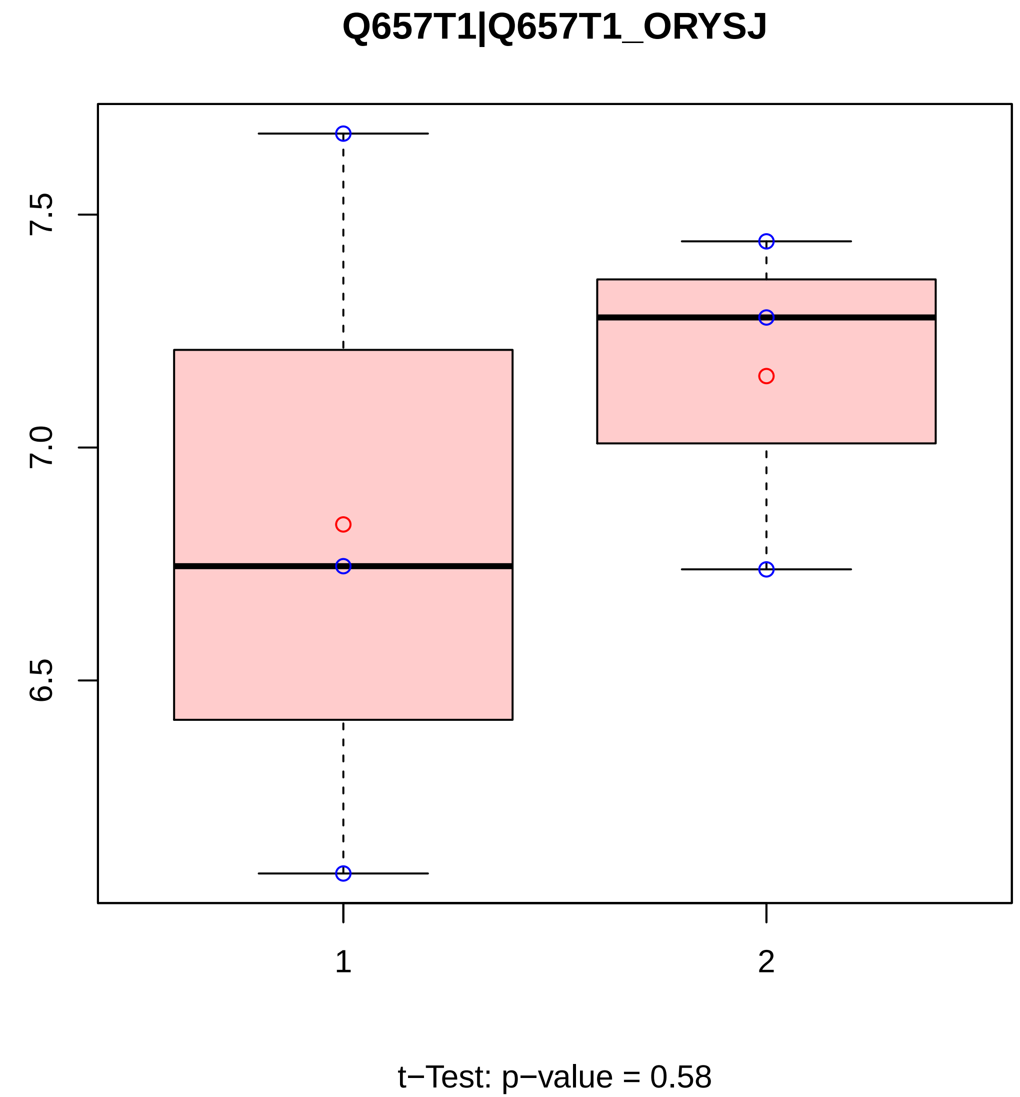
Q657T1|Q657T1_ORYSJ
7.5
7.0
6.5
1
2
t−Test: p−value = 0.58

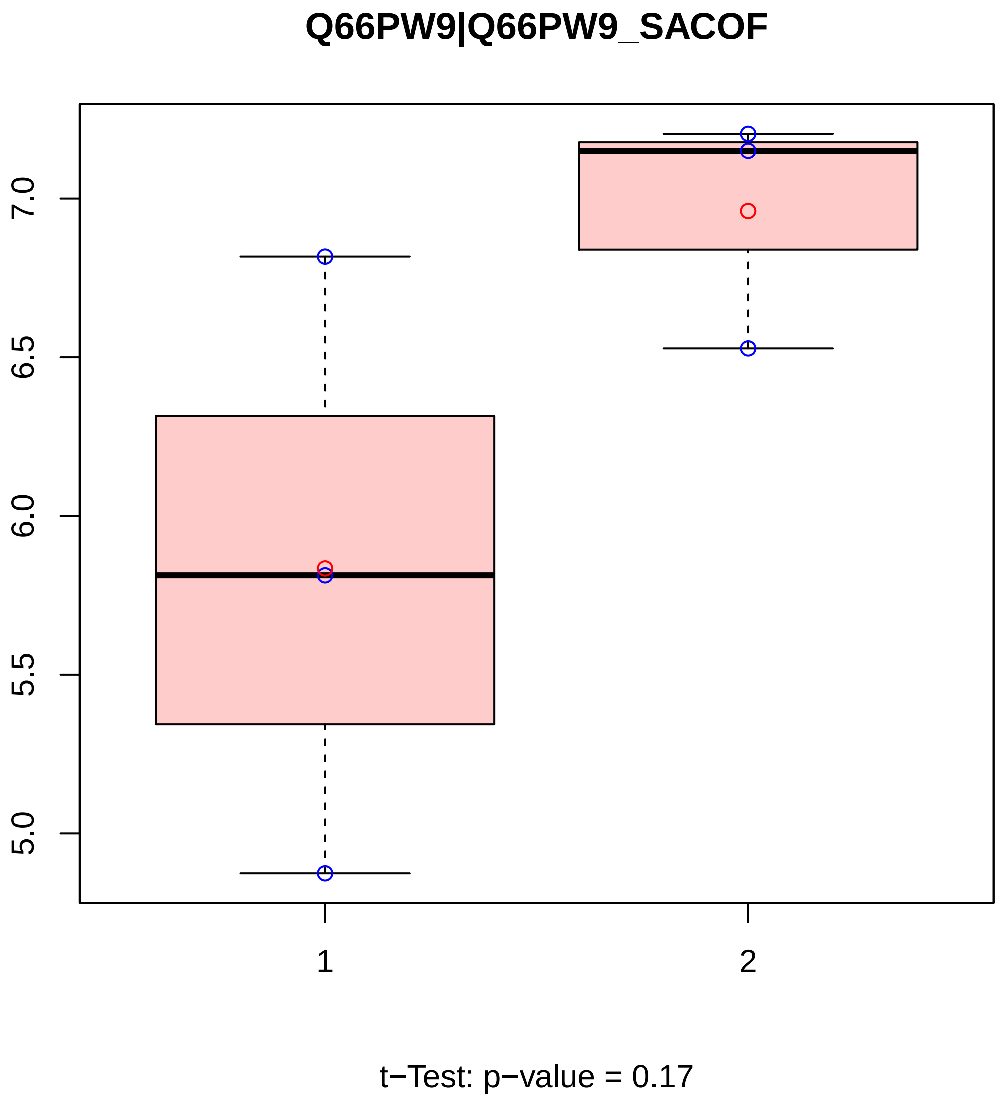
Q66PW9|Q66PW9_SACOF
7.0
6.5
6.0
5.5
5.0
1
2
t−Test: p−value = 0.17

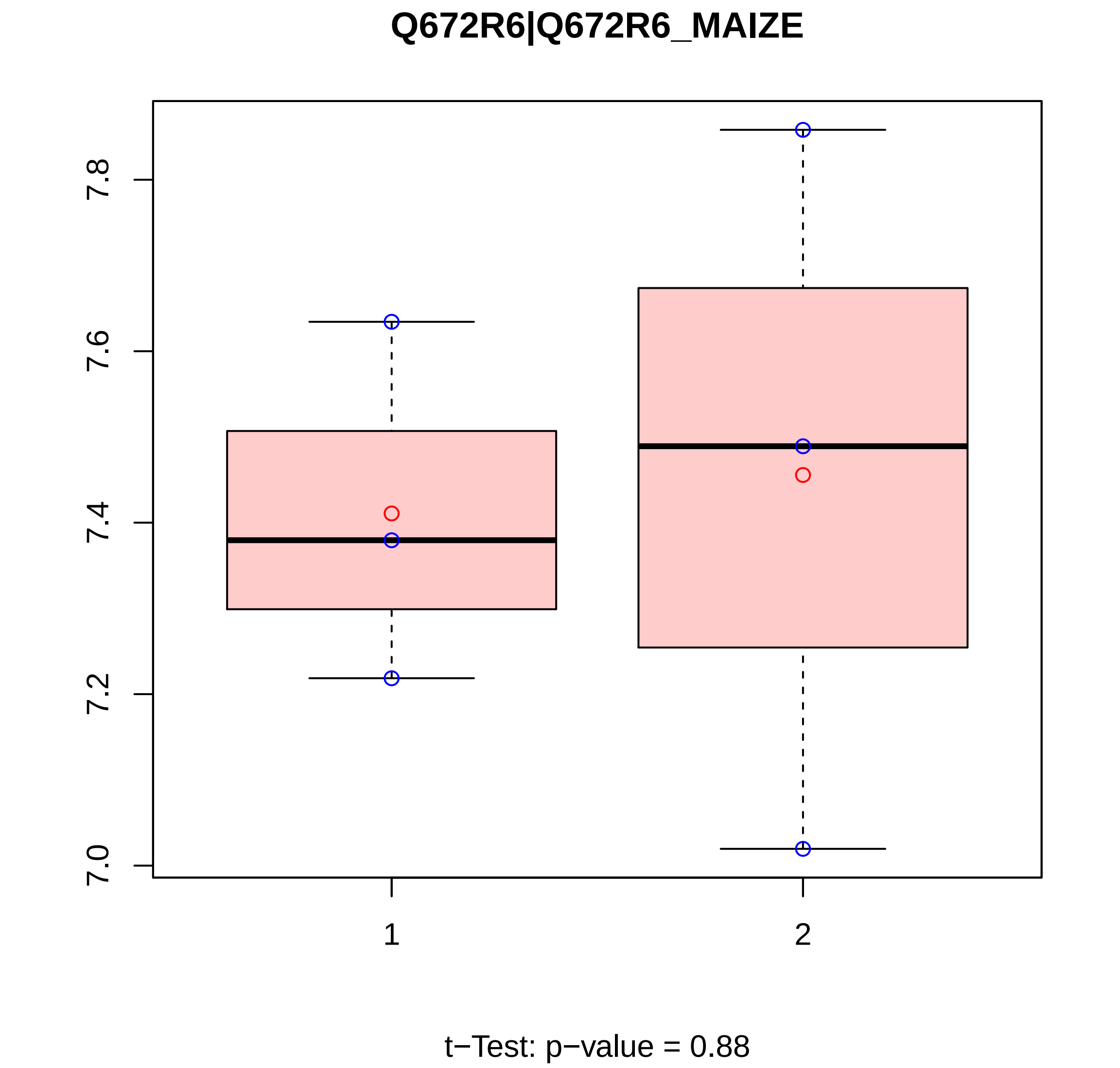
Q672R6|Q672R6_MAIZE
7.0
7.2
7.4
7.6
7.8
1
2
t−Test: p−value = 0.88

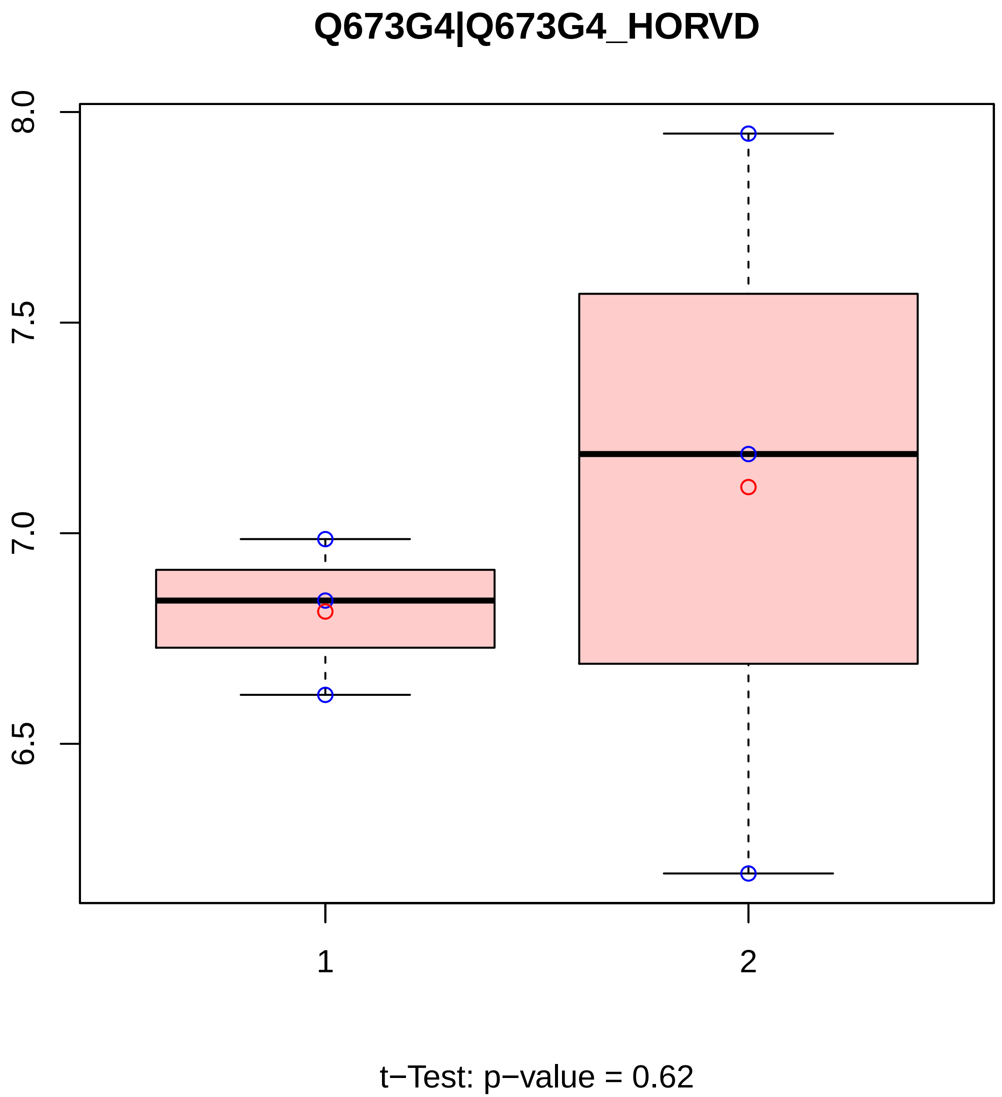

Q673G4|Q673G4_HORVD
8.0
7.5
7.0
6.5
1
2
t−Test: p−value = 0.62

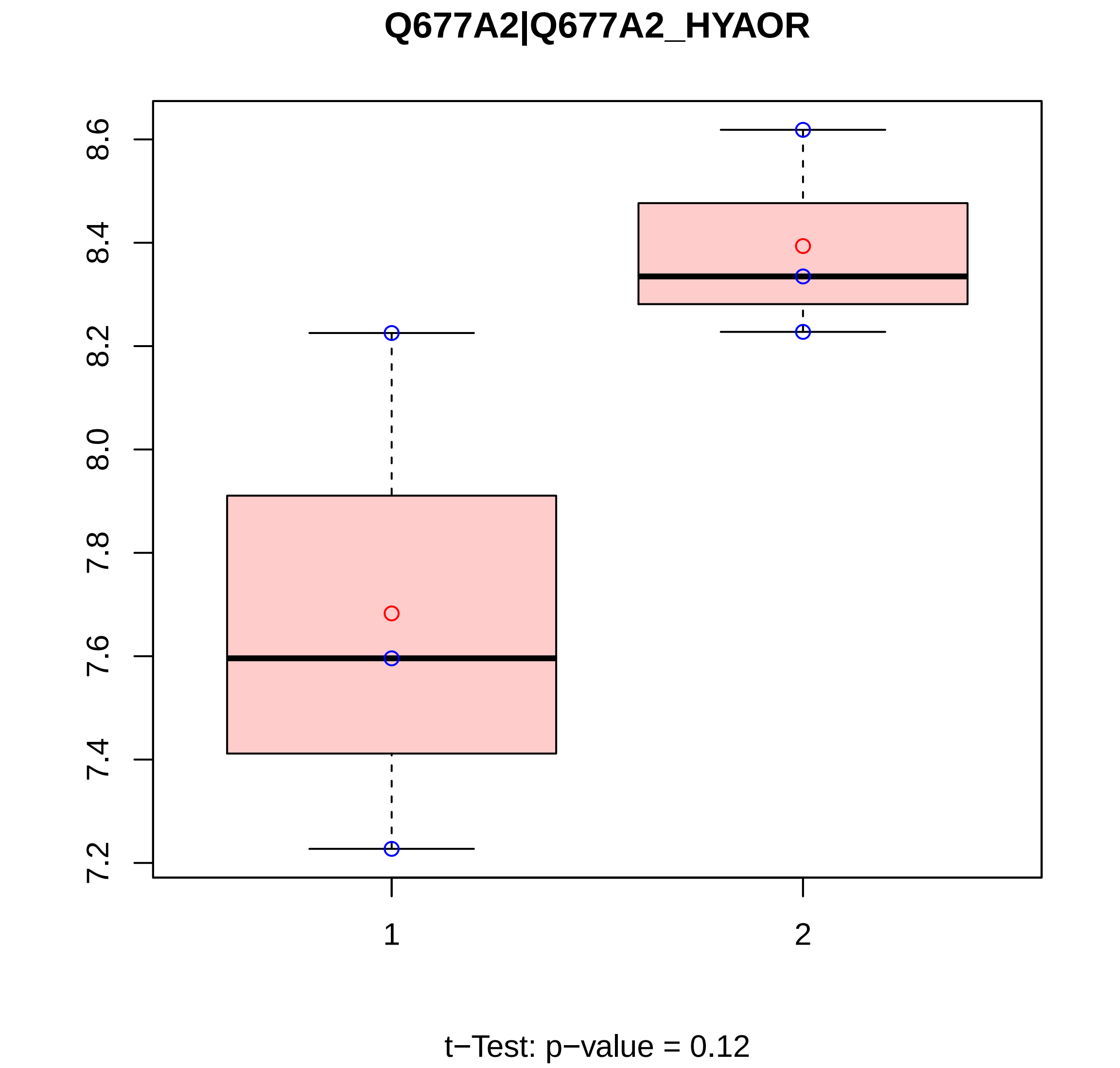
Q677A2|Q677A2_HYAOR
8.6
8.4
8.2
8.0
7.8
7.6
7.4
7.2
1
2
t−Test: p−value = 0.12

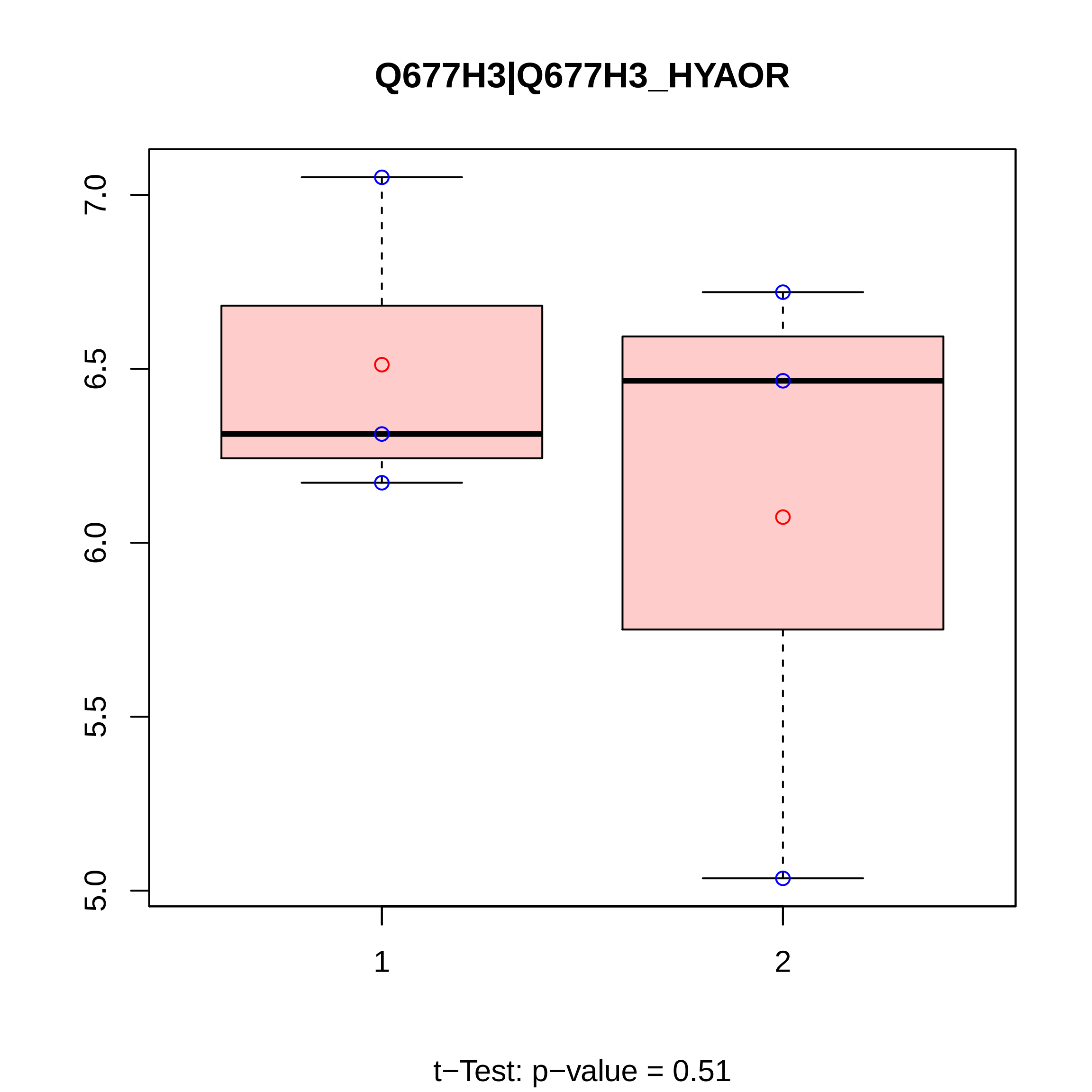
Q677H3|Q677H3_HYAOR
7.0
6.5
6.0
5.5
5.0
1
2
t−Test: p−value = 0.51

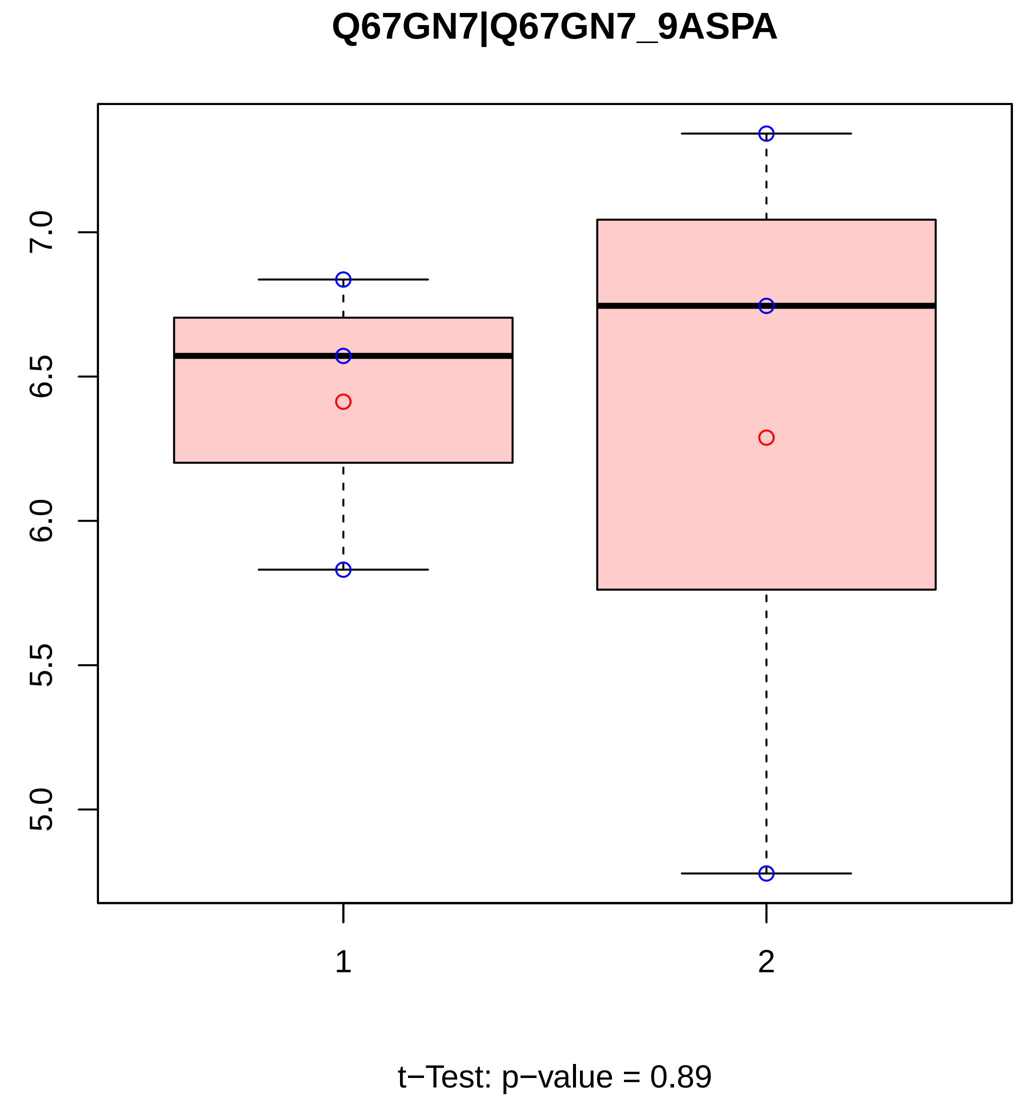

Q67GN7|Q67GN7_9ASPA
7.0
6.5
6.0
5.5
5.0
1
2
t−Test: p−value = 0.89

Q67VZ1|Q67VZ1_ORYSJ

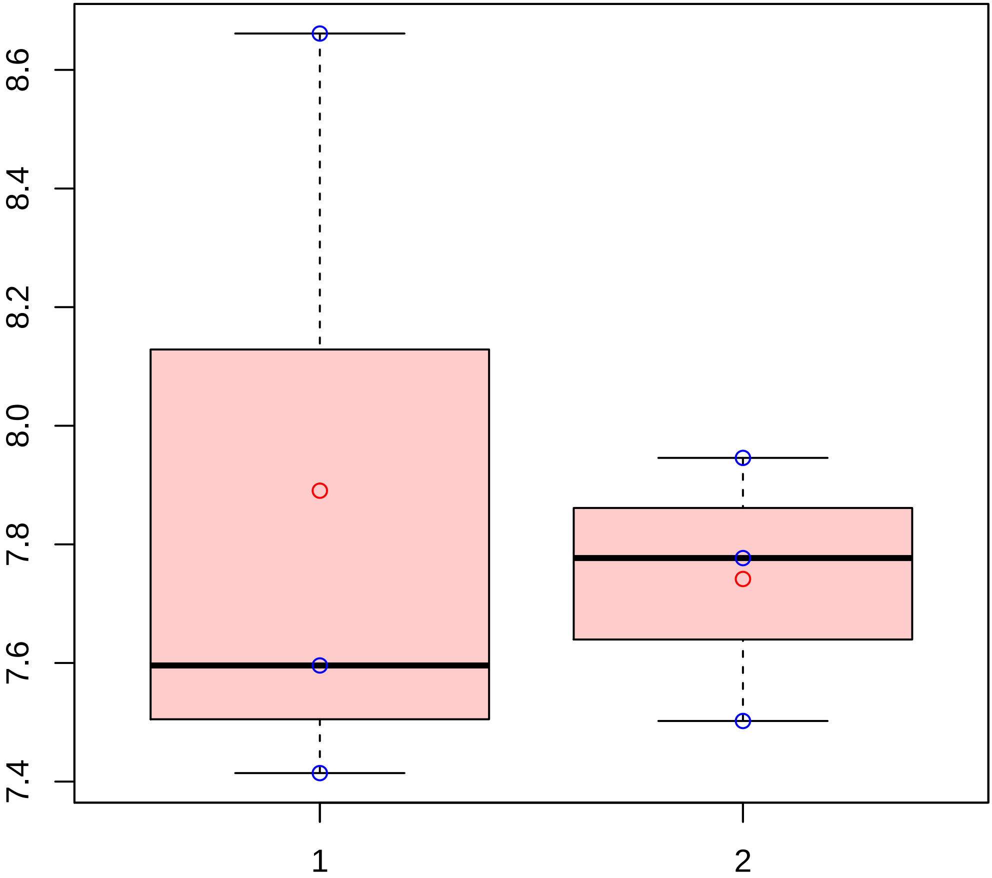

t−Test: p−value = 0.75

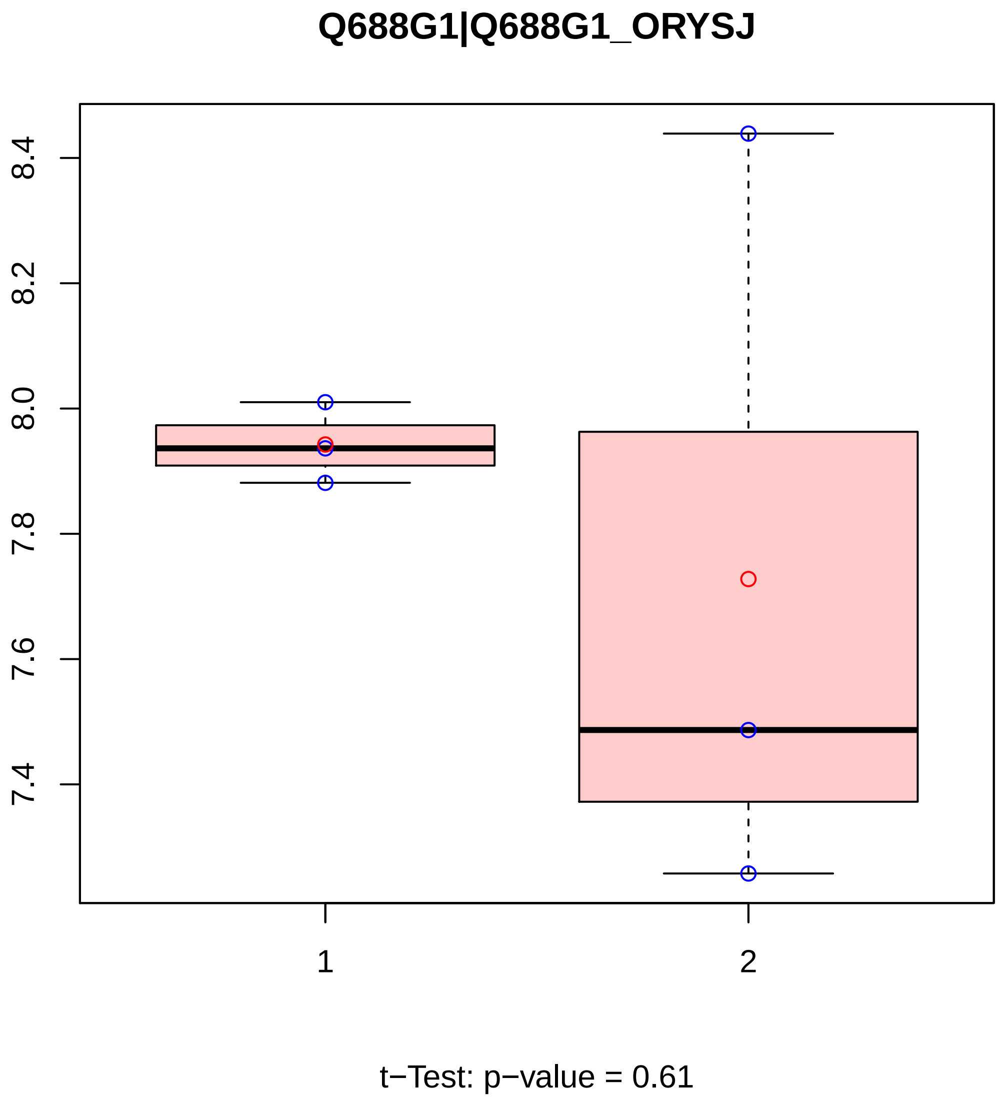
Q688G1|Q688G1_ORYSJ
8.4
8.2
8.0
7.8
7.6
7.4
1
2
t−Test: p−value = 0.61

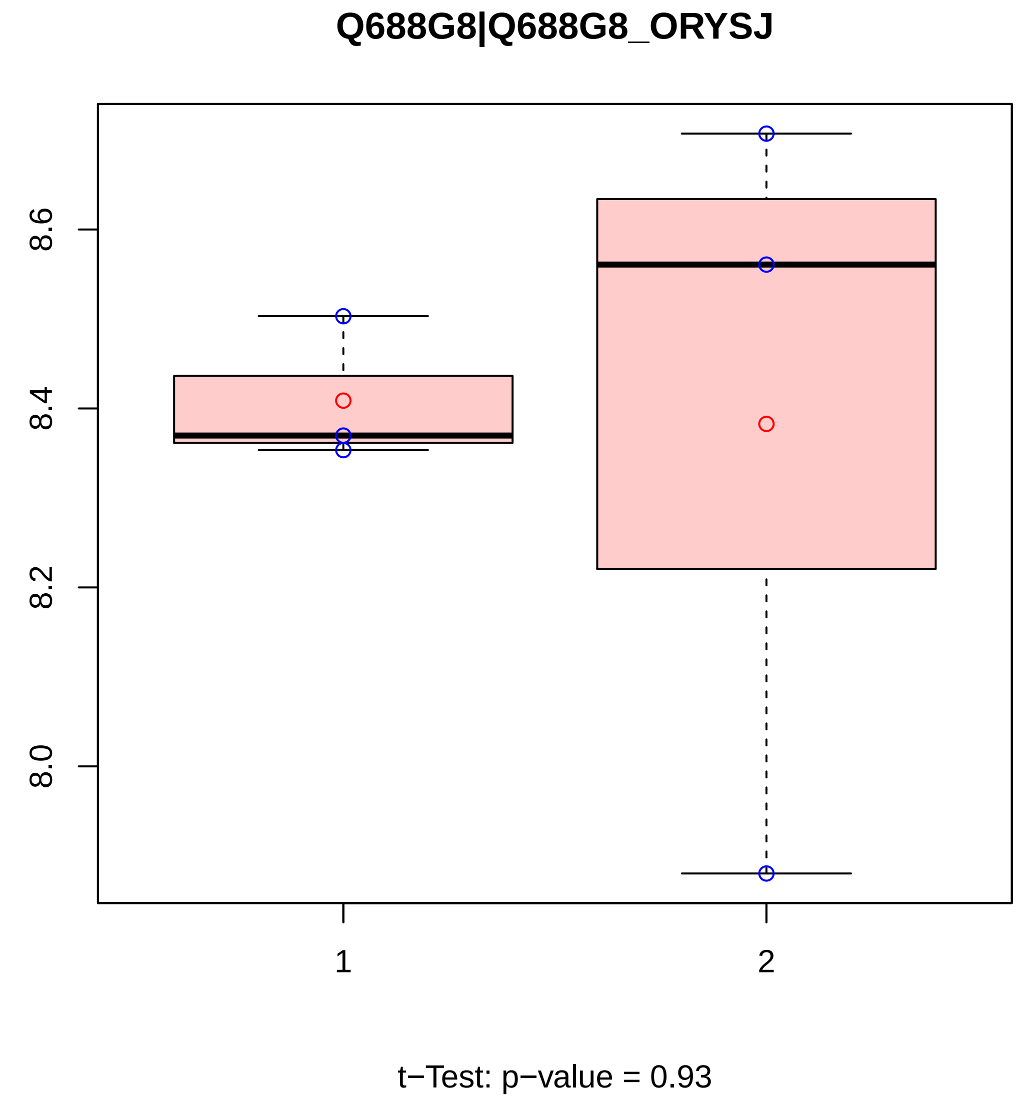
Q688G8|Q688G8_ORYSJ
8.0
8.2
8.4
8.6
1
2
t−Test: p−value = 0.93

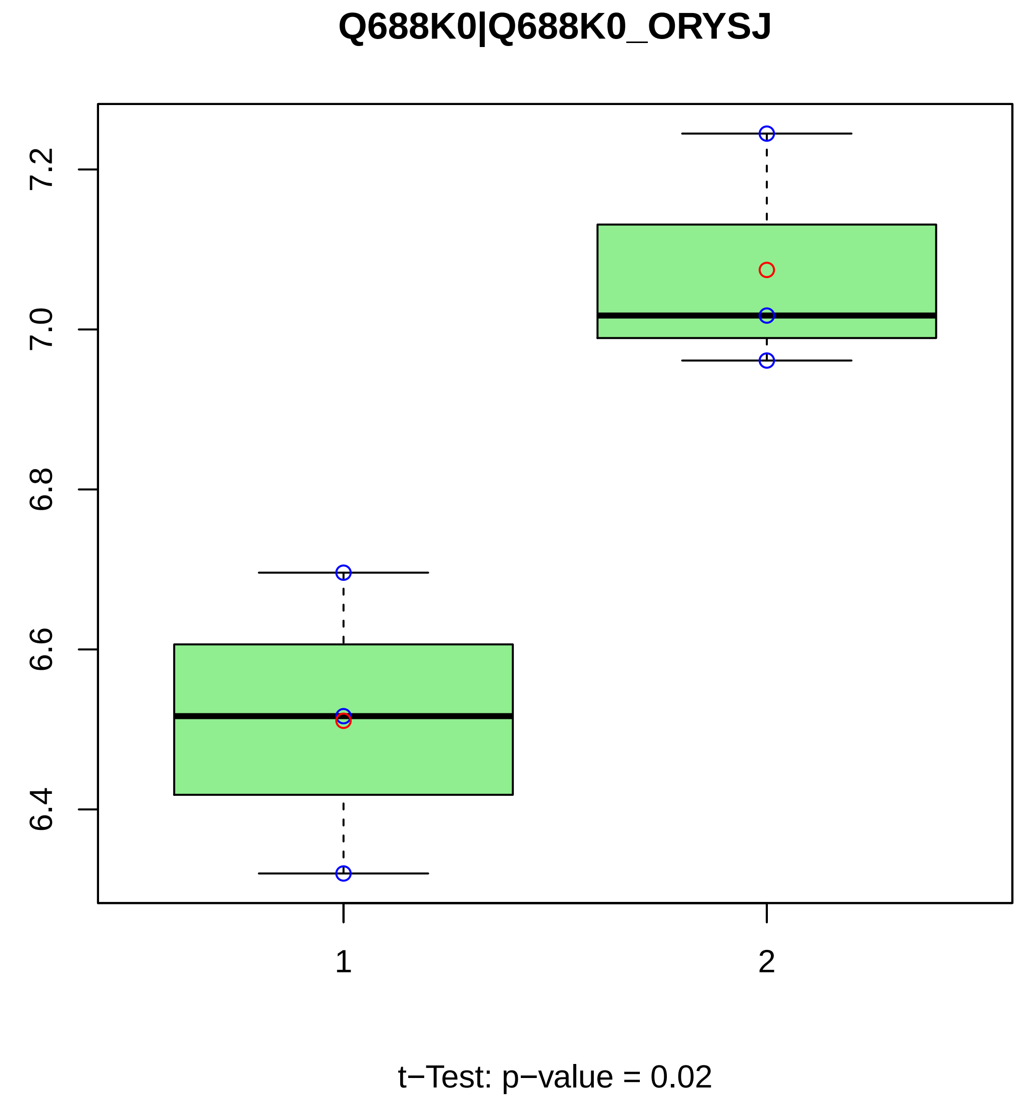

Q688K0|Q688K0_ORYSJ
7.2
7.0
6.8
6.6
6.4
1
2
t−Test: p−value = 0.02

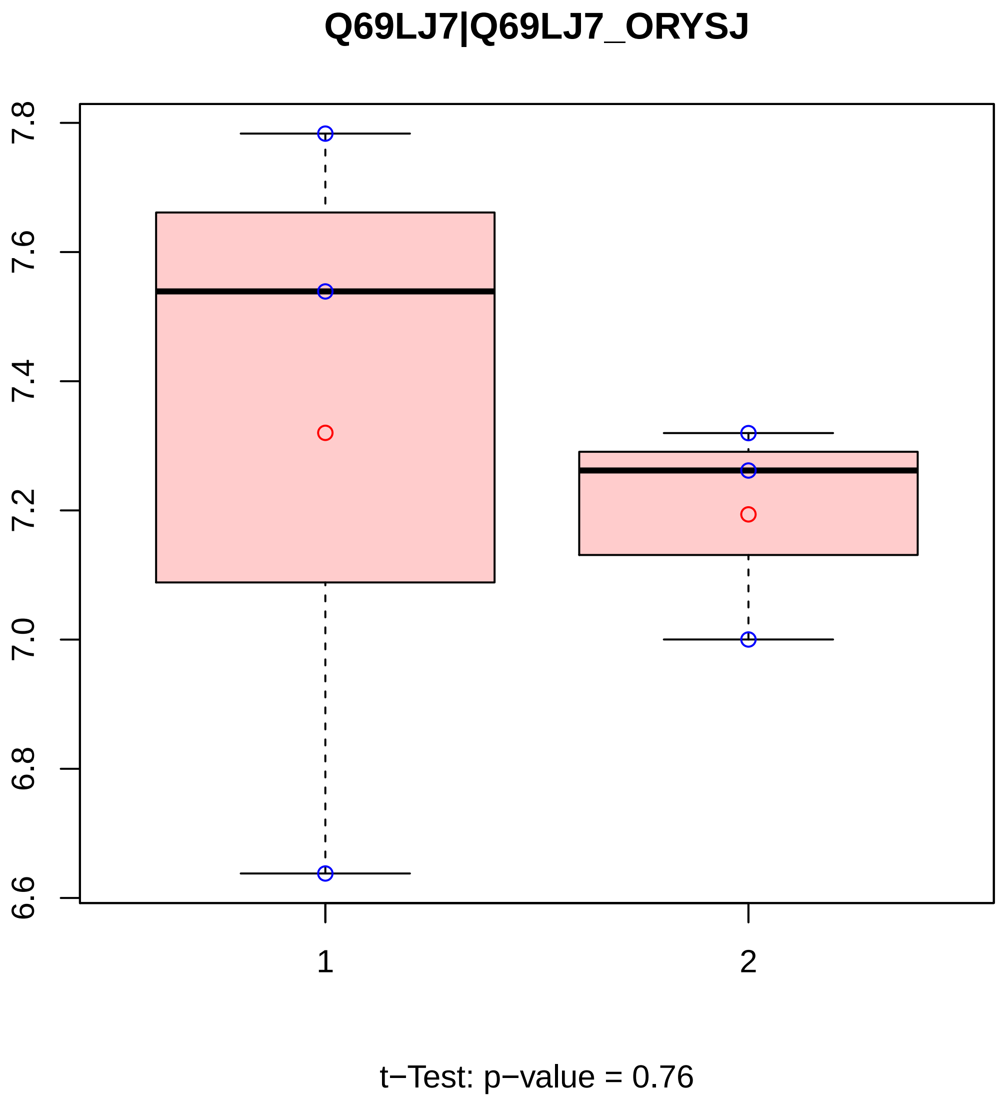
Q69LJ7|Q69LJ7_ORYSJ
7.8
7.6
7.4
7.2
7.0
6.8
6.6
1
2
t−Test: p−value = 0.76

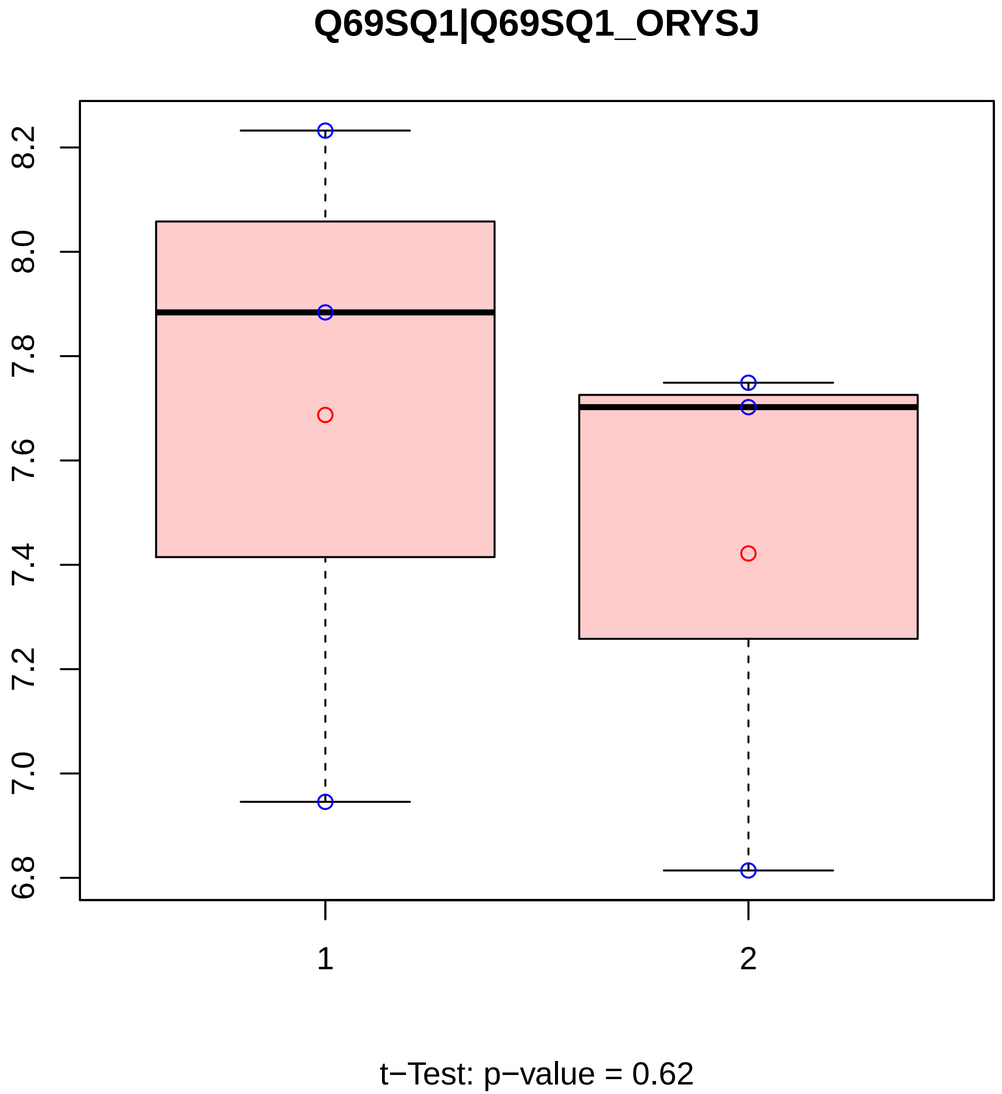
Q69SQ1|Q69SQ1_ORYSJ
8.2
8.0
7.8
7.6
7.4
7.2
7.0
6.8
1
2
t−Test: p−value = 0.62

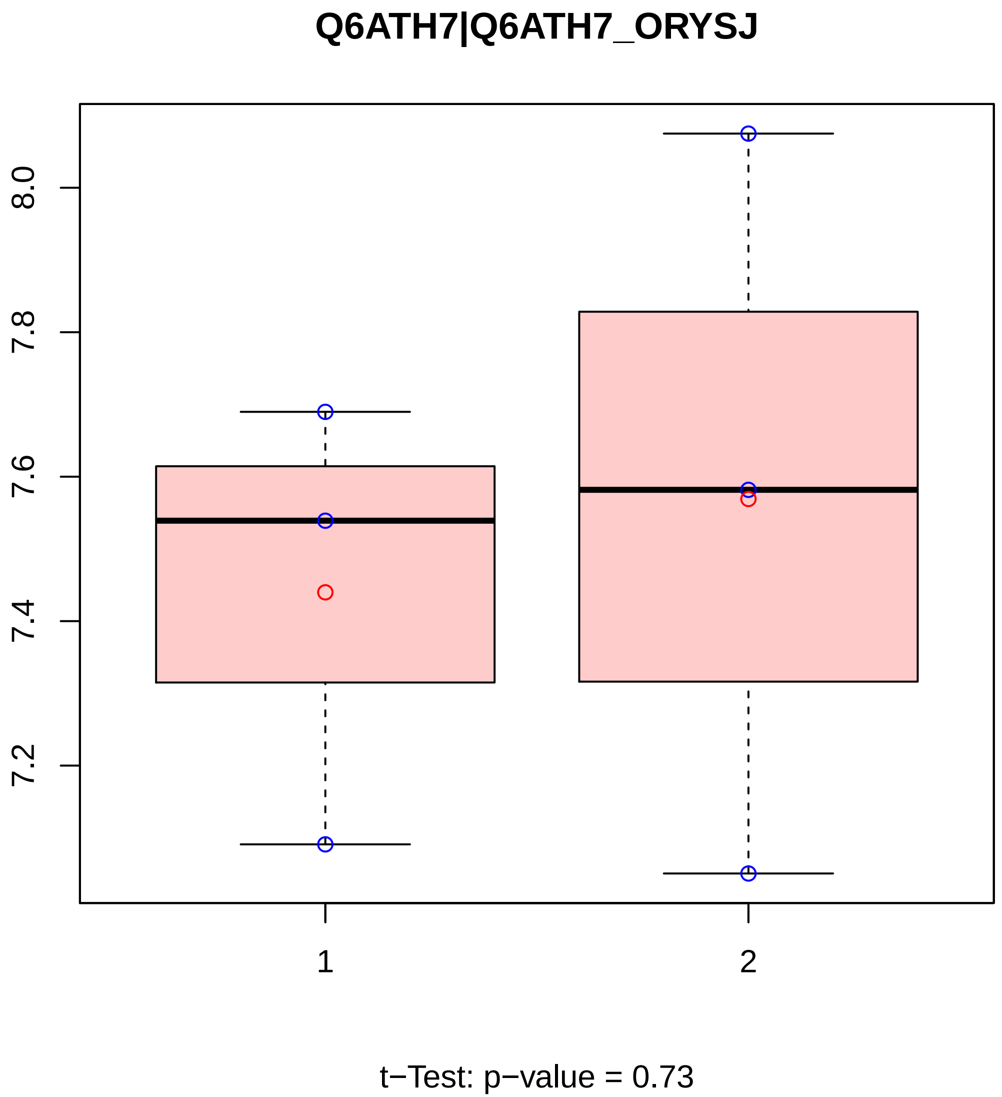

Q6ATH7|Q6ATH7_ORYSJ
8.0
7.8
7.6
7.4
7.2
1
2
t−Test: p−value = 0.73

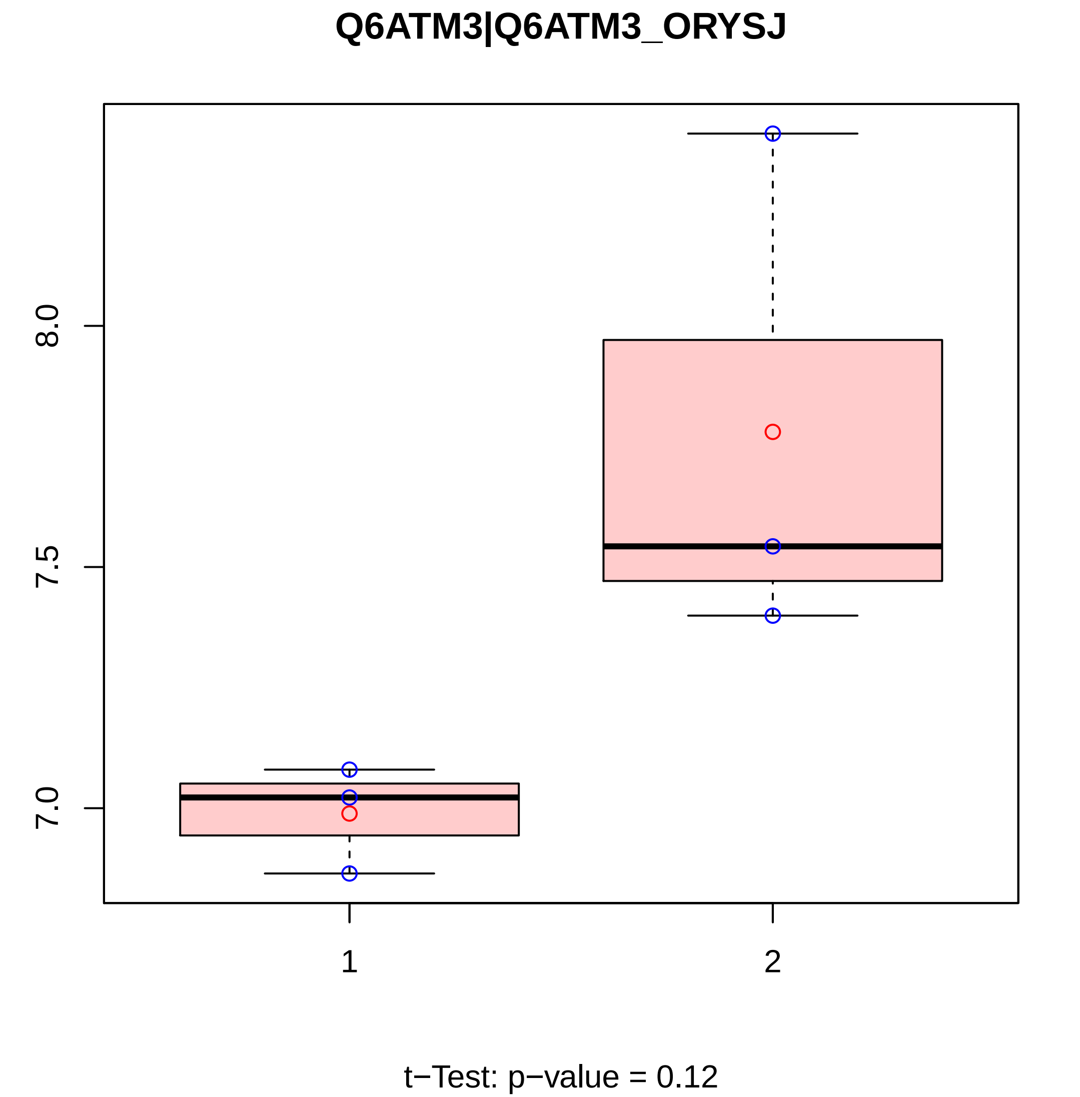
Q6ATM3|Q6ATM3_ORYSJ
8.0
7.5
7.0
1
2
t−Test: p−value = 0.12

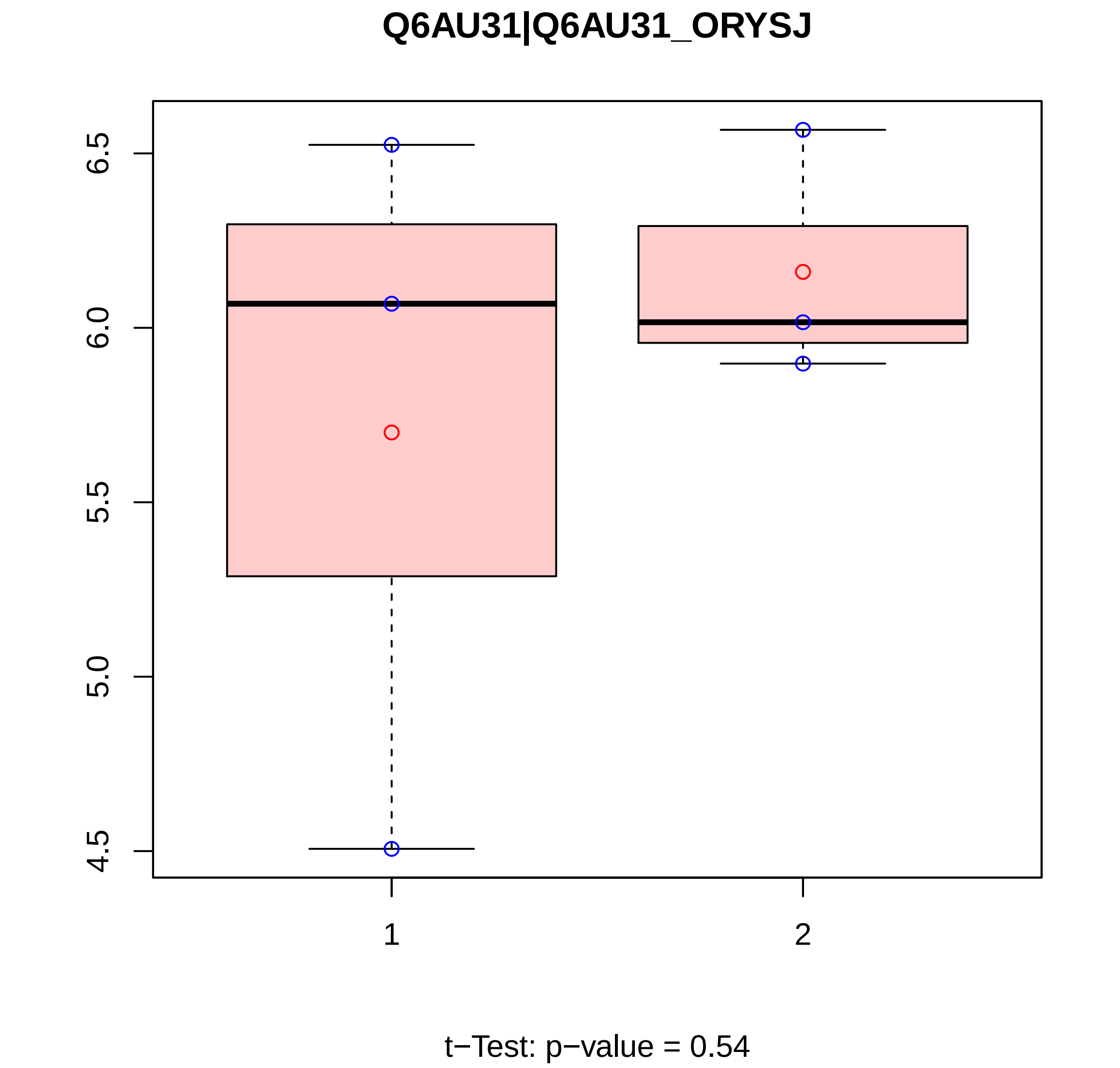
Q6AU31|Q6AU31_ORYSJ
6.5
6.0
5.5
5.0
4.5
1
2
t−Test: p−value = 0.54

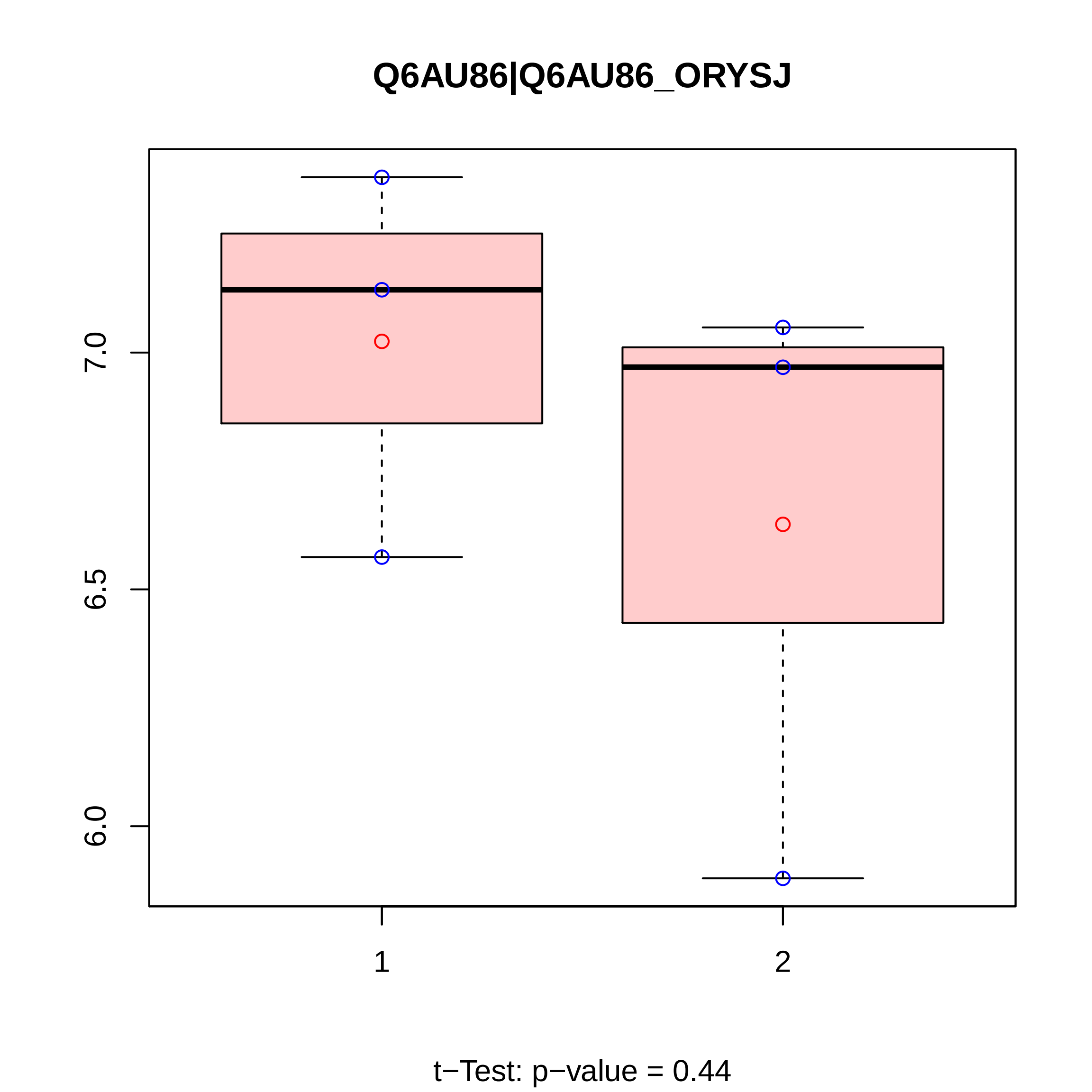

Q6AU86|Q6AU86_ORYSJ
7.0
6.5
6.0
1
2
t−Test: p−value = 0.44

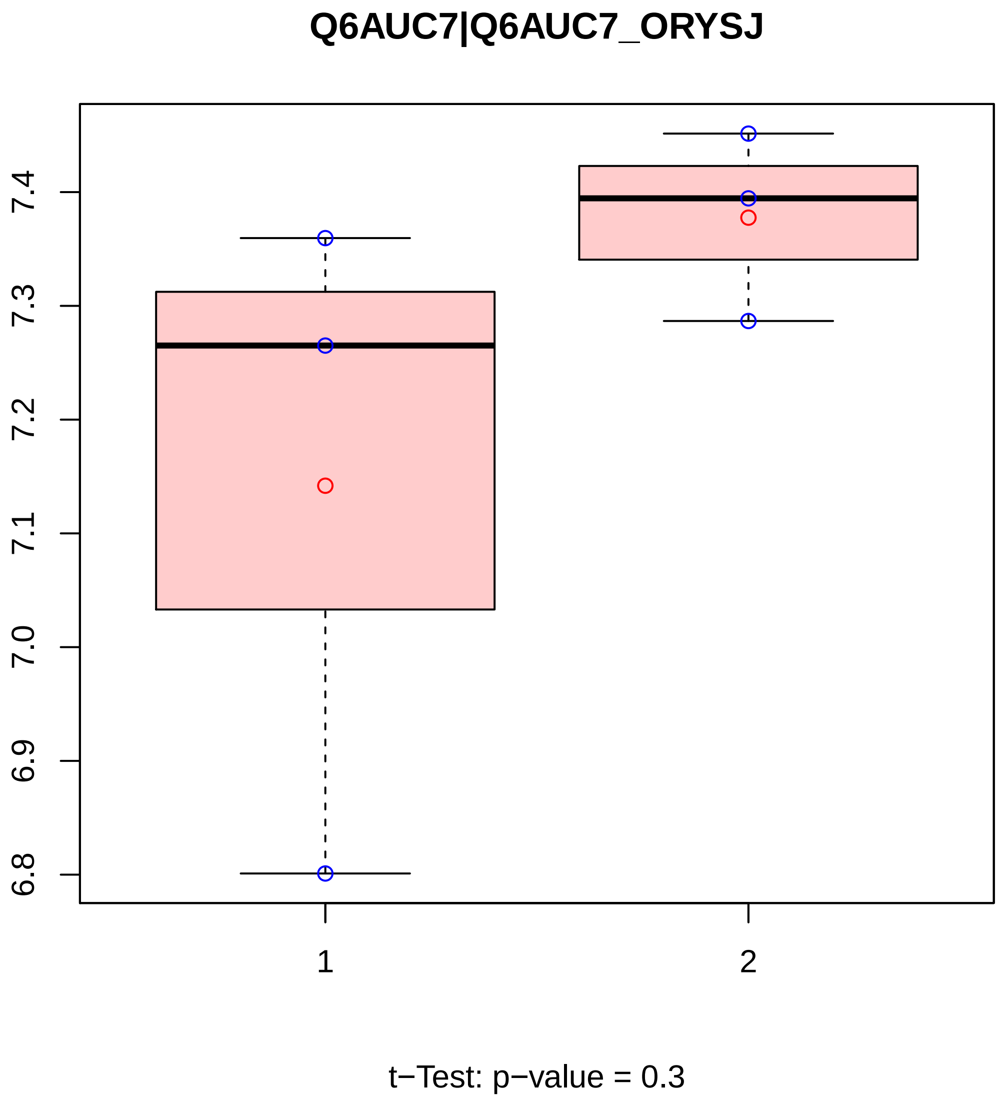
Q6AUC7|Q6AUC7_ORYSJ
6.8
6.9
7.0
7.1
7.2
7.3
7.4
1
2
t−Test: p−value = 0.3

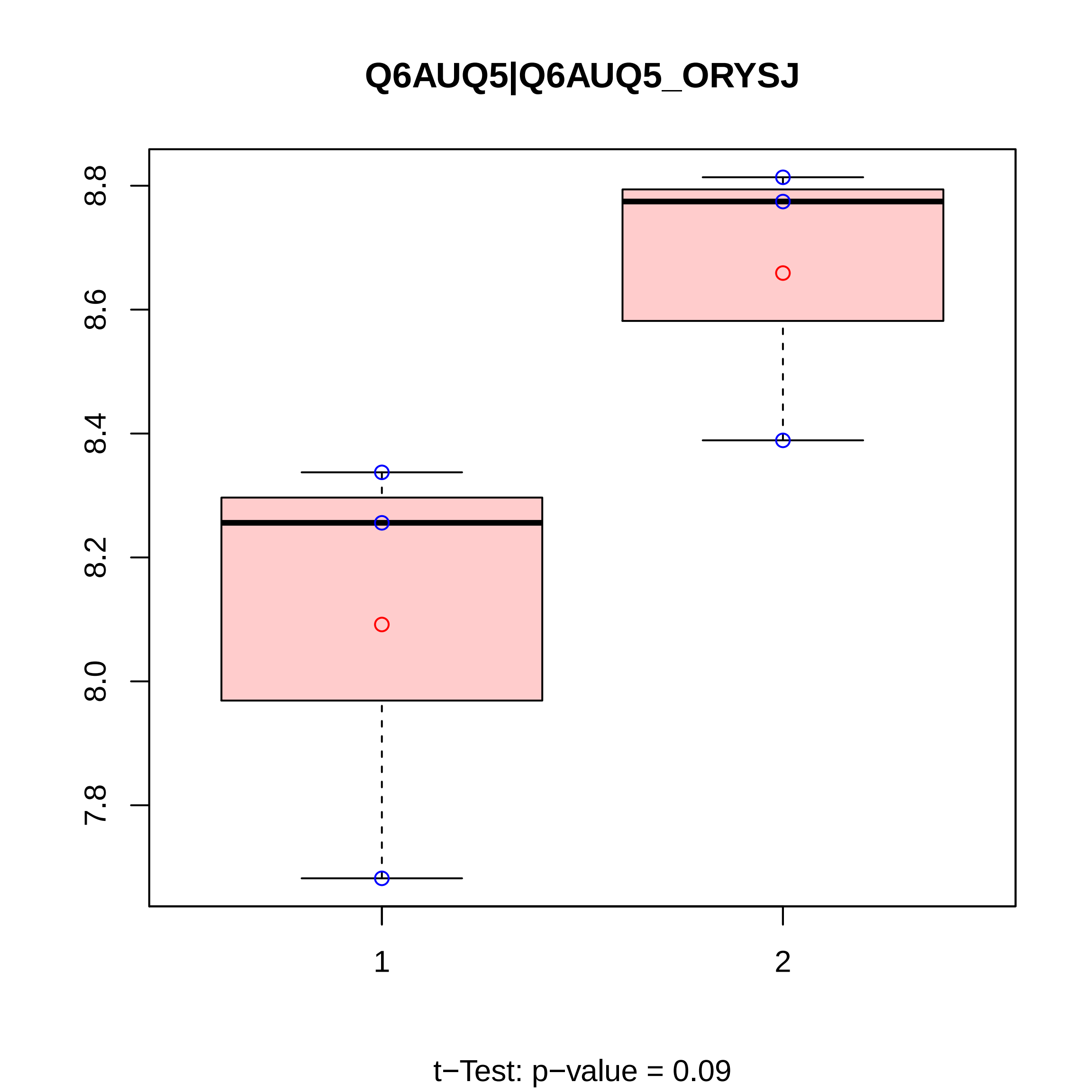
Q6AUQ5|Q6AUQ5_ORYSJ
8.8
8.6
8.4
8.2
8.0
7.8
1
2
t−Test: p−value = 0.09

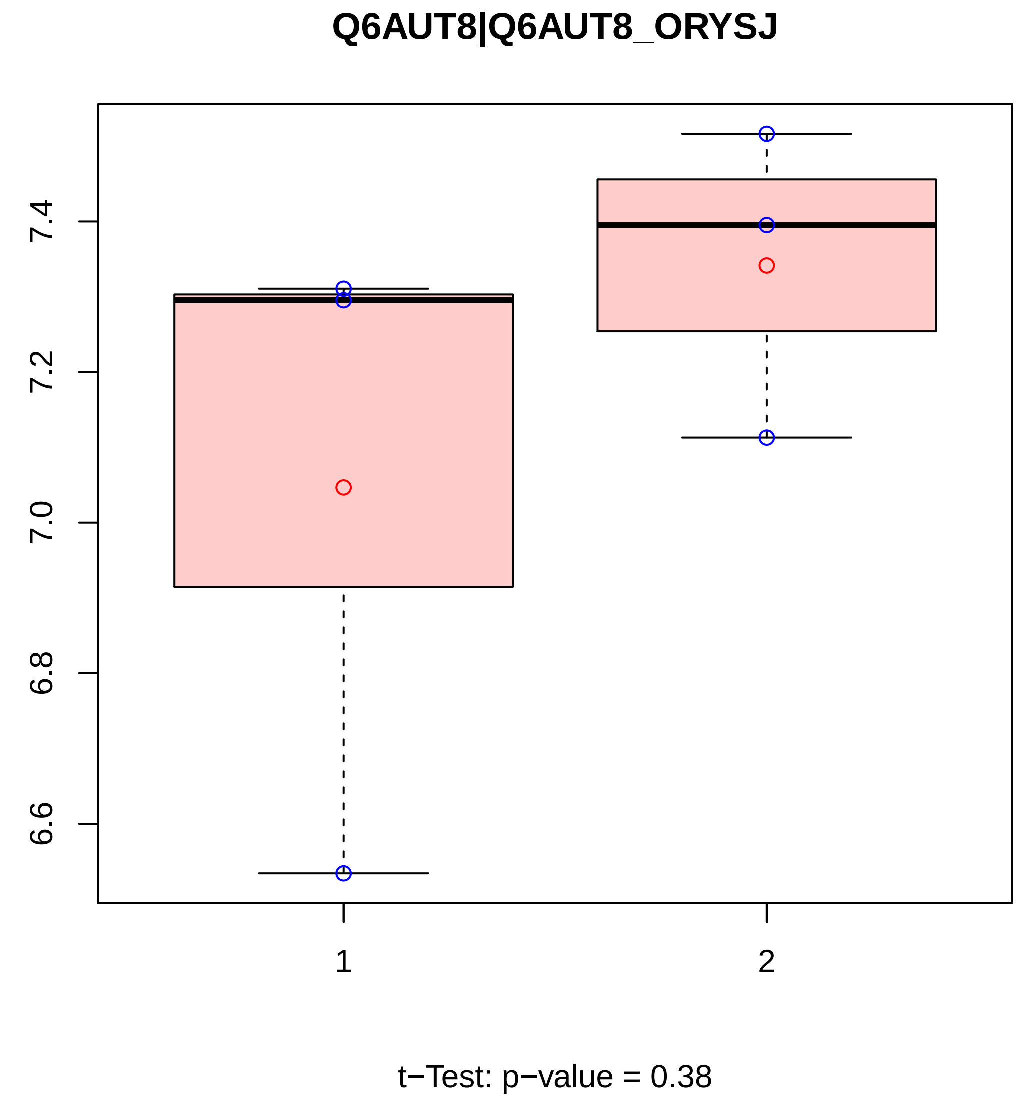
Q6AUT8|Q6AUT8_ORYSJ
7.4
7.2
7.0
6.8
6.6
1
2
t−Test: p−value = 0.38

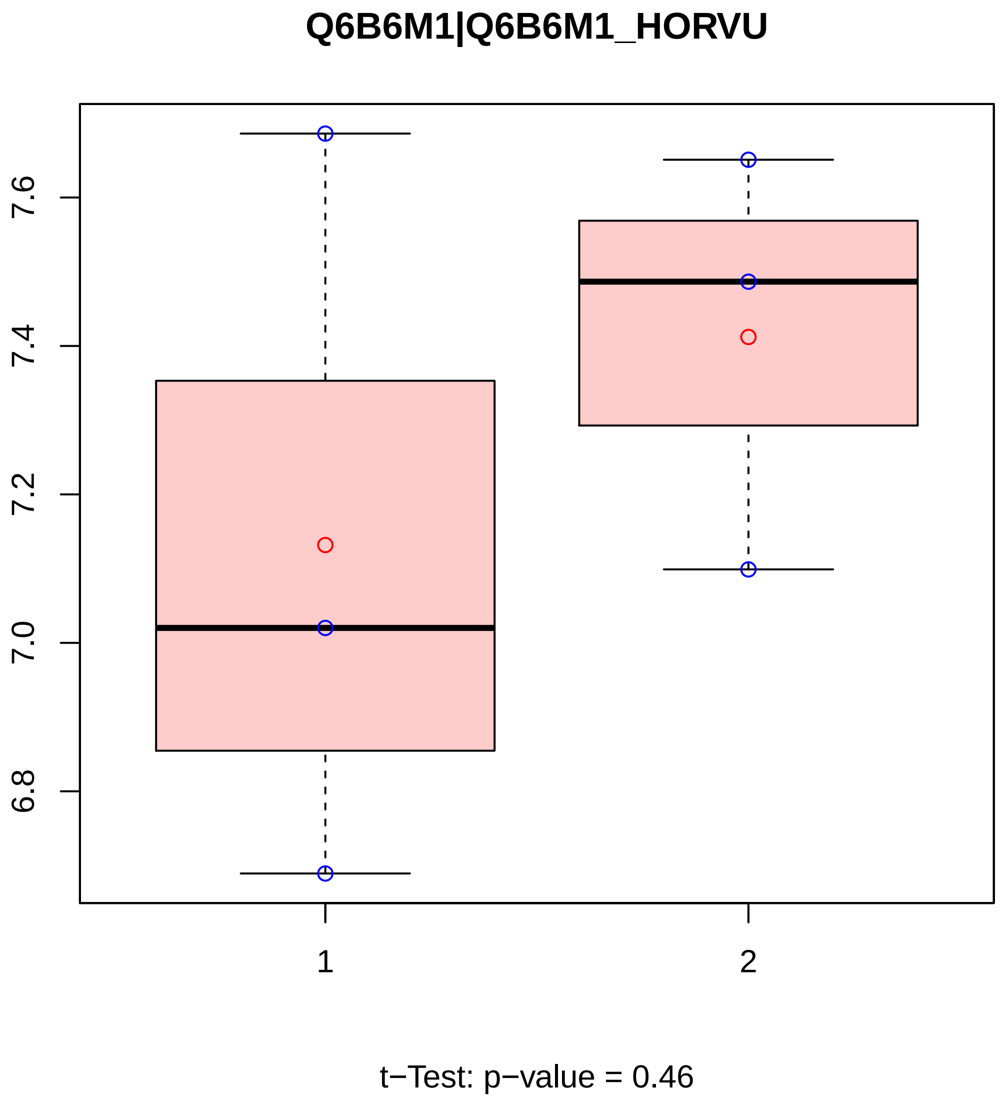

Q6B6M1|Q6B6M1_HORVU
6.8
7.0
7.2
7.4
7.6
1
2
t−Test: p−value = 0.46

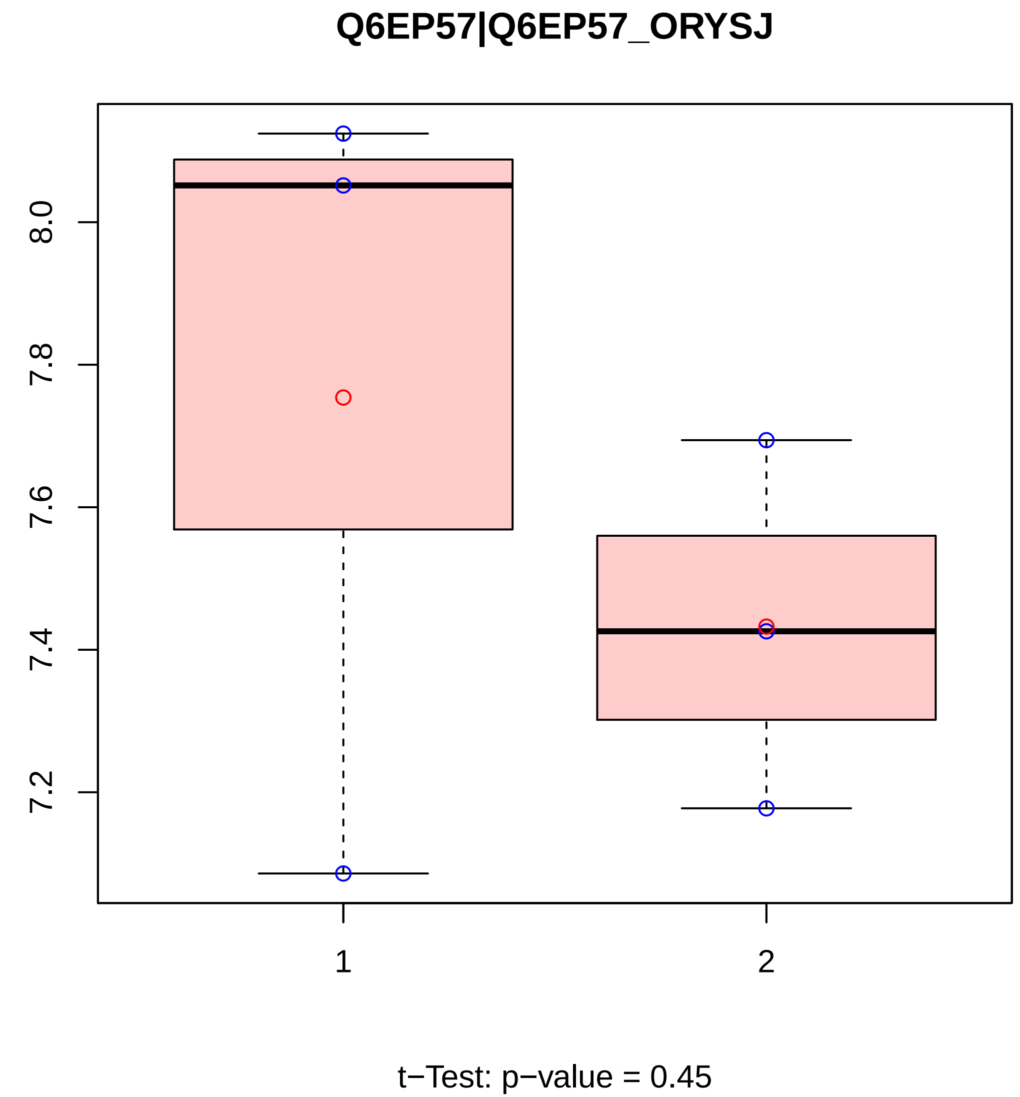
Q6EP57|Q6EP57_ORYSJ
8.0
7.8
7.6
7.4
7.2
1
2
t−Test: p−value = 0.45

Q6ERW2|Q6ERW2_ORYSJ

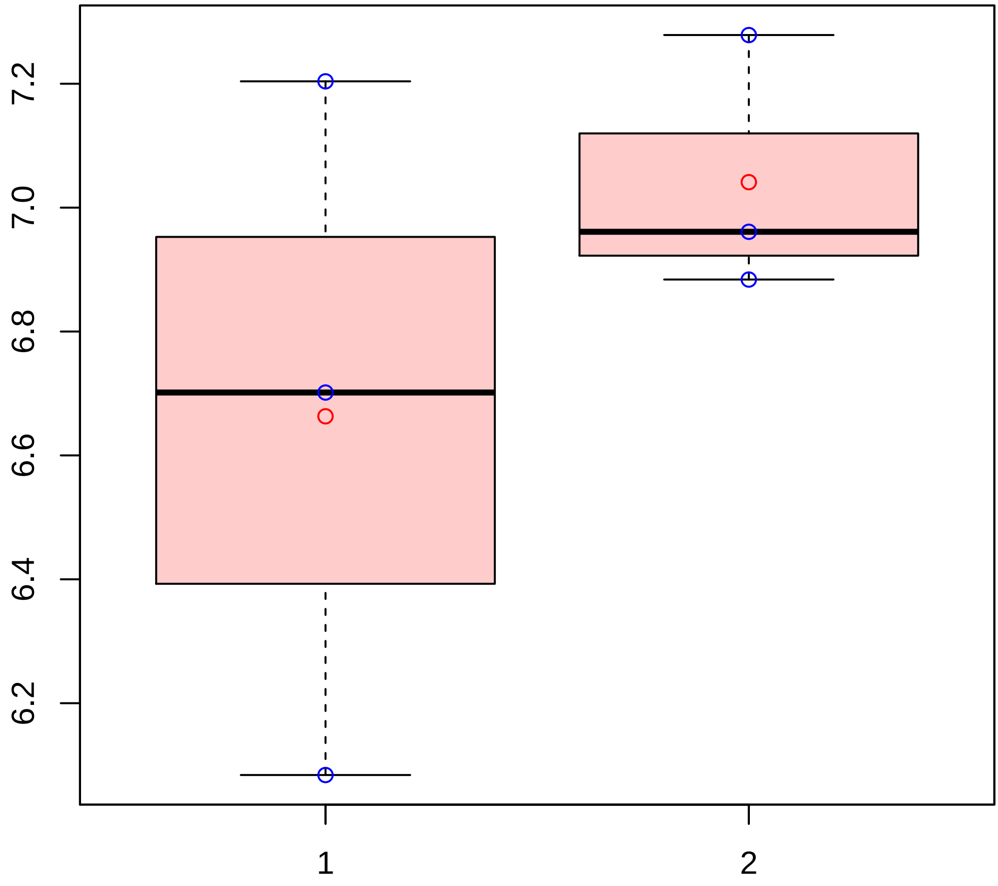

t−Test: p−value = 0.37

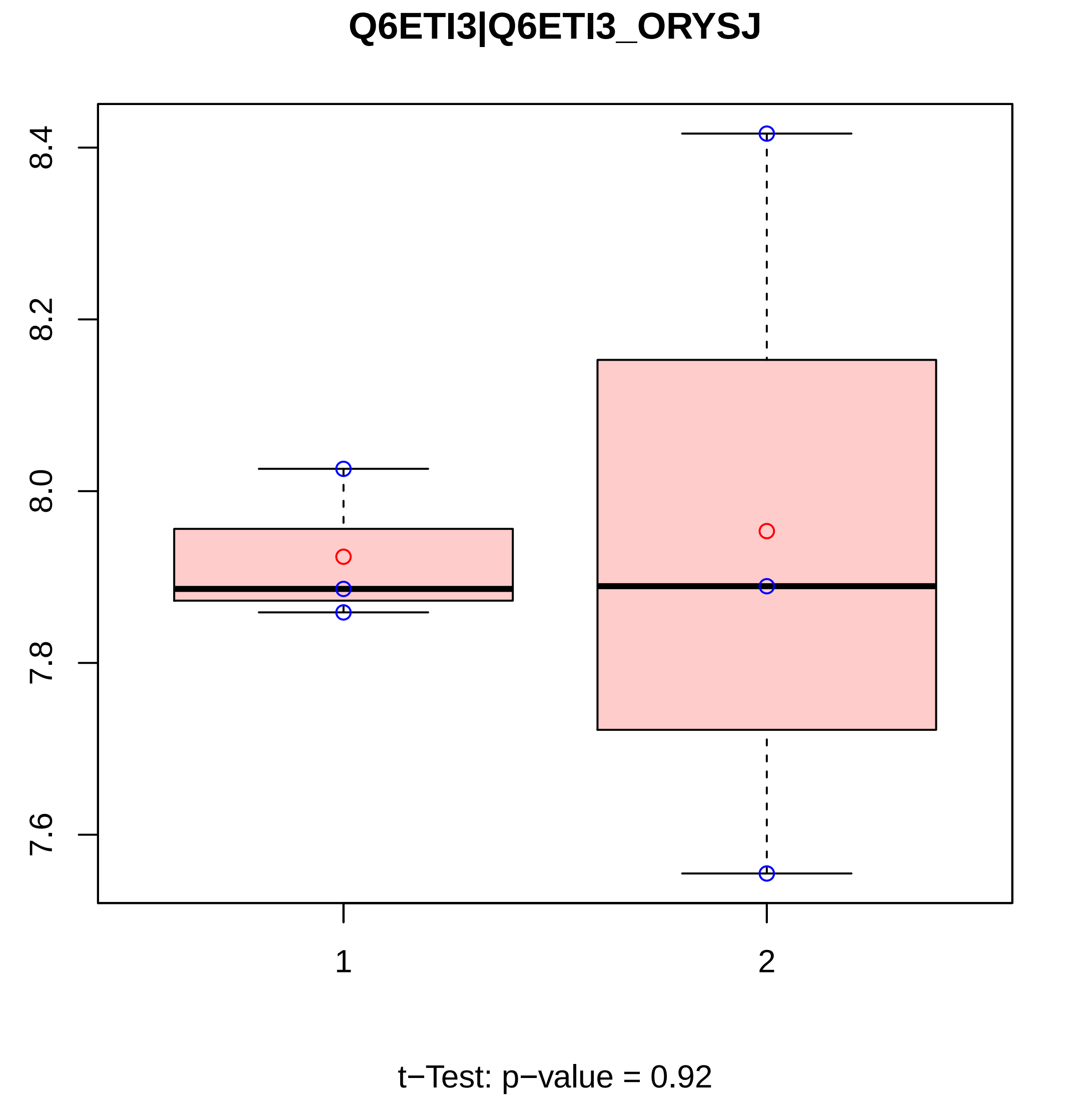
Q6ETI3|Q6ETI3_ORYSJ
8.4
8.2
8.0
7.8
7.6
1
2
t−Test: p−value = 0.92

Q6EUQ9|Q6EUQ9_ORYSJ

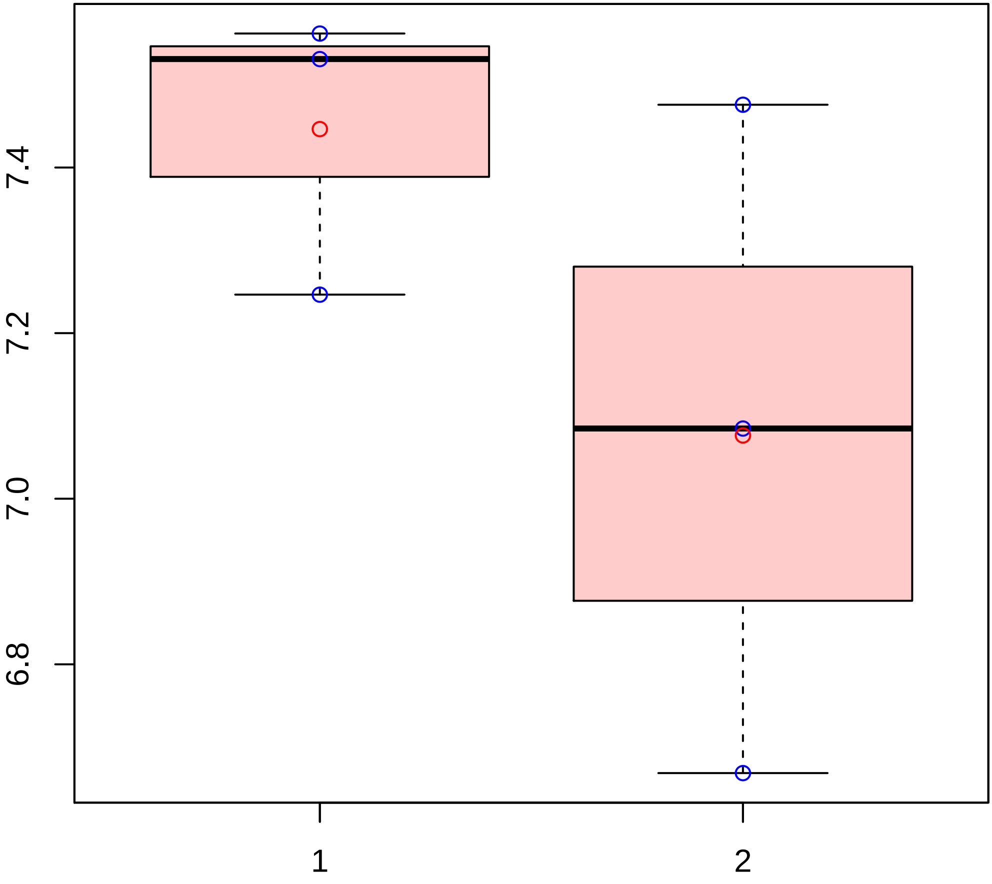

t−Test: p−value = 0.25

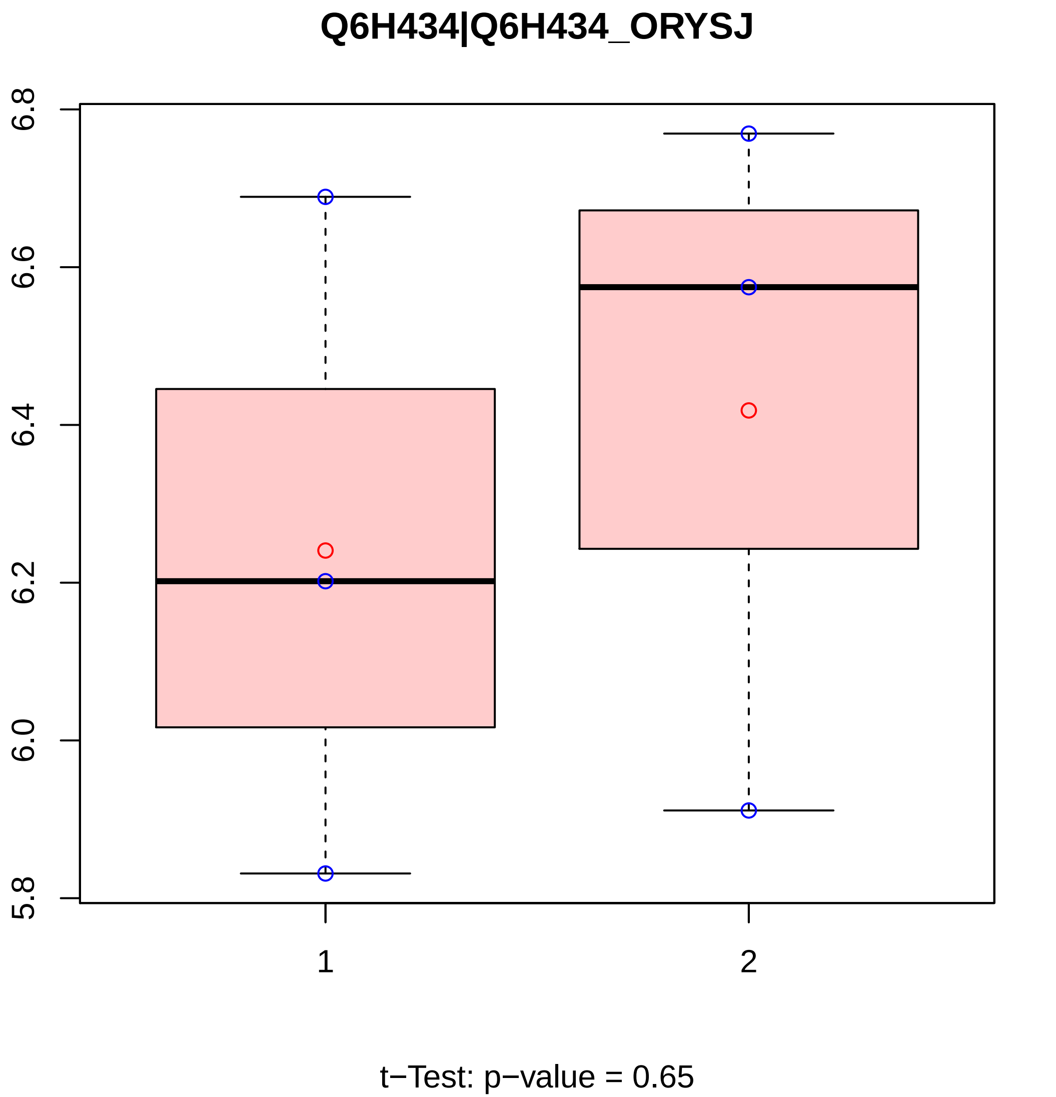
Q6H434|Q6H434_ORYSJ
6.8
6.6
6.4
6.2
6.0
5.8
1
2
t−Test: p−value = 0.65

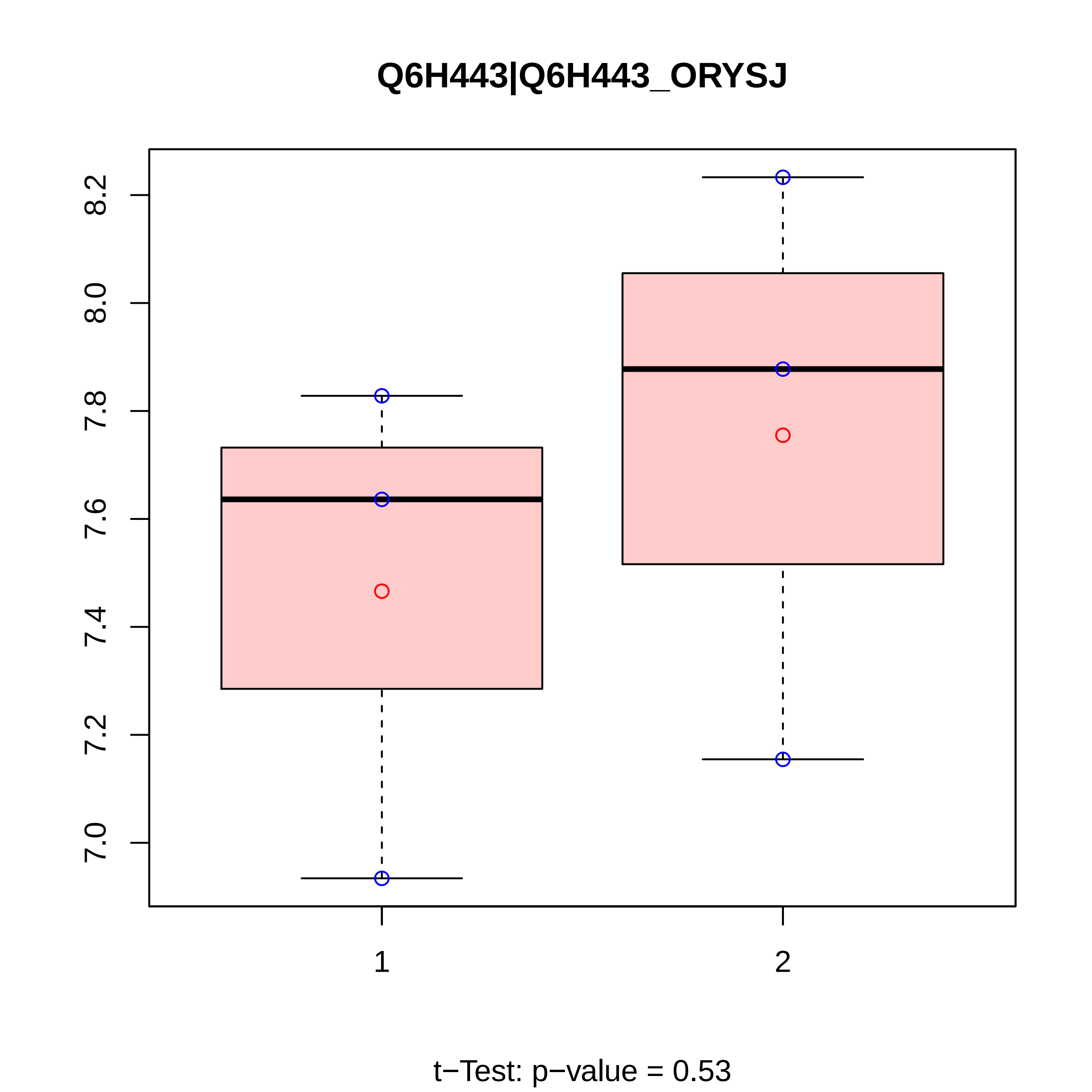
Q6H443|Q6H443_ORYSJ
8.2
8.0
7.8
7.6
7.4
7.2
7.0
1
2
t−Test: p−value = 0.53

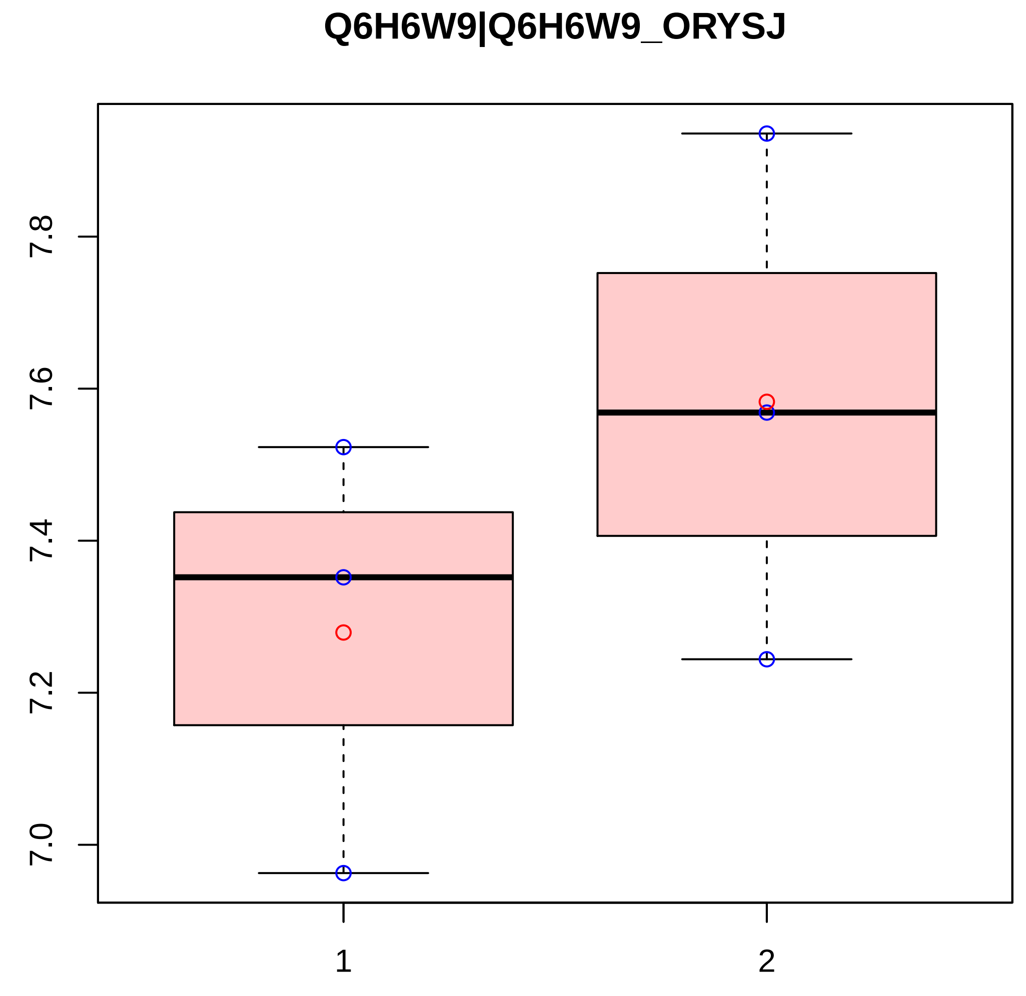

t−Test: p−value = 0.31

Q6H801|Q6H801_ORYSJ

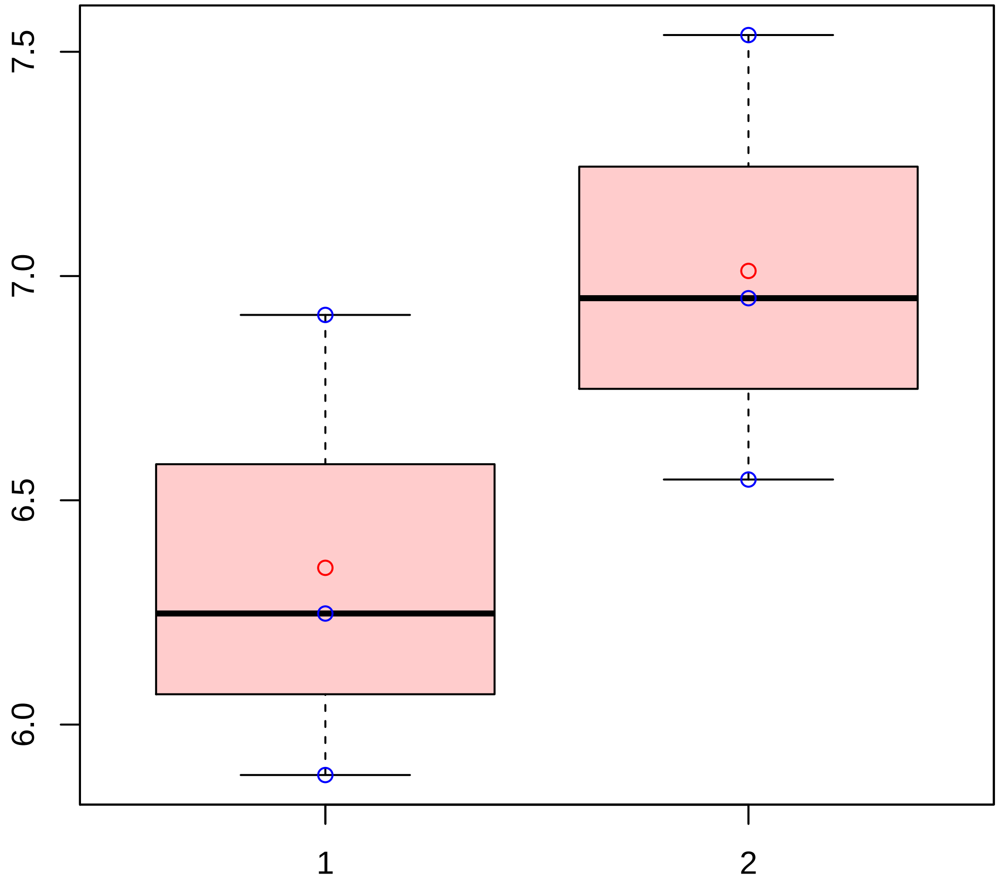

t−Test: p−value = 0.19

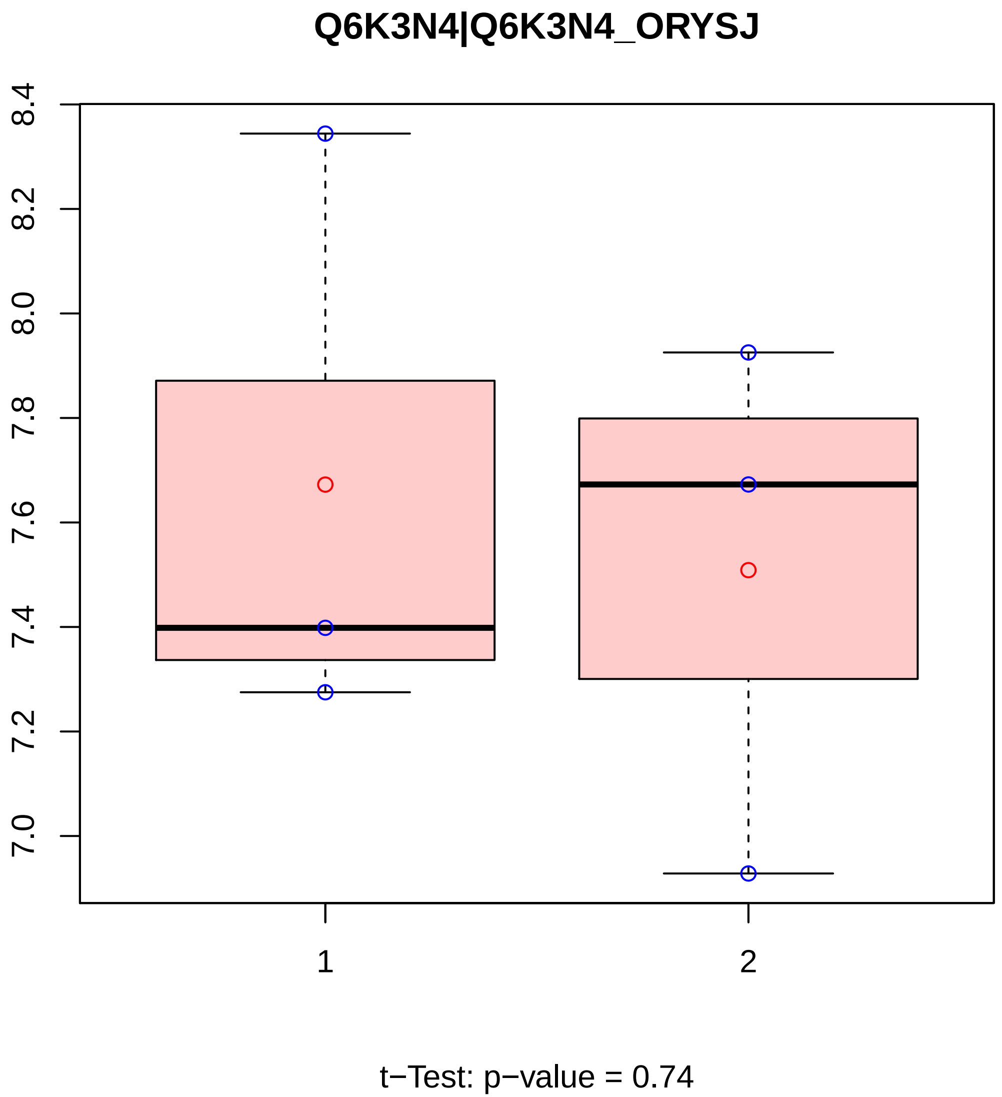
Q6K3N4|Q6K3N4_ORYSJ
8.4
8.2
8.0
7.8
7.6
7.4
7.2
7.0
1
2
t−Test: p−value = 0.74

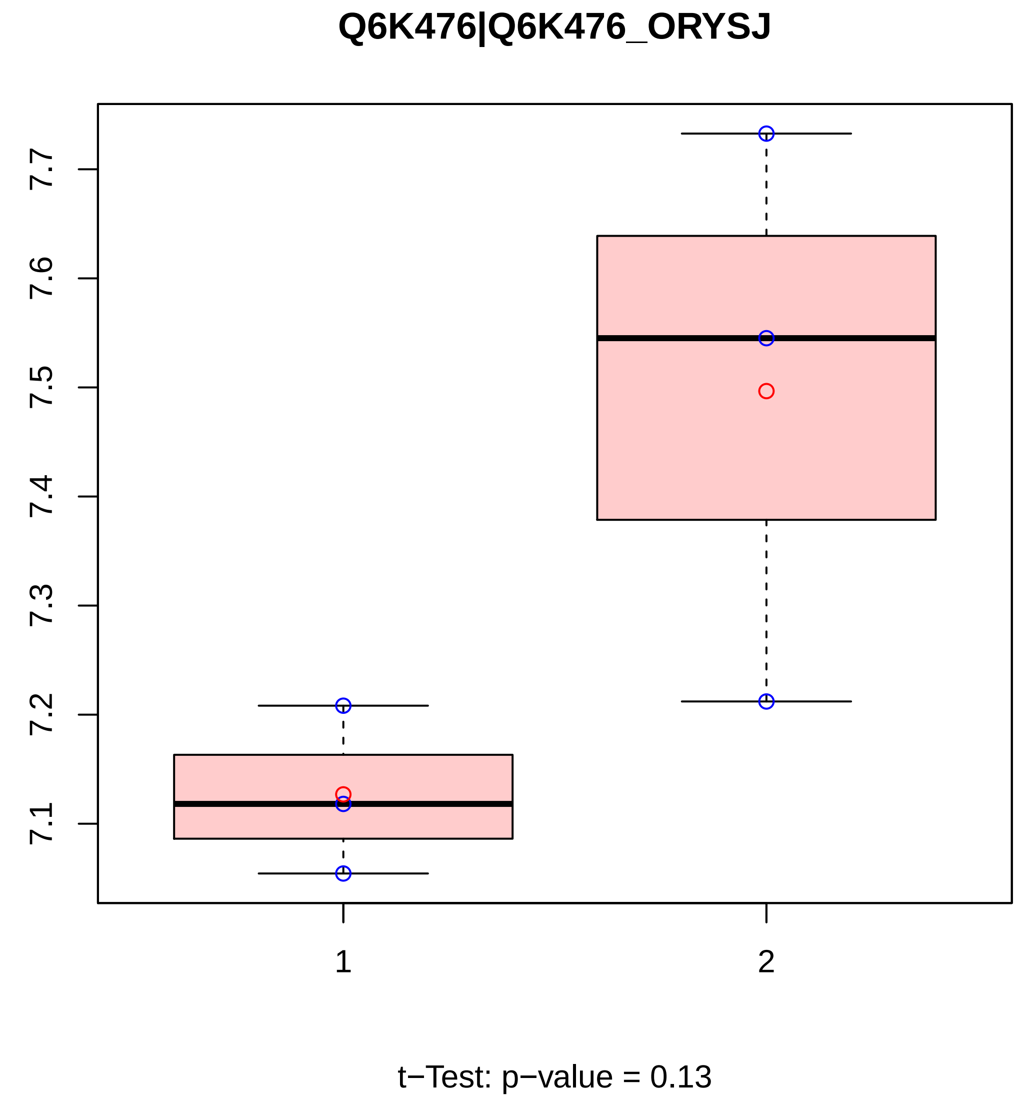
Q6K476|Q6K476_ORYSJ
7.1
7.2
7.3
7.4
7.5
7.6
7.7
1
2
t−Test: p−value = 0.13

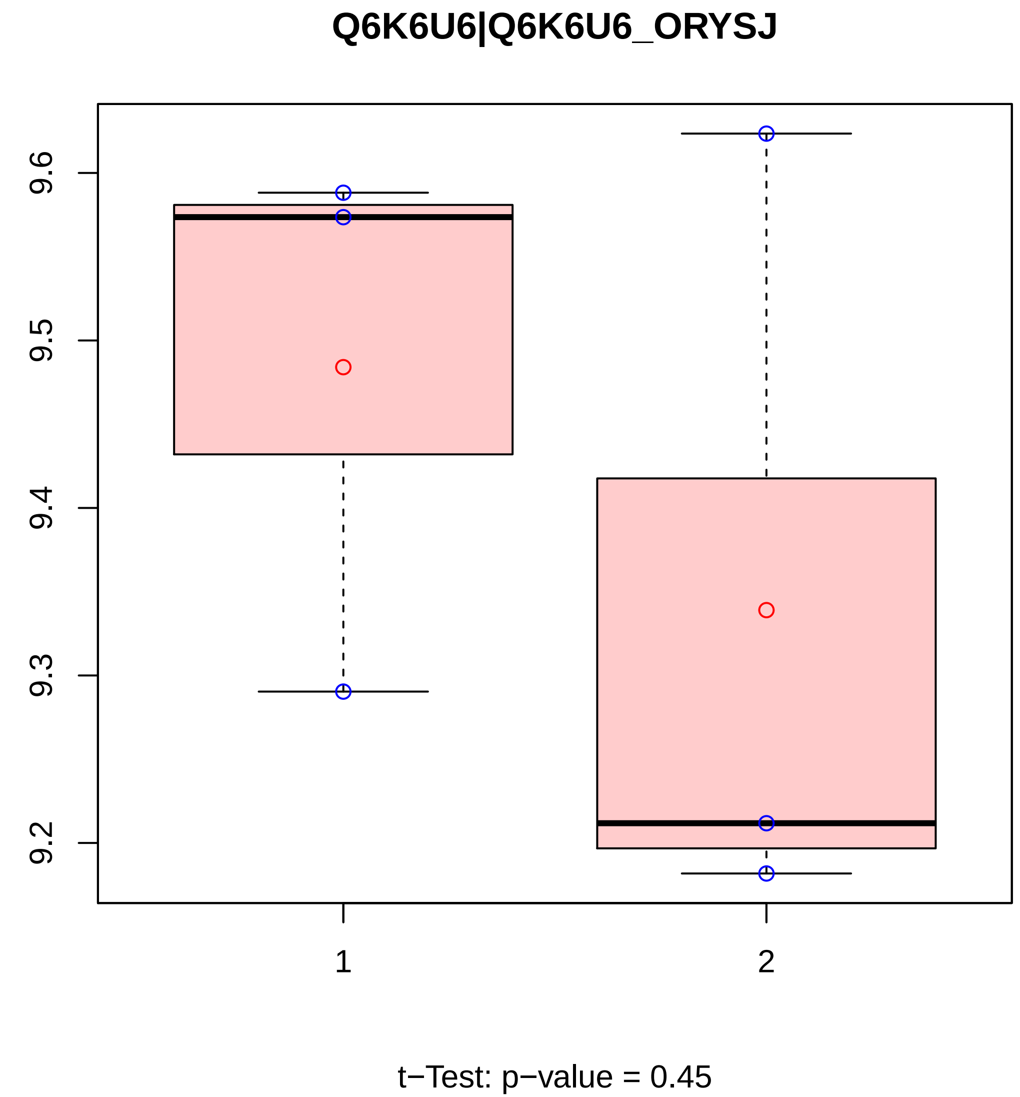
Q6K6U6|Q6K6U6_ORYSJ
9.2
9.3
9.4
9.5
9.6
1
2
t−Test: p−value = 0.45

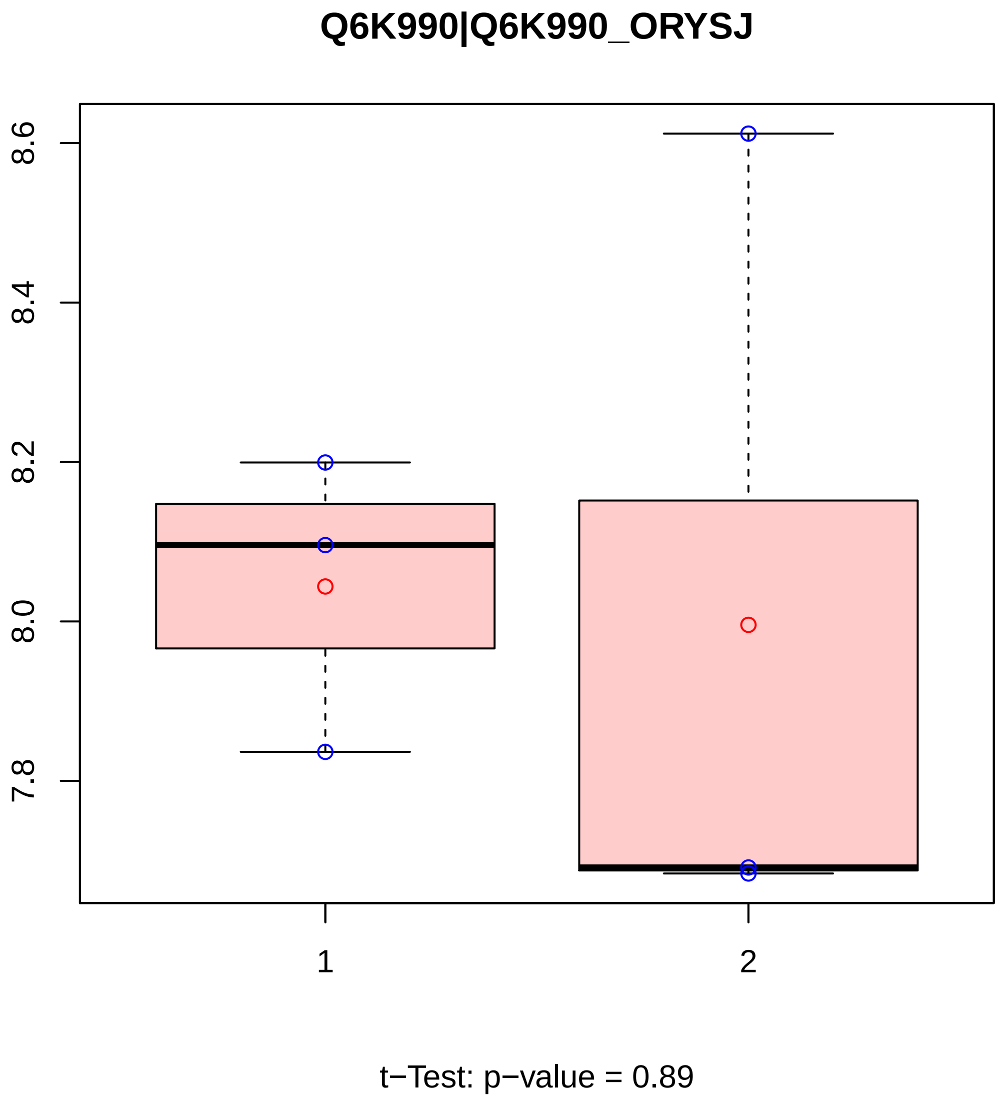
Q6K990|Q6K990_ORYSJ
8.6
8.4
8.2
8.0
7.8
1
2
t−Test: p−value = 0.89

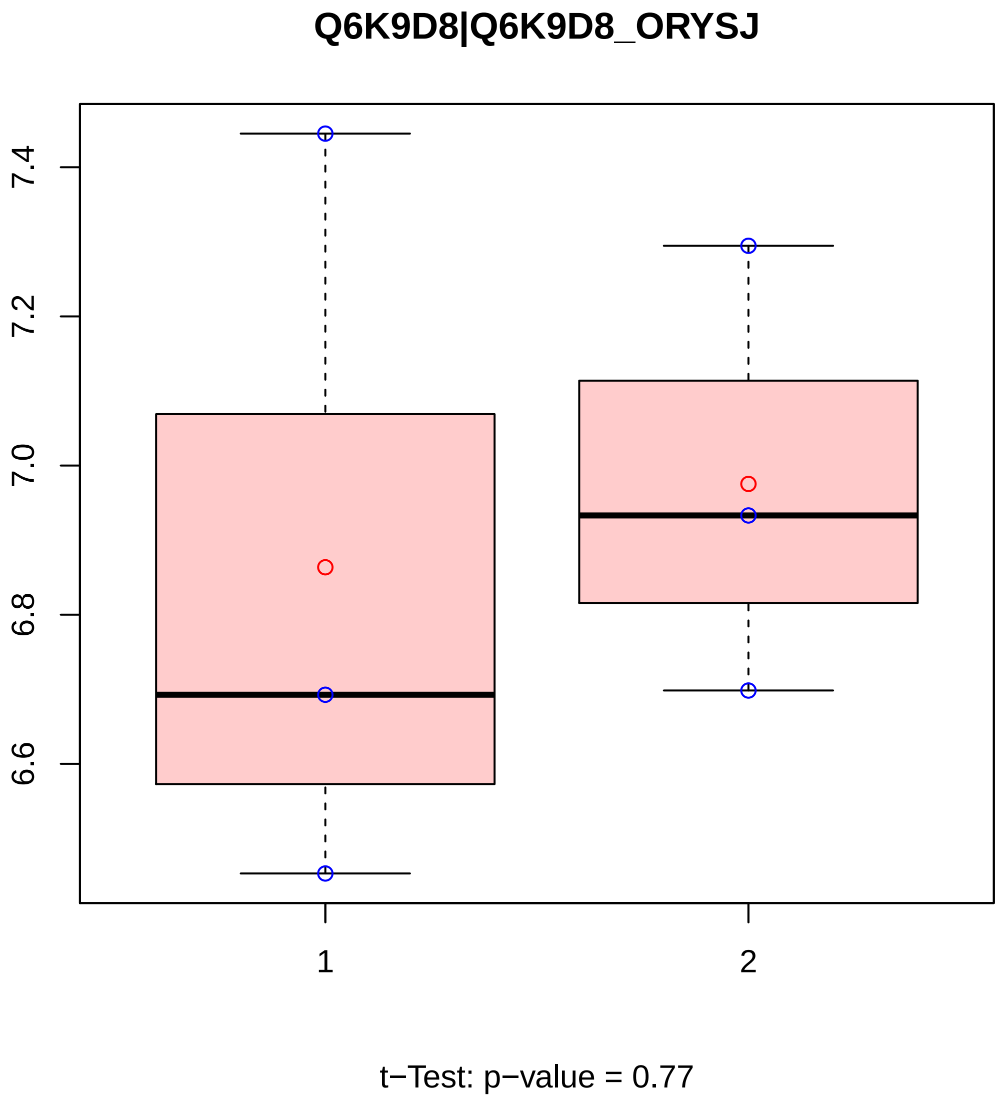
Q6K9D8|Q6K9D8_ORYSJ
7.4
7.2
7.0
6.8
6.6
1
2
t−Test: p−value = 0.77

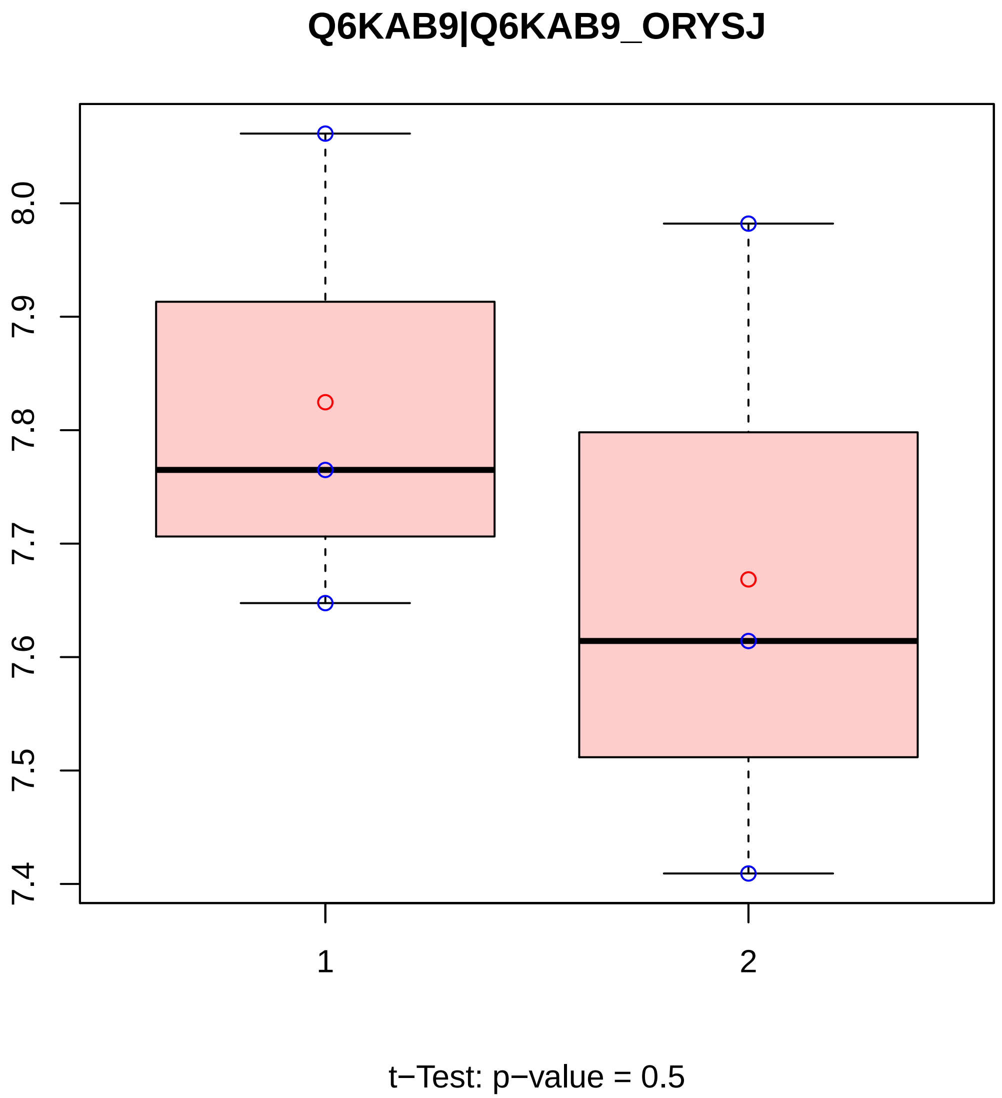
Q6KAB9|Q6KAB9_ORYSJ
8.0
7.9
7.8
7.7
7.6
7.5
7.4
1
2
t−Test: p−value = 0.5

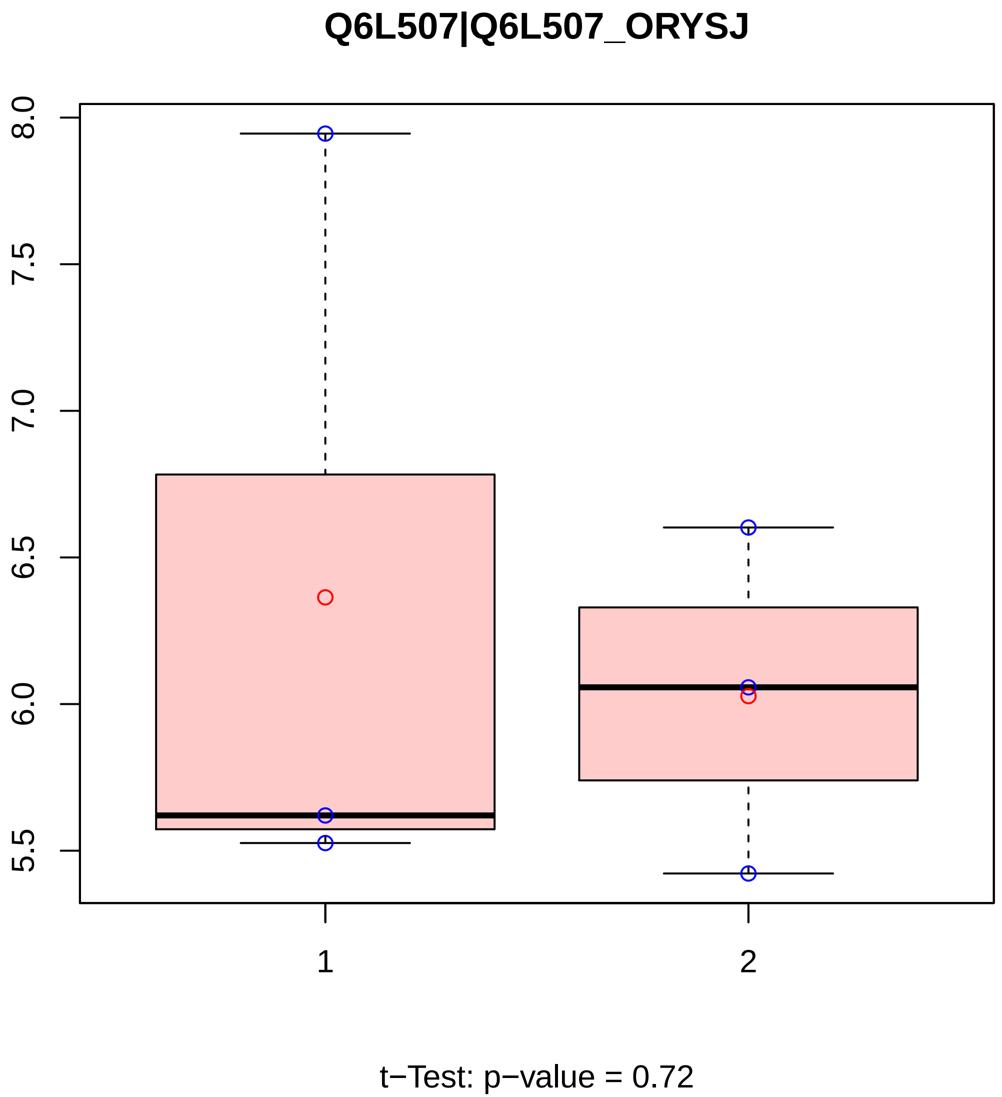

Q6L507|Q6L507_ORYSJ
8.0
7.5
7.0
6.5
6.0
5.5
1
2
t−Test: p−value = 0.72

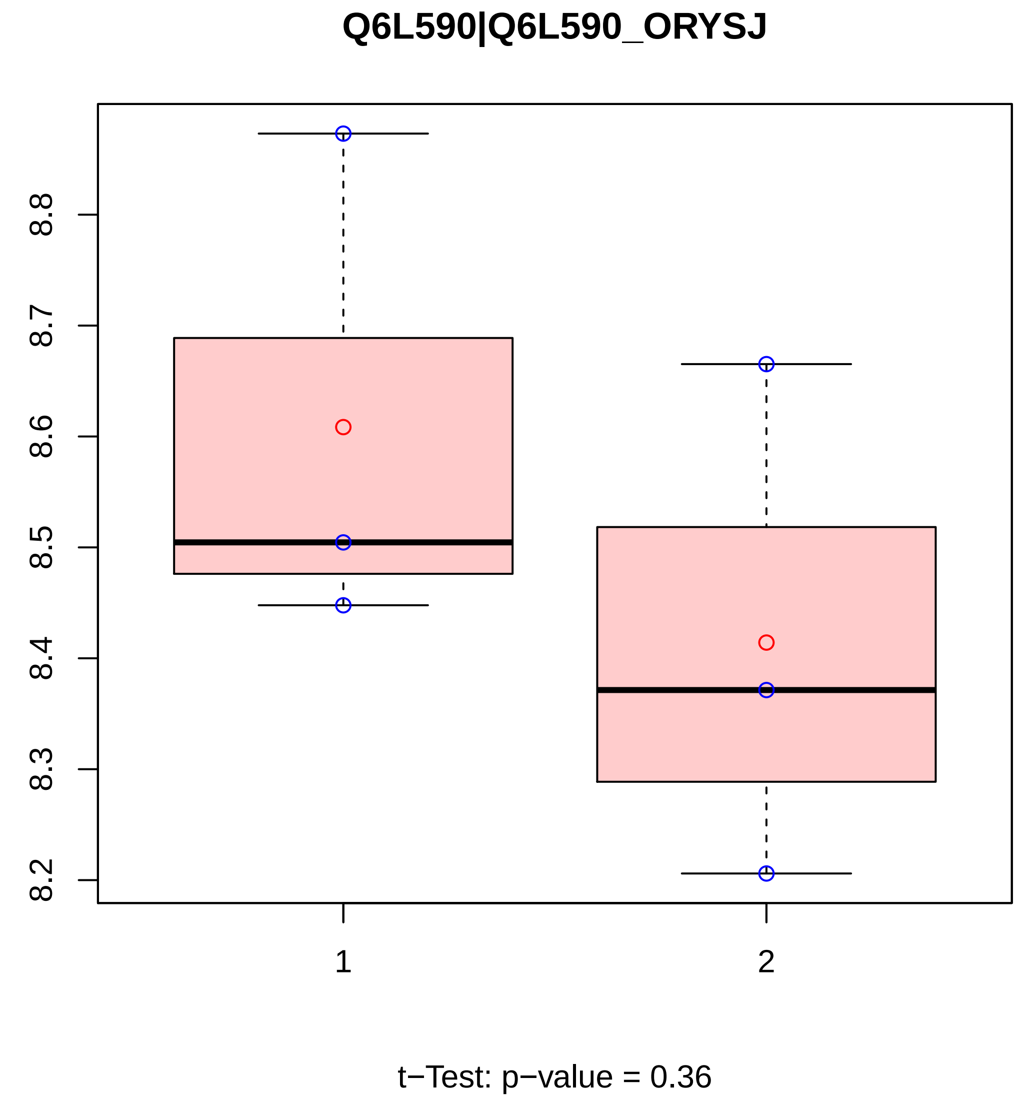
Q6L590|Q6L590_ORYSJ
8.2
8.3
8.4
8.5
8.6
8.7
8.8
1
2
t−Test: p−value = 0.36

Q6Q297|Q6Q297_MAIZE

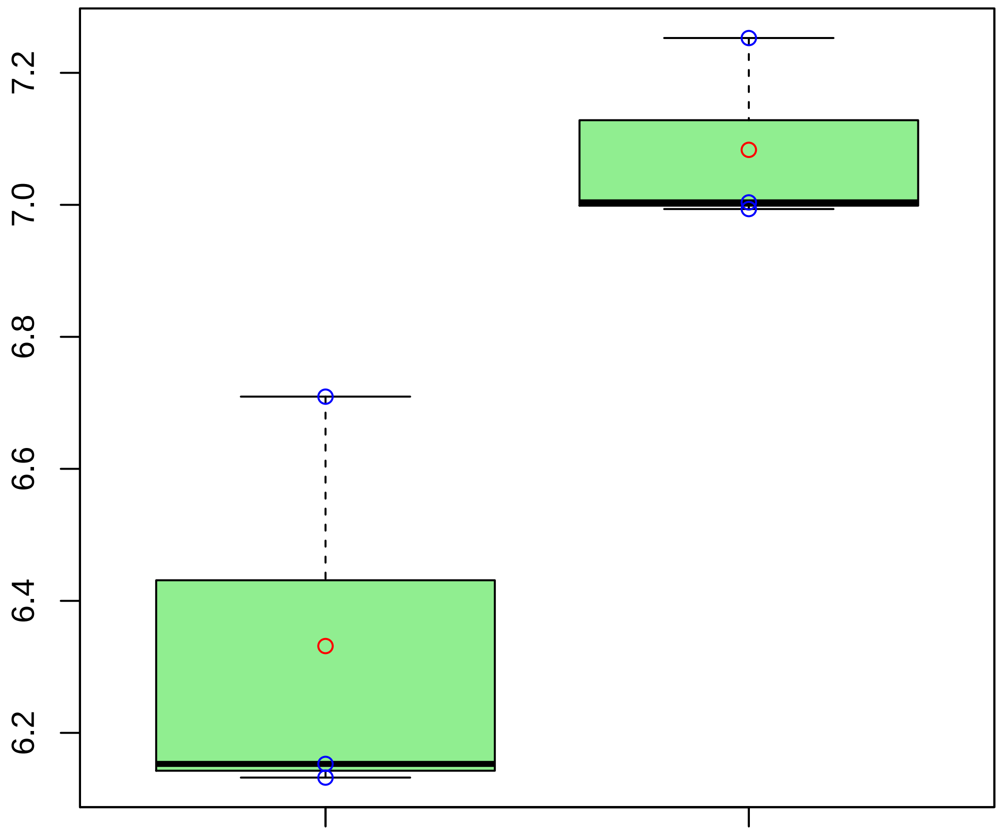

t−Test: p−value = 0.04

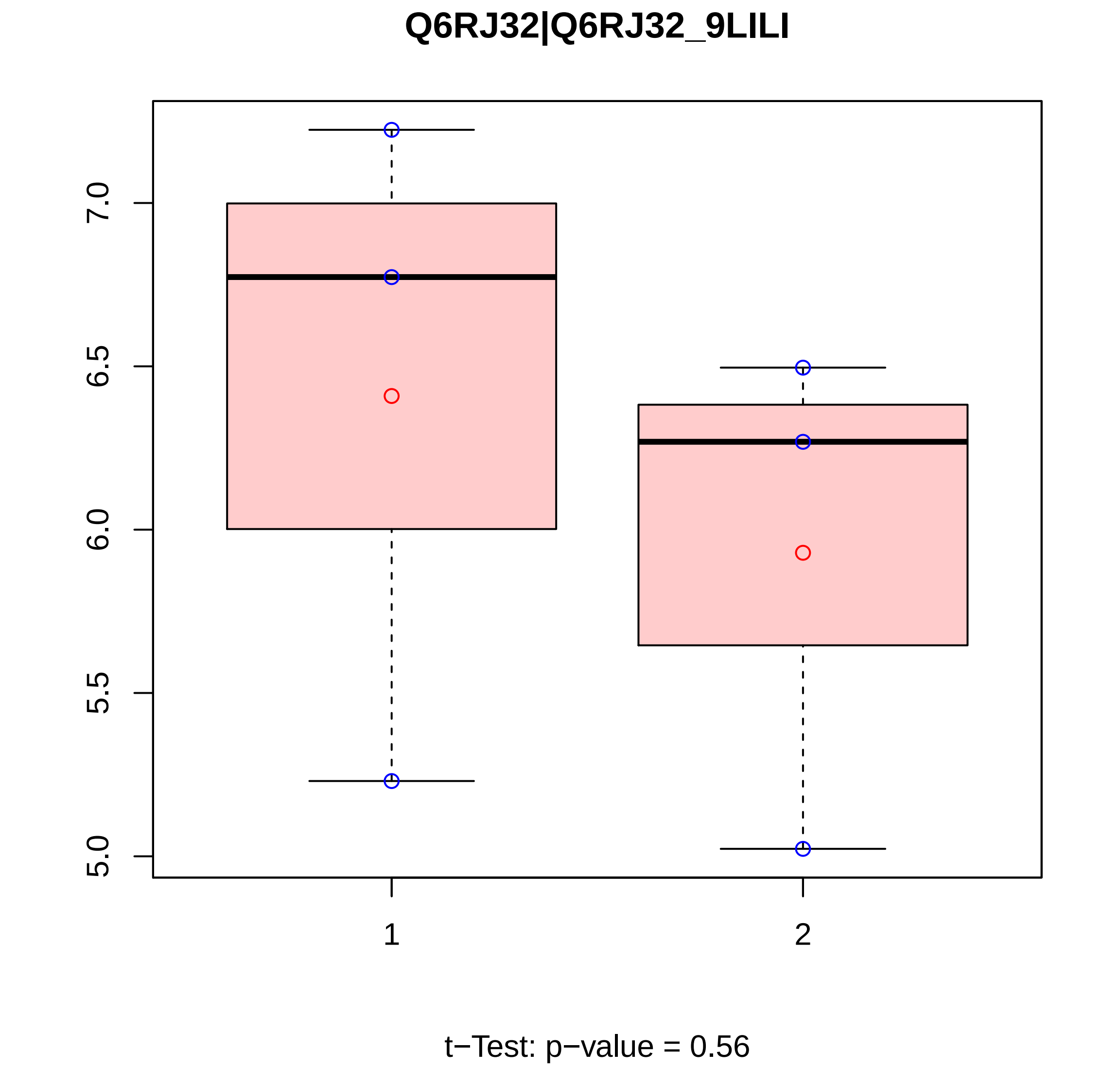
Q6RJ32|Q6RJ32_9LILI
7.0
6.5
6.0
5.5
5.0
1
2
t−Test: p−value = 0.56

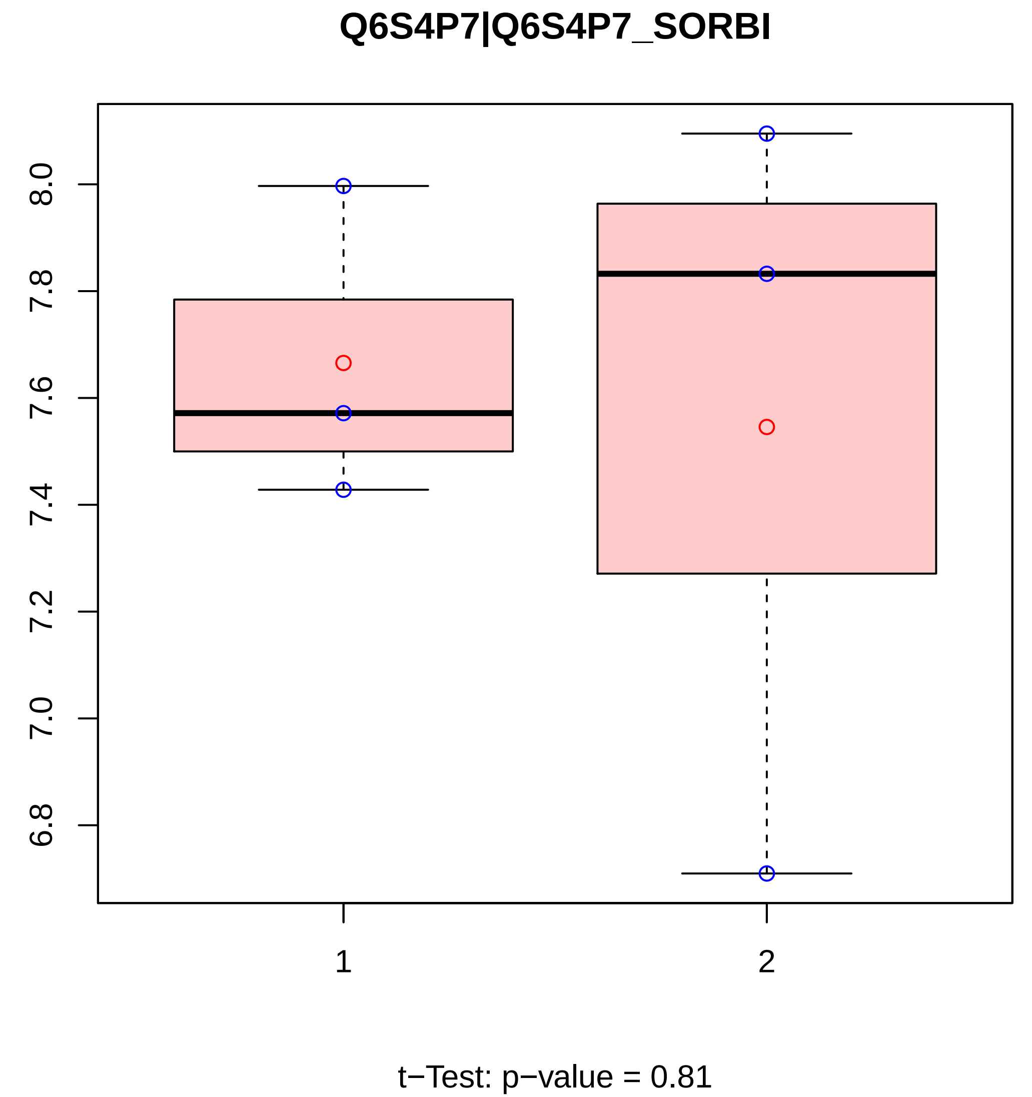
Q6S4P7|Q6S4P7_SORBI
8.0
7.8
7.6
7.4
7.2
7.0
6.8
1
2
t−Test: p−value = 0.81

Q6TUC6|Q6TUC6_ORYSJ

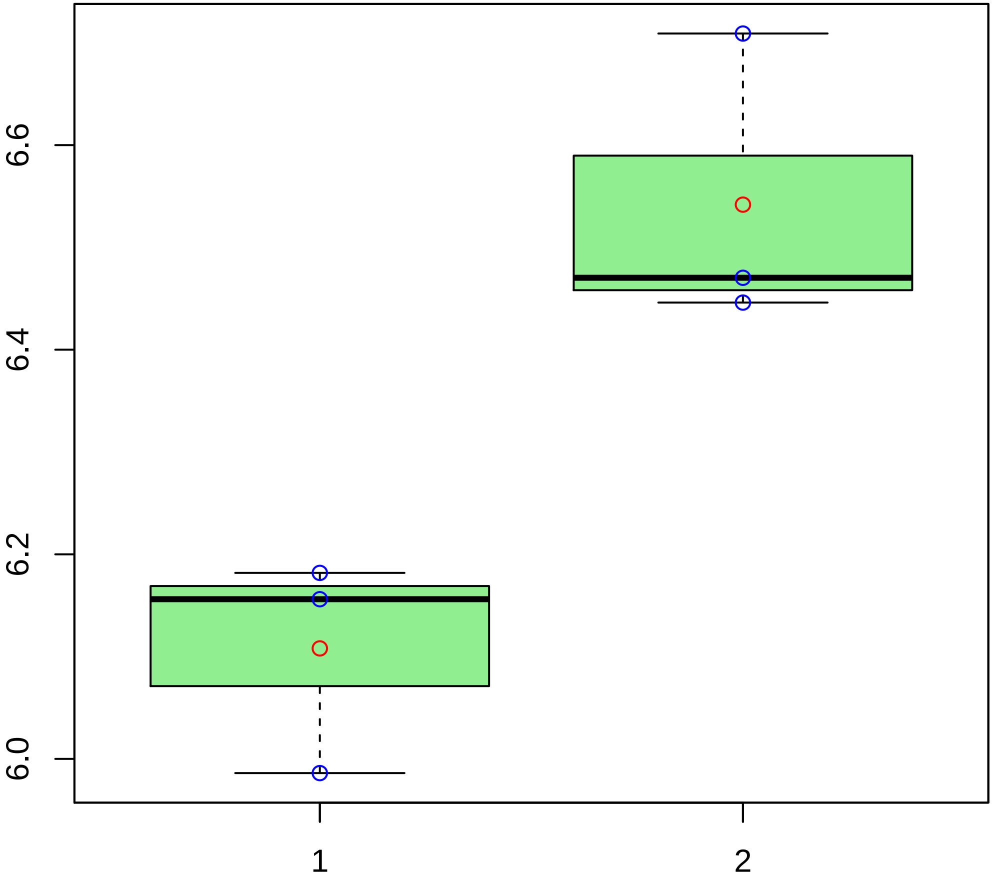

t−Test: p−value = 0.02

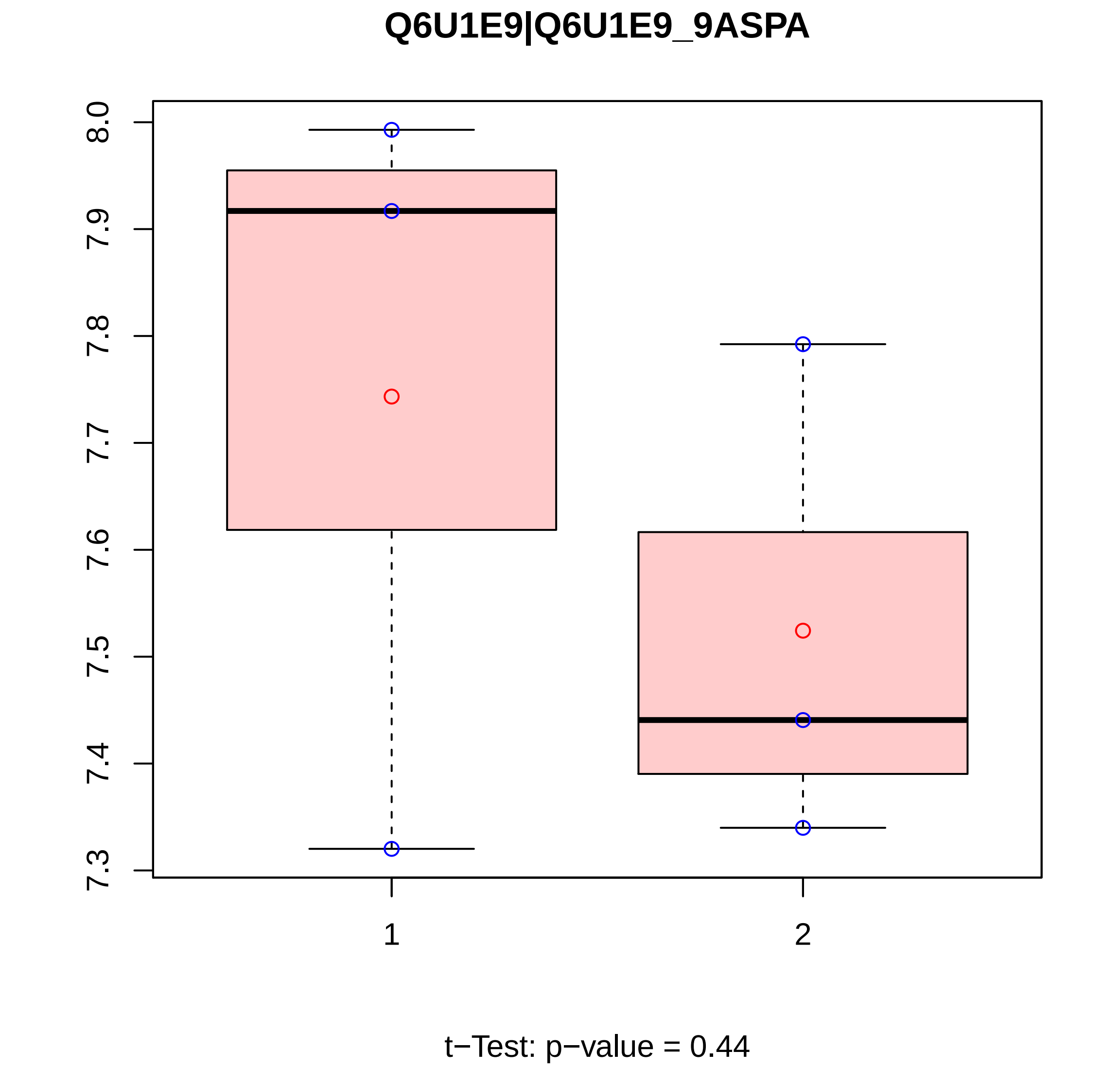

Q6U1E9|Q6U1E9_9ASPA
8.0
7.9
7.8
7.7
7.6
7.5
7.4
7.3
1
2
t−Test: p−value = 0.44

Q6UB39|Q6UB39_ORYSJ

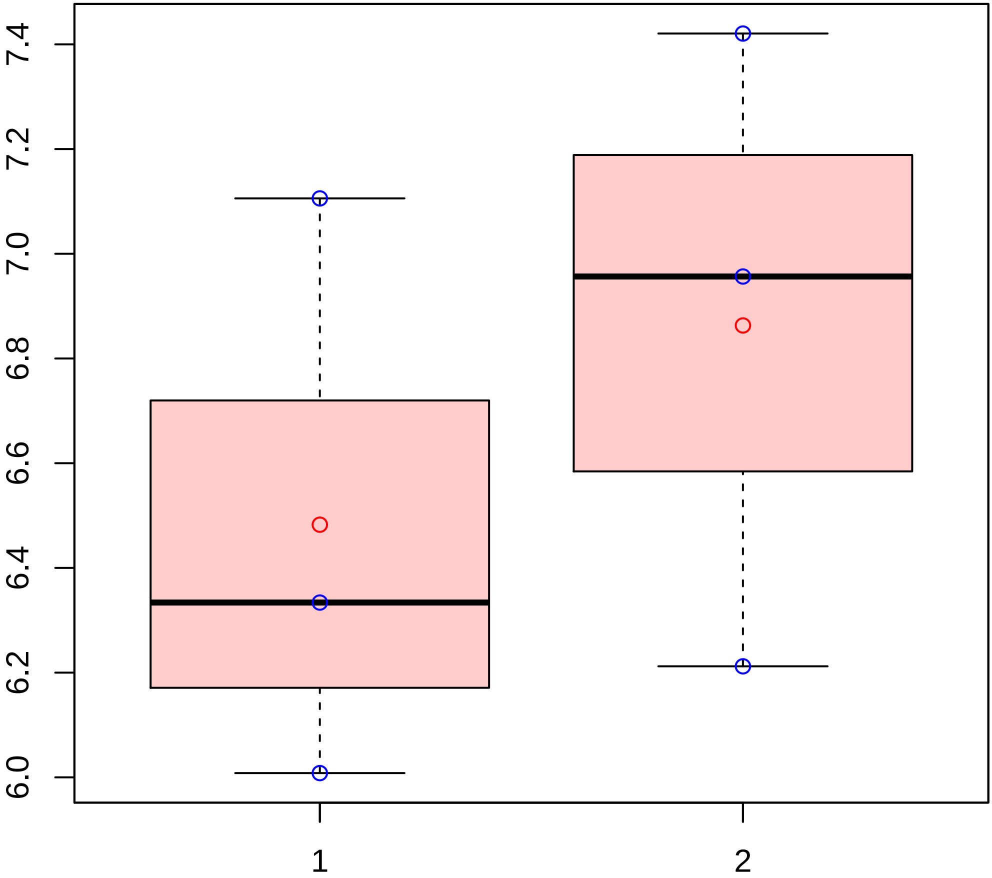

t−Test: p−value = 0.47

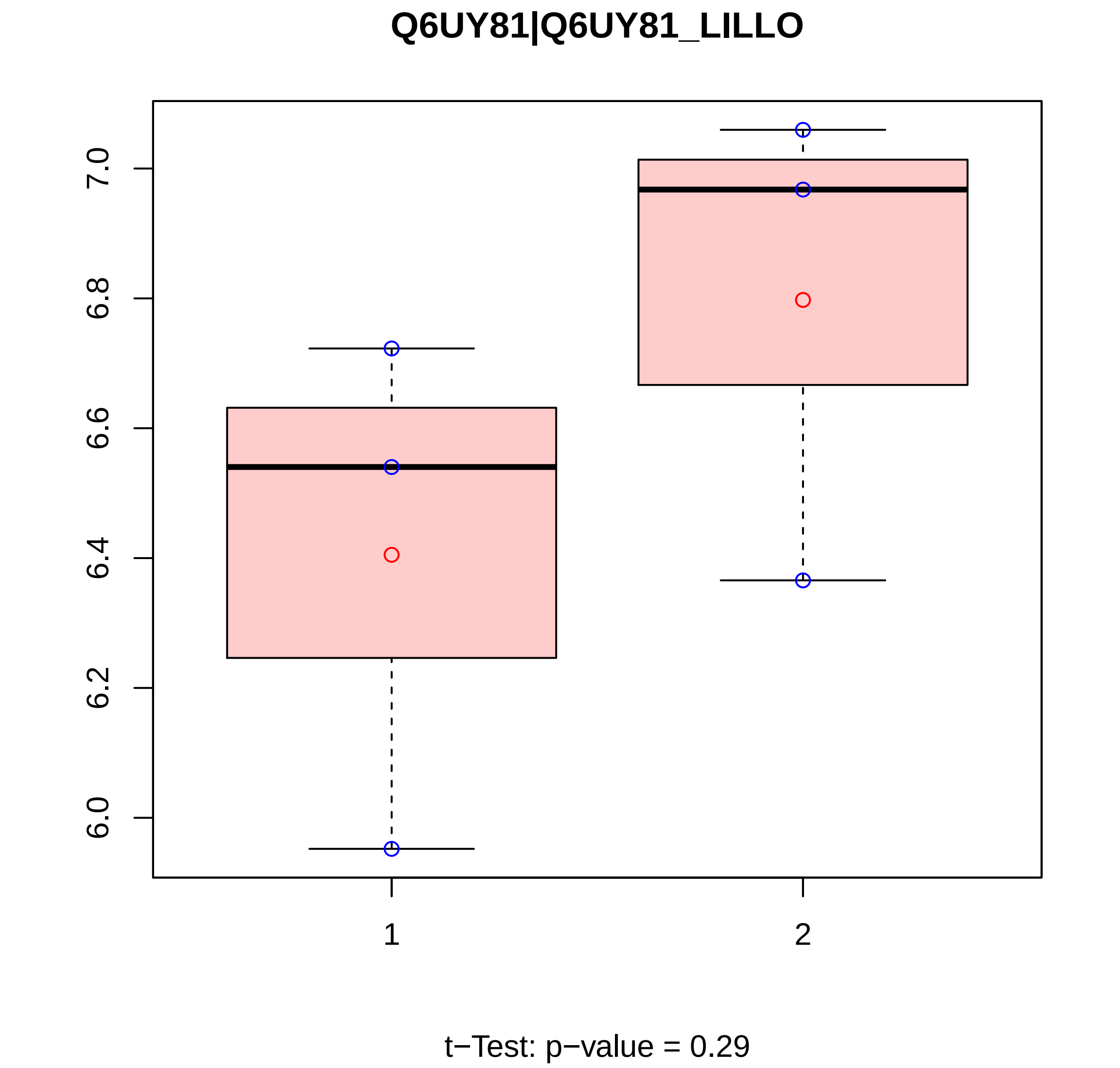
Q6UY81|Q6UY81_LILLO
7.0
6.8
6.6
6.4
6.2
6.0
1
2
t−Test: p−value = 0.29

Q6V9T1|Q6V9T1_ORYSJ

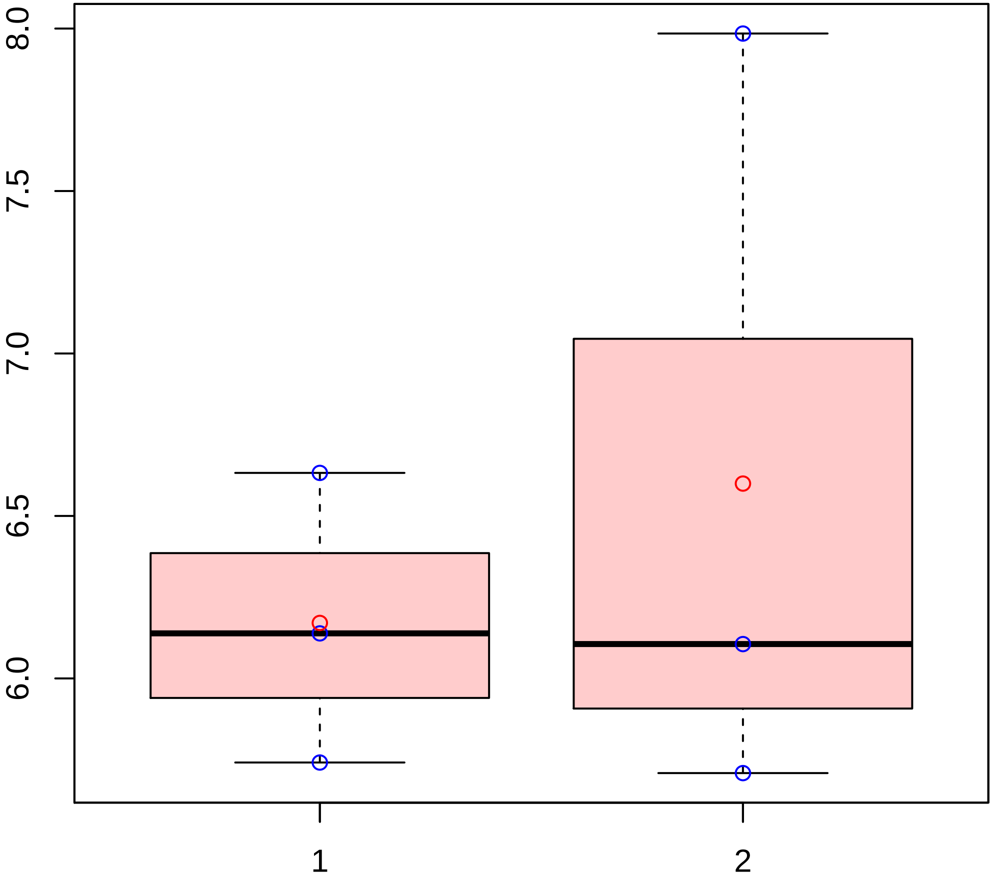

t−Test: p−value = 0.61

Q6YKA9|Q6YKA9_WHEAT

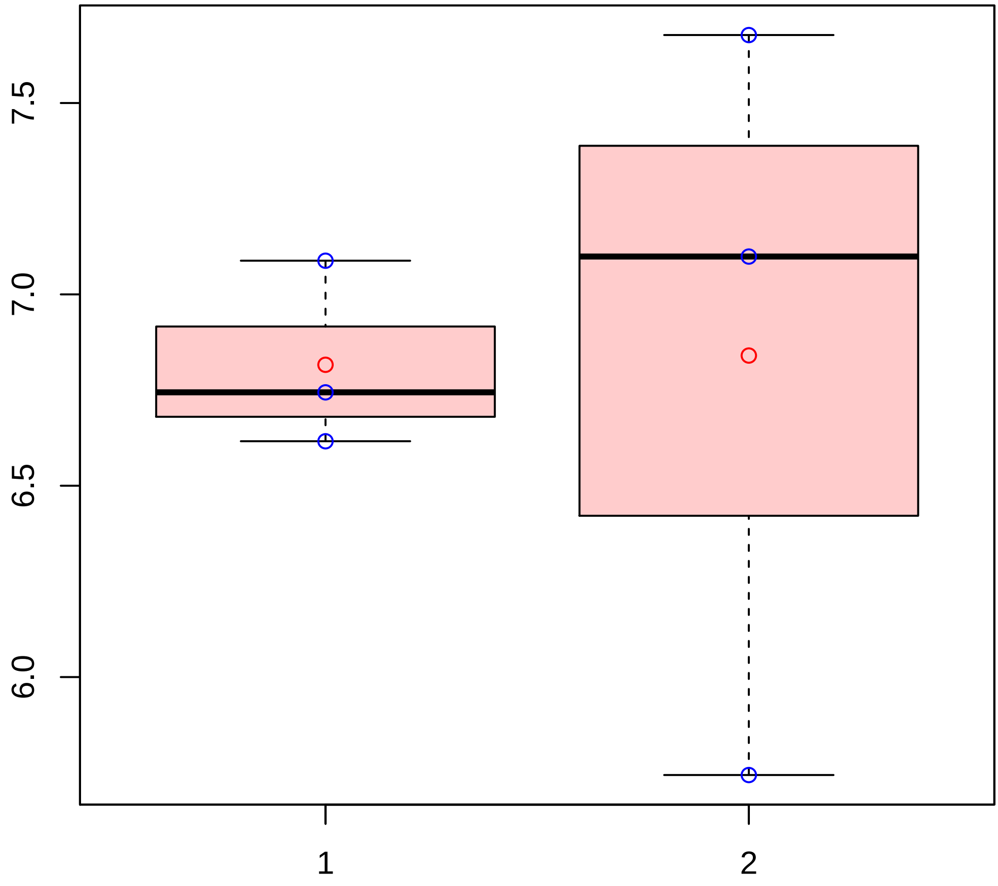

t−Test: p−value = 0.97

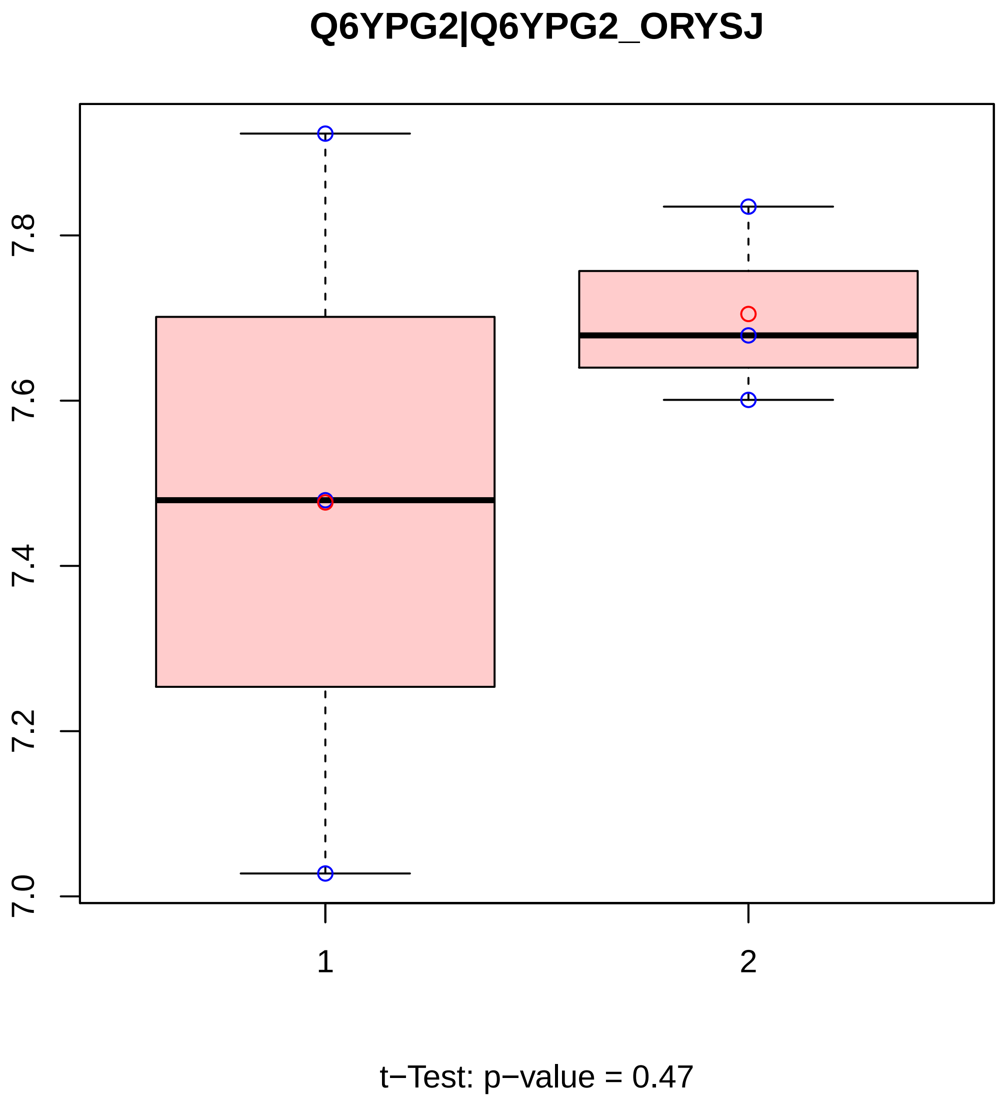

Q6YPG2|Q6YPG2_ORYSJ
7.0
7.2
7.4
7.6
7.8
1
2
t−Test: p−value = 0.47

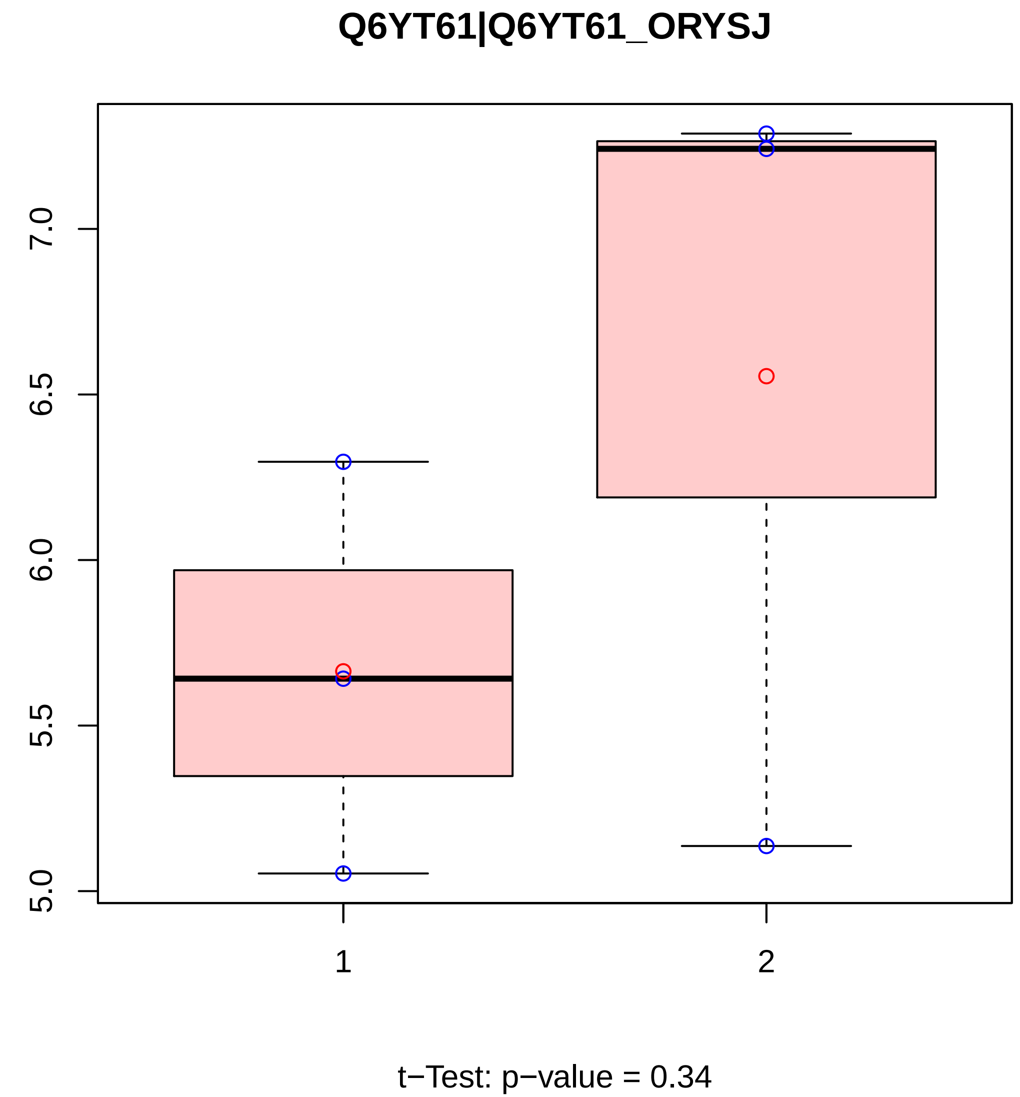
Q6YT61|Q6YT61_ORYSJ
7.0
6.5
6.0
5.5
5.0
1
2
t−Test: p−value = 0.34

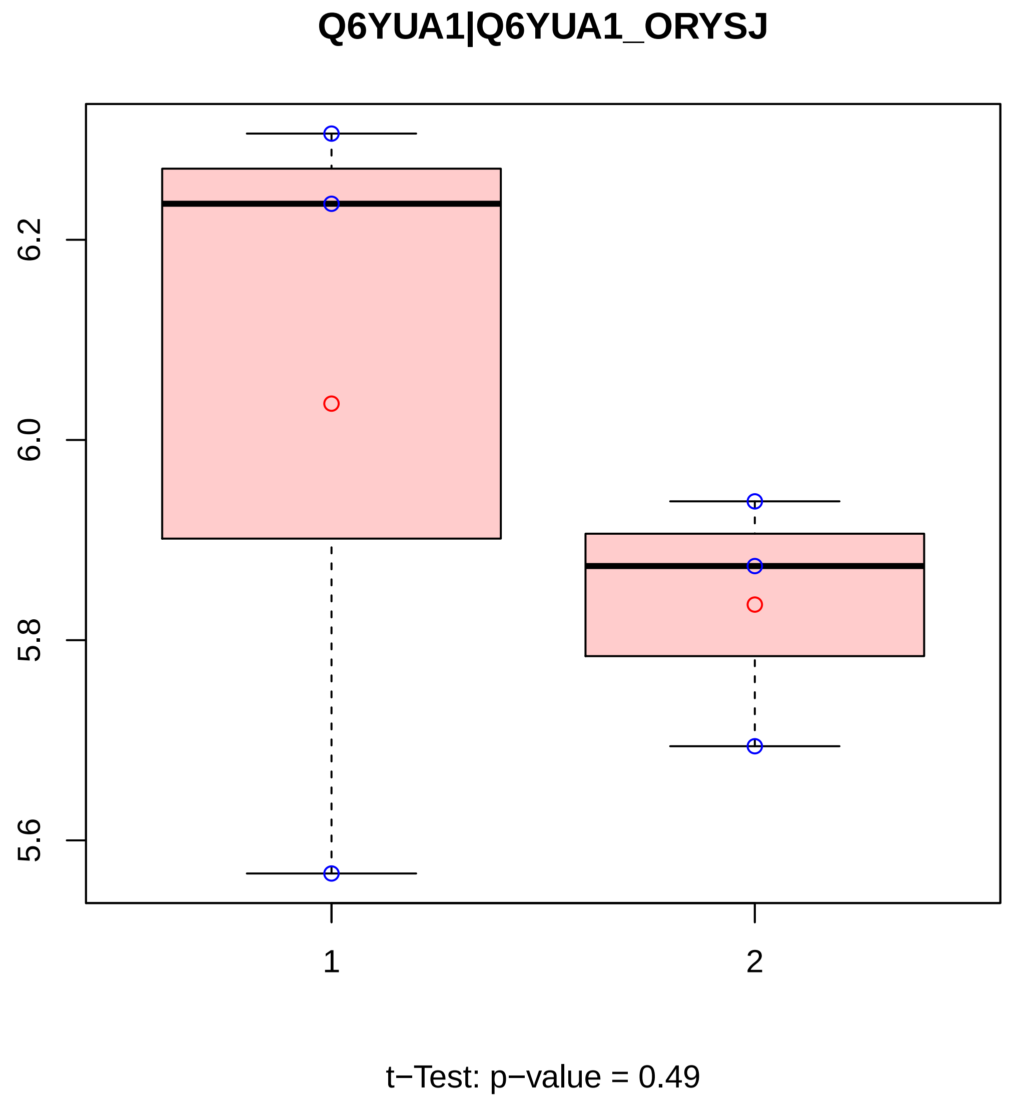

Q6YUA1|Q6YUA1_ORYSJ
6.2
6.0
5.8
5.6
1
2
t−Test: p−value = 0.49

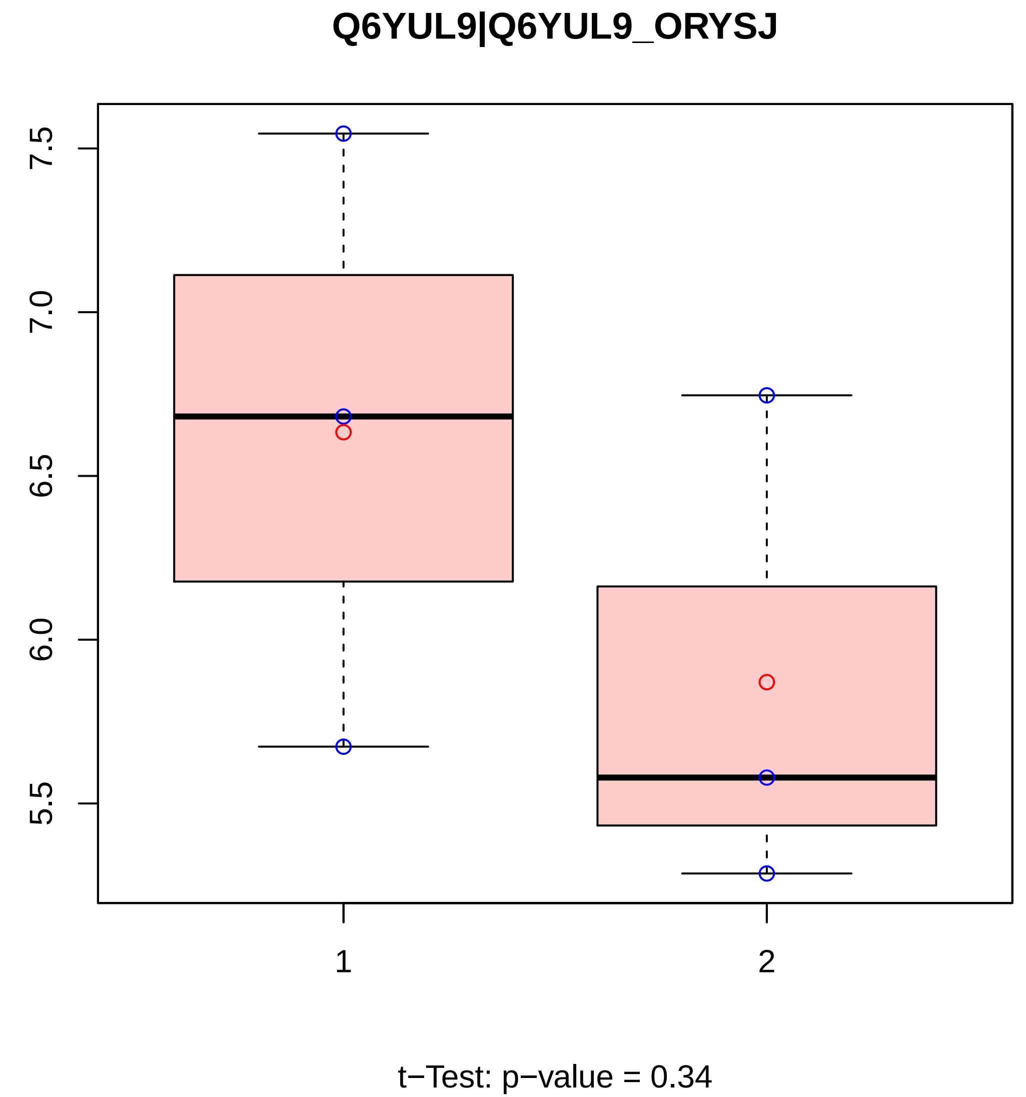
Q6YUL9|Q6YUL9_ORYSJ
7.5
7.0
6.5
6.0
5.5
1
2
t−Test: p−value = 0.34

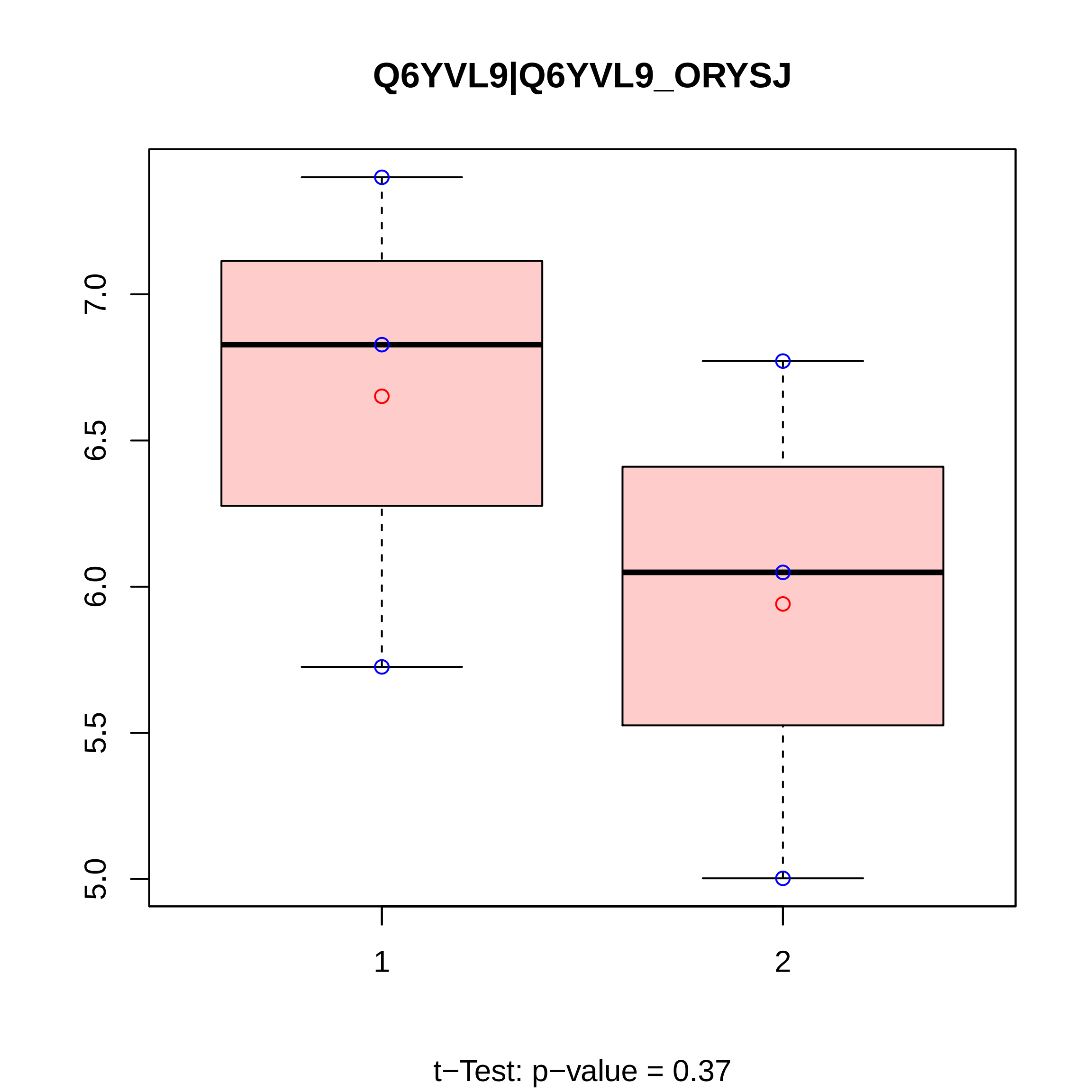
Q6YVL9|Q6YVL9_ORYSJ
7.0
6.5
6.0
5.5
5.0
1
2
t−Test: p−value = 0.37

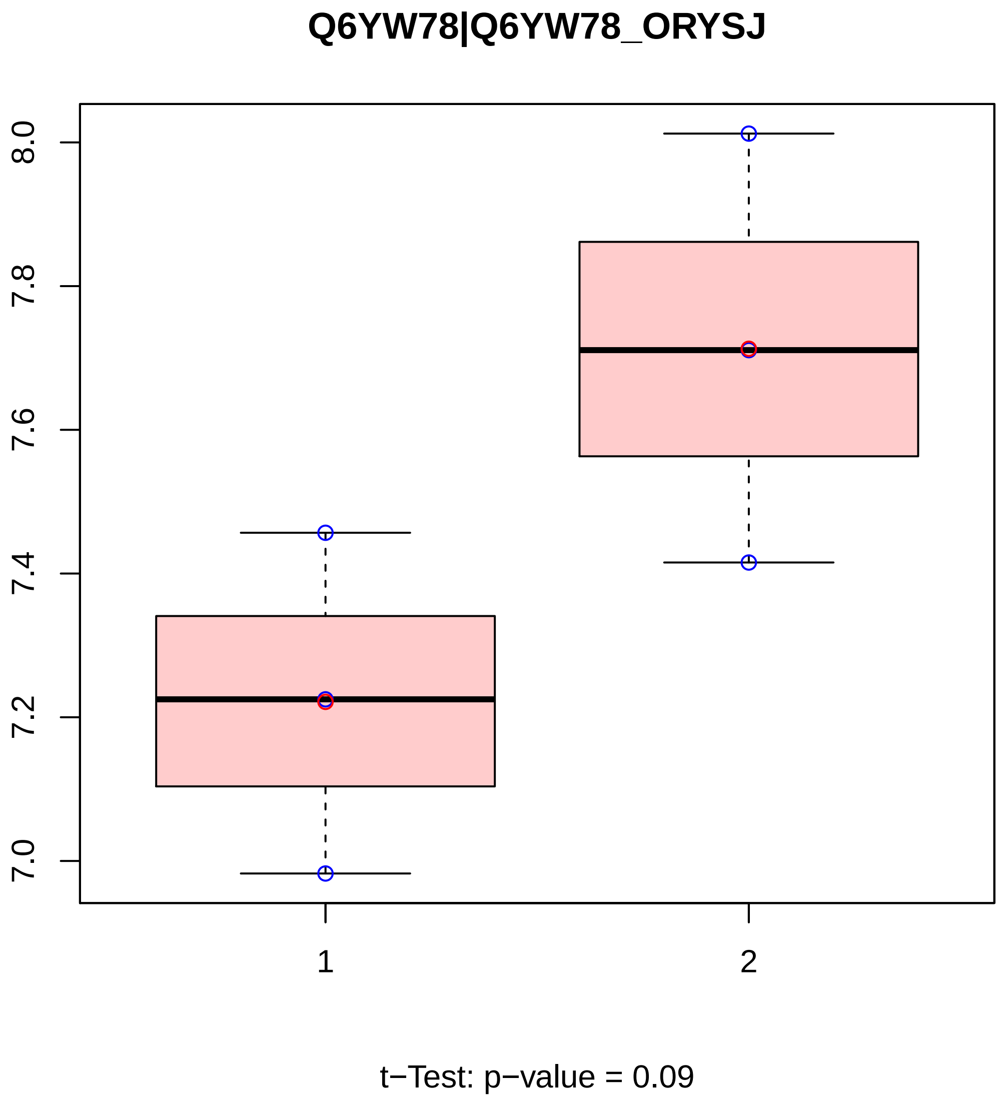
Q6YW78|Q6YW78_ORYSJ
8.0
7.8
7.6
7.4
7.2
7.0
1
2
t−Test: p−value = 0.09

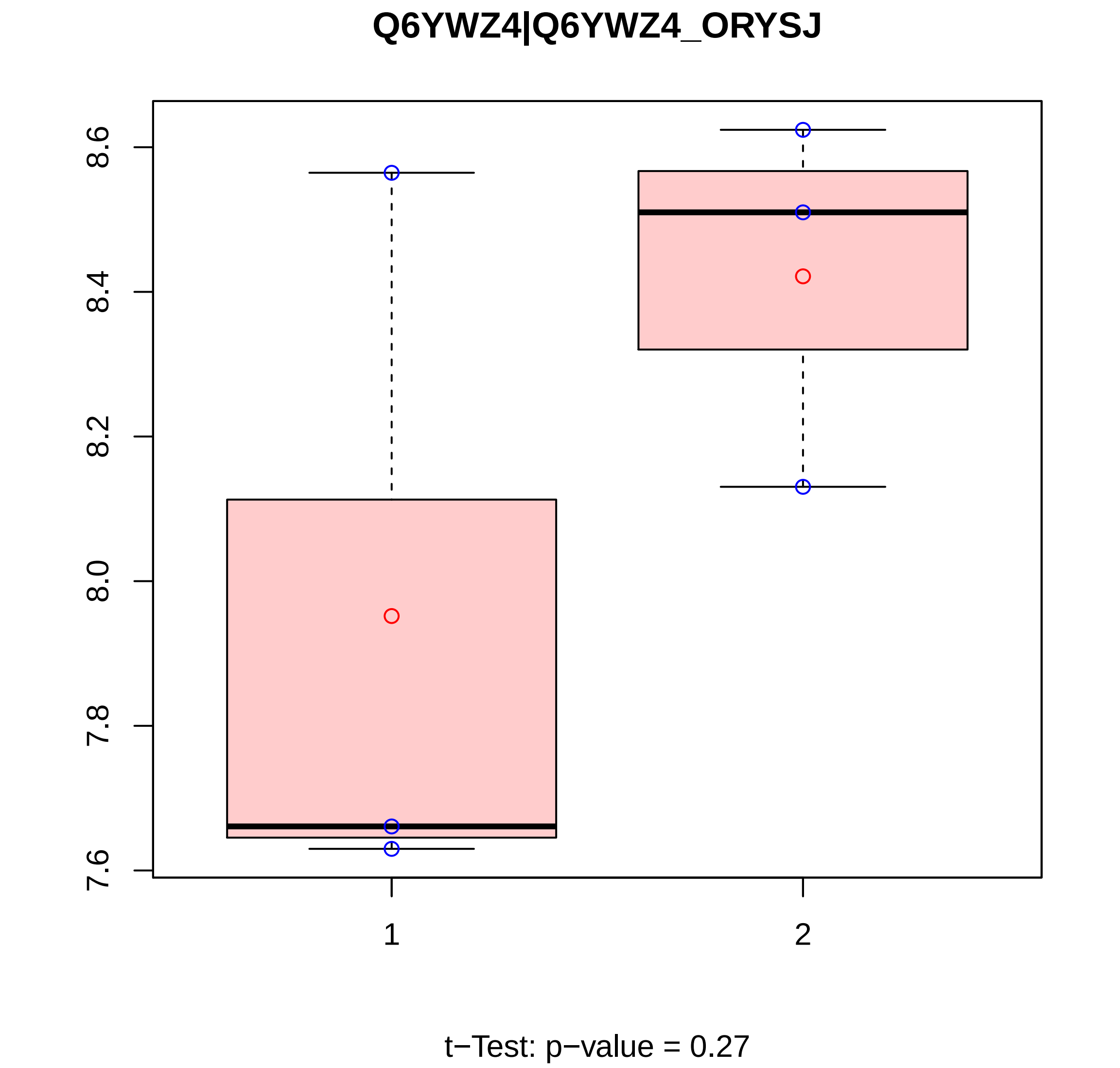

Q6YWZ4|Q6YWZ4_ORYSJ
8.6
8.4
8.2
8.0
7.8
7.6
1
2
t−Test: p−value = 0.27

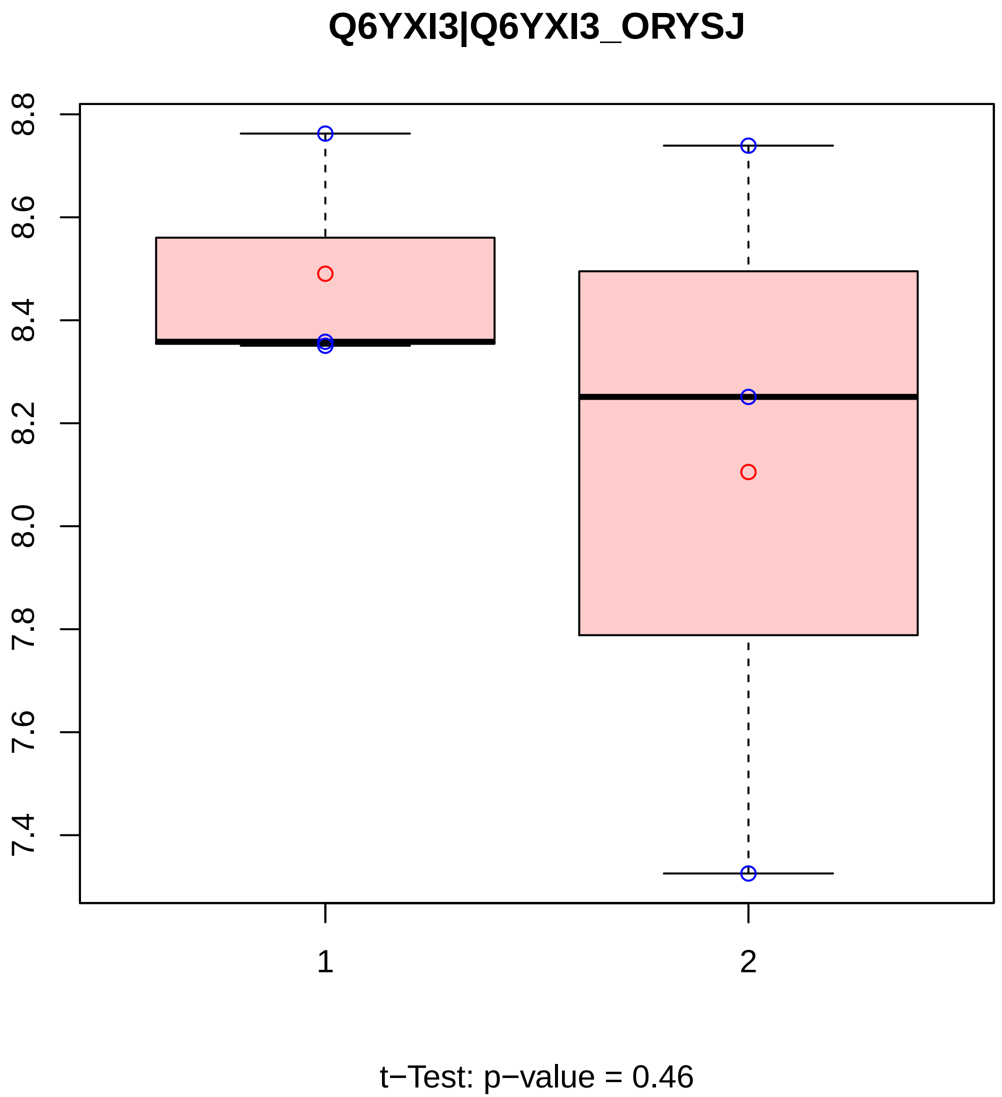
Q6YXI3|Q6YXI3_ORYSJ
8.8
8.6
8.4
8.2
8.0
7.8
7.6
7.4
1
2
t−Test: p−value = 0.46

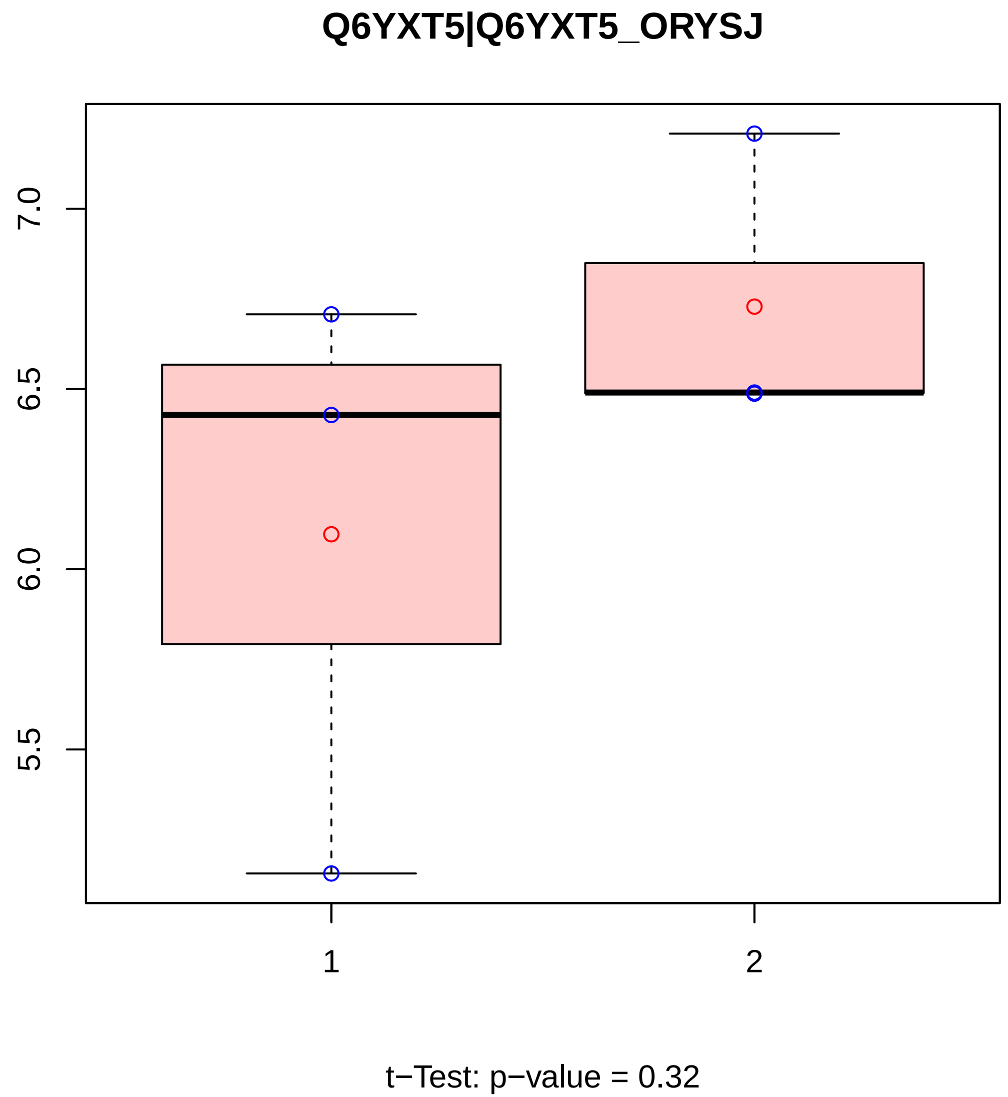

Q6YXT5|Q6YXT5_ORYSJ
7.0
6.5
6.0
5.5
1
2
t−Test: p−value = 0.32

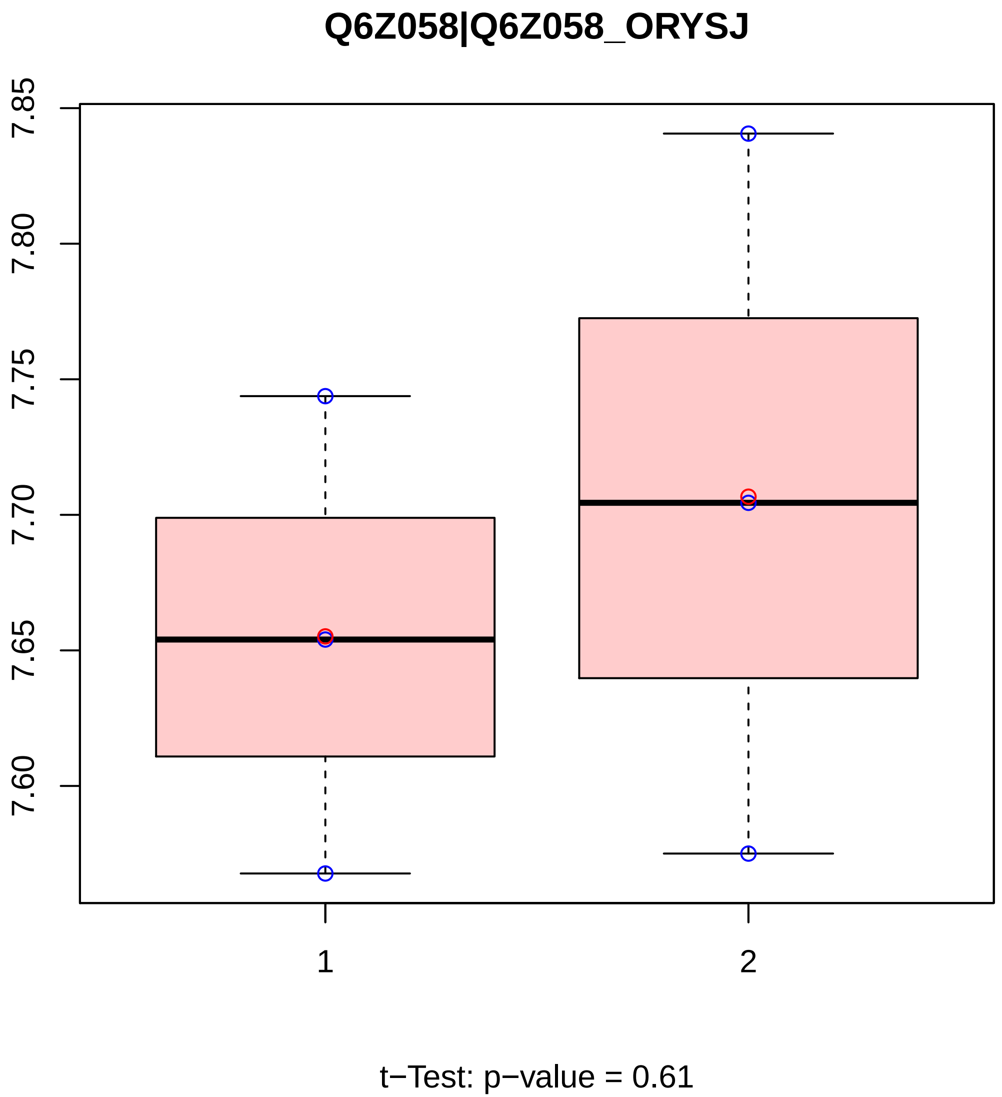

Q6Z058|Q6Z058_ORYSJ
7.85
7.80
7.75
7.70
7.65
7.60
1
2
t−Test: p−value = 0.61

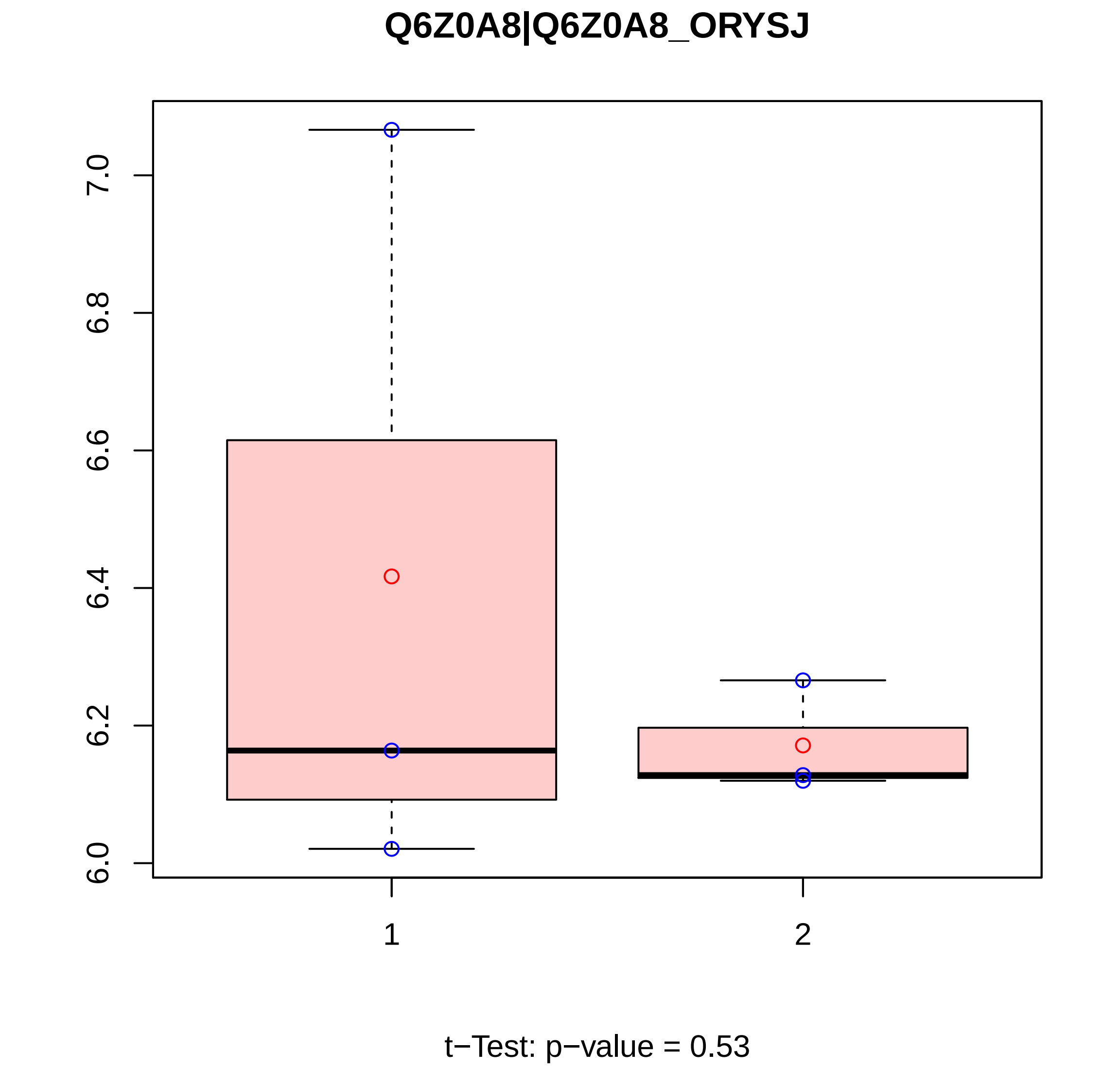
Q6Z0A8|Q6Z0A8_ORYSJ
7.0
6.8
6.6
6.4
6.2
6.0
1
2
t−Test: p−value = 0.53

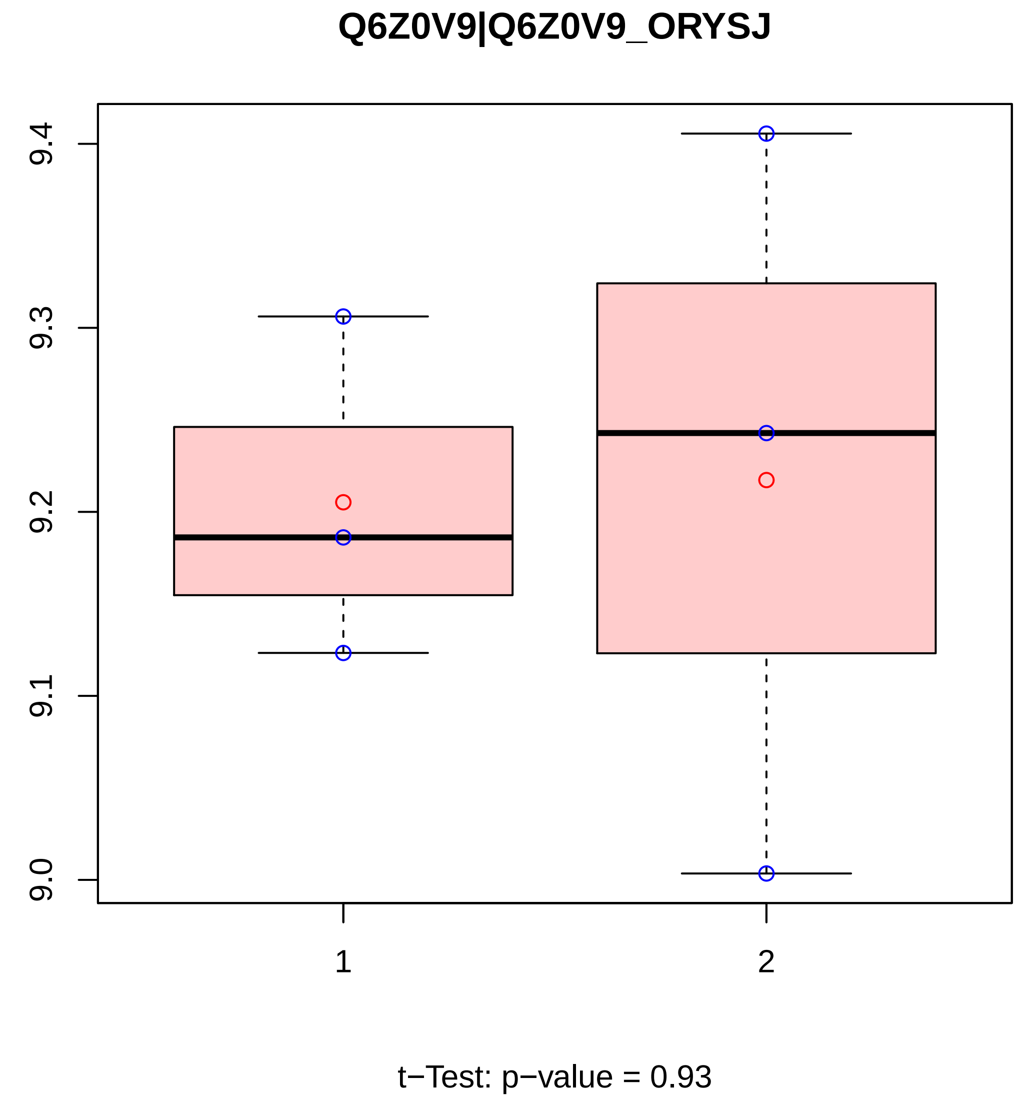

Q6Z0V9|Q6Z0V9_ORYSJ
9.0
9.1
9.2
9.3
9.4
1
2
t−Test: p−value = 0.93

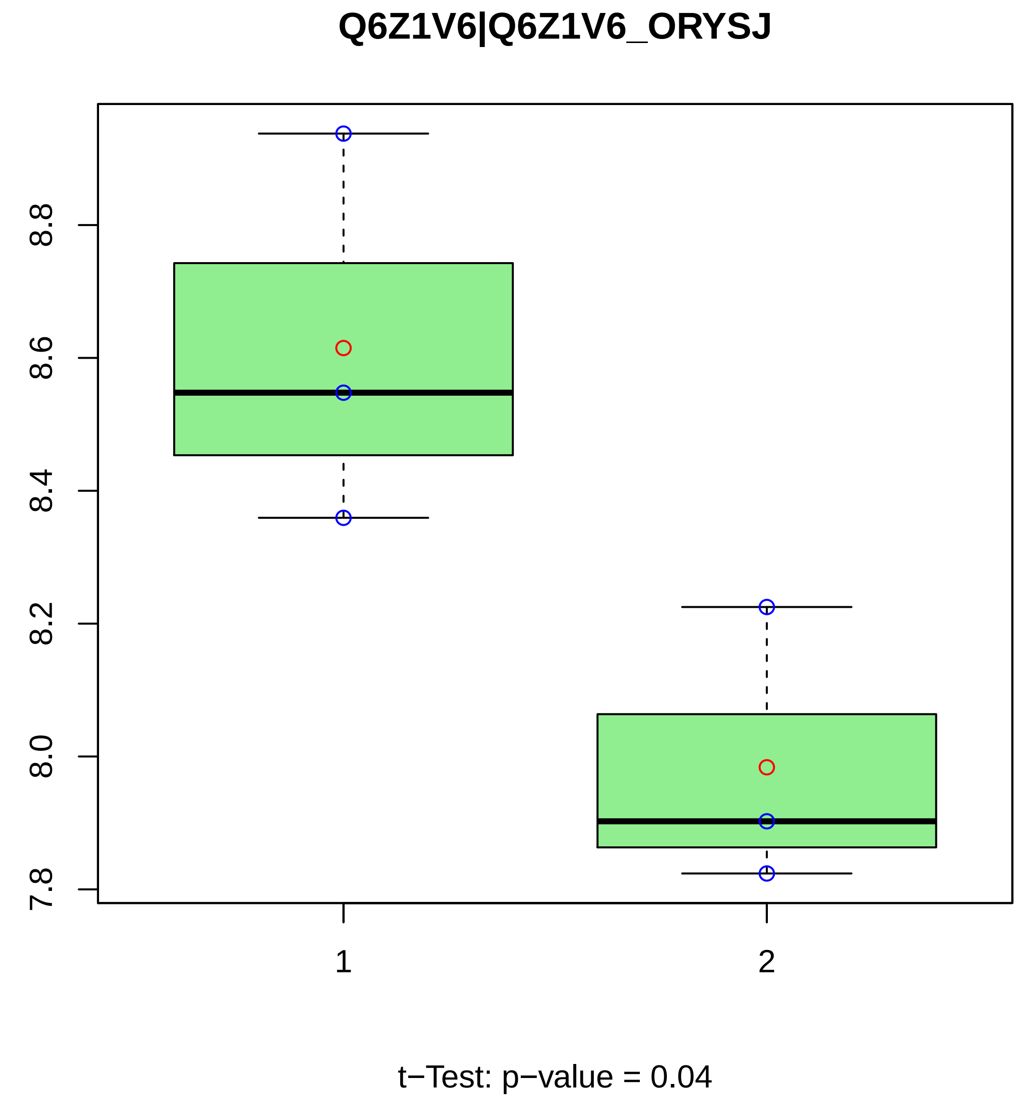
Q6Z1V6|Q6Z1V6_ORYSJ
8.8
8.6
8.4
8.2
8.0
7.8
1
2
t−Test: p−value = 0.04

Q6Z3L5|Q6Z3L5_ORYSJ

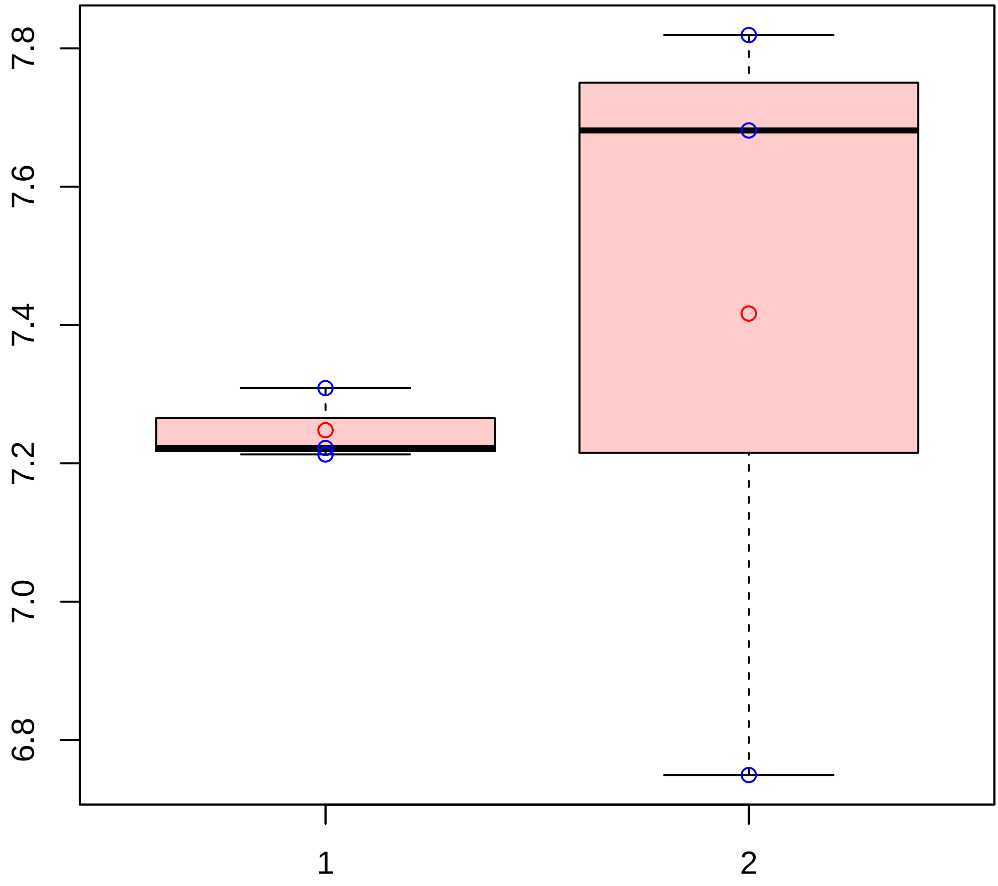

t−Test: p−value = 0.67

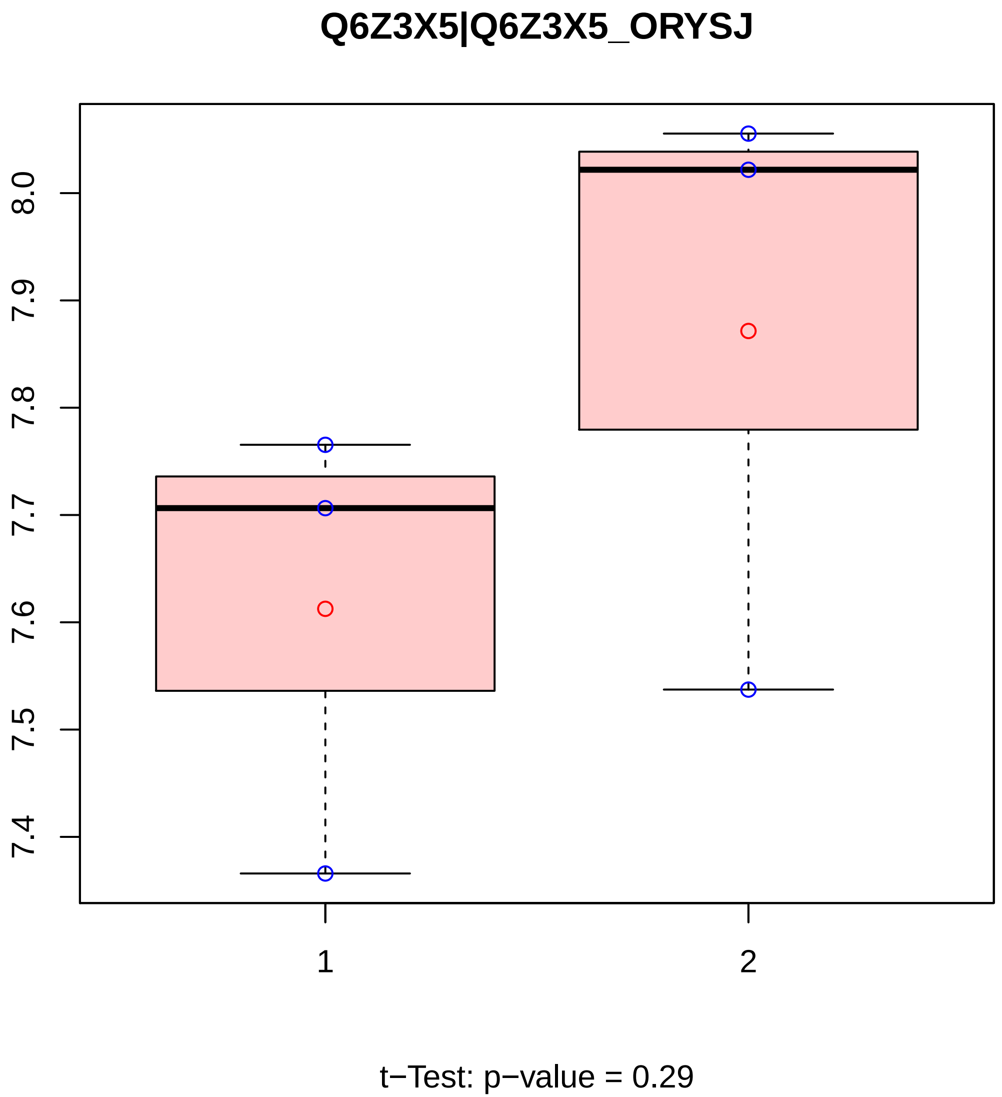
Q6Z3X5|Q6Z3X5_ORYSJ
8.0
7.9
7.8
7.7
7.6
7.5
7.4
1
2
t−Test: p−value = 0.29

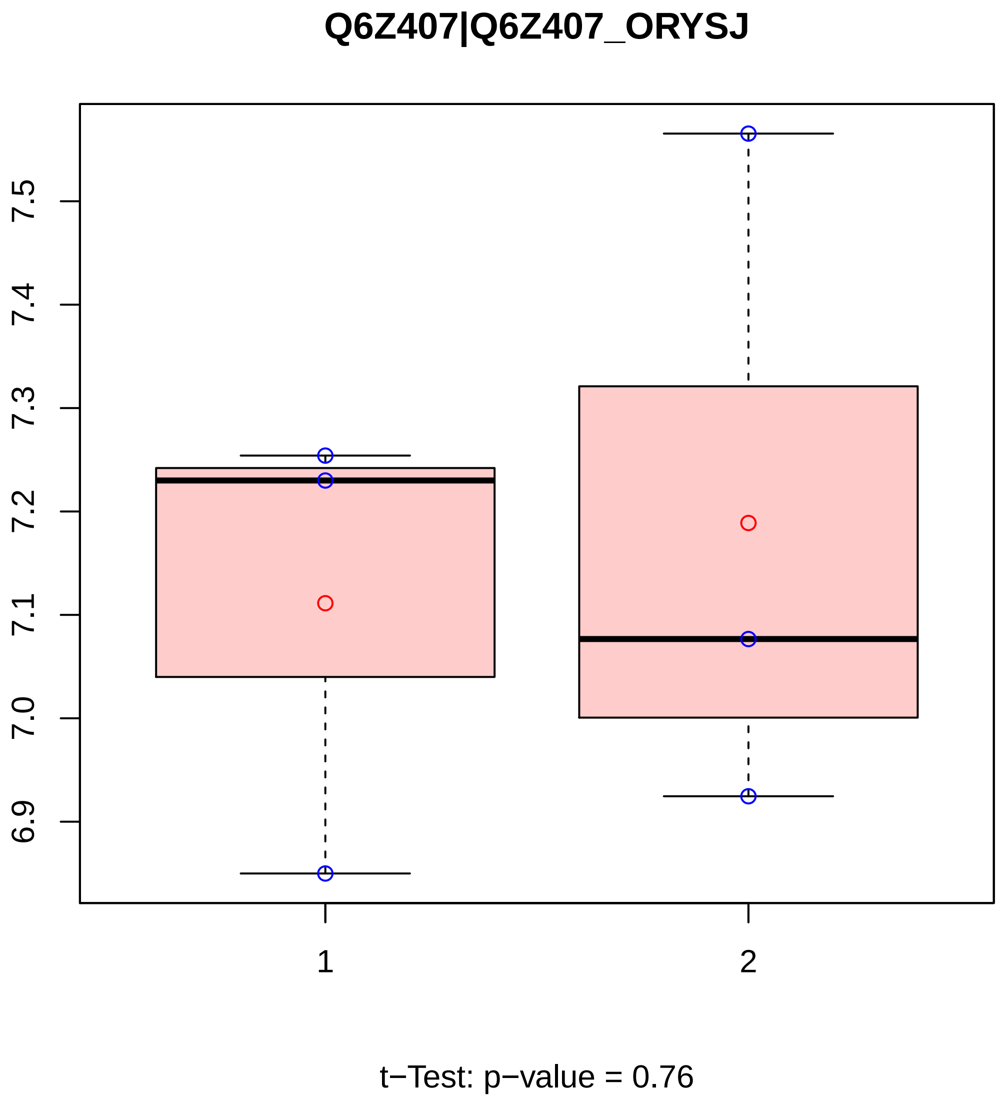
Q6Z407|Q6Z407_ORYSJ
7.5
7.4
7.3
7.2
7.1
7.0
6.9
1
2
t−Test: p−value = 0.76

Q6Z7F0|Q6Z7F0_ORYSJ

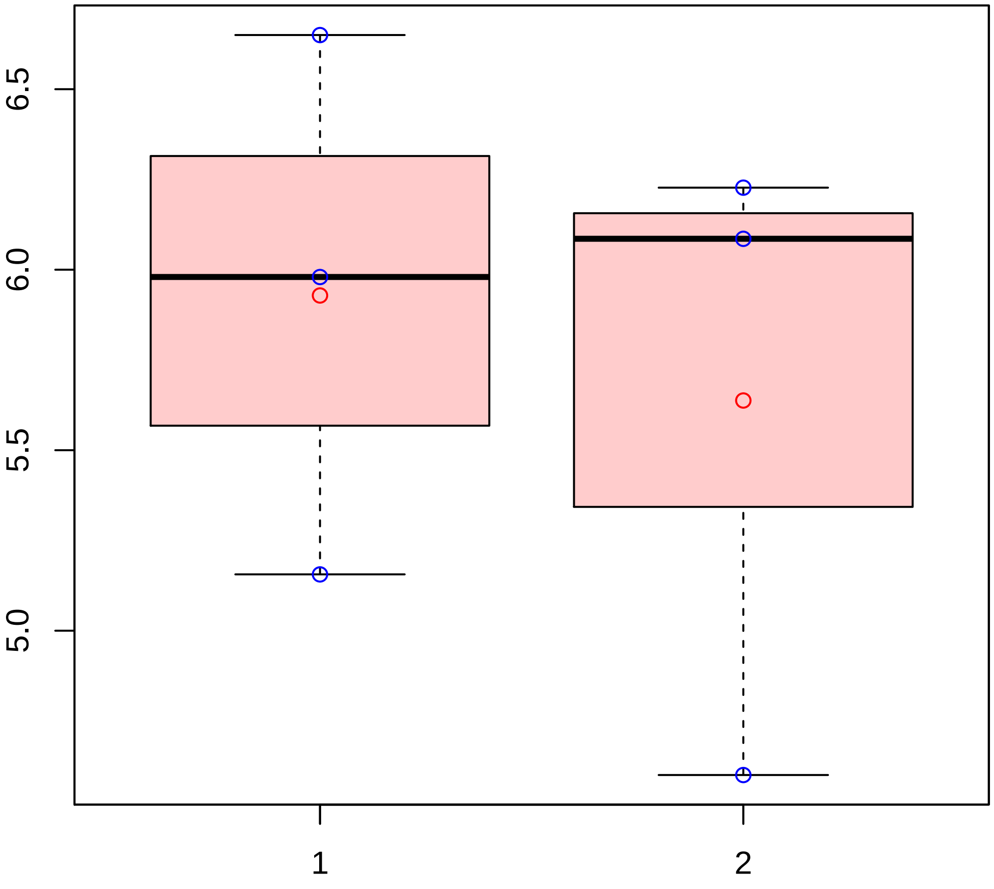

t−Test: p−value = 0.69

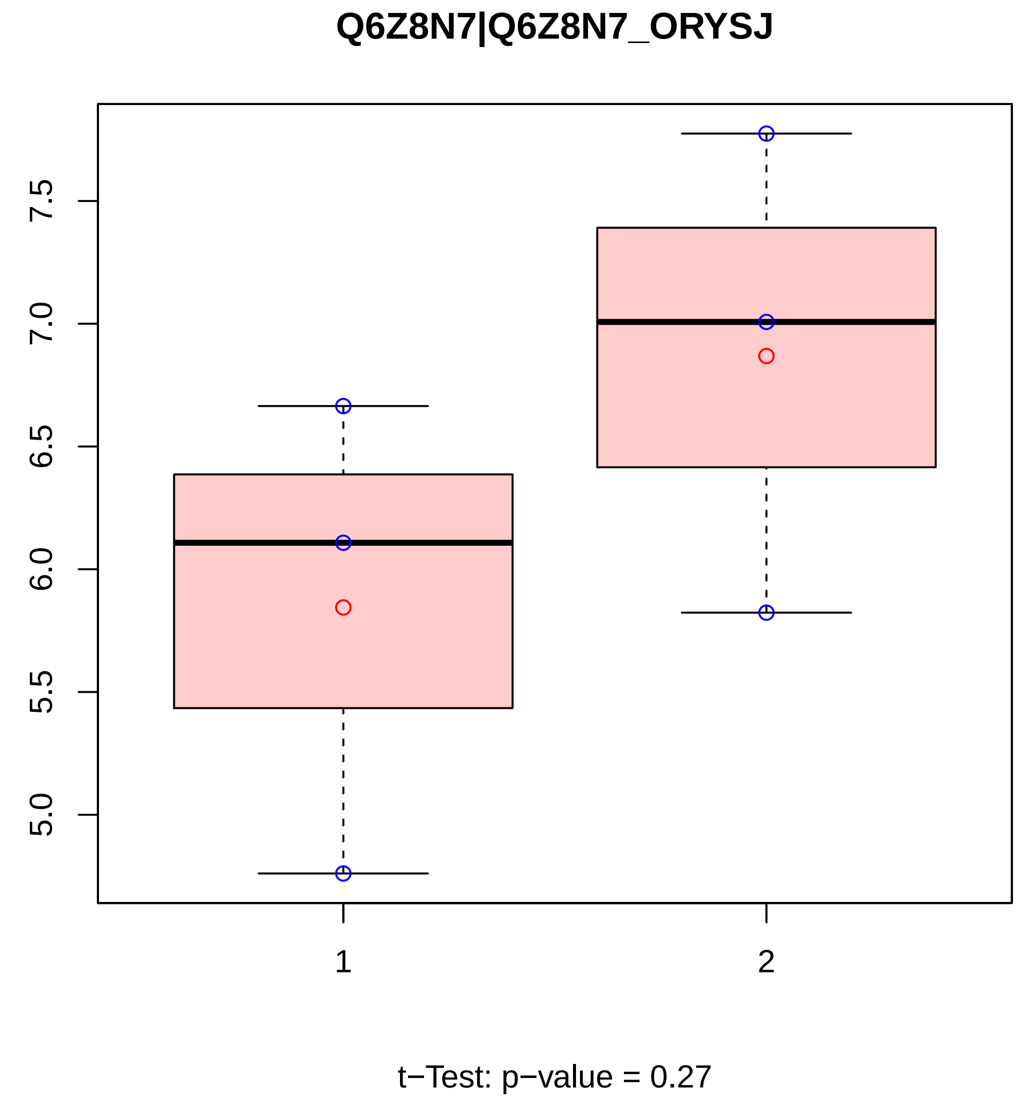
Q6Z8N7|Q6Z8N7_ORYSJ
7.5
7.0
6.5
6.0
5.5
5.0
1
2
t−Test: p−value = 0.27

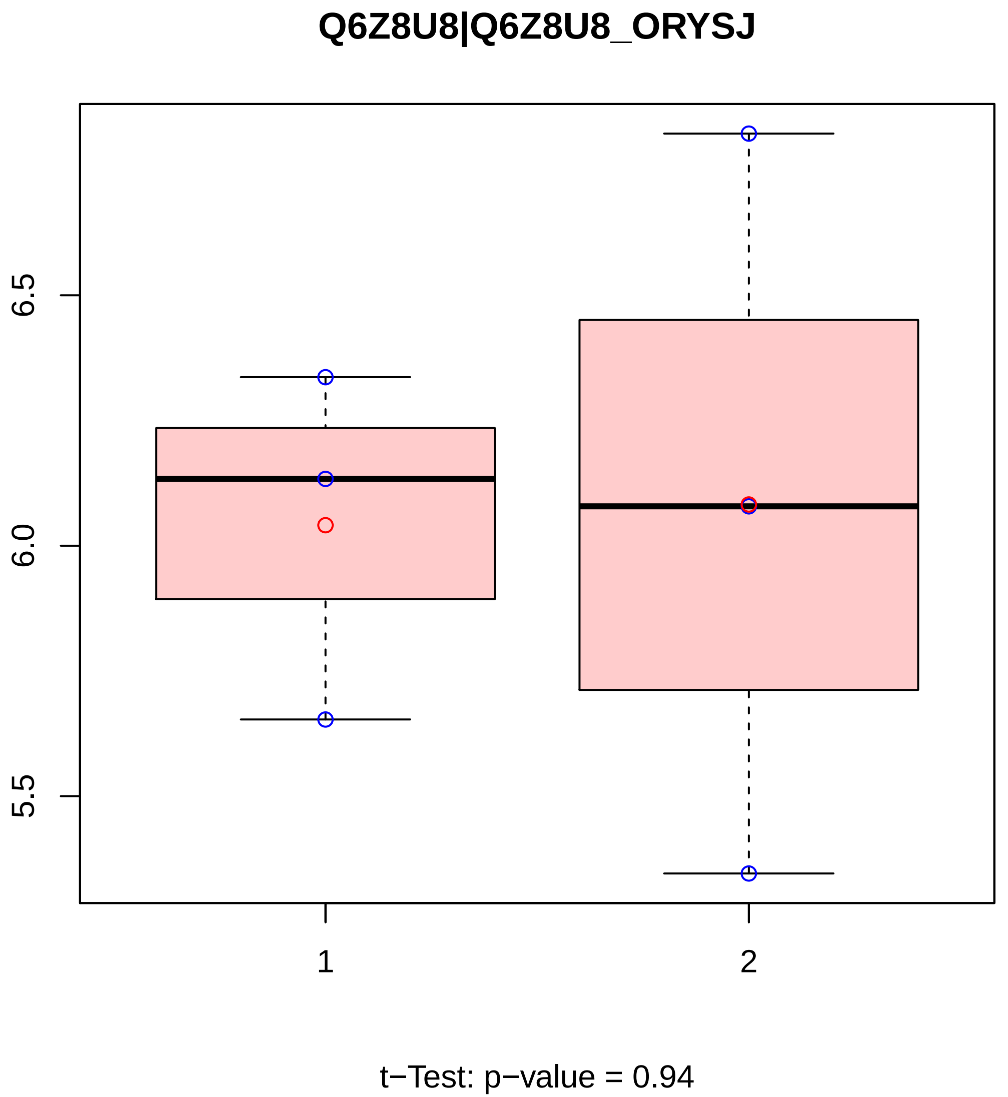

Q6Z8U8|Q6Z8U8_ORYSJ
6.5
6.0
5.5
1
2
t−Test: p−value = 0.94

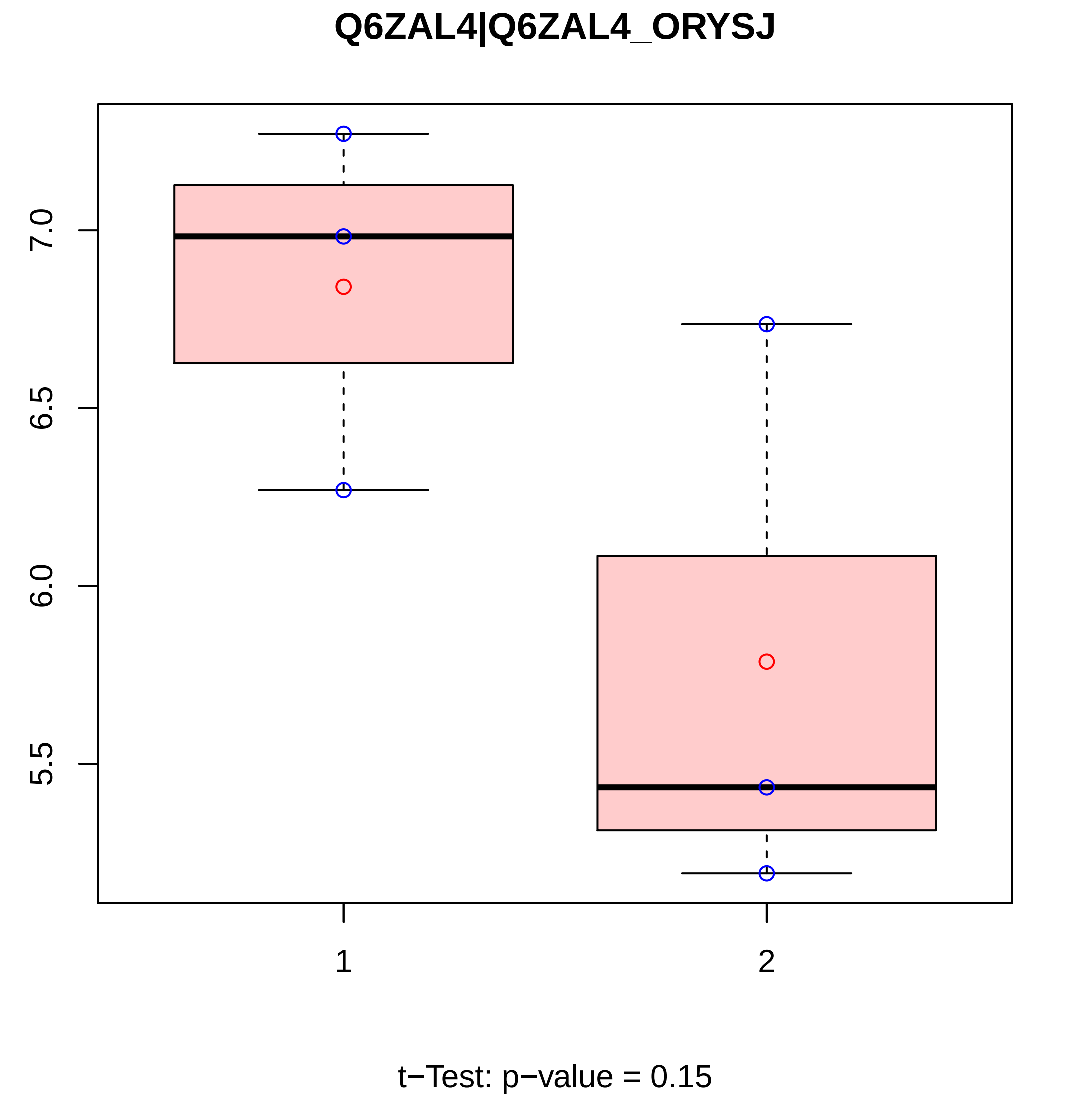
Q6ZAL4|Q6ZAL4_ORYSJ
7.0
6.5
6.0
5.5
1
2
t−Test: p−value = 0.15

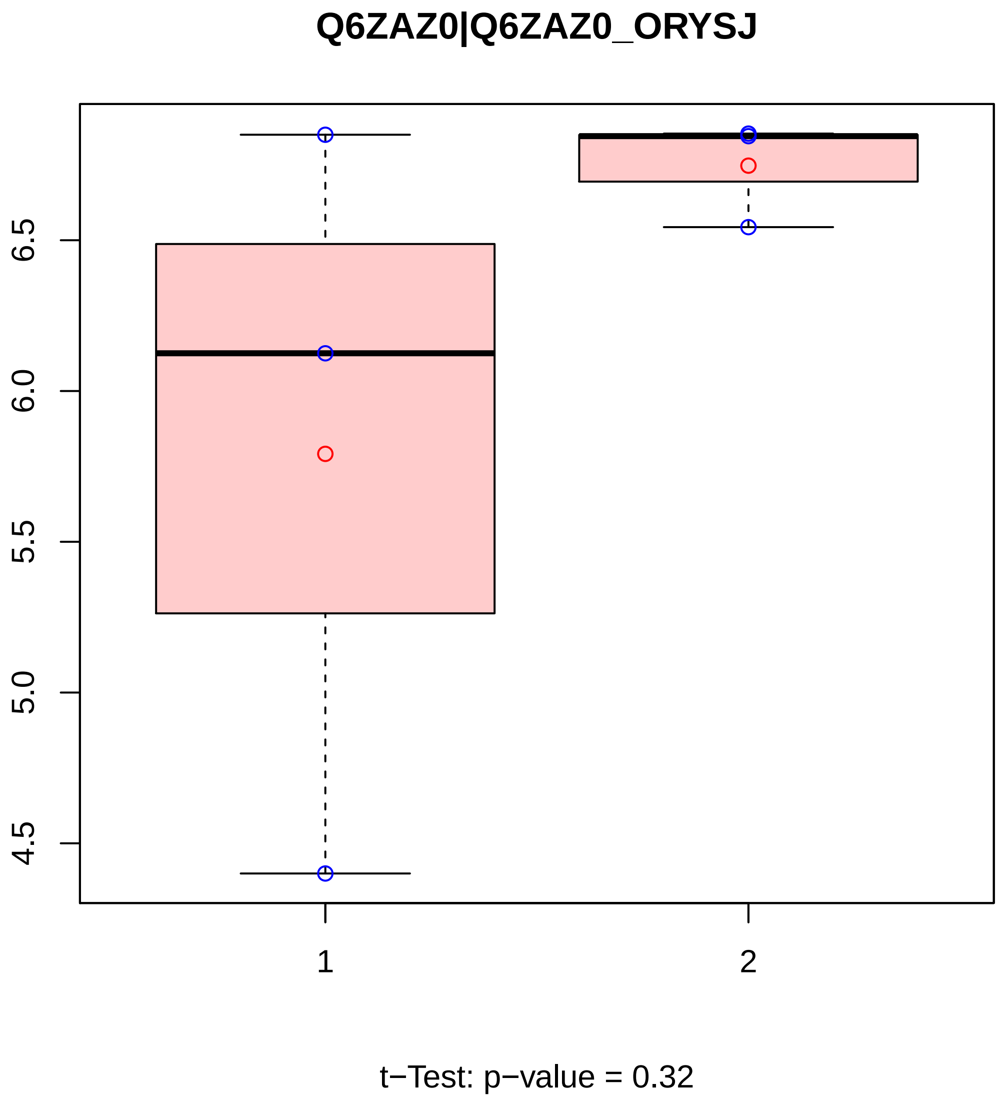

Q6ZAZ0|Q6ZAZ0_ORYSJ
6.5
6.0
5.5
5.0
4.5
1
2
t−Test: p−value = 0.32

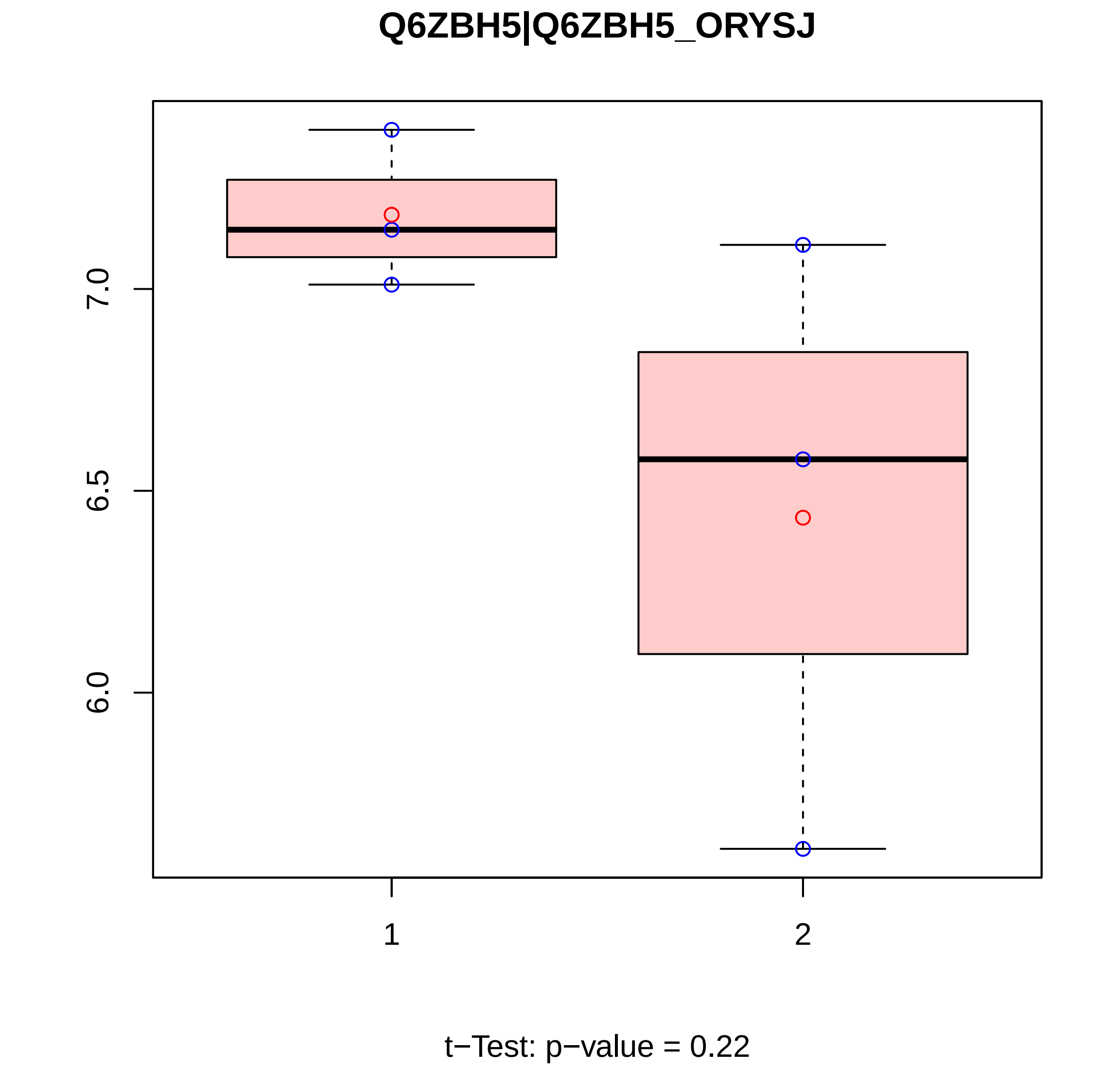
Q6ZBH5|Q6ZBH5_ORYSJ
7.0
6.5
6.0
1
2
t−Test: p−value = 0.22

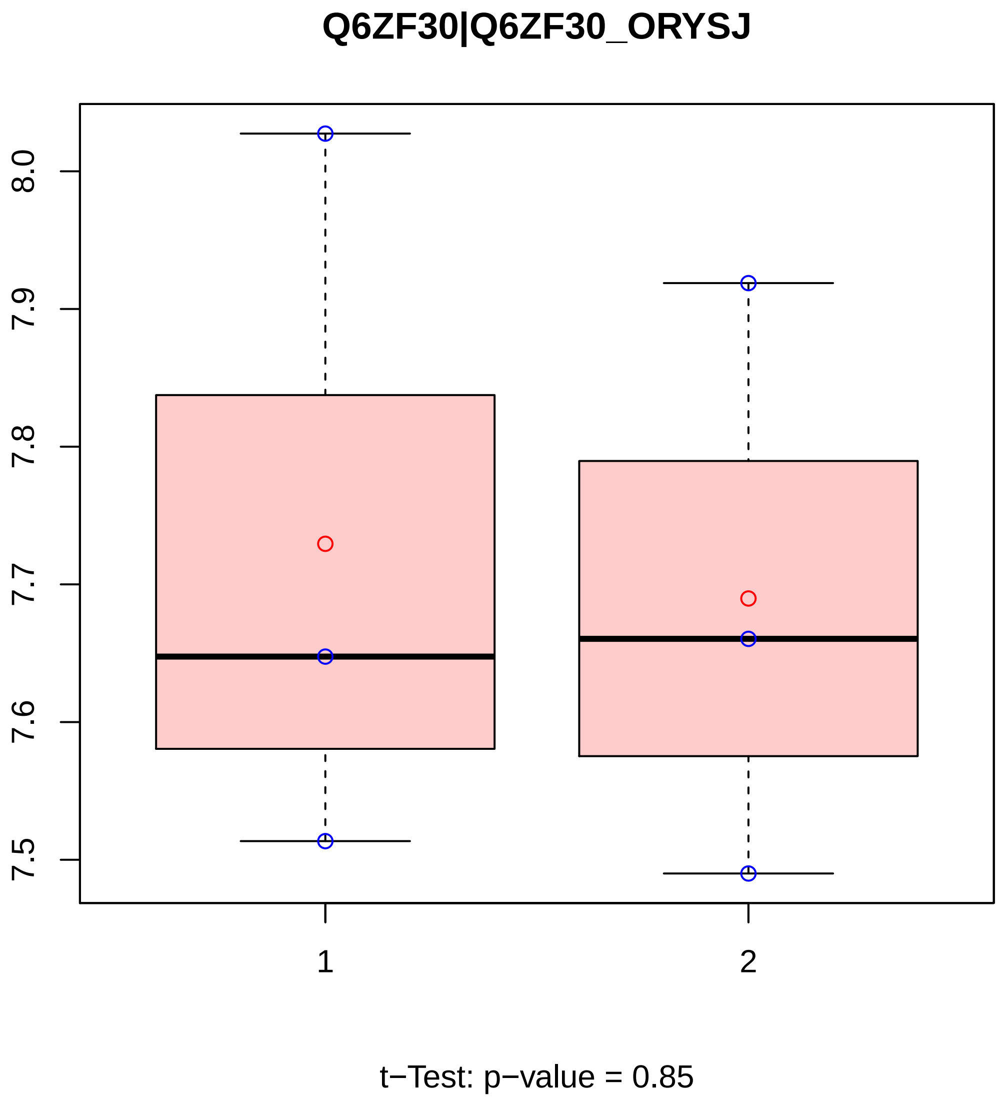
Q6ZF30|Q6ZF30_ORYSJ
8.0
7.9
7.8
7.7
7.6
7.5
1
2
t−Test: p−value = 0.85

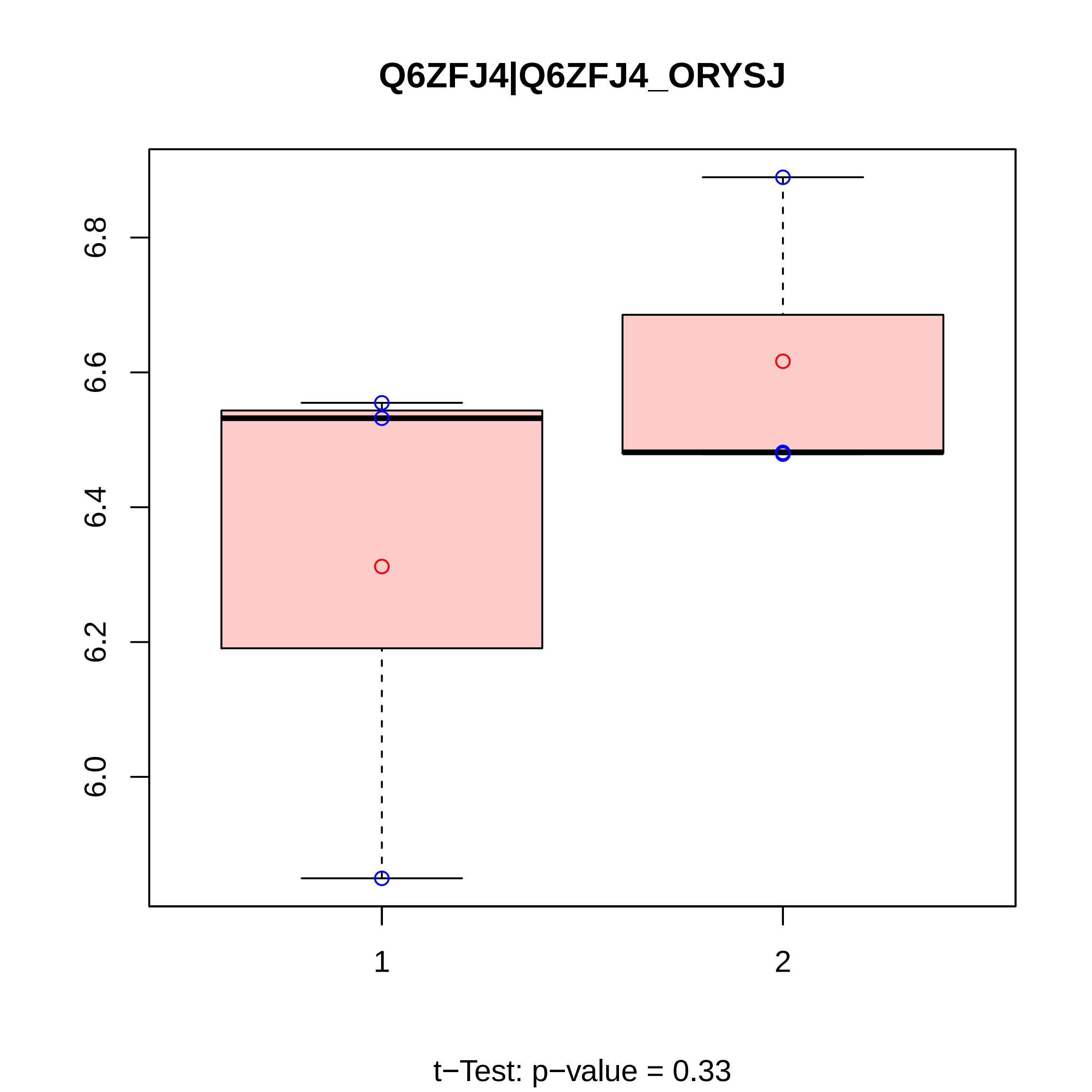
Q6ZFJ4|Q6ZFJ4_ORYSJ
6.0
6.2
6.4
6.6
6.8
1
2
t−Test: p−value = 0.33

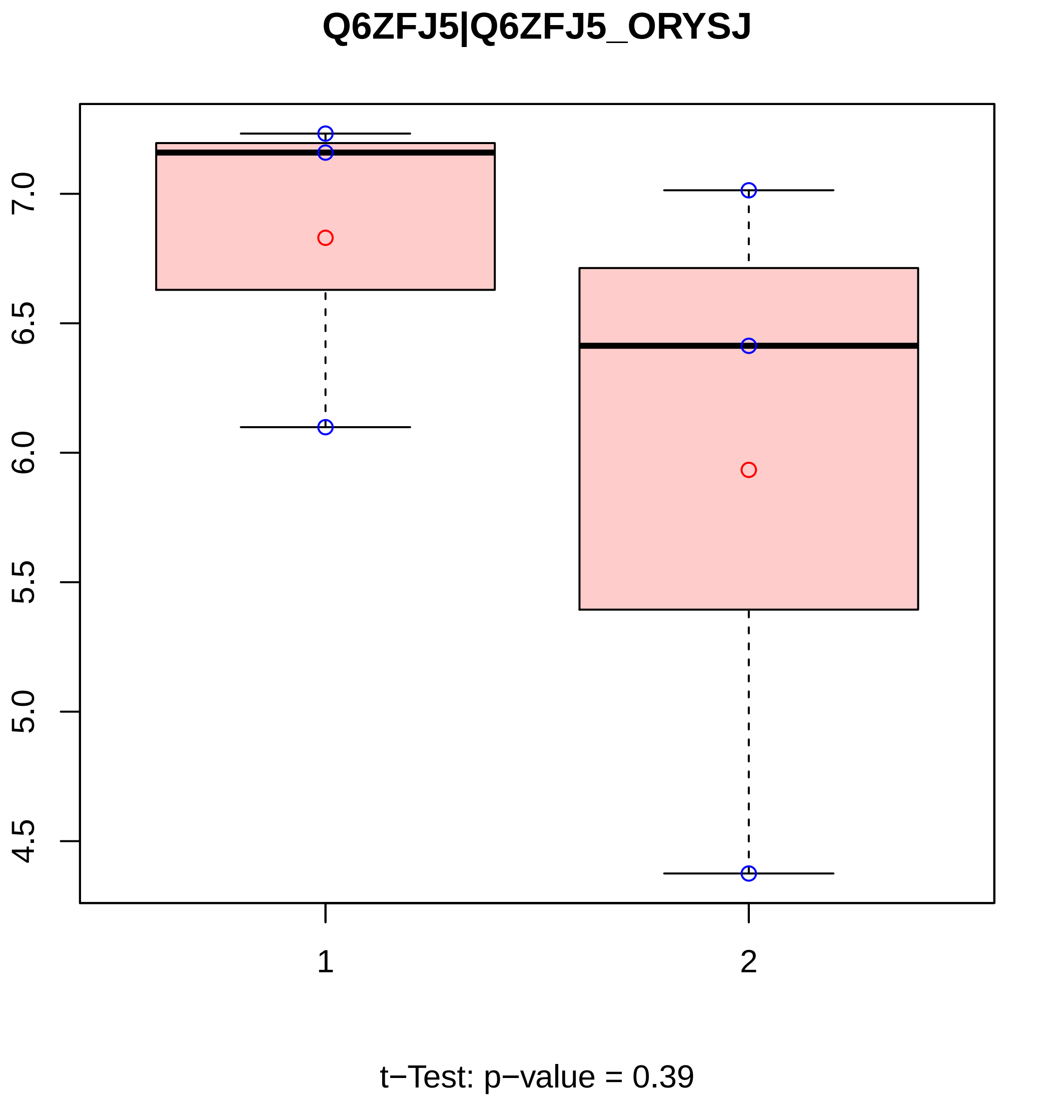
Q6ZFJ5|Q6ZFJ5_ORYSJ
7.0
6.5
6.0
5.5
5.0
4.5
1
2
t−Test: p−value = 0.39

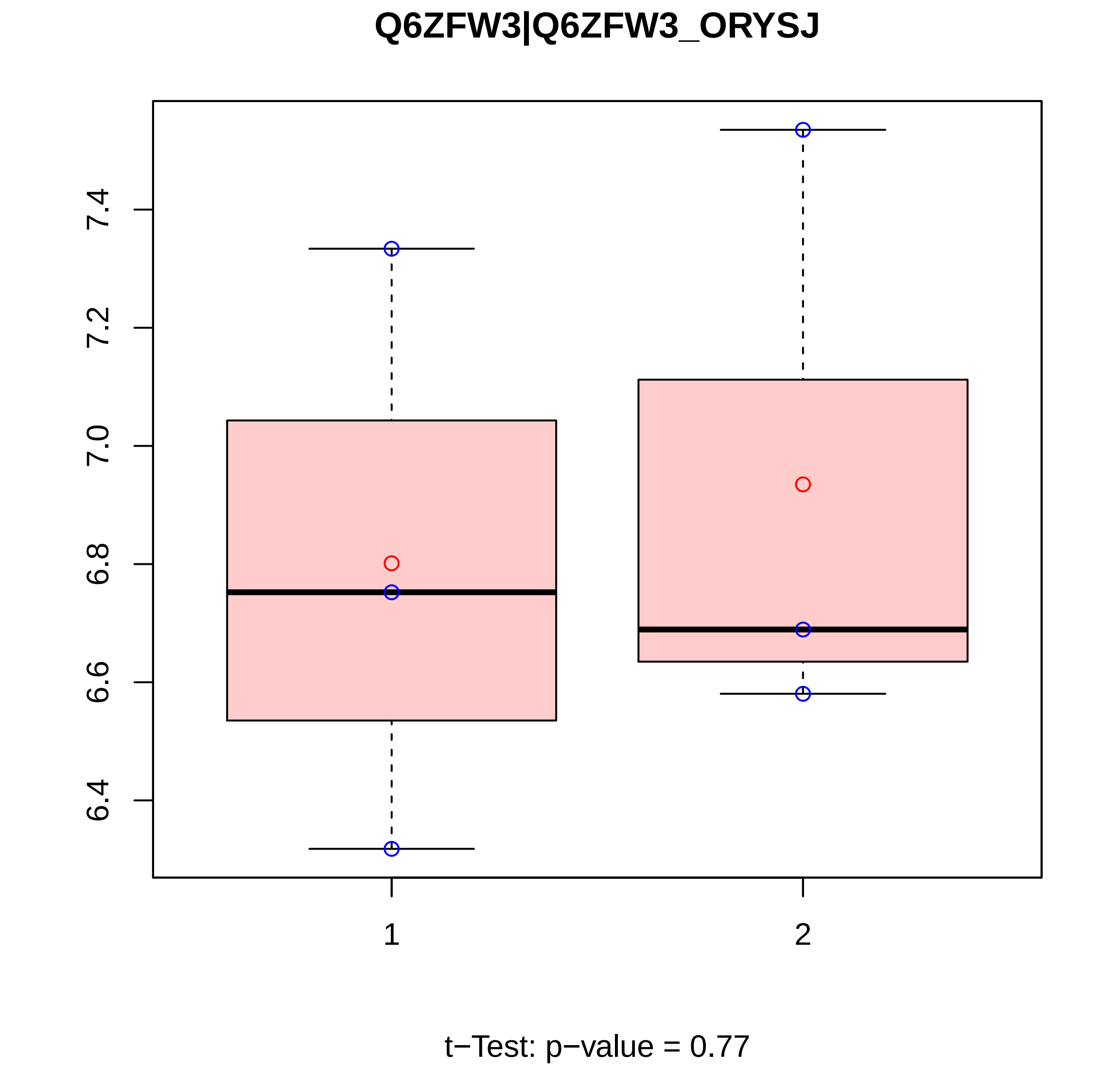
Q6ZFW3|Q6ZFW3_ORYSJ
7.4
7.2
7.0
6.8
6.6
6.4
1
2
t−Test: p−value = 0.77

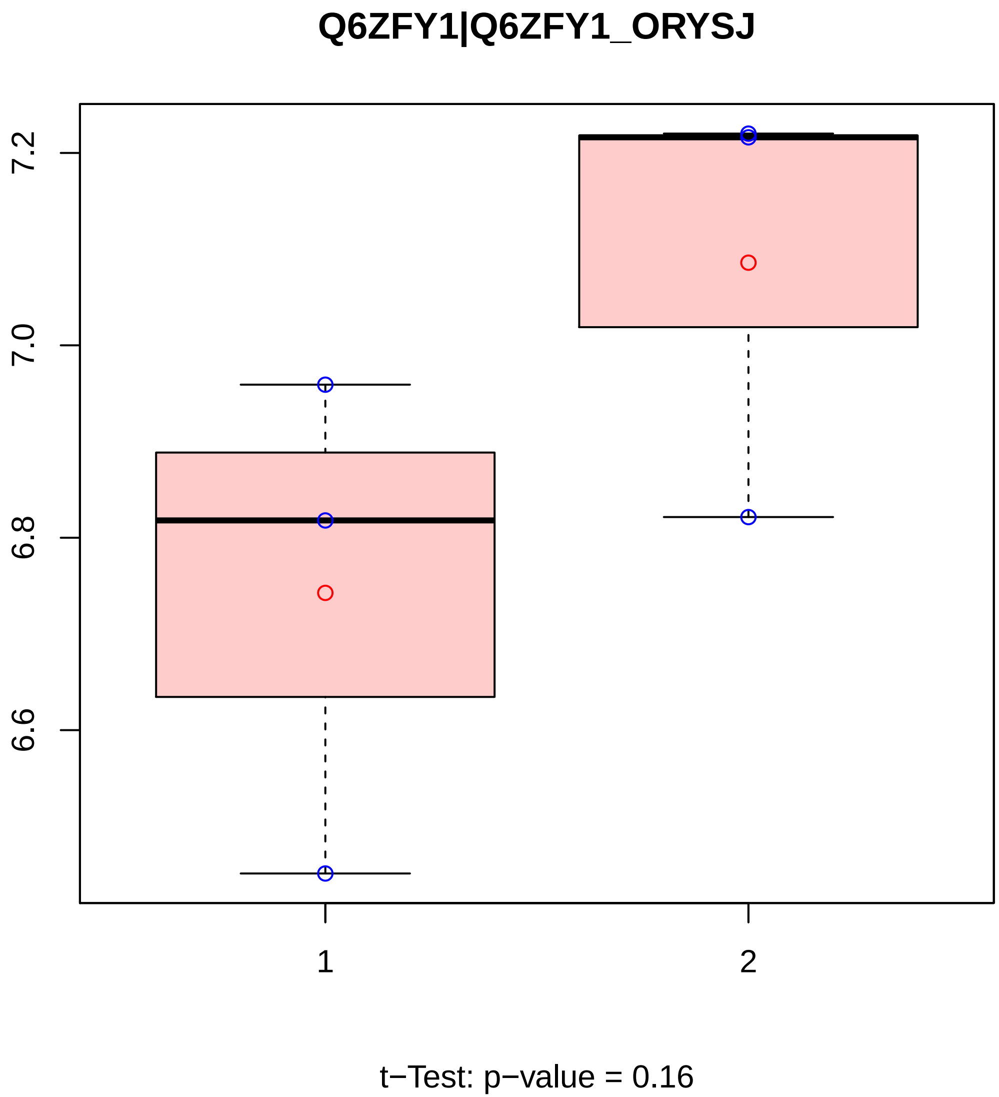
Q6ZFY1|Q6ZFY1_ORYSJ
7.2
7.0
6.8
6.6
1
2
t−Test: p−value = 0.16

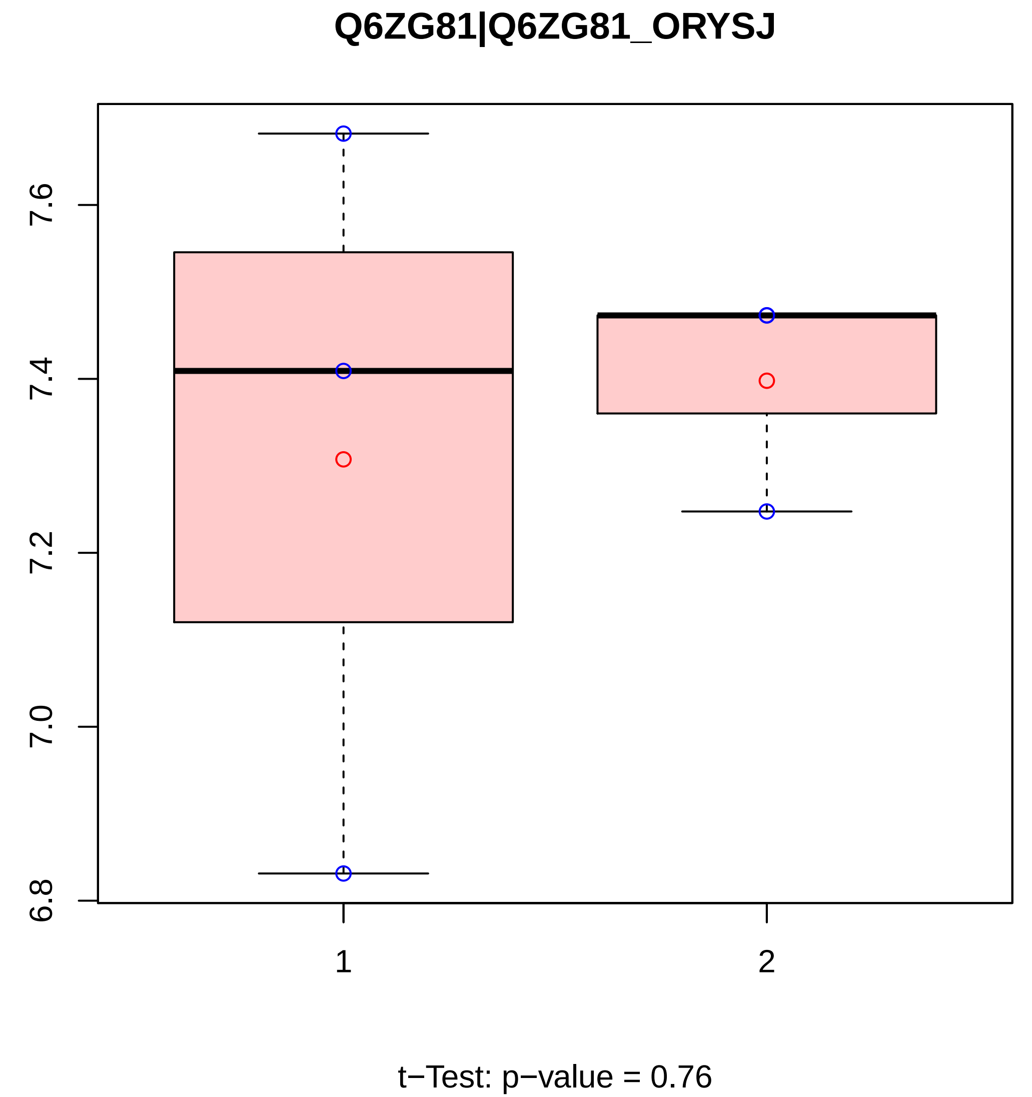
Q6ZG81|Q6ZG81_ORYSJ
7.6
7.4
7.2
7.0
6.8
1
2
t−Test: p−value = 0.76

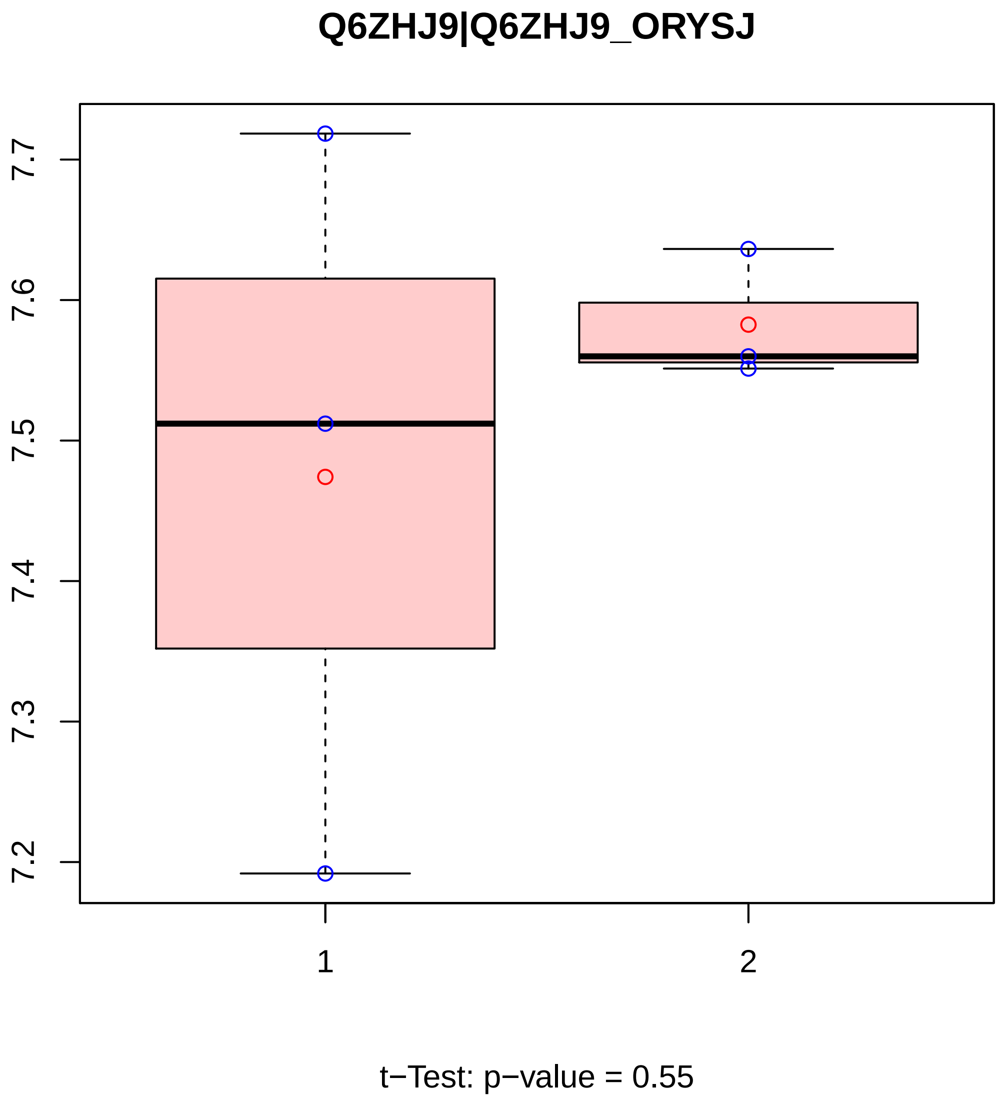
Q6ZHJ9|Q6ZHJ9_ORYSJ
7.2
7.3
7.4
7.5
7.6
7.7
1
2
t−Test: p−value = 0.55

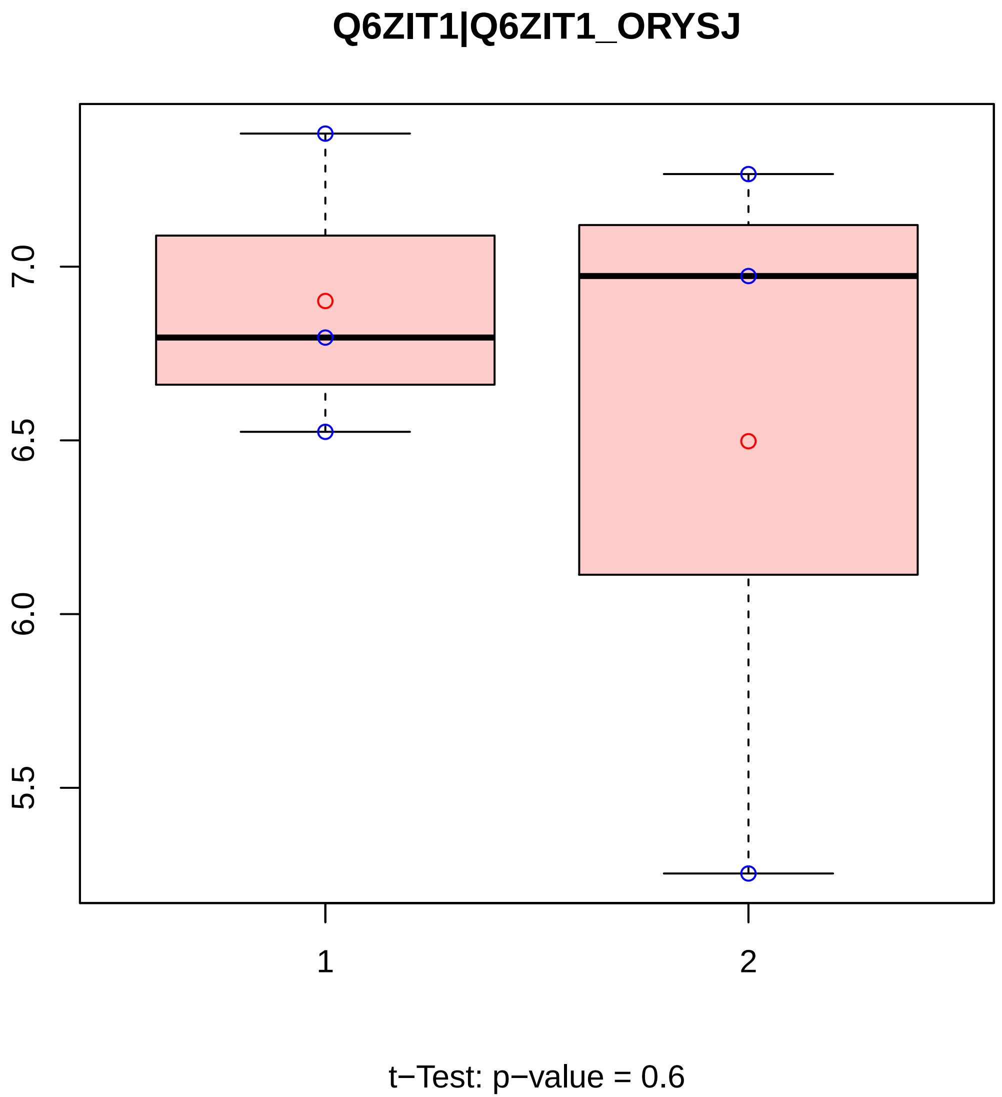
Q6ZIT1|Q6ZIT1_ORYSJ
7.0
6.5
6.0
5.5
1
2
t−Test: p−value = 0.6

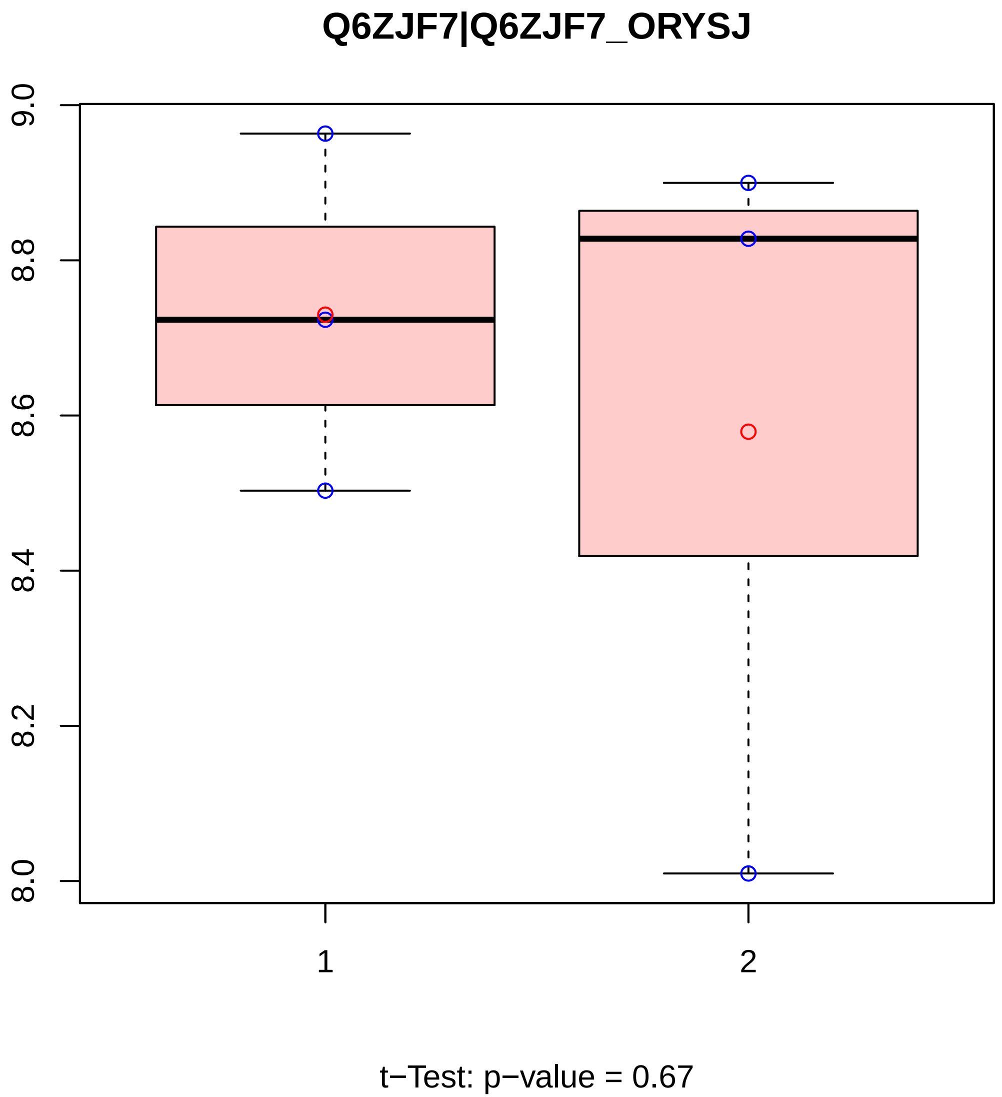

Q6ZJF7|Q6ZJF7_ORYSJ
9.0
8.8
8.6
8.4
8.2
8.0
1
2
t−Test: p−value = 0.67

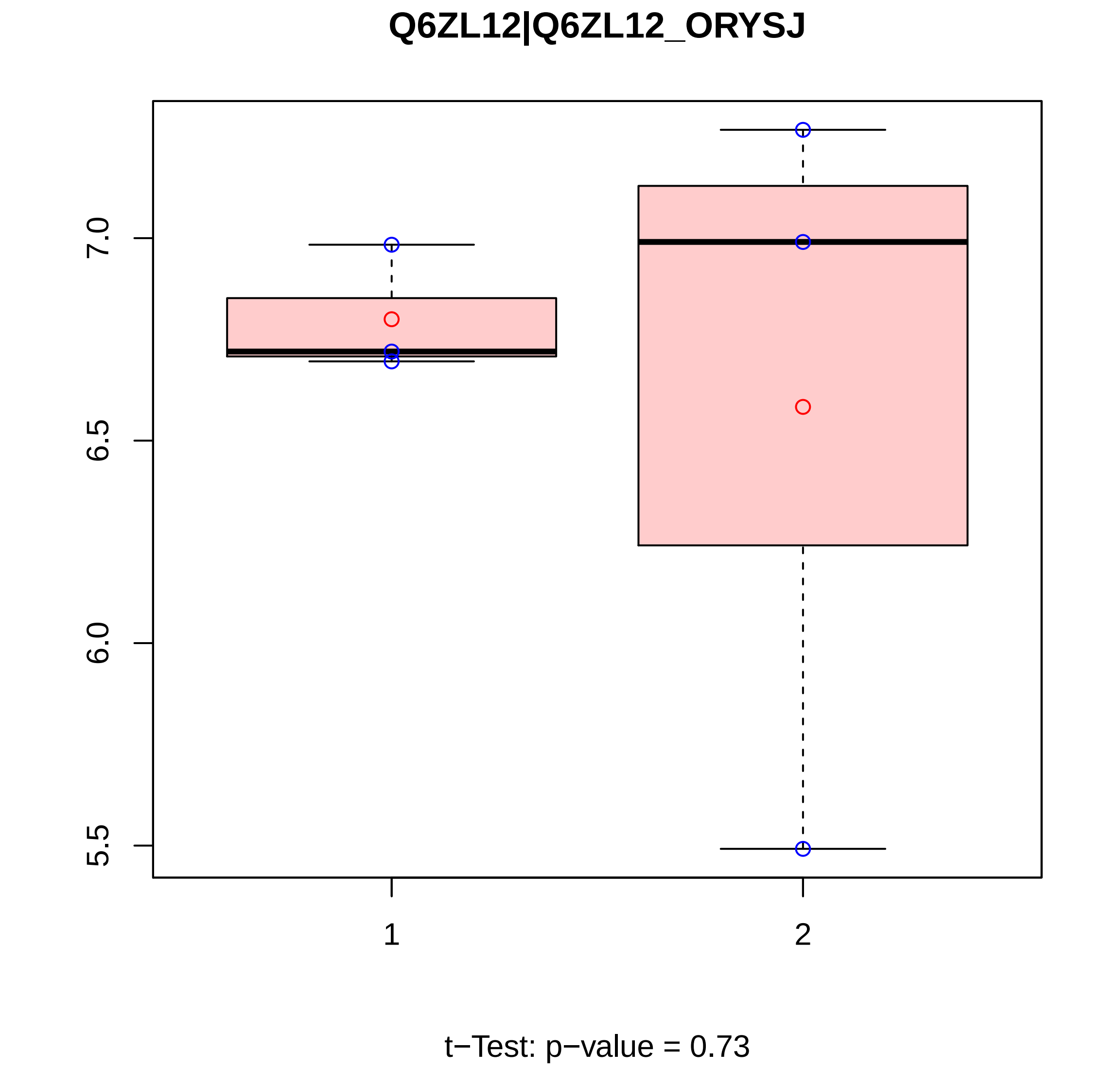
Q6ZL12|Q6ZL12_ORYSJ
7.0
6.5
6.0
5.5
1
2
t−Test: p−value = 0.73

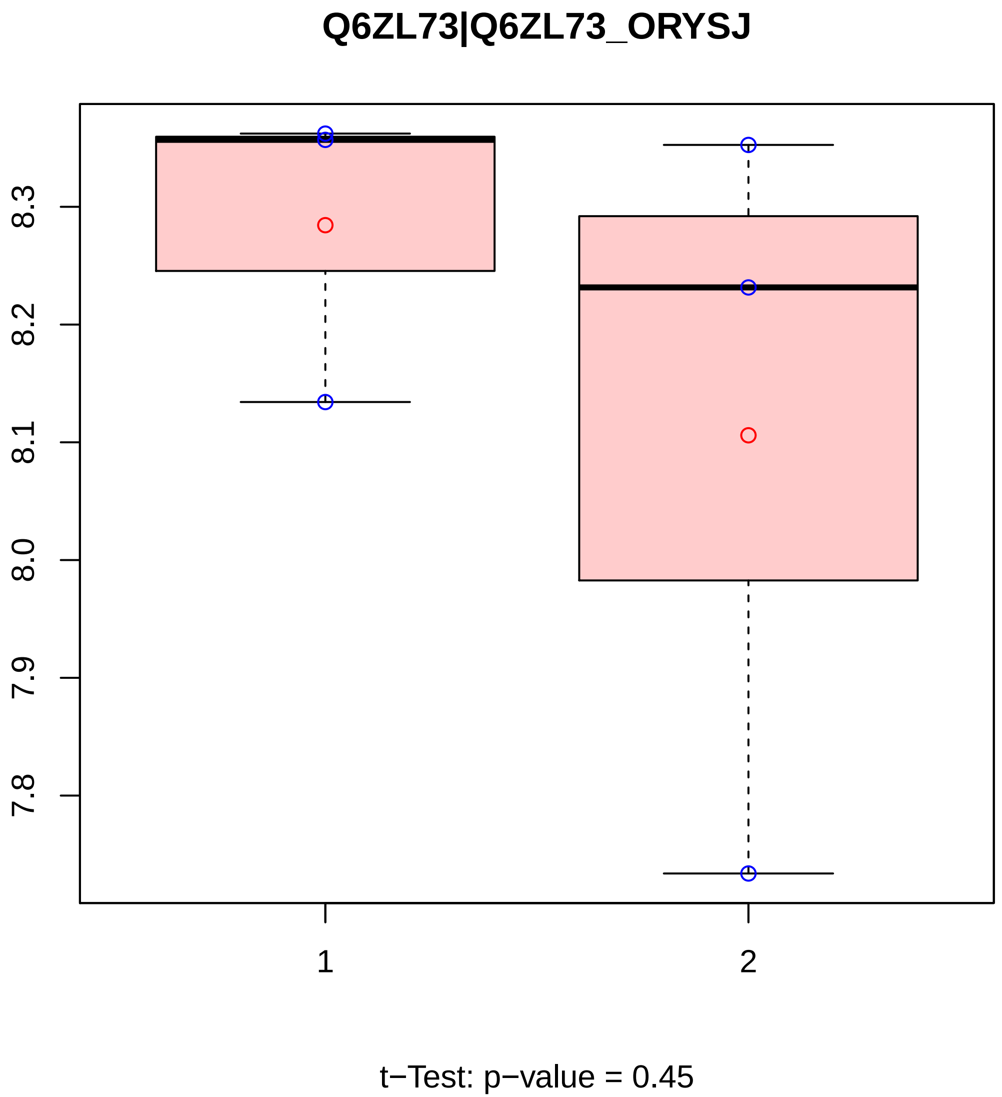

Q6ZL73|Q6ZL73_ORYSJ
8.3
8.2
8.1
8.0
7.9
7.8
1
2
t−Test: p−value = 0.45

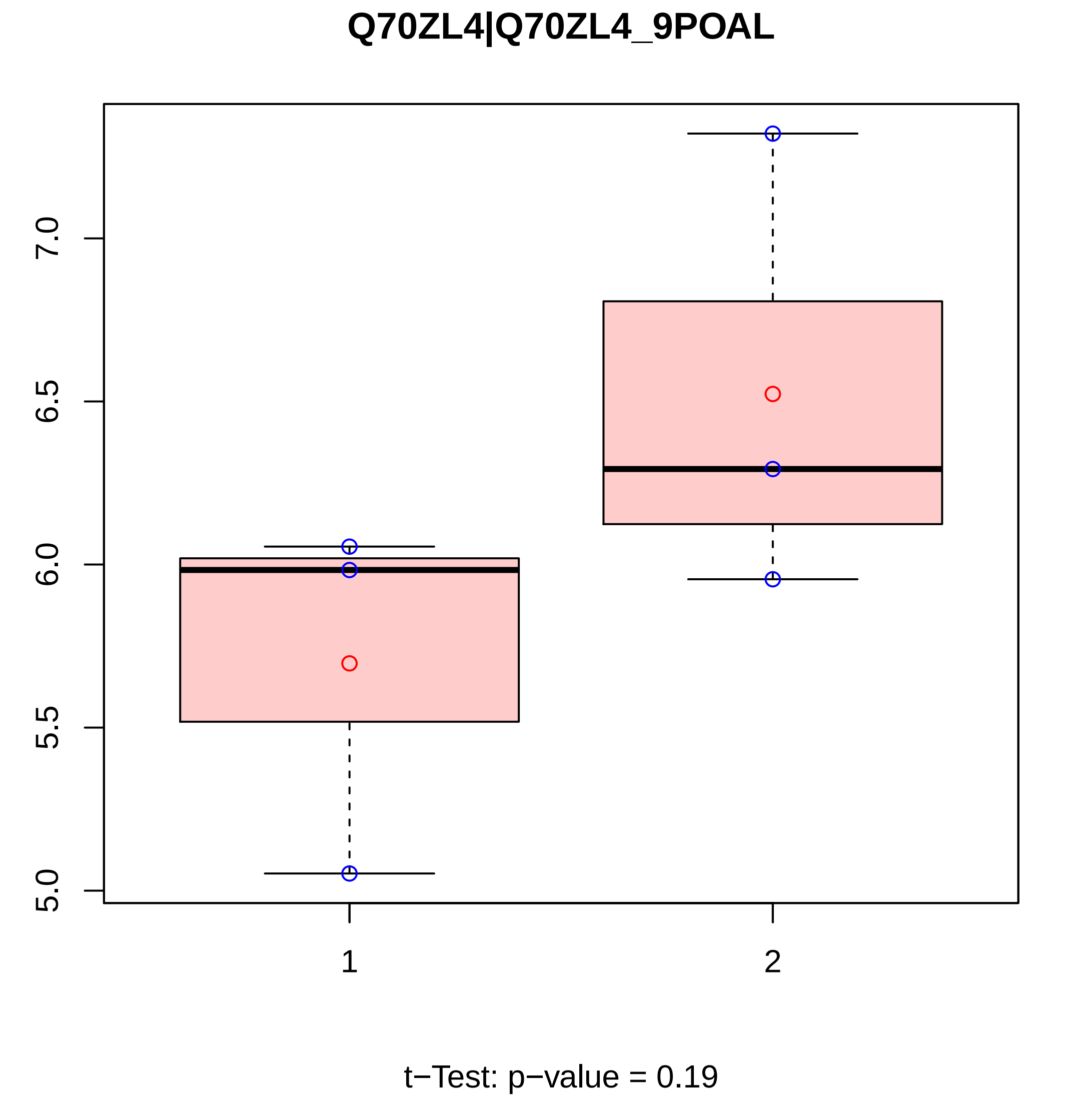

Q70ZL4|Q70ZL4_9POAL
7.0
6.5
6.0
5.5
5.0
1
2
t−Test: p−value = 0.19

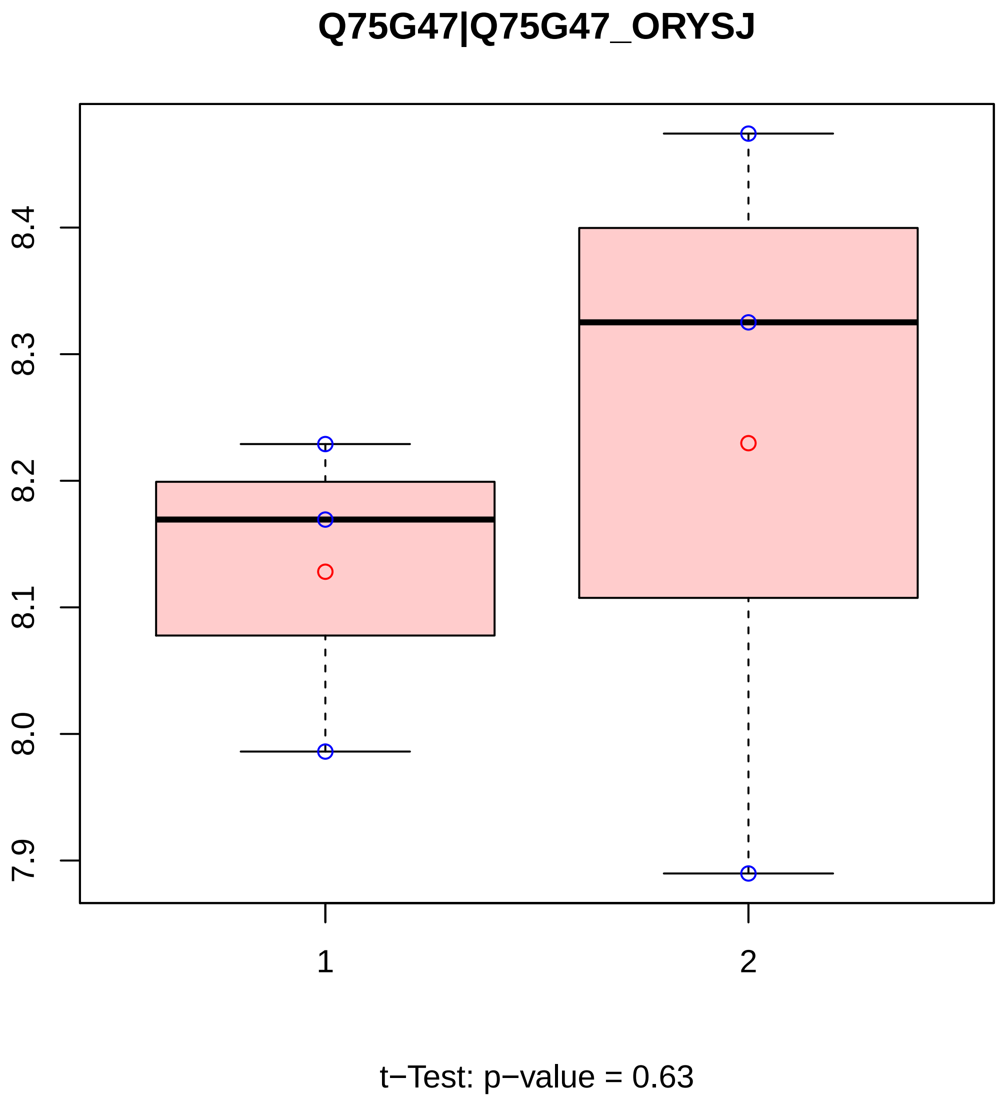

Q75G47|Q75G47_ORYSJ
8.4
8.3
8.2
8.1
8.0
7.9
1
2
t−Test: p−value = 0.63

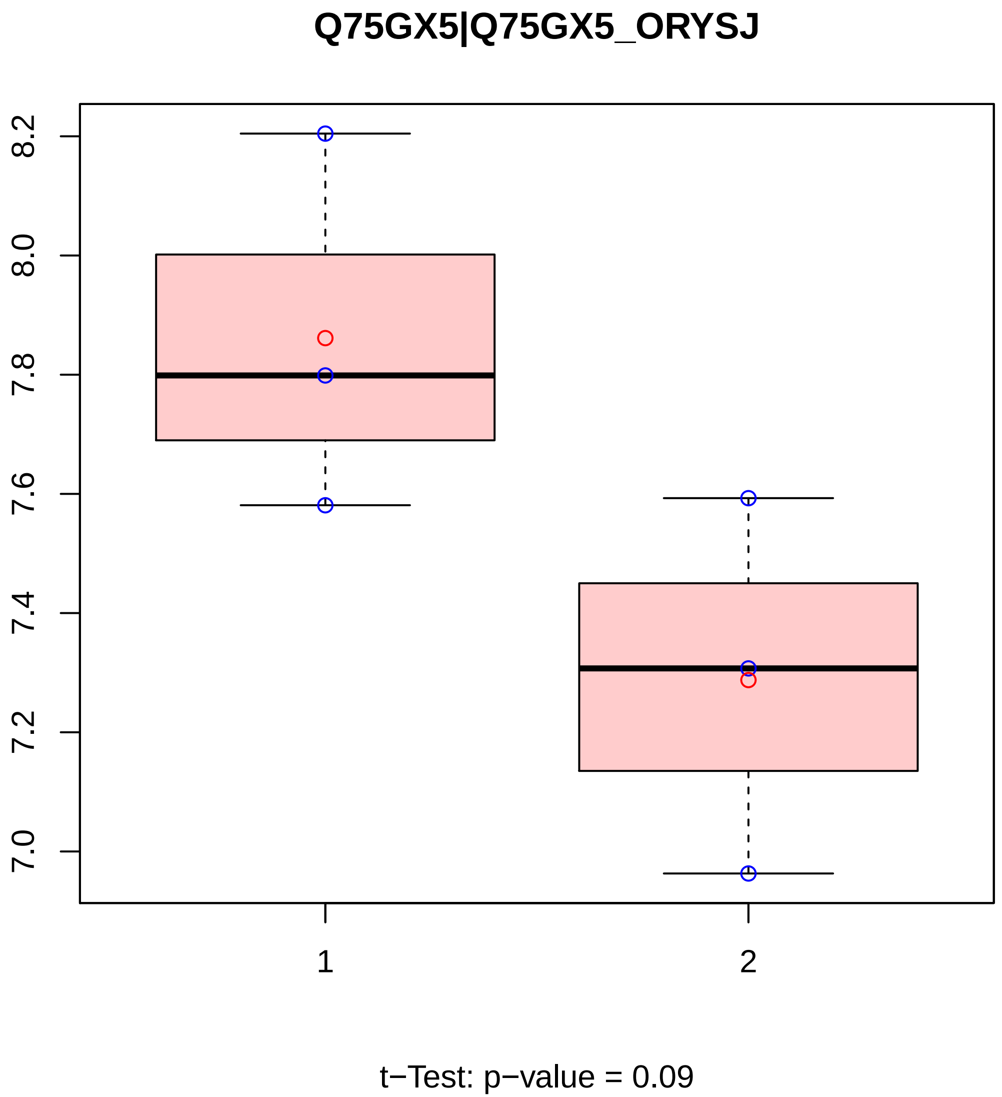

Q75GX5|Q75GX5_ORYSJ
8.2
8.0
7.8
7.6
7.4
7.2
7.0
1
2
t−Test: p−value = 0.09

Q75HK1|Q75HK1_ORYSJ

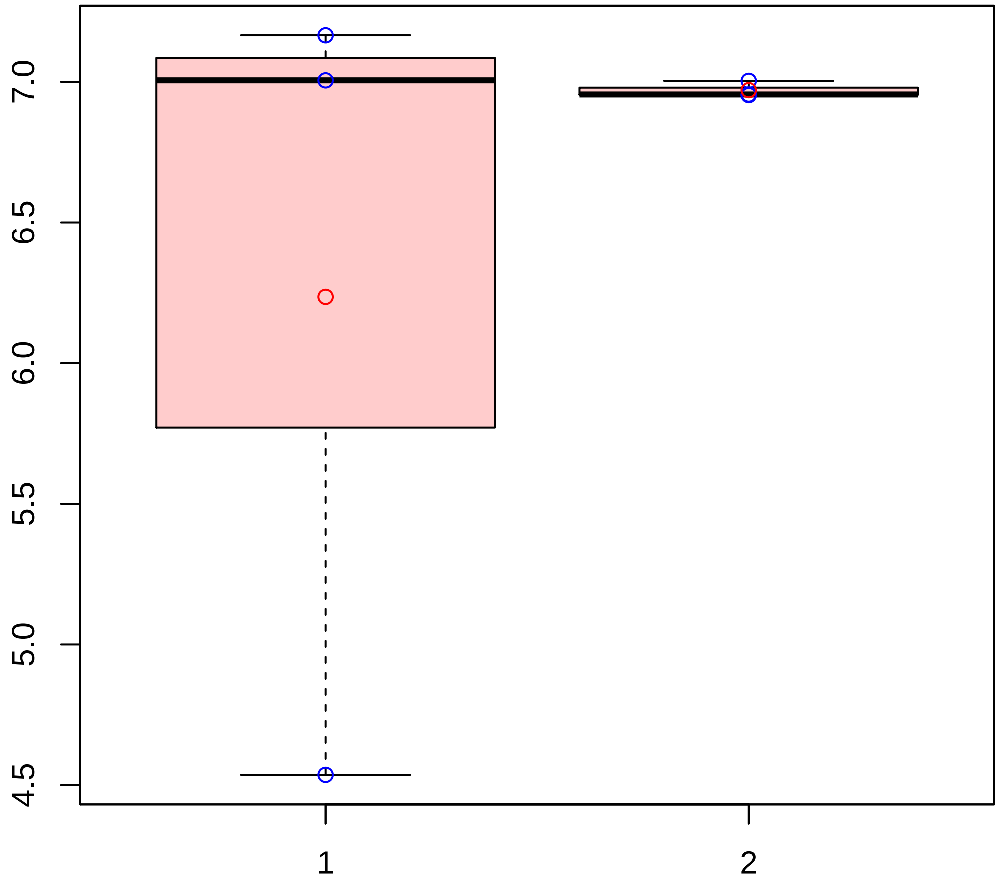

t−Test: p−value = 0.48

Q75I16|Q75I16_ORYSJ

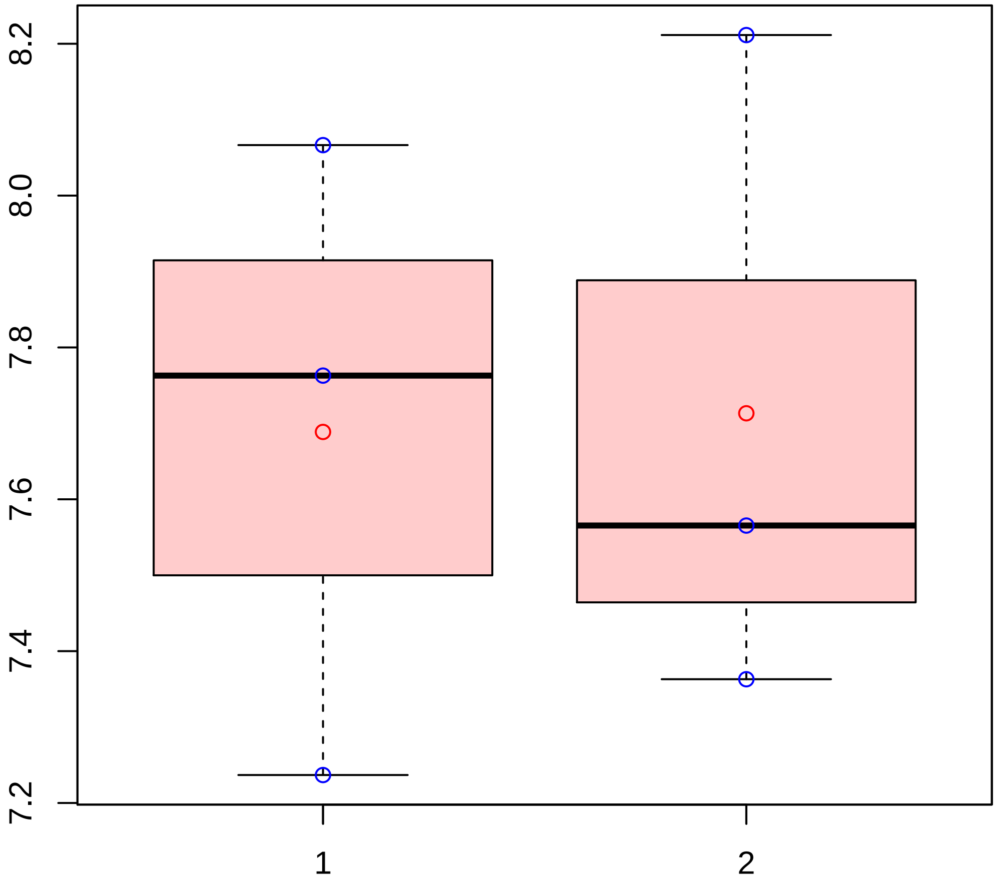

t−Test: p−value = 0.95

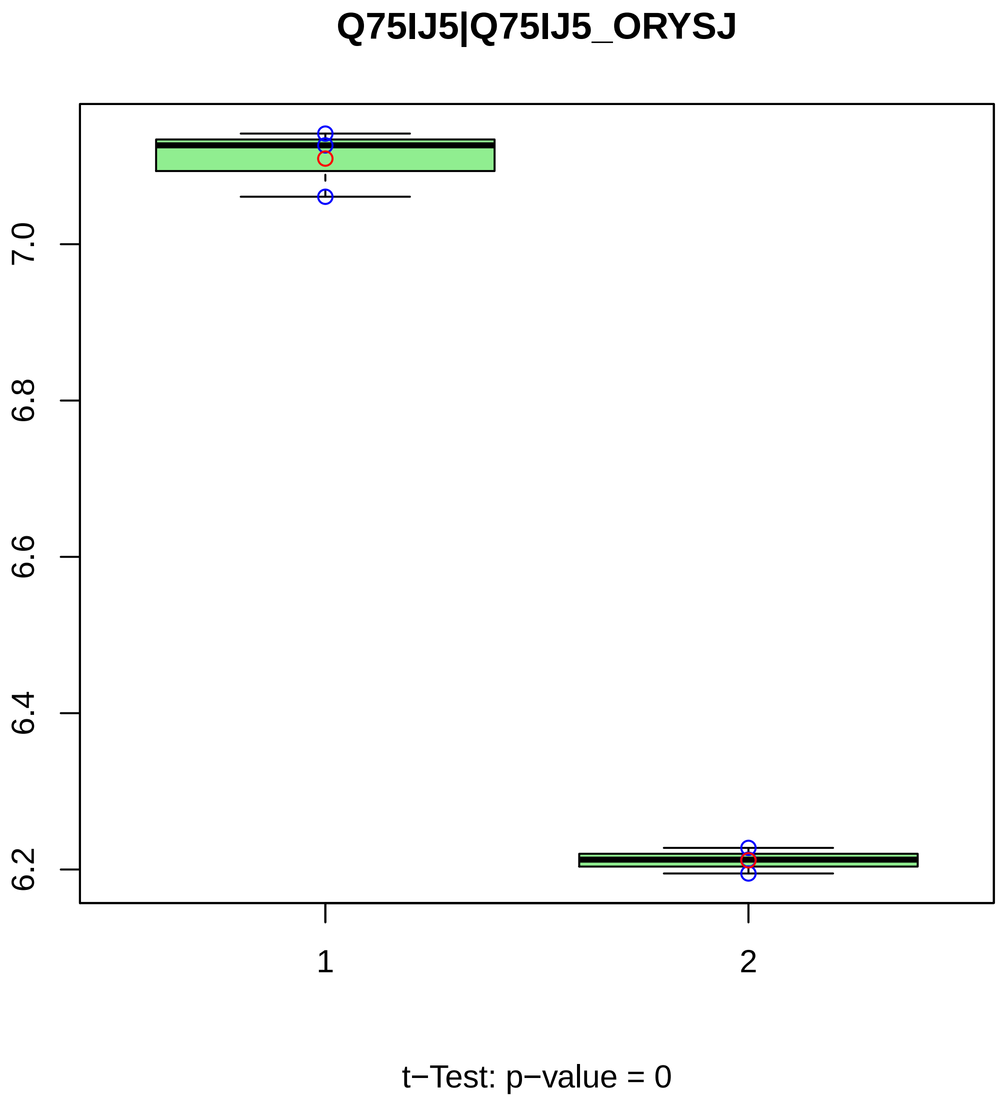
Q75IJ5|Q75IJ5_ORYSJ
7.0
6.8
6.6
6.4
6.2
1
2
t−Test: p−value = 0

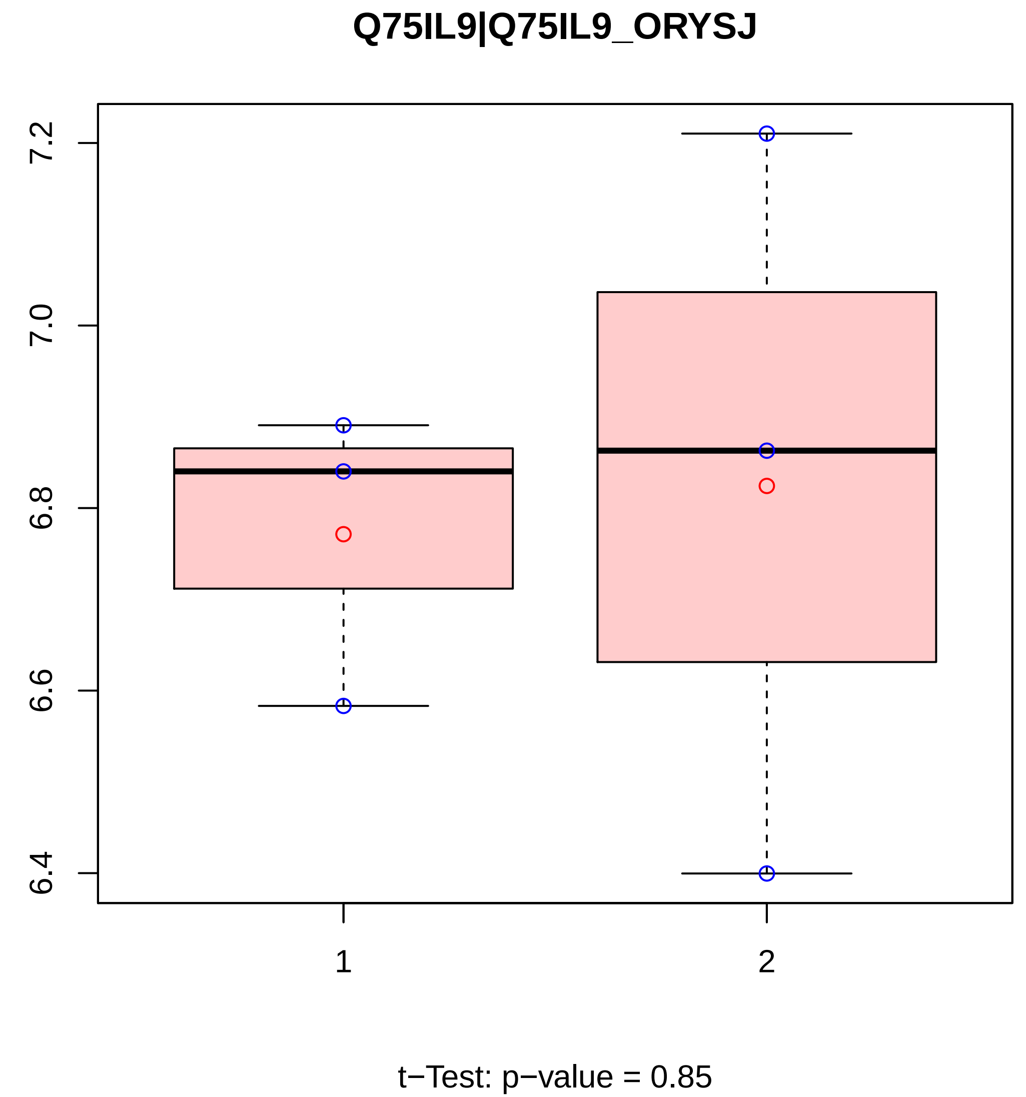
Q75IL9|Q75IL9_ORYSJ
7.2
7.0
6.8
6.6
6.4
1
2
t−Test: p−value = 0.85

Q75KU6|Q75KU6_ORYSJ

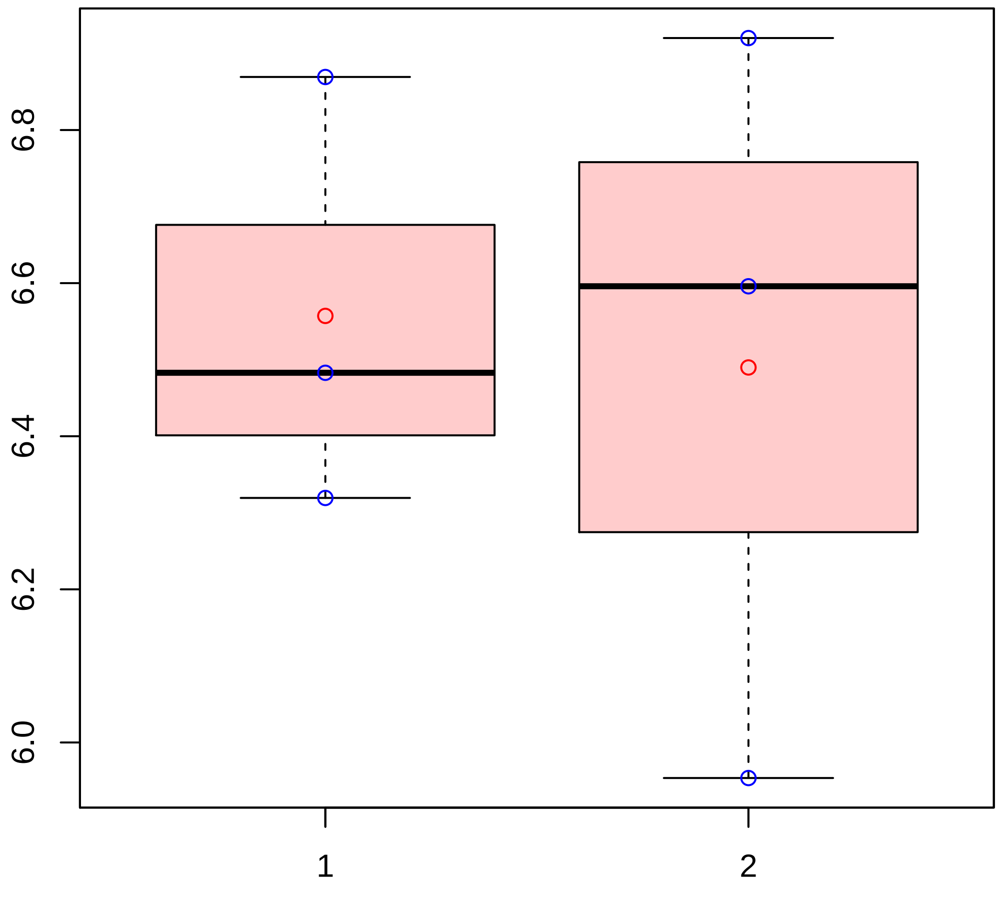

t−Test: p−value = 0.85

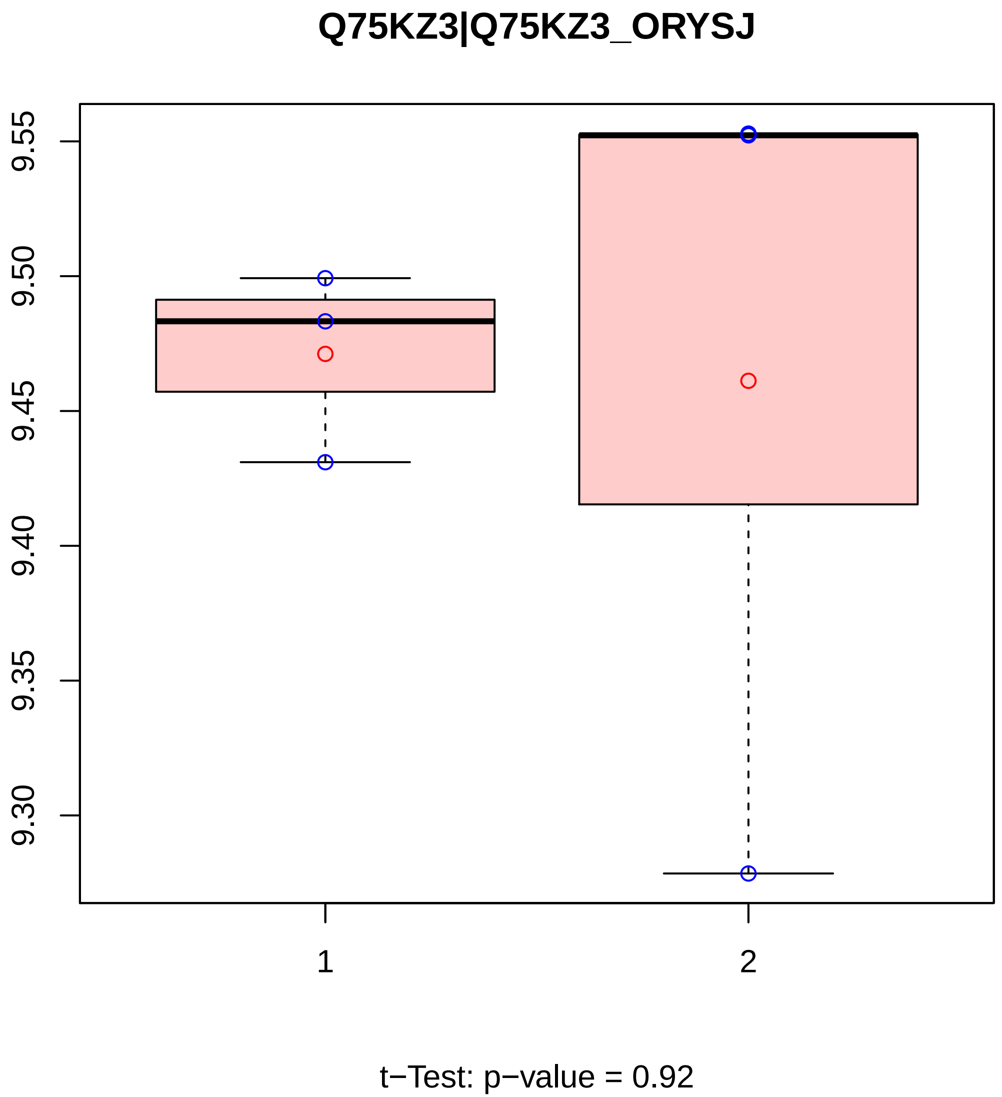

Q75KZ3|Q75KZ3_ORYSJ
9.55
9.50
9.45
9.40
9.35
9.30
1
2
t−Test: p−value = 0.92

Q75M32|Q75M32_ORYSJ

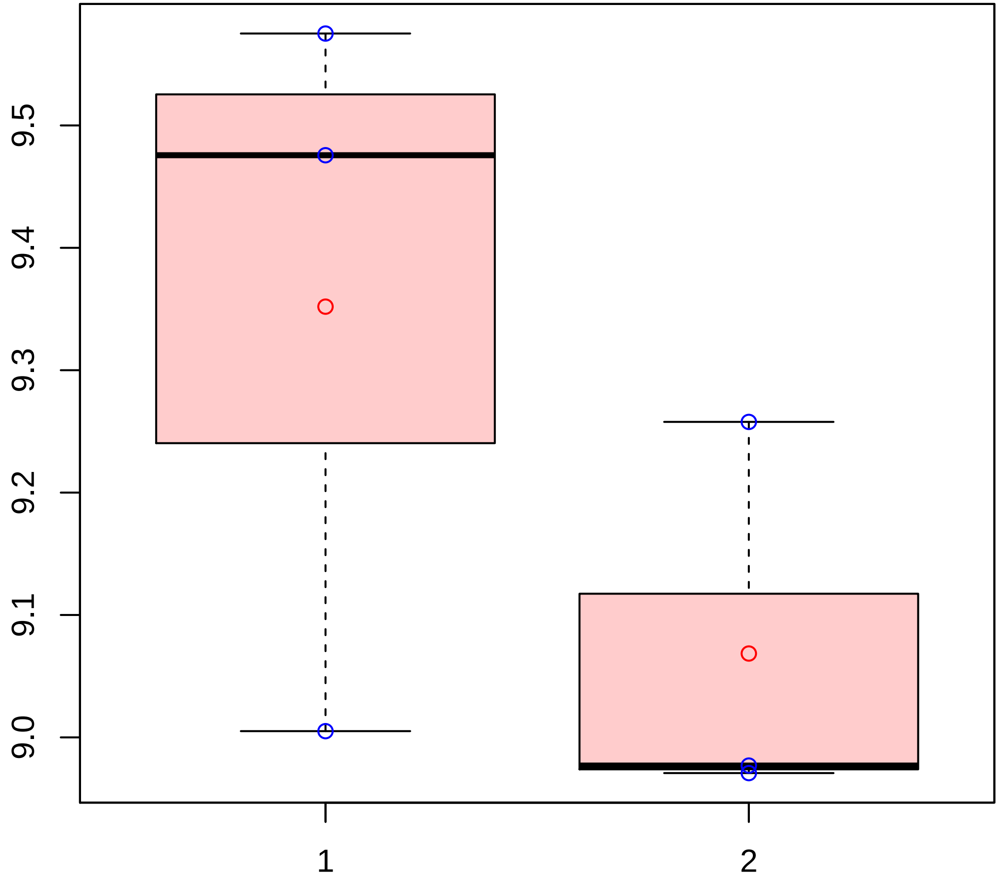

t−Test: p−value = 0.25

Q75U52|Q75U52_ORYSJ

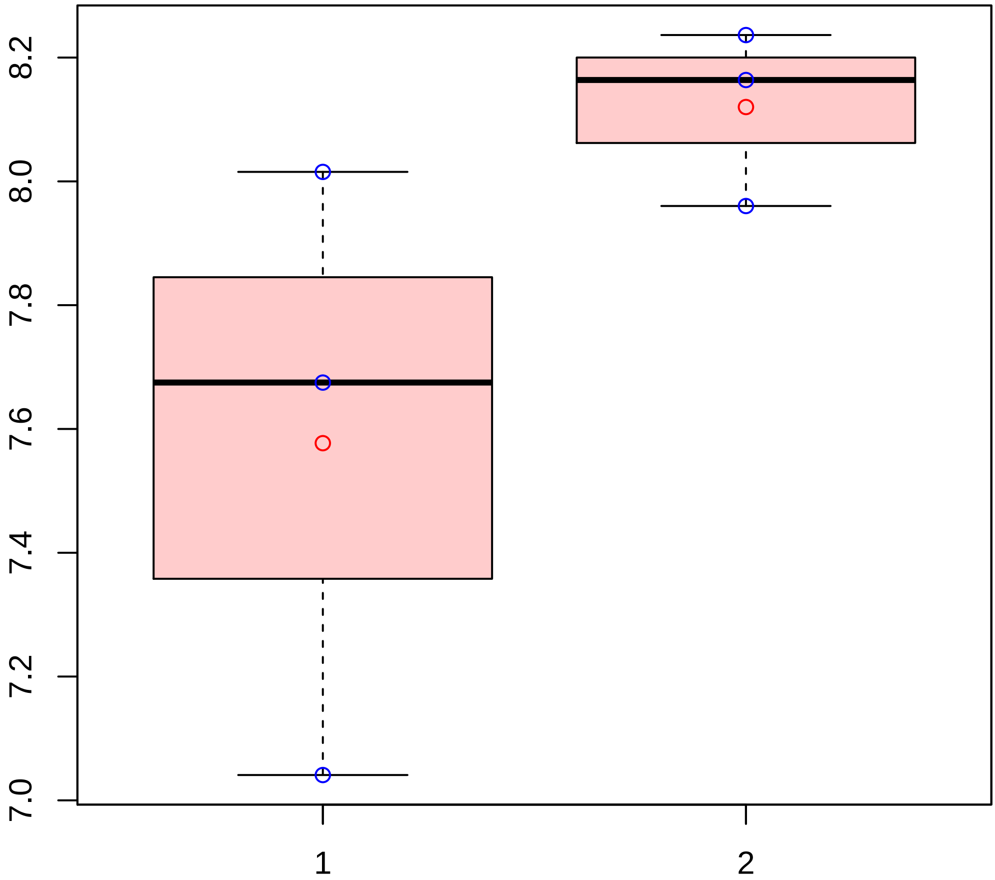

t−Test: p−value = 0.19

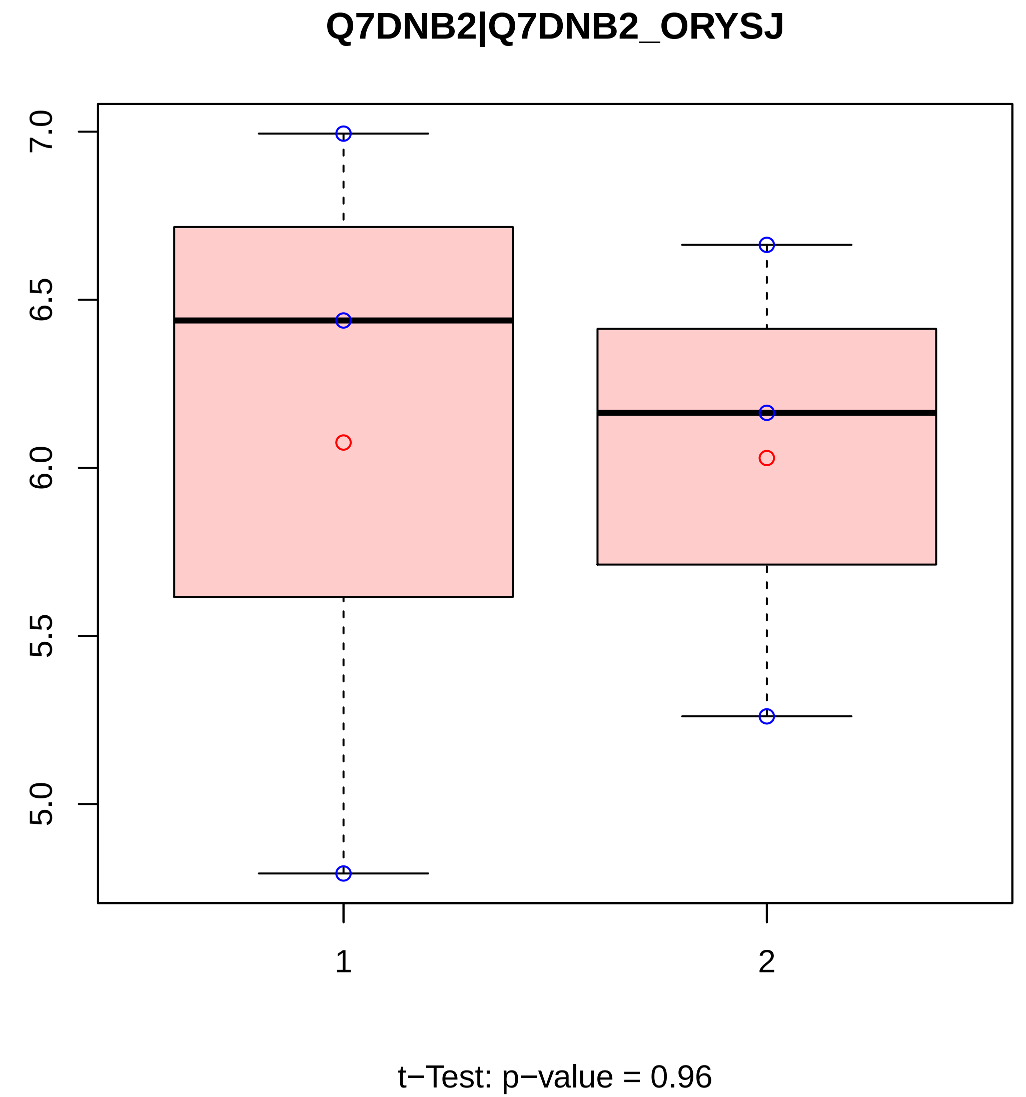
Q7DNB2|Q7DNB2_ORYSJ
7.0
6.5
6.0
5.5
5.0
1
2
t−Test: p−value = 0.96

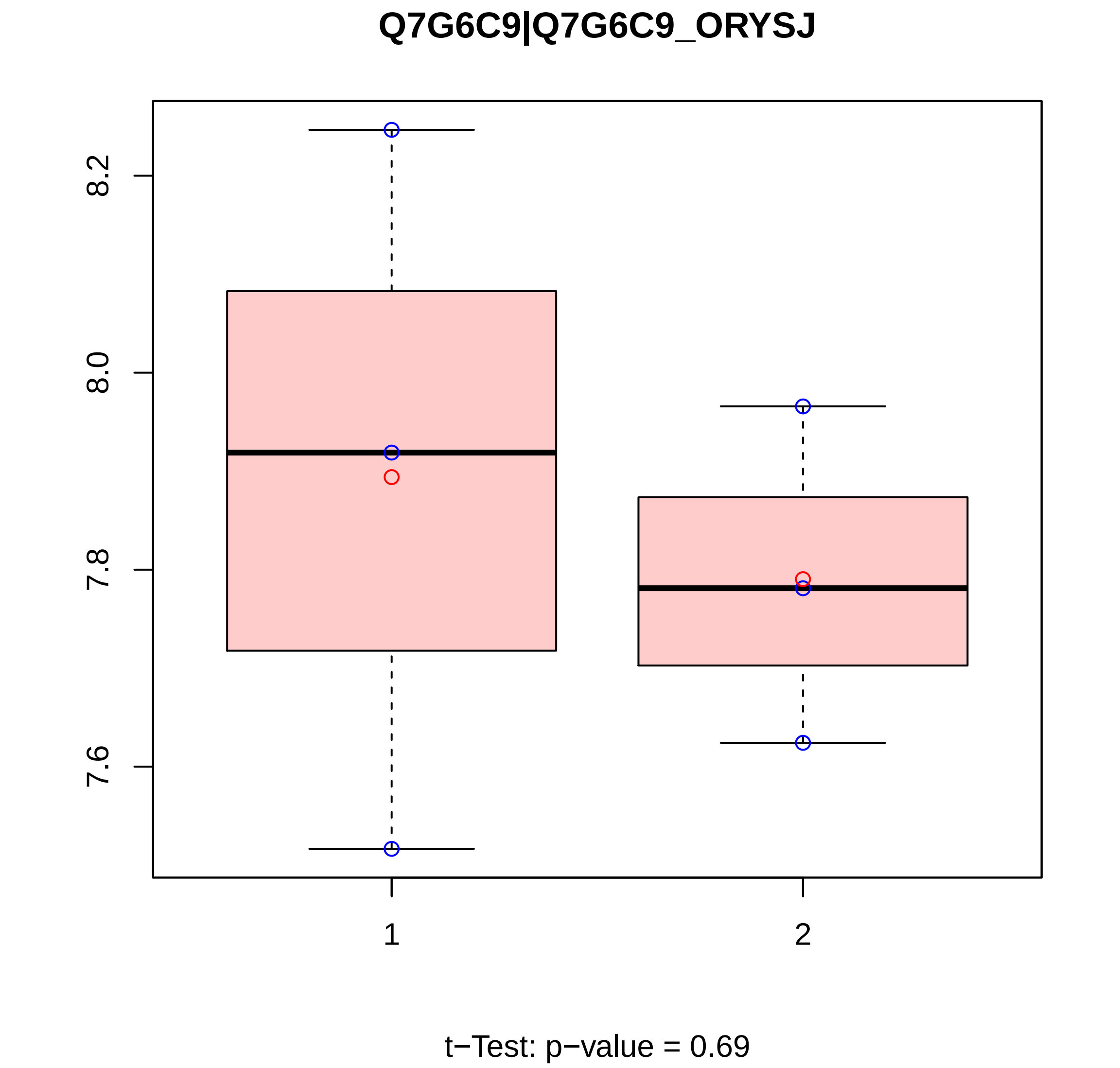
Q7G6C9|Q7G6C9_ORYSJ
8.2
8.0
7.8
7.6
1
2
t−Test: p−value = 0.69

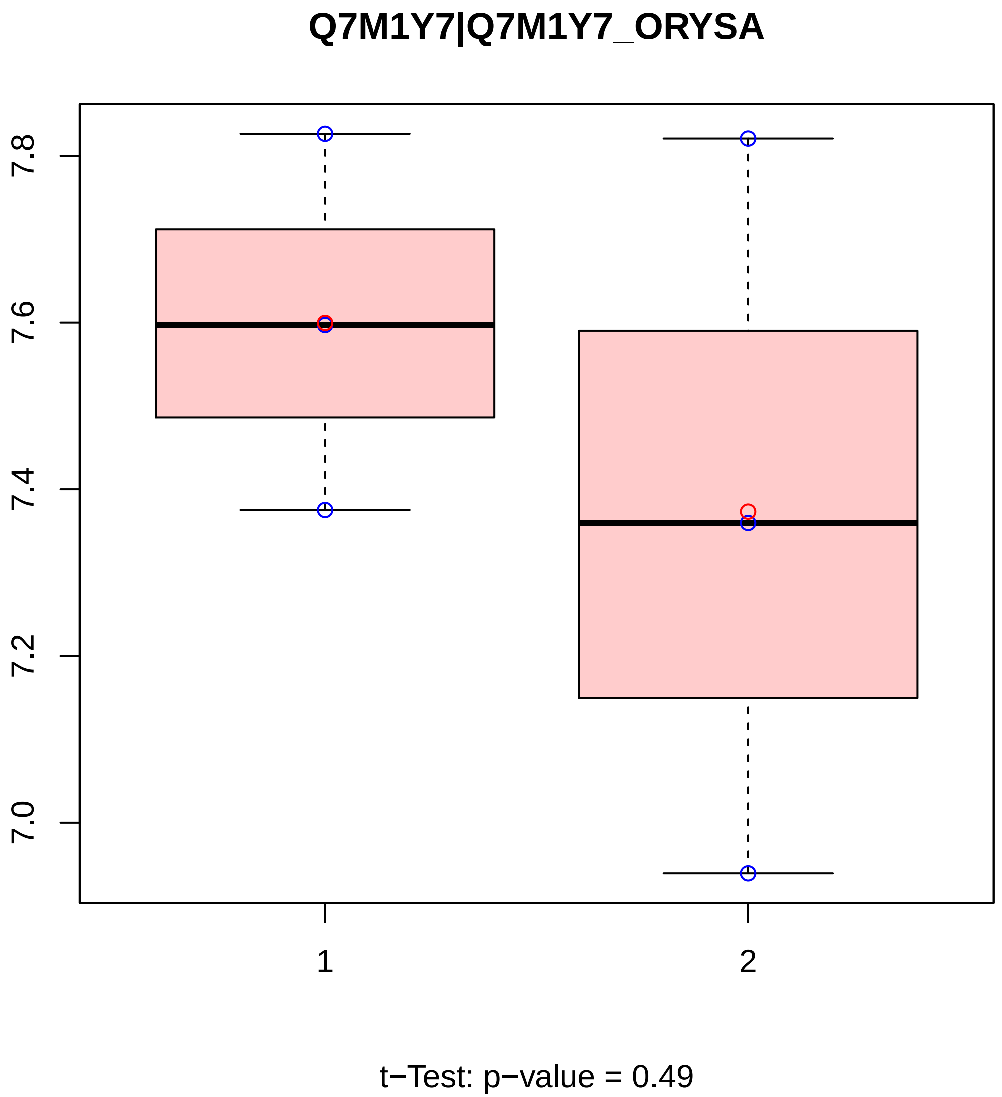
Q7M1Y7|Q7M1Y7_ORYSA
7.8
7.6
7.4
7.2
7.0
1
2
t−Test: p−value = 0.49

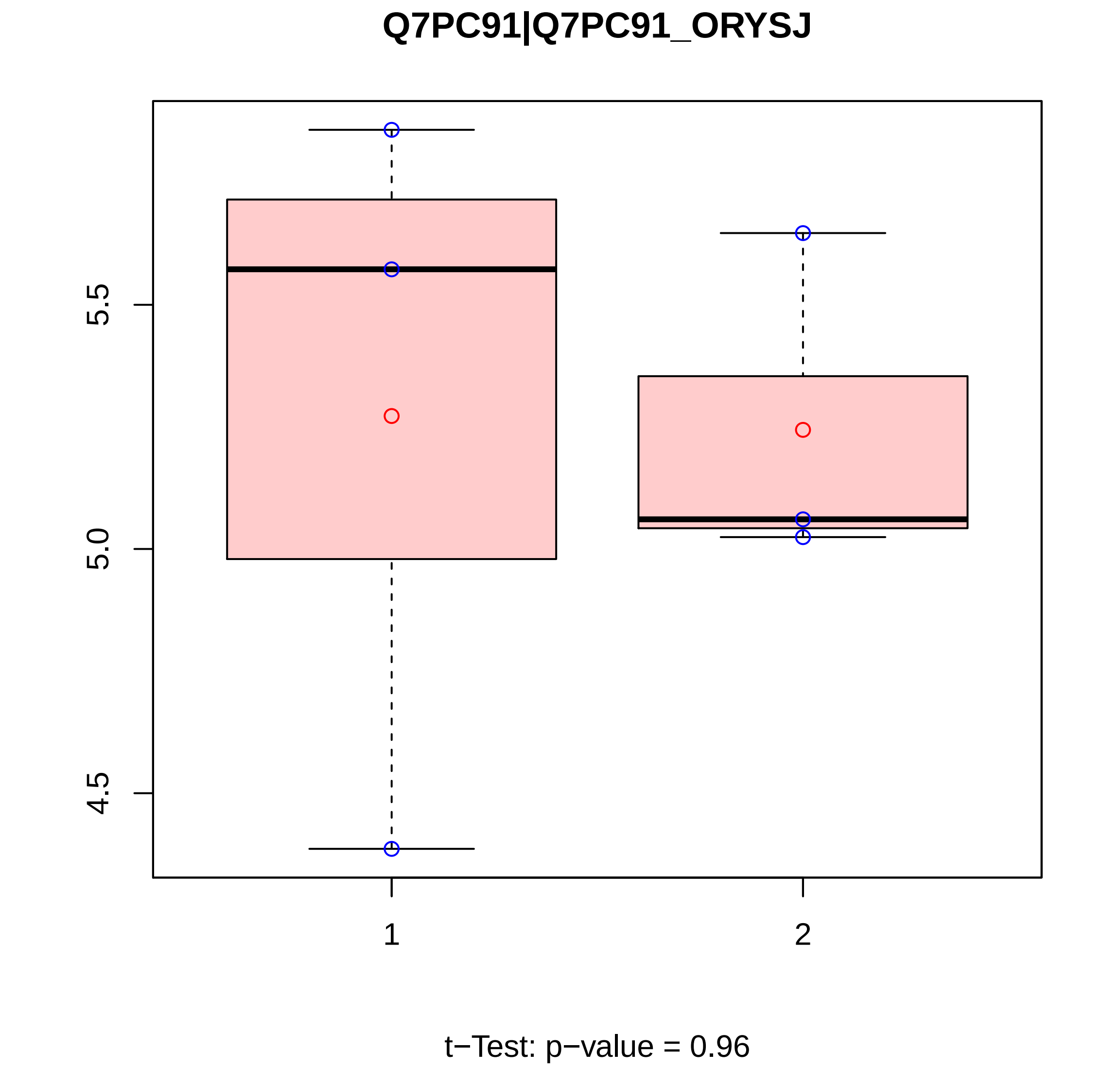

Q7PC91|Q7PC91_ORYSJ
4.5
5.0
5.5
1
2
t−Test: p−value = 0.96

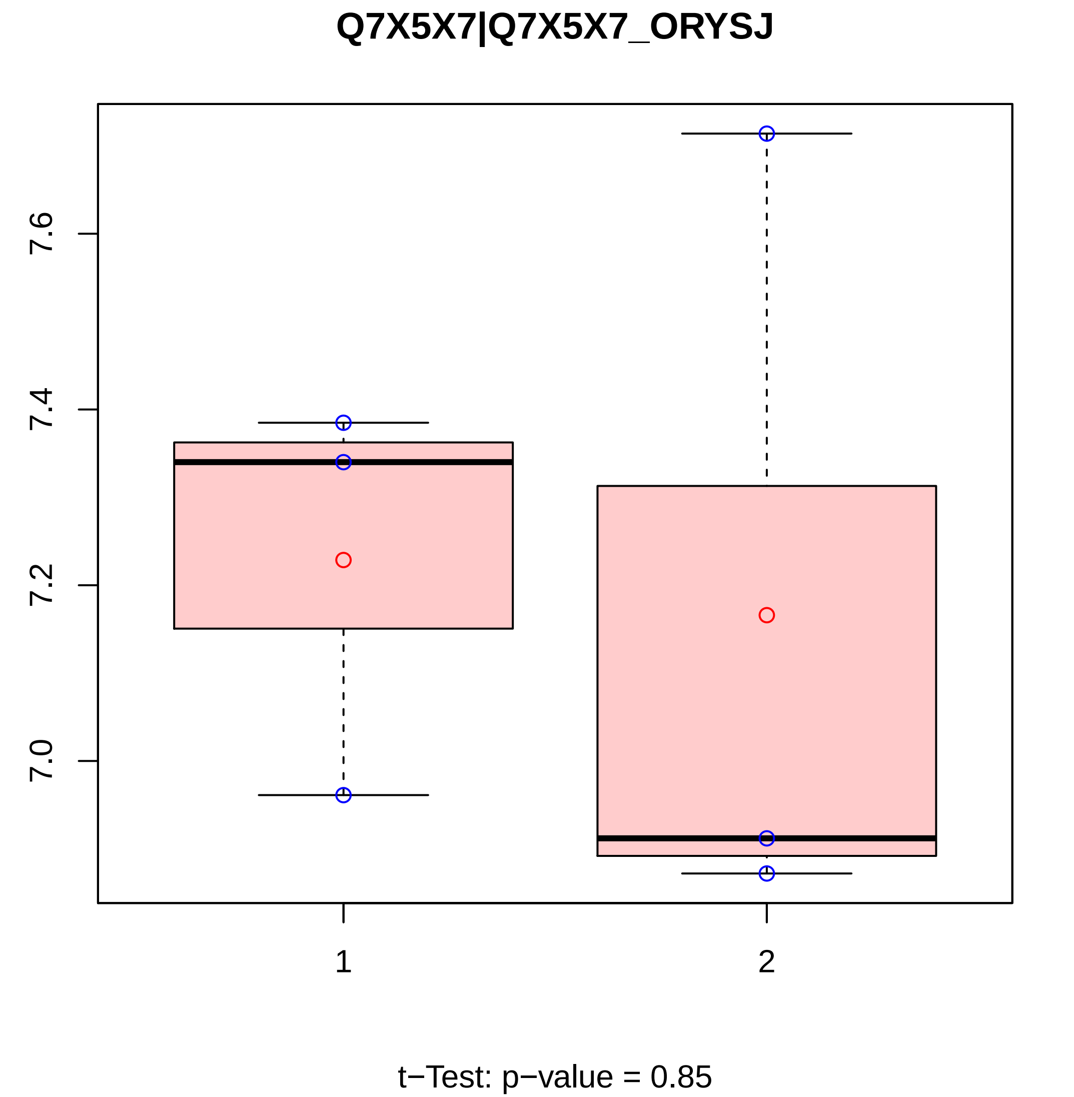
Q7X5X7|Q7X5X7_ORYSJ
7.0
7.2
7.4
7.6
1
2
t−Test: p−value = 0.85

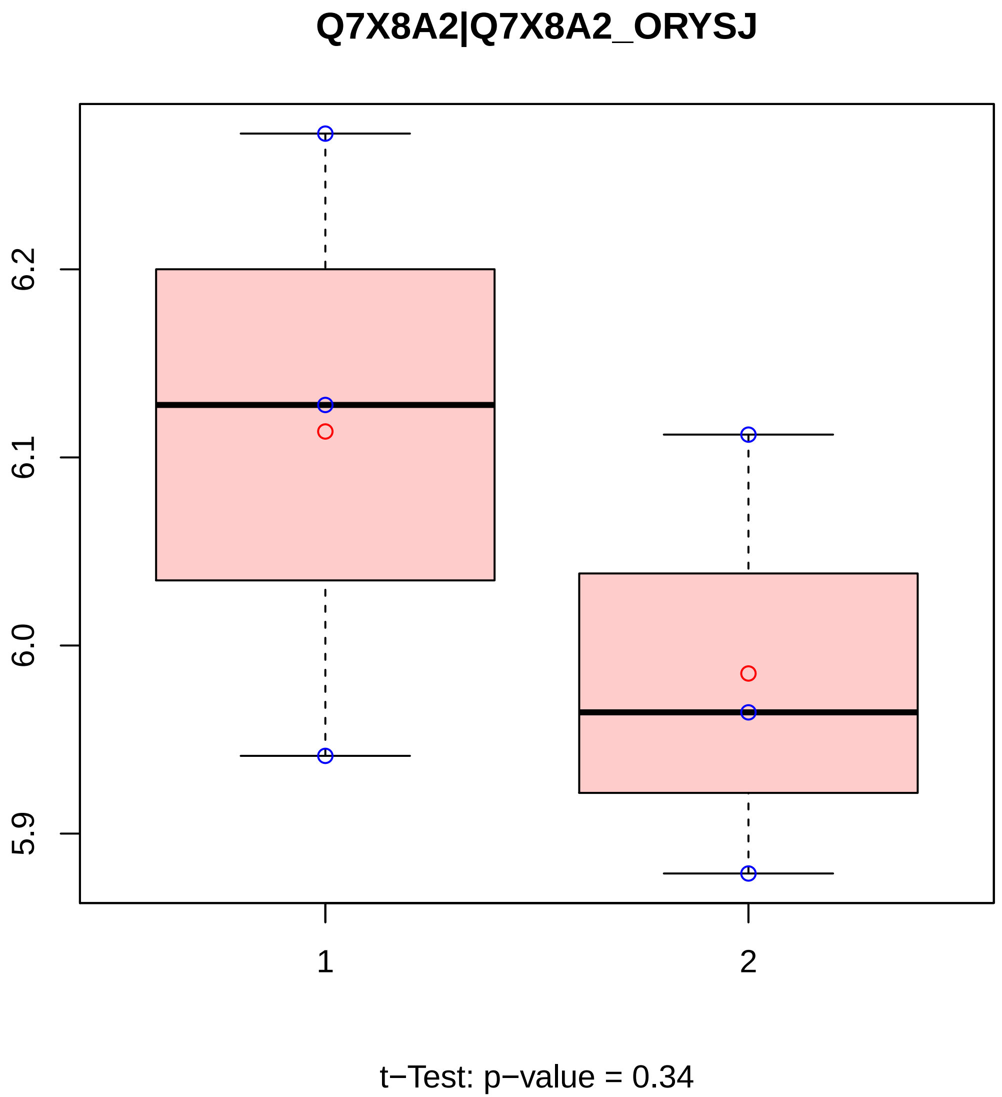
Q7X8A2|Q7X8A2_ORYSJ
6.2
6.1
6.0
5.9
1
2
t−Test: p−value = 0.34

Q7X8G3|Q7X8G3_ORYSJ

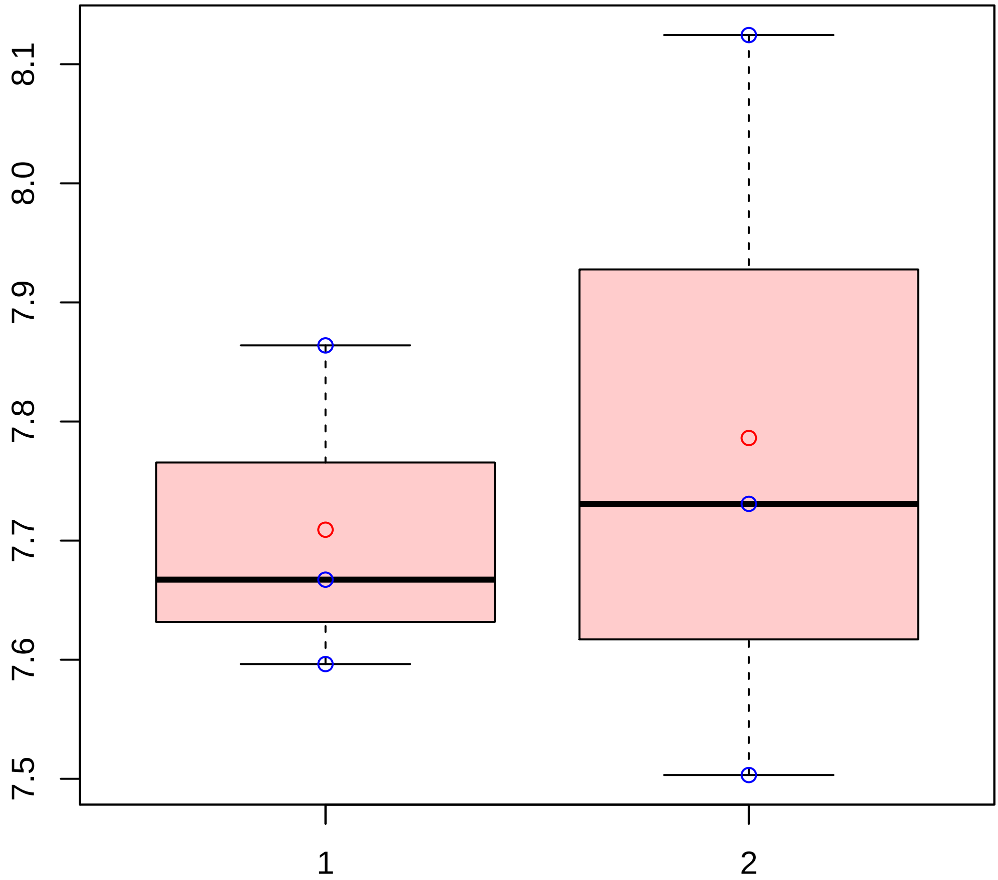

t−Test: p−value = 0.73

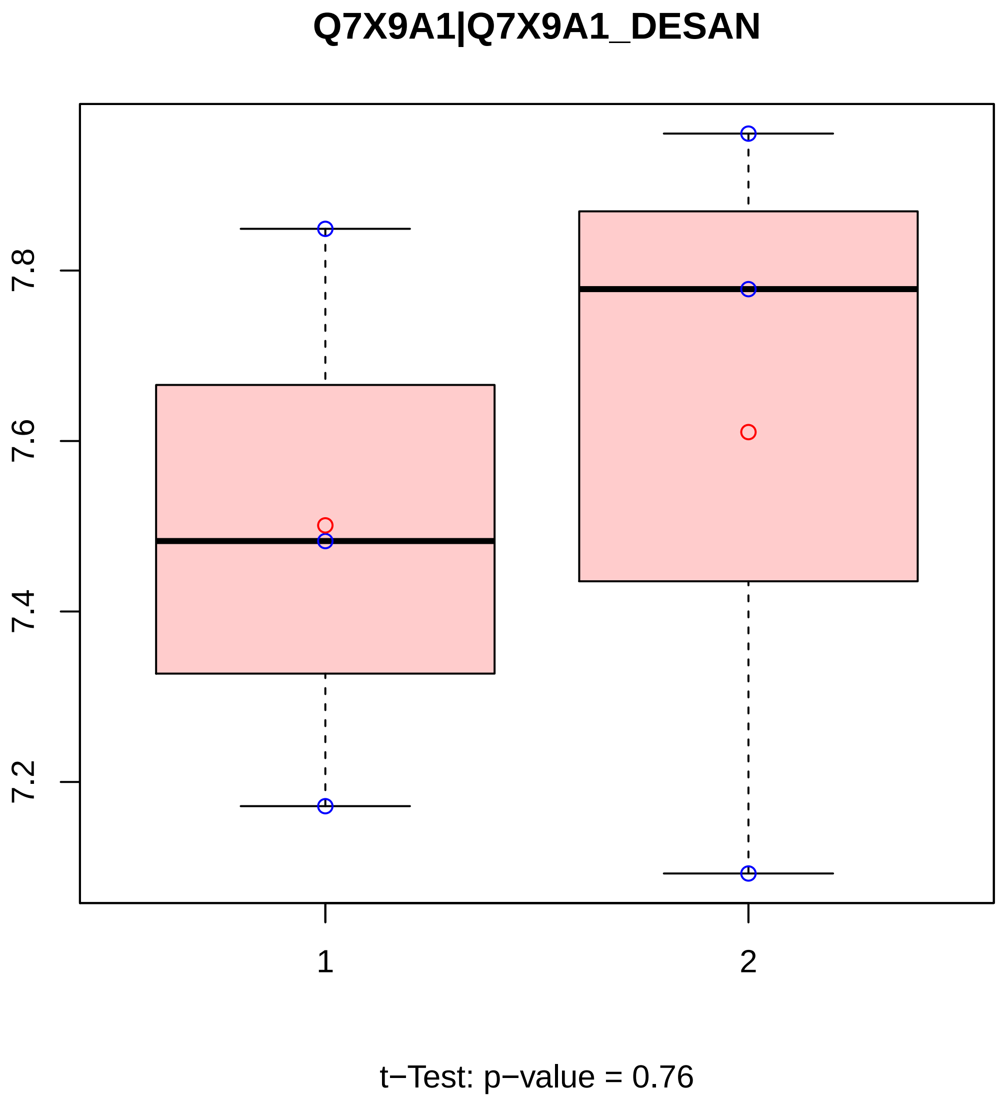
Q7X9A1|Q7X9A1_DESAN
7.2
7.4
7.6
7.8
1
2
t−Test: p−value = 0.76

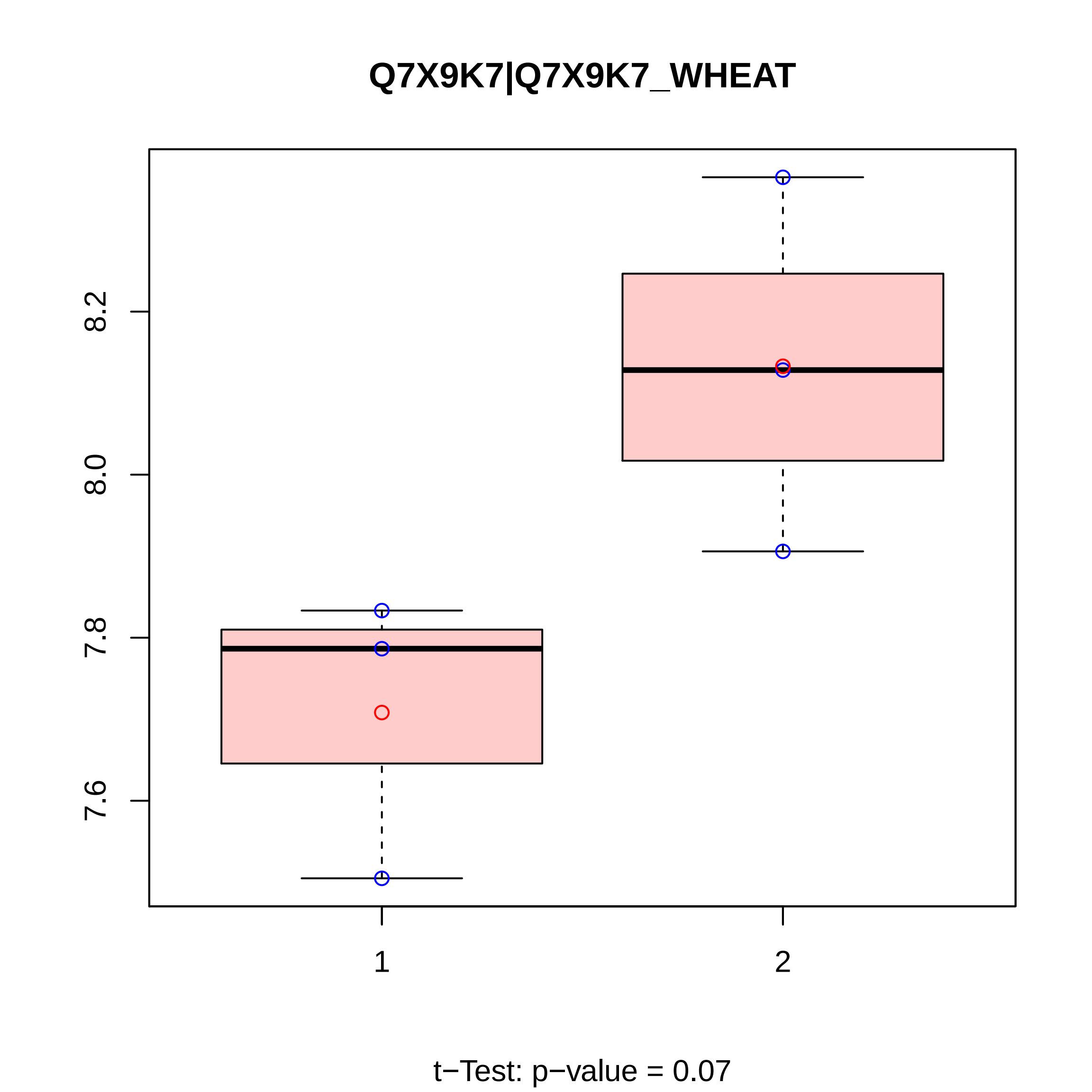
Q7X9K7|Q7X9K7_WHEAT
8.2
8.0
7.8
7.6
1
2
t−Test: p−value = 0.07

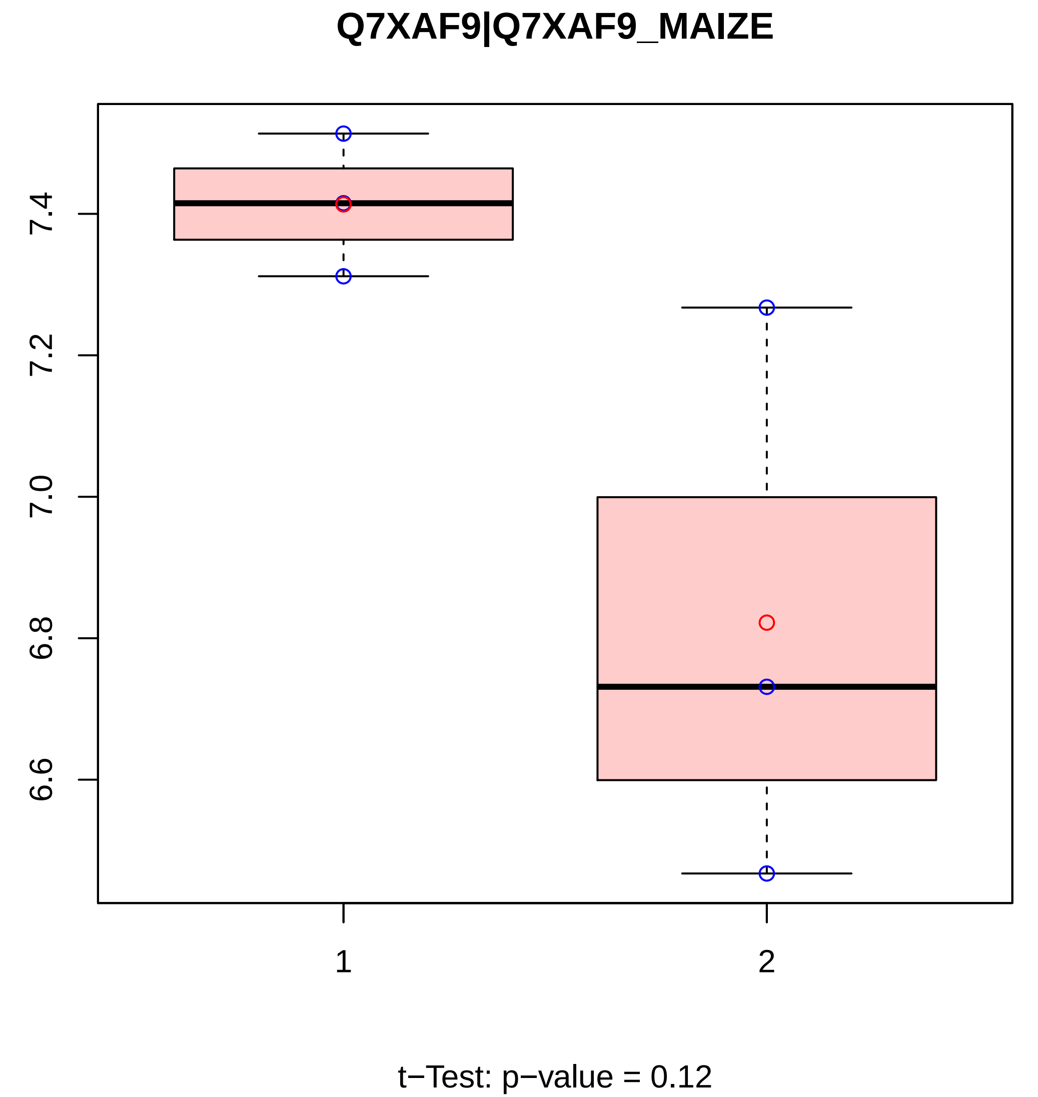

Q7XAF9|Q7XAF9_MAIZE
7.4
7.2
7.0
6.8
6.6
1
2
t−Test: p−value = 0.12

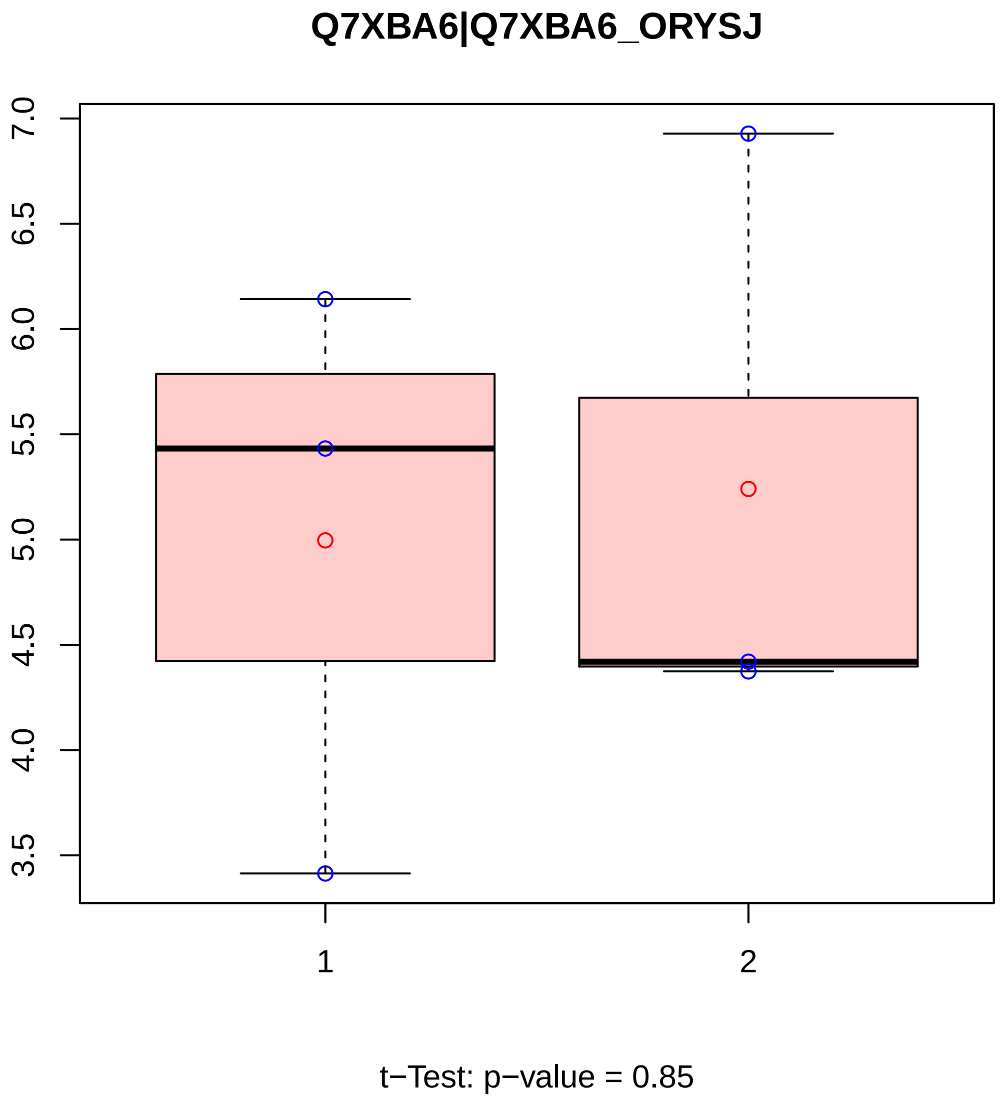

Q7XBA6|Q7XBA6_ORYSJ
7.0
6.5
6.0
5.5
5.0
4.5
4.0
3.5
1
2
t−Test: p−value = 0.85

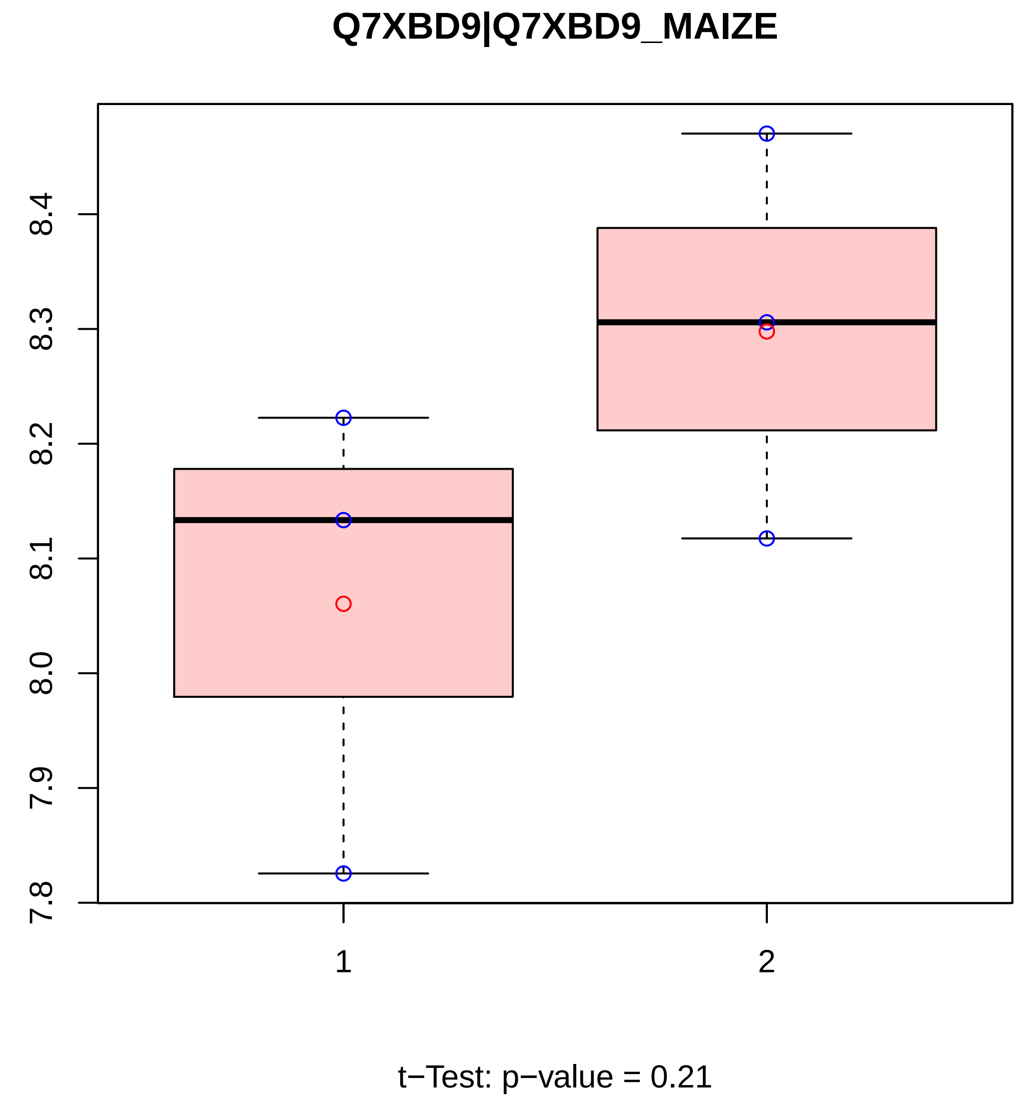
Q7XBD9|Q7XBD9_MAIZE
8.4
8.3
8.2
8.1
8.0
7.9
7.8
1
2
t−Test: p−value = 0.21

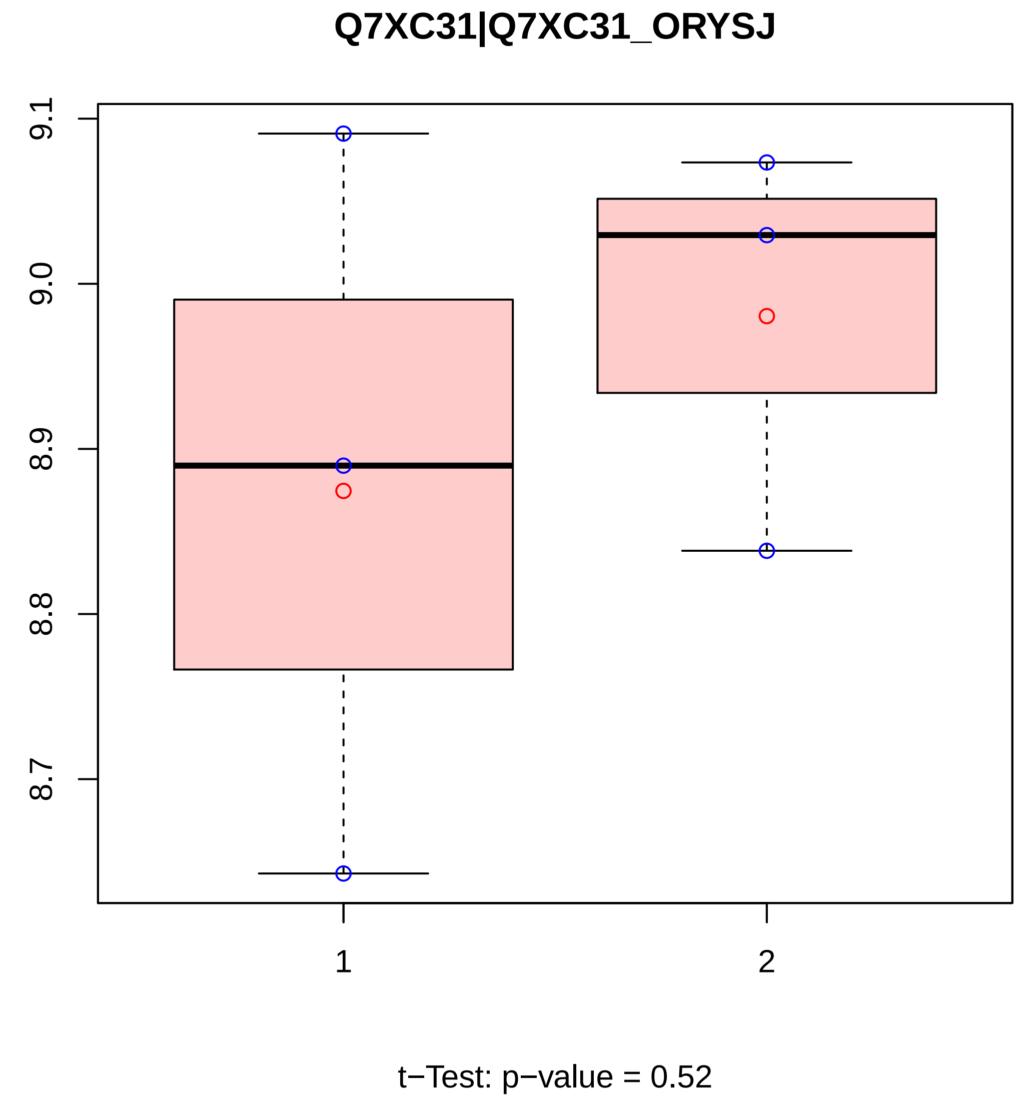

Q7XC31|Q7XC31_ORYSJ
9.1
9.0
8.9
8.8
8.7
1
2
t−Test: p−value = 0.52

Q7XC87|Q7XC87_ORYSJ

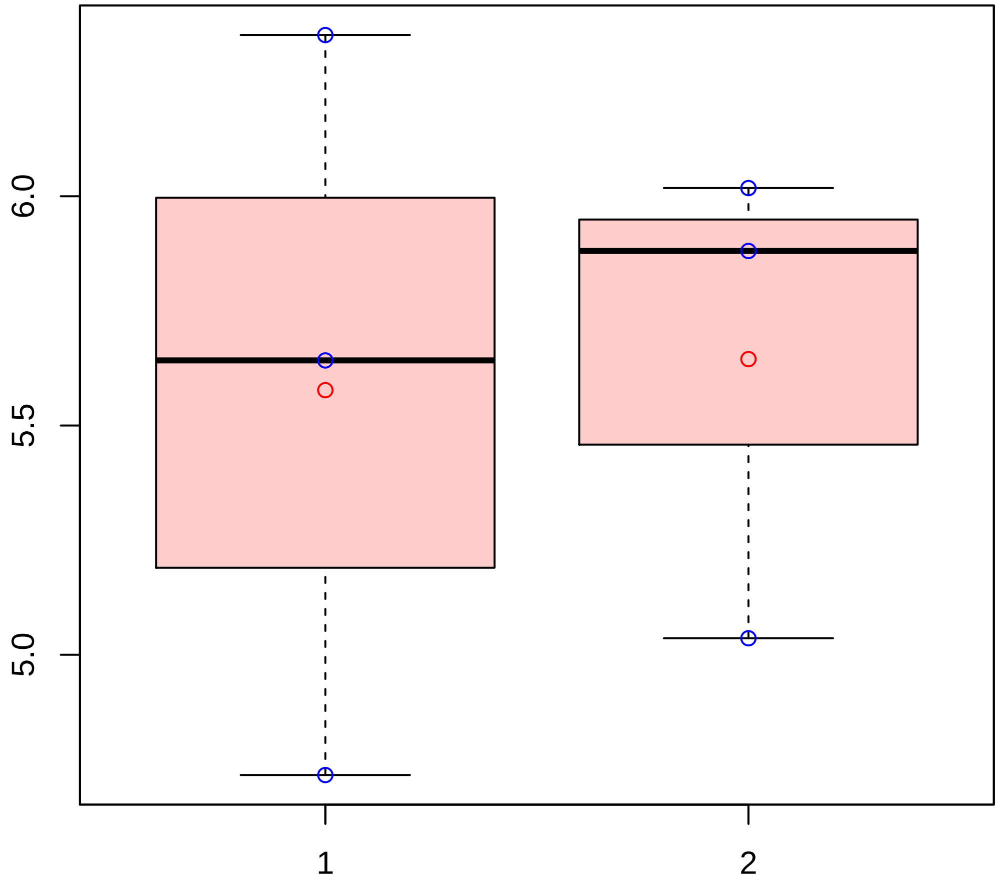

t−Test: p−value = 0.91

Q7XDP7|Q7XDP7_ORYSJ

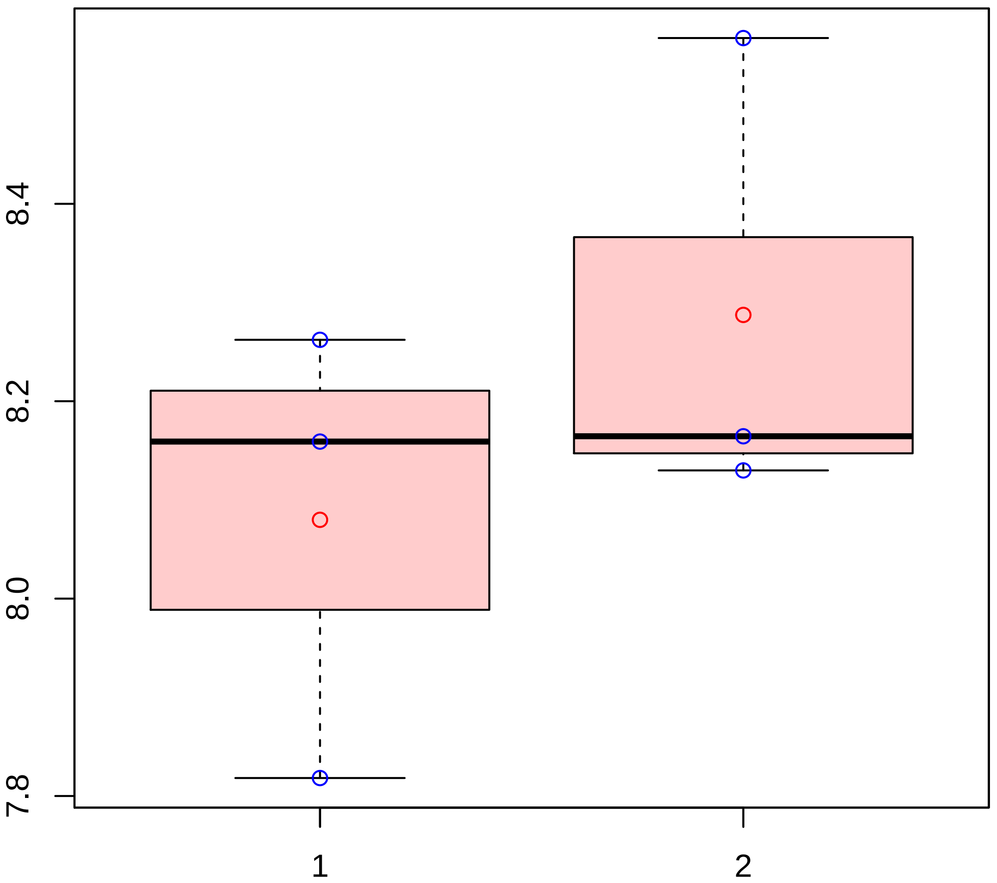

t−Test: p−value = 0.35

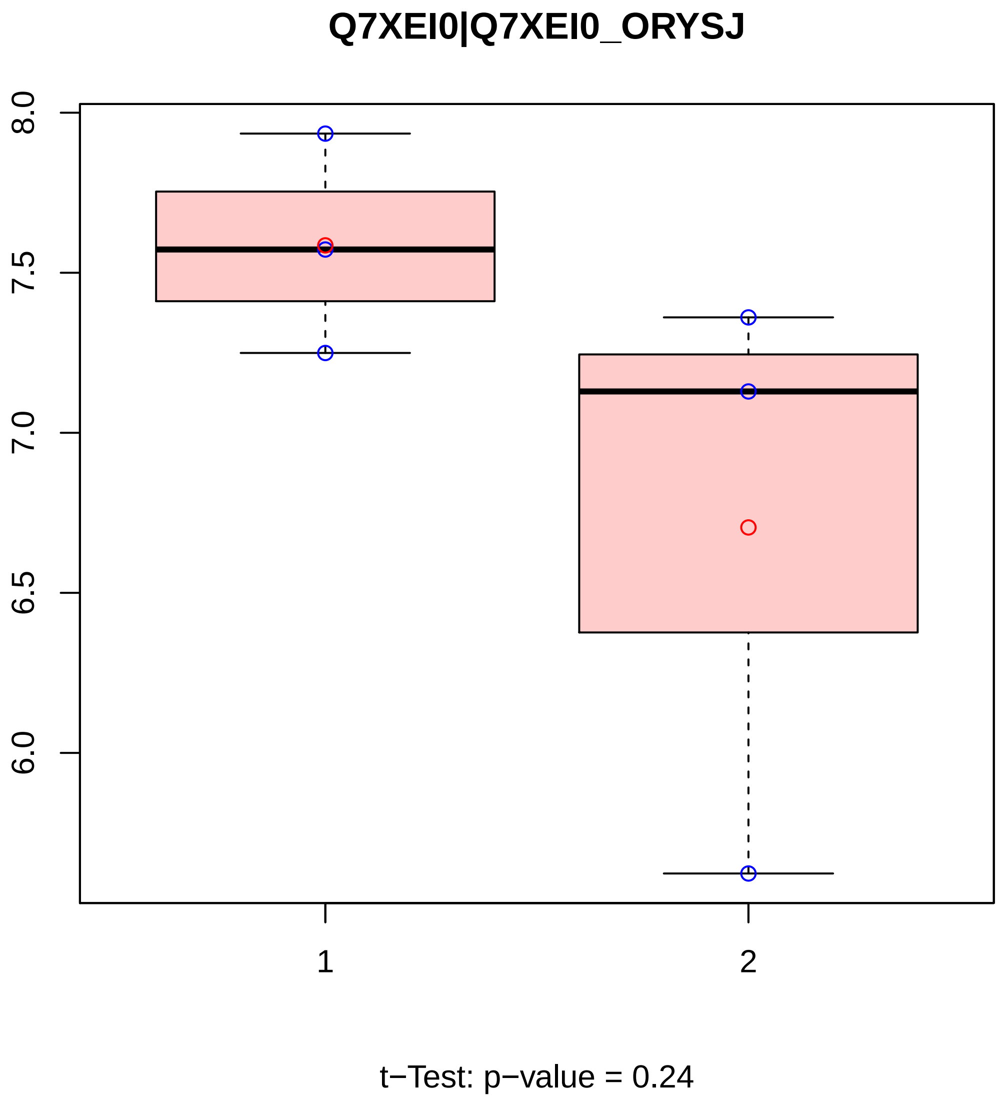
Q7XEI0|Q7XEI0_ORYSJ
8.0
7.5
7.0
6.5
6.0
1
2
t−Test: p−value = 0.24

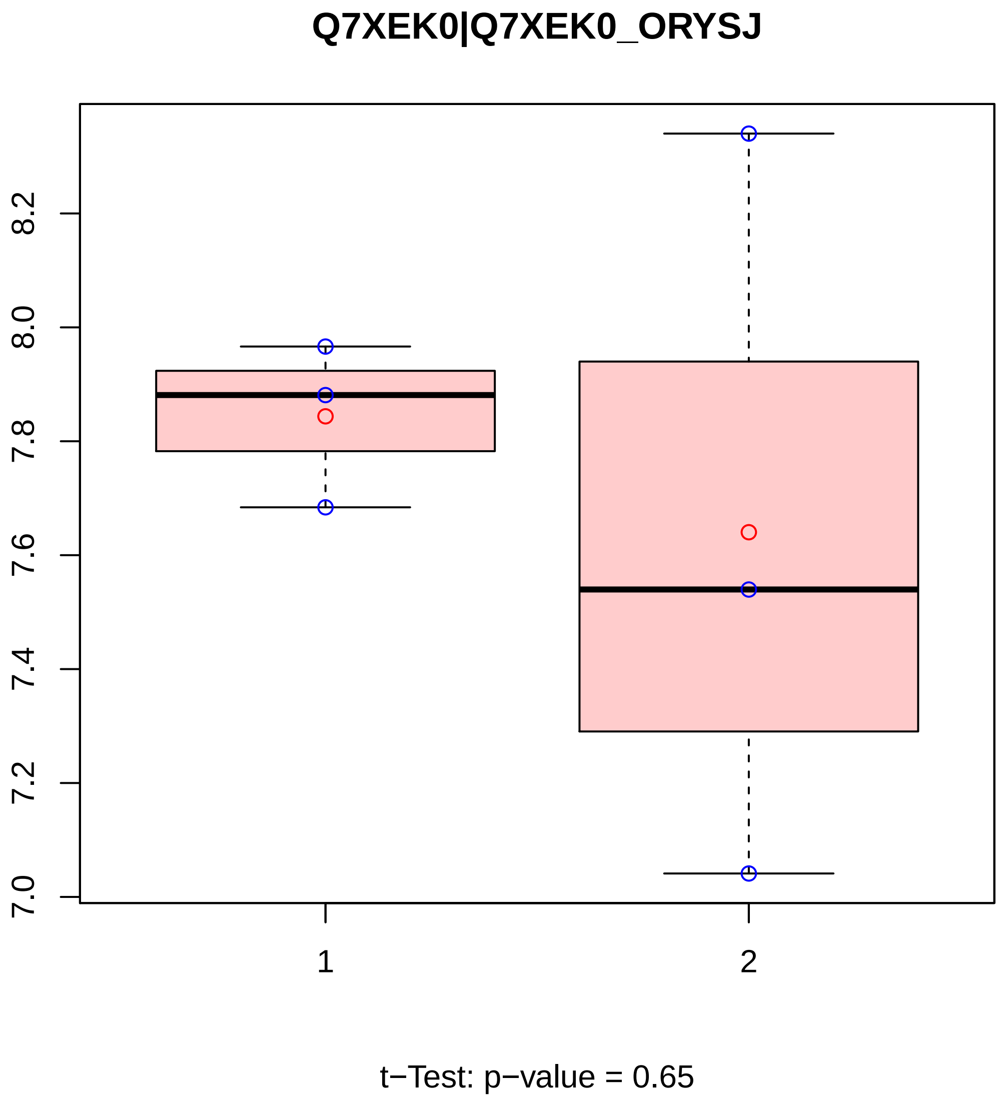
Q7XEK0|Q7XEK0_ORYSJ
8.2
8.0
7.8
7.6
7.4
7.2
7.0
1
2
t−Test: p−value = 0.65

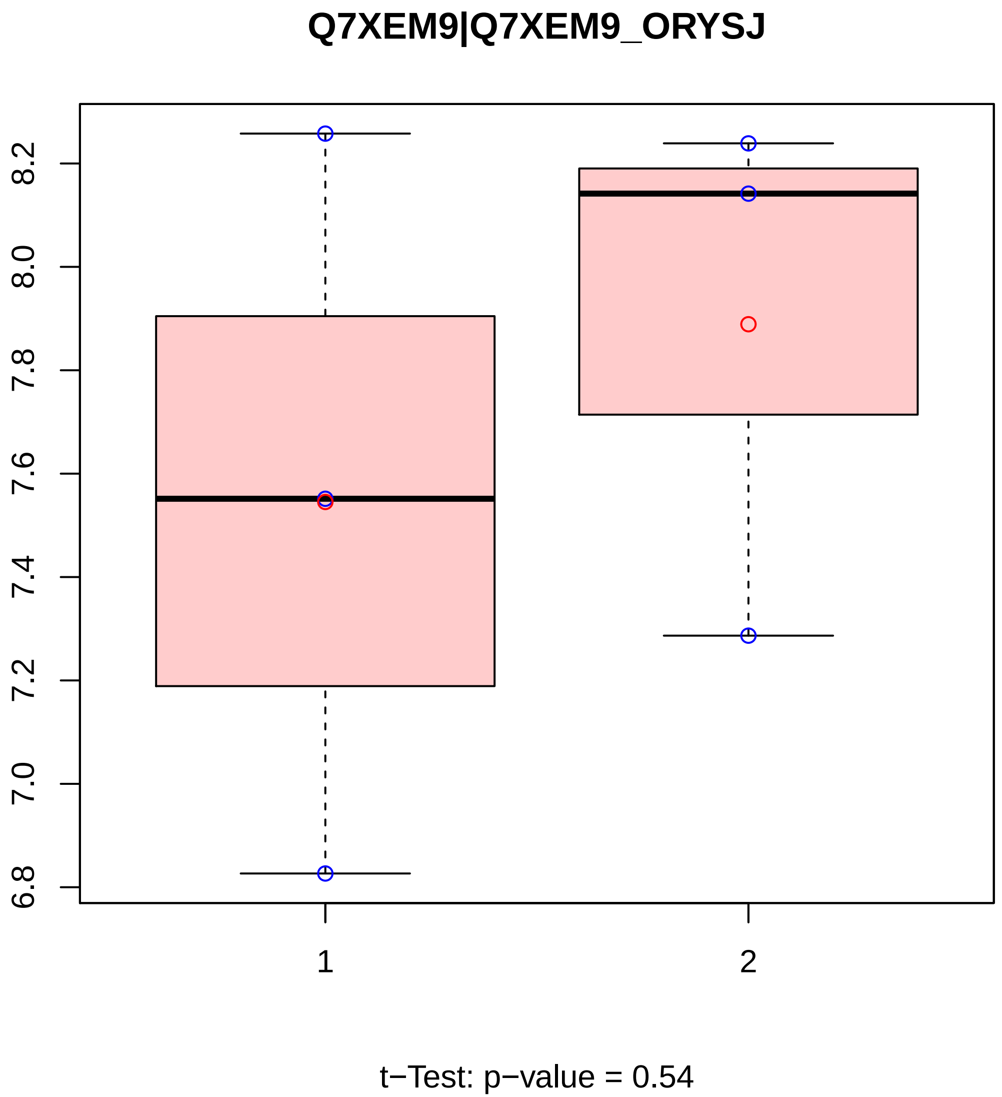
Q7XEM9|Q7XEM9_ORYSJ
8.2
8.0
7.8
7.6
7.4
7.2
7.0
6.8
1
2
t−Test: p−value = 0.54

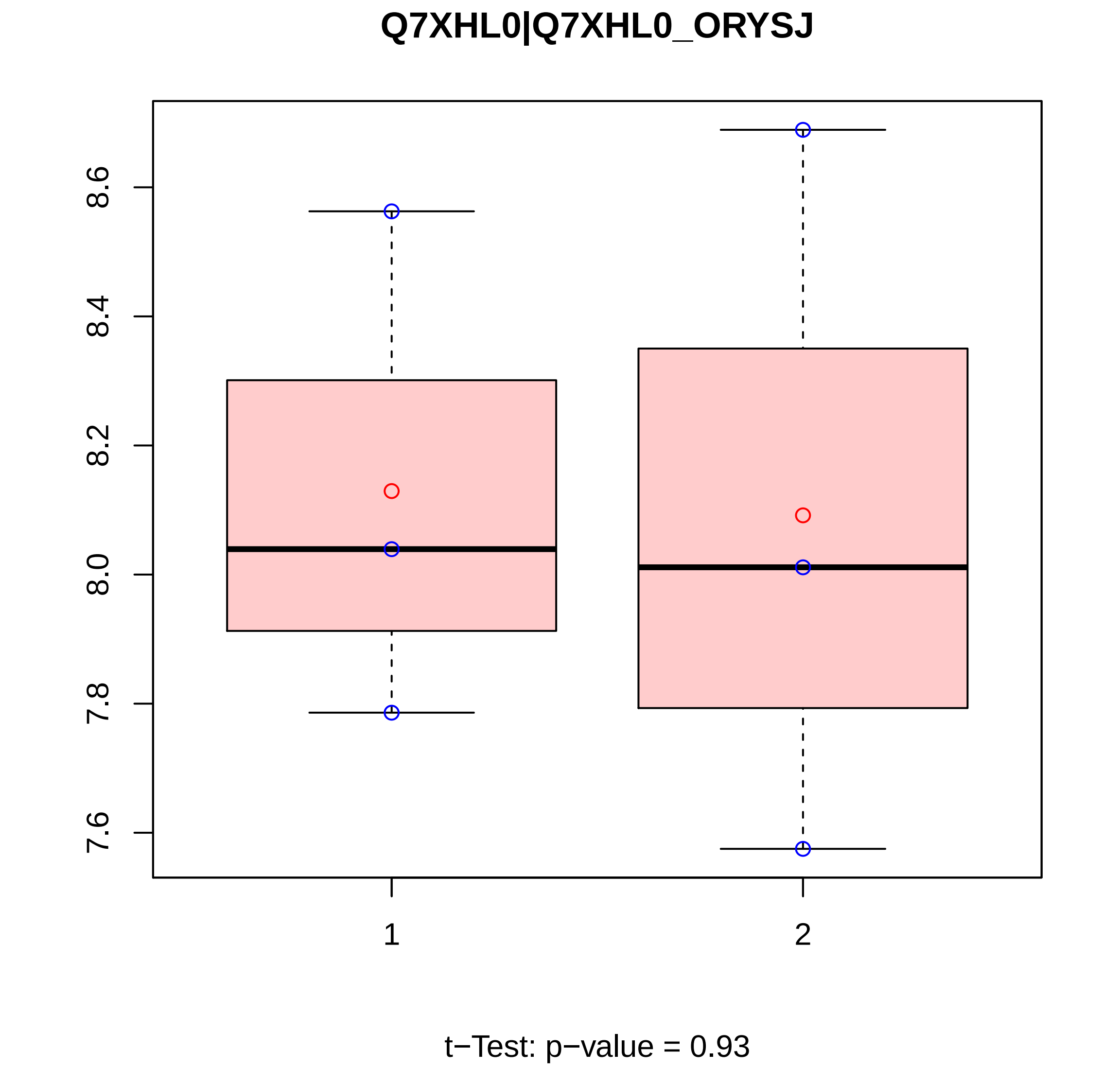
Q7XHL0|Q7XHL0_ORYSJ
8.6
8.4
8.2
8.0
7.8
7.6
1
2
t−Test: p−value = 0.93

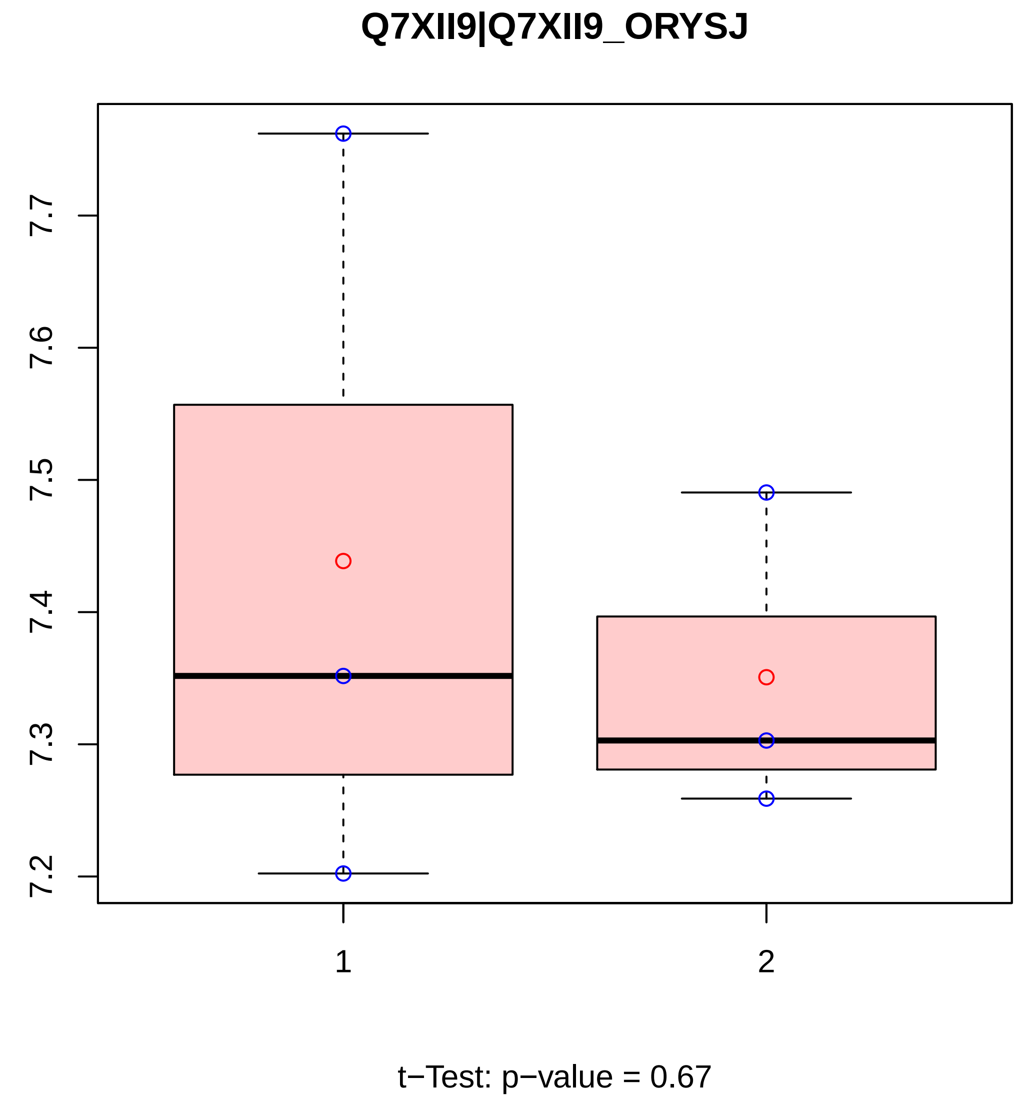
Q7XII9|Q7XII9_ORYSJ
7.2
7.3
7.4
7.5
7.6
7.7
1
2
t−Test: p−value = 0.67

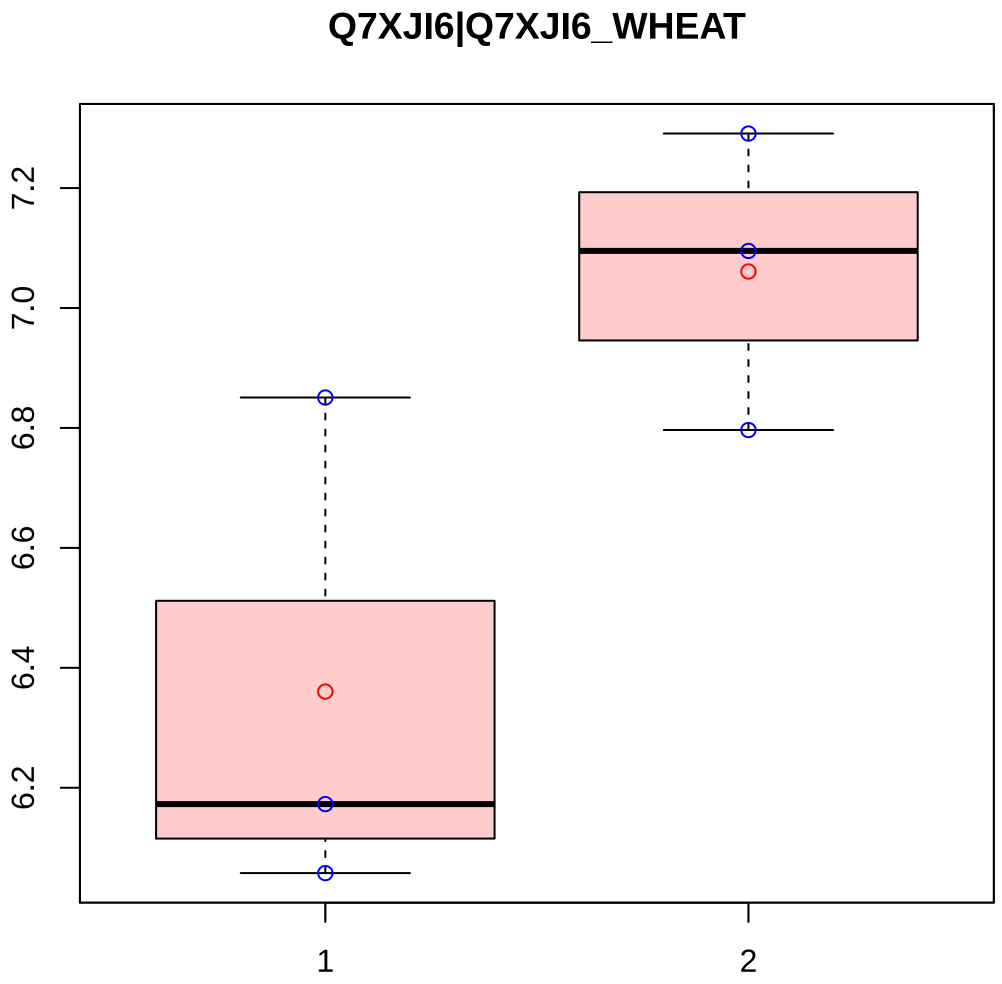

t−Test: p−value = 0.09

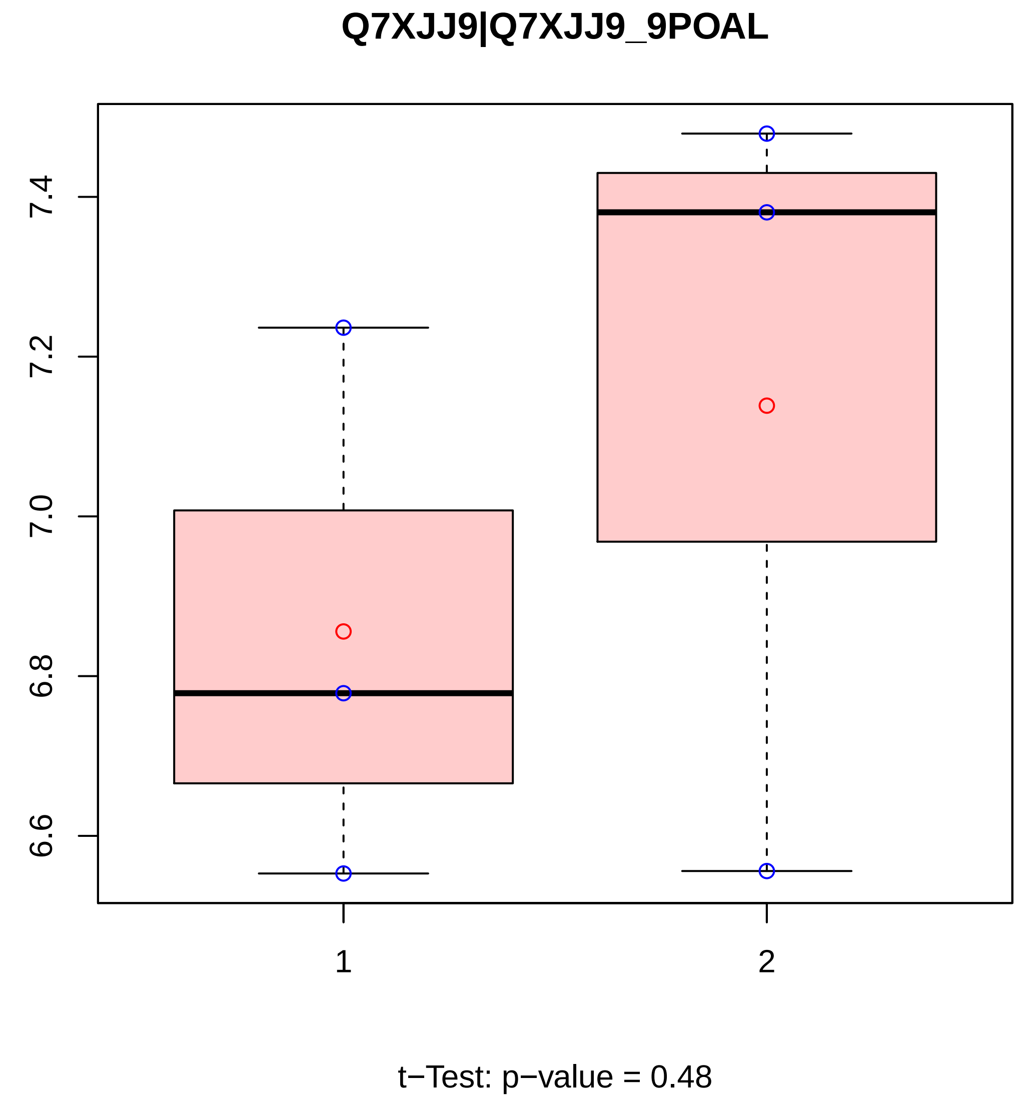
Q7XJJ9|Q7XJJ9_9POAL
7.4
7.2
7.0
6.8
6.6
1
2
t−Test: p−value = 0.48

Q7XJK1|Q7XJK1_9POAL

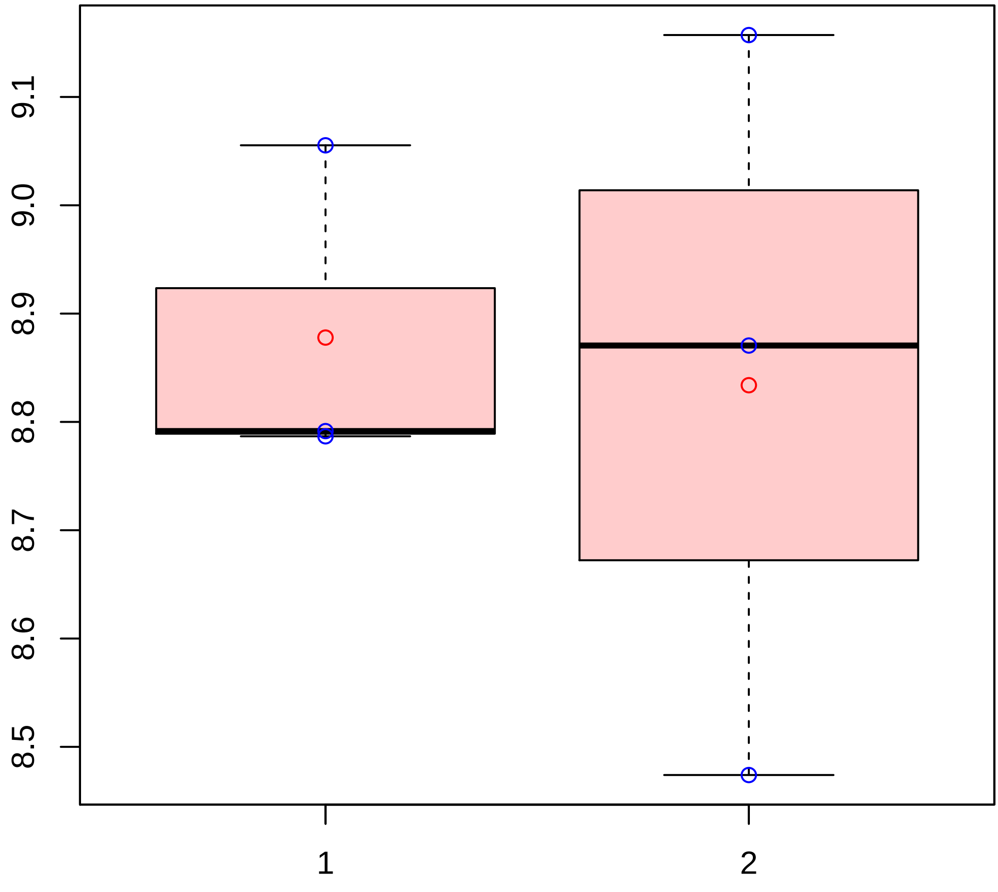

t−Test: p−value = 0.85

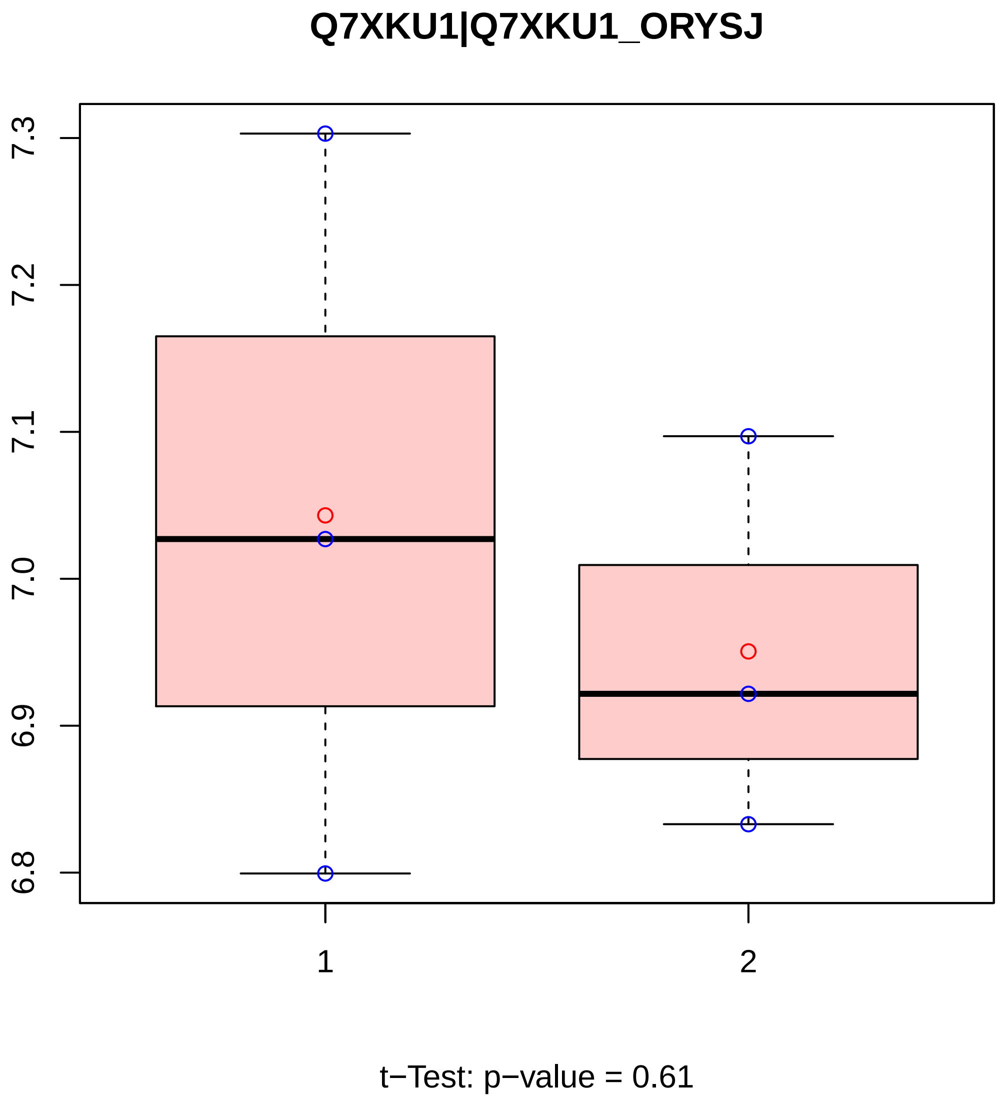
Q7XKU1|Q7XKU1_ORYSJ
7.3
7.2
7.1
7.0
6.9
6.8
1
2
t−Test: p−value = 0.61

Q7XLA7|Q7XLA7_ORYSJ

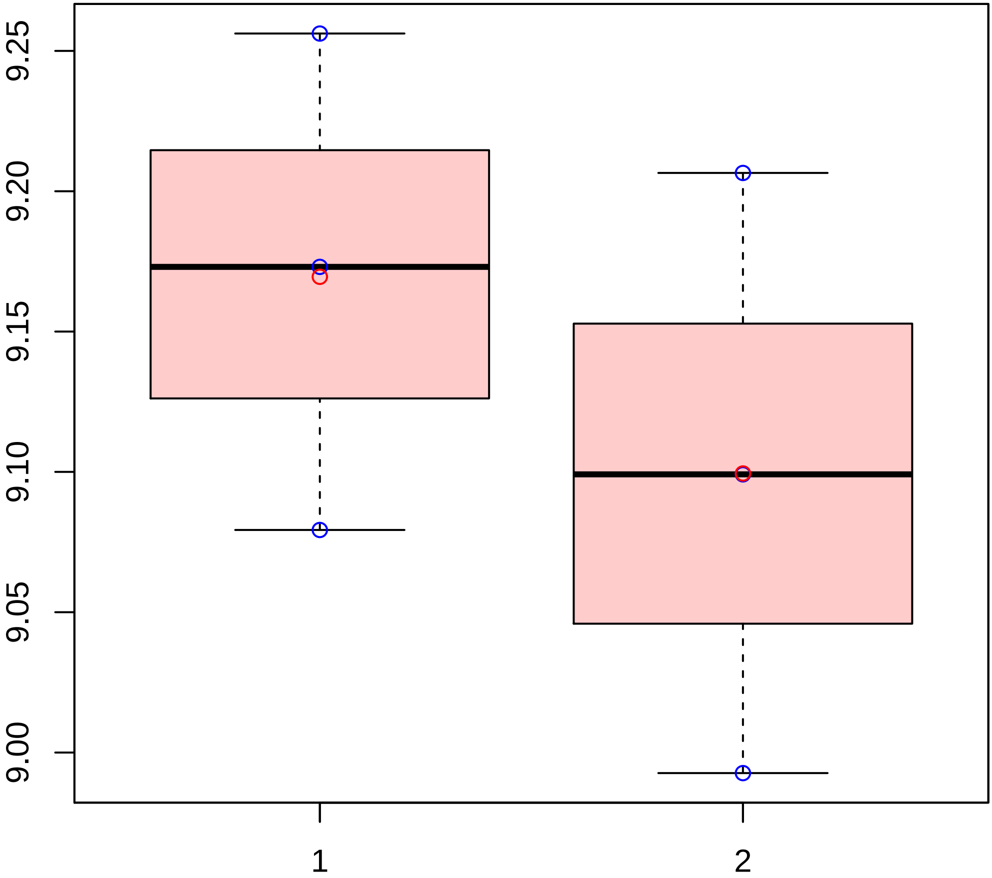

t−Test: p−value = 0.43

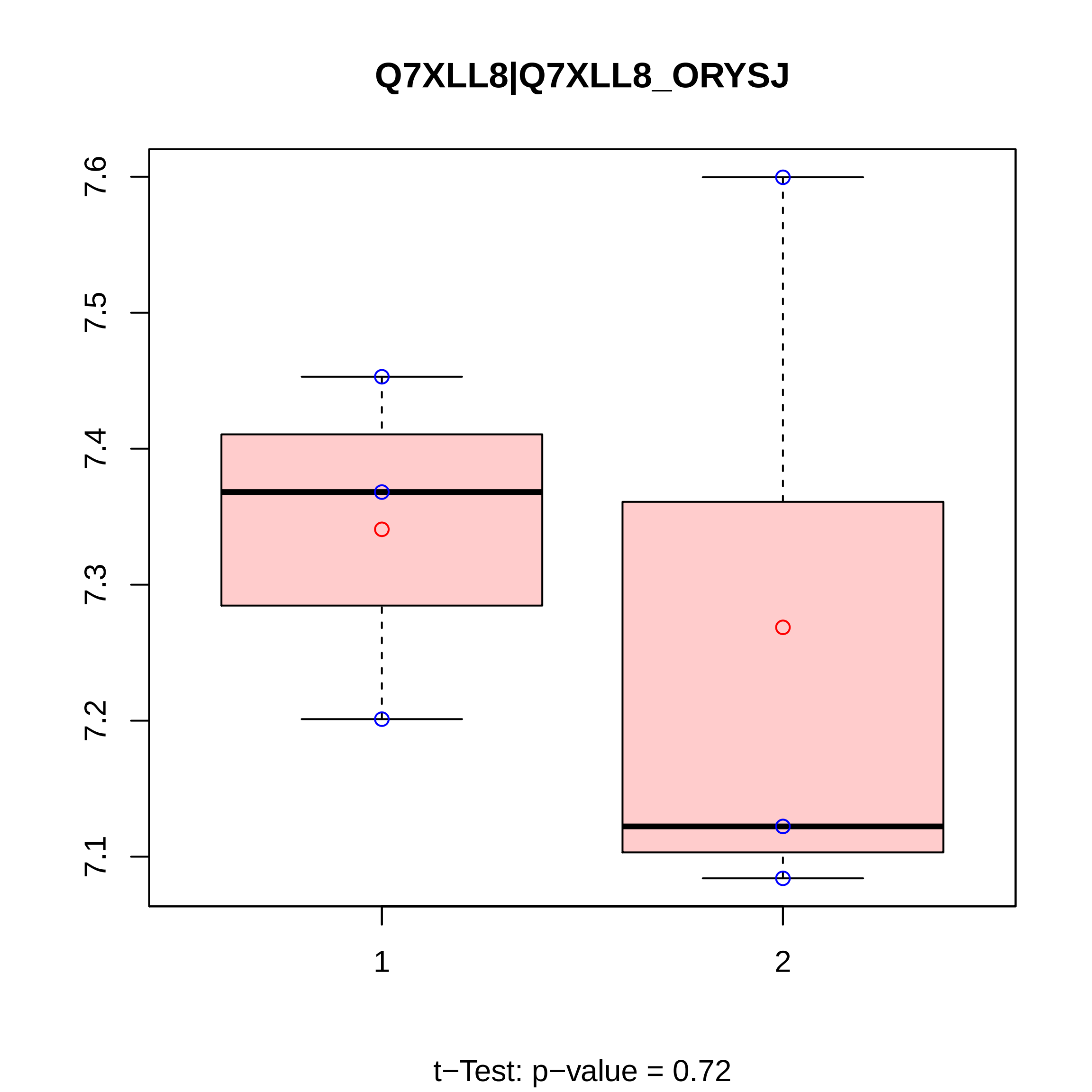
Q7XLL8|Q7XLL8_ORYSJ
7.6
7.5
7.4
7.3
7.2
7.1
1
2
t−Test: p−value = 0.72

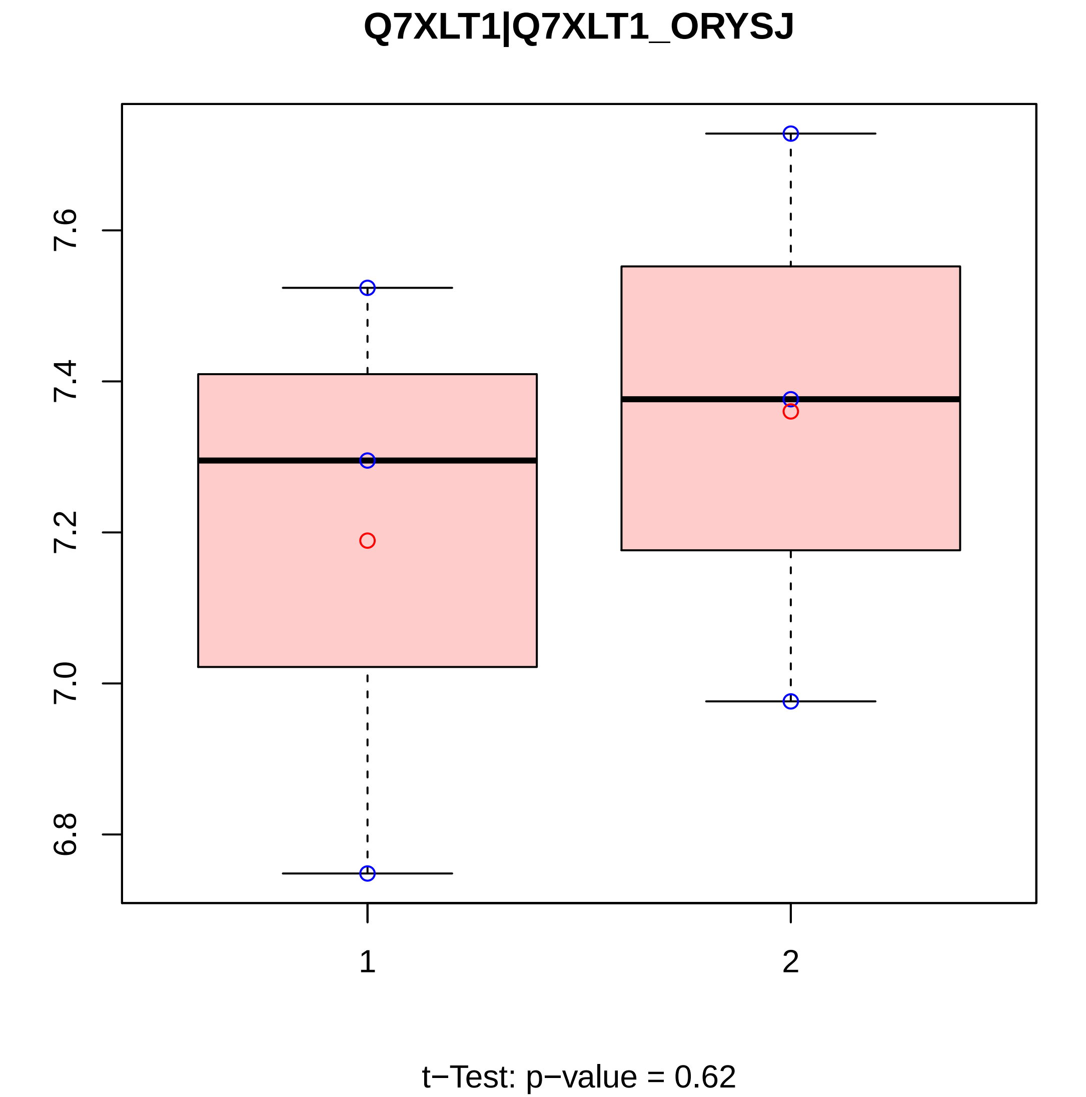

Q7XLT1|Q7XLT1_ORYSJ
6.8
7.0
7.2
7.4
7.6
1
2
t−Test: p−value = 0.62

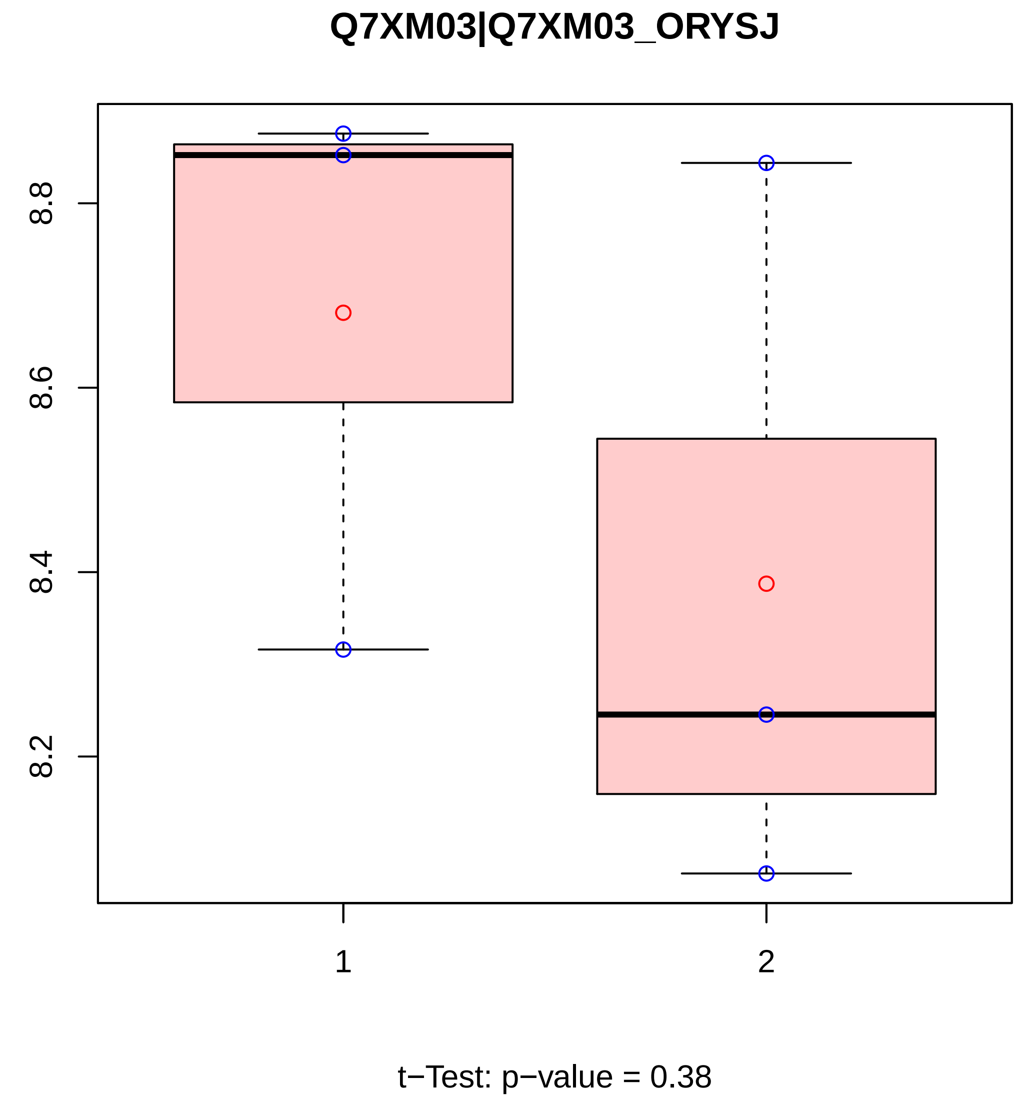
Q7XM03|Q7XM03_ORYSJ
8.8
8.6
8.4
8.2
1
2
t−Test: p−value = 0.38

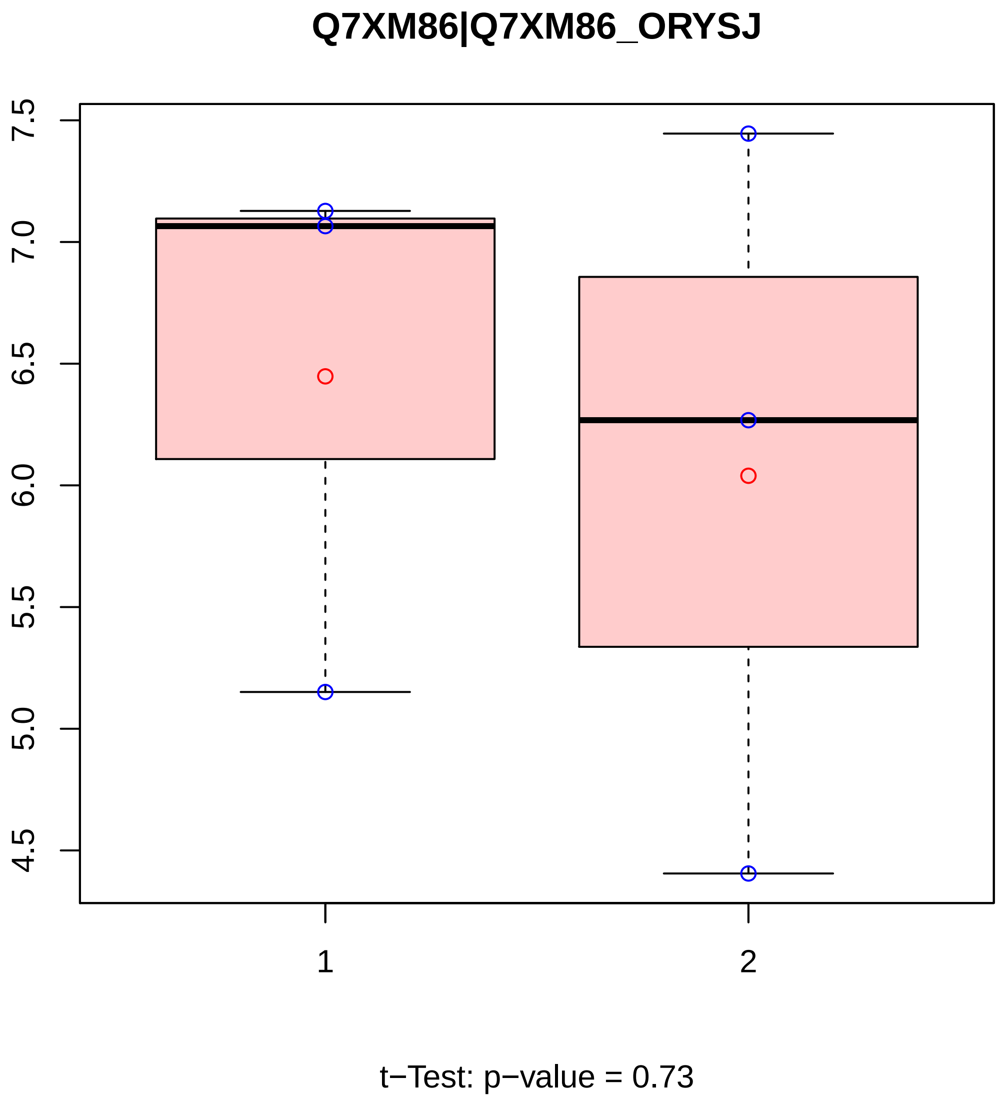
Q7XM86|Q7XM86_ORYSJ
7.5
7.0
6.5
6.0
5.5
5.0
4.5
1
2
t−Test: p−value = 0.73

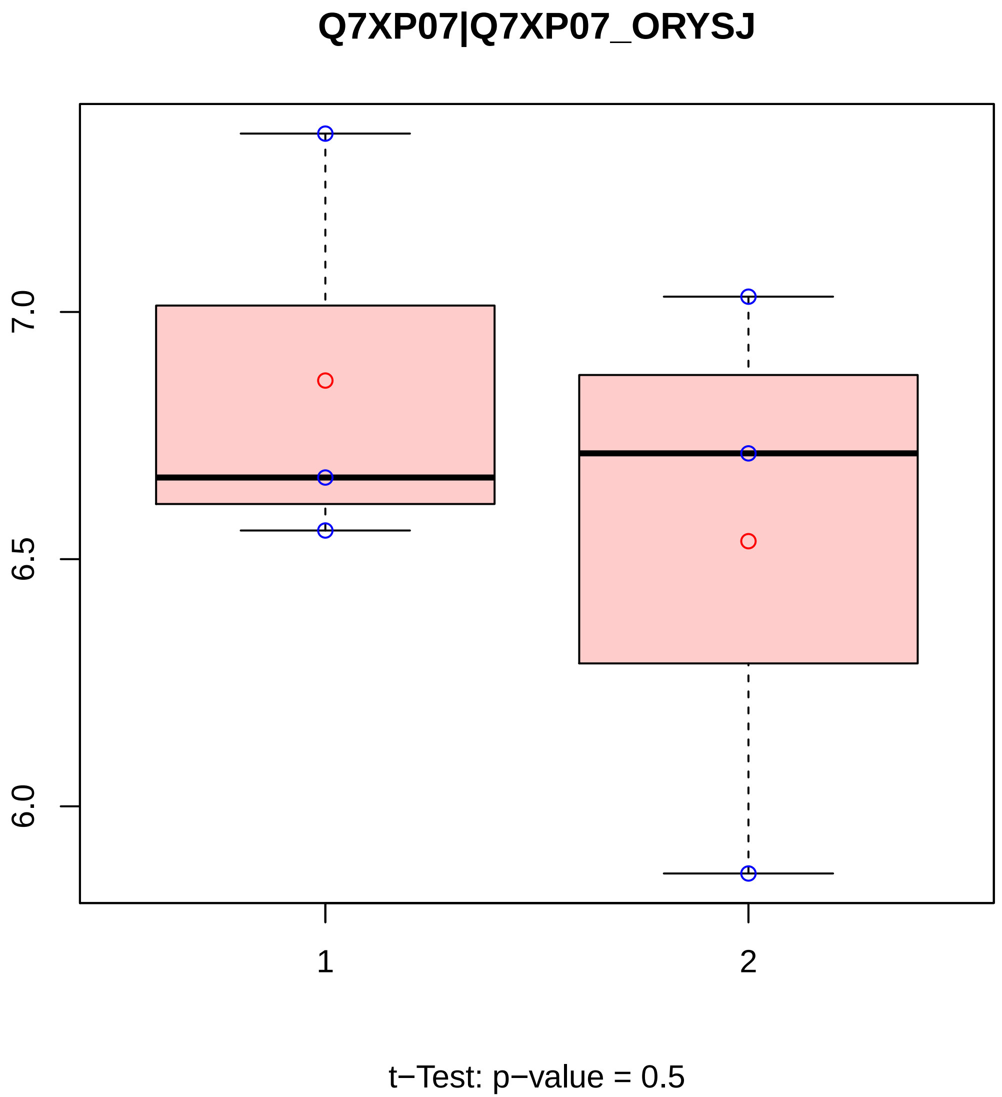
Q7XP07|Q7XP07_ORYSJ
7.0
6.5
6.0
1
2
t−Test: p−value = 0.5

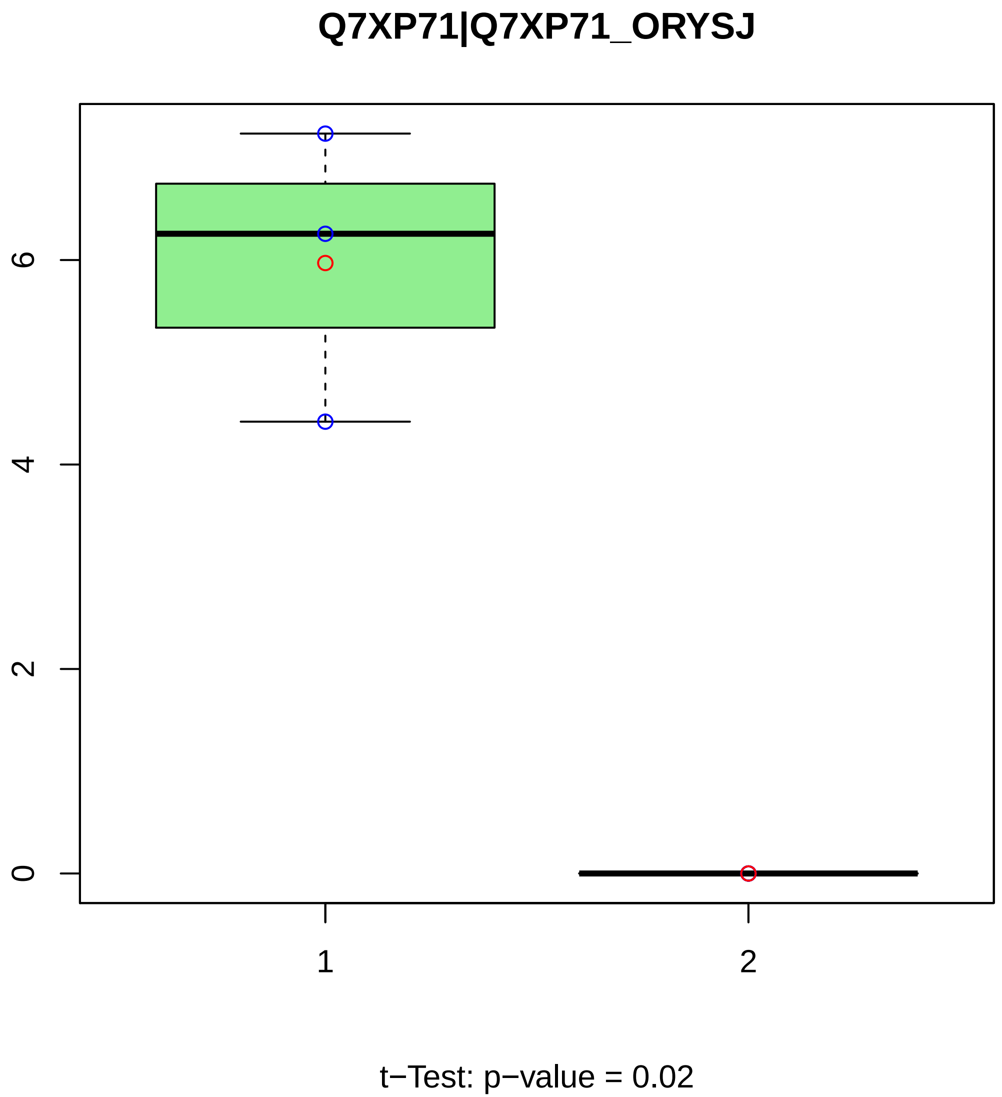

Q7XP71|Q7XP71_ORYSJ
0
2
4
6
1
2
t−Test: p−value = 0.02

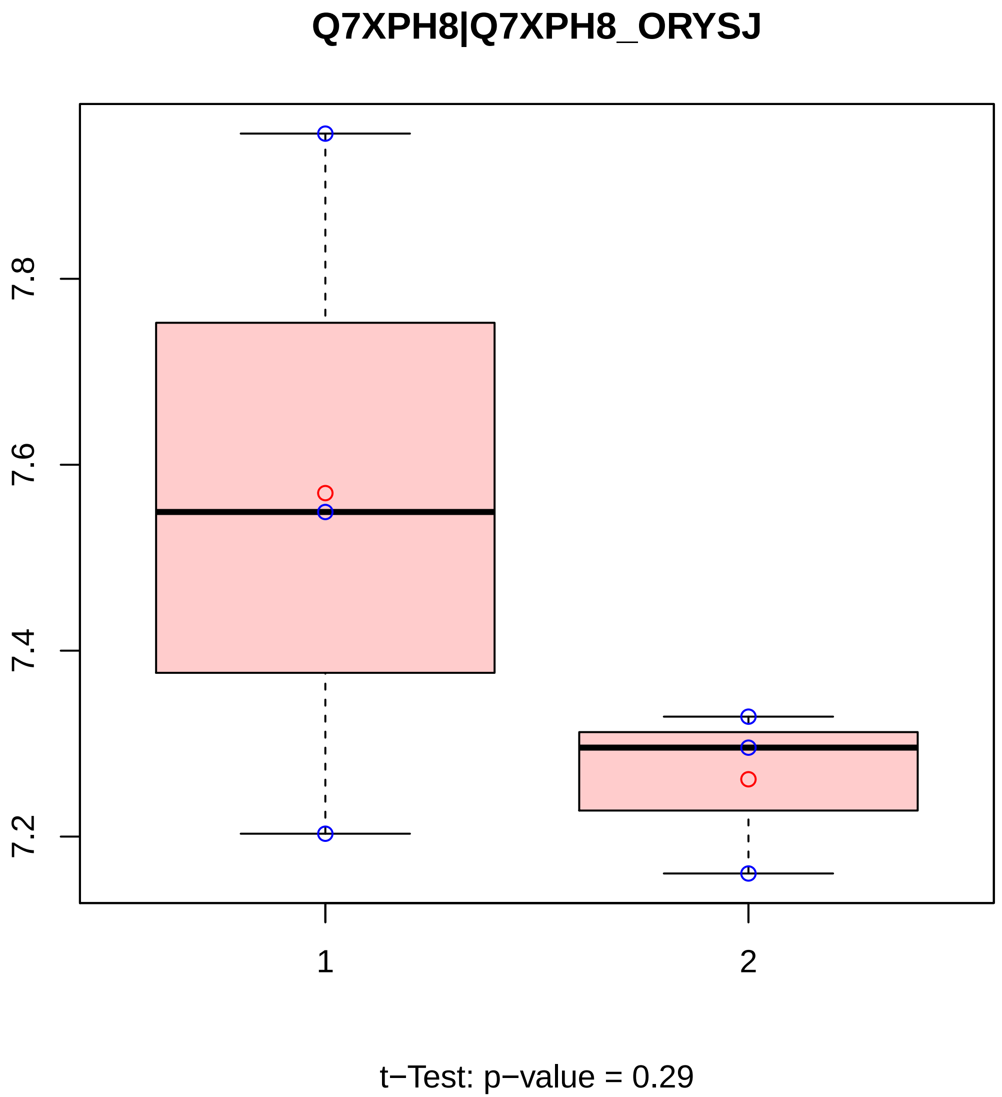
Q7XPH8|Q7XPH8_ORYSJ
7.2
7.4
7.6
7.8
1
2
t−Test: p−value = 0.29

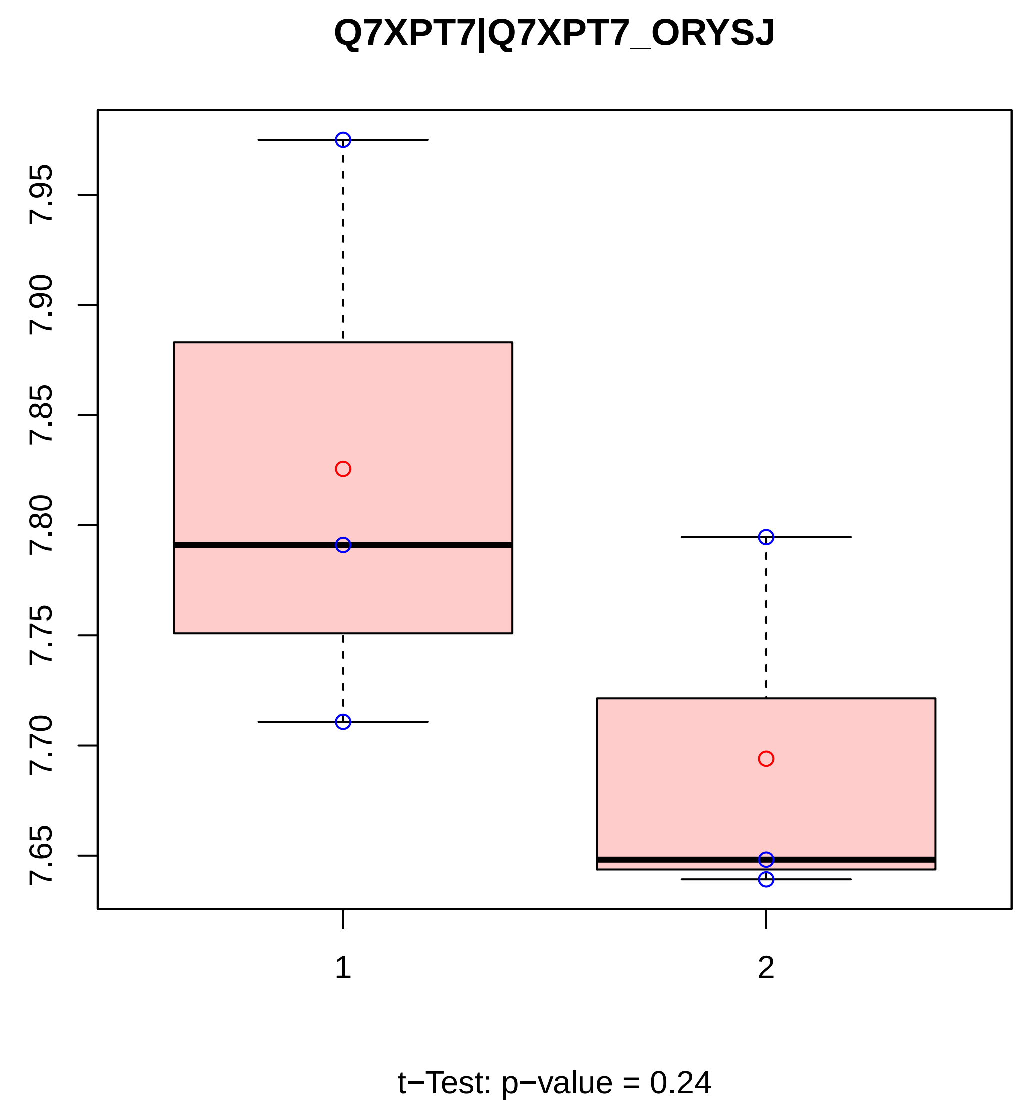

Q7XPT7|Q7XPT7_ORYSJ
7.65
7.70
7.75
7.80
7.85
7.90
7.95
1
2
t−Test: p−value = 0.24

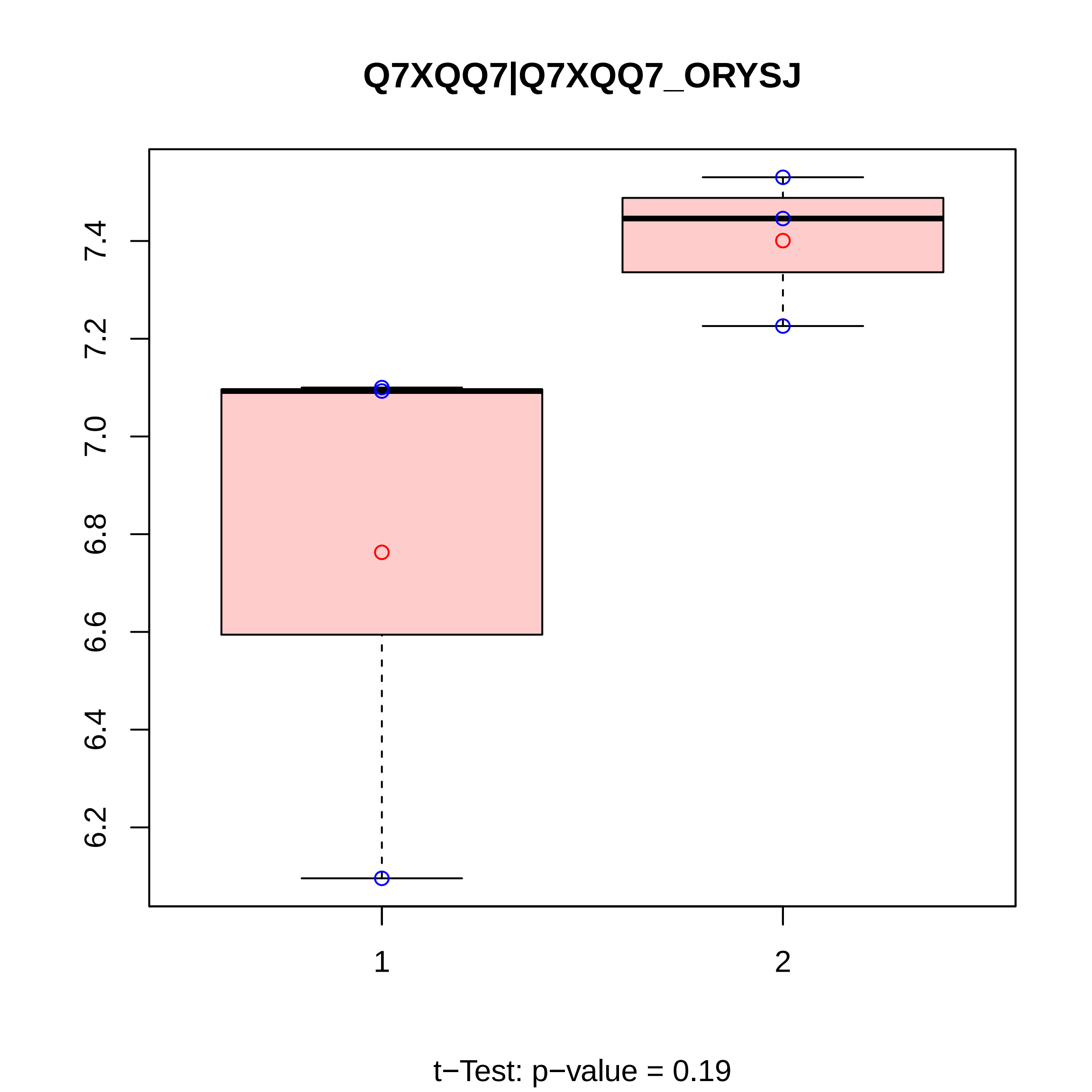
Q7XQQ7|Q7XQQ7_ORYSJ
7.4
7.2
7.0
6.8
6.6
6.4
6.2
1
2
t−Test: p−value = 0.19

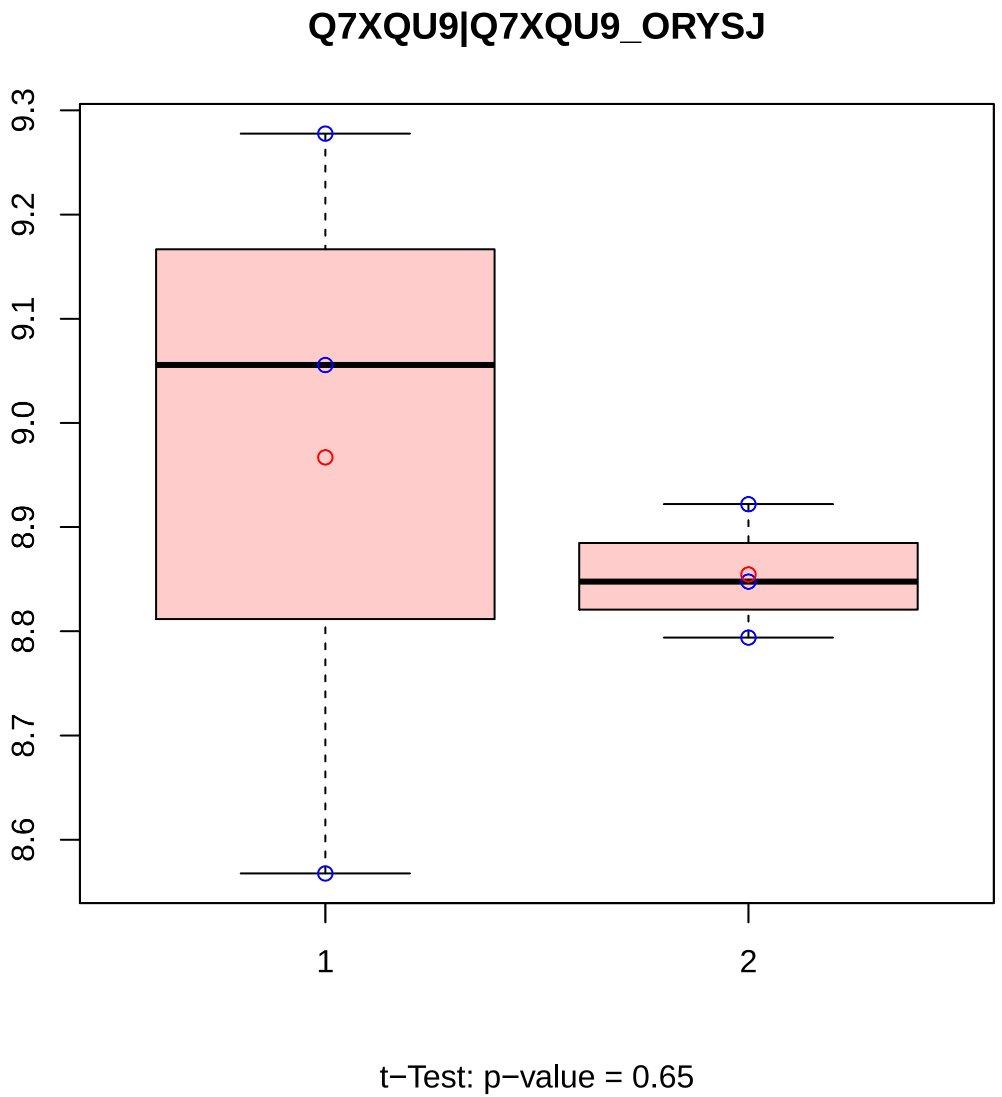
Q7XQU9|Q7XQU9_ORYSJ
9.3
9.2
9.1
9.0
8.9
8.8
8.7
8.6
1
2
t−Test: p−value = 0.65

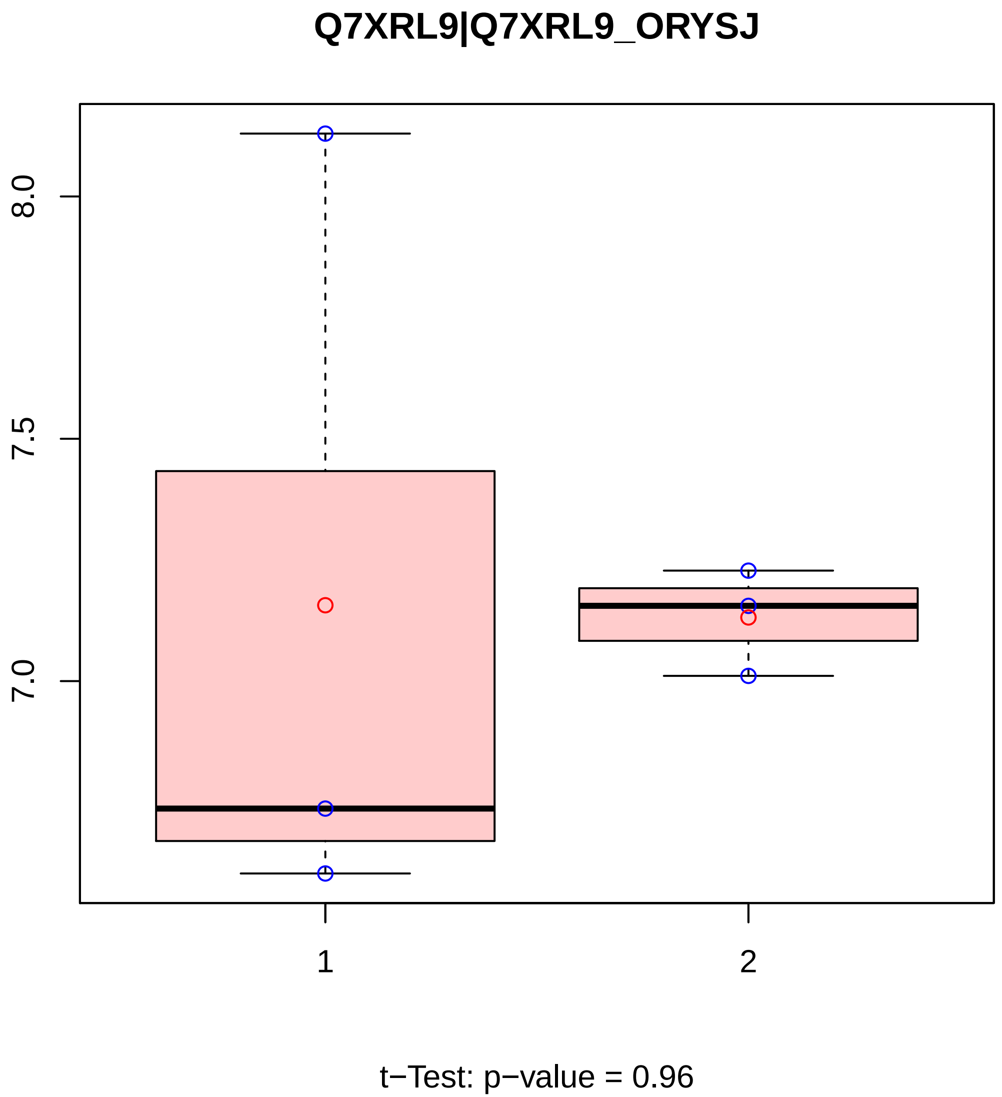

Q7XRL9|Q7XRL9_ORYSJ
8.0
7.5
7.0
1
2
t−Test: p−value = 0.96

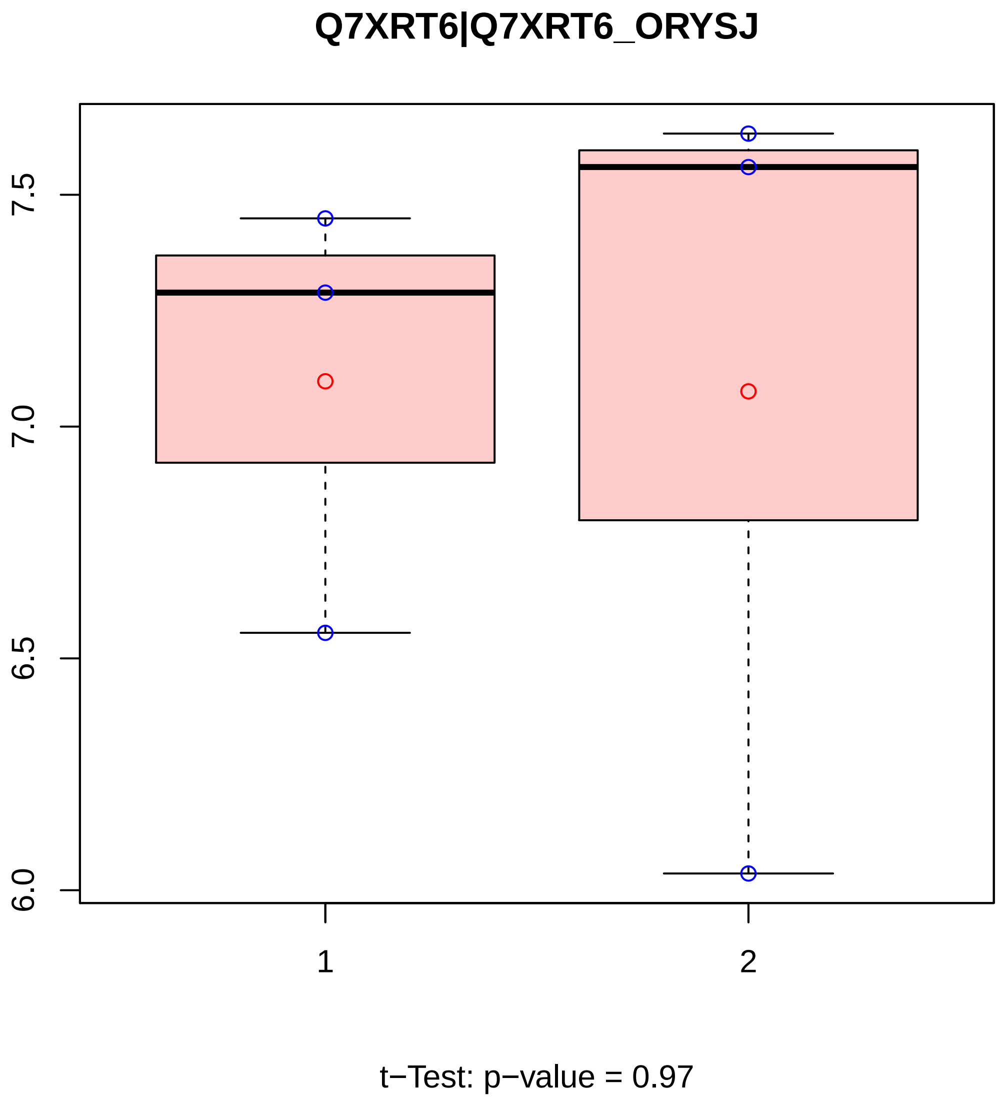
Q7XRT6|Q7XRT6_ORYSJ
7.5
7.0
6.5
6.0
1
2
t−Test: p−value = 0.97

Q7XRV6|Q7XRV6_ORYSJ

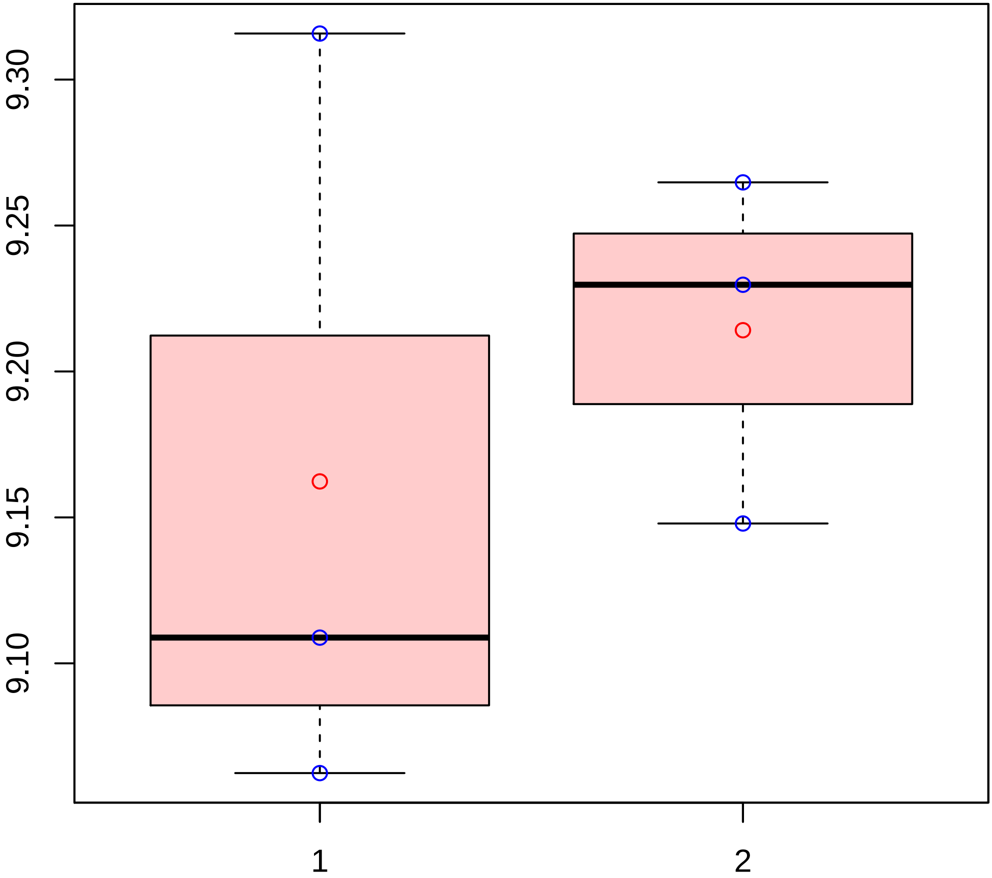

t−Test: p−value = 0.59

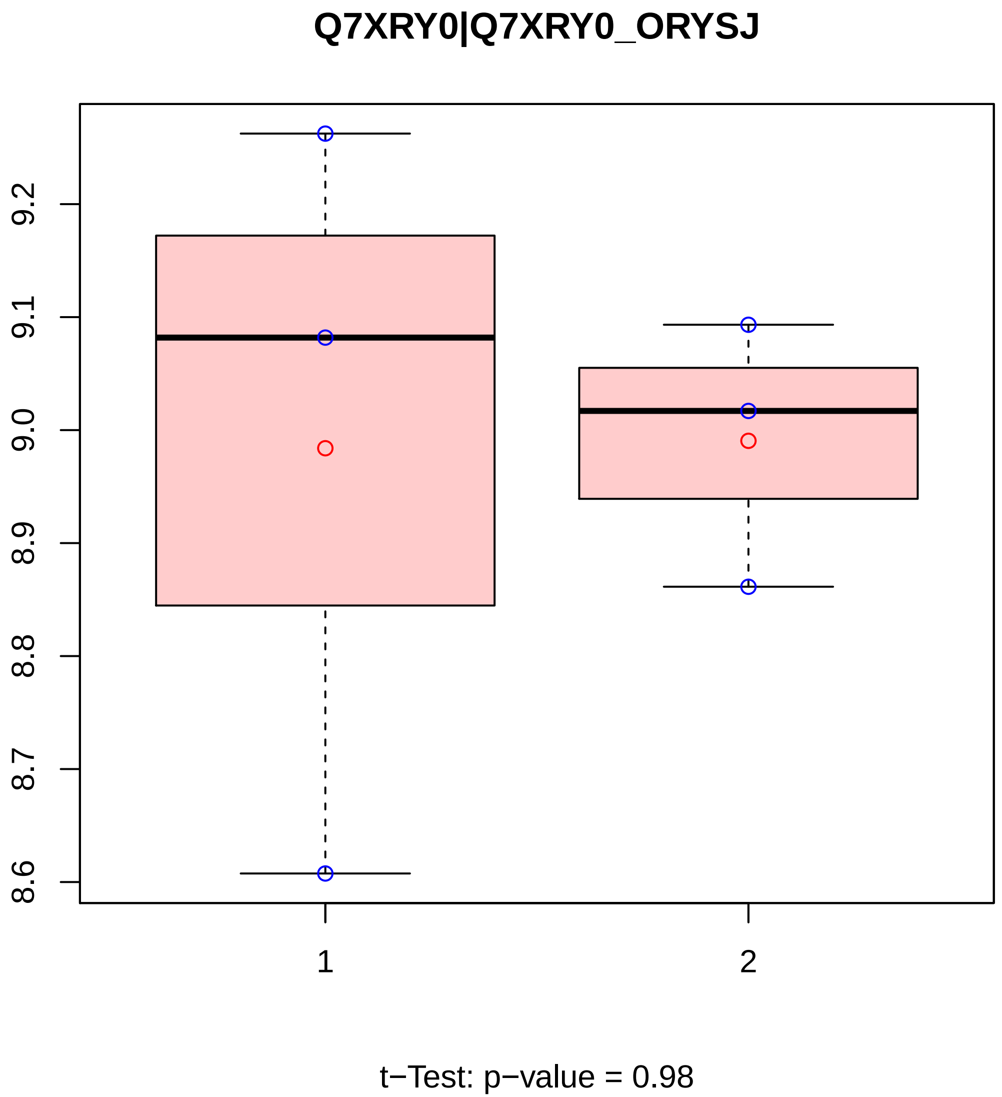
Q7XRY0|Q7XRY0_ORYSJ
9.2
9.1
9.0
8.9
8.8
8.7
8.6
1
2
t−Test: p−value = 0.98

Q7XSS2|Q7XSS2_ORYSJ

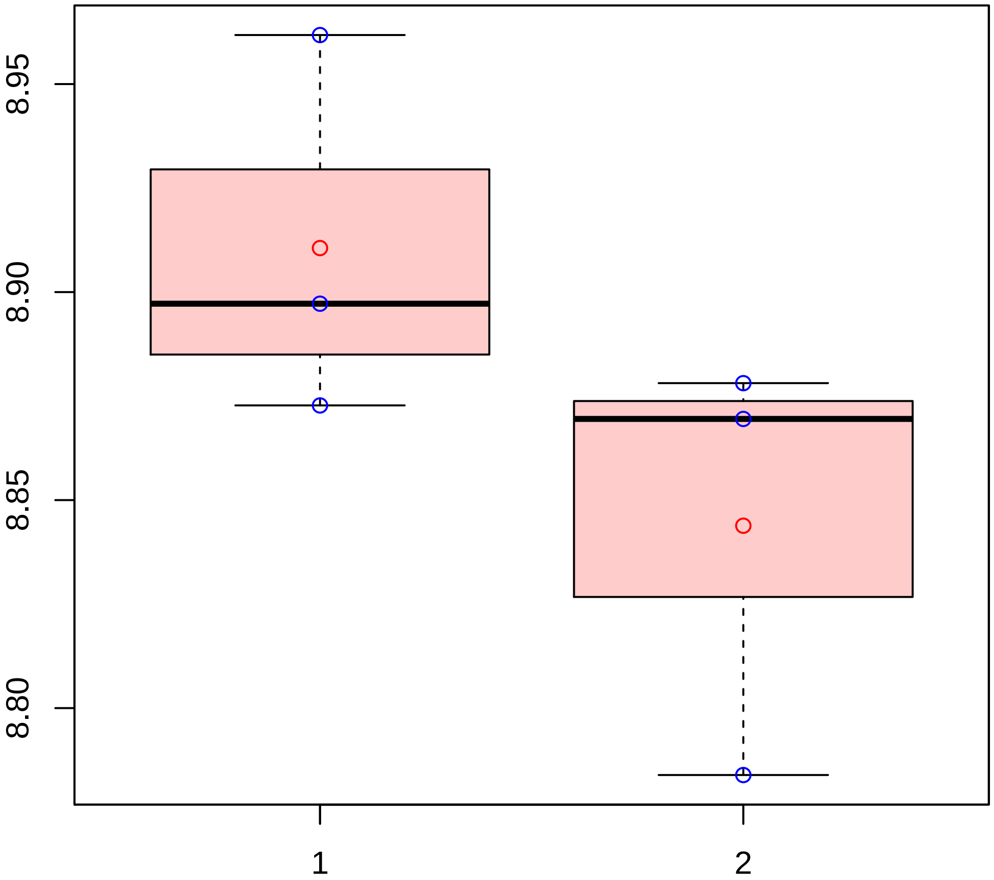

t−Test: p−value = 0.17

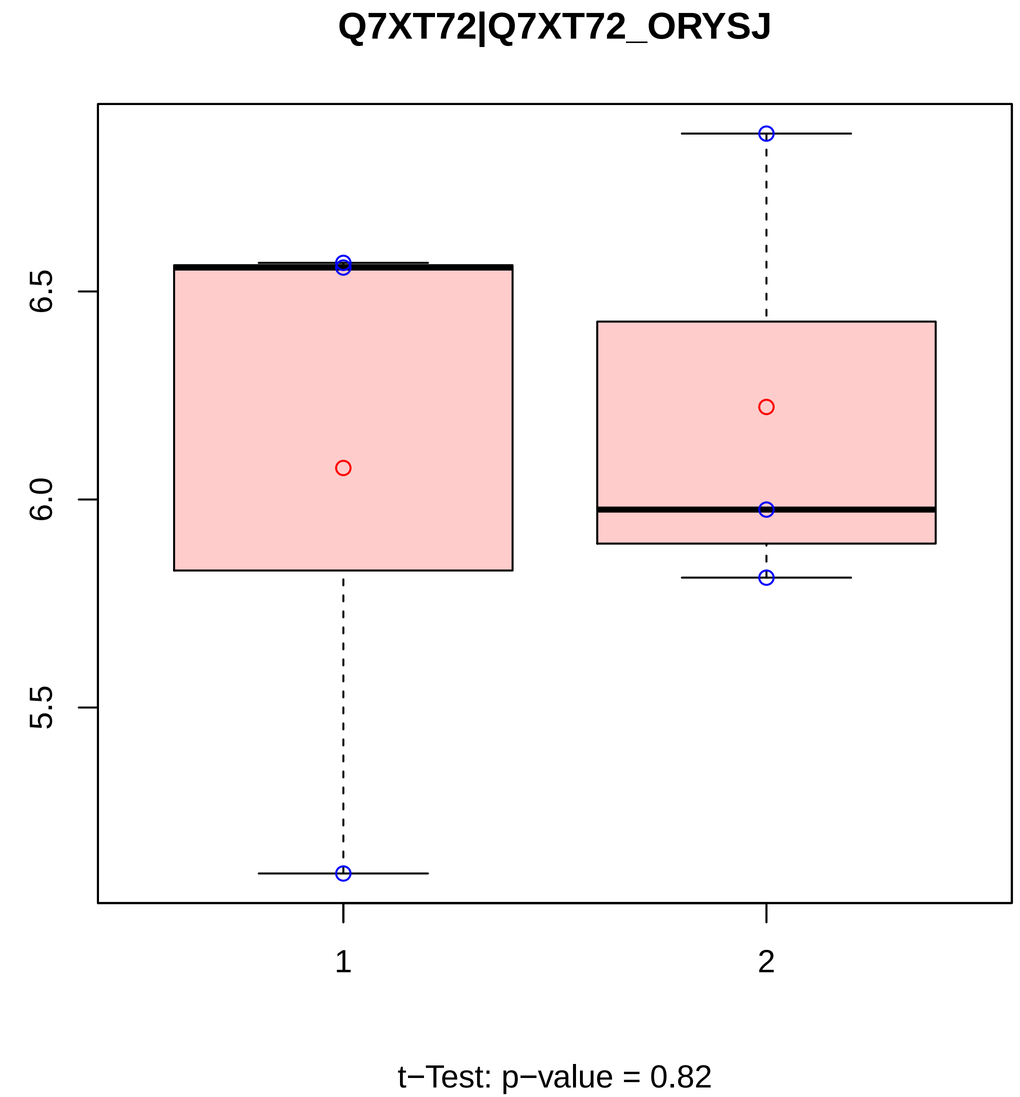
Q7XT72|Q7XT72_ORYSJ
6.5
6.0
5.5
1
2
t−Test: p−value = 0.82

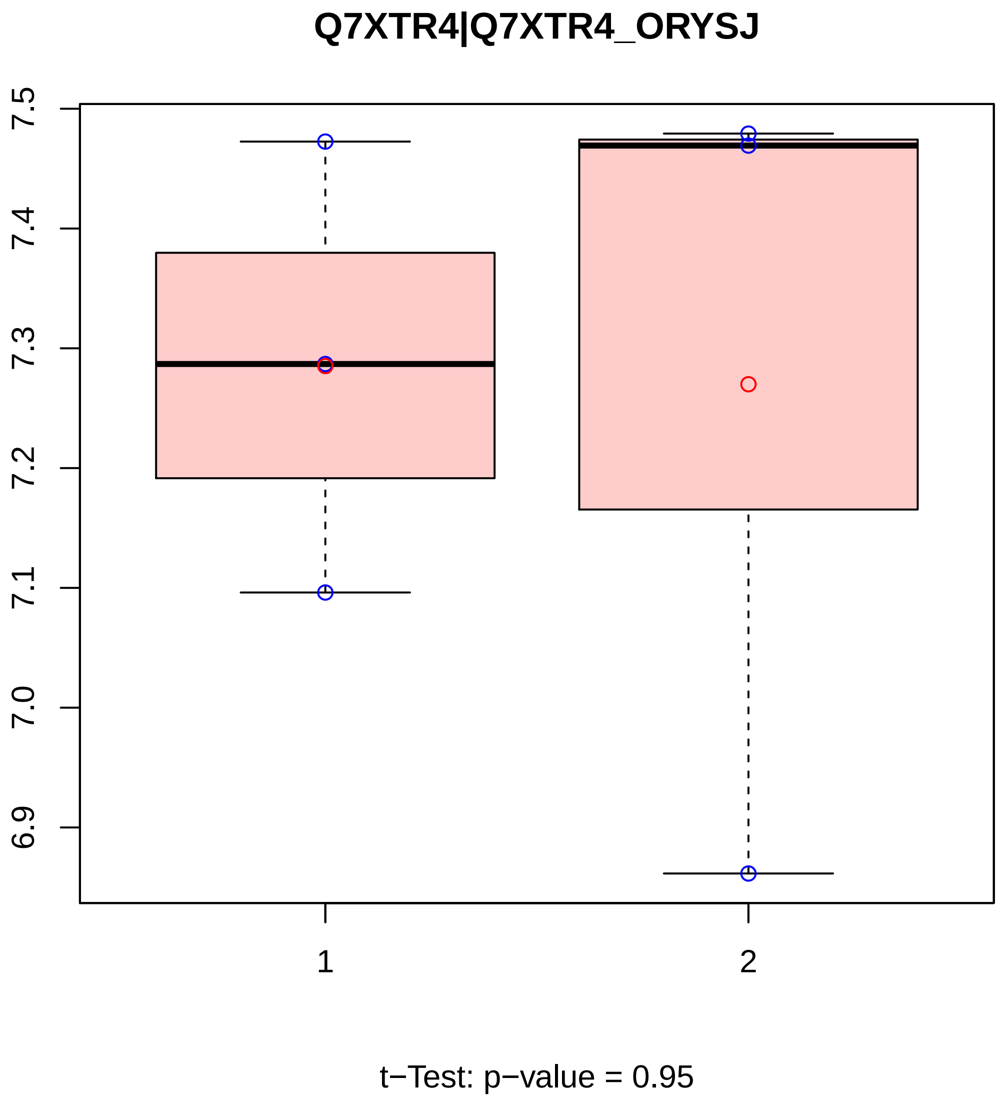
Q7XTR4|Q7XTR4_ORYSJ
7.5
7.4
7.3
7.2
7.1
7.0
6.9
1
2
t−Test: p−value = 0.95

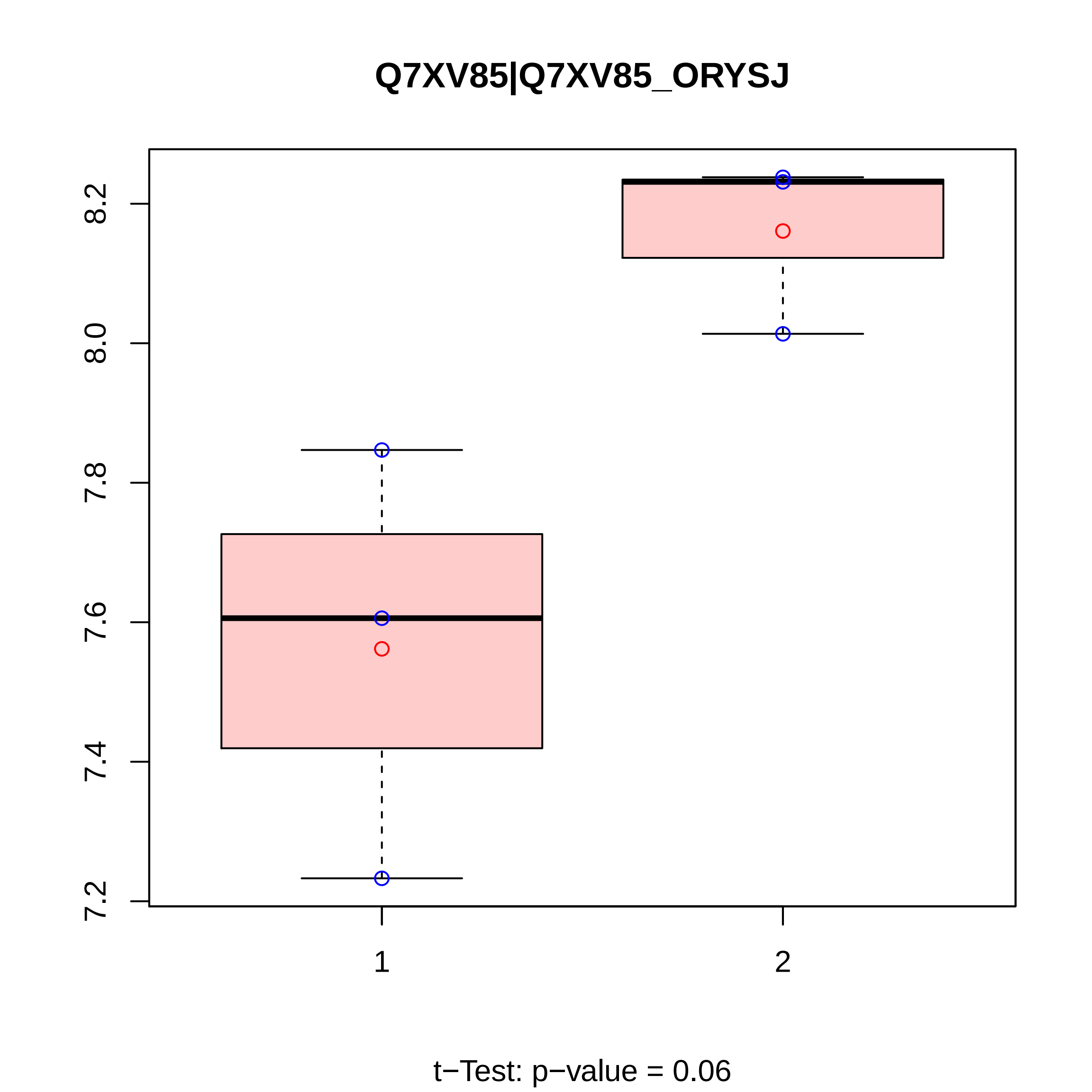

Q7XV85|Q7XV85_ORYSJ
7.2
7.4
7.6
7.8
8.0
8.2
1
2
t−Test: p−value = 0.06

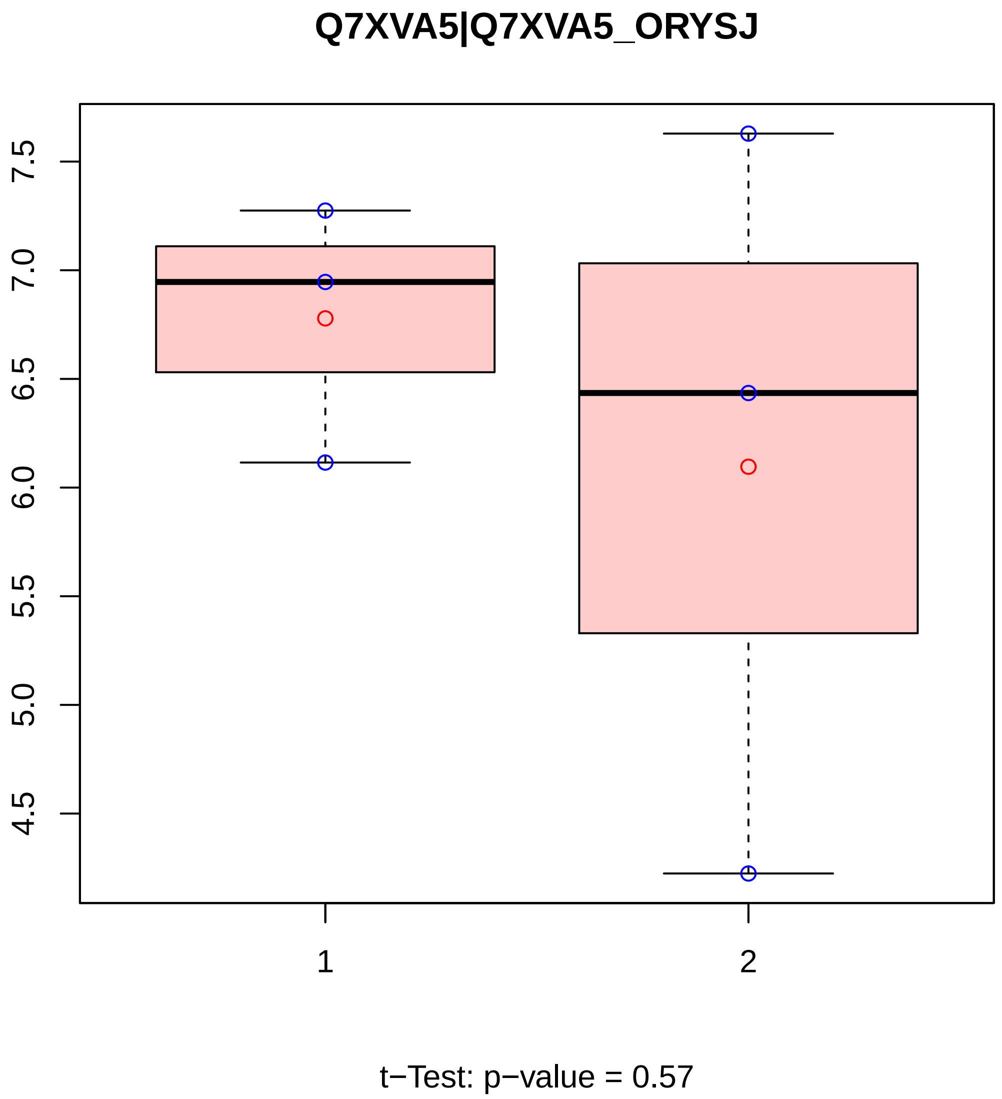
Q7XVA5|Q7XVA5_ORYSJ
7.5
7.0
6.5
6.0
5.5
5.0
4.5
1
2
t−Test: p−value = 0.57

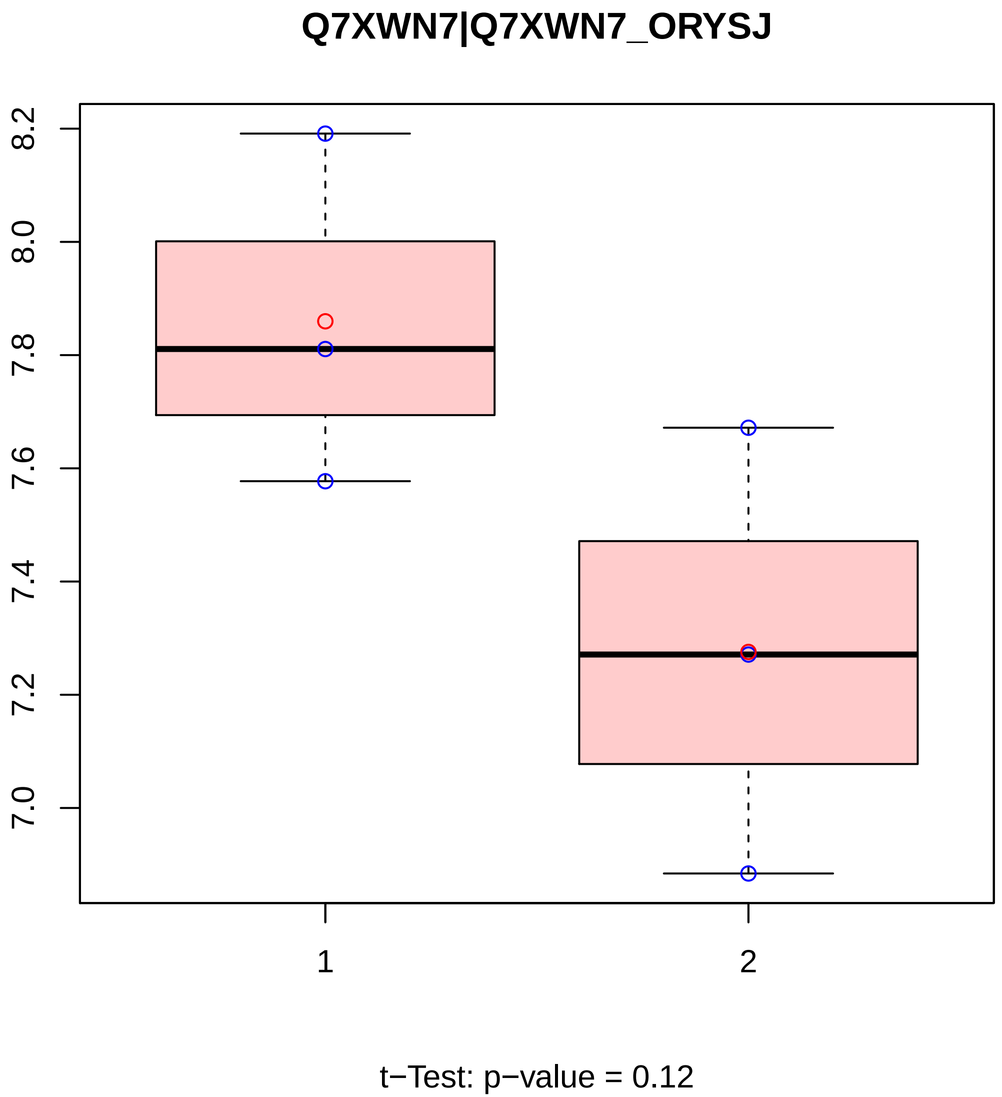

Q7XWN7|Q7XWN7_ORYSJ
8.2
8.0
7.8
7.6
7.4
7.2
7.0
1
2
t−Test: p−value = 0.12

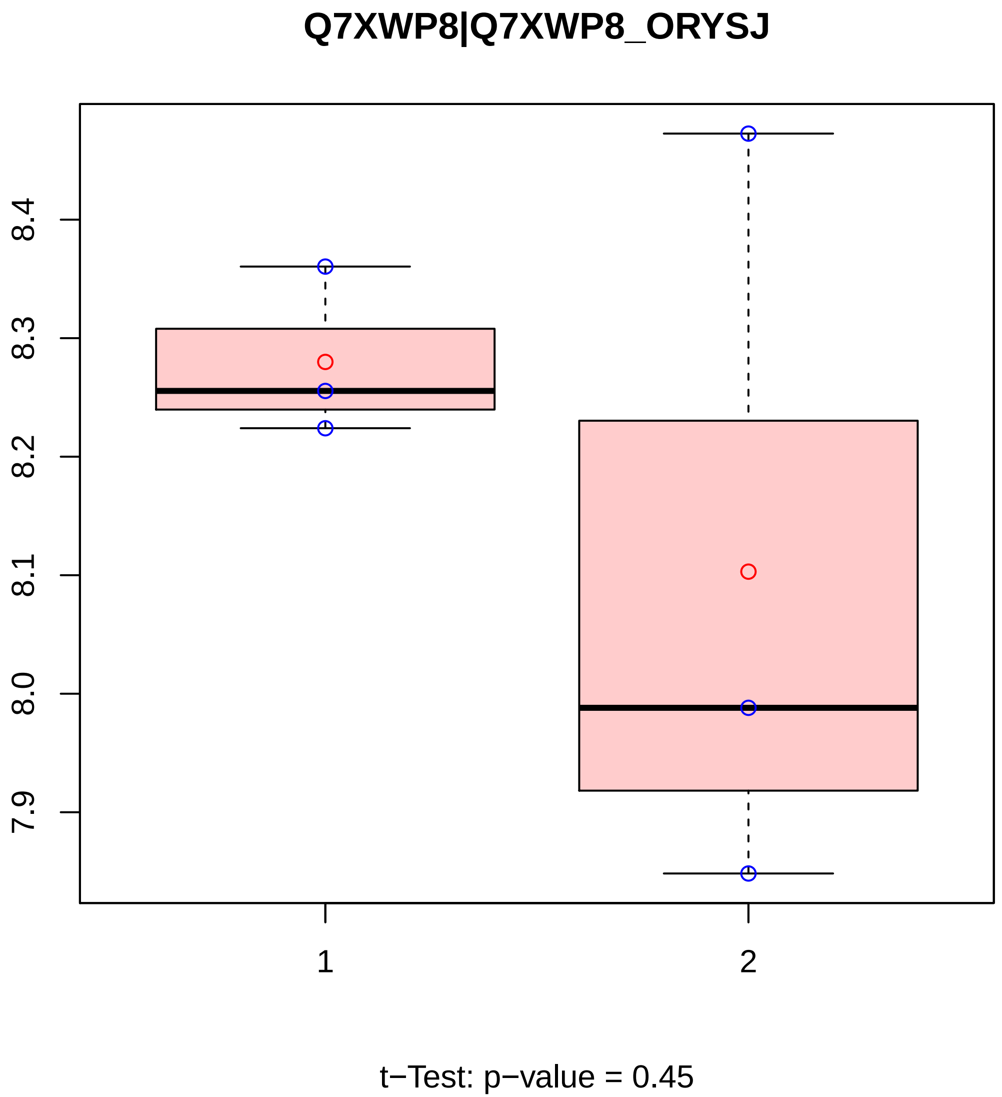
Q7XWP8|Q7XWP8_ORYSJ
8.4
8.3
8.2
8.1
8.0
7.9
1
2
t−Test: p−value = 0.45

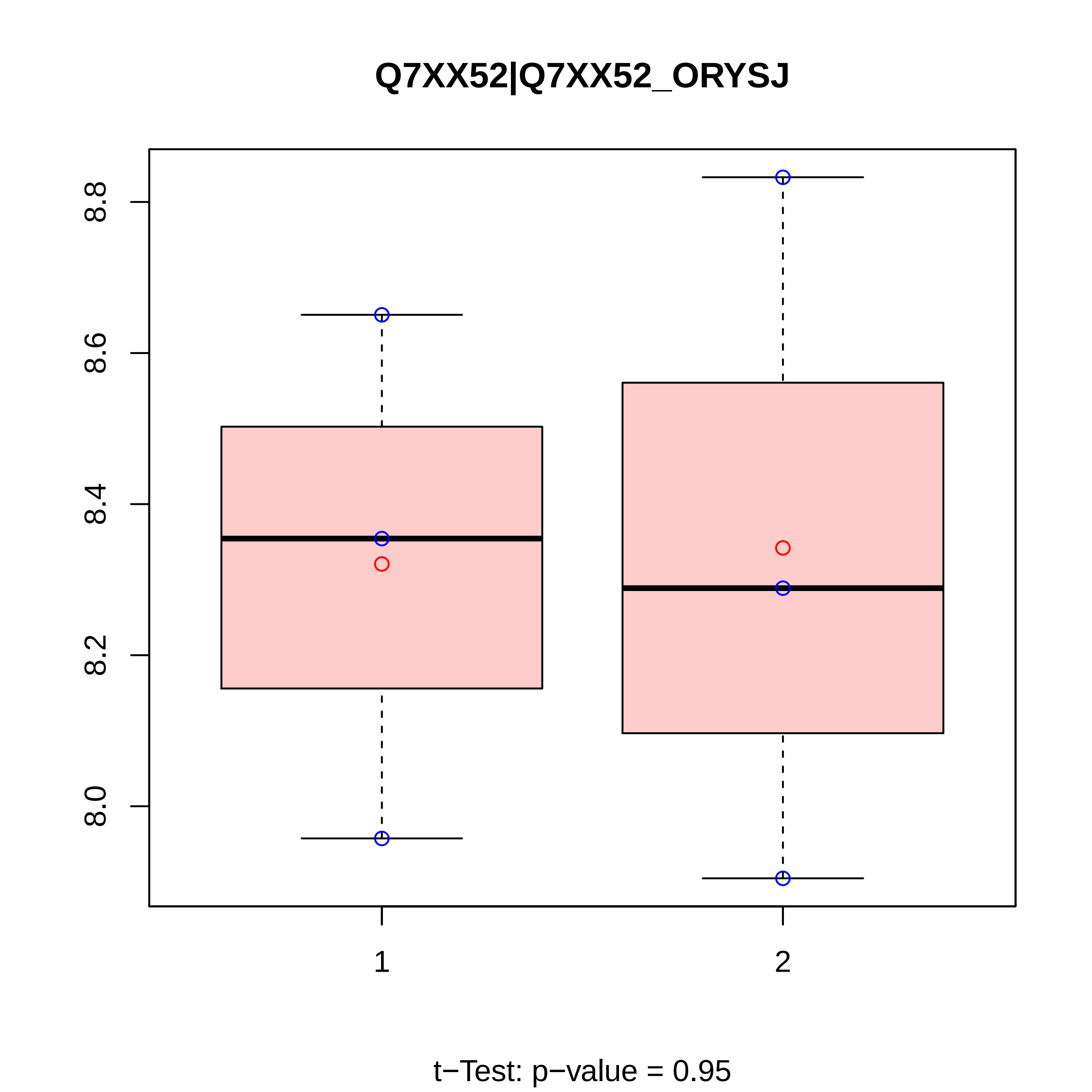

Q7XX52|Q7XX52_ORYSJ
8.0
8.2
8.4
8.6
8.8
1
2
t−Test: p−value = 0.95

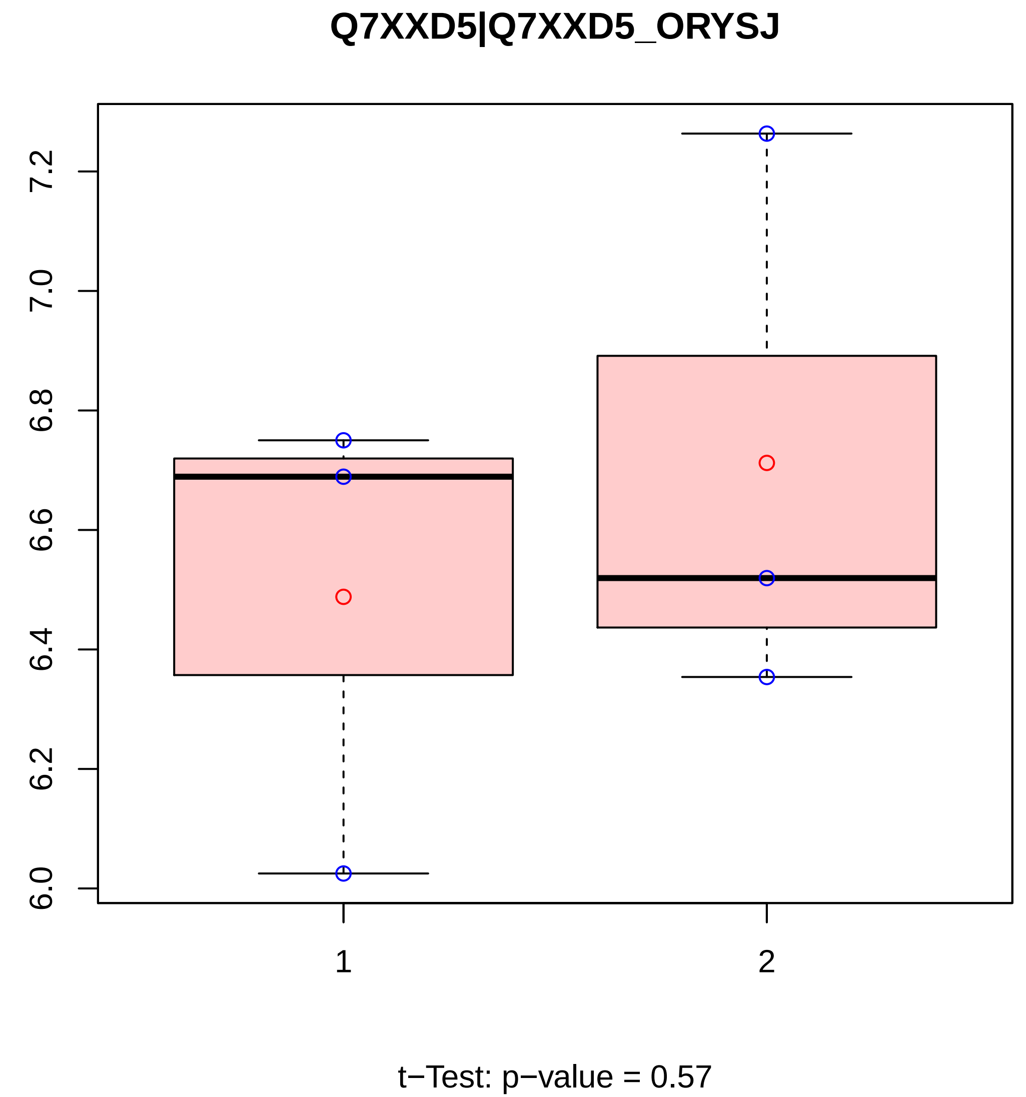
Q7XXD5|Q7XXD5_ORYSJ
6.0
6.2
6.4
6.6
6.8
7.0
7.2
1
2
t−Test: p−value = 0.57

Q7Y1C6|Q7Y1C6_PENAM

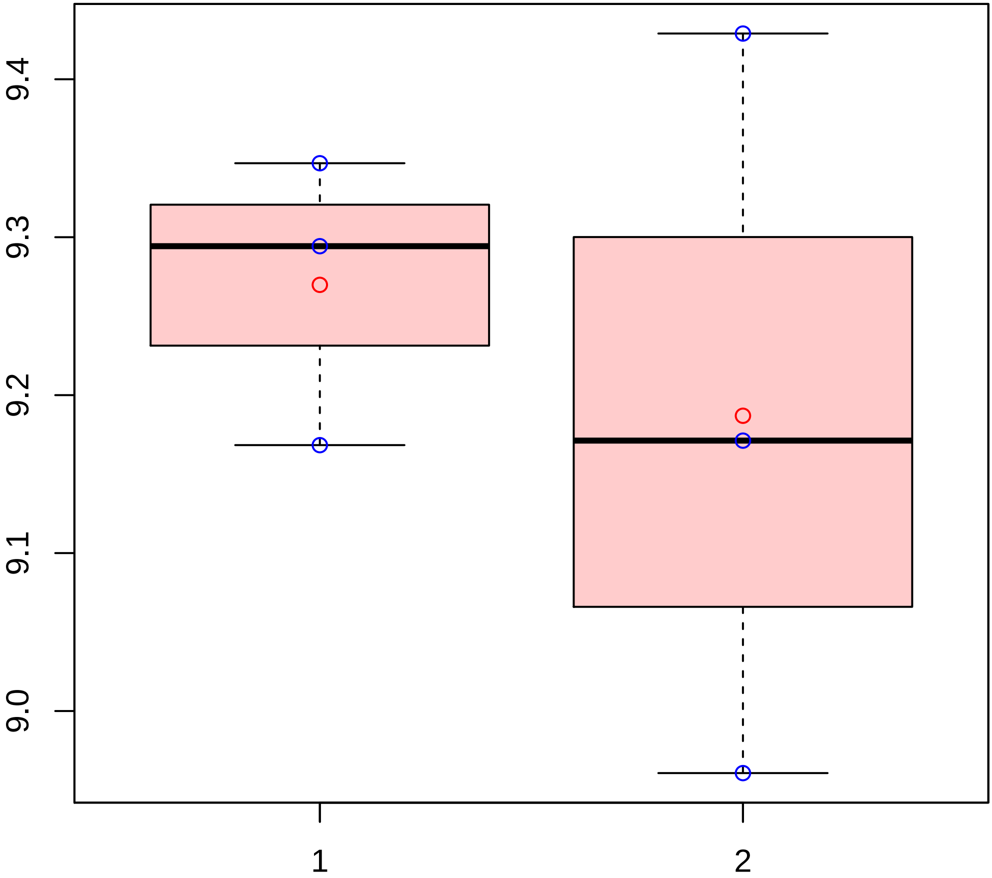

t−Test: p−value = 0.61

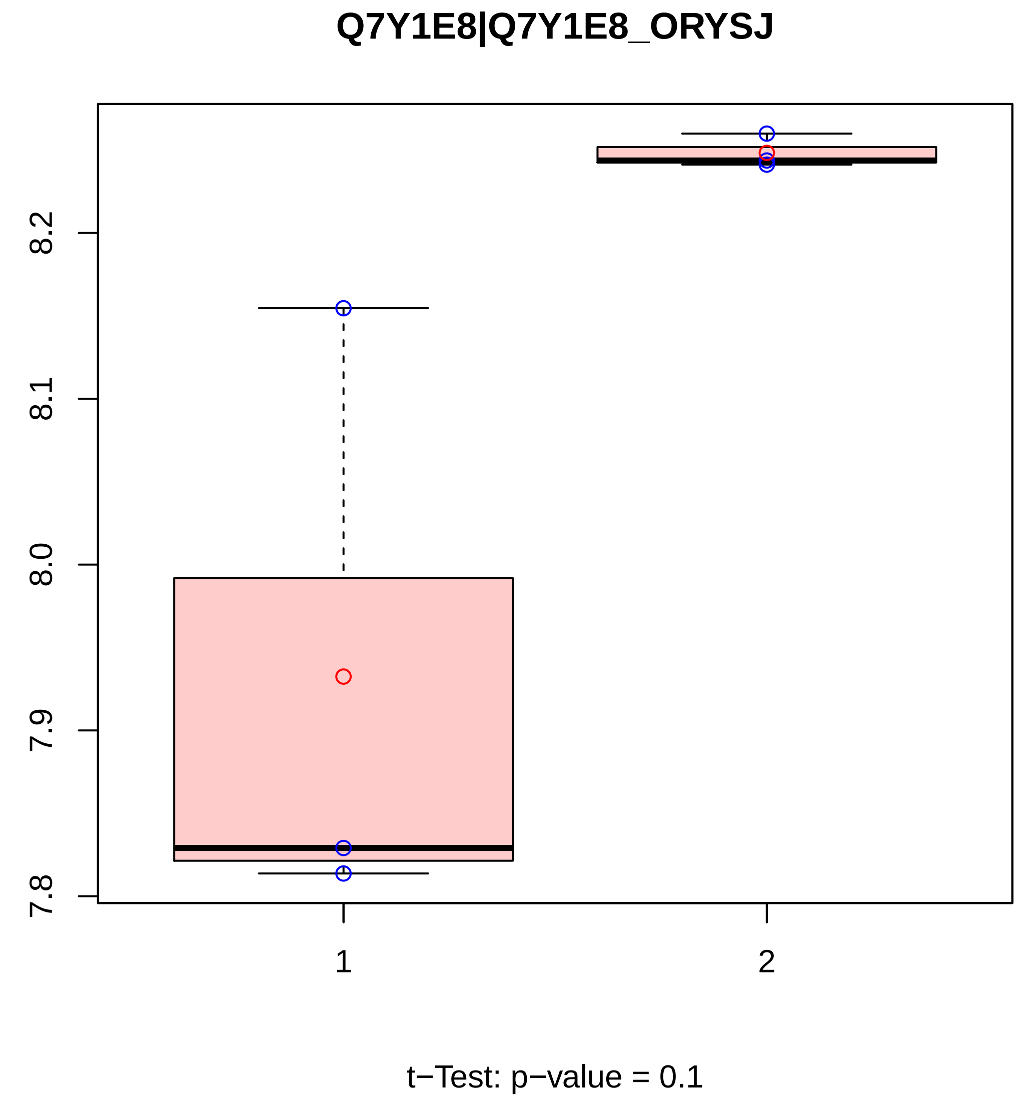

Q7Y1E8|Q7Y1E8_ORYSJ
8.2
8.1
8.0
7.9
7.8
1
2
t−Test: p−value = 0.1

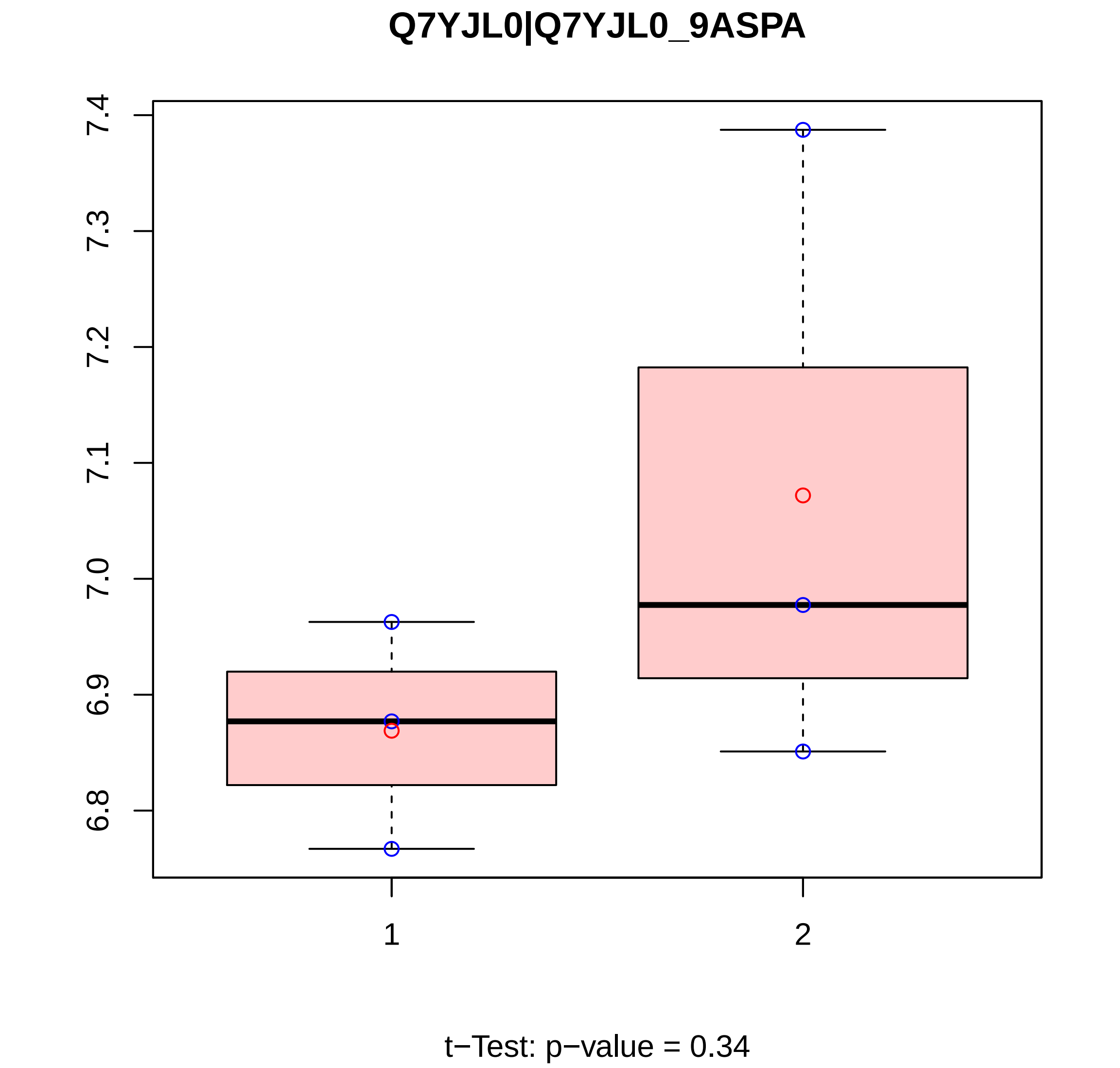

Q7YJL0|Q7YJL0_9ASPA
7.4
7.3
7.2
7.1
7.0
6.9
6.8
1
2
t−Test: p−value = 0.34

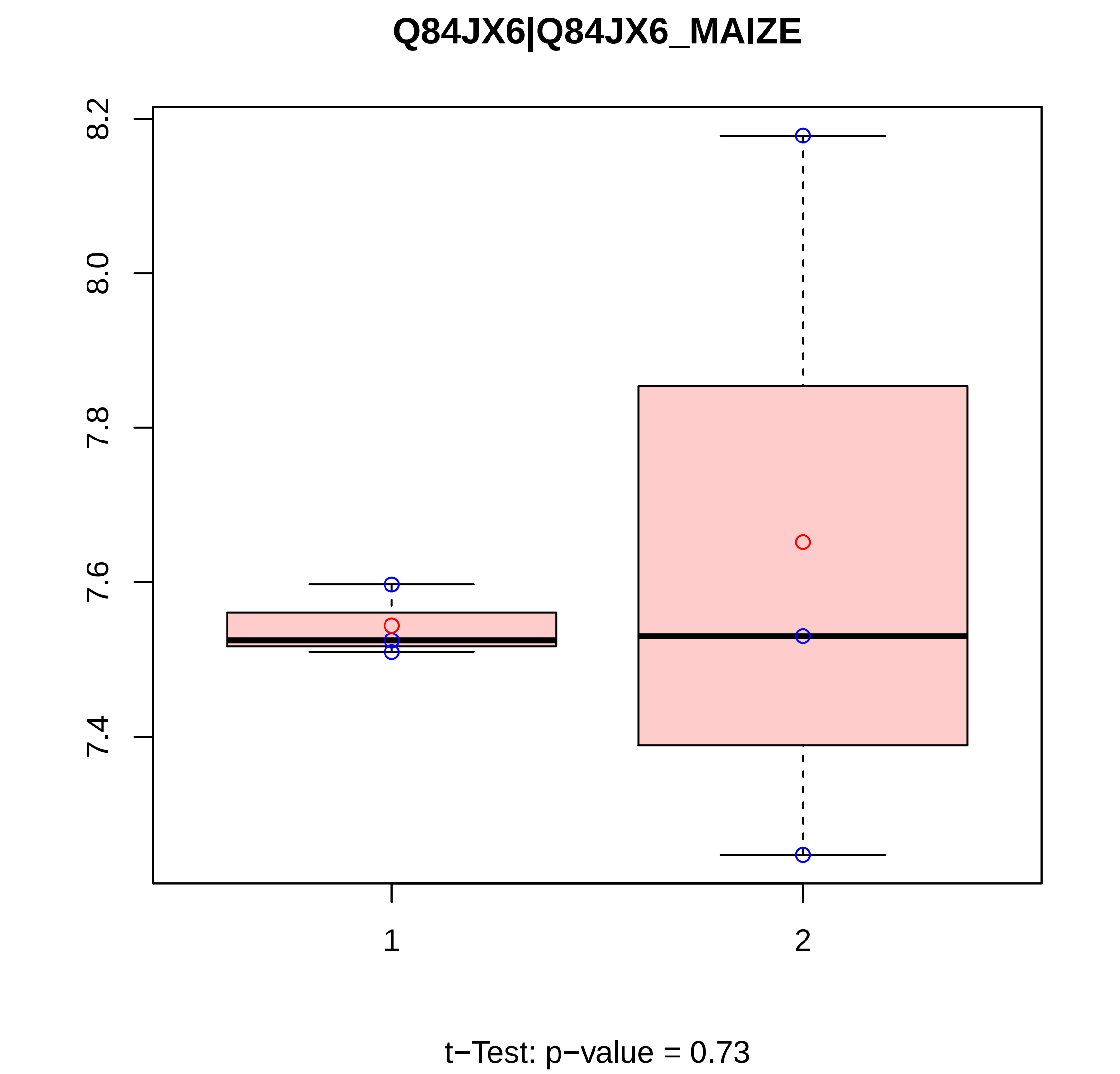
Q84JX6|Q84JX6_MAIZE
8.2
8.0
7.8
7.6
7.4
1
2
t−Test: p−value = 0.73

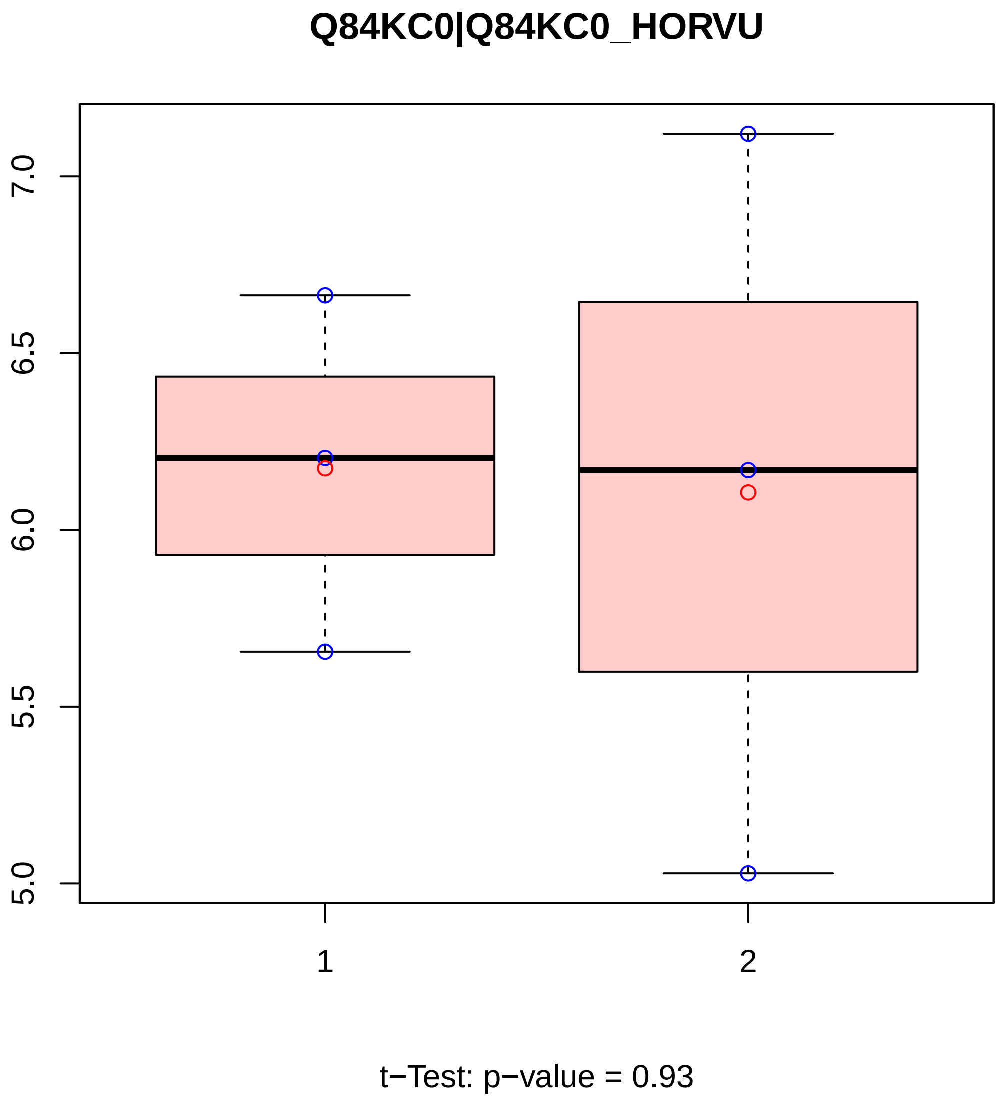
Q84KC0|Q84KC0_HORVU
7.0
6.5
6.0
5.5
5.0
1
2
t−Test: p−value = 0.93

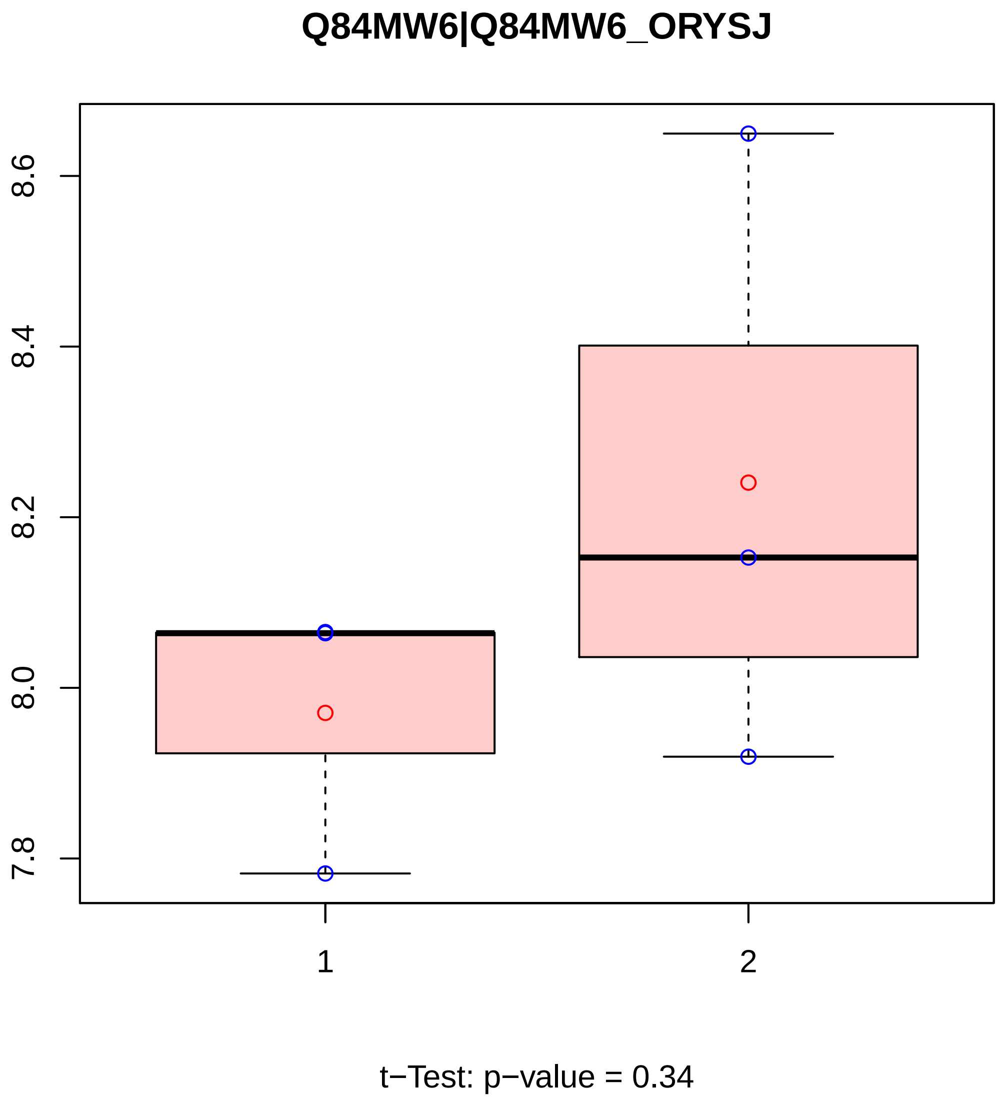
Q84MW6|Q84MW6_ORYSJ
8.6
8.4
8.2
8.0
7.8
1
2
t−Test: p−value = 0.34

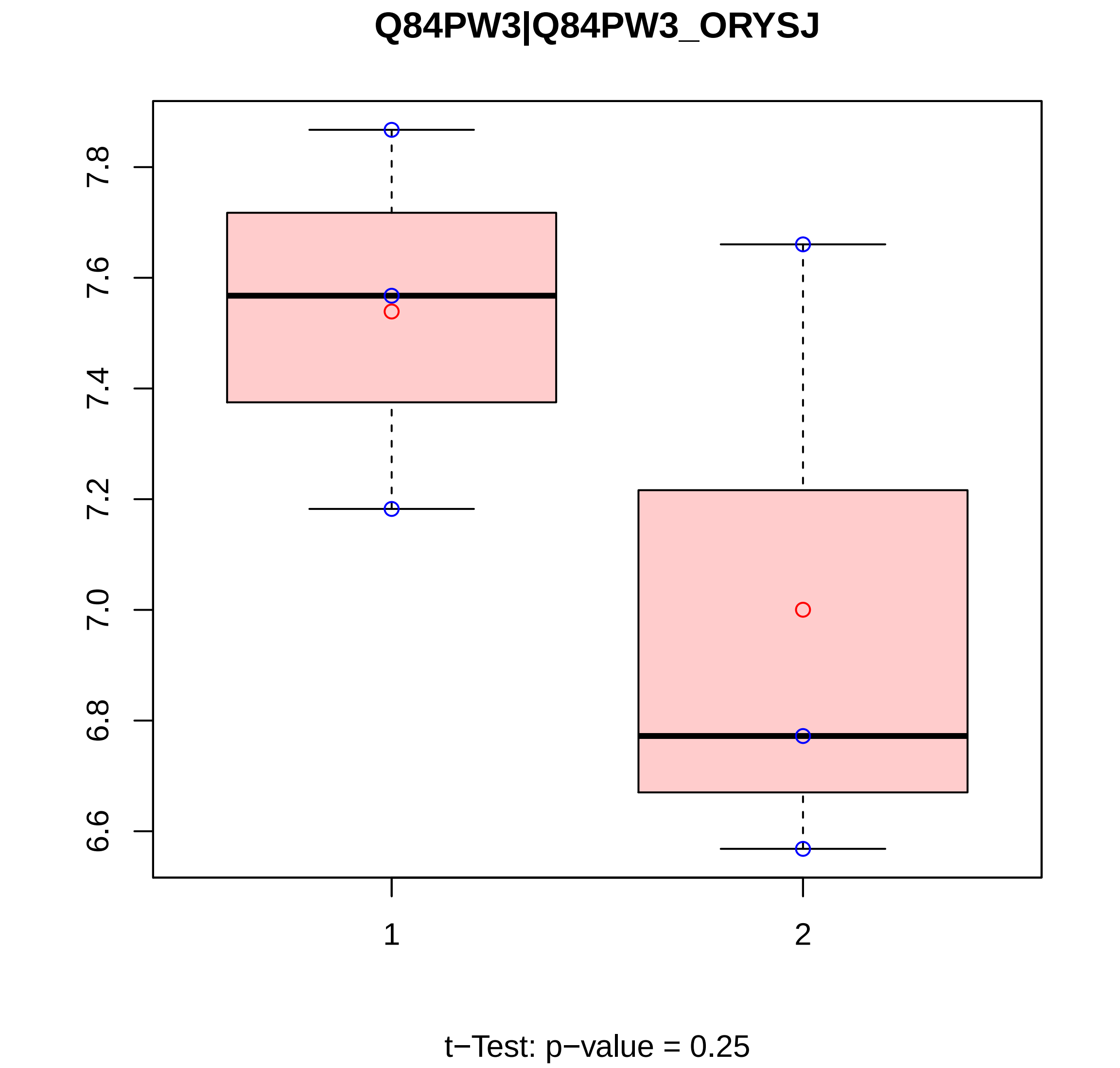
Q84PW3|Q84PW3_ORYSJ
7.8
7.6
7.4
7.2
7.0
6.8
6.6
1
2
t−Test: p−value = 0.25

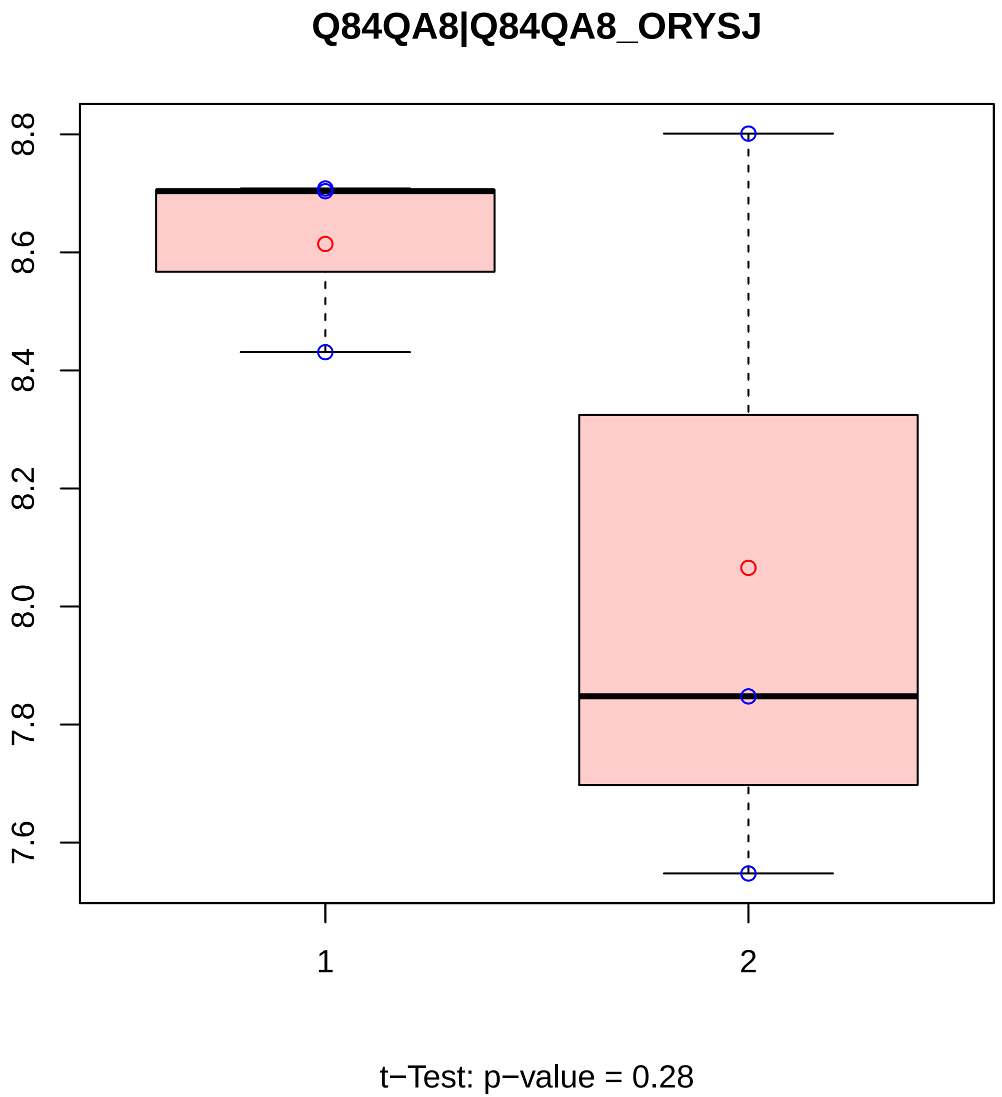

Q84QA8|Q84QA8_ORYSJ
8.8
8.6
8.4
8.2
8.0
7.8
7.6
1
2
t−Test: p−value = 0.28

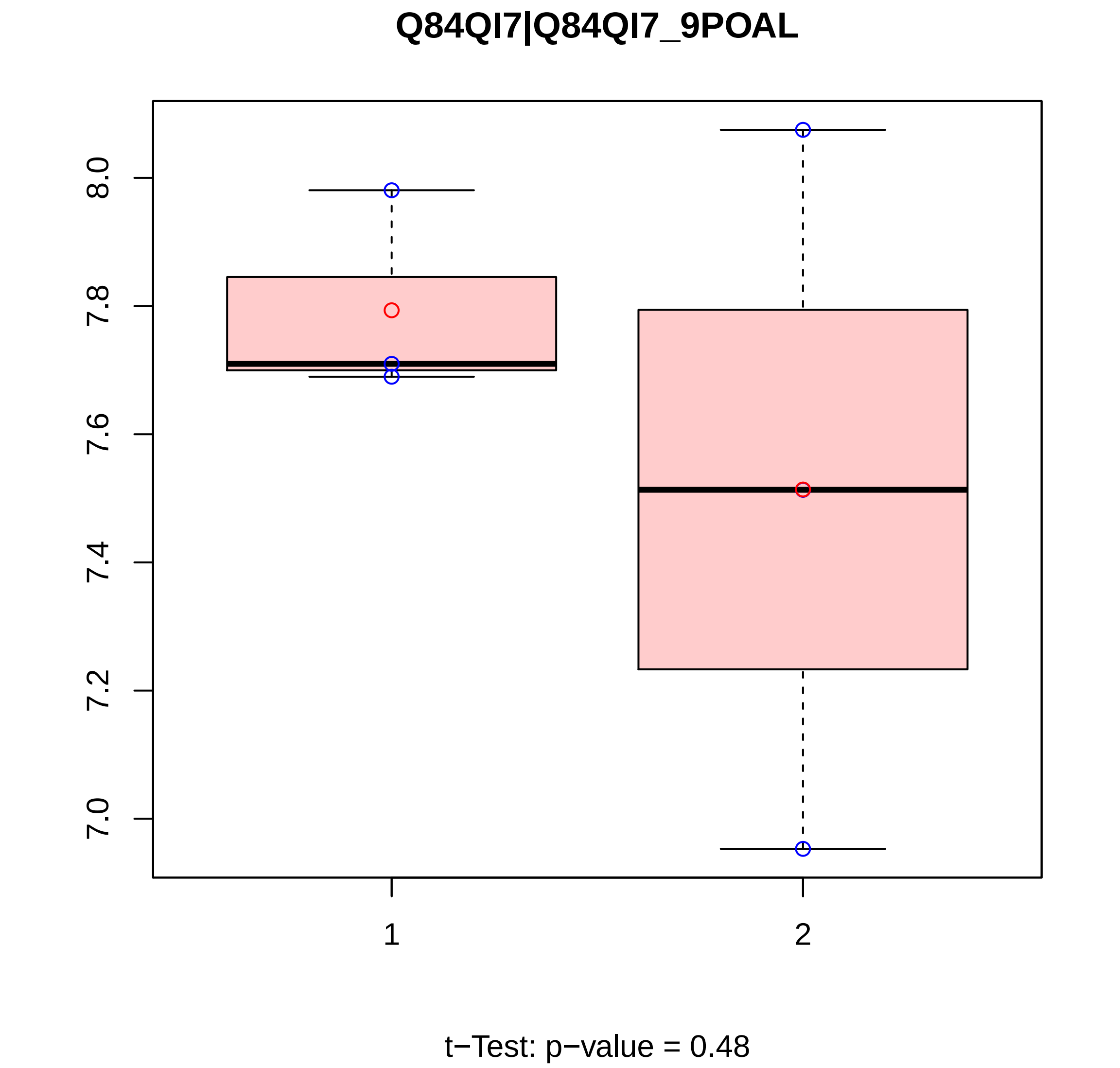
Q84QI7|Q84QI7_9POAL
8.0
7.8
7.6
7.4
7.2
7.0
1
2
t−Test: p−value = 0.48

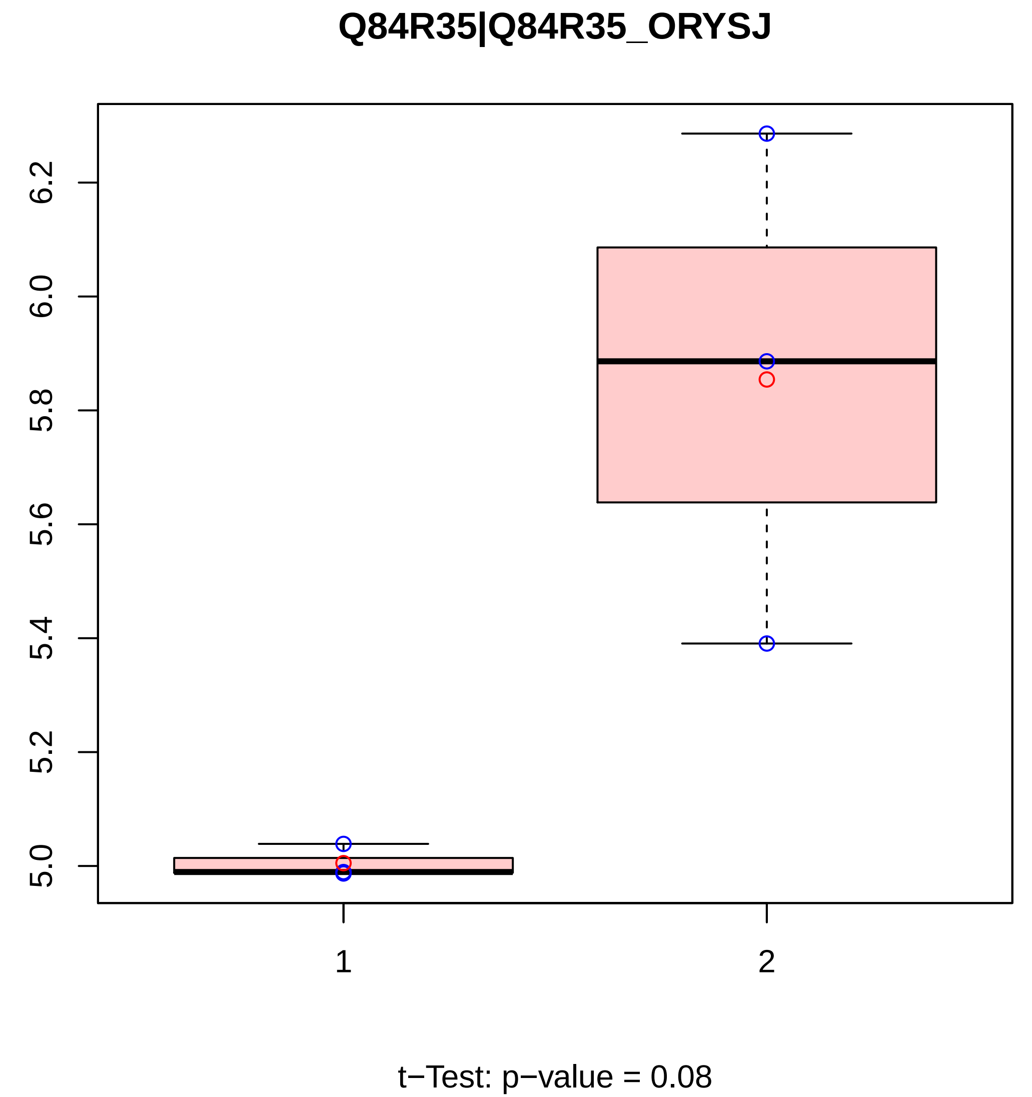
Q84R35|Q84R35_ORYSJ
5.0
5.2
5.4
5.6
5.8
6.0
6.2
1
2
t−Test: p−value = 0.08

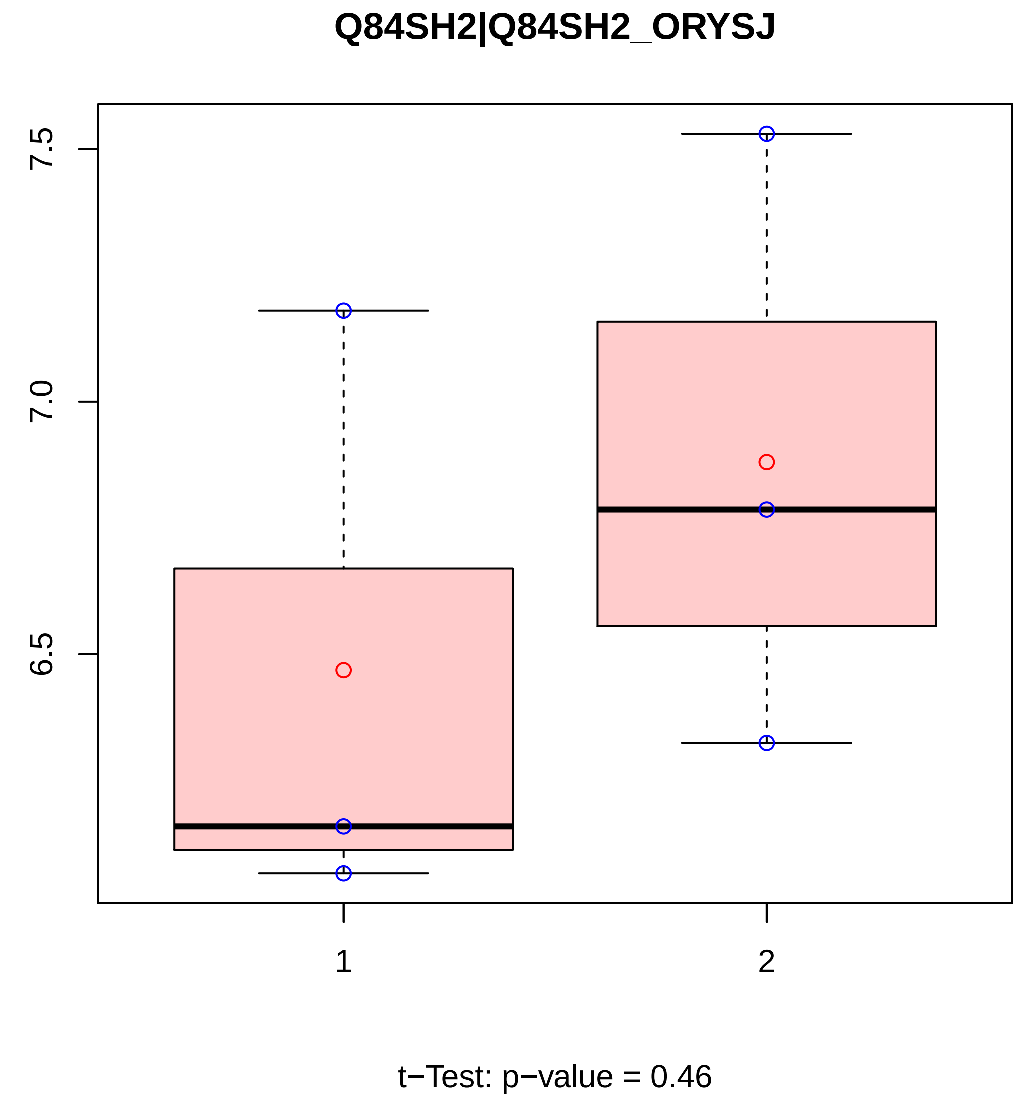
Q84SH2|Q84SH2_ORYSJ
7.5
7.0
6.5
1
2
t−Test: p−value = 0.46

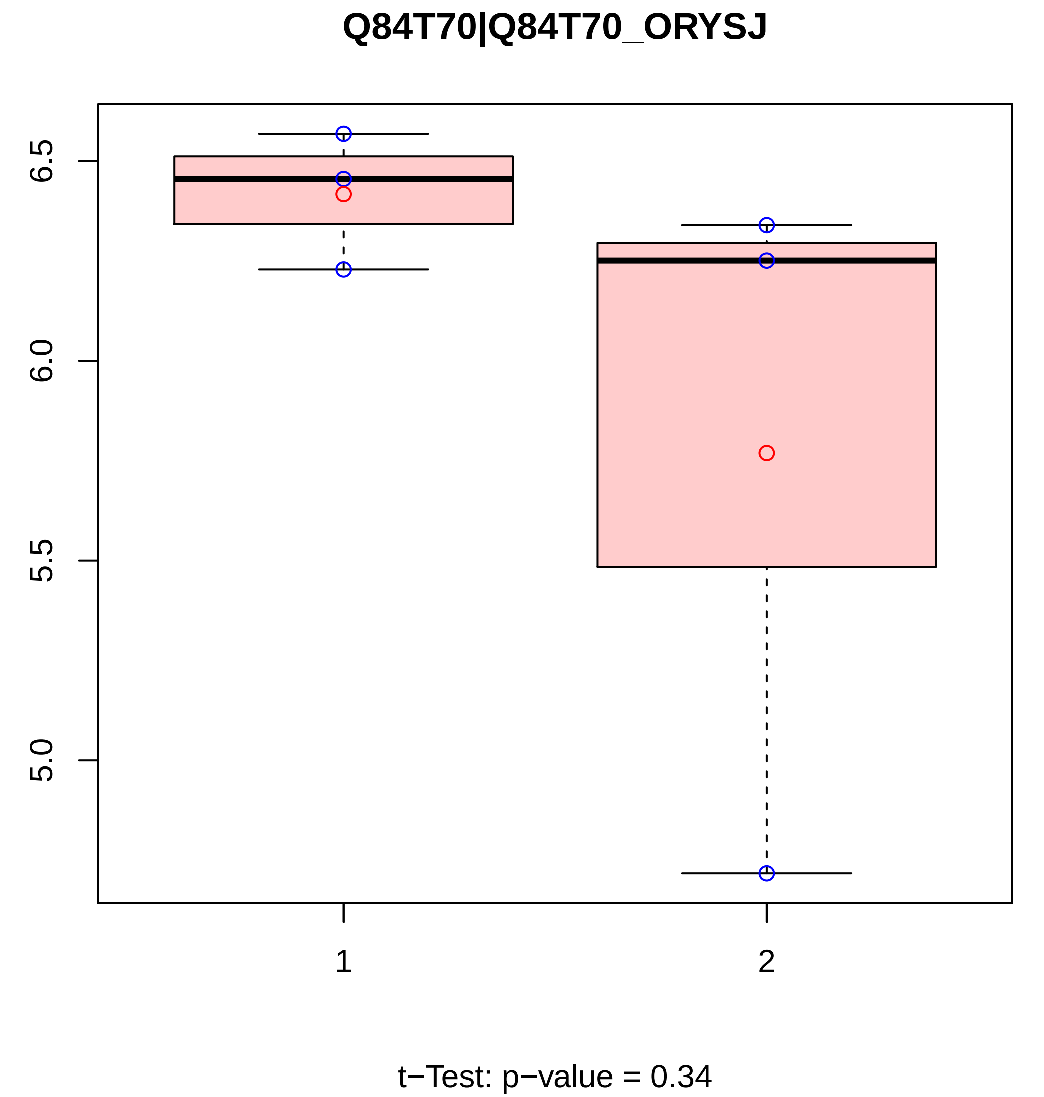

Q84T70|Q84T70_ORYSJ
6.5
6.0
5.5
5.0
1
2
t−Test: p−value = 0.34

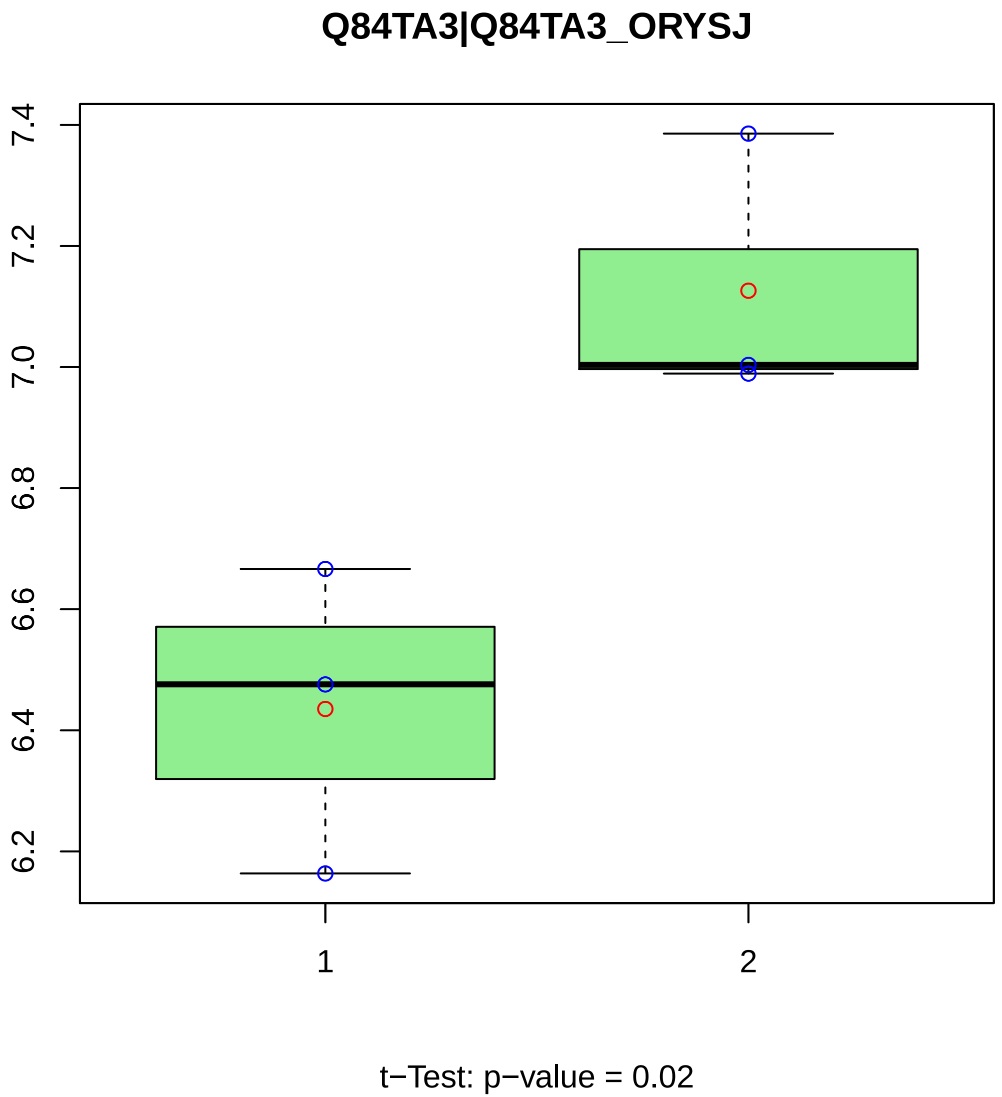
Q84TA3|Q84TA3_ORYSJ
7.4
7.2
7.0
6.8
6.6
6.4
6.2
1
2
t−Test: p−value = 0.02

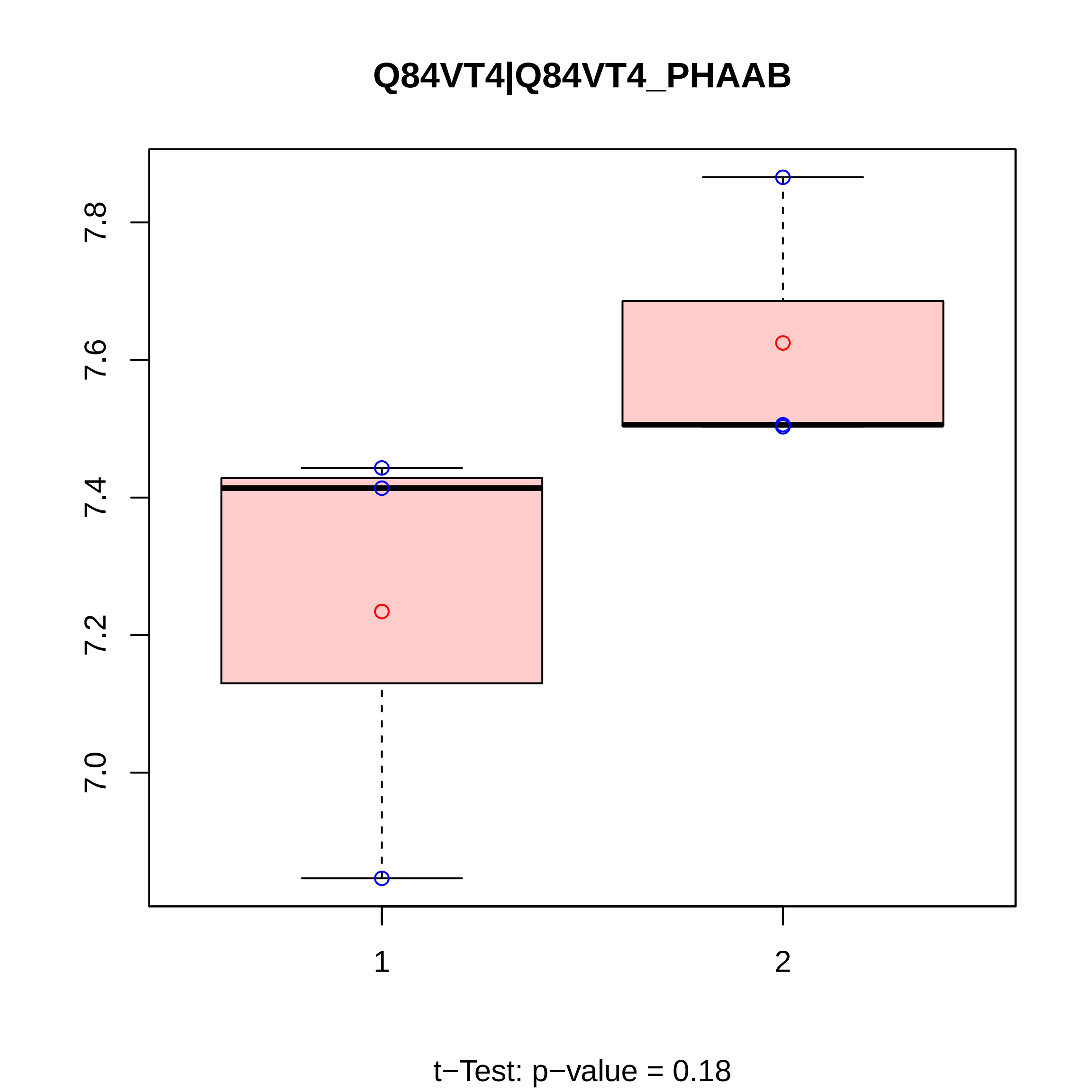

Q84VT4|Q84VT4_PHAAB
7.0
7.2
7.4
7.6
7.8
1
2
t−Test: p−value = 0.18

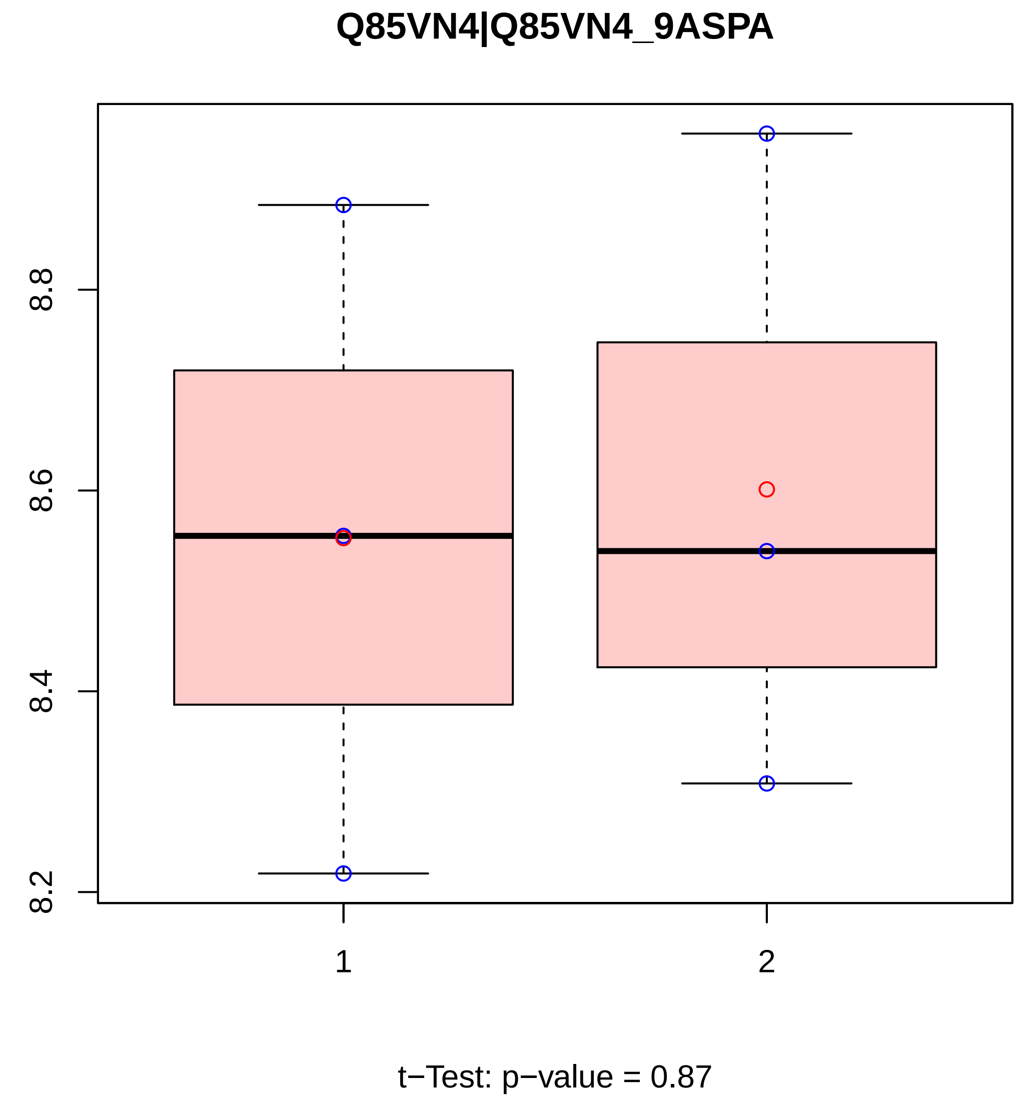
Q85VN4|Q85VN4_9ASPA
8.2
8.4
8.6
8.8
1
2
t−Test: p−value = 0.87

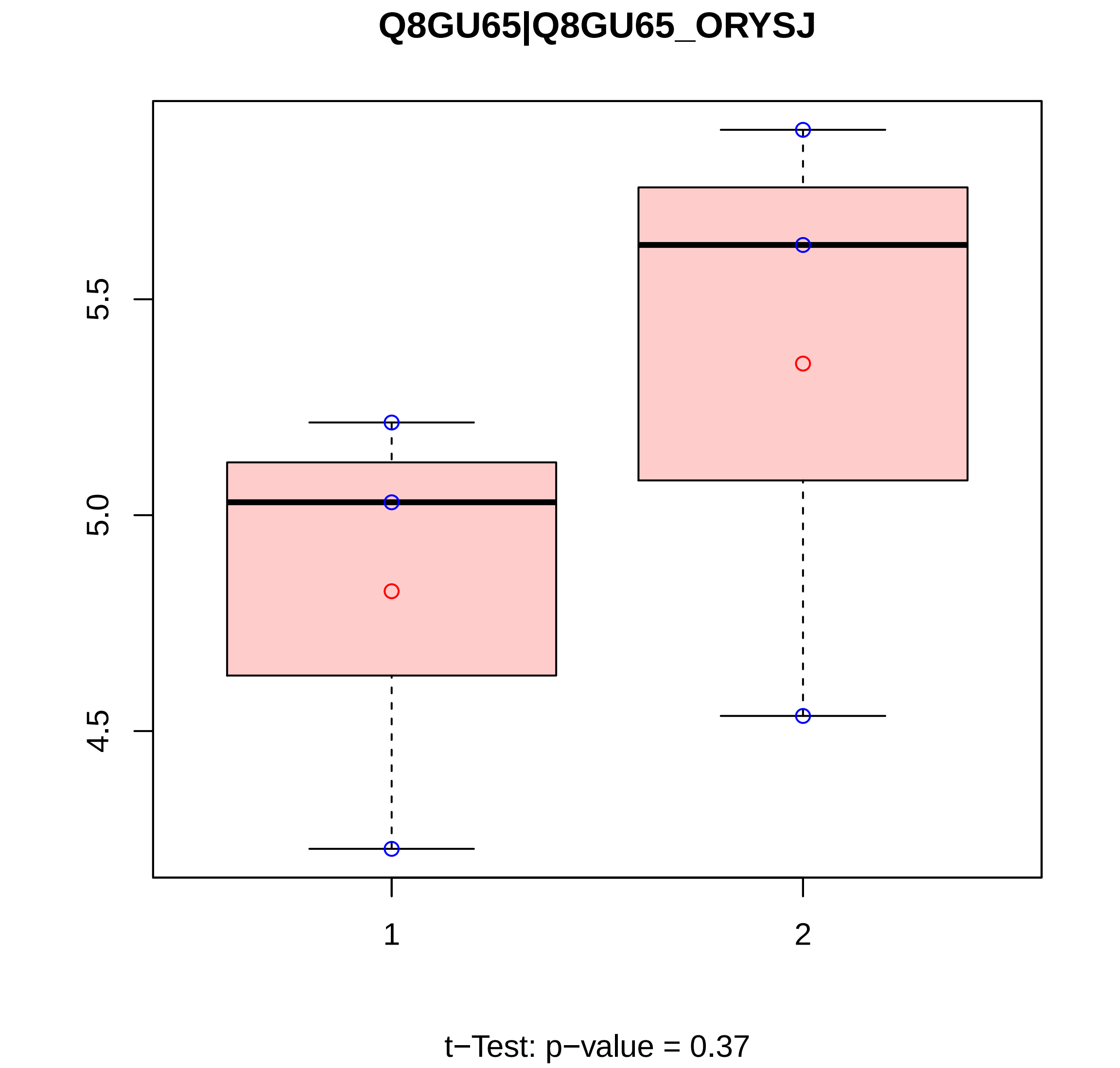
Q8GU65|Q8GU65_ORYSJ
4.5
5.0
5.5
1
2
t−Test: p−value = 0.37

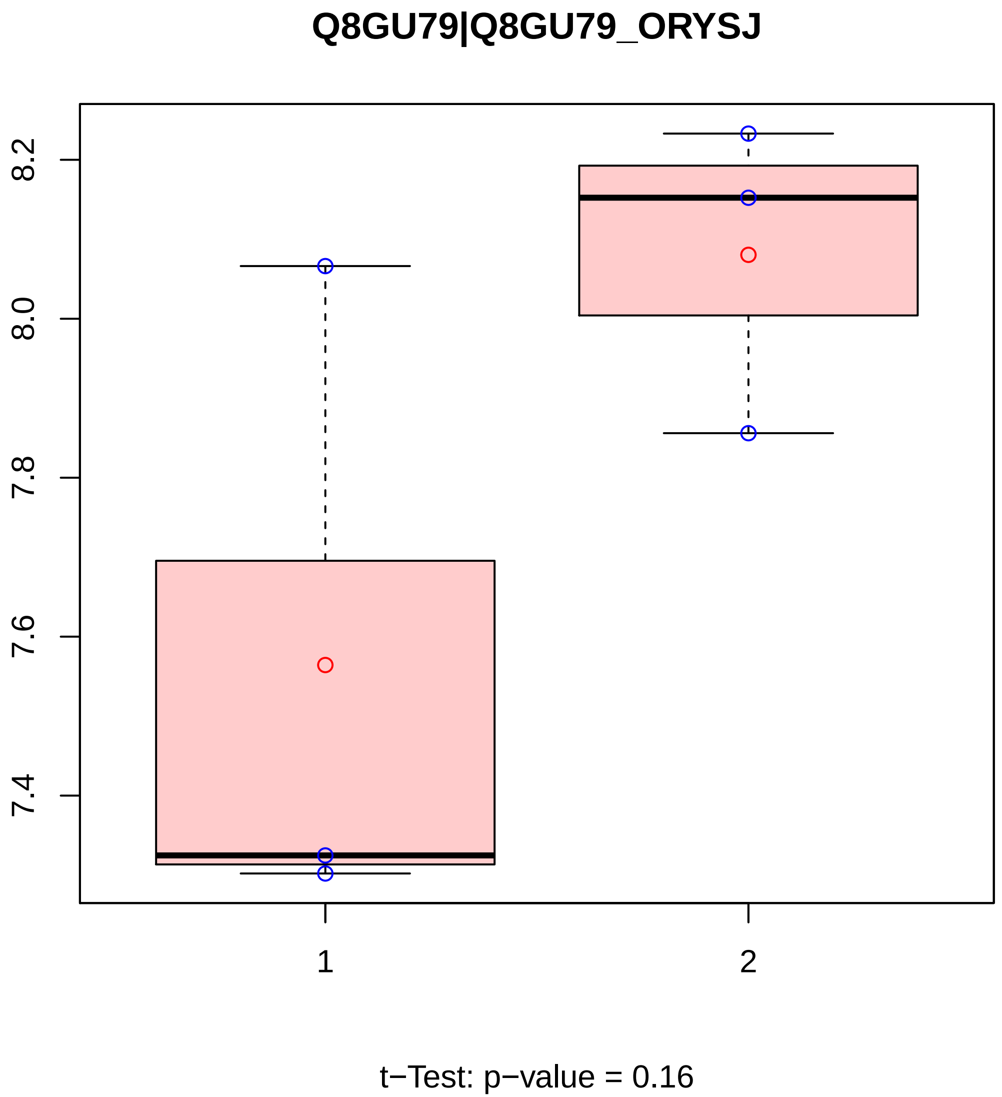
Q8GU79|Q8GU79_ORYSJ
8.2
8.0
7.8
7.6
7.4
1
2
t−Test: p−value = 0.16

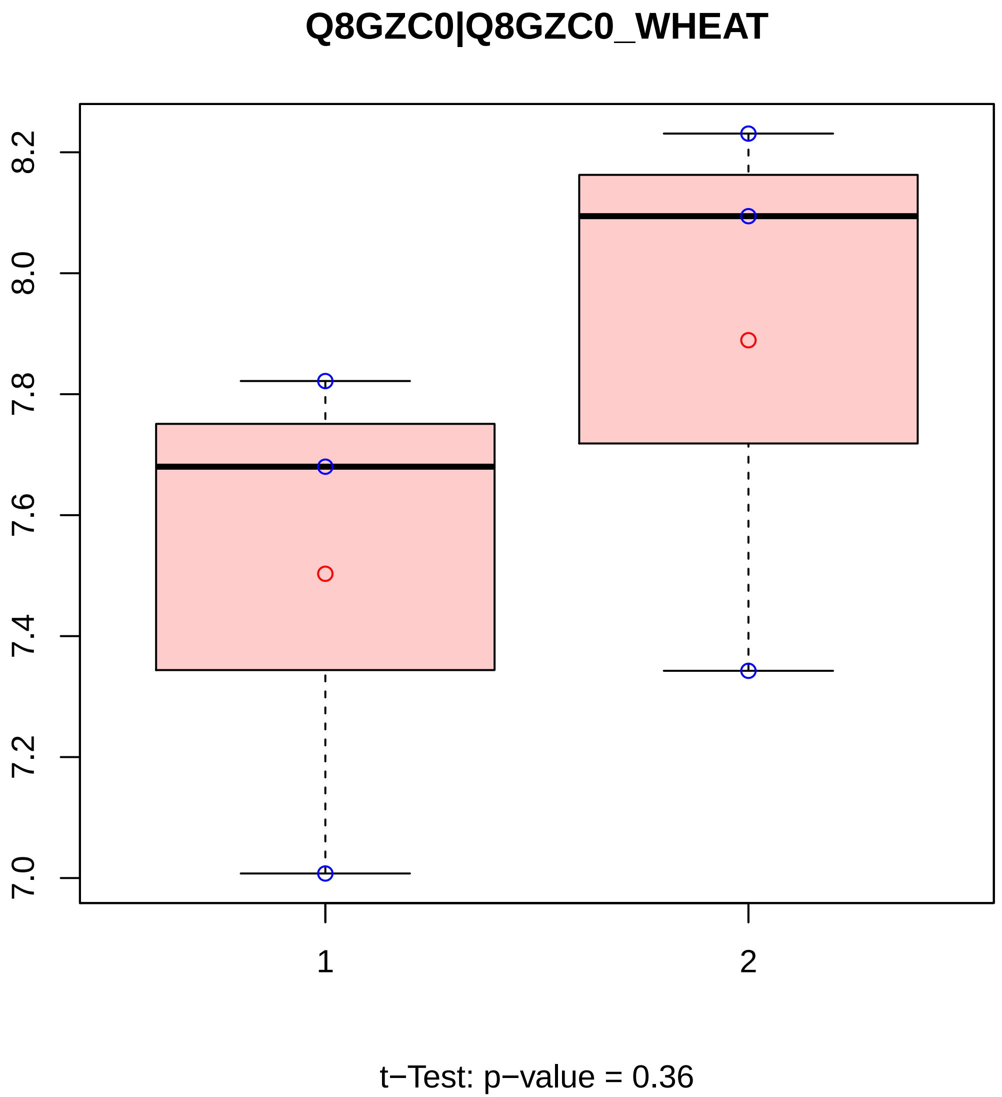

Q8GZC0|Q8GZC0_WHEAT
8.2
8.0
7.8
7.6
7.4
7.2
7.0
1
2
t−Test: p−value = 0.36

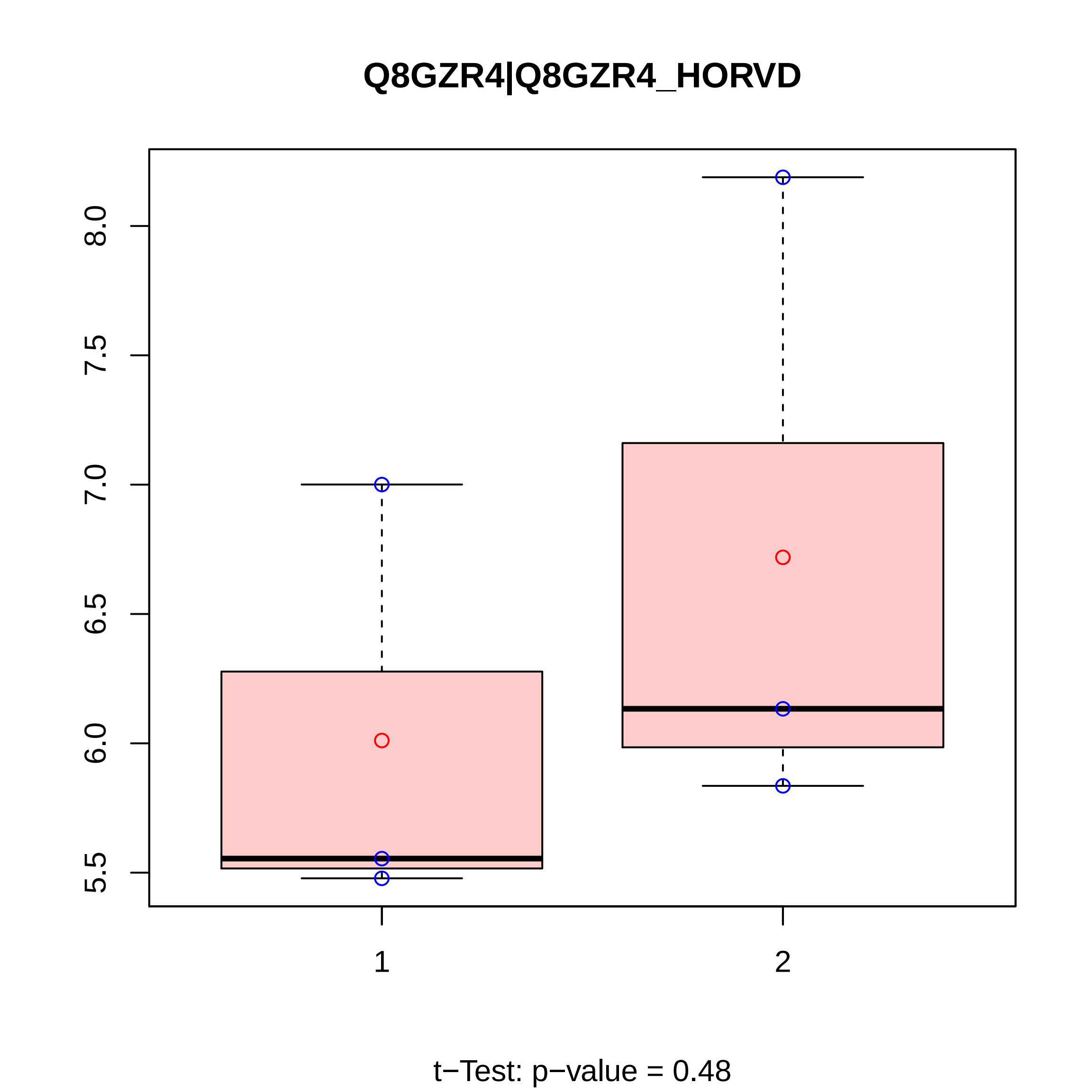
Q8GZR4|Q8GZR4_HORVD
8.0
7.5
7.0
6.5
6.0
5.5
1
2
t−Test: p−value = 0.48

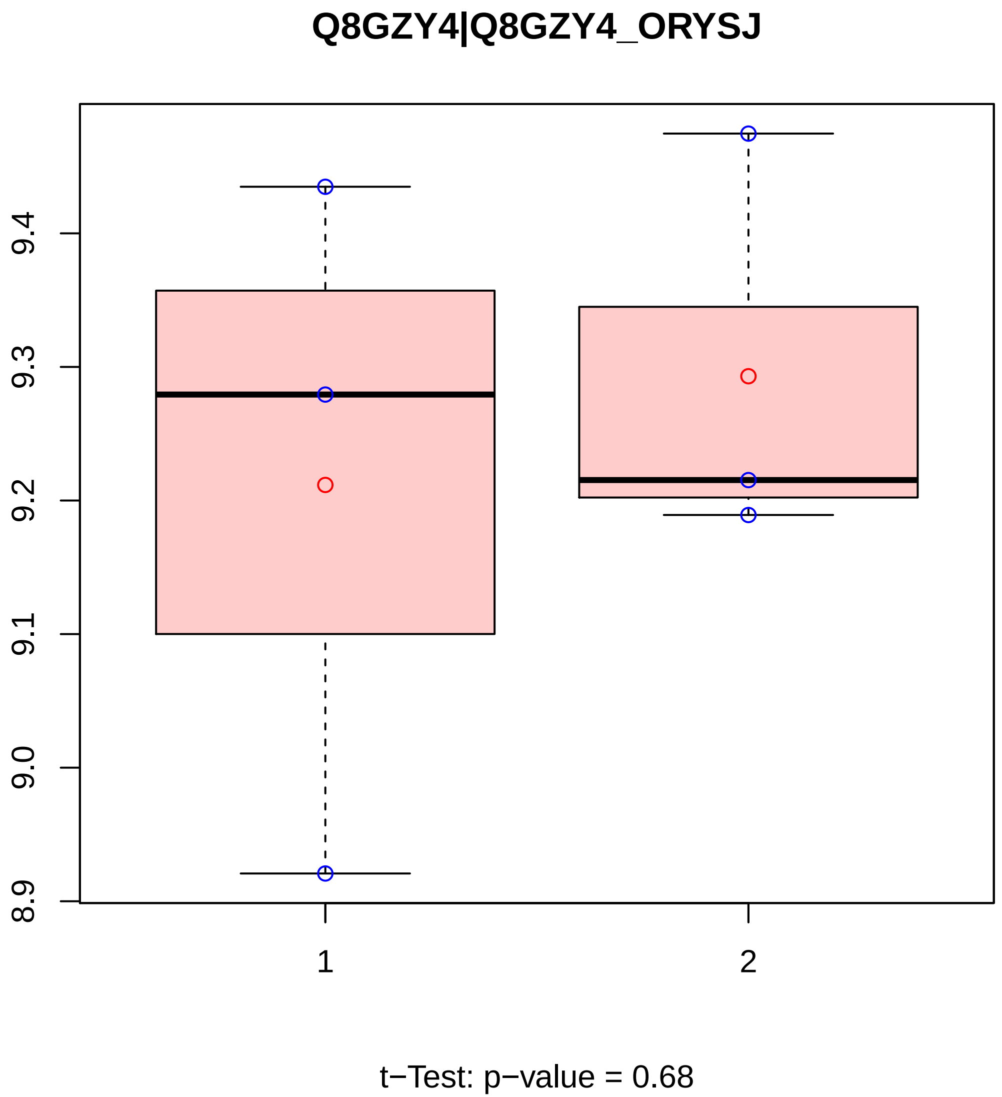
Q8GZY4|Q8GZY4_ORYSJ
9.4
9.3
9.2
9.1
9.0
8.9
1
2
t−Test: p−value = 0.68

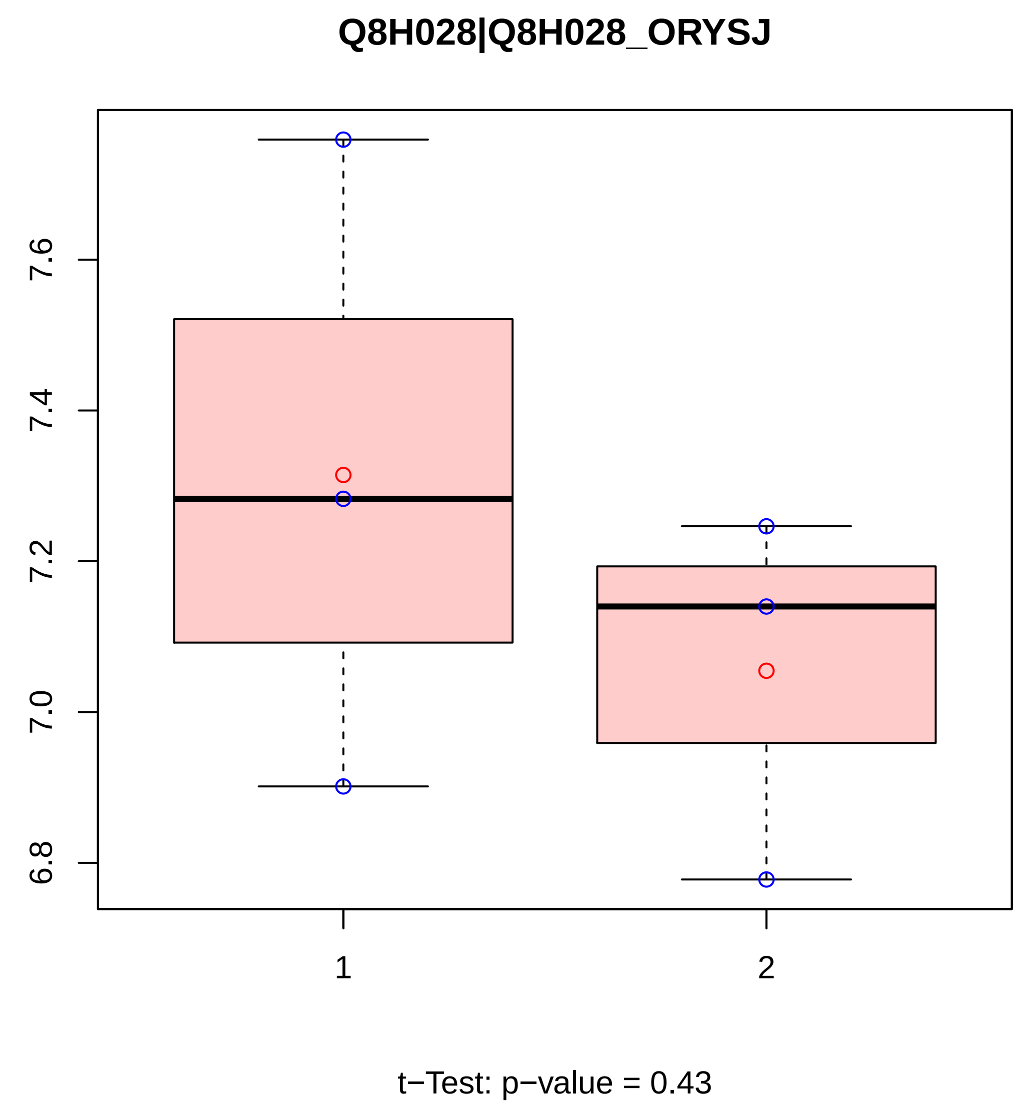
Q8H028|Q8H028_ORYSJ
7.6
7.4
7.2
7.0
6.8
1
2
t−Test: p−value = 0.43

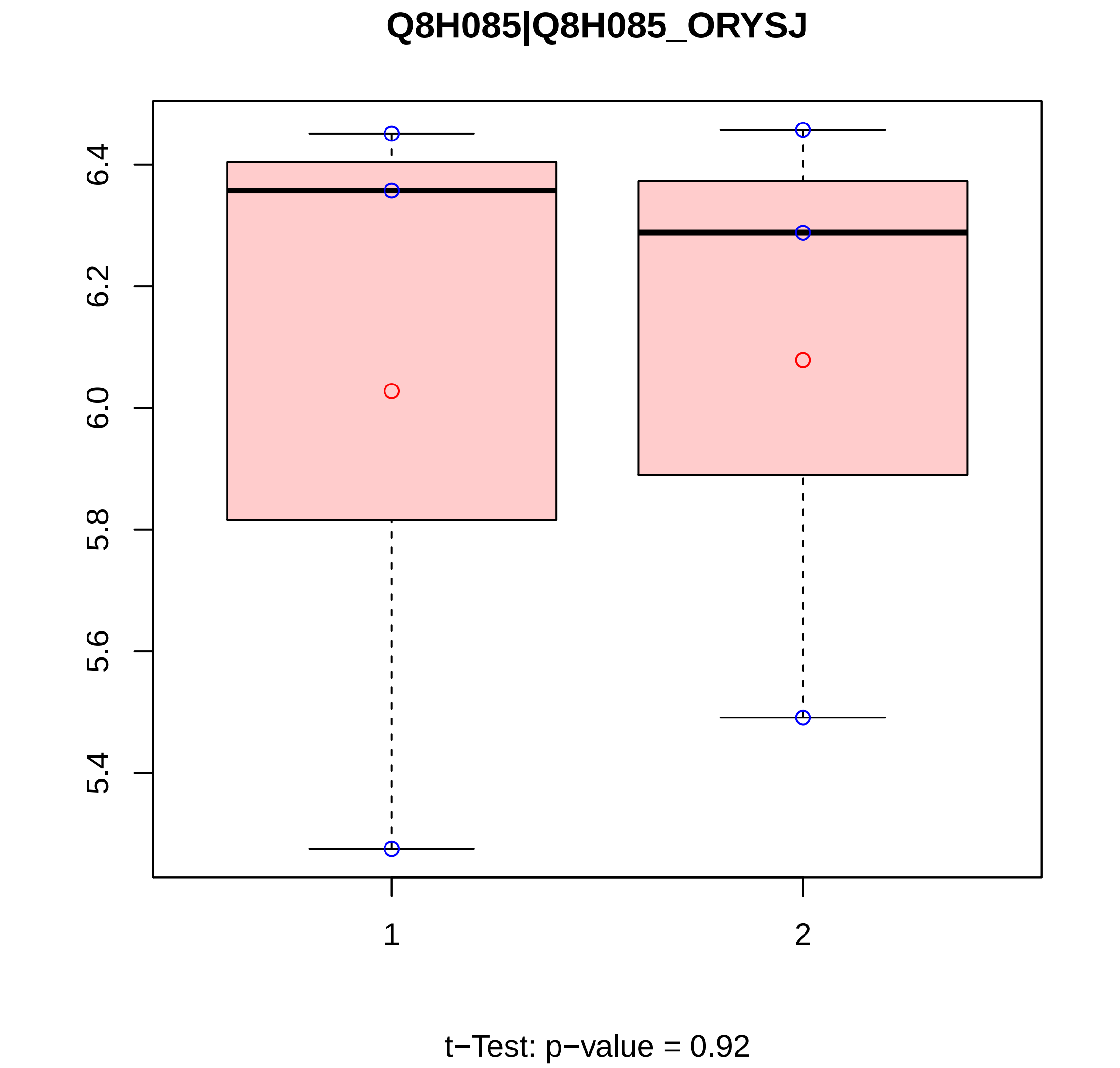
Q8H085|Q8H085_ORYSJ
6.4
6.2
6.0
5.8
5.6
5.4
1
2
t−Test: p−value = 0.92

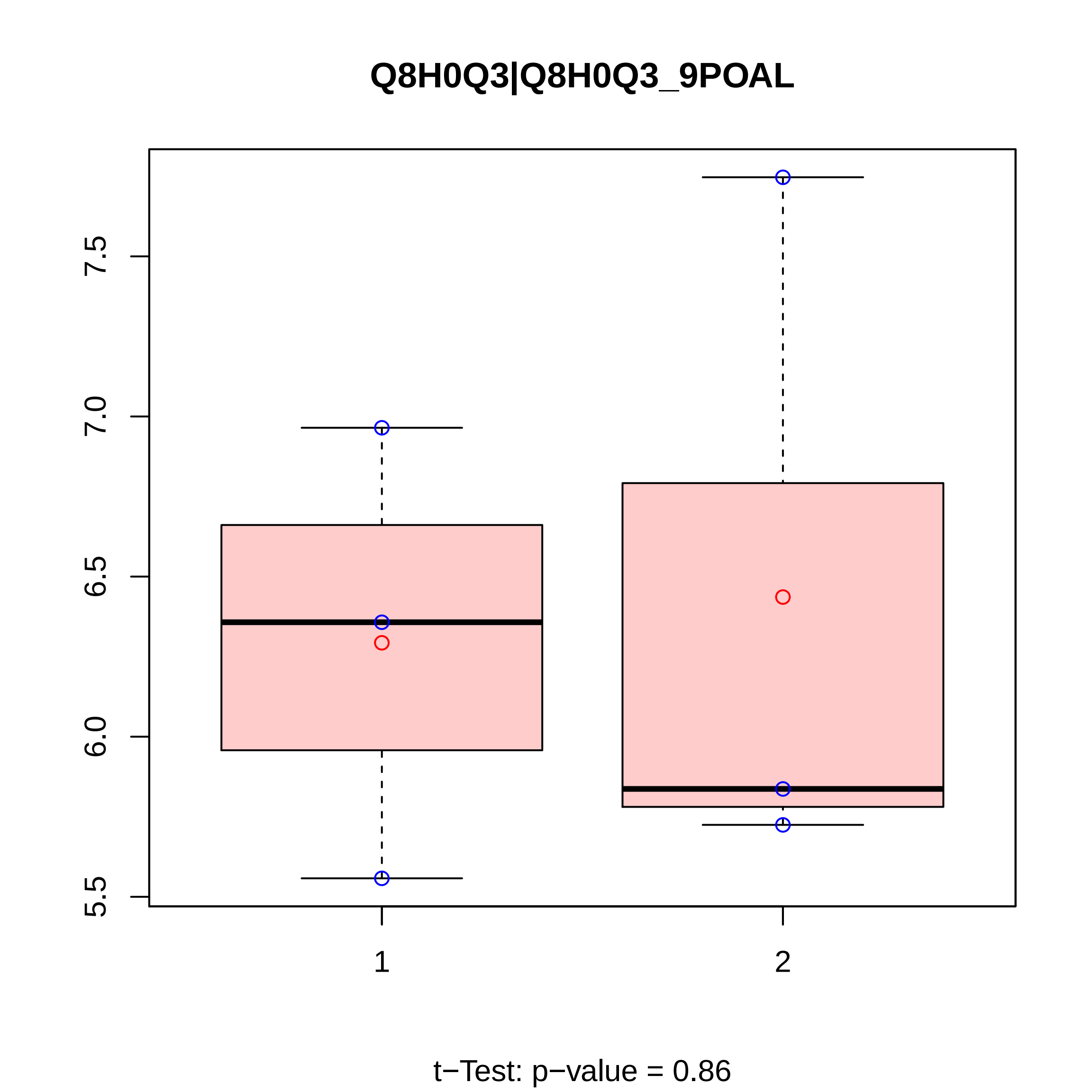
Q8H0Q3|Q8H0Q3_9POAL
7.5
7.0
6.5
6.0
5.5
1
2
t−Test: p−value = 0.86

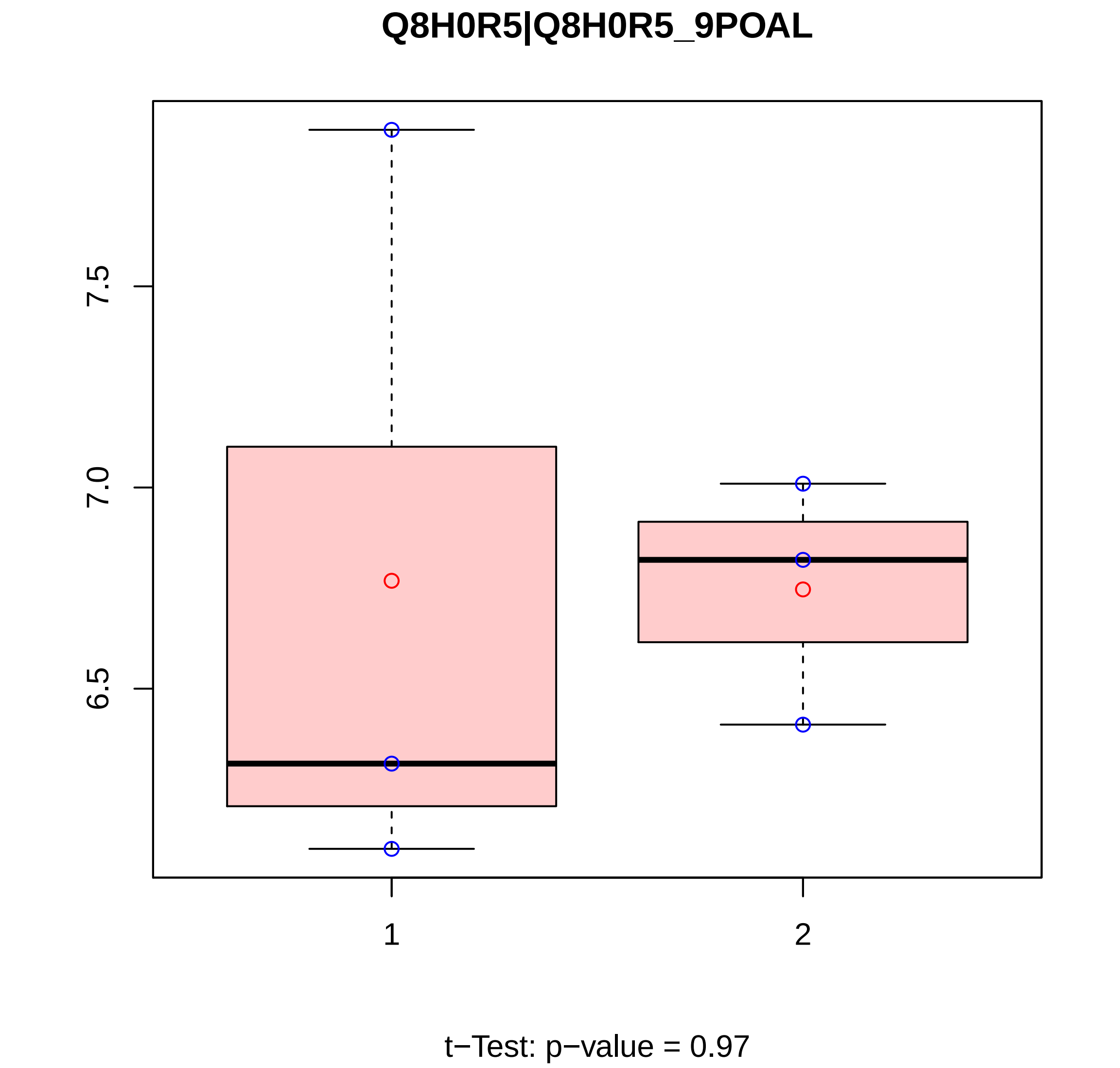
Q8H0R5|Q8H0R5_9POAL
7.5
7.0
6.5
1
2
t−Test: p−value = 0.97

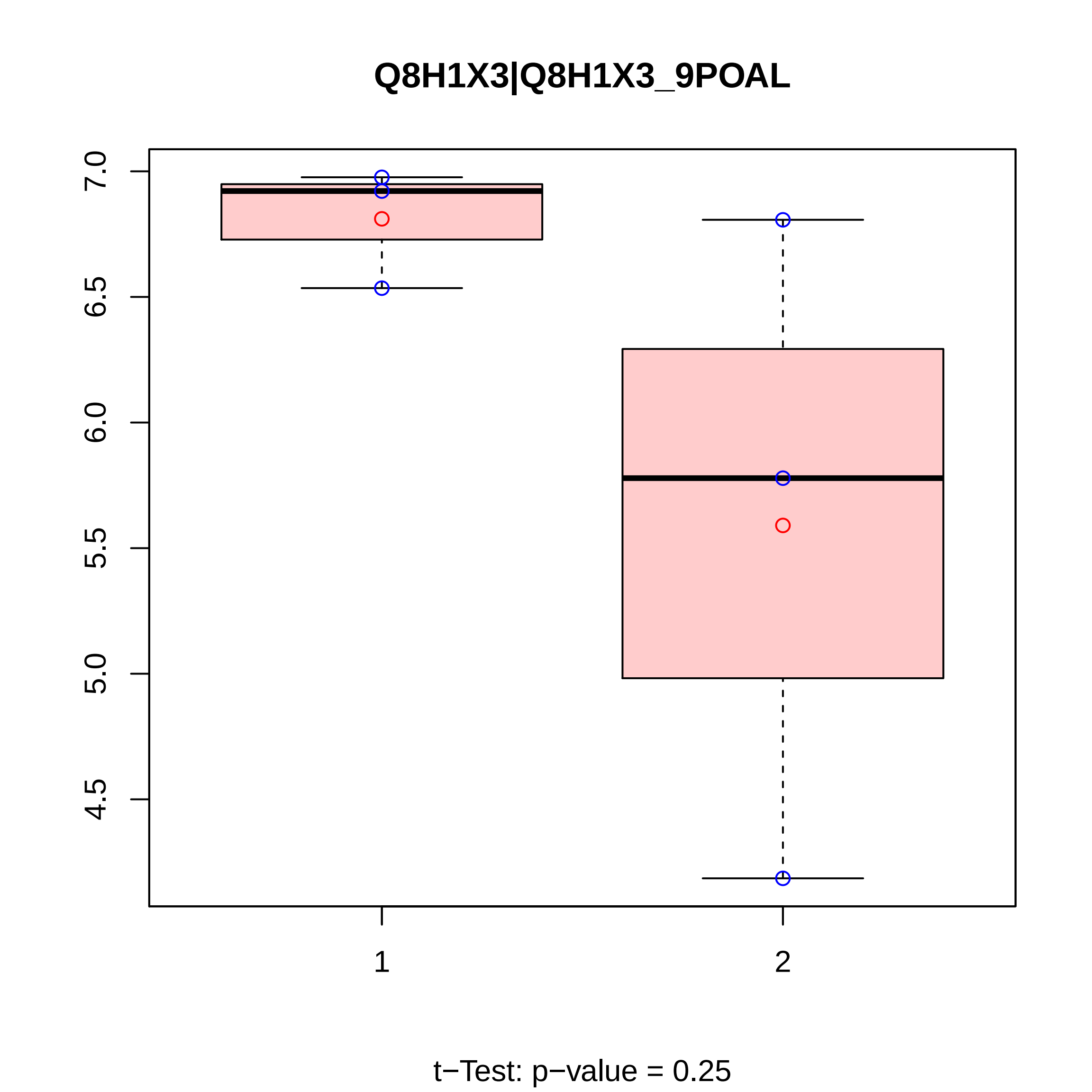
Q8H1X3|Q8H1X3_9POAL
7.0
6.5
6.0
5.5
5.0
4.5
1
2
t−Test: p−value = 0.25

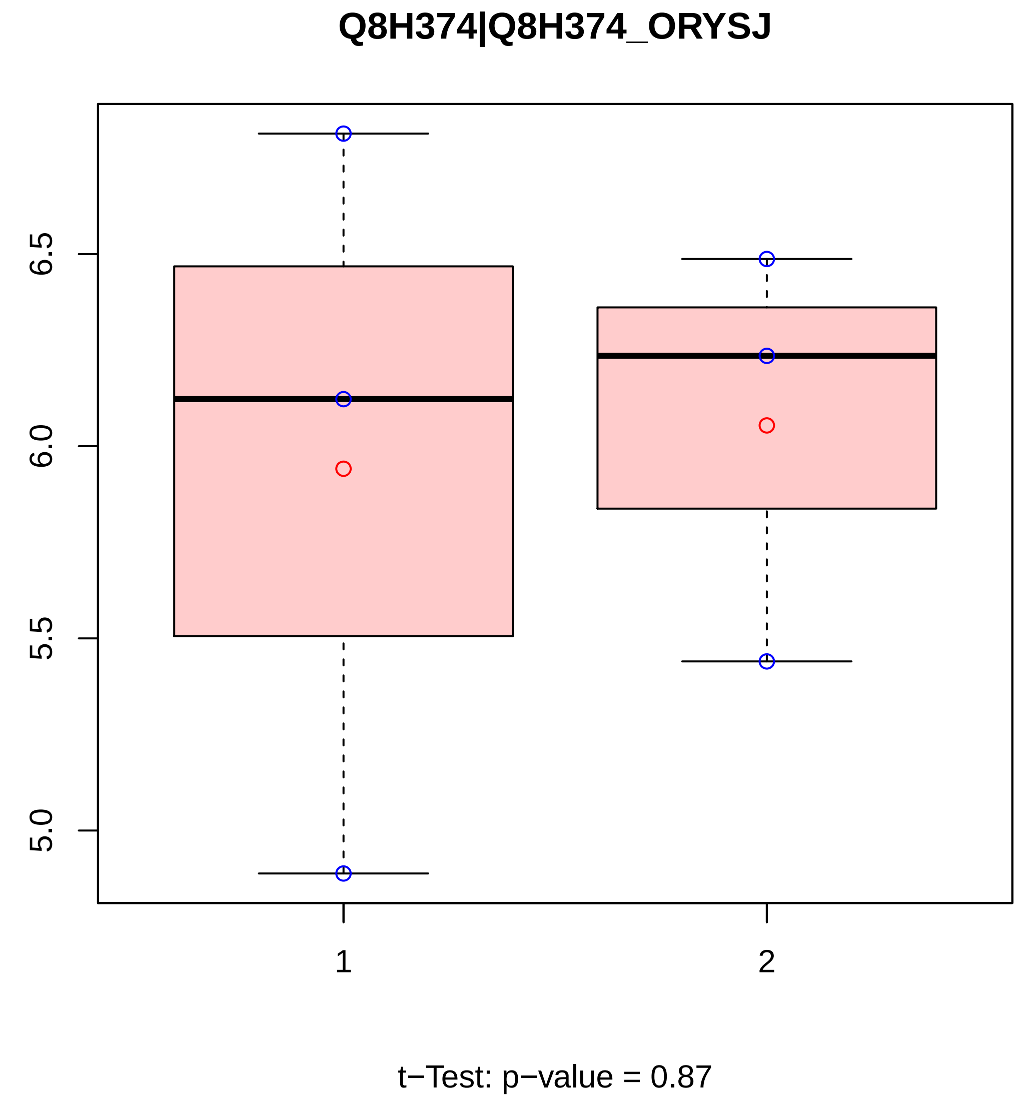
Q8H374|Q8H374_ORYSJ
6.5
6.0
5.5
5.0
1
2
t−Test: p−value = 0.87

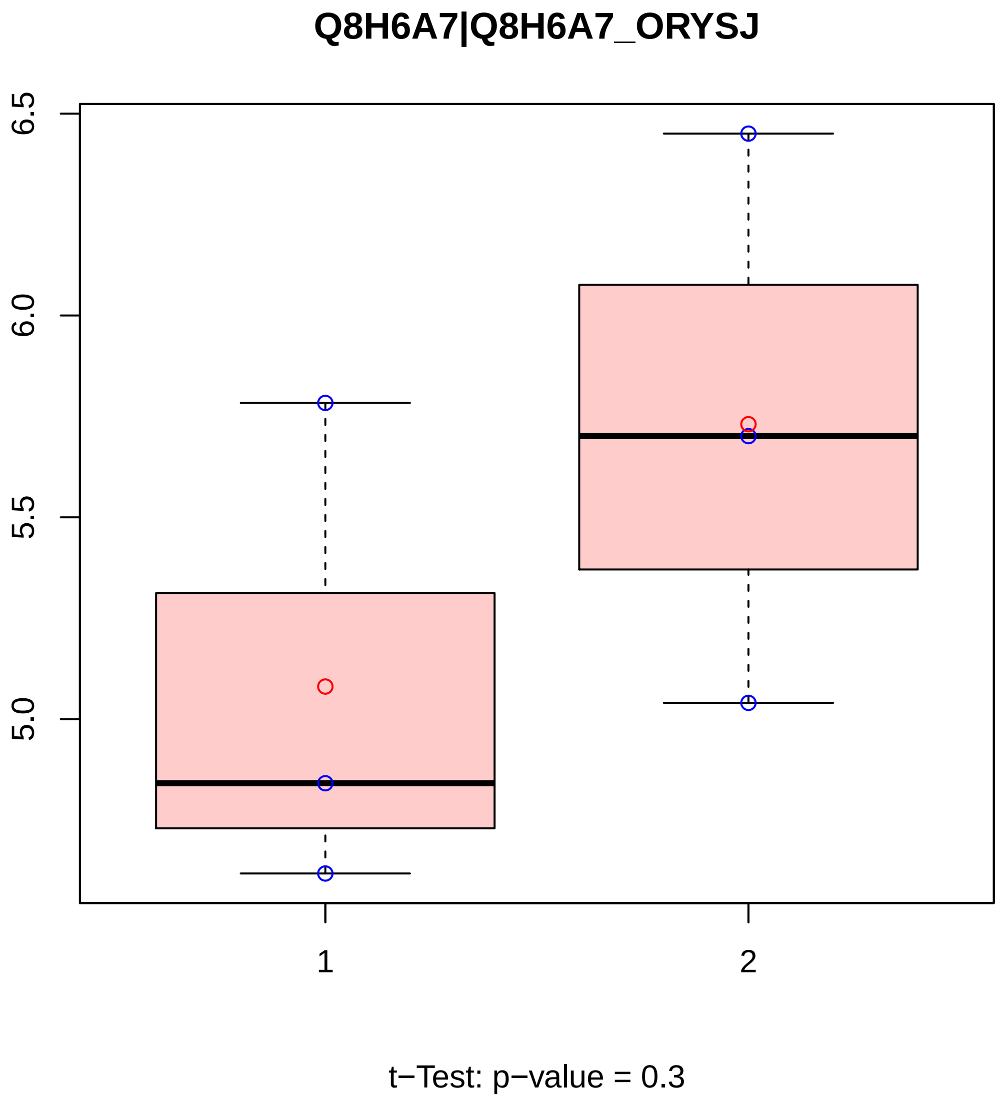
Q8H6A7|Q8H6A7_ORYSJ
6.5
6.0
5.5
5.0
1
2
t−Test: p−value = 0.3

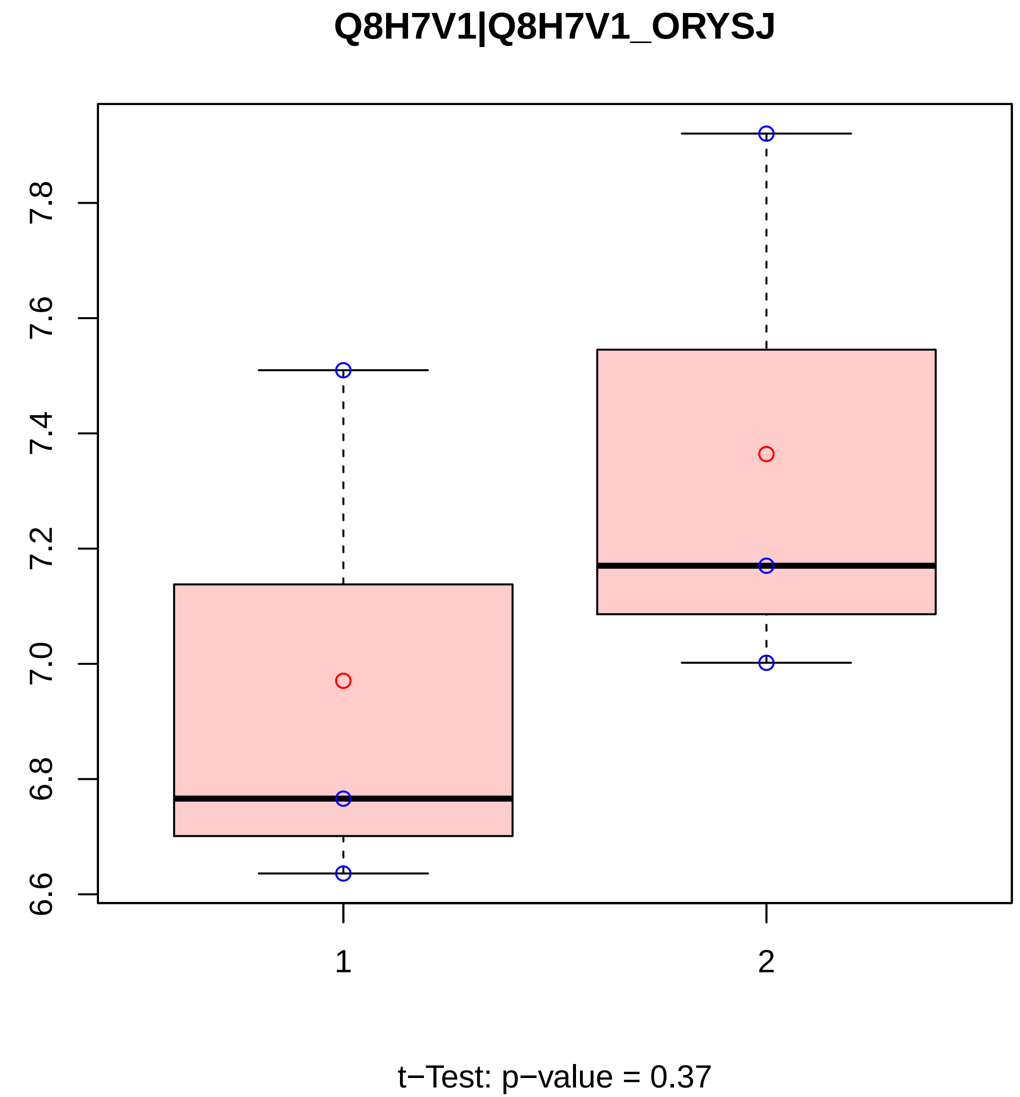

Q8H7V1|Q8H7V1_ORYSJ
7.8
7.6
7.4
7.2
7.0
6.8
6.6
1
2
t−Test: p−value = 0.37

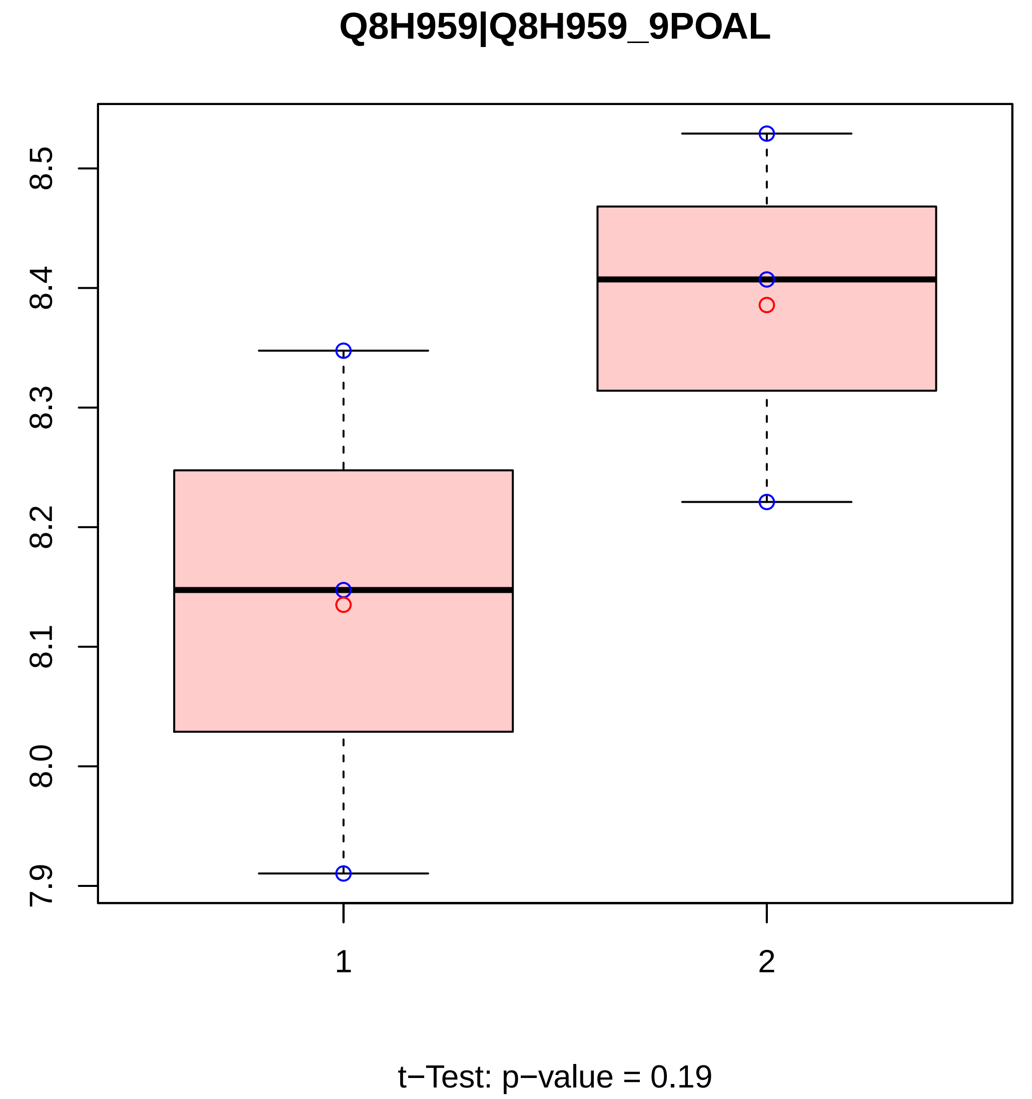
Q8H959|Q8H959_9POAL
7.9
8.0
8.1
8.2
8.3
8.4
8.5
1
2
t−Test: p−value = 0.19

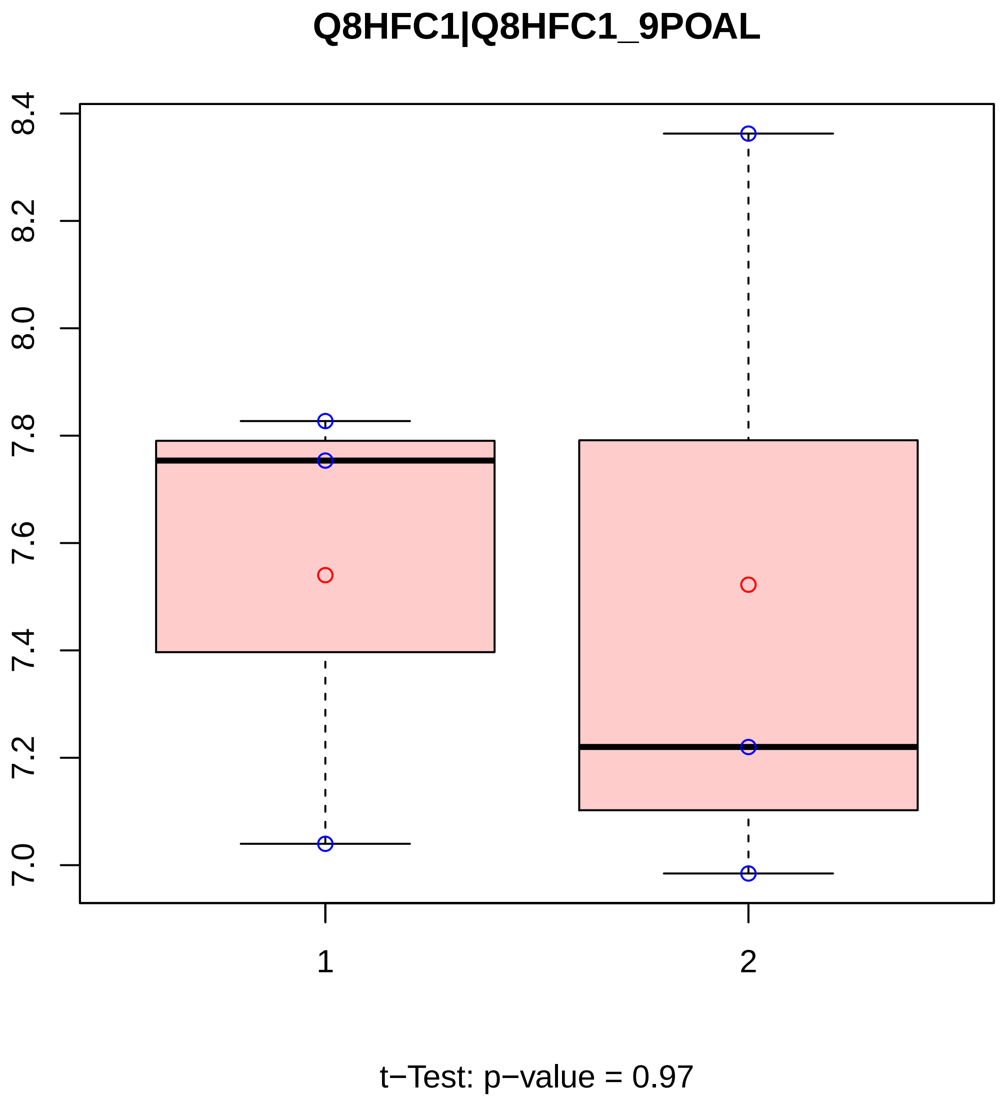
Q8HFC1|Q8HFC1_9POAL
8.4
8.2
8.0
7.8
7.6
7.4
7.2
7.0
1
2
t−Test: p−value = 0.97

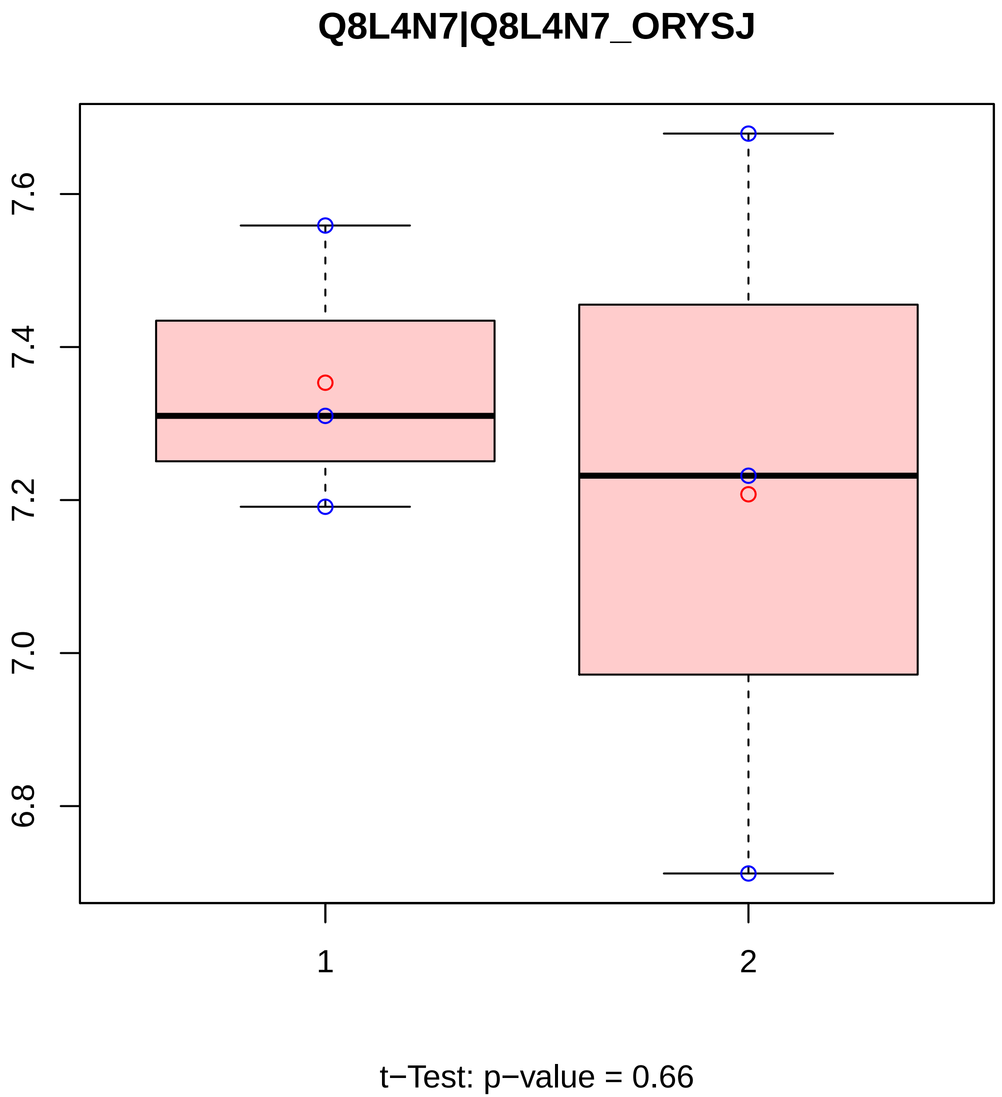
Q8L4N7|Q8L4N7_ORYSJ
7.6
7.4
7.2
7.0
6.8
1
2
t−Test: p−value = 0.66

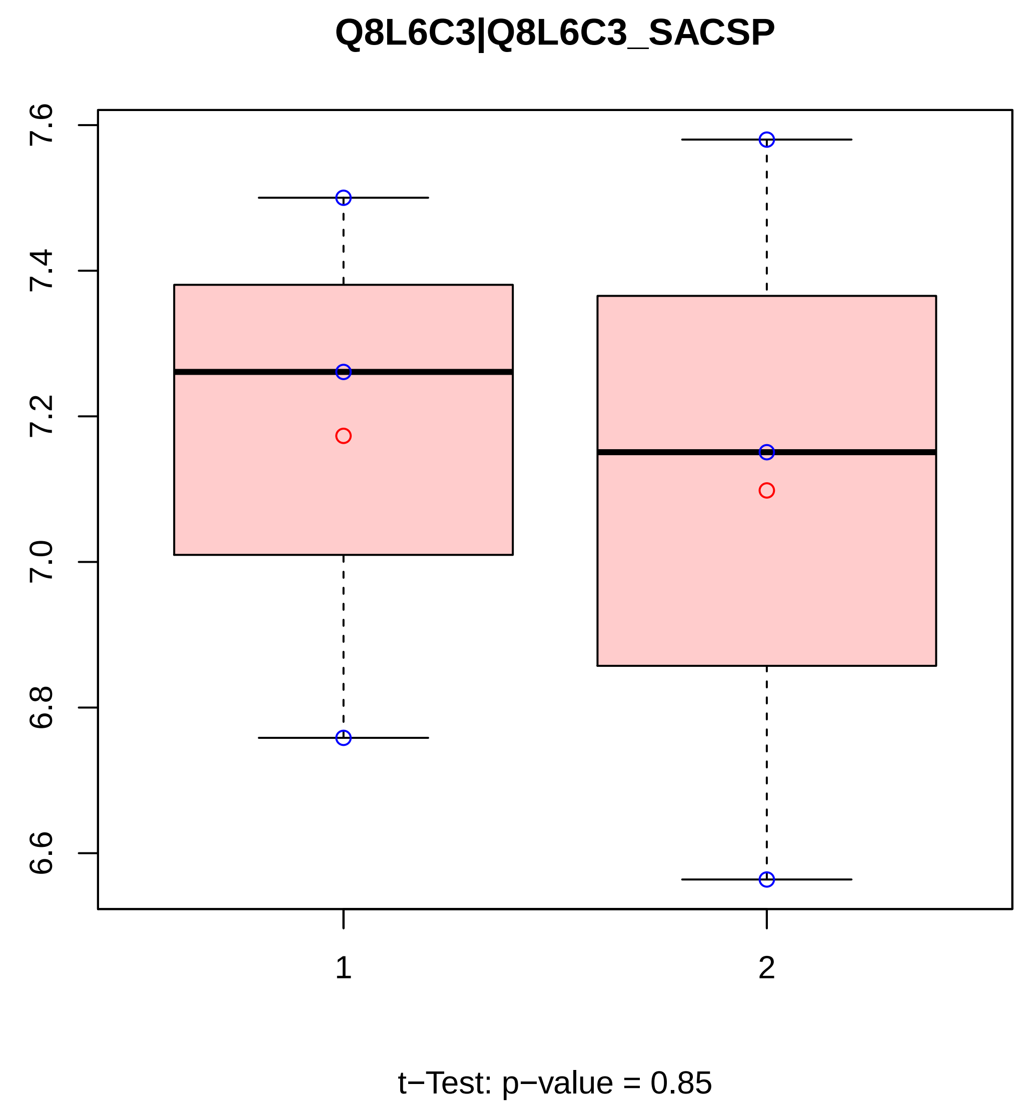

Q8L6C3|Q8L6C3_SACSP
7.6
7.4
7.2
7.0
6.8
6.6
1
2
t−Test: p−value = 0.85

Q8L6I0|Q8L6I0_ORYSJ

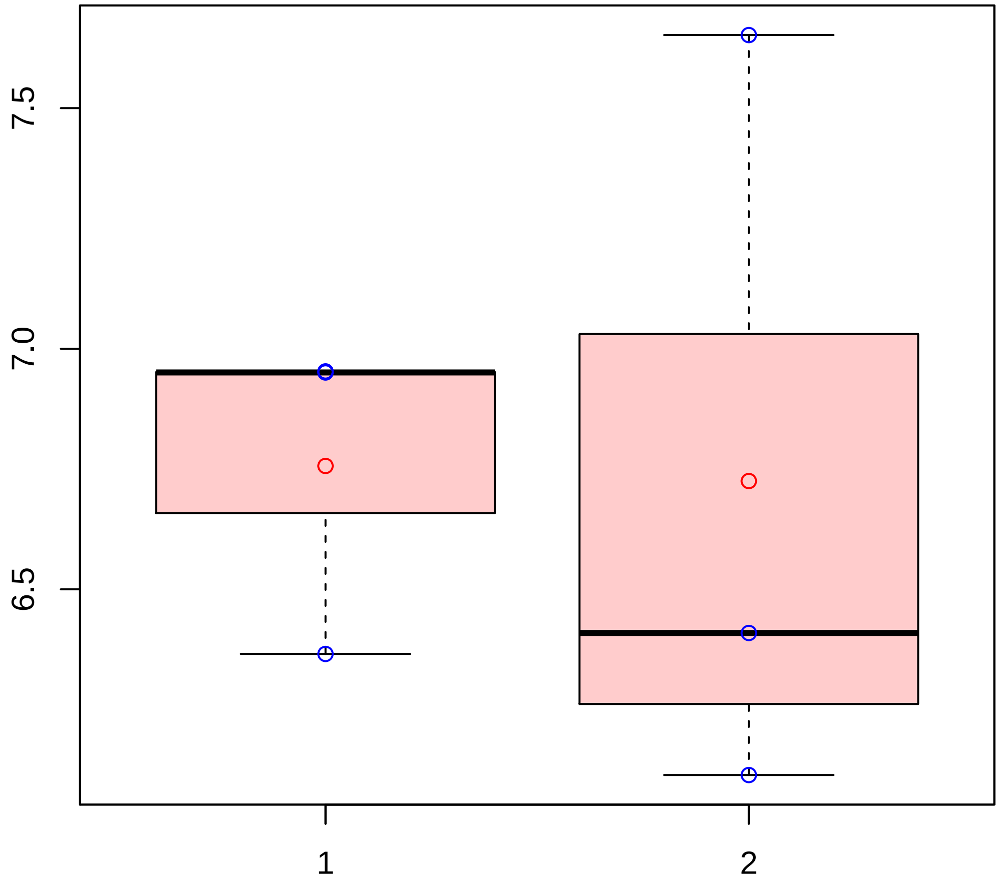

t−Test: p−value = 0.96

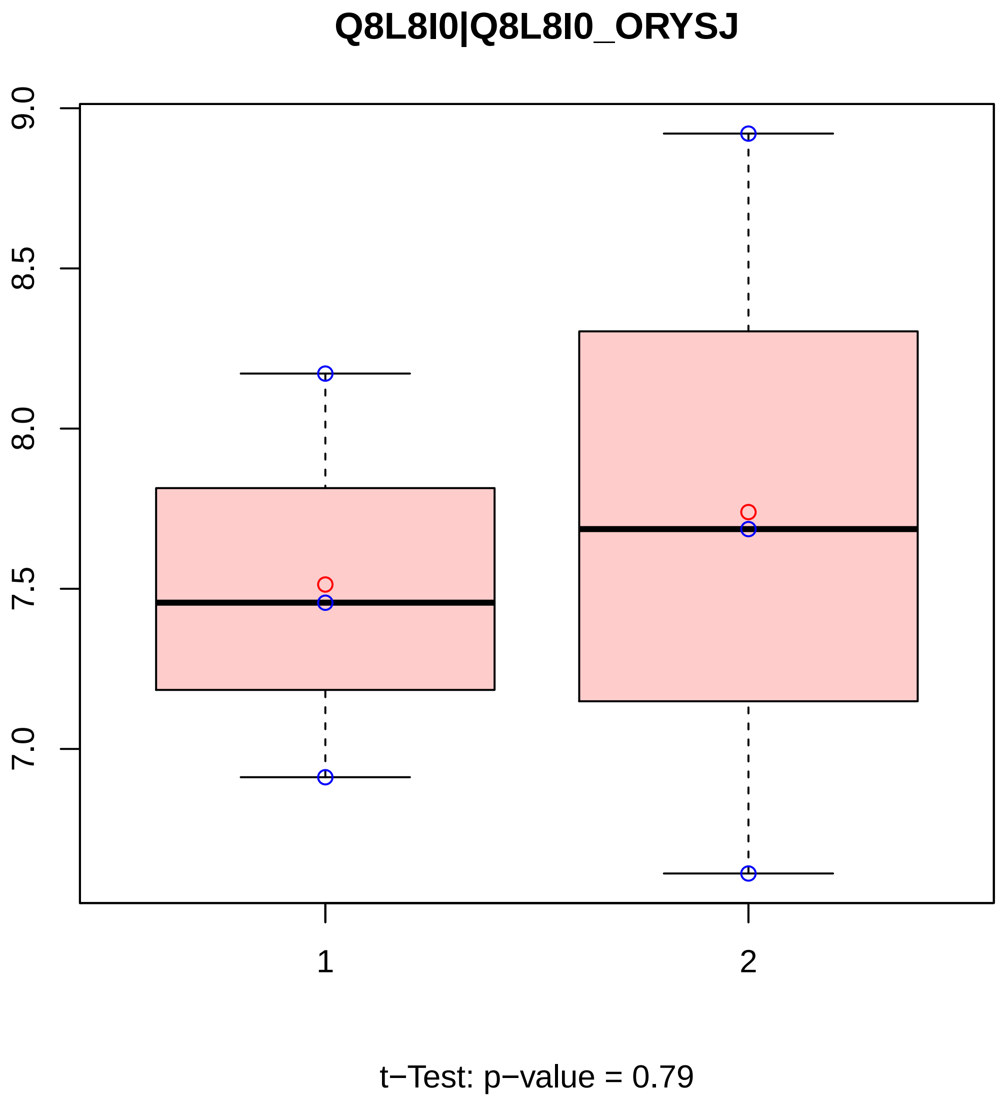

Q8L8I0|Q8L8I0_ORYSJ
9.0
8.5
8.0
7.5
7.0
1
2
t−Test: p−value = 0.79

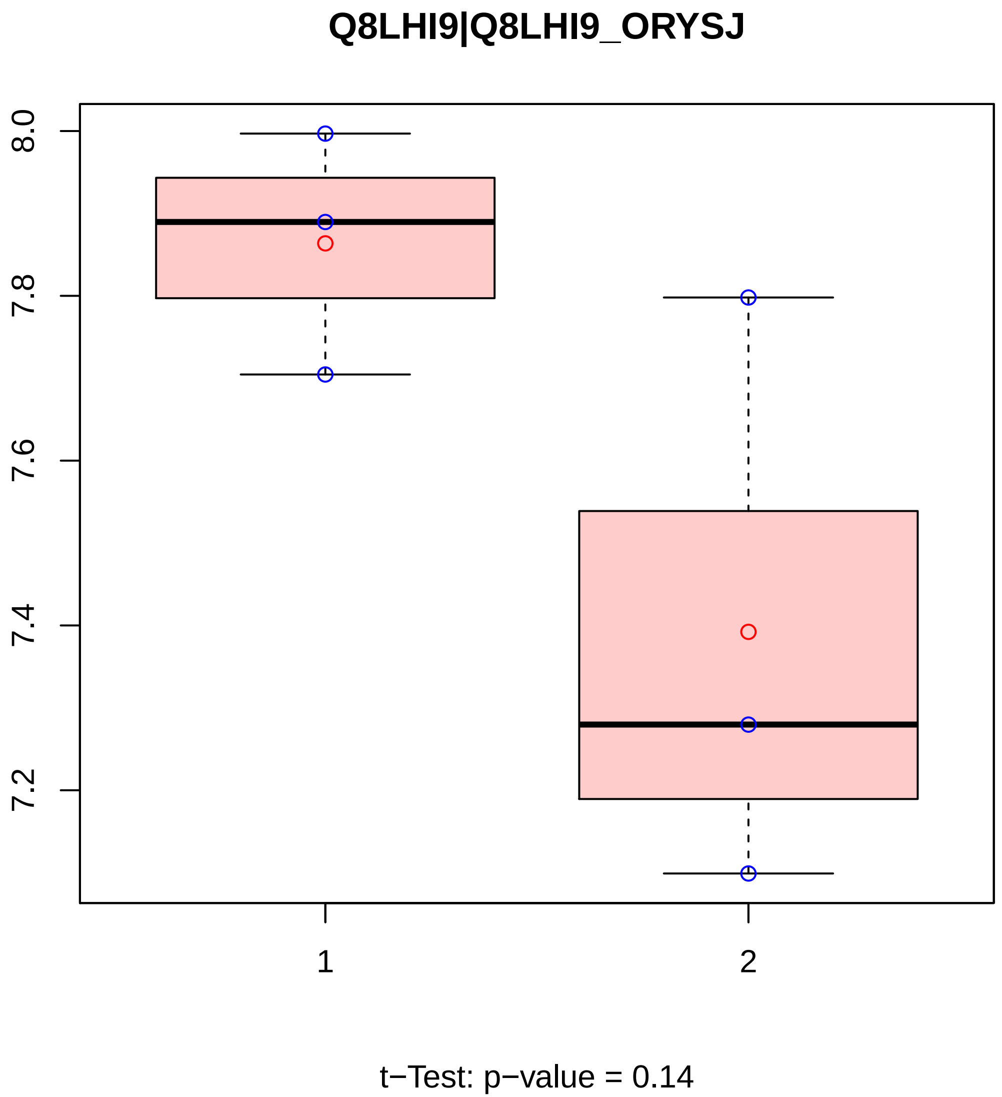
Q8LHI9|Q8LHI9_ORYSJ
8.0
7.8
7.6
7.4
7.2
1
2
t−Test: p−value = 0.14

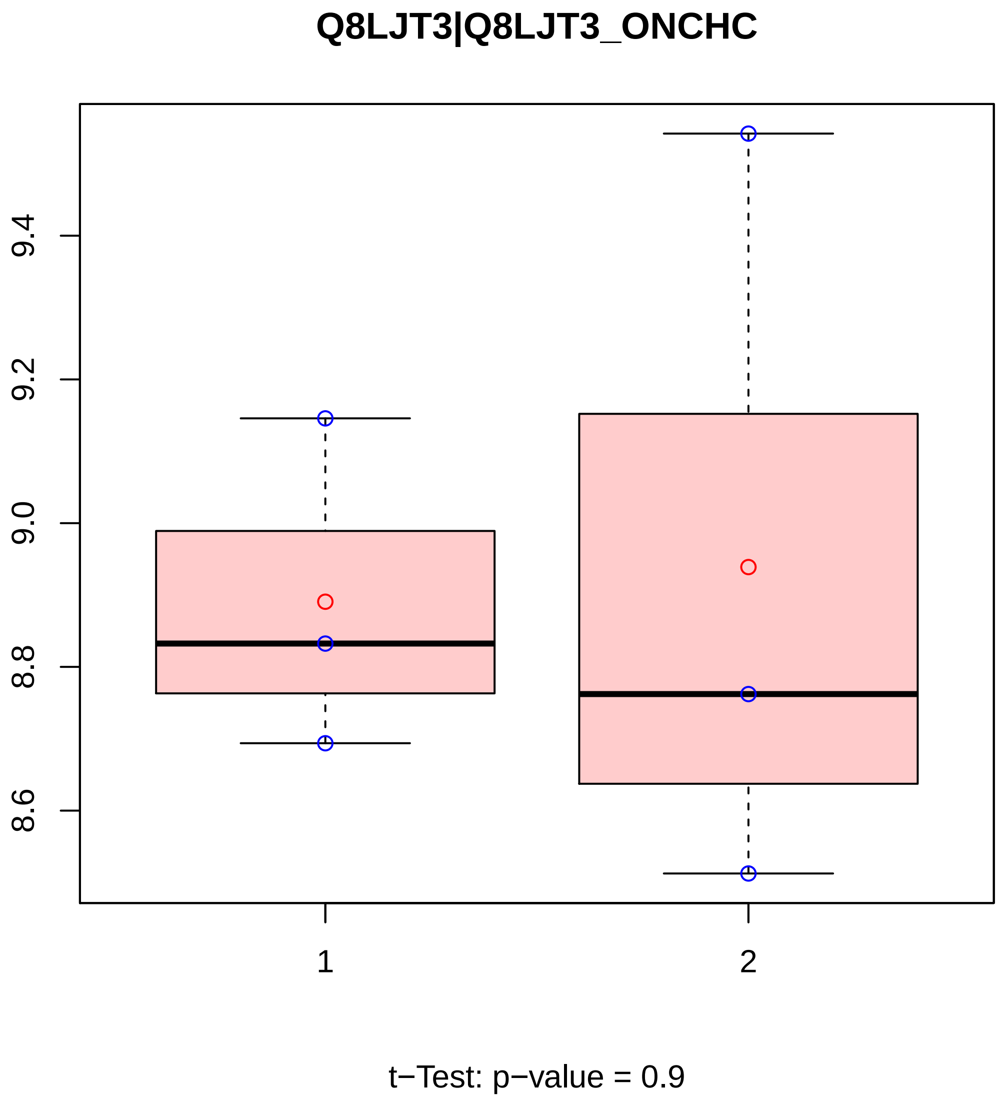

Q8LJT3|Q8LJT3_ONCHC
8.6
8.8
9.0
9.2
9.4
1
2
t−Test: p−value = 0.9

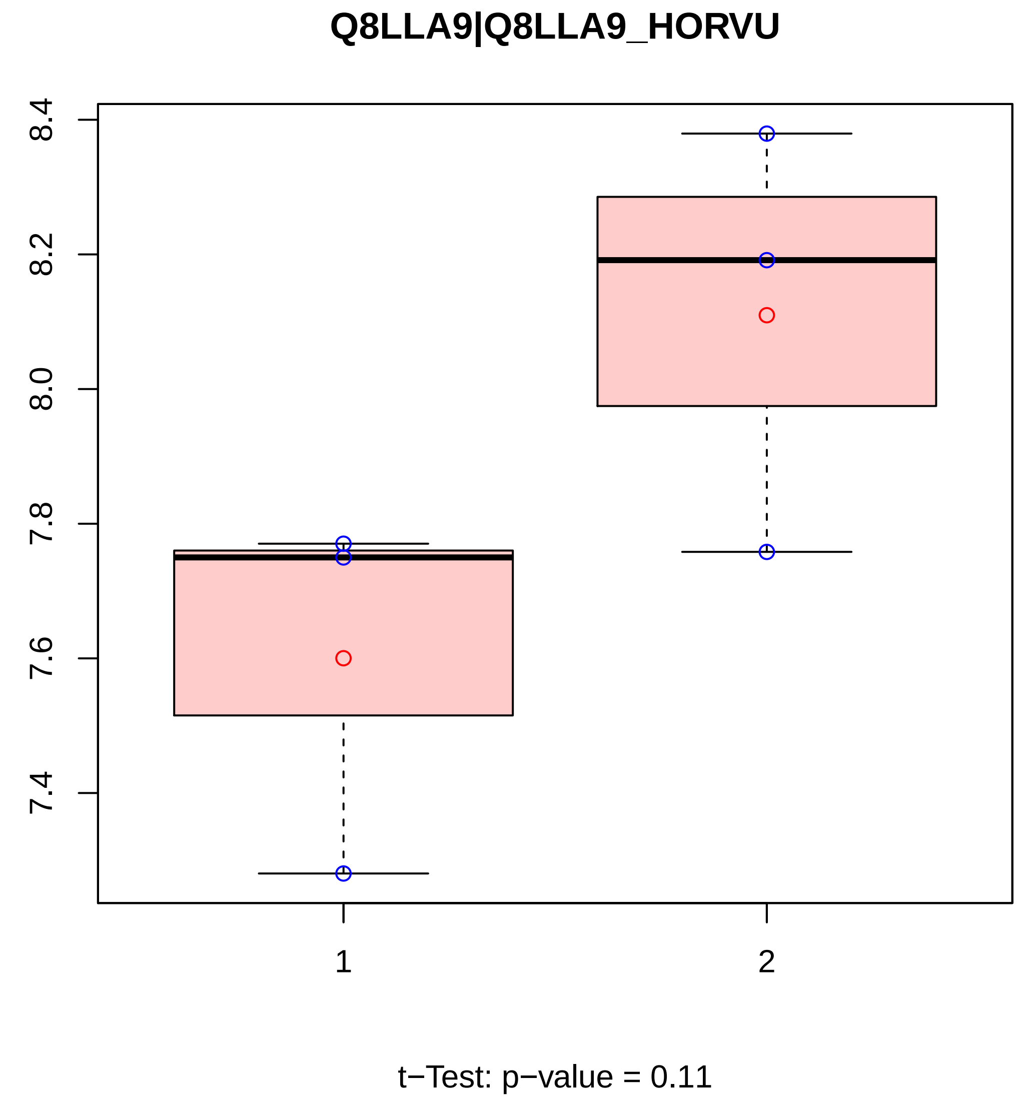
Q8LLA9|Q8LLA9_HORVU
8.4
8.2
8.0
7.8
7.6
7.4
1
2
t−Test: p−value = 0.11

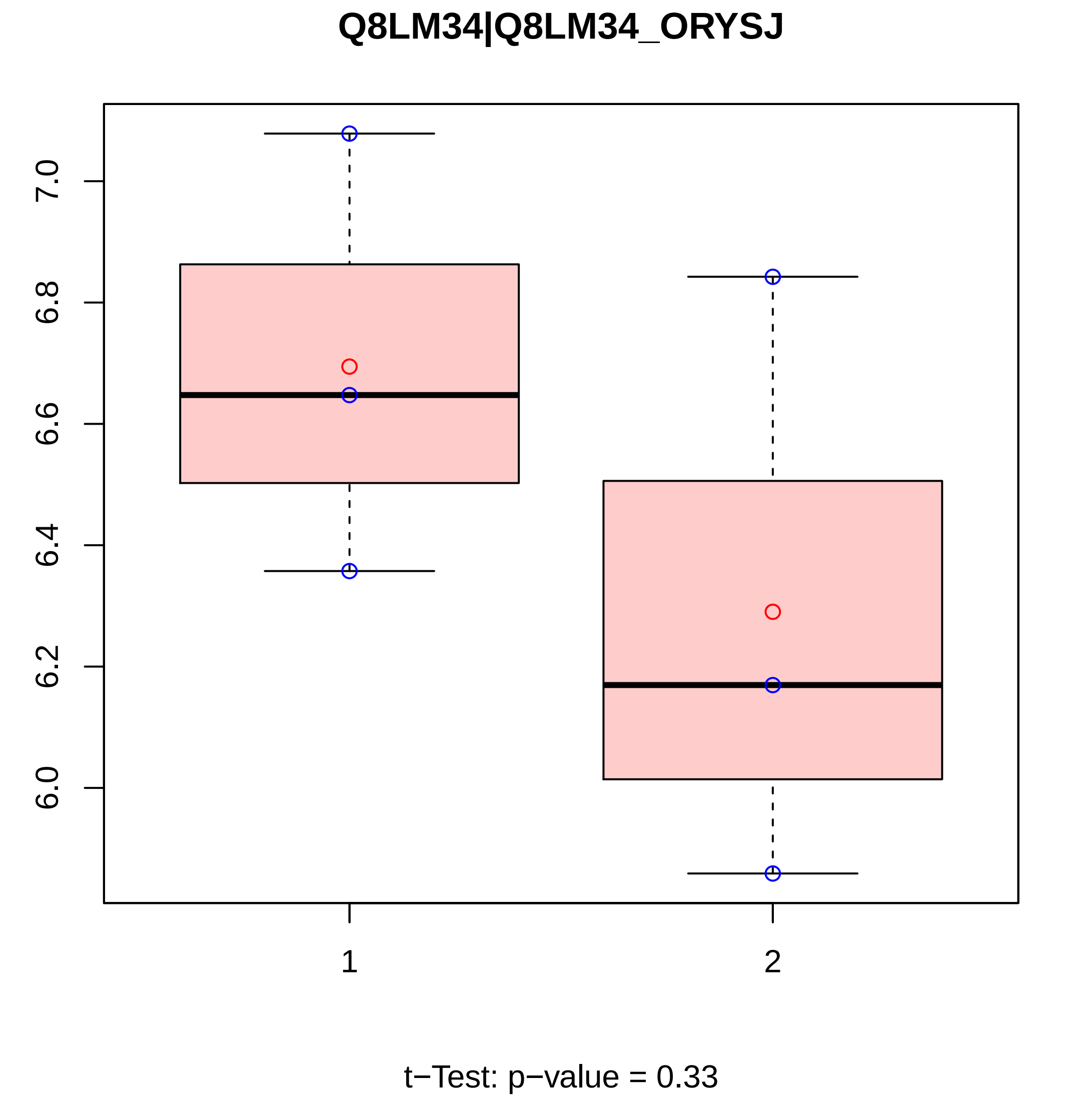
Q8LM34|Q8LM34_ORYSJ
7.0
6.8
6.6
6.4
6.2
6.0
1
2
t−Test: p−value = 0.33

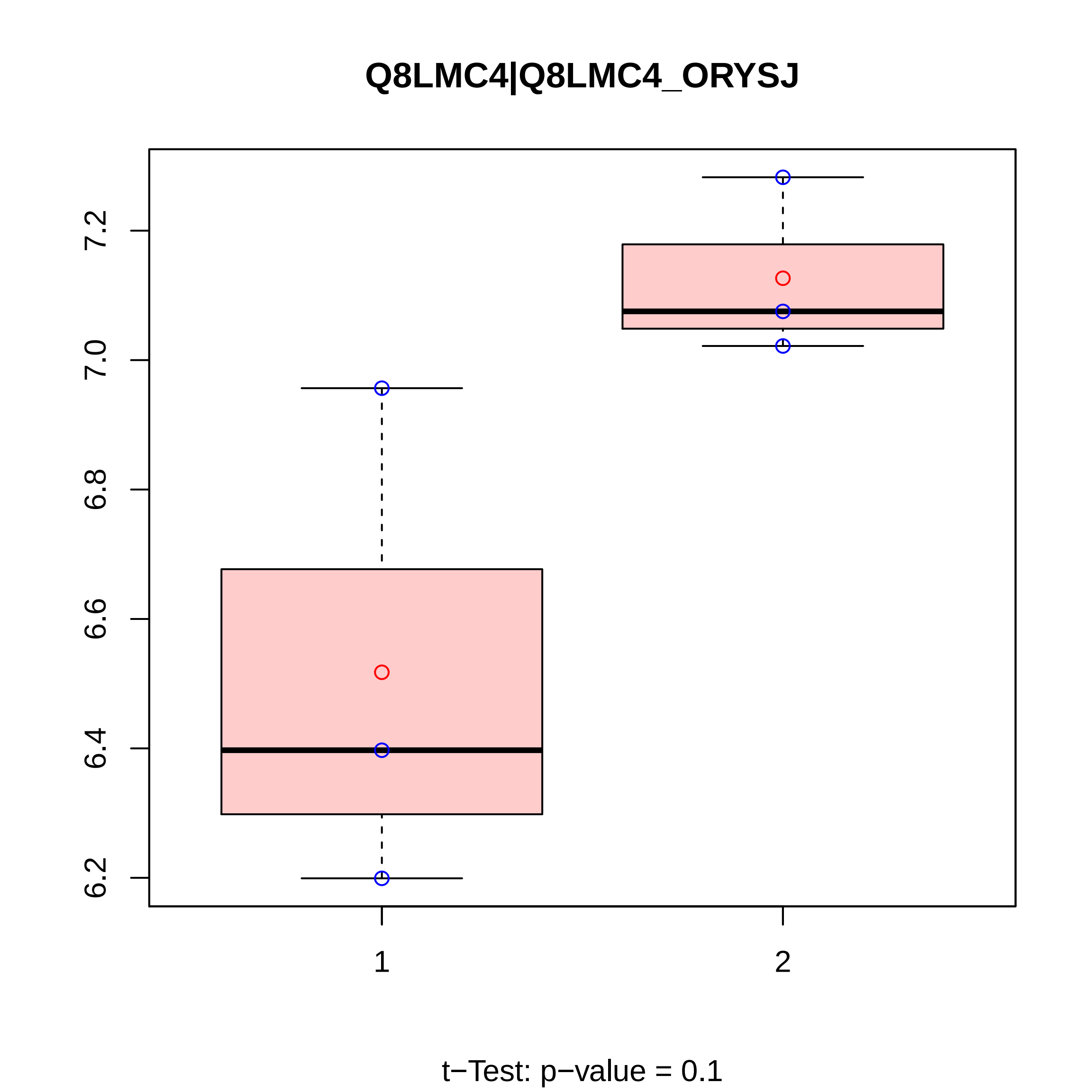

Q8LMC4|Q8LMC4_ORYSJ
7.2
7.0
6.8
6.6
6.4
6.2
1
2
t−Test: p−value = 0.1

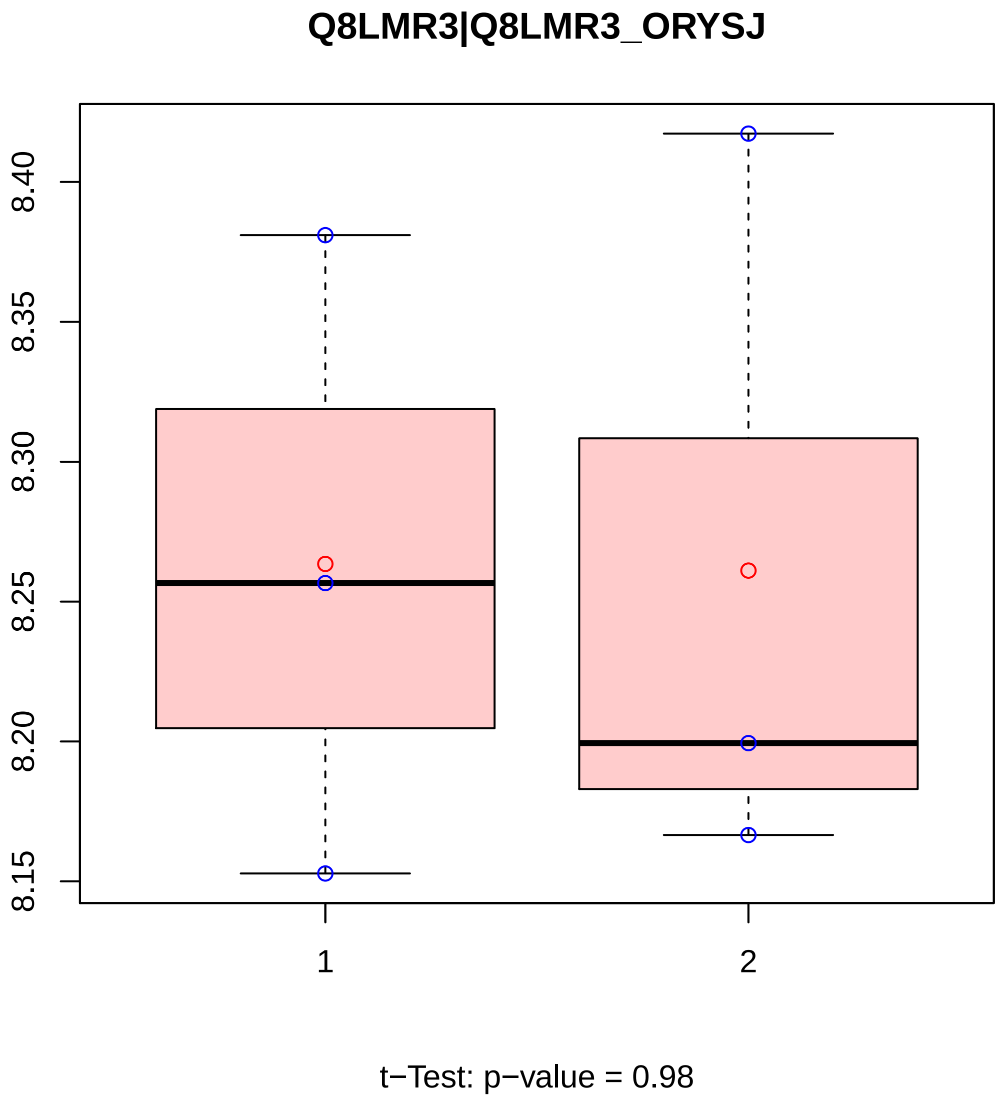

Q8LMR3|Q8LMR3_ORYSJ
8.15
8.20
8.25
8.30
8.35
8.40
1
2
t−Test: p−value = 0.98

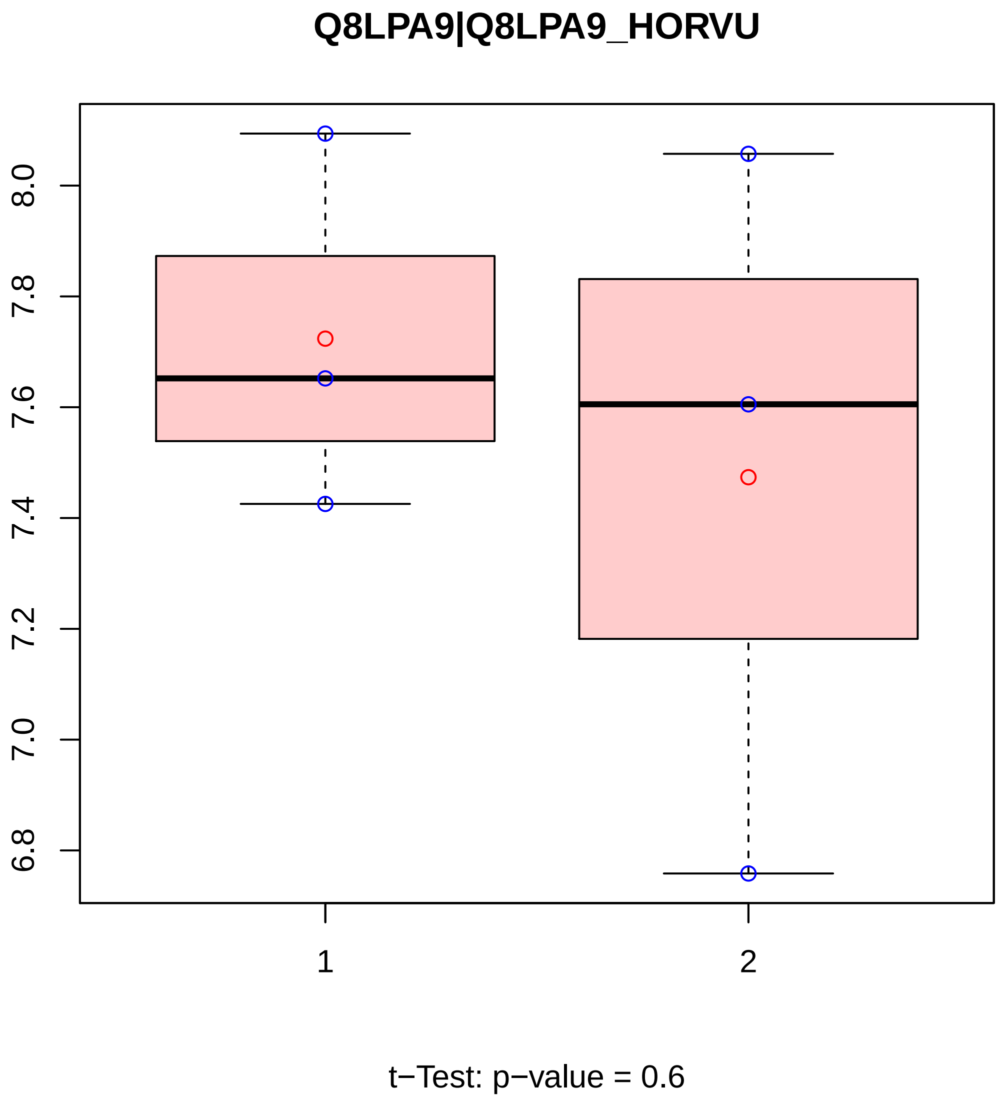

Q8LPA9|Q8LPA9_HORVU
8.0
7.8
7.6
7.4
7.2
7.0
6.8
1
2
t−Test: p−value = 0.6

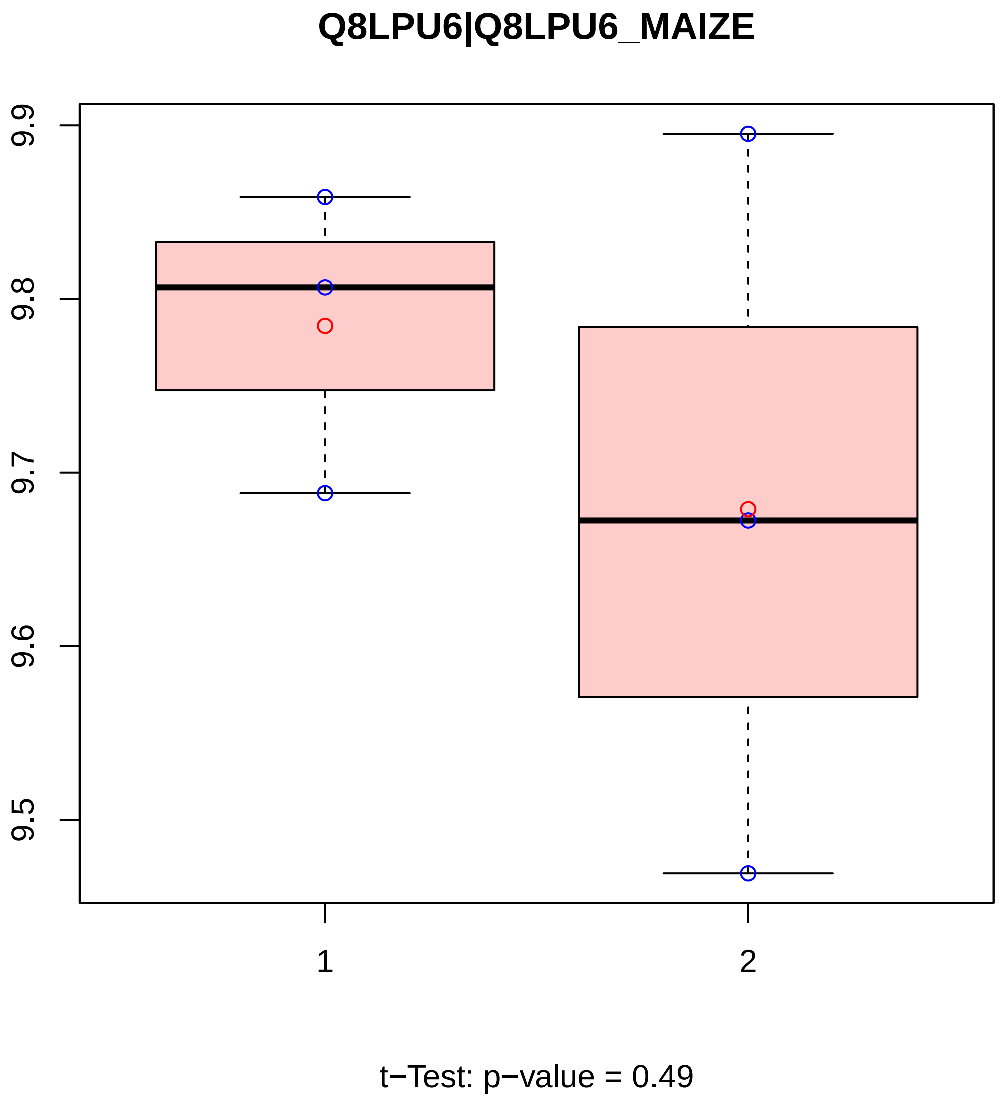
Q8LPU6|Q8LPU6_MAIZE
9.9
9.8
9.7
9.6
9.5
1
2
t−Test: p−value = 0.49

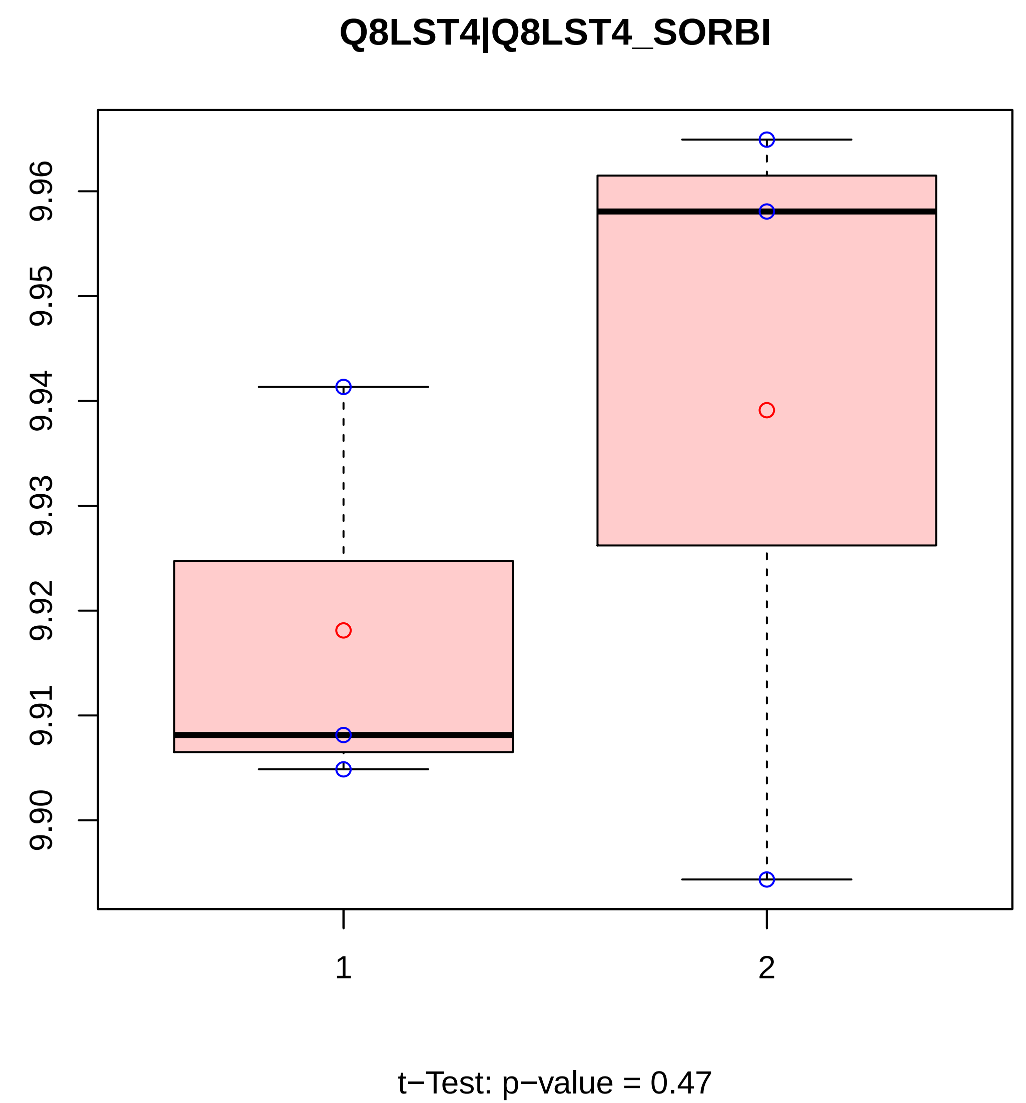
Q8LST4|Q8LST4_SORBI
9.90
9.91
9.92
9.93
9.94
9.95
9.96
1
2
t−Test: p−value = 0.47

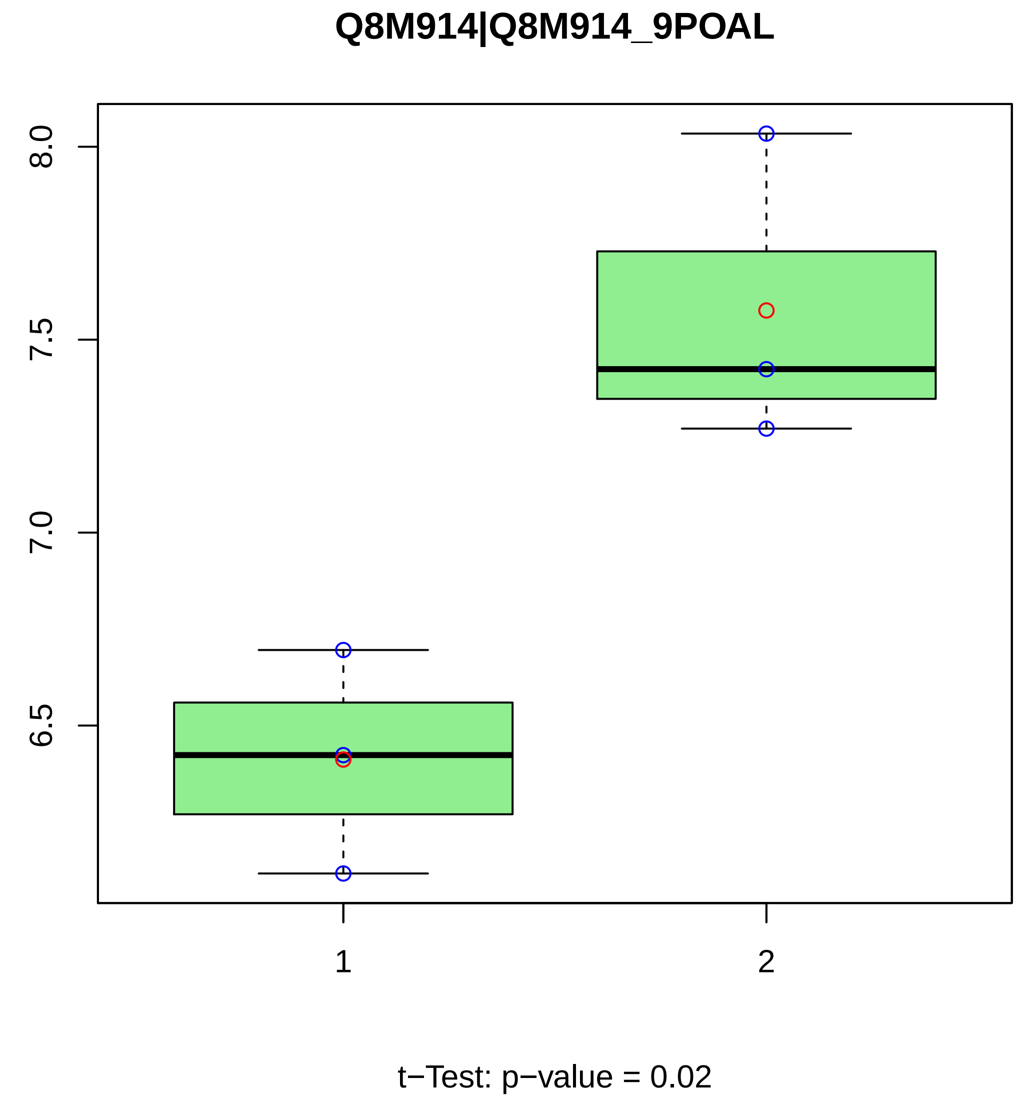
Q8M914|Q8M914_9POAL
8.0
7.5
7.0
6.5
1
2
t−Test: p−value = 0.02

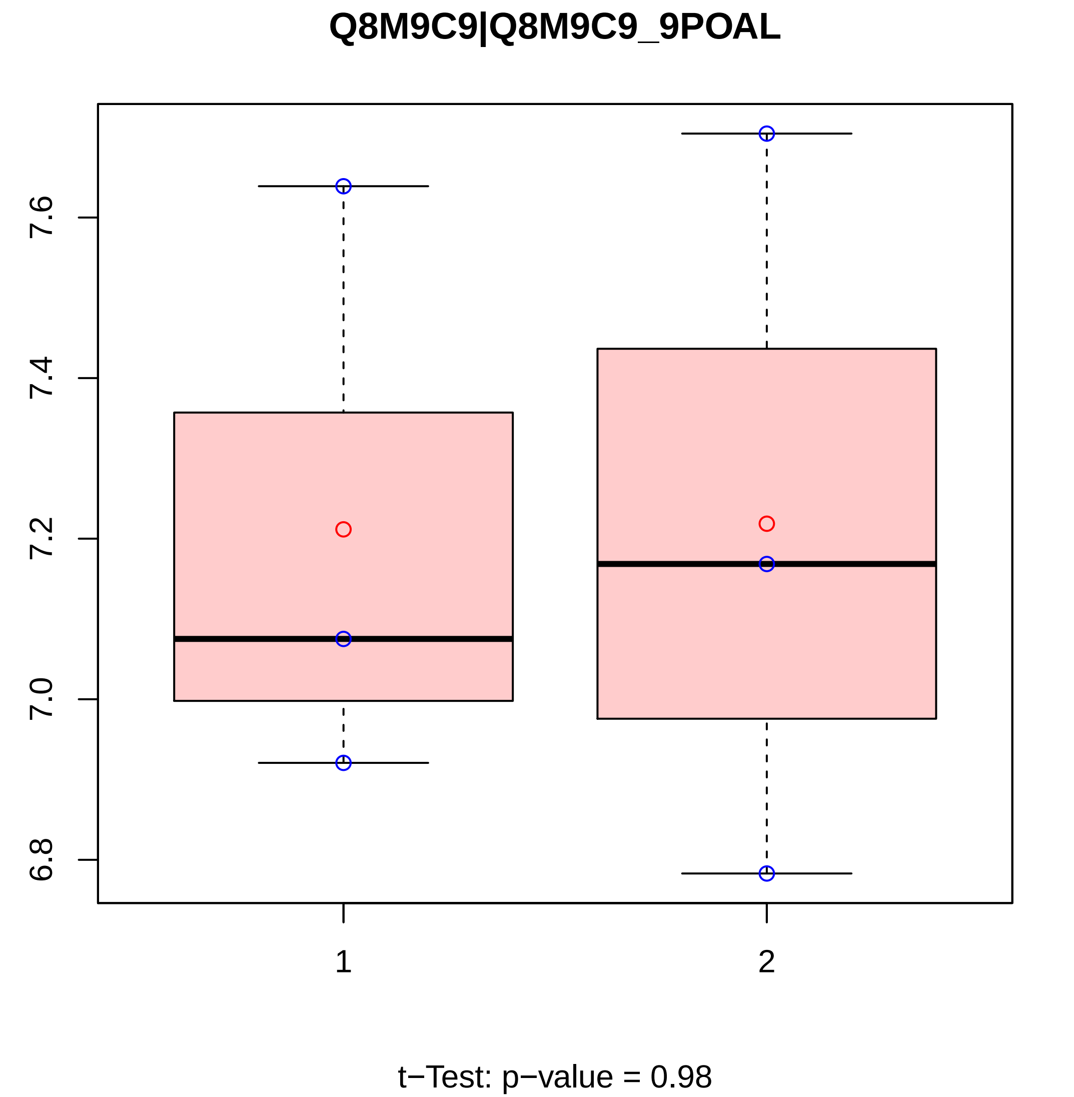
Q8M9C9|Q8M9C9_9POAL
6.8
7.0
7.2
7.4
7.6
1
2
t−Test: p−value = 0.98

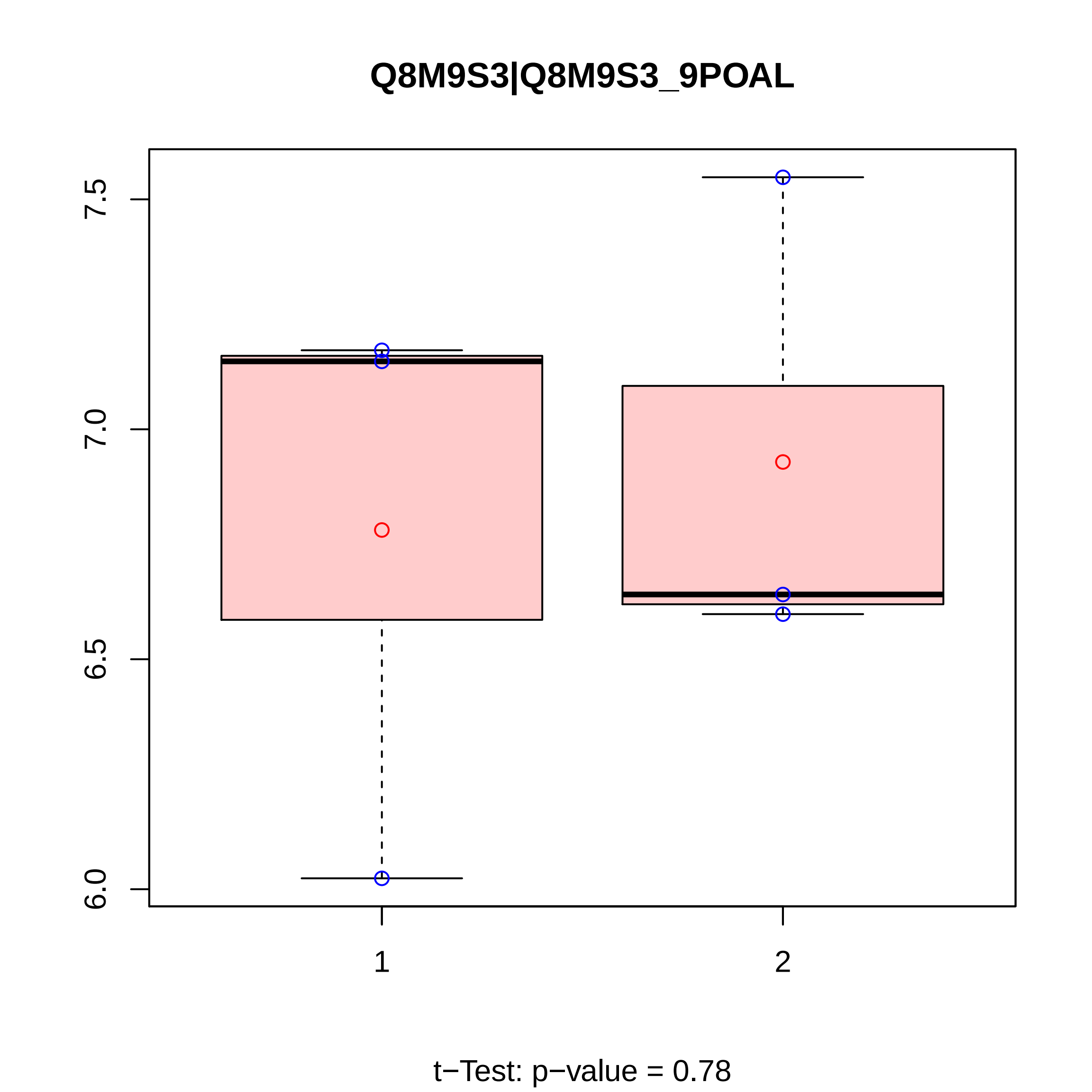
Q8M9S3|Q8M9S3_9POAL
7.5
7.0
6.5
6.0
1
2
t−Test: p−value = 0.78

Q8MA62|Q8MA62_9POAL

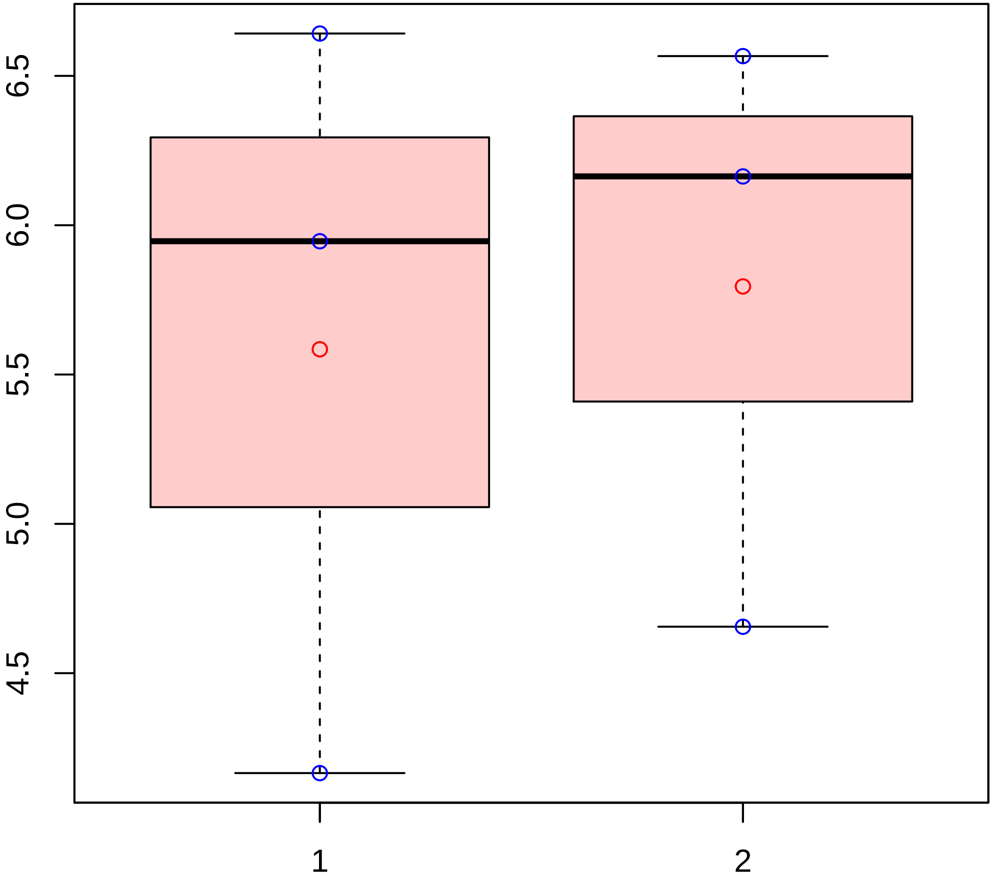

t−Test: p−value = 0.83

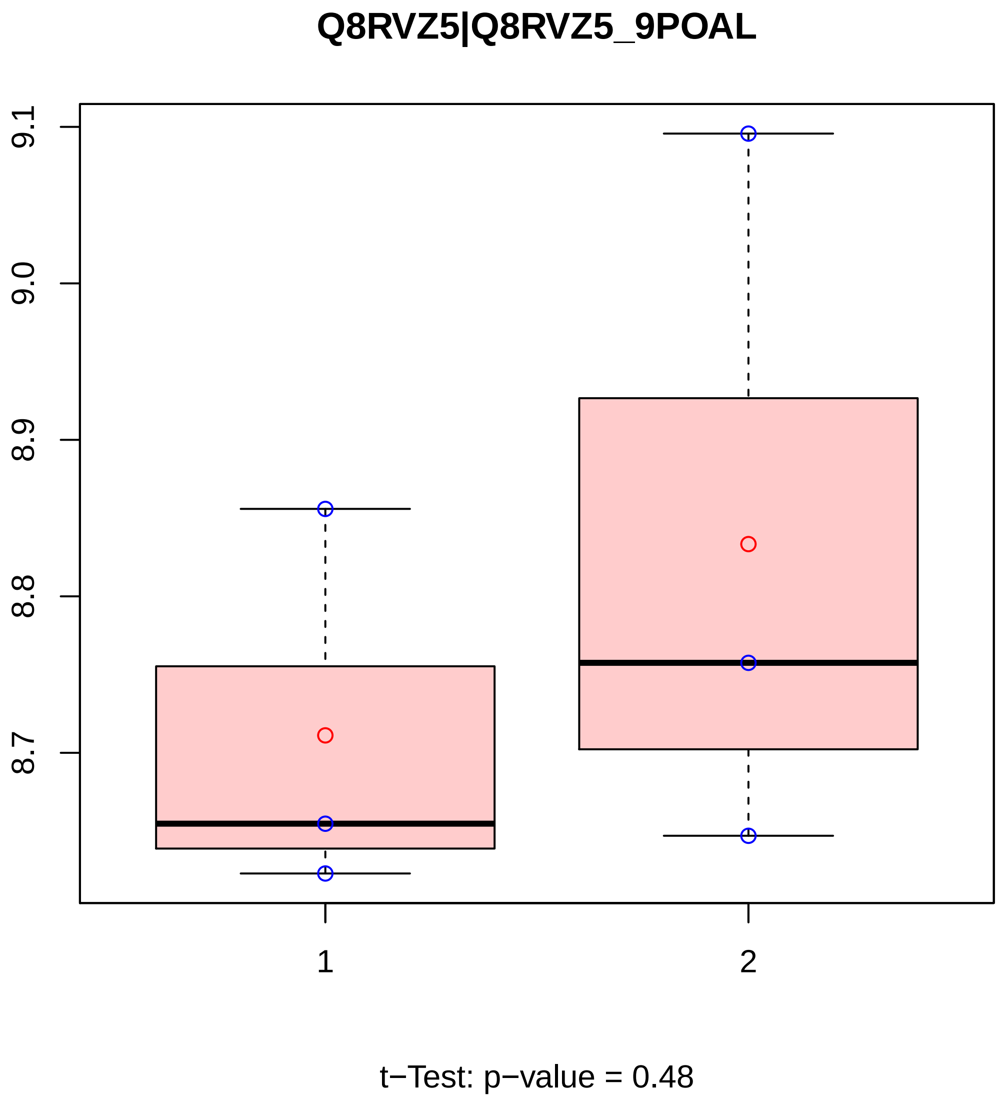
Q8RVZ5|Q8RVZ5_9POAL
9.1
9.0
8.9
8.8
8.7
1
2
t−Test: p−value = 0.48

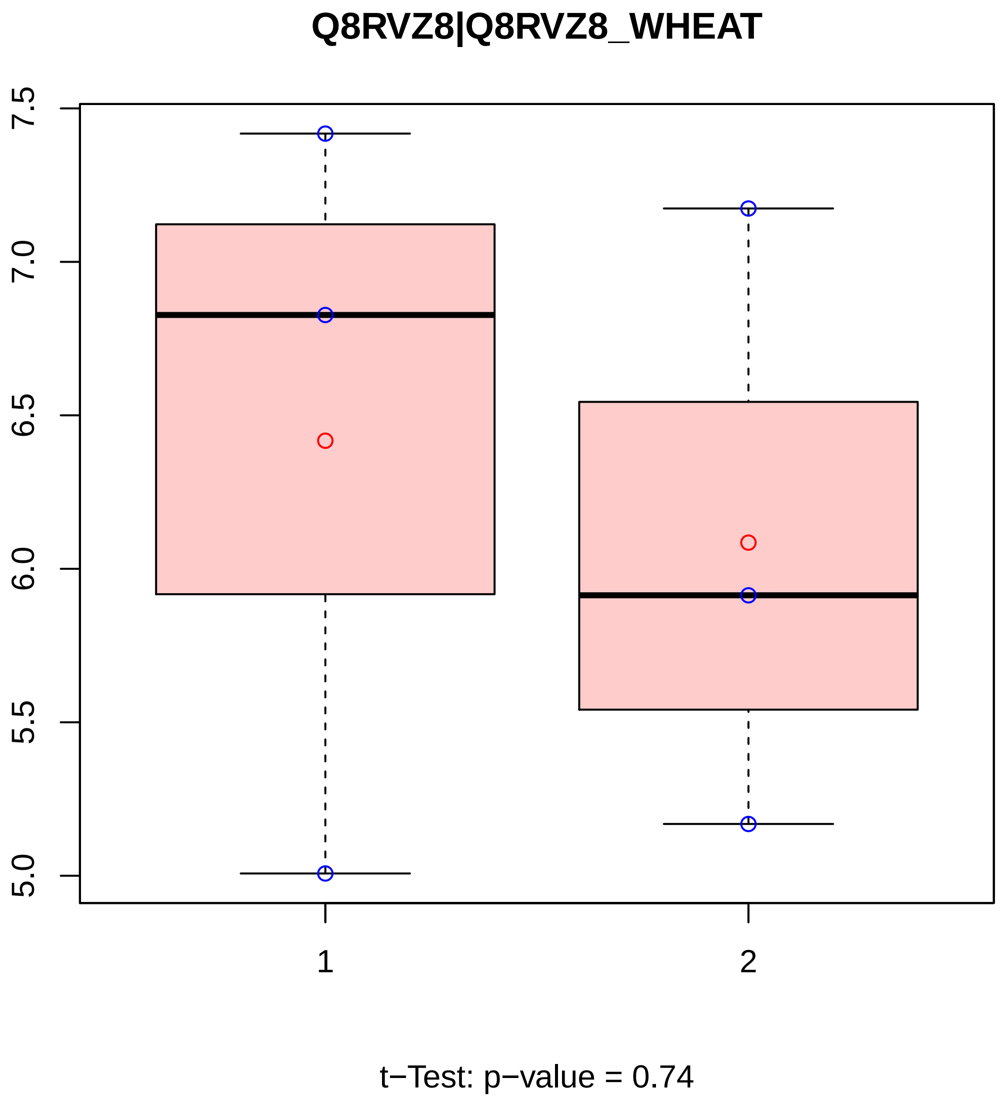

Q8RVZ8|Q8RVZ8_WHEAT
7.5
7.0
6.5
6.0
5.5
5.0
1
2
t−Test: p−value = 0.74

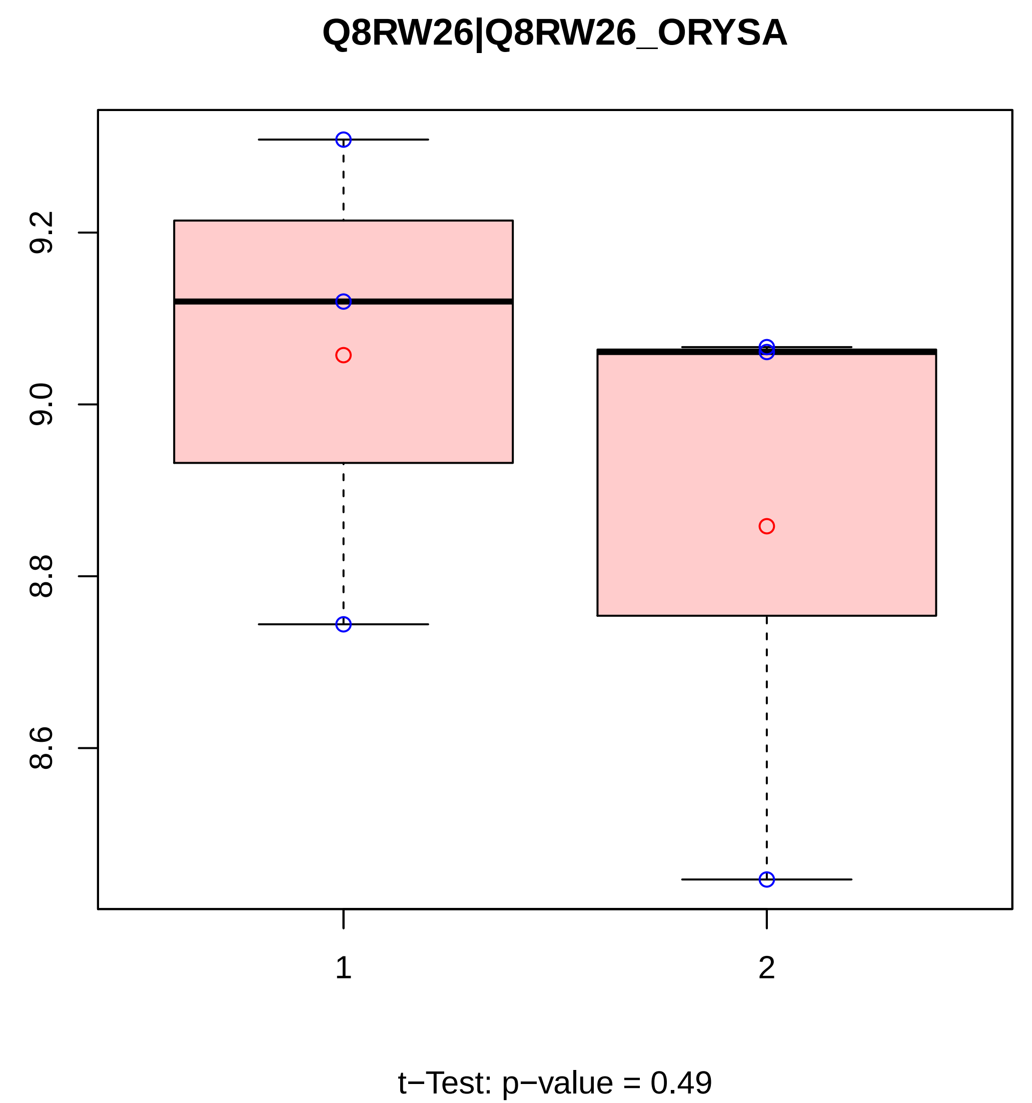
Q8RW26|Q8RW26_ORYSA
9.2
9.0
8.8
8.6
1
2
t−Test: p−value = 0.49

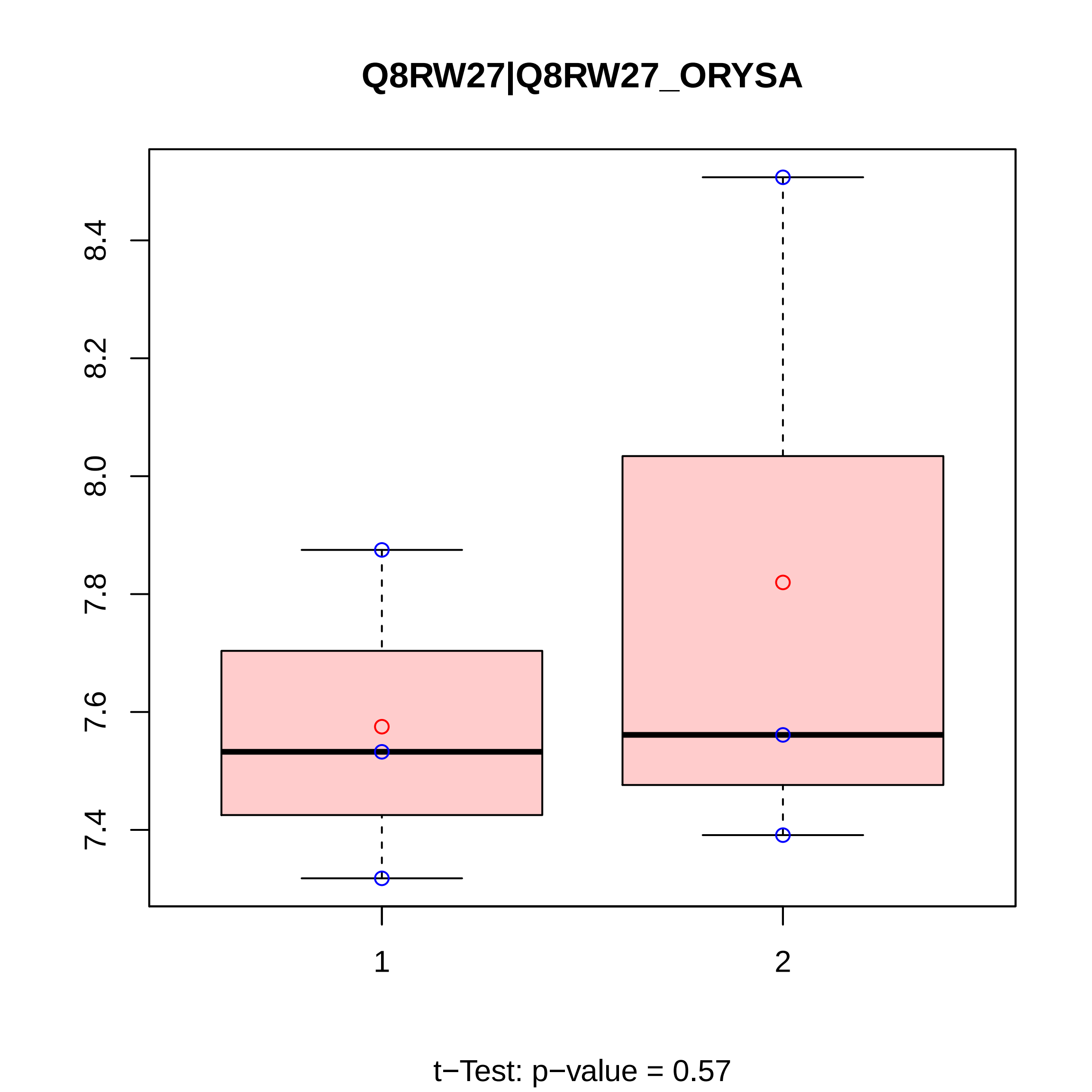

Q8RW27|Q8RW27_ORYSA
8.4
8.2
8.0
7.8
7.6
7.4
1
2
t−Test: p−value = 0.57

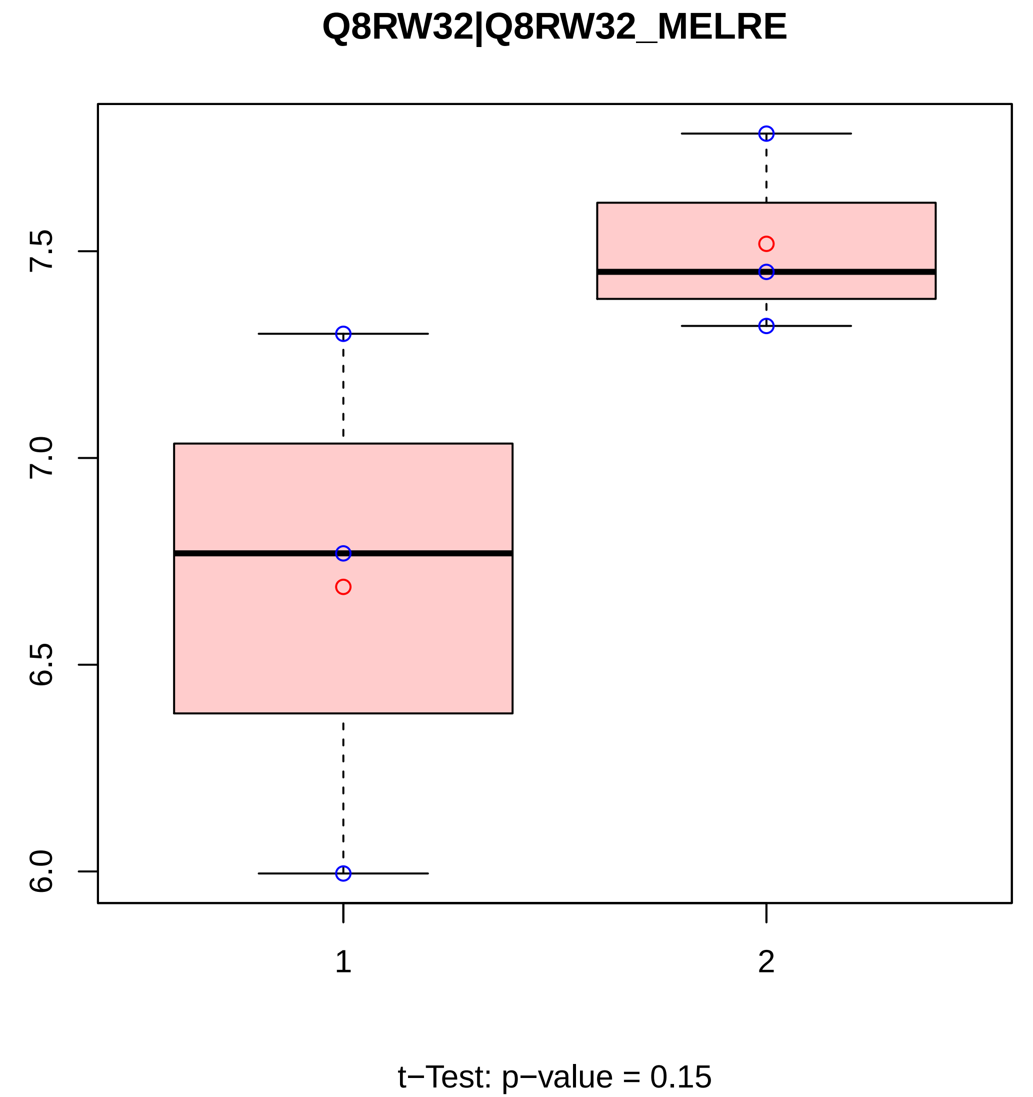
Q8RW32|Q8RW32_MELRE
7.5
7.0
6.5
6.0
1
2
t−Test: p−value = 0.15

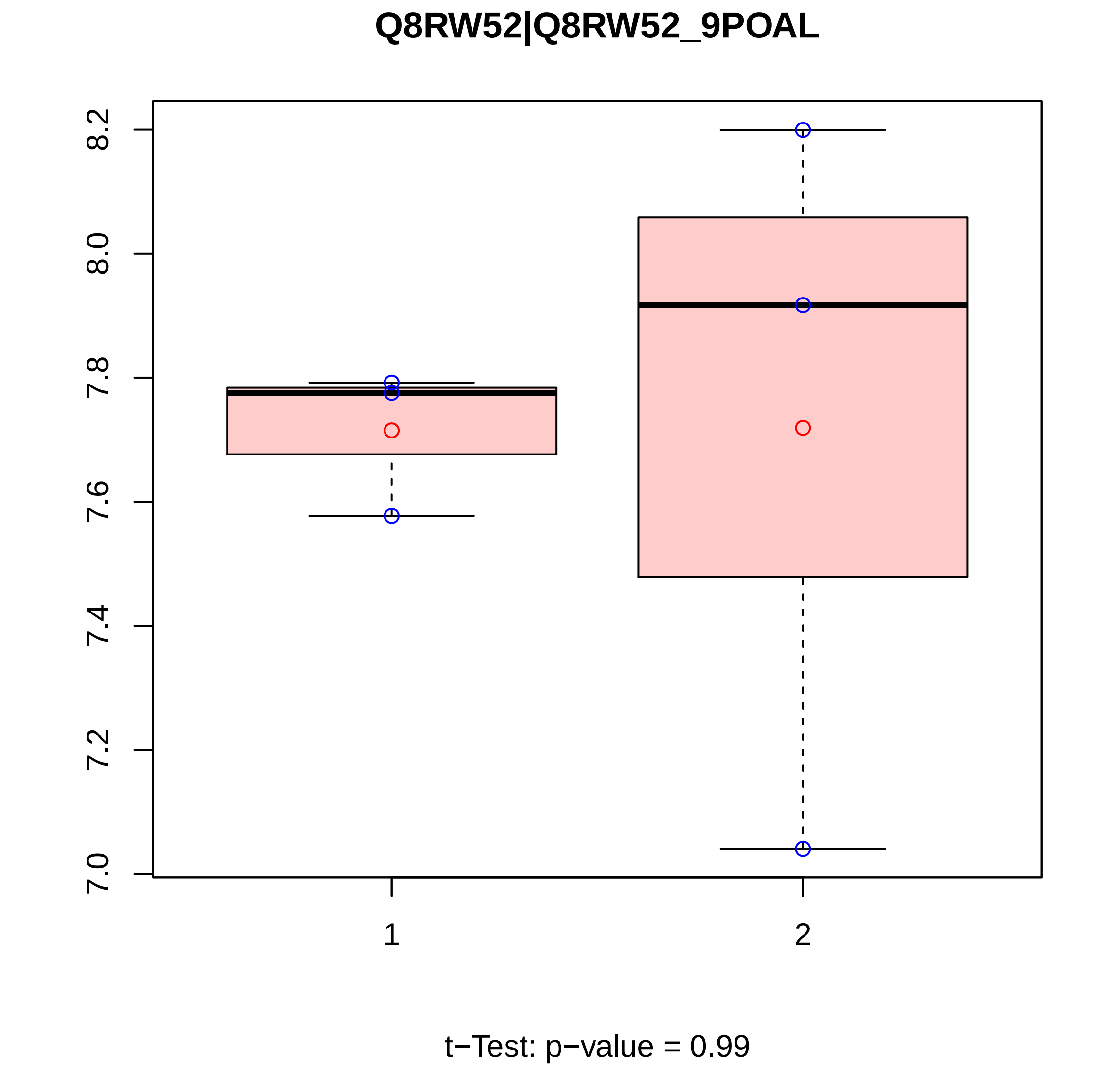
Q8RW52|Q8RW52_9POAL
8.2
8.0
7.8
7.6
7.4
7.2
7.0
1
2
t−Test: p−value = 0.99

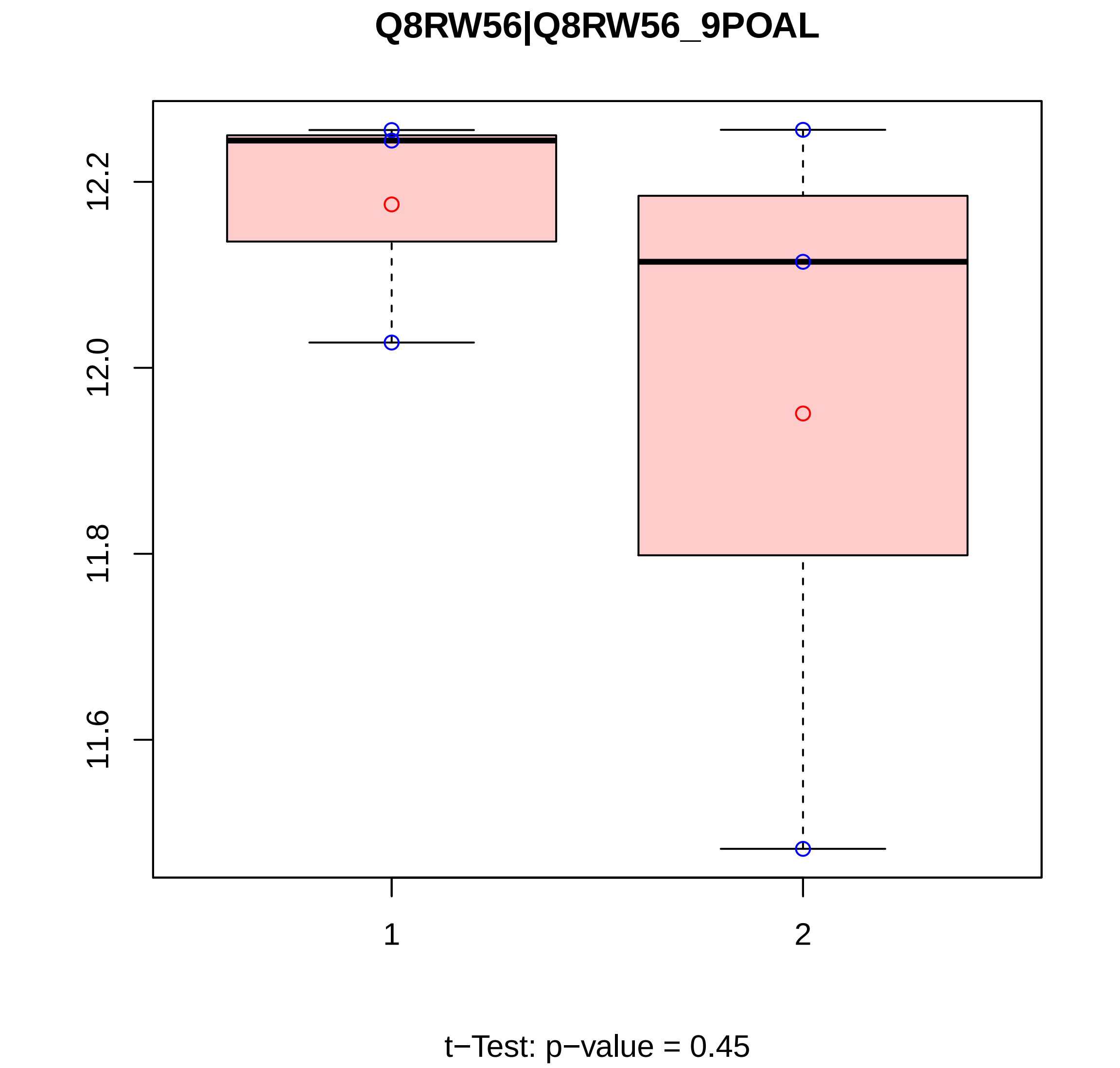

Q8RW56|Q8RW56_9POAL
12.2
12.0
11.8
11.6
1
2
t−Test: p−value = 0.45

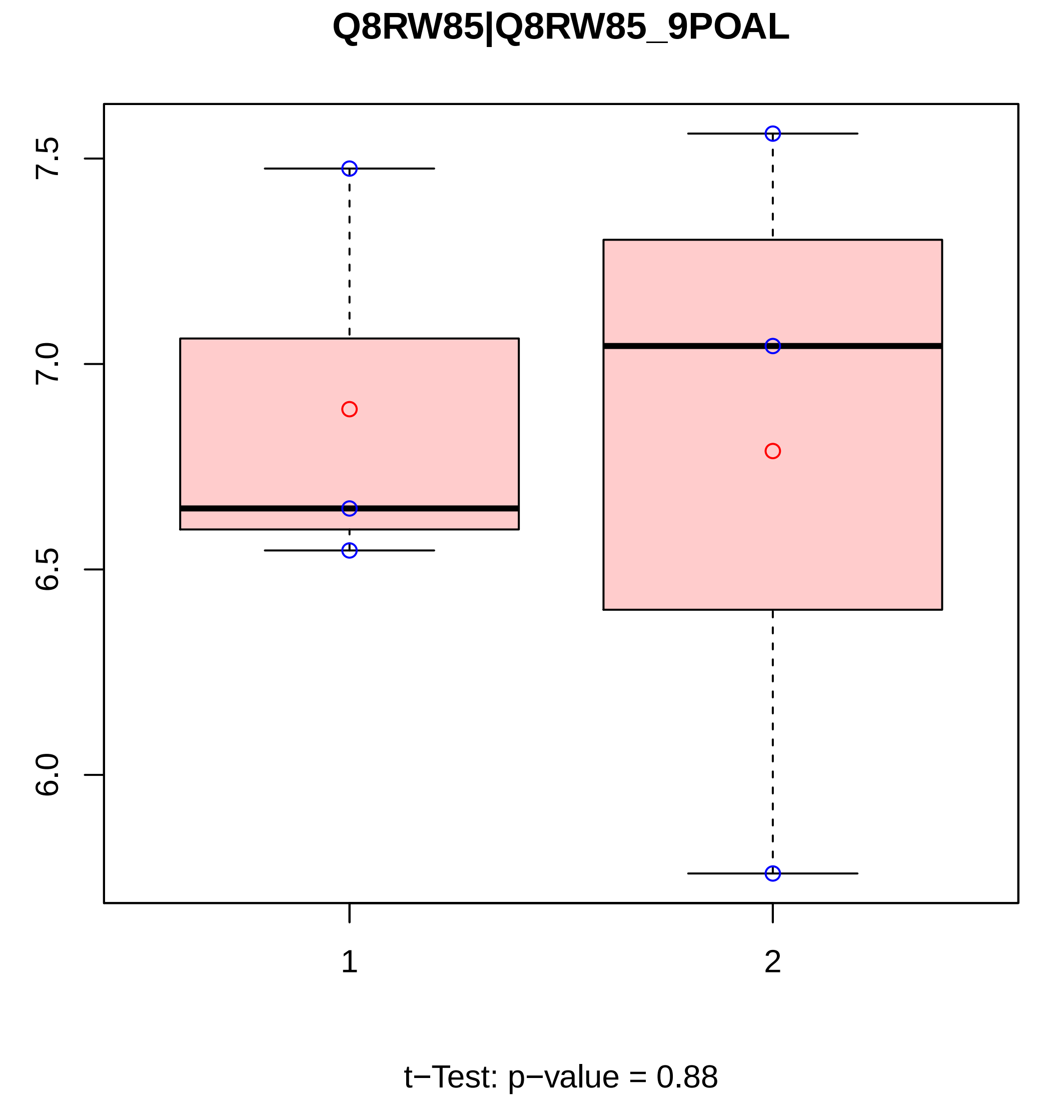
Q8RW85|Q8RW85_9POAL
7.5
7.0
6.5
6.0
1
2
t−Test: p−value = 0.88

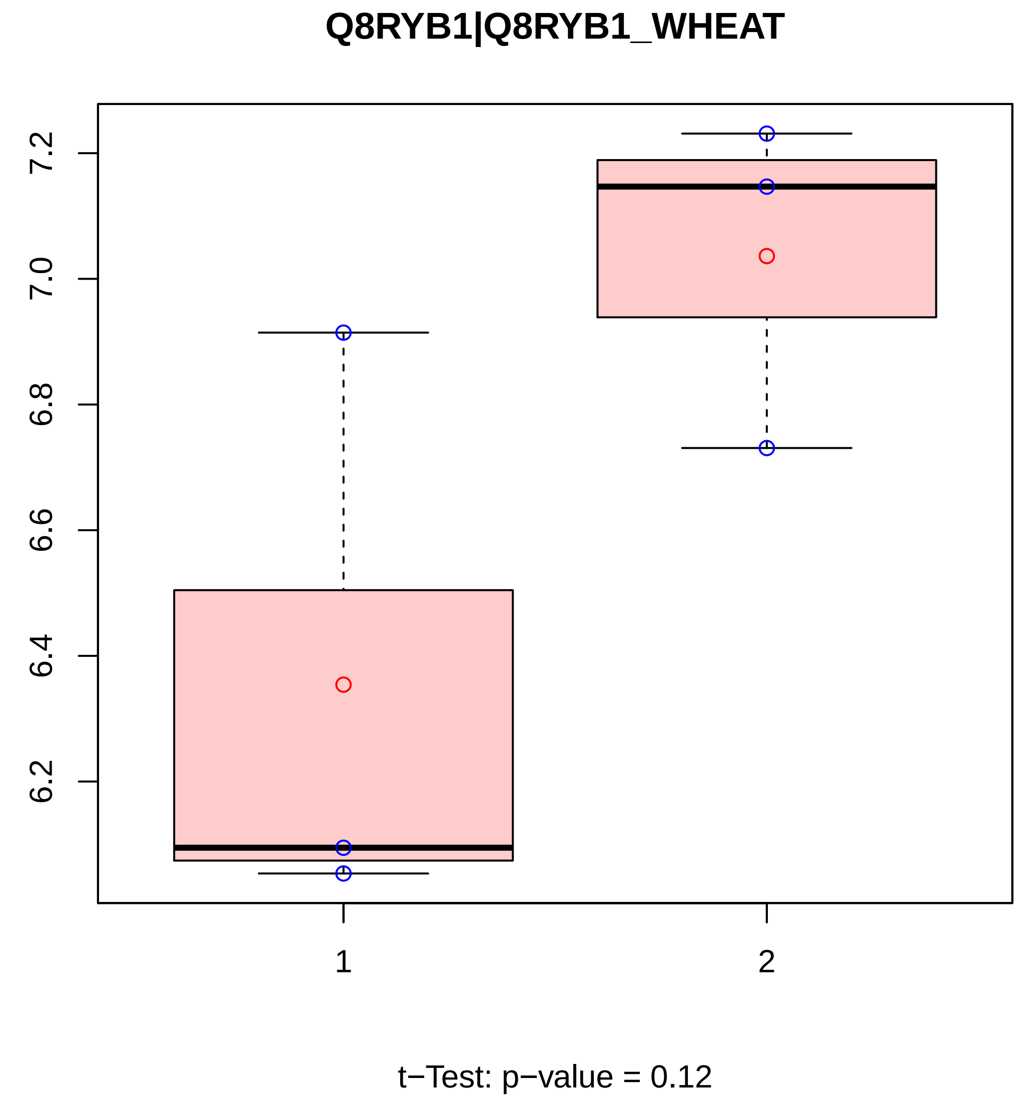
Q8RYB1|Q8RYB1_WHEAT
7.2
7.0
6.8
6.6
6.4
6.2
1
2
t−Test: p−value = 0.12

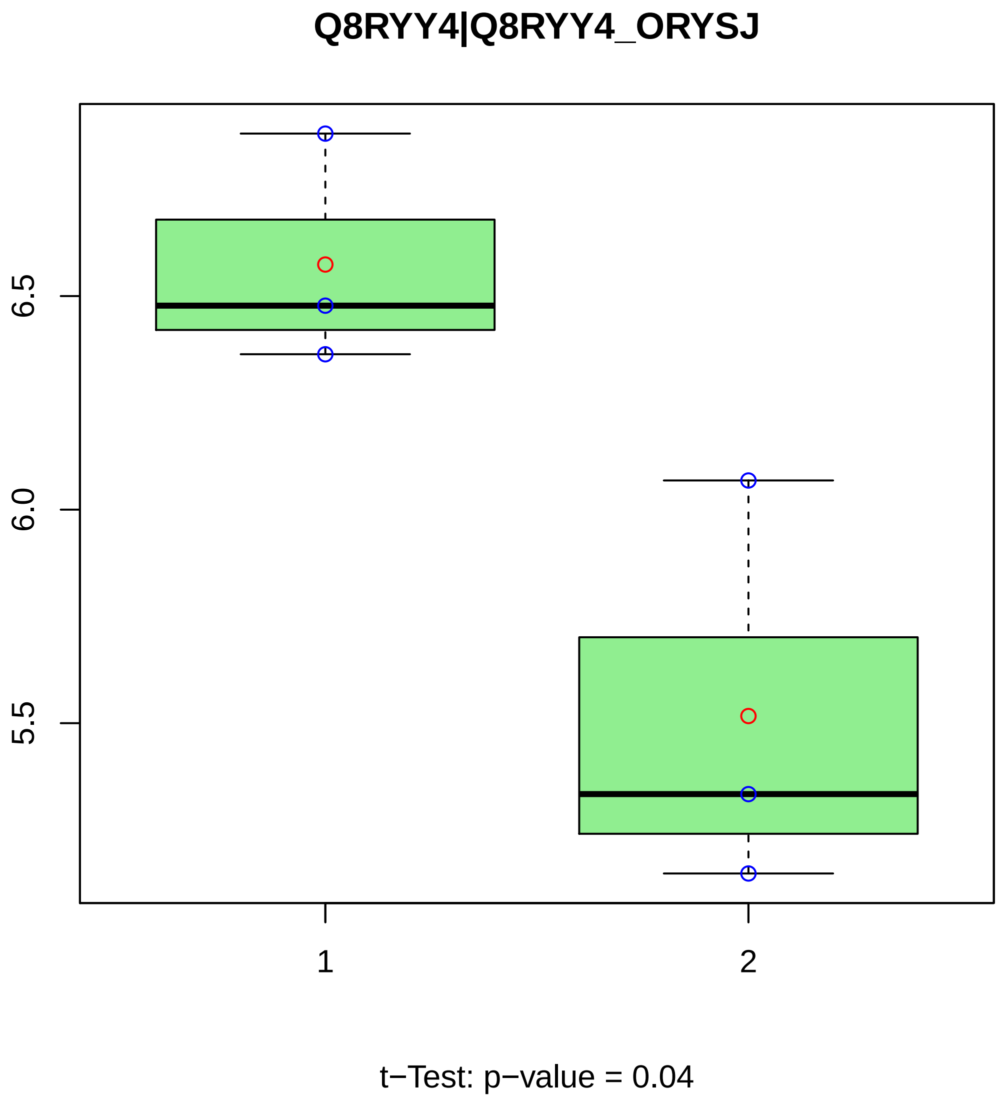
Q8RYY4|Q8RYY4_ORYSJ
6.5
6.0
5.5
1
2
t−Test: p−value = 0.04

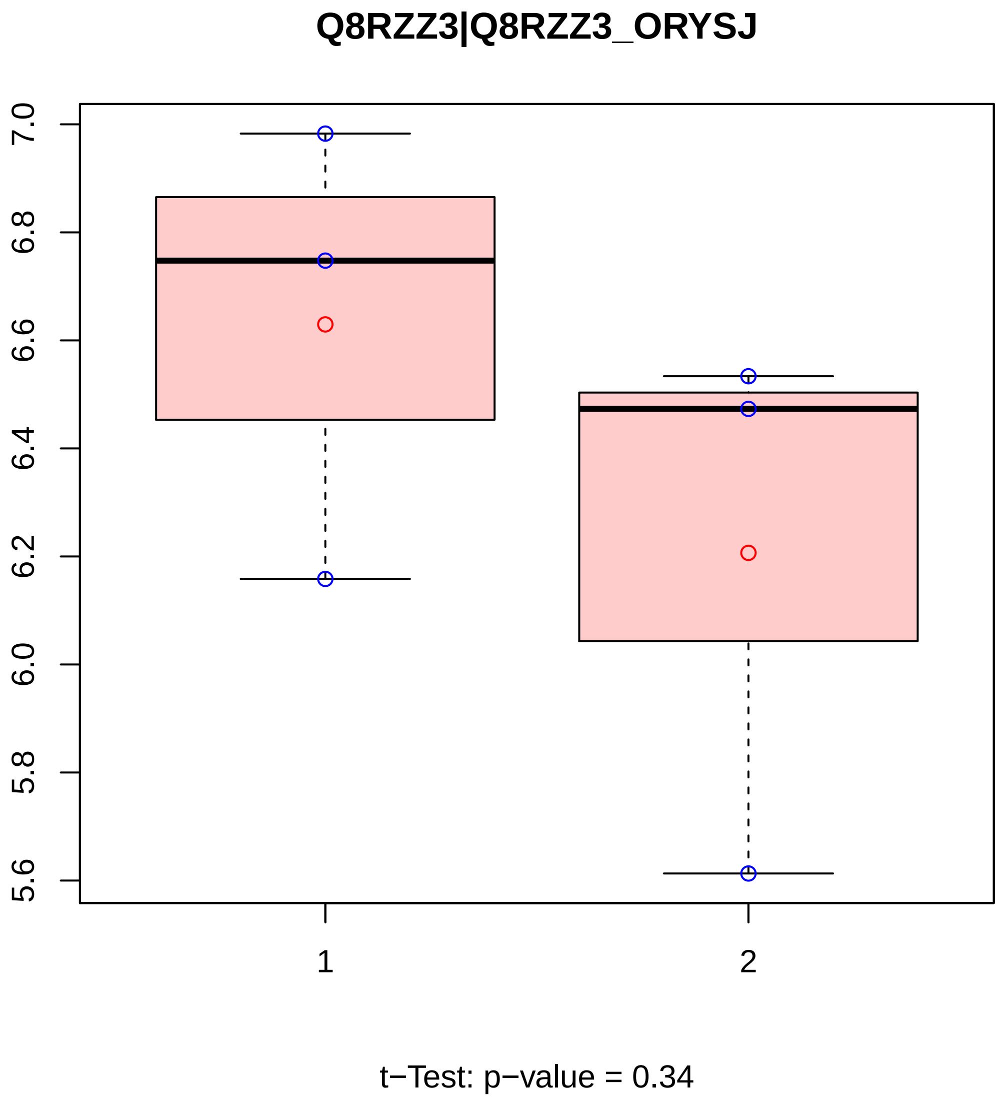
Q8RZZ3|Q8RZZ3_ORYSJ
7.0
6.8
6.6
6.4
6.2
6.0
5.8
5.6
1
2
t−Test: p−value = 0.34

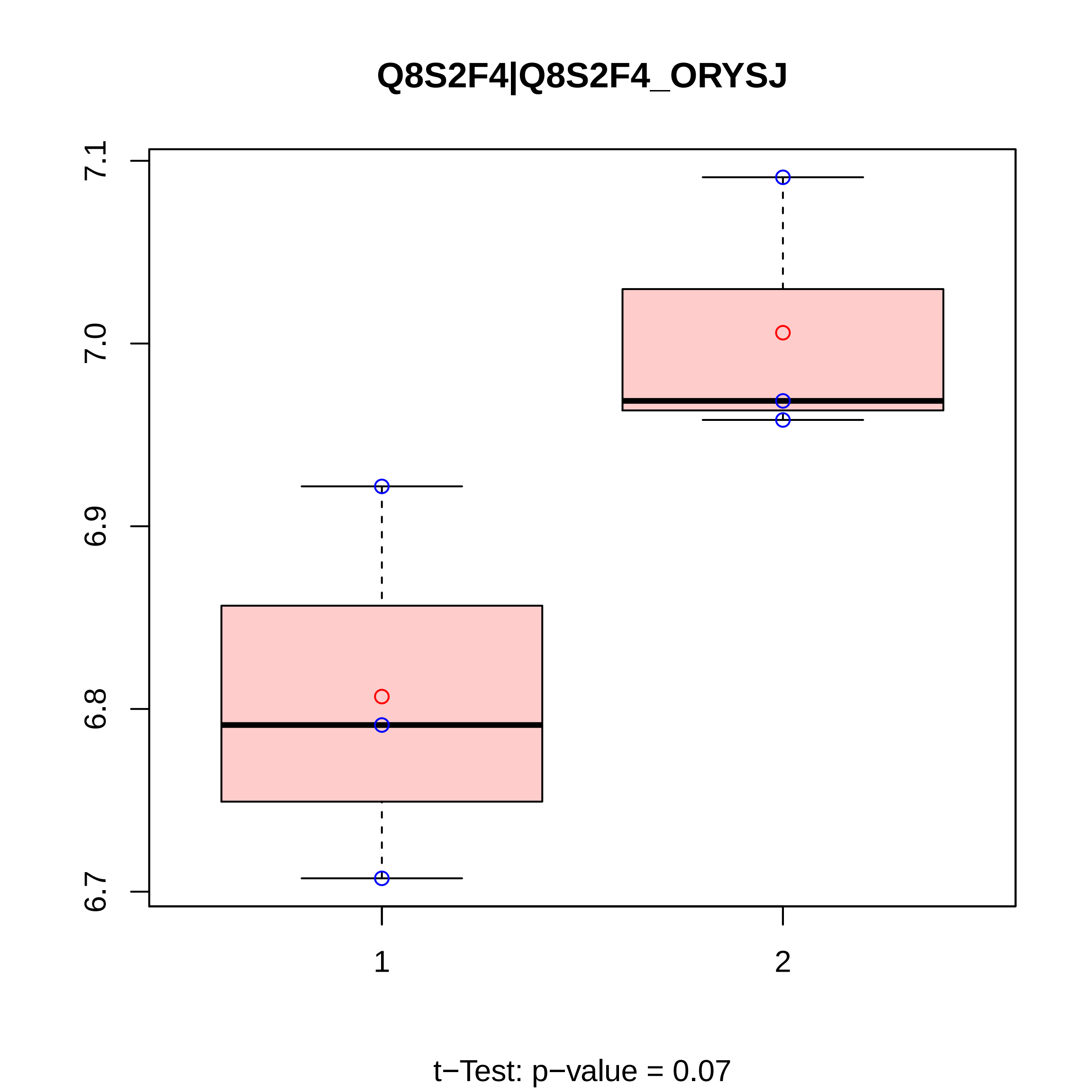
Q8S2F4|Q8S2F4_ORYSJ
7.1
7.0
6.9
6.8
6.7
1
2
t−Test: p−value = 0.07

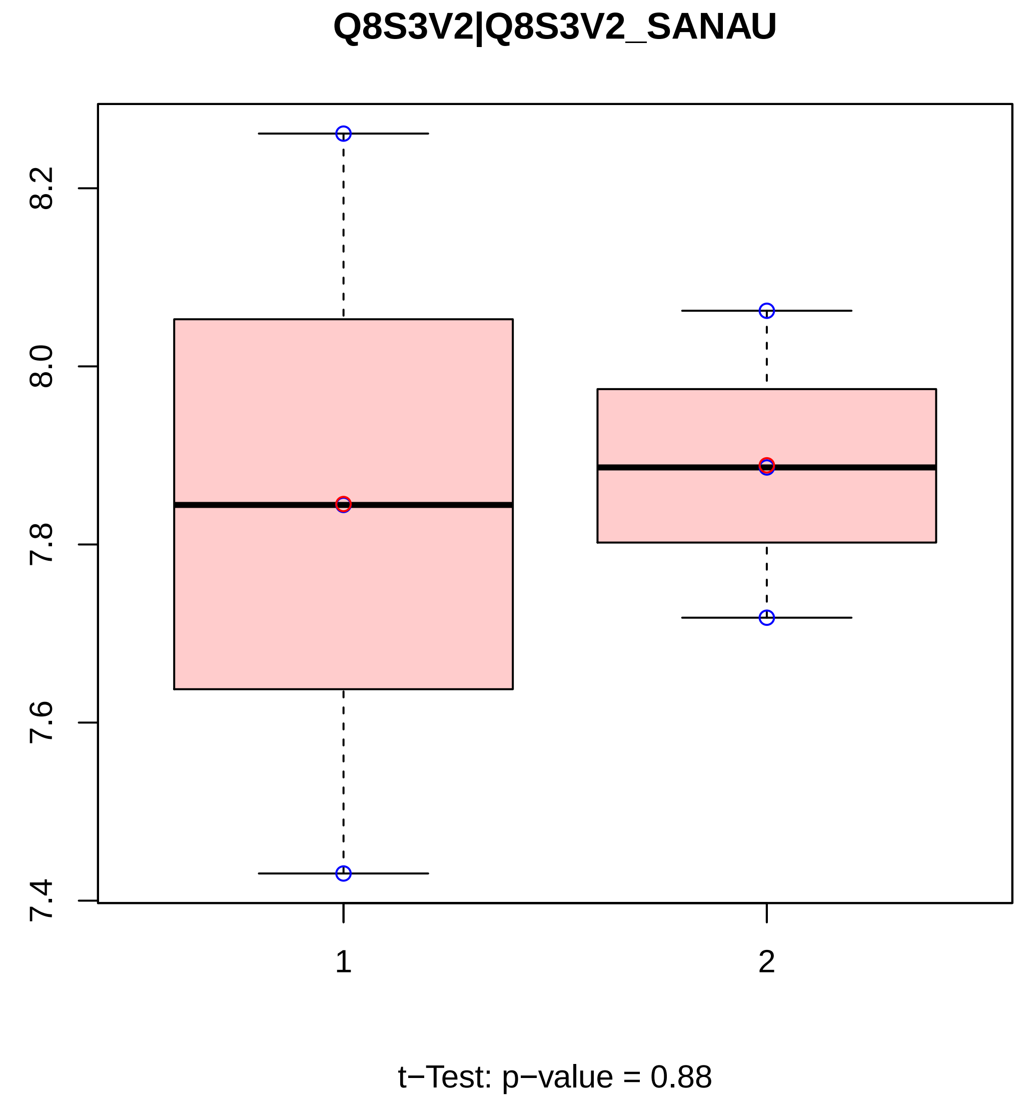

Q8S3V2|Q8S3V2_SANAU
8.2
8.0
7.8
7.6
7.4
1
2
t−Test: p−value = 0.88

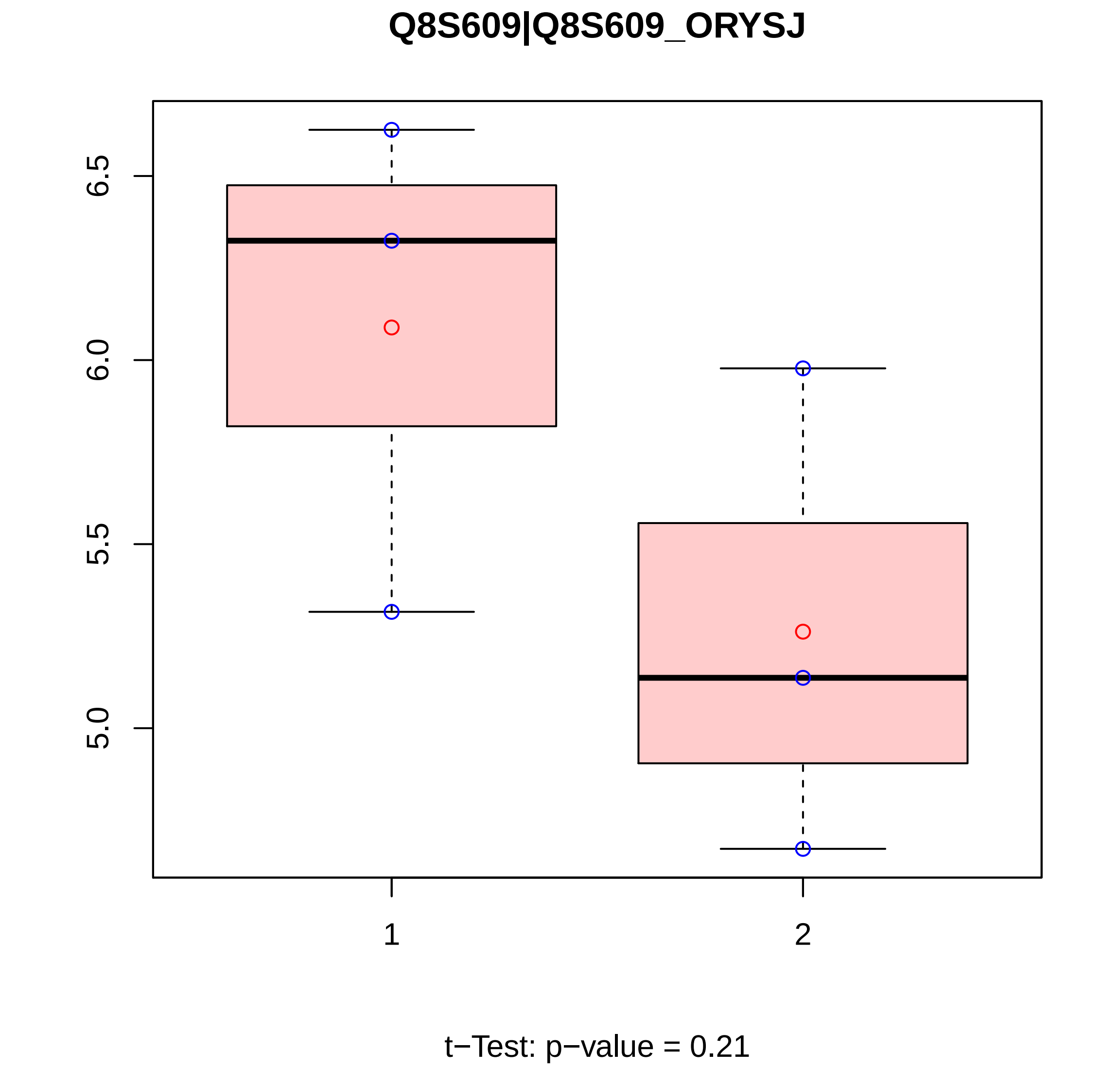
Q8S609|Q8S609_ORYSJ
6.5
6.0
5.5
5.0
1
2
t−Test: p−value = 0.21

Q8S6P5|Q8S6P5_ORYSJ

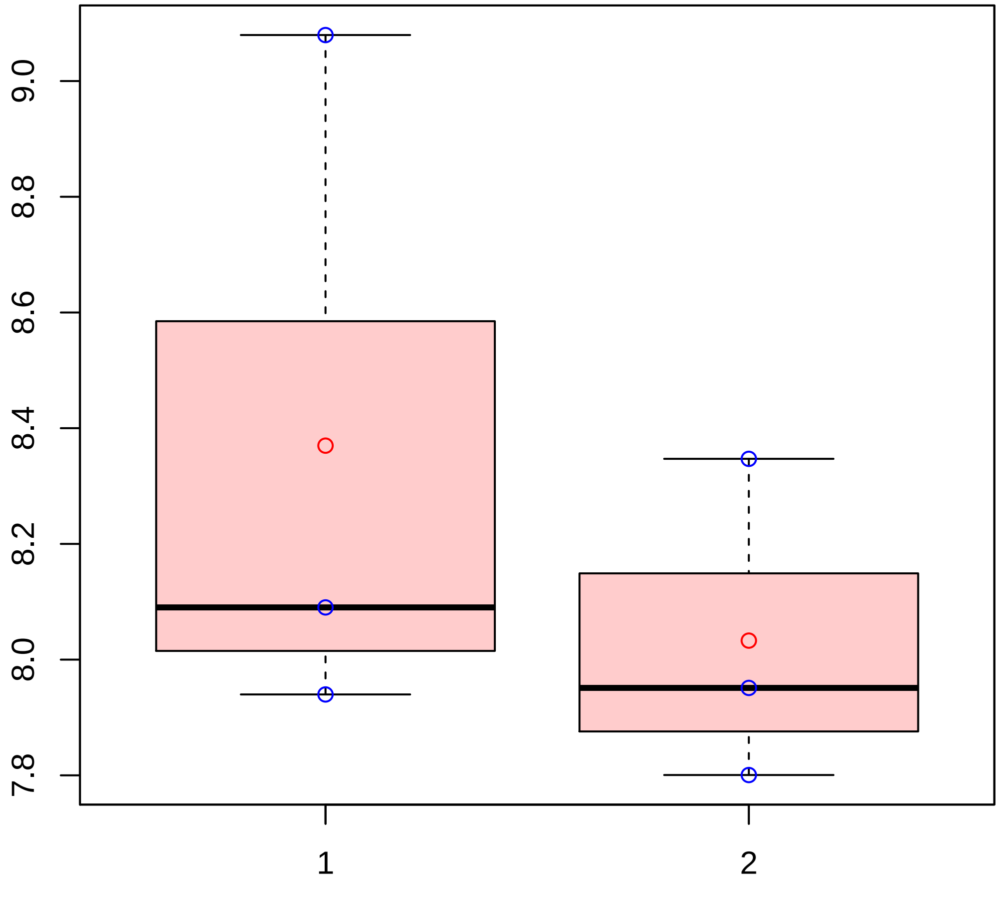

t−Test: p−value = 0.46

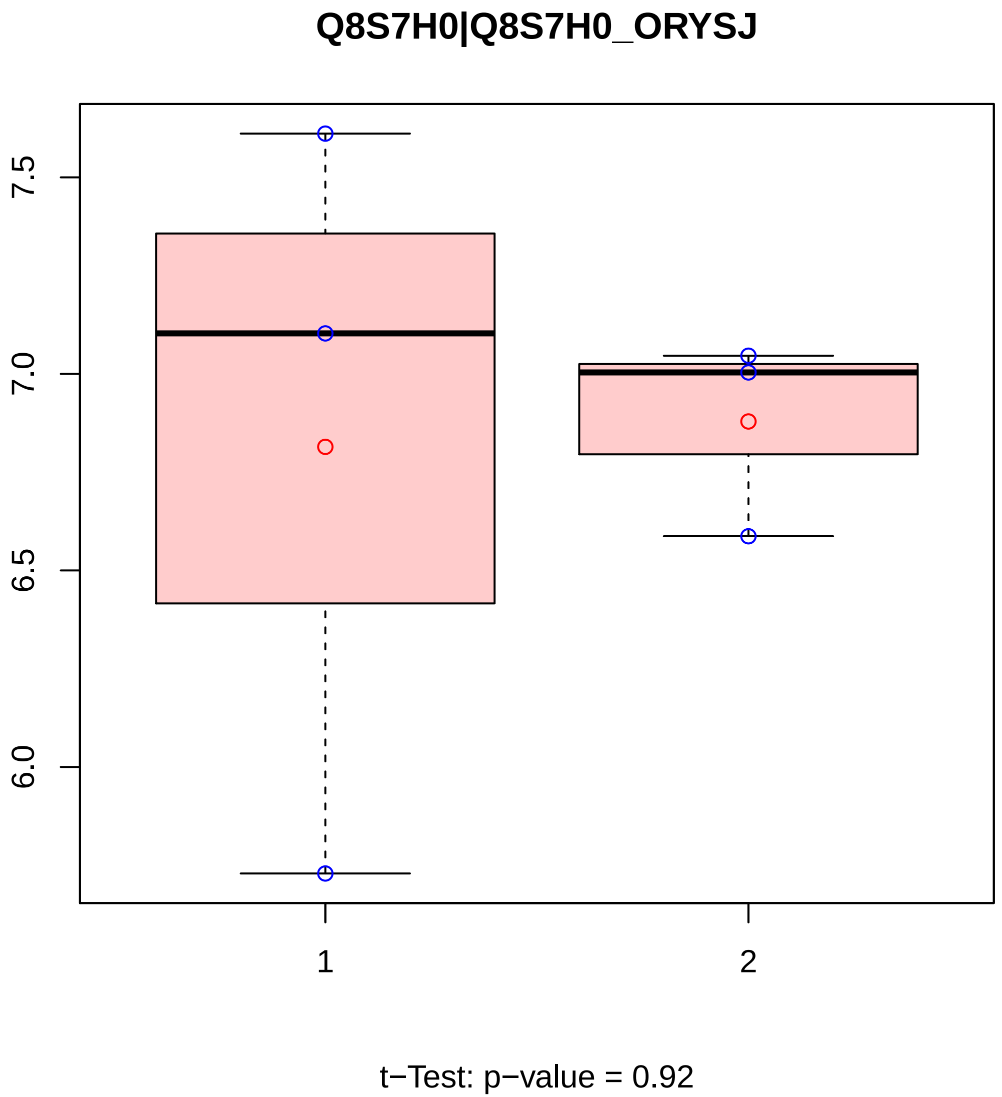
Q8S7H0|Q8S7H0_ORYSJ
7.5
7.0
6.5
6.0
1
2
t−Test: p−value = 0.92

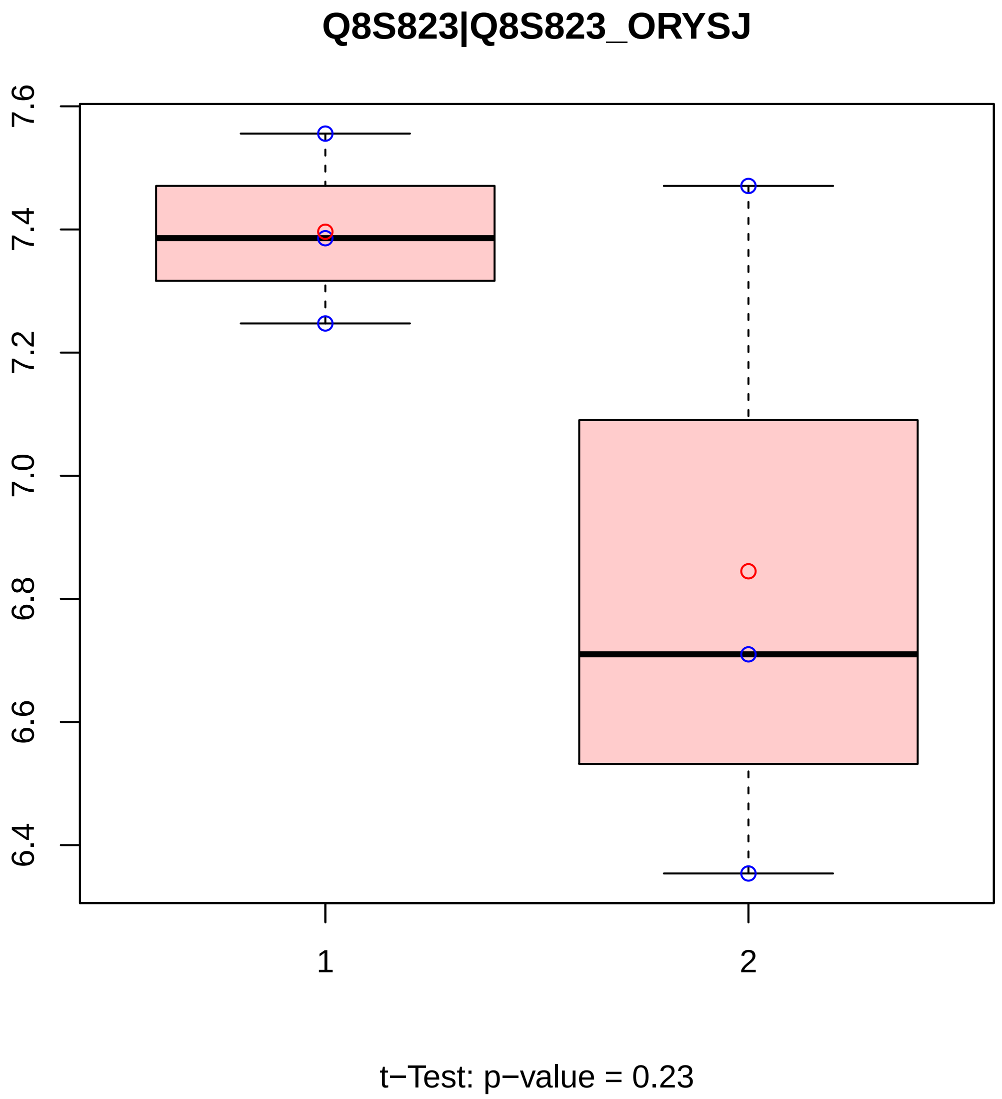
Q8S823|Q8S823_ORYSJ
7.6
7.4
7.2
7.0
6.8
6.6
6.4
1
2
t−Test: p−value = 0.23

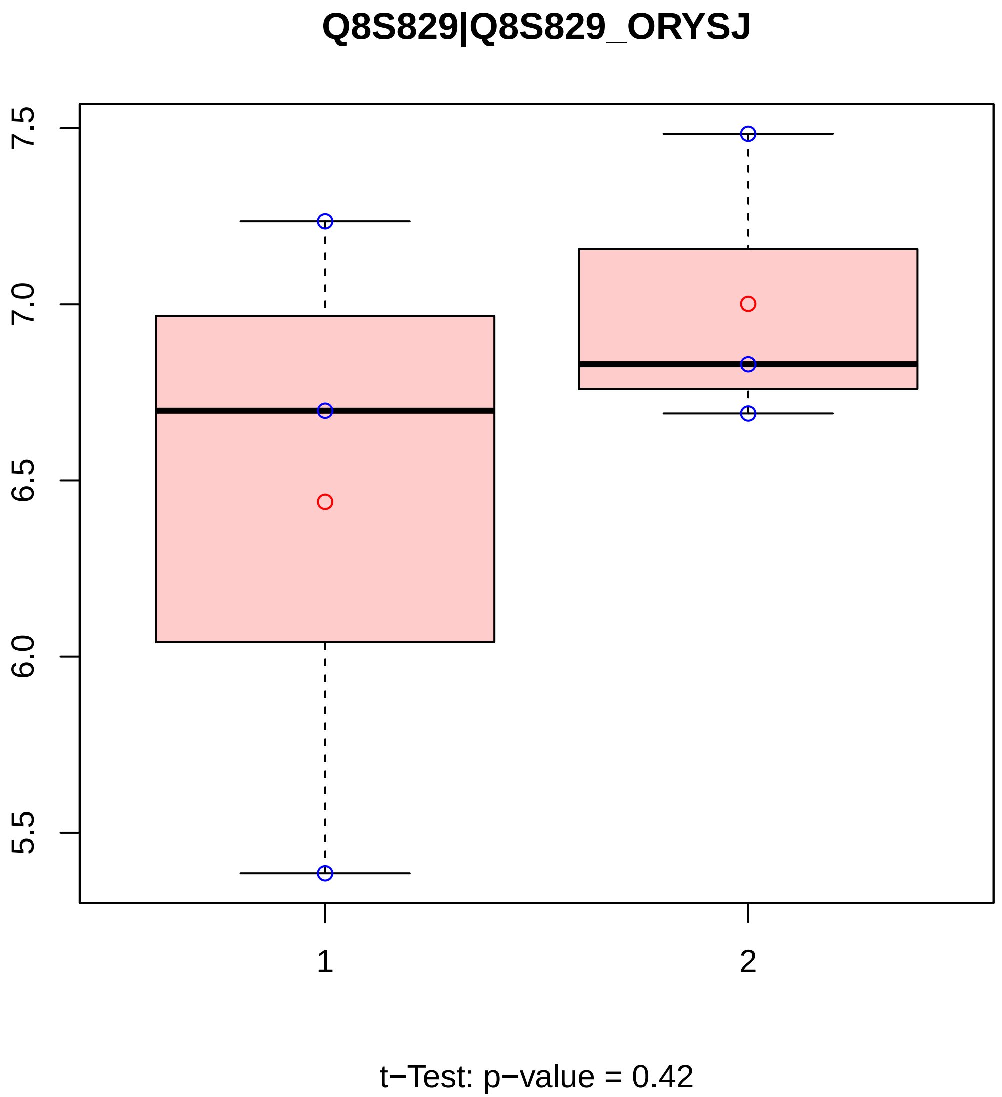
Q8S829|Q8S829_ORYSJ
7.5
7.0
6.5
6.0
5.5
1
2
t−Test: p−value = 0.42

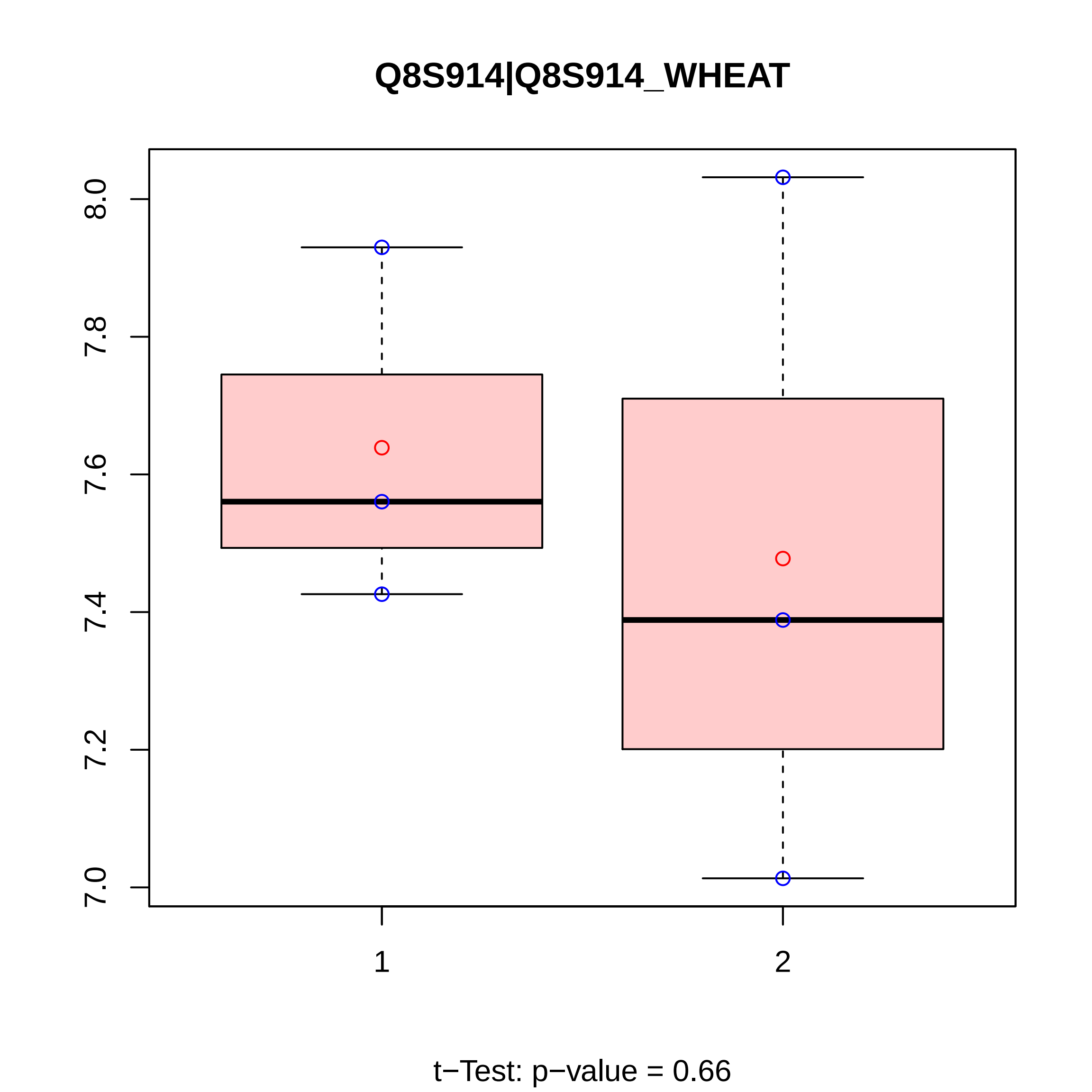

Q8S914|Q8S914_WHEAT
8.0
7.8
7.6
7.4
7.2
7.0
1
2
t−Test: p−value = 0.66

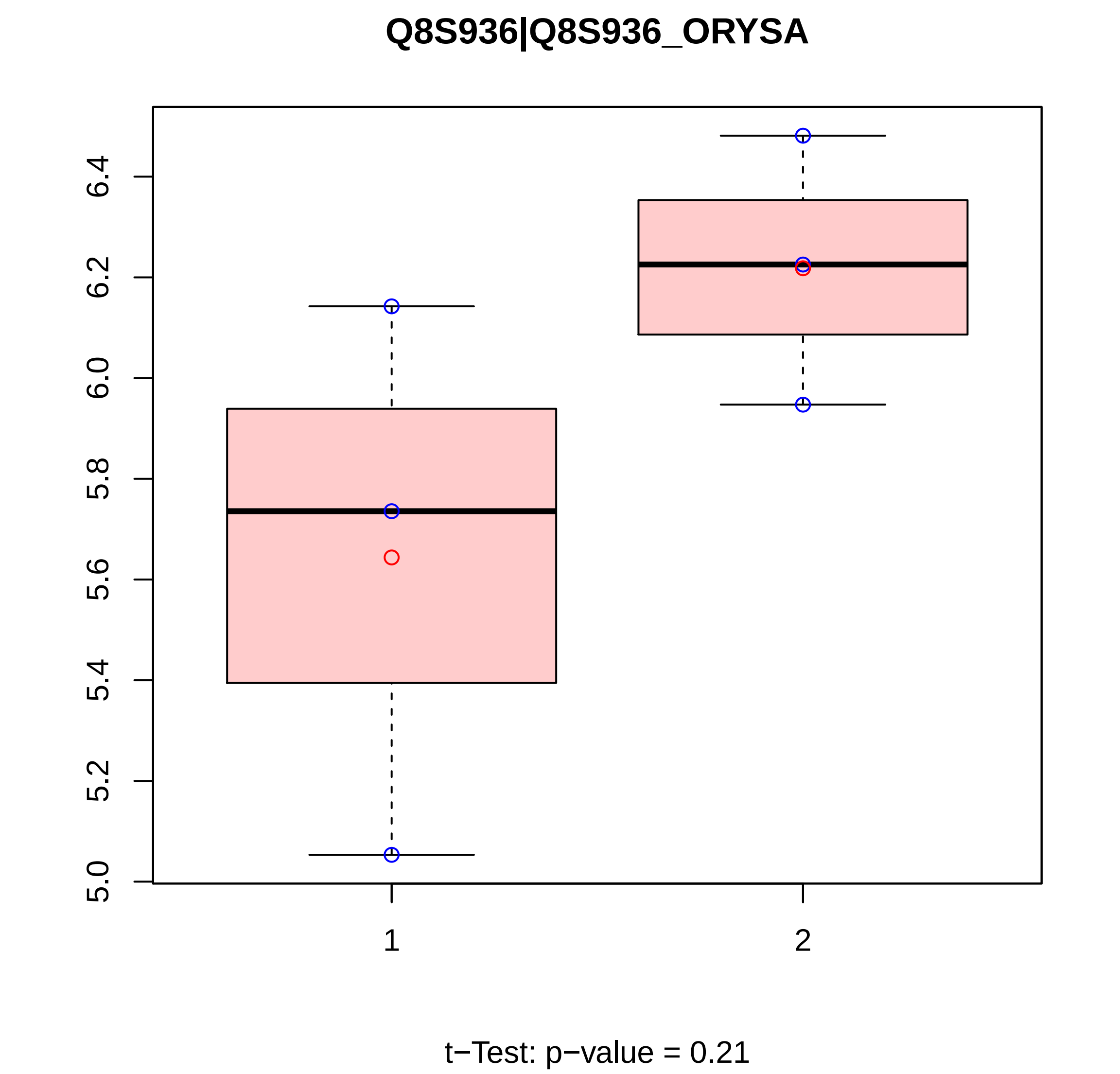

Q8S936|Q8S936_ORYSA
6.4
6.2
6.0
5.8
5.6
5.4
5.2
5.0
1
2
t−Test: p−value = 0.21

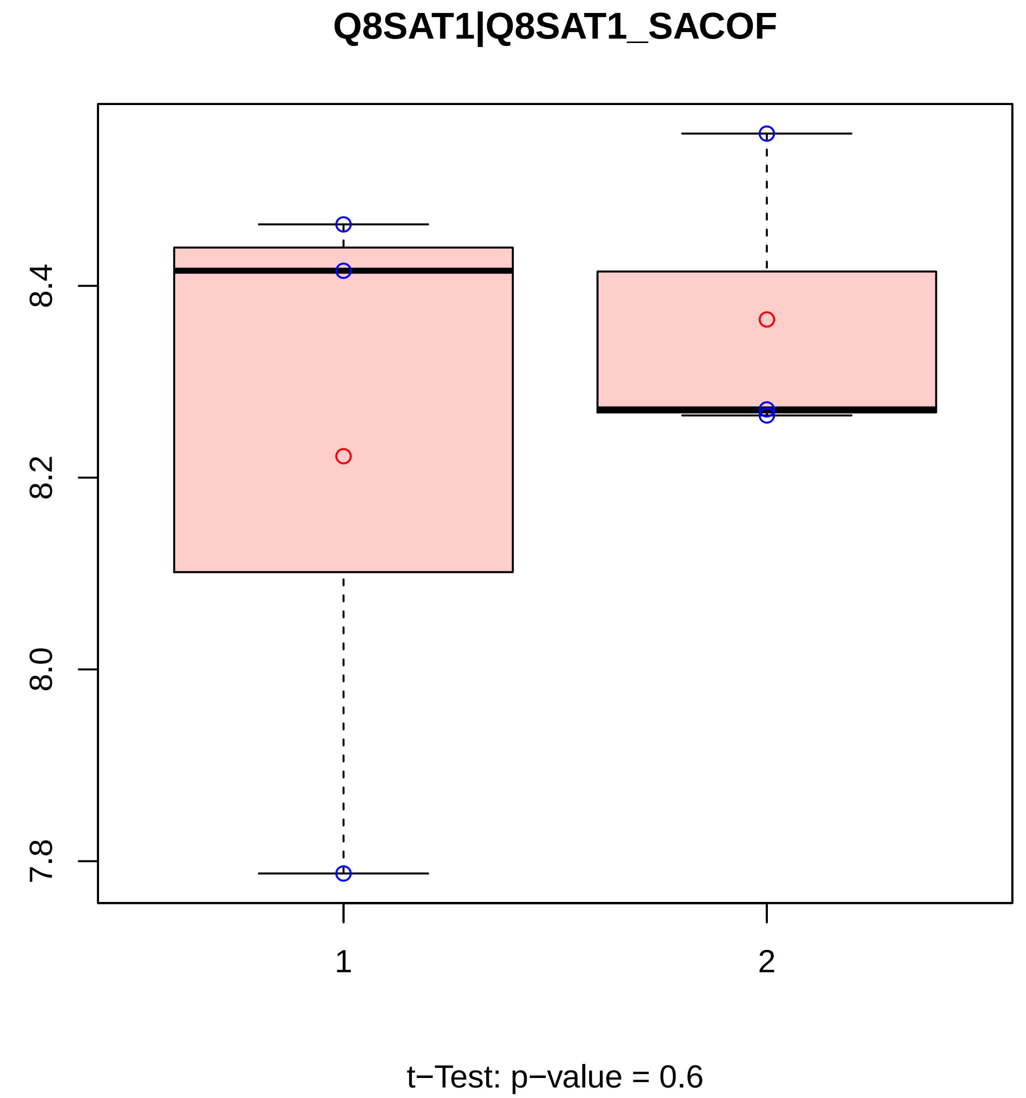
Q8SAT1|Q8SAT1_SACOF
8.4
8.2
8.0
7.8
1
2
t−Test: p−value = 0.6

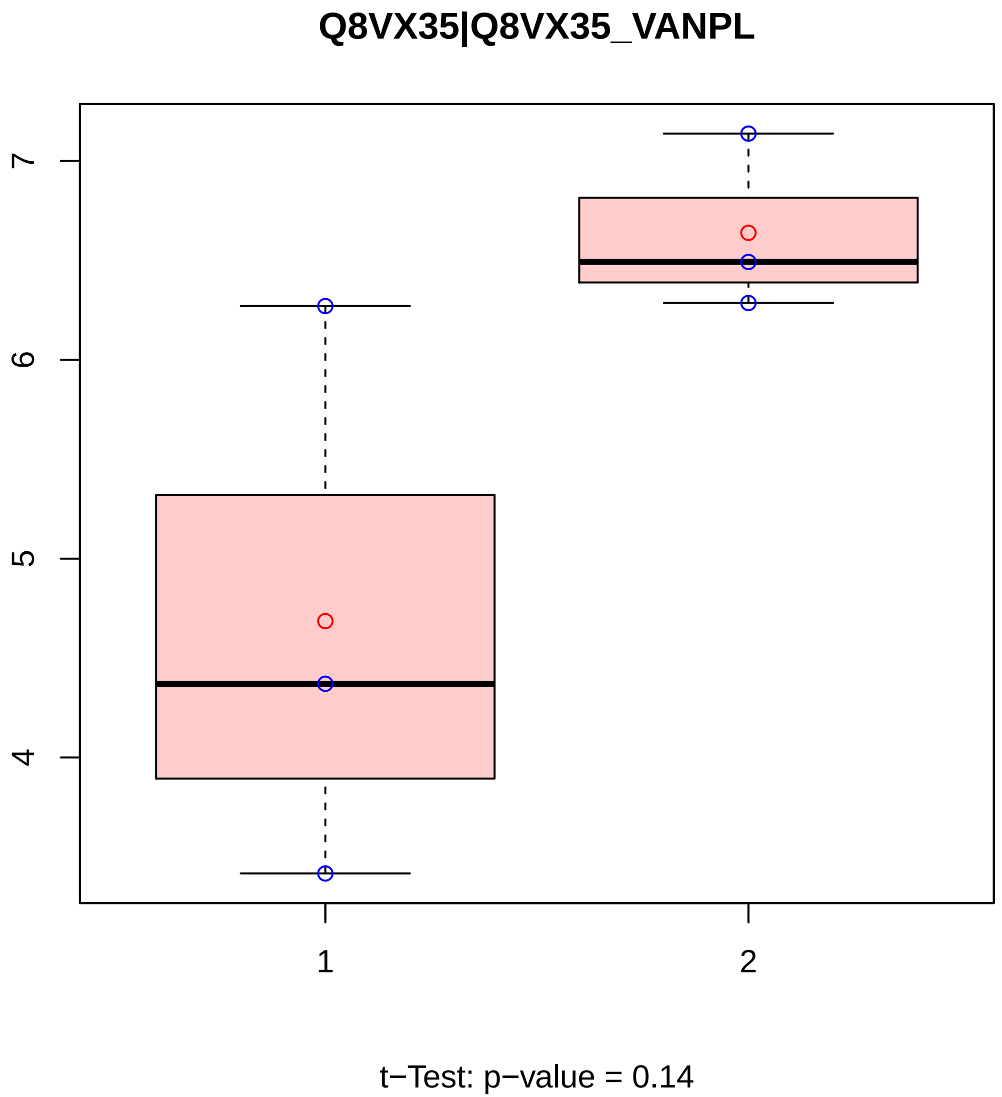
Q8VX35|Q8VX35_VANPL
4
5
6
7
1
2
t−Test: p−value = 0.14

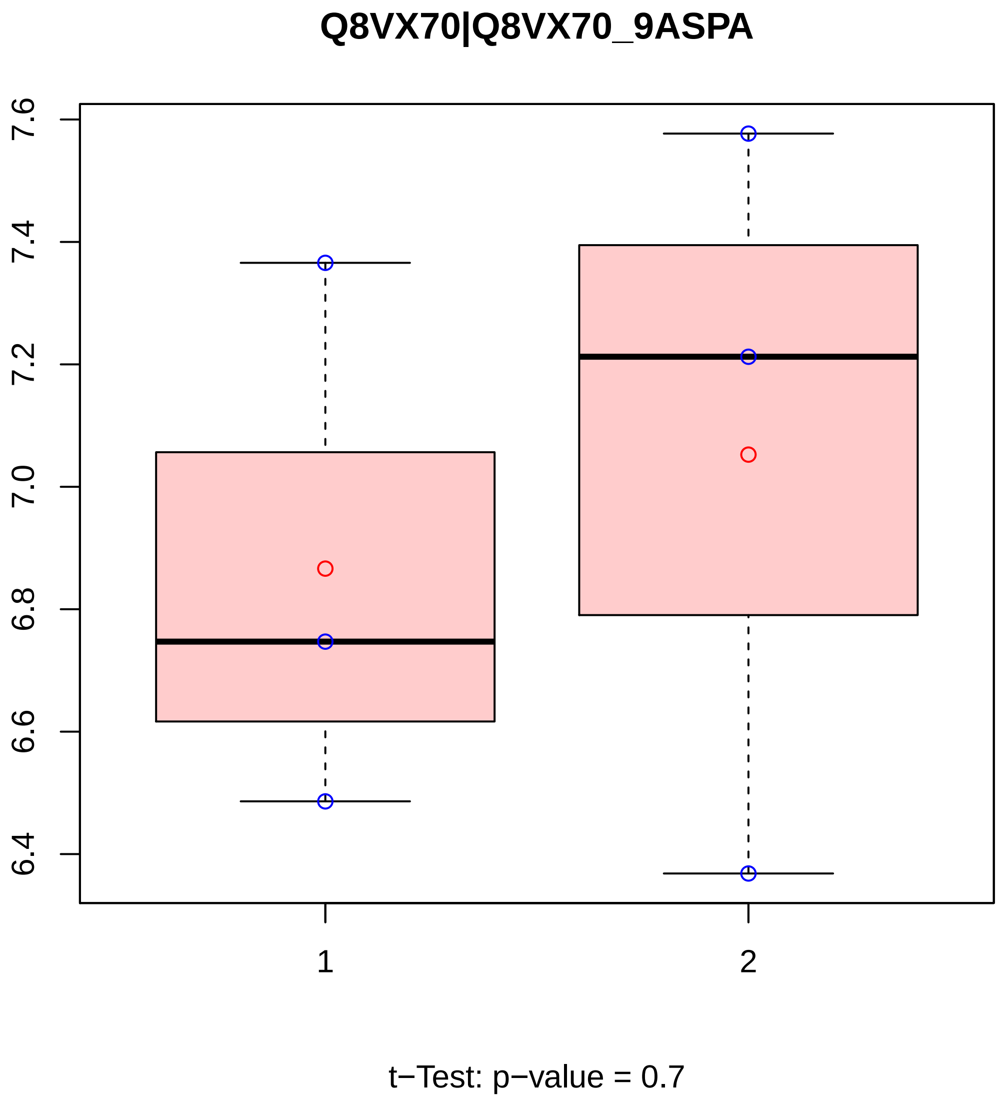

Q8VX70|Q8VX70_9ASPA
7.6
7.4
7.2
7.0
6.8
6.6
6.4
1
2
t−Test: p−value = 0.7

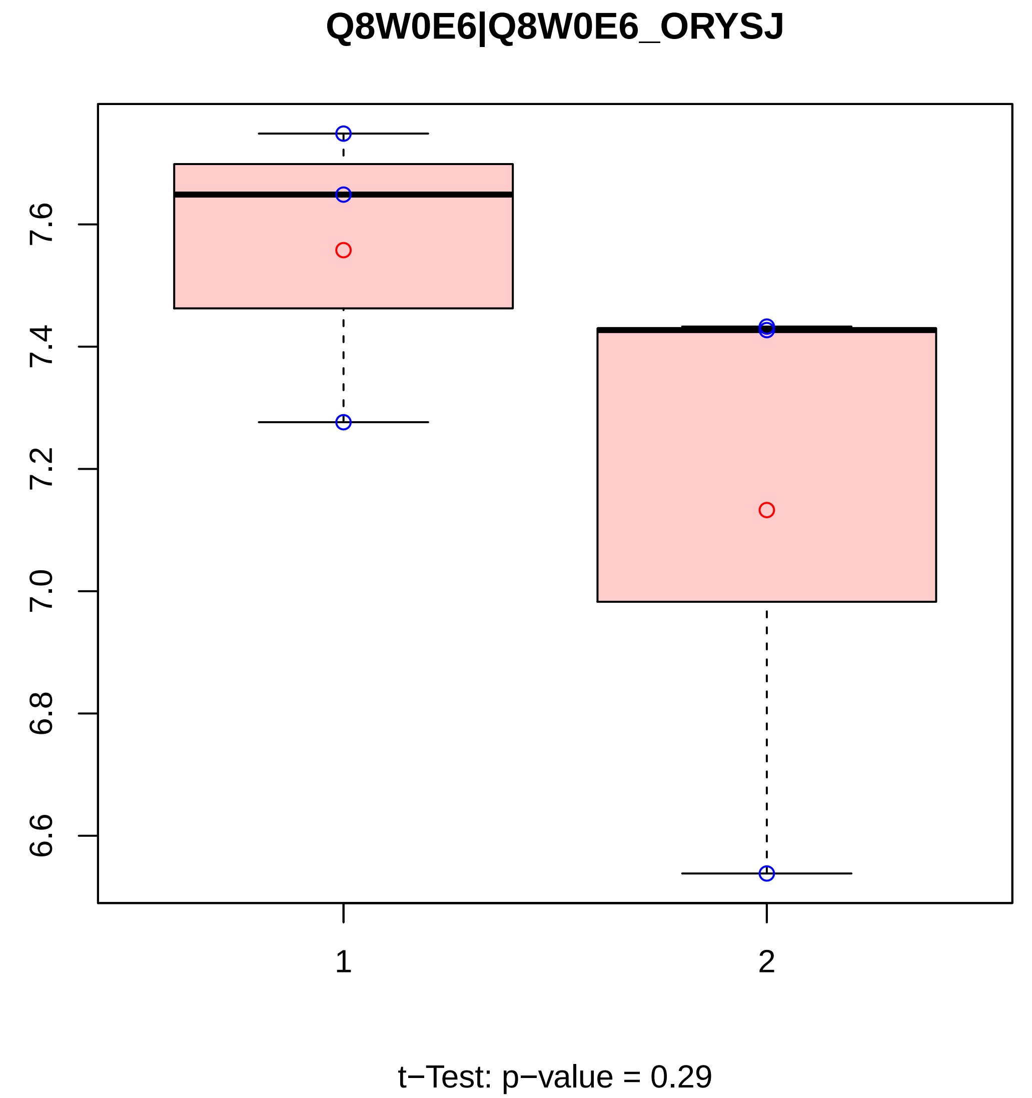
Q8W0E6|Q8W0E6_ORYSJ
6.6
6.8
7.0
7.2
7.4
7.6
1
2
t−Test: p−value = 0.29

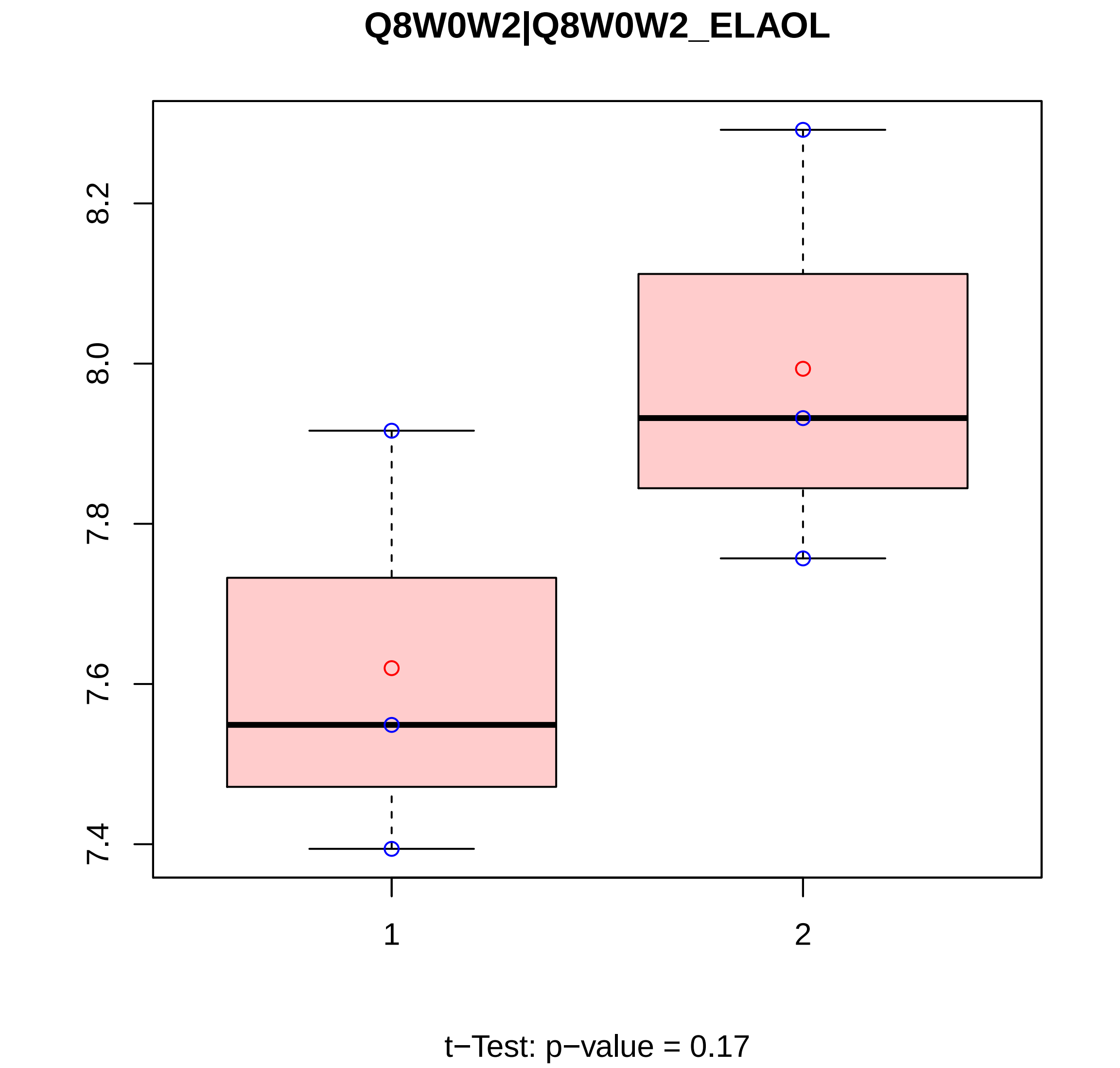
Q8W0W2|Q8W0W2_ELAOL
8.2
8.0
7.8
7.6
7.4
1
2
t−Test: p−value = 0.17

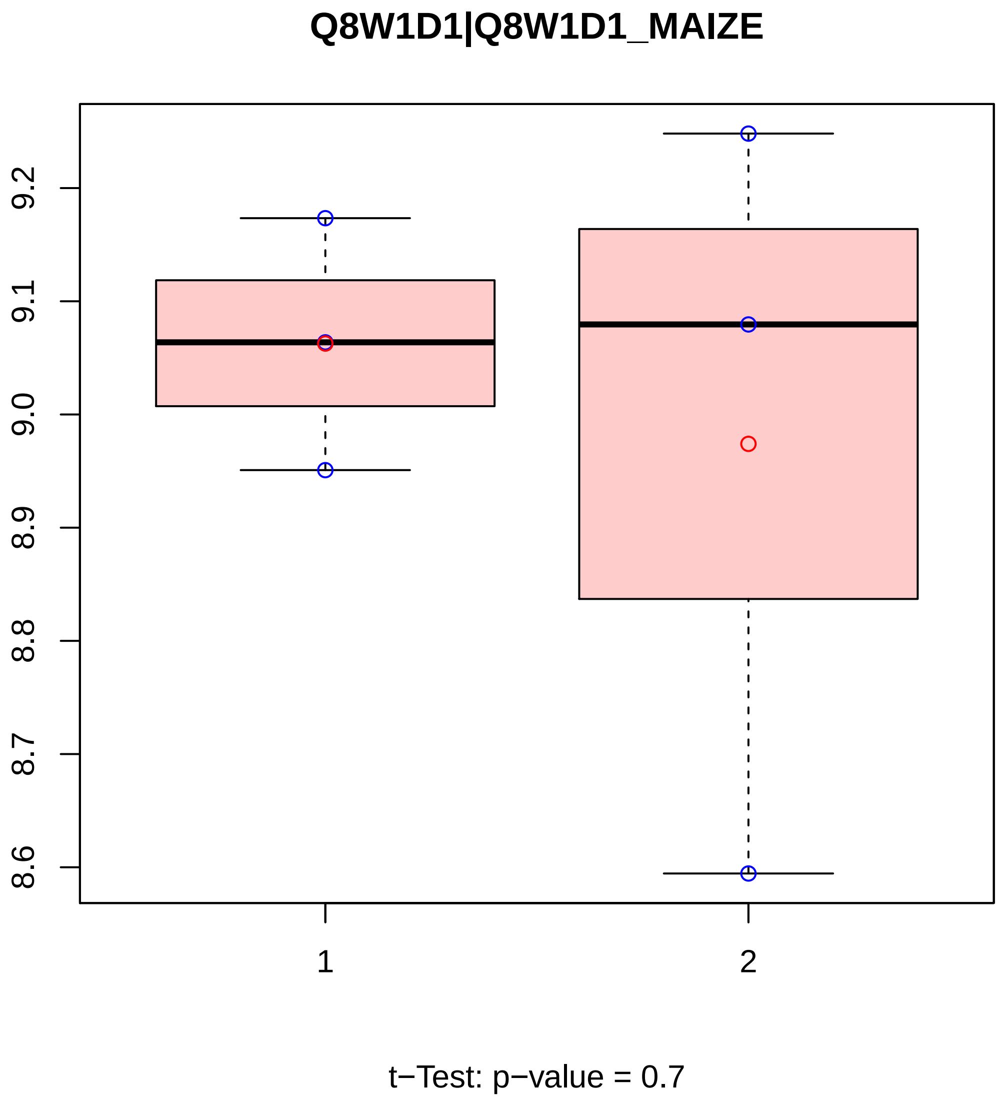

Q8W1D1|Q8W1D1_MAIZE
9.2
9.1
9.0
8.9
8.8
8.7
8.6
1
2
t−Test: p−value = 0.7

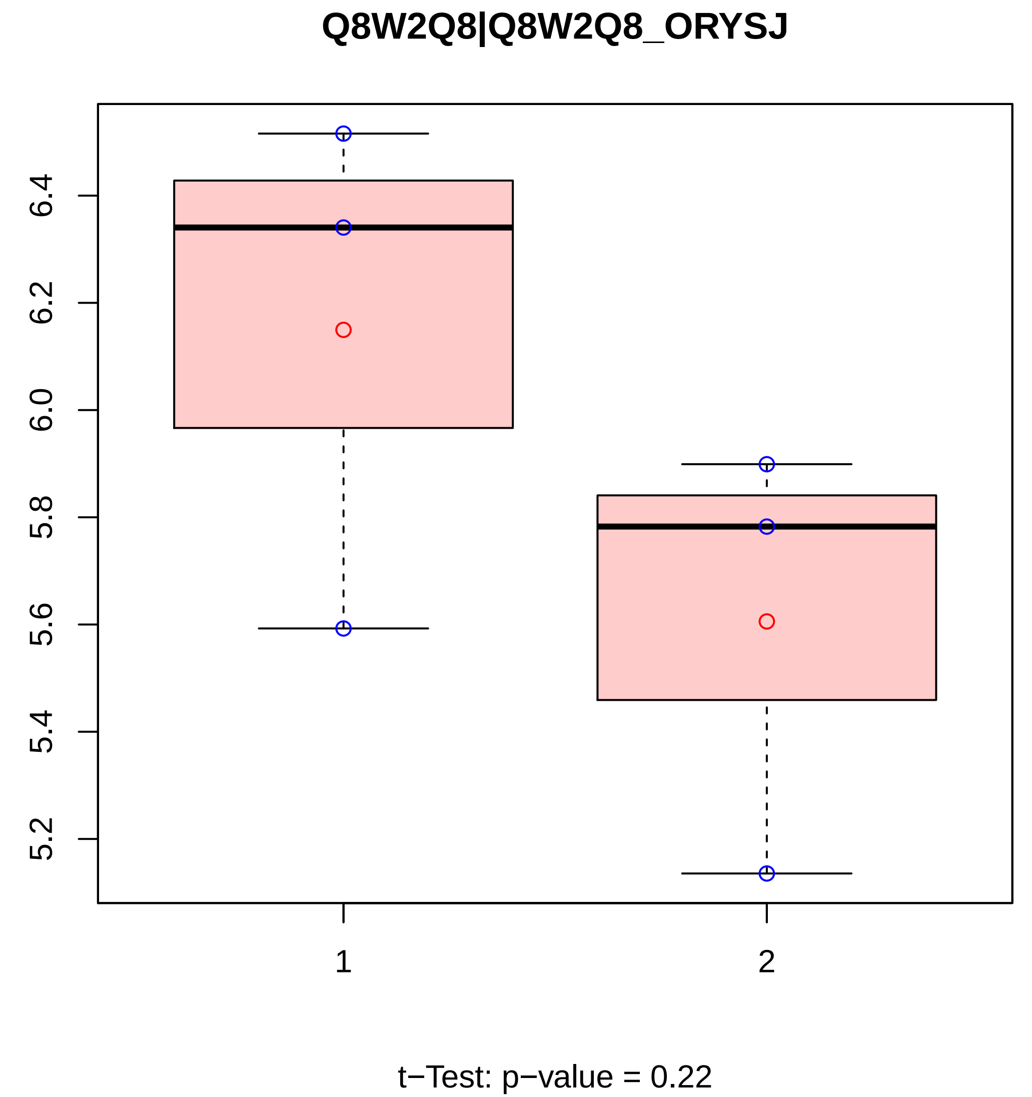
Q8W2Q8|Q8W2Q8_ORYSJ
6.4
6.2
6.0
5.8
5.6
5.4
5.2
1
2
t−Test: p−value = 0.22

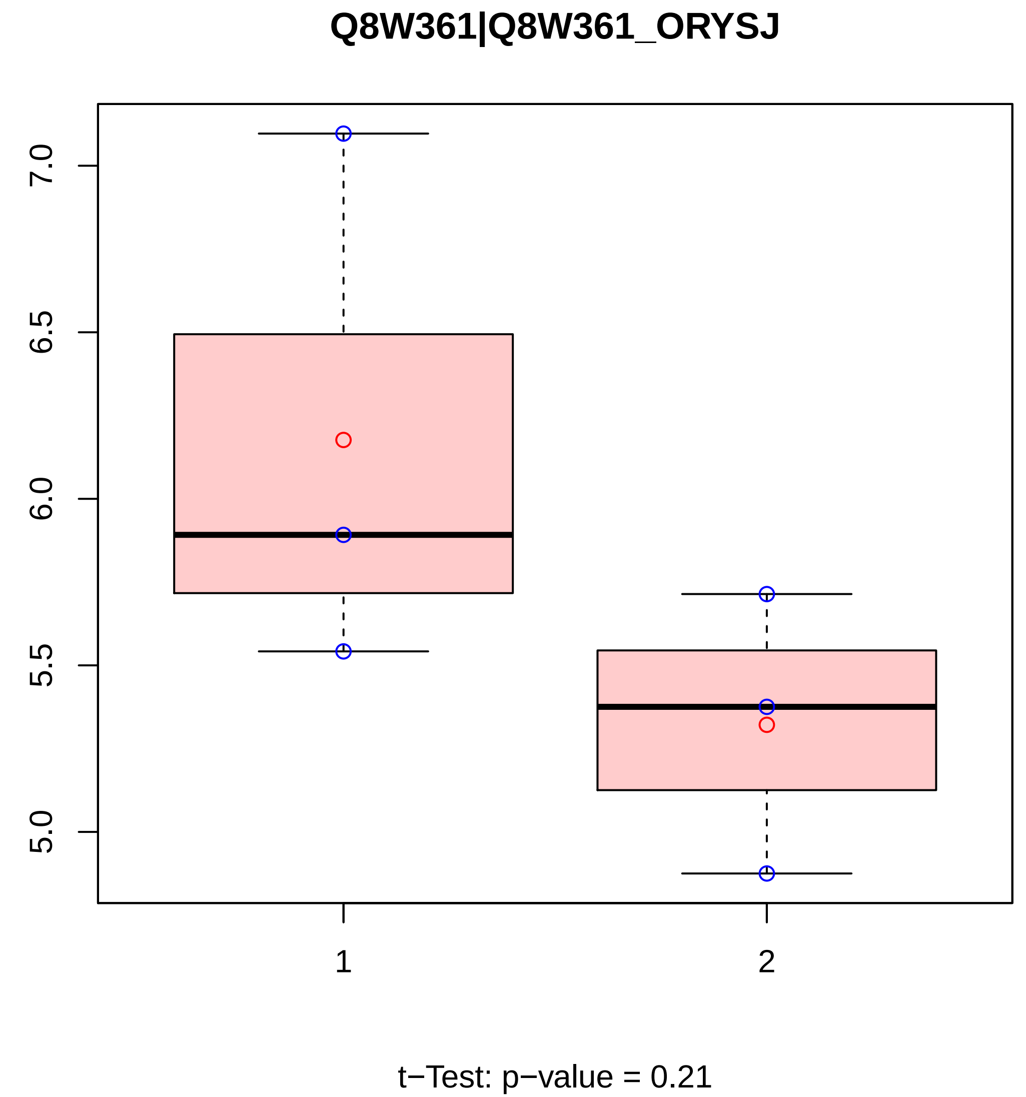
Q8W361|Q8W361_ORYSJ
7.0
6.5
6.0
5.5
5.0
1
2
t−Test: p−value = 0.21

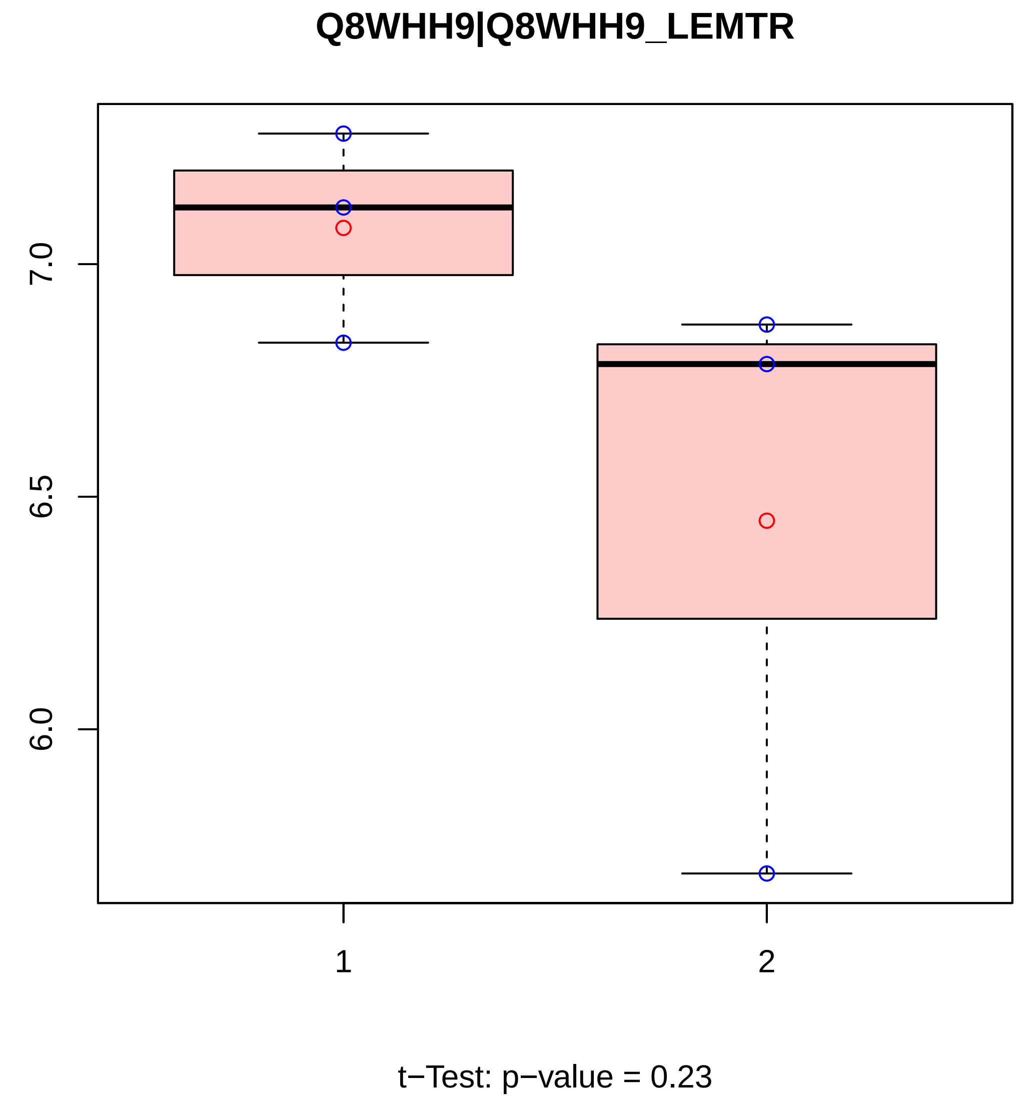
Q8WHH9|Q8WHH9_LEMTR
7.0
6.5
6.0
1
2
t−Test: p−value = 0.23

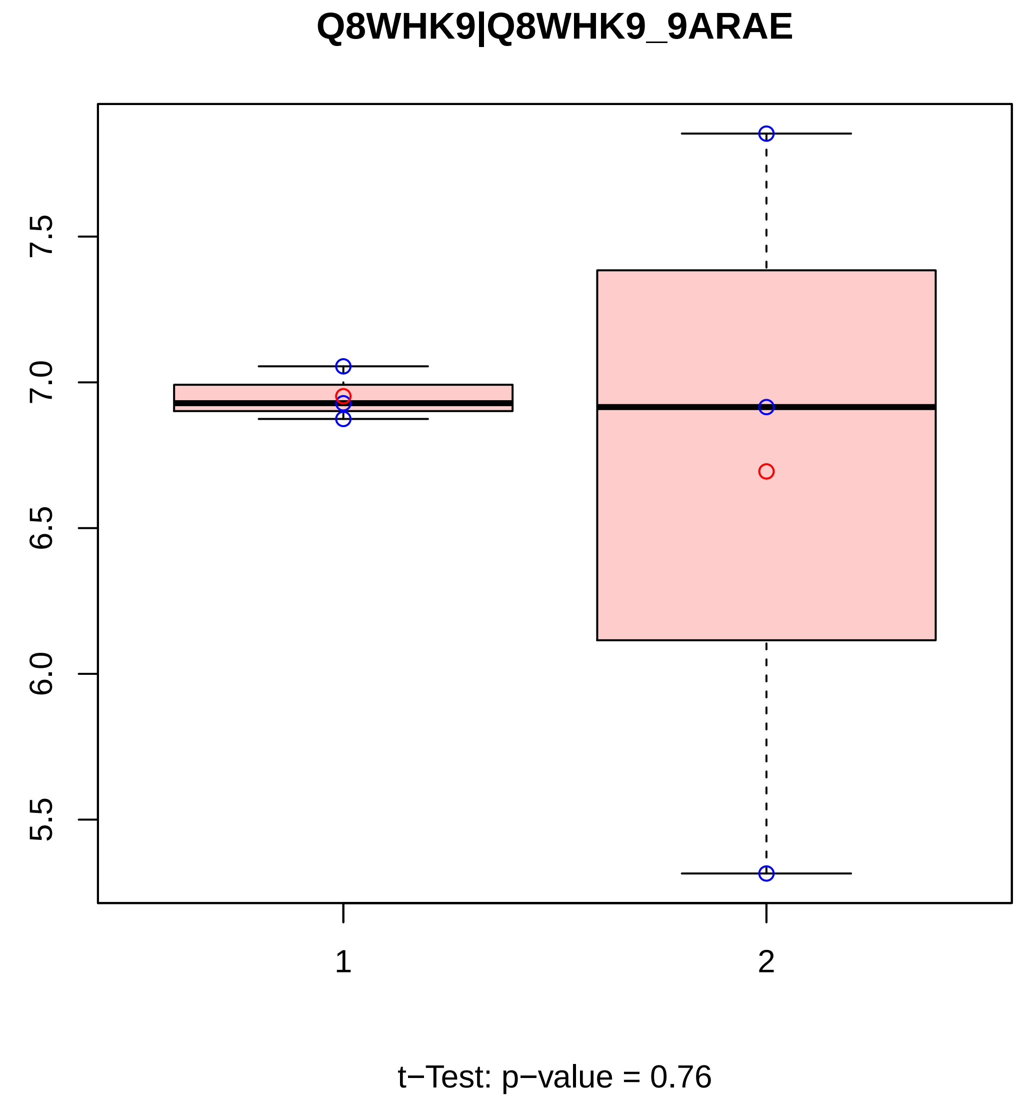
Q8WHK9|Q8WHK9_9ARAE
7.5
7.0
6.5
6.0
5.5
1
2
t−Test: p−value = 0.76

Q8WJX3|Q8WJX3_AREBU

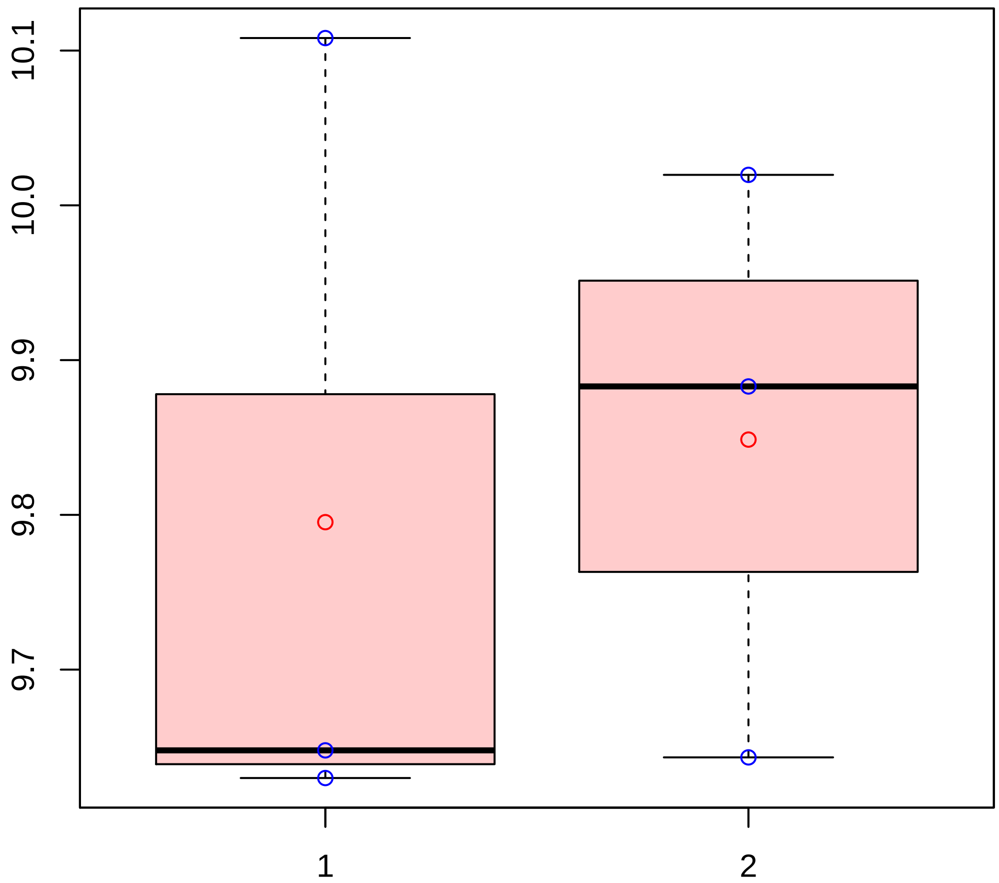

t−Test: p−value = 0.8

Q8WKN9|Q8WKN9_9ASPA

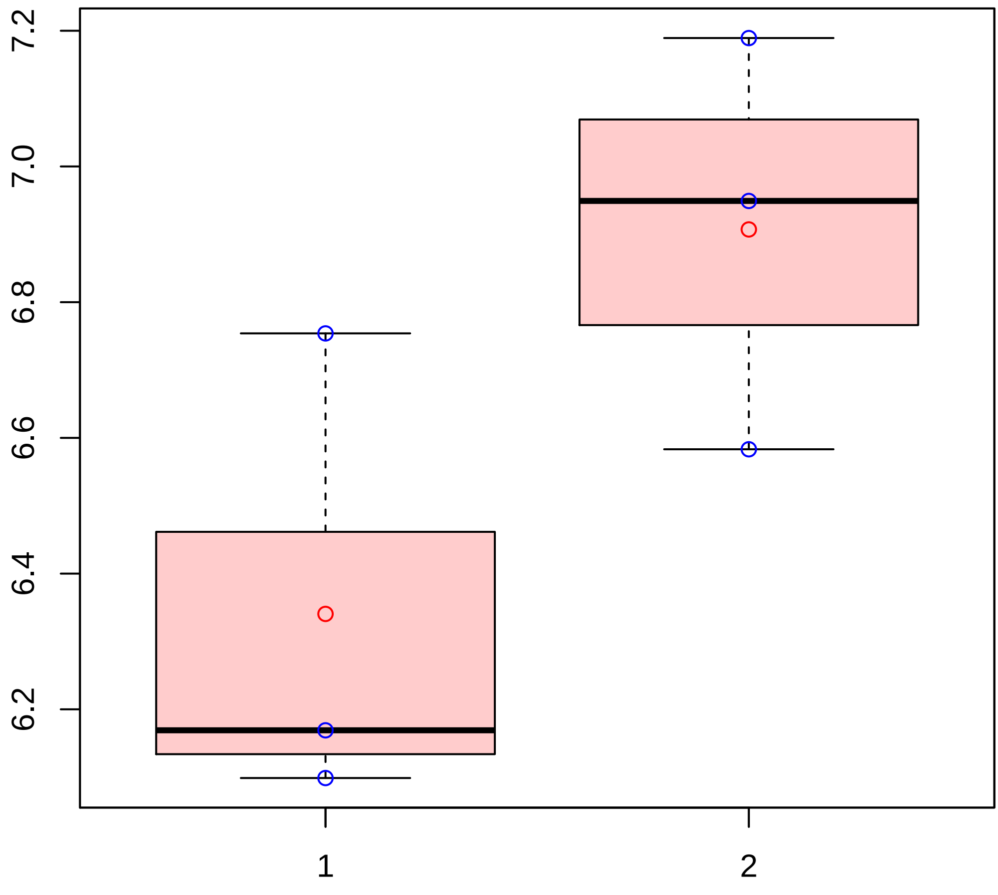

t−Test: p−value = 0.11

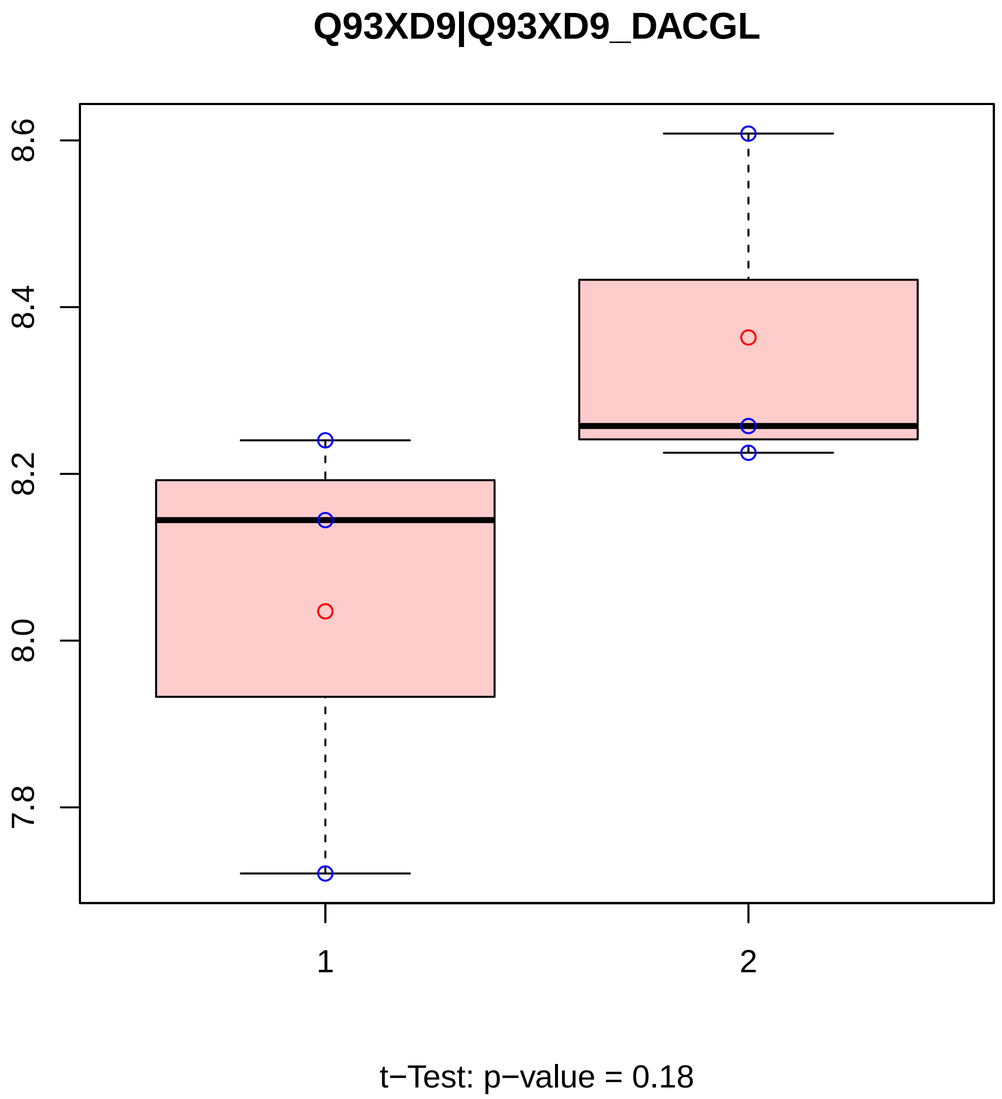
Q93XD9|Q93XD9_DACGL
8.6
8.4
8.2
8.0
7.8
1
2
t−Test: p−value = 0.18

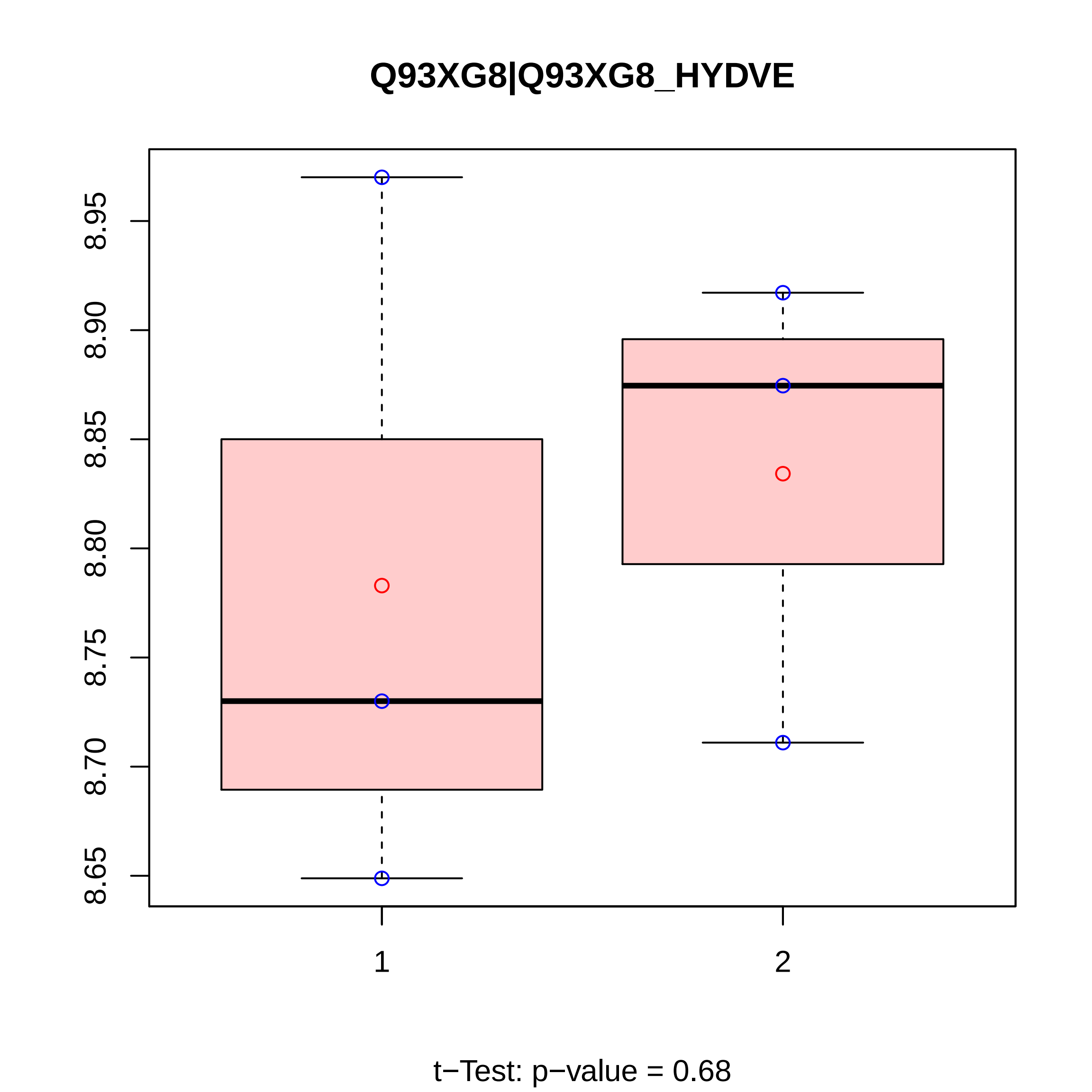
Q93XG8|Q93XG8_HYDVE
8.65
8.70
8.75
8.80
8.85
8.90
8.95
1
2
t−Test: p−value = 0.68

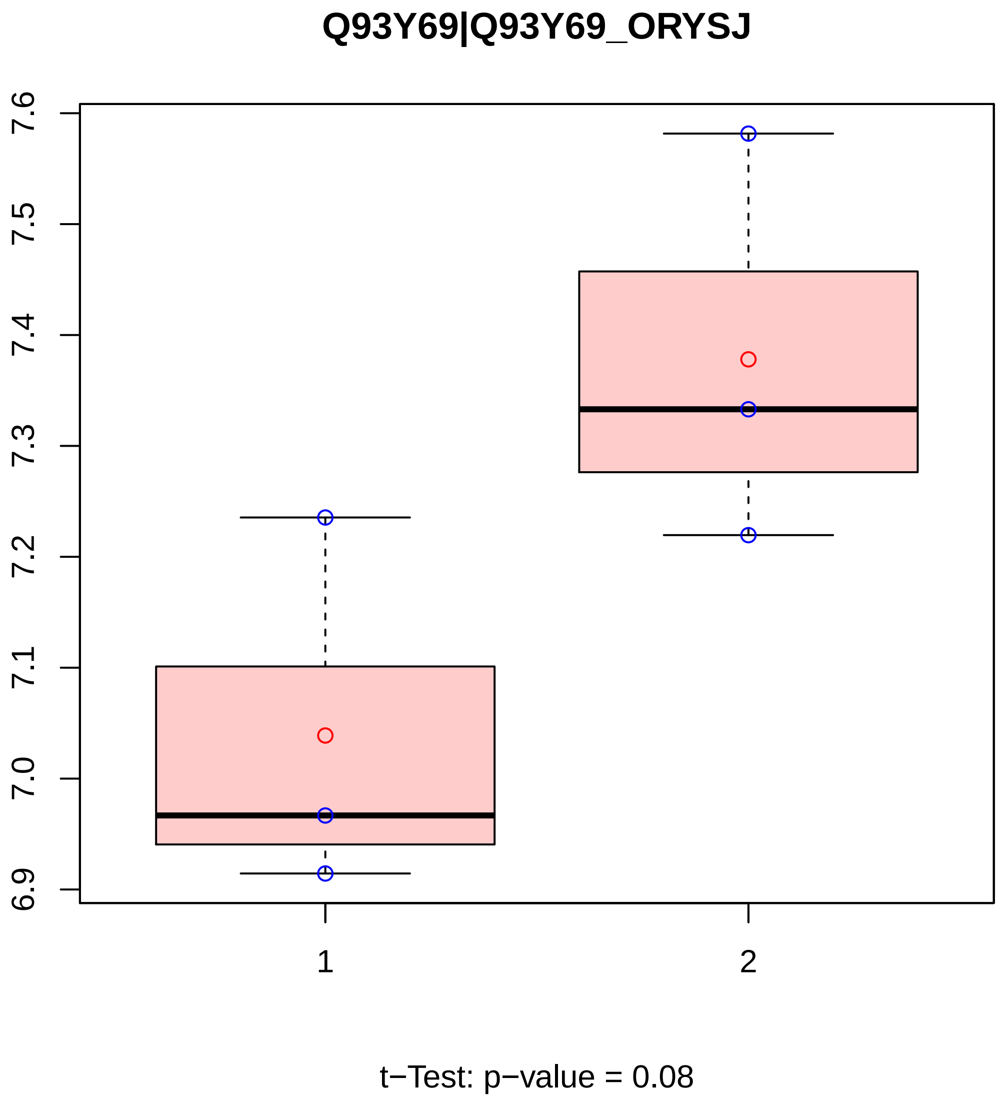
Q93Y69|Q93Y69_ORYSJ
7.6
7.5
7.4
7.3
7.2
7.1
7.0
6.9
1
2
t−Test: p−value = 0.08

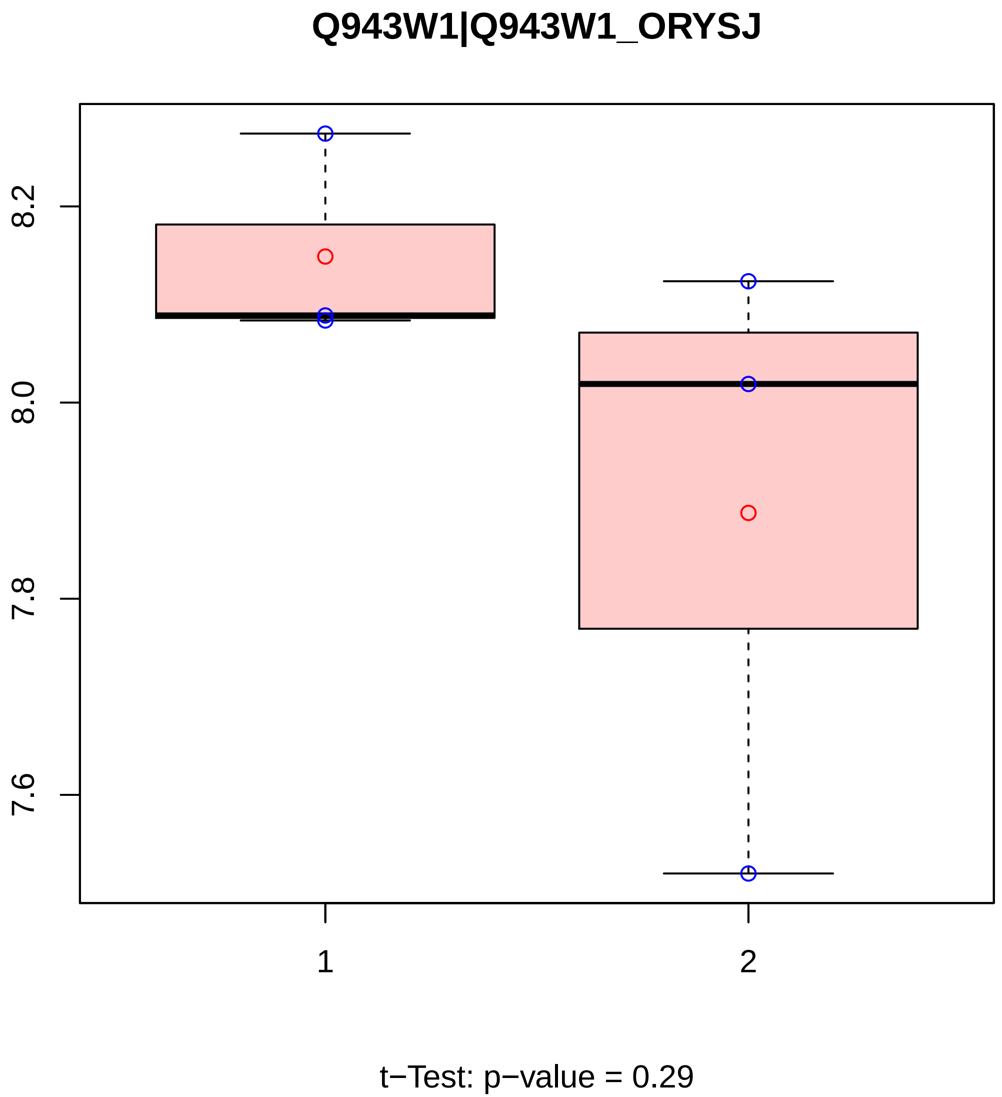

Q943W1|Q943W1_ORYSJ
8.2
8.0
7.8
7.6
1
2
t−Test: p−value = 0.29

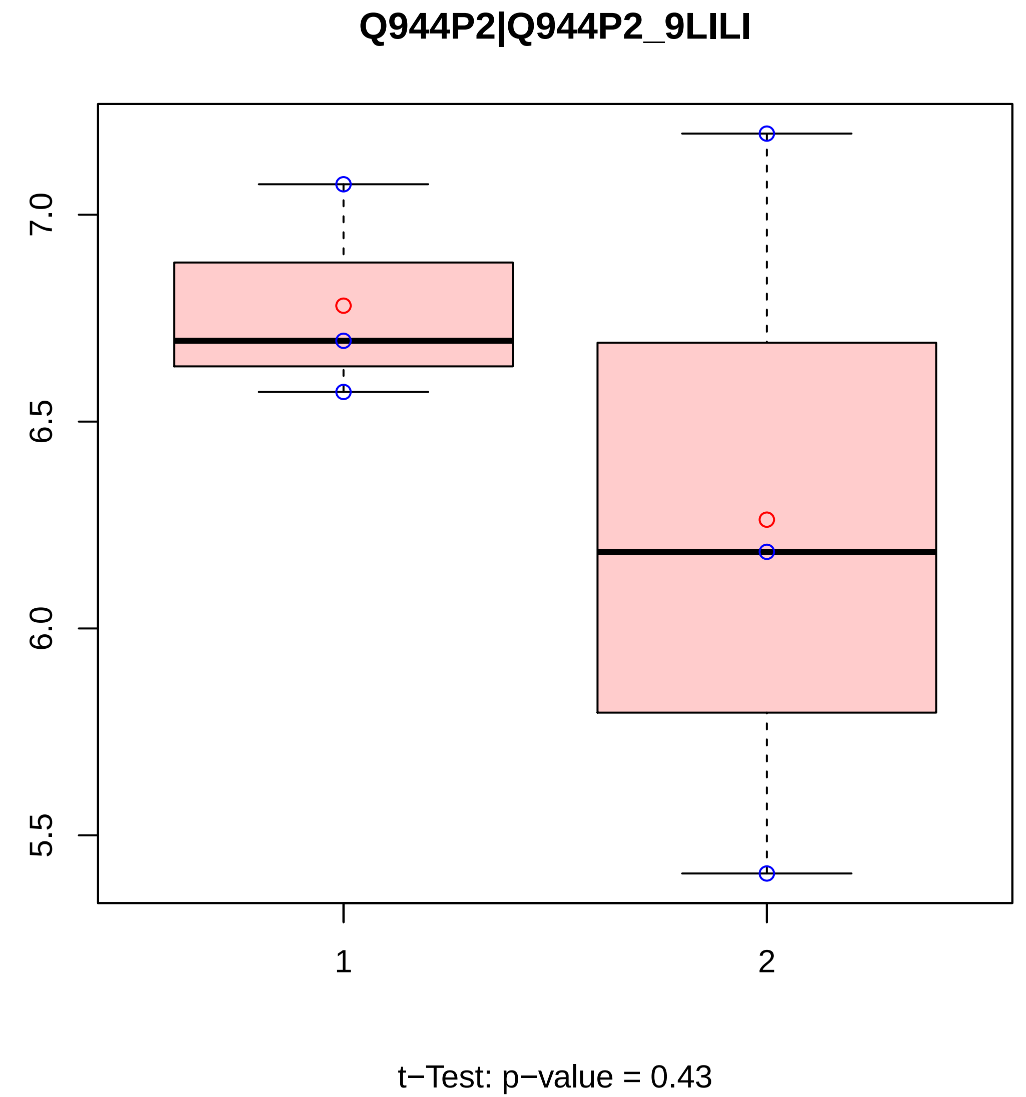
Q944P2|Q944P2_9LILI
7.0
6.5
6.0
5.5
1
2
t−Test: p−value = 0.43

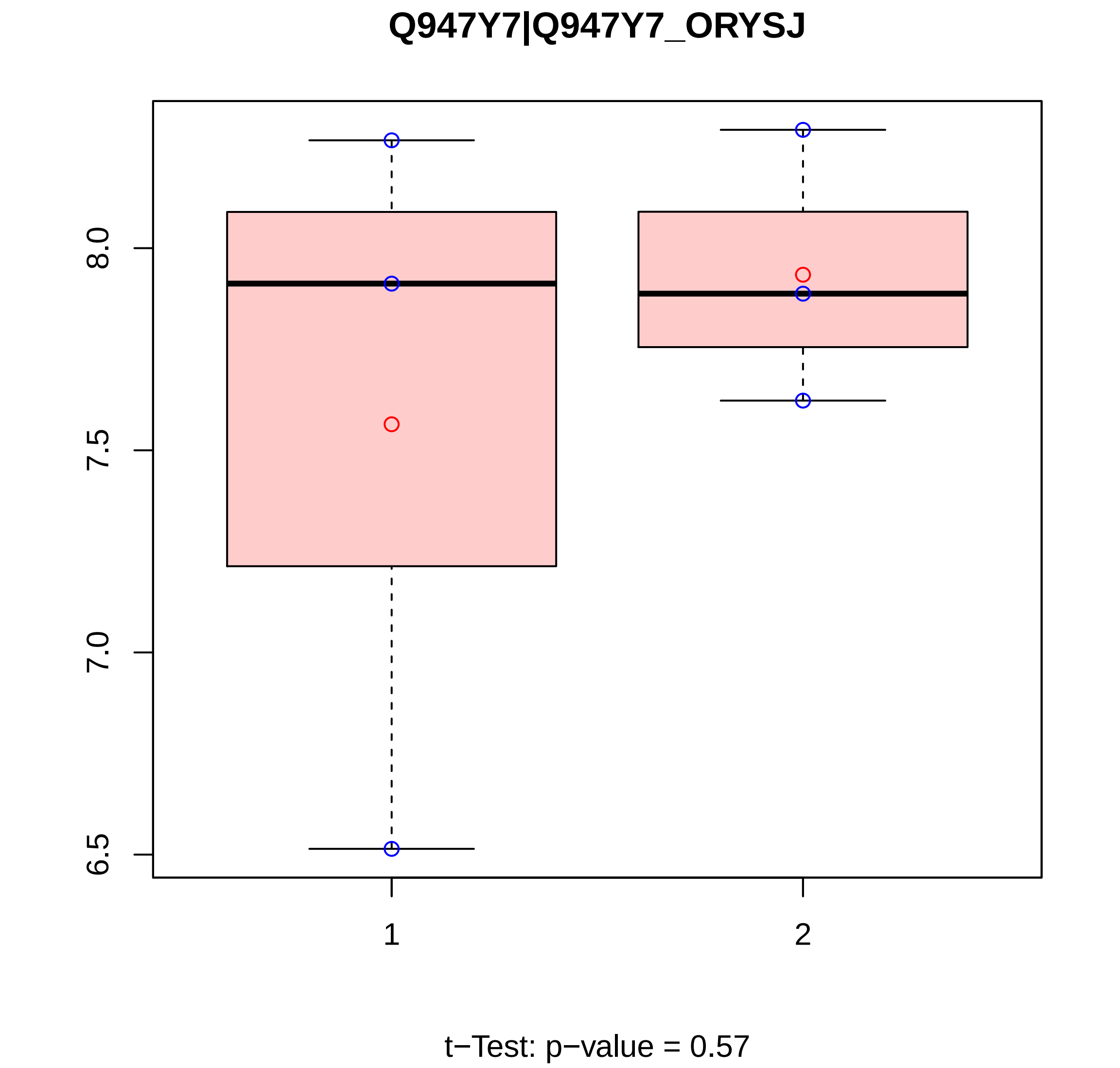
Q947Y7|Q947Y7_ORYSJ
8.0
7.5
7.0
6.5
1
2
t−Test: p−value = 0.57

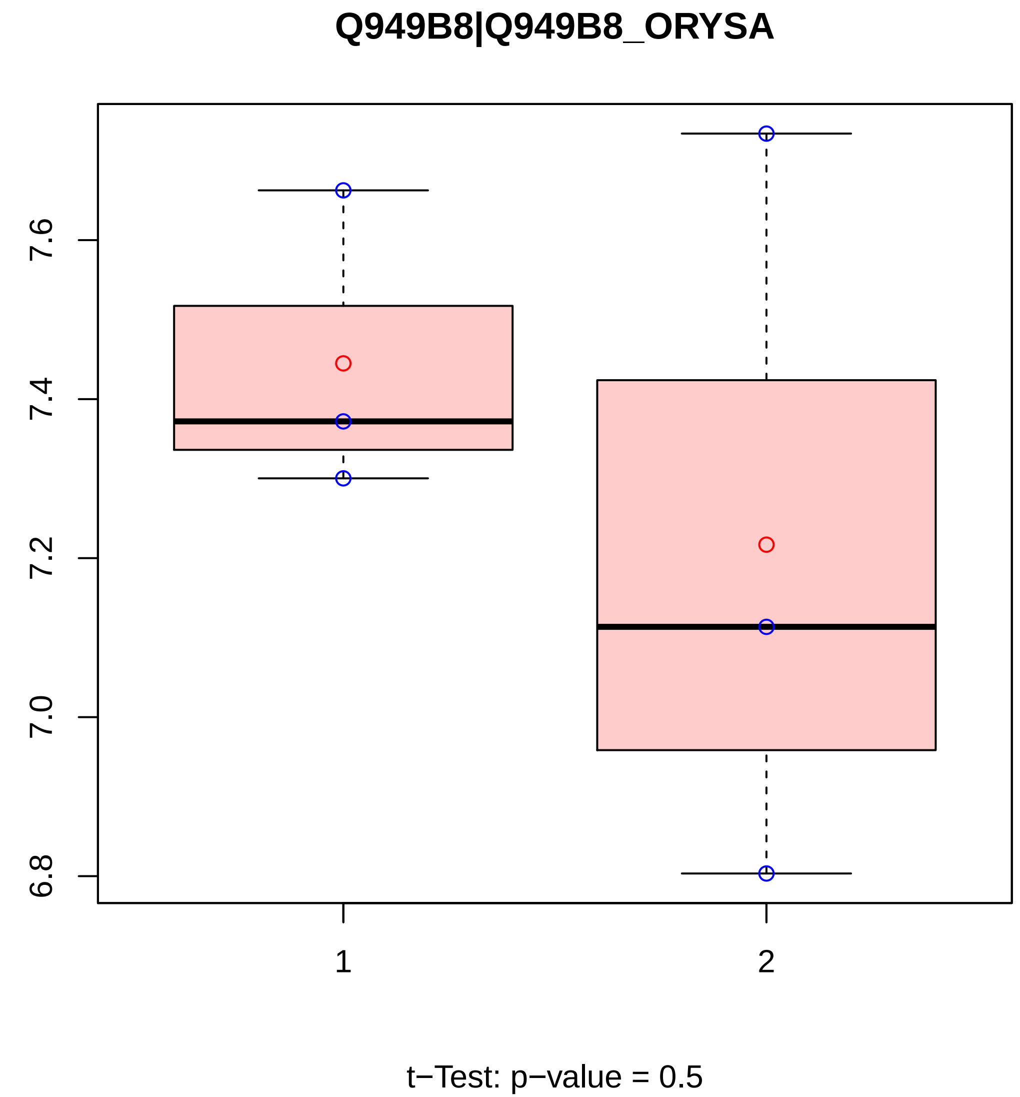

Q949B8|Q949B8_ORYSA
6.8
7.0
7.2
7.4
7.6
1
2
t−Test: p−value = 0.5

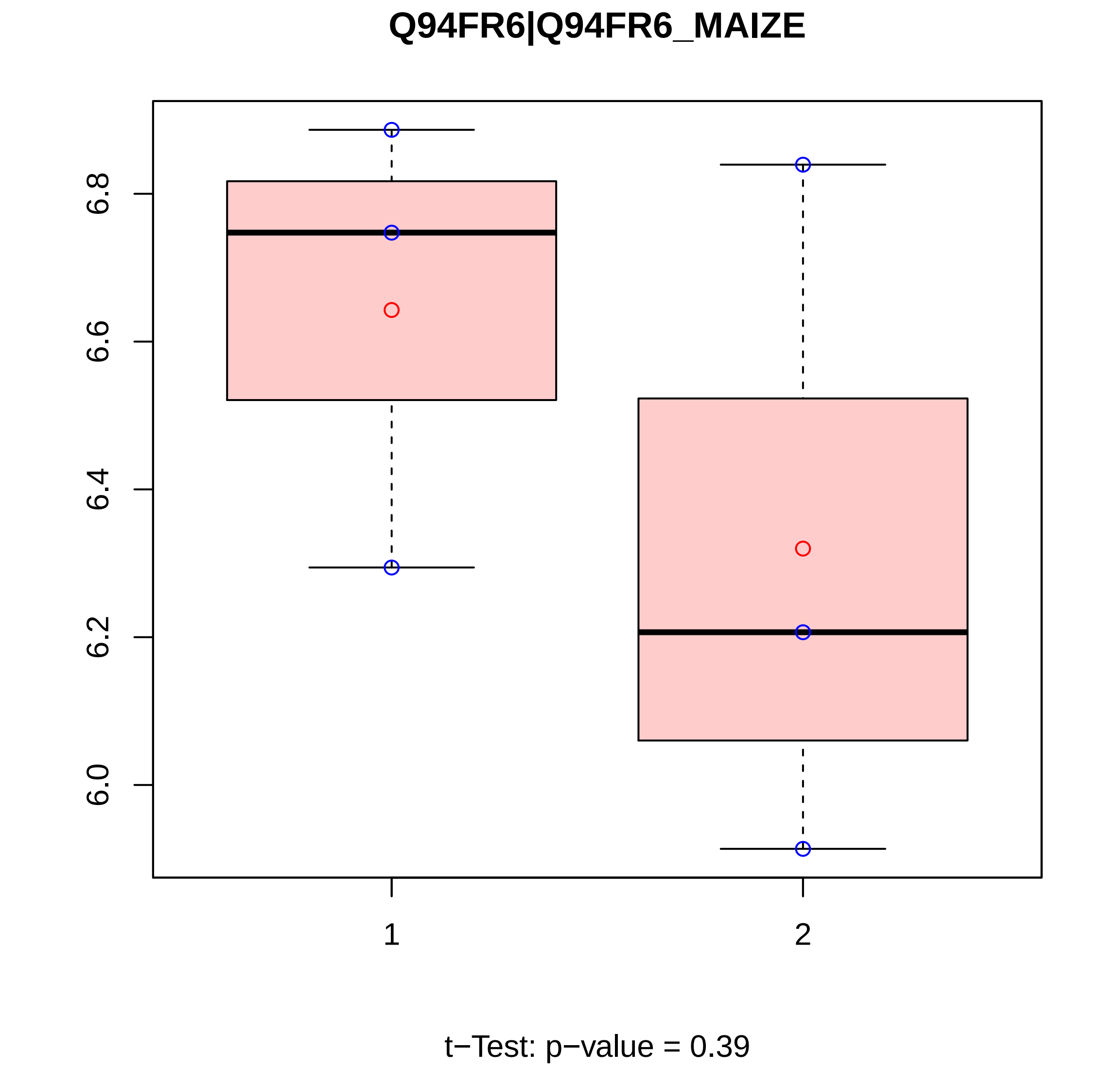

Q94FR6|Q94FR6_MAIZE
6.0
6.2
6.4
6.6
6.8
1
2
t−Test: p−value = 0.39

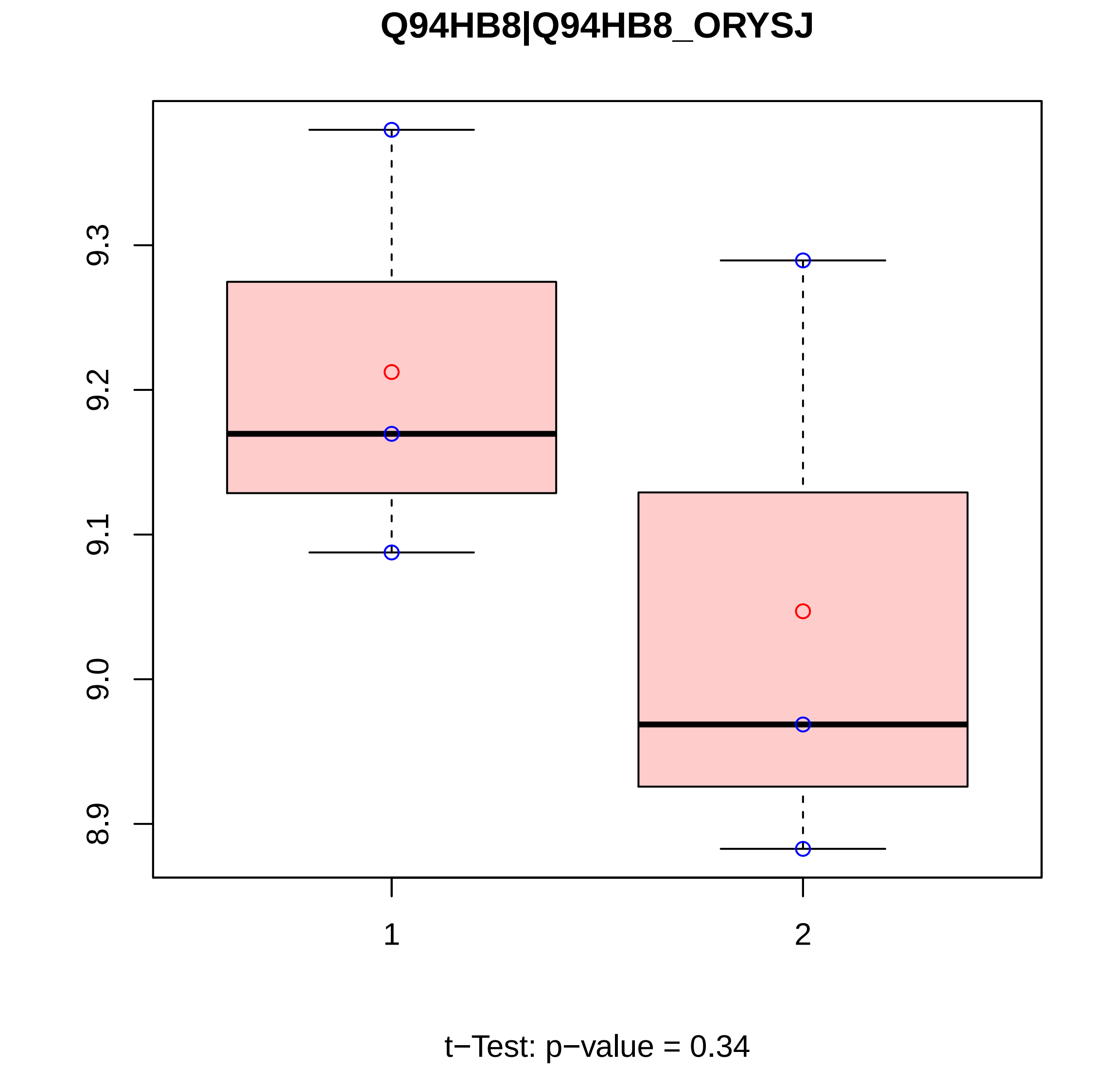
Q94HB8|Q94HB8_ORYSJ
9.3
9.2
9.1
9.0
8.9
1
2
t−Test: p−value = 0.34

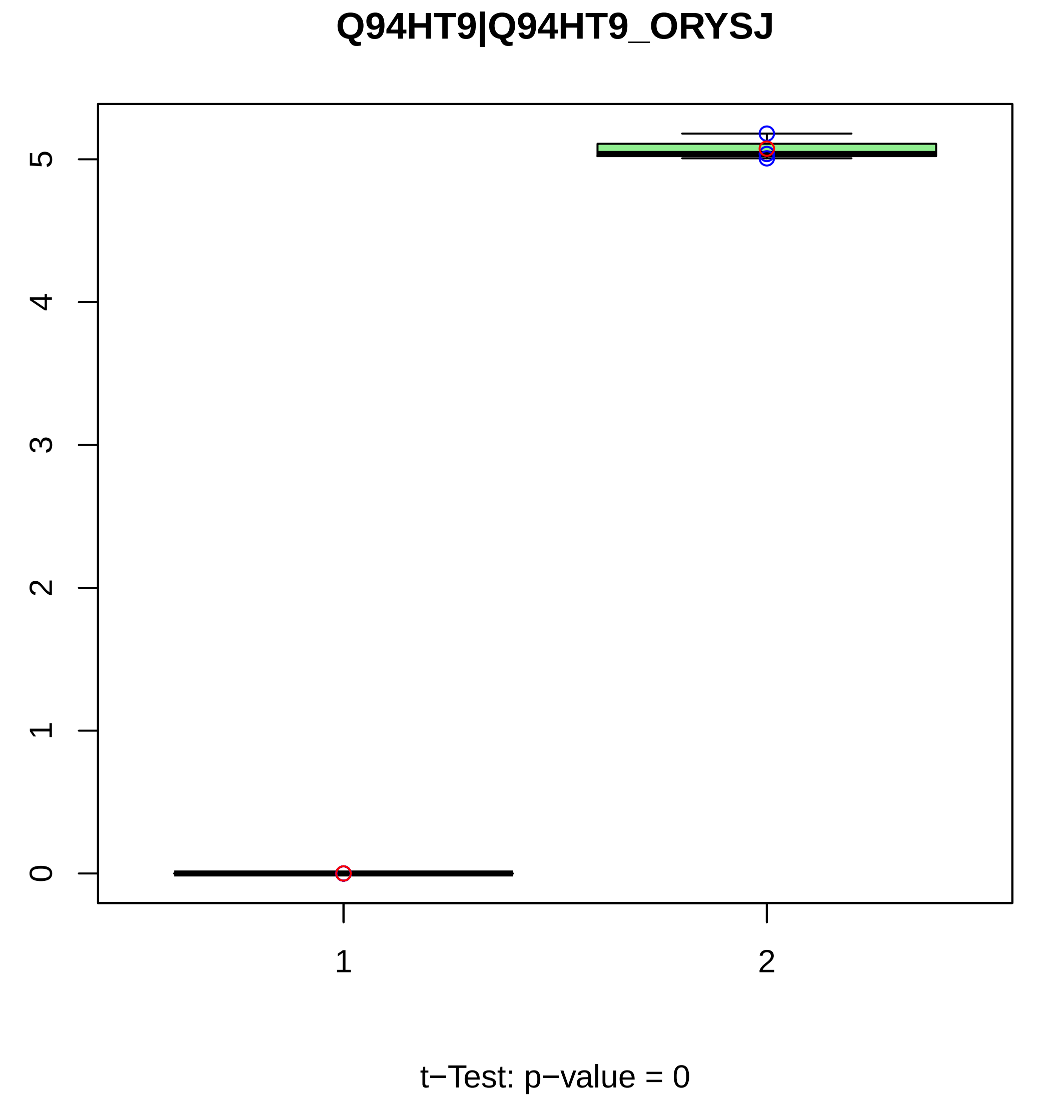

Q94HT9|Q94HT9_ORYSJ
0
1
2
3
4
5
1
2
t−Test: p−value = 0

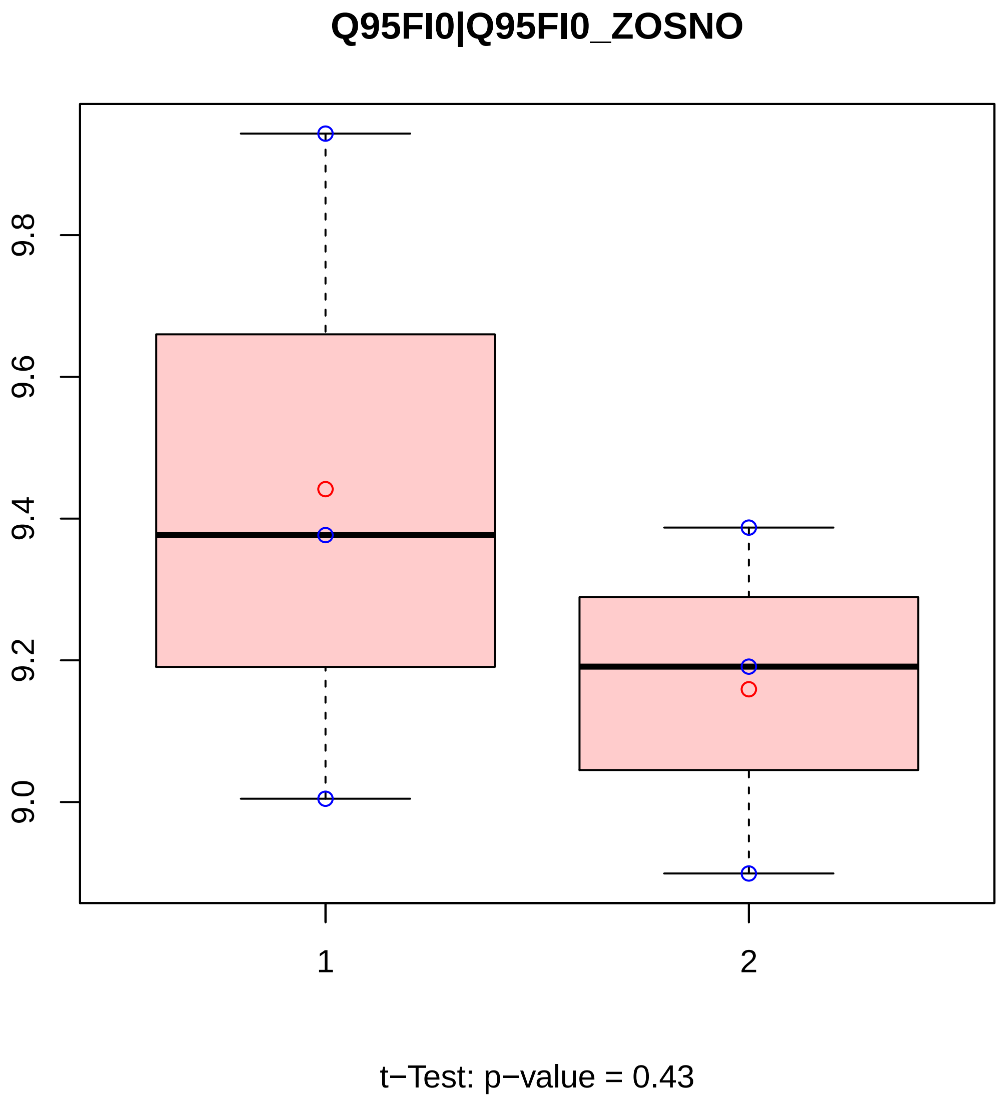
Q95FI0|Q95FI0_ZOSNO
9.0
9.2
9.4
9.6
9.8
1
2
t−Test: p−value = 0.43

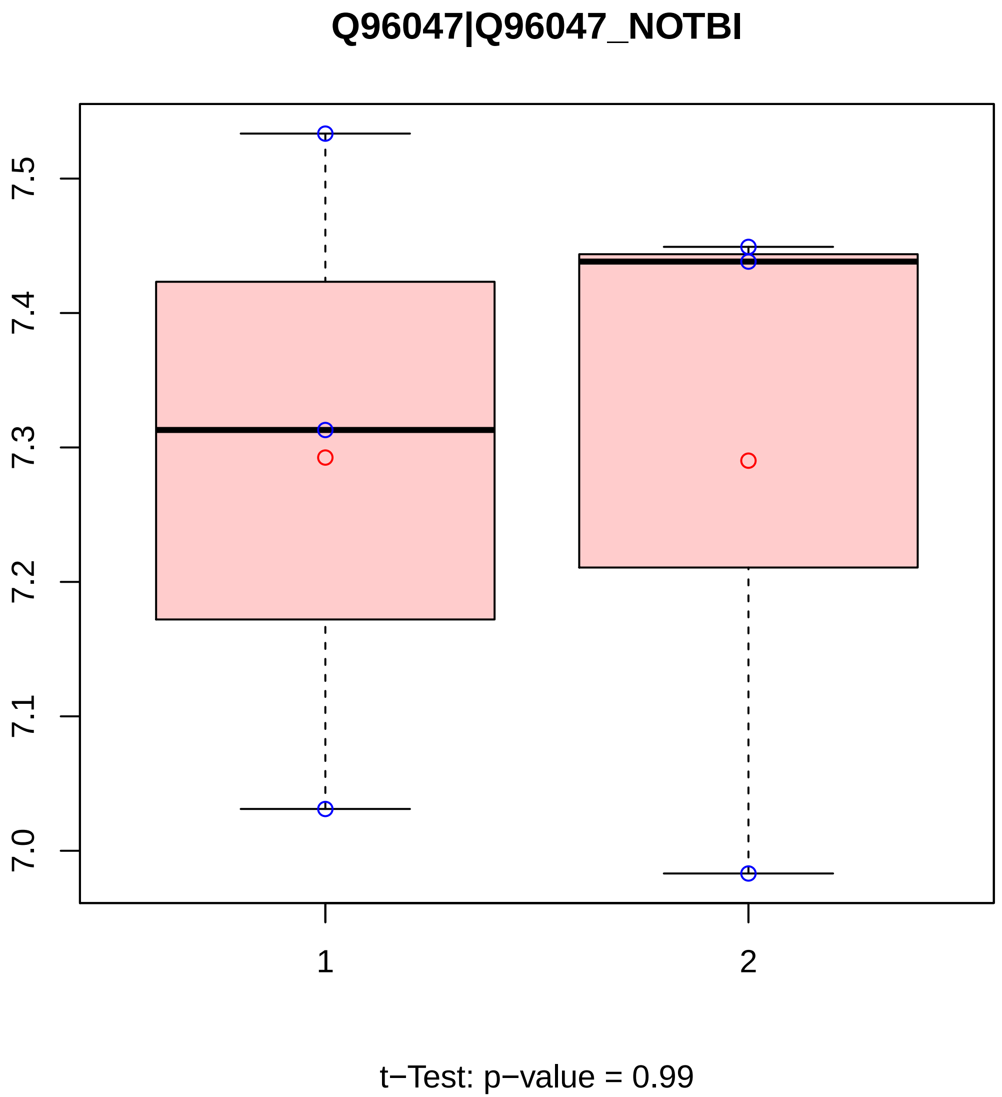
Q96047|Q96047_NOTBI
7.0
7.1
7.2
7.3
7.4
7.5
1
2
t−Test: p−value = 0.99

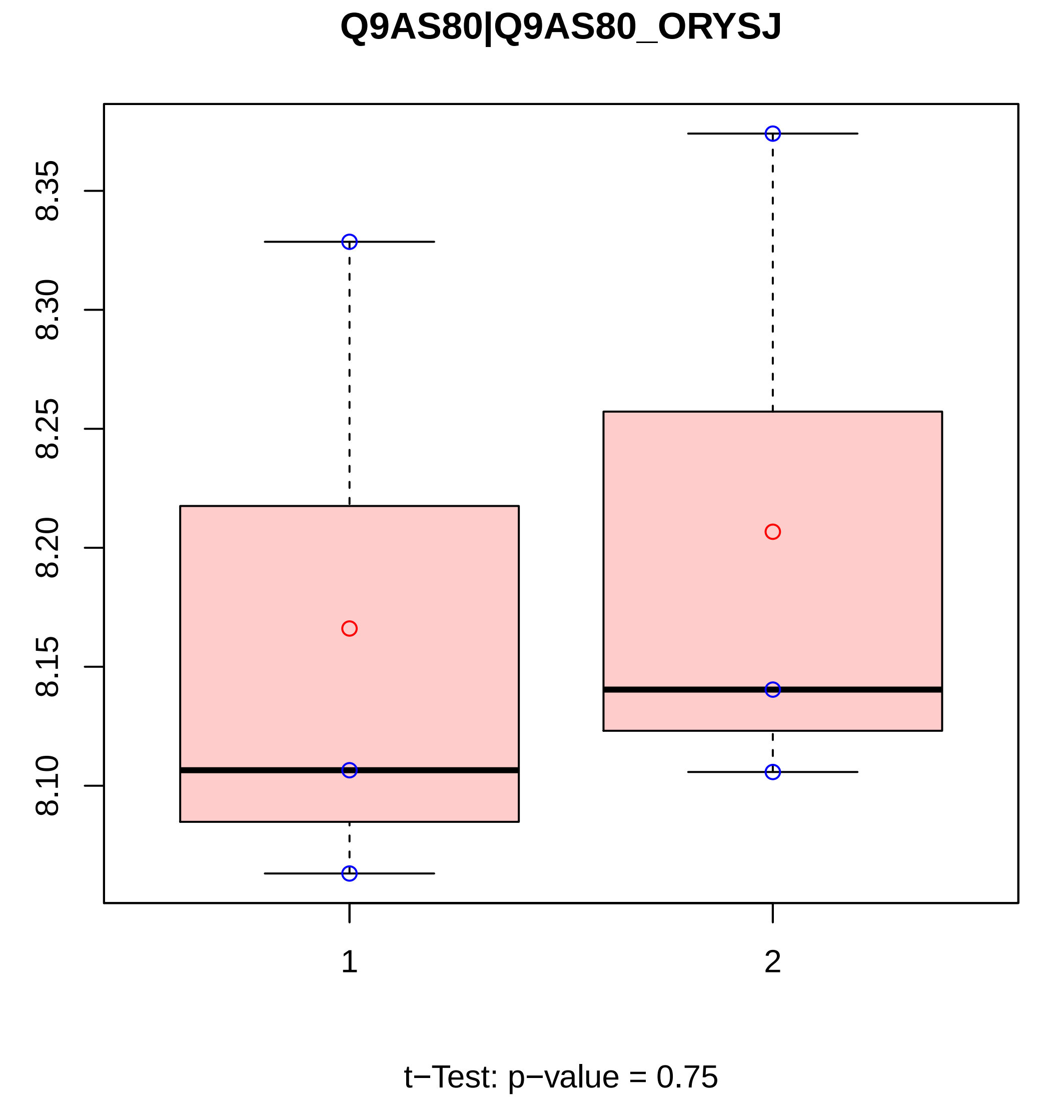
Q9AS80|Q9AS80_ORYSJ
8.10
8.15
8.20
8.25
8.30
8.35
1
2
t−Test: p−value = 0.75

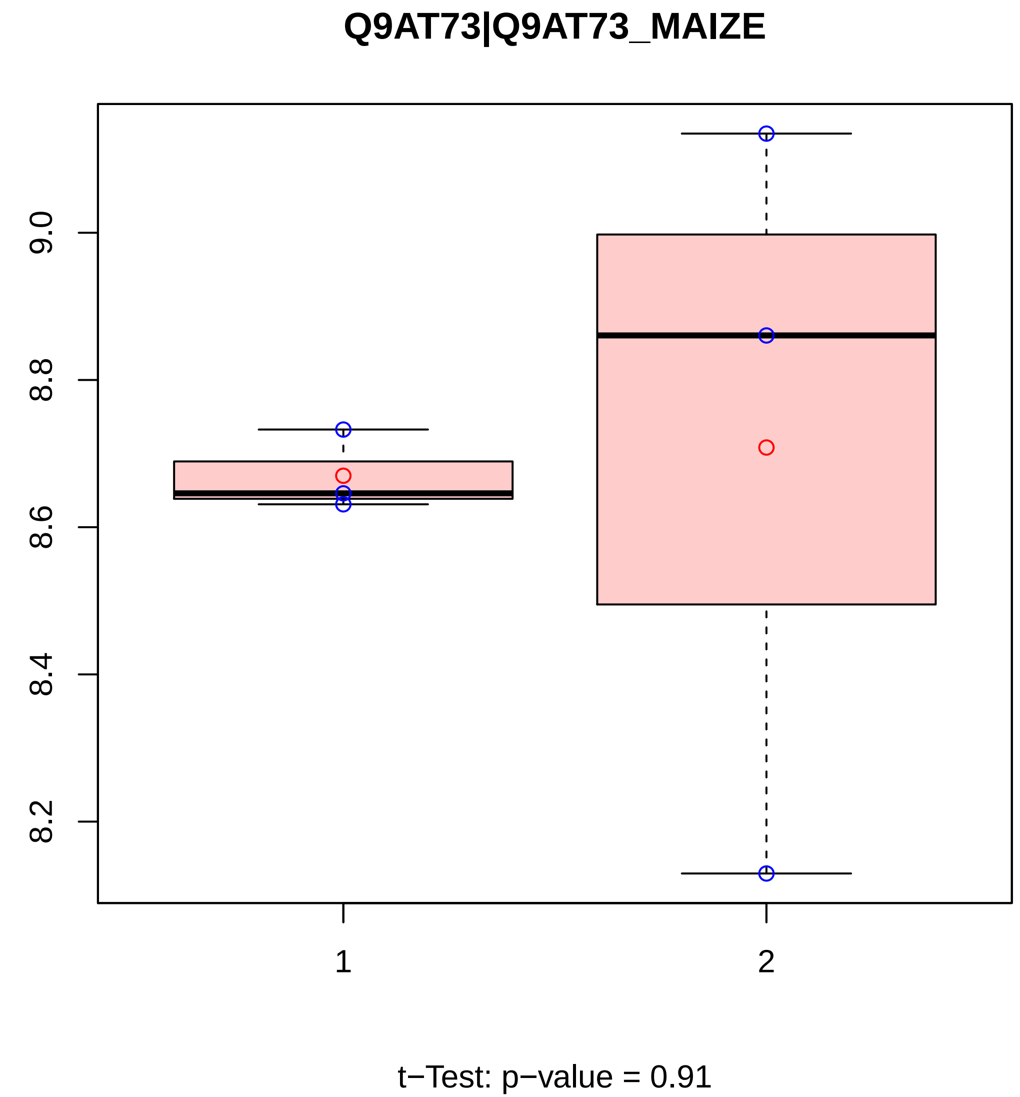

Q9AT73|Q9AT73_MAIZE
9.0
8.8
8.6
8.4
8.2
1
2
t−Test: p−value = 0.91

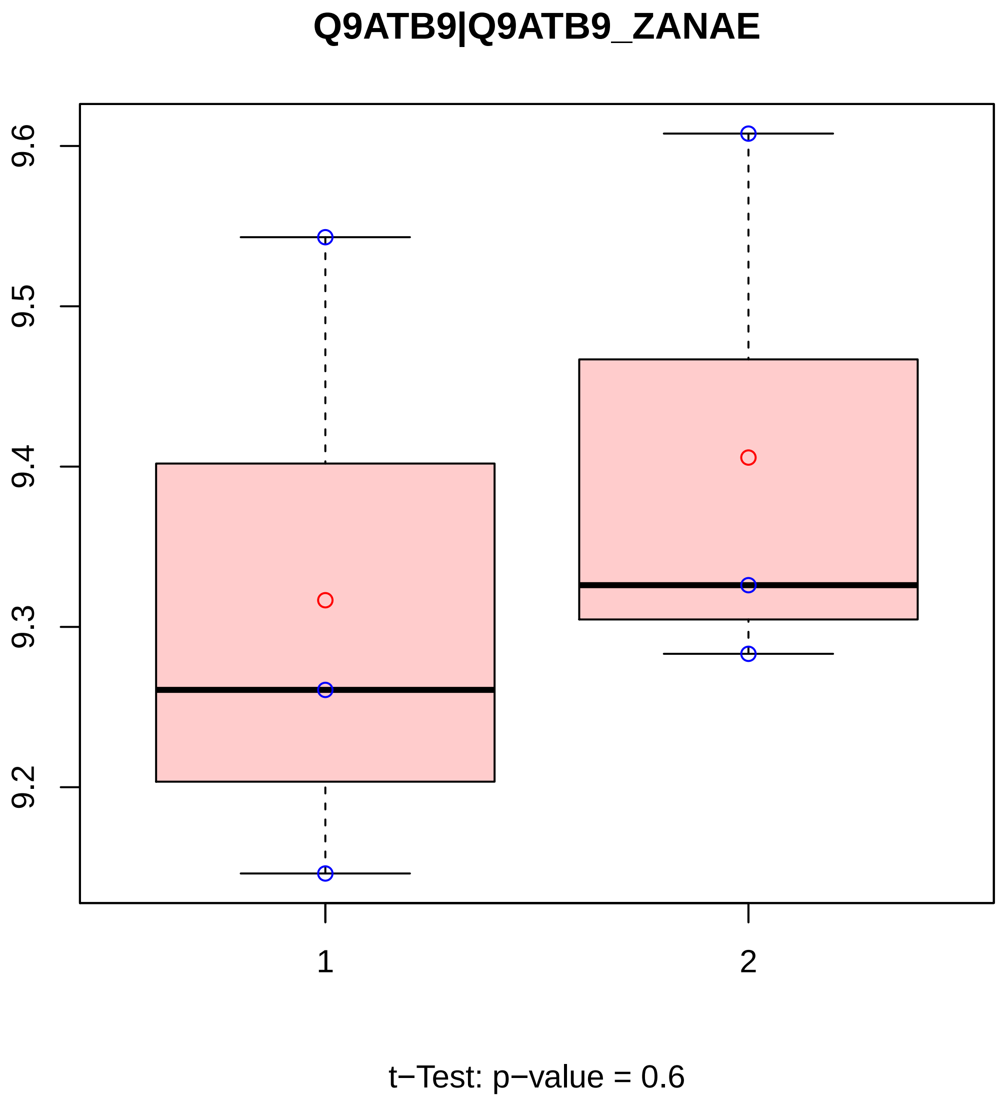

Q9ATB9|Q9ATB9_ZANAE
9.2
9.3
9.4
9.5
9.6
1
2
t−Test: p−value = 0.6

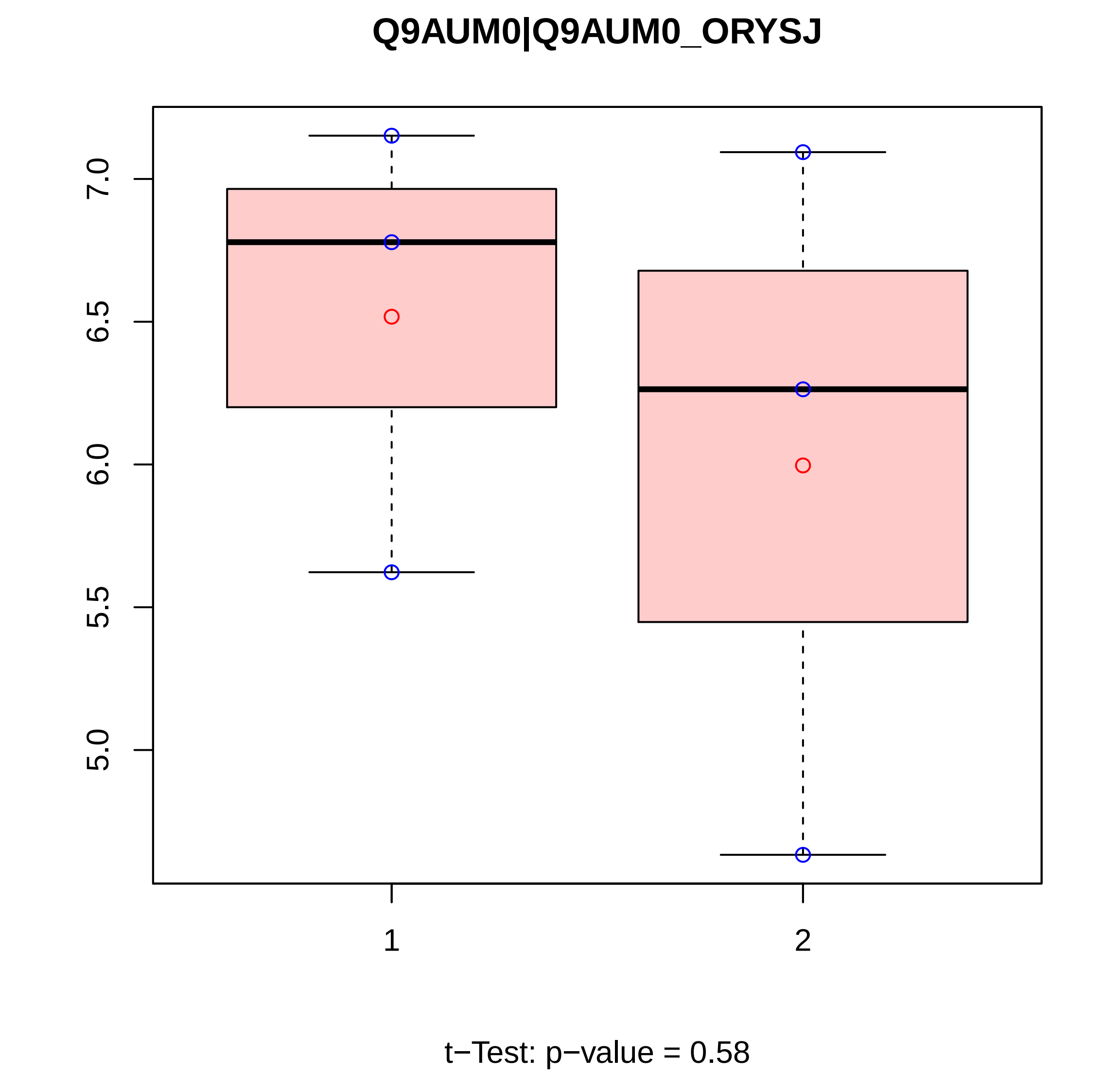
Q9AUM0|Q9AUM0_ORYSJ
7.0
6.5
6.0
5.5
5.0
1
2
t−Test: p−value = 0.58

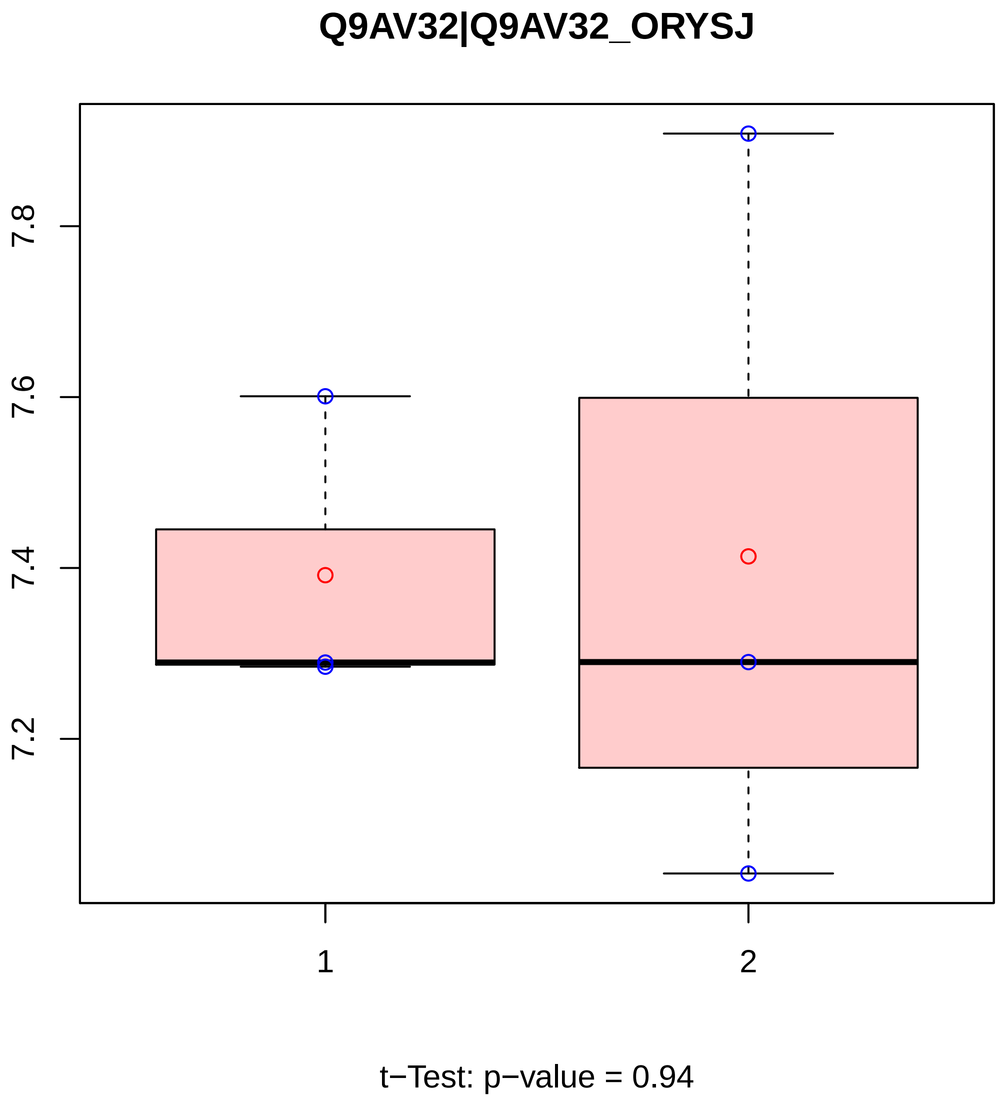
Q9AV32|Q9AV32_ORYSJ
7.2
7.4
7.6
7.8
1
2
t−Test: p−value = 0.94

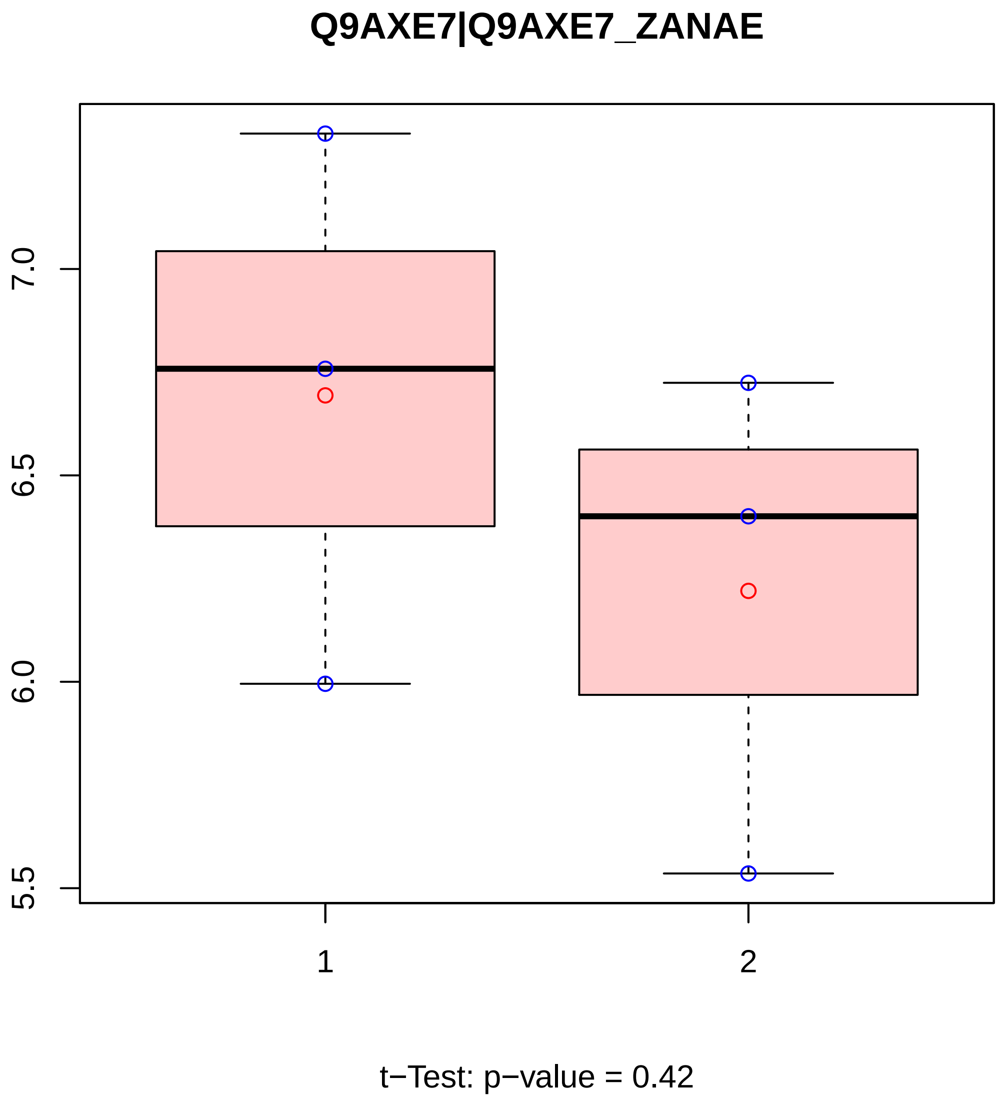

Q9AXE7|Q9AXE7_ZANAE
7.0
6.5
6.0
5.5
1
2
t−Test: p−value = 0.42

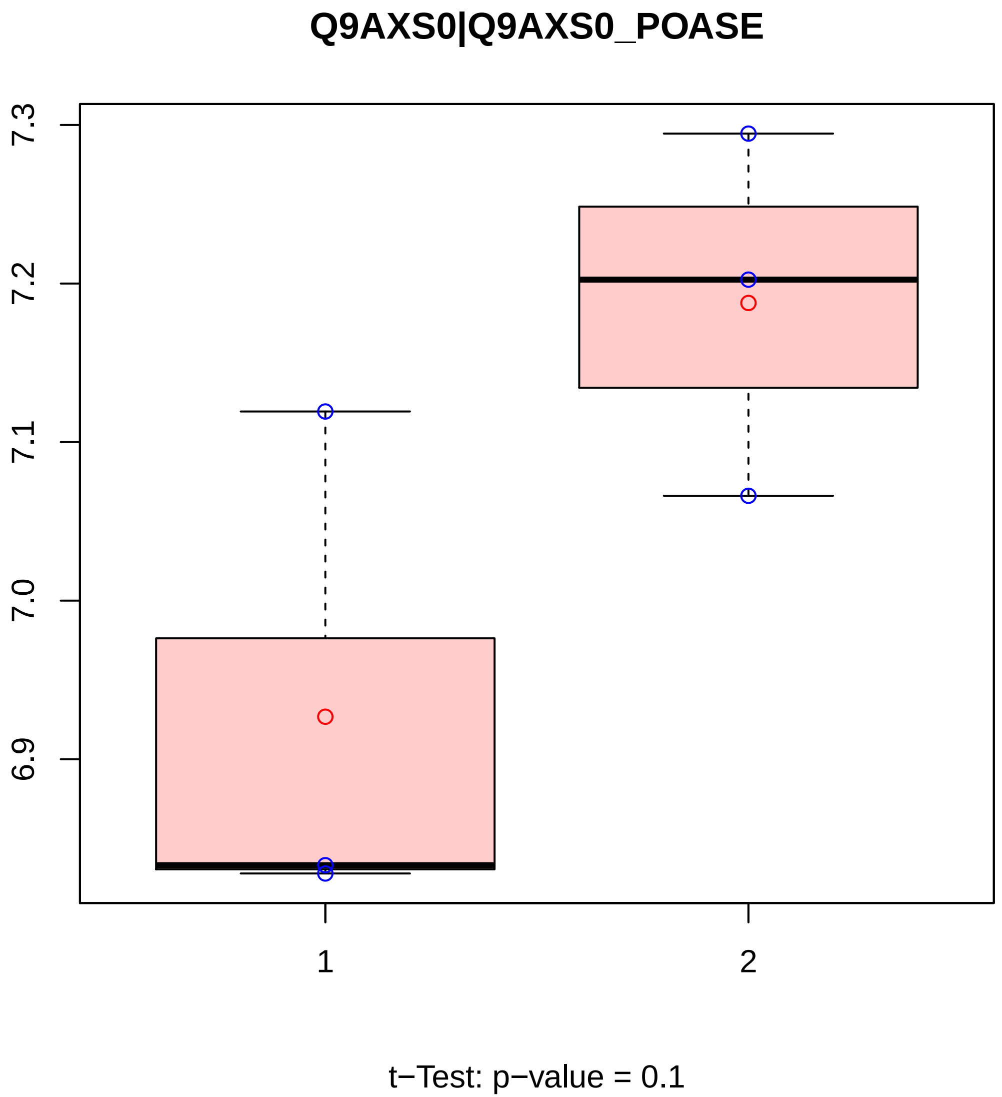

Q9AXS0|Q9AXS0_POASE
7.3
7.2
7.1
7.0
6.9
1
2
t−Test: p−value = 0.1

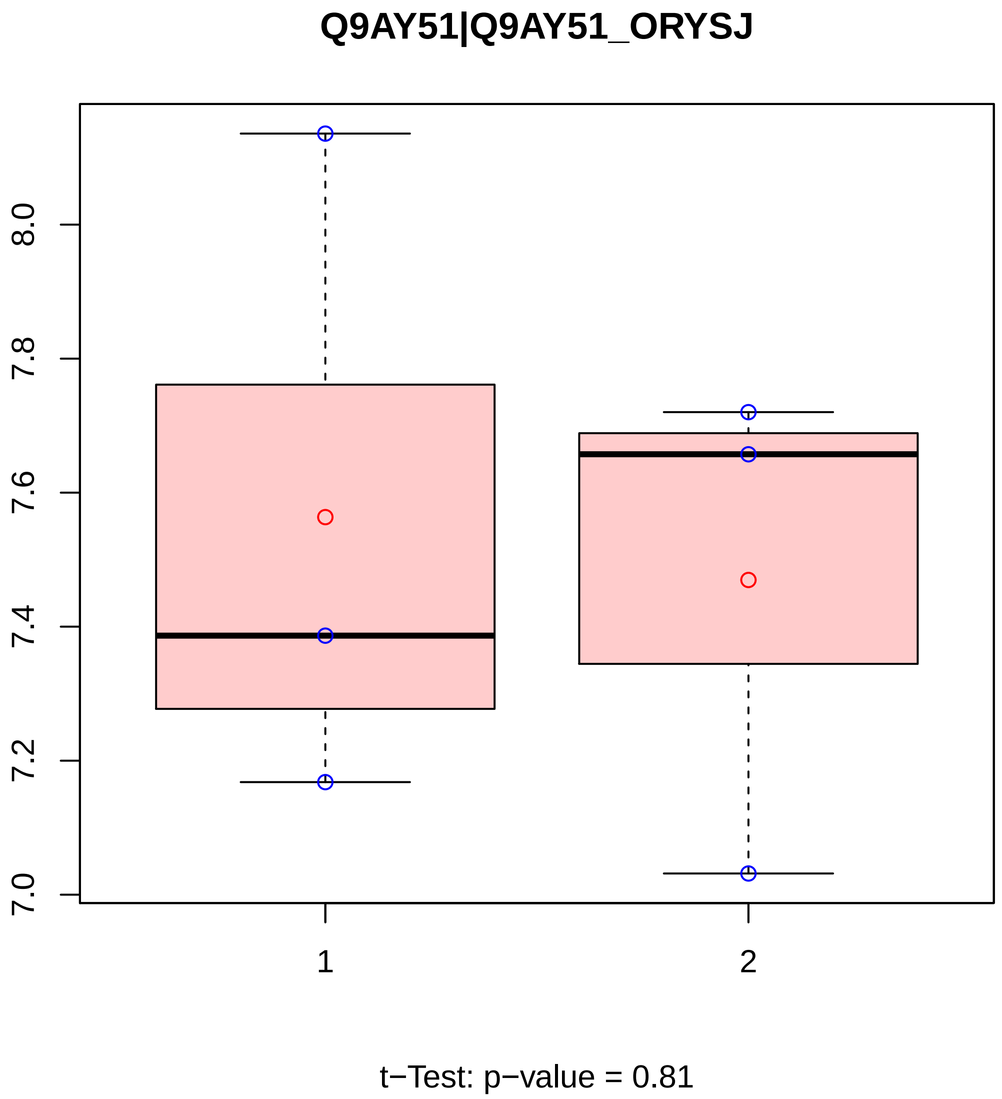
Q9AY51|Q9AY51_ORYSJ
8.0
7.8
7.6
7.4
7.2
7.0
1
2
t−Test: p−value = 0.81

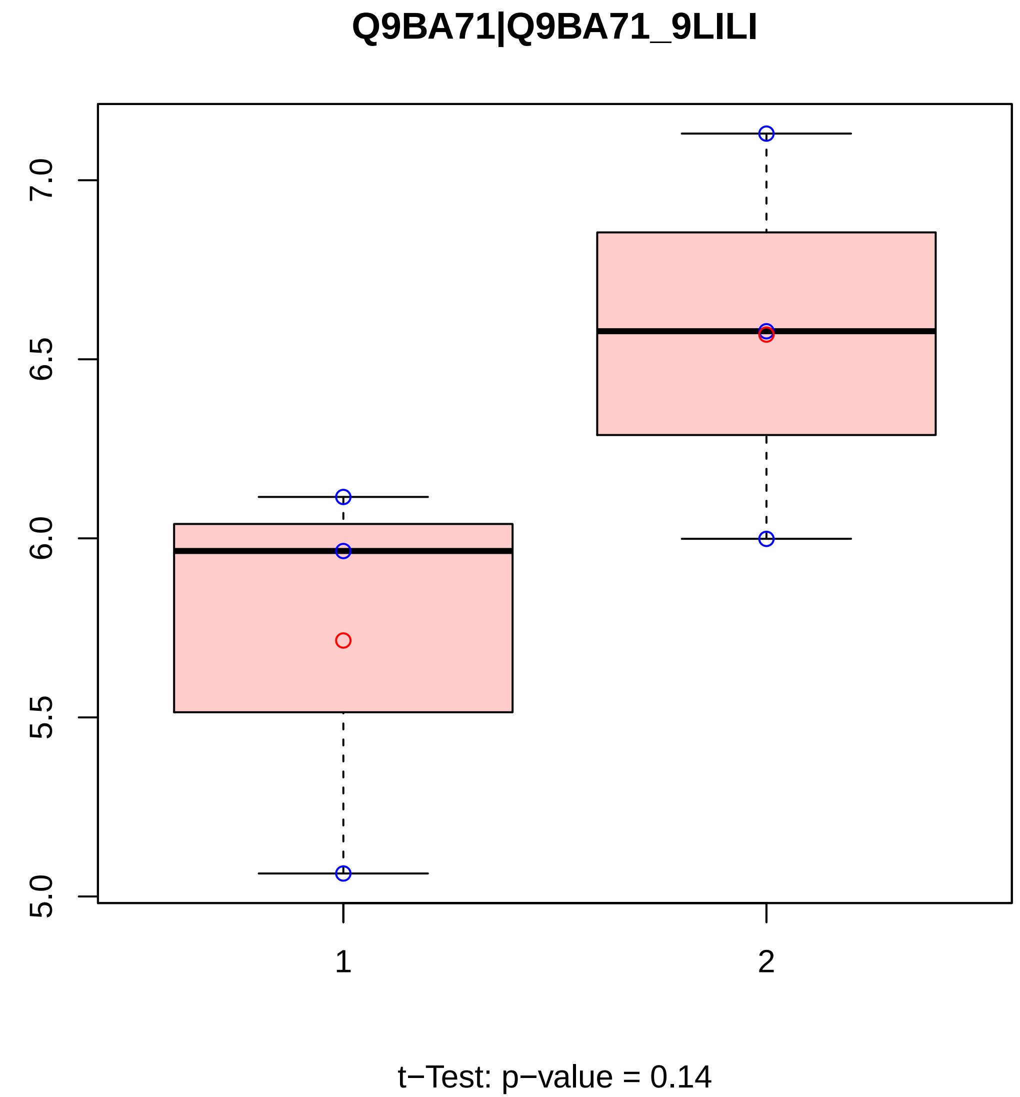
Q9BA71|Q9BA71_9LILI
7.0
6.5
6.0
5.5
5.0
1
2
t−Test: p−value = 0.14

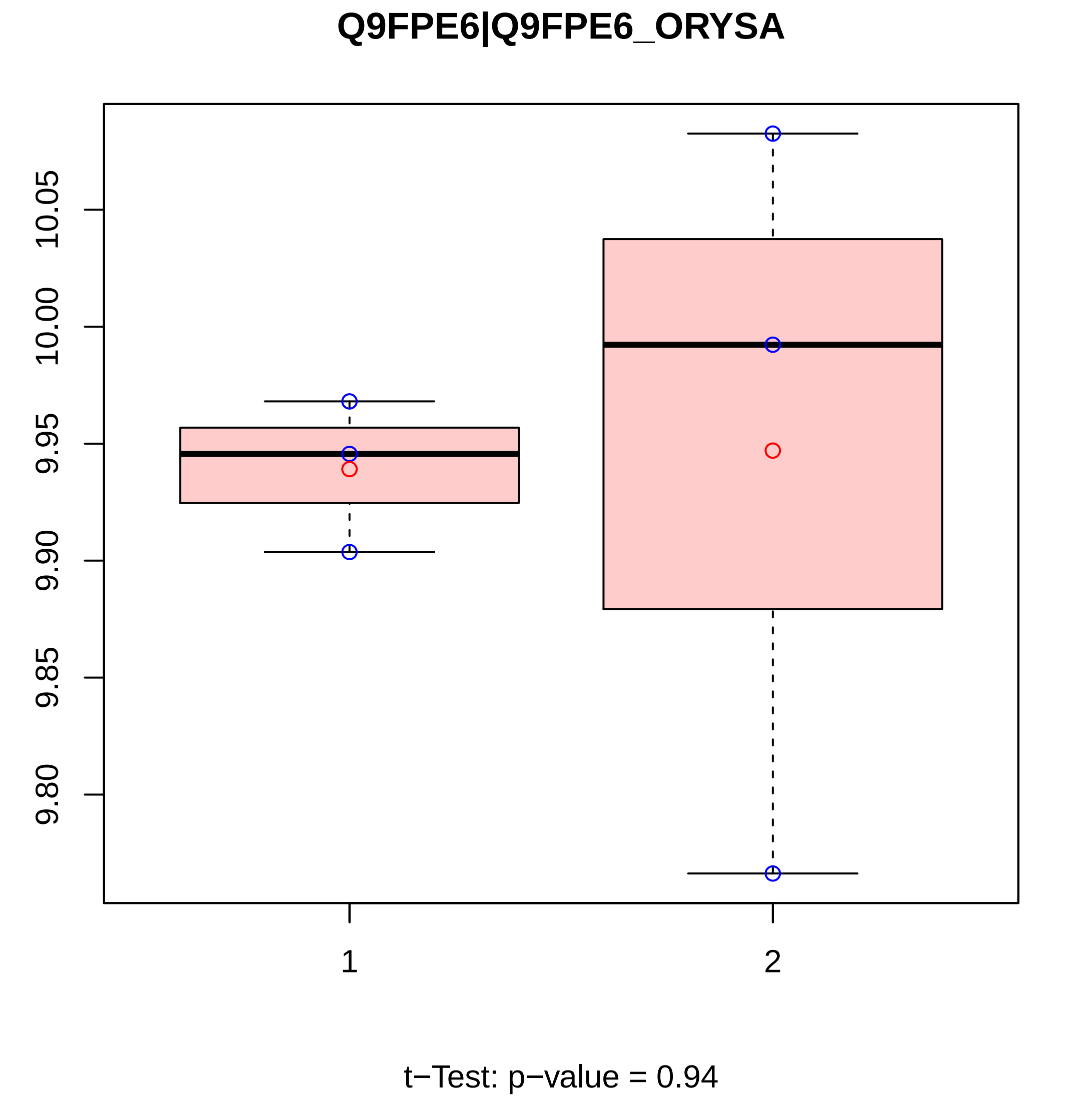
Q9FPE6|Q9FPE6_ORYSA
10.05
10.00
9.95
9.90
9.85
9.80
1
2
t−Test: p−value = 0.94

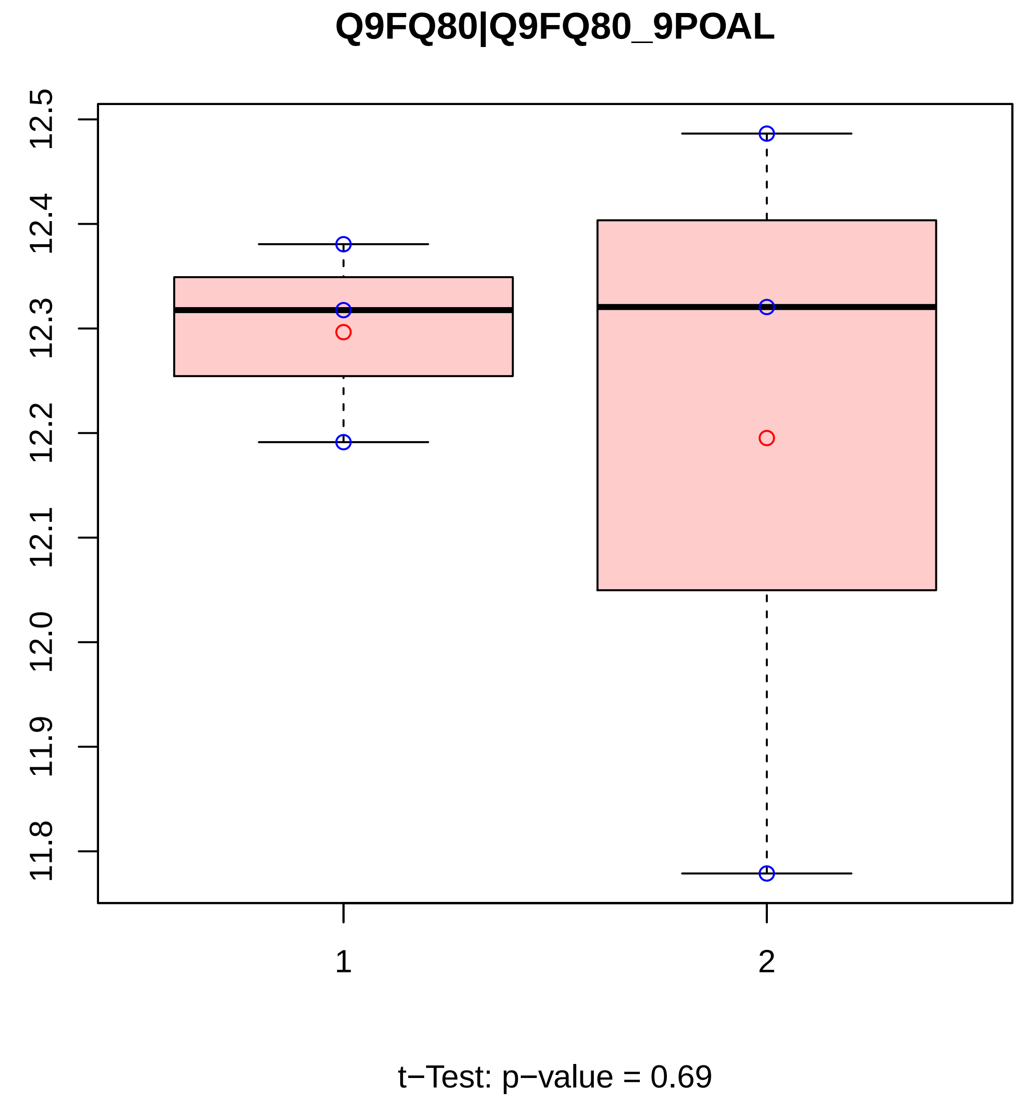
Q9FQ80|Q9FQ80_9POAL
12.5
12.4
12.3
12.2
12.1
12.0
11.9
11.8
1
2
t−Test: p−value = 0.69

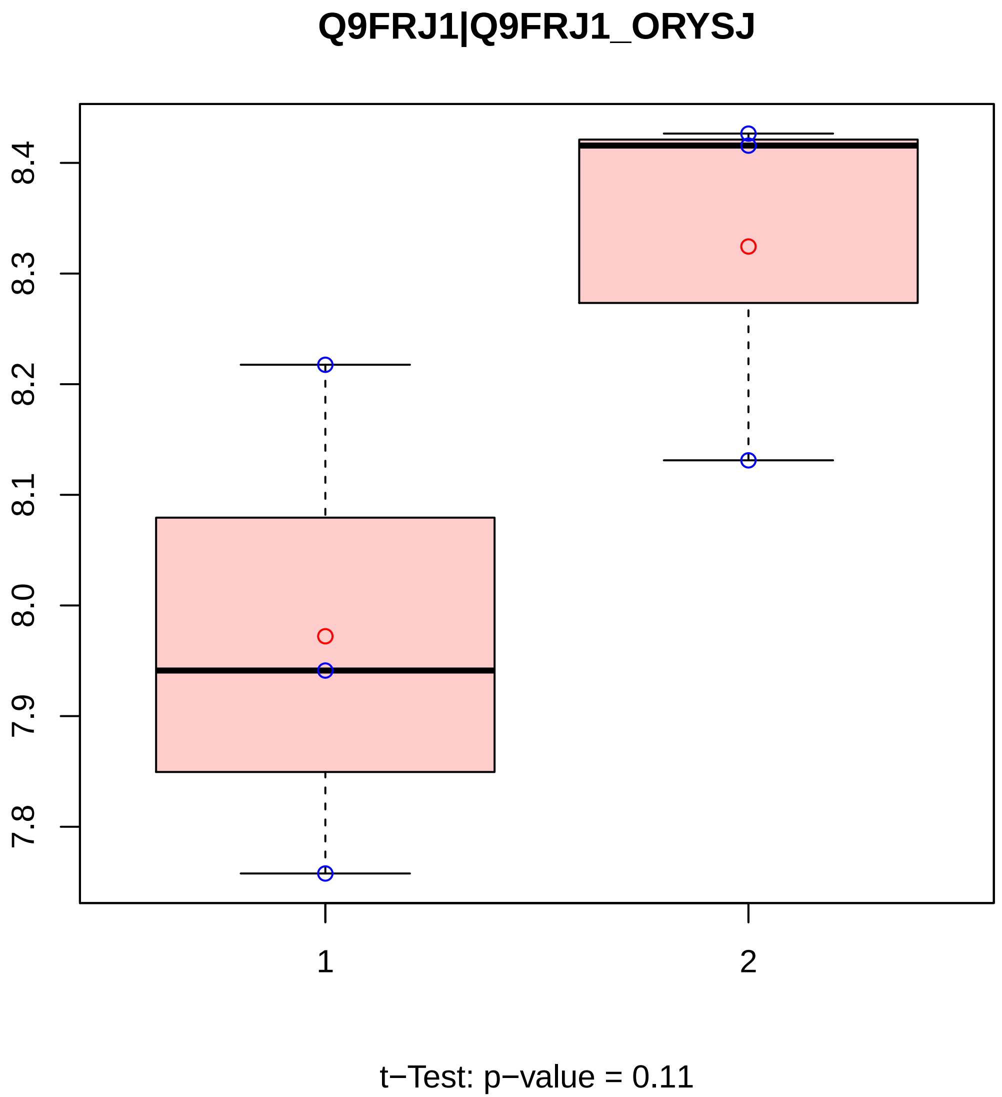
Q9FRJ1|Q9FRJ1_ORYSJ
8.4
8.3
8.2
8.1
8.0
7.9
7.8
1
2
t−Test: p−value = 0.11

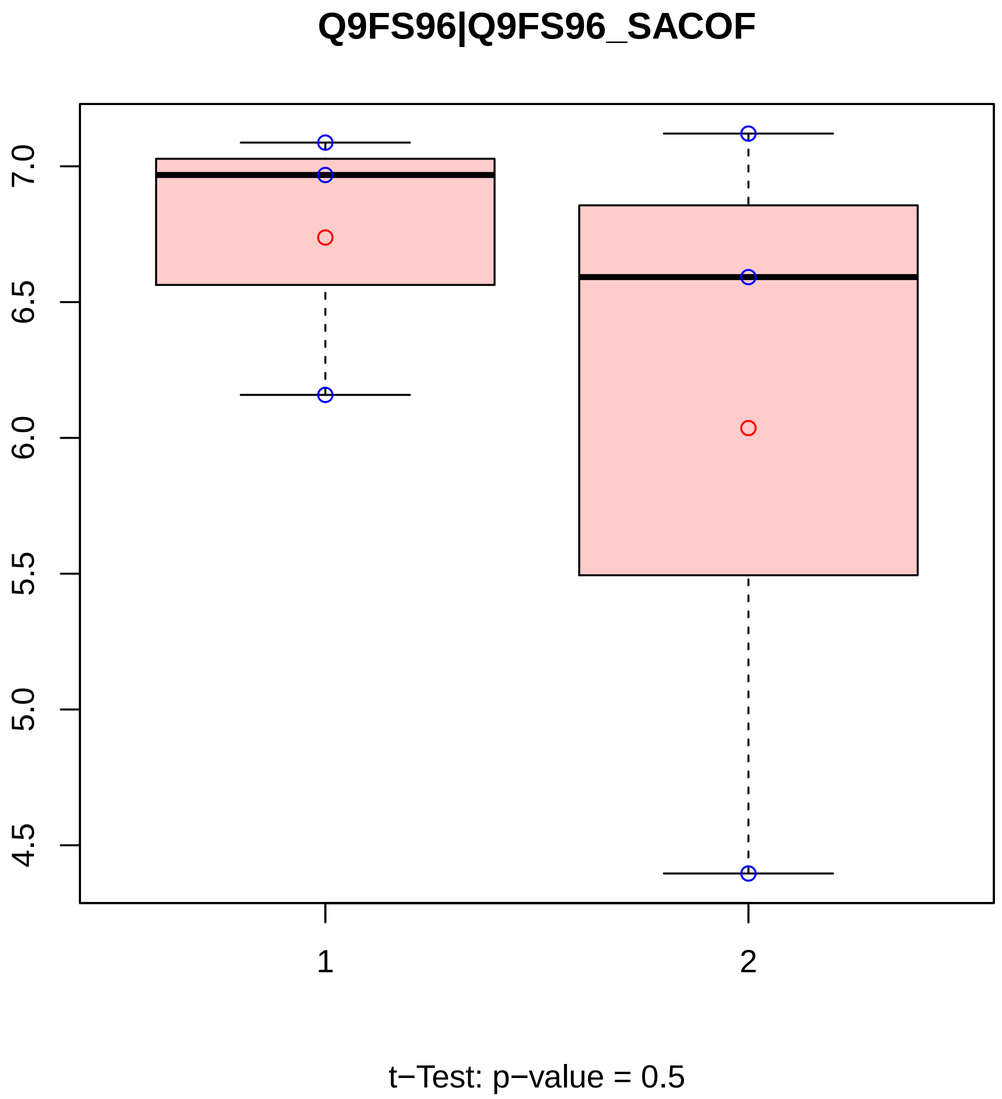

Q9FS96|Q9FS96_SACOF
7.0
6.5
6.0
5.5
5.0
4.5
1
2
t−Test: p−value = 0.5

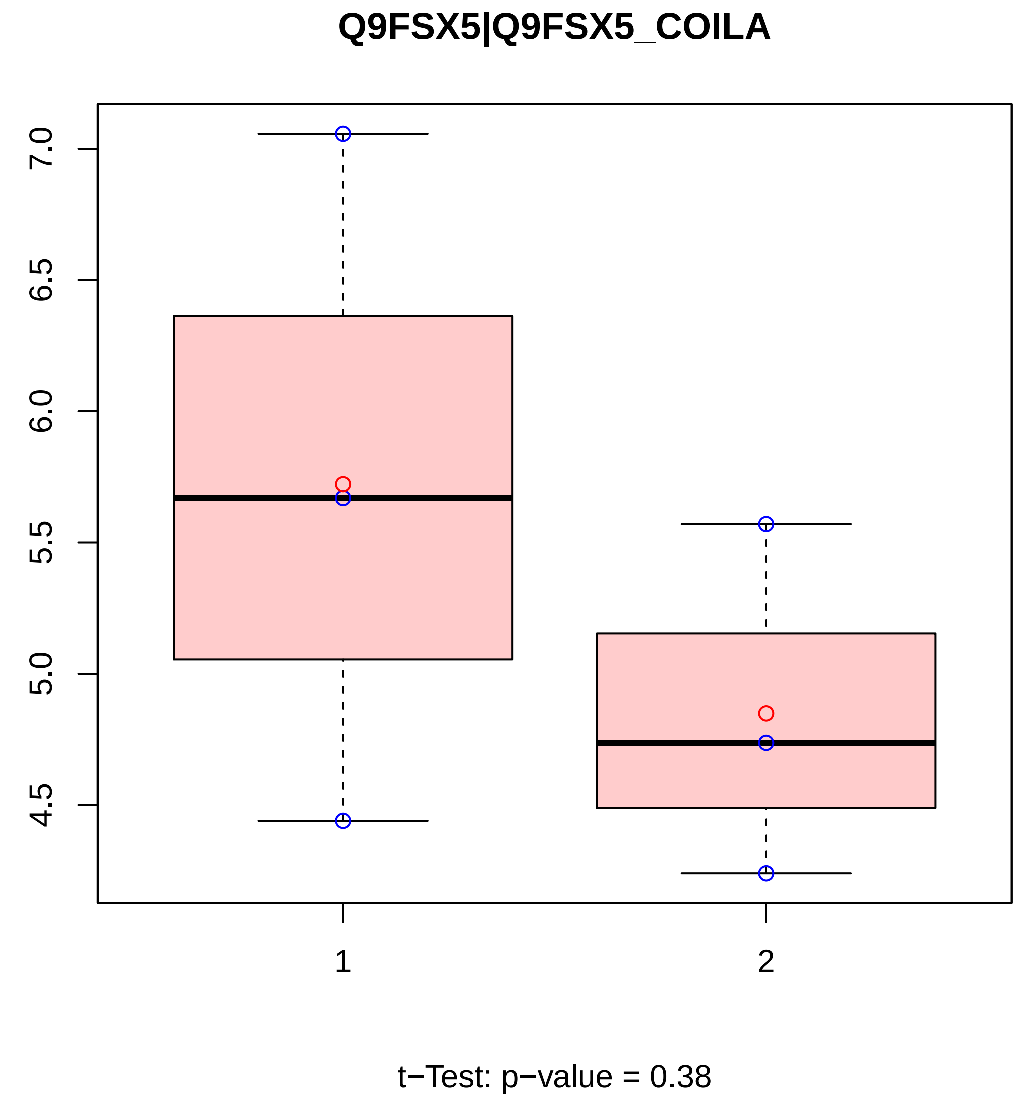
Q9FSX5|Q9FSX5_COILA
7.0
6.5
6.0
5.5
5.0
4.5
1
2
t−Test: p−value = 0.38

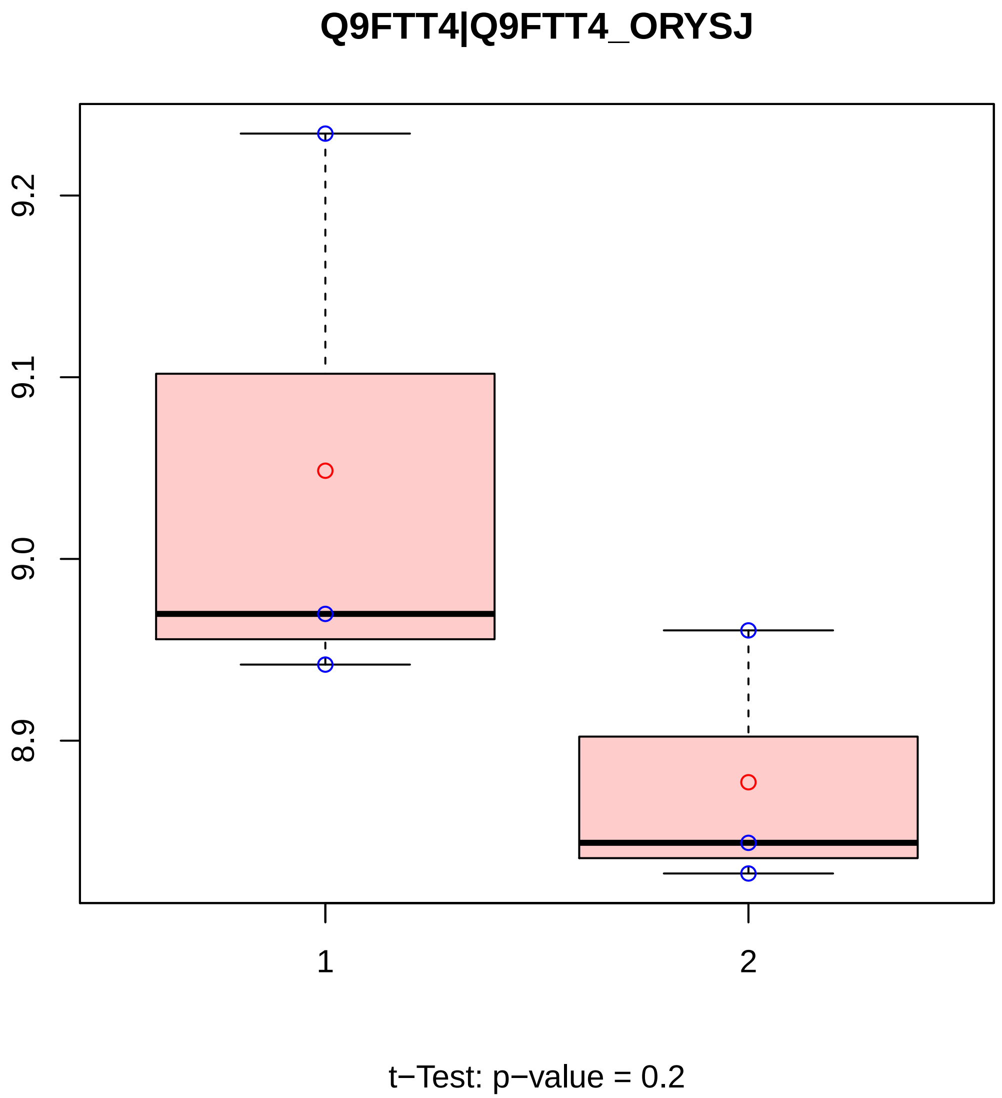
Q9FTT4|Q9FTT4_ORYSJ
9.2
9.1
9.0
8.9
1
2
t−Test: p−value = 0.2

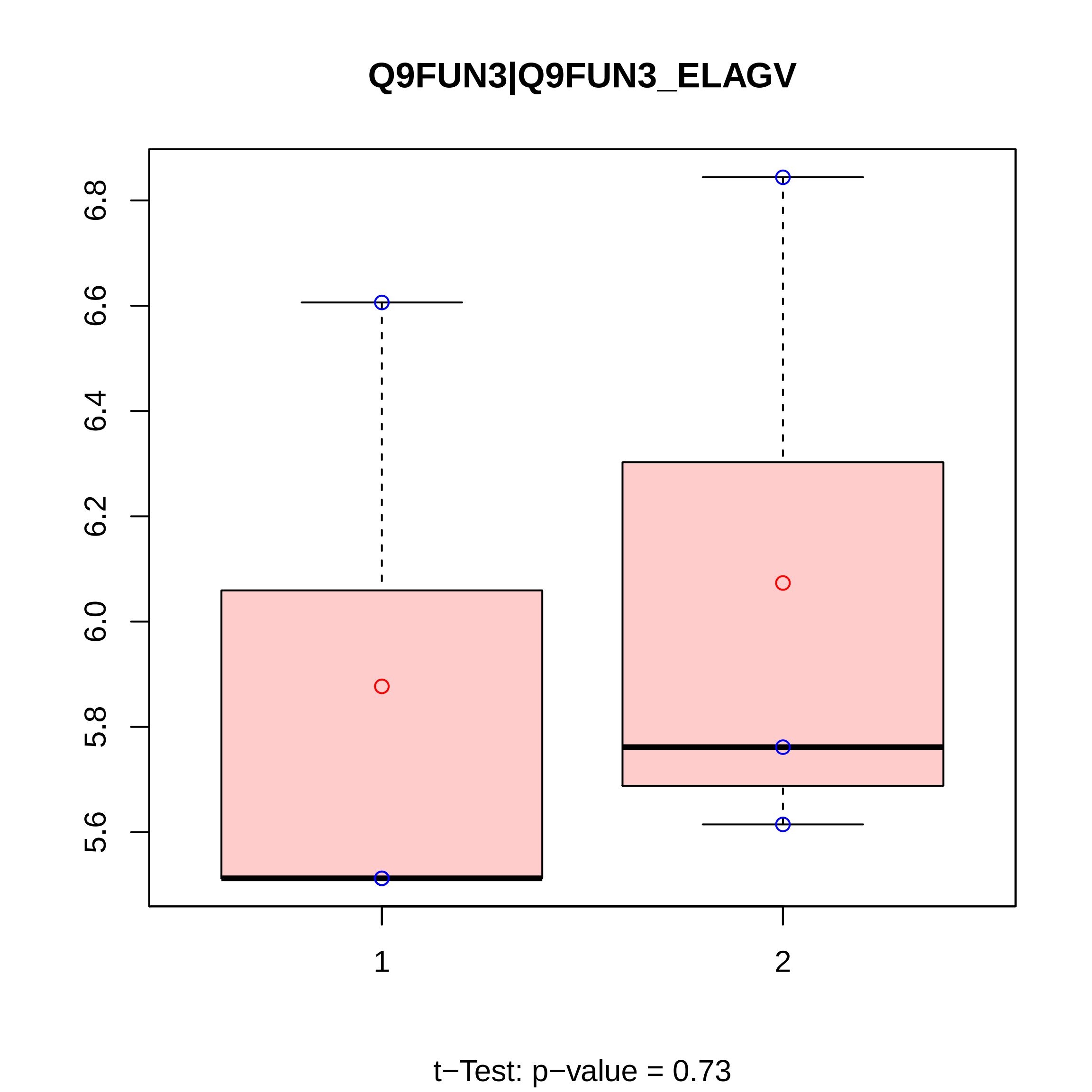
Q9FUN3|Q9FUN3_ELAGV
6.8
6.6
6.4
6.2
6.0
5.8
5.6
1
2
t−Test: p−value = 0.73

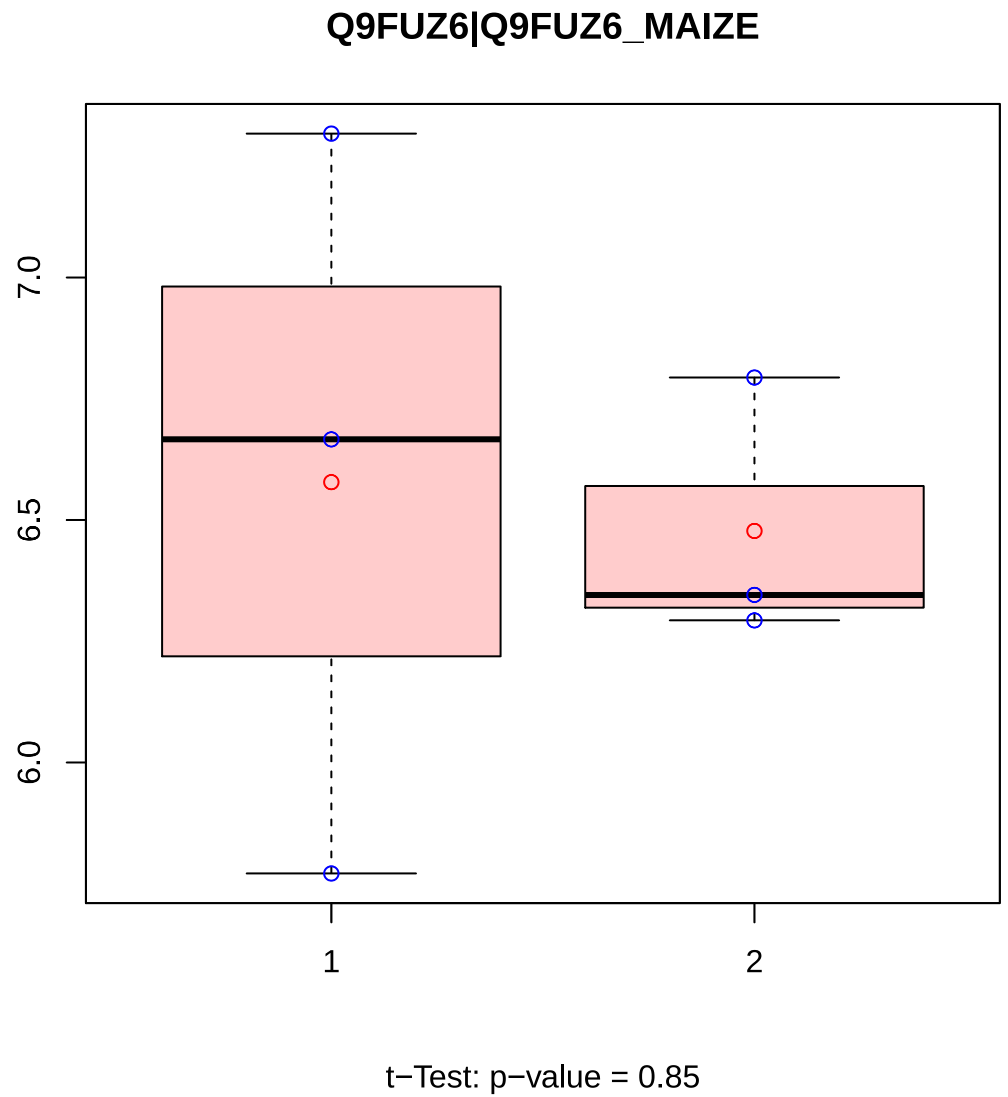
Q9FUZ6|Q9FUZ6_MAIZE
7.0
6.5
6.0
1
2
t−Test: p−value = 0.85

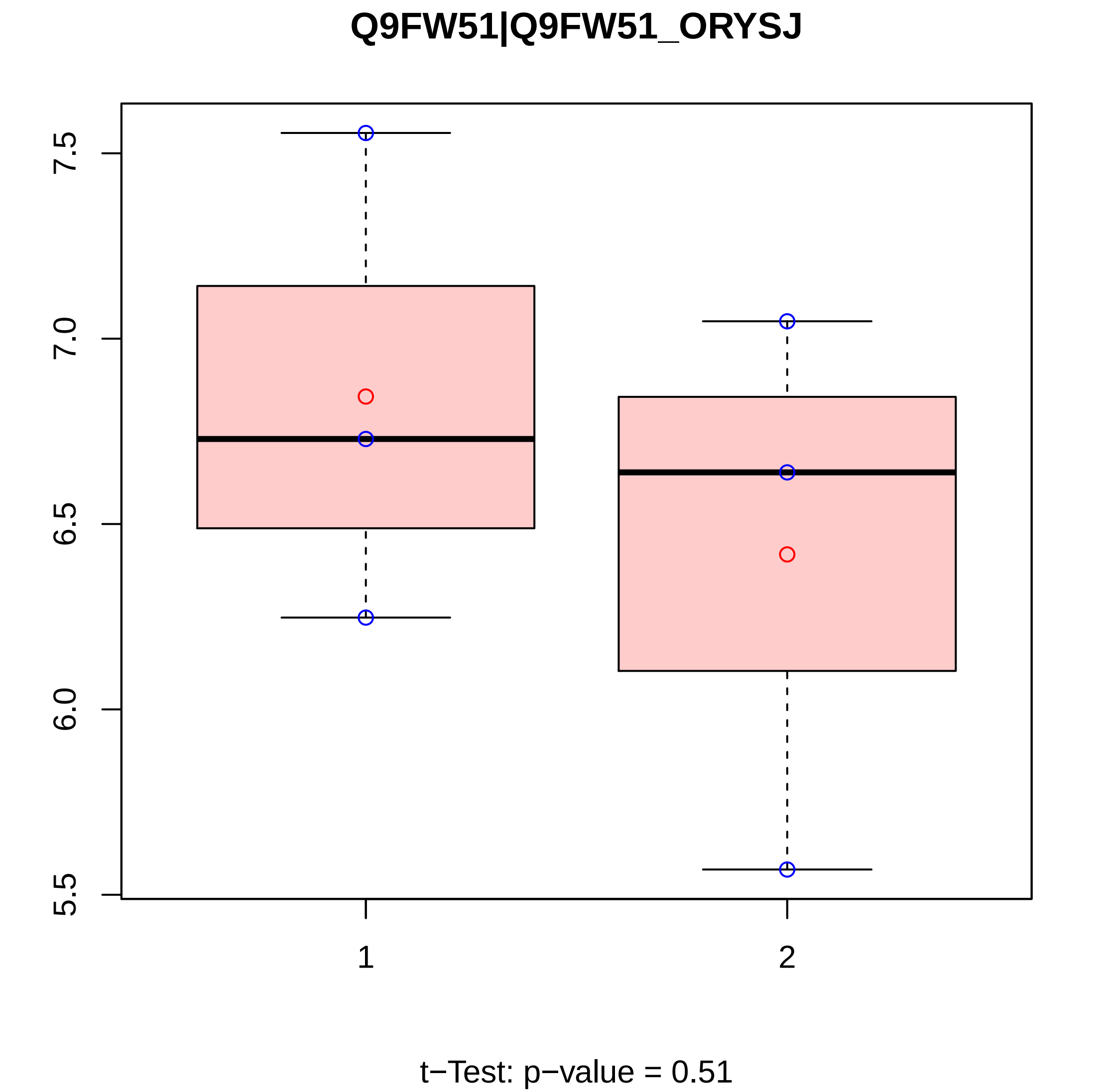

Q9FW51|Q9FW51_ORYSJ
7.5
7.0
6.5
6.0
5.5
1
2
t−Test: p−value = 0.51

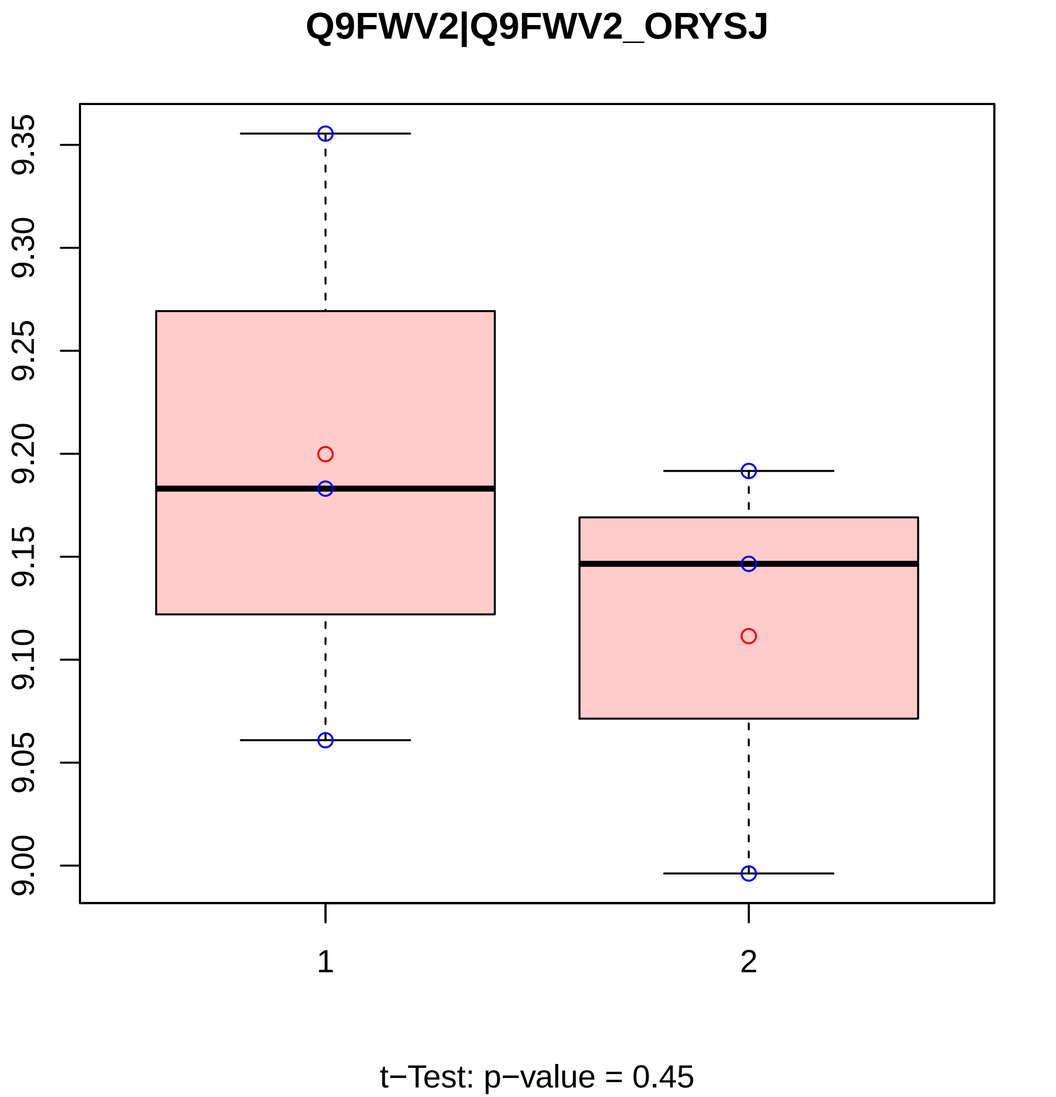

Q9FWV2|Q9FWV2_ORYSJ
9.00
9.05
9.10
9.15
9.20
9.25
9.30
9.35
1
2
t−Test: p−value = 0.45

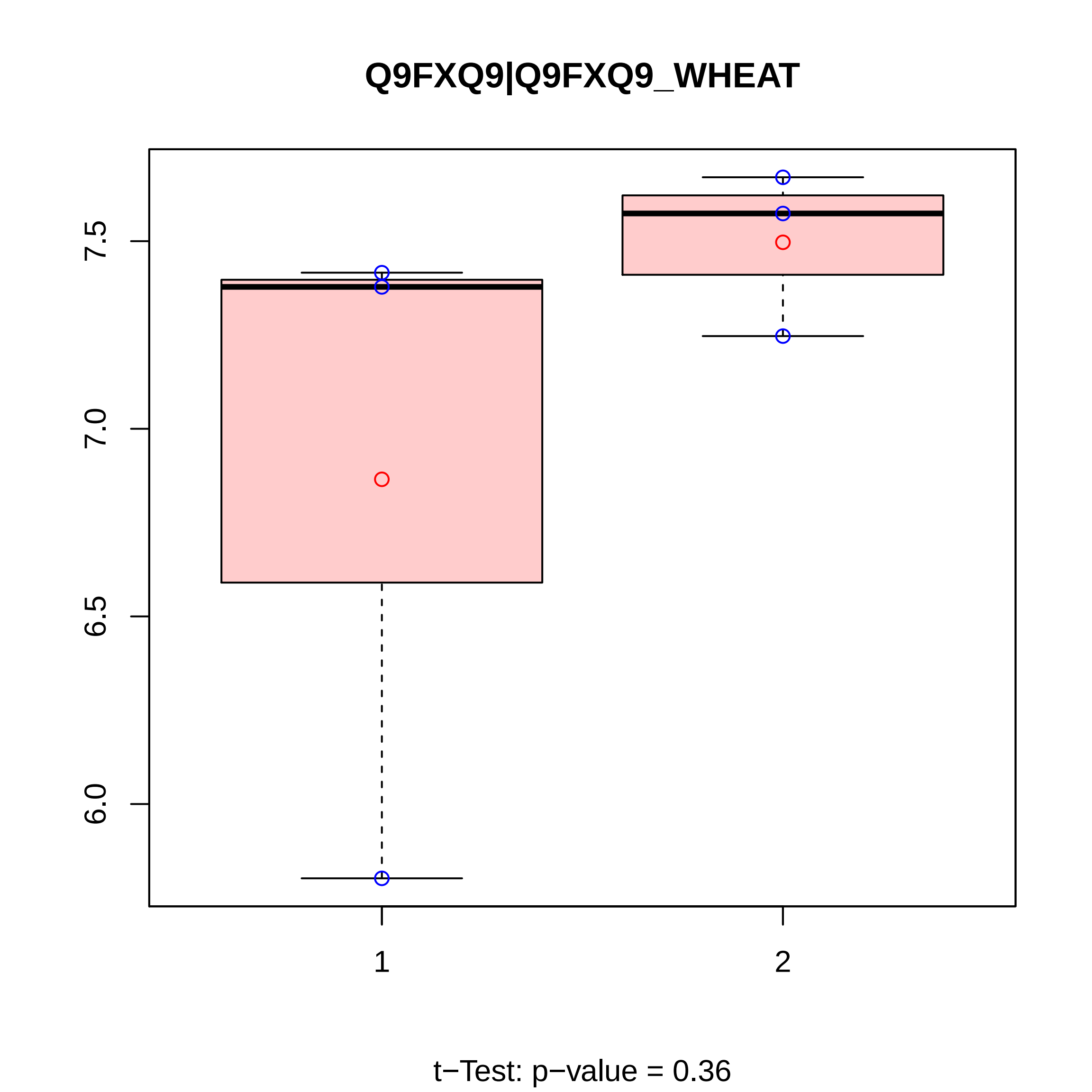

Q9FXQ9|Q9FXQ9_WHEAT
6.0
6.5
7.0
7.5
1
2
t−Test: p−value = 0.36

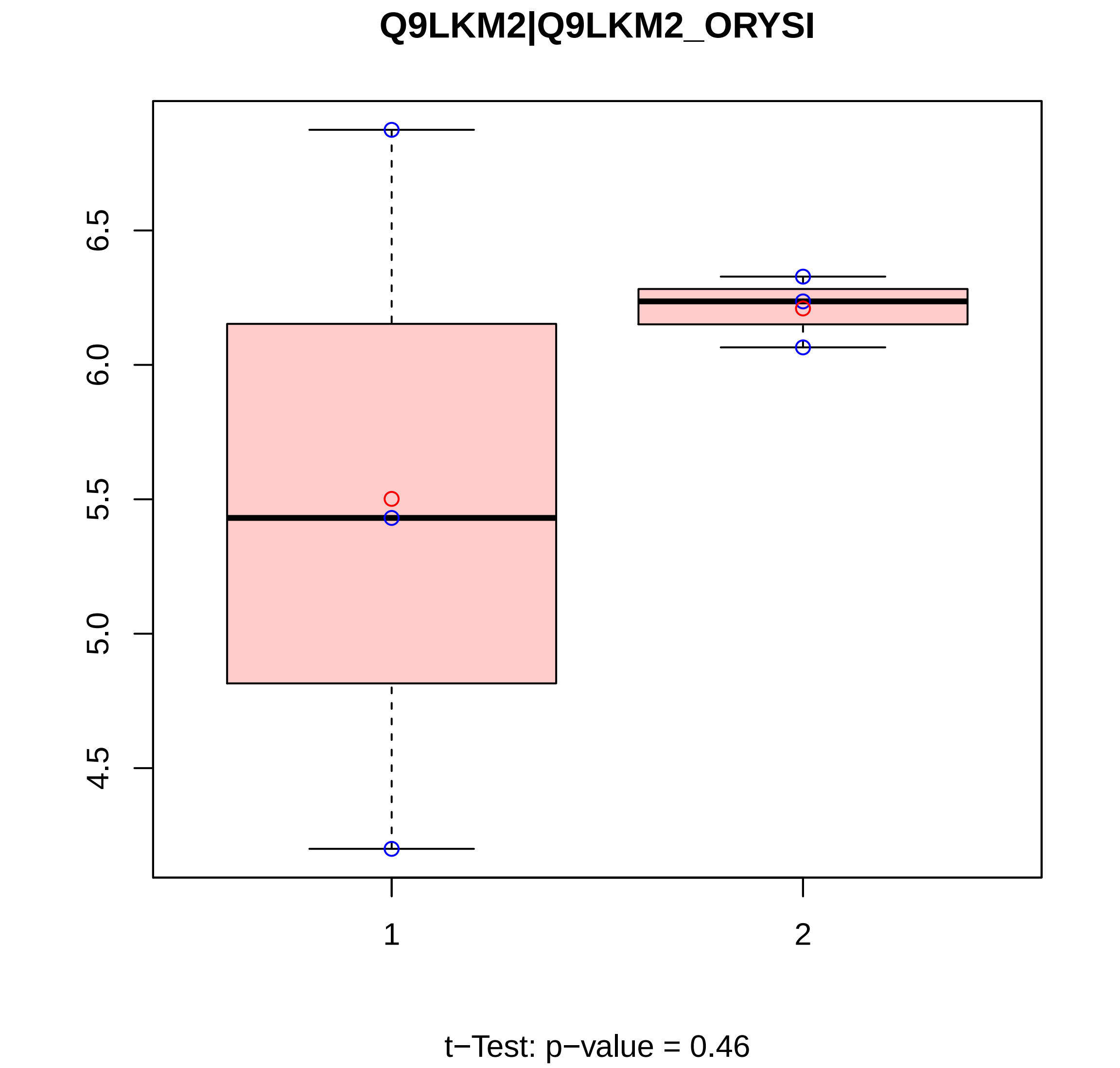
Q9LKM2|Q9LKM2_ORYSI
6.5
6.0
5.5
5.0
4.5
1
2
t−Test: p−value = 0.46

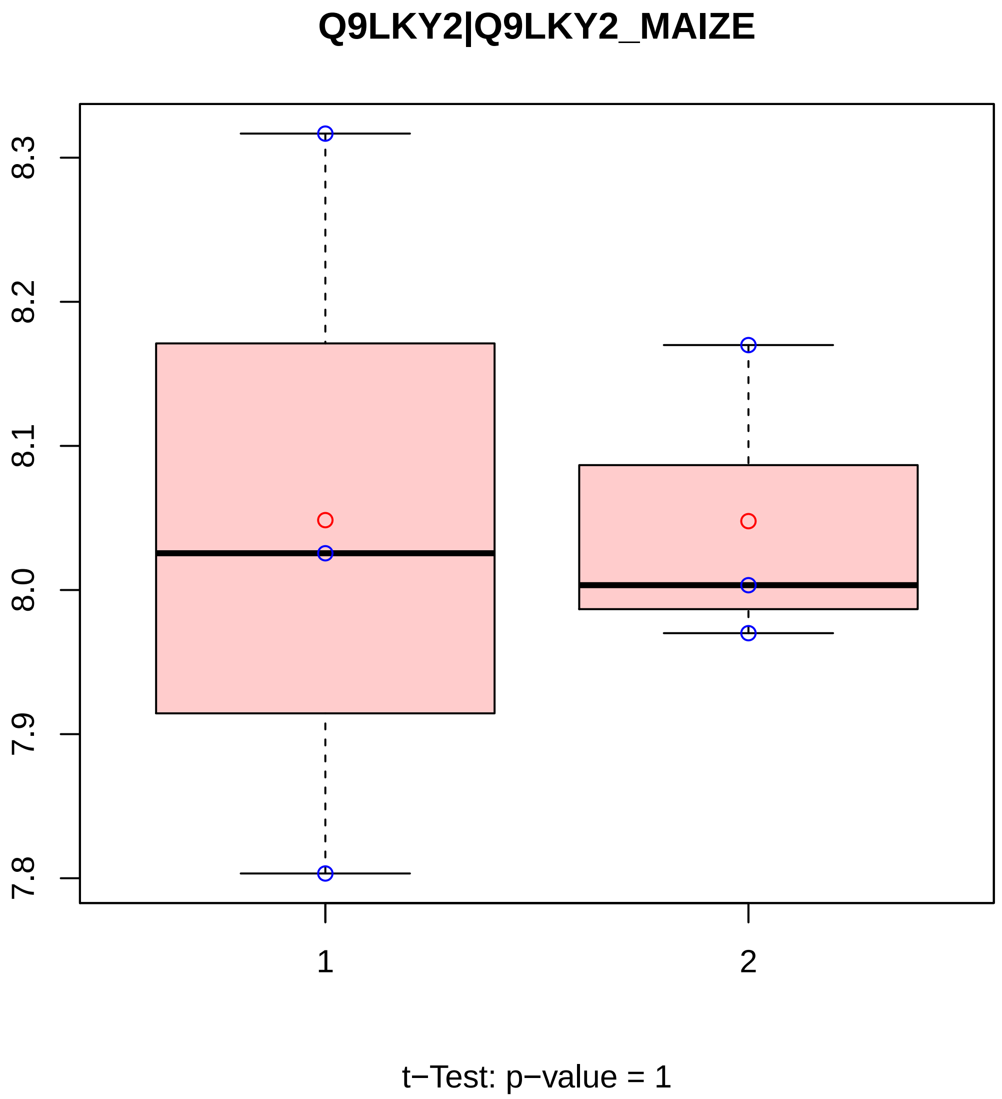

Q9LKY2|Q9LKY2_MAIZE
8.3
8.2
8.1
8.0
7.9
7.8
1
2
t−Test: p−value = 1

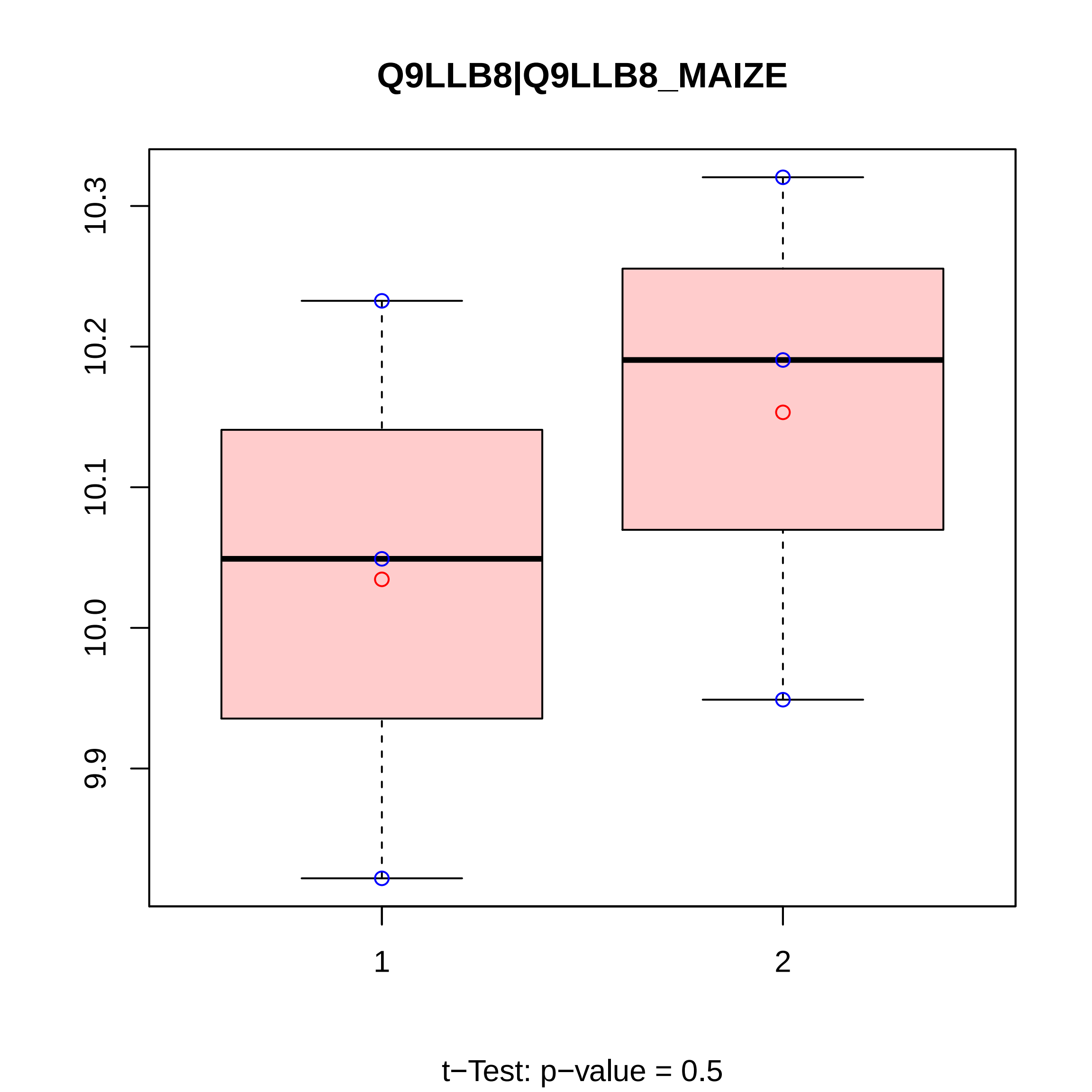

Q9LLB8|Q9LLB8_MAIZE
10.3
10.2
10.1
10.0
9.9
1
2
t−Test: p−value = 0.5

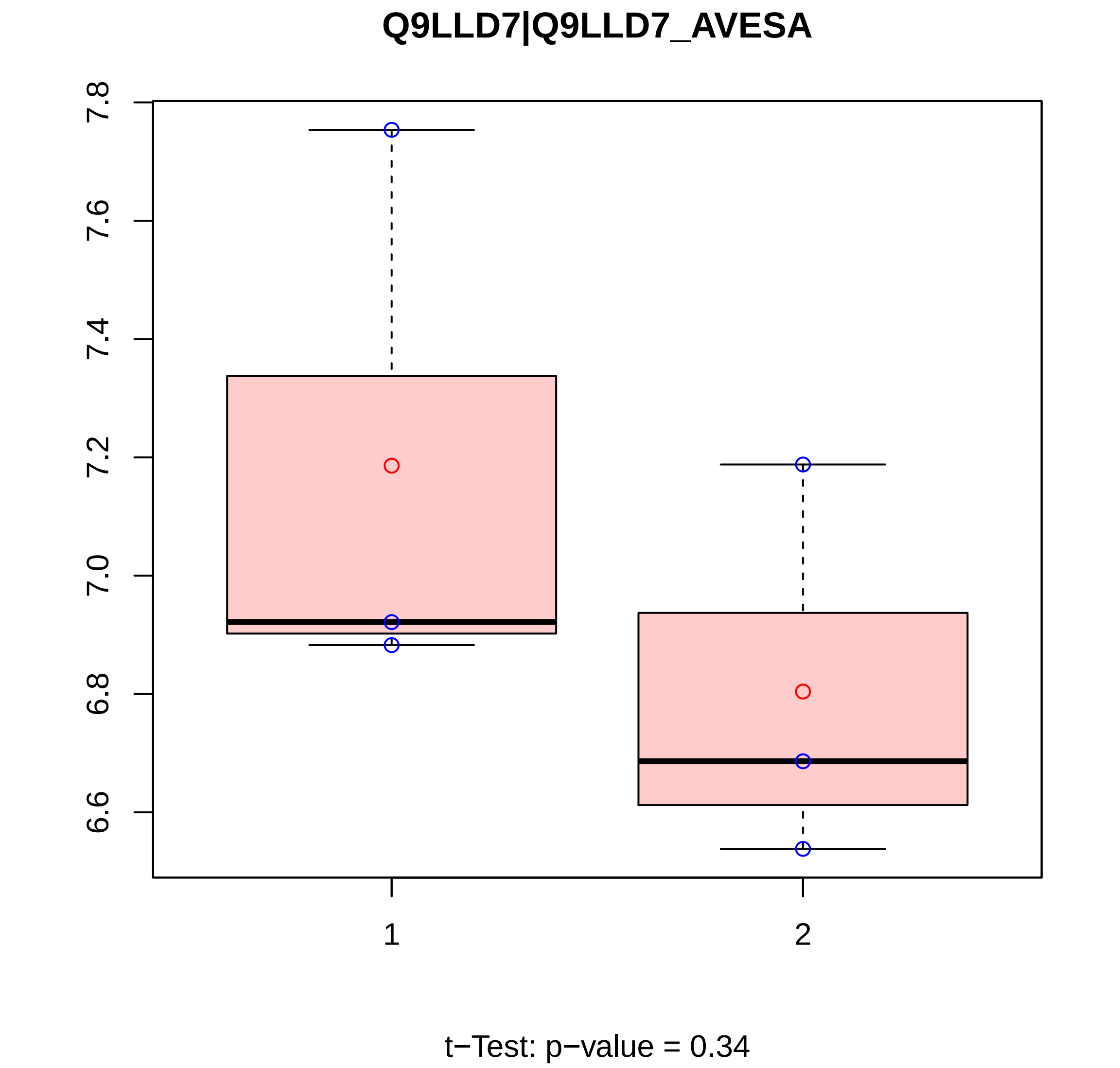
Q9LLD7|Q9LLD7_AVESA
7.8
7.6
7.4
7.2
7.0
6.8
6.6
1
2
t−Test: p−value = 0.34

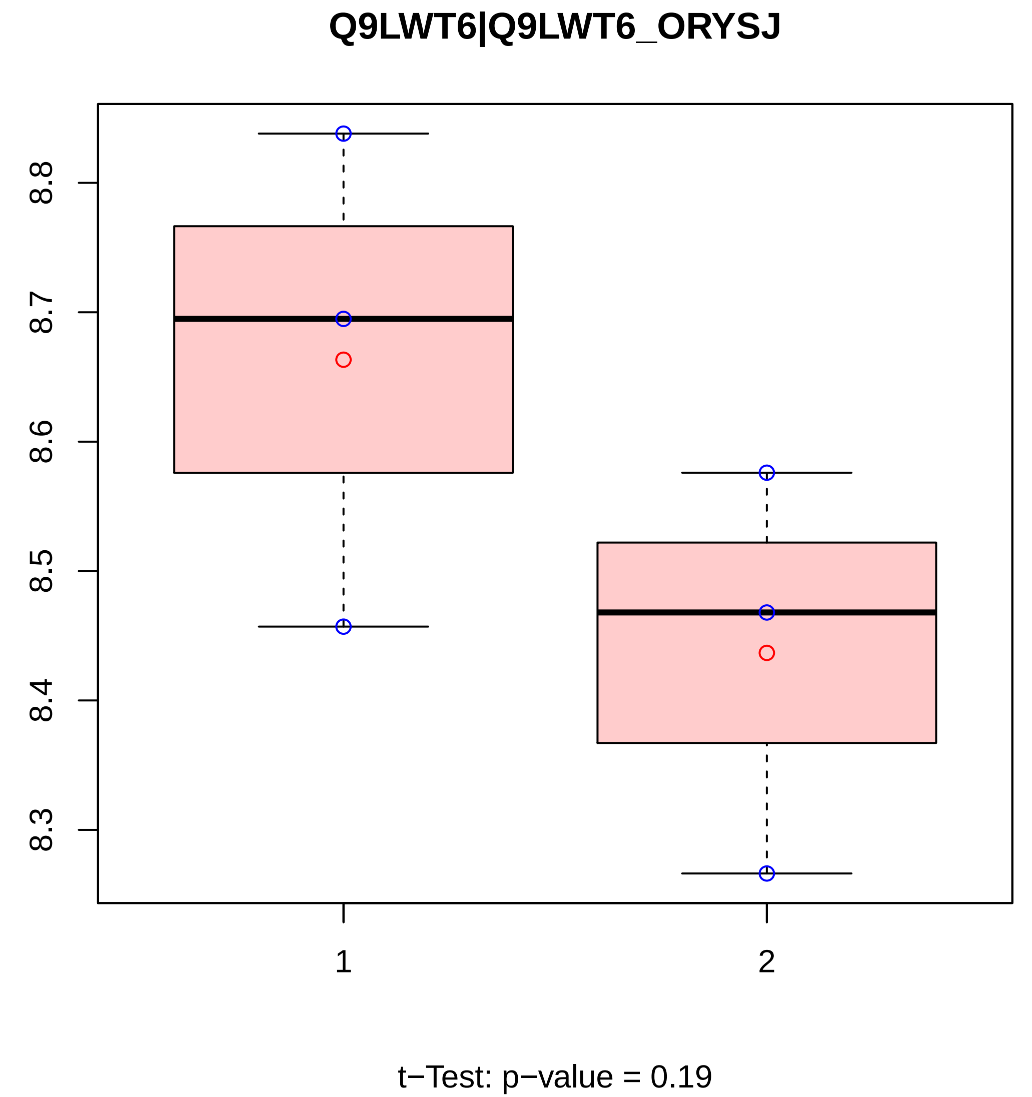
Q9LWT6|Q9LWT6_ORYSJ
8.3
8.4
8.5
8.6
8.7
8.8
1
2
t−Test: p−value = 0.19

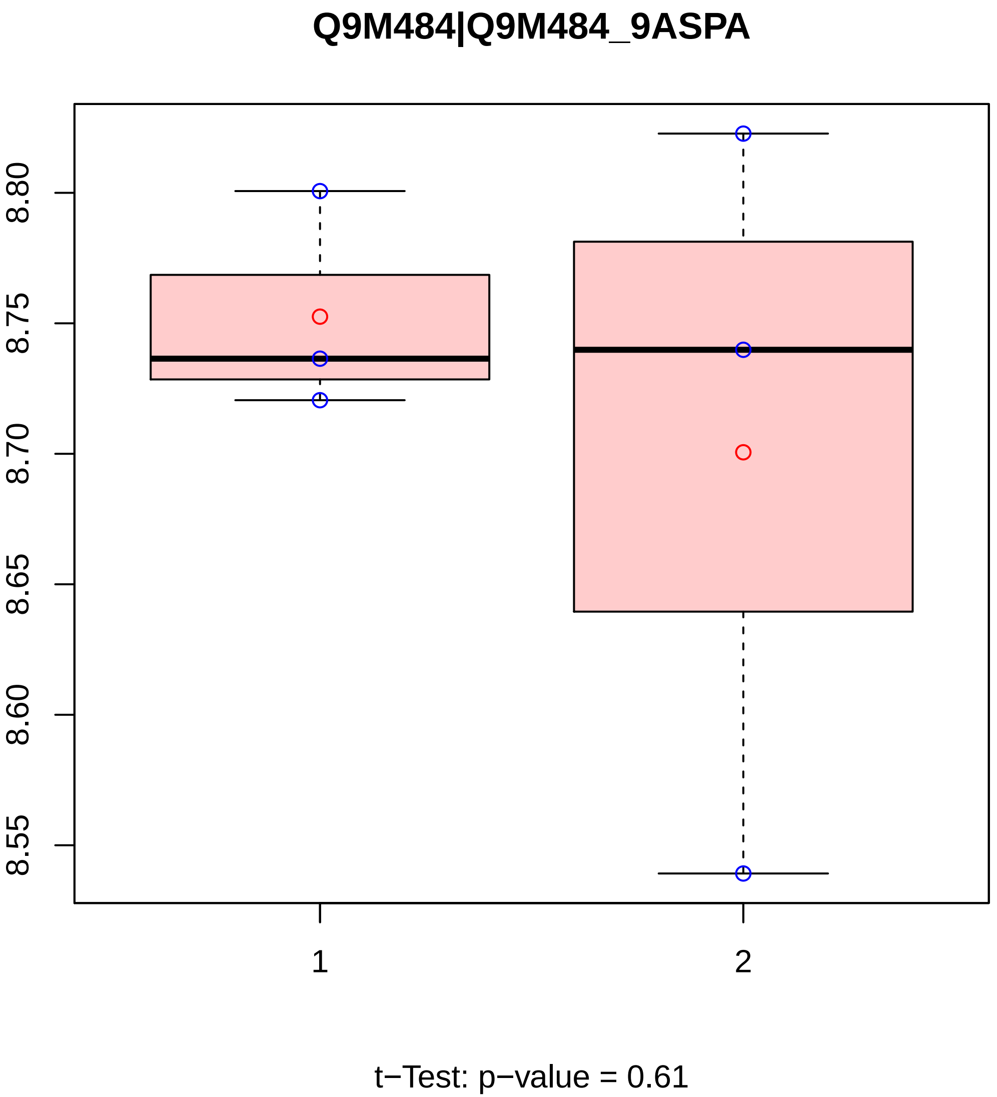

Q9M484|Q9M484_9ASPA
8.80
8.75
8.70
8.65
8.60
8.55
1
2
t−Test: p−value = 0.61

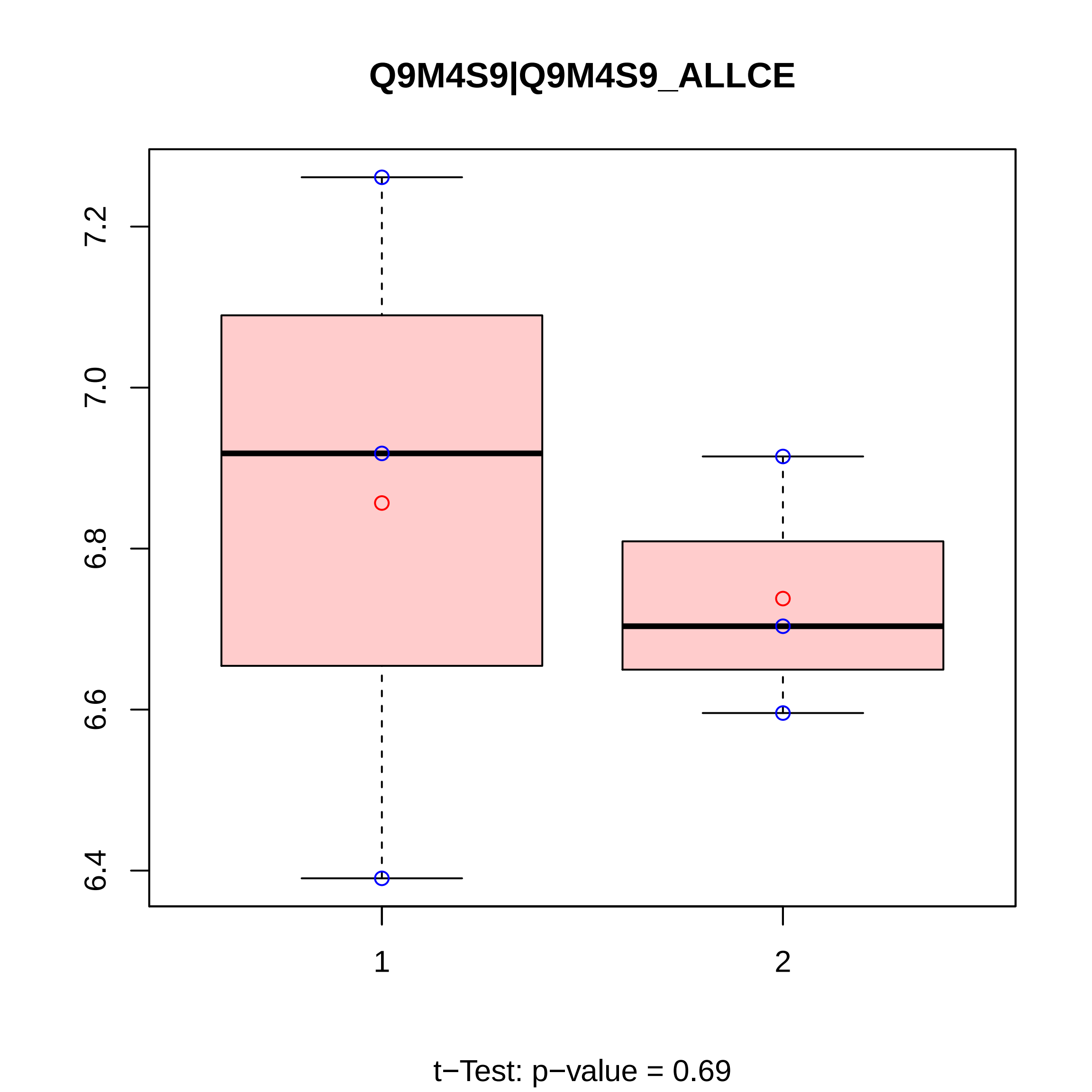
Q9M4S9|Q9M4S9_ALLCE
7.2
7.0
6.8
6.6
6.4
1
2
t−Test: p−value = 0.69

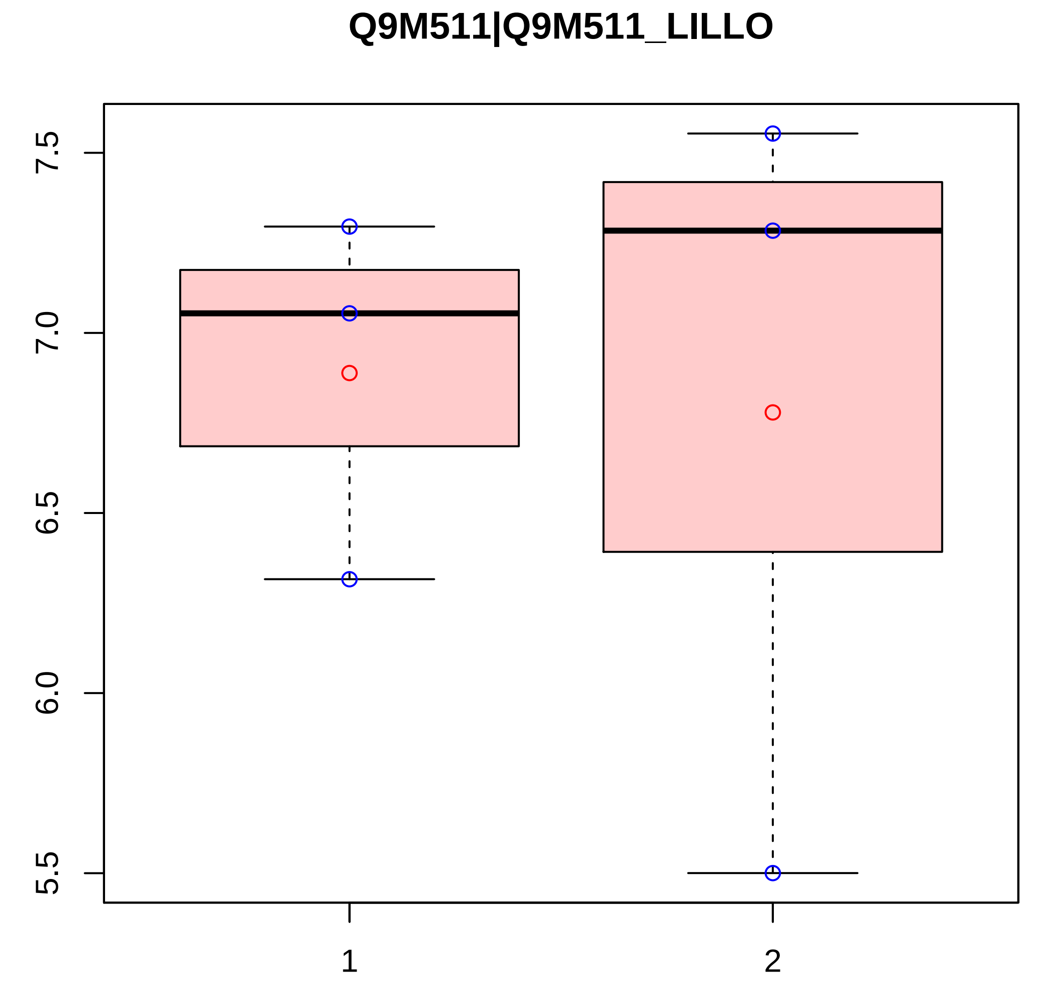

t−Test: p−value = 0.89

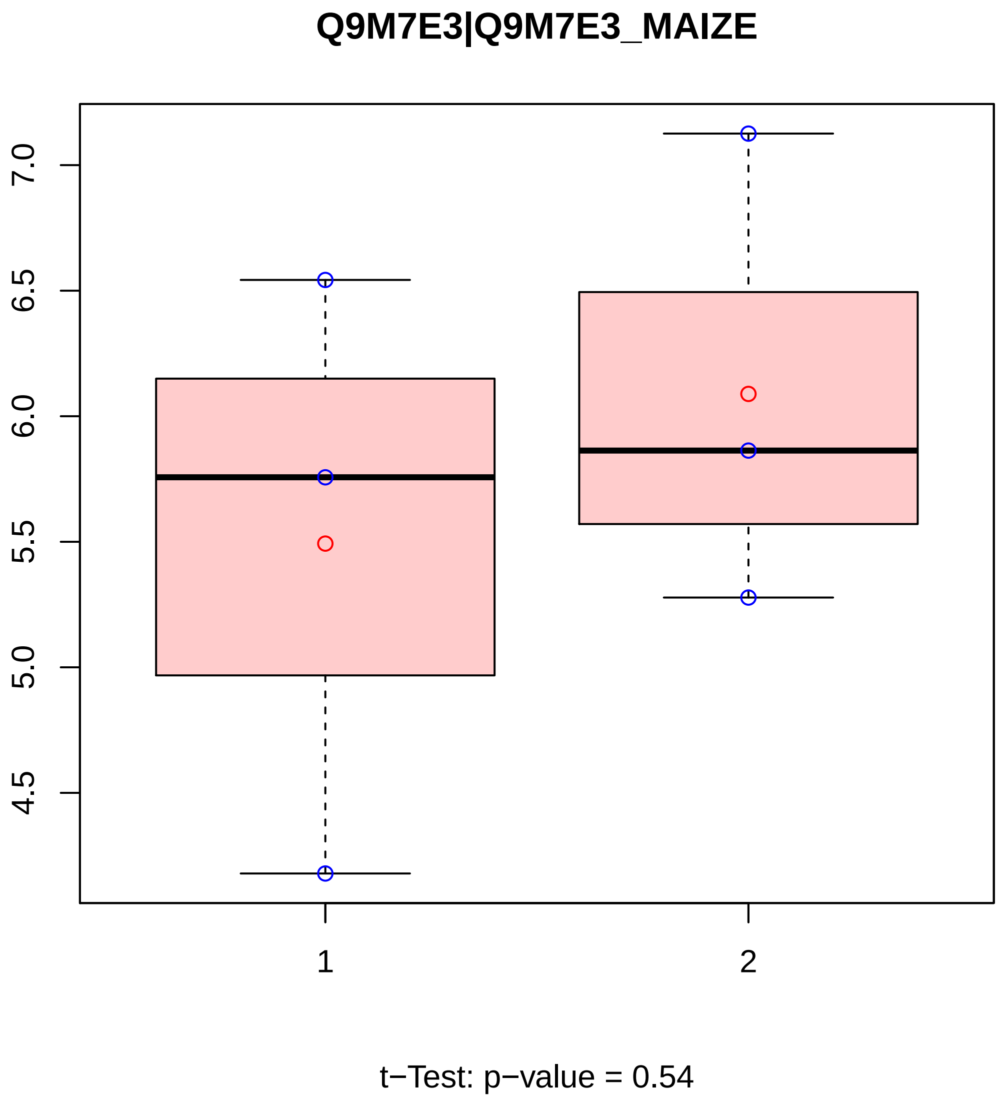
Q9M7E3|Q9M7E3_MAIZE
7.0
6.5
6.0
5.5
5.0
4.5
1
2
t−Test: p−value = 0.54

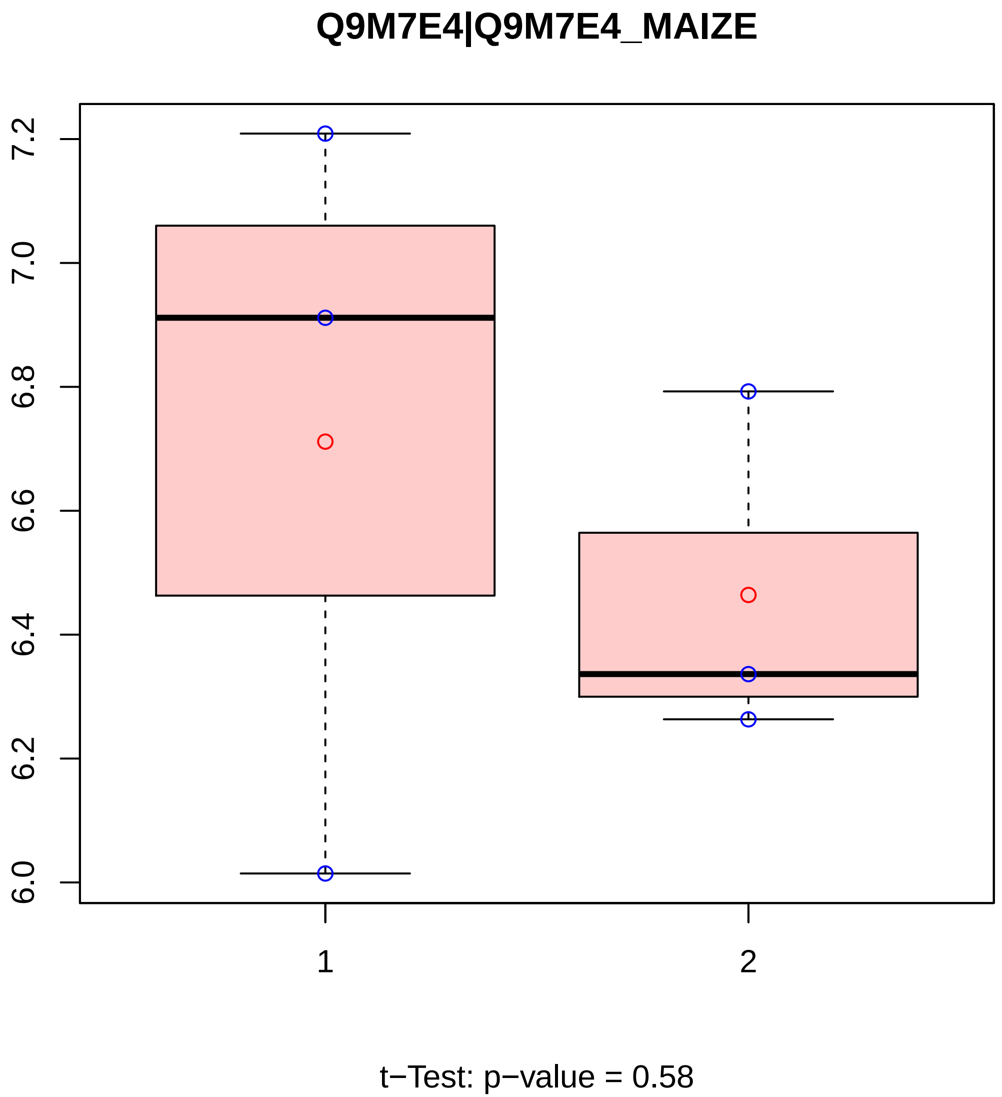
Q9M7E4|Q9M7E4_MAIZE
7.2
7.0
6.8
6.6
6.4
6.2
6.0
1
2
t−Test: p−value = 0.58

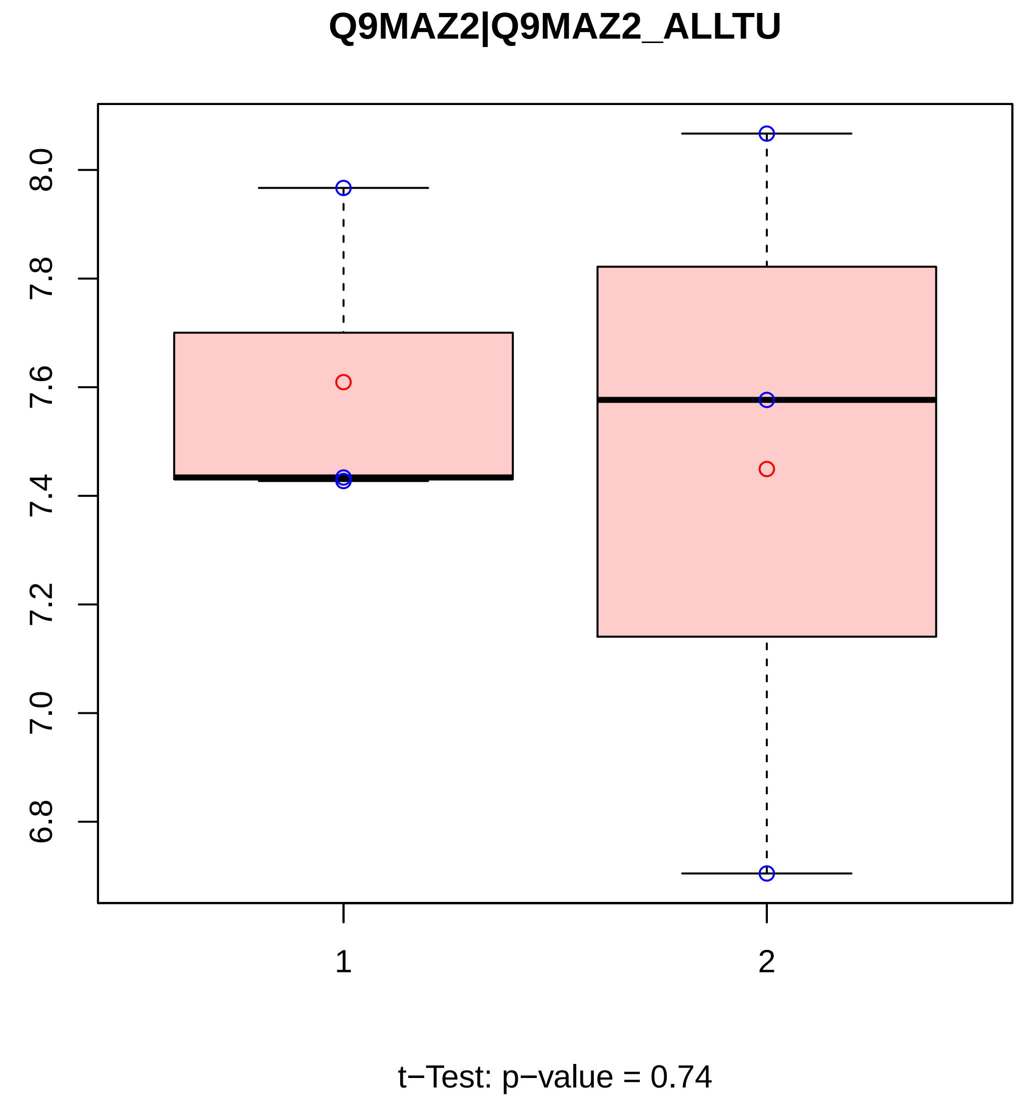
Q9MAZ2|Q9MAZ2_ALLTU
8.0
7.8
7.6
7.4
7.2
7.0
6.8
1
2
t−Test: p−value = 0.74

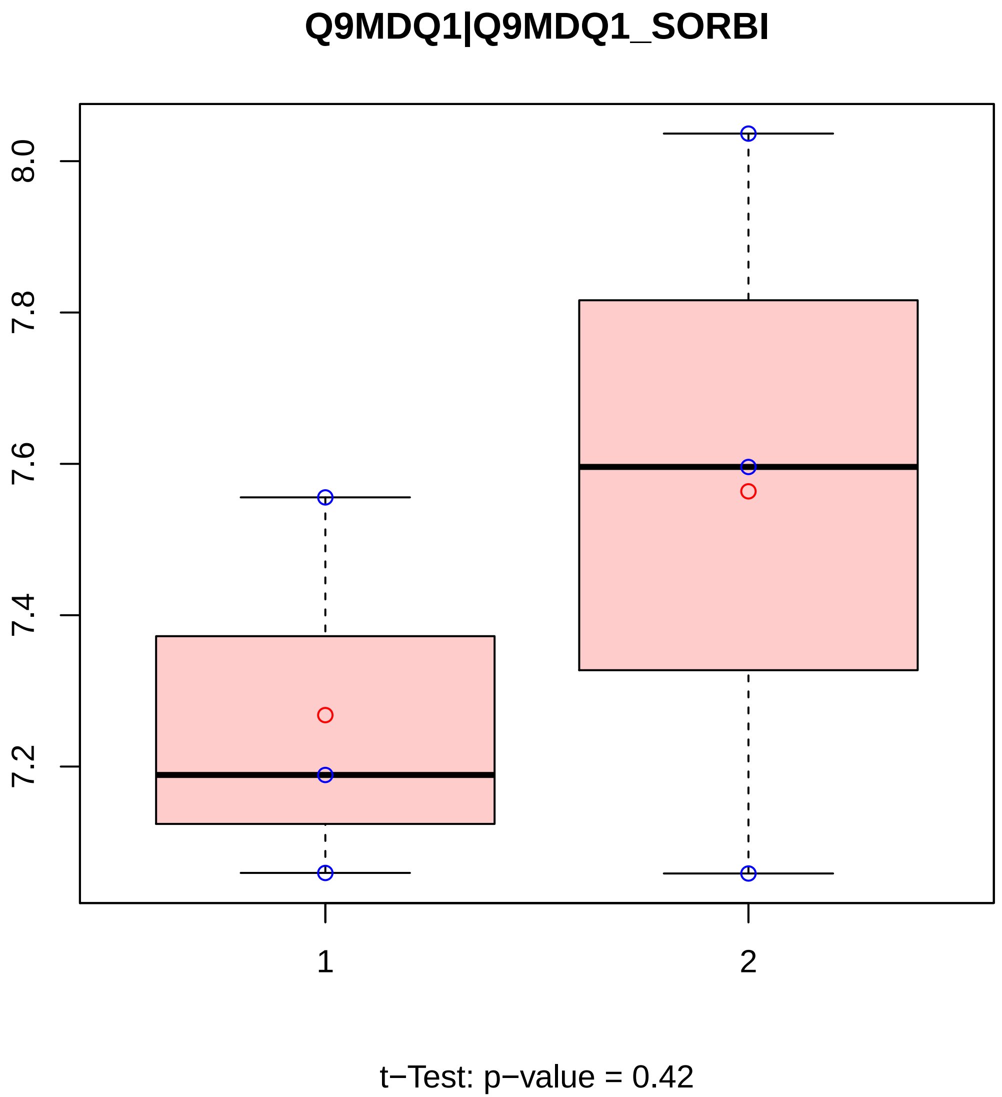
Q9MDQ1|Q9MDQ1_SORBI
8.0
7.8
7.6
7.4
7.2
1
2
t−Test: p−value = 0.42

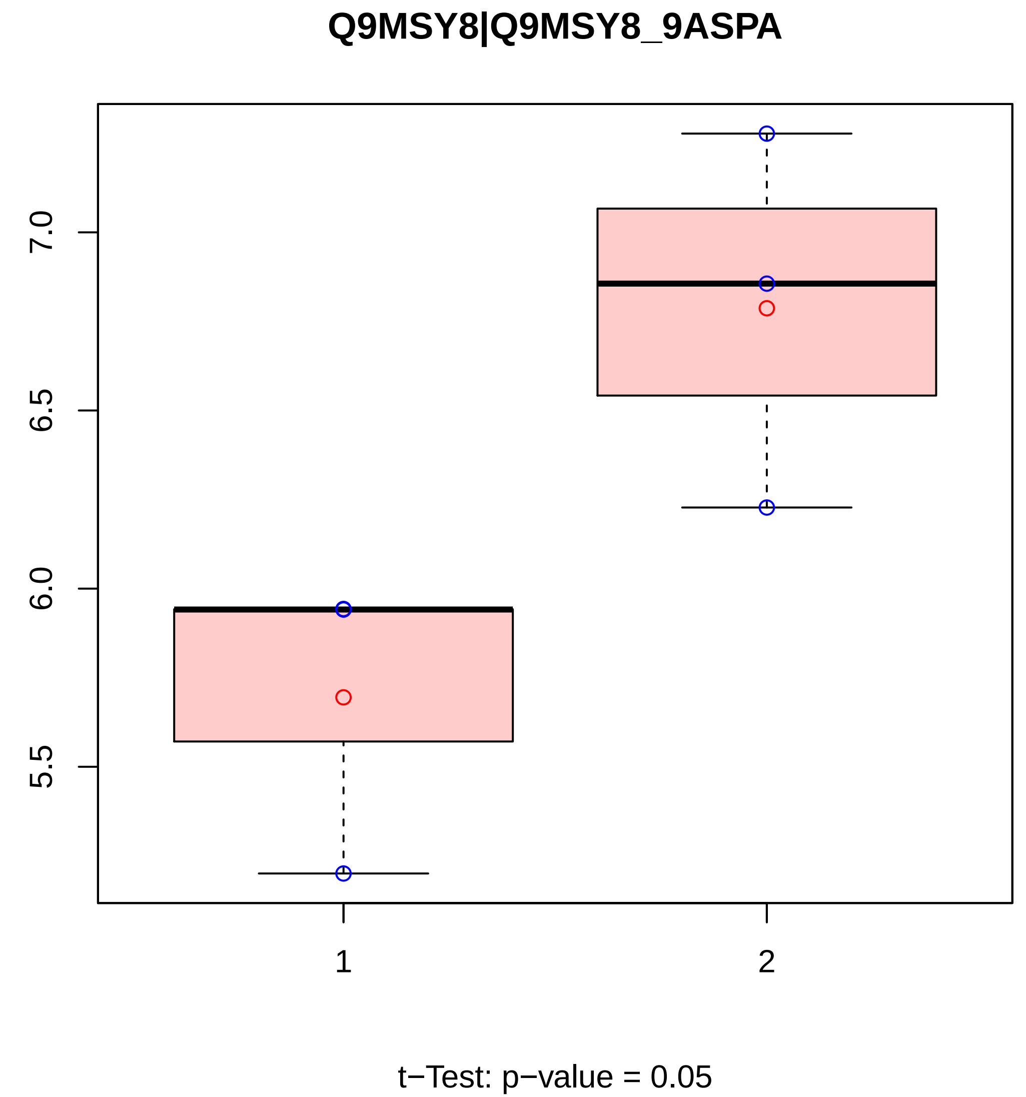
Q9MSY8|Q9MSY8_9ASPA
7.0
6.5
6.0
5.5
1
2
t−Test: p−value = 0.05

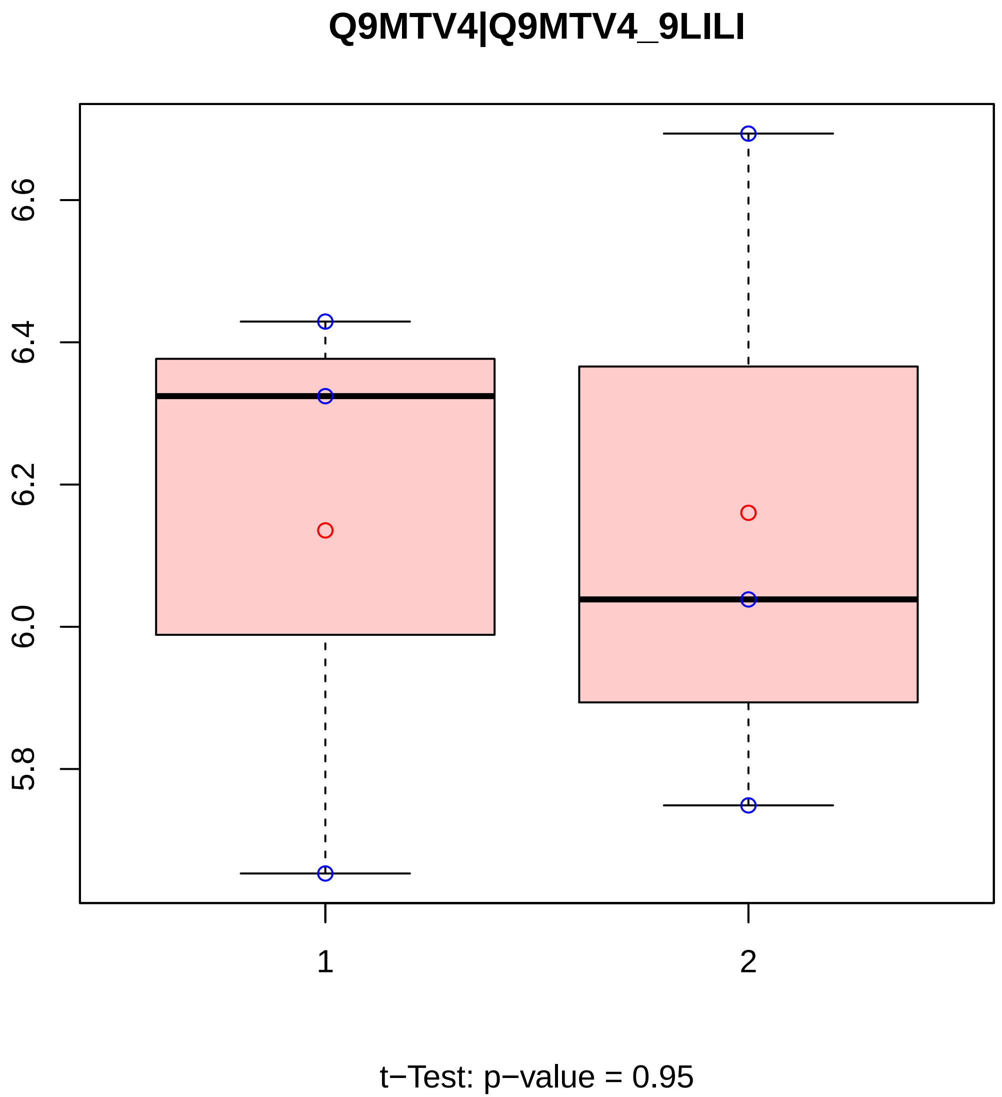
Q9MTV4|Q9MTV4_9LILI
6.6
6.4
6.2
6.0
5.8
1
2
t−Test: p−value = 0.95

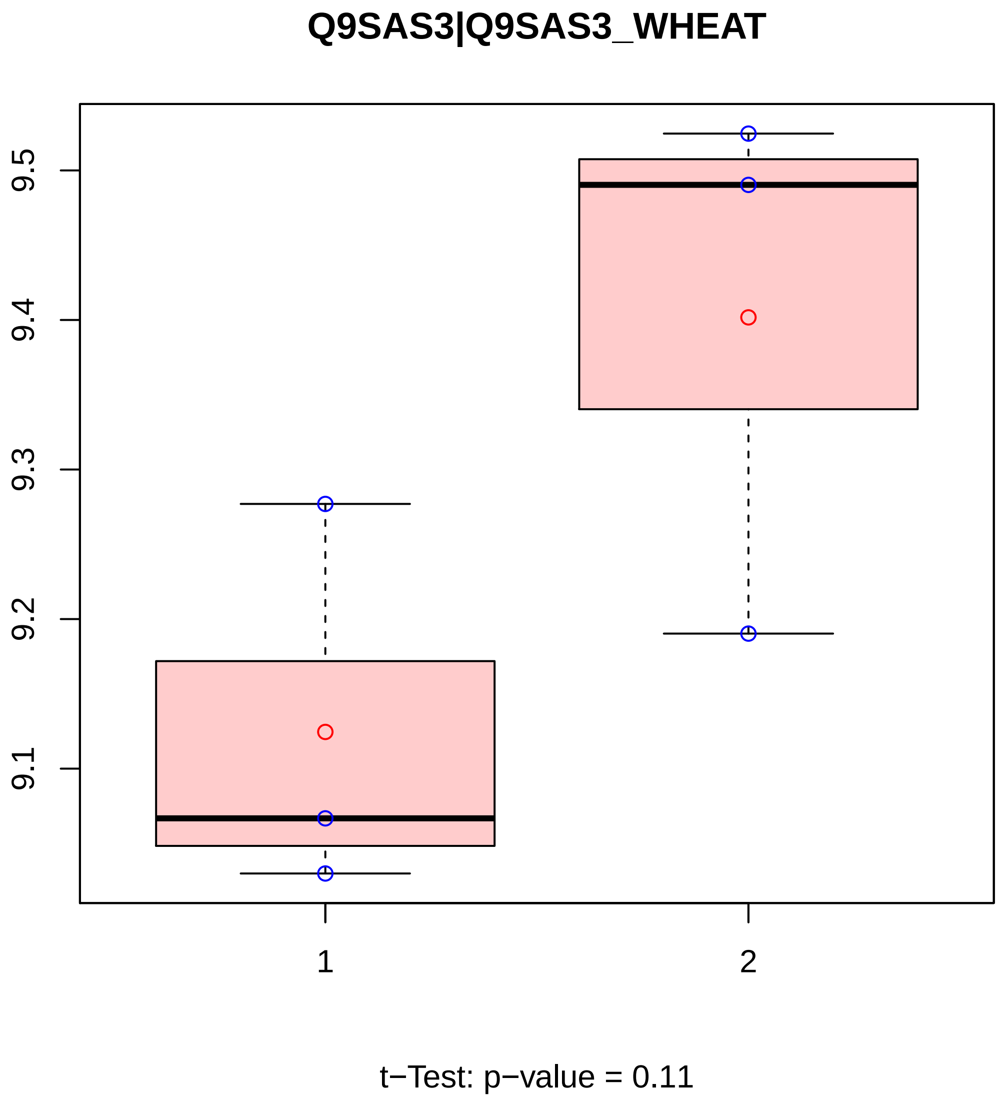
Q9SAS3|Q9SAS3_WHEAT
9.1
9.2
9.3
9.4
9.5
1
2
t−Test: p−value = 0.11

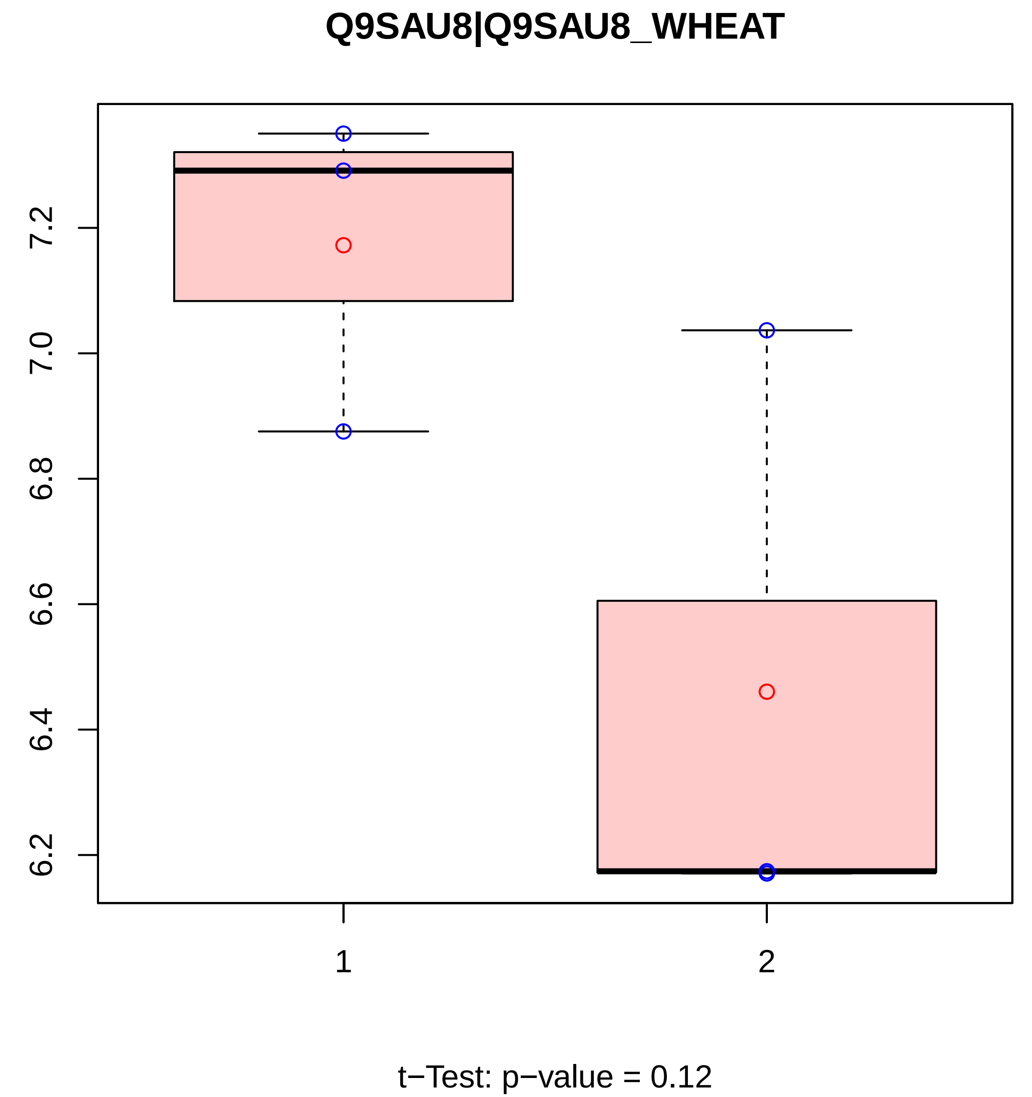
Q9SAU8|Q9SAU8_WHEAT
7.2
7.0
6.8
6.6
6.4
6.2
1
2
t−Test: p−value = 0.12

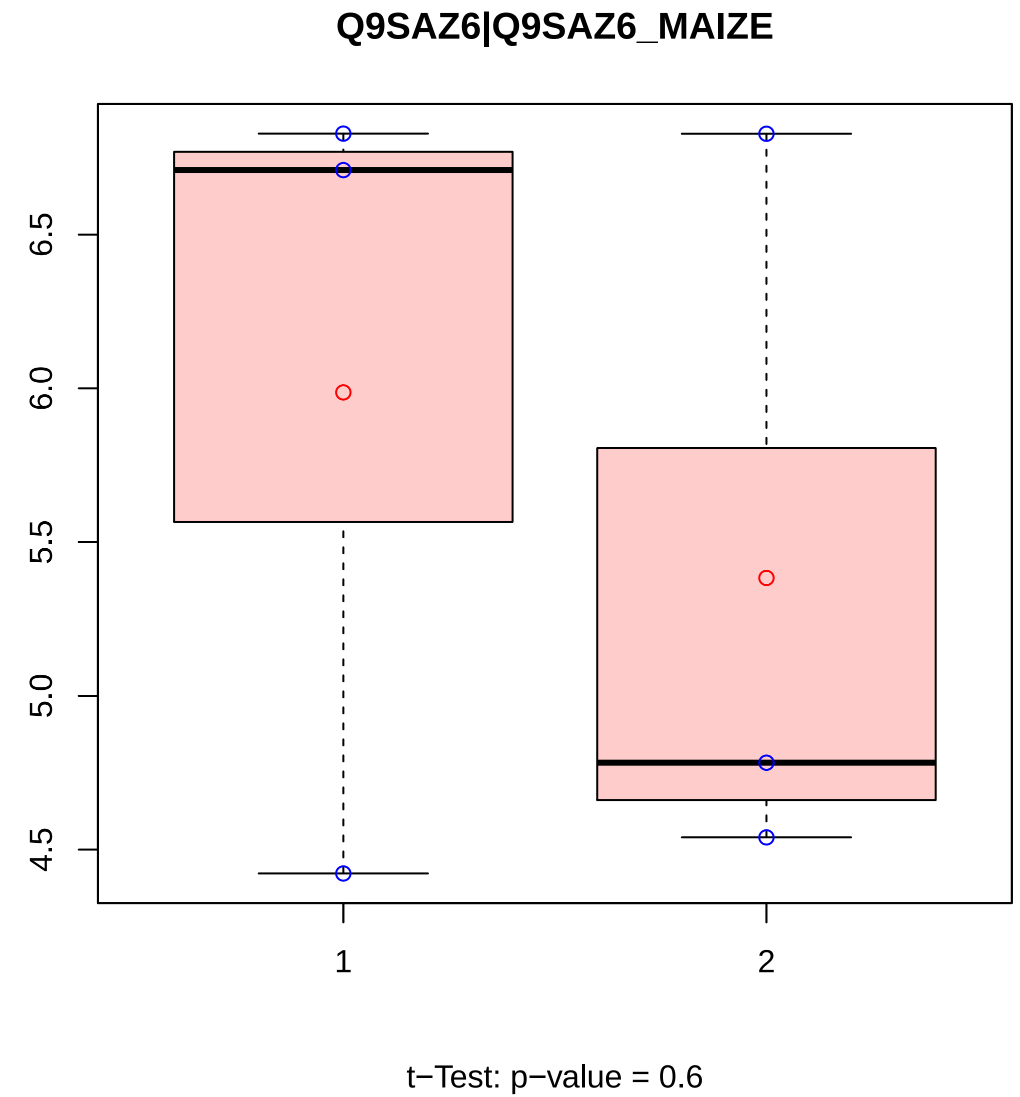

Q9SAZ6|Q9SAZ6_MAIZE
6.5
6.0
5.5
5.0
4.5
1
2
t−Test: p−value = 0.6

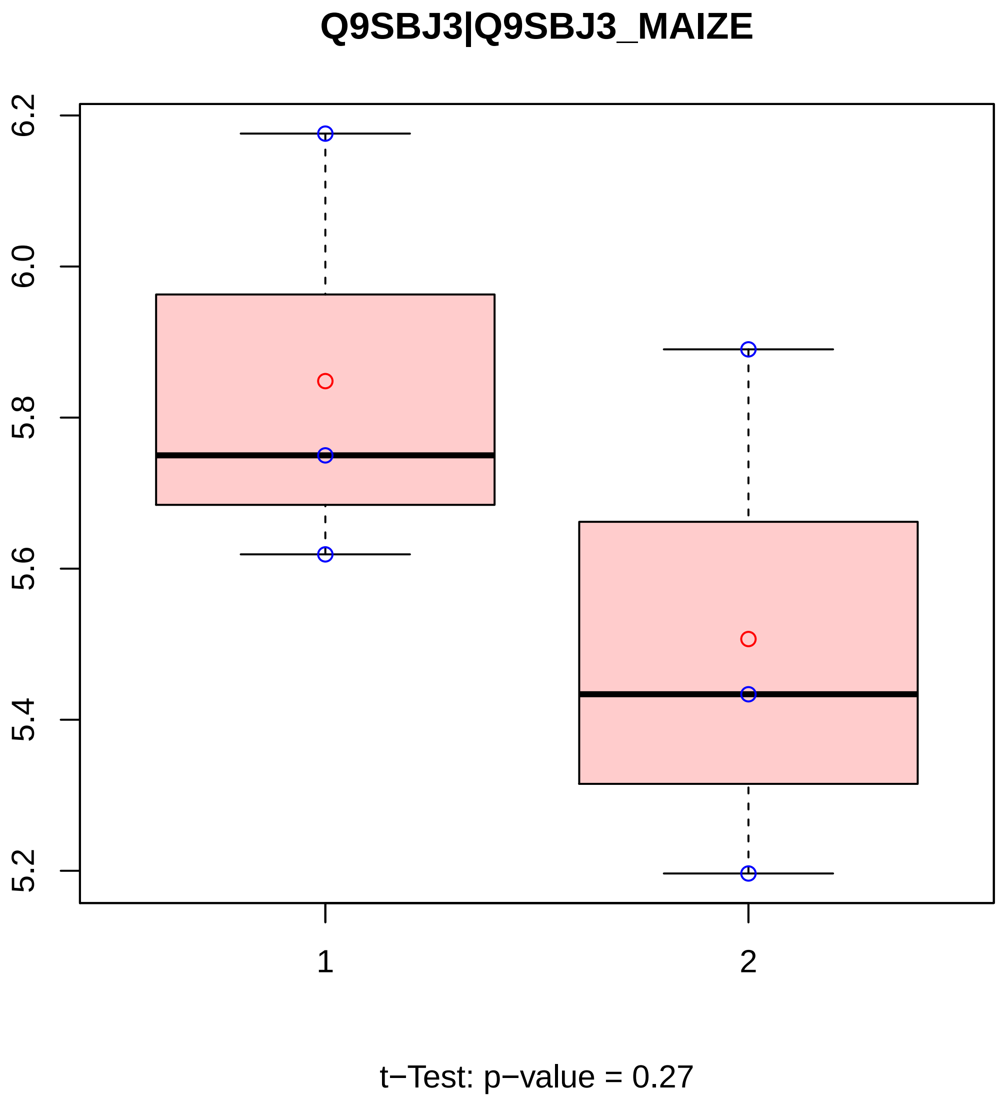
Q9SBJ3|Q9SBJ3_MAIZE
6.2
6.0
5.8
5.6
5.4
5.2
1
2
t−Test: p−value = 0.27

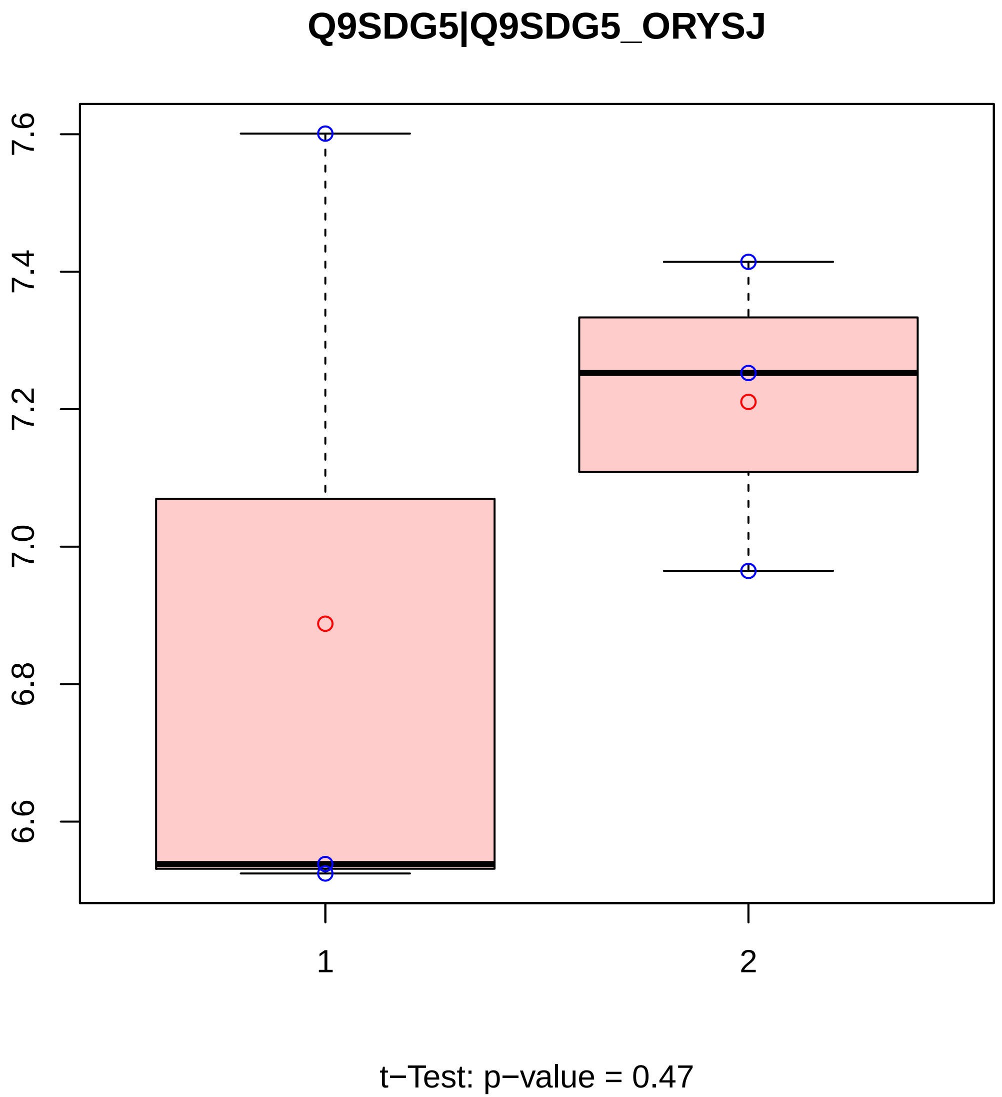

Q9SDG5|Q9SDG5_ORYSJ
7.6
7.4
7.2
7.0
6.8
6.6
1
2
t−Test: p−value = 0.47

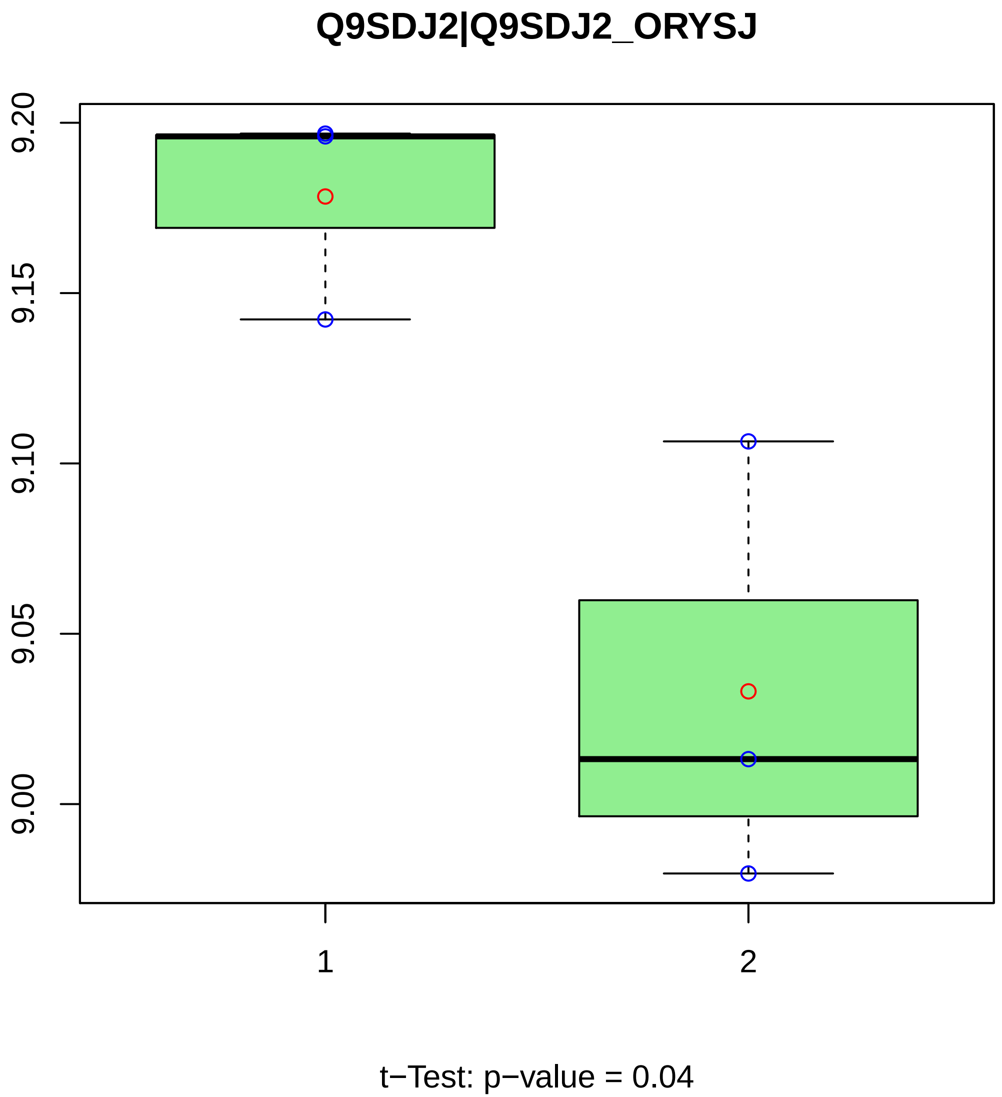
Q9SDJ2|Q9SDJ2_ORYSJ
9.20
9.15
9.10
9.05
9.00
1
2
t−Test: p−value = 0.04

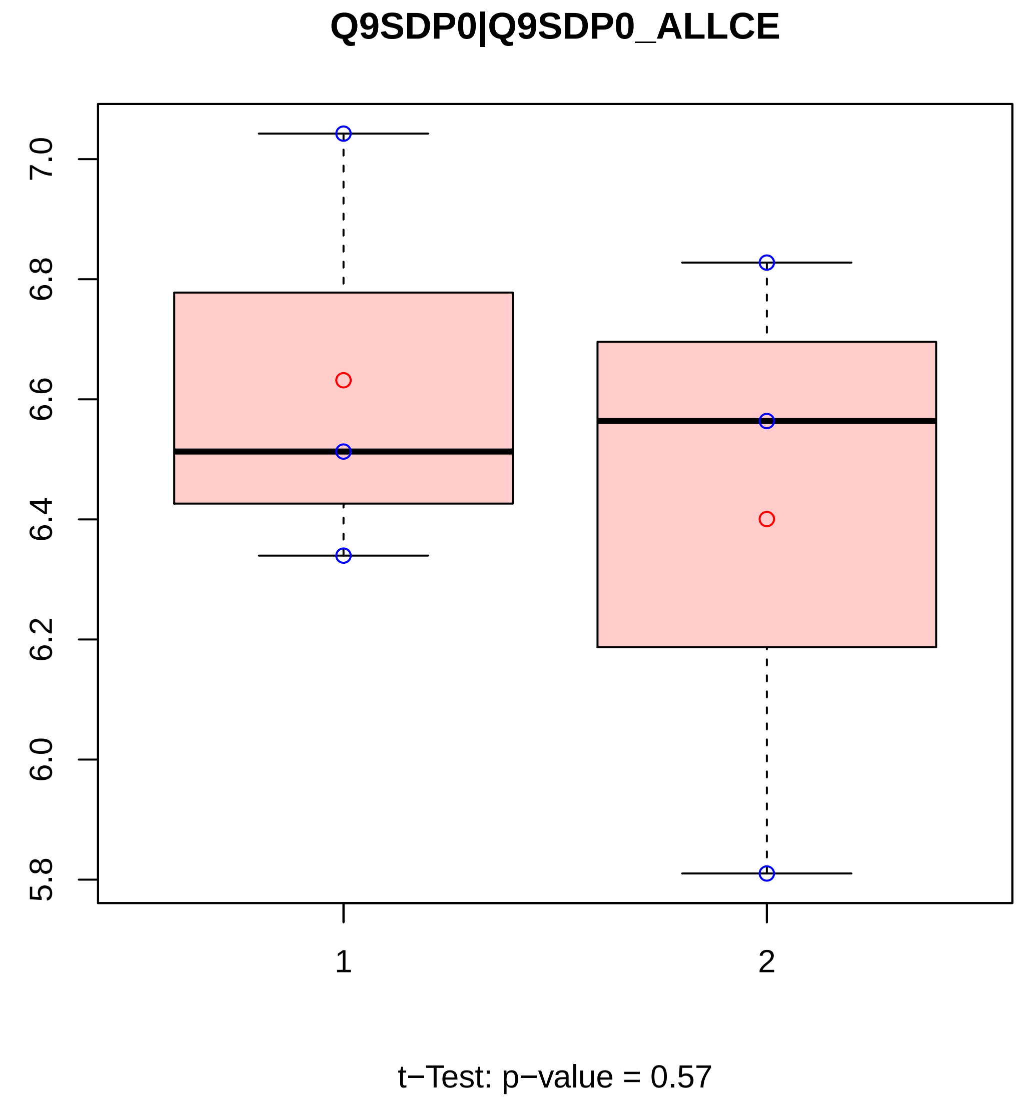
Q9SDP0|Q9SDP0_ALLCE
7.0
6.8
6.6
6.4
6.2
6.0
5.8
1
2
t−Test: p−value = 0.57

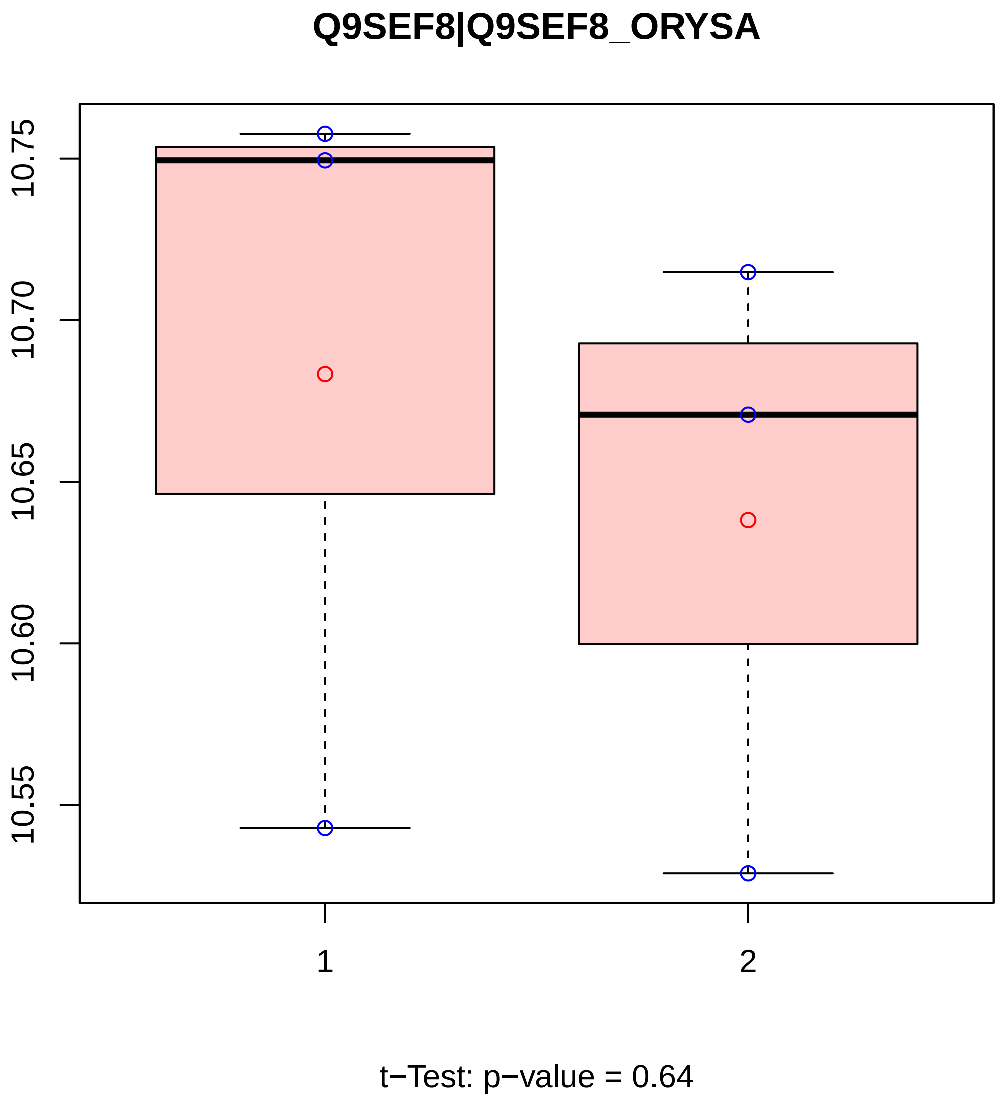
Q9SEF8|Q9SEF8_ORYSA
10.55
10.60
10.65
10.70
10.75
1
2
t−Test: p−value = 0.64

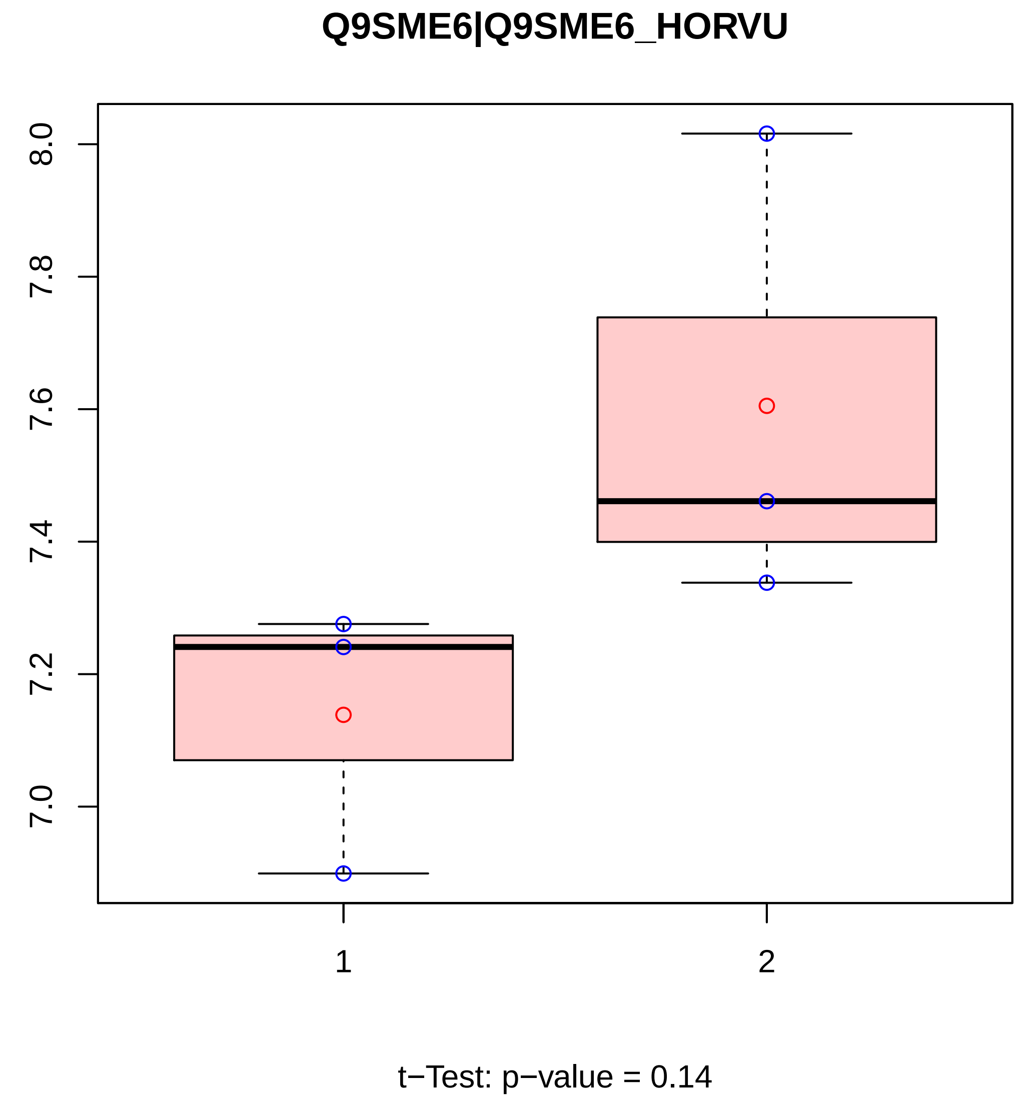
Q9SME6|Q9SME6_HORVU
8.0
7.8
7.6
7.4
7.2
7.0
1
2
t−Test: p−value = 0.14

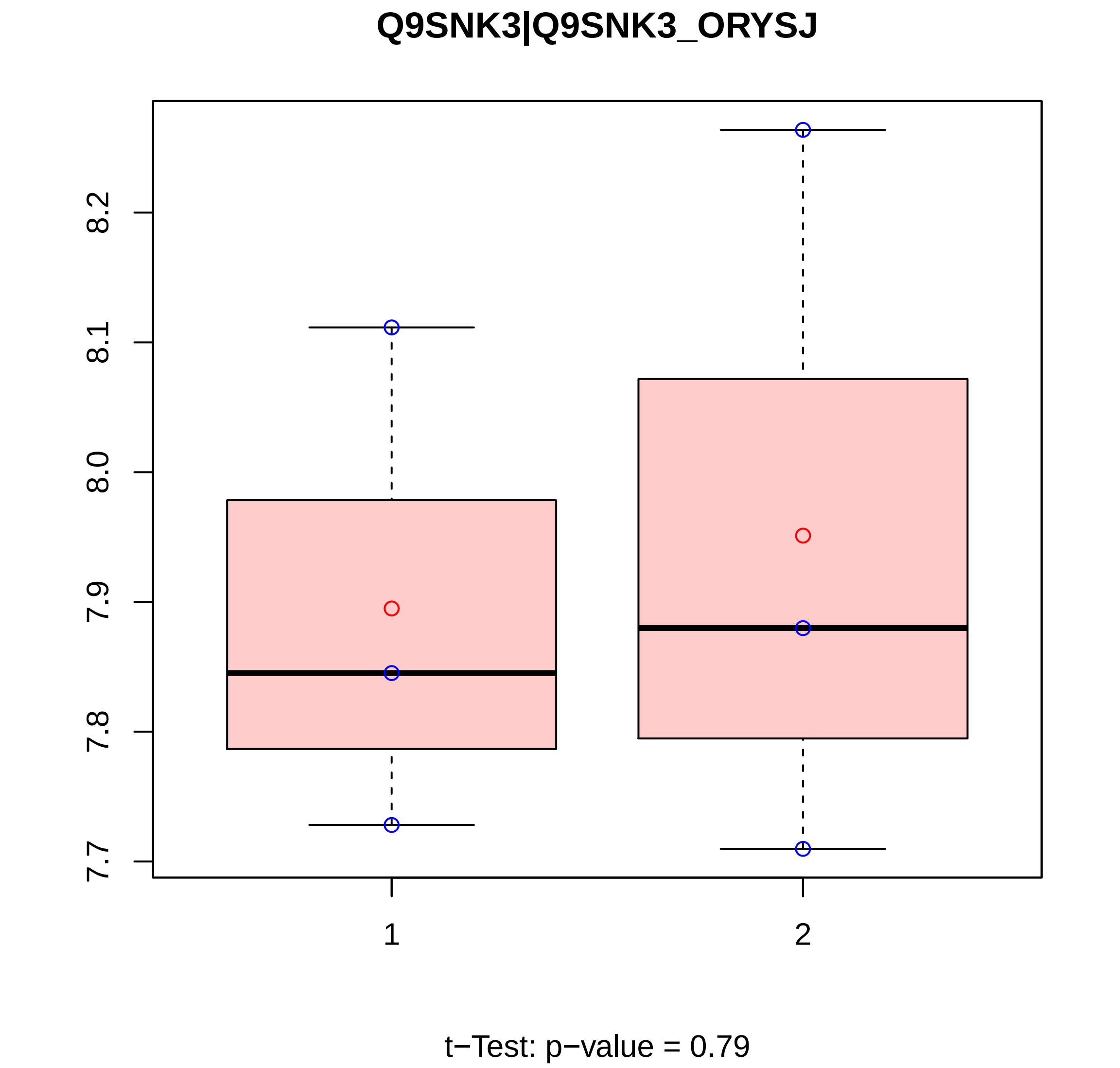
Q9SNK3|Q9SNK3_ORYSJ
8.2
8.1
8.0
7.9
7.8
7.7
1
2
t−Test: p−value = 0.79

Q9SPA1|Q9SPA1_LILLO

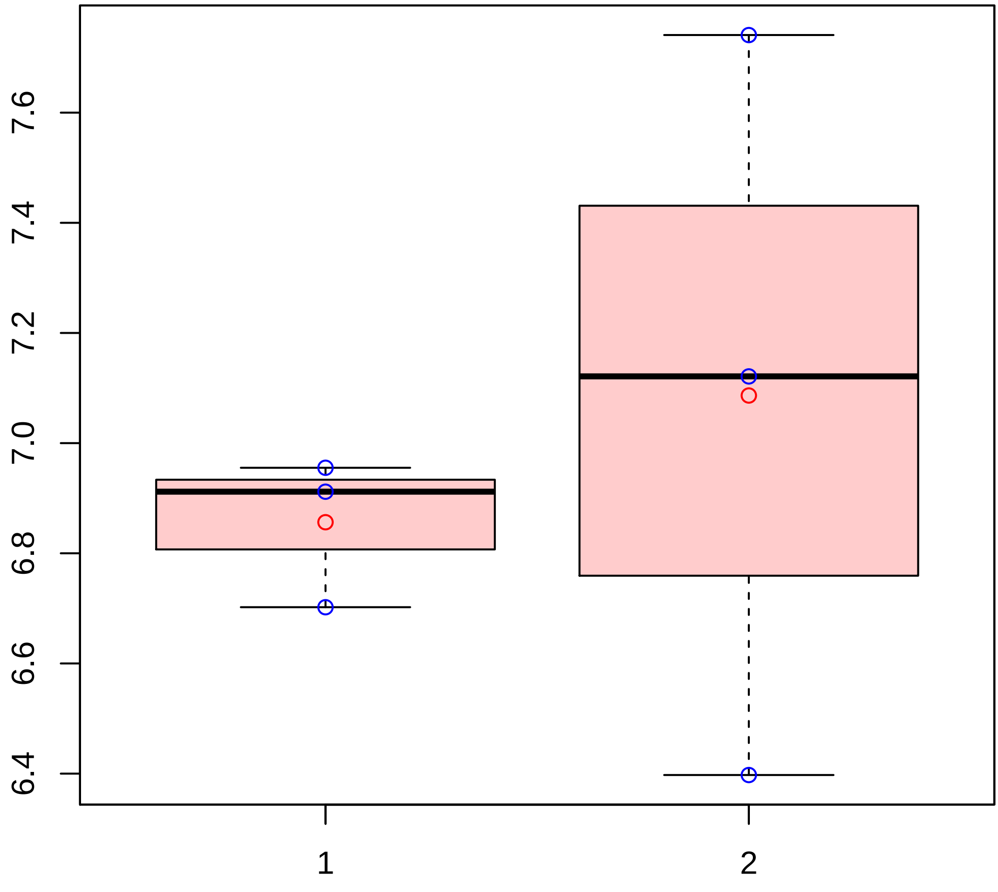

t−Test: p−value = 0.62

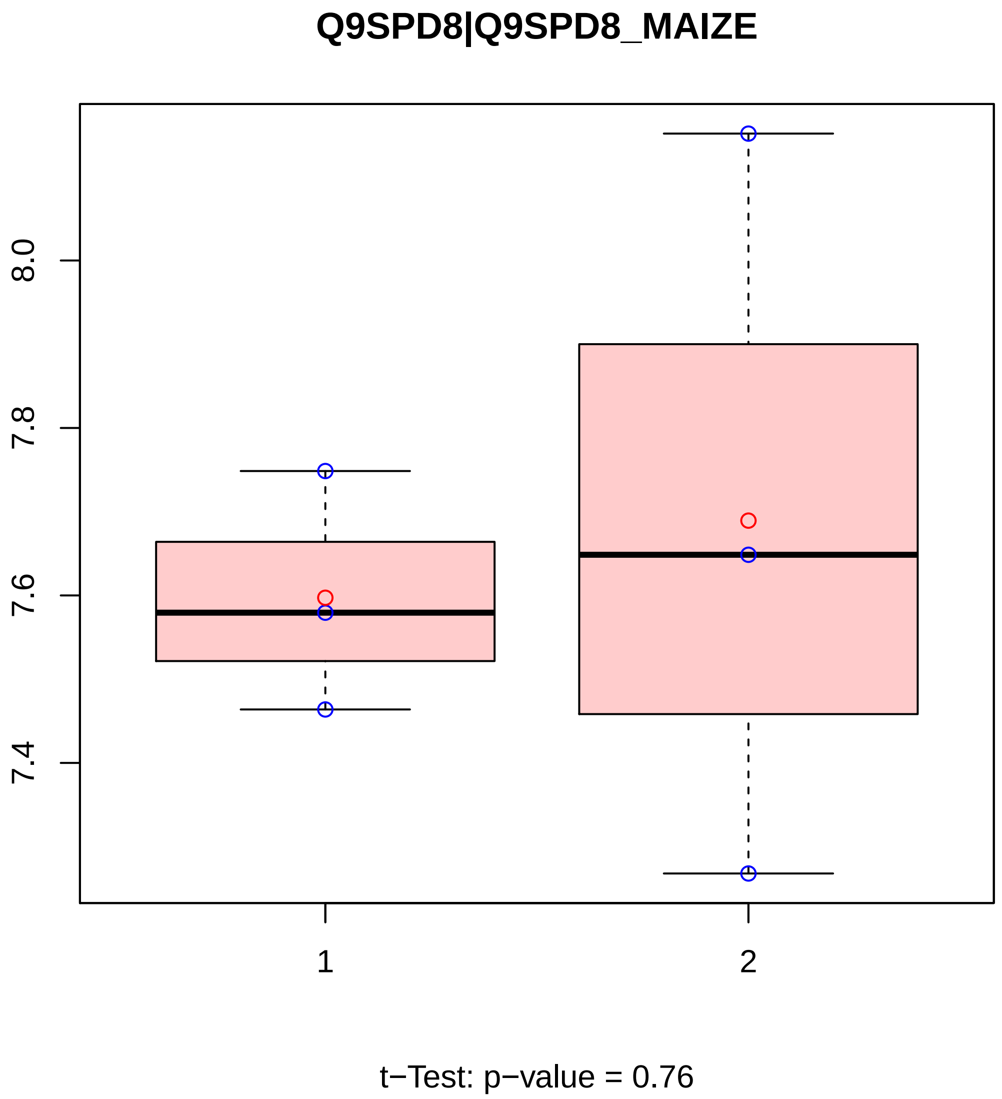
Q9SPD8|Q9SPD8_MAIZE
8.0
7.8
7.6
7.4
1
2
t−Test: p−value = 0.76

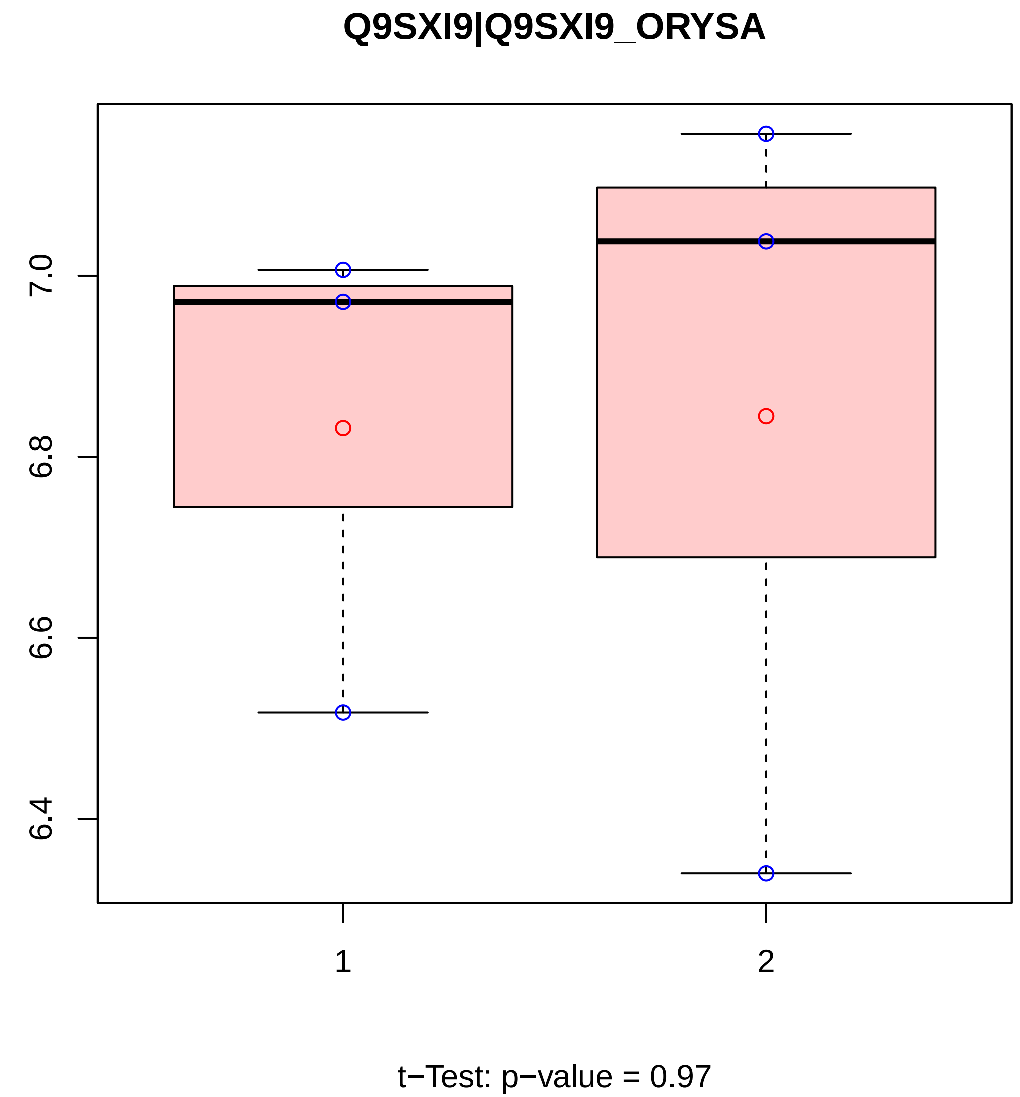
Q9SXI9|Q9SXI9_ORYSA
7.0
6.8
6.6
6.4
1
2
t−Test: p−value = 0.97

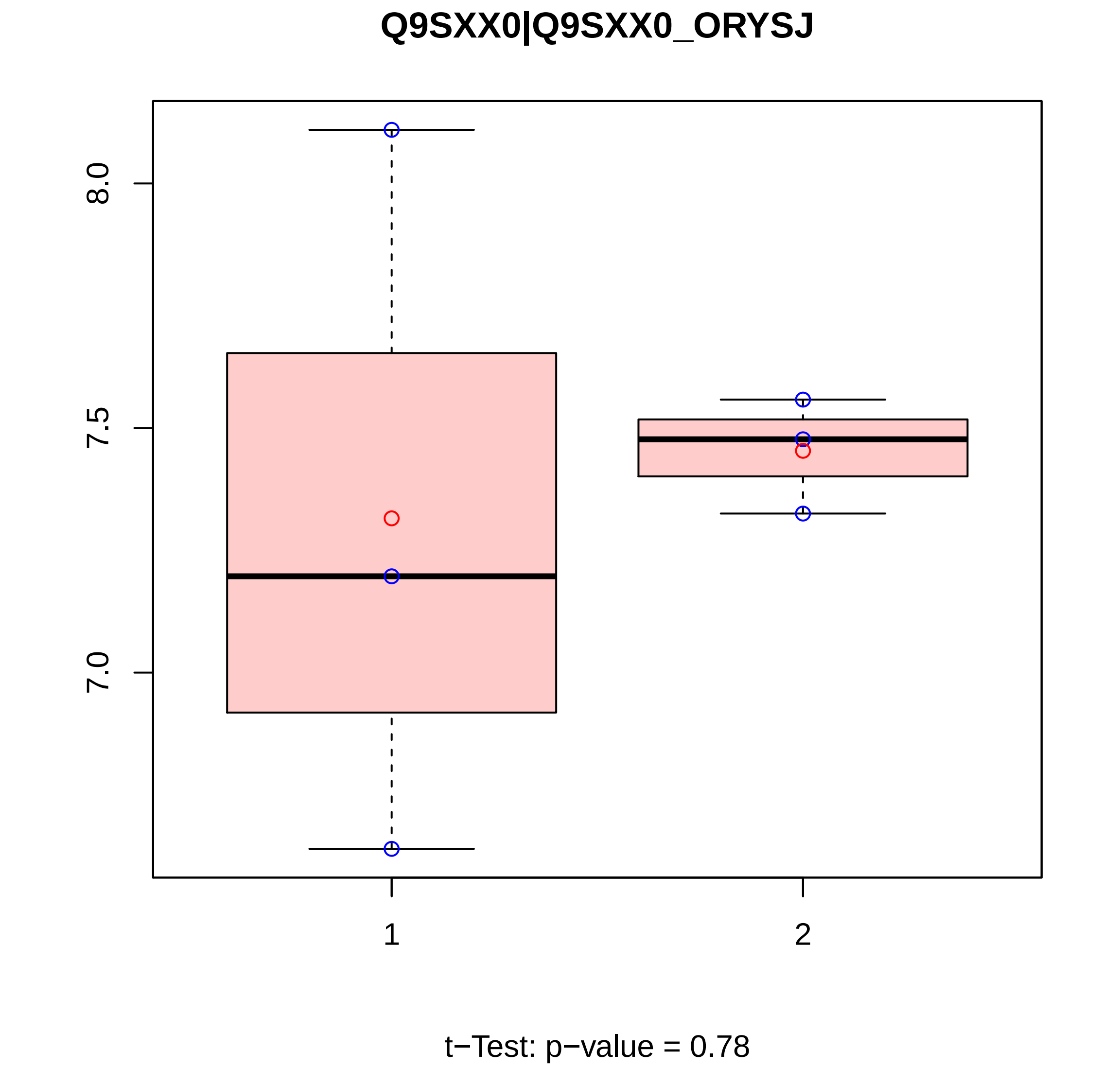
Q9SXX0|Q9SXX0_ORYSJ
8.0
7.5
7.0
1
2
t−Test: p−value = 0.78

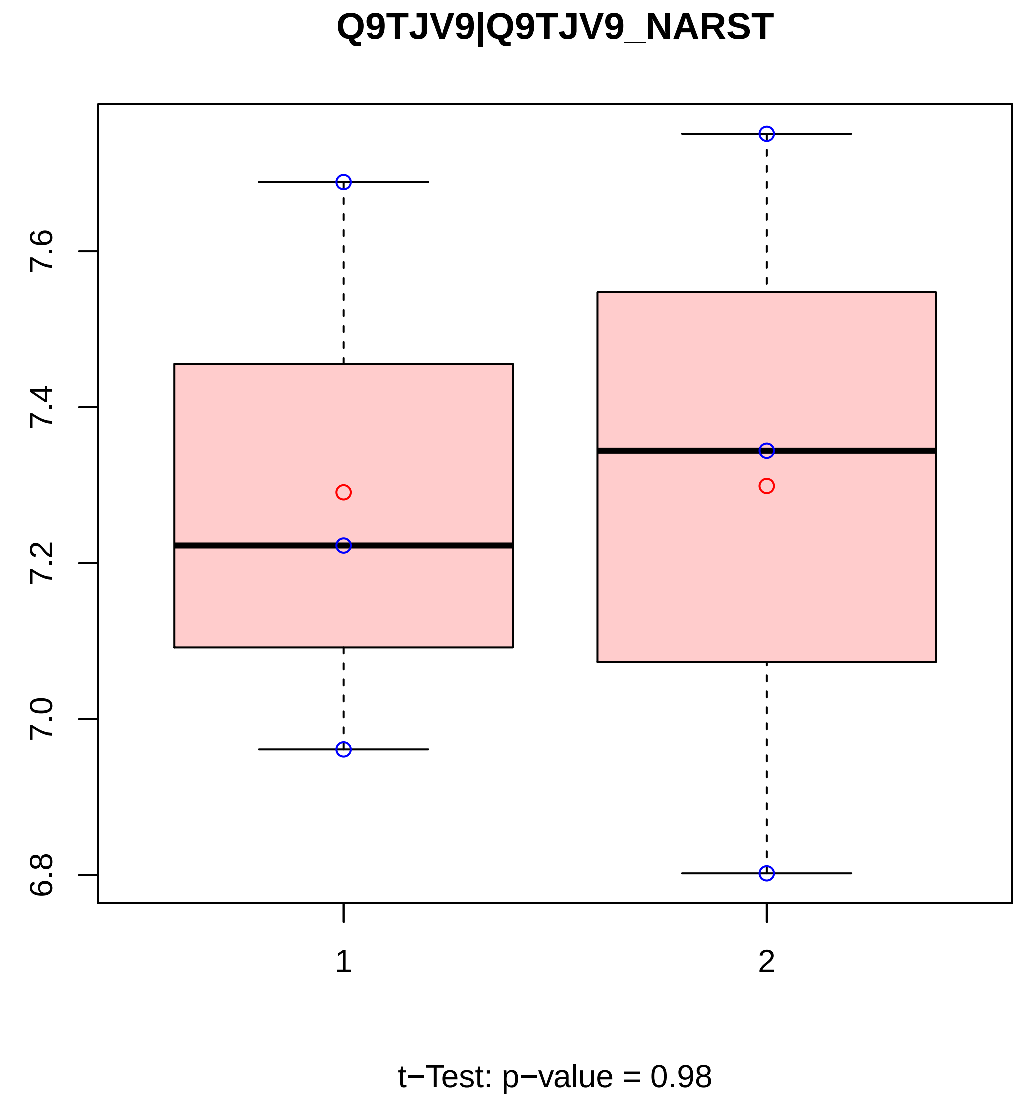

Q9TJV9|Q9TJV9_NARST
7.6
7.4
7.2
7.0
6.8
1
2
t−Test: p−value = 0.98

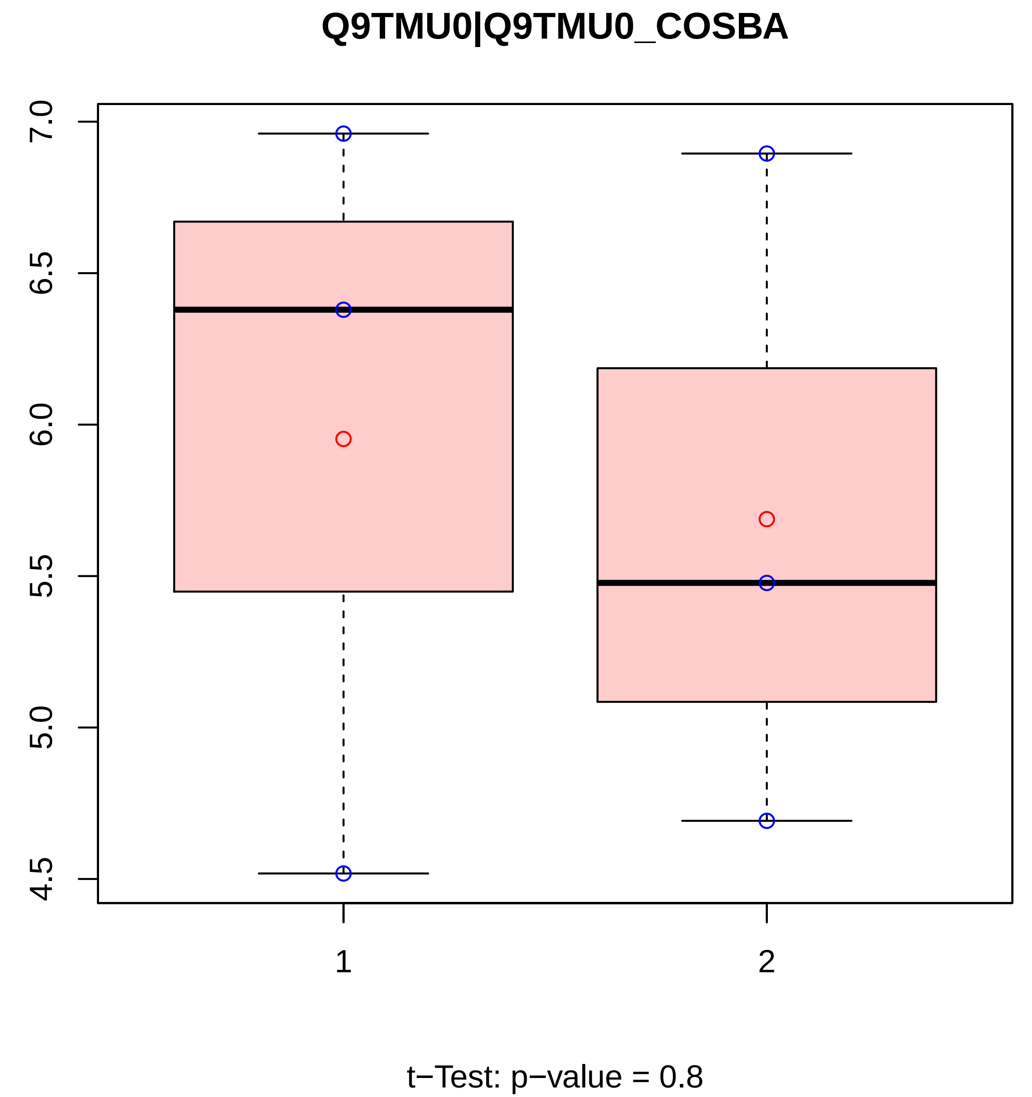
Q9TMU0|Q9TMU0_COSBA
7.0
6.5
6.0
5.5
5.0
4.5
1
2
t−Test: p−value = 0.8

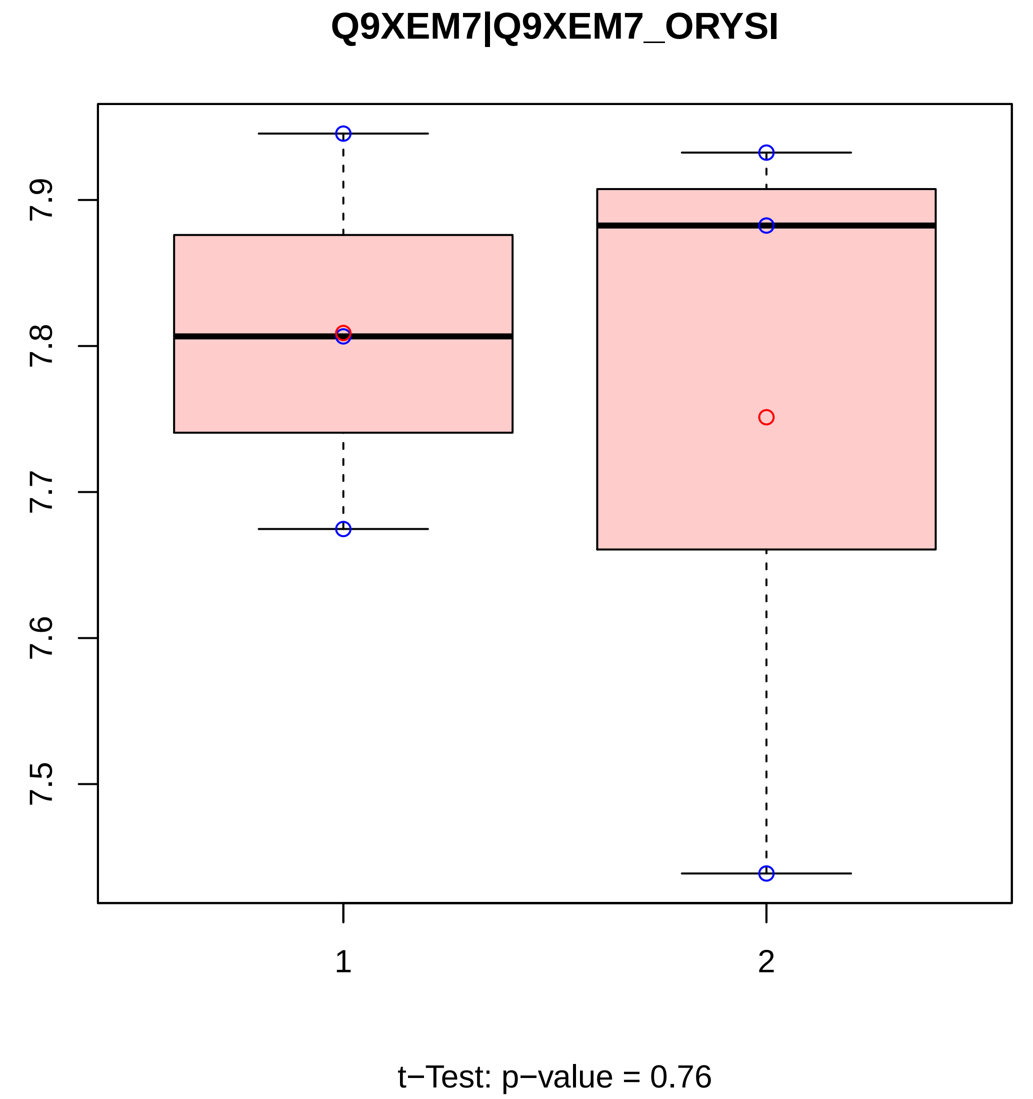
Q9XEM7|Q9XEM7_ORYSI
7.5
7.6
7.7
7.8
7.9
1
2
t−Test: p−value = 0.76

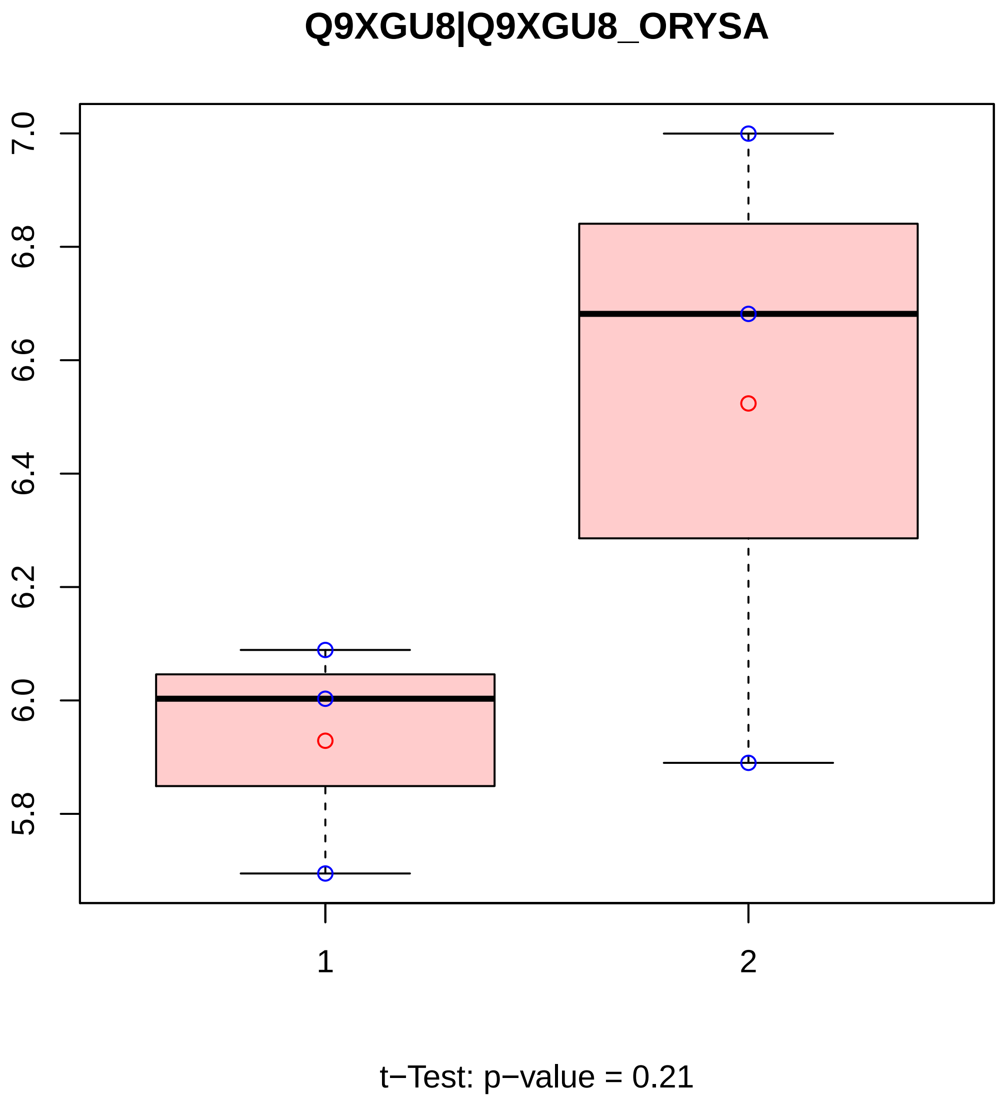
Q9XGU8|Q9XGU8_ORYSA
7.0
6.8
6.6
6.4
6.2
6.0
5.8
1
2
t−Test: p−value = 0.21

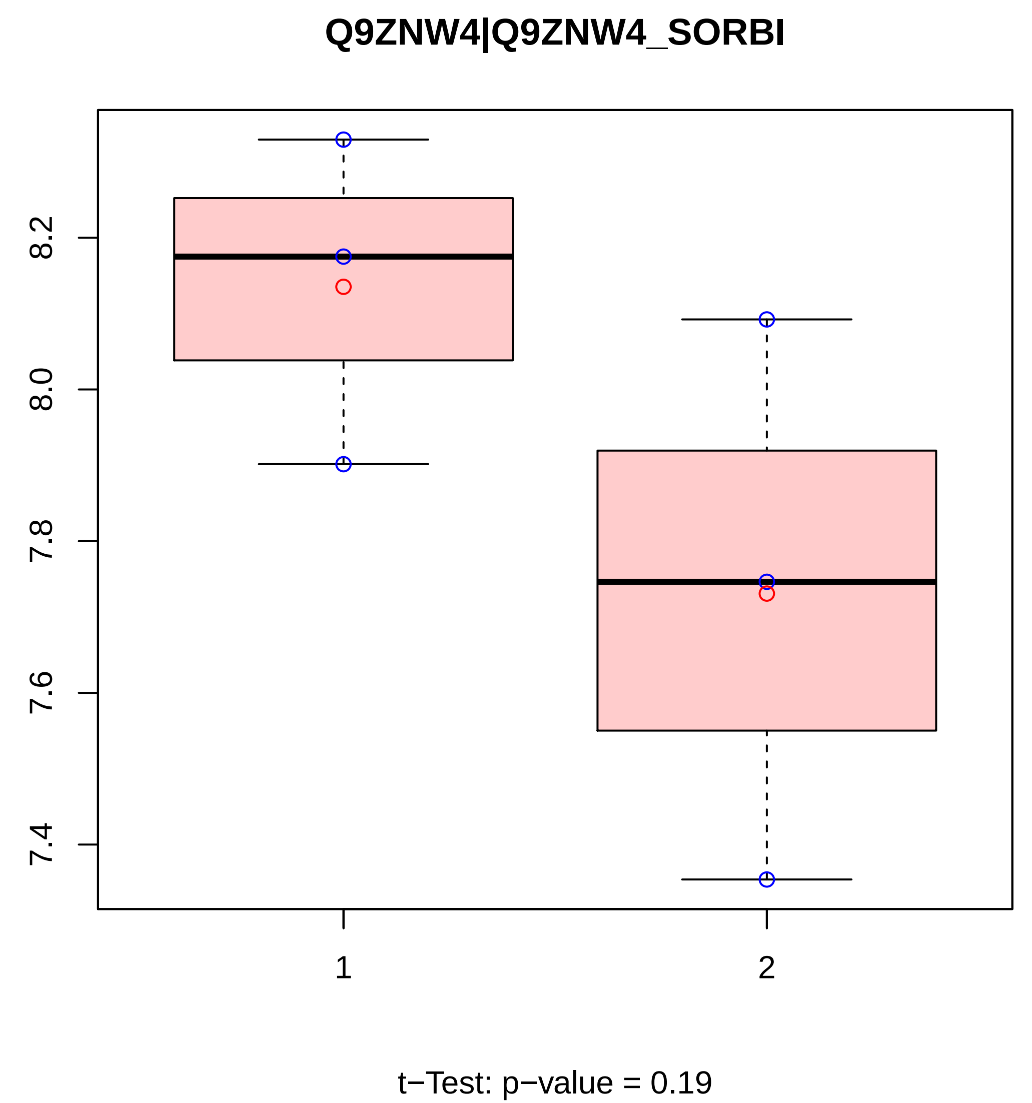
Q9ZNW4|Q9ZNW4_SORBI
8.2
8.0
7.8
7.6
7.4
1
2
t−Test: p−value = 0.19

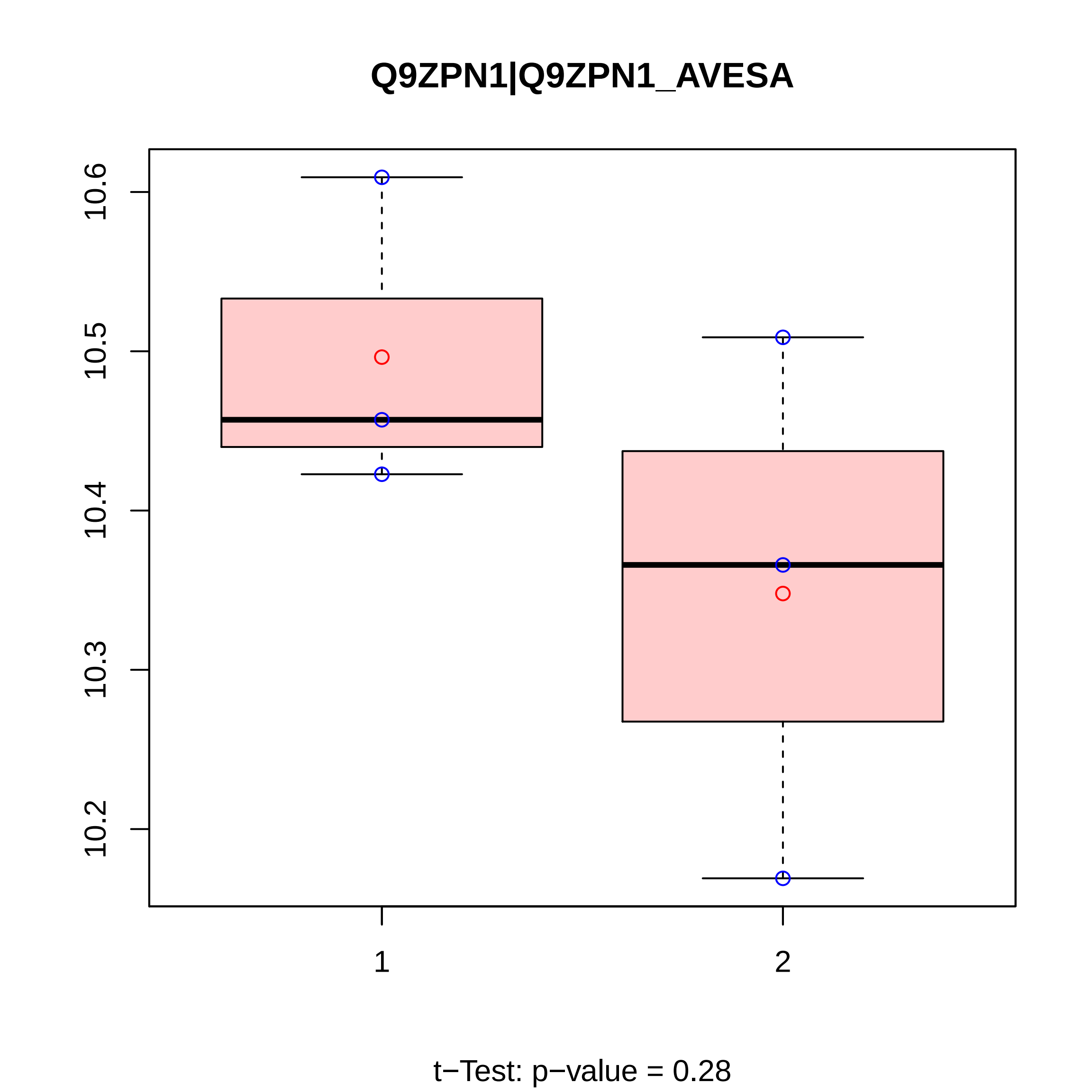

Q9ZPN1|Q9ZPN1_AVESA
10.6
10.5
10.4
10.3
10.2
1
2
t−Test: p−value = 0.28

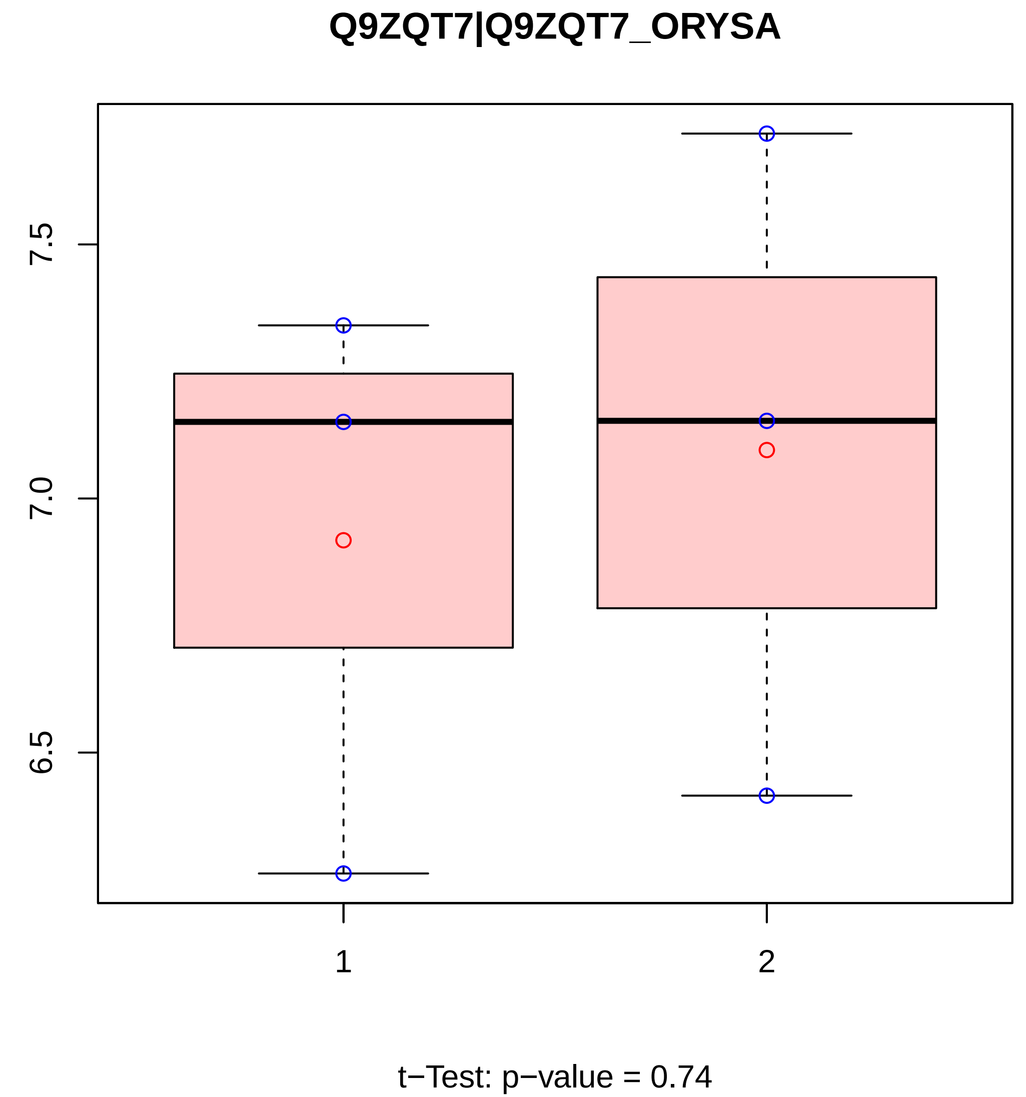
Q9ZQT7|Q9ZQT7_ORYSA
7.5
7.0
6.5
1
2
t−Test: p−value = 0.74

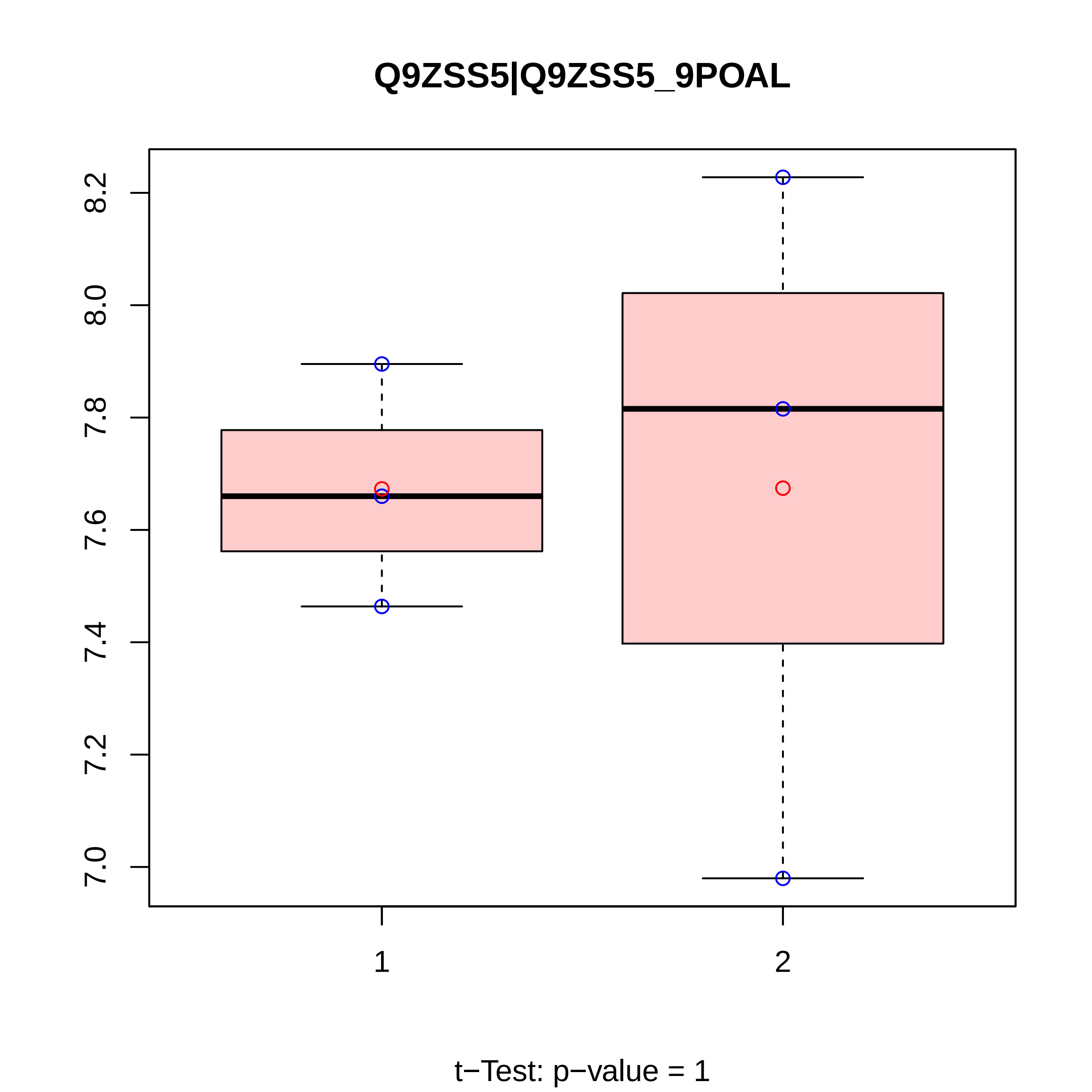
Q9ZSS5|Q9ZSS5_9POAL
8.2
8.0
7.8
7.6
7.4
7.2
7.0
1
2
t−Test: p−value = 1

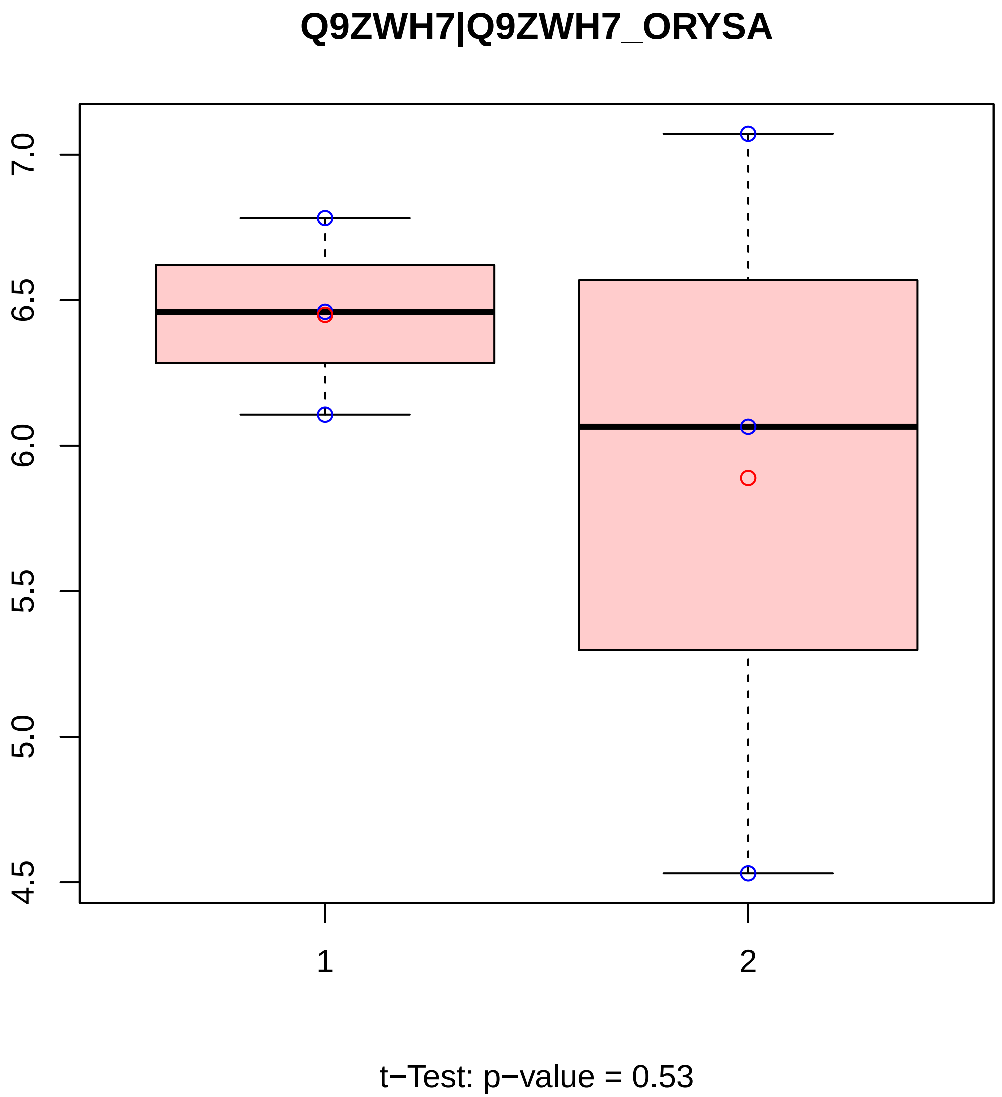
Q9ZWH7|Q9ZWH7_ORYSA
7.0
6.5
6.0
5.5
5.0
4.5
1
2
t−Test: p−value = 0.53

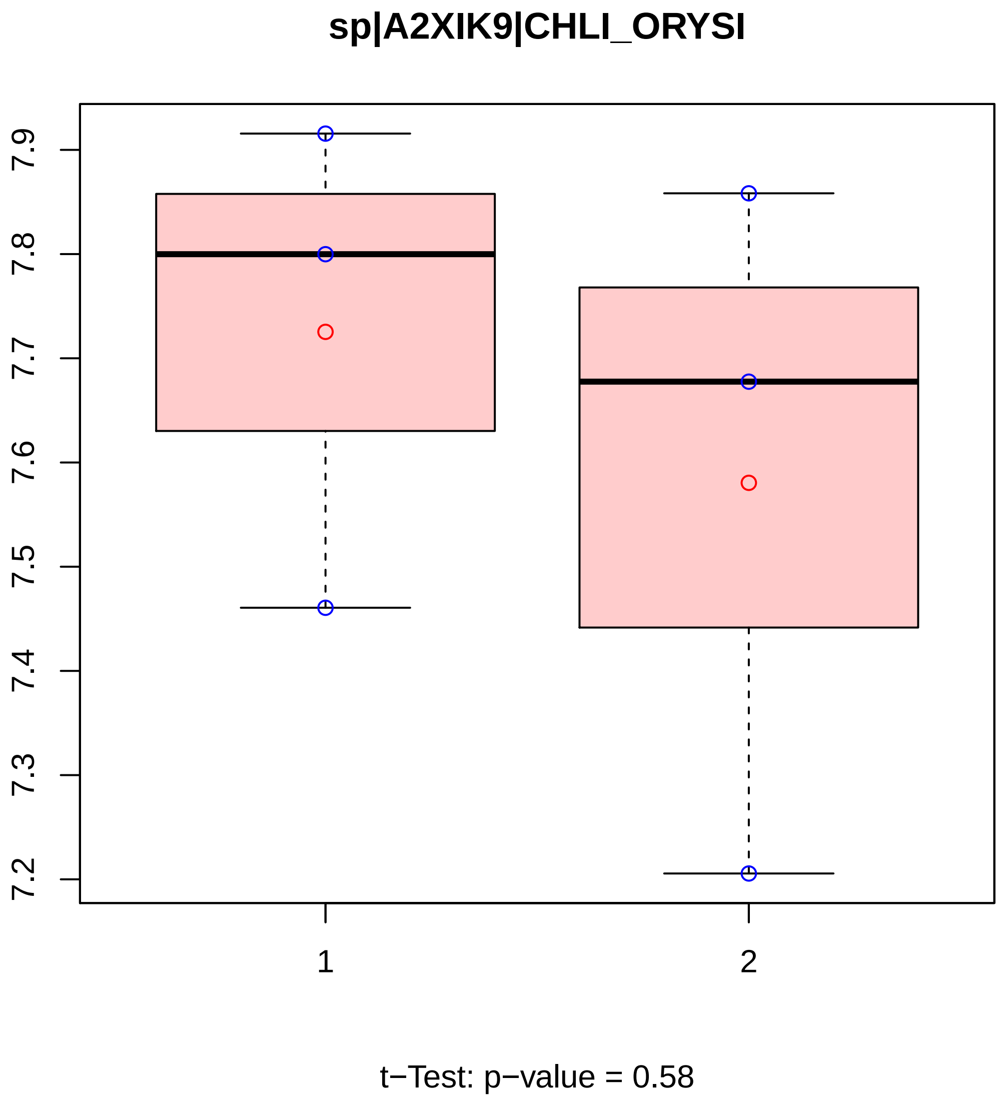

sp|A2XIK9|CHLI_ORYSI
7.9
7.8
7.7
7.6
7.5
7.4
7.3
7.2
1
2
t−Test: p−value = 0.58

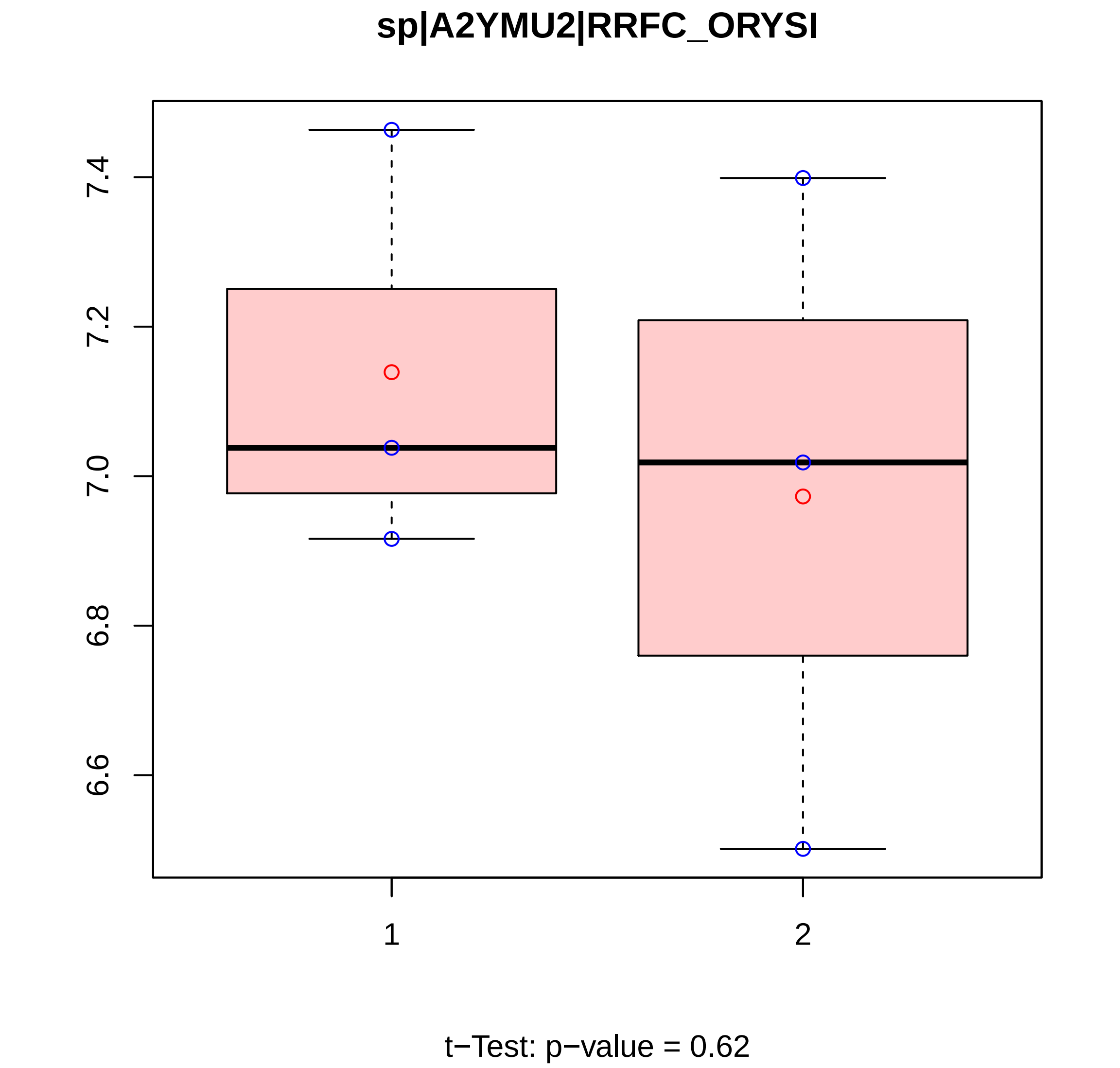

sp|A2YMU2|RRFC_ORYSI
7.4
7.2
7.0
6.8
6.6
1
2
t−Test: p−value = 0.62

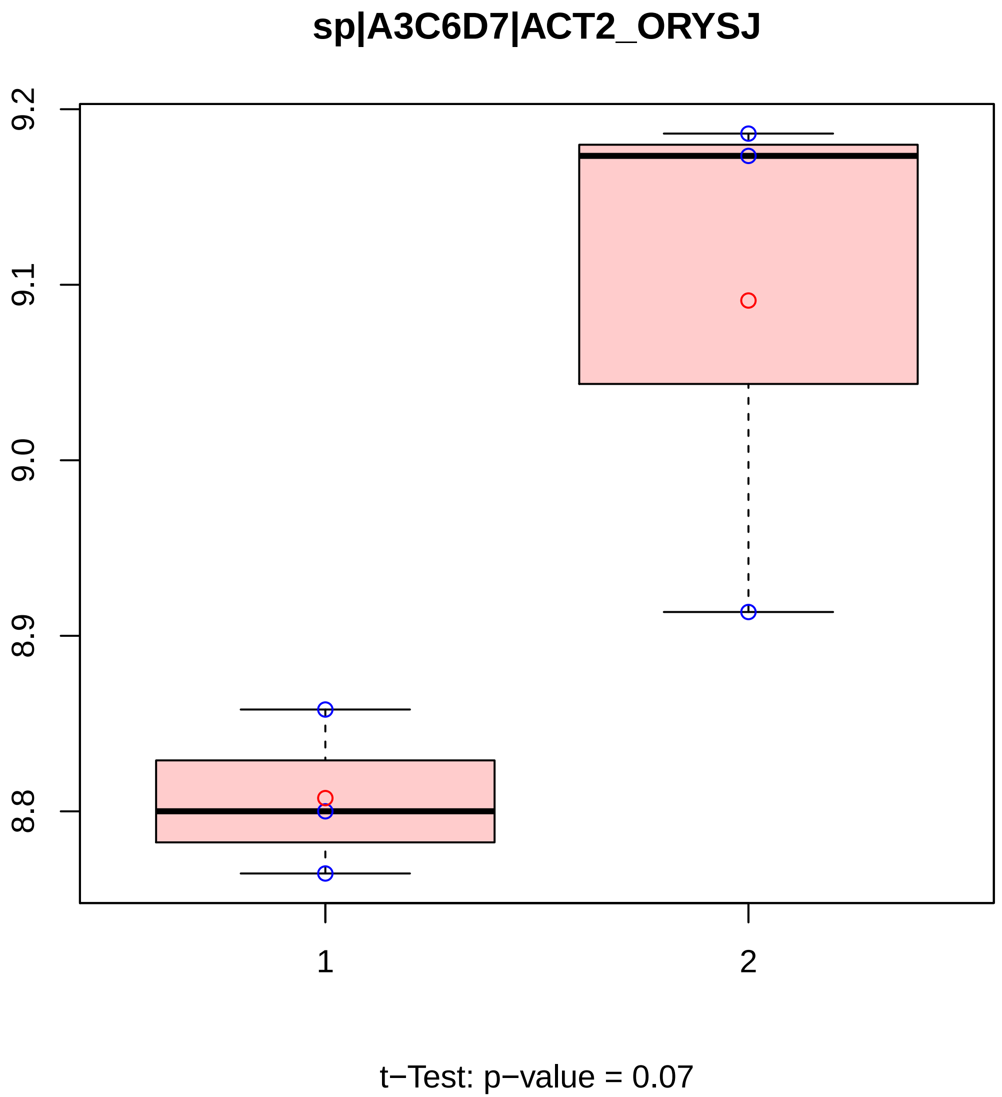
sp|A3C6D7|ACT2_ORYSJ
9.2
9.1
9.0
8.9
8.8
1
2
t−Test: p−value = 0.07

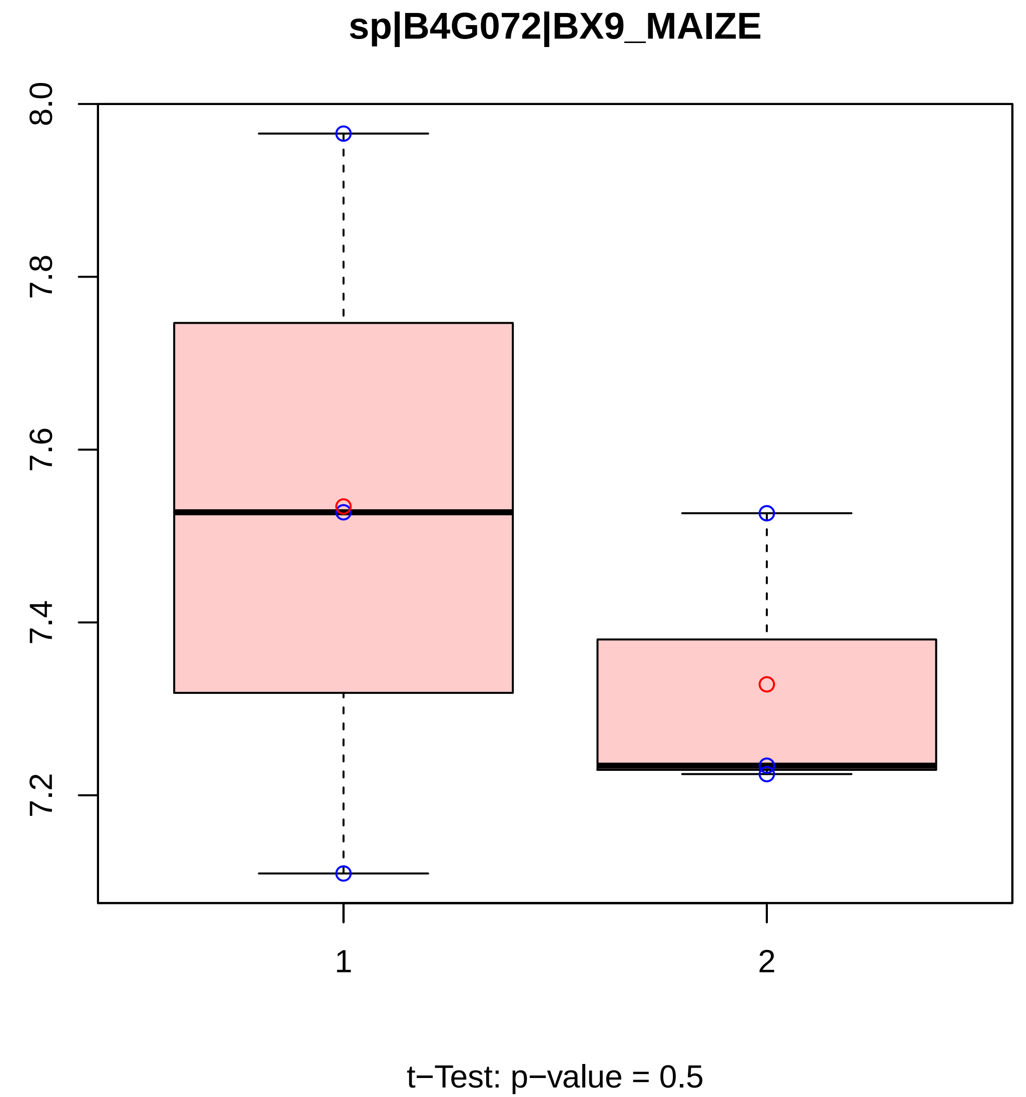
sp|B4G072|BX9_MAIZE
8.0
7.8
7.6
7.4
7.2
1
2
t−Test: p−value = 0.5

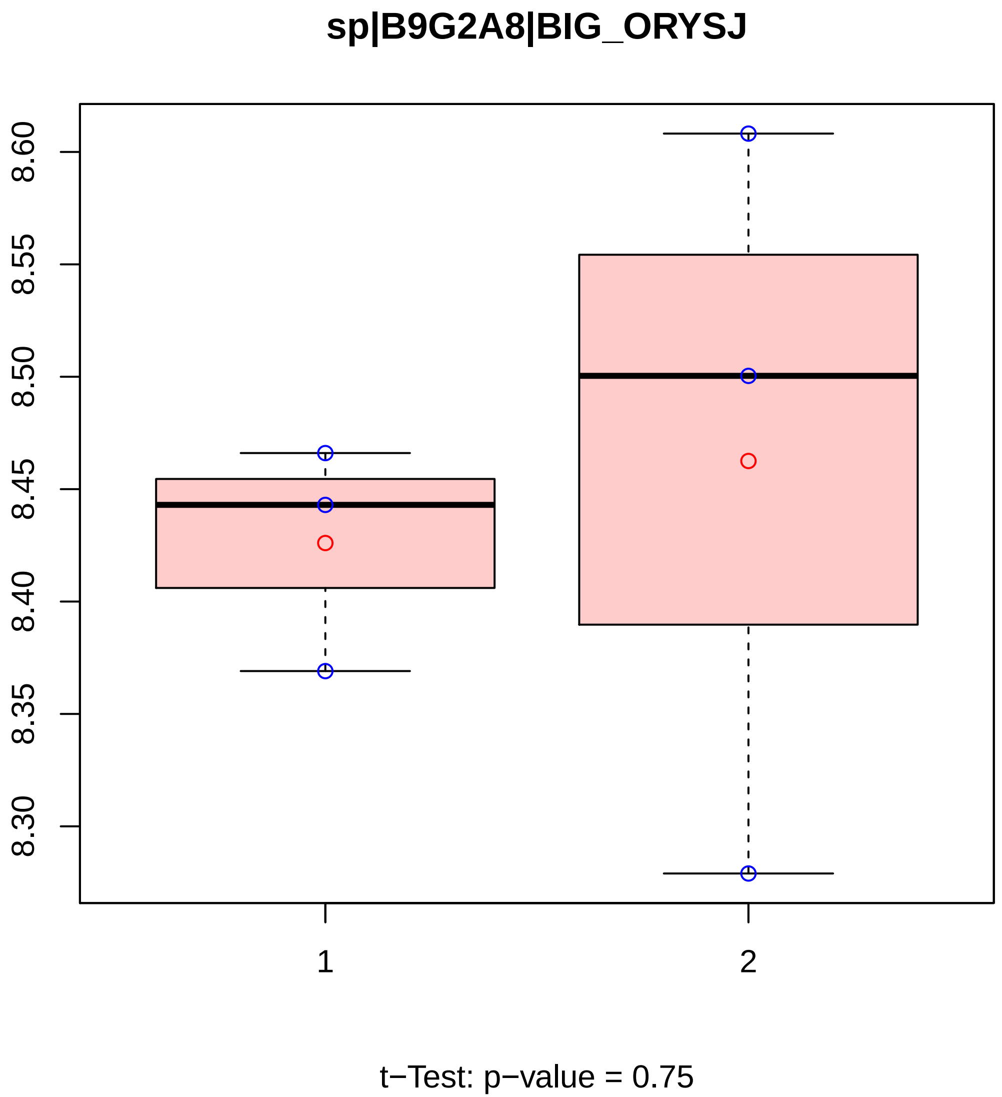
sp|B9G2A8|BIG_ORYSJ
8.30
8.35
8.40
8.45
8.50
8.55
8.60
1
2
t−Test: p−value = 0.75

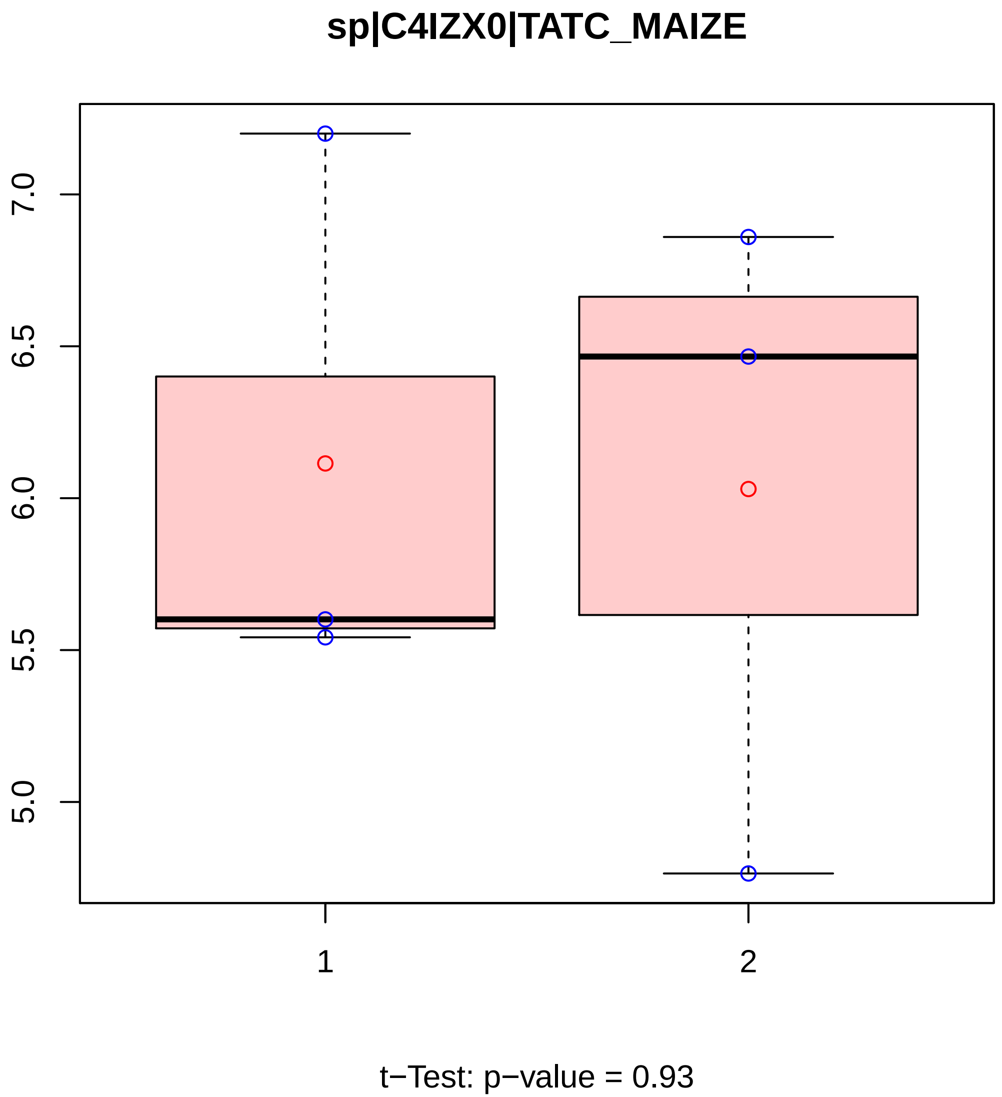

sp|C4IZX0|TATC_MAIZE
7.0
6.5
6.0
5.5
5.0
1
2
t−Test: p−value = 0.93

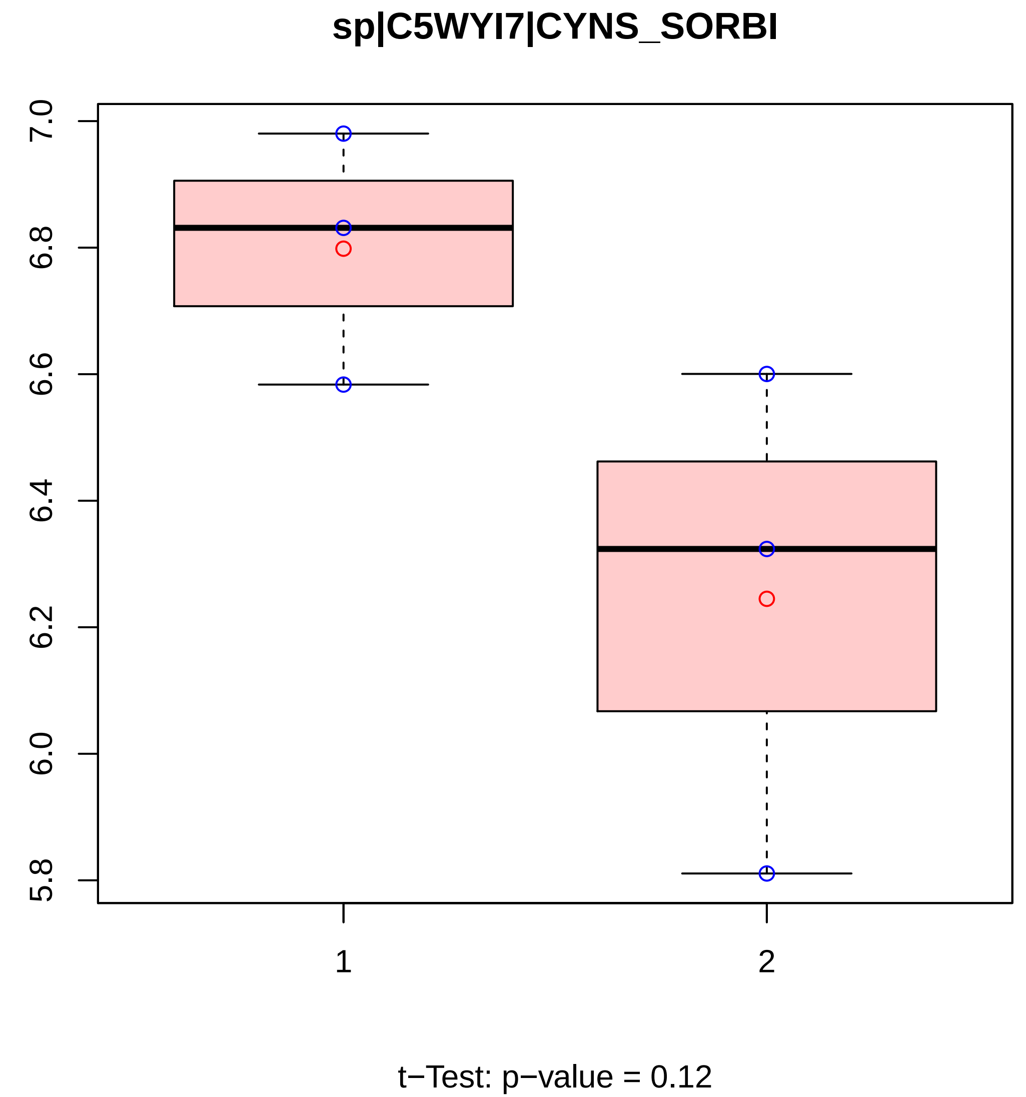
sp|C5WYI7|CYNS_SORBI
7.0
6.8
6.6
6.4
6.2
6.0
5.8
1
2
t−Test: p−value = 0.12

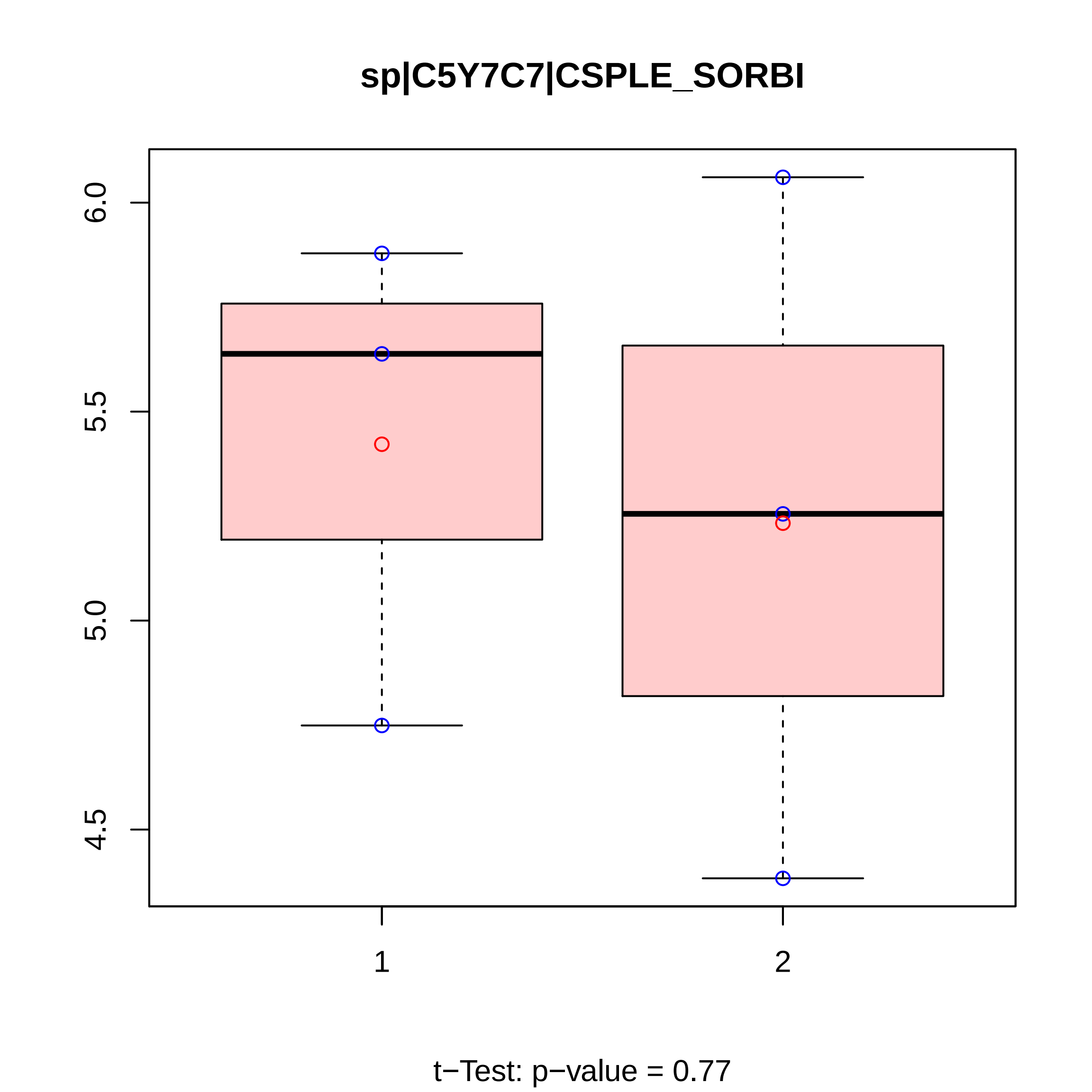
sp|C5Y7C7|CSPLE_SORBI
6.0
5.5
5.0
4.5
1
2
t−Test: p−value = 0.77

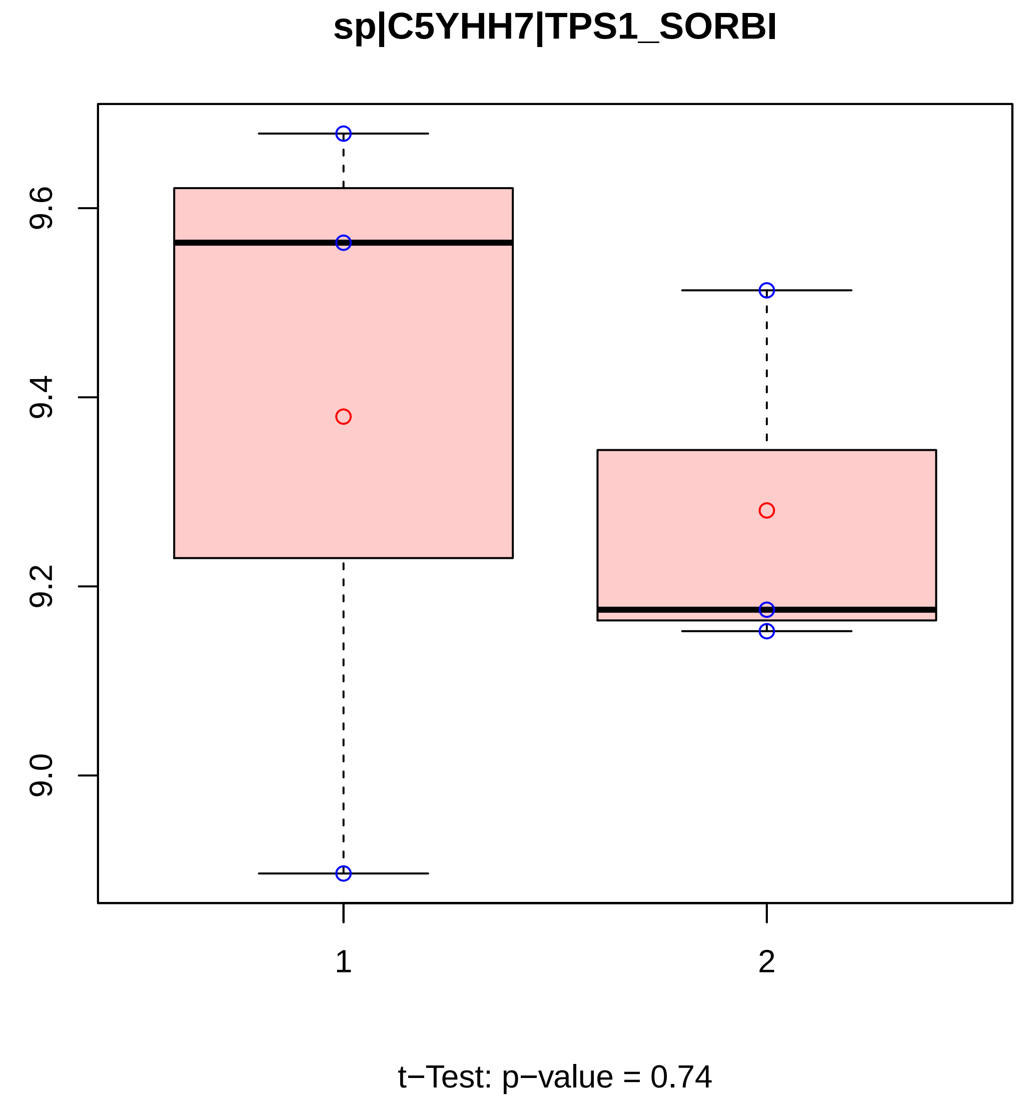
sp|C5YHH7|TPS1_SORBI
9.0
9.2
9.4
9.6
1
2
t−Test: p−value = 0.74

sp|O23813|SIR_MAIZE

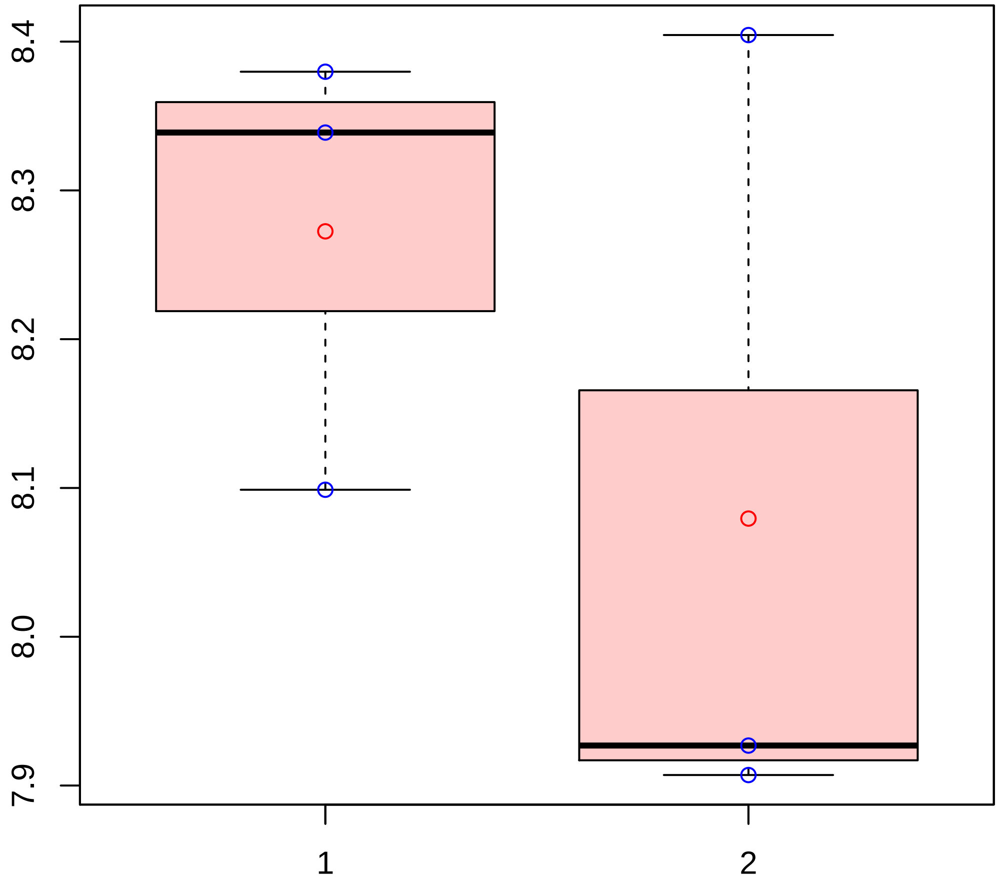

t−Test: p−value = 0.37

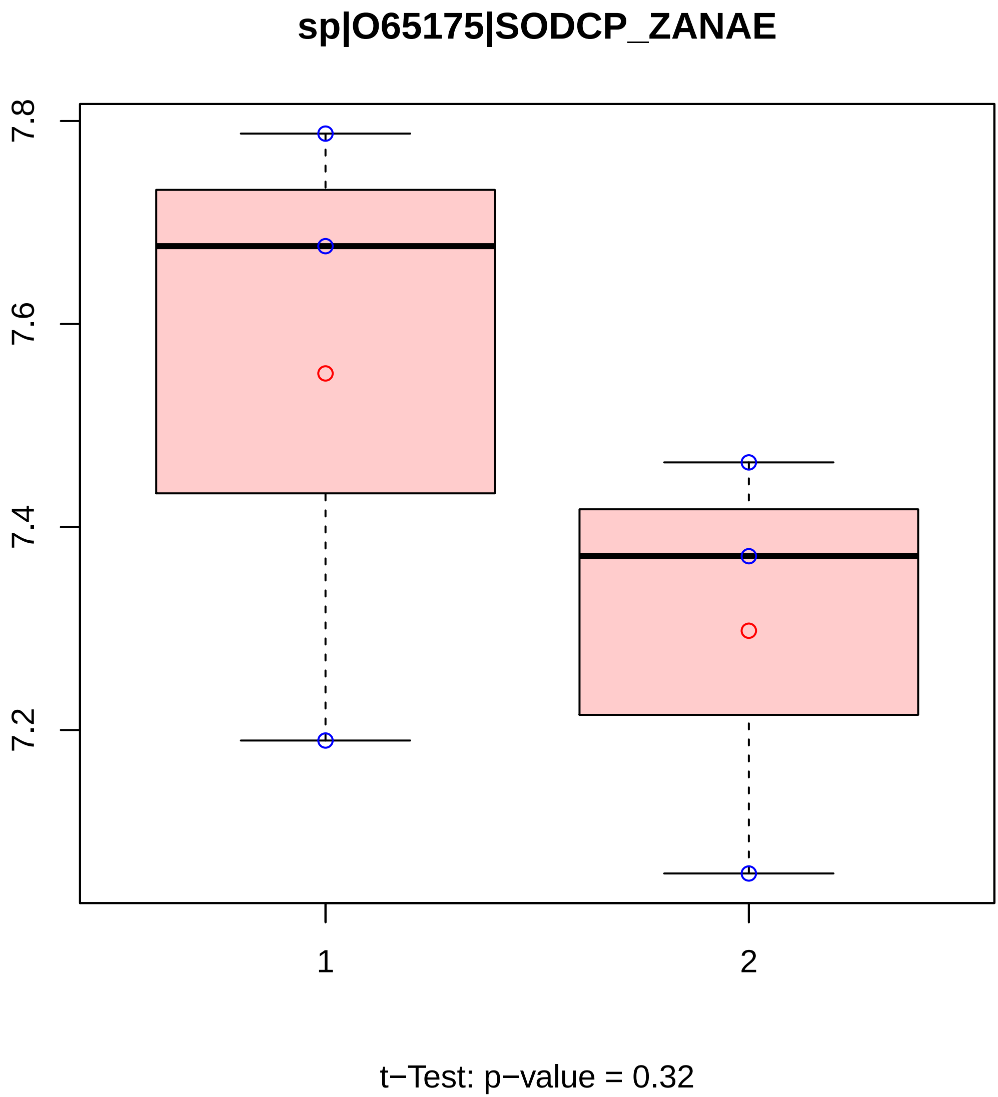
sp|O65175|SODCP_ZANAE
7.8
7.6
7.4
7.2
1
2
t−Test: p−value = 0.32

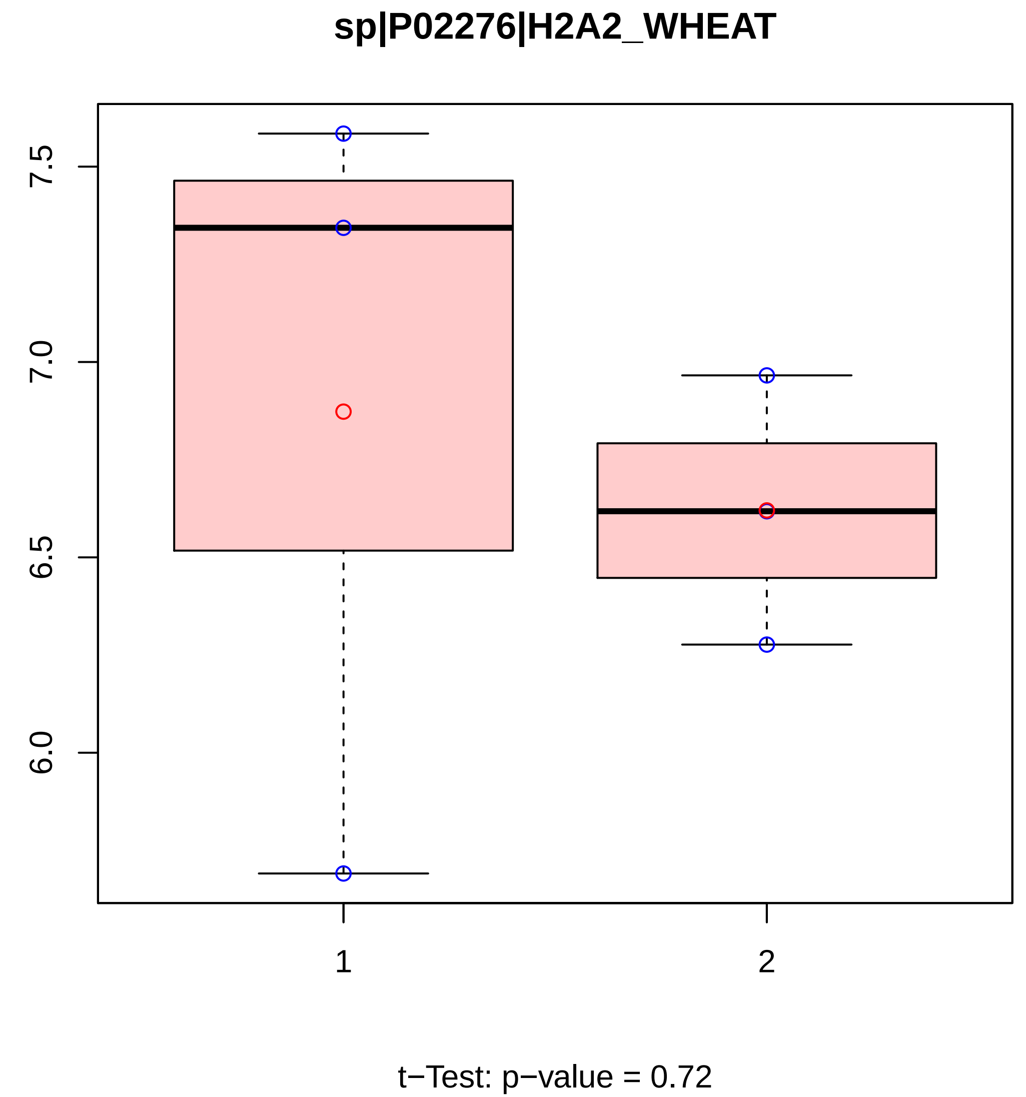
sp|P02276|H2A2_WHEAT
7.5
7.0
6.5
6.0
1
2
t−Test: p−value = 0.72

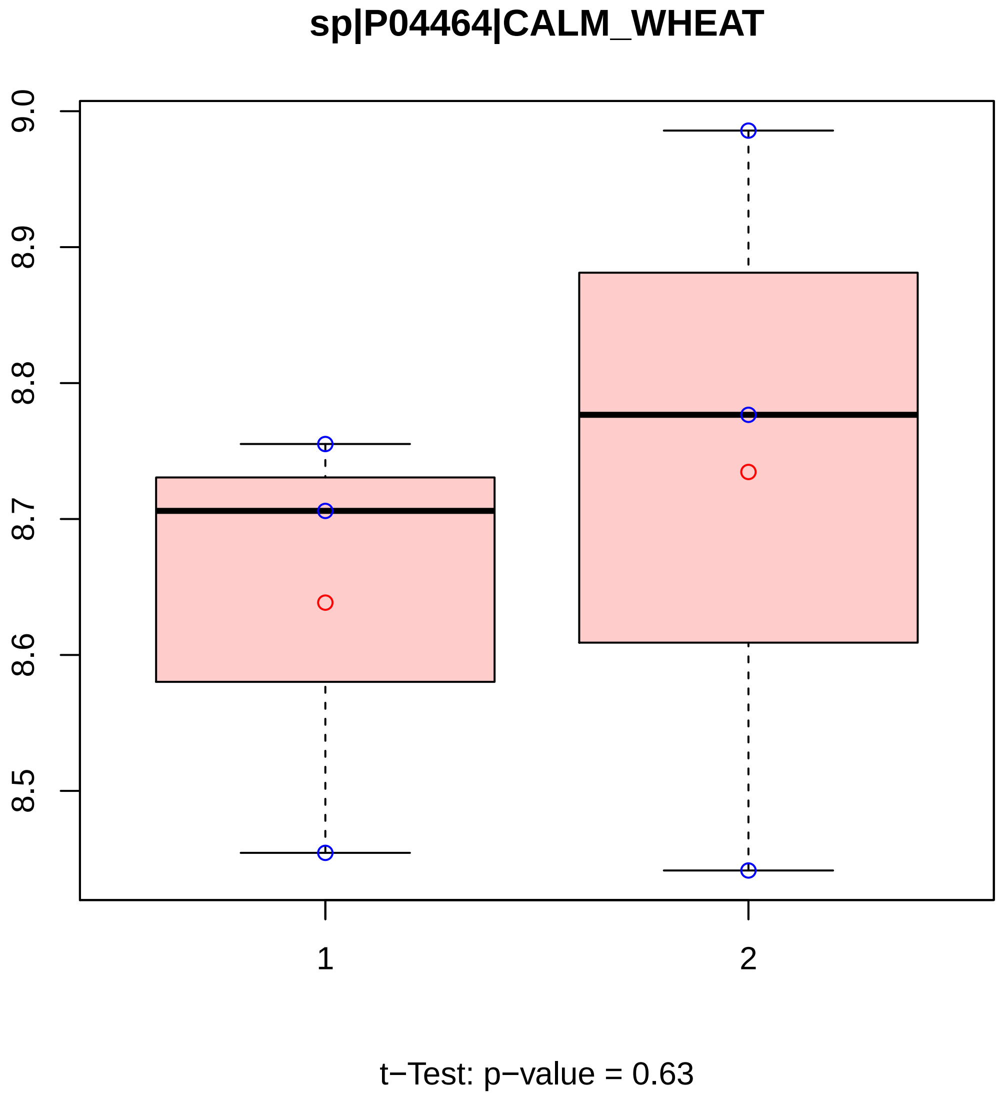
sp|P04464|CALM_WHEAT
9.0
8.9
8.8
8.7
8.6
8.5
1
2
t−Test: p−value = 0.63

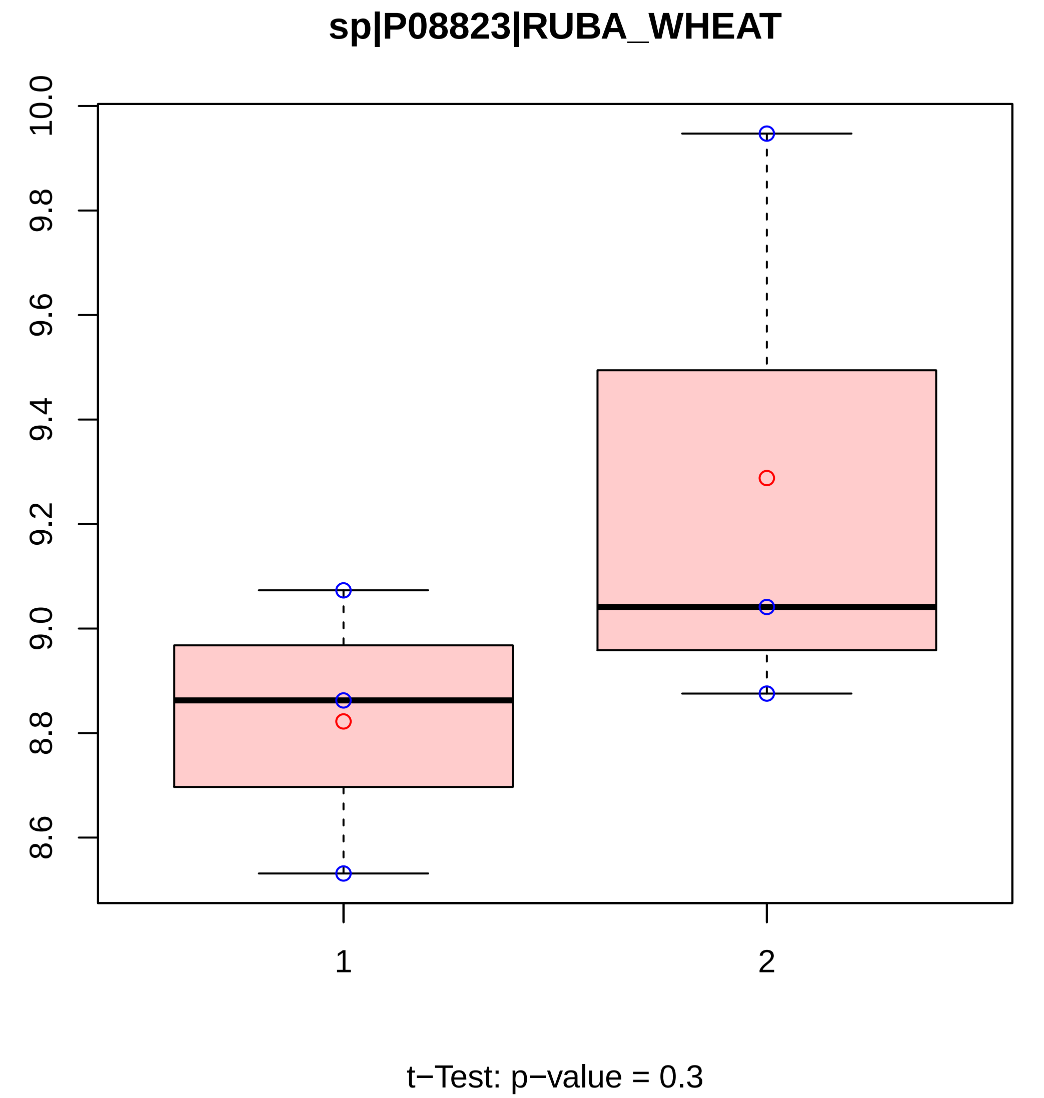
sp|P08823|RUBA_WHEAT
10.0
9.8
9.6
9.4
9.2
9.0
8.8
8.6
1
2
t−Test: p−value = 0.3

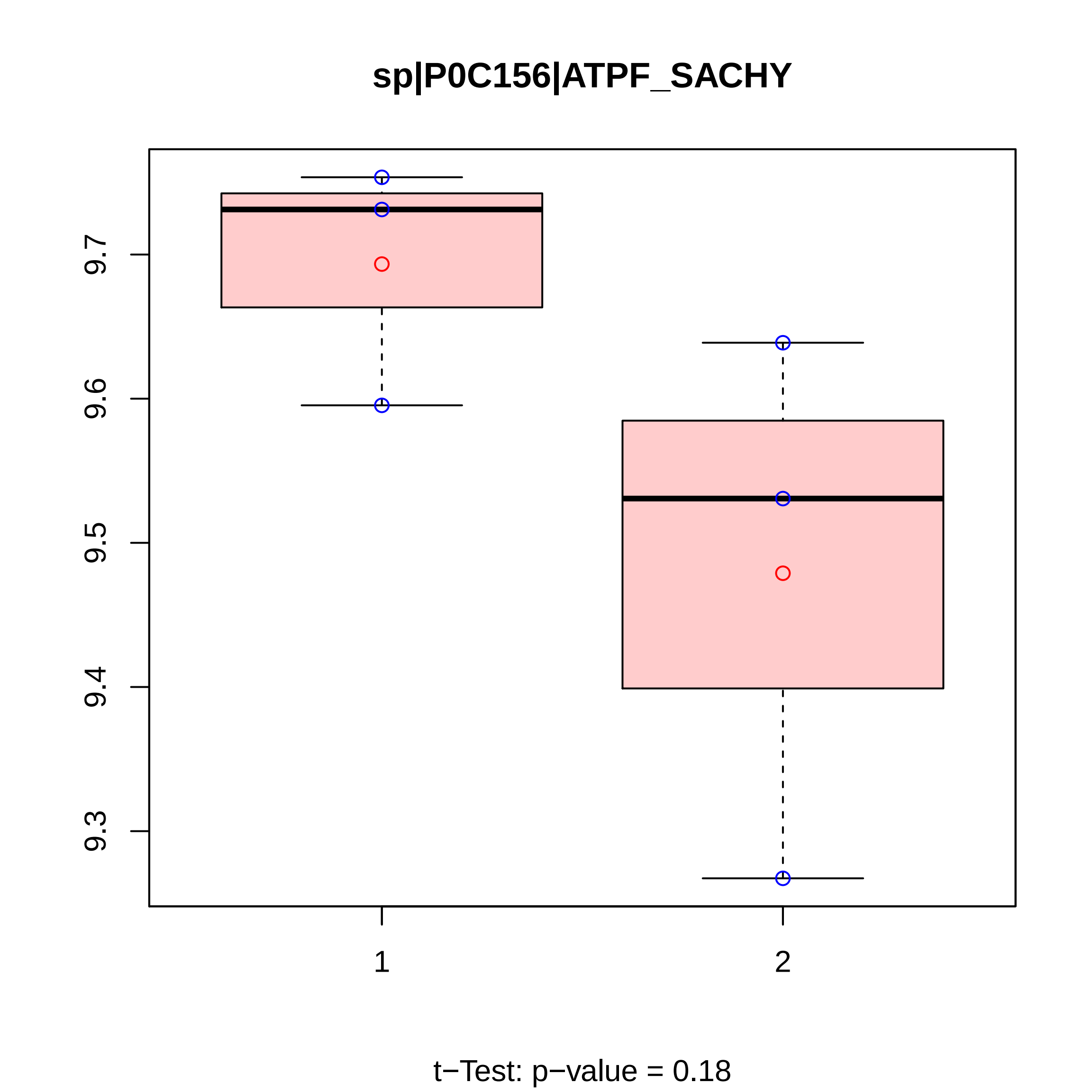
sp|P0C156|ATPF_SACHY
9.3
9.4
9.5
9.6
9.7
1
2
t−Test: p−value = 0.18

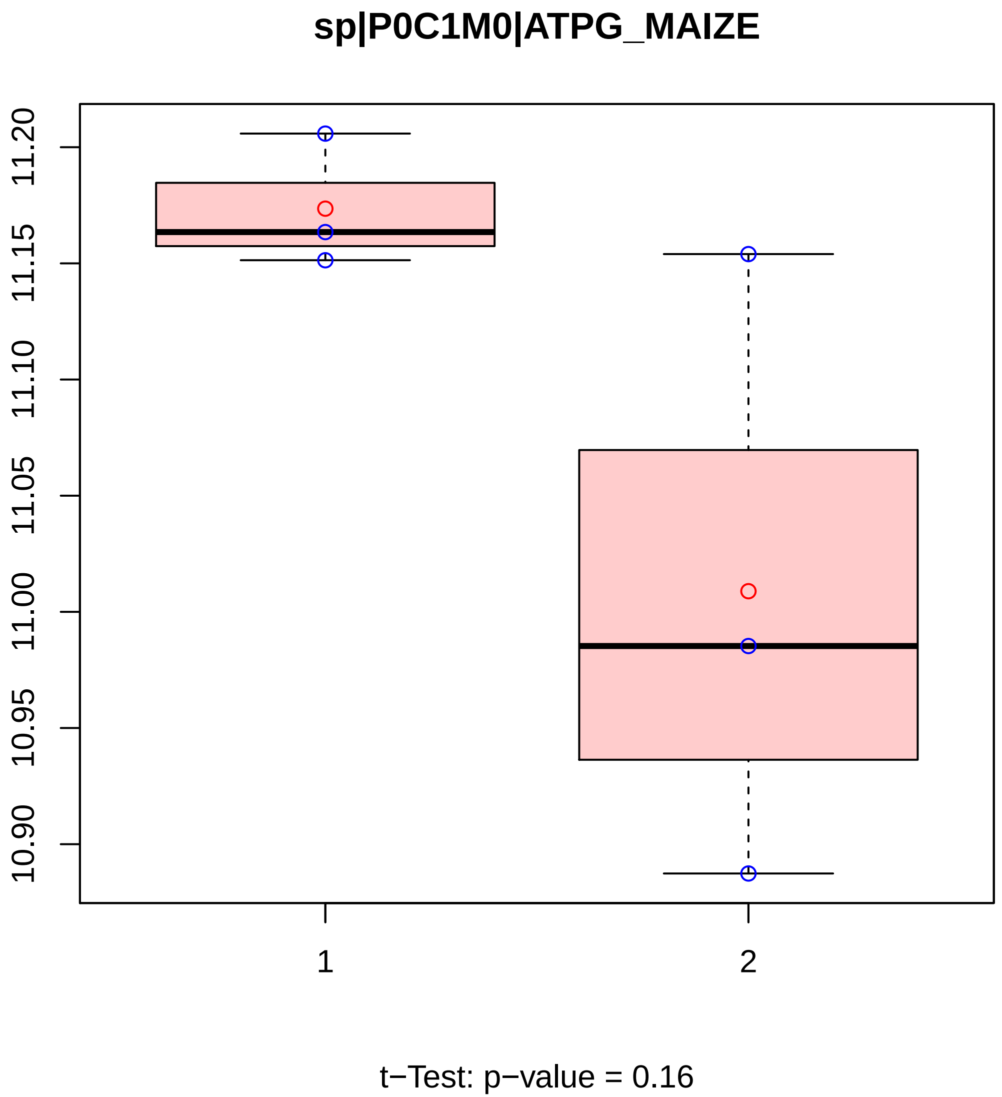
sp|P0C1M0|ATPG_MAIZE
11.20
11.15
11.10
11.05
11.00
10.95
10.90
1
2
t−Test: p−value = 0.16

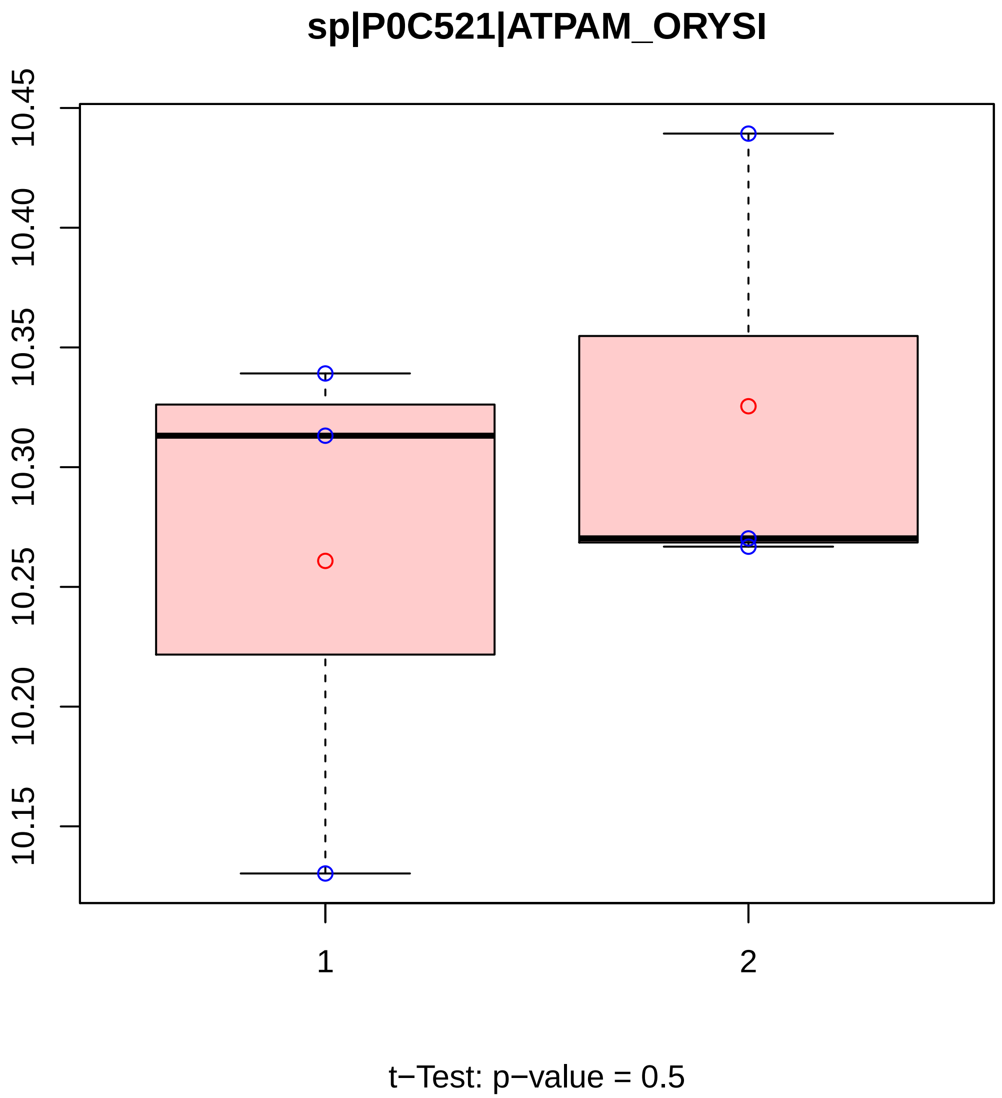

sp|P0C521|ATPAM_ORYSI
10.15
10.20
10.25
10.30
10.35
10.40
10.45
1
2
t−Test: p−value = 0.5

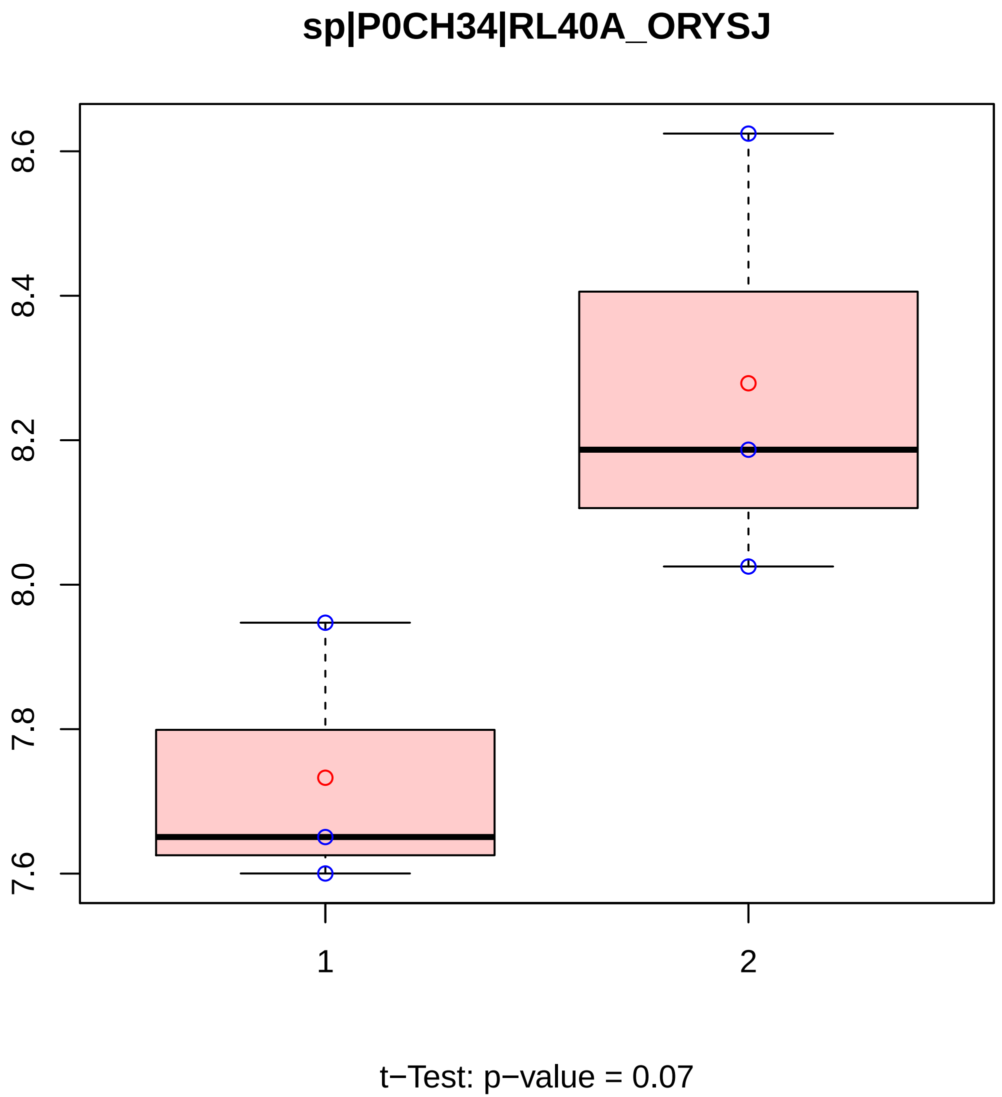
sp|P0CH34|RL40A_ORYSJ
8.6
8.4
8.2
8.0
7.8
7.6
1
2
t−Test: p−value = 0.07

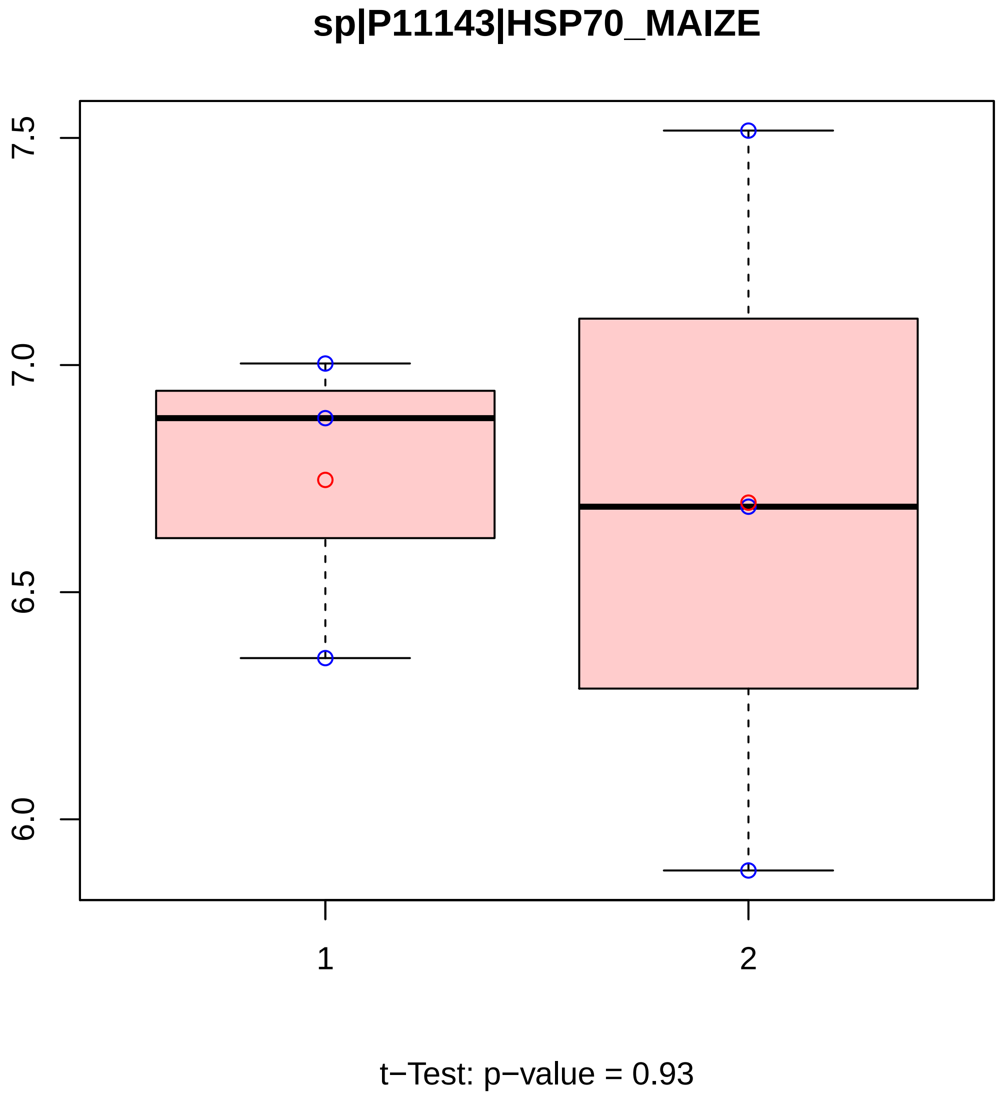
sp|P11143|HSP70_MAIZE
7.5
7.0
6.5
6.0
1
2
t−Test: p−value = 0.93

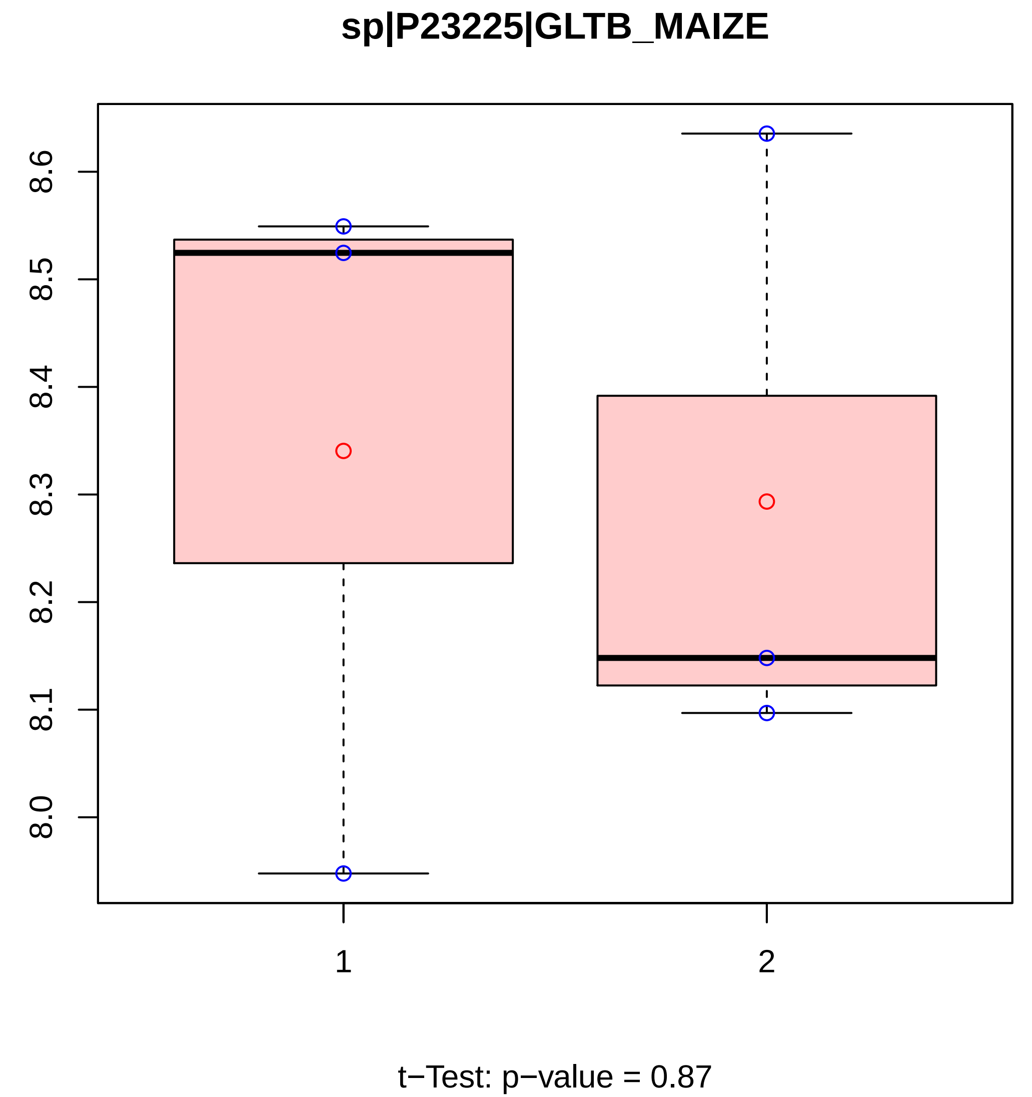
sp|P23225|GLTB_MAIZE
8.0
8.1
8.2
8.3
8.4
8.5
8.6
1
2
t−Test: p−value = 0.87

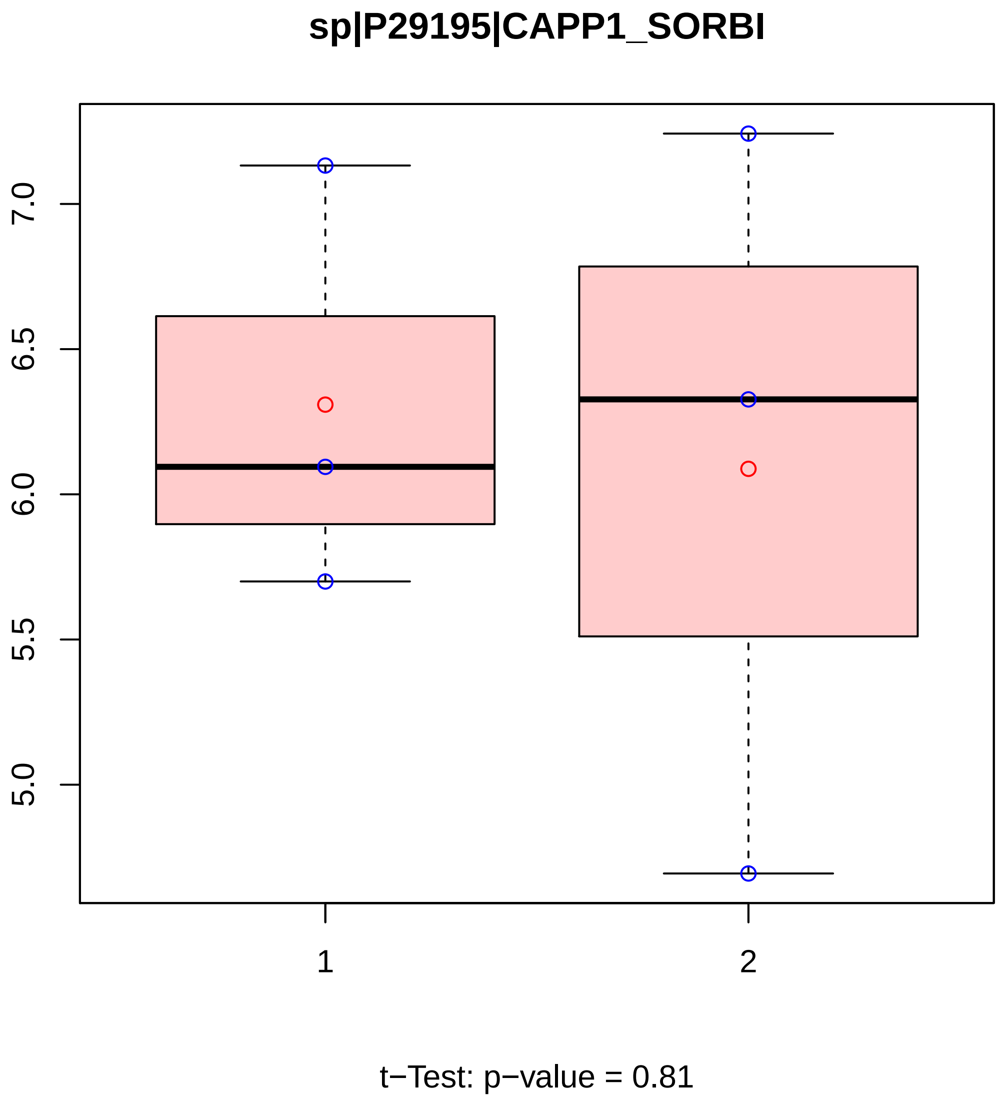
sp|P29195|CAPP1_SORBI
7.0
6.5
6.0
5.5
5.0
1
2
t−Test: p−value = 0.81

sp|P34106|ALA2_PANMI

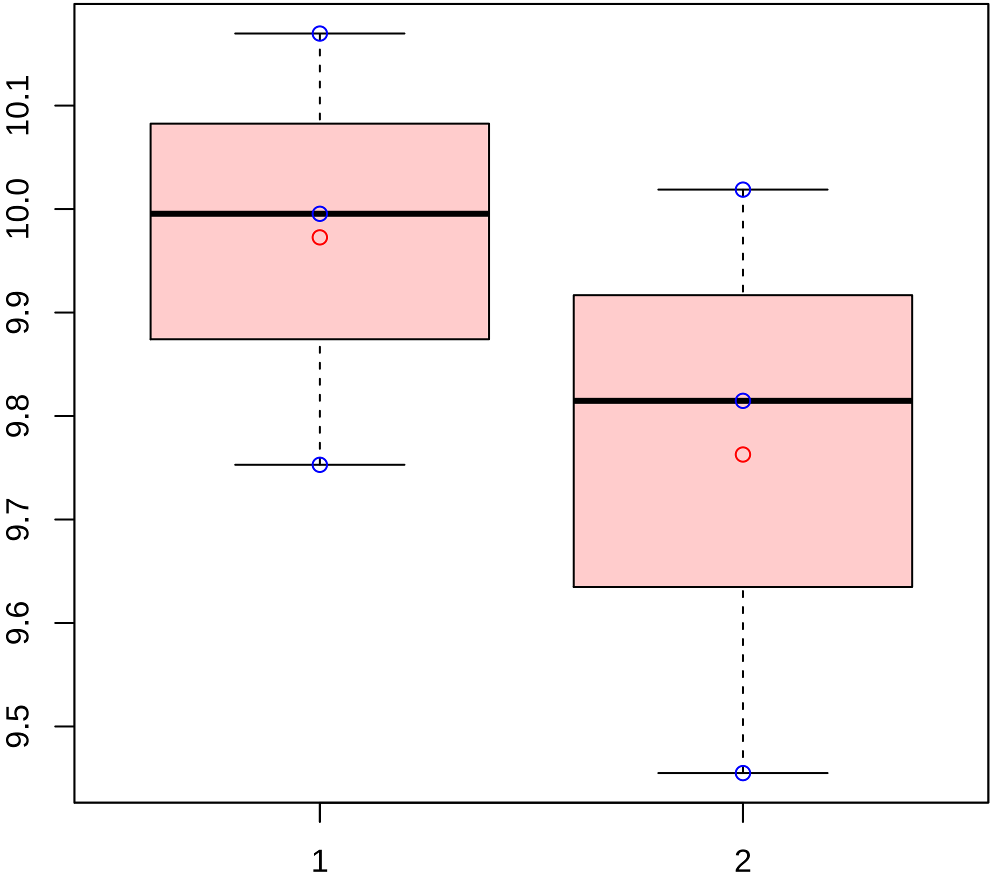

t−Test: p−value = 0.37

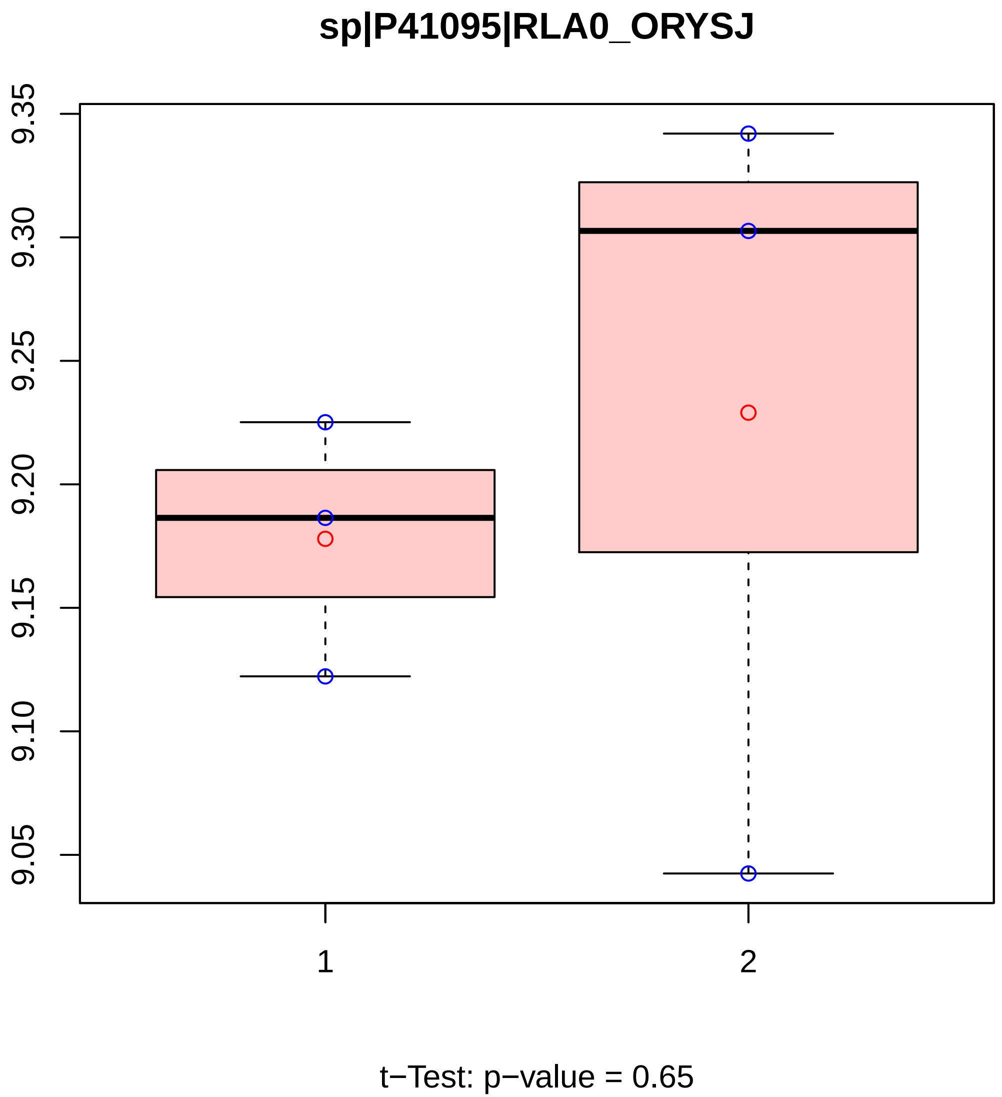
sp|P41095|RLA0_ORYSJ
9.35
9.30
9.25
9.20
9.15
9.10
9.05
1
2
t−Test: p−value = 0.65

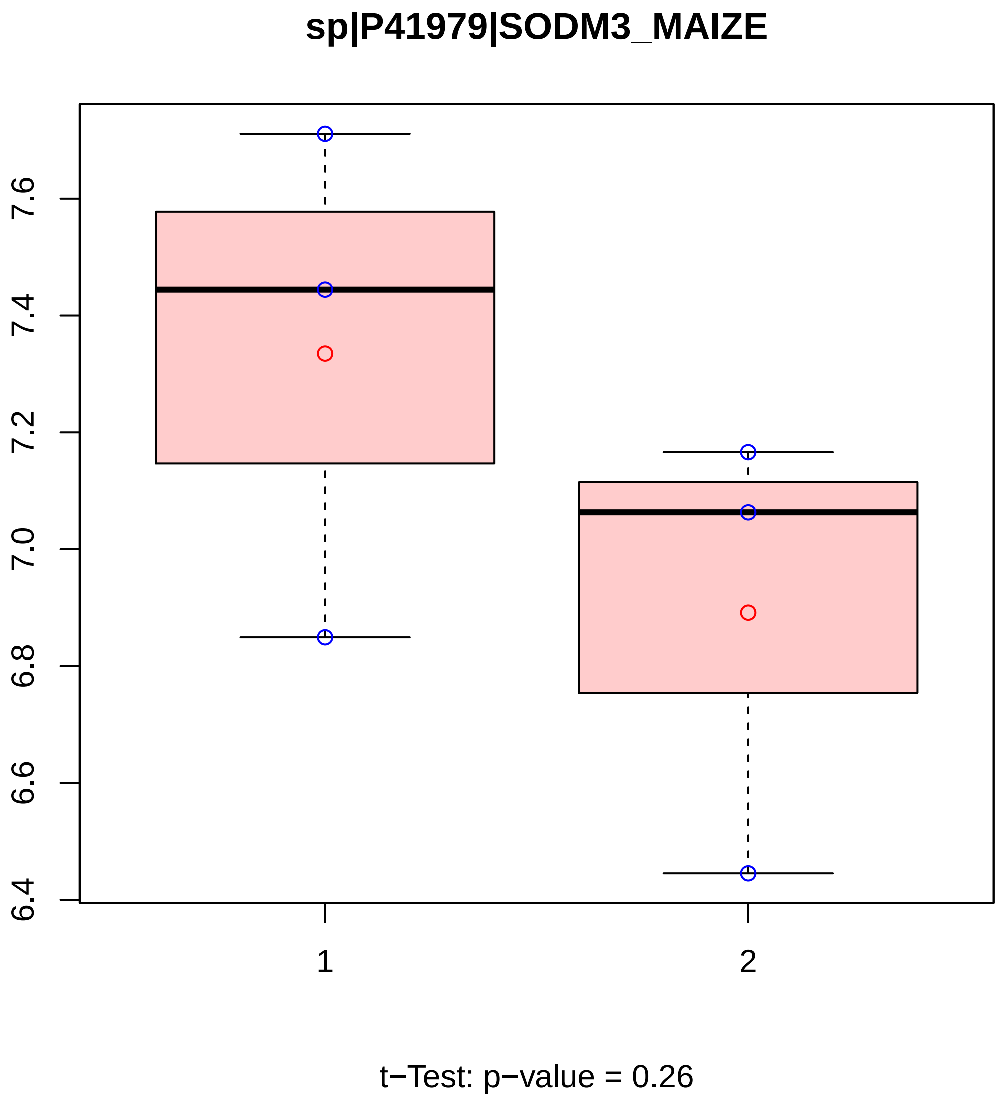

sp|P41979|SODM3_MAIZE
7.6
7.4
7.2
7.0
6.8
6.6
6.4
1
2
t−Test: p−value = 0.26

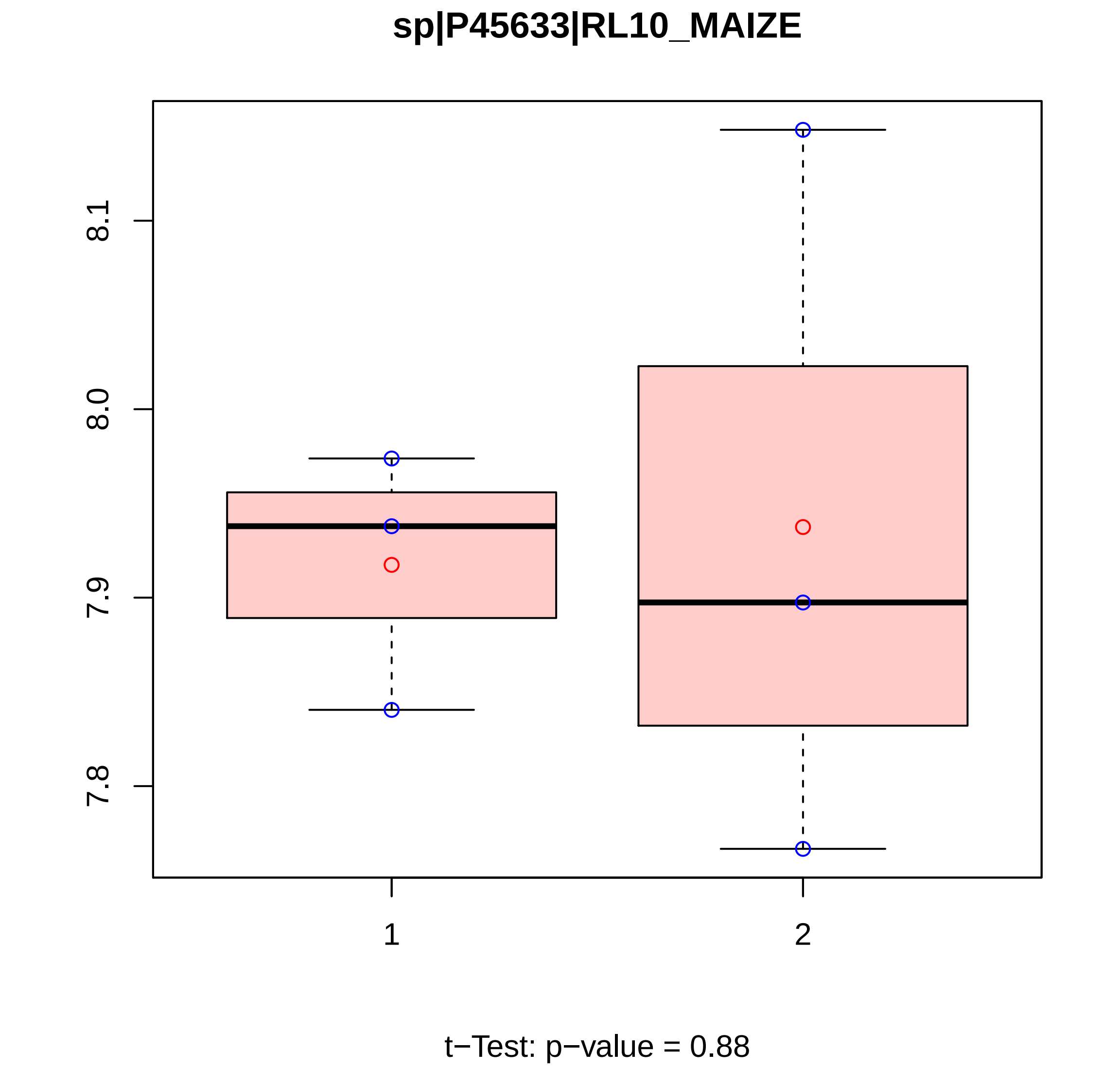
sp|P45633|RL10_MAIZE
8.1
8.0
7.9
7.8
1
2
t−Test: p−value = 0.88

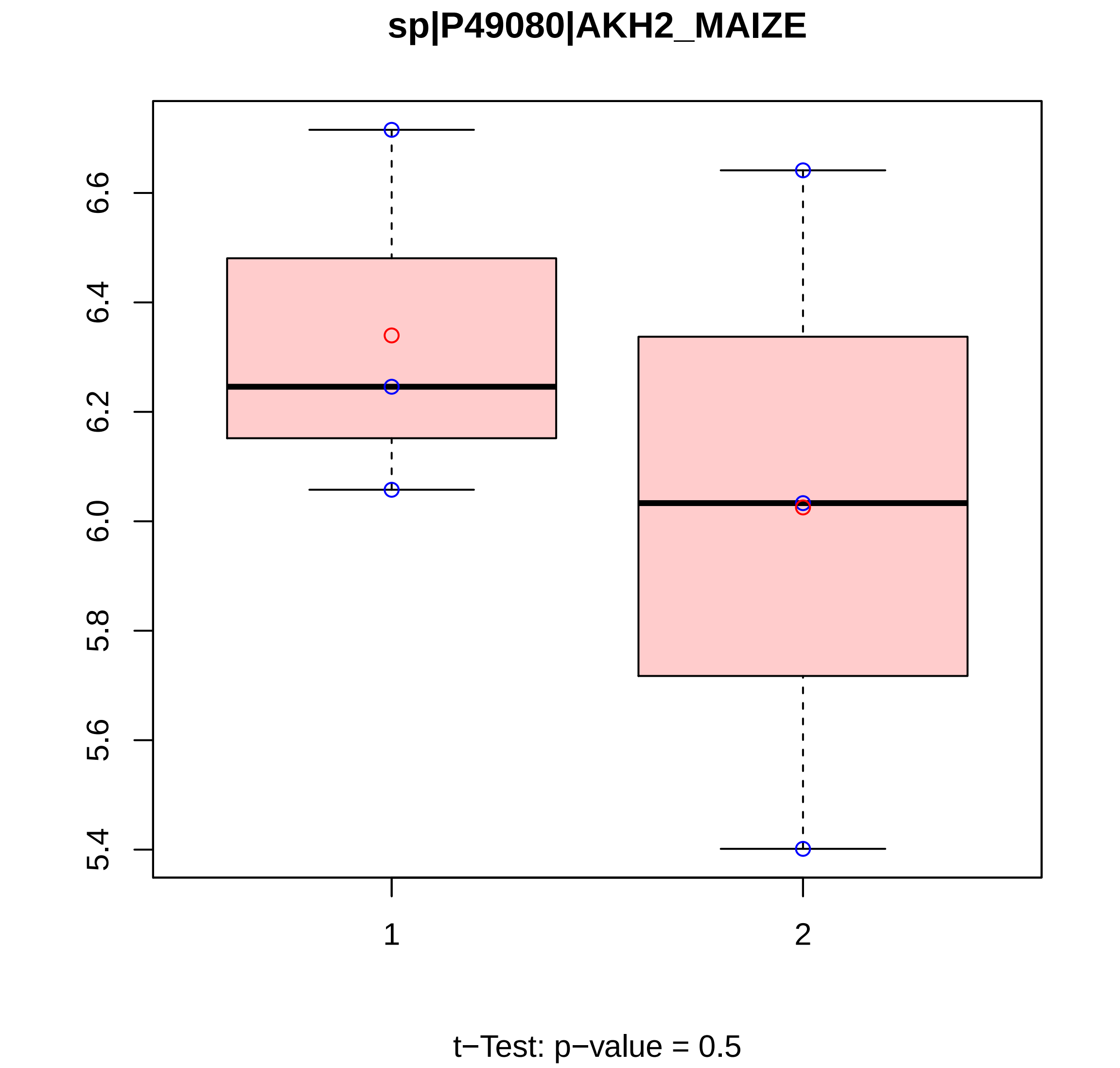
sp|P49080|AKH2_MAIZE
6.6
6.4
6.2
6.0
5.8
5.6
5.4
1
2
t−Test: p−value = 0.5

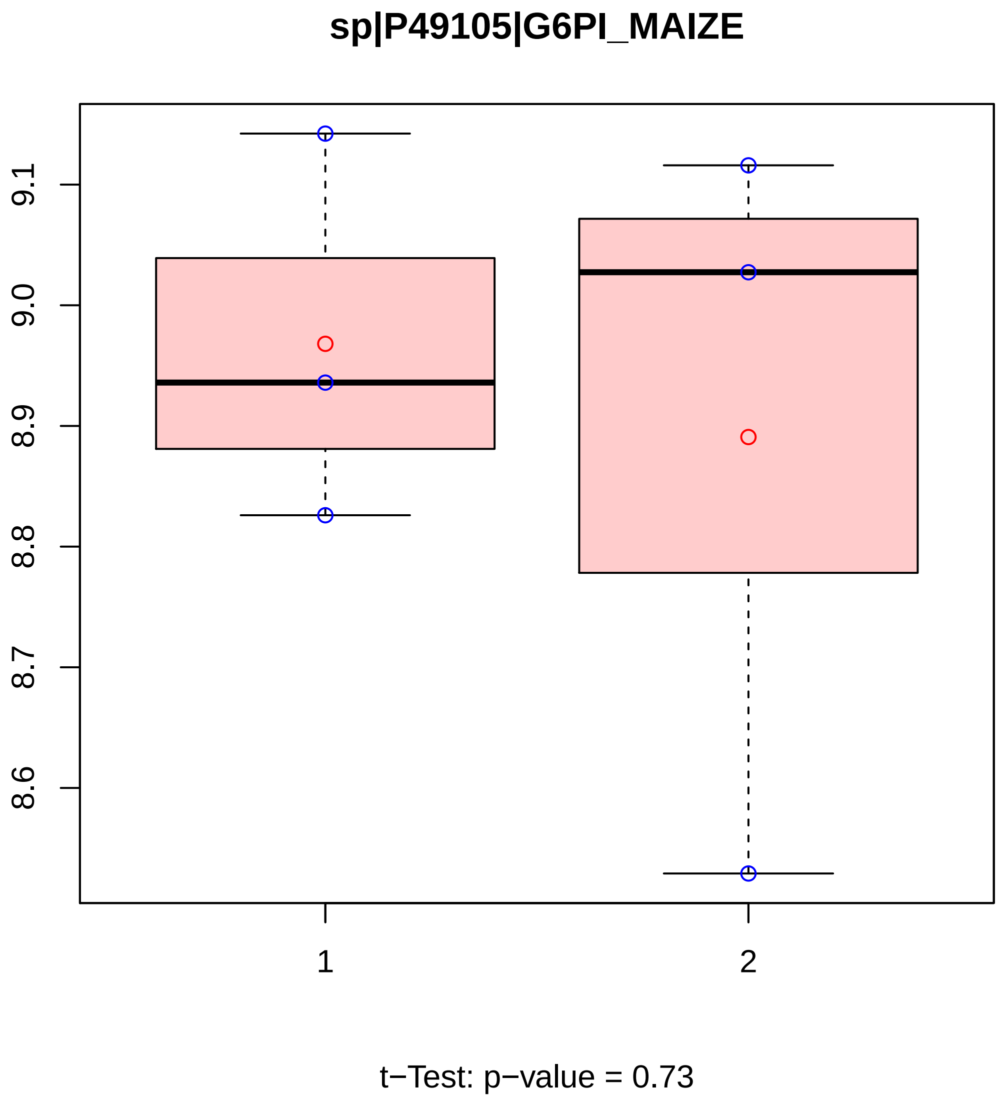
sp|P49105|G6PI_MAIZE
9.1
9.0
8.9
8.8
8.7
8.6
1
2
t−Test: p−value = 0.73

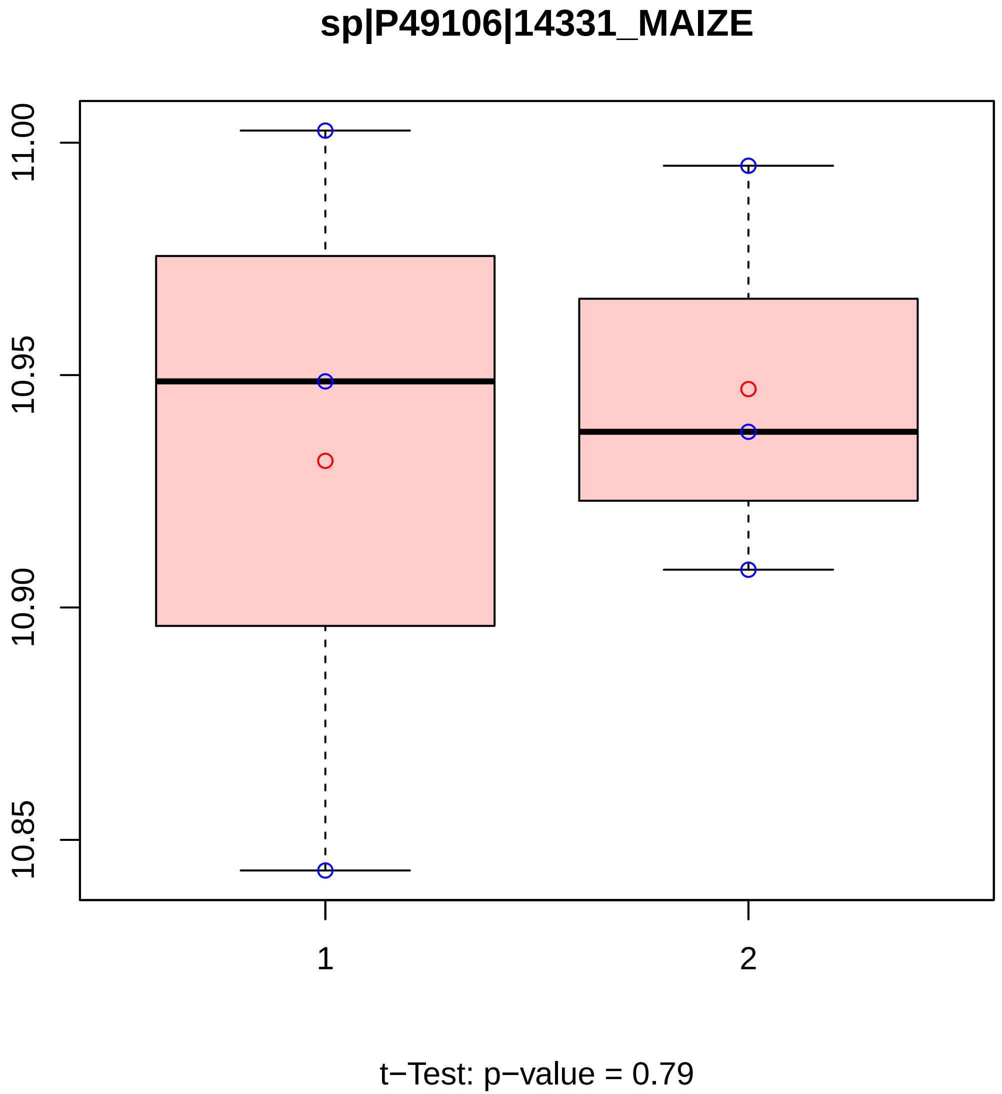
sp|P49106|14331_MAIZE
11.00
10.95
10.90
10.85
1
2
t−Test: p−value = 0.79

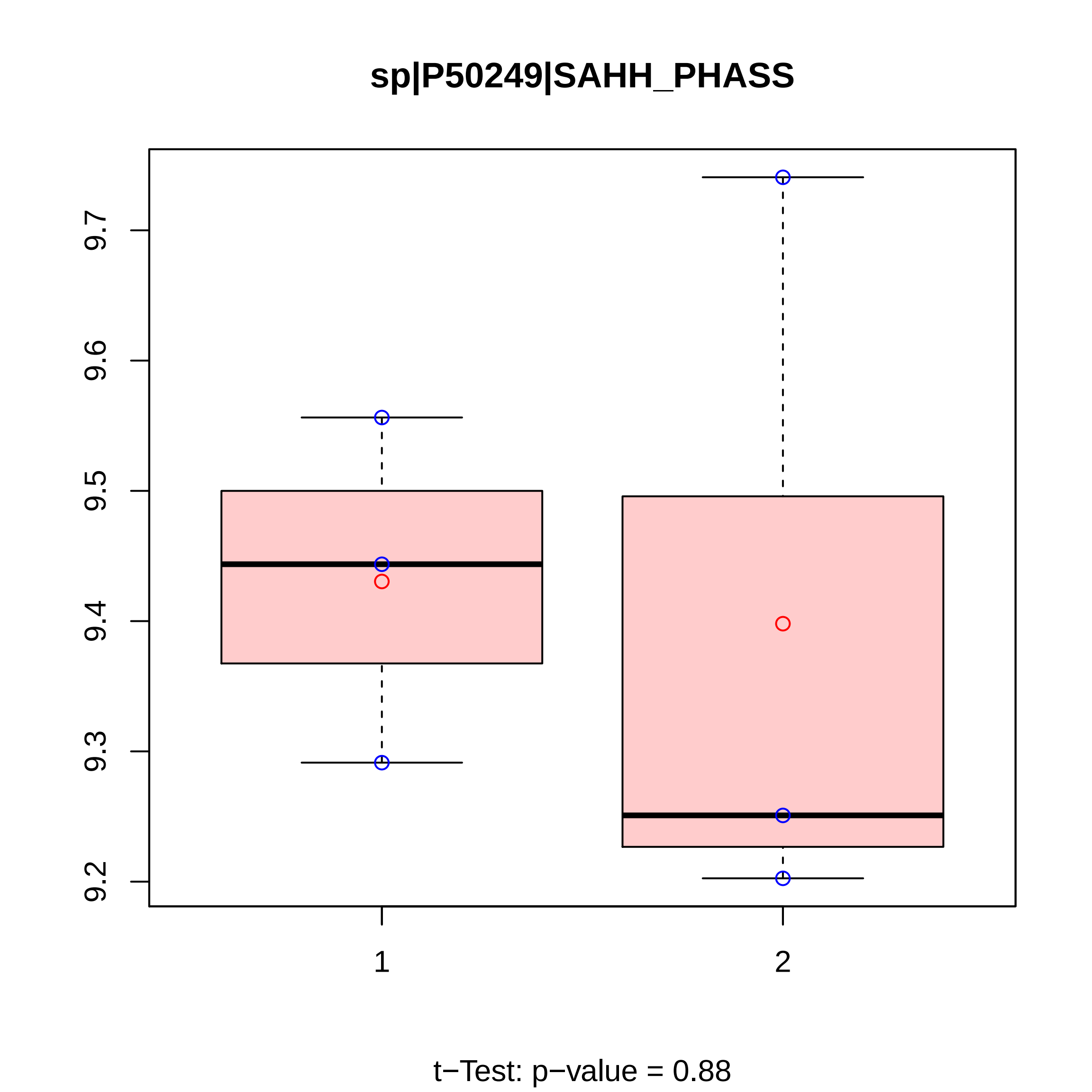
sp|P50249|SAHH_PHASS
9.2
9.3
9.4
9.5
9.6
9.7
1
2
t−Test: p−value = 0.88

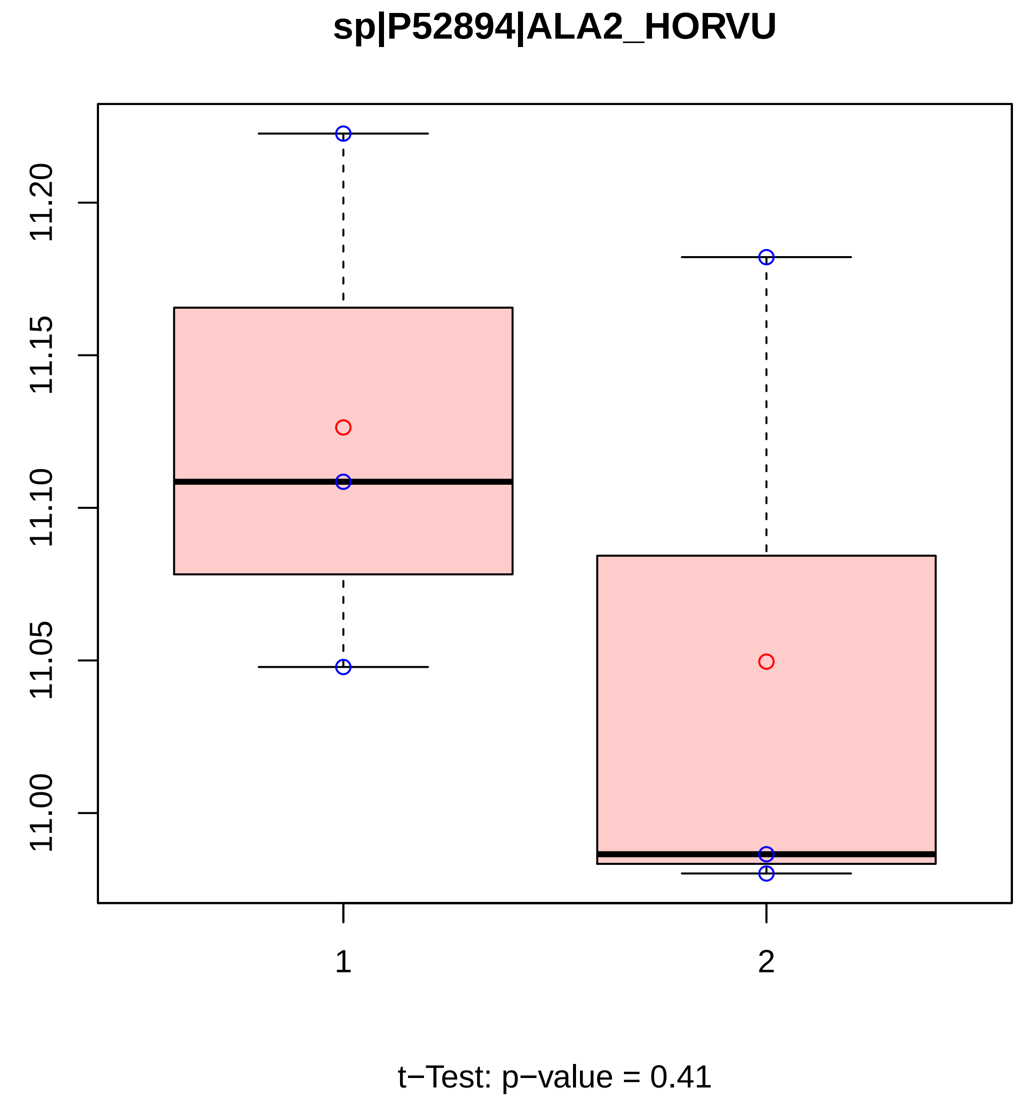
sp|P52894|ALA2_HORVU
11.20
11.15
11.10
11.05
11.00
1
2
t−Test: p−value = 0.41

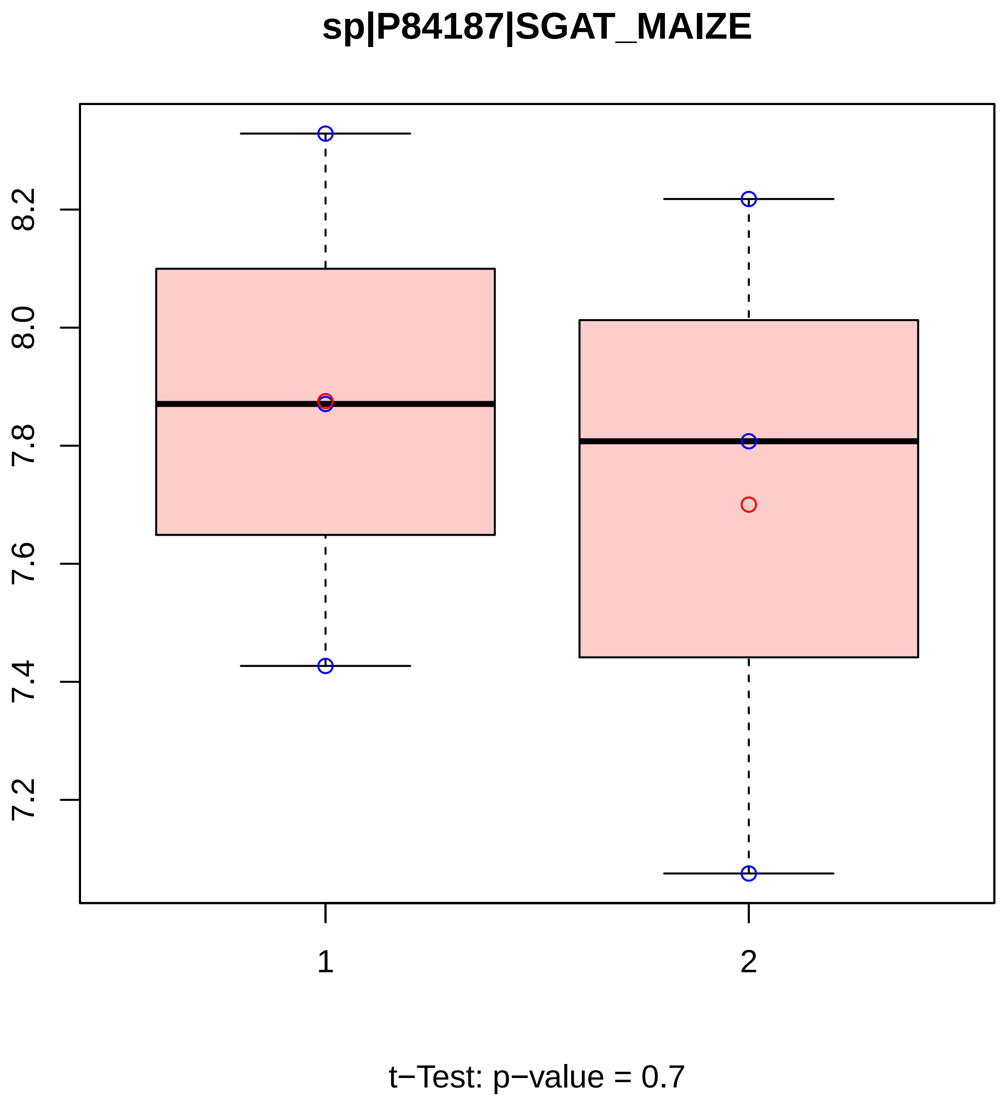
sp|P84187|SGAT_MAIZE
8.2
8.0
7.8
7.6
7.4
7.2
1
2
t−Test: p−value = 0.7

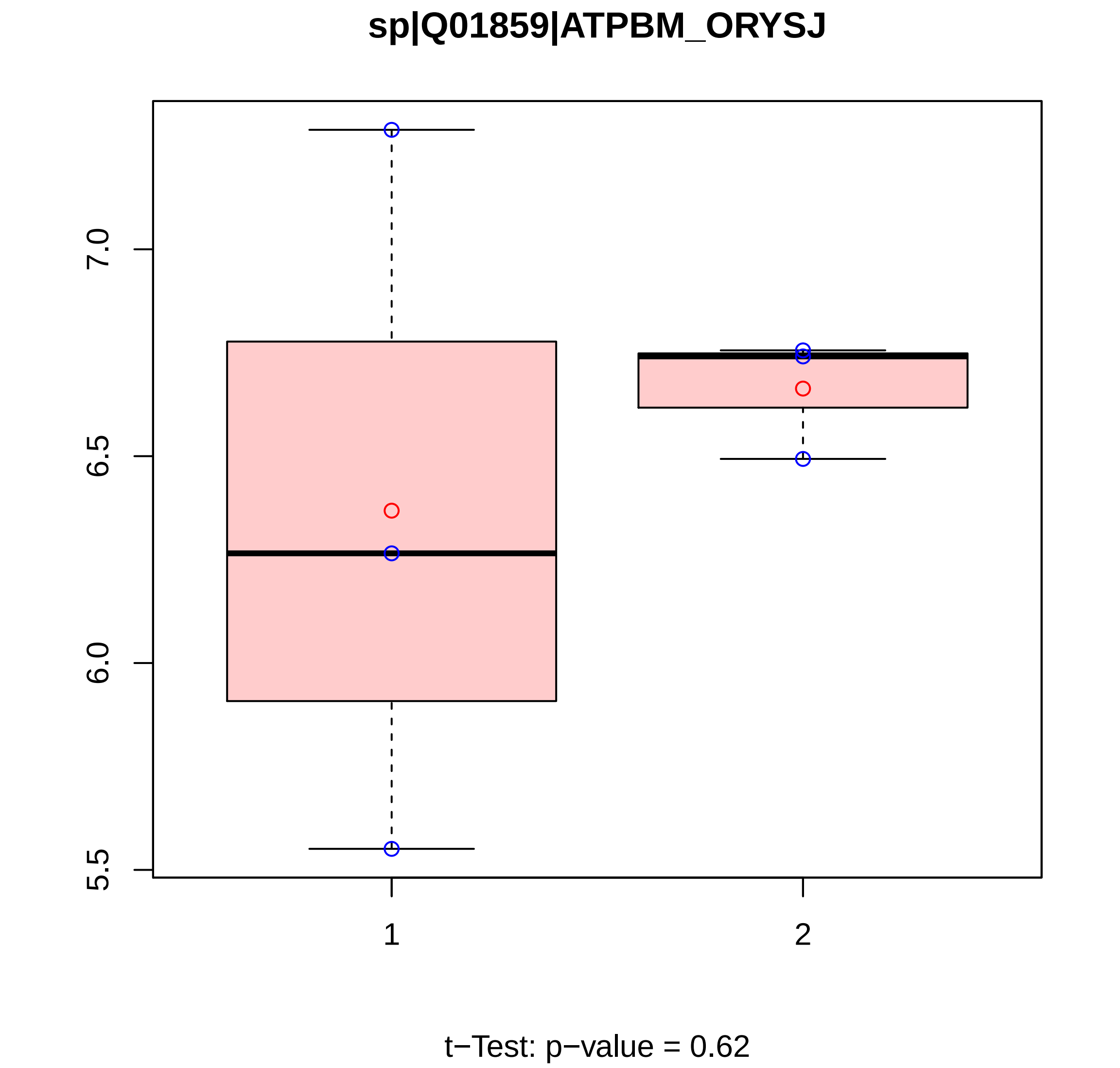
sp|Q01859|ATPBM_ORYSJ
7.0
6.5
6.0
5.5
1
2
t−Test: p−value = 0.62

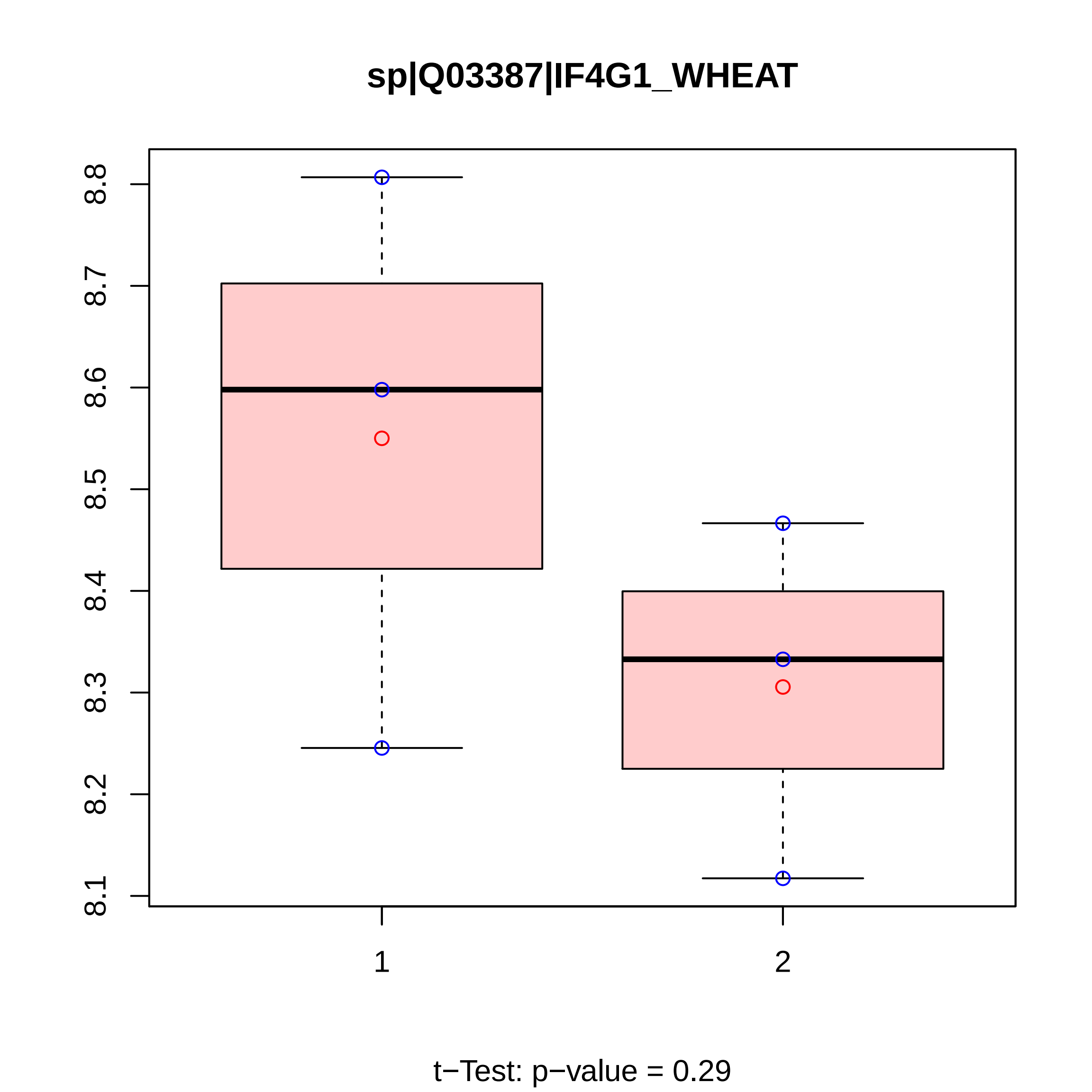
sp|Q03387|IF4G1_WHEAT
8.8
8.7
8.6
8.5
8.4
8.3
8.2
8.1
1
2
t−Test: p−value = 0.29

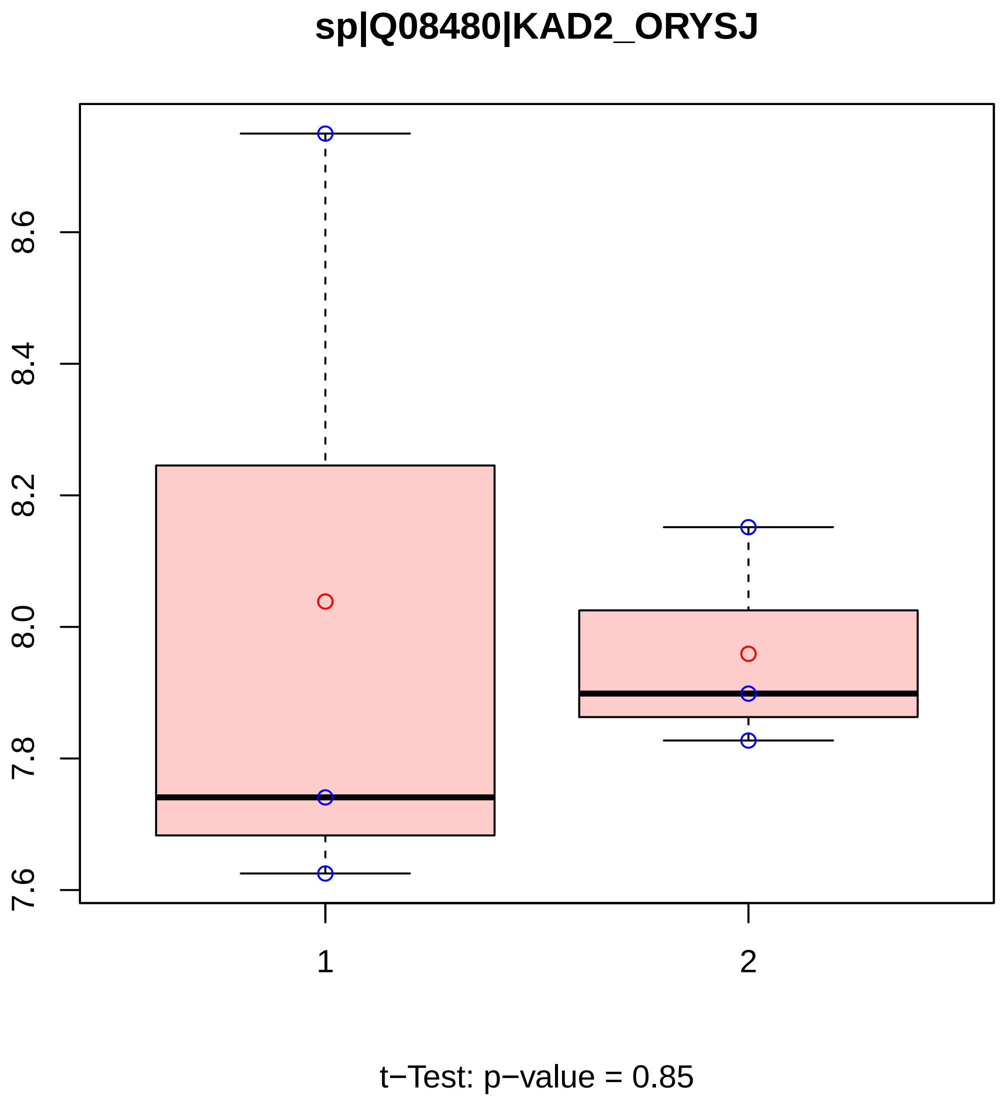
sp|Q08480|KAD2_ORYSJ
8.6
8.4
8.2
8.0
7.8
7.6
1
2
t−Test: p−value = 0.85

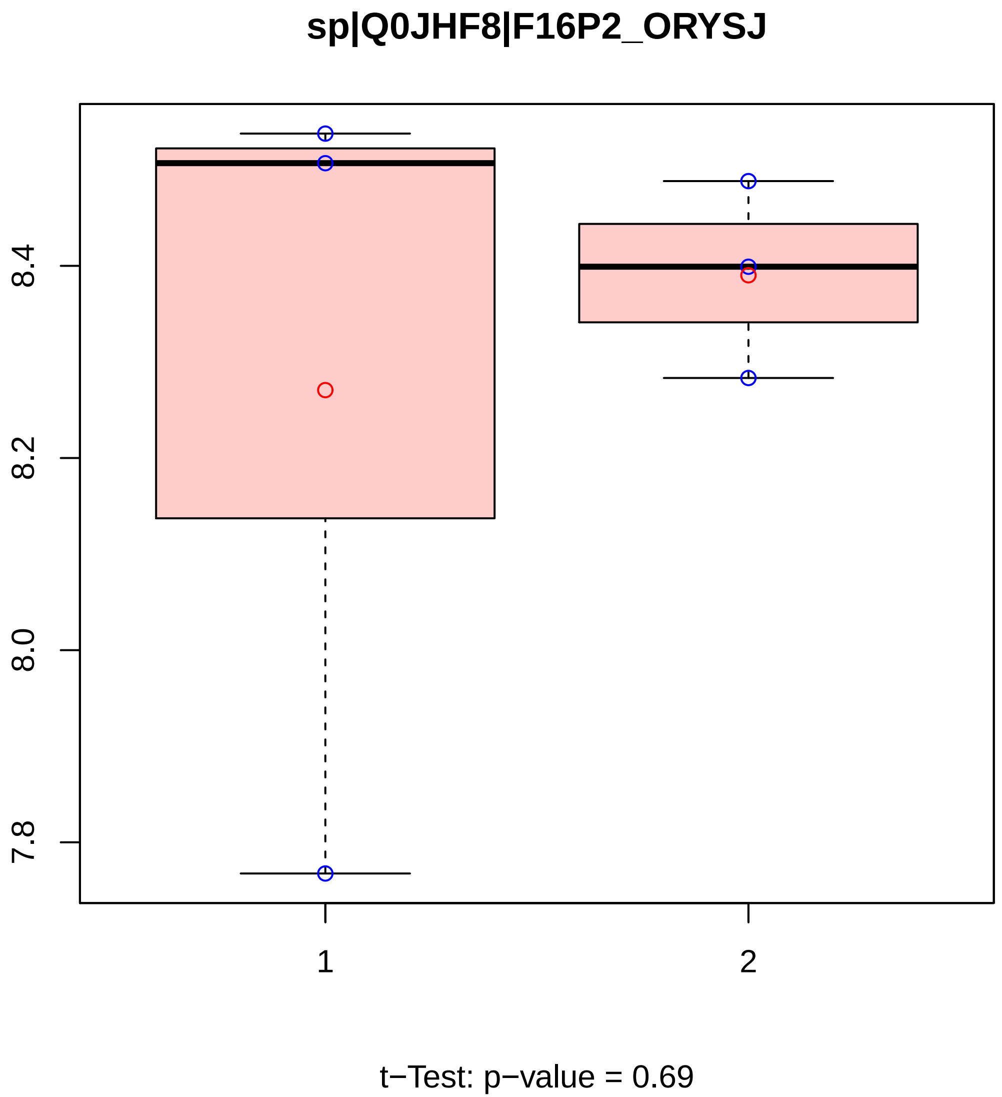
sp|Q0JHF8|F16P2_ORYSJ
8.4
8.2
8.0
7.8
1
2
t−Test: p−value = 0.69

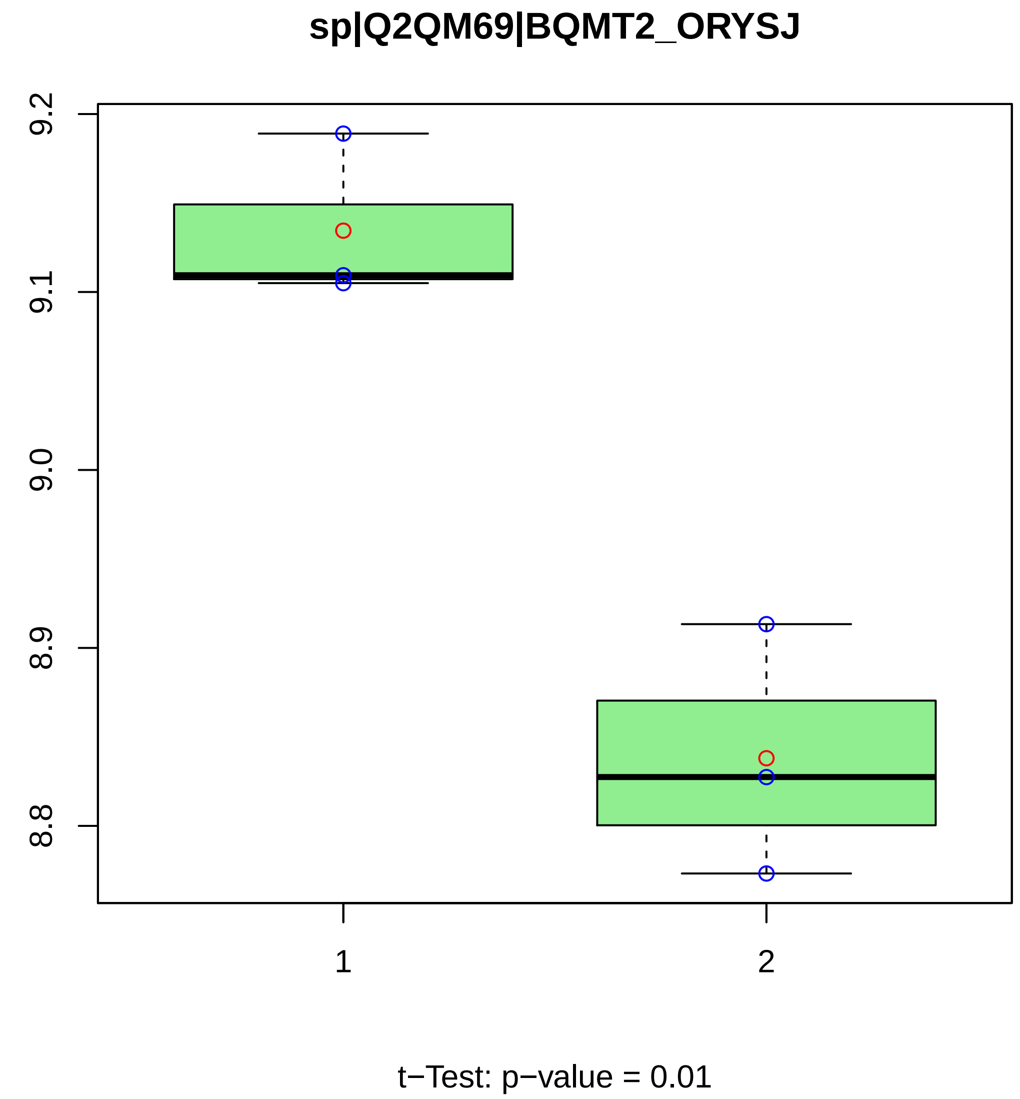
sp|Q2QM69|BQMT2_ORYSJ
9.2
9.1
9.0
8.9
8.8
1
2
t−Test: p−value = 0.01

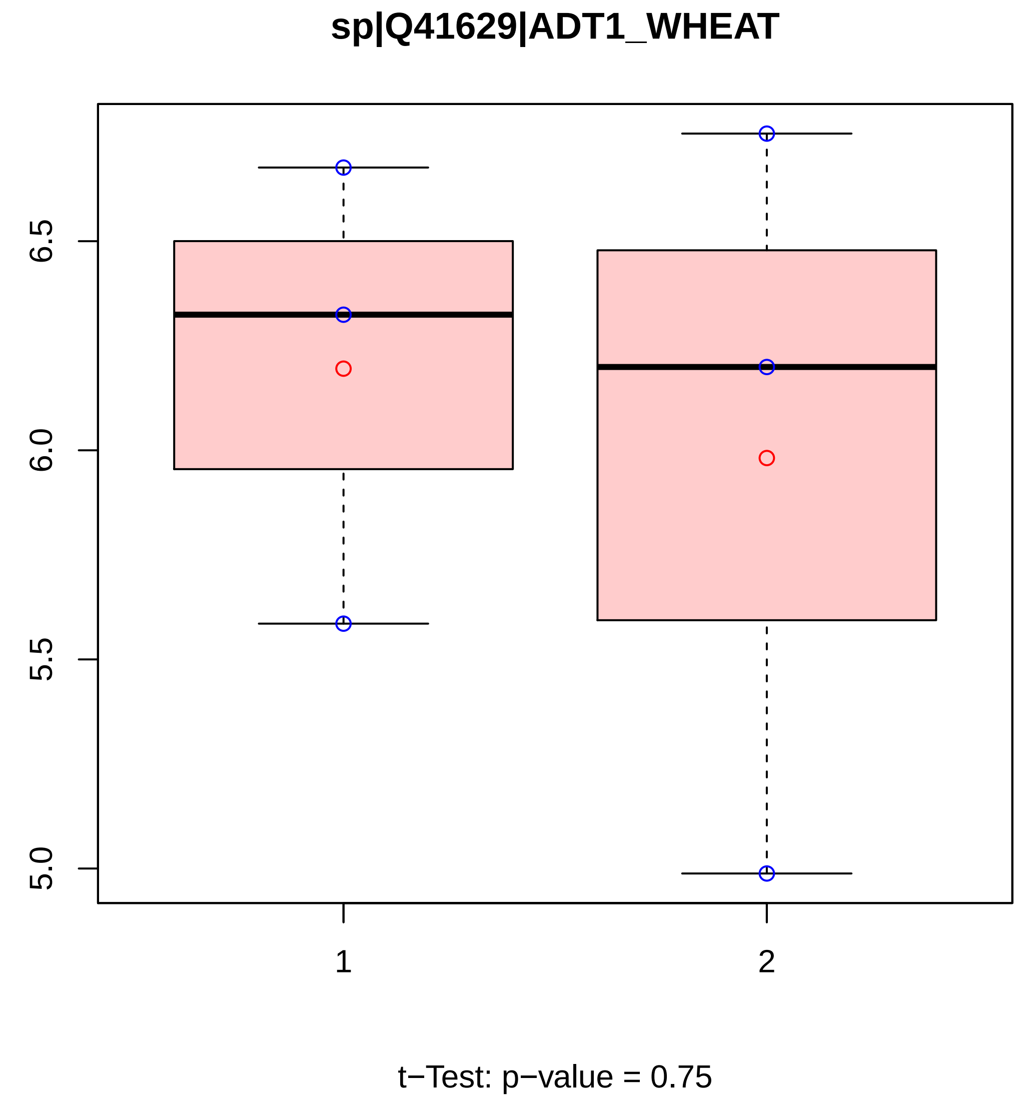
sp|Q41629|ADT1_WHEAT
6.5
6.0
5.5
5.0
1
2
t−Test: p−value = 0.75

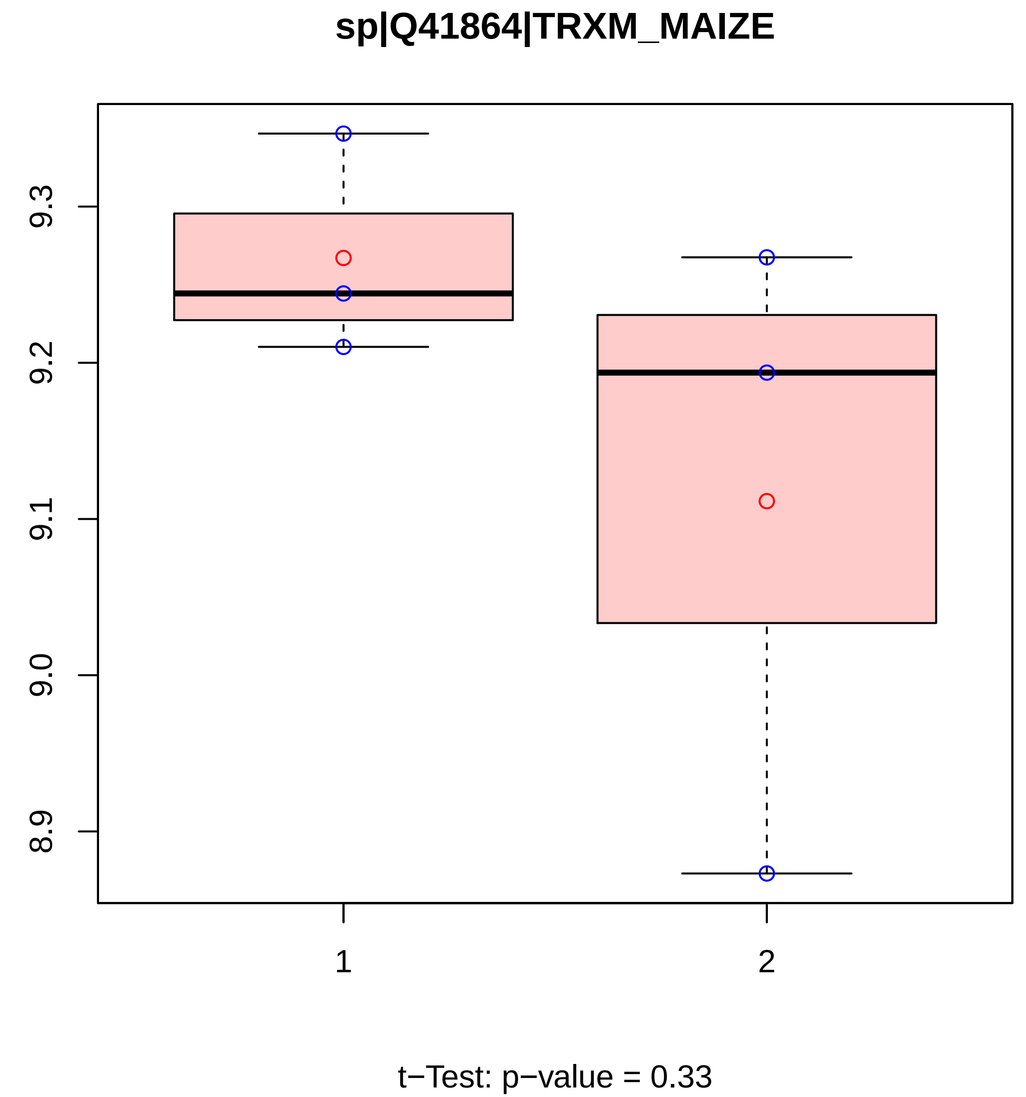
sp|Q41864|TRXM_MAIZE
9.3
9.2
9.1
9.0
8.9
1
2
t−Test: p−value = 0.33

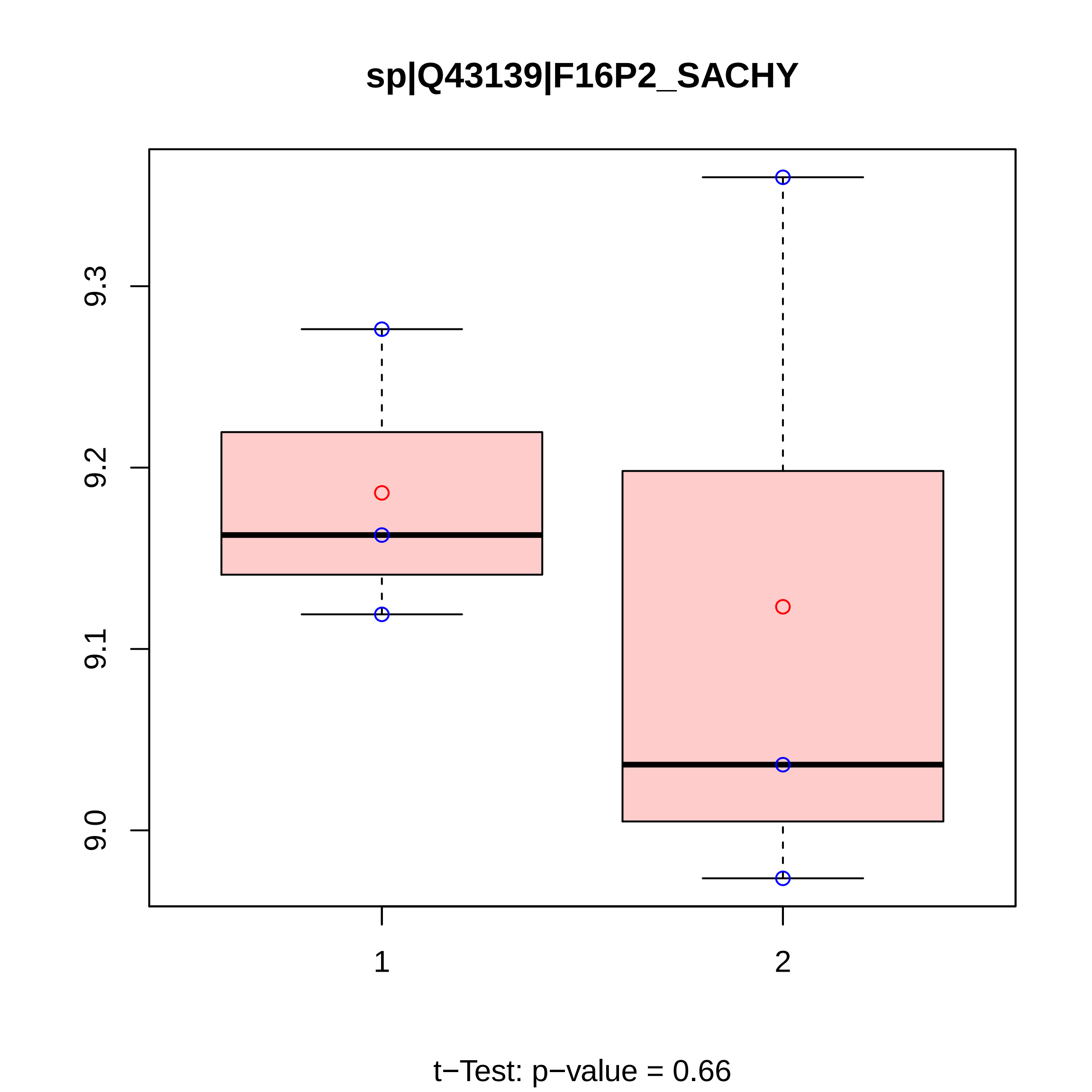
sp|Q43139|F16P2_SACHY
9.0
9.1
9.2
9.3
1
2
t−Test: p−value = 0.66

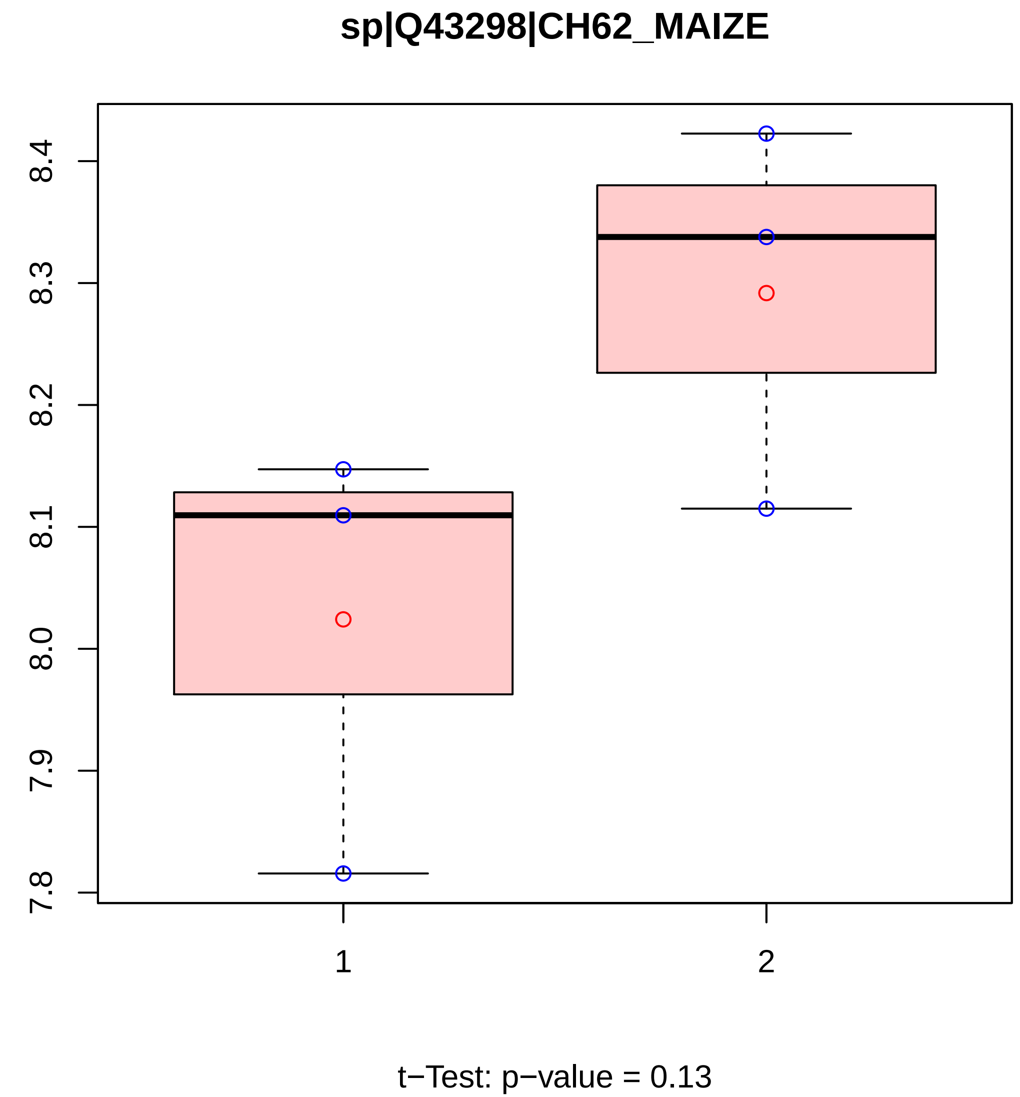
sp|Q43298|CH62_MAIZE
8.4
8.3
8.2
8.1
8.0
7.9
7.8
1
2
t−Test: p−value = 0.13

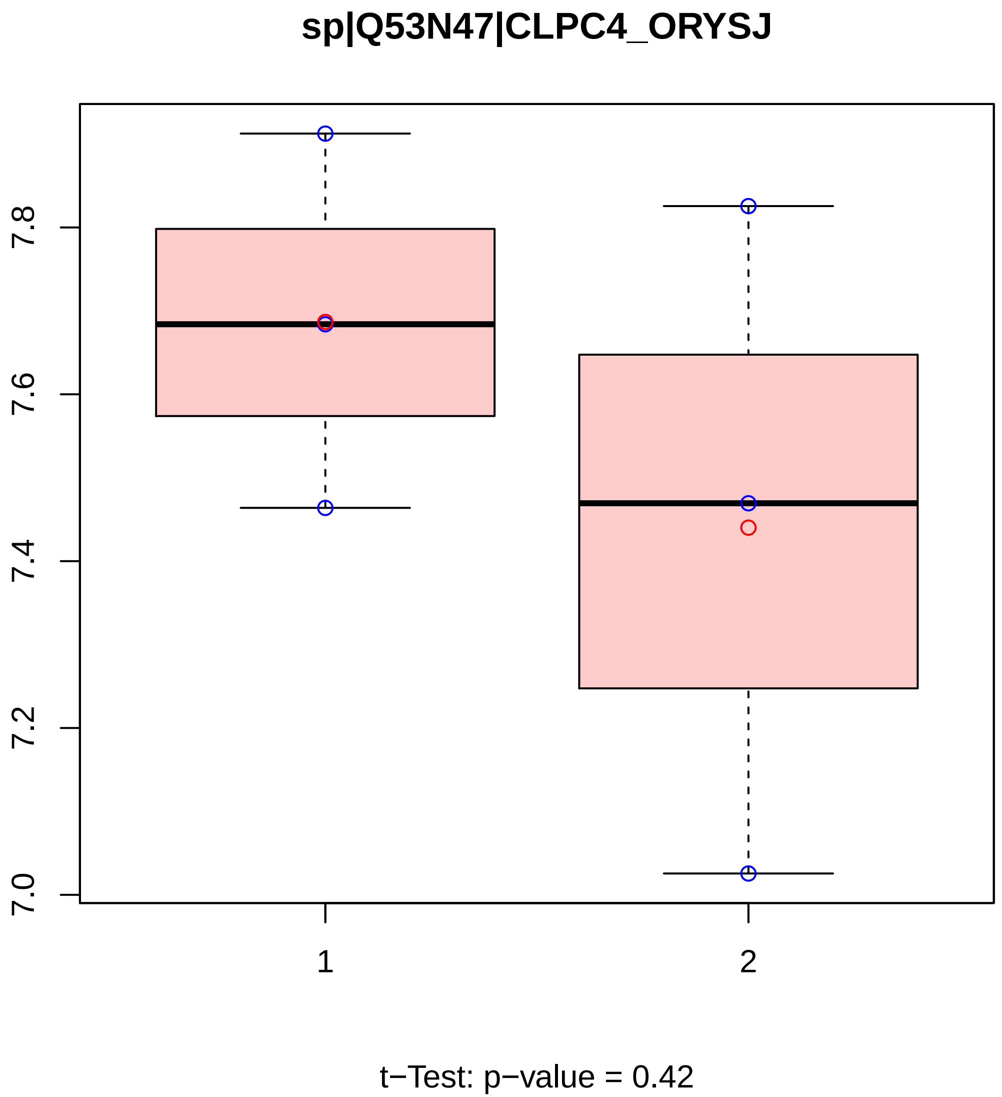
sp|Q53N47|CLPC4_ORYSJ
7.0
7.2
7.4
7.6
7.8
1
2
t−Test: p−value = 0.42

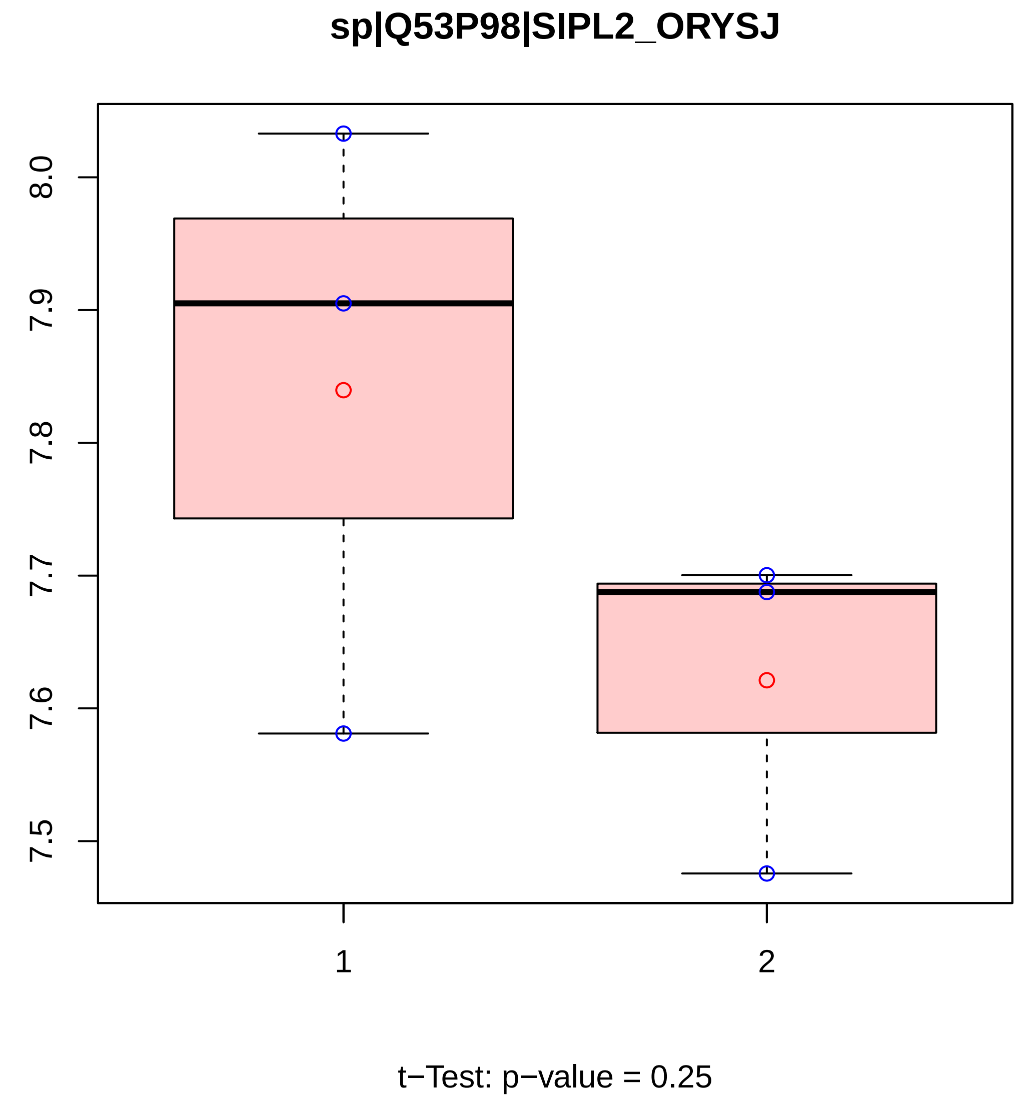
sp|Q53P98|SIPL2_ORYSJ
8.0
7.9
7.8
7.7
7.6
7.5
1
2
t−Test: p−value = 0.25

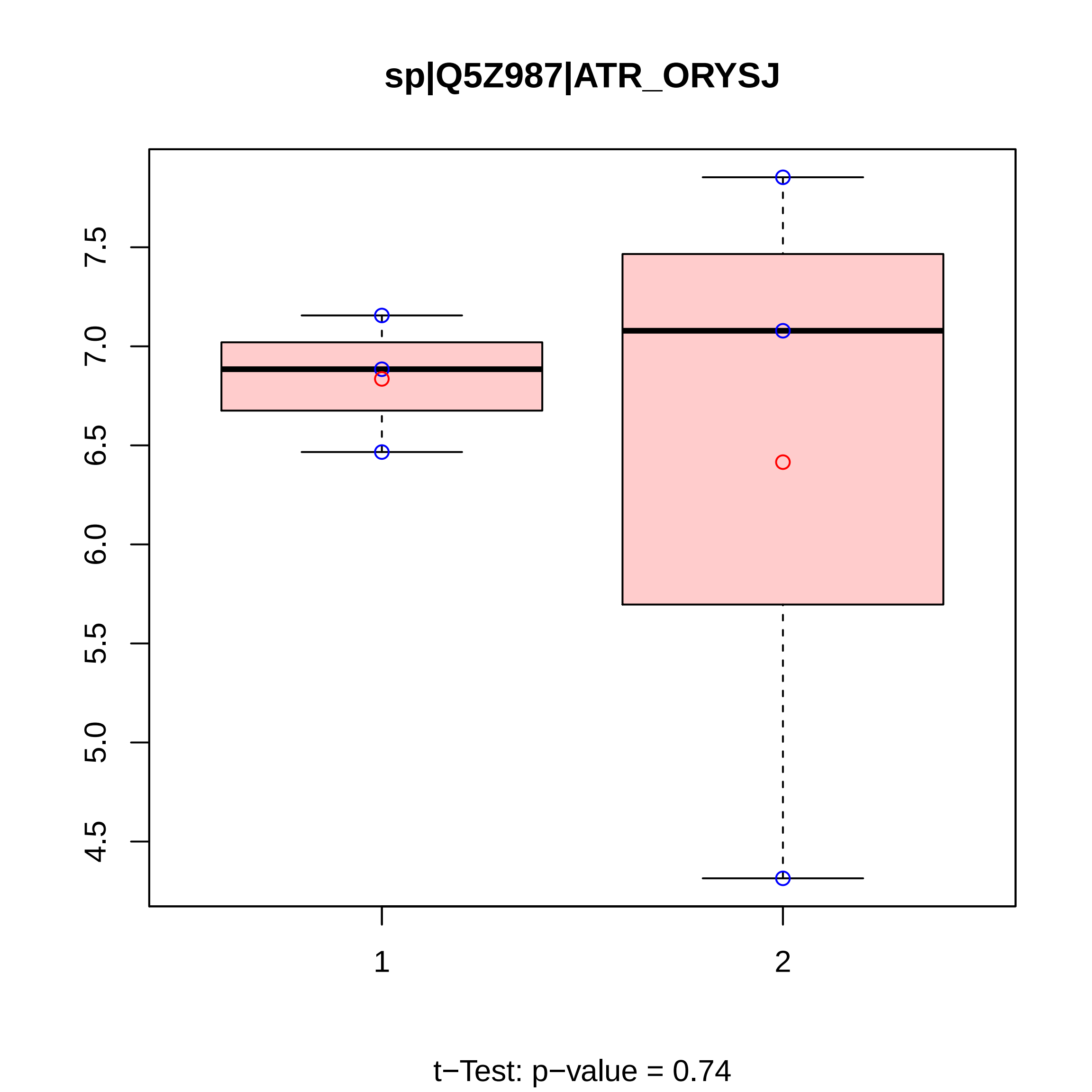
sp|Q5Z987|ATR_ORYSJ
7.5
7.0
6.5
6.0
5.5
5.0
4.5
1
2
t−Test: p−value = 0.74

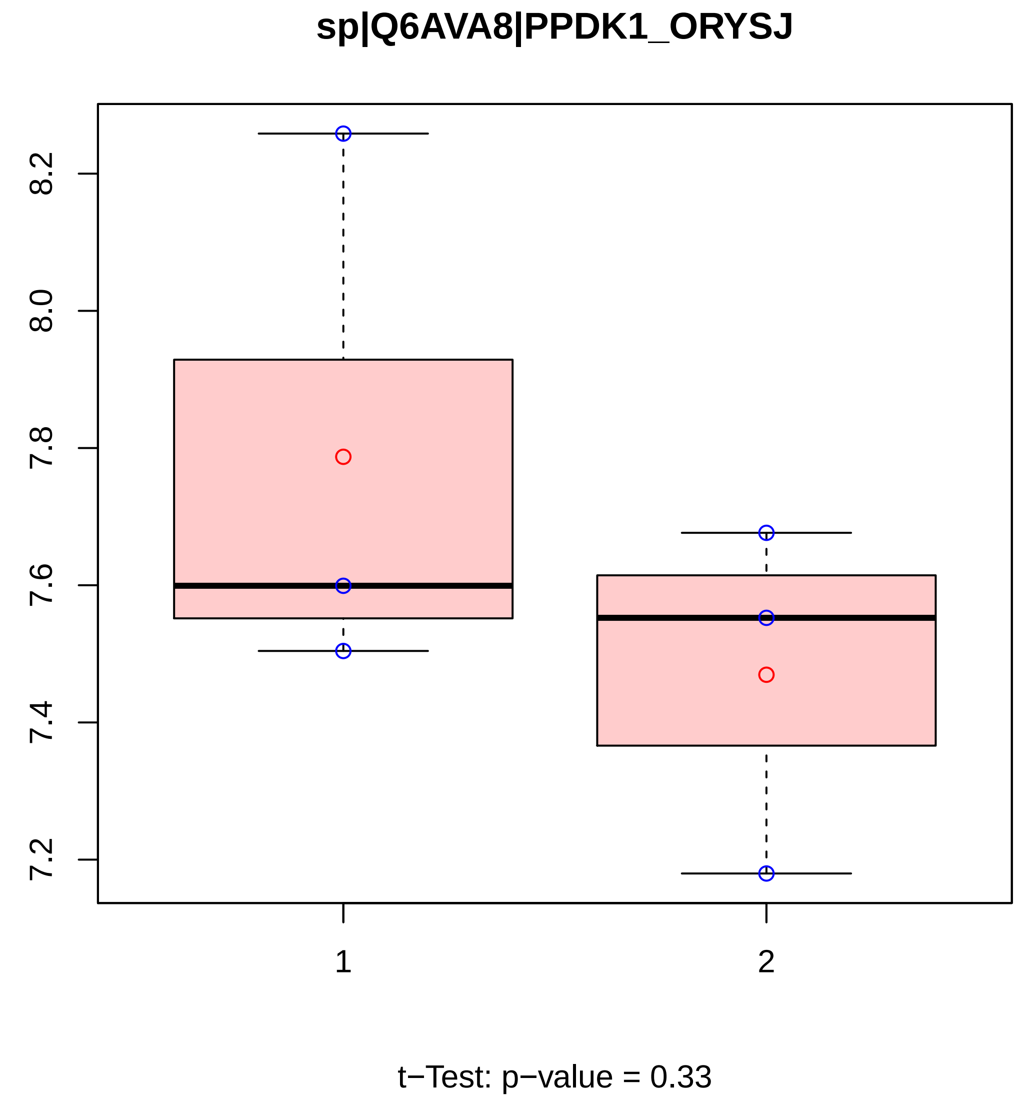

sp|Q6AVA8|PPDK1_ORYSJ
8.2
8.0
7.8
7.6
7.4
7.2
1
2
t−Test: p−value = 0.33

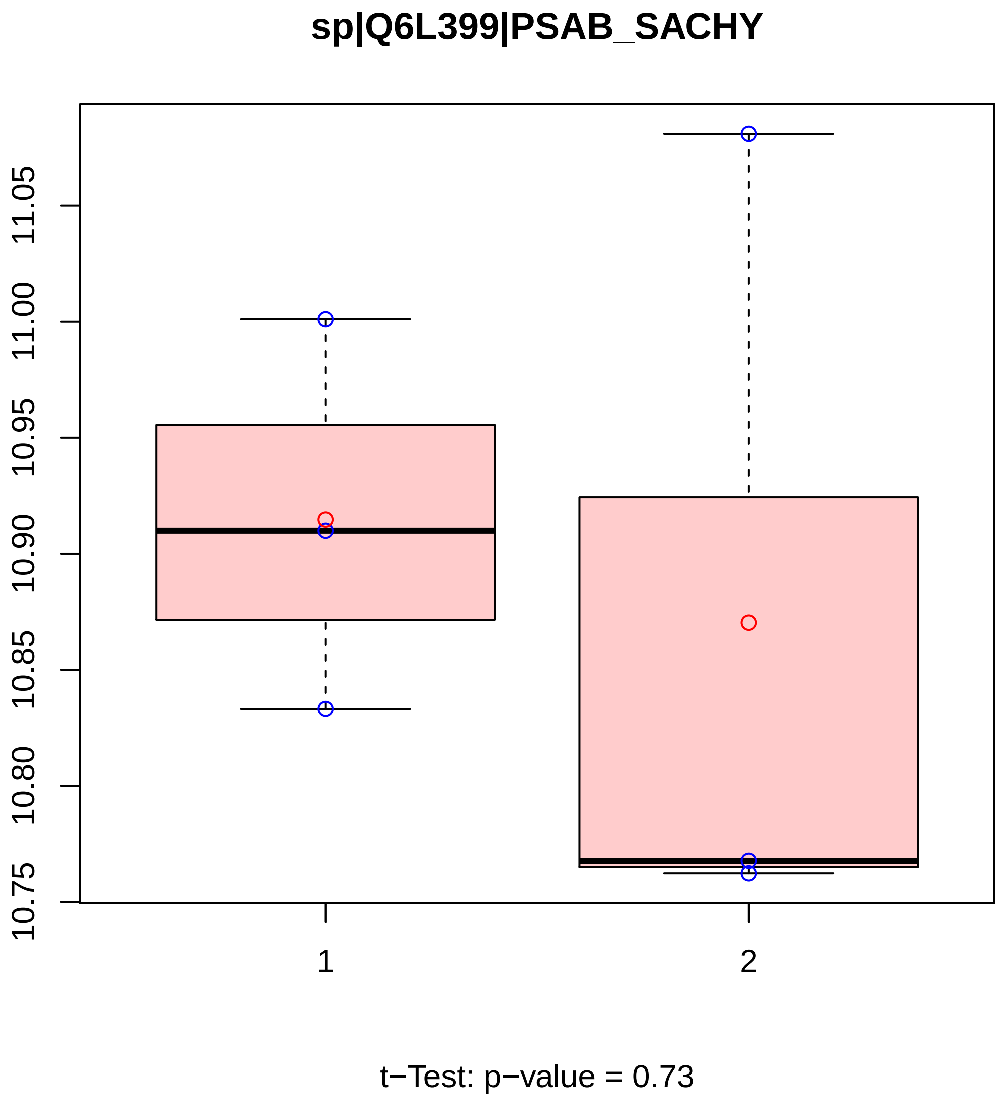
sp|Q6L399|PSAB_SACHY
10.75
10.80
10.85
10.90
10.95
11.00
11.05
1
2
t−Test: p−value = 0.73

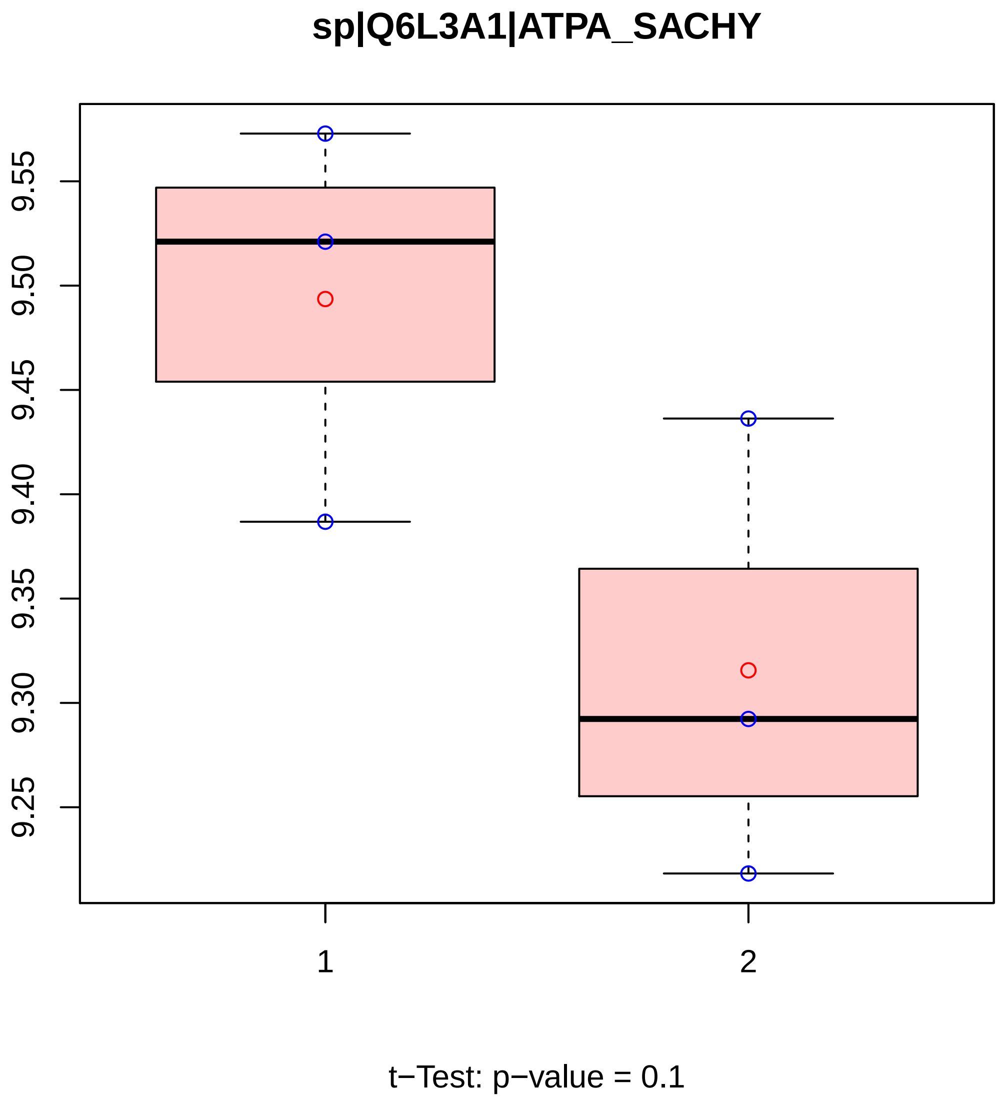
sp|Q6L3A1|ATPA_SACHY
9.55
9.50
9.45
9.40
9.35
9.30
9.25
1
2
t−Test: p−value = 0.1

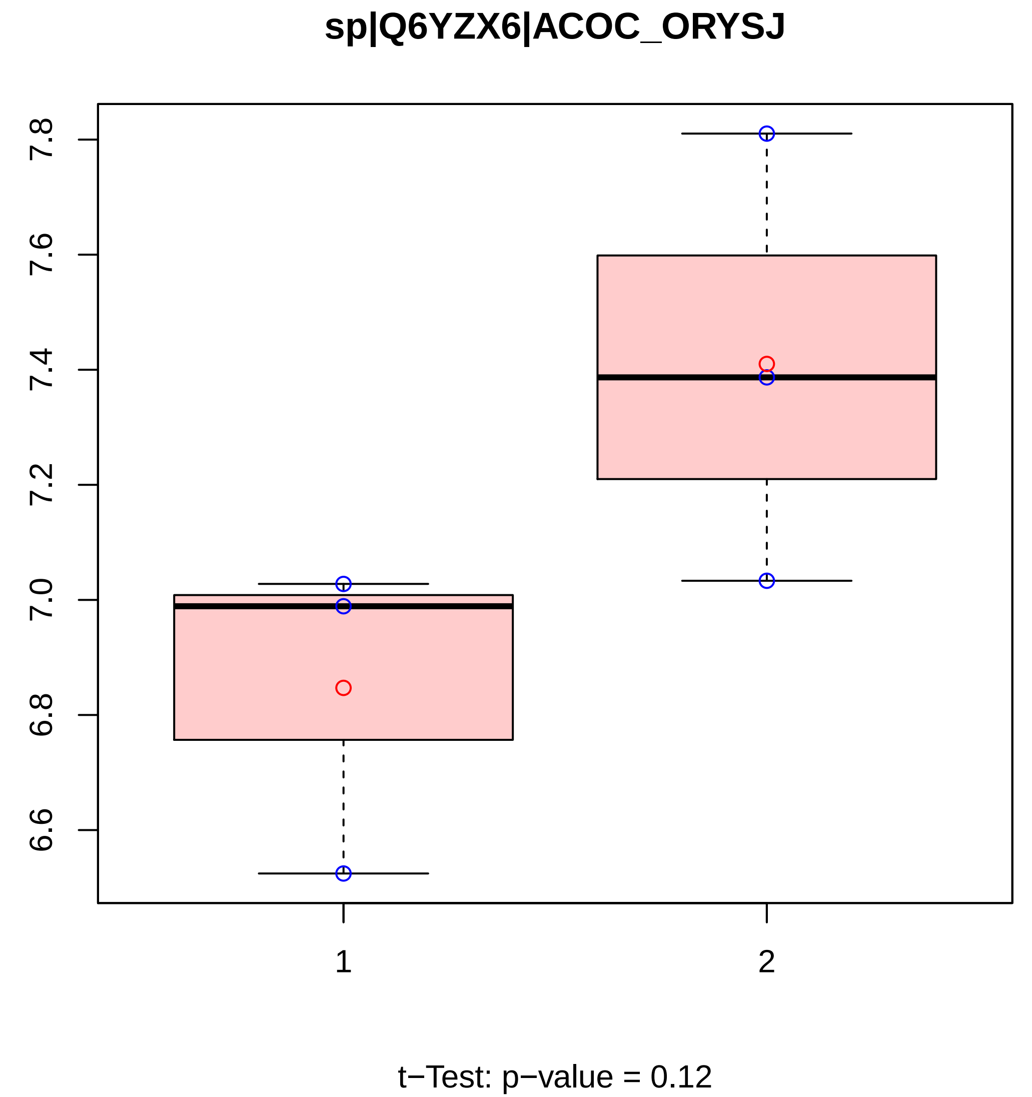
sp|Q6YZX6|ACOC_ORYSJ
7.8
7.6
7.4
7.2
7.0
6.8
6.6
1
2
t−Test: p−value = 0.12

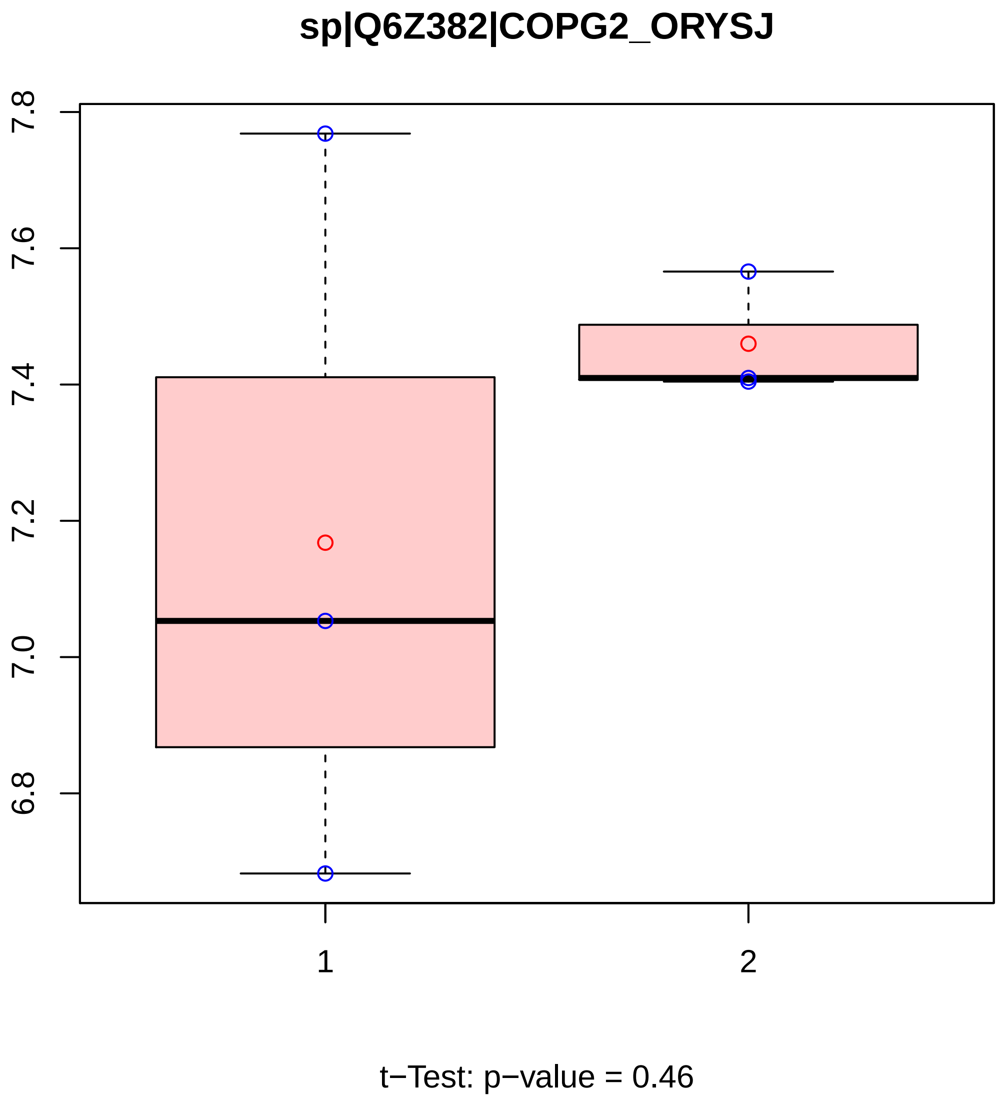
sp|Q6Z382|COPG2_ORYSJ
7.8
7.6
7.4
7.2
7.0
6.8
1
2
t−Test: p−value = 0.46

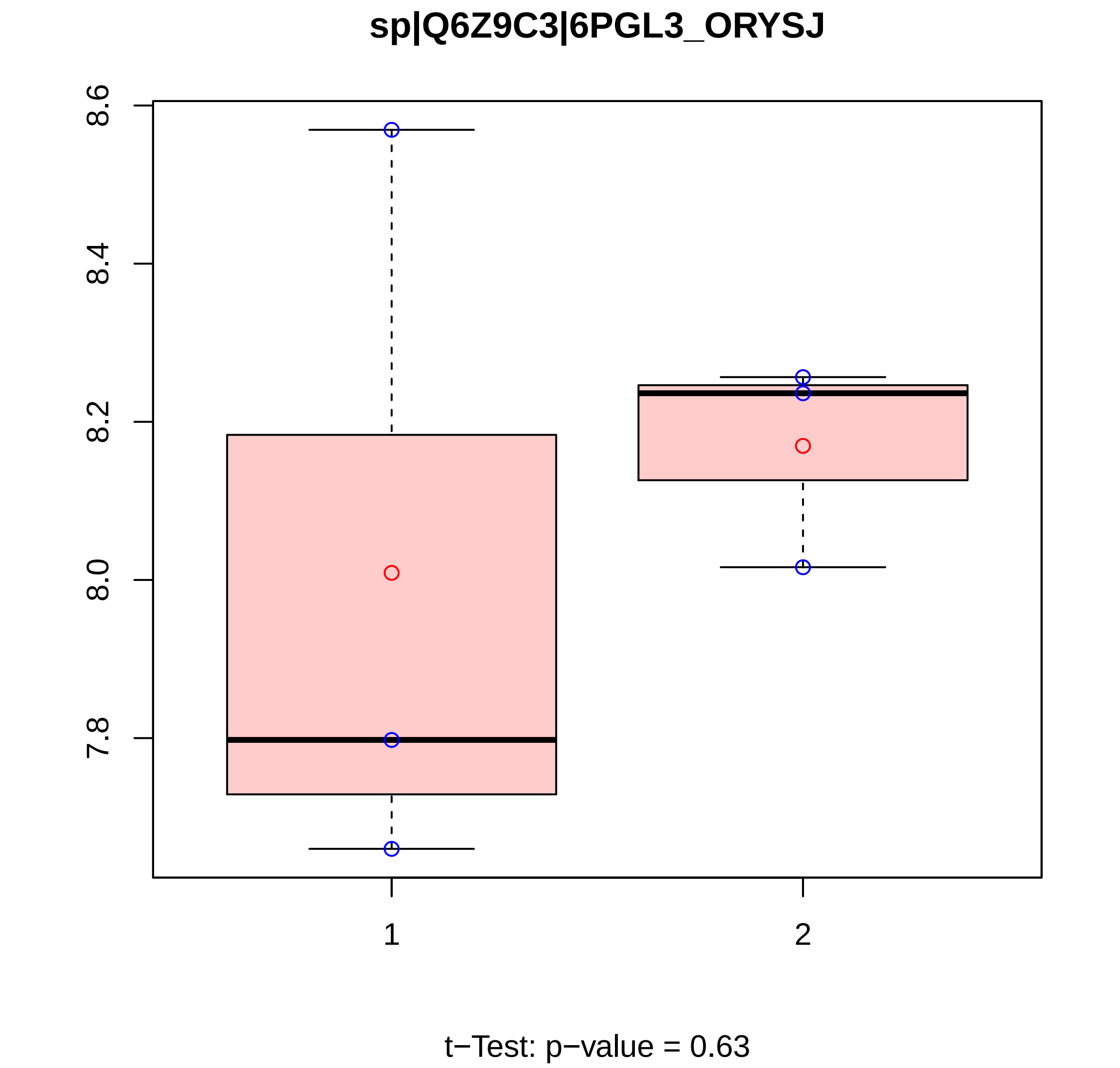

sp|Q6Z9C3|6PGL3_ORYSJ
8.6
8.4
8.2
8.0
7.8
1
2
t−Test: p−value = 0.63

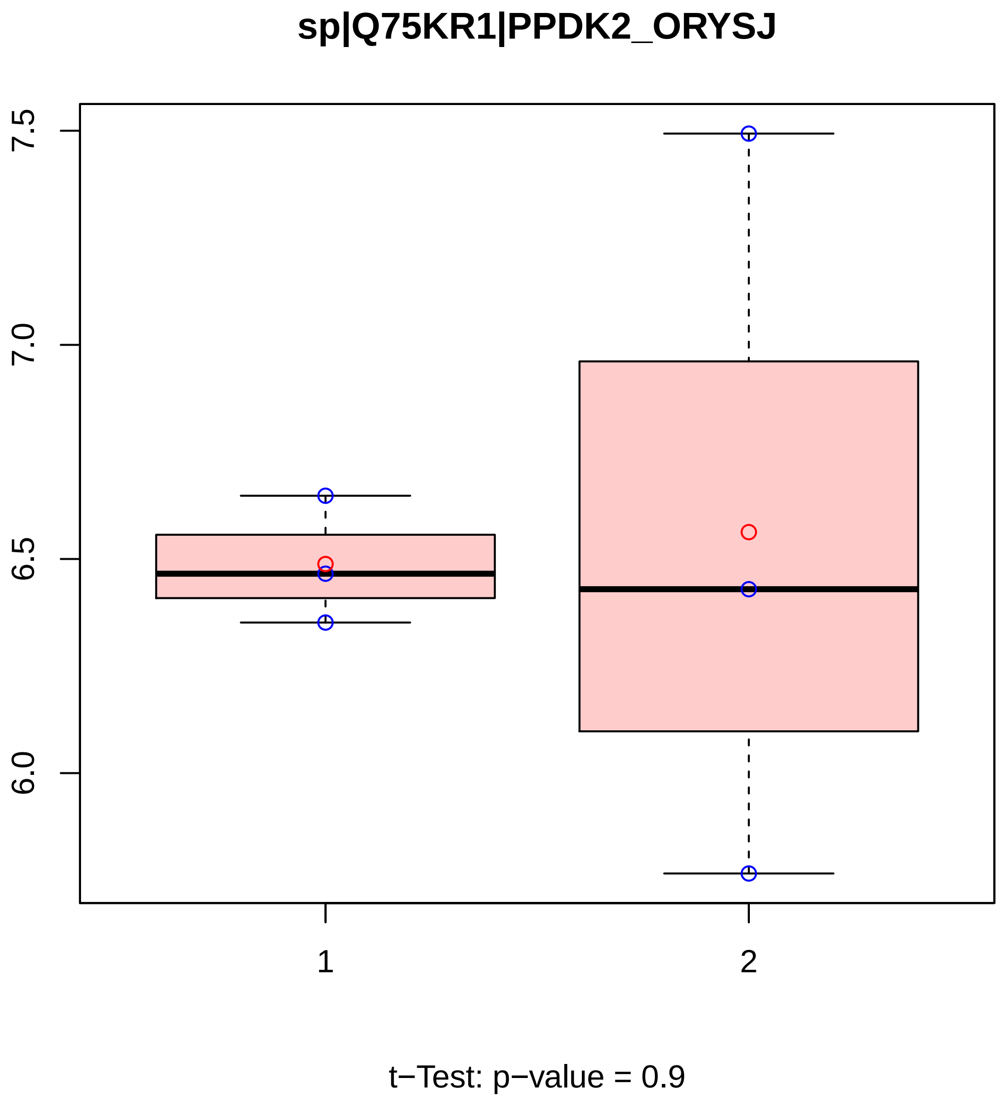
sp|Q75KR1|PPDK2_ORYSJ
7.5
7.0
6.5
6.0
1
2
t−Test: p−value = 0.9

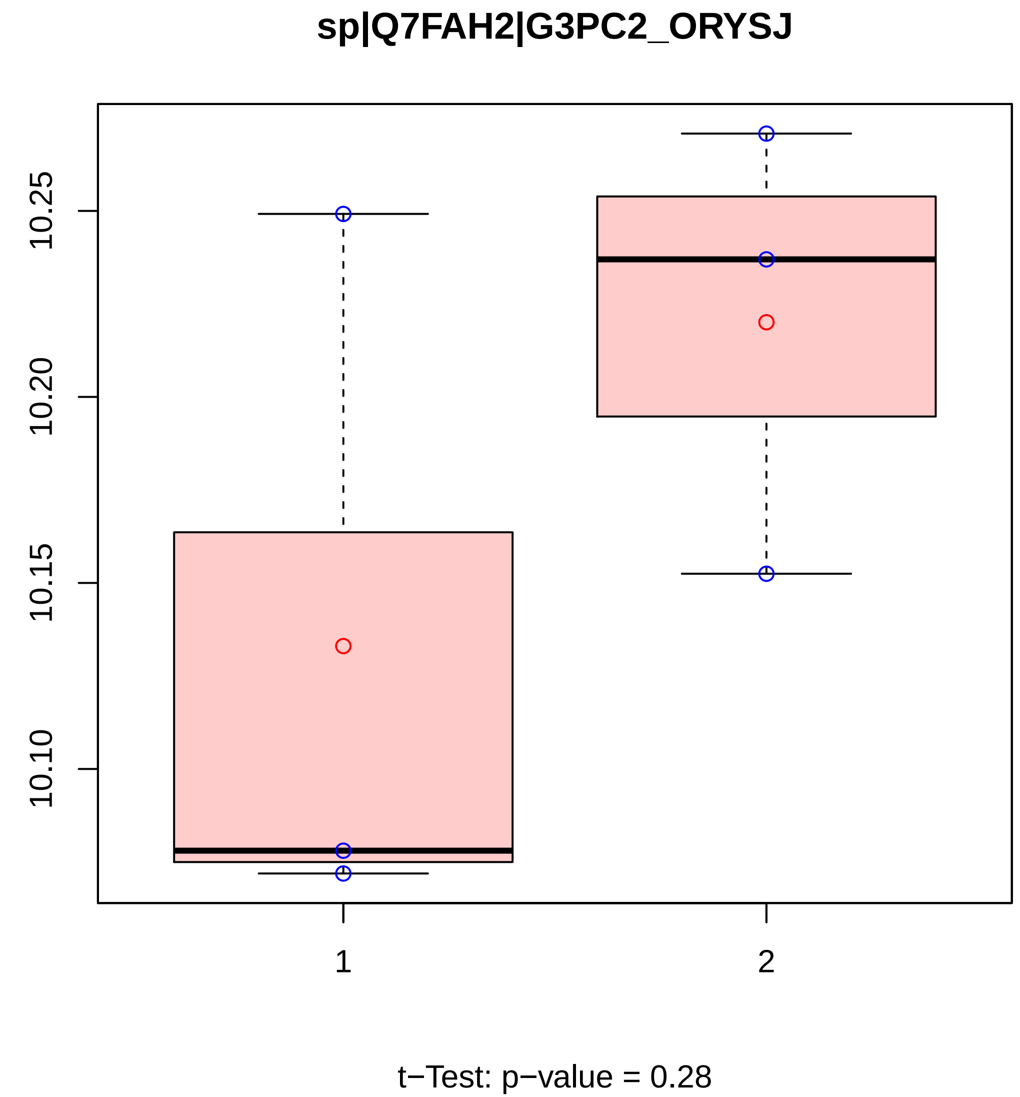
sp|Q7FAH2|G3PC2_ORYSJ
10.10
10.15
10.20
10.25
1
2
t−Test: p−value = 0.28

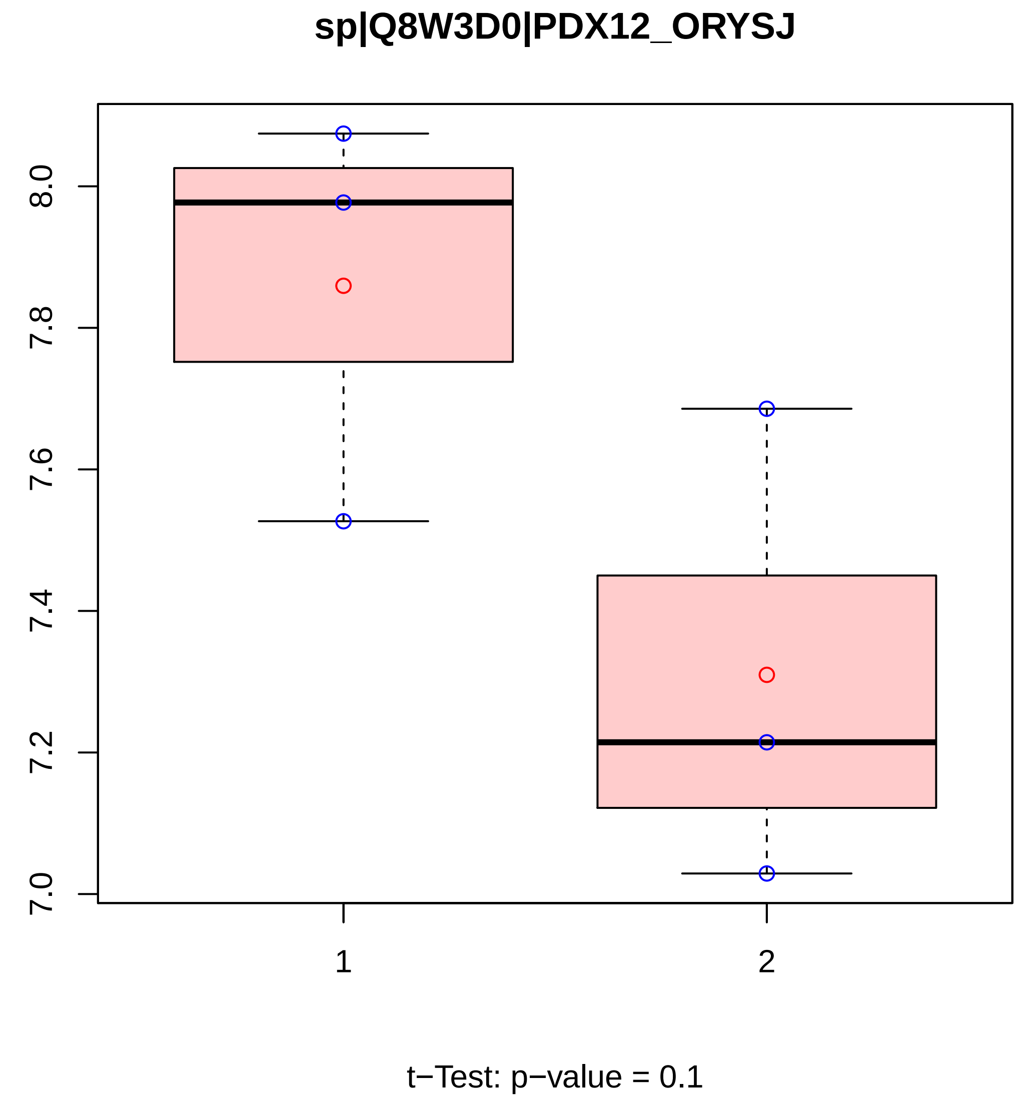

sp|Q8W3D0|PDX12_ORYSJ
8.0
7.8
7.6
7.4
7.2
7.0
1
2
t−Test: p−value = 0.1

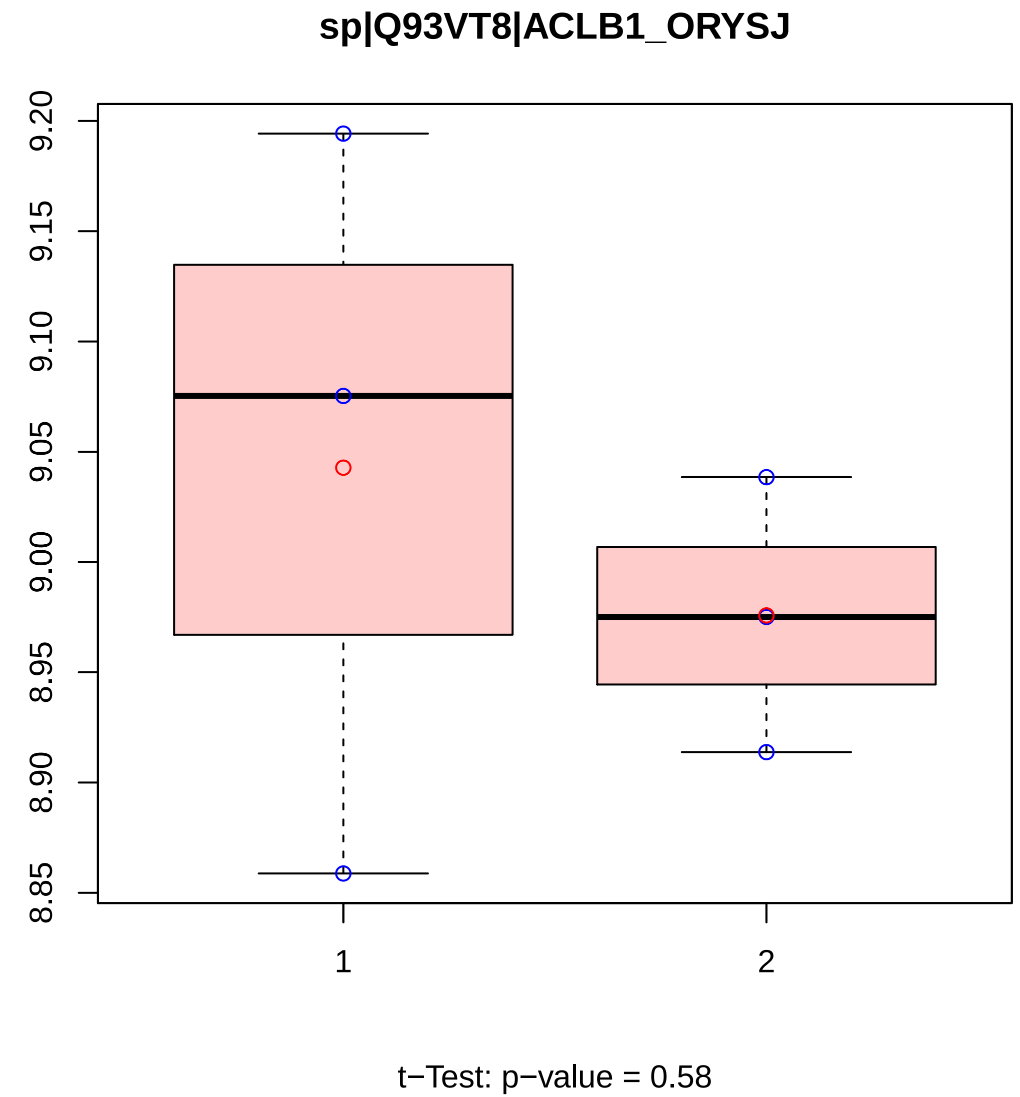
sp|Q93VT8|ACLB1_ORYSJ
9.20
9.15
9.10
9.05
9.00
8.95
8.90
8.85
1
2
t−Test: p−value = 0.58

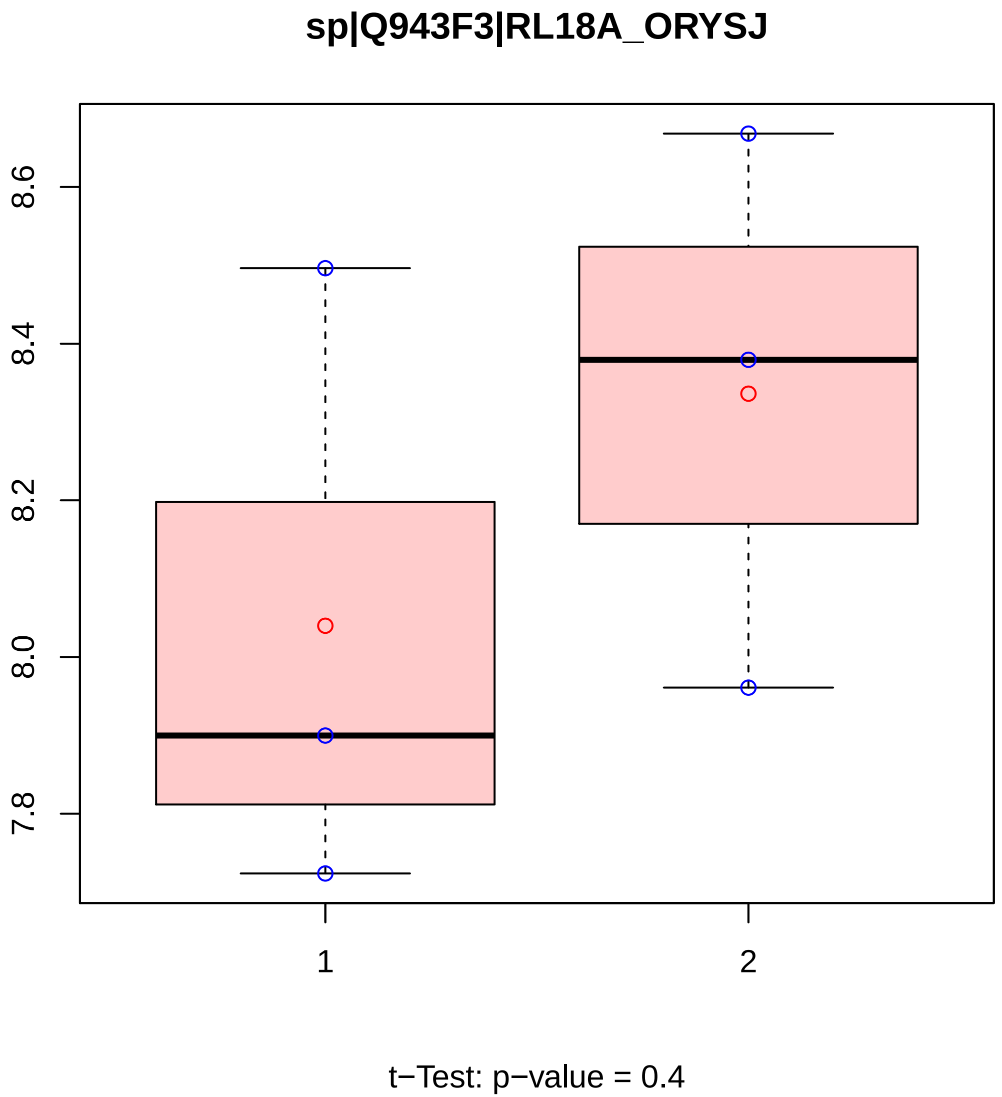
sp|Q943F3|RL18A_ORYSJ
8.6
8.4
8.2
8.0
7.8
1
2
t−Test: p−value = 0.4

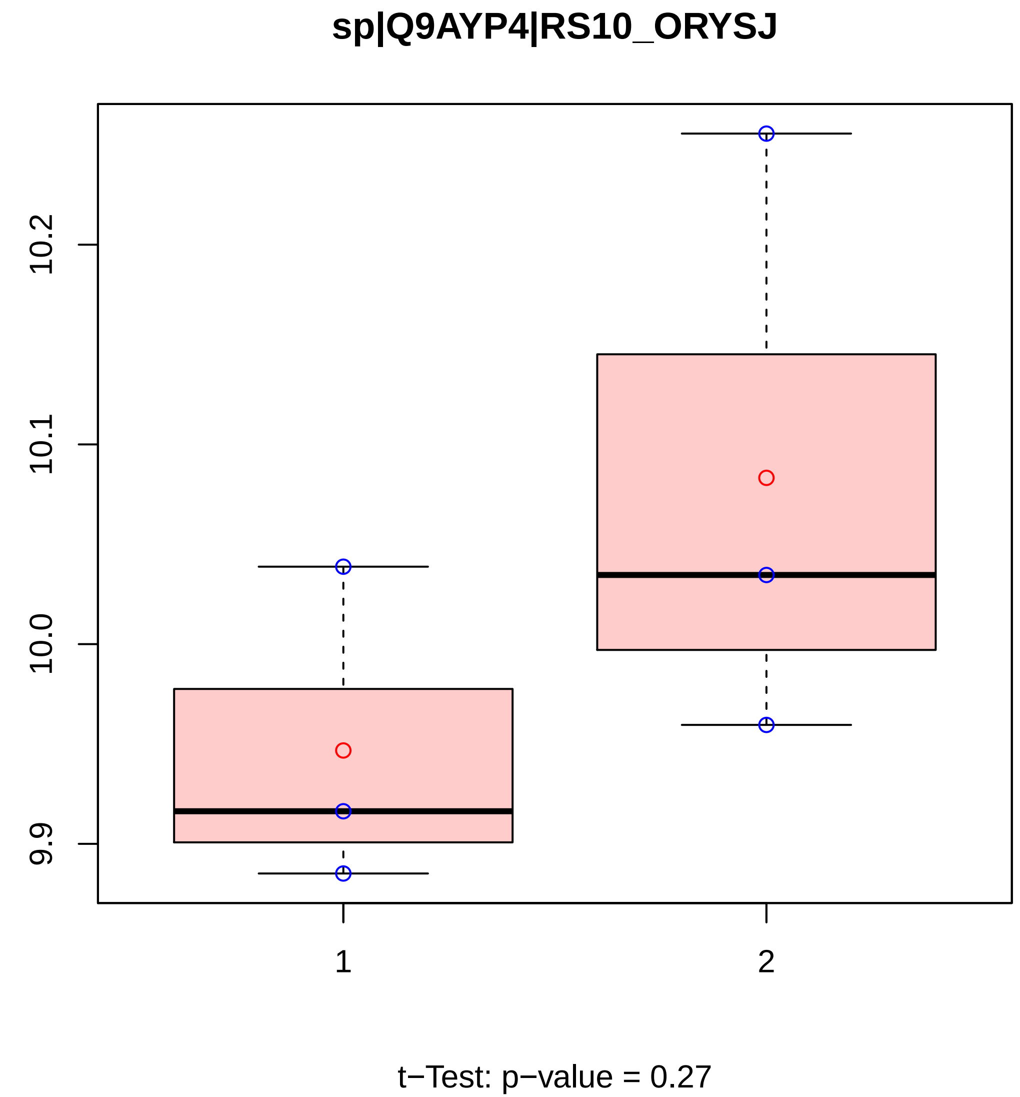
sp|Q9AYP4|RS10_ORYSJ
10.2
10.1
10.0
9.9
1
2
t−Test: p−value = 0.27

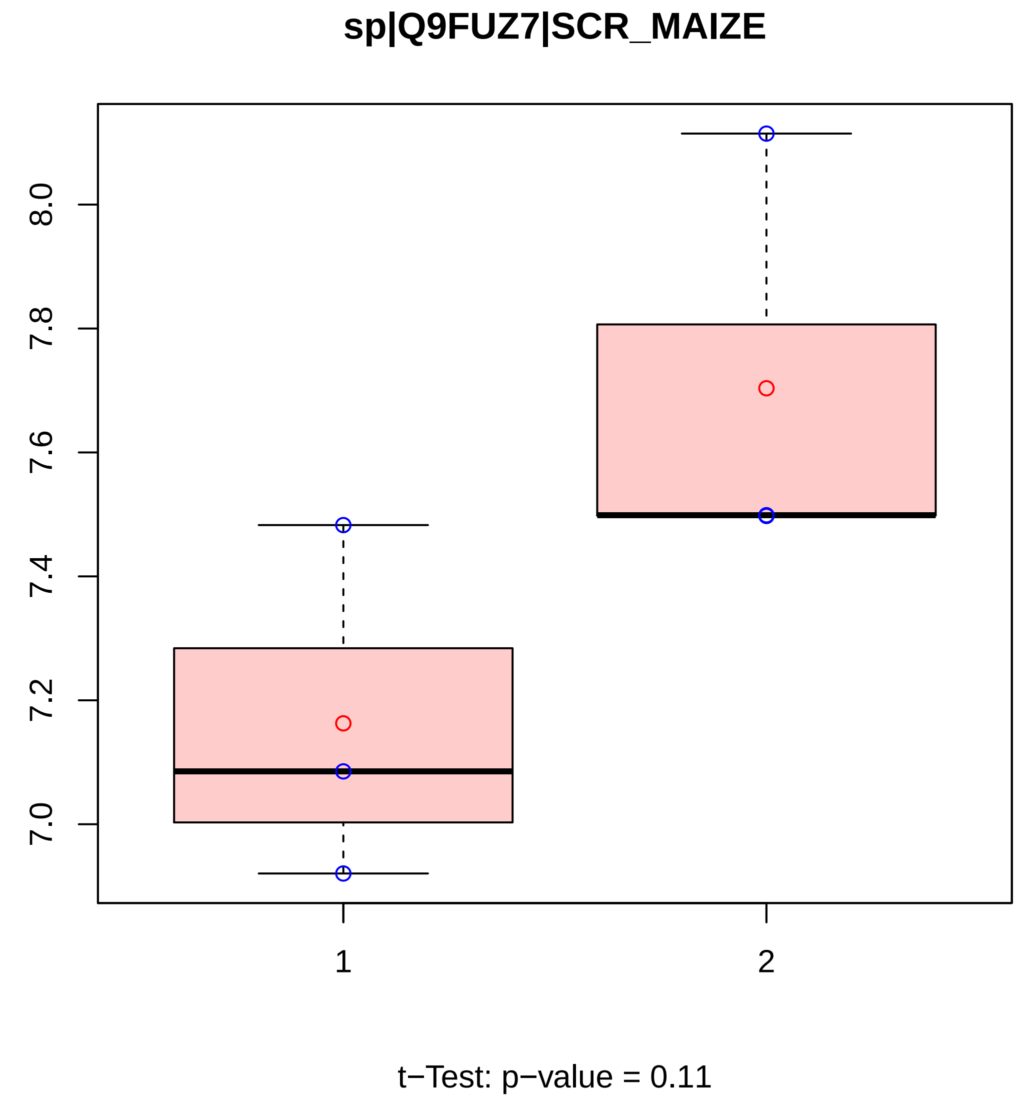
sp|Q9FUZ7|SCR_MAIZE
8.0
7.8
7.6
7.4
7.2
7.0
1
2
t−Test: p−value = 0.11

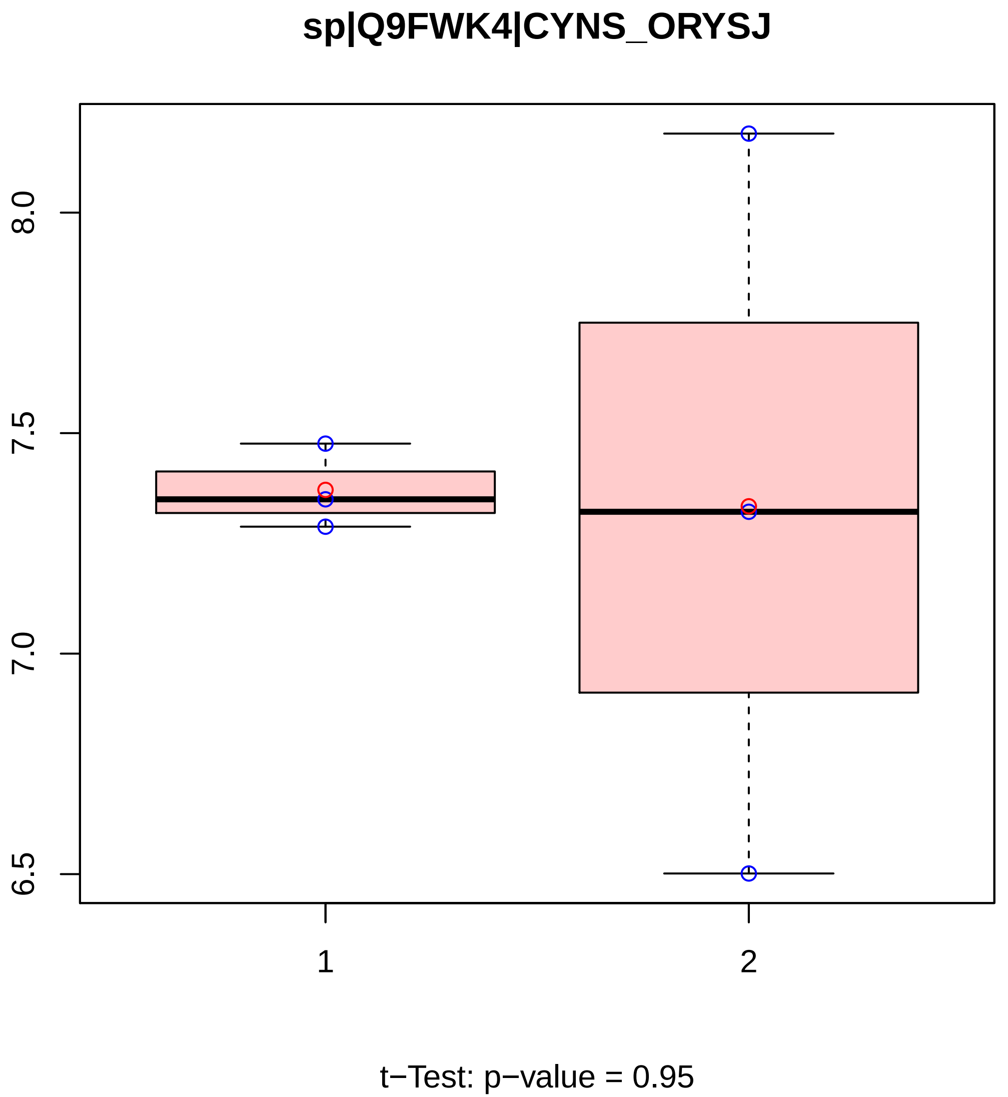
sp|Q9FWK4|CYNS_ORYSJ
8.0
7.5
7.0
6.5
1
2
t−Test: p−value = 0.95

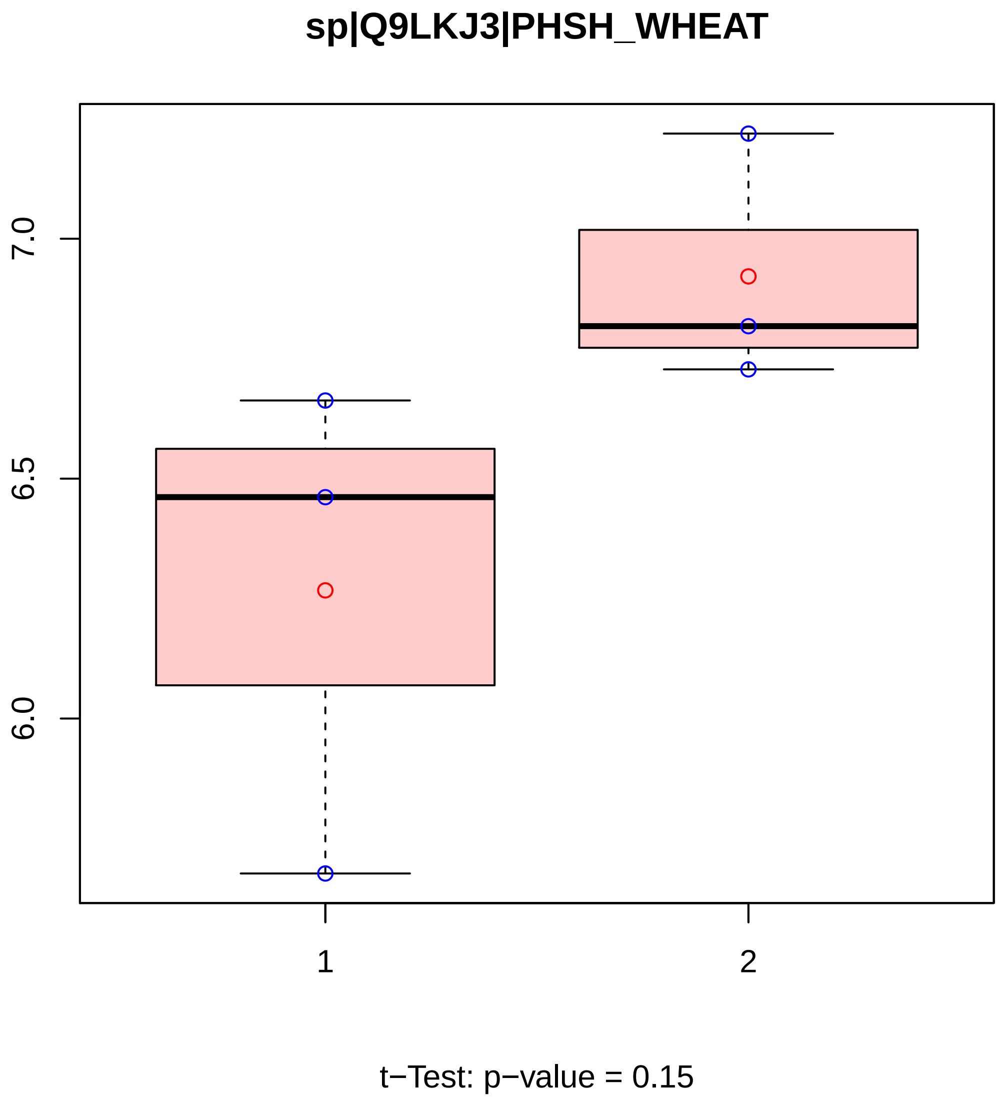
sp|Q9LKJ3|PHSH_WHEAT
7.0
6.5
6.0
1
2
t−Test: p−value = 0.15

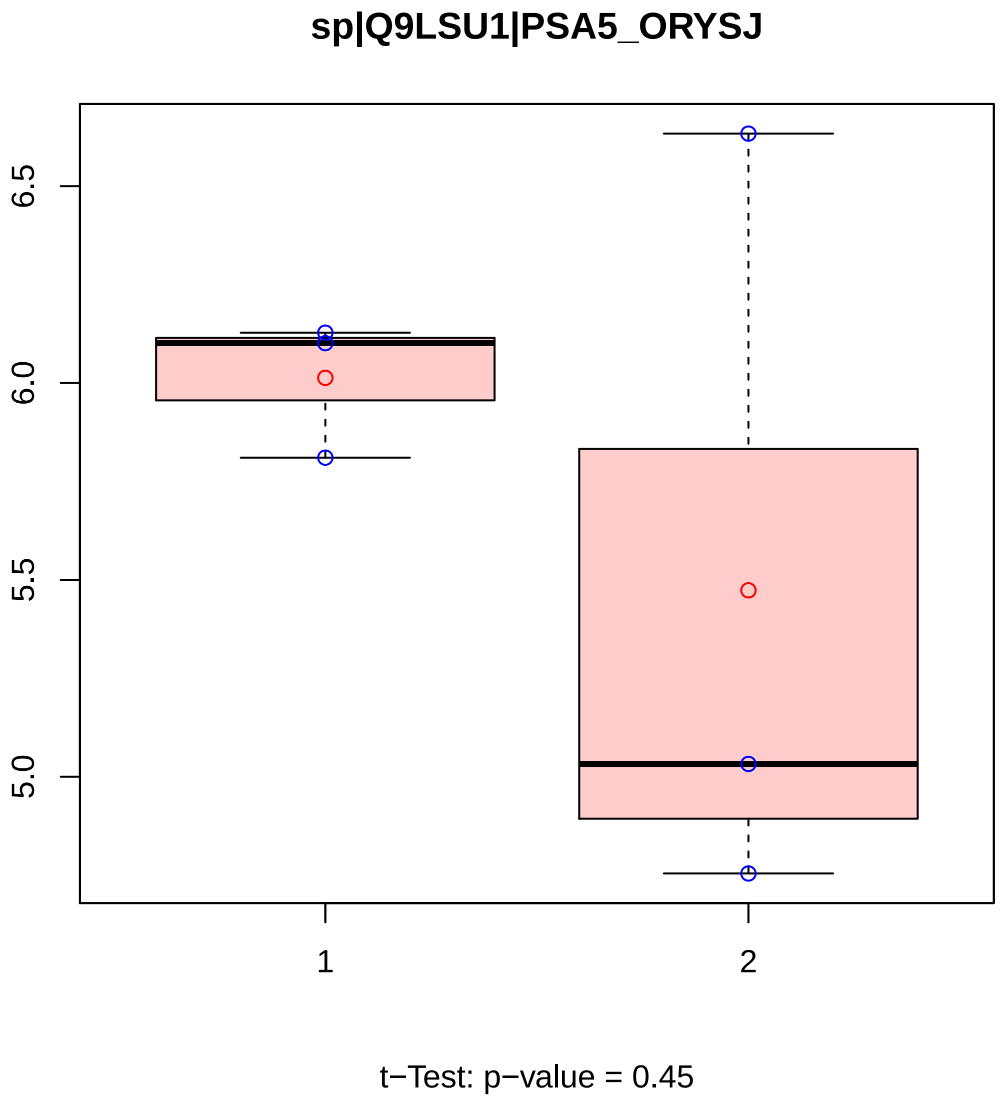

sp|Q9LSU1|PSA5_ORYSJ
6.5
6.0
5.5
5.0
1
2
t−Test: p−value = 0.45

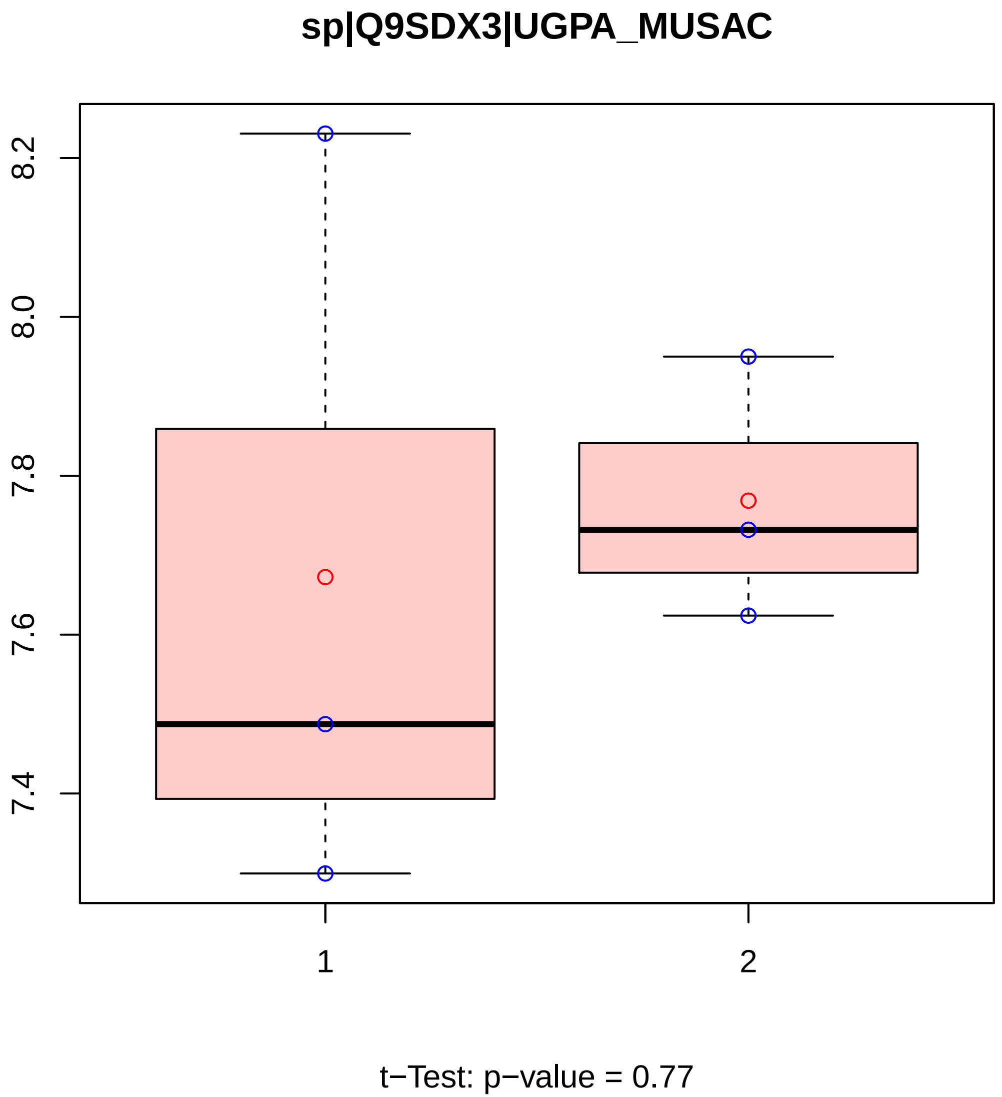
sp|Q9SDX3|UGPA_MUSAC
8.2
8.0
7.8
7.6
7.4
1
2
t−Test: p−value = 0.77

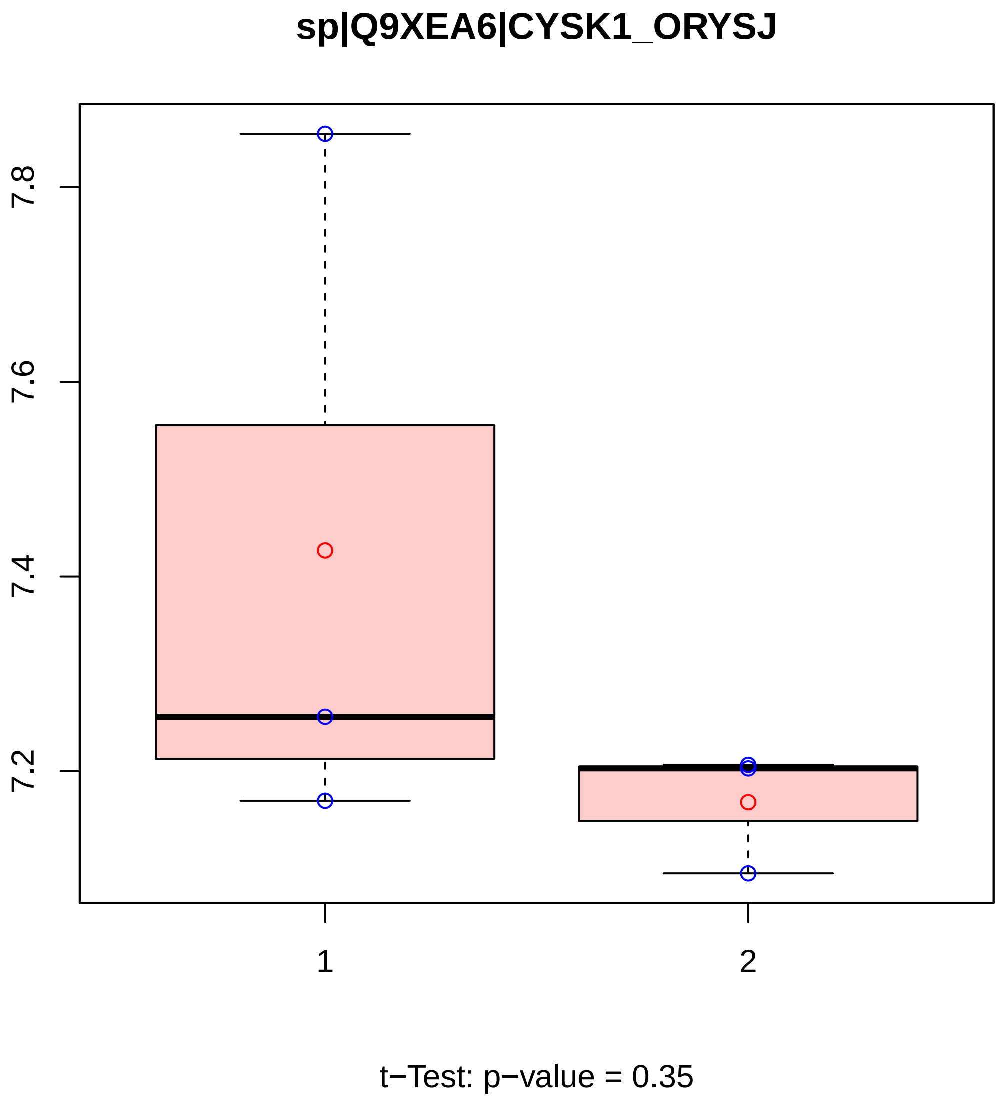

sp|Q9XEA6|CYSK1_ORYSJ
7.8
7.6
7.4
7.2
1
2
t−Test: p−value = 0.35
