# Supplementary material for: A Proteomic Approach to Investigate the Drought Response in the Orphan Crop Eragrostis tef
Source: Proteomes. 2017 Nov 15;5(4):32. doi: 10.3390/proteomes5040032 (PMC5748567; doi:10.3390/proteomes5040032)
Supplement: Supplementary file 1 [file proteomes-05-00032-s001.zip › proteomes-208511 supplementary final/Supplementary Figures and tables.doc]

**Supplementary Figures**

**Figure S1** iTRAQ analysis workflow of tef biological protein samples in preparation for mass spectrometry analysis using an 8-plex iTRAQ labelling system. Hydrated protein samples (H1-H3) represented by iTRAQ labels 115-117, respectively and dehydrated protein samples (D1-D3) represented by iTRAQ labels 118-121, respectively. Labels 113 and 114 were used as internal control standards containing equal amounts of both hydrated and dehydrated pooled protein.

**Figure S2** Steps to tef iTRAQ data pre-processing, refinement and statistical analysis, showing tools used to achieve each step. Data refinement and statistical analysis performed as follows: **(A)** peptides with only 1 or 2 expression values present (of the 3 for each hydrated or dehydrated treatment) in the labelled channels (115 to 121) were removed, peptides with a zero were kept if the zero values were found consecutively in the hydrated (115-117) or dehydrated (118-121) labelled channels (i.e. 115-117 had values of 0 while 118-121 had quantitative expression values or vice versa), these peptide expression values of zero were then changed to 1 to avoid later numerical errors; **(B)** refined non-normalised peptide list subjected to quantile normalisation and **(C)** inverse hyperbolic sine (arcsinh) transformation; **(D)** two-group comparison and statistical analysis of normalised, transformed peptide quantitative expression values; **(E)** conversion of respective peptides to corresponding proteins, displayed as box-plots.

**Figure S3** Sanity Check (Q-Q plots) of all labels (113-121) in iTRAQ experiment that have been quantile normalised and archsinh (inverse hyperbolic sine) transformed to observe reporter ion channel (label) distributions. The last figure is a boxplot for all individual channels showing normalised channels.

**Figure S4** Heat map (cluster analysis) of the labelled channels in iTRAQ experiment after data refinement steps. Channels 1 and 2 represent labels 113 and 114 (internal controls), respectively. Channels 3, 4 and 5 represent the hydrated labelled peptides 115, 116 and 117, respectively, while channels 6, 7 and 8 represent dehydrated labelled peptides 118, 119 and 121, respectively.

**Table S1**. Tef differentially regulated proteins based on highly significant fold change and p-values*.

| **Protein ID** | **Protein Description** | **Hydrated** | **Dehydrated** | **Fold change** | **p-value** |
| --- | --- | --- | --- | --- | --- |
| ***A: Up-regulated proteins*** | | | | | |
| **CL3156Contig1** | ---NA--- | 0.69 | 6.05 | 8.73 | 0.004 |
| **CL6974Contig4** | ---NA--- | 0.69 | 6.05 | 8.73 | 0.004 |
| **CL3894Contig4** | leucoanthocyanidin dioxygenase | 0.69 | 5.49 | 7.92 | 0.021 |
| **CL3374Contig5** | ---NA--- | 0.69 | 5.41 | 7.80 | 0.016 |
| **CL57Contig23** | ---NA--- | 0.69 | 5.41 | 7.80 | 0.016 |
| **CL8690Contig2** | ---NA--- | 0.69 | 5.34 | 7.70 | 0.005 |
| **CL3629Contig1** | poly polymerase i | 0.69 | 5.13 | 7.41 | 0.013 |
| **CL3629Contig2** | poly polymerase i | 0.69 | 5.13 | 7.41 | 0.013 |
| **CL7746Contig3** | ---NA--- | 0.69 | 5.04 | 7.28 | 0.013 |
| **CL873Contig3** | cyclin-p4-1 | 0.69 | 5.04 | 7.28 | 0.013 |
| **CL2976Contig3** | ---NA--- | 0.69 | 4.94 | 7.12 | 0.007 |
| **CL68Contig6** | peroxidase 3-rare cold-inducible protein | 0.66 | 4.49 | 6.82 | 0.008 |
| **CL8983Contig3** | ---NA--- | 0.66 | 4.49 | 6.82 | 0.008 |
| **CL4104Contig2** | gras family protein 2 | 0.69 | 4.68 | 6.75 | 0.003 |
| **CL1942Contig1** | ---NA--- | 0.69 | 4.53 | 6.54 | 0.006 |
| **CL2699Contig6** | monodehydroascorbate cytoplasmic isoform 2 | 0.69 | 4.53 | 6.54 | 0.006 |
| **isotig02308** | probable monodehydroascorbate cytoplasmic isoform 2 | 0.69 | 4.53 | 6.54 | 0.006 |
| ***B: Down-regulated proteins*** | | | | | |
| **CL3227Contig1** | ---NA--- | 5.69 | 0.69 | 0.12 | 0.018 |
| **CL3227Contig2** | ---NA--- | 5.69 | 0.69 | 0.12 | 0.018 |
| **CL58Contig14** | ---NA--- | 5.69 | 0.69 | 0.12 | 0.018 |
| **CL58Contig2** | ---NA--- | 5.69 | 0.69 | 0.12 | 0.018 |
| **CL14686Contig1** | alliin lyase 1 | 5.51 | 0.69 | 0.13 | 0.015 |
| **CL1456Contig11** | s-adenosylmethionine decarboxylase proenzyme | 5.33 | 0.69 | 0.13 | 0.007 |
| **CL1456Contig8** | s-adenosylmethionine decarboxylase proenzyme | 5.33 | 0.69 | 0.13 | 0.007 |
| **isotig10649** | s-adenosylmethionine decarboxylase proenzyme | 5.33 | 0.69 | 0.13 | 0.007 |
| **CL1Contig5286** | metal tolerance protein 5 | 5.00 | 0.69 | 0.14 | 0.029 |
| **CL1Contig5699** | metal tolerance protein 6 | 5.00 | 0.69 | 0.14 | 0.029 |
| **CL1Contig8303** | metal tolerance protein 5 | 5.00 | 0.69 | 0.14 | 0.029 |
| **CL1595Contig2** | ---NA--- | 4.99 | 0.69 | 0.14 | 0.009 |
| **CL11972Contig1** | metal tolerance protein 5 | 6.23 | 1.19 | 0.19 | 0.004 |

* Protein ID: protein identifier; protein description: identified protein; hydrated: averaged quantitative expression value of group 1 (hydrated labels 115-117); dehydrated: averaged quantitative expression value of group 2 (dehydrated labels 118-121); fold change: change in quantitative expression of proteins with dehydration stress (group 2/group 1) where values > 2 display a significant increase in protein expression and values < 0.5 display a significant decrease in protein expression; p-value: associated p-value during two-group analysis for statistical significance (p-value ≤ 0.05).

**Table S2** TEU up-regulated proteins in response to dehydration stress*.

| **Protein ID** | **Protein description** | **Hydrated (Group 1)** | **Dehydrated (Group 2)** | **Fold change** | **p-value** |
| --- | --- | --- | --- | --- | --- |
| **CL856Contig3** | 40s ribosomal protein s28 | 9.17 | 9.33 | 1.02 | 0.001 |
| **CL1127Contig6** | morc family cw-type zinc finger protein 4 | 6.62 | 7.33 | 1.11 | 0.002 |
| **CL4104Contig2** | gras family protein 2 | 0.88 | 4.68 | 5.32 | 0.002 |
| **CL1Contig10662** | beta-glucosidase 10 | 5.90 | 6.63 | 1.12 | 0.004 |
| **CL14878Contig1** | ---NA--- | 6.19 | 6.81 | 1.10 | 0.005 |
| **CL5492Contig2** | fructose-bisphosphate aldolase cytoplasmic isozyme | 10.69 | 10.87 | 1.02 | 0.005 |
| **CL714Contig6** | gdp-mannose -epimerase 1 | 4.93 | 6.35 | 1.29 | 0.005 |
| **CL124Contig7** | ---NA--- | 8.03 | 8.57 | 1.07 | 0.006 |
| **CL6Contig61** | ---NA--- | 0.88 | 5.10 | 5.80 | 0.006 |
| **CL1Contig8614** | magnesium-protoporphyrin ix monomethyl ester | 7.93 | 8.30 | 1.05 | 0.007 |
| **CL2976Contig3** | ---NA--- | 0.88 | 4.95 | 5.63 | 0.007 |
| **CL8163Contig2** | actin-1 | 6.48 | 7.47 | 1.15 | 0.007 |
| **CL243Contig5** | serine threonine-protein kinase 11-interacting protein | 8.07 | 8.72 | 1.08 | 0.008 |
| **CL5983Contig4** | udp-glucose:cinnamate glucosyltransferase | 0.88 | 4.76 | 5.41 | 0.01 |
| **CL7060Contig1** | eukaryotic translation initiation factor 3 subunit h | 7.78 | 8.01 | 1.03 | 0.011 |
| **CL1Contig7473** | protoporphyrinogen chloroplastic | 0.88 | 5.93 | 6.74 | 0.014 |
| **CL394Contig12** | ---NA--- | 7.82 | 8.39 | 1.07 | 0.014 |
| **CL1322Contig3** | ubiquinol oxidase mitochondrial | 6.26 | 6.92 | 1.11 | 0.015 |
| **CL1Contig4551** | ---NA--- | 0.88 | 5.40 | 6.14 | 0.016 |
| **CL1Contig4553** | chlorophyll a-b binding protein chloroplastic | 6.71 | 7.42 | 1.11 | 0.017 |
| **CL7068Contig1** | ---NA--- | 0.88 | 5.54 | 6.29 | 0.017 |
| **CL8759Contig1** | proteasome subunit beta type-3 | 4.72 | 6.50 | 1.38 | 0.018 |
| **CL1533Contig11** | uncharacterized oxidoreductase chloroplastic | 7.33 | 7.77 | 1.06 | 0.019 |
| **CL3894Contig4** | leucoanthocyanidin dioxygenase | 0.88 | 5.50 | 6.25 | 0.021 |
| **SPEE4_ERATE** | spermine synthase | 5.28 | 6.38 | 1.21 | 0.021 |
| **CL1317Contig7** | glyceraldehyde-3-phosphate dehydrogenase cytosolic | 0.88 | 4.92 | 5.59 | 0.023 |
| **CL136Contig17** | ---NA--- | 5.03 | 6.62 | 1.32 | 0.023 |
| **CL1498Contig7** | guanosine nucleotide diphosphate dissociation inhibitor 2 | 5.51 | 6.78 | 1.23 | 0.024 |
| **CL5895Contig4** | calcium sensing chloroplastic | 7.72 | 7.95 | 1.03 | 0.024 |
| **CL4852Contig2** | ---NA--- | 8.90 | 9.52 | 1.07 | 0.025 |
| **CL4000Contig1** | monodehydroascorbate reductase | 9.93 | 10.23 | 1.03 | 0.027 |
| **CL61Contig20** | probable ufm1-specific protease | 7.49 | 8.09 | 1.08 | 0.028 |
| **CL13655Contig1** | ---NA--- | 5.12 | 6.54 | 1.28 | 0.03 |
| **CL1Contig3898** | photosystem ii 10 kda chloroplastic | 7.54 | 8.13 | 1.08 | 0.03 |
| **CL1Contig7889** | ---NA--- | 8.41 | 9.03 | 1.07 | 0.031 |
| **CL433Contig3** | atpase family aaa domain-thorase | 8.39 | 8.55 | 1.02 | 0.033 |
| **CL1030Contig10** | nucleolar gtp-binding protein nsn1 | 5.79 | 7.17 | 1.24 | 0.037 |
| **CL102Contig20** | ---NA--- | 6.33 | 7.08 | 1.12 | 0.038 |
| **CL2Contig153** | splicing factor u2af large subunit a | 6.14 | 6.98 | 1.14 | 0.038 |
| **CL9427Contig2** | ---NA--- | 8.88 | 9.07 | 1.02 | 0.039 |
| **CL1371Contig3** | nadph--cytochrome p450 reductase | 5.76 | 6.58 | 1.14 | 0.041 |
| **CL1Contig167** | extracellular lipase | 8.56 | 8.92 | 1.04 | 0.042 |
| **CL1Contig10732** | chlorophyll a-b binding protein chloroplastic | 9.10 | 9.22 | 1.01 | 0.045 |
| **Locus_11848_5_6** | lim domain-containing protein wlim1 | 7.21 | 7.68 | 1.07 | 0.05 |

* Protein ID: protein identifier; protein description: identified protein; hydrated: averaged quantitative expression value of group 1 (hydrated labels 115-117) ; dehydrated: averaged quantitative expression value of group 2 (dehydrated labels 118-121) ; fold change: change in quantitative expression of proteins with dehydration stress ( group 2/group 1), where values > 1 display an increase in protein expression; p-value: associated p-value during two-group analysis for statistical significance (p-value ≤ 0.05).

**Table S3** TEU down-regulated proteins in response to dehydration stress *.

| **Protein ID** | **Protein description** | **Hydrated (Group 1)** | **Dehydrated (Group 2)** | **Fold change** | **p-value** |
| --- | --- | --- | --- | --- | --- |
| **CL111Contig6** | ---NA--- | 6.04 | 0.88 | 0.15 | 0.001 |
| **CL2546Contig3** | 40s ribosomal protein s2-4 | 7.82 | 7.36 | 0.94 | 0.001 |
| **CL5604Contig1** | 2-methyl-6-phytyl- -hydroquinone methyltransferase chloroplastic | 9.37 | 8.93 | 0.95 | 0.001 |
| **CL8805Contig2** | ---NA--- | 8.94 | 7.98 | 0.89 | 0.001 |
| **CL1042Contig2** | alpha-glucan water chloroplastic precursor | 9.22 | 8.76 | 0.95 | 0.002 |
| **CL2878Contig1** | xanthine dehydrogenase | 5.42 | 0.88 | 0.16 | 0.002 |
| **CL6845Contig2** | ---NA--- | 5.34 | 0.88 | 0.16 | 0.002 |
| **CL1Contig3694** | ---NA--- | 5.52 | 0.88 | 0.16 | 0.005 |
| **CL323Contig2** | 187-kda microtubule-associated protein air9 | 8.65 | 8.23 | 0.95 | 0.006 |
| **CL2349Contig3** | protein dek | 9.76 | 9.29 | 0.95 | 0.007 |
| **CL682Contig1** | nuclease harbi1 | 7.22 | 5.73 | 0.79 | 0.01 |
| **CL8133Contig1** | electron transfer flavoprotein subunit mitochondrial s | 7.44 | 6.18 | 0.83 | 0.01 |
| **CL1013Contig3** | probable plastid-lipid-associated protein chloroplastic | 7.27 | 6.05 | 0.83 | 0.011 |
| **CL1Contig962** | ---NA--- | 6.14 | 0.88 | 0.14 | 0.012 |
| **SECA9_ERATE** | protein translocase subunit chloroplastic short | 9.92 | 9.68 | 0.98 | 0.013 |
| **CL16131Contig1** | ---NA--- | 8.34 | 7.87 | 0.94 | 0.014 |
| **CL3513Contig2** | btb poz and math domain-containing protein 3 | 8.26 | 7.9 | 0.96 | 0.014 |
| **Locus_2288_7_9** | ---NA--- | 9.04 | 8.6 | 0.95 | 0.014 |
| **CL6495Contig2** | polyamine oxidase | 7.43 | 6.7 | 0.90 | 0.015 |
| **CL1670Contig2** | intron-binding protein aquarius | 7.4 | 6.28 | 0.85 | 0.016 |
| **CL1Contig8969** | ---NA--- | 10.54 | 10.33 | 0.98 | 0.016 |
| **CL7215Contig2** | ---NA--- | 8.94 | 8.42 | 0.94 | 0.016 |
| **CL14686Contig1** | alliin lyase 1 | 5.4 | 0.88 | 0.16 | 0.017 |
| **CL14873Contig1** | ---NA--- | 4.94 | 0.88 | 0.18 | 0.017 |
| **CL1759Contig3** | photosynthetic ndh subunit of subcomplex b chloroplastic | 5.78 | 0.88 | 0.15 | 0.017 |
| **CL7716Contig2** | ---NA--- | 7.74 | 7.25 | 0.94 | 0.018 |
| **CL2491Contig2** | ---NA--- | 4.94 | 0.88 | 0.18 | 0.019 |
| **CL2706Contig2** | probable gtp-binding protein | 7.8 | 7 | 0.90 | 0.019 |
| **CL4711Contig1** | e3 ubiquitin-protein ligase ring1a | 8.01 | 7.61 | 0.95 | 0.019 |
| **CL1Contig8961** | ubiquitin-nedd8-like protein rub1 | 8.18 | 7.74 | 0.95 | 0.021 |
| **CL316Contig3** | ---NA--- | 5.7 | 0.88 | 0.15 | 0.022 |
| **CL3294Contig6** | nad h-quinone oxidoreductase subunit chloroplastic | 9.37 | 9.17 | 0.98 | 0.024 |
| **CL1Contig3562** | ---NA--- | 8.14 | 7.54 | 0.93 | 0.026 |
| **CL227Contig12** | beta-amylase chloroplastic | 7.19 | 6.6 | 0.92 | 0.026 |
| **CL154Contig18** | ankyrin repeat domain-containing protein chloroplastic a | 9.84 | 9.61 | 0.98 | 0.027 |
| **CL1662Contig3** | ---NA--- | 7.44 | 6.7 | 0.90 | 0.027 |
| **CL1805Contig7** | protein dj-1 homolog b | 8.1 | 7.57 | 0.93 | 0.027 |
| **CL338Contig6** | ---NA--- | 8.44 | 8.16 | 0.97 | 0.03 |
| **CL612Contig6** | inactive ubiquitin carboxyl-terminal hydrolase 54 | 7.16 | 6.77 | 0.95 | 0.03 |
| **CL714Contig5** | gdp-mannose -epimerase 1 | 7.2 | 6.93 | 0.96 | 0.032 |
| **CL7534Contig1** | probable mannan synthase 9 | 7.17 | 6.54 | 0.91 | 0.033 |
| **CL3204Contig2** | ---NA--- | 6.44 | 5.77 | 0.90 | 0.034 |
| **CL909Contig5** | phytoene chloroplastic flags | 7 | 6.25 | 0.89 | 0.034 |
| **CL44Contig17** | ras-related protein ric2 | 8.08 | 7.91 | 0.98 | 0.035 |
| **CL5788Contig2** | ---NA--- | 8.64 | 8.56 | 0.99 | 0.036 |
| **CL10469Contig1** | nadp-dependent d-sorbitol-6-phosphate dehydrogenase | 6.5 | 5.54 | 0.85 | 0.038 |
| **CL811Contig3** | protein phr1-like 1 | 7.45 | 6.96 | 0.93 | 0.038 |
| **CL1Contig4635** | protease do-like 14 | 6.8 | 5.87 | 0.86 | 0.039 |
| **CL2948Contig2** | haloalkane dehalogenase | 7.98 | 7.56 | 0.95 | 0.039 |
| **CL52Contig6** | ---NA--- | 7.03 | 6.39 | 0.91 | 0.039 |
| **CL1035Contig6** | phosphoglycerate chloroplastic | 9.75 | 9.57 | 0.98 | 0.04 |
| **CL5563Contig3** | ---NA--- | 9.04 | 8.76 | 0.97 | 0.04 |
| **Locus_49_80_82** | chlorophyll a-b binding protein of lhcii type chloroplastic | 10.48 | 10.36 | 0.99 | 0.04 |
| **CL1Contig1301** | activating signal cointegrator 1 complex subunit 3 | 8.45 | 8.01 | 0.95 | 0.041 |
| **CL7698Contig2** | 50s ribosomal protein chloroplastic | 9.53 | 9.172 | 0.96 | 0.041 |
| **CL1Contig4227** | nad h-quinone oxidoreductase subunit chloroplastic | 8.44 | 7.94 | 0.94 | 0.042 |
| **CL424Contig8** | ---NA--- | 6.74 | 5.89 | 0.87 | 0.043 |
| **Locus_471_2_7** | glutamine chloroplastic | 10.33 | 10.1 | 0.98 | 0.043 |
| **CL321Contig12** | ---NA--- | 8.24 | 7.66 | 0.93 | 0.044 |
| **CL10226Contig1** | ---NA--- | 8.04 | 7.08 | 0.88 | 0.045 |
| **CL36Contig12** | nad-dependent malic enzyme 59 kda mitochondrial | 8.87 | 8.57 | 0.97 | 0.046 |
| **CL3496Contig11** | chlorophyll a-b binding protein 1b | 10.14 | 9.84 | 0.97 | 0.047 |
| **CL9878Contig2** | ---NA--- | 8.54 | 8.27 | 0.97 | 0.047 |
| **CL113Contig3** | 40s ribosomal protein s20-2 | 7.87 | 7.27 | 0.92 | 0.048 |
| **CL1Contig2108** | ---NA--- | 9.07 | 8.32 | 0.92 | 0.048 |
| **CL3757Contig1** | ankyrin repeat-containing protein | 8.2 | 7.54 | 0.92 | 0.048 |
| **CL2414Contig3** | ---NA--- | 6.24 | 4.58 | 0.73 | 0.05 |

* Protein ID: protein identifier; protein description: identified protein; hydrated: averaged quantitative expression value of group 1 (hydrated labels 115-117); dehydrated: averaged quantitative expression value of group 2 (dehydrated labels 118-121); fold change: change in quantitative expression with dehydration stress (group 2/group 1), where values < 1 display a decrease in protein expression; p-value: associated p-value during two-group analysis for statistical significance (p-value ≤ 0.05).

**Table S4** MU up-regulated proteins in response to dehydration stress *.

| **Protein ID** | **Protein description** | **Hydrated (Group 1)** | **Dehydrated (Group 2)** | **Fold change** | **p-value** |
| --- | --- | --- | --- | --- | --- |
| **A2Y6W7** | 30s ribosomal protein s1 | 6.33 | 7.3 | 1.15 | 0.001 |
| **H6WCP2** | glutamine synthetase cytosolic isozyme 1-2 | 5.67 | 6.92 | 1.22 | 0.002 |
| **I1R1T6** | ---NA--- | 5.93 | 7.07 | 1.19 | 0.002 |
| **M0SLX3** | probable nad h dehydrogenase fqr1-like 2 | 1 | 5.81 | 5.81 | 0.002 |
| **M0TFQ1** | phosphoglucose isomerase | 7.89 | 8.44 | 1.07 | 0.003 |
| **J3L4S9** | ---NA--- | 8.3 | 8.84 | 1.07 | 0.004 |
| **J3NA21** | probable lrr receptor-like serine threonine-protein kinase | 4.94 | 6.35 | 1.29 | 0.004 |
| **K3Z4Y8** | serine threonine-protein kinase chloroplastic | 6.11 | 6.94 | 1.14 | 0.004 |
| **F2EE28** | chaperonin cpn60- mitochondrial | 1 | 5.27 | 5.27 | 0.006 |
| **B8YAB7** | ---NA--- | 1 | 5.47 | 5.47 | 0.007 |
| **K7UL12** | ribulose bisphosphate carboxylase large chain | 6.59 | 7.66 | 1.16 | 0.007 |
| **M0S163** | spermidine synthase 1 short | 8.17 | 8.67 | 1.06 | 0.007 |
| **I1PWQ2** | bisphosphoglycerate-independent phosphoglycerate mutase | 6.7 | 7.52 | 1.12 | 0.008 |
| **Q09EN6** | atp synthase subunit mitochondrial | 8.23 | 8.69 | 1.06 | 0.008 |
| **C5WRI0** | phospholipase a1- chloroplastic | 1 | 5 | 5.00 | 0.009 |
| **C5XWE5** | glycerophosphodiester phosphodiesterase gdpdl3 | 9.76 | 9.95 | 1.02 | 0.009 |
| **M8AZH0** | enolase 1 a | 10.8 | 10.95 | 1.01 | 0.009 |
| **K3XM31** | 60s ribosomal protein l13-2 | 8.96 | 9.35 | 1.04 | 0.01 |
| **K3YEI2** | v-type proton atpase 16 kda proteolipid subunit | 7.19 | 7.66 | 1.07 | 0.01 |
| **M7ZWS2** | red chlorophyll catabolite reductase | 6.92 | 7.54 | 1.09 | 0.01 |
| **I1P1A2** | udp-glucuronate:xylan alpha-glucuronosyltransferase 1 | 7.34 | 7.84 | 1.07 | 0.011 |
| **K4APG4** | abc transporter i family member chloroplastic | 1 | 5.25 | 5.25 | 0.011 |
| **K7V3Y4** | bisphosphoglycerate-independent phosphoglycerate mutase | 7.96 | 8.17 | 1.03 | 0.011 |
| **M8B2N0** | peroxidase 3 -rare cold-inducible protein | 8.33 | 8.53 | 1.02 | 0.011 |
| **C5XWJ8** | elongation factor ef-tu chloroplastic | 1 | 5.44 | 5.44 | 0.012 |
| **M8BP49** | atp synthase f1 sector subunit alpha | 1 | 5.38 | 5.38 | 0.012 |
| **B8BH08** | ---NA--- | 6.21 | 7.17 | 1.15 | 0.013 |
| **J3LQZ7** | ---NA--- | 6.83 | 7.58 | 1.11 | 0.013 |
| **B5AMJ8** | alpha-glucan h isozyme | 7.82 | 8.51 | 1.09 | 0.015 |
| **Q2EZ09** | probable lrr receptor-like serine threonine-protein kinase | 5.56 | 6.74 | 1.21 | 0.015 |
| **I1HPZ8** | cytosolic isocitrate dehydrogenase | 5.03 | 6.77 | 1.35 | 0.016 |
| **F2D6T1** | 4-diphosphocytidyl-2-c-methyl-d-erythritol chloroplastic | 8.28 | 8.62 | 1.04 | 0.017 |
| **I1PYX0** | ---NA--- | 8.99 | 9.27 | 1.03 | 0.017 |
| **J3MU66** | heat shock protein 81-1 | 8.52 | 9.12 | 1.07 | 0.017 |
| **Q688K0** | guanosine nucleotide diphosphate dissociation inhibitor 1 | 6.51 | 7.07 | 1.09 | 0.017 |
| **Q6TUC6** | glycine hydroxymethyltransferase | 6.11 | 6.54 | 1.07 | 0.017 |
| **J3LZ24** | glyceraldehyde-3-phosphate dehydrogenase cytosolic | 7.57 | 7.85 | 1.04 | 0.018 |
| **H6T2S0** | dna-directed rna polymerase subunit gamma | 7.66 | 8.19 | 1.07 | 0.019 |
| **Q8M914** | atp synthase subunit chloroplastic a | 6.41 | 7.58 | 1.18 | 0.019 |
| **B4FQ90** | vacuolar-processing enzyme | 7.08 | 7.65 | 1.08 | 0.02 |
| **J3LEH7** | glyceraldehyde-3-phosphate dehydrogenase cytosolic | 6.6 | 7.02 | 1.06 | 0.02 |
| **K4A8N1** | serine methylase flags | 7.65 | 7.98 | 1.04 | 0.021 |
| **C5YBL4** | heat shock protein 82 | 7.67 | 8.35 | 1.09 | 0.022 |
| **I1Q436** | homeobox protein knotted-1-like 11 | 9.04 | 9.32 | 1.03 | 0.022 |
| **J3KZC5** | s-adenosylmethionine synthase 2 | 7.83 | 8.32 | 1.06 | 0.022 |
| **K3Y5D4** | elongation factor g- chloroplastic | 8.37 | 8.68 | 1.04 | 0.022 |
| **K3XG60** | protein iq-domain 14 | 7.92 | 8.43 | 1.06 | 0.023 |
| **F2E4B4** | elongation factor 2 -ef-2 | 5.68 | 6.8 | 1.20 | 0.025 |
| **Q84TA3** | leukotriene a-4 hydrolase homolog | 6.44 | 7.13 | 1.11 | 0.025 |
| **A3BY14** | mitochondrial outer membrane protein porin 1 | 7.69 | 8.31 | 1.08 | 0.027 |
| **M0SYI2** | f-box protein skip23 | 8.37 | 8.86 | 1.06 | 0.028 |
| **C5WYH9** | disease resistance rpp13-like protein 1 | 7.22 | 7.65 | 1.06 | 0.029 |
| **F2CTM7** | proline--trna ligase | 8.1 | 8.37 | 1.03 | 0.029 |
| **K3YQN8** | luminal-binding protein 3 | 10.32 | 10.5 | 1.02 | 0.031 |
| **K3Z4G5** | heat shock cognate 70 kda protein 2 | 10.76 | 10.9 | 1.01 | 0.032 |
| **Q5SBT2** | atp synthase subunit chloroplastic | 6.37 | 7.19 | 1.13 | 0.032 |
| **K3XUT7** | arf guanine-nucleotide exchange factor gnom | 4.86 | 6.29 | 1.29 | 0.033 |
| **B8A2B4** | guanine nucleotide-binding protein subunit beta-like protein a | 5.08 | 7.12 | 1.40 | 0.034 |
| **K7TM76** | serine threonine-protein kinase | 1 | 5.13 | 5.13 | 0.034 |
| **M0TC33** | cytochrome p450 714b2 | 5.79 | 7.17 | 1.24 | 0.037 |
| **M7Z031** | splicing factor u2af large subunit a | 6.13 | 6.98 | 1.14 | 0.037 |
| **M7ZWJ0** | mixed-linked glucan synthase 2 a | 7.68 | 8.04 | 1.05 | 0.037 |
| **F1DI22** | phosphoenolpyruvate carboxylase 1 s | 9.17 | 9.76 | 1.06 | 0.038 |
| **J3NDJ0** | 40s ribosomal protein s9-2 | 9.81 | 9.85 | 1.00 | 0.038 |
| **K3ZIK0** | ribulose bisphosphate carboxylase oxygenase chloroplastic | 7.43 | 7.81 | 1.05 | 0.038 |
| **K3ZRY2** | sodium proton antiporter | 5.86 | 6.64 | 1.13 | 0.038 |
| **M0U0C4** | serine protease chloroplastic | 7.23 | 7.63 | 1.06 | 0.038 |
| **N1QQ42** | probable rna helicase sde3 | 5.07 | 6.37 | 1.26 | 0.04 |
| **B6SYK7** | ---NA--- | 7.05 | 7.69 | 1.09 | 0.041 |
| **K3YJA1** | 14-3-3-like protein gf14-c | 9.5 | 9.75 | 1.03 | 0.041 |
| **K7UU03** | probable histone h2a variant 3 | 6.74 | 7.32 | 1.09 | 0.041 |
| **Q6Q297** | probable 4-coumarate-- ligase 3 | 6.33 | 7.08 | 1.12 | 0.041 |
| **J3NCI1** | tropinone reductase-like 2 | 6.38 | 7.09 | 1.11 | 0.042 |
| **M7ZF75** | alpha-l-arabinofuranosidase 1 | 6.83 | 7.86 | 1.15 | 0.042 |
| **C0PP08** | ---NA--- | 5.98 | 7.03 | 1.18 | 0.043 |
| **J3L4R5** | ---NA--- | 8.42 | 8.93 | 1.06 | 0.043 |
| **Q5JK51** | ---NA--- | 7.27 | 7.89 | 1.09 | 0.043 |
| **Q0D574** | probable indole-3-acetic acid-amido synthetase | 6.82 | 7.63 | 1.12 | 0.045 |
| **B6TV07** | glutamate decarboxylase | 6.76 | 7.45 | 1.10 | 0.046 |
| **E9KJF9** | atp synthase subunit chloroplastic | 6.78 | 7.83 | 1.15 | 0.046 |
| **K4AD01** | cholesterol dehydrogenase | 7.66 | 8.21 | 1.07 | 0.046 |
| **M8C7G4** | cell division cycle protein 48 homolog mj1156 | 9.99 | 10.25 | 1.03 | 0.047 |
| **K7AF06** | 14-3-3-like protein gf14-d | 5.34 | 6.84 | 1.28 | 0.048 |
| **J3MGQ3** | atp-dependent zinc metalloprotease ftsh chloroplastic | 6.98 | 7.86 | 1.13 | 0.049 |
| **Q94HT9** | transposon tf2-12 polyprotein | 1 | 5.07 | 5.07 | 0.05 |

* Protein ID: protein identifier; protein description: identified protein; hydrated: averaged quantitative expression value of group 1 (hydrated labels 115-117) ; dehydrated: averaged quantitative expression value of group 2 (dehydrated labels 118-121) ; fold change: change in quantitative expression of proteins with dehydration stress ( group 2/group 1), where values > 1 display an increase in protein expression; p-value: associated p-value during two-group analysis for statistical significance (p-value ≤ 0.05).

**Table S5** MU down-regulated proteins in response to dehydration stress*.

| **Protein ID** | **Protein description** | **Hydrated (Group 1)** | **Dehydrated (Group 2)** | **Fold change** | **p-value** |
| --- | --- | --- | --- | --- | --- |
| **M0RYP4** | plasma membrane atpase | 8.97 | 7.98 | 0.89 | 0.002 |
| **A2YVN3** | probable lrr receptor-like serine threonine-protein kinase | 7.16 | 6.49 | 0.91 | 0.003 |
| **I1GKL4** | chromosome region maintenance 1 protein homolog | 5.48 | 1 | 0.18 | 0.004 |
| **J3LTP8** | 60s ribosomal protein l4-1 | 4.66 | 1 | 0.21 | 0.004 |
| **J7F224** | 50s ribosomal protein chloroplastic | 6.12 | 1 | 0.16 | 0.004 |
| **K3ZXX1** | 40s ribosomal protein s12 | 5.7 | 1 | 0.18 | 0.004 |
| **K7V3R8** | ribonucleoprotein complex subunit 1-like protein 1 | 7.4 | 6.14 | 0.83 | 0.004 |
| **Q40693** | heat shock cognate 70 kda protein 2 | 5.57 | 1 | 0.18 | 0.004 |
| **A7DX42** | phosphoenolpyruvate carboxylase 1 | 8.1 | 7.29 | 0.90 | 0.005 |
| **I1HY72** | ---NA--- | 6.14 | 1 | 0.16 | 0.005 |
| **K3YHA5** | glutamate decarboxylase | 4.78 | 1 | 0.21 | 0.005 |
| **Q2QM69** | 2-methyl-6-phytyl- -hydroquinone methyltransferase chloroplastic | 9.13 | 8.84 | 0.97 | 0.006 |
| **H6UDT9** | light-harvesting complex i 11 kda protein | 5.3 | 1 | 0.19 | 0.008 |
| **K3XID0** | ---NA--- | 4.91 | 1 | 0.20 | 0.01 |
| **K3Z686** | protease do-like chloroplastic | 9.35 | 9.1 | 0.97 | 0.01 |
| **Q2R2U7** | transposon tf2-12 polyprotein | 5.49 | 1 | 0.18 | 0.01 |
| **C0P5W4** | electron transfer flavoprotein subunit mitochondrial | 7.44 | 6.19 | 0.83 | 0.011 |
| **D6C788** | phosphoenolpyruvate | 5.2 | 1 | 0.19 | 0.011 |
| **J3KWV6** | nadh-cytochrome b5 reductase-like protein | 6.93 | 6.22 | 0.90 | 0.011 |
| **K7TWX2** | ---NA--- | 6.77 | 5.29 | 0.78 | 0.011 |
| **M0YEW4** | ---NA--- | 7.4 | 6.83 | 0.92 | 0.011 |
| **B9EUA8** | dual specificity protein kinase pom1 | 6.98 | 5.74 | 0.82 | 0.012 |
| **B9F6D9** | protein stichel-like 3 | 7 | 5.95 | 0.85 | 0.012 |
| **B9G402** | heat stress transcription factor b-1 | 8.32 | 7.87 | 0.95 | 0.012 |
| **J3N642** | signal recognition particle 54 kda chloroplastic | 6.42 | 4.8 | 0.75 | 0.012 |
| **M0WSZ3** | mannose-1-phosphate guanyltransferase alpha | 7.65 | 6.98 | 0.91 | 0.012 |
| **Q0DF89** | ferredoxin--nadp leaf chloroplastic | 5.49 | 1 | 0.18 | 0.012 |
| **I1HQV5** | ---NA--- | 4.93 | 1 | 0.20 | 0.013 |
| **B4FM07** | 2-cys peroxiredoxin chloroplastic | 7.65 | 7.14 | 0.93 | 0.014 |
| **F4Y5B3** | heat shock protein 90-1 | 7.34 | 5.96 | 0.81 | 0.014 |
| **A2X358** | 50s ribosomal protein chloroplastic | 9.01 | 8.38 | 0.93 | 0.015 |
| **Q0IPN8** | ---NA--- | 5.24 | 1 | 0.19 | 0.015 |
| **C5YTE5** | disease resistance rpp8-like protein 3 | 5.71 | 1 | 0.18 | 0.016 |
| **B4FAE5** | cytoplasmic 1 short | 5.19 | 1 | 0.19 | 0.018 |
| **F2D6S1** | mitochondrial substrate carrier family protein b | 7.81 | 7 | 0.90 | 0.018 |
| **K4AI98** | photosystem ii repair protein psb27- chloroplastic | 8.66 | 8.42 | 0.97 | 0.018 |
| **M0U0B7** | enolase chloroplastic | 6.63 | 5.53 | 0.83 | 0.018 |
| **I1NU41** | ---NA--- | 5.66 | 1 | 0.18 | 0.019 |
| **M7Z1H8** | auxin efflux carrier component 8 | 7.19 | 6.35 | 0.88 | 0.019 |
| **Q7XP71** | gypsy retrotransposon integrase-like protein 1 | 5.97 | 1 | 0.17 | 0.019 |
| **I1I0K8** | programmed cell death protein | 6.82 | 5.75 | 0.84 | 0.02 |
| **K3XE49** | protein translocase subunit chloroplastic | 9.44 | 9.1 | 0.96 | 0.02 |
| **F2EBB5** | histone | 8.22 | 7.72 | 0.94 | 0.021 |
| **B8AKV0** | haloacid dehalogenase-like hydrolase domain-containing protein | 7.35 | 6.98 | 0.95 | 0.022 |
| **M8CMG6** | germin-like protein 1 | 5.07 | 1 | 0.20 | 0.022 |
| **C5XKF4** | ---NA--- | 6.74 | 5.99 | 0.89 | 0.023 |
| **I1IP77** | glucose-1-phosphate adenylyltransferase small chloroplastic amyloplastic | 6.64 | 5.63 | 0.85 | 0.023 |
| **A7DX76** | phosphoenolpyruvate carboxylase 3 | 6.47 | 4.96 | 0.77 | 0.024 |
| **E0WCS9** | phosphoenolpyruvate carboxylase 1 | 7.96 | 7.02 | 0.88 | 0.024 |
| **K4AMW7** | disease resistance rpp13-like protein 4 | 7.24 | 6.7 | 0.93 | 0.026 |
| **M0YAF5** | ---NA--- | 7.78 | 6.8 | 0.87 | 0.026 |
| **K3XDR1** | ddt domain-containing protein ddb | 7.77 | 7.2 | 0.93 | 0.028 |
| **B8Y2W7** | 30s ribosomal protein chloroplastic | 8.99 | 8.52 | 0.95 | 0.029 |
| **K6Z6J1** | ascorbate peroxidase cytosolic | 7.41 | 6.7 | 0.90 | 0.029 |
| **M0SUI3** | e3 ubiquitin-protein ligase | 7.9 | 7.44 | 0.94 | 0.03 |
| **C5Z2G8** | peroxidase 5 | 6.08 | 4.65 | 0.76 | 0.031 |
| **K7TZY7** | chlorophyll a-b binding protein chloroplastic | 10.37 | 10.22 | 0.99 | 0.032 |
| **C5YNB9** | nad h azoreductase | 8.99 | 8.71 | 0.97 | 0.036 |
| **C7J7J1** | beta-galactosidase 14 | 9.35 | 8.99 | 0.96 | 0.036 |
| **I1HEZ1** | probable sarcosine oxidase | 6.62 | 5.64 | 0.85 | 0.036 |
| **C5YHN2** | glycine--trna ligase mitochondrial | 6.9 | 4.9 | 0.71 | 0.037 |
| **J3NAA0** | disease resistance protein rpm1 | 8.01 | 7.51 | 0.94 | 0.037 |
| **K3YTT8** | nadp-dependent d-sorbitol-6-phosphate dehydrogenase | 6.58 | 5.53 | 0.84 | 0.037 |
| **B9F8C3** | fumarate hydratase chloroplastic | 9.42 | 5.19 | 0.55 | 0.038 |
| **K3YWM5** | 60s ribosomal protein l14-1 | 9.77 | 9.64 | 0.99 | 0.038 |
| **M0TZF6** | elongation factor 1-gamma 2 | 8.29 | 7.9 | 0.95 | 0.038 |
| **B3SHC6** | atp synthase subunit chloroplastic | 8.04 | 7.53 | 0.94 | 0.039 |
| **C5WWE2** | nad-dependent malic enzyme 59 kda mitochondrial | 6.39 | 4.96 | 0.78 | 0.04 |
| **M8BG98** | trna (cytosine -c )-methyltransferase | 6.66 | 6.08 | 0.91 | 0.04 |
| **F8U875** | glucose phosphomutase 1 | 7.37 | 6.41 | 0.87 | 0.041 |
| **I1I2I7** | photosynthetic ndh subunit of subcomplex b chloroplastic | 9.16 | 8.88 | 0.97 | 0.041 |
| **K3YL45** | translocon at the inner envelope membrane of chloroplasts 62 | 10.01 | 9.91 | 0.99 | 0.041 |
| **M0SDW7** | fructose-bisphosphate cytoplasmic isozyme 1 | 8.99 | 8.37 | 0.93 | 0.042 |
| **Q8RYY4** | ---NA--- | 6.57 | 5.52 | 0.84 | 0.043 |
| **I1IF76** | atp synthase delta chloroplastic | 9.89 | 9.71 | 0.98 | 0.044 |
| **Q9SDJ2** | magnesium-protoporphyrin ix monomethyl ester | 9.18 | 9.03 | 0.98 | 0.044 |
| **F2CXC6** | ---NA--- | 8.01 | 7.09 | 0.89 | 0.045 |
| **M0VAF3** | patellin-3 | 7.26 | 6.69 | 0.92 | 0.045 |
| **Q5MD10** | glutamine synthetase root isozyme 5 | 10.01 | 9.69 | 0.97 | 0.045 |
| **Q6Z1V6** | nad h-quinone oxidoreductase subunit chloroplastic | 8.61 | 7.98 | 0.93 | 0.045 |
| **C5X3Z0** | gamma carbonic anhydrase mitochondrial | 7.67 | 7.06 | 0.92 | 0.046 |
| **M0U3I3** | cysteine synthase | 7.06 | 6.17 | 0.87 | 0.046 |
| **M0WBU8** | squamosa promoter-binding-like protein 15 | 8.07 | 7.67 | 0.95 | 0.047 |
| **J3KZJ0** | ---NA--- | 8.6 | 7.86 | 0.91 | 0.048 |
| **J3M442** | importin subunit alpha-1b | 8.46 | 7.82 | 0.92 | 0.048 |
| **M0RV12** | ribulose bisphosphate carboxylase oxygenase activase chloroplastic | 8.95 | 8.55 | 0.96 | 0.048 |
| **M0S1D8** | chloroplastic | 7.69 | 6.72 | 0.87 | 0.049 |
| **M7ZWK6** | transaminase a | 8.62 | 8.19 | 0.95 | 0.049 |
| **K3Z133** | homoserine kinase | 9.83 | 9.63 | 0.98 | 0.05 |

* Protein ID: protein identifier; protein description: identified protein; hydrated: averaged quantitative expression value of group 1 (hydrated labels 115-117); dehydrated: averaged quantitative expression value of group 2 (dehydrated labels 118-121); fold change: change in quantitative expression with dehydration stress (group 2/group 1), where values < 1 display a decrease in protein expression; p-value: associated p-value during two-group analysis for statistical significance (p-value ≤ 0.05).

**Table S6** Functional enrichment analysis of GO-terms allocated to proteins differentially expressed by being up-regulated **(A)** or down-regulated **(B)** in response to dehydration stress *.

| **GO-ID** | **Term** | **Ontology** | **FDR** | **P-value** | **Over/Under** |
| --- | --- | --- | --- | --- | --- |
| **A: Up-regulated** | | | | | |
| **GO:0031430** | M band | CC | 4.06E-2 | 8.76E-5 | Over |
| **GO:0016656** | monodehydroascorbate reductase (NADH) activity | MF | 1.40E-3 | 1.37E-7 | Over |
| **GO:0005093** | Rab GDP-dissociation inhibitor activity | MF | 1.55E-3 | 3.59E-7 | Over |
| **GO:0005097** | Rab GTPase activator activity | MF | 1.55E-3 | 1.06E-6 | Over |
| **GO:005092** | GDP-dissociation inhibitor activity | MF | 1.55E-3 | 1.06E-6 | Over |
| **GO:0090315** | negative regulation of protein targeting to membrane | BP | 1.55E-3 | 1.06E-6 | Over |
| **GO:0090313** | regulation of protein targeting to membrane | BP | 1.55E-3 | 1.06E-6 | Over |
| **GO:0050771** | negative regulation of axonogenesis | BP | 1.55E-3 | 1.06E-6 | Over |
| **GO:0032851** | positive regulation of Rab GTPase activity | BP | 2.08E-3 | 2.45E-6 | Over |
| **GO:0032483** | regulation of Rab protein signal transduction | BP | 2.08E-3 | 2.45E-6 | Over |
| **GO:0032313** | regulation of Rab GTPase activity | BP | 2.08E-3 | 2.45E-6 | Over |
| **GO:0050768** | negative regulation of neurogenesis | BP | 2.08E-3 | 2.45E-6 | Over |
| **GO:0010721** | negative regulation of cell development | BP | 2.08E-3 | 2.45E-6 | Over |
| **GO:0043903** | regulation of symbiosis, encompassing mutualism through parasitism | BP | 4.26E-3 | 5.42E-6 | Over |
| **GO:0031345** | negative regulation of cell projection organization | BP | 6.27E-3 | 8.60E-6 | Over |
| **GO:0019321** | pentose metabolic process | BP | 2.55E-2 | 3.76E-5 | Over |
| **GO:0048227** | plasma membrane to endosome transport | BP | 2.96E-2 | 4.64E-5 | Over |
| **GO:0009610** | response to symbiotic fungus | BP | 3.11E-2 | 5.74E-5 | Over |
| **GO:0009608** | response to symbiont | BP | 3.11E-2 | 5.74E-5 | Over |
| **GO:0090317** | negative regulation of intracellular protein transport | BP | 3.11E-2 | 6.42E-5 | Over |
| **GO:0032387** | negative regulation of intracellular transport | BP | 3.11E-2 | 6.42E-5 | Over |
| **GO:0051224** | negative regulation of protein transport | BP | 3.11E-2 | 6.42E-5 | Over |
| **B: Down-regulated** | | | | | |
| **GO:0010598** | NAD(P)H dehydrogenase complex (plastoquinone) | CC | 5.06E-4 | 1.48E-7 | Over |
| **GO:0030076** | light-harvesting complex | CC | 1.16E-2 | 2.16E-5 | Over |
| **GO:0005576** | extracellular region | CC | 1.21E-2 | 2.47E-5 | under |
| **GO:0051139** | metal ion: proton antiporter activity | MF | 1.93E-3 | 1.13E-6 | Over |
| **GO:0010486** | manganese: proton antiporter activity | MF | 1.93E-3 | 1.13E-6 | Over |
| **GO:0005384** | manganese ion transmembrane transporter activity | MF | 2.67E-3 | 2.61E-6 | Over |
| **GO:0008810** | cellulase activity | MF | 3.47E-3 | 4.76E-6 | Over |
| **GO:0046524** | sucrose-phosphate synthase activity | MF | 6.24E-3 | 9.18E-6 | Over |
| **GO:0048038** | quinone binding | MF | 9.07E-3 | 1.51E-5 | Over |
| **GO:0015491** | cation: cation antiporter activity | MF | 9.07E-3 | 1.51E-5 | Over |
| **GO:0015299** | solute: proton antiporter activity | MF | 1.61E-2 | 3.47E-5 | Over |
| **GO:0015298** | solute: cation antiporter activity | MF | 1.61E-2 | 3.47E-5 | Over |
| **GO:0016168** | chlorophyll binding | MF | 3.11E-2 | 7.94E-5 | Over |
| **GO:0010258** | NADH dehydrogenase complex (plastoquinone) assembly | BP | 7.53E-5 | 7.37E-9 | Over |
| **GO:0010257** | NADH dehydrogenase complex assembly | BP | 1.30E-4 | 2.54E-8 | Over |
| **GO:0044248** | cellular catabolic process | BP | 1.93E-3 | 1.13E-6 | under |
| **GO:0009768** | photosynthesis, light harvesting in photosystem I | BP | 2.67E-3 | 2.07E-6 | Over |
| **GO:0055071** | manganese ion homeostasis | BP | 2.67E-3 | 2.61E-6 | Over |
| **GO:0030026** | cellular manganese ion homeostasis | BP | 2.67E-3 | 2.61E-6 | Over |
| **GO:0051275** | beta-glucan catabolic process | BP | 3.47E-3 | 4.76E-6 | Over |
| **GO:0042547** | cell wall modification involved in multidimensional cell growth | BP | 3.47E-3 | 4.76E-6 | Over |
| **GO:0030245** | cellulose catabolic process | BP | 3.47E-3 | 4.76E-6 | Over |
| **GO:0071421** | manganese ion transmembrane transport | BP | 1.06E-2 | 1.88E-5 | Over |
| **GO:0006828** | manganese ion transport | BP | 2.06E-2 | 4.65E-5 | Over |
| **GO:0044712** | single-organism catabolic process | BP | 2.54E-2 | 5.97E-5 | under |
| **GO:0010042** | response to manganese ion | BP | 2.79E-2 | 6.84E-5 | Over |
| **GO:0009117** | nucleotide metabolic process | BP | 4.39E-2 | 1.20E-4 | under |
| **GO:0006753** | nucleoside phosphate metabolic process | BP | 4.39E-2 | 1.20E-4 | under |

*Enrichment of GO-terms has been selected on the basis of false discovery rate (FDR < 0.05) and p-value (p-value < 0.01). GO-ID: the ID number of the GO-term. Term: description of the GO-term. Ontology: GO-terms categorization in cellular component (CC), molecular function (MF) biological processes (BP) groups. FDR: the proportion of false positives that was determined by calculating the false discovery rate corresponding to each enrichment score. P-value: p-value indicating the statistical significant difference between the fraction of proteins assigned to GO-term retrieved and the fraction of all proteins within the background set assigned to the same GO-term. Over/under: GO terms under or over-represented in the test set in response to dehydration stress.
